# Age-related ceRNA networks in adult *Drosophila* ageing

**DOI:** 10.3389/fgene.2023.1096902

**Published:** 2023-02-28

**Authors:** Deying Yang, Feng Xiao, Jiamei Li, Siqi Wang, Xiaolan Fan, Qingyong Ni, Yan Li, Mingwang Zhang, Taiming Yan, Mingyao Yang, Zhi He

**Affiliations:** ^1^ College of Animal Science and Technology, Sichuan Agricultural University, Chengdu, China; ^2^ Farm Animal Genetic Resources Exploration and Innovation Key Laboratory of Sichuan Province, Sichuan Agricultural University, Chengdu, China

**Keywords:** *Drosophila*, ageing, ceRNA network, correlation of mRNA/protein, circRNA, lncRNA

## Abstract

As *Drosophila* is an extensively used genetic model system, understanding of its regulatory networks has great significance in revealing the genetic mechanisms of ageing and human diseases. Competing endogenous RNA (ceRNA)-mediated regulation is an important mechanism by which circular RNAs (circRNAs) and long non-coding RNAs (lncRNAs) regulate ageing and age-related diseases. However, extensive analyses of the multiomics (circRNA/miRNA/mRNA and lncRNA/miRNA/mRNA) characteristics of adult *Drosophila* during ageing have not been reported. Here, differentially expressed circRNAs and microRNAs (miRNAs) between 7 and 42-day-old flies were screened and identified. Then, the differentially expressed mRNAs, circRNAs, miRNAs, and lncRNAs between the 7- and 42-day old flies were analysed to identify age-related circRNA/miRNA/mRNA and lncRNA/miRNA/mRNA networks in ageing *Drosophila*. Several key ceRNA networks were identified, such as the dme_circ_0009500/dme_miR-289-5p/*CG31064*, dme_circ_0009500/dme_miR-289-5p/*frizzled*, dme_circ_0009500/dme_miR-985-3p/*Abl*, and XLOC_027736/dme_miR-985-3p/*Abl* XLOC_189909/dme_miR-985-3p/*Abl* networks. Furthermore, real-time quantitative PCR (qPCR) was used to verify the expression level of those genes. Those results suggest that the discovery of these ceRNA networks in ageing adult *Drosophila* provide new information for research on human ageing and age-related diseases.

## 1 Introduction

*Drosophila melanogaster* is an extensively used genetic model system that has been used for more than 100 years to study various aspects of the life sciences. In particular, fruit flies have been widely utilized to study ageing (Gubina et al., 2019) and human diseases, such as cancer (Enomoto et al., 2018), neurodegenerative disease (Cha et al., 2019), obesity and diabetes (Musselman et al., 2019), sterile inflammation (Nainu et al., 2019), and regeneration (Fox et al., 2020). *D. melanogaster* has also been utilized to study complex behavioural and developmental biology topics, including exercise (Watanabe and Riddle, 2019), courtship (Liu et al., 2019), and foraging (Khodaei and Long, 2019). Recently, mounting evidence has suggested that *Drosophila* is an outstanding model for studying ageing and age-related diseases (Surguchov et al., 2019; Brenman-Suttner et al., 2020). Ageing is a physiologic/pathologic process featuring declines in normal physiological functions and progressive impairment of cellular functions (Stallone et al., 2019). The ageing phenomenon has been conserved during biological evolution; even yeast other single-celled eukaryotes experience ageing (He et al., 2019). Thus, in-depth study of the regulatory mechanism of ageing in *Drosophila* can inform the study of human ageing and disease.

Current studies suggest that non-coding RNAs (ncRNAs) are involved in organismal ageing (Kim and Lee, 2019; Tower, 2019). With the development of sequencing technology, dynamic changes in the transcriptome [including changes in mRNA, long non-coding RNA (lncRNA), microRNA (miRNA), and circular RNA (circRNA) (Yang et al., 2016; Perry et al., 2017; Barter et al., 2019; Kinser and Pincus, 2020); the proteome (Brown et al., 2018); and the metabolome (Song et al., 2017)] have been described in the context of *Drosophila* ageing. Most miRNAs, mRNAs, and the proteins in fruit flies are evolutionarily conserved up to humans and regulate similar signalling pathways across organisms, such as the NF-κB, AMPK, mTOR, P53, PGC1α, and FoxO pathways (Zha et al., 2019; Kinser and Pincus, 2020; Wang et al., 2020). Large studies have demonstrated that the circRNA/miRNA/mRNA and axis the lncRNA/miRNA/mRNA axis play vital roles in ageing and age-related disease (Ruan et al., 2020; Wang et al., 2020).

As *Drosophila* is a workhorse model organism, thoroughly studying the characteristics of adult *Drosophila* from a multiomics perspective is necessary. Understanding how these conserved protein genes regulate ageing through ceRNA mechanisms in *Drosophila* is important. However, extensive analyses of the multiomics (circRNA/miRNA/mRNA lncRNA/miRNA/mRNA) characteristics of adult *Drosophila* ageing have not been reported. In the present study, we investigated the circRNA/miRNA/mRNA the lncRNA/miRNA/mRNA axis in adult *Drosophila* at two age points (day 7 and day 42) and determined the regulatory network of key differentially expressed (DE) genes in *Drosophila* ageing. The results provide knowledge on the gene regulation network of adult *Drosophila* ageing and a solid foundation for understanding the mechanisms of human ageing and age-related diseases.

## 2 Results

### 2.1 Overview of multiomics data

The DE genes and proteins in *Drosophila* between day 7 and day 42 were identified, and their networks were analysed (Table 1). A total of 537 DE mRNAs and 43 DE lncRNAs were obtained from a previous study in our laboratory (Supplementary Data Sheet S1). A total of 6,003 circRNAs and 226 miRNAs were identified at day 7 and day 42 (Supplementary Data Sheet S1). Ultimately, 29 DE circRNAs and 24 DE miRNAs were found (Supplementary Data Sheet S1). The merged sequences of novel circRNAs (Supplementary Data Presentation S1–S3) and lncRNAs (Supplementary Data Sheet S3) are shown in supplementary files.

**TABLE 1:** DE mRNA, proteins, lncRNAs, circRNAs, miRNAs at day 42 compared to day 7.

	mRNA	LncRNAs	CircRNAs	miRNAs
Total	537	43	29	24
Upregulated	194	15	21	11
Downregulated	343	28	8	13

### 2.2 DE circRNAs and miRNAs in *Drosophila* between day 7 day 42

The DE circRNAs and miRNAs between 7 and 42-day old flies were analysed. Between day 7 and day 42, 29 DE circRNAs in *Drosophila* were identified, including 21 upregulated and 8 downregulated circRNAs at day 42 (Table 2). The circRNAs were derived from different source genes. These source genes were found to be involved in multiple molecular functions (Table 2). Evidently, the biological processes of the short lifespan-related source genes *arm* and *pan* were involved in the Wnt signalling pathway and had similar molecular functions, such as binding, protein binding, and transcription factor binding, transcription regulator activity. In addition, different circRNAs were observed to originate from the same mRNA transcript. For example, and dme_circ_0008175 and dme_circ_0008173 originated from the *Nlg1* gene, and dme_circ_0009514 dme_circ_0009500 were derived from the *pan* gene.

**TABLE 2:** DE circRNAs of *Drosophila* between day 7 days 42.

ID	CircBase ID	Fold change	*p*-value	Source gene	Portion of biological process term(s) from flybase database
Dme_circ_0009372	Dme_circ_0005033	4.5034↑	0.007635	*Asator*	Protein serine/threonine kinase activity; ATP binding
Dme_circ_0006708	Dme_circ_0005241	4.2791↑	0.012415	*Dad*	TGF-beta signalling pathway; negative regulation of BMP signalling pathway
Dme_circ_0004259	Dme_circ_0002098	4.2083↑	0.014401	*shot*	Cytoplasmic microtubule organization; wound healing; branching involved in open tracheal system development; cilium organization
Dme_circ_0002070	Dme_circ_0002195	3.9032↑	0.026301	*Scp1*	-
Dme_circ_0010408	Dme_circ_0003710	3.8118↑	0.031385	*CoRest*	Negative regulation of transcription by RNA polymerase II; positive regulation of DNA methylation-dependent heterochromatin assembly; negative regulation of histone H4-K16 acetylation; negative regulation of histone H3-K27 methylation
Dme_circ_0003904	Dme_circ_0003738	3.5948↑	0.045349	*CG33144*	Ubiquitin-dependent protein catabolic process
Dme_circ_0008175	Dme_circ_0001709	3.5948↑	0.045412	*Nlg1*	Neuromuscular junction development; cellular process
Dme_circ_0005030	Dme_circ_0002884	3.5765↑	0.046714	*Ccn*	Negative regulation of cell death; signal transduction; cell adhesion
Dme_circ_0006667	Dme_circ_0001321	3.5765↑	0.046714	*gish*	Positive regulation of Wnt-TCF hedgehog signalling pathways; negative regulation of Hippo signalling pathway
Dme_circ_0010536	Dme_circ_0000629	3.559↑	0.048096	*slgA*	Arginine proline metabolism
Dme_circ_0008383	Dme_circ_0004519	2.6054↑	0.043863	*mura*	Protein ubiquitination; long-term memory
Dme_circ_0010134	Dme_circ_0002087	1.1067↑	0.039466	*Stim*	Developmental process; cellular homeostasis
Dme_circ_0009358	-	0.74476↑	0.002481	*CaMKI*	Protein phosphorylation
Dme_circ_0000626	-	3.7656↑	0.03384	*CG17646*	Triglyceride metabolic process; transmembrane transport
Dme_circ_0011075	-	3.7618↑	0.03415	*Trf2*	Post-embryonic development; response to organic cyclic compound; programmed cell death; respiratory system development; response to oxygen-containing compound
Dme_circ_0006956	-	3.5884↑	0.045804	*GluClalpha*	Cellular process; transport; localization; establishment of localization; biological regulation
Dme_circ_0009667	-	3.559↑	0.048096	*dlg1*	Hippo signalling pathway-fly
Dme_circ_0009514	-	4.0161↑	0.021197	*pan*	Canonical Wnt signalling pathway
Dme_circ_0006334	-	3.8925↑	0.026799	*CG42402*	-
Dme_circ_0008173	-	3.881↑	0.027405	*Nlg1*	Neuromuscular junction development; cellular process
Dme_circ_0004404	-	0.61768↑	0.020721	*Dbp80*	Poly(A)+ mRNA export from nucleus
Dme_circ_0003891	Dme_circ_0001623	−3.8065↓	0.033572	*psq*	Anterior/posterior axis specification, embryo; DNA binding
Dme_circ_0006619	Dme_circ_0003501	−4.3746↓	0.010954	*srp*	Autophagy; cell fate commitment; midgut development
Dme_circ_0004913	Dme_circ_0004913	−0.52833↓	0.047568	CG34347	Actomyosin structure organization
Dme_circ_0004843	-	−0.48662↓	0.02228	*CG15715*	
Dme_circ_0010498	-	−0.73454↓	0.04614	*CG1304*	Proteolysis
Dme_circ_0006913	-	−2.7169↓	0.042802	*Cyp12a5*	Oxidation-reduction process
Dme_circ_0010310	-	−3.8065↓	0.033572	*arm*	Wnt signalling pathway
Dme_circ_0009500	-	−4.0543↓	0.021183	*pan*	Canonical Wnt signalling pathway

Notes: “↑” “↓” indicate upregulation downregulation at day 42 compared to day 7, respectively.

**FIGURE 1:**
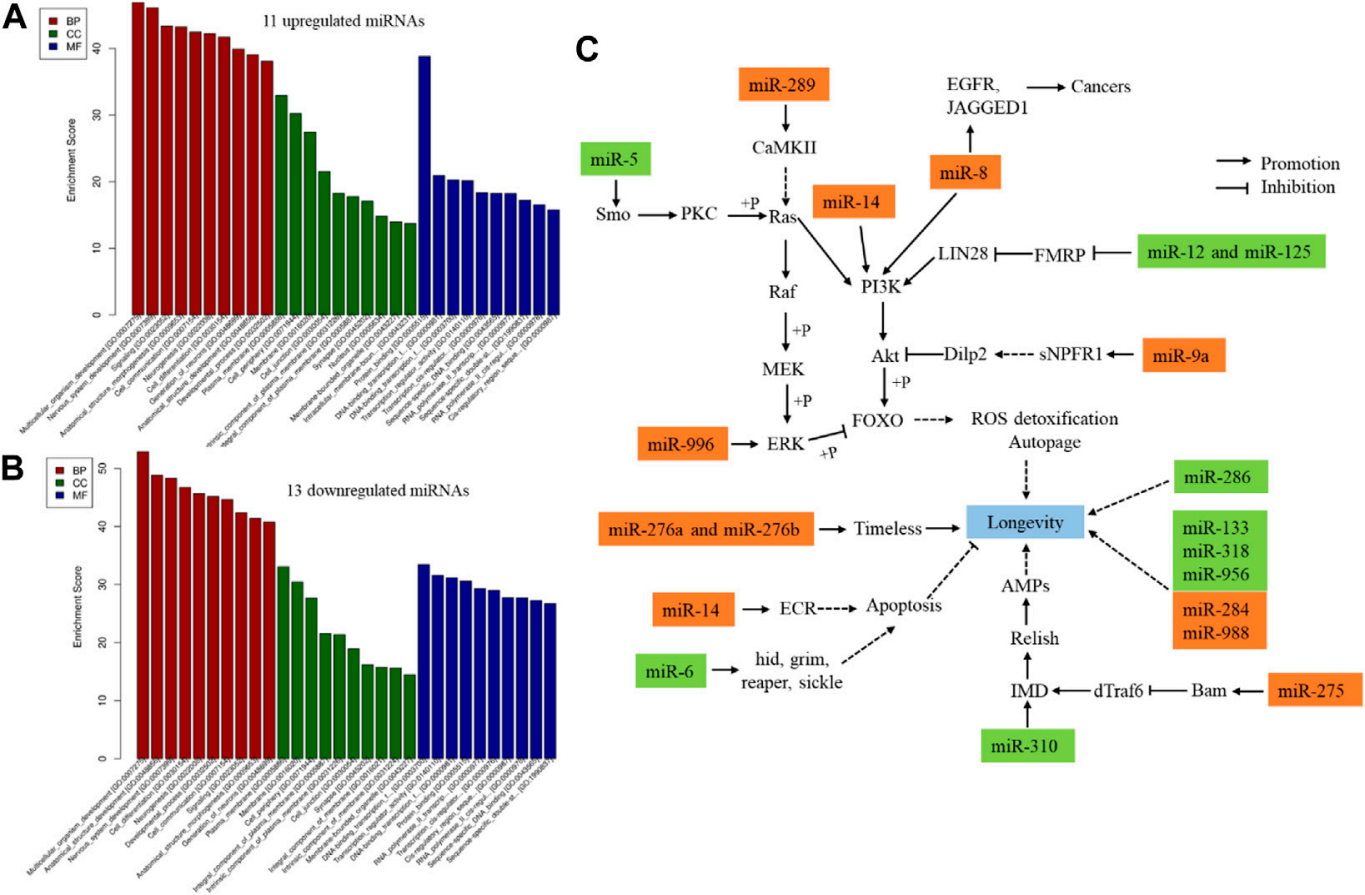
GO annotations age-related pathways of DE miRNAs in *Drosophila* between day 7 day 42. Twenty-four DE miRNAs were identified between 7 and 42-day old flies. **(A)**, GO annotations of 11 upregulated miRNAs. **(B)**, GO annotations of 13 downregulated miRNAs. **(C)**, 20 DE miRNA affected the *Drosophila* agineg; rust colored frame, upregulated in 42 days when compared to 7 days (*p* < 0.05); green colored frame, downregulated in 42 days when compared to 7 days (*p* < 0.05). The references were followed as miR-289 (Chen et al., 2014; Nesler et al., 2016), miR-5 (Leaman et al., 2005; Chen et al., 2014), miR-8 (Soler Beatty et al., 2021; Chen et al., 2022), miR-12 (Yang et al., 2009), miR-125 (Bushey et al., 2009; Gendron and Pletcher, 2017; Luhur et al., 2017), miR-9a (Chen et al., 2014; Suh et al., 2015), miR-275 (Chen et al., 2014; Ji et al., 2019), miR-310 (Robins et al., 2005; Chen et al., 2014), miR-6 (Leaman et al., 2005; Chen et al., 2014), miR-14 (Xu et al., 2003; Varghese and Cohen, 2007), miR-276a (Chen et al., 2014; Zhang et al., 2021a), miR-276b (Chen et al., 2014; Ulgherait et al., 2020; Zhang et al., 2021b), miR-996 (Sun et al., 2015; Duan et al., 2018). BP, biological process; CC, celluar component; MF, molecular function.

Furthermore, 24 DE miRNAs (11 upregulated and 13 downregulated) were identified at day 42 compared to day 7 (Supplementary Data Sheet S1). Dme_miR-9a-3p and dme_miR-985-3p were identified as canonical specific fruit fly miRNAs. Then, dme_miR-956-3p, dme_miR-284-3p, and dme_miR-289-5p were identified as non-canonical miRNAs. The remaining 19 DE miRNAs were canonically conserved miRNAs. Then, functional annotation was carried out for the DE miRNAs. The Gene Ontology (GO) annotations of age-related DE miRNAs based on their targets were investigated. 11 upregulated and 13 downregulated miRNAs targeted to 3,292 mRNAs and 2,951 mRNAs, respectively (Supplementary Data Sheet S1), which mainly enriched in biological process (1,314 terms and 1,233 terms, respectively) (Supplementary Data Sheet S1). The bar plot shows the top ten enrichment score value of the significant enrichment terms (Figures 1A, B), such as multicellular organism development, nervous system development, neurogenesis, development process, and cell differentiation. Specifically, 20 DE miRNAs were related to *Drosophila* aging based on previous reports, including 11 upregulated and 9 downregulated miRNAs in 42 days when compared to 7 days (Figure 1C). 13 DE miRNAs involved in the age-signalling pathways by target genes on post-transcriptional level (Figure 1C). Their tagets involved in regulation of ROS detoxification, autopage, circadian rhythm, apoptosis, and immunity biological process. Otherwise, three out of 24 total miRNAs were conserved in *Drosophila*, human and mouse, containing dme-miR-8-5p, dme-miR-133-3p, and dme-miR-10-5p.

### 2.3 DE circRNA/miRNA/mRNA networks in *Drosophila* ageing

**FIGURE 2:**
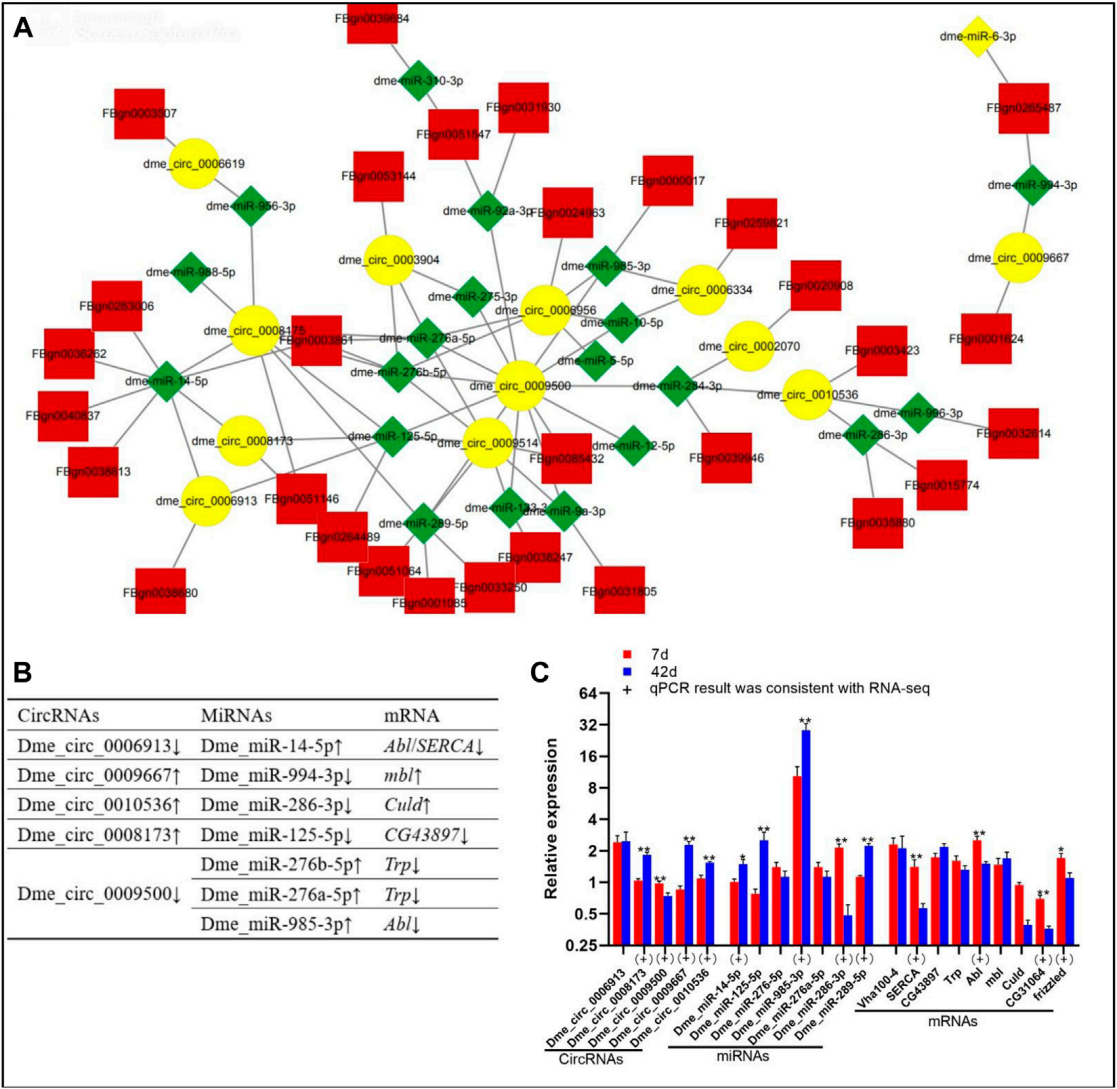
DE circRNA/miRNA/mRNA networks of *Drosophila* at day 7 day 42. **(A)**, circRNA/miRNA/mRNA interaction networks; yellow, circRNAs; red, mRNAs; green, miRNAs. **(B)**, Specific circRNA/miRNA/mRNA networks; “↑” “↓” indicate upregulation downregulation on day 42 compared to day 7 in RNA-seq data, respectively. **(C)**, Data on the relative expression of circRNA, miRNA, mRNA detected by qPCR analysis. The relative gene expression levels were calculated by the cycle threshold values that were identified as 2^−ΔΔCT^. *The ribosomal protein L32* (*rp 49*) gene was used as the reference gene to calculate the relative mRNA, miRNA, circRNA levels. “*” above the bars indicates a significant difference at the 0.05 level, “**” indicates a significant difference at the 0.01 level. (+) represents the qPCR results were consistent with the RNA-seq data.

In this section**,** the DE circRNA/miRNA/mRNA networks are analysed. Through the ceRNA mechanism, miRNAs can negatively regulate mRNA expression. Overall, 12 DE circRNAs, 21 DE miRNAs, and 30 DE mRNAs had interactions (Figure 2A, Supplementary Data Sheet S4). The binding sites between circRNA vs. miRNA (Supplementary Data Sheet S5) and miRNA vs. mRNA (Supplementary Data Sheet S6) are shown. According to the trends in the expression quantity changes, five DE circRNAs (dme_circ_0006913, dme_circ_0008173, dme_circ_0009500, dme_circ_0009667, and dme_circ_0010536) targeted the DE miRNAs with opposite expression trends (Figure 2B). Based on qPCR results, the expression patterns of four circRNAs (dme_circ_0008173, dme_circ_0009500, dme_circ_0009667, and dme_circ_0010536), and four miRNAs (dme_miR-289-5p, dme_miR-985-3p, dme_miR-286-3p, dme_miR-14-5p), four mRNAs (*frizzled*, CG31064, *Abl* and *SERCA*) were consistent with the RNA-seq data (Figure 2C). Importantly, the expression trends of dme_circ_0009500/dme_miR-289-5p/*CG31064*, dme_circ_0009500/dme_miR-289-5p/*frizzled*, and dme_circ_0009500/dme_miR-985-3p/*Abl* conformed to the ceRNA mechanism.

### 2.4 DE lncRNA/miRNA/mRNA networks in *Drosophila* ageing

**FIGURE 3:**
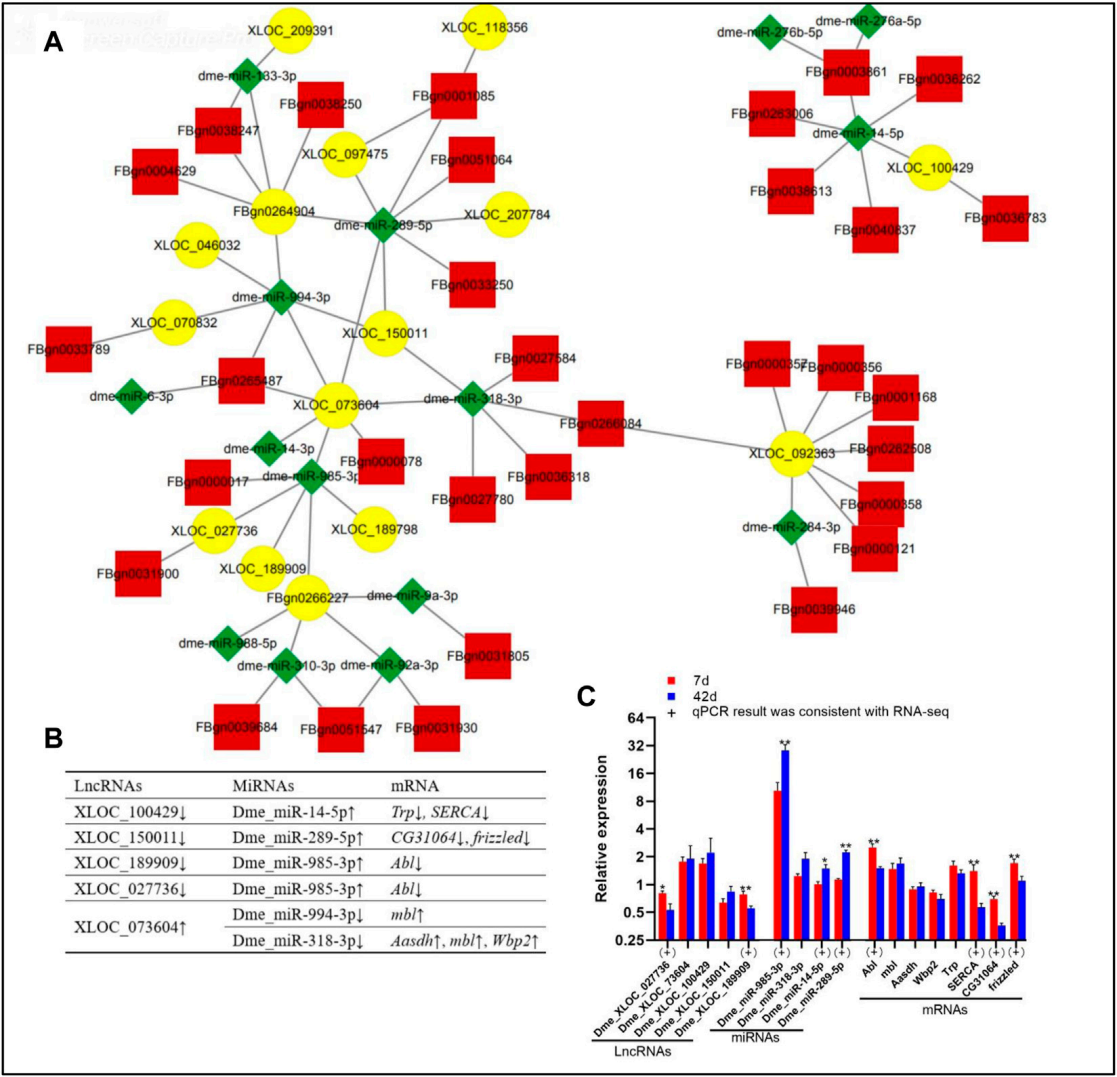
DE lncRNA/miRNA/mRNA networks in *Drosophila* at day 7 day 42. **(A)**, lncRNA/miRNA/mRNA interaction networks; yellow, lncRNAs; red, mRNAs; green, miRNAs. **(B)**, Specific lncRNA/miRNA/mRNA networks; “↑” “↓” indicate upregulation downregulation on day 42 compared to day 7, respectively. **(C)**, Data on the relative expression of lncRNAs, miRNAs, mRNAs by qPCR analysis. 7 days, 7 days; 42 days, 42 days. The relative gene expression levels were calculated using the cycle threshold values that were identified as 2^−ΔΔCT^. *The ribosomal protein L32* (*rp 49*) gene was used as the reference gene to calculate the relative mRNA, miRNA, lncRNA levels. “*” above the bars indicates a significant difference at the 0.05 level, “**” indicates a significant difference at the 0.01 level. (+) represents the qPCR results were consistent with the RNA-seq data.

According to the functional patterns of lncRNAs competing with mRNAs for binding to miRNAs, the interaction networks of DE lncRNAs/miRNAs/mRNAs were identified (Supplementary Data Sheet S7). Then, the binding sites between lncRNAs and miRNAs were included in Supplementary Data Sheet S8. In addition, DE miRNAs targeted DE mRNAs with opposite expression trends. Based on the DE genes in our database, 15 lncRNAs, 15 miRNAs, and 32 mRNAs had interactions (Figure 3A). Based on qPCR results, the expression patterns of two lncRNAs (XLOC_027736 and XLOC_189909), three miRNAs (dme_miR-289-5p, dme_miR-985-3p, and dme_miR-14-5p), and three mRNAs (*frizzled*, *CG31064*, and *Abl*) were consistent with the RNA-seq data (Figure 3A). Several specific lncRNA/miRNA/mRNA networks, including XLOC_027736/dme_miR-985-3p/*Abl*, XLOC_073604/dme_miR-994-3p-3p/*mbl*, XLOC_189909/dme_miR-985-3p/*Abl* (Figure 3B), were found. In the XLOC_027736/dme_miR-985-3p/*Abl* and XLOC_189909/dme_miR-985-3p/*Abl* networks, the expression trend of these genes was determined by qPCR analysis to be consistent with the RNA-seq data from our study (Figure 3C).

### 2.5 Functional annotation of DE circRNA/lncRNA-associated networks

**FIGURE 4:**
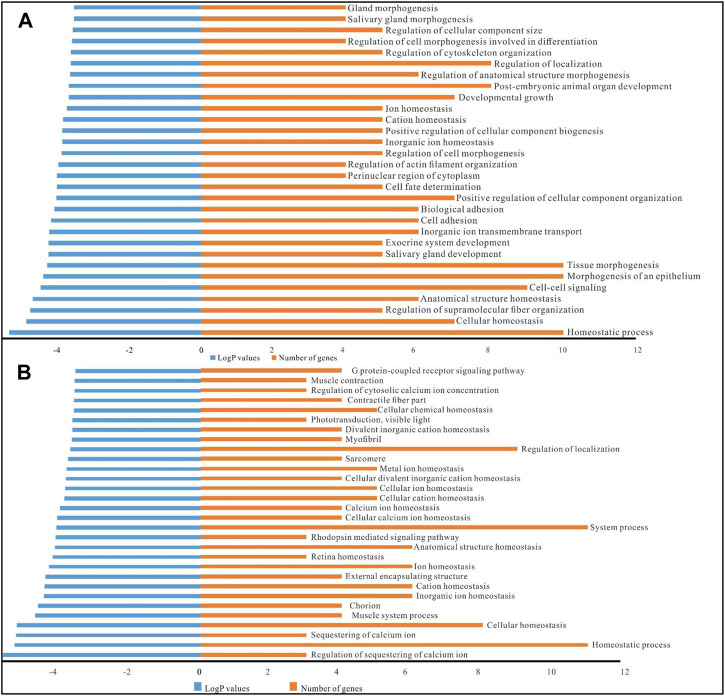
GO annotations of the DE circRNA-/lncRNA-associated networks. **(A)**, GO annotations of the DE circRNA/miRNA/mRNA networks. **(B)**, GO annotations of the DE lncRNA/miRNA/mRNA networks. LogP values indicate the enrichment degree of targets in the corresponding GO term, a smaller value represents a higher enrichment degree. The number of genes represents the number of target genes enriched in GO terms.

GO functional annotation of DE circRNAs/lncRNAs/mRNAs was carried out based on 74 target mRNA genes (Supplementary Data Sheet S9). The first 30 GO terms based on the lowest *p* values are listed (Figure 4). In circRNA/mRNA GO terms, there were just two major GO categories in the circRNA-associated networks, including biological processes (29 GO terms) and cellular components (1 GO term, perinuclear region of cytoplasm). Similarly, the GO annotations of the lncRNA-associated networks consisted of 25 GO terms in biological processes and 5 GO terms in the cellular component.

**FIGURE 5:**
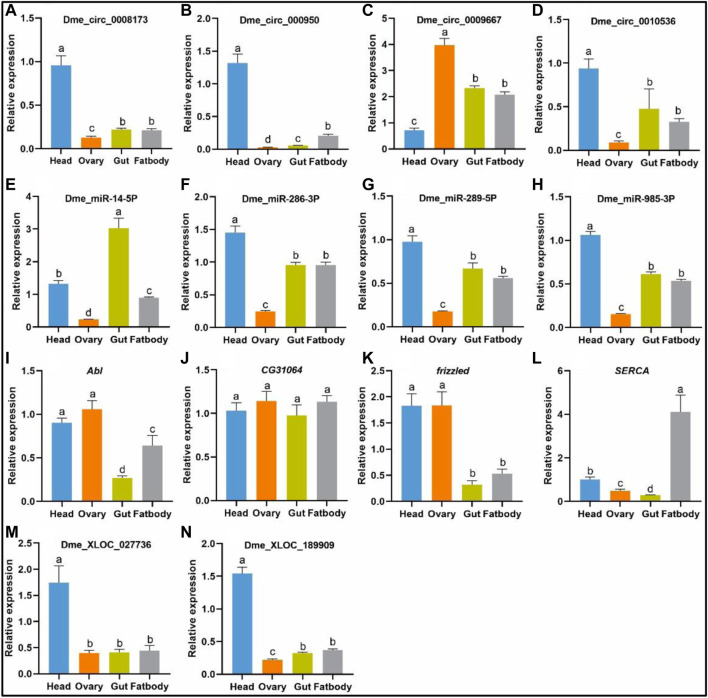
Tissue-specific expression patterns of the specific DE lncRNAs, circRNAs, miRNAs, mRNAs in the head, ovary, gut, fat body of flies. The expression levels of the DE genes in the circRNA/miRNA/mRNA lncRNA/miRNA/mRNA networks, as determined through qPCR, were consistent with the RNA-seq data. **(A–D)**, The relative expression level of four DE circRNAs; **(E–H)**, the relative expression level of four DE miRNAs; **(I–L)**, the relative expression level of four DE mRNAs; **(M,N)**, the relative expression level of two DE lncRNAs. The relative gene expression levels were calculated by the cycle threshold values that were identified as 2^−ΔΔCT^. *The ribosomal protein L32* (*rp 49*) gene was used as the reference gene to calculate the relative mRNA, miRNA, lncRNA levels. The different letters above the bars indicate significant differences at the 0.05 level. (+) represents the qPCR results were consistent with the RNA-seq data.

In the GO annotations of the circRNA-associated networks, there were several ageing-related biological processes in GO terms, such as homeostatic process (10 DE genes), cellular homeostasis (7 DE genes), cation homeostasis (5 DE genes), ion homeostasis (5 DE genes), cell-cell signalling (9 DE genes), cell fate determination (5 DE genes), developmental growth (7 DE genes) (Figure 5A). Furthermore, 13 out of the first 30 GO terms of the lncRNA-associated networks were related to homeostatic processs, including cellular homeostasis, retinal homeostasis, ion homeostasis, calcium ion homeostasis, and cellular cation homeostasis (Figure 5B). In the ageing-related ceRNA networks dme_circ_0009500/dme_miR-985-3p/*Abl*, XLOC_027736/dme_miR-985-3p/*Abl* and XLOC_189909/dme_miR-985-3p/*Abl* in *Drosophila*, *Abl* was involved in multiple GO terms, such as regulation of cell morphogenesis, developmental growth, regulation of cell differentiation, and regulation of neuron differentiation. Furthermore, *frizzled* was involved in the positive regulation of developmental growth, developmental growth involved in morphogenesis, cell-cell signalling, cell fate determination, and the Wnt signalling pathway in the dme_circ_0009500/dme_miR-289-5p/*frizzled* network.

### 2.6 Tissue expression pattern analysis

The tissue-specific expression patterns of the DE lncRNAs, circRNAs, miRNAs, and mRNAs were analysed in the head, ovary, gut, and fat body, the qPCR results of which were consistent with the RNA-seq data in the DE circRNA/miRNA/mRNA networks and lncRNA/miRNA/mRNA networks (Figure 5). The results showed that the tissue-specific expression patterns of dme_circ_0009500/dme_miR-289-5p/*CG31064*, dme_circ_0009500/dme_miR-289-5p/*frizzled*, and dme_circ_0009500/dme_miR-985-3p/*Abl* were mainly expressed in the head. Specifically, dme_circ_0009667 was mainly located in the ovary, and dme_miR-14-5p was mainly expressed in the gut. Similar results were found in the lncRNA/miRNA/mRNA networks. The tissue patterns of the XLOC_027736/dme_miR-985-3p/*Abl* and XLOC_189909/dme_miR-985-3p/*Abl* networks were also mainly expressed in the head.

### 2.7 Binding sites of specific ceRNAs

**FIGURE 6:**
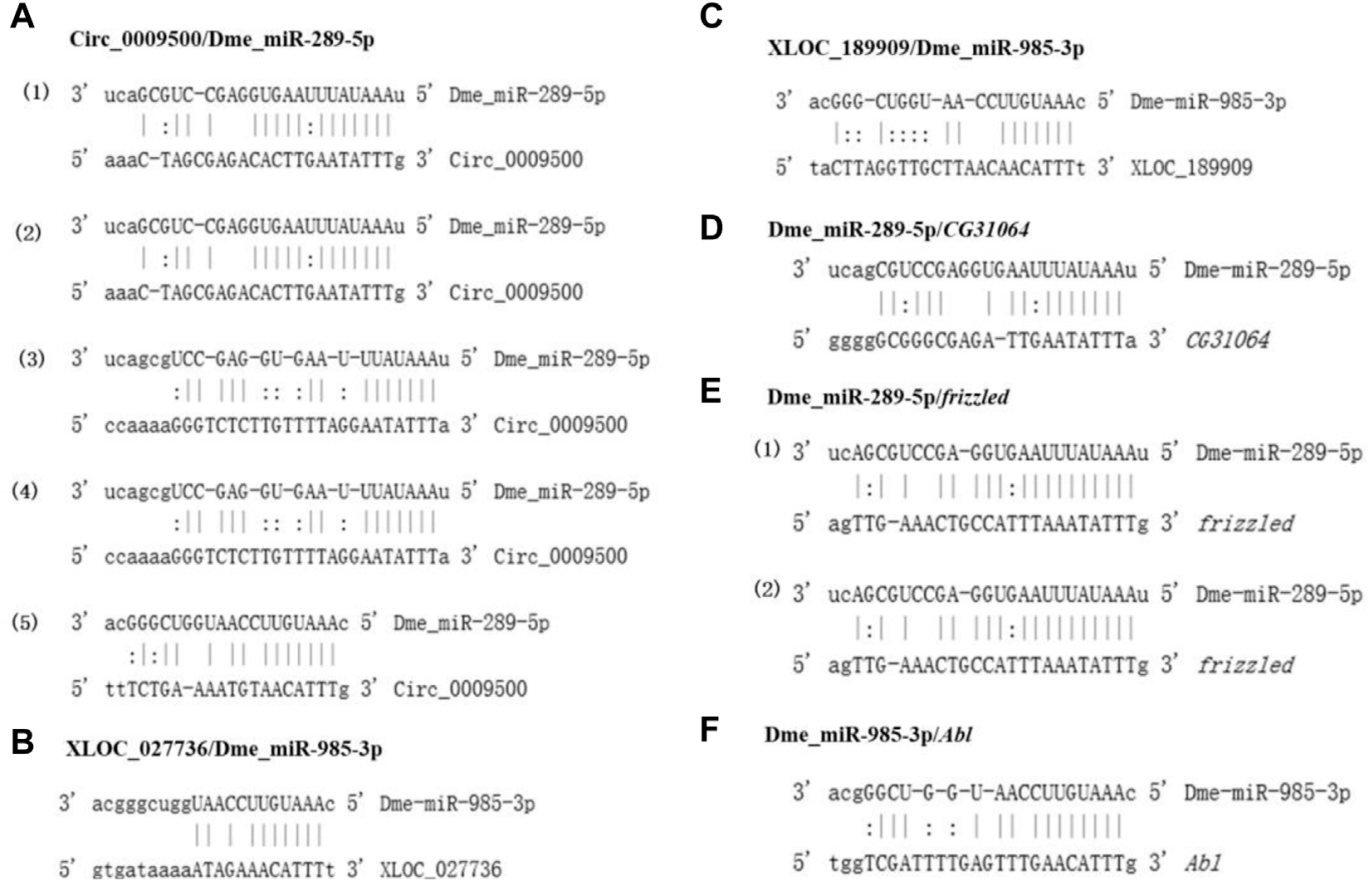
The binding sites of specific ceRNAs. **(A)**, the binding sites between circ_0009500/Dme_miR-289-5p; **(B)**, the binding sites between XLOC_027736 dme_miR-985-3p; **(C)**, the binding sites between XLOC_189909 dme_miR-985-3p; **(D)**, the binding sites between dme_miR-289-5p *CG31064*; **(E)**, the binding sites between dme_miR-289-5p *frizzled*; **(F)**, the binding sites between dme_miR-985-3p *Abl*.

The binding sites of dme_circ_0009500/dme_miR-289-5p/*CG31064*, dme_circ_0009500/dme_miR-289-5p/*frizzled*, dme_circ_0009500/dme_miR-985-3p/*Abl*, XLOC_027736/dme_miR-985-3p/*Abl*, and XLOC_189909/dme_miR-985-3p/*Abl* were analyzed (Figure 6). Specificly, there were five binding sites between dme_circ_0009500 and dme_miR-289-5p with the higher binding free energy from -15.23 to -20.30 (Figure 6A; and Supplementary Data Sheet S5). Furthermore, 3′UTR sequence of *frizzled* had the two binding site with miR-289-5p, and also had the higher binding free energy -23.31 (Figure 6E and Supplementary Data Sheet S6).

## 3 Discussion

*Drosophila* is an ideal model for genetics, and the multiple age-related researches were carried out by Dahomey strain (Mołoń et al., 2020; De Groef et al., 2021). Previous study has reported the trend of wild-type female lifespan (Dahomey, Canton S, Oregon R) changes were similar (Sanz et al., 2010). Furthermore, several studies just used one wildtype strain in multiple-omics research (Shi et al., 2020; Wang et al., 2022). Thus, the wild-type female of Dahomey strain was utilized in our manuscript. Increasing evidence has suggested that ceRNA networks play key roles in a variety of biological processes, such as cancer (Wang et al., 2018; Abdollahzadeh et al., 2019), Alzheimer’s disease (AD) (Zhang et al., 2019), skeletal muscle myogenesis (Yue et al., 2019), and ageing (Zhao et al., 2019; Chen et al., 2020). miRNAs are the core molecules of the ceRNA regulatory system (Yue et al., 2019; Chen et al., 2020). Thus, screening DE miRNAs is essential for research on ceRNA networks related to *Drosophila* ageing. miRNAs generally induce mRNA degradation or repress translation of target transcripts through sequence-specific binding to the transcript 3′UTR (Bushati and Cohen, 2007; Chen et al., 2019). Each transcript can be targeted by multiple miRNAs, and each miRNA can target hundreds of different transcripts (mRNA, circRNA, and lncRNA transcripts) (Dori and Bicciato, 2019; Zhou et al., 2019; Kinser and Pincus, 2020). Thus, the miRNA regulatory network is far-reaching (Kinser and Pincus, 2020). Previous studies have verified that miRNAs are important small regulatory ncRNA molecules that control a fairly large number of biological processes; their important functions have generated interest in their use as biomarkers and their roles as regulators of ageing (Lai et al., 2019; Kinser and Pincus, 2020) and a number of cancer types (Liang et al., 2020; Mishan et al., 2020; Wang et al., 2020). Recent studies have also suggested that miRNAs are involved in the regulation of age-associated processes and pathologies in multiple mammalian tissues, including the brain, heart, bones, and muscles (Chen et al., 2020; John et al., 2020; Kinser and Pincus, 2020; Ullah et al., 2020).

In our study, more than 80% (20 out of 24 total miRNAs) of DE miRNAs between day 7 and day 42 could affect fruit fly ageing. Among these miRNAs, miR-14 as a cell death suppressor, regulates fat metabolism, insulin production and metabolism through its targets (Xu et al., 2003; Nelson et al., 2014). In addition, knockout of five DE miRNAs (miR-133, miR-284, miR-286, miR-318, miR-956, and miR-988) decreased the *Drosophila* lifespan, and miR-286 KO increased female lifespan (Chen et al., 2014). The consreved miR-8 was the homolog of vertebrate miR-200 family. It is worth noting that miR-8 acted through U-shaped to activate PI3K and thereby promoted fat cell growth cell-autonomously and the Insulin-like Receptor signaling pathway (Soler Beatty et al., 2021; Chen et al., 2022). Then, two targets of miR-200 (dme-miR-8 homolog) in human were JAGGED1 (JAG1) (Xue et al., 2021) and epidermal growth factor receptor (EGFR) (Xue et al., 2019). In a lung cancer metastasis, overexpression of the miR-200 decreased JAG1 protein levels and impeded cell growth (Xue et al., 2021). Furthermore, Dmel\ EGFR is orthologous to human gene ERBB2, which has also been implicated in multiple cancers (Xue et al., 2019). The results indicate that these miRNAs may act as sponges through the circRNA/miRNA/mRNA and lncRNA/miRNA/mRNA networks involved in the regulation of *Drosophila* ageing. In a previous study, global identification of functional miRNA-mRNA interactions in *Drosophila* was performed (Wessels et al., 2019). However, circRNA/miRNA/mRNA and lncRNA/miRNA/mRNA interactions in fly ageing have remained unclear.

Most of the DE circRNAs accumulated in 42-day-old *Drosophila*, which has been reported in a previous study (Westholm et al., 2014) in flies and other model organisms (Knupp and Miura, 2018; Kim and Lee, 2019). circRNAs are highly stable molecules that play important roles in ageing (Weigelt et al., 2020) and age-related diseases (Ren et al., 2020; Wang P. et al., 2020). Additionally, six source genes of circRNAs were related to fruit fly ageing, including *asator* (short-lived, *Asator* ^GD7323^) (Neely et al., 2010), *dad* (short-lived, *Dad* ^GD1335^) (Kamiya et al., 2008), *gish* (short-lived, *gish* ^GD10588^) (Neely et al., 2010; Fulford et al., 2019), *pan* (short-lived, pan^ΔN.UAS^ ) (Buchon et al., 2013; Franz et al., 2017), *psq* (long-lived, *psq* ^BG01031^) (Magwire et al., 2010; Bonchuk et al., 2011), and *arm* (short-lived, *arm* ^S10. UAS. Tag:MYC^) (Dupont et al., 2012). In the present study, multiple networks of DE circRNAs/miRNAs/mRNAs and DE lncRNAs/miRNAs/mRNAs in *Drosophila* between day 7 and day 42 were identified. GO annotations of the DE circRNA/miRNA/mRNA and DE lncRNA/miRNA/mRNA networks demonstrated that the DE circRNAs and lncRNAs may take part in *Drosophila* ageing *via* various biological processes. Multiple GO terms enriched in the DE circRNA/miRNA/mRNA networks, such as cellular homeostasis (Hartl, 2016) and developmental growth (Clarke et al., 2020), are clearly related to ageing. Specifically, multiple GO terms, including inorganic ion homeostasis, cation homeostasis, and cellular calcium ion homeostasis, are involved in regulating the sequestration of calcium ions. Ca^2+^ dyshomeostasis is associated with several ageing-related neurodegenerative diseases, such as AD, Huntington’s disease (HD), Parkinson’s disease (PD), and amyotrophic lateral sclerosis (ALS), with an altered Ca^2+^ buffering capacity, an altered regulation of Ca^2+^ channels and pumps, and an altered neuronal excitability (Bezprozvanny, 2010). A previous study demonstrated that ageing was closely linked to the dysregulation of Ca^2+^ homeostasis, resulting in a chronically elevated level of cytosolic Ca^2+^ in experimental models of neuronal ageing (Thibault et al., 2007; Duncan et al., 2010). The results suggest that the DE circRNA/miRNA/mRNA and DE lncRNA/miRNA/mRNA networks may play a significant role in *Drosophila* ageing.

Based on the same expression trend between the RNA-seq and qPCR detection results in our study, some specific ceRNA networks, including the dme_circ_0009500/dme_miR-289-5p/*CG31064*, dme_circ_0009500/dme_miR-289-5p/*frizzled*, dme_circ_0009500/dme_miR-985-3p/*Abl*, XLOC_027736/dme_miR-985-3p/*Abl* and XLOC_189909/dme_miR-985-3p/*Abl* networks, were identified. Thus, we determined that these networks merited further investigation. In our study, miR-289-5p was mainly expressed in the *Drosophila* head and upregulated in 42-day-old flies compared to 7-day-old flies. In previous studies, miR-289-5p, which may be responsible for silencing the expression of candidate genes during the diapause of *Sarcophaga bullata*, was overexpressed in diapausing pupae (Reynolds et al., 2017). Then, miR-289 participates in the control of a diverse array of pleiotropic cellular processes during *Drosophila* development (Nesler et al., 2013). It has been reported that miR-289 is downregulated in adult-onset AD *Drosophila* brains (Kong et al., 2014). Neuronal misexpression of miR-289 suppresses activity-dependent synaptic growth (Nesler et al., 2013). The target *CG31064* of miR-289-5p was located in the adult head in our study in previous research (Aradska et al., 2015), which enabled a small GTPase binding activity (Gillingham et al., 2014). Another target, *frizzled* of miR-289-5p, has been reported to be involved in the regulation of the mTOR and signalling pathway the Wnt signalling pathway (Zeng et al., 2018). The GO *frizzled* terms were related to positive regulation of developmental growth, developmental growth involved in morphogenesis, cell-cell signalling, cell fate determination, and the Wnt signalling pathway. Previous study has reported that *frizzled* in involved in regulation of pro-survival processes in human PD through Wnt1/Fzd-1/β-catenin astrocyte-dopamine autoprotective loop (L'Episcopo et al., 2011). Therefore, dme_circ_0009500/dme_miR-289-5p/*CG31064* and dme_circ_0009500/dme_miR-289-5p/*frizzled* may play roles in brain ageing in fruit flies.

Furthermore, the lncRNAs XLOC_027736 and XLOC_189909, the dme_circ_0009500 and the mRNA *Abl* were predicted to competitively bind miR-985-3p, which was mainly expressed in the *Drosophila* head. *Abl* phosphorylates cell adhesion and cytoskeletal proteins and acts as a scaffold in a signalling complex to regulate both epithelial and nervous system morphogenesis (Zhu and Bhat, 2011; Liu and Wu, 2014). The age-related phenotype associated with the *Abl* mutant led to a shorter lifespan for flies with three specific alleles (*Abl* ^l2^, *Abl* ^l3^, and *Abl* ^GD1344^) (Belote et al., 1990; Huang et al., 2007; Neely et al., 2010). Furthermore, human ABL1 (*Drosophila Abl* homolog) protein kinases play many important roles in neuron development, maintenance, and signalling (Manley et al., 2022). In future studies, it will be necessary to determine the biological role of the lncRNAs XLOC_027736 XLOC_189909, the circRNA dme_circ_0009500, and miR-985-3p in the fly ageing process.

In our study, DESeq2 and EdgeR were used to analyse the DE circRNAs and miRNAs, respectively. DESeq2 and EdgeR are efficient tools for differential analysis of RNA-seq data with the more than 80% overlapping, both of which use the negative binomial distribution (Liu et al., 2021). Then, DESeq2 can more accurately identify DE genes for small samples to reduce false positives (Love et al., 2014). Then, two biological replicates were utilized to analysis DE circRNAs. Thus, DESeq2 was chosen to identify the DE circRNAs. The above results suggest that our data are also available. To date, DE circRNAs and miRNAs cannot be reanalysed only by DESeq2 or EdgeR in the present study. This would bring some questions to our subsequent analysis. For example, some DE circRNAs and miRNAs may be lost, which may result that part ceRNA networks could not be recognized. More experiments will be needed to verify these networks in our future study.

In this study, several DE miRNAs were identified between day 7 and day 42, such as the dme_miR-289, dme_miRNA-14, and the conserved miR-8. Then, the potential ceRNA networks may play a role in *Drosophila* aging, for example, dme_circ_0009500/dme_miR-289-5p/*frizzled* and XLOC_027736/dme_miR-985-3p/*Abl* networks. Therefore, the result of DE miRNAs, circRNA/miRNA/mRNA, and lncRNA/miRNA/mRNA interactions provides an important foundation to parse the genetic process of *Drosophila* ageing.

## 4 Materials and methods

### 4.1 Sample collection and preparation

Female flies (Dahomey^WT^) that had mated with male flies for 48 h after hatching were bred under a 12 h on/off light cycle at 25°C in 50% humidity. Sample collection of adult flies at day 7 and day 42 was performed as described by Yang et al. (2016). RNA-seq (mRNA, lncRNA, and circRNA) was conducted on two biological replicates, while miRNA sequencing was conducted on five biological replicates. All samples were stored at −80°C until use.

### 4.2 LncRNA, mRNA, circRNA data from *Drosophila* at days 7 and 42

RNA-seq data for the lncRNAs and mRNAs of *Drosophila* at day 7 and day 42 were obtained by Yang et al. (2016). The circRNA analysis was based on the RNA-seq data for *Drosophila* at day 7 and day 42 from Yang et al. (2016). Overall, 95.79% clean reads were obtained from 26.7 GB of raw sequence data (SRP073695) then aligned to the *D. melanogaster* genome from FlyBase (Dmel_Release_6, http://FlyBase.org/). The find_circ (Memczak et al., 2013) and CIRI2 (Gao et al., 2015) software tools were utilized to identify circRNAs. Then, the overlapping *Drosophila* circRNAs identified by both software programs were selected. The input data for the circRNA differential expression analysis were readCount data obtained from the circRNA expression level analysis. Then, paired differential expression analysis of circRNAs between day 7 and day 42 was conducted with DESeq2 (Varet et al., 2016) based on a negative binomial distribution. The *p*-value was adjusted using Hochberg and Benjamini’s methods (Hinkley et al., 2018) to control the error discovery rate. A *p*-value <0.05 was considered to indicate a DE circRNA. These original data were from our laboratory.

### 4.3 MiRNAs in *Drosophila* at day 7 and day 42

TRIzol Reagent (Invitrogen, CA) was used to extract the total RNA from fruit flies at day 7 and day 42. Agarose gel electrophoresis was performed to verify the integrity of the total RNA samples. A NanoDrop ND-1000 instrument was used to accurately measure the concentrations and protein contamination of the total RNA samples. miRNA sequencing libraries were generated using rRNA-depleted RNA with a NEBNext^®^ Ultra™ Multiplex Small RNA Library Prep Set Kit for Illumina^®^ (New EnglBiolabs, United States) following the manufacturer’s recommendations. Subsequently, an Agilent 2,100 Bioanalyser and an Agilent DNA 1000 chip kit (Agilent, part #5067-1504) were utilized to accurately assess the quality and concentration of the sequencing libraries. The libraries were sequenced using an Illumina NextSeq 500. MiRNA fragment sequencing was performed by the Aksomics company.

Clean reads were generated from the raw sequence data from the Illumina NextSeq instrument through real-time base calling and quality filtering. The clean reads were recorded in FASTQ format and contained read information, sequences and quality encoding. Subsequently, the 5′- and 3′-adapter sequences were trimmed from the clean reads by Cutadapt, and reads with lengths shorter than 14 nt or longer than 40 nt were discarded. The trimmed reads were collapsed into FASTA format. The raw data has been uploaded to NCBI database (PRJNA716466). The trimmed reads that did not map to mature or precursor tRNA sequences were aligned with an allowance of only one mismatch to miRNA reference sequences with miRDeep2 (Friedlander et al., 2012). The expression profiles of miRNAs were determined based on the counts of the reads mapped. The DE miRNAs were identified with the R package EdgeR based on the count values (Robinson et al., 2010). A fold change cut-off of 1.5 and a *p*-value cut-off of 0.05 were applied only when replicates were used for screening DE miRNAs. The gene prediction of DE miRNA integrates two algorithms, miRanda (Enright et al., 2003) and TargetScan (Garcia et al., 2011). GO enrichment analysis of the targets of DE miRNAs was implemented with the GOseq R package (Young et al., 2010).

### 4.4 CeRNA analysis of lncRNA/circRNA-miRNA-mRNA

The lncRNAs, circRNAs, and miRNAs showed significantly different expression levels between day 7 and day 42 and were thus analysed. The potential ceRNAs were searched based on the sequences of the lncRNAs, circRNAs, and mRNAs. The offline software MiRanda (Enright et al., 2003) was utilized to predict miRNA binding seed sequence sites, and overlap of the same miRNA binding sites on lncRNAs/circRNAs-miRNAs and miRNAs-mRNAs was taken to indicate a lncRNA/circRNA-miRNA-mRNA interaction. Then, the “clusterProfiler” R package was utilized to perform Gene Ontology (GO) enrichment of ceRNA networks based on the mRNAs.

The DE mRNAs associated with ageing and the corresponding ceRNA networks (including the circRNAs, lncRNAs, miRNAs, and mRNAs) were selected to detect the expression level by qPCR. In total, 9 miRNAs, 10 mRNAs, 5 lncRNAs, and 5 circRNAs were selected to qualify the expression level. Samples (three biological duplicates) from 7- and 42-day-old fruit flies were used to isolate the total RNA using TRIzol™ LS reagent (Thermo Fisher) according to the manufacturer’s instructions. The total RNA (1 μg) from each sample was reverse transcribed with random primers using a RevertAid First-strand cDNA Synthesis Kit (Thermo Fisher) according to the manufacturer’s protocol, which was utilized to detect the expression levels of mRNA, lncRNA, and circRNA. In addition, the total RNA (1 μg) used for miRNA expression detection in each sample was reverse transcribed using a TaqMan™ MicroRNA Reverse Transcription Kit (Thermo Fisher) according to the kit instructions. All gene primers (Supplementary Data Sheet S10) were designed using the Primer 5.0 software and purchased from Sangon Biotech. The SYBR green method was used for qRT-PCR with TransStart® Green qPCR SuperMix (TransGen Biotech) following the manufacturer’s instructions. All tested samples were three biological replicates. The relative gene expression levels were calculated using cycle threshold values and the 2-ΔΔCT method. Ribosomal protein L32 (*rp 49*) was used as the reference gene to calculate the relative mRNA, miRNA, lncRNA, and circRNA levels. Differential expression levels were compared by independent-samples t-tests between groups.

### 4.5 Tissue-specific expression pattern

Based on the same expression pattern between day 7- and day 42-day-old flies, as determined by the RNA-seq data and qPCR tests on 7-day and 42-day-old flies, the tissue-specific expression model of four circRNAs (dme_circ_0008173, dme_circ_000950, dme_circ_0009667, and dme_circ_0010536), four miRNAs (dme_miR-289P, dme_miR-985-3P, dme_miR-286-3P, and dme_miR-14-5P), four mRNA (SERCA, frizzled, Abl, and CG31064), and two lncRNAs (XLOC_027736 and XLOC_189909) were selected to analyse the tissue specificity of the head, ovary, gut and fat body of female fruit flies at 7 days. Each sample included three biological replicates. The expression levels of these genes were detected by qRT-PCR as described above. Differential expression levels were compared by independent-samples t-tests between groups.

## Data Availability

The original contributions presented in the study are publicly available. This data can be found here: https://www.ncbi.nlm.nih.gov/ (Accession number PRJNA716466).
