# Supplementary material for: Age-related ceRNA networks in adult Drosophila ageing
Source: Front Genet. 2023 Feb 28;14:1096902. doi: 10.3389/fgene.2023.1096902 (PMC10012872; doi:10.3389/fgene.2023.1096902)
Supplement: Supplementary file 1 [file DataSheet6.docx]

#miRNA mRNA Score Energy miRNA_position mRNA_position Align_len miRNA_align_rate mRNA_align_rate Pairing target and miRNA

>dme-miR-6-3p FBgn0265487 171.00 -21.39 2 21 552 575 22 77.27% 77.27%

Query: 3' uuUUUC-UUG-U-CGGUGACACUAu 5'

|||| ||| | | ||||||||

Ref: 5' agAAAGAAACGAAG-AACTGTGATa 3'

>dme-miR-6-3p FBgn0265487 171.00 -21.39 2 21 552 575 22 77.27% 77.27%

Query: 3' uuUUUC-UUG-U-CGGUGACACUAu 5'

|||| ||| | | ||||||||

Ref: 5' agAAAGAAACGAAG-AACTGTGATa 3'

>dme-miR-6-3p FBgn0265487 171.00 -21.39 2 21 552 575 22 77.27% 77.27%

Query: 3' uuUUUC-UUG-U-CGGUGACACUAu 5'

|||| ||| | | ||||||||

Ref: 5' agAAAGAAACGAAG-AACTGTGATa 3'

>dme-miR-6-3p FBgn0265487 171.00 -21.39 2 21 552 575 22 77.27% 77.27%

Query: 3' uuUUUC-UUG-U-CGGUGACACUAu 5'

|||| ||| | | ||||||||

Ref: 5' agAAAGAAACGAAG-AACTGTGATa 3'

>dme-miR-6-3p FBgn0265487 171.00 -21.39 2 21 552 575 22 77.27% 77.27%

Query: 3' uuUUUC-UUG-U-CGGUGACACUAu 5'

|||| ||| | | ||||||||

Ref: 5' agAAAGAAACGAAG-AACTGTGATa 3'

>dme-miR-6-3p FBgn0265487 171.00 -21.39 2 21 552 575 22 77.27% 77.27%

Query: 3' uuUUUC-UUG-U-CGGUGACACUAu 5'

|||| ||| | | ||||||||

Ref: 5' agAAAGAAACGAAG-AACTGTGATa 3'

>dme-miR-6-3p FBgn0265487 171.00 -21.39 2 21 552 575 22 77.27% 77.27%

Query: 3' uuUUUC-UUG-U-CGGUGACACUAu 5'

|||| ||| | | ||||||||

Ref: 5' agAAAGAAACGAAG-AACTGTGATa 3'

>dme-miR-310-3p FBgn0051547 167.00 -24.66 2 20 94 115 18 72.22% 88.89%

Query: 3' uuuCCGGCCCUUCACACGUUAu 5'

| ::||||: ||||||||

Ref: 5' cttGATTGGGAGTTGTGCAATg 3'

>dme-miR-310-3p FBgn0051547 167.00 -24.66 2 20 94 115 18 72.22% 88.89%

Query: 3' uuuCCGGCCCUUCACACGUUAu 5'

| ::||||: ||||||||

Ref: 5' cttGATTGGGAGTTGTGCAATg 3'

>dme-miR-310-3p FBgn0039684 165.00 -21.44 2 18 23 44 16 81.25% 87.50%

Query: 3' uuuccGGCCCUUCACACGUUAu 5'

|| |:||||||||||

Ref: 5' taaacCCAAGGAGTGTGCAATt 3'

>dme-miR-996-3p FBgn0032614 163.00 -22.39 2 21 449 472 22 68.18% 77.27%

Query: 3' ucUG-C-UCGUAC-UUUAGAUCAGu 5'

|: | |||| | :||||||||

Ref: 5' agATAGTAGCA-GTTGATCTAGTCa 3'

>dme-miR-996-3p FBgn0032614 163.00 -22.39 2 21 449 472 22 68.18% 77.27%

Query: 3' ucUG-C-UCGUAC-UUUAGAUCAGu 5'

|: | |||| | :||||||||

Ref: 5' agATAGTAGCA-GTTGATCTAGTCa 3'

>dme-miR-289-5p FBgn0001085 183.00 -23.31 2 25 994 1019 24 75.00% 83.33%

Query: 3' ucAGCGUCCGA-GGUGAAUUUAUAAAu 5'

|:| | || |||:||||||||||

Ref: 5' agTTG-AAACTGCCATTTAAATATTTg 3'

>dme-miR-289-5p FBgn0051064 169.00 -20.81 2 23 54 78 21 71.43% 80.95%

Query: 3' ucagCGUCCGAGGUGAAUUUAUAAAu 5'

||:||| | ||:|||||||

Ref: 5' ggggGCGGGCGAGA-TTGAATATTTa 3'

>dme-miR-289-5p FBgn0033250 166.00 -21.78 2 23 1214 1242 24 66.67% 75.00%

Query: 3' ucagCGUCCGAG-GU-G-AAUUUAUAAAu 5'

||:||||| : | ||||||||

Ref: 5' gggaGCGGGCTCGGGTCCGGAAATATTTt 3'

>dme-miR-289-5p FBgn0033250 166.00 -21.78 2 23 1273 1301 24 66.67% 75.00%

Query: 3' ucagCGUCCGAG-GU-G-AAUUUAUAAAu 5'

||:||||| : | ||||||||

Ref: 5' gggaGCGGGCTCGGGTCCGGAAATATTTt 3'

>dme-miR-289-5p FBgn0001085 183.00 -23.31 2 25 901 926 24 75.00% 83.33%

Query: 3' ucAGCGUCCGA-GGUGAAUUUAUAAAu 5'

|:| | || |||:||||||||||

Ref: 5' agTTG-AAACTGCCATTTAAATATTTg 3'

>dme-miR-289-5p FBgn0051064 169.00 -20.81 2 23 54 78 21 71.43% 80.95%

Query: 3' ucagCGUCCGAGGUGAAUUUAUAAAu 5'

||:||| | ||:|||||||

Ref: 5' ggggGCGGGCGAGA-TTGAATATTTa 3'

>dme-miR-284-3p FBgn0039946 158.00 -21.81 2 20 757 784 25 68.00% 72.00%

Query: 3' acGA-CC--U-UAG-UU-C-AACGACUg 5'

|| || | ||| |: | |||||||

Ref: 5' ttCTGGGACATATCTAGTGGTTGCTGAa 3'

>dme-miR-276b-5p FBgn0003861 168.00 -24.99 2 21 31 53 19 73.68% 84.21%

Query: 3' gcaUCCUUGAGAUAUGGAGCGAc 5'

|||| :|| : ||||||||

Ref: 5' tgcAGGACTTCGGGACCTCGCTc 3'

>dme-miR-9a-3p FBgn0031805 156.00 -20.03 2 18 35 55 16 75.00% 81.25%

Query: 3' auugaAGCCAUUCGAUCGAAAu 5'

||||| : |||||||

Ref: 5' gctagTCGGT-GTATAGCTTTa 3'

>dme-miR-14-5p FBgn0263006 164.00 -25.76 2 21 196 218 20 70.00% 80.00%

Query: 3' ucACUCAG-GGGCAGAGCGAGGg 5'

||:| | |:| ||||||||

Ref: 5' gaTGGGCCGACTGACTCGCTCCt 3'

>dme-miR-14-5p FBgn0040837 161.00 -28.70 2 19 71 92 18 72.22% 83.33%

Query: 3' ucacUCAGGG-GCAGAGCGAGGg 5'

:|||:| || |||||||

Ref: 5' tcccGGTCTCGCG-GTCGCTCCc 3'

>dme-miR-14-5p FBgn0038613 158.00 -22.87 2 21 13 35 22 68.18% 72.73%

Query: 3' ucACUCAGG-GGCA-G-AGCGAGGg 5'

| || || :||| |||||||

Ref: 5' gaT-AG-CCATCGTGATTCGCTCCt 3'

>dme-miR-14-5p FBgn0036262 155.00 -21.06 2 21 297 318 20 65.00% 75.00%

Query: 3' ucACUCAG-GGGCAGAGCGAGGg 5'

|| || |:: | |||||||

Ref: 5' ggTG-TTCACTTCTATCGCTCCa 3'

>dme-miR-14-5p FBgn0003861 156.00 -28.82 2 21 29 55 24 62.50% 70.83%

Query: 3' ucAC-U-CAGGGG-C-A-GAGCGAGGg 5'

|| | | |::| | ||||||||

Ref: 5' ggTGCAGGACTTCGGGACCTCGCTCCt 3'

>dme-miR-14-5p FBgn0036262 155.00 -21.06 2 21 297 318 20 65.00% 75.00%

Query: 3' ucACUCAG-GGGCAGAGCGAGGg 5'

|| || |:: | |||||||

Ref: 5' ggTG-TTCACTTCTATCGCTCCa 3'

>dme-miR-125-5p FBgn0264489 152.00 -20.46 2 21 1123 1147 22 63.64% 68.18%

Query: 3' agUGUUC-A-AUC-CCAGAGUCCCu 5'

||:|| | || |||||||

Ref: 5' ggACGAGCAGCCGCGGACTCAGGGc 3'

>dme-miR-125-5p FBgn0264489 152.00 -20.46 2 21 1123 1147 22 63.64% 68.18%

Query: 3' agUGUUC-A-AUC-CCAGAGUCCCu 5'

||:|| | || |||||||

Ref: 5' ggACGAGCAGCCGCGGACTCAGGGc 3'

>dme-miR-125-5p FBgn0264489 152.00 -20.46 2 21 1123 1147 22 63.64% 68.18%

Query: 3' agUGUUC-A-AUC-CCAGAGUCCCu 5'

||:|| | || |||||||

Ref: 5' ggACGAGCAGCCGCGGACTCAGGGc 3'

>dme-miR-125-5p FBgn0264489 152.00 -20.46 2 21 1123 1147 22 63.64% 68.18%

Query: 3' agUGUUC-A-AUC-CCAGAGUCCCu 5'

||:|| | || |||||||

Ref: 5' ggACGAGCAGCCGCGGACTCAGGGc 3'

>dme-miR-125-5p FBgn0264489 152.00 -20.46 2 21 1123 1147 22 63.64% 68.18%

Query: 3' agUGUUC-A-AUC-CCAGAGUCCCu 5'

||:|| | || |||||||

Ref: 5' ggACGAGCAGCCGCGGACTCAGGGc 3'

>dme-miR-125-5p FBgn0264489 152.00 -20.46 2 21 1123 1147 22 63.64% 68.18%

Query: 3' agUGUUC-A-AUC-CCAGAGUCCCu 5'

||:|| | || |||||||

Ref: 5' ggACGAGCAGCCGCGGACTCAGGGc 3'

>dme-miR-125-5p FBgn0264489 152.00 -20.46 2 21 1123 1147 22 63.64% 68.18%

Query: 3' agUGUUC-A-AUC-CCAGAGUCCCu 5'

||:|| | || |||||||

Ref: 5' ggACGAGCAGCCGCGGACTCAGGGc 3'

>dme-miR-125-5p FBgn0264489 152.00 -20.46 2 21 1123 1147 22 63.64% 68.18%

Query: 3' agUGUUC-A-AUC-CCAGAGUCCCu 5'

||:|| | || |||||||

Ref: 5' ggACGAGCAGCCGCGGACTCAGGGc 3'

>dme-miR-125-5p FBgn0264489 152.00 -20.46 2 21 1123 1147 22 63.64% 68.18%

Query: 3' agUGUUC-A-AUC-CCAGAGUCCCu 5'

||:|| | || |||||||

Ref: 5' ggACGAGCAGCCGCGGACTCAGGGc 3'

>dme-miR-125-5p FBgn0264489 152.00 -20.46 2 21 1123 1147 22 63.64% 68.18%

Query: 3' agUGUUC-A-AUC-CCAGAGUCCCu 5'

||:|| | || |||||||

Ref: 5' ggACGAGCAGCCGCGGACTCAGGGc 3'

>dme-miR-125-5p FBgn0264489 152.00 -20.46 2 21 1123 1147 22 63.64% 68.18%

Query: 3' agUGUUC-A-AUC-CCAGAGUCCCu 5'

||:|| | || |||||||

Ref: 5' ggACGAGCAGCCGCGGACTCAGGGc 3'

>dme-miR-125-5p FBgn0264489 152.00 -20.46 2 21 1123 1147 22 63.64% 68.18%

Query: 3' agUGUUC-A-AUC-CCAGAGUCCCu 5'

||:|| | || |||||||

Ref: 5' ggACGAGCAGCCGCGGACTCAGGGc 3'

>dme-miR-125-5p FBgn0264489 152.00 -20.46 2 21 1123 1147 22 63.64% 68.18%

Query: 3' agUGUUC-A-AUC-CCAGAGUCCCu 5'

||:|| | || |||||||

Ref: 5' ggACGAGCAGCCGCGGACTCAGGGc 3'

>dme-miR-125-5p FBgn0264489 152.00 -20.46 2 21 2094 2118 22 63.64% 68.18%

Query: 3' agUGUUC-A-AUC-CCAGAGUCCCu 5'

||:|| | || |||||||

Ref: 5' ggACGAGCAGCCGCGGACTCAGGGc 3'

>dme-miR-125-5p FBgn0264489 152.00 -20.46 2 21 2094 2118 22 63.64% 68.18%

Query: 3' agUGUUC-A-AUC-CCAGAGUCCCu 5'

||:|| | || |||||||

Ref: 5' ggACGAGCAGCCGCGGACTCAGGGc 3'

>dme-miR-125-5p FBgn0264489 152.00 -20.46 2 21 1123 1147 22 63.64% 68.18%

Query: 3' agUGUUC-A-AUC-CCAGAGUCCCu 5'

||:|| | || |||||||

Ref: 5' ggACGAGCAGCCGCGGACTCAGGGc 3'

>dme-miR-125-5p FBgn0264489 152.00 -20.46 2 21 1123 1147 22 63.64% 68.18%

Query: 3' agUGUUC-A-AUC-CCAGAGUCCCu 5'

||:|| | || |||||||

Ref: 5' ggACGAGCAGCCGCGGACTCAGGGc 3'

>dme-miR-994-3p FBgn0265487 176.00 -21.43 2 22 20 44 23 78.26% 78.26%

Query: 3' uaGAUUUUC-U-U-UGUCGUUGACAc 5'

||||||| | ||| |||||||

Ref: 5' atCTAAAAGTCCAAACA-CAACTGTa 3'

>dme-miR-994-3p FBgn0265487 176.00 -21.43 2 22 20 44 23 78.26% 78.26%

Query: 3' uaGAUUUUC-U-U-UGUCGUUGACAc 5'

||||||| | ||| |||||||

Ref: 5' atCTAAAAGTCCAAACA-CAACTGTa 3'

>dme-miR-994-3p FBgn0265487 176.00 -21.43 2 22 20 44 23 78.26% 78.26%

Query: 3' uaGAUUUUC-U-U-UGUCGUUGACAc 5'

||||||| | ||| |||||||

Ref: 5' atCTAAAAGTCCAAACA-CAACTGTa 3'

>dme-miR-994-3p FBgn0265487 176.00 -21.43 2 22 20 44 23 78.26% 78.26%

Query: 3' uaGAUUUUC-U-U-UGUCGUUGACAc 5'

||||||| | ||| |||||||

Ref: 5' atCTAAAAGTCCAAACA-CAACTGTa 3'

>dme-miR-994-3p FBgn0265487 176.00 -21.43 2 22 20 44 23 78.26% 78.26%

Query: 3' uaGAUUUUC-U-U-UGUCGUUGACAc 5'

||||||| | ||| |||||||

Ref: 5' atCTAAAAGTCCAAACA-CAACTGTa 3'

>dme-miR-994-3p FBgn0265487 176.00 -21.43 2 22 20 44 23 78.26% 78.26%

Query: 3' uaGAUUUUC-U-U-UGUCGUUGACAc 5'

||||||| | ||| |||||||

Ref: 5' atCTAAAAGTCCAAACA-CAACTGTa 3'

>dme-miR-994-3p FBgn0265487 176.00 -21.43 2 22 20 44 23 78.26% 78.26%

Query: 3' uaGAUUUUC-U-U-UGUCGUUGACAc 5'

||||||| | ||| |||||||

Ref: 5' atCTAAAAGTCCAAACA-CAACTGTa 3'

>dme-miR-133-3p FBgn0038247 163.00 -20.75 2 21 56 78 21 71.43% 76.19%

Query: 3' ugUCGA-CC-AACUUCCCCUGGUu 5'

||:| | ||| | |||||||

Ref: 5' taAGTTCAGCTTGTA-GGGACCAt 3'

>dme-miR-985-3p FBgn0000017 159.00 -20.31 2 20 4723 4748 22 63.64% 77.27%

Query: 3' acgGGCU-G-G-U-AACCUUGUAAAc 5'

:||| : : | || ||||||||

Ref: 5' tggTCGATTTTGAGTTTGAACATTTg 3'

>dme-miR-985-3p FBgn0000017 159.00 -20.31 2 20 4723 4748 22 63.64% 77.27%

Query: 3' acgGGCU-G-G-U-AACCUUGUAAAc 5'

:||| : : | || ||||||||

Ref: 5' tggTCGATTTTGAGTTTGAACATTTg 3'

>dme-miR-286-3p FBgn0015774 150.00 -21.57 2 22 215 238 23 65.22% 69.57%

Query: 3' ucGU-GCUCACAAGCC--AGAUCAGu 5'

|| | :| | ||| |||||||

Ref: 5' tcCATCCGG-G-GCGGTCTCTAGTCt 3'

>dme-miR-286-3p FBgn0035880 156.00 -23.51 2 22 57 83 24 62.50% 75.00%

Query: 3' ucGUGCU-C-ACAAGCC--AGAUCAGu 5'

:::|| || |||| |||||||

Ref: 5' ggTGTGATTCTGGTCGGATTCTAGTCa 3'

>dme-miR-286-3p FBgn0035880 156.00 -23.51 2 22 746 772 24 62.50% 75.00%

Query: 3' ucGUGCU-C-ACAAGCC--AGAUCAGu 5'

:::|| || |||| |||||||

Ref: 5' ggTGTGATTCTGGTCGGATTCTAGTCa 3'

>dme-miR-286-3p FBgn0035880 156.00 -23.51 2 22 966 992 24 62.50% 75.00%

Query: 3' ucGUGCU-C-ACAAGCC--AGAUCAGu 5'

:::|| || |||| |||||||

Ref: 5' ggTGTGATTCTGGTCGGATTCTAGTCa 3'

>dme-miR-286-3p FBgn0035880 156.00 -23.51 2 22 2472 2498 24 62.50% 75.00%

Query: 3' ucGUGCU-C-ACAAGCC--AGAUCAGu 5'

:::|| || |||| |||||||

Ref: 5' ggTGTGATTCTGGTCGGATTCTAGTCa 3'

>dme-miR-286-3p FBgn0015774 150.00 -21.57 2 22 215 238 23 65.22% 69.57%

Query: 3' ucGU-GCUCACAAGCC--AGAUCAGu 5'

|| | :| | ||| |||||||

Ref: 5' tcCATCCGG-G-GCGGTCTCTAGTCt 3'

>dme-miR-286-3p FBgn0015774 150.00 -21.57 2 22 215 238 23 65.22% 69.57%

Query: 3' ucGU-GCUCACAAGCC--AGAUCAGu 5'

|| | :| | ||| |||||||

Ref: 5' tcCATCCGG-G-GCGGTCTCTAGTCt 3'

>dme-miR-286-3p FBgn0015774 150.00 -21.57 2 22 215 238 23 65.22% 69.57%

Query: 3' ucGU-GCUCACAAGCC--AGAUCAGu 5'

|| | :| | ||| |||||||

Ref: 5' tcCATCCGG-G-GCGGTCTCTAGTCt 3'

>dme-miR-286-3p FBgn0015774 150.00 -21.57 2 22 215 238 23 65.22% 69.57%

Query: 3' ucGU-GCUCACAAGCC--AGAUCAGu 5'

|| | :| | ||| |||||||

Ref: 5' tcCATCCGG-G-GCGGTCTCTAGTCt 3'

>dme-miR-286-3p FBgn0015774 150.00 -21.57 2 22 215 238 23 65.22% 69.57%

Query: 3' ucGU-GCUCACAAGCC--AGAUCAGu 5'

|| | :| | ||| |||||||

Ref: 5' tcCATCCGG-G-GCGGTCTCTAGTCt 3'

>dme-miR-286-3p FBgn0015774 150.00 -21.57 2 22 215 238 23 65.22% 69.57%

Query: 3' ucGU-GCUCACAAGCC--AGAUCAGu 5'

|| | :| | ||| |||||||

Ref: 5' tcCATCCGG-G-GCGGTCTCTAGTCt 3'

>dme-miR-318-3p FBgn0036318 156.00 -22.35 2 19 193 212 17 64.71% 88.24%

Query: 3' acucUAUUUGUUUCGGGUCACu 5'

| |::| ::||||||||

Ref: 5' aaccA-AGGC-GGGCCCAGTGg 3'

>dme-miR-318-3p FBgn0036318 156.00 -22.35 2 19 186 205 17 64.71% 88.24%

Query: 3' acucUAUUUGUUUCGGGUCACu 5'

| |::| ::||||||||

Ref: 5' aaccA-AGGC-GGGCCCAGTGg 3'

>dme-miR-318-3p FBgn0266084 168.00 -24.20 2 21 43 66 21 76.19% 76.19%

Query: 3' acUCUAUU-U-GUUUCGGGUCACu 5'

|||||| | || |||||||

Ref: 5' cgAGATAATACCATCCCCCAGTGa 3'

>dme-miR-318-3p FBgn0266084 168.00 -24.20 2 21 290 313 21 76.19% 76.19%

Query: 3' acUCUAUU-U-GUUUCGGGUCACu 5'

|||||| | || |||||||

Ref: 5' cgAGATAATACCATCCCCCAGTGa 3'

>dme-miR-318-3p FBgn0266084 168.00 -24.20 2 21 193 216 21 76.19% 76.19%

Query: 3' acUCUAUU-U-GUUUCGGGUCACu 5'

|||||| | || |||||||

Ref: 5' cgAGATAATACCATCCCCCAGTGa 3'

>dme-miR-318-3p FBgn0266084 168.00 -24.20 2 21 193 216 21 76.19% 76.19%

Query: 3' acUCUAUU-U-GUUUCGGGUCACu 5'

|||||| | || |||||||

Ref: 5' cgAGATAATACCATCCCCCAGTGa 3'

>dme-miR-318-3p FBgn0266084 168.00 -24.20 2 21 43 66 21 76.19% 76.19%

Query: 3' acUCUAUU-U-GUUUCGGGUCACu 5'

|||||| | || |||||||

Ref: 5' cgAGATAATACCATCCCCCAGTGa 3'

>dme-miR-318-3p FBgn0266084 168.00 -24.20 2 21 43 66 21 76.19% 76.19%

Query: 3' acUCUAUU-U-GUUUCGGGUCACu 5'

|||||| | || |||||||

Ref: 5' cgAGATAATACCATCCCCCAGTGa 3'

>dme-miR-318-3p FBgn0036318 156.00 -22.35 2 19 59 78 17 64.71% 88.24%

Query: 3' acucUAUUUGUUUCGGGUCACu 5'

| |::| ::||||||||

Ref: 5' aaccA-AGGC-GGGCCCAGTGg 3'

>dme-miR-318-3p FBgn0036318 156.00 -22.35 2 19 91 110 17 64.71% 88.24%

Query: 3' acucUAUUUGUUUCGGGUCACu 5'

| |::| ::||||||||

Ref: 5' aaccA-AGGC-GGGCCCAGTGg 3'

>dme-miR-318-3p FBgn0266084 168.00 -24.20 2 21 43 66 21 76.19% 76.19%

Query: 3' acUCUAUU-U-GUUUCGGGUCACu 5'

|||||| | || |||||||

Ref: 5' cgAGATAATACCATCCCCCAGTGa 3'

>dme-miR-318-3p FBgn0266084 168.00 -24.20 2 21 43 66 21 76.19% 76.19%

Query: 3' acUCUAUU-U-GUUUCGGGUCACu 5'

|||||| | || |||||||

Ref: 5' cgAGATAATACCATCCCCCAGTGa 3'

>dme-miR-318-3p FBgn0027584 170.00 -22.49 2 20 276 296 18 77.78% 88.89%

Query: 3' acuCUAUUUGUUUCGGGUCACu 5'

|||:|: ||| |||||||

Ref: 5' tgcGATGAG-AAAACCCAGTGa 3'

>dme-miR-318-3p FBgn0027780 156.00 -21.38 2 21 1049 1074 23 60.87% 73.91%

Query: 3' acUC-U-AUUUGUUU-C-GGGUCACu 5'

|| | |: |:|:| |||||||

Ref: 5' cgAGAAGTGCATAGACTCCCCAGTGa 3'

>dme-miR-276a-5p FBgn0003861 168.00 -24.99 2 21 31 53 19 73.68% 84.21%

Query: 3' gcaUCCUUGAGAUAUGGAGCGAc 5'

|||| :|| : ||||||||

Ref: 5' tgcAGGACTTCGGGACCTCGCTc 3'

>dme-miR-92a-3p FBgn0051547 155.00 -22.86 2 20 94 115 18 66.67% 77.78%

Query: 3' uauCCGGCCCUGUUCACGUUAc 5'

| ::|||| |||||||

Ref: 5' cttGATTGGGAGTTGTGCAATg 3'

>dme-miR-92a-3p FBgn0051547 155.00 -22.86 2 20 94 115 18 66.67% 77.78%

Query: 3' uauCCGGCCCUGUUCACGUUAc 5'

| ::|||| |||||||

Ref: 5' cttGATTGGGAGTTGTGCAATg 3'

>dme-miR-92a-3p FBgn0031930 159.00 -23.40 2 21 1 29 26 65.38% 73.08%

Query: 3' uaUC-CGG-CCC--U-G-UU-CACGUUAc 5'

|| ||: ||| | : || |||||||

Ref: 5' taAGTGCTGGGGAAATTCAATGTGCAATa 3'
