# Supplementary material for: Age-related ceRNA networks in adult Drosophila ageing
Source: Front Genet. 2023 Feb 28;14:1096902. doi: 10.3389/fgene.2023.1096902 (PMC10012872; doi:10.3389/fgene.2023.1096902)
Supplement: Supplementary file 8 [file DataSheet3.doc]

211000022278174 Cufflinks exon 617 1151 . + . gene_id "XLOC_000043"; transcript_id "TCONS_00000097"; exon_number "1"; gene_type "lincRNA";

211000022278174 Cufflinks exon 617 1145 . - . gene_id "XLOC_000044"; transcript_id "TCONS_00000098"; exon_number "1"; gene_type "lincRNA";

211000022278224 Cufflinks exon 13 1507 . + . gene_id "XLOC_000071"; transcript_id "TCONS_00000138"; exon_number "1"; gene_type "lincRNA";

211000022278227 Cufflinks exon 6 1350 . + . gene_id "XLOC_000080"; transcript_id "TCONS_00000149"; exon_number "1"; gene_type "lincRNA";

211000022278227 Cufflinks exon 1 1350 . - . gene_id "XLOC_000081"; transcript_id "TCONS_00000150"; exon_number "1"; gene_type "lincRNA";

211000022278231 Cufflinks exon 48 259 . - . gene_id "XLOC_000084"; transcript_id "TCONS_00000155"; exon_number "1"; gene_type "lincRNA";

211000022278312 Cufflinks exon 633 1010 . - . gene_id "XLOC_000138"; transcript_id "TCONS_00000233"; exon_number "1"; gene_type "lincRNA";

211000022278312 Cufflinks exon 1120 1244 . - . gene_id "XLOC_000138"; transcript_id "TCONS_00000233"; exon_number "2"; gene_type "lincRNA";

211000022278312 Cufflinks exon 681 873 . - . gene_id "XLOC_000138"; transcript_id "TCONS_00000234"; exon_number "1"; gene_type "lincRNA";

211000022278312 Cufflinks exon 931 1010 . - . gene_id "XLOC_000138"; transcript_id "TCONS_00000234"; exon_number "2"; gene_type "lincRNA";

211000022278312 Cufflinks exon 1120 1258 . - . gene_id "XLOC_000138"; transcript_id "TCONS_00000234"; exon_number "3"; gene_type "lincRNA";

211000022278327 Cufflinks exon 22 256 . + . gene_id "XLOC_000176"; transcript_id "TCONS_00000318"; exon_number "1"; gene_type "lincRNA";

211000022278411 Cufflinks exon 1403 1611 . + . gene_id "XLOC_000218"; transcript_id "TCONS_00000404"; exon_number "1"; gene_type "lincRNA";

211000022278413 Cufflinks exon 1031 1235 . - . gene_id "XLOC_000223"; transcript_id "TCONS_00000408"; exon_number "1"; gene_type "lincRNA";

211000022278421 Cufflinks exon 1 2697 . - . gene_id "XLOC_000236"; transcript_id "TCONS_00000426"; exon_number "1"; gene_type "lincRNA";

211000022278435 Cufflinks exon 3 1886 . + . gene_id "XLOC_000241"; transcript_id "TCONS_00000443"; exon_number "1"; gene_type "lincRNA";

211000022278451 Cufflinks exon 4 1527 . - . gene_id "XLOC_000246"; transcript_id "TCONS_00000460"; exon_number "1"; gene_type "lincRNA";

211000022278465 Cufflinks exon 2 1723 . - . gene_id "XLOC_000275"; transcript_id "TCONS_00000520"; exon_number "1"; gene_type "lincRNA";

211000022278470 Cufflinks exon 457 669 . - . gene_id "XLOC_000286"; transcript_id "TCONS_00000560"; exon_number "1"; gene_type "lincRNA";

211000022278477 Cufflinks exon 825 1030 . - . gene_id "XLOC_000300"; transcript_id "TCONS_00000590"; exon_number "1"; gene_type "lincRNA";

211000022278487 Cufflinks exon 78 284 . + . gene_id "XLOC_000326"; transcript_id "TCONS_00000636"; exon_number "1"; gene_type "lincRNA";

211000022278487 Cufflinks exon 65 302 . - . gene_id "XLOC_000328"; transcript_id "TCONS_00000637"; exon_number "1"; gene_type "lincRNA";

211000022278634 Cufflinks exon 34 1729 . - . gene_id "XLOC_000504"; transcript_id "TCONS_00000996"; exon_number "1"; gene_type "lincRNA";

211000022278640 Cufflinks exon 995 1233 . - . gene_id "XLOC_000512"; transcript_id "TCONS_00001011"; exon_number "1"; gene_type "lincRNA";

211000022278652 Cufflinks exon 137 447 . - . gene_id "XLOC_000534"; transcript_id "TCONS_00001045"; exon_number "1"; gene_type "lincRNA";

211000022278655 Cufflinks exon 7 216 . + . gene_id "XLOC_000541"; transcript_id "TCONS_00001064"; exon_number "1"; gene_type "lincRNA";

211000022278655 Cufflinks exon 7 318 . - . gene_id "XLOC_000544"; transcript_id "TCONS_00001068"; exon_number "1"; gene_type "lincRNA";

211000022278774 Cufflinks exon 477 698 . - . gene_id "XLOC_000634"; transcript_id "TCONS_00001258"; exon_number "1"; gene_type "lincRNA";

211000022278833 Cufflinks exon 128 341 . + . gene_id "XLOC_000724"; transcript_id "TCONS_00001440"; exon_number "1"; gene_type "lincRNA";

211000022278853 Cufflinks exon 770 1157 . + . gene_id "XLOC_000756"; transcript_id "TCONS_00001506"; exon_number "1"; gene_type "lincRNA";

211000022278882 Scripture exon 192 641 . + . gene_id "XLOC_000794"; transcript_id "TCONS_00001565"; exon_number "1"; gene_type "lincRNA";

211000022278911 Cufflinks exon 47 259 . + . gene_id "XLOC_000836"; transcript_id "TCONS_00001647"; exon_number "1"; gene_type "lincRNA";

211000022278921 Scripture exon 26 1423 . + . gene_id "XLOC_000847"; transcript_id "TCONS_00001667"; exon_number "1"; gene_type "lincRNA";

211000022278928 Cufflinks exon 467 666 . + . gene_id "XLOC_000860"; transcript_id "TCONS_00001686"; exon_number "1"; gene_type "lincRNA";

211000022278928 Cufflinks exon 453 670 . - . gene_id "XLOC_000861"; transcript_id "TCONS_00001685"; exon_number "1"; gene_type "lincRNA";

211000022278942 Cufflinks exon 1730 1773 . + . gene_id "XLOC_000873"; transcript_id "TCONS_00001721"; exon_number "1"; gene_type "lincRNA";

211000022278942 Cufflinks exon 1838 2375 . + . gene_id "XLOC_000873"; transcript_id "TCONS_00001721"; exon_number "2"; gene_type "lincRNA";

211000022278976 Cufflinks exon 1095 1297 . - . gene_id "XLOC_000902"; transcript_id "TCONS_00001759"; exon_number "1"; gene_type "lincRNA";

211000022279006 Cufflinks exon 21 608 . + . gene_id "XLOC_000916"; transcript_id "TCONS_00001782"; exon_number "1"; gene_type "lincRNA";

211000022279016 Cufflinks exon 128 460 . + . gene_id "XLOC_000928"; transcript_id "TCONS_00001833"; exon_number "1"; gene_type "lincRNA";

211000022279056 Cufflinks exon 453 472 . + . gene_id "XLOC_000957"; transcript_id "TCONS_00001892"; exon_number "1"; gene_type "lincRNA";

211000022279056 Cufflinks exon 540 560 . + . gene_id "XLOC_000957"; transcript_id "TCONS_00001892"; exon_number "2"; gene_type "lincRNA";

211000022279056 Cufflinks exon 678 764 . + . gene_id "XLOC_000957"; transcript_id "TCONS_00001892"; exon_number "3"; gene_type "lincRNA";

211000022279056 Cufflinks exon 832 1009 . + . gene_id "XLOC_000957"; transcript_id "TCONS_00001892"; exon_number "4"; gene_type "lincRNA";

211000022279056 Cufflinks exon 1076 1187 . + . gene_id "XLOC_000957"; transcript_id "TCONS_00001892"; exon_number "5"; gene_type "lincRNA";

211000022279056 Cufflinks exon 1249 1602 . + . gene_id "XLOC_000957"; transcript_id "TCONS_00001892"; exon_number "6"; gene_type "lincRNA";

211000022279056 Cufflinks exon 621 764 . + . gene_id "XLOC_000957"; transcript_id "TCONS_00001897"; exon_number "1"; gene_type "lincRNA";

211000022279056 Cufflinks exon 832 1009 . + . gene_id "XLOC_000957"; transcript_id "TCONS_00001897"; exon_number "2"; gene_type "lincRNA";

211000022279056 Cufflinks exon 1076 1557 . + . gene_id "XLOC_000957"; transcript_id "TCONS_00001897"; exon_number "3"; gene_type "lincRNA";

211000022279066 Cufflinks exon 63 305 . - . gene_id "XLOC_000970"; transcript_id "TCONS_00001917"; exon_number "1"; gene_type "lincRNA";

211000022279078 Cufflinks exon 1517 1729 . + . gene_id "XLOC_001000"; transcript_id "TCONS_00001986"; exon_number "1"; gene_type "lincRNA";

211000022279078 Cufflinks exon 1501 1730 . - . gene_id "XLOC_001003"; transcript_id "TCONS_00001987"; exon_number "1"; gene_type "lincRNA";

211000022279081 Cufflinks exon 9 301 . - . gene_id "XLOC_001013"; transcript_id "TCONS_00002005"; exon_number "1"; gene_type "lincRNA";

211000022279081 Cufflinks exon 415 1060 . - . gene_id "XLOC_001014"; transcript_id "TCONS_00002006"; exon_number "1"; gene_type "lincRNA";

211000022279082 Cufflinks exon 1339 1541 . + . gene_id "XLOC_001019"; transcript_id "TCONS_00002026"; exon_number "1"; gene_type "lincRNA";

211000022279103 Cufflinks exon 720 1001 . - . gene_id "XLOC_001059"; transcript_id "TCONS_00002123"; exon_number "1"; gene_type "lincRNA";

211000022279218 Cufflinks exon 796 1015 . - . gene_id "XLOC_001145"; transcript_id "TCONS_00002309"; exon_number "1"; gene_type "lincRNA";

211000022279246 Cufflinks exon 9 1536 . + . gene_id "XLOC_001163"; transcript_id "TCONS_00002348"; exon_number "1"; gene_type "lincRNA";

211000022279356 Cufflinks exon 151 490 . + . gene_id "XLOC_001276"; transcript_id "TCONS_00002606"; exon_number "1"; gene_type "lincRNA";

211000022279388 Cufflinks exon 807 1260 . + . gene_id "XLOC_001297"; transcript_id "TCONS_00002630"; exon_number "1"; gene_type "lincRNA";

211000022279430 Cufflinks exon 366 599 . - . gene_id "XLOC_001318"; transcript_id "TCONS_00002669"; exon_number "1"; gene_type "lincRNA";

211000022279501 Cufflinks exon 774 1061 . + . gene_id "XLOC_001372"; transcript_id "TCONS_00002822"; exon_number "1"; gene_type "lincRNA";

211000022279542 Cufflinks exon 45 327 . + . gene_id "XLOC_001439"; transcript_id "TCONS_00002990"; exon_number "1"; gene_type "lincRNA";

211000022279605 Cufflinks exon 6 1085 . + . gene_id "XLOC_001501"; transcript_id "TCONS_00003126"; exon_number "1"; gene_type "lincRNA";

211000022279605 Cufflinks exon 1 1081 . - . gene_id "XLOC_001502"; transcript_id "TCONS_00003125"; exon_number "1"; gene_type "lincRNA";

211000022279810 Scripture exon 17 203 . - . gene_id "XLOC_001635"; transcript_id "TCONS_00003463"; exon_number "1"; gene_type "lincRNA";

211000022279810 Scripture exon 279 555 . - . gene_id "XLOC_001635"; transcript_id "TCONS_00003463"; exon_number "2"; gene_type "lincRNA";

211000022279991 Cufflinks exon 87 330 . - . gene_id "XLOC_001821"; transcript_id "TCONS_00003895"; exon_number "1"; gene_type "lincRNA";

211000022280302 Cufflinks exon 1247 1502 . - . gene_id "XLOC_002137"; transcript_id "TCONS_00004570"; exon_number "1"; gene_type "lincRNA";

211000022280453 Cufflinks exon 1337 1548 . + . gene_id "XLOC_002192"; transcript_id "TCONS_00004723"; exon_number "1"; gene_type "lincRNA";

211000022280534 Cufflinks exon 1394 1599 . + . gene_id "XLOC_002322"; transcript_id "TCONS_00005150"; exon_number "1"; gene_type "lincRNA";

211000022280577 Cufflinks exon 2200 3011 . - . gene_id "XLOC_002415"; transcript_id "TCONS_00005282"; exon_number "1"; gene_type "lincRNA";

211000022280593 Cufflinks exon 694 922 . + . gene_id "XLOC_002454"; transcript_id "TCONS_00005369"; exon_number "1"; gene_type "lincRNA";

211000022280607 Cufflinks exon 828 1087 . - . gene_id "XLOC_002495"; transcript_id "TCONS_00005456"; exon_number "1"; gene_type "lincRNA";

211000022280607 Cufflinks exon 4282 4513 . - . gene_id "XLOC_002499"; transcript_id "TCONS_00005450"; exon_number "1"; gene_type "lincRNA";

211000022280641 Cufflinks exon 125 433 . + . gene_id "XLOC_002565"; transcript_id "TCONS_00005649"; exon_number "1"; gene_type "lincRNA";

211000022280687 Cufflinks exon 1941 2152 . + . gene_id "XLOC_002657"; transcript_id "TCONS_00005958"; exon_number "1"; gene_type "lincRNA";

211000022280687 Cufflinks exon 1931 2167 . - . gene_id "XLOC_002658"; transcript_id "TCONS_00005959"; exon_number "1"; gene_type "lincRNA";

2L Cufflinks exon 289056 289256 . + . gene_id "XLOC_002734"; transcript_id "TCONS_00112415"; exon_number "1"; gene_type "anti-sense_lncRNA";

2L Cufflinks exon 683569 683770 . + . gene_id "XLOC_002994"; transcript_id "TCONS_00031530"; exon_number "1"; gene_type "intronic_lncRNA";

2L Cufflinks exon 720829 721057 . + . gene_id "XLOC_003026"; transcript_id "TCONS_00031557"; exon_number "1"; gene_type "lincRNA";

2L Cufflinks exon 1027251 1027466 . + . gene_id "XLOC_003284"; transcript_id "TCONS_00078072"; exon_number "1"; gene_type "intronic_lncRNA";

2L Cufflinks exon 1215916 1216119 . + . gene_id "XLOC_003385"; transcript_id "TCONS_00031817"; exon_number "1"; gene_type "lincRNA";

2L Cufflinks exon 1556965 1557174 . + . gene_id "XLOC_003865"; transcript_id "TCONS_00006952"; exon_number "1"; gene_type "intronic_lncRNA";

2L Cufflinks exon 1693342 1693550 . + . gene_id "XLOC_003980"; transcript_id "TCONS_00007046"; exon_number "1"; gene_type "intronic_lncRNA";

2L Cufflinks exon 1721834 1722055 . + . gene_id "XLOC_004055"; transcript_id "TCONS_00097366"; exon_number "1"; gene_type "intronic_lncRNA";

2L Cufflinks exon 1947956 1948204 . + . gene_id "XLOC_004207"; transcript_id "TCONS_00058159"; exon_number "1"; gene_type "lincRNA";

2L Cufflinks exon 1970657 1970866 . + . gene_id "XLOC_004233"; transcript_id "TCONS_00032309"; exon_number "1"; gene_type "lincRNA";

2L Cufflinks exon 1989461 1989841 . + . gene_id "XLOC_004242"; transcript_id "TCONS_00097418"; exon_number "1"; gene_type "lincRNA";

2L Cufflinks exon 1989913 1990377 . + . gene_id "XLOC_004242"; transcript_id "TCONS_00097418"; exon_number "2"; gene_type "lincRNA";

2L Cufflinks exon 2086149 2086362 . + . gene_id "XLOC_004337"; transcript_id "TCONS_00032387"; exon_number "1"; gene_type "intronic_lncRNA";

2L Cufflinks exon 2651980 2652188 . + . gene_id "XLOC_004877"; transcript_id "TCONS_00078895"; exon_number "1"; gene_type "anti-sense_lncRNA";

2L Cufflinks exon 2688582 2688799 . + . gene_id "XLOC_004927"; transcript_id "TCONS_00078927"; exon_number "1"; gene_type "intronic_lncRNA";

2L Cufflinks exon 2710072 2710288 . + . gene_id "XLOC_004965"; transcript_id "TCONS_00032840"; exon_number "1"; gene_type "intronic_lncRNA";

2L Cufflinks exon 3136157 3136370 . + . gene_id "XLOC_005199"; transcript_id "TCONS_00058733"; exon_number "1"; gene_type "intronic_lncRNA";

2L Cufflinks exon 3220338 3220545 . + . gene_id "XLOC_005278"; transcript_id "TCONS_00033022"; exon_number "1"; gene_type "lincRNA";

2L Cufflinks exon 3285345 3285544 . + . gene_id "XLOC_005376"; transcript_id "TCONS_00079127"; exon_number "1"; gene_type "lincRNA";

2L Cufflinks exon 3381288 3381493 . + . gene_id "XLOC_005423"; transcript_id "TCONS_00033106"; exon_number "1"; gene_type "lincRNA";

2L Cufflinks exon 3423026 3423125 . + . gene_id "XLOC_005483"; transcript_id "TCONS_00058837"; exon_number "1"; gene_type "anti-sense_lncRNA";

2L Cufflinks exon 3432215 3432389 . + . gene_id "XLOC_005483"; transcript_id "TCONS_00058837"; exon_number "2"; gene_type "anti-sense_lncRNA";

2L Cufflinks exon 3476360 3477011 . + . gene_id "XLOC_005540"; transcript_id "TCONS_00097953"; exon_number "1"; gene_type "lincRNA";

2L Cufflinks exon 3477077 3477577 . + . gene_id "XLOC_005540"; transcript_id "TCONS_00097953"; exon_number "2"; gene_type "lincRNA";

2L Cufflinks exon 3616305 3616516 . + . gene_id "XLOC_005682"; transcript_id "TCONS_00008130"; exon_number "1"; gene_type "lincRNA";

2L Cufflinks exon 3616668 3617915 . + . gene_id "XLOC_005682"; transcript_id "TCONS_00008130"; exon_number "2"; gene_type "lincRNA";

2L Cufflinks exon 3667186 3667409 . + . gene_id "XLOC_005708"; transcript_id "TCONS_00079303"; exon_number "1"; gene_type "intronic_lncRNA";

2L Cufflinks exon 3846468 3846671 . + . gene_id "XLOC_005873"; transcript_id "TCONS_00008298"; exon_number "1"; gene_type "lincRNA";

2L Cufflinks exon 3857792 3857992 . + . gene_id "XLOC_005893"; transcript_id "TCONS_00008305"; exon_number "1"; gene_type "lincRNA";

2L Cufflinks exon 4585796 4586053 . + . gene_id "XLOC_006488"; transcript_id "TCONS_00008703"; exon_number "1"; gene_type "intronic_lncRNA";

2L Cufflinks exon 4746680 4746929 . + . gene_id "XLOC_006681"; transcript_id "TCONS_00079808"; exon_number "1"; gene_type "intronic_lncRNA";

2L Cufflinks exon 5508950 5509159 . + . gene_id "XLOC_007184"; transcript_id "TCONS_00034298"; exon_number "1"; gene_type "lincRNA";

2L Cufflinks exon 5847352 5847551 . + . gene_id "XLOC_007506"; transcript_id "TCONS_00059968"; exon_number "1"; gene_type "intronic_lncRNA";

2L Cufflinks exon 5874603 5874811 . + . gene_id "XLOC_007531"; transcript_id "TCONS_00034532"; exon_number "1"; gene_type "intronic_lncRNA";

2L Cufflinks exon 6079737 6079834 . + . gene_id "XLOC_007686"; transcript_id "TCONS_00080319"; exon_number "1"; gene_type "lincRNA";

2L Cufflinks exon 6079892 6080022 . + . gene_id "XLOC_007686"; transcript_id "TCONS_00080319"; exon_number "2"; gene_type "lincRNA";

2L Cufflinks exon 6086716 6086982 . + . gene_id "XLOC_007695"; transcript_id "TCONS_00080323"; exon_number "1"; gene_type "intronic_lncRNA";

2L Cufflinks exon 6319133 6319344 . + . gene_id "XLOC_007983"; transcript_id "TCONS_00009707"; exon_number "1"; gene_type "intronic_lncRNA";

2L Cufflinks exon 6321249 6321460 . + . gene_id "XLOC_007987"; transcript_id "TCONS_00080487"; exon_number "1"; gene_type "intronic_lncRNA";

2L Cufflinks exon 6547315 6547335 . + . gene_id "XLOC_008083"; transcript_id "TCONS_00034923"; exon_number "1"; gene_type "lincRNA";

2L Cufflinks exon 6551852 6552374 . + . gene_id "XLOC_008083"; transcript_id "TCONS_00034923"; exon_number "2"; gene_type "lincRNA";

2L Cufflinks exon 6703102 6703330 . + . gene_id "XLOC_008196"; transcript_id "TCONS_00009824"; exon_number "1"; gene_type "intronic_lncRNA";

2L Cufflinks exon 6707458 6707681 . + . gene_id "XLOC_008202"; transcript_id "TCONS_00080585"; exon_number "1"; gene_type "intronic_lncRNA";

2L Scripture exon 6866329 6867063 . + . gene_id "XLOC_008357"; transcript_id "TCONS_00182447"; exon_number "1"; gene_type "intronic_lncRNA";

2L Scripture exon 6869002 6869354 . + . gene_id "XLOC_008357"; transcript_id "TCONS_00182447"; exon_number "2"; gene_type "intronic_lncRNA";

2L Cufflinks exon 6872724 6873058 . + . gene_id "XLOC_008357"; transcript_id "TCONS_00035084"; exon_number "1"; gene_type "lincRNA";

2L Cufflinks exon 6873130 6873680 . + . gene_id "XLOC_008357"; transcript_id "TCONS_00035084"; exon_number "2"; gene_type "lincRNA";

2L Cufflinks exon 7113473 7113720 . + . gene_id "XLOC_008508"; transcript_id "TCONS_00060452"; exon_number "1"; gene_type "intronic_lncRNA";

2L Cufflinks exon 7142284 7142503 . + . gene_id "XLOC_008538"; transcript_id "TCONS_00010013"; exon_number "1"; gene_type "intronic_lncRNA";

2L Cufflinks exon 7212016 7212223 . + . gene_id "XLOC_008606"; transcript_id "TCONS_00080730"; exon_number "1"; gene_type "intronic_lncRNA";

2L Cufflinks exon 7262264 7262463 . + . gene_id "XLOC_008645"; transcript_id "TCONS_00035218"; exon_number "1"; gene_type "intronic_lncRNA";

2L Cufflinks exon 7350486 7350691 . + . gene_id "XLOC_008775"; transcript_id "TCONS_00010106"; exon_number "1"; gene_type "intronic_lncRNA";

2L Scripture exon 7423857 7423976 . + . gene_id "XLOC_008833"; transcript_id "TCONS_00182517"; exon_number "1"; gene_type "lincRNA";

2L Scripture exon 7425172 7426218 . + . gene_id "XLOC_008833"; transcript_id "TCONS_00182517"; exon_number "2"; gene_type "lincRNA";

2L Scripture exon 7423859 7423991 . + . gene_id "XLOC_008833"; transcript_id "TCONS_00169868"; exon_number "1"; gene_type "lincRNA";

2L Scripture exon 7425172 7426420 . + . gene_id "XLOC_008833"; transcript_id "TCONS_00169868"; exon_number "2"; gene_type "lincRNA";

2L Scripture exon 7423859 7424363 . + . gene_id "XLOC_008833"; transcript_id "TCONS_00010148"; exon_number "1"; gene_type "lincRNA";

2L Scripture exon 7425172 7426420 . + . gene_id "XLOC_008833"; transcript_id "TCONS_00010148"; exon_number "2"; gene_type "lincRNA";

2L Cufflinks exon 7555063 7555271 . + . gene_id "XLOC_008925"; transcript_id "TCONS_00010185"; exon_number "1"; gene_type "intronic_lncRNA";

2L Cufflinks exon 7602217 7602426 . + . gene_id "XLOC_008975"; transcript_id "TCONS_00010213"; exon_number "1"; gene_type "lincRNA";

2L Cufflinks exon 7844653 7844874 . + . gene_id "XLOC_009157"; transcript_id "TCONS_00060730"; exon_number "1"; gene_type "lincRNA";

2L Cufflinks exon 8236903 8237144 . + . gene_id "XLOC_009321"; transcript_id "TCONS_00035599"; exon_number "1"; gene_type "intronic_lncRNA";

2L Cufflinks exon 8295799 8296008 . + . gene_id "XLOC_009352"; transcript_id "TCONS_00010440"; exon_number "1"; gene_type "intronic_lncRNA";

2L Cufflinks exon 8330052 8330280 . + . gene_id "XLOC_009373"; transcript_id "TCONS_00035611"; exon_number "1"; gene_type "intronic_lncRNA";

2L Cufflinks exon 8389310 8389553 . + . gene_id "XLOC_009412"; transcript_id "TCONS_00010485"; exon_number "1"; gene_type "anti-sense_lncRNA";

2L Cufflinks exon 8394446 8394779 . + . gene_id "XLOC_009418"; transcript_id "TCONS_00010487"; exon_number "1"; gene_type "anti-sense_lncRNA";

2L Cufflinks exon 8433619 8433820 . + . gene_id "XLOC_009431"; transcript_id "TCONS_00060826"; exon_number "1"; gene_type "lincRNA";

2L Cufflinks exon 8470053 8470271 . + . gene_id "XLOC_009446"; transcript_id "TCONS_00035657"; exon_number "1"; gene_type "intronic_lncRNA";

2L Cufflinks exon 8760501 8760702 . + . gene_id "XLOC_009600"; transcript_id "TCONS_00010624"; exon_number "1"; gene_type "lincRNA";

2L Cufflinks exon 8882107 8882310 . + . gene_id "XLOC_009773"; transcript_id "TCONS_00035868"; exon_number "1"; gene_type "lincRNA";

2L Cufflinks exon 8906540 8908534 . + . gene_id "XLOC_009798"; transcript_id "TCONS_00115887"; exon_number "1"; gene_type "intronic_lncRNA";

2L Cufflinks exon 8908596 8909815 . + . gene_id "XLOC_009798"; transcript_id "TCONS_00115887"; exon_number "2"; gene_type "intronic_lncRNA";

2L Cufflinks exon 8974760 8974973 . + . gene_id "XLOC_009834"; transcript_id "TCONS_00035907"; exon_number "1"; gene_type "anti-sense_lncRNA";

2L Cufflinks exon 9104007 9104212 . + . gene_id "XLOC_009989"; transcript_id "TCONS_00134944"; exon_number "1"; gene_type "lincRNA";

2L Cufflinks exon 9180811 9181053 . + . gene_id "XLOC_010097"; transcript_id "TCONS_00010899"; exon_number "1"; gene_type "intronic_lncRNA";

2L Cufflinks exon 9377053 9377261 . + . gene_id "XLOC_010226"; transcript_id "TCONS_00010981"; exon_number "1"; gene_type "intronic_lncRNA";

2L Cufflinks exon 9470327 9470345 . + . gene_id "XLOC_010279"; transcript_id "TCONS_00011030"; exon_number "1"; gene_type "lincRNA";

2L Cufflinks exon 9470420 9470628 . + . gene_id "XLOC_010279"; transcript_id "TCONS_00011030"; exon_number "2"; gene_type "lincRNA";

2L Cufflinks exon 9625958 9626163 . + . gene_id "XLOC_010378"; transcript_id "TCONS_00011098"; exon_number "1"; gene_type "lincRNA";

2L Cufflinks exon 9820467 9820666 . + . gene_id "XLOC_010524"; transcript_id "TCONS_00011188"; exon_number "1"; gene_type "intronic_lncRNA";

2L Cufflinks exon 10043952 10044187 . + . gene_id "XLOC_010702"; transcript_id "TCONS_00061447"; exon_number "1"; gene_type "lincRNA";

2L Cufflinks exon 10120001 10120202 . + . gene_id "XLOC_010814"; transcript_id "TCONS_00011350"; exon_number "1"; gene_type "anti-sense_lncRNA";

2L Scripture exon 10297705 10297936 . + . gene_id "XLOC_010902"; transcript_id "TCONS_00170440"; exon_number "1"; gene_type "anti-sense_lncRNA";

2L Cufflinks exon 10524283 10524496 . + . gene_id "XLOC_010976"; transcript_id "TCONS_00061576"; exon_number "1"; gene_type "lincRNA";

2L Cufflinks exon 10570384 10570587 . + . gene_id "XLOC_011037"; transcript_id "TCONS_00036670"; exon_number "1"; gene_type "intronic_lncRNA";

2L Cufflinks exon 10695075 10698184 . + . gene_id "XLOC_011186"; transcript_id "TCONS_00011579"; exon_number "1"; gene_type "anti-sense_lncRNA";

2L Cufflinks exon 10827435 10827639 . + . gene_id "XLOC_011296"; transcript_id "TCONS_00061685"; exon_number "1"; gene_type "intronic_lncRNA";

2L Cufflinks exon 10936427 10936629 . + . gene_id "XLOC_011398"; transcript_id "TCONS_00036893"; exon_number "1"; gene_type "intronic_lncRNA";

2L Cufflinks exon 11243822 11244028 . + . gene_id "XLOC_011626"; transcript_id "TCONS_00135659"; exon_number "1"; gene_type "intronic_lncRNA";

2L Cufflinks exon 11289657 11289857 . + . gene_id "XLOC_011650"; transcript_id "TCONS_00011834"; exon_number "1"; gene_type "lincRNA";

2L Cufflinks exon 11354327 11354528 . + . gene_id "XLOC_011760"; transcript_id "TCONS_00082086"; exon_number "1"; gene_type "lincRNA";

2L Cufflinks exon 11736431 11736801 . + . gene_id "XLOC_012280"; transcript_id "TCONS_00012211"; exon_number "1"; gene_type "lincRNA";

2L Cufflinks exon 11736871 11737546 . + . gene_id "XLOC_012280"; transcript_id "TCONS_00012211"; exon_number "2"; gene_type "lincRNA";

2L Cufflinks exon 11758267 11758474 . + . gene_id "XLOC_012306"; transcript_id "TCONS_00012230"; exon_number "1"; gene_type "lincRNA";

2L Cufflinks exon 12198516 12198722 . + . gene_id "XLOC_012722"; transcript_id "TCONS_00082524"; exon_number "1"; gene_type "intronic_lncRNA";

2L Cufflinks exon 12344804 12345010 . + . gene_id "XLOC_012860"; transcript_id "TCONS_00037829"; exon_number "1"; gene_type "intronic_lncRNA";

2L Cufflinks exon 12394918 12395155 . + . gene_id "XLOC_012921"; transcript_id "TCONS_00012616"; exon_number "1"; gene_type "intronic_lncRNA";

2L Cufflinks exon 12527734 12527979 . + . gene_id "XLOC_012991"; transcript_id "TCONS_00037883"; exon_number "1"; gene_type "intronic_lncRNA";

2L Cufflinks exon 12568811 12569011 . + . gene_id "XLOC_013027"; transcript_id "TCONS_00136321"; exon_number "1"; gene_type "lincRNA";

2L Cufflinks exon 12593363 12593580 . + . gene_id "XLOC_013075"; transcript_id "TCONS_00117402"; exon_number "1"; gene_type "intronic_lncRNA";

2L Cufflinks exon 12606425 12606646 . + . gene_id "XLOC_013097"; transcript_id "TCONS_00037933"; exon_number "1"; gene_type "intronic_lncRNA";

2L Cufflinks exon 12830079 12830281 . + . gene_id "XLOC_013338"; transcript_id "TCONS_00038104"; exon_number "1"; gene_type "lincRNA";

2L Cufflinks exon 12910232 12910470 . + . gene_id "XLOC_013460"; transcript_id "TCONS_00136492"; exon_number "1"; gene_type "lincRNA";

2L Cufflinks exon 12990164 12990374 . + . gene_id "XLOC_013567"; transcript_id "TCONS_00062743"; exon_number "1"; gene_type "lincRNA";

2L Cufflinks exon 13041365 13041579 . + . gene_id "XLOC_013589"; transcript_id "TCONS_00038255"; exon_number "1"; gene_type "intronic_lncRNA";

2L Cufflinks exon 13052111 13052316 . + . gene_id "XLOC_013599"; transcript_id "TCONS_00117656"; exon_number "1"; gene_type "intronic_lncRNA";

2L Cufflinks exon 13174104 13175267 . + . gene_id "XLOC_013739"; transcript_id "TCONS_00013087"; exon_number "1"; gene_type "intronic_lncRNA";

2L Scripture exon 13211905 13212024 . + . gene_id "XLOC_013744"; transcript_id "TCONS_00170779"; exon_number "1"; gene_type "anti-sense_lncRNA";

2L Scripture exon 13215898 13216408 . + . gene_id "XLOC_013744"; transcript_id "TCONS_00170779"; exon_number "2"; gene_type "anti-sense_lncRNA";

2L Cufflinks exon 13479250 13479481 . + . gene_id "XLOC_013901"; transcript_id "TCONS_00136741"; exon_number "1"; gene_type "intronic_lncRNA";

2L Cufflinks exon 14116519 14116718 . + . gene_id "XLOC_014300"; transcript_id "TCONS_00038793"; exon_number "1"; gene_type "lincRNA";

2L Cufflinks exon 14173601 14173862 . + . gene_id "XLOC_014367"; transcript_id "TCONS_00038856"; exon_number "1"; gene_type "intronic_lncRNA";

2L Cufflinks exon 14190778 14191011 . + . gene_id "XLOC_014386"; transcript_id "TCONS_00063287"; exon_number "1"; gene_type "intronic_lncRNA";

2L Cufflinks exon 14439924 14440131 . + . gene_id "XLOC_014628"; transcript_id "TCONS_00137147"; exon_number "1"; gene_type "lincRNA";

2L Cufflinks exon 14564404 14564608 . + . gene_id "XLOC_014801"; transcript_id "TCONS_00039190"; exon_number "1"; gene_type "lincRNA";

2L Cufflinks exon 14669297 14669512 . + . gene_id "XLOC_014892"; transcript_id "TCONS_00063559"; exon_number "1"; gene_type "intronic_lncRNA";

2L Cufflinks exon 14670327 14670526 . + . gene_id "XLOC_014893"; transcript_id "TCONS_00013969"; exon_number "1"; gene_type "intronic_lncRNA";

2L Cufflinks exon 14679226 14679425 . + . gene_id "XLOC_014901"; transcript_id "TCONS_00101573"; exon_number "1"; gene_type "intronic_lncRNA";

2L Cufflinks exon 14890997 14891215 . + . gene_id "XLOC_015184"; transcript_id "TCONS_00039454"; exon_number "1"; gene_type "lincRNA";

2L Cufflinks exon 14896363 14896573 . + . gene_id "XLOC_015191"; transcript_id "TCONS_00101660"; exon_number "1"; gene_type "lincRNA";

2L Cufflinks exon 15156904 15157113 . + . gene_id "XLOC_015430"; transcript_id "TCONS_00014280"; exon_number "1"; gene_type "lincRNA";

2L Cufflinks exon 15408534 15408744 . + . gene_id "XLOC_015726"; transcript_id "TCONS_00063962"; exon_number "1"; gene_type "lincRNA";

2L Cufflinks exon 15468610 15468813 . + . gene_id "XLOC_015807"; transcript_id "TCONS_00014498"; exon_number "1"; gene_type "lincRNA";

2L Cufflinks exon 15554289 15554495 . + . gene_id "XLOC_015916"; transcript_id "TCONS_00101948"; exon_number "1"; gene_type "intronic_lncRNA";

2L Cufflinks exon 15626685 15626886 . + . gene_id "XLOC_015986"; transcript_id "TCONS_00118999"; exon_number "1"; gene_type "lincRNA";

2L Scripture exon 15746455 15746460 . + . gene_id "XLOC_016010"; transcript_id "TCONS_00170941"; exon_number "1"; gene_type "intronic_lncRNA";

2L Scripture exon 15746519 15747205 . + . gene_id "XLOC_016010"; transcript_id "TCONS_00170941"; exon_number "2"; gene_type "intronic_lncRNA";

2L Cufflinks exon 15869400 15869600 . + . gene_id "XLOC_016136"; transcript_id "TCONS_00064192"; exon_number "1"; gene_type "lincRNA";

2L Cufflinks exon 15997316 15997523 . + . gene_id "XLOC_016284"; transcript_id "TCONS_00084288"; exon_number "1"; gene_type "lincRNA";

2L Cufflinks exon 16066862 16067084 . + . gene_id "XLOC_016359"; transcript_id "TCONS_00014877"; exon_number "1"; gene_type "intronic_lncRNA";

2L Cufflinks exon 16181539 16181774 . + . gene_id "XLOC_016509"; transcript_id "TCONS_00014965"; exon_number "1"; gene_type "intronic_lncRNA";

2L Cufflinks exon 16397494 16397694 . + . gene_id "XLOC_016706"; transcript_id "TCONS_00040532"; exon_number "1"; gene_type "lincRNA";

2L Cufflinks exon 16562806 16563021 . + . gene_id "XLOC_016861"; transcript_id "TCONS_00138189"; exon_number "1"; gene_type "intronic_lncRNA";

2L Cufflinks exon 16986193 16986433 . + . gene_id "XLOC_017131"; transcript_id "TCONS_00138362"; exon_number "1"; gene_type "intronic_lncRNA";

2L Cufflinks exon 17338675 17338880 . + . gene_id "XLOC_017629"; transcript_id "TCONS_00064956"; exon_number "1"; gene_type "lincRNA";

2L Cufflinks exon 17355800 17356005 . + . gene_id "XLOC_017662"; transcript_id "TCONS_00041211"; exon_number "1"; gene_type "lincRNA";

2L Cufflinks exon 17552461 17552662 . + . gene_id "XLOC_017768"; transcript_id "TCONS_00065036"; exon_number "1"; gene_type "lincRNA";

2L Cufflinks exon 17582129 17582341 . + . gene_id "XLOC_017811"; transcript_id "TCONS_00015770"; exon_number "1"; gene_type "lincRNA";

2L Cufflinks exon 17642517 17642716 . + . gene_id "XLOC_017897"; transcript_id "TCONS_00065100"; exon_number "1"; gene_type "lincRNA";

2L Cufflinks exon 18316258 18316465 . + . gene_id "XLOC_018647"; transcript_id "TCONS_00041812"; exon_number "1"; gene_type "lincRNA";

2L Cufflinks exon 18404807 18405029 . + . gene_id "XLOC_018677"; transcript_id "TCONS_00041836"; exon_number "1"; gene_type "lincRNA";

2L Cufflinks exon 18406448 18406649 . + . gene_id "XLOC_018680"; transcript_id "TCONS_00085369"; exon_number "1"; gene_type "lincRNA";

2L Cufflinks exon 18439973 18440232 . + . gene_id "XLOC_018729"; transcript_id "TCONS_00041868"; exon_number "1"; gene_type "lincRNA";

2L Cufflinks exon 18440356 18440621 . + . gene_id "XLOC_018729"; transcript_id "TCONS_00041868"; exon_number "2"; gene_type "lincRNA";

2L Cufflinks exon 18455518 18455963 . + . gene_id "XLOC_018733"; transcript_id "TCONS_00016255"; exon_number "1"; gene_type "anti-sense_lncRNA";

2L Cufflinks exon 18470869 18470931 . + . gene_id "XLOC_018733"; transcript_id "TCONS_00016255"; exon_number "2"; gene_type "anti-sense_lncRNA";

2L Cufflinks exon 18471154 18471197 . + . gene_id "XLOC_018733"; transcript_id "TCONS_00016255"; exon_number "3"; gene_type "anti-sense_lncRNA";

2L Cufflinks exon 18471259 18473030 . + . gene_id "XLOC_018733"; transcript_id "TCONS_00016255"; exon_number "4"; gene_type "anti-sense_lncRNA";

2L Cufflinks exon 18455518 18455963 . + . gene_id "XLOC_018733"; transcript_id "TCONS_00016254"; exon_number "1"; gene_type "anti-sense_lncRNA";

2L Cufflinks exon 18470869 18470928 . + . gene_id "XLOC_018733"; transcript_id "TCONS_00016254"; exon_number "2"; gene_type "anti-sense_lncRNA";

2L Cufflinks exon 18471154 18471197 . + . gene_id "XLOC_018733"; transcript_id "TCONS_00016254"; exon_number "3"; gene_type "anti-sense_lncRNA";

2L Cufflinks exon 18471259 18473030 . + . gene_id "XLOC_018733"; transcript_id "TCONS_00016254"; exon_number "4"; gene_type "anti-sense_lncRNA";

2L Scripture exon 18455690 18455814 . + . gene_id "XLOC_018733"; transcript_id "TCONS_00178308"; exon_number "1"; gene_type "lincRNA";

2L Scripture exon 18455869 18457956 . + . gene_id "XLOC_018733"; transcript_id "TCONS_00178308"; exon_number "2"; gene_type "lincRNA";

2L Cufflinks exon 18455740 18455814 . + . gene_id "XLOC_018733"; transcript_id "TCONS_00085390"; exon_number "1"; gene_type "lincRNA";

2L Cufflinks exon 18455869 18455963 . + . gene_id "XLOC_018733"; transcript_id "TCONS_00085390"; exon_number "2"; gene_type "lincRNA";

2L Cufflinks exon 18470869 18470931 . + . gene_id "XLOC_018733"; transcript_id "TCONS_00085390"; exon_number "3"; gene_type "lincRNA";

2L Cufflinks exon 18471154 18471197 . + . gene_id "XLOC_018733"; transcript_id "TCONS_00085390"; exon_number "4"; gene_type "lincRNA";

2L Cufflinks exon 18471259 18472537 . + . gene_id "XLOC_018733"; transcript_id "TCONS_00085390"; exon_number "5"; gene_type "lincRNA";

2L Cufflinks exon 18468447 18468650 . + . gene_id "XLOC_018744"; transcript_id "TCONS_00016268"; exon_number "1"; gene_type "lincRNA";

2L Cufflinks exon 18574494 18574504 . + . gene_id "XLOC_018797"; transcript_id "TCONS_00041909"; exon_number "1"; gene_type "intronic_lncRNA";

2L Cufflinks exon 18579323 18579674 . + . gene_id "XLOC_018797"; transcript_id "TCONS_00041909"; exon_number "2"; gene_type "intronic_lncRNA";

2L Cufflinks exon 18760608 18760808 . + . gene_id "XLOC_018917"; transcript_id "TCONS_00065582"; exon_number "1"; gene_type "lincRNA";

2L Cufflinks exon 18903158 18903358 . + . gene_id "XLOC_019064"; transcript_id "TCONS_00016478"; exon_number "1"; gene_type "lincRNA";

2L Cufflinks exon 18906364 18906568 . + . gene_id "XLOC_019070"; transcript_id "TCONS_00016481"; exon_number "1"; gene_type "lincRNA";

2L Cufflinks exon 19109065 19109227 . + . gene_id "XLOC_019196"; transcript_id "TCONS_00016579"; exon_number "1"; gene_type "lincRNA";

2L Cufflinks exon 19109419 19109493 . + . gene_id "XLOC_019196"; transcript_id "TCONS_00016579"; exon_number "2"; gene_type "lincRNA";

2L Cufflinks exon 19212744 19212962 . + . gene_id "XLOC_019229"; transcript_id "TCONS_00016630"; exon_number "1"; gene_type "lincRNA";

2L Cufflinks exon 19316810 19317009 . + . gene_id "XLOC_019411"; transcript_id "TCONS_00065852"; exon_number "1"; gene_type "lincRNA";

2L Cufflinks exon 19812655 19813509 . + . gene_id "XLOC_019637"; transcript_id "TCONS_00120716"; exon_number "1"; gene_type "intronic_lncRNA";

2L Cufflinks exon 20248371 20248577 . + . gene_id "XLOC_019952"; transcript_id "TCONS_00086079"; exon_number "1"; gene_type "lincRNA";

2L Cufflinks exon 20512903 20513105 . + . gene_id "XLOC_020146"; transcript_id "TCONS_00042899"; exon_number "1"; gene_type "lincRNA";

2L Scripture exon 20647534 20647667 . + . gene_id "XLOC_020319"; transcript_id "TCONS_00198337"; exon_number "1"; gene_type "lincRNA";

2L Scripture exon 20648198 20648479 . + . gene_id "XLOC_020319"; transcript_id "TCONS_00198337"; exon_number "2"; gene_type "lincRNA";

2L Scripture exon 20648785 20648860 . + . gene_id "XLOC_020319"; transcript_id "TCONS_00198337"; exon_number "3"; gene_type "lincRNA";

2L Scripture exon 20647534 20647788 . + . gene_id "XLOC_020319"; transcript_id "TCONS_00194944"; exon_number "1"; gene_type "lincRNA";

2L Scripture exon 20648189 20648479 . + . gene_id "XLOC_020319"; transcript_id "TCONS_00194944"; exon_number "2"; gene_type "lincRNA";

2L Scripture exon 20648785 20648860 . + . gene_id "XLOC_020319"; transcript_id "TCONS_00194944"; exon_number "3"; gene_type "lincRNA";

2L Scripture exon 21628391 21628586 . + . gene_id "XLOC_020714"; transcript_id "TCONS_00178714"; exon_number "1"; gene_type "anti-sense_lncRNA";

2L Scripture exon 21628651 21629720 . + . gene_id "XLOC_020714"; transcript_id "TCONS_00178714"; exon_number "2"; gene_type "anti-sense_lncRNA";

2L Cufflinks exon 21765344 21765551 . + . gene_id "XLOC_020802"; transcript_id "TCONS_00017789"; exon_number "1"; gene_type "intronic_lncRNA";

2L Cufflinks exon 21803935 21804148 . + . gene_id "XLOC_020838"; transcript_id "TCONS_00066738"; exon_number "1"; gene_type "lincRNA";

2L Cufflinks exon 22044658 22044896 . + . gene_id "XLOC_021142"; transcript_id "TCONS_00066939"; exon_number "1"; gene_type "lincRNA";

2L Cufflinks exon 22179090 22179566 . + . gene_id "XLOC_021247"; transcript_id "TCONS_00121651"; exon_number "1"; gene_type "intronic_lncRNA";

2L Cufflinks exon 22179632 22180206 . + . gene_id "XLOC_021247"; transcript_id "TCONS_00121651"; exon_number "2"; gene_type "intronic_lncRNA";

2L Cufflinks exon 22193380 22193581 . + . gene_id "XLOC_021259"; transcript_id "TCONS_00043692"; exon_number "1"; gene_type "intronic_lncRNA";

2L Cufflinks exon 22522685 22522888 . + . gene_id "XLOC_021399"; transcript_id "TCONS_00067132"; exon_number "1"; gene_type "lincRNA";

2L Cufflinks exon 22707348 22707566 . + . gene_id "XLOC_021458"; transcript_id "TCONS_00043856"; exon_number "1"; gene_type "intronic_lncRNA";

2L Scripture exon 71915 73642 . - . gene_id "XLOC_021826"; transcript_id "TCONS_00172241"; exon_number "1"; gene_type "anti-sense_lncRNA";

2L Scripture exon 73755 74175 . - . gene_id "XLOC_021826"; transcript_id "TCONS_00172241"; exon_number "2"; gene_type "anti-sense_lncRNA";

2L Cufflinks exon 521600 521823 . - . gene_id "XLOC_022002"; transcript_id "TCONS_00044283"; exon_number "1"; gene_type "intronic_lncRNA";

2L Cufflinks exon 973800 974002 . - . gene_id "XLOC_022440"; transcript_id "TCONS_00044585"; exon_number "1"; gene_type "lincRNA";

2L Cufflinks exon 993376 993589 . - . gene_id "XLOC_022456"; transcript_id "TCONS_00067825"; exon_number "1"; gene_type "lincRNA";

2L Cufflinks exon 1097685 1097903 . - . gene_id "XLOC_022488"; transcript_id "TCONS_00019052"; exon_number "1"; gene_type "intronic_lncRNA";

2L Cufflinks exon 1338192 1338401 . - . gene_id "XLOC_022732"; transcript_id "TCONS_00087600"; exon_number "1"; gene_type "lincRNA";

2L Cufflinks exon 1432569 1432775 . - . gene_id "XLOC_022860"; transcript_id "TCONS_00044841"; exon_number "1"; gene_type "lincRNA";

2L Cufflinks exon 1478296 1478514 . - . gene_id "XLOC_022930"; transcript_id "TCONS_00068070"; exon_number "1"; gene_type "lincRNA";

2L Cufflinks exon 1693334 1693553 . - . gene_id "XLOC_023194"; transcript_id "TCONS_00045022"; exon_number "1"; gene_type "intronic_lncRNA";

2L Cufflinks exon 1743071 1743271 . - . gene_id "XLOC_023209"; transcript_id "TCONS_00087856"; exon_number "1"; gene_type "intronic_lncRNA";

2L Cufflinks exon 1760307 1760521 . - . gene_id "XLOC_023227"; transcript_id "TCONS_00068227"; exon_number "1"; gene_type "lincRNA";

2L Cufflinks exon 1959074 1959275 . - . gene_id "XLOC_023375"; transcript_id "TCONS_00045132"; exon_number "1"; gene_type "intronic_lncRNA";

2L Cufflinks exon 2086145 2086378 . - . gene_id "XLOC_023497"; transcript_id "TCONS_00105203"; exon_number "1"; gene_type "intronic_lncRNA";

2L Cufflinks exon 2221470 2221679 . - . gene_id "XLOC_023560"; transcript_id "TCONS_00019720"; exon_number "1"; gene_type "intronic_lncRNA";

2L Cufflinks exon 2259079 2259295 . - . gene_id "XLOC_023585"; transcript_id "TCONS_00122970"; exon_number "1"; gene_type "lincRNA";

2L Cufflinks exon 2283862 2284325 . - . gene_id "XLOC_023623"; transcript_id "TCONS_00019761"; exon_number "1"; gene_type "lincRNA";

2L Cufflinks exon 2284767 2284985 . - . gene_id "XLOC_023623"; transcript_id "TCONS_00019761"; exon_number "2"; gene_type "lincRNA";

2L Cufflinks exon 2432488 2432701 . - . gene_id "XLOC_023715"; transcript_id "TCONS_00045446"; exon_number "1"; gene_type "intronic_lncRNA";

2L Cufflinks exon 2526642 2526842 . - . gene_id "XLOC_023841"; transcript_id "TCONS_00019911"; exon_number "1"; gene_type "lincRNA";

2L Cufflinks exon 2728014 2728217 . - . gene_id "XLOC_024098"; transcript_id "TCONS_00141751"; exon_number "1"; gene_type "intronic_lncRNA";

2L Cufflinks exon 3269111 3269315 . - . gene_id "XLOC_024460"; transcript_id "TCONS_00045895"; exon_number "1"; gene_type "lincRNA";

2L Cufflinks exon 3369321 3370138 . - . gene_id "XLOC_024513"; transcript_id "TCONS_00020275"; exon_number "1"; gene_type "intronic_lncRNA";

2L Cufflinks exon 4132612 4132876 . - . gene_id "XLOC_025288"; transcript_id "TCONS_00046403"; exon_number "1"; gene_type "lincRNA";

2L Cufflinks exon 4171982 4172197 . - . gene_id "XLOC_025345"; transcript_id "TCONS_00046460"; exon_number "1"; gene_type "lincRNA";

2L Cufflinks exon 4235973 4236181 . - . gene_id "XLOC_025395"; transcript_id "TCONS_00020835"; exon_number "1"; gene_type "lincRNA";

2L Cufflinks exon 4317143 4317344 . - . gene_id "XLOC_025509"; transcript_id "TCONS_00046583"; exon_number "1"; gene_type "intronic_lncRNA";

2L Cufflinks exon 4337550 4337764 . - . gene_id "XLOC_025525"; transcript_id "TCONS_00020903"; exon_number "1"; gene_type "intronic_lncRNA";

2L Cufflinks exon 4427769 4427989 . - . gene_id "XLOC_025595"; transcript_id "TCONS_00046632"; exon_number "1"; gene_type "intronic_lncRNA";

2L Scripture exon 5264696 5265280 . - . gene_id "XLOC_026015"; transcript_id "TCONS_00173024"; exon_number "1"; gene_type "anti-sense_lncRNA";

2L Scripture exon 5267804 5268747 . - . gene_id "XLOC_026015"; transcript_id "TCONS_00173024"; exon_number "2"; gene_type "anti-sense_lncRNA";

2L Cufflinks exon 5096795 5097022 . - . gene_id "XLOC_026025"; transcript_id "TCONS_00124089"; exon_number "1"; gene_type "intronic_lncRNA";

2L Cufflinks exon 5565501 5565755 . - . gene_id "XLOC_026404"; transcript_id "TCONS_00021463"; exon_number "1"; gene_type "lincRNA";

2L Cufflinks exon 5697177 5697390 . - . gene_id "XLOC_026581"; transcript_id "TCONS_00089519"; exon_number "1"; gene_type "lincRNA";

2L Cufflinks exon 6057393 6057631 . - . gene_id "XLOC_026845"; transcript_id "TCONS_00089616"; exon_number "1"; gene_type "anti-sense_lncRNA";

2L Cufflinks exon 6085015 6085245 . - . gene_id "XLOC_026858"; transcript_id "TCONS_00069949"; exon_number "1"; gene_type "intronic_lncRNA";

2L Cufflinks exon 6274027 6274229 . - . gene_id "XLOC_027072"; transcript_id "TCONS_00047546"; exon_number "1"; gene_type "intronic_lncRNA";

2L Cufflinks exon 6281746 6281961 . - . gene_id "XLOC_027078"; transcript_id "TCONS_00143016"; exon_number "1"; gene_type "intronic_lncRNA";

2L Cufflinks exon 6741082 6741299 . - . gene_id "XLOC_027400"; transcript_id "TCONS_00070196"; exon_number "1"; gene_type "lincRNA";

2L Scripture exon 7048840 7048958 . - . gene_id "XLOC_027607"; transcript_id "TCONS_00173240"; exon_number "1"; gene_type "intronic_lncRNA";

2L Scripture exon 7049176 7049747 . - . gene_id "XLOC_027607"; transcript_id "TCONS_00173240"; exon_number "2"; gene_type "intronic_lncRNA";

2L Cufflinks exon 7163019 7163254 . - . gene_id "XLOC_027736"; transcript_id "TCONS_00070378"; exon_number "1"; gene_type "lincRNA";

2L Cufflinks exon 7284729 7284935 . - . gene_id "XLOC_027829"; transcript_id "TCONS_00048017"; exon_number "1"; gene_type "lincRNA";

2L Cufflinks exon 7311020 7311249 . - . gene_id "XLOC_027869"; transcript_id "TCONS_00090094"; exon_number "1"; gene_type "intronic_lncRNA";

2L Cufflinks exon 7513065 7513285 . - . gene_id "XLOC_028049"; transcript_id "TCONS_00090155"; exon_number "1"; gene_type "intronic_lncRNA";

2L Cufflinks exon 7513954 7514242 . - . gene_id "XLOC_028051"; transcript_id "TCONS_00090156"; exon_number "1"; gene_type "intronic_lncRNA";

2L Cufflinks exon 7597298 7597521 . - . gene_id "XLOC_028096"; transcript_id "TCONS_00048177"; exon_number "1"; gene_type "lincRNA";

2L Cufflinks exon 7668518 7668740 . - . gene_id "XLOC_028192"; transcript_id "TCONS_00090230"; exon_number "1"; gene_type "anti-sense_lncRNA";

2L Cufflinks exon 7838539 7838806 . - . gene_id "XLOC_028285"; transcript_id "TCONS_00048334"; exon_number "1"; gene_type "lincRNA";

2L Cufflinks exon 7955909 7956199 . - . gene_id "XLOC_028369"; transcript_id "TCONS_00048381"; exon_number "1"; gene_type "intronic_lncRNA";

2L Cufflinks exon 8347408 8347613 . - . gene_id "XLOC_028493"; transcript_id "TCONS_00070780"; exon_number "1"; gene_type "intronic_lncRNA";

2L Cufflinks exon 8610425 8610658 . - . gene_id "XLOC_028635"; transcript_id "TCONS_00022887"; exon_number "1"; gene_type "intronic_lncRNA";

2L Cufflinks exon 8697988 8698480 . - . gene_id "XLOC_028708"; transcript_id "TCONS_00070852"; exon_number "1"; gene_type "intronic_lncRNA";

2L Cufflinks exon 8910189 8910418 . - . gene_id "XLOC_028952"; transcript_id "TCONS_00048703"; exon_number "1"; gene_type "intronic_lncRNA";

2L Cufflinks exon 8936578 8936623 . - . gene_id "XLOC_028969"; transcript_id "TCONS_00070985"; exon_number "1"; gene_type "lincRNA";

2L Cufflinks exon 8936726 8936948 . - . gene_id "XLOC_028969"; transcript_id "TCONS_00070985"; exon_number "2"; gene_type "lincRNA";

2L Cufflinks exon 9011258 9011498 . - . gene_id "XLOC_029007"; transcript_id "TCONS_00023105"; exon_number "1"; gene_type "anti-sense_lncRNA";

2L Cufflinks exon 9194466 9194692 . - . gene_id "XLOC_029256"; transcript_id "TCONS_00071102"; exon_number "1"; gene_type "intronic_lncRNA";

2L Cufflinks exon 9195623 9195843 . - . gene_id "XLOC_029257"; transcript_id "TCONS_00023229"; exon_number "1"; gene_type "intronic_lncRNA";

2L Cufflinks exon 9202694 9202912 . - . gene_id "XLOC_029264"; transcript_id "TCONS_00125494"; exon_number "1"; gene_type "intronic_lncRNA";

2L Cufflinks exon 9206273 9206480 . - . gene_id "XLOC_029269"; transcript_id "TCONS_00090670"; exon_number "1"; gene_type "intronic_lncRNA";

2L Cufflinks exon 9263617 9263839 . - . gene_id "XLOC_029306"; transcript_id "TCONS_00071115"; exon_number "1"; gene_type "intronic_lncRNA";

2L Cufflinks exon 9742085 9742311 . - . gene_id "XLOC_029665"; transcript_id "TCONS_00049115"; exon_number "1"; gene_type "intronic_lncRNA";

2L Cufflinks exon 9777463 9777663 . - . gene_id "XLOC_029699"; transcript_id "TCONS_00071335"; exon_number "1"; gene_type "lincRNA";

2L Scripture exon 9894067 9894132 . - . gene_id "XLOC_029850"; transcript_id "TCONS_00180134"; exon_number "1"; gene_type "anti-sense_lncRNA";

2L Scripture exon 9894331 9894401 . - . gene_id "XLOC_029850"; transcript_id "TCONS_00180134"; exon_number "2"; gene_type "anti-sense_lncRNA";

2L Scripture exon 9894576 9894725 . - . gene_id "XLOC_029850"; transcript_id "TCONS_00180134"; exon_number "3"; gene_type "anti-sense_lncRNA";

2L Scripture exon 9894980 9895380 . - . gene_id "XLOC_029850"; transcript_id "TCONS_00180134"; exon_number "4"; gene_type "anti-sense_lncRNA";

2L Scripture exon 9894067 9894401 . - . gene_id "XLOC_029850"; transcript_id "TCONS_00180132"; exon_number "1"; gene_type "anti-sense_lncRNA";

2L Scripture exon 9894576 9894725 . - . gene_id "XLOC_029850"; transcript_id "TCONS_00180132"; exon_number "2"; gene_type "anti-sense_lncRNA";

2L Scripture exon 9894980 9895380 . - . gene_id "XLOC_029850"; transcript_id "TCONS_00180132"; exon_number "3"; gene_type "anti-sense_lncRNA";

2L Cufflinks exon 10041612 10041813 . - . gene_id "XLOC_029951"; transcript_id "TCONS_00023695"; exon_number "1"; gene_type "lincRNA";

2L Cufflinks exon 10148583 10148785 . - . gene_id "XLOC_030095"; transcript_id "TCONS_00023768"; exon_number "1"; gene_type "anti-sense_lncRNA";

2L Cufflinks exon 10480107 10480314 . - . gene_id "XLOC_030222"; transcript_id "TCONS_00049508"; exon_number "1"; gene_type "intronic_lncRNA";

2L Cufflinks exon 10896353 10896573 . - . gene_id "XLOC_030600"; transcript_id "TCONS_00024077"; exon_number "1"; gene_type "lincRNA";

2L Scripture exon 11272302 11272595 . - . gene_id "XLOC_030868"; transcript_id "TCONS_00173925"; exon_number "1"; gene_type "anti-sense_lncRNA";

2L Scripture exon 11277346 11277375 . - . gene_id "XLOC_030868"; transcript_id "TCONS_00173925"; exon_number "2"; gene_type "anti-sense_lncRNA";

2L Cufflinks exon 11329470 11329680 . - . gene_id "XLOC_030946"; transcript_id "TCONS_00071927"; exon_number "1"; gene_type "lincRNA";

2L Cufflinks exon 11481372 11481573 . - . gene_id "XLOC_031187"; transcript_id "TCONS_00024437"; exon_number "1"; gene_type "lincRNA";

2L Cufflinks exon 11552867 11553091 . - . gene_id "XLOC_031248"; transcript_id "TCONS_00050135"; exon_number "1"; gene_type "lincRNA";

2L Cufflinks exon 11656122 11656324 . - . gene_id "XLOC_031382"; transcript_id "TCONS_00050210"; exon_number "1"; gene_type "lincRNA";

2L Cufflinks exon 11682376 11682576 . - . gene_id "XLOC_031429"; transcript_id "TCONS_00126476"; exon_number "1"; gene_type "lincRNA";

2L Cufflinks exon 11931891 11932091 . - . gene_id "XLOC_031676"; transcript_id "TCONS_00072293"; exon_number "1"; gene_type "lincRNA";

2L Cufflinks exon 12211936 12212176 . - . gene_id "XLOC_031908"; transcript_id "TCONS_00024909"; exon_number "1"; gene_type "intronic_lncRNA";

2L Cufflinks exon 12267118 12267256 . - . gene_id "XLOC_032003"; transcript_id "TCONS_00072447"; exon_number "1"; gene_type "intronic_lncRNA";

2L Cufflinks exon 12270247 12270548 . - . gene_id "XLOC_032003"; transcript_id "TCONS_00072447"; exon_number "2"; gene_type "intronic_lncRNA";

2L Cufflinks exon 12360739 12360960 . - . gene_id "XLOC_032088"; transcript_id "TCONS_00091941"; exon_number "1"; gene_type "intronic_lncRNA";

2L Cufflinks exon 12527723 12527952 . - . gene_id "XLOC_032178"; transcript_id "TCONS_00072501"; exon_number "1"; gene_type "intronic_lncRNA";

2L Cufflinks exon 12533900 12534179 . - . gene_id "XLOC_032183"; transcript_id "TCONS_00025030"; exon_number "1"; gene_type "intronic_lncRNA";

2L Cufflinks exon 12835196 12835399 . - . gene_id "XLOC_032520"; transcript_id "TCONS_00050882"; exon_number "1"; gene_type "lincRNA";

2L Cufflinks exon 12840429 12840643 . - . gene_id "XLOC_032531"; transcript_id "TCONS_00050888"; exon_number "1"; gene_type "lincRNA";

2L Cufflinks exon 12847008 12847208 . - . gene_id "XLOC_032544"; transcript_id "TCONS_00025208"; exon_number "1"; gene_type "lincRNA";

2L Cufflinks exon 12982364 12982576 . - . gene_id "XLOC_032748"; transcript_id "TCONS_00072707"; exon_number "1"; gene_type "lincRNA";

2L Cufflinks exon 13056739 13056999 . - . gene_id "XLOC_032785"; transcript_id "TCONS_00092172"; exon_number "1"; gene_type "intronic_lncRNA";

2L Cufflinks exon 13122942 13123009 . - . gene_id "XLOC_032876"; transcript_id "TCONS_00051078"; exon_number "1"; gene_type "lincRNA";

2L Cufflinks exon 13129668 13129816 . - . gene_id "XLOC_032876"; transcript_id "TCONS_00051078"; exon_number "2"; gene_type "lincRNA";

2L Cufflinks exon 13294166 13294399 . - . gene_id "XLOC_032999"; transcript_id "TCONS_00092260"; exon_number "1"; gene_type "intronic_lncRNA";

2L Scripture exon 13399193 13399660 . - . gene_id "XLOC_033083"; transcript_id "TCONS_00127122"; exon_number "1"; gene_type "anti-sense_lncRNA";

2L Scripture exon 13399764 13400146 . - . gene_id "XLOC_033083"; transcript_id "TCONS_00127122"; exon_number "2"; gene_type "anti-sense_lncRNA";

2L Cufflinks exon 13756990 13757195 . - . gene_id "XLOC_033348"; transcript_id "TCONS_00092421"; exon_number "1"; gene_type "intronic_lncRNA";

2L Cufflinks exon 14119229 14119439 . - . gene_id "XLOC_033643"; transcript_id "TCONS_00073131"; exon_number "1"; gene_type "lincRNA";

2L Cufflinks exon 14158490 14158722 . - . gene_id "XLOC_033697"; transcript_id "TCONS_00025861"; exon_number "1"; gene_type "anti-sense_lncRNA";

2L Cufflinks exon 14191834 14192040 . - . gene_id "XLOC_033721"; transcript_id "TCONS_00092580"; exon_number "1"; gene_type "intronic_lncRNA";

2L Cufflinks exon 14475656 14475864 . - . gene_id "XLOC_034019"; transcript_id "TCONS_00051750"; exon_number "1"; gene_type "lincRNA";

2L Cufflinks exon 14665682 14665911 . - . gene_id "XLOC_034222"; transcript_id "TCONS_00051895"; exon_number "1"; gene_type "intronic_lncRNA";

2L Cufflinks exon 14708891 14709093 . - . gene_id "XLOC_034292"; transcript_id "TCONS_00051927"; exon_number "1"; gene_type "lincRNA";

2L Cufflinks exon 14801731 14801935 . - . gene_id "XLOC_034428"; transcript_id "TCONS_00052018"; exon_number "1"; gene_type "lincRNA";

2L Cufflinks exon 15013839 15014040 . - . gene_id "XLOC_034692"; transcript_id "TCONS_00052180"; exon_number "1"; gene_type "lincRNA";

2L Cufflinks exon 15153279 15153492 . - . gene_id "XLOC_034824"; transcript_id "TCONS_00093118"; exon_number "1"; gene_type "lincRNA";

2L Cufflinks exon 15323100 15323310 . - . gene_id "XLOC_034999"; transcript_id "TCONS_00093215"; exon_number "1"; gene_type "lincRNA";

2L Cufflinks exon 15430018 15430245 . - . gene_id "XLOC_035160"; transcript_id "TCONS_00052519"; exon_number "1"; gene_type "lincRNA";

2L Cufflinks exon 15465232 15465432 . - . gene_id "XLOC_035202"; transcript_id "TCONS_00052549"; exon_number "1"; gene_type "lincRNA";

2L Cufflinks exon 15468653 15468870 . - . gene_id "XLOC_035209"; transcript_id "TCONS_00073957"; exon_number "1"; gene_type "lincRNA";

2L Cufflinks exon 15531045 15531246 . - . gene_id "XLOC_035307"; transcript_id "TCONS_00026826"; exon_number "1"; gene_type "lincRNA";

2L Cufflinks exon 15640665 15640870 . - . gene_id "XLOC_035445"; transcript_id "TCONS_00146528"; exon_number "1"; gene_type "lincRNA";

2L Cufflinks exon 15810018 15810018 . - . gene_id "XLOC_035510"; transcript_id "TCONS_00093415"; exon_number "1"; gene_type "lincRNA";

2L Cufflinks exon 15810075 15810286 . - . gene_id "XLOC_035510"; transcript_id "TCONS_00093415"; exon_number "2"; gene_type "lincRNA";

2L Cufflinks exon 16055546 16055772 . - . gene_id "XLOC_035828"; transcript_id "TCONS_00027118"; exon_number "1"; gene_type "intronic_lncRNA";

2L Cufflinks exon 16220471 16220673 . - . gene_id "XLOC_036061"; transcript_id "TCONS_00053124"; exon_number "1"; gene_type "lincRNA";

2L Cufflinks exon 16490105 16490323 . - . gene_id "XLOC_036282"; transcript_id "TCONS_00074464"; exon_number "1"; gene_type "anti-sense_lncRNA";

2L Cufflinks exon 16490386 16490542 . - . gene_id "XLOC_036282"; transcript_id "TCONS_00074464"; exon_number "2"; gene_type "anti-sense_lncRNA";

2L Cufflinks exon 16490616 16490697 . - . gene_id "XLOC_036282"; transcript_id "TCONS_00074464"; exon_number "3"; gene_type "anti-sense_lncRNA";

2L Cufflinks exon 16490796 16490992 . - . gene_id "XLOC_036283"; transcript_id "TCONS_00027387"; exon_number "1"; gene_type "lincRNA";

2L Cufflinks exon 16491055 16491228 . - . gene_id "XLOC_036283"; transcript_id "TCONS_00027387"; exon_number "2"; gene_type "lincRNA";

2L Cufflinks exon 16578856 16579057 . - . gene_id "XLOC_036347"; transcript_id "TCONS_00027420"; exon_number "1"; gene_type "intronic_lncRNA";

2L Scripture exon 16719645 16720785 . - . gene_id "XLOC_036462"; transcript_id "TCONS_00174328"; exon_number "1"; gene_type "anti-sense_lncRNA";

2L Scripture exon 16721107 16721196 . - . gene_id "XLOC_036462"; transcript_id "TCONS_00174328"; exon_number "2"; gene_type "anti-sense_lncRNA";

2L Scripture exon 16719679 16720785 . - . gene_id "XLOC_036462"; transcript_id "TCONS_00180760"; exon_number "1"; gene_type "anti-sense_lncRNA";

2L Scripture exon 16720988 16721198 . - . gene_id "XLOC_036462"; transcript_id "TCONS_00180760"; exon_number "2"; gene_type "anti-sense_lncRNA";

2L Cufflinks exon 16856929 16857134 . - . gene_id "XLOC_036533"; transcript_id "TCONS_00053410"; exon_number "1"; gene_type "intronic_lncRNA";

2L Cufflinks exon 16881861 16882081 . - . gene_id "XLOC_036557"; transcript_id "TCONS_00093825"; exon_number "1"; gene_type "intronic_lncRNA";

2L Cufflinks exon 17085707 17085951 . - . gene_id "XLOC_036845"; transcript_id "TCONS_00147064"; exon_number "1"; gene_type "lincRNA";

2L Cufflinks exon 17119155 17119377 . - . gene_id "XLOC_036908"; transcript_id "TCONS_00053615"; exon_number "1"; gene_type "lincRNA";

2L Cufflinks exon 17170168 17170376 . - . gene_id "XLOC_036968"; transcript_id "TCONS_00110025"; exon_number "1"; gene_type "anti-sense_lncRNA";

2L Cufflinks exon 17316823 17317047 . - . gene_id "XLOC_037209"; transcript_id "TCONS_00053751"; exon_number "1"; gene_type "lincRNA";

2L Cufflinks exon 17574224 17574425 . - . gene_id "XLOC_037392"; transcript_id "TCONS_00094083"; exon_number "1"; gene_type "lincRNA";

2L Cufflinks exon 17692372 17692587 . - . gene_id "XLOC_037538"; transcript_id "TCONS_00028060"; exon_number "1"; gene_type "intronic_lncRNA";

2L Cufflinks exon 17951938 17951999 . - . gene_id "XLOC_037870"; transcript_id "TCONS_00129268"; exon_number "1"; gene_type "lincRNA";

2L Cufflinks exon 17953414 17953561 . - . gene_id "XLOC_037870"; transcript_id "TCONS_00129268"; exon_number "2"; gene_type "lincRNA";

2L Cufflinks exon 18075739 18075940 . - . gene_id "XLOC_037996"; transcript_id "TCONS_00054312"; exon_number "1"; gene_type "lincRNA";

2L Cufflinks exon 18370786 18371002 . - . gene_id "XLOC_038269"; transcript_id "TCONS_00110521"; exon_number "1"; gene_type "intronic_lncRNA";

2L Cufflinks exon 18397124 18397327 . - . gene_id "XLOC_038291"; transcript_id "TCONS_00054513"; exon_number "1"; gene_type "lincRNA";

2L Cufflinks exon 18427460 18427668 . - . gene_id "XLOC_038333"; transcript_id "TCONS_00054543"; exon_number "1"; gene_type "lincRNA";

2L Cufflinks exon 19576956 19577180 . - . gene_id "XLOC_039180"; transcript_id "TCONS_00129944"; exon_number "1"; gene_type "intronic_lncRNA";

2L Cufflinks exon 19625097 19625317 . - . gene_id "XLOC_039217"; transcript_id "TCONS_00029269"; exon_number "1"; gene_type "intronic_lncRNA";

2L Cufflinks exon 19684478 19684701 . - . gene_id "XLOC_039248"; transcript_id "TCONS_00110919"; exon_number "1"; gene_type "intronic_lncRNA";

2L Cufflinks exon 19736984 19737455 . - . gene_id "XLOC_039276"; transcript_id "TCONS_00029276"; exon_number "1"; gene_type "anti-sense_lncRNA";

2L Cufflinks exon 19832445 19832649 . - . gene_id "XLOC_039345"; transcript_id "TCONS_00148225"; exon_number "1"; gene_type "intronic_lncRNA";

2L Cufflinks exon 19937719 19937927 . - . gene_id "XLOC_039470"; transcript_id "TCONS_00095165"; exon_number "1"; gene_type "intronic_lncRNA";

2L Cufflinks exon 19982936 19983141 . - . gene_id "XLOC_039498"; transcript_id "TCONS_00076066"; exon_number "1"; gene_type "intronic_lncRNA";

2L Cufflinks exon 20002336 20002545 . - . gene_id "XLOC_039529"; transcript_id "TCONS_00076088"; exon_number "1"; gene_type "intronic_lncRNA";

2L Cufflinks exon 20243570 20243867 . - . gene_id "XLOC_039656"; transcript_id "TCONS_00076165"; exon_number "1"; gene_type "lincRNA";

2L Cufflinks exon 20436206 20436407 . - . gene_id "XLOC_039827"; transcript_id "TCONS_00076259"; exon_number "1"; gene_type "intronic_lncRNA";

2L Cufflinks exon 20477316 20477526 . - . gene_id "XLOC_039855"; transcript_id "TCONS_00055549"; exon_number "1"; gene_type "lincRNA";

2L Cufflinks exon 20596435 20596665 . - . gene_id "XLOC_040034"; transcript_id "TCONS_00095479"; exon_number "1"; gene_type "lincRNA";

2L Cufflinks exon 20625894 20626118 . - . gene_id "XLOC_040081"; transcript_id "TCONS_00111268"; exon_number "1"; gene_type "lincRNA";

2L Cufflinks exon 20636299 20638070 . - . gene_id "XLOC_040096"; transcript_id "TCONS_00076397"; exon_number "1"; gene_type "lincRNA";

2L Cufflinks exon 20638152 20638378 . - . gene_id "XLOC_040096"; transcript_id "TCONS_00076397"; exon_number "2"; gene_type "lincRNA";

2L Cufflinks exon 21190496 21190705 . - . gene_id "XLOC_040305"; transcript_id "TCONS_00029984"; exon_number "1"; gene_type "intronic_lncRNA";

2L Cufflinks exon 21323201 21323406 . - . gene_id "XLOC_040357"; transcript_id "TCONS_00076577"; exon_number "1"; gene_type "lincRNA";

2L Cufflinks exon 21327588 21327791 . - . gene_id "XLOC_040366"; transcript_id "TCONS_00030034"; exon_number "1"; gene_type "lincRNA";

2L Cufflinks exon 21398746 21401435 . - . gene_id "XLOC_040413"; transcript_id "TCONS_00076601"; exon_number "1"; gene_type "lincRNA";

2L Cufflinks exon 21401488 21401533 . - . gene_id "XLOC_040413"; transcript_id "TCONS_00076601"; exon_number "2"; gene_type "lincRNA";

2L Cufflinks exon 21683731 21683940 . - . gene_id "XLOC_040496"; transcript_id "TCONS_00055998"; exon_number "1"; gene_type "lincRNA";

2L Cufflinks exon 21879710 21879918 . - . gene_id "XLOC_040644"; transcript_id "TCONS_00030234"; exon_number "1"; gene_type "lincRNA";

2L Cufflinks exon 21900510 21900713 . - . gene_id "XLOC_040676"; transcript_id "TCONS_00130720"; exon_number "1"; gene_type "lincRNA";

2L Cufflinks exon 22081228 22081443 . - . gene_id "XLOC_040912"; transcript_id "TCONS_00111678"; exon_number "1"; gene_type "lincRNA";

2L Cufflinks exon 22239165 22239357 . - . gene_id "XLOC_041079"; transcript_id "TCONS_00096033"; exon_number "1"; gene_type "lincRNA";

2L Cufflinks exon 22239427 22239437 . - . gene_id "XLOC_041079"; transcript_id "TCONS_00096033"; exon_number "2"; gene_type "lincRNA";

2L Cufflinks exon 22543195 22544498 . - . gene_id "XLOC_041081"; transcript_id "TCONS_00056464"; exon_number "1"; gene_type "lincRNA";

2L Cufflinks exon 22546344 22546757 . - . gene_id "XLOC_041081"; transcript_id "TCONS_00056464"; exon_number "2"; gene_type "lincRNA";

2L Cufflinks exon 22543195 22544498 . - . gene_id "XLOC_041081"; transcript_id "TCONS_00056463"; exon_number "1"; gene_type "lincRNA";

2L Cufflinks exon 22546070 22546210 . - . gene_id "XLOC_041081"; transcript_id "TCONS_00056463"; exon_number "2"; gene_type "lincRNA";

2L Cufflinks exon 22546344 22546757 . - . gene_id "XLOC_041081"; transcript_id "TCONS_00056463"; exon_number "3"; gene_type "lincRNA";

2L Cufflinks exon 22923846 22924055 . - . gene_id "XLOC_041273"; transcript_id "TCONS_00111844"; exon_number "1"; gene_type "lincRNA";

2R Cufflinks exon 125561 125768 . + . gene_id "XLOC_045560"; transcript_id "TCONS_00290493"; exon_number "1"; gene_type "lincRNA";

2R Cufflinks exon 460178 460385 . + . gene_id "XLOC_045613"; transcript_id "TCONS_00204032"; exon_number "1"; gene_type "intronic_lncRNA";

2R Cufflinks exon 386583 386797 . + . gene_id "XLOC_045634"; transcript_id "TCONS_00204010"; exon_number "1"; gene_type "lincRNA";

2R Cufflinks exon 900261 900469 . + . gene_id "XLOC_045911"; transcript_id "TCONS_00228619"; exon_number "1"; gene_type "lincRNA";

2R Scripture exon 999434 1000181 . + . gene_id "XLOC_046011"; transcript_id "TCONS_00204181"; exon_number "1"; gene_type "lincRNA";

2R Scripture exon 1000263 1000435 . + . gene_id "XLOC_046011"; transcript_id "TCONS_00204181"; exon_number "2"; gene_type "lincRNA";

2R Cufflinks exon 1023155 1023386 . + . gene_id "XLOC_046032"; transcript_id "TCONS_00252866"; exon_number "1"; gene_type "lincRNA";

2R Cufflinks exon 1359323 1359664 . + . gene_id "XLOC_046208"; transcript_id "TCONS_00204311"; exon_number "1"; gene_type "intronic_lncRNA";

2R Cufflinks exon 1485414 1485622 . + . gene_id "XLOC_046344"; transcript_id "TCONS_00306592"; exon_number "1"; gene_type "lincRNA";

2R Cufflinks exon 2145283 2145511 . + . gene_id "XLOC_046673"; transcript_id "TCONS_00229030"; exon_number "1"; gene_type "intronic_lncRNA";

2R Cufflinks exon 2184868 2185074 . + . gene_id "XLOC_046685"; transcript_id "TCONS_00253197"; exon_number "1"; gene_type "intronic_lncRNA";

2R Cufflinks exon 2255770 2255975 . + . gene_id "XLOC_046721"; transcript_id "TCONS_00253209"; exon_number "1"; gene_type "intronic_lncRNA";

2R Cufflinks exon 2701919 2702141 . + . gene_id "XLOC_046894"; transcript_id "TCONS_00204717"; exon_number "1"; gene_type "lincRNA";

2R Cufflinks exon 3129531 3129735 . + . gene_id "XLOC_047118"; transcript_id "TCONS_00307035"; exon_number "1"; gene_type "intronic_lncRNA";

2R Scripture exon 4100018 4100551 . + . gene_id "XLOC_047626"; transcript_id "TCONS_00361296"; exon_number "1"; gene_type "lincRNA";

2R Cufflinks exon 4160855 4161057 . + . gene_id "XLOC_047651"; transcript_id "TCONS_00229678"; exon_number "1"; gene_type "lincRNA";

2R Cufflinks exon 4204285 4204500 . + . gene_id "XLOC_047689"; transcript_id "TCONS_00273346"; exon_number "1"; gene_type "lincRNA";

2R Cufflinks exon 4272586 4273035 . + . gene_id "XLOC_047731"; transcript_id "TCONS_00307364"; exon_number "1"; gene_type "lincRNA";

2R Cufflinks exon 4273097 4274016 . + . gene_id "XLOC_047731"; transcript_id "TCONS_00307364"; exon_number "2"; gene_type "lincRNA";

2R Cufflinks exon 4272637 4273035 . + . gene_id "XLOC_047731"; transcript_id "TCONS_00253752"; exon_number "1"; gene_type "lincRNA";

2R Cufflinks exon 4273094 4274111 . + . gene_id "XLOC_047731"; transcript_id "TCONS_00253752"; exon_number "2"; gene_type "lincRNA";

2R Cufflinks exon 4871340 4871847 . + . gene_id "XLOC_047973"; transcript_id "TCONS_00205546"; exon_number "1"; gene_type "lincRNA";

2R Cufflinks exon 4871936 4872012 . + . gene_id "XLOC_047973"; transcript_id "TCONS_00205546"; exon_number "2"; gene_type "lincRNA";

2R Cufflinks exon 5008100 5008300 . + . gene_id "XLOC_048112"; transcript_id "TCONS_00230141"; exon_number "1"; gene_type "intronic_lncRNA";

2R Scripture exon 5049278 5050976 . + . gene_id "XLOC_048157"; transcript_id "TCONS_00393498"; exon_number "1"; gene_type "lincRNA";

2R Scripture exon 5051185 5051802 . + . gene_id "XLOC_048157"; transcript_id "TCONS_00393498"; exon_number "2"; gene_type "lincRNA";

2R Cufflinks exon 5097836 5098074 . + . gene_id "XLOC_048205"; transcript_id "TCONS_00254116"; exon_number "1"; gene_type "lincRNA";

2R Cufflinks exon 5136670 5136875 . + . gene_id "XLOC_048228"; transcript_id "TCONS_00230245"; exon_number "1"; gene_type "lincRNA";

2R Cufflinks exon 5138683 5138743 . + . gene_id "XLOC_048229"; transcript_id "TCONS_00230246"; exon_number "1"; gene_type "lincRNA";

2R Cufflinks exon 5138796 5139147 . + . gene_id "XLOC_048229"; transcript_id "TCONS_00230246"; exon_number "2"; gene_type "lincRNA";

2R Cufflinks exon 5365580 5366737 . + . gene_id "XLOC_048336"; transcript_id "TCONS_00205887"; exon_number "1"; gene_type "lincRNA";

2R Cufflinks exon 5366804 5367738 . + . gene_id "XLOC_048336"; transcript_id "TCONS_00205887"; exon_number "2"; gene_type "lincRNA";

2R Cufflinks exon 5720461 5720677 . + . gene_id "XLOC_048545"; transcript_id "TCONS_00308005"; exon_number "1"; gene_type "intronic_lncRNA";

2R Cufflinks exon 5835254 5835457 . + . gene_id "XLOC_048653"; transcript_id "TCONS_00230587"; exon_number "1"; gene_type "intronic_lncRNA";

2R Cufflinks exon 6072782 6073004 . + . gene_id "XLOC_048832"; transcript_id "TCONS_00274169"; exon_number "1"; gene_type "lincRNA";

2R Cufflinks exon 6110854 6111063 . + . gene_id "XLOC_048848"; transcript_id "TCONS_00230771"; exon_number "1"; gene_type "intronic_lncRNA";

2R Cufflinks exon 6140312 6140549 . + . gene_id "XLOC_048866"; transcript_id "TCONS_00230780"; exon_number "1"; gene_type "intronic_lncRNA";

2R Cufflinks exon 6142713 6142919 . + . gene_id "XLOC_048868"; transcript_id "TCONS_00292221"; exon_number "1"; gene_type "intronic_lncRNA";

2R Cufflinks exon 6323817 6330291 . + . gene_id "XLOC_048931"; transcript_id "TCONS_00206377"; exon_number "1"; gene_type "lincRNA";

2R Cufflinks exon 6338087 6342255 . + . gene_id "XLOC_048931"; transcript_id "TCONS_00206377"; exon_number "2"; gene_type "lincRNA";

2R Scripture exon 6872938 6873265 . + . gene_id "XLOC_049126"; transcript_id "TCONS_00372319"; exon_number "1"; gene_type "anti-sense_lncRNA";

2R Scripture exon 6875982 6876197 . + . gene_id "XLOC_049126"; transcript_id "TCONS_00372319"; exon_number "2"; gene_type "anti-sense_lncRNA";

2R Scripture exon 6876273 6876431 . + . gene_id "XLOC_049126"; transcript_id "TCONS_00372319"; exon_number "3"; gene_type "anti-sense_lncRNA";

2R Cufflinks exon 6926881 6927088 . + . gene_id "XLOC_049172"; transcript_id "TCONS_00274499"; exon_number "1"; gene_type "intronic_lncRNA";

2R Cufflinks exon 6948706 6948923 . + . gene_id "XLOC_049182"; transcript_id "TCONS_00231137"; exon_number "1"; gene_type "intronic_lncRNA";

2R Cufflinks exon 7333794 7333910 . + . gene_id "XLOC_049403"; transcript_id "TCONS_00206996"; exon_number "1"; gene_type "intronic_lncRNA";

2R Cufflinks exon 7334401 7334513 . + . gene_id "XLOC_049403"; transcript_id "TCONS_00206996"; exon_number "2"; gene_type "intronic_lncRNA";

2R Cufflinks exon 7599728 7599935 . + . gene_id "XLOC_049537"; transcript_id "TCONS_00231483"; exon_number "1"; gene_type "intronic_lncRNA";

2R Cufflinks exon 7615368 7615571 . + . gene_id "XLOC_049545"; transcript_id "TCONS_00292765"; exon_number "1"; gene_type "intronic_lncRNA";

2R Cufflinks exon 7861133 7861379 . + . gene_id "XLOC_049617"; transcript_id "TCONS_00231527"; exon_number "1"; gene_type "intronic_lncRNA";

2R Cufflinks exon 8523686 8523891 . + . gene_id "XLOC_050043"; transcript_id "TCONS_00275089"; exon_number "1"; gene_type "intronic_lncRNA";

2R Cufflinks exon 8562881 8563092 . + . gene_id "XLOC_050100"; transcript_id "TCONS_00275120"; exon_number "1"; gene_type "lincRNA";

2R Scripture exon 9094936 9095339 . + . gene_id "XLOC_050502"; transcript_id "TCONS_00362500"; exon_number "1"; gene_type "anti-sense_lncRNA";

2R Scripture exon 9095578 9095629 . + . gene_id "XLOC_050502"; transcript_id "TCONS_00362500"; exon_number "2"; gene_type "anti-sense_lncRNA";

2R Scripture exon 9095898 9095958 . + . gene_id "XLOC_050502"; transcript_id "TCONS_00362500"; exon_number "3"; gene_type "anti-sense_lncRNA";

2R Scripture exon 9096150 9096202 . + . gene_id "XLOC_050502"; transcript_id "TCONS_00362500"; exon_number "4"; gene_type "anti-sense_lncRNA";

2R Scripture exon 9096383 9096438 . + . gene_id "XLOC_050502"; transcript_id "TCONS_00362500"; exon_number "5"; gene_type "anti-sense_lncRNA";

2R Scripture exon 9096671 9096735 . + . gene_id "XLOC_050502"; transcript_id "TCONS_00362500"; exon_number "6"; gene_type "anti-sense_lncRNA";

2R Scripture exon 9096926 9097045 . + . gene_id "XLOC_050502"; transcript_id "TCONS_00362500"; exon_number "7"; gene_type "anti-sense_lncRNA";

2R Scripture exon 9095062 9095339 . + . gene_id "XLOC_050502"; transcript_id "TCONS_00373238"; exon_number "1"; gene_type "lincRNA";

2R Scripture exon 9095578 9095629 . + . gene_id "XLOC_050502"; transcript_id "TCONS_00373238"; exon_number "2"; gene_type "lincRNA";

2R Scripture exon 9095898 9095958 . + . gene_id "XLOC_050502"; transcript_id "TCONS_00373238"; exon_number "3"; gene_type "lincRNA";

2R Scripture exon 9096150 9096202 . + . gene_id "XLOC_050502"; transcript_id "TCONS_00373238"; exon_number "4"; gene_type "lincRNA";

2R Scripture exon 9096383 9096438 . + . gene_id "XLOC_050502"; transcript_id "TCONS_00373238"; exon_number "5"; gene_type "lincRNA";

2R Scripture exon 9096671 9096905 . + . gene_id "XLOC_050502"; transcript_id "TCONS_00373238"; exon_number "6"; gene_type "lincRNA";

2R Scripture exon 9095221 9095629 . + . gene_id "XLOC_050502"; transcript_id "TCONS_00362502"; exon_number "1"; gene_type "anti-sense_lncRNA";

2R Scripture exon 9095898 9095958 . + . gene_id "XLOC_050502"; transcript_id "TCONS_00362502"; exon_number "2"; gene_type "anti-sense_lncRNA";

2R Scripture exon 9096150 9096202 . + . gene_id "XLOC_050502"; transcript_id "TCONS_00362502"; exon_number "3"; gene_type "anti-sense_lncRNA";

2R Scripture exon 9096383 9096438 . + . gene_id "XLOC_050502"; transcript_id "TCONS_00362502"; exon_number "4"; gene_type "anti-sense_lncRNA";

2R Scripture exon 9096671 9096735 . + . gene_id "XLOC_050502"; transcript_id "TCONS_00362502"; exon_number "5"; gene_type "anti-sense_lncRNA";

2R Scripture exon 9096926 9097044 . + . gene_id "XLOC_050502"; transcript_id "TCONS_00362502"; exon_number "6"; gene_type "anti-sense_lncRNA";

2R Scripture exon 9095822 9095958 . + . gene_id "XLOC_050502"; transcript_id "TCONS_00381778"; exon_number "1"; gene_type "anti-sense_lncRNA";

2R Scripture exon 9096150 9096202 . + . gene_id "XLOC_050502"; transcript_id "TCONS_00381778"; exon_number "2"; gene_type "anti-sense_lncRNA";

2R Scripture exon 9096383 9096438 . + . gene_id "XLOC_050502"; transcript_id "TCONS_00381778"; exon_number "3"; gene_type "anti-sense_lncRNA";

2R Scripture exon 9096671 9096735 . + . gene_id "XLOC_050502"; transcript_id "TCONS_00381778"; exon_number "4"; gene_type "anti-sense_lncRNA";

2R Scripture exon 9096926 9097045 . + . gene_id "XLOC_050502"; transcript_id "TCONS_00381778"; exon_number "5"; gene_type "anti-sense_lncRNA";

2R Cufflinks exon 9226664 9226892 . + . gene_id "XLOC_050563"; transcript_id "TCONS_00275387"; exon_number "1"; gene_type "lincRNA";

2R Scripture exon 9284662 9285195 . + . gene_id "XLOC_050593"; transcript_id "TCONS_00381851"; exon_number "1"; gene_type "anti-sense_lncRNA";

2R Scripture exon 9285424 9285538 . + . gene_id "XLOC_050593"; transcript_id "TCONS_00381851"; exon_number "2"; gene_type "anti-sense_lncRNA";

2R Cufflinks exon 9332334 9332582 . + . gene_id "XLOC_050615"; transcript_id "TCONS_00207928"; exon_number "1"; gene_type "intronic_lncRNA";

2R Cufflinks exon 9620482 9620693 . + . gene_id "XLOC_050761"; transcript_id "TCONS_00232399"; exon_number "1"; gene_type "intronic_lncRNA";

2R Cufflinks exon 9927728 9927943 . + . gene_id "XLOC_050966"; transcript_id "TCONS_00208187"; exon_number "1"; gene_type "intronic_lncRNA";

2R Cufflinks exon 9953450 9953649 . + . gene_id "XLOC_050993"; transcript_id "TCONS_00256114"; exon_number "1"; gene_type "intronic_lncRNA";

2R Scripture exon 10031085 10031244 . + . gene_id "XLOC_051040"; transcript_id "TCONS_00381930"; exon_number "1"; gene_type "anti-sense_lncRNA";

2R Scripture exon 10031306 10031446 . + . gene_id "XLOC_051040"; transcript_id "TCONS_00381930"; exon_number "2"; gene_type "anti-sense_lncRNA";

2R Cufflinks exon 10202186 10202393 . + . gene_id "XLOC_051111"; transcript_id "TCONS_00232605"; exon_number "1"; gene_type "lincRNA";

2R Cufflinks exon 10394703 10394909 . + . gene_id "XLOC_051242"; transcript_id "TCONS_00309773"; exon_number "1"; gene_type "intronic_lncRNA";

2R Scripture exon 10473269 10474111 . + . gene_id "XLOC_051268"; transcript_id "TCONS_00382069"; exon_number "1"; gene_type "anti-sense_lncRNA";

2R Scripture exon 10474172 10474791 . + . gene_id "XLOC_051268"; transcript_id "TCONS_00382069"; exon_number "2"; gene_type "anti-sense_lncRNA";

2R Scripture exon 10478360 10478863 . + . gene_id "XLOC_051270"; transcript_id "TCONS_00362900"; exon_number "1"; gene_type "anti-sense_lncRNA";

2R Scripture exon 10539300 10541773 . + . gene_id "XLOC_051283"; transcript_id "TCONS_00362915"; exon_number "1"; gene_type "anti-sense_lncRNA";

2R Scripture exon 10541841 10543116 . + . gene_id "XLOC_051283"; transcript_id "TCONS_00362915"; exon_number "2"; gene_type "anti-sense_lncRNA";

2R Cufflinks exon 10758642 10758882 . + . gene_id "XLOC_051395"; transcript_id "TCONS_00208498"; exon_number "1"; gene_type "intronic_lncRNA";

2R Cufflinks exon 10767772 10767984 . + . gene_id "XLOC_051403"; transcript_id "TCONS_00208502"; exon_number "1"; gene_type "intronic_lncRNA";

2R Cufflinks exon 10787818 10788026 . + . gene_id "XLOC_051423"; transcript_id "TCONS_00232796"; exon_number "1"; gene_type "intronic_lncRNA";

2R Cufflinks exon 10861377 10861594 . + . gene_id "XLOC_051480"; transcript_id "TCONS_00208533"; exon_number "1"; gene_type "intronic_lncRNA";

2R Cufflinks exon 10933293 10933493 . + . gene_id "XLOC_051552"; transcript_id "TCONS_00328740"; exon_number "1"; gene_type "lincRNA";

2R Cufflinks exon 11045739 11045958 . + . gene_id "XLOC_051653"; transcript_id "TCONS_00232904"; exon_number "1"; gene_type "intronic_lncRNA";

2R Cufflinks exon 12014938 12015091 . + . gene_id "XLOC_052472"; transcript_id "TCONS_00233456"; exon_number "1"; gene_type "intronic_lncRNA";

2R Cufflinks exon 12015189 12015490 . + . gene_id "XLOC_052472"; transcript_id "TCONS_00233456"; exon_number "2"; gene_type "intronic_lncRNA";

2R Cufflinks exon 12102971 12103312 . + . gene_id "XLOC_052548"; transcript_id "TCONS_00233501"; exon_number "1"; gene_type "intronic_lncRNA";

2R Cufflinks exon 12103528 12103649 . + . gene_id "XLOC_052548"; transcript_id "TCONS_00233501"; exon_number "2"; gene_type "intronic_lncRNA";

2R Cufflinks exon 12769077 12769295 . + . gene_id "XLOC_052958"; transcript_id "TCONS_00209510"; exon_number "1"; gene_type "lincRNA";

2R Scripture exon 12875842 12876024 . + . gene_id "XLOC_053065"; transcript_id "TCONS_00363419"; exon_number "1"; gene_type "anti-sense_lncRNA";

2R Scripture exon 12876082 12876207 . + . gene_id "XLOC_053065"; transcript_id "TCONS_00363419"; exon_number "2"; gene_type "anti-sense_lncRNA";

2R Cufflinks exon 13432672 13432875 . + . gene_id "XLOC_053564"; transcript_id "TCONS_00209864"; exon_number "1"; gene_type "intronic_lncRNA";

2R Cufflinks exon 13464307 13464509 . + . gene_id "XLOC_053574"; transcript_id "TCONS_00209867"; exon_number "1"; gene_type "intronic_lncRNA";

2R Cufflinks exon 13475929 13476129 . + . gene_id "XLOC_053580"; transcript_id "TCONS_00310893"; exon_number "1"; gene_type "intronic_lncRNA";

2R Scripture exon 13538198 13538353 . + . gene_id "XLOC_053605"; transcript_id "TCONS_00363523"; exon_number "1"; gene_type "anti-sense_lncRNA";

2R Scripture exon 13549110 13549536 . + . gene_id "XLOC_053605"; transcript_id "TCONS_00363523"; exon_number "2"; gene_type "anti-sense_lncRNA";

2R Cufflinks exon 13611093 13611309 . + . gene_id "XLOC_053676"; transcript_id "TCONS_00276836"; exon_number "1"; gene_type "lincRNA";

2R Cufflinks exon 13638251 13638452 . + . gene_id "XLOC_053722"; transcript_id "TCONS_00234237"; exon_number "1"; gene_type "intronic_lncRNA";

2R Cufflinks exon 13659450 13659654 . + . gene_id "XLOC_053751"; transcript_id "TCONS_00209995"; exon_number "1"; gene_type "intronic_lncRNA";

2R Cufflinks exon 13692272 13692490 . + . gene_id "XLOC_053793"; transcript_id "TCONS_00210033"; exon_number "1"; gene_type "anti-sense_lncRNA";

2R Cufflinks exon 13791417 13791637 . + . gene_id "XLOC_053919"; transcript_id "TCONS_00234431"; exon_number "1"; gene_type "lincRNA";

2R Cufflinks exon 14129542 14129749 . + . gene_id "XLOC_054082"; transcript_id "TCONS_00277065"; exon_number "1"; gene_type "intronic_lncRNA";

2R Cufflinks exon 14143126 14143329 . + . gene_id "XLOC_054095"; transcript_id "TCONS_00234512"; exon_number "1"; gene_type "lincRNA";

2R Cufflinks exon 14265882 14266086 . + . gene_id "XLOC_054141"; transcript_id "TCONS_00311167"; exon_number "1"; gene_type "intronic_lncRNA";

2R Cufflinks exon 14318767 14319014 . + . gene_id "XLOC_054177"; transcript_id "TCONS_00210294"; exon_number "1"; gene_type "intronic_lncRNA";

2R Cufflinks exon 14337288 14337526 . + . gene_id "XLOC_054195"; transcript_id "TCONS_00234583"; exon_number "1"; gene_type "intronic_lncRNA";

2R Cufflinks exon 14338176 14338420 . + . gene_id "XLOC_054197"; transcript_id "TCONS_00210311"; exon_number "1"; gene_type "intronic_lncRNA";

2R Cufflinks exon 14339549 14339914 . + . gene_id "XLOC_054199"; transcript_id "TCONS_00210313"; exon_number "1"; gene_type "intronic_lncRNA";

2R Cufflinks exon 14407966 14408176 . + . gene_id "XLOC_054268"; transcript_id "TCONS_00210369"; exon_number "1"; gene_type "lincRNA";

2R Cufflinks exon 14419041 14419251 . + . gene_id "XLOC_054275"; transcript_id "TCONS_00210379"; exon_number "1"; gene_type "lincRNA";

2R Cufflinks exon 14456832 14457040 . + . gene_id "XLOC_054332"; transcript_id "TCONS_00330060"; exon_number "1"; gene_type "intronic_lncRNA";

2R Scripture exon 14621524 14621750 . + . gene_id "XLOC_054386"; transcript_id "TCONS_00363918"; exon_number "1"; gene_type "lincRNA";

2R Scripture exon 14621960 14622176 . + . gene_id "XLOC_054386"; transcript_id "TCONS_00363918"; exon_number "2"; gene_type "lincRNA";

2R Scripture exon 14622240 14622294 . + . gene_id "XLOC_054386"; transcript_id "TCONS_00363918"; exon_number "3"; gene_type "lincRNA";

2R Scripture exon 14622478 14622531 . + . gene_id "XLOC_054386"; transcript_id "TCONS_00363918"; exon_number "4"; gene_type "lincRNA";

2R Scripture exon 14622713 14622766 . + . gene_id "XLOC_054386"; transcript_id "TCONS_00363918"; exon_number "5"; gene_type "lincRNA";

2R Scripture exon 14622945 14623004 . + . gene_id "XLOC_054386"; transcript_id "TCONS_00363918"; exon_number "6"; gene_type "lincRNA";

2R Scripture exon 14623189 14623245 . + . gene_id "XLOC_054386"; transcript_id "TCONS_00363918"; exon_number "7"; gene_type "lincRNA";

2R Scripture exon 14623417 14623469 . + . gene_id "XLOC_054386"; transcript_id "TCONS_00363918"; exon_number "8"; gene_type "lincRNA";

2R Scripture exon 14623687 14623731 . + . gene_id "XLOC_054386"; transcript_id "TCONS_00363918"; exon_number "9"; gene_type "lincRNA";

2R Scripture exon 14623920 14623978 . + . gene_id "XLOC_054386"; transcript_id "TCONS_00363918"; exon_number "10"; gene_type "lincRNA";

2R Scripture exon 14624157 14624220 . + . gene_id "XLOC_054386"; transcript_id "TCONS_00363918"; exon_number "11"; gene_type "lincRNA";

2R Scripture exon 14624403 14624617 . + . gene_id "XLOC_054386"; transcript_id "TCONS_00363918"; exon_number "12"; gene_type "lincRNA";

2R Scripture exon 14621524 14621750 . + . gene_id "XLOC_054386"; transcript_id "TCONS_00363920"; exon_number "1"; gene_type "lincRNA";

2R Scripture exon 14621812 14622020 . + . gene_id "XLOC_054386"; transcript_id "TCONS_00363920"; exon_number "2"; gene_type "lincRNA";

2R Scripture exon 14622240 14622294 . + . gene_id "XLOC_054386"; transcript_id "TCONS_00363920"; exon_number "3"; gene_type "lincRNA";

2R Scripture exon 14622478 14622531 . + . gene_id "XLOC_054386"; transcript_id "TCONS_00363920"; exon_number "4"; gene_type "lincRNA";

2R Scripture exon 14622713 14622766 . + . gene_id "XLOC_054386"; transcript_id "TCONS_00363920"; exon_number "5"; gene_type "lincRNA";

2R Scripture exon 14622945 14623004 . + . gene_id "XLOC_054386"; transcript_id "TCONS_00363920"; exon_number "6"; gene_type "lincRNA";

2R Scripture exon 14623189 14623245 . + . gene_id "XLOC_054386"; transcript_id "TCONS_00363920"; exon_number "7"; gene_type "lincRNA";

2R Scripture exon 14623417 14623469 . + . gene_id "XLOC_054386"; transcript_id "TCONS_00363920"; exon_number "8"; gene_type "lincRNA";

2R Scripture exon 14623687 14623731 . + . gene_id "XLOC_054386"; transcript_id "TCONS_00363920"; exon_number "9"; gene_type "lincRNA";

2R Scripture exon 14623920 14623978 . + . gene_id "XLOC_054386"; transcript_id "TCONS_00363920"; exon_number "10"; gene_type "lincRNA";

2R Scripture exon 14624157 14624220 . + . gene_id "XLOC_054386"; transcript_id "TCONS_00363920"; exon_number "11"; gene_type "lincRNA";

2R Scripture exon 14624403 14624617 . + . gene_id "XLOC_054386"; transcript_id "TCONS_00363920"; exon_number "12"; gene_type "lincRNA";

2R Scripture exon 14621524 14621750 . + . gene_id "XLOC_054386"; transcript_id "TCONS_00363915"; exon_number "1"; gene_type "lincRNA";

2R Scripture exon 14621960 14622020 . + . gene_id "XLOC_054386"; transcript_id "TCONS_00363915"; exon_number "2"; gene_type "lincRNA";

2R Scripture exon 14622240 14622294 . + . gene_id "XLOC_054386"; transcript_id "TCONS_00363915"; exon_number "3"; gene_type "lincRNA";

2R Scripture exon 14622713 14622766 . + . gene_id "XLOC_054386"; transcript_id "TCONS_00363915"; exon_number "4"; gene_type "lincRNA";

2R Scripture exon 14622945 14623004 . + . gene_id "XLOC_054386"; transcript_id "TCONS_00363915"; exon_number "5"; gene_type "lincRNA";

2R Scripture exon 14623189 14623245 . + . gene_id "XLOC_054386"; transcript_id "TCONS_00363915"; exon_number "6"; gene_type "lincRNA";

2R Scripture exon 14623417 14623469 . + . gene_id "XLOC_054386"; transcript_id "TCONS_00363915"; exon_number "7"; gene_type "lincRNA";

2R Scripture exon 14623687 14623731 . + . gene_id "XLOC_054386"; transcript_id "TCONS_00363915"; exon_number "8"; gene_type "lincRNA";

2R Scripture exon 14623920 14623978 . + . gene_id "XLOC_054386"; transcript_id "TCONS_00363915"; exon_number "9"; gene_type "lincRNA";

2R Scripture exon 14624157 14624220 . + . gene_id "XLOC_054386"; transcript_id "TCONS_00363915"; exon_number "10"; gene_type "lincRNA";

2R Scripture exon 14624403 14624617 . + . gene_id "XLOC_054386"; transcript_id "TCONS_00363915"; exon_number "11"; gene_type "lincRNA";

2R Scripture exon 14621524 14621750 . + . gene_id "XLOC_054386"; transcript_id "TCONS_00277228"; exon_number "1"; gene_type "lincRNA";

2R Scripture exon 14621960 14622020 . + . gene_id "XLOC_054386"; transcript_id "TCONS_00277228"; exon_number "2"; gene_type "lincRNA";

2R Scripture exon 14622240 14622294 . + . gene_id "XLOC_054386"; transcript_id "TCONS_00277228"; exon_number "3"; gene_type "lincRNA";

2R Scripture exon 14622478 14622531 . + . gene_id "XLOC_054386"; transcript_id "TCONS_00277228"; exon_number "4"; gene_type "lincRNA";

2R Scripture exon 14622713 14622766 . + . gene_id "XLOC_054386"; transcript_id "TCONS_00277228"; exon_number "5"; gene_type "lincRNA";

2R Scripture exon 14622945 14623004 . + . gene_id "XLOC_054386"; transcript_id "TCONS_00277228"; exon_number "6"; gene_type "lincRNA";

2R Scripture exon 14623189 14623245 . + . gene_id "XLOC_054386"; transcript_id "TCONS_00277228"; exon_number "7"; gene_type "lincRNA";

2R Scripture exon 14623417 14623469 . + . gene_id "XLOC_054386"; transcript_id "TCONS_00277228"; exon_number "8"; gene_type "lincRNA";

2R Scripture exon 14623687 14623731 . + . gene_id "XLOC_054386"; transcript_id "TCONS_00277228"; exon_number "9"; gene_type "lincRNA";

2R Scripture exon 14623920 14623978 . + . gene_id "XLOC_054386"; transcript_id "TCONS_00277228"; exon_number "10"; gene_type "lincRNA";

2R Scripture exon 14624157 14624220 . + . gene_id "XLOC_054386"; transcript_id "TCONS_00277228"; exon_number "11"; gene_type "lincRNA";

2R Scripture exon 14624403 14624617 . + . gene_id "XLOC_054386"; transcript_id "TCONS_00277228"; exon_number "12"; gene_type "lincRNA";

2R Cufflinks exon 14621578 14621750 . + . gene_id "XLOC_054386"; transcript_id "TCONS_00234717"; exon_number "1"; gene_type "lincRNA";

2R Cufflinks exon 14621960 14622020 . + . gene_id "XLOC_054386"; transcript_id "TCONS_00234717"; exon_number "2"; gene_type "lincRNA";

2R Cufflinks exon 14622240 14622294 . + . gene_id "XLOC_054386"; transcript_id "TCONS_00234717"; exon_number "3"; gene_type "lincRNA";

2R Cufflinks exon 14622478 14622531 . + . gene_id "XLOC_054386"; transcript_id "TCONS_00234717"; exon_number "4"; gene_type "lincRNA";

2R Cufflinks exon 14622713 14622766 . + . gene_id "XLOC_054386"; transcript_id "TCONS_00234717"; exon_number "5"; gene_type "lincRNA";

2R Cufflinks exon 14622945 14623004 . + . gene_id "XLOC_054386"; transcript_id "TCONS_00234717"; exon_number "6"; gene_type "lincRNA";

2R Cufflinks exon 14623189 14623245 . + . gene_id "XLOC_054386"; transcript_id "TCONS_00234717"; exon_number "7"; gene_type "lincRNA";

2R Cufflinks exon 14623417 14623657 . + . gene_id "XLOC_054386"; transcript_id "TCONS_00234717"; exon_number "8"; gene_type "lincRNA";

2R Cufflinks exon 14659476 14659682 . + . gene_id "XLOC_054440"; transcript_id "TCONS_00311320"; exon_number "1"; gene_type "lincRNA";

2R Cufflinks exon 14779817 14780022 . + . gene_id "XLOC_054561"; transcript_id "TCONS_00258025"; exon_number "1"; gene_type "intronic_lncRNA";

2R Cufflinks exon 14912441 14912645 . + . gene_id "XLOC_054668"; transcript_id "TCONS_00210606"; exon_number "1"; gene_type "intronic_lncRNA";

2R Cufflinks exon 15232819 15233023 . + . gene_id "XLOC_054932"; transcript_id "TCONS_00277442"; exon_number "1"; gene_type "intronic_lncRNA";

2R Cufflinks exon 15235393 15235600 . + . gene_id "XLOC_054934"; transcript_id "TCONS_00210787"; exon_number "1"; gene_type "intronic_lncRNA";

2R Cufflinks exon 15372370 15372570 . + . gene_id "XLOC_055018"; transcript_id "TCONS_00295122"; exon_number "1"; gene_type "lincRNA";

2R Cufflinks exon 15419562 15420310 . + . gene_id "XLOC_055037"; transcript_id "TCONS_00295157"; exon_number "1"; gene_type "intronic_lncRNA";

2R Cufflinks exon 15701253 15701462 . + . gene_id "XLOC_055307"; transcript_id "TCONS_00258442"; exon_number "1"; gene_type "anti-sense_lncRNA";

2R Cufflinks exon 15805931 15806143 . + . gene_id "XLOC_055396"; transcript_id "TCONS_00330567"; exon_number "1"; gene_type "anti-sense_lncRNA";

2R Cufflinks exon 15889507 15889741 . + . gene_id "XLOC_055459"; transcript_id "TCONS_00235410"; exon_number "1"; gene_type "intronic_lncRNA";

2R Cufflinks exon 16067882 16068085 . + . gene_id "XLOC_055542"; transcript_id "TCONS_00211236"; exon_number "1"; gene_type "lincRNA";

2R Cufflinks exon 16352280 16352375 . + . gene_id "XLOC_055665"; transcript_id "TCONS_00258655"; exon_number "1"; gene_type "lincRNA";

2R Cufflinks exon 16352464 16352578 . + . gene_id "XLOC_055665"; transcript_id "TCONS_00258655"; exon_number "2"; gene_type "lincRNA";

2R Cufflinks exon 16356390 16356603 . + . gene_id "XLOC_055673"; transcript_id "TCONS_00211340"; exon_number "1"; gene_type "lincRNA";

2R Cufflinks exon 16358633 16358843 . + . gene_id "XLOC_055675"; transcript_id "TCONS_00235543"; exon_number "1"; gene_type "lincRNA";

2R Cufflinks exon 16509682 16509908 . + . gene_id "XLOC_055866"; transcript_id "TCONS_00211467"; exon_number "1"; gene_type "intronic_lncRNA";

2R Cufflinks exon 16638842 16639043 . + . gene_id "XLOC_055924"; transcript_id "TCONS_00258821"; exon_number "1"; gene_type "intronic_lncRNA";

2R Cufflinks exon 16707047 16707250 . + . gene_id "XLOC_055956"; transcript_id "TCONS_00211553"; exon_number "1"; gene_type "intronic_lncRNA";

2R Cufflinks exon 16805676 16805756 . + . gene_id "XLOC_056042"; transcript_id "TCONS_00295630"; exon_number "1"; gene_type "lincRNA";

2R Cufflinks exon 16807099 16807240 . + . gene_id "XLOC_056042"; transcript_id "TCONS_00295630"; exon_number "2"; gene_type "lincRNA";

2R Scripture exon 16829100 16830805 . + . gene_id "XLOC_056059"; transcript_id "TCONS_00364335"; exon_number "1"; gene_type "lincRNA";

2R Scripture exon 16831170 16831557 . + . gene_id "XLOC_056059"; transcript_id "TCONS_00364335"; exon_number "2"; gene_type "lincRNA";

2R Cufflinks exon 16911457 16911661 . + . gene_id "XLOC_056107"; transcript_id "TCONS_00235899"; exon_number "1"; gene_type "lincRNA";

2R Cufflinks exon 17140581 17140869 . + . gene_id "XLOC_056155"; transcript_id "TCONS_00235964"; exon_number "1"; gene_type "anti-sense_lncRNA";

2R Cufflinks exon 17140927 17141488 . + . gene_id "XLOC_056155"; transcript_id "TCONS_00235964"; exon_number "2"; gene_type "anti-sense_lncRNA";

2R Scripture exon 17145622 17145860 . + . gene_id "XLOC_056155"; transcript_id "TCONS_00364439"; exon_number "1"; gene_type "lincRNA";

2R Scripture exon 17145915 17146645 . + . gene_id "XLOC_056155"; transcript_id "TCONS_00364439"; exon_number "2"; gene_type "lincRNA";

2R Cufflinks exon 17338353 17338586 . + . gene_id "XLOC_056494"; transcript_id "TCONS_00211931"; exon_number "1"; gene_type "intronic_lncRNA";

2R Scripture exon 17700420 17700433 . + . gene_id "XLOC_056633"; transcript_id "TCONS_00389227"; exon_number "1"; gene_type "lincRNA";

2R Scripture exon 17700635 17700703 . + . gene_id "XLOC_056633"; transcript_id "TCONS_00389227"; exon_number "2"; gene_type "lincRNA";

2R Scripture exon 17700901 17700942 . + . gene_id "XLOC_056633"; transcript_id "TCONS_00389227"; exon_number "3"; gene_type "lincRNA";

2R Scripture exon 17701134 17701321 . + . gene_id "XLOC_056633"; transcript_id "TCONS_00389227"; exon_number "4"; gene_type "lincRNA";

2R Cufflinks exon 17700439 17700703 . + . gene_id "XLOC_056633"; transcript_id "TCONS_00212103"; exon_number "1"; gene_type "lincRNA";

2R Cufflinks exon 17700901 17700942 . + . gene_id "XLOC_056633"; transcript_id "TCONS_00212103"; exon_number "2"; gene_type "lincRNA";

2R Cufflinks exon 17701134 17701334 . + . gene_id "XLOC_056633"; transcript_id "TCONS_00212103"; exon_number "3"; gene_type "lincRNA";

2R Cufflinks exon 17700446 17700703 . + . gene_id "XLOC_056633"; transcript_id "TCONS_00236246"; exon_number "1"; gene_type "lincRNA";

2R Cufflinks exon 17700901 17700942 . + . gene_id "XLOC_056633"; transcript_id "TCONS_00236246"; exon_number "2"; gene_type "lincRNA";

2R Cufflinks exon 17701134 17701192 . + . gene_id "XLOC_056633"; transcript_id "TCONS_00236246"; exon_number "3"; gene_type "lincRNA";

2R Cufflinks exon 17701369 17701543 . + . gene_id "XLOC_056633"; transcript_id "TCONS_00236246"; exon_number "4"; gene_type "lincRNA";

2R Scripture exon 17698939 17699168 . + . gene_id "XLOC_056713"; transcript_id "TCONS_00236245"; exon_number "1"; gene_type "lincRNA";

2R Scripture exon 17699351 17699449 . + . gene_id "XLOC_056713"; transcript_id "TCONS_00236245"; exon_number "2"; gene_type "lincRNA";

2R Scripture exon 17699858 17699897 . + . gene_id "XLOC_056713"; transcript_id "TCONS_00236245"; exon_number "3"; gene_type "lincRNA";

2R Scripture exon 17700081 17700148 . + . gene_id "XLOC_056713"; transcript_id "TCONS_00236245"; exon_number "4"; gene_type "lincRNA";

2R Scripture exon 17698939 17699168 . + . gene_id "XLOC_056713"; transcript_id "TCONS_00312582"; exon_number "1"; gene_type "lincRNA";

2R Scripture exon 17699351 17699449 . + . gene_id "XLOC_056713"; transcript_id "TCONS_00312582"; exon_number "2"; gene_type "lincRNA";

2R Scripture exon 17699858 17699897 . + . gene_id "XLOC_056713"; transcript_id "TCONS_00312582"; exon_number "3"; gene_type "lincRNA";

2R Scripture exon 17700085 17700148 . + . gene_id "XLOC_056713"; transcript_id "TCONS_00312582"; exon_number "4"; gene_type "lincRNA";

2R Scripture exon 17698939 17699286 . + . gene_id "XLOC_056713"; transcript_id "TCONS_00374735"; exon_number "1"; gene_type "lincRNA";

2R Scripture exon 17699351 17699449 . + . gene_id "XLOC_056713"; transcript_id "TCONS_00374735"; exon_number "2"; gene_type "lincRNA";

2R Scripture exon 17699858 17699897 . + . gene_id "XLOC_056713"; transcript_id "TCONS_00374735"; exon_number "3"; gene_type "lincRNA";

2R Scripture exon 17700085 17700148 . + . gene_id "XLOC_056713"; transcript_id "TCONS_00374735"; exon_number "4"; gene_type "lincRNA";

2R Scripture exon 17698939 17699286 . + . gene_id "XLOC_056713"; transcript_id "TCONS_00374736"; exon_number "1"; gene_type "lincRNA";

2R Scripture exon 17699351 17699449 . + . gene_id "XLOC_056713"; transcript_id "TCONS_00374736"; exon_number "2"; gene_type "lincRNA";

2R Scripture exon 17699858 17699897 . + . gene_id "XLOC_056713"; transcript_id "TCONS_00374736"; exon_number "3"; gene_type "lincRNA";

2R Scripture exon 17700081 17700148 . + . gene_id "XLOC_056713"; transcript_id "TCONS_00374736"; exon_number "4"; gene_type "lincRNA";

2R Scripture exon 17698939 17699286 . + . gene_id "XLOC_056713"; transcript_id "TCONS_00374733"; exon_number "1"; gene_type "lincRNA";

2R Scripture exon 17699351 17699444 . + . gene_id "XLOC_056713"; transcript_id "TCONS_00374733"; exon_number "2"; gene_type "lincRNA";

2R Scripture exon 17699858 17699897 . + . gene_id "XLOC_056713"; transcript_id "TCONS_00374733"; exon_number "3"; gene_type "lincRNA";

2R Scripture exon 17700081 17700148 . + . gene_id "XLOC_056713"; transcript_id "TCONS_00374733"; exon_number "4"; gene_type "lincRNA";

2R Scripture exon 17698939 17699168 . + . gene_id "XLOC_056713"; transcript_id "TCONS_00236244"; exon_number "1"; gene_type "lincRNA";

2R Scripture exon 17699351 17699444 . + . gene_id "XLOC_056713"; transcript_id "TCONS_00236244"; exon_number "2"; gene_type "lincRNA";

2R Scripture exon 17699858 17699897 . + . gene_id "XLOC_056713"; transcript_id "TCONS_00236244"; exon_number "3"; gene_type "lincRNA";

2R Scripture exon 17700081 17700148 . + . gene_id "XLOC_056713"; transcript_id "TCONS_00236244"; exon_number "4"; gene_type "lincRNA";

2R Scripture exon 17698953 17699168 . + . gene_id "XLOC_056713"; transcript_id "TCONS_00389221"; exon_number "1"; gene_type "lincRNA";

2R Scripture exon 17699351 17699897 . + . gene_id "XLOC_056713"; transcript_id "TCONS_00389221"; exon_number "2"; gene_type "lincRNA";

2R Scripture exon 17700081 17700143 . + . gene_id "XLOC_056713"; transcript_id "TCONS_00389221"; exon_number "3"; gene_type "lincRNA";

2R Scripture exon 17698953 17699168 . + . gene_id "XLOC_056713"; transcript_id "TCONS_00389220"; exon_number "1"; gene_type "lincRNA";

2R Scripture exon 17699351 17699897 . + . gene_id "XLOC_056713"; transcript_id "TCONS_00389220"; exon_number "2"; gene_type "lincRNA";

2R Scripture exon 17700085 17700143 . + . gene_id "XLOC_056713"; transcript_id "TCONS_00389220"; exon_number "3"; gene_type "lincRNA";

2R Scripture exon 17698953 17699168 . + . gene_id "XLOC_056713"; transcript_id "TCONS_00212102"; exon_number "1"; gene_type "lincRNA";

2R Scripture exon 17699351 17699449 . + . gene_id "XLOC_056713"; transcript_id "TCONS_00212102"; exon_number "2"; gene_type "lincRNA";

2R Scripture exon 17699858 17700143 . + . gene_id "XLOC_056713"; transcript_id "TCONS_00212102"; exon_number "3"; gene_type "lincRNA";

2R Scripture exon 17701636 17701649 . + . gene_id "XLOC_056714"; transcript_id "TCONS_00348878"; exon_number "1"; gene_type "anti-sense_lncRNA";

2R Scripture exon 17701834 17701894 . + . gene_id "XLOC_056714"; transcript_id "TCONS_00348878"; exon_number "2"; gene_type "anti-sense_lncRNA";

2R Scripture exon 17702075 17702125 . + . gene_id "XLOC_056714"; transcript_id "TCONS_00348878"; exon_number "3"; gene_type "anti-sense_lncRNA";

2R Scripture exon 17702315 17702369 . + . gene_id "XLOC_056714"; transcript_id "TCONS_00348878"; exon_number "4"; gene_type "anti-sense_lncRNA";

2R Scripture exon 17702547 17702598 . + . gene_id "XLOC_056714"; transcript_id "TCONS_00348878"; exon_number "5"; gene_type "anti-sense_lncRNA";

2R Scripture exon 17702779 17702841 . + . gene_id "XLOC_056714"; transcript_id "TCONS_00348878"; exon_number "6"; gene_type "anti-sense_lncRNA";

2R Scripture exon 17703021 17703064 . + . gene_id "XLOC_056714"; transcript_id "TCONS_00348878"; exon_number "7"; gene_type "anti-sense_lncRNA";

2R Scripture exon 17703244 17705190 . + . gene_id "XLOC_056714"; transcript_id "TCONS_00348878"; exon_number "8"; gene_type "anti-sense_lncRNA";

2R Cufflinks exon 17709237 17709450 . + . gene_id "XLOC_056716"; transcript_id "TCONS_00236248"; exon_number "1"; gene_type "intronic_lncRNA";

2R Scripture exon 17868956 17870490 . + . gene_id "XLOC_056809"; transcript_id "TCONS_00364548"; exon_number "1"; gene_type "anti-sense_lncRNA";

2R Scripture exon 17871794 17872027 . + . gene_id "XLOC_056809"; transcript_id "TCONS_00364548"; exon_number "2"; gene_type "anti-sense_lncRNA";

2R Cufflinks exon 17936888 17937088 . + . gene_id "XLOC_056884"; transcript_id "TCONS_00236376"; exon_number "1"; gene_type "lincRNA";

2R Cufflinks exon 17944021 17944221 . + . gene_id "XLOC_056895"; transcript_id "TCONS_00212265"; exon_number "1"; gene_type "lincRNA";

2R Cufflinks exon 18499651 18499855 . + . gene_id "XLOC_057410"; transcript_id "TCONS_00278699"; exon_number "1"; gene_type "lincRNA";

2R Cufflinks exon 18526554 18526904 . + . gene_id "XLOC_057432"; transcript_id "TCONS_00212576"; exon_number "1"; gene_type "lincRNA";

2R Cufflinks exon 18527044 18527089 . + . gene_id "XLOC_057432"; transcript_id "TCONS_00212576"; exon_number "2"; gene_type "lincRNA";

2R Cufflinks exon 18889717 18889918 . + . gene_id "XLOC_057649"; transcript_id "TCONS_00212738"; exon_number "1"; gene_type "intronic_lncRNA";

2R Cufflinks exon 18917314 18917556 . + . gene_id "XLOC_057682"; transcript_id "TCONS_00212768"; exon_number "1"; gene_type "intronic_lncRNA";

2R Cufflinks exon 19042416 19042658 . + . gene_id "XLOC_057806"; transcript_id "TCONS_00313148"; exon_number "1"; gene_type "lincRNA";

2R Cufflinks exon 19094174 19094386 . + . gene_id "XLOC_057847"; transcript_id "TCONS_00212932"; exon_number "1"; gene_type "anti-sense_lncRNA";

2R Cufflinks exon 19087615 19087817 . + . gene_id "XLOC_057880"; transcript_id "TCONS_00259807"; exon_number "1"; gene_type "intronic_lncRNA";

2R Cufflinks exon 19550743 19550980 . + . gene_id "XLOC_058108"; transcript_id "TCONS_00259962"; exon_number "1"; gene_type "intronic_lncRNA";

2R Cufflinks exon 19810354 19810555 . + . gene_id "XLOC_058346"; transcript_id "TCONS_00213248"; exon_number "1"; gene_type "lincRNA";

2R Cufflinks exon 19821398 19821616 . + . gene_id "XLOC_058358"; transcript_id "TCONS_00213262"; exon_number "1"; gene_type "lincRNA";

2R Cufflinks exon 19922371 19922570 . + . gene_id "XLOC_058488"; transcript_id "TCONS_00213354"; exon_number "1"; gene_type "lincRNA";

2R Cufflinks exon 20184724 20184928 . + . gene_id "XLOC_058859"; transcript_id "TCONS_00213589"; exon_number "1"; gene_type "lincRNA";

2R Cufflinks exon 20202140 20202346 . + . gene_id "XLOC_058885"; transcript_id "TCONS_00260370"; exon_number "1"; gene_type "lincRNA";

2R Cufflinks exon 20302483 20302575 . + . gene_id "XLOC_058981"; transcript_id "TCONS_00237819"; exon_number "1"; gene_type "lincRNA";

2R Cufflinks exon 20302632 20303583 . + . gene_id "XLOC_058981"; transcript_id "TCONS_00237819"; exon_number "2"; gene_type "lincRNA";

2R Cufflinks exon 20389870 20390076 . + . gene_id "XLOC_059066"; transcript_id "TCONS_00213712"; exon_number "1"; gene_type "lincRNA";

2R Cufflinks exon 20438629 20438842 . + . gene_id "XLOC_059143"; transcript_id "TCONS_00237951"; exon_number "1"; gene_type "intronic_lncRNA";

2R Cufflinks exon 20497776 20497978 . + . gene_id "XLOC_059217"; transcript_id "TCONS_00213833"; exon_number "1"; gene_type "lincRNA";

2R Cufflinks exon 20518471 20518675 . + . gene_id "XLOC_059225"; transcript_id "TCONS_00213855"; exon_number "1"; gene_type "lincRNA";

2R Cufflinks exon 20531694 20532061 . + . gene_id "XLOC_059259"; transcript_id "TCONS_00260588"; exon_number "1"; gene_type "lincRNA";

2R Cufflinks exon 20532525 20533004 . + . gene_id "XLOC_059259"; transcript_id "TCONS_00260588"; exon_number "2"; gene_type "lincRNA";

2R Cufflinks exon 20656906 20657120 . + . gene_id "XLOC_059335"; transcript_id "TCONS_00213917"; exon_number "1"; gene_type "intronic_lncRNA";

2R Cufflinks exon 20680686 20680904 . + . gene_id "XLOC_059354"; transcript_id "TCONS_00297148"; exon_number "1"; gene_type "lincRNA";

2R Cufflinks exon 20892308 20892518 . + . gene_id "XLOC_059587"; transcript_id "TCONS_00238283"; exon_number "1"; gene_type "intronic_lncRNA";

2R Cufflinks exon 21663164 21663360 . + . gene_id "XLOC_060157"; transcript_id "TCONS_00214497"; exon_number "1"; gene_type "lincRNA";

2R Cufflinks exon 21663425 21663921 . + . gene_id "XLOC_060157"; transcript_id "TCONS_00214497"; exon_number "2"; gene_type "lincRNA";

2R Cufflinks exon 21675570 21675780 . + . gene_id "XLOC_060162"; transcript_id "TCONS_00238670"; exon_number "1"; gene_type "intronic_lncRNA";

2R Cufflinks exon 21750190 21750391 . + . gene_id "XLOC_060211"; transcript_id "TCONS_00280192"; exon_number "1"; gene_type "lincRNA";

2R Cufflinks exon 21856708 21856913 . + . gene_id "XLOC_060348"; transcript_id "TCONS_00214608"; exon_number "1"; gene_type "lincRNA";

2R Cufflinks exon 22522456 22522657 . + . gene_id "XLOC_060935"; transcript_id "TCONS_00215007"; exon_number "1"; gene_type "intronic_lncRNA";

2R Cufflinks exon 22557413 22557622 . + . gene_id "XLOC_060962"; transcript_id "TCONS_00261530"; exon_number "1"; gene_type "intronic_lncRNA";

2R Cufflinks exon 22623597 22623807 . + . gene_id "XLOC_061001"; transcript_id "TCONS_00239259"; exon_number "1"; gene_type "intronic_lncRNA";

2R Scripture exon 22643343 22643416 . + . gene_id "XLOC_061011"; transcript_id "TCONS_00383572"; exon_number "1"; gene_type "anti-sense_lncRNA";

2R Scripture exon 22643479 22643653 . + . gene_id "XLOC_061011"; transcript_id "TCONS_00383572"; exon_number "2"; gene_type "anti-sense_lncRNA";

2R Cufflinks exon 22904836 22905058 . + . gene_id "XLOC_061197"; transcript_id "TCONS_00239411"; exon_number "1"; gene_type "intronic_lncRNA";

2R Scripture exon 22982327 22982879 . + . gene_id "XLOC_061262"; transcript_id "TCONS_00261713"; exon_number "1"; gene_type "anti-sense_lncRNA";

2R Scripture exon 22982988 22983232 . + . gene_id "XLOC_061262"; transcript_id "TCONS_00261713"; exon_number "2"; gene_type "anti-sense_lncRNA";

2R Scripture exon 22983329 22984255 . + . gene_id "XLOC_061262"; transcript_id "TCONS_00261713"; exon_number "3"; gene_type "anti-sense_lncRNA";

2R Scripture exon 22982348 22982879 . + . gene_id "XLOC_061262"; transcript_id "TCONS_00390042"; exon_number "1"; gene_type "anti-sense_lncRNA";

2R Scripture exon 22982988 22983227 . + . gene_id "XLOC_061262"; transcript_id "TCONS_00390042"; exon_number "2"; gene_type "anti-sense_lncRNA";

2R Scripture exon 22983324 22984287 . + . gene_id "XLOC_061262"; transcript_id "TCONS_00390042"; exon_number "3"; gene_type "anti-sense_lncRNA";

2R Cufflinks exon 23016442 23016668 . + . gene_id "XLOC_061314"; transcript_id "TCONS_00280774"; exon_number "1"; gene_type "anti-sense_lncRNA";

2R Cufflinks exon 23112494 23112721 . + . gene_id "XLOC_061439"; transcript_id "TCONS_00280833"; exon_number "1"; gene_type "lincRNA";

2R Cufflinks exon 23291840 23292043 . + . gene_id "XLOC_061699"; transcript_id "TCONS_00239717"; exon_number "1"; gene_type "lincRNA";

2R Cufflinks exon 23629786 23629988 . + . gene_id "XLOC_062003"; transcript_id "TCONS_00281105"; exon_number "1"; gene_type "lincRNA";

2R Scripture exon 23905605 23905760 . + . gene_id "XLOC_062152"; transcript_id "TCONS_00365855"; exon_number "1"; gene_type "anti-sense_lncRNA";

2R Scripture exon 23905819 23905996 . + . gene_id "XLOC_062152"; transcript_id "TCONS_00365855"; exon_number "2"; gene_type "anti-sense_lncRNA";

2R Cufflinks exon 24322838 24323043 . + . gene_id "XLOC_062373"; transcript_id "TCONS_00315557"; exon_number "1"; gene_type "lincRNA";

2R Cufflinks exon 24643771 24643972 . + . gene_id "XLOC_062581"; transcript_id "TCONS_00216230"; exon_number "1"; gene_type "lincRNA";

2R Cufflinks exon 24688776 24689000 . + . gene_id "XLOC_062609"; transcript_id "TCONS_00262486"; exon_number "1"; gene_type "lincRNA";

2R Cufflinks exon 24691149 24691357 . + . gene_id "XLOC_062613"; transcript_id "TCONS_00240429"; exon_number "1"; gene_type "lincRNA";

2R Cufflinks exon 25244356 25244564 . + . gene_id "XLOC_063048"; transcript_id "TCONS_00298734"; exon_number "1"; gene_type "intronic_lncRNA";

2R Cufflinks exon 25248152 25248360 . + . gene_id "XLOC_063052"; transcript_id "TCONS_00240747"; exon_number "1"; gene_type "intronic_lncRNA";

2R Cufflinks exon 25268988 25269131 . + . gene_id "XLOC_063063"; transcript_id "TCONS_00262727"; exon_number "1"; gene_type "lincRNA";

2R Cufflinks exon 25269226 25270064 . + . gene_id "XLOC_063063"; transcript_id "TCONS_00262727"; exon_number "2"; gene_type "lincRNA";

2R Cufflinks exon 693092 693298 . - . gene_id "XLOC_063439"; transcript_id "TCONS_00334401"; exon_number "1"; gene_type "intronic_lncRNA";

2R Cufflinks exon 1263440 1263642 . - . gene_id "XLOC_063780"; transcript_id "TCONS_00263004"; exon_number "1"; gene_type "lincRNA";

2R Cufflinks exon 1368942 1369083 . - . gene_id "XLOC_063814"; transcript_id "TCONS_00263069"; exon_number "1"; gene_type "intronic_lncRNA";

2R Cufflinks exon 1369151 1369205 . - . gene_id "XLOC_063814"; transcript_id "TCONS_00263069"; exon_number "2"; gene_type "intronic_lncRNA";

2R Cufflinks exon 1369385 1369569 . - . gene_id "XLOC_063814"; transcript_id "TCONS_00263069"; exon_number "3"; gene_type "intronic_lncRNA";

2R Cufflinks exon 3342061 3342270 . - . gene_id "XLOC_064852"; transcript_id "TCONS_00241679"; exon_number "1"; gene_type "lincRNA";

2R Cufflinks exon 3344654 3344854 . - . gene_id "XLOC_064854"; transcript_id "TCONS_00299324"; exon_number "1"; gene_type "lincRNA";

2R Cufflinks exon 3352841 3353060 . - . gene_id "XLOC_064863"; transcript_id "TCONS_00241695"; exon_number "1"; gene_type "lincRNA";

2R Cufflinks exon 4004758 4004966 . - . gene_id "XLOC_065108"; transcript_id "TCONS_00299484"; exon_number "1"; gene_type "lincRNA";

2R Cufflinks exon 4675932 4675964 . - . gene_id "XLOC_065402"; transcript_id "TCONS_00217785"; exon_number "1"; gene_type "lincRNA";

2R Cufflinks exon 4676018 4676187 . - . gene_id "XLOC_065402"; transcript_id "TCONS_00217785"; exon_number "2"; gene_type "lincRNA";

2R Cufflinks exon 4677124 4677840 . - . gene_id "XLOC_065402"; transcript_id "TCONS_00242137"; exon_number "1"; gene_type "lincRNA";

2R Scripture exon 4782738 4783049 . - . gene_id "XLOC_065442"; transcript_id "TCONS_00384195"; exon_number "1"; gene_type "lincRNA";

2R Scripture exon 4783108 4783286 . - . gene_id "XLOC_065442"; transcript_id "TCONS_00384195"; exon_number "2"; gene_type "lincRNA";

2R Cufflinks exon 4860007 4860247 . - . gene_id "XLOC_065483"; transcript_id "TCONS_00317146"; exon_number "1"; gene_type "lincRNA";

2R Scripture exon 5257850 5258483 . - . gene_id "XLOC_065797"; transcript_id "TCONS_00366730"; exon_number "1"; gene_type "anti-sense_lncRNA";

2R Scripture exon 5259769 5259819 . - . gene_id "XLOC_065797"; transcript_id "TCONS_00366730"; exon_number "2"; gene_type "anti-sense_lncRNA";

2R Cufflinks exon 5466100 5466364 . - . gene_id "XLOC_065917"; transcript_id "TCONS_00218199"; exon_number "1"; gene_type "lincRNA";

2R Cufflinks exon 5466423 5466477 . - . gene_id "XLOC_065917"; transcript_id "TCONS_00218199"; exon_number "2"; gene_type "lincRNA";

2R Cufflinks exon 5967463 5967559 . - . gene_id "XLOC_066349"; transcript_id "TCONS_00218438"; exon_number "1"; gene_type "lincRNA";

2R Cufflinks exon 5967679 5967984 . - . gene_id "XLOC_066349"; transcript_id "TCONS_00218438"; exon_number "2"; gene_type "lincRNA";

2R Cufflinks exon 6164586 6164834 . - . gene_id "XLOC_066405"; transcript_id "TCONS_00264388"; exon_number "1"; gene_type "intronic_lncRNA";

2R Cufflinks exon 6200322 6200523 . - . gene_id "XLOC_066439"; transcript_id "TCONS_00218523"; exon_number "1"; gene_type "lincRNA";

2R Cufflinks exon 6500659 6501595 . - . gene_id "XLOC_066509"; transcript_id "TCONS_00242858"; exon_number "1"; gene_type "lincRNA";

2R Cufflinks exon 6501673 6501707 . - . gene_id "XLOC_066509"; transcript_id "TCONS_00242858"; exon_number "2"; gene_type "lincRNA";

2R Cufflinks exon 6562934 6563136 . - . gene_id "XLOC_066572"; transcript_id "TCONS_00317725"; exon_number "1"; gene_type "intronic_lncRNA";

2R Cufflinks exon 6612103 6612179 . - . gene_id "XLOC_066627"; transcript_id "TCONS_00218638"; exon_number "1"; gene_type "intronic_lncRNA";

2R Cufflinks exon 6612253 6612457 . - . gene_id "XLOC_066627"; transcript_id "TCONS_00218638"; exon_number "2"; gene_type "intronic_lncRNA";

2R Cufflinks exon 6714518 6714857 . - . gene_id "XLOC_066648"; transcript_id "TCONS_00242937"; exon_number "1"; gene_type "intronic_lncRNA";

2R Cufflinks exon 6821909 6822115 . - . gene_id "XLOC_066696"; transcript_id "TCONS_00218720"; exon_number "1"; gene_type "lincRNA";

2R Cufflinks exon 6822243 6822763 . - . gene_id "XLOC_066696"; transcript_id "TCONS_00218720"; exon_number "2"; gene_type "lincRNA";

2R Cufflinks exon 6891311 6891514 . - . gene_id "XLOC_066746"; transcript_id "TCONS_00218763"; exon_number "1"; gene_type "lincRNA";

2R Cufflinks exon 7584366 7584632 . - . gene_id "XLOC_067206"; transcript_id "TCONS_00300558"; exon_number "1"; gene_type "intronic_lncRNA";

2R Cufflinks exon 8330149 8330391 . - . gene_id "XLOC_067583"; transcript_id "TCONS_00265105"; exon_number "1"; gene_type "intronic_lncRNA";

2R Cufflinks exon 8437578 8437798 . - . gene_id "XLOC_067699"; transcript_id "TCONS_00219412"; exon_number "1"; gene_type "anti-sense_lncRNA";

2R Cufflinks exon 8563251 8563445 . - . gene_id "XLOC_067790"; transcript_id "TCONS_00219509"; exon_number "1"; gene_type "lincRNA";

2R Cufflinks exon 8563518 8563566 . - . gene_id "XLOC_067790"; transcript_id "TCONS_00219509"; exon_number "2"; gene_type "lincRNA";

2R Cufflinks exon 8650800 8651053 . - . gene_id "XLOC_067857"; transcript_id "TCONS_00265273"; exon_number "1"; gene_type "intronic_lncRNA";

2R Cufflinks exon 8651251 8651808 . - . gene_id "XLOC_067857"; transcript_id "TCONS_00265273"; exon_number "2"; gene_type "intronic_lncRNA";

2R Scripture exon 8669850 8670047 . - . gene_id "XLOC_067865"; transcript_id "TCONS_00390973"; exon_number "1"; gene_type "intronic_lncRNA";

2R Scripture exon 8670116 8670698 . - . gene_id "XLOC_067865"; transcript_id "TCONS_00390973"; exon_number "2"; gene_type "intronic_lncRNA";

2R Scripture exon 8669850 8670047 . - . gene_id "XLOC_067865"; transcript_id "TCONS_00390972"; exon_number "1"; gene_type "intronic_lncRNA";

2R Scripture exon 8670140 8670698 . - . gene_id "XLOC_067865"; transcript_id "TCONS_00390972"; exon_number "2"; gene_type "intronic_lncRNA";

2R Cufflinks exon 8785334 8785552 . - . gene_id "XLOC_067962"; transcript_id "TCONS_00265327"; exon_number "1"; gene_type "lincRNA";

2R Cufflinks exon 8795225 8795460 . - . gene_id "XLOC_067973"; transcript_id "TCONS_00265333"; exon_number "1"; gene_type "lincRNA";

2R Cufflinks exon 9004732 9004950 . - . gene_id "XLOC_068108"; transcript_id "TCONS_00244019"; exon_number "1"; gene_type "intronic_lncRNA";

2R Cufflinks exon 9982044 9982261 . - . gene_id "XLOC_068631"; transcript_id "TCONS_00244428"; exon_number "1"; gene_type "lincRNA";

2R Cufflinks exon 10052655 10052985 . - . gene_id "XLOC_068645"; transcript_id "TCONS_00220182"; exon_number "1"; gene_type "intronic_lncRNA";

2R Cufflinks exon 10076124 10076149 . - . gene_id "XLOC_068653"; transcript_id "TCONS_00318888"; exon_number "1"; gene_type "lincRNA";

2R Cufflinks exon 10076246 10076469 . - . gene_id "XLOC_068653"; transcript_id "TCONS_00318888"; exon_number "2"; gene_type "lincRNA";

2R Cufflinks exon 10129966 10130229 . - . gene_id "XLOC_068681"; transcript_id "TCONS_00220198"; exon_number "1"; gene_type "intronic_lncRNA";

2R Cufflinks exon 10138225 10138426 . - . gene_id "XLOC_068685"; transcript_id "TCONS_00244452"; exon_number "1"; gene_type "anti-sense_lncRNA";

2R Cufflinks exon 10263371 10263617 . - . gene_id "XLOC_068812"; transcript_id "TCONS_00220288"; exon_number "1"; gene_type "lincRNA";

2R Cufflinks exon 10263681 10263750 . - . gene_id "XLOC_068812"; transcript_id "TCONS_00220288"; exon_number "2"; gene_type "lincRNA";

2R Cufflinks exon 10438413 10438614 . - . gene_id "XLOC_068910"; transcript_id "TCONS_00220327"; exon_number "1"; gene_type "intronic_lncRNA";

2R Cufflinks exon 10791751 10791954 . - . gene_id "XLOC_069078"; transcript_id "TCONS_00284894"; exon_number "1"; gene_type "anti-sense_lncRNA";

2R Cufflinks exon 11042082 11042294 . - . gene_id "XLOC_069301"; transcript_id "TCONS_00220604"; exon_number "1"; gene_type "intronic_lncRNA";

2R Cufflinks exon 11443242 11443465 . - . gene_id "XLOC_069640"; transcript_id "TCONS_00266259"; exon_number "1"; gene_type "intronic_lncRNA";

2R Cufflinks exon 11477720 11477940 . - . gene_id "XLOC_069656"; transcript_id "TCONS_00220887"; exon_number "1"; gene_type "intronic_lncRNA";

2R Cufflinks exon 11483054 11483277 . - . gene_id "XLOC_069663"; transcript_id "TCONS_00245039"; exon_number "1"; gene_type "intronic_lncRNA";

2R Cufflinks exon 11766114 11766324 . - . gene_id "XLOC_069904"; transcript_id "TCONS_00221034"; exon_number "1"; gene_type "intronic_lncRNA";

2R Cufflinks exon 12320539 12320821 . - . gene_id "XLOC_070390"; transcript_id "TCONS_00221333"; exon_number "1"; gene_type "lincRNA";

2R Cufflinks exon 12320879 12321086 . - . gene_id "XLOC_070390"; transcript_id "TCONS_00221333"; exon_number "2"; gene_type "lincRNA";

2R Cufflinks exon 12776970 12777174 . - . gene_id "XLOC_070666"; transcript_id "TCONS_00221467"; exon_number "1"; gene_type "lincRNA";

2R Cufflinks exon 12890743 12890964 . - . gene_id "XLOC_070763"; transcript_id "TCONS_00338076"; exon_number "1"; gene_type "intronic_lncRNA";

2R Cufflinks exon 12987127 12987397 . - . gene_id "XLOC_070832"; transcript_id "TCONS_00338112"; exon_number "1"; gene_type "lincRNA";

2R Cufflinks exon 13255539 13255709 . - . gene_id "XLOC_071035"; transcript_id "TCONS_00266909"; exon_number "1"; gene_type "lincRNA";

2R Cufflinks exon 13255783 13255834 . - . gene_id "XLOC_071035"; transcript_id "TCONS_00266909"; exon_number "2"; gene_type "lincRNA";

2R Scripture exon 13255951 13255982 . - . gene_id "XLOC_071036"; transcript_id "TCONS_00368470"; exon_number "1"; gene_type "anti-sense_lncRNA";

2R Scripture exon 13256154 13256697 . - . gene_id "XLOC_071036"; transcript_id "TCONS_00368470"; exon_number "2"; gene_type "anti-sense_lncRNA";

2R Cufflinks exon 13273299 13273527 . - . gene_id "XLOC_071049"; transcript_id "TCONS_00285757"; exon_number "1"; gene_type "intronic_lncRNA";

2R Cufflinks exon 13274091 13274296 . - . gene_id "XLOC_071050"; transcript_id "TCONS_00221741"; exon_number "1"; gene_type "intronic_lncRNA";

2R Cufflinks exon 13303271 13303473 . - . gene_id "XLOC_071078"; transcript_id "TCONS_00221771"; exon_number "1"; gene_type "lincRNA";

2R Cufflinks exon 13315268 13315476 . - . gene_id "XLOC_071101"; transcript_id "TCONS_00302084"; exon_number "1"; gene_type "lincRNA";

2R Cufflinks exon 13386417 13386617 . - . gene_id "XLOC_071173"; transcript_id "TCONS_00221856"; exon_number "1"; gene_type "lincRNA";

2R Cufflinks exon 13557220 13558159 . - . gene_id "XLOC_071213"; transcript_id "TCONS_00267024"; exon_number "1"; gene_type "anti-sense_lncRNA";

2R Cufflinks exon 13799551 13799763 . - . gene_id "XLOC_071472"; transcript_id "TCONS_00267125"; exon_number "1"; gene_type "intronic_lncRNA";

2R Cufflinks exon 14145370 14145858 . - . gene_id "XLOC_071586"; transcript_id "TCONS_00246235"; exon_number "1"; gene_type "lincRNA";

2R Cufflinks exon 14147173 14147234 . - . gene_id "XLOC_071586"; transcript_id "TCONS_00246235"; exon_number "2"; gene_type "lincRNA";

2R Cufflinks exon 14330323 14330529 . - . gene_id "XLOC_071668"; transcript_id "TCONS_00267210"; exon_number "1"; gene_type "intronic_lncRNA";

2R Cufflinks exon 14455055 14455262 . - . gene_id "XLOC_071802"; transcript_id "TCONS_00246352"; exon_number "1"; gene_type "intronic_lncRNA";

2R Cufflinks exon 14825054 14825258 . - . gene_id "XLOC_072148"; transcript_id "TCONS_00246580"; exon_number "1"; gene_type "lincRNA";

2R Cufflinks exon 14998730 14998955 . - . gene_id "XLOC_072226"; transcript_id "TCONS_00338729"; exon_number "1"; gene_type "lincRNA";

2R Cufflinks exon 15238855 15239240 . - . gene_id "XLOC_072476"; transcript_id "TCONS_00222635"; exon_number "1"; gene_type "intronic_lncRNA";

2R Cufflinks exon 15417858 15418079 . - . gene_id "XLOC_072583"; transcript_id "TCONS_00246807"; exon_number "1"; gene_type "intronic_lncRNA";

2R Cufflinks exon 15598303 15598503 . - . gene_id "XLOC_072749"; transcript_id "TCONS_00302659"; exon_number "1"; gene_type "lincRNA";

2R Cufflinks exon 16119824 16120048 . - . gene_id "XLOC_073098"; transcript_id "TCONS_00223011"; exon_number "1"; gene_type "anti-sense_lncRNA";

2R Cufflinks exon 16734259 16734460 . - . gene_id "XLOC_073515"; transcript_id "TCONS_00247351"; exon_number "1"; gene_type "lincRNA";

2R Scripture exon 17142490 17143695 . - . gene_id "XLOC_073604"; transcript_id "TCONS_00386221"; exon_number "1"; gene_type "lincRNA";

2R Scripture exon 17145622 17145746 . - . gene_id "XLOC_073604"; transcript_id "TCONS_00386221"; exon_number "2"; gene_type "lincRNA";

2R Cufflinks exon 17028378 17028577 . - . gene_id "XLOC_073762"; transcript_id "TCONS_00223431"; exon_number "1"; gene_type "intronic_lncRNA";

2R Cufflinks exon 17207116 17207321 . - . gene_id "XLOC_073900"; transcript_id "TCONS_00286961"; exon_number "1"; gene_type "lincRNA";

2R Cufflinks exon 17355018 17355241 . - . gene_id "XLOC_074061"; transcript_id "TCONS_00268352"; exon_number "1"; gene_type "intronic_lncRNA";

2R Cufflinks exon 17815574 17816455 . - . gene_id "XLOC_074300"; transcript_id "TCONS_00247796"; exon_number "1"; gene_type "intronic_lncRNA";

2R Cufflinks exon 17819744 17819821 . - . gene_id "XLOC_074300"; transcript_id "TCONS_00247796"; exon_number "2"; gene_type "intronic_lncRNA";

2R Cufflinks exon 17874080 17874288 . - . gene_id "XLOC_074333"; transcript_id "TCONS_00287123"; exon_number "1"; gene_type "intronic_lncRNA";

2R Cufflinks exon 17957069 17957273 . - . gene_id "XLOC_074442"; transcript_id "TCONS_00247875"; exon_number "1"; gene_type "lincRNA";

2R Cufflinks exon 17970661 17970867 . - . gene_id "XLOC_074465"; transcript_id "TCONS_00268552"; exon_number "1"; gene_type "lincRNA";

2R Cufflinks exon 18127328 18127537 . - . gene_id "XLOC_074614"; transcript_id "TCONS_00268657"; exon_number "1"; gene_type "intronic_lncRNA";

2R Cufflinks exon 18323165 18323383 . - . gene_id "XLOC_074800"; transcript_id "TCONS_00224035"; exon_number "1"; gene_type "intronic_lncRNA";

2R Cufflinks exon 18341060 18341277 . - . gene_id "XLOC_074823"; transcript_id "TCONS_00303457"; exon_number "1"; gene_type "intronic_lncRNA";

2R Cufflinks exon 18379006 18379218 . - . gene_id "XLOC_074860"; transcript_id "TCONS_00224064"; exon_number "1"; gene_type "lincRNA";

2R Cufflinks exon 18402655 18402874 . - . gene_id "XLOC_074883"; transcript_id "TCONS_00287344"; exon_number "1"; gene_type "lincRNA";

2R Cufflinks exon 18404115 18404314 . - . gene_id "XLOC_074884"; transcript_id "TCONS_00287345"; exon_number "1"; gene_type "lincRNA";

2R Cufflinks exon 18696456 18696699 . - . gene_id "XLOC_075084"; transcript_id "TCONS_00268867"; exon_number "1"; gene_type "anti-sense_lncRNA";

2R Cufflinks exon 18766289 18766516 . - . gene_id "XLOC_075143"; transcript_id "TCONS_00224213"; exon_number "1"; gene_type "intronic_lncRNA";

2R Cufflinks exon 18916450 18916665 . - . gene_id "XLOC_075259"; transcript_id "TCONS_00287476"; exon_number "1"; gene_type "intronic_lncRNA";

2R Cufflinks exon 19009080 19009288 . - . gene_id "XLOC_075351"; transcript_id "TCONS_00268976"; exon_number "1"; gene_type "lincRNA";

2R Cufflinks exon 19247690 19247896 . - . gene_id "XLOC_075567"; transcript_id "TCONS_00269081"; exon_number "1"; gene_type "lincRNA";

2R Cufflinks exon 19548831 19549135 . - . gene_id "XLOC_075759"; transcript_id "TCONS_00248621"; exon_number "1"; gene_type "intronic_lncRNA";

2R Cufflinks exon 19989975 19990180 . - . gene_id "XLOC_076198"; transcript_id "TCONS_00248954"; exon_number "1"; gene_type "lincRNA";

2R Cufflinks exon 20065864 20065910 . - . gene_id "XLOC_076307"; transcript_id "TCONS_00249034"; exon_number "1"; gene_type "lincRNA";

2R Cufflinks exon 20065967 20067081 . - . gene_id "XLOC_076307"; transcript_id "TCONS_00249034"; exon_number "2"; gene_type "lincRNA";

2R Cufflinks exon 20158246 20158454 . - . gene_id "XLOC_076429"; transcript_id "TCONS_00249115"; exon_number "1"; gene_type "lincRNA";

2R Cufflinks exon 20180578 20180816 . - . gene_id "XLOC_076474"; transcript_id "TCONS_00269583"; exon_number "1"; gene_type "lincRNA";

2R Cufflinks exon 20816469 20816676 . - . gene_id "XLOC_077149"; transcript_id "TCONS_00249539"; exon_number "1"; gene_type "lincRNA";

2R Cufflinks exon 20909505 20909711 . - . gene_id "XLOC_077247"; transcript_id "TCONS_00249621"; exon_number "1"; gene_type "anti-sense_lncRNA";

2R Cufflinks exon 20934832 20935042 . - . gene_id "XLOC_077291"; transcript_id "TCONS_00249637"; exon_number "1"; gene_type "intronic_lncRNA";

2R Cufflinks exon 21047692 21048847 . - . gene_id "XLOC_077394"; transcript_id "TCONS_00225554"; exon_number "1"; gene_type "lincRNA";

2R Cufflinks exon 21048963 21049424 . - . gene_id "XLOC_077394"; transcript_id "TCONS_00225554"; exon_number "2"; gene_type "lincRNA";

2R Cufflinks exon 21114189 21114389 . - . gene_id "XLOC_077440"; transcript_id "TCONS_00270036"; exon_number "1"; gene_type "intronic_lncRNA";

2R Scripture exon 21297554 21298192 . - . gene_id "XLOC_077552"; transcript_id "TCONS_00370101"; exon_number "1"; gene_type "intronic_lncRNA";

2R Scripture exon 21298258 21298285 . - . gene_id "XLOC_077552"; transcript_id "TCONS_00370101"; exon_number "2"; gene_type "intronic_lncRNA";

2R Scripture exon 21297554 21298192 . - . gene_id "XLOC_077552"; transcript_id "TCONS_00370100"; exon_number "1"; gene_type "intronic_lncRNA";

2R Scripture exon 21298263 21298285 . - . gene_id "XLOC_077552"; transcript_id "TCONS_00370100"; exon_number "2"; gene_type "intronic_lncRNA";

2R Scripture exon 21329768 21330313 . - . gene_id "XLOC_077561"; transcript_id "TCONS_00379772"; exon_number "1"; gene_type "anti-sense_lncRNA";

2R Scripture exon 21330616 21331256 . - . gene_id "XLOC_077561"; transcript_id "TCONS_00379772"; exon_number "2"; gene_type "anti-sense_lncRNA";

2R Cufflinks exon 21663267 21663484 . - . gene_id "XLOC_077741"; transcript_id "TCONS_00270168"; exon_number "1"; gene_type "lincRNA";

2R Cufflinks exon 21947919 21948125 . - . gene_id "XLOC_078044"; transcript_id "TCONS_00270306"; exon_number "1"; gene_type "lincRNA";

2R Cufflinks exon 21972098 21972308 . - . gene_id "XLOC_078095"; transcript_id "TCONS_00225875"; exon_number "1"; gene_type "lincRNA";

2R Cufflinks exon 22197133 22197369 . - . gene_id "XLOC_078295"; transcript_id "TCONS_00226010"; exon_number "1"; gene_type "intronic_lncRNA";

2R Cufflinks exon 22261829 22262033 . - . gene_id "XLOC_078348"; transcript_id "TCONS_00250270"; exon_number "1"; gene_type "intronic_lncRNA";

2R Scripture exon 22317661 22320622 . - . gene_id "XLOC_078401"; transcript_id "TCONS_00370195"; exon_number "1"; gene_type "anti-sense_lncRNA";

2R Scripture exon 22320782 22324606 . - . gene_id "XLOC_078401"; transcript_id "TCONS_00370195"; exon_number "2"; gene_type "anti-sense_lncRNA";

2R Cufflinks exon 22384773 22384983 . - . gene_id "XLOC_078451"; transcript_id "TCONS_00304633"; exon_number "1"; gene_type "intronic_lncRNA";

2R Cufflinks exon 22501766 22501980 . - . gene_id "XLOC_078545"; transcript_id "TCONS_00250359"; exon_number "1"; gene_type "intronic_lncRNA";

2R Cufflinks exon 22590169 22590472 . - . gene_id "XLOC_078622"; transcript_id "TCONS_00250391"; exon_number "1"; gene_type "intronic_lncRNA";

2R Cufflinks exon 22903246 22903463 . - . gene_id "XLOC_078847"; transcript_id "TCONS_00226363"; exon_number "1"; gene_type "intronic_lncRNA";

2R Cufflinks exon 22905537 22905736 . - . gene_id "XLOC_078850"; transcript_id "TCONS_00250564"; exon_number "1"; gene_type "intronic_lncRNA";

2R Cufflinks exon 23215038 23215256 . - . gene_id "XLOC_079236"; transcript_id "TCONS_00270912"; exon_number "1"; gene_type "lincRNA";

2R Cufflinks exon 23436352 23436553 . - . gene_id "XLOC_079479"; transcript_id "TCONS_00251042"; exon_number "1"; gene_type "lincRNA";

2R Cufflinks exon 23459131 23459330 . - . gene_id "XLOC_079519"; transcript_id "TCONS_00226814"; exon_number "1"; gene_type "intronic_lncRNA";

2R Cufflinks exon 23710576 23710811 . - . gene_id "XLOC_079725"; transcript_id "TCONS_00251169"; exon_number "1"; gene_type "lincRNA";

2R Scripture exon 23713744 23714160 . - . gene_id "XLOC_079728"; transcript_id "TCONS_00392861"; exon_number "1"; gene_type "anti-sense_lncRNA";

2R Scripture exon 23714270 23714742 . - . gene_id "XLOC_079728"; transcript_id "TCONS_00392861"; exon_number "2"; gene_type "anti-sense_lncRNA";

2R Cufflinks exon 23729267 23729477 . - . gene_id "XLOC_079735"; transcript_id "TCONS_00226923"; exon_number "1"; gene_type "intronic_lncRNA";

2R Cufflinks exon 23819666 23819883 . - . gene_id "XLOC_079790"; transcript_id "TCONS_00251198"; exon_number "1"; gene_type "lincRNA";

2R Cufflinks exon 24055370 24055473 . - . gene_id "XLOC_079862"; transcript_id "TCONS_00289512"; exon_number "1"; gene_type "anti-sense_lncRNA";

2R Cufflinks exon 24055559 24056042 . - . gene_id "XLOC_079862"; transcript_id "TCONS_00289512"; exon_number "2"; gene_type "anti-sense_lncRNA";

2R Cufflinks exon 24059448 24059735 . - . gene_id "XLOC_079864"; transcript_id "TCONS_00227038"; exon_number "1"; gene_type "anti-sense_lncRNA";

2R Cufflinks exon 24059814 24060010 . - . gene_id "XLOC_079864"; transcript_id "TCONS_00227038"; exon_number "2"; gene_type "anti-sense_lncRNA";

2R Cufflinks exon 24081952 24082172 . - . gene_id "XLOC_079875"; transcript_id "TCONS_00227050"; exon_number "1"; gene_type "anti-sense_lncRNA";

2R Cufflinks exon 24083273 24083481 . - . gene_id "XLOC_079877"; transcript_id "TCONS_00271201"; exon_number "1"; gene_type "anti-sense_lncRNA";

2R Cufflinks exon 24340818 24341017 . - . gene_id "XLOC_080084"; transcript_id "TCONS_00227179"; exon_number "1"; gene_type "lincRNA";

2R Cufflinks exon 24724061 24724265 . - . gene_id "XLOC_080329"; transcript_id "TCONS_00289773"; exon_number "1"; gene_type "intronic_lncRNA";

2R Cufflinks exon 24724972 24725194 . - . gene_id "XLOC_080329"; transcript_id "TCONS_00251603"; exon_number "1"; gene_type "intronic_lncRNA";

2R Cufflinks exon 25205540 25205744 . - . gene_id "XLOC_080695"; transcript_id "TCONS_00227672"; exon_number "1"; gene_type "intronic_lncRNA";

3Cen_mapped_Scaffold_31_D1643_D1653_D1791 Cufflinks exon 29123 29332 . + . gene_id "XLOC_085509"; transcript_id "TCONS_00412437"; exon_number "1"; gene_type "lincRNA";

3L Cufflinks exon 89384 90024 . + . gene_id "XLOC_085618"; transcript_id "TCONS_00529500"; exon_number "1"; gene_type "lincRNA";

3L Cufflinks exon 90385 90501 . + . gene_id "XLOC_085618"; transcript_id "TCONS_00529500"; exon_number "2"; gene_type "lincRNA";

3L Cufflinks exon 90739 91722 . + . gene_id "XLOC_085618"; transcript_id "TCONS_00529500"; exon_number "3"; gene_type "lincRNA";

3L Cufflinks exon 91904 92561 . + . gene_id "XLOC_085618"; transcript_id "TCONS_00440139"; exon_number "1"; gene_type "lincRNA";

3L Cufflinks exon 93021 94362 . + . gene_id "XLOC_085618"; transcript_id "TCONS_00440139"; exon_number "2"; gene_type "lincRNA";

3L Cufflinks exon 616470 616693 . + . gene_id "XLOC_085825"; transcript_id "TCONS_00468239"; exon_number "1"; gene_type "lincRNA";

3L Cufflinks exon 621414 621619 . + . gene_id "XLOC_085832"; transcript_id "TCONS_00468243"; exon_number "1"; gene_type "anti-sense_lncRNA";

3L Cufflinks exon 638516 640186 . + . gene_id "XLOC_085839"; transcript_id "TCONS_00412803"; exon_number "1"; gene_type "lincRNA";

3L Cufflinks exon 640296 644500 . + . gene_id "XLOC_085839"; transcript_id "TCONS_00412803"; exon_number "2"; gene_type "lincRNA";

3L Cufflinks exon 639130 640186 . + . gene_id "XLOC_085839"; transcript_id "TCONS_00468255"; exon_number "1"; gene_type "lincRNA";

3L Cufflinks exon 640296 640686 . + . gene_id "XLOC_085839"; transcript_id "TCONS_00468255"; exon_number "2"; gene_type "lincRNA";

3L Cufflinks exon 640892 643629 . + . gene_id "XLOC_085839"; transcript_id "TCONS_00468255"; exon_number "3"; gene_type "lincRNA";

3L Scripture exon 681857 682211 . + . gene_id "XLOC_085884"; transcript_id "TCONS_00608876"; exon_number "1"; gene_type "anti-sense_lncRNA";

3L Scripture exon 682276 682594 . + . gene_id "XLOC_085884"; transcript_id "TCONS_00608876"; exon_number "2"; gene_type "anti-sense_lncRNA";

3L Cufflinks exon 739585 739792 . + . gene_id "XLOC_085949"; transcript_id "TCONS_00440403"; exon_number "1"; gene_type "intronic_lncRNA";

3L Cufflinks exon 1119507 1119726 . + . gene_id "XLOC_086247"; transcript_id "TCONS_00440611"; exon_number "1"; gene_type "lincRNA";

3L Cufflinks exon 1182273 1182511 . + . gene_id "XLOC_086319"; transcript_id "TCONS_00491042"; exon_number "1"; gene_type "lincRNA";

3L Cufflinks exon 1480884 1480995 . + . gene_id "XLOC_086607"; transcript_id "TCONS_00512224"; exon_number "1"; gene_type "intronic_lncRNA";

3L Cufflinks exon 1481082 1481680 . + . gene_id "XLOC_086607"; transcript_id "TCONS_00512224"; exon_number "2"; gene_type "intronic_lncRNA";

3L Cufflinks exon 1481721 1481752 . + . gene_id "XLOC_086607"; transcript_id "TCONS_00468721"; exon_number "1"; gene_type "intronic_lncRNA";

3L Cufflinks exon 1481816 1482516 . + . gene_id "XLOC_086607"; transcript_id "TCONS_00468721"; exon_number "2"; gene_type "intronic_lncRNA";

3L Cufflinks exon 1482633 1483490 . + . gene_id "XLOC_086608"; transcript_id "TCONS_00491199"; exon_number "1"; gene_type "intronic_lncRNA";

3L Cufflinks exon 1483792 1483875 . + . gene_id "XLOC_086608"; transcript_id "TCONS_00491199"; exon_number "2"; gene_type "intronic_lncRNA";

3L Cufflinks exon 1487509 1487596 . + . gene_id "XLOC_086610"; transcript_id "TCONS_00440863"; exon_number "1"; gene_type "anti-sense_lncRNA";

3L Cufflinks exon 1487652 1487945 . + . gene_id "XLOC_086610"; transcript_id "TCONS_00440863"; exon_number "2"; gene_type "anti-sense_lncRNA";

3L Cufflinks exon 1488457 1489530 . + . gene_id "XLOC_086610"; transcript_id "TCONS_00413373"; exon_number "1"; gene_type "anti-sense_lncRNA";

3L Scripture exon 2088433 2089139 . + . gene_id "XLOC_087009"; transcript_id "TCONS_00413624"; exon_number "1"; gene_type "intronic_lncRNA";

3L Scripture exon 2089196 2091129 . + . gene_id "XLOC_087009"; transcript_id "TCONS_00413624"; exon_number "2"; gene_type "intronic_lncRNA";

3L Cufflinks exon 2142921 2143125 . + . gene_id "XLOC_087030"; transcript_id "TCONS_00413633"; exon_number "1"; gene_type "intronic_lncRNA";

3L Cufflinks exon 2192364 2192564 . + . gene_id "XLOC_087085"; transcript_id "TCONS_00468935"; exon_number "1"; gene_type "lincRNA";

3L Cufflinks exon 2221651 2221850 . + . gene_id "XLOC_087129"; transcript_id "TCONS_00441159"; exon_number "1"; gene_type "lincRNA";

3L Cufflinks exon 2319907 2320109 . + . gene_id "XLOC_087213"; transcript_id "TCONS_00441239"; exon_number "1"; gene_type "lincRNA";

3L Cufflinks exon 2860790 2860994 . + . gene_id "XLOC_087548"; transcript_id "TCONS_00441466"; exon_number "1"; gene_type "intronic_lncRNA";

3L Cufflinks exon 2876836 2877064 . + . gene_id "XLOC_087566"; transcript_id "TCONS_00441473"; exon_number "1"; gene_type "intronic_lncRNA";

3L Cufflinks exon 2951786 2951991 . + . gene_id "XLOC_087644"; transcript_id "TCONS_00414052"; exon_number "1"; gene_type "intronic_lncRNA";

3L Cufflinks exon 3407759 3407989 . + . gene_id "XLOC_087912"; transcript_id "TCONS_00469400"; exon_number "1"; gene_type "intronic_lncRNA";

3L Cufflinks exon 3409994 3410206 . + . gene_id "XLOC_087913"; transcript_id "TCONS_00414278"; exon_number "1"; gene_type "intronic_lncRNA";

3L Cufflinks exon 3763317 3763522 . + . gene_id "XLOC_088134"; transcript_id "TCONS_00551937"; exon_number "1"; gene_type "intronic_lncRNA";

3L Scripture exon 3940007 3940710 . + . gene_id "XLOC_088268"; transcript_id "TCONS_00592240"; exon_number "1"; gene_type "anti-sense_lncRNA";

3L Scripture exon 3941112 3941304 . + . gene_id "XLOC_088268"; transcript_id "TCONS_00592240"; exon_number "2"; gene_type "anti-sense_lncRNA";

3L Cufflinks exon 4077037 4077247 . + . gene_id "XLOC_088385"; transcript_id "TCONS_00442114"; exon_number "1"; gene_type "intronic_lncRNA";

3L Scripture exon 4256560 4257052 . + . gene_id "XLOC_088473"; transcript_id "TCONS_00592268"; exon_number "1"; gene_type "anti-sense_lncRNA";

3L Scripture exon 4257146 4257256 . + . gene_id "XLOC_088473"; transcript_id "TCONS_00592268"; exon_number "2"; gene_type "anti-sense_lncRNA";

3L Cufflinks exon 4345558 4346288 . + . gene_id "XLOC_088539"; transcript_id "TCONS_00512991"; exon_number "1"; gene_type "intronic_lncRNA";

3L Cufflinks exon 4346809 4348225 . + . gene_id "XLOC_088539"; transcript_id "TCONS_00512991"; exon_number "2"; gene_type "intronic_lncRNA";

3L Scripture exon 4345850 4346284 . + . gene_id "XLOC_088539"; transcript_id "TCONS_00552109"; exon_number "1"; gene_type "intronic_lncRNA";

3L Scripture exon 4346809 4348321 . + . gene_id "XLOC_088539"; transcript_id "TCONS_00552109"; exon_number "2"; gene_type "intronic_lncRNA";

3L Cufflinks exon 4363593 4363822 . + . gene_id "XLOC_088552"; transcript_id "TCONS_00414700"; exon_number "1"; gene_type "intronic_lncRNA";

3L Cufflinks exon 4422894 4423103 . + . gene_id "XLOC_088590"; transcript_id "TCONS_00442259"; exon_number "1"; gene_type "intronic_lncRNA";

3L Cufflinks exon 4634710 4634913 . + . gene_id "XLOC_088760"; transcript_id "TCONS_00442374"; exon_number "1"; gene_type "anti-sense_lncRNA";

3L Cufflinks exon 4734483 4734730 . + . gene_id "XLOC_088845"; transcript_id "TCONS_00414868"; exon_number "1"; gene_type "intronic_lncRNA";

3L Cufflinks exon 4764185 4764409 . + . gene_id "XLOC_088879"; transcript_id "TCONS_00414917"; exon_number "1"; gene_type "intronic_lncRNA";

3L Cufflinks exon 4853779 4854035 . + . gene_id "XLOC_088956"; transcript_id "TCONS_00414982"; exon_number "1"; gene_type "lincRNA";

3L Cufflinks exon 5027354 5027595 . + . gene_id "XLOC_089157"; transcript_id "TCONS_00492446"; exon_number "1"; gene_type "intronic_lncRNA";

3L Cufflinks exon 5245646 5245862 . + . gene_id "XLOC_089344"; transcript_id "TCONS_00442690"; exon_number "1"; gene_type "intronic_lncRNA";

3L Cufflinks exon 5800765 5800966 . + . gene_id "XLOC_089920"; transcript_id "TCONS_00443025"; exon_number "1"; gene_type "intronic_lncRNA";

3L Cufflinks exon 5853212 5853442 . + . gene_id "XLOC_089961"; transcript_id "TCONS_00492752"; exon_number "1"; gene_type "lincRNA";

3L Scripture exon 6232264 6232792 . + . gene_id "XLOC_090350"; transcript_id "TCONS_00615467"; exon_number "1"; gene_type "anti-sense_lncRNA";

3L Scripture exon 6232853 6233721 . + . gene_id "XLOC_090350"; transcript_id "TCONS_00615467"; exon_number "2"; gene_type "anti-sense_lncRNA";

3L Cufflinks exon 6312676 6312883 . + . gene_id "XLOC_090440"; transcript_id "TCONS_00443382"; exon_number "1"; gene_type "lincRNA";

3L Cufflinks exon 6666292 6666499 . + . gene_id "XLOC_090775"; transcript_id "TCONS_00443560"; exon_number "1"; gene_type "lincRNA";

3L Cufflinks exon 6723997 6724204 . + . gene_id "XLOC_090846"; transcript_id "TCONS_00470835"; exon_number "1"; gene_type "lincRNA";

3L Cufflinks exon 6759183 6759386 . + . gene_id "XLOC_090864"; transcript_id "TCONS_00443609"; exon_number "1"; gene_type "intronic_lncRNA";

3L Cufflinks exon 6816607 6816813 . + . gene_id "XLOC_090948"; transcript_id "TCONS_00443671"; exon_number "1"; gene_type "lincRNA";

3L Cufflinks exon 6877022 6877231 . + . gene_id "XLOC_091048"; transcript_id "TCONS_00416162"; exon_number "1"; gene_type "lincRNA";

3L Cufflinks exon 7392441 7392647 . + . gene_id "XLOC_091568"; transcript_id "TCONS_00514089"; exon_number "1"; gene_type "intronic_lncRNA";

3L Cufflinks exon 7714885 7715112 . + . gene_id "XLOC_091820"; transcript_id "TCONS_00532449"; exon_number "1"; gene_type "anti-sense_lncRNA";

3L Cufflinks exon 7792647 7792872 . + . gene_id "XLOC_091887"; transcript_id "TCONS_00532471"; exon_number "1"; gene_type "intronic_lncRNA";

3L Cufflinks exon 8294336 8294536 . + . gene_id "XLOC_092148"; transcript_id "TCONS_00416824"; exon_number "1"; gene_type "intronic_lncRNA";

3L Cufflinks exon 8672262 8672474 . + . gene_id "XLOC_092363"; transcript_id "TCONS_00444543"; exon_number "1"; gene_type "lincRNA";

3L Cufflinks exon 9158812 9159021 . + . gene_id "XLOC_092607"; transcript_id "TCONS_00553737"; exon_number "1"; gene_type "intronic_lncRNA";

3L Cufflinks exon 9236542 9236748 . + . gene_id "XLOC_092705"; transcript_id "TCONS_00417270"; exon_number "1"; gene_type "anti-sense_lncRNA";

3L Scripture exon 9510282 9510624 . + . gene_id "XLOC_092909"; transcript_id "TCONS_00593240"; exon_number "1"; gene_type "anti-sense_lncRNA";

3L Cufflinks exon 9528697 9528901 . + . gene_id "XLOC_092935"; transcript_id "TCONS_00444909"; exon_number "1"; gene_type "lincRNA";

3L Cufflinks exon 9598444 9598652 . + . gene_id "XLOC_092986"; transcript_id "TCONS_00417458"; exon_number "1"; gene_type "intronic_lncRNA";

3L Cufflinks exon 9737783 9738028 . + . gene_id "XLOC_093037"; transcript_id "TCONS_00417512"; exon_number "1"; gene_type "intronic_lncRNA";

3L Cufflinks exon 10160725 10160940 . + . gene_id "XLOC_093358"; transcript_id "TCONS_00514888"; exon_number "1"; gene_type "intronic_lncRNA";

3L Cufflinks exon 10280473 10280682 . + . gene_id "XLOC_093525"; transcript_id "TCONS_00472230"; exon_number "1"; gene_type "lincRNA";

3L Cufflinks exon 10332227 10332800 . + . gene_id "XLOC_093566"; transcript_id "TCONS_00554193"; exon_number "1"; gene_type "lincRNA";

3L Cufflinks exon 10333078 10333497 . + . gene_id "XLOC_093566"; transcript_id "TCONS_00554193"; exon_number "2"; gene_type "lincRNA";

3L Cufflinks exon 10332236 10332800 . + . gene_id "XLOC_093566"; transcript_id "TCONS_00417821"; exon_number "1"; gene_type "lincRNA";

3L Cufflinks exon 10348193 10348243 . + . gene_id "XLOC_093566"; transcript_id "TCONS_00417821"; exon_number "2"; gene_type "lincRNA";

3L Cufflinks exon 10332236 10332800 . + . gene_id "XLOC_093566"; transcript_id "TCONS_00417822"; exon_number "1"; gene_type "lincRNA";

3L Cufflinks exon 10348193 10348230 . + . gene_id "XLOC_093566"; transcript_id "TCONS_00417822"; exon_number "2"; gene_type "lincRNA";

3L Cufflinks exon 10358991 10360695 . + . gene_id "XLOC_093566"; transcript_id "TCONS_00417822"; exon_number "3"; gene_type "lincRNA";

3L Cufflinks exon 10376588 10376836 . + . gene_id "XLOC_093613"; transcript_id "TCONS_00554232"; exon_number "1"; gene_type "lincRNA";

3L Cufflinks exon 10428557 10428782 . + . gene_id "XLOC_093697"; transcript_id "TCONS_00533400"; exon_number "1"; gene_type "lincRNA";

3L Cufflinks exon 10437399 10437600 . + . gene_id "XLOC_093709"; transcript_id "TCONS_00417926"; exon_number "1"; gene_type "lincRNA";

3L Cufflinks exon 10605081 10605284 . + . gene_id "XLOC_093908"; transcript_id "TCONS_00418013"; exon_number "1"; gene_type "lincRNA";

3L Cufflinks exon 10699721 10699951 . + . gene_id "XLOC_093971"; transcript_id "TCONS_00445532"; exon_number "1"; gene_type "lincRNA";

3L Cufflinks exon 10784598 10784626 . + . gene_id "XLOC_094117"; transcript_id "TCONS_00418109"; exon_number "1"; gene_type "lincRNA";

3L Cufflinks exon 10784892 10785563 . + . gene_id "XLOC_094117"; transcript_id "TCONS_00418109"; exon_number "2"; gene_type "lincRNA";

3L Cufflinks exon 10917820 10918094 . + . gene_id "XLOC_094301"; transcript_id "TCONS_00494783"; exon_number "1"; gene_type "lincRNA";

3L Cufflinks exon 10921584 10921783 . + . gene_id "XLOC_094311"; transcript_id "TCONS_00418209"; exon_number "1"; gene_type "lincRNA";

3L Cufflinks exon 10950402 10950634 . + . gene_id "XLOC_094362"; transcript_id "TCONS_00445762"; exon_number "1"; gene_type "lincRNA";

3L Cufflinks exon 11094386 11094610 . + . gene_id "XLOC_094517"; transcript_id "TCONS_00418308"; exon_number "1"; gene_type "anti-sense_lncRNA";

3L Cufflinks exon 11297983 11298459 . + . gene_id "XLOC_094638"; transcript_id "TCONS_00418384"; exon_number "1"; gene_type "lincRNA";

3L Cufflinks exon 11301677 11302286 . + . gene_id "XLOC_094638"; transcript_id "TCONS_00418384"; exon_number "2"; gene_type "lincRNA";

3L Cufflinks exon 11338193 11338399 . + . gene_id "XLOC_094695"; transcript_id "TCONS_00445979"; exon_number "1"; gene_type "lincRNA";

3L Cufflinks exon 11385869 11386070 . + . gene_id "XLOC_094773"; transcript_id "TCONS_00446034"; exon_number "1"; gene_type "lincRNA";

3L Cufflinks exon 11652504 11652780 . + . gene_id "XLOC_095050"; transcript_id "TCONS_00418598"; exon_number "1"; gene_type "intronic_lncRNA";

3L Cufflinks exon 11652843 11652872 . + . gene_id "XLOC_095050"; transcript_id "TCONS_00418598"; exon_number "2"; gene_type "intronic_lncRNA";

3L Cufflinks exon 11656516 11656551 . + . gene_id "XLOC_095055"; transcript_id "TCONS_00418600"; exon_number "1"; gene_type "intronic_lncRNA";

3L Cufflinks exon 11656637 11657091 . + . gene_id "XLOC_095055"; transcript_id "TCONS_00418600"; exon_number "2"; gene_type "intronic_lncRNA";

3L Cufflinks exon 11745300 11745501 . + . gene_id "XLOC_095116"; transcript_id "TCONS_00533999"; exon_number "1"; gene_type "intronic_lncRNA";

3L Cufflinks exon 11918461 11918661 . + . gene_id "XLOC_095287"; transcript_id "TCONS_00534058"; exon_number "1"; gene_type "intronic_lncRNA";

3L Cufflinks exon 12094807 12095006 . + . gene_id "XLOC_095439"; transcript_id "TCONS_00473088"; exon_number "1"; gene_type "lincRNA";

3L Cufflinks exon 12377401 12377620 . + . gene_id "XLOC_095689"; transcript_id "TCONS_00446593"; exon_number "1"; gene_type "intronic_lncRNA";

3L Cufflinks exon 12459652 12459862 . + . gene_id "XLOC_095776"; transcript_id "TCONS_00473219"; exon_number "1"; gene_type "lincRNA";

3L Cufflinks exon 12606151 12606362 . + . gene_id "XLOC_095882"; transcript_id "TCONS_00495420"; exon_number "1"; gene_type "lincRNA";

3L Cufflinks exon 12667682 12667749 . + . gene_id "XLOC_095955"; transcript_id "TCONS_00473323"; exon_number "1"; gene_type "lincRNA";

3L Cufflinks exon 12668208 12668347 . + . gene_id "XLOC_095955"; transcript_id "TCONS_00473323"; exon_number "2"; gene_type "lincRNA";

3L Cufflinks exon 13125555 13125773 . + . gene_id "XLOC_096381"; transcript_id "TCONS_00419376"; exon_number "1"; gene_type "lincRNA";

3L Cufflinks exon 13216056 13216268 . + . gene_id "XLOC_096535"; transcript_id "TCONS_00473649"; exon_number "1"; gene_type "lincRNA";

3L Cufflinks exon 13345517 13345722 . + . gene_id "XLOC_096654"; transcript_id "TCONS_00473706"; exon_number "1"; gene_type "lincRNA";

3L Cufflinks exon 13750722 13750934 . + . gene_id "XLOC_097070"; transcript_id "TCONS_00496018"; exon_number "1"; gene_type "intronic_lncRNA";

3L Cufflinks exon 13877102 13877315 . + . gene_id "XLOC_097197"; transcript_id "TCONS_00473916"; exon_number "1"; gene_type "intronic_lncRNA";

3L Cufflinks exon 14114703 14114922 . + . gene_id "XLOC_097308"; transcript_id "TCONS_00496113"; exon_number "1"; gene_type "intronic_lncRNA";

3L Cufflinks exon 14153239 14153464 . + . gene_id "XLOC_097367"; transcript_id "TCONS_00535010"; exon_number "1"; gene_type "lincRNA";

3L Cufflinks exon 14272007 14272083 . + . gene_id "XLOC_097475"; transcript_id "TCONS_00419973"; exon_number "1"; gene_type "lincRNA";

3L Cufflinks exon 14272211 14272819 . + . gene_id "XLOC_097475"; transcript_id "TCONS_00419973"; exon_number "2"; gene_type "lincRNA";

3L Cufflinks exon 14436834 14437042 . + . gene_id "XLOC_097531"; transcript_id "TCONS_00474059"; exon_number "1"; gene_type "intronic_lncRNA";

3L Cufflinks exon 14440375 14440575 . + . gene_id "XLOC_097533"; transcript_id "TCONS_00420014"; exon_number "1"; gene_type "intronic_lncRNA";

3L Cufflinks exon 14515392 14515594 . + . gene_id "XLOC_097567"; transcript_id "TCONS_00420019"; exon_number "1"; gene_type "intronic_lncRNA";

3L Cufflinks exon 14902632 14902853 . + . gene_id "XLOC_097778"; transcript_id "TCONS_00555953"; exon_number "1"; gene_type "lincRNA";

3L Cufflinks exon 15356646 15356860 . + . gene_id "XLOC_098254"; transcript_id "TCONS_00420466"; exon_number "1"; gene_type "lincRNA";

3L Cufflinks exon 15478398 15478634 . + . gene_id "XLOC_098465"; transcript_id "TCONS_00516571"; exon_number "1"; gene_type "lincRNA";

3L Cufflinks exon 15483132 15483332 . + . gene_id "XLOC_098472"; transcript_id "TCONS_00448495"; exon_number "1"; gene_type "lincRNA";

3L Cufflinks exon 15490485 15490696 . + . gene_id "XLOC_098483"; transcript_id "TCONS_00448502"; exon_number "1"; gene_type "lincRNA";

3L Cufflinks exon 15643824 15644030 . + . gene_id "XLOC_098579"; transcript_id "TCONS_00448575"; exon_number "1"; gene_type "intronic_lncRNA";

3L Cufflinks exon 15766122 15766329 . + . gene_id "XLOC_098747"; transcript_id "TCONS_00420757"; exon_number "1"; gene_type "lincRNA";

3L Cufflinks exon 16012320 16012529 . + . gene_id "XLOC_098904"; transcript_id "TCONS_00516723"; exon_number "1"; gene_type "lincRNA";

3L Cufflinks exon 16018587 16018803 . + . gene_id "XLOC_098915"; transcript_id "TCONS_00474734"; exon_number "1"; gene_type "lincRNA";

3L Cufflinks exon 16245012 16245220 . + . gene_id "XLOC_099075"; transcript_id "TCONS_00556569"; exon_number "1"; gene_type "lincRNA";

3L Cufflinks exon 16535250 16535477 . + . gene_id "XLOC_099270"; transcript_id "TCONS_00421079"; exon_number "1"; gene_type "intronic_lncRNA";

3L Scripture exon 16653040 16654034 . + . gene_id "XLOC_099333"; transcript_id "TCONS_00594481"; exon_number "1"; gene_type "intronic_lncRNA";

3L Scripture exon 16656646 16659007 . + . gene_id "XLOC_099334"; transcript_id "TCONS_00611097"; exon_number "1"; gene_type "intronic_lncRNA";

3L Cufflinks exon 16707868 16708067 . + . gene_id "XLOC_099362"; transcript_id "TCONS_00421129"; exon_number "1"; gene_type "intronic_lncRNA";

3L Cufflinks exon 17447667 17447875 . + . gene_id "XLOC_099880"; transcript_id "TCONS_00449360"; exon_number "1"; gene_type "intronic_lncRNA";

3L Cufflinks exon 17709072 17709277 . + . gene_id "XLOC_100034"; transcript_id "TCONS_00475278"; exon_number "1"; gene_type "intronic_lncRNA";

3L Cufflinks exon 17914053 17914449 . + . gene_id "XLOC_100197"; transcript_id "TCONS_00517195"; exon_number "1"; gene_type "lincRNA";

3L Cufflinks exon 17914646 17914704 . + . gene_id "XLOC_100197"; transcript_id "TCONS_00517195"; exon_number "2"; gene_type "lincRNA";

3L Cufflinks exon 17948396 17948610 . + . gene_id "XLOC_100229"; transcript_id "TCONS_00421706"; exon_number "1"; gene_type "lincRNA";

3L Cufflinks exon 18077597 18078107 . + . gene_id "XLOC_100337"; transcript_id "TCONS_00449668"; exon_number "1"; gene_type "lincRNA";

3L Cufflinks exon 18081315 18081341 . + . gene_id "XLOC_100337"; transcript_id "TCONS_00449668"; exon_number "2"; gene_type "lincRNA";

3L Cufflinks exon 18188469 18188697 . + . gene_id "XLOC_100429"; transcript_id "TCONS_00449713"; exon_number "1"; gene_type "lincRNA";

3L Cufflinks exon 18241422 18241650 . + . gene_id "XLOC_100524"; transcript_id "TCONS_00449769"; exon_number "1"; gene_type "lincRNA";

3L Cufflinks exon 18540292 18540496 . + . gene_id "XLOC_100962"; transcript_id "TCONS_00475734"; exon_number "1"; gene_type "lincRNA";

3L Scripture exon 18896409 18896794 . + . gene_id "XLOC_101234"; transcript_id "TCONS_00611278"; exon_number "1"; gene_type "intronic_lncRNA";

3L Scripture exon 18896852 18897017 . + . gene_id "XLOC_101234"; transcript_id "TCONS_00611278"; exon_number "2"; gene_type "intronic_lncRNA";

3L Cufflinks exon 18976211 18976440 . + . gene_id "XLOC_101327"; transcript_id "TCONS_00422264"; exon_number "1"; gene_type "lincRNA";

3L Cufflinks exon 19155581 19155784 . + . gene_id "XLOC_101485"; transcript_id "TCONS_00497920"; exon_number "1"; gene_type "intronic_lncRNA";

3L Cufflinks exon 19186567 19186768 . + . gene_id "XLOC_101526"; transcript_id "TCONS_00422348"; exon_number "1"; gene_type "intronic_lncRNA";

3L Cufflinks exon 19305010 19305222 . + . gene_id "XLOC_101637"; transcript_id "TCONS_00450395"; exon_number "1"; gene_type "intronic_lncRNA";

3L Scripture exon 19989350 19989998 . + . gene_id "XLOC_102031"; transcript_id "TCONS_00631041"; exon_number "1"; gene_type "anti-sense_lncRNA";

3L Scripture exon 19990295 19990304 . + . gene_id "XLOC_102031"; transcript_id "TCONS_00631041"; exon_number "2"; gene_type "anti-sense_lncRNA";

3L Cufflinks exon 19989373 19989670 . + . gene_id "XLOC_102031"; transcript_id "TCONS_00450634"; exon_number "1"; gene_type "anti-sense_lncRNA";

3L Cufflinks exon 19989769 19990791 . + . gene_id "XLOC_102031"; transcript_id "TCONS_00450634"; exon_number "2"; gene_type "anti-sense_lncRNA";

3L Cufflinks exon 20166754 20166960 . + . gene_id "XLOC_102169"; transcript_id "TCONS_00450718"; exon_number "1"; gene_type "intronic_lncRNA";

3L Cufflinks exon 20453225 20453516 . + . gene_id "XLOC_102302"; transcript_id "TCONS_00422816"; exon_number "1"; gene_type "lincRNA";

3L Cufflinks exon 20453902 20454300 . + . gene_id "XLOC_102302"; transcript_id "TCONS_00422816"; exon_number "2"; gene_type "lincRNA";

3L Cufflinks exon 20490097 20490317 . + . gene_id "XLOC_102314"; transcript_id "TCONS_00450828"; exon_number "1"; gene_type "intronic_lncRNA";

3L Cufflinks exon 20492869 20493072 . + . gene_id "XLOC_102315"; transcript_id "TCONS_00476375"; exon_number "1"; gene_type "anti-sense_lncRNA";

3L Cufflinks exon 20624496 20624697 . + . gene_id "XLOC_102439"; transcript_id "TCONS_00476438"; exon_number "1"; gene_type "lincRNA";

3L Cufflinks exon 20670615 20670819 . + . gene_id "XLOC_102500"; transcript_id "TCONS_00498395"; exon_number "1"; gene_type "lincRNA";

3L Cufflinks exon 20677908 20678108 . + . gene_id "XLOC_102507"; transcript_id "TCONS_00537325"; exon_number "1"; gene_type "lincRNA";

3L Cufflinks exon 20919753 20919990 . + . gene_id "XLOC_102706"; transcript_id "TCONS_00451132"; exon_number "1"; gene_type "lincRNA";

3L Cufflinks exon 20929865 20930081 . + . gene_id "XLOC_102711"; transcript_id "TCONS_00423104"; exon_number "1"; gene_type "intronic_lncRNA";

3L Cufflinks exon 20932221 20932421 . + . gene_id "XLOC_102713"; transcript_id "TCONS_00423105"; exon_number "1"; gene_type "intronic_lncRNA";

3L Cufflinks exon 20948955 20949157 . + . gene_id "XLOC_102723"; transcript_id "TCONS_00451141"; exon_number "1"; gene_type "intronic_lncRNA";

3L Cufflinks exon 20971377 20971580 . + . gene_id "XLOC_102749"; transcript_id "TCONS_00537461"; exon_number "1"; gene_type "anti-sense_lncRNA";

3L Scripture exon 21195186 21195646 . + . gene_id "XLOC_102876"; transcript_id "TCONS_00616999"; exon_number "1"; gene_type "anti-sense_lncRNA";

3L Scripture exon 21195787 21196117 . + . gene_id "XLOC_102876"; transcript_id "TCONS_00616999"; exon_number "2"; gene_type "anti-sense_lncRNA";

3L Cufflinks exon 21268045 21268384 . + . gene_id "XLOC_102910"; transcript_id "TCONS_00451306"; exon_number "1"; gene_type "anti-sense_lncRNA";

3L Cufflinks exon 21268470 21268531 . + . gene_id "XLOC_102910"; transcript_id "TCONS_00451306"; exon_number "2"; gene_type "anti-sense_lncRNA";

3L Cufflinks exon 21356211 21356442 . + . gene_id "XLOC_102948"; transcript_id "TCONS_00498706"; exon_number "1"; gene_type "anti-sense_lncRNA";

3L Cufflinks exon 21687167 21687373 . + . gene_id "XLOC_103149"; transcript_id "TCONS_00423500"; exon_number "1"; gene_type "lincRNA";

3L Cufflinks exon 22003930 22004156 . + . gene_id "XLOC_103359"; transcript_id "TCONS_00498996"; exon_number "1"; gene_type "lincRNA";

3L Cufflinks exon 22012459 22012665 . + . gene_id "XLOC_103375"; transcript_id "TCONS_00499003"; exon_number "1"; gene_type "lincRNA";

3L Cufflinks exon 22143573 22143780 . + . gene_id "XLOC_103506"; transcript_id "TCONS_00477240"; exon_number "1"; gene_type "intronic_lncRNA";

3L Cufflinks exon 22479533 22479734 . + . gene_id "XLOC_103724"; transcript_id "TCONS_00424012"; exon_number "1"; gene_type "lincRNA";

3L Cufflinks exon 23149530 23150329 . + . gene_id "XLOC_104224"; transcript_id "TCONS_00424441"; exon_number "1"; gene_type "anti-sense_lncRNA";

3L Cufflinks exon 23150453 23150547 . + . gene_id "XLOC_104224"; transcript_id "TCONS_00424441"; exon_number "2"; gene_type "anti-sense_lncRNA";

3L Cufflinks exon 23315195 23315897 . + . gene_id "XLOC_104271"; transcript_id "TCONS_00538488"; exon_number "1"; gene_type "lincRNA";

3L Cufflinks exon 23315967 23316051 . + . gene_id "XLOC_104271"; transcript_id "TCONS_00538488"; exon_number "2"; gene_type "lincRNA";

3L Cufflinks exon 23397715 23397917 . + . gene_id "XLOC_104301"; transcript_id "TCONS_00424537"; exon_number "1"; gene_type "lincRNA";

3L Cufflinks exon 23427955 23428156 . + . gene_id "XLOC_104335"; transcript_id "TCONS_00499657"; exon_number "1"; gene_type "lincRNA";

3L Cufflinks exon 24388341 24388546 . + . gene_id "XLOC_104748"; transcript_id "TCONS_00478177"; exon_number "1"; gene_type "intronic_lncRNA";

3L Cufflinks exon 24648875 24649085 . + . gene_id "XLOC_104896"; transcript_id "TCONS_00500019"; exon_number "1"; gene_type "lincRNA";

3L Cufflinks exon 24729495 24729701 . + . gene_id "XLOC_104956"; transcript_id "TCONS_00538898"; exon_number "1"; gene_type "intronic_lncRNA";

3L Cufflinks exon 25200320 25200472 . + . gene_id "XLOC_105149"; transcript_id "TCONS_00453057"; exon_number "1"; gene_type "lincRNA";

3L Cufflinks exon 25200536 25200992 . + . gene_id "XLOC_105149"; transcript_id "TCONS_00453057"; exon_number "2"; gene_type "lincRNA";

3L Cufflinks exon 25380907 25381131 . + . gene_id "XLOC_105239"; transcript_id "TCONS_00453112"; exon_number "1"; gene_type "intronic_lncRNA";

3L Cufflinks exon 25671163 25671188 . + . gene_id "XLOC_105392"; transcript_id "TCONS_00478527"; exon_number "1"; gene_type "lincRNA";

3L Cufflinks exon 25708394 25708930 . + . gene_id "XLOC_105392"; transcript_id "TCONS_00478527"; exon_number "2"; gene_type "lincRNA";

3L Cufflinks exon 25673583 25673685 . + . gene_id "XLOC_105392"; transcript_id "TCONS_00478530"; exon_number "1"; gene_type "lincRNA";

3L Cufflinks exon 25673744 25673943 . + . gene_id "XLOC_105392"; transcript_id "TCONS_00478530"; exon_number "2"; gene_type "lincRNA";

3L Cufflinks exon 25708517 25708572 . + . gene_id "XLOC_105392"; transcript_id "TCONS_00500406"; exon_number "1"; gene_type "lincRNA";

3L Cufflinks exon 25708624 25708978 . + . gene_id "XLOC_105392"; transcript_id "TCONS_00500406"; exon_number "2"; gene_type "lincRNA";

3L Cufflinks exon 25709037 25709408 . + . gene_id "XLOC_105392"; transcript_id "TCONS_00500406"; exon_number "3"; gene_type "lincRNA";

3L Cufflinks exon 26277782 26277983 . + . gene_id "XLOC_105583"; transcript_id "TCONS_00453321"; exon_number "1"; gene_type "lincRNA";

3L Cufflinks exon 26564918 26565123 . + . gene_id "XLOC_105747"; transcript_id "TCONS_00539486"; exon_number "1"; gene_type "lincRNA";

3L Cufflinks exon 26566323 26566578 . + . gene_id "XLOC_105748"; transcript_id "TCONS_00453416"; exon_number "1"; gene_type "lincRNA";

3L Cufflinks exon 26662733 26664070 . + . gene_id "XLOC_105769"; transcript_id "TCONS_00560115"; exon_number "1"; gene_type "lincRNA";

3L Cufflinks exon 26695186 26695262 . + . gene_id "XLOC_105769"; transcript_id "TCONS_00560115"; exon_number "2"; gene_type "lincRNA";

3L Cufflinks exon 26694703 26694909 . + . gene_id "XLOC_105777"; transcript_id "TCONS_00539519"; exon_number "1"; gene_type "lincRNA";

3L Cufflinks exon 26819147 26819347 . + . gene_id "XLOC_105842"; transcript_id "TCONS_00539599"; exon_number "1"; gene_type "lincRNA";

3L Cufflinks exon 28059797 28060553 . + . gene_id "XLOC_106174"; transcript_id "TCONS_00479043"; exon_number "1"; gene_type "lincRNA";

3L Cufflinks exon 28076528 28077430 . + . gene_id "XLOC_106174"; transcript_id "TCONS_00479043"; exon_number "2"; gene_type "lincRNA";

3L Cufflinks exon 110203 110260 . - . gene_id "XLOC_106247"; transcript_id "TCONS_00426177"; exon_number "1"; gene_type "lincRNA";

3L Cufflinks exon 119375 119826 . - . gene_id "XLOC_106247"; transcript_id "TCONS_00426177"; exon_number "2"; gene_type "lincRNA";

3L Cufflinks exon 122016 122215 . - . gene_id "XLOC_106250"; transcript_id "TCONS_00426183"; exon_number "1"; gene_type "lincRNA";

3L Cufflinks exon 296791 296997 . - . gene_id "XLOC_106314"; transcript_id "TCONS_00426238"; exon_number "1"; gene_type "intronic_lncRNA";

3L Cufflinks exon 557328 557566 . - . gene_id "XLOC_106404"; transcript_id "TCONS_00426289"; exon_number "1"; gene_type "intronic_lncRNA";

3L Cufflinks exon 588493 588712 . - . gene_id "XLOC_106418"; transcript_id "TCONS_00501049"; exon_number "1"; gene_type "intronic_lncRNA";

3L Cufflinks exon 1184220 1184425 . - . gene_id "XLOC_106991"; transcript_id "TCONS_00501331"; exon_number "1"; gene_type "lincRNA";

3L Cufflinks exon 1482011 1482250 . - . gene_id "XLOC_107292"; transcript_id "TCONS_00454525"; exon_number "1"; gene_type "intronic_lncRNA";

3L Cufflinks exon 1565640 1565999 . - . gene_id "XLOC_107309"; transcript_id "TCONS_00479739"; exon_number "1"; gene_type "anti-sense_lncRNA";

3L Cufflinks exon 1711543 1711748 . - . gene_id "XLOC_107416"; transcript_id "TCONS_00426870"; exon_number "1"; gene_type "intronic_lncRNA";

3L Cufflinks exon 1796902 1797385 . - . gene_id "XLOC_107428"; transcript_id "TCONS_00454621"; exon_number "1"; gene_type "anti-sense_lncRNA";

3L Cufflinks exon 1974468 1974691 . - . gene_id "XLOC_107577"; transcript_id "TCONS_00521060"; exon_number "1"; gene_type "lincRNA";

3L Cufflinks exon 1993779 1993989 . - . gene_id "XLOC_107615"; transcript_id "TCONS_00454736"; exon_number "1"; gene_type "lincRNA";

3L Cufflinks exon 2299255 2299492 . - . gene_id "XLOC_107864"; transcript_id "TCONS_00454932"; exon_number "1"; gene_type "anti-sense_lncRNA";

3L Cufflinks exon 2385521 2385760 . - . gene_id "XLOC_107963"; transcript_id "TCONS_00501758"; exon_number "1"; gene_type "intronic_lncRNA";

3L Cufflinks exon 2386529 2386732 . - . gene_id "XLOC_107964"; transcript_id "TCONS_00521221"; exon_number "1"; gene_type "intronic_lncRNA";

3L Cufflinks exon 2462837 2463052 . - . gene_id "XLOC_108011"; transcript_id "TCONS_00427268"; exon_number "1"; gene_type "intronic_lncRNA";

3L Cufflinks exon 2765495 2765717 . - . gene_id "XLOC_108182"; transcript_id "TCONS_00427396"; exon_number "1"; gene_type "intronic_lncRNA";

3L Cufflinks exon 2848693 2848896 . - . gene_id "XLOC_108244"; transcript_id "TCONS_00427467"; exon_number "1"; gene_type "intronic_lncRNA";

3L Cufflinks exon 2956850 2957057 . - . gene_id "XLOC_108369"; transcript_id "TCONS_00480301"; exon_number "1"; gene_type "intronic_lncRNA";

3L Cufflinks exon 3005540 3005739 . - . gene_id "XLOC_108436"; transcript_id "TCONS_00455282"; exon_number "1"; gene_type "intronic_lncRNA";

3L Cufflinks exon 3240259 3240468 . - . gene_id "XLOC_108554"; transcript_id "TCONS_00455354"; exon_number "1"; gene_type "lincRNA";

3L Cufflinks exon 3412028 3412285 . - . gene_id "XLOC_108693"; transcript_id "TCONS_00541090"; exon_number "1"; gene_type "intronic_lncRNA";

3L Cufflinks exon 3501710 3501910 . - . gene_id "XLOC_108755"; transcript_id "TCONS_00455464"; exon_number "1"; gene_type "intronic_lncRNA";

3L Cufflinks exon 3661258 3661482 . - . gene_id "XLOC_108871"; transcript_id "TCONS_00541168"; exon_number "1"; gene_type "lincRNA";

3L Scripture exon 3813117 3813165 . - . gene_id "XLOC_108960"; transcript_id "TCONS_00597106"; exon_number "1"; gene_type "anti-sense_lncRNA";

3L Scripture exon 3813261 3813437 . - . gene_id "XLOC_108960"; transcript_id "TCONS_00597106"; exon_number "2"; gene_type "anti-sense_lncRNA";

3L Cufflinks exon 3828695 3828899 . - . gene_id "XLOC_108968"; transcript_id "TCONS_00427863"; exon_number "1"; gene_type "intronic_lncRNA";

3L Cufflinks exon 3830381 3830587 . - . gene_id "XLOC_108969"; transcript_id "TCONS_00480572"; exon_number "1"; gene_type "intronic_lncRNA";

3L Cufflinks exon 3888843 3889045 . - . gene_id "XLOC_109044"; transcript_id "TCONS_00480602"; exon_number "1"; gene_type "lincRNA";

3L Cufflinks exon 3892867 3893332 . - . gene_id "XLOC_109049"; transcript_id "TCONS_00427904"; exon_number "1"; gene_type "anti-sense_lncRNA";

3L Cufflinks exon 4274562 4274782 . - . gene_id "XLOC_109296"; transcript_id "TCONS_00502388"; exon_number "1"; gene_type "anti-sense_lncRNA";

3L Cufflinks exon 4531096 4531325 . - . gene_id "XLOC_109513"; transcript_id "TCONS_00480852"; exon_number "1"; gene_type "intronic_lncRNA";

3L Cufflinks exon 4741410 4741642 . - . gene_id "XLOC_109670"; transcript_id "TCONS_00502528"; exon_number "1"; gene_type "intronic_lncRNA";

3L Cufflinks exon 4987586 4987790 . - . gene_id "XLOC_109945"; transcript_id "TCONS_00456159"; exon_number "1"; gene_type "intronic_lncRNA";

3L Cufflinks exon 5285075 5285283 . - . gene_id "XLOC_110162"; transcript_id "TCONS_00428546"; exon_number "1"; gene_type "lincRNA";

3L Cufflinks exon 5388489 5388720 . - . gene_id "XLOC_110305"; transcript_id "TCONS_00428609"; exon_number "1"; gene_type "lincRNA";

3L Cufflinks exon 5641211 5641416 . - . gene_id "XLOC_110585"; transcript_id "TCONS_00456580"; exon_number "1"; gene_type "intronic_lncRNA";

3L Cufflinks exon 5664590 5664791 . - . gene_id "XLOC_110607"; transcript_id "TCONS_00541918"; exon_number "1"; gene_type "intronic_lncRNA";

3L Cufflinks exon 5724294 5724505 . - . gene_id "XLOC_110661"; transcript_id "TCONS_00456613"; exon_number "1"; gene_type "intronic_lncRNA";

3L Cufflinks exon 5735496 5735696 . - . gene_id "XLOC_110668"; transcript_id "TCONS_00481350"; exon_number "1"; gene_type "intronic_lncRNA";

3L Cufflinks exon 5876377 5876583 . - . gene_id "XLOC_110780"; transcript_id "TCONS_00456672"; exon_number "1"; gene_type "lincRNA";

3L Cufflinks exon 5891896 5892104 . - . gene_id "XLOC_110802"; transcript_id "TCONS_00456692"; exon_number "1"; gene_type "lincRNA";

3L Cufflinks exon 6161494 6161717 . - . gene_id "XLOC_111102"; transcript_id "TCONS_00456868"; exon_number "1"; gene_type "anti-sense_lncRNA";

3L Cufflinks exon 6364117 6364322 . - . gene_id "XLOC_111262"; transcript_id "TCONS_00456993"; exon_number "1"; gene_type "lincRNA";

3L Cufflinks exon 6391759 6392007 . - . gene_id "XLOC_111306"; transcript_id "TCONS_00429187"; exon_number "1"; gene_type "lincRNA";

3L Cufflinks exon 6837068 6837271 . - . gene_id "XLOC_111694"; transcript_id "TCONS_00503408"; exon_number "1"; gene_type "lincRNA";

3L Cufflinks exon 6984673 6984877 . - . gene_id "XLOC_111875"; transcript_id "TCONS_00457373"; exon_number "1"; gene_type "lincRNA";

3L Cufflinks exon 7169800 7170011 . - . gene_id "XLOC_112044"; transcript_id "TCONS_00457468"; exon_number "1"; gene_type "intronic_lncRNA";

3L Cufflinks exon 7218584 7218799 . - . gene_id "XLOC_112121"; transcript_id "TCONS_00503590"; exon_number "1"; gene_type "lincRNA";

3L Cufflinks exon 7562262 7562482 . - . gene_id "XLOC_112458"; transcript_id "TCONS_00457721"; exon_number "1"; gene_type "lincRNA";

3L Cufflinks exon 7831846 7832402 . - . gene_id "XLOC_112603"; transcript_id "TCONS_00429962"; exon_number "1"; gene_type "intronic_lncRNA";

3L Cufflinks exon 7864733 7865477 . - . gene_id "XLOC_112603"; transcript_id "TCONS_00457855"; exon_number "1"; gene_type "intronic_lncRNA";

3L Cufflinks exon 7793406 7793693 . - . gene_id "XLOC_112621"; transcript_id "TCONS_00429931"; exon_number "1"; gene_type "intronic_lncRNA";

3L Cufflinks exon 7874660 7874870 . - . gene_id "XLOC_112666"; transcript_id "TCONS_00457856"; exon_number "1"; gene_type "intronic_lncRNA";

3L Cufflinks exon 8226234 8226489 . - . gene_id "XLOC_112870"; transcript_id "TCONS_00457963"; exon_number "1"; gene_type "lincRNA";

3L Cufflinks exon 8280645 8280849 . - . gene_id "XLOC_112938"; transcript_id "TCONS_00457982"; exon_number "1"; gene_type "anti-sense_lncRNA";

3L Scripture exon 8339876 8340171 . - . gene_id "XLOC_112986"; transcript_id "TCONS_00583802"; exon_number "1"; gene_type "anti-sense_lncRNA";

3L Scripture exon 8340347 8340426 . - . gene_id "XLOC_112986"; transcript_id "TCONS_00583802"; exon_number "2"; gene_type "anti-sense_lncRNA";

3L Scripture exon 8339876 8340171 . - . gene_id "XLOC_112986"; transcript_id "TCONS_00563304"; exon_number "1"; gene_type "anti-sense_lncRNA";

3L Scripture exon 8340351 8340426 . - . gene_id "XLOC_112986"; transcript_id "TCONS_00563304"; exon_number "2"; gene_type "anti-sense_lncRNA";

3L Cufflinks exon 8589280 8589498 . - . gene_id "XLOC_113137"; transcript_id "TCONS_00458079"; exon_number "1"; gene_type "intronic_lncRNA";

3L Cufflinks exon 8727960 8728730 . - . gene_id "XLOC_113215"; transcript_id "TCONS_00543099"; exon_number "1"; gene_type "anti-sense_lncRNA";

3L Cufflinks exon 8865721 8865929 . - . gene_id "XLOC_113301"; transcript_id "TCONS_00430351"; exon_number "1"; gene_type "intronic_lncRNA";

3L Cufflinks exon 9036318 9036526 . - . gene_id "XLOC_113388"; transcript_id "TCONS_00504191"; exon_number "1"; gene_type "lincRNA";

3L Scripture exon 9094722 9095113 . - . gene_id "XLOC_113403"; transcript_id "TCONS_00458278"; exon_number "1"; gene_type "anti-sense_lncRNA";

3L Scripture exon 9095192 9095345 . - . gene_id "XLOC_113403"; transcript_id "TCONS_00458278"; exon_number "2"; gene_type "anti-sense_lncRNA";

3L Scripture exon 9094722 9095116 . - . gene_id "XLOC_113403"; transcript_id "TCONS_00458277"; exon_number "1"; gene_type "anti-sense_lncRNA";

3L Scripture exon 9095192 9095345 . - . gene_id "XLOC_113403"; transcript_id "TCONS_00458277"; exon_number "2"; gene_type "anti-sense_lncRNA";

3L Scripture exon 9094722 9095116 . - . gene_id "XLOC_113403"; transcript_id "TCONS_00430449"; exon_number "1"; gene_type "anti-sense_lncRNA";

3L Scripture exon 9095192 9095291 . - . gene_id "XLOC_113403"; transcript_id "TCONS_00430449"; exon_number "2"; gene_type "anti-sense_lncRNA";

3L Scripture exon 9095398 9095473 . - . gene_id "XLOC_113403"; transcript_id "TCONS_00430449"; exon_number "3"; gene_type "anti-sense_lncRNA";

3L Cufflinks exon 9094757 9095113 . - . gene_id "XLOC_113403"; transcript_id "TCONS_00430450"; exon_number "1"; gene_type "anti-sense_lncRNA";

3L Cufflinks exon 9095192 9095291 . - . gene_id "XLOC_113403"; transcript_id "TCONS_00430450"; exon_number "2"; gene_type "anti-sense_lncRNA";

3L Cufflinks exon 9095398 9096255 . - . gene_id "XLOC_113403"; transcript_id "TCONS_00430450"; exon_number "3"; gene_type "anti-sense_lncRNA";

3L Cufflinks exon 9173335 9173542 . - . gene_id "XLOC_113442"; transcript_id "TCONS_00458319"; exon_number "1"; gene_type "intronic_lncRNA";

3L Cufflinks exon 9253919 9254119 . - . gene_id "XLOC_113558"; transcript_id "TCONS_00523258"; exon_number "1"; gene_type "intronic_lncRNA";

3L Cufflinks exon 9289282 9289516 . - . gene_id "XLOC_113590"; transcript_id "TCONS_00482806"; exon_number "1"; gene_type "lincRNA";

3L Cufflinks exon 9290969 9291187 . - . gene_id "XLOC_113593"; transcript_id "TCONS_00458418"; exon_number "1"; gene_type "lincRNA";

3L Cufflinks exon 9339089 9339290 . - . gene_id "XLOC_113679"; transcript_id "TCONS_00482839"; exon_number "1"; gene_type "intronic_lncRNA";

3L Cufflinks exon 9583290 9583527 . - . gene_id "XLOC_113804"; transcript_id "TCONS_00430685"; exon_number "1"; gene_type "intronic_lncRNA";

3L Cufflinks exon 9584155 9584359 . - . gene_id "XLOC_113805"; transcript_id "TCONS_00563655"; exon_number "1"; gene_type "intronic_lncRNA";

3L Cufflinks exon 10048153 10048393 . - . gene_id "XLOC_114084"; transcript_id "TCONS_00458649"; exon_number "1"; gene_type "intronic_lncRNA";

3L Cufflinks exon 10107542 10107764 . - . gene_id "XLOC_114141"; transcript_id "TCONS_00458678"; exon_number "1"; gene_type "intronic_lncRNA";

3L Cufflinks exon 10230317 10230518 . - . gene_id "XLOC_114315"; transcript_id "TCONS_00483121"; exon_number "1"; gene_type "lincRNA";

3L Cufflinks exon 10278305 10278508 . - . gene_id "XLOC_114386"; transcript_id "TCONS_00430999"; exon_number "1"; gene_type "lincRNA";

3L Cufflinks exon 10421489 10421690 . - . gene_id "XLOC_114581"; transcript_id "TCONS_00543696"; exon_number "1"; gene_type "lincRNA";

3L Cufflinks exon 10448359 10448560 . - . gene_id "XLOC_114633"; transcript_id "TCONS_00459014"; exon_number "1"; gene_type "lincRNA";

3L Cufflinks exon 10465390 10465590 . - . gene_id "XLOC_114658"; transcript_id "TCONS_00459029"; exon_number "1"; gene_type "anti-sense_lncRNA";

3L Cufflinks exon 10506731 10506987 . - . gene_id "XLOC_114658"; transcript_id "TCONS_00459029"; exon_number "2"; gene_type "anti-sense_lncRNA";

3L Cufflinks exon 10734992 10735192 . - . gene_id "XLOC_114943"; transcript_id "TCONS_00431261"; exon_number "1"; gene_type "lincRNA";

3L Cufflinks exon 10962313 10962515 . - . gene_id "XLOC_115282"; transcript_id "TCONS_00431422"; exon_number "1"; gene_type "lincRNA";

3L Cufflinks exon 11163852 11164238 . - . gene_id "XLOC_115416"; transcript_id "TCONS_00523822"; exon_number "1"; gene_type "anti-sense_lncRNA";

3L Cufflinks exon 11273467 11273692 . - . gene_id "XLOC_115471"; transcript_id "TCONS_00431572"; exon_number "1"; gene_type "lincRNA";

3L Scripture exon 11349428 11349840 . - . gene_id "XLOC_115569"; transcript_id "TCONS_00505067"; exon_number "1"; gene_type "lincRNA";

3L Scripture exon 11351399 11351478 . - . gene_id "XLOC_115569"; transcript_id "TCONS_00505067"; exon_number "2"; gene_type "lincRNA";

3L Cufflinks exon 11515438 11515638 . - . gene_id "XLOC_115820"; transcript_id "TCONS_00431743"; exon_number "1"; gene_type "intronic_lncRNA";

3L Cufflinks exon 11908790 11908997 . - . gene_id "XLOC_116150"; transcript_id "TCONS_00459920"; exon_number "1"; gene_type "intronic_lncRNA";

3L Cufflinks exon 12111354 12111553 . - . gene_id "XLOC_116307"; transcript_id "TCONS_00460038"; exon_number "1"; gene_type "lincRNA";

3L Cufflinks exon 12132455 12133035 . - . gene_id "XLOC_116328"; transcript_id "TCONS_00432099"; exon_number "1"; gene_type "anti-sense_lncRNA";

3L Cufflinks exon 12133304 12133604 . - . gene_id "XLOC_116328"; transcript_id "TCONS_00432099"; exon_number "2"; gene_type "anti-sense_lncRNA";

3L Cufflinks exon 12299964 12300172 . - . gene_id "XLOC_116420"; transcript_id "TCONS_00484109"; exon_number "1"; gene_type "lincRNA";

3L Cufflinks exon 12333119 12333325 . - . gene_id "XLOC_116471"; transcript_id "TCONS_00460150"; exon_number "1"; gene_type "intronic_lncRNA";

3L Cufflinks exon 12407436 12407720 . - . gene_id "XLOC_116525"; transcript_id "TCONS_00505522"; exon_number "1"; gene_type "intronic_lncRNA";

3L Cufflinks exon 12481201 12481413 . - . gene_id "XLOC_116597"; transcript_id "TCONS_00505567"; exon_number "1"; gene_type "intronic_lncRNA";

3L Cufflinks exon 12575833 12576045 . - . gene_id "XLOC_116619"; transcript_id "TCONS_00432296"; exon_number "1"; gene_type "lincRNA";

3L Cufflinks exon 12806337 12806337 . - . gene_id "XLOC_116859"; transcript_id "TCONS_00505678"; exon_number "1"; gene_type "anti-sense_lncRNA";

3L Cufflinks exon 12806853 12808606 . - . gene_id "XLOC_116859"; transcript_id "TCONS_00505678"; exon_number "2"; gene_type "anti-sense_lncRNA";

3L Cufflinks exon 12848096 12848313 . - . gene_id "XLOC_116867"; transcript_id "TCONS_00432415"; exon_number "1"; gene_type "intronic_lncRNA";

3L Cufflinks exon 13198195 13198406 . - . gene_id "XLOC_117324"; transcript_id "TCONS_00505900"; exon_number "1"; gene_type "lincRNA";

3L Cufflinks exon 13207816 13208023 . - . gene_id "XLOC_117344"; transcript_id "TCONS_00544914"; exon_number "1"; gene_type "lincRNA";

3L Cufflinks exon 13232519 13232720 . - . gene_id "XLOC_117377"; transcript_id "TCONS_00544931"; exon_number "1"; gene_type "intronic_lncRNA";

3L Cufflinks exon 13453239 13453442 . - . gene_id "XLOC_117622"; transcript_id "TCONS_00524644"; exon_number "1"; gene_type "intronic_lncRNA";

3L Cufflinks exon 13500532 13500733 . - . gene_id "XLOC_117650"; transcript_id "TCONS_00484671"; exon_number "1"; gene_type "intronic_lncRNA";

3L Cufflinks exon 13610031 13610248 . - . gene_id "XLOC_117735"; transcript_id "TCONS_00432965"; exon_number "1"; gene_type "intronic_lncRNA";

3L Cufflinks exon 13786414 13786637 . - . gene_id "XLOC_118002"; transcript_id "TCONS_00461261"; exon_number "1"; gene_type "intronic_lncRNA";

3L Cufflinks exon 13944262 13944469 . - . gene_id "XLOC_118104"; transcript_id "TCONS_00524881"; exon_number "1"; gene_type "intronic_lncRNA";

3L Cufflinks exon 13947147 13947376 . - . gene_id "XLOC_118108"; transcript_id "TCONS_00484933"; exon_number "1"; gene_type "intronic_lncRNA";

3L Cufflinks exon 14036723 14036924 . - . gene_id "XLOC_118143"; transcript_id "TCONS_00506348"; exon_number "1"; gene_type "anti-sense_lncRNA";

3L Cufflinks exon 14256901 14257100 . - . gene_id "XLOC_118356"; transcript_id "TCONS_00485049"; exon_number "1"; gene_type "lincRNA";

3L Cufflinks exon 14530977 14531197 . - . gene_id "XLOC_118535"; transcript_id "TCONS_00525028"; exon_number "1"; gene_type "intronic_lncRNA";

3L Cufflinks exon 14570484 14570688 . - . gene_id "XLOC_118567"; transcript_id "TCONS_00461636"; exon_number "1"; gene_type "lincRNA";

3L Cufflinks exon 14613211 14613350 . - . gene_id "XLOC_118627"; transcript_id "TCONS_00461690"; exon_number "1"; gene_type "lincRNA";

3L Cufflinks exon 14613409 14613623 . - . gene_id "XLOC_118627"; transcript_id "TCONS_00461690"; exon_number "2"; gene_type "lincRNA";

3L Cufflinks exon 14684993 14685193 . - . gene_id "XLOC_118666"; transcript_id "TCONS_00506577"; exon_number "1"; gene_type "anti-sense_lncRNA";

3L Cufflinks exon 14839909 14840132 . - . gene_id "XLOC_118740"; transcript_id "TCONS_00461726"; exon_number "1"; gene_type "intronic_lncRNA";

3L Cufflinks exon 14967302 14967543 . - . gene_id "XLOC_118872"; transcript_id "TCONS_00461810"; exon_number "1"; gene_type "lincRNA";

3L Scripture exon 15097715 15098787 . - . gene_id "XLOC_118990"; transcript_id "TCONS_00598661"; exon_number "1"; gene_type "anti-sense_lncRNA";

3L Scripture exon 15098844 15098865 . - . gene_id "XLOC_118990"; transcript_id "TCONS_00598661"; exon_number "2"; gene_type "anti-sense_lncRNA";

3L Scripture exon 15099261 15099340 . - . gene_id "XLOC_118990"; transcript_id "TCONS_00598661"; exon_number "3"; gene_type "anti-sense_lncRNA";

3L Cufflinks exon 15319294 15319497 . - . gene_id "XLOC_119226"; transcript_id "TCONS_00462023"; exon_number "1"; gene_type "lincRNA";

3L Cufflinks exon 15395032 15395238 . - . gene_id "XLOC_119340"; transcript_id "TCONS_00433988"; exon_number "1"; gene_type "lincRNA";

3L Cufflinks exon 15396755 15396973 . - . gene_id "XLOC_119345"; transcript_id "TCONS_00433989"; exon_number "1"; gene_type "lincRNA";

3L Cufflinks exon 15418074 15418273 . - . gene_id "XLOC_119380"; transcript_id "TCONS_00434007"; exon_number "1"; gene_type "lincRNA";

3L Cufflinks exon 15538989 15539387 . - . gene_id "XLOC_119540"; transcript_id "TCONS_00462230"; exon_number "1"; gene_type "intronic_lncRNA";

3L Cufflinks exon 15539486 15539743 . - . gene_id "XLOC_119540"; transcript_id "TCONS_00462230"; exon_number "2"; gene_type "intronic_lncRNA";

3L Cufflinks exon 15624460 15624663 . - . gene_id "XLOC_119575"; transcript_id "TCONS_00525381"; exon_number "1"; gene_type "intronic_lncRNA";

3L Cufflinks exon 15735577 15735786 . - . gene_id "XLOC_119709"; transcript_id "TCONS_00434225"; exon_number "1"; gene_type "lincRNA";

3L Cufflinks exon 16144942 16145218 . - . gene_id "XLOC_120008"; transcript_id "TCONS_00462534"; exon_number "1"; gene_type "intronic_lncRNA";

3L Cufflinks exon 16216211 16216421 . - . gene_id "XLOC_120093"; transcript_id "TCONS_00485868"; exon_number "1"; gene_type "intronic_lncRNA";

3L Cufflinks exon 16530661 16530919 . - . gene_id "XLOC_120328"; transcript_id "TCONS_00462752"; exon_number "1"; gene_type "intronic_lncRNA";

3L Cufflinks exon 16831417 16831620 . - . gene_id "XLOC_120488"; transcript_id "TCONS_00507445"; exon_number "1"; gene_type "lincRNA";

3L Cufflinks exon 16987219 16987421 . - . gene_id "XLOC_120586"; transcript_id "TCONS_00525828"; exon_number "1"; gene_type "intronic_lncRNA";

3L Cufflinks exon 18029613 18029836 . - . gene_id "XLOC_121417"; transcript_id "TCONS_00463438"; exon_number "1"; gene_type "intronic_lncRNA";

3L Cufflinks exon 18250931 18251140 . - . gene_id "XLOC_121666"; transcript_id "TCONS_00463600"; exon_number "1"; gene_type "lincRNA";

3L Cufflinks exon 18347809 18348013 . - . gene_id "XLOC_121829"; transcript_id "TCONS_00463701"; exon_number "1"; gene_type "lincRNA";

3L Cufflinks exon 18952427 18952632 . - . gene_id "XLOC_122417"; transcript_id "TCONS_00508369"; exon_number "1"; gene_type "intronic_lncRNA";

3L Cufflinks exon 19221348 19221549 . - . gene_id "XLOC_122645"; transcript_id "TCONS_00487287"; exon_number "1"; gene_type "intronic_lncRNA";

3L Cufflinks exon 19394600 19394799 . - . gene_id "XLOC_122819"; transcript_id "TCONS_00464424"; exon_number "1"; gene_type "intronic_lncRNA";

3L Cufflinks exon 19511172 19511378 . - . gene_id "XLOC_122872"; transcript_id "TCONS_00436307"; exon_number "1"; gene_type "lincRNA";

3L Cufflinks exon 19580593 19580794 . - . gene_id "XLOC_122949"; transcript_id "TCONS_00436355"; exon_number "1"; gene_type "intronic_lncRNA";

3L Scripture exon 20023953 20024059 . - . gene_id "XLOC_123129"; transcript_id "TCONS_00614000"; exon_number "1"; gene_type "anti-sense_lncRNA";

3L Scripture exon 20024124 20024287 . - . gene_id "XLOC_123129"; transcript_id "TCONS_00614000"; exon_number "2"; gene_type "anti-sense_lncRNA";

3L Cufflinks exon 20091955 20092159 . - . gene_id "XLOC_123216"; transcript_id "TCONS_00526951"; exon_number "1"; gene_type "lincRNA";

3L Scripture exon 20315398 20315538 . - . gene_id "XLOC_123324"; transcript_id "TCONS_00599310"; exon_number "1"; gene_type "anti-sense_lncRNA";

3L Scripture exon 20316891 20317416 . - . gene_id "XLOC_123324"; transcript_id "TCONS_00599310"; exon_number "2"; gene_type "anti-sense_lncRNA";

3L Scripture exon 20390268 20390435 . - . gene_id "XLOC_123339"; transcript_id "TCONS_00607702"; exon_number "1"; gene_type "anti-sense_lncRNA";

3L Scripture exon 20390500 20390634 . - . gene_id "XLOC_123339"; transcript_id "TCONS_00607702"; exon_number "2"; gene_type "anti-sense_lncRNA";

3L Cufflinks exon 20415172 20415375 . - . gene_id "XLOC_123350"; transcript_id "TCONS_00436690"; exon_number "1"; gene_type "intronic_lncRNA";

3L Cufflinks exon 20442187 20442386 . - . gene_id "XLOC_123365"; transcript_id "TCONS_00464789"; exon_number "1"; gene_type "lincRNA";

3L Cufflinks exon 20740589 20740811 . - . gene_id "XLOC_123636"; transcript_id "TCONS_00548007"; exon_number "1"; gene_type "intronic_lncRNA";

3L Cufflinks exon 20795174 20795369 . - . gene_id "XLOC_123676"; transcript_id "TCONS_00487912"; exon_number "1"; gene_type "lincRNA";

3L Cufflinks exon 20795452 20795521 . - . gene_id "XLOC_123676"; transcript_id "TCONS_00487912"; exon_number "2"; gene_type "lincRNA";

3L Cufflinks exon 20853695 20853902 . - . gene_id "XLOC_123692"; transcript_id "TCONS_00527173"; exon_number "1"; gene_type "intronic_lncRNA";

3L Cufflinks exon 20921382 20921479 . - . gene_id "XLOC_123796"; transcript_id "TCONS_00437045"; exon_number "1"; gene_type "lincRNA";

3L Cufflinks exon 20921569 20921806 . - . gene_id "XLOC_123796"; transcript_id "TCONS_00437045"; exon_number "2"; gene_type "lincRNA";

3L Cufflinks exon 21238232 21238436 . - . gene_id "XLOC_124031"; transcript_id "TCONS_00465277"; exon_number "1"; gene_type "intronic_lncRNA";

3L Cufflinks exon 21401727 21401950 . - . gene_id "XLOC_124135"; transcript_id "TCONS_00568311"; exon_number "1"; gene_type "intronic_lncRNA";

3L Cufflinks exon 21407317 21407522 . - . gene_id "XLOC_124142"; transcript_id "TCONS_00437271"; exon_number "1"; gene_type "intronic_lncRNA";

3L Cufflinks exon 21602427 21602629 . - . gene_id "XLOC_124275"; transcript_id "TCONS_00465445"; exon_number "1"; gene_type "intronic_lncRNA";

3L Cufflinks exon 22011182 22011383 . - . gene_id "XLOC_124621"; transcript_id "TCONS_00509535"; exon_number "1"; gene_type "lincRNA";

3L Cufflinks exon 22197349 22197550 . - . gene_id "XLOC_124833"; transcript_id "TCONS_00509639"; exon_number "1"; gene_type "intronic_lncRNA";

3L Cufflinks exon 22924365 22924573 . - . gene_id "XLOC_125307"; transcript_id "TCONS_00438081"; exon_number "1"; gene_type "lincRNA";

3L Cufflinks exon 22926021 22926223 . - . gene_id "XLOC_125308"; transcript_id "TCONS_00438083"; exon_number "1"; gene_type "lincRNA";

3L Cufflinks exon 22982598 22982809 . - . gene_id "XLOC_125377"; transcript_id "TCONS_00509998"; exon_number "1"; gene_type "lincRNA";

3L Cufflinks exon 23010149 23010372 . - . gene_id "XLOC_125402"; transcript_id "TCONS_00488931"; exon_number "1"; gene_type "intronic_lncRNA";

3L Cufflinks exon 23189022 23189237 . - . gene_id "XLOC_125517"; transcript_id "TCONS_00489013"; exon_number "1"; gene_type "lincRNA";

3L Cufflinks exon 24144969 24145201 . - . gene_id "XLOC_126021"; transcript_id "TCONS_00438557"; exon_number "1"; gene_type "intronic_lncRNA";

3L Cufflinks exon 24674693 24674912 . - . gene_id "XLOC_126259"; transcript_id "TCONS_00510490"; exon_number "1"; gene_type "lincRNA";

3L Cufflinks exon 24911602 24911816 . - . gene_id "XLOC_126404"; transcript_id "TCONS_00510600"; exon_number "1"; gene_type "lincRNA";

3L Cufflinks exon 25022431 25022641 . - . gene_id "XLOC_126464"; transcript_id "TCONS_00438855"; exon_number "1"; gene_type "lincRNA";

3L Scripture exon 25181118 25182156 . - . gene_id "XLOC_126532"; transcript_id "TCONS_00599692"; exon_number "1"; gene_type "lincRNA";

3L Cufflinks exon 25282147 25282390 . - . gene_id "XLOC_126599"; transcript_id "TCONS_00489666"; exon_number "1"; gene_type "intronic_lncRNA";

3L Cufflinks exon 25385773 25386014 . - . gene_id "XLOC_126656"; transcript_id "TCONS_00439008"; exon_number "1"; gene_type "intronic_lncRNA";

3L Cufflinks exon 25610806 25611144 . - . gene_id "XLOC_126816"; transcript_id "TCONS_00439055"; exon_number "1"; gene_type "intronic_lncRNA";

3L Cufflinks exon 25626779 25627376 . - . gene_id "XLOC_126816"; transcript_id "TCONS_00439055"; exon_number "2"; gene_type "intronic_lncRNA";

3L Cufflinks exon 25665366 25665585 . - . gene_id "XLOC_126868"; transcript_id "TCONS_00439069"; exon_number "1"; gene_type "intronic_lncRNA";

3L Cufflinks exon 26160812 26163035 . - . gene_id "XLOC_127001"; transcript_id "TCONS_00439186"; exon_number "1"; gene_type "lincRNA";

3L Cufflinks exon 26163515 26163603 . - . gene_id "XLOC_127001"; transcript_id "TCONS_00439186"; exon_number "2"; gene_type "lincRNA";

3L Cufflinks exon 26245170 26245390 . - . gene_id "XLOC_127034"; transcript_id "TCONS_00549970"; exon_number "1"; gene_type "lincRNA";

3L Cufflinks exon 27091336 27091920 . - . gene_id "XLOC_127353"; transcript_id "TCONS_00439399"; exon_number "1"; gene_type "lincRNA";

3L Cufflinks exon 27091976 27092347 . - . gene_id "XLOC_127353"; transcript_id "TCONS_00439399"; exon_number "2"; gene_type "lincRNA";

3L Cufflinks exon 27680951 27681161 . - . gene_id "XLOC_127510"; transcript_id "TCONS_00511053"; exon_number "1"; gene_type "lincRNA";

3R Cufflinks exon 451809 452082 . + . gene_id "XLOC_132147"; transcript_id "TCONS_00705485"; exon_number "1"; gene_type "lincRNA";

3R Cufflinks exon 727338 730181 . + . gene_id "XLOC_132298"; transcript_id "TCONS_00637545"; exon_number "1"; gene_type "intronic_lncRNA";

3R Cufflinks exon 972753 972956 . + . gene_id "XLOC_132501"; transcript_id "TCONS_00705783"; exon_number "1"; gene_type "intronic_lncRNA";

3R Cufflinks exon 1058120 1058341 . + . gene_id "XLOC_132558"; transcript_id "TCONS_00807262"; exon_number "1"; gene_type "intronic_lncRNA";

3R Cufflinks exon 1059989 1060192 . + . gene_id "XLOC_132562"; transcript_id "TCONS_00759563"; exon_number "1"; gene_type "intronic_lncRNA";

3R Cufflinks exon 1210666 1210879 . + . gene_id "XLOC_132683"; transcript_id "TCONS_00705935"; exon_number "1"; gene_type "intronic_lncRNA";

3R Cufflinks exon 2211032 2211255 . + . gene_id "XLOC_133399"; transcript_id "TCONS_00706539"; exon_number "1"; gene_type "intronic_lncRNA";

3R Cufflinks exon 3118211 3118277 . + . gene_id "XLOC_134140"; transcript_id "TCONS_00672734"; exon_number "1"; gene_type "intronic_lncRNA";

3R Cufflinks exon 3119282 3119906 . + . gene_id "XLOC_134140"; transcript_id "TCONS_00672734"; exon_number "2"; gene_type "intronic_lncRNA";

3R Cufflinks exon 3118220 3118277 . + . gene_id "XLOC_134140"; transcript_id "TCONS_00760330"; exon_number "1"; gene_type "intronic_lncRNA";

3R Cufflinks exon 3119056 3119389 . + . gene_id "XLOC_134140"; transcript_id "TCONS_00760330"; exon_number "2"; gene_type "intronic_lncRNA";

3R Cufflinks exon 3119456 3119782 . + . gene_id "XLOC_134140"; transcript_id "TCONS_00760330"; exon_number "3"; gene_type "intronic_lncRNA";

3R Cufflinks exon 3118240 3118277 . + . gene_id "XLOC_134140"; transcript_id "TCONS_00638919"; exon_number "1"; gene_type "intronic_lncRNA";

3R Cufflinks exon 3119456 3119906 . + . gene_id "XLOC_134140"; transcript_id "TCONS_00638919"; exon_number "2"; gene_type "intronic_lncRNA";

3R Cufflinks exon 3118254 3118277 . + . gene_id "XLOC_134140"; transcript_id "TCONS_00638918"; exon_number "1"; gene_type "intronic_lncRNA";

3R Cufflinks exon 3119056 3119906 . + . gene_id "XLOC_134140"; transcript_id "TCONS_00638918"; exon_number "2"; gene_type "intronic_lncRNA";

3R Cufflinks exon 3199214 3199418 . + . gene_id "XLOC_134207"; transcript_id "TCONS_00672769"; exon_number "1"; gene_type "intronic_lncRNA";

3R Cufflinks exon 3321351 3321551 . + . gene_id "XLOC_134305"; transcript_id "TCONS_00760385"; exon_number "1"; gene_type "lincRNA";

3R Cufflinks exon 3635607 3635808 . + . gene_id "XLOC_134412"; transcript_id "TCONS_00706969"; exon_number "1"; gene_type "lincRNA";

3R Cufflinks exon 3845738 3845813 . + . gene_id "XLOC_134471"; transcript_id "TCONS_00707053"; exon_number "1"; gene_type "lincRNA";

3R Cufflinks exon 3845880 3846149 . + . gene_id "XLOC_134471"; transcript_id "TCONS_00707053"; exon_number "2"; gene_type "lincRNA";

3R Cufflinks exon 4162527 4162731 . + . gene_id "XLOC_134674"; transcript_id "TCONS_00639341"; exon_number "1"; gene_type "lincRNA";

3R Cufflinks exon 4189350 4189444 . + . gene_id "XLOC_134692"; transcript_id "TCONS_00639369"; exon_number "1"; gene_type "anti-sense_lncRNA";

3R Cufflinks exon 4189525 4189692 . + . gene_id "XLOC_134692"; transcript_id "TCONS_00639369"; exon_number "2"; gene_type "anti-sense_lncRNA";

3R Cufflinks exon 4229932 4230076 . + . gene_id "XLOC_134703"; transcript_id "TCONS_00782450"; exon_number "1"; gene_type "lincRNA";

3R Cufflinks exon 4230132 4230186 . + . gene_id "XLOC_134703"; transcript_id "TCONS_00782450"; exon_number "2"; gene_type "lincRNA";

3R Cufflinks exon 4375138 4377695 . + . gene_id "XLOC_134762"; transcript_id "TCONS_00639454"; exon_number "1"; gene_type "lincRNA";

3R Scripture exon 4393828 4395728 . + . gene_id "XLOC_134764"; transcript_id "TCONS_00857551"; exon_number "1"; gene_type "anti-sense_lncRNA";

3R Scripture exon 4395890 4396036 . + . gene_id "XLOC_134764"; transcript_id "TCONS_00857551"; exon_number "2"; gene_type "anti-sense_lncRNA";

3R Scripture exon 4396118 4396303 . + . gene_id "XLOC_134764"; transcript_id "TCONS_00857551"; exon_number "3"; gene_type "anti-sense_lncRNA";

3R Scripture exon 4396380 4396828 . + . gene_id "XLOC_134764"; transcript_id "TCONS_00857551"; exon_number "4"; gene_type "anti-sense_lncRNA";

3R Cufflinks exon 4575552 4575752 . + . gene_id "XLOC_134897"; transcript_id "TCONS_00639546"; exon_number "1"; gene_type "lincRNA";

3R Cufflinks exon 4616216 4616438 . + . gene_id "XLOC_134965"; transcript_id "TCONS_00782582"; exon_number "1"; gene_type "intronic_lncRNA";

3R Cufflinks exon 4866350 4866554 . + . gene_id "XLOC_135104"; transcript_id "TCONS_00782664"; exon_number "1"; gene_type "intronic_lncRNA";

3R Cufflinks exon 5014899 5015971 . + . gene_id "XLOC_135203"; transcript_id "TCONS_00673476"; exon_number "1"; gene_type "lincRNA";

3R Cufflinks exon 5021070 5021077 . + . gene_id "XLOC_135203"; transcript_id "TCONS_00673476"; exon_number "2"; gene_type "lincRNA";

3R Cufflinks exon 5074608 5074812 . + . gene_id "XLOC_135254"; transcript_id "TCONS_00639804"; exon_number "1"; gene_type "lincRNA";

3R Cufflinks exon 5103368 5103588 . + . gene_id "XLOC_135280"; transcript_id "TCONS_00673545"; exon_number "1"; gene_type "lincRNA";

3R Scripture exon 5463731 5463872 . + . gene_id "XLOC_135503"; transcript_id "TCONS_00857753"; exon_number "1"; gene_type "anti-sense_lncRNA";

3R Scripture exon 5465182 5465618 . + . gene_id "XLOC_135503"; transcript_id "TCONS_00857753"; exon_number "2"; gene_type "anti-sense_lncRNA";

3R Scripture exon 5465682 5465731 . + . gene_id "XLOC_135503"; transcript_id "TCONS_00857753"; exon_number "3"; gene_type "anti-sense_lncRNA";

3R Scripture exon 5599280 5599414 . + . gene_id "XLOC_135547"; transcript_id "TCONS_00890910"; exon_number "1"; gene_type "anti-sense_lncRNA";

3R Scripture exon 5599488 5600157 . + . gene_id "XLOC_135547"; transcript_id "TCONS_00890910"; exon_number "2"; gene_type "anti-sense_lncRNA";

3R Cufflinks exon 5931507 5931733 . + . gene_id "XLOC_135733"; transcript_id "TCONS_00673876"; exon_number "1"; gene_type "intronic_lncRNA";

3R Cufflinks exon 6103252 6103464 . + . gene_id "XLOC_135935"; transcript_id "TCONS_00708009"; exon_number "1"; gene_type "lincRNA";

3R Cufflinks exon 6967593 6967796 . + . gene_id "XLOC_136812"; transcript_id "TCONS_00640905"; exon_number "1"; gene_type "intronic_lncRNA";

3R Cufflinks exon 7158110 7158331 . + . gene_id "XLOC_136969"; transcript_id "TCONS_00809841"; exon_number "1"; gene_type "lincRNA";

3R Cufflinks exon 7623614 7623834 . + . gene_id "XLOC_137512"; transcript_id "TCONS_00675115"; exon_number "1"; gene_type "intronic_lncRNA";

3R Cufflinks exon 7680393 7680593 . + . gene_id "XLOC_137582"; transcript_id "TCONS_00761984"; exon_number "1"; gene_type "intronic_lncRNA";

3R Cufflinks exon 7690391 7690593 . + . gene_id "XLOC_137597"; transcript_id "TCONS_00641379"; exon_number "1"; gene_type "intronic_lncRNA";

3R Cufflinks exon 7992529 7992729 . + . gene_id "XLOC_137771"; transcript_id "TCONS_00762103"; exon_number "1"; gene_type "intronic_lncRNA";

3R Cufflinks exon 8211237 8211460 . + . gene_id "XLOC_137916"; transcript_id "TCONS_00784249"; exon_number "1"; gene_type "lincRNA";

3R Cufflinks exon 8478410 8478612 . + . gene_id "XLOC_138115"; transcript_id "TCONS_00810451"; exon_number "1"; gene_type "lincRNA";

3R Cufflinks exon 8918598 8918797 . + . gene_id "XLOC_138409"; transcript_id "TCONS_00709385"; exon_number "1"; gene_type "intronic_lncRNA";

3R Cufflinks exon 8929072 8929275 . + . gene_id "XLOC_138415"; transcript_id "TCONS_00709387"; exon_number "1"; gene_type "intronic_lncRNA";

3R Cufflinks exon 9147309 9147542 . + . gene_id "XLOC_138531"; transcript_id "TCONS_00709431"; exon_number "1"; gene_type "intronic_lncRNA";

3R Cufflinks exon 9378810 9379022 . + . gene_id "XLOC_138636"; transcript_id "TCONS_00642142"; exon_number "1"; gene_type "intronic_lncRNA";

3R Cufflinks exon 9833369 9833581 . + . gene_id "XLOC_138829"; transcript_id "TCONS_00709544"; exon_number "1"; gene_type "intronic_lncRNA";

3R Cufflinks exon 9885682 9885895 . + . gene_id "XLOC_138870"; transcript_id "TCONS_00676087"; exon_number "1"; gene_type "intronic_lncRNA";

3R Cufflinks exon 9887464 9887670 . + . gene_id "XLOC_138871"; transcript_id "TCONS_00784762"; exon_number "1"; gene_type "intronic_lncRNA";

3R Cufflinks exon 9913099 9913365 . + . gene_id "XLOC_138887"; transcript_id "TCONS_00642328"; exon_number "1"; gene_type "intronic_lncRNA";

3R Cufflinks exon 10564654 10564890 . + . gene_id "XLOC_139271"; transcript_id "TCONS_00737557"; exon_number "1"; gene_type "intronic_lncRNA";

3R Cufflinks exon 10681371 10681663 . + . gene_id "XLOC_139404"; transcript_id "TCONS_00676392"; exon_number "1"; gene_type "lincRNA";

3R Cufflinks exon 10683623 10684103 . + . gene_id "XLOC_139404"; transcript_id "TCONS_00676392"; exon_number "2"; gene_type "lincRNA";

3R Cufflinks exon 10757266 10758280 . + . gene_id "XLOC_139483"; transcript_id "TCONS_00642725"; exon_number "1"; gene_type "anti-sense_lncRNA";

3R Cufflinks exon 10825142 10825385 . + . gene_id "XLOC_139530"; transcript_id "TCONS_00676474"; exon_number "1"; gene_type "lincRNA";

3R Scripture exon 10859595 10860734 . + . gene_id "XLOC_139557"; transcript_id "TCONS_00869960"; exon_number "1"; gene_type "anti-sense_lncRNA";

3R Scripture exon 10860804 10861044 . + . gene_id "XLOC_139557"; transcript_id "TCONS_00869960"; exon_number "2"; gene_type "anti-sense_lncRNA";

3R Cufflinks exon 10902459 10902680 . + . gene_id "XLOC_139576"; transcript_id "TCONS_00676500"; exon_number "1"; gene_type "lincRNA";

3R Cufflinks exon 10936661 10936870 . + . gene_id "XLOC_139631"; transcript_id "TCONS_00737713"; exon_number "1"; gene_type "lincRNA";

3R Cufflinks exon 11075274 11075473 . + . gene_id "XLOC_139781"; transcript_id "TCONS_00676686"; exon_number "1"; gene_type "lincRNA";

3R Cufflinks exon 11280596 11280821 . + . gene_id "XLOC_139999"; transcript_id "TCONS_00710260"; exon_number "1"; gene_type "lincRNA";

3R Cufflinks exon 11477782 11478009 . + . gene_id "XLOC_140178"; transcript_id "TCONS_00710385"; exon_number "1"; gene_type "lincRNA";

3R Scripture exon 11637070 11637738 . + . gene_id "XLOC_140295"; transcript_id "TCONS_00858908"; exon_number "1"; gene_type "anti-sense_lncRNA";

3R Scripture exon 11637799 11637936 . + . gene_id "XLOC_140295"; transcript_id "TCONS_00858908"; exon_number "2"; gene_type "anti-sense_lncRNA";

3R Scripture exon 11638015 11638552 . + . gene_id "XLOC_140295"; transcript_id "TCONS_00858908"; exon_number "3"; gene_type "anti-sense_lncRNA";

3R Cufflinks exon 11691413 11691525 . + . gene_id "XLOC_140306"; transcript_id "TCONS_00677078"; exon_number "1"; gene_type "anti-sense_lncRNA";

3R Cufflinks exon 11691620 11693372 . + . gene_id "XLOC_140306"; transcript_id "TCONS_00677078"; exon_number "2"; gene_type "anti-sense_lncRNA";

3R Cufflinks exon 12619328 12619527 . + . gene_id "XLOC_140999"; transcript_id "TCONS_00738492"; exon_number "1"; gene_type "lincRNA";

3R Cufflinks exon 12777074 12777285 . + . gene_id "XLOC_141129"; transcript_id "TCONS_00738573"; exon_number "1"; gene_type "intronic_lncRNA";

3R Cufflinks exon 12876314 12876513 . + . gene_id "XLOC_141288"; transcript_id "TCONS_00738632"; exon_number "1"; gene_type "lincRNA";

3R Cufflinks exon 13376909 13377805 . + . gene_id "XLOC_141653"; transcript_id "TCONS_00644278"; exon_number "1"; gene_type "anti-sense_lncRNA";

3R Cufflinks exon 13524193 13524418 . + . gene_id "XLOC_141831"; transcript_id "TCONS_00644373"; exon_number "1"; gene_type "lincRNA";

3R Cufflinks exon 13596847 13597060 . + . gene_id "XLOC_141945"; transcript_id "TCONS_00812283"; exon_number "1"; gene_type "lincRNA";

3R Cufflinks exon 13604966 13605165 . + . gene_id "XLOC_141957"; transcript_id "TCONS_00763884"; exon_number "1"; gene_type "lincRNA";

3R Cufflinks exon 13721972 13722205 . + . gene_id "XLOC_142035"; transcript_id "TCONS_00786405"; exon_number "1"; gene_type "intronic_lncRNA";

3R Cufflinks exon 13737623 13737868 . + . gene_id "XLOC_142055"; transcript_id "TCONS_00678240"; exon_number "1"; gene_type "intronic_lncRNA";

3R Cufflinks exon 13844025 13844229 . + . gene_id "XLOC_142171"; transcript_id "TCONS_00644576"; exon_number "1"; gene_type "lincRNA";

3R Cufflinks exon 13880677 13880884 . + . gene_id "XLOC_142235"; transcript_id "TCONS_00644612"; exon_number "1"; gene_type "lincRNA";

3R Cufflinks exon 13909718 13909917 . + . gene_id "XLOC_142295"; transcript_id "TCONS_00678382"; exon_number "1"; gene_type "lincRNA";

3R Cufflinks exon 14230495 14230698 . + . gene_id "XLOC_142597"; transcript_id "TCONS_00764093"; exon_number "1"; gene_type "lincRNA";

3R Cufflinks exon 14257964 14258171 . + . gene_id "XLOC_142613"; transcript_id "TCONS_00644833"; exon_number "1"; gene_type "intronic_lncRNA";

3R Cufflinks exon 14405585 14405799 . + . gene_id "XLOC_142700"; transcript_id "TCONS_00739236"; exon_number "1"; gene_type "intronic_lncRNA";

3R Cufflinks exon 14568867 14568995 . + . gene_id "XLOC_142856"; transcript_id "TCONS_00644952"; exon_number "1"; gene_type "anti-sense_lncRNA";

3R Cufflinks exon 14569148 14569924 . + . gene_id "XLOC_142856"; transcript_id "TCONS_00644952"; exon_number "2"; gene_type "anti-sense_lncRNA";

3R Cufflinks exon 14568974 14569013 . + . gene_id "XLOC_142856"; transcript_id "TCONS_00678700"; exon_number "1"; gene_type "anti-sense_lncRNA";

3R Cufflinks exon 14569148 14569994 . + . gene_id "XLOC_142856"; transcript_id "TCONS_00678700"; exon_number "2"; gene_type "anti-sense_lncRNA";

3R Cufflinks exon 14587154 14587353 . + . gene_id "XLOC_142872"; transcript_id "TCONS_00644967"; exon_number "1"; gene_type "intronic_lncRNA";

3R Cufflinks exon 14599488 14599716 . + . gene_id "XLOC_142888"; transcript_id "TCONS_00711789"; exon_number "1"; gene_type "intronic_lncRNA";

3R Cufflinks exon 14676122 14676330 . + . gene_id "XLOC_142916"; transcript_id "TCONS_00645002"; exon_number "1"; gene_type "intronic_lncRNA";

3R Cufflinks exon 14694882 14695110 . + . gene_id "XLOC_142928"; transcript_id "TCONS_00678739"; exon_number "1"; gene_type "intronic_lncRNA";

3R Cufflinks exon 14718275 14718482 . + . gene_id "XLOC_142945"; transcript_id "TCONS_00678745"; exon_number "1"; gene_type "intronic_lncRNA";

3R Cufflinks exon 14860533 14860742 . + . gene_id "XLOC_142997"; transcript_id "TCONS_00786807"; exon_number "1"; gene_type "intronic_lncRNA";

3R Cufflinks exon 14912881 14913128 . + . gene_id "XLOC_143049"; transcript_id "TCONS_00645064"; exon_number "1"; gene_type "lincRNA";

3R Cufflinks exon 15009845 15010060 . + . gene_id "XLOC_143201"; transcript_id "TCONS_00711942"; exon_number "1"; gene_type "lincRNA";

3R Cufflinks exon 15023429 15023637 . + . gene_id "XLOC_143219"; transcript_id "TCONS_00812816"; exon_number "1"; gene_type "lincRNA";

3R Cufflinks exon 15118233 15118442 . + . gene_id "XLOC_143287"; transcript_id "TCONS_00645194"; exon_number "1"; gene_type "intronic_lncRNA";

3R Cufflinks exon 15130884 15131429 . + . gene_id "XLOC_143294"; transcript_id "TCONS_00678952"; exon_number "1"; gene_type "intronic_lncRNA";

3R Cufflinks exon 15157150 15157382 . + . gene_id "XLOC_143314"; transcript_id "TCONS_00711996"; exon_number "1"; gene_type "intronic_lncRNA";

3R Cufflinks exon 15700175 15700380 . + . gene_id "XLOC_143690"; transcript_id "TCONS_00645537"; exon_number "1"; gene_type "lincRNA";

3R Cufflinks exon 15701170 15701495 . + . gene_id "XLOC_143690"; transcript_id "TCONS_00645537"; exon_number "2"; gene_type "lincRNA";

3R Cufflinks exon 15724544 15724751 . + . gene_id "XLOC_143721"; transcript_id "TCONS_00679293"; exon_number "1"; gene_type "lincRNA";

3R Cufflinks exon 15726114 15726315 . + . gene_id "XLOC_143723"; transcript_id "TCONS_00679297"; exon_number "1"; gene_type "lincRNA";

3R Cufflinks exon 15876259 15876481 . + . gene_id "XLOC_143848"; transcript_id "TCONS_00679363"; exon_number "1"; gene_type "lincRNA";

3R Cufflinks exon 16078103 16078311 . + . gene_id "XLOC_143946"; transcript_id "TCONS_00645734"; exon_number "1"; gene_type "intronic_lncRNA";

3R Cufflinks exon 16161332 16161554 . + . gene_id "XLOC_144031"; transcript_id "TCONS_00645792"; exon_number "1"; gene_type "intronic_lncRNA";

3R Scripture exon 16352089 16352345 . + . gene_id "XLOC_144119"; transcript_id "TCONS_00879946"; exon_number "1"; gene_type "anti-sense_lncRNA";

3R Scripture exon 16356796 16357113 . + . gene_id "XLOC_144119"; transcript_id "TCONS_00879946"; exon_number "2"; gene_type "anti-sense_lncRNA";

3R Cufflinks exon 16384595 16384822 . + . gene_id "XLOC_144154"; transcript_id "TCONS_00645896"; exon_number "1"; gene_type "intronic_lncRNA";

3R Cufflinks exon 16413456 16413656 . + . gene_id "XLOC_144194"; transcript_id "TCONS_00787481"; exon_number "1"; gene_type "lincRNA";

3R Scripture exon 16428264 16429179 . + . gene_id "XLOC_144220"; transcript_id "TCONS_00645936"; exon_number "1"; gene_type "lincRNA";

3R Cufflinks exon 16523453 16523652 . + . gene_id "XLOC_144283"; transcript_id "TCONS_00764808"; exon_number "1"; gene_type "intronic_lncRNA";

3R Cufflinks exon 16797719 16797919 . + . gene_id "XLOC_144545"; transcript_id "TCONS_00712741"; exon_number "1"; gene_type "lincRNA";

3R Cufflinks exon 17228315 17228515 . + . gene_id "XLOC_144948"; transcript_id "TCONS_00765046"; exon_number "1"; gene_type "lincRNA";

3R Cufflinks exon 17342075 17342283 . + . gene_id "XLOC_145108"; transcript_id "TCONS_00646533"; exon_number "1"; gene_type "intronic_lncRNA";

3R Cufflinks exon 17827938 17828162 . + . gene_id "XLOC_145537"; transcript_id "TCONS_00646783"; exon_number "1"; gene_type "intronic_lncRNA";

3R Cufflinks exon 18065143 18065342 . + . gene_id "XLOC_145820"; transcript_id "TCONS_00646931"; exon_number "1"; gene_type "lincRNA";

3R Cufflinks exon 18085387 18085644 . + . gene_id "XLOC_145860"; transcript_id "TCONS_00765336"; exon_number "1"; gene_type "lincRNA";

3R Cufflinks exon 18233020 18233225 . + . gene_id "XLOC_146029"; transcript_id "TCONS_00740879"; exon_number "1"; gene_type "lincRNA";

3R Cufflinks exon 18311542 18311744 . + . gene_id "XLOC_146074"; transcript_id "TCONS_00647076"; exon_number "1"; gene_type "intronic_lncRNA";

3R Cufflinks exon 18343673 18343904 . + . gene_id "XLOC_146118"; transcript_id "TCONS_00740907"; exon_number "1"; gene_type "intronic_lncRNA";

3R Cufflinks exon 18436094 18436304 . + . gene_id "XLOC_146172"; transcript_id "TCONS_00713482"; exon_number "1"; gene_type "intronic_lncRNA";

3R Cufflinks exon 18545920 18546132 . + . gene_id "XLOC_146295"; transcript_id "TCONS_00740943"; exon_number "1"; gene_type "lincRNA";

3R Scripture exon 18760699 18760905 . + . gene_id "XLOC_146407"; transcript_id "TCONS_00859929"; exon_number "1"; gene_type "anti-sense_lncRNA";

3R Scripture exon 18760969 18761154 . + . gene_id "XLOC_146407"; transcript_id "TCONS_00859929"; exon_number "2"; gene_type "anti-sense_lncRNA";

3R Cufflinks exon 18865632 18865848 . + . gene_id "XLOC_146486"; transcript_id "TCONS_00681099"; exon_number "1"; gene_type "lincRNA";

3R Cufflinks exon 19278161 19278378 . + . gene_id "XLOC_146759"; transcript_id "TCONS_00741182"; exon_number "1"; gene_type "lincRNA";

3R Cufflinks exon 19345218 19345425 . + . gene_id "XLOC_146848"; transcript_id "TCONS_00647580"; exon_number "1"; gene_type "lincRNA";

3R Cufflinks exon 19722619 19722841 . + . gene_id "XLOC_146959"; transcript_id "TCONS_00765862"; exon_number "1"; gene_type "lincRNA";

3R Cufflinks exon 19792925 19793141 . + . gene_id "XLOC_147034"; transcript_id "TCONS_00647734"; exon_number "1"; gene_type "lincRNA";

3R Cufflinks exon 20188731 20188931 . + . gene_id "XLOC_147368"; transcript_id "TCONS_00714110"; exon_number "1"; gene_type "lincRNA";

3R Cufflinks exon 20519560 20519783 . + . gene_id "XLOC_147669"; transcript_id "TCONS_00681889"; exon_number "1"; gene_type "lincRNA";

3R Scripture exon 20574162 20574372 . + . gene_id "XLOC_147703"; transcript_id "TCONS_00766163"; exon_number "1"; gene_type "anti-sense_lncRNA";

3R Scripture exon 20574629 20575826 . + . gene_id "XLOC_147703"; transcript_id "TCONS_00766163"; exon_number "2"; gene_type "anti-sense_lncRNA";

3R Scripture exon 20585137 20585284 . + . gene_id "XLOC_147708"; transcript_id "TCONS_00871170"; exon_number "1"; gene_type "anti-sense_lncRNA";

3R Scripture exon 20585781 20585875 . + . gene_id "XLOC_147708"; transcript_id "TCONS_00871170"; exon_number "2"; gene_type "anti-sense_lncRNA";

3R Cufflinks exon 20712742 20712943 . + . gene_id "XLOC_147780"; transcript_id "TCONS_00814917"; exon_number "1"; gene_type "intronic_lncRNA";

3R Scripture exon 20827025 20827338 . + . gene_id "XLOC_147861"; transcript_id "TCONS_00860314"; exon_number "1"; gene_type "anti-sense_lncRNA";

3R Scripture exon 20827926 20830690 . + . gene_id "XLOC_147861"; transcript_id "TCONS_00860314"; exon_number "2"; gene_type "anti-sense_lncRNA";

3R Scripture exon 20911449 20911538 . + . gene_id "XLOC_147932"; transcript_id "TCONS_00682061"; exon_number "1"; gene_type "anti-sense_lncRNA";

3R Scripture exon 20912124 20912345 . + . gene_id "XLOC_147932"; transcript_id "TCONS_00682061"; exon_number "2"; gene_type "anti-sense_lncRNA";

3R Scripture exon 20912422 20913139 . + . gene_id "XLOC_147932"; transcript_id "TCONS_00682061"; exon_number "3"; gene_type "anti-sense_lncRNA";

3R Scripture exon 20911478 20911709 . + . gene_id "XLOC_147932"; transcript_id "TCONS_00741762"; exon_number "1"; gene_type "anti-sense_lncRNA";

3R Scripture exon 20911771 20912345 . + . gene_id "XLOC_147932"; transcript_id "TCONS_00741762"; exon_number "2"; gene_type "anti-sense_lncRNA";

3R Scripture exon 20912422 20913081 . + . gene_id "XLOC_147932"; transcript_id "TCONS_00741762"; exon_number "3"; gene_type "anti-sense_lncRNA";

3R Scripture exon 20911484 20912345 . + . gene_id "XLOC_147932"; transcript_id "TCONS_00648284"; exon_number "1"; gene_type "anti-sense_lncRNA";

3R Scripture exon 20912422 20913153 . + . gene_id "XLOC_147932"; transcript_id "TCONS_00648284"; exon_number "2"; gene_type "anti-sense_lncRNA";

3R Scripture exon 21296827 21297144 . + . gene_id "XLOC_148093"; transcript_id "TCONS_00871508"; exon_number "1"; gene_type "lincRNA";

3R Scripture exon 21297810 21298141 . + . gene_id "XLOC_148093"; transcript_id "TCONS_00871508"; exon_number "2"; gene_type "lincRNA";

3R Cufflinks exon 21600962 21601181 . + . gene_id "XLOC_148381"; transcript_id "TCONS_00741962"; exon_number "1"; gene_type "intronic_lncRNA";

3R Cufflinks exon 21659100 21659308 . + . gene_id "XLOC_148407"; transcript_id "TCONS_00815232"; exon_number "1"; gene_type "intronic_lncRNA";

3R Cufflinks exon 21671809 21672013 . + . gene_id "XLOC_148413"; transcript_id "TCONS_00682366"; exon_number "1"; gene_type "lincRNA";

3R Cufflinks exon 21861176 21861382 . + . gene_id "XLOC_148566"; transcript_id "TCONS_00766530"; exon_number "1"; gene_type "lincRNA";

3R Cufflinks exon 21924817 21925036 . + . gene_id "XLOC_148628"; transcript_id "TCONS_00648749"; exon_number "1"; gene_type "lincRNA";

3R Cufflinks exon 22394887 22395095 . + . gene_id "XLOC_148870"; transcript_id "TCONS_00682670"; exon_number "1"; gene_type "lincRNA";

3R Cufflinks exon 22503773 22503982 . + . gene_id "XLOC_148940"; transcript_id "TCONS_00682732"; exon_number "1"; gene_type "intronic_lncRNA";

3R Cufflinks exon 22717935 22718135 . + . gene_id "XLOC_149064"; transcript_id "TCONS_00649078"; exon_number "1"; gene_type "intronic_lncRNA";

3R Cufflinks exon 22859830 22860042 . + . gene_id "XLOC_149176"; transcript_id "TCONS_00715107"; exon_number "1"; gene_type "lincRNA";

3R Cufflinks exon 22877451 22877660 . + . gene_id "XLOC_149212"; transcript_id "TCONS_00649177"; exon_number "1"; gene_type "lincRNA";

3R Cufflinks exon 23158426 23158628 . + . gene_id "XLOC_149505"; transcript_id "TCONS_00683142"; exon_number "1"; gene_type "anti-sense_lncRNA";

3R Cufflinks exon 23392832 23393034 . + . gene_id "XLOC_149669"; transcript_id "TCONS_00742579"; exon_number "1"; gene_type "lincRNA";

3R Cufflinks exon 23634097 23634312 . + . gene_id "XLOC_149947"; transcript_id "TCONS_00742691"; exon_number "1"; gene_type "intronic_lncRNA";

3R Cufflinks exon 23661200 23661413 . + . gene_id "XLOC_149966"; transcript_id "TCONS_00742693"; exon_number "1"; gene_type "intronic_lncRNA";

3R Scripture exon 23826333 23826414 . + . gene_id "XLOC_150011"; transcript_id "TCONS_00861063"; exon_number "1"; gene_type "intronic_lncRNA";

3R Scripture exon 23827889 23828950 . + . gene_id "XLOC_150011"; transcript_id "TCONS_00861063"; exon_number "2"; gene_type "intronic_lncRNA";

3R Scripture exon 23826339 23826430 . + . gene_id "XLOC_150011"; transcript_id "TCONS_00886918"; exon_number "1"; gene_type "intronic_lncRNA";

3R Scripture exon 23827874 23828296 . + . gene_id "XLOC_150011"; transcript_id "TCONS_00886918"; exon_number "2"; gene_type "intronic_lncRNA";

3R Scripture exon 23826339 23826476 . + . gene_id "XLOC_150011"; transcript_id "TCONS_00886919"; exon_number "1"; gene_type "intronic_lncRNA";

3R Scripture exon 23827878 23828656 . + . gene_id "XLOC_150011"; transcript_id "TCONS_00886919"; exon_number "2"; gene_type "intronic_lncRNA";

3R Scripture exon 23826339 23826454 . + . gene_id "XLOC_150011"; transcript_id "TCONS_00861064"; exon_number "1"; gene_type "intronic_lncRNA";

3R Scripture exon 23827887 23828656 . + . gene_id "XLOC_150011"; transcript_id "TCONS_00861064"; exon_number "2"; gene_type "intronic_lncRNA";

3R Scripture exon 23826438 23826466 . + . gene_id "XLOC_150011"; transcript_id "TCONS_00871909"; exon_number "1"; gene_type "intronic_lncRNA";

3R Scripture exon 23827887 23828780 . + . gene_id "XLOC_150011"; transcript_id "TCONS_00871909"; exon_number "2"; gene_type "intronic_lncRNA";

3R Scripture exon 23826438 23826466 . + . gene_id "XLOC_150011"; transcript_id "TCONS_00861066"; exon_number "1"; gene_type "intronic_lncRNA";

3R Scripture exon 23827889 23828780 . + . gene_id "XLOC_150011"; transcript_id "TCONS_00861066"; exon_number "2"; gene_type "intronic_lncRNA";

3R Cufflinks exon 24361003 24361210 . + . gene_id "XLOC_150236"; transcript_id "TCONS_00683554"; exon_number "1"; gene_type "anti-sense_lncRNA";

3R Cufflinks exon 24998605 24998824 . + . gene_id "XLOC_150680"; transcript_id "TCONS_00683879"; exon_number "1"; gene_type "intronic_lncRNA";

3R Cufflinks exon 25089264 25089471 . + . gene_id "XLOC_150724"; transcript_id "TCONS_00683932"; exon_number "1"; gene_type "lincRNA";

3R Cufflinks exon 25497310 25497513 . + . gene_id "XLOC_150930"; transcript_id "TCONS_00790782"; exon_number "1"; gene_type "lincRNA";

3R Cufflinks exon 25668619 25668821 . + . gene_id "XLOC_151037"; transcript_id "TCONS_00650513"; exon_number "1"; gene_type "intronic_lncRNA";

3R Cufflinks exon 25770980 25771204 . + . gene_id "XLOC_151131"; transcript_id "TCONS_00684279"; exon_number "1"; gene_type "lincRNA";

3R Cufflinks exon 26313180 26313399 . + . gene_id "XLOC_151622"; transcript_id "TCONS_00816862"; exon_number "1"; gene_type "lincRNA";

3R Cufflinks exon 26788914 26789154 . + . gene_id "XLOC_152015"; transcript_id "TCONS_00651247"; exon_number "1"; gene_type "lincRNA";

3R Cufflinks exon 26958749 26958953 . + . gene_id "XLOC_152062"; transcript_id "TCONS_00743896"; exon_number "1"; gene_type "lincRNA";

3R Cufflinks exon 27071773 27071977 . + . gene_id "XLOC_152163"; transcript_id "TCONS_00743945"; exon_number "1"; gene_type "lincRNA";

3R Cufflinks exon 27109116 27111012 . + . gene_id "XLOC_152205"; transcript_id "TCONS_00651381"; exon_number "1"; gene_type "anti-sense_lncRNA";

3R Cufflinks exon 27324463 27324672 . + . gene_id "XLOC_152349"; transcript_id "TCONS_00685209"; exon_number "1"; gene_type "lincRNA";

3R Cufflinks exon 27427492 27427710 . + . gene_id "XLOC_152460"; transcript_id "TCONS_00717073"; exon_number "1"; gene_type "intronic_lncRNA";

3R Cufflinks exon 27494934 27495137 . + . gene_id "XLOC_152551"; transcript_id "TCONS_00685335"; exon_number "1"; gene_type "lincRNA";

3R Cufflinks exon 27537847 27538897 . + . gene_id "XLOC_152599"; transcript_id "TCONS_00817349"; exon_number "1"; gene_type "lincRNA";

3R Cufflinks exon 27735261 27735463 . + . gene_id "XLOC_152713"; transcript_id "TCONS_00651723"; exon_number "1"; gene_type "lincRNA";

3R Cufflinks exon 27753720 27753924 . + . gene_id "XLOC_152721"; transcript_id "TCONS_00817414"; exon_number "1"; gene_type "lincRNA";

3R Cufflinks exon 28032364 28032583 . + . gene_id "XLOC_152985"; transcript_id "TCONS_00651899"; exon_number "1"; gene_type "intronic_lncRNA";

3R Cufflinks exon 28132439 28132647 . + . gene_id "XLOC_153123"; transcript_id "TCONS_00652010"; exon_number "1"; gene_type "lincRNA";

3R Cufflinks exon 28173320 28173723 . + . gene_id "XLOC_153190"; transcript_id "TCONS_00652054"; exon_number "1"; gene_type "intronic_lncRNA";

3R Cufflinks exon 28181101 28181178 . + . gene_id "XLOC_153190"; transcript_id "TCONS_00652054"; exon_number "2"; gene_type "intronic_lncRNA";

3R Cufflinks exon 28439594 28439808 . + . gene_id "XLOC_153537"; transcript_id "TCONS_00717679"; exon_number "1"; gene_type "intronic_lncRNA";

3R Cufflinks exon 29021948 29022033 . + . gene_id "XLOC_153975"; transcript_id "TCONS_00686459"; exon_number "1"; gene_type "lincRNA";

3R Cufflinks exon 29022544 29023871 . + . gene_id "XLOC_153975"; transcript_id "TCONS_00686459"; exon_number "2"; gene_type "lincRNA";

3R Cufflinks exon 29086948 29087270 . + . gene_id "XLOC_153998"; transcript_id "TCONS_00818073"; exon_number "1"; gene_type "intronic_lncRNA";

3R Scripture exon 29214569 29214959 . + . gene_id "XLOC_154050"; transcript_id "TCONS_00872967"; exon_number "1"; gene_type "lincRNA";

3R Scripture exon 29215015 29217513 . + . gene_id "XLOC_154050"; transcript_id "TCONS_00872967"; exon_number "2"; gene_type "lincRNA";

3R Scripture exon 29214569 29214959 . + . gene_id "XLOC_154050"; transcript_id "TCONS_00872966"; exon_number "1"; gene_type "lincRNA";

3R Scripture exon 29215020 29218411 . + . gene_id "XLOC_154050"; transcript_id "TCONS_00872966"; exon_number "2"; gene_type "lincRNA";

3R Cufflinks exon 29775629 29776000 . + . gene_id "XLOC_154747"; transcript_id "TCONS_00718363"; exon_number "1"; gene_type "intronic_lncRNA";

3R Scripture exon 29783987 29785675 . + . gene_id "XLOC_154756"; transcript_id "TCONS_00872992"; exon_number "1"; gene_type "intronic_lncRNA";

3R Cufflinks exon 30088263 30088506 . + . gene_id "XLOC_154896"; transcript_id "TCONS_00653188"; exon_number "1"; gene_type "intronic_lncRNA";

3R Cufflinks exon 30127745 30127955 . + . gene_id "XLOC_154907"; transcript_id "TCONS_00687027"; exon_number "1"; gene_type "anti-sense_lncRNA";

3R Cufflinks exon 30150353 30150576 . + . gene_id "XLOC_154929"; transcript_id "TCONS_00687036"; exon_number "1"; gene_type "lincRNA";

3R Cufflinks exon 30227248 30227452 . + . gene_id "XLOC_155010"; transcript_id "TCONS_00718498"; exon_number "1"; gene_type "lincRNA";

3R Cufflinks exon 30719437 30719660 . + . gene_id "XLOC_155424"; transcript_id "TCONS_00687327"; exon_number "1"; gene_type "lincRNA";

3R Scripture exon 30744581 30744960 . + . gene_id "XLOC_155461"; transcript_id "TCONS_00718729"; exon_number "1"; gene_type "anti-sense_lncRNA";

3R Scripture exon 30745994 30746448 . + . gene_id "XLOC_155461"; transcript_id "TCONS_00718729"; exon_number "2"; gene_type "anti-sense_lncRNA";

3R Cufflinks exon 31995266 31995474 . + . gene_id "XLOC_156372"; transcript_id "TCONS_00793708"; exon_number "1"; gene_type "intronic_lncRNA";

3R Cufflinks exon 1951674 1951879 . - . gene_id "XLOC_157617"; transcript_id "TCONS_00719898"; exon_number "1"; gene_type "intronic_lncRNA";

3R Cufflinks exon 2775830 2776037 . - . gene_id "XLOC_158295"; transcript_id "TCONS_00655217"; exon_number "1"; gene_type "intronic_lncRNA";

3R Cufflinks exon 3180729 3180940 . - . gene_id "XLOC_158647"; transcript_id "TCONS_00720304"; exon_number "1"; gene_type "intronic_lncRNA";

3R Cufflinks exon 3287074 3287284 . - . gene_id "XLOC_158757"; transcript_id "TCONS_00655491"; exon_number "1"; gene_type "lincRNA";

3R Cufflinks exon 4151389 4151554 . - . gene_id "XLOC_159174"; transcript_id "TCONS_00655833"; exon_number "1"; gene_type "lincRNA";

3R Cufflinks exon 4151625 4151804 . - . gene_id "XLOC_159174"; transcript_id "TCONS_00655833"; exon_number "2"; gene_type "lincRNA";

3R Cufflinks exon 4825433 4825638 . - . gene_id "XLOC_159552"; transcript_id "TCONS_00747796"; exon_number "1"; gene_type "lincRNA";

3R Cufflinks exon 5024320 5024562 . - . gene_id "XLOC_159701"; transcript_id "TCONS_00690176"; exon_number "1"; gene_type "lincRNA";

3R Cufflinks exon 5045614 5045820 . - . gene_id "XLOC_159719"; transcript_id "TCONS_00771483"; exon_number "1"; gene_type "lincRNA";

3R Cufflinks exon 5518378 5518600 . - . gene_id "XLOC_159959"; transcript_id "TCONS_00690421"; exon_number "1"; gene_type "anti-sense_lncRNA";

3R Cufflinks exon 5624605 5624889 . - . gene_id "XLOC_159991"; transcript_id "TCONS_00771662"; exon_number "1"; gene_type "intronic_lncRNA";

3R Scripture exon 5835839 5835911 . - . gene_id "XLOC_160095"; transcript_id "TCONS_00893283"; exon_number "1"; gene_type "anti-sense_lncRNA";

3R Scripture exon 5835991 5836169 . - . gene_id "XLOC_160095"; transcript_id "TCONS_00893283"; exon_number "2"; gene_type "anti-sense_lncRNA";

3R Cufflinks exon 6172153 6172367 . - . gene_id "XLOC_160411"; transcript_id "TCONS_00690794"; exon_number "1"; gene_type "lincRNA";

3R Cufflinks exon 6216083 6216283 . - . gene_id "XLOC_160459"; transcript_id "TCONS_00656929"; exon_number "1"; gene_type "anti-sense_lncRNA";

3R Cufflinks exon 6350486 6350686 . - . gene_id "XLOC_160637"; transcript_id "TCONS_00821225"; exon_number "1"; gene_type "anti-sense_lncRNA";

3R Cufflinks exon 6829761 6829965 . - . gene_id "XLOC_161086"; transcript_id "TCONS_00748772"; exon_number "1"; gene_type "intronic_lncRNA";

3R Cufflinks exon 7017095 7017314 . - . gene_id "XLOC_161301"; transcript_id "TCONS_00796313"; exon_number "1"; gene_type "lincRNA";

3R Cufflinks exon 7035519 7035724 . - . gene_id "XLOC_161334"; transcript_id "TCONS_00748921"; exon_number "1"; gene_type "lincRNA";

3R Cufflinks exon 7054437 7054701 . - . gene_id "XLOC_161360"; transcript_id "TCONS_00722158"; exon_number "1"; gene_type "anti-sense_lncRNA";

3R Cufflinks exon 7179672 7179885 . - . gene_id "XLOC_161423"; transcript_id "TCONS_00691480"; exon_number "1"; gene_type "intronic_lncRNA";

3R Cufflinks exon 7295480 7295686 . - . gene_id "XLOC_161533"; transcript_id "TCONS_00691558"; exon_number "1"; gene_type "intronic_lncRNA";

3R Cufflinks exon 7299695 7299899 . - . gene_id "XLOC_161541"; transcript_id "TCONS_00657729"; exon_number "1"; gene_type "intronic_lncRNA";

3R Cufflinks exon 7425734 7425985 . - . gene_id "XLOC_161715"; transcript_id "TCONS_00657848"; exon_number "1"; gene_type "lincRNA";

3R Cufflinks exon 7946901 7947123 . - . gene_id "XLOC_162115"; transcript_id "TCONS_00722613"; exon_number "1"; gene_type "intronic_lncRNA";

3R Cufflinks exon 8090364 8090565 . - . gene_id "XLOC_162210"; transcript_id "TCONS_00692072"; exon_number "1"; gene_type "lincRNA";

3R Cufflinks exon 8116107 8116370 . - . gene_id "XLOC_162216"; transcript_id "TCONS_00658214"; exon_number "1"; gene_type "intronic_lncRNA";

3R Cufflinks exon 8119258 8119478 . - . gene_id "XLOC_162218"; transcript_id "TCONS_00692075"; exon_number "1"; gene_type "intronic_lncRNA";

3R Scripture exon 8562734 8562865 . - . gene_id "XLOC_162630"; transcript_id "TCONS_00888293"; exon_number "1"; gene_type "anti-sense_lncRNA";

3R Scripture exon 8562935 8563329 . - . gene_id "XLOC_162630"; transcript_id "TCONS_00888293"; exon_number "2"; gene_type "anti-sense_lncRNA";

3R Scripture exon 8563670 8563883 . - . gene_id "XLOC_162630"; transcript_id "TCONS_00888293"; exon_number "3"; gene_type "anti-sense_lncRNA";

3R Scripture exon 8562741 8562865 . - . gene_id "XLOC_162630"; transcript_id "TCONS_00874068"; exon_number "1"; gene_type "anti-sense_lncRNA";

3R Scripture exon 8562935 8563329 . - . gene_id "XLOC_162630"; transcript_id "TCONS_00874068"; exon_number "2"; gene_type "anti-sense_lncRNA";

3R Scripture exon 8563409 8564082 . - . gene_id "XLOC_162630"; transcript_id "TCONS_00874068"; exon_number "3"; gene_type "anti-sense_lncRNA";

3R Scripture exon 8821384 8822155 . - . gene_id "XLOC_162778"; transcript_id "TCONS_00863384"; exon_number "1"; gene_type "anti-sense_lncRNA";

3R Scripture exon 8822614 8822666 . - . gene_id "XLOC_162778"; transcript_id "TCONS_00863384"; exon_number "2"; gene_type "anti-sense_lncRNA";

3R Cufflinks exon 8933044 8933093 . - . gene_id "XLOC_162782"; transcript_id "TCONS_00749742"; exon_number "1"; gene_type "lincRNA";

3R Cufflinks exon 8933153 8933392 . - . gene_id "XLOC_162782"; transcript_id "TCONS_00749742"; exon_number "2"; gene_type "lincRNA";

3R Scripture exon 8934082 8934823 . - . gene_id "XLOC_162782"; transcript_id "TCONS_00863441"; exon_number "1"; gene_type "anti-sense_lncRNA";

3R Scripture exon 8935013 8935199 . - . gene_id "XLOC_162782"; transcript_id "TCONS_00863441"; exon_number "2"; gene_type "anti-sense_lncRNA";

3R Cufflinks exon 9337475 9340225 . - . gene_id "XLOC_162948"; transcript_id "TCONS_00658779"; exon_number "1"; gene_type "anti-sense_lncRNA";

3R Cufflinks exon 9340827 9341449 . - . gene_id "XLOC_162948"; transcript_id "TCONS_00658779"; exon_number "2"; gene_type "anti-sense_lncRNA";

3R Cufflinks exon 9337475 9338644 . - . gene_id "XLOC_162948"; transcript_id "TCONS_00658778"; exon_number "1"; gene_type "anti-sense_lncRNA";

3R Cufflinks exon 9338725 9341449 . - . gene_id "XLOC_162948"; transcript_id "TCONS_00658778"; exon_number "2"; gene_type "anti-sense_lncRNA";

3R Scripture exon 9349675 9350450 . - . gene_id "XLOC_162952"; transcript_id "TCONS_00874227"; exon_number "1"; gene_type "intronic_lncRNA";

3R Scripture exon 9350580 9350706 . - . gene_id "XLOC_162952"; transcript_id "TCONS_00874227"; exon_number "2"; gene_type "intronic_lncRNA";

3R Cufflinks exon 9421165 9421412 . - . gene_id "XLOC_162966"; transcript_id "TCONS_00658804"; exon_number "1"; gene_type "intronic_lncRNA";

3R Cufflinks exon 9436588 9436804 . - . gene_id "XLOC_162982"; transcript_id "TCONS_00658813"; exon_number "1"; gene_type "intronic_lncRNA";

3R Scripture exon 9632107 9632341 . - . gene_id "XLOC_163042"; transcript_id "TCONS_00863693"; exon_number "1"; gene_type "anti-sense_lncRNA";

3R Scripture exon 9632447 9632544 . - . gene_id "XLOC_163042"; transcript_id "TCONS_00863693"; exon_number "2"; gene_type "anti-sense_lncRNA";

3R Cufflinks exon 9779409 9780171 . - . gene_id "XLOC_163080"; transcript_id "TCONS_00658946"; exon_number "1"; gene_type "anti-sense_lncRNA";

3R Cufflinks exon 9856061 9856271 . - . gene_id "XLOC_163118"; transcript_id "TCONS_00723302"; exon_number "1"; gene_type "intronic_lncRNA";

3R Cufflinks exon 9940163 9940422 . - . gene_id "XLOC_163208"; transcript_id "TCONS_00659028"; exon_number "1"; gene_type "intronic_lncRNA";

3R Cufflinks exon 9948672 9948886 . - . gene_id "XLOC_163217"; transcript_id "TCONS_00749962"; exon_number "1"; gene_type "intronic_lncRNA";

3R Cufflinks exon 9969720 9969946 . - . gene_id "XLOC_163238"; transcript_id "TCONS_00723355"; exon_number "1"; gene_type "intronic_lncRNA";

3R Cufflinks exon 10225086 10225292 . - . gene_id "XLOC_163344"; transcript_id "TCONS_00750012"; exon_number "1"; gene_type "anti-sense_lncRNA";

3R Cufflinks exon 10439179 10439385 . - . gene_id "XLOC_163467"; transcript_id "TCONS_00797484"; exon_number "1"; gene_type "lincRNA";

3R Cufflinks exon 10526584 10526806 . - . gene_id "XLOC_163585"; transcript_id "TCONS_00797550"; exon_number "1"; gene_type "intronic_lncRNA";

3R Cufflinks exon 10567637 10567848 . - . gene_id "XLOC_163653"; transcript_id "TCONS_00750118"; exon_number "1"; gene_type "intronic_lncRNA";

3R Cufflinks exon 10634112 10634320 . - . gene_id "XLOC_163725"; transcript_id "TCONS_00693079"; exon_number "1"; gene_type "intronic_lncRNA";

3R Cufflinks exon 11016169 11016369 . - . gene_id "XLOC_164090"; transcript_id "TCONS_00659500"; exon_number "1"; gene_type "intronic_lncRNA";

3R Cufflinks exon 11315275 11315478 . - . gene_id "XLOC_164419"; transcript_id "TCONS_00823312"; exon_number "1"; gene_type "lincRNA";

3R Cufflinks exon 11338445 11338673 . - . gene_id "XLOC_164446"; transcript_id "TCONS_00823327"; exon_number "1"; gene_type "intronic_lncRNA";

3R Cufflinks exon 12135261 12135465 . - . gene_id "XLOC_164904"; transcript_id "TCONS_00693954"; exon_number "1"; gene_type "lincRNA";

3R Cufflinks exon 12167853 12168060 . - . gene_id "XLOC_164933"; transcript_id "TCONS_00693994"; exon_number "1"; gene_type "lincRNA";

3R Cufflinks exon 12170812 12171011 . - . gene_id "XLOC_164936"; transcript_id "TCONS_00750818"; exon_number "1"; gene_type "lincRNA";

3R Cufflinks exon 12247941 12248143 . - . gene_id "XLOC_165026"; transcript_id "TCONS_00694075"; exon_number "1"; gene_type "lincRNA";

3R Scripture exon 12441521 12443275 . - . gene_id "XLOC_165154"; transcript_id "TCONS_00888884"; exon_number "1"; gene_type "intronic_lncRNA";

3R Scripture exon 12443485 12444042 . - . gene_id "XLOC_165154"; transcript_id "TCONS_00888884"; exon_number "2"; gene_type "intronic_lncRNA";

3R Cufflinks exon 12585172 12585399 . - . gene_id "XLOC_165248"; transcript_id "TCONS_00694265"; exon_number "1"; gene_type "intronic_lncRNA";

3R Cufflinks exon 12637317 12637531 . - . gene_id "XLOC_165294"; transcript_id "TCONS_00751034"; exon_number "1"; gene_type "anti-sense_lncRNA";

3R Cufflinks exon 12656326 12656573 . - . gene_id "XLOC_165315"; transcript_id "TCONS_00751045"; exon_number "1"; gene_type "intronic_lncRNA";

3R Cufflinks exon 12723515 12723732 . - . gene_id "XLOC_165348"; transcript_id "TCONS_00694343"; exon_number "1"; gene_type "lincRNA";

3R Scripture exon 12981468 12982493 . - . gene_id "XLOC_165693"; transcript_id "TCONS_00874886"; exon_number "1"; gene_type "intronic_lncRNA";

3R Scripture exon 12982550 12983325 . - . gene_id "XLOC_165693"; transcript_id "TCONS_00874886"; exon_number "2"; gene_type "intronic_lncRNA";

3R Cufflinks exon 13346904 13347104 . - . gene_id "XLOC_165931"; transcript_id "TCONS_00660763"; exon_number "1"; gene_type "lincRNA";

3R Cufflinks exon 13464061 13464399 . - . gene_id "XLOC_166031"; transcript_id "TCONS_00660838"; exon_number "1"; gene_type "anti-sense_lncRNA";

3R Cufflinks exon 13465707 13466325 . - . gene_id "XLOC_166031"; transcript_id "TCONS_00660838"; exon_number "2"; gene_type "anti-sense_lncRNA";

3R Cufflinks exon 13508943 13509149 . - . gene_id "XLOC_166109"; transcript_id "TCONS_00694835"; exon_number "1"; gene_type "lincRNA";

3R Cufflinks exon 13721965 13722190 . - . gene_id "XLOC_166353"; transcript_id "TCONS_00725079"; exon_number "1"; gene_type "intronic_lncRNA";

3R Scripture exon 13782113 13782808 . - . gene_id "XLOC_166389"; transcript_id "TCONS_00874930"; exon_number "1"; gene_type "anti-sense_lncRNA";

3R Scripture exon 13782861 13782996 . - . gene_id "XLOC_166389"; transcript_id "TCONS_00874930"; exon_number "2"; gene_type "anti-sense_lncRNA";

3R Cufflinks exon 13896901 13897118 . - . gene_id "XLOC_166557"; transcript_id "TCONS_00661140"; exon_number "1"; gene_type "lincRNA";

3R Cufflinks exon 13899970 13900172 . - . gene_id "XLOC_166562"; transcript_id "TCONS_00661144"; exon_number "1"; gene_type "lincRNA";

3R Cufflinks exon 13911356 13911559 . - . gene_id "XLOC_166575"; transcript_id "TCONS_00725189"; exon_number "1"; gene_type "lincRNA";

3R Cufflinks exon 13925759 13925959 . - . gene_id "XLOC_166598"; transcript_id "TCONS_00695139"; exon_number "1"; gene_type "lincRNA";

3R Cufflinks exon 13955547 13955757 . - . gene_id "XLOC_166650"; transcript_id "TCONS_00725227"; exon_number "1"; gene_type "lincRNA";

3R Cufflinks exon 14132592 14133095 . - . gene_id "XLOC_166755"; transcript_id "TCONS_00661332"; exon_number "1"; gene_type "lincRNA";

3R Cufflinks exon 14133183 14133635 . - . gene_id "XLOC_166755"; transcript_id "TCONS_00661332"; exon_number "2"; gene_type "lincRNA";

3R Cufflinks exon 14547653 14547860 . - . gene_id "XLOC_167076"; transcript_id "TCONS_00695500"; exon_number "1"; gene_type "intronic_lncRNA";

3R Cufflinks exon 14552251 14552456 . - . gene_id "XLOC_167084"; transcript_id "TCONS_00725474"; exon_number "1"; gene_type "intronic_lncRNA";

3R Cufflinks exon 14581290 14581491 . - . gene_id "XLOC_167103"; transcript_id "TCONS_00725487"; exon_number "1"; gene_type "intronic_lncRNA";

3R Cufflinks exon 14598841 14599043 . - . gene_id "XLOC_167121"; transcript_id "TCONS_00661585"; exon_number "1"; gene_type "intronic_lncRNA";

3R Cufflinks exon 14721726 14722037 . - . gene_id "XLOC_167175"; transcript_id "TCONS_00695572"; exon_number "1"; gene_type "intronic_lncRNA";

3R Cufflinks exon 15699215 15699473 . - . gene_id "XLOC_167989"; transcript_id "TCONS_00752426"; exon_number "1"; gene_type "lincRNA";

3R Cufflinks exon 16718509 16718725 . - . gene_id "XLOC_168717"; transcript_id "TCONS_00662607"; exon_number "1"; gene_type "intronic_lncRNA";

3R Cufflinks exon 16747537 16747748 . - . gene_id "XLOC_168759"; transcript_id "TCONS_00696640"; exon_number "1"; gene_type "lincRNA";

3R Cufflinks exon 16898237 16898446 . - . gene_id "XLOC_168941"; transcript_id "TCONS_00696760"; exon_number "1"; gene_type "lincRNA";

3R Cufflinks exon 17053724 17053957 . - . gene_id "XLOC_169077"; transcript_id "TCONS_00662860"; exon_number "1"; gene_type "lincRNA";

3R Scripture exon 17081244 17081472 . - . gene_id "XLOC_169100"; transcript_id "TCONS_00864670"; exon_number "1"; gene_type "intronic_lncRNA";

3R Scripture exon 17082331 17082507 . - . gene_id "XLOC_169100"; transcript_id "TCONS_00864670"; exon_number "2"; gene_type "intronic_lncRNA";

3R Cufflinks exon 17182397 17182599 . - . gene_id "XLOC_169163"; transcript_id "TCONS_00696933"; exon_number "1"; gene_type "intronic_lncRNA";

3R Cufflinks exon 17522914 17523122 . - . gene_id "XLOC_169593"; transcript_id "TCONS_00697215"; exon_number "1"; gene_type "lincRNA";

3R Cufflinks exon 17585925 17586151 . - . gene_id "XLOC_169674"; transcript_id "TCONS_00776007"; exon_number "1"; gene_type "lincRNA";

3R Cufflinks exon 17764528 17764732 . - . gene_id "XLOC_169811"; transcript_id "TCONS_00726963"; exon_number "1"; gene_type "lincRNA";

3R Cufflinks exon 17899585 17899728 . - . gene_id "XLOC_169913"; transcript_id "TCONS_00663367"; exon_number "1"; gene_type "intronic_lncRNA";

3R Cufflinks exon 17900384 17901040 . - . gene_id "XLOC_169913"; transcript_id "TCONS_00663367"; exon_number "2"; gene_type "intronic_lncRNA";

3R Cufflinks exon 17899585 17899728 . - . gene_id "XLOC_169913"; transcript_id "TCONS_00727045"; exon_number "1"; gene_type "intronic_lncRNA";

3R Cufflinks exon 17900773 17901159 . - . gene_id "XLOC_169913"; transcript_id "TCONS_00727045"; exon_number "2"; gene_type "intronic_lncRNA";

3R Cufflinks exon 17936079 17936283 . - . gene_id "XLOC_169953"; transcript_id "TCONS_00697446"; exon_number "1"; gene_type "intronic_lncRNA";

3R Cufflinks exon 18124649 18124922 . - . gene_id "XLOC_170198"; transcript_id "TCONS_00727141"; exon_number "1"; gene_type "intronic_lncRNA";

3R Cufflinks exon 18356563 18356771 . - . gene_id "XLOC_170381"; transcript_id "TCONS_00663580"; exon_number "1"; gene_type "intronic_lncRNA";

3R Cufflinks exon 18511498 18511699 . - . gene_id "XLOC_170537"; transcript_id "TCONS_00727317"; exon_number "1"; gene_type "intronic_lncRNA";

3R Cufflinks exon 18782506 18782747 . - . gene_id "XLOC_170723"; transcript_id "TCONS_00697883"; exon_number "1"; gene_type "intronic_lncRNA";

3R Cufflinks exon 18916877 18917115 . - . gene_id "XLOC_170883"; transcript_id "TCONS_00727495"; exon_number "1"; gene_type "intronic_lncRNA";

3R Cufflinks exon 18950515 18950734 . - . gene_id "XLOC_170911"; transcript_id "TCONS_00753914"; exon_number "1"; gene_type "intronic_lncRNA";

3R Cufflinks exon 19123683 19123904 . - . gene_id "XLOC_171007"; transcript_id "TCONS_00753935"; exon_number "1"; gene_type "intronic_lncRNA";

3R Cufflinks exon 19470606 19470806 . - . gene_id "XLOC_171292"; transcript_id "TCONS_00754055"; exon_number "1"; gene_type "intronic_lncRNA";

3R Cufflinks exon 19766253 19766463 . - . gene_id "XLOC_171476"; transcript_id "TCONS_00698276"; exon_number "1"; gene_type "intronic_lncRNA";

3R Cufflinks exon 20175955 20176160 . - . gene_id "XLOC_171766"; transcript_id "TCONS_00664404"; exon_number "1"; gene_type "lincRNA";

3R Cufflinks exon 20226030 20226232 . - . gene_id "XLOC_171836"; transcript_id "TCONS_00664435"; exon_number "1"; gene_type "intronic_lncRNA";

3R Cufflinks exon 20252468 20252673 . - . gene_id "XLOC_171868"; transcript_id "TCONS_00698536"; exon_number "1"; gene_type "lincRNA";

3R Cufflinks exon 20356383 20356583 . - . gene_id "XLOC_171949"; transcript_id "TCONS_00727917"; exon_number "1"; gene_type "lincRNA";

3R Cufflinks exon 20790455 20790656 . - . gene_id "XLOC_172298"; transcript_id "TCONS_00664767"; exon_number "1"; gene_type "intronic_lncRNA";

3R Cufflinks exon 21102153 21102973 . - . gene_id "XLOC_172496"; transcript_id "TCONS_00664917"; exon_number "1"; gene_type "anti-sense_lncRNA";

3R Cufflinks exon 21705488 21705688 . - . gene_id "XLOC_172924"; transcript_id "TCONS_00777293"; exon_number "1"; gene_type "intronic_lncRNA";

3R Cufflinks exon 22049869 22050072 . - . gene_id "XLOC_173192"; transcript_id "TCONS_00728634"; exon_number "1"; gene_type "intronic_lncRNA";

3R Cufflinks exon 22060206 22060431 . - . gene_id "XLOC_173201"; transcript_id "TCONS_00699435"; exon_number "1"; gene_type "intronic_lncRNA";

3R Cufflinks exon 22080751 22080954 . - . gene_id "XLOC_173218"; transcript_id "TCONS_00665365"; exon_number "1"; gene_type "intronic_lncRNA";

3R Cufflinks exon 22338587 22338811 . - . gene_id "XLOC_173368"; transcript_id "TCONS_00755046"; exon_number "1"; gene_type "intronic_lncRNA";

3R Cufflinks exon 22400952 22401153 . - . gene_id "XLOC_173416"; transcript_id "TCONS_00699544"; exon_number "1"; gene_type "lincRNA";

3R Cufflinks exon 22809693 22809897 . - . gene_id "XLOC_173672"; transcript_id "TCONS_00699667"; exon_number "1"; gene_type "lincRNA";

3R Cufflinks exon 22970021 22970221 . - . gene_id "XLOC_173926"; transcript_id "TCONS_00802783"; exon_number "1"; gene_type "lincRNA";

3R Cufflinks exon 22974473 22974677 . - . gene_id "XLOC_173933"; transcript_id "TCONS_00755266"; exon_number "1"; gene_type "lincRNA";

3R Cufflinks exon 23040264 23040491 . - . gene_id "XLOC_173999"; transcript_id "TCONS_00729003"; exon_number "1"; gene_type "lincRNA";

3R Cufflinks exon 23146432 23146643 . - . gene_id "XLOC_174088"; transcript_id "TCONS_00699956"; exon_number "1"; gene_type "lincRNA";

3R Cufflinks exon 23171014 23171237 . - . gene_id "XLOC_174111"; transcript_id "TCONS_00665905"; exon_number "1"; gene_type "intronic_lncRNA";

3R Cufflinks exon 23171359 23172198 . - . gene_id "XLOC_174111"; transcript_id "TCONS_00665905"; exon_number "2"; gene_type "intronic_lncRNA";

3R Cufflinks exon 23172717 23173885 . - . gene_id "XLOC_174111"; transcript_id "TCONS_00665905"; exon_number "3"; gene_type "intronic_lncRNA";

3R Cufflinks exon 23324607 23324816 . - . gene_id "XLOC_174192"; transcript_id "TCONS_00665998"; exon_number "1"; gene_type "intronic_lncRNA";

3R Cufflinks exon 23330029 23330257 . - . gene_id "XLOC_174202"; transcript_id "TCONS_00700034"; exon_number "1"; gene_type "intronic_lncRNA";

3R Scripture exon 23359159 23359259 . - . gene_id "XLOC_174232"; transcript_id "TCONS_00865561"; exon_number "1"; gene_type "anti-sense_lncRNA";

3R Scripture exon 23359589 23359693 . - . gene_id "XLOC_174232"; transcript_id "TCONS_00865561"; exon_number "2"; gene_type "anti-sense_lncRNA";

3R Cufflinks exon 23417990 23418194 . - . gene_id "XLOC_174304"; transcript_id "TCONS_00700107"; exon_number "1"; gene_type "intronic_lncRNA";

3R Cufflinks exon 24304707 24304925 . - . gene_id "XLOC_174737"; transcript_id "TCONS_00700450"; exon_number "1"; gene_type "intronic_lncRNA";

3R Cufflinks exon 24468826 24469070 . - . gene_id "XLOC_174896"; transcript_id "TCONS_00729585"; exon_number "1"; gene_type "lincRNA";

3R Cufflinks exon 24592292 24592491 . - . gene_id "XLOC_174999"; transcript_id "TCONS_00729622"; exon_number "1"; gene_type "intronic_lncRNA";

3R Cufflinks exon 24771876 24772091 . - . gene_id "XLOC_175104"; transcript_id "TCONS_00755859"; exon_number "1"; gene_type "lincRNA";

3R Cufflinks exon 24831648 24831854 . - . gene_id "XLOC_175138"; transcript_id "TCONS_00755880"; exon_number "1"; gene_type "lincRNA";

3R Cufflinks exon 24845094 24845303 . - . gene_id "XLOC_175153"; transcript_id "TCONS_00666703"; exon_number "1"; gene_type "intronic_lncRNA";

3R Cufflinks exon 25140878 25141085 . - . gene_id "XLOC_175344"; transcript_id "TCONS_00700836"; exon_number "1"; gene_type "lincRNA";

3R Scripture exon 25266849 25267697 . - . gene_id "XLOC_175464"; transcript_id "TCONS_00666900"; exon_number "1"; gene_type "anti-sense_lncRNA";

3R Scripture exon 25269923 25270974 . - . gene_id "XLOC_175464"; transcript_id "TCONS_00666900"; exon_number "2"; gene_type "anti-sense_lncRNA";

3R Scripture exon 25268039 25268212 . - . gene_id "XLOC_175464"; transcript_id "TCONS_00876505"; exon_number "1"; gene_type "lincRNA";

3R Scripture exon 25268269 25269713 . - . gene_id "XLOC_175464"; transcript_id "TCONS_00876505"; exon_number "2"; gene_type "lincRNA";

3R Cufflinks exon 25303887 25304092 . - . gene_id "XLOC_175477"; transcript_id "TCONS_00729842"; exon_number "1"; gene_type "anti-sense_lncRNA";

3R Cufflinks exon 25341528 25341744 . - . gene_id "XLOC_175490"; transcript_id "TCONS_00666927"; exon_number "1"; gene_type "anti-sense_lncRNA";

3R Cufflinks exon 25661782 25662740 . - . gene_id "XLOC_175631"; transcript_id "TCONS_00666995"; exon_number "1"; gene_type "anti-sense_lncRNA";

3R Scripture exon 25718627 25718788 . - . gene_id "XLOC_175663"; transcript_id "TCONS_00884159"; exon_number "1"; gene_type "intronic_lncRNA";

3R Scripture exon 25719791 25719887 . - . gene_id "XLOC_175663"; transcript_id "TCONS_00884159"; exon_number "2"; gene_type "intronic_lncRNA";

3R Cufflinks exon 25864471 25864681 . - . gene_id "XLOC_175776"; transcript_id "TCONS_00730021"; exon_number "1"; gene_type "intronic_lncRNA";

3R Cufflinks exon 26104094 26104289 . - . gene_id "XLOC_175972"; transcript_id "TCONS_00667283"; exon_number "1"; gene_type "lincRNA";

3R Cufflinks exon 26104353 26104409 . - . gene_id "XLOC_175972"; transcript_id "TCONS_00667283"; exon_number "2"; gene_type "lincRNA";

3R Cufflinks exon 26121647 26121868 . - . gene_id "XLOC_175980"; transcript_id "TCONS_00701303"; exon_number "1"; gene_type "intronic_lncRNA";

3R Cufflinks exon 26153273 26153499 . - . gene_id "XLOC_176022"; transcript_id "TCONS_00756340"; exon_number "1"; gene_type "lincRNA";

3R Cufflinks exon 26368034 26368234 . - . gene_id "XLOC_176225"; transcript_id "TCONS_00701454"; exon_number "1"; gene_type "lincRNA";

3R Cufflinks exon 26419569 26419774 . - . gene_id "XLOC_176288"; transcript_id "TCONS_00667462"; exon_number "1"; gene_type "lincRNA";

3R Cufflinks exon 26529648 26530802 . - . gene_id "XLOC_176390"; transcript_id "TCONS_00667501"; exon_number "1"; gene_type "intronic_lncRNA";

3R Cufflinks exon 26531019 26531366 . - . gene_id "XLOC_176391"; transcript_id "TCONS_00667502"; exon_number "1"; gene_type "intronic_lncRNA";

3R Cufflinks exon 26557173 26557382 . - . gene_id "XLOC_176402"; transcript_id "TCONS_00667506"; exon_number "1"; gene_type "intronic_lncRNA";

3R Cufflinks exon 26786700 26786910 . - . gene_id "XLOC_176601"; transcript_id "TCONS_00701735"; exon_number "1"; gene_type "lincRNA";

3R Cufflinks exon 27070626 27070834 . - . gene_id "XLOC_176743"; transcript_id "TCONS_00730507"; exon_number "1"; gene_type "lincRNA";

3R Cufflinks exon 27084950 27085155 . - . gene_id "XLOC_176762"; transcript_id "TCONS_00667763"; exon_number "1"; gene_type "intronic_lncRNA";

3R Cufflinks exon 27324459 27324672 . - . gene_id "XLOC_176897"; transcript_id "TCONS_00667911"; exon_number "1"; gene_type "lincRNA";

3R Cufflinks exon 27535686 27537486 . - . gene_id "XLOC_177109"; transcript_id "TCONS_00668122"; exon_number "1"; gene_type "lincRNA";

3R Cufflinks exon 27537686 27537922 . - . gene_id "XLOC_177109"; transcript_id "TCONS_00668122"; exon_number "2"; gene_type "lincRNA";

3R Cufflinks exon 27610946 27611158 . - . gene_id "XLOC_177160"; transcript_id "TCONS_00668143"; exon_number "1"; gene_type "intronic_lncRNA";

3R Cufflinks exon 27710902 27711113 . - . gene_id "XLOC_177219"; transcript_id "TCONS_00779021"; exon_number "1"; gene_type "lincRNA";

3R Cufflinks exon 28268505 28268704 . - . gene_id "XLOC_177860"; transcript_id "TCONS_00731153"; exon_number "1"; gene_type "lincRNA";

3R Cufflinks exon 28271614 28271813 . - . gene_id "XLOC_177867"; transcript_id "TCONS_00702625"; exon_number "1"; gene_type "lincRNA";

3R Cufflinks exon 28279549 28279753 . - . gene_id "XLOC_177879"; transcript_id "TCONS_00702631"; exon_number "1"; gene_type "lincRNA";

3R Cufflinks exon 28355861 28356066 . - . gene_id "XLOC_177961"; transcript_id "TCONS_00702696"; exon_number "1"; gene_type "lincRNA";

3R Cufflinks exon 28373123 28373344 . - . gene_id "XLOC_177987"; transcript_id "TCONS_00757283"; exon_number "1"; gene_type "lincRNA";

3R Cufflinks exon 28958335 28958564 . - . gene_id "XLOC_178542"; transcript_id "TCONS_00757520"; exon_number "1"; gene_type "intronic_lncRNA";

3R Cufflinks exon 28978776 28979004 . - . gene_id "XLOC_178569"; transcript_id "TCONS_00668986"; exon_number "1"; gene_type "lincRNA";

3R Cufflinks exon 29054321 29055370 . - . gene_id "XLOC_178613"; transcript_id "TCONS_00669009"; exon_number "1"; gene_type "anti-sense_lncRNA";

3R Cufflinks exon 29261223 29261423 . - . gene_id "XLOC_178742"; transcript_id "TCONS_00703157"; exon_number "1"; gene_type "lincRNA";

3R Cufflinks exon 29410720 29410962 . - . gene_id "XLOC_178836"; transcript_id "TCONS_00703321"; exon_number "1"; gene_type "anti-sense_lncRNA";

3R Cufflinks exon 29455059 29455087 . - . gene_id "XLOC_178836"; transcript_id "TCONS_00703321"; exon_number "2"; gene_type "anti-sense_lncRNA";

3R Cufflinks exon 29455180 29455849 . - . gene_id "XLOC_178836"; transcript_id "TCONS_00703321"; exon_number "3"; gene_type "anti-sense_lncRNA";

3R Cufflinks exon 29454823 29455087 . - . gene_id "XLOC_178836"; transcript_id "TCONS_00779686"; exon_number "1"; gene_type "intronic_lncRNA";

3R Cufflinks exon 29455180 29455535 . - . gene_id "XLOC_178836"; transcript_id "TCONS_00779686"; exon_number "2"; gene_type "intronic_lncRNA";

3R Cufflinks exon 29691934 29692140 . - . gene_id "XLOC_179234"; transcript_id "TCONS_00731904"; exon_number "1"; gene_type "lincRNA";

3R Cufflinks exon 29927692 29927900 . - . gene_id "XLOC_179391"; transcript_id "TCONS_00779850"; exon_number "1"; gene_type "anti-sense_lncRNA";

3R Cufflinks exon 30255836 30256039 . - . gene_id "XLOC_179631"; transcript_id "TCONS_00669639"; exon_number "1"; gene_type "lincRNA";

3R Cufflinks exon 30269859 30270068 . - . gene_id "XLOC_179655"; transcript_id "TCONS_00758142"; exon_number "1"; gene_type "lincRNA";

3R Cufflinks exon 30391206 30391415 . - . gene_id "XLOC_179707"; transcript_id "TCONS_00732135"; exon_number "1"; gene_type "lincRNA";

3R Cufflinks exon 30405125 30405146 . - . gene_id "XLOC_179716"; transcript_id "TCONS_00703841"; exon_number "1"; gene_type "lincRNA";

3R Cufflinks exon 30405329 30406008 . - . gene_id "XLOC_179716"; transcript_id "TCONS_00703841"; exon_number "2"; gene_type "lincRNA";

3R Cufflinks exon 30545080 30545295 . - . gene_id "XLOC_179824"; transcript_id "TCONS_00669766"; exon_number "1"; gene_type "intronic_lncRNA";

3R Cufflinks exon 30762307 30762634 . - . gene_id "XLOC_179981"; transcript_id "TCONS_00806033"; exon_number "1"; gene_type "anti-sense_lncRNA";

3R Cufflinks exon 30781157 30781421 . - . gene_id "XLOC_179981"; transcript_id "TCONS_00806033"; exon_number "2"; gene_type "anti-sense_lncRNA";

3R Cufflinks exon 30781477 30781754 . - . gene_id "XLOC_179981"; transcript_id "TCONS_00806033"; exon_number "3"; gene_type "anti-sense_lncRNA";

3R Cufflinks exon 30783166 30783464 . - . gene_id "XLOC_179981"; transcript_id "TCONS_00806033"; exon_number "4"; gene_type "anti-sense_lncRNA";

3R Cufflinks exon 30783538 30783910 . - . gene_id "XLOC_179981"; transcript_id "TCONS_00806033"; exon_number "5"; gene_type "anti-sense_lncRNA";

3R Scripture exon 30779211 30780630 . - . gene_id "XLOC_179981"; transcript_id "TCONS_00704133"; exon_number "1"; gene_type "intronic_lncRNA";

3R Scripture exon 30781157 30781421 . - . gene_id "XLOC_179981"; transcript_id "TCONS_00704133"; exon_number "2"; gene_type "intronic_lncRNA";

3R Scripture exon 30781477 30781754 . - . gene_id "XLOC_179981"; transcript_id "TCONS_00704133"; exon_number "3"; gene_type "intronic_lncRNA";

3R Scripture exon 30783166 30783464 . - . gene_id "XLOC_179981"; transcript_id "TCONS_00704133"; exon_number "4"; gene_type "intronic_lncRNA";

3R Scripture exon 30783538 30783650 . - . gene_id "XLOC_179981"; transcript_id "TCONS_00704133"; exon_number "5"; gene_type "intronic_lncRNA";

3R Cufflinks exon 30780722 30781421 . - . gene_id "XLOC_179981"; transcript_id "TCONS_00669989"; exon_number "1"; gene_type "intronic_lncRNA";

3R Cufflinks exon 30781477 30781754 . - . gene_id "XLOC_179981"; transcript_id "TCONS_00669989"; exon_number "2"; gene_type "intronic_lncRNA";

3R Cufflinks exon 30783166 30783464 . - . gene_id "XLOC_179981"; transcript_id "TCONS_00669989"; exon_number "3"; gene_type "intronic_lncRNA";

3R Cufflinks exon 30783538 30783647 . - . gene_id "XLOC_179981"; transcript_id "TCONS_00669989"; exon_number "4"; gene_type "intronic_lncRNA";

3R Scripture exon 30780980 30781421 . - . gene_id "XLOC_179981"; transcript_id "TCONS_00895425"; exon_number "1"; gene_type "intronic_lncRNA";

3R Scripture exon 30781477 30781729 . - . gene_id "XLOC_179981"; transcript_id "TCONS_00895425"; exon_number "2"; gene_type "intronic_lncRNA";

3R Scripture exon 30783166 30783464 . - . gene_id "XLOC_179981"; transcript_id "TCONS_00895425"; exon_number "3"; gene_type "intronic_lncRNA";

3R Scripture exon 30783538 30783650 . - . gene_id "XLOC_179981"; transcript_id "TCONS_00895425"; exon_number "4"; gene_type "intronic_lncRNA";

3R Cufflinks exon 30830139 30830356 . - . gene_id "XLOC_180008"; transcript_id "TCONS_00704139"; exon_number "1"; gene_type "intronic_lncRNA";

3R Cufflinks exon 30891622 30892152 . - . gene_id "XLOC_180073"; transcript_id "TCONS_00670054"; exon_number "1"; gene_type "lincRNA";

3R Cufflinks exon 30893374 30893499 . - . gene_id "XLOC_180073"; transcript_id "TCONS_00670054"; exon_number "2"; gene_type "lincRNA";

3R Cufflinks exon 30891748 30892152 . - . gene_id "XLOC_180073"; transcript_id "TCONS_00670053"; exon_number "1"; gene_type "lincRNA";

3R Cufflinks exon 30893069 30893499 . - . gene_id "XLOC_180073"; transcript_id "TCONS_00670053"; exon_number "2"; gene_type "lincRNA";

3R Scripture exon 30891749 30891811 . - . gene_id "XLOC_180073"; transcript_id "TCONS_00806098"; exon_number "1"; gene_type "lincRNA";

3R Scripture exon 30891909 30892152 . - . gene_id "XLOC_180073"; transcript_id "TCONS_00806098"; exon_number "2"; gene_type "lincRNA";

3R Scripture exon 30893374 30893499 . - . gene_id "XLOC_180073"; transcript_id "TCONS_00806098"; exon_number "3"; gene_type "lincRNA";

3R Cufflinks exon 30891783 30891811 . - . gene_id "XLOC_180073"; transcript_id "TCONS_00732432"; exon_number "1"; gene_type "lincRNA";

3R Cufflinks exon 30891909 30892583 . - . gene_id "XLOC_180073"; transcript_id "TCONS_00732432"; exon_number "2"; gene_type "lincRNA";

3R Cufflinks exon 31217362 31217657 . - . gene_id "XLOC_180380"; transcript_id "TCONS_00670281"; exon_number "1"; gene_type "intronic_lncRNA";

3R Cufflinks exon 31419806 31420010 . - . gene_id "XLOC_180542"; transcript_id "TCONS_00704510"; exon_number "1"; gene_type "intronic_lncRNA";

3R Cufflinks exon 31483431 31483635 . - . gene_id "XLOC_180589"; transcript_id "TCONS_00670388"; exon_number "1"; gene_type "lincRNA";

3R Scripture exon 31587997 31588471 . - . gene_id "XLOC_180681"; transcript_id "TCONS_00884502"; exon_number "1"; gene_type "anti-sense_lncRNA";

3R Scripture exon 31592016 31593032 . - . gene_id "XLOC_180681"; transcript_id "TCONS_00884502"; exon_number "2"; gene_type "anti-sense_lncRNA";

3R Scripture exon 31589346 31590283 . - . gene_id "XLOC_180681"; transcript_id "TCONS_00866737"; exon_number "1"; gene_type "anti-sense_lncRNA";

3R Scripture exon 31590341 31592330 . - . gene_id "XLOC_180681"; transcript_id "TCONS_00866737"; exon_number "2"; gene_type "anti-sense_lncRNA";

3R Cufflinks exon 32010638 32010675 . - . gene_id "XLOC_180802"; transcript_id "TCONS_00704757"; exon_number "1"; gene_type "intronic_lncRNA";

3R Cufflinks exon 32010776 32010997 . - . gene_id "XLOC_180802"; transcript_id "TCONS_00704757"; exon_number "2"; gene_type "intronic_lncRNA";

4 Cufflinks exon 56921 57411 . + . gene_id "XLOC_186025"; transcript_id "TCONS_00911200"; exon_number "1"; gene_type "anti-sense_lncRNA";

4 Cufflinks exon 59976 60570 . + . gene_id "XLOC_186025"; transcript_id "TCONS_00911200"; exon_number "2"; gene_type "anti-sense_lncRNA";

4 Cufflinks exon 56921 57411 . + . gene_id "XLOC_186025"; transcript_id "TCONS_00911199"; exon_number "1"; gene_type "anti-sense_lncRNA";

4 Cufflinks exon 59976 60415 . + . gene_id "XLOC_186025"; transcript_id "TCONS_00911199"; exon_number "2"; gene_type "anti-sense_lncRNA";

4 Cufflinks exon 60570 60570 . + . gene_id "XLOC_186025"; transcript_id "TCONS_00911199"; exon_number "3"; gene_type "anti-sense_lncRNA";

4 Scripture exon 454949 455942 . + . gene_id "XLOC_186036"; transcript_id "TCONS_00927213"; exon_number "1"; gene_type "anti-sense_lncRNA";

4 Scripture exon 460056 460520 . + . gene_id "XLOC_186036"; transcript_id "TCONS_00927213"; exon_number "2"; gene_type "anti-sense_lncRNA";

4 Scripture exon 457890 459907 . + . gene_id "XLOC_186036"; transcript_id "TCONS_00918065"; exon_number "1"; gene_type "anti-sense_lncRNA";

4 Scripture exon 460056 460563 . + . gene_id "XLOC_186036"; transcript_id "TCONS_00918065"; exon_number "2"; gene_type "anti-sense_lncRNA";

4 Scripture exon 460574 460608 . + . gene_id "XLOC_186036"; transcript_id "TCONS_00918067"; exon_number "1"; gene_type "anti-sense_lncRNA";

4 Scripture exon 460729 461619 . + . gene_id "XLOC_186036"; transcript_id "TCONS_00918067"; exon_number "2"; gene_type "anti-sense_lncRNA";

4 Cufflinks exon 285960 286170 . + . gene_id "XLOC_186100"; transcript_id "TCONS_00912264"; exon_number "1"; gene_type "intronic_lncRNA";

4 Cufflinks exon 380790 380811 . + . gene_id "XLOC_186132"; transcript_id "TCONS_00912278"; exon_number "1"; gene_type "intronic_lncRNA";

4 Cufflinks exon 381045 381367 . + . gene_id "XLOC_186132"; transcript_id "TCONS_00912278"; exon_number "2"; gene_type "intronic_lncRNA";

4 Cufflinks exon 427064 427711 . + . gene_id "XLOC_186174"; transcript_id "TCONS_00913096"; exon_number "1"; gene_type "lincRNA";

4 Cufflinks exon 429437 432017 . + . gene_id "XLOC_186174"; transcript_id "TCONS_00913096"; exon_number "2"; gene_type "lincRNA";

4 Cufflinks exon 427432 427711 . + . gene_id "XLOC_186174"; transcript_id "TCONS_00911366"; exon_number "1"; gene_type "lincRNA";

4 Cufflinks exon 429497 432009 . + . gene_id "XLOC_186174"; transcript_id "TCONS_00911366"; exon_number "2"; gene_type "lincRNA";

4 Cufflinks exon 550046 550273 . + . gene_id "XLOC_186240"; transcript_id "TCONS_00912364"; exon_number "1"; gene_type "intronic_lncRNA";

4 Cufflinks exon 553949 554151 . + . gene_id "XLOC_186241"; transcript_id "TCONS_00911430"; exon_number "1"; gene_type "intronic_lncRNA";

4 Scripture exon 878851 879943 . + . gene_id "XLOC_186340"; transcript_id "TCONS_00918514"; exon_number "1"; gene_type "anti-sense_lncRNA";

4 Scripture exon 884564 884698 . + . gene_id "XLOC_186340"; transcript_id "TCONS_00918514"; exon_number "2"; gene_type "anti-sense_lncRNA";

4 Scripture exon 878851 879450 . + . gene_id "XLOC_186340"; transcript_id "TCONS_00918513"; exon_number "1"; gene_type "anti-sense_lncRNA";

4 Scripture exon 884564 884740 . + . gene_id "XLOC_186340"; transcript_id "TCONS_00918513"; exon_number "2"; gene_type "anti-sense_lncRNA";

4 Scripture exon 883143 883497 . + . gene_id "XLOC_186340"; transcript_id "TCONS_00924714"; exon_number "1"; gene_type "anti-sense_lncRNA";

4 Scripture exon 883570 884120 . + . gene_id "XLOC_186340"; transcript_id "TCONS_00924714"; exon_number "2"; gene_type "anti-sense_lncRNA";

4 Scripture exon 884046 884705 . + . gene_id "XLOC_186340"; transcript_id "TCONS_00924717"; exon_number "1"; gene_type "intronic_lncRNA";

4 Scripture exon 1027677 1028328 . + . gene_id "XLOC_186391"; transcript_id "TCONS_00912513"; exon_number "1"; gene_type "anti-sense_lncRNA";

4 Scripture exon 1028710 1030062 . + . gene_id "XLOC_186391"; transcript_id "TCONS_00912513"; exon_number "2"; gene_type "anti-sense_lncRNA";

4 Scripture exon 1027715 1027882 . + . gene_id "XLOC_186391"; transcript_id "TCONS_00911614"; exon_number "1"; gene_type "anti-sense_lncRNA";

4 Scripture exon 1028710 1030062 . + . gene_id "XLOC_186391"; transcript_id "TCONS_00911614"; exon_number "2"; gene_type "anti-sense_lncRNA";

4 Cufflinks exon 1255484 1255720 . + . gene_id "XLOC_186465"; transcript_id "TCONS_00912590"; exon_number "1"; gene_type "intronic_lncRNA";

4 Cufflinks exon 1278768 1280424 . + . gene_id "XLOC_186478"; transcript_id "TCONS_00915770"; exon_number "1"; gene_type "lincRNA";

4 Cufflinks exon 1280494 1280856 . + . gene_id "XLOC_186478"; transcript_id "TCONS_00915770"; exon_number "2"; gene_type "lincRNA";

4 Cufflinks exon 1280916 1281456 . + . gene_id "XLOC_186478"; transcript_id "TCONS_00915770"; exon_number "3"; gene_type "lincRNA";

4 Cufflinks exon 1278768 1280424 . + . gene_id "XLOC_186478"; transcript_id "TCONS_00912597"; exon_number "1"; gene_type "lincRNA";

4 Cufflinks exon 1280494 1280670 . + . gene_id "XLOC_186478"; transcript_id "TCONS_00912597"; exon_number "2"; gene_type "lincRNA";

4 Cufflinks exon 1280761 1280856 . + . gene_id "XLOC_186478"; transcript_id "TCONS_00912597"; exon_number "3"; gene_type "lincRNA";

4 Cufflinks exon 1280916 1281456 . + . gene_id "XLOC_186478"; transcript_id "TCONS_00912597"; exon_number "4"; gene_type "lincRNA";

4 Cufflinks exon 1278784 1279560 . + . gene_id "XLOC_186478"; transcript_id "TCONS_00911709"; exon_number "1"; gene_type "lincRNA";

4 Cufflinks exon 1279625 1279635 . + . gene_id "XLOC_186478"; transcript_id "TCONS_00911709"; exon_number "2"; gene_type "lincRNA";

4 Cufflinks exon 1295028 1295260 . + . gene_id "XLOC_186484"; transcript_id "TCONS_00911712"; exon_number "1"; gene_type "lincRNA";

4 Cufflinks exon 181439 181516 . - . gene_id "XLOC_186547"; transcript_id "TCONS_00912651"; exon_number "1"; gene_type "lincRNA";

4 Cufflinks exon 181569 182300 . - . gene_id "XLOC_186547"; transcript_id "TCONS_00912651"; exon_number "2"; gene_type "lincRNA";

4 Cufflinks exon 181439 181509 . - . gene_id "XLOC_186547"; transcript_id "TCONS_00911767"; exon_number "1"; gene_type "lincRNA";

4 Cufflinks exon 181569 182300 . - . gene_id "XLOC_186547"; transcript_id "TCONS_00911767"; exon_number "2"; gene_type "lincRNA";

4 Cufflinks exon 1125443 1125671 . - . gene_id "XLOC_186922"; transcript_id "TCONS_00916781"; exon_number "1"; gene_type "lincRNA";

4 Cufflinks exon 1247312 1247517 . - . gene_id "XLOC_186959"; transcript_id "TCONS_00916090"; exon_number "1"; gene_type "intronic_lncRNA";

Unmapped_Scaffold_28_D1723 Cufflinks exon 8937 9216 . + . gene_id "XLOC_187412"; transcript_id "TCONS_00937083"; exon_number "1"; gene_type "lincRNA";

Unmapped_Scaffold_29_D1705 Cufflinks exon 3842 4147 . - . gene_id "XLOC_187421"; transcript_id "TCONS_00937092"; exon_number "1"; gene_type "lincRNA";

Unmapped_Scaffold_37_D1608 Cufflinks exon 3017 3528 . - . gene_id "XLOC_187439"; transcript_id "TCONS_00937175"; exon_number "1"; gene_type "lincRNA";

Unmapped_Scaffold_37_D1608 Cufflinks exon 13557 15866 . - . gene_id "XLOC_187439"; transcript_id "TCONS_00937175"; exon_number "2"; gene_type "lincRNA";

X Scripture exon 258893 259690 . + . gene_id "XLOC_187707"; transcript_id "TCONS_01082600"; exon_number "1"; gene_type "anti-sense_lncRNA";

X Scripture exon 259754 263005 . + . gene_id "XLOC_187707"; transcript_id "TCONS_01082600"; exon_number "2"; gene_type "anti-sense_lncRNA";

X Cufflinks exon 544274 544492 . + . gene_id "XLOC_187919"; transcript_id "TCONS_00938312"; exon_number "1"; gene_type "intronic_lncRNA";

X Cufflinks exon 673000 673223 . + . gene_id "XLOC_187974"; transcript_id "TCONS_00938372"; exon_number "1"; gene_type "lincRNA";

X Cufflinks exon 865385 865588 . + . gene_id "XLOC_188047"; transcript_id "TCONS_00960819"; exon_number "1"; gene_type "intronic_lncRNA";

X Cufflinks exon 874224 874424 . + . gene_id "XLOC_188056"; transcript_id "TCONS_00960826"; exon_number "1"; gene_type "intronic_lncRNA";

X Cufflinks exon 1083772 1083994 . + . gene_id "XLOC_188187"; transcript_id "TCONS_00938545"; exon_number "1"; gene_type "intronic_lncRNA";

X Cufflinks exon 1084690 1084943 . + . gene_id "XLOC_188189"; transcript_id "TCONS_01019375"; exon_number "1"; gene_type "intronic_lncRNA";

X Cufflinks exon 1088274 1088476 . + . gene_id "XLOC_188198"; transcript_id "TCONS_00938549"; exon_number "1"; gene_type "intronic_lncRNA";

X Cufflinks exon 1128786 1128985 . + . gene_id "XLOC_188242"; transcript_id "TCONS_00960962"; exon_number "1"; gene_type "intronic_lncRNA";

X Cufflinks exon 1314475 1314676 . + . gene_id "XLOC_188361"; transcript_id "TCONS_01019484"; exon_number "1"; gene_type "intronic_lncRNA";

X Cufflinks exon 1570802 1571019 . + . gene_id "XLOC_188491"; transcript_id "TCONS_00961187"; exon_number "1"; gene_type "intronic_lncRNA";

X Cufflinks exon 1708139 1708343 . + . gene_id "XLOC_188619"; transcript_id "TCONS_00938938"; exon_number "1"; gene_type "lincRNA";

X Scripture exon 2069876 2070203 . + . gene_id "XLOC_188797"; transcript_id "TCONS_01082857"; exon_number "1"; gene_type "anti-sense_lncRNA";

X Scripture exon 2070273 2071580 . + . gene_id "XLOC_188797"; transcript_id "TCONS_01082857"; exon_number "2"; gene_type "anti-sense_lncRNA";

X Cufflinks exon 2320624 2320833 . + . gene_id "XLOC_188883"; transcript_id "TCONS_00939205"; exon_number "1"; gene_type "intronic_lncRNA";

X Cufflinks exon 2332356 2332564 . + . gene_id "XLOC_188898"; transcript_id "TCONS_00939222"; exon_number "1"; gene_type "anti-sense_lncRNA";

X Cufflinks exon 2351874 2352099 . + . gene_id "XLOC_188908"; transcript_id "TCONS_00961507"; exon_number "1"; gene_type "intronic_lncRNA";

X Cufflinks exon 2353435 2353645 . + . gene_id "XLOC_188910"; transcript_id "TCONS_00961508"; exon_number "1"; gene_type "intronic_lncRNA";

X Cufflinks exon 2496639 2496850 . + . gene_id "XLOC_189007"; transcript_id "TCONS_00939283"; exon_number "1"; gene_type "intronic_lncRNA";

X Cufflinks exon 2757373 2757578 . + . gene_id "XLOC_189118"; transcript_id "TCONS_00961656"; exon_number "1"; gene_type "intronic_lncRNA";

X Cufflinks exon 2958152 2958368 . + . gene_id "XLOC_189357"; transcript_id "TCONS_00939575"; exon_number "1"; gene_type "anti-sense_lncRNA";

X Cufflinks exon 2991150 2991356 . + . gene_id "XLOC_189396"; transcript_id "TCONS_00939597"; exon_number "1"; gene_type "intronic_lncRNA";

X Cufflinks exon 3012475 3012676 . + . gene_id "XLOC_189424"; transcript_id "TCONS_00961875"; exon_number "1"; gene_type "intronic_lncRNA";

X Cufflinks exon 3301079 3301301 . + . gene_id "XLOC_189716"; transcript_id "TCONS_00939787"; exon_number "1"; gene_type "intronic_lncRNA";

X Cufflinks exon 3347141 3347195 . + . gene_id "XLOC_189747"; transcript_id "TCONS_00985240"; exon_number "1"; gene_type "lincRNA";

X Cufflinks exon 3348663 3348907 . + . gene_id "XLOC_189747"; transcript_id "TCONS_00985240"; exon_number "2"; gene_type "lincRNA";

X Cufflinks exon 3522550 3522791 . + . gene_id "XLOC_189798"; transcript_id "TCONS_00939948"; exon_number "1"; gene_type "anti-sense_lncRNA";

X Cufflinks exon 3530322 3530386 . + . gene_id "XLOC_189798"; transcript_id "TCONS_00939948"; exon_number "2"; gene_type "anti-sense_lncRNA";

X Cufflinks exon 3543264 3543466 . + . gene_id "XLOC_189909"; transcript_id "TCONS_01034267"; exon_number "1"; gene_type "intronic_lncRNA";

X Cufflinks exon 3562963 3563164 . + . gene_id "XLOC_189941"; transcript_id "TCONS_00985378"; exon_number "1"; gene_type "anti-sense_lncRNA";

X Cufflinks exon 4087585 4087807 . + . gene_id "XLOC_190248"; transcript_id "TCONS_01003980"; exon_number "1"; gene_type "intronic_lncRNA";

X Cufflinks exon 4573104 4573175 . + . gene_id "XLOC_190700"; transcript_id "TCONS_01051683"; exon_number "1"; gene_type "lincRNA";

X Cufflinks exon 4573271 4573534 . + . gene_id "XLOC_190700"; transcript_id "TCONS_01051683"; exon_number "2"; gene_type "lincRNA";

X Cufflinks exon 4592409 4592608 . + . gene_id "XLOC_190728"; transcript_id "TCONS_01051698"; exon_number "1"; gene_type "lincRNA";

X Cufflinks exon 4742038 4742246 . + . gene_id "XLOC_190810"; transcript_id "TCONS_00963121"; exon_number "1"; gene_type "lincRNA";

X Cufflinks exon 5019676 5019883 . + . gene_id "XLOC_191121"; transcript_id "TCONS_01004478"; exon_number "1"; gene_type "lincRNA";

X Cufflinks exon 5215737 5216192 . + . gene_id "XLOC_191170"; transcript_id "TCONS_00941013"; exon_number "1"; gene_type "intronic_lncRNA";

X Cufflinks exon 5464966 5465242 . + . gene_id "XLOC_191398"; transcript_id "TCONS_00941175"; exon_number "1"; gene_type "intronic_lncRNA";

X Cufflinks exon 6024947 6025151 . + . gene_id "XLOC_191850"; transcript_id "TCONS_00986570"; exon_number "1"; gene_type "lincRNA";

X Cufflinks exon 6033189 6033418 . + . gene_id "XLOC_191865"; transcript_id "TCONS_00963876"; exon_number "1"; gene_type "lincRNA";

X Cufflinks exon 6736394 6736686 . + . gene_id "XLOC_192320"; transcript_id "TCONS_00986763"; exon_number "1"; gene_type "intronic_lncRNA";

X Cufflinks exon 6748740 6748940 . + . gene_id "XLOC_192342"; transcript_id "TCONS_00964122"; exon_number "1"; gene_type "intronic_lncRNA";

X Cufflinks exon 6750627 6750882 . + . gene_id "XLOC_192347"; transcript_id "TCONS_00941729"; exon_number "1"; gene_type "intronic_lncRNA";

X Cufflinks exon 6800860 6801059 . + . gene_id "XLOC_192371"; transcript_id "TCONS_00964141"; exon_number "1"; gene_type "intronic_lncRNA";

X Cufflinks exon 6979235 6979443 . + . gene_id "XLOC_192536"; transcript_id "TCONS_00986870"; exon_number "1"; gene_type "intronic_lncRNA";

X Cufflinks exon 7375990 7376250 . + . gene_id "XLOC_192828"; transcript_id "TCONS_00986987"; exon_number "1"; gene_type "intronic_lncRNA";

X Cufflinks exon 7404668 7404900 . + . gene_id "XLOC_192871"; transcript_id "TCONS_00964439"; exon_number "1"; gene_type "intronic_lncRNA";

X Cufflinks exon 7521252 7521467 . + . gene_id "XLOC_193050"; transcript_id "TCONS_00942143"; exon_number "1"; gene_type "intronic_lncRNA";

X Cufflinks exon 8331423 8331663 . + . gene_id "XLOC_193775"; transcript_id "TCONS_00987475"; exon_number "1"; gene_type "intronic_lncRNA";

X Cufflinks exon 8353467 8353669 . + . gene_id "XLOC_193808"; transcript_id "TCONS_00965027"; exon_number "1"; gene_type "intronic_lncRNA";

X Cufflinks exon 8638117 8638323 . + . gene_id "XLOC_194015"; transcript_id "TCONS_00942665"; exon_number "1"; gene_type "intronic_lncRNA";

X Cufflinks exon 8651283 8651506 . + . gene_id "XLOC_194038"; transcript_id "TCONS_00942673"; exon_number "1"; gene_type "lincRNA";

X Cufflinks exon 8771703 8771910 . + . gene_id "XLOC_194202"; transcript_id "TCONS_00942738"; exon_number "1"; gene_type "intronic_lncRNA";

X Cufflinks exon 9126383 9126596 . + . gene_id "XLOC_194524"; transcript_id "TCONS_00942865"; exon_number "1"; gene_type "intronic_lncRNA";

X Scripture exon 9133940 9134190 . + . gene_id "XLOC_194531"; transcript_id "TCONS_01108968"; exon_number "1"; gene_type "anti-sense_lncRNA";

X Scripture exon 9134983 9135211 . + . gene_id "XLOC_194531"; transcript_id "TCONS_01108968"; exon_number "2"; gene_type "anti-sense_lncRNA";

X Cufflinks exon 9528188 9528413 . + . gene_id "XLOC_194801"; transcript_id "TCONS_00943003"; exon_number "1"; gene_type "intronic_lncRNA";

X Cufflinks exon 9689009 9689219 . + . gene_id "XLOC_194878"; transcript_id "TCONS_01053371"; exon_number "1"; gene_type "lincRNA";

X Cufflinks exon 9764496 9764707 . + . gene_id "XLOC_194996"; transcript_id "TCONS_00965635"; exon_number "1"; gene_type "lincRNA";

X Cufflinks exon 9869414 9869625 . + . gene_id "XLOC_195065"; transcript_id "TCONS_00965671"; exon_number "1"; gene_type "lincRNA";

X Cufflinks exon 9870778 9870985 . + . gene_id "XLOC_195066"; transcript_id "TCONS_00965672"; exon_number "1"; gene_type "lincRNA";

X Cufflinks exon 10181342 10181542 . + . gene_id "XLOC_195394"; transcript_id "TCONS_00965886"; exon_number "1"; gene_type "intronic_lncRNA";

X Cufflinks exon 10655921 10656125 . + . gene_id "XLOC_195671"; transcript_id "TCONS_01037006"; exon_number "1"; gene_type "intronic_lncRNA";

X Cufflinks exon 10753873 10754074 . + . gene_id "XLOC_195738"; transcript_id "TCONS_01053755"; exon_number "1"; gene_type "intronic_lncRNA";

X Cufflinks exon 10893210 10893440 . + . gene_id "XLOC_195802"; transcript_id "TCONS_01006460"; exon_number "1"; gene_type "lincRNA";

X Cufflinks exon 10893507 10893690 . + . gene_id "XLOC_195802"; transcript_id "TCONS_01006460"; exon_number "2"; gene_type "lincRNA";

X Cufflinks exon 11079376 11079604 . + . gene_id "XLOC_196001"; transcript_id "TCONS_00966240"; exon_number "1"; gene_type "intronic_lncRNA";

X Cufflinks exon 11189965 11190185 . + . gene_id "XLOC_196039"; transcript_id "TCONS_00943779"; exon_number "1"; gene_type "lincRNA";

X Cufflinks exon 11416697 11416901 . + . gene_id "XLOC_196193"; transcript_id "TCONS_01006651"; exon_number "1"; gene_type "anti-sense_lncRNA";

X Scripture exon 11805106 11805840 . + . gene_id "XLOC_196309"; transcript_id "TCONS_01109389"; exon_number "1"; gene_type "intronic_lncRNA";

X Scripture exon 11806047 11806610 . + . gene_id "XLOC_196309"; transcript_id "TCONS_01109389"; exon_number "2"; gene_type "intronic_lncRNA";

X Cufflinks exon 11875815 11876393 . + . gene_id "XLOC_196331"; transcript_id "TCONS_00943984"; exon_number "1"; gene_type "anti-sense_lncRNA";

X Cufflinks exon 11876464 11876999 . + . gene_id "XLOC_196331"; transcript_id "TCONS_00943984"; exon_number "2"; gene_type "anti-sense_lncRNA";

X Cufflinks exon 12085009 12085211 . + . gene_id "XLOC_196488"; transcript_id "TCONS_01037408"; exon_number "1"; gene_type "intronic_lncRNA";

X Cufflinks exon 12182190 12182411 . + . gene_id "XLOC_196643"; transcript_id "TCONS_01022786"; exon_number "1"; gene_type "intronic_lncRNA";

X Cufflinks exon 12383048 12383253 . + . gene_id "XLOC_196960"; transcript_id "TCONS_00966836"; exon_number "1"; gene_type "lincRNA";

X Cufflinks exon 12387319 12387524 . + . gene_id "XLOC_196963"; transcript_id "TCONS_00944271"; exon_number "1"; gene_type "lincRNA";

X Cufflinks exon 12423559 12423782 . + . gene_id "XLOC_197022"; transcript_id "TCONS_00966876"; exon_number "1"; gene_type "lincRNA";

X Cufflinks exon 12622257 12622364 . + . gene_id "XLOC_197151"; transcript_id "TCONS_01022977"; exon_number "1"; gene_type "lincRNA";

X Cufflinks exon 12622424 12622829 . + . gene_id "XLOC_197151"; transcript_id "TCONS_01022977"; exon_number "2"; gene_type "lincRNA";

X Cufflinks exon 12837912 12838178 . + . gene_id "XLOC_197228"; transcript_id "TCONS_00988991"; exon_number "1"; gene_type "intronic_lncRNA";

X Cufflinks exon 12890240 12890440 . + . gene_id "XLOC_197281"; transcript_id "TCONS_01007090"; exon_number "1"; gene_type "intronic_lncRNA";

X Cufflinks exon 13228577 13228805 . + . gene_id "XLOC_197575"; transcript_id "TCONS_00944647"; exon_number "1"; gene_type "intronic_lncRNA";

X Cufflinks exon 13327036 13327247 . + . gene_id "XLOC_197639"; transcript_id "TCONS_00944688"; exon_number "1"; gene_type "intronic_lncRNA";

X Cufflinks exon 13604468 13604670 . + . gene_id "XLOC_197920"; transcript_id "TCONS_00944855"; exon_number "1"; gene_type "lincRNA";

X Cufflinks exon 13604898 13605222 . + . gene_id "XLOC_197920"; transcript_id "TCONS_00944855"; exon_number "2"; gene_type "lincRNA";

X Scripture exon 13755253 13755470 . + . gene_id "XLOC_197949"; transcript_id "TCONS_01093661"; exon_number "1"; gene_type "anti-sense_lncRNA";

X Scripture exon 13755850 13755980 . + . gene_id "XLOC_197949"; transcript_id "TCONS_01093661"; exon_number "2"; gene_type "anti-sense_lncRNA";

X Cufflinks exon 13887487 13887686 . + . gene_id "XLOC_197979"; transcript_id "TCONS_00967478"; exon_number "1"; gene_type "intronic_lncRNA";

X Cufflinks exon 13937546 13937773 . + . gene_id "XLOC_198032"; transcript_id "TCONS_00967501"; exon_number "1"; gene_type "intronic_lncRNA";

X Cufflinks exon 13983199 13983430 . + . gene_id "XLOC_198073"; transcript_id "TCONS_00967525"; exon_number "1"; gene_type "intronic_lncRNA";

X Cufflinks exon 14070655 14070858 . + . gene_id "XLOC_198169"; transcript_id "TCONS_00967571"; exon_number "1"; gene_type "lincRNA";

X Cufflinks exon 14091813 14092030 . + . gene_id "XLOC_198208"; transcript_id "TCONS_00967593"; exon_number "1"; gene_type "lincRNA";

X Cufflinks exon 14371581 14371781 . + . gene_id "XLOC_198495"; transcript_id "TCONS_00945202"; exon_number "1"; gene_type "intronic_lncRNA";

X Cufflinks exon 14414573 14414802 . + . gene_id "XLOC_198572"; transcript_id "TCONS_00967841"; exon_number "1"; gene_type "intronic_lncRNA";

X Cufflinks exon 14733721 14733939 . + . gene_id "XLOC_198996"; transcript_id "TCONS_01007775"; exon_number "1"; gene_type "intronic_lncRNA";

X Cufflinks exon 14787651 14787881 . + . gene_id "XLOC_199057"; transcript_id "TCONS_00945426"; exon_number "1"; gene_type "anti-sense_lncRNA";

X Cufflinks exon 15209600 15209802 . + . gene_id "XLOC_199392"; transcript_id "TCONS_00968360"; exon_number "1"; gene_type "lincRNA";

X Cufflinks exon 15614118 15614328 . + . gene_id "XLOC_199781"; transcript_id "TCONS_00968537"; exon_number "1"; gene_type "intronic_lncRNA";

X Cufflinks exon 15630015 15630687 . + . gene_id "XLOC_199797"; transcript_id "TCONS_00945880"; exon_number "1"; gene_type "intronic_lncRNA";

X Cufflinks exon 15630754 15630984 . + . gene_id "XLOC_199797"; transcript_id "TCONS_00945880"; exon_number "2"; gene_type "intronic_lncRNA";

X Cufflinks exon 16205891 16206093 . + . gene_id "XLOC_200175"; transcript_id "TCONS_00968766"; exon_number "1"; gene_type "lincRNA";

X Cufflinks exon 16261399 16261629 . + . gene_id "XLOC_200245"; transcript_id "TCONS_00968821"; exon_number "1"; gene_type "lincRNA";

X Scripture exon 16293565 16293831 . + . gene_id "XLOC_200259"; transcript_id "TCONS_00968826"; exon_number "1"; gene_type "anti-sense_lncRNA";

X Scripture exon 16293902 16294040 . + . gene_id "XLOC_200259"; transcript_id "TCONS_00968826"; exon_number "2"; gene_type "anti-sense_lncRNA";

X Cufflinks exon 16831873 16832079 . + . gene_id "XLOC_200490"; transcript_id "TCONS_00946308"; exon_number "1"; gene_type "intronic_lncRNA";

X Cufflinks exon 17762864 17763079 . + . gene_id "XLOC_201255"; transcript_id "TCONS_00969385"; exon_number "1"; gene_type "lincRNA";

X Cufflinks exon 17786542 17786763 . + . gene_id "XLOC_201289"; transcript_id "TCONS_00969415"; exon_number "1"; gene_type "lincRNA";

X Cufflinks exon 18053533 18053756 . + . gene_id "XLOC_201516"; transcript_id "TCONS_00969526"; exon_number "1"; gene_type "intronic_lncRNA";

X Cufflinks exon 18154625 18154843 . + . gene_id "XLOC_201602"; transcript_id "TCONS_00969590"; exon_number "1"; gene_type "lincRNA";

X Cufflinks exon 18401749 18401950 . + . gene_id "XLOC_201889"; transcript_id "TCONS_01008914"; exon_number "1"; gene_type "intronic_lncRNA";

X Cufflinks exon 18702563 18702720 . + . gene_id "XLOC_202177"; transcript_id "TCONS_00947279"; exon_number "1"; gene_type "lincRNA";

X Cufflinks exon 18702896 18703395 . + . gene_id "XLOC_202177"; transcript_id "TCONS_00947279"; exon_number "2"; gene_type "lincRNA";

X Cufflinks exon 18723593 18723842 . + . gene_id "XLOC_202210"; transcript_id "TCONS_00947292"; exon_number "1"; gene_type "intronic_lncRNA";

X Cufflinks exon 18912346 18912500 . + . gene_id "XLOC_202310"; transcript_id "TCONS_00970043"; exon_number "1"; gene_type "lincRNA";

X Cufflinks exon 18912585 18912976 . + . gene_id "XLOC_202310"; transcript_id "TCONS_00970043"; exon_number "2"; gene_type "lincRNA";

X Cufflinks exon 19073129 19073337 . + . gene_id "XLOC_202399"; transcript_id "TCONS_00970111"; exon_number "1"; gene_type "lincRNA";

X Cufflinks exon 19130891 19131100 . + . gene_id "XLOC_202491"; transcript_id "TCONS_00970168"; exon_number "1"; gene_type "lincRNA";

X Cufflinks exon 19229510 19229720 . + . gene_id "XLOC_202552"; transcript_id "TCONS_00991528"; exon_number "1"; gene_type "intronic_lncRNA";

X Cufflinks exon 19299094 19299299 . + . gene_id "XLOC_202580"; transcript_id "TCONS_01040090"; exon_number "1"; gene_type "lincRNA";

X Cufflinks exon 19355117 19355352 . + . gene_id "XLOC_202620"; transcript_id "TCONS_01024902"; exon_number "1"; gene_type "intronic_lncRNA";

X Cufflinks exon 19453586 19453876 . + . gene_id "XLOC_202649"; transcript_id "TCONS_00970275"; exon_number "1"; gene_type "intronic_lncRNA";

X Cufflinks exon 20263239 20263448 . + . gene_id "XLOC_203202"; transcript_id "TCONS_00970559"; exon_number "1"; gene_type "lincRNA";

X Cufflinks exon 20375043 20375264 . + . gene_id "XLOC_203380"; transcript_id "TCONS_01056768"; exon_number "1"; gene_type "lincRNA";

X Cufflinks exon 20483957 20484163 . + . gene_id "XLOC_203455"; transcript_id "TCONS_01040409"; exon_number "1"; gene_type "intronic_lncRNA";

X Cufflinks exon 20498981 20499203 . + . gene_id "XLOC_203465"; transcript_id "TCONS_00948022"; exon_number "1"; gene_type "intronic_lncRNA";

X Cufflinks exon 20736933 20737138 . + . gene_id "XLOC_203863"; transcript_id "TCONS_00992105"; exon_number "1"; gene_type "lincRNA";

X Cufflinks exon 20873631 20873870 . + . gene_id "XLOC_204018"; transcript_id "TCONS_01056965"; exon_number "1"; gene_type "intronic_lncRNA";

X Cufflinks exon 20895299 20895506 . + . gene_id "XLOC_204041"; transcript_id "TCONS_00971019"; exon_number "1"; gene_type "intronic_lncRNA";

X Cufflinks exon 21283888 21284111 . + . gene_id "XLOC_204398"; transcript_id "TCONS_00948433"; exon_number "1"; gene_type "lincRNA";

X Cufflinks exon 21303724 21303927 . + . gene_id "XLOC_204401"; transcript_id "TCONS_00971248"; exon_number "1"; gene_type "intronic_lncRNA";

X Scripture exon 21396535 21397033 . + . gene_id "XLOC_204433"; transcript_id "TCONS_01101112"; exon_number "1"; gene_type "anti-sense_lncRNA";

X Scripture exon 21397121 21397396 . + . gene_id "XLOC_204433"; transcript_id "TCONS_01101112"; exon_number "2"; gene_type "anti-sense_lncRNA";

X Cufflinks exon 21594201 21594443 . + . gene_id "XLOC_204656"; transcript_id "TCONS_01009963"; exon_number "1"; gene_type "lincRNA";

X Cufflinks exon 21747042 21747701 . + . gene_id "XLOC_204687"; transcript_id "TCONS_00948616"; exon_number "1"; gene_type "lincRNA";

X Cufflinks exon 21916791 21917606 . + . gene_id "XLOC_204747"; transcript_id "TCONS_00948665"; exon_number "1"; gene_type "lincRNA";

X Cufflinks exon 22362491 22363010 . + . gene_id "XLOC_204874"; transcript_id "TCONS_00992557"; exon_number "1"; gene_type "lincRNA";

X Cufflinks exon 22408789 22408936 . + . gene_id "XLOC_204881"; transcript_id "TCONS_00971535"; exon_number "1"; gene_type "lincRNA";

X Cufflinks exon 22413264 22413341 . + . gene_id "XLOC_204881"; transcript_id "TCONS_00971535"; exon_number "2"; gene_type "lincRNA";

X Cufflinks exon 22559441 22559645 . + . gene_id "XLOC_204968"; transcript_id "TCONS_00992617"; exon_number "1"; gene_type "lincRNA";

X Cufflinks exon 22657348 22657659 . + . gene_id "XLOC_205119"; transcript_id "TCONS_00992663"; exon_number "1"; gene_type "lincRNA";

X Cufflinks exon 22692941 22692999 . + . gene_id "XLOC_205119"; transcript_id "TCONS_00992663"; exon_number "2"; gene_type "lincRNA";

X Cufflinks exon 22740542 22740748 . + . gene_id "XLOC_205119"; transcript_id "TCONS_00992663"; exon_number "3"; gene_type "lincRNA";

X Cufflinks exon 22702734 22702939 . + . gene_id "XLOC_205170"; transcript_id "TCONS_00971715"; exon_number "1"; gene_type "lincRNA";

X Cufflinks exon 22752145 22752353 . + . gene_id "XLOC_205239"; transcript_id "TCONS_01041157"; exon_number "1"; gene_type "lincRNA";

X Cufflinks exon 23006805 23007010 . + . gene_id "XLOC_205478"; transcript_id "TCONS_00971947"; exon_number "1"; gene_type "lincRNA";

X Cufflinks exon 23007669 23007798 . + . gene_id "XLOC_205480"; transcript_id "TCONS_00949096"; exon_number "1"; gene_type "anti-sense_lncRNA";

X Cufflinks exon 23008921 23009533 . + . gene_id "XLOC_205480"; transcript_id "TCONS_00949096"; exon_number "2"; gene_type "anti-sense_lncRNA";

X Scripture exon 23206133 23206258 . + . gene_id "XLOC_205597"; transcript_id "TCONS_01094784"; exon_number "1"; gene_type "lincRNA";

X Scripture exon 23214889 23215025 . + . gene_id "XLOC_205597"; transcript_id "TCONS_01094784"; exon_number "2"; gene_type "lincRNA";

X Scripture exon 23278002 23278145 . + . gene_id "XLOC_205613"; transcript_id "TCONS_01110074"; exon_number "1"; gene_type "lincRNA";

X Scripture exon 23441780 23441891 . + . gene_id "XLOC_205613"; transcript_id "TCONS_01110074"; exon_number "2"; gene_type "lincRNA";

X Scripture exon 23278002 23278137 . + . gene_id "XLOC_205613"; transcript_id "TCONS_01010419"; exon_number "1"; gene_type "lincRNA";

X Scripture exon 23441773 23441898 . + . gene_id "XLOC_205613"; transcript_id "TCONS_01010419"; exon_number "2"; gene_type "lincRNA";

X Cufflinks exon 111887 112076 . - . gene_id "XLOC_205670"; transcript_id "TCONS_01025969"; exon_number "1"; gene_type "lincRNA";

X Cufflinks exon 112144 112896 . - . gene_id "XLOC_205670"; transcript_id "TCONS_01025969"; exon_number "2"; gene_type "lincRNA";

X Cufflinks exon 84572 84962 . - . gene_id "XLOC_205680"; transcript_id "TCONS_00949201"; exon_number "1"; gene_type "lincRNA";

X Cufflinks exon 85541 85576 . - . gene_id "XLOC_205680"; transcript_id "TCONS_00949201"; exon_number "2"; gene_type "lincRNA";

X Scripture exon 496601 497040 . - . gene_id "XLOC_205888"; transcript_id "TCONS_01086889"; exon_number "1"; gene_type "anti-sense_lncRNA";

X Scripture exon 497172 498826 . - . gene_id "XLOC_205888"; transcript_id "TCONS_01086889"; exon_number "2"; gene_type "anti-sense_lncRNA";

X Scripture exon 498887 500086 . - . gene_id "XLOC_205888"; transcript_id "TCONS_01086889"; exon_number "3"; gene_type "anti-sense_lncRNA";

X Scripture exon 763222 763233 . - . gene_id "XLOC_205909"; transcript_id "TCONS_01087005"; exon_number "1"; gene_type "intronic_lncRNA";

X Scripture exon 763441 763806 . - . gene_id "XLOC_205909"; transcript_id "TCONS_01087005"; exon_number "2"; gene_type "intronic_lncRNA";

X Cufflinks exon 985092 985365 . - . gene_id "XLOC_206110"; transcript_id "TCONS_00949531"; exon_number "1"; gene_type "lincRNA";

X Cufflinks exon 985417 985596 . - . gene_id "XLOC_206110"; transcript_id "TCONS_00949531"; exon_number "2"; gene_type "lincRNA";

X Cufflinks exon 985198 985360 . - . gene_id "XLOC_206110"; transcript_id "TCONS_00993376"; exon_number "1"; gene_type "lincRNA";

X Cufflinks exon 985417 985600 . - . gene_id "XLOC_206110"; transcript_id "TCONS_00993376"; exon_number "2"; gene_type "lincRNA";

X Cufflinks exon 1177136 1177340 . - . gene_id "XLOC_206238"; transcript_id "TCONS_01026204"; exon_number "1"; gene_type "lincRNA";

X Cufflinks exon 1370092 1370305 . - . gene_id "XLOC_206314"; transcript_id "TCONS_00993508"; exon_number "1"; gene_type "anti-sense_lncRNA";

X Cufflinks exon 1387990 1388206 . - . gene_id "XLOC_206323"; transcript_id "TCONS_00972620"; exon_number "1"; gene_type "intronic_lncRNA";

X Cufflinks exon 1582932 1583137 . - . gene_id "XLOC_206390"; transcript_id "TCONS_01041904"; exon_number "1"; gene_type "anti-sense_lncRNA";

X Cufflinks exon 1534457 1534660 . - . gene_id "XLOC_206407"; transcript_id "TCONS_00993567"; exon_number "1"; gene_type "intronic_lncRNA";

X Cufflinks exon 1539386 1539590 . - . gene_id "XLOC_206419"; transcript_id "TCONS_00972685"; exon_number "1"; gene_type "intronic_lncRNA";

X Cufflinks exon 1574240 1574449 . - . gene_id "XLOC_206452"; transcript_id "TCONS_00972710"; exon_number "1"; gene_type "intronic_lncRNA";

X Cufflinks exon 1599475 1599686 . - . gene_id "XLOC_206488"; transcript_id "TCONS_00949834"; exon_number "1"; gene_type "anti-sense_lncRNA";

X Cufflinks exon 1623764 1623991 . - . gene_id "XLOC_206523"; transcript_id "TCONS_00972744"; exon_number "1"; gene_type "anti-sense_lncRNA";

X Cufflinks exon 1646918 1647130 . - . gene_id "XLOC_206545"; transcript_id "TCONS_01058129"; exon_number "1"; gene_type "anti-sense_lncRNA";

X Cufflinks exon 1768773 1768979 . - . gene_id "XLOC_206644"; transcript_id "TCONS_01041986"; exon_number "1"; gene_type "lincRNA";

X Scripture exon 2177151 2177521 . - . gene_id "XLOC_206803"; transcript_id "TCONS_01087185"; exon_number "1"; gene_type "anti-sense_lncRNA";

X Scripture exon 2177587 2177623 . - . gene_id "XLOC_206803"; transcript_id "TCONS_01087185"; exon_number "2"; gene_type "anti-sense_lncRNA";

X Scripture exon 2178337 2178697 . - . gene_id "XLOC_206804"; transcript_id "TCONS_01087186"; exon_number "1"; gene_type "anti-sense_lncRNA";

X Scripture exon 2178787 2179262 . - . gene_id "XLOC_206804"; transcript_id "TCONS_01087186"; exon_number "2"; gene_type "anti-sense_lncRNA";

X Cufflinks exon 2196548 2196776 . - . gene_id "XLOC_206814"; transcript_id "TCONS_00950069"; exon_number "1"; gene_type "intronic_lncRNA";

X Cufflinks exon 2365155 2365356 . - . gene_id "XLOC_206897"; transcript_id "TCONS_00950128"; exon_number "1"; gene_type "intronic_lncRNA";

X Cufflinks exon 2516488 2516804 . - . gene_id "XLOC_206957"; transcript_id "TCONS_01026576"; exon_number "1"; gene_type "intronic_lncRNA";

X Cufflinks exon 2762166 2762391 . - . gene_id "XLOC_207062"; transcript_id "TCONS_00973099"; exon_number "1"; gene_type "intronic_lncRNA";

X Cufflinks exon 2864848 2865051 . - . gene_id "XLOC_207196"; transcript_id "TCONS_00950329"; exon_number "1"; gene_type "intronic_lncRNA";

X Cufflinks exon 3090777 3091021 . - . gene_id "XLOC_207402"; transcript_id "TCONS_00994106"; exon_number "1"; gene_type "intronic_lncRNA";

X Cufflinks exon 3120187 3120435 . - . gene_id "XLOC_207433"; transcript_id "TCONS_00950446"; exon_number "1"; gene_type "intronic_lncRNA";

X Cufflinks exon 3519452 3519653 . - . gene_id "XLOC_207784"; transcript_id "TCONS_01011540"; exon_number "1"; gene_type "intronic_lncRNA";

X Cufflinks exon 3552688 3552887 . - . gene_id "XLOC_207837"; transcript_id "TCONS_00950621"; exon_number "1"; gene_type "intronic_lncRNA";

X Cufflinks exon 3554573 3554777 . - . gene_id "XLOC_207841"; transcript_id "TCONS_00994272"; exon_number "1"; gene_type "intronic_lncRNA";

X Cufflinks exon 3842363 3842570 . - . gene_id "XLOC_208065"; transcript_id "TCONS_00973638"; exon_number "1"; gene_type "intronic_lncRNA";

X Scripture exon 3879007 3879315 . - . gene_id "XLOC_208075"; transcript_id "TCONS_01087427"; exon_number "1"; gene_type "anti-sense_lncRNA";

X Scripture exon 3880464 3880811 . - . gene_id "XLOC_208075"; transcript_id "TCONS_01087427"; exon_number "2"; gene_type "anti-sense_lncRNA";

X Cufflinks exon 4235411 4235617 . - . gene_id "XLOC_208295"; transcript_id "TCONS_00973783"; exon_number "1"; gene_type "lincRNA";

X Cufflinks exon 4267388 4267587 . - . gene_id "XLOC_208343"; transcript_id "TCONS_00973814"; exon_number "1"; gene_type "lincRNA";

X Cufflinks exon 4502852 4503061 . - . gene_id "XLOC_208604"; transcript_id "TCONS_00994633"; exon_number "1"; gene_type "lincRNA";

X Cufflinks exon 4610947 4611242 . - . gene_id "XLOC_208720"; transcript_id "TCONS_01042914"; exon_number "1"; gene_type "lincRNA";

X Cufflinks exon 4611385 4611446 . - . gene_id "XLOC_208720"; transcript_id "TCONS_01042914"; exon_number "2"; gene_type "lincRNA";

X Cufflinks exon 4616665 4616870 . - . gene_id "XLOC_208726"; transcript_id "TCONS_00994723"; exon_number "1"; gene_type "lincRNA";

X Cufflinks exon 5133881 5134105 . - . gene_id "XLOC_209106"; transcript_id "TCONS_00974347"; exon_number "1"; gene_type "intronic_lncRNA";

X Cufflinks exon 5200637 5200849 . - . gene_id "XLOC_209158"; transcript_id "TCONS_01027330"; exon_number "1"; gene_type "intronic_lncRNA";

X Cufflinks exon 5258341 5258550 . - . gene_id "XLOC_209188"; transcript_id "TCONS_01027335"; exon_number "1"; gene_type "lincRNA";

X Cufflinks exon 5442911 5443117 . - . gene_id "XLOC_209391"; transcript_id "TCONS_01012182"; exon_number "1"; gene_type "lincRNA";

X Cufflinks exon 5446097 5446308 . - . gene_id "XLOC_209396"; transcript_id "TCONS_00994939"; exon_number "1"; gene_type "lincRNA";

X Cufflinks exon 5464611 5464815 . - . gene_id "XLOC_209426"; transcript_id "TCONS_01059328"; exon_number "1"; gene_type "intronic_lncRNA";

X Cufflinks exon 5538943 5539152 . - . gene_id "XLOC_209519"; transcript_id "TCONS_01012235"; exon_number "1"; gene_type "intronic_lncRNA";

X Cufflinks exon 5579531 5579731 . - . gene_id "XLOC_209596"; transcript_id "TCONS_00995023"; exon_number "1"; gene_type "lincRNA";

X Cufflinks exon 5790965 5791624 . - . gene_id "XLOC_209717"; transcript_id "TCONS_01043340"; exon_number "1"; gene_type "lincRNA";

X Cufflinks exon 5834641 5834870 . - . gene_id "XLOC_209775"; transcript_id "TCONS_00995124"; exon_number "1"; gene_type "intronic_lncRNA";

X Cufflinks exon 5840911 5841113 . - . gene_id "XLOC_209779"; transcript_id "TCONS_00974745"; exon_number "1"; gene_type "lincRNA";

X Cufflinks exon 5898233 5898827 . - . gene_id "XLOC_209835"; transcript_id "TCONS_00995168"; exon_number "1"; gene_type "lincRNA";

X Cufflinks exon 5899055 5899946 . - . gene_id "XLOC_209835"; transcript_id "TCONS_00995168"; exon_number "2"; gene_type "lincRNA";

X Cufflinks exon 5922724 5922933 . - . gene_id "XLOC_209848"; transcript_id "TCONS_00951784"; exon_number "1"; gene_type "intronic_lncRNA";

X Cufflinks exon 5987988 5988459 . - . gene_id "XLOC_209891"; transcript_id "TCONS_01027556"; exon_number "1"; gene_type "lincRNA";

X Cufflinks exon 5988610 5988734 . - . gene_id "XLOC_209891"; transcript_id "TCONS_01027556"; exon_number "2"; gene_type "lincRNA";

X Cufflinks exon 6548661 6548874 . - . gene_id "XLOC_210443"; transcript_id "TCONS_00952124"; exon_number "1"; gene_type "intronic_lncRNA";

X Scripture exon 6758240 6758953 . - . gene_id "XLOC_210553"; transcript_id "TCONS_01087631"; exon_number "1"; gene_type "intronic_lncRNA";

X Scripture exon 6759425 6759894 . - . gene_id "XLOC_210553"; transcript_id "TCONS_01087631"; exon_number "2"; gene_type "intronic_lncRNA";

X Cufflinks exon 6863415 6863618 . - . gene_id "XLOC_210593"; transcript_id "TCONS_00952190"; exon_number "1"; gene_type "intronic_lncRNA";

X Cufflinks exon 7226347 7226553 . - . gene_id "XLOC_210998"; transcript_id "TCONS_00975352"; exon_number "1"; gene_type "intronic_lncRNA";

X Cufflinks exon 7353568 7353784 . - . gene_id "XLOC_211140"; transcript_id "TCONS_01012915"; exon_number "1"; gene_type "intronic_lncRNA";

X Cufflinks exon 7450195 7450395 . - . gene_id "XLOC_211287"; transcript_id "TCONS_00975558"; exon_number "1"; gene_type "lincRNA";

X Cufflinks exon 7808215 7808439 . - . gene_id "XLOC_211738"; transcript_id "TCONS_00996083"; exon_number "1"; gene_type "lincRNA";

X Cufflinks exon 8368123 8368337 . - . gene_id "XLOC_212147"; transcript_id "TCONS_00976153"; exon_number "1"; gene_type "lincRNA";

X Cufflinks exon 8383622 8383854 . - . gene_id "XLOC_212182"; transcript_id "TCONS_00953172"; exon_number "1"; gene_type "lincRNA";

X Cufflinks exon 8533962 8534171 . - . gene_id "XLOC_212261"; transcript_id "TCONS_00953236"; exon_number "1"; gene_type "intronic_lncRNA";

X Cufflinks exon 8705563 8705843 . - . gene_id "XLOC_212448"; transcript_id "TCONS_00976311"; exon_number "1"; gene_type "lincRNA";

X Cufflinks exon 8809849 8810061 . - . gene_id "XLOC_212620"; transcript_id "TCONS_00976409"; exon_number "1"; gene_type "lincRNA";

X Cufflinks exon 8816550 8816753 . - . gene_id "XLOC_212633"; transcript_id "TCONS_00953410"; exon_number "1"; gene_type "lincRNA";

X Cufflinks exon 8865125 8865397 . - . gene_id "XLOC_212726"; transcript_id "TCONS_00976469"; exon_number "1"; gene_type "intronic_lncRNA";

X Cufflinks exon 8919635 8920134 . - . gene_id "XLOC_212730"; transcript_id "TCONS_00996514"; exon_number "1"; gene_type "intronic_lncRNA";

X Cufflinks exon 8920257 8920756 . - . gene_id "XLOC_212730"; transcript_id "TCONS_00996514"; exon_number "2"; gene_type "intronic_lncRNA";

X Cufflinks exon 9108392 9108775 . - . gene_id "XLOC_212786"; transcript_id "TCONS_01013619"; exon_number "1"; gene_type "anti-sense_lncRNA";

X Cufflinks exon 9108846 9109260 . - . gene_id "XLOC_212786"; transcript_id "TCONS_01013619"; exon_number "2"; gene_type "anti-sense_lncRNA";

X Cufflinks exon 9139063 9139272 . - . gene_id "XLOC_212798"; transcript_id "TCONS_00976527"; exon_number "1"; gene_type "lincRNA";

X Cufflinks exon 9140382 9140613 . - . gene_id "XLOC_212801"; transcript_id "TCONS_01013629"; exon_number "1"; gene_type "lincRNA";

X Cufflinks exon 9140697 9140827 . - . gene_id "XLOC_212801"; transcript_id "TCONS_01013629"; exon_number "2"; gene_type "lincRNA";

X Cufflinks exon 9293349 9293555 . - . gene_id "XLOC_212880"; transcript_id "TCONS_00953581"; exon_number "1"; gene_type "intronic_lncRNA";

X Cufflinks exon 9590246 9590450 . - . gene_id "XLOC_213018"; transcript_id "TCONS_01013724"; exon_number "1"; gene_type "lincRNA";

X Cufflinks exon 9748741 9748944 . - . gene_id "XLOC_213136"; transcript_id "TCONS_00976724"; exon_number "1"; gene_type "intronic_lncRNA";

X Cufflinks exon 9758361 9758574 . - . gene_id "XLOC_213147"; transcript_id "TCONS_01044789"; exon_number "1"; gene_type "lincRNA";

X Cufflinks exon 9841850 9842081 . - . gene_id "XLOC_213226"; transcript_id "TCONS_00996737"; exon_number "1"; gene_type "intronic_lncRNA";

X Cufflinks exon 10101506 10102742 . - . gene_id "XLOC_213498"; transcript_id "TCONS_00953905"; exon_number "1"; gene_type "intronic_lncRNA";

X Cufflinks exon 10147430 10147696 . - . gene_id "XLOC_213543"; transcript_id "TCONS_01013942"; exon_number "1"; gene_type "intronic_lncRNA";

X Cufflinks exon 10244594 10244626 . - . gene_id "XLOC_213630"; transcript_id "TCONS_01061009"; exon_number "1"; gene_type "intronic_lncRNA";

X Cufflinks exon 10249895 10250393 . - . gene_id "XLOC_213630"; transcript_id "TCONS_01061009"; exon_number "2"; gene_type "intronic_lncRNA";

X Cufflinks exon 10449941 10450142 . - . gene_id "XLOC_213735"; transcript_id "TCONS_00996986"; exon_number "1"; gene_type "intronic_lncRNA";

X Cufflinks exon 10997136 10997335 . - . gene_id "XLOC_214057"; transcript_id "TCONS_00954270"; exon_number "1"; gene_type "intronic_lncRNA";

X Cufflinks exon 11067011 11067215 . - . gene_id "XLOC_214144"; transcript_id "TCONS_00997171"; exon_number "1"; gene_type "intronic_lncRNA";

X Cufflinks exon 11204227 11204454 . - . gene_id "XLOC_214217"; transcript_id "TCONS_01014272"; exon_number "1"; gene_type "lincRNA";

X Cufflinks exon 11212143 11212351 . - . gene_id "XLOC_214230"; transcript_id "TCONS_00997205"; exon_number "1"; gene_type "lincRNA";

X Cufflinks exon 11420296 11420326 . - . gene_id "XLOC_214348"; transcript_id "TCONS_00977500"; exon_number "1"; gene_type "lincRNA";

X Cufflinks exon 11420895 11421098 . - . gene_id "XLOC_214348"; transcript_id "TCONS_00977500"; exon_number "2"; gene_type "lincRNA";

X Cufflinks exon 11970669 11970946 . - . gene_id "XLOC_214594"; transcript_id "TCONS_00997457"; exon_number "1"; gene_type "intronic_lncRNA";

X Cufflinks exon 11971043 11971381 . - . gene_id "XLOC_214594"; transcript_id "TCONS_00997457"; exon_number "2"; gene_type "intronic_lncRNA";

X Cufflinks exon 11975270 11975484 . - . gene_id "XLOC_214639"; transcript_id "TCONS_00997459"; exon_number "1"; gene_type "intronic_lncRNA";

X Cufflinks exon 12058759 12058977 . - . gene_id "XLOC_214710"; transcript_id "TCONS_00977701"; exon_number "1"; gene_type "intronic_lncRNA";

X Cufflinks exon 12140010 12140221 . - . gene_id "XLOC_214801"; transcript_id "TCONS_00977737"; exon_number "1"; gene_type "intronic_lncRNA";

X Cufflinks exon 12349294 12349499 . - . gene_id "XLOC_214998"; transcript_id "TCONS_00997599"; exon_number "1"; gene_type "lincRNA";

X Cufflinks exon 12414470 12414679 . - . gene_id "XLOC_215104"; transcript_id "TCONS_00977867"; exon_number "1"; gene_type "lincRNA";

X Cufflinks exon 12480697 12480899 . - . gene_id "XLOC_215166"; transcript_id "TCONS_00997677"; exon_number "1"; gene_type "lincRNA";

X Scripture exon 12595901 12596091 . - . gene_id "XLOC_215262"; transcript_id "TCONS_01088588"; exon_number "1"; gene_type "anti-sense_lncRNA";

X Scripture exon 12596306 12596612 . - . gene_id "XLOC_215262"; transcript_id "TCONS_01088588"; exon_number "2"; gene_type "anti-sense_lncRNA";

X Cufflinks exon 12631917 12632119 . - . gene_id "XLOC_215288"; transcript_id "TCONS_00955020"; exon_number "1"; gene_type "intronic_lncRNA";

X Cufflinks exon 12657828 12658402 . - . gene_id "XLOC_215299"; transcript_id "TCONS_00977988"; exon_number "1"; gene_type "anti-sense_lncRNA";

X Cufflinks exon 12658477 12659179 . - . gene_id "XLOC_215299"; transcript_id "TCONS_00977988"; exon_number "2"; gene_type "anti-sense_lncRNA";

X Cufflinks exon 12704923 12705493 . - . gene_id "XLOC_215309"; transcript_id "TCONS_00977997"; exon_number "1"; gene_type "intronic_lncRNA";

X Cufflinks exon 12752881 12753428 . - . gene_id "XLOC_215340"; transcript_id "TCONS_00955045"; exon_number "1"; gene_type "lincRNA";

X Cufflinks exon 12753510 12753543 . - . gene_id "XLOC_215340"; transcript_id "TCONS_00955045"; exon_number "2"; gene_type "lincRNA";

X Cufflinks exon 12887247 12887597 . - . gene_id "XLOC_215441"; transcript_id "TCONS_00955134"; exon_number "1"; gene_type "intronic_lncRNA";

X Cufflinks exon 12923440 12923664 . - . gene_id "XLOC_215471"; transcript_id "TCONS_00978090"; exon_number "1"; gene_type "intronic_lncRNA";

X Cufflinks exon 13220722 13221003 . - . gene_id "XLOC_215727"; transcript_id "TCONS_00955256"; exon_number "1"; gene_type "intronic_lncRNA";

X Cufflinks exon 13390363 13390562 . - . gene_id "XLOC_215817"; transcript_id "TCONS_00955302"; exon_number "1"; gene_type "lincRNA";

X Cufflinks exon 13395902 13395973 . - . gene_id "XLOC_215817"; transcript_id "TCONS_00955302"; exon_number "2"; gene_type "lincRNA";

X Cufflinks exon 13390434 13390562 . - . gene_id "XLOC_215817"; transcript_id "TCONS_00955301"; exon_number "1"; gene_type "lincRNA";

X Cufflinks exon 13394466 13394698 . - . gene_id "XLOC_215817"; transcript_id "TCONS_00955301"; exon_number "2"; gene_type "lincRNA";

X Cufflinks exon 13472761 13472964 . - . gene_id "XLOC_215912"; transcript_id "TCONS_00955350"; exon_number "1"; gene_type "intronic_lncRNA";

X Cufflinks exon 13657661 13657878 . - . gene_id "XLOC_216061"; transcript_id "TCONS_00998103"; exon_number "1"; gene_type "intronic_lncRNA";

X Cufflinks exon 13671455 13671673 . - . gene_id "XLOC_216065"; transcript_id "TCONS_01061984"; exon_number "1"; gene_type "intronic_lncRNA";

X Cufflinks exon 13909032 13909331 . - . gene_id "XLOC_216154"; transcript_id "TCONS_00978434"; exon_number "1"; gene_type "intronic_lncRNA";

X Cufflinks exon 13928503 13928766 . - . gene_id "XLOC_216175"; transcript_id "TCONS_00998157"; exon_number "1"; gene_type "intronic_lncRNA";

X Cufflinks exon 13981746 13981953 . - . gene_id "XLOC_216227"; transcript_id "TCONS_00978462"; exon_number "1"; gene_type "intronic_lncRNA";

X Cufflinks exon 13982883 13983158 . - . gene_id "XLOC_216228"; transcript_id "TCONS_00955482"; exon_number "1"; gene_type "intronic_lncRNA";

X Cufflinks exon 13983294 13983509 . - . gene_id "XLOC_216229"; transcript_id "TCONS_00955483"; exon_number "1"; gene_type "intronic_lncRNA";

X Cufflinks exon 13984878 13985159 . - . gene_id "XLOC_216230"; transcript_id "TCONS_00978463"; exon_number "1"; gene_type "intronic_lncRNA";

X Cufflinks exon 14078433 14078633 . - . gene_id "XLOC_216342"; transcript_id "TCONS_00955533"; exon_number "1"; gene_type "lincRNA";

X Cufflinks exon 14156517 14156727 . - . gene_id "XLOC_216479"; transcript_id "TCONS_00998281"; exon_number "1"; gene_type "lincRNA";

X Cufflinks exon 14409771 14409970 . - . gene_id "XLOC_216716"; transcript_id "TCONS_00978707"; exon_number "1"; gene_type "intronic_lncRNA";

X Cufflinks exon 14649825 14650119 . - . gene_id "XLOC_217046"; transcript_id "TCONS_01015352"; exon_number "1"; gene_type "intronic_lncRNA";

X Cufflinks exon 14674397 14674596 . - . gene_id "XLOC_217082"; transcript_id "TCONS_00978893"; exon_number "1"; gene_type "lincRNA";

X Cufflinks exon 14770976 14771234 . - . gene_id "XLOC_217224"; transcript_id "TCONS_01015460"; exon_number "1"; gene_type "lincRNA";

X Cufflinks exon 14881474 14881676 . - . gene_id "XLOC_217298"; transcript_id "TCONS_00955985"; exon_number "1"; gene_type "anti-sense_lncRNA";

X Scripture exon 15082676 15083046 . - . gene_id "XLOC_217416"; transcript_id "TCONS_01088871"; exon_number "1"; gene_type "anti-sense_lncRNA";

X Scripture exon 15083107 15083336 . - . gene_id "XLOC_217416"; transcript_id "TCONS_01088871"; exon_number "2"; gene_type "anti-sense_lncRNA";

X Cufflinks exon 15129763 15129964 . - . gene_id "XLOC_217453"; transcript_id "TCONS_00998614"; exon_number "1"; gene_type "intronic_lncRNA";

X Cufflinks exon 15168338 15168547 . - . gene_id "XLOC_217497"; transcript_id "TCONS_00979114"; exon_number "1"; gene_type "lincRNA";

X Cufflinks exon 15237919 15238123 . - . gene_id "XLOC_217593"; transcript_id "TCONS_01046599"; exon_number "1"; gene_type "lincRNA";

X Scripture exon 15334990 15335228 . - . gene_id "XLOC_217712"; transcript_id "TCONS_01088877"; exon_number "1"; gene_type "anti-sense_lncRNA";

X Scripture exon 15335289 15335480 . - . gene_id "XLOC_217712"; transcript_id "TCONS_01088877"; exon_number "2"; gene_type "anti-sense_lncRNA";

X Cufflinks exon 15566973 15567187 . - . gene_id "XLOC_217887"; transcript_id "TCONS_00956368"; exon_number "1"; gene_type "lincRNA";

X Cufflinks exon 15646188 15646394 . - . gene_id "XLOC_217952"; transcript_id "TCONS_00979451"; exon_number "1"; gene_type "lincRNA";

X Cufflinks exon 15680587 15680797 . - . gene_id "XLOC_218005"; transcript_id "TCONS_00979480"; exon_number "1"; gene_type "lincRNA";

X Cufflinks exon 15869671 15869876 . - . gene_id "XLOC_218066"; transcript_id "TCONS_01030475"; exon_number "1"; gene_type "lincRNA";

X Cufflinks exon 15974306 15974512 . - . gene_id "XLOC_218098"; transcript_id "TCONS_01046868"; exon_number "1"; gene_type "anti-sense_lncRNA";

X Cufflinks exon 15977013 15977217 . - . gene_id "XLOC_218102"; transcript_id "TCONS_00998977"; exon_number "1"; gene_type "anti-sense_lncRNA";

X Cufflinks exon 16130910 16131114 . - . gene_id "XLOC_218163"; transcript_id "TCONS_00956585"; exon_number "1"; gene_type "intronic_lncRNA";

X Cufflinks exon 16136094 16136310 . - . gene_id "XLOC_218172"; transcript_id "TCONS_00956590"; exon_number "1"; gene_type "intronic_lncRNA";

X Cufflinks exon 16158805 16159008 . - . gene_id "XLOC_218203"; transcript_id "TCONS_00979626"; exon_number "1"; gene_type "lincRNA";

X Cufflinks exon 16204844 16205048 . - . gene_id "XLOC_218296"; transcript_id "TCONS_00999068"; exon_number "1"; gene_type "lincRNA";

X Scripture exon 16253573 16253710 . - . gene_id "XLOC_218361"; transcript_id "TCONS_00956697"; exon_number "1"; gene_type "lincRNA";

X Scripture exon 16253859 16254102 . - . gene_id "XLOC_218361"; transcript_id "TCONS_00956697"; exon_number "2"; gene_type "lincRNA";

X Cufflinks exon 16325001 16325208 . - . gene_id "XLOC_218392"; transcript_id "TCONS_00956721"; exon_number "1"; gene_type "intronic_lncRNA";

X Cufflinks exon 16645141 16646548 . - . gene_id "XLOC_218536"; transcript_id "TCONS_00956864"; exon_number "1"; gene_type "intronic_lncRNA";

X Cufflinks exon 16815381 16815612 . - . gene_id "XLOC_218591"; transcript_id "TCONS_00999248"; exon_number "1"; gene_type "intronic_lncRNA";

X Cufflinks exon 16815743 16816647 . - . gene_id "XLOC_218591"; transcript_id "TCONS_00999248"; exon_number "2"; gene_type "intronic_lncRNA";

X Cufflinks exon 17052626 17052830 . - . gene_id "XLOC_218805"; transcript_id "TCONS_00957088"; exon_number "1"; gene_type "intronic_lncRNA";

X Cufflinks exon 17145214 17145685 . - . gene_id "XLOC_218862"; transcript_id "TCONS_00957121"; exon_number "1"; gene_type "intronic_lncRNA";

X Cufflinks exon 17231627 17231833 . - . gene_id "XLOC_218911"; transcript_id "TCONS_00980106"; exon_number "1"; gene_type "lincRNA";

X Cufflinks exon 17371241 17371447 . - . gene_id "XLOC_219083"; transcript_id "TCONS_01047398"; exon_number "1"; gene_type "lincRNA";

X Cufflinks exon 17381191 17381391 . - . gene_id "XLOC_219100"; transcript_id "TCONS_00999535"; exon_number "1"; gene_type "lincRNA";

X Cufflinks exon 17561584 17561803 . - . gene_id "XLOC_219274"; transcript_id "TCONS_00999628"; exon_number "1"; gene_type "lincRNA";

X Cufflinks exon 17586821 17587033 . - . gene_id "XLOC_219288"; transcript_id "TCONS_00957374"; exon_number "1"; gene_type "intronic_lncRNA";

X Scripture exon 17827931 17828709 . - . gene_id "XLOC_219476"; transcript_id "TCONS_01089468"; exon_number "1"; gene_type "anti-sense_lncRNA";

X Scripture exon 17830799 17830956 . - . gene_id "XLOC_219476"; transcript_id "TCONS_01089468"; exon_number "2"; gene_type "anti-sense_lncRNA";

X Cufflinks exon 18003385 18003590 . - . gene_id "XLOC_219568"; transcript_id "TCONS_01047670"; exon_number "1"; gene_type "intronic_lncRNA";

X Cufflinks exon 18005007 18005222 . - . gene_id "XLOC_219571"; transcript_id "TCONS_00999827"; exon_number "1"; gene_type "intronic_lncRNA";

X Cufflinks exon 18028427 18028646 . - . gene_id "XLOC_219591"; transcript_id "TCONS_00980592"; exon_number "1"; gene_type "intronic_lncRNA";

X Cufflinks exon 18141710 18141915 . - . gene_id "XLOC_219686"; transcript_id "TCONS_01016708"; exon_number "1"; gene_type "lincRNA";

X Cufflinks exon 18175785 18175995 . - . gene_id "XLOC_219725"; transcript_id "TCONS_00957707"; exon_number "1"; gene_type "lincRNA";

X Cufflinks exon 18260275 18260474 . - . gene_id "XLOC_219828"; transcript_id "TCONS_00957768"; exon_number "1"; gene_type "lincRNA";

X Cufflinks exon 18681773 18682674 . - . gene_id "XLOC_220259"; transcript_id "TCONS_00958086"; exon_number "1"; gene_type "lincRNA";

X Cufflinks exon 18682740 18683824 . - . gene_id "XLOC_220259"; transcript_id "TCONS_00958086"; exon_number "2"; gene_type "lincRNA";

X Cufflinks exon 18724009 18724220 . - . gene_id "XLOC_220312"; transcript_id "TCONS_01017050"; exon_number "1"; gene_type "intronic_lncRNA";

X Cufflinks exon 19044854 19045056 . - . gene_id "XLOC_220521"; transcript_id "TCONS_00981216"; exon_number "1"; gene_type "lincRNA";

X Cufflinks exon 19367985 19368187 . - . gene_id "XLOC_220791"; transcript_id "TCONS_01064078"; exon_number "1"; gene_type "intronic_lncRNA";

X Cufflinks exon 19406313 19406541 . - . gene_id "XLOC_220822"; transcript_id "TCONS_00958391"; exon_number "1"; gene_type "intronic_lncRNA";

X Cufflinks exon 19423373 19423591 . - . gene_id "XLOC_220839"; transcript_id "TCONS_00981396"; exon_number "1"; gene_type "intronic_lncRNA";

X Cufflinks exon 19537878 19538081 . - . gene_id "XLOC_220896"; transcript_id "TCONS_01048306"; exon_number "1"; gene_type "intronic_lncRNA";

X Cufflinks exon 19602071 19602293 . - . gene_id "XLOC_220956"; transcript_id "TCONS_00958436"; exon_number "1"; gene_type "lincRNA";

X Cufflinks exon 19602354 19602385 . - . gene_id "XLOC_220956"; transcript_id "TCONS_00958436"; exon_number "2"; gene_type "lincRNA";

X Cufflinks exon 19666884 19668134 . - . gene_id "XLOC_220979"; transcript_id "TCONS_00958455"; exon_number "1"; gene_type "anti-sense_lncRNA";

X Cufflinks exon 20086149 20086352 . - . gene_id "XLOC_221328"; transcript_id "TCONS_00981660"; exon_number "1"; gene_type "lincRNA";

X Cufflinks exon 20097171 20097387 . - . gene_id "XLOC_221345"; transcript_id "TCONS_00981668"; exon_number "1"; gene_type "lincRNA";

X Cufflinks exon 20238590 20238797 . - . gene_id "XLOC_221434"; transcript_id "TCONS_00981704"; exon_number "1"; gene_type "lincRNA";

X Cufflinks exon 20319463 20319663 . - . gene_id "XLOC_221525"; transcript_id "TCONS_01048502"; exon_number "1"; gene_type "intronic_lncRNA";

X Cufflinks exon 20342092 20342299 . - . gene_id "XLOC_221562"; transcript_id "TCONS_00958731"; exon_number "1"; gene_type "lincRNA";

X Cufflinks exon 20371715 20371915 . - . gene_id "XLOC_221617"; transcript_id "TCONS_00981815"; exon_number "1"; gene_type "lincRNA";

X Cufflinks exon 20415440 20415653 . - . gene_id "XLOC_221658"; transcript_id "TCONS_01000806"; exon_number "1"; gene_type "lincRNA";

X Cufflinks exon 20556102 20556307 . - . gene_id "XLOC_221766"; transcript_id "TCONS_00981890"; exon_number "1"; gene_type "intronic_lncRNA";

X Cufflinks exon 20567459 20567685 . - . gene_id "XLOC_221781"; transcript_id "TCONS_00958823"; exon_number "1"; gene_type "lincRNA";

X Cufflinks exon 20703202 20703426 . - . gene_id "XLOC_221996"; transcript_id "TCONS_01064459"; exon_number "1"; gene_type "lincRNA";

X Cufflinks exon 21575627 21575830 . - . gene_id "XLOC_222862"; transcript_id "TCONS_01001433"; exon_number "1"; gene_type "lincRNA";

X Cufflinks exon 22414526 22414753 . - . gene_id "XLOC_223166"; transcript_id "TCONS_01001530"; exon_number "1"; gene_type "lincRNA";

X Scripture exon 22487058 22487209 . - . gene_id "XLOC_223214"; transcript_id "TCONS_01090081"; exon_number "1"; gene_type "anti-sense_lncRNA";

X Scripture exon 22487666 22487854 . - . gene_id "XLOC_223214"; transcript_id "TCONS_01090081"; exon_number "2"; gene_type "anti-sense_lncRNA";

X Scripture exon 22487058 22487209 . - . gene_id "XLOC_223214"; transcript_id "TCONS_01103555"; exon_number "1"; gene_type "anti-sense_lncRNA";

X Scripture exon 22490720 22491021 . - . gene_id "XLOC_223214"; transcript_id "TCONS_01103555"; exon_number "2"; gene_type "anti-sense_lncRNA";

X Cufflinks exon 22514592 22514681 . - . gene_id "XLOC_223233"; transcript_id "TCONS_00982858"; exon_number "1"; gene_type "lincRNA";

X Cufflinks exon 22514812 22515068 . - . gene_id "XLOC_223233"; transcript_id "TCONS_00982858"; exon_number "2"; gene_type "lincRNA";

X Cufflinks exon 22559408 22559637 . - . gene_id "XLOC_223267"; transcript_id "TCONS_01018195"; exon_number "1"; gene_type "lincRNA";

X Cufflinks exon 22564177 22564428 . - . gene_id "XLOC_223273"; transcript_id "TCONS_00959661"; exon_number "1"; gene_type "lincRNA";

X Cufflinks exon 22572838 22573334 . - . gene_id "XLOC_223273"; transcript_id "TCONS_00959661"; exon_number "2"; gene_type "lincRNA";

X Cufflinks exon 22564559 22564595 . - . gene_id "XLOC_223274"; transcript_id "TCONS_01018199"; exon_number "1"; gene_type "lincRNA";

X Cufflinks exon 22575218 22575464 . - . gene_id "XLOC_223274"; transcript_id "TCONS_01018199"; exon_number "2"; gene_type "lincRNA";

X Cufflinks exon 22594732 22594947 . - . gene_id "XLOC_223315"; transcript_id "TCONS_01049267"; exon_number "1"; gene_type "intronic_lncRNA";

X Cufflinks exon 22614520 22614748 . - . gene_id "XLOC_223349"; transcript_id "TCONS_00982917"; exon_number "1"; gene_type "intronic_lncRNA";

X Cufflinks exon 22674137 22674342 . - . gene_id "XLOC_223443"; transcript_id "TCONS_00982960"; exon_number "1"; gene_type "lincRNA";

X Cufflinks exon 22729446 22729657 . - . gene_id "XLOC_223496"; transcript_id "TCONS_01018283"; exon_number "1"; gene_type "lincRNA";

X Cufflinks exon 22816860 22817065 . - . gene_id "XLOC_223613"; transcript_id "TCONS_00983038"; exon_number "1"; gene_type "lincRNA";

X Cufflinks exon 22856924 22857012 . - . gene_id "XLOC_223628"; transcript_id "TCONS_00983049"; exon_number "1"; gene_type "intronic_lncRNA";

X Cufflinks exon 22857075 22857199 . - . gene_id "XLOC_223628"; transcript_id "TCONS_00983049"; exon_number "2"; gene_type "intronic_lncRNA";

X Cufflinks exon 22857504 22857715 . - . gene_id "XLOC_223630"; transcript_id "TCONS_00983051"; exon_number "1"; gene_type "intronic_lncRNA";

X Cufflinks exon 23157049 23157250 . - . gene_id "XLOC_223845"; transcript_id "TCONS_01001793"; exon_number "1"; gene_type "lincRNA";

X Cufflinks exon 23192073 23192281 . - . gene_id "XLOC_223880"; transcript_id "TCONS_00983190"; exon_number "1"; gene_type "lincRNA";

X Scripture exon 23216312 23216429 . - . gene_id "XLOC_223902"; transcript_id "TCONS_01032484"; exon_number "1"; gene_type "lincRNA";

X Scripture exon 23278007 23278082 . - . gene_id "XLOC_223902"; transcript_id "TCONS_01032484"; exon_number "2"; gene_type "lincRNA";

X Scripture exon 23441718 23441890 . - . gene_id "XLOC_223902"; transcript_id "TCONS_01032484"; exon_number "3"; gene_type "lincRNA";

X Scripture exon 23278002 23278082 . - . gene_id "XLOC_223902"; transcript_id "TCONS_01018477"; exon_number "1"; gene_type "lincRNA";

X Scripture exon 23441718 23441898 . - . gene_id "XLOC_223902"; transcript_id "TCONS_01018477"; exon_number "2"; gene_type "lincRNA";

Y Cufflinks exon 3169345 3169911 . + . gene_id "XLOC_228599"; transcript_id "TCONS_01123998"; exon_number "1"; gene_type "lincRNA";

Y Cufflinks exon 1794035 1794282 . - . gene_id "XLOC_228709"; transcript_id "TCONS_01123886"; exon_number "1"; gene_type "intronic_lncRNA";

Y_mapped_Scaffold_18_D1698 Scripture exon 11177 11339 . - . gene_id "XLOC_228846"; transcript_id "TCONS_01124502"; exon_number "1"; gene_type "lincRNA";

Y_mapped_Scaffold_18_D1698 Scripture exon 11422 11836 . - . gene_id "XLOC_228846"; transcript_id "TCONS_01124502"; exon_number "2"; gene_type "lincRNA";

Y_mapped_Scaffold_18_D1698 Scripture exon 28523 28667 . - . gene_id "XLOC_228846"; transcript_id "TCONS_01124502"; exon_number "3"; gene_type "lincRNA";

Y_mapped_Scaffold_18_D1698 Scripture exon 11178 11339 . - . gene_id "XLOC_228846"; transcript_id "TCONS_01124509"; exon_number "1"; gene_type "lincRNA";

Y_mapped_Scaffold_18_D1698 Scripture exon 11422 11836 . - . gene_id "XLOC_228846"; transcript_id "TCONS_01124509"; exon_number "2"; gene_type "lincRNA";

Y_mapped_Scaffold_18_D1698 Scripture exon 11904 12048 . - . gene_id "XLOC_228846"; transcript_id "TCONS_01124509"; exon_number "3"; gene_type "lincRNA";

Y_mapped_Scaffold_18_D1698 Scripture exon 28729 28810 . - . gene_id "XLOC_228846"; transcript_id "TCONS_01124509"; exon_number "4"; gene_type "lincRNA";

Y_mapped_Scaffold_18_D1698 Scripture exon 11178 11339 . - . gene_id "XLOC_228846"; transcript_id "TCONS_01124432"; exon_number "1"; gene_type "lincRNA";

Y_mapped_Scaffold_18_D1698 Scripture exon 11422 11836 . - . gene_id "XLOC_228846"; transcript_id "TCONS_01124432"; exon_number "2"; gene_type "lincRNA";

Y_mapped_Scaffold_18_D1698 Scripture exon 11897 12048 . - . gene_id "XLOC_228846"; transcript_id "TCONS_01124432"; exon_number "3"; gene_type "lincRNA";

Y_mapped_Scaffold_18_D1698 Scripture exon 28729 28810 . - . gene_id "XLOC_228846"; transcript_id "TCONS_01124432"; exon_number "4"; gene_type "lincRNA";

Y_mapped_Scaffold_18_D1698 Cufflinks exon 11179 11339 . - . gene_id "XLOC_228846"; transcript_id "TCONS_01124466"; exon_number "1"; gene_type "lincRNA";

Y_mapped_Scaffold_18_D1698 Cufflinks exon 11422 12349 . - . gene_id "XLOC_228846"; transcript_id "TCONS_01124466"; exon_number "2"; gene_type "lincRNA";

Y_mapped_Scaffold_18_D1698 Scripture exon 11179 11298 . - . gene_id "XLOC_228846"; transcript_id "TCONS_01124497"; exon_number "1"; gene_type "lincRNA";

Y_mapped_Scaffold_18_D1698 Scripture exon 28103 28143 . - . gene_id "XLOC_228846"; transcript_id "TCONS_01124497"; exon_number "2"; gene_type "lincRNA";

Y_mapped_Scaffold_18_D1698 Scripture exon 28226 28428 . - . gene_id "XLOC_228846"; transcript_id "TCONS_01124497"; exon_number "3"; gene_type "lincRNA";

Y_mapped_Scaffold_18_D1698 Scripture exon 11179 11339 . - . gene_id "XLOC_228846"; transcript_id "TCONS_01124496"; exon_number "1"; gene_type "lincRNA";

Y_mapped_Scaffold_18_D1698 Scripture exon 28226 28428 . - . gene_id "XLOC_228846"; transcript_id "TCONS_01124496"; exon_number "2"; gene_type "lincRNA";

Y_mapped_Scaffold_18_D1698 Scripture exon 11179 11339 . - . gene_id "XLOC_228846"; transcript_id "TCONS_01124433"; exon_number "1"; gene_type "lincRNA";

Y_mapped_Scaffold_18_D1698 Scripture exon 11422 11836 . - . gene_id "XLOC_228846"; transcript_id "TCONS_01124433"; exon_number "2"; gene_type "lincRNA";

Y_mapped_Scaffold_18_D1698 Scripture exon 28516 28667 . - . gene_id "XLOC_228846"; transcript_id "TCONS_01124433"; exon_number "3"; gene_type "lincRNA";

Y_mapped_Scaffold_18_D1698 Scripture exon 28729 28806 . - . gene_id "XLOC_228846"; transcript_id "TCONS_01124433"; exon_number "4"; gene_type "lincRNA";

Y_mapped_Scaffold_18_D1698 Cufflinks exon 11250 11339 . - . gene_id "XLOC_228846"; transcript_id "TCONS_01124471"; exon_number "1"; gene_type "lincRNA";

Y_mapped_Scaffold_18_D1698 Cufflinks exon 11422 11836 . - . gene_id "XLOC_228846"; transcript_id "TCONS_01124471"; exon_number "2"; gene_type "lincRNA";

Y_mapped_Scaffold_18_D1698 Cufflinks exon 28516 29799 . - . gene_id "XLOC_228846"; transcript_id "TCONS_01124471"; exon_number "3"; gene_type "lincRNA";

Y_mapped_Scaffold_18_D1698 Cufflinks exon 25939 26816 . - . gene_id "XLOC_228846"; transcript_id "TCONS_01124458"; exon_number "1"; gene_type "lincRNA";

Y_mapped_Scaffold_18_D1698 Cufflinks exon 26933 27351 . - . gene_id "XLOC_228846"; transcript_id "TCONS_01124458"; exon_number "2"; gene_type "lincRNA";

Y_mapped_Scaffold_18_D1698 Cufflinks exon 27408 27929 . - . gene_id "XLOC_228846"; transcript_id "TCONS_01124458"; exon_number "3"; gene_type "lincRNA";

Y_mapped_Scaffold_18_D1698 Cufflinks exon 27989 28143 . - . gene_id "XLOC_228846"; transcript_id "TCONS_01124458"; exon_number "4"; gene_type "lincRNA";

Y_mapped_Scaffold_18_D1698 Cufflinks exon 28226 28423 . - . gene_id "XLOC_228846"; transcript_id "TCONS_01124458"; exon_number "5"; gene_type "lincRNA";

Y_mapped_Scaffold_18_D1698 Cufflinks exon 15873 18894 . - . gene_id "XLOC_228850"; transcript_id "TCONS_01124436"; exon_number "1"; gene_type "lincRNA";

Y_mapped_Scaffold_18_D1698 Cufflinks exon 18990 19638 . - . gene_id "XLOC_228850"; transcript_id "TCONS_01124436"; exon_number "2"; gene_type "lincRNA";

Y_mapped_Scaffold_18_D1698 Cufflinks exon 15889 18894 . - . gene_id "XLOC_228850"; transcript_id "TCONS_01124445"; exon_number "1"; gene_type "lincRNA";

Y_mapped_Scaffold_18_D1698 Cufflinks exon 18981 19630 . - . gene_id "XLOC_228850"; transcript_id "TCONS_01124445"; exon_number "2"; gene_type "lincRNA";

Y_mapped_Scaffold_18_D1698 Scripture exon 19146 19465 . - . gene_id "XLOC_228850"; transcript_id "TCONS_01124503"; exon_number "1"; gene_type "lincRNA";

Y_mapped_Scaffold_18_D1698 Scripture exon 19527 19644 . - . gene_id "XLOC_228850"; transcript_id "TCONS_01124503"; exon_number "2"; gene_type "lincRNA";

Y_mapped_Scaffold_23_D1638 Cufflinks exon 7843 8044 . - . gene_id "XLOC_228857"; transcript_id "TCONS_01124579"; exon_number "1"; gene_type "lincRNA";

Y_mapped_Scaffold_9_D1573 Cufflinks exon 25559 25864 . + . gene_id "XLOC_228897"; transcript_id "TCONS_01124640"; exon_number "1"; gene_type "lincRNA";

rDNA Scripture exon 48335 48776 . + . gene_id "XLOC_228922"; transcript_id "TCONS_01124904"; exon_number "1"; gene_type "lincRNA";

rDNA Scripture exon 48832 48843 . + . gene_id "XLOC_228922"; transcript_id "TCONS_01124904"; exon_number "2"; gene_type "lincRNA";

rDNA Scripture exon 48335 48455 . + . gene_id "XLOC_228922"; transcript_id "TCONS_01124751"; exon_number "1"; gene_type "lincRNA";

rDNA Scripture exon 72560 72951 . + . gene_id "XLOC_228922"; transcript_id "TCONS_01124751"; exon_number "2"; gene_type "lincRNA";

3L FlyBase exon 3934718 3935992 0 + . gene_id "FBgn0004911"; gene_symbol "Eip63F-2"; transcript_id "FBtr0073162"; transcript_symbol "Eip63F-2-RA"; gene_type "ncRNA";

3R FlyBase exon 13465707 13466106 9 - . gene_id "FBgn0040554"; gene_symbol "CR17025"; transcript_id "FBtr0309273"; transcript_symbol "CR17025-RB"; gene_type "ncRNA";

3R FlyBase exon 13457763 13458545 9 - . gene_id "FBgn0040554"; gene_symbol "CR17025"; transcript_id "FBtr0309273"; transcript_symbol "CR17025-RB"; gene_type "ncRNA";

3R FlyBase exon 13455511 13456267 9 - . gene_id "FBgn0040554"; gene_symbol "CR17025"; transcript_id "FBtr0309273"; transcript_symbol "CR17025-RB"; gene_type "ncRNA";

3R FlyBase exon 13465707 13466106 0 - . gene_id "FBgn0040554"; gene_symbol "CR17025"; transcript_id "FBtr0309274"; transcript_symbol "CR17025-RC"; gene_type "ncRNA";

3R FlyBase exon 13462723 13464399 0 - . gene_id "FBgn0040554"; gene_symbol "CR17025"; transcript_id "FBtr0309274"; transcript_symbol "CR17025-RC"; gene_type "ncRNA";

3R FlyBase exon 13465707 13466106 0 - . gene_id "FBgn0040554"; gene_symbol "CR17025"; transcript_id "FBtr0346963"; transcript_symbol "CR17025-RD"; gene_type "ncRNA";

3R FlyBase exon 13459671 13464399 0 - . gene_id "FBgn0040554"; gene_symbol "CR17025"; transcript_id "FBtr0346963"; transcript_symbol "CR17025-RD"; gene_type "ncRNA";

X FlyBase exon 12091264 12091518 1 + . gene_id "FBgn0040859"; gene_symbol "CR32658"; transcript_id "FBtr0073626"; transcript_symbol "CR32658-RA"; gene_type "ncRNA";

X FlyBase exon 12091570 12091742 1 + . gene_id "FBgn0040859"; gene_symbol "CR32658"; transcript_id "FBtr0073626"; transcript_symbol "CR32658-RA"; gene_type "ncRNA";

X FlyBase exon 12091806 12092070 1 + . gene_id "FBgn0040859"; gene_symbol "CR32658"; transcript_id "FBtr0073626"; transcript_symbol "CR32658-RA"; gene_type "ncRNA";

X FlyBase exon 12092143 12092272 1 + . gene_id "FBgn0040859"; gene_symbol "CR32658"; transcript_id "FBtr0073626"; transcript_symbol "CR32658-RA"; gene_type "ncRNA";

X FlyBase exon 12091264 12091492 1 + . gene_id "FBgn0040859"; gene_symbol "CR32658"; transcript_id "FBtr0073627"; transcript_symbol "CR32658-RB"; gene_type "ncRNA";

X FlyBase exon 12091570 12091742 1 + . gene_id "FBgn0040859"; gene_symbol "CR32658"; transcript_id "FBtr0073627"; transcript_symbol "CR32658-RB"; gene_type "ncRNA";

X FlyBase exon 12091806 12092083 1 + . gene_id "FBgn0040859"; gene_symbol "CR32658"; transcript_id "FBtr0073627"; transcript_symbol "CR32658-RB"; gene_type "ncRNA";

X FlyBase exon 12092143 12092272 1 + . gene_id "FBgn0040859"; gene_symbol "CR32658"; transcript_id "FBtr0073627"; transcript_symbol "CR32658-RB"; gene_type "ncRNA";

X FlyBase exon 12091264 12091518 1 + . gene_id "FBgn0040859"; gene_symbol "CR32658"; transcript_id "FBtr0073628"; transcript_symbol "CR32658-RC"; gene_type "ncRNA";

X FlyBase exon 12091580 12091742 1 + . gene_id "FBgn0040859"; gene_symbol "CR32658"; transcript_id "FBtr0073628"; transcript_symbol "CR32658-RC"; gene_type "ncRNA";

X FlyBase exon 12091806 12092070 1 + . gene_id "FBgn0040859"; gene_symbol "CR32658"; transcript_id "FBtr0073628"; transcript_symbol "CR32658-RC"; gene_type "ncRNA";

X FlyBase exon 12092143 12092272 1 + . gene_id "FBgn0040859"; gene_symbol "CR32658"; transcript_id "FBtr0073628"; transcript_symbol "CR32658-RC"; gene_type "ncRNA";

2L FlyBase exon 9984486 9984743 9 - . gene_id "FBgn0040965"; gene_symbol "CR13130"; transcript_id "FBtr0344476"; transcript_symbol "CR13130-RB"; gene_type "ncRNA";

2L FlyBase exon 9983671 9984245 9 - . gene_id "FBgn0040965"; gene_symbol "CR13130"; transcript_id "FBtr0344476"; transcript_symbol "CR13130-RB"; gene_type "ncRNA";

2R FlyBase exon 21803402 21803566 1 + . gene_id "FBgn0046297"; gene_symbol "CR9284"; transcript_id "FBtr0308944"; transcript_symbol "CR9284-RD"; gene_type "ncRNA";

2R FlyBase exon 21803615 21804338 1 + . gene_id "FBgn0046297"; gene_symbol "CR9284"; transcript_id "FBtr0308944"; transcript_symbol "CR9284-RD"; gene_type "ncRNA";

2R FlyBase exon 21803402 21803566 9 + . gene_id "FBgn0046297"; gene_symbol "CR9284"; transcript_id "FBtr0344884"; transcript_symbol "CR9284-RE"; gene_type "ncRNA";

2R FlyBase exon 21803622 21804338 9 + . gene_id "FBgn0046297"; gene_symbol "CR9284"; transcript_id "FBtr0344884"; transcript_symbol "CR9284-RE"; gene_type "ncRNA";

3R FlyBase exon 31767973 31768320 9 + . gene_id "FBgn0046756"; gene_symbol "pncr017:3R"; transcript_id "FBtr0091956"; transcript_symbol "pncr017:3R-RA"; gene_type "ncRNA";

3R FlyBase exon 31768379 31769423 9 + . gene_id "FBgn0046756"; gene_symbol "pncr017:3R"; transcript_id "FBtr0091956"; transcript_symbol "pncr017:3R-RA"; gene_type "ncRNA";

X FlyBase exon 7149782 7149850 9 - . gene_id "FBgn0047092"; gene_symbol "CR32730"; transcript_id "FBtr0071016"; transcript_symbol "CR32730-RA"; gene_type "ncRNA";

X FlyBase exon 7147849 7148467 9 - . gene_id "FBgn0047092"; gene_symbol "CR32730"; transcript_id "FBtr0071016"; transcript_symbol "CR32730-RA"; gene_type "ncRNA";

X FlyBase exon 7145600 7146767 9 - . gene_id "FBgn0047092"; gene_symbol "CR32730"; transcript_id "FBtr0071016"; transcript_symbol "CR32730-RA"; gene_type "ncRNA";

3L FlyBase exon 6976322 6977006 9 + . gene_id "FBgn0047205"; gene_symbol "CR32385"; transcript_id "FBtr0076899"; transcript_symbol "CR32385-RA"; gene_type "ncRNA";

3L FlyBase exon 6977169 6977479 9 + . gene_id "FBgn0047205"; gene_symbol "CR32385"; transcript_id "FBtr0076899"; transcript_symbol "CR32385-RA"; gene_type "ncRNA";

3L FlyBase exon 6977647 6979222 9 + . gene_id "FBgn0047205"; gene_symbol "CR32385"; transcript_id "FBtr0076899"; transcript_symbol "CR32385-RA"; gene_type "ncRNA";

2R FlyBase exon 10263681 10263827 9 - . gene_id "FBgn0050009"; gene_symbol "CR30009"; transcript_id "FBtr0088361"; transcript_symbol "CR30009-RA"; gene_type "ncRNA";

2R FlyBase exon 10261315 10263617 9 - . gene_id "FBgn0050009"; gene_symbol "CR30009"; transcript_id "FBtr0088361"; transcript_symbol "CR30009-RA"; gene_type "ncRNA";

2R FlyBase exon 10263681 10263827 9 - . gene_id "FBgn0050009"; gene_symbol "CR30009"; transcript_id "FBtr0303450"; transcript_symbol "CR30009-RB"; gene_type "ncRNA";

2R FlyBase exon 10262599 10263617 9 - . gene_id "FBgn0050009"; gene_symbol "CR30009"; transcript_id "FBtr0303450"; transcript_symbol "CR30009-RB"; gene_type "ncRNA";

2R FlyBase exon 12589767 12590339 9 + . gene_id "FBgn0050055"; gene_symbol "CR30055"; transcript_id "FBtr0345021"; transcript_symbol "CR30055-RD"; gene_type "ncRNA";

2R FlyBase exon 12590439 12590705 9 + . gene_id "FBgn0050055"; gene_symbol "CR30055"; transcript_id "FBtr0345021"; transcript_symbol "CR30055-RD"; gene_type "ncRNA";

2R FlyBase exon 12589239 12590339 9 + . gene_id "FBgn0050055"; gene_symbol "CR30055"; transcript_id "FBtr0303918"; transcript_symbol "CR30055-RB"; gene_type "ncRNA";

2R FlyBase exon 12590439 12590705 9 + . gene_id "FBgn0050055"; gene_symbol "CR30055"; transcript_id "FBtr0303918"; transcript_symbol "CR30055-RB"; gene_type "ncRNA";

2R FlyBase exon 12589239 12590339 9 + . gene_id "FBgn0050055"; gene_symbol "CR30055"; transcript_id "FBtr0303919"; transcript_symbol "CR30055-RC"; gene_type "ncRNA";

2R FlyBase exon 12590439 12591395 9 + . gene_id "FBgn0050055"; gene_symbol "CR30055"; transcript_id "FBtr0303919"; transcript_symbol "CR30055-RC"; gene_type "ncRNA";

2R FlyBase exon 18741199 18741658 9 - . gene_id "FBgn0050121"; gene_symbol "CR30121"; transcript_id "FBtr0308920"; transcript_symbol "CR30121-RB"; gene_type "ncRNA";

2R FlyBase exon 18739131 18740969 9 - . gene_id "FBgn0050121"; gene_symbol "CR30121"; transcript_id "FBtr0308920"; transcript_symbol "CR30121-RB"; gene_type "ncRNA";

3R FlyBase exon 29214562 29219667 1 + . gene_id "FBgn0051044"; gene_symbol "CR31044"; transcript_id "FBtr0085391"; transcript_symbol "CR31044-RA"; gene_type "ncRNA";

3R FlyBase exon 29214562 29214959 8 + . gene_id "FBgn0051044"; gene_symbol "CR31044"; transcript_id "FBtr0303426"; transcript_symbol "CR31044-RB"; gene_type "ncRNA";

3R FlyBase exon 29215015 29215181 8 + . gene_id "FBgn0051044"; gene_symbol "CR31044"; transcript_id "FBtr0303426"; transcript_symbol "CR31044-RB"; gene_type "ncRNA";

3R FlyBase exon 29218465 29219667 8 + . gene_id "FBgn0051044"; gene_symbol "CR31044"; transcript_id "FBtr0303426"; transcript_symbol "CR31044-RB"; gene_type "ncRNA";

3R FlyBase exon 29214562 29214959 1 + . gene_id "FBgn0051044"; gene_symbol "CR31044"; transcript_id "FBtr0303427"; transcript_symbol "CR31044-RC"; gene_type "ncRNA";

3R FlyBase exon 29215015 29217852 1 + . gene_id "FBgn0051044"; gene_symbol "CR31044"; transcript_id "FBtr0303427"; transcript_symbol "CR31044-RC"; gene_type "ncRNA";

3R FlyBase exon 29218465 29219667 1 + . gene_id "FBgn0051044"; gene_symbol "CR31044"; transcript_id "FBtr0303427"; transcript_symbol "CR31044-RC"; gene_type "ncRNA";

3R FlyBase exon 11305544 11305608 9 - . gene_id "FBgn0051386"; gene_symbol "CR31386"; transcript_id "FBtr0308921"; transcript_symbol "CR31386-RB"; gene_type "ncRNA";

3R FlyBase exon 11295228 11295251 9 - . gene_id "FBgn0051386"; gene_symbol "CR31386"; transcript_id "FBtr0308921"; transcript_symbol "CR31386-RB"; gene_type "ncRNA";

3R FlyBase exon 11287252 11287547 9 - . gene_id "FBgn0051386"; gene_symbol "CR31386"; transcript_id "FBtr0308921"; transcript_symbol "CR31386-RB"; gene_type "ncRNA";

3R FlyBase exon 11277630 11277841 9 - . gene_id "FBgn0051386"; gene_symbol "CR31386"; transcript_id "FBtr0308921"; transcript_symbol "CR31386-RB"; gene_type "ncRNA";

3R FlyBase exon 11269415 11271580 9 - . gene_id "FBgn0051386"; gene_symbol "CR31386"; transcript_id "FBtr0308921"; transcript_symbol "CR31386-RB"; gene_type "ncRNA";

3R FlyBase exon 23983503 23984565 9 + . gene_id "FBgn0051451"; gene_symbol "CR31451"; transcript_id "FBtr0308922"; transcript_symbol "CR31451-RC"; gene_type "ncRNA";

3R FlyBase exon 23983503 23985065 9 + . gene_id "FBgn0051451"; gene_symbol "CR31451"; transcript_id "FBtr0308923"; transcript_symbol "CR31451-RD"; gene_type "ncRNA";

2L FlyBase exon 5439228 5439277 9 - . gene_id "FBgn0051647"; gene_symbol "CR31647"; transcript_id "FBtr0308924"; transcript_symbol "CR31647-RC"; gene_type "ncRNA";

2L FlyBase exon 5436767 5437101 9 - . gene_id "FBgn0051647"; gene_symbol "CR31647"; transcript_id "FBtr0308924"; transcript_symbol "CR31647-RC"; gene_type "ncRNA";

2L FlyBase exon 5435582 5436711 9 - . gene_id "FBgn0051647"; gene_symbol "CR31647"; transcript_id "FBtr0308924"; transcript_symbol "CR31647-RC"; gene_type "ncRNA";

2L FlyBase exon 16869462 16870997 9 + . gene_id "FBgn0051781"; gene_symbol "CR31781"; transcript_id "FBtr0308925"; transcript_symbol "CR31781-RD"; gene_type "ncRNA";

2L FlyBase exon 16877497 16878300 9 + . gene_id "FBgn0051781"; gene_symbol "CR31781"; transcript_id "FBtr0308925"; transcript_symbol "CR31781-RD"; gene_type "ncRNA";

2L FlyBase exon 16869462 16870997 1 + . gene_id "FBgn0051781"; gene_symbol "CR31781"; transcript_id "FBtr0308926"; transcript_symbol "CR31781-RE"; gene_type "ncRNA";

2L FlyBase exon 16876150 16876250 1 + . gene_id "FBgn0051781"; gene_symbol "CR31781"; transcript_id "FBtr0308926"; transcript_symbol "CR31781-RE"; gene_type "ncRNA";

2L FlyBase exon 16877497 16878300 1 + . gene_id "FBgn0051781"; gene_symbol "CR31781"; transcript_id "FBtr0308926"; transcript_symbol "CR31781-RE"; gene_type "ncRNA";

2L FlyBase exon 16869462 16870997 9 + . gene_id "FBgn0051781"; gene_symbol "CR31781"; transcript_id "FBtr0308927"; transcript_symbol "CR31781-RF"; gene_type "ncRNA";

2L FlyBase exon 16875387 16875508 9 + . gene_id "FBgn0051781"; gene_symbol "CR31781"; transcript_id "FBtr0308927"; transcript_symbol "CR31781-RF"; gene_type "ncRNA";

2L FlyBase exon 16876150 16876250 9 + . gene_id "FBgn0051781"; gene_symbol "CR31781"; transcript_id "FBtr0308927"; transcript_symbol "CR31781-RF"; gene_type "ncRNA";

2L FlyBase exon 16877497 16878300 9 + . gene_id "FBgn0051781"; gene_symbol "CR31781"; transcript_id "FBtr0308927"; transcript_symbol "CR31781-RF"; gene_type "ncRNA";

2L FlyBase exon 16870490 16870997 1 + . gene_id "FBgn0051781"; gene_symbol "CR31781"; transcript_id "FBtr0308928"; transcript_symbol "CR31781-RG"; gene_type "ncRNA";

2L FlyBase exon 16875387 16875508 1 + . gene_id "FBgn0051781"; gene_symbol "CR31781"; transcript_id "FBtr0308928"; transcript_symbol "CR31781-RG"; gene_type "ncRNA";

2L FlyBase exon 16877497 16878300 1 + . gene_id "FBgn0051781"; gene_symbol "CR31781"; transcript_id "FBtr0308928"; transcript_symbol "CR31781-RG"; gene_type "ncRNA";

2L FlyBase exon 16869462 16870997 1 + . gene_id "FBgn0051781"; gene_symbol "CR31781"; transcript_id "FBtr0308929"; transcript_symbol "CR31781-RH"; gene_type "ncRNA";

2L FlyBase exon 16877500 16878300 1 + . gene_id "FBgn0051781"; gene_symbol "CR31781"; transcript_id "FBtr0308929"; transcript_symbol "CR31781-RH"; gene_type "ncRNA";

2L FlyBase exon 16869346 16870997 1 + . gene_id "FBgn0051781"; gene_symbol "CR31781"; transcript_id "FBtr0346590"; transcript_symbol "CR31781-RI"; gene_type "ncRNA";

2L FlyBase exon 16876150 16876250 1 + . gene_id "FBgn0051781"; gene_symbol "CR31781"; transcript_id "FBtr0346590"; transcript_symbol "CR31781-RI"; gene_type "ncRNA";

2L FlyBase exon 16877497 16878300 1 + . gene_id "FBgn0051781"; gene_symbol "CR31781"; transcript_id "FBtr0346590"; transcript_symbol "CR31781-RI"; gene_type "ncRNA";

2L FlyBase exon 14023722 14024099 0 - . gene_id "FBgn0051840"; gene_symbol "CR31840"; transcript_id "FBtr0345758"; transcript_symbol "CR31840-RB"; gene_type "ncRNA";

2L FlyBase exon 13777195 13777835 8 + . gene_id "FBgn0051845"; gene_symbol "CR31845"; transcript_id "FBtr0307578"; transcript_symbol "CR31845-RB"; gene_type "ncRNA";

2L FlyBase exon 13777898 13778124 8 + . gene_id "FBgn0051845"; gene_symbol "CR31845"; transcript_id "FBtr0307578"; transcript_symbol "CR31845-RB"; gene_type "ncRNA";

3L FlyBase exon 18841579 18842148 1 + . gene_id "FBgn0052027"; gene_symbol "CR32027"; transcript_id "FBtr0307116"; transcript_symbol "CR32027-RB"; gene_type "ncRNA";

3L FlyBase exon 18842214 18842266 1 + . gene_id "FBgn0052027"; gene_symbol "CR32027"; transcript_id "FBtr0307116"; transcript_symbol "CR32027-RB"; gene_type "ncRNA";

3L FlyBase exon 18842346 18842786 1 + . gene_id "FBgn0052027"; gene_symbol "CR32027"; transcript_id "FBtr0307116"; transcript_symbol "CR32027-RB"; gene_type "ncRNA";

3L FlyBase exon 12627934 12628407 9 + . gene_id "FBgn0052111"; gene_symbol "CR32111"; transcript_id "FBtr0344477"; transcript_symbol "CR32111-RC"; gene_type "ncRNA";

3L FlyBase exon 12633329 12633945 9 + . gene_id "FBgn0052111"; gene_symbol "CR32111"; transcript_id "FBtr0344477"; transcript_symbol "CR32111-RC"; gene_type "ncRNA";

3L FlyBase exon 12642047 12642878 9 + . gene_id "FBgn0052111"; gene_symbol "CR32111"; transcript_id "FBtr0344477"; transcript_symbol "CR32111-RC"; gene_type "ncRNA";

3L FlyBase exon 12644802 12645040 9 + . gene_id "FBgn0052111"; gene_symbol "CR32111"; transcript_id "FBtr0344477"; transcript_symbol "CR32111-RC"; gene_type "ncRNA";

3L FlyBase exon 12645113 12645211 9 + . gene_id "FBgn0052111"; gene_symbol "CR32111"; transcript_id "FBtr0344477"; transcript_symbol "CR32111-RC"; gene_type "ncRNA";

3L FlyBase exon 12645272 12645306 9 + . gene_id "FBgn0052111"; gene_symbol "CR32111"; transcript_id "FBtr0344477"; transcript_symbol "CR32111-RC"; gene_type "ncRNA";

3L FlyBase exon 12645363 12648280 9 + . gene_id "FBgn0052111"; gene_symbol "CR32111"; transcript_id "FBtr0344477"; transcript_symbol "CR32111-RC"; gene_type "ncRNA";

3L FlyBase exon 12627934 12628407 1 + . gene_id "FBgn0052111"; gene_symbol "CR32111"; transcript_id "FBtr0344478"; transcript_symbol "CR32111-RD"; gene_type "ncRNA";

3L FlyBase exon 12633329 12633811 1 + . gene_id "FBgn0052111"; gene_symbol "CR32111"; transcript_id "FBtr0344478"; transcript_symbol "CR32111-RD"; gene_type "ncRNA";

3L FlyBase exon 12633871 12633945 1 + . gene_id "FBgn0052111"; gene_symbol "CR32111"; transcript_id "FBtr0344478"; transcript_symbol "CR32111-RD"; gene_type "ncRNA";

3L FlyBase exon 12644802 12645040 1 + . gene_id "FBgn0052111"; gene_symbol "CR32111"; transcript_id "FBtr0344478"; transcript_symbol "CR32111-RD"; gene_type "ncRNA";

3L FlyBase exon 12645113 12645211 1 + . gene_id "FBgn0052111"; gene_symbol "CR32111"; transcript_id "FBtr0344478"; transcript_symbol "CR32111-RD"; gene_type "ncRNA";

3L FlyBase exon 12645272 12645306 1 + . gene_id "FBgn0052111"; gene_symbol "CR32111"; transcript_id "FBtr0344478"; transcript_symbol "CR32111-RD"; gene_type "ncRNA";

3L FlyBase exon 12645363 12645798 1 + . gene_id "FBgn0052111"; gene_symbol "CR32111"; transcript_id "FBtr0344478"; transcript_symbol "CR32111-RD"; gene_type "ncRNA";

3L FlyBase exon 16308940 16309517 9 + . gene_id "FBgn0052160"; gene_symbol "CR32160"; transcript_id "FBtr0308930"; transcript_symbol "CR32160-RB"; gene_type "ncRNA";

3L FlyBase exon 16309759 16311318 9 + . gene_id "FBgn0052160"; gene_symbol "CR32160"; transcript_id "FBtr0308930"; transcript_symbol "CR32160-RB"; gene_type "ncRNA";

3L FlyBase exon 18087037 18087058 9 - . gene_id "FBgn0052194"; gene_symbol "CR32194"; transcript_id "FBtr0306733"; transcript_symbol "CR32194-RC"; gene_type "ncRNA";

3L FlyBase exon 18085305 18086961 9 - . gene_id "FBgn0052194"; gene_symbol "CR32194"; transcript_id "FBtr0306733"; transcript_symbol "CR32194-RC"; gene_type "ncRNA";

3L FlyBase exon 19423379 19424183 9 - . gene_id "FBgn0052205"; gene_symbol "CR32205"; transcript_id "FBtr0343097"; transcript_symbol "CR32205-RC"; gene_type "pseudogene";

3L FlyBase exon 19423285 19423323 9 - . gene_id "FBgn0052205"; gene_symbol "CR32205"; transcript_id "FBtr0343097"; transcript_symbol "CR32205-RC"; gene_type "pseudogene";

3L FlyBase exon 19423774 19424183 9 - . gene_id "FBgn0052205"; gene_symbol "CR32205"; transcript_id "FBtr0343096"; transcript_symbol "CR32205-RB"; gene_type "pseudogene";

3L FlyBase exon 19423385 19423567 9 - . gene_id "FBgn0052205"; gene_symbol "CR32205"; transcript_id "FBtr0343096"; transcript_symbol "CR32205-RB"; gene_type "pseudogene";

3L FlyBase exon 19423774 19424183 9 - . gene_id "FBgn0052205"; gene_symbol "CR32205"; transcript_id "FBtr0075000"; transcript_symbol "CR32205-RA"; gene_type "pseudogene";

3L FlyBase exon 19423379 19423567 9 - . gene_id "FBgn0052205"; gene_symbol "CR32205"; transcript_id "FBtr0075000"; transcript_symbol "CR32205-RA"; gene_type "pseudogene";

3L FlyBase exon 19422838 19423323 9 - . gene_id "FBgn0052205"; gene_symbol "CR32205"; transcript_id "FBtr0075000"; transcript_symbol "CR32205-RA"; gene_type "pseudogene";

3L FlyBase exon 19895216 19896391 9 - . gene_id "FBgn0052218"; gene_symbol "CR32218"; transcript_id "FBtr0304110"; transcript_symbol "CR32218-RA"; gene_type "ncRNA";

3L FlyBase exon 20207866 20208801 0 - . gene_id "FBgn0052224"; gene_symbol "CR32224"; transcript_id "FBtr0347178"; transcript_symbol "CR32224-RC"; gene_type "ncRNA";

X FlyBase exon 15836115 15836208 9 + . gene_id "FBgn0052582"; gene_symbol "CR32582"; transcript_id "FBtr0347267"; transcript_symbol "CR32582-RD"; gene_type "ncRNA";

X FlyBase exon 15836282 15836397 9 + . gene_id "FBgn0052582"; gene_symbol "CR32582"; transcript_id "FBtr0347267"; transcript_symbol "CR32582-RD"; gene_type "ncRNA";

X FlyBase exon 15836525 15836674 9 + . gene_id "FBgn0052582"; gene_symbol "CR32582"; transcript_id "FBtr0347267"; transcript_symbol "CR32582-RD"; gene_type "ncRNA";

X FlyBase exon 15836738 15836923 9 + . gene_id "FBgn0052582"; gene_symbol "CR32582"; transcript_id "FBtr0347267"; transcript_symbol "CR32582-RD"; gene_type "ncRNA";

X FlyBase exon 15836115 15836208 0 + . gene_id "FBgn0052582"; gene_symbol "CR32582"; transcript_id "FBtr0347268"; transcript_symbol "CR32582-RE"; gene_type "ncRNA";

X FlyBase exon 15836282 15836397 0 + . gene_id "FBgn0052582"; gene_symbol "CR32582"; transcript_id "FBtr0347268"; transcript_symbol "CR32582-RE"; gene_type "ncRNA";

X FlyBase exon 15836457 15836674 0 + . gene_id "FBgn0052582"; gene_symbol "CR32582"; transcript_id "FBtr0347268"; transcript_symbol "CR32582-RE"; gene_type "ncRNA";

X FlyBase exon 15836738 15836923 0 + . gene_id "FBgn0052582"; gene_symbol "CR32582"; transcript_id "FBtr0347268"; transcript_symbol "CR32582-RE"; gene_type "ncRNA";

X FlyBase exon 15835601 15835623 9 + . gene_id "FBgn0052582"; gene_symbol "CR32582"; transcript_id "FBtr0347269"; transcript_symbol "CR32582-RF"; gene_type "ncRNA";

X FlyBase exon 15836116 15836208 9 + . gene_id "FBgn0052582"; gene_symbol "CR32582"; transcript_id "FBtr0347269"; transcript_symbol "CR32582-RF"; gene_type "ncRNA";

X FlyBase exon 15836282 15836674 9 + . gene_id "FBgn0052582"; gene_symbol "CR32582"; transcript_id "FBtr0347269"; transcript_symbol "CR32582-RF"; gene_type "ncRNA";

X FlyBase exon 15836738 15836923 9 + . gene_id "FBgn0052582"; gene_symbol "CR32582"; transcript_id "FBtr0347269"; transcript_symbol "CR32582-RF"; gene_type "ncRNA";

X FlyBase exon 13332374 13333857 9 + . gene_id "FBgn0052636"; gene_symbol "CR32636"; transcript_id "FBtr0347127"; transcript_symbol "CR32636-RC"; gene_type "ncRNA";

X FlyBase exon 12412103 12412181 1 - . gene_id "FBgn0052652"; gene_symbol "CR32652"; transcript_id "FBtr0347270"; transcript_symbol "CR32652-RB"; gene_type "ncRNA";

X FlyBase exon 12411874 12411991 1 - . gene_id "FBgn0052652"; gene_symbol "CR32652"; transcript_id "FBtr0347270"; transcript_symbol "CR32652-RB"; gene_type "ncRNA";

X FlyBase exon 12411644 12411816 1 - . gene_id "FBgn0052652"; gene_symbol "CR32652"; transcript_id "FBtr0347270"; transcript_symbol "CR32652-RB"; gene_type "ncRNA";

X FlyBase exon 12411365 12411589 1 - . gene_id "FBgn0052652"; gene_symbol "CR32652"; transcript_id "FBtr0347270"; transcript_symbol "CR32652-RB"; gene_type "ncRNA";

X FlyBase exon 12411018 12411310 1 - . gene_id "FBgn0052652"; gene_symbol "CR32652"; transcript_id "FBtr0347270"; transcript_symbol "CR32652-RB"; gene_type "ncRNA";

X FlyBase exon 12087211 12087276 1 + . gene_id "FBgn0052657"; gene_symbol "CR32657"; transcript_id "FBtr0073625"; transcript_symbol "CR32657-RA"; gene_type "ncRNA";

X FlyBase exon 12087351 12087439 1 + . gene_id "FBgn0052657"; gene_symbol "CR32657"; transcript_id "FBtr0073625"; transcript_symbol "CR32657-RA"; gene_type "ncRNA";

X FlyBase exon 12087498 12087695 1 + . gene_id "FBgn0052657"; gene_symbol "CR32657"; transcript_id "FBtr0073625"; transcript_symbol "CR32657-RA"; gene_type "ncRNA";

X FlyBase exon 11920410 11920574 9 - . gene_id "FBgn0052660"; gene_symbol "CR32660"; transcript_id "FBtr0343098"; transcript_symbol "CR32660-RC"; gene_type "ncRNA";

X FlyBase exon 11920244 11920337 9 - . gene_id "FBgn0052660"; gene_symbol "CR32660"; transcript_id "FBtr0343098"; transcript_symbol "CR32660-RC"; gene_type "ncRNA";

X FlyBase exon 11920004 11920186 9 - . gene_id "FBgn0052660"; gene_symbol "CR32660"; transcript_id "FBtr0343098"; transcript_symbol "CR32660-RC"; gene_type "ncRNA";

X FlyBase exon 11920410 11920474 9 - . gene_id "FBgn0052660"; gene_symbol "CR32660"; transcript_id "FBtr0331750"; transcript_symbol "CR32660-RB"; gene_type "ncRNA";

X FlyBase exon 11920244 11920337 9 - . gene_id "FBgn0052660"; gene_symbol "CR32660"; transcript_id "FBtr0331750"; transcript_symbol "CR32660-RB"; gene_type "ncRNA";

X FlyBase exon 11920004 11920186 9 - . gene_id "FBgn0052660"; gene_symbol "CR32660"; transcript_id "FBtr0331750"; transcript_symbol "CR32660-RB"; gene_type "ncRNA";

X FlyBase exon 10204400 10204491 9 + . gene_id "FBgn0052690"; gene_symbol "CR32690"; transcript_id "FBtr0303456"; transcript_symbol "CR32690-RB"; gene_type "ncRNA";

X FlyBase exon 10204557 10205289 9 + . gene_id "FBgn0052690"; gene_symbol "CR32690"; transcript_id "FBtr0303456"; transcript_symbol "CR32690-RB"; gene_type "ncRNA";

X FlyBase exon 4393761 4394040 0 - . gene_id "FBgn0052773"; gene_symbol "CR32773"; transcript_id "FBtr0344479"; transcript_symbol "CR32773-RC"; gene_type "ncRNA";

X FlyBase exon 4393325 4393604 0 - . gene_id "FBgn0052773"; gene_symbol "CR32773"; transcript_id "FBtr0344479"; transcript_symbol "CR32773-RC"; gene_type "ncRNA";

X FlyBase exon 4387676 4388251 0 - . gene_id "FBgn0052773"; gene_symbol "CR32773"; transcript_id "FBtr0344479"; transcript_symbol "CR32773-RC"; gene_type "ncRNA";

X FlyBase exon 4610851 4611385 0 - . gene_id "FBgn0052773"; gene_symbol "CR32773"; transcript_id "FBtr0344480"; transcript_symbol "CR32773-RD"; gene_type "ncRNA";

X FlyBase exon 4562605 4562650 0 - . gene_id "FBgn0052773"; gene_symbol "CR32773"; transcript_id "FBtr0344480"; transcript_symbol "CR32773-RD"; gene_type "ncRNA";

X FlyBase exon 4459955 4460105 0 - . gene_id "FBgn0052773"; gene_symbol "CR32773"; transcript_id "FBtr0344480"; transcript_symbol "CR32773-RD"; gene_type "ncRNA";

X FlyBase exon 4393325 4393604 0 - . gene_id "FBgn0052773"; gene_symbol "CR32773"; transcript_id "FBtr0344480"; transcript_symbol "CR32773-RD"; gene_type "ncRNA";

X FlyBase exon 4387676 4388251 0 - . gene_id "FBgn0052773"; gene_symbol "CR32773"; transcript_id "FBtr0344480"; transcript_symbol "CR32773-RD"; gene_type "ncRNA";

2R FlyBase exon 22953422 22953800 9 - . gene_id "FBgn0052835"; gene_symbol "CR32835"; transcript_id "FBtr0308940"; transcript_symbol "CR32835-RC"; gene_type "ncRNA";

2R FlyBase exon 22951988 22952060 9 - . gene_id "FBgn0052835"; gene_symbol "CR32835"; transcript_id "FBtr0308940"; transcript_symbol "CR32835-RC"; gene_type "ncRNA";

2R FlyBase exon 22951148 22951918 9 - . gene_id "FBgn0052835"; gene_symbol "CR32835"; transcript_id "FBtr0308940"; transcript_symbol "CR32835-RC"; gene_type "ncRNA";

2R FlyBase exon 22953422 22953800 9 - . gene_id "FBgn0052835"; gene_symbol "CR32835"; transcript_id "FBtr0308941"; transcript_symbol "CR32835-RD"; gene_type "ncRNA";

2R FlyBase exon 22951988 22952060 9 - . gene_id "FBgn0052835"; gene_symbol "CR32835"; transcript_id "FBtr0308941"; transcript_symbol "CR32835-RD"; gene_type "ncRNA";

2R FlyBase exon 22951574 22951918 9 - . gene_id "FBgn0052835"; gene_symbol "CR32835"; transcript_id "FBtr0308941"; transcript_symbol "CR32835-RD"; gene_type "ncRNA";

X FlyBase exon 2330159 2330355 9 + . gene_id "FBgn0053218"; gene_symbol "CR33218"; transcript_id "FBtr0308931"; transcript_symbol "CR33218-RC"; gene_type "ncRNA";

X FlyBase exon 2330413 2330826 9 + . gene_id "FBgn0053218"; gene_symbol "CR33218"; transcript_id "FBtr0308931"; transcript_symbol "CR33218-RC"; gene_type "ncRNA";

X FlyBase exon 2330159 2330355 1 + . gene_id "FBgn0053218"; gene_symbol "CR33218"; transcript_id "FBtr0308932"; transcript_symbol "CR33218-RD"; gene_type "ncRNA";

X FlyBase exon 2330506 2330826 1 + . gene_id "FBgn0053218"; gene_symbol "CR33218"; transcript_id "FBtr0308932"; transcript_symbol "CR33218-RD"; gene_type "ncRNA";

X FlyBase exon 12659462 12660632 9 + . gene_id "FBgn0053963"; gene_symbol "CR33963"; transcript_id "FBtr0100004"; transcript_symbol "CR33963-RA"; gene_type "ncRNA";

2L FlyBase exon 22019316 22020254 9 + . gene_id "FBgn0053987"; gene_symbol "CR33987"; transcript_id "FBtr0100032"; transcript_symbol "CR33987-RA"; gene_type "ncRNA";

2L FlyBase exon 22019208 22020254 9 + . gene_id "FBgn0053987"; gene_symbol "CR33987"; transcript_id "FBtr0335390"; transcript_symbol "CR33987-RB"; gene_type "ncRNA";

3R FlyBase exon 27217726 27218184 0 - . gene_id "FBgn0054006"; gene_symbol "CR34006"; transcript_id "FBtr0344481"; transcript_symbol "CR34006-RC"; gene_type "ncRNA";

3R FlyBase exon 27217188 27217482 0 - . gene_id "FBgn0054006"; gene_symbol "CR34006"; transcript_id "FBtr0344481"; transcript_symbol "CR34006-RC"; gene_type "ncRNA";

3R FlyBase exon 27216304 27217136 0 - . gene_id "FBgn0054006"; gene_symbol "CR34006"; transcript_id "FBtr0344481"; transcript_symbol "CR34006-RC"; gene_type "ncRNA";

3R FlyBase exon 25931401 25931593 1 + . gene_id "FBgn0054024"; gene_symbol "CR34024"; transcript_id "FBtr0308933"; transcript_symbol "CR34024-RB"; gene_type "ncRNA";

3R FlyBase exon 25931675 25932016 1 + . gene_id "FBgn0054024"; gene_symbol "CR34024"; transcript_id "FBtr0308933"; transcript_symbol "CR34024-RB"; gene_type "ncRNA";

3R FlyBase exon 13714121 13714204 0 - . gene_id "FBgn0054044"; gene_symbol "CR34044"; transcript_id "FBtr0308934"; transcript_symbol "CR34044-RB"; gene_type "ncRNA";

3R FlyBase exon 13713314 13713626 0 - . gene_id "FBgn0054044"; gene_symbol "CR34044"; transcript_id "FBtr0308934"; transcript_symbol "CR34044-RB"; gene_type "ncRNA";

3R FlyBase exon 13712469 13713266 0 - . gene_id "FBgn0054044"; gene_symbol "CR34044"; transcript_id "FBtr0308934"; transcript_symbol "CR34044-RB"; gene_type "ncRNA";

3R FlyBase exon 31709275 31709380 1 + . gene_id "FBgn0054046"; gene_symbol "CR34046"; transcript_id "FBtr0308935"; transcript_symbol "CR34046-RB"; gene_type "ncRNA";

3R FlyBase exon 31709451 31709788 1 + . gene_id "FBgn0054046"; gene_symbol "CR34046"; transcript_id "FBtr0308935"; transcript_symbol "CR34046-RB"; gene_type "ncRNA";

3L FlyBase exon 5099544 5099795 9 - . gene_id "FBgn0054047"; gene_symbol "CR34047"; transcript_id "FBtr0308936"; transcript_symbol "CR34047-RB"; gene_type "ncRNA";

3L FlyBase exon 5098376 5099438 9 - . gene_id "FBgn0054047"; gene_symbol "CR34047"; transcript_id "FBtr0308936"; transcript_symbol "CR34047-RB"; gene_type "ncRNA";

X FlyBase exon 2322125 2322264 9 - . gene_id "FBgn0054052"; gene_symbol "CR34052"; transcript_id "FBtr0308938"; transcript_symbol "CR34052-RB"; gene_type "ncRNA";

X FlyBase exon 2321855 2322060 9 - . gene_id "FBgn0054052"; gene_symbol "CR34052"; transcript_id "FBtr0308938"; transcript_symbol "CR34052-RB"; gene_type "ncRNA";

X FlyBase exon 2322129 2322264 0 - . gene_id "FBgn0054052"; gene_symbol "CR34052"; transcript_id "FBtr0308939"; transcript_symbol "CR34052-RC"; gene_type "ncRNA";

X FlyBase exon 2321758 2322060 0 - . gene_id "FBgn0054052"; gene_symbol "CR34052"; transcript_id "FBtr0308939"; transcript_symbol "CR34052-RC"; gene_type "ncRNA";

3L FlyBase exon 24011233 24011363 9 - . gene_id "FBgn0058053"; gene_symbol "CR40053"; transcript_id "FBtr0334764"; transcript_symbol "CR40053-RB"; gene_type "ncRNA";

3L FlyBase exon 24009931 24011160 9 - . gene_id "FBgn0058053"; gene_symbol "CR40053"; transcript_id "FBtr0334764"; transcript_symbol "CR40053-RB"; gene_type "ncRNA";

2L FlyBase exon 20248928 20249722 0 - . gene_id "FBgn0058172"; gene_symbol "CR40172"; transcript_id "FBtr0344578"; transcript_symbol "CR40172-RC"; gene_type "ncRNA";

X FlyBase exon 122493 122706 9 + . gene_id "FBgn0058469"; gene_symbol "CR40469"; transcript_id "FBtr0307364"; transcript_symbol "CR40469-RA"; gene_type "ncRNA";

2R FlyBase exon 18711391 18711485 9 + . gene_id "FBgn0062961"; gene_symbol "pncr016:2R"; transcript_id "FBtr0091953"; transcript_symbol "pncr016:2R-RA"; gene_type "ncRNA";

2R FlyBase exon 18711544 18711951 9 + . gene_id "FBgn0062961"; gene_symbol "pncr016:2R"; transcript_id "FBtr0091953"; transcript_symbol "pncr016:2R-RA"; gene_type "ncRNA";

3L FlyBase exon 7406323 7406617 9 + . gene_id "FBgn0063083"; gene_symbol "pncr015:3L"; transcript_id "FBtr0091959"; transcript_symbol "pncr015:3L-RA"; gene_type "ncRNA";

3R FlyBase exon 6700018 6700164 9 - . gene_id "FBgn0063127"; gene_symbol "pncr002:3R"; transcript_id "FBtr0091948"; transcript_symbol "pncr002:3R-RA"; gene_type "ncRNA";

3R FlyBase exon 6699560 6699917 9 - . gene_id "FBgn0063127"; gene_symbol "pncr002:3R"; transcript_id "FBtr0091948"; transcript_symbol "pncr002:3R-RA"; gene_type "ncRNA";

4 FlyBase exon 975168 975562 9 + . gene_id "FBgn0083990"; gene_symbol "sphinx"; transcript_id "FBtr0111044"; transcript_symbol "sphinx-RA"; gene_type "ncRNA";

4 FlyBase exon 976766 977261 9 + . gene_id "FBgn0083990"; gene_symbol "sphinx"; transcript_id "FBtr0111044"; transcript_symbol "sphinx-RA"; gene_type "ncRNA";

4 FlyBase exon 975168 975906 9 + . gene_id "FBgn0083990"; gene_symbol "sphinx"; transcript_id "FBtr0111045"; transcript_symbol "sphinx-RB"; gene_type "ncRNA";

4 FlyBase exon 976766 977261 9 + . gene_id "FBgn0083990"; gene_symbol "sphinx"; transcript_id "FBtr0111045"; transcript_symbol "sphinx-RB"; gene_type "ncRNA";

4 FlyBase exon 975168 975810 9 + . gene_id "FBgn0083990"; gene_symbol "sphinx"; transcript_id "FBtr0111046"; transcript_symbol "sphinx-RC"; gene_type "ncRNA";

4 FlyBase exon 976795 977261 9 + . gene_id "FBgn0083990"; gene_symbol "sphinx"; transcript_id "FBtr0334622"; transcript_symbol "sphinx-RD"; gene_type "ncRNA";

2R FlyBase exon 5253945 5255295 0 + . gene_id "FBgn0084008"; gene_symbol "CR41443"; transcript_id "FBtr0308117"; transcript_symbol "CR41443-RB"; gene_type "ncRNA";

2R FlyBase exon 5253945 5254465 0 + . gene_id "FBgn0084008"; gene_symbol "CR41443"; transcript_id "FBtr0335256"; transcript_symbol "CR41443-RC"; gene_type "ncRNA";

2R FlyBase exon 5254521 5255295 0 + . gene_id "FBgn0084008"; gene_symbol "CR41443"; transcript_id "FBtr0335256"; transcript_symbol "CR41443-RC"; gene_type "ncRNA";

2R FlyBase exon 5253945 5254465 0 + . gene_id "FBgn0084008"; gene_symbol "CR41443"; transcript_id "FBtr0335257"; transcript_symbol "CR41443-RD"; gene_type "ncRNA";

2R FlyBase exon 5254529 5255295 0 + . gene_id "FBgn0084008"; gene_symbol "CR41443"; transcript_id "FBtr0335257"; transcript_symbol "CR41443-RD"; gene_type "ncRNA";

2R FlyBase exon 1071247 1071319 1 - . gene_id "FBgn0085582"; gene_symbol "CR41257"; transcript_id "FBtr0344616"; transcript_symbol "CR41257-RB"; gene_type "ncRNA";

2R FlyBase exon 1070657 1071044 1 - . gene_id "FBgn0085582"; gene_symbol "CR41257"; transcript_id "FBtr0344616"; transcript_symbol "CR41257-RB"; gene_type "ncRNA";

3R FlyBase exon 4138510 4138652 1 - . gene_id "FBgn0085812"; gene_symbol "CR41601"; transcript_id "FBtr0114267"; transcript_symbol "CR41601-RA"; gene_type "ncRNA";

3R FlyBase exon 4138263 4138446 1 - . gene_id "FBgn0085812"; gene_symbol "CR41601"; transcript_id "FBtr0114267"; transcript_symbol "CR41601-RA"; gene_type "ncRNA";

3R FlyBase exon 4137894 4138180 1 - . gene_id "FBgn0085812"; gene_symbol "CR41601"; transcript_id "FBtr0114267"; transcript_symbol "CR41601-RA"; gene_type "ncRNA";

2L FlyBase exon 20253816 20254449 9 - . gene_id "FBgn0250817"; gene_symbol "CR40465"; transcript_id "FBtr0100850"; transcript_symbol "CR40465-RA"; gene_type "ncRNA";

X FlyBase exon 763441 763810 9 - . gene_id "FBgn0259993"; gene_symbol "CR42491"; transcript_id "FBtr0300446"; transcript_symbol "CR42491-RA"; gene_type "ncRNA";

X FlyBase exon 762615 762991 9 - . gene_id "FBgn0259993"; gene_symbol "CR42491"; transcript_id "FBtr0300446"; transcript_symbol "CR42491-RA"; gene_type "ncRNA";

2L FlyBase exon 20248039 20248423 8 + . gene_id "FBgn0259996"; gene_symbol "CR40341"; transcript_id "FBtr0344577"; transcript_symbol "CR40341-RC"; gene_type "ncRNA";

2L FlyBase exon 20248504 20248872 8 + . gene_id "FBgn0259996"; gene_symbol "CR40341"; transcript_id "FBtr0344577"; transcript_symbol "CR40341-RC"; gene_type "ncRNA";

3R FlyBase exon 9551766 9551806 9 - . gene_id "FBgn0260722"; gene_symbol "CR42549"; transcript_id "FBtr0301176"; transcript_symbol "CR42549-RA"; gene_type "ncRNA";

3R FlyBase exon 9548104 9549810 9 - . gene_id "FBgn0260722"; gene_symbol "CR42549"; transcript_id "FBtr0301176"; transcript_symbol "CR42549-RA"; gene_type "ncRNA";

3R FlyBase exon 9551316 9551357 1 - . gene_id "FBgn0260722"; gene_symbol "CR42549"; transcript_id "FBtr0301248"; transcript_symbol "CR42549-RB"; gene_type "ncRNA";

3R FlyBase exon 9548104 9549810 1 - . gene_id "FBgn0260722"; gene_symbol "CR42549"; transcript_id "FBtr0301248"; transcript_symbol "CR42549-RB"; gene_type "ncRNA";

3R FlyBase exon 9551701 9551748 1 - . gene_id "FBgn0260722"; gene_symbol "CR42549"; transcript_id "FBtr0301249"; transcript_symbol "CR42549-RC"; gene_type "ncRNA";

3R FlyBase exon 9548104 9549810 1 - . gene_id "FBgn0260722"; gene_symbol "CR42549"; transcript_id "FBtr0301249"; transcript_symbol "CR42549-RC"; gene_type "ncRNA";

3R FlyBase exon 9551571 9551748 1 - . gene_id "FBgn0260722"; gene_symbol "CR42549"; transcript_id "FBtr0301250"; transcript_symbol "CR42549-RD"; gene_type "ncRNA";

3R FlyBase exon 9548104 9549810 1 - . gene_id "FBgn0260722"; gene_symbol "CR42549"; transcript_id "FBtr0301250"; transcript_symbol "CR42549-RD"; gene_type "ncRNA";

2R FlyBase exon 5363253 5363495 9 + . gene_id "FBgn0261429"; gene_symbol "CR42646"; transcript_id "FBtr0302350"; transcript_symbol "CR42646-RA"; gene_type "ncRNA";

2R FlyBase exon 5364271 5364375 9 + . gene_id "FBgn0261429"; gene_symbol "CR42646"; transcript_id "FBtr0302350"; transcript_symbol "CR42646-RA"; gene_type "ncRNA";

2R FlyBase exon 5364455 5364576 9 + . gene_id "FBgn0261429"; gene_symbol "CR42646"; transcript_id "FBtr0302350"; transcript_symbol "CR42646-RA"; gene_type "ncRNA";

2R FlyBase exon 5364651 5365205 9 + . gene_id "FBgn0261429"; gene_symbol "CR42646"; transcript_id "FBtr0302350"; transcript_symbol "CR42646-RA"; gene_type "ncRNA";

2R FlyBase exon 5365291 5365560 9 + . gene_id "FBgn0261429"; gene_symbol "CR42646"; transcript_id "FBtr0302350"; transcript_symbol "CR42646-RA"; gene_type "ncRNA";

2R FlyBase exon 5365620 5366092 9 + . gene_id "FBgn0261429"; gene_symbol "CR42646"; transcript_id "FBtr0302350"; transcript_symbol "CR42646-RA"; gene_type "ncRNA";

2R FlyBase exon 5366469 5366737 9 + . gene_id "FBgn0261429"; gene_symbol "CR42646"; transcript_id "FBtr0302350"; transcript_symbol "CR42646-RA"; gene_type "ncRNA";

2R FlyBase exon 5366804 5367357 9 + . gene_id "FBgn0261429"; gene_symbol "CR42646"; transcript_id "FBtr0302350"; transcript_symbol "CR42646-RA"; gene_type "ncRNA";

2R FlyBase exon 5363253 5363495 1 + . gene_id "FBgn0261429"; gene_symbol "CR42646"; transcript_id "FBtr0335261"; transcript_symbol "CR42646-RB"; gene_type "ncRNA";

2R FlyBase exon 5364210 5364375 1 + . gene_id "FBgn0261429"; gene_symbol "CR42646"; transcript_id "FBtr0335261"; transcript_symbol "CR42646-RB"; gene_type "ncRNA";

2R FlyBase exon 5364455 5364576 1 + . gene_id "FBgn0261429"; gene_symbol "CR42646"; transcript_id "FBtr0335261"; transcript_symbol "CR42646-RB"; gene_type "ncRNA";

2R FlyBase exon 5364651 5365205 1 + . gene_id "FBgn0261429"; gene_symbol "CR42646"; transcript_id "FBtr0335261"; transcript_symbol "CR42646-RB"; gene_type "ncRNA";

2R FlyBase exon 5365291 5365560 1 + . gene_id "FBgn0261429"; gene_symbol "CR42646"; transcript_id "FBtr0335261"; transcript_symbol "CR42646-RB"; gene_type "ncRNA";

2R FlyBase exon 5365620 5366092 1 + . gene_id "FBgn0261429"; gene_symbol "CR42646"; transcript_id "FBtr0335261"; transcript_symbol "CR42646-RB"; gene_type "ncRNA";

2R FlyBase exon 5366469 5366737 1 + . gene_id "FBgn0261429"; gene_symbol "CR42646"; transcript_id "FBtr0335261"; transcript_symbol "CR42646-RB"; gene_type "ncRNA";

2R FlyBase exon 5366804 5367357 1 + . gene_id "FBgn0261429"; gene_symbol "CR42646"; transcript_id "FBtr0335261"; transcript_symbol "CR42646-RB"; gene_type "ncRNA";

X FlyBase exon 9688189 9688489 1 + . gene_id "FBgn0261522"; gene_symbol "CR42657"; transcript_id "FBtr0344595"; transcript_symbol "CR42657-RB"; gene_type "ncRNA";

X FlyBase exon 9688645 9688764 1 + . gene_id "FBgn0261522"; gene_symbol "CR42657"; transcript_id "FBtr0344595"; transcript_symbol "CR42657-RB"; gene_type "ncRNA";

X FlyBase exon 9688829 9689295 1 + . gene_id "FBgn0261522"; gene_symbol "CR42657"; transcript_id "FBtr0344595"; transcript_symbol "CR42657-RB"; gene_type "ncRNA";

2L FlyBase exon 21326660 21327114 0 + . gene_id "FBgn0261586"; gene_symbol "CR42696"; transcript_id "FBtr0344486"; transcript_symbol "CR42696-RD"; gene_type "ncRNA";

2L FlyBase exon 21326660 21326775 8 + . gene_id "FBgn0261586"; gene_symbol "CR42696"; transcript_id "FBtr0344487"; transcript_symbol "CR42696-RE"; gene_type "ncRNA";

2L FlyBase exon 21326845 21327114 8 + . gene_id "FBgn0261586"; gene_symbol "CR42696"; transcript_id "FBtr0344487"; transcript_symbol "CR42696-RE"; gene_type "ncRNA";

2R FlyBase exon 14639361 14639430 1 + . gene_id "FBgn0261632"; gene_symbol "CR42715"; transcript_id "FBtr0347197"; transcript_symbol "CR42715-RB"; gene_type "ncRNA";

2R FlyBase exon 14639496 14639762 1 + . gene_id "FBgn0261632"; gene_symbol "CR42715"; transcript_id "FBtr0347197"; transcript_symbol "CR42715-RB"; gene_type "ncRNA";

3L FlyBase exon 616571 616646 0 - . gene_id "FBgn0261636"; gene_symbol "CR42719"; transcript_id "FBtr0344638"; transcript_symbol "CR42719-RB"; gene_type "ncRNA";

3L FlyBase exon 615627 616494 0 - . gene_id "FBgn0261636"; gene_symbol "CR42719"; transcript_id "FBtr0344638"; transcript_symbol "CR42719-RB"; gene_type "ncRNA";

2L FlyBase exon 6271024 6271468 0 + . gene_id "FBgn0261699"; gene_symbol "CR42735"; transcript_id "FBtr0333241"; transcript_symbol "CR42735-RB"; gene_type "ncRNA";

2R FlyBase exon 18377487 18377644 0 + . gene_id "FBgn0261700"; gene_symbol "CR42736"; transcript_id "FBtr0340530"; transcript_symbol "CR42736-RB"; gene_type "ncRNA";

2R FlyBase exon 18377709 18377945 0 + . gene_id "FBgn0261700"; gene_symbol "CR42736"; transcript_id "FBtr0340530"; transcript_symbol "CR42736-RB"; gene_type "ncRNA";

2R FlyBase exon 18378050 18378196 0 + . gene_id "FBgn0261700"; gene_symbol "CR42736"; transcript_id "FBtr0340530"; transcript_symbol "CR42736-RB"; gene_type "ncRNA";

2L FlyBase exon 12543176 12543598 9 + . gene_id "FBgn0261709"; gene_symbol "CR42746"; transcript_id "FBtr0303215"; transcript_symbol "CR42746-RA"; gene_type "ncRNA";

2L FlyBase exon 12543940 12544343 9 + . gene_id "FBgn0261709"; gene_symbol "CR42746"; transcript_id "FBtr0303215"; transcript_symbol "CR42746-RA"; gene_type "ncRNA";

3L FlyBase exon 11383966 11384258 1 - . gene_id "FBgn0261813"; gene_symbol "CR42755"; transcript_id "FBtr0344645"; transcript_symbol "CR42755-RB"; gene_type "ncRNA";

3L FlyBase exon 11382808 11383611 1 - . gene_id "FBgn0261813"; gene_symbol "CR42755"; transcript_id "FBtr0344645"; transcript_symbol "CR42755-RB"; gene_type "ncRNA";

3R FlyBase exon 13438587 13439209 9 - . gene_id "FBgn0261814"; gene_symbol "CR42756"; transcript_id "FBtr0303300"; transcript_symbol "CR42756-RA"; gene_type "ncRNA";

3R FlyBase exon 26325000 26325054 9 - . gene_id "FBgn0261833"; gene_symbol "CR42765"; transcript_id "FBtr0346734"; transcript_symbol "CR42765-RC"; gene_type "ncRNA";

3R FlyBase exon 26324499 26324791 9 - . gene_id "FBgn0261833"; gene_symbol "CR42765"; transcript_id "FBtr0346734"; transcript_symbol "CR42765-RC"; gene_type "ncRNA";

3R FlyBase exon 26325000 26325294 9 - . gene_id "FBgn0261833"; gene_symbol "CR42765"; transcript_id "FBtr0344656"; transcript_symbol "CR42765-RB"; gene_type "ncRNA";

3R FlyBase exon 26324499 26324791 9 - . gene_id "FBgn0261833"; gene_symbol "CR42765"; transcript_id "FBtr0344656"; transcript_symbol "CR42765-RB"; gene_type "ncRNA";

X FlyBase exon 9558324 9558466 9 - . gene_id "FBgn0261835"; gene_symbol "CR42767"; transcript_id "FBtr0303397"; transcript_symbol "CR42767-RA"; gene_type "ncRNA";

X FlyBase exon 9557539 9558261 9 - . gene_id "FBgn0261835"; gene_symbol "CR42767"; transcript_id "FBtr0303397"; transcript_symbol "CR42767-RA"; gene_type "ncRNA";

2L FlyBase exon 15863200 15863369 0 + . gene_id "FBgn0261924"; gene_symbol "CR42791"; transcript_id "FBtr0342854"; transcript_symbol "CR42791-RB"; gene_type "ncRNA";

2L FlyBase exon 15863424 15863663 0 + . gene_id "FBgn0261924"; gene_symbol "CR42791"; transcript_id "FBtr0342854"; transcript_symbol "CR42791-RB"; gene_type "ncRNA";

X FlyBase exon 5697123 5697224 0 + . gene_id "FBgn0261927"; gene_symbol "CR42794"; transcript_id "FBtr0344580"; transcript_symbol "CR42794-RB"; gene_type "ncRNA";

X FlyBase exon 5697288 5697660 0 + . gene_id "FBgn0261927"; gene_symbol "CR42794"; transcript_id "FBtr0344580"; transcript_symbol "CR42794-RB"; gene_type "ncRNA";

3R FlyBase exon 20139849 20139921 0 - . gene_id "FBgn0262025"; gene_symbol "CR42836"; transcript_id "FBtr0303855"; transcript_symbol "CR42836-RA"; gene_type "ncRNA";

3R FlyBase exon 20139624 20139734 0 - . gene_id "FBgn0262025"; gene_symbol "CR42836"; transcript_id "FBtr0303855"; transcript_symbol "CR42836-RA"; gene_type "ncRNA";

3R FlyBase exon 20139148 20139542 0 - . gene_id "FBgn0262025"; gene_symbol "CR42836"; transcript_id "FBtr0303855"; transcript_symbol "CR42836-RA"; gene_type "ncRNA";

3R FlyBase exon 20139624 20139921 9 - . gene_id "FBgn0262025"; gene_symbol "CR42836"; transcript_id "FBtr0303856"; transcript_symbol "CR42836-RB"; gene_type "ncRNA";

3R FlyBase exon 20139148 20139542 9 - . gene_id "FBgn0262025"; gene_symbol "CR42836"; transcript_id "FBtr0303856"; transcript_symbol "CR42836-RB"; gene_type "ncRNA";

3R FlyBase exon 16006404 16008073 1 - . gene_id "FBgn0262028"; gene_symbol "CR42839"; transcript_id "FBtr0309032"; transcript_symbol "CR42839-RB"; gene_type "ncRNA";

3R FlyBase exon 16004321 16005672 1 - . gene_id "FBgn0262028"; gene_symbol "CR42839"; transcript_id "FBtr0309032"; transcript_symbol "CR42839-RB"; gene_type "ncRNA";

3R FlyBase exon 16006997 16008073 9 - . gene_id "FBgn0262028"; gene_symbol "CR42839"; transcript_id "FBtr0303861"; transcript_symbol "CR42839-RA"; gene_type "ncRNA";

3R FlyBase exon 16004620 16005672 9 - . gene_id "FBgn0262028"; gene_symbol "CR42839"; transcript_id "FBtr0303861"; transcript_symbol "CR42839-RA"; gene_type "ncRNA";

2L FlyBase exon 9304848 9305007 0 - . gene_id "FBgn0262033"; gene_symbol "CR42844"; transcript_id "FBtr0342852"; transcript_symbol "CR42844-RB"; gene_type "ncRNA";

2L FlyBase exon 9304437 9304780 0 - . gene_id "FBgn0262033"; gene_symbol "CR42844"; transcript_id "FBtr0342852"; transcript_symbol "CR42844-RB"; gene_type "ncRNA";

2L FlyBase exon 18946299 18947086 9 + . gene_id "FBgn0262095"; gene_symbol "CR42848"; transcript_id "FBtr0342924"; transcript_symbol "CR42848-RC"; gene_type "ncRNA";

2L FlyBase exon 5380393 5381074 1 - . gene_id "FBgn0262097"; gene_symbol "CR42850"; transcript_id "FBtr0342943"; transcript_symbol "CR42850-RC"; gene_type "ncRNA";

2L FlyBase exon 5379095 5380332 1 - . gene_id "FBgn0262097"; gene_symbol "CR42850"; transcript_id "FBtr0342943"; transcript_symbol "CR42850-RC"; gene_type "ncRNA";

2L FlyBase exon 5379095 5381074 9 - . gene_id "FBgn0262097"; gene_symbol "CR42850"; transcript_id "FBtr0342944"; transcript_symbol "CR42850-RD"; gene_type "ncRNA";

3R FlyBase exon 9824842 9824864 8 - . gene_id "FBgn0262105"; gene_symbol "CR42858"; transcript_id "FBtr0304027"; transcript_symbol "CR42858-RA"; gene_type "ncRNA";

3R FlyBase exon 9824085 9824741 8 - . gene_id "FBgn0262105"; gene_symbol "CR42858"; transcript_id "FBtr0304027"; transcript_symbol "CR42858-RA"; gene_type "ncRNA";

2L FlyBase exon 2245230 2245494 1 + . gene_id "FBgn0262106"; gene_symbol "CR42859"; transcript_id "FBtr0309033"; transcript_symbol "CR42859-RB"; gene_type "ncRNA";

2L FlyBase exon 2245551 2246207 1 + . gene_id "FBgn0262106"; gene_symbol "CR42859"; transcript_id "FBtr0309033"; transcript_symbol "CR42859-RB"; gene_type "ncRNA";

2L FlyBase exon 2245230 2245494 1 + . gene_id "FBgn0262106"; gene_symbol "CR42859"; transcript_id "FBtr0304029"; transcript_symbol "CR42859-RA"; gene_type "ncRNA";

2L FlyBase exon 2245545 2245901 1 + . gene_id "FBgn0262106"; gene_symbol "CR42859"; transcript_id "FBtr0304029"; transcript_symbol "CR42859-RA"; gene_type "ncRNA";

2L FlyBase exon 2245230 2245494 1 + . gene_id "FBgn0262106"; gene_symbol "CR42859"; transcript_id "FBtr0335160"; transcript_symbol "CR42859-RC"; gene_type "ncRNA";

2L FlyBase exon 2245545 2247383 1 + . gene_id "FBgn0262106"; gene_symbol "CR42859"; transcript_id "FBtr0335160"; transcript_symbol "CR42859-RC"; gene_type "ncRNA";

X FlyBase exon 13744928 13744944 1 + . gene_id "FBgn0262108"; gene_symbol "CR42861"; transcript_id "FBtr0304030"; transcript_symbol "CR42861-RA"; gene_type "ncRNA";

X FlyBase exon 13745017 13745476 1 + . gene_id "FBgn0262108"; gene_symbol "CR42861"; transcript_id "FBtr0304030"; transcript_symbol "CR42861-RA"; gene_type "ncRNA";

X FlyBase exon 13744940 13745476 9 + . gene_id "FBgn0262108"; gene_symbol "CR42861"; transcript_id "FBtr0304031"; transcript_symbol "CR42861-RB"; gene_type "ncRNA";

X FlyBase exon 13744940 13744969 1 + . gene_id "FBgn0262108"; gene_symbol "CR42861"; transcript_id "FBtr0343987"; transcript_symbol "CR42861-RC"; gene_type "ncRNA";

X FlyBase exon 13745024 13745476 1 + . gene_id "FBgn0262108"; gene_symbol "CR42861"; transcript_id "FBtr0343987"; transcript_symbol "CR42861-RC"; gene_type "ncRNA";

3L FlyBase exon 224106 224183 1 + . gene_id "FBgn0262109"; gene_symbol "CR42862"; transcript_id "FBtr0309035"; transcript_symbol "CR42862-RB"; gene_type "ncRNA";

3L FlyBase exon 224973 225140 1 + . gene_id "FBgn0262109"; gene_symbol "CR42862"; transcript_id "FBtr0309035"; transcript_symbol "CR42862-RB"; gene_type "ncRNA";

3L FlyBase exon 225726 229244 1 + . gene_id "FBgn0262109"; gene_symbol "CR42862"; transcript_id "FBtr0309035"; transcript_symbol "CR42862-RB"; gene_type "ncRNA";

3L FlyBase exon 229589 230964 1 + . gene_id "FBgn0262109"; gene_symbol "CR42862"; transcript_id "FBtr0309035"; transcript_symbol "CR42862-RB"; gene_type "ncRNA";

3L FlyBase exon 224106 224183 9 + . gene_id "FBgn0262109"; gene_symbol "CR42862"; transcript_id "FBtr0309036"; transcript_symbol "CR42862-RC"; gene_type "ncRNA";

3L FlyBase exon 224973 225140 9 + . gene_id "FBgn0262109"; gene_symbol "CR42862"; transcript_id "FBtr0309036"; transcript_symbol "CR42862-RC"; gene_type "ncRNA";

3L FlyBase exon 225726 227459 9 + . gene_id "FBgn0262109"; gene_symbol "CR42862"; transcript_id "FBtr0309036"; transcript_symbol "CR42862-RC"; gene_type "ncRNA";

3L FlyBase exon 227518 230964 9 + . gene_id "FBgn0262109"; gene_symbol "CR42862"; transcript_id "FBtr0309036"; transcript_symbol "CR42862-RC"; gene_type "ncRNA";

3L FlyBase exon 224106 224183 1 + . gene_id "FBgn0262109"; gene_symbol "CR42862"; transcript_id "FBtr0309037"; transcript_symbol "CR42862-RD"; gene_type "ncRNA";

3L FlyBase exon 224973 225140 1 + . gene_id "FBgn0262109"; gene_symbol "CR42862"; transcript_id "FBtr0309037"; transcript_symbol "CR42862-RD"; gene_type "ncRNA";

3L FlyBase exon 225726 227764 1 + . gene_id "FBgn0262109"; gene_symbol "CR42862"; transcript_id "FBtr0309037"; transcript_symbol "CR42862-RD"; gene_type "ncRNA";

3L FlyBase exon 227821 230964 1 + . gene_id "FBgn0262109"; gene_symbol "CR42862"; transcript_id "FBtr0309037"; transcript_symbol "CR42862-RD"; gene_type "ncRNA";

3L FlyBase exon 224106 224183 9 + . gene_id "FBgn0262109"; gene_symbol "CR42862"; transcript_id "FBtr0309038"; transcript_symbol "CR42862-RE"; gene_type "ncRNA";

3L FlyBase exon 224973 225140 9 + . gene_id "FBgn0262109"; gene_symbol "CR42862"; transcript_id "FBtr0309038"; transcript_symbol "CR42862-RE"; gene_type "ncRNA";

3L FlyBase exon 225726 225849 9 + . gene_id "FBgn0262109"; gene_symbol "CR42862"; transcript_id "FBtr0309038"; transcript_symbol "CR42862-RE"; gene_type "ncRNA";

3L FlyBase exon 225910 230959 9 + . gene_id "FBgn0262109"; gene_symbol "CR42862"; transcript_id "FBtr0309038"; transcript_symbol "CR42862-RE"; gene_type "ncRNA";

3L FlyBase exon 224106 224183 9 + . gene_id "FBgn0262109"; gene_symbol "CR42862"; transcript_id "FBtr0304059"; transcript_symbol "CR42862-RA"; gene_type "ncRNA";

3L FlyBase exon 224973 225140 9 + . gene_id "FBgn0262109"; gene_symbol "CR42862"; transcript_id "FBtr0304059"; transcript_symbol "CR42862-RA"; gene_type "ncRNA";

3L FlyBase exon 225726 230959 9 + . gene_id "FBgn0262109"; gene_symbol "CR42862"; transcript_id "FBtr0304059"; transcript_symbol "CR42862-RA"; gene_type "ncRNA";

2R FlyBase exon 22666359 22666543 0 + . gene_id "FBgn0262142"; gene_symbol "CR42868"; transcript_id "FBtr0343003"; transcript_symbol "CR42868-RB"; gene_type "ncRNA";

2R FlyBase exon 22666613 22666764 0 + . gene_id "FBgn0262142"; gene_symbol "CR42868"; transcript_id "FBtr0343003"; transcript_symbol "CR42868-RB"; gene_type "ncRNA";

2L FlyBase exon 1989450 1989841 0 + . gene_id "FBgn0262148"; gene_symbol "CR42874"; transcript_id "FBtr0304139"; transcript_symbol "CR42874-RA"; gene_type "ncRNA";

2L FlyBase exon 1989913 1990894 0 + . gene_id "FBgn0262148"; gene_symbol "CR42874"; transcript_id "FBtr0304139"; transcript_symbol "CR42874-RA"; gene_type "ncRNA";

3R FlyBase exon 7128087 7128297 1 + . gene_id "FBgn0262149"; gene_symbol "CR42875"; transcript_id "FBtr0304140"; transcript_symbol "CR42875-RA"; gene_type "ncRNA";

3R FlyBase exon 7128464 7128647 1 + . gene_id "FBgn0262149"; gene_symbol "CR42875"; transcript_id "FBtr0304140"; transcript_symbol "CR42875-RA"; gene_type "ncRNA";

3R FlyBase exon 7128703 7129067 1 + . gene_id "FBgn0262149"; gene_symbol "CR42875"; transcript_id "FBtr0304140"; transcript_symbol "CR42875-RA"; gene_type "ncRNA";

3R FlyBase exon 7128087 7128297 1 + . gene_id "FBgn0262149"; gene_symbol "CR42875"; transcript_id "FBtr0304141"; transcript_symbol "CR42875-RB"; gene_type "ncRNA";

3R FlyBase exon 7128464 7129067 1 + . gene_id "FBgn0262149"; gene_symbol "CR42875"; transcript_id "FBtr0304141"; transcript_symbol "CR42875-RB"; gene_type "ncRNA";

2L FlyBase exon 16945220 16945374 8 - . gene_id "FBgn0262355"; gene_symbol "CR43053"; transcript_id "FBtr0343811"; transcript_symbol "CR43053-RB"; gene_type "ncRNA";

2L FlyBase exon 16944723 16945171 8 - . gene_id "FBgn0262355"; gene_symbol "CR43053"; transcript_id "FBtr0343811"; transcript_symbol "CR43053-RB"; gene_type "ncRNA";

2L FlyBase exon 19910648 19911811 1 - . gene_id "FBgn0262543"; gene_symbol "CR43097"; transcript_id "FBtr0304883"; transcript_symbol "CR43097-RA"; gene_type "ncRNA";

2L FlyBase exon 19911704 19911811 1 - . gene_id "FBgn0262543"; gene_symbol "CR43097"; transcript_id "FBtr0332244"; transcript_symbol "CR43097-RB"; gene_type "ncRNA";

2L FlyBase exon 19910648 19911637 1 - . gene_id "FBgn0262543"; gene_symbol "CR43097"; transcript_id "FBtr0332244"; transcript_symbol "CR43097-RB"; gene_type "ncRNA";

2R FlyBase exon 16737717 16737885 8 - . gene_id "FBgn0262564"; gene_symbol "CR43104"; transcript_id "FBtr0347233"; transcript_symbol "CR43104-RB"; gene_type "ncRNA";

2R FlyBase exon 16736813 16737442 8 - . gene_id "FBgn0262564"; gene_symbol "CR43104"; transcript_id "FBtr0347233"; transcript_symbol "CR43104-RB"; gene_type "ncRNA";

2R FlyBase exon 16737492 16737885 0 - . gene_id "FBgn0262564"; gene_symbol "CR43104"; transcript_id "FBtr0347234"; transcript_symbol "CR43104-RC"; gene_type "ncRNA";

2R FlyBase exon 16736813 16737442 0 - . gene_id "FBgn0262564"; gene_symbol "CR43104"; transcript_id "FBtr0347234"; transcript_symbol "CR43104-RC"; gene_type "ncRNA";

3R FlyBase exon 8820770 8820862 0 + . gene_id "FBgn0262604"; gene_symbol "CR43130"; transcript_id "FBtr0344597"; transcript_symbol "CR43130-RC"; gene_type "ncRNA";

3R FlyBase exon 8820919 8821098 0 + . gene_id "FBgn0262604"; gene_symbol "CR43130"; transcript_id "FBtr0344597"; transcript_symbol "CR43130-RC"; gene_type "ncRNA";

3R FlyBase exon 8819618 8819637 0 + . gene_id "FBgn0262604"; gene_symbol "CR43130"; transcript_id "FBtr0344598"; transcript_symbol "CR43130-RD"; gene_type "ncRNA";

3R FlyBase exon 8820778 8820862 0 + . gene_id "FBgn0262604"; gene_symbol "CR43130"; transcript_id "FBtr0344598"; transcript_symbol "CR43130-RD"; gene_type "ncRNA";

3R FlyBase exon 8820919 8821098 0 + . gene_id "FBgn0262604"; gene_symbol "CR43130"; transcript_id "FBtr0344598"; transcript_symbol "CR43130-RD"; gene_type "ncRNA";

X FlyBase exon 15689490 15689531 0 + . gene_id "FBgn0262606"; gene_symbol "CR43132"; transcript_id "FBtr0347271"; transcript_symbol "CR43132-RB"; gene_type "ncRNA";

X FlyBase exon 15690687 15690773 0 + . gene_id "FBgn0262606"; gene_symbol "CR43132"; transcript_id "FBtr0347271"; transcript_symbol "CR43132-RB"; gene_type "ncRNA";

X FlyBase exon 15690848 15691146 0 + . gene_id "FBgn0262606"; gene_symbol "CR43132"; transcript_id "FBtr0347271"; transcript_symbol "CR43132-RB"; gene_type "ncRNA";

2L FlyBase exon 21657659 21657776 8 + . gene_id "FBgn0262620"; gene_symbol "CR43144"; transcript_id "FBtr0342855"; transcript_symbol "CR43144-RD"; gene_type "ncRNA";

2L FlyBase exon 21657833 21658249 8 + . gene_id "FBgn0262620"; gene_symbol "CR43144"; transcript_id "FBtr0342855"; transcript_symbol "CR43144-RD"; gene_type "ncRNA";

2L FlyBase exon 21657659 21657741 1 + . gene_id "FBgn0262620"; gene_symbol "CR43144"; transcript_id "FBtr0342856"; transcript_symbol "CR43144-RE"; gene_type "ncRNA";

2L FlyBase exon 21657833 21658249 1 + . gene_id "FBgn0262620"; gene_symbol "CR43144"; transcript_id "FBtr0342856"; transcript_symbol "CR43144-RE"; gene_type "ncRNA";

2L FlyBase exon 21657625 21657667 0 + . gene_id "FBgn0262620"; gene_symbol "CR43144"; transcript_id "FBtr0342857"; transcript_symbol "CR43144-RF"; gene_type "ncRNA";

2L FlyBase exon 21657833 21658249 0 + . gene_id "FBgn0262620"; gene_symbol "CR43144"; transcript_id "FBtr0342857"; transcript_symbol "CR43144-RF"; gene_type "ncRNA";

3L FlyBase exon 13282393 13282854 0 + . gene_id "FBgn0262622"; gene_symbol "CR43146"; transcript_id "FBtr0344571"; transcript_symbol "CR43146-RC"; gene_type "ncRNA";

3L FlyBase exon 13282913 13283410 0 + . gene_id "FBgn0262622"; gene_symbol "CR43146"; transcript_id "FBtr0344571"; transcript_symbol "CR43146-RC"; gene_type "ncRNA";

3L FlyBase exon 13282393 13282854 8 + . gene_id "FBgn0262622"; gene_symbol "CR43146"; transcript_id "FBtr0344572"; transcript_symbol "CR43146-RD"; gene_type "ncRNA";

3L FlyBase exon 13282916 13283410 8 + . gene_id "FBgn0262622"; gene_symbol "CR43146"; transcript_id "FBtr0344572"; transcript_symbol "CR43146-RD"; gene_type "ncRNA";

2L FlyBase exon 21624679 21626190 9 + . gene_id "FBgn0262631"; gene_symbol "CR43148"; transcript_id "FBtr0309039"; transcript_symbol "CR43148-RB"; gene_type "ncRNA";

2L FlyBase exon 21624679 21625388 9 + . gene_id "FBgn0262631"; gene_symbol "CR43148"; transcript_id "FBtr0305496"; transcript_symbol "CR43148-RA"; gene_type "ncRNA";

3L FlyBase exon 157818 158211 8 - . gene_id "FBgn0262681"; gene_symbol "CR43151"; transcript_id "FBtr0344639"; transcript_symbol "CR43151-RB"; gene_type "ncRNA";

X FlyBase exon 12974519 12974615 0 - . gene_id "FBgn0262682"; gene_symbol "CR43152"; transcript_id "FBtr0309992"; transcript_symbol "CR43152-RB"; gene_type "ncRNA";

X FlyBase exon 12973825 12974460 0 - . gene_id "FBgn0262682"; gene_symbol "CR43152"; transcript_id "FBtr0309992"; transcript_symbol "CR43152-RB"; gene_type "ncRNA";

X FlyBase exon 11919525 11919589 1 - . gene_id "FBgn0262687"; gene_symbol "CR43157"; transcript_id "FBtr0305608"; transcript_symbol "CR43157-RA"; gene_type "ncRNA";

X FlyBase exon 11919359 11919452 1 - . gene_id "FBgn0262687"; gene_symbol "CR43157"; transcript_id "FBtr0305608"; transcript_symbol "CR43157-RA"; gene_type "ncRNA";

X FlyBase exon 11919125 11919306 1 - . gene_id "FBgn0262687"; gene_symbol "CR43157"; transcript_id "FBtr0305608"; transcript_symbol "CR43157-RA"; gene_type "ncRNA";

X FlyBase exon 11919525 11919657 1 - . gene_id "FBgn0262687"; gene_symbol "CR43157"; transcript_id "FBtr0343099"; transcript_symbol "CR43157-RB"; gene_type "ncRNA";

X FlyBase exon 11919359 11919452 1 - . gene_id "FBgn0262687"; gene_symbol "CR43157"; transcript_id "FBtr0343099"; transcript_symbol "CR43157-RB"; gene_type "ncRNA";

X FlyBase exon 11919125 11919306 1 - . gene_id "FBgn0262687"; gene_symbol "CR43157"; transcript_id "FBtr0343099"; transcript_symbol "CR43157-RB"; gene_type "ncRNA";

2R FlyBase exon 20896994 20897309 9 + . gene_id "FBgn0262690"; gene_symbol "CR43160"; transcript_id "FBtr0305615"; transcript_symbol "CR43160-RA"; gene_type "ncRNA";

2R FlyBase exon 20897375 20897754 9 + . gene_id "FBgn0262690"; gene_symbol "CR43160"; transcript_id "FBtr0305615"; transcript_symbol "CR43160-RA"; gene_type "ncRNA";

2R FlyBase exon 20897831 20898629 9 + . gene_id "FBgn0262690"; gene_symbol "CR43160"; transcript_id "FBtr0305615"; transcript_symbol "CR43160-RA"; gene_type "ncRNA";

3R FlyBase exon 24826381 24826471 0 - . gene_id "FBgn0262722"; gene_symbol "CR43166"; transcript_id "FBtr0347198"; transcript_symbol "CR43166-RB"; gene_type "ncRNA";

3R FlyBase exon 24825791 24825944 0 - . gene_id "FBgn0262722"; gene_symbol "CR43166"; transcript_id "FBtr0347198"; transcript_symbol "CR43166-RB"; gene_type "ncRNA";

4 FlyBase exon 467497 467693 1 + . gene_id "FBgn0262731"; gene_symbol "pncr013:4"; transcript_id "FBtr0333916"; transcript_symbol "pncr013:4-RE"; gene_type "ncRNA";

4 FlyBase exon 467751 467994 1 + . gene_id "FBgn0262731"; gene_symbol "pncr013:4"; transcript_id "FBtr0333916"; transcript_symbol "pncr013:4-RE"; gene_type "ncRNA";

4 FlyBase exon 468790 468998 1 + . gene_id "FBgn0262731"; gene_symbol "pncr013:4"; transcript_id "FBtr0333916"; transcript_symbol "pncr013:4-RE"; gene_type "ncRNA";

4 FlyBase exon 469157 469330 1 + . gene_id "FBgn0262731"; gene_symbol "pncr013:4"; transcript_id "FBtr0333916"; transcript_symbol "pncr013:4-RE"; gene_type "ncRNA";

4 FlyBase exon 470155 470214 1 + . gene_id "FBgn0262731"; gene_symbol "pncr013:4"; transcript_id "FBtr0333916"; transcript_symbol "pncr013:4-RE"; gene_type "ncRNA";

4 FlyBase exon 471014 471476 1 + . gene_id "FBgn0262731"; gene_symbol "pncr013:4"; transcript_id "FBtr0333916"; transcript_symbol "pncr013:4-RE"; gene_type "ncRNA";

4 FlyBase exon 467497 467693 9 + . gene_id "FBgn0262731"; gene_symbol "pncr013:4"; transcript_id "FBtr0091952"; transcript_symbol "pncr013:4-RA"; gene_type "ncRNA";

4 FlyBase exon 467751 468693 9 + . gene_id "FBgn0262731"; gene_symbol "pncr013:4"; transcript_id "FBtr0091952"; transcript_symbol "pncr013:4-RA"; gene_type "ncRNA";

4 FlyBase exon 467497 467693 9 + . gene_id "FBgn0262731"; gene_symbol "pncr013:4"; transcript_id "FBtr0303020"; transcript_symbol "pncr013:4-RC"; gene_type "ncRNA";

4 FlyBase exon 467751 467994 9 + . gene_id "FBgn0262731"; gene_symbol "pncr013:4"; transcript_id "FBtr0303020"; transcript_symbol "pncr013:4-RC"; gene_type "ncRNA";

4 FlyBase exon 469157 469330 9 + . gene_id "FBgn0262731"; gene_symbol "pncr013:4"; transcript_id "FBtr0303020"; transcript_symbol "pncr013:4-RC"; gene_type "ncRNA";

4 FlyBase exon 471014 471476 9 + . gene_id "FBgn0262731"; gene_symbol "pncr013:4"; transcript_id "FBtr0303020"; transcript_symbol "pncr013:4-RC"; gene_type "ncRNA";

4 FlyBase exon 467497 467693 9 + . gene_id "FBgn0262731"; gene_symbol "pncr013:4"; transcript_id "FBtr0303021"; transcript_symbol "pncr013:4-RD"; gene_type "ncRNA";

4 FlyBase exon 467751 467994 9 + . gene_id "FBgn0262731"; gene_symbol "pncr013:4"; transcript_id "FBtr0303021"; transcript_symbol "pncr013:4-RD"; gene_type "ncRNA";

4 FlyBase exon 471014 471476 9 + . gene_id "FBgn0262731"; gene_symbol "pncr013:4"; transcript_id "FBtr0303021"; transcript_symbol "pncr013:4-RD"; gene_type "ncRNA";

3R FlyBase exon 28586048 28591386 9 + . gene_id "FBgn0262741"; gene_symbol "MRE23"; transcript_id "FBtr0309075"; transcript_symbol "MRE23-RB"; gene_type "ncRNA";

3R FlyBase exon 28586048 28588127 9 + . gene_id "FBgn0262741"; gene_symbol "MRE23"; transcript_id "FBtr0305123"; transcript_symbol "MRE23-RA"; gene_type "ncRNA";

X FlyBase exon 20864813 20865331 8 + . gene_id "FBgn0262822"; gene_symbol "CR43193"; transcript_id "FBtr0347238"; transcript_symbol "CR43193-RB"; gene_type "ncRNA";

X FlyBase exon 20865386 20865731 8 + . gene_id "FBgn0262822"; gene_symbol "CR43193"; transcript_id "FBtr0347238"; transcript_symbol "CR43193-RB"; gene_type "ncRNA";

3R FlyBase exon 17382503 17382767 8 - . gene_id "FBgn0262825"; gene_symbol "CR43196"; transcript_id "FBtr0344657"; transcript_symbol "CR43196-RB"; gene_type "ncRNA";

3R FlyBase exon 17382129 17382406 8 - . gene_id "FBgn0262825"; gene_symbol "CR43196"; transcript_id "FBtr0344657"; transcript_symbol "CR43196-RB"; gene_type "ncRNA";

X FlyBase exon 7450667 7450904 8 + . gene_id "FBgn0262828"; gene_symbol "CR43199"; transcript_id "FBtr0346195"; transcript_symbol "CR43199-RB"; gene_type "ncRNA";

X FlyBase exon 7450986 7451651 8 + . gene_id "FBgn0262828"; gene_symbol "CR43199"; transcript_id "FBtr0346195"; transcript_symbol "CR43199-RB"; gene_type "ncRNA";

X FlyBase exon 7450383 7450904 8 + . gene_id "FBgn0262828"; gene_symbol "CR43199"; transcript_id "FBtr0346196"; transcript_symbol "CR43199-RC"; gene_type "ncRNA";

X FlyBase exon 7450986 7452199 8 + . gene_id "FBgn0262828"; gene_symbol "CR43199"; transcript_id "FBtr0346196"; transcript_symbol "CR43199-RC"; gene_type "ncRNA";

2L FlyBase exon 20478569 20478920 0 - . gene_id "FBgn0262879"; gene_symbol "CR43234"; transcript_id "FBtr0344576"; transcript_symbol "CR43234-RC"; gene_type "ncRNA";

2L FlyBase exon 20477660 20478284 0 - . gene_id "FBgn0262879"; gene_symbol "CR43234"; transcript_id "FBtr0344576"; transcript_symbol "CR43234-RC"; gene_type "ncRNA";

2L FlyBase exon 20476711 20477577 0 - . gene_id "FBgn0262879"; gene_symbol "CR43234"; transcript_id "FBtr0344576"; transcript_symbol "CR43234-RC"; gene_type "ncRNA";

3R FlyBase exon 30746685 30747172 0 - . gene_id "FBgn0262883"; gene_symbol "CR43238"; transcript_id "FBtr0306291"; transcript_symbol "CR43238-RA"; gene_type "ncRNA";

2L FlyBase exon 22131221 22131447 1 + . gene_id "FBgn0262886"; gene_symbol "CR43241"; transcript_id "FBtr0306295"; transcript_symbol "CR43241-RA"; gene_type "ncRNA";

2L FlyBase exon 22131503 22131956 1 + . gene_id "FBgn0262886"; gene_symbol "CR43241"; transcript_id "FBtr0306295"; transcript_symbol "CR43241-RA"; gene_type "ncRNA";

2L FlyBase exon 22131221 22131447 9 + . gene_id "FBgn0262886"; gene_symbol "CR43241"; transcript_id "FBtr0306296"; transcript_symbol "CR43241-RB"; gene_type "ncRNA";

2L FlyBase exon 22131503 22131699 9 + . gene_id "FBgn0262886"; gene_symbol "CR43241"; transcript_id "FBtr0306296"; transcript_symbol "CR43241-RB"; gene_type "ncRNA";

2L FlyBase exon 22132233 22132435 9 + . gene_id "FBgn0262886"; gene_symbol "CR43241"; transcript_id "FBtr0306296"; transcript_symbol "CR43241-RB"; gene_type "ncRNA";

2L FlyBase exon 22131221 22131447 1 + . gene_id "FBgn0262886"; gene_symbol "CR43241"; transcript_id "FBtr0306297"; transcript_symbol "CR43241-RC"; gene_type "ncRNA";

2L FlyBase exon 22131503 22131699 1 + . gene_id "FBgn0262886"; gene_symbol "CR43241"; transcript_id "FBtr0306297"; transcript_symbol "CR43241-RC"; gene_type "ncRNA";

2L FlyBase exon 22132118 22132435 1 + . gene_id "FBgn0262886"; gene_symbol "CR43241"; transcript_id "FBtr0306297"; transcript_symbol "CR43241-RC"; gene_type "ncRNA";

2L FlyBase exon 23069005 23069261 9 + . gene_id "FBgn0262887"; gene_symbol "CR43242"; transcript_id "FBtr0306299"; transcript_symbol "CR43242-RA"; gene_type "ncRNA";

2L FlyBase exon 23069892 23070724 9 + . gene_id "FBgn0262887"; gene_symbol "CR43242"; transcript_id "FBtr0306299"; transcript_symbol "CR43242-RA"; gene_type "ncRNA";

2R FlyBase exon 21607687 21608176 8 + . gene_id "FBgn0262888"; gene_symbol "CR43243"; transcript_id "FBtr0344613"; transcript_symbol "CR43243-RB"; gene_type "ncRNA";

2R FlyBase exon 21608250 21608477 8 + . gene_id "FBgn0262888"; gene_symbol "CR43243"; transcript_id "FBtr0344613"; transcript_symbol "CR43243-RB"; gene_type "ncRNA";

3L FlyBase exon 15332246 15332381 8 + . gene_id "FBgn0262892"; gene_symbol "CR43247"; transcript_id "FBtr0344641"; transcript_symbol "CR43247-RB"; gene_type "ncRNA";

3L FlyBase exon 15332451 15332877 8 + . gene_id "FBgn0262892"; gene_symbol "CR43247"; transcript_id "FBtr0344641"; transcript_symbol "CR43247-RB"; gene_type "ncRNA";

3L FlyBase exon 17033370 17033676 8 - . gene_id "FBgn0262895"; gene_symbol "CR43250"; transcript_id "FBtr0344640"; transcript_symbol "CR43250-RB"; gene_type "ncRNA";

3L FlyBase exon 17033119 17033152 8 - . gene_id "FBgn0262895"; gene_symbol "CR43250"; transcript_id "FBtr0344640"; transcript_symbol "CR43250-RB"; gene_type "ncRNA";

3R FlyBase exon 6904066 6904682 8 - . gene_id "FBgn0262897"; gene_symbol "CR43252"; transcript_id "FBtr0344599"; transcript_symbol "CR43252-RB"; gene_type "ncRNA";

3R FlyBase exon 6903442 6903907 8 - . gene_id "FBgn0262897"; gene_symbol "CR43252"; transcript_id "FBtr0344599"; transcript_symbol "CR43252-RB"; gene_type "ncRNA";

3L FlyBase exon 18085017 18085310 8 - . gene_id "FBgn0262898"; gene_symbol "CR43253"; transcript_id "FBtr0344581"; transcript_symbol "CR43253-RB"; gene_type "ncRNA";

3L FlyBase exon 18084817 18084926 8 - . gene_id "FBgn0262898"; gene_symbol "CR43253"; transcript_id "FBtr0344581"; transcript_symbol "CR43253-RB"; gene_type "ncRNA";

3L FlyBase exon 18081121 18081544 8 - . gene_id "FBgn0262898"; gene_symbol "CR43253"; transcript_id "FBtr0344581"; transcript_symbol "CR43253-RB"; gene_type "ncRNA";

2R FlyBase exon 5127249 5127342 1 + . gene_id "FBgn0262901"; gene_symbol "CR43256"; transcript_id "FBtr0306314"; transcript_symbol "CR43256-RA"; gene_type "ncRNA";

2R FlyBase exon 5127403 5127523 1 + . gene_id "FBgn0262901"; gene_symbol "CR43256"; transcript_id "FBtr0306314"; transcript_symbol "CR43256-RA"; gene_type "ncRNA";

2R FlyBase exon 5128973 5129296 1 + . gene_id "FBgn0262901"; gene_symbol "CR43256"; transcript_id "FBtr0306314"; transcript_symbol "CR43256-RA"; gene_type "ncRNA";

2R FlyBase exon 5128467 5128513 8 + . gene_id "FBgn0262901"; gene_symbol "CR43256"; transcript_id "FBtr0306315"; transcript_symbol "CR43256-RB"; gene_type "ncRNA";

2R FlyBase exon 5128973 5129296 8 + . gene_id "FBgn0262901"; gene_symbol "CR43256"; transcript_id "FBtr0306315"; transcript_symbol "CR43256-RB"; gene_type "ncRNA";

3R FlyBase exon 19329736 19329782 8 + . gene_id "FBgn0262903"; gene_symbol "CR43258"; transcript_id "FBtr0306319"; transcript_symbol "CR43258-RA"; gene_type "ncRNA";

3R FlyBase exon 19329842 19330836 8 + . gene_id "FBgn0262903"; gene_symbol "CR43258"; transcript_id "FBtr0306319"; transcript_symbol "CR43258-RA"; gene_type "ncRNA";

3R FlyBase exon 19329736 19329782 0 + . gene_id "FBgn0262903"; gene_symbol "CR43258"; transcript_id "FBtr0306320"; transcript_symbol "CR43258-RB"; gene_type "ncRNA";

3R FlyBase exon 19329842 19330088 0 + . gene_id "FBgn0262903"; gene_symbol "CR43258"; transcript_id "FBtr0306320"; transcript_symbol "CR43258-RB"; gene_type "ncRNA";

3R FlyBase exon 19330241 19330836 0 + . gene_id "FBgn0262903"; gene_symbol "CR43258"; transcript_id "FBtr0306320"; transcript_symbol "CR43258-RB"; gene_type "ncRNA";

3R FlyBase exon 25906662 25906766 8 - . gene_id "FBgn0262904"; gene_symbol "CR43259"; transcript_id "FBtr0306321"; transcript_symbol "CR43259-RA"; gene_type "ncRNA";

3R FlyBase exon 25906072 25906567 8 - . gene_id "FBgn0262904"; gene_symbol "CR43259"; transcript_id "FBtr0306321"; transcript_symbol "CR43259-RA"; gene_type "ncRNA";

3R FlyBase exon 25905710 25906006 8 - . gene_id "FBgn0262904"; gene_symbol "CR43259"; transcript_id "FBtr0306321"; transcript_symbol "CR43259-RA"; gene_type "ncRNA";

3R FlyBase exon 25906662 25906766 8 - . gene_id "FBgn0262904"; gene_symbol "CR43259"; transcript_id "FBtr0306322"; transcript_symbol "CR43259-RB"; gene_type "ncRNA";

3R FlyBase exon 25906072 25906567 8 - . gene_id "FBgn0262904"; gene_symbol "CR43259"; transcript_id "FBtr0306322"; transcript_symbol "CR43259-RB"; gene_type "ncRNA";

3R FlyBase exon 25905970 25906006 8 - . gene_id "FBgn0262904"; gene_symbol "CR43259"; transcript_id "FBtr0306322"; transcript_symbol "CR43259-RB"; gene_type "ncRNA";

3R FlyBase exon 25904219 25904610 8 - . gene_id "FBgn0262904"; gene_symbol "CR43259"; transcript_id "FBtr0306322"; transcript_symbol "CR43259-RB"; gene_type "ncRNA";

3R FlyBase exon 25902846 25903212 8 - . gene_id "FBgn0262904"; gene_symbol "CR43259"; transcript_id "FBtr0306322"; transcript_symbol "CR43259-RB"; gene_type "ncRNA";

X FlyBase exon 19485353 19485423 8 - . gene_id "FBgn0262905"; gene_symbol "CR43260"; transcript_id "FBtr0342869"; transcript_symbol "CR43260-RB"; gene_type "ncRNA";

X FlyBase exon 19484858 19485293 8 - . gene_id "FBgn0262905"; gene_symbol "CR43260"; transcript_id "FBtr0342869"; transcript_symbol "CR43260-RB"; gene_type "ncRNA";

2L FlyBase exon 8242055 8242118 8 + . gene_id "FBgn0262943"; gene_symbol "CR43262"; transcript_id "FBtr0306547"; transcript_symbol "CR43262-RA"; gene_type "ncRNA";

2L FlyBase exon 8242180 8242435 8 + . gene_id "FBgn0262943"; gene_symbol "CR43262"; transcript_id "FBtr0306547"; transcript_symbol "CR43262-RA"; gene_type "ncRNA";

2L FlyBase exon 1217485 1217785 0 - . gene_id "FBgn0262944"; gene_symbol "CR43263"; transcript_id "FBtr0306546"; transcript_symbol "CR43263-RA"; gene_type "ncRNA";

2L FlyBase exon 1216854 1217384 0 - . gene_id "FBgn0262944"; gene_symbol "CR43263"; transcript_id "FBtr0306546"; transcript_symbol "CR43263-RA"; gene_type "ncRNA";

X FlyBase exon 5831898 5832215 1 - . gene_id "FBgn0262945"; gene_symbol "CR43264"; transcript_id "FBtr0306548"; transcript_symbol "CR43264-RA"; gene_type "ncRNA";

2L FlyBase exon 17919112 17919131 0 - . gene_id "FBgn0262963"; gene_symbol "CR43274"; transcript_id "FBtr0306710"; transcript_symbol "CR43274-RA"; gene_type "ncRNA";

2L FlyBase exon 17918552 17919050 0 - . gene_id "FBgn0262963"; gene_symbol "CR43274"; transcript_id "FBtr0306710"; transcript_symbol "CR43274-RA"; gene_type "ncRNA";

X FlyBase exon 7493110 7493276 9 - . gene_id "FBgn0262967"; gene_symbol "CR43278"; transcript_id "FBtr0347239"; transcript_symbol "CR43278-RC"; gene_type "ncRNA";

X FlyBase exon 7489550 7489725 9 - . gene_id "FBgn0262967"; gene_symbol "CR43278"; transcript_id "FBtr0347239"; transcript_symbol "CR43278-RC"; gene_type "ncRNA";

X FlyBase exon 7488953 7489483 9 - . gene_id "FBgn0262967"; gene_symbol "CR43278"; transcript_id "FBtr0347239"; transcript_symbol "CR43278-RC"; gene_type "ncRNA";

X FlyBase exon 7493110 7493276 1 - . gene_id "FBgn0262967"; gene_symbol "CR43278"; transcript_id "FBtr0347240"; transcript_symbol "CR43278-RD"; gene_type "ncRNA";

X FlyBase exon 7491417 7491610 1 - . gene_id "FBgn0262967"; gene_symbol "CR43278"; transcript_id "FBtr0347240"; transcript_symbol "CR43278-RD"; gene_type "ncRNA";

X FlyBase exon 7489550 7489725 1 - . gene_id "FBgn0262967"; gene_symbol "CR43278"; transcript_id "FBtr0347240"; transcript_symbol "CR43278-RD"; gene_type "ncRNA";

X FlyBase exon 7488953 7489483 1 - . gene_id "FBgn0262967"; gene_symbol "CR43278"; transcript_id "FBtr0347240"; transcript_symbol "CR43278-RD"; gene_type "ncRNA";

X FlyBase exon 7494544 7494847 1 + . gene_id "FBgn0262968"; gene_symbol "CR43279"; transcript_id "FBtr0306719"; transcript_symbol "CR43279-RA"; gene_type "ncRNA";

X FlyBase exon 7494910 7495048 1 + . gene_id "FBgn0262968"; gene_symbol "CR43279"; transcript_id "FBtr0306719"; transcript_symbol "CR43279-RA"; gene_type "ncRNA";

X FlyBase exon 7495114 7495274 1 + . gene_id "FBgn0262968"; gene_symbol "CR43279"; transcript_id "FBtr0306719"; transcript_symbol "CR43279-RA"; gene_type "ncRNA";

X FlyBase exon 7495337 7495497 1 + . gene_id "FBgn0262968"; gene_symbol "CR43279"; transcript_id "FBtr0306719"; transcript_symbol "CR43279-RA"; gene_type "ncRNA";

X FlyBase exon 7494544 7494851 1 + . gene_id "FBgn0262968"; gene_symbol "CR43279"; transcript_id "FBtr0343183"; transcript_symbol "CR43279-RB"; gene_type "ncRNA";

X FlyBase exon 7494910 7495048 1 + . gene_id "FBgn0262968"; gene_symbol "CR43279"; transcript_id "FBtr0343183"; transcript_symbol "CR43279-RB"; gene_type "ncRNA";

X FlyBase exon 7495114 7495274 1 + . gene_id "FBgn0262968"; gene_symbol "CR43279"; transcript_id "FBtr0343183"; transcript_symbol "CR43279-RB"; gene_type "ncRNA";

X FlyBase exon 7495337 7495497 1 + . gene_id "FBgn0262968"; gene_symbol "CR43279"; transcript_id "FBtr0343183"; transcript_symbol "CR43279-RB"; gene_type "ncRNA";

3L FlyBase exon 18440730 18440951 8 + . gene_id "FBgn0262969"; gene_symbol "CR43280"; transcript_id "FBtr0306721"; transcript_symbol "CR43280-RA"; gene_type "ncRNA";

3L FlyBase exon 18441007 18441871 8 + . gene_id "FBgn0262969"; gene_symbol "CR43280"; transcript_id "FBtr0306721"; transcript_symbol "CR43280-RA"; gene_type "ncRNA";

3L FlyBase exon 18440730 18440951 0 + . gene_id "FBgn0262969"; gene_symbol "CR43280"; transcript_id "FBtr0345002"; transcript_symbol "CR43280-RB"; gene_type "ncRNA";

3L FlyBase exon 18441007 18441178 0 + . gene_id "FBgn0262969"; gene_symbol "CR43280"; transcript_id "FBtr0345002"; transcript_symbol "CR43280-RB"; gene_type "ncRNA";

3L FlyBase exon 18441601 18441871 0 + . gene_id "FBgn0262969"; gene_symbol "CR43280"; transcript_id "FBtr0345002"; transcript_symbol "CR43280-RB"; gene_type "ncRNA";

3R FlyBase exon 19921137 19921310 8 - . gene_id "FBgn0262971"; gene_symbol "CR43282"; transcript_id "FBtr0306726"; transcript_symbol "CR43282-RA"; gene_type "ncRNA";

3R FlyBase exon 19920859 19921089 8 - . gene_id "FBgn0262971"; gene_symbol "CR43282"; transcript_id "FBtr0306726"; transcript_symbol "CR43282-RA"; gene_type "ncRNA";

3R FlyBase exon 19921150 19921310 0 - . gene_id "FBgn0262971"; gene_symbol "CR43282"; transcript_id "FBtr0306727"; transcript_symbol "CR43282-RB"; gene_type "ncRNA";

3R FlyBase exon 19920597 19921089 0 - . gene_id "FBgn0262971"; gene_symbol "CR43282"; transcript_id "FBtr0306727"; transcript_symbol "CR43282-RB"; gene_type "ncRNA";

3R FlyBase exon 19921141 19921310 0 - . gene_id "FBgn0262971"; gene_symbol "CR43282"; transcript_id "FBtr0308067"; transcript_symbol "CR43282-RC"; gene_type "ncRNA";

3R FlyBase exon 19920597 19921089 0 - . gene_id "FBgn0262971"; gene_symbol "CR43282"; transcript_id "FBtr0308067"; transcript_symbol "CR43282-RC"; gene_type "ncRNA";

3R FlyBase exon 19921187 19921310 0 - . gene_id "FBgn0262971"; gene_symbol "CR43282"; transcript_id "FBtr0308068"; transcript_symbol "CR43282-RD"; gene_type "ncRNA";

3R FlyBase exon 19920597 19921089 0 - . gene_id "FBgn0262971"; gene_symbol "CR43282"; transcript_id "FBtr0308068"; transcript_symbol "CR43282-RD"; gene_type "ncRNA";

3R FlyBase exon 11255435 11256123 9 - . gene_id "FBgn0262972"; gene_symbol "CR43283"; transcript_id "FBtr0306728"; transcript_symbol "CR43283-RA"; gene_type "ncRNA";

3R FlyBase exon 11252551 11255339 9 - . gene_id "FBgn0262972"; gene_symbol "CR43283"; transcript_id "FBtr0306728"; transcript_symbol "CR43283-RA"; gene_type "ncRNA";

3R FlyBase exon 11255435 11256123 1 - . gene_id "FBgn0262972"; gene_symbol "CR43283"; transcript_id "FBtr0306729"; transcript_symbol "CR43283-RB"; gene_type "ncRNA";

3R FlyBase exon 11254558 11255339 1 - . gene_id "FBgn0262972"; gene_symbol "CR43283"; transcript_id "FBtr0306729"; transcript_symbol "CR43283-RB"; gene_type "ncRNA";

3R FlyBase exon 11247631 11250933 1 - . gene_id "FBgn0262972"; gene_symbol "CR43283"; transcript_id "FBtr0306729"; transcript_symbol "CR43283-RB"; gene_type "ncRNA";

3R FlyBase exon 11255522 11256123 1 - . gene_id "FBgn0262972"; gene_symbol "CR43283"; transcript_id "FBtr0345159"; transcript_symbol "CR43283-RC"; gene_type "ncRNA";

3R FlyBase exon 11247631 11255339 1 - . gene_id "FBgn0262972"; gene_symbol "CR43283"; transcript_id "FBtr0345159"; transcript_symbol "CR43283-RC"; gene_type "ncRNA";

3R FlyBase exon 23825352 23825907 0 - . gene_id "FBgn0262974"; gene_symbol "CR43285"; transcript_id "FBtr0344658"; transcript_symbol "CR43285-RB"; gene_type "ncRNA";

X FlyBase exon 7707775 7708439 0 + . gene_id "FBgn0262979"; gene_symbol "CR43287"; transcript_id "FBtr0344596"; transcript_symbol "CR43287-RB"; gene_type "ncRNA";

X FlyBase exon 7708531 7708587 0 + . gene_id "FBgn0262979"; gene_symbol "CR43287"; transcript_id "FBtr0344596"; transcript_symbol "CR43287-RB"; gene_type "ncRNA";

X FlyBase exon 18621498 18621678 0 + . gene_id "FBgn0262981"; gene_symbol "CR43289"; transcript_id "FBtr0344563"; transcript_symbol "CR43289-RB"; gene_type "ncRNA";

X FlyBase exon 18621757 18621877 0 + . gene_id "FBgn0262981"; gene_symbol "CR43289"; transcript_id "FBtr0344563"; transcript_symbol "CR43289-RB"; gene_type "ncRNA";

X FlyBase exon 8724674 8725109 0 + . gene_id "FBgn0262989"; gene_symbol "CR43297"; transcript_id "FBtr0306827"; transcript_symbol "CR43297-RA"; gene_type "ncRNA";

X FlyBase exon 8725493 8725901 0 + . gene_id "FBgn0262989"; gene_symbol "CR43297"; transcript_id "FBtr0306827"; transcript_symbol "CR43297-RA"; gene_type "ncRNA";

X FlyBase exon 4001507 4001607 0 - . gene_id "FBgn0262990"; gene_symbol "CR43298"; transcript_id "FBtr0306828"; transcript_symbol "CR43298-RA"; gene_type "ncRNA";

X FlyBase exon 4001210 4001424 0 - . gene_id "FBgn0262990"; gene_symbol "CR43298"; transcript_id "FBtr0306828"; transcript_symbol "CR43298-RA"; gene_type "ncRNA";

3R FlyBase exon 13827968 13828107 0 + . gene_id "FBgn0262992"; gene_symbol "CR43300"; transcript_id "FBtr0306832"; transcript_symbol "CR43300-RA"; gene_type "ncRNA";

3R FlyBase exon 13828207 13828427 0 + . gene_id "FBgn0262992"; gene_symbol "CR43300"; transcript_id "FBtr0306832"; transcript_symbol "CR43300-RA"; gene_type "ncRNA";

3R FlyBase exon 9338223 9339992 0 - . gene_id "FBgn0262993"; gene_symbol "CR43301"; transcript_id "FBtr0306833"; transcript_symbol "CR43301-RA"; gene_type "ncRNA";

3R FlyBase exon 9338725 9339992 0 - . gene_id "FBgn0262993"; gene_symbol "CR43301"; transcript_id "FBtr0306834"; transcript_symbol "CR43301-RB"; gene_type "ncRNA";

3R FlyBase exon 9338223 9338635 0 - . gene_id "FBgn0262993"; gene_symbol "CR43301"; transcript_id "FBtr0306834"; transcript_symbol "CR43301-RB"; gene_type "ncRNA";

3R FlyBase exon 8197679 8197711 0 - . gene_id "FBgn0262994"; gene_symbol "CR43302"; transcript_id "FBtr0306836"; transcript_symbol "CR43302-RA"; gene_type "ncRNA";

3R FlyBase exon 8197320 8197595 0 - . gene_id "FBgn0262994"; gene_symbol "CR43302"; transcript_id "FBtr0306836"; transcript_symbol "CR43302-RA"; gene_type "ncRNA";

3R FlyBase exon 8197320 8197711 0 - . gene_id "FBgn0262994"; gene_symbol "CR43302"; transcript_id "FBtr0306837"; transcript_symbol "CR43302-RB"; gene_type "ncRNA";

3R FlyBase exon 8201480 8201594 0 + . gene_id "FBgn0262995"; gene_symbol "CR43303"; transcript_id "FBtr0306835"; transcript_symbol "CR43303-RA"; gene_type "ncRNA";

3R FlyBase exon 8201658 8201994 0 + . gene_id "FBgn0262995"; gene_symbol "CR43303"; transcript_id "FBtr0306835"; transcript_symbol "CR43303-RA"; gene_type "ncRNA";

2L FlyBase exon 17541854 17541934 8 + . gene_id "FBgn0262996"; gene_symbol "CR43304"; transcript_id "FBtr0306838"; transcript_symbol "CR43304-RA"; gene_type "ncRNA";

2L FlyBase exon 17541996 17542383 8 + . gene_id "FBgn0262996"; gene_symbol "CR43304"; transcript_id "FBtr0306838"; transcript_symbol "CR43304-RA"; gene_type "ncRNA";

3L FlyBase exon 18294604 18294751 1 + . gene_id "FBgn0262998"; gene_symbol "CR43306"; transcript_id "FBtr0306840"; transcript_symbol "CR43306-RA"; gene_type "ncRNA";

3L FlyBase exon 18295272 18295541 1 + . gene_id "FBgn0262998"; gene_symbol "CR43306"; transcript_id "FBtr0306840"; transcript_symbol "CR43306-RA"; gene_type "ncRNA";

3L FlyBase exon 18295598 18295800 1 + . gene_id "FBgn0262998"; gene_symbol "CR43306"; transcript_id "FBtr0306840"; transcript_symbol "CR43306-RA"; gene_type "ncRNA";

3L FlyBase exon 21830044 21830219 1 - . gene_id "FBgn0263001"; gene_symbol "CR43309"; transcript_id "FBtr0306844"; transcript_symbol "CR43309-RA"; gene_type "ncRNA";

3L FlyBase exon 21829254 21829995 1 - . gene_id "FBgn0263001"; gene_symbol "CR43309"; transcript_id "FBtr0306844"; transcript_symbol "CR43309-RA"; gene_type "ncRNA";

2L FlyBase exon 18308601 18308684 8 - . gene_id "FBgn0263085"; gene_symbol "CR43353"; transcript_id "FBtr0342850"; transcript_symbol "CR43353-RB"; gene_type "ncRNA";

2L FlyBase exon 18308088 18308507 8 - . gene_id "FBgn0263085"; gene_symbol "CR43353"; transcript_id "FBtr0342850"; transcript_symbol "CR43353-RB"; gene_type "ncRNA";

2L FlyBase exon 12174576 12174638 1 - . gene_id "FBgn0263088"; gene_symbol "CR43356"; transcript_id "FBtr0307106"; transcript_symbol "CR43356-RA"; gene_type "ncRNA";

2L FlyBase exon 12174090 12174524 1 - . gene_id "FBgn0263088"; gene_symbol "CR43356"; transcript_id "FBtr0307106"; transcript_symbol "CR43356-RA"; gene_type "ncRNA";
[truncated: 551,510 more chars]
