# Supplementary material for: Age-related ceRNA networks in adult Drosophila ageing
Source: Front Genet. 2023 Feb 28;14:1096902. doi: 10.3389/fgene.2023.1096902 (PMC10012872; doi:10.3389/fgene.2023.1096902)
Supplement: Supplementary file 10 [file DataSheet8.docx]

#miRNA mRNA Score Energy miRNA_position mRNA_position Align_len miRNA_align_rate mRNA_align_rate Pairing target and miRNA

>dme-miR-310-3p FBgn0266227 152.00 -11.65 2 13 2161 2182 11 90.91% 90.91%

Query: 3' uuuccggcccUUCACACGUUAu 5'

|| ||||||||

Ref: 5' aagcatataaAAATGTGCAATg 3'

>dme-miR-289-5p XLOC_073604 164.00 -12.48 2 25 1009 1039 28 60.71% 71.43%

Query: 3' ucA-GCG-UCCGA-G-G-UGAAUUUAUAAAu 5'

| ||: || || : :| |||||||||

Ref: 5' taTACGTAAGACTATAGTGCATAAATATTTg 3'

>dme-miR-289-5p XLOC_097475 152.00 -7.20 2 17 311 336 15 66.67% 86.67%

Query: 3' ucagcguccgAGGUGAAUUUAUAAAu 5'

|::|: | |||||||

Ref: 5' tggaaaaaaaTTTATATCAATATTTa 3'

>dme-miR-289-5p FBgn0264904 157.00 -16.26 2 18 676 704 19 68.42% 78.95%

Query: 3' ucagcguccGAG-G-UGAAU-UUAUAAAu 5'

||| ::||| |||||||

Ref: 5' agaacctcaCTCGGGGTTTACAATATTTa 3'

>dme-miR-289-5p FBgn0264904 153.00 -9.18 2 22 736 764 23 56.52% 73.91%

Query: 3' ucagcGUCCGAG-G-UGA-AUUUAUAAAu 5'

:|| :|: |:| ||||||||

Ref: 5' aaaaaTAGTTTTAAGATTCGAAATATTTt 3'

>dme-miR-289-5p XLOC_150011 180.00 -26.29 2 25 371 398 25 68.00% 84.00%

Query: 3' ucAG-CGU-CCGAGGUGAAUUUAUAAAu 5'

|| ||| ||:|:|:: ||||||||

Ref: 5' agTCAGCACGGTTTCGTGCAAATATTTa 3'

>dme-miR-289-5p XLOC_150011 180.00 -26.29 2 25 407 434 25 68.00% 84.00%

Query: 3' ucAG-CGU-CCGAGGUGAAUUUAUAAAu 5'

|| ||| ||:|:|:: ||||||||

Ref: 5' agTCAGCACGGTTTCGTGCAAATATTTa 3'

>dme-miR-289-5p XLOC_150011 180.00 -26.29 2 25 318 345 25 68.00% 84.00%

Query: 3' ucAG-CGU-CCGAGGUGAAUUUAUAAAu 5'

|| ||| ||:|:|:: ||||||||

Ref: 5' agTCAGCACGGTTTCGTGCAAATATTTa 3'

>dme-miR-289-5p XLOC_150011 180.00 -26.29 2 25 320 347 25 68.00% 84.00%

Query: 3' ucAG-CGU-CCGAGGUGAAUUUAUAAAu 5'

|| ||| ||:|:|:: ||||||||

Ref: 5' agTCAGCACGGTTTCGTGCAAATATTTa 3'

>dme-miR-289-5p XLOC_150011 180.00 -26.29 2 25 396 423 25 68.00% 84.00%

Query: 3' ucAG-CGU-CCGAGGUGAAUUUAUAAAu 5'

|| ||| ||:|:|:: ||||||||

Ref: 5' agTCAGCACGGTTTCGTGCAAATATTTa 3'

>dme-miR-289-5p XLOC_150011 180.00 -26.29 2 25 438 465 25 68.00% 84.00%

Query: 3' ucAG-CGU-CCGAGGUGAAUUUAUAAAu 5'

|| ||| ||:|:|:: ||||||||

Ref: 5' agTCAGCACGGTTTCGTGCAAATATTTa 3'

>dme-miR-289-5p XLOC_207784 162.00 -9.89 2 25 139 166 26 61.54% 76.92%

Query: 3' ucAGCGUCCGAG-GUGA--AUUUAUAAAu 5'

|| | |:|: :|:| |||||||||

Ref: 5' ctTC-AATGTTTATATTGCTAAATATTTt 3'

>dme-miR-284-3p XLOC_092363 149.00 -17.20 2 20 61 80 19 63.16% 73.68%

Query: 3' acG-ACCUUAGUUCAACGACUg 5'

| | |::| | |||||||

Ref: 5' gtCGTCGGGT-A-TTTGCTGAc 3'

>dme-miR-9a-3p FBgn0266227 148.00 -8.75 2 13 352 376 14 78.57% 78.57%

Query: 3' auugaagccaUU-C-G-AUCGAAAu 5'

|| | | |||||||

Ref: 5' aaccacataaAACGTCATAGCTTTt 3'

>dme-miR-14-5p XLOC_100429 163.00 -31.29 2 21 157 177 19 73.68% 78.95%

Query: 3' ucACUCAGGGGCAGAGCGAGGg 5'

|| :|||| ||||||||

Ref: 5' tcTGCCATCCCG-CTCGCTCCc 3'

>dme-miR-994-3p XLOC_046032 165.00 -11.97 2 22 121 148 25 68.00% 72.00%

Query: 3' uaGA-UU-UU-C-U-UUGUCGUUGACAc 5'

:| || || | |||| |||||||

Ref: 5' gtTTCAATAACGCTTAACAACAACTGTc 3'

>dme-miR-994-3p XLOC_070832 142.00 -13.07 2 19 126 149 18 61.11% 66.67%

Query: 3' uagauUUUCUUUG-UCGUUGACAc 5'

||| :| |||||||

Ref: 5' cgtccAAATCCGCTTTCAACTGTg 3'

>dme-miR-994-3p XLOC_073604 141.00 -9.89 2 10 1075 1098 9 88.89% 88.89%

Query: 3' uagauuuucuuuguC-GUUGACAc 5'

| |||||||

Ref: 5' gttgcctagtttttGTCAACTGTc 3'

>dme-miR-994-3p FBgn0264904 145.00 -18.22 2 14 339 362 13 61.54% 92.31%

Query: 3' uagauuuucuUUGUC-GUUGACAc 5'

::::| |||||||

Ref: 5' gaccatctccGGTGGCCAACTGTg 3'

>dme-miR-994-3p XLOC_150011 148.00 -17.77 2 21 931 958 24 54.17% 70.83%

Query: 3' uagAUUUU-CU-U-U-G-UCGUUGACAc 5'

||||: | : : :||||||||

Ref: 5' aaaTAAAGCCATCTGCTTGGCAACTGTg 3'

>dme-miR-994-3p XLOC_150011 148.00 -17.77 2 21 878 905 24 54.17% 70.83%

Query: 3' uagAUUUU-CU-U-U-G-UCGUUGACAc 5'

||||: | : : :||||||||

Ref: 5' aaaTAAAGCCATCTGCTTGGCAACTGTg 3'

>dme-miR-994-3p XLOC_150011 148.00 -17.77 2 21 880 907 24 54.17% 70.83%

Query: 3' uagAUUUU-CU-U-U-G-UCGUUGACAc 5'

||||: | : : :||||||||

Ref: 5' aaaTAAAGCCATCTGCTTGGCAACTGTg 3'

>dme-miR-14-3p XLOC_073604 160.00 -15.60 2 21 74 99 23 65.22% 73.91%

Query: 3' uaUC-CUCUCUC-U-U-UUUCUGACu 5'

|| | || || | : :|||||||

Ref: 5' ctAGTGTGAAAGTATGTGAAGACTGa 3'

>dme-miR-12-5p XLOC_228709 151.00 -14.99 2 18 14 34 16 75.00% 75.00%

Query: 3' uggucaUGGACUACAUUAUGAGu 5'

|| || | |||||||

Ref: 5' tggacaACAGGA-G-AATACTCa 3'

>dme-miR-133-3p FBgn0264904 152.00 -16.83 2 21 320 344 22 63.64% 68.18%

Query: 3' ugUCGA-CCAAC-UU-CCCCUGGUu 5'

| || ||| |: |||||||

Ref: 5' ctAACTCACTTGTAGCTGGGACCAt 3'

>dme-miR-133-3p XLOC_209391 148.00 -22.04 2 21 152 176 22 63.64% 63.64%

Query: 3' ugUCG-A-CCAACU-UCCCCUGGUu 5'

||| | | | | |||||||

Ref: 5' caAGCACAGCTCCACATGGGACCAg 3'

>dme-miR-985-3p XLOC_027736 149.00 -7.13 2 14 25 46 12 83.33% 83.33%

Query: 3' acgggcuggUAACCUUGUAAAc 5'

|| | |||||||

Ref: 5' gtgataaaaATAGAAACATTTt 3'

>dme-miR-985-3p XLOC_073604 151.00 -5.56 2 17 666 687 16 75.00% 75.00%

Query: 3' acgggcUGGUA-ACCUUGUAAAc 5'

| ||| | |||||||

Ref: 5' agaattA-CATATAAAACATTTt 3'

>dme-miR-985-3p FBgn0266227 147.00 -15.45 2 17 175 199 18 72.22% 72.22%

Query: 3' acgggcUGG-UAAC--CUUGUAAAc 5'

||| | | ||||||||

Ref: 5' ttttaaACCAAGAGACGAACATTTg 3'

>dme-miR-985-3p XLOC_189798 146.00 -6.91 2 20 258 278 18 61.11% 72.22%

Query: 3' acgGGCUGGUAACCUUGUAAAc 5'

| |:: | | |||||||

Ref: 5' cagCAGGTGAAT-CAACATTTt 3'

>dme-miR-985-3p XLOC_189909 148.00 -6.89 2 21 74 98 22 50.00% 77.27%

Query: 3' acGGG-CUGGU-AA-CCUUGUAAAc 5'

|:: |:::: || |||||||

Ref: 5' taCTTAGGTTGCTTAACAACATTTt 3'

>dme-miR-318-3p XLOC_073604 156.00 -20.88 2 21 195 218 21 61.90% 76.19%

Query: 3' acUC-UAUUUGU-UUCGGGUCACu 5'

|| :|:|: | ||||||||

Ref: 5' gcAGCGTGAGGATTTGCCCAGTGt 3'

>dme-miR-318-3p XLOC_150011 141.00 -15.83 2 18 997 1018 16 62.50% 68.75%

Query: 3' acucuAUUUGUUUCGGGUCACu 5'

| | |: |||||||

Ref: 5' aaatcTTATCGCCTCCCAGTGa 3'

>dme-miR-988-5p FBgn0266227 156.00 -12.31 2 21 82 107 23 60.87% 73.91%

Query: 3' uaGU-GA-A-ACG-AUGUUUAGUGUg 5'

:| || | | | | ::|||||||

Ref: 5' ttTATCTATATACTTTTGAATCACAt 3'

>dme-miR-92a-3p FBgn0266227 148.00 -13.07 2 13 2160 2182 12 83.33% 83.33%

Query: 3' uauccggcccUGUU-CACGUUAc 5'

| || |||||||

Ref: 5' gaagcatataAAAATGTGCAATg 3'

>dme-miR-8-5p XLOC_071213 151.00 -5.79 2 20 824 846 19 63.16% 73.68%

Query: 3' agaUUA-CGACGGGCCAUUCUAc 5'

||| | | : : |||||||

Ref: 5' tgcAATAGATTTATTGTAAGATc 3'
