# Supplementary material for: Age-related ceRNA networks in adult Drosophila ageing
Source: Front Genet. 2023 Feb 28;14:1096902. doi: 10.3389/fgene.2023.1096902 (PMC10012872; doi:10.3389/fgene.2023.1096902)
Supplement: Supplementary file 4 [file Presentation1.pdf]

>dme_circ_0000001

CTAACACATTAGGTACATTGACGGTGCTGTATTCGGCTTGTGGAGTTTTGCTGCAGTTTTTCCGCGGAGA

AGATGATCATATAAACACAGTAATTGCGGGCTCTGCCACAGGACTATTATACAAGTCAACAGG

>dme_circ_0000001_junction_seq

CCACAGGACTATTATACAAGTCAACAGGCTAACACATTAGGTACATTGACGGTGCT

>dme_circ_0000002

GTTACTTAATCACATTATGAAGCAAGGTTCCGGCACAGCTAACACATTAGGTACATTGACGGTGCTGTAT

TCGGCTTGTGGAGTTTTGCTGCAGTTTTTCCGCGGAGAAGATGATCATATAAACACAG

>dme_circ_0000002_junction_seq

CGCGGAGAAGATGATCATATAAACACAGGTTACTTAATCACATTATGAAGCAAGGT

>dme_circ_0000003

AGTTCATTTTTCCCGAAGGGGCCAACAAGCAGCGTGGACGCTTCGAGTTGGCCTTCTCTCAGATAGGCAC

TTCGGTAATGATTGGCGGTGGAATTGGCGGCCTAGCAGGTGTTTATAATGGTTTAAAAGTCACAAAAGCA

CTCGAGCAGAAGGGAAAAGTTCGTCGAACACAG

>dme_circ_0000003_junction_seq

GCAGAAGGGAAAAGTTCGTCGAACACAGAGTTCATTTTTCCCGAAGGGGCCAACAA

>dme_circ_0000004

gttttctaaacgatcgaacattccacgtctctactgatggattcaaatcgtcaacgaagacaattgcacc

tagagttccccaaggaagcgtcctagtccttaccAATTGCTTCATGCCGAACGGCTCATCCAAACGTAGC

CACAATATTTTATATAGCTTTGCgaggagtcttaagactctccacaaatctcgtgtaggagtggcctagc

aacagccgcgatgcagggcggccagggcaaagcggggagaccgtgcagtaacggtaccccgttaggcctt

ggattcgagttgaccaagggcacagaggtcgaccggtagtggtgcgtgcacaaagagaacgcaccgtgaa

ctaacggtacaactgttggaccttgtacccaagcgggccgtgcgcaaaaggggatataacgggatcccca

cggcctataaagggacggcgcaagtgcaactcgaggcagtcagtCTCAACACGCGAGGAGCCGTACGTAC

GCGAGGATAGTCGAGGAGAATATCGGCGCGAGTCGCGCGGTCAGCGTGCAGTCGGAACGGAGTTTGAAGg

cggaggagtacggttcagtcagccagaccgcgctcggaacaagatagtgagacggagcgttgaaggaagg

agttcgggagtgagaacgcgGACATGGACAGATCAAAAGAAACGCTTCTGTGTATACCTTAACATACTTC

TAAACGAATCTTTTGTACTCGGACATCCTCAG

>dme_circ_0000004_junction_seq

CGAATCTTTTGTACTCGGACATCCTCAGgttttctaaacgatcgaacattccacgt

>dme_circ_0000005

GTGATTCCAAAAACTGTAACTGTGCAATATTTCAGAAAATATCAATGGAGAATGGGCACAAGAAGGCGAA

TTAATTTGAAATGCTGTATAGTCATaaaaaaaaTAATGAGGCATGGCTACAAAGAAATATTAATGCAAGC

TCAAAACAAAATAAAAG

>dme_circ_0000005_junction_seq

TTAATGCAAGCTCAAAACAAAATAAAAGGTGATTCCAAAAACTGTAACTGTGCAAT

>dme_circ_0000006

GTTTCTGAATATCTTAGCATGTTGGAGTCACAAAAACATTTGCCTGTTGGAGCTGTAAGTTTTCAACGAA

TTTCTCCCAACACACTAATGGAGTCGGCCGTATCGGATGATGTGCTAAGTCCCGGCGAAGATGGTATCTG

CCTAGGAAATCATTTCACTGAAACTGGGTTGAAGGCCTTGCTGGAAGAAGCCTCCAATTCTTTTCAAGTT

GCTGGCAT

>dme_circ_0000006_junction_seq

CCTCCAATTCTTTTCAAGTTGCTGGCATGTTTCTGAATATCTTAGCATGTTGGAGT

>dme_circ_0000007

AATTTTGTACACTTCGTTCATTGCTTCATACATGCCAGCAACTTGAAAAGAATTGGAGGCTTCTTCCAGC
AAGGCCTTCAACCCAGTTTCAGTGAAATGATTTCCTAGGCAGATACCATCTTCGCCGGGACTTAGCACAT
CATCCGATACGGCCGACTCCATTAGTGTGTTGGGAGAAATTCGTTGAAAACTTACAGCTCCAACAGGCAA
ATG
>dme_circ_0000007_junction_seq
GAAAACTTACAGCTCCAACAGGCAAATGAATTTTGTACACTTCGTTCATTGCTTCA
>dme_circ_0000008
GGTTTACCAAGGCTGGGACTCCTTCTACAGCACAACAAAAGCAACATTGTGAAGGAGGCTGCCTGGACGG
TCAGCAACATCACAGCAGGTAACCAGAAGCAGATCCAGGCTGTGATTCAGGCCGGCATCTTCCAGCAGCT
GCGCACCGTGCTGGAGAAGG
>dme_circ_0000008_junction_seq
CAGCAGCTGCGCACCGTGCTGGAGAAGGGGTTTACCAAGGCTGGGACTCCTTCTAC
>dme_circ_0000009
TATGTGTCAATGATGGCGTAGGCCTTCTTGTAGACCTCCTCGTTCTCGTGCTGCTGCAGAGTTTCCAGCT
TGTCTAGACCGCCCATCTCCTCGACCATCAAGCATAGGTTCTCGGTGCCACCAAGTTTCTCCGCCAGGGC
AAACAGATTGGATAGGCCCGTCTGCACCACCTTGATGGTACGCGGATCCTTTGTGTCCAGCAAATCGATA
AAAGGCTTCAATATTTTGTACTTCTCAATTAGATCGACGATCTGTTCGGGAGTGCCAGATGTCGTGGTGT
TTGTCACCGCCCAGGCAGCCTCTTTTTGAGCCTTGAAATCACCCTTCTCCAGCACGGTGCGCAGCTGCTG
GAAGATGCCGGCCTGAATCACAGCCTGGATCTGCTTCTGGTTACCTGCTGTGATGTTGCTGACCG
>dme_circ_0000009_junction_seq
TGGTTACCTGCTGTGATGTTGCTGACCGTATGTGTCAATGATGGCGTAGGCCTTCT
>dme_circ_0000010
TAATTTGAATGTAAGCCTTATCGGGATCCAAGCTATTAATATCAACGGTATTGGAATCTTTAATGATATG
CACAGAGTCCGGTCCGAATCGTTCAGTGTAAAAGTTCTAACGTATGTTGAAACAAAATTGATTAGTTCTA
TTTTTTCCAATTTGCAATATATACCTGAAGCCGACTAAATATTTCGGGCAACTTCGTCAATGTTGGCTCT
TTATAAATGAATTCCTGCTGATCCAAGTCCCCAAATTTTCCGCCATAGAAGCCAACACGAAAG
>dme_circ_0000010_junction_seq
TTTTCCGCCATAGAAGCCAACACGAAAGTAATTTGAATGTAAGCCTTATCGGGATC
>dme_circ_0000011
GCGAGGAAGCCGAGGATGCCTCGAAATGGAACAATTTTGTGGAATCAATTAAGTCGAAATTAACAGTCAA
ACAGGTGGTGGATGGAGTTCGAGCCGGAGGATCCTTGTCCTTTGACTACTTGCTGCTAATCGTTACAGCC
GAGTGAGTACACTGAACGCAATTCGAAATGCAATTAAAAGAATTCAAAGTATTTTTATAAAATTTGAAAG
TAATATTATGAAATTCCTTAAATTTTGTCAAATAATATAACTTGATACTGAAATGGAATATGAAATTTTT
AGCATTATCACCTTCTTAGTTTACAGTGTAGCTAGTGAAAGAGTTGGCAGTATTAATTTCGCAAATTCCA
CCAGCTGACGGTGGGTCAGCAGTAAAAGTAATCATTATGGTACTCTCTCCTTTTTCCCAGCTCTTTGGCC
GCCCTGGGCCTCGTGGAGAATAATGCCCCCAATATAGTGGCTGCCATGTTAGTTTCGCCGCTTATGGGTC
CCGTAATGTCGATAACATTCGGCGCAATTATCTCCGACAAAGAACTAATGGTAAGTGCAGTTTCCCCTTA
TGTCAGTGGCTTTTTTCGACCAACAGCAAATTGTGTAGCTCCGCGAAAAAGCCATTCAAAACCATTGACG
TTGTGGCCAGCGAAAAGAAAAGCATACATTTGAACTTATATGAGTGTATGGAAAACTacacacacacaca
catacacacCCAACAGTGAAAAATGGAATAGTGAAAATAGTTGTCAGGGTGATGATGCGGAGGGATGTGG
AggggggggAGGTACTGCCAAAAATCCATTTCACAGCGCGTTAAATGAAATCGTCAACACTCTTGCCCAA
CGCGACTCTTCGACTGGTTCTATGTGGTACTTTTGAGTGATTTATGAGCAAATGACATTTGGAAAATAAT
AAAAAATTGCACCGAAAATTGCTATGCCAGTACGTCGTCCGTCGTGGCGTTCTATGTCCTGTATTCCAGC
TGTCAAAATTCGCATGCAGCCAACAGAAATATGAGCGGGGTTTCGttttttttttttttggttttttGG

GTTATTCCAGTCAAATGCATAGCGACATAGCGCTGATGTTTTGCAAATTTGCATCGCTTTTGTGGTAAAT
GGCAAAGTTGCTGAATTGTTTATTGACAGCGTTGGCCTGAAAAGGGGCGTGGCCCTATGAAatatatata
tatatataAATGTTTTTGTCCACCGAAAATTGGATGAAGTTGTAACGCCAGCGAGTGTTTGTGATATACA
TACAACACCTGGTGCATTCCTTTACAGGCCTGCTGAATAGCAAAATATTTCCATCGCCCGTTTATGGGGA
ATTAATAAAATGGAAACGAGAACTTTGAAGCTTGCACTGCAGAAGGACATAATACAAAGCCTTGTGCTGT
ATATTGCTGGGCTTAAAACCACACCCTGCGGCCGGGCAATCATTTTGTTCGCTCTTCTAGCTGACAGTCT
GAAAACTAAGTTAAGCTACTTAGAATTAAAGGACGCAGCACTTGGGACCGCATTCAGCGCAAAGCCAATA
AAACTGAACTTTAACTGCAGTCGCCAGACGGCAGCTGCTCTCCAAAGTGTCATAATTGTCACCGCCAGGT
GGCGGGTTGGTGGTACAAAAGTAAGGGCGGCTTATGCTGCGAGATAAGAACGTCTGTGTGTATAGTTTGC
TCGGCATCAAAGACGGCGGCTTGTGCTTCAGGAAGCACAGATACATAGCTTTTAATGTGCAAGCTGAGTG
GTAAATGGATGGTGGATGGTGCACaaaaaaaagggaccacaacaaataaaataagtgaaataaaaatata
aGGTTTAGATCTATATAATCATCATTCCAACCATTTCTAATGAAGAGACATCTCTGTGCTTCTGGCCCCA
GAGGCAACTTGTTGTCTGCTGACACAATTTTCACCGCACTCTTGGCcacaacacacacatacacacacac
acacacacacacTTGCAGGCACAGAGACTGGATTCAGGATTCAGGAATCCAGGTGTCTTGCCGGCTGTTG
ACACCTGCGTCGTGTACTTCAATTAAATTTTAATTGAAATGAATATATTCGTGTGCGAACACATGCTAGT
GTGCATCTTTTTTGTCGCCTCCTTTCTTTGTGAAGTGTCTCCAGCTGCGTCATCATAATCTTTGATAAAT
CCGGCAAGGAAGTTGAATCTGCAGGCAGCATTTGCCATATATTATGGTCACAAAAATGTCCAGGCAAACA
GATATTAATGCAATGCCAGCTTTATTAAATTTACTTGCTTAATGCCAAATAATCAATACATATGCATGCC
CTTTACAATTTGTGACTTTTGGCTTTAAATTTGAGCTGATAAAATACTATATGAAATATTAAAAGCTCAC
TGTTAAATTCATAAAACTCCAGATCCTTGAGTTGTTTTGATTCCATTACATCACCATGAAGTGCACAAAA
GGGCAGGAAAAAATGTTTTTAAAATACGCAAAAAGCATGCGAACCGTTTGCGGCCAAAATTCATATGCAA
AAGTGTTTAAACAATTTCACACAAAAATGCCAATTACAAACGATGACAAAAACGGCAACATCGAATGGGG
CCAGTCAAAATTGATGGCAAACTATTAAAAATAATTGGCAGGaaaaaaaaCTGCACCGGAATCCCTATAA
TATAGTGAAAACGAGTTGGCTTTGCACATATTGCCAGCCGAGTTATTGAATAGCGATTTTTGCAAATATT
TGCACGCCATTTAAAGTTGATTCGGACATGAATGTAATTTGTTTGCGCATTACGCATACGCAGTGTGGGC
GCAATTGCATTGGGGTGGCTCACATTTGTATGCAAATTTTAAAGCTGCCATTTTCAAATGCTTATGAGGA
GCACTGATGttttttttttgtttttttttttCAAAGAAAAGGATCTTATGGCCAGCTCATTTGCTTTATT
CTTATACGTTTAATTGAATTTTAAAGTAAATTTATTAGCTGAGAATAGTCATCATTTTCGAACACCCTAA
TATGCATATGCATAACCTGCTAGTAGAAGAGCGAGCAATAAACAAGAAATTATCAAAAGTTTTCGCACCC
CACTTAAATTGTTTTGCCAACTCTTTAAAGAGCATTTAATACTGGAGAGTGCGAACTTTCATCCCGCAAT
ACATGCACATATTTTCAGCATTCTTGCAAGTTGTTAGCCGTGTGCTTTAAGCCTCGAGACAAGTTACTTT
ATGTGTGCCGCAGTTGTGTTTGACTCGTAAaatgaggggaatgaggggaaAAGACACCACCCACAAAATA
CCCAACCAACTTGGAGTAGCAAGACGACATCATGTGGCTGACATTAGAGCGTTTTTTCTTTTGTGTGTGC
AGCTGCCAACAACAGTTTGCAGCTTGCGTGGCTGCAAATTTGGGTTAACATGTCACGAATCTACTCGCAG
TTCCTTGGCGCTTCAAAGTATGCAGCATTATCGCTTAAAGTGTCGTTGGCTGGGGTCCTTTGGCATTTAA
CTCGCTGTCTCGCCCCTAATTTCGGCTTCGCATTCTCTATTCTCCATTTTCCATTTAACGTTCTCCATTT
TCCATTTGGTATCCTTCCAGGGTAAGGTCATTTGAGTAACAAAACAGAAAATAGGGCCCAAATCAATGTC
AATCAGGCCGAACAATTGGGTTAGATTGCGCAAAATGCCGCACGTTCGAATCAAATAGCTAAACCGCCAG
AAAGCATAAATTACAGTCCCATCGACATATTGATTGTAATCAAATATGAACGATATTGCACTAGATGCGG
TGTACGATAAACATTAAAATTTCCAATTTCGGTTTAGAATTTAATTAAGACATATTTTCGATTTGCGTGC
GCCATAATTCTGTGAACTCATAGGCATTCCAGTGAAACGGACGACTGCTGTCTGTTTATTTAAATTAATC
AAAGGAATAGCTAAGCAGCAAAAGGTATGCAAGTGACACTCTAATAGAGAATTACCCTGGAAATCTACAA
ATATCCTTTAATGTGCCAATCACAAAAACTGACAAAAGCAAAATACTAAAATTCTGACAACGATGTGAGT
AACTCTTTAGATCTTTGATTAAAGCAGTGAAAAAAAGACAGACGTTTAAATCAATAACGCTAAGCGATAT

AAATTTTCTCATTTTAATTAAGAAGCATTATATCACAAGTCATTCATTTCACAGAGTCATGAGTTGGGCG
CACTCTCAAATCCTTCGACTTTCGAAAGTGAATTAGGAGTTTGTCGAGAATCCAAAATCCATTAAGTATG
TTAATTAACCAGTGTCTCGCACAGCCACTTGCTTCACAGTAAGTCGGCTGCTAAATCTATGAGTGCGAAT
GTAACAAATTACTTGCAAATAATTCAAATAATCTAGGGCCCCGCAGAAATGTGAGGCGTGTGCTCATTAG
AGGGCCTGCAAGACTTTCTAGGACACTCTTCCACTTCCCCCTCCCCCCaaaaaaaaaCAACGCCATTCTC
CACCATTTCCCAGTCTCTCAGTCACTTGAGTCTATATATATGGGCATACATTTATTACGGCATCGTTTTT
TGCCAAAGAAATCTTTAAGTCGCCACGCGCCGTCTGTGTAAATCTCCTCCAGCATTGGCATTGGGTTTTG
GTTTGGTTTTAGATCCACAATGGGGGAGCTTAGAGTAGGACACAACACATACACGCCCCCTGGCGGCTGA
ACAATAAATTAAGCTCAGCCACAGAATTTGCCGGAAGTTGCGTGAAATGAATGTGTGTCGGCAATTGTGT
TAATTGCACAAAATAAATTGTTAAAATAATAATACACAATGCCAGTTGTTCGCCTGGTCGACTGTTCAGG
CCTGACATCTAAatatatatattgtttatatgtatatatCTCTCGGCCAATCTCTATAATTAAGTGCCTT
GGGTCCATTCGGATGCTTTGTTGTTGGCCAAGGCCAGTCAAGTGGCTCTGTCCAGACGCCACTCTCCATC
TGAATCGctccatcttcatttccatctccatctctatcATAATAATGGGCACACATAATGAATTTCTCCA
GATTGCGTGTGCCTATCCACCGGGTCGACCATGCAATCAACGTGGATTTCCAATGGTCATCCCGAAATTT
CATCCGCCCCGTTGTCATCGCTCACTGGCCTCAATAATTGTGTCACGTGGCGCGTGAAAATACGGCCTTG
TCATTCGGTCCATTGGATTCCTGATGATGACCGTGCTGATCCGCCCACATAAGTAGAAGGTCCTTCAAGT
ATCTCTACCTTGCATATATGCATCTCATTGCGCTAAAATTAATTTCATATTTGTCTGCGTATCGTTACTT
GTCGTTTCGTTTCTAAATAAAGACTGAAATGCTTTCCACGATAAGCTTACGTTTCAATTTGTTGATGAAA
GCCTTAATTACATTAAAATTGATACACCAAACCGGACAGAGTAATGCTATTTTAACTAGACAACCAACTG
CCCACACATTTGGCTCACTCTATTCGCCATAAATAACCGCACGGGGCCAAATTAAATTTATGCTACTTAA
CTGCGAGGGCGTGGCGACACGCCCACATACATACATTGCAACAGTCGGCAAATGCCACATGCAAACCGGC
GGAAAATAATCAGTGCAAATGTCGTCGTGGGATGTTGCAAGGATATACAAAAGGTGGATGGGGTTTTGTC
TAGTTTGCAAATAGCTAAATATATATTTGACTACTATTCTGCACGAATAGAATGGGGAATTTCAGCACAT
TTTACGGCTGCCAACGAGCTCTGTTGATTATTACATTTTGAATGCAGAGCAAACGAAATTCCCATGGTCA
GCTTAGTTAAATAAATCGCAATTAAAAGCAGAGCGTCTGTTCTGGGCGTTTTCCATTTGCCCTCGACGTG
GACAGCAAGTGGCAGAAGCCGAAGGGGTTTAAACGATTTTTTATCCAACACTAGAATCTGACCCGCTGCG
ACCCACATCATGGCAATCTAGATGCTGTTTTTGGTCTCATTTGCATTGTTTTCAATCTACGAATTTCCAC
CATAAACAATTTGCTGCAACAAATTTATGTATACGACTTGGCTAATGTTTGCGGCTATAATTGAGCGTAA
ATTGGACGACGTATAATAAAGTTGTTTTATGCATAAAAGTTAAATACTTGTATAACTCAAACGGCGGCGT
TCATAAATAAAACTCACACTTTCGTTGGCGCAAAAACCCACGGAAGCAAAGTAAAAGCGCCATTAATCAG
TGAAAAGTTGGTCCCAGGTAAAAATCCCCACTTTTCATATATGTAAGCGAAAGGCAGGGTCACAAATGAG
GCGAATGAATCAAAAAATGAGAGAGTTACGCAATTTGGGATAGCCAAAGTCGAAAATACCCTTGCACCCG
TTGCAAAGATTTAGGGATTTCAAGATTAGGCAAATTTGTCGTTGTAGAGTGAACCTTGTTATTAATTCAA
TTTTCAAAATATTTATATATATCTAGAAGAGCTATCTATGCATTATAGAGTCAGTGCTTAAGCATTTAAA
CATTTAGGATAACACCTATATTTATGGTTATCCAATTCAGCTTGTGTTTTCTAGCCAAACAAAGTTGAAT
AGATCAAATATGGAAAATCAACACTGTCAGCTCAACACTGCGTATGAGTGATAAATATTGCGATGCAAAC
TGTTAGTACAAAAACCCCGCAGGCTTAGCAGAAGTTTGGAATTATGTCTAATCAGCGACTCAACTTCGTT
CAGAGGGTTGGAGTCAACTTATCGGACCAATTTGTAATACCCCAAAAGAAGGGTATAAGGGCCAGGCGCA
GTGAAAAGTTTGCCAACATTGCTCACCATTAAAGGACTCGTAAATGCCATGGGCGACCAGGCCGGTTTGG
CGGAAGCGATAACAAAGCCGCTGACACTTGACAAACTTGCGCGAGTGTCaaaaaaaaatctccgtgaaat
gggcaaaggttccaaaggaaagagaaaaaaaagaaatgcaaaagaTGCCATCACCTGGCTTCCACGAAAT
GAAGCATTTGGCCCACCTGCTGACCCTGACACCAAACGCCAGATAAGCAGTGTCTGCCACAGTTTCAGTC
GCCTGGCTGGCCAAACATTCATCTTGATGTGACTTAATTTGGCGCAATTGTGAATAGCATTTAGCTGCTG
GGCGGGCATTTGTCATCCGCCGGCTGTTGTGtttttaattaatttttttaacattttGTTCCCACTGTT

TCGATGGAAATTTCACAGTTGATTTTTCAATAATTTCCACCTCCCTCTTCGATTTGCAGGGGGCAATGTG
CGCGCCCTTTGGATGGGCGTTTTGTGGGCGTTAACATCTGGCACTGGCGTGGCAGTGGCCTTGCTCCAGG
GTTCCGCCGGTCCGCTGATCGGGGTCGCCATATCCGCCTCCCTGCTCCCGCCCGTCGTCAATTGCCGTAA
GTAACCCACGGGGCTGAATTAAATCAAATATCCAAAGGACCGAAAATAAATCATTTTGGAGGCCTATGGG
AATGGGATTTATTAAAAGATTGTTACTAGAGCAAAACAGTTGGAAAGTAAGCCAGAGAACCATATGCTTA
GAAACCAATTTTATTATAATATTAAAACTAATAGTTTAATTTAAGTAAGCAAGTTATTTAAGTACTTCAC
TAAATAACTAAAATTATGATGAAATTTAAACTTAAATAAGTAGTCATTCACTTTGAACTTAACTAAGCCA
ATTAACATACTATTTTGGTAATATTTCTAAACAATGAGTGTAGAAAGCAAAAGCTTGTTTGCCTAATTAC
GAAATTGATTGTGCAGACAAACGTGGAAGTCCTTAACCACACCATAGCATCTCTTCATCAAAATATTAAT
TATATAAAGTGTACATTTAGATTTACCTTCATTGATAATCCGTTACCTCCAGCAAAGAAAACAGTACGAG
TATTCAGCATTTGTGAGTGTACTCAAAACTCATATTTACCCATAATGCCACATATACTCGTATGTCGATG
CAGAAGCCCATTTAATAAGTAATTTTCCTTGGCCTTGAAATCGGCTTCTCCATGGCAATTTTCCTCCCAT
TAGCATGCTATTAACAAAAGCTTCGATTGCGCAGTGGATGTTTCATCATGAGTGAGCAGGTGCCGGAAAT
GACTTTGCCAGCAATATTTATGGCCATGCAGAGGCCCAAAAGCATTCCTCAAAATGCGAATAGAACGGGC
TTCTACAATTCCAATCGAATATATATGTATGTACATATATGTATACATACACACTTCGGTCAGTCGCATG
GAAAATTGCGCAATTCCCATTTATATTATGCTCCGTTTATATTTTGATTCCACTCGAATTTTCTACCATC
GATGATCATCGCTATAGGCCAAatatatatatatattatatCCTTGAACAGCCGCAAACATCGTCGAAAC
TTCCAAGCGGCCAGAGTACCCGAAGTACTCGATGTAACGCACTTAAAGACCAACATGCAAAACGGATCTA
GAAGTTTTTGGGCTTGGCTAATATATTTTTGGGGGCTCTTCGAGTGAGTTTCACCATAAATTAGGCCCAA
ATACAATTTGCTGATTAATCTGAAGTGCATTGCCCAAAAACTTTCGCCCATAAATCTATCCTGCCGGCAG
GGCAATCGCAGTCTGGCTATGAAGAAGGTTCTCAGCTGTTTTGCCACTTGGTGTGGCGCAACCGCAGCGA
GTGCATAAATTTCGACCCAATTTTCAAGTGCAAATTGCGAATCCGCATCTGAAGCTGAAACTGAATCCCC
AAGCTGCTGGCGGATGTTGCGTAAAATTATAAAGCAAGTACGAGGCCGAGTCGGAGTCGACAACTCCACC
CCAAAAAGGGAAGTGCATGGAAAAACTAGGAAAAAGCACAGGAACCGGCAGGCCAAGTGCCACACAAACT
GGCAGACAAACAGCCCGGCTGGATGGCATTTATCAATGGTGACAGTGGGACAAAGGCGCCGCCGGGAGGC
GTGTTCCACAACCAAGACAAAATGATGCAAAGGGCGAATTATGGACACCGGGTAAGGGGCATTCCACCAC
ATAGCGTCCAACAACTTTAACCGGCCAAAACAATGGCGCTGAAATCGCTTAATCATGCCGGCCGAGCTGC
GGTCGAGCATGTGTGCAAGTGTCGCTTCAACTTCGCGTCCTTATCCTGTGAAAAGTATACGATACCATGC
CATACCATGCCATACATATGAGCTGCATAAATCAGTGGCACGCACTGAGGCCGAAGGAGCAACCTTTGTT
GTCCATGTTTTCTCAGCTCCATTTCCTTATTTTACTCCAGCAGCGAGGAGCGTGTTACAATTAATTGCCT
GCTGTCTGGCCCTAAGGCAGTGTCGCGGCCAACAGAGCGTATGAGTAATTTCCACTACCCGCCAACTACT
CGCCATTCACATGGCGAGGTGCCTCGCACATTTCAGCTGGGAAAGTAAACTTGCAGCACAGCGGCGCAAT
AATTGCAACTCTTAATTTTGGCTTATTAAATTGCCAGCGAGCCAATTTATGTGGCATGTATACATGAACT
CCACAATGCCATAAAGCGGTCTGCAACGCCTTTACTTTCAAATCCGAGCACGCAATAATTTAGCCCGAAA
CGGTCGTAAATTCGAATAATAGCGAAAATAGAAAGTAAGTCGGGCAAAGAAAAGTCATGAGCTCGCACAC
AAATGGAAAAACAAACACACACTTCACAGATTTGTGGATTTTACAGCGGAGGTGGCGTTATTAGCAGCAG
TATGATATAAATTTCAGGAGTTCAAAGTTCAAGACAAGAAATGATCTCTTTAAAATTTCTCATTATGAAA
TGttttttttttCATTATGCAAGTTTAACTTCCTTTTTACTGCAAACTTTTATTCACCTTTTTATAAAAAA
ATATGAACATCTAATGCTAttttttttAGTACACACATCTAATAAGCTTTAAAAGCAAAGCGATTAAGAC
AGCAGTACCTAAGTTTATAGGATTATATTCTTAAGATGGTATAATGATGAGCTGACCGCAATAGTACGTG
GGTTTATATAGAAAAGGCACGCACTTCCGGACTTGCTGAGCAATGTGCTGAGTCATTATAATTTATAATA
GCAGAGAGAAGGATACAGAGaaaaatcgaaaaaaaaaaaaaTAACCGAAGAGCGAATGGCAGAAGGAGGA
GAATGAACTGAAAATGCGACAATGGAGTTCTATCTAGTACTAGTGGGTGGTGGTGGGTGTTCTTGGGGCG
ATTGTGTGGCGCCCATGCTCACACATATGCTGCAAAGTTATAGCATTTCAAGGCCGGCTGCCACTGTTTG

GTATGCCAACGGGAACGCCATCTAACCGGAACTGGGCACGCGGCGACCTTATGACCTCGAACCGAACcac
acacacacacacacacacacacacaTGACCATGGGAATGGAACTTCAAAGTCACAGCTACCCACCCATATGC
TCTTCTTCTTTTTTGCGCCTGTGTTGCAGGGGTCTCTTCTGGTCACTGGCCTGCATTTGGCTCATCTATC
CGGAGAAGCGGATTCCGCACTTGAAGAACGAGGCGATGAACGCGACTAGTGCATACCCCTTCCTCTACAC
CAACTATCTGCCCACCGAGTTCCTGATCAACGGCATCGTGTCCGCCTGCCTGACCATCGTGAATGTCATT
TGCATATTTATCACGGCCATAATCGTATTGAAAATTAAAGAGGTCTCGGCGCCATATACGGCCAGTCCGG
ATTTAAAGCGGGTGAGTAGGCAGTTCTGTTTTTAAGTCGATGACGACATGTAGTCGATGCTCGTCTGGGA
AAACTTTTAGGGTGGACCTTGCttttttttttattatttaaatgaaatttattatagtaaaaagaaaaaat
taatacatttataaaatTAAATACGGGCAGCAAATATTAAGAACTAATAATATAATATATAAAACAGGAA
TGTCTATTTAGTGTTTTTAAAATTTTTGTAAGAATTAAATAAATTAAtgcacatgcagattttgatttgg
gtgtttgtaagtacccgcgatgcgagtgttgagtggcacccgcactcaaaattgttgagtgtgagtcgcg
tggcaaaaaaaatgccacccttgcagttcATACTACAAATAGCAGCAAATTAAAAACGTACACAGCGAGAT
GCGCTGGAGTGTGTTTAATGATGGCGAAGCAAGTGCTTTTGAGCTTATCATTTCCGTAATGTACGCTTGG
TCAGTTGGCCATTAGTAAGTGTAATTAATAACGACAACAGACAGACGCAGAGACAAAATCACAATGGTCA
ACAAAATGGGTTGTTCGGCGAGAATTCCCAGTTAATAACCATTTAAAATGCACATCTATTAGTATGCGAA
TTATATATTACGTCAACATTGCCATTGACTTAATCTACGATGGGTTAATAAATCAGATCTTGTCTGCATT
GTGTATTAAGTCCATCTTAGATTTCTCCATTCAAACAATGTGCAAACACATATGACCAACTAGTTTTGGA
GGAAATTAGCCCACATAGCTCAACACTTGCCATCCTGCATAGTACAAATCAAGAAAGGAGCTGAGTCCTG
TGGCGGAATTCGAACATAGGATTCTAAAGTTGTAATTTTGGGGCAAGCATTTCCCTTTTGACTTTGTATG
TACTCTTAGACACTCCGATTTGTAGCCAATGCTTTTTGATTTGCTCTCCGTACGATAATCCTTAGAAATG
TCTGTCTGCGCAAGTGTTTCGCTCCAAAAATGATATATCGCGTAATTAACAGACTGATTTCGCGTTTTTA
TTCCGCCAGCGCCGAGTGACCGAGTGGCAATCCAAATGAAAACGAATGCCCCACATCGCCAGCTCGGATG
ACAAATGGGGATCTAAGCTCGAAGTCTGCATCTTAGTCCGCTCCAGACATGTGTCTTTAATTTACCCAAA
AGGGGCGCATTAAATTGCTTTAGGCGAGGACTATTCGTAGGCTTTCGTCAACAACAcacacagcatacaa
gcatacatacatatacatacatgtactcatacatacacaCTCGGCCCGCATAAACGGAAAGGCCCGCGAA
AGTGTGCTACAATTTTATTTCTCTGCCGCCGTTTGCGGCATCCTTTTTATTATTATTATAGTGCACATAC
ATATGTATGCATGAATGTCCTGTGACTGTTTCAACGTTTATTTAAGCACGCTCACGCTCGTTTGCCCATT
TGTTTGACTTGTTTGCGTAGCTTTTAGAAATTGTGTGTATTTTGTGCATACCCTTCGCCAAGGATACCAG
CATTTTGATCTGATTCCTGCACTACAGCCAGTCGAATATTGACCATTTGTTGTTCACAAAAAAATAGAAA
ACAAAGGCACTCGAATCGGATTTCAATATAAGCTGGCCACTAGAAGGAACGGCTATGCAGCAATTCGAAT
CCGATACCATTTGTTGGGCTCACACGCTGCCACTCTTAGCCGAGTGTCCTGGTTCTGGAAAAGCCTGGGC
AGAGAACAGGACCAAAAaccaaagcagaaaagagaccaacagcagagaaagggacTCCCATGGCCGGGCT
CTTGCCGCAGGCTGTTGCATTACGCATAAAGTGCATGATTTCATATTTATTTGTTGTTACTCATCGTATA
CTAGAGACCCATCCAGTTTGCTCGACTCTGGCGAATTGGTTTTGTTTTCATTGCTGCGGTTTTTCTTTTG
ACAGCGGTTTATGGTTGCGAATTTCTTGTCTCCGCCAATTCAAAATGATTTTTAGGGGTTTTCTGTCCGA
CGAGAGAGCAACTCCTATGCCAACATAAATGGGGACAGCTTCTGCCGGGAGCCCGAAGGTTATTTCCTAT
AATAGAAAAAGTTTTCCGACGGGCACCGAGTAAAATTGAACTAAAAGTTTTCCaaaaaaaaaaGAGTCGT
GCATTGCCAATCATTCGAAATATAATTTTACAGCTCTTTTACAGTGCAGTCTTAGTGGCGAAAGTGGGAA
ATTACTATACTTAATTAAATGAGTCATATTTTACAGGCAGCTTAATATGAAAAACAGAAACGTAGGATCC
ATATCATAGTAGTATTAAAAACCCATATTGAAAGGCttctaagaatttgtagaatttctagaattGTTTG
AACTAATCATTAGAAGATATATAATTATTTTAACAAAAAGTTTAAACATTCATACCTAATAGCTGTTGAA
TCGTTTGTCACTCAACTCTTCTTCTATGCATACAACGAAAAATACATTGCTTTTACAATAAATTTCCCCC
CTTTTCTCACAGGATTTTGGGAAACAGATCTTCGCACCGTACGCAAAACGAATCGCTCAACAATGCGGCA
TCCTCGCAGCCGCAGCTCGATGGCCGGAGGTCCTGGAGGACATGGAGGGATGGGTACCGTCAGGGGCATG

GACAAGTTCAGCGG
>dme_circ_0000011_junction_seq
CCGTCAGGGGCATGGACAAGTTCAGCGGGCGAGGAAGCCGAGGATGCCTCGAAATG
>dme_circ_0000012
CTCGTATGGCTGCTCCTGTTTTCCCGGCTTCAGCGGCAGTGACTGTGGTCATGGACCCCTGTGCAAGGAT
CTGCACACCAACGTTTGCCAAAACGGCGGCACTTGCAAGTAAGTAACAATTGTTTTGCTAATTGGGTGAA
ATCGAAGAAAATCACTTGGCAGAGGTGGTCTCAAATTAAAGCTTTCTGCTATACTTTACTTCCACATATG
AGTTGATGATATGTAACGCATTTTTATTCACTGCCATCATCATCATCATCAATTGCAGGCATATCGGCGA
CGCCGCCATAACTTGCCATTGTCCGAAGGGCTTCAAGGGGACCAGATGCGAAATACCCGAAATAAACGAG
ACTACGCAGGGTAAGTGCAATAATAAATGTTATTTCCGCGATATATACACATACAGCCGGGTTCAGCAGT
CTGGGCTACATGCAGGCTAATAGGCGCCAGGACACCCTaaaaaaaaaaaaaaataaaaTAAAGGGGGTGA
GGGAAACATAAAAAGCTGAAAAAGTTGTATGGAAAAACGGAACAAAGGGGCGCTGCTGTTAAAGTGAAAA
TGTAATTAAGGAAATAATTAGCATTAAAAATGCACTCATTGCCAGCTGAGGTACCCGCGCAGACACTGTG
CAGACAAATTGAAGGGAAAGGCGACGGGCGGTGGCGTTACTCCAACCCCCACTCACTTTTCCCTCCTCGC
TCGCTTTTCCACCACCCCCACCCATCTGGAAAATTCTCGCGCCCCATTTAATCGGCAAGTGCGTGGGAAA
ACAATAAAAATGCATAACAACTGCAATGGAGAGCATTAAAAGGGAACACGGTGGAAAACCTCAGGTTGTC
ACTGCCCGGGCTCGGTGCCAATTTCCATACGGCTGCCGTTTTCCGCGGTGGAAAACCCAGGACATGAACA
ACTGCGAACATGTCCCCAGTCAGCGGCAACAATGTTTAAGCTTTAAGTTGTGCAGTTCATTGAATCGCAA
AGTGCCTGCAATGGTGCAATGGTAATGAGAAATGGTAGGAGAACAGGTCCTGAATCCTGCAAGAGGATTG
CGAGTTGCTCCTTTTCAttttttttttGCTTCGGTCCTTTGGCATTTCCCAAAAAATGTGGACAAAGCCT
TTTACAGTAGAGTGAAATTTTTGGTAAGCATTTAATATTTTATCGGCTGGGTCTTAAATAAATTTTCATG
GAATAAACACTTCAAAGAGCTGACGAAAAATGTTAAAGCAACTAGTTTaaaaaaaaaaaTGTGCACCGAG
AAATGTTCACCAAAAGCGGATGTCTACTGAAAAATTCCAATTGAATCGATTTAGAGACTCCATACCtttt
tttttttGGCTATGACAAAAATGAATAAGAGCAGCTTGATGGGATGGTTAAGAACCGAGAGATTATCCAA
ACGAATACCTGGCACATGCCTCTCATATTTTTGCTGCATGATGTTATGTCCTTTATTCAGCTCCATGCCC
CAAGGAGATTTAAAGGATTCTTCTTGTGGCAATTGCAAGAGCATCTCCATCCACTCACTGGACGGGGCAG
GGGTTAATGTTGCCACAGGTGTCGGTCCACATAGGTACACATACACATCCATGTCTTAGCTTCGACCTTT
TATGGAAGCAATTACCAAATGTTATCCATCCTTTCAGGGCTGCACCAGCAACTCCAGCTCCATCGAATGC
CACCAGGCCTGCGACTTTGACAATTTCGGCCGCTCCATCTCACCTCCTGGCCAGAGTTATTCCGCATCCA
AAGGCAATCGGAATGCGGCACGCAGTGGAAAAACCCGATACGAGGTAACCATGCGACTGGGCGCCAATCT
TACAGGATACTATCGCCATGCCGACCGTGAGCAGTTGTCGGGCGTTATGGAAAGACAGG
>dme_circ_0000012_junction_seq
CAGTTGTCGGGCGTTATGGAAAGACAGGCTCGTATGGCTGCTCCTGTTTTCCCGGC
>dme_circ_0000013
GCTGCCCGCCGTGCCGGAATCGAAGCTGAAGTTCAGCAAGAAACAAATCTCCAAGCGAGTCGCCGAGTCG
AAACGTCGCCTGAAGAAGGCCGCCGTGATTGCCCTGCGCAAGAAGGAGAACCTGGTCCGCGCCGAGAAGT
ACCAGAATGAGTACATCAAGGCGGAACAGCGCGAGATCAAGCTGCGTCGCTTGGCCAAGAAGCGCAACCA
GTTCTAC
>dme_circ_0000013_junction_seq
CTTGGCCAAGAAGCGCAACCAGTTCTACGCTGCCCGCCGTGCCGGAATCGAAGCTG
>dme_circ_0000014
GAAGTTGGAGGCGTACTTGAAGTTGGGCCCCACGGTGAAGATCTCATGGACAAGATCCTCGACGCACTGA
ATTTGGTGCGCCTGACGCAGCTTCCGCTCGATCACGAAGTTATCGGTGATGGGCACGCGCTGGCGGTTAT
GCTTCACGAATCCACGCTTGTAGATCAGCTCCCGCACGGACTTCAGATTGGGATAGCCCCAGG

>dme_circ_0000014_junction_seq
ACGGACTTCAGATTGGGATAGCCCCAGGGAAGTTGGAGGCGTACTTGAAGTTGGGC
>dme_circ_0000015
GGCCACAGGAAGTTGGAGGCGTACTTGAAGTTGGGCCCCACGGTGAAGATCTCATGGACAAGATCCTCGA
CGCACTGAATTTGGTGCGCCTGACGCAGCTTCCGCTCGATCACGAAGTTATCGGTGATGGGCACGCGCTG
GCGGTTATGCTTCACGAATCCACGCTTG
>dme_circ_0000015_junction_seq
GCGGTTATGCTTCACGAATCCACGCTTGGGCCACAGGAAGTTGGAGGCGTACTTGA
>dme_circ_0000016
TCCTTAGACCATCTTGCGCAGCAGACGGTTGATCTGGTCCTCGCGGTTGCCGAAGTCACCACCGTTGACA
TAATGGTTGGCCTTCTTGCGCCAGCCGCCGGTGGGGGTGTTTAGCTTGAAGGGCCACAGGAAGTTGGAGG
CGTACTTGAAG
>dme_circ_0000016_junction_seq
CCACAGGAAGTTGGAGGCGTACTTGAAGTCCTTAGACCATCTTGCGCAGCAGACGG
>dme_circ_0000017
GCTAAACACCCCCACCGGCGGCTGGCGCAAGAAGGCCAACCATTATGTCAACGGTGGTGACTTCGGCAAC
CGCGAGGACCAGATCAACCGTCTGCTGCGCAAGATGGTCTAAGGACTCCTTTTGATTTATGTGCTGAAAC
GACAACTGCAGCGGTTCGAT
>dme_circ_0000017_junction_seq
GCTGAAACGACAACTGCAGCGGTTCGATGCTAAACACCCCCACCGGCGGCTGGCGC
>dme_circ_0000018
AATTGAATGCTCAAGATGAATGGCACGTTTTACCATCATTATATCTAGATCTACGTGTTGAACTATTGTA
AATGATCTGTAAATGGATGCTCGACGGATACTTTTATCTCTTTTATCATTTCTTGTATTGTAATATATGT
ACGTGGAACTTTCTTTACTTTAGAGATAGTTGAAAATATGTTATGCATTTTACAAAAATAAATG
>dme_circ_0000018_junction_seq
TATGTTATGCATTTTACAAAAATAAATGAATTGAATGCTCAAGATGAATGGCACGT
>dme_circ_0000019
GCTGGGTGTTGATGAGGACGACGTGACTGCATTCTGTTCCTGCTCCTCATCGCTATTCTGATCCTTTCCA
CTGCCAGCGCCTCGCTGTAACAACTCGTCGACCATGGCCTGCAGGCACACGGACATCAGCATGGCCTGAT
CACTGTTAATAGTGATCCAGCGCAGCGTCTGCTTGCCAATCAGATATTCAAAGGCCAGCTGGAGATGC
>dme_circ_0000019_junction_seq
CAGATATTCAAAGGCCAGCTGGAGATGCGCTGGGTGTTGATGAGGACGACGTGACT
>dme_circ_0000020
AATATTTGAGCAGTAGTCGATCCTTAATGGACTCGCGCAGCAAAACGCCGTCGGTAAGGAATCGAATCTT
TGTGGCCCTTGAGGTGACATCCTCGAAACGCACCGTATAGCCCACCGTGTCGCCAATGGTTCCGTTCAAC
TCCTGGGCCACGCGACGTGCCACTG
>dme_circ_0000020_junction_seq
AACTCCTGGGCCACGCGACGTGCCACTGAATATTTGAGCAGTAGTCGATCCTTAAT
>dme_circ_0000021
GGTTTTCTTGTAAAATAACAATTTCTTAACTTAACTATGGATTAAAATGTCTAAGAAGTTAAATCAATGG
AAATTATACGCCCAGTGCCATTGAAAGGCGGAGCCGCGGAAATCGCATCAAACGTACTACTCTCAGTAGT
CTGATTTGTAGTGCTTTTTTTCGAATTAGCCGAATGAGCGTACCAGTCTGTGAGTGCGACCAAGTTCTTA
GAAAAATTGCA
>dme_circ_0000021_junction_seq

GTGCGACCAAGTTCTTAGAAAAATTGCAGGTTTTCTTGTAAAATAACAATTTCTTA
>dme_circ_0000022
GGTCGAAGCGAAAAAACCAGAAGCGAACGACGACGATGACGATGTGGATCTATTTGGATCGGACAGCGAG
GAGGAGGATGGAGAGGCCGCCCGCATAAGGGAGGAAAGGCTGGCAGCCTATGCTGCCAAGAAGGCCAAGA
AAGTTCAAATTATTGCCAA
>dme_circ_0000022_junction_seq
AGGCCAAGAAAGTTCAAATTATTGCCAAGGTCGAAGCGAAAAAACCAGAAGCGAAC
>dme_circ_0000023
GGAGGAGGATGGAGAGGCCGCCCGCATAAGGGAGGAAAGGCTGGCAGCCTATGCTGCCAAGAAGGCCAAG
AAAGTTCAAATTATTGCCAAGTCGAACATCATTCTGGATGTTAAGCCGTGGGACGATGAGACCGATCTGA
AGGTTATGGAGACGGAAATCCGCAAAATCACCCAGGACGGTCTACTGTGGGGAGCTTCCAAATTCGTGCC
AGTTGCCTTTGGCATTCAGAAACTGAGCATTTCATGCGTG
>dme_circ_0000023_junction_seq
CATTCAGAAACTGAGCATTTCATGCGTGGGAGGAGGATGGAGAGGCCGCCCGCATA
>dme_circ_0000024
GGATGGAGAGGCCGCCCGCATAAGGGAGGAAAGGCTGGCAGCCTATGCTGCCAAGAAGGCCAAGAAAGTT
CAAATTATTGCCAAGTCGAACATCATTCTGGATGTTAAGCCGTGGGACGATGAGACCGATCTGAAG
>dme_circ_0000024_junction_seq
GCCGTGGGACGATGAGACCGATCTGAAGGGATGGAGAGGCCGCCCGCATAAGGGAG
>dme_circ_0000025
GCAGTATATGAAAATCGGTGAATACAGTGACTTCATTAGTCAGCCGAAAGCAAAAGAGAGCGAAATACAT
TTTTTAAACAAACTAAACTAAAATTTCACTGTGAATAATGATGACTGAAAAGTTAAATTCTGGGCACACT
AATTTAACAAGCAAGGG
>dme_circ_0000025_junction_seq
CTGGGCACACTAATTTAACAAGCAAGGGGCAGTATATGAAAATCGGTGAATACAGT
>dme_circ_0000026
GGATGTGACCGATACGCGAGGCAATGAATTCGAGGAGTTTTGCCTTAAAAGAGAACTGCTTATGGGTATA
TTCGAGAAGGGATGGGAGCGCCCTTCGCCAATTCAGGAAGCAGCTATTCCTATAGCATTAAGTGGAAAAG
ATGTGCTGGCTCGGGCCAAGAACGGCACAGGCAAGACAGGAGCCTACTGCATCCCAGTTTTAGAACAAAT
CGACCCAACCAAGGACTACATTCAGGCACTTGTGATGGTCCCCACCCGAGAGTTGGCGCTACAAACCTCA
CAGATATGTATTGAGCTGGCGAAACATCTTGATATCCGGGTAATGGTCACAACGGGAGGTACCATTCTAA
AGGATGACATTCTTCGTATTTATCAAAAAG
>dme_circ_0000026_junction_seq
GATGACATTCTTCGTATTTATCAAAAAGGGATGTGACCGATACGCGAGGCAATGAA
>dme_circ_0000027
GGCAAGACAGGAGCCTACTGCATCCCAGTTTTAGAACAAATCGACCCAACCAAGGACTACATTCAGGCAC
TTGTGATGGTCCCCACCCGAGAGTTGGCGCTACAAACCTCACAGATATGTATTGAGCTGGCGAAACATCT
TGATATCCGGGTAATGGTCACAACGGGAG
>dme_circ_0000027_junction_seq
GATATCCGGGTAATGGTCACAACGGGAGGGCAAGACAGGAGCCTACTGCATCCCAG
>dme_circ_0000028
GGAGCCTACTGCATCCCAGTTTTAGAACAAATCGACCCAACCAAGGACTACATTCAGGCACTTGTGATGG
TCCCCACCCGAGAGTTGGCGCTACAAACCTCACAGATATGTATTGAGCTGGCGAAACATCTTGATATCCG
GGTAATGGTCACAACGGGAG

>dme_circ_0000028_junction_seq
GATATCCGGGTAATGGTCACAACGGGAGGGAGCCTACTGCATCCCAGTTTTAGAAC
>dme_circ_0000029
AATGGTACCTCCCGTTGTGACCATTACCCGGATATCAAGATGTTTCGCCAGCTCAATACATATCTGTGAG
GTTTGTAGCGCCAACTCTCGGGTGGGGACCATCACAAGTGCCTGAATGTAGTCCTTGG
>dme_circ_0000029_junction_seq
ATCACAAGTGCCTGAATGTAGTCCTTGGAATGGTACCTCCCGTTGTGACCATTACC
>dme_circ_0000030
GAAGCATTTACGCGAGCCTTACGAGATCAACCTGATGGAGGAGCTAACACTGAAGGGTGTCACTCAGTAC
TATGCATTCGTACAGGAGCGCCAGAAAGTACACTGCTTGAATACGCTCTTTTCGAAGCTGCAGATCAATC
AGTCTATCATATTCTGCAATTCCACGCAACGTGTCGAGCTATTGGCCAAAAAGATCACAGAGCTTGGATA
TTGCTGTTACTATATACACGCTAAGATGGCCCAGGCACACAGAAATCGAGTATTCCACGATTTCCGCCAA
GGACTCTGTCGCAATCTGGTGTGCTCCGACCTGTTCACCCGTG
>dme_circ_0000030_junction_seq
CTGGTGTGCTCCGACCTGTTCACCCGTGGAAGCATTTACGCGAGCCTTACGAGATC
>dme_circ_0000031
GATGGCCCAGGCACACAGAAATCGAGTATTCCACGATTTCCGCCAAGGACTCTGTCGCAATCTGGTGTGC
TCCGACCTGTTCACCCGTGGTATCGACGTGCAGGCCGTGAATGTAGTAATCAATTTCGATTTCCCACGAA
TGGCAGAGACATACTTGCATCGAATTG
>dme_circ_0000031_junction_seq
ATGGCAGAGACATACTTGCATCGAATTGGATGGCCCAGGCACACAGAAATCGAGTA
>dme_circ_0000032
ATTAAAGATTTGAATTTCCCCTCAAGGTCGTCGTCTCGCATATCCCAGCCGGAGCTGCACTACGCCAGCC
TAGATCTTCCCCATTGCAGTGGCCAAAATCCAGCTAAATACCTGAAGAGAGGATCACGCGAATCGCCGCC
GGTGTCCGCATGCCCGGAGGATGGGAATACCTATGCGAAAATCGACTTTGACCAATCCGACTCCTCTTCC
TCCTCATCGAACATATTTAATACGTAAAG
>dme_circ_0000032_junction_seq
CCTCATCGAACATATTTAATACGTAAAGATTAAAGATTTGAATTTCCCCTCAAGGT
>dme_circ_0000033
GGAAACAGCACACCGCCCCGTATCATTTCGCCCATAATGCAACCGACGGACCATATGTCCACGTTCTCTG
TGTATCCCATGCCCAAAATGACCTCTGGAGCCCTGTAATATCGGGTGACCACATAGGGAGTCATCATAAA
GGTAGTTCCTGCAGTACGTGCCAGACCGAAATCCAAAATTTTTAGAGTGCAGTCGGCCTTTACAACTATA
TTGGATGGCTTTAAGTCCTGCAAATGAAGTATTTCATATATTTCATACTCATTTTAAAGATGTATTCATA
CTGTACGTACTCTGTGAATAATTCCTGCTGAGTGTAAATGTTTTATTCCGCACAACATTTGATAAAGCAA
ATAGGACATTCTGTCGTGATCCAAGTCCATTTGGATGACCTGGCAGAGATTAGCGTCCATCAGCTCCATG
ACCAGGTAGACATCCTGAAACTCTTCCAAGTTCCTTTGCGGCGTAAAAGCATTAAGTAAGCCAATTATCT
ACGGATTAAATACCTATGTTTAGTAGAAATTTGTTAAAGTTAAAGGTGCAAGTTTCATACGTTTTTATGG
TTAACAAGCTTCATTAGCTTGAATTCCCTATATGCTCGCTTGGCATGGGTTACATTTTGGAATGGTCGAG
ATAGTTTCTTAATCGCCACATTTTGCTGGGTGATAGTATCGTAAGCGGCGCTGAAATGCATTAAATTTGT
TAtttttttttttCATATTCCAGCTAATCCCATCTAATCTAATCTAATATACAATGGAGCGTTACAAATA
TGACTTCATACTCATGCGAAATATCTTAATTGTTTAAGCCAAAAAATTGTTAAATGTATTATATGTAATA
ACATATTGATAACCACTTACCATACTATTCCTTGGGCACCTGATCCTATGGGCCGTAGATTAATGTACCG
ACTGTGGATGGTGAAGTTGGTGTCCCCCACCTCGACGGTGTAGTGTTGGTGCTGAGCTGTCGTCATTGTT
CACTGATTTTATTTATTACTAGTTGAGAGCAAGGAGCATATCGGCCTTTTCAAGAGGGTAAATAAGATAA

ATCAATACGGTTTTACAGTGTGATTCTGTTTTATATAAGGAGCAAAGACGTCCGTATGTATGGCTCCATT
CTGAATGGGTGGAGGCAGTTTTCAGACAACTTCGTCTGGAATTAGGGGGTAGGAAAACATTAACTTTGCC
TGGCCTTATTTCGGCAGAATGTTATGGCCATTAAGTGGGTCTCTGTTGTCGCGAGGGGTGAAATTTACTG
GCTAGTGTGTGGCGAGTTTGTTGCCAAACCACGAGCAAAAAATATCGAGTTCCTTCAAAAAGATACAATT
GCATAACGCAGTCACTTATTTGAGCCGACTTCGAATGCCGAACCCCCTTTTCTATTGATAAGCATTTAAA
TGTTTGCCGAACACATGTCGAATATGGTTGGAAAAACACCCAACAGAAATAACCGAGCGTTTCGTTCTTT
GAGCAAAGTGTTTAGCGGCACGCACAGGaaaaaaaaCCGATTTAAAATCGATATGTCCGGCTTAAACATA
AAAGTACGGAATGTTGGACCACAGCGATGTACATAGCGTTCATGTGTATGCATGCCGTTGCCTGTGTAAA
TATGTACATACACACGATTTGTATGCGGATGAAAACTGTAAACTTCTACTGGTATACATATGTGTAAGAT
GTAAGCCACATACATACATATGCACATACAGCCACACAAATGTGGGTCGATAATTTAGTAACTTTTCAAA
CTATCTCTCAGATAAAAGATATTCCCCGTATTCGTACATCGAAAAAGCGCTCGTTTGCGCTGCAGATAAT
AAAGCGGTCTACGCTTCGGGTAGTGTCGATATGTAAACGTTAGACATTTGCGCATACATAGGTACATACA
CTCTTTGGGCAGTTGACATTTTATGTTAAATATCAATAATTTTTTTCATAACCGGCACAACTATAATTGC
TTTCAATCGATTATGGCACGTTTTGCAGGAGTGTTTCGGTGATTCGATATTTGATGTGAATGGAACGTAA
TAGAATATTTATTTACCATTTAACGAAGAAGTACAGAATTTCTTGTGAAAATATAAAATCGAtttttttt
CTATTTTATTCTACACACTGGGCACTCAAAGTGGCGAGCATTTTAAACTAT
>dme_circ_0000033_junction_seq
ACTCAAAGTGGCGAGCATTTTAAACTATGGAAACAGCACACCGCCCCGTATCATTT
>dme_circ_0000034
TGGAATTAAAAAATCCGCCGACGAGGCATTAGCGTGGTATGATGAGAAGCGTACAAAGGACCGAAAAGGT
GTGACAATCCCATCTCAGCGTCGATATGTTCAGTATTTTTCCAAACTAGTTTGTTCAAG
>dme_circ_0000034_junction_seq
AGTATTTTTCCAAACTAGTTTGTTCAAGTGGAATTAAAAAATCCGCCGACGAGGCA
>dme_circ_0000035
GAAGAAGATGCAGGTGGCCAAGTTTGCAAAGAGCGTTAACGTAGTGTTCATCCTGGTGTTTTTCTTCAAT
GATGGACACCTTTTCCTTGGCATCTTGCTCGAAAGGTTTGGTGTCGTCATTGTTTCGGGAAGATGTGTTT
TCCATATGTTCACCTGCTTGAGTTGATGGCTCAAACTTTT
>dme_circ_0000035_junction_seq
CCTGCTTGAGTTGATGGCTCAAACTTTTGAAGAAGATGCAGGTGGCCAAGTTTGCA
>dme_circ_0000036
GCGGCCAACTTTTTCAAAGGGAACACTTCATTGAACCCATCACTGAAGGCCTTCATTTGCTGAGCGATTC
CGTCCTGCAGGATGAAGTTCATCAGCAGTTCGCAGTACGCTTCCAGGTTGTCGATGGTGACGTTCACTGA
CGATCCATTGGGCAGCAATTCGGCCTGGGTGTAACCATAGATCGAGGAACTCGGCAGATACGTGAAC
>dme_circ_0000036_junction_seq
GATCGAGGAACTCGGCAGATACGTGAACGCGGCCAACTTTTTCAAAGGGAACACTT
>dme_circ_0000037
ACCAGGCAGTTGTACTTCAACTGCACATCGTTCGGAGCATCGCGCAGCATAGTATGCCTACAGTCGCAGC
GAGATGTCACCGTGGAACGGCAGAGGATACCCATCATGAGTCCGCGGCGGGATGATCACGAATTCCGCAT
CGGGCGTTTAAAGCACGAACGCGTTAAAGTGCCGCGAAACGAGGACCTGCTCATGTGGGCCATGCAGGTG
ATGAAGACTCACTGCAATCGAAAGTCCGTGCTGGAGGTGGAGTTTTTGGATGAGGAAGGAACTGGTTTGG
GACCAACCTTGGAGTTCTACGCCTTGGTGGCCGCAGAAATCCAGCGTTCCGACCTCTGTATGTGGCTGTG
TGACGATGACTTGGGCGAGGACACGGAAAACTCGACGCAGAGTGCGGAAGGAAACTCAAAACCAGTTGGG
TATTACG
>dme_circ_0000037_junction_seq

GGAAACTCAAAACCAGTTGGGTATTACGACCAGGCAGTTGTACTTCAACTGCACAT
>dme_circ_0000038
CGAAAACATAGCTCCGTCGAACCCTTCAAATACCTCTGCGGGACAGCAATCGGCATCAACGATTAATCAT
ACCCTAACCATGAGTCTAACATCCACATCGAGTGACAGTGAGCAAGTATCGCTGGAAGACTTTTTGGAGA
GCTGCAGAGCGCCGGCGTTGTTGGGTGACTTGGACGATGAGGACGACATGGATGAGGACAACGATGAAGA
GGAGAATGAGGACGAGTACGAAGAAGTTGGAAACACACTGTTGCAAGTGATGGTCTCACGCAACTTGCTT
ACCTTTATGGATGACGAGGCGATGGAGAACCGATTGGTGGGTGTGACCAAGCGCAAGTCCTGGGACGATG
AGTTTGTACTAAAGAGGCAGTTCTCCGCGTTGATACCAGCATTCGATCCACGCCCGGGCCGCACAAACGT
CAATCAAACGTCTGATTTGGAAATATCCCCCCTTGGCGCCGAGCTACCGAAACCCCAGCAGAGTGGTGGA
CCTGAGACTATCGAACAGCCCTTGCTGGGCCTTAAATTGCGTGGTCCTGGAATTGGTGGTATACCCGAAG
TGGAAATCGACCTGAGCAACACCGACTGGACCATATTCAGAGCGGTACAGGAATTACTTCAGTGCAGTCA
GTTGAACAAGCTGGACAAATTCCGAAAGATATGGGAGCCCACATACACCATCGTGTACCGGGAGGTATCA
CCCGAGGCCCAGGAGAGCACTTGCTTGGAATCGGAGGAGTTCCCGCAAACGCCCGATGTGTCCTCCAAGA
GTGGTGCATCCACTTTGTCCCCCAACTCGCCCATGCACATCGGTTTCAACGTGGCCGATAATAACCTGTG
CTCCGTTGACGATGTACTCGAGCTGCTCACTCAAATCAATGGTCTCAATCAGTCCGAAATCGATTCGGAT
GTCAAGGAGCACGGTGTATCGGTGTTGTCCGAAGATCTCTTCATCAGCAAGAAAATAACCAACAAACTGC
AGCAGCAAATTCAGGATCCATTGGTGCTAGCAAGCAACGCGCTGCCGAATTGGTGCGAGAACCTAAACCA
ATCCTGTCCCTTTCTGTTTCCGTTTGAGACCAGGCAGTTGTACTTCAACTGCACATCGTTCGGAGCATCG
CGCAGCATAGTATGCCTACAGTCGCAGCGAGATGTCACCGTGGAACGGCAGAGGATACCCATCATGAGTC
CGCGGCGGGATGATCACGAATTCCGCATCGGGCGTTTAAAGCACGAACGCGTTAAAGTGCCGCGAAACGA
GGACCTGCTCATGTGGGCCATGCAGGTGATGAAGACTCACTGCAATCGAAAGTCCGTGCTGGAGGTGGAG
TTTTTGGATGAGGAAGGAACTGGTTTGGGACCAACCTTGGAGTTCTACGCCTTGGTGGCCGCAGAAATCC
AGCGTTCCGACCTCTGTATGTGGCTGTGTGACGATGACTTGGGCGAGGACACGGAAAACTCGACGCAGAG
TGCGGAAGGAAACTCAAAACCAGTTGGGTATTACG
>dme_circ_0000038_junction_seq
GGAAACTCAAAACCAGTTGGGTATTACGCGAAAACATAGCTCCGTCGAACCCTTCA
>dme_circ_0000039
GGTCCTTGGATATCTTATTCACCGAGTTGTTGGCGTTCATCTTGTTGTTTTCCTCAATGTTGGATATGTT
TTTGATATCGTTCTCTCGCGGCGACGAACTCTGCACATCCGGATGACTTGAGCTCTGACTCGCAGTAACG
CTCTCAGCGGGTTTGTAGGAAGGCGTTAGGACCCTGCATATATTCTCTTTGGACAATTCAGTTAGTGATA
GGTTCTCCGAAATGGTGGCTAG
>dme_circ_0000039_junction_seq
GTGATAGGTTCTCCGAAATGGTGGCTAGGGTCCTTGGATATCTTATTCACCGAGTT
>dme_circ_0000040
GACTCTTGTCATCCACATGTGTGCTGAGGGTGGTCCAAGTTGAGCCATCCTTGGAGCCTTGAAGTAGCCA
GTTTCTCAAAGCCGATCTTCCATAACCGCGGGCATGGCGCAGCGTGTAAGCAGTGGGAATTATATAGACA
CCCAAGTCTATGGCAAACCAAGCCTTCTTGTTGTCCTTG
>dme_circ_0000040_junction_seq
GGCAAACCAAGCCTTCTTGTTGTCCTTGGACTCTTGTCATCCACATGTGTGCTGAG
>dme_circ_0000041
GTTGAGATAGCTTCATTAGGGCGTCCGTAAGTGTGGTCTCCCAGTTTCCATTTGGTGCCATGTGCTGCTC
CTCAAAGGATAGCTCAATTTGCTTTACGATATCCGTAAGTATGGCTACGGCGCCCCGAGGAATTATTTGC
GCAGACTTGAAGTACTTGTTATAAATTTCCCGCGATAG
>dme_circ_0000041_junction_seq

AGTACTTGTTATAAATTTCCCGCGATAGGTTGAGATAGCTTCATTAGGGCGTCCGT
>dme_circ_0000042
GTCCAAGCCCTGATGGACACCGATGCGGAGGAATTGTATGTACAATTACCAGGGACCGTAGAGGAACCAG
CCGCAGCGCAAAGATCGTCGACCAGTGTAGTCGTTGCTCCTAGGCCAACATCAGGTATATTCAATATATT
TGATTTGAAACCAATTCTTATCGATCGGACCATCCCATGTAGATGATCCTATGGAGGACGCGAAGGAGAT
ATTGCAAGGGAAACCCTATCACTGGCGCGAGTGGAGCATTTGCAGAGGCCGGGATTGTTTGTACGTCTGG
TCAGATTCTGTGGCCCTTGAGCTG
>dme_circ_0000042_junction_seq
CTGGTCAGATTCTGTGGCCCTTGAGCTGGTCCAAGCCCTGATGGACACCGATGCGG
>dme_circ_0000043
GAAGTAACAACCCCAATCAGGGACAGTTGGCGCCTCGACCAAGGCGTAATAATACCAACACCGATCGCAC
GCATCGCCAGCTCATTGATTGCATACGATCAAAGGATTCGGAAGCCCTTCGTGAGGCCATCGAATCGGGT
GGCATTGACGTCAATTG
>dme_circ_0000043_junction_seq
TCGAATCGGGTGGCATTGACGTCAATTGGAAGTAACAACCCCAATCAGGGACAGTT
>dme_circ_0000044
GTAACAGCAATAGATCCGCAAATCGCATGCAATCCAACACGCATCGCTCATCGCCCTGCAGAGCTCTTTC
GAGGGCATCAGCCAATTGGGATCGCAAAATGTCATGGGTAATCGAGGGTGATCCGCGACAAAGTGTGGAC
AACAAGGAAATCGTTGTTGATATCGATTGCGGCGAGGCGGCGGCATCAGATGACTTAACCTTGGTGCTGT
TGGATATGGTTGCAGTTCCAGCAACGTTTTCATTAATGGCATCCGA
>dme_circ_0000044_junction_seq
CAGCAACGTTTTCATTAATGGCATCCGAGTAACAGCAATAGATCCGCAAATCGCAT
>dme_circ_0000045
GCATTTCATTCTGCACGACAGCTTCATTTGCGCCGGCGGTGAGAAGGACAAGGACACCTGCAAGGGTGAT
GGTGGCTCCCCACTGGTGTGTCCAATTGCTGGCCAGAAGAACCGCTTCAAGTCCGCCGGCATTGTTGCCT
GGGGCATCGGATGCGGCGAGGTGAACATTCCCGGAGTGTACGCCAGCGTGGCCAAGCTTCGTCCGTGGAT
CGACGCCAAGTTGAAGATTTGGAGTATTGACCCCAGGCACTATACACCCTAAGAGCAAAATTGTCACTTG
AATTTGTGGG
>dme_circ_0000045_junction_seq
GAGCAAAATTGTCACTTGAATTTGTGGGGCATTTCATTCTGCACGACAGCTTCATT
>dme_circ_0000046
GATTATTTCCTTGACATATCGGTCTTCGTGCCTTCTGATCTCAGTCTGCGTTTGCGTATCCCATTCGCCA
GCTCTCACTAAATGCCAGTTCATAATTAGATCTcatagatggcatagatggcatGTACACTTACCAACAA
TGCTACTGGGCTGTTTGTTGTGAACGCAATGCGCCGCGGTCAGAACCACATTGGGAGCTATCAATGCTCC
TCCACATTCGTACAG
>dme_circ_0000046_junction_seq
CTATCAATGCTCCTCCACATTCGTACAGGATTATTTCCTTGACATATCGGTCTTCG
>dme_circ_0000047
ACAAGAAAATGGAAGTTGACGTTGTCAAACGCAATGAACCCAACGAAGAGAAGAAGGACGTTAAGTACGA
GCAGTTCTGGTTCGACGACGACTTGGGCAGCAACAATTCGGGATACTATACGCTGCAGGCGGTGCTCACG
CACAAGGGACGCTCCAGTTCGTCGGGTCATTATGTGGCCTGGGTTCGCTCCAGCGGCGATGTGTGGTTCA
AG
>dme_circ_0000047_junction_seq
TCGCTCCAGCGGCGATGTGTGGTTCAAGACAAGAAAATGGAAGTTGACGTTGTCAA

>dme_circ_0000048
TTCTGGTTCGACGACGACTTGGGCAGCAACAATTCGGGATACTATACGCTGCAGGCGGTGCTCACGCACA
AGGGACGCTCCAGTTCGTCGGGTCATTATGTGGCCTGGGTTCGCTCCAGCGGCGATG
>dme_circ_0000048_junction_seq
GTGGCCTGGGTTCGCTCCAGCGGCGATGTTCTGGTTCGACGACGACTTGGGCAGCA
>dme_circ_0000049
GTTTTGCTGGTGCTGATGGAGTTGTTTAGTAAATTACACTATACATCCAGTATGGCACCGAAGTATCGAC
TCAGATTTTTGCTTTCGGACGCAGGCGTCCTACTAAACTTCCAAGGCTCCAAGAAGTGGCTCGAGGTTGA
TGATAATGCGCTGCAG
>dme_circ_0000049_junction_seq
GCTCGAGGTTGATGATAATGCGCTGCAGGTTTTGCTGGTGCTGATGGAGTTGTTTA
>dme_circ_0000050
GATCAATCTAGCAGACACCAAACTGGCCTGGGAGCACGAGCGGTTCAGCATAAAGCGCTATCCCTCGTTC
ACTCTATCGTCTGTAAAGAGTCCAAGAAGCCCCATTCGTACAACCATTTTCAAAAACGACGAGAGTCGTC
TAGTGGAGCACACTCTTAACACGACGCGAATTATTGCGGAAGCTCTGGCTA
>dme_circ_0000050_junction_seq
ACGCGAATTATTGCGGAAGCTCTGGCTAGATCAATCTAGCAGACACCAAACTGGCC
>dme_circ_0000051
GCTTCTTGAAATCATACGTCTTCTCCGGCGTTACAAACATGTTGAGGGCAATGGAGGCCAAGTTGCAGAC
TGCTATCTCATCCGGTGCGGAGTATTCTACGATTTCCGTACACAAGTTGCTGCACTTGATTGTGCCCACA
TTCTGCTGGTTGCTCTTCCTGTTGCAAGC
>dme_circ_0000051_junction_seq
TCTGCTGGTTGCTCTTCCTGTTGCAAGCGCTTCTTGAAATCATACGTCTTCTCCGG
>dme_circ_0000052
GTTTATTCAAATCAATTACTATGAAAAGCGATCAAAAGAGCAAAGCAATGTCTGGATCCTCACACAGCCA
GTTTGATTCAAAACTGCAAAAGGACTCGGATATCGAGAAATATGAAAATCTGCAGCAGCAGCTTGAAACG
GCTGCCGTTTTAATGGATATAAGCAAAAAAATA
>dme_circ_0000052_junction_seq
CGTTTTAATGGATATAAGCAAAAAAATAGTTTATTCAAATCAATTACTATGAAAAG
>dme_circ_0000053
GTATCATTAAACGCCGGCAAAGAATTTGACTTCTTACGCCTTGATAAAATATCTTTAAGTGCTGCTGCGG
CTGGGGTCTCGGTATCATCAATTTCCTTTTTGAATTTCTAAAATAAAAACATATTAGAAAATTGAAGTTT
AAGTGTATTTAAATCTTATGCAAATAGATCTAGTTGATCAATCTACTTATCAAGACTTTAAAGTAATAGT
GCAGATACATACATTTAAAACAAGGCCCGAAATGGCTAGAGATTCCCTAGCCGTCAAGAAATCTTGCTTC
GTTGTTTCTATTGAAGAACATGATGACATTTGTTTGTGTTCTGCAAAAGAAAGTGTATATTTATAATTTT
CGCAAATAAATCATACAATTAATAATGAAAGGCTCGTAGATATTTATTCAAACTTGAGCAGTTTAACGAC
TTAACAGGCACGTCCAATGCCATCTACAACAATGACATCATAAAATATGATTGGACAAGCAAGAAAATAT
TTTGGGTGGGTACGCGGTTAATATTCCGATTGCTAAATCAATAGCTTAATGAACATATTAACAGAAAAAT
AAATTGTTTTGGCAAGTTACATTTAATTACCAACAATAAAATGGTCAGGGAACATaaaaaaaaaaCATTA
AGTAAACAAATTGTTTTCATTGTTAGATACTCAAAAAATAAATAAATCTATGGGTCACAGAGGGCTAGCT
TTCGAATCGAAAAATGTAGATAAATTTCGAACTCTCACTCACTCTTTTTAGATCGCTCCAATTCTTTAGG
ACGCCTGCGTCTGGTGTGTATGG
>dme_circ_0000053_junction_seq
TTAGGACGCCTGCGTCTGGTGTGTATGGGTATCATTAAACGCCGGCAAAGAATTTG

>dme_circ_0000054
CTGTTCCAGGCCTTGGTGCAAACACAGCTGTCTGGCCTGGACTATCATTCGCTCGTTTCGAAGCTAATGG
CACCAGTTCTGGGTGGCAACGGTGACGTCAAATCCAGAGCCACTGCCGGCGCTCCCAGTGAGGTGGTGCA
GCTGCACAAGCAGGCCTATCACTCATCGGCCAAGTGCATTGCTGCCTTAACACAGCAGTGTCCCCAGGTG
GCGACCCCATTGGCCACAAAGCTGATAACGGATCTGCAGAAGCGTAACGATACGGAAATCATATTCTGTT
TGCTCACCATCGGTGAGATTGGCAGGCACTTTGATCTGAGCTCCATACAGGTGCTGCCGCAGACAATTAT
TGAATGCTTTGGGGCCACCTCGGAGGATGTAAAGGCTGCAGCCTCGCATGCTCTCGGCGCTGTGTCCGTG
GGCAGCCTGCAGACATACCTGCCTCTGATTCTGCACGAGATTGAGGTGCAGCCGAAGAGACAGTATTTGC
TGCTACATTCGCTTAAGGAGGTCATTTCATCGCTATCGGTATCGCCCAGCGGACTCGCCCAGCTCCTACC
CTCGGTGCCATCCATTTGGGATCAGCTCTTCAAGCATTGCGAGTGTTCGGAGGAGGGATCACGCAACGTG
GTGGCCGAGTGCCTGGGCAAACTGGTGCTGGTCAATCCCGACGAGCTGCTGCCGCAACTGCAGCAGGCAT
TGCGTTCCGAAAGCGCCACCATGCGCACCGTTGTTG
>dme_circ_0000054_junction_seq
GAAAGCGCCACCATGCGCACCGTTGTTGCTGTTCCAGGCCTTGGTGCAAACACAGC
>dme_circ_0000055
GATGATGAGTACGTCGATAGTGAGGAGTACAGTGACGACGACGACATGAGCTGGAAGGTTCGTCGCGCTG
CAGCCAAGTGTCTGGAGGTCTTGATATCCACACGGCAGGAGCTCGTCGAGGACTTCTATCGCAGCCTGAG
TCCGGCTTTGATTGCGCGCTTCAAGGAGCGTGAGGAGAATGTTAAGTCCGACATATTTCACGCCTATGTG
GCCCTGCTAAAGAACACACGCCTCACCGACGACGTGGCCAACGACCACGACTCAATGGATCAGGTATCGG
GTCCGACCTCGCTGTTAATTGAGCAGCTGCCACTCATTGTGAAAGCCATACAGCCG
>dme_circ_0000055_junction_seq
GCCACTCATTGTGAAAGCCATACAGCCGGATGATGAGTACGTCGATAGTGAGGAGT
>dme_circ_0000056
GAAACATTACCTTTTTACGATTAAATTTCACGCCATCAAGTGCAAGCTTTTATTTATTTTCGTTTGTTAT
TTGCAAAATAGTAGTTTTGTTTCATTTTGATCTTTTACAATCTGATGAGTGACTAAATTTAGAGGAAATC
TAAAGATTTACTTATTCCTTATGCGCATGGGATGTGATCGAAAA
>dme_circ_0000056_junction_seq
CCTTATGCGCATGGGATGTGATCGAAAAGAAACATTACCTTTTTACGATTAAATTT
>dme_circ_0000057
GTGCGCTGGGATAAACTTAAGGCTCTGGATCTTAGATTCAACCCGTGGAATTGCGACGAATCCAATGACT
TCTTGATCAACGTCTTGATAGATCGAATCAACAAGACCACGCCCGTTCTGGCCAAAGATGTCAAATGTGG
CGGTCCGAACAAGTTAAACGAT
>dme_circ_0000057_junction_seq
ATGTGGCGGTCCGAACAAGTTAAACGATGTGCGCTGGGATAAACTTAAGGCTCTGG
>dme_circ_0000058
GCTGGGCATCCTGAATATGGCCGTCGAGGTGATCATGACGCTGGAGCAGCAGGTGCGCGAGCGCAACCTG
AATCCGAAGGCGGCGTGCCTGAAGCGGCGCGAGGAGGAGAAGGCCGAGGATGGTCCCAAGCTAAGTGCCC
AGCATCACATGATACCGCAGCCGCAGCAG
>dme_circ_0000058_junction_seq
GCATCACATGATACCGCAGCCGCAGCAGGCTGGGCATCCTGAATATGGCCGTCGAG
>dme_circ_0000059
AACATCATTATTTGGGGCAACCACTCCTCCACCCAGTATCCCGATGCTGGACAGGCCAAGGTGACCGCCA
ATGGTACCGTTAAGTCCGTGGTGGATGCGATCAACGATAATGGCTACCTGCAGGGATCCTTCGTGGAAAC
CGTTCAGAAGCGTGGAGCCGCTGTCATTGCGGCCCGCAAAATGTCATCGGCCATGTCGGCGGCGAAGGCC

GCTTGCGATCACATGCACGACTGGTGGAACGGCACTGCTCCCGGCCAGTTCGTCTCCATGGGTGTCTTCT
CCGACGGCAGCTACGATTCGCCCAAGGATGTGATCTTCTCGTTCCCCGTCGAGATCAAGAATAAGCAATG
GAAAATCGTCTCTGGCCTGACCCTCAGCGATTTCGCTAAGACAAAGTTAAGCGTTACCGGCAAGGAGCTG
CAGGAGGAGAAGGACGAGGCTCTGTCCGTGCTGGACTCGAATGTGTCCAACTTGTAATGCGATCCTTAAA
CGTTAATTACTAACATCATAGTTGTGCATTTAGGAACAAAGTTCATGGCAATAAAAG
>dme_circ_0000059_junction_seq
TTAGGAACAAAGTTCATGGCAATAAAAGAACATCATTATTTGGGGCAACCACTCCT
>dme_circ_0000060
GATGTCTCGGCCGCTTTCCTCGTGGGCGCCATGCCCCGCAAGGAGGGAATGGAGCGAAAGGATCTGCTGT
CCGCCAACGTGAAGATCTTCAGGACCCAGGGCCAGGCTCTGGACAAGTTCGCCAAGAAGGACGTCAAGGT
GCTGGTTGTGGGCAACCCGGCCAATACCAATGCCCTGG
>dme_circ_0000060_junction_seq
GGCAACCCGGCCAATACCAATGCCCTGGGATGTCTCGGCCGCTTTCCTCGTGGGCG
>dme_circ_0000061
GTGGGAAAAGTAAACGGGAAAAGCTGCGCAACGAGAAGAGGCACGAGAAGAACAAGGCGAAGAATAAGAA
CGGCAGCAATACAAACGCCAATGCCTCGAACAAGGAGAACTATGTTCCCGAAACGAGAAGCAGCACAAGT
ACTGAGACATTCGCAGAGGCTACGGCGGATGCACCGGCTTCCACCAAGGCAGAACCGCCGCAAGCCTCTA
GCAATAGAACGAGGGCGGATC
>dme_circ_0000061_junction_seq
GCCTCTAGCAATAGAACGAGGGCGGATCGTGGGAAAAGTAAACGGGAAAAGCTGCG
>dme_circ_0000062
GATAACTTACCACAAAAGAGATTAGCGGGAACAAACGTTCAAAGATCTGTGAGCTCTTGTAGCGAAAATA
GCGAAGGACACGTCTCTGAGAGTAGCTTAAGTGAGAAGAGTTTAACTGGTGATTATGTGGAGGAAAAGTG
CAATAGTGTGAATTCGGAAAGCCAGCAAGAAAGTGTAAA
>dme_circ_0000062_junction_seq
ATTCGGAAAGCCAGCAAGAAAGTGTAAAGATAACTTACCACAAAAGAGATTAGCGG
>dme_circ_0000063
GTATATGGAACGCCATCGCAAGCTGTATGGCCGGCGATTGGATTACGAGGAACGGAAACGCAAGAAGGAA
GCGCGTCTTCCCAAGGACCGAGCACGAAAGGCTCGCAAGTTGCGCGGCATCAAGGCCAAACTCTTTAATA
AGGAGCGACGCAATGAAAAGATTCAGATTAAGAAGAAGATCCAGGCCCACGAAGAGAAGAAGGTCAAAAA
GCAGGAGGAGAAGGTCGAGGATGGAGCCCTGCCGCATTATCTGCTCGACAGAGGCATCCAGTCCAGCGCC
AAGGTCCT
>dme_circ_0000063_junction_seq
GAGGCATCCAGTCCAGCGCCAAGGTCCTGTATATGGAACGCCATCGCAAGCTGTAT
>dme_circ_0000064
ATTGAAGGTGGCTTTCAGTTCTGGATGCGTAACGTGAGCCTTCTTCATGCGCAGACCCATGGGTCGAATG
AAACGCTCGAACTTTGGTGGCTTGCGTGTGAAGTTCTCACCGACGAATGTGACTTTTGTGACCATGCGCT
TCCATGCCTTTCGCTTTGTCTTTCCGGTCTTTAGTACTTTGAAGACCTCAGCATCTGACTGAGCGCGTAC
TTTGGGAATGGGCACG
>dme_circ_0000064_junction_seq
CTGAGCGCGTACTTTGGGAATGGGCACGATTGAAGGTGGCTTTCAGTTCTGGATGC
>dme_circ_0000065
GTTTGGTGACTGGAATACAAGATGCAGATCTTCGTTAAGACCCTCACGGGCAAGACCATCACTCTTGAGG
TCGAACCGTCGGATACTATCGAAAATGTCAAAGCCAAGATTCAGGACAAAGAAGGAATTCCTCCAGATCA

GCAGCGTCTGATCTTCGCTGGCAAGCAGCTGGAAGATGGCCGCACCCTCTCTGACTATAACATTCAGAAG
GAGTCCACTTTGCACTTGGTGCTCCGCCTGCGTGGTGGTGCCAAGAAGCGCAAGAAGAAGAACTACTCCA
CTCCCAAGAAAATCAAGCACAAGAGGAAGAAGGTCAAGCTAGCTGTCCTGAAATACTACAAGGGTACACA
CAACAATCTACCGACACCATACCGAAAACATTCTGATCTGTTCTTGGAATTCTTGAGTCTTGGTCTTGTG
CTCATTGGCTTAAGATTTGGATATCAGAGTGACTTTACCAGTGCCCTATATTGAATGAACATGTGTACGA
TCTCAGTATTTAATCACTTTCTATGCTCTTTCCTTTCCATAGGGTTGACGAGAACGGCAAGATCCACCGT
CTCCGTCGCGAGTGCCCTGGTGAGAACTGCGGCGCCGGCGTCTTCATGGCTGCCCACGAAGATCGTCACT
ACTGCGGCAAGTGCAACCTGACCTTTGTCTTCAGCAAACCAGAGGAAAAGTAATTTTGCTACATAAGATC
AT
>dme_circ_0000065_junction_seq
GAAAAGTAATTTTGCTACATAAGATCATGTTTGGTGACTGGAATACAAGATGCAGA
>dme_circ_0000066
GTTTGGTGACTGGAATACAAGATGCAGATCTTCGTTAAGACCCTCACGGGCAAGACCATCACTCTTGAGG
TCGAACCGTCGGATACTATCGAAAATGTCAAAGCCAAGATTCAGGACAAAGAAGGAATTCCTCCAGATCA
GCAGCGTCTGATCTTCGCTGGCAAGCAGCTGGAAGATGGCCGCACCCTCTCTGACTATAACATTCAGAAG
GAGTCCACTTTGCACTTGGTGCTCCGCCTGCGTGGTGGTGCCAAGAAGCGCAAGAAGAAGAACTACTCCA
CTCCCAAGAAAATCAAGCACAAGAGGAAGAAGGTCAAGCTAGCTGTCCTGAAATACTACAAGGGTACACA
CAACAATCTACCGACACCATACCGAAAACATTCTGATCTGTTCTTGGAATTCTTGAGTCTTGGTCTTGTG
CTCATTGGCTTAAGATTTGGATATCAGAGTGACTTTACCAGTGCCCTATATTGAATGAACATGTGTACGA
TCTCAGTATTTAATCACTTTCTATGCTCTTTCCTTTCCATAGGGTTGACGAGAACGGCAAGATCCACCGT
CTCCGTCGCGAGTGCCCTGGTGAGAACTGCGGCGCCGGCGTCTTCATGGCTGCCCACGAAGATCGTCACT
ACTGCGGCAAGTGCAACCTGACCTTTGTCTTCAGCAAACCAGAGGAAAAGTAATTTTGCTACATAAGATC
ATGTACGTTTCCAGAAATCAAATAAAG
>dme_circ_0000066_junction_seq
CATGTACGTTTCCAGAAATCAAATAAAGGTTTGGTGACTGGAATACAAGATGCAGA
>dme_circ_0000067
GATGCAGATCTTCGTTAAGACCCTCACGGGCAAGACCATCACTCTTGAGGTCGAACCGTCGGATACTATC
GAAAATGTCAAAGCCAAGATTCAGGACAAAGAAGGAATTCCTCCAGATCAGCAGCGTCTGATCTTCGCTG
GCAAGCAGCTGGAAGATGGCCGCACCCTCTCTGACTATAACATTCAGAAGGAGTCCACTTTGCACTTGGT
GCTCCGCCTGCGTGGTGGTGCCAAGAAGCGCAAGAAGAAGAACTACTCCACTCCCAAGAAAATCAAGCAC
AAGAGGAAGAAGGTCAAGCTAGCTGTCCTGAAATACTACAAGGGTACACACAACAATCTACCGACACCAT
ACCGAAAACATTCTGATCTGTTCTTGGAATTCTTGAGTCTTGGTCTTGTGCTCATTGGCTTAAGATTTGG
ATATCAGAGTGACTTTACCAGTGCCCTATATTGAATGAACATGTGTACGATCTCAGTATTTAATCACTTT
CTATGCTCTTTCCTTTCCATAGGTTGACGAGAACGGCAAGATCCACCGTCTCCGTCGCGAGTGCCCTGGT
GAGAACTGCGGCGCCGGCGTCTTCATGGCTGCCCACGAAGATCGTCACTACTGCGGCAAGTGCAACCTGA
CCTTT
>dme_circ_0000067_junction_seq
CTACTGCGGCAAGTGCAACCTGACCTTTGATGCAGATCTTCGTTAAGACCCTCACG
>dme_circ_0000068
TAGTTCTTCTTCTTGCGCTTCTTGGCACCACCACGCAGGCGGAGCACCAAGTGCAAAGTGGACTCCTTCT
GAATGTTATAGTCAGAGAGGGTGCGGCCATCTTCCAGCTGCTTGCCAGCGAAGATCAGACGCTGCTGATC
TGGAGGAATTCCTTCTTTGTCCTGAATCTTGGCTTTGACATTTTCGATAGTATCCGACGGTTCGACCTCA
AGAGTGATGGTCTTGCCCGTGAGGG
>dme_circ_0000068_junction_seq

TCAAGAGTGATGGTCTTGCCCGTGAGGGTAGTTCTTCTTCTTGCGCTTCTTGGCAC
>dme_circ_0000069
TGGAGTAGTTCTTCTTCTTGCGCTTCTTGGCACCACCACGCAGGCGGAGCACCAAGTGCAAAGTGGACTC
CTTCTGAATGTTATAGTCAGAGAGGGTGCGGCCATCTTCCAGCTGCTTGCCAGCGAAGATCAGACGCTGC
TGATCTGGAGGAATTCCTTCTTTGTCCTGAATCTTGGCTTTGACATTTTCGATAGTATCCGACGGTTCGA
CCTCAAGAGTGATGGTCTTGCCCG
>dme_circ_0000069_junction_seq
TCGACCTCAAGAGTGATGGTCTTGCCCGTGGAGTAGTTCTTCTTCTTGCGCTTCTT
>dme_circ_0000070
TAGTGACGATCTTCGTGGGCAGCCATGAAGACGCCGGCGCCGCAGTTCTCACCAGGGCACTCGCGACGGA
GACGGTGGATCTTGCCGTTCTCGTCAACCTATGGAAAGGAAAGAGCATAGAAAGTGATTAAATACTGAGA
TCGTACACATGTTCATTCAATATAGGGCACTGGTAAAGTCACTCTGATATCCAAATCTTAAGCCAATGAG
CACAAGACCAAGACTCAAGAATTCCAAGAACAGATCAGAATGTTTTCGGTATGGTGTCGGTAGATTGTTG
TGTGTACCTTGTAGTATTTCAGGACAGCTAGCTTGACCTTCTTCCTCTTGTGCTTGATTTTCTTGGGAGT
GGAGTAGTTCTTCTTCTTGCGCTTCTTGGCACCACCACGCAGGCGGAGCACCAAGTGCAAAGTGGACTCC
TTCTGAATGTTATAGTCAGAGAGGGTGCGGCCATCTTCCAGCTGCTTGCCAGCGAAGATCAGACGCTGCT
GATCTGGAGGAATTCCTTCTTTGTCCTGAATCTTGGCTTTGACATTTTCGATAGTATCCGACGGTTCGAC
CTCAAGAG
>dme_circ_0000070_junction_seq
ATAGTATCCGACGGTTCGACCTCAAGAGTAGTGACGATCTTCGTGGGCAGCCATGA
>dme_circ_0000071
TAGTTCTTCTTCTTGCGCTTCTTGGCACCACCACGCAGGCGGAGCACCAAGTGCAAAGTGGACTCCTTCT
GAATGTTATAGTCAGAGAGGGTGCGGCCATCTTCCAGCTGCTTGCCAGCGAAGATCAGACGCTGCTGATC
TGGAGGAATTCCTTCTTTGTCCTGAATCTTGGCTTTGACATTTTCGATAGTATCCGACGG
>dme_circ_0000071_junction_seq
CTTTGACATTTTCGATAGTATCCGACGGTAGTTCTTCTTCTTGCGCTTCTTGGCAC
>dme_circ_0000072
TGGAGTAGTTCTTCTTCTTGCGCTTCTTGGCACCACCACGCAGGCGGAGCACCAAGTGCAAAGTGGACTC
CTTCTGAATGTTATAGTCAGAGAGGGTGCGGCCATCTTCCAGCTGCTTGCCAGCGAAGATCAGACGCTGC
TGATCTGGAGGAATTCCTTCTTTGTCCTGAATCTTGGCTTTGACATTTTCGATAG
>dme_circ_0000072_junction_seq
TGAATCTTGGCTTTGACATTTTCGATAGTGGAGTAGTTCTTCTTCTTGCGCTTCTT
>dme_circ_0000073
GATTCAGGACAAAGAAGGAATTCCTCCAGATCAGCAGCGTCTGATCTTCGCTGGCAAGCAGCTGGAAGAT
GGCCGCACCCTCTCTGACTATAACATTCAGAAGGAGTCCACTTTGCACTTGGTGCTCCGCCTGCGTGGTG
GTGCCAAGAAGCGCAAGAAGAAGAACTACTCCACTCCCAAGAAAATCAAGCACAAGAGGAAGAAGGTCAA
GCTAGCTGTCCTGAAATACTACAAG
>dme_circ_0000073_junction_seq
CAAGCTAGCTGTCCTGAAATACTACAAGGATTCAGGACAAAGAAGGAATTCCTCCA
>dme_circ_0000074
GGAATTCCTCCAGATCAGCAGCGTCTGATCTTCGCTGGCAAGCAGCTGGAAGATGGCCGCACCCTCTCTG
ACTATAACATTCAGAAGGAGTCCACTTTGCACTTGGTGCTCCGCCTGCGTGGTGGTGCCAAGAAGCGCAA
GAAGAAGAACTACTCCACTCCCAAGAAAATCAAGCACAAGAGGAAGAAG
>dme_circ_0000074_junction_seq

CAAGAAAATCAAGCACAAGAGGAAGAAGGGAATTCCTCCAGATCAGCAGCGTCTGA
>dme_circ_0000075
GCTAGCTGTCCTGAAATACTACAAGGTACACACAACAATCTACCGACACCATACCGAAAACATTCTGATC
TGTTCTTGGAATTCTTGAGTCTTGGTCTTGTGCTCATTGGCTTAAGATTTGGATATCAGAGTGACTTTAC
CAGTGCCCTATATTGAATGAACATGTGTACGATCTCAGTATTTAATCACTTTCTATGCTCTTTCCTTTCC
ATAGGGTTGACGAGAACGGCAAGATCCACCGTCTCCGTCGCGAGTGCCCTGGTGAGAACTGCGGCGCCGG
CGTCTTCATGGCTGCCCACGAAGATCGTCACTACTGCGGCAAGTGCAACCTGACCTTTGTCTTCAGCAAA
CCAGAGGAAAAGTAATTTTGCTACATAAGATCATGTACGTTTCCAGAAATCAAATAAAG
>dme_circ_0000075_junction_seq
CATGTACGTTTCCAGAAATCAAATAAAGGCTAGCTGTCCTGAAATACTACAAGGTA
>dme_circ_0000076
TACAAATCGAAATCTATGCGGTTGCTTGTATAGAAATTTGTGTTTTCGCCGCACTTTCGCACATAGACTC
CGTTTTTAAAGTAGTTCTCCTGGGACCATTTCAACATTTTCTCCGTGGAAAAGGGTCCCTGGATATCAGT
CTCATCCTGCGACCATTTAAACTCCCAGG
>dme_circ_0000076_junction_seq
TCATCCTGCGACCATTTAAACTCCCAGGTACAAATCGAAATCTATGCGGTTGCTTG
>dme_circ_0000077
GTTTAAATGGTCGCAGGATGAGACTGATATCCAGGGACCCTTTTCCACGGAGAAAATGTTGAAATGGTCC
CAGGAGAACTACTTTAAAAACGGAGTCTATGTGCGAAAGTGCGGCGAAAACACAAATTTCTATACAAGCA
ACCGCATAGATTTCGATTTGTACTTGTAGGCCTTAGTTAACAAATGTTAAATTCTTTGGGTTTTAACATG
TTTTCTGCAAATACTTTGTTCTATAATTGTATACTGTATACTTTACAAGAGGTTTCAATTA
>dme_circ_0000077_junction_seq
CTGTATACTTTACAAGAGGTTTCAATTAGTTTAAATGGTCGCAGGATGAGACTGAT
>dme_circ_0000078
TTATGGCATCGTGATGCTGCATTATTCCGAGCGTTTCCCTCATGAAGGTCAAGTGGGGATCGAATTCTTC
GGGTTTCTTGGGCGCCAGGGCACTCAACTGCTTGCAGACCTGCAGGAAATGATTGCCATCCCGTTCGAAT
CGCTTCAAGGTGGGTCGCGATGTGAAATATCCCGTCCAGTAAGCATGAGGATCGGAAGCGTATGGGAAGA
AGTCGTCGCTCTTGGTTGGCCAGGTGATACCGGCATCGTGCAGGGATTTCAGATAGCACGATGGAGTGGA
G
>dme_circ_0000078_junction_seq
GGATTTCAGATAGCACGATGGAGTGGAGTTATGGCATCGTGATGCTGCATTATTCC
>dme_circ_0000079
GAATCCTCACATACTCATTAACCGTGTGAGCCAGTGGATTGTAGAGGGTTAGAGCAAATCTATCGTTCGC
CTCGGACACGGGACACGAGGTGATATTTAGTAGGGCACACGTTTTGAACTCAAACACATATTTGGCCGAC
GTATCCTTGACATTATCCTTCGATTGCACCGTCAATTGATTCAAGGCATTACGAGTGG
>dme_circ_0000079_junction_seq
GTCAATTGATTCAAGGCATTACGAGTGGGAATCCTCACATACTCATTAACCGTGTG
>dme_circ_0000080
GGTGTTACTCTGGAAAGCCAAGCTGTTCCGATTCCCCAAGTGCTGATCGACATTAAGCACAGAAATTCCA
CAGCCAAATACGAAATTGTTTTCCTAGCCACCAATATTCCAGCTTTGGGATACCGTACATACTATGTTGA
AAAACTGGATAGTACCGAGG
>dme_circ_0000080_junction_seq
TATGTTGAAAAACTGGATAGTACCGAGGGGTGTTACTCTGGAAAGCCAAGCTGTTC
>dme_circ_0000081

GTAAATATTATCGTATAACGCATTAACAAAAGCGTACGCATGCTATCCAATCTTAAAGGCACATCTTTTA
TGTATGATATATAGAATTTTGTTTGATTTTGGTCTGTTTGGTTGCTAACATATGCACTATTGTATACTCA
TGCCGAGTCTAAGTGGTCCCAATCGTTGCGGGCCCAATTCG
>dme_circ_0000081_junction_seq
TGGTCCCAATCGTTGCGGGCCCAATTCGGTAAATATTATCGTATAACGCATTAACA
>dme_circ_0000082
GGTCATTGGGCTTGATGCAGCGCACATAGAAGGGTTCCTTCTTCAGCAGAGTAACCACCAGATCAGCCAT
CGATCTCTGGAACAAGGTGCCGGCTGTCAATGGTCTCTTGGTGGTTTTCTTGATATCCTGTGCTCCTTCG
GGCCACATCTCGCTCAGGTTGGCATCCTTAGAGTTGTGCAGCAAGCGCTTAAA
>dme_circ_0000082_junction_seq
CCTTAGAGTTGTGCAGCAAGCGCTTAAAGGTCATTGGGCTTGATGCAGCGCACATA
>dme_circ_0000083
GATGCAAGACAGCGGCAATTGTGCGCCAAATGGTCTGCACCTCATCCGTGGAGAAGCCCAGGGTCTTGAA
GGCATTGCAGGTGCCCTTGTAGTCGGACTTCTCCGTCAGTATGTCCATGCTGCCCTGGTTCAAGTAGTGA
TACTTGCCCGTTTCCTTCTGCAGTTCATATTGCCGCAGCTCGTTGTCGTTGGCTCCACGCAGCAGCTATA
AATGGATGAATTGGTTGGGGGTATTAATTAAGCGATTGCAACTATTTTAAATTAACCCAACTTGGTCAGT
GCAACATGACCGTCAGTCCATGTGCCAAGAATTTAGCACGCCAGACAATTAGCAGACGTGCTAAGCGTAA
TTAAACTTCCGTTTCCTGTCGAGCATTTGGCAGTTGCCTTTTAAACTTTAACAACACATGTTATTAAATT
GTATCTTTTAACATAAAGttaactacatttctatttttcccattaactacatttctatttttcccattaa
TTCATCACAGATAGGTAAATTGATATCATATTAGTTGAGTAAGATATTTTCAAGATCTTTGATGTTGGGA
AACCCGTAAACTTGGTAATCAATAAAAATCTACATACATGCCTATCGGCGTTTTTGCCGGTGTGAGTGTT
AATTGAACATTGTTTATTTCAAGATAGCCTGGCTAGTCATAAATTATATATTAGCCAAGTTGGAAATTTA
TGACCGATGCAGACAAATTGTAGACCCATTCCCAAAGCAAGCATTCCATAATACGGGCAAATATTTATTT
TGACTCACTTTAAAAATAAACAACTTGCACTTTCCAACAAATAAACAAATCGTTTAAACTCGAACAAAAG
CCAGATGAAGTATTTGACTTGATTAAGTGTTTCCAGATGATAAATGTGCTAAAAGTGTTTCGTGTTGCTC
AAATAGATATGCATGCATCGGAAATAGTGATCAATTATCACCTCAGCAGGTTTAATTTACCAGATGAATG
GATCATATAATATATAAGTTAACGCCTTGAATGATGAATAAGCGATTGAGTTGTGACGTGATATTTTTTT
CTAGTTTATTGGATGAGTCTTGGCTACGCCTATGTTATCATTTTGCCAGTGATATCATCCAATTCTGGAT
ACCTAAAAAGAAGTGAGCAATGTAGGCCTGCAGCGGGAAGCAAATTTAATTAAATTAATTAACAGTTTTA
GACtttttttttATAACTGGCAGAGGAATCTCCAATAATGATTATACATAAATTACTTAGGCATGTGTCA
TTTGCTTATATATACATTTATTTAAATATATCTATAAAATAAAGGAGATCATGACCAGCTTTCATCTGTT
GCCAAACAACTTAGTCATACGACTTGAAAGTAATAAAGTTATATTAATACTAGGCGGCCGCACACAACGT
ATGTGTATTCCCCTCATAAAACTGTCTCAACACTCAATTTAAATGCCCACAATTGTTTACAACGAAAGCC
CGCACCCACAAAAAGAGCCAGCGACAATAACTGTCTAACCAGCCGAAAAACCAGAAGAAAAAAAGAGCAA
CTTCATCACGGCCACAACAACCTGACACCCCCAGGTAATGGACGCCCCACTGCCATGGCACCCAACCCAT
GAGCTGGAACACCTTTCGTCACCTGGCACGCGTCACGCTGACTCACTTGGTAAATAATCGTGTACACTTA
ATGCCACACACGACTAGTTCGGAACGATTCGTATCCGTATGCCACTCACCTGATAGAAACTATGGAAGTT
CCGCTCGCCGGGCTGCTGCTGCACCACGCGCGACTTCTCGAGCAGATAGTTGGTGATGATGCCACCCACG
GGATCCGCCTTGTAGTCGAACTCAATGTCCATGTACTTTCCGAAGCGGCTGGAGTTGTCGTTGCGGTTAG
TCTTTGCGTTGCCGAAGGTTTCCAGAATTGCATTGCTCTGGATCAAAACGTTCTTGACTCTGGAAAAATA
ATATATTTATTATTATATATGTACTTAAGAACTTCTTCCTATAAAATCATTCTCTTATAAAATTTTTAAT
TAATTTCCATGCCAGCAAGACTATAAAGACAACCCTCATTAAAATCAAATCGACATAATTAACAAACGAA
ATCAACACAATAAATACGCTATTAGTGCTTCGTAAATTTTTCTCCCCTCGGTGGGCAAGCAATTTGGAAA
ATGGCACTCACCCTAATCCCCGAAACTATGTTAAATGTCGTCTCCCATTTCATTCAAGGCACTAAAAATT

GGCAATTTAAAAGACCATTGTGTTCCGGGGCTTGGCCATGGCGGCTTTAGCGCTTTTATGGAGCACCTTA
AACTACACCCAAGTGATAAAAAGTTCCTTTGGACAAAGTGGTTTGCAATTAGTGGGGCAATTAATTTGGC
AACTTAAACTGCAAGTCAACAGACGACTGATGTCATCCGGGGTCATGGGTCACGCGATGACCATGCCAGC
GATTATGACCCATCTGGGAAATGCTTCATTAGCCACTGCCACCTGGCAACCGCGTCTGAAAATCTTCCCT
CAATGAGAATTCCATTGCGCATTTGCATGCGAAACAATGGTGGATTTTCTCTGGGACCTATGATGTACAT
ACATGTTGTTATATAGATCGGTTTGAGGTTCCCATTGTCTGGTTCACAGTATTGTGATAAGGCCGCAAGC
GTTCCATCAACCTATGTAAATATGTGGCCAAATTGATCAACTAATTTACTATGTAATCAGAGCAAACGGC
TTGGGAACCTTTGAGCTAGATAAACATATTTGCATTACTTTGTATTTGGTGCTATTAACTTGATTTCCCC
AAGACAATTGCTAATTAAACTTGTCCTTCTTGAAGATTTAATCAAGAATTTTCCTATCTAAACTAACATT
TTGTGTCTAATCATTAAACAATATATAAGCGTATTTATTGGGTATATGGTTTCATCCATCATTCAGCTCA
TCCATCCACTTACCTCTCGATTTCGTTCTGACCCTGGGCATTCGTCACCGCCGCAATGTACTTCATGATG
ATCTTGGAGGCCTCCGTTTTGCCCGCACCGGACTCACCGGAAATTAGGATGCAGGTGTCTTGTTGGCGCT
GCTTGAGCACCCTGTAGGCGGAATCGGCAATGGCGAACAAATGTGGGGCATTCTCGAAGAGCTCCCGACC
CTTGTATTTGCGAATGGTTTCCGGACCGTAGATGTTCATCTGTCTGTAGGGATTCATCGAGACGCACACT
TCGCCGAT
>dme_circ_0000083_junction_seq
GATTCATCGAGACGCACACTTCGCCGATGATGCAAGACAGCGGCAATTGTGCGCCA
>dme_circ_0000084
ATTCCAAAACGGATCCATCTACACATACATCGGCGAAGTGTGCGTCTCGATGAATCCCTACAGACAGATG
AACATCTACGGTCCGGAAACCATTCGCAAATACAAGGGTCGGGAGCTCTTCGAGAATGCCCCACATTTGT
TCGCCATTGCCGATTCCGCCTACAGGGTGCTCAAGCAGCGCCAACAAGACACCTGCATCCTAATTTCCGG
TGAGTCCGGTGCGGGCAAAACGGAGGCCTCCAAGATCATCATGAAGTACATTGCGGCGGTGACGAATGCC
CAGGGTCAGAACGAAATCGAGAGGGTAAGTGGATGGATGAGCTGAATGATGGATGAAACCATATACCCAA
TAAATACGCTTATATATTGTTTAATGATTAGACACAAAATGTTAGTTTAGATAGGAAAATTCTTGATTAA
ATCTTCAAGAAGGACAAGTTTAATTAGCAATTGTCTTGGGGAAATCAAGTTAATAGCACCAAATACAAAG
TAATGCAAATATGTTTATCTAGCTCAAAGGTTCCCAAGCCGTTTGCTCTGATTACATAGTAAATTAGTTG
ATCAATTTGGCCACATATTTACATAGGTTGATGGAACGCTTGCGGCCTTATCACAATACTGTGAACCAGA
CAATGGGAACCTCAAACCGATCTATATAACAACATGTATGTACATCATAGGTCCCAGAGAAAATCCACCA
TTGTTTCGCATGCAAATGCGCAATGGAATTCTCATTGAGGGAAGATTTTCAGACGCGGTTGCCAGGTGGC
AGTGGCTAATGAAGCATTTCCCAGATGGGTCATAATCGCTGGCATGGTCATCGCGTGACCCATGACCCCG
GATGACATCAGTCGTCTGTTGACTTGCAGTTTAAGTTGCCAAATTAATTGCCCCACTAATTGCAAACCAC
TTTGTCCAAAGGAACTTTTTATCACTTGGGTGTAGTTTAAGGTGCTCCATAAAAGCGCTAAAGCCGCCAT
GGCCAAGCCCCGGAACACAATGGTCTTTTAAATTGCCAATTTTTAGTGCCTTGAATGAAATGGGAGACGA
CATTTAACATAGTTTCGGGGATTAGGGTGAGTGCCATTTTCCAAATTGCTTGCCCACCGAGGGGAGAAAA
ATTTACGAAGCACTAATAGCGTATTTATTGTGTTGATTTCGTTTGTTAATTATGTCGATTTGATTTTAAT
GAGGGTTGTCTTTATAGTCTTGCTGGCATGGAAATTAATTAAAAATTTTATAAGAGAATGATTTTATAGG
AAGAAGTTCTTAAGTACATATATAATAATAAATATATTATTTTTCCAGAAGTCAAGAACGTTTTGATCCA
GAGCAATGCAATTCTGGAAACCTTCGGCAACGCAAAGACTAACCGCAACGACAACTCCAGCCGCTTCGGA
AAGTACATGGACATTGAGTTCGACTACAAGGCGGATCCCGTGGGTGGCATCATCACCAACTATCTGCTCG
AGAAGTCGCGCGTGGTGCAGCAGCAGCCCGGCGAGCGGAACTTCCATAGTTTCTATCAGG
>dme_circ_0000084_junction_seq
GAGCGGAACTTCCATAGTTTCTATCAGGATTCCAAAACGGATCCATCTACACATAC
>dme_circ_0000085
TTCTGGAAGCACGACATAAAGCTGGCGCGTAATGGCAAAGTGGAAACGGAGTCGGAGATACTCAACGAGC

TAATAGCCAATCTGGCCACCGAAGACAAGCACGCACTGGGCTCCATCAATCGTCAGTTCCTGCGCCATCT
GGACGAATCAAACTACCAGCACACTTGGTCGCCGCTCAGCAACCGACATAGTTTCTCAATGCCACAACCG
CCTGGCGCCGGCATCTCCACCATCCATCGCCTGGAACAATTGTATGCGTCGAGAATAGGGTTTAATCCAC
CAGCAGTAGAACGACGCCAACGCCACAGCGTAGTAAGCAGACCATCGCAACGGAGCCACGAGGTCTCCGT
TCAGCTGAGTCCTCCACAGCCAGCGTCCACGATTCGCGAGCATGCCATCGCAAGTGGGAAAGGACGAGCC
AGCTAGGGATGCGGCCATCCAAGTCGCGAGCTCCAAGAGATTTACGGTAACACCAGCTCGAGTGGACGAG
CAGACCTAGAATGTTGTCAAG
>dme_circ_0000085_junction_seq
GGACGAGCAGACCTAGAATGTTGTCAAGTTCTGGAAGCACGACATAAAGCTGGCGC
>dme_circ_0000086
TTCTACGGCGCCACCGAGGGTAATGCGAACATCATGAATCATGACAACACGGTGGGCGCCATCGGCTTTG
TGTCGCGCATCCTGCCCAAGATCTACCCAATCTCGATCATTCGCGCCGATCCGGACACCGGAGAGCCCAT
TAGAGATAGGAATGGCCTATGCCAACTGTGCGCTCCCAACGAGCCAGGCGTATTCATCGGCAAGATCGTC
AAAGGAAATCCTTCTCGCGAATTCCTCGGATACGTCGATGAAAAGGCCTCCGCGAAGAAGATTG
>dme_circ_0000086_junction_seq
GATGAAAAGGCCTCCGCGAAGAAGATTGTTCTACGGCGCCACCGAGGGTAATGCGA
>dme_circ_0000087
GATAACCGCAGCCTTTGGCAATCCTGTGGTGCCGGAGGTGTAGATGTAGACCAGCTTGTCGTGGTGGTTA
ACCTGCGTCTTGTTAGGCTTCTCATAGCTGGCCGTGGTCAGCAGCGCGTTCAGATTCTTGGCCTGCGGTA
TGTTCTTTTCCGTCTCGCTGTTGTTGTTCTCGTTGTTGAACTGGAAGAGTGTGAG
>dme_circ_0000087_junction_seq
TCTCGTTGTTGAACTGGAAGAGTGTGAGGATAACCGCAGCCTTTGGCAATCCTGTG
>dme_circ_0000088
AAATCTTTGACTAATAGCTAAAAATGTACACCATCTGATTCAATCAGGAAAAGATTATATTGAAGAGCAA
CTTCAGTTCAGCAGATGCACTGCACCTAGAGTCACAGAGGACCACATCCGCGTAGATCTGATGCTCACGA
CTGTATGTGAGG
>dme_circ_0000088_junction_seq
GATCTGATGCTCACGACTGTATGTGAGGAAATCTTTGACTAATAGCTAAAAATGTA
>dme_circ_0000089
GGTAATTTTCCAACGATTTCATCCATTCAATACCCAAATTAAGTGCGTAAGTTGAAATGTCTTAAGTGGC
ACAACCCAACCAGTCACTTACCTATAAATCAATAAAACAATGACAAGAGACCCTAAGAAGAGTAGTCTCC
TTCGTTGGTCTTCCTAGTTCTTAGGTGGCTGAAAGTGCTCTGCCATATATCACTTGACTATTCGGCTGAT
TGCCATCGCTTAAACGGCCAGCGGAGCATTTTATTTAACTGCCTTCCCGGTTAACGCTGCGTATACGTAC
TGTGTACTCCCTTCATATTCGTGGCATACATACGCACAGGCCTACTTACTTATGGTAGTTGTGATATGTG
GAATTGATGTTGAGTGCGGTCGCCAAAGGAATTGGAGGCAGCGTTTCCATTTAATAGGCGCACATGAGAC
TCCCGACACATAAAAACCAATTTCGCGTAAGTGATAAAAATGCAATGAGCACTCGCCTTCAAGTCCCACA
Cgattgggattgtgattgcgattgggatgatcatcgtcatgatgatgattatgatgatgatgatgacaat
catCAGCTGGTGACGCAGACGCCTATGAATTAAATTTACGATGGCACACAAGTAGTTTTGACACATATAT
CTTAAAGAACATCGTGGCGCCATAAAAATCTGTGGCCAGCCCTAAATGGAAGTATGAGATGGGTTGGGGA
TGGGCTGGTGTGGGGGGCCTCTGATGTGGGGATACAACACAGCCTGTTTGCCTTTTAATTGTTTAATTCG
TATGCCGACATCAGCGAGCGGATGGCACAAGATTACAACTAGAGATGAGCTAGTATTCTACATATACAAT
GCTATATGTACTTCTTGTAAATGTACTTTGGTGATGAGAAAATGTCAGGAACTTTGCATATGAAATGGAA
TAATCTAAAATAATATAAGATCATTTTCTCACAAAAATGTGTCGAAATATATAGCTTAGATATAAATGTA
TCTATTATCCGCAAATGTCGTGAGCATCACGCTGTCATGTCCAATAATTCCTTGCCTTTCCAACCGATGC

AGCTTTCAATCATCATCAATTTCACATAAATGTGTCGCCCACACGACGCAACGTGCAGACGTCTTTGCCC
CCCGAGATCCGCCTTGGAACCCGGGGACTATGGTGTCTGCCTTTGGCGACATAATGACGAGATCATATCG
ACAACAGGGAGTCGTCGGAGTCCCGGAGTCGTGGCATTTTATTTTCTTCTGCTGCTGCCGCTGGCTTGGC
TGGCTTTTGTGTAATCGTGAGAGCCTTGTCATTGTCATTGCGATGTCGTCTGTCTGCTGTGCACTTTTCA
AGTTCAATTTCAATCCCGGCTCTGCCCTCCAACCACTTGCAAATCAATCTGGTGGACACAGCGATGgcca
gccagtcagtcagtcagtcagtccgtcagttattcagtcagctagtcattcagtcagctagacattGGCA
AATGACCGGCAGCTCGTTTTATTGTGCGGGCCCTGTCATATTTCCTGTCTGCAAAGTGTTGGGCGAAAGT
TTTATGACCCTTTGATAGTTTCCATTGATGCGGCCCTCCTCTCCAGGAGTTTATATTACAGGAAATATCT
CACGGCAGACCTGCGAAATTGATGAGTCTCGCGCAAAGTTTTCAACGTGATTTGTGTAAAGTTTCGCACA
TGCCACTTATGTTTCCGCTTGCCAGCACAACGTGTTTACATCAAACTAGTTACTAAGAATATTTAATTTA
TAATAGTTTAAAGCCTAGAAAACTATGCAAATGTATTTAAAGATGAATTTTATTTTGTTTAGTATCTCCT
CTTACAGTGCTTATTCATCTTGATAGCTAGACTTCGATTGATATGTATTTTGAGCCTTGAACCTTGGCTT
ATTCTACTGAAATTCTAACACACTTAAGTGAAAGAGCTTATCTAGAATGGGTCCCAACTTACATTCGATT
TATTGGTAAACTGAGTGCTTTATTCCTACGAAATCCGCGCCTCGGGCGGCTGACAACTGTCAGCAAATTG
TGGGCCAACATTTGAGCCCTAACAACTATAATTACCCTGTAGACAATCAATCCGCCCAGAGAATGTGGAT
GTGGATGCGGATGCCACAATTGAAGGCGTTGCCAATTCTGCAATCGATAGAGCTTTACAGGAAAATTGAC
ACATTTGTTGAAACGCTGAAATAAAATCGGATTCGAGTACTTTTTTAGCCCGCGGCAAAGGGCATAATT
GAAATTGACATATTTCATTGTCGGAACGGTGCTGCAATCCAAATTGCCAAAGTAAATTTTCAATTTCGAA
TTTTCATCAATTTTTTAGAGGGGAGTCCAAAGCTATTGCCTCGCATTGATTTGTCTTGATTTTGGGATGG
GAGTCAGATTTTGATTTCCATTTCAACGCCCGCTGTCATCGCTTTTGGCCTTTATTTACCATTTTGCTTT
GTTGTAAGCCGCCGCAGGGGCGGTTTGGTAGGAGGGGGCGTGGCCCGTGGGCTTAAGCCTGAAGCTTTGT
CTGTCTGTTGCTAAGCGCCTGCCACGAGTGTCCGTCCGTCCGCCACTTTGAGCCCAGCTTCTCCTCTTCC
TCCTCATCCGGACCATCTACGTTTTGTGCGTCACATCGAAGAAAAACGACGCAGATTTTTGGCAGCTTTG
AACGGATTAGCCGCCCGTGGAGCGATTCCCACTCACCACCATGGCGATCAATTCTTTGGTATTTGGAAAG
TTTAAAGTTTTCCTTAAGCGGAAAAATCATGTGAAACTCCCAGTTCAAGTATCCCTCAAGTTTCAGTATT
ACTGTACAACTTTCCTGCTGAATTAAATTTTAAAGCCAGTGGAACAATATGAAGTGAATACCAACGGACA
CCTTTTGTTCACTTCGCTGCTCCACTTTTCTCTTGGCTGCTGCGACCCCAAAAAAGCCCCACCACCATCA
CTTTCGCAGTCCATAAATTACAATACCAAAGGAGCAAAATTACAATAGAGCCAGGTATTTGGGTCAATTC
TCATTGTCTTTGCTTTTATTTCGACATGCAAAACAGAAGTGGCGAAAATTTAAGGTTTGTGGCATATACA
AAGCACAGAAATACTGTTGCATACCGCGGGGCGTTCAGAGTGGTGACTGTACCATGGAAGCTCAGTAAAT
GAGATACAGATGCACTTCTTCAAGATGCAAAGAAGCAAGTGCACACAATGAATGCAAGAGGTACAGTCCC
ATTGAATCCCCCTTTCGGCAGGTCGTCCCACTTCGTAGCTATGATTTCGGGGCTCGGGTTCTAGGTTATA
TTATATTCTTCTAGATTTGGAAAGCCCGGCAACACTTTTGTGGATATTTTTTAGAAAATGAAGTAGGGTG
TATGTTGTAAAAATTGTGCTTGTACTGAGTGTGTAGTGTAGTGAGCAGTAAGTAGCAGCAGTAGCAGGAA
TAACAATCCTTTCATTGTAGATAAATGTAGTGTGTTTAGTTAATTTTGATCATTACTTTTGTGTGAGTCT
GTGCTAGATGGAGCAACGATCTAGAAACTGATGGTGTGATATTATCCTATCTTTGGTGGTGGATCCACTT
ATTCCTCTTTTGCGTCTAGCATCTCTTTTAAAGGGGGGCTATTTAACGTCTGGTTCTTAATGGAGTGATA
GTGGATATACAGTTGCTGGGCCTCGCTTTTGTAGTTGTTTTTCATGTCATTTTTCAGCTGCTTTAGCAGC
TCCAATCGATAATTGTAGTTCTCCTTAAGGACATACATTTTCCTTTTGAAGTCCGCCTTGGCGGTGCGCT
GTGGTATGGCTTGTACGGCGGATTTTGGCGGCTTACCCGAAGATGTGCTGTGACTACTGTAAAGAAATAT
AAGTTGAAGTATTATATGCCATATGGTATAAACCATATATGCTTACGTAGATGTAGATAAGGTTGTGTGG
GTCACTGATAGAAGCATTCCCTTCCAGGTTTTCTTTCGCTGATCCTCTCTTAAAATGACTTCACCTTCCT
TTGCACTCAGCTCGTCGCGAATTGTCTTCTTGACCTCCAGAGCCTGAATGCCACTAAAATATTCTTCCAT
GTCCTTCCTGATTTTTTCCCTTGGGTCAATGTATTCCTCTTTGCCAGGCATCACTTCGTTTGGCTTTTTC

ATTTCCGCCTCCTGAGCTGCCGATGAAGTACTTGCCTCCATTTGATCCAGTTTTTCGATTTTCACTTCCT
CGGATTGCGGTATGATGGTATCCTGTTCCTTGGCTTCTTGAACTTCTTCCTTTACTGGTTTTTCTGGTGA
GCTTGGTAAATTTTCCACGACATTTGAGCTTTCTTCTTTGGGACTATTTAACGTTTCTTCTGCAATTAAT
TGCTTTGATTTTCGGGCATTCTCAATG
>dme_circ_0000089_junction_seq
TTGCTTTGATTTTCGGGCATTCTCAATGGGTAATTTTCCAACGATTTCATCCATTC
>dme_circ_0000090
GATGCGGCTGTAGAAGAGGACTTTCAACCAGGAGGTACACGAGATTGAAGGAACCCACATAGGAGTCATG
TCGCAGCAAGTGTTCTCGGCGCATCCAGCGCTGGCGGTGGACCAGCTGCAGCAGGCGGAACAAGAGGAGC
ACAATACCAGTAGTAATCATCACCAGCAGAGATCGGCGACACAGAGCCGCCGGCATACCAAGGCCGCACC
CAAGAAGTTCACCCTGAGCAGGAGTTGCGCCGGCGGAGGATCGGGAACCCTGAGCGGAGTCCACCAGCAA
GTCAAGCCCAGTCCTTCAAACCCCGCCATCAGTCCGGAGTGCAGGAAAACACTGCCCGTGCGAACAAACT
ATGCGGCGGTGGATGACGACGATGACATCGAGTGCGAGGATGTGGACGAGGTGAACTTTGGCCAGCAGGA
GAAGGAGCGGGAGCGGGAGACCCGGCAACCAACCAAAGACTGTGGCACCGATGAGACGGATCATGTGCAG
CAGCGCCACAAAAACACAATGACGACCTCGGCGACGGCGGCGAGCAGGCACCACCACCAGGACGGAGGAG
GAGGTGACCAGAGCGATCTGAGCAGCGTGATCAGCTCGCCGTCGGTCAGCACAGTGTCCTCGCCCCTCTC
CACGCCCACCAGGCTGCCACAGGCGCTGCAACAGCAGCTACACTGCTGCCAGAAGTCCACGGGCATGGAG
TCCCGGGCGAGAACATCTCCGCAACAGATccagcatccgcacagacagcaccaccagcagcagcaccatc
accaccaccaccatcaccacctcacGGCAGCGGGTTGTACGGGCGGAGGAGGAGGAGGCGGTGGCTCTGG
AGGATCGGGATCCTGCAAGGCGAAGAAGCTCGATCCCCGACTGAATCCCTCGCCCTATCGCCAGCTATTG
CCCATCGCCCTGTGCCTGCTCTCCTTCGCGGCAGTCTTCGCCACATTAATAGTTTACATGGACACGACAG
G
>dme_circ_0000090_junction_seq
CATTAATAGTTTACATGGACACGACAGGGATGCGGCTGTAGAAGAGGACTTTCAAC
>dme_circ_0000091
GACGGAGCTCTCACACAATCTGCAACAATTGTCGGAGAAGGCACGTTCCACGACCGAGTTCATCCAGCGG
CTCAAAGGCATGAGCGACAAAGTCACGGTAAGTGGGAAAAAACCGGCGAAAAAGTCCGCAATTCGGCTAA
ATCTCCGTATCTCATCCGTAATCAGGAATCCTGTATGGAGTTCGAGCGCCTCGTCCACGCTCAGTGTGAG
GCCCTCATTCAGGCCATCCACGACAGACGCGAATATCTGCTGGAGGCCATTCGCATGGACAAGGACACCA
AGATCAGGATACTCAAGGTATGTTGTTTCTTCAAGAAGCTATTATAAATAACTAACATATACAAACCCAA
AGTATATGAAAGTTAAATAATGATTTTGTAAATTATTATAATTTAATAAATGTACAATATTTTACTTAAA
CAACAGGACCAACAATCGAATTGCACTGGCAAGTTGCAGCAAACCACAGGACTAATTCAGTTCTGCATTG
AAGCTCTAAAAGAGACTGACAGCGCCGCCTTTCTTCAGGTGGGTTTTTTGACTTCATTAATTAATGTGTT
CGTCAGTTAATAAAATACTTTTTATTCTGCTGTGCAGGTGGGCTCCATGCTCATCAATCGAGTGACCAAC
ACGGACATGACCTGGCACCAGGAGGTCACCAATGCCGCCCCTCGAGTCTCGCCCATTGTTGACTTGACCC
TGGATGATGCTGCTCTGGCACGTGCCATAGATAACTTGAACTTTATCCAGATGAGAG
>dme_circ_0000091_junction_seq
GATAACTTGAACTTTATCCAGATGAGAGGACGGAGCTCTCACACAATCTGCAACAA
>dme_circ_0000092
GACGGAGCTCTCACACAATCTGCAACAATTGTCGGAGAAGGCACGTTCCACGACCGAGTTCATCCAGCGG
CTCAAAGGCATGAGCGACAAAGTCACGGTAAGTGGGAAAAAACCGGCGAAAAAGTCCGCAATTCGGCTAA
ATCTCCGTATCTCATCCGTAATCAGGAATCCTGTATGGAGTTCGAGCGCCTCGTCCACGCTCAGTGTGAG
GCCCTCATTCAGGCCATCCACGACAGACGCGAATATCTGCTGGAGGCCATTCGCATGGACAAGGACACCA
AGATCAGGATACTCAAGGGTGGGCTCCATGCTCATCAATCGAGTGACCAACACGGACATGACCTGGCACC

AGGAGGTCACCAATGCCGCCCCTCGAGTCTCGCCCATTGTTGACTTGACCCTGGATGATGCTGCTCTGGC
ACGTGCCATAGATAACTTGAACTTTATCCAGATGAGAGGGTAAGTGCGAGGGCACCTAATGAAAAATGCT
TAAAATATTTACTCTAATAGCTATGTTTGCTAGCCCACGTAATAACATTATGCATAGGTATAGGTAAAAC
AATTATAGAATCTAAAATTAATAGATATTTATACGTAACTAGAACTCCTTGAGTATTTCTATGTAGAACA
ATTTCCCACCAATTTCGAGCTGCAGACTTCTTGTTCAATATTATTGTATCCTAACCAGCCGTGAATTTTT
GAGCCCAAATGTATGTGCCAGGCAAACCCACGTCATAAATATTGTCACTAATTACGCACAAACACACAGA
CCCACCCAATTGGCCAAAGGTGTGACTTTGGGCCACCCAATAATAAGCACAAGCACAAATATAAACAAAA
ACAGAGCCCGCACTTGAAACAAAAGGGGAAAGAGCCCTTCATTCGAGGGTCGTTTAAGAGTTCCGACTGA
AGGGCTTCAAAAATAGTTTTGGGAACTCCCCCGGGAGTTGGGTTTTCAAATTTAGAACTGGTTGAGTGTT
GGGAAAAAAGGCAAAAAGAGTTTCTGTTGACAACTTCTGGGGGAAATGAACACCCAAAATAGGTGCCCCAT
AATTAATGTGAAAGTGTTGAAATCGGATAGTGCACACACAGACTGCGGACTAACTATTAAGATACATTAA
ACATCTGATATGGCTAACATATTTGTGCTTTTCTTGCATCTTCCTGTCATTCTGAATGTGCCGCTTGCTA
TCATTTAAATGAATATCGAATATACCCATAAAAACCAAACAAATCATCTAAAGCCGTCAAAGATGGAGAT
GAAAGATGTCCGGCAGGTGAGCAAAAACCACAATCACTTGAATGCAAAAATATCAACTGAAAAAATCAAT
ACACAAATTAAGTTTTCGTACACAAGACAGAAACACATGAATCTTGatttcataatttttgttcagtttt
ttttGGGGAAAACTGATTTTTTGGTGCTTTTGGACTTCTTCCCTCTCTCacacacactcacacacgctca
ttgactcactcacacacacacaTATCCACCTGGCTGTCGGCCATTTTGTTTTCATGGTTTTTGGCCATTG
CTTTCTACCTTTGTTTTCCGTTCTTTTACGTTCGACGGGCACACGCCTACGCATAATAATTTTTCATTTT
CATTTAAGCCACTAGCTGGAAATACAAAATACCCGAAAATGCGTCCAAATATTTTCGGCTTCACTTCCGT
TTCCTGGCGAATGTCAAAAAACTGTCAACTTGTGGGTGGGGGCGGTACCACTGGAGGGGCGTGGTCGGCG
ATGTCATTTCATGTGACATAATTTTTTACGCCACGCTTTTGCCTTTGCCCGTCGCTTTTCATTTGACTAC
TCTTTCTCATCTTcattatttcatgcttaagcttttatttcgcatttttcgttttatatgtttattattt
ttatgctacccttgctgctttttttctggtattttCCCTCGCAATTGACCCTCTTCAAGCGGGCTGCTTAG
GCTTATCCCTTACACTTTTAGCCCGATTGCCGAGGCACCTTGAATTCAACTTTTTACTTTGTGCCTCGCT
TTTTGTTTCGGTGACATTTGACAGCTTTTGGCGCCATTTTAATTCGATGCCTGTTCAGGGGAGCGGAAAT
GCCCGGAGTGTCGTCGTTTTTATGTTTATTTTGCGGCATAATTTTAGGCGCTTTACTTCGGACTCGCCAT
TTGTGTTGTGTCATGAATTTTTATTACCAGGCGTTGGTTTTATGCCCCGATTTTATGACACCTGGGCGTG
GCCCCCCTCAGGGCAATTAATAAAAAATGAAACTTGAACAAACGAGCATATCTTTTTGTTGACAGATTCG
TTTTGGTTAGTCTTGGGATCAATTGGTTGTCCTGTGGTCCTCTCATCCATCGAAACTCTTTTATTGGTTG
TGGTATTGTCTTAGTTTTACTGATAATTTGGTTTACTTTTATTTCACTACCTCTATACCAGTACTAACAC
TTATTTGGGTCTTTTATTCATCGTTTTCCCATTTCCCATCTAAAGATAACTTTTTTTGTTCTTCCCACAG
GCACCCATGACACCAACTATACTGCCATCGGATTGCTCGGCCGAGAATAATTCGGTGACCGTTGCGTGGC
AGCCACCAAATCACTCTTTCGTCGAAGGATATGTCCTGGAATTGGATGATGGCAGCGGCGGTGAATTTAG
GGGAAGTCTACTGCGGCAAGGAAACAATTTGCACCGTGGACGGGCTGCACTTCAACTCGATGTACAATGC
CCGGGTGAAGGCCTTCAATTCCGCCGGAGAGGGTGAATACTCGGAGCTGATTGGACTGCAGACTGCCGAA
G
>dme_circ_0000092_junction_seq
GAGCTGATTGGACTGCAGACTGCCGAAGGACGGAGCTCTCACACAATCTGCAACAA
>dme_circ_0000093
GACGGAGCTCTCACACAATCTGCAACAATTGTCGGAGAAGGCACGTTCCACGACCGAGTTCATCCAGCGG
CTCAAAGGCATGAGCGACAAAGTCACGGTAAGTGGGAAAAAACCGGCGAAAAAGTCCGCAATTCGGCTAA
ATCTCCGTATCTCATCCGTAATCAGGAATCCTGTATGGAGTTCGAGCGCCTCGTCCACGCTCAGTGTGAG
GCCCTCATTCAGGCCATCCACGACAGACGCGAATATCTGCTGGAGGCCATTCGCATGGACAAGGACACCA
AGATCAGGATACTCAAGGGTGGGCTCCATGCTCATCAATCGAGTGACCAACACGGACATGACCTGGCACC

AGGAGGTCACCAATGCCGCCCCTCGAGTCTCGCCCATTGTTGACTTGACCCTGGATGATGCTGCTCTGGC
ACGTGCCATAGATAACTTGAACTTTATCCAGATGAGAGGGTAAGTGCGAGGGCACCTAATGAAAAATGCT
TAAAATATTTACTCTAATAGCTATGTTTGCTAGCCCACGTAATAACATTATGCATAGGTATAGGTAAAAC
AATTATAGAATCTAAAATTAATAGATATTTATACGTAACTAGAACTCCTTGAGTATTTCTATGTAGAACA
ATTTCCCACCAATTTCGAGCTGCAGACTTCTTGTTCAATATTATTGTATCCTAACCAGCCGTGAATTTTT
GAGCCCAAATGTATGTGCCAGGCAAACCCACGTCATAAATATTGTCACTAATTACGCACAAACACACAGA
CCCACCCAATTGGCCAAAGGTGTGACTTTGGGCCACCCAATAATAAGCACAAGCACAAATATAAACAAAA
ACAGAGCCCGCACTTGAAACAAAAGGGGAAAGAGCCCTTCATTCGAGGGTCGTTTAAGAGTTCCGACTGA
AGGGCTTCAAAAATAGTTTTGGGAACTCCCCCGGGAGTTGGGTTTTCAAATTTAGAACTGGTTGAGTGTT
GGGAAAAAGGCAAAAAGAGTTTCTGTTGACAACTTCTGGGGGAAATGAACACCCAAAATAGGTGCCCCAT
AATTAATGTGAAAGTGTTGAAATCGGATAGTGCACACACAGACTGCGGACTAACTATTAAGATACATTAA
ACATCTGATATGGCTAACATATTTGTGCTTTTCTTGCATCTTCCTGTCATTCTGAATGTGCCGCTTGCTA
TCATTTAAATGAATATCGAATATACCCATAAAAACCAAACAAATCATCTAAAGCCGTCAAAGATGGAGAT
GAAAGATGTCCGGCAGGTGAGCAAAAACCACAATCACTTGAATGCAAAAATATCAACTGAAAAAATCAAT
ACACAAATTAAGTTTTCGTACACAAGACAGAAACACATGAATCTTGatttcataatttttgttcagtttt
ttttGGGGAAAACTGATTTTTTGGTGCTTTTGGACTTCTTCCCTCTCTCacacacactcacacacgctca
ttgactcactcacacacacacaTATCCACCTGGCTGTCGGCCATTTTGTTTTCATGGTTTTTGGCCATTG
CTTTCTACCTTTGTTTTCCGTTCTTTTACGTTCGACGGGCACACGCCTACGCATAATAATTTTTCATTTT
CATTTAAGCCACTAGCTGGAAATACAAAATACCCGAAAATGCGTCCAAATATTTTCGGCTTCACTTCCGT
TTCCTGGCGAATGTCAAAAAACTGTCAACTTGTGGGTGGGGGCGGTACCACTGGAGGGGCGTGGTCGGCG
ATGTCATTTCATGTGACATAATTTTTTACGCCACGCTTTTGCCTTTGCCCGTCGCTTTTCATTTGACTAC
TCTTTCTCATCTTcattatttcatgcttaagcttttatttcgcatttttcgttttatatgtttattattt
ttatgctacccttgctgctttttttctggtattttCCCTCGCAATTGACCCTCTTCAAGCGGGCTGCTTAG
GCTTATCCCTTACACTTTTAGCCCGATTGCCGAGGCACCTTGAATTCAACTTTTTACTTTGTGCCTCGCT
TTTTGTTTCGGTGACATTTGACAGCTTTTGGCGCCATTTTAATTCGATGCCTGTTCAGGGGAGCGGAAAT
GCCCGGAGTGTCGTCGTTTTTATGTTTATTTTGCGGCATAATTTTAGGCGCTTTACTTCGGACTCGCCAT
TTGTGTTGTGTCATGAATTTTTATTACCAGGCGTTGGTTTTATGCCCCGATTTTATGACACCTGGGCGTG
GCCCCCCTCAGGGCAATTAATAAAAAATGAAACTTGAACAAACGAGCATATCTTTTTGTTGACAGATTCG
TTTTGGTTAGTCTTGGGATCAATTGGTTGTCCTGTGGTCCTCTCATCCATCGAAACTCTTTTATTGGTTG
TGGTATTGTCTTAGTTTTACTGATAATTTGGTTTACTTTTATTTCACTACCTCTATACCAGTACTAACAC
TTATTTGGGTCTTTTATTCATCGTTTTCCCATTTCCCATCTAAAGATAACTTTTTTTGTTCTTCCCACAG
GGTATGTTTAATCAAAACAAAAATAATATATAAATATAACAAATGCTTCAATGGCAACAGTTGTAAATCC
TTTATATATATCTTGTTCAAAACGATTGCTTTtaaagttgaaaatgtaatgaaaatgtaaataaaataat
gtatgtaaatgAGCCAAGTCAAGTGAATGCCATATCAGCTAATGAATGGCTATACGAATCGTTCGATCCA
AGTGCTGATTGGCTGAGTGAATGGCTGTCATCTTCCAATTTATTAACCCAGAACTTTAATTTGATTGCCT
GCCCCCATTCAGGGTGAGTGCCGCTTTTCACGTTTCCAGCTCTACTAGCTTTTTCAATTAAGCTCCACCA
CGCTCGCCGCTGAGCTACTTTCCCAACTGTTTTATAATTTAATCCTTTTCGGTCATGCGCCACCCCAATC
CATATCCACCTACCGAGTTCCCAGCTCGGACTGCTTCTGGCTTCCCGTTCGTGCTTTGCATGTCAAACAG
CCATTGACCCCTTTTGCGCCTGCAGGGCAGGAAAATGTGAATGGAAATGGGTCTGGAGGTGGTCGCCATG
GGCGGAGGATGTCTTCTGCAATCCAATTCGCACTTGATTGATGCATGCGCCACCTGACAGCAGCTCCTAT
TCCTACAGGAGGCACATCGACTCCTGCTTCCACTCCCACTGCTCACCACAAACGGAACGCCGCGTAAAGT
TGTTTAATTTATAAAATTAACAGAGCGCTCTAGGCAACATTTACTTTCCGTCGTTTTATTAGTTGGGCGC
CATTAATTTGCAACTTTTAATTGATTATTCCTCGCTGTCTACGAGTTGCCTTCAAAGGGGTCACAGGCGA
TTTAAGCGTATCCATCCGAACCCGAAACCGAGCCGATCCCACTGCAAATTGTCATTTTATAAAGTGTTTA

CAACGAGCGCGTGTCCCTAAGTAATTAACGGAAAACCAGTTAACAGTTTATGGCTCGTGACGTTTGCGGG
TGGTCGGCACTAATGGGTTAAGTCGGACACCTTAAGGACATTAGGGAGACAGTTTACAAGACGTCAGTCT
TGGGATGTGAGACGGTTCAAGTAATTTCAATTAAGCCAGCGATGATGCGGTCGTTTGGTTACGTGACAAA
TTGAGAAACTCAGTTCAGAAATAAAGAAATCCATACATTAAACCATAGCAGATGCGTATCTTAAAGTTAT
CAAACTAAACCTAATAAACCAAGGCGATTAAAGACCACTTCTAATCCTTTTTAATAGACTCCTAATCCCA
ACTTCAGACGCATTCATTTGGCAAATATTTTAGCTCGGTCTGCCTCTTGGTTATCCAATTAAGAAACTGG
GAACAGTTTACAACAACTAATTAAATGCCTGGGCATCCAGGACTCTCGGTTTATGGCGCCTGGCTCAAAT
CTCTCATAGTTTTAATCGCAAAGTCATATGGCCGACATAAACAAAACAATGGCCAGCCGGAGTCCTCCTG
GCCCAGATGCTTGCTTTGTGGCTTGTTTGGCCACACAATTGCCTTTGGTGGCCAATAAAACAGGCAACGT
CATCATCGTCAGCAGACCGAGAAGTGGAGCCATGACTCTCTTTATATATACATAGATGTATAGCCGGTCC
CAGCTGATTCCGGACTAGCCTTGGCCACTTAATGCGGCCACAACAATAAAAAAGGAAATTTAATAAAGCA
CTCCACTCGACTCGCCACTCGTTGCCTACTTTGCGGCGCATAACAGCATGAACTGCAAGGAACCACCAAC
TTGGACTCCGAATTGCACTGGCCACTTGAGCTTTGACAATGGGGAAAATTGCAACCGGTCTGGCTTTGTT
TTTCTTTCCACTTTTCACTGCCCCGTGCACTGGGAAAAGATGATGGTCTTAAATTTAGAAATAATAACTC
GGCGGTAATGGGATACTATTGGACCGTAATCAGCTGAATAGGGGTAGTGGAAAATAATCTTATCTCATTT
AAGGAATCCAATTGAGCTCGTTTGTTATCCAACATGTTTTGTAACCAAAAGTAATCTAGCGATTCGGTTA
ACTGGGTGGTATTGCCAAGATATTTCTATCTGGAATTCTTTCAACTTTCCAAGAGTTAATGTGCCAAGTG
TATTTATCGTCTAAATTTTTCTTCATTCCATTGCAGGTGGCCTGGTTCACGTTTGATCCCGTTCTGAGCG
GTGGAGCCGGTTCTGGCTTAATCTTTAGCAAAAACAACGCCACCGTGAGCGTCGAGGGCTGGGAACACCG
GGTGGCCCTCGGCTCGGTGGGATTCTCCCGCGGCGTCCACTACTGGGAGTTCACCATCGACAACTATACG
GCGGACACGGACCCGGCCTTCGGAGTGGCCCGCATCGACGTGGCCCGCAACAAAATGCTGG
>dme_circ_0000093_junction_seq
ATCGACGTGGCCCGCAACAAAATGCTGGGACGGAGCTCTCACACAATCTGCAACAA
>dme_circ_0000094
GTGGCCTGGTTCACGTTTGATCCCGTTCTGAGCGGTGGAGCCGGTTCTGGCTTAATCTTTAGCAAAAACA
ACGCCACCGTGAGCGTCGAGGGCTGGGAACACCGGGTGGCCCTCGGCTCGGTGGGATTCTCCCGCGGCGT
CCACTACTGGGAGTTCACCATCGACAACTATACGGCGGACACGGACCCGGCCTTCGGAGTGGCCCGCATC
GACGTGGCCCGCAACAAAATGCTGG
>dme_circ_0000094_junction_seq
ATCGACGTGGCCCGCAACAAAATGCTGGGTGGCCTGGTTCACGTTTGATCCCGTTC
>dme_circ_0000095
GACACCAGCGCTGTTTCTATCCCTTAAAACCCAAATGTTCCTATATAATTTGAATTATTTATCCAAAATA
AAGACCATTTTAATATACAATTTATTAAATGGGGACGGACCAATTTATCGGTATTTGTGTTTATACGGAC
GAACAGAAGTAATTAATCGGCATGTTATCCAGGTTGCGTTTTAATATTTTACAATCGGCCTTAAATGTCA
ACTTTTATCCTTTGTACCTTTCTACAAATATCATGTTTCGTTTCATCATACGGGTAAAGGGTATAAAGTC
ACTACGCGAAAATACATGAAAACATTACATAAAATCATAAATAATTATATGGTTTTGGAACAACAACATC
GCTTAAGCCAAATAAAAAATGAATATTTTATATTATCATTCTGTCATTTCTGTTTGTATGTAAATCGCGT
TTAATTTAATTTAATTTTTTTGCGTTCCTATTTTGCTGTCCCCGTTCGATTTTTAGTGAGACATGTTTGT
AATTATAGATTAAATTACATCAGGACTAGTACTCGTTTGCGTCGTATTTCTGTAAAGGTATAAAATAATT
AGTAG
>dme_circ_0000095_junction_seq
TTCTGTAAAGGTATAAAATAATTAGTAGGACACCAGCGCTGTTTCTATCCCTTAAA
>dme_circ_0000096
gcggaggcggcggtggcggctacggAATTGGACCTGCAGTCTCCTTAGGTGGCGGACCAGGCGGTGGTgg

tagctacggaggtggctacggaggtggctccggagCCTCTGCTTCAGCCAGTGCCAGTGCCTCAGCAGGA
GCAGGCGGCGGCTATCATGGCTAGTCTGGATTACCCAAAGCCCAATATACCTGTAATTCTCCCAG
>dme_circ_0000096_junction_seq
AAGCCCAATATACCTGTAATTCTCCCAGgcggaggcggcggtggcggctacggAAT
>dme_circ_0000097
GGTATATTGGGCTTTGGGTAATCCAGACTAGCCATGATAGCCGCCGCCTGCTCCTGCTGAGGCACTGGCA
CTGGCTGAAGCAGAGGctccggagccacctccgtagccacctccgtagctaccACCACCGCCTGGTCCGC
CACCTAAGGAGACTGCAGGTCCAATTccgtagccgccaccgccgcctccgcctccgccgccgccgccgcc
>dme_circ_0000097_junction_seq
cgcctccgcctccgccgccgccgccgccGGTATATTGGGCTTTGGGTAATCCAGAC
>dme_circ_0000098
gtggccattctggtggaggtagcggaatcggtggcggccctggatttggtggcggaatcggcggcggagg
tggccattctggtggaggcggtggaatcggcggcggtcctggatttggtggaggaatcggcggATCCGGT
AGCTCTGCATCATCTTCAGTTGGAGTTATTGGTggaggtcatggcggcggaggtcacggcggaggaggct
tcggacctggtggaggaggtggaggaggcttcggaccaggtggaggaggctttggacccggcattggagg
tggcggtggaggctttggccccggcattggaggcggatccggtggtggacactttggtggtggtcttagc
cacaagcacaaaggccatggaggcggaggcggcttcaaaggcggatacggcggcggcggcggcggaggcg
gaggcggcggtggcggctacggAATTGGACCTGCAGTCTCCTTAGGTGGCGGACCAGGCGGTGGTggtag
ctacggaggtggctacggagg
>dme_circ_0000098_junction_seq
GTggtagctacggaggtggctacggagggtggccattctggtggaggtagcggaat
>dme_circ_0000099
gaggctttggacccggcattggaggtggcggtggaggctttggccccggcattggaggcggatccggtgg
tggacactttggtggtggtcttagccacaagcacaaaggccatggaggcggaggcggcttcaaaggcgga
tacggcggcggcggcggcggaggcggaggcggcggtggcggctacggAATTGGACCTGCAGTCTCCTTAG
GTGGCGGACCAGGCGGTGGTggtagctacggagg
>dme_circ_0000099_junction_seq
GACCAGGCGGTGGTggtagctacggagggaggctttggacccggcattggaggtgg
>dme_circ_0000100
gaggcttcggacctggtggaggaggtggaggaggcttcggaccaggtggaggaggctttggacccggcat
tggaggtggcggtggaggctttggccccggcattggaggcggatccggtggtggacactttggtggtggt
cttagccacaagcacaaaggccatggaggcggaggcggcttcaaaggcggatacggcggcggcggcggcg
gaggcggaggcggcggtggcggctacggAATTGGACCTGCAGTCTCCTTAGGTGGCGGACCAGGCGGTGG
Tggtagctacggagg
>dme_circ_0000100_junction_seq
GACCAGGCGGTGGTggtagctacggagggaggcttcggacctggtggaggaggtgg
>dme_circ_0000101
gctttggccccggcattggaggcggatccggtggtggacactttggtggtggtcttagccacaagcacaa
aggccatggaggcggaggcggcttcaaaggcggatacggcggcggcggcggcggaggcggaggcggcggt
ggcggctacggAATTGGACCTGCAGTCTCCTTAGGTGGCGGACCAGGCGGTGGTgg
>dme_circ_0000101_junction_seq
CCTTAGGTGGCGGACCAGGCGGTGGTgggctttggccccggcattggaggcggatc
>dme_circ_0000102
gtcatggcggcggaggtcacggcggaggaggcttcggacctggtggaggaggtggaggaggcttcggacc

aggtggaggaggctttggacccggcattggaggtggcggtggaggctttggccccggcattggaggcgga
tccggtggtggacactttggtggtgg
>dme_circ_0000102_junction_seq
gatccggtggtggacactttggtggtgggtcatggcggcggaggtcacggcggagg
>dme_circ_0000103
gaatcggcggATCCGGTAGCTCTGCATCATCTTCAGTTGGAGTTATTGGTggaggtcatggcggcggagg
tcacggcggaggaggcttcggacctggtggaggaggtggaggaggcttcggaccaggtggaggaggcttt
ggacccggcattggaggtggcggtggaggctttggccccggcattggaggcggatccggtggtggacact
ttggtggtgg
>dme_circ_0000103_junction_seq
gatccggtggtggacactttggtggtgggaatcggcggATCCGGTAGCTCTGCATC
>dme_circ_0000104
gtggccattctggtggaggcggtggaatcggcggcggtcctggatttggtggaggaatcggcggATCCGG
TAGCTCTGCATCATCTTCAGTTGGAGTTATTGGTggaggtcatggcggcggaggtcacggcggaggaggc
ttcggacctggtggaggaggtggaggaggcttcggaccaggtggaggaggctttggacccggcattggag
gtggcggtggaggctttggccccggcattggaggcggatccggtggtggacactttggtgg
>dme_circ_0000104_junction_seq
gcggatccggtggtggacactttggtgggtggccattctggtggaggcggtggaat
>dme_circ_0000105
gcggtggaatcggcggcggtcctggatttggtggaggaatcggcggATCCGGTAGCTCTGCATCATCTTC
AGTTGGAGTTATTGGTggaggtcatggcggcggaggtcacggcggaggaggcttcggacctggtggagga
ggtggaggaggcttcggaccaggtggaggaggctttggacccggcattggaggtggcggtggaggctttg
gccccggcattggaggcggatccggtggtggacactttgg
>dme_circ_0000105_junction_seq
gaggcggatccggtggtggacactttgggcggtggaatcggcggcggtcctggatt
>dme_circ_0000106
GCggattcggtggcggtcccggattcggatcaggtggtcattccggtggaggaatcggtgacggccctgg
atttggatcaggtggatattccggaggaggtggcggaatcggcggtggaggtggccattctggtggaggt
agcggaatcggtggcggccctggatttggtggcggaatcggcggcggaggtggccattctggtggaggcg
gtggaatcggcggcggtcctggatttggtggaggaatcggcggATCCGGTAGCTCTGCATCATCTTCAGT
TGGAGTTATTGGTggaggtcatggcggcggaggtcacggcggaggaggcttcggacctggtggaggaggt
ggaggaggcttcggaccaggtggaggaggctttggacccggcattggagg
>dme_circ_0000106_junction_seq
gaggaggctttggacccggcattggaggGCggattcggtggcggtcccggattcgg
>dme_circ_0000107
GTGGAGGCggattcggtggcggtcccggattcggatcaggtggtcattccggtggaggaatcggtgacgg
ccctggatttggatcaggtggatattccggaggaggtggcggaatcggcggtggaggtggccattctggt
ggaggtagcggaatcggtggcggccctggatttggtggcggaatcggcggcggaggtggccattctggtg
gaggcggtggaatcggcggcggtcctggatttggtggaggaatcggcggATCCGGTAGCTCTGCATCATC
TTCAGTTGGAGTTATTGGTggaggtcatggcggcggaggtcacggcggaggaggcttcggacctggtgga
ggaggtggaggaggcttcggaccagg
>dme_circ_0000107_junction_seq
gaggaggtggaggaggcttcggaccaggGTGGAGGCggattcggtggcggtcccgg
>dme_circ_0000108

gtggccattctggtggaggcggtggaatcggcggcggtcctggatttggtggaggaatcggcggATCCGG
TAGCTCTGCATCATCTTCAGTTGGAGTTATTGGTggaggtcatggcggcggaggtcacggcggaggaggc
ttcggacctggtggaggagg
>dme_circ_0000108_junction_seq
gaggaggcttcggacctggtggaggagggtggccattctggtggaggcggtggaat
>dme_circ_0000109
gtagcggaatcggtggcggccctggatttggtggcggaatcggcggcggaggtggccattctggtggagg
cggtggaatcggcggcggtcctggatttggtggaggaatcggcggATCCGGTAGCTCTGCATCATCTTCA
GTTGGAGTTATTGGTggagg
>dme_circ_0000109_junction_seq
CATCTTCAGTTGGAGTTATTGGTggagggtagcggaatcggtggcggccctggatt
>dme_circ_0000110
gtggccattctggtggaggtagcggaatcggtggcggccctggatttggtggcggaatcggcggcggagg
tggccattctggtggaggcggtggaatcggcggcggtcctggatttggtggaggaatcggcggATCCGGT
AGCTCTGCATCATCTTCAGTTGGAGTTATTGGTggagg
>dme_circ_0000110_junction_seq
CATCTTCAGTTGGAGTTATTGGTggagggtggccattctggtggaggtagcggaat
>dme_circ_0000111
GCggattcggtggcggtcccggattcggatcaggtggtcattccggtggaggaatcggtgacggccctgg
atttggatcaggtggatattccggaggaggtggcggaatcggcggtggaggtggccattctggtggaggt
agcggaatcggtggcggccctggatttggtggcggaatcggcggcggaggtggccattctggtggaggcg
gtggaatcggcggcggtcctggatttggtggaggaatcggcggATCCGGTAGCTCTGCATCATCTTCAGT
TGGAGTTATTGGTggagg
>dme_circ_0000111_junction_seq
CATCTTCAGTTGGAGTTATTGGTggaggGCggattcggtggcggtcccggattcgg
>dme_circ_0000112
GTGGAGGCggattcggtggcggtcccggattcggatcaggtggtcattccggtggaggaatcggtgacgg
ccctggatttggatcaggtggatattccggaggaggtggcggaatcggcggtggaggtggccattctggt
ggaggtagcggaatcggtggcggccctggatttggtggcggaatcggcggcggaggtggccattctggtg
gaggcggtggaatcggcggcggtcctggatttggtggaggaatcggcggATCCGGTAGCTCTGCATCATC
TTCAGTTGGAGTTATTGG
>dme_circ_0000112_junction_seq
CTGCATCATCTTCAGTTGGAGTTATTGGGTGGAGGCggattcggtggcggtcccgg
>dme_circ_0000113
gtggcggaatcggcggtggaggtggccattctggtggaggtagcggaatcggtggcggccctggatttgg
tggcggaatcggcggcggaggtggccattctggtggaggcggtggaatcggcggcggtcctggatttgg
>dme_circ_0000113_junction_seq
gtggaatcggcggcggtcctggatttgggtggcggaatcggcggtggaggtggcca
>dme_circ_0000114
GTGGAGGCggattcggtggcggtcccggattcggatcaggtggtcattccggtggaggaatcggtgacgg
ccctggatttggatcaggtggatattccggaggaggtggcggaatcggcggtggaggtggccattctggt
ggaggtagcggaatcggtggcggccctggatttggtggcggaatcggcggcggaggtggccattctggtg
gaggcggtggaatcggcggcggtcctggatttgg
>dme_circ_0000114_junction_seq

gtggaatcggcggcggtcctggatttggGTGGAGGCggattcggtggcggtcccgg
>dme_circ_0000115
GACTTGGAGGCGGCGGTGTCGGAGGAGGTCCCTTCGCAGGTGGCCATGCTGGCGGTGGAGTGATCTCCGG
CGGAGGTCATTCTGGAGGTGGAGGCggattcggtggcggtcccggattcggatcaggtggtcattccggt
ggaggaatcggtgacggccctggatttggatcaggtggatattccggaggaggtggcggaatcggcggtg
gaggtggccattctggtggaggtagcggaatcggtggcggccctggatttggtggcggaatcggcggcgg
aggtggccattctggtggaggcggtggaatcggcggcggtcctggatttgg
>dme_circ_0000115_junction_seq
gtggaatcggcggcggtcctggatttggGACTTGGAGGCGGCGGTGTCGGAGGAGG
>dme_circ_0000116
gttatggatctggtggtcacggttctggaggacttggatctggcggtttcggctctggcggtGATTTAGG
ACTTGGAGGCGGCGGTGTCGGAGGAGGTCCCTTCGCAGGTGGCCATGCTGGCGGTGGAGTGATCTCCGGC
GGAGGTCATTCTGGAGGTGGAGGCggattcggtggcggtcccggattcggatcaggtggtcattccggtg
gaggaatcggtgacggccctggatttggatcaggtggatattccggaggaggtggcggaatcggcggtgg
aggtggccattctggtggaggtagcggaatcggtggcggccctggatttggtggcggaatcggcggcgga
ggtggccattctggtggaggcggtggaatcggcggcgg
>dme_circ_0000116_junction_seq
ctggtggaggcggtggaatcggcggcgggttatggatctggtggtcacggttctgg
>dme_circ_0000117
GCggattcggtggcggtcccggattcggatcaggtggtcattccggtggaggaatcggtgacggccctgg
atttggatcaggtggatattccggaggaggtggcggaatcggcggtggaggtggccattctggtggaggt
agcggaatcggtggcggccctggatttggtggcggaatcggcggcggaggtggccattctggtggaggcg
g
>dme_circ_0000117_junction_seq
gcggaggtggccattctggtggaggcggGCggattcggtggcggtcccggattcgg
>dme_circ_0000118
gaatggccacctccgccgccgattccgccaccaaatccagggccgccaccgattccgctacctccaccag
aatggccacctccaccgccgattccgccacctcctccggaatatccacctgatccaaatccagggccgtc
accgattcctccaccggaatgaccacctgatccgaatccgggaccgccaccgaatccGCCTCCACCTCCA
GAATGACCTCCGCCGGAGATCACTCCACCGCCAGCATGGCCACCTGCGAAGGGACCTCCTCCGACACCGC
CGCCTCCAA
>dme_circ_0000118_junction_seq
GGACCTCCTCCGACACCGCCGCCTCCAAgaatggccacctccgccgccgattccgc
>dme_circ_0000119
gtggtcattccggtggaggaatcggtgacggccctggatttggatcaggtggatattccggaggaggtgg
cggaatcggcggtggaggtggccattctggtggaggtagcggaatcggtggcggccctggatttggtggc
ggaatcggcggcggagg
>dme_circ_0000119_junction_seq
gatttggtggcggaatcggcggcggagggtggtcattccggtggaggaatcggtga
>dme_circ_0000120
GCggattcggtggcggtcccggattcggatcaggtggtcattccggtggaggaatcggtgacggccctgg
atttggatcaggtggatattccggaggaggtggcggaatcggcggtggaggtggccattctggtggaggt
agcggaatcggtggcggccctggatttggtggcggaatcggcggcggagg
>dme_circ_0000120_junction_seq

gatttggtggcggaatcggcggcggaggGCggattcggtggcggtcccggattcgg
>dme_circ_0000121
gttatggatctggtggtcacggttctggaggacttggatctggcggtttcggctctggcggtGATTTAGG
ACTTGGAGGCGGCGGTGTCGGAGGAGGTCCCTTCGCAGGTGGCCATGCTGGCGGTGGAGTGATCTCCGGC
GGAGGTCATTCTGGAGGTGGAGGCggattcggtggcggtcccggattcggatcaggtggtcattccggtg
gaggaatcggtgacggccctggatttggatcaggtggatattccggaggaggtggcggaatcggcggtgg
aggtggccattctggtggaggtagcggaatcggtggcggccctggatttggtggcggaatcggcggcgga
gg
>dme_circ_0000121_junction_seq
gatttggtggcggaatcggcggcggagggttatggatctggtggtcacggttctgg
>dme_circ_0000122
GCggattcggtggcggtcccggattcggatcaggtggtcattccggtggaggaatcggtgacggccctgg
atttggatcaggtggatattccggaggaggtggcggaatcggcggtggaggtggccattctggtggagg
>dme_circ_0000122_junction_seq
gcggtggaggtggccattctggtggaggGCggattcggtggcggtcccggattcgg
>dme_circ_0000123
GTGGAGGCggattcggtggcggtcccggattcggatcaggtggtcattccggtggaggaatcggtgacgg
ccctggatttggatcaggtggatattccggaggaggtggcggaatcggcggtggaggtggccattctgg
>dme_circ_0000123_junction_seq
gaatcggcggtggaggtggccattctggGTGGAGGCggattcggtggcggtcccgg
>dme_circ_0000124
TGATCTCCGGCGGAGGTCATTCTGGAGGTGGAGGCggattcggtggcggtcccggattcggatcaggtgg
tcattccggtggaggaatcggtgacggccctggatttggatcaggtggatattccggaggaggtggcgga
atcggcggtggagg
>dme_circ_0000124_junction_seq
gaggaggtggcggaatcggcggtggaggTGATCTCCGGCGGAGGTCATTCTGGAGG
>dme_circ_0000125
gggccgtcaccgattcctccaccggaatgaccacctgatccgaatccgggaccgccaccgaatccGCCTC
CACCTCCAGAATGACCTCCGCCGGAGATCACTCCACCGCCAGCATGGCCACCTGCGAAGGGACCTCCTCC
GACACCGCCGCCTCCAA
>dme_circ_0000125_junction_seq
GGACCTCCTCCGACACCGCCGCCTCCAAgggccgtcaccgattcctccaccggaat
>dme_circ_0000126
GACTTGGAGGCGGCGGTGTCGGAGGAGGTCCCTTCGCAGGTGGCCATGCTGGCGGTGGAGTGATCTCCGG
CGGAGGTCATTCTGGAGGTGGAGGCggattcggtggcggtcccggattcggatcaggtggtcattccgg
>dme_circ_0000126_junction_seq
ccggattcggatcaggtggtcattccggGACTTGGAGGCGGCGGTGTCGGAGGAGG
>dme_circ_0000127
GTGGTGGTGGTGGACACGCCGGAGCATCCGCTTCCTCGTCCGCTTCTGCCGGTGCGATTGGCGGCGGTGA
TGGAGGCGGTAAGCTCGGCggaggttatggatctggtggtcacggttctggaggacttggatctggcggt
ttcggctctggcggtGATTTAGGACTTGGAGGCGGCGGTGTCGGAGGAGGTCCCTTCGCAGGTGGCCATG
CTGGCGGTGGAGTGATCTCCGGCGGAGGTCATTCTGGAGGTGGAGGCggattcgg
>dme_circ_0000127_junction_seq
GTCATTCTGGAGGTGGAGGCggattcggGTGGTGGTGGTGGACACGCCGGAGCATC

>dme_circ_0000128
gacttggatctggcggtttcggctctggcggtGATTTAGGACTTGGAGGCGGCGGTGTCGGAGGAGGTCC
CTTCGCAGGTGGCCATGCTGGCGGTGGAGTGATCTCCGGCGGAGGTCATTCTGGAGG
>dme_circ_0000128_junction_seq
TGATCTCCGGCGGAGGTCATTCTGGAGGgacttggatctggcggtttcggctctgg
>dme_circ_0000129
gttatggatctggtggtcacggttctggaggacttggatctggcggtttcggctctggcggtGATTTAGG
ACTTGGAGGCGGCGGTGTCGGAGGAGGTCCCTTCGCAGGTGGCCATGCTGGCGGTGGAGTGATCTCCGGC
GGAGGTCATTCTGGAGG
>dme_circ_0000129_junction_seq
TGATCTCCGGCGGAGGTCATTCTGGAGGgttatggatctggtggtcacggttctgg
>dme_circ_0000130
gttatggatctggtggtcacggttctggaggacttggatctggcggtttcggctctggcggtGATTTAGG
ACTTGGAGGCGGCGGTGTCGGAGGAGGTCCCTTCGCAGGTGGCCATGCTGGCGGTGGAGTGATCTCCGGC
GGAGG
>dme_circ_0000130_junction_seq
CTGGCGGTGGAGTGATCTCCGGCGGAGGgttatggatctggtggtcacggttctgg
>dme_circ_0000131
CTGCAGGCAAAGGTTCCGATGCGTCTGTTTATAGTGCTGAGTGTTTGTGTGGCGTTAGCTTCCGCCGGAG
GTTACGGCGGTGGAGGCGGAGGTGGTGGTGGTGGACACGCCGGAGCATCCGCTTCCTCGTCCGCTTCTGC
CGG
>dme_circ_0000131_junction_seq
CATCCGCTTCCTCGTCCGCTTCTGCCGGCTGCAGGCAAAGGTTCCGATGCGTCTGT
>dme_circ_0000132
TCTGGGAGGAAATGGTCTAAGCCTTGGTGGCAATCTCGGCGGGAACTCTGGAAGTGCCGGAATTGGATTG
GGTGGCGCTCACCTTGGAGGATCCATTAACCGTGGCGGCGGCCTTTTCCTTGGCTAATTTTAAG
>dme_circ_0000132_junction_seq
GGCGGCCTTTTCCTTGGCTAATTTTAAGTCTGGGAGGAAATGGTCTAAGCCTTGGT
>dme_circ_0000133
GAGTTCCCGCCGAGATTGCCACCAAGGCTTAGACCATTTCCTCCCAGACTGATACTTCCCTGGCGATATC
CTCCTTGGCTGGCAGATCCGCTCGATCCCAAGGAGGCAGTGCTACCACCGAGAGAGAGACTTCCCGAACC
CACAGCAGGGGTCAGTATACCACTGGCGACTCCATGGGCCAGAGACGCAGCCTCGTGGGCACCGGCTAGT
CCAATCGATCCAGCTGTGTTAGAGTTGTGTCCGTTTGCTCCTATTGACAGGCCATTTGTTCCAA
>dme_circ_0000133_junction_seq
GCTCCTATTGACAGGCCATTTGTTCCAAGAGTTCCCGCCGAGATTGCCACCAAGGC
>dme_circ_0000134
gatgtccgacaccaatagatgcagcttgagaggaaccggtacttccagcagattggatatttagatgacc
agcttcgatggaggcagaacctgatccggtgtgcagggaactagatcccgatgactcaactttcttgtgc
tctgtcttgatgtgggcagatccagtgtgtgatccagaggctccagaaccagcagatcctgagattccag
ctgatccgatgctcaa
>dme_circ_0000134_junction_seq
ctgagattccagctgatccgatgctcaagatgtccgacaccaatagatgcagcttg
>dme_circ_0000135
ggcagaacctgatccggtgtgcagggaactagatcccgatgactcaactttcttgtgctctgtcttgatg

tgggcagatccagtgtgtgatccagaggctccagaaccagcagatcctgagattccagctgatccgatgc
tcaagtggccagatgaaccgagggcagaggatcccttagaggcggaatcagttgctccaatgttcaggtg
tcctacaccaatagatgc
>dme_circ_0000135_junction_seq
tgttcaggtgtcctacaccaatagatgcggcagaacctgatccggtgtgcagggaa
>dme_circ_0000136
TTGCCAGTGGCGTTGTCAACGGTGCAACCTCTGGTTCCGGAGCCAATTCTGGTCACTTGAGCATCGGATC
ATCTGGAATTTCCGGATCCAGCTCAGGCTCCCTTGGCAATGCCAAAATCACTGGATCTTCGTCAACTGG
>dme_circ_0000136_junction_seq
CCAAAATCACTGGATCTTCGTCAACTGGTTGCCAGTGGCGTTGTCAACGGTGCAAC
>dme_circ_0000137
GGAGCTGACGTGATCCTTGGCCAGCTGAAGGAGGGCGTCAGCCGGAGGCGAGTGGGTCTCCAGATGTTGG
GCACCAAGCCACCGCCAGCTCGTTCCGGAGTGGCCATCTTTAGTCAGGGTCAGCAGGTGGGTCAGGTGAC
TAGTGGCTGCCCAAGTCCCAGTGCTGGACGCAACATTGCTATGGGATATGTGGCAGAGAATCTAAAAGCA
CCCGGCACAAAGGTGGAGTTCAAG
>dme_circ_0000137_junction_seq
AGCACCCGGCACAAAGGTGGAGTTCAAGGGAGCTGACGTGATCCTTGGCCAGCTGA
>dme_circ_0000138
atggatggttggttggatggatggatggCGAAGGTGTGTTGTGTAGGAAATGATACGGATACCGGGCGAA
ATAAAGTCGGAAGAAGTCTTCAGCACAACGAAAATAAAAGCTGGGAACAGTGGCGTAGGCGGTGGGGTAA
GATCAGGGG
>dme_circ_0000138_junction_seq
GGCGTAGGCGGTGGGGTAAGATCAGGGGatggatggttggttggatggatggatgg
>dme_circ_0000139
CTAGCTGTGGATGCCGCCCTGCAGGCGGGCTTGGATGTGGAACAGATCACACGCGTCACCGTGCAGAATA
TTCGCCTCGCACATCAGCCACTCGGGGAATTCGGTGAGCCGCAGTCCGGCGAGATCTCTGCAATCGATCA
GCGCAAGATCTCAGCATTGGAGTGGCTAATCCATTTGCCGGAGCAGCGCGGTGAG
>dme_circ_0000139_junction_seq
AATCCATTTGCCGGAGCAGCGCGGTGAGCTAGCTGTGGATGCCGCCCTGCAGGCGG
>dme_circ_0000140
GATGACGATGGAGCAGATCTTCGAGGAACTGAACGTTGCCAAGGATGCGTCGGTGCGGGATTTCGCCCAA
AGTCAACTGGGCATTATCCAGCGTCATTTAATCCTGGATACTTGCGGCGAGCTGATCCAACACATGGTGC
GCTGGGTGGAGAAGGATACCTCCCAACAATCGCCCCATCAATTGCGCTTTATGGCCCACATAGTGCTCTT
CCTGCGTCAAATCGGCCGTGTGGAGCAGGAGCGACAGGCGGAGAAGATCGTAGCCGCATATGTGGAGGCA
TTGATTGCCAGGGGCGAACCGCAGCTAATAGCCTACTATACTGCATCGCTTTCGAATCCGCTGCAGGTTC
AGCTCTACTCGCGGTTTCTGGAGCAAGTGGAGCAGAAGCGTCCGCGGGAGCTAGCTGTGGATGCCGCCCT
GCAGGCGGGCTTGGATGTGGAACAGATCACACGCGTCACCGTGCAGAATATTCGCCTCGCACATCAGCCA
CTCGGGGAATTCGGTGAGCCGCAGTCCGGCGAGATCTCTGCAATCGATCAGCGCAAGATCTCAGCATTGG
AG
>dme_circ_0000140_junction_seq
CGATCAGCGCAAGATCTCAGCATTGGAGGATGACGATGGAGCAGATCTTCGAGGAA
>dme_circ_0000141
GCGAGCACCGAAAACAGGAAGCAAGTTATTCGCATTTCGCTGGCGGCGCATAAAATGGAACGCAGTGGAC
GAATGTGGACGAGAGCCCTGAGAGAAACACAAAAGCACGGAACCTGAGCAACAGCACACACCAGTGATAT

AATAGTAGTCTACGGAACCCCTGATCCACGGCACTTCGCATCTAATCTCACCCAAG

>dme_circ_0000141_junction_seq

CGGCACTTCGCATCTAATCTCACCCAAGGCGAGCACCGAAAACAGGAAGCAAGTTA

>dme_circ_0000142

TACATGTCAGAGTCTAGCAGGATCTGTTCCTCCTGCAGCCCGAGAATGGGATAGCCCGGATTGGTGAGAG
CGTCCTCTGCTGCCAGCACTTCCGTTTTCACCTCCGCCGGCTCCTCTGGTTCATCTTGTTCCTCCTCCTC
ATCCGGCGGTTCCTCCGCTTCGTCGGGCTCTTCTTCGTCCTCGCCGG

>dme_circ_0000142_junction_seq

TCGTCGGGCTCTTCTTCGTCCTCGCCGGTACATGTCAGAGTCTAGCAGGATCTGTT

>dme_circ_0000143

GGTGAAAACGGAAGTGCTGGCAGCAGAGGACGCTCTCACCAATCCGGGCTATCCCATTCTCGGGCTGCAG
GAGGAACAGATCCTGCTAGACTCTGACATGTACTACGAGGACTTCGGCGACATGGACGTGGGCTGTCAGG
AGGAG

>dme_circ_0000143_junction_seq

CGACATGGACGTGGGCTGTCAGGAGGAGGGTGAAAACGGAAGTGCTGGCAGCAGAG

>dme_circ_0000144

GTCCAGAACCATACTTTTTAGATACTGTATCCAAACTCCGGGCAATCCGCTGCCGCTTTATAAACAAACA
GTTAAACAAACCGACCGCTCGAACGTCGCCgtgtgtgtgtgtgcctgtgtgCTTTTCGCCCTCATTGTGC
TCTCGTGCAAATGAAAATTTCATTGAGCAGAAAGTCgcagcagcagaagcagcagcagcagcagTAGAAA
AGTGGAAAATCCTAAAGCGGCGCCAGCCTCAGCaaaaaaagaaaataaattaaaaatCTCGGCTAGTGAA
ATTTCAG

>dme_circ_0000144_junction_seq

ttaaaaatCTCGGCTAGTGAAATTTCAGGTCCAGAACCATACTTTTTAGATACTGT

>dme_circ_0000145

GTTAAATAATTTCTCCTTCGTTTATGTTGCGACTTATTCGATGATTTTCAAATGTGGGGGACAATGGTTG
ATTTTGTTTTCAGTGCACTTAGCTCAGTACGTATAAATTAGAAGTATAACTAAGCAATATGGATTGCCAG
ATAATGGAAACATTTCGTTATTGCATTTGGGGAGCCCAGCTTTGCTAATCTGCTAATCATACATTAGTTA
GTTAATGATTACGTTCGTAAAGTTGATAAATTCAAAGGTCGTTCTATGCAGTCTATGGAAATGATAATGG
AATCTTCTTCCCCTTTCTTCTGGCTACTTAAACCTAACAATCTGCCGCCATTCTGCTCACTTGAATGGTT
GGATATTCGCTGCTTTGCAGGCCACTAGGCCCCATGGTGTCC

>dme_circ_0000145_junction_seq

TTGCAGGCCACTAGGCCCCATGGTGTCCGTTAAATAATTTCTCCTTCGTTTATGTT

>dme_circ_0000146

GAGCTAGAGCTCATCCGACGGGACATTCAGTCGCGCCTGTGGTGCACATATCGCCATGGATTTTCGCCGT
TGGGAGAAGTCCAGCTCACCACGGACAAGGGCTGGGGCTGCATGCTGCGCTGTGGTCAGATGGTTCTCGC
CCAGGCCCTGATCGACTTACATTTGGGACGCGACTGGTTCTGGACGCCGGACTGCCGTGATGCCACCTAC
CTCAAGATCGTGAACCGTTTTGAAGACGTAAGGAATAGCTTCTACTCCATACACCAGATTGCCCAGATGG
GAGAGTCCCAGAACAAAGCGGTCGGCGAGTGGCTGGGACCGAATACAGTAGCCCAGATTCTCAAGATGCG
TCGTGCCGAGAAGGAGGCTCGTGGAAGCCACTGCTGCTCATAATCCCCCTGCGCCTGGGCATCACAGACA
TTAACCCGCTTTACGTGCCTGCCTTAAAGCGCTGCCTTGAACTAGACAGCAGTTGTGGCATGATCGGAGG
TCGACCAAACCAGGCTCTGTACTTCCTGGGCTATGTGGACGATGAGGTTCTCTACCTAGATCCTCATACC
ACGCAGAGAACGGGCGCCGTGGCACAGAAAACTGCTGCGGCAGAACAGGACTACGACGAGACCTACCATC
AAAAGCATGCTGCTCGCCTGAACTTCTCCGCCATGGACCCCTCGCTGGCGGTTTGTTTCCTCTGCAAGAC
CAGTGATAGTTTCGAGTCCCTGTTAACCAAGTTGAAGGAGGAGGTGCTCAGCCTCTGCTCGCCTGCGCTC

TTCGAGATCAGTCAAACCCGCGCGGTCGATTGGGAcaccacggaggacatcgactggcccaccatgccgg
acatcgattggccTGCGGGCACCTCGGATTCGGACTCTTTTGCCATAGTTG
>dme_circ_0000146_junction_seq
TCGGATTCGGACTCTTTTGCCATAGTTGGAGCTAGAGCTCATCCGACGGGACATTC
>dme_circ_0000147
TTGTCAGCCGGCCATCACCACATCCGCCCATTCCCTGCCCCAGCCACGCGAATCTGCAGGAGAGACACTG
CCAAACAAACGATGCAGGGGGATGGATGGGTGGTGCAATCGATGGGTCGGTCAGGTCAG
>dme_circ_0000147_junction_seq
GGTGCAATCGATGGGTCGGTCAGGTCAGTTGTCAGCCGGCCATCACCACATCCGCC
>dme_circ_0000148
GTTCTTGCCATCGACCAGCGTGGACAGGGTCAGGGTGACTCCGTCGCGCAACTTCTGCTGGTAGCCCAGA
CCCACCTGGCTGGCGTTGTTCACCTTAGCGCGCACGCTGGCATCATCATCCAGCTGATACTTGGCGCCGA
TGGCGAACTTGGTGTTGCTGGTGCCGCTGGCCCACGACAGCTGTACACCCACATCCAGCTTGTCCGAAGT
GCGTTGGAAGATCGAGCCGCTGAACTCCTGGCCATC
>dme_circ_0000148_junction_seq
AGATCGAGCCGCTGAACTCCTGGCCATCGTTCTTGCCATCGACCAGCGTGGACAGG
>dme_circ_0000149
GCTCCACCAACAATTTAGCCGACCGGAACTCGGGTTATAGCACTGCTCCCCCATTGCCCCTTCAAACTTC
GAGTTACATATTACAAACTACCCATCAACATGCCGCAAAAGACAAACGGACATAGCGCCAACGGATGCAA
CGGCAGCAATGGCAACTCATATGACATGGAAGACGGAGCTACATTCCTGTTCACTTCCGAGTCCGTTGGC
GAGGGTCATCCAGGGTGAGTGCTTCATTGCATCTGCGGCGCCTCCTATTGCATCATACCGATAGTGTCAG
TGTAAAAAAAGAAACAATCTCAAATGCCCCTTCGGGCGTGGTGAGAGATGTTGCCTGTCAGTTCTCATAA
CCTGACCAAACTGATTCTCTAGCGTCTGAGGCCCTTCTGTCTGAAGTCCCTTTCCCTGTATACCCAGTTC
CACACCTCCACCGTCTACTCACTTCTGCTTACCGCCTGAACTCTAACAAAATGGCGACGAAATTATGTCC
ATACCTTTGTTgatttctacccaatttgcacgacttgaacagaggctgagaacgggaagaagagctagTA
AAACGTGCCTCTGTTGCATCGCCCGGTGACCAAGCCGGAAAGCTGGGACCACGGAGAACAGCTGGCTTCT
CTTGAAATCCCCGCACAACAGCGGGCTAAACCTTGTGCTTGGATGGGCAGACGCAATTAATAGACTGGTT
CTTTATCGTTTGCGGCTCTCACATATGTATGTATGTGTGTACAGAGAGACTACGTCATGCGCACCATGTG
CTTGTGGCTCATGGGAGATGCGAAATTCAAGTATTCTCTACGTTGTTCTACGTTGTCCACGCACTTGTTC
ATTTGATCGCAGATGGCGATCAAAACAACGGCCAACACAAACATACCTTACATTTTTCTAGACCGCTGTC
AAAGCTTATTTTTTTCACCGAAATTGCCAACCACGTGTTTTGCTCGAGCTCGATCTTTTCGCCCCTCCCA
GAGTTTGTTTATATCAGCAGTGGCGACCAGTATAGTAGTGTGTTTGACAACAGCTGTTGATAAGCCGAGC
GACCTTTTATACTACGCATTGATTACTTTTACTATTCTATACTATTTTATTTTATAATGCTAATTAAGTC
GGCTTGGcaagctagccctagacaatctagccctatacaatcGTGATGATAAGCGCCGAGTGCCATTCAG
AAGAATACACCCCAAATGGTTGACATTAATATCGCCTGTCTTTTTGTAATAAATGAGTGACGGACTTGTT
AGAATAGATAATTAGACTAGCCGGAAATTGGAAACCCTGAAACCCATTTCAGAGTAGCTACTTTAAACCC
TTGACCTAACTAGTTTCCTCGCAATCATTATCCCAGCCGAAATCGATACCATGCGCCATTTCTATGAGTC
AACAGTTTTGGTAATTTATGTTGCTATTGCTGACGCATGTTCGAGTTCAATAACGCCTTTACTACGAAAA
GTAGCCGAATGTTGACGGACTCCAATTACGCGCCAAGTTTTGAGGCAAGCGCGAGCAACCCGACTGGTAT
CAGATGAATCCGACTCCCAGGATTTCATGGACGATTCGGCTTCAAATGTCATCATTTTGTGTTTGGACAA
CACTACGGTCGCCTTATGCTCTTGTTCTGGGAATTGAATTTCGCTGTCAATAGCAGACAGATTAAATGAA
ACCGCATGGCCCAGCAAATTTTGATCCGTCGCACTGAATAACATTTTGGCCCGTTCCTCCTTCCACTCCC
CGACAAAAATGCTTTCCCCTCCTTCTTCGCCCACCTCTGGCTTACCTCTTGCTTACCTTAtttttttttAG
GGTAAGTGTTATGAAACCTGGAGTACATGACTGCTCTGACCCAAAATCAGATTTATATTAAATTCGGCCA

ATGAAGGTGCGCTGACCAACAATAAATAATCTCATTCATAGAATTAATCAAGAATCACCGTGAGTGCCAT
TCTTTTTGAAGTTGACTTATTCGATAGCGCCAACATAAAAAAACAAGGTCAAACCACCGGCAAATCTGTT
TTTGGATTACCTGAAAAAGTTGCCGAAATTTCTTTTAACACATTCCATAATATTTAATACAGAAACCGTC
GCCAAGACGGGCATGATTCTGCTGTGCGGCGAGATCACGTCTAAGGCTGTGGTCGACTACCAGAAGGTTG
TTCGTGAGACAGTGCAGCACATTGGTTATGATGACTCCTCCAAGG
>dme_circ_0000149_junction_seq
CACATTGGTTATGATGACTCCTCCAAGGGCTCCACCAACAATTTAGCCGACCGGAA
>dme_circ_0000150
ATTAATGGCGTCGACGAGGTCGCTGGACAACTGCGTCAGTTGAGCGTGCTTGCGGTCGACGCGTTCCAGC
TCGGCATCGATCAGCGGTCCCATCTGATGGACCTCCTGCTCCAGCTGCAGCATTTCGTCGCTGTCCTGCG
AGGGATCTTCGGGATTCGCCTCGTGCAGCAAATGCAGCAGCCGG
>dme_circ_0000150_junction_seq
CGCCTCGTGCAGCAAATGCAGCAGCCGGATTAATGGCGTCGACGAGGTCGCTGGAC
>dme_circ_0000151
TGGCCCCCTTGATGGAAAAGTGCTTGCAGACACAGCATTTAATAATGTAAGACTGCCCTAAGACCTGCCG
TTCTGTAATTACACGCGAGTTGCGGAGCAACCGAGGCAGCCACGCCACTCGACTGCGCCAGGACGCCAAG
AAAGGAGACGAAGCCGAGGACCAACAAGTTGCCAGCCCAGTCAGGAACCCCCGGATTCCCAAGGAAGCGA
AAGCAGCAAAACGGCAGCCGTGGATACCAAGGCACTGCGCAGCTCAGCCACTGTGAAGGTGACGTCGGCG
GCCACGCCCCCGCACCCGCCCCCGCTGACAACGACGACGACGCCCCTGACAATTTGTCAGCCGCCACCGC
CCACTCCGCCCTACCAGCGGCTCACCAAGGCGCTGCAATGCGATCCGCGCTGCGGCCACGAGG
>dme_circ_0000151_junction_seq
CAATGCGATCCGCGCTGCGGCCACGAGGTGGCCCCCTTGATGGAAAAGTGCTTGCA
>dme_circ_0000152
GGAATGATTAATAACGCTCGAGCCGCCTGGAGGATAGTTCGGGGCAGGCCCTGATCCCGGGCCGCTGGCA
GGCTGCTGTGCCTGAGGCTGATTGGGGATCGCATTTGGGTTGTTAACTGGATTCTGTCCGGGAGCCCCCT
GCGGTGGATAGCCGCCTGGATATCCGGACGGGTCGGGTGGATACATTGATGGTGGCGGAGCTTG
>dme_circ_0000152_junction_seq
GTGGATACATTGATGGTGGCGGAGCTTGGGAATGATTAATAACGCTCGAGCCGCCT
>dme_circ_0000153
GTTTTTGCATTTTCTCCGGTGAGAGACCCATGCTGCTATAAAGAACATTGTAGCTGGTCGGCGACACTGT
ACCCTGACGAACTTGTTGCGAGACCAGATAAAAGTCGTATCTCTCGGGCAGAGTTATAACGTCATCAACT
ATAGTACCAGGCGGAGGATTTTGTCCGTTGAGGAAGAAGCGCGTGTTCATGGATCTGGTTACCACAATAT
AAGCTAATTGCGGTGGGCTTAGCTGGACGCGGGCGTATTCAGTTTTCAACTTCTCAATGATGTCCTTGAC
TTCAAATTCAAAAAGCTGCTTTAGAGAGCCGGAGCTCACACCGTCTCGATAAAATACGATTCGAGATGGC
AGCTTCCTATGCTCATGTTGATATTGGCGCAGGGCCTTTGCTATCATCGGCCAAAGGGTGTTAGCGAGCA
CATCAAAGGCGCTGCACTCCGTGACTGTGCTGAAGTACGTGGA
>dme_circ_0000153_junction_seq
ACTCCGTGACTGTGCTGAAGTACGTGGAGTTTTTGCATTTTCTCCGGTGAGAGACC
>dme_circ_0000154
CGGGTGAAAGTATTCAGAGGATCTTCATCAGGTGACCCGAGAGCGGATCCTCGTATAGAGGCTTCAAGAG
AGAGAAGAGCTCTCGAGGAAGCTCCCAGGCGTGAAGGTGGCCCGACAGAGCGAAAGCCGTGGGGTGACCA
ATATGATTACCTGAATACCCGTCCGGCTGAGCTGGTATCCAAGAAGGGAACCGATGGCGTCCCGGTCATG
CTGCAGACGAACTTTTTCCGATTAAAAACCAAGCCGGAATGGCGGATCGTTCATTATCACGTGGAGTTTG
AGCCGAGCATCGAGAATCCTCGTGTCCGTATGGGAGTTTTGTCCAATCATGCTAACCTTCTGGGATCAGG

CTATCTATTCGATGGACTGCAACTGTTCACCACCAGGAAATTCGAGCAGGAAATCACGGTGCTCAGCGGA
AAGTCGAAGCTGGACATTGAATACAAGATATCCATAAAGTTCGTTGGATTCATATCGTGTGCTGAGCCCC
GCTTTTTGCAAGTCTTAAATCTAATATTGCGCCGCTCCATGAAGGGCCTAAATTTGGAATTAGTTGGCCG
TAATCTCTTTGATCCCCGAGCTAAGGATCGAAATAAGGGAGTTCAAAATGGAGCTATGGCCGGGCTATGA
GACATCGATTCGTCAGCACGAAAAAGATATTTTATTGGGCACCGAAATAACTCACAAAGTTATGCGCACC
GAGACGATCTACGACATAATGCGACGTTGCTCACACAATCCGGCTCGTCATCAGGACGAAGTACGGGTAA
ATGTTTTGGACTTGATTGTCCTTACGGATTACAATAACAGAACTTATCGTATCAATGATGTCGACTTTGG
ACAAACTCCGAAATCAACATTCAGTTGCAAGGGTAGAGATATCAGTTTCGTGGAATACTATCTCACTGAA
ATATAATATACGCATTCGCGACCACAATCAGCCGCTGCTGATCTCCAAAAATAGGGACAAGGCTCTAAAA
ACTAACGCTAGCGAATTAGTGGTACTAATTCCTGAGCTCTGCCGAGTGACTGGGCTCAATGCCGAGATGC
GCTCAAACTTTCAG
>dme_circ_0000154_junction_seq
CAATGCCGAGATGCGCTCAAACTTTCAGCGGGTGAAAGTATTCAGAGGATCTTCAT
>dme_circ_0000155
AAGGGAACCGATGGCGTCCCGGTCATGCTGCAGACGAACTTTTTCCGATTAAAAACCAAGCCGGAATGGC
GGATCGTTCATTATCACGTGGAGTTTGAGCCGAGCATCGAGAATCCTCGTGTCCGTATGGGAG
>dme_circ_0000155_junction_seq
ATCGAGAATCCTCGTGTCCGTATGGGAGAAGGGAACCGATGGCGTCCCGGTCATGC
>dme_circ_0000156
GCTCGGAACGCCCGGTCCAGTATCAGACGACAAACCAGCACTATCAGCACTATTTTTCGTACTTCTACAA
CCGTGGAGCGGAGAAACTGTCCAAGCCAACGTTTGCCGCTGCCAATAGCCGCAAAGTGCCAATGAGTAAA
TTTCAAAAGGCCGGCGCCAGTCGCAGGGATTCGCGAGAAGTCGATGGCACAGTTATCATTGGACGAAGGG
ACAATTCCGAGGACAGGACCTGGCAGGGTCAAAAGTATGAGAAGCACGTGCTAAAGGAAAAGCTCCCAAG
CAATGATTTAAAAGCCGATTACCTAGACAATGGCTGGGGAGGAGACCCTGGAAGCGAAGATGACTACAGG
GACATTAAAGATAGCTATGACCCCGTTGTTGTAATTGACAATGGGCTGGAGCAGGTCGGAGTACATCGGC
GCTACTGCAGAATGACCGAAAATACGGTCCTGGATGGCTCCGGAGACACGCTTATTAATAACAACAATAA
TGAATTAAAGATTATGCGTAAGTCGACGGATCAGCCCATGCCACGGTTGCCCATGACGCCAAAGAGGGCC
AAAGGTGGGACCGTGTCTACCAAAAAGCCAATGCCCACGGTTCACCGAGTCCTGCTGTACAGCACTCAGA
GTCCACGGCTCGGCCGCAAGCAGCAGTTCAGCACCCAGCGGCTAAGTGTAAAGCAGCAGAGCTCCAAGGT
ACTGGGTGCAGGTGATGAAAGACAGACGGTCTTATTGCTGGAGCCGGAACACAAACAGGAAGATCACCGT
AGTGCCATTGACTTCGATCGTAATGACGACTTCGACGAAGATGATGATCTCGACAATTGTATCAAATTTC
GACGAAAGCGACACGGCGAGTGTTTTGTCAGACACTGAGGACGGTTATCGTGCCCATGCCTTCAAGCTTA
CCCACTCTGTGGATCGATTTTCATCGCCCCAAAGCACGCCCAGCTTCTTGTCTTCGCAGTCGTCTGAGGA
TGATCTGAAAAATCCGGATTCCCTTCTGGTTCCCAGTTTGTTTCCCTACGTGCCCCCTTATTTGTCTTTT
TCGTCGAACACAAAAAGAGGTCCACGCGTGCCGCCGGATCTTCATCGCGTTTTGAAGTGGCGCATTACAA
ACATAATGCCAAAGGTAGTTCGTCTGATTCTGGCCAACAGCGGAATGCGAATGCTAAAGAGAAACCAACG
ACTGGATGGGCGTATGGGGAAAGCATTTAAAGTCACCGTGTTTTAAGGCCATTCGTTCGTATCAGAAAAT
TAATCACTTGCCCGGTTCATTTCGCATCGGCCGAAAGGATTCGTGCTGGAAGAATCTGCAAAGGCAGATG
GGTAAGCACAGCAACAAGGAATTCGGGTTCATGCCACGCACATACATTATTCCCAATGATCTGGGAGCGC
TGCGCCGGCACTGGCCGAAATATGCCCAGCGAAATACTAAGTGGATTATAAAGCCACCAGCTAGTGCTCG
GGGTGCTGGCATCAGGGTTATCAACCGATGGGGCCAGATACCCAAGAGAAGGCCCCTTATCGTACAAAAG
GTATATAGAGCGACCGCTTCTAATAAATGGTAGTAAATTTGATCTGCGTCTGTATGTCTTGGTTACATCA
GTGAATCCCTTGCGAGTGTTCATGTACCACAACGGATTGGCACGGTTCGCTTCAGGTAAAGTACAGCGCT
AAAACGGACACTTTGAATGATCGGTGTATGCATTTGACCAACTACAGCATCAATAAGTTTTCCTCGAACT

ATTCGAAGAACGAAGACGTCAATGCCTGCCACGGACATAAGTGGACTATCAAGTCGCTGTGGACGTATTT
GGCGAATCGTGGAGTGCGTACCGACTGTCTGTGGGAGGCTCTGAGGAGTTTGGTGCTTCGGACAATTCTT
GCTGGCGAGAATGGCATTAATAGCATGATTAGAGCAAATGTTGAATCAAAGTACAGCTGCTTCGAGCTAT
TCGGCTTCGATGTTATTCTGGACTCTGATCTTGTGCCTTGGCTGTTGGAAGTTAATATTTCGCCCAGTTT
ACACTCGGAACTGCCCCTAGATGCTCATGTTAAAGCGCCTTTAGTGCAGGGCGTTCTTAATACAGCGCTG
TACAACGGTACCTCCAAAACTATCGCTTGATAAGCAAAAAGAACTGGCCGCAGAATTTTCCTTTCCTCCC
GGCACACAAATGTGCTATGACAAGCGGCTTTACATTAACTACTTGTCGCGCGAGGAAAAGATCAAACATA
ACACATTTACCCGAAAGTCTATGGAAGACCGGAATGAGGTATGTGGATGCGATCCTAAATAATCTGACCC
CCGATGATGTGAGGTGCCTTATCATAGCCGAGGATGAACTAGCGCGATGTGCACCGCTGGAGCGCATCTT
TCCAACTGATCAAACCCATAAGTACCTTAAATACAACGACACTCCGCGGTATTACAATCGCCTTTTGGAT
GCCTGGGAATCCCGCTACGCTAACAATCGCACAGAAGGGATAGCGTTGTTGCGCGATTATTGTCAAAACA
AATATCATCTTCAAGTCCCAACTCCTCCAGCCAAAAAG
>dme_circ_0000156_junction_seq
TCAAGTCCCAACTCCTCCAGCCAAAAAGGCTCGGAACGCCCGGTCCAGTATCAGAC
>dme_circ_0000157
GGACCTGGCAGGGTCAAAAGTATGAGAAGCACGTGCTAAAGGAAAAGCTCCCAAGCAATGATTTAAAAGC
CGATTACCTAGACAATGGCTGGGGAGGAGACCCTGGAAGCGAAGATGACTACAGGGACATTAAAGATAGC
TATGACCCCGTTGTTGTAATTGACAATGGGCTGGAGCAG
>dme_circ_0000157_junction_seq
TGTTGTAATTGACAATGGGCTGGAGCAGGGACCTGGCAGGGTCAAAAGTATGAGAA
>dme_circ_0000158
GAAATCAAGCATCCGGAGGCAATGGTGGTGGTGGTGATGCTCAAGTGGGTCCCTCGATAGAGAAATCCAG
TTTGAGCGCGGTGCAAATGCACAAGTCCGAGGGTTAGTAGTTTTATTTGGTTTTGCTCAAAAAAATTTGt
tttttttttttCGAACCCAGCTTCTGTGAGCTGTTGTTATTCTCATTTGTTTTCAATGCGTTCGTAATT
AATTAGTAATTTAATTCAGGCGATCCGCGTGGCTCCGTGCGCGGCCGTAGGCTAATAACGGATTTGGTGT
ATTCACGTCCTCCAGGGATGACCAGCAAGAAAGGAGTGGTTGGCACTCATATTACCGTGCAGGCAAACTA
TTTTAAGG
>dme_circ_0000158_junction_seq
ATTACCGTGCAGGCAAACTATTTTAAGGGAAATCAAGCATCCGGAGGCAATGGTGG
>dme_circ_0000159
TTCATGATAACTTGAGATTGAAAGCACTACAAAAGGACCTTTAGGAAGAGATCCCGCTTAAGGCAGAGGT
AGTCCTCTGCTGGTTCCCTCTGGCACACATATGGAAACTGAACCTTGCACTCTATATCGTGATACCCAAA
GGCTCCATTGTAATTGGCGAATCCAACACAATCCTCTTCGGGGGAAGCGTTGTTCGGTTCCAGGGGTACC
CAGTTTAGGTACGGCACAGTTTCGCCGTTCCTGGCGAGAAGAAAGGTGCGCCGGTTACCCAAGCAATTGA
TGCCAGCCCAAACGGAGTGATGCCAGCTTTGGGTGAAGGATAGCCCCATACTCTTTAGGAACGTGGTTGT
GAGGAGTTTGTCCTCTTGATTG
>dme_circ_0000159_junction_seq
GGTTGTGAGGAGTTTGTCCTCTTGATTGTTCATGATAACTTGAGATTGAAAGCACT
>dme_circ_0000160
GGTTGAAACACATTCCAAAAGTCTAAGACTCTAGCTTCACAACGGTCGTCTTCTCGGACACGTACAGACC
GTCAAGGAACTTACGGATATCCTTGTTCTTGACGGTCGTGGACTGCTGGATGAGGGCGGCAGATCCGGAG
ACAGACTCAATATCGTTTCCCTCCACGATAAGTTCGTCCTTCTGGGCA
>dme_circ_0000160_junction_seq
CTCCACGATAAGTTCGTCCTTCTGGGCAGGTTGAAACACATTCCAAAAGTCTAAGA

>dme_circ_0000161
GGTTGAAACACATTCCAAAAGTCTAAGACTCTAGCTTCACAACGGTCGTCTTCTCGGACACGTACAGACC
GTCAAGGAACTTACGGATATCCTTGTTCTTGACGGTCGTGGACTGCTGGATGAGGGCGGCAGATCCGGAG
ACAGACTCAATATCGTTTCCCTCCACGATAAGTTCGTCCTTCTGGGCAGTGGA
>dme_circ_0000161_junction_seq
CGATAAGTTCGTCCTTCTGGGCAGTGGAGGTTGAAACACATTCCAAAAGTCTAAGA
>dme_circ_0000162
ATCCGTAACTTCTTGGGTGAGAAGTACATCCGTCGTGTGGAGATGGCTCCTGGCGTCACCGTGGTCAACT
CCACTGCCCAGAAGGACGAACTTATCGTGGAGGGAAACGATATTGAGTCTGTCTCCGGATCTGCCGCCCT
CATCCAGCAGTCCACGACCGTCAAGAACAAGGATATCCGTAAGTTCCTTGACGGTCTGTACGTGTCCGAG
AAGACGACCGTTGTGAAGCTAGAGTCTTAGACTTTTGGAATGTG
>dme_circ_0000162_junction_seq
AGCTAGAGTCTTAGACTTTTGGAATGTGATCCGTAACTTCTTGGGTGAGAAGTACA
>dme_circ_0000163
GCTTCACAACGGTCGTCTTCTCGGACACGTACAGACCGTCAAGGAACTTACGGATATCCTTGTTCTTGAC
GGTCGTGGACTGCTGGATGAGGGCGGCAGATCCGGAGACAGACTCAATATCGTTTCCCTCCACGATAA
>dme_circ_0000163_junction_seq
GACTCAATATCGTTTCCCTCCACGATAAGCTTCACAACGGTCGTCTTCTCGGACAC
>dme_circ_0000164
GCTTCACAACGGTCGTCTTCTCGGACACGTACAGACCGTCAAGGAACTTACGGATATCCTTGTTCTTGAC
GGTCGTGGACTGCTGGATGAGGGCGGCAGATCCGGAGACAGACTCAATATCGTTTCCCTCCACGATAAGT
TCGTCCTTCTGGGCAGTGGAGTTGACCACG
>dme_circ_0000164_junction_seq
GTCCTTCTGGGCAGTGGAGTTGACCACGGCTTCACAACGGTCGTCTTCTCGGACAC
>dme_circ_0000165
GACAGACTCAATATCGTTTCCCTCCACGATAAGTTCGTCCTTCTGGGCAGTGGAGTTGACCACGGTGACG
CCAGGAGCCATCTCCACACGACGGATGTACTTCTCACCCAAGAAGTTACGGATCTCAATGACCGTGTTGT
TCTCGGAG
>dme_circ_0000165_junction_seq
GATCTCAATGACCGTGTTGTTCTCGGAGGACAGACTCAATATCGTTTCCCTCCACG
>dme_circ_0000166
GTGGAGAAGTGGTTCGGAACCAAGAAGGAGTTGGCCGCCGTCCGCACCGTCTGCAGTCACATCGAGAACA
TGATCAAGGGTGCGTACAGCTAGGGTAAACACAACAGGAACACTTTCACAGCCAGGCCTTTCATTTGCCA
ATCACACGAGAATTTAATAATAATATCTGGGAAGACGATTTACTAAACTCCATTCATAGATATCAATTTT
CTTTACAAATTACCTAAAAATGCTATATAGTAGTTAGTAAATATTTTCAGTGTGTGCTGGCAAATTAGAT
TCGGCATTAGGTTCCTAAAGACATTTGCATTCAAAGTACCACACAATTAATTGCAATTTCTCTCTCGCCA
GGGAGTCACGTTTGGATTCCAGTACAAGATGCGTGCTGTGTACGCCCATTTCCCCATCAACTGTGTCACC
TCCGAGAACAACACGGTCATTGAGATCCGTAACTTCTTGGGTGAGAAGTACATCCGTCGTG
>dme_circ_0000166_junction_seq
TTCTTGGGTGAGAAGTACATCCGTCGTGGTGGAGAAGTGGTTCGGAACCAAGAAGG
>dme_circ_0000167
GACAATGACCTCAATGGGCGTAGTGAGAACCAGGAGGTACTTGACGTCCTGCACAAAGACGCCGGGTTTT
GGTTTCACCAGGCCAACGCTTACGATCAGATGACTGAGGCCATCGTAGTACGCCACATCCCTCGTCTGAT
TGAACGTCCAGATG

>dme_circ_0000167_junction_seq
ATCCCTCGTCTGATTGAACGTCCAGATGGACAATGACCTCAATGGGCGTAGTGAGA
>dme_circ_0000168
GGAACATATGCTGCGAACCACCCTGCATATGTGCAAGGAAGCTGCTCCCACATTGCCACTGCATAGTATT
TGCATGCAATTCATATCGGCTGACTTCTTTGAGGGAGTGATCGAGCTTTCGGCTGTTTGTGCCTCAAAAA
GTGATCCAGAGGAGGTTGGTGTTCATTTCTATAACAATGGGGAACCCGCCGATGATCGCGAGGGTTACAC
TTGCTTTGCCACAAGGGATGGCCTACTACAAGGAAGTGCAACTTATGCTGGACCATATCTACCAGAGAGT
ATGCAACAAGACGCACGTTCAGGATAAGTCTATAAATCCACTTAAAGGTACGGCTAAAGCGTCCGATGCA
AAAAACGGTGCTACGCAAACGATTCCAAAAATAGTGGCCCATACCCTAAAGGTGAAAGACCCGCTCATTC
ATATAACGCTCTACGAATGGCTGCTGGCCCACGACATGCTCAAAGAGCTTTTAGACGTGGTTGAGCCGTC
ATTAGGAGAGTTCTTGCGGCGCAGTGTGTCGCAAAAT
>dme_circ_0000168_junction_seq
GTTCTTGCGGCGCAGTGTGTCGCAAAATGGAACATATGCTGCGAACCACCCTGCAT
>dme_circ_0000169
GAGCAGTTCAATGTCCAAATTCATACTCACCAGTTTTTCCGGCACTATGCCCTCGGGAAATCGCAGTTGG
CAGGCTTTAACTTCCAGTTCCCTTATGAGAAAGGCAAAGGGAAAGCAAACTCCAGACTCGCCATACTCCC
TTACCAAGATTTCGATTTTCGTGAATAACCTATTGCAGCGCTCACTAGTTGTGCCCGGCTTATCCACAAC
GCTGTTGATAATCTGGCCCCAAACGGACTCGATCAGTAGGGGGTCGTTGTGATGCGAGCAGTTGAGTATG
GACAATTGGCACTCCCAAAGGTCGAAGGGCTCCGCAAAGTGCTGG
>dme_circ_0000169_junction_seq
AAGGTCGAAGGGCTCCGCAAAGTGCTGGGAGCAGTTCAATGTCCAAATTCATACTC
>dme_circ_0000170
GCTGCCAAGAAGATGAAGATCGACACGTGCGTCCTGCGATTCGCCAAACTCACCGAGAATGCTTTGGAGC
CGGTGAGGGGATCCGCCAAAGCGGCGGGAGTTGACCTGCGCAGCGCCTACGACGTTGTGGTGCCGGCACG
CGGAAAGGCCATTGTCAAGACCGATCTGCAA
>dme_circ_0000170_junction_seq
AAAGGCCATTGTCAAGACCGATCTGCAAGCTGCCAAGAAGATGAAGATCGACACGT
>dme_circ_0000171
TTCCTCGTTTACGTAGCAACAGGAGCCGGAGCAGCTCCCTCCGGTTCGGCAGCCTTTTCTCCGTTGCCGT
TCTGCGCCTTGGCTGCCGGCAGATCCTTGACTCCGGTGGAACCGAATCCTGCCTCGCCGCGCTCGGTGTC
CTCCAGTTTGTCCACCATCACCAG
>dme_circ_0000171_junction_seq
TGTCCTCCAGTTTGTCCACCATCACCAGTTCCTCGTTTACGTAGCAACAGGAGCCG
>dme_circ_0000172
GTAACGCACCCACCGACATACTGCAGCAGAAGCCAGCCAAGGCCACCACCGCCATTGTGGGCGGTCGCAG
CGCAGCCCAACCGGCGACCCACGACGACCAGGAAGCGGCCACGACCGCTTCCACAGCCACCAAGCGGAAG
CATCTCGAGCGGTTGGCCAGCGATCTGCGGCAGCTGGGCAAGAAGGGCAGGAGGAGTCGATCGGTTAGTC
CCTGTAGTAGATATCCCGCCGTTGCGGAAACGGCCAGAATGCTTAGCGAACGATCGCTGCTGCTCAGCGG
ATATGGAGCCATTGAGAGCAGTGCCAAGAAGCTGAGCGAAGCGGCGACCggtggtgcgggtggtggtggt
ggtggtgCGGCCGGTCAGGCTGCCACGCCCACTACCAGCCAACTAAGTGCACCCGCCTCCTCAGCATCCT
CCTCATCCATGTCGTCCTCCTCTGCCGGCAGCACTTGCACCACCAATGCAAATACGGCATCGGTGGCAGC
CGCCTATGCTGCTTTCTATTTGGAGAAGGTCAAGCATGAGAAGATGGACAATCATAAGGATGCACCGATG
GTCACCATacacaataacaacaacaacaacaccatcaacaacaacaacagcagtagcagcagcagcagca
gcatcatcaacaaccatcaatcgagtcaaCCAAGTCTGGGCCACAGCGTCAAGAGGGAGCGGCTGAGTCC

AGGGAGCAATAGCAGTAGCCACAACGGCGAACTCACCGTCAGCTCATTTGCCAGGGTGagtggaagtgga
agtggaagtTCGGGGGTACACTCCGGAGAGACATGGCTTTattgaatcattgattcattgcatcactgat
tcGTGATATAGCCGTGACCCATGTGATCTGTTCGAATGATTCATTAGAGCAGCGCTCAACCGATCACATA
ACAAATTGGTTTGTCCACTCATATTGATTATAGGGAAAGATTTGGTAAAAGTACTTTATGCTAATTTAAT
TGAAAGGTAGTTTCGGTAGTTTTAATTaaaaattaaaaaaaaaTGAAACCAATCTCTATTCCGGCTTGCA
ATTGGCTTCACACTTTGGGTGATTCTCCGTGTGATCTTCATCCTCCGTATCCGCCATGTTGCATTGCTCG
CTATGGCTGACGTAGTTGTAGTGCTCACGCCGCAACTCGAGAAACCTCATTCTTCGCCGGAGTCGCTCCT
CCGGAGTTTCCTCGGTCACGACGAGGGTCTCGCTACTTGGCAACGCAATGACAACCGAGGGGGCACTTGA
AATATCAGGACGGGGAGCGTTGGGGTGTCGCAGAATCGAGGGGGATGCACTGATGGTAGGATCGGAATAC
TGATCTCGAACTGCTCTCTGGTTGGCATCGTCGTCACTGGAATCATCATCCAACTGTTCCGGTTTATCTT
TCTTATCCTTGTCAGACATATTCTAGAGACTTACATGGAATCGCTATTAAAATACGTAAATTTCTGAGCC
ATGTTGATTTGGAAAAGTAAAGAGAGATATCATTTCCCATTCGAACTTGCCACTATTTTGGACTATTTAT
CAAGCGAATTCAACGCCTGTCGACGCCTGACAAATTAATTAACATTTTAATAAATTAATACCTCGAAGAG
AAACCAAACCGATGCGGCTTCTGTTCGCAGCCAATGGTAAATTAATCAAAACCACGAAACAAAAAAGCGG
CCAACACGAATGTGTCTACACAAGAGCACGCTTTTGCTGTCaaaaagaggcaataacaaagaacccgcca
agaaaagaaaaagaaaaacaacttgaaaaacttgaatttaaaaacaaaTAAGACGaaaaaaaaaaaTATA
ATAAGGCCCAGACGGCGACTAAATGTGATTTGACAAGCGAAATCAGCAGCAATTAATACGCGCAACTAAA
TTAATGGTGTTAAAGATTATATATTAACTAATATAAATAAAGCCAATTTATCTTCCGAGCGCaaaaaaaa
aaaaagagaagaaaaaaaccaaaaCGAAATACCTTAACAAGCCTTAGGCCTTAGCAGaaagatacaaaga
tacatagatacaaagatacTAGTTGGGCCATATGATTTGGGCGAACGGCTGACCATATTAATATTAATGA
GACATTCTAGACTATCGGCACCTGGGGTGTGGAAATTGAGTGGTGCGACCACGACAGATTCGGATTTACG
TATAGTCGATTCGCATCTGGCCGTGGGTTCACTCAATTCGTCGGGGTGGTCGTCTGAAGAGAGATTGGAT
AAGCCGCATCATTTTCATAATTTATAATTGTTTATACTCAAATTAGAGTGGCTTATTAAGCCAGCCTTAA
TGCGATATGACAAATGAATGTTTAAGGGTCGGACAAATAACTGGTTTGAAGACTGTGTTTATATCATAAA
TACATTATGCGATTTTGGACTTTTGAAGAATTAATGTAAATTCGATGCGAATTCTTCTCACCTGTCGAAT
ATAACTGATAAAATGTATCCCCTGACGAAATCACGAATGTTAAATAAATATAGTTATACACATAACTTGA
TTTTGCTACGTGACCATTGACAGAAATACGATGTCTGTCTGCTTCATTCAGTTTGTAttttttttttttt
gtttttATTTATAAACTTAATTTGCATATTTAATACATGTTCTTAATAGTATTGGAAGTTTTAAAAGATT
TGGCTAGACTTTTTCCCTAGGCTTGGTCCAATGGCTTTTTAAGTTCttttgttttttttttCACGTCAAC
CAATACTCTTTGGACATATGCGAAATCTTGTATCTTTTACAGATGCATACAGCGCGTTTCAAAACCATAA
ATGTTCTTTTGGAAATAAAAATGGTATTACTTTTTAAAATCTAATTAATATATTAGTTTAAAAAGTGCCT
TACATATGCGTGGTTTTGACCTTAAGGTATTAATACCAAGTGGGTCTAGTTTCTATCGATGAAATTTTTG
CTAAGAGCGCTTTGTCAGTTTCGAACCGCACCGTATTCACATACATTTATTTTTATTAGGGACTTTTTGG
GTCTTGTGTAGGCGTAACAATAGACACCATTTGCATACCGAGGCGGGCACATGTTGCCGGTTGTATAATA
AAAACCAATTAGGCATGCCACCGATCTAAAGATCTTAGACAACCAGAGCACCCAAACCGTTAGACACACA
TCGAGATTGGGTGAAGTCGGAACTGGGAGGGCGATCGGTGGCAGTAGCCAACATAATCGCCACCATAAAC
CATTTTTGCAATGCTTTTTTGCTTCCACTACttttttttttGGGTTGATCTGGTTGCTGTTCAAACAATT
GCCAAGAACAAGCAACAAAACACAACTAAGTAGCTGCTACCAATCGCCAAAGCAATTTGCAATAATCAAC
ATTTTTAACaaaaccgaaaaaaaaattactaaaaaagatacaacagaaaaaaatgagacaaaaaagagcaa
caaaaaaaaaaGTTGTAAGCAGCAAGAGGCAACAACGCCGAACCGGCAAACGAACAAAATAACAGCAAAA
AATCGACAACCAACAGATCACGATAAATCAATGGCAACAACAACGCAGGGAGCCAGCTGAATGGGCAAAG
GGAAGGATGGGGAGCGGTACGAGGAGGCAAGTTAGGCGGCAAGCTGCTATATATAAATTTGGTGCCGCAG
CCTCCGAAAGAGCGACACGACATGACCAACGACCAGCGACGACGACGACGTGCAGCACGAGTTACTCTGC
CATAGTCGGGCCATGCCCCCAAACCACCTAGCCATGTGGCATGCAACCAGTGATGGCCAAAAACAAAACA

GCCAAAATGGAGCTGGAAACCAGAAGAAACTATAATATTATTGTTCATTCGTTAAGaaaaaaaaacaaca
aaatatagtttctaaattataaaaactatagtataaaacaacaaatCTATTATAAAAGGATGCGTCCTTA
TTTCAATATTATAATAATTCATATATTATTTTATTGACATTATAACATAAAAAGTGCAACTGCCAGCTCT
GTTGCAATGCAGCGCACAGCGCTTTCGACGAGCTGAAATAGAAGCAGGCACTTGCAGCTGCCACTGGCTG
CGGCATGGTTCGGTTTAGGAGGTGACCGCTGCCGCCACTGCGGATCTGGCCAGAAATCTATGCTGATTAT
TTTCTTTTCGGCCAGCATTGAGTTCGTTTGGCTCGTGTTGCAAAGCTCTAGGAATGGCTAATGGCTGGCT
ATTGCAGCAAGGGGCAAGGTGGAGCATTCGACTGCGCCTTGCTGGTGGCATGGTAGTTGCATATGAGTTA
TGGCCATGCCAGCCAGATTGTAGTCCACAAATGCTCAACATTGGTTAACATGAAATGTTTCGTTTCGAAC
CGAGAGCATCTGAAATGAAATTGTTTGGGAAATGTTCCGTGATCGTCCTAACAAGATTGAAAGTTACTGA
AAAGAATTATGAGAGATACACAGGCATACGGTTGTAAAGAAGATACATTTTAGTGaaaaaaaaTAGTTCG
ATTCTTGTGTTTCTTCTTAAATTCTGCAAAAAGTAATCACAATGGAATTGTACATATTTACCACCTTACT
TTATAATTTGTATCTATAAGCCACTCTGTTCAACAACTATATACCAGTTGGAATTAAAACTAAGTGTGAA
CTCTCCCAGGCTTCTCATTTGGATACCAGTTATTGGACTCCTTTCGCCAGGGAACTCCTTGCAAAACAGT
TCCCATGTCAAATCGCATTTGTAATCGCATTATGACCAGAGTCTTGCCACTTCTCCAAAGAATTAAACTA
AATATCAAACTTCCACAAGACGTAAATCGTGGTCGCAGCTGGCTGAAAAACTTGGATGCTTTTTTCTTCA
TAGCAGAGAAGATCTCACGGAAGCCACTCATTAGCATGTCAAATAAGCCAAGAGAACTTGGCTCGGATGC
TGTGCGGAATGACAAATAATCCAATTGTATGCGACACCTTTTCTGTCGTTGAATGGGAACTCGAGATAAT
TCAATATAAATGCAAATTTACGAGAAATCATGGGGTTAGGTTATAGTAAATATATCATGCTGATGTCACA
CTCATCCACTTATGCAAAACCTGCTAAATGTTCTCTTTAACATAACTAATCCGTATGAAtttttggcatt
tttcttacccttttttttctGACAGAATCTCGCAGCGCCACGCCCTCGTCGTCATCGTATCGTGGTGCCG
GCGGTGGTGCTGGTGGTGGTGTCTCACCcagccaccagcagcagcagcaacaacaacaacagtccctgca
gcagcaacaacgcctaagtccgcccagtggcccatcgatggcaacagcaaccactacatcatcattatca
tcatcatcatctgcatcggcatcggcatcggcatcagcaagtgcagcCACACACATGCCGCTGCACAATT
TGTCAACGAATTTGCTAAATAGCATTcagcaggctgccgccagtcagcagcaactgcgcagcagcaacag
caacagcagcagcaacatcagcggcagcagcaacaacaccaAATCGGATGGTGGTGCCGCATCGAATGCC
ACGGATTTCTCCACCCGCAACTATTCGGATATTATGCGATGCCTGGCGGCCAAGTACAACAATGCCAATC
CCAATGAGTAAGTTTCGAGTTAGCCCTGAAATAACTTTATATTTTCCTAGGCCATATTCCCACCGAGAAA
AAAATGTGGGAATGTCGTGGTAAGTTTCGAGTTAGCCCTGAAATAACTTTATATTTTCCTAGGCCATATT
CCCACCGAGAAAAAAATGTGGGAATGTCGTGTGAGTTATACACGTTTAAACTTAAGATCAATATGATATG
ATAAACCATACTTCGAAACTGAGAAGAACCTTCGGAAATGCGTAAAAATATTAAGATCATAATGAAATGC
TTTTTTGGTAAACTATTAACTAAGTTGTACAACATTTTCGTTCCGTGCAGCCAATAACGCAACTCGACGC
AATTCCTACTATGACGGCAGCACTTCTGCGACCACAGCCACTGGAGGAGCCGGCGGTGGAGGCGGAGGAG
CAGGAGGATCAAACTCTGCCCCAAGCAGCGCCCTCAAGACGGCTAGCAGCTCCACCGGCAGCACTACGAC
CACTGCCAAGAAGCTCTCTCCATCGGCTGCATCGGCGGCGAACAGTGCTGCCGTCAGTCTGTCCAAGGAG
TCCAAAGTGAGTGCGGTCGGTGGTGCAGGTGTGGGTGGTGCAGTAGCCAGTAGTGGTGGCGGTGGTTCGT
CCATGTCGCAACTGGCTCCACCACCCACCGCTCCATCGCCCACGGAGCAGGCCAAGAGCCAAATGGCCGC
CGTGGTGGCAGCAGCAAGTGTTTCGCCGTTTATGACCAACTTTCTGCCCTTCTCGTCTGGCATTTTCCCG
CCGCTGATAGACATGAGCAGCACCCAGGCACTGCTGACACTGGGTGAGTAGCATTAACGAGTAGTAGTTG
TTCCATTGGTGGTCAACAAAAAATGTTACCAAATAATGGGGGTTCCTCTTTAATGGGATATTTAGTTTGC
CACCTGTAGGCAGTACCACTTTATTAACAGCAAAGTATGAAATACATACTACCGTACACCCACACTAAGC
TTCGTTAATCCAAGTAATTATTTTCTGTTTTACTTAATCCCTTCTCttttttttttCTGTACCATTTCAT
TCAACCCGATCTCCTCAGGGCGCGTGCTGTGAAAGATGCCGAAATCCAGGAGATTCTGCGCAGCAAACAG
CAGCGCTCCGGTTCGAATGCCTCGTCGCCGAGTGCGAGTGCGGCGGGCACGCCACGTTCCAGTTCCACCC
TGAATGCGGCACTGCATCAGGCAGCCCAATTTGTAACACCCGCTCTGATCTACTCGGCCcagttgcaaca

gcaacaacagcagcagcagcagcaacatcaacggcagcagcagcagcaatcgcagcacGCTTCGCGGCAG
TCTAGTCCACGTTCCACCAATGATTCCACGACGGTGGCGCCCATCGGTGACGGCGGCGGTGGCTCCTCGA
TTGCGTCGGCGGCGCCATTGGATTTGAGCTCCCAGCCGCCGGCCGCCAAGCGCTTCAAGGCAGAGCGTCG
TGCCAGCTCTACGGCCACCACAGCAACAACAGCCACCACAGGCTCATCCACGTCCACACCATCCCCGCCA
CCGTTGGAGCAGCACGACGATATCAATTCTGGCAACGCCAACGCGACACTAGGCACGAGATCGGGAACGG
GAACGGGTGAGAGTTCTGCAGCGGGATCGGGAGCAGGAGTTGCCAAGGCCCGAAGACGATGTCAGGCGCA
GAGCGAAGAGGTTAACTCCTGGTCTGTGGACGATGTGTGCGGCTTTGTGGGCGGAATTGATATATGCGCC
GAATACGTTCAGG
>dme_circ_0000172_junction_seq
ATTGATATATGCGCCGAATACGTTCAGGGTAACGCACCCACCGACATACTGCAGCA
>dme_circ_0000173
GTAACGCACCCACCGACATACTGCAGCAGAAGCCAGCCAAGGCCACCACCGCCATTGTGGGCGGTCGCAG
CGCAGCCCAACCGGCGACCCACGACGACCAGGAAGCGGCCACGACCGCTTCCACAGCCACCAAGCGGAAG
CATCTCGAGCGGTTGGCCAGCGATCTGCGGCAGCTGGGCAAGAAGGGCAGGAGGAGTCGATCGGTTAGTC
CCTGTAGTAGATATCCCGCCGTTGCGGAAACGGCCAGAATGCTTAGCGAACGATCGCTGCTGCTCAGCGG
ATATGGAGCCATTGAGAGCAGTGCCAAGAAGCTGAGCGAAGCGGCGACCggtggtgcgggtggtggtggt
ggtggtgCGGCCGGTCAGGCTGCCACGCCCACTACCAGCCAACTAAGTGCACCCGCCTCCTCAGCATCCT
CCTCATCCATGTCGTCCTCCTCTGCCGGCAGCACTTGCACCACCAATGCAAATACGGCATCGGTGGCAGC
CGCCTATGCTGCTTTCTATTTGGAGAAGGTCAAGCATGAGAAGATGGACAATCATAAGGATGCACCGATG
GTCACCATacacaataacaacaacaacaacaccatcaacaacaacaacagcagtagcagcagcagcagca
gcatcatcaacaaccatcaatcgagtcaaCCAAGTCTGGGCCACAGCGTCAAGAGGGAGCGGCTGAGTCC
AGGGAGCAATAGCAGTAGCCACAACGGCGAACTCACCGTCAGCTCATTTGCCAGG
>dme_circ_0000173_junction_seq
CGAACTCACCGTCAGCTCATTTGCCAGGGTAACGCACCCACCGACATACTGCAGCA
>dme_circ_0000174
AAGATCGGCGAGATATACAGTTCGTATTGTGAATTGTGGGGCAGCGGGATGTCGCAGGATAACACGCGTC
AGGCTTGGCAGCGTCTCGTGTACGAGCAGCGGGAGAGTGGACCAAGCATGGATCTGCTGGAGG
>dme_circ_0000174_junction_seq
AGTGGACCAAGCATGGATCTGCTGGAGGAAGATCGGCGAGATATACAGTTCGTATT
>dme_circ_0000175
CCATTTCCAAGCGCAAGAGAAGCAGTGTTGCCGCGTTATTAGATCCAATAAGACCAAGGTCCTCACATCC
GGCGGCGTTCACACACGCGGTTCCTCCAAGGTCAAGCTAAAGAACATCGTCGACTACAAGTGGGAGCGCA
AGTACTACTATCCGGGTCACCTGGTCGCTGTCCACCGCGATGGCAAGCACCTGGCGTACGCCATTAATGG
>dme_circ_0000175_junction_seq
GCAAGCACCTGGCGTACGCCATTAATGGCCATTTCCAAGCGCAAGAGAAGCAGTGT
>dme_circ_0000176
GAAAGTATAACAAATTATAAGGAATGTTAAATTTTGGTTCGAGACACTTAAAATGGTCGCAAAAATACAC
ACACAGGCAAACGAAAGTCACATATCGCCCAAAAATAAATCGATTCACCCTCATTCACATGATCATTTTC
GCTGCTTAAAC
>dme_circ_0000176_junction_seq
TTCACATGATCATTTTCGCTGCTTAAACGAAAGTATAACAAATTATAAGGAATGTT
>dme_circ_0000177
GGTTATGCTCACCATTGACGGATCCCTTATCGCCGGGCGCATACTGAAACGACGGATATGGCAGATGCCA
GCCCTGTGAGATGAGCATCCAGTCGGCGGAGCGACCCAGATTATCGCGTTCCTTAAACCACACCTCGTAG

CTGAGAAGGCACTGCGTGGATTTGGGGTGTTCCATCCAGCTAATGAACACCTCACGCTGAGTCACCTCTG
CGATGGAAAGTTGCTGCGGTCGTCGCAGTTTGGGCCAAGATGCGGAACACACCCGGAGCAGCATCGCCCA
GGGCAGTGGGATGCTGGCGGAGTTGATAACTAGCTCGCTGTTGTACTGCCAGATGGGTCCCGTCTTGTAG
AGTCTAGGTGCTTGGGCTCTTCGCATCGCTTCCCTTAGAGTGGCGTTGGGATAAGCCGGACTTCCAGCTT
GCTGCCATATGTAGTACGGATCCGTTTCCTTTGGCACCAGGAGCTCGGTGACGAAGGCCGACTCATTCGC
TATCGACATGTTTAGCGTGTACTTGTGATGAAAGGCTGTCATCTTTGGACCCGCCTCCTCGGGACTCAGA
AATATTGTGGCCATATACTGACCAGGACCGCCTAATCCACCGGATACTGTACGCAGGACCTGGACGCTAT
GTTTAGTATCCATATTAACCATTTCCACGTCCGCAGCCCGGGTGCCCAGCTTCGCCAGCATTCCCAGAGC
AGCGTATACGGGTTTCTGAACGAGCTGCGAGTGCGGTGGTTTGGTTTCGTTCATCCTGAAGTGGGCTAGA
AGGGTGCGCTGTGTGAACTCGTGGGGATGGTAGCTCAGGAAGGCGTTGTCATGACTAATCGACTCCAAGC
GACTCAGTGGACCACCGGCCAGCTTGGCATGCCAGAACTGCATTACCGTAGATATCAGTGTGATTCCATA
CCGCACATCCGCCTGAAAATCCCGGGAGGTGCTCCAACCGGCCACAGGATCGGCTTCACTGAGAGATTGA
AAAGTAAGGATTTCAATAAGGACTACCATGTATTTCTAGCATATTTTTTGATAATTTAGTTCTTACTCAT
TGGCCACCGGCAGCTGCTTGAGATTGGGATACTCCTCATAGATTTTGGCCATCAAGGAAAGCGAACCATT
TACGATTTCCGTGGCAGTGCCTTCGATTCCCTTTCGGTGGAAAGTCAGTATGTCGATGGGACAGTAAACC
ACTCTTTGACTGCATAGCTCCAATAGATTCCAGCAGAGCGGATGATTCGAGTCCTTAAAGAGTCCTGCAG
GGCCGCGCAGGGAGCGATACATGGGCAGTGGAACCTTTCCGTCCTGGTTGTCCAGGTTTCCAGCTTTACT
CAAACCCCGCCGCACAGCTTGAACGTAATCCAGAAAG
>dme_circ_0000177_junction_seq
CCGCACAGCTTGAACGTAATCCAGAAAGGGTTATGCTCACCATTGACGGATCCCTT
>dme_circ_0000178
AGAACGAGTGTCCCCGCGAAAGTTTCAATGCCGCGGTGTACAGGCCGGAACTAGCCAGCAGGTATGATAG
CTCCTCGGGTGTGGCCCGCTCGTAGGCAGCTGTATCTTTGCGGTAGAAGGAAAGCTCACGTAAAGCTTCT
GCGTGCACCAGTTCCCTTCGGATATCAGCCAGCTCTAGAACAACCACCTCCTGGCCTCTCTTGTCCTCGT
CACATTTGGGCTCACCATCG
>dme_circ_0000178_junction_seq
GTCCTCGTCACATTTGGGCTCACCATCGAGAACGAGTGTCCCCGCGAAAGTTTCAA
>dme_circ_0000179
ATTCCATGGGACCAGGACGACACGGCAAGACACCGGCGGCGGCGGCCAACAAGGAGAAGCGAAAGCCATT
CTTCAAGAAACAGGAGACGGCCAGTCCCTACGACGTGGTGCCCTCGATGCGTCCCGTCGTCCTGGTGGGG
CCCAGTTTGAAGGGCTACGAGGTGACCGACATGATGCAGAAAGCCCTCTTCGACTTCCTCAAGCACCGCT
TCGAGGGCCGCATCATCATCACCCGGGTGATGGCCGACATCTCGCTGGCCAAGCGCTCCATCATGAACAA
TCCCTCGAAGCGGGCCATCATGGAGCGATCCTCCAGCCGGAGCGACTGCCTCGGCAAGGTGCAGGAGGAG
ATCGAGCGCATCTTCGAGCTGGCCCGCTCGCTCCAGCTGGTCGTGCTCGATTGTGATACAATCAATCATC
CGTCACAACTAGCGAAAACTTCATTAGCGCCAACAATAGTGTACTTAAAGATTAGTAGTAGTAAGGGTTT
TACAGCGTTTGATCAAATCACGTGGCAAGTCGCAGGCGAAAAACCTCTCCGTTCAAATGGTTGCCGCCGA
GAAGCTAGCCCAATGTCCACCGGATATGTTCGATGTAATCCTAGACGAGAATCAGCTGGAAGACGCCTGC
GAGCACATTGCCGAGTACTTGGAGGTGGGTTAAAGGACATATGGGTTAGATTCGCAAATATATTATATAT
TAAAATATTACGTATACGCACATTGGGACTTAGGATTTATCATGTATGTATCTAAACTAATCTATAATGT
CTTGCAGACGTATTGGAAGGCCACCCACCCGGACATTCGAGCAGTGCCGCCCATCGCCCGCCCCCTGCCG
CAGGAGGCCTCGCCCTCCGCAGATCCCGCGCGTCTGGGACCGACGCCTCCAGG
>dme_circ_0000179_junction_seq
CCGCGCGTCTGGGACCGACGCCTCCAGGATTCCATGGGACCAGGACGACACGGCAA
>dme_circ_0000180

TGCCATCAGCAGCCAATATGTCGATCGCCAGTGTTCACGGTCCCCAAATCGCCGACATCTGCAGTCCCCT
GTCGTAAGTATTTTAATTTTGGATGCCCATCTTCAGATCTACAGACGTACATTCATGATTCATTTAGCTG
GAAGCAGCGATTCGATATAATTCGCCTCCATTTCCGTTTTCGCATTTGCTAAATATCGATGTAAACAATG
TCAGGGCTGATTTGTGGGGGAATACGAAGGGGCGTGAGTTGGTGGTGGTGGTGGCTCATTCATGATTTTA
GCAATCTTATCGGGCGGGGAATGGCGACTGGGCCAGGGTGCAGAGTACACACAGCCACAATACCACTCAA
TACAACACAGTGTCGCAGTAGAAACCACACCTGCAATTTGGCCACGACTTTGGCCCATTAATGAAGGGGG
CCAGCACCTTCACCTCAACAGTTCGGCGTCCGTCTGTGTTTGTATACAGCCATAAACATATATACATATG
TATATATACAAGTTCGCAGTCGTATCATTATCATTTCGCATTTTGGGGCCGCACAAAAGCAACAAAAGCC
CGTCGCCATCGCTCACATGATCGGGCGAAAGTCGCGCCGTAGAGTCGGGTACAGTTGCCAATCATTGCCC
TCGTCCCCATCCCCGCATCCCAATCGGGCCATCCCAAATCCCATTCCACCACCGGCCCACCGGCACGCAG
CTCATTATCATAATCACGCCAGCGCACGCCGCCACCCACTTGGCAAGGTCACGGTTGCCCGACGCCAGCC
GCAATTTGTCACTATTACTAATCAAGAATTCCCCGCTTCGGCCTAATCTCTTCCAGGGCATCACAATCTC
GGTCTGTCTGCCTCGCTGCCAGATCTGGTGGGCAGTCCCCTGGAGATCAGCATGGACAACGTGCTGACCA
TTGAGGAGCTGCGCCAGCACATGGGCTCCTGCTTCACCTGCGGCGTCTCCTGGACGGACGACCATGTGTC
CCTCGACTGCAGCGAATGCGGTGGCTACAGCCTGGAGCGTCCCTGTCCCCTGTGCGACGGCCAGTGCGGT
GTCCAATGGAAACGTGATTTCGCCATGG
>dme_circ_0000180_junction_seq
GTCCAATGGAAACGTGATTTCGCCATGGTGCCATCAGCAGCCAATATGTCGATCGC
>dme_circ_0000181
GTGCTTTGCTTGCCCACTTGGTCTTTCGGCTCTGGAGAAAACTACTCGCATCGAATTGAATTGAATCTGG
GGAAAATCAGTCCGAGTCCCAGCTACACAGTTCAGTTTCGCTTCCCAGTCCAACTATAAAAG
>dme_circ_0000181_junction_seq
GTTTCGCTTCCCAGTCCAACTATAAAAGGTGCTTTGCTTGCCCACTTGGTCTTTCG
>dme_circ_0000182
TTATCTCACCCTCTATGGCTTAATCATACGTATTTTGCTTATGTAACTCTATGAATTAATGTTATTATTC
TTGTGGTTTTTAGTTCTTCGGTTTCGTTAGCTTTTACTTTAATACTGCACATGCTTAACTGGAGCGTTTT
TTGGAACCGAGACCTACAGATTTGATCTCCATCAGCTAGTGTAAAGGAATTCAGAGTTTGAGATTTACTT
TTGCCTTAGG
>dme_circ_0000182_junction_seq
AGAGTTTGAGATTTACTTTTGCCTTAGGTTATCTCACCCTCTATGGCTTAATCATA
>dme_circ_0000183
GTCTCCATTTCTGCCTGGTGCTGGACAATCAAATTGTTTCTTTCCTCCACAGCCTGAGAAATATTTTCTT
TATGAGCAGATTTAAGCGCTTCCAACTCCGCCTGACAACGAACCGTAACGCTGGAGAGTGTCTCCTCTAC
CTGCAATAGCTTCTCCTTTAACTCGGCCACATTGCTCTCATAGCGATCCTTAGTCTCCTGCAATTCAGCG
TAGAACTTGGCTTGGTTCTCTTTCTCATTCGTTTCAATTTGGAGCAGGAACTCCATTCGCAAAGCACTTA
TCTCATCATCTTTTATCCTTCGCACATCGTGTAGCTCATCTTTGGCTGCATTTAGCAATTGATTAA
>dme_circ_0000183_junction_seq
TCTTTGGCTGCATTTAGCAATTGATTAAGTCTCCATTTCTGCCTGGTGCTGGACAA
>dme_circ_0000184
GTGGAGTGCGAAGAGGTGCAGGGTTTAAAAGAAAAGCTGGCCGAGGTTACAGCACAGCGGGATAATTTAG
AGCAAGAATCGTTAGCCGAAAAGGAGCGGTGAGTTAATGAGGTGTTTTCTGTCAAGGAATCTGTCGTCCA
AAACATAGTGCCTGCATCGTAAAAAACGATGTAAACGTAACTCGACTTTTGTGCTTTAAGCAGTCAACCC
AAAATCTAACATACCTATTCATACAAATCTGTAAAACTAGTGCAGCGATATTCTATTCCATTCTATAAAA
ACAAAACACTTAAGTATATGTTTCAGTTTTTAACAGTAAATTTTGTTTATAGAACACCACTCCACAATAC

CTGTTTGTTTGATTTATTTTATTATAGTTTTAGTGTTTAACAATAGTTTACTACTTGTGTATCTTCTTAT
AGTTATGATGCTCTGGAAAAGGAAGTAACCAGCCTAAGAGCGGATAATGAGGCTGCGAATTCAAAAATCA
GCGAATTGGAAGAGAAACTGAGTACTCTAAAGCAGACTATGAGAATAATGGAAG
>dme_circ_0000184_junction_seq
CTAAAGCAGACTATGAGAATAATGGAAGGTGGAGTGCGAAGAGGTGCAGGGTTTAA
>dme_circ_0000185
AGGCGCCTGCCACCGGAGTAGTCGGTGAGCTCAATCTGTTGGATGGACTTCGACAGCTTGGTGAACTGTT
GCAGCAGATCAGTGTCCAATTCGTTTTCCGGATCACTGCACTCAGTGCCTACACCCGAACTCTTCATGGA
TTCCAAATCGGAGTTCGTTGTTCGTTGCTTGTGCTCCTCTCGGAGTGCATCGCACTCTTGAATAG
>dme_circ_0000185_junction_seq
TCTCGGAGTGCATCGCACTCTTGAATAGAGGCGCCTGCCACCGGAGTAGTCGGTGA
>dme_circ_0000186
AAATTTCCTTATCGCGCTCCTCCTCCGCTTGCCTTAGTTTCTCCTTGTAAGAAGATTCCTTCTCCTTCAG
CGTTTCCCTGATTTTTTCAAGCTCTGCCTTGTGTCGGACAGCCAATTCGTTTCTTTCCTTCTCGGCCTGA
GTAATCTTTTCTTGAAGAGATCTTTTAATGACATCCAAATGCACCTGACACTGAGTCGTAACACTGGCGA
GCTTTTCATTGGTCTGCTGTAGCTGCTCTTCGTAGCGTTTCTGGGTTTCCTTTAAATCCAGGCAGTATGC
ACGGAATTTGGAGCGGTTTTCATTCTCGCTCGTCTCAATTTTCGCCATGTACTCAAGCTGAAGGCTTTTG
TTCTCATCTGTCTTTCGCTTCTCCAGAATACTTAATTCCTCTTGTGCCCTTTGTAATGATTCTTCAAGTT
GCTCGATTCGGCGTTCATATTCGGTGGTAAGAGTTCG
>dme_circ_0000186_junction_seq
GGCGTTCATATTCGGTGGTAAGAGTTCGAAATTTCCTTATCGCGCTCCTCCTCCGC
>dme_circ_0000187
GGGGCGAGAAAGCAGAACTGCTATCGGAGATCCAGAAAATCAACGGACAGCACTCGAATACCATAAAAAA
ACTTGAGGAAATCGAAGCGGAAATGATTACACTAACCACTCAAAAGGAGTTGGAGAG
>dme_circ_0000187_junction_seq
CACTAACCACTCAAAAGGAGTTGGAGAGGGGGCGAGAAAGCAGAACTGCTATCGGA
>dme_circ_0000188
GGGGCGAGAAAGCAGAACTGCTATCGGAGATCCAGAAAATCAACGGACAGCACTCGAATACCATAAAAAA
ACTTGAGGAAATCGAAGCGGAAATGATTACACTAACCACTCAAAAGGAGTTGGAGAGGTGTGAAATTGCA
GAAAAGCTGGAGACTTTCAAGTCCAAGGAGGCAGATATCAAAGAAGCTCTGCACTGTGCCCAGCTCAGGC
TTCATGCCTACGATAAGCTGGT
>dme_circ_0000188_junction_seq
TCAGGCTTCATGCCTACGATAAGCTGGTGGGGCGAGAAAGCAGAACTGCTATCGGA
>dme_circ_0000189
GGAAGCTCAAACTAATGATTTCCTAAAAGAAAGGGAAACTTTGAACTTGACTATATCCGATCTTCGGTTG
CATAATGAGCAATTGCTAGAAACCTCGAAAAATTATCTATCAGACATAACAGCAGCAAATAATTTAAATC
TCGAAATGAAGAAGAATCTTCACGATCTCACAAAAGAGTGCAAGAGCTTACGTTCCGATCTG
>dme_circ_0000189_junction_seq
AGAGTGCAAGAGCTTACGTTCCGATCTGGGAAGCTCAAACTAATGATTTCCTAAAA
>dme_circ_0000190
GTAGTCATTCCCGCCAAATACATTGATGCCAACACCATTGTCCACATCAATCCTTGTGGGCTTTTTGTAA
TCGGTGGACCAATGGGTGACGCTGGGCTTACAGGAAGAAAAATCATTGTAGATACTTACGGAGGCTGGGG
CGCCCACGGCGGCGGTGCATTCTCTGGCAAGGACTTCACCAAGGTTGACAGATCAGCTGCATATGCAGCA
CGCTGGGTGGCCAAATCACTGGTTAAGGCCGGTCTCTGCAAGCGCTGCCTTGTCCAAGTGTCCTACGCCA

TAGGTCTTGCTGAGCCTCTCTCCATAACTGTGTTCGACTACGGAACTTCTCACAAGTCACAAAAAGAACT
TCTGGATATCATCCGTCGAAACTTTGACCTCAGGCCCGGCAAGATTGTCAAGGGACCTAAACCTGCGCCA
GCCGATATACCAGCGAACAAGCACTTATGGACACTTCGGTCGCGCGGGCTTCTCGTGGGAGGAGGCCAAG
CCTCTGGAGATTGACAACTGACTAGACGCTGAAGCGTTGCAACCGCAGTCACCATATGCTAAAAG
>dme_circ_0000190_junction_seq
TGCAACCGCAGTCACCATATGCTAAAAGGTAGTCATTCCCGCCAAATACATTGATG
>dme_circ_0000191
GATAGCTTCGACCTTTTAGCATATGGTGACTGCGGTTGCAACGCTTCAGCGTCTAGTCAGTTGTCAATCT
CCAGAGGCTTGGCCTCCTCCCACGAGAAGCCCGCGCGACCGAAGTGTCCATAAGTGCTTGTTCGCTGGTA
TATCGGCTGGCGCAGGTTTAGGTCCCTTAAAACAAAAATATAGACAATTGTTATGAAATATTTTTTAGCA
TCTAGAGTTTGACACTTACTTGACAATCTTGCCGGGCCTGAGGTCAAAGTTTCGACGGATGATATCCAGA
AGTTCTTTTTGTGACTTGTGAGAAGTTCCGTAGTCGAACACAGTTATGGAGAGAGGCTCAGCAAGACCTA
TGGCGTAGGACACTTGGACAAGGCAGCGCTTGCAGAGACCGGCCTTAACCAGTGATTTGGCCACCCAGCG
TGCTGCATATGCAGCTGATCTGTCAACCTTGGTGAAGTCCTTGCCAGAGAATGCACCGCCGCCGTGGGCG
CCCCAGCCTCCGTAAGTATCTACAATGATTTTTCTTCCTGTAAGCCCAGCGTCACCCATTGGTCCACCGA
TTACAAAAAGCCCACAAGGATTGATGTGGACAATGGTG
>dme_circ_0000191_junction_seq
CCCACAAGGATTGATGTGGACAATGGTGGATAGCTTCGACCTTTTAGCATATGGTG
>dme_circ_0000192
AAAGAAACGCACTGCATCCGGCACAGGAAACTCTGTGGCCGAGTTCTCTACCAGCTCGTAAACGGAGACG
GGCAGGCACAGGGCCTGTGATACCTTGTTTGCCTCCTCCTGGCTGCGCGGTGTATCATTCTGCTTGCCGT
ACAGCGCCTTCAGATAGTCCGTCTTGAAGGAAGTTGGTGTCTTGAGCGCGTAG
>dme_circ_0000192_junction_seq
GAAGGAAGTTGGTGTCTTGAGCGCGTAGAAAGAAACGCACTGCATCCGGCACAGGA
>dme_circ_0000193
CATGAGGTTGCAGTCCAGATGGAATGCCGTGGACAGGTGGCCGGCGTTGTATTGCTCGGCCGGGCGGCAG
TCAACGAGAAAGAAACGCACTGCATCCGGCACAGGAAACTCTGTGGCCGAGTTCTCTACCAGCTCGTAAA
CGGAGACGGGCAGGCACAGGGCCTGTGATACCTTGTTTGCCTCCTCCTGGCTGCGCGGTGTATCATTCTG
CTTGCCGTACAGCGCCTTCAGATAGTCCGTCTTGAAGGAAG
>dme_circ_0000193_junction_seq
GCCTTCAGATAGTCCGTCTTGAAGGAAGCATGAGGTTGCAGTCCAGATGGAATGCC
>dme_circ_0000194
TTATGGAGAGAGGCTCAGCAAGACCTATGGCGTAGGACACTTGGACAAGGCAGCGCTTGCAGAGACCGGC
CTTAACCAGTGATTTGGCCACCCAGCGTGCTGCATATGCAGCTGATCTGTCAACCTTGGTGAAGTCCTTG
CCAGAGAATGCACCGCCGCCGTGGGCGCCCCAGCCTCCGTAAG
>dme_circ_0000194_junction_seq
CCGCCGTGGGCGCCCCAGCCTCCGTAAGTTATGGAGAGAGGCTCAGCAAGACCTAT
>dme_circ_0000195
CTTCGACCTTTTAGCATATGGTGACTGCGGTTGCAACGCTTCAGCGTCTAGTCAGTTGTCAATCTCCAGA
GGCTTGGCCTCCTCCCACGAGAAGCCCGCGCGACCGAAGTGTCCATAAGTGCTTGTTCGCTGGTATATCG
GCTGGCGCAGGTTTAGGTCCCTTAAAACAAAAATATAGACAATTGTTATGAAATATTTTTTAGCATCTAG
AGTTTGACACTTACTTGACAATCTTGCCGGGCCTGAGGTCAAAGTTTCGACGGATGATATCCAGAAGTTC
TTTTTGTGACTTGTGAGAAGTTCCGTAGTCGAACACAGTTATGGAGAGAGGCTCAGCAAGACCTATGGCG
TAGGACACTTGGACAAGGCAGCGCTTGCAGAGACCGGCCTTAACCAGTGATTTGGCCACCCAGCGTGCTG

CATATGCAGCTGATCTGTCAACCTTGG
>dme_circ_0000195_junction_seq
GCATATGCAGCTGATCTGTCAACCTTGGCTTCGACCTTTTAGCATATGGTGACTGC
>dme_circ_0000196
CATATGGTGACTGCGGTTGCAACGCTTCAGCGTCTAGTCAGTTGTCAATCTCCAGAGGCTTGGCCTCCTC
CCACGAGAAGCCCGCGCGACCGAAGTGTCCATAAGTGCTTGTTCGCTGGTATATCGGCTGGCGCAGGTTT
AGGTCCCTTAAAACAAAAATATAGACAATTGTTATGAAATATTTTTTAGCATCTAGAGTTTGACACTTAC
TTGACAATCTTGCCGGGCCTGAGGTCAAAGTTTCGACGGATGATATCCAGAAGTTCTTTTTGTGACTTGT
GAGAAGTTCCGTAGTCGAACACAGTTATGGAGAGAGGCTCAGCAAGACCTATGGCGTAGGACACTTGGAC
AAGGCAGCGCTTGCAGAGACCGGCCTTAACCAGTGATTTGGCCACCCAGCGTGCTGCATATGCAGCTGAT
CTG
>dme_circ_0000196_junction_seq
CCAGCGTGCTGCATATGCAGCTGATCTGCATATGGTGACTGCGGTTGCAACGCTTC
>dme_circ_0000197
GTCGCGGTGTCGCTTCTTGGCCGTGATCTTCACGATGCTCGCCAGCGGGCGACGCACCATTATCTGCGCC
TGGCCGCGTCCCAGTTCCCGCAGCACCACCAGTTGCCCGGGCGTGATGATCAAATGACTGTCGTACATGT
AGCCGCTCATGTGCACCTCCTGGCACTTGAAGGCGTTGATGATG
>dme_circ_0000197_junction_seq
CTCCTGGCACTTGAAGGCGTTGATGATGGTCGCGGTGTCGCTTCTTGGCCGTGATC
>dme_circ_0000198
GCTGCATATGCAGCACGCTGGGTGGCCAAATCACTGGTTAAGGCCGGTCTCTGCAAGCGCTGCCTTGTCC
AAGTGTCCTACGCCATAGGTCTTGCTGAGCCTCTCTCCATAACTGTGTTCGACTACGGAACTTCTCACAA
GTCACAAAAAGAACTTCTGGATATCATCCGTCGAAACTTTGACCTCAGGCCCGGCAAGATTGTCAAGGGA
CCTAAACCTGCGCCAGCCGATATACCAGCGAACAAGCACTTATGGACACTTCGGTCGCGCGGGCTTCTCG
TGGGAGGAGGCCAAGCCTCTGGAGATTGACAACTGACTAGACGCTGAAGCGTTGCAACCGCAGTCACCAT
ATGCTAAAAG
>dme_circ_0000198_junction_seq
TGCAACCGCAGTCACCATATGCTAAAAGGCTGCATATGCAGCACGCTGGGTGGCCA
>dme_circ_0000199
GATTTCATGTTTTTCCAGCAGCGGAAGATTGCAGGTTGTGGTGCGATTCTGTGGATCCTGGAGACCATTG
ACTTCGTTGACAAGATTTTGAGCCTGCGCAAGTTGTTTGCTCCGATGGGTGCGATATAACCAGGTCGCCG
AAACAAGTAAGGCGACCAGCAATATGGCTGCGCACACAGCTACTACTACTACGACTACCATTGGGTAATC
TCGGACTGCTTCCAGCTTTTTCTGATTCAGCTCCAGTTTGCCCACGATCGCCAGGA
>dme_circ_0000199_junction_seq
AGCTCCAGTTTGCCCACGATCGCCAGGAGATTTCATGTTTTTCCAGCAGCGGAAGA
>dme_circ_0000200
GACTCTTCTCCTTCACTTGACGCAAGGTGCAGACTTCGGAAGACTGGTTCCGTATGGCCGCCAGGGAGCA
GGCAAAACTGTCATCCCCAGTCCCGTAGATAATGCGGTTGCGCCTGCAGATGAACCTTTCTTCGCCCAAC
TTGATGCAGTCGTCCAAGCTCTGAAGCGGAAAATACCTGTCCATATGATCGTTGATGCCCAGATAAGGTG
TTTCGGTGTCCACCA
>dme_circ_0000200_junction_seq
CCCAGATAAGGTGTTTCGGTGTCCACCAGACTCTTCTCCTTCACTTGACGCAAGGT
>dme_circ_0000201
GATACCCAAGCACCAACGTAATTTTTAGTGTCGTTTTTAAAGCCTAAAGTCAGAGGAGAAGGCGGCATAG

GCTTGCCAACTCTTATGGGCTTTCTTCTCTAACTTATGCTGTGCTCTTCGCTATATAATTTAAACCATTC
TGATGTGTGTAGTCTTCTCACACGGAACTAGTTTAGTCGTCTTAATACCCGCATATATACCTAATAGAG
AGCTCCAAAAGAATTGATGAGCTTTA
>dme_circ_0000201_junction_seq
AGAGCTCCAAAAGAATTGATGAGCTTTAGATACCCAAGCACCAACGTAATTTTTAG
>dme_circ_0000202
GGGTGCAGTCCATGTTCAGACTCCAGCGGCAATTGGGATCAGGAGGTGAGCGCTTCCGATCGAGAGGATA
ATGTGTTCAATATTTCGCAACCGCATCAATTACCGTGGAACTGGTGGCACCAGGAGCGGCAGCGGGGAGc
gggagagggaccgagatcgggacagggatcgtgatcgagatcgggaaagggacagagacACCCTGCTGGC
CAAGGAACTGGATGCGGATAGCAATAACAATGGTACCGAGCCACAAATGGAGGCGGAAGCAGTGCCGGAA
TCTGATACGGAAAGAGAAACGGCCGAGGAGCGCGGCGAGGAACAGGAGCCGGAGAACAGTAACGAGGCCC
TGGATCTCTCACTGATTTCGTCCGGCGGTCGGGAATCACTGCCGGGCAGTGGTCACGTGTCGCTGGAGGC
TCTACAACATACCAAAGTGGCGGTGGCCCAGTTTGCGGCCACTGCGATGGCGGGAAACCACCAGTCCGCC
GATCTGGCCATGGTGCAGTCCACCATCTTTAATGTGCAGCGGCAGCATCTGATGCAGTTACAGCTCATCC
AACACCTGCAGAGTCAATTGAAAAGGGCCGAGGCAGCCGCCTTGGGTAGGCACTCTCACAGCGATGAGGA
GGAGGAAGAGCCGGAGCCAGAGCCGGAACCTAAGAAGCAGCCCACCAATGGCCTGAAGGAGGAGTTGGAG
CTGGAGCAGGGTCCGGAGTCAGAGCAGGATCAGGAGTCGCGGCGGGAGGAGAATTCAAAGACTGACAAGA
GGGGAACTGAGGACAGAAAGGCAGAGCCAGAGGGTTACCAATCGATGATGTGCGACATAAGCTCGAGTCT
GGCATCGAGCATTATCACCAACCACGATCCCCCGCCAGCCCCCAACGAGCCCAATTGCCTGGAGATGTTG
CAACGGAGAACGGAGGAGGTCCTCGACTCCGCATCCCAGAGCTTACACGCTGCTCAGATGCAAGAGGAAT
ACTCGGAGTATGCCTCCAAAGAGGCTCAAAGTCGCGGTGAGATCTTTAAGCATCGATGCAAGTACTGTGG
CAAGATCTTTGGCAGCTACTCGGCCCTGCAGATACATCTGAGATCACATACCGGTGAACGCCCATTCGTT
TGTAATGTTTGCGGAAGTAAATTCACCACGAAAGGCAATCTGAAGGTTCACTACCAGCGGCATACGCAAA
TCTTTCCGCCAATGCTTCTGCCACCAGGAGTGGCGCCCAACGTGGGTCATTCGGGTCAGGGGCAAGTCCA
GGGCGAGCAGTATCCCATTCGCCTGCCTTTTGCGCCACCAGTAGCACCTGTGGGTCAGGAACAGCATCAG
AATCAAGTTGAGGAGCCGGAGGAGATTAGACAGGAGATTCCAGTTCCGCAGGCAGAAGATCTCAGCAAGC
CAATGGTTAAGGAAAAGGAGAAATCCCACTCGCCCGTGGAGCGTGTCAAGACTCCCAAAGAAGTTAAGAC
AGATGCAGCGCTGCCGTCATCAGAGAAGCCTGAAAAAGAGATCTCCAAGCCGGTGGTGACCTCGTCACGA
CGAAATGGATCTGTGCGGAAGCGACAAACAAGTGCTGTTAGCCCGCCGCAAGAGGATAGGGAACGGGATC
TTGTCGAGCACTTGCACATAGCCAAGCTGGTGAGACGCTCCTCTGCCAGCAGAGAATCACAGCCGGCGGA
GTACTCGCTCGCGCAAATGGAGCGCATTATCGACAAGTCATGGGAGGATCTGATTGAGATCGACAAGACT
TCGGAGACCTCGAAGCTGCAACAGTTGGTGGACAACATCGAGAACAAGCTGACCGATCCTAACCAGTGCA
TCTTCTGCCAGAAGGTTATGTCCTGTCGCAGTTCCCTGCAAATGCATATTCGCACCCATACCGGAGAGCG
TCCGTTCCGTTGCAAGATCTGCGGCAGGGCTTTCGCCACGAAGGGCAATCTTAAAGCCCACATGAGCATA
CACAAGATCAAGCCGCCGATGAGGAGTCAGTTCAAGTGTCCGGTGTGCCATCAGAAGTTCTCCAACGGCA
TCATCCTGCAGCAGCACATTCGCATCCACACCATGGACGATGGCAGCGGTGGTCAGGGAGCCCCTGCCGC
CAATCCTGGCGAGGCCGAACGGCTGGGTATCGAAGATCAAAACTCGAACAAGTCCCTGGGCACTTCGGAT
ACATTGGATTTCTCCACCACCATATCGGATCACTCGGGTCAAAGATCTGAGTCCTCGCAAGGCGGAGATT
TTGATGAGTTTATGACCATGGACAGCACGGATGACTCCAGGGATAACTCAAGTGCGGCCACAGCTACGCC
TCATCCTCTAGAAAGAGAGAGGGATAGGGAGAAGGAGCGGGAGAGAAGGATTCCCAACGATTGCTCTGAT
GAGAGATCGCATTCCAATCCTGATTTAACTGGAGGCCGCAGCGAGAGTGGCGAAATGCCCGCCATGGATC
TCTCTTCACCCTCTTCCAACTCGGGTCGTATCTTCGCAACTGGACTGGCTAACGGAGCTGCTGGTGGAGG
ATCTGGAAATGGTGGTCTTCCCATGCTCGGAATGCCCATGCCTCCAAATCTTCTGCTCATGGCAGCTGCT
CGCGAAGAAATGCACGCTCTAGGTCATGCTCACGCCAAATTTCCCCTGCTGCCCTTTGGTCCACTTGGAT

TTATGGGTAAGCTATAAATATTCTATAATATTAATATTTTTTTGGGGGCTTATTTTATAAAATTTTCCGA
AAATTGTCCGAAAAATATCATTGTCTGATACATATAAGGAGCTTTGAGAAATCTTATTTGCTTTGGTACT
TCACTATGAATTTACCCAGCTAATTAAAAGCAAAATATAGAGCTTATAGTTTGCTCTTTGTCGCGTCCAA
CAATATAAAAATAAACCCGGCAAATTGCGTATCTTTTGGCCGCCACACTTGGCATTAACTTTCTTCTCCG
TTCTGTTCGTGGATGCGAGGTGACAAAGCCCCTTTGTCTGCCACCTTTGACCCCGTCAGCGTTTGCCAAG
CAACTCGCAGTGGAAGTGCGATAAATCTGCGATCTGGGCGCGACATAACTCAATTGGACCGAGACAAAGC
ATCCGCCGAGCACTTCGCACCGCTCCACCCAGCTCCATCCCAATCCATGTCTGATATGCCTATGCCAAAT
TCCGCAATTTATTTATAAACATTGGCGCATTCCCCGAATCGTGGCGCAAAAGGCGCAAGCGATCGGATCG
AGTAGCGATCTGTCGCCTCATTATGTCACAACCTTTTACGCGGATGGATATTTATTATATCTCCGATCGC
AAAGAGCCACATTCGGCAGCACGTGGCGGGAATTATGTGTACCCCACTGACCTCGCAAGTAACTCTTTGT
CTGCATCCCGCCCTCATCCGCAGGTCTGCATCCGCCCCCAAATGTGTGCAATCTCTGCTTTAAGATGCTG
CCCAGTTTGGCCGCCTTGGAGAGTCATTTGCAGAGCGAACATGCCAAAGAACCGGCGACGGGCCACGCCC
AACGCCCACAGTGCAGCGATGCTGGATCGCCGTATGGAGCAAAG
>dme_circ_0000202_junction_seq
CGATGCTGGATCGCCGTATGGAGCAAAGGGGTGCAGTCCATGTTCAGACTCCAGCG
>dme_circ_0000203
GGGTGCAGTCCATGTTCAGACTCCAGCGGCAATTGGGATCAGGAGGTGAGCGCTTCCGATCGAGAGGATA
ATGTGTTCAATATTTCGCAACCGCATCAATTACCGTGGAACTGGTGGCACCAGGAGCGGCAGCGGGGAGc
gggagagggaccgagatcgggacagggatcgtgatcgagatcgggaaagggacagagacACCCTGCTGGC
CAAGGAACTGGATGCGGATAGCAATAACAATGGTACCGAGCCACAAATGGAGGCGGAAGCAGTGCCGGAA
TCTGATACGGAAAGAGAAACGGCCGAGGAGCGCGGCGAGGAACAGGAGCCGGAGAACAGTAACGAGGCCC
TGGATCTCTCACTGATTTCGTCCGGCGGTCGGGAATCACTGCCGGGCAGTGGTCACGTGTCGCTGGAGGC
TCTACAACATACCAAAGTGGCGGTGGCCCAGTTTGCGGCCACTGCGATGGCGGGAAACCACCAGTCCGCC
GATCTGGCCATGGTGCAGTCCACCATCTTTAATGTGCAGCGGCAGCATCTGATGCAGTTACAGCTCATCC
AACACCTGCAGAGTCAATTGAAAAGGGCCGAGGCAGCCGCCTTGGGTAGGCACTCTCACAGCGATGAGGA
GGAGGAAGAGCCGGAGCCAGAGCCGGAACCTAAGAAGCAGCCCACCAATGGCCTGAAGGAGGAGTTGGAG
CTGGAGCAGGGTCCGGAGTCAGAGCAGGATCAGGAGTCGCGGCGGGAGGAGAATTCAAAGACTGACAAGA
GGGGAACTGAGGACAGAAAGGCAGAGCCAGAGGGTTACCAATCGATGATGTGCGACATAAGCTCGAGTCT
GGCATCGAGCATTATCACCAACCACGATCCCCCGCCAGCCCCCAACGAGCCCAATTGCCTGGAGATGTTG
CAACGGAGAACGGAGGAGGTCCTCGACTCCGCATCCCAGAGCTTACACGCTGCTCAGATGCAAGAGGAAT
ACTCGGAGTATGCCTCCAAAGAGGCTCAAAGTCGCGGTGAGATCTTTAAGCATCGATGCAAGTACTGTGG
CAAGATCTTTGGCAGCTACTCGGCCCTGCAGATACATCTGAGATCACATACCGGTGAACGCCCATTCGTT
TGTAATGTTTGCGGAAGTAAATTCACCACGAAAGGCAATCTGAAGGTTCACTACCAGCGGCATACGCAAA
TCTTTCCGCCAATGCTTCTGCCACCAGGAGTGGCGCCCAACGTGGGTCATTCGGGTCAGGGGCAAGTCCA
GGGCGAGCAGTATCCCATTCGCCTGCCTTTTGCGCCACCAGTAGCACCTGTGGGTCAGGAACAGCATCAG
AATCAAGTTGAGGAGCCGGAGGAGATTAGACAGGAGATTCCAGTTCCGCAGGCAGAAGATCTCAGCAAGC
CAATGGTTAAGGAAAAGGAGAAATCCCACTCGCCCGTGGAGCGTGTCAAGACTCCCAAAGAAGTTAAGAC
AGATGCAGCGCTGCCGTCATCAGAGAAGCCTGAAAAAGAGATCTCCAAGCCGGTGGTGACCTCGTCACGA
CGAAATGGATCTGTGCGGAAGCGACAAACAAGTGCTGTTAGCCCGCCGCAAGAGGATAGGGAACGGGATC
TTGTCGAGCACTTGCACATAGCCAAGCTGGTGAGACGCTCCTCTGCCAGCAGAGAATCACAGCCGGCGGA
GTACTCGCTCGCGCAAATGGAGCGCATTATCGACAAGTCATGGGAGGATCTGATTGAGATCGACAAGACT
TCGGAGACCTCGAAGCTGCAACAGTTGGTGGACAACATCGAGAACAAGCTGACCGATCCTAACCAGTGCA
TCTTCTGCCAGAAGGTTATGTCCTGTCGCAGTTCCCTGCAAATGCATATTCGCACCCATACCGGAGAGCG
TCCGTTCCGTTGCAAGATCTGCGGCAGGGCTTTCGCCACGAAGGGCAATCTTAAAGCCCACATGAGCATA

CACAAGATCAAGCCGCCGATGAGGAGTCAGTTCAAGTGTCCGGTGTGCCATCAGAAGTTCTCCAACGGCA
TCATCCTGCAGCAGCACATTCGCATCCACACCATGGACGATGGCAGCGGTGGTCAGGGAGCCCCTGCCGC
CAATCCTGGCGAGGCCGAACGGCTGGGTATCGAAGATCAAAACTCGAACAAGTCCCTGGGCACTTCGGAT
ACATTGGATTTCTCCACCACCATATCGGATCACTCGGGTCAAAGATCTGAGTCCTCGCAAGGCGGAGATT
TTGATGAGTTTATGACCATGGACAGCACGGATGACTCCAGGGATAACTCAAGTGCGGCCACAGCTACGCC
TCATCCTCTAGAAAGAGAGAGGGATAGGGAGAAGGAGCGGGAGAGAAGGATTCCCAACGATTGCTCTGAT
GAGAGATCGCATTCCAATCCTGATTTAACTGGAGGCCGCAGCGAGAGTGGCGAAATGCCCGCCATGGATC
TCTCTTCACCCTCTTCCAACTCGGGTCGTATCTTCGCAACTGGACTGGCTAACGGAGCTGCTGGTGGAGG
ATCTGGAAATGGTGGTCTTCCCATGCTCGGAATGCCCATGCCTCCAAATCTTCTGCTCATGGCAGCTGCT
CGCGAAGAAATGCACGCTCTAGGTCATGCTCACGCCAAATTTCCCCTGCTGCCCTTTGGTCCACTTGGAT
TTATGGGGTAAGCTATAAATATTCTATAATATTAATATTTTTTTGGGGGCTTATTTTATAAAATTTTCCG
AAAATTGTCCGAAAAATATCATTGTCTGATACATATAAGGAGCTTTGAGAAATCTTATTTGCTTTGGTAC
TTCACTATGAATTTACCCAGCTAATTAAAAGCAAAATATAGAGCTTATAGTTTGCTCTTTGTCGCGTCCA
ACAATATAAAAATAAACCCGGCAAATTGCGTATCTTTTGGCCGCCACACTTGGCATTAACTTTCTTCTCC
GTTCTGTTCGTGGATGCGAGGTGACAAAGCCCCTTTGTCTGCCACCTTTGACCCCGTCAGCGTTTGCCAA
GCAACTCGCAGTGGAAGTGCGATAAATCTGCGATCTGGGCGCGACATAACTCAATTGGACCGAGACAAAG
CATCCGCCGAGCACTTCGCACCGCTCCACCCAGCTCCATCCCAATCCATGTCTGATATGCCTATGCCAAA
TTCCGCAATTTATTTATAAACATTGGCGCATTCCCCGAATCGTGGCGCAAAAGGCGCAAGCGATCGGATC
GAGTAGCGATCTGTCGCCTCATTATGTCACAACCTTTTACGCGGATGGATATTTATTATATCTCCGATCG
CAAAGAGCCACATTCGGCAGCACGTGGCGGGAATTATGTGTACCCCACTGACCTCGCAAGTAACTCTTTG
TCTGCATCCCGCCCTCATCCGCAGGGTCTGCATCCGCCCCCAAATGTGTGCAATCTCTGCTTTAAGATGC
TGCCCAGTTTGGCCGCCTTGGAGAGTCATTTGCAGAGCGAACATGCCAAAGAACCGGCGACGGGCCACGC
CCAACGCCCACAGTGCAGCGATGCTGGATCGCCGTATGGAGCAAAGGGTAAGTGCAATAATATCGAATTG
GATATTGGATCACTATGTTTGCTAATCCACGCCATGACAGTAAGCAACTTATCGCCATGATCCAATTTAA
TAAAAGTCCTAAAGAATTCGCGTAAGTTTTGTGAAAGCAATATTTAAATGGTTTATAGAAATGCTTGCGG
AATACATGACAGTTCTCTTTATTCTCAATAATAAGATGAACTACTCGGAAATTGGTACTTCATTACGTGT
CACTGACTCCGAACTCAACTTATTTTTCGCGGTGCAAAACCCACTAATCACTTCCATAGTTAACCATGGG
GCAGCATTCCCATGCCCCAATATAGTAGCACTTACATCACATCCAACTAGCCTAATCTCACCTCAACTCT
CGCTTTGAGAACCAGTGCCATCTGGCAGTGAGAGTCTTGCCTTGGTCATGATTAATTAAAATGTTCACGG
GTTCAGCTCGTTCCTTGGGGTTGGCTTGATTTGGCCAAATATGGTTGGAGCAAACGGCCCGTTTTGGCCG
CGACCATACAAATTATGAGCACATTATTAATTGCCCGACGTGTTCGTCACTGGATGGGTATTACTGTGAA
AGAAATTGTGGCCAACTTGCATAGCTTTGCCTTAATTGTTGGCGTTCCGCTTTGCAGACTCCAATCGACC
AAGTTGTTGGGGCTGGCAACCCAGACTCACGGGTTTAAGTGCCCGGATTACATCGCCCAACAATGCCAAT
TATTTATTAAAGTGCGTTCGCTGCTCCGATGTTCGGATGGAGTGGTTTTCTGCAGTGGCTGTCCATAACT
TAAGTACAAATGCGAGTATTAATATTTATTTCTGGCCGGAGCATGCAGTGCGGCATTGTCCCTCTATGAC
TAATGACATCGTTCATTATGGCATACATTCTTGGAGTAATTCCAAAACAGAAACTTAGTGTCTCCGCACA
GGGATGGAATCTGAACAAATTTCGCAGCTGGGGGCTTTTAGTTGGATAAGGATGCATTTTGAACAGAATG
TTTGATTTTTGAGAAATATACTTAAGAGAGCTAGCTCTTTGTATTTCACAATCCCTTGAACCGCTCCTGT
GCTTCCTATTGTGGCCAAAATGCAAGTGCAGTGTAAATGTCATAACATTTTCAGTTGGTAATCAGCAAGT
CTCTGCTGGGATGCCCTCCTTTCCCCAATACCCCCCAAGTTTTGCGACAGACTGCATTTGTACATCTTTG
GCTGCGCAGCGGCTACGTTCCGTTTTGCTCGCTTAGCACATTGTTGATGATCTAGAGCAGACGAGCATCG
GATCGAGTTGGTCCGGGTATGGGGTTATCCCATGCAGATGGTTGCAGGGGGATTGTGACAGGCAATGGAG
GTGGGGCTGGGAATCGGCATCATCTTTTGGGCTTGAAATTTCTAAATGATTCAAAAATCTTAAGTAATTA
CGAGAATGCATTATGATGATGCGAAATTTACGACTCCGTCTGGTATGTTTTCACTCTGGATCGTGGCGAA

ATGATGAAAACGAAGGGCTATTCGGGGGCTATAGAGGATCGGGTTGGTTTGAGAGATATAGAGGAGCCTG
TGCCGGATCGGTGACGGTGTGTGCGATGCATTCAATGCAAATAAATTATGCTACGCTTTATGCAAAATTA
TTTAATCTTTTGTTCGTCGCGTCTGCTGGGGACACCAGAACAGAGCACTACATAGTTGGATTCGGCTCCC
CTCGCACACAGATTCCCCTCTTTTTGGCGAACAATTGTTTTGCCATAAAAATATGAGACCCGGCGGCATC
GTCTGCGCGAGATAATTAGTTGGCATTATTACACGCGATGGTATATAGAAAAATTGTAAATTATGTTTTT
GGCTCTCACTCTCCGCGAAAATAAGATTATTAAGAGGCGCGAGTCTGCCGCATCGGAATGAACGGCACAT
TTAGCCGAAAATGCGCAGAAATTAGCGCCCGTCGCTGAAAGTATCTCGCCCTATAATATTGTACATCATC
GCATTTCAGGCTCACTCTCAATCCAAATCTGTTCGCAAAGAAGCCGCCTTCGTCGTCGTCCTCGTCGTCG
GGTGAAAAGTTGCCGGAGTCATCCAATCCGCCCTTTCCCGCTGAAAATCCCCCAGCAACGCCCATCAAAG
AGGATCCCGATCAGGAGCAGTTGATGGTGGAGGAGGGTGCTTCGGCTGGAGAAGGATCTGGCACCGGAGC
CACCTCGAATTATCCCCAGGAGGCCGGCGATGCCGAGCAGTCACTGATGAAGATGCAGCTCCGTAAGTCG
CTAGCGAGGATGACTGAGCTCCTTTCCGCAATCCTAAGAGTTTCTCTCCATTAGATGCCCATCGTTTTCC
TGCCAGTCCCCTCGACTTTCAGCAGGCTCTGATGTCCGCAGGACCACCGACTTCTAGTTTGGATCCGCCG
GTGAATAACAAGCACTTTTGCCACGTCTGCCGGAGGAACTTCAGTTCGAGTTCCGCTCTCCAGATCCACA
TGCGCACCCACACGGGCGACAAGCCCTTCCAGTGCAATGTGTGCCAGAAGGCTTTCACCACCAAGGGGAA
TCTCAAGGTACAGTCAAGAATTTCCGTCTGTCCCTCCAGCATTTTACTCAACTGAGTTCCTGCTTGTCCC
TATTTTTGGGTTTCTATATTTTCTAGGTCCATATGGGTACTCACATGTGGACGAATCCGACTTCGCGGCG
GGGTCGTCGAATGTCCTTGGAGCTACCCATGCGACCTGGTCCAAACTCCGGCCAAGGTCATCCTGGATCC
AGTGCAGAGCAGGAGTTCATGCAGAGACGACCAGAGCTCTTTTTTCCCTACCTGCCGCCCTTCTTTAACG
GATTGCCACCAAAG
>dme_circ_0000203_junction_seq
GCCCTTCTTTAACGGATTGCCACCAAAGGGGTGCAGTCCATGTTCAGACTCCAGCG
>dme_circ_0000204
GCTCACTCTCAATCCAAATCTGTTCGCAAAGAAGCCGCCTTCGTCGTCGTCCTCGTCGTCGGGTGAAAAG
TTGCCGGAGTCATCCAATCCGCCCTTTCCCGCTGAAAATCCCCCAGCAACGCCCATCAAAGAGGATCCCG
ATCAGGAGCAGTTGATGGTGGAGGAGGGTGCTTCGGCTGGAGAAGGATCTGGCACCGGAGCCACCTCGAA
TTATCCCCAGGAGGCCGGCGATGCCGAGCAGTCACTGATGAAGATGCAGCTCCGATGCCCATCGTTTTCC
TGCCAGTCCCCTCGACTTTCAGCAGGCTCTGATGTCCGCAGGACCACCGACTTCTAGTTTGGATCCGCCG
GTGAATAACAAGCACTTTTGCCACGTCTGCCGGAGGAACTTCAGTTCGAGTTCCGCTCTCCAGATCCACA
TGCGCACCCACACGGGCGACAAGCCCTTCCAGTGCAATGTGTGCCAGAAGGCTTTCACCACCAAGGGGAA
TCTCAAGGGTCCATATGGGTACTCACATGTGGACGAATCCGACTTCGCGGCGGGGTCGTCGAATGTCCTT
GGAGCTACCCATGCGACCTGGTCCAAACTCCGGCCAAGGTCATCCTGGATCCAGTGCAGAGCAGGAGTTC
ATGCAGAGACGACCAGAGCTCTTTTTTCCCTACCTGCCGCCCTTCTTTAACGGATTGCCACCAAAG
>dme_circ_0000204_junction_seq
GCCCTTCTTTAACGGATTGCCACCAAAGGCTCACTCTCAATCCAAATCTGTTCGCA
>dme_circ_0000205
GTTTTAGTCGTCTTAATACCCGCATATATACCTAATAGAGAGCTCCAAAAGAATTGATGAGCTTTAGTTA
TGCATATACATAGTATAGTGTCGGTCAGTTCAGTAAAAATTAAAGCAGTAGTTAGTACCTCCTTCAATTA
TCATGCAACTTAATTATGTTGGT
>dme_circ_0000205_junction_seq
AATTATCATGCAACTTAATTATGTTGGTGTTTTAGTCGTCTTAATACCCGCATATA
>dme_circ_0000206
GTTTTAGTCGTCTTAATACCCGCATATATACCTAATAGAGAGCTCCAAAAGAATTGATGAGCTTTAGTTA
TGCATATACATAGTATAGTGTCGGTCAGTTCAGTAAAAATTAAAGCAGTAGTTAGTACCTCCTTCAATTA

TCATGCAACTTAATTATGTTGGTGTCTATGGAAATTGTATAAAACAATTAAATCCGTTGCCTAGAAATTA
TGCAACATTCTCATGTCCCCATTGTAAACTTATTTTTGTCTCTTTTAACATTTCTATTTGTTGTATGCGT
TGTATAT
>dme_circ_0000206_junction_seq
ATTTCTATTTGTTGTATGCGTTGTATATGTTTTAGTCGTCTTAATACCCGCATATA
>dme_circ_0000207
GAGGCGGTGGAGGAGGAAGTGGCCGCGGTTCGCCCATGCCGCAGGTCCATGTGGAAGTCCTTAGCCATGG
AGGCGGCGGAAGTGGCAAGCGCAACTCCAATGTGCCTGCGGATTTTCTCTGCCCCGGAGACCTTAGAAGA
GTCACCATCGACAAGCGCGACAAGTCGCTAGGTATCACCATTCAGTGCAACAACAACGGCGGCGGAATAT
TTGTGTCAACCGTCGCTGACAAAAGCACGGCGATGCGTGCCGGTCTCCAGGTGGGCGATCAACTCTTGGA
AG
>dme_circ_0000207_junction_seq
CTCCAGGTGGGCGATCAACTCTTGGAAGGAGGCGGTGGAGGAGGAAGTGGCCGCGG
>dme_circ_0000208
GTTATCCTCTGGTGTGCCTGGTTTGCTGAACTTAAGCTTTCGGAAGAAAAATTCCGCGAACCTGGACGGA
TGTTTGGTGGCGCTACTGGGCCCACTTCCTCCACCTCCCCCGGCACTTGAGACATGCTGCATGGAGTCAA
TGGAATCTGTCTGCGCCGACGAGGTGGTCATTCCCGGGGGTAACTGATCCTGTAC
>dme_circ_0000208_junction_seq
TCATTCCCGGGGGTAACTGATCCTGTACGTTATCCTCTGGTGTGCCTGGTTTGCTG
>dme_circ_0000209
AAGTGGATCATCGAGTACCAGAAGAACAACACCGGGCTGTTGGTGGAGAACGCCGAGATGAACAACGTGG
TGTATGTGTTCAAGTGTGAGGGATCCACTCTGACCGTCAAGGGCAAGGTGAACAACATCGTCTTCGACTC
CTGCAAGAAGTGCTCCCTGCTGTTCGATTCCGTGGTGGCCTCCG
>dme_circ_0000209_junction_seq
CTGCTGTTCGATTCCGTGGTGGCCTCCGAAGTGGATCATCGAGTACCAGAAGAACA
>dme_circ_0000210
TGCCCGGTCCGGATCCTAAAGATCCTACCAAGACTATTGAGTTGGATCAGATCGCGTGCTATCCAGCACC
GCCACCGGAGACTGCTACTCCAGCCAATGGAACGGCACCAGCCGTGGAGGGTGCAGTACCCCCTCCCGTT
TCCGCTCCCGAATCCGTTCAACTGGCTCCCATTCAGCCGACCACTCCTGTGGCCATTCCCGAGGGATCTG
>dme_circ_0000210_junction_seq
ACTCCTGTGGCCATTCCCGAGGGATCTGTGCCCGGTCCGGATCCTAAAGATCCTAC
>dme_circ_0000211
GGTGCCGCTGGATCAACGGGAGCAGGTGGCGATGGACCTAGAGGCGCCAGTGGAACTGATCCTGCAACTG
GGGCTGGAGGAACGGGTAGAGCTTGAGAGTCAGTAACAGGTGGAGCGGCATAAACAG
>dme_circ_0000211_junction_seq
TCAGTAACAGGTGGAGCGGCATAAACAGGGTGCCGCTGGATCAACGGGAGCAGGTG
>dme_circ_0000212
GGGCACTCAGATCCAGCATGGGCGGCGGTGGGGGCAGACCACCGGGTGGCGGTGGTGGCGGGGCACCTCC
GGCGGGGGCCGCTCCCTTGCCGGACCACACCAAGCCCGTGGTGTGGTACTGCCGGATGTAGGCCTGCAGT
TCGGTGAGCGTCTGCACCCAGGCGCGGGCCCACTCCACATGC
>dme_circ_0000212_junction_seq
CACCCAGGCGCGGGCCCACTCCACATGCGGGCACTCAGATCCAGCATGGGCGGCGG
>dme_circ_0000213
TTGGCCAGAGTCTTCAGGAAGCCGAACTCCGGCTCCGACTCCTTTTCGGCGTAGGCCTCCAGGAAGCCCG

TGATCGAGGTGTTGCCAAAGCCGAGGCCGCAGTCCGTAAAGACAACCACCTGCAGGATGTTCTGGTTGCC
CCACGGCGACATTATGGACACCACCGCCTTGAGCATGCTCATCAGGCAGGCCTTGTCCACGGGCTCCACC
TTCTTGACCGCCTGCCGCACCTGGTCGTAG
>dme_circ_0000213_junction_seq
CTTGACCGCCTGCCGCACCTGGTCGTAGTTGGCCAGAGTCTTCAGGAAGCCGAACT
>dme_circ_0000214
GGTCTGGAAACCTGAGCGCAGCAGCCAAGCCACCGAAGCTCAATCTAGATACCAGCAATCCCAACTACGA
GCTGGAGAAGCTGGAAGCCGATATCAAAGAGTTTTACGCCAAGGATTCAAAAGACACGGAGGAAAGTGGC
GACGACGATGTGACCATT
>dme_circ_0000214_junction_seq
GGAAAGTGGCGACGACGATGTGACCATTGGTCTGGAAACCTGAGCGCAGCAGCCAA
>dme_circ_0000215
GATCAGTCCGTGTCCCAAGTCACCGAACATCACGGCGAACAGAAAGGGGAATGTGATGCAGGCGTAAAGA
GCCGGATTCACTTCTCTATATGAGGCCATTCCATACGAGTCAACCAGGTTCTGGAAGCCATTGGTGAATT
TATTGG
>dme_circ_0000215_junction_seq
TTCTGGAAGCCATTGGTGAATTTATTGGGATCAGTCCGTGTCCCAAGTCACCGAAC
>dme_circ_0000216
GGAAAAGCAATTGTCTTCGATCAAGGAGGAGATCTTCAACATATTCTTCGGCGGCCGATACATCATCTTC
CTCATGGGAATTTTCTCCATTTACACCGGATTCATTTACAACGATGTGTTCTCAAAGTCCATGAATATAT
TCGGCTCCGCTTGGCACATGAACTATACAAGGGACGTGGTTGAGGATGAAAACCTTAAATATATCACCCT
CAGGCCGAACGATACTGTTTACAAGACATATCCCTTCGGCATGGATCCCATTTGGCAACTGGCCGACAAC
AAGATCATCTTTCTAAACACCTTTAAGATGAAGCTGTCGATCATTGTGGGTGTGATCCACATGATCTTCG
GCGTCTCCATGTCGGTGGTTAACTTTGCCTACTACAAGAAGTACGCAAGTATTTTCCTAGAGTTCCTGCC
TCAAGTCCTGTTCCTTCTCCTGTTGTTCGGTTACATG
>dme_circ_0000216_junction_seq
GTTCCTTCTCCTGTTGTTCGGTTACATGGGAAAAGCAATTGTCTTCGATCAAGGAG
>dme_circ_0000217
GTATGAGGCCGTGTGGGAGACCGTACTCAGGACATATTCGATGGTGTGGATGGCCTGATGGATGAAGATC
TCGCCCATCTCATCGTCCTCTCCATGACCCTCCCCTCCACCGCTTTGTGGTTTTGGTGGGGGAGCTCCGT
TCGTCTTGCGTTTGATCATGATGTACAGAGGCTTGCCCAAAAGCATCCAAGGAATGCAAATGATGGCAAC
GACGACAAAGACCTGTTGGATGCTCTTCTGACCGTCGAACATGAACTCCTTGCAGGGCTCAGGAGTGTCC
TGGCTGCCCTGCAGTATCATGTTGATGAACAGGATCAGGATAGAAGGAGCACAAGCCGGTGAAAGTGGTC
CTTCAACGGTGTCGTTGTAAACGACCCACTTGAAGAACATCATGAAGACCATGTAACCGAACAACAGGAG
AAGGAACAGGACTTGAGGCAGGAACTCTAGGAAAATACTTGCGTACTTCTTGTAGTAGGCAAAGTTAACC
ACCGACATGGAGACGCCGAAGATCATGTGGATCACACCCACAATGATCGACAGCTTCATCTTAAAGGTGT
TTAGAAAGATGATCTTGTTGTCGGCCAGTTGCCAAATGGGATCCATGCCGAAGGGATATGTCTTGTAAAC
AGTATCGTTCGGCCTGAGGGTGATATATTTAAGGTTTTCATCCTCAACCACGTCCCTTGTATAGTTCATG
TGCCAAGCGGAGCCGAATATATTCATGGACTTTGAGAACACATCGTTGTAAATGAATCCGGTGTAAATGG
AGAAAATTCCCATGAGGAAGATGATG
>dme_circ_0000217_junction_seq
GGAGAAAATTCCCATGAGGAAGATGATGGTATGAGGCCGTGTGGGAGACCGTACTC
>dme_circ_0000218
GAAAATACTTGCGTACTTCTTGTAGTAGGCAAAGTTAACCACCGACATGGAGACGCCGAAGATCATGTGG

ATCACACCCACAATGATCGACAGCTTCATCTTAAAGGTGTTTAGAAAGATGATCTTGTTGTCGGCCAGTT
GCCAAATGGGATCCATGCCGAAGGGATATGTCTTGTAAACAGTATCGTTCGGCCTGAGGGTGATATATTT
AAGGTTTTCATCCTCAACCACGTCCCTTGTATAG
>dme_circ_0000218_junction_seq
TTCATCCTCAACCACGTCCCTTGTATAGGAAAATACTTGCGTACTTCTTGTAGTAG
>dme_circ_0000219
AAGGAACAGGACTTGAGGCAGGAACTCTAGGAAAATACTTGCGTACTTCTTGTAGTAGGCAAAGTTAACC
ACCGACATGGAGACGCCGAAGATCATGTGGATCACACCCACAATGATCGACAGCTTCATCTTAAAGGTGT
TTAGAAAGATGATCTTGTTGTCGGCCAGTTGCCAAATGGGATCCATGCCGAAGGGATATGTCTTGTAAAC
AGTATCGTTCGGCCTGAGGGTGATATATTTAAGGTTTTCATCCTCAACCACGTCCCTTG
>dme_circ_0000219_junction_seq
AGGTTTTCATCCTCAACCACGTCCCTTGAAGGAACAGGACTTGAGGCAGGAACTCT
>dme_circ_0000220
GAAAATACTTGCGTACTTCTTGTAGTAGGCAAAGTTAACCACCGACATGGAGACGCCGAAGATCATGTGG
ATCACACCCACAATGATCGACAGCTTCATCTTAAAGGTGTTTAGAAAGATGATCTTGTTGTCGGCCAGTT
GCCAAATGGGATCCATGCCGAAGGGATATGTCTTGTAAACAG
>dme_circ_0000220_junction_seq
ATGCCGAAGGGATATGTCTTGTAAACAGGAAAATACTTGCGTACTTCTTGTAGTAG
>dme_circ_0000221
GCTATATTGGCGGCATTCTCATCTACGTATTCTTTGGAGCCTGGGCGTTGCTCACAGTCGGTATCCTGGT
GCTCATCGAAGGACTCTCGGCCTTCTTGCACACACTGCGTTTGCACTGGTGAGTTGCGAATAATGAGGAC
ACAATTCAGATGCATTTTAACCTGCGATTTGCTCTTTCAGGGTTGAGTTCATGAGCAAGTTCTACGAGGG
CGCTGGTTACGCCTTTGAGCCTTTTGCCTTTAAGACCATTTTGGATGTCAGCGAGGATGACTAAGGAGCG
CGGCACGAGAG
>dme_circ_0000221_junction_seq
AGGATGACTAAGGAGCGCGGCACGAGAGGCTATATTGGCGGCATTCTCATCTACGT
>dme_circ_0000222
ACGGCACAGCATGGCTTTCCTCATAAGCCTTCGGCTCTTGCGTATGATCCAGTTTTGAAACTTATGGCAA
TAGGGACGCAAACAGGGGCTTTAAAAGTTTTCGGTCAACCCGGAGTTGAATTGTACGGTCAGCATACTTT
GTTAAACAATTCAGCATCGGAGCTTAATGTACAATTACTTGAATGGGTGTATGGAACTGGTCGCATACTT
TCGTTGACGGCAGCGAATCAATTAATTCTATGGGAGCCAGTTGGAGCAACGTTGCTGCCAATCAAAACAC
TACCGTTTGACGGCAAACTTAAAAAAGTTTCATCGCTGTGCTGTTCTCTCAGTAAGGATCTGCTATGGAT
TGGAACAGAAGGTGGAAACATCTATCAACTGGATTTACATACATTTACCATTAAGGAGCCTGTAATTTAC
CATGACGTTGTGCTAGAGCAGGTGCCACCAGCCTACAAGCTAAATCCTGGTGCAATTGAGTCAATCCGCC
AACTTCCAAACTCCCCTAGCAAACTTCTAGTTGCATACAATCGCGGCCTTTGTGTTTTGTGGGATTTTGA
AAGCGCATCTGTCCAGCGAGCATACATAGCCCCTGGACATGGACAGAGCGTTGGTCTTACAGTGAACTTC
GAAGGATCTGAATTTACCTGGTACCACGCTGATGGTTCATACGCCACTTGGAGCATAGATAACCCAGAAC
CGCCGTCGAATGTTAATTATGTGCCTTATGGACCTGATCCATGCAAAAGCATAAATCGACTGTACAAAGG
CAAGCGAAGGATCCAACGATGTAATTGTTTTTTCCGGCGGCATGCCACGGTCAGCATATGGTGATCACAA
TTGTGTGTCCGTTCACGCCAGCGATGGACACAAAGTGTGTCTTGACTTTACGTCTAAAGTGATTGACTTT
TTTGTGACCTTTGAAAATAATAGAGATGTCGCTGAAGTTCTTGTTGTACTACTTGAAGAGGAACTCTGCG
CTTACGATCTTACTGACCCTAATATTTGTGCTATCAAAGCGCCATATCTTCACTCTGTCCATGCATCAGC
TGTCACTTGCAATTACCTTGCTTCTGAAGTCGTACAGTCGGTATATGAAAGTATTTTAAGAGCTGGAGAT
GAACAAGACATTGACTATAGCAATATTAGCTGGCCTATCACTGGCGGTACTCTCCCGGATAACTTAGAAG

AATCTGTAGAAGAGGACGCGACTAAGCTTTATGAGATTTTGTTAACTGGTCACGAAGATGGTTCTGTTAA
ATTTTGGGACTGCACTGGAGTGTTGCTTAAACCAATTTATAATTTTAAAACTAGCAGCATTTTTGGAAGT
GAGTCAGACTTCCGAGATGACGCAGCTGCAGATATGAGTGCGGAACAAGTCGATGAAGGAGAACCGCCAT
TTCGGAAATCAGGACTTTTTGATCCTTATTCAGATGACCCTCGTTTAGCAGTGAAGAAAATAGCATTCTG
CCCAAAAAACCGGACAACTTATTGTTGGTGGCACAGCGGGCCAAATAGTTATAGCCGACTTCATAGACTTA
CCCGAAAAAGTGTCTTTAAAATACATTTCAATGAATTTGGTCAGCGATCGTGATGGATTTGTGTGGAAGG
GTCACGATCAGTTAAACGTGCGATCGAACTTATTAGACGGAGAAGCAATTCCTACGACGGAACGTGGTGT
AAATATATCGGGAGTACTGCAAGTTTTGCCGCCAGCCAGCATAACATGCATGGCACTCGAAGCAAGCTGG
GGCCTAGTATCTGGTGGGACTGCGCACGGCTTAGTTCTCTTTGACTTCAAAAACTTTGTTCCAGTATTTC
ATCGCTGCACTTTAAACCCAAATGATCTTACTGGAGCAGGAGAGCAGCTGTCTCGTCGAAAGTCTTTTAA
GAAATCATTGAGGGAGTCATTTAGAAAGCTTCGCAAGGGTCGATCGACCAGGACCAACCAGAGCAATCAA
GTACCAACAACGGGTAATCAACATTTTGTACTGTTATTAGTTTTGATGTAACGATaaaaaaaaaaaaaGG
TAACGGAAATAGTCTCTGAACCACGGCCTCGCGAGAATTGAACCCAAAAGCTAAAAACATACAGTGCTTT
TCATTTTGGGACGCCCAGAGCTTTAATTATATTTTTGTGCAACAAATTTTTATGCATTGGATATATTGAT
CAAAGTTTTAAAGGTATATGTATTTATGATCAGTTTAAACAGCCGACTACATTTGACTATGTCCGTCCTT
AGAAAGAAATTCAGTCATTACAATATTCCTTGCTAGTCTATCGATATGCTATAAGTGTTAAAAGTGCTTT
TGCATTTACTCTGGTTTGACAATATATTTTATTGATATTGATTTCGTTTCATTTACTAGTTGGAAAACAG
TTCTTAAACTAATTAAATCATAGTCTTTTTGTTTTCGCGATTTCGATCTATATGGCAAATTTTGTTTTTA
AAATTTCACCTATCCTCCTACTATTTCTGTAATGGGTAACTCGACTGTAGCGTCTATTCTTTTGGTGTTC
TGTATATGTACATCTAATTTGTTGTATAATTTTTACATTTTCAGTTCAACATAACGTCGCCAACTTTGTG
GTCAGCAACAAATGCCAGTACAGTCTCGGTTTTCCTTCTGCATTTGCCACCAGCGCAGACCGCGGCAACT
GCCGTCCCGTCGGCAAGTGGCAATGCACCACCACACATGCCCCGCCGAATTTCTGCGCAGCTTGCTAAAG
AAATACAATTAAAACATCGTGCTCCTGTGGTGGGTATTTCTATTTTTGATCAGGCGGGTAGCCCTGTCGA
TCAGCTGAACGCCGGTGAAAACGGGAGTCCACCGCATCGTGTACTTATTGCTTCCGAGGAACAGTTCAAG
GTGTTTTCACTTCCGCAACTAAAGCCGATTAACAAATATAAGCTTACCGCTAACGAAGGTGCTCGGATTC
GCCGCATCCATTTTGGTTCGTTTAGTTGTCGCATATCCCCGGAAACACTGCAGAGTATGCACGGTTGTAG
CCCAACTAAGTCCACGCGTTCACATGGCGATGGAGAGGCGGATCCTAATATCAGTGGAAGCTTGGCTGTA
AGTCGTGGAGATGTATATAACGAAACAGCATTGATATGTTTAACGAATATGGGCGATATCATGGTTTTAT
CAGTACCTGAATTAAAAAGACAGCTGAATGCCGCAGCAGTGCGACGGGAAGACATTAAGTGGAGTTTCGT
CACTTTGCTTTACAAACTCTGGAGAAGCACTGTATATGATGTCTTCTTCTGAACTGCAGCGTATTGCTTT
AGCCACGTCCAGAGTCGTGCAACCCACTGGCGTTGTTCCAGTAGAACCATTAGAAAATGAAGAGTCTGTG
TTGGAAGAAAATGATGCAGAGAATAATAAGGAAACCTACGCATGTGATGAAGTTGTGAATACATATGAAA
TTAAAAATCCATCAGGCATTTCAATATGCACAAGGCCTGCAGAGGAAAACGTTGGAAGAAATAGTGTTCA
GCAAGTTAATGGAGTCAACATTTCAAATTCACCTAATCAAGCTAACGAGACTATCAGCAGCTCTATTGGC
GATATTACCGTTGACTCGGTGCGCGACCATTTAAATATGACGACCACCACTTTGTGTTCTATTAATACAG
AGGAAACCATTGG
>dme_circ_0000222_junction_seq
GTTCTATTAATACAGAGGAAACCATTGGACGGCACAGCATGGCTTTCCTCATAAGC
>dme_circ_0000223
TTTAGAATAAATACGGCCACAATTAGTATACATATTTATTCATCGAAACCAACCCACCACAAGAGCATAA
CCAATTATGTTAAAGTTTATCAGAGGAAAAGGGCAGCAGCCCAGTGCTGACAGACACCGCCTACAGAAGG
ACCTTTTTGCTTATCGTAAGGTACAACAAACAATTTTCTCTTTCCATTCTAtttatttttttttttCG
TATATGTAGCCGATTGGTATATTTTGTGGGGTGTCATTTATTTTGATCCTGGGTGGTAAATACATTTTTA
TGGAAGTTAGAAGAAAGCTTATAATTTATTAAGAATAAATAAATACCTCCTTTTCAATCTTTTGTTATGT

CTTCTCCTATGTGCATACTTTTGGGCATTAACATTTAGGTAAATTTTTTCCACCGCAACTACAAGTAAAC
CGCATACACACACAGTATTTTGTAGACTCGCGCCACAAAGCACCGCATAAAAAAGGTTACGGTGCTTTTT
TGCTCGTTTCTTTTGTGCACTCCTTCTATGCGGTGCCAAATATGAGGAGGTACCTGAAGCTTTGTTGATT
TTGTGAGTGTCTTTTTCCACCGCCTGTTTTTGGCTACTTTAAAAATTCTATTATATCGTTGTGCTCGTAA
TGTTGTCCATTCCTTTTAAATATTTTCGAATTAAATATACCATAGATGTTTCCCTATTACCATAATAGTT
TAATAAATATACGTATATGAAATTTTATAAGAAttttttttATCCCATGTTAGTATAAACATGTTATTAA
ACAAATAAAAGTAGGTCAGAGGTAATCGCCATCCAGCTCCAGAAAGAGGGGGGAGCTGATAAGATTGCTT
TTATGTGCTTAATGGGTTGGGTAAATGGCTTGGTTTCACAAACACCCTTATTTCAGGCACAAAATAAGTA
TGTCATAGACGGCGTAAAATTGATAAGATTTTAAAGTCAAAAGAAAAAGAATTTATTCGCCTGTAACTGC
GTTAGAAATCCAAATACACCTGCAATATGAATCAAACAAAATCAACTCATAACGAGATCTGATAGCAACT
TCTTAAAACTTTTTTTGTATAAAAATACAATTTATACAATTAGATAAGATTTACCAAACCTTTTTTCGAT
CAATGTAAATATAGTATGATAGTAGGGACTGCTGTCTACAGTGATGTTTATAAGAGACATACTTAAGAGC
AGCATTTAAAGCTTTGGGCGCTTTTTGAACTTAATTTAAATTCAAACCTCAATATTTATTGAGGTTTtta
ttcccgttaattgtaaagaaaaatgctgtattagattcggtaaaagtatgtaaatggtttaagaaagcgt
tcccgactctataaagtGGCAAGTAATCTGACGATTTACCAGATCTTGGGAACTGAAAAAACTAGAATGT
TTTGGAAAACCCTTTTACAATTAAGAAATGAACCAAACAATTTTGAATTATTAAGAATCTGTAATTGCtt
tttgaaactttttaatattttttttcttttAGAACGGCACAGCATGGCTTTCCTCATAAGCCTTCGGCTCT
TGCGTATGATCCAGTTTTGAAACTTATGGCAATAGGGACGCAAACAGGGGCTTTAAAAGTTTTCGGTCAA
CCCGGAGTTGAATTGTACGGTCAGCATACTTTGTTAAACAATTCAGCATCGGAGCTTAATGTACAATTAC
TTGAATGGGTGTATGGAACTGGTCGCATACTTTCGTTGACGGCAGCGAATCAATTAATTCTATGGGAGCC
AGTTGGAGCAACGTTGCTGCCAATCAAAACACTACCGTTTGACGGCAAACTTAAAAAAGTTTCATCGCTG
TGCTGTTCTCTCAGTAAGGATCTGCTATGGATTGGAACAGAAGGTGGAAACATCTATCAACTGGATTTAC
ATACATTTACCATTAAGGAGCCTGTAATTTACCATGACGTTGTGCTAGAGCAGGTGCCACCAGCCTACAA
GCTAAATCCTGGTGCAATTGAGTCAATCCGCCAACTTCCAAACTCCCCTAGCAAACTTCTAGTTGCATAC
AATCGCGGCCTTTGTGTTTTGTGGGATTTTGAAAGCGCATCTGTCCAGCGAGCATACATAGCCCCTGGAC
ATGGACAGAGCGTTGGTCTTACAGTGAACTTCGAAGGATCTGAATTTACCTGGTACCACGCTGATGGTTC
ATACGCCACTTGGAGCATAGATAACCCAGAACCGCCGTCGAATGTTAATTATGTGCCTTATGGACCTGAT
CCATGCAAAAGCATAAATCGACTGTACAAAGGCAAGCGAAGGATCCAACGATGTAATTGTTTTTTCCGGC
GGCATGCCACGGTCAGCATATGGTGATCACAATTGTGTGTCCGTTCACGCCAGCGATGGACACAAAGTGT
GTCTTGACTTTACGTCTAAAGTGATTGACTTTTTTGTGACCTTTGAAAATAATAGAGATGTCGCTGAAGT
TCTTGTTGTACTACTTGAAGAGGAACTCTGCGCTTACGATCTTACTGACCCTAATATTTGTGCTATCAAA
GCGCCATATCTTCACTCTGTCCATGCATCAGCTGTCACTTGCAATTACCTTGCTTCTGAAGTCGTACAGT
CGGTATATGAAAGTATTTTAAGAGCTGGAGATGAACAAGACATTGACTATAGCAATATTAGCTGGCCTAT
CACTGGCGGTACTCTCCCGGATAACTTAGAAGAATCTGTAGAAGAGGACGCGACTAAGCTTTATGAGATT
TTGTTAACTGGTCACGAAGATGGTTCTGTTAAATTTTGGGACTGCACTGGAGTGTTGCTTAAACCAATTT
ATAATTTTAAAACTAGCAGCATTTTTGGAAGTGAGTCAGACTTCCGAGATGACGCAGCTGCAGATATGAG
TGCGGAACAAGTCGATGAAGGAGAACCGCCATTTCGGAAATCAGGACTTTTTGATCCTTATTCAGATGAC
CCTCGTTTAGCAGTGAAGAAAATAGCATTCTGCCCAAAAACCGGACAACTTATTGTTGGTGGCACAGCGG
GCCAAATAGTTATAGCCGACTTCATAGACTTACCCGAAAAAGTGTCTTTAAAATACATTTCAATGAATTT
GGTCAGCGATCGTGATGGATTTGTGTGGAAGGGTCACGATCAGTTAAACGTGCGATCGAACTTATTAGAC
GGAGAAGCAATTCCTACGACGGAACGTGGTGTAAATATATCGGGAGTACTGCAAGTTTTGCCGCCAGCCA
GCATAACATGCATGGCACTCGAAGCAAGCTGGGGCCTAGTATCTGGTGGGACTGCGCACGGCTTAGTTCT
CTTTGACTTCAAAAACTTTGTTCCAGTATTTCATCGCTGCACTTTAAACCCAAATGATCTTACTGGAGCA
GGAGAGCAGCTGTCTCGTCGAAAGTCTTTTAAGAAATCATTGAGGGAGTCATTTAGAAAGCTTCGCAAGG

GTCGATCGACCAGGACCAACCAGAGCAATCAAGTACCAACAACGGGTAATCAACATTTTGTACTGTTATT
AGTTTTGATGTAACGATaaaaaaaaaaaaaGGTAACGGAAATAGTCTCTGAACCACGGCCTCGCGAGAAT
TGAACCCAAAAGCTAAAAACATACAGTGCTTTTCATTTTGGGACGCCCAGAGCTTTAATTATATTTTTGT
GCAACAAATTTTTATGCATTGGATATATTGATCAAAGTTTTAAAGGTATATGTATTTATGATCAGTTTAA
ACAGCCGACTACATTTGACTATGTCCGTCCTTAGAAAGAAATTCAGTCATTACAATATTCCTTGCTAGTC
TATCGATATGCTATAAGTGTTAAAAGTGCTTTTGCATTTACTCTGGTTTGACAATATATTTTATTGATAT
TGATTTCGTTTCATTTACTAGTTGGAAAACAGTTCTTAAACTAATTAAATCATAGTCTTTTTGTTTTCGC
GATTTCGATCTATATGGCAAATTTTGTTTTTAAAATTTCACCTATCCTCCTACTATTTCTGTAATGGGTA
ACTCGACTGTAGCGTCTATTCTTTTGGTGTTCTGTATATGTACATCTAATTTGTTGTATAATTTTTACAT
TTTCAGTTCAACATAACGTCGCCAACTTTGTGGTCAGCAACAAATGCCAGTACAGTCTCGGTTTTCCTTC
TGCATTTGCCACCAGCGCAGACCGCGGCAACTGCCGTCCCGTCGGCAAGTGGCAATGCACCACCACACAT
GCCCCGCCGAATTTCTGCGCAGCTTGCTAAAGAAATACAATTAAAACATCGTGCTCCTGTGGTGGGTATT
TCTATTTTTGATCAGGCGGGTAGCCCTGTCGATCAGCTGAACGCCGGTGAAAACGGGAGTCCACCGCATC
GTGTACTTATTGCTTCCGAGGAACAGTTCAAGGTGTTTTCACTTCCGCAACTAAAGCCGATTAACAAATA
TAAGCTTACCGCTAACGAAGGTGCTCGGATTCGCCGCATCCATTTTGGTTCGTTTAGTTGTCGCATATCC
CCGGAAACACTGCAGAGTATGCACGGTTGTAGCCCAACTAAGTCCACGCGTTCACATGGCGATGGAGAGG
CGGATCCTAATATCAGTGGAAGCTTGGCTGTAAGTCGTGGAGATGTATATAACGAAACAGCATTGATATG
TTTAACGAATATGGGCGATATCATGGTTTTATCAGTACCTGAATTAAAAAGACAGCTGAATGCCGCAGCA
GTGCGACGGGAAGACATTAAGTGGAGTTTCGTCACTTTGCTTTACAAACTCTGGAGAAGCACTGTATATG
ATGTCTTCTTCTGAACTGCAGCGTATTGCTTTAGCCACGTCCAGAGTCGTGCAACCCACTGGCGTTGTTC
CAGTAGAACCATTAGAAAATGAAGAGTCTGTGTTGGAAGAAAATGATGCAGAGAATAATAAGGAAACCTA
CGCATGTGATGAAGTTGTGAATACATATGAAATTAAAAATCCATCAGGCATTTCAATATGCACAAGGCCT
GCAGAGGAAAACGTTGGAAGAAATAGTGTTCAGCAAGTTAATGGAGTCAACATTTCAAATTCACCTAATC
AAGCTAACGAGACTATCAGCAGCTCTATTGGCGATATTACCGTTGACTCGGTGCGCGACCATTTAAATAT
GACGACCACCACTTTGTGTTCTATTAATACAGAGGAAACCATTGG
>dme_circ_0000223_junction_seq
GTTCTATTAATACAGAGGAAACCATTGGTTTAGAATAAATACGGCCACAATTAGTA
>dme_circ_0000224
GTTACAGTTTGTTAAATTAAATTAAAATTGTATTTTAAGCATTTAATTCTCGATAATTAAATGTATAAAA
TGTTGCATTTAAAAGGAAGCATAATGTGTGGGTCGTGTGTCTGTCAGTTTGTCTGTGTATTGTTTTCTTA
CTAAATTATTAAACTTTGACTCGTGTTTAATATACttttttttagatttctcttcctttttttGTAAAATT
TAAAGCAGATATGTTGTTGCTTGTGGCGCTTGGGGATTTCTCGTTAATTGTTTCACTTTCTTTTATTTCG
TTACGGCTTAAACTTGTTCTTAATGTTTGTTGTTTTCAACACAAGCACAGTTAAGGGTTATAATTAATTT
TAGAGTTGTGCCTAGTCGTATATATGTATATGTATGGGTTGTTGTTGTTGTTGCTTGTTAACTTTAATAG
TGGGGTAATAACTTGTTGTTATAGGTTTA
>dme_circ_0000224_junction_seq
GGGGTAATAACTTGTTGTTATAGGTTTAGTTACAGTTTGTTAAATTAAATTAAAAT
>dme_circ_0000225
GTCAAACGCCGCACCAGTGCGATGTCTGCGGCAAGAAATATACGCGCAAGGAGCACCTAGCCAATCATAT
GCGATCCCATACCAACGAGACGCCGTTCCGTTGCGAGATCTGCGGCAAGAGCTTTAGCCGCAAGGAGCAC
TTCACCAATCACATACTTTGGCATACAGGTAAAGAGACAAAACGAATCGTAAGCCCGCTTCCTGCACCTC
GATCCCGATCGAATCCTACCCAGCCTCCAGCTACAGTCCACGACACATaaaaaaaaaCCCTGTATACACA
GACACTTGGCGGCGCAGCGATAACGAACTCTGGCTAGGATTGGGGTAAAAGCTAGTTGCACTCCACTGTT
CTTTGTGGATCAATTACTTTTCCAACTTTACTTTTAACATTCTTGTTAAAAAGAGATTAATCATTTCTAA

TATAACTAACTTTTGATTTAGAATGTAGTCTTAGAATTACTTACAGCAATTGTATGTTTTGATAGTCATT
TTCAGCGTAACTCTCTATAAAAATATGTTTTGATAGGACTTAACTTAGAAGTCAGCAAGTCTTTAAATTA
TTTTCTTTAATCCttttttttttAATGTTTAAAAGCTGATTGATTCTAGCTTTTAAATTCTTCTACTTCT
CTTTAATCCCTTGAAACTCAAGGTTTAGACATATGGTTTGTTCTTGTAGTTAGTTTTCCTAAAATCCAGA
TCGCGGGGACGGTGAACACACGATTGCAACACGCAGCTCTTTTCCCCCGTCGCCTAGAGGAGATAAACAA
CACAAGACCAATCCGCGCCAGGTGTCCAGTGACCCGCAACCAGCCAGACCAAACCATGACCGCCCCCCCT
GTTCCATGGCGATGATTACTAATCTCTAAACATTTTCTCGTCCCATTCGCGCGACACCCACACATTTGCC
ACGCAAGCAGGGCGAGACGCCGCACCGGTGCGACTTTTGCTCCaagacgtttacgcgaaaggagcacttg
cttaaccacgtgcgccagcatacgggagagtcgccacaccgctgctcctactgcatgaagacgttcacgc
gcaaggagcacctggtcaaccacatacgccagcacacgggtgagacaccgttcaagtgcacgtactgcac
gaaagcgttcacgcgcaaagatcacatggttaatcatgtacggcaacatacaggcgagtcgccgcacaag
tgcacgtactgcaccaagacgttcacgcgcaaggagcacctgacgaaccatgtgcgccagcacacgggcg
actccccgcaccgctgctcctactgcaagaagaccttcacgcgcaaggagcacctgacgaaccatgtgcg
cctgcacacgggcgactcgccgcacaagtgcgagtactgccagaagacgtttacgcggaaggagcacctc
aacaatcatatgcgccagcattcgagcgacaatccgcattgctgcaacgtttgcaacaagccgtttacgc
gcaaggaacacctgatcaaccatATGTCGCGGTGCCACACCGGTGACCGGCCCTTCACCTGCGAGACGTG
CGGCAAATCGTTCCCGCTCAAGGGCAATCTGCTCTTCCATCAGCGCAGCCATACCAAGGGCCAGGAGATG
GAGCGGCCATTCGCCTGCGAGAAGTGCCCCAAGAACTTCATCTGCAAAGGGTGAGTGAGCTCTATGATTG
TTGGCTGTGCGTTGCATGCATCCAGTGGAGGCACGCGTGGCGTGGCGTCAGCATAGTGTCCCAGCTACAG
TCATAATCCAAATACCTTAATCCCGATCCTCTTCTCCAGTTGCTGTTGTGGGGATTGCTTTCACAGCCTC
CAAATGTCGTTTTCGTGGGTCGATTCTAGTCAGAAACCAAATCAAAAATCGTTTAGTTTTTCAGAAGATT
CATGTTTAAAAACTTGTTTTATTATTTTAATATATGTAGCTATTTTAATTAAACAGTAAAGAAAGTAAAG
AATTGCAAATCATATAGAAAGGAATTAAATAGATAATGTGTACATAATTCTTGATTTAATACATTTATTC
ATTTTATGATCAGTGCAAAACTTGTTGCATTGAAAAGCAAATTATGTAAATACTTATTAGATAACTTCTA
GCATTATTTAAGATTTCGGTTTCTTGTTAACAAAAATGATGACTAGACATTTACCTCTTGAGCCTAATTT
GCATGCCGACAACGACTGTGAGAGCAAGCCCCCAAAACTTACCTGAGCCGTTTTCGAAATTCAAGATCCC
CGACGATCAGATCTTCATGTCGCTTCCATTGTCCGTTTCGCAGGTCACTTGGTCTCGCACATGCGCTCCC
ATTCGGGTGAGAAACCACACGCGTGCACACTGTGCAGCAAGGCGTTCGTCGAGCGCGGCAATTTGAAGCG
CCACATGAAGATGAATCACCCGGATGCTATGATGCCGCCACCACCCGTGCATCCGCATCCGCAAATACCG
GCTGGTGTGCTGACGCAAGTCAAGCAGGAAGTGAAACCGATCATAAGTGAGTCGGCAAACACGACTGCGC
TAGCGCAAGCGAGTCTTCCCGTGCCCAAGTCGCCCAACTTAACCGGTCCTCCTACCGTTTCAGTTTCCCC
ATCACTCGGCGACCACCACAATGCACACCATTCAGCAGATCACGGCGGGTGCGGCGGGCGGAGCCGGTGC
GGTCCAGTTAACACCGGGTCTGGTGCCCCTGGTTACCTCTACGCTCATCTCACATAATGCGgctgctcaa
cagcagtcgcagaagcagcaagcagccgccgcagcagctgcacagcaacaggccgcagccgccgctgctg
cccaacagcaagcagctcagcaacaggcggctgctgcacatcagcagcatcaacaacaagtggccgcgca
acatcaacagcaggctgcagtggctgcacaccagcagcagcaacagcagttgcagcagcagcaacaactg
cttcagttgtccatccaacaggcggctcaccatcatcagcaggagcagcatcgtcaacagcagcaacagc
aacaccagcagcaacaacagcaacagcatcaccagcagcaacagcagGGTCATCCACAGGCCCCGCCACC
GCAGCAGCAACAGCAGCCGCCGCCCATCGCCTTGATCAGTGACCCAAGTGCTCTGGCACGTGCCGCCATC
CAGCTGCAGCATCTGCCAGCGAACGTGGAACAGCACCCGGTTGTTTACTAACAGTAGCCCCTCCTGCACG
GCTACACTCCCACCACAGCCCACTCCAGCAGCACGACCAGCACCTCAATAGCTGCCACAGCGGCCACTGC
GTCGTCGCAAGCGGCGTGGTGAAGGCATGCGTGCCACCAGTGAGCCAGTGCTAGTGAGCCGAGCTGACGC
GACTTCTCTAGATAGGACACACATCATAACAAGCGCACCCCATATAAGCAGCTAGTTG
>dme_circ_0000225_junction_seq

AAGCGCACCCCATATAAGCAGCTAGTTGGTCAAACGCCGCACCAGTGCGATGTCTG
>dme_circ_0000226
GCGAGACGCCGCACCGGTGCGACTTTTGCTCCaagacgtttacgcgaaaggagcacttgcttaaccacgt
gcgccagcatacgggagagtcgccacaccgctgctcctactgcatgaagacgttcacgcgcaaggagcac
ctggtcaaccacatacgccagcacacgggtgagacaccgttcaagtgcacgtactgcacgaaagcgttca
cgcgcaaagatcacatggttaatcatgtacggcaacatacaggcgagtcgccgcacaagtgcacgtactg
caccaagacgttcacgcgcaaggagcacctgacgaaccatgtgcgccagcacacgggcgactccccgcac
cgctgctcctactgcaagaagaccttcacgcgcaaggagcacctgacgaaccatgtgcgcctgcacacgg
gcgactcgccgcacaagtgcgagtactgccagaagacgtttacgcggaaggagcacctcaacaatcatat
gcgccagcattcgagcgacaatccgcattgctgcaacgtttgcaacaagccgtttacgcgcaaggaacac
ctgatcaaccatATGTCGCGGTGCCACACCGGTGACCGGCCCTTCACCTGCGAGACGTGCGGCAAATCGT
TCCCGCTCAAGGGCAATCTGCTCTTCCATCAGCGCAGCCATACCAAGGGCCAGGAGATGGAGCGGCCATT
CGCCTGCGAGAAGTGCCCCAAGAACTTCATCTGCAAAGG
>dme_circ_0000226_junction_seq
AGTGCCCCAAGAACTTCATCTGCAAAGGGCGAGACGCCGCACCGGTGCGACTTTTG
>dme_circ_0000227
GTCAAACGCCGCACCAGTGCGATGTCTGCGGCAAGAAATATACGCGCAAGGAGCACCTAGCCAATCATAT
GCGATCCCATACCAACGAGACGCCGTTCCGTTGCGAGATCTGCGGCAAGAGCTTTAGCCGCAAGGAGCAC
TTCACCAATCACATACTTTGGCATACAGGTAAAGAGACAAAACGAATCGTAAGCCCGCTTCCTGCACCTC
GATCCCGATCGAATCCTACCCAGCCTCCAGCTACAGTCCACGACACATaaaaaaaaaCCCTGTATACACA
GACACTTGGCGGCGCAGCGATAACGAACTCTGGCTAGGATTGGGGTAAAAGCTAGTTGCACTCCACTGTT
CTTTGTGGATCAATTACTTTTCCAACTTTACTTTTAACATTCTTGTTAAAAAGAGATTAATCATTTCTAA
TATAACTAACTTTTGATTTAGAATGTAGTCTTAGAATTACTTACAGCAATTGTATGTTTTGATAGTCATT
TTCAGCGTAACTCTCTATAAAAATATGTTTTGATAGGACTTAACTTAGAAGTCAGCAAGTCTTTAAATTA
TTTTCTTTAATCCttttttttttAATGTTTAAAAGCTGATTGATTCTAGCTTTTAAATTCTTCTACTTCT
CTTTAATCCCTTGAAACTCAAGGTTTAGACATATGGTTTGTTCTTGTAGTTAGTTTTCCTAAAATCCAGA
TCGCGGGGACGGTGAACACACGATTGCAACACGCAGCTCTTTTCCCCCGTCGCCTAGAGGAGATAAACAA
CACAAGACCAATCCGCGCCAGGTGTCCAGTGACCCGCAACCAGCCAGACCAAACCATGACCGCCCCCCCT
GTTCCATGGCGATGATTACTAATCTCTAAACATTTTCTCGTCCCATTCGCGCGACACCCACACATTTGCC
ACGCAAGCAGGGCGAGACGCCGCACCGGTGCGACTTTTGCTCCaagacgtttacgcgaaaggagcacttg
cttaaccacgtgcgccagcatacgggagagtcgccacaccgctgctcctactgcatgaagacgttcacgc
gcaaggagcacctggtcaaccacatacgccagcacacgggtgagacaccgttcaagtgcacgtactgcac
gaaagcgttcacgcgcaaagatcacatggttaatcatgtacggcaacatacaggcgagtcgccgcacaag
tgcacgtactgcaccaagacgttcacgcgcaaggagcacctgacgaaccatgtgcgccagcacacgggcg
actccccgcaccgctgctcctactgcaagaagaccttcacgcgcaaggagcacctgacgaaccatgtgcg
cctgcacacgggcgactcgccgcacaagtgcgagtactgccagaagacgtttacgcggaaggagcacctc
aacaatcatatgcgccagcattcgagcgacaatccgcattgctgcaacgtttgcaacaagccgtttacgc
gcaaggaacacctgatcaaccatATGTCGCGGTGCCACACCGGTGACCGGCCCTTCACCTGCGAGACGTG
CGGCAAATCGTTCCCGCTCAAGGGCAATCTGCTCTTCCATCAGCGCAGCCATACCAAGGGCCAGGAGATG
GAGCGGCCATTCGCCTGCGAGAAGTGCCCCAAGAACTTCATCTGCAAAGG
>dme_circ_0000227_junction_seq
AGTGCCCCAAGAACTTCATCTGCAAAGGGTCAAACGCCGCACCAGTGCGATGTCTG
>dme_circ_0000228
GTGAACAATACAAATTGGTTGTGCGCCAATTAAACACCCAAATGGATCAACTGTTACAGTAGCAGCCACA

ACATCAGCACAACGGGAGCCAGAGCAAGAGGCAGCAATTTCATGGTAATTGTCCCCAGTCAGCGTCGATA
AGAAAGACCTCGAAAAATACCTGAGCAGCCAGAGACTAAGTGACACCACCTCCACCTTCCACTTGCATTT
TTTGTTTGTTGTAGCCACGCTCAGCGAAAGCAGTCGACAGCAGCAACAACAGCAATATGTAGGCGCTCCT
CAGCAGCAACACATGAAATACAACAGCCAAGAACAGCAGCAACATCAACAACAACTACTCCCAACAAACC
AATAACTCCCaaaacaaaaaaaaaaaCAACACAACAACAAGAACATCATTAGGATATAGGAATCAGGGAAC
GAGCAACGCCACAACGATCGCCCGGATCTGGACACAAGAATGCAGCACGTGAGCGCTGCCAGCTCGGTGC
CATCAGTAGTAACTCCTGTTGTGACCACTGGTGGGACAACGATCACATTGGGCGGCCCACCACCGCTCCC
TAAATCCGAGCACAAAGAGGATGGCAAGCCGCCGCACGGCATCGAAATGTACAAGGTGAACATTGAGGAC
ATTTCGCAGCTCTTCACCTACCACGAGGTCTTTGGCAAAATCCACGGCGATGTGGTCAATCATCAATTAG
CAGCGGCCCACGGCGGCCAGTTGCCACCACCTCCTCCGCTGCCGCCGCAGGTTACCAGTCATGCGGCGAG
TGCAGCGGCAGCCGCAGCAGCGGCGTCCACGAATAATGCCGCCGTTGCAGCGGTAATGGCATCAGCGAAT
GCAGCAGCGGCCGCGGCGGCAGCTGCATCGGCGGGGGGAGGACTACCGCCGGCCACCAGCGGCAATGGGG
GCCAGCAGGTGACGGTGACGACGACCAGCAGCTCGACTAGCAGCGGCGGGAGCACCACCAGTGGGGGCAC
CACGACCACGGCGGGTGAGTTGCTTATGCCTAAAATGGAGGGCGGAATTCATGGCGTGGACGGCAGCGGC
AATGGCGGCAATGGTGGCGGACAAAACGTGGCGCTGGCGCCAGACGGTACGCCCATTGCGACGGGGACGC
ACGTCTGCGACATCTGCGGCAAGATGTTCCAGTTCCGGTACCAGCTGATCGTGCACCGGCGCTACCACAG
CGAACGGAAGCCGTTCATGTGCCAGGTGTGCGGCCAGGGGTTCACCACGTCGCAGGATTTGACGCGCCAC
GGCAAGATCCATATTGGCGGGCCCATGTTCACCTGCATCGTGTGCTTCAATGTGTTCGCGAACAATACGA
GCCTGGAGCGGCACATGAAACGGCACTCGACGGACAAACCGTTCGCCTGCACCATTTGCCAAAAGACCTT
TGCCCGCAAAGAGCATCTGGACAATCACTTCCGCTCGCACACGGGCGAAACGCCCTTCCGTTGCCAGTAC
TGCGCCAAGACGTTCACGCGCAAGGAGCACATGGTCAACCATGTGCGCAAACACACGGGTGAGACGCCAC
ATCGTTGCGATATTTGTAAGAAGTCCTTTACGCGCAAGGAACACTATGTTAACCACTACATGTGGCACAC
TGGGTGAGTTGCTTATGCCTAAAATGGAGGGCGGAATTCATGGCGTGGACGGCAGCGGCAATGGCGGCAA
TGGTGGCGGACAAAACGTGGCGCTGGCGCCAGACGGTACGCCCATTGCGACGGGGACGCACGTCTGCGAC
ATCTGCGGCAAGATGTTCCAGTTCCGGTACCAGCTGATCGTGCACCGGCGCTACCACAGCGAACGGAAGC
CGTTCATGTGCCAGGTGTGCGGCCAGGGGTTCACCACGTCGCAGGATTTGACGCGCCACGGCAAGATCCA
TATTGGCGGGCCCATGTTCACCTGCATCGTGTGCTTCAATGTGTTCGCGAACAATACGAGCCTGGAGCGG
CACATGAAACGGCACTCGACGGACAAACCGTTCGCCTGCACCATTTGCCAAAAGACCTTTGCCCGCAAAG
AGCATCTGGACAATCACTTCCGCTCGCACACGGGCGAAACGCCCTTCCGTTGCCAGTACTGCGCCAAGAC
GTTCACGCGCAAGGAGCACATGGTCAACCATGTGCGCAAACACACGGGTGAGACGCCACATCGTTGCGAT
ATTTGTAAGAAGTCCTTTACGCGCAAGGAACACTATGTTAACCACTACATGTGGCACACTGG
>dme_circ_0000228_junction_seq
ATGTTAACCACTACATGTGGCACACTGGGTGAACAATACAAATTGGTTGTGCGCCA
>dme_circ_0000229
GAATCACACGAAATCATATTGAGTTATTAAGAAAACATGGATGTAAGGTAAACAAATTAAATTCGTAACA
AATGTACAGGGAAATGtttttcttttcttttttGGGCGCGGACGCGCTAAGTAAATGACAATTTGGTGG
CCGTGGCCAACCGTTGAGATTAAATTGCTTAGATGCCAGCTGGATTAGGTGGCCAAGTCAATTACAATAT
TATTTAAGCCGTTTTGTACAAACAATTCTCTACTGTTCGGTTTAGGTTTCTGTATCTCTTTCTGCTTCTG
TGATCTTTCGTTTGGCAATTGGTTACGCGTTGCTGTAGTAGTACTTCTGTGTCTCTGTTTAAAATCATCA
TCCAAATCCATGACTGCATCGGTAGTTTCGCTTACATACATCGATCGCATGTACGTATATGTAAAAAGTG
GTCCTTAATTCGAGACGTGTGGTTAAAACGCTTTTCTTCGT
>dme_circ_0000229_junction_seq
GACGTGTGGTTAAAACGCTTTTCTTCGTGAATCACACGAAATCATATTGAGTTATT
>dme_circ_0000230

GGTTTCTGTATCTCTTTCTGCTTCTGTGATCTTTCGTTTGGCAATTGGTTACGCGTTGCTGTAGTAGTAC
TTCTGTGTCTCTGTTTAAAATCATCATCCAAATCCATGACTGCATCGGTAGTTTCGCTTACATACATCGA
TCGCATGTACGTATATGTAAAAAGTGGTCCTTAATTCGAGACGTGTGGTTAAAACGCTTTTCTTCGTGTA
TGTATATATTGTATATAAGACTGTGATTCTTTCTGCACGTATTTGGGT
>dme_circ_0000230_junction_seq
CTGTGATTCTTTCTGCACGTATTTGGGTGGTTTCTGTATCTCTTTCTGCTTCTGTG
>dme_circ_0000231
GCGCCTGGCCGACGAGGACGATGAGCTGTCGGAGGTGCAGCCGGATGCAGTGCCGCCGGAGGTGCGCGAG
TGGCTGGCCTCCACCTTCACCCGCCAGATGGCCACCAGCCGGCGCAAGAGCGACGAGAAGCCCAAGTTCC
GTTCGGTGGCCCACGCCATCCGTGCTGGCATCTTTGTGGACCGCATGTATCGCCGCGTCTCCAGCTCGGC
CCTAACGGCCTTCCCACCGGACGTGGTCAGGCTGCTCAAGGAACCTGGACGACTGGACCTTCGATGTGTT
CGCCCTGACGGAGGCAGCCAGCGGCCAGGTGGTCAAGTACGTGGCCTATGAGCTCTTCAACCGCTACGGC
TCGATTCACAAGTTCAAGATTGCGCCGGGAATTCTGGAGGCGTTCCTGCATCGCGTGGAGGAGGGCTACT
GCCGGTATCGGAATCCGTATCATAACAATCTGCACGCCGTGGACGTGATGCAGACGATCCACTACTGCCT
GTGCAACACGGGTCTGATGAACTGGCTGACGGACCTGGAGATCTTTGCCTCGCTGCTGGCCGCCCTGCTC
CACGATTACGAGCACACCGGCACCACCAATAACTTCCATGTGATGTCCGGTTCGGAGACGGCACTGCTGT
ACAACGATCGGGCTGTGCTGGAGAATCATCATGCCAGCGCCAGTTTCCGGCTGCTGCGCGAGGATGAGTA
CAACATACTGTCGCATTTGTCGCGCGAGGAGTTCCGCGAATTGCGCGGCCTGGTTATCGAAATGGTACTC
GGCACCGATATGACCAATCACTTTCAGCAGATGAAGGCCATGCGCCAGCTGCTGACGCTCCAGGAGGCGA
CCATTGACAAGCAGAAGGTGCTGTCCCTGGTTCTCCACTGCTGCGACATCTCGCATCCGGCCAAGCAGTG
GGGCGTCCATCATCGCTGGACGATGCTACTGCTGGAGGAGTTCTTCCGCCAGGGTGACCTCGAGAAGGAG
CTGGGTTTGCCCTTCAGTCCGCTGTGTGATCGCAACAATACGCTGGTGGCCGAGTCTCAGATCTGTTTCA
TCGATTTCATCGTGGAGCCCAGCATGGGCGTCATGTCCGACATGCTGGAGCTCATACTGGCGCCAATTGC
GCCGATGAACAAGTCCAAGCCCGCCACTCTGGTGGAGCACGAGACGACCGCTAACTCCACGACAAACTCG
GCGATCGTCATACCCAATTCGGGAATCACGCCCAGCATGGACAAGCCACGGGATCATCGCACCGAGGCCA
AGACCACCGCCGCCGAGTGCCTCGCACGCAAGAGTGTCACCGGCACCACCGCCTCCAAGTTCAACATACC
CAAGCCGTGGCTCACCTGTCTGGTGGAGAACAAGCGGATATGGAAGGAGCAGGCCGTCAAAGG
>dme_circ_0000231_junction_seq
GGATATGGAAGGAGCAGGCCGTCAAAGGGCGCCTGGCCGACGAGGACGATGAGCTG
>dme_circ_0000232
GACACGGCTCACTGACGATTATCCGACGCACCAATAGCCGCAACTTTACCCAATTCGACGGCCAACTGGC
ATCGCCCTCATCCACCGCCTGCAGCAGCACAGCGCCGGGAATTGGCTTCGCCCAGACGCCGCTCAGTGAA
CCACTTGCGGAGCGACTGAGCTTTCGTGCTTCCGGTGGATCCAACGGATCCGCGTCCGCCGGCGACAGCC
GATCCAGCAATCAAGGCCCATCAAACTCCGGACCAGCCGCCTCCGCCGCCGTGAAAATGCAGCCATCATC
GCCGAATGCAACCAACTATCTGGCCGATAATATACAGATCTCGTCGGCCAATCTATCGCAAACCGAAATG
GTCGTGGGTCGCGATTCGGCCGATTATACCGCCATGCATTCGATTAACGTTGGCGTCGGCAATTCGTTCC
TGCGGGGGGACACCGATATACCGCAGGAGTCGGGACACTCCTTCGAAACGCCCTCGAATATGTCGTTCAC
CGCCGGGCAATGGGATACGGAAAGTTTGCCGCCAGTCGACACGCCAGATGCTCTTAACAAAGCGGCCGGA
AGGGTAAGTTCCTATCAAATAACCAATAACTATAGTAGGTATGCGATGTATGAGCAAACGAGGGATCACT
GTACTTTAATATAAATCCAAGCCCACTTATACCGCGGTACAAAGGCGAAAACGATCGCATATTTAACATA
CAGCCATTGTGACGAACCTTTTTCGCTTTCTCCAAGAAGAGTATAAAAGTTTTTTTCTTATAACTCCAAA
ATATATTTAAAAAGTATACTTTTGATTACTAGCATCTTCCTTTCTTGACAAAATTAACATTTTCATGCCA
GGCTTTACATATGAGAAAGCATAAGTGAGGGTAGGTGATGTTGTCGGATCCGCAGACAGGCTCAGAGATT
TTAGGACACGGGCAACGAGATTTTTGGGCCTCAGTCAGGACCAAAACTGCAAGTAGGCACACAGCGAAAA

CAACTTTGCAATGCATTGTATTTTATTTTCACTGATTAGATACGAATACAACTTTCAAATCCGATGGATA
CTGTTTCTTAAGGGAATAAGCAAAGGGAATTCATCTAAAGCGATACATTTTAATTTCACGTTTTATGACA
TTGATTTATTCGTGGTATCGGCTTTGACTTTTAACGTTTCACTACAGTTAAtatttatttttattgtatt
tcatattttatatttaatttttagattatttttaatatatttatttatatttatttgttatCAAATGCTA
CTACTTGTGTGATGTTTTTTTATCTATATTTATTATTTGTATATCTGATACTAGCACCTTCCGGCCTTGA
CCACATAAATCAATTTAATTTGCGCTGCGCATTTAAGTTCGCATGAGTTGGTGTAGGTGACGTGGTCCGA
TCCGCAGACAGGAAAGTAGATTCTTGGGCACGGGCAAAGGGGTCTTGTGACTTGGGCTTCAGTTAGGACG
ACAAATGCCAGGAGGCACAGTGCAATGAAAATTGGGCTATACATGGTATAAGTTTTTGGAGTGATCCGAT
GCGAATGAGACTTTCAGTCGATCGACTCGCAAATATATTTGTATATGCAACAGGAAATGtttttttttt
tGTAAGCGATACATTTCAATTTACCGTATCAGGGTATTGCAGTGACTTAATTTAAGTTAATCAGACTGAA
ACGTCTGGACTCAAAAGTGGTTTGGTTTAGTCTAATAGATGCAGGTCATGATGAAAATGTAAAGGAAATA
ATGATTAGTCTTGTTCTTTAGATACTATGGATGCAATTAGATCAAAAGAAGAACATCTGATCGACAAATA
AGTCCATGTAAATCTTAAATTTCACTTTGCTTTATACATACACAATAGTTCGTAACTCATTAATTTCTAA
TCCCCaaaaaaaaaTGTACATAAAAGTGAGTTAAACAAGGAAATATTTTAATGTCAACGTGACTTTGCAT
ATATTTTTACTCTTTATGACATATTCGTAGACATGAGTATATTTTTTAAGTTAGGAGTAATACTTGTATT
TGTACATACCCAAAGTTAGCTGTTCCAACTGGttaattattattattttttttaaggcttattttattt
aaattatGTACATAACAACTCCCCTCTTACTGTCATACTTGAATTGAAAATTTGAGCTTCACAATTCTGT
AACCATTATTATTATTATTTTTTTATCACAACATACACATAAGCGTATATGCTAAAGTTACAAATTTATA
GTTACACAAATAAAACTAATGTTTTCTTTATTTCCACAGGATACGCAGTCTCCTCCGCCGCATGGATCAT
GAGACCGTTGCCTACGAGGACATGCAGCGGAATCTGCACTACGCGGCTCGTGTGCTCGAGGCGGTCTTCA
TAGACGAATCAAGG
>dme_circ_0000232_junction_seq
CGAGGCGGTCTTCATAGACGAATCAAGGGACACGGCTCACTGACGATTATCCGACG
>dme_circ_0000233
TTGCCAGCACGCCGGAAAGTTTGCCCAGCAAGCGGGACGAGGCAAGTGGTGGGCCGGGCTCCAGTGGCGG
CACAAGTGGTGCGGCAACCAGCAGCAGCAGCAGCGGTGGTGGTGCAGCGAGCGGTGCAGCGAGTGGGTCA
GTTGGCCCTGCAAGCGGAACGGCAACCGGGACGGGATCGGCGTCCGGAGGAGCAGGTGGTGTCAGTACGC
CGGCACAAGAAGCCGCCAGTCCCACGGTCTCCGAGCTCAGTGGCAGCGGCTCAAATGCGACAATAAATGG
TCAAGTGGCAAGGTAAGTAAAGCGCAGCTCATGGCATTTCGCAGCCCAGTTGTGTCCTCGCTGTTGTGTT
TCATTATTTTTGCATATTTCATCATCTAAACCTAGTCCTCCTAGTCCTGGAGGTCCTGTGGGGAGGTGGC
ATTATAATTTCCATTTTGAGGACAGGATACTCCGTGCATAATCCATCCAGCATCAGCTGCGGAGATTGCG
GTGGCTAAGTCGGTTTAACATCGCTCGGAAGTTGCAGGAATTTAGATGAGATGGCAATTCGTGGATTGTT
GTGGTTCGAAACCAAATCACCTAATTGAAATTGCATCGCAGCGCAGTGGTCTTAATTACATTTTTGTGCT
ATTGTTTTGCTTTATTAACCCAATTAATTTGACCAGATATAGAGAGCTTTTTAAAAACTCTTTCTGCGGC
TAGAAAATTTTGGAAATAGCATTAACAGCTTTAGGCAGTAATTTTATTGTAACATTTCGGATACAATAGT
CAACATGTTAATATACACATAACTTATTGCAAACTACTGGAATTACTGACCCTAATGTTTTTATTTTTCG
TAAAATATTCTTATGCGGCTTGTTTTACAACCTGATCGTTGATCTATCCGCATCTATCTTCCGTAGGTCC
AAACATTCGCATTGCTATCCCAGCACCATCCTCTATAGTTAGGTACACAAAAAGTAGATCCTTCCTTTGG
ATTCAACGCGTTGGTCAAATCTAATAAAATAATCACGCAAAGCGCATTTAAATGATAGAAATGCATGGCC
ACAAAATATAGTGAACAGCAACTGAGATTTATTTATATATATCTATAAATGAGGAAATCATTACCACCTA
CAGCGTTAAATTGTAATTTTCCCACTACATTACAAGAGATTTCTATTGAACGTGAAGCGTTAACTTACAT
TTTAATTGTCCGTAAATATACTATTTCGAAGAAGTAATGTTATTTACTTTAAAGTTTATATCATACCTTG
TAAGTATTAAATCTACTTACATAGCTTACAAAAAGAAAATAACCAACTTTATTATCGAAAGTTACTTGGT
ATCATGATTACAAGTCACAAGCGTTTATCGCATCTACTTTCCCGTGGGCCACATATTAAATCTTTTATCC

CAGCATACACCATTAAAATAGGATACACAATAGGTAGAACTTTCCTTTTCGTTCACCCCGTTTGTTAATT
CCAAAAGAGCGAACAAGCAAAACGCAATAGGAATATAACAATGCATCGTTGCAGAATATAATGAATACAA
CCTGATAGTAAGTTTGATTATATACTCATTAGCCAGGAAATCCATAGAGCGTTAAATCCATAATTTCGCA
CTATATTTACAGTTTTTAATGTATCAAATTTTTTGGGCTAGCAAATAAAGCACCAGATAGgctttagtcg
agttccccgaatataagatacccattagttaagtagtggaagtgcaaacgagaaattAAAATGTTCCATA
CGGACGGACAGACGCACTAAATATTTACTTATTTATTATACAGTAGTTTACAAAACGAATAAATATATTT
ATGATTTGCACTGCAGTAGATGGAAACCTAAACTGTCTTAGTGTATAAAAAAGATTGATGGTTGGCCATC
GATCATGTTACCAGTTGCGAAACGCACTTTATGCGGCCATCAGACCATTTAATTGCCGGCAATTTCCATC
GCCGTGCTCACATGGGTCAAAACTACATACGGAATTGGGTTTTTAGTCGTAAGGCGATGCCAGGTGGGGA
TGCCAGCAGAGCACTTTCCTCCGGTTTCCCCAACATTGTGGGGTAAAATGAAGCGTTGAAAGTCATTAAG
GACGTGAAGAGGCGCCAGTAAAAGGAGCATAAAACATTTCATCCCaaaaaaaaCTCGACTCGATAAAGAG
CCCGCGTATCTAGCAGTTAAGCGAAGGAATCTCGGGTGTAACATAAATTTAAGTGCAGCATAAAGCGGAT
TTTATGACTGCCCCATCACCTTTTCTACCGAGGTGCTTGCACTGTCAAAATAAATGGAATGCAGTTAAGA
TTCTCGCAGCCAGTACGAAGACACTCCGCTCTTCTTGGGGCAGCAATCCCTGTGGGGTTGGGGTATTTGC
AGATAGATTGCTCCCTGCAAACCCCCAACTTGCCACTTGACAAACACACACAGATAAATCCTTTGCACTT
TTTCGACACGCCCGGGATGGCAACTTAGGGCCAACTTAGGATGATGCCACTTAGCGAGCTCAAAGAGGGG
GATTGGTGCGGATgggggggggggtggggCCAGGACATGCCTTTGGGGGAGAGCAACTTCTTTGGGGCTT
TTGTTCGCTGCTCGTTAAGCATTTTGCCCTCACTGGGGGCTAAACTAAATAAAGACTGCCATGCTACAAT
GGCCTCAAATGATGGGAATAAATTATTGATTTCTTGTCCATTGCGTCCAACGATTTTTGATCGAACAACG
GTGGAGAAATGCAAAGTTAAAATTaaaaaaaaaaGTTGTTTTCAGAACCACAACTTGATTAAAATGATGT
ATATTAATATAAAAACAAGTGGGGTAATGAAATAAATCACAATTTAGAATTATTAATAAAAAGAAATTTT
TATTTAAAATTTCTTCACACAATTCTATACCTGTTATCCTAATTTTGCATATGGTTCGCATAGCTCTGTT
ACCACTGTACTGGTTTCACCCAAACCCTTCTTGCATTTTCAAGACCTTTGGGGCTGCCTTGTGTGCTTCG
TTAGATCCCAGTAGGTCCTTAATCAATGTGTCAACAGTTAGAGTGATATAACTATATATATAAGCAACCG
ATTCAGTTCTTCGCCCACTGAATTCTCAACGGCTTTCTTGCAGGGACACGGCTCACTGACGATTATCCGA
CGCACCAATAGCCGCAACTTTACCCAATTCGACGGCCAACTGGCATCGCCCTCATCCACCGCCTGCAGCA
GCACAGCGCCGGGAATTGGCTTCGCCCAGACGCCGCTCAGTGAACCACTTGCGGAGCGACTGAGCTTTCG
TGCTTCCGGTGGATCCAACGGATCCGCGTCCGCCGGCGACAGCCGATCCAGCAATCAAGGCCCATCAAAC
TCCGGACCAGCCGCCTCCGCCGCCGTGAAAATGCAGCCATCATCGCCGAATGCAACCAACTATCTGGCCG
ATAATATACAGATCTCGTCGGCCAATCTATCGCAAACCGAAATGGTCGTGGGTCGCGATTCGGCCGATTA
TACCGCCATGCATTCGATTAACGTTGGCGTCGGCAATTCGTTCCTGCGGGGGGACACCGATATACCGCAG
GAGTCGGGACACTCCTTCGAAACGCCCTCGAATATGTCGTTCACCGCCGGGCAATGGGATACGGAAAGTT
TGCCGCCAGTCGACACGCCAGATGCTCTTAACAAAGCGGCCGGAAGGGTAAGTTCCTATCAAATAACCAA
TAACTATAGTAGGTATGCGATGTATGAGCAAACGAGGGATCACTGTACTTTAATATAAATCCAAGCCCAC
TTATACCGCGGTACAAAGGCGAAAACGATCGCATATTTAACATACAGCCATTGTGACGAACCTTTTTCGC
TTTCTCCAAGAAGAGTATAAAAGTTTTTTTCTTATAACTCCAAAATATATTTAAAAAGTATACTTTTGAT
TACTAGCATCTTCCTTTCTTGACAAAATTAACATTTTCATGCCAGGCTTTACATATGAGAAAGCATAAGT
GAGGGTAGGTGATGTTGTCGGATCCGCAGACAGGCTCAGAGATTTTAGGACACGGGCAACGAGATTTTTG
GGCCTCAGTCAGGACCAAAACTGCAAGTAGGCACACAGCGAAAACAACTTTGCAATGCATTGTATTTTAT
TTTCACTGATTAGATACGAATACAACTTTCAAATCCGATGGATACTGTTTCTTAAGGGAATAAGCAAAGG
GAATTCATCTAAAGCGATACATTTTAATTTCACGTTTTATGACATTGATTTATTCGTGGTATCGGCTTTG
ACTTTTAACGTTTCACTACAGTTAAtatttatttttattgtatttcatattttatatttaatttttagat
tatttttaatatatttatttatatttatttgttatCAAATGCTACTACTTGTGTGATGTTTTTTTATCTA
TATTTATTATTTGTATATCTGATACTAGCACCTTCCGGCCTTGACCACATAAATCAATTTAATTTGCGCT

GCGCATTTAAGTTCGCATGAGTTGGTGTAGGTGACGTGGTCCGATCCGCAGACAGGAAAGTAGATTCTTG
GGCACGGGCAAAGGGGTCTTGTGACTTGGGCTTCAGTTAGGACGACAAATGCCAGGAGGCACAGTGCAAT
GAAAATTGGGCTATACATGGTATAAGTTTTTGGAGTGATCCGATGCGAATGAGACTTTCAGTCGATCGAC
TCGCAAATATATTTGTATATGCAACAGGAAATGttttttttttttGTAAGCGATACATTTCAATTTACCG
TATCAGGGTATTGCAGTGACTTAATTTAAGTTAATCAGACTGAAACGTCTGGACTCAAAAGTGGTTTGGT
TTAGTCTAATAGATGCAGGTCATGATGAAAATGTAAAGGAAATAATGATTAGTCTTGTTCTTTAGATACT
ATGGATGCAATTAGATCAAAAGAAGAACATCTGATCGACAAATAAGTCCATGTAAATCTTAAATTTCACT
TTGCTTTATACATACACAATAGTTCGTAACTCATTAATTTCTAATCCCCaaaaaaaaaTGTACATAAAAG
TGAGTTAAACAAGGAAATATTTTAATGTCAACGTGACTTTGCATATATTTTTACTCTTTATGACATATTC
GTAGACATGAGTATATTTTTTAAGTTAGGAGTAATACTTGTATTTGTACATACCCAAAGTTAGCTGTTCC
AACTGGttaattattattattttttttttaaggcttattttatttaaattatGTACATAACAACTCCCCTC
TTACTGTCATACTTGAATTGAAAATTTGAGCTTCACAATTCTGTAACCATTATTATTATTATTTTTTTAT
CACAACATACACATAAGCGTATATGCTAAAGTTACAAATTTATAGTTACACAAATAAAACTAATGTTTTC
TTTATTTCCACAGGATACGCAGTCTCCTCCGCCGCATGGATCATGAGACCGTTGCCTACGAGGACATGCA
GCGGAATCTGCACTACGCGGCTCGTGTGCTCGAGGCGGTCTTCATAGACGAATCAAGG
>dme_circ_0000233_junction_seq
CGAGGCGGTCTTCATAGACGAATCAAGGTTGCCAGCACGCCGGAAAGTTTGCCCAG
>dme_circ_0000234
GGCTACAGTACTTCAAGGATACGGGCATCACGTCCGTATGGTTGAGTCCCATTTATGAGTCACCAATGGT
AGACTTTGGATACGATATATCTAACTATACAAATATACAGCCGGAATATGGCACCCTTGAGGACTTTGAC
GCCTTGATAGCCAAGGCCAATGAACTGGGCGTGAAAGTTATTTTGGACTTTGTTCCCAATCACAGCTCAA
ATAAGCATCCCTGGTTCATAAAGTCAGTAGCCCGAGAGCCAGGGTACGAGGATTTCTAT
>dme_circ_0000234_junction_seq
CCGAGAGCCAGGGTACGAGGATTTCTATGGCTACAGTACTTCAAGGATACGGGCAT
>dme_circ_0000235
ATAGTACTGCTGCCTCTCATCGTTCCACATCCAAGCGGATCCGGAGAACACCGACAGCCAATTGTTGGGC
GGCACACGAGTTCCGTTCTCCAGGAGAATACCATCCTCCCACACATAGAAATCCTCGTACCCTGGCTCTC
GGGCTACTGACTTTATGAACCAGGGATGCTTATTTGAGCTGTGATTGGGAACAAAGTCCAAAATAACTTT
CACGCCCAGTTCATTGGCCTTGGCTATCAAGGCGTCAAAGTCCTCAAGGGTGCCATATTCCGGCTG
>dme_circ_0000235_junction_seq
AGTCCTCAAGGGTGCCATATTCCGGCTGATAGTACTGCTGCCTCTCATCGTTCCAC
>dme_circ_0000236
TTGCGACACCGAAGCATAACCCTCGGTCATCATTATCCTCAATGGCCCATCGTGGTTAGCTGTATAATTA
TCCAGAAGTTGCCGCCAATGCTGAAGTAGACCGTAATCCTCAGGCTGATTTCTGGTATAGATGTGGCTCA
AGTAGGCCTCATTATTTGGATCATCGGTAG
>dme_circ_0000236_junction_seq
TAGGCCTCATTATTTGGATCATCGGTAGTTGCGACACCGAAGCATAACCCTCGGTC
>dme_circ_0000237
TATTCCATTAGTTGCGACACCGAAGCATAACCCTCGGTCATCATTATCCTCAATGGCCCATCGTGGTTAG
CTGTATAATTATCCAGAAGTTGCCGCCAATGCTGAAGTAGACCGTAATCCTCAGGCTGATTTCTGGTATA
GATGTGGCTCAAGTAGGCCTCATTATTTGGATCATCGGTAG
>dme_circ_0000237_junction_seq
TAGGCCTCATTATTTGGATCATCGGTAGTATTCCATTAGTTGCGACACCGAAGCAT
>dme_circ_0000238

TTGCGACACCGAAGCATAACCCTCGGTCATCATTATCCTCAATGGCCCATCGTGGTTAGCTGTATAATTA
TCCAGAAGTTGCCGCCAATGCTGAAGTAGACCGTAATCCTCAGGCTGATTTCTGGTATAGATGTGGCTCA
AGTAGGCCTCATTATTTGGATCATCGG
>dme_circ_0000238_junction_seq
AAGTAGGCCTCATTATTTGGATCATCGGTTGCGACACCGAAGCATAACCCTCGGTC
>dme_circ_0000239
ACAAAGTCCGCAGCTGTCGAATTGGCATTCAGTTCGGTGATGAAGTCAAAGTTGAAGGGAAACTGGGGGC
CCTGTACACCATTCGAATCTTCATAGTATTCCATTAGTTGCGACACCGAAGCATAACCCTCGGTCATCAT
TATCCTCAATGGCCCATCGTGGTTAGCTGTATAATTATCCAGAAGTTGCCGCCAATGCTGAAGTAGACCG
TAATCCTCAGGCTGATTTCTGGTATAGATGTGGCTCAAGTAGGCCTCATTATTTGGATCATCGG
>dme_circ_0000239_junction_seq
AAGTAGGCCTCATTATTTGGATCATCGGACAAAGTCCGCAGCTGTCGAATTGGCAT
>dme_circ_0000240
GCATTGGCGGCAACTTCTGGATAATTATACAGCTAACCACGATGGGCCATTGAGGATAATGATGACCGAG
GGTTATGCTTCGGTGTCGCAACTAATGGAATACTATGAAGATTCGAATGGTGTACAGGGCCCCCAGTTTC
CCTTCAACTTTGACTTCATCACCGAACTGAATGCCAATTCGACAGCTGCGGACTTTGTCTTCTATATCTC
CAG
>dme_circ_0000240_junction_seq
CTGCGGACTTTGTCTTCTATATCTCCAGGCATTGGCGGCAACTTCTGGATAATTAT
>dme_circ_0000241
GATTGTCGTGATTTCCCATCACCCAGTTGGCCACATGACCATGTGGCATATAGATGAGCCACCTGGAGAT
ATAGAAGACAAAGTCCGCAGCTGTCGAATTGGCATTCAGTTCGGTGATGAAGTCAAAGTTGAAGGGAAAC
TGGGGGCCCTGTACACCATTCGAATCTTCATAGTATTCCATTAG
>dme_circ_0000241_junction_seq
CATTCGAATCTTCATAGTATTCCATTAGGATTGTCGTGATTTCCCATCACCCAGTT
>dme_circ_0000242
ATTTCTCACCGAATCGTGATGCCACTCGAGGATTGTCGTGATTTCCCATCACCCAGTTGGCCACATGACC
ATGTGGCATATAGATGAGCCACCTGGAGATATAGAAGACAAAGTCCGCAGCTGTCGAATTGGCATTCAGT
TCGGTGATGAAGTCAAAGTTGAAGGGAAACTGGGGGCCCTGTACACCATTCGAATCTTCATAG
>dme_circ_0000242_junction_seq
GCCCTGTACACCATTCGAATCTTCATAGATTTCTCACCGAATCGTGATGCCACTCG
>dme_circ_0000243
TAATACCAATTCCTGGCAATGTCATCAGCAGCATATTCATGGCGTCCACAGATTTCTCACCGAATCGTGA
TGCCACTCGAGGATTGTCGTGATTTCCCATCACCCAGTTGGCCACATGACCATGTGGCATATAGATGAGC
CACCTGGAGATATAGAAGACAAAGTCCGCAGCTGTCGAATTGGCATTCAGTTCGGTGATGAAGTCAAAGT
TGAAGGGAAACTGGGGGCCCTGTACACCATTCGAATCTTCATAG
>dme_circ_0000243_junction_seq
GCCCTGTACACCATTCGAATCTTCATAGTAATACCAATTCCTGGCAATGTCATCAG
>dme_circ_0000244
AAATCGTCTTGTAGTTGTCGATTCCAGCCTCACAAGCGGGCTGATCCACCGTATCGCTCCAGCTGATGTC
CCTGTAGTCAGTCATGCCCAACTCCTCGCCCTAAAGTAATGGAAAGTCTGTATAAAATTATCCTTTAATA
ATGAGTGTTCTGTACCCACATTATAAGTAATACCAATTCCTGGCAATGTCATCAGCAGCATATTCATGGC
GTCCACAGATTTCTCACCGAATCGTGATGCCACTCGAGGATTGTCGTGATTTCCCATCACCCAGTTGGCC
ACATGACCATGTGGCATATAGATGAGCCACCTGGAGATATAGAAGACAAAGTCCGCAGCTGTCGAATTGG

CATTCAGTTCGGTGATGAAGTCAAAGTTGAAGGGAAACTGGGGGCCCTGTACACCATTCGAATCTTCATA
G
>dme_circ_0000244_junction_seq
GCCCTGTACACCATTCGAATCTTCATAGAAATCGTCTTGTAGTTGTCGATTCCAGC
>dme_circ_0000245
TAATACCAATTCCTGGCAATGTCATCAGCAGCATATTCATGGCGTCCACAGATTTCTCACCGAATCGTGA
TGCCACTCGAGGATTGTCGTGATTTCCCATCACCCAGTTGGCCACATGACCATGTGGCATATAGATGAGC
CACCTGGAGATATAGAAGACAAAGTCCGCAGCTGTCGAATTGGCATTCAGTTCGGTGATGAAG
>dme_circ_0000245_junction_seq
CGAATTGGCATTCAGTTCGGTGATGAAGTAATACCAATTCCTGGCAATGTCATCAG
>dme_circ_0000246
ATCTTGTAATGACTTCGCCTCGCCTGCTGCTGATTCCGAAGATTAAGTTCCTTATAATTCGGATTGACAG
GCAACCAAGTGCGATCGGCGGAGGAGAATCCTGCATTCACATCACTACTCCATTGCATGGGAGTTCGCTC
AGGATCTCTAGAAATCGTCTTGTAGTTGTCGATTCCAGCCTCACAAGCGGGCTGATCCACCGTATCGCTC
CAGCTGATGTCCCTGTAGTCAG
>dme_circ_0000246_junction_seq
TCGCTCCAGCTGATGTCCCTGTAGTCAGATCTTGTAATGACTTCGCCTCGCCTGCT
>dme_circ_0000247
TTCGCCGCTTACTTTCGCTTATTCAGCTGAATAACTAATCCCTCGTTGGGCGCCAATTCAATTGTCTCGG
CCTTAAGTCGATCCCCCACCCGGTGTTGCGAGTCCACTCCCGCCACAAGGACACTCAATCGATTGGGCTG
TTCTATAAAGTCCGCGATGTCAACCAGTTCAG
>dme_circ_0000247_junction_seq
ATAAAGTCCGCGATGTCAACCAGTTCAGTTCGCCGCTTACTTTCGCTTATTCAGCT
>dme_circ_0000248
GATCTTTTGCGATAGTAGAGCAGTGAAACAGCCAGAGAGAACGACAAGATGCATGTGCCATTATAAGGAT
AAAGGGAAGGATAGCAACTAAGCAGGTCCTCGTTATATATGGAAACCCAAAACTGATATTATTGTAGGAT
ACTAGACTACAGTGAACTTGAGAGTCAACTCTTTACCCACATACTTAGAACCCACTCGCCCCCGCCCCCA
GAAGTAACCTGCAATGGGTAAGGACTACTACAAAATCCTGGGTCTGCCCAAAACTGCCACCGACGATGAG
ATCAAAAAGGCCTACAGGAAACTGGCCCTGCGTTATCATCCGGACAAAAACAAAGCCGCCAACGCCGAGG
ACAAGTTCAAGGAGGTCGCCGAGGCGTACGAGGTTCTTTCGGACAAGAGCAAGCGGGAGGTGTACGACAA
ATACGGCGAGGATGGTCTCAAGAGCGGAGGGCACACGAAACGGCGGTCCGTCGAGCAACTCGTTTACATA
TCAGTTCCACGGCGATCCGCGGGCCACCTTCGCCCAGTTCTTTGGCAACAGCAATCCGTTCGCCTCGTTC
TTCGACATGGGCGACAACCTGTTCGACAAGAAGGTCTTCGATCTGGACACGGAACCCGATTTCTTCTCCT
CGCCCTTTGGCGGCATTGGGTCGCGACATGGTCTGGGAAGTGGCTTCAGGGCCCTCTTTCAGATCACACT
CCTTCAATGTGCACACGCCGTTCAAGAAGGAGCAGAAGCAGGATCCGCCCGTCGAACACGATCTCTATGT
GACGCTGGAGGAGATCTACCACGGCTGTGTTAAGAAAATGAAAATCTCCCGGCGCATCGTTCAGGCGGAC
GGCAGTTCCCGCAAGGAGGAGAAGTTCCTGGCGATATCCATCAAGCCCGGCTGGAAGTCCGGCACCAAGG
TCACCTTCCAGAAGGAGGGCGACCAGGCGCCCGGAAAAATTCCCGCCGACATTGTGTTCATCATCCGGGA
CAAGCCGCACGCCATGTTCAAGCGCGAGGGCAGCGATCTGCGCTACACGGCGAGACTGACGCTCAAGCAG
>dme_circ_0000248_junction_seq
CTACACGGCGAGACTGACGCTCAAGCAGGATCTTTTGCGATAGTAGAGCAGTGAAA
>dme_circ_0000249
AGTCTGTGTTGGAAGAAAATGATGCAGAGAATAATAAGGAAACCTACGCATGTGATGAAGTTGTGAATAC
ATATGAAATTAAAAATCCATCAGGCATTTCAATATGCACAAGGCCTGCAGAGGAAAACGTTGGAAGAAAT

AGTGTTCAGCAAG
>dme_circ_0000249_junction_seq
AACGTTGGAAGAAATAGTGTTCAGCAAGAGTCTGTGTTGGAAGAAAATGATGCAGA
>dme_circ_0000250
GTCTTTCCTGCGCCCTTCTCTACTTAAATTTGGCTATTGCGGAGCCACTGACACCTGGTACATTTTTGTT
GTTGCCACTGAGCACCACGTAAGCGGTCTTCGTCTCCTTTTGCTTCTGCTGCAACATGGTCAGTGTGCCC
AAGGGTGTGTCTGTCGAACTCATG
>dme_circ_0000250_junction_seq
GCCCAAGGGTGTGTCTGTCGAACTCATGGTCTTTCCTGCGCCCTTCTCTACTTAAA
>dme_circ_0000251
GTCAGCCGAAAAAGCGCAAGAACTTTGTGATTGGTGAGAATATGGATGAGGAGCGGGAGCACAGACGCAA
GCTCCTGGAGATTAAGACCAAATTAGCCGGCAAGCGTTTGGAAACCTTGGAGGACACCAATCTGCAGCTA
GCATCGGACACTTACAGTGCAGAAGAGTTGGCCAAGATTTAAAAAGCCAAAGAAAAAGGTTAAGAAACTT
CGGCAGAAACTTAAAGCCGAGGATCTTGCGCCTTTGGCGATGGGCGAAGCATCAGGTTCTTCAGACTTTG
GTAGTCGAGGTGGACGTTATCGTGATCACGAAGATGCGGAAATGGAAGAGTTTAAACCCCAAATAAAAGT
GGAAGTAGAGGATGATGATTTGGAACG
>dme_circ_0000251_junction_seq
TGGAAGTAGAGGATGATGATTTGGAACGGTCAGCCGAAAAAGCGCAAGAACTTTGT
>dme_circ_0000252
GATGATCACGGCCAGCCGAGTATACTCCCACAACGTCACCTGGGCACGGAACTCCGGACCCTGCAATATG
TAGTAGTTGACCAGCTGCCACAGGATGTAGCTCCATCCGACCACCTGGCCGGCGTTGTACGCAAACAGGT
ACAATTTAGTGACCGCCGACGGCTCCTTGGAAGCGCCTGGTTTGCTGGACTTGGACACTGCCTTTGCTGA
CATGTTGGAGCGGAGATGTGTGAACTTTCACCTTGCCGATCGCCGCTG
>dme_circ_0000252_junction_seq
TGAACTTTCACCTTGCCGATCGCCGCTGGATGATCACGGCCAGCCGAGTATACTCC
>dme_circ_0000253
CGGCAGTTAAATCGAAGAATGTCTGCCTTACTCTTGTGGATCAGCACCGGAATCTCAGCCAGCGCGGCAT
CGTCCAGCTCTTTGCAAGCGCACATCCTCCACGTCGATATGCGACGCCCTGGTTTAGTAGAACCGCACCC
AGGCTAAGCCAGCTGTACGCCAGTCCTGCAAAACG
>dme_circ_0000253_junction_seq
GCCAGCTGTACGCCAGTCCTGCAAAACGCGGCAGTTAAATCGAAGAATGTCTGCCT
>dme_circ_0000254
GGCGCCTGATCACCGATTGCTTGATAACTACCGTCTGCGCTACCATGCTGGCTTGCATGGGCGTACATGA
GATCCAGATGGACATCGCCCTTGTTCAGGCGATACTTGCTCTTTGTCAAATCCCGCCATGCGCCTCCTCC
GCGTCCACTAAAGTCAGTAACCCAGGGCATGATGTG
>dme_circ_0000254_junction_seq
TAAAGTCAGTAACCCAGGGCATGATGTGGGCGCCTGATCACCGATTGCTTGATAAC
>dme_circ_0000255
CGAGGAGTAAAGACCGTTGGCCTTGATCGAGAACTGAAGAACGTCGGCCATGTACTTCGGCAGATCCGTC
ACCATGATGTAGAAGCCCCAATCGTGACCAATCTGGGCGGCCACCAGGGCAAACATGGGCAGGTTGGTGA
GGATGGCCTTCCAGGGAGTGGGGGGCAGG
>dme_circ_0000255_junction_seq
GATGGCCTTCCAGGGAGTGGGGGGCAGGCGAGGAGTAAAGACCGTTGGCCTTGATC
>dme_circ_0000256

GTACATGGCCGACGTTCTTCAGTTCTCGATCAAGGCCAACGGTCTTTACTCCTCGCTGCCCTACGTCATG
ATGTGGATCGTTTCCGTGGGCAGTGGCTTTGTGGCCGATTGGATGATCCGCCGTGGCGTGTTGAGCACCA
CAAACACCCGAAAGGTGATGACTGGTCTGGCTGCCTTCGGTCCGGCCATCTTCATGGTGGGCGCCTCCTA
CGCCGGCTGCGATCGTGTCCTCGTCGTGGTGCTCTTCACCATCTGCATGGGTCTGATGGGCGCCTACTAT
GCGGGCATGAAGCTGAGTCCCCTGGACATGAGTCCGAACTATGCCGGAACGCTCATGGCCATCACAAACG
GAATTGGAGCAATCACCGGAGTGATCACGCCGTACCTGGTGGGCGTGATGACACCAAATGGCCTCGCTGC
TGGAGTGGCGTCTTGTGTTCTGGGTGGCCTTTGGCGTGCTCTGCTTCACTGCCGTGATCTACTGCATTTG
GGCATCCGGAGAGGTGCAGCCGTTCAATAACGCACCCATCCAGCCGCGCTCCGTCGACTTTGAGGCACAG
GAGCGCAAGGTGGGTGGTGAAAAGACAAGTGGTCTGGAGCAGAGCTCGTAAGGTGGATCGCCaaaaaaaa
GTGCCATACATATATGTATACATTGTAATCTAGTTAAAGTGCTGTCTACGCGGATTCTGGAATCCGTAAT
CTGGGATCCGAGTGGTTCCCAAAAGCAGTGTCATGCTGTATACGCAGTGGCTGTTGTTCAAATTGCTAGT
TTTATAGTATTCCCGATCTGGCCGATCCCTGGCATCAGCGCCAACTAATTAATTTAAGCGAATTTACTTA
CACTCCCGAACGTCCGCAGTGCTACCATGTTTATCCCTTTTGTTAGCTAGGAtaatccttagcctaatcc
taatcctaaACTATACCCCTATGCCGCGCACGCAACGTACTCTCTTAATTATTATTTAGTATTTAAACAA
ACTGCGATTTCGCATTAAGGATTTCGTGTACCAGAGCTCGTATTGCCGTATTGTAAATCAAATGATGAAA
TAATTTGTACATTTTAAATTGTTGATCAATGTGTAAAGTAAAGCGTGTTTTTAGCTGTTCTGTTAAGACA
TTACTGTTTTTATGATGTTAGTCAAATAAAGTGTATAAAGCTAATTAAAACTATAAATCTAAAATCCAGT
TTGTAAAAATGTAGATCTGGAGGTGCGACAGCGCTGGGTTCTCTGCTGCTTCACATTTTTGGCGATCATT
AATGCCTACACAATGCGTTTGTGCTTGGACTGTACGCTTAACCGAATCATATTGGAATGTGGTGGTCATA
GGGTGGACAACGTTCCTCAAGTACTAAATCCAAAGGCACTGCCCAACTGGCGTATTGAACTGGCCATGGC
ACTGAATCACACGGAATATCAACTGAGAAGCCGCCTAAAAGCGGGAAAGGAGCAACCAGATCTCAACCAG
ATAAGCGCCAAAGGAACGCGGGATAGAGAACCTGCCGGTGAGAAACGTCCTGGTCAAGAGGACTCTCAAG
AACGTTTGAGCTGCTCCGAGCAGTGGCCTCGCCAAACCCAATCCCTTGTAGTAATGGCCTTCTATGCCGG
CTATGTGCTCAGTCATGTTCCGGGCGGACGGCTGGCGGAGAGGTATGGTGGAAAGTGGGTGCTCAGTGCG
GCCATACTCACGTCAGCGGTGCTGACCCTGCTAACTCCCACTGCAGTGCGACAGGGCGGTCTGTATGCCC
TTGTCGCTGTCCGTCTATTGGTCGGAATTTGTGAGGGCCCTTGCTTTCCGGCCGTGTGTGCACTCCTGGC
CCAGTGGGTTCCGGAACAAGAGCGCGGAATGCTGGCCAGCTGCGTACTCAGTGGCGGTGAGATCGGCATC
ACGATGGTCCAGTTGGTCAGCGGGTTGCTTATTGCGGAGCAGGATTGGCCCGTGTTCTTCTATCTGGTTG
GTGGAGGAGCCGTGGCCTGGTTCTTGGGATTTGACCTTAGTTTGCTATAGCACTCCGGATCATTGTCCGT
TTATTCAGAGCGAAGAAAGGGAATACATAAGGTGCAATACTAGCAACTCCTTTTTGTTGACCACTGGCAG
AGAAAGAGAGGAGATGGACGGGGAAGATGGATATGAAGGTGAGGATAGGGAGCACCGCAGAGAAGTGGAA
GCAACGTGCAACACTGCACCTTGGCGGAGCATGCTTAACAGCACGCCACTCTGGGCCCTGGTTTCCACCT
CCATGCAGCAGGAATTCCAACAGAAATTGCCCCAGGAGCTGCAAATTGCGCTAGAGGAAGTAAGAGCCCG
TGGCACCAGTTTCTCAGAACTAACCACCATCATCGAAACGATAGCACCGTCCGTTGGAAACTGGATAGCT
TCACTGACAACAGGGCGGCTGAGCGACGTGCTAATCGAGCAGCAGATTCTCACGCGCACTCAGACACGCC
GCCTCATGTCCTGGTTGGTCTTCCTATGCGGATCCATGTACATGCTACAGATCAAGATGAGCGGAGCACG
AATCTGGAGCGTTCTTGGCATGGGAGCCTACTATGCGAGCATTAAGCTGCTACCGCTGGATATGAGTCCC
AACTATGCTGGCACTTTGATGGGCATTTCGGGCGGCATGGGCGCGCTTCCAGCACTTCTGATGCCCTATC
TGGAACAACTGGAGACCGACTACAAGCTGGTGAGCAGTGTGAGGGCCGCCATGTGGGTAATCGGCGCCAG
CTACATATCCGGCGATGTCCAGGCGTTTAACCAACCGGAACGGGAGCCACAATGAGGAGACTTGCTTGTT
TTAAGTTTAATCTTACGCTTAAACATAAGCTACTGGATTTGTCTTAGTGGATTCCTTAGGCAACAGATTC
TAGTTTACATATGTACCATAATACATGACAATTTCTATGCTAAAGCTAGGAGCATAAACGTAAACAAGCT
TtatatatatatatataAATAACAGGTATGGTCGATATTAACTTAATTAGAGTAGGACTGCTGGCTAAAT
GCTCCGCGCTGGCTCTTATTTTCTCGGGATATCAATCCCAACACACACCGGTTTCATACTCTATGCAACG

CCCTGGGCCATCAATCCGGATTGGGCCATTGCCGACACTGAGTTCATGTCGATACCCAGCTTTTTGGTGA
CGTTGATTTGGGCCACTGCCGCGTTGCGCAGCGAGTAAACGTGCACCAACTCCGCCTGACTCTTGGCATA
AAGCAGTGCCTTTTCAAAAAGCTCGACGGCCCGTGTGAGTTGCGCCCGCTGAACTTCGACTGTTCCGAGT
GTCTCGTAGGCCAGCTCGCATTTGGGATCTACTTCGATCGCCTTGTTGAGCAGCTGGACAGCCAAATTAA
TGTCTCCGCGCCACTGAAGCACCATGATGGCCTGGTGAACGATAAGTGCAGGATTTGTTGGCGCCAATAC
CATGGCCTTTTTGTAGTATTCCTCCGCCTGGGTAAACTGTTGCTGGTCGGCCAACACCTGAGCCGTTAGA
CTATAGCACTCAACGCAGCTCGGGAAACGCTCGATGGCATTTTGGAAGGTGTGCATTACGCTCTCCAAGC
GCCGCTGGTCGCCGGCCAGGAGGGATAGTCGGTACTCGGCATAGCACTTCTGGACGAAGGCGATGGCGTG
GTTGGGTGCAATGCTCACCGCCTTTTCGAACTCGGCCAAGGCGGGCTCTATCTGCTCCAGCAGCAGAAGT
ATCTGGGCGCGCTGGTGGTACACATCTGGGTTCTCTGGGTTCAGGCGTTCCGCCTCAGCAAAATCGGCTA
TACCCTTTTCGCGCTGATCCAGCTGGATGTAGAGAGCTGCCCGCTTTATGTAGGCATACGCACGCAAGTT
GGGATCAGCATAGTCTGTAGTGAAATAAAGTTAATAGAATAGAAATCAAAAAACAACTGAAGAAAACCGA
CTAATTAATGAATTAAACTTAAACAAATTCTTACCATTAGCTATAATAGCATCAAAGTCCTGCTGGCTTT
CTACATAGGAGCCGCACAGCAGATGGAAAGTTCCCCGCATAAGCAGAGCCTCCACTTTGTACTGTGCTTC
GGCCTCGGAGGATTCGATCTCTTCGGTGCAGGCGGGAATAATTTCCTCGTACTTCTCCTCGAGGAAAGCG
AGGTGGGCGCGCAGGAAACCCTTTGGGGGTGCATCGGATTTAGGGGCTGGGACTTTCATGGTCTGCAGCG
GATCCGCGATGAAGGAACGCGTGTAGGTGTTTACGAAGCAAGCCGACGGCACGACTGGCACCCGATTGCG
CATACCCTTCTCGGCGTCCAGTCGGCCGGTCTCCTTGAGCACACGGTCAGCGAACATGATAGTTTGATTA
TTTTGGAACATCTCCAGGATACAGGTGGCTGTGACATCGTCCAAGCACTCGTTCATATCCTTGGTGGCTT
CGTGAGCACGGGCACGACGATAATATGCCTTGGCGTAACGAGGATTGAACTCCAGCGAGGCTGTGCAGTC
CTCCTTGACGTTGGACCACTTCTTGAGCATCTCGTAAGAGGCGGCGCGGTTCTGGTAGAAGATGGCCATG
TCAGTGCGGTGCTCCTTGGGACACTTGTCGATGGCCTTGTCATAGAATTTAATGGCCTCATCGTACTTCC
CATTCCTGTAGCAGTTGTTACCCTCCGTCTTGTAGTTGTTGGCCTCCTTAAGAGGCGACAGCTTCTCGCC
CAGCTCAGCGGACTTTTGATTGCGCTCTAGCTCCTGGTCCGGTGCGGTGCCGTCCAGGGAGATGGCCTGC
TTCTCGATCTTGGCCTTTGGTCTCTTCTTCTCCCCGTCCAACTCCTTGGACGCAGCCGTCCAGCTCTTCA
CCGCATAGGTGCCCAGTCCAATGGCCAGGGGAGTTCCAAGGATGAGCGCCAGCTGCCACTTTGTCAGCTT
CATCGAGCCAATGTTCAGAGTCATCGCCATTGAGCCGGTCTTTTTATGGATGTGTGTTATGTGTGATGAA
CTGCAAGAAAAGAGCTGAATCAGTTTGGGTGCCGCCACAAATTGTGTAATACTCCGCTGGCTTATCGCTC
AGCGCCTTTTGAGCTTGCTAAATGCCGGGCGGGGGGCACGTTTCTGCAGTACCTAACCCCCAACCCAATT
GATTTTTCACACCCCCAACTGGCCGGGATGTGAATCAGTGCAGTCAGCGAACGGAACTTACACTTTTTA
AAACTTTACACTGCTGTGAATTTGGCAATTGTTAAACGTCGATTGTTCTATTTTCAGTTAGAACTCGGGA
AACTTAGATTCTATTGTATATCAGCCTGCTGAGCACACGACATCGAAAAACGATAGACATCGATAGGTAT
GGAGTAATAACGGCACTGCGAATGCTTATTATGGAAGAAGAAGATCAGTGAGAGAATAAATTGTGTGAAT
AGTTTaaaaaaaaaCTAAGGAGTTCGAATATGATGTATTTGTAATAAAGCCTGAATTTATACTTTACGAA
TACTGTACGAATAAATATTTTAGATCTCTATATTACATTCTATCAGACCGAAACAGAGTTCTTAAATTTT
AAAATCGGTTTCATCCACTTTACAAATTGAAATACAAATGCATTGTAATATTACAATCAATGTATTTATA
GGAATTCACTAGAATACACGGACAGCCTATGGGTGATTCTCCTCATCTGTGCTGGAAGTTGGAGCTGGAG
CAGGAGCAGAAGCGGAACTGGTAGTCGACTTATCGGTTTCCGTAACCAAGATCTTGTCCAGATTCTCCAG
TTTAACTAGGCCCTGGGAATCGGCGTTGTGGATGAGATCACATATTGTGGGCGACTCCACGACCAGGATA
TACTGGCACGTCTTCGGCTCAAGCAGGTACATGGATACAGCGGTGCCGCTGTTCGTTACAGGTGTGCAGG
TTAACTTCACGTCCACTTCGCGGGGCACTCCAATCTGTTCGCAGTGGGTTCCCTTGCCATAATGGTGCCA
GATGGATGTCGTGAAGCCGGAACGGCGTGCACCCTTGTCCGGGTTGGCGTTGGACCAAGCACGATGCGCT
TCCTCTGAGAAGTAGCCAAGAAAGAGCTCCACTTCGCTGGTCTTATCTTTGTGGAACTGACGCACATGTC
GGCCATAGCAGAACTCGTACTTCCACCAGCCATTGCCCTaaaaaaaaGAGTATTATTGAAGCAAATGCAT

GGGTCAGCTGCATGTTATTCCTACCCCAGTTAGGCAGTTCTTGCCCGATATAAAGTCCTTAAGGGGCGTC
AGATCAGTGATGGGTGTTGGTCCAGTTTCAGAGGCTGCTATAGACAGCAGGAGCTGCTGAAAGTCTCCGT
CTACCTTCAGGTCACCATTCCTGAAATGCTCGAGCAGGACTTTTTCCATATCACCGCCATAGTTAAGGGT
GACCAAATCGCCATCCGTCTTGGGCCACAGATTTTCCGTAGCCTTTTCCTTGAAAACCAGCAGATCCGCC
TCGGATTCAACCCATTCCTTTAGCTCCGACCGCAGCATGCTCAGCGGTCTGTGCGGTTTCGATTCGGAGT
TGAAACATTGTATGGATAGTTCCTTGGTCTCTTCGGCATGAAGGGCGCGAATTGGGCACAGCGCCGAGGA
AAGTATAATGGCCTCATAATTGCACGAGGATGTCTCCTTGAAGGAGTAGATATCGTTCTTACCATGGGGA
TAGCACACATAACGCACCATTGTGGTGCGAGGAGCGTTAATTATGTCGCACATAGTGCCATCGCTGTATT
CCATCTCAAAGTATGGATACCGAGTGTTGTCGATCTTCAGGCTTTTGTATTTCGGCTTAACGCCCGCCTT
GATGTCTGCTTGCCAGGCTTTTGTCAGTATTTCCATCTTTTCATCGGTCCATTTGCCCAGGTAGTACTCC
TGGAACTTGACGTTCTTTCCTTCGCGCTCCTCGTGATACTGACGCACATGGTGGCCATGGCAGATTTCGT
ACGACCAGTAGGCCTCGATGCGGTACGTGCAAGTCAGGGCGGAGAAAATGGGCTGCAGCAGAGTAATTGG
AGCTGCGGAATTCGGAAATTATGTATAATCCAATGGGATGTTAGTTGCAATGCCAATTTCTTACACAACT
CTGGCTTGTCGGACTTTTCCTCCTCTTTTTGGTGTTCCAAAGTGGGAATCAGGCAGTCGTACTTCTCCTT
ATCCGGCGTGTAGAATGTTCGCAGCTGATTGCCCAAGCTCTAAACAGCATTACTTAAGGAAATTTGTTTA
TGTCTGCATTAATAAATTTGTACTCACTGGCTGCACATCTAAATCCGGCACTTCGAAATCAATTTTATAC
AAAATTGAATCGTCAAAATCTTTGGCTTCGTGGGAAACCGCTGCAATTATTAAATTCAGCAGCAATGCTA
TTCTCGCAATTATCACGTAGGACTTGCCCATTTTCGTGTTTAAGGCAGCGATTTCTTTACATGAGTATTT
TCGTGACAATATACAAATAGTTGCAAATGCTACGTGTTGTTTTTCTCAAAAAAATAAGGAACATATGTTG
ATTGTGTGTGCGGCACTGTGAATGGTTACTGCGAAAGAAGAAGAAAATCTGCTGGCCTGCATGGGCATTT
TAGCTTGGAAATAGCTAAATATCAAAAAATAACAGTCGCAGTCGACGGCACATACAAATTTTTTGAATTC
AAATTCTTTAAAATCTAAGTTTTGCATTTAAATCTAGTCATTGAATATTTCTCATTAACAAAATACAAAA
TTTTATCTCTTAAACTTAACTGAATGTTTTCGTATTTATTGCAGATTTAATGGTCATTCATCATAAAATC
TTTTAAGAATCTGTATGAAAATCCAAAATAAGTCGAGAAACTAAAGCCACGTCCTCCAACAAATTAAACT
CAGTTCCCAGTTTATCTTCAACATAATTATATTTTCCTTAACAAATATCGCATATGGTAGTCAGTTCCCT
ATCTCATTCCCAGCGCCGGCGTGTCCTTTTGCGCCGTGGACCAGCCGAGCTCCTTGTCCTTATCATGATC
CACTGGCACCTTGTCGATGGCATCCAGTATAAACTGGATGGAATGCGCCGTCCGTGGCAGCCACTCCATG
ATACGAGAGGTGGTCGAGACGGGCGGTGTCGCTGCATCGCCGTCATCCACGAAATCCGCATCTCCGCCGG
ACAACGAACGAACTCCACCCAGGTAATCCGTGTAGTCGTAATCACTGGTCAGCGGATTCACCTTGGAGTA
GTAAATGGCAGCGAACGTCGAGTAGATAATCAGCAGTGCTGTGCCCACCTATAAGTTTTATATACATATT
TAAAACGATTATATTACACATTGCACTCATATACTTTATGTATTTTCACTCACCTGAAAGACGGCCCAGA
ACTTGTAGGAGCCCTCCGTGATGACGAAATCGTAGTATCCTTTGAGAGGATCACCCTTGATATAGCGACC
CTCGCCCACATCCGTGTCGGCCTTGTCCTCCGCCTCGGCATCGCGTCTTTGGCGCAGTCCACGCACCGAT
CTCAGATTTCCCTTTTCCCCCTTTAGTGCCGAGACTTTGGTGGCGGGTGCCGATGGTGTGACTGTCTCAA
CTTTCTCCACTGTCTTCTGCGACTGTtcctcctcctccgcttcctcctcctcctcATCAGAGACATCTGG
AGCAGCTGTCGTCGTCGTCGTCGTGGTTGCCTTAGTTGTCGTTGTAGCCGGCCTCTTGTTCTGGTTCTTT
TTGTTTCCCTCGATGCGCGGCGGTTTTTTGGAGTTTATCCTGTTCTTCTGCTGCTGCTGCTGACGCAGTT
GCTCCTTCTCGGCTTTCTTCGCCGCCTGCTCTTGGCGGAACTTCTCAGTGCTCTGCTGATGGCGTTCGCT
GACCGCGTTCCAGGCACTCATCAGTGCCTGATACAGATCCCAGATCTGCTTGAGGCTCTTCAGCACGGTG
ACCAGTGTGGATTCACTTTGGATTTGGACTACTGCATTGTCGGACGTGGGCTGAGATTGTTTGGCCATCG
CTGTTTCCGGATGTGGGCGCGGCTGGAAGCCCATGGGATAGTCGTACATCTCATCGTAGGCGGGATAAGG
CGATCTGTAGAAAAATACATACGTTTCGTTTATGTTGTTGTAATAATTTCATCCAACACCCATGACCCAG
ATCTAGTTGGATTAAGCATTTCTCACCTGCTAGGAGCTAAAGACGCCTGAAGCTTTCTATTATTGCCCTC
GGCAGCGGGATGAGTAGCGACTGGAGCTGGCTGCTGTTTATATGACCACGGAGCGTAGAAGGAATCGTAG

GGACGTGCCTGCTGTGACCTGCCCTCACCGGTGTATTCCTTATCATAGAAACGCTCATCCTCTTCCGACT
CCTGCTTGGACTCTGCACTGGGATATCGTTCAGGTCCGTAGTCATCGGCCACATCGTAGTACGGATCCTG
CTCGTAGGAATCCTCGCGACTCTGCGGCGGACCGTAGGCATCCTGGCGTTGTCCCAACACGGGAGAATCC
ATGCTCTCTGCATTCACCACCACCGCCTGCTGCTCCTCGTAGAAGTTTCTGCCCTGCTGCGATGCGGATG
CACTCGGATTGCTTGCATTTCCGGCATCCCTAGCAATTCGCTCCACCGTGGCAATTTCTGTATCATTATC
GGCACCGGACTTGAAATGATTCCACATGCGCGTAAGGATTAGGAGATCGCTTTCTCCGCCCCGCGTTGCTC
TGTGCCCGTCCAGCTTCGATCACGGCCAGCGCCGTTAAGGTGGTGGCAAAGAGAAGAAACGTGACCACCA
GAAACCATATGGGTGAGAGGCGTCGTGGCGCCTTCGCCGGCATTTTCTCGTAGGCAGACATTATCCGTTT
GTCACTATTGATTCTATTGTTTCGAATCCTGGTTCAGCACACACCGCACGGCCAGTTGGCTCGGAGTTAA
GTCGACAACTAACCGAAGATGTCCCAATGCTGAGCCAGAAAAAACTCCGCCCGGCAATGGTGCTAATGGCG
GGTTTTTGGGTATCTAGAACATCCATCCGTAGTGGCTCGTTGAAAGGTGCTAATCCGCGGGCCGCGTGCA
TCATCGTTATTGGGCCAGTGCTCCAGTTTCGATTTAACGGTTCATCCAAGCCACCAAGTGCCGCGGCAAT
TAGCACCATCCATATCACCAATGTAGCCAGTACTACGATCCCATCCCATCCGATGCTAACTTCCATTCTC
CATTCAGTTATGCATAATTCCCGATTTTGTTGTTCGCCACAAATTCGttttttttttttCGCGGTGAAAGT
GTGCAATATGATTCTCTTTCCAGAAGATGGACTATGGAATTTAGGAAATCGTAATTGGAATGGCTTTGTG
CCGTTGGAGCACAAAGATCCACGCCTGTGGAGTGGCCGCCATCGAGGTCCGTCGTCGGTCGTGTGCCAGG
CGAAGATCACGATCTAGGAAGCTGCATACATGCCCATATTTAGACACAAATCGTGGCAAGCGTGCGACTT
TCATTAATGGGGTTGAAAAGAAATCTATTTCTACAATTAAGATACGTTAAGTCAACATCAATGGGGTGTT
TGTACATTGGTGAGATCGCCTTAACGATTCGAAATTTGAAGACAACACCATTTTAAAAATGCTAATACAC
AGtttttttttGGGAAATTGGTATACTACACAAAGTAACCAAAAAGAAAGGATATCGTATTAGAATATTGT
ATACACATATGAGCTTATCATAGTTTATTATATAGATACATATAGGTAGCCACCTATTAAAACTCATATC
TTAGAAAAGAAAGCGATGTTAACAATTTTATTCTTTAATGCGAATTGAAATGAAATGAAAATTGCTCATG
TTATGAAGTTAGTATTTAATCTTAAGAGCGCAACTAGAAAAGTGCAATCACGAAgtacatatgtacatat
gtacatatgtatacatatgtatgtatgtatattcgatgtactatcttaccttcaaatcttacatacacat
aaatatgaatgtatgtatgtatgtaaatatgtatAGACCTCAACTTCTATCGATTTCTAGACTTTACTGA
ACTATTAATGCGTCATATGTTTACCGGCGAAACGCAGCGTGGCAAACTAGTTTCGTTACCCCCAAAATTG
TTTACTCGTTTTTTAGACCTCTTGCTTGTTTTCCTCTAAAAAGCTCGATTGTAAATTTTATTTGTTGATA
TTGCTTTGTTTGAAACCGCGGCGATAATGTTTATCTATCGATTTTGTTTTACTTTTGTAATGCGAGTTTA
ACGAATCTTTAATTTAGAAATACATTTAATAAAAATAAAAAACCGATTCTAAGGCACTTTTAACACGCAA
TATATTGCAATGTTTAATTTTATAAAAAAACAGGTTTTTCATCACCTTCTATTTGTTTGAAGTTTGGAAC
ACCACATTTTTAGATAGATCAATGATTCAGACGAAATGCTATTTAATGAAGATAAGGAATATTTATGACT
AACCAAAATGCGAATTTAAACATACATAAGAGTCTTGGAATTTTAATTGTCAATATGTTCGAACAATTTC
ATTAATATGCATTATAGCAAAACCACCTGATCGAGAGTCTGTTTTATTGTTTACCAAATATCAACAACGC
AATTCAAAAAGCCGGCAAATGGCGTTTATagagagagagagagcgagagagaggagagaATTCCATTAGA
GTGGACCTTTCTGGAAAAGTTTGTTCGGTTTATTTTTGTACGTGTGCTTGTTTGTTTGTGAGTGGAAAAT
AACATAGCTTTTGTAATTTTGGACACCTAATGTAAAAATATCGTTTAGTATTGAAAGTCAATAAAAGCGG
CGCAATATTCATAAATGCAACCTTACACGCAAATTTAAACCGGAAGAAATGATGCAGGAATATGTGTGTG
CAAGTTTAATTACCTGTGTAACTGGTGAACTTTCAATGGAAGGTCTCGGGAATATTTCCGTTTGCAAACT
AACATACATACATTACATACACAAATAAATATGTTCATACATGTTCTCTTTTTGGTCGATCGCTGTAAAC
AAAGAAAAAAGTCGACTGCAACTGCTTTTGCGATAATCGAGGAGCTGAGTGCAAATGAGTTCAACTAAAA
GGCTATAAATATGCACAACAGCACACCAACGGCATTCAAAAAGCTCTCTCGCTTGGAAAGGTGCGCTGCA
AATGCAAATGAAAATAAGACGACCAGGCTATTCAGTTTTGGTTTTCTCTTTATTTTATATGTATTACCTA
AGTCAAACTTCATTATTTGAAACGCTCTAAGGCTTAGCAACTAAAACTTGCAAGTCTGAATTAGTTTAGG
AATATATTAGAAGCTAAGAGTAGTATGTGCCTTTCTTTTTTTGTGTTTGTGAAATTATAAGCGAAATAAA

ATTATTATAATGTTCACAAAGTTTAGCGCCGCATGAAAAGCCAATGCACAATACAAATGCAATGTGTATT
TCCAgctggcttttgtttttctttctttccttccagcaaaactgctgcttgttcttctttttcttctctc
ctttgcttttgctttcctttttAAATTCGAAGATTTCTTTTCTCCTTTCAGCGCGGCCCCGTTACTTCTTT
CTCTCCTGTTCCACCGCCTCCGTCGATGCCCTGCATCAGTTCTTGCAGGCGGCGGCTTTCTTGTTGTTGT
TGTTGGTGTTCATCAGCTTGGTCAGAATCTTGCGGGAATGACCGGAGGGATCGAAGTGGTTGGTGTGCTC
ATTGGCCTCCCTCTTGTTGCGCTGCTTGCCGCTGTGTCCAGAGAAGATGTGCTT
>dme_circ_0000256_junction_seq
TGCCGCTGTGTCCAGAGAAGATGTGCTTGTACATGGCCGACGTTCTTCAGTTCTCG
>dme_circ_0000257
CGTTTCCCGAGCTGCGTTGAGTGCTATAGTCTAACGGCTCAGGTGTTGGCCGACCAGCAACAGTTTACCC
AGGCGGAGGAATACTACAAAAAGGCCATGGTATTGGCGCCAACAAATCCTGCACTTATCGTTCACCAGGC
CATCATGG
>dme_circ_0000257_junction_seq
GCACTTATCGTTCACCAGGCCATCATGGCGTTTCCCGAGCTGCGTTGAGTGCTATA
>dme_circ_0000258
GCTCCACTTCGCTGGTCTTATCTTTGTGGAACTGACGCACATGTCGGCCATAGCAGAACTCGTACTTCCA
CCAGCCATTGCCCTaaaaaaaaGAGTATTATTGAAGCAAATGCATGGGTCAGCTGCATGTTATTCCTACC
CCAGTTAGGCAGTTCTTGCCCGATATAAAGTCCTTAAGGGGCGTCAGATCAGTGATGGGTGTTGGTCCAG
TTTCAGAGGCTGCTATAGACAGCAGGAGCTGCTGAAAGTCTCC
>dme_circ_0000258_junction_seq
TAGACAGCAGGAGCTGCTGAAAGTCTCCGCTCCACTTCGCTGGTCTTATCTTTGTG
>dme_circ_0000259
GTTAAGGGTGACCAAATCGCCATCCGTCTTGGGCCACAGATTTTCCGTAGCCTTTTCCTTGAAAACCAGC
AGATCCGCCTCGGATTCAACCCATTCCTTTAGCTCCGACCGCAGCATGCTCAGCGGTCTGTGCGGTTTCG
ATTCGGAGTTGAAACATT
>dme_circ_0000259_junction_seq
TGCGGTTTCGATTCGGAGTTGAAACATTGTTAAGGGTGACCAAATCGCCATCCGTC
>dme_circ_0000260
GTTAAGGGTGACCAAATCGCCATCCGTCTTGGGCCACAGATTTTCCGTAGCCTTTTCCTTGAAAACCAGC
AGATCCGCCTCGGATTCAACCCATTCCTTTAGCTCCGACCGCAGCATGCTCAGCGGTCTGTGCGGTTTCG
ATTCGGAGTTGAAACATTGTATGGATAGTTCCTTG
>dme_circ_0000260_junction_seq
GTTGAAACATTGTATGGATAGTTCCTTGGTTAAGGGTGACCAAATCGCCATCCGTC
>dme_circ_0000261
GTTAAGGGTGACCAAATCGCCATCCGTCTTGGGCCACAGATTTTCCGTAGCCTTTTCCTTGAAAACCAGC
AGATCCGCCTCGGATTCAACCCATTCCTTTAGCTCCGACCGCAGCATGCTCAGCGGTCTGTGCGGTTTCG
ATTCGGAGTTGAAACATTGTATGGATAGTTCCTTGGTCTCTTCGGCATGAAGGGCGCGAATTGGGCACAG
CGCCGAGGAAAGTATAATGGCCTCATAATTGCACGAGGATGTCTCCTTGAAGGAGTAGATATC
>dme_circ_0000261_junction_seq
AGGATGTCTCCTTGAAGGAGTAGATATCGTTAAGGGTGACCAAATCGCCATCCGTC
>dme_circ_0000262
GGGTGACCAAATCGCCATCCGTCTTGGGCCACAGATTTTCCGTAGCCTTTTCCTTGAAAACCAGCAGATC
CGCCTCGGATTCAACCCATTCCTTTAGCTCCGACCGCAGCATGCTCAGCGGTCTGTGCGGTTTCGATTCG
GAGTTGAAACATTGTATGGATAGTTCCTTGGTCTCTTCGGCATGAAGGGCGCGAATTGGGCACAGCGCCG

AGGAAAGTATAATGGCCTCATAATTGCACGAGGATGTCTCCTTGAAGGAGTAGATATC
>dme_circ_0000262_junction_seq
AGGATGTCTCCTTGAAGGAGTAGATATCGGGTGACCAAATCGCCATCCGTCTTGGG
>dme_circ_0000263
GATATCGTTCTTACCATGGGGATAGCACACATAACGCACCATTGTGGTGCGAGGAGCGTTAATTATGTCG
CACATAGTGCCATCGCTGTATTCCATCTCAAAGTATGGATACCGAGTGTTGTCGATCTTCAGGCTTTTGT
ATTTCGGCTTAACGCCCGCCTTGAT
>dme_circ_0000263_junction_seq
TGTATTTCGGCTTAACGCCCGCCTTGATGATATCGTTCTTACCATGGGGATAGCAC
>dme_circ_0000264
GCACACATAACGCACCATTGTGGTGCGAGGAGCGTTAATTATGTCGCACATAGTGCCATCGCTGTATTCC
ATCTCAAAGTATGGATACCGAGTGTTGTCGATCTTCAGGCTTTTGTATTTCGGCTTAACGCCCGCCTTGA
TGTCTGCTTGCCAGGCTTTTGTCAGTATTTCCATCTTTTCATCG
>dme_circ_0000264_junction_seq
TTTTGTCAGTATTTCCATCTTTTCATCGGCACACATAACGCACCATTGTGGTGCGA
>dme_circ_0000265
GCTTGGTCAGAATCTTGCGGGAATGACCGGAGGGATCGAAGTGGTTGGTGTGCTCATTGGCCTCCCTCTT
GTTGCGCTGCTTGCCGCTGTGTCCAGAGAAGATGTGCTTGTCATGGTCGAAGTTGTAGTGCTCGTACTCA
TCGAAGTGGGCCTCGGACATAATGGCGATTTAAGGTTGCAAAACTAACTGCGTTCTTG
>dme_circ_0000265_junction_seq
TAAGGTTGCAAAACTAACTGCGTTCTTGGCTTGGTCAGAATCTTGCGGGAATGACC
>dme_circ_0000266
GGGATCGAAGTGGTTGGTGTGCTCATTGGCCTCCCTCTTGTTGCGCTGCTTGCCGCTGTGTCCAGAGAAG
ATGTGCTTGTCATGGTCGAAGTTGTAGTGCTCGTACTCATCGAAGTGGGCCTCGGACATAATGGCGATTT
AAGGTTGCAAAACTAACTGCGTTCTTGGTTGGT
>dme_circ_0000266_junction_seq
TGCAAAACTAACTGCGTTCTTGGTTGGTGGGATCGAAGTGGTTGGTGTGCTCATTG
>dme_circ_0000267
TTAACAGGTTTTTGCAAAACCCCCGCCTTTTTGGCCCACTTCCCACAAAAGTTTACTTTTTATGTCTATG
GTTAGGTGCAACAATCCGAATTCCTTTGTTAAAACAATCAGGAAATAAGTGAATGCGCTTATATGGGAAC
AAACTACGAATATGTAACATGTTTAGGGAAGCTACACAGCTAGCAG
>dme_circ_0000267_junction_seq
ATGTTTAGGGAAGCTACACAGCTAGCAGTTAACAGGTTTTTGCAAAACCCCCGCCT
>dme_circ_0000268
GATTATCGGTAACGACAGCCAGGCGTCCACCAGGTGCCACAGACAAGGTCAGTCCATCTCTCTTGCCGTC
ATATAGACGACAGCGAATCCGCATCGGTGCTTCTTTCGTGGGCACCGGCAGTGGTGATTCCTCGGGCGAT
GGCAGGGGCTCTGGAGCGCGTCGAAAGATTCCGCCCAGCAGGTTCTT
>dme_circ_0000268_junction_seq
GTCGAAAGATTCCGCCCAGCAGGTTCTTGATTATCGGTAACGACAGCCAGGCGTCC
>dme_circ_0000269
ATGACAACTGGTTAAACGCGATAAACCATGCCATTTCACCAACTGGGGAACTGATTGCCATGGCGCAGGG
TGAAAAGTTGGCATTCCTCAGCACCTGCTGGAGCAGCCATGGCAATGGCAACACCTACGTTCTTGGCTGG
TGCGGCGAGCTGGAGGATCCCAACCAAATCGTGACTAGCCTCACCTGCTTGCCGATGACTCAGAACAAAA
GCACCGATGGAGCCATCGAATGGACCTGCGTGGCAGTTGGCCTGTGCTCTGGCATGGTTACCTTCTACAC

GGACTCGGGTGTAAAACTCTTCTCGCAATGGTTGCCAAGAGGATCCAGTAATTGGAGTCAAGCTGCAGTC
CGCTCCGCGACATTCTGAGGCAGATTCACTGCTCTACATCATCTATCCTCGCTGCCTGTGCTTCATCCAG
GGCCAGGACATTTTGCCCACGCTGAACAATTGCCGGCACAATGTGCAAAGAGGTGCATTGGAGCG
>dme_circ_0000269_junction_seq
ACAATGTGCAAAGAGGTGCATTGGAGCGATGACAACTGGTTAAACGCGATAAACCA
>dme_circ_0000270
GGTGATGGTCGTTCCAGTGGGTCCTGCCTATGATCAGTACGCCCAGTCAGTGCGGGATCAGTTGCACGAT
GCTGGATTCATGAGCGAGGCGGATTGCGATGCCGGCGACACGATGAACAAGAAGATACGCAATGCTCAGT
TGGCCCAGTTCAACTTCATTCTG
>dme_circ_0000270_junction_seq
TCAGTTGGCCCAGTTCAACTTCATTCTGGGTGATGGTCGTTCCAGTGGGTCCTGCC
>dme_circ_0000271
AAGCTGTCCTCGTTGGCGATGAATTCGTCGCGAATCTTTTGCAGCTTTGTGATGAGTTCCGCCACGGAGA
CCTCGCCGTGCACCTTGTTGTCCCGAGTGCGCACGTTAACGGTATTCGAGGAGCGCTCCTTGTCGCCCAC
CACCAGAATGAAGTTGAACTGGGCCAACTGAGCATTGCG
>dme_circ_0000271_junction_seq
AGTTGAACTGGGCCAACTGAGCATTGCGAAGCTGTCCTCGTTGGCGATGAATTCGT
>dme_circ_0000272
AAAGAACAAAAATGCGTACTTGCTTTGCTATGCAAATGTTAAGTATACTTTTGATAAATAGTTCGAAATA
GAGTCTTAGAAGCTGTCCTCGTTGGCGATGAATTCGTCGCGAATCTTTTGCAGCTTTGTGATGAGTTCCG
CCACGGAGACCTCGCCGTGCACCTTGTTGTCCCGAGTGCGCACGTTAACGGTATTCGAGGAGCGCTCCTT
GTCGCCCACCACCAGAATGAAGTTGAACTGGGCCAACTGAGCATTGCG
>dme_circ_0000272_junction_seq
AGTTGAACTGGGCCAACTGAGCATTGCGAAAGAACAAAAATGCGTACTTGCTTTGC
>dme_circ_0000273
TTCGAAATAGAGTCTTAGAAGCTGTCCTCGTTGGCGATGAATTCGTCGCGAATCTTTTGCAGCTTTGTGA
TGAGTTCCGCCACGGAGACCTCGCCGTGCACCTTGTTGTCCCGAGTGCGCACGTTAACGGTATTCGAGGA
GCGCTCCTTGTCGCCCACCACCAGAATGAAG
>dme_circ_0000273_junction_seq
CTCCTTGTCGCCCACCACCAGAATGAAGTTCGAAATAGAGTCTTAGAAGCTGTCCT
>dme_circ_0000274
AATTCATTTCTTTTGGTTGGCTGCTATCCATGTTATGAACGAGGAGACCTGGACATACACCGACGGAGAG
TTGGGCTGACCACATGGAAGTTTTCCCCAAGACACGATGCCGATTAGAACGTTTCCCTGGACCAGCGGAC
CACCGGAGTCCGAGGTGCAAAAGCTGGTGCCGCCGGTGAGAGGACCTGTGCACAGGTTCGTGGTGTGCAC
ATCCTGACCCTTAGAGCCAAGTGCCGCAGCGCAGGAGTCGAGGCTTATGATGGGTATGTTCTTCGCCTCC
TGGAGGGTTTTCGGATATGAGGGGCTATTCGTTTTGCTAGTACTGCCCCAGCCAAAGAGGTCAGCCTTTC
CAGTTGGCCTCACGCCGGACGATGGCAGCTTCACCGGAGCCACTGCAGCGGTCCAAGTGAAGGCGGTGGG
CGTG
>dme_circ_0000274_junction_seq
AGCGGTCCAAGTGAAGGCGGTGGGCGTGAATTCATTTCTTTTGGTTGGCTGCTATC
>dme_circ_0000275
GTTGCAAATGCGAAAACAACAATGAAATGCTGGCACAATGTGGTGAGGCGCAGAAGGAAGAAGCCGACGG
CAGCGGCGGCGGCGAAAGGATCAAAGGGGTCGCATGGAGGCGTGGCAAGTCCTCGAGATGAAGATGCCAT
CTGCCAGCGGCAGCAACAAGTGGATACCTACACTTGGCTAACAACACAAGCTGTCCCTCTGCTGTGGCTG

CACTTGTTGCTCCTTGTCGCATTTGCAAAACATGCGGCTGCAGCCACAACAATTGCCACAAATCCCGAGG
CCACCACAACAACGGTGAGCAACTACACGGTGACGACCACGGCACTATCATCATCCACGACAACCACTTC
ATCGGGATCAGCTCCAGCTTTATCATCTGCGGCGGATAAGTTCGCCGGCAACTGGGCCAACTTGGAGGAT
TCGTTCCGGAATATCGTGCGGATTGGTGATGGGAACACGGTGACACGGGGTAAGTCATTAAAATTCACTG
AATTTGCTTACCCGAAAACACATTGATGAAGAACAGACTCCACTTAATCCAAAATTATAAGACTAAAGTG
GATTATTTCACGTATTTATATATACTTTATATACAATTAATTTGTGTGGTTTACAGAATACTTGAAATAG
ATAGAAAAGGGTCTTTATTTCTTTAAAATTGAGATCTTCTCGATTACTTTCCGACATTTGGGCATATAGA
AAGATATATTCCTCGTTGTCAGCCACTCCATGTTGCCTAGTGCTAACCCCAATTAAGCTGCACTTACATA
GGAATGCAATTGAAATAATGCGAATAAGTGTTGCTCAAGAGCGGGAATGAGTGGCTTTAAATGTGGAGCT
GAGTTCAAGGTGCGTTCCGCAGCTTGGGGCACTCGGCTGAATTTAATTGAATTGAGAATGCAGACGGAAG
CCCACTTGAGCCCCTCAACCCCCTTCCACTACAAAATTAATAAAGTGGCTATTTAGTGTGCACTTAATGA
TGCTCAATGCGGACGATAAGAGCTGTCAAATGCAGTTTGCTAGGCAAAATATTATATGTTCTTGGTGAAA
TTACACAGGAAAACAAAATAGTATTTACACAGCATAGCGAATTTTAAGTGCTTGGTAATAAACAGACAAA
AACCATTTAATTTCttttttttttAAATACCACAATTAACCCAAGTTGATTTCGGCAACAATTTTGGCGT
ATTTTCTTGTGGTTTTTGTGCACTTAATGAGCGCAGTTAATTAGGTTAAATGCTTAGCAGTTGAGAAAAG
CGAATTTTGATGTAAATACTTGGCGAGAAAAAATCCTTAGAAAAAACTTGAGTGTAACTAAGCTTCCGCA
CTTGGGCTCCATTTCTTTTGGCCAAAACTTTATTGTCTTGGTTTGTCAAGCCAAACACAAAACACTTGTT
TAACAATGGAAGCTTCAAGTGAAAATTCTCAGAAAAGTTTTCAATTCAATTTCTTTCACTTTTCTTTAGC
AATTTaaagtctaaaacaaaaccgaaccaaccaacgaaaaattcaaaaataaaaagaaaaGTCAATAACG
TAATTTTCCAATCAAACGATAAAAGGGTCTTAACAAATGATGGTCACCATGAGTTTTTGTTCCGCCTAAA
ATATATATATTTTTAATGCGTTTGCCAGCGAAACAATCGCTTGAATAGACaaaaaaaaaGTGATTTGGGT
AGCCCCAAAATATGTAAATGCACTCGAAAAAATTATGTTGGAAAATAGAATGAAACACCAATAAAAAATC
AACTTTAAGAAGCATtataattgattaaaaatatatatataaataaaataaataaatatatGTATTGTAG
CGAGATTTTAAAAGTGTTAGATGTGTTTTAATATTTTCAAATTTGATTAACACAAATATTTTTTGATTCT
TTCAGTACAGTACTTTCCACCCCCCTTTTAAGCCTACTTAAATAACAATAATGACCAACTCCCCAACCAT
TTACAGGACGTCCATTGAACAATCGCTGGTGACGATACACGACATTGCCACCGAGAACTTGGGGACATTG
TGCATCTCGACCCTATATCGCCCCCTACAAGTGCCCATCAACTCGGAACGATATGAGAGCTCTAGACAGA
AAACCGATTTGGCCGCCTCGATACTCCAGGAAGTGGGCATCATTCGGCACGGCG
>dme_circ_0000275_junction_seq
CAGGAAGTGGGCATCATTCGGCACGGCGGTTGCAAATGCGAAAACAACAATGAAAT
>dme_circ_0000276
GTGCTTTTGGTATGAGTGATAAGAGCAGCAGTGCAACGAACAGCCTGCCGAATAGTCCCATACACAGCAA
CAACAATAATCCATCGCTGCTGAACAACAAcaacaataacagcaacggcaccagcagcaacaacagctta
aacgttggcaacaacaacagcaaTCCGAGCTTGGGCGGCAGCAACAGCAACGCACTGGTTTCCGTAGGCA
GCAACGGTATAATGTCCGCCGCCGGTTTGGTCAACAACAATAACAATCCGTGCAGCGCCAACCGCAATGT
GGTCGCCATGGTCGACGATGACGCCTGCTTCCGTCTGGATACGGACGCCACGGTGACGTACGGCGAGAAG
GAACCGGATCCGGACAACATCAAGATGTTCGTCGGCCAGGTGCCCAAGTCGATGGACGAGTCGCAGCTGC
GCGAGATGTTCGAGGAGTACGGCGCCGTGCACTCGATCAACGTTCTGCGGGACAAGGCTACCGGAATTAG
CAAGG
>dme_circ_0000276_junction_seq
CGGGACAAGGCTACCGGAATTAGCAAGGGTGCTTTTGGTATGAGTGATAAGAGCAG
>dme_circ_0000277
GAACGCAAACTCTTTGTGGGTATGCTAAACAAGAAGCTGAACGAGAACGACGTTCGCAAGCTATTTGAAG
TCCACGGAGCCATCGAGGAGTGCACCGTCCTTCGGGACCAAAACGGACAGAGCAAGGGTTGTGCCTTCGT

CACATTCGCCACCAAGCACGCTGCCATTTCGGCCATTAAAGTGGACCCTGAGCCAGAACAAGATCATGGA
GGGCTGCACATCGCCGCTGGTCGTCAAGTTCGCCGATACGCAAAAAGAGAAGGAGCAGAAGAAGATTCAG
CAAATTCAGGCCAATCTCTGGAACCTGGCCAGCAACATCAACATACCTCTGGGCCAAACGGCCACCAGCG
TGACCACGCCCATCCTGCCCAATCCGCCGCAGCAGCCTTCGCCGGTTCTCGGCGCAGATGCCATTACCCC
GGCCTCCATTCAACTGCTGCAGCAGCTGCAGGCGGTGGGACTGCAGCACCAGTTGTTGCAAGGCCCTCAC
AGGACTGGGAGCCCAGCAGAGCAGCTCGGCGACGGACACCAGCGCTGTGGCGGCCGCAGGACTTTTGACC
CCGATGACCGTCCAGAATCTAGCGGCCATTGCGGCCATGACGCAGCCGAGTCTCTCGAATGCCGCCGCGG
CAGCCGCCGCTGCCACATCGCCTGGAAGCGCCCAACTGACCAACACGGCTGCGCTGCTCT
>dme_circ_0000277_junction_seq
CAACTGACCAACACGGCTGCGCTGCTCTGAACGCAAACTCTTTGTGGGTATGCTAA
>dme_circ_0000278
GGACCCGAGGGCTGCAACCTTTTCATCTACCATCTGCCGCAGGAGTTCACCGATACGGATCTGGCCTCCA
CGTTCCTGCCCTTCGGCAATGTGATCTCGGCCAAGGTGTTCATCGACAAACAGACGAGCCTGTCGAAGTG
CTTCGGGTTTGTCTCCTTCGACAATCCGGACTCGGCCCAGGTGGCCATAAAGGCGATGAACGGCTTCCAG
GTGGGCACCAAGCGTCTCAAGGTGCAGCTGAAGAAGCCCAAGGACTCGAAGCCCTACTAGAGGCCACCAT
CGGCGGCAAGGTTAAACGGACACATCGAGTAGAGGAACACTGAGATTTGTGAAATATGGAGTAGTCAATG
AATGAAGAGTTGCCCGTGTAGAGCGCGTTGCAAGATTTAAACGATGATGCGAATGCCAACGTTGTACGTA
TCATaaaaaaaaaaacaaaaacaaaagcagaaaaaGACATGAAATCTGTGCCaaaaaaaaaGACAGAATC
ACTTATGATTTAGCACGTTAATTGTTGAATTAGaaaaaaaaaaaaaaCGAAAATCAAATACCTAAAAACA
AGATCAAAAAGAAACGAATAACACAACACTTAGACCGTAGTTTAGTTAACTTTCTGTTGAAGTTCCCTTC
GAACCCAATCTTTAACGACTCGTTTTCCTAATTTTAAACTGTCGTACGAGTTGGTTGAACCATTGCCTTT
TATTAAATATTTTGTATACATCCTCATGACTCACAAACGAAAGaaaaaaataaaaaacaaacaaaaaaaa
TAATTACAAAGATCTGTTCGAGTGTTAGGTTTAAGTTTTTGATATAACTAATAATTTAATACATACTCTT
TCTCTGTTTGATTTGTTCATCTCTCATGTTGCAAGTCACAATGAAGTTCTTTATTATAAATATATACCCA
CATGATATATATATTCTAGTTATAGTTACAAGTTACTCGTTTTGTTCACTGGATTATCTCTTTTTATATA
TCTAAACCTATGCTGACTCGCATAATTAGTTTTAGTTATCTGATTTGTTACACCCCCAAACTAACTCAC
ATATTTTATTTGTAATGAAAAAATGAAAATGTTTTTGTTAACCACATTATAAGTAATACCTTTTTAGTTT
TATAATTAGTTGAACACACAAATTCACATACGCTTCTCAGTGTTATTTATGCGATTTGTAAAGGAAACGA
ACTATGCCTaaaaaacaaaccaaatgaaatcgaaaaaaagaaaaCTTGTCATTAATGGAGtttattattt
tattttttagtttctattatttGCTACATCATCCAAATTTACACAAATCTGGTATTATATTATATAACAT
ATGATTCATTTCATTGTAAATTTCTTGTTTTAGTTGAAGGTTAATTGGCTATTTGTCGTTGCACATCTTG
AAGTAGATAAGTGCCTTTTGTAGCATACTTTGTTTATGTAAAAATCATGCACATATGTAAACACTCATAC
ACCACAGCATACATATCTAAATAAAGTTTTGTTCTCTGTTTCAAATGCACAAAAACATGACTTAAACAGC
GTGCTTAAAATTTTATTTGTTATTTTGGTTTTAAATAAAAGAAAGGTAATTATTTGTGATTTATTCTTTG
CAAACATTACTCGCTTTATTGACACCCCCTGATACTAACTTTATTAAATGAAGACTAATTTATAAACTTC
GCTTATATTCCTTAGATGGTAATGTTCTTTGCAAAGTTTCGCCATTTATTTCATTTTTGGAAATGCGATG
TCAAGTGGTAGCTGATGCAAGCCGCCAGTTGAGAGGTAAGTTCTTAATTAAATAAATTGTTTGGATATTA
AAAACTAATTTCAAATCCATTTCAGTTTTATGCAAATCGTTCTTACAAGACGCATTGGTCCCTGAAATAT
GTAAATAATATCCGCTTGAGTCTCGAGAATCCTACTAAAGATTGCCTATCCCACTGCTGTCGCATaaaaa
tttccaaaaact
>dme_circ_0000278_junction_seq
TGCTGTCGCATaaaaatttccaaaaactGGACCCGAGGGCTGCAACCTTTTCATCT
>dme_circ_0000279
GATCACAATTAGGGCCACAATGTCGAGTGTAAAGTTAGCGTTAACCATGTCCAGTTCCTCCAGCGTGGTA

ATAGGTGATTTAAAATGGCAGTAAGTCTGGAAGCACCTAAGTTTCTCCCGTCCGTAACCGTAGGTGGCCA
GAGCTGTGCCCTCGAATCCATATCGTATGTACGACAAATAT
>dme_circ_0000279_junction_seq
GAATCCATATCGTATGTACGACAAATATGATCACAATTAGGGCCACAATGTCGAGT
>dme_circ_0000280
GTTCCTCCAGCGTGGTAATAGGTGATTTAAAATGGCAGTAAGTCTGGAAGCACCTAAGTTTCTCCCGTCC
GTAACCGTAGGTGGCCAGAGCTGTGCCCTCGAATCCATATCGTATGTACGACAAATATGTGATCCACCTT
AA
>dme_circ_0000280_junction_seq
TGTACGACAAATATGTGATCCACCTTAAGTTCCTCCAGCGTGGTAATAGGTGATTT
>dme_circ_0000281
GGTACTGATAAAACCATGATATCGCCCATATTCGTTAAACATATCAATGCTGTTTCGTTATATACATCTC
CACGACTTACAGCCAAGCTTCCACTGATATTAGGATCCGCCTCTCCATCGCCATGTGAACGCGTGGACTT
AGTTGGGCTACAACCGTGCATACTCTGCAGTGTTTCCGGGGATATGCGACAACTAAACGAACCAAAATGG
ATGCGGCGAATCCGAGCACCTTCGTTAGCGGTAAGCTTATATTTGTTAATCGGCTTTAGTTGCGGAAGTG
AAAACACCTTGAACTGTTCCTCGGAAGCAATAAGTACACGATGCGGTGGACTCCCGTTTTCACCGGCGTT
CAGCTGATCGACAGGGCTACCCGCCTGATCAAAAATAGAAATACCCACCACAGGAGCACGATGTTTTAAT
TGTATTTCTTTAGCAAGCTGCGCAGAAATTCGGCGGGGCATGTGTGGTGGTGCATTGCCACTTGCCGACG
GGACGGCAGTTGCCGCGGTCTGCGCTGGTGGCAAATGCAGAAGGAAAACCGAGACT
>dme_circ_0000281_junction_seq
TGGCAAATGCAGAAGGAAAACCGAGACTGGTACTGATAAAACCATGATATCGCCCA
>dme_circ_0000282
gctattttctgcgttatctacgtgtcgatcgtgtattatttcacttcacaaccttgggagctattccgct
tttccatgttcttGTCGGCCTGCCTTCTCATTTCTTTCGTTGCTCAGTCCGTGGGTCTGGTGGTAGGCGC
TGCAATGAATGTTCAGAACGGTGTCTTCCTGGCCCCAGTGATGTCCGTGCCCTTCCTTCTGTTCTCCGGC
TTCTTTGTCTCCTTTGACGCTATACCAGTATACTTAAGG
>dme_circ_0000282_junction_seq
CTTTGACGCTATACCAGTATACTTAAGGgctattttctgcgttatctacgtgtcga
>dme_circ_0000283
GTATACTGGTATAGCGTCAAAGGAGACAAAGAAGCCGGAGAACAGAAGGAAGGGCACGGACATCACTGGG
GCCAGGAAGACACCGTTCTGAACATTCATTGCAGCGCCTACCACCAGACCCACGGACTGAGCAACGAAAG
AAATGAGAAGGCAGGCCGACaagaacatggaaaagcggaatagctcccaaggttgtgaagtgaaataata
cacgatcgacac
>dme_circ_0000283_junction_seq
gtgaagtgaaataatacacgatcgacacGTATACTGGTATAGCGTCAAAGGAGACA
>dme_circ_0000284
GTGTTTTCACTTCCGCAACTAAAGCCGATTAACAAATATAAGCTTACCGCTAACGAAGGTGCTCGGATTC
GCCGCATCCATTTTGGTTCGTTTAGTTGTCGCATATCCCCGGAAACACTGCAGAGTATGCACGGTTGTAG
CCCAACTAAGTCCACGCGTTCACATGGCGATGGAGAGGCGGATCCTAATATCAGTGGAAGCTTGGCTGTA
AGTCGTGGAGATGTATATAACGAAACAGCATTGATATGTTTAACGAATATGGGCGATATCATGG
>dme_circ_0000284_junction_seq
TGTTTAACGAATATGGGCGATATCATGGGTGTTTTCACTTCCGCAACTAAAGCCGA
>dme_circ_0000285
GCTAAAACTCCAGCTCCACCCTTTATAAAAGTTAAAAAACAAAAAGCGTAAACGCAGAGGCTAATTGTGA

AAACGAAAATAAAAACACTGTGATAAAGAAGGCATAAACAAAAGGGGGACTGAACTAAACACAGAAAGAG
GACCCTCCTGCCGGCAGCAAAATAATATACAATTTTAATAAAACTAAAACCCGAAGCAAATTAAAAAGCA
AGCGACGCCAAATGCGGCACTAAAAGTAATTAGCGACAGGAGCTGCAGGAGTTGGCAATAAAAACAAAGC
AGAGGTGTAATAAATGATGTTGCAATCCTTGAGTCTGCACGCGGATAAACTaagcggcaattcaccagca
gcagcagcagcaactggagcagcagcagcaacatcagaggcaacagcagtagcatccgcagcagcagcaa
caacaatgcttgcaacggcacctgttgcccatgttgctaatggcaacaacagcaattcgagtgccggcag
cagcagcaacagcaacagcagcagcaacaaccacagcctctacagcaataacaatttaaatacaggcaac
aatagccagcagcagcagcagcagcagcagcaacatccgcaaaTACTGCCGGTCAAGTACGAGTATTTAG
AGAATCTAACGAGCTCGGGCGCAACTTCAGGTGCCATTGCAGCGACAACAACTTTCACGGGAGCTGCGAA
AAGTTTTCATCCCTATTTGCGGCCCACCGGcagtggcagcaacagcaacaacagcatcgcagcattatct
acaacaacggcagcaacagcggctaaaatgtcgacaacatTGTTTGAGACACTCTTGCACAACCAAATAA
ATTCCAGCCcaatagcactgccagcagcaacggcaatcgcagcaatatctgcagcagcaacagcagcaac
agcaacaccaggcaccgcagcaaccgcagcagctgccgctgcagcagcaccgtcaacgcagcTGAATTCC
AGCATTAATTCCAGAACTTCATTAGGCGACCAGGGTGGTGCAATTGTTGTTGTTACCCCCGCACCACCCG
TTGCTGCACCACCAACTGGCGGCCCAAATCG
>dme_circ_0000285_junction_seq
CTGCACCACCAACTGGCGGCCCAAATCGGCTAAAACTCCAGCTCCACCCTTTATAA
>dme_circ_0000286
GATGCACCATCCCATTCAGATGAAGCCGGCGGATAGTGAGAACAGAAATGGTAAGTTCCTTTTTAGACAT
CGATTCCGAGGATATTTGTTCTTCTTCCCTCTCTGTCCCACACTCCTGACTCTGTCTCCGTTCTGTTGAT
TGCTAGTTCGTGCtttttttttATCAGTGTGCATGAGCCTTTTGGGCTTAATCACAAGCAAGGATAAGAG
GTGGTGGGATGGGGTGCCTGTATTTGATTACACAAAACAACACATGGAAAAGAAATTCGAACAGAGAATG
CGGCAATGAAAATGAATAATGCTCAAACTGACAGGACGGGTGGATGGAAAAAAGCGGAATGGAGAAGGCT
AACTGGCTACAATCGTGTATTAGTTGGATTGTGTGAGACCAATAAATCGATTAGG
>dme_circ_0000286_junction_seq
ATTGTGTGAGACCAATAAATCGATTAGGGATGCACCATCCCATTCAGATGAAGCCG
>dme_circ_0000287
GAACGTAAACTTTTCGTTGGCATGCTGAACAAAAAGTACACCGAGGCCGATGTGAGGCAGCTCTTCACGG
GCCACGGAACCATCGAGGAGTGCACAGTGCTCCGGGATCAGGCTGGTCAGAGCAAGGGCTGCGCCTTCGT
CACATTCGCCACTAAGCAGAACGCCATTGGAGCTATAAAGGGTAAGCTTCCAAAATGCATCaaaaaaaaG
GGTATTCAAATAGTGTTTGGTAATGAGAATTTTCCAAAATGTATGAGAATTAATATAAAATCCGCTTTAA
GTCGTCTTAAATTTCAAATTTCAAATATTCTTTAACAATTTAACACAGTGTATCAGATCGTTTGACAATT
CATAGTGGGTTGACATACAGTTCAGAGCCTTAGCAAAGAAGATTGATAAAAGAAATGATGAGCGAATGGC
GAAACGATCACCTTAAAGTCACTTTAGCTCTTCAAGCGCAGCATTGCCTTTTGGTCCTGTGGCTAGTGTG
GGAAAAACTGTCGCAATGATTAATTGCAGCGGCGGCAAGAGTCGCGCATATAAAACACGTTTCTTTACGG
CACACATACCAAAACAGAGTCGGTGacaccacacacacacacacacacacaTGTGCAGGCATACTAGCAC
AGCTTCATTCCACAATGGCGGCGGCAGCAGCAACAAAAACCGCATGCAACTGCAAACAGGAAGCTGGAAC
GCAAGCAAGACGCCGCCTCTGGCAGCGGAAGAAGTCAACGGTAATCCTGGCAACCAAGTAACCCCCATCC
TGCTTCCTCCACATTCACATTCCGCTCCTGCCACGCCCACTTATAAGCAAACACTCACACACACTCACAC
TAGCGCACTAGCGGAGACTAATGCAAGTTTTGTGCCGGAAACAGGCAAAATTACAAACTTCCGACAGCCA
AACACAAACACATGCAGGGTGTGGGAGGAAATGAGCGGGCAGAGCGAGAGCAGGACAGTCCGAGCATGCT
GTCTCTGTCCTGTGTATATTGCACATAATTTGTCGCCTTTTGCAAGATGAATGTTGTTGTGGGCGGACTG
CCATTATAAAGCCATACATGCACACTGCAGAGACACAGATATACCACTACACACACCAACATGAGAGTAC
AGTATGAGCAGGCTACAACATGCCTGAAGGATATTTTCTTTTGCCTATTAAATAAACATGATAGACTGGC

CAGCCCCATAATAAAACTTAAGAAGTTTCTGAAATATTTTAAATTAGTTCTCTTATTTGGCTTACCGGAA
TAAAAATGTTTATACCTAAAAAGGGAATAATAAAATCATTTCCTTTTACCACAGTTATCACTGTACCTTC
ACATGTGTGTATGTGTGGCAGCGATTTTAATGCATTTGAAGTCAAATTACGGGATGCTAGGCACGCACAC
GCCAGCAATAAGCTGGGGCCAAGTGGTCCAAGGATATTAAGCAGGAAAAGGAACCAGGGAGCAGCCTGTC
CAGGACCAAATGATTTGGATCGACGCAGTGAAGCAAGGGTTGCAAAGTCATTGCAGTCTATTGACGCCTG
AAAGTATGCAGCCCCATGAAATGCAATGCAAAAGCTGTGCAACTTGCCCATCAAAATACGAAGACCCAGG
CGAAAGACGCTTCAGCTGAAGCTGCTGGCAAAGTGCATTTAATATTAATGTGCCATGTTTATAGCCCGAC
AATTTGTTCTCGGCTTCTGCTCCTCCATTAAGTCTCTTTTACTTTACAAAAACCCAAATGAATGCAAAAA
ATGCGAGCTGAAACGGGTGGGTGGCCAAATGATGATAGCATTTTTCAACGCATGCAGATTTTATTTTTCG
CATTCGTCTCGCTAATTTGTCAAGTGGTTGACGCTGAGTTCTGGCCAACTTGGGAATGCGGTTGAGTCTC
CAGTGCCCACTGTTGCCCAACGGGTCATTTAACCCGTTTTTTGCTGCCCCAGAGGGAAGTGAGATGTTAG
GCCACGCTGTGAAAAATGACTGAGGATTAGTAGGTGACTTGGGAATGGTTCAAGTAAAAAATTTTAAAGA
AATGAAGTGTTTTCAAGTGTCAAGAAGCTAAGTTGGAACACGATGAAAAATAATGTAATAAAAATAAGAA
ACTTTCGTATTAATCGttttttttttttttGTGGACAATGCCATTCGTCGTTACACCATTTTTTATACCA
TACGCATAAAATTTAAAGCTTAAATACACGACGCTTAATATTTTGTCAGGATTTCTTTTCTCTACTCTAT
CGACCGGAAACAAAGGGAAATCACGAAACTGTCTTGATAAAATCCTTTAACATTTACTTCTCTCAGGTGT
CCTTGATGCAAAACCACGACAACTTCTATACAAGCACCACCCACAAATTCGCGGCATTTAGAGGTCGAAA
ACAGTTTTGATAAGTCTTATGGCAGCGGCAAGTCGGCTTACAGCATCAGAATAATGACGCGCACTTAAGC
CGCCTTCGCATGTccacccgactatcaaaccacccactttacaaaccacccaTCCAGCCATGCCCACCCA
ACATCATACCCCCCAGATAGTTTAGGCCACACAGCCATTTGTCCGCTTAGGCGACTTAATTTACACCTTT
CTGCTAAGCCCGAAAACAAACAAAATGGCACGGGAACGGGAATGGGAATGGGGCGGAGAATCGGGGAGGC
TGGCAACTCTGGGCGCGTCACGAAAACCAGGCCAAACATTTAGCGAAGGCATTTAGGCTGCGGTCTGATG
AGGGGGTTGCCGGGCATTTGGGGCATTCGAGGGAGGGGTTTGGCAAGCCACTTTACTTATTTTCCGGTTT
ATGTAGACTTTATCATTGAAACTCAGCCGGGGCCATGGCGACCCGGGCCGAAACTTCCGCTCAATTGTGG
CGACGGTGGCAACTCTGGCCTGCATCCTATCCTGCATCCCATTCGCCTGTATCGTGGCAGCCACCAGAAG
TGCTCTTTAATTACATTGGCTATAATTCTTGCGGCTCTGGCGCGACTTTTATGGCCGGCGCATTGTTTTG
CCCCACCCCGTTCACCAGCCATCGCGACACTGCTGCTCTTGGCTACAAAGTAATTAGGGAAATTTCCGAG
TTTTTAAACAGGTCGCCATGGCTGCACAGTTGAAGCAGAGTTTTTCACAGAGTGAGGTTGCTGGATTTTT
TTGGACTGCAAGAGCATGGAATAAAGAAGAATTTAAGATTATGAAATTATTATATAAAATAAGATAACTt
tttattttatttacattttctttatcgaatttactttacacaaccattttttaAGGAACGACAACACTGCG
GATGAGTAATATAATCCAAAGTACTAAATGTGCAAGGTGAACTTCGAGTTAGCTGACAAAGTATACAAAG
TATTACTAACACAGAAGTAGGCTTTAAAAGTCACAAATGTTTGGAGCAATAAACAACATCTAATGACTAG
AACACACCTCTTTTGGTGCCAACGTTGCGTATAAGTAATAAGAATAAATAAACATTTTGTATACGCCAGG
TGTGCAAACTAAAAACCTTAAAACCAGTAATTAAAGATCAAAACTATATTTCTTTTAGCCAGCTCTAAAT
AATATAGTTACACACAAAATTTGATTGTTAATTATTATGATTATTTCCTATACTTTTTAGGGCCCTCCAT
CAAAGTCAGACCATGGAAGGCTGTTCAGCTCCGCTGGTTGTGAAATTCGCCGACACGCAAAAGGAAAAAG
ACCAAAAGAAGATGCAGCAGATTCACGCATTCTGTGGCATCAATACGCCGAGCGGTGCGACAGCAGGTGC
GGCCACGCCCACCATTAATGCCGCAACGGCTCTAATAGCTGCTCCGCCCTCGGCGGGACGTACCAATCCG
TCGATGGCGGCCGCACTGGCGGCAGTGCCTCAGGTTCAGCAGGCTGGTTCTGCAGCAACGGCACCAACCA
CCCTCGTTCCCCTGAATTCCACCACCGCCCTCAGTGCGTCCTTGACGCCCAATCTCCTAGCCACCAATGC
CGCCCACCAGGGGGCGGCAGCTGCGGCCGCCTATTTGGGGGCAGATCCCGCTGCGGCGGCCCACCTTCAG
CTGTACCAACAACTACACGGATACGGACTGAGTCCGGCACACTATCTGCCAG
>dme_circ_0000287_junction_seq
GGACTGAGTCCGGCACACTATCTGCCAGGAACGTAAACTTTTCGTTGGCATGCTGA

>dme_circ_0000288
GGCCCTCCATCAAAGTCAGACCATGGAAGGCTGTTCAGCTCCGCTGGTTGTGAAATTCGCCGACACGCAA
AAGGAAAAAGACCAAAAGAAGATGCAGCAGATTCACGCATTCTGTGGCATCAATACGCCGAGCGGTGCGA
CAGCAGGTGCGGCCACGCCCACCATTAATGCCGCAACGGCTCTAATAGCTGCTCCGCCCTCGGCGGGACG
TACCAATCCGTCGATGGCGGCCGCACTGGCGGCAGTGCCTCAGGTTCAGCAGGCTGGTTCTGCAGCAACG
GCACCAACCACCCTCGTTCCCCTGAATTCCACCACCGCCCTCAGTGCGTCCTTGACGCCCAATCTCCTAG
CCACCAATGCCGCCCACCAGGGGGCGGCAGCTGCGGCCGCCTATTTGGGGGCAGATCCCGCTGCGGCGGC
CCACCTTCAGCTGTACCAACAACTACACGGATACGGACTGAGTCCGGCACACTATCTGCCAG
>dme_circ_0000288_junction_seq
GGACTGAGTCCGGCACACTATCTGCCAGGGCCCTCCATCAAAGTCAGACCATGGAA
>dme_circ_0000289
ATGCCCGTGCTGGGATTCCGGAATCCCTCCACTCGCGCATCGCGATCAAAGAGTCTCGCGAAGATCACGG
GATCATCGACGGGCGGCGCCTTCGACTGCTTGCCACCCACTATCCGCTTCACGCGAACGTGCGCATCCCC
GCCAGACTTGCAGCTGGCCAAGATCACCACCGAAAG
>dme_circ_0000289_junction_seq
TGCAGCTGGCCAAGATCACCACCGAAAGATGCCCGTGCTGGGATTCCGGAATCCCT
>dme_circ_0000290
TCTCGGTTTTCCTTCTGCATTTGCCACCAGCGCAGACCGCGGCAACTGCCGTCCCGTCGGCAAGTGGCAA
TGCACCACCACACATGCCCCGCCGAATTTCTGCGCAGCTTGCTAAAGAAATACAATTAAAACATCGTGCT
CCTGTGGTGGGTATTTCTATTTTTGATCAGGCGGGTAGCCCTGTCGATCAGCTGAACGCCGGTGAAAACG
GGAGTCCACCGCATCGTGTACTTATTGCTTCCGAGGAACAGTTCAAGGTGTTTTCACTTCCGCAACTAAA
GCCGATTAACAAATATAAGCTTACCGCTAACGAAGGTGCTCGGATTCGCCGCATCCATTTTGGTTCGTTT
AGTTGTCGCATATCCCCGGAAACACTGCAGAGTATGCACGGTTGTAGCCCAACTAAGTCCACGCGTTCAC
ATGGCGATGGAGAGGCGGATCCTAATATCAGTGGAAGCTTGGCTGTAAGTCGTGGAGATG
>dme_circ_0000290_junction_seq
GGAAGCTTGGCTGTAAGTCGTGGAGATGTCTCGGTTTTCCTTCTGCATTTGCCACC
>dme_circ_0000291
GACCGGTGTACAAAAGGCTGGCTGGTGACTTCAATGCCACCAAACATGGCCCACCTTGTATACAGCCGCA
TCCGCAGTTTCCTCAGAGGATTATCGGCGATGAGGATTGCCTGCTGCTGAACGTCTACACCCCGCAAATG
CCGGATGAGACCACAGGGCTGCCAGTGTTCGTTTGGATTCACCCGG
>dme_circ_0000291_junction_seq
CTGCCAGTGTTCGTTTGGATTCACCCGGGACCGGTGTACAAAAGGCTGGCTGGTGA
>dme_circ_0000292
TGATCCCAGGCGATACTGAGGAGCCACCACGATTGCGCCCCTTTGGGCCATAGGCGTGGCATCGTACTGG
GCCGCCGATCCATATCGATAGCCACCCGGGTGAATCCAAACGAACACTGGCAGCCCTGTGGTCTCATCCG
GCATTTGCGGGGTGTAGACGTTCAGCAGCAGGCAATCCTCATCGCCGATAATCCTCTGAGGAAACTGCGG
ATGCGGCTGTATACAAGG
>dme_circ_0000292_junction_seq
GAAACTGCGGATGCGGCTGTATACAAGGTGATCCCAGGCGATACTGAGGAGCCACC
>dme_circ_0000293
GAGCCACCACGATTGCGCCCCTTTGGGCCATAGGCGTGGCATCGTACTGGGCCGCCGATCCATATCGATA
GCCACCCGGGTGAATCCAAACGAACACTGGCAGCCCTGTGGTCTCATCCGGCATTTGCGGGGTGTAGACG
TTCAGCAGCAGGCAATCCTCATCGCCGATAATCCTCTGAGGAAACTGCGGATGCGGCTG
>dme_circ_0000293_junction_seq

TCCTCTGAGGAAACTGCGGATGCGGCTGGAGCCACCACGATTGCGCCCCTTTGGGC

>dme_circ_0000294

AGAGCGGTTCCCGACATGGCCACCACTCCGTGAACATCGCCAGCGCTTCGCGACGTCGGCGACATCGATA
GATACATGGCACTGGCTGCTCCGGATCCGTGGCCTATGGCCTGGACCTGCTTGGGATTGCCCCCGAAATA
GGAGATGTAATCGGTGACCCAACGGAGAGCGGCGGCCAAGTCGAACATGGCCAGG

>dme_circ_0000294_junction_seq

AGCGGCGGCCAAGTCGAACATGGCCAGGAGAGCGGTTCCCGACATGGCCACCACTC

>dme_circ_0000295

CGCGCAACGATCCACAGGACGATGCGGAAGTGATTAAAGTACCATCACGCCCTCAGCCCGAATCGGATTT
CCATAACTTCCGCGGCGTCATCGATACCGGCTCTGGTTATCCCTTCCTTCAGCCGAATCCTTCGAGCTTC
AATGTGGGCATCTTCGACTCCTTCGATGGGTGAGTACAGTGCAGCACACGTGGCGTATGCGTGATGAGGC
GCTTAATCGGCTTGAGCATTTATAATGATATTTTAATTAAACATTATTTTAATGTGGCCTTAAAAACTGC
ATGCAACATGCAATTACAATTGCCTCGCATGCATTTAATGTCGTCATTATTATTCTGCTTAACGATGAGC
GAACGTTTGACATATTACGTATACGTATTATTGTCGCGGGTGAGCAGAGCATTTCGGTTTTGAGGAAAAC
TTTGTAACAACAACAATACCACGATTTAATACTATTTCTAACTTAATTATTCATCATGCTGCCAAAAAAT
CGTGTTTTTAATTCTATTTTGTGTAGGTAAACAGAGTAGAAGAGCGGTTGACTTAAATGTTTATTATTGT
TGGCCATGGCTTAAAAGCACATCCCCAGGGCCAAGCCAATTAGCCAATGTTCTTTGGAGACTTTCTAACT
TAGCCATCTCGTTTTCTGCCTATTTCCCACTCCATGTCATGGAAGTGAAATGAAAAACAATTTATCAAAA
TATTATGTCAAGTTCTTGTAGCCAGACAAAGTTTATTTATGCTCTTGTATTTATTTCTATATTTATGGTG
GACCCAAGAGAGTGGCGTCGAGTAGGTCAAACAAGTCTGTTGACCATTAAAAGTGAAAAGTTTTGCGCCT
CTTCTTGCCAGTTTGTGCTTAACTGAGACCGTAAGACGTCGCAGATGACGACGATGAAGCTGAAACTTCA
TAAATCTTCTCCTAATACGCTCGAGGTCGAGGGGTAGGATAAAAACTTCCAAAAGCCTTGGGCATTGTAC
ATGCATAAAAGTTGTTATGTTCTTTGTTGTCTTATTTTTTGGTTTGGGAAATTGAAGATTGCAGGTCAAT
GATAACGAAGCGTAGAAAATGGCATATAGACAAATGGAAAGTTGAACGTTGCGGTGGCCAGCAAAGTTTC
TTGTCAGCGAATTATTTTTGAAACACCAGGATCGATTTCTTAGAACTGAAAAACGAAAAATGTGCGAAAG
TTATAAGATATAAGCTTTAAAGTGTGTCTTGTAATCTTTCAAGTAGTCCAGAATCTCGAATTTTTGAGGC
GAAGAGCTTATTCAGTGCCGTAAACTATTTCACGCAGCTATTTAAACGCATTAAAATGCAGCAAGCAAAT
GAATTTGTCGACAAACGGCAATCAGCAACCGGGCCAAAACAAAGTCGTGCAAACAATGCGGTCAAGAATT
TATGGCAAGGTTGAAGACGACaaaaaaaaaGCTACTGAAAAAGGCCAAAGAGCCGAGGAGAAGATAGACT
TATAGACCAGAACAGGGAGGGGTTGCAGCCAGGGGTTGAGAATTACAAAAGTGCAGACAAGAAAATGACA
GAGACGCCGGCGACACAGAAAACGCTTTTTAGTGCGTTTACGGATTAGGGATTTTCAAACTGAAAAATAC
ATTAGCAGCAGGCTGGGACGACAACGGAATGTCTCTCCCTGCTCAAGGATTGAAATATAGATAGAGACTC
AAGGGAAATTCCATGAGCCAGTCTAGACCAGAAACACCTGCTCTGTTTCCCACCGGATGTTTGAATTTCT
TTAGTTTTAGCAGTGCGTTGCGTTGCTTCTTAGATTAAATCAATTGAAAATAATCTTTAAAAATGTTAGA
CAAAATATGCAAttttttttAAGGATGAGATATTATGATAATTTAATGTTTAATTAAATATTTTCTAAAG
AATCTCTTCCGCCGTCTGCGCACCCGTTTGTGGCCTGTAATTGGCAGGGATTCCGGTGAGGAGGGCGCTT
CTCCGACTGGCGACTCTGATGATTCTAGTGATGGCAGCGGAGCAGGCTTTCCATTTGGAGTGCGTCCTCT
GATTCCGCTGGACTCGAAGAACGCCAACACCACTTCCACCATTAAGGGTTGTCAATGGCCATAAGGTTGA
GATCAACGAGACTGTGTATGGTGATAGCAACAGCGTCTTTAAGGTCCGCTTGGTGAATGTGCGTCCTTTG
GAGAGCGGCGAGGAGGTGGCCCAGGGAGTGCACACCAGTGGAGGTGACTTCCAGCCGGCGTTGGAGCCCA
GCACCTCCGCCCCGCCCAAGAAGTTCGAGGAGTTCGACGAGGAGGACGACGATGATCGCCGCGAGCCGCT
GGAGAAGCAGCCACAGGATAACGAGGTGCGCGACATTGATGAGCCCAAGG

>dme_circ_0000295_junction_seq

GAGGTGCGCGACATTGATGAGCCCAAGGCGCGCAACGATCCACAGGACGATGCGGA

>dme_circ_0000296

GCTATTCTCGATAAGAACGACGCTTCAAGTGTCAGCGGTCGCAAGTACGAGGACAATTTGACCTTGAACA
ACGGTTTGCTCAACGGCATGGTCAATGATATTGTGAAGGAGGGCAACGTTAGCTCTGCCTTGGTCACTAC
CAATGAAAGGGGAGATGCCATGATCGATGTGGAGAAGTCCGTAAACTGCACCACTGCCTTGCTTACGGAG
GAGATAACATCACCAGAGCGATATCCAACATCGCAGTTTCACCAGTTCTGGGTGGTTCTTAAGCGAACTT
TACTCTTCAGTTACCGGGATTGGG

>dme_circ_0000296_junction_seq

ACTTTACTCTTCAGTTACCGGGATTGGGGCTATTCTCGATAAGAACGACGCTTCAA

>dme_circ_0000297

ATGCCATGATCGATGTGGAGAAGTCCGTAAACTGCACCACTGCCTTGCTTACGGAGGAGATAACATCACC
AGAGCGATATCCAACATCGCAGTTTCACCAGTTCTGGGTGGTTCTTAAGCGAACTTTACTCTTCAG

>dme_circ_0000297_junction_seq

TGGTTCTTAAGCGAACTTTACTCTTCAGATGCCATGATCGATGTGGAGAAGTCCGT

>dme_circ_0000298

TTACTGCTCTTGAGCTGCTGCTCCGGTTCTCCAACTATCAAACTGGGCAATGCTCTACCATCGTCCTCGC
TCTCGATGTGAGGCTGGAAGCCCACGTTTCCAGTTAGTCCTTTGACCAGGGCACGTCCTGCCAAACGCTC
CGTTTGAACTTTATCATCGTTCTTGATTATG

>dme_circ_0000298_junction_seq

TTGAACTTTATCATCGTTCTTGATTATGTTACTGCTCTTGAGCTGCTGCTCCGGTT

>dme_circ_0000299

AAAACTCGTCAGCTTGTTCAGAGAATCACTTAGTGATCCCAGGAACTGCTCCGCCGATCCAAAGACCTTT
TCTATCGTTTCCACAGTGACGGAATTTGCTGTCTCGTGCTTGGTGACTCCAGTCAGCAGGGGAATCCCCG
AGAAGTTACTGCTCTTGAGCTGCTGCTCCGGTTCTCCAACTATCAAACTGGGCAATGCTCTACCATCGTC
CTCGCTCTCGATGTGAGGCTGGAAGCCCACGTTTCCAGTTAG

>dme_circ_0000299_junction_seq

GAGGCTGGAAGCCCACGTTTCCAGTTAGAAAACTCGTCAGCTTGTTCAGAGAATCA

>dme_circ_0000300

CACGTCGGTCGTTGATTCCACCACCTTGGAAAGTACTTGATCCACATTCAGCGCCTGCGGCACCTTCCAA
ACGTCCTGGAGAGTGGGAGTCAGGACACTCGTGAGGCCCGGCAGTTCCGGTTTGGCGATCTGACCGGTTA
GCTTGTCGATTTTCAGAAAACTCG

>dme_circ_0000300_junction_seq

GTTAGCTTGTCGATTTTCAGAAAACTCGCACGTCGGTCGTTGATTCCACCACCTTG

>dme_circ_0000301

GCGTGGCTTTTCCCGTAACACTCTGGAAAATGGAACTCTTTCCGCCCTCTTCCTTGTCCTTATTGGCAAA
CTTGACCAACAGATCGATGAGGTTGTTGCGCACTTTGAGATCCTCGGCGCTGGTCAAACGAGTCTCCTCC
ATGGGATTCCCGAATATGTCATTTGCATCGAACATG

>dme_circ_0000301_junction_seq

CCCGAATATGTCATTTGCATCGAACATGGCGTGGCTTTTCCCGTAACACTCTGGAA

>dme_circ_0000302

GAGGGCGGAAAGAGTTCCATTTTCCAGAGTGTTACGGGAAAAGCCACGCCTTTTATTAAGATAGATACCA
AACTGCAGACCTCCAATGATTTCCGCTTCTGCGAATTGTCCGTTCTGGGCGCTTCCCTCTCTCCGCTAAG
TTCCACCAGCTGTGCGGGATTGGGTAACTTGCTAGGTCAACTTGGAAGCCTGGGAGGCGGATTGGGAGGC
ACCCTGGGAGGAGTGGGCAGCACCTTGGGATTGGGTGGCAATGGGGGAGGTGGTAAACGGGGTGGTCTCG
GTCTTGGAATTTTGTAGGTATAGTGTTACCTCTATCACATTGTTGTGTACTTAAGGAATAATAAAT

>dme_circ_0000302_junction_seq
ATTGTTGTGTACTTAAGGAATAATAAATGAGGGCGGAAAGAGTTCCATTTTCCAGA
>dme_circ_0000303
GATACCAAACTGCAGACCTCCAATGATTTCCGCTTCTGCGAATTGTCCGTTCTGGGCGCTTCCCTCTCTC
CGCTAAGTTCCACCAGCTGTGCGGGATTGGGTAACTTGCTAGGTCAACTTGGAAGCCTGGGAGGCGGATT
GGGAGGCACCCTGGGAGGAGTGGGCAGCACCTTGGGATTGGGTGGCAATGGGGGAGGTG
>dme_circ_0000303_junction_seq
TTGGGATTGGGTGGCAATGGGGGAGGTGGATACCAAACTGCAGACCTCCAATGATT
>dme_circ_0000304
GTTCCACCAGCTGTGCGGGATTGGGTAACTTGCTAGGTCAACTTGGAAGCCTGGGAGGCGGATTGGGAGG
CACCCTGGGAGGAGTGGGCAGCACCTTGGGATTGGGTGGCAATGGGGGAGGTGGTAAACGGGGTGGTCTC
G
>dme_circ_0000304_junction_seq
GGGGGAGGTGGTAAACGGGGTGGTCTCGGTTCCACCAGCTGTGCGGGATTGGGTAA
>dme_circ_0000305
GGGCTTACGGTAAGTTGGCTAGCAGGGGTTTGGTAGGCAACTGTTGCAAGTGTACACATTCAATCTAGCA
TCCTTTTGCTAAAGAAGATGTACAGACTGAAAGCTGTTACACATTCAAAAGGGGCAAATAATGCAG
>dme_circ_0000305_junction_seq
ACACATTCAAAAGGGGCAAATAATGCAGGGGCTTACGGTAAGTTGGCTAGCAGGGG
>dme_circ_0000306
GATGTAGTATTCTCTGTACGTTGGCTAGTAAATGTTGAGTAGGCAACGGTTGCAAGTGAACACATTTGTT
CTGGCACCATTTTGCTACTGAAAGTGTACAGATTAGCAACTGATACACAATCGATGATAGCAAATAATGC
AAATA
>dme_circ_0000306_junction_seq
CAATCGATGATAGCAAATAATGCAAATAGATGTAGTATTCTCTGTACGTTGGCTAG
>dme_circ_0000307
GGGCAAGACCGTCGAGGTGGGACGAGCCTTCTTCGAAACGGACCGAAAGCATTTCACTATCCTCGACGCA
CCCGGTCACAAGAGCTTCGTGCCTAACATGATCGGTGGCGCGGCGCAGGCCGATCTCGCAGTGCTGGTCA
TCTCGGCGCGAAAGG
>dme_circ_0000307_junction_seq
GCAGTGCTGGTCATCTCGGCGCGAAAGGGGGCAAGACCGTCGAGGTGGGACGAGCC
>dme_circ_0000308
GCCTTCAGGTTTTGCACCTTTACCAAATCGTTGCGAGCCTTGAGGCCGGCATAGTCTGCTGATGAACGAA
CGTCCCGCTTGCCGTTGTCAATAGCATCGACCAGTTTGCGCGTATGATCTCCGTGCTCCCCGCAGGACAC
CTCAATCACGTAGCTTGCCGGATTGTGGTAACTGGGGCACTCCAGGTTTAGCGTAGACAAGAAGGGCACC
AGTTGCTTTGTACTGCCTTGATAAACGCACTGTCCATCTGCGAGAGTGTACAGCTGGTCGAACATCTCGA
AGAGACGAGCGGACGGTTGATGGATGGTGCATATGACTGTGCGTCCGCCAGCAGCTAGCATTTTTAGCAG
ATGAATGCACTGAAAACAG
>dme_circ_0000308_junction_seq
TTTTAGCAGATGAATGCACTGAAAACAGGCCTTCAGGTTTTGCACCTTTACCAAAT
>dme_circ_0000309
GGTTTTGCACCTTTACCAAATCGTTGCGAGCCTTGAGGCCGGCATAGTCTGCTGATGAACGAACGTCCCG
CTTGCCGTTGTCAATAGCATCGACCAGTTTGCGCGTATGATCTCCGTGCTCCCCGCAGGACACCTCAATC
ACGTAGCTTGCCGGATTGTGGTAACTGGGGCACTCCAGGTTTAGCGTAGACAAGAAGGGCACCA

>dme_circ_0000309_junction_seq
AGGTTTAGCGTAGACAAGAAGGGCACCAGGTTTTGCACCTTTACCAAATCGTTGCG
>dme_circ_0000310
GGATATAAAAGACCGCCGAGAGGATGTTGAAGATCAGCAGCTTGTTGCGCAGCAGTCTCATAAGGGCACG
TGGAAAGTCCTTTAGCTTGGGCAGATCTGCATTGGCACCGGCCGCTGCTCCAATGGTATCCAGAGCGGCG
TTGGAGGAAAAGCGGCTGCTCAGTGAGAGGTTCTCCTCCCGCTTAAGCTCCTCCTTCGTCTGACGGAGTG
CCAACGGCAAATGGGAGTTT
>dme_circ_0000310_junction_seq
ACGGAGTGCCAACGGCAAATGGGAGTTTGGATATAAAAGACCGCCGAGAGGATGTT
>dme_circ_0000311
GATGAAGGTGCGCAGTCGGGCATATTTCTGGCATCCGGGACCGCGACACCATCCCAGACCCTTGATGCCA
CAATCGACGCTGTCTGTTTGGTGGTGGGAAGGGGGTAGAAATTCTATATCAAGTATACGTAAAGGGGAAA
TCAGGGATTTGGGTCACCCACCATCGGGTATATTGTTGGCATTGACAGCTATCTGCGATAAATCGGAGTT
GCGCGTATTATGCGCCATATTCCGTATTCGATTCGATTTCGGATTCGTTTATG
>dme_circ_0000311_junction_seq
ATTCGATTCGATTTCGGATTCGTTTATGGATGAAGGTGCGCAGTCGGGCATATTTC
>dme_circ_0000312
GTGCTTGGCGTTGGTCTCACCCATGTGGGTTGGTGGCTCGGTTGGGTGATACTGGCACCCTTCATTTTCG
TGGGCGCTGTGCTCCTGGGACTGTTTCCCAAACGACTGCCAAAGACCGTCATTCACCAGGCGGCGCAACG
GATCATTGAGCAGTCCAGCATGCGTAG
>dme_circ_0000312_junction_seq
GGATCATTGAGCAGTCCAGCATGCGTAGGTGCTTGGCGTTGGTCTCACCCATGTGG
>dme_circ_0000313
GGATTAAAACCAAATATGATACTCCAACTCCAAGTGCCGAAAACTAGCTGTCTACAGGGCAAACACCAAT
GGACAGACAAATCAGATAGACGGACGGACAGGACAGACTAATGTCACATCCCCCGAACACAGTAATTCTG
TTGAAAAGTATTTCCCTTTATGATTTTTG
>dme_circ_0000313_junction_seq
TGAAAAGTATTTCCCTTTATGATTTTTGGGATTAAAACCAAATATGATACTCCAAC
>dme_circ_0000314
GGAATTTTCCAACTAAGAGTAATCCAAAATTATCGACTAAATAATTTTAACAAAAAATAGGACGAGTTTG
TCTTCATCTTACAATTTTAGCTTTTCCATCATGTTGATTTTGTGTGATTTAAATTTTTGTTGCAATGTAT
CTTCCTCGTCGGCGACTTTCGAAGTTTGTTCTCAATTTTAGTTTTTAGTTTTACACAATATTTACAGATA
tttttgggtttttttgtttgcagatagtttttagagtttAACAAACAattttgataagctttctttttctg
tttggctgttgttttctttctttttttttttGAATAGCTATACAATCTTTGAAATATAGCTTGAGAATAT
GCTAGAATTACTATAATTTACTACttttttttttGTTATCCATTGTTTAATTTGAAACATATTCATATT
TATGATGTAGTTAATATTTCCCCTTGTATTTTTACAATAAAAGTTAGGATTAAAGGTAAATTATAAAAAT
AGCAGCTGGCCTTTTCTTGTTTCATTAAATTGTATTTGTATTTAATTTATCGATCAATGTTGAATGTTTG
GAAATTTGAATCCTACTTTCCTGTCCTTTGCTTCTCGGGTTTGCttttgtttttttttttACAATTTAC
ATCTTATGTCTCTGACTGCACAATGCAAATTATCACATATTATTTCATTTATTTTCCCTTCTGTCCCTAT
TCATTTTCTGTGTGTTTTCCCTGGCACTCTA
>dme_circ_0000314_junction_seq
TTTTCTGTGTGTTTTCCCTGGCACTCTAGGAATTTTCCAACTAAGAGTAATCCAAA
>dme_circ_0000315
CTGCTATTTTTATAATTTACCTTTAATCCTAACTTTTATTGTAAAAATACAAGGGGAAATATTAACTACA

TCATAAATATGAATATGTTTCAAATTAAACAATGGATAACaaaaaaaaaaaGTAGTAAATTATAGTAATT
CTAGCATATTCTCAAGCTATATTTCAAAGATTGTATAGCTATTCaaaaaaaaaaaagaaagaaaacaacag
ccaaacagaaaaagaaagcttatcaaaatTGTTTGTTaaactctaaaaactatctgcaaacaaaaaaccc
aaaaaTATCTGTAAATATTGTGTAAAACTAAAAACTAAAATTGAGAACAAACTTCGAAAGTCGCCGACGA
GGAAGATACATTGCAACAAAAATTTAAATCACACAAAATCAACATGATGGAAAAGCTAAAATTGTAAGAT
GAAGACAAACTCG
>dme_circ_0000315_junction_seq
CTAAAATTGTAAGATGAAGACAAACTCGCTGCTATTTTTATAATTTACCTTTAATC
>dme_circ_0000316
GGTATTAAACAAAAATGTGGAGGCGCAGCAGCGCGATATTTAAAAGAACTCCTATACTCGCTTCGAGTTA
ACAGCGGACGTTAACAGCGCCAGCCGCAGCCGCTGTTAGCGTGCAgtctatctgtgtgtttgtgtgtgta
tttcgtatggatgtgtgtgtaaatgcgtatgagtgtgtgtgtgtgtggtggtctgtatgt
>dme_circ_0000316_junction_seq
gtgtgtgtgtgtgtggtggtctgtatgtGGTATTAAACAAAAATGTGGAGGCGCAG
>dme_circ_0000317
GGCGCAGCAGCGCGATATTTAAAAGAACTCCTATACTCGCTTCGAGTTAACAGCGGACGTTAACAGCGCC
AGCCGCAGCCGCTGTTAGCGTGCAgtctatctgtgtgtttgtgtgtgtatttcgtatggatgtgtgtgta
aatgcgtatgagtgtgtgtgtgtgtggtg
>dme_circ_0000317_junction_seq
atgcgtatgagtgtgtgtgtgtgtggtgGGCGCAGCAGCGCGATATTTAAAAGAAC
>dme_circ_0000318
GGTTTAGCGTAGACAAGAAGGGCACCAGTTGCTTTGTACTGCCTTGATAAACGCACTGTCCATCTGCGAG
AGTGTACAGCTGGTCGAACATCTCGAAGAGACGAGCGGACGGTTGATGGATGGTGCATATGACTGTGCGT
CCGCCAGCAGCTAGCATTTTTAGCAGATGAATGCACTGAAAACAGGTAGAGCTGTCCAGGCCGGATGTGG
GTTCGTCAAAGAACATTATGGGAGGATTGCTGACCA
>dme_circ_0000318_junction_seq
AAGAACATTATGGGAGGATTGCTGACCAGGTTTAGCGTAGACAAGAAGGGCACCAG
>dme_circ_0000319
GGGCAATCGAGAGCCGCTTTTTCTGACCGCCGGACAAATTTCGAGTCATCGTATATCGGTGCTCGCTCAG
GCTGAGAGTAAGCAAAATGTCATCGATCTGGAAGAGGGTGTTAAGCAGGTTATTAGCACGTTTAAGATAA
AACCCAATACACAAGACTGTCGAATCAAATTGTCTACTGGATTGGCTACTTATGATTAATCTTTAAAGTT
ATCAAATTCTTCCTCTCGGGCttttttaatttttttCAACTTGCCATTGAATGCTTCTCGGGCTTCGAAAA
CTTCTTGCTGAGCTTCAGATTCGTTGCCACAGTCATTGCTTCCTGGACGGTAAG
>dme_circ_0000319_junction_seq
CCACAGTCATTGCTTCCTGGACGGTAAGGGGCAATCGAGAGCCGCTTTTTCTGACC
>dme_circ_0000320
GTGAGTTTTATTGAATAAACGTTGTTATGGTAAATCATGTCGCAGAGGGTAAGCTTTAAAGGAAACCCAG
AATTATTTGGAAAAAGTAATGCAAAGTATTGGTAAGAAAGTCCAGGCCAATGTTTCTCTCGGATAAGTGT
AAACATAGATCTTTTATGGGCTTATCGACAGAGCCCCACCCATAAACCAAACAAAAATCAGCTGATTTCA
AACCCCATAACCCATACAATATATTTAATATTCCACAATGCAACAGCGTGGGAAAACCGTGAAATGCGTA
TGGAAAATCTACAGTTAGCACACGGGTTATTGAGCCTTCAACGTGGCAACTTGTCATAGTCAAGTGGCAT
AGAGAGTGTCTTGCTGTCCCTTTCTGTCGCTGATGCTGCTGCTGCTGCATCGCTTCACTGGATTTCAAAA
GTTCTTTTGCCGTTACAGCAAACATTCGCTCTTCGGCATTTGCTCTCTGCTGACAAAGTCGCATTTGTAT
GGTTTCGCTCTCTTTGTCTGAATTCAGCTAAAAAGCTTTTGAAAAAGCAGCCATGTTGCTTTTTTATCG

TCCATGTGTGTGTGTGGGTGTCTTGTCTGTTGGTATCTCACTCTCTCTCCTCTCATAAATTCACTGATTG
GAAAGATTCGTTCCTCTGtttttttttttttttGGTTTCCACAACCttttttcaatgtttatatcgcttg
tttgtttatttttcatgcatttttgtttGCCTTTAACGCGGCATTGCATTGCCTCGCTGTCGAGAAAAG
GGTTCCGTAATAAAGATCGCAGACAAATGTGTACAGTGAAGCCTATGTGCAACGATTTCAATTTGCTGCA
ATTTGGATAACCAAAATACTAATGGGTCAGCTAAAGAAAATTTTGAGTATATAGTACACTTAACACGACT
TACATAAACCCTACAAGCATAACTCGTATAGCCATTTAAATTTTTATACCACAATCCCTACGGTGCTTAT
TTTGATATTTATGAGGTGTTTAGTGATTTTTATTACAAGCTTCACTTTACCTGCCAAGCTTTGCTTTGTT
GTGCTCTCAGTGGCTTTGCATTTGTGCAAAAACAACAGCTTAGTAAAACTCCCTCTCTCTCGCTCTGGGC
TTCTCTTCCACCAACTTGGAAACTTCAAAAAGCCACCACCGAAAAGCTGTTGTAAACATTGTTGTTGTTG
CTGCTGCTGCACGTTCAGCTCTCTTTTTGGCTCTCGCATTGGCATCGTATTGACTGCGAAGCGCAGCAGC
GACGTCAACATTTTCAGTTATGCTGAGGAGCTCTTTCCATTCAAACACGAAAATCGCCTAGAAGTTAAAA
CTTCGAGTCGCTTTTGTCGCTTGGCCTTTTATTTTATTTTGTTGCTCAATTGTTGTTAATTAATTGTGTG
AAAATTACCTATTTTTATCTGTGCGCCTGTGTTGCAATTCGTGTAAAAGAGAGAGGGAGAGTGGAGAATA
GACAAAGCGAAATTGTTGTTAACCGAAAAAGAACAAAAAGCTGGCGAAATTGTTTACACCAGCAGAGTCT
AAACAATTCGCAAAAGTTCGCCAGACACTGAATATCAAAATCCAGAGAAAAGGAAAAACTGCGAAAACGT
CTCGCGCGCATTTTTAACTTTTTCACTCAGTGAGTGAGTCCAGGAGAAAGCAGAGAACGTGAGAGAGGGA
GTTGCTGAATAACGAAAGCAGCCGCATTCAGCAAAAGAGAGGACCACTCTTTCGATTTTTATCGCTCTCT
GAGCACACAAATAAAAGAGCTCCCCAGTTCGTTGTTTTTGTTTGTTCGGTGTGTTAAGAAGTTAAAACAT
TAAAAAGGCCTACATAACTCGAACACCAAAAATACATGTGTGAATGCAAGTGAAACTCATTAAAAGGGAA
GCTGGTGAAAAGAGAACCGAATTAACTGTTTGCACTAAAACCAGTGTTCGTGCTCTCCCAACTGTTTGTG
TGTATATCTAATATCTAATAAGAACAAGCAGCTGGGACCTCCGCAAACAAATTTGTCAAATTAAACAAGC
GTGAAAAACCAAAGTCCTAAGCAATTTTTAAACATATCGCTACTGAGCATTTAAAGTATAAAGTTCTTCC
AACTTTAAATAAGAATTACGGTAGGGAAAGAAGTCGTTTTTAATTATCGTAATATCGATAATGAAAACTG
AAACCGGCAGCAATAATAATAATACCACAGTCGTCAATATGGATTTCGATATGTATCCAAGTATTTCGGG
CAAGCAACAGGATCCCGTTCGCGAAGTCGTTATGAAATATATCGATTACTTTCTGCCAGAG
>dme_circ_0000320_junction_seq
GAAATATATCGATTACTTTCTGCCAGAGGTGAGTTTTATTGAATAAACGTTGTTAT
>dme_circ_0000321
GTAAGCGTGTTTTCATTTCAACCCAACGCAAAAGTCAGTAAGTTAAACGGGGTTAATTAGGCAGTTGAAC
ATAAAGCAATTAATAATAAAAATAGGTTGTTATTATAAtttttttttttCGTTAAAATAAAATCCTTATC
TTTCTCAAATACTTCCTTTCCCGTATCCAAAATCATCTCATATTGCCTAACGTTGAGTAATGTCCTATAT
TTAGAATATGACTAAACAATACCGAATAATGCCGGGCTTATCGCTCGACTATATAAATTATCAGCTCGGC
GATAAGAGTTTCCTTGATGAGAACGTTCAACTGACAGCTGTCCAGTCGCAATGACTTTCAAGCAACTGCA
ATTAAACGCCAATTAGACATGTGTTGTACGACTGTTTGCATTCATATATAATACACTTTAATATAGATAG
ATATAGATGAGTTTTGGTGAACGAAGGCAGCGCACAAAAAATATACCAAGCATAAATTTTGCTTTAGCTG
CAAACTATTTTTGCCGCGACGCTTTAACGTTTGCTCAACTCTAATGAGCCAATAAATTCACATTACGTAT
ACGCGCAGGTGGCCAGCTGCTGTAATTTGCTTAAATCAGAAAGCAAAACTGTATCGTTTGCATTTTTTCC
TTTGCATCGCTTTTCTCTGACTTTTCTTTCACTCAGACGCGGACTGTTGTTGGCTATAAATTTCACGCTA
ACTCGTCACTTGTCGCAACCTATTTCCTCTAATTTTCTTGGTCTCCCCTGCAATTTGCAGCATGGGTTAT
GAAATCGTATTCCTTTGCCTGTTCATAATTTGCCATGAAATCGGTGTTCAGTCAGATGATCATACAGAAC
ATTCGATAAGTCATTTGAACATGTGTGCTGCCCAGGCATTTTGTATTTGCATTTGACTTTTCGCATTTCC
ATATTTATTTGGTTAGAGTTGGTTAATCGAGTGGCTTATAATCAAAGTGCACACGCTTTTCCTGTCAGTT
GATAGCCATTGTTTGTTTTGTGTGACGTCAGTGATTAGTTCTTATGTTAATTCTGTTGAGGTAGAGTTCA
GTAATCGTGATTGGAAAGTATCTATTAGTATAGATAGATTAGTATAGAAAGTATCTATTAGATAAATTCA

TTGAATATCATTTTATTAAAGTCATTTTGCATTCTTTTTCAAGTTTAAAAACGGCGATCTTTGAATTGGC
GGTTACTTCAATACAAATTGAATGTTATCAGTTCTATTTCCTTTTCATTTGCAAAGCGTATTATTAATTG
ATTTTTTGCTTGTCTTTCTTATCAGACTCATTTACATATGGGCTTTATATTCTTGAAACCAACCGAGACT
TTAAATTTTAAGACACATTAAGACCCATTTATTGACTGGCTGATAATGTTTTGGCAAGATAAAGTTGTCT
CAAAGGCGGCGAATCGTGAGGAGAGCGAATAATTTACACTGAAAATATTGTTTTTAGAAGCGGCAATATT
GGTGATAAGTTATATCTTCAGTTAAGATTCGGTAATGCAGATTCAGCTGAGGACAAAACATGTTTTTAAA
CTGCATTTAGCCATATATATTCTTGTAGTAAGCAAGTGATTAGCACGTTGCATCAATTGAATTGAATTTT
TTGAAAGCAGCTTCCACGTGTTTTGTTTCCTAGCTCGCTTCCTCTAAGAAACTTCCATCCAGTTCCTCTG
CTTCCATTGGATTCCTCACTTTTTCTCTACACCCACCTATCACTCAATGCTAATTATCAGACGGCGATTA
GATAGAGTTATATTTGATAAGGGGCACACGACGGTTCTCCGATGTAATCTACTTTGTTTTTTTCGCCTGT
CGAGTATGGTGTTTTTTTCCCCCTTATCGTTCATCGGTGCTCCAAATaaaaaaaaaaaagtgaaataaaa
tataaaaaaaaataGCTTCGGCTCCGGTCGTGTGTGTAAATTTTGTTTCTCGCCTATTATTCGGCGCTTT
CGTCGCCGTCGTCTTCCCAAAACTAAAGAGCGAAGAAACGCAGAAAAAAATGTATTTTTGAAAATAATTT
TATTGCTAGCGTTGAGCGCAGCCACCACTGTCGACGTCGCTGCCTTTTCttttttttttttGTTCGTAT
TCCGCTGGTCGTTTTTGGATATTGCGTAAAATTCAGTTTTTGGTTCGAATTCAGTAAACAAAAACTTGTC
GCCGGCGTGAGACGCAAACGCGAGCAACAAAGGTTGTTCACCAACAAAAAGTTACAACCGAAGATCAACG
AAAGAGTAAAGACCGAATAGTATTTGTTTAAACAATTTGCTATAGTTTAAGTAGCAGCTCGGCTCTCCAT
CCGTCCCGCGTTTCTGTCTATCTATCTATCTGTCGCTATTTGTATCTTACGGATACGCGTCGAGTGTTGA
TGACGCGCGTTAATTGTTGCCGCTGTTCATTTTCGTTGTCGATTTCAAATGTCGATGGCTCGGTACCCTG
ACCTTGTTACTGACTGATTGTGAATTCTTTTGGTTATTTATTTACCGAACCGCGTTGGGATTTCTATATA
CTTACGATTGAGATACAAATCTATCTATCTATCTGCCTATCTATGTAGTTGGCGATCGCACGCACAATTT
GTCTAAATCGAATTCGTCTAAAAATAAGACGCGAGCGAAAACAAAATGAGGGAAAACGGCAAAAACAAAT
TGTGGAATAAATTTTTGGGTGGTTTCAAGAGTTTTGCCTTACATTCGGTTTTCTTGGTGGGAGATCGCTT
AATTTGTTTTCACATTCGAATGGAGCCAGAAATGGTCCGGAAAAAGTCCAAATATATTATACAAAAAGTG
GAAGAATTTCAAAAACTTAATTTGCAAGCGTAGAAGAGTGAGTGGCCAAGATAAGTTTAACTACAAATGA
AATTAAATTATCGTTTGATTATATATTCTAATGCATTTACATAACTTAAAAAAAATGTTAATTACATAAA
TATAACTTGCAACaaaaaaaaaaCTCTAGAATACACAGCAGAATCACGTTTTGCCATTTTGTACACAAAT
TCAACAAACTCGACTATCGCAGGCGCTCCTCTGCTAGGCAAACACTAAATTTGTCTGCAATTTCAACTTT
GTATCTTTCGAGTGCTCGTGTGCGGCTGCTGTGCGTATCTTAATAGATAAATTTAATTGGTTAATATTTA
TTTTTAGTCCAGACTTGATTAATTGCCACCAGAGAGGAGTATTCCTCCGTTCGGTTCGGGGAATTCTCTA
ACCGCCGTGTTCCCTCATATTAGTTTTTGCTATTTGGCACGCGCTACCAGTTCCAGACAGCGCTTAACAA
TTTATCAGAGAGAAATACTGAAAGTAACACAACAACAAAAATATACATATCAAGAAACGGCGACAACAAC
AATGTATGTAAAATATATAAATAAAATTATAAATACAATTGGCCAATGTATTGGGAGCCGTTGCGACGTG
TTTGTTGTCCAAGTTGTTGAAGTGCATGTTTCTCTCGGCTTTTTTCTCTCTGTATGGCTCTCACTCTCGG
TCGCGAGAAAAATAAAATTAATGCAAAATTCGAAAATTCAAAGCAATTTTTGTTTTTAGTTGTCGTTCGA
CCGGCTACACAATTCGTTTGCTGTTTCTCTACCCATTGAATAAATATCCAATATATTTGACCGTTTTTAT
ATAACAGCAGAAAATCAATAATAACCAAAAGGGTCAAATATACCAAACAAGTTGTCCAAGGCGCTAATTT
ATTAGCTCAAAAATAAATAAACGCTAAGTGAAAAAGTAAATATTTACTTAAGGCTAGACCATAAATTGTA
CTAAACAAAAATTAAGAAGTGAATTAAAAAAGTGTGTGAAAATGATCAAGTATCCGCGTACCACATCGTC
AACTTCATCGGGTTACTTTGATGATGATGAGGCCATGATGAATCCCTATCAAACGCCGCCAGCATCGCCG
CAATCCGTGCCCCAGTATCTGCAGCGTCACATGGTGCGAATGTTCAGCACTGAAGTTGATAAG
>dme_circ_0000321_junction_seq
GCGAATGTTCAGCACTGAAGTTGATAAGGTAAGCGTGTTTTCATTTCAACCCAACG
>dme_circ_0000322

CTTTATAACCGCATCGAATCCTGGCGGAAATCGAAAGAGGGCCTTTAGTAATCCGCACATAGGAGGGCCC
AATGATCCGTCGTTTATGCATCGATTGAATCCGCAGCTCTACTACTACAACAAATCGCAGTCGGGTAGAA
GTTCCTTTTGCGTGGACGAATCACTGTGGCCCACAAACAATCCGAATGGAGCGGTCAGCGATACGAATTT
GTACATGGGTTCCAGTACGGATGAGGACTATGAGGAGGCCATAGATCAGTTCTCGCCAACGATATACACC
ACTCGATCGGCAAGTCGTGCGATTCCCATACCAAATAGTGCCGGCAGCAGTCCACAGCATCAGATGCATC
ATTCGCAGCCAAGGATTGCGATCGGAATTAATCAAATGGAAGGAGGAGGTCCAACACGGAGCACGGGTGC
ATTCAGTGCCAGTCCCTCGATTTATGCCTATCCGCATTCGCCATTCTATGCCAGTTCGCCGGAAACGTCG
TTTGGCAGTGCCGCAATGCCAGGACATCCGGCGGCCAGTTCGCGTATATCCTTCAGCTATGATCCTGCCT
TTCAGCGATTGCAAGTGCCGAACGCATCCGGTGATCGTAGACCACGATCACCGCTGGAATGTGCCAGCGT
TTTTGCCGCCGTTGCGGCGGCTGCAACATGCGGTGATGCAGCCGGTGGTGCAGCGGATACCGTTATCAGC
AGG
>dme_circ_0000322_junction_seq
TGGTGCAGCGGATACCGTTATCAGCAGGCTTTATAACCGCATCGAATCCTGGCGGA
>dme_circ_0000323
TTCTTATCTCCCAATTTGCTTGCCcagcagcaacagcagcagcagcagcTCTTCGATAGCGTCAATGCCA
ATGCTGCCAGTTCACCAAATCCCGCCGGTGACCCCAACAACATGGACTATGCGCGCACGGCGGCGATGCA
ATTGCATCAAACGTTGCAGCAGCTCAAGCAGCGCGAGGACGCTATGGACGTGCCGCCAGGGGCAGGAGGT
TATGCCAACTATCAAAACGGTGGTGATTCAGCTGTTGGAGCGGCTAGCAACAACAACAGCGCTGCAGCTG
CAACTGGAGAATCgcagctgagcaccagctacgttgagcaacagcagcagcagcagcagcCATTATCCCC
AGCACCACTAACGCCCCAGGCTGCACCCACATTCGCAGCTGTTGCTGCCGGACAGTCGCCCAACTTCCAA
TTGGagcaacagcagcagcagcagcaagcaaCATCGCAGATAGATGGAATAGTCCCACAGCCATTTAACC
CACAAcagcagcagcagcagaccccacagcaatcaacagctcagcaagcagcagcagcagcaaatgcaac
atctgcagTGACTGCACCACCACCACAACAGACTTCAAACACTTCCAATGCAGCGGTAACCACTGGCCAG
GGTCAGACAATGCCGCTGTTATCACACATGACCAGCTACGagcagcagcagcctaatctaggagcagcag
cagcagcagcagccgcaggaggcactgcagcaacatcggtggctgcaccgcaagcgattcccacgctgca
attgcagagtgctccctcaacgattgccgatccgcagcaattgatggtgccacagcaacagcagcagcag
cagcatcaggaggagcagcaacagcagccgcaacaacaacaacaaccgctTCCGCCAGCAAATATCGCGA
GTGCTAGTGCCAATAATAGTAATCTAAACCTAACGAATACGAATGTTGTGGCCACCGGCGAGGCAACAAC
AAACGCGCTGACCTTGACCGATGAACAGGCGACCGCGGCACTTGCAGCTGCATTTGCGACCGGCGCAGCA
GCAGCAGCAACTGGAGCCACATCGGCAGCGGCAGCAACGCAGCAGCAAATCCAACAGCTACAACAGCAAC
CAAATGCAGAGTCCGAGACTGAAAGG
>dme_circ_0000323_junction_seq
ACCAAATGCAGAGTCCGAGACTGAAAGGTTCTTATCTCCCAATTTGCTTGCCcagc
>dme_circ_0000324
GATTTCATTTCTACAATTTACTGCAATGTGCTAGTAAAACTGAACCGATGAAATTGATTAATTCTACGAT
AAAGATATAGCTGAATTAAACCAACAAATTGAAAACCTATGCGTAACCGTGTGACGACAAAGTGGAGAAG
TGCAACAAATTTATGAATATAGAAGTAGATGAATAGAAATTCCAAGTTAACCAAGATCACCAAAATATTT
TCATAATTTCTAAGAAATATTTTAAGTGTGATACCAAGTTTTGAAACCACGTTTAAGCAACAAGCCTACT
TTCGAAAACGAATCATCCAACTGTAGAAACTGAATCAAATCGAATTGATTCGATTAACAGCTGTGCTGTA
CACATGCACCGGTGTGTGTGAGAGGCTGCGCGCGTGCGATTAACAGCTGTGCTGTACACATGCACCGGTG
TGTGTGAGAGGCTGCGCGCGTGTGCGTATACGTGATAGATCCgtgttcgtgtgtgtgtgtgagtgctg
cttatgtgttagtgtgtgtGCGTCGGTGTTGTTGGCGAAAATTGAAACGCCCGCTAAACAAAGTCAAGCG
CTAGATGGCGCTCTCGCCTGTGACCAGTGCtgttgttgttgttgtcggtgtgccacacactgttgtagtt
gttgttgATGCCGCTGCAGCATCAACAAACCAAACGCGGAAAGTCAAATTATAACCGCCCACCAGCCGCC

CGCAACACaaaaaaaaGAGACCACCTAGCCCCCAATAAATTGTAATTTAACAGCCTAACTAAATCAACTC
ACCCAACACAAATAACATACACAAATTAATAAAGGAAAAATAGTAGTAATAGTCAAGAGTTTCGCATTAC
CCATCGTCGGTCGGTCGGTTCAAATTGCGTCGCGTCGGGTCGCGAACGTCGCTAGTCCtcggatcggatc
gtatcgtatcgtatcgtatcggatcggatcggatcggaACAGAACGGAACCCTATAGAAGGGACGGTTAC
GGATTAGATTGGATCAGTTGGCAGTCAGAGGCACCTGACAACGCAACGGTAACCGCTATTATGCATCGGC
GTCGTCGGTTGGCCAGTGCAACAATCGCATTCGAATTGGCAGGCGTGCAGCAGCTAATGCAACAACTGAC
AGAACTTAATTAAACAGTCTAATAACTATCATCATAGTCCGTAGTGGATAACTAAGAAATCGAAACTAAC
CGCAAGTTGGTATAGTTTATAACTTTTTCCGTGGCCCCAAATAATCCAAGAATCGTTAGCAATCCTGGAT
TATTTGCTAAAGACTTTGCGCACGCTGAAATCCAATTCGATTATGGCCGAGAATCAAAGTGCGGCCAGCA
AAGATTCCGGtcaccaacagcaacatcagcagcaacagcaacagcaacaccagcaacatcagcaaccact
agccaccacatcagtcacggccgcttcaaccacatcggtcttagctaaCCAATCGCCCACTAACTCGCAG
GCCTCATCCCCTGAAAATTCCCAGGAAGCACTGCCCTTGCTCCGACGCcagcaatcagccgcagcagcca
cagtagcagcagcagcagcaacagttgcagccacaaccagcggaacatcgcagcagcagcagcaacagca
tcgcaacagcatcagcaacatGTTCGATCGCACGGTGAATGCCAAGTTCAAGCCGGCTTCCTCAAATGCC
GGACCTGGCAACAATCCGGTCCGTCGGAACTCGATGCTGACACCATCCCGTGGAGTCACTATCGGTGGCA
CTGGCGGTAACATACGCAAGCTGACCAAGGTCAGCTCACTGACCAGCAACCATCACTTTGCTGTGTGCTA
TCCACCCAGCAACATCTATCAGAATAGCAACAATGCCGGCAGCAATTCGGCTTTGCAGCGCACCACCAGC
GAAAGTTTGCGTTTGAACATGATGAGTCGCGTGGCAGCTGGAGCGACACCCACAACGGTGTCCAGGGCCA
GTAGTAACTCCAGCCTGGCCACATCCACCTCCACATCGCTGGCACCCAAGTCcagcagcagcagtggtgg
cagcaattcgacaccgcagcagcaacagcagcagctagtgagcagtaacaacagcagtagcagcagcaac
aacagcTTCACCAAGGCCAGTTCGCCCAACAATAATGGAGCACGTTCGGTGGGCGGAGCAGCTACGTCAG
CTGCTACTGGCACAACCGCTGCAGCAGGCAGtcatcatcatcagccacatcaccaccatcatcatcatca
tcaccaccatcagcatcacaaccaccagcagcagcagcagcaacagACTAGCCTTAGCCAAGGACACGCT
TCGTTAACCGTTGCCGGTGGATCAGCTTCAGCTGGTGGTGGTGGTGGCGGCGGCAGTGGAAGCTCCTCGG
GGACCGCCGCTGGTGGCACCAATCGCAAACCAAAGACGACTTCCTCATTCGAGATCACCTCGGTGACGGT
GGGCCATCCCAAGTTGAATGCTGCCGGCGATACTGGAGACGAGTCGGCCGATGATCTGGACGAATCTCAC
ACGGATGACAATAGTCGGATAACGGACCTGGAGAACGAGACGCCCTCCATGTCGGAAGATACGTTCTCCA
AGGAGGAGGTGTACTATGCCAACAATGCCCTCAGCACCAATGCACCGGTGATACCGACTAGCTCGCAATA
CGGCCTTGTCGTGGTGGATCCCATCGCCCCTTCGTTGGGCCAGACGATCCAGAATGTCCAGGTCAATGTA
TCGGATAACATTATCAATGTGGTGAGCGGTGCCGTCACACCGGGCGGCACCAAGAAGAAGGACGACATCA
AGGAGACGCAGCACCGCAGTGAGCGGTTCAAGGTGGTCAAAATTGAGTCCACGGAGCCGTTTAAACGAGG
CCGTTGGATGTGCATGGATTACCTGGATCACTCGAGTGTGGGTAATGGCGGTAATAACAACGAAAAGACT
GGCTCTTCTACTTCCGAGGCGCACGCCGCAACAACGGATGGCGGCGCAGCAGGTGTTGGCGCTGGATCTG
AGGCTCCGGCCCACAAGACCACCCAAAGTATGATTCTGCCACCCACCCAGAAGCTGAACGAGAATCATTT
GGAGGCCAACTCGACCGATGCCAACTGGAACTATGCCGagcagcagcagcaacagcagcagcagcagcaa
caacaacaAACAATAGTTGGCAACGCCCTGACCAAGACATTGCCCGTGGCTCTTCGCAATGGGTAAGTAA
ATCCCCTGTTTACTTAACACTTCCATACGGTTAATTGTTTTCAGTAGCAGTTGTTTACTTAAATTCAAAT
AAATATTGAAATCGTAGAAATGTTCTGCGAACGAACTCTTTATTAGTCTTAAGAAACTTGACAACAGACA
CCGCTAACAATATATTTGTGTCTGTCCTTCGCTCGTTTCTTTTCATGCCACCACCATCATGCAACAGTTT
CTTATCTCCCAATTTGCTTGCCcagcagcaacagcagcagcagcagcTCTTCGATAGCGTCAATGCCAAT
GCTGCCAGTTCACCAAATCCCGCCGGTGACCCCAACAACATGGACTATGCGCGCACGGCGGCGATGCAAT
TGCATCAAACGTTGCAGCAGCTCAAGCAGCGCGAGGACGCTATGGACGTGCCGCCAGGGGCAGGAGGTTA
TGCCAACTATCAAAACGGTGGTGATTCAGCTGTTGGAGCGGCTAGCAACAACAACAGCGCTGCAGCTGCA
ACTGGAGAATCgcagctgagcaccagctacgttgagcaacagcagcagcagcagcagcCATTATCCCCAG

CACCACTAACGCCCCAGGCTGCACCCACATTCGCAGCTGTTGCTGCCGGACAGTCGCCCAACTTCCAATT
GGagcaacagcagcagcagcagcaagcaaCATCGCAGATAGATGGAATAGTCCCACAGCCATTTAACCCA
CAAcagcagcagcagcagaccccacagcaatcaacagctcagcaagcagcagcagcagcaaatgcaacat
ctgcagTGACTGCACCACCACCACAACAGACTTCAAACACTTCCAATGCAGCGGTAACCACTGGCCAGGG
TCAGACAATGCCGCTGTTATCACACATGACCAGCTACGagcagcagcagcctaatctaggagcagcagca
gcagcagcagccgcaggaggcactgcagcaacatcggtggctgcaccgcaagcgattcccacgctgcaat
tgcagagtgctccctcaacgattgccgatccgcagcaattgatggtgccacagcaacagcagcagcagca
gcatcaggaggagcagcaacagcagccgcaacaacaacaacaaccgctTCCGCCAGCAAATATCGCGAGT
GCTAGTGCCAATAATAGTAATCTAAACCTAACGAATACGAATGTTGTGGCCACCGGCGAGGCAACAACAA
ACGCGCTGACCTTGACCGATGAACAGGCGACCGCGGCACTTGCAGCTGCATTTGCGACCGGCGCAGCAGC
AGCAGCAACTGGAGCCACATCGGCAGCGGCAGCAACGCAGCAGCAAATCCAACAGCTACAACAGCAACCA
AATGCAGAGTCCGAGACTGAAAGG
>dme_circ_0000324_junction_seq
ACCAAATGCAGAGTCCGAGACTGAAAGGGATTTCATTTCTACAATTTACTGCAATG
>dme_circ_0000325
GACCAGCCTTCGTCCACTTGACCAACTTGCGGCTATCGGCTCTAATCAAGGTTCCTGAGGTGTTTTTCGA
GAACCGTTTTCAACTGCTATCTGCTCCGGAGAGTGATTACCTATGTGTTCTTTGCTGTTGCTGCTGTTGG
CACAGTTAGCCGTAAACACTGATTATGCCATTGTTGCTAGCAAACTACTATcagcagcaacttcagcaac
ggcagcagcgtgttgctagcaacaacggcatgttacttgaatacaaaagccattgcaatacaaatcgggg
cagcaacaattgcagcagcaacaacagcagcagcaggaggaacagcaacagcaacaacaaccagcgacag
ctgttcgcagcggaagcactaacaATGTCAACGCCGACAACAACATCGAAATACAAAATCAATGGCGAGG
CCACCGAAGGCGTTTcagcatccacactgctgtatcatcatccgcatcacagccatcgtcatcatcacca
tcagcagtcgcaacagcagcagctacatcagcagcagcagcagcagcaacatcatcatcagcagcagcac
cagttgctgctgcagcaacagctccttcagcaacatgtggccaacagTTCACCACTGCAACATCTGAGCA
ATCTGTTTGGCCAACATTTGCCTACGCACAGCCTGCAGCACTTGATTGCCGCCGAGTACTGgcagcagca
gcagcaacagccagcagcaACAATTAAAAGCGAGCCAACAACTCAATCAGTGGCAGCAACATCGGTCTTT
AGCTTCACGCCGCAGCAACACGTCAATTTTGCCGCCTCATTAGTCGCATTACAGCAAAATGATGCCTTGC
CCGAGGAAACAATGGCAACGGCAACAGCAACACCGACACCGGCGAcaactgcatcaggagcggcaactgc
agcagcagtcgcaacAAGCCCTGCAGCAGCGACAACATTGAAGGCGCCAACAGCAACACAGGCGGCAGCA
ACAATGGACGACATCGATGATGATGATGACGACAGCAACAACGGCGGCCACATGCAAAATGCAGGCG
>dme_circ_0000325_junction_seq
AACGGCGGCCACATGCAAAATGCAGGCGGACCAGCCTTCGTCCACTTGACCAACTT
>dme_circ_0000326
GTTTATATCTTCTTGTGCTCCGGCATCGGATCTCTGACTGTGATGTCCTGCAAGGCATTGGGCCTAGCCA
TACGGCAGACGCTGAATAACGGAGGCAACGTCTTCCTCACATGGATGCCATGGTTCCTGATCCTGGTCAC
CGTCACTTTCATAGCCATCCAGATGAACTATCTGAACAAGGCGCTGGATATATTCAATACTAGTATAGTT
ACTCCAGTGTACTAT
>dme_circ_0000326_junction_seq
TACTAGTATAGTTACTCCAGTGTACTATGTTTATATCTTCTTGTGCTCCGGCATCG
>dme_circ_0000327
CAACCTTGTCTTCAGGTCAGCCGCTGCAAGGGATGTAACTAGACCTGGTAGCGCTGCTTGTGTCAGGCCT
TCCGGAAGATGTCGCTGGATCCGTAGGACAGCCGCCGTTCCTTGGTATTGCTGGTGACCAGCACCTCCTC
GTCGAACTGCGACACCCGCTGCATTTTCGG
>dme_circ_0000327_junction_seq

CGAACTGCGACACCCGCTGCATTTTCGGCAACCTTGTCTTCAGGTCAGCCGCTGCA
>dme_circ_0000328
GAGGGAAAGACAGTGCGATGGTCTTTCCATTCGGTTTCGCTGATTGCCTATAAAAAGCAAGGTCGGAGTT
AGTTAGTATGAAATAGGCTGCATGACTATGTTACCAACTTACCACTAGCTCGTCGAAAGCCTGTTTGGCC
AGCCAGTGGAAGCATGGACTCTTCCATTCGCGCGCAATCGCCTGCTGATCCTTGACGTCGTCCTCGGCGG
TGGACTTGGAATTG
>dme_circ_0000328_junction_seq
GTCGTCCTCGGCGGTGGACTTGGAATTGGAGGGAAAGACAGTGCGATGGTCTTTCC
>dme_circ_0000329
GCTTTATTGAGGAATCTGATGCCAAGGTGGACAAGAACCAGGTTTGCAAGTACCCGTCCGTGATTGCCAA
CCGTTGGCTGGCCATTCGTTTGGAGATGGTGGGCAATCTGATTATTCTGTTCGCATCGCTTTTCGCCGTC
CTGGGAGGTCAAACCAATCCCGGCCTGGTGGGTCTGTCGGTAAGCTACGCCCTGCAGGTGACCCAAACCC
TCAACTGGCTG
>dme_circ_0000329_junction_seq
GCAGGTGACCCAAACCCTCAACTGGCTGGCTTTATTGAGGAATCTGATGCCAAGGT
>dme_circ_0000330
GCTTTAAGCCATTTGGCACTGACAGCTCTACCTCATGGCTAGAACAAGCAACTATGAGACAAAGCAAGCA
AACAAACACAACAATGGAACAATAAGCGAAAGAAATTAGCAAATACCAACTAAATGTTCATTAGACTAAG
CGAAATAATTCAAGCCAGTTGCGCCTTCGTCGGTTACGCTATGTTCGTTTTTATTATGTATATGTATGTA
TTTACATAGTCAAGTGTATTTTTCTATTGTCTATT
>dme_circ_0000330_junction_seq
AGTCAAGTGTATTTTTCTATTGTCTATTGCTTTAAGCCATTTGGCACTGACAGCTC
>dme_circ_0000331
GCAAGCTGCGCAGAAATTCGGCGGGGCATGTGTGGTGGTGCATTGCCACTTGCCGACGGGACGGCAGTTG
CCGCGGTCTGCGCTGGTGGCAAATGCAGAAGGAAAACCGAGACTGTACTGGCATTTGTTGCTGACCACAA
AGTTGGCGACGTTATGTTGACTGAAAATGTAAAAATTATACAACAAATTAGATGTACATATACAGAACAC
CAAAAGAATAGACGCTACAGTCGAGTTACCCATTACAGAAATAGTAGGAGGATAGGTGAAATTTTAAAAA
CAAAATTTGCCATATAGATCGAAATCGCGAAAACAAAAAGACTATGATTTAATTAGTTTAAGAACTGTTT
TCCAACTAGTAAATGAAACGAAATCAATATCAATAAAATATATTGTCAAACCAGAGTAAATGCAAAAGCA
CTTTTAACACTTATAGCATATCGATAGACTAGCAAGGAATATTGTAATGACTGAATTTCTTTCTAAGGAC
GGACATAGTCAAATGTAGTCGGCTGTTTAAACTGATCATAAATACATATACCTTTAAAACTTTGATCAAT
ATATCCAATGCATAAAAATTTGTTGCACAAAAATATAATTAAAGCTCTGGGCGTCCCAAAATGAAAAGCA
CTGTATGTTTTTAGCTTTTGGGTTCAATTCTCGCGAGGCCGTGGTTCAGAGACTATTTCCGTTACCtttt
tttttttttATCGTTACATCAAAACTAATAACAGTACAAAATGTTGATTACCATTAGTAACATAAGTTTT
GGCAAATAGTAAACATCGCACCATGGATCCTAGCCCGTCATCTGCACAACGAGCCTCTATTTGCCTCTCG
ACGGGTCTTGCTTCCAGCTGTAGGATTGTGAATTTAATATAAAAGACTAGTTGAGTATATATTATCTTAC
TAACCGTTGTTGGTACTTGATTGCTCTGGTTGGTCCTGGTCGATCGACCCTTGCGAAGCTTTCTAAATGA
CTCCCTCAATGATTTCTTAAAAGACTTTCGACGAGACAGCTGCTCTCCTGCTCCAGTAAGATCATTTGGG
TTTAAAGTGCAGCGATGAAATACTGGAACAAAGTTTTTGAAGTCAAAGAGAACTAAGCCGTGCGCAGTCC
CACCAGATACTAGGCCCCAGCTTGCTTCGAGTGCCATGCATGTTATGCTGGCTGGCGGCAAAACTTGCAG
TACTCCCGATATATTTACACCACGTTCCGTCGTAGGAATTGCTTCTCCGTCTAATAA
>dme_circ_0000331_junction_seq
TCGTAGGAATTGCTTCTCCGTCTAATAAGCAAGCTGCGCAGAAATTCGGCGGGGCA
>dme_circ_0000332

GTGTACTCCATAGCGGACAGCCAGCCGATGACTGAGGATGGCTACATGCCCCCCAGCCAGCACTTTCCGC
CCACCCACTCCGACTTGGATCCCCCCGCCCAGCAGCAGAGCACCTGCCAAACGGTTTGCGCCTGCAAGTG
GAAGGGTGGCAAGCAGACGGTGGAGTGCATCGATCGCCACCTCATCCAGATACCCGAGCACATCGATCCC
AATACCCAGGTGCTGGACATGTCCGGTAATAAGCTGCAGACCCTCTCCAACGAGCAGTTCATCCGTGCGA
ATCTGCTAAATCTGCAGAAGCTGTATTTGAGGAACTGCAAGATCGGCGAAATCGAGCGGGAGACCTTCAA
GGGACTGACCAATCTGGTGGAGTTGGATCTGTCACATAATCTGCTGGTTACCGTGCCCAGTTTGGCCCTG
GGCCACATACCCTCACTGCGCGAACTCACCCTGGCCTCCAATCACATACACAAAATCGAGAGCCAGGCCT
TCGGGAACACACCATCGCTGCACAAATTGGATCTGTCGCATTGCGATATTCAGACCATTTCCGCCCAGGC
ATTTGGTGGCCTCCAAGGATTGACGTTGCTCCGATTGAATGGCAATAAACTGAGCGAGCTTTTGCCCAAG
ACAATTGAGACCCTGAGTCGTCTTCATGGCATCGAACTGCACGACAATCCCTGGCTCTGTGATTGTCGAT
TGAGGGACACGAAGCTCTGGCTGATGAAGAGGAACATACCCTATCCGGTGGCACCGGTTTGCTCGGGTGG
CCCCGAAAGGATTATCGATCGCAGCTTTGCGGATCTGCATGTGGATGAGTTTGCCTGCCGACCGGAGATG
TTGCCCATATCGCATTATGTGGAGGCGGCCATGGGCGAGAATGCCTCGATTACATGTCGAGCTCGAGCGG
TTCCAGCTGCGAATATCAACTGGTACTGGAACGGACGGCTGCTGGCCAACAATTCCGCCTTCACCGCGTA
CCAGAGGATACACATGTTGGAGCAGGTGGAAGGTGGATTCGAAAAGCGATCCAAACTGGTGCTGACCAAC
GCACAGGAAACGGATTCCAGTGAGTTCTACTGCGTGGCCGAGAATCGAGCTGGGATGGCCGAGGCCAACT
TCACCCTGCACGTGAGCATGAGAGCTGCGGGCATGGCCTCCCTGGGTAGTGGCCAAATTGTGGGTCTGAG
TGCCGCCCTGGTTGCTCTGATTGTGTTTGCCCTTGGGGTTATCATGTGCCTGCTCCTGAGGGTAAAACGG
CAGCCGTATGTCGATAGCAAGACGCCCAATCACATGGAGG
>dme_circ_0000332_junction_seq
GATAGCAAGACGCCCAATCACATGGAGGGTGTACTCCATAGCGGACAGCCAGCCGA
>dme_circ_0000333
TCTTGCCGCAACCGAAAGCTCCGGGAATGGCGGTGGTACCGCCCTGGACACAGGGGAAGAGCGAGTCGAG
CACACGCTGTCCGGTGAGCAGGGGGTGGTTGGCGGGCAGCTTCTCGGTCACGGGACGTGGCTGACGCACT
GGCCACACCTGCAACATGGTGTGCTTGGTGATCTCTCCATCGAACTCGGTCTCCAGGACGACATCGTCGA
CCTTGTAGTTGCCGGAGGGGGCGATG
>dme_circ_0000333_junction_seq
GACCTTGTAGTTGCCGGAGGGGGCGATGTCTTGCCGCAACCGAAAGCTCCGGGAAT
>dme_circ_0000334
GTTCGATGGAGAGATCACCAAGCACACCATGTTGCAGGTGTGGCCAGTGCGTCAGCCACGTCCCGTGACC
GAGAAGCTGCCCGCCAACCACCCCCTGCTCACCGGACAGCGTGTGCTCGACTCGCTCTTCCCCTGT
>dme_circ_0000334_junction_seq
GCGTGTGCTCGACTCGCTCTTCCCCTGTGTTCGATGGAGAGATCACCAAGCACACC
>dme_circ_0000335
GTTCGATGGAGAGATCACCAAGCACACCATGTTGCAGGTGTGGCCAGTGCGTCAGCCACGTCCCGTGACC
GAGAAGCTGCCCGCCAACCACCCCCTGCTCACCGGACAGCGTGTGCTCGACTCGCTCTTCCCCTGTGTCC
AGGGCGGTACCACCGCCATTCCCGGAGCTTTCGGTTGCGGCAAGACTGTGATCTCGCAGGGCTCTGTCCA
AGTACTCCAACTCCGATGTCATCATCTACGTCGGTTGCGGTGAGCGTGGTAACGAGATGTCTGAGGTACT
GCGTGACTTCCCCGAGCTGTCCGTGGAGATCGACGGTGTCACCGA
>dme_circ_0000335_junction_seq
TGTCCGTGGAGATCGACGGTGTCACCGAGTTCGATGGAGAGATCACCAAGCACACC
>dme_circ_0000336
AAATTTCACGAAGAGCCTCAGCCCAACGGGAGGTGGAATCAGCCATCATGGACACGTTGTAACCCATATC
ACGGAAGTATTCGGACAAGGTGATACCAGTGTAGATGGAGGCCTCACGAGCAGCCACAGGCATGTTGGAG

GTGTTGGCCACAAGGGCGGTACGCTTCATGATGGACTCGGTGACACCGTCGATCTCCACGGACAGCTCGG
GGAAGTCACGCAG
>dme_circ_0000336_junction_seq
CCACGGACAGCTCGGGGAAGTCACGCAGAAATTTCACGAAGAGCCTCAGCCCAACG
>dme_circ_0000337
TAGGAGATGAGCCAGTTGATCGAGGGGAAGTGCTTGCGCTGGGCCAACTTCTTGTCGAGACCCCAGAACA
CCTGCACGATACCCAGAGTGGCGGAGGTCACGGGATCGGAGAAGTCACCACCAGGAGGAGACACAGCTCC
GACAATGGACACGGATCCCTCGCGCTCGGGGTTACCCAAGCACTTAACGCGACCGGCACGCTCGTAGAAG
GAGGCCAGACGGGCTCCCAAGTAGGCTGGGTAGCCGGAATCGGCAGGCATCTCAGCGAGACGACCAGAAA
TTTCACGAAGAGCCTCAGCCCAACGGGAGG
>dme_circ_0000337_junction_seq
TCACGAAGAGCCTCAGCCCAACGGGAGGTAGGAGATGAGCCAGTTGATCGAGGGGA
>dme_circ_0000338
CTGGTACATAATGTTGCCCATTGCCTCACGAATCACGTTCCAGGTGATCTTGTTCTCAGACTGAGCCGTA
GACTCCACGGAGTGACGGGCCATGTCGTAGAAGTCGATGATGTTCCTCAACATGCCCACGGTCTTGTAGA
AGGGGCAGAAGCGATCGTACGAGGAGTAGGAGTTCTGCTGCAGGAAATCGTCCTTCAGCAGCTTGGCCAC
CTCCAGCGTGATCTTGTCGGTTTCGGCCAGAGAGGCCTTGCCGACCAGTTGCACGATCTCAGACAGATCC
TCCTCCTCCTGCAGGATCTCCTTGACCTTGGTACGCAGCGGCACGAATTCGGGGAAGTTCTTGTCATAGA
AGTCATCCAGAGCACGCATGTACTTCGAGTAGGAGATGAGCCAGTTGATCGAGGGGAAGTGCTTGCGCTG
GGCCAACTTCTTGTCGAGACCCCAGAACACCTGCACGATACCCAGAGTGGCGGAGGTCACGGGATCGGAG
AAGTCACCACCAGGAGGAGACACAGCTCCGACAATGGACACGGATCCCTCGCGCTCGGGGTTACCCAAGC
ACTTAACGCGACCGGCACGCTCGTAGAAGGAGGCCAGACGGGCTCCCAAGTAGGCTGGGTAGCCGGAATC
GGCAGGCATCTCAGCGAGACGACCAGAAATTTCACGAAGAGCCTCAGCCCAACGGGAGG
>dme_circ_0000338_junction_seq
TCACGAAGAGCCTCAGCCCAACGGGAGGCTGGTACATAATGTTGCCCATTGCCTCA
>dme_circ_0000339
TTGATCGAGGGGAAGTGCTTGCGCTGGGCCAACTTCTTGTCGAGACCCCAGAACACCTGCACGATACCCA
GAGTGGCGGAGGTCACGGGATCGGAGAAGTCACCACCAGGAGGAGACACAGCTCCGACAATGGACACGGA
TCCCTCGCGCTCGGGGTTACCCAAGCACTTAACGCGACCGGCACGCTCG
>dme_circ_0000339_junction_seq
CAAGCACTTAACGCGACCGGCACGCTCGTTGATCGAGGGGAAGTGCTTGCGCTGGG
>dme_circ_0000340
TTGCACGATCTCAGACAGATCCTCCTCCTCCTGCAGGATCTCCTTGACCTTGGTACGCAGCGGCACGAAT
TCGGGGAAGTTCTTGTCATAGAAGTCATCCAGAGCACGCATGTACTTCGAGTAGGAGATGAGCCAG
>dme_circ_0000340_junction_seq
CATGTACTTCGAGTAGGAGATGAGCCAGTTGCACGATCTCAGACAGATCCTCCTCC
>dme_circ_0000341
GAAATCGTCCTTCAGCAGCTTGGCCACCTCCAGCGTGATCTTGTCGGTTTCGGCCAGAGAGGCCTTGCCG
ACCAGTTGCACGATCTCAGACAGATCCTCCTCCTCCTGCAGGATCTCCTTGACCTTGGTACGCAGCGGCA
CGAATTCGGGGAAGTTCTTGTCATAGAAGTCATCCAGAGCACGCATGTACTTCGAGTAGGAGATGAGCCA
G
>dme_circ_0000341_junction_seq
CATGTACTTCGAGTAGGAGATGAGCCAGGAAATCGTCCTTCAGCAGCTTGGCCACC
>dme_circ_0000342

TAGGAGTTCTGCTGCAGGAAATCGTCCTTCAGCAGCTTGGCCACCTCCAGCGTGATCTTGTCGGTTTCGG
CCAGAGAGGCCTTGCCGACCAGTTGCACGATCTCAGACAGATCCTCCTCCTCCTGCAGGATCTCCTTGAC
CTTGGTACGCAGCGGCACGAATTCGGGGAAG
>dme_circ_0000342_junction_seq
GGTACGCAGCGGCACGAATTCGGGGAAGTAGGAGTTCTGCTGCAGGAAATCGTCCT
>dme_circ_0000343
AAGTCGATGATGTTCCTCAACATGCCCACGGTCTTGTAGAAGGGGCAGAAGCGATCGTACGAGGAGTAGG
AGTTCTGCTGCAGGAAATCGTCCTTCAGCAGCTTGGCCACCTCCAGCGTGATCTTGTCGGTTTCGGCCAG
AGAGGCCTTGCCGACCAGTTGCACGATCTCAGACAGATCCTCCTCCTCCTGCAGGATCTCCTTGACCTTG
GTACGCAGCGGCACGAATTCGGGGAAG
>dme_circ_0000343_junction_seq
GGTACGCAGCGGCACGAATTCGGGGAAGAAGTCGATGATGTTCCTCAACATGCCCA
>dme_circ_0000344
GGGCAGAAGCGATCGTACGAGGAGTAGGAGTTCTGCTGCAGGAAATCGTCCTTCAGCAGCTTGGCCACCT
CCAGCGTGATCTTGTCGGTTTCGGCCAGAGAGGCCTTGCCGACCAGTTGCACGATCTCAGACAGATCCTC
CTCCTCCTGCAGGATCTCCTTGACCTTGG
>dme_circ_0000344_junction_seq
TCCTCCTGCAGGATCTCCTTGACCTTGGGGGCAGAAGCGATCGTACGAGGAGTAGG
>dme_circ_0000345
TGTAAAAGTAGGGCCAGCGCGGTCTCTAGTCCTCCAGATTTCTGAAGGCCTGCTGCAGGTCCTCGTGCAG
CTGCTCGAAGTCAGCCTTGATCTTGGCCTCACCATCCTTAACGGGGTCCTGAAATGGATTGAAAAATGTG
GTCACCCAGTGTCTAGGCAATGGATAAATTTGCGTGTTAACCCACCTTGAACTTCATGGATGACAGCTGG
TACATAATGTTGCCCATTGCCTCACGAATCACGTTCCAGGTGATCTTGTTCTCAGACTGAGCCGTAGACT
CCACGGAGTGACGGGCCATGTCGTAGAAG
>dme_circ_0000345_junction_seq
CACGGAGTGACGGGCCATGTCGTAGAAGTGTAAAAGTAGGGCCAGCGCGGTCTCTA
>dme_circ_0000346
CGCGGTCTCTAGTCCTCCAGATTTCTGAAGGCCTGCTGCAGGTCCTCGTGCAGCTGCTCGAAGTCAGCCT
TGATCTTGGCCTCACCATCCTTAACGGGGTCCTGAAATGGATTGAAAAATGTGGTCACCCAGTGTCTAGG
CAATGGATAAATTTGCGTGTTAACCCACCTTGAACTTCATGGATGACAGCTGGTACATAATGTTGCCCAT
TGCCTCACGAATCACGTTCCAGGTGATCTTGTTCTCAGACTGAGCCGTAGACTCCACGGAGTGACGGGCC
ATG
>dme_circ_0000346_junction_seq
CGTAGACTCCACGGAGTGACGGGCCATGCGCGGTCTCTAGTCCTCCAGATTTCTGA
>dme_circ_0000347
GGATGGTGAGGCCAAGATCAAGGCTGACTTCGAGCAGCTGCACGAGGACCTGCAGCAGGCCTTCAGAAAT
CTGGAGGACTAGAGACCGCGCTGGCCCTACTTTTACACTCTAATCTTATATTTGTTATATAGTTAAC
>dme_circ_0000347_junction_seq
CTAATCTTATATTTGTTATATAGTTAACGGATGGTGAGGCCAAGATCAAGGCTGAC
>dme_circ_0000348
GGATGGTGAGGCCAAGATCAAGGCTGACTTCGAGCAGCTGCACGAGGACCTGCAGCAGGCCTTCAGAAAT
CTGGAGGACTAGAGACCGCGCTGGCCCTACTTTTACACTCTAATCTTATATTTGTTATATAGTTAACGTT
TAAAAATGAAAGCAGTCAAAAACCATCCGAAAAAGCCTAATCAAACACCAACAATTCCGTGCTGCATTCG
ATGAAAAACAAAAGTCCAACAAATACCACAACTTCTTGGTGCCTGCGAGAGATGTAAACATTCCGGCCTG

CGGTTAATACTTTCCCCTAACCACGCCCCCTCCGCCCCTTGAAGGGCAACTCTAGGCAACAGCAACTACA
ACGTCCTGCTATGTACTTCCATTTACAACAACAACACCAACATACACTTGAATAAAAGTACACGGACACT
GGCGCACACACAACACATACATAAAAGACACAAATACAAATGCATGCATAAATAGTATTATT
>dme_circ_0000348_junction_seq
TACAAATGCATGCATAAATAGTATTATTGGATGGTGAGGCCAAGATCAAGGCTGAC
>dme_circ_0000349
GTTAACGTTTAAAAATGAAAGCAGTCAAAAACCATCCGAAAAAGCCTAATCAAACACCAACAATTCCGTG
CTGCATTCGATGAAAAACAAAAGTCCAACAAATACCACAACTTCTTGGTGCCTGCGAGAGATGTAAACAT
TCCGGCCTGCGGTTAATACTTTCCCCTAACCACGCCCCCTCCGCCCCTTGAAGGGCAACTCTAGGCAACA
GCAACTACAACGTCCTGCTATGTACTTCCATTTACAACAACAACACCAACATACACTTGAATAAAAGTAC
ACGGACACTGGCGCACACACAACACATACATAAAAGACACAAATACAAATGCATGCATAAATAGTATTAT
TGTTTAATGAATGGAAATTCTTGTTTATTTGTGAAAAAAGTCAT
>dme_circ_0000349_junction_seq
ATTCTTGTTTATTTGTGAAAAAAGTCATGTTAACGTTTAAAAATGAAAGCAGTCAA
>dme_circ_0000350
TACATAGCAGGACGTTGTAGTTGCTGTTGCCTAGAGTTGCCCTTCAAGGGGCGGAGGGGGCGTGGTTAGG
GGAAAGTATTAACCGCAGGCCGGAATGTTTACATCTCTCGCAGGCACCAAGAAGTTGTGGTATTTGTTGG
ACTTTTGTTTTTCATCGAATGCAGCACGGAATTGTTGGTGTTTGATTAGGCTTTTTCGGATGGTTTTTGA
CTGCTTTCATTTTTAAACG
>dme_circ_0000350_junction_seq
GGTTTTTGACTGCTTTCATTTTTAAACGTACATAGCAGGACGTTGTAGTTGCTGTT
>dme_circ_0000351
AAAACATGACTTTTTTCACAAATAAACAAGAATTTCCATTCATTAAACAATAATACTATTTATGCATGCA
TTTGTATTTGTGTCTTTTATGTATGTGTTGTGTGTGCGCCAGTGTCCGTGTACTTTTATTCAAGTGTATG
TTGGTGTTGTTGTTGTAAATGGAAGTACATAGCAGGACGTTGTAGTTGCTGTTGCCTAGAGTTGCCCTTC
AAGGGGCGGAGGGGGCGTGGTTAGGGGAAAGTATTAACCGCAGGCCGGAATGTTTACATCTCTCGCAGGC
ACCAAGAAGTTGTGGTATTTGTTGGACTTTTGTTTTTCATCGAATGCAGCACGGAATTGTTGGTGTTTGA
TTAGGCTTTTTCGGATGGTTTTTGACTGCTTTCATTTTTAAACG
>dme_circ_0000351_junction_seq
GGTTTTTGACTGCTTTCATTTTTAAACGAAAACATGACTTTTTTCACAAATAAACA
>dme_circ_0000352
AATTTCCATTCATTAAACAATAATACTATTTATGCATGCATTTGTATTTGTGTCTTTTATGTATGTGTTG
TGTGTGCGCCAGTGTCCGTGTACTTTTATTCAAGTGTATGTTGGTGTTGTTGTTGTAAATGGAAGTACAT
AGCAGGACGTTGTAGTTGCTGTTGCCTAGAGTTGCCCTTCAAGGGGCGGAGGGGGCGTGGTTAGGGGAAA
G
>dme_circ_0000352_junction_seq
GGGCGGAGGGGGCGTGGTTAGGGGAAAGAATTTCCATTCATTAAACAATAATACTA
>dme_circ_0000353
ACGCTCGCCGTGCTGAATCCGCTGCGCGGTACCGACCAGCAAATCCTCCAGGATACTGACATGGCGGGTC
CACTGGTCTTTTGCCTCACACTTGGCGGCTTCCTGCTGCTGGTAAGTTTGACAGAGATGAACACCACATC
ATTGTATTTTAAATGCGGTTGAAATCAATTGTAATCAATGTCTTAAGTAAGAATTGCCGTTTAAAATTTT
ATATTGTGCAATATTGCCTTCGTTATATCACCTATCTTATTTATACTTATATTTCTTTTATTCACAGAGC
GGTAAGGTGACGTTCTCGTACATCTACGGCATCGGCGTAATGGGCTGCATCTTCTTCTACTGCCTGCTGT
CCCTGATGGTCAGTCGGTCGCAGGTTACCTTCGGAGCAGTGGCCTCCGTGCTGGGCTATTGTCTGCTGCC

CATGGTGGTGCTGTCGGGCATCAACATTTTAATCACCATTCAG

>dme_circ_0000353_junction_seq

GGGCATCAACATTTTAATCACCATTCAGACGCTCGCCGTGCTGAATCCGCTGCGCG

>dme_circ_0000354

GTTCCTGGAGGCCAGCAGTTCGTCGAGTATGGGTTCCCAGCTGAATACTCTAGTGCCGCGGCTAAAGGTG
CCCTTCATTGACAAGCTCGGCAGCACCCAAACGCATTTGGGTGAGGGAGAGCATCACAAAGGAGTTGCCA
AGTTCGCGAGCCGTGAGTCTAACGATCAAATACCTCCACACACGAATGTATTTGTCATTTTCATTTATTT
ACCTTTTGGTTTACGTAGTATGTACTTTACATGTATCTAAAAACCAAGCGATGCCTGACGGGAGGAAATC
AGCTGACCTAATTATAGTTTAAAAATTGTGTAAAATGTAGCAGTAGAGTGTGATTCGTTACCAGTAGTAA
TTGTGGGGCGGCAATCATTTAGACAAGCATGTTTGTATTAAAATAGTATATTTTCCATTTGTTCATTACA
AAAAAGATATTTTAATGTTTGTTAATTTAAAATACAAACTATTTAGTTATGTTTAAGCAAGTAACTAATA
ATATCGACATCCAGCAGCACAACGTACTGGAGTCCGCATATGCTCAGGAGATCCTTCAGGAGCACTACAC
CTCCATTTGCGACAATGTTTTGGAGCAGCGAACTTCGAAGTCTCCGTAT

>dme_circ_0000354_junction_seq

GGAGCAGCGAACTTCGAAGTCTCCGTATGTTCCTGGAGGCCAGCAGTTCGTCGAGT

>dme_circ_0000355

GCGCAAAGATGCTTGACCATTTGGTGATAAGCACGCCCAGGCTAATATCCTCTTACATGAATCCGATCCT
CAAAGCCTTAGTGCCCAAGTTGCATGAGCCGGAATCGAATCCAGGAGTTATTTTAAATGTGCTTCGCACA
ATTGGTGATTTAGCAGAG

>dme_circ_0000355_junction_seq

GCTTCGCACAATTGGTGATTTAGCAGAGGCGCAAAGATGCTTGACCATTTGGTGAT

>dme_circ_0000356

TTTTATGGGACAAGTGCAGGGAGCCGCTCTTCCTGCACAGACTAGCGTACTTCAGCCAGGTATGTATGTC
CTCGTGCGGTTTTACCACCAGCGAGTGCACCTGGATGATCCTTCGCCAGTCTTCTACTAGTCGCTGGCCT
CCTTGAAGACGCTTCCACCACATGGTCTTTAACGGCTCTCGCCTCTCGGGAATGAGCTTGTATTGGATGA
CCTCCTCCAGCTCCGCCAGCATTTG

>dme_circ_0000356_junction_seq

TGACCTCCTCCAGCTCCGCCAGCATTTGTTTTATGGGACAAGTGCAGGGAGCCGCT

>dme_circ_0000357

TTGCAAGATATCCGCTGGAAATGCCGAGTTGTCCATTGAAACTGTAGTCGGTCCaaaaaaaaGTGCGGAT
GAGCAAAACGAACAATTGCTCTCCCGCCTACTGAATGCCACGTGGAATGAGTACGGTAGCGGGTCCATCA
TTTGTGCCCAGAGTGCCAGCTTCCTGGAGCAGCAGCCACACCGCAGATTCGACTTCGAGTGCATTGTGGC
CAATGTGCTCATGGAGGCTGTTTTGAAGATCTACAACGG

>dme_circ_0000357_junction_seq

TGGAGGCTGTTTTGAAGATCTACAACGGTTGCAAGATATCCGCTGGAAATGCCGAG

>dme_circ_0000358

ATGTGGATATGACGACAGCGCTCAGCCTGCCAACAGCCGAGGCCACCAAAAGGAGTGTGTTGCAGAGTTG
CAAGATATCCGCTGGAAATGCCGAGTTGTCCATTGAAACTGTAGTCGGTCCaaaaaaaaGTGCGGATGAG
CAAAACGAACAATTGCTCTCCCGCCTACTGAATGCCACGTGGAATGAGTACGGTAGCGGGTCCATCATTT
GTGCCCAGAGTGCCAGCTTCCTGGAGCAGCAGCCACACCGCAGATTCGACTTCGAGTGCATTGTGGCCAA
TGTGCTCATGGAGGCTG

>dme_circ_0000358_junction_seq

ATTGTGGCCAATGTGCTCATGGAGGCTGATGTGGATATGACGACAGCGCTCAGCCT

>dme_circ_0000359

GCGGAGGAGGAGTTCGATCTGGCCAAGACAATCGCCGAGGCGGAGGATTACCTCAAGCAGCACCCGTTGA
CATACGTGCAGCGCGTCGAACAGAACGGCGAGCGGCTGCAGGATGGACTGCTCAGCATCGAGGATATGCT
AAACGAAGTCGGCGAGGAGG
>dme_circ_0000359_junction_seq
GATATGCTAAACGAAGTCGGCGAGGAGGGCGGAGGAGGAGTTCGATCTGGCCAAGA
>dme_circ_0000360
GTTAGATTCTGTATACATATCGCTTGTTTGCGCTATATCTTCTGGTTTTGATTTGCTTTGTTTTCAAATG
TTTCCTTGCATTAAGTTTAAACTATTTAAATATGGCCCCGCTATTCCACATGTGTTCTTTTTGTTTGCTT
ATAGTTTAATGATAACATTTTGTTTGTAGTGTTAACGCCTACACTGTGCTACAAAGACGCCGTTTGCTGG
AAAACATTCGCACTAATGATGAGAACATAACTAATCCGTA
>dme_circ_0000360_junction_seq
CTAATGATGAGAACATAACTAATCCGTAGTTAGATTCTGTATACATATCGCTTGTT
>dme_circ_0000361
GGTGGTGGTGGTGTTGTGGTATTGTTGGTGGATGAGGCGACGTAGACGGCATCCAGCCGGAGTCCATTTC
CCACAACCGAGTTACTGTTAGCGTTAGAAGGATGATTTCCATTTCTGGCACTGCGCGGTGAGGTGAGCAG
ATCCTGCAGCGATCTGGCGGTGGTGTCTTTGGTGGTTCTACTAGTGGTTGAGTTC
>dme_circ_0000361_junction_seq
TTTGGTGGTTCTACTAGTGGTTGAGTTCGGTGGTGGTGGTGTTGTGGTATTGTTGG
>dme_circ_0000362
GGACGAACAGCGGCACCAGGAAGCAGACTTCATCTACGCTGAAAGTGCCCAGGATGAGCCACAAATCGAG
GCAGAGATACCGCGCCTGCAGGAGCCACAACCACATGCACTAAGCCGTCTGGCACTGTACGCCCTCAAAG
TGAGACGACTGTCCGTCCTCGTCTTCCTGCTGTC
>dme_circ_0000362_junction_seq
GACTGTCCGTCCTCGTCTTCCTGCTGTCGGACGAACAGCGGCACCAGGAAGCAGAC
>dme_circ_0000363
TTTGTAGGGGGACTGGGGATGCAATAGGAAATGGACAACCTAAATTTATACGACGTTCTTAAAGTGGCTC
CGGATGCAACTGATGAGGAAATAAAAAAGGTATGTAACAGTTCCAATTCGATATGTTGGTAAATAGTTTG
TATTCACCTTTTCACCAATAGAACTACCGAAAATTGGCAAAAGAGTTCCATCCAGACAAGAATCCCGATG
CGGGCGACAAGTTTAAAGAAATATCCTTCGCCTACGAAGTGCTGTCCGATCCCGAGAAGCGGCGCATCTA
CGACCGATATGGATTGAAGGGCCTGCAAGAGGGCGCCGAGGGATTCTCTGATGCCTCCGAATTCTTCGCC
CAGTGGTTCCCCTTCGATCGAGTCTCGTCCGAAGGTCGGGGCAGGCGCAATGGCAAAG
>dme_circ_0000363_junction_seq
GAAGGTCGGGGCAGGCGCAATGGCAAAGTTTGTAGGGGGACTGGGGATGCAATAGG
>dme_circ_0000364
GTTTACTCAATTCGAGGCTTTAGAGATCTGACCCTCGTTGCTTTACCACCAACCCCTTATGCCCAATCCC
GTTTTGATATTTTTGAAAAACTCTTAGTTTATTTATTATCTCTCTTTTTCAAAACGAAACGCTGTAAATG
TTA
>dme_circ_0000364_junction_seq
TTTTCAAAACGAAACGCTGTAAATGTTAGTTTACTCAATTCGAGGCTTTAGAGATC
>dme_circ_0000365
GTTATTGTCCCTCTGACCGTACACTTGGTCGTCATCGTCCACGACCGCCTCCATGGCCATGCCAACCATG
CCGTTCTCCACATCGTTATCCATTGCCTCCGCATAGTTCTGGAAGACTGCCGCCGCCGCCGACTGCTGGA
GCAGTTGCTGCTGCAGGCCGCCCATGTGGGCAAATCC
>dme_circ_0000365_junction_seq

GCTGCAGGCCGCCCATGTGGGCAAATCCGTTATTGTCCCTCTGACCGTACACTTGG
>dme_circ_0000366
GCTTTTTCATACAGATCGTGTGTGTTATGTCTTAAAAATAATGCTAAATTGCGATCAACCCATTTCACAA
CTTTTTCCAGCAAATTTGAGACTTCTCTCAGTTGGTTGCATCGACTTCCTCTCATAAATTCATTGTTTAG
TTCTG
>dme_circ_0000366_junction_seq
CCTCTCATAAATTCATTGTTTAGTTCTGGCTTTTTCATACAGATCGTGTGTGTTAT
>dme_circ_0000367
GTTTTGATGCTTTAAACTCGTTAAGTAATGAAATCACAAATGGGGGGCTCTCTTTTCAAGTTTTATTGAC
GTGATGATACCTCAGCGATAGAATCCAGCTGTGAATAGATCTCAATTTTAAATTCATTAAGCTTGGTTTT
TGATCAGCTCATCGGTCGGTGTAATC
>dme_circ_0000367_junction_seq
TTTGATCAGCTCATCGGTCGGTGTAATCGTTTTGATGCTTTAAACTCGTTAAGTAA
>dme_circ_0000368
GGCATTGTAAAGCAATAGCTGAGGTTCCATTTTAAAATCGTGCTCCAACTGCTTTTGCTGATCCAGATTC
TCATCAATGTAGTTGATGTGGATGGGGGAGTTCTGCAGGTTAAGTTGCGAGCTATGCAGCAAGTCATCGT
CTGGTGGG
>dme_circ_0000368_junction_seq
GCTATGCAGCAAGTCATCGTCTGGTGGGGGCATTGTAAAGCAATAGCTGAGGTTCC
>dme_circ_0000369
GTCATCGTCTGGTGGGGTATAGTAAAGCGACGCCTTGCTGGCTGAGTAATTAGCAGATAGGCGCGCCTTA
ATTTTTTGTATGTGCAGCTCCTTGGAGTCACCAAAGTCCATTTGGCCTTTGAAGTAGCAGTCTATGTCTG
ATGaaaaaaaaaGGAGTAAATTTAATATATATACGACTTAAAGGTAGTTTGTTATAATATTTTACCCTCG
GCAATGTCTTCCTCACATCGTCCGCGACAATTCTCAAAGTAGAAGCTAGGAAGGTTCTCCAGCACCTTGT
TCAGCGTCTGCTGTTGGTATTTGTATCCCTCGCGGAGGCGCAATACCTGATGCGCACTAAACTTCCGGAT
TTTGTTCCTCTGGAAGACGTAGTTTTCCCGCACCCAGCTCAACTGGCCTGTGGAGTAGTCGCGCACCTTC
TTCACCTGATCGTAGTATTGGTCCTTCAGCGTGGTCAGATGATTCGAGCCTATGTCACGGAACTCGCGAA
TATGCTTGGCCTGGCTGGTGTAGCTGCTCTGGATCCACTCCACCTGCTGGGCGCAGTTCTCCCGGATGCG
GTGCACCTGCTGCGTATAGTTCTCTCTCAGCCTCTCCAATTGCTGACTCTTGTAGGTCTCGATGCTGTCC
AGCATAGCGTATATTTGGTGTTTGCGCGGTGACTTCTTCTTACGATTGACGCAACAATAGAAACGGTTAC
AGATGCCCCAACGTGACAGGCAGCTCCTCAATCCCTGAACAATCAGGGTTAGCAGCAGGAATGCGGTAGC
CAGAGCCGTGCCAAACAGAATGCTACCGATTTTAACTCGGTAGAACACAATGGGATCGATGTACAGTCTG
CGAGAAAAGGaaaaaaaaaaCCCTTTGAAGAAGTATTTAAACATAATAGTCGCCATTATTCTCACCGTAA
TCCTGCAGAGGCTTTGCCCATCACATTGAAGGCATAACAAGTGTAGAGGCCACTGTCGATTCTCGAAATA
TTGTGCACCAGCAGCGATCCGTTCTCCACCAGCACCACTCGACGACCCACTAGACTATTCTGACGTGTCC
AGTTCAACGACTGAATGTAGCTCTCGTCCATCAGAGCAGCTAGCTCCTGGGCACTCGGTGGCTGGTGAGC
ATCCTCCTTGCTGTCGATGATGATCGGGCGCTTGTCAGGATCAGCATGGTGGCGTAAAATCTTATTGCGC
GGTGTCACCTGATGAGGGGTAAACGATTAACTCTGTGCCTCATAATAAGTTCATGGAAGAAAAATACATT
TAGTCACCTCTAGGAAAGCTAAAGGATTAACTATTAAAAGGGCATAATAAGTTCCGATATCATGGGATAT
TTAAATATATAATAATATGCAATGCTATATGAACATATTCTTTTAACATTAAGTTACTCCTAAACGAACA
CGATAAACTCAAAATATGAGTACAATACAAGGATGTTATACTCACCCAGATGATATCCGGCACTGGGCTC
CCCGAAATCTGGCACTCCAGCTTGGCGGTGGAGAGAAGCTTGTGCATCTTTGGCTCGGAGAACTGCACCA
CCGTGGGCACCGAGCAGTTGAGTTTGCTTAGCACGCGGAGCATATCGCCGCGATAACCATACTGACACTT
GAGCGCATCGATTTGATGATCGATACTCTCGTACGAGTCCAGTGGAGTCTGATTGGTACTGCCATTGCTA

TGGTTCTGTGCCTGGGGCATCTGGTGGCGTAGGCTTGTGCTGGTATTCGCCAGCCAGGCAGTCAGCCAAT
ACATGCTGCAGTCGCACTGATACTCGTTGCCCAATATATTAAGGTAGTGCAGCTGTCTGAGCTCTTTGAT
ATTTTCGGGCAGCGATCCCAAGCTATTTCCTCGCACACTGAGTCGCTGCAATCGTGACAGCCCTGAGATC
TGCGTGGGCAGCTGCAGCAATCCGTTCTCGGCCAGCAGCAGCTCCCTTATATTCGGAGTATTGGCGAACA
AGGTGGCGGGTAAATACGTTAGTTTTCGATTCTGCTGCACGGAAAGGTAACGCAAACGATGAAGCACTTG
CGGATCCACAAGGGACTCGTCATAGCGCGATATCCAGTTGGAGAACTGCTCTATTGGCGTCTCATCAATG
CGCAGCAGCACCAGGGAGCGCTGTCTCTGAAACAGGCCACTGGGCAGCTGCTTGAACTGATTACGGCTGA
GATCCAACTGCCTGAGGGCATGCAGCGACTGGAAGATGATGGATGACAGCAGGGTCAG
>dme_circ_0000369_junction_seq
GGAAGATGATGGATGACAGCAGGGTCAGGTCATCGTCTGGTGGGGTATAGTAAAGC
>dme_circ_0000370
ATTACATACTGAAAAATGGAAGCAATATCGAAGATTTCCTTGATATTGTGTGCCTTGTTTGTCGGGCTCA
AGGCCGCAGCGGCCATCAGTGGTGACAGCACCCACTGTCTGGCCACATACAGCAGTGCCGAGGCGTACCT
GGCCCAAATCCCGCAGCAACATCGTCCACAGATCAGGCCCAGGATAAGGACCTGGCAGGAGCACGAGTTC
TCACTGCTGGGCTACAAGTTCCACCTACCGTTTGTGGGCCATGCCGTCGACTCGGATCTAGATGATAGTG
ACAGTGATGAGGGTCTGTGGCTGGATGCGGCGGACGCCGGATCAGAGAGCGTAGAGGTGGAGGAGCATGA
ACTTCCTTCGGTCGGTCACGTCGATCCCACGGGCAATGTTTTTAAGCTTAACTGCGAGCATGTGGACCTG
AGGCGAGTTAACCAGGAACTGCTCTCCCAGCGAAGCTCGCACATTAATTACAACCAGCTGATGCTGGCCC
ATGTACCTGCGGATCGCAGTAATCCCCTGAAGCTGCCACAGCTTGAAAGTCTGAGGGAATTCAGCTGGCA
GAGTAGTGAACTTAAAGATGAAACGCTCATGGAGCTCTTCACCCGCCAGCCGCGCTCCTTTGAGTATATG
GAACGGCTCAATCTCGCCGAGAATCGCCTGGAGTGCCTTCATTGGGCAATTCCGCTGGCCGTGCGGCGCG
TGAAAG
>dme_circ_0000370_junction_seq
ATTCCGCTGGCCGTGCGGCGCGTGAAAGATTACATACTGAAAAATGGAAGCAATAT
>dme_circ_0000371
GGATGTGCAGGATATCGCCGTCGATTGTGTGCAGCACAACGGATTTTTGCTTGACCTCATTTAACCCGAG
CTCGGGTCGAAACATGGCCTCGAAGTAGGCACTGGCTGCACTCAGGACAGCCCGGTGGGCACTCAATGTT
GCCATTCCGGCGATGATCTCCACGTCGCAGAAACGGGACTGCTCGCGCAGTTG
>dme_circ_0000371_junction_seq
CGCAGAAACGGGACTGCTCGCGCAGTTGGGATGTGCAGGATATCGCCGTCGATTGT
>dme_circ_0000372
GTTTCGAGTGCAAAAAACGGAATTTATGGGAATAAAATAAATAAATAGTAGCTGAATCGTGTAAGAATCT
GTGCTTGCCAGCACCAGAAATACGCAAAATGTCCAAGTCAAGCGCAGGTGGGTTTATAACATCGTGAAAA
CGAAACAAACTGAACAGACGCTTCCGGGTGCGAAACAAAAATAAAGACCTAAAAAATATCACACACTTTA
AAAATAATGAACTCACCACCGAAAATTACAGCACGCTGGGTAAAATTCTTCAATGCGGCCGGCATCCCAT
CTCCTGCGGCTGCCGGCTATGCCCACGTCTTCGTGGAGAACCGCATCCAGGACGACATGCTGTTGGATCT
TAACAAGGAGTATCTCCGGGAAATGGGCATAACCTTGATGGGCGATATAATCGCCATCCTGCGGCACTCA
AAGACCGTCTGCGAACAGAATGCCCGGGAACAGGTCCTCACAACGGAGGTGGTTCAATCAGTTGTCCGAC
CGGTATCGCCAAAACCCAAAGCGCCGGTTGCAACTCCAAAAGCAAAG
>dme_circ_0000372_junction_seq
AGCGCCGGTTGCAACTCCAAAAGCAAAGGTTTCGAGTGCAAAAAACGGAATTTATG
>dme_circ_0000373
GGGTGACATCAACATCAGCGAGTGCATCAAGCAGGGCCTCCAGCAGATAACGCCACGCATGAAGTACGGC
ATCAGCGAGCTGAACATTCCGCCACTCGATCCCTTCGAGATGGGAAAGAGCTCCTATAGCTACACCTCCG

GCCTGCTCCAGGGACGGATTTCCATGAAGAACGTTGTGATCCACGGCCTCAGCGAGGGCATC
>dme_circ_0000373_junction_seq
TGTGATCCACGGCCTCAGCGAGGGCATCGGGTGACATCAACATCAGCGAGTGCATC
>dme_circ_0000374
GGTCCAAGACCATCGGCGGCTAGCGTTGAAAGCAGCTACAGTCCTTACACTCCGACTCCGCAAGTGCCGC
CCGTGGCCAAGAGTCCTCCGGTGCAATATCAACAGCCAACGCCACCGgcaacaccgccgcagcagcagca
gcaacagtcggagcagcagcctgcaacacgcccggagTTCCGCAGTGTGCCCATGCCCACATCGCCAGCG
GTGAACGTCTACACACGTCAATCGGACAGTCCCAGATCGCCTTTCGagccgcagcaacagcagcagcagc
agcaacccacagcgatccactgagagccccttccggtttgcacagcagcaacagcaacagtcaccgcagca
gcgtccaccaacagcgatatcgccgctggctcaggtgcagcagcagcagcaacagcagttgcaacagcag
cagcaacagcagttgcaacagcagcagcaacagcagttgcaacagcagcagcaacagctccagcagctgc
agcaacagcagttccagcagcaacaattgcagcaacaacagttccagcaacaacagttgcagcagcagca
acagcagcaacagcagcaacagcagcagcaacagcagcagcaacCGGCTGTTCAATCAGTACCATGGCGC
ACTCAACGTGCTCAGCCTGGAGCACAACAGCAACAGGATTCGCATCCACAACCAATCTACAACAATGTTC
AGCAGCAGCAACAAAGATCTCGCGATGTCTTCAGTCCGGCAAGGAATGAAACATCGGCAGCAAACACGTT
CAATTCACAGCAGCAACAAAACCAATTTGGTGGAGCAGCAAAGCCGGGTAAGTGATCTATAAAATAGatt
tttatttattttattctttttaCTCTGGAATCTGCAATGCAATTAATCTTACTATTCAAGATTGCCCCAC
TTATTTTTGCGTTATTTCCATATCGAATTGACCTTTAACTATATTACTCATACGACATTGAGCATCATTG
TGCAAGTATCTGTGTGGCCTATGAGTAATTCCTAAGTAAACCAGTCACAGCTAATTTGACAAATACCCAT
ATATGCTAAGTAATATGAAGTGTTCACGTTGCTACAATGCAAATAAATGGAACGCCTATAATCACTTACA
TTCTGTTTTCGCGGAAGAACATTAGGATATTTATAAACCAAAATCTTTGCGAGAGAATGCTCGAACATTG
AATGCATTTACCAAGTGCAGTGTCTAAAATTAAGCGCAACCAAAGGCCAAACACGAATTTTAAACTTGAG
GCGACTGACGAGCGCAAAACTTCATGGGGAAATCTAAAAACCAGAATAATCATGTTAAGAAAACATTGGT
GATTAACAATTGCTAACCAAATGTTGTATTAtttttcctttttttttCAATGTGCAGGACCAACGTTGGA
TCGCTTTACATAGCTCCACTGGCCCAGCCCACTGAACCGCAGGCTCAACGAATCCTTTTGCAACAGCAGC
AGCAGTCATCTGCTCGGGATTCTCCCATGCGCCAACTTCCACAGCAGCAGCCGCAGACCAATCAACCGAT
GAGATGGCTCAGTTCACAGCCGGCTAGCAAGGAGCAGGCTCCATGGGCTCGTCTCGAGGAGAATGGCAAC
GTCCTGCCCTCCACCTTGCGTCAGACTACCCCGCAGCCCCAAGTCGTCCCTcagcagcaaccacagcagc
agcagcagccacagcagcagcagcagcccCACCTTCTACCAGCCGCAGTTGGTCCAAGGTAATGGCTACGG
ACCAACACCGGTTTCAGCCGCTCCCATCAGTCTGCAGAACTTCGGATCAAATCCACAGCCTGGAGGACTA
CGTTTGCAGATCAACTTAAACACCAATGGCAACAGCAGCAATAACACAAATCAAAGTGCTCCACGGGTGA
GTTTAAAACTAGAGCATTTTGATGATTATCACAACATGCCCTGTATACGACGAGTTCATGTCGGCATATG
GAGTATTAGTGTATAATTATCATAAATAAGTCTGACATATTTCTAACTATGTTTTGATCCGCTTTTATAC
TTTCCTTAGAAAATGTTGTATTTAATATATAATTATCTTTCTTTTATAGGAGCGTATCATACCGATAACT
CTAGAGCAGACGCCGACATATGCCGCAGCCCAGCCCAACTTTGGTG
>dme_circ_0000374_junction_seq
TATGCCGCAGCCCAGCCCAACTTTGGTGGGTCCAAGACCATCGGCGGCTAGCGTTG
>dme_circ_0000375
ATTGTGTTGTGTGGCCAAAACCAGCTGAGGAACTGGTTTTCGAATTTTTTAGCATTTCTTGGTGACTTCT
TGAGTCGTTTTCGTTCATGTTTCTTATTTTCGTTCGCCCACCAATGGGGCTTAAAGTCGCACGGCAAACA
TCGATTAAAAATGCACAGCTAACGGG
>dme_circ_0000375_junction_seq
CATCGATTAAAAATGCACAGCTAACGGGATTGTGTTGTGTGGCCAAAACCAGCTGA
>dme_circ_0000376

GCTATCGGGAAACATTCTGCAACAGCAGCAGGGTAGTTTATCGAGCTCGAGCAGCGGCGGAGACTCGACC
AAGCCAATGATGTTTGGCAACAGGATGCGTCGACCGGAGGGATGGCGGGAATGGGAGGCGGTGCGAGCAC
TGGCCCTGTGCCACAAT
>dme_circ_0000376_junction_seq
GGTGCGAGCACTGGCCCTGTGCCACAATGCTATCGGGAAACATTCTGCAACAGCAG
>dme_circ_0000377
GTTTATCGAGCTCGAGCAGCGGCGGAGACTCGACCAAGCCAATGATGTTTGGCAACAGGATGCGTCGACC
GGAGGGATGGCGGGAATGGGAGGCGGTGCGAGCACTGGCCCTGTGCCACAATGTTACACCGGTAAGCGAT
GACGAGGACAACCGATCCGTGTCCACGGCATCGACCGTTACCGGCGGTAACAACTCGCCAACCAAATCTG
TGATAAACATCGAGGCACCTGGCAGTACGGACACAGAGCATCA
>dme_circ_0000377_junction_seq
CACCTGGCAGTACGGACACAGAGCATCAGTTTATCGAGCTCGAGCAGCGGCGGAGA
>dme_circ_0000378
GATAACGTTTTATTTGAAGGGTGCCGACGTGGTTATGTCCAGTATTGTGCAGTACAACGACTGGCTAAGC
GAGGAGTCAGGAAACATGGCTAGAGAGGGTCTTCGCACTCTTGTTGTGGCTAAGAAGGTTCTCACCGAGG
AACAGTACTCTGACTTTGAGACGCGCTACAATGCTGCTCGACTGAGCATCACCGATCGTGTCGCCAAAGT
GGCGGCAGTCACTGAATCCCTCGAGCGTGAACTGGAACTGCTCTGTTTGACAGGAGTGGAGGATCGTCTG
CAGGAGAACGTGCGTCCAACGCTGGAGCTACTGCGCAATGCTGGAGTTCGCGTTTGGATGCTAACGGGCG
ACAAGCTGGAGACTGCTTGCTGTATTGCCAAGTCCTCACAGCTGATTGGTCGGAACCAGGGACTCCATGT
TTTGCGTTCCGTAAAGACGCGTACCGATGCTCACCAGGAGCTGAACAGCTTCCGGCGTAAGCAGGGTCAT
GCACTGGTCATCTCAGGCGAATCCCTCGAGGTTTGCCTGCAGTATTATAGACCAGAGTTCCTGGAGCTGG
CCACGGCCTCGCCGGCCGTC
>dme_circ_0000378_junction_seq
GGAGCTGGCCACGGCCTCGCCGGCCGTCGATAACGTTTTATTTGAAGGGTGCCGAC
>dme_circ_0000379
GATAACGTTTTATTTGAAGGGTGCCGACGTGGTTATGTCCAGTATTGTGCAGTACAACGACTGGCTAAGC
GAGGAGTCAGGAAACATGGCTAGAGAGGGTCTTCGCACTCTTGTTGTGGCTAAGAAGGTTCTCACCGAGG
AACAGTACTCTGACTTTGAGACGCGCTACAATGCTGCTCGACTGAGCATCACCGATCGTGTCGCCAAAGT
GGCGGCAGTCACTGAATCCCTCGAGCGTGAACTGGAACTGCTCTGTTTGACAGGAGTGGAGGATCGTCTG
CAGGAGAACGTGCGTCCAACGCTGGAGCTACTGCGCAATGCTGGAGTTCGCGTTTGGATGCTAACGGGCG
ACAAGCTGGAGACTGCTTGCTGTATTGCCAAGTCCTCACAGCTGATTGGTCGGAACCAGGGACTCCATGT
TTTGCGTTCCGTAAAGACGCGTACCGATGCTCACCAGGAGCTGAACAGCTTCCGGCGTAAGCAGGGTCAT
GCACTGGTCATCTCAGGCGAATCCCTCGAGGTTTGCCTGCAGTATTATAGACCAGAGTTCCTGGAGCTGG
CCACGGCCTCGCCGGCCGTCGTTTGCTGTCGCTGCTCGCCCACACAGAAAGCACAGGTGGTGGCGCTCAT
ACAAAAGCACACAGGCAAACGAACCTGTGCCGTGGGTGACGGTGGCAACGATGTCAGCATGATTCAGCAG
GCGGACGCTGGAGTTGGAATTGAAGGACGAGAGGGAAGACAAGCCTCTCTGGCCGGCGATTTTAGCATTC
CGCAGTTTTCGCACATCGCCAAGCTCCTGCTTATCCACGGTCGCAGGAGCTACAAGCGATCGGCGGCACT
ATCCCAGTTCGTTATCCACCGCGGCTTGATCATAACCACATTGCAGGCGGTCTTTTCAGCGGTATTCTAC
CTCAGCTCGGTTGCTTTGTATCAGGGCTTTCTCATGGTCGGTTACTCGACGCTGTACACTATGTTCCCGG
TGTTCTCATTAGTGCTAGATCAGGATATCACATCGGAGACGGCCGTCACATACCCAGAGCTTTACAAGGA
TTTGTCAAAAGGACGCAGTCTAAGCTACAAGACCTTCTTTATATGGGTACTAATCAGCATATACCAAGGC
GGCGTCATCATGTACGGAGCATTAATACTCTTTGTGGATGAGTTTATCCACATTGTGGCGATCAGTTTCT
CGGCATTAATCATGACTGAGCTCATCATGGTGGCTCTCACCGTGCGTACTTGGCATAGGTGAGTTTTGTA
AACTGCATTTAATCTTTATTGTTTATTAACGTTGCTGCTTTGTTCACAGGTTAATGGTGCTAGCGGAACT

ATTCAGTTTAGCGCTTTATCTCATCTCATTGGCCGTGTTACATGAGTATTTCGGTACGTAAATCGTTAAT
TTTGCCAATAACAATATAATAATATTTCTGTTTTTTTCAGACTGGGAGTTTATTTGGTCGTATGATTTTC
TGTGGAAGGTTTCTCTCATCACTCTGGTCTCCTGCTTACCATT
>dme_circ_0000379_junction_seq
TCATCACTCTGGTCTCCTGCTTACCATTGATAACGTTTTATTTGAAGGGTGCCGAC
>dme_circ_0000380
CAAACGACGGCCGGCGAGGCCGTGGCCAGCTCCAGGAACTCTGGTCTATAATACTGCAGGCAAACCTCGA
GGGATTCGCCTGAGATGACCAGTGCATGACCCTGCTTACGCCGGAAGCTGTTCAGCTCCTGGTGAGCATC
GGTACGCGTCTTTACGGAACGCAAAACATGGAGTCCCTGGTTCCGACCAATCAGCTGTGAGGACTTGGCA
ATACAGCAAGCAGTCTCCAGCTTGTCGCCCGTTAGCATCCAAACGCGAACTCCAGCATTGCGCAGTAGCT
CCAGCGTTGGACGCACGTTCTCCTGCAGACGATCCTCCACTCCTGTCAAACAGAGCAGTTCCAGTTCACG
CTCGAGGGATTCAGTGACTGCCGCCACTTTGGCGACACGATCGGTGATGCTCAGTCGAGCAGCATTGTAG
CGCGTCTCAAAGTCAGAGTACTGTTCCTCGGTGAGAACCTTCTTAGCCACAACAAGAGTGCGAAGACCCT
CTCTAGCCATGTTTCCTGACTCCTCGCTTAGCCAGTCGTTGTACTGCACAATACTGGACATAACCACGTC
GGCACCCTTCAAATAAAACG
>dme_circ_0000380_junction_seq
ACCACGTCGGCACCCTTCAAATAAAACGCAAACGACGGCCGGCGAGGCCGTGGCCA
>dme_circ_0000381
GAGTATTTCGCCATAATCCAGAAGAAGTGGTACCAATGGTCTGACGCCCAATTGGAACGAGTGGATAAGC
TGACGAATGGAGTTGGCAACGAGGAAGCCGAGGAGATCATAAATGAGCTACTCTCCCGGTATTCCGGATA
TCTGCACAGCTGCCTGGAGGAGTTGCAAGCGCAGCAAGCCACGTTTGAGCAAAAT
>dme_circ_0000381_junction_seq
AGCGCAGCAAGCCACGTTTGAGCAAAATGAGTATTTCGCCATAATCCAGAAGAAGT
>dme_circ_0000382
GCTGGCTTTTCTTCTTCACCTTCGTCTGGTCATCTTTGTTCTGCTGTGGCGGTTCTTCCTTTTTTGCTTT
CTTCGACTTGGGCTCGGCCTCCTTGACCTCCTCTACTACCCATGCGTTGTTTATGCTAGAACGCACCTTT
GCATTGTAATTCACCTTCTTCTGCAGCTCAGGAGGCAGAGCCTTGCTAATAACCTCATC
>dme_circ_0000382_junction_seq
GAGGCAGAGCCTTGCTAATAACCTCATCGCTGGCTTTTCTTCTTCACCTTCGTCTG
>dme_circ_0000383
GTCTTTGCAACCGGAAGAAAACTGAAGAAGCTCAACAAATCCAAACGCAAACGCCTACTGCAGTCCATTA
ACTTTGAGGAGGTGGACGAGCACAACTACGATGAGGTTATTAGCAAGGCTCTGCCTCCTGAGCTGCAGAA
GAAGGTGAATTACAATGCAAAGGTGCGTTCTAGCATAAACAACGCATGGGTAGTAGAGGAGG
>dme_circ_0000383_junction_seq
ATAAACAACGCATGGGTAGTAGAGGAGGGTCTTTGCAACCGGAAGAAAACTGAAGA
>dme_circ_0000384
TAAAAAACGATGGCCCAAGTTCCCTTGGCAATCGTCTCGCACAAGCGCCAGGTGTGCAGCCTGTACAAGC
GAGCACTGCGCAACTTAGAGTCGTGGTACGACCGACGGTGAGTGCACTCGGCGCAGAATTCCCAAATATT
ATATAATGAGCAAGTGCATCTTGGTTGTTTCTTAATGCCCATCAGCAATGTCTACCGCTACCGCGCTGTG
CAGCTGCGTGCTCGCTTCGACGAAAACCGCTCCAAAGATCTGGGCGAGGGCATCCGCCTCCTAGCCTGTG
GACAAAGGGAGCTTTTCGAGACAAGGCATTTCCAGCCCCGAAACTGTTGCCAACAGTGCCGGTGGCTGTG
CTTTCGAACGAGAGGTGATTCCGCCCGACTGGGTCCTGGACTACTGGCATCCCCTGGAGAAGGCCCAGTA
CCCCGAGTACTTTGCCAAGCGGGAGCAGCGCAAGAAGGAGTTTG
>dme_circ_0000384_junction_seq

AAGCGGGAGCAGCGCAAGAAGGAGTTTGTAAAAAACGATGGCCCAAGTTCCCTTGG
>dme_circ_0000385
GTACAAAAAATTAACGTAGAGTACAATGTTTTTATTATTTAGATTAAAGCAATGATAGATATACGTAGT
TTGTGAGATATATGGCAAAGTATTCATGAGGCCCCATCGCTTTAAGGAAATTTCGCTTTAGCTTAGTTCT
TCTTGCCACCCACCCACGTTCTTAAATTCTAAGCGCACGCTTTTCGTGCCTCGGTCTCCTGTCTTTTTG
CACAAGCAAACTAAATTTTGCTTCAGTTCAATGTAAATATAACCAAATGATCGGATACGCTTCACAGTTC
CATAAAACATAATG
>dme_circ_0000385_junction_seq
ACGCTTCACAGTTCCATAAAACATAATGGTACAAAAAATTAACGTAGAGTACAATG
>dme_circ_0000386
GTCTCATGGGAGCGGGAGCAGCCAGGTATATAATGGTAAAGGAGTTAACGACCTGCTTCGGGTTTACTAT
TGACCTTGCGCCGGCTCAAACTGCCCAACCAACTGCCGCGTTGCTCATTAGCCGTATATTCGTCATTGAT
AAGCGCGAATCCG
>dme_circ_0000386_junction_seq
ATATTCGTCATTGATAAGCGCGAATCCGGTCTCATGGGAGCGGGAGCAGCCAGGTA
>dme_circ_0000387
GATGACATGCAGATCCGTGTGTTCAACTACAACACCCTGGAGAAGGTGCACTCGTTCGAGGCGCACTCGG
ACTATCTGCGCTGCATCGCGGTGCATCCCACACAGCCGCTGGTGCTGACCAGCAGTG
>dme_circ_0000387_junction_seq
ACACAGCCGCTGGTGCTGACCAGCAGTGGATGACATGCAGATCCGTGTGTTCAACT
>dme_circ_0000388
GTAAATACGATTGTCCTTGGGCACGTAGCCCAGCAGATACATTGTGCGGTCTAAGTGGGATACTGTCACA
ATCTCGCCGCCCACGTAGTAGTTGATGCGGTTCACCGAGTTGGTATAGATGAAGCAGTCTCCCACCCAAA
GTCCTGTTTTGACGCACTCCGAAACCTCGCCTGCAACACAAATGCATAATACAATACAATTATACTTTCT
TGCTATTATGTGGTAAATGGAAATATTTTTTTAAATTTAAATTTAAAATAGTTTGCTCTCATGGTGAAAT
ACAGAGTAAACCATTGTTGTATGCTCTCTATAGGAATATTGAAAGCGGAATCTCATTTGAGAAATCGTGT
TTGAGACACGTATTCTAAAGATAAGATACATATTAGATAATTGGTATCAACTTACCCAGCACATTGAAAG
CGCTCTCCACACCATCATCCTCCAGTCCCTCCTTGGTCTCTACGGCATTGGCAACCTGGGCAGTGTCCAC
CCCCAGGACAAAGTAGGAGTCATCTGTGGCCAAGCAGACCAGACTGCCACTTTCGTTCCAAAACACATTC
TTTGGCTGCACCTCGATGCGCCTCACCAGTTGCAACGTCTCCCAGTCATAGAAGGCCAATCCGGATGAGG
TTTTTACACCGAAATAG
>dme_circ_0000388_junction_seq
TCCGGATGAGGTTTTTACACCGAAATAGGTAAATACGATTGTCCTTGGGCACGTAG
>dme_circ_0000389
GGTTTCGGAGTGCGTCAAAACAGGACTTTGGGTGGGAGACTGCTTCATCTATACCAACTCGGTGAACCGC
ATCAACTACTACGTGGGCGGCGAGATTGTGACAGTATCCCACTTAGACCGCACAATGTATCTGCTGGGCT
ACGTGCCCAAGGACAATC
>dme_circ_0000389_junction_seq
CTGCTGGGCTACGTGCCCAAGGACAATCGGTTTCGGAGTGCGTCAAAACAGGACTT
>dme_circ_0000390
AGACATGTTGTTCTTGCTAGACGCCACATCTGCCAGCTGTGACCACTTCTGGGAGTTCTCCGACTCACGG
GCCAGCTTTAAGGCGATTTCCAAATCTCCGATCTGTAAGGCCAAGTCAAACTTATGGTCGGCATCGGTAG
AGACTTGCAGGGCCTGCGACTTAAAGCCCTGTTTCTCCAGAAAATGTGCCACCCTAGTGCGGTGCTCCTT
GGGAATGGTCGGTAGCACAACGTCTGCGCGCTCAAAGTCTCTTCGCATAACCGCCGTCTGGTACTCCAGC

ACGGACAGCTGCAGACAGAAACTGATCACGTTCAACTCCTTATCGCCGAGG
>dme_circ_0000390_junction_seq
GATCACGTTCAACTCCTTATCGCCGAGGAGACATGTTGTTCTTGCTAGACGCCACA
>dme_circ_0000391
GTCGCTGGCCGATCCGGCACAGTATACAAATCTCTTCCCTGGCCTGGGCGATGCGCTGCGGGTGGAGCAG
CATTTGCAGGAGGAGCGAGCTCGTAAGGCGCCGGCACGCCTCGCTGCGAATCTACCACTGAATAGCGAAC
GTCATCCTCTGCAAGAACTATTTGCCGCCGAGCAGGGTGCCGCCGGCCAGCATTTGGAGGAGAAGGTGAA
GCCAGCTTAC
>dme_circ_0000391_junction_seq
TTTGGAGGAGAAGGTGAAGCCAGCTTACGTCGCTGGCCGATCCGGCACAGTATACA
>dme_circ_0000392
ATATATATTTAAACAACATTCAATCAAATCAAATGTGAATGCATGCGCGGAATTCTGTGGCTCGAAATGC
ACGAAATGTGGGACTCCCGTAGCAGTAAACGATAGTTTAAATGGTATTCTAGATCTGGCTGTGATCTTCC
GTCCTAATCGTCGCTTAGAAAATCGTCATCGAGGTTCACATCTGTCGTATCAATGTTATCATCCAACG
>dme_circ_0000392_junction_seq
TCTGTCGTATCAATGTTATCATCCAACGATATATATTTAAACAACATTCAATCAAA
>dme_circ_0000393
AAAAAGTCCGCATCCTCGGATGAGGAAACTGGGCGCGGCAACAACCAATCTAACGCCAATTCCAGTGTCA
ATTCGAGCAGCAATGCCGGGCAAGGTAGCAAAAAGAAAAAGTCCAAGGTAAATCAAGAAAAAGAAACTCG
CAAGGGGGGCGCTCAAAAGG
>dme_circ_0000393_junction_seq
GAAACTCGCAAGGGGGGCGCTCAAAAGGAAAAAGTCCGCATCCTCGGATGAGGAAA
>dme_circ_0000394
GTCGCAAAAAAACCAAGGACAGCAAGACGACGGGCAAAAAGAAAAAGTCCGCATCCTCGGATGAGGAAAC
TGGGCGCGGCAACAACCAATCTAACGCCAATTCCAGTGTCAATTCGAGCAGCAATGCCGGGCAAGG
>dme_circ_0000394_junction_seq
TCAATTCGAGCAGCAATGCCGGGCAAGGGTCGCAAAAAAACCAAGGACAGCAAGAC
>dme_circ_0000395
AAGCGCACCCGCCGCACCCAGAAGTTCCAGCGCGCCATCGTCGGTGCCTCGCTGGCCGAGATTCTGGCCA
AGCGTAACATGAAGCCCGAGGTGCGCAAGGCGCAGCGCGACCAGGCCATCAAGGTGGCCAAGGAGCAGAA
GCGTGCCGTCAAGGCCGCCAAGAAGGCTGCTGCCCCCGCTCCCGCTAAGAAGTCGGCGCCCAAGCAGAAG
GCCGCCAAGG
>dme_circ_0000395_junction_seq
TCGGCGCCCAAGCAGAAGGCCGCCAAGGAAGCGCACCCGCCGCACCCAGAAGTTCC
>dme_circ_0000396
GCAGCCTTCTTGGCGGCCTTGACGGCACGCTTCTGCTCCTTGGCCACCTTGATGGCCTGGTCGCGCTGCG
CCTTGCGCACCTCGGGCTTCATGTTACGCTTGGCCAGAATCTCGGCCAGCGAGGCACCGACGATGGCGCG
CTGGAACTTCTGGGTGCGGCGG
>dme_circ_0000396_junction_seq
GGCGCGCTGGAACTTCTGGGTGCGGCGGGCAGCCTTCTTGGCGGCCTTGACGGCAC
>dme_circ_0000397
GTTCTAGAACTAAACTATGCTGTGCCACCAGCCAAGGACTCTACGTCGAGTAATCTGACCGATGTGTCGC
CACCATATTCCGTTAACATAGCGCGGAACTCGGTATTCCATGCCGACTACAAGGCAACGCCGGTGAAGCC
GTCTATCCTCGAACAACTGCCAGCGACTTCGGGTAGTGCAAACACCAGTTTCGAGCTGGACAGCGCCGAT

GAGCAGGCTATGGAGCGGGTGCGACGTCAGACGGATCAGCGCTTGTCGCGCTATCTCAAGAACTACGGCG
ATCTGGCCACCAAGCAGCAACAAAAATTAAAGCAGTCGGAGCTGCATCGTAGCAAGAGTGAGTCCCGTCA
TCGGGGCACTCAAACGACACCGCAGTCATTGGCCAGGAGAGATGTTGTTCTTAGGGAGGCTGCCGGCCCC
AATCTATCGATGCCGCTGCTGCGCGAGATGCCCAAAATGACGCGACAGGAGACAGGTGGACCCTTGGCTG
AAAACAGCGGACAGCGGAAGATTGAAAATCTAAAAAAGCGTTGGGAAGc
>dme_circ_0000397_junction_seq
TTGAAAATCTAAAAAAGCGTTGGGAAGcGTTCTAGAACTAAACTATGCTGTGCCAC
>dme_circ_0000398
GGAAAAGTTTGGCAGCCATGAAAACCCCCGACTTTGATAACAAACTGCGCGTATAAACTGCAATGGGAAA
TGAGCACACAGGGAGAAGCCATTGTGAAACTGTGAAAGTGTAATCAAATAAATTCCCTCGCTGAATAATC
CGAGTCGCAGAATAAAGGTCAAATCGTAGAAAATGTGCATAAAAACCTGCAAAGACATTAAATTCGGTGA
ACATTGCGATGGAGGCCATGAAGGTGGAGAGAACATAGTCATCCGCACATCCAAATCTGATAACGAGCAC
TTCCAACTCCAACTGCAGCTGCAGGCCAGGCTAAACAAACAAAAGCACAAAGGCTACAAATTGCCCAACT
TTAAGATTCTCATGTGTTGTTGCTACTTCATCATCATTGTATTGATGATATGAGAACGGAAGGCACAAAC
TATCATGCAATGGAACTCAATCTCCAACCAAAAATGCTTCAATTATATAGATAAATGCCAACATATTTAA
ACCACAAAAGCGAACCAGAAATTATTGTTCAACTAAACTCATAGAAAAGGTAAACATTTTGTGTTGCCTA
CTTCTGGGCGGAAATAGTTTATAAGCGGTATAATTTAGTTATTGCAAAATATTGAATCTGAAACCTGTGC
ATACACTTATAATTAATTTTAAGTTTTAGTTAATAGCCAGTGAAACCGTTTATGTTGTGCAATATATTAT
TTAAGTATTTTTTAACATGTGTTCTTTGCGACTATATTGATTTTGTTTAACGGGCTTACTATCAATAAAC
CATGAAGAATAGAAACTACCTGATTTACTGGGCTACACTGCTAGCAAGGTCTTCTAAAAATAGTCACGGG
TTTTAAAATGCTAGACCAGTTAGCATGGGTTACTCCACCATATTTTCTACTTATCGAACTATATCAATTG
GCATTTTACGGGGCTGTATGGTTTATTGAAATTAGCGCTAAATTGATTTATACCGCAATATGTTGCCGCC
GTCAATATACGAATTACGCATACAGTTTGCTTTTTGTTTGTACCCTGGCTTTGTTTGCTTGGCTCGTACC
CACTGATTACTCATACGACGCGTGGCACATTTTATGAAAATTGATTTTACACTGCGACAAAGCTCGAAGT
ACACACACACTTCGCCACGCCCCACCCCCTACACTCTTCCCCCATGTGTGGGTTGAGTATGTGGTATTTG
TTTTTGTTTTTATTCTGATTTTCAATTCACATTGCTGACCTACATTTTCGGTTTGCGTGGGTGAGGCTCT
GGTAttttttttGTATTATTGTTGGTTTTTGGGCCACAGGTTTCTGGAAAAAAATAAGGAGAAAACAGCCA
GGGGCTATAATTTATTCGTTTACTGCCAGGAAGTTAATTAATCAACATAGCCACAATTTTTAAATATTCC
CAATGAATAATTAAATGTGCCTTTAAATTTTGACGAGTGTCTGAACGGTTTACGAATTATTTATTCAACA
ATTGCAGTTTGAACGATTTTTTAGGCGCATAAATATTTAAACGATATTTTTGCTGGCCTCTATAAATATA
TTTTGGCATAAAAATATTAAATGAAATTCCATTTCCATTCATGGAAAATAGAGAGCAGCCTGGCTGGTTT
GTATATTTTGAAAAACCATAAGCCCCTCAAGTCGGAAAAGTGTAATTTCACCTTAAATGATTTTCTTTTA
TTTTTATTCCTTTGTATGGTCGTAAGCAGCGTATTTACAGCCGAGGCAACTCGTAAATGGAGGCAGTCGT
TATTGCGCTGATATTGTCAGCTTTGAATTTATTTTTATATTATCGGACTTTATGGCTACCGTAAACATGC
AGTTCGGGTGCTTTCTTCGGCTCAAGGATTTTTACGCGAGTTTTAAATCTTTGAAAGGAACATGCTCTAT
AGCTAGCAATCTTATATATGTATATGTAAATATTTGTTCCCATATGTACTCATATGTCAGCTGAAGTGAA
TTTCGAAATATTGTGAACATAAATGAAACGTGCAGTAAATGGAAAAAATGGGAACTCAATCTCGTTTCCC
TTCGGCCTTTGCCGCAGATGCTTCCATTAGGTAGATAAAATACAAAGCATTAACCAAGTAAATGAGACTT
TGAGAAAAATGGTTATTTATATGCAGAAATAAGGCGAATGAAAATGTGTTCATGTTATACGTTTGTAGTA
AAGGTGTATATTATTTTCCTCCAAATCATTAAGCATTAATTTACAGCAACAAGTTTACCTTTCTAACTTT
GTTGTTCTTTAAAAAATGATTTTGTATTGATCTTAGTTTTTTAAGTTTTATAAAAAAGGTCGTATACTTA
GTGTGTCTATTAGACAGACTGACAAGCAAAGCACTCCCGAATAAGCCTATAGTGTCCTGTTAAATAAAAT
AGATTTTTCACTCTACTCAGATATATATTTTCGATTCTCTACCACTGTGCAACACGAAACATTTTTATGA
AAATGTACAAACAAACACATGTGACCCGATACATACTCTTTGTCATTTGTATGCGTAGCTAAACCCTTTA

TTATCCTTTATGTGTTTTTATACCAATTTACACACACACAATCACTGACCACGTAAGCATATCCTGTCCA
AATGTTTTTGTAGCTGGTGTCTAGCCATTGTGCAATCAAATTGTTCGGTGGGCGTTGCTTTTGGGTTAGC
GCCGCTTACGCATGCATTTACAACAAATTATACGAATGGAATTGTTATTAATGCTTTTGGCAAGGTCACG
GAATAGCTTTTTGGAAAATTAGATAAAGGGTTTTAGGTGGGTGGGCGCGAATGTGACTGAAATATAAAAA
AGAGACTTCTGTATTCATATCCATCCCACAACTAATTTACTTGTTTTTGTTCTATTTTCAGGATCTCATT
CGATTGCTGTCTATAGTATAAACCATCAGTTATTTCAATACATGTGTTACAACAAATTAAATGAATTAAa
tatatatatatatatatatataaatatatGTGTATAGTTCAATAACGAAGTTAAATATTTTCAAATCTAG
CTTAAAGAGTGCTATAAGCTAATGTTTTTGTGTGTGTGTAAAGTGCAATTGTTAAGCCATAATCATTGAA
AAGCACATGTTTAACTGCAAAGCAATCAATAATTCAAATAACCTGTAGCTGCAATAATAAACAACAATAT
TCAGCAATGAAATCGACGAATGGCTTCAGCAAGTGGCATAATTTATGAAAACAATACCTTCGTGAA
>dme_circ_0000398_junction_seq
TAATTTATGAAAACAATACCTTCGTGAAGGAAAAGTTTGGCAGCCATGAAAACCCC
>dme_circ_0000399
GATCTCATTCGATTGCTGTCTATAGTATAAACCATCAGTTATTTCAATACATGTGTTACAACAAATTAAA
TGAATTAAatatatatatatatatatatataaatatatGTGTATAGTTCAATAACGAAGTTAAATATTTT
CAAATCTAGCTTAAAGAGTGCTATAAGCTAATGTTTTTGTGTGTGTGTAAAGTGCAATTGTTAAGCCATA
ATCATTGAAAAGCACATGTTTAACTGCAAAGCAATCAATAATTCAAATAACCTGTAGCTGCAATAATAAA
CAACAATATTCAGCAATGAAATCGACGAATGGCTTCAGCAAGTGGCATAATTTATGAAAACAATACCTTC
GTGAA
>dme_circ_0000399_junction_seq
TAATTTATGAAAACAATACCTTCGTGAAGATCTCATTCGATTGCTGTCTATAGTAT
>dme_circ_0000400
GTGGAAAACGATTCAATAAATAGTGTAAGAAAAGCCTAGTGTTACCAAAAGAAAAAACCCTGTATAAAAA
TGAAGCAATACTCACAAAATAGCTATGATACTAGTTGTAAAACCTCTCTGACAAAGCAACAATGCGACAG
CAACAGGTTCAGTCAGTGTGTGTGATAGTAGCTAAGTAACGAGGGTACTTGCTATCTGGATATCGATG
>dme_circ_0000400_junction_seq
GAGGGTACTTGCTATCTGGATATCGATGGTGGAAAACGATTCAATAAATAGTGTAA
>dme_circ_0000401
GATATTGAGCTTCTCAATGGTCAATGCAAGCAACGACCTGATTTGCTGGCCAGCCACAATCACAATGACG
CCATCAGTGAGGCAGGTACCGAGCTGGAAGATGTGGAGAATCAGACCCCACCTCCACAGTCCTTCATCAC
GGAGAAGCTGTACAACGAGTTTCACGTGAAGACCAGGCAGTATAGCAAGTCATCG
>dme_circ_0000401_junction_seq
GAAGACCAGGCAGTATAGCAAGTCATCGGATATTGAGCTTCTCAATGGTCAATGCA
>dme_circ_0000402
GTGGAGCAGCCGTTGGCGCCCATCACGGCCACCATTCGGCGGCTGGGGCGCGCAAGCCCAAACTGCGGCG
CTTCAACTCCCACGACACCAGTTCGAATATGTTCTCGGTGGCCGATTTCGAGAACGCCCGCCTAGCTCGC
CGCAACGAGATCGAGCTGAACCAGCGGAGGGCACGACGCGTGCGCGGCAGTGCCAACAACAGCACCTACG
GCCTGGGATCGGGCTCAGGATTGCTGGGAGGTGCCCATTCTGGATACTGTGACGCACTCAATGGCAGCGG
CGATTACTCCACCGGGGACAGCAAGGCCTCCAAGGGCAGCAGCGAAGGTAAGATTGAATTACACTATACC
TATATATACACAAATCTACGTCAAACCAATTTCAAATAGTAACATAATCTGATGGCAAAAAATTCGAATA
ACGATAGAAACGGGTTTTTAAAATCCCCCAAAGTTTGTTCTACAAATACAATATATCATAAAAGCAGATT
AACGTAGATTAGGATTAAAATAAAATATAGAATATACATTTTTTTATATTAAATTTTCTCGTGTACATGA
CGTCAATAAGTTTTCTAATCGCGCAAAAATGTTGGTTAATTATTCGTTGTCGATTTGTAAGTTTCCTAAC
CACATTCGAAATTATTTAAATAAACTCTAACTTCAACTAATAAACAATGCATCTTACAGGGGTGCGCTGA

CAGCCATTGAGTTCCTTGAGCGGAATTCCCTGCCACGAGTCGTCCGCATTCTGCATGCGAGCAAGAGCTC
AAACGAAACGCTGCAGTCCAGCTCATCGCATGGCTCCTCCACGGCGGCATCGAGCAGCTCCTCGACGGGA
GCGAATCCGTTTGGTTCTGGTGGCAGTGGTGGTGGAAACATTTCCAGTGGCAACATTGACACCGGCACCC
ATTCATCGTCCAGCGGAGGCAGCGCCACCGGAAACGGAActggctctggtcctggtcttagttcgggctc
gggttctggttctggttctggtcaccagaacaacaacggtcgccacagcagcaacggtGGCACTCCACCC
GGCCACGATGAGCTTTTTCTGCTCTACAGGCTGGTGCGACAGCGCAACATCTACCACGGACACAACGCCA
AGACTCAGGCATCGCAGCGCAAGAAGACTCTGCTCATTCCACAGGAGTTTCCGGG
>dme_circ_0000402_junction_seq
CTCTGCTCATTCCACAGGAGTTTCCGGGGTGGAGCAGCCGTTGGCGCCCATCACGG
>dme_circ_0000403
CTACAACTACAAATCCTGGCTCCACAGTGGGTGGAGCAGGCGCTGGCCCAGTGGGCGGCAACGGGGTCGC
AAcagcagcagcagcgggcgcacccagcaacagcgtcgcagctgcagcagcagctgcatGTAACGGGTAA
GCTTAGCGCGAAGAGTGGAAGTGGGTGCGGAGGAGGGGTGGTGGGGAAGGTTGACAAGGCACGCGCTTCA
AGCgagcgtgagagagagagaggagagagcgggagagcgCAATGGGTAATTGTTGCTCAATGACGTCGC
CTTTGCTTTTATTTTGTAAATGCTCTGCATTTAGGGTATGGTAATTCAGATCATCCTTATATACACTTAA
TAATCCTGCGAAGGGAATTGAATGTACTAAAAAGATAAGACCTAATACACTCAAAAAATGATTAAACTGA
AGACTTAATTTACACCATTTCGAAAATGAAAATGTTTTTGCGTTGATTCCTTTAGGAAGTTTGATAAATA
CAACTTTATGAGACTGAAACAGTAGTGGCACTAACTATTTTCACACAATACAGTATGGTAATTACGAGCA
ATTGGTTGCTGCTGTAAAATAATGAATTTAATTTTTTACAACAAGCACCCAATTATTTAAAAATGAGTGT
ATTTTCTACGTCATTTTCCGTAAAAGGTGCGCCGTTACAAGATTTTACAGCACGTAATAAAAGATCCAAG
TTTACAACTACAGGTAAACTTCAGTAAGTGAAACTAATGATTTGAAAATGGATTTGTGCAATATATTAAC
GAAGTTGTAATCAATTTCAATGGTGGTGTTATACGAAAAAAGTTCAAAAGAGATTGCAGTATGATTTAAA
GTTCTTGATTATCGAATTTGCATCTAAATTAATTAATTTGATAGCCAACTGTACTGGTCTTTGGGATAAG
CGAATCCAAATCGTATAAATATTTATTTTTGTGCAAATCGAATTCGAGCAGAACGTTTAAAAGTGAAAAC
CGAAACATGTGCTATTTTTATGGCCAGCTAGTTTGGATGGAATACCAATCAACACTCTGAACAATAAAGA
GATGAAGTGATTAACATTACTCACTTAAATTTTAAAATATATGGTTGATAAGATGACCTAGAAAGACTTA
TTTTTGTCTACCCATTCAAAAATATGAATGGTTCTAAAGAAATCTCGACCATTAAAGACTAAGATGTGTA
GATTAAGTCATATTGTTGATAAGTATGTGTATCTGCGGTAAATTCCGGAAAGGGTAAGATCAGAAGAAGG
GTTTTTAGATTGGATTGAAAGGGTACAACAGTTTCGTTACCAGAAGTAGttttttttttGTCTAACTGGT
GGTTGTGTTGTCTGTATGGGGATTTCTTTTCGCAAAAGGCGCGCGCGTCGATTCGCATAATTTCACACGA
AACGTGACAATCTCACACCCACCCCCCTTCATCCATTCTATCGCTTTCTCTCTCTATTtatatgtatata
tatatgtatgtatgtatatgGCTTACTCTCTCGCTCGCTGGCTGAAAGTCTACTCTGCAATTGATATTTC
ACGAAAGATAAGAGCAATTTCACAAAACGTTTTAGCCCCTTCCCGGTGTTATGCTGTGATTTCTGCCCTC
GCCTGCATTTATTAAATGTGTGTTGTGTGTGGGGCAAAAAATTAAGCCAAATGTGCGCAATTAAAGTGAG
TAATGCGCCGTCGACCACCAATCAGCGGATCCAAATCGCACCATGGGCCGCATTCGAACAATGAGCAATC
AAAAATCGCGATATCTCTTTTTAATTGCAACGCTCGAAGTTCGCTTATTTACACAACTACATACATACCT
ATATATTTCTAAAGCAACACAAAAAATCGGGGAAAAAAACGAATATGGCTAAGTGATTTCATGCGGTATT
GTTTGGGGCGACAGACAATTGCTTTTTTGGATGTCGGCCCGATAACGATATAATACAATATATGTTGCCC
ATGAGGGAGGTGTTATCTTTATGTGTTGTCTACGTACTTGTGTGCCGTTAATCGCCTCCAGTTGTTGACT
CTTTTATTAGTGCCTCGATATCCGATCCGATTGGATCTTTCAACCAAAAATACAATTCGACCTTAAAATT
GTCAAGTCATAGACCCCCGCAGTTCTTCCTCGATATTAACTCAAGTGCATTCGAATCTAACAAAAAGTTC
TCAAAACTTTTTAAACAAGTAATCAATAACCCGGAAATGCGAATCGAAGTGACCTAATCGATAATAAAAT
ACGTTGATTAATAGCTTACACATAGATCATTGACCTGATAACGAATTTTTATGCTTGGAAATAAATCAAA
AATGTTTTCACAAGTTTTTAAGCAATTTTCAAGCATTTCATAAACCAAGCAGGTATTTTTAATGACCCAT

GGCAAACGTCAATAGCAAAATTTAGTATCAATTAGCTGATAGTCAATATATTTGTATTGCAAATAAAGTC
TTGTTAGTGACAAATTTGTTTGCGTAAACGTTGTTAAAACGCAAAAATAAGCACTGATTAGTAAACTTTG
AATTATAGAATCGAGTGCAAATAGAGCAGTTTGAGTTTTGTGTATACCTACGTTTTTAGTATTTGCATGT
TATTATTAGATAGAGATAGATTGCCTTTCTGGCCAGTTGAACTTGAAGTTCGTAGATTCCAAATCATATA
CAAAACTTTGGCTAAAAGATTTTCATTTATATTTAGCCTTGAAACGATGTTTAACAAAGCCGAGCAGGCA
AGAATGTTGTGTACAGGTGCAGCTATTCAGTGCATTAAATTTATTGTGGTCTAAAAATGGCCGTCTTGAA
CAATAAAAGTTCGATTTTCCCCACTTTAAATTCAGTGCAAGATCTTTCGACATTCCTTCCTGTCTATTTC
GAAAATTCTTGTTAATTTTGCGGTTACGCTTTGGGGCGATATTTGCCCACTGTGTGTCATCGTCTCGTTG
ACCGTTGACTGAAAATTAGCTGCGCTTAGCAACAGTTTTGAACCACTTGCCTTAttttttttatattttt
tttttGTTGTTGCGTCACGCCAACTGAAATTCGCTTGGGGAATATACACTCATTATAGACGAAGCTCTGC
CACTGTACTCGTAGATGTGGAAGTTGATACGACGGTGTGTGGGTTTTTCCCACTGCAGACAACGGGGTGG
CAATTAGACACTCACATGTGCATTTATATTTATGCGATTACCTCACCGATGCACAATGTGCTACCTCACT
CACTCACGACTCCTCCGCGTTGCGTTGCGTTTCTTTATTCTTTATTTTAATTTTTTTCGTACCTTTTGCT
CTTGCCACTGCAGGGTGGAGCAGCCGTTGGCGCCCATCACGGCCACCATTCGGCGGCTGGGGCGCGCAAG
CCCAAACTGCGGCGCTTCAACTCCCACGACACCAGTTCGAATATGTTCTCGGTGGCCGATTTCGAGAACG
CCCGCCTAGCTCGCCGCAACGAGATCGAGCTGAACCAGCGGAGGGCACGACGCGTGCGCGGCAGTGCCAA
CAACAGCACCTACGGCCTGGGATCGGGCTCAGGATTGCTGGGAGGTGCCCATTCTGGATACTGTGACGCA
CTCAATGGCAGCGGCGATTACTCCACCGGGGACAGCAAGGCCTCCAAGGGCAGCAGCGAAGGTAAGATTG
AATTACACTATACCTATATATACACAAATCTACGTCAAACCAATTTCAAATAGTAACATAATCTGATGGC
AAAAAATTCGAATAACGATAGAAACGGGTTTTTAAAATCCCCCAAAGTTTGTTCTACAAATACAATATAT
CATAAAAGCAGATTAACGTAGATTAGGATTAAAATAAAATATAGAATATACATTTTTTTATATTAAATTT
TCTCGTGTACATGACGTCAATAAGTTTTCTAATCGCGCAAAAATGTTGGTTAATTATTCGTTGTCGATTT
GTAAGTTTCCTAACCACATTCGAAATTATTTAAATAAACTCTAACTTCAACTAATAAACAATGCATCTTA
CAGGGGTGCGCTGACAGCCATTGAGTTCCTTGAGCGGAATTCCCTGCCACGAGTCGTCCGCATTCTGCAT
GCGAGCAAGAGCTCAAACGAAACGCTGCAGTCCAGCTCATCGCATGGCTCCTCCACGGCGGCATCGAGCA
GCTCCTCGACGGGAGCGAATCCGTTTGG
>dme_circ_0000403_junction_seq
GCTCCTCGACGGGAGCGAATCCGTTTGGCTACAACTACAAATCCTGGCTCCACAGT
>dme_circ_0000404
ACGGCACAGCATGGCTTTCCTCATAAGCCTTCGGCTCTTGCGTATGATCCAGTTTTGAAACTTATGGCAA
TAGGGACGCAAACAGGGGCTTTAAAAGTTTTCGGTCAACCCGGAGTTGAATTGTACGGTCAGCATACTTT
GTTAAACAATTCAGCATCGGAGCTTAATGTACAATTACTTGAATGGGTGTATGGAACTGGTCGCATACTT
TCGTTGACGGCAGCGAATCAATTAATTCTATGGGAGCCAGTTGGAGCAACGTTGCTGCCAATCAAAACAC
TACCGTTTGACGGCAAACTTAAAAAAGTTTCATCGCTGTGCTGTTCTCTCAGTAAGGATCTGCTATGGAT
TGGAACAGAAGGTGGAAACATCTATCAACTGGATTTACATACATTTACCATTAAGGAGCCTGTAATTTAC
CATGACGTTGTGCTAGAGCAGGTGCCACCAGCCTACAAGCTAAATCCTGGTGCAATTGAGTCAATCCGCC
AACTTCCAAACTCCCCTAGCAAACTTCTAGTTGCATACAATCGCGGCCTTTGTGTTTTGTGGGATTTTGA
AAGCGCATCTGTCCAGCGAGCATACATAGCCCCTGGACATGGACAGAGCGTTGGTCTTACAGTGAACTTC
GAAGGATCTGAATTTACCTGGTACCACGCTGATGGTTCATACGCCACTTGGAGCATAGATAACCCAGAAC
CGCCGTCGAATGTTAATTATGTGCCTTATGGACCTGATCCATGCAAAAGCATAAATCGACTGTACAAAGG
CAAGCGAAGGATCCAACGATGTAATTGTTTTTTCCGGCGGCATGCCACGGTCAGCATATGGTGATCACAA
TTGTGTGTCCGTTCACGCCAGCGATGGACACAAAGTGTGTCTTGACTTTACGTCTAAAGTGATTGACTTT
TTTGTGACCTTTGAAAATAATAGAGATGTCGCTGAAGTTCTTGTTGTACTACTTGAAGAGGAACTCTGCG
CTTACGATCTTACTGACCCTAATATTTGTGCTATCAAAGCGCCATATCTTCACTCTGTCCATGCATCAGC

TGTCACTTGCAATTACCTTGCTTCTGAAGTCGTACAGTCGGTATATGAAAGTATTTTAAGAGCTGGAGAT
GAACAAGACATTGACTATAGCAATATTAGCTGGCCTATCACTGGCGGTACTCTCCCGGATAACTTAGAAG
AATCTGTAGAAGAGGACGCGACTAAGCTTTATGAGATTTTGTTAACTGGTCACGAAGATGGTTCTGTTAA
ATTTTGGGACTGCACTGGAGTGTTGCTTAAACCAATTTATAATTTTAAAACTAGCAGCATTTTTGGAAGT
GAGTCAGACTTCCGAGATGACGCAGCTGCAGATATGAGTGCGGAACAAGTCGATGAAGGAGAACCGCCAT
TTCGGAAATCAGGACTTTTTGATCCTTATTCAGATGACCCTCGTTTAGCAGTGAAGAAAATAGCATTCTG
CCCAAAAACCGGACAACTTATTGTTGGTGGCACAGCGGGCCAAATAGTTATAGCCGACTTCATAGACTTA
CCCGAAAAAGTGTCTTTAAAATACATTTCAATGAATTTGGTCAGCGATCGTGATGGATTTGTGTGGAAGG
GTCACGATCAGTTAAACGTGCGATCGAACTTATTAGACGGAGAAGCAATTCCTACGACGGAACGTGGTGT
AAATATATCGGGAGTACTGCAAGTTTTGCCGCCAGCCAGCATAACATGCATGGCACTCGAAGCAAGCTGG
GGCCTAGTATCTGGTGGGACTGCGCACGGCTTAGTTCTCTTTGACTTCAAAAACTTTGTTCCAGTATTTC
ATCGCTGCACTTTAAACCCAAATGATCTTACTGGAGCAGGAGAGCAGCTGTCTCGTCGAAAGTCTTTTAA
GAAATCATTGAGGGAGTCATTTAGAAAGCTTCGCAAGGGTCGATCGACCAGGACCAACCAGAGCAATCAA
GTACCAACAACGGTTAGTAAGATAATATATACTCAACTAGTCTTTTATATTAAATTCACAATCCTACAGC
TGGAAGCAAGACCCGTCGAGAGGCAAATAGAGGCTCGTTGTGCAGATGACGGGCTAGGATCCATGGTGCG
ATGTTTACTATTTGCCAAAACTTATGTTACTAATGG
>dme_circ_0000404_junction_seq
TATTTGCCAAAACTTATGTTACTAATGGACGGCACAGCATGGCTTTCCTCATAAGC
>dme_circ_0000405
GGTCATGAACGACTTAACGAATACATATCCCACTATGAAACACTCAACTATGATCACGAGCACATCCGAG
CTAGTCACAATAGAGCGCGACGATCAGTGACCAAAGATCAATATGTACATTTAAAGTTTGCATCACATGG
AAGAGACTTCCATCTTAGATTAAAACGTGATTTAAATACATTTAGCAATAAGTTAGACGTTTTATGATAG
CAAAGGTCCCATTGATGTCTCCACGGATCATATCTATGAGGGCGAAGTGATAGGGGATCGTAATAGTTAT
GTATTTGGTTCCATACACAATGGGGTATTCGAGGGTAAAATTATAACGGAACGTGATGCCTATTATGTTG
AACATGCCAAACATTATTTTCCCACAAATCGCACGGCGACAACAACACCACCATCGACTTCAACGACATC
CTCAGCAACAACAGCCACAAAAAGCACACAACCAACACGGCCTTTGGCCAAAAGCAACACCAGTACTACT
GCCGTTAATAGTAAGACAGAAAACTTTATAAAGAAAATTGCTGAATCCACAACGACGAGCCAGCAGCTTC
CAGAGTATACCGAATCGTCGTCGTCGTCGACAACAACATTCCCACCCACAACAGAGTATTTCGAGGACGA
AAAGGAGCGTAATGCCGAGGACGAACTTGATTTTCACTCCATTATCTACAAGGAGTCACATGTCGAGGAC
GCCTACGAAAATGTGCGCGAAG
>dme_circ_0000405_junction_seq
GAGGACGCCTACGAAAATGTGCGCGAAGGGTCATGAACGACTTAACGAATACATAT
>dme_circ_0000406
GGTCACGTGGCCGGCTGTGGCATCACGGATGAGGTCTCTCAGTGGATGGAGAACATACAAAATTCAGCCG
TCGAAGAGTTGCCGGAGCCCATGTCAAAGGACTATCAAAAGCTCCACCGGAAGCAGCTGCACAAAAAGTC
CGCCCCACAGCAACAACAGCAGCCCCATCCGCCGAAGAAGTACATCAGCGGGGATGAGGACTTCAAGTAT
CCCCACCAGAAGTACACGAAGGAAGCTAACTTCGCCGAGGGTGCATTCTACGATCCATCGACCGGACGTC
GCCTGGGCTCATCCGCCAACGTGGCCGACTGGCATCAGCTCGTCCACGAGCGCGTCCGCCGCGCCACCGA
CAATGGTGCTGGGGATAGGGGCTCATCCGGTGGATCTGGACGCGGTCGCGAGGACAACAAGAATACCTGC
TCGCTCTACATTCAAACGGATCCATTGATATGGCGCCACATACGCGAAGGCATTGCTGAC
>dme_circ_0000406_junction_seq
GCGCCACATACGCGAAGGCATTGCTGACGGTCACGTGGCCGGCTGTGGCATCACGG
>dme_circ_0000407
TTACAGCACACAGACATTCACAAGAACAACAAGATTTAACTATCGATATCGTAGCGTTCAGATGCCATCA

CCCAATGCACTAAATTGCAACACTTGAACTTGCCTCGCGGTTTGCACTGCAGGTGCTCTTTGATGTACTG
GCAGGGGCACTGGAACTGTCCGCAGTGGCGTCCCGGCTCCAAGGGGGTCACGTCCACCAGGCCCCTCAGG
GCCTCCATGCCCCATCGGGATCCGTAGCGCAGGAACAGCTGCAGGCCGCGCTCCTGCGGAGATTCGGGGG
AGATCGGGTGGTGAGACACGGGGTAAACAGATAGGGCTGGGTGCATGTGGATGGTGCTGGTGAGTTGGGT
TAGGAACGTGGGCAATGATGATTGTATGGTGCATGAGGTTAGTAAGCCGGAATTACACTCTAAGGATGCT
GCATACTTTCCGGCAGCAGCTGAAATTCGTACAAATTTCATACGTAGGTAAGTGAATACAATGCATAAGA
TACAATGGGATATAAATGCAATAGATACATTACATAAATAAACACGCTTACATAATGTTAATACTATTAA
AACAATCGCATAATATACAGTCGGAAAACTTATTTAAAAACTTTATACTACTATCTATACATTCTATTAA
TCAATTATTTGAAAATGTACTTGGGAAACACTATTTTGCATTCTCATAAGCTTTGGAATTCCTTTAAAAA
TAATAATGTTTTTAAAAACATATGATATATGTTTAAGCTTTAAATTATTAAAAATATTATAAATTAGTTT
ACTATTACTGGAAAACAAAAGAAACTTCGATTATTGTATACATAAAAGAGAATATTGGTAATAAAATAAC
CATTTTAATCTTTTTTGGCTAGGCATTTAATAGATTCTCGGCAATCTAAGAAACGAAACATTTTTTGAGG
ATTACATGTCAGGGATTAGAGCCAGCAGCCCATGCAGTTGATGCCGCAGCAGTGGCGGTGCTGCAGGCTG
ATCTTGATCTTGTTGCGCAACAACTCCTCCTCGTATTGGCACGGACAGATGGACGATTGCAGATGGCGGG
GCGTCTCGTGGACGCCGGCCTCCTGGACAGTCCAATCCAGACGGCGGCGCAGGAAGTCCTTGCCCCAGCG
CTGCAGGTAGCTCAGAAACAGCTCCTGGGCCACGGCCTAAAGAGAGTAGTTGGACATTTGGATCATGAGG
GGCGTGAGTGAGAGAAAGACGTGGTAATTTGGGTTGCGAGTGCGATCCCGGGAGATGAAGATGAAGTCAA
CTACCCCTACTTAGAAACAACCCCCTCCGATAGTTAACGTAAACTTCTAGCTCACCTGCTTGCGGGGCAT
TCGCACCGGCTGACTGCCCTGGATAACAATATCGTTTAGGTCAATGTCCAGCTTCGCCTCCCAGCCCGCC
TTGATGGCATCGCCACCAGTTTGCTCCG
>dme_circ_0000407_junction_seq
TTGATGGCATCGCCACCAGTTTGCTCCGTTACAGCACACAGACATTCACAAGAACA
>dme_circ_0000408
TAACAGACAACCATGCAATTTTCCGCAAGTAACCAAGCAGAAGAACAATATAAAATGCAGTTGCTAAACA
CAGGGGTGGATTCAAAACGGATAAGGTATAACAAGTCAATTGATTTATATCACTATCTTGGTTTGGCTAG
GATAAGATTTATACAGCTTGACATGATGCTAAATTATTGTATAATAACAATTTAAAAAGAGGAGCTGGCA
ATTAGTCCCCTCGTTGAAAAAGCCTAATTGCCCACATATGCAATATTTCAAGAAAAGAACAAAACCGAGT
GAAGTATAATGGATAAAAGCAAAAGCAAGGCCACAATGGAAGAAAACGAGAG
>dme_circ_0000408_junction_seq
GCAAGGCCACAATGGAAGAAAACGAGAGTAACAGACAACCATGCAATTTTCCGCAA
>dme_circ_0000409
AGTACGTGAAATATAGCTGCGTCTCTCCGTTCGGATTGACCAGCTGCGGTCGGGCGCTGTCTGGGAAGTT
GCAGGTGTCCCCATGGAATTTCAGCTCTTTGTGACTGACCGAAACGGTTTCCACCTCAAAGCCAACCACA
CGATACTTATCCTTGGAGTGCATGTGGTAGGATAGGATAAACTTCAGGTGG
>dme_circ_0000409_junction_seq
GTGGTAGGATAGGATAAACTTCAGGTGGAGTACGTGAAATATAGCTGCGTCTCTCC
>dme_circ_0000410
AAATCGTCTCGAGATGGGATCCACACCGGTGGTAGGTTCGTCCAGGAGCACCAGTGCGGGTGCACCAATC
ATGGCCATTGCCGCCAGAAGCTTGCGTTTCGTGCCGCCCGAATAGTGGCGCACTTGCACCTTTTGATATT
TGGTCAAATGCATCTTCTCTAGCCAGTACTTCACGTGCTCCTTGCAGCTGGCTGGTCCATCGGTCCTTCG
GCGGCTCAGGCCGCGCAGCAATGCCATGTAGTATAGGCACTGCTCGGCGGTCATGAATTTATTTAGTGCA
TCGTACTGAGGGCAGTAGCCAAAG
>dme_circ_0000410_junction_seq
TGCATCGTACTGAGGGCAGTAGCCAAAGAAATCGTCTCGAGATGGGATCCACACCG

>dme_circ_0000411
GATGAAGAACAACTCCGGTATGCTTCAACAGGCGAAACCCACTGGAAAGCAGGCCTTCATAACGGAGAAA
CGCAGAAAGCTGTTGTGGAACATGGAGATGGAGGTCATCTCGCTGACGGCCACCAATCTAATGCAGTCAG
TTTCGCACGTCAAGTCACCCTTCACCTCAGCGAAACACTTGGAGCATGTCCGGCCCATGTTCAAAATGGC
TTGGACACCATTTCTGGCCGCTTTCTCTGTGGGTCTCCAGGACTGCGACGATCCTGAGATTGCTACACTC
TGCTTGGATGGTATACGTTGTGCTATTCGAATCGCATGCATCTTCCACATGTCCCTGGAGCGAGATGCCT
ATGTACAAGCCCTGGCCAGGTTTACTCTCCTGAATGCTAACTCGCCCATCAACGAAATGAAGGCCAAGAA
TATCGATACCATCAAGACGCTTATAATGGTAGCCCACACGGATGGCAATTATCTGGGCAGCAGCTGGCTG
GATATAGTGAAGTGCATTAGCCAGTTGGAGCTGGCCCAACTGATCGGCACTGGGGTGCGGCCCCAGTTTC
TTTCTGGAGCGCAGACAACGCTCAAGGACTCGCTTAATCCCAGCGTGAAAGAACACATCGGCGAGACGAG
CAGCCAGAGCGTGGTGGTCGCAGTCGATCGTATTTTCACCGGCTCAATGCGACTGGATGGCGATGCTATC
GTGGACTTCGTGAAGGCCCTGTGCCAGGTGTCTGTGGATGAGCTTCAGCAGCAGCAACCGAGGATGTTCT
CCTTGCAAAAGATAGTGGAAATTAGTTACTACAACATGGAGCGTATTCGTCTGCAGTGGTCACGCATTTG
GCAAGTTTTGGGTGAGCACTTTAACGCGGTCGGATGCAATAGCAACGAGGAGATCTCATTTTTCGCCCTG
GACTCACTGCGTCAGTTGTCGATGAAGTTCATGGAGAAGGGCGAGTTCAGTAATTTCCGCTTCCAGAAGG
ATTTCCTGCGTCCCTTTGAGCATATCATGAAGAAAAACGCATCGCCGGCAATACGAGATATGGTGGTGCG
CTGCATTGCCCAGATGGTAAACTCACAGGCGCATAACATCCGTTCCGGCTGGAAGAATATCTTTAGCATT
TTCCACCTGGCAGCGGGAGACAACGAAGAGCCAATTGTGGAGCTGGCCTTCCAAACCACGGGCAAAATCA
TCGGTGATCTGTACAAGCGTCAGTTCGCCATTATGGTGGACTCGTTCCAGGATGCGGTCAAGTGCCTGTC
AGAGTTCGCCACCGCCAGATTCCCCGATACCAGCATGGAAGCCATACGTCTGGTCCGTACCTGCGCGCAG
TGCGTCCACGAGGCACCACAACTGTTTGCGGAGCATGCCGGCATGGAGAACGACGCCTCGGTGGCCGAGG
AGGATCGAGTCTGGGTGCGCGGCTGGTTTCCGATGCTATTCTCGCTTTCCTGCGTGGTCAATCGCTGCAA
ATTGGATGTGCGTACTCGCGCCTTAACC
>dme_circ_0000411_junction_seq
ATTGGATGTGCGTACTCGCGCCTTAACCGATGAAGAACAACTCCGGTATGCTTCAA
>dme_circ_0000412
GGTTTACTCTCCTGAATGCTAACTCGCCCATCAACGAAATGAAGGCCAAGAATATCGATACCATCAAGAC
GCTTATAATGGTAGCCCACACGGATGGCAATTATCTGGGCAGCAGCTGGCTGGATATAGTGAAGTGCATT
AGCCAGTTGGAGCTGGCCCAACTGATCGGCACTGGGGTGCGGCCCCAGTTTCTTTCTGGAGCGCAGACAA
CGCTCAAGGACTCGCTTAATCCCAGCGTGAAAGAACACATCGGCGAGACGAGCAGCCAGAGCGTGGTGGT
CGCAGTCGATCGTATTTTCACCGGCTCAATGCGACTGGATGGCGATGCTATCGTGGACTTCGTGAAGGCC
CTGTGCCAG
>dme_circ_0000412_junction_seq
CGTGGACTTCGTGAAGGCCCTGTGCCAGGGTTTACTCTCCTGAATGCTAACTCGCC
>dme_circ_0000413
ACGAGAATTGAACAATGGAGATGTGCCGGTCCAGCTCGTGGCGTACGGATGTGATGTGTGGGTGCGAAAA
GCTAATCATACGACACTCGAATCCGTTGACTATCCAAGGACTTAGTTGTCCAACATCCACCGACTTAGTG
GCCCCCGATCTCGCGCAGCTCACGCTTGCCCAGCGGCAGCGGAGGACGGAAGTTGTTGATGACCTTGCCG
TTGAACTCGTTGCAGGGGCACTCCTCCTTCACGTCGTAGGCATTGAGATTGCGCATGGCGTTCAGCTGGT
CGTCGGACACTTCGATGGGCGTCTTGAACACGATCCAGATGACCGACTCCGAGCACGGTGGAG
>dme_circ_0000413_junction_seq
CAGATGACCGACTCCGAGCACGGTGGAGACGAGAATTGAACAATGGAGATGTGCCG
>dme_circ_0000414
GGTGATCAACTCGAAGAAACGCAAGTCTGTCCTGCCCACGGAAAAGGTGCACACGCTGCTCCAGAAGGAT

GTGTTGCAGTACAAGATCGATAGCTCCGTGTCCGCCTTCCTGGTGGCCGTGCTCGAGTACATCTCAGCGG
ACATACTCAAAATGGCCGGCGACTAT
>dme_circ_0000414_junction_seq
GGACATACTCAAAATGGCCGGCGACTATGGTGATCAACTCGAAGAAACGCAAGTCT
>dme_circ_0000415
GCTTTCCATCGGCAGTCAGACTGTAGAGCAGTGGCACACAGCTATAGCCGGCCACCACGACGGACGTGGG
CGAGACCCATTCGCATGACAGGAAGGGCAGATAGCCGGTGCGGCATCGAATGACCGTATTGCCATTGGTA
GCGTCCGCGATGCTCACGCAGCTGTCGTGGCCCACCCAGCAGACCTT
>dme_circ_0000415_junction_seq
AGCTGTCGTGGCCCACCCAGCAGACCTTGCTTTCCATCGGCAGTCAGACTGTAGAG
>dme_circ_0000416
GTGCTCCAGTGCGCGATGTGGTTCCACTAGGAAGTCCGTGGTTAAATGTAGCGTAGGACCCTTTATCCCA
AATAGGGAATTGCTAGTGGGACCAGAACAAGTGCTTTGATGTTTCAGAAGAGTGGGACATTTGGCCTTTC
GAGGATGATCTTTGACGAGTTTTTGGAAATTGTTACGGAATTGCTGGCTTTGTGGAAAGGAAAAACGCTG
GAT
>dme_circ_0000416_junction_seq
GGCTTTGTGGAAAGGAAAAACGCTGGATGTGCTCCAGTGCGCGATGTGGTTCCACT
>dme_circ_0000417
GGGAGTAGCTAATTCTACCCTGAATGTTCAGATCCTCGAGAGCAAAGAAGGCGCCACCGTTGCGCTTGAT
GCGATAGCCACTGCCCATGTCCATCTCGTAATCGGTGGTGAAGTTAAGGCTGGCAAAGTTGATGTTGAAG
GTCAAGCGACTCCTGATCACGTCCATGTTCATG
>dme_circ_0000417_junction_seq
GCGACTCCTGATCACGTCCATGTTCATGGGGAGTAGCTAATTCTACCCTGAATGTT
>dme_circ_0000418
CGTTCTGGACGACATCAGCATCGCCGAGATCATCTCCGGTGCCGGTGGTGATGGTGAGGGTGGAGAGAAG
GAGGCCTGCATTCCCCCCGAGTTCGAGTGGTAAACAAATCATCGGTCATTAAACAACCG
>dme_circ_0000418_junction_seq
AAACAAATCATCGGTCATTAAACAACCGCGTTCTGGACGACATCAGCATCGCCGAG
>dme_circ_0000419
GGCCATCAATGGACTGCTGAACCAAATGCCTTGTGGTTGGCCCCAATATGGAATTCCACCACTGGATCCC
TACACCAATGCTGATCTCCGCATCCACTTGGCCGAGTCGGTTGTCGAGTGAGTTATAATTAATTGGAGTG
ATTGTATCAGTGGATCTAATAGTCATCCTTAGTACCCTGCTGCAGTTCCTGCGCTTCCGTTTCGATGGCC
TGGAAGGCATGGAGATCAAGAAGATGAAGGTCAGCTACACCTTTAGCAAGAAG
>dme_circ_0000419_junction_seq
GAAGGTCAGCTACACCTTTAGCAAGAAGGGCCATCAATGGACTGCTGAACCAAATG
>dme_circ_0000420
ATCTTGTGACCCGTGAGATGCGCATTGGCCAGCGGCACAAAGATCTCCTCGATCTTGGCGCTGATCTGTT
CCTGGTTGCCATTGATGAAGGCCGGGATCTCCTTGTCGAGCATGTCGTTGATGAACTCATTGATCCTTCC
ATTTCCCATTACACCGCCAATGTTCGAGGATACACCGCCCAAGCCGACGGCGCAACTGAACTTG
>dme_circ_0000420_junction_seq
GCCCAAGCCGACGGCGCAACTGAACTTGATCTTGTGACCCGTGAGATGCGCATTGG
>dme_circ_0000421
GGAGCGGGAGTGGGATTGCAACTGCCAGTGGTGGCGGACAGCAAGCTAAAGAGATACCAGATCTTGTGAC
CCGTGAGATGCGCATTGGCCAGCGGCACAAAGATCTCCTCGATCTTGGCGCTGATCTGTTCCTGGTTGCC

ATTGATGAAGGCCGGGATCTCCTTG
>dme_circ_0000421_junction_seq
GCCATTGATGAAGGCCGGGATCTCCTTGGGAGCGGGAGTGGGATTGCAACTGCCAG
>dme_circ_0000422
GTGATCTTGGCGGAACCCCACAGCATGGGAAGCTTGTACTTCAGAGTGCCAGCAATACGCAGGTTGACCA
AATCGAACAACAGCTCTCCGGATCCCTCGTACTCCACGGACAGACCCAACTGGCGCAGGGCATCGATCAG
CGTATCAGTGTCGTACTTCTGGGCGGTGGTATCGATGTTCTTGAACAGAAAATCGAAGGTAATCTTGCTG
G
>dme_circ_0000422_junction_seq
AACAGAAAATCGAAGGTAATCTTGCTGGGTGATCTTGGCGGAACCCCACAGCATGG
>dme_circ_0000423
TGATGTCCGAAGTCACAGACTCCAACGAGATGGTGGTCTTCAGCGAGGTGATCTTGGCGGAACCCCACAG
CATGGGAAGCTTGTACTTCAGAGTGCCAGCAATACGCAGGTTGACCAAATCGAACAACAGCTCTCCGGAT
CCCTCGTACTCCACGGACAGACCCAACTGGCGCAGGGCATCGATCAGCGTATCAGTGTCGTACTTCTGGG
CGGTGG
>dme_circ_0000423_junction_seq
GTATCAGTGTCGTACTTCTGGGCGGTGGTGATGTCCGAAGTCACAGACTCCAACGA
>dme_circ_0000424
TGTTTATTCACGGCTGTCCCATAGTTTAGGCCCAGGGATCGGCGGAGGGATCGCAATCAACGACAATCGG
ATCGTCCTCGCTCTCACCGTCGCTGCTGGCCAGGATCAGGTCCACGACGGTCCAGAAGTCCTTGCCCTTC
AGCATCTTG
>dme_circ_0000424_junction_seq
CCAGAAGTCCTTGCCCTTCAGCATCTTGTGTTTATTCACGGCTGTCCCATAGTTTA
>dme_circ_0000425
GTTTCCCAATTCATGGAGGAGCTCATTGTTCCGCCCATGAATTTGGTTCTGGATAATCTGGCCTGGTATG
AGATTACCGCTATCATTTTGGGCCTGGCCGAGGGAATTTTGCCGGTGGAGCCAATTTGCTGAAGTGCTGA
CAAAAACGATTGCCAGGTGTTCCCTGTGCTTAAAT
>dme_circ_0000425_junction_seq
GATTGCCAGGTGTTCCCTGTGCTTAAATGTTTCCCAATTCATGGAGGAGCTCATTG
>dme_circ_0000426
GCGCGACGAGTGGGTAGCGGCCGTCGAGCAGGAGATCTTCAAGAGCCTGCAGAGCATCGAGAGCAGCAAG
ACCAAACAGGCCACCAGCACTGATCTCGCTGCCATGCTGGCCATCCGTCAGCGGGTTCCTGGCAACGGCT
TCTGC
>dme_circ_0000426_junction_seq
TCAGCGGGTTCCTGGCAACGGCTTCTGCGCGCGACGAGTGGGTAGCGGCCGTCGAG
>dme_circ_0000427
ATAACGACAGGCATGACCTGAGCGGTTCACGGACAGAAGATCTTCGTGGAACAGTTGGGAGAGCGGAATG
ACGGCGACCAGGACGGATCGACCTTCTAATCGGTTATTTGCTTGATGTACCATGTCGTATTCAAGATTTC
GCATTTCCTGTGTCGGGCTGGACTATGGATCCAAAAACTCTTGATCTTTCCTGCAGTTGTGTTTCGTTCT
CGG
>dme_circ_0000427_junction_seq
CTTTCCTGCAGTTGTGTTTCGTTCTCGGATAACGACAGGCATGACCTGAGCGGTTC
>dme_circ_0000428
GATGCTTGGCTCCGGAACGCGCAGCTGCTGGGAGCACAGGATCTTGCGTAGATCAATCAGCACACAGTGG

CACAGAGCGTTAAAGTCCGGCACTTCCTTGTCGAAGAAAAATATGTCCACAATCAGATAGAAACGGGCAA
AGAAGTCCTCGATGTTGAAGTCTAGTTTTTCTGGATCCATACGCTCTAGGTATTCGGT
>dme_circ_0000428_junction_seq
CTGGATCCATACGCTCTAGGTATTCGGTGATGCTTGGCTCCGGAACGCGCAGCTGC
>dme_circ_0000429
GGCGGTGAGGAAGAGCTGGTTGATGCGAGCCTCGTCATCGCGCACATAATCCTTCAACAGGCCAATCTTC
TCGAGATGTTTGGGTGTCGATACGGACAGGGAGAGCACATCACCGACAGCTTCGTGGAAACCGGGATTGG
CACCAGTGCGATAGACGAAGGGTTGGTGTTGATACTGCAGGAAATACTGAATGTGTCCCAG
>dme_circ_0000429_junction_seq
ACTGCAGGAAATACTGAATGTGTCCCAGGGCGGTGAGGAAGAGCTGGTTGATGCGA
>dme_circ_0000430
GTCGTGTGCACGTACTTGAGGACCGCAGTCTGAAGCTGGACGACGTTACTCTGGAGGACATGGGCGAGTA
CACTTGCGAGGCGGACAATGCGGTGGGCGGCATCACGGCCACTGGCATCCTCACCGTTCACGGTAAGTTA
ATTGCCCGAATGCTGCGGCCAGCTTCTGTAGTGCACTTGAAAAAACATTGGGCGGCAGTTTCAATTTTCT
AAACGCTGGGCGCCAAATATTCAACAATAAATGCTTAGCGTCGTTGTTTTAAGTGTACTAACTTCCTTAT
TTTCTTCATCATTTCAGCTCCCCCCAAATTTGTGATACGCCCCAAGAATCAGCTGGTGGAGATCGGTGAT
GAAGTGCTGTTCGAGTGCCAAGCGAATGGACATCCCCGACCAACGCTCTACTGGTCGGTGGAGGGCAACA
GCTCCCTGCTGCTCCCCGGCTATCGGGATGGCCGCATGGAAGTGACCCTGACGCCCGAGGGGCGCTCGGT
GCTCTCGATAGCTCGATTTGCCCGTGAGGATTCCGGAAAGGTGGTCACTTGCAACGCCCTGAACGCCGTG
GGCAGCGTCAGCAGTCGGACTGTGGTCAGTGTGGATACGCAATTCGAGCTGCCACCGCCGATTATCGAAC
AGGGGCCCGTGAATCAAACGTTGCCCGTTAAATCAATTGTGGTTCTGCCATGCCGAACTCTGGGCACTCC
AGTGCCACAGGTCTCTTGGTACCTGGATGGCATACCCATCGATGTGCAGGAGCACGAGCGGCGGAATCTT
TCGGACGCTGGAGCCTTAACCATTTCGGATCTTCAGCGCCACGAGGATGAAGGCTTGTACACCTGCGTGG
CCAGCAATCGCAACGGAAAATCCTCTTGGAGTGGTTACCTTCGTCTGGACACCCCGACAAATCCGAATAT
CAAGTTCTTCAGAGCCCCAGAACTTTCCACCTACCCAGGGCCGCCAGGAAAACCGCAAATGGTGGAGAAG
GGCGAAAATTCGGTGACTCTCAGCTGGACGAGGAGCAACAAGGTGGGCGGCTCCAGTCTGGTGGGCTATG
TAATCGAGATGTTTGGCAAAAACGAAACGGATGGCTGGGTGGCTGTGGGCACTAGGGTGCAAAATACCAC
GTTTACCCAAACGGGTCTGCTGCCGGGTGTGAATTACTTCTTTCTAATTCGAGCCGAGAACTCCCATGGC
TTATCACTGCCCAGTCCGATGTCGGAACCCATTACGGTGGGAACGGATCATCAATGGCAAATACGTCGAG
GGCTTCTATGTCTATGCGAGACAGTTGCCAAATCCAATAGTCAACAATCCGGCGCCCGTTACTAGCAATA
CCAATCCGCTGCTGGGCTCTAcatccacatccgcatccgcatccgcctcggcatcggcatTGATTTCGAC
AAAGCCAAATATTGCAGCTGCCGGCAAACGTGATGGGGAGACAAACCAGAGTGGAGGAGGAGCTCCGACC
CCACTGAACACCAAGTATCGCATGCTAACGATTCTCAATGGCGGTGGCGCCTCATCCTGCACCATCACCG
GGCTCGTCCAGTACACGCTGTATGAATTTTTCATCGTGCCATTTTACAAATCCGTCGAGGGCAAGCCGTC
GAATTCGCGCATCGCTCGCACCCTTGAAGATGG
>dme_circ_0000430_junction_seq
CGCGCATCGCTCGCACCCTTGAAGATGGGTCGTGTGCACGTACTTGAGGACCGCAG
>dme_circ_0000431
GTGAAAATCCACGCATCATCGAGCATCCCATGGACACGACGGTGCCAAAAAATGATCCATTTACGTTTAA
TTGCCAGGCCGAGGGCAATCCAACACCAACCATTCAATGGTTTAAGGACGGTCGCGAACTGAAGACGGAT
ACGGGTTCGCATCGCATAATGCTGCCCGCCGGGGGTCTATTCTTTCTCAAGGGTACGCATCATAACCAAT
CAACTCATTCCGTATCGGCTCTATCATTATGGCCCTTACAGCTGTCGAAGGCGGTGGCCGAGGAGGCGGA
TGAGGCGTGGAATTCCTTTATTTGGGTTCCCAGGCCGAGTCCTACTCCTCGAGTCCCATGTGAAACGCAA
CATTTGTGCATAAGCAGTTCGTGTGTCTTCTGCTGTTTCTGCTTTTTTCGGCCTGCCCATATTATTTTAT

GGGCTTCCGTCTGGTTGCACTTTAGTTGATAGGGAAAACAACCTGATGAACAAACAGAGATCTCGTTGGG
GTTAGACGACGCCGACCAACCCCTATATCATTTCGTGCGTGCACATTGCGCCATTTCAGCTAACGAGACT
CCTCGAAATCCCCAAACGCCGATGAAAAAGGGGTGTCGTCGTCTGTCTGTCTCTCTATCAGGCATTGCAA
TTTCCTCGGACTCTGTTTATATTAAATTCCTACTCGTTTTTCTTTCTTTCTCCGCTATTTTCATTAAATG
CAACAAATTCACTGTGCTTTTGTTATTGTTTTTCTGCAGATTAATAATGCCTCAGTGGGTGCTTGAATTT
GTGTTAAATTTTAAATTTAAATGTaaaaaaaaaaCAACCTACTAGATGCCCTCGAGTAATTCCACGCAAA
TTCTCAAGTTGAACCTCGAGGCGAAGCTCAAGTTCAGCAATTCACTTTTATTATTGTCCATAACAATTTG
TAtttttttttGGGGGTAAGTCACCCCGACCCCCAAATGAGATTCGGTTTGTTGCCAATTAAAATTGAAGT
GGCCGAGTGATTAAGGCCCAACCAAGCCCAGAGAAGGATGCTACCCCTTGGGCTGGCTTGAATGTATTTT
TTAATTAAATTAAATTGCGTATACGCAGACTGGACAATGGGCCTATGGCACGTTTCCCTTTTATTTGATT
TCGGATGAGCGATTTTGAGACTTTTTGATCTGACACGTGACCCGGACCGGCCAAAGCTGCACCCAAAAAA
CTACTTAAATCTCTTAGCTCGTGTGTCTAATAATCTCTTTCCCCTCGACTCCCCGCAGGTTATCCACTCA
CGTAGAGAGAGCGATGCGGGCACTTACTGGTGCGAGGCCAAAAACGAGTTTGGAGTGGCACGGTCCAGGA
ATGCAACGTTGCAAGTGGCATG
>dme_circ_0000431_junction_seq
CCAGGAATGCAACGTTGCAAGTGGCATGGTGAAAATCCACGCATCATCGAGCATCC
>dme_circ_0000432
ACGGCTGGCATCAAGACACGGCAGCCGCCATCGGGAAACCTGCAGCTTGCCGGCAATAGCAGTGAGTCGG
GATGGAGGAGCTACAACCAGAGCAGCTACGGTTGGAGCACTCAAAACCAGAGCAACTACGCCTGGAACCA
GCAGAACCATGTGGAACAGGGATCCGCTGGATTTGTGCGCGCCGAGG
>dme_circ_0000432_junction_seq
GGATCCGCTGGATTTGTGCGCGCCGAGGACGGCTGGCATCAAGACACGGCAGCCGC
>dme_circ_0000433
GCGATCGAAAATGGTTCTGATTACTTTGACCCTGGTTTCGTTGGTGGTGGGCCTGCTGTATGCCGTGCTG
GTATGGAACTATGACTATTGGAGGAAACGGGGCGTTCCTGGTCCCAAGCCAAAGCTATTGTGCGGAAACT
ATCCAAACATGTTTACTATGAAGCGGCATGCAATCTACGATCTAGACGATATATATACAG
>dme_circ_0000433_junction_seq
CAATCTACGATCTAGACGATATATATACAGGCGATCGAAAATGGTTCTGATTACTTTG
>dme_circ_0000434
GCCAGCAATTGACGATTGTCCAGGTTGCGCTTCTCACCCAGTTGCAAGATGTAGTCCAGGAAGTCGGACC
GCTCGAACTGCTTTCCCGCCGCCAACTGGGCACGACGTGTCTCTACCGCACTGCCCATCAGGTCGACAAA
GAAGCGTTCCACATCCACTGGCACGAAGCGTAGCTTAATCAAATGACTTAGCGATGGGAAGGAACTAGTC
AGTATAAAGAACAGCACAAAGGTCCAGGGCTGGTTGAAGAGATCATTGATTTTCGACATTATGGGTGTGG
GCTTATCCGAAAAGCTTTCAGCGCCCAGACCCAAAACACAATCGGTGACCATCTCTGTTGTGAAGCAGAG
ACTCATCTGTGGACGATAAAGTAGCaaaaaaaaaaaaaaaaCAGGTTTGGTTTCATAGAATACGTACAAC
AGATAGTATAGATGGAATCTTACGTGCTTGGCATCGATGCCGTCTTTAGAACCCAGACGCAATTGCTTCT
CCACCCACTCGGTCAACTTTTGGCACACCTTGTTTGTCACCGGATAGACTGTCTTTATCTGTAATTATGT
ATATTGGTTGGATCAGTACTTTTCGGGCTTGCCACTGGATGAACTGCCTCACCCGTCCCATCGTAAGACC
AGGCGTTACGTCCGCCCTTCGAGTTTTCCACTTTTCGCCAGTTAACGAGAACGGATTGTTGGCGAAAATG
AAATCGG
>dme_circ_0000434_junction_seq
AACGGATTGTTGGCGAAAATGAAATCGGGCCAGCAATTGACGATTGTCCAGGTTGC
>dme_circ_0000435
GTGTAGACCAGGCAGCAGCACTCGTTGATCGCCACATTCTCCAAACGGGTCATGTACTGATCCTCCACGT

CGCTAACGTTCATCGACTCGATCTCCGACCACTGAGGATGATGAGTTACAAAATTAGATATATCCTAGAC
TTTTTAGGGTTCCTCACTCACCCTGTAGTAGCCATCCTCCTTCTTCAAGTAGGGGGAATAGGGCTCCTGG
ATCTGAATGGCGGCCTTGAGCTTGGGCAGCTTGTCGCGAATGGCGTGAATCTTGTCCATTTGCTTGGCGT
CGTCGACGACCACGATTTGGGCATGTGAGCTCTCCAGAACGTGCTGCACTGCATCGGCGGAATTGGTGG
>dme_circ_0000435_junction_seq
TGCTGCACTGCATCGGCGGAATTGGTGGGTGTAGACCAGGCAGCAGCACTCGTTGA
>dme_circ_0000436
GGTGTGCCCGGCAAACGTAACGAAATCTATTACAACTGCTGCCCGGAACCCTATATAGACATCACCTTCG
CCATCATCATCCGCCGACGAACACTGTACTATTTCTTCAACCTGATCATACCTTGTGTACTGATTGCCTC
CATGGCCTTGCTCGGATTCACTCTGCCGCCAGATTCGGGTGAAAAATTATCACTGGGGTAAGTTGGGCTA
GCATAGCATTCCAGAGGCACACCAAACTGGAGGGGGAAGGCAAGTGCCACATTAATTTCAAACCACTTAC
GCAACGCAAATACTGGGGTATAACAAGCTGCCAACACACATGCAAACAGCTTGAGAGCCGCTCGAAAAGT
ATTTGCCAAGTACTTTTTAAATAATGACAGCCTAGTTGGCGGCGAAAGCAGCGAATTTCAAATTAGAAGC
TCCTCATAATCGTGTCGATAGGAATGAGCTACAGCTCCCCAGCCAGCTGCCTTCTCACTTTGTAACCGAC
TGACTAACCGACTGACATGGCACATTATCCATTATGGCCATCGTTACCCACTCCGCCAGTTTGGGCCCGC
TGATGACGCCACAGTCTCAGCGATGGTGAGCAAACTATTGAACTTTTAATGCTGCCATCGGAGCTGGGAG
CTGTGTAAAGTGTTCAACGAGTCGAGGAATGCTCCTTAAAGTGTGCTGCTGTCTGTCGACAGGACATGGC
ATTAGTGGCTCGGAAAATCAGTTGGGACTCGTCCTAGCCGACGACTCGGCTCCTTGAGTACACAAAACTG
AATGGCTAGTGAGGTCGAGTTGAAAAGTGCATGATACTAAAAGCTTTTGGGTAGGTTGCACCTACTCAAG
CAGTGTTAATGATGGGTCATGTCTTTCCAAATGATTTGGAAAACAATTAGTCAGAGAAGCAGACTTTAAG
AACTGTTAGAAAATGGTGAGAAATGGTTTGGCTAGGAACACAAAACTAAATAAAACGAAGTTAACACATT
TTAATTAAAACTGAAACCAAAATGGACTCTAAAAATGTAATGCAATACTTAAGAACAACAAGTAATTGCA
TCACTATCTGAAACTGCCTTGATTTTCCATGCACTTACTTTACTTAATCGAAACTTTTCATCTGATATCT
AGGGTAAGATCATTTTGTTTACCAATAGTAGTCGATGGTAGAGCTTTTCGTATATGTAACCTGTATCATG
TTATCGCTGTTCCTACTTATGCTTTTCAATCATTAACTTAACATTTATACCGTAATACTTTCATATACCT
ATCGTTTATGAATATCTATAAAGTTGCAGTTGCATACTCATTTTCTATATATCCATGCATTTCTAAAATG
ATTTTAAAATCCGAATAAAATCCGATGATCTTATAATATTAAAACTTTGACACACAATTAAAAACCCAAG
CGAATTTGGAGAGCACAGCTAACGACTTGAGTTATTTACTTTGGCAGCAAAGTCAAATGTTGTACATAAA
TATTTGGCTTTGAAGTCTAAAGCGAATATACGTGCTGATGCAACCAAAATAAATGTTTCCAAAAAAAGAA
GTAGCGATCGAAAAACTAATTAACCATAACTAGAGGGGCATATAATTAGATTGTATAGCCTTAACAAATT
TGCGAAGAACAAATTAATAACATTGTAACCCACTATATTATATGAACCTTAAAGACCAAAAGCTGATCAA
AACCTAAATTATACCACGAGTTAGTTTGAACAATGCACACATATTTGACAACGAGTTTCTTGCACGTTAT
AACTATTTTTAGTTTCGGTTGTCAAGGTATCTGAATTTTAAATACAGTTGGGTTGTTCCTTTTTGAGCGA
ATAAAATCGGATAATACAAAAGAGTCGTTAATCTCTAGTTTCTCGACTTTGAGATATCCACTACTCAGGC
AAAAAAGTCCTAGTGAAATTAAGATAGAGAGCAGACAGTTTCCAAAACTGCAGTTCTATAAATCGTAAAT
CTTTTTAAAATTGCTCAGATCAGAAAACATTTTTGTATTTAGTTAGATATTGATAGAAATCCATATGTTA
TTACTGCCTTTTCTACACTGACTCGGCTAGTGATCCTGTTCTTTAGATCCttttttttATACTTAGAGAA
GAATAAAAAGGAGCAACCCCACTTTATAGTTAAAACCAAATTATAGTCAACCGAACTAAAttttttttcca
cttttccataattttgtttttCAAAATTAAGCAATCCTCAAATCCGAATACTTACTTATCTTTCAAAAAG
AGCTAACAAAATTAAAATTGAAATTATATTGTTGAATTTCTCTAATTCCGTTCTTGGCGTACACTTTTTG
GTGTCTAACTTCTATATTTACCTAACCTATGTCTAAAGTATACAAGCTAAAGTTAACCTCGTGTGTGTGT
GAATAAAAACAGAATATAAATCTATATGTATTTTTCCTGAAAATAACATTTGCTATGCCTGTTAAAAACA
GAACGATTAAGAAGCTAACAAAACGACAATTTTTTGCATTTAATGTACTAACTAAAATATTTTGTACATA
TTTCTTGCCTTTTTCTTTCTTTCAAACAACTGTGTACCCTCCGCAAAAATGATATGTGCACACAAAATGC

GTATAAACGAAACGAAACCATACACTATGAATAATATCTGAACTTTGAAATATGCGTTACCCCAAAACGA
ACTAACACAACAACAACTTCCAATCCAACATAATAATAATGACAATGAAACTAACCAAACTAACCACGTG
CACCACTACAACAATTACAATATATTCCCACACTAAAcataataaatatatataatatttgcatatatat
atatatatTGACAACACGCCCAACCAACCAACAGGTACATATTTCAATTGCATAATGTTTATGGTAGCTT
CATCCGTTGTGTCAACGATTTTAATATTAAATTATCATCATCGAAATGCTGATACGCACGAAATGTCCGA
ATGG
>dme_circ_0000436_junction_seq
TGCTGATACGCACGAAATGTCCGAATGGGGTGTGCCCGGCAAACGTAACGAAATCT
>dme_circ_0000437
TTACAACCAAGACGGAAACGGATAGCTCGGGACGCAAGGTGACCACCACAACTAGAACTTATAGCGGCAT
TCTGGTCCCAGGCAGTAATGTATTCAAGCAGATATTCCCCGACATCTCCAGAGAGCCTGGTCCAATTAAC
ACAGACTCAAACCATCCAGCCGAGAATCCTAACACCTCAGTCTTTTCCCCATCTCGTTCCCCATCTACTG
GACCTATTGATTTCAACTATGTATATAATTCCCGACCAAGCCAAAGGGAACCCGTCTTTGTGCCAGTGAT
TGACTCAACCACCACGCAGCGGACTTCAGTTCCCCGGTTCAGTTCAGCATTACCAAGAGACGACGGATCA
GATCGAGTCATAGATGATTATTTGAAACAAGTGAATCTGAGTGCCTCAGAAATTGAGAATTCGAATGGTG
AAGTGGTCAAGACTATTGTGGATAAGAATGGCAGAATTCTAACTGCCAGATTTGTTCTAAGCTCAGTCAA
AGGAACCAACGAAGGATCTCAGCAGCAGAGGCCGACCAAG
>dme_circ_0000437_junction_seq
AGGATCTCAGCAGCAGAGGCCGACCAAGTTACAACCAAGACGGAAACGGATAGCTC
>dme_circ_0000438
AACTTATAGCGGCATTCTGGTCCCAGGCAGTAATGTATTCAAGCAGATATTCCCCGACATCTCCAGAGAG
CCTGGTCCAATTAACACAGACTCAAACCATCCAGCCGAGAATCCTAACACCTCAGTCTTTTCCCCATCTC
GTTCCCCATCTACTGGACCTATTGATTTCAACTATG
>dme_circ_0000438_junction_seq
TCTACTGGACCTATTGATTTCAACTATGAACTTATAGCGGCATTCTGGTCCCAGGC
>dme_circ_0000439
GACATAAAGTTGGTGACTAGCGACGAACGGGACGTAAATCGCGTCAAGGAGCTGCTGGACATTTTCCAGG
CTTTTCAAGCTAGCAACGATGTGGACGACGGCCCATTCACTACCCTCGTGCACGGAGACCTGTGGATCAA
CAATATGATGCTAAAATACGGTATGAGGGGTTAGTTCTTAAAGCAAAACAGGCTATATTAATACTTTAAG
AATTTTGTCAGGTGAGGAAGGCACTCCACTCAAGGTGAAAATTGTTGACTTTCAAATCGCGCAGTACGGA
TCATTGG
>dme_circ_0000439_junction_seq
TTTCAAATCGCGCAGTACGGATCATTGGGACATAAAGTTGGTGACTAGCGACGAAC
>dme_circ_0000440
GCAGTGGAATTGTGGGTGCCATGTCTTCCAAATACGGCAGCTCAACGTTGGCGCACAACCATCACAATGT
GACGCACCACAATGCATCGACTGCCACCATTGCAACGACCACGCACCACCATCACCACCATGGTGGGCAG
CAGCAACAGCAGTCGTCGTCGGGAGCATCCACCATGGCCATG
>dme_circ_0000440_junction_seq
GTCGTCGGGAGCATCCACCATGGCCATGGCAGTGGAATTGTGGGTGCCATGTCTTC
>dme_circ_0000441
TTGGCACCCAACAAGCAAAACAAATGCAAATGTGCGCCCCAAAGACATATTACCCCGAAAGTGATCAATA
ATTGTTTGCCGGAAGAGGAGATACAACTCAACCAGTTAAGCACCGGACTCAGGGTGACTACTCTTCGTGG
ACACGATTCTCTTTTGGCTAAAACGACAACTCAGCGAAGTCGACACAATGTTGGATCGATGCGAAATGCC
GGGTAAGTAGATGGGATCGATTACGTGCGGGCTGTAAACAAAACGTTAATAACCTGGGATAAATGAAACT

AGATTTGAAACCTTAAACTAGATTGGGAACATACTGTCAAGAATGTATTTGATATATTTGTACAAGTAAA
ATATTCATTGAACAAGTATAATTTACAATGATGAAATGGAAGAAGATACGCAAACGTTTGGTTAATAAGG
AATTTAGCCAGAAAGTATGCTTATTTTTGCGCAACGGAATCACGGGCTATAAGCTTCTTCAGTCCGACCT
CGTTTGGTGCCCGGGAGTATGCTATACAATACCATTACTCATCCGAACTTCACTAATCATTGGAATGCTT
ACTTTCCCCATGCAGAATCCAGCTGGACAACGAGAAGTTGCGGCGGGATGTTCGATTGTCGGGCTCTCGA
CTTGATTTGCCACAACTCTGTAATGGATCCCGTCGGTTGGATGGCCACAACAACCATGTGGCTGCCAATG
AGAACACGGTTACAACGACATCCCTGaatggaaatggcaacggcaatggcaatagcaatagcaaCAACAA
CAACAACATCGGATCGCCCGTCTCCTCATCGACAACAAACAGCAGTAATGGCGGCAACGAGCGCGGCTCC
TCCACAAAGTCCAATAGCAGCAGTGGAAGTGGCAGCTCGGGAAACTCGGCCAGCAGCACGGGAAGTGGCG
AGCTCAAATGCAATACGCCCATGACGCCATCTGGGTAAGCAGGAGCATCACCATTAACCCTTTAATGCCG
GCTGGTGCTTTAAGTGTTATTTTTAGTTTTAAACCACAGAAAGAAAACCCTTGCCATTTATAGAGTGTTA
TAAACAGTATTTAATTCttttttttAAGCTTTTACATATTACTAGATCTTTGTTTGTTAGTCTGAAGAGT
GTAAATCCTGAGTGGGTTAAAAACACCCTCCATAAGCTTTGGATAGAGAGACTGTCTATTTGGGGCCAGA
GCTCAGGGATGCTTTGTGCGTTTGCTGGCTTGCATCGCTGCAGCCCAATGCCAATTAGTTGAGCACACCC
TTCGGGCATTTGGTGGCCACACTCCATCATTCCACAGCTCTCTCTAATTAATTTTATATAAATTTCAGTA
AAAAAGCAAGTGAAATGGCATTGAGGTCAGGGTCAATGGGGTTTTAGAGTGTGGCAGTTGCCACATGGGA
ACATTTTCAATTACCTGTTCACTTGGTTACAAAATGGGTAAAAGGACGAGCTGTTTCGGTGGAAAGTAGT
AAGCACGAAAAAGCTTTTAGCCATGACGGAAATTAAATTTCAGATGCAGATGCAAATAAAATGAGAACTG
CATATTTCACTTACCATATATTGAGTTATTTTTAAAAACTATTGATTTCTTATGTGTACAACATTTGTTT
TCATTTTTAACAGAAGCTGGTGAAGAAGTTCCGCAACTACCTAACCGATCTTGAGTTCGAAGAGCTCAAG
GTGTACAAGGAAGTGTGGTATTTTGGCCAGCACGCCAGCAAGAACTACAACAAACCCGCCCCCACAGCCA
ATACCACAAATCTGGGCTATGACGATGATAATGGCAACTACAAGATCGATCGAACACGATCACATAGCTT
TTCGTTACGAGATTCTGGAGGTAATTGGCAAGGGCAGTTTTGGCCAGGTCATCAGGGCGCTGGATCACAA
GACCAACACACACGTGGCCATCAAGATCATAAGGAACAAGAAGCGCTTCCTCAACCAGGCCGTCGTCGAA
CTGAATATCCTCGATGAGCTGCGCGAAAAGGACGCGGATGGATCTCATAATGTCATACACATGCTCGACT
ATACATATTTCCGCAAGCATTTGTGTATTACTTTTGAGCTAATGAGG
>dme_circ_0000441_junction_seq
TTTGTGTATTACTTTTGAGCTAATGAGGTTGGCACCCAACAAGCAAAACAAATGCA
>dme_circ_0000442
TTGGCACCCAACAAGCAAAACAAATGCAAATGTGCGCCCCAAAGACATATTACCCCGAAAGTGATCAATA
ATTGTTTGCCGGAAGAGGAGATACAACTCAACCAGTTAAGCACCGGACTCAGGGTGACTACTCTTCGTGG
ACACGATTCTCTTTTGGCTAAAACGACAACTCAGCGAAGTCGACACAATGTTGGATCGATGCGAAATGCC
GGGTAAGTAGATGGGATCGATTACGTGCGGGCTGTAAACAAAACGTTAATAACCTGGGATAAATGAAACT
AGATTTGAAACCTTAAACTAGATTGGGAACATACTGTCAAGAATGTATTTGATATATTTGTACAAGTAAA
ATATTCATTGAACAAGTATAATTTACAATGATGAAATGGAAGAAGATACGCAAACGTTTGGTTAATAAGG
AATTTAGCCAGAAAGTATGCTTATTTTTGCGCAACGGAATCACGGGCTATAAGCTTCTTCAGTCCGACCT
CGTTTGGTGCCCGGGAGTATGCTATACAATACCATTACTCATCCGAACTTCACTAATCATTGGAATGCTT
ACTTTCCCCATGCAGAATCCAGCTGGACAACGAGAAGTTGCGGCGGGATGTTCGATTGTCGGGCTCTCGA
CTTGATTTGCCACAACTCTGTAATGGATCCCGTCGGTTGGATGGCCACAACAACCATGTGGCTGCCAATG
AGAACACGGTTACAACGACATCCCTGaatggaaatggcaacggcaatggcaatagcaatagcaaCAACAA
CAACAACATCGGATCGCCCGTCTCCTCATCGACAACAAACAGCAGTAATGGCGGCAACGAGCGCGGCTCC
TCCACAAAGTCCAATAGCAGCAGTGGAAGTGGCAGCTCGGGAAACTCGGCCAGCAGCACGGGAAGTGGCG
AGCTCAAATGCAATACGCCCATGACGCCATCTGG
>dme_circ_0000442_junction_seq

AATGCAATACGCCCATGACGCCATCTGGTTGGCACCCAACAAGCAAAACAAATGCA
>dme_circ_0000443
GTTCAATATTTTCTGTACGGGCGCATTGTTTGAGGCTCGTTTGGAGAACTGCAGGCGTTCAAAACACGGA
ATCGAAATCGAATAACATAGCCCCCTCCACAAAACCAAATCAATCCAAATTCAAATCGTTCGCGAGTCGA
AAGTGAAATTCGTCGCAGCACAACGGTAAATCAAAGCCAGACGCGATTTTTG
>dme_circ_0000443_junction_seq
GGTAAATCAAAGCCAGACGCGATTTTTGGTTCAATATTTTCTGTACGGGCGCATTG
>dme_circ_0000444
GACTTTTCAAATATTTTCCGTGTGGCTAAAATCGGCCAACTCTCCGCGTCCAGCCTCATGGATACTGGAA
AAGTCCCTGGATGGGATTAACTTTGAGCCATGGCAGTATTTCGGATTGAGCGATGCAGATTGTCAGCGAC
GATGGAATTTATCCGGGCAAAATGGCAAATACGTCTTCCAGAACGATACAGAAATCATTTGCTCCACTCA
GTTTTCGAAGCCGGGTCCTTTGGAGAACGGCGTCTTGCATGCTTCGCTGCTGAAGAATCGCCCTGGAGCC
ACGGATCAAAGTCCGGAGCTAATGAAGTTCATAACGACTCGGTACATAAGGATTCGCCTGCAGGGCATGC
ACTCCACGGCCAATCAGGACAACAGTCTGGACTGGCTGCTGGACTCTCCGTCGCTGGAGAAGCACAGCTT
CTACTCGCTGTCCCAGTTGAAGGTGAGTGCCCGTCTGGATTGTAATGGGCACGCCAATCGCTCCCACGAG
TCGCCAGATGACCCTCTGATGCAGTGCATCTGCCAGCACAATACCTGTGGTGCTCAGTGCGAACAGTGCT
GTCCGCTTTTCCAGGATCGCCCCTATCAAATGGGCGGAGAGTGCGAGATATGCCAATGCTATGGTCACGC
CGAGAGCTGCACCTATGATCCCTTCCTGGACAAGGGCATTTGCCAGAGCTGCTCGAATAACACGGCTGGC
ATTGAGTGCGAGTTTTGCGAGATGGGATTTTACAGGGAGCTGGATGCACCGCTCACCGATCCCTGTTTGC
CCTGCTCCTGTAATCCTGCTCGTTCCACGGGTGGTTGCCAGTCGGATGGAGGAAGCTGTAACTGCTTGGA
GGGTTTCCAGGGCAAGAACTGCGAGGAATGTGCTCCGGGCTACTACGGAGACGATTGCAAACGGTGTGAG
TGCGACGAAAGGGGAAGTCTTGGCTCCACGGGTTCCTGCTCGGGTGTGTGCCAGTGCAAGCTCAATGTGG
AGGGCAGCACCTGCTCCGAGTGTGCGCCCGGATATTTTGATCTAAGCGCAGAGAATGCAGAGGGATGCAC
CAGCTGTTGGTGCTCAGGTGTCTCGCAAACATGTCACAGTGCCAAACTGCAGACATTGGCCTTTGAAACC
CTCAACGATTGGAAAATAACCGATATTCAAAGAGTGAAACCAATTTCAATACCAGTTGATGCCGAAACAA
ACCGTTTGATCTTTGCCAACGAACTGGATGAAGTGGAGGCCATTTACTGGCAGGCTTCGTTGGGTTACTT
GGGAAATCGCTTGACCAGCTATGGGTCGAGACTGCAGCTGGTGTTGTCGTGGGATGTGATAAGAGGCGAT
AGATCGGGAAAGCCCACGACGGGGCCCAATGTCATCCTAGTCGGCAAGAATGGACTGAAGATTGCCTTCG
GCGATGAATCCTTGGACGGATTGGGAATCAACTTGAATGTAACCCTAACAGAGGTTGGCTGGTATCACGT
GCCACCCACTGTCGTTGATATAAAAACAAGACTTCGGCGAACCGAGGGCGGTGACTATCACGGAGAATCT
GTTACCCGTTCCCAGTTTCTCTCCGTTCTCGTTTCCCTCGATGCTGTCCTTATTAGAGCGGCTTTTCACA
CGGATCAAGGGGAAACATCACTCGAGAGAGCAGTCATCTATTCAGGAGGTGTGGAGTTGGGCGGAAAGTC
TTCCAGCCAAGTGGAGCAATGCCTCTGCCCAGCTGGCTATACCGGTCTGTCCTGCGAGGGCTGCGCCTTT
GGATTCAAGCGGATTTACGAGAACACCTCGGACCATCAGATTCTGAGCAAGTGCATCCCGTGTCCCTGCA
ACGGACACTCAAACTCCTGCGATCTGCAGAGCGGAAATTGCGGCGACTGCATGCACAACACTTTTGGAGA
TCGGTTGTGAGCGTTGCCAGTTGGGTTACTACGGGAATCCCCTGCAGGGAACCCCCAATGACTGCAAGCG
ATGCGCCTGTCCCCTGTCCGAGGATTCCAACAACTTCTCGCCCAGTTGTCAGCTAAAGAGCTACAACTAC
ATGGACCTGAATCCGCAGTTCGAGCTGATAGAGCATGCGGAGTACATATGCACCCAATGTCCGGAAGGTT
ATACGGGTGATCATTGTCAGGTGTGCGACGATGGGTACTTCGGGAATCCACGCCAGCCGGGAAGCAGTTG
TCAGCGCTGCGATTGCGCCGGAGGGCCCTGCAACGTGACCACCGGGGAGTGCATCACGTGCCGCGGAAAC
ACAGAGGGTTGGCACTGCGAGCGCTGCAAGCTGGGCTACTGGGGTGATCCGGCCGTGGGCTGTGATCCCT
GTCATTGCCACACGGAAGGATCGGAGAGCGGACTATGCGACAGCACCGATGGTCAATGCCTGTGTAAGCC
ACGTTATGCTGGACAGAAGTGCGATGAATGTGACGTGGGCTATGCTAATGTTGAGCTACGCTGTCCATCC
TGCAACTGCGATCCATTGGGCTCGTTGGTCCAGGACAGATGTGATCCCCACACTGGACAATGCCATTGTA

AGGAGGGCGTTATGGGTGCCAAATGCCACGAGTGCCAGGATGGCTACTTCGGCATGAATGCTGTGGCCTA
TCGAATGGATGACCTTGCTGCACTGCGTCAAAACTCCGATAGCGACGATGACGAATGGGAATTGGTTCCA
GATACTGAAGATCCCAATAGCGAATCCACGGTGGCCTGCGAGG
>dme_circ_0000444_junction_seq
AATAGCGAATCCACGGTGGCCTGCGAGGGACTTTTCAAATATTTTCCGTGTGGCTA
>dme_circ_0000445
GCTACCTAGAGCAGCAGGCCGAGAAGGAGCAACAGGTGTTCGAGGTGTGGAACAACAGCATGGCCCAGCA
ACTGCAGCAGCTCCGGGATCAAATAGCCAAGGCCAGACATGCAGCAGAAGCGGTACGTATGTCATACTTA
ATATACGGCCAGTTATTTAAATAAAAATACTTGTTACAGATCGATGTTTCCCTGGAATCGCTGGGTCCTA
AATGCATTCGATCCTATTTGCCCGCCTCCTACGGTTTGAGTACCTCGAATAAGATTCGTATGAGCTTCGC
CTTATCCAATCACCTCGAGAGCTCTCCATTGATTCACCTGGCCAGCAGCGAGGGGCGTCACATCACCCTG
GAGCTGTACAAACGTCGTGTGCGCCTGGTGTGGAATCTTGGTGGCACCACCGCAACCGTTACGCATCCCA
TGGTGGTGCATACTCGTGACCCCAAGTACGACGATGCCTG
>dme_circ_0000445_junction_seq
CTCGTGACCCCAAGTACGACGATGCCTGGCTACCTAGAGCAGCAGGCCGAGAAGGA
>dme_circ_0000446
TTAACCCAAACATGCAATTGACCATCGACCAGGCTAATGGAGAAGTTGCCCTTAATGTCCTTAGCATCAT
AGTCGCCCAGGATCTCAGGCGGATAGGCAGCAAGGAGCAGCAGGCAATCGGGCTGAAGGGTGCGGAACAC
CAAAGCGGTATTGGAGCGCTTTCGTAGTGACTGCGATGGCAGCTCCAAG
>dme_circ_0000446_junction_seq
TCGTAGTGACTGCGATGGCAGCTCCAAGTTAACCCAAACATGCAATTGACCATCGA
>dme_circ_0000447
TTAATGGTCCGATTGTTGAACACCACATCCCGAATGGCTCCTTCTAGATTTACAAAAGTCGGCGCCAATG
GCGTATAACTTTCGTGCGGCGGAGCTCCACCTATATACAGGCCACCTGCATCCCTCGGCAGGCTGACCAC
CGTGGGATTGCCGTTTAGATTGCGAATCTCTTGCAGCTCATCGTCCACCATCAGCTCCAGCTTGCGACCG
GTCTTGATCAAATGTACCACG
>dme_circ_0000447_junction_seq
GCGACCGGTCTTGATCAAATGTACCACGTTAATGGTCCGATTGTTGAACACCACAT
>dme_circ_0000448
GGAACTGGCTTATGATGATCCCCGACTGGCTTATGGCCGTTTCGGTTACCTGGAAGGCCACGAATACCGC
ATGTACAACACCTACGATGTCCATTTCTATGCATCGCCGGCTCTGGCCCACCTGTGGCCCAATCTTCAGG
TCAGTCTGCAGTATGACTTCAAGGATGCCATTGCCGCAGAACTAAACGATACTCGTAAAATGCTTTACGA
TGGCAAGGTGATGCCGCGCAAG
>dme_circ_0000448_junction_seq
TTACGATGGCAAGGTGATGCCGCGCAAGGGAACTGGCTTATGATGATCCCCGACTG
>dme_circ_0000449
CTGCAGGTGACCAAGCCGCCGTACAGCATCTCCGGACCCTCCCATGTGCGCTCGGATCAAAAGCTGACCC
TGACGCTGGGCGGCGATGAGTTCCTTGGCTTCATGATCCAGGCACGCGATGGCCAGAACCGCGTCGTCGG
CCAGTTCCAGGTGGTGGACTCCGTGCACTCGCAGACCTTGGACTGCTCCGGCAAGGACGACACCATCACC
CATCTGAGTGCGCAAAAGGGCAAGCCGCTGACCGGCATCACCTTCGACTGGATTCCGCCAGCAGGCTACA
AGGGCAACG
>dme_circ_0000449_junction_seq
ATTCCGCCAGCAGGCTACAAGGGCAACGCTGCAGGTGACCAAGCCGCCGTACAGCA
>dme_circ_0000450

GCCAAGGAACTCAAGAAGCGCGTGGAGGTGGCTCATGTGGCGGAGTTGCACGCGGACGAGGCACAGCAGC
GCGAGGAAAGCGATAATGACGAGGACCTATTTGGCTTCGAGAAGAGCGATGGCGAAACGAAGGATGACAG
CGGAGATGAGGCCTCATCTGACGATGATGACCGAAGGGAGTATG
>dme_circ_0000450_junction_seq
TCTGACGATGATGACCGAAGGGAGTATGGCCAAGGAACTCAAGAAGCGCGTGGAGG
>dme_circ_0000451
GTTATCCGGGAGAGTACCGCCAGTGATAGGCCAGCTAATATTGCTATAGTCAATGTCTTGTTCATCTCCA
GCTCTTAAAATACTTTCATATACCGACTGTACGACTTCAGAAGCAAGGTAATTGCAAGTGACAGCTGATG
CATGGACAGAGTGAAGATATGGCGCTTTGATAGCACAAATATTAGGGTCAGTAAGATCGTAAGCGCAGAG
TTCCTCTTCAAGTA
>dme_circ_0000451_junction_seq
TCGTAAGCGCAGAGTTCCTCTTCAAGTAGTTATCCGGGAGAGTACCGCCAGTGATA
>dme_circ_0000452
GATAAAAGCACAAAACGATTGCAAAGGCTGACACATCTCTGGCTTTTGCGCTATTCTGTGATACTAAACT
GTAGTTTAAAGAGCGCTAGTTACACTTAAGTTTCGCAAACAGAAAATAATCAACCACCTAAAGAACAGTA
GTAGCCGCAAAAATAGCAGGATCACTCTCTTCTATCATCGTAAATGTCTACATTGTTAG
>dme_circ_0000452_junction_seq
CTATCATCGTAAATGTCTACATTGTTAGGATAAAAGCACAAAACGATTGCAAAGGC
>dme_circ_0000453
CGCTGGCAAAGAAGATGGTTTGTTCTCTATGATGATGGCGAGCTGACGTACTCGGTTGATGATTATGTAA
GTAATTCATTTGAACATATTTTAAACCATTTCCATTTTTATAGCTAATAATAAAATTAAAACGAATTTTT
GAGTTTGTAATAGCTAATCAATCCATAGGCATAATTGTGTTTTTCTGTTTTAAATACCGTCTCGTTGGTT
CTCCAGTGATAACaaaaaaataaaaagataataaaaCTTTCTGTTATGGGGTGGTCTGAATTTATAAGAT
AACAAAGCTCATAAAATATTATACAATCCCAAAAGAGCTTCTAATTATGGTATTATAGCATTGCTGGACG
TTATCGGTAGAACCAATAAGTGTGCTGGCGGTAAAAACAACAATAAATGTACCTTTCCTTAACCAGCCGA
TTATTTATGAAGTGAAAGAATTAAAGTGGCTTCTTAAATTGCCACCAGATTGATTTACTTTTACTAACTT
ACTGAACTATTAATTAGATTTTATATTATTTATTAGTCATTAAATTAAAAAATGTATATTATTACGCGAT
AGGCCGAGTTCATTTGATTACCGATTAATTAATTCTCCTAATTAAAATCTTAAAATACAACTTGTTTACT
CTTTAATTTACATATATTTTCTATAGCCCCGAAACCATACCGCAGGCGTGCGTTGATATGACCAAAGTGC
TGGAGGTTACCAGCGCCGTGGAGGTGACAGGTCACCCCAACTCCATCGCCATAACCGCTCCCGAGCGCGT
GACCTTTGTGAAGGGCACCAGTTCAGAGGAATCCCAGTGGTGGCTCAACATCCTGGCTGCCTTTCCCAAG
TCGAAGGGTCGCCACAAGAGAAGCGCCACCCTGCCCGGTGGTCAGGTGGTGGGCAGTCTACGCCCAACGA
GCAACGGCGACCTAACACTATCCACTAAGCTGGGCAATCGGCACAGCTCCTATCACAAGGACACCCTAAC
GTCTTCCCAGTCGGCAGGCAATCTGTTGAGCAGCCTTGATCTGGGTCCCAGCTCCACGAAGACCGCTGGT
TCGCCTCTGACCACCACGCAATCAGCTCTCGCCGATGATGAGGAGGACGACGGCGTGGAAACGGGCGAGG
ATGTCGACGAGGACGAGGAGGATGAGACGTCGGTGCCCAGAAAGTCAAAGACGGTCAGCATGGGGGGCCA
GGACGAGAACAATCGTAATGCCGGCAACGAGATCACAAATCGGGG
>dme_circ_0000453_junction_seq
ATGCCGGCAACGAGATCACAAATCGGGGCGCTGGCAAAGAAGATGGTTTGTTCTCT
>dme_circ_0000454
GTGCAGTTGTCAGTTGCAGTTCAGCAGACGGGCTAACGAGTACTTGCATCTCTTCAAATTTACTTAATTG
ATCAAGTAAGTAGCAAAAGGGCACACAATTGAAGGAAATTCTTGTTTAATTGAATTTATTATGCAAGTGC
GGAAATAAAATGACAGTATTAAATAGTAAATATTTTGTAAAATCATATATAATCAAATTTATTCAATCAG
AACTAATTCAAGCTGTCACAAGTAGTGCGAACTCAATTAATTGGCATCGAATTAAAATTTGGAGGCCTGT

TCCGCATATTCCTCTTGGAAAATCACCTGTTAGTTAACTTCTAAAAATAGGAATTTTAACATAACTCGTC
CCTGTTAATCGGCGCCGTGCCTTCGTTAGCTATCTCAAAAGCGAGCGCGTGCAGACGAGCAGTAATTTTC
CAAGCATCAGGCatataatatactaatactaatactaatactaatataagaatactaatataGAAAAAGC
TTTGCCGGTacaaaatcccaaacaaaaacaaaccgtgtgtgccgaaaaataaaaataaaccataaaCTAG
GCAGCGCTGCCGTCGCCGGCTGAGCAGCCTGCGTACATAGCCGAGATCGCGTAACGGTAGATAATGAAAA
GCTCTACGTAACCGAAGCTTCTGCTGTACGGATCTTCCTATAAATACGGGGCCGACACGAACTGGAAACC
AACAACTAACGGAGCCCTCTTCCAATTGAAACAGGGCAGCGCTGCCGTCGCCGGCTGAGCAGCCTGCGTA
CATAGCCGAGATCGCGTAACGGTAGATAATGAAAAGCTCTACGTAACCGAAGCTTCTGCTGTACGGATCT
TCCTATAAATACGGGGCCGACACGAACTGGAAACCAACAACTAACGGAGCCCTCTTCCAATTGAAACAGA
TCGAAAGAGCCTGCTAAAGCAAAAAAGAAGTCACCATGTCGTTTACTTTGACCAACAAGAACGTGATTTT
CGTTGCCGGTCTGGGAGGCATTGGTCTGGACACCAGCAAGGAGCTGCTCAAGCGCGATCTGAAG
>dme_circ_0000454_junction_seq
CAAGGAGCTGCTCAAGCGCGATCTGAAGGTGCAGTTGTCAGTTGCAGTTCAGCAGA
>dme_circ_0000455
GTGACGGTCACCTTTGGATTGATTGCCTTCAGCTCGGCAATGGCAGCCGGGTTCTCAATGCGGTCGAGGA
TCACCAGGTTCTAGGATTGAATACACGAGATTAACCTCCATCGCTGCATGGAGCCTGTGGGCATCGCATA
GTTACCTTCAGATCGCGCTTGAGCAGCTCCTTGCTGGTGTCCAGACCAATGCCTCCCAGACCGGCAACGA
AAATCACGTTCTTGTTGGTCAAAGTAAACGACATGG
>dme_circ_0000455_junction_seq
TTCTTGTTGGTCAAAGTAAACGACATGGGTGACGGTCACCTTTGGATTGATTGCCT
>dme_circ_0000456
GGGTAGAAGGTGACGGTCACCTTTGGATTGATTGCCTTCAGCTCGGCAATGGCAGCCGGGTTCTCAATGC
GGTCGAGGATCACCAGGTTCTAGGATTGAATACACGAGATTAACCTCCATCGCTGCATGGAGCCTGTGGG
CATCGCATAGTTACCTTCAGATCGCGCTTGAGCAGCTCCTTGCTGGTGTCCAGACCAATGCCTCCCAGAC
CGGCAACGAAAATCACGTTCTTGTTGGTCAAAG
>dme_circ_0000456_junction_seq
ACGAAAATCACGTTCTTGTTGGTCAAAGGGGTAGAAGGTGACGGTCACCTTTGGAT
>dme_circ_0000457
CTCCGTTGATCAGGACATCGACGGTCTTCAGCTGGGCGAAGATGGTCTTCAGCAGCTTGGTGGTCTCGGC
AATGGGCACGGTCACATCATAGGGGTAGAAGGTGACGGTCACCTTTGGATTGATTGCCTTCAGCTCGGCA
ATGGCAGCCGGGTTCTCAATGCGGTCGAGGATCACCAGG
>dme_circ_0000457_junction_seq
GTTCTCAATGCGGTCGAGGATCACCAGGCTCCGTTGATCAGGACATCGACGGTCTT
>dme_circ_0000458
GAACCCGGCTGCCATTGCCGAGCTGAAGGCAATCAATCCAAAGGTGACCGTCACCTTCTACCCCTATGAT
GTGACCGTGCCCATTGCCGAGACCACCAAGCTGCTGAAGACCATCTTCGCCCAGCTGAAGACCGTCGATG
TCCTGATCAACGGAGCTGGTATCCTGGACGATCACCAGATCGAGCGCACCATTGCCGTCAACTACACTGG
CCTGGTCAACACCACGACGGCCATTCTGGACTTCTGGGACAAGCGCAAGGGCGGTCCCGGTGGTATCATC
TGCAACATTGGATCCGTCACTGGATTCAATGCCATCTACCAG
>dme_circ_0000458_junction_seq
CGTCACTGGATTCAATGCCATCTACCAGGAACCCGGCTGCCATTGCCGAGCTGAAG
>dme_circ_0000459
GGTGACCGTCACCTTCTACCCCTATGATGTGACCGTGCCCATTGCCGAGACCACCAAGCTGCTGAAGACC
ATCTTCGCCCAGCTGAAGACCGTCGATGTCCTGATCAACGGAGCTGGTATCCTGGACGATCACCAGATCG

AGCGCACCATTGCCGTCAACTACACTGGCCTGGTCAACACCACGACGGCCATTCTGGACTTCTGGGACAA
GCGCAAGGGCGGTCCCGGTGGTATCATCTGCAACATTGGATCCGTCACTGGATTCAATGCCATCTACCAG
>dme_circ_0000459_junction_seq
CGTCACTGGATTCAATGCCATCTACCAGGGTGACCGTCACCTTCTACCCCTATGAT
>dme_circ_0000460
TTGACGGCAATGGTGCGCTCGATCTGGTGATCGTCCAGGATACCAGCTCCGTTGATCAGGACATCGACGG
TCTTCAGCTGGGCGAAGATGGTCTTCAGCAGCTTGGTGGTCTCGGCAATGGGCACGGTCACATCATAGGG
GTAGAAGG
>dme_circ_0000460_junction_seq
GGCACGGTCACATCATAGGGGTAGAAGGTTGACGGCAATGGTGCGCTCGATCTGGT
>dme_circ_0000461
AATGGCCGTCGTGGTGTTGACCAGGCCAGTGTAGTTGACGGCAATGGTGCGCTCGATCTGGTGATCGTCC
AGGATACCAGCTCCGTTGATCAGGACATCGACGGTCTTCAGCTGGGCGAAGATGGTCTTCAGCAGCTTGG
TGGTCTCGGCAATGGGCACGGTCACATCATAGGGG
>dme_circ_0000461_junction_seq
GGCAATGGGCACGGTCACATCATAGGGGAATGGCCGTCGTGGTGTTGACCAGGCCA
>dme_circ_0000462
GAGCTTCTCGGCAACCTGAGGCTCAACATCCAACCAGGAGTTGAACGTGTGCACCAGGGTGGTGCGGGTG
ATGCCGGGGTTCACAGTGTAAGCCGTCACGCCGGTAATGGGGGCCAGTTTCTAAAGGTGTTATAAATCAA
ATTAGTTTTGTTTTTTCTTGAAAACTTTGCGTTTCCTTTGATCAACTTACCGCCAGGGAGCTGGTGAAGT
TGACCACGGCGGCCTTGGTGCCGGAGTAGACGGGCACCTGGTAGATGGCATTGAATCCAGTGACGGATCC
AATGTTGCAGATGATACCACCGGGACCGCCCTTGCGCTTGTCCCAGAAGTCCAGAATGGCCGTCGTGGTG
TTGACCAGGCCAGTGTAGTTGACGGCAATGGTGCGCTCGATCTGGTGATCGTCCAGGATACCAGCTCCGT
TGATCAGGACATCGACGGTCTTCAGCTGGGCGAAGATGGTCTTCAGCAGCTTGGTGG
>dme_circ_0000462_junction_seq
GCGAAGATGGTCTTCAGCAGCTTGGTGGGAGCTTCTCGGCAACCTGAGGCTCAACA
>dme_circ_0000463
ATGATACCACCGGGACCGCCCTTGCGCTTGTCCCAGAAGTCCAGAATGGCCGTCGTGGTGTTGACCAGGC
CAGTGTAGTTGACGGCAATGGTGCGCTCGATCTGGTGATCGTCCAGGATACCAGCTCCGTTGATCAGGAC
ATCGACGGTCTTCAGCTGGGCGAAGATGGTCTTCAGCAGCTTGG
>dme_circ_0000463_junction_seq
TGGGCGAAGATGGTCTTCAGCAGCTTGGATGATACCACCGGGACCGCCCTTGCGCT
>dme_circ_0000464
TTGACCACGGCGGCCTTGGTGCCGGAGTAGACGGGCACCTGGTAGATGGCATTGAATCCAGTGACGGATC
CAATGTTGCAGATGATACCACCGGGACCGCCCTTGCGCTTGTCCCAGAAGTCCAGAATGGCCGTCGTGGT
GTTGACCAGGCCAGTGTAGTTGACGGCAATGGTGCGCTCGATCTGGTGATCG
>dme_circ_0000464_junction_seq
GGCAATGGTGCGCTCGATCTGGTGATCGTTGACCACGGCGGCCTTGGTGCCGGAGT
>dme_circ_0000465
TATCACTTCTTAGATGCCGGAGTCCCAGTGCTTGGTCCACTGGATGGCCTCCAGGGTGCCCAAGTCCAGT
TTCCAGATGGCTCCGTTCTGGTTCAGCTCGATAGCCTTGACGAAGTTCTCGGCGCAGGCCAACGAGGGCT
GGGTGGGATGAGCCAGGAGCTTCTCGGCAACCTGAGGCTCAACATCCAACCAGGAGTTGAACGTGTGCAC
CAGGGTGGTGCGGGTGATGCCGGGGTTCACAGTGTAAGCCG
>dme_circ_0000465_junction_seq

GTGATGCCGGGGTTCACAGTGTAAGCCGTATCACTTCTTAGATGCCGGAGTCCCAG
>dme_circ_0000466
TCCCAGTGCTTGGTCCACTGGATGGCCTCCAGGGTGCCCAAGTCCAGTTTCCAGATGGCTCCGTTCTGGT
TCAGCTCGATAGCCTTGACGAAGTTCTCGGCGCAGGCCAACGAGGGCTGGGTGGGATGAGCCAGGAGCTT
CTCGGCAACCTGAGGCTCAACATCCAACCAGGAGTTGAACGTGTGCACCAGGGTGGTGCGGGTGATGCCG
GGGTTCACAG
>dme_circ_0000466_junction_seq
GTGGTGCGGGTGATGCCGGGGTTCACAGTCCCAGTGCTTGGTCCACTGGATGGCCT
>dme_circ_0000467
TCCAGTTTCCAGATGGCTCCGTTCTGGTTCAGCTCGATAGCCTTGACGAAGTTCTCGGCGCAGGCCAACG
AGGGCTGGGTGGGATGAGCCAGGAGCTTCTCGGCAACCTGAGGCTCAACATCCAACCAGGAGTTGAACG
>dme_circ_0000467_junction_seq
GGCTCAACATCCAACCAGGAGTTGAACGTCCAGTTTCCAGATGGCTCCGTTCTGGT
>dme_circ_0000468
ATGCCGGAGTCCCAGTGCTTGGTCCACTGGATGGCCTCCAGGGTGCCCAAGTCCAGTTTCCAGATGGCTC
CGTTCTGGTTCAGCTCGATAGCCTTGACGAAGTTCTCGGCGCAGGCCAACGAGGGCTGGGTGGGATGAGC
CAGGAGCTTCTCGGCAACCTGAGGCTCAACATCCAACCAGGAGTTGAACG
>dme_circ_0000468_junction_seq
GGCTCAACATCCAACCAGGAGTTGAACGATGCCGGAGTCCCAGTGCTTGGTCCACT
>dme_circ_0000469
TATCACTTCTTAGATGCCGGAGTCCCAGTGCTTGGTCCACTGGATGGCCTCCAGGGTGCCCAAGTCCAGT
TTCCAGATGGCTCCGTTCTGGTTCAGCTCGATAGCCTTGACGAAGTTCTCGGCGCAGGCCAACGAGGGCT
GGGTGGGATGAGCCAGGAGCTTCTCGGCAACCTGAGGCTCAACATCCAACCAGGAGTTGAACG
>dme_circ_0000469_junction_seq
GGCTCAACATCCAACCAGGAGTTGAACGTATCACTTCTTAGATGCCGGAGTCCCAG
>dme_circ_0000470
TCAGATTGCCAGACACGACCTGTGCGAGCGAGTGTATGTGCGATGTCTATCGCCCAAAAGTCGAAAAAGA
GTGGAGTTGAGTTTGAGTCCCCGGCAACTTGCAAACTCATGTGCGGCaataaaaataaaaataaaaataa
gaaatacatactcgtacagctggaaaagaaaaagaaacaCAATCAAATGCAAACATATGCTGCTGCCGCT
GCTGCTGCGGCGCACAAGAAGCCGAAAACGTTGAAGGTGATTTTTACTTTTACTTACTGAAATTCATGTG
CACGATTTTCCACCATCGGACGTCGATTTCAAATGCTGAGCTTTCAAATGAAGAGGCCACCACCACGGAG
CAGCATCCACATCCATCCACGCAGTACGTCTCTACGAATCCAGATGCGAAAGATGATGAAGCTACCAATA
AAAGCCTCCACCTAGCCCTTCTTCATCTCCGCAAACCGATTCATTGAAAGTGAAATGTTGCCTTTAAACT
ATTGTTTTGTAATTTCTTGTTTTCTCGTGGCTGCCCCGTGCGTTGTTTTCCCATTTGGAAATCAATTTCT
GACAGTTTTTCTATTGTAATTTCCTAGCCATCACAATAGTAAAAGGATTGCATTTTCCGCCTTAGTGTGT
GGCCACTGGTTGAATGACTAGATTTTTATATCATTTATTTTATTTACTTCACTATGACTTGTTTGGCTTG
GCTTTTACGAGTTACCCACGGAGTTTTAATTAGTTTAGTCTGGTCGAGGTTTCCTTTTGAGCAATTGTAA
TCTTGACTTTAATTGTTGCCTTATGGGTTTGTTGCTTTGAATTTGCGGTTATTCTTTAATTGTGGTAACC
ATAATGCGTAAATCGACGGAAATCGCAATTGGATCAATTACAAAAAGATCTGAAGGGGCCGTTGATTGTT
ATTAATTGATGTTATGTTTGttttttttttttttttAAGGGAAAAGGTTTCCTTCTATCAGATAATATAG
CTAGCCAATAGCTTTATATAGAAAAATGAATAAAATGGAGAGGAAACCTATAAAGCATCTTCATTTCCTT
ATGTTGCGTTTCATACTCAATTTCTCCACTCATAATGAAGACTAATTACCGGTCCTAAACGATGTCACCC
ATGCAGGTGATCTCATCCAAGTCGATTCCAAGCCATTCCCGCACAGCATGATGCACACATGCACTTCCCA
CAGAGCGTGCAACGAAAGTGAAACTGGAAAGAGAAAGACGCGAGGTGTAGGAAACTGAAATTTCCAATAT

TTTTGTTTTTGTCTTTCTTAAATATTACACAATGCAGCCGAACGCCCATCCAAGTCAATATTATGTTATG
TTAATAACTCGGAGGCCGCCGATGGGACTGCAACAATGTTGGGACCCAAACTTTCCCACTCTAGCTGGGC
GATGCAGTCGAAATTCTTGGCATGCAATGCAGAGAACTGGTCGGATGTTGGGGATACGTTGACTCGCCTC
GAATCGGATTCGATCGCTGGCAGCGGTTCCGGATCGGATCGGATCGGTTCGCGGACAAACAGACATACTG
TCTGCTTGCCCCTCAGTATCGCCAATGCGATGGTCGTATCTCTTTGGCCCTCGCTTCGATCCGCtttttt
tttCGTAAATGGAGCTTGAAGCGCGACCGCGCACCGCAGGCGATGATCGAGCGATTGCACTGAGAAAAAT
TACAAAAGATGGTAATTCTACTATATAGCTAGCTTCTTTTTCTGTAGTTTTTCGAATCAAATTTTGGACA
AAGAACTGTTGAAAAATCCGTAAATTTCATAGTATTTTGGAATTGTTATtgttcacacatgaacacgaat
atatttaaagacttacaattttgggctccgttcatatcttatgtaaatgaatcgagagcgataaattata
tttaggattttgttatctaaggcgacatgggtgcattgctcaaaaacatgtaatttaagtgcacactaca
tgagtcagtcacttgagatcgttccccgcctcctaaaatagtcccttagtgggagaccacagataaggtc
ctcgccgctcaagataggcagatgtgcccgagcgtgggacctcgataaggcggggactatttacttaggc
ctctgcgtaggccatttactttaagatgcgattctcatgtcacctatttaaaccgaagatatttccaaat
aaaatcagtttcttacaaaaactcaacgagtaaagtcttcttatttgggattttacatttggtcaatcga
gcctttaatcgactctgcagtttcccccctaccaaaggtaaggaactcagagaaaggccagctcctttaag
catcttacagctaaaggtagcaaaaataagtgactcttgtttcccccctaccaaaggtaaggaacagagta
taaaaataaaaagcaaaagatacaaaagaatcttttatgttttaaaacaagcaccttatagtctatagct
aaaggttgctttgtgtaccattataaattgtggtaaggcgtgcttgaggccatacatcagcaattgtgaa
attaaaaagtgcataacaaaagtgccttataaatgctctaatagcattaaatcagctcataaatagagtg
cagtgtatatgccataagagcataaattaaataaaaagtgcctgaaaacagtgccttataaatgctctaa
tagcattaaatcagctcataaatagagtgcagtgtatatgccaaaagagcataaatgccgaaataaatgg
ctaaaaaacaaaaaatctgactggactacaaaaataataaaacgtgccaaaaaaaaaaaaaaaaaaatca
tctttaaacatcgacggagccttaaagaagagaaggaagtcaaattcaaaggagcctctaccagcagcag
aagcagcaacaacagcagcagcagaagcagcaacagcagtagcaacagcagcaacaacagcagcaacagc
agcagcaacaacaacgacatcagctaagtcaaaacaagaattttctgtttatccaaacacacatatatat
ataaatacatataaaatacatatacacgtactatatatattaagaaattacaaaaaattttcaaaatgat
gtcagaaaagactattcaattccttaagaagcagtccgaaattattttggaaattagaaagttggaagta
aaaccaacattaacagatgtagaaattctaaaattaaatgagcttcaaaaatgtttcattgctaatcata
gcaatttgttaaagatcggcgttgtcgatcatgaatattttaacgcgaagcagtatgatttaataatgat
ggtgttagaaaaaattaaaaataaaaatgaaaaaattaagggcgagtcggtagaaaacactttccctaaa
tcaaacactgtccctaaatcaaaccctcccccctacattaaaccttgaaatgcgtggtcaccctgaaaaag
agggtatagcacaaaacaacgctttaaaagtagagcaggcatttcgtaataatgttggccaatttcgagt
atatctagaagatacgtctaaactaatagacagtagtccagatttccttaaaataaggaaaaataaaatt
gaatttttatggcataaaatagataacctgattgaacaggtgaatagtcattttgagagttcgctattcg
aagaagaaattagcgaacttgaatttgacaaacaaaatattcttacagccattaatagtcgactcagtgg
cacaataaataaagctgaaatgtcgacggttgttaaggcggaggagttaccaaccctgcctaaaatacag
attcccaccttctttggtgattccaaagaatgggatctttttaatgaactctttacagagctcatacatg
tgagagaggatctcagtccttctctcaaatttaattatctaaagtcagcattaaaaggagaagccagaaa
tgtggttactcatttactgctcggctctggagaaaattatgaagccacttgggagtttttgaccaagcga
tatgagaataaaagaaacatattctcagatcatatgaataggcttatggatatgccaaatttaaatttag
aatccaataagcaaataaagacatttattgacacgattaacgagtcaatttatattataaaattaaaggc
acaattaccagaagatgtggatgcaattttcgctcacataattcttcggaaattcaataaagaatcactc
aatttatatgaaagccatgttaaaaagacaaaagaaatacaggcactttctgatgtcatggactttttag
agcaaaggctcaattctatatcatcattctcacaggaagtaaaacctgtaaagaaaatgattaataataa

caagaataaaaattatagtgacaattgtgcatattgcaaactaccagggcattatttaattcaatgccat
aaatttaaaataatgaatccagcagaacggtctgactgggtaagaaaaaatgggatttgcctaagatgtc
tgaggcatccgtttggtaaaaaatgtataagcgagcagctttgttcgacttgtcgtaaacctcaccacac
gttacttcactttgcaggtcataatccagaaaaagtgaatacgtgtagaacaacaggtcaagccttgttg
gccacggccttgattcaagtaaagtcgaggtatggaggctttgaacaattaagagcattgattgatagtg
gctctcaaagcacaattatttcagaagagtctgcacagattctaaaattgaaaaaatttcggtctcatac
tgaaataagtggagtatcttccacaggaacgtgcatctccaagcacaaagcggttatttcgataagaaat
tctccgaaaaatttagaaattgaagcaattattctcccaaaacttatgaaggcacttccagtcaacacga
ttaatgttgatcagaaaaaaatggaagaactttaaattagccgaccccgattttaataaaccgggtcgcat
tgatttaatcattggagcagacgtatatactcacattctgcaaaatggagttataaaaatagacggtctc
cttgggcaaaaaactgatttcgggtggatagtttctggatgtaaaaaatccaaaggaaaagaaaccattg
tagccacaacaatagaaataaaagagttagatcgctactgggaagtggaagaagaagaaaaagatgatat
cgagtctgaaatctgtgaaaataaatttatcaaaacgacaaaaaaagattcagatgggcgatacattgtg
tcaattccattcaaggaggatgtcaccttaggagattcaaagaaacaagcgatagctcgttacatgaatc
tggagaaaaaactaaaaagaaatgaaaaacttaaggttgactacactaaattcatgaatgaatacatgga
tttaggacacatgattgaagtgagtgatgaaggcaaatattttttaccgcaccaggcagtgattagagat
tcaagccttacgaccaaattgagagtagtttttgatgcttcagcaaaaactacgaataacaaaagtttga
acgacataatgtgggttgggccacgagttcaaaaagatatttttgacattattattaaatggagaaaatg
ggaatttgttgtttcggcagacattgaaaagatgtaccgacaaattaaaatagataataatgatcaaaaa
tatcaatatattttatggagaaattctccaaaagaaaaaattaaaacatataaattaaccacagtcactt
acggaactgcatctgcaccatatttggctaccagggttctggtagatattgcagataaatgtaaaaacca
agttattagtgcaataattaggaatgatttctatatggatgacctaatgactggagctgattcggtagaa
gaagctaataaattaataacattaattccccatgaattgcagaaagttggattcaacttaaggaaatgga
tttccaacaattccaaaatattaaccactgtggaggacacaggggacaataaggttctcaatattatcga
aaatgaatgtgttaaaactttaggactaaaatgggaacctcaaaaggatttatttaagttcagcgtaaat
tgtaatgatgaatcaaaaaatataaataagcgcgttgtgttatcaacgctagcaaaaatatttgatccgt
taggatggttggcaccagtcacggtttcaggaaaactttttattcaaaaactttggataaataaaagtga
atgggatcaggaattatccatagaagataaaaattattgggaaaaatataaagaaaatttattattgtta
gagaatattcgaatcccaaggtggattaattcaaacagttcttcagtcattcagattcacggatttgcgg
acgcctccgaaaaagcatatgctgcagtagtctatgctaaagtaggacctcatgttaatataatagctag
caaaagtagagtcaaccctataaaaaataggaagacaattcccaaactcgagctgtgtgcagctcacctg
cttagtgaattaatccaaagactaaaaggatcaattgacaatataatggagatctatgcttggagtgatt
ccacgattaccttagcatggattaacagtggtcaaagtaagatcaaatttataaaaagaagaacggatga
cattcggaaattaaaaaatactgaatggaatcatgttaagtcagaggataatccagcagatttagcatcc
aggggagtggattctaaccagttgatcaactgtgatttttggtggaaaggtccgaaatggctagcagacc
caaaagaactttggcctcggcagcagtctgtagaagaacctgtcttaataaatacggtattaaatgacaa
aatagatgatcctatttacgaattaatagaaaggtattccagtatagaaaaacttatacgtataatagca
tacataaatagattcgtgcagatgaaaacaagaaataaagcctattcatcaattatttcagtaaaggaga
taagaatagcggaaacagttgttattaagaaacaacaagaataccagtttaggcaagagataaagtgcct
taaaatcaaaaaggaaatcaagacaaataataaaatattgtcattgaatccatttttggacaaggatggg
gttctaagagttggaggaagattgcaaaattccaatgcagaatttaatgttaaacatccaatcattttag
aaaaatgccacctaacaagcttattaataaaaaatgctcataaggaaacattgcatggagggataaacct
aatgcgaaactatatccaaagaaagtattggattttcgggttgaaaaattcgttgaaaaagtatttaaga
gaatgtgtaacgtgtgcaaggtataaacaaaatacagctcagcaaataatgggtaacttgccaaaatata

gagtgacgatgacattcccgtttcttaatactggaatagattacgcaggtccttattatgttaaatgttc
aaaaaatcgtggccaaaaaacatttaaaggatacgttgctgtattcgtttgcatggccaccaaagccata
cacttagaaatggtaagcgatctaacttcagacgcatttttagcagcactcagaagatttattgctagac
ggggaaaatgttccaatatctattcagacaacggaacaaattttgtaggagctgcaagaaaattagatca
agagttatttaatgcaatacaagaaaatataacgattgcagcgcagcttgaaaaggacaggattgattgg
cattttattcccccggcaggacctcacttcggaggtatttgggaagctggagttaagtcaatgaaatacc
atttaaagcgtataatcggcgacactattttgacttacgaagaaatgtcaactcttttatgtcaaataga
agcatgcttaaattcaaggccattatacactatagttagtgagaaggaccaacaagaagttttaacacca
ggtcatttttttaattggaagaccacctttagaaatagtcgaaccaatggaagatgaaaaaatcggaaatt
tggataggtggagacttatccaaaaaatgaagaaagatttctgggttaagtggaaaagtgaatatttgca
tacgctccagcaaaggaataaatggaaaaaggaaattcctaatatagaagaagggcaaatagttttatta
aaggatgagaattgtcatcctgcaagatggcctttaggaaaggtggaaaaggtgcataaggggaatgatg
ataaggtccgagtggctaaagtaaagatgcaggaaggatatatcactagacccattactaaaatttgtcc
cttggaaggaataaagtctgttgacaaaaatgaggctgaccaagagccaaaaagacgaactagagcgaca
tcgggaatgtccaagatcggaatcattatggcaatgttgttgtttgtgttaagttgtcaagtttctagcg
cattacctaaagatatagcaccaagatattctatagacaaaataaataaaacctcagcaatatatctaga
cccgctaggagatgttgagattgtgagtacttcttggaatttggttatctattataaaatggatccatat
tttaaaatgttaacaaagggtaatgcgcttatacaaagtatgaggaaagtttgcgaaagacttcatagct
ttgaagagcaatgtagtctagtcttagataatatgcaaagtcagttatcggaacttgaagaaaacaataa
attgtttatgatacagtctagatctagaagcaagcgtgctcctttcgaatttatgggttccttgtatcat
attttatttggtataatggatgaagatgatagagagcaattagaagaaaatatgaagaatttgttagata
accagaacaaccttgataaactaattcaaaaacaaacatctgtggttgattcaacttctaatctattaaa
gagaacaacagaagatgttaactccaattttagaagtatgcaaataagaattgagaacatgacagaagtt
cttaaagaaaattattatgtttataaggaatcaataaaattctttatgattacgaaacagctacactcat
tgattgaagaaggcgaaaaaattcaagcaggcattataagcctgttgattgatattaatcacggtaggct
aaatacaaatattctcaggccaaatcagcttaaaaaagaaattgccaaaattcagcagagtctttcggag
aacctagtaattccaggaaaacggtcaggtacggaacttaaggaggtgtatacactgttaacagccaggg
gtttattcatcgacgataaattgatcattagtgcaaaagtgcctctgtttagcaggcatccatccaaatt
gttcaggcttattccggtgccaattcgaaatgaagatcggataataatggtgcatacaacgtccgaatat
ttaatttataattttgagatagattcctatcacataatgacggaagccacattaaatcaatgtcagaaat
ggcaactaaataagagaatatgcaaaggaagttggccctggaattcagcgaatgataatgcatgtgagat
tcagcctctaaagccagataaagcggcgaactgcatctataaaacagtagtcgactctaaaggttactgg
gtagagttagaaaagaaaagtagttggttgtttaaggttcctgcgaattcaaaagtccgtctgcaatgta
ctggctctcaaattgaattgtttgatttgcctcagcaaggagttttaagcattgcgccatattgtacggc
aagaaccgacgataaaattctagttgcccaccataacattcagtccgaaagtgaagaattattatcaaca
ccttatataggagaagttagtgaagcgccgaagattatttgggatccgctgaaactatcaatattaaatc
atactgaggaatttgaacgattgaataatgaaattaaatttatgaaagagaaccatcaaaaattgaaaga
tttacatttccatcatatttccggacatgctggattaattattgctttaatactaatgatagtattaata
atatatttcatacggaaatgtgctgtgcaacaaagaatgcaagcaataacctttgcaggtccgttgccag
tactataaatatcaatagtaaataaacaataaaataatataacaaataaaaatatacagtccactatatc
gttgtttaatagaaaatgtacttctacatagaaaaagcaaaatgtttaaaataagttaattgagtacaaa
ttgttgaattaaaaataatataaaccataattgtaatccaataaaattaaaagccagaaaaactaggccc
attgaaatcttagttgcaaaataaatgaacatatatcaaataaatacagtccactactgttataaatgca
actaatatactaatgtacatctcagcttgctggccctttggcagaatgttcacacatgaacacgaatata

tttaaagacttacaattttgggctccgttcatatcttatgtaaatgaatcgagagcgataaattatattt
aggattttgttatctaaggcgacatgggtgcattgctcaaaaacatgtaatttaagtgcacactacatga
gtcagtcacttgagatcgttccccgcctcctaaaatagtcccttagtgggagaccacagataaggtcctc
gccgctcaagataggcagatgtgcccgagcgtgggacctcgataaggcggggactatttacttaggcctc
tgcgtaggccatttactttaagatgcgattctcatgtcacctatttaaaccgaagatatttccaaataaa
atcagtttcttacaaaaactcaacgagtaaagtcttcttatttgggattttacaGTTATACTCTCTTGAA
AAATTTTTGTTTTATTTTTCATGGTGCACTAAACCGTCGACACACAACCGCTAAATCGACGACATTCGCT
TTCATTTCCAAATGCAGTTGCCTCTCAGCTGCTGGTCAGTCACACTTGCTACCCAGTGCCATAGCACACA
ATATTTTCATGCCATGCCGTGCCATGCACTTGCCACTCGGACCCCCCTGCACCCGTTGCACTCCACGCCA
CAGCGCCCCTCCCCCTTCCCTTCCCCTTCCGACGAGGATCTGCCCCTTCTCGGCTCGGCGGTTCATCGGT
TTGTTGCACTTGATGTTCGGCCTCTCTGTTTCTGTTCAAGTTTCGTTTGTGTTGCGCAGTTTCGCTTTCG
ATTTCTTTTGTACTTTCGATTTTTCATACCCTTGAATAAGTTGTCAAAGGTGCATCAGAAGTTTTGGTTT
TGATAAtaataaatattaagattatttttgatataaagatatgttctaaaaaataggataatatatatat
CATTTAAAATTAAATAATTCTTAGTTTGTTAATGGTTTATTAAGATTAATCGCTTATTTTACCAATATGT
TTCATATGGAGAGATTAGTTTAATAAACTGTATAAATTGTATATTTTCCCATACTGTTTATTTTTAAACT
ACGTCGATACTAGGAGTAAAGCTTACCCACAGAAATTTATTTAAATATGCTGATGGCATTAACCCATGTC
CCAACCCTCTTGAAGTCAATCAACCTGTGTCTTGGCGGATCGCAAACCACTTTCTTCTCCCAGTTCGTTG
CCTCTTGCTTTTTGATCAGAGTCTGGGGGACTTAATAATCGTGCGAAACTTATTAATCATATTTCATGTC
GGAGACGCTTCCATGCGTGGTGTGTAAAGGCACatatatatatatatatGTTTATAGTTATGAATGAGAA
GAGATTAGCCGTCATTGAGTTAATCGGGCCTAAGATCGGCGACGGCCCTTTATGGCCAACAAATCGAGGG
CCACTTTCATTTTTGTTTCAGGCGCAGTCTGTGTCGGAATCCGAAACACTGAGGCGCTTCGATTCATGGT
CAGCGAAGAAAGTTGTTCTCACACATTAATTATGCTCCTCGGAAACCTCCCCATCGATTGATTGATACGA
TTTATAACCGGAAAAAAAGACGGTTTCTGGTAAAAATTGTTTTCATTCATTGATGGCCAGTCAAGTAATA
CCAATTGTCTCACTCGATCTTGCTTTTCTCGATCGCTGGCCCACTTTACTGAAGGTATGAACTGTATGAC
AAAAGCCCAGACTTTGCTGGACTTCATCCACTTTACAATTTGATGGTTCAATTATAAGAATTGATGGTCT
GTAATTTTCATGTCCCATATATAAGCATTACGCCAATGAAATGCCAAAAATGCCGCCTTGGTTTCGCTTT
CTGAAGAATAGTTTATTGTTAAATATTTATTTATTTAAAATAAACTTTGGGGATTCTCCAGCGCTTACT
GTGAATATTTTGTTTTTGTCGCCGTGTTGTTTTCATGGAATCCACTTAATTAGCATTTGTGTCAGAGTTC
TGCAGAAATGATTGAAAATTCCAAAATGCTAATAAAAAATTCAGCAATCTCTAACAGATTGCGTGAGATG
ATGCTCAAACGCTGCAAAACAAAATATAATTTTTGTTTATTTGCAATTTTTTAATCAAAGAATATTAATC
ATTTGACTTCTATTAATGAATTATGCGTGTAGTATGCGCGTGTTTTTTGTGTTTGAATTTAATGGCTTCA
TTGAATGGGAAATCAAATTCAAAATACTATTCGATAACTTTAGCAGAAACTGTTTATAGCATGATGGGAT
GGTTGAAAAACCTGTTGCCATCTCTTTGCCAACTGGATGGATGTGGCTAATGATGATTTAGTCGGTTGTT
GTTGTTACTGTGGTTGAGGATGTTGGTTGTTGTGGCTTGCCTATTAAGCCGCAACTTAGAAACTCGAAGC
GAAACAACAACTGCAATGGCAAAGATTGAAAATACACATGTATAAATAAACACAAAATAAATAAACAAAC
AAAGCTAGGCAGCTAAAGCAGGCTGAAATTGAGTTAAAAGCCCAAGAGATTCGACGAGATTCAAGAGATT
GAAGCTGCAAACAACTTTCCACTCCAACTCGAAGTTGGACCAGGTTTGTTTGGGACTTGACTTGGCATGG
CTTGACTTTGACTTGGACTTGGATGTGGATCCACGTGTGCGTGCGACTCACTAACTAAACACGGTATACC
ATATGTGCAGATAGATAGATGTATGTGATACTCGTATGTGGAGGGGATGGATGCCACAAATTGTTTATTT
GTCAATCGGTTTTTGGCTTATTAGCCGCTGCGGCAGCAAAAGTGAAATTTTCCACAACTCTGCAAGTGGA
GCTCAGCCGGCAGCCGATTTATGGCCAGCGGTACGATTCAGTTAATAGCCCCGACTGCTGCCAATAAATC
AGCTAGCTTGGCGGCGAGTGAAAAACGTGACTTTTGATTTGACATTTCACAGTCATAAGTATGCGACGAC
TACGGACGACCCGTCTCGCCCCGGGTTTCTTATCAAATTAAATCGAAAACAAGTCGGTCTCATGCAATTT
AAATTCATTTGGCTGCCGCTGCAACTAAGGGGTAAGATGGGGTTCGGGCTCATCTCGAGCGTCTTGAATG

TCATCTCAAATGTGGCTAAATGTCAAAATGCATCACCATCAGCCTCTCATCTGAGACATCTTCCCCGCTG
CTAATTGAGTCATTTCGTTCGTCTTCACCCCTTCTCTTTTTCTATAACTATTTAATTCACCAACTAACCG
AATGCATTCTCTATTCGTATCTTGCACGCTTGCGCAATAGCGAACCTCGTCGAATCTGGCAGAACCAGTA
AGTATAAGACTGCATTTATTGCGGATAAGTATGATCTAAGATCCATCGATCGCCTATAGTTTTCTTGCCC
GTGTCTCTCATACATAATTGCGTTTGCATGACTTCATATCGAGTGTTTTCAGTGTGTGATTCTTTTGTAA
AAACGTTTTTGAGTTCTACAGGAATGTATTTAATCACGCATTTCGTTTTACATAACTCTTTGGGTTTCAA
AACACATTACACATAGTTACCTGTTAAAATGTTGAATATATAATACTTTAAATAGAAATCATCACAGGTT
ATATAAAGAGAATTTCATTGCTTAAATTTTGTTTGTTAAAATGTGCTCGAACATATGTAGGTTGAGAAAA
TCCGCTAGATTGGTAATGGTTTTCAATTAAAAATTTTTTGTAAACTTCTTAAATTACCTTGAAGCCGAAT
ACATACGCGATTCTCAACAGTCACAACCTTTTGATTTGTCATTAGCATTCGTATTTGTGGCTGAGTTACT
CACTCGGAGCGATCAATCAGTTGGGCTGTGGTTTTATTTCTGTCTGTGTCGGTCCACATTTGCACGGCGC
ACTTTGCTTCATTAGCCAGCGCCTGGGGGCGTTGCCACGCCCACCATTCCAGCGGCTCACTCAAAATTCT
GCACGCGCAGCCCCAAAAGccccccccccTCCAGTTCCACGTGGTTTGCCCCATTTCATTTGAAAATCTC
CCATTTGTGTGTCAGCAGCGACATGCGCGATGCATAAATATTCAGCCTGGCTTGGTGGCTTGTTTTTGAT
TTGAAATACCCTTAAACAAGAAGTTGGCTTGGCTCCTGGATCTTAAAAGTGAAGAAATTACTAAAAAACA
TGTAGACACAACTTAAAAATATAACACATCAATTCACACAGCAATACAATAAGCATATTCATTCACTTTA
CTTTGGCCAAACATATCACAGTTCACATATTTTTCTCATCTCGTAGCCTTATTGTTATTTTTAATTAAAA
TAACTTACTGCCAATTATGTTGCCATTTTTTGTATTTTCAAAGGTGAACCTACAATCTTAATCATAGTGC
ATGACTGACCTTATGAGAGGCAAATGTTTTCCCACAGCTATCAATTGCCAAACAATTATTAAAAACTATT
TACTGAGGGTATACTGGCATCCATACACACAAAGTCTTTACTTATGTGTTTGATCAATTCTTTTGGGGAC
ACCGAAAATGGAAAACAGTTCGCACAATTTAGAAGACAATTGTGACTCATTTAGTCACGAAttttgccaa
tttttttcgcctgtagttttcgtaaaagtttttattttttttttCCCCCAAACAAGCAAAACAGCGACCA
GGCTGGCATTGATAATTTATGTTTGAATGGCTGAATGTCTGCCAGTGCCAGTGGAGTCGGTCTTGTGGGC
TACGTGGTCGGGTATTACGAGTAGCCAACGGTATTTTTGAAACCCCCTCTTTTTTTCCATCGCATCCATC
GGAGTCCCCACACGTCGCACAGTTTAGCAGTGGCCGTAATGTGACAAAAGTAGATCGCAATTTGGTGATC
GTGTGAGCCAGCTATCGGTAAACGGGTCGTTACTTAGAAGGTGCTACCTTAGCTACCCACTAAACCTGAA
CCTATTTCTGTCACTTAACTTTTCGGACCCATTGAAATTTATGTGGAAAACGTGTCGACTGACTTAGCCT
GATGAAAGCCTGACAgcagctgcaagctgcaactgcaactgcaactTGCGATTGCAATTGCGGCTACTGT
TGCTGCAACGGTTGCAAGTCTCTCACTCATTCTCTCTCTCCCCCAGTTATTTGAATACAAACGTGCGACT
TTTCACATAAATCAATGTTAATGGCGCATTTAAACGACCCACCACCGACTACAACAGCACCAACCACAGA
AAATATCATAAAATGCaaaaaaaaaaaaaCAATCCAAGACAGATAAATAAAATTAGCCAGCGTAATTGCA
AACCAGACAGCTGGAGACCCCAAAAAAATAAGAAAACATGTATATAGAGTACCCCGATTAGACCTCCGTG
GCAGAGGAGTGGGAGGCATTTTTTTCTCTTTTGACCAACTTTGTTAAGTTGTTGGATAGTTTTGGAGGCG
AGTGAGTGGCCGGGAACTACCGTATTACACAtttcgtttttgggctttgattttcgtttttgctatttta
ttgttttgtAAACATTTTATGGTGTCAGCTTACAAGACATGATCGGGGTCGGTCGGGATCGAGTCCTAGG
CGATGCTTAAAGTCGTTGCCAATGGCAAAAGGTGGCTTAATTGGTGAGCGTTTTCAAAGTTATGTTGTGT
GGGAATAGAAACTGTGAGGCCACTAAAAAATTTGGAATTTAAAGTATTTTCCTTTTTTTATGATATATTC
CTTTTTATGCTAAAATTCGTCTGATATGTAGATTTGATAAAATCCAATATTGGTTTGATTTGATCAACTA
AAACTTTTAAATAATGTGGCCAATAATGTATTTATTATATTGTCTTAAAAGCCTGTTATAAATTCTACAA
ATACATACTGTTTTCTTTACTTAGTTGGCGTTTATATGTTTCATTGTTTTTAATTTAAAGATTGTCTCAC
TTTCTTTAATTAGAAGCTGATTGGCGACCTTAAGCCACCGTCACAGTTGGCTCAAATTAAACGCCAGTTC
AGCTGGGTTCAATTGGTGGGAGCATTGTGCTATCCCCCCATTTGGCACTCTCGTTTGCAGCTTTGTCAAA
TCGAAAATATATATGTACACAATACACAATATGTATGTACCAACCACCCACCCAAAGCAGACCCAAAACA
ATCCCAAACAAGAGACCGACAGCAGCCTAATGGCGGGCGCGACTAACGACTGAAATCCACTTTAAATGCG

GCCCAATTGTCGCTTATTGTTCCAGCTCTAGACCAGAGGTGTGCAAAGGCCAAGACCCGTTGAAACCCCA
CCAATGTGTGGCAGCCAAGTGGTCTGGTTTTCATAGTAGTTTCTACTTGTAATTTATTTTATTTACTTTA
ACCCCTCCaaaaataaaaaaaaactaaaaGCGAGAACCAAAAATACCACGCAAACAGTTGAATTGGCGCAC
GTCATTAACATTAGTCACGGGTTAGACGAATAGCTTTGCTAAAAAACTATATAAATGTGTGCAGATATAC
CTGGTTGCGGTATAGGTAAACATAACACGTGCGCGATAAGCAGACAACTTTAAAGCCAAACAAAAAAGGA
GGCTACGAAAATTTGACGTTTTTTAATTAAAAATACATATAAATTTTGATTTCTTTTACGGTCAGGAGAT
AAGCGCGATTTGATAACTCTGTTCATTTTCTTTTTCGATTATTTTTCGCCAATGCCATTTCCGAATAAAT
TTGTTTTGCTAATTAAGATTAAATAGTTTTGGTAATTTATTGATCATTTGTTATCAAAGAGCTAGAAAAT
ATTTTCATAATTCACTAGACATTCGAGCCAAAATTATATAATTTATATTCGAAAAGCAATCAGCATTGAA
ATGTTTTTATTAGGCTTATGTGACACTTTTGTTTACCACAAATATTAACATAACCATTTTTTCTATTTCA
ACATCTGCAGGCATCGCGAAAGGTCTCCAAATGCGGATATCTGTTTGTGGCGCCAGATTGGGACTTTAGC
AATCCATTGTACAGAACAAAGG
>dme_circ_0000470_junction_seq
TTTAGCAATCCATTGTACAGAACAAAGGTCAGATTGCCAGACACGACCTGTGCGAG
>dme_circ_0000471
CTTCGATGTGCGCGATAAGGAGAGTCGGGAGCAGCCACGACATTGGTCAGCCCCGGAGGTTTTTGGCTCA
CGTGCCAAATTCCATTTTGACTCGCAGCCAGCAACCTTGGAAATTAAGGTAAGATTTAACCGTGGGAATC
GTGAATCCGTACCCATTAATTTGGCTCAGTGTGCTGCGGGGCGGATGGGCTGTTGCTTGATAATGAAATA
AATGCGGGGATTTAACATTTCAAATTACCTGGAATTAAATAATGCATTCAATCGAAATGCACCTGAAAGG
ATGCATTCTCTGCAGACttttttttGGGCCACTGGTCATGCTTATTTGCCTATATAAAAACTGTTAGCAT
GAATGCACATTTTAAATAAATAAATGTCCAACCAGAAAATGAAAAATTAATGCAGAAAAAAGGAATAGGA
ATAACACAAAGTGTTTCCATTTAAACATGTTGGCCTGATATAATACATATATATACACAAATCGGAAGTG
AATGTTTCATTTTGAATAGCACATTTCGGCTTAAAGTCTTCCCATTTCTTGGCCAGACAAATGCAATTTG
AGATACAATTCCAACGAAGCGGTGACTGTTATACAGTTAATGAAAATCTGAAATGCGTACGAAAAACATC
AAAAAGGAAGAAGTACAGATTTGGCCAAAAACAGAGAATCAACCAACTAGCGGAAGCAGCAATGGCGAAA
ACATGGAAGAAGAGCCGAGGAGCTGAATGGAATGTGAAAAACAAATTTAGATATCAAAAGTTGTTAAATT
GCCTGGCGCACGCGAAATGTTTATTAATGTCTTGCATGTTTACAGATAACTTGATTTAGCGTTTCTGTCA
TGCAatacatagatacatacatacatacCAACAAATATATATCGCACCGTACTTCGTCCTTATGCAATGT
GTTTGCTACTTCAATTCGTCGTATCGCATCGTCTCGCCTTGGATTTCCTTGCCCTGGGCTTGTCTCTGCC
CTGGCATTTGACTTTTTAAAGGCCAACTTAGTTAAGGTCTTAAGTGAGTTAAGCAATAAACAGCCAGCTT
GGTGGAAATGTGGGGGTTTTTCGGGGACTCCGTTAACGAGCTGCATTTCGAAATGAATGTGAAATGCACA
CGAAGAGTCGGCCACCATAAATCCGCCAGGCGAAAAGTCCTTGCAATGTTTAGCAGGGTGTGCGAAATGT
AAGGAGTCTGCAAAAATTGTTATTTATGAATTATGTGCTAAAATTGGTCTCGGAGTGTGGGGCGTGGCTT
TGGGTCTGAGCTTATAAATGAATTTTAATTGCGTTTCAATTGCAAGCCAATTAACTTGAAGCAATTTATA
AGCGGGTAGCAAACACAGGAGTCTAGGATATATAAGGGGGATGGGTGCTATCTACTTCCCATATAATCAA
TAGCCCTTCATAGTGTTCGCTTTCAAAGAGAAGTAAAGCAGGTCAAAGAGTCTGCTCACGCATCGAGAAT
CAAACTAAATGGACGAATACGTATTTCCATTGGGTGTCATCTGTTCACGAATTTTGTAATTAATAGTTTA
CGCATGAATAACTTGATCTATTCAATCCTAACTGTACCAACAAAAAGGAGTAGCTCCTGAAAAACGGTTA
AAAATTAAATCTATAATTCAATTGCTTAGCATTTATTTAAAATTGAAATGTGAATTATTAAAAATCCTCC
TTACCTGATTGCATCTTAGAAAAATGTCAAAAAATAATCCGCATTGAATGTGCGTAGTATCTGAATCACT
TCTGCAATATTATATTTAAAGAAGCGGATTAACAACCATAGATTTTGAGATTCAATTCTAAGCAGCAGag
aaaaatcacaaaagaagaaatcacaaaaTATTGGAAGGAGTCCTTCCTCGCTCAATTGAATTATTAAATA
AGTAAATGAATTCGAGCAAAAAGAGCAAAGTCTCTGGGAGAACAATATGCTGAAAAATTGTATTTATTGA
AAACTACACAGGTTTTGCAAAGGCCCATAAGGACTCTTGTGACAATGTGACAAACGACAACTTCTAGTCT

GGCATTTGAAGCCCACACGAGGGAAAACCGCAAAAGCTCAAGTGTCATGCAATAATTGAAAATTATACGG
TTGGGAAACCAGTTTTTCTGGTTGATTGTATGCATGCTAATTCTCCGTTTACTCACTTGCATAAAAACAC
AAAATCCACAAATAATAACTTACAATTTTTGCAGGATATCAAGCGTAAGTTAAGCTGACTGACTACAAAA
GACGAACTTAACCCCAATACTCCGatacacacatacatacacacaagagaaacacactcgcccacacaca
cacacacacacacagagagacacccacatacatccatacGAGCCATATCCGTATGCTCGGCTAGACAATT
TCCTGTTAATTTCCTTAAAAGCTTTGACCGGCAAAAAAGCAAAGAAAAAGTGCTGCAAATGAAAATATCA
CAAAGTCTGTGCTCTGCTTTTGGGTTGGACTACTTTTTGGCTCAGGCTTGGCTTGTGGTAtttttttcaag
cttgactttttaatttgcaccatttttcgtttttACAACCAGTTTACTAACTTCTTGTCCCCCtttcttt
tttatttttgttttttttttgtttttgGTATTCCAATAGGGTCGGCCGCAACCGCAAGTCCGTTGGCTCG
TAAATGGACTGCTTGTCGACAATCAAAACGAGCACAATTCCGGCGATGTTATCGAGAACCGTCTGTTGTG
GCCATCGGTGCAGCGCAACGATTTGAATTCCGTATTCACATGTCAGGCACTGAATACGCAATTAGATAAA
CCCAAGGAGAAGAGCTTCATACTGGATATGCACTGGTAAGTCGCGTTGATTGTTGCCCTAATATATTTGT
TAAAAGTCGGACAAATTGGACGGAAAGCCACCATCCTGTGAAATACCTTGGCACCAATATCTTAAATACC
TAAAAGTTAGGATGCAATCGAAAGATTATTTCCACTAAATATATACCTGTTCCATAAATCTTTACCAATA
TATTCGGATATTTTGTTAGCCTACATTTTCCAATTTCGGATTGATGTAATTGTATTCTAAATTGCACAAC
GGAGTGCAAAATTATTTAAAACAAAAAAATCGATTTGCTCATAAATATGTATGTAGGCTCTAATTTAAAG
GCCTTCTGCTAAAGAAAATTCAAAATTTCAAGCGGCCAAGCCGTTAGCATCACTTTCCGGCCAACGCACA
CTTGCCATAATGCTAAATGTAGCATTATAAGGGCCATTAAATAATTATATGCCAACTGACGAGAAAACAA
CATAGTCGATGGGGGGCGTCAACTCTCGAATGTCAGGCCCTCGTGTTCGCAGAAGGCCGTGGCCTCAGTC
CACTCCATCCATCCATCCAGGTGGAACACAATGCAGCAGGAGCTCAAGGAGTCCAGTCATCAACGAGCAG
CAGACACGGCATCGGAAGGCCGGACAAAGTCATAATCCAGCCAGGAAGTGCAGCACTTGAGAGCTGTCGA
GTCGTAATAGACACAAATTGTTGTTGATACTGTGGGGCATAGAGCACACAGCCCTAGCTTTGTACGAAGT
AAACGGGAAgggggggggggggggTCCAAAATGATTGCCTTACAAAACGAACTGCAATTATGAGACCATG
AAATATTATGGTAAACAGTTTTAATTACAATTGCATTGAATTGAATGTTCTTCCTGTCCAATTACAGTGT
TAGTGGAAGTATATACATACATATATATTTGGATGCATGTATAGCTATGAAGGAACTTTCGATGGAGTGG
AGTGACCGCAATTGACGCAGCTAGTTGGCTGTTTCTGTCCCTGCCTCCGGTTCCATGCTGCTGTTGACTG
ACTCACGTTCAGCTCGATAACTTATTGTGTTTGCACAGTCATGCCAAACAAACGGAGCTCGTAACTCCTG
ACCCACCCACGTACCCTGTCCAAAGTCGGTTGGATACCATAGTGCAGAGACAGACAGAACCAGAACCAGA
ACTACAGACCACCACCCACTTGTTGTCCCTGGGCGTGGTTCACATTCGCAGCTGGGTCCTAGTCCAGTGC
CCCCCAGCCAACCCCACAACTTGGTTAGAATAAACACAGATAATGTGATATAAACTCCACAATACACAAA
CGATTCTAGTTCGCTTTGCATTTTGCTTTGGCCCACCCACttttcttttttttttGTTTGTACCCTTTCAA
CGACTGCTTTCTGTCCAGCAAACTAAACTCAGTTTCAGGTTTCACCATAGTTTTTAAAGTTATTTTGCTT
AAGTCTTCTAAACATGTCTTAAATGCATTATATATTTTATAAGTTAGATAAATTGAAAGACTATGtttat
ttatttttttatttatttCTCAGTTAGTTATTGTAAACTATGAACCATCTATTGCATATATAAACCATTTT
TAAAAAGGATACATAAATTTAGCGATTTTTCTTGAAAAATTAAACACGTTTGTTAGGGTATCCCAAAACA
TTCGTAGTGAAAACGAGtttttctgtttgttgttttcttcctttgcttttttttttCTCTTCAACTTTGG
ACAAATTTATGGGAGTTTTGGCGCAATTTTGTTGCAATTCATTTTTGTGTGTCGGGACCtcgagttgagt
cgagccgagtcgagtcCTGGTTAGCTATATCCTAACCCGGGCCTCTACGGTCTGGGCTGTTGTTTATGTC
CATGTCTATCCGCTTATATGCATATACGCTGATTGCTTTGCCGTTTTGGCCAGCTCCTGGGCCAACCAAT
GTTTTAAAGACCGGAAATGTTTTTCAGTAGTTTTCATGTATAGCATAAAGTTGCCGTAATGCGGCACAGG
TCCCGATTCCTCTGCCCATATGCCGCTGTCTGATGTCCTCGGTCCTGCCATCCCACATGTTTTACATGCC
TTTTCGTACCCTACTTTGGGGTTTCTAATATATGTCTGTGTCTGCCTCCTTTTGCCAAGTTGTGGGTGGG
ATCAGGCGGAGTGTTTTAGCTGTGGAAGCCTCAGGCATTAATCAAAGCCCCGCAGACGGTCTAATTGAGA
AGTTTGGCCCATGAATTGAAATAGCTTGTGCAAAGGGAACGAGCATGTTAAAAAATGGAAATTTTTTCAA

TATTAAACTGATACAGTTTATGGAGGTCCTTAAAAACGCCGGAGGTTCTAAAAAATTCTATTTACTGATT
AATTAAAAAGTTAAGCTGGATACCACGCCAAAGTTTATTTTTTATGTGAATTGTGATTTCGCATACTCAA
TTCAGTCCATTAGGCTTTGGTTACATTTGCTATATTTCTTAGAGTGCTGAAATCGTCACGCTATATTCAT
CCCCGTAAAGTGACCCCAAATTGTTTCAAATTAAATCCAATCGATCATATTTCGCCCCCTAAAGACAAGG
ATTTATATTCCCGCTAACTGCCGGCACTTTAACACCCCACCACCAAATAACCCGAACACCAAATCACATA
TCTGGCAAATGCCTTGCCTTGGAGCGATTTGAGCCATGTGTAAAGTGCTAACAAAGCGAGCCACATGGCG
AGGGATGGGGGAAAAACACTTTATGGCCATACCAACTATATGGCGGAGGTTGGAGCATCGACTAATTGTG
TGACGCCTGTTGTTGGTGTCCCTGTCTGCCATTAAATTTATAATACCCCAAAACACACACACTCACGAAG
ACAACCACCCACACGTGGAGCACACATAAAACCCGAACAAACATTCCAATAAATTTTAAATTGCAAACAT
AAAATATTTCGACAAATTTGCACGCAAAAAACAGAAGGACAACCAACTTTTCCTCAAGGCAAGGTCTTAG
CCAGGGTCTTAGGCACTGGCTGGCGGGAAAACTTAATTAATGCAGCAACATTTTCTTACAttttttgtga
tttaccttattttcttgtgtcctttttttttGTTTGCAGGGACGACATATCAAAGAACTCGACACGCTCCG
AGCTGAGCTTTGTGCCGACCACAGACGACGATGGCAAATCGATAACATGCCGTGCGGAAAATCCAAATGT
GAATGGCCTGTATTTGGAGACCATGTGGAAATTGAATGTCGTTTG
>dme_circ_0000471_junction_seq
AGACCATGTGGAAATTGAATGTCGTTTGCTTCGATGTGCGCGATAAGGAGAGTCGG
>dme_circ_0000472
TGAAAACGAAGGACATTGATGCCGTCGAGGGAAAATCGGTGTCGCTGCCCTGTCCCATCACAGAGCCACT
GGATAATGTGTATATGGTGCTCTGGTTCCGCGATAATGCCGGCATACCGCTCTATAGGTGAGTCCTAGAG
GACGACATCTCAATTGGACTATTGTCTCGGCCTGGGCACACATATAATTAAATGCAAACGAGAGTCTCGA
GAGTTGGTACCTTTTTTAAATCGTTTGGGGCGCTGGCGCACATAAGTATGCTATGATTTTCCCCTCTTTA
AGTATGCCTGTATAtgtgtgtgtgtttgattgtgtgtgtgtttgccagtgtgCGTTTGAAGGATGTGTGA
TTTAGCTTAGCGTCCCCTGtttttgtttcatttttttcccctcgttttttttttgcgtttgacttttgttt
ttCCTCTGTCTGCGCCTGGGTATTAACTTAATTATGTTTACCTAATTTAGAGTGATTAACCTCGGGCCAT
GACAAACCACAGGAATTATGTGCCGGTGAAAATATTCAAGTAGAACGTCGACGGGCACCGAAGATTAAAT
TACGGTAAAATTGCCAAGTAAACGCAGGTCAGATCGCAAGTCAACCAAAGTCGGGTCCATAATAAAGTGG
CCGTAATTGACGGCCCTTGGCTGAGAACCTGCACTGTGTTAACTTCTAATTGCATTCACGGCATTTCACG
CCACGTTGCTCCCTTTGGTTGTCAACCATAGATTTTTTCCTCTACATAATTGCTTTTGCCTTCGCTTAGG
GTATTTTAGGCTGTGTCGCAGCGGCTTGAATTGATTTGAACGAGGTTGACTAATTTATTACTCCTAGTTA
AGGTAACCAATAAAATGTCAATCATAATGCCACCCTAAAATACAATTTGAATAAAGCATCCCCTAAGCCA
TAAAGCAGGTTAATCTAGTGTAACCCATTTGAAATAGTTTACTGTGGTAAAATTTTATTTACTTTCGAAG
GCTTTAGTCCACATGAACTCGATTTCCTACTTGCGCCGCAACGGCAACTCGCTTATCCTTTAGGGTATTC
CCAAATTCAATCAGTCTGCGCAGTTTACATCCTGTTCCGTTCTCATCGTCCTTTTTTCTTAGTTTCCAGC
ACCGCCTCCTGGAGCCGCCGGTGCATTTTTATTACACTCAATTTGCATGCAGTCGCGTCACTGGCAGCCC
AGTTCGTTGCGTACTATTTAGGCATAAACATTTGCTGCATAGGCTGCCGTGGAACTATTATTAACTTGGG
CACTCGCTTTGCAATTTCGCTTTCGCACACGCTCTCCTTCTGTCGCGCTTCTGCTGCCATTTAAGCAAAG
TGCAACTGTTTTTTAGTTTTTAATTAAAACGGCGTCGCAAAGGACGACGAGAAATaaaaaaaaaaaaaaa
CAGCGATTAAAATTTGGGTTAACGTTGGCCCAggggggggggCGGGTGGTGCGTTTTGGCCCTCATTCTG
CTCTTGAGTCGGCTGCTGGCCGTGGCGCAATTAAGTTGTTGCATTTGCAACGAGTGCAAGTTCGACGGCT
TCCCGCATAAATTATTGTGTGTTGCATACAAAAGGGCGCTTAACGCTCAGCTCGCCGCAGAAATATTCGA
AAGTGGCTGCAATCGCAGCTGGAATTGCAACAGCAGTAGCAACAATTTGGTAGAAAGCGAAAGAGCCATT
CAGAAAGCagagagcgagagagggagagagagagagaCAGCGGGTCAGAGTGGCTGAAAAGGACCTCTTC
TGATTGAGAGACCTCAGTCTGGTTTTAAATTTGAGGATACGAATTTTTTCCCAACCGAAATGGCCGGAAC
GAAAAACGTTAATTGACAGAGTTGGCTTTAATGAAATTAAATGAAAACCAATGTAACGTTGCAAATATTT

CACGTATTTTAATTATTTACATTTAAAGTTATTTGCTTAAAGTGACTATAATCACAATTGAAATCGTTGA
AAGTGGGTGTGCATTTTAGCCGACAGTGCTCTTTTTATCATGTTTATTTATTTATTTTAAAAAGTTTACA
AATGCTTTAATTACGAAAAATAGCCCTAATAAACTATGGCATCtttttttatttgcatttttttgtatgtat
tttCAACGGCTCTCGTTCCAAGGTATATTGTCTTTTAAATACCGAAGTACTTGGAAAGTTGGAAACTTAG
CCCATTAAATGCAGTGCTGGGGCTTAAATCTGCAGAACAATTTGAGGAGCAAAATGCTTGAAAAGCAAAA
CAAAAAATAATTTTTATCTGCAGCACATAAATTGCATTTGGCAAGAGGCCTTACACACAAACGCCGTTCG
CCATCGCATTTTCCACCCAGCCTAATGAGCAAACCAAAATAAATTACGCTAATTTTCGTGCGAAAAGTTC
AGAGGCGTGTGGAAAACGCGGGCATGAGCTGAGGTTGAATTATATTGGCTATGGATTTCATTAATTTCCG
AGCTGGACGAAATTTTGGGGCTTGATTTAATTCGCATTGCCCGCCCACATTAAATATTAATGGAGCATGG
GCGGGGCATTTTTCAAGGCTTCATGTTATTTAATGTGCTTGTGTGtttttcatctcttttctcggttttt
ttttgttttATTTCTCTACCCCCCAGCTTCGATGTGCGCGATAAGGAGAGTCGGGAGCAGCCACGACATT
GGTCAGCCCCGGAGGTTTTTGGCTCACGTGCCAAATTCCATTTTGACTCGCAGCCAGCAACCTTGGAAAT
TAAGG
>dme_circ_0000472_junction_seq
TCGCAGCCAGCAACCTTGGAAATTAAGGTGAAAACGAAGGACATTGATGCCGTCGA
>dme_circ_0000473
GTTCCAGTATCGGCGATTGCCTGGCATCCGCTGGAGATCACGGTTCCATTCCAGGTGATGTTGTTCACAG
CGAACTGCCAGTAGCCCTGCTGAGTGACGGGTGTGTAGGTCATTTCACCGGCGATGAGGTTCTGGTCGG
>dme_circ_0000473_junction_seq
ATTTCACCGGCGATGAGGTTCTGGTCGGGTTCCAGTATCGGCGATTGCCTGGCATC
>dme_circ_0000474
GCCACGATTCCCTTCACGGAGCCCGATCCGTAGGTGATGCTAAATGGAGTTCCACTTGACCGAAAGGATT
TGGAATTGGACTTCCGGAAAAACTTGTTCCCGCACGTTTTGATGCAGAATCTGCAATGGGAACTGGGCAC
CCAAAAGTCCGAGGAACCCGTGTCGAATTGCATGG
>dme_circ_0000474_junction_seq
TCCGAGGAACCCGTGTCGAATTGCATGGGCCACGATTCCCTTCACGGAGCCCGATC
>dme_circ_0000475
CGATTCCCATTGGAAACTGGACACCGCCGGATATAGACTACAACGAACGCTTCACCTGGGAGGGAGCCAA
TGACATGAGTGCCAACTATCGTCACGCCCTCCAGCGGGGTCTCCCCTTTCCCATCCTGACCGTTGCCGAA
TACTTTAGCCTGGGCCGTGAGGGATTCTCGTGGGGTGGACAGTACCGGGCTGCGGGATATTTCGCGAGCA
TAATGCTCTGGGCCTCGCTGGCCTCGTGGCTGCTGATGAACCTGCTGCTGATAGCGGTACCCCGATATGG
GGCCTATATGAAGGCTTTGACAGGTGCCTTGCTGGTCTGCACCACGGTGGGCTATCATTGCCTGCTCCCG
AAGAGGCCTTTGTCCATTCACCTCGAAGGCGGACGCCTGGAGTTTCATTTCGGATGGTGCTACTGGCTGG
TCTTGGTGGCAGGGCATTCTCTGCTTTATTGCGGGAGTTCTGATCTCCATTATTGACTTGGTTTGGCCGC
ACACCTTCTCCACTGTGCTGGAGGTATACTACGGCACGCCATACGATCGCCATGTGATTCTGGAGGAGTC
CAGTGATGTGCGTTATCGAAAGCCGCGCAACAGTCGCAGCTTAGAGGATCCACCTGGACTGGGCTCCCGC
ATCCTGCGCCGCCTCAGCTCCAAGGCACGTGACTTGCAACCTGGCACGGCACCGCGTCGCGATAGTCCAG
CCGGAGTGTCCAGCAGCGGTGGAGTTGAAAACAAGGGCTTCCAGTCGGATGCACCAAAGAGTCCTTGGAG
ATATCCCTTCCGAAGGTCgcagcaaatggcgcagcagcagcattcgcatccactgcatcagcatccgttg
cagcagcatcatcagcaccaccagcaccaccatcagcagcagctgcagtTTGTGGGCGGTCCGGTGGTGC
AGCATCCCATGCATCATATGCAGCGCACCATGTCGCAGGATTCAGGATCCAGCATTGCCTCGGCAGCCGT
GCAAATCTCCCCGCTGCACAAGCATGCTTTGGCGCGGATGTTGCCG
>dme_circ_0000475_junction_seq
CAAGCATGCTTTGGCGCGGATGTTGCCGCGATTCCCATTGGAAACTGGACACCGCC

>dme_circ_0000476
ATCCCAAAACCCCGACATCAGCAACAGCAACCAGCATGAAAGGCTGGTTCGACGCGTTTCGAGACGATGG
GGGACCAACGCTATATTCCTTCTCAAATCGAACACCCGTAACCGGAGATGTCTCAATCGTAGCCGTCTCA
GTGCTATTTGCCACATTCTATGTAGCATTTTTAGTCATCTTTCCGGGTGTCAGAAAACAGGGTGAGTATA
GAGCCCATAGAAGACATTTAATAATAAGGCTCATATTTCAAGATTGCATTTAATATCTGACTAACTTAAT
TTGACGTAAAATATTGGTAATAAAAGTCAAGAAAGCTCGTTGGTTCAAATTAATCAATTTATATTACTTT
AAAAATGCCACGTTATTGAGTATTTATTACCGTATCGTGTACTAGTCAGACTGGAATCAATTTTCTCACG
CTGTTGCCAGCTGGTCTGTTTAATTGAATTGTGCCAACTACTTGGCATCGTCGTAATCGCCGGCACGTTT
CCAACAGAAGTCAGATAAATCTTAAAGCAATATATATATATAagttaacgcaagttccacttgcatcagg
gttctcggcactcgctgccgcatacctcaccttgatatgacaaccaacatgctgtggttctccaaataat
agtcaagatagttaagagggttcgagtttacgattagctttattttaagattcagttcgcgatctgctag
cggttccgttatgtgtccgttcgagactgggacctcttctcccgccccgctgcttttatatccgcttccc
ccgttgacccctcaccctgagtgcgaccaacggcacttgttccgaatgcacacatacacaaacatgttcc
tagacaggccccgagtcgcctgctgacggggcccgaaaacacgagagccgagcgctggtgagtcctgacg
aagatcgcaagaagagggttcgtaacttacacgaacatgttactagacaggccccgagtcgcctgctgac
ggggcccgaaaacacgagagccgagcgctggtgagtcctgacgaagagcgcaagaagagggttcttaact
cctccccccctcaagatctgcgtcccgcagattagtgagccttgttcagctcggtttggcccaatgcttct
aagtttgcggcaatctcgcggacatcgggcatgcctttggtggacatcagcttcttccataggaaataaa
agcctaggaatatgcagattgcgagtgccgcctcggtgagaagagaggctagtagctcactcgccatgtt
ggacatcttttccaaattcttcatgctcacgtcgtggacgtattccagagaaagttgaaagttgctggcc
gttatgtggctcaacacggccggcatcgccattagatgactgaccgagtaactggaatagttttgaccgt
tgaccattatcgtctcattgtcgaattggatgataaaggagccgatcaggtcgtagttctctgcagccgt
tcttagagttccgttgaagttggtcaggaggagcattccatcgtcgacttgcttaaccactcgttggtta
cttcagtagccgagggatacagctagcctcctccagtttttgcagttcctcgaagccgtcgcttgcgcgg
ccaagacggcgttccaatcagcagcatctggagatccagctacccatttccaagccgaccccagccagtc
cagtgagcgggatttgcgccgcatgcagaaatcgcgcttacaaaaagacaattcgtaaatatctggtcgt
ctaagttgttctaagactacgtagtgtgtgggggtacggtacattttgtgtacttatatttttattttt
tatatttcttatttcttatttcttataatctgtatttttttatattatttttttatagtttttttgta
tttctttttgtatttcttaactcattttttttttttttttttttttttttttttttttttattaatgt
tttttttatatagggtatgcatgggtggaatatttcattagctgccacaacgtacttttattactttcgt
tattctttttcgcgtttctttttttaaatgtgaaagccacgtctcaatttcttagatcagatttgtggaaa
atttttcctgatctggttttaactgttatcttgttgtcttcctgtaccttttcgcatctgaacctcgctt
tttcctttgtttttatttgcttatttgcaacaaaaacttcatctcccggcccataagactttggctcgtc
cctatttttattccgagccatattacctctaatttgcttatactcaattaagcccttgatgtcctctaaa
gtctgccgccggaaatctgtcagaccctgatagtttacccttgacgagcggtcgaaaaaaacgtctgctg
gttttcgattcgttaccgagtgaacggaagtgttgtagcggtccactgctatgtgtaccagctcaacggg
tttgaaggtagggagctcatctttaaggcaacgataaatttctaggaacgtagagtggaatctctcgact
tgaccatttacttcgctcttctgggttggagcataatacagatcgatatctagagaccgaagatagttga
gcactgtggggcataacaaccctcgctcgttatccgaaaccaagaccttaggggcggtgaagtaatgtag
ggcctccaccaaagtttctcgcaaatgcacagatgcttttgactgcagatggaaaagtttggcaaacttg
ctaaatttgtcaatacaacttaggtataacctttttttcgagcgcaaaaatgtcgatgtgaagtatttcac
atgggtagttaggaattggcgtaggttgtaggtttggtttgttagggtgtctctcgtacttgtagagttt
gcaacactgacatgaggaagtttgcagacggatcgtactggacattcgcgggaaataatatttttctaaa
agttggagacgaatctccgtagggcccctatgtgctctacggtgttctttttcaattatttcacaaattt

cctctgcgccagacacgtccgccactaggcgctgcgttatccgaattttgtataaaagaaaattcgctaa
gcagatggactgaaagcgttgcaaatgtgcttccgggattttgacgccgttaattattacaggtcgtaga
cacgattgtaacgagttggttaaatcggcaagtgatccgtctttgagaggaatcagatggcgagtataac
ctgggaacgggtgctcgcataagtattttgacctggttgtgtcaaaaatgagttgattcttgaaaacgtt
gattggagattcaacgtggggaatcaatcgtgaactgtcatgtaaagcgctatgggcagtagccaaagac
tgcatgtcatcctcgggattagcatctaaatcggtgctcaactggttaagctgaggcggaatgcgtgaaa
gcgcgtcagccaccacattagatttcccaggcttgtagatgagttcgcagttgtattcctctatacgagc
cttccagcgttttagcttcgcattgaaatttctgttgcctagggcaaacgttagaggttgatggtcagta
tatactttaatagtaccagcgccatataagtaagcccgaagattgtccaatgaccaaattatcgcgagca
tttccttttcgatagtagcgtagttttcctccgtcttatttaatgaacgggaaatgtacgctatcggcct
atctctaccctggtcgtcctgtgagaggacagctccgatggcccagttagaagcgtccgtggttagatgg
aaaggtttagtgaaacatgggaacgccagtatttcagaagaacagagaattgattttaaatcattaaaag
actgtagggccgtctcgtctaatgtaattggcactttgcttgattgtgaagactttatattagcgtacaa
tccacgcgtcaagtttgtaaggggctttgctaccttcgcatagtcctgaatgaacttcctgtagtacgag
gtcatgcctagaaatctttttaactccttaacagaggtcggaggaggcatttcgctaatcgctctgacct
ttttcggatctgccttaatgccatcggccgtgacgatatatcctaaaaattctacctgcgtgtctaaaaa
atgcgacttctcaaggttcacttggaggttagcttttgataaactcgctaataccaatcggagatttttc
cagtgtgtgtcataatcttcactgaagacgatgatatcgtcaatataaacgtagcagaccttgccaatat
gctcgcgcaaaatatcatcgatcattctttggaagattgcaggtgcattcttcaaaccgaatggtagacg
gaggaactcgtactttccatttagagtagagaaagctgtctttggaatgtcgctttccttcatgtggatt
tgatggaatccagaagtcaaatctagggtggtaaagtatttggcattgccaaggctggctagcgtagcgt
ttatatctgggatggggtaagtgtcgggtatggtgacggtatttaaccgcttgaaatcgactaccatgcg
atattgtttttctccgtttggtttaggtttcttcgggactatccagataggggaattgtaagggctatta
gagggtcgaattataccgtcctgcagcagttcatcgatttgacgttcgacttctccgcgcatgttgactg
ggtaaggataacttttagcatagatcgggtcttgtgtgtttgtccggatttcagccttgacggccgtctc
cacggacattccagataagggggctcgaagatgcggggaaattccccgagaagggaattcaaaatttct
tgtgtaccatctgggtgttcggcggcgagtagcgggttaacgtttattgaagctctcgccaaaagaggga
ttttaattcctggggttattatcaaacaattgtttttcctatccactatggcttttaagtctttgagagt
atcgtcgccaatgataccatcaaaggaatgtaaattcggtagtacgaaaaaggtaatatccgagtcgttc
cctagtggtttgaagaatttaccaactaggcggtgcgttatcatgatgttacccgcagcggacagtacgg
agaagggtttacggactgtggtacacccggctgcaagtcttcggctaataaagtttttgttcgcccccgt
gtcgatcaaaaacttcagcctttctcccctctccgtagcatactctaagtacggtacactggagaggcgt
tggtcataaaatttgcccctccctcagtctcaaggttatgtggagcatctgtaactccttccacgttttt
ctgcggaggataattgttgcttctctcgacagcttgagactcgtcatcatagtaaccctcgtgtgaacgt
ccttggttatctccactagccgccggggctagcgggggtggtgccaattccatgtggtacaatctttgtg
ccttttggaatgggttgtttccttcggacattctcttaataccggagccttcttcctggcgaccactgta
tcctgattggtaagattgacccgaccgattggattccattttatggcctgtctaggaccctgagttctg
taggagttttgcgaaaatctgcctgaaccaggagaaaaaggggttcggaacggtggcctggcctgatgtt
gatatgggcctgcgaatcttggggcacctgacggttgatagttctgagctgtgaaatttcttgacattac
attctgcaatcccatacaaaaagagtaagcctctggtagattcttcgggctgcggacaagtaagtgtttg
gaacaatctctccccactcctcggataaaaacatccaaagccttgtctctataggtttctatcaaagcgt
tgactacttcacgagggtggtttctggccttcaagttatttaagatcaacgagagatggccattaacgtc
catatagaacctatctagtggtctagaaccttggcacatagcgccaagctcatgctcaagcgtacgtaag
tctcgtttgtctgcgtaatgcagcgccaagactcgttttatgtcgtcccaatcgtcgtcttggacgccat

aggccgccaaggccatgtcggcgtgtccccttatttctgtctaatatgcaagacaattgccctatacag
gggtctggttttaatcaagtcatattcggtcagtattgactgtgccctgtttatccaagacaggtaagcc
tcctgccgtccttcgaacgcctgcagttctttgacgcagtcagggaggaccgatatctctctcagctcat
actcggtgcgtgcgcgcaccaggggttgcggttggtcgaccgtagggtcagcgttcccgcgctcctgttc
taaaatagccaacctatcattatttttcttaatatcttccttaatgcctgtcaccacgttggtcagggca
ttgagggactcgctaagagcacgcagggtctcttccattttgatttttagtttgcgttggaaagatgcag
gaagctttcaaggcaacagaattcgttattactttacggaaatgtgagacaacggttttaacccgataaa
aaagaatatctgttcttctttgctttttctttatttctctaaatttagcaaatatggcaataaagtttaa
tgaaatatttcactcacccttgtatgctctttattagttttcgtcgtttaggttgtacccctacagcatg
aatattttgttttcctttgaattgtggtgttgtgcatgtgtttatacaatttttgtgcgctattccattt
tgcaatatttttttccttgcgtgcgtgctgttacagtttttttattgatgacctctaatttatttcggcact
gggcacggtggttccgtagtatcccttttggctggttgcctactaaggtacggccgccacacaacttgtt
gggcggttttttttgggtgcattccacaccacttaacgtatccaacaagctgccacacaccaccttatca
ctctgggcgctcatattactcgtactgccgcaggtaaactgctgcaatggttaattattggtttattata
cacaatcttttgcactcgcgaattggcaaggagagaaaaaaaaaaaaatttcccacaagccgtaccgcc
gcagtgggttgatcgtgttttaattatgttataggtatcgtacaacttttgcgctcggcatgggcgacgt
ggcgtcctccggtagcgggtcgttgttttattaatttaaattaggtcttggcgcaacataggtttacatt
tttattttagaaattttacaccttttattgaattatgcaatatttgttgcgccccgggatgggcgtagtt
ccccccggcagcgggtaattgctattttatcattttatacacagctcacgcagcacttctttttcattta
tggatttcagatcttaatctgacttatgcacaccaccactatttttacgcacaaatttccacaatttttt
cgtatcacttttatcggccggacattgccggtccctgttcgggcgccagttaacgcaagttccacttgca
tcagggttctcggcactcgctgccgcatacctcaccttgatatgacaaccaacatgctgtggttctccaa
ataatagtcaagatagttaagagggttcgagtttacgattagctttattttaagattcagttcgcgatct
gctagcggttccgttatgtgtccgttcgagactgggacctcttctcccgccccgctgctttatatccgc
ttcccccgttgacccctcaccctgagtgcgaccaacggcacttgttccgaatgcacacatacacaaacat
gttcctagacaggccccgagtcgcctgctgacggggcccgaaaacacgagagccgagcgctggtgagtcc
tgacgaagatcgcaagaagagggttcgtaacttacacgaacatgttactagacaggccccgagtcgcctg
ctgacggggcccgaaaacacgagagccgagcgctggtgagtcctgacgaagagcgcaagaagagggttct
taacttatatatatatatatatatatatatataAACCTCTAATGAGAGAGTTAAAAGCAAACCAGTTTTCTA
TAGGCGCGCCCTTTTTTCATGTCCATTGGTCTCTATTTATTTGTTCGTTTTATTGGAGCGTTGCAAACTC
TGAATGCATTTTGTCTTGTAGCTTGATTAAGTTTAATTTGTCTTAAATATTATCCCGTTCGCAGAAATTC
ACAACGTTTTCGACAGTCACATTGAGCCTTTTTGTGGGTCTAGTCATATTAAGTGAGTATAGAAGTGACC
TTCAATCAGCACAATATTATATTTGATTTGCATTAAAATCAACAACAATGAACAACCTGGGAAACGCTAA
CGTTCCACGTCTTTATCCAACAGTCACTCGCCTGGGATCCGCGTGGCATGTGGCACATGCAACTATCATC
GCGCCATACAAAGCCTTCTCACGCGAGAAGCTCCCAGCGCGCATCGGCACCCACATTGGCCTCATGCATG
TCAATGTGACGCTGACGGG
>dme_circ_0000476_junction_seq
TCATGCATGTCAATGTGACGCTGACGGGATCCCAAAACCCCGACATCAGCAACAGC
>dme_circ_0000477
GAGTCGAAACCATCGGATCAGCAGGCGCATTCCTGGCGTGACTGCGGTGCATTAAATCACCCGGAAAATC
GCGGCGACGAACAGCAACCGGAAGTTCGGCGCGAAACGAGCAACTGGTGGTCACCAGAATGGAGGTTTAC
GCCTGGGGTAGGAGCCGCAGTAGAACTCTCGATAAGGAGCCGACCACTGAAATACCCTTGCAGAGAACGA
GTTGGTTCGAATTAATCGTATCTCTGTGCCACCTGTAGATCCAAAACCAATATCTTACCTAAAGGCCAAC
TACTACGTAGTACTTCCTATAACATATCATTCAAGTCCTCGTTAAACCTTCGAAATCATTGTTTTACCAC

TTTTCTTATACTATATAAAAAGCCTATCTGAGAAGCCTAATATCTTTATATCTCTTAGCCATTCTAACGA
TAATTGCTTATCAACTTGCGTCTGAAACTACATTACTCTCTGCAAGGGTATGACAAGAAAGAAACAGAGA
CATCGCCACGGTGCATTTGCATTGCAAATTAATGAGTGATTTCCTTGGCTTGGGTTCTCGCCCCTTTTTT
CGCCATGTCTTAGGGGTGCCAATTCTCACGGCCAGCTCGGCCTTGGCTTTGAGTCCGAATTGTGCATGAC
ACCGCAACGGGTGGCCAAGTGCTCCTTCGCCCCCGCCCAGGTTCGCTTGATTCGTGGAGGTGGTGGCCAT
GTTCTCATCCTCGACACAAGCGGACGGATTCATGCCTGCGGATGGAACAATCGTGG
>dme_circ_0000477_junction_seq
TTCATGCCTGCGGATGGAACAATCGTGGGAGTCGAAACCATCGGATCAGCAGGCGC
>dme_circ_0000478
GCTGGAGGAGAAGTTCTCGTGGAAGCAACTGGCCTTCGATTGGCCCACTCCGGAGGCCGAGGCGGAGGCC
AAGTCGAATGGCCACTACATCGTGGAGAACAACCTGCCGCTGGGCGTGGAGCGTTGGCAAAACAGAATCT
TTGTCACTGTGCCAAG
>dme_circ_0000478_junction_seq
AAAACAGAATCTTTGTCACTGTGCCAAGGCTGGAGGAGAAGTTCTCGTGGAAGCAA
>dme_circ_0000479
ACCAGAGTGTGCGAATCGGAATCGATTAGCCCCTGGGTGTCCGGAGTGTATGGACGCTTGATGTTCCAAC
AAGCAATGGCGTCTTTGTTCACCTGCGTATAGAAGATGACGCCGGTCTCGGGGTCAAAGACCTCGGCGGT
GGACTGGCCATTCATGCCACGGTCACCAACGTATTTAAAATCG
>dme_circ_0000479_junction_seq
GCCACGGTCACCAACGTATTTAAAATCGACCAGAGTGTGCGAATCGGAATCGATTA
>dme_circ_0000480
GTCCACCGCCGAGGTCTTTGACCCCGAGACCGGCGTCATCTTCTATACGCAGGTGAACAAAGACGCCATT
GCTTGTTGGAACATCAAGCGTCCATACACTCCGGACACCCAGGGGCTAATCGATTCCGATTCGCACACTC
TGGTCTTTCCAAATGACATGAAAATCGACAATGAAGGCACTATTTGGGTGCT
>dme_circ_0000480_junction_seq
TCGACAATGAAGGCACTATTTGGGTGCTGTCCACCGCCGAGGTCTTTGACCCCGAG
>dme_circ_0000481
GATATATGAAGTCAGCAGATCGTCCATCTTGTAGCCCAGCGACGTTTCACATAACAATTTCGAGCCCCTA
ACCAGATTTCCGATGGTCATGTGGAAATATGTATTGCCCGATGACCAATTCGAGATGCGTGTAAATGGAT
GCGTAACCAAAATGTCCTGAAGTTTTAAAATGTATCATGTTCTTGGTTTATATTGTAATAGATTTATATA
GTTTATAGTTCTCACCTTGGTCACTGGATGTATGAGACTCACGCCGTGTTTGTTAATGGCTATCAATAGC
ATTTCGGGATAATTCGGTTCGGTGGTTTGCTTCACCTCAAAGAAAGCGGAGCCAAAAGTTGGCCATCT
>dme_circ_0000481_junction_seq
AGAAAGCGGAGCCAAAAGTTGGCCATCTGATATATGAAGTCAGCAGATCGTCCATC
>dme_circ_0000482
GGCCTGCAGAACCAAAGCAGCCCAACGTTCAATGGCCTTGTCCACTCCGCTGAGACAAAAGTCGGGTATA
AAGTTGGGCAGAAGGGTAAACAGCCGCTCCATTGACATATCGGTTGAGTATTCGATGAAATATTGCTGCG
CAGCTATCATGGCCAAGTCTTCCTCCTTGTCGCAGCGATATTCGCCAAACTTAACGCCTCTCACCACCTG
CTGGTAAATAAGATTG
>dme_circ_0000482_junction_seq
TCTCACCACCTGCTGGTAAATAAGATTGGGCCTGCAGAACCAAAGCAGCCCAACGT
>dme_circ_0000483
GCATGATGGGCTTTTTGGATTTGGTGGCCTGCAACTCCAGCCACGAAGGCGGTTGATTGCGAGTTCCATT
GTTGAAAGTACGCTTCAGTCGCTCCTCGCAGTACGGTGCATATCCAGGTGGACCCTCACGGATAAAGGCC

CTCAAGTA
>dme_circ_0000483_junction_seq
GACCCTCACGGATAAAGGCCCTCAAGTAGCATGATGGGCTTTTTGGATTTGGTGGC
>dme_circ_0000484
GGCCGTGATGTTGAATATTATATATTTTATCTTTAGTTTATTTTATTCGCCTTGATCACTGGTTAGCGAC
TGTAATTTGTTTTTATAAGGTAGGATGATTTTTTGATTAGGTGAGTGGTAATAACATAAAAATAAACAAC
TTCACATCAGGTTTTGGTTGATGCAATAAAACAATTTCCATGACCGCCCCTGCCGTTTTAGAAAAAATCT
CGAAACAAATCTCGTATTGTGAACAGCTACTAGATATAAA
>dme_circ_0000484_junction_seq
CGTATTGTGAACAGCTACTAGATATAAAGGCCGTGATGTTGAATATTATATATTTT
>dme_circ_0000485
GTGAGGACACCACACAATGGAAGAAAATGGTCGTCCTAACTAGCAACAAGAAGAAGGAGAACGACAGCGA
AGAGGAGCTCGAGTACGACCCTGCCCTTTATCCACAGCGTGTGGGCAGGCAACAGCGCGTCCTGGACATT
CAGTTCAATTTCAACGATGGTCGTCGTGGCGGACCTGGCGGTTTCGGTGGACGCGGTGGCCGTGGAGGCC
CCAGACCCGGTGGATTTGGAGGCGGACCACGCAgcgagggtggcaaccgcgatggcggcaatcg
>dme_circ_0000485_junction_seq
agggtggcaaccgcgatggcggcaatcgGTGAGGACACCACACAATGGAAGAAAAT
>dme_circ_0000486
GGTAAATTCAGATCCTTCGAAGTTCACTGTAAGACCAACGCTCTGTCCATGTCCAGGGGCTATGTATGCT
CGCTGGACAGATGCGCTTTCAAAATCCCACAAAACACAAAGGCCGCGATTGTATGCAACTAGAAGTTTGC
TAGGGGAGTTTGGAAGTTGGCGGATTGACTCAATTGCACCAGGATTTAGCTTGTAGGCTGGTGGCACCTG
CTCTAGCACAACGTCATG
>dme_circ_0000486_junction_seq
GTGGCACCTGCTCTAGCACAACGTCATGGGTAAATTCAGATCCTTCGAAGTTCACT
>dme_circ_0000487
AGCAAAAAGGCAATTAAAGCGGCAGCCAGCTAGCAACGAAAAACACACCCACCCACAAAATCATGGACAG
CGCCGGTAAAAATCGTTATGAACTTTTGTTCATGGACGACGATGTCAGCGATCCATTAGATAATCTTGTG
GCGCCGACGGCCGCTGCTGCCGCAGCAGCAGCTGGCAAGAAGAAGCAACCGTCAGCTGCTGCTGCAGCGG
CCACCAAAACGACCGCCAACAAGGTGGCCAACAGCAATAACAAGGCGAACGCAGGATCCAATATTGGTGG
ACCCAATGCCAAGAAACCAAATCAGGCCGAAAAGGAGAACAAGCCCAATAATGCGTTAAACAAAACCGAT
GGCAAGAAGTTCACTCCATCGGCCGACAACAAACAGCAGCAGTTCAATAATAACGCCAGCAGCAATTACA
AACAACAGGGTGCTCCACGGCAAGGAGGTGGCGCCAACCGAACACGTGAATTTGGTAGCGGTCAAGGACA
GGGTCAAGGACAAGGTGGCCAGCAGCAGCGCAGCGTAAACTTCCGTCAACAGAATGGCAATGCCGAGACT
CGTGAGCAGCGCAACAATCGCCGCAATGTCCGCGAAAACGTCGGAGCACCCGATGGCCAGCAGTCGCGAC
CTTACCGCGGACCAGGTGGAGGCCCAGGTGCTGGCGGAGATCGTCCCCAGCGTCAGAACCGCAACTACGA
TGGCCAGAACAGGAAGCGCGAGTTCGACCGCCAGTCGGGTTCCGATAGAACTGGGTGAGTGTGTGGTCAT
TTCATTAAGGCATTAAGGTACCAAATGGGTTAGGGGTCAAGTCAAGTAGATACAGTGTAGATCACATTAA
AGTCTTTACAGAGAAGTATACTTAGTAAAAAGGATAGAGAAGATGGGATATTTAGGGAGTAAATCGTTTA
GATTAATGACCATTTTTTCGCATATGAATAGTTAATGTTTCTGTTGATAAGATCGAAACTAAAGTTGAAC
CACCAAGGGGTAGACATAACCATAAAACGAGCGCGCCCATACGAAAAAATTAACAAATAAACCAAAAGCC
ACACTCCATGACAACCAGAAAATAAAACTCAGCAGGCAACGCAGGAAAAACAAATTCCATCCACAGTTTA
CGCGTAACCAATTACCATGTTTATAGTCATGAGAAATTATTAGTCGCCTTCAAGCAACATGATTCCAATC
TTCTGTGTACACAACAAATATTTAAAAGAAACTGGAATTCGTTCGGTGTTTGAAATGCGATTCCTTTTAA
TAAACTATGTGAAATTTCCATCAATTGATTTAAGATTTCTATAGCTAATCTAAATCATGAATTTAATGTA

TTTCAAAGTATAAAATATTTATTAGCCTGCTCCGAGCAGATTGCCAATCTTATTTAATGGATAGTAATAT
TTAAACAGTTGCATTATAAATCAGTTTAAATATATATTGTTTATGCCAAGCAATTTCACGAAAAGTGCGG
GAGAACGGTGTAAACCAATTTGTGGCACACAAAATCCCGATGAACTTATGGGCAATGGTCAAACAACACA
GGCAAATCTCCCACTCACGTGGGGGATAAGTGAGTCAAATCAATACTAATGGTCGGACAGAACATTCAAC
AAGCTACATCACACTTCTTTCCATTGAATGAAATCTATTTCGCCGCGTTTTCCCCCGTTTTCCACTGCCT
GCTTAGACCGACCGCTTCGTTTTTGGAATATCCAAAATTATAATATCACAGCGGCCGGCAGCAACAGAAA
CAAGAAGCACTGCGGCACAAATCGAAAGAGAAAATGTGTTAAAAATAGTTCGCCCATCTTCTTGGCGGCA
TTCCCGTCTAACGCAAGTGCTTGTAGGAGGCCTTTGCAGGCATAATCATTGGGGGGAAACAGGGGTTGGA
ATAGTTCCCCCACTGAATCTGATTACAGTGAACTGGGGCTGCTACTGTCGGCTTATCTGTGACTATGCGA
GCGAGGGCAGTGAATATAGTCCCCCTATCGTGGTTACTTCTCAATTGACCTAATTAGTGGGGTATTGATA
AAATAGACCATTATTTGGCTAATAATTAAAGTATTCAGTTAATAAATTAAGCAGAAAAACTTACTTTAGG
GCTTGCAGGCAAGTTGCACTATAGCTGTGGCTAAAATTTGCAAATATAGAAGCTCAGATTTTCCGCTTCT
TGACGTTTCTGAATTTAAGCAGACATAAATCATTATTAATATTTGATACTTTCTTTATGCACAGGGTGTG
AAGTCAATTGACAAACGCGATGGAGCTGGCTCCCACAACTGGGGATCGGTCAAGGAGGCTATTGATGATG
TGAACAAGAACGAAAGCGAAACCAATGTGACCAATGCCGAGGGCGGCGCCAAGGCCGATGAATCTGGCAC
TGAACCACAAAACGAGCAGGCTACCGCCGAGGAGGAAGCCAAAGAGCTGACCTTGGACGAGTGGAAGGCA
CAGCAAGGACAGCGCATCAAGCCAACCTTTAATATCCGCAAGGCTGGAGAAGG
>dme_circ_0000487_junction_seq
CCTTTAATATCCGCAAGGCTGGAGAAGGAGCAAAAAGGCAATTAAAGCGGCAGCCA
>dme_circ_0000488
CATGGGCGTTCAGCTGCAGCATGGCCATTCGCATGGCCTGGGCGGTGGGCATGGTCACAGCCATGGTGGT
TCCAAGAACGCATCCCATGTGCAGGCCACATCCACACCATGCTCCGATTCGCCCAGCCAGCGTATTGAGG
GCGGTGTGGCCTACGCGCCCGAGGATGCCGAATTGCCTGGCGGCGGGCTGCCAACGTTCTCCTACCAAAA
TACCAAGTTGGTCGATCCCACACTGGATTTGGAAATTGCTGCCGTTCTGGCCGAAACGGCACCCGGATCA
CATCATCACGGCGGACCGGTTGGACGCGAGGCCGTCAACATGAATGTCCGGGCAGCCCTCATCCATGTGA
TTGGCGATGTCATCCAGAGCGTGGGAGTTTTTGTCGCCGCTGGCGTGATTTACTTTTGGCCAGAGTACTC
CATCGTTGATCCCATTTGCACGTTTGTGTTCTCCATCATTGTGCTCTTCACCACGTTCACGATCATGAAG
GATGCGTTGCTGGGTGCTCATGGAGGGGACTCCCAACTATATGCACTACGCCGAGGTACTGCAGATTTTC
CAAGGCATCGAGGGCGTTGAGCGGGTGCACAATTTGCGCATCTGGGCGCTGAGCATTAATAAAGTGGCGC
TATCTGCACATTTGGCCATTGG
>dme_circ_0000488_junction_seq
TGGCGCTATCTGCACATTTGGCCATTGGCATGGGCGTTCAGCTGCAGCATGGCCAT
>dme_circ_0000489
GATGTCCTTCGGCTGGTATCGGGCTGAGGTTATTGGCGCCATGGCCTCCGTCTTCATGATCTGGGTCATC
ACGGGCATCCTCGTTTGGCTGGCCATCGGACGACTCATCAGCGGCGACTACGAGGTGAATGCGAAGATCA
TGCTGATCACATCCGGCCTGGCCATATTGGTGAATGTCATG
>dme_circ_0000489_junction_seq
CGGCCTGGCCATATTGGTGAATGTCATGGATGTCCTTCGGCTGGTATCGGGCTGAG
>dme_circ_0000490
TCCGGGATCATTGTCATCGTGCCCGCAGCGAGGGCGTGGATGTGAAGGCTCGCCGAAAACTGATCATTGC
GAGCATTTTGTGTCTGGTTTTTATGATTGCCGAAATCGTGGGTAAGTAGAGTTAGTAATCATAAAAAGGG
CTATATGCATCTTTGGAGCCACCAATTGGACGAGTTCACAAAGGACTCTTTTCCCCCCTTCGATTTCTTT
TTGAAATATTTCTTTTCCGTCTGCCTTTCAATTCCCAAACTAATTTTACTCACCTCTGAGAGAATCTGAG
CCAACAAATAAACAAAACGTATAAAGTGAAAAGCGAAAGAAATAAGGTAAAGTCAAACAATGTGGGCAGC

GAGGAATAGGGAAACGGGAAAAGGACACGACGAGAGGACGACGACGAGACGATTAAAAACTGAGGACCGG
AAGTGCAACAATATGGCGACCGGAAGAGCTTACTGTCTGCCATCAGCCCTCCTCCATTTATGACGGCACC
GGGGTTGGTTATACAATAAACATTTAATTTTCGAGTGGCGACGATGTCGACGGAGCTTCGACGGCTGTTG
TCGTTAATCGCTTTGCAATTACCTACAATGTTCGCTGGGAAAAGGACAAAGGGCCAGGACTGAGGACCAC
GACTAGGCGGCTACCAATTCCCAGTTGCCAGGAAAGGATTTCATTTTTGCGCCCCTTGCTTTACGTTTTG
CACTCTTTTTTCCTTTCTTTCGCTTTCAAATCGAAATTGTTGCTGCGATAATGGGAAATAGAGAGAAAGG
GCTCCAACTCTCCACCCTTTTAATGTTTTCCGTCTGAGGCCGGGGGTATTGTCACTCGGCAGCCGTTTTG
GGGGGTAATCCCCCAAAATCAGCATCCACAAGCCCCCCCACAGAATGTCGAGTTAATTTTTTGCGCATTT
ATTTACTTTCTCCCCAACCCGAACAGGTGGCGTCCTATCGAACAGCCTGGCCATCGCAACAGACGCCGCT
CATTTGCTAACGGATTTCGCCAGTTTTATGATCTCGTTGTTTGCCATCTGGATTGCCGGGCGACCATCTA
CGCAGCGG
>dme_circ_0000490_junction_seq
GATTGCCGGGCGACCATCTACGCAGCGGTCCGGGATCATTGTCATCGTGCCCGCAG
>dme_circ_0000491
TCGCCACCGGAATCCGAAGATAAATCGAAGAAAGATGCTTCCAATTCAAACAAAAAGGAAGCCTCGAAAA
AGAAATCCCCCAGTCCCACCAAAAAGCCAACAACGGCAAACACTTCCATGCAACTGTTCGACGAGGAGAG
TGCCGAATCGTCCGACGAGGAGGAAAAGCTGGACATGCTGCGGCGGAAGGTGATCGAGTCGGACAACGAC
TCCGATCAGGAAAAGGCCTCGTCCTCCAAGCGGCGACGAATCAGCGACTCTGAGGACGAGGAGCAGCCGC
CCAAGAAGTCCGCCGATGAAGAGACCATCGCATTGGATGAGAAAATGGATACGGAACCGGCCAATGAAAC
CTATCTGGATGAAGATGGCTTCGTCATTACGCAGCGTAAACCAACAAAAGCCCAGCCTGCCAACAAAAAG
G
>dme_circ_0000491_junction_seq
ACAAAAGCCCAGCCTGCCAACAAAAAGGTCGCCACCGGAATCCGAAGATAAATCGA
>dme_circ_0000492
GACCTGTGGCGGTACACCAGCGGGATAGGAATCGGAACACTCATCCGGAATCATTCTAGTCCGAGTATGC
GATGGCAGCGGGAAGAGCAGCCGCTACGCTGGAGGCTCCGGGACCGCCCAGCGGTCAGGACATAGCCAGC
GACAACAGCGCCCAGCGCCGCACGCTGGCGACGAAGGTGCGTCGAAAAGGGCCACGCCCCCAACGGCGCC
TGCACCCGCCCCTGCGCCCTCGCCTGCCGCTCCATTTGCACCTGCTACTCTGGCTGCTGTGCTGCTGTTC
TCAGCTGGGCCAGCTGAGGGCCGAGTGTCCAGCGGTGTGCGAGTGCAAGTGGAAGAGTGGCAAGGAGTCC
GTCTTGTGCCTTAACGCCAACCTAACCCACATCCCGCAGCCGCTGGACGCGGGAACTCAGTTGCTGGACC
TTAGCGGCAATGAGATCCAACTAATACCCGACGATAGCTTCGCAACCGCCCAGTTGCTCAACCTACAGAA
GGTGTACCTGGCCAGGTGTCACCTCCGGCTTATCGAACGCCATGCCTTCCGTAAGCTGATCAATCTAGTG
GAACTGGATCTAAGCCAGAACCTGCTCTCGGCAATACCCTCATTGGCGCTCTACCATGTCTCAGAGCTAA
GGGAGCTCCGACTGAGTGGCAATCCCATACTGAGAGTGCCAGACGATGCATTTGGTCATGTCCCACAATT
GGTGAAGCTGGAGCTGAGCGACTGCCGCCTTTCGCACATCGCTGTGCGAGCATTTGCCGGGCTGGAGAGC
AGTCTGGAGTGGCTAAAACTGGATGGGAATCGGCTGAGCGAGGTCAGGAGTGGCACGATCACCTCGCTGG
CTTCACTGCATGGTCTGGAGTTGGCGCGCAATACCTGGAATTGCAGCTGCTCCTTGCGTCCTTTGAGGGC
CTGGATGCTGCAGCAGAATATACCGAGTGGCATACCGCCAACATGTGAGTCTCCTCCTAGATTGTCCGGG
AGGGCTTGGGATAAGCTCGATGTAGATGACTTTGCGTGCGTTCCACAAATTGTGGCCACGGACACCACAG
CGCATGGAGTGGAGGGCAGGAACATAACCATGAGCTGCTACGTGGAAGGAGTACCCCAACCGGCTGTCAA
GTGGCTGCTTAAAAACCGACTGATAGCCAATCTCAGTGCTGGCGGGGATGGTGACTCCGATTCGGAGCCC
AGGACAGCGGCAGCAACTCAGGGTAGGAAGACCTATGTGGTCAACATGCTGAGAAATGCCTCGAACCTGA
CCATTCTCACGGCTGACATGCAGGATGCCGGGATCTATACGTGTGCGGCGGAAAATAAGGCTGGAAAAGT
GGAGGCCAGTGTGACTCTGGCAGTATCCCGTAGACCCCCGGAAGCTCCGTGGGGCGTAAGAATTATTCTG

CTGGGGGCGGTAGCCGCTCTGCTCCTCGTCGGTGGATCCTCCTTTGCGGCCATTTGCTTGTGTTCCCTAC
AAAGGCGAAGAAAGCTGCGTCTCTGGAACTCTGTACCTCCTGTGAGGAGAAGCGAGAGCTACGAAAAGAT
CGAGATGACGGCCAGAACGAGACCGGATCTGGGAGGAGGGGCTAGTTGCGGAGGCGGCAGTGCCACGGGC
GCCGGACTCTTTCACGATGCCGAGGAGCAGGGCTATCTGCGGGCAGCTCATACGCCACTAAATGACAACG
ATGCCGGGCAGGCGGCGGCCATCGTAAATCCGAGTGCAGGAAGTGCACAGCGAAGAAATGGAGACTACCT
GCACGTGTCCACCCACTGCGATGATGAGGAGGAGGACCAACAGCTGCATCACCACCCACAACAGCAGCCC
GCGAGCCAGCACCACCCACATCCCAATCAGCAGCAGCATCAGCAAAGGAAGGGCTCCCAGGGCCATGTTG
TCTCCGCATCCGGGGCGAATAATTCAGCACCGCTGGAGGAAACGGATCTGCACATACCGCGCCTCATCGA
CATCGGCGG
>dme_circ_0000492_junction_seq
ACATACCGCGCCTCATCGACATCGGCGGGACCTGTGGCGGTACACCAGCGGGATAG
>dme_circ_0000493
TCGTGACCGATTTCGAAGTTGGTTACGACTACGATAGCTGTCTTCATTATCGACCAGGAGCCTTCTCTAT
CAACGGCGAGGATACTATAGTTCCCCTAGATTCCAGTGCGGTAATTGGACAGCGAGTGGGTCTTAGCTCC
AAGGACATCGATAAGATCAACATTATGTACAAG
>dme_circ_0000493_junction_seq
CATCGATAAGATCAACATTATGTACAAGTCGTGACCGATTTCGAAGTTGGTTACGA
>dme_circ_0000494
GAATCCCAAGGCGTGCATAAACTCATGCAGGATCGTTCCGGGCCGGAAACAGCCTTCGCCGAGCGGGTAT
ATTTCCAGATTCATTTCCTGCGGGGCACCTTGGTAGCCGACGGCAGTGTAGCATCCTCCAGACTTGGCCG
TAACCGTCAAATAGGCCTTATCCTCATCGGTGGCTTCCCTAAACCTCAAACAAGT
>dme_circ_0000494_junction_seq
CGGTGGCTTCCCTAAACCTCAAACAAGTGAATCCCAAGGCGTGCATAAACTCATGC
>dme_circ_0000495
GTGAAGGGAGTATCTTTTTAATATAGCCAAACATTCGTATAAGCTAAAAGTAATCTAAGTTATCGCTCTT
CTCGACCTCAATTATTTACTATTCAAATCTATATGAGATAATTACAAATTTAAAGTCTAATCATTTATAT
TGGCAAAATATAAAATGTCATATTTCAAATCAACATCAACTATCGCAGTCGTCATAGCTGTTTACTAAAT
ATTTATTAAGAAAGTCTACTTCATGCACTTTTTATTGGCAACATAAAATTATGTTTGTATTCAAAATATT
TTCTTAGCCTACTACTATTTTAAGTACAACATGTAATGCACTTAGCCCCCAAACGATATTTTAGAGAGCC
CACAACATGGCGGCCAAAATGCCCTTGAAAACTTCAAT
>dme_circ_0000495_junction_seq
CGGCCAAAATGCCCTTGAAAACTTCAATGTGAAGGGAGTATCTTTTTAATATAGCC
>dme_circ_0000496
ATAAAGTCTCCTTCGGGGGACAAGGACATTCCGGTAATCGTATCCGAATGCCCTCGCAGATGGTGCAAGA
CAGCCTGTTTTCGAATGTCCCAGATTTTGACTTCGTTGTCGATCCCGCCGCTAATTACTTGTTCTCCAGT
GTCACCAAAACAAACAGCTGTGACTTGAAAAGGGGATTCCAAAG
>dme_circ_0000496_junction_seq
GCTGTGACTTGAAAAGGGGATTCCAAAGATAAAGTCTCCTTCGGGGGACAAGGACA
>dme_circ_0000497
ACCTTCTACTTGGCCGTCGACTACCTGGATCGCTATTTGCATGTGGCGCATAAGGTGCAGAAGACGCACT
TGCAGTTGATCGGCATCACCTGCCTGTTTGTGGCCGCCAAGGTAGAGGAGATTTATCCGCCAAAGATCGG
GGAG
>dme_circ_0000497_junction_seq
GGAGATTTATCCGCCAAAGATCGGGGAGACCTTCTACTTGGCCGTCGACTACCTGG

>dme_circ_0000498
TGTTTGTTCTACCAGCAGCACTGAGCCAAATGGCAGCATAGTCACAACCGCCCCCAGCAACGGAGAAGTG
agcagcagcatagtcgtcgtcgtcagcagcagtagtatcagtagcagcagcgatagCCCAATCGCTATCC
TTCCACATCCAGATCCAATCCCATCAACATCGTTCTCTTCAGCGTCGCAACGAAGTGAAGAGGAGCTACC
AGGAACCTCAGCAGCCTCCAGAACCGACGAGATGTGCTCTTGTGATAGCCAGAATCTTGCAGCATCCACA
GCCGCGACTTCCAATGGCAATAAACGTAAACGGCGTTTAAGCAGCGATTCAAACGAGG
>dme_circ_0000498_junction_seq
CGGCGTTTAAGCAGCGATTCAAACGAGGTGTTTGTTCTACCAGCAGCACTGAGCCA
>dme_circ_0000499
TCCTCTTTAAATTGTACATTTCATGCAAAACATTTCTGCTTTTGACGAGCAGCTTAAAAACAAGATTCAG
GATAAAGAACCTTGACGGCTTCTCAAGGCCGTACAATTATGGGTTTAAATGCCAAGAGGTAAGCTTATGA
GTACTTTATTCATTTTAAATTCTACTGGCTTACTTTTAAATAATTTAATTTAATTTTCGATGTTTTAAGA
GGTAAATTATTCTTGCAAACCCCAAGTTCGATTTGAGTTTGTAAAAATCTTAGTTTCAAGCGAACCTGCA
TGTTTTTCTTGACATCTCTTATGATCTTAGACATTTCCTTTTCCCATCAAACACTTTTCGTGACTTCCAC
TTCCTCCGCAGCCCTGACCAAACTAATTAGCACCCCACATGAAAGCCAAGAGGCGACTGACTCCCCAACG
GACTGAGCTACCATTTGTTTCTAACGCTCGGCGACTTCCTGTGGCGACATCCGATCGACTTCCTTTTTAA
CCACTAACAACACTATCCTGGTAGGCAGCGCGTCTCCCTTTAATTCTCTTAACCAAAAGTGATGTTTATC
TAATAGCTATTTCGTAATTATAAAGCGACAGAGCCCAACTGGTTATGCCCGAATACGCCGCGGGAAAGTT
GGACCCGCAGGAGCTATAACCACTCGGGATCCTACTGTTCCAACCTTCTGACTCAAGTGCGATAATCTAA
AATGCATTTTCACTGTCAATTTTCaaaacaaaaaaaaaaaaGCCATCTGTTTGTAGGTGTTAAAGTCGAG
GCAGCGTTGATAACCAAGTGCCGCATTCCTCAACACTCTTGCATACCTTTACAGGACCTTTCCGAGGTCC
TGCGTGTGATTAGTATATAATTGCGCGCGAAAATGTCATTAGAAAAGTGTTTAGTTGTGGGACGGTTCAG
GTCAGTTTGGTCATTCAGGGGGTTGGGTTATGAAGGGGGTTTGTTTGGTCGGCTCTTGATAATGCTGTCA
CTGCAGACACACACTTTTTGTGGCTTTTGCAGTCATGTCCTGGACAAAAATTAATTGAGTTTAATATGTT
TTCACCCTGCACTATATATTCATTCTTTTTGCTTGTAGTAGCTGCCTGCGCATGACCATAATTGCATAAT
TGCCTAGTCATTTGGTTTTGTCAGTTGCGTCCCCGTTTTGGCATtcaacatcatcatcatcatcatcatc
atcaCCCACTGATCACTGCGATCACTTAAGTGATAGTCCATTTTATGGACATATTTCGTGGTCAGCTTGT
GTGCGACATAATTATTGGTATGTGTTGCGAAGCTATTTCCCTGCCATCTATAACGGAATTTATCAAAAAA
ATGTCACGGAAGTTCAGGTTTTCCAGATTCCATTGCATACTTTTGAGGGCATGGTAGTACAAGTAGTACA
AGGAACTCACATAAAAATGTTCAAAAAATGACAAAATTCTTGTAACCTTAAGAGTTTAGGATCAAACACA
TAAATTCCGAAACATAACTGTTTCGTTGATACCAAGTTAAACGCATAAATAACTAATTTCTAATCGAAAA
GTCGCCGTTGCTTTATAATTTATCATGTGAGAAAGATCGATCCTTCGATAGTCCACTTATTATCCACTGG
ACCAGATTCCAGGAAATACTTCTTCGAATTTTTTCATCATATCATCTCCGAGATAGTCACCCTAGCCTTT
TGCTACCTACAGGGATTCGGATTTGAAACGGAATAGAAAAGGGGAAACGAAACCGAAACAAAGTATCTGC
ATTTGCCGCAAAGGAAAGGAAACTTTCACCTCGAAATGACAAATTTCAGTTTGAACCTTAAGCCGCGGAG
ATGACTGGAAACCCCTGACCCCGAAACCCTGAGTACAATGAAAGAAAGCGGGAAAAAGTTGCAGGCCGCG
TGCACGTACGCTCCATTAGTCCTTTCTCGCCATAACTCATTTTTAAAGCGATTTCATTTCCTCGTAATTT
ACTACGTGATTACTTTTTCTTGTTCATTAAACCATTAAACCCGATCGATAGTGCAGGGCAAGGATCAAAA
CAGGAGGCTGCAATGCCGAGCCTCTGGCCAAGAAAGAAATGGCTAAAGCATTTGAATTTATGTATGGGCA
GCAGCCCttttttatttttttttttCTATTATCCCTTTTCACGAGTTCCTTAAGAAGGGTTTTTGGTCCCA
AGGGTTAGATTTCGtttttttttCTCTCGTTCTCCTGAGCTCCTCGCTCAAATTGCAAAAATTCGCGCAT
CGAATTGCTTAAAGTGCAGTTGCTTGGATCGCAACACTTGAGAGTTGCGACGCAAGGATATAACATCTGT
CCTTTGGAAAGTATGGAAAATTGTATTCTTCCCGTTTGCCAGCCTCCTGCATTATGCTAATGTGCATGCA
AATTTGCCAAAATTGCTCAATCTCGAGGAGGAAAGGCTTTCCCCGGGGCCCTCACAACGATCccatttcc

atttccatttccccccccGATCAGTCATCTCGATTTCAATTTCCCGGATGTTAAATATTAATATATCCGT
TGGGCGCCTTAATCTCGAGTCTTGCATCGCGATTCGAAATTCGAATGCCGTGTCAAAATTCAATGAATTT
ATGTTCGTTTCGTTTGTATCTATTTTAGAACGCCCCTATGTGATTTGCTGCCCTGCCTCCGTGGCATATT
GAATTTCTCTATTTTGCaaaaaaaaaaaaaaaGTGGAGAAATTGGAAATAACTGCGCTCCAGAGTTTGGG
TTTGGGTTTACAAAAATATGGGGGATTCTTTAAATGGAAGGGTTTTTGATACAATCATGTCTTTTAAATC
TACAAAAATATAGGCAGCTATGTTGAAGTATTCTATCAAATAGCTGCTCTTTGGGGACATTCAACTATAT
AATTTAAATTATTTAGTATTATTATTTAGTACCCCACTGCTAATTCCACTCACTTTCTTTTGCAGTTGTT
TGTTCTACCAGCAGCACTGAGCCAAATGGCAGCATAGTCACAACCGCCCCCAGCAACGGAGAAGTGagca
gcagcatagtcgtcgtcgtcagcagcagtagtatcagtagcagcagcgatagCCCAATCGCTATCCTTCC
ACATCCAGATCCAATCCCATCAACATCGTTCTCTTCAGCGTCGCAACGAAGTGAAGAGGAGCTACCAGGA
ACCTCAGCAGCCTCCAGAACCGACGAGATGTGCTCTTGTGATAGCCAGAATCTTGCAGCATCCACAGCCG
CGACTTCCAATGGCAATAAACGTAAACGGCGTTTAAGCAGCGATTCAAACGAGG
>dme_circ_0000499_junction_seq
CGGCGTTTAAGCAGCGATTCAAACGAGGTCCTCTTTAAATTGTACATTTCATGCAA
>dme_circ_0000500
GTGACTCATATTGCGATCGTTATCCGTCCACATATTGCGCTGCAGATGCTCCTTTTTGGGAAAGAACTCG
GTGTCCATGAAGGGAGCTCCGGTGAGGAAATAACGTTCGATGTCGTGTGGATACTTTCGCCACTGTGGCA
GATTCAGTGCCTCCACAGTGGTGTTCAGTACGTACATATAAACGG
>dme_circ_0000500_junction_seq
GTGGTGTTCAGTACGTACATATAAACGGGTGACTCATATTGCGATCGTTATCCGTC
>dme_circ_0000501
GCGAGTCCTGGCTAGCGTTCACACAATCAGTAATGCCACTGAGCGAGATGCCCGCTTTCAGGCGCTGGCT
GCCGATACCCGCGTGGTGATCATCACACTGCTGCAGCGCAGGAAACAGAACATCGGGCAACTGGGCGAGC
TCATCACCCTGTACCGACAGAGCTGTATAGACTTCAACACCTTTTTGGAATACGACAAGTTCGCTGAGGA
GCTGAAGAAGATCGCGCTGGCCAAGAATCGCAGCGAATTCTGGCAAGACGTTATGGTCGACAAGCAATTG
GGCCCTTTGGTGCTTGGCGAGCCGACGCTGGACGACGAATTGGCTCTAAAAGCCTATTACACCATCGAGA
ATTGGGTGGAGAGTGGGGCGAACGATATGGAAGACGTTGAGATG
>dme_circ_0000501_junction_seq
GGCGAACGATATGGAAGACGTTGAGATGGCGAGTCCTGGCTAGCGTTCACACAATC
>dme_circ_0000502
GTAGCATGATTATTACTATAATAAAATATTTTGTGTGGAGATACAAGATAGAAATGCATGCAATCCATTC
CATCTATTCTGCTGTTTATAATTGTGAGAAGATCTCTGTTGGATTTCTAATTTGATTTGATTTAAATAAC
TTATCTACTTTTATGTTTTGGTACACTTGACTTAGACAAACGCCGTACTTTCT
>dme_circ_0000502_junction_seq
CTTGACTTAGACAAACGCCGTACTTTCTGTAGCATGATTATTACTATAATAAAATA
>dme_circ_0000503
AATTTCCGCTGGCTTTCCCGACACGCGCACCTGCCTCCTCCACCAGAAACTACAGATGCTTAACGTTTGC
GTGGAGCGTCGCGTGCAACGGGAGGCGAATAGCAAAAGAAAATCCGAGGGAATGGTGGGAAAAGCTAGTT
CCGAGGAGGAAGAGGATGAGGATGACGACGAGGGCGAGTTCTTTGACTGCGATGATCTGACTGCCGGTGC
GGGCTCACCCACAAAAGCAGTTCTTAGTCTGAAGCCCGAGGGTCGCCTGAGGCGTTTGAACAACGAACGA
TTGCTGGAGGAGCCCGATGAGTATCTGTATATCCCAGATACCCAGGAGCCCGTTCCAAAGACCGAAGATC
AGCTGCAGGACGACGCAGAAGTTATGCTGAAACTTGGACCCGGTTCGGGCCTGACCACCCAAATGATGTG
TACTTCATTGCTCTCCGATATGGAAGCCTTCAAGGCCGCCAATCCCAGGGGCATCATGGAGGACTTTATA
CGCTGGTATAGCCCCAAAGACTGGGAGGAGGTGACTGATGGAACTGGGCCAGGTGAAGCATCAACTTAGT

ATACGGATGACCACTGAGGGCAACACCTGGCAGAAGGTCTGGGAACAGGCTCAAGCCGTTCCAGTTAGCA
GGCAAAAAAGGCTATTCGACGACACCAACGAAGCTCTGAAAGTTCTTCACTATTTGGAGACACGAAAAAT
GCACGAGATCTACAATCTGACAGTAATTCCACTACTGCATTCAGCAATACTCAAACTAGCTGGACATTCT
AAGCAATGCCGAACTGGAAGATTTGTTCAGTTCCCAGATCGAGAAGCTTCTCAGCGATCTCTGCCGTCTC
TCACGATCCCATTCGGATGAACTTCCGTCAATAAAACCCTTATTGGATGACCTGGCCGAGTTGGAGCGAC
GGTTCTACCAGTTCAAGTGTTTCGAACGACTATCGGGATATCCGAAAAGAAGCTCTCTTCAGCAAGTGAA
ACTCCAGTTCGAGGAGATCCTTCGCAATGACAACTGTTGCACGATTGTAAATCGACGACTGACTGCAGCT
GGCGATGGCACTTTGTATGACATACTCATACCCAAACTGGAGGAGGACATGGCTGACCGACTGATAAGCA
AGGATTACATCATTAGATTGGATGGGGACACCAAGACCACAGAGAAGGGTCTATATTTGGGCCCACAGTT
CATGAGGGCCATTGTAACCGGGGAGAAGCTGAGACTTTGTGGAGCGTTTACAGAAAG
>dme_circ_0000503_junction_seq
TGAGACTTTGTGGAGCGTTTACAGAAAGAATTTCCGCTGGCTTTCCCGACACGCGC
>dme_circ_0000504
GATTTTATTGAAGCACTCAAGGATGTGTCGGTAATGATACCGCAGGCGGTGAAACGTGGCAGCAATGCGC
TATTCACTTGTAATTACGATATGGAAAACGATACTCTATATTCGGTTAAATGGTATAAGGGCAAGCGTGA
GTTTTATCGCTATACGCCCAAGGAGAATCCGGCGATGAAGGTCTTTGCCATGACAAGTGGTCTCAATGTC
GAGGGTAAGTACAGCAGTGCCCAGACACTGCCTCTCGTTGATAATGTAGATGAGGATGTGGATATGGATA
TGGATAATTTCCCTTAACTCCGTGTGTCCCCATAACGCGCACATGTTACTGCAGTTGCGTGTAAATTATA
TCCATAATGGCTGATTGCCAGGACCCCGGCTGTATTCGTTCTCATAACGACATTTGCCGGCTTAAATGTC
CTGTCCCACTGCCCGGTGTAATTATGCCAGTCCATCCGGAGACGGATCCGTTAAGAGTTGTTGGGCGTGG
CCAATTAGTCGCGCTTGCGATTGACTTGCGGCTCATTAAAAAACAATATATAGAGATTGCATACCCCGTA
AAAAGAAAAACTACACCAGAGAACACGAAAATACACTCGGTCCACACAAATTTTAAATTAATAAAATACA
GTTTCAGTTTAAAATCATTGTTAGATACATAAAATAGTTAAAATTGTATCTAAACAAATAGCTGCTTACC
TCCAATTCTTACCAGTTTAATAAGATATCCTAACAGACCCGTTGGCAGGGTATGATGACAACCCTTAATT
ATTACGGCGAAATACACAAGGGCCAATTAGGTATCAGCAGCAACAAATTGGGTTAAACATTCGCACGAGC
CTCAGCCATTGATCCTTTGGAAGTAGCAACGACCTTTGCCCCCACTTGCCACACTTAATTTCGCCTTCTC
CATTGCATTTATGTGACATTTATTCTGAAAATAAAGCACAACATTAAATGCAATTTATTAAATTTGATTT
TTATTAAGTGAATTGTGTTATGTGAAGTTTCAACGTTTCCTCTTCAAGAGATTAAATTTCGTCAGCTTAA
CAAAAAATATATATTCTCATTTATGATTGGAAAGCTTAAAATGGCCGATGTTAAAATGATGGTTAAACAA
AAGCTGCTAAGCCATTATAAAAATTTACTTTAAAAGGCAATTATTTATAATAAACGTGTAGAAACAAATA
ACTGGAGTAAAGGGAGTTTTAAGTTAcgtttatatattattatatttaatttttttgtttttatcctatat
gtattttctataGCTTTATGTTTAGGTATTAAGTAACAAAGCTTTTAAGAACTAAATAAGTTTTTGCTTA
TTATTTATTAAATGTAAACAACAAGTGCTTTAAGTGGTCAAGACCAGAGACCTTAAGATAGTTGAAGTTT
TTCCCCTTGCTTTCGTCTTATTAAAAGAAAAGTATGGGAATAAGGATTACACTCGTGAATCaaaaaaaaT
CTGCCACTCATAGCCCCATGGCAAGTTGCTTTGACTCAAAGAAAAACTTAATCAAATCGAGATGACCCCT
TCAGCTTTAAAATCAACCCTTCACCCTTCACCTTTGTGCCCCCAATTTGCAGTCACTTTCCAGCAAGCTG
CTGCGCTTTTCATCATTCAGGACCAAGGCAAAATTTTCATATTTTTCCCATAGCATTTTTCACTTTCTGC
TCGCGCTCTGTCGCAGGTTATATTCAATTTCTATGCACCGCGTCCAGCCAATTTTGGGAGCAACTAATTT
TTATTACGTTCTTTATTTCCCCGCCGCTGATTTTCTCCATTCGCTTTATCCTTTCTCCAGCGCAACCTTT
CGAATCAGAGCCACGTCGTCCTGCAGTCGGTGCCGCTGAATATTTCGGGGAAATTTACATGCGAAATATC
GGTGGAGGCGCCCACTTTTCAAACGGCCATGGTGTCCGGCGAAATGGAGGTGGTTGGTAAGTGAAAAATC
CAAAAATGtacatacatacatacatacgcatatatatatatCCAAATCCAGATATAGCGTTAAGAATTGC
AATGGACTAATGGTAATGTAAAGGCAAACCGGCGGTCGGGGCAAGCAAATTAATTAGAACAAATCTATTT
GGGCCTGCCACCGGTCAGGTCCTTTTCGGCTAATTTTACATTTCCATTCCTTGGCTAACTACTAACGACT

GTTTCCCCTCCTTTTTGGCCAACTTTTTGCAGAGCTGCCGGAAGAGCACACTGTGGTTACCGGGATACAG
GCACGTTATCGCATCGGCGATTTGGTCGACGGCAATTGCTCAATTAAATACTCGAAACCGGCTGCTAATT
TGACATGGACTATTAATGGTATTGTG
>dme_circ_0000504_junction_seq
TTTGACATGGACTATTAATGGTATTGTGGATTTTATTGAAGCACTCAAGGATGTGT
>dme_circ_0000505
GTATGCAATTTTGGACGCGTGGAGAATATGACCTGAAAGGAGGAAAAATCTTTAAAATATATTCAATAAT
AATTAAGAGCAACGAAAAAACTGTTTACAAAAATCCTTGGTTGTAATTTAAGGTTGCCAAATTCGAAAAT
AACGTTTAAGTAACTTTATTACTTTAAATGCGTTCTAAATCACTTAAAAATATATAATAATAACACAACT
GCAAAATTAGCCTAATGTAACATCAGTTGATAAAATTATAATGGAGCAATATATAATCACAACTCTCAAG
GGTTGGGaaaaaaaaaTTTGACATTTTCTATACCCCCTCTTAAATGTATGTATGAATGTATATTACAAAA
TCTTACCTAAATTAGTTAAACTAATCAAGCCATTATAGTCAAATGCAAATAATATTTGGTAACATGCCAT
GATTAATTTATAATTAATTTATAATCTTACACTTAGTTGACTCCGACATCTGGAACTCACCTGAACATTG
AACTTGGTCTCGATCATCTTGATGTAGGGAGTCTCGTGGTCGGGCTGCGGCTTGTTGGGTCCCATTACGG
GCATCTCGAAGGAGATGAGCAGCGGTGTGGAGAGCCGAACGAGAGCCCGGGCTCGCTCCACGCCCTCTAG
GCTGCCGCACAGCGACACCTGGTTGGATTTCTCCGTGGGATTGGAGCGATTCGAGTCGGGGAAGTGGATA
TGGG
>dme_circ_0000505_junction_seq
CGATTCGAGTCGGGGAAGTGGATATGGGGTATGCAATTTTGGACGCGTGGAGAATA
>dme_circ_0000506
GATGGAGATCTCGCCGCAGCATCACGAAATCGTCAAGGGCAAGAACAACGTGAATCTGCTGAGCATTATG
GAGCGAACCCAGACCAAGATCATATTCCCCGATCTGAGCGACATGAACGTGAAGCCCCTGAAGAAGTCCC
AG
>dme_circ_0000506_junction_seq
GAACGTGAAGCCCCTGAAGAAGTCCCAGGATGGAGATCTCGCCGCAGCATCACGAA
>dme_circ_0000507
ATCGAGATCAAGCTGTCCGACATCCCGGAGGGCAAGTCGGTTACTTTCAAGTGGCGCGGAAAGCCCCTGT
TCATCCGCCACCGCACGGCCGCGGAAATCGAGACCGAGCGAAATGTGCCCACATCCACGCTGCGCGATCC
GGAGGCTGATGATGTAGGTACCGTGGAGTGGGTCGATTCGCTAATGCTGTTACCCATTTCCTCTTTAGCA
ACGTGTGATCAAGCCCGAGTGGCTGGTGGTCATCGGAGTGTGCACGCATCTGGGCTGTGTGCCCATCGCG
AACGCCGGCGACTGGGGTGGCTACTACTGCCCCTGCCACGGCTCCCACTACGACGCCTCCGGAAGGATCC
GCAAGGGACCCGCGCCCCTCAACTTGGAGGTGCCCACCCACGAGTTCCCCAACGAGGGTCTTCTCGTGGT
CGGCTAGAGGAGCTGTTATCGTTTAAGCCCCCAACTGCATAGTGAATGGATCGATG
>dme_circ_0000507_junction_seq
CCCCAACTGCATAGTGAATGGATCGATGATCGAGATCAAGCTGTCCGACATCCCGG
>dme_circ_0000508
GTAGCCACCCCAGTCGCCGGCGTTCGCGATGGGCACACAGCCCAGATGCGTGCACACTCCGATGACCACC
AGCCACTCGGGCTTGATCACACGTTGCTAAAGAGGAAATGGGTAACAGCATTAGCGAATCGACCCACTCC
ACGGTACCTACATCATCAGCCTCCGGATCGCGCAGCGTGGATGTGGGCACATTTCGCTCGGTCTCGATTT
CCGCGGCCGTGCGGTGGCGGATGAACAGGGGCTTTCCGCGCCACTTGAAA
>dme_circ_0000508_junction_seq
GAACAGGGGCTTTCCGCGCCACTTGAAAGTAGCCACCCCAGTCGCCGGCGTTCGCG
>dme_circ_0000509
GGGCGATAAGTACTGCACTCTCCCAATTGATGAAGGACGCCTTTGATGTTAAGGCCTTACGTGCCTTTCG

AGTGCTACGTCCACTGCGACTTGTATCGGGTGTACCAAGTAAGGCCTTCATTGCACCATCGAACATATTC
TAAAACGAAAGAATCCCTCCAAAATCCTCTGGCCTGGTAGAGCGGAAATCTAGGAACTACGGATGGATTC
GTAGTTAAAATACAGCACAGATAAAGCACAATAATTGTTATAGCTAAACAAAAAACCTTGAAATGAAAAT
GCTGATTGTCCGATAGATTGTCCGAAATTTTTTCTGCTATGCTATTTTTTCTGTGACGTATTTTCTGCTG
AACTTTGATTGTTCGCACTAGATTTTTAGCCGCCCACCATGCCCACAATAACCCAATCGAATATTGGCAA
TAAATAACGCTATTCAACAGGGTCTACAGGTTGTGCTGAATTCAATTTTAAAGGCCATGGTGCCACTGTT
TCACATTGCACTCCTGGTCCTATTCGTAATCATAATCTATGCGATAATTGGCCTAGAGCTCTTCTCTGGC
AAATTGCACAAGGCGTGTCGCGATGAGATCACAG
>dme_circ_0000509_junction_seq
CACAAGGCGTGTCGCGATGAGATCACAGGGGCGATAAGTACTGCACTCTCCCAATT
>dme_circ_0000510
GTGGATGAAGAAGGCATGATTACAGCACGACCCCGACGTATGTCTGAGGTTAATACGGCAACGAAAATTC
TACCCATACCGCCGGGCACATcatttttttcttttctcacaaacgaacaggtttttatgtttgagttttat
ttttCCCCCAAATATTGAAAGTGTCCGAAAATACtatatatatatatatatatTTGTGTGTATGTGTGCG
TGCGCGTAGATTAtttttttttAAAACATAATTTCAATAATCATGTATTTGTCCTTTTGTTTAACCATGT
TTTGTATATTGTCATAAGAAATAATATACGGTTTTATTTTGAGTTTGATTAGACTATAGAAATCCATTAT
GGTAGAAAAAATTTATTTCCACTCTTAGCTGCTTTTTATTCCATAATTTCGTTTATAACTTGAATTGTAA
AACTTCTAACTGCATGCTAAAAATATCTTTGTTTTTTCTGTGATTTTACTTTTGTGATTTATAAAACTTT
GAGAGCACGTTTCTTAACACTCTGCTCTATGATATTTCAACAGAGTAAGTAGCTCCAGTTCCGATCAAAA
CTAATAAATCAGCGTCAAATGAACTAAAAAGACTTGTAAGTTACGATTGAAAATCGCAAAGTGCATATAT
TAGTTTTCACCTCTAGTAGAAACACGTTCCATTCGATGTTCTTCACAACTGTCCATTTGTTTTTGCAGGG
TGCTCAATAAATTTGATTATTTTTTCACGGCAGTTTTCACAATAGAACTGATTCTGAAATTGATTTCATA
CGGCTTCGTATTACACGACGGAGCCTTTTGCAGATCCGCATTTAATCTATTAGATTTACTTGTGGTCTGC
GTGTCATTGATTTCTCTAGTGTCCAGGGTAAGAATTCATTCAAAACCTCTTCAATTTCGCTTCCTACTTT
CTGACACATAATAAAATGCTTCAACAGAAAATTGTTGTACTTCTTCTTTGAGTTTCAGTTTTATTATTTC
AATTTTTAGTTCATTACGAAGAGCACAATCAATGACAATATTTATCTCAAACTAATATGCAAGTTAAACA
TTTTAAATGATAATAATCCCAAGAATCTTGGGCCTTCAATTAACTTTAGAGTAAGCCAAACAAAAAACCT
TGAAATGCTGGGTGTAGGGTGCACCTCAATAAAGTTATGAAAATATAAATTAAGCAATATAAAAAGTATA
TTCATGAACTACACACAGCTGACAAATATATTTCTTGATTTCGAGTTTTACTCGTAACACCCTAACGAAA
ATGGGCGTGGAATAGGAATGTATGGGCGTGGCTTGGGCATAGTGAACTCTATGTAATAACCCCTGATACT
AAAAACACATGGCCAGCAATATGTTCGCGCAAAATATGGAAGTCAGAAGCCAAACATGCCTTAATATAAT
TAGACTTATATATTAAATAGTGTTTTTATTATAACTATATATCGTCGTATCACATACAGCATGTTGTTCA
ATGTGTCATAGTCGCTGTTAAGACTATCGGAAATATTGTGCTCGTCACATGCCTACTGCAGTTCATGTTT
GCCGTAATAGGAGTCCAATTGTTTAAG
>dme_circ_0000510_junction_seq
TGCCGTAATAGGAGTCCAATTGTTTAAGGTGGATGAAGAAGGCATGATTACAGCAC
>dme_circ_0000511
GCATATAAAAAAGAAGGTTCTAATATAAATAAATGTATTTTTTACTATTGAAAATTAAAACTCAAATAAG
TGTTCAGATATATGCCTTCACCCATTGATAGCGAAAAGTGGACGAAGTGAAAAGAAGATAAAAAACAAAA
TATAAATAATGCAAATTTTTGAGGGCTGAGCTTCAAACTAATATGCGATTAAGTACATTTTACAGTTTTA
GAATATAGATGTTAA
>dme_circ_0000511_junction_seq
TTTTACAGTTTTAGAATATAGATGTTAAGCATATAAAAAAGAAGGTTCTAATATAA
>dme_circ_0000512

TTGTGTAAAATTTGTATGTATTAATTAATGTAATTTATTGTTATTTTACAATTCGATTCAGCCACCCCAT
AGCGAGCTaaaaaaaaaaCAATTTAATTTTGTACCTACAGCATATATAAACAGACTTATACGCATATATT
TAATTATGAACGGCCCGAATACCCCATTAAATGGTTGTAGAGGGCTTGTCGAAGTAGTTGAAAAATAAAC
AACATGTTTAAATGTAAAATG
>dme_circ_0000512_junction_seq
AATAAACAACATGTTTAAATGTAAAATGTTGTGTAAAATTTGTATGTATTAATTAA
>dme_circ_0000513
AAATCAAACTACTTCCGTTAAAAGAAACCAATCAAATCTTTGTGAAAACGAAACAAAAACCACACCTCAA
GATTGTAAGAAAACAGACTTAAGTGCACACGAGCCAGATTAAGTCCTTCAGAACAGCTTTTGTAATTGTT
TATGAGCATCACCATGCGTCAAAAAGATCTGCGCCCAGCGCCCGCTCTAATTGAGTACAAGGGCATGAAG
TTCCTAATCACCGATCGACCATCAGACATAACCATTAATCACTACATTATGGGAGCTAAAGAAGAACAAT
GTCAACACTGTGGTGCGCGTGTGCGAACCTAGCTATAATACCGACGAGCTCGAGACACAAGGCATTACCG
TCAAGGACCTGGCCTTTGAGGACGGCACCTTCCCGCCTCAGCAAGTCGTTGACGAGTGGTTTGAGGTCTT
AAAGGATAAG
>dme_circ_0000513_junction_seq
CGAGTGGTTTGAGGTCTTAAAGGATAAGAAATCAAACTACTTCCGTTAAAAGAAAC
>dme_circ_0000514
TTGGTTAGACCTGTTCCCACATCTATGGAGTTTCATCTAGACAGTGCAGAATACTGCCAGGCACAGCACA
AATAAAAAGTGAGTATGGATTATTTTTGTAGGGTATATGGACTTTTCGTTTAATTAATTAGCTCAAGTGG
CACGGGCGGTACACTTGCTGGCCATGGAAAGGTCACTTGACTTACAGTGAACAAAGCACTGTGACCTTTC
TGGACAATTGACGGCTTGACCCGCCCCGAAGAACATCGCGGATAAAAAGAGAGTGTCGTGCGAGACGAAA
ATGGCGAAAGTCTCAATTATTTCATTGCGTGTAATTGAGCTAAATTGTTTTTTAGTTTCCCACGAATTGT
TTTCAATTAAATGAACAATGGGAGTCGCGAGAAAGAAGCTCAAACCGACTTGCGCCAGATTTGTCTGGAA
TATTGAACTCATTCTAGTCTCACTCTTTGCAGGTAAGCAATCCTACAAAAAGGCGGAGATAGCCTGAGTA
AACACAGTCACCACTTCGGAAGTCGCCATCGATAGATAAGTGGTGTGGATACCATCAGAATAATACCGTC
AACGGATCAGCTGAAATAGCGGCGACAGCAGCGGCATCAATTAGCCAAAGACTGTCGATCTATGGGAGAG
AAAGAAGAATACTAAATTCAAAGCATCGCGGTGATCACAGATCATATCACAGCAAGGATATATTTAAATT
TTTAACAACGAAACCTTGAAAAAGTTGTAATATTTTCGAAAACAAAACGGGAACTGAGCCCAAATCATTT
TTGTTAGCTTAAAAAATACGTGTGACACTTAACAGGGAACCCTTTTTTCAGTCAAAGAAAATTGATTGAT
TTTCTTTGTGATAGCAGAAAAAAATCCATAATTGTACTTAATTGAAAAGAAAAAGAACTAATTTTACTTAA
ACGATACTTAAACTAAGTTTGTGTTTTGGTGTAAAACATTTAAGATACGATACGAAAAGAGTTAattata
tttatatttatatttatttttttataCATAACTCTAGCCTAAACAAGATTCAATTGTAAGCCTCTAACACG
CTGAATTTTTAATCTAGAGTAAGCAATCCTACAAAAAGGCGGAGATAGCCTGAGTAAACACAGTCACCAC
TTCGGAAGTCGCCATCGATAGATAAGTGGTGTGGATACCATCAGAATAATACCGTCAACGGATCAGCTGA
AATAGCGGCGACAGCAGCGGCATCAATTAGCCAAAGACTGTCGATCTATGGGAGAGAAAGAAGAATACTA
AATTCAAAGCATCGCGGTGATCACAGATCATATCACAGCAAGGATATATTTAAATTTTTAACAACGAAAC
CTTGAAAAAGTTGTAATATTTTCGAAAACAAAACGGGAACTGAGCCCAAATCATTTTTGTTAGCTTAAAA
AATACGTGTGACACTTAACAGGGAACCCTTTTTTCAGTCAAAGAAAATTGATTGATTTTCTTTGTGATAG
CAGAAAAAAATCCATAATTGTACTTAATTGAAAAGAAAAAGAACTAATTTTACTTAAACGATACTTAAACT
AAGTTTGTGTTTTGGTGTAAAACATTTAAGATACGATACGAAAAGAGTTAattatatttatatttatatt
tattttttataCATAACTCTAGCCTAAACAAGATTCAATTGTAAGCCTCTAACACGCTGAATTTTTAATC
TAGAAATCAAACTACTTCCGTTAAAAGAAACCAATCAAATCTTTGTGAAAACGAAACAAAAACCACACCT
CAAGATTGTAAGAAAACAGACTTAAGTGCACACGAGCCAGATTAAGTCCTTCAGAACAGCTTTTGTAATT
GTTTATGAGCATCACCATGCGTCAAAAAGATCTGCGCCCAGCGCCCGCTCTAATTGAGTACAAGGGCATG

AAGTTCCTAATCACCGATCGACCATCAGACATAACCATTAATCACTACATTATGGGAGCTAAAGAAGAAC
AATGTCAACACTGTGGTGCGCGTGTGCGAACCTAGCTATAATACCGACGAGCTCGAGACACAAGGCATTA
CCGTCAAGGACCTGGCCTTTGAGGACGGCACCTTCCCGCCTCAGCAAGTCGTTGACGAGTGGTTTGAGGT
CTTAAAGGATAAG
>dme_circ_0000514_junction_seq
CGAGTGGTTTGAGGTCTTAAAGGATAAGTTGGTTAGACCTGTTCCCACATCTATGG
>dme_circ_0000515
GCTCATCCTCGAGGAGTCGCCGCAGACTGCGCTCCTTCTCCAACTGCTTCTTCGACTCATGCAGCTCCTT
GGCCGCCAGAAGAGTAGCAGCATTGCTCAGTTTGGTGGCCGCCGCTCCGCCGGCTGTCAGCAGAGTCATC
GGCTCTGGAGACATCGATCCCAGACCTTCGTCGGAACTGTCACTAATCGCCTGGAT
>dme_circ_0000515_junction_seq
CGTCGGAACTGTCACTAATCGCCTGGATGCTCATCCTCGAGGAGTCGCCGCAGACT
>dme_circ_0000516
GCGGCCATCCTACAACAGACCTTCCAATACATTGTGGAACTGGAGAACCAGAAGACGCAGCTGCTGACGC
AGAACAGTGAACTGAAGCGACAGGTGGGCGAGCACGAGGCCGGCGGTTCTGGCGATGGTGGTGGTACCAA
CTCCGGCGGAAACTACAATGATTCCAATGCCAGCGGGGGCAATTCCACGGCGG
>dme_circ_0000516_junction_seq
AATGCCAGCGGGGGCAATTCCACGGCGGGCGGCCATCCTACAACAGACCTTCCAAT
>dme_circ_0000517
ATTGCAACTAGAGGATTCAGGGGGTGAGAACTATCACATCGAGTTCGCCAGCGAGTCAGCTCATCAGGGT
TCTCAGCGTCGACAGTCGAGCATGAGCGGTACGAATACCTCCAAGGGTGGAGGAGGAGGGGGCAGCGGGG
CCTTGAGCGATGGAGACGGTTCGGGAAAGCCCCTTGTGGACAGCGAGAAGCGAATGCGTCGGGAAATCGC
CAACAGCAACGAGCGCAGACGCATGCAGAGCATCAATGCCGGATTCCAGAGTCTGCGATCCCTGCTCCCG
AGACACGAAGGCGAGAAGTTGAGCAAGG
>dme_circ_0000517_junction_seq
AGACACGAAGGCGAGAAGTTGAGCAAGGATTGCAACTAGAGGATTCAGGGGGTGAG
>dme_circ_0000518
ACGGCACTCGGACTGGAGGGAGAAGTATTTGTCGTATATGATCATGGCATCGCTTTGCGCAGCCTTGCGA
TCCTCTTCCTTCTCCTCGCCACAAGCAAAGCTCTTGCGGAAGTTAATGGCGGTGATCCAGAACTCCAGGC
ATTCACGCTCTTGTCGCTGCTCCAGGTACTCGGTGAAG
>dme_circ_0000518_junction_seq
TTGTCGCTGCTCCAGGTACTCGGTGAAGACGGCACTCGGACTGGAGGGAGAAGTAT
>dme_circ_0000519
GGAAACGGAACCCGAACCAGAAATATTCGTCATGGCCGCACGCGATCGAACGGGCGAGTTCGCCAATGCG
ATTCGTTCCCTGCAGGCTCGAAATATCACCCGGGCAGTGAATATACGCGATCCCCGCAAAGCTAAGCAG
>dme_circ_0000519_junction_seq
TATACGCGATCCCCGCAAAGCTAAGCAGGGAAACGGAACCCGAACCAGAAATATTC
>dme_circ_0000520
GAGAACGCTGTGTGCATTTGGCCGCCGAGGCGGGTCACATTGATATCTTACGCATTTTGGTATCCCATGG
AGCAGACATTAACGCCAGGGTAAGTCTCAGAAATGCATAAAATTTTCAAGTTACTAATGATGCCAATTTT
TGATTGCCAGGAGGGAAAATCGGGCCGCACACCGCTGCACATTGCGATCGAGGGCTGCAACGAGGATCTG
GCCAACTTCCTGCTCGATGAGTGCGAGAAGCTGAACCTGGAAACGGCCACCTACGCCGGCTTGACGGCCT
ACCAGTTTGCGTGCATCATGAACAAGTCGCGCATGCAGAACATCCTGGAGAAGCGCGGAGCGGAGACCGT
AACGCCTCCGGACAGTGATTACGACAGTAGCGATATCGAAGATCTAGACGATACGAAGG

>dme_circ_0000520_junction_seq
GATATCGAAGATCTAGACGATACGAAGGGAGAACGCTGTGTGCATTTGGCCGCCGA
>dme_circ_0000521
ACCTTTGCACCTGGCCTGTATTTCGGGATCAGTGGACGTGGTGGCCGCCCTGATTCGCATGGCTCCTCAT
CCGTGCCTGCTCAACATCCAGAACGATGTGGCCCAGACACCCCTTCATCTGGCCGCTCTTACTGCCCAGC
CGAACATCATGAGGATACTTTTACTCGCCGGAGCTGAGGGTAATATTCAACAAAGATCGAATAAGAAGAT
TTAGATATTTGCATATTTGTAAAATCTAGCTTAATCCCCTATTGACGAATGTAATGTCATATCATCTTGA
TTTTCTAACAAAAGCTAATTTAAGTATTTATATTTGTAACAAAAGATTCCCATTACTCATTTGAATAGCC
CCACGGTGCGCGATCGTCATGGAAACACAGCTCTGCATCTTTCCTGCATCGCCGGAGAGAAACAGTGCGT
CCGTGCCTTGACAGAGAAGTTCGGCGCCACGGAAATCCATGAGGCCCACCGACAATATGGACACCGATCC
AACGACAAAGCGGTCAGTTCCCTGAGCTACGCCTGCCTGCCCGCCGATCTGGAGATTCGCAACTACGATG
G
>dme_circ_0000521_junction_seq
CCGATCTGGAGATTCGCAACTACGATGGACCTTTGCACCTGGCCTGTATTTCGGGA
>dme_circ_0000522
CAGCAGCACCCAAATCACCGGTCGCAGCAAGGTTCAATCCTCCACAGCCAGCACGGCGAACGCGAATCCT
TCGGGATCGGGAGCAACATCATCCGCTCCGCCCAGCAGCATTAATATCATGAACGCCTGGGAGCAGTTCT
ATCAACAAAACGACGACGGCGACACG
>dme_circ_0000522_junction_seq
CTATCAACAAAACGACGACGGCGACACGCAGCAGCACCCAAATCACCGGTCGCAGC
>dme_circ_0000523
CTAATTTGCGCCAGAAGGTATGCAACCGCAGTCGGACAAACAAAGCGCCAGCAGACTCGCAACAACAACT
AGCCACTCAACAGCAGCAGCTTCCGCAACAGCAGCAACTCCTCCTAAAGTAAAGGTCATAAAGAGCAAAC
GCCCCCTGTGCCACTTTAAGTTCTACCTGGACATTTGCGATCATCAGTTAGCAAAGCGCATTGAGTCCGA
CATCAAAGCATTGGGTGGACATCTCGAGTTCTTTCTCAGTGACGATATAACACATTTTGTCACTGATAAG
CCAGAGGTCATAGGCGGAACCTCCGGAACACCAGGTACACCTAGCACACCCGGCACCCCCACGAGTCACT
ACCAGCAAAATGATGGTTCGGCAAGAAAACCGAATCAGAGGCAATCAAGAGCGGATGCAATACTGAGTCG
CGTGCGGCGGAGCACGGTTGGAGTCGTAAACAGTGGCAATAGCACACCCACGACATCCCTAAAACGGAGC
TATACCATCTGGCAGACGGATTATGCCCAACGCTTTATAAAGCGCATTCAGACCGAGCTTAAGCAATACC
TTGAGGGTAAGAAGGAAGGTGGAGGAGGATCAACATCAGCTAGCCCCCATCATATACAGCTAAAAAAGCA
GTATGTGAAGATCGAATCCGTCAAGCGTAATTATAGACCTTACTACCATCTTATCAAACAGCCAGACGAC
TGGCCAAAGATAGATTTAAGCAGCGAAGACGGCGCCTTTCGACTACTAACAAAGTCGAAGACCAAGGATA
AGGAGCATAGCATGACCCGCAAGCCTTTGGGTTCACGCACATCACAGAAAGACAAACAAGCTGCTGGGGA
AGCAAAGCCACTCCAGCATCCTTCTCTGCAGGAACTTAAAAAACAGTCTGCGATTCCGAATAGTCCGCGT
TCCAATTGTCGCGAACCAATAGATTCGAGTGAAAAACAAGGTGGAGTGTGTGAGATCTGTAAATTGGAAT
ACGATATCCTGAATATCCATTTGCAGAGCAAAGATCACGAGCTGTTTGCAAAGAATTCGGATAATTTTCT
GGCTCTAGACACTCTAATTCAAAGTTCGGCGGATGTGAATAGGTTCCTGGAGGAGGAGCCCGTAGAGAGT
GAACTTGACATGGATGTCGATGAATCGTTGAGTAATGAGGAGTTGCAGAGTCCCCGTCAGAGGCCATCGC
CAGCGTTGCGGGAGAAGTCCAAAAGGATAACCAAGGGAAAGCATTCGTCAGAAAAGTTTCAAGGAGTTGC
AGTGGCATCACCTCAAACGCCATTCCCTGGTGCCAAGAAGGTTCAAGGAAATTCACCTGGTAGTCTTTCT
GAACTTCAGCGTCAGGAACATCCAACAACAGCCGCAGCAACGCCAACAACGAATTCAGGACGAAGGAAAA
CACAGAACTCCGGTCTGTCACCCCCTAAGAGAGCCATGTTGCCGCCTTCTTCTATTTATAAAGTAGTAGA
AACTAGGGAGGAATGTGCTACGCCTCCCAGAGGGCGAGGAAGACCTCCCAACCAAGTGGATTCGCCCTCG
CTGATTGTAAAGTTCCAAAAGATCCGACAAACAGAGTTACAACGACTCAATGGAGAGGCAGAGAACTTTA

TGTTTCCCAGAACAGCGGTGCCAACTACAAGGAGCAGCAGTGAGCTGCCCACGGATGTCGATCGACAAAC
CACCTCGGATGTAAGAGGCAGATACAGTATCTCCTCCGCCAGCCTAGACACTAGCACCAGCGAAGCAGAA
ACTAAGGAATCTTCAGGATTACCTACTAGCATACGCAAGCGAGCTCAAGCAGTGGGAAGGCGAAGAAAAG
TAGGAGGAGCAGCGGCGCAGGATGTTTTCCAACGCCAATTATCAACGGGGAGTAGTAGCAGCAATAGTAA
CCAGCAGCGCTTTCCAAGCGCCCCCATCCAGCCAGAAGAAGGGCCTCAACCGCAGCCAAAACCGCAGCTA
AAGATCAAGATAAAGCAGGAGCAATTGGTGGCCACCAGGAAAAGCAGTCGCACAGCTACGGCTATTGTGA
CAGCAGCCACCGCCAGCAGTCATCAGCAGCAGCAGCTAAGGCAGACAACTTGCCGGAAAATGGCCAATAA
ACTGGAGGATAGGATGGGGGAATTAGTAAAGCCCAAAATAAAAATAAAGAAGGAAGTGATCGAAGAGCAG
AAAGTGAAGGAGTTGGAAGATCTGGAAGAGATATTAGATAAAGAATTAGATGAGGAGGTGGACAGTAGTT
GCAGTAGTGGTAGCGATGAAGACTACATTGCTGGTAGCCAACGAAGAATCACGGCAGCTCCTCGTAAATC
AACAGATACTCGTGAACAAAGAGCAGCTAGGCGGCTTTCCAGATTAACCATAAATCGTAGTGCCGGAGAG
CTGGAGTTAACGGAGGTCAAAACGTCTCCTTCGAAAAGCCGCACTAAGATCCAGAAACCGTCAAGTCCCA
CGAAAAATAAAGTTAAACAAACGAAGGCAGTGCCACCAGCGATAGATTTATTCTTTGATTGCAGTAAAAG
CGAAAGACTTCGGGAAATGCAGTATACCTTCGAATCGTTGCCAAGTGGGGAGCTTTGGAATAGAGTTTTT
CTGCGACAGGATGCGGGTGAAGAGAACTATTACACGTATTATGGTAGCACTAATTATAGGAAACTGCCCT
ATGAAATGGGTCCCATACCCATGGCCAAAACATTGCCAGCACACAGTTGTGCATTATGCCGCGAGGCAAG
CGAAGTAAAGCAGGATAAGGGAGAGCAGATCAAGCTGGAAGATCAGAAGCCCGCGCCCAAGAAAGAAGTT
AAGAAGGAGGAGGAAGTCCAGTCCTCTTCCTCATCCGCGACCTACAAGAACAAAAAGCTCCATCTGCTCC
AACGTTATCAGCAGGAACAGGAGCAACTGCAGCAGTTGGAGGGAAATTCCTTGGCCACTGCCGGAGCCAA
ATGTGATAGCAAGGCGAGCACACCAGAATTACTGGAAAGGGAGTTTGCCTCGGGTTCTATGGGGGATAGA
GTGCAGCTGATAGAGAGAGTGAGGAGCACATCCAGTTCAAGCTGCAGTAACAGTCAACGGAGCGGCATAA
CCTGCAGGAATAAGCAGCTGGCCCGGATTGCCGAATTACCACCAAGAAAATCCCCTAGAGAACATGCTTC
TACTCTGGCTTTGGTCAGTTGCATTATACGGCAGCGTCAGGACTCACAAAGTAAAACGAACTCAGAAGCA
GAAGAGCCACCTCCTCCTGTAGCCGCACCAAAGCTTAAAACTCCGATAAAACAGGAACCAGTAGCACCTT
CATCGCCCCGAACGACTCGTTCTCAAGCTGCGACTCCTGTTGAGGAACTACGATTTGCCACCGAGATTAG
CGAGACAGTAAAGAGAATGAGGCGGGGGCAAAACAAATATGATCATTCCCCGCCAGCGCCAGTTCCTACC
CCCGCAACTTCTTCACCCGTTAGAAGTCGCCGCCTGACACCAGCGGCTCGTAATCAGAGTCAGATTTATT
CGCGCCGATTGGAATTCGCAACCAGTCAAAGGGAATCCTCAGCAAGTGCGCTTCTGGGTAAGCGTAAAAG
AAGAGTTAATCCTTCCGTTGCAGGCACAGTGCGACCGACTACCCAAAATCTTCCCGGAACGGGTGCGTAT
CGTGGAGTCCGCAAATTGCCCAGCAAGAAGGGATTGCTCGAATACGAAATGGAAACGTGTGCCCTTAAAG
CTTTGGATCAAGCCCGTCAGTATTGCAATCCTGGCTTCGTTGCCTGGCAGTTAGATAAATATCTGGAGCT
AGCTGGCAAGGAGTACGATATAGAATTTGACCAGATATCACCGGAGGTGGAGTCTGAAGGACGAGAAGAA
AGACTTGTTAACACACCACAAACTCCACCACCTACTGATTGCTTTACAAGCGAGTTTGACTTGTGCGATC
TAATTATGGGTAGTGCAGGGAGTGGAGATGATGATGAAGATGTATCCAGAGGAAACCCACCTGGTTCCGG
TCGACGAATGAGTAATCTCAATCTATACGCCAGCTATTATAGGAAAAGGAAGAGCCTGAAGTCCAATCGC
ACGGGTTGGCCTAAAGCTCAGAGAAGACGGAACGCAGGCGGATTAGGTGGCAGTCGAGCTTTGCCAGATG
AGCGAATTAATTTCCAGAAAATGGGTCTTGCTGAGCTACACCCTATAAAGCAGGAGCCAATGGAAACGGA
AGAGGAACAGACCACAACCACTACCACAACTACTACGACTTCTGCGACGAGAGGAAATCTCCTGAGCAAA
GACGATGAAGATGATGAAGGAGGCGGAAACTCGCCCTCTGGTGGATCACCTGCTGATGATAAACAGAATT
CCCGCGAAGATGCCGTGATGACTCCGCCAGCGACCGATGTTGACGAGCAGGCTGAACCGCAGGCGGATGA
AATGGAAAGTCTGCCCGACGAAGATGAAACAATGGCGGATTCTGTGGATCAGCAGCAGGATGTTGAGGCG
GAAATCGAAGCAACTGATGCGGATGTCGAGGAAGAAGAAGAGGAGGAGGACGAGGACGAAGATGTTTTTG
AAGATGCCTACGAAGAGCAGGACATGGGCATTCAGAAAACTGAGCCTCATGAAAAAAGAGCTCGAATACC
TTCCATATCGGTAACCACGCCGCCTGAAGACTCTTCACAGGGCAAAAAACTCTTGCTTACTCTGCATAAT

GGCCAGCGACTACAGGCCACCTCCACACCAAGTACCGGGCAAGTTCAACAGCATCAGAGAAGAACTCCCC
AGCTGAACGGAAGTCTAGGCAGCTGTATTTCGCCAAGTGAGAAACTGGGTGACAATTCCGACATATTCAC
GGTATCATCTGACGGCTTGGATACCGATTTGGATTTAAGCAACACCCAGGCAGGCGATTCGCATGAGCAT
TGCCCACACCAGACGACGCCGAAGCGGAAGTTTGACATCTCCAAGTATGCGCCACCGAATAGTGGAAAGG
CGGCGAGCAGTTGTGCCGCCGAAGCGGCGACGGCTGCCGTGAAGTCATTGGCCATCTCGCAGTTCCTGAA
GAAGGAGG
>dme_circ_0000523_junction_seq
GCCATCTCGCAGTTCCTGAAGAAGGAGGCTAATTTGCGCCAGAAGGTATGCAACCG
>dme_circ_0000524
AGCCTGAAGTCCAATCGCACGGGTTGGCCTAAAGCTCAGAGAAGACGGAACGCAGGCGGATTAGGTGGCA
GTCGAGCTTTGCCAGATGAGCGAATTAATTTCCAGAAAATGGGTCTTGCTGAGCTACACCCTATAAAGCA
GGAGCCAATGGAAACGGAAGAGGAACAGACCACAACCACTACCACAACTACTACGACTTCTGCGACGAGA
GGAAATCTCCTGAGCAAAGACGATGAAGATGATGAAGGAGGCGGAAACTCGCCCTCTGGTGGATCACCTG
CTGATGATAAACAGAATTCCCGCGAAGATGCCGTGATGACTCCGCCAGCGACCGATGTTGACGAGCAGGC
TGAACCGCAGGCGGATGAAATGGAAAG
>dme_circ_0000524_junction_seq
CTGAACCGCAGGCGGATGAAATGGAAAGAGCCTGAAGTCCAATCGCACGGGTTGGC
>dme_circ_0000525
TTAAGAAGGAGGAGGAAGTCCAGTCCTCTTCCTCATCCGCGACCTACAAGAACAAAAAGCTCCATCTGCT
CCAACGTTATCAGCAGGAACAGGAGCAACTGCAGCAGTTGGAGGGAAATTCCTTGGCCACTGCCGGAGCC
AAATGTGATAGCAAGGCGAGCACACCAGAATTACTGGAAAGGGAGTTTGCCTCGGG
>dme_circ_0000525_junction_seq
AATTACTGGAAAGGGAGTTTGCCTCGGGTTAAGAAGGAGGAGGAAGTCCAGTCCTC
>dme_circ_0000526
GCTCCCCACTTGGCAACGATTCGAAGGTATACTGCATTTCCCGAAGTCTTTCGCTTTTACTGCAATCAAA
GAATAAATCTATCGCTGGTGGCACTGCCTTCGTTTGTTTAACTTTATTTTTCGTGGGACTTGACGGTTTC
TGGATCTTAGTGCGGCTTTTCGAAGGAGACGTTTTGACCTCCGTTAACTCCAGCTCTCCGGCACTACGAT
TTATGGTTAATCTGGAAAGCCGCCTAGCTGCTCTTTGTTCACGAGTATCTGTTGATTTACGAGGAGCTGC
CGTGATTCTTCGTTGGCTACCAGCAATGTA
>dme_circ_0000526_junction_seq
TGATTCTTCGTTGGCTACCAGCAATGTAGCTCCCCACTTGGCAACGATTCGAAGGT
>dme_circ_0000527
ATATTAGATAAAGAATTAGATGAGGAGGTGGACAGTAGTTGCAGTAGTGGTAGCGATGAAGACTACATTG
CTGGTAGCCAACGAAGAATCACGGCAGCTCCTCGTAAATCAACAGATACTCGTGAACAAAGAGCAGCTAG
GCGGCTTTCCAGATTAACCATAAATCGTAGTGCCGGAGAGCTGGAGTTAACGGAGGTCAAAACGTCTCCT
TCGAAAAGCCGCACTAAGATCCAGAAACCGTCAAGTCCCACGAAAAATAAAGTTAAACAAACGAAGGCAG
TGCCACCAGCGATAGATTTATTCTTTGATTGCAG
>dme_circ_0000527_junction_seq
CAGCGATAGATTTATTCTTTGATTGCAGATATTAGATAAAGAATTAGATGAGGAGG
>dme_circ_0000528
ATAAAGAATTAGATGAGGAGGTGGACAGTAGTTGCAGTAGTGGTAGCGATGAAGACTACATTGCTGGTAG
CCAACGAAGAATCACGGCAGCTCCTCGTAAATCAACAGATACTCGTGAACAAAGAGCAGCTAGGCGGCTT
TCCAGATTAACCATAAATCGTAGTGCCGGAGAGCTGGAGTTAACGGAGGTCAAAACGTCTCCTTCGAAAA
GCCGCACTAAGATCCAGAAACCGTCAAGTCCCACGAAAAATAAAG

>dme_circ_0000528_junction_seq
AAACCGTCAAGTCCCACGAAAAATAAAGATAAAGAATTAGATGAGGAGGTGGACAG
>dme_circ_0000529
GCGAAGAAAAGTAGGAGGAGCAGCGGCGCAGGATGTTTTCCAACGCCAATTATCAACGGGGAGTAGTAGC
AGCAATAGTAACCAGCAGCGCTTTCCAAGCGCCCCCATCCAGCCAGAAGAAGGGCCTCAACCGCAGCCAA
AACCGCAGCTAAAGATCAAGATAAAGCAGGAGCAATTGGTGGCCACCAGGAAAAGCAGTCGCACAGCTAC
GGCTATTGTGACAGCAGCCACCGCCAGCAGTCATCAGCAGCAGCAGCTAAGGCAGACAACTTGCCGGAAA
ATGGCCAATAAACTGGAGGATAGGATGGGGGAATTAGTAAAGCCCAAAATAAAAATAAAGAAGGAAGTGA
TCGAAGAGCAGAAAG
>dme_circ_0000529_junction_seq
AAGAAGGAAGTGATCGAAGAGCAGAAAGGCGAAGAAAAGTAGGAGGAGCAGCGGCG
>dme_circ_0000530
GTTTATTGGCCATTTTCCGGCAAGTTGTCTGCCTTAGCTGCTGCTGCTGATGACTGCTGGCGGTGGCTGC
TGTCACAATAGCCGTAGCTGTGCGACTGCTTTTCCTGGTGGCCACCAATTGCTCCTGCTTTATCTTGATC
TTTAGCTGCGGTTTTGGCTGCGGTTGAGGCCCTTCTTCTGGCTGGATGGGGGCGCTTGGAAAGCGCTGCT
G
>dme_circ_0000530_junction_seq
GGATGGGGGCGCTTGGAAAGCGCTGCTGGTTTATTGGCCATTTTCCGGCAAGTTGT
>dme_circ_0000531
TAGAAACTAGGGAGGAATGTGCTACGCCTCCCAGAGGGCGAGGAAGACCTCCCAACCAAGTGGATTCGCC
CTCGCTGATTGTAAAGTTCCAAAAGATCCGACAAACAGAGTTACAACGACTCAATGGAGAGGCAGAGAAC
TTTATGTTTCCCAGAACAGCGG
>dme_circ_0000531_junction_seq
GAGAACTTTATGTTTCCCAGAACAGCGGTAGAAACTAGGGAGGAATGTGCTACGCC
>dme_circ_0000532
TTGCAGAGTCCCCGTCAGAGGCCATCGCCAGCGTTGCGGGAGAAGTCCAAAAGGATAACCAAGGGAAAGC
ATTCGTCAGAAAAGTTTCAAGGAGTTGCAGTGGCATCACCTCAAACGCCATTCCCTGGTGCCAAGAAGG
>dme_circ_0000532_junction_seq
CAAACGCCATTCCCTGGTGCCAAGAAGGTTGCAGAGTCCCCGTCAGAGGCCATCGC
>dme_circ_0000533
GAAAATTATCCGAATTCTTTGCAAACAGCTCGTGATCTTTGCTCTGCAAATGGATATTCAGGATATCGTA
TTCCAATTTACAGATCTCACACACTCCACCTTGTTTTTCACTCGAATCTATTGGTTCGCGACAATTGGAA
CGCGGACTATTCGGAATCGCAGACTGTTTTTTAAGTTCCTGCAGAGAAGGATGCTGGAGTGGCTTTGCTT
CCCCAGCAGCTTGTTTGTCTTTCTGTGATGTGCGTGAACCCAAAGGCTTGCGGGTCATGCTATGCTCCTT
ATCCTTGGTCTTCGACTTTGTTAGTAGTCGAAAGGCGCCGTCTTCGCTGCTTAAATCTATCTTTGGCCAG
TCGTCTGGCTGTTTGATAAGATGGTA
>dme_circ_0000533_junction_seq
AGTCGTCTGGCTGTTTGATAAGATGGTAGAAAATTATCCGAATTCTTTGCAAACAG
>dme_circ_0000534
GATGATAATGGAGATTTTGGCGTATGGCTGAGCTTCGATGATCCGGATTTTGCTGGAATAAAGGCAGTAT
ATGCCAAGGGCAAAGGACTCGGCGGAATTGCCCTTTTCGATTTAAGTTACGATGACTTCCGTGGACTTTG
CACTGGCCAAAAGTACCCCATACTGCGGTCAATTAAGTACTTTATGGGATAAATGATTTGTTTGTTAAGC
CTAACAAAGCCATATATTTTAAGTTTTCTAATAAAT
>dme_circ_0000534_junction_seq

GCCATATATTTTAAGTTTTCTAATAAATGATGATAATGGAGATTTTGGCGTATGGC
>dme_circ_0000535
TCATAGGCCGATCGGATGAAACCAATTTGACGAACCTTCTCGCCCTCCAGCAGATCGATATACTTGTTGG
GATGATCAGGGTCGATGTCATGATCGCCGCCCACACTCAACAAAACTTTCAGGTGGGGATACTTCCTCTT
GAGGGAGGTAACCTCGGAGAACTGATGCTTG
>dme_circ_0000535_junction_seq
GGAGGTAACCTCGGAGAACTGATGCTTGTCATAGGCCGATCGGATGAAACCAATTT
>dme_circ_0000536
GGTTCACGGTGATCTTGGACTGGCATGGAAGAGTATTAAGAAGCTGTTCACCGGCGATTTCATAGTGGAT
CCACACGCAGCCCTTCATAAGGAGCAGTTTACCGCTTTAGTAAGGGATGTAAAGGACTCCTTGAGAGCTG
ATGGATTTCTGCTCAGTCTGACTGTGCTGCCCAAT
>dme_circ_0000536_junction_seq
TCTGCTCAGTCTGACTGTGCTGCCCAATGGTTCACGGTGATCTTGGACTGGCATGG
>dme_circ_0000537
CATAGCTTAGTAATCCAGGCTTTTGGGACTGGAAGCCCTCGGGTGCTGGTCCACTTGTCTCCGGCACAAC
AGGAACTCCTTCGAGACCCGAGTCCTTGGTAAGTTTCCATGCATTACCATAGGTGGCAACTCCCAAATTG
ATCTTATTGGATGGGAATCCCTGAGATAGCCAGTACTCCACCTGGAAATCAGCGTTAAGGTGTGCCAACC
TATCCTTCGATCCATCGGGATGGTAGATGGGAGCACTGTAGTCCGCCTCCTCGGGATTACGGGCTGGTGT
CAGGAAATCGAAGGTAGCCAGGTTCACAAAGTCCACCAGGCCATTCAGAGCGGGAATATCAAAG
>dme_circ_0000537_junction_seq
CAGGCCATTCAGAGCGGGAATATCAAAGCATAGCTTAGTAATCCAGGCTTTTGGGA
>dme_circ_0000538
GCTCCTGGAATCGGGACAACAGGGCCACAGACGCTTCATCGAATCTGCTCGAGATTTGGTGAGACGTTAT
AATTTCGATGGTCTGGACCTGGCTCTCCAGCTTCCCAGAAATAAGCCACGCAAAGTTCATGGGGATGTGG
GCTCTGCGTGGAAGAGTTTCAAGAAGTTCTTCACCGGCGACTTTATT
>dme_circ_0000538_junction_seq
CAAGAAGTTCTTCACCGGCGACTTTATTGCTCCTGGAATCGGGACAACAGGGCCAC
>dme_circ_0000539
GATAAACATTGGCATTGCCACCTATGGCAGGTCGTGGAAGATGAGCAAGGATTCGGGAGACTCTGGTATG
CCAGTGGTTCCCTCCACCCAGGGACCTGCTCCAGCTGGACCTCAGAGCAAACAGGAGGGTCTGCTCAACT
GGGCTGAGATCTGCTCACTGATGCCCAACCCCAGTAACTCCAATGCCCGAGGTCCTAATGCTCCGGTGAA
AAGGGTTGTGGATCCCACCAAACGTTATGGCAGCTACGCCTTCCGTGCCGCCGATGAAAATGGTGACCAT
GGACTGTGGATCAGCTACGATGATCCGGACTCTGCCTCCAGCAAGGCTATGTACGCCAGGGCAAGGAATC
TCGGTGGAGTGGCTCTGTTTGATCTGACCCAAGACGATTTCCGTGGTCA
>dme_circ_0000539_junction_seq
ATCTGACCCAAGACGATTTCCGTGGTCAGATAAACATTGGCATTGCCACCTATGGC
>dme_circ_0000540
GGATTCGGGAGACTCTGGTATGCCAGTGGTTCCCTCCACCCAGGGACCTGCTCCAGCTGGACCTCAGAGC
AAACAGGAGGGTCTGCTCAACTGGGCTGAGATCTGCTCACTGATGCCCAACCCCAGTAACTCCAATGCCC
GAGGTCCTAATGCTCCGGTGAAAAGGGTTGTGGATCCCACCAAAC
>dme_circ_0000540_junction_seq
GTGAAAAGGGTTGTGGATCCCACCAAACGGATTCGGGAGACTCTGGTATGCCAGTG
>dme_circ_0000541
AGCCACTCCACCGAGATTCCTTGCCCTGGCGTACATAGCCTTGCTGGAGGCAGAGTCCGGATCATCGTAG

CTGATCCACAGTCCATGGTCACCATTTTCATCGGCGGCACGGAAGGCGTAGCTGCCATAACGTTTGGTGG
GATCCACAACCCTTTTCACCGGAGCATTAGGACCTCGGGCATTGGAGTTACTGGGGTTGGGCATCAGTGA
GCAGATCTCAGCCCAGTTGAGCAGACCCTCCTGTTTGCTCTGAGGTCCAGCTGGAGCAGG
>dme_circ_0000541_junction_seq
GTTTGCTCTGAGGTCCAGCTGGAGCAGGAGCCACTCCACCGAGATTCCTTGCCCTG
>dme_circ_0000542
GCTGGAGAAGACGCCGGAGATTAAGCGAATGCTGCGCGAGGCCAAGTTTGCGTTGAAGAAGTCGAAGCGA
AAGGACTACTACAAGATCCTGGGCATTGGACGCAATGCGTCCGACGACGAGATCAAGAAGGCGTATCGCA
AAAAGGCGCTGGTACATCATCCGGATCGACACGCAAACAGCAGTGCCGAGGAGCGCAAGGAGGAGGAGCT
CAAGTTCAAGGAG
>dme_circ_0000542_junction_seq
CAAGGAGGAGGAGCTCAAGTTCAAGGAGGCTGGAGAAGACGCCGGAGATTAAGCGA
>dme_circ_0000543
GTGCCTTTCTCAACGGTTAGCTTTTCTCGGGAACATGAGTTTCAGTGATGCCCAAATTGGAGCTTGACTT
TTTAGCAGCCGcttttccttgtctttacgctagttttgcttacttttcctcttctttatt
>dme_circ_0000543_junction_seq
gttttgcttacttttcctcttctttattGTGCCTTTCTCAACGGTTAGCTTTTCTC
>dme_circ_0000544
CACCACCCACTGTACCAGGTGGCGGAGCACCTGGCTCACAGACCGTCATGCAGCACAGACGCATCAAGAC
CAAAGAGCTGAAATATTTCGGAGCGGTAAGCGAATATTCTAGTTTAAACAAATTCCATTAACTTCCATCG
GTGCATGAGAAAACAAATGACGCACGTCAAGCACAAAAGGTTCGAAAAAATCCCCAATAGGAATTAAACA
AAATGTTGGTTATATATAATTATCAATTTATTTGATTAAAGACAAATCAATGGGATTTTTATACTGTTT
ATAAAAATGGTTCGACTTATAGATGATCAGTTTTCTCAATAGTTACTGTTTTTTACATGTACATATGTAT
TATGTTGAAAAATTTTAATACTCATCCAATTACTCAAGGGTGATCAATTGTAGAAAATCTTTTTTGAGTG
TGCCCCAATAATTTGTTAAAGATTTTTATTTCATTGAGTAGAGTGCCGTTCTTATCGTATTTACACACAC
ATTCGTGTGTATTATTTACGAAAACCAAAGTCCACAATGTGAAAAATTAACCAGAATTAAAACAGATCTT
AACCCAAGGAGGTTAAAATTTAAAAACACACACAAACGGTATTAAGCAAACATTTTCCACGttttttttt
tttATTGTTTTCCAGTGAAAAAATTTTACTTCTACAAAACTGAAAAGTGCTATTTGTAAAATCACATGGA
AACCAAGGGAAGTCGCCTTTTTTTGCGAAATCGTTGGACATTTCATTCGAAATATCCGTATTTCATTATA
TTCCTTAGAACTCTTATCACAATCAACTTCAATAGAAAAAGTTTTCAGCATTAGAGGAGCACACACGTGT
GACGTGTGACCAATTTTTCGGCTTAGCAACCAATATTTATTTTACACATTTCATTTTATATTTTCGGAAG
CTTATCACTTCAATTGAATATTTTAAAAGCAAACATGAATTTCTAGTGTTAATTTCAATCAAAACAACGG
CTTCGAGTTAACTGTGAAATAAGATGGAGAGTCGGCGTAAGAGAGCGACCACGGTCTGCACTCTCTTTGG
CGCTAGCGCTCAGGCAAACATGTGCGCAAGTGACTTGCTATTTCTGCACTGGCCCCACTCTCTGGCGCTC
TCTTATTGGCGGTAGTGTTAGTGTGCTCGGCTTGTTTGTGCTCttttttttGTTGTTGTTGACGATCAAA
ACAGAATTAAAATTTTACGCATAAATTTTTCTTAGAAATGCGCAGTGGTGTGTGTGCGAGGCATTGGCCC
CAGCTCTCTCCCGCGCCTCCTCCCTCTCCATCAGCAAATCTTCCCCTCCCTCTCGCACTGTCTGCCTGCT
TGTTGTTGGTATTTTTGTTGCTTTTATATGCGTTGTCGCGCCAGGCAAGCAAAAAGTGTGCGAGGATGAG
GAAGGGGGTGTCTTGTGGCTGGGGGGCCCTGACATTTCGTCGCCGAACTCTTCTTCGTCTTCTCCTTCGC
CTGTTTTTGCTGTTATTGTTGTTGTTGTTTCTGTTTCTCTGAATGATGAAATGTTTGTTTTGCACAGCGA
AATGTGTTGGCGACTGCGACGCCAGCAGAGGAGCAGCAGCACTTGCTATGGTGATTTTTTCACCAGTTTC
ATTTTCTTCTGTTTGTTATTTTGAGTCCCCTAACTGTAAGGTTGAGATTACCTGTGGGGTTATCAATATG
CATATCCATTTTATAGGCACTACCTGTTAAAAAAGTTTTCCATTGCATTCTACACCAAATGAACATACAT
ATTTAACTTTAATAACACTTAGACTTAATTAAGATAGTAACTGAAAACTAGAAATTAGAAATGTGCTGGA

GCAGCCCATTTTGCTAGCTAACTTGAAAAGAATAATGCCAAACTAGTTTGCTCAAATAATACTTTCTTTA
ATTATTTTCAAGTGGTGTATTTAAATTAATTAACAAAAAACCACCGTAGCTATTGACTTTTAACCAATTA
ATGATTCATACTAACTGGCAACTTCGGGTCTCGAAACTCCGCTACGAACTGGCTCTTTGTTTTGCTGTTG
CTGTAGTGACGTTTTGTTTTGTGGTTACCCAGTGGACACCACCTTATGCCCCAACCAACCCCAAGTTTCC
ACAAACCCCCCTCCCACCCCTTTGCGCCGTGATTCGCTGGAAGCTTTTGTTTTTACTTGGCTCTGTTGTT
TGTCAGACTTATCAAAACAAAATGCCAGGAGTTGTTGCTGGCTGCTGCCGTTGCTGTTCGCCGACTTTGA
CTCTCTTCTGGCTGTTTTCGCTTGTTTTTTGCTGCCGTTTCTGGAAAACAAATGAAATTTTTGCAAGCCA
AAGAATATAAAACTTGGCGCCTGGACTTTTTCTCAGCTTTTTGTTTTTACTTTTCCTCGTACTGCGGATT
GAGTAgggcgggagggggagggggggcggaaggcatggggcgatcgggggcgTGGCTTGTCTAAATGCATG
CAACTTGTTTGTCCAAGTCGTGTCACCTTTGGTTTTCTGGTTTTGCGGTCTCAATACAAATTTCGGCACT
TACGGTCATCAAAATAGAGCTGTTtaaaaagtaaaataaaaaaattccttaaaatattcttaaaaGAACT
ATTTTTTTAAGCTATAATATTCTAATTTGCAAATAATTTGTTCTTAAATGAAATGAAAATAAATTTTATT
ATGTTTTATGTACGTACATATTATTTATGTATGTACACATAAGCTTGTTTTAAACCTGCTAAGAATGTTA
AGTTATTGCATCATTTTTTATTTATAATAGATAAAATATAAAATTGTATTATTTCTTATATGATGCATTA
TTTTCAAATATATAGTATATTTTGTTTGTTTCTTGCTCTTTGAAAGGGCGTAGTTATCCATGAGCTAATT
CGTTTAATGTCTGtttttttgttgtgattttttttGTATCTCACTTTGGACAGGATAAAATCGGTTCACA
CTGGACGTGCTCCCATTTAATCCACTTCCTATGGCAtttttttaaaaaactttttcttcttcttctaccg
ttgctccgtttttttatgttttgGTTTGGCTTTGGTCTCGTGTTGTTTTTCTATTTCTGCGTCATTATTAT
TTGCGTGTGCAGGCAGCACTAACAACGACGCCCACGCCCCCTTTTCAGAACGCCCACCTACGAACAGAGC
ACCCCGCTCCAccaccacccacgcccaccgcccacctcccacTTATTGTTATGTATTTTTGTTCATATTT
TGCAATTGTTTTGCTCTGCGGTATTTTCCTCAAGACAAGTGAACAGTAAAAACAAAAACAAGATGATGAA
GCCGAAGAGGAGCTGGTGTTTTTGTTATTATTGTTGTTTCGGTTGTTGTCCCGCAATGTTGTGGCTAGCA
AACGACTCACGCACCGTCTCACATTTACCACCCACAATTTGCAGCACCCAAATTGAAACACCTGCaaaaa
aataaaaaGTTATTAGCTTGAAAGTAGCCTATTTATTGGAGATATTGGCAAAACGCGTTTTCTTTAGTTT
AGGTACGAAGTAAAGTTGATACTTCATTATTTACAGGTTAAGTTGAGTTTTTTAATTTAAAATTGATTTT
AAGTACCTTAGTAATTTACCTTTGTTTACATCACCTTTATAGTTTAAATATGAACTCTTCCCTTGAGATA
TCACGTAAACTGTGTTCTTCATTAAGATATTAAATTAATTGAAAGTTGTTGTAAATATCTAAACTAAAGT
CCTTGTCATCAATAATTAAATTAAATGTAAGTCTTAAACACAGATCGATCTGTAGTGTGTAAATTGTTGT
CATTAGGCATGTGCATCCCCAATATACAATAGATAAAAAAATATATATAATAAGTATGATTCTTCACTAA
ATCACTGTTAAAATTCGAAATATTGCTTTGATTTTGAGACTTCTTAACGGTTTTATGTTTGAATCGACCT
CGATTTTGAAGTTAATTTATGTTTTGCACGTCCACGACAGCTTGAACTTGACTCGTTTCTCGGCAATGTG
TTTTCGAGTGTAGTTTTCTCTTGTTTGTGTTTTCCTACTGTTGTTGTAGCAGTAGCCGAGTATCAAGCTG
TTGTTGTAATTCGCGTTGTTTTGTTTTCCCTCGCATTGCCTATAAACCACCCACTACCCACTACCCGCCC
CCGCGGTGGTGGAATGAAAAGTGCAAGGCAAATATGCAGCAACTGCGACGCCGGCAGCGAAGTCAGCGTT
TGTTTTTGTTTACTCATTCATTGCAAAGTTGTTTAGAGCCGAGCGCGCAGCGTATGGtttttttttCTAT
GCAtatgtacatatgtatgtatgtatgtacatacatatgtgtgtagtatgtagatggtaatgatgATAGT
GATGATGATAGGGTATAGGGTTTGTTGTAGTGGGCGGCAAATCTGGGGCTTTTTGGTGGGAAATTTGCTT
GGAATTCGCCAAAGTAGTGATTCTAAATTTAAGCAAGAATTTTGAAGTTTTGGACACAGGAGTGCTAAAA
AATTTAATGGCCCTGACAACTCTAGAATTTGATCCTGTGTAATAGTGACAAAAAAGGAGTTTAAGGTTTT
TGAAAGTTTTTGAAAAGATGTTCAATATTATGATATTGAAAGATTTGAAAGACCTGGCAACTCTAGAAAT
CGTACCTGTAAATAGATAAAAAAATAAACAGTTTCAATTCGGGTTGAGGCGCAGTCAGACAGTTCGAACA
ATAGGAAAAAGCAGATGAAGTTCCCGGAAATAAGTGCTTTTAAGTTATTTTCTTTGGCTTTGGCAACTCT
AGAAATCGCACCTGCGTTTTAGTGAGAAAATACAAGACGTTCAAATCCACGTTCGAATACAAATACAAAA
ATATGTGGGAATAGTGGGGAACTTTCCCAGCAACTAGGGAGCAAAGCTTAATGGGGATTAACCCGTTAAC

CGAAACACTTGGCAACTCTGGTATTGGCACCTGCCAGGTGACGAAGCCATCTTacacacacacacacacC
GCAATGCATTTTCAATTGCAATTTGTTCCACTGTTAATTTTACGACTCCCCTTTCCAGTGTAAATCCCCC
ATTTTAGATGGTGGTAGTGTGATGGAAGACGAGGGGTTTGGGGGCTTTGTTTGCGGCGCTTGCGCTTGCG
GTTTTCATGGCAGTTTCATTAATGTTAGTGTAGCAGTTATTTTTGTTGTTGTTGCGGTGTATGCGCTTTT
GCGAAAATATCGCCGTCGTACTTCAAAAACATTGGCGTGAGCAGCCGGATAGATACAGTATAACTTTGGT
AATGGAAACTTCTTCGAAATACTCCTTAATATATTTGGTAAAATAACTTGAATTTTTCAAATAAATATGC
CAGATAGAATTTCCTGAGATATGTATATTATATAAATACAttttttttATTTCCGAACAGAAAAGATATT
TTTTCAATAATCCTCTAATATTTTTTAATCTAAAGAGCAGAGTAGTAACTTCAATATCTTTGTTTTATTA
ATAGTGTTTTTTCCAGATTAAATGAATATTAGCTTTTAAAATCCAATAAAGTCACTGATAATAGCTCCAG
TCCACAGAAATGTTTTAATATTTATGATAAAAACTAAGGCAACGGAATATTGATCTTGTTAGTGTTGAGC
GAAAAGAGTTTGGAATGCTTTACTGTAGCAGTGGAAAGAGGTAGCTTGCGACAATTGTGAATGGGGCGGT
GTGGAGAAGGGGGGCGTTGGTGATGAGTGAGAGAggtgtgcggggtgtgtggtgtgtggtgtgggCAAGA
GGGGGAGGCAGTAGGACTGGGGCGGATTTCGTGCCAAAGGAGTAGAAGCAGCGACGACGACGACGACGCG
TAGTTTGTGTAGTGTGTTATTTATTCATTGGATTGGATGATAATCAGTTTGTTTACATTTTTCAGTTGCA
CACACAATCCGAGCGAAAGAGCGAGCGGGCGCGAGTGTATGCGCGAGCGAGAGCGGGCGAGTTCATTCTC
TTGTCTGTTTTATTTTATTTGCCTTAACAAAATAATAATACAGACATGCAAacacgcacacagcgcactc
actcacacacacacgcacgcacactcTGCTCCGAATGCGTAAACAACAAAAGCAGCTGCAACAACAATAG
CAACAAGCAGCGGAAAAGAGATGGCGATAGCGGTGGAATGATGGGAAAGGGGGGATTGGGCGGCGTAGTT
GAGGAAACTTAAAAACAGCAGCGGGAGCAACAACAAATAAACAACCCAGGTTGAACTCTTATTCTCGGGA
TGCCCCAATAGGCAACATTCGATTTTTGACATTTGTAGGCGTTTGACGgaaaataatggaaaatcgaaaa
tggaaaaagaaaaatatgtacaaaatcaTACATTTTAATTTTCAATAGGCAATGTCGGAAGTCTTAGATC
GACACTTTTGACAGCTATGTGTTTAGATACATGTGGACATACCATATAACGATAGAAAAGTAATTTCGTA
AAAAATAGAGCATATTGTTATTCGGGTTTTCCAAAATGTTGAGGCCCATTTGCTTTGTCGCAGCATTTTG
ATGGAAAGTTCTAGGCGCCACAACCCAGACAGACAGAAAGGCAAAATCAAAGAGATCGTCGCAGACAGAA
AGGGATGGCGCTAGCCAGGGTGGAGCGGGCAGAAAGAGAGGGAGAGCCCGCTTTGATCGCCGCTGACCAC
ATTAAACTTTTTATTGTAGTTGTTGCTGAATTATTTTCGTGTGAGGCCCCAAACGTGGGGTAGGTTAATG
CTAATGCCGCGCCTGACCATTAAAAACCCTCTCCGGTTAACTCTCCATCTTTCTGTCCCACTCTTTGGCT
TCTTCTAGAAGAAGAATTCTTCGTGCGCTCTCTTTCGCGTGTCAGAAGAGTCTTTGTGAAATAACGCTGA
CATTTGCAAAAGTTTCTACAAAGAAGAACAACCAAGATGCAGTCTTTACATATCTTTGAATCTAATAATT
CAATTAGAAATGCGttttttttttAGCTaaaaaaaaGAATATTACATTACCAAATGCATTTTAAAAGTAT
CCAAAAATAAGTAGAGTTTTGAATCAACAAATTTAATTTGTTACGTTTGTTTAGTTATTTCCTTAATTTT
CGATAAATAGACCGTTAATTGCCGTTCTATCGGGCTCTTAATGAGTCACCCGGACTTTGCTTAAAAAAGC
ACGCTTCTCTTAAATCGTTCTCTTCAGGCCGCAGCTTTTTActctctctctctctTGGTCGTTCACTCTC
TTCCGCGCTCGCACGTTTCGGCTCTTCGAAAATTTTTACTACGCCGCTAGAATAATTTCGGACAAAAATA
TTACGAGTTCGAGTCGCGTTGTCGTCGCTTTCGCGCACAGCAAACaaaaaaaagtctagctacaacaagt
gtaaatagcgatcccaagcaaacaaaaaaaaaaaaaaaaaaaaacaaaaGCCGAAATGCATTATCAAAACa
ataataataataataataatGTCATTAATCTGAGATCCCCTGCAATTCCACACAGAATTTCCCCGGAATC
CGTAGCAACTACAAATTCTTCAAACGTAAGTTTCTGCATTATTCGATTTGCCGGGGGGAGCTGAGGGGCT
CTTTCACAAGGCGATTGTTTGGAAAGTTCGCGCACTTTGCCACCTGTGTGCGTGTTTTGATGATACCCTT
CCCGAAGGGTAGACCGATTTATTCAGAACATCGATCAAAAATTATGAACAAGTTCAGGCTTATTTGGTGC
TTATATTAGCATGTAGGTTCATAAAATAAATATTTTATTTAATATAATACAAATACAAAACATCGTTAAC
ATTATTTAATGTCCATGATTCGTTACGGTTAAATATTATTTTTATACCTAGAAGAGCATACAAACATCGC
CACTCTTAACTTTCTTTCATGTTGTTTTGCTTTTTTCAACTGTGTGCGTGCGTGTGTCTGCTAATTTGTT
TTGATCGCTTCGTGTGTTTGTCCATATATGGATATACATTCATACATCTATTGGGTAAAAGCAACAAAAA

CACGATTTGAGCGCAGTCAAATTATTATTTATTTACCATTATTATCATAATTCGGTACGACTTCGCACTC
TTTCGGTTTTAATTTATGTTTCGAATTCACTCAGAACAATGACAAAAACAACTTTTCGCCGAGGATTTGA
CCGGCATTGGAATGCGATGAGaaaaaaaaaaaaCAGAATCCGAAATTTCTAATTCAATTCGTTTTGGGGA
TATCAATTAGTATTATTATATGCAAATTGCTTTCGACTTTTAACGCGACAGTGCAGCAGGTATGCAAAAC
GAAATGTTCTCAACAATTTGTTAAGAAAAAGACGTTAATCTCTTGTTTTCCATCGAATTCTTTAATAAAA
CAAAGTAGAATCGAGTTTGATATAAATTTGGTTCTTATCAAGATATTGTTAAGAATTGTCTTTAATACTT
TATTTGTACAagttaataattacagttatcgatttgattttgaagatcgcaagcgaccgtttattgcaat
ttatcatttgaaactaaatctagcgtacaaaatgtttccctaagtccctagcaatcaagtgaagtcgtcg
gcagcggcgcagcaggcgtcggccgcggcgcagcgcagaagtgtcgatgtcgcgcttaaccgttcgttgg
cgttgatggcagcggagactatgtggaaccacaagatgttagagaatcaattgcagggcaataactcctc
ccctcttaattgacgcttgtcctcgagcggtcatcataaggatgggatgtttttgagtcgcatcgttggt
ggggtgattcgtggtagtttgggcacagcattaatggtttgaatgccaggtgtttattgttgcgtgcgtt
cgaggtttgtgcaactcggatgtgttctgcattgacaacttgattgcttctattaaaattctggtttta
ctaaattactaatgaatttatatctacatatttttagaccattggaacctttgcagaaggcattgattcc
gtacatttcgtctttaattggtaaattttattattattcaaattaaattattatttatttattattatta
gtttcctttactgatcatgttttaaaatataagaaataaggaattaactgattcaatatggagatttcaa
tggcattcaaattgtagttccgttgtgtaaagcgagaacgtgtgaaagttttaacatttatttgaccgtc
aaggcagaaatcggtatattgttttcaagtaggctactggtctcattcaatttaaactcgctccgcagaa
acgcattcacaacgcaatctcttctttcacgagcccaatctaattctgcaaacctatggccaactcgtct
gtcaaagtcgaatcgtttcaaatttaattctcgtggttacgttaatcagtatgtttaatgtttctatgtt
ctacttgtctatcgatgtggccacagtaattattctgaagcaatcatgatcttatgttacttttatgtac
taatttccctgattctgttaatattgtggtacttcggtcttccttaactagttccttcctaaatctactt
gataacttagaacctaatcttgtattaaccctaacgaaaatttcttgccctttgatataggtttttatgg
gctttcttgtccggttgtggtattctatgtcggtttcctgtttttgcctaagtcggtctatattgtccag
tctagcttgttcatatttttctggagctacggtagctatcctgccgaagaacacctctagaggtcgtttt
tttgtgacagaatggacagtgtagttatattcatagatggccctatcgagaagttcctcgaagcccctat
gtgttccatctcctttcaaacatctcattatttcagagagtgtggagtgaaatctttctatctgtccatt
tactgtactcttatacggaggtgctttgtagagctcaatacccagctggtctgtcaacatgaatttgata
gaggctgagttgagggacttttcattgtccattactattaatttaggcactccataataaaacacgatat
ctcttagggctttcctaatgtcttctacagctttggatttgataactcttcccatggccagtttggaaaa
tttatcaatcgccgtgagcaccagatgtcgttctgtcgagtagatgtcaatatgaataatttgtccgggg
tattctggcaagggagtttgtcttatttctggatgcgtgggatgtctgtcatatttagcagtcttacaca
ccaaacactgattcacgatagccgaaactttttcctcattttaggaaagtatactctttcggacaactg
agctttattttccacagcatttctgtgtgctctgttatgtgtcctaagtatttcttcctcttgttcggct
tcgttaacaaggtctttgactttgctttggcagaatctaatcttgaaaccctgaaaatggatggggtaga
gaatttgaattttccccattacatcctcggatgtgaaaattccattaatcacggatgggttaagatattc
tttcaaatctgacaagagactatcgggtgtgaacaagttgcgatctactaaatgcctttgaaatgtcggg
aatggaatgctaaatgagtagttctctgactcggactccctgaagaaaatttgattcttgaatgcgttta
tcggggcttcaacgctaggtatcagcccatggctcgaactgtcagagctgtgcattgttgaggctactgt
gttaatttgttcgactgtcggtcctctggacagagcgtcggctacagtattttctctgcctggcttgtag
aaaatttcgtagtcatattcctcaaggtatgctttccatctcttaatcctcgcatttccattccaactac
tgagagaatgggtcaaaggctgatggtcagtaaagattttcacctttgctttaccgtaaaggtaaatttt
tagctttttttagagcccagataatggctaacatttccttctcattcgtggcataattttcttccgccttc
gagagtgttctcgataaaaatgagatgggtctgttctcttgtgaaagaacagcacctactgcgaaattgg

aagcatccgttgttaggtgaaattctttcttaaagtccgggtagtggagtattacgtctggagaaaccaa
gctgctcttcaatttcttgaaggcttctattgcttcgcgattaagggagacggatttttttgacgataat
gtcctggaaattcgaccatcctcccctctcaaaagcgagctaagtggttttgctaacttagcgtagttag
gaataaatcgcctgtaatatccggataatcccaagaatgatctcagttctttgagtgtccttggaattgg
aaagtctgagattgcctgtaccttggttgggctggtttcaatgcccttgtcggacacgacgaagcctagg
aactccacttttctcttcatgaactcgcatttatccaactgacatttcatgttggcttgctgaagagttc
tgaaaatagtgtcaaggttctggtaatgtgtttcatcatctttactaaagatgatgatgtcgtcaatata
aatgaaacatatcttaccgatatgttcgtgaagaatatcgtccagtgcgcgctggaaaattgacggtgca
tttttcagaccgaatgggagtcgtgtaaactcatattttccattattgatggagaaggcggtcttttcga
tatcagattcctttaaaagaatctgatgaaacccacttttcagatcgagcactgagaaaatcttgttgtc
tcccaattgggccaacacttcattaatgtcagggatagggtatctgtccgctaccgttaccatgttaagt
tttcgatagtcaattaccaccctgtatttttcttgccagaggagtcgagtttttttggtacaatccaca
ctggtgaattgtaaggtgaccttgagggtcgaatgattccatcgtgtaaaagttcggaaatttgtttgtg
tacctcgtctttaagagacattgggtactgatagaattttgagtatattggcgtatccgatatagttcgg
attgctgcccttacacttgttgtgtaggttagtttttgattcgggtctgcgaagagacctggatacgaat
caattattttattcaatatcattctctgttccacctctaagtgttttattctcggactaattgtgctaac
agcctcgaactgtttttctttgagaacaactcttttcccgttttccagtgtcatggttaggttcttcaaa
tcaatttgcgctcccattcccttcattgtgtctttgccaagtatggcgtcaaaagtcttcagcgttggta
acaaaaaaaatttatttcgaaatcgaaaaggcttgctcttttgtaatgcgatatttttacatcaccgcc
tggagtatcagctatgtaaggcttgttatttggtatcgcgttcgtcaccaaattgggctggatgtaattt
ttattagagcctgtgtcaatcaacacccttaaaacctttccactccccactctacattcgaagtatggta
gtgaggagtcttctactctaaaaaatgcaacacgtggtctcgttgttcgctgtccaaattgtctatttgt
ccatcacagtattcatttagtgtgccgtttatattgttctgggtttcataatcttgcatcgaccgttgat
agcctgtcatgcttgaactggacccagttacttcaatacttggctgtctcatgttcactgtttggatgtt
gtagttcttttgtcgtttgtttatgtccgagtgtggtctattcatataattaatgtttcgcgtctgtaca
ctgccgtccacgtccattggttctggtcttggttgtggtttggtcgcaaagggacggggtggggcattgt
attgttgccattgctgttgatagttttgtcggggtggaatgaacggtgcattaccgaagttagacacgtg
ttgccttggcgccgggaatggctggcctaaatattgtggaggtctcctcatgggatgtcgtggagggacc
ggtggaggctgtcgatggccaaaggcattcgacggctgaaaattgcggggtgccggaatgggtttttcgt
ttcctctgaaactcgactgttgacccttattcgccgcatggtttgacctgaaagtttgattttcaagttt
aaggcaataatgtaaagctgaggggagatcagctggctcttttattgccaaaaggcgaggtaaatctcct
cgaaggcctctgataaaagtatccagagctttttctctgtacattttgtaacaaagtgctcggattctc
ggctcatttgcatgcagccgactttatttagaataagcgacagatttttgtagacgtcttggtggaagtc
ttccaccgactgctggtggccttgaaccaaagttgacatttggtattccaaggtagttatgtcccgttta
tctgcgtaatgcagagtgagacatctcgacatggccttccagtccagcggaatgctgtaggactcgagtg
ccacatcagcacttccaacaattttatttcttatggtatgaagtataccataatattttggagtacccac
aaatggggtataagtctccatgattctatcaacgctctttttccaagacccgaattctgctggatttcca
gagaattccctgatcgattttacgatatcaggcaccctgtcaaagtccgctaaattatttctgtattcgg
gctcgacgacctggtcgctcacgtcagtaaagtcaagagtattgttgttcatttgttttatcaatccagg
taaaacttgggccaccgtttggccaattaatgctgtcaactgttcgacactcatgtctacgcgatgctgt
tcaggcaggtcgtgtctttctgagggcaccggtctttcgtttgaactaaaagctgatggtcttattatat
tattaggatttgccatactgatttttaattcggacaacacctgcacaaaacaagcctaaaccaatttgaa
cttaacaaaagttgtgggcagaaaatatggaatgcgtgtgtatgtgtttgcttatttatcttttgttttt
gtttttttatttttttatttttatatttttttttttatttttgcatgcaataaatttagctcaccgcggtt

attttgctttgagaacagtgtaccagaaaggaacagtaaataggaaaattttcctttcagaagatctcta
ggaactgggttgaaaatatagtattaataataatatcaataataataataaaataataataataatataa
taataataatgtaataataataatataataataatatataataataataatataatactaataatataa
taataaaaatataataataataataatattataataataatataataataataatatacttaaattttt
ttttttttttttttttggtatacttatgttttattatgggtggatgcctgaatttcaaattgctgatg
ttttttccagtgtactttccaggttcttgtagtctctctgcctcctcttcggttgtttcagcttcagctt
cttcgtcgtcaacgtgttcttattcctgttctttttggcgggctgctgctgctgctacggctgtgctgtt
gttgttcttttttttccttcttgtctgcactttggttcacgcagccgttctctgccgttcgtcgtcttt
acctctgccgttacgttttgggttgcactgttaactgttatccgtgttgctcttctgtagttccgttatt
ctcggcgttctacttctacttttccttctcctttcgatgaaattgctgttcttcgttttgcatttattt
aacatagaattgttgcggtcactcctcctggctgggtgaatgttgtcctttttgcctgggctactgggga
atccacgtcagcactccaaagaaggttttctgcgggctggcctgacagaaactgtgatccaccaatgggc
aatgctatgccgataagtattttcctcttccggaaaatctgtttttttctcaaacaattttattttatag
ccgaccgtgaattttgtttgttaataactcgcgggagtttcttcggcacaatttaaccaacgaatatttt
ttagcaaaattttgattttgctccgcgacttagaattttgtctttcgttcgcgtttattcgctggtccct
gttcgggcgccagttaataattacagttatcgatttgattttgaagatcgcaagcgaccgtttattgcaa
tttatcatttgaaactaaatctagcgtacaaaatgtttccctaagtccctagcaatcaagtgaagtcgtc
ggcagcggcgcagcaggcgtcggccgcggcgcagcgcagaagtgtcgatgtcgcgcttaaccgttcgttg
gcgttgatggcagcggagactatgtggaaccacaagatgttagagaatcaattgcagggcaataactTAC
ATGTACAAAAGAACTAAAATATTAAAAAGAAACTCGAAGGCCTCTTTAAGTCAGTAATTTTCCTATCAAA
CATCAATGCGCCTATCCATGATGATTGGGCAAAAATATAGTAAGAGACCATAAATATTGCTTAGCCATAA
TGATTTCACTTAACAATCATCGTTACGCTTTAGTTTTAAAAATCGAATTTTTCTGCTATCTCGacaattt
gttagaatcacaattatggcagaatcacaattatgttCCACTAAATTTTCTTAAAATTTACGAAATTGAA
GATAAATATATATATTTTAAAAATATTGTGTATATTTGATTCGATTTTGAAACCGGATTCCCTTTTTATC
GGGAGCAAAGCTCTTAATTAAACAATTTCGTACTTAATTACTTTATGAGCTCCGCATTATAGTTCTCAAG
TAGCCAGTGTCCCTGTGTTGGTTATCTTGATCGTTCAATGTTTTGGACTGCGTCACGATGACGAAGTGAG
GAACCTTTGAACACCCCACCCGACTGCAAATGCACTATCTCTAATCTCCGCGTACTCGTTCCGTTTCTCT
ACTTCCAGAATGGGCCTGAACATACCCATGTTGATAGCGAGCAGCCAGACGCTGGACAGTTGGAATACGG
CGGGTCAGTACTCGCCACCGATATCGGGATCCGGCGGCGGTGGATCCGAGCGAGGAGGAAGTGCTGGAGG
CGGCGGAGTGCCGCCACAGCAACTGCGAGTACCGCTGCCGATGAGCTATCATGGCGGAGCTGGTGGTGGA
AGCAGCAGTAGTGATTTCCAGCAGAGTCCCCTGAAGACCATGGACACCTCGGACAATGAGGATAATAACA
ATACCAAGGAGGATG
>dme_circ_0000544_junction_seq
GAGGATAATAACAATACCAAGGAGGATGCACCACCCACTGTACCAGGTGGCGGAGC
>dme_circ_0000545
TTAATGAAAGATAGTGAACACAGTGCTAAATGAAAGATAATGAGACCCAGTCGACAGGACCCAAGAAGAC
GATGTTGATTGAGTGCATTTCGCGTGACATTCCCTGAGAGTCAGCTTCGCAAATCGCCAGAAAGCAGAAG
TAAAGCTCAAGTGTCGAGCTCGGCTGCACAGATCTGTACatatatatatatatatataaacatatacata
tataAGGCAATCAACCATCCAACGATCGGTTGAGAGGCGTGGCAAATGAACTTCAATCCGGCCAGCGTGG
TAGCCGCCGCAGCAGCAGCCGCACATCAGACGACccatcttcaccaccatcaccatcatcaTCCGTATTT
AAGCCATCACCAGAGCAGCATCACCCAACCACAAGGACAGCAACAACAGCTGATCCCACTGCCTGTCGGC
GGAGGAGGCGGAAGTTTACCAGTGGGTGGCAGTGGCGGTGGCGATACATCGCCCAAGATCATGGAGTGGA
CCAACGACGATGCTCTGGAGCTGATTGAGCAGTATCGCTGCCATACAGAGCTATGGAATCGTGCCGATCC
CAAGTACAAGGATAAACTATGTCGATTCCGGGCATGGTCGGAAATAGCCGAGCGTTTCGGTTGTAGCAAG

GCGGAAGTGGAGCGGAAGATGAACGTACTGCTCACACAGTATCGCCGGGAGAAGCACAAGATGTTCGTGA
AGATATACCAGGGCATACAGCCCAATCCGTCCAAATGGTATGCCTTCAAGCGATTCGATTTCATGGAGGC
GGGCGGCGGCAGCCTGAGCGGCGGTGGGTCCGTGGGATCCGGTGTTAATGCCGCCAGCGGAAACCTGGTG
CCGAATCCCGTTTCCTCATCCGCTGCCGCTGCCGCCCACAACGGGCTAAACGGACTGGCTGCCGCGGCAG
CGGCCTACGAAAGCGGTACGATTGCGGCGGCGGGCAACGGAGGAGCACCACCCACTGTACCAGGTGGCGG
AGCACCTGGCTCACAGACCGTCATGCAGCACAGACGCATCAAGACCAAAGAGCTGAAATATTTCGGAGCG
G
>dme_circ_0000545_junction_seq
ACCAAAGAGCTGAAATATTTCGGAGCGGTTAATGAAAGATAGTGAACACAGTGCTA
>dme_circ_0000546
GCCAAATGTCAATGTGAAAATGTCTCAACGAGCAAATTTTAGGAAAGTCAAGAAAAACATTCAACGACCT
CAAAACCAGCGAATGCTACAAAATGCGAATCAAGAACATTTAACCATCTAAATATTCAAAATCAATTTAA
TTGAATCAAATAACGCGaaattgcaacaaatcaacctaaaaatgaactgaaaacaaaaacatttcgaaaa
acaacgcaaaaTTCTAAAATTGGCAACATAAGTTTTGGTCTTGAATTAAATACAACAAAAATAAATTAAC
AAAAAACGATTTTTGGCAACAAAACATCATACATAAAATATACACAAATTGTAATTATCTCAAAACGTCC
ACACGTGAAAATGATTTGCAACTAAGATGACAATCTAATAAAATCAATGTAAATTTTTAACGCAACAAAA
GGAAAACCAAAACCGTCAGCAAAAACCAGCCCAAAATCAGTTGAAAAGCCGAGGGAAATCTTTTTAGAAC
AAACGCAGAAAACCGCAAAAATTTAAGCCACCAAATTGGTAAACTATTCAAAATTATTGGTTCGGACATA
CGCAATACTAaacacagcaacacaacaacaacaacaacaacagcaacaacagcagccatagcaacTACTA
CAGTGATCACTACAGTAAAAGCAACCGCAACAGAAGCAGCAGATAAAACGTGTGTGTTAGTTTGCATAAA
AATTTCGTTTCgataaaagcaaagcaaaatcatttaacaaaataaataaagaataaagtaaaaaaaaaaa
TTACCAATCGTAATACATGTATTTATAAAATATATACCTATACATATATCTATGCTTACGAATTATATCG
GAGAATATTTGGTAAACAATTTTTGATAAAAACAACTCGAGTATATATCCTAACTAATTAGCAACAAAAA
GAACTAGAACATTTGGCCTAAGTGaaaaaaaaagtgaagtgagaaaaactaaaaagaaaaaCTTATAAAT
TCAAAATTACCACTTGTGAAAGGTGTTACAACTGAGGATTCTGGATTTGGATTAATGCGTAAATTTGCAA
TTGAATATTAAG
>dme_circ_0000546_junction_seq
ATGCGTAAATTTGCAATTGAATATTAAGGCCAAATGTCAATGTGAAAATGTCTCAA
>dme_circ_0000547
GCTACCGAAAAAGTATTTTTTATCAGGATTTCCTTTCTGTGACGCCCACCCATTCAGGAAAATATAGGCT
AAGTGGCGCTTACAGCTTTTCGAGTTTTGCGTTTTGATATCTACCCTTAAAATAATGGTTATAC
>dme_circ_0000547_junction_seq
ATATCTACCCTTAAAATAATGGTTATACGCTACCGAAAAAGTATTTTTTATCAGGA
>dme_circ_0000548
GGCATTGCCAATCCGAAAGGTGTAAATAATTGAGGCCTAAAACTTGGCCCCTGTGTTTATCCAAGTTCAA
CTTGTGATCGCCCATTTCACTTTAAGCAACATGACACTTTTTCCGACAATTTTTAAAGGCAATCTCAACA
ACTCGCAACTGTTGTCAAAACAGAATCTCAGCTTGCACTCTCTGATTACTACGATGAAAATCAGCTATTA
CTAATCAACGATAAATAAGAGAGTTTCGCCTGaaaaaaaaaTGGCAGCTGCTGTCTTGGAAATTAACGA
AAACCAATTGGAGCTGGTTTGCGATGAATGCGATTATTACGGCGCCACAGACACCATATTTATTGATAAT
AGTACCCAAACCGATGGCTATGATGATGACGAGTCTGAGCTAAAGCCTTTGAAACCCAGACTGAGTCAGA
TTGTGGAAGAGGAAGCGGAAATGGAAGGGACAGTCAAAGAGGAAATGGACACGCAAGTGGAAACAAACGG
TATACAAAGCGATAGCAATGGCGTTAGTGATGTGAAAACCGCGATTAATGGCAATGAATCGATCGTGGCG
GAGGAGATCTCTGAGGAGGAGCCTCCAGTTGAAGCGAGTGTACCACATGTTGTCCAGGAACTAGTCGATT
CCGATGATGAGGTGGAAATCGACGACGTGACCAGGCTAGCAGGCTTGACTTTGGGCATGGGCTATGCTCG

CATTATAGACTACGATAACTTGCGCATTATCGAGCATGTTATCGAAAGCGACGATGAGGACGATAACAAT
GTTGCAGCAGCTGGTACAGCGGCAACATCAGCGGAATTGCAGCAGGCCTGCCATGATTTGGTGACTTTCA
TCGGTGAAAGCTATCAGGTAATCGAGAGGTCAGGTCATAGTCGTGAACCCACCGCAACTTTGTCGGTGGT
GGACAACAACAATAATAATCGAAGTAGCGAAGTATCCACAAACGGTCGTGGCGCCATCACCGATAGATCA
AAATCAACCCGGAAAGTGCACAACGGGTTGCAAGTAAATCAAAACGCATCCACTAGtgcaacagcaacca
gcagcagcaacagcaaaagcaacagcagcaacaacactgctgcaacatctaacagcagTGCGTTGATAAA
GGCTTACAAATCGAATCTTACCCCCTTGGCGCCGCCCTTTCAGCCCGCCGCCTTGAAGGCCAAGCAGCTG
CAACAATCGTCGCTGCCCAGCGGCAACATGTCCTCGGTGACGACGACAACAACCACAGCTTTGGGCTATA
CCGCCTATGAGCAGACAACGGTCTACTATcagcagcaagttcaacagcagcagcagcaacacccacaaca
gcagcaacagttgtcacccaagcagcagcagcagcagcagcagcagccgcagcaacagcagccaatctgc
gctcaacatggaggcttgccgcatattcatggcgcccaactgctgcccgtgcagttgatagccctgccac
cgaatggagtaacagcagcagctgcaacagcagcagtagcagcagcagcaggagcaactgtggcagcggc
cggaactccagcagcCGCAGCAGCGGGTGGTGCCGCAGCAACATCTTTGACTGGCGCCGCTTGGCCACTT
GTGGAGGCGCCAATTTACATCGACATCAATGGCGAATATCGCAGCTATTGCCCGGCACATGGACCACCGG
CCGTGGCAGTCCCAAATGGCGCCATCGGCCATCATCCCCACATTCATGCCCATCCTCCGCACACCCATTC
GCATGCCCATCCTACACATTCGCATCACgcacagcagcaacagcaacagcagcagcagcagcagcagcag
cttcatcatcaccatcagcaacatgggcagcatcagcaacatttgacgccatcgccagtgacaaatgtta
atggaaatatagcacaggcagcagcagcagcagcttcagcgcctagattgctgctgcaactggacgcagc
aactgtccagcagcaacagcaacagcagcatcagcagcaacaacagcatcagcagcaacagcagcaacaa
ATCGTGGCCGCTGGCAAGCGTAGCAAAGTTTATCGAG
>dme_circ_0000548_junction_seq
GCTGGCAAGCGTAGCAAAGTTTATCGAGGGCATTGCCAATCCGAAAGGTGTAAATA
>dme_circ_0000549
GCTATCAGGTAATCGAGAGGTCAGGTCATAGTCGTGAACCCACCGCAACTTTGTCGGTGGTGGACAACAA
CAATAATAATCGAAGTAGCGAAGTATCCACAAACGGTCGTGGCGCCATCACCGATAGATCAAAATCAACC
CGGAAAGTGCACAACGGGTTGCAA
>dme_circ_0000549_junction_seq
AACCCGGAAAGTGCACAACGGGTTGCAAGCTATCAGGTAATCGAGAGGTCAGGTCA
>dme_circ_0000550
GGTCCCGCCGTTCAGATGGTCTCCCAGAACCGACCTCCACCACCGATGCCGGTGCCCGTCCAGGTGCCGC
AGTACGTCAACGAGAACGGAACGCTGACCCACGTGATGCTCTCGCCGCAGCAGTACCAGCAACTGCATCC
TGGCCAGGGACACCTCCATCCAGCGCCATTC
>dme_circ_0000550_junction_seq
CCAGGGACACCTCCATCCAGCGCCATTCGGTCCCGCCGTTCAGATGGTCTCCCAGA
>dme_circ_0000551
GATTCATGGCACTCAGTTTAAGGCCCGCTGGGATGCACCCAGCGATTCCGGTGGTGCTGAGATCCTGTGC
TACCACTTGGAGTTAAGTGCCGCTGGGCCAGCTTTCGAAAGGATCTACAGTGGCGCCGAAACAGAAGCCA
TGTGCGAAAGACTTCAGCCGGGTACCACATACGCTCTGCGAGCCTGTTGCGAAGGTCCAGCTGGTCAGAG
TCC
>dme_circ_0000551_junction_seq
GTTGCGAAGGTCCAGCTGGTCAGAGTCCGATTCATGGCACTCAGTTTAAGGCCCGC
>dme_circ_0000552
GTGGCTTAATGCCGGAAACCGGCTACAAGTTCCGTGTTCAAGCGGTCAACGCCATTGGTGCCGGAGCTTT
TAGTGCCTATGCTAAGCTGACCACCCAACCAGCTCCTCCGGCACCACCTCGCTTAGAGTGCAGCGGTGCT

GGCCACAATTACATAAAGCTCAAGTGGGGTGAGAACGGAAACGGCAAA
>dme_circ_0000552_junction_seq
CAAGTGGGGTGAGAACGGAAACGGCAAAGTGGCTTAATGCCGGAAACCGGCTACAA
>dme_circ_0000553
CCATTGCCGGCGGACAGTTTTCGGCTGCTTCTCTGGCTGGAGGCCATCGCCGGTGTTGCGGTGGAGCCGT
GCAGGTGGTGATGACAGGTCGCCGAGCTAGGATCCAGTTCACTGGGCACCTGGTAGCGCAACGGACTGGA
CGGCTGACCGTACAGCAG
>dme_circ_0000553_junction_seq
ACGGACTGGACGGCTGACCGTACAGCAGCCATTGCCGGCGGACAGTTTTCGGCTGC
>dme_circ_0000554
AAAGCTCCTGCTGCAGTCGTCCAATTGCCACGGGATCTGCTCCCACGCCGCTCGCTCCACTTATTGCCGA
CGCACTTACCGATCGCATGTGCAGACCGTTGGTTGAGCCATTGCCGGCGGACAGTTTTCGGCTGCTTCTC
TGGCTGGAGGCCATCGCCGGTGTTGCGGTGGAGCCGTGCAGGTGGTGATGACAGGTCGCCGAGCTAGGAT
CCAGTTCACTGGGCACCTGG
>dme_circ_0000554_junction_seq
GCTAGGATCCAGTTCACTGGGCACCTGGAAAGCTCCTGCTGCAGTCGTCCAATTGC
>dme_circ_0000555
CTCCTGCTGCAGTCGTCCAATTGCCACGGGATCTGCTCCCACGCCGCTCGCTCCACTTATTGCCGACGCA
CTTACCGATCGCATGTGCAGACCGTTGGTTGAGCCATTGCCGGCGGACAGTTTTCGGCTGCTTCTCTGGC
TGGAGGCCATCGCCGGTGTTGCGGTGGAGCCGTGCAGGTGGTGATGACAGG
>dme_circ_0000555_junction_seq
GTGGAGCCGTGCAGGTGGTGATGACAGGCTCCTGCTGCAGTCGTCCAATTGCCACG
>dme_circ_0000556
GAAAGTGCCCACTAGGAAGGAGACCACAATGCACACGGCCTTTTGTTGGTCGCTAAGACGCCTCCGGTTG
GAGTACAGTGAAGTAACTTTACAAACGATCCGCCTTAATCCTCCCGCCTGGGCCAAACTGGAGGATATGG
CCGCCGCCGTTGAGGAGCTGGAGTTCGAAGAGCTACCGCCCATTCCGGTGTGCGGATGGTGGGCGTGGTG
CATG
>dme_circ_0000556_junction_seq
GGTGTGCGGATGGTGGGCGTGGTGCATGGAAAGTGCCCACTAGGAAGGAGACCACA
>dme_circ_0000557
CACTGAATACTTGTAGCTAGTAGTCTAATAAATTCATACTTTTCGTTTAATTATTCGTTCTGCATTTGCC
CTCTGGCCTCAATAAGCGCCCCCCTTCCACCATTCGTTTAtttttttcttttttttAGCTACTTAGGAGT
GTTTTTAGATGGTGCAATTTGTTGACCAGCTATCTACAATCGTTTTCTTGGAATACATTTTACAAAAAGT
CTTGTAAAAAAATCATGCGCTGCGGGCGGGGCCACGCCCACATGCTAGTTCTACACGTATTATACACGAA
TTTTACAATCCTGCCGGGAGGGCAAATGCAACTTTTTTTGGGGCCACACAGAAAGTAAAAGCTTGATAAG
CATTTCGAAACAGGTTTTGACAAGTACTAGTGAGCCTTTAGTTTCATTTGCTGGCTGGTCTCTTTTGTTT
ATAGCACATATGGAGATTAGACTTTCGTTTTGTGACAACAAATATTATAAG
>dme_circ_0000557_junction_seq
TTCGTTTTGTGACAACAAATATTATAAGCACTGAATACTTGTAGCTAGTAGTCTAA
>dme_circ_0000558
GATATGCACGAGGTGGCGAAAGCTGAGCTAGAATCGGGCGGCAGATGACAAGGACAACGCCACGCACATG
GCGGCAAGGCTACAACAAGGATACAGGATACGGCCAACCGGATATTAAACCATCAACACTAGCCAGCACA
GCAAAGCAAAATTAAGGAACAACTGCAATCCCGGTACACCAGTACACCATGGCTGCAACGCTGACGGAAG
CGGGAACAGGTCCTGCGGCCACCAGGGAGTTCGTCGAGGGATGGACTTTGGCCCAAACTCTGGGCGAAGG

TGCCTACGGCGA
>dme_circ_0000558_junction_seq
AAACTCTGGGCGAAGGTGCCTACGGCGAGATATGCACGAGGTGGCGAAAGCTGAGC
>dme_circ_0000559
GATATGCACGAGGTGGCGAAAGCTGAGCTAGAATCGGGCGGCAGATGACAAGGACAACGCCACGCACATG
GCGGCAAGGCTACAACAAGGATACAGGATACGGCCAACCGGATATTAAACCATCAACACTAGCCAGCACA
GCAAAGCAAAATTAAGGAACAACTGCAATCCCGGTACACCAGTACACCATGGCTGCAACGCTGACGGAAG
CGGGAACAGGTCCTGCGGCCACCAGGGAGTTCGTCGAGGGATGGACTTTGGCCCAAACTCTGGGCGAAGG
TGCCTACGGCGAAGTGAGTGTCTCCGAACTACATACATATAAAATTACAAATTCCATCACAGACCAGTAG
ACCTTTAAAAGGCCGAAAAATACCATTAAACGATTACCAATCTTAAAAGTGATTTTATATTTCAAAAACT
TTTGTAATCGGTTGTAAATAATGCTCGTGAGCATGTTTCTTATACGCTTTAATTTATAGTTTCTCAGCTT
CAAGTTTAAAAACATTCTTTATAACCATAGTTCGAGGGGTTTAATGACTTTAAATTACCCATGACATTGA
GATGCAATTAATTTTATTTTATGATACAAATTCGCAAGTTCGTGCGTTTTGTTGTGCGTCTGTAtgtgtg
tgtgtgtgCGTGGAAGTCAATAAACCGGAAATGCTGCAGAGCAGCATGAAAAAGAATAAGTACTAAGTGA
AACGCACTTTTCCGGCGTATTCGCTGTATACATTCATCCCGAAAGCACCCGCTGCGCTGCCCCTTTTTTA
AGGGACTCCTCAGTGCGCATGTCCCCGGTGTCGTCCGGGTCCGGGGAATTCGAGGAATACGGGAGTACGA
CCAAAGACAGACAGACGGCATACGGCGCACTCAATGCGCCCGGACGGAGAGCAGTCGATACACAGACACC
AGCGTTGCAGCTGCAGTTGCAGTTCGTCCTGCAGCCGTCGTATATCCGTTACTAATCCCCGAACTATTGT
ATATCCGATATTTGGATGCAGGGGTAAAGCTACTAATCAACCGGCAGACTGGCGAGGCTGTGGCCATGAA
AATGGTGGATCTAAAAAAACATCCGGATGCAGCGAACTCGGTGCGAAAGGAGGTATGCATACAGAAGATG
CTCCAGGATAAGCATATCCTCCGATTTTTCGGCAAACGTTCGCAAGGCAGTGTGGAGTACATATTCCTGG
AATACGCCGCCGGCGGAGAGCTATTCGATCGAATAG
>dme_circ_0000559_junction_seq
GCCGGCGGAGAGCTATTCGATCGAATAGGATATGCACGAGGTGGCGAAAGCTGAGC
>dme_circ_0000560
GGTATGCATACAGAAGATGCTCCAGGATAAGCATATCCTCCGATTTTTCGGCAAACGTTCGCAAGGCAGT
GTGGAGTACATATTCCTGGAATACGCCGCCGGCGGAGAGCTATTCGATCGAATAGGTGAGTTCGAATTTA
ATTTATATAATTATGATTTTCAGTCCAGGTGATCAAGGTGCTACCACTTTATTCGACTTTTCTAAGCAAG
TTTCGATATGCAAGCCACAAAAATGTAGTGTTAGGAACTAAAGGAACTAATAACTTCTTGGCCACAACTA
AGATTTTGAAATTAAACTTCAAATTGTATAAAAATATATAGGTTGGGGTGATGGACTGCAATACGTTGGC
TTTTAAAGCTTATATTTTCAAAAGGAGCTAAACCAGCCCCAATTGTTGTCCGAGTCAGTACCAACTGAGC
TTTTTATCATAGCAAAAACCTCTTGACGGAGGAGCTACTTAAAGTTTGTTGGCCACTCAACTTGCTGTAA
GCCACTATGCGGCATAAAAGTTGTCCACCGCAGAAATCCAATAAATAAAATGATCACCCAAAGCGTGCTT
TGAAATAGTTAAGAATATGGCTCAAACCTCGTTGGCAAATGGCGATTATAAGACTTAATAATAAAAGCAA
ACTACTTTAAATAGCTTAACTGTGTGCAGTTATAAACTTGCCTAACATACGCACGTTTAAGAATATTTCC
ATTTATACGTCATTTAAAATTGGTTTTCTATTAGCTCTTTATTCCGAATATGATTGGATAAGAATGCTCC
TGTTTATGGTTCATTACTATTCCTGCCTCCGTGCTGGCATCCTACATCCGAAACTCATCCAAGTCGTAAA
TGAGATAGGTGGAGCCAGCGGCTTGGCGCAGATGCCACATAGTCATATAGACGTCGGCCAGACGGGGCAA
ATATGCCAATatgcgacgcgatgcgatgcgatgcgaGAACTGGCAGACGACGGACAAACAAACCGACTGC
TGACAGCGTTGTGCTATGATTTTGTTGTACTTTTGTGGGTCCAACTTGAAGTTCGTCCTGCCATCCGGTG
AATCGAAGTGGAacacacacatacacacacatatatatatatatTTCAAAAGCAGCGGGAGCGACAAATC
AGACTGACAAGATACGTATCTTGATTACGCCAGCTTTCTGTCTCTTTTGCAATGACAATTTGCCAAATGC
AtgggcgggggggaaatggaaacgcggggcaaggggaaaattggggggaCAGTAGGTGCCAAGCTCGTGG
CATTTTCATAAATATTAACAGCTTGGCAAAAGGTCAGTAGAAGAGCAGCGAGCGAGTATGGAAAGGAAGC

GAGATTGCCCCTAATGGAAGTGGCGTCCTTGCATTGATTGCCACCACTCATTCTGGTGCTGGTCCACCCC
CGATTTCGCCTATTCCCAATCACCATCATCATCATTTTGCTCCGCCCTCTCCGCAGGGGATGTTGCATGG
GAAGCTGGTGTAATAATCAATGTGGCAGCCATATTTAGTCCCTGGTTGCTAAGCCATTTTTAATGATATC
TAGAGATGTTGCGAAGCCTGAGTGATGGCCGATGCGAACCTAATGGCTTTAAATATTTATCAATAAGTAT
GGTGCGATTTTTGTTCGACTCACATTGAGTGTGTAGTTTAGTTAACGAACTATGCTTTTATTCCACTTAA
CACCAATTAACATCCTGTCGGATAAATTTTCAGTAAATAAAATTTGACTCCCTTACTGATAATTGGGCTG
TCAGATATTTTTCTGTAAATCAAATATATTGTGATGACAGCTGAATGAGCATGTTAAATGCAGTCAATTA
AGCGAACGCCATTATGGCATCTTATCACCTATTTTATTTTACTACTGCATATATGTACATGGACAGTTCT
AGAGATTGAGATACGAGTTAGTTACAAAAATGCTTATGGTTTTCTACTTACTAACTCACTTTGAAATTTA
ACAAAAATTATAGAACAAATGCTTGTTAATAATTCAAAAGTTTGTGAAAGATTGGACATTGAAATGGACG
ACCACTTCCATCGCCAATTATGTGGCCACTCACGAAATCAGATCATCATTGCATATCACGCTTGCATCTC
TGCTTTCGATTTTAGCGGTCTAGCGCCTGTTGGGTTTGGCAACAAAACATCCTTTTCCCAGCCACGATGG
GAAATTGGTAAGGGAAGGGGTGGGGTGGTCGTGGAATAGGCGGAGGGACAGAGGGGAGCCGAGTGCAATG
AATGGCTGCCATAAATAGGTTCAATATGCAAATGAAACATAATGGAGATGCACACATGCAACCAGATGTC
CTCGACTACGAACGTGCGTGTGTGCGAGTGTTTAATTGTCTGTTTGCTCAGAAACGCATTCATTAAGCTT
GTACGCCAAATGGCGAGATGAAAggggggggggggggggTATTCAGAGGGTGGGAGAAATGGGGGCTTAT
AGATTCGCAACCGATTTGGCCACTCTCTCAATCTGAGAAGGGGATTCGACATAAATTATGCTTCAATTAC
AAATGAAATTTTCGAGTAATTTCCCTCCATTTCCATTGCATTGCATAATTTTTGTACTGCTTGGGTGGGA
ATGCTTCTCTCCCACTCAGTGAAATGCATTTAATTATGCTAAAATGTTACTGACGGCCAGCAATTTTATT
GCTTTCATCTTATTTTCTCGGCTTCATGTATGCAAATTTATACTTTTTCAATTCAATGTGTATATATTTG
TGCGCTACTCGAACTTAAATTCAGTCAATATGTCCGTTTATTCTTTAGCATATAAGCCTTTGCATACAGA
AACTGGAAGAAAAGCGGTGTGAATTTCAGAATACTTATGGTAAAAGTGTAGATTTCGTACATTTCTTTGC
TATTCTCAAAAGAAACCAGATTAGCATTCCAAAAATTGGATGCAAATTCATTAGTGAATGAAAATTTTAA
TTAATTGTTTTCCTGAGTGCATGATGCACTTGCCTTCGTTTAATTTTACCTGTGGCCAAAGAGTTGTGCA
AATGGCGCCAACATGCCACATGATAAATGAATTGCAAACAATGGGCACCATGGAATGGGAGGCCCACAGA
TTGGGGCTGGGCTTATAGACGTGGGCGTGGCCCAATTTTATCTCCTGCAAAATCAACATTAAAGGTTTTA
CACGTTGAGAGTGACAAGAAGAAACCAAAGGTAAAAAAGGAGTAATATTATTaaaaaaaaaTGTGTGAGA
AAAGCTCGATAGAAACTAAAGCTGCTGAAAATAATTACTTGAGTTTCTGCTTACCGCTTCTATCGTTCGA
ATTGATAAAACAGAAAAGAATGCAAATTACTCTTATAGTATTTCTTTCTTTCACGTTTAACGCGTTTATT
GTTGGTAATGAAAACTGTATTTAAAATTTTCATTGACAGCTATATTTCTGAGTTTTCCGATTTTGATTAA
AATTATACCTTGTGAAGAAAATAGCATATGTCAAATAATAAAATATTGTTATAAAAAAACAGAGGTGAAG
CCCTTTCACCTTaaaaaaaaaaaaTTTCACTCCACAAATGTAAAATTTATTCAATCAAAATGGCAAATTC
CATCAACTCGATGCAAATTTTATAAACATAATATTGCTTTCCCTGTTCACCTTTGAACCGCCCCGACCAA
AGTGCTGGAGTCAAGAGCAGAAACAAGAGGGTTAAAGGTAAAATCGATGAGAGGCGGATGAATGGGGGAA
ATGAGATGATGATGTTTGATATCGCTTGCAGGATGCGGCTGCCTTTGACAGTCAATCAGGTGAAAGGCAT
AGGAAATACAATCTGTCTAGCTATTGCTAACCCCAGGCGATGGCCTGGCCTCCTCTTCCTTGTGCCTTTC
ATTGTTTCTCCTTTCGTTTGACTATTTCCATGCACTTCCGAGTGGCACTCTTACGCCAAAGTGGCTGGCG
AGCTAAGAAAACTTTAAAGCCTTTCGTATCAGTAATCCCAGTACTACAGAACTATCTCTATATGACACGT
AACTACTTTAATTCTTCTTAAAGTAAAAGATAGTGCTCCTATGTTGCATAGTGACTTCAAGACAGGCAGC
CAAAAGTTAAGTGGCGTTTTATATTTGTTGTCTACCTAACCAACAGGTAAACCTAACTAAATAAACATTT
TGACATTGTTTGCGCTCGGGCCTTGATTAAAATTGCATAAAACTGTTGATTGTGCTGCGGCTGTTTCATC
CGGGGACTTTCGTTGTTCCGTGAAAGTAGGCGCAACCAGCTAGGCTCATAAATCCTTTACACACACGCGC
AACAGAATGGATGGATTGCATTTGTTTTTCACCTTTCCTCCAATGGGTTTGGTCGGTTGCACATTTTTCC
CATTTTCCAAAAGTTCAGTTCCGCTGAAAAATAACCATAAAGAAAATACAATAAATATATGCATAAAAG

GGGTGGAGGAGAGTATTGTTCAACATACCTGTATAAATTGCATTGCAAAATGTGTCGAAAATTGTAATTC
AATTTCTGCGAAATGTTTGttttttttttGCATTTTGATCTGACATTTCTCAATTGTATGCAGCATGCAG
GCTTTAAAACCGTTATTTGTTTCttttttttACAGGAACCAGACGTGGGAATGCCGCAGCATGAGGCTCA
AAGGTATTTTACACAGCTCCTGTCCGGACTCAATTACCTGCATCAGCGTGGGATCGCTCATCGGGATCTG
AAGCCGGAAAATCTGCTGCTTGACGAGCATGACAACGTGAAAATATCGGACTTTGGCATGGCTACTATGT
TTAG
>dme_circ_0000560_junction_seq
CGGACTTTGGCATGGCTACTATGTTTAGGGTATGCATACAGAAGATGCTCCAGGAT
>dme_circ_0000561
GAACCAGACGTGGGAATGCCGCAGCATGAGGCTCAAAGGTATTTTACACAGCTCCTGTCCGGACTCAATT
ACCTGCATCAGCGTGGGATCGCTCATCGGGATCTGAAGCCGGAAAATCTGCTGCTTGACGAGCATGACAA
CGTGAAAATATCGGACTTTGGCATGGCTACTATGTTTAGGGTATTAGGTACAACATTATAGAATAATTCC
AATTAAAAGGCAAAGAATGATACTTAAAAGTATATTCGAGTTATTTAAAGGATTTATTTAAATAGCATCT
TATTTAGAAATTAAATGCGCTCTGCATGCGAACGTACAAAATCCATTAACGATGGCTAACCGGTAGTTAA
AAGTTTTTCTGCACAAAACTGACCGACAAGGAGAATGGTTAATTGAAAGCGCTGCGGGCACAGCATCCAA
ACAAAACATGCCCCTGGTGGTTTTTAGTTTTCAACTAAACAAAAACTAATTTAAATCGTTAGCCAGTCTG
AATTAGAAAGTTAAAAATAGTTGCAAGAAAAACAAGTGTGCAAATATAAGATTCGTACCAAACTTTATTT
AAATCATAAATCTAAATAGAAAAGCGTATCTGGTTTCGCTGATTATTCAGCTTTGGATTCataatatatt
tttaatattttcttaatcaatgaaactttttttatattttatatTAAGGTGTTTCCAGTAACAATATTTTA
TACCATATTTTATTTGAATTTTTATTGCAAATCATATACTTGTTAGGCGAATTTTTTATTTATTTAGCAC
TTTTATATAGTCATGTATTATTCTCTCAGTGTTACGACTTACAGAAGCCCAAAACTTATTCTCAATTCCT
TATGCTCTCTTTTCCCTTCTTTCTCGCAATAAACGACGACTGCGATCTGGTTGTAATCACGTCATTACCA
TGGATCAGGGTGCAAGGGCAAGGAGCGACTGCTGGACAAACGCTGCGGCACCTTGCCGTATGTGGCGCCC
GAGGTGCTACAGAAGGCATATCACGCCCAGCCGGCGGATCTCTGGTCGTGTGGCGTTATATTGGTGACAA
TGCTGGCGGGTG
>dme_circ_0000561_junction_seq
GTTATATTGGTGACAATGCTGGCGGGTGGAACCAGACGTGGGAATGCCGCAGCATG
>dme_circ_0000562
GAGCTGCCCTGGGATCAGCCGTCCACCAATTGCACGGAGTTCACCAACTGGAGGGATAACGATCACTGGC
AACTGCAGACTCCTTGGAGCAAACTGGACACCTTGGCTATTTCGCTGCTTCGCAAGCTGCTGGCCACCAG
TCCTGGCACGCGTTTGACCCTGGAGAAAACCCTGGATCACAAATGGTGCAACATGCAGTTTGCAGACAAT
G
>dme_circ_0000562_junction_seq
TGGTGCAACATGCAGTTTGCAGACAATGGAGCTGCCCTGGGATCAGCCGTCCACCA
>dme_circ_0000563
ATCATCAAGCAAAGCTGGTTGCGAGAAGGAGTAACTGAGCCGAGCCTCCTGGGCCAACGTCTGGCGGTCG
CCTCCATCCTCCTTGGATCGGCCACTGCCCAGTCTGACATTGAAGTCATCGTCGCTGCGCATTGTGGGCA
TGGGTTGAGAGCAGTAGTTCCGGGAGATGGAG
>dme_circ_0000563_junction_seq
TTGAGAGCAGTAGTTCCGGGAGATGGAGATCATCAAGCAAAGCTGGTTGCGAGAAG
>dme_circ_0000564
TCGTGCCAAAAGAACAGGAAATGGACGGTGGCCATAGTGGCCATTATGGCCATCGGGATATTGCACATTG
GCACCCCTTGGTTCCCTGCACTCAGCGTAATTGTTTGTTTGAAAATGTATTGATATG
>dme_circ_0000564_junction_seq

ATTGTTTGTTTGAAAATGTATTGATATGTCGTGCCAAAAGAACAGGAAATGGACGG
>dme_circ_0000565
GTGCAATACCAGTTGATCTTTTGCCTGTGGGTGCTGACCTTCAATCCCCTGCTGGCCGCCAAGATGAATA
AGTTCAGCGTGATCCCCATCCTGGCTGATATCCTCAGCGATTGTGCCAAGGAGAAGGTGACACGCATTAT
CCTCGCGGTCTTCCGCAATCTGATCGAGAAGCCGGAGGATTCATCGGTGGCCAAGGACCATTGCATCGCC
ATGGTCCAGTGCAAGGTGCTGAAGCAGCTATCCATCCTGGAGCAGCGTCGCTTCGACGACGAGGACATTA
CCGCCGACGTAGAGTACCTGAGCGAGAAGCTCCAGAATTCGG
>dme_circ_0000565_junction_seq
TACCTGAGCGAGAAGCTCCAGAATTCGGGTGCAATACCAGTTGATCTTTTGCCTGT
>dme_circ_0000566
GTGCTCATCGGAAGTTCCAGGTGGTGTGTCCAGTAGCAGGAGATCCAGATTTCCCCAGTCCACCTCGCTC
AGAAACTGCCTGATCATGCCATTCTTCTTGGGTCCTCGCCAAATAATGGCATCGTCCACAGAGCCCAGAA
GAAATCCGATGGACATCAGACACACGTTGTCTTCGATTCCCACAGG
>dme_circ_0000566_junction_seq
GACACACGTTGTCTTCGATTCCCACAGGGTGCTCATCGGAAGTTCCAGGTGGTGTG
>dme_circ_0000567
GCCGAGTTAACTAAAACCACTGCACCGCTTACCGAGAAGCGCCTTAAGCTCAGTGACGAACTGGTGGGCC
TTAAGGAGAAGGTCAACACGGCTAAAGGAGAGGTTCAAGTGTTCGAGTCGCAGCTCAAGATCCTTAAACA
GGCAGAGACCACTGAG
>dme_circ_0000567_junction_seq
GATCCTTAAACAGGCAGAGACCACTGAGGCCGAGTTAACTAAAACCACTGCACCGC
>dme_circ_0000568
GGCTCTCCAATTTCTTGTTGCAATCCTCGATCTCGCGCTGATTTTTCTCCGGCAACTTGTGCAGATCCTC
CAGCTCTTTTTCGTTCTTTTCTATCTGGGCTTTGTCCTTCTTGCGCTGCTTGTTCGT
>dme_circ_0000568_junction_seq
CTTTGTCCTTCTTGCGCTGCTTGTTCGTGGCTCTCCAATTTCTTGTTGCAATCCTC
>dme_circ_0000569
GATGCCGCTCCCTGGGCACATGCAGCTTATGCAGCCGATGCTGAGACCGGCCATGGCCATTGGACCCCAT
GGAACTCTCATCGGTGGGAATATGCTTAGAGCCGGTGGCCCCGGATTACACGGCATGCCTGCAGGTATGT
GACGAGATCAACGATTGCTTGTTGTAAATGGAGCAGCAGTTGCTAACTATCTAAAGATCTGAATATTTAA
AGATATACATTATGATTGCGGTTTAGGTCTACTGCCGATGCAGATTGGACTACCGGGAATGCCGGGTGCC
TTACCAGCAGGAGCGATGGCAATGCCTGGAATGGGCGTGATGCTGCAAGGACATCCCAATATGCTGCAAA
TGATGCAGCCGCGCTTTAGATGATGTCTGCCCGCAGCGCCGGGTCTGTTGCAGGCGCCAGCAGGCCAGCC
GCCAACGCACCTGCAACCGCAGCCGCATCCTCAGCACGTGCAGCCAGCCCGGTCCTTTTCCCGGACCACG
GCCGCTCTGCAGGCTCAGGCACCACTGTTGAGCCTGCCGCTGCCACTGCTTTTCCATGAGTGAGGCACGG
ATCAAGCTTATGATTGCAAACAGCAAACAGCGCGCACTCAGCAAGCAAGTTCTTTTATAAAATCAAACCA
AA
>dme_circ_0000569_junction_seq
GCAAGTTCTTTTATAAAATCAAACCAAAGATGCCGCTCCCTGGGCACATGCAGCTT
>dme_circ_0000570
GATGCCGCTCCCTGGGCACATGCAGCTTATGCAGCCGATGCTGAGACCGGCCATGGCCATTGGACCCCAT
GGAACTCTCATCGGTGGGAATATGCTTAGAGCCGGTGGCCCCGGATTACACGGCATGCCTGCAGGTATGT
GACGAGATCAACGATTGCTTGTTGTAAATGGAGCAGCAGTTGCTAACTATCTAAAGATCTGAATATTTAA
AGATATACATTATGATTGCGGTTTAGGTCTACTGCCGATGCAGATTGGACTACCGGGAATGCCGGGTGCC

TTACCAGCAGGAGCGATGGCAATGCCTGGAATGGGCGTGATGCTGCAAGGACATCCCAATATGCTGCAAA
TGATGCAGCCGCGCTTTAGATGATGTCTGCCCGCAGCGCCGGGTCTGTTGCAGGCGCCAGCAGGCCAGCC
GCCAACGCACCTGCAACCGCAGCCGCATCCTCAGCACGTGCAGCCAGCCCGGTCCTTTTCCCGGACCACG
GCCGCTCTGCAGGCTCAGGCACCACTGTTGAGCCTGCCGCTGCCACTGCTTTTCCATGAGTGAGGCACGG
ATCAAGCTTATGATTGCAAACAGCAAACAGCGCGCACTCAGCAAGCAAGTTCTTTTATAAAATCAAACCA
AAGTCAGTTTTTTTCAATGTATAACAGTTTTTGAGACACGGTACCCTAAAAACACACATACACACTTCTT
GGCCACACGCTACCCACTAATCAGCGGTTATGCCTAGTAAACATGTCGATGTCATAGatatatatatata
tTTCCTTCATCTAAAGCACTCCATTTTAGCTTTTGTTTTAAACCAAATTGCCTATTTTTTCCAATCCGAC
CTTCTCGATGTGCGTGCATAAATACATCTTTTAGATATATTTTTCGTACTATTTGTATAACACAATGTAT
CACAACGCTAAACCACACACACACTAACACCATCCATCTGAAGCTATTTTGTTGTGCTGTAGAAATCTAT
CTAATATCTGTATCTATATCTGTATAATCAACAAATTAATCGTTGACACATAACCTATACAATTTGTTGt
tttttaacattctttttctttttttttAGTTTCATTTAGTGAGCATATACTTTAAACACATCCACACCCA
CACACTCTCAAGTTGTTTAATAATATCTCCTACaaaacaaatgaaatccgaaaagaaaaaaaccttcgaa
atcacacaaaaccaaatgcaaaaccaaaaaaCATGAACGCTCAAAGCCCTGGGTATGTTGACAATTGCCA
TAAGCAGCATTTTTGTGTTGTTTTTGTGTTATTCGTGCGTAATTATACTTTCGATTCTTTATGTCCATGT
GAAAAGCTCTGCAAACTGTTATAATGTTTTAAAATTCATAAAACTTCTGGCCAGGCCCCATGTTGTCTAG
ACCTTCGTTCAATCGTTCAGTTGCGGCTGGCAAAAA
>dme_circ_0000570_junction_seq
TCAATCGTTCAGTTGCGGCTGGCAAAAAGATGCCGCTCCCTGGGCACATGCAGCTT
>dme_circ_0000571
GTTTCATTTAGTGAGCATATACTTTAAACACATCCACACCCACACACTCTCAAGTTGTTTAATAATATCT
CCTACaaaacaaatgaaatccgaaaagaaaaaaaccttcgaaatcacacaaaaccaaatgcaaaaccaaa
aaaCATGAACGCTCAAAGCCCTGGGTATGTTGACAATTGCCATAAGCAGCATTTTTGTGTTGTTTTTGTG
TTATTCGTGCGTAATTATACTTTCGATTCTTTATGTCCATGTGAAAAGCTCTGCAAACTGTTATAATGTT
TTAAAATTCATAAAACTTCTGGCCAGGCCCCATGTTGTCTAGACCTTCGTTCAATCGTTCAGTTGCGGCT
GGCAAAAA
>dme_circ_0000571_junction_seq
TCAATCGTTCAGTTGCGGCTGGCAAAAAGTTTCATTTAGTGAGCATATACTTTAAA
>dme_circ_0000572
GTATTCAAATAATATGCATTCCCGACGAAAGTAAAGCAAGAGTGGTAAGTAAAAGCAACTCTACAAAATG
AAATGCGTCGATCAAATATTTACAAGTCTTCGCCATACAATTACCAGAGAAAGAGAATATGTATTCTACG
AGTACGATTATAACGACTGTGTACAGATGCGAGCTTAGTCGAGGAGTGTGTGAATCGTATACGAGTAAGT
AATGCCACCAAATCTGCGATATTGTCTGTCTTGCATTGAGGTACTTTCGTCTGAAGCACGCCG
>dme_circ_0000572_junction_seq
TTGAGGTACTTTCGTCTGAAGCACGCCGGTATTCAAATAATATGCATTCCCGACGA
>dme_circ_0000573
GATGCAGGGAGAGACCAACCAGAAGACCGGTGAGGAGCTCCAGGCCGCCGAGGACAAGATCAACCACTTG
AACAAGGTTAAGGCCAAGCTCGAGCAGACCCTCGATGAACTGGAGGATTCGCTGGAGCGCGAGAAGAAG
>dme_circ_0000573_junction_seq
GGAGGATTCGCTGGAGCGCGAGAAGAAGGATGCAGGGAGAGACCAACCAGAAGACC
>dme_circ_0000574
GGAACTGCGACCACTTGAGATCACCGCGGTCGATAATCGTGTCCACCTGGAAGAGACCCCAGTCACGATC
CTCACGATTGGCATCACCCTCGTGATAGATTTCCGGATCGTGATGCGCCGCATTCAGACCAATCAAGCAG
AAGCTAAAGCTGGCTATCGACGTCATCGCGAGCCACTGGCGCACGCAGATCCAAATGCCTAAAGATCCCC

CGGTTCCCAGGTATATGGCAATGG
>dme_circ_0000574_junction_seq
CCCCCGGTTCCCAGGTATATGGCAATGGGGAACTGCGACCACTTGAGATCACCGCG
>dme_circ_0000575
GATGCCCAGCGACAGGACTTCGATTGGGGTCTTTACCAGGTGGACACGATTATAGATCGCGGAGACATCA
AGTGGTCGGATCTTCTGGTGCTCACACACTTTGGTGAGCATGCTTTGCATCACCTATTTCCCACACTGGA
TCATGGAGTGCTAAAGCATCTGTATCCTGAGTTAAGGAAAACCATGAAAGAGTTCGACGTGGAACTGCGG
GAGATCAATCACTGGAGTCACATAAAGGGACAGAATCAGCAATTGCTAAGAACAGAGAAGAATCCAATTC
CGCCGGGTA
>dme_circ_0000575_junction_seq
ACAGAGAAGAATCCAATTCCGCCGGGTAGATGCCCAGCGACAGGACTTCGATTGGG
>dme_circ_0000576
GATCGCGGAGACATCAAGTGGTCGGATCTTCTGGTGCTCACACACTTTGGTGAGCATGCTTTGCATCACC
TATTTCCCACACTGGATCATGGAGTGCTAAAGCATCTGTATCCTGAGTTAAGGAAAACCATGAAAGAGTT
CGACGTGGAACTGCGGGAGATCAATCACTGGAGTCACATAAAGGGACAGAATCAGCAATTGCTAAGAACA
GAGAAGAATCCAATTCCGCCGGGTAGTAAAAA
>dme_circ_0000576_junction_seq
AGAATCCAATTCCGCCGGGTAGTAAAAAGATCGCGGAGACATCAAGTGGTCGGATC
>dme_circ_0000577
GTCAGTTGGGCAAATGCCACTTGATAGCCCGCATCGGGATCTTCGGCAACTCCAGCTGTCTCTCCTATTT
CGAGACCCATCAATTTCTCTGGTGGTCGTTCAAAAAGATCGATTAGAGAATGCAGCAGTCGTGGCCAGAA
AGTAGCGTACTGTTGCTGCAATATCTCAGGAGTTTCTGTAA
>dme_circ_0000577_junction_seq
TGCTGCAATATCTCAGGAGTTTCTGTAAGTCAGTTGGGCAAATGCCACTTGATAGC
>dme_circ_0000578
GCAAATTTACAGCTGCTGGCCGGTTATTTGCAGCAGACGCTGAGCGCCGATCCCAATGTCCGCCGGCCGG
GTGAGTGTGTGTGTGTAGGAATGCATGTGCACACACACAACTGCACATGCGTGCAGGGGTGCTGATGTGT
ATGTGCGCGGGAAATGGTTTTTGGCGACGCCAAAGAAAAATACGGTTTTTAAATCCATTTGAAGTTGTCT
ACAAGTTTTCCCTATGTCAAAGAATTTATACAGTCACGCTTTCTATTAATTGAATCGAGATTAGCTGATC
TTGATATGACTGTTGCCAAGCCACCTTTCAAAACCATAGACCTAGTTTTAGGATTATTTAAGGCATACCA
AGCCTATAGACTCCGCACCACCATAACCAAGCTTTTGAAAATATTGTATAAAAAACTTATTGCTTGAATT
GAAAATATGTTTAGTCTAACGAGAAACACTGTAAGCTCCTATCGGTTCCTTATATATTTGTATTTGCAAC
ATCCGATTTACATAAGTACAATAGTTCTAACAAACACACCATTTCCTTCTCTACTTTTATAGCCCGAAAA
ACTTTTGGAGTCCACGGAACTACAGCAGAATTACCCGATCCTACTGCTCAATCTGATAGACAAAGCCCAA
ATGGACATGACTACGCGGGTGGCGGGAGCCATTGCCTTTAAGAACTATATTAAGCGGAATTGGGCAGCCC
ACCTTGACAGCGACGGACCGGATCGCATCCACGAAAGTGACAGGAATACAATTAAGACCTTGATCGTTAC
GCTAATGCTCCACTCACCGGTGGCATTGCAAAAACAACTAAGCGACGCGGTCAGCATCATAGGCAAATAT
GATTTTCCCAAAAAATGGCCGCAGCTTATCGACGAGATGGTGGAGAGATTTGCCTCTGGTGATTTCAATG
TCATCAACGGCGTTTTGCAAACCGCACACTCGCTTTTCAAGCGCTATCGTTATGAGTTCAAGTCGCAGGC
TTTGTGGGAGGAGATCAAATTTGTACTGGATCGTATGGCTAAACCTCTGACAGACCTGCTCCAGGCCACA
ATGCAGTTGACTAAGG
>dme_circ_0000578_junction_seq
CTCCAGGCCACAATGCAGTTGACTAAGGGCAAATTTACAGCTGCTGGCCGGTTATT
>dme_circ_0000579

GAACCCTTTGGAGAAGTCATCAGCGCCTTCAGCTGCAACAGCAACTCGTGCACAGTGCATCCAGTGGAGG
TTTCTTGGCGGATAGGCACATTCGGCATCGAAGCACCCCGGACTCGTTGTTCTCGGCGAAAGAACCGGCT
GATTGCG
>dme_circ_0000579_junction_seq
TTCTCGGCGAAAGAACCGGCTGATTGCGGAACCCTTTGGAGAAGTCATCAGCGCCT
>dme_circ_0000580
TTTCTCCTGCACATCCTTTGTTGGGCCAAATTTGCGCACGAACAACGTACCCTTGAACTCCTTGCCTAGG
CTTTCGTAATGCTTTCGGGCAGCTCCATTCCAAAAGGCTTCACTGGCTCGCACTACAATGGCATAAGCGC
CAAGATCATCG
>dme_circ_0000580_junction_seq
TACAATGGCATAAGCGCCAAGATCATCGTTTCTCCTGCACATCCTTTGTTGGGCCA
>dme_circ_0000581
GGACTGTTAAAAAGAAGGTATAGAGCGGCGACTTAGTAACATTACGGATAACGAATTAGCATTTTACGTG
TAAGCTCTGTGGTCAGGACGGAGGATGGCAGCTGGCGTGGAAATTGGTCGGATATTCAGATAACACGACA
TTCGGGTCGGGTCAATTAACTAACGAATCCCATTTTATACACACAGTTGTATACCAAAAAG
>dme_circ_0000581_junction_seq
TTTATACACACAGTTGTATACCAAAAAGGGACTGTTAAAAAGAAGGTATAGAGCGG
>dme_circ_0000582
TGAAAATACCGAGTAATTCGAGATGGTAAGAGGGTCATTTCTTTAGCGGCAATTAAGAGTCAAATAATTC
ACCCGGCACCCAGACACGTAGATTTTCAACACCTACAGGCCGCAGTAAAAGGCAACAAAATGAAGTTTAC
AATTTTATGAAATAATCAACCGACAAAGAGCTGAG
>dme_circ_0000582_junction_seq
TGAAATAATCAACCGACAAAGAGCTGAGTGAAAATACCGAGTAATTCGAGATGGTA
>dme_circ_0000583
GGAAATGCTTTTTGCCATGCTGATAAGCGCAACTTATGGTGTTGTAAAAGATAGTGAAGGAAAATGCATT
ATACAGTTTTATTAAATAAAGGCAGAGCATGTAAAAACAACTTCATGCAATTATCTGTGAAAAATCTAGA
AGCCATAAAATGTATTTGTTCTTGAAACAGATAAATTAAATAGATAACAACGTATTAGAGCTGTGTTGAA
TATGCAATTATCATTTATAAATTAAAAAATATGTAAGTaaaaaaaaaTAAGTTTCATAGCAAGCTTGTCA
TATTTTAGCGCAGTAATTATTTTTCCAAAATCACTCCTAGAGTATTTAGCTAAATGCAAAAGGAAAAACA
GGTAGAGGACTATCTGTACAAATAAACTGTCTCAATTGACAAAATTTGCTTCAAATCCCATTAtaaaaaa
ataaataaaattaaaatttaaaCATATACTTAGACAACAAAATATAGCACACCTTTTGGCGGGTTTTTGA
GCCGACAACTGGTTTGAAGCTTCGCTAAAAAGTTTGCAATTTATATTACACTGTGTTGCAAAATTAGACT
TTTATTGTACTATGGGAACAAAAAACATATAGACttcgatgtacttatgtacttatttctattctattac
tattcgatgtacttattactattcTATTCACATATGATTTTAAATAAAttttttttttttAATTTAATAA
AGAAAATAGTTAATTGTTAAAGAGACAGACTGCATTTTGTGGTAATAAGAGAAATAAATATCGCCTTCCA
ACTGCACTGGCCTTATCATTTAAAGCCAGTTTAGTCATGGCCGTGTACACAATCTCTTGAGTACGCTCTT
ATCACCCAGTCAAAAGTTCGCTTATTCGTTCCTATATAAAGAGAACTCTTTTCGGCACGCAGTCAGTTCT
TAAAAGAAGAAATTATATTAAGTGAAACATGGCTCTTATTTTGCAAGTGATTTCTACAGGAATCTACATA
GTGGGTTCTCTTAGCATCGTCGCTTATCTCTACGAAAACTTAAAGTCCTTGTTCAGCATTATCAAGTCTG
TTGTGGAACCGTTCTTCAGGCCGAATTTGCCAAAGACTTTGGCGGAGAAATTCGGAAATTGGGCAGGGTG
AGTGTTTCGATCGCAAGGCCCATTATATCCATGAGTTTCGCTTTATTCACCGAATGTTTCCATGTTATTC
AATCCGGTATTTGCATACTTTAACCCCAGGGGTCTGTCAATATTTGAGGCATGTGTTTTCGGTTGATTCA
CTGTCGCGAATGCAAATTGTTTACCTTATTTCTTAAGGAGTGTTTTTGCTATATCCAGGCAGCATCCAGC
TTTTCTCAGAATTGAATGACAACAGCTATCTCAGGTCCCCATAATAGCTATATATCACGATTTGCCTGAT

AATTCTTTAAATGCCAATAATTTCAATATAAATTAGGCTTTAATGAGAGTATATTGACTTTCTTTACATG
TTAGCATAATAAATTTGAGTAATAATAATTTGAGCATATATATTTTTTTATGATAAATTAAATTATCGAT
TTATACGTTAATTTCACCTCATGTACTACATTACCAAGTTTATGATCAAATTAATTGTTGAGCGATAGAA
TCTATAGTTACCGGGTTTGGGAAAAGCGGTTACAAAATCCATTTATGATAAGATAAGAAACCCGTTACAA
TTATGAATTTCCATTATTATTTATACCAACGAAATGAACAAAATTGTATTTCTTATCTGAGTGTTATTGA
GCGTATTGCTGATGGTTATTTTTAGTTAAGCTTAGATATTAAATGAATTCACATATTTGTGAATCATAAA
AAATAATATCAGACTATAAAAGATATTCTTGCTAAATTATCCAATACAATTTATGTTCAATTGTTAAGTT
CAATATTATGAATTAAAAAAGGTTTAACTTATTCCACAAATCCCTAAATAAAATAAAATAAATATAATTT
ATTTTCAAATTGTCTGCTGATTCATTATAATTTGGCATCCAATAACTAAATTATTCAAACAACTTGATTT
GTTTACAATTTCATTTATGGACCAGACAAAGTCATTCAACTCGTAAACCGGCTTGCGATTTTCTCGTTTG
TTTCGCACATTGCGATCGCTTTGTTTTTGCTTTATTAACTAATTAATTCGCTTTCATTGACTAGAGTCTC
GTGCTGCTCTTCGTGGTCAAACAGTTATAAATTTAACTATCGCTTCTTGAGTTTATTGCTTAATTAATGG
GTGACCTTAGGTGTGAGATCGCTACAATTTACCAAATTGAATTATGGATTTTATACAACTGCTTTTATTG
CTCACCAATCACAAAGTGCAAAATCTTTGCCAAACTATAGCAGTCATAATAGTTGAAAAATAAATAGAAA
AATATTTTCTTATTATAAAATAAATTATAACTTATGGACTTTTTAGTCTTAAAAAGATTGGACAGTATTT
TTGAAAATTTGTAATATGATAGTTGTTTAACCAACCAGTGAAGGAAATTTATTGCCTTAAATATATTTTT
CGATAAGAAACCAATGTGCTAATCATTCTGACTTTCTGTTTAAACTACCGTTGTCAGCATGAACTTACTT
ATCTTTCGATATTTTCCGATTTCCAACTGATAACCGCGGTTGACGTCCACTTTGTGACGAGGCACACGAT
GAAAGTGTGACGGTACTAAAGAAAAGAACTAGAAAATGGATCAAAATTAATTACGGACTCGGCTTTTTGA
CCTTATTAGAACAGATTAAAAATATCTCAATCATTGTGACTCTAGATCACAATTTGAATTGTCAGCATGA
GTAGTAGTTTTCATTAAATTTATCTATCGATTGGGGCTTTTCTGGTTTCCAATAGCGATAATATAGCACT
ATAAAATATTTAATCCGTTTCAGGTTGTGGAGATTTAAatatattaatatatatataatatataaatcta
tgtaataaatctaaaatctaacaaaaactaaatttatttatcTAATTTAAGTTCAACAATGTGGGCACGA
TTCACGACCCGGAAAGCCTTGACAAGGTATCGGAGGACATGTTGTGGGACCTTCTCACCGTCAACGTGGG
ATCGGTTACCATGCTCACCCGCAAGATCCTGCCCCAGATGATTAGTCGTCGAAAGGGAGCAATTGTTAAC
CTCGGATCCTCCTCCGAACTCCAGCCCCATCCCAATTTGACTGCTTATGCGGCTACCAAGAAGTTCGTTA
CCCATTTTACTAAAGGTTTAGAATATGAGGTGGCCGAACACAATATCCATGTGCAGTTGGTTATGCCAGC
CTTTGTGGCCACCAATATGAATTCCTATTCCGATAAAGTGAGGCAAGGAGGACTGCTTTTCCCCAACGCT
TATTCCTATGCCCGGTCCGCAGTCTTTACACTGGGAAAGACGAGCGAGACCAACGGTTTCTGGGTTCATG
GATTACAGGTACGCCTTCATGAAGTTGGCTCCCATGGATATTCGTACTTACTTCGGATACCAACTCTTTA
AACGAATGAGAATCGAGGCCATGGAGCACAGACTGAAAAACCAAAAACTTTCTTAGGTTCCTAGCAGCCA
TGTAAATATTTGGCAAAATAAATACCTTAGAGTTTACGGCAGTTTTTAACAGTGGAAATAATATTTTAAA
TTTTTGAGAATATGTTATGCTGAAAATAAAATATATTACACTTATTGACGGGTTCATGTATTGGTTGGTA
TTGGTTCGTGACTTTGCTAAGTCACAAATTTTCAAGTTTAAATATTGGCTTAATAAACTTTCTATTTTTG
TGGGGTTTTATACCGTTAAAAACTTTCGATAATTTAGAACAAGTTGTAAGTAAAATAATTGCATGTGTTA
TTTAGGAATTGTGGGCACAGAAGACGTAGCATGATAAAACCCAATTTAGCTTAAAAACGGCTATTACGAC
AACACTATTTACTATTTAGAACGTTTGGGAAAAGCTTGTGGACTTCCTCTTTGAACTTTTCCCGTCAGTC
TGAAATAAATATTCGGCTTGGCAGGAGGTTACTAATAGCCCTTCACTCATACAATATCGATTTATATTTA
TAGCGTTTGAAAACTCGTACTGTTTTGTTCGCTGACAGCATG
>dme_circ_0000583_junction_seq
TCGTACTGTTTTGTTCGCTGACAGCATGGGAAATGCTTTTTGCCATGCTGATAAGC
>dme_circ_0000584
TGGTGACCGGTGCGACCGATGGAATCGGAAAGGAATATGCCAGGGAGTTGGCTCGTCAGGGTCTTAACCT
GGTGCTCGTTTCGCGGAAAGAGGAAAAGCTGATTGCAGTCACCAACGAGATTGGTAGGCAATGTTATTCA

ACTAAAAGATAAAAGTGTTTATTCCATTTCGAAACGATTATTTAGGGAGCCAATATAATGTGAAAATCA
AGTGGATTGTTGCGGACTTTGCAAAGGGACGCGAGGTGTACGCTCATATCGAAAAGGAACTGAATGGAAT
CGAAGTTGGAATTTTGGGTAAATATGTGAATTGGCATAGAAATACATGATTAATATATATAACAAATATA
CTGGTCATTAACATATAGTTTATAGTTTACTGCTTACGAAGATACGATTATTTGATTGTTAAAAAAATTA
AATGCCTTTCATTTTAACAGTCAACAATGTGGGCACGATTCACGACCCGGAAAGCCTTGACAAGGTATCG
GAGGACATGTTGTGGGACCTTCTCACCGTCAACGTGGGATCGGTTACCATGCTCACCCGCAAGATCCTGC
CCCAGATGATTAGTCGTCGAAAGGGAGCAATTGTTAACCTCGGATCCTCCTCCGAACTCCAGCCCCATCC
CAATTTGACTGCTTATGCGGCTACCAAGAAGTTCGTTACCCATTTTACTAAAGGTTTAGAATATGAGGTG
GCCGAACACAATATCCATGTGCAGTTGGTTATGCCAGCCTTTGTGGCCACCAATATGAATTCCTATTCCG
ATAAAGTGAGGCAAGGAGGACTGCTTTTCCCCAACGCTTATTCCTATGCCCGGTCCGCAGTCTTTACACT
GGGAAAGACGAGCGAGACCAACGGTTTCTGGGTTCATGGATTACAGGTCAGTTCTTAAAAGAAGAAATTA
TATTAAGTGAAACATGGCTCTTATTTTGCAAGTGATTTCTACAGGAATCTACATAGTGGGTTCTCTTAGC
ATCGTCGCTTATCTCTACGAAAACTTAAAGTCCTTGTTCAGCATTATCAAGTCTGTTGTGGAACCGTTCT
TCAGGCCGAATTTGCCAAAGACTTTGGCGGAGAAATTCGGAAATTGGGCAGG
>dme_circ_0000584_junction_seq
TGGCGGAGAAATTCGGAAATTGGGCAGGTGGTGACCGGTGCGACCGATGGAATCGG
>dme_circ_0000585
GTGGAGGCCCTGTACTCGCTGAAGAAGTACCGAAGTGTCAAGCAGCGCTATAGTTACAATGACTAAGTGG
GCGGAACGTAAATCGGCGGGCGGGAGGCGTGGCAGCGATGCGGAAATCGCTGCAAAAACTCAAGGACTGA
TGCTGTGGATCGGGGGGAGCACGAAGAAGGAGG
>dme_circ_0000585_junction_seq
TGGATCGGGGGGAGCACGAAGAAGGAGGGTGGAGGCCCTGTACTCGCTGAAGAAGT
>dme_circ_0000586
GATATGGTTGCTTGCGTCGCTCCTGACCACGATCACCTGGGTGACGTATATCACCTTTTACAATTCTCGT
GTGATTGGCATGCTGATAACGAAGATCGCCAACCGTTGGTTCATCAAGGGCGCGTACTTCAAGATCGGTT
CTGTTGCTCTAAATCCGCTGGCAGGAAAGATAATGTTCCGAGACTTCGTGTACATCACGTATGACTACAC
AGTGCGAGCGCAGGATGGATACTTCATATTCCGCTGGTGGCGCTCCTATGTACCCAAGGATGTCTCAGAA
GGATCTTTCCCACTCGGACACGCGCCTATCGGTCCAGCTAAATGGCTACGAGCTGCACATATACAACAGA
TCGGATTTGTACGACACGCTGGAAAAGACGTTTGGTCTGGAACCATCGCTGCTTATACCGACAGATGCGG
CTAGCAATGAGGAGAGAAACAAGCTGAAGGAGCATCATATGAACCTGGAGAATGCACGGCAGAGCCAGAG
GATTCAGAATGTGAAAAACTCGGAGGCTATGCAGGCCACAACCTGGCGGGATTTAATACCAGTGATAAAA
ATTGACGTTTGCTCCGGTCGCTTTGTGTTTGGAAACCGATTAACGCCGACTACACTATCGATTTCCGTGG
AAGAGGCCCATTGCACCTACAGTACAAAGCCAGCTGTCTGTAGATTGGATCACTTTATGCACTTTGTGAA
GGCTAAAGTCGAAAATGCGAAAGTGCTCTTCTGTCCGAGTCCCAAGTATACTGGTTTGATTGATGAACCA
CCGCGATATATGGGCGAAGGCTTTGTGGTGATGATGTCCAATCAAATGGATTTGTACTTCTACATGGACG
AGCCAGGCGTTGTGCCGGAACATCCTGTACAGATTGTCCTGCCCAACGGTGATGTTGTGGAGCCTTCGCC
TCCGGTCTGGGGCATAAATGCCAGGTGTCTCAGAGGCACCGATTTCAGCTACGGACCGTGGGCAGATCGG
CAAAGAGATCATTTGTACAGGTACTTTTATCCTTCTGACTGGAAGGAGGCAGAAGTTACACCCACACCCC
AGCCAGGGGAACTTCGCAGCTATCAATCCTTCGATGTGACATTGTGCGTGCTAAATGAGGCAACTATTGA
CATACTCTTTTCCAAAGAAAAAGAGACGAATGCAATGCACATTACCGTGGGACCTGCTTCGTATGTAGAG
ATGACAATTCCCTGGGTCACTCAACCGGATGGTTACACATCGAAGATCCAGGGACAGCTCTTCCACGTGG
AGGCCACCACATCGCTGCAGTATCGCAGCCTGGCAGAGTTCGAGTCCTTGGAGTACAAGGTGCGCATACA
CTACCCGACCAAGTGGAACGCACCGCAGGATTGGAGCATATCTCTCTCCGGCTGCAAGACGAGTGCTTTC
ATCGTGTACAAACACAAGTGCTTCTTCCAGGATCTCATCGAGGATTGGGCTAACAAGGCCAGGCCGGATA

TACTCAGTTTTGTTCCGTATACCTGTAACTTTAGCATCCGCCTGCATGAGTTCGAAATCCTAATGCTGTG
CAACGAGTACAATTGGATCGATTGCAGTAGTGCCAACCAGGAGAACAATCATTTGGCTTTCTGTGGAGAC
GTCTTTGAGATGAGTTTCGCCCTGCCATTTGACGATTTCCTGCCCAAGACAGTTACGCTAAAGTTTTGGA
TCCACGGAGAGGGTTTGGATTTGAGCTTATACGTACCGGAAGTGTCCTCGGTGCGTCCTATTGTTTTAGC
CATAGATGAGAACGCAAGACTGCTGACCAGGGAGGGCAAGCTCATCCGGCGCCCAGAGTTGTACTCGAAG
AAGTGGCGCAAGATTTGCCAGCGCTCTGCCGGTTGGATCGACTGTTGGGCAGTGCCCATTCTGGCACTGT
CAATTCAGTATGTTTATCATCCAGTTCCACCACTAGGACCTGATCCTCAAGCGGATATAACTACGCCAGA
AAAAGAAGAGATCTTACTGAGTCCTATGAGGATTCCAAAAGTGAGAAAATCACCTGTTAGTAGCTGGCAG
CAGCCACCTGAACAGTATAGTAAATTTGATCCTGGAACGCTGGCCGCGGACCACGTTACTGTGGAACTGG
AGATTGGAAGCTCCGTGCTTATGGCTTATGGAAATGTGCTGCGCAACTTTATTAGCCTGAAGGAAAATAT
TTTCGGCGAGGATCAGAATTTCACAGACATGGAGCAATCGAATGTAAATATGAAGGAGCCCGGCGTTGCC
CAAGTAAATCCCAAGGACCAACTGCTGGCCAAAGAAAAGGAGCTGGCCAATAAATCCATTTCAGAGACTC
AACCGCCCGAGGAAAAGCGCAAACCCTTCGATCCTCGTCTTTACCGTCCTCTTGAGGTGATGGTTTCCGT
GATTGTACACGACATTCAGGCGCACGTAATGAAGAATTGCAATGAAGATGATCCGCCCTGTCCGGTCGTT
CTAATAGAAAGATTCGGCTTCGAAATGAATAAGAAGTATCATGAAACCACTCTGCAAGTGTTAGTAAGTC
CGTCGTATTTACTTACCTCGGATTGCCTGCAGCGATCGCAGAGGGAGCAGCATATTAACCAGGGTCACCT
CATGCTCTCCGCAGTCCAGGTTAGAGGTCATGCCATGTTCAGTAATGAGGGCTGTGCTCTGGACGAGGAC
ACTCTAGAATATTCCTGGCTCGTGGAGGTTCAGCTGGGCAAACTCACCGGAAAGCTTACTCTACCACAGC
TTGTAAACGTCGTTACAGGTCTGGAGACACTTATACTCTTGGCCATTGATCCGGAGAACTGTTTAAAGTC
ACCTAAGACAGTAAGAAACTGCCATCACGGAGTGCCCAGCAATCTGTGCCCCCAAACCAAGGAAGAGAAG
AAATACAAGTGTCCGTCATCCGAGGATATAAAATACAAAATGACGCGCGTTTCGGTGGATGCTGTGGATG
TATATCTGATAGAAAGTGGCACCGCTTTGCATGCCTGGATCTCTCCTATTCGTTTGGCAAATTGTAATCT
ACACGGCCAGCGGGTAAAGTCAGGGATCTCTGGGCTACTGCCCTCCATTCTATTGCGTTTGTTTATGCTC
CACACGACAAACTCGACATTCAACACCAACACCACGGGCAGTAATCGTTCTGGCAAGCTGAGACGAGCTG
ATCAGGATTCCCTAAAGTCTCAGGATGCAGGAGGATCTCATTACGCTTCACATGGAAAGACCGGAAAGCG
AAGCTCCAACTCCTTCTCACGGCGAGATTCTCGGGAGGAGGCCACGCGGAAGCTTAGGGGAAGTTTCTCG
GAAACGCATACCAAACGTACACCAGAAACAGAGATAACTGAAAATTGGGTGGAGGTGGGCTGCACTTCGC
TGGGGCCTATTTTGTTGGAGGGAGCGTCGGCTCTACCCATTCCGGATCACGAACTGCATCTCGTGCAGCA
CAAGTTTTCTGCGTGAGCACGATGCCAAATTCAAGCGACTTTGGTTCCTTTGGTCGAATAACGGAAGTGC
ATTGTCCTCGGGCAGTGAGATCTCGCGTTGTGGCTGTATTGGTGGATGTGCCTTTTTCGGAAGCAATCGA
AATGGACAAAAATTCTTTAAGCCAACGGCGCAGGATGCCCACGATAACTACAATATAGCGCGATACTTGC
ATTATTAACAACAATAAGGACTTTGGTTTCGGCGAAAGCATTCTGCACCAGGGTCAACTTGTCTTCCACA
CCCCGCCCTACAGTCTTCATTGTGTTTCGCTGTACGATACAGCGGATTTTAATGGCAAAGGCCGCCTGTA
CAGACCAGCAGGAGATCTGCGAAACGGAAGTCTGAAGAAGACGGATCTCTGCTCTCTGCCAGATGGCACT
AAGTTTAAGATCGGTGCAAGTGGTACCACTGGAGTTGAGAAACCTGAGCCCAACTGCCGAAATAAGTCTC
GAGAGAGCATTGGATCCCCGAATACGTTGGAAAGGAGGACCAAGCGCTATCCATGTACGAGACAAACTTC
CGTGGATGTACCCTATGCCCGCCTGCTCGATAGTCCCTCCAAAAAGCTACAGCTGCAGCACGAGGCGTCT
GCTGGCGATGCGGGAAGTAGCCATCGACGCGGTTCCGATTCCAATAGGCTGCGGGTTTCACCACCTAAGA
CTAGCATTTCCGACTCCAGACTGACGGGCGATGTCCTCGATGATGAAACAGAGGATACCGGATGAAATGT
CGCACTCGGCACCTCATTCACATCCGTTGGAATTCGAAATAGCCGATATTGCGCTTCACGTCCAGGGACT
AGGTGGAGATCAGTTGCCACGCGAAGTGCAACGCACTATATCGCTGACGTCGGAGAATCCCTCAGAGATG
TTCTTCTCTGCCGAAGAAGATATATCCAATGTGCTGTCTCAGCGGGGATCCATGAAGCAGCGAAACAGTG
TAAACAGTGGATCCGTGGTGCTGTCCGGAAAGAAGAGATTCTCATCGGATCTAAGGTGCCTATATAAAAG
TTATACCCAACCCCAACATATTTTGACTAACAATATATCTTCAAAACTAGCATTGGAGCTCAAAACGATA

ATGGAAGTCACACATTACCCACATATCGCAGCGATCTAGAGATCCACGCTCCCGACGGCAACAAAACACT
TCCAAAAAGGCCGCAGAGCACAACAGAACTG

>dme_circ_0000586_junction_seq

AAAAAGGCCGCAGAGCACAACAGAACTGGATATGGTTGCTTGCGTCGCTCCTGACC

>dme_circ_0000587

GAAGACATTCAGTTGACCTTTTAGCCTGGCCACCACCGACGTACAGACCGCTCCTCCCTCAGCCTGCAGA
CTCATGAACTCGCCATCATCAAATCCGTTCGTGGTCTTGTCCGATTCCTCCTCTTTCAAGAGCACACTGG
GGTCCCAGGGATGAGACTTCGGTCCACCCATAAATG

>dme_circ_0000587_junction_seq

GGATGAGACTTCGGTCCACCCATAAATGGAAGACATTCAGTTGACCTTTTAGCCTG

>dme_circ_0000588

GATATTGTCGTTGTTCACCTTGGCGATGCAGGACGTGTGGATGAAGTTTACCACCGAGAGCAGGTGCATT
GATTGAATTGTCGGGACGGCGGCCTCCACCATTCTACAAGACCAATAATGATTAAATAAAGCGAAATAAC
TTATAATTATTATTCAATAATTTTAGTTTATATCATTTTTTATAACTTTATTAAATATTTTTGTTGTCTA
AATATGCAGACAACATAAATATCCAAAAATAGGTTTTTTAAGAAATAATTCAAAAATTCTTAAATTTAAT
TTTAATTTGCATTACATTTTTTCAAATGTTTGTGATTTAAATAATAAAAACGGTATATTCGGTATATATT
TTATCTTGAACATTTCAGTATTTTTATTTTGAACGCATGGTATTTTTAACGGTCAAGGGCGGGCACACTT
TTGGGCCACCACACGTGTAGAAAAAACATATTATTGATAAAGTGCTAATTTGTGCAAGAATGAAAACACC
AAAAGCGAAAGAATCAGAAAAGGCTGAATTAAAGCCGATTAAAAAAGAGCCAGGCGTCGTGGAGGATGGT
AAAGTGACAAAGAAATCAAAGACGAAGCCCAAAAAGAAGACGGTTAAAGTGCCAAAAGATGAAGTAAAGG
CCGCTGTCCAGGAAAGTCCCAAAAAATTGTCCGCCAAAGACCTGGCCAAGATCGAAAAAAAGAAGGCCAA
GAAGCAGGCACAGAAGCTGAAGAAGCAGCAAAACAAACTGGCGCCCAAGGAGATCAAAACGGAACCAGAA
GCGCAGGTTAAAGTCGAAAGTAAGGCCACCAAAAAGGAAAAAGTGGAGAAGGAACCAACGACTGCTccaa
atgaaaaaccaaaaggaaaagtgtctaaaaaagccaaagccaatgccaatAACAAGGACGAAGGAAGGAG
TTAAGCGGATCCGCAATCCTGCAGAAGAGGCGTCCACCGTTTTCGTGGGGAATTTGCCAATCAACACCAA
ACGTGTACAGCTGGTGAAATTGTTCCAGCCATATGGGTTGGTTCAATCCATCCGGCTGCGCACCGCCGGT
GGTAAGCAGCTTTTCAAGCACAAACAGCGCAAGGTAGCCGGCTCCCTGAACGCCTACGTGGTGCTCGAGA
AACCCGAGATTGCCCAGCAAGCGCTGGCTCTTAATGGCTCCGAGTTCAAGGAAAATCACCTGCGGGTGAC
TCCCGCGTCTATGGCGGAGAAATTCGGCCAGGCAAAGGATAAGCAGCCCAGTGACAAGGATGCCAAGCGA
ACCATTTTCGTTGGCAGCCTAAAGTACTGCGGCCACCGAGGAGCAGCTGCGAGAGATCTTCTCCAGTTGC
GGCGAAATAGACTACATTCGCTGCCTGCAGGATGGCGACAAGGGCTGCAAAGGAGTAGCATACGTCTGTT
TCCAAAAGCCCGATGCTGTGGGACTAGCTCTTGAACTTAATCAGACGCTTCTAGATGATAGGCCTATCAA
CGTGGAGCGCTATCAAGTCAAAAAACTGGGTGCCAAGCAAGTGCGCGATGCTGCCGCCGCCTCAGCTGCT
TCGAAAACGTCCTCAAAGACCAAAGCTAAGAACCAGAATTCTGCGGGCGCaaaaaaaaGGCTAGACAAGA
AGAAGGGCAAGGAGAACGGTAGTTTGAAAAAAGAAGGAGCACCTACTGGTCAAAAGAAGAAGTCAGAGTA
CCGGGGCGTCAAAGTCGACGGCATCAAGAAGGCTAAGAAGCCTAAAAAGAAAAGCAATGACCAACAAACG
GCTCTTGCCAAAAAAATAGCACCCAAGCAAAAGAGTTAATATACATTTAGACTAGGCATAAGGTTTGAAA
TAAGTTGAAACTCAATTCAATGAGATTCGTTTGACTTTCTGTTACGAAGTTAACCACCGGATCAGTTTCC
GTAGCGGACACTAGATTGATTATGTTGACCAAGTAATCGTTGAAAGATTACTCAGAGAACAACTTAAAAA
TAAAATGCAAAATTTTAGAATCCCAGGCCGATTCCAAGGATTTGAGTGTCCTGAATGCTCCCTTGACCGA
TCCTGCCGACTATCCCGACCTCACAAATGGTCTTACCGATGCGGAGGTACAGGAGAGCCTCAAGGATCTA
ACCTTAGATGATCTTAACTCCTTGAACAAGCTACTTGATGAGCGCCTTGGCCGAGATGAAGATGTCGACT
CTGACGGCGAGTCTAACAACACATGGTAGACAAGCGATGGCCAATATACGCAAGCAGGACTTTCAGTCCA
TGGAAGTAGGTCTCGATGATAACTGCCGGGATGAGGAGGATTCCGATGAAACGAAGCCTAATTTGTGCAC

AAAGAGACCCACTTGCCCGACTACCAAGCGTTGCTCAAGGAAAAGCACTTGTGTGCCGAAAACCACTCGA
ATGTGCTCAACTAAAAGCGCCTACGATAGTTGCAAGCCAAAATCCGGTGGCTTTATGGACGACGATCTTA
TAAGCGAAACTACCAAATCTCCGAAGCCAAAGAACAAAAAGTGCAAGAAACCTAAGGATGACTTCGAATG
CGAGGCAGATGACTTTCTGTGCCAGGCCAGCAGGCGATCAAGGGGCAAGGAAAGTAGAAACACACTTTTG
GATGAAGAATATATTCCAGGGCGGCCGTTGACTGGAAATGCGCAGCTGGGTGATGAGCTCACATCAGCAT
TAAATGATCAAGGTGAACCATTGAATGATGCACAAATGGAACTATCAAACTCCGAGTTTGACAACAACTT
AGGCCTAGAAAATCCAGATAGTTTGGAGCAAGGTCAGCCTTTTGACTTAGGAAAGGATAGCAAGATTAAT
ACAGAGCAGAAACATCAAACTAATTTAAAAAACGAGTATAGTCGTGAAAATCTTATGCAGGATTACCCCA
AAAACTTGAAGCACGAACCTTCAGGCCAATTGAAAGAAGAAGATCAGGCCAACTTGGATCAGAACTATAA
TGCTAAGATTGATCAAGAACAATCTACTGGCTTAGAGCTAAAAAGCCAGTCCACTTTGGATCAAGAAGAT
CAGTTTAATTTAAACAAGGAATATCAGCCAAACTTAGATTTTACCAATCCGGTTAATTTCGAGCAGGAGC
ATCAGTCTAATTTCGATTCAGAATatcagaacaactttcaaaaagatcatctgaccaactgggagaaaga
acatctggcaaacttCGATGCTGGACAATCAGCTAGTTTGCATCAAGGTGAACAGCAGTTCAATCGGAAT
CAGAAGGCACCTAATGTGGAGGGAGTCGACAGGAGTCGACAAAACGAACCGATAGCGAATGAAGCAGATC
CGCTTGATCCTAACGATCGGCATCAGTCCTCCAGGACCTCACTTCGTAAGAGAGAACAAACAGAGAAGCA
ATTTGAGGAGCACATGCCCAATGAAGATGATAATAAACAGGCGAGTATCAAGGATGAGCTGATGCCACTT
AACACTGATCTGCCCAAGCAGGAACATCTCTTCGATAGCGAAAGGTTTTATTGCCAATAATGCTCGGGAT
CCACCTCGTTATTTGATTCAAATGCAAGACGACTATATACAAAGTGAAAATGATAGTGGCGAGCGTCGAT
TGCTTGAGGATCGAAGGGAAAGCGAGGCGTTAAACATGGACAAACTCACCGAGGAGGAGCCCAGAACAAT
GGAGGAATTCCATTTGGATTCCGAGTACAGGAGGAAAAAGCGCGAGAATAAGAATACTGATGACTCTCCC
GAGGAACTCCAAAGAAACATATATAAAGATGCTGATGGACTCTTTTCCGCGGGATCAGGGTGGCCGGATC
AACGCTCATGCGGCATTGAGGCAGTCCAATCTCGCCCATTTACGCATCAAGCGAAGTTGAATTATATATG
TATATTCAACGCTATACTTGATAATTTCCATTATATATATATTTTATATGCACCCATAAAATGATAATCA
AACTGCGATTTTGTTGTTCCTACGATTTGTAATTGCCAGCAAATGGCTCTTGTTTAAATATTATGCTGGT
AATGAAATTGAAATGAAATTATCGTATCATCATTTCCACTTACCTTTGTAATCCCTCCAGTAGAAGCGGT
GTAAGGAAGACATTCAGTTGACCTTTTAGCCTGGCCACCACCGACGTACAGACCGCTCCTCCCTCAGCCT
GCAGACTCATGAACTCGCCATCATCAAATCCGTTCGTGGTCTTGTCCGATTCCTCCTCTTTCAAGAGCAC
ACTGGGGTCCCAGGGATGAGACTTCGGTCCACCCATAAATG
>dme_circ_0000588_junction_seq
GGATGAGACTTCGGTCCACCCATAAATGGATATTGTCGTTGTTCACCTTGGCGATG
>dme_circ_0000589
GCATTTGGCGTGTTCGGAGTCGGATTCAGTTTTGAGATTCGGTGCTCTCACAGTCTCTGGTTAAGTTTCT
ATGCCAACGCCGACACAAAACTTATCAGCTACGATTAAAGCAGCGGAAGTGATTAGAGAACCGGAACGAT
TCGCACCGAAATCGGCGACGCACAAGAAATTAACTAAACAATTGCAAAATTCCCTTTGGCTAAGGATTTG
CAGCGGCCGTATTGCTTTTATGCTCTGCTGGCAGCTACAAAATTCCACGAAATTCCATTTTGGCAAACCG
AAGACAAACAAATCAATTTGCAGAGCCGCTGAAAAAcagcagcagcatcagcagcatcagcagcCCCATT
ACCAAAATTCGAAAATAATTGGAAAATATCGAAAGCAGCGTCGTCGTTGTCGCCGTTCTGGCGCCCTTCG
AACAGCTTGGATCCTGagagctaaggacaccgatacggacagagatacggacaaagatacggCCACGGAT
CGGGACAAGGCTATAGCATCACTGCTGGCACCATGGATCCGCAGCAGCAGTTCTGCCTCAAATGGAACAG
TTTCTCGTCTAACTTGGCAATAACATTCTCCAATCTGTTCAAATCGGATCTACTGGCCGATGTCATATTA
TCCTGCGATG
>dme_circ_0000589_junction_seq
CTGGCCGATGTCATATTATCCTGCGATGGCATTTGGCGTGTTCGGAGTCGGATTCA
>dme_circ_0000590

CCAATGCTTTCGATGCCCGTGCTCTCCGGCATTTTCAGGCATTCAAACAGCACTTTGCTTTTGTGTTCTG
GAATGGCAGTGACAATGATGGGCGAGTCCTGGGTGCAAGACTGTCCCTCTGTCTTCAGGCGTCGCAGTTG
AGCATGCGCTCTCCCAATATCGAGCGTTATGACCACCTCCTCCAACTGCTTCTCCGATGTCTCGATAAGA
ATGGGCTCTCCTTGCACATTG
>dme_circ_0000590_junction_seq
GATAAGAATGGGCTCTCCTTGCACATTGCCAATGCTTTCGATGCCCGTGCTCTCCG
>dme_circ_0000591
TAAGATTGTGTCGACCAGGGAGTCAAAGATCTGGATAGCACCCACCAGCAGCTACTATCTAACTGCTTTG
ATACCCAAGTCACGTAGAATTCAGGAGGTAACGCCGATTTGTGTGCTGAAGGCCATTAAGAACGATGTCG
AGATCGCAGGCTTCATCAACAGCCACATTCGCGATGGAGTCGCTTTGTGTCAGTATTTCGCCTGGCTTGA
GGATCAAGTGAATAAGGGTGCGGAAGTCGATGAAATGTCTGGTGCCGATAAACTCGAGTCCTTCCGGTCG
ACAAAGGATAAG
>dme_circ_0000591_junction_seq
CGAGTCCTTCCGGTCGACAAAGGATAAGTAAGATTGTGTCGACCAGGGAGTCAAAG
>dme_circ_0000592
ATAAGCGTTTTGCCTTACGCCTCAATTGGCATTGAAATCAGTAAGATTGTGTCGACCAGGGAGTCAAAGA
TCTGGATAGCACCCACCAGCAGCTACTATCTAACTGCTTTGATACCCAAGTCACGTAGAATTCAGGAGG
>dme_circ_0000592_junction_seq
ATACCCAAGTCACGTAGAATTCAGGAGGATAAGCGTTTTGCCTTACGCCTCAATTG
>dme_circ_0000593
GTGGTGTGCCAGCTTCAAGTGTAAAATCTCATAAGCGGGAAAAAAGCATAGGCAACGATTGGGGCAGTCC
AACTCCCTCGAGAAGGCAGAGGGACAAGAGCAAGCTGCGCTACAATGCAAATGCATTGCTGGCGGATCTC
ACCATCTTCCACATCCCAGGATTGGATGTAAAACTTCATTATCAGTCAAAAACACTGGCCGAGCAACTAA
CCGGAGATCAGTTGCCAACTGGACGACGAATGGGCAGCAAACGAGCAACTTTGTGCGCTTGGATGACTTT
ACAGA
>dme_circ_0000593_junction_seq
ACTTTGTGCGCTTGGATGACTTTACAGAGTGGTGTGCCAGCTTCAAGTGTAAAATC
>dme_circ_0000594
ATTAACACCTGTGTTGTGGGAGGGAGTTGGCGGAACACTACTCGATTGCCGAGCCGGAATGGGTTCTAGA
GTCTGCTCTAGGAATTCCAAAATATGGGGTGATATGATTGTTTCCTCTGGTATACTCTGTAAAGTCATCC
AAGCGCACAAAGTTGCTCGTTTGCTGCCCATTCGTCGTCCAGTTGGCAACTGATCTCCGG
>dme_circ_0000594_junction_seq
CGTCGTCCAGTTGGCAACTGATCTCCGGATTAACACCTGTGTTGTGGGAGGGAGTT
>dme_circ_0000595
GTGACCCCGAAGCATGGACCACAAATCACTGGCGGACAGCCGCGGTACCAAATTGGCGACATGGTCCGCG
TCAATTGCACCTCGGCGGCATCAAGGCCGGTCTGCCATCTCAGCTGGCTGATTAACGGGATGCACGCGAA
CCGTTCGCTGCTGCGACCTTATGAACCACTGATTGTGGGTCGCGAGGGTCTGGAGGTCGCCCGATTGGGC
CTGGAGTTCCGGGTGAGAGGATGACATTTCAAGCATGGCGACATGAAACTAAAGGGTAAGTTAGTGGTGA
AGTTTTCTCAATGCAGTTGGAAAATCTCTCTTTTCTAAACATTATGATGCAATGGTGTTTCTAGGaaaaa
aaaaTGTCCATTGTATTGCCTTATAGGTAATATCATTTTAAGGCAGATGTTTGAAGTTTGAAAGTTAGAA
GATttaaaatagtaaatcaaaaagttcataaaaaagaaaaatgtgcaaacttaaaaaGGTTACCAAATTG
ATTAAAAGTTAAATGTACCCCATTTAAGAGCCACAATAAAAGTTCCTGCCAAGGATATGCAATAAGCAGC
TTATGGGCAGCAGGAAAGGCTATGAAAATTGATACATTCATCAGCTATCCATCTCATACATATTTGCCAG
CGCACTGTTGCTCTGATTCAATTTGCGACAACATAACATGGCAGCTCATGTTGATTATTATCCATTCGGA

CCCAGTTGGCCAGCCACTCATCCACCGGCCATCACATCCCGCTGATCCGCTCCACCGAGCCAACCACCCA
AACCACTGCGAGTGCAAATGTGTGTTGAACAAGCCACAAAATTTCATGCGAACGCGGGCGGCAGACGAAG
TGCTCTTATGACATGTTCAGTATGCACAGTGGTTCCAAATAAAGTCACCTATTAATAGTCACAACTCTTA
TTAAATTTTTTATTTATTAATTGGTGTCTTAAAGCAGTTAATACAAATGTGAATAAATTCAGGGCCTTTG
ATACTAAATTGATCTGAATAAACATTTAAAAAGTTATAAACCTATCAATTTAGAAgatttcaacggtttc
aacgatttcTGCTACCATATAAAGTAACAAGCCTACCATAGTAAATTCTTGGTCAGTTTCAAAAGCCGAG
TCTATATAGACATGTCTGGATGTTCGTCTGTCCATTTGTCCGTCTGTTTCAACGCAAGCTGGTCCCTCAG
TTCTGCAGCGATCTGCATGAAACTACTCTAACAGGTCCCATTATATTGCATGTAGTATATATGTCGGAAC
GGATAGGATGGGACCACTATATCACATAGCTGTCAAAGGAAAAGAAATTAAAAGGAATATACCTAAAGTG
TTTTAAAACATAAAATGCTTGCTAGTTTCCTTTCTTGTTTATTTTAAATTTCCGTAAATaaaaaaaaaTA
TCTGTAAATATTATTTGAAGACATGAGTAACCACTGTGCAACGACCTTTCGGTCACCGTGTGGGTCACAG
TCCGTTTGCATATGTGGTGACGCAGGGTGTGCTGGTGTGCATACTTGTGTGTGTGCTGCGGAGATGTATG
CGGGTCATTTTCGGGTTATTTGGGCGAAAACGGGGGGAGTTAATGAAAGCATAAAACTTTTACTACGCCG
TCAAAAGGATGGCCAATCCGCCATGGCCCCCAATTCCCACAGCCCCCCACACATTTATCAATGTACACAG
ATTTGATGAAAGTTTTCCCTGCTCTTTCCATCTTGCATTTCCCGGCCATAATCCGACTGCAGGTGTGTCG
CCAAAATCTCCTCCGTGTACTGGCAGAGCAACGAGGAAAGCGTGGAGAGCGACAAGCACCAGAGGATTCC
GGTGCTGGAGTCGCGGGAGACGGTCATGTCCAAGTCCCGTCAGTCCATGGACAAGGCTCAGCTCGAAG
>dme_circ_0000595_junction_seq
CAGTCCATGGACAAGGCTCAGCTCGAAGGTGACCCCGAAGCATGGACCACAAATCA
>dme_circ_0000596
ATCATGGCCATTTCACACTGCGGCATCTCATCCACAACGCCCAAATCCATGAGCATCGTGCAGTGCCTGC
GGTAAGTTCACCCTAGCTTCCATCAACTGCCAGTTACTATAATGCATCGAGCTGCTCCTGCAGGCACATT
GTCCAGAACGAGGGTCCACGCGCCCTGTTCAAGGGTCTGGGTCCGAATCTCGTGGGCGTGGCCCCATCCC
GTGCCATTTACTTCTGCACCTACTCGCAAACCAAAAACACGCTCAACAGTCTGGGGCTTTGTTGAACGTG
ACTCGCCACTGGTGCACATCATGAGTGCAGCGAGTGCCGGCTTTGTCTCCTCCACGGCAACGAATCCCAT
TTGGTTTGTGAAGACGCGAATGCAGCTGGACTACAACTCCAAGGTGCAGATGACCGTGAGGCAGTGCATC
GAGAGGGTGTACGCCCAGGGAGGCGTGGCGGCCTTCTACAAGGGCATCACGGCCAGCTACTTTGGAATCT
GCGAGACGATGGTGCACTTTGTCATCTACGAGTTTATCAAGTCCAAGTTGGCTAGAGCAGCGAAATCAGC
GGCATACGGACACCAAGGGATCCCGCGACTTTCTGGAGTTCATGATGGCTGGCGCTGTTTCCAAAACCAT
CGCCTCCTGCATCGCCTATCCCCACGAAGTGGCACGCACTCGTCTGCGGGAGGAGGGCAACAAATACAAC
TCCTTCTGGCAGACCCTGCACACTGTTTGGAAGGAGGAGGGCAGAGCAGGACTCTACAGG
>dme_circ_0000596_junction_seq
GGAGGAGGGCAGAGCAGGACTCTACAGGATCATGGCCATTTCACACTGCGGCATCT
>dme_circ_0000597
TTTATTAAGGACTTCCCCATCGTCTCGATTGAGGATCCCTTTGACCAGGACCACTGGGAGGCCTGGTCCA
ACCTGACCGGCTGCACCGACATCCAGATCGTGGGCGATGATCTGACCGTGACCAACCCCAAGCGTATTGC
CACCGCCGTGGAGAAGAAGGCCTGCAACTGCCTGCTCCTGAAGGTCAACCAGATCGGCACCGTCACCGAG
TCCATTGCCGCCCATCTGCTGGCCAAGAAGAACGGCTGGGGCACCATGGTCTCCCACCGTTCCGGTGAGA
CCGAGGACTCG
>dme_circ_0000597_junction_seq
CCACCGTTCCGGTGAGACCGAGGACTCGTTTATTAAGGACTTCCCCATCGTCTCGA
>dme_circ_0000598
CTGGCCAACCTGTACCAGGAGTTTATTAAGGACTTCCCCATCGTCTCGATTGAGGATCCCTTTGACCAGG
ACCACTGGGAGGCCTGGTCCAACCTGACCGGCTGCACCGACATCCAGATCGTGGGCGATGATCTGACCG

>dme_circ_0000598_junction_seq
ATCCAGATCGTGGGCGATGATCTGACCGCTGGCCAACCTGTACCAGGAGTTTATTA
>dme_circ_0000599
GCCTCCTTGTTGGACTGGATGTTGGGGGCGAAGCCGCCCTCATCGCCCACAGCGGTGGCGTCCAGACCGA
ACTTGGCCTTGATCACATTCTTCAGGTGGTGGTACACCTCGGAGCCCATCTTCATGGCCTCTGTGAAGCT
GGTGGCGCCGGTGGGCAGGATCATGAACTCCTGCATGGCCAGCTTGTTGCCGGCGTGGCTGCCGCCGTTG
ATCACGTTGAAAGCGGGCACCGGCAGGATGATCTCCTTGTTGCCAGCCAGATCGGCAATGTGTTTGTACA
GCGGCACGCCCTTCTTGGCGGCTCCAGCCTTGGCCACGGCCAGGGAAACGCCCAGGATGGCGTTGGCTCC
GAACTTGCTCTTGTTCTCGGTGCCGTCCAACTTGATCATGAAGTT
>dme_circ_0000599_junction_seq
CGGTGCCGTCCAACTTGATCATGAAGTTGCCTCCTTGTTGGACTGGATGTTGGGGG
>dme_circ_0000600
AACAAGAGCAAGTTCGGAGCCAACGCCATCCTGGGCGTTTCCCTGGCCGTGGCCAAGGCTGGAGCCGCCA
AGAAGGGCGTGCCGCTGTACAAACACATTGCCGATCTGGCTGGCAACAAGGAGATCATCCTGCCGGTGCC
CGCTTTCAACGTGATCAACGGCGGCAGCCACGCCGGCAACAAGCTGGCCATGCAGGAGTTCATGATCCTG
CCCACCGGCGCCACCAGCTTCACAGAGGCCATGAAGATGGGCTCCGAGG
>dme_circ_0000600_junction_seq
ACAGAGGCCATGAAGATGGGCTCCGAGGAACAAGAGCAAGTTCGGAGCCAACGCCA
>dme_circ_0000601
GGGTGTCGTTCACATGGCCCACGGCCTTCAGCACCGACTTGCCATGGTAGTTGGCCTTGTCGTTGTCGCG
CAGCTCCAGGGCCTCGTGGACGCCAGTGGAGGCGCCGGAGGGCACGGCGGCTCGGAAGAGTCCCAGCTCG
GTGGTCAG
>dme_circ_0000601_junction_seq
CTCGGAAGAGTCCCAGCTCGGTGGTCAGGGGTGTCGTTCACATGGCCCACGGCCTT
>dme_circ_0000602
GCAATTGGAGCAAGTGCAAGAGGAATGCCTAGCTGCAGCTCGCGGACTGCTGGCCACCAAGGACGTGCTG
GTCATTCAAATGGACATGCTCGATCTCGACGAGCATAAGACGCATCTCAACACGGTGCTCAATCACTTTC
ATCGACTGGAT
>dme_circ_0000602_junction_seq
GGTGCTCAATCACTTTCATCGACTGGATGCAATTGGAGCAAGTGCAAGAGGAATGC
>dme_circ_0000603
TATAAAAGATGACTGAGAGCCGTTTGGAGCAGGAATAGGGAAGGGGCGAGGCACACCGGGGTTGTTCTCA
CAGTTTGCCCTCGCGAATCTTGTTCAGCGTCTTCTCGGTCATCAATTGAGCCAGAATCTTGCTCAGCGTG
GGATTCCGGGCGCAGTAGGCCAAGAGG
>dme_circ_0000603_junction_seq
GGGATTCCGGGCGCAGTAGGCCAAGAGGTATAAAAGATGACTGAGAGCCGTTTGGA
>dme_circ_0000604
GATACTTTGAGTGCAAGCCCAAGTACGGGGTGTTTGTACCTATTGCGAAGGTTTCACTGTCGCCGTCGTC
CAAGAAAACGCGTCTTTCCAGGACCGGATCAAGGGAGTCGCTCACCTCGATTGGCACCATGAACAGCATC
GCCACCACGGCCACGTCGCGCATGCGCATGAATGCTCAGGTATAAGCGGTGACCTCGAAAGGATCACCCT
CACCACCCAGTTCTCCCACAGAAAGAACACCCACAGTACCTTaaaaaaaaaaaGCAAACACAAATACCGT
TTAAAGTTCTGGTTCTGTTCTTTGTTAATTTTTATATTTTACCCATTAACCTACCTGTTACTTTTTATTT
GAGCCCTTTTACTAACGCGTTCCACATCTAATCGCAAATCCCTTCCGTTCCTAGCAGCGCAAGTCGAGCA
CGCCCGTTAAGCCAATTTTAGCGACGCCGAAAAGCCAATTTTCCATGCAGGTATGCTAACTCATCTACAA

GATCCACAAATAAATTGTATGGTATTCGCTGTTGGTTTTTCCTTTTCGCTCTTAAAAACTATTCGTTTGC
CGAGCTGTAGTCGCTGTTAGCAAACCCAATCCATTAACGTTAAAATTTTGCAAAATATTGTACGCAGAAT
TAATGGTTTGGCTATTAAATTAGGTTAAACAAAAAATGTATAAAGATTTTGACGTTAATGGATCTGTGCC
CTCTTCTGTATAATTGTGGTTTAACCCTTAGTTTGTAAGCATTTAACTAACATAATacacactaacccac
acaaaacacaacacaaaagacacaccccaaaaacaaaaaaaaactcccaccaaaacaaaCTATGTAAATT
AATAATAAATTTATGCCTATTAAGTCTCAATGCATTTTATTATGTCATGTGTTTGAGTTGGTTTATATAT
ATCACAATGTACAATAAAAATATTTAAATACTTCCTGCGGTATAAGCGGTGACCTCGAAAGGATCACCCT
CACCACCCAGTTCTCCCACAGAAAGAACACCCACAGTACCTTaaaaaaaaaaaGCAAACACAAATACCGT
TTAAAGTTCTGGTTCTGTTCTTTGTTAATTTTTATATTTTACCCATTAACCTACCTGTTACTTTTTATTT
GAGCCCTTTTACTAACGCGTTCCACATCTAATCGCAAATCCCTTCCGTTCCTAGCAGCGCAAGTCGAGCA
CGCCCGTTAAGCCAATTTTAGCGACGCCGAAAAGCCAATTTTCCATGCAGGTATGCTAACTCATCTACAA
GATCCACAAATAAATTGTATGGTATTCGCTGTTGGTTTTTCCTTTTCGCTCTTAAAAACTATTCGTTTGC
CGAGCTGTAGTCGCTGTTAGCAAACCCAATCCATTAACGTTAAAATTTTGCAAAATATTGTACGCAGAAT
TAATGGTTTGGCTATTAAATTAGGTTAAACAAAAAATGTATAAAGATTTTGACGTTAATGGATCTGTGCC
CTCTTCTGTATAATTGTGGTTTAACCCTTAGTTTGTAAGCATTTAACTAACATAATacacactaacccac
acaaaacacaacacaaaagacacaccccaaaaacaaaaaaaaactcccaccaaaacaaaCTATGTAAATT
AATAATAAATTTATGCCTATTAAGTCTCAATGCATTTTATTATGTCATGTGTTTGAGTTGGTTTATATAT
ATCACAATGTACAATAAAAATATTTAAATACTTCCTGCACGCTTTCGCTGTTCAAGTGTTGATTGTTCAA
TGTTTTTGTTAAATTTGCTTCTATTTGCATGTGTGTGCGTTCGCTGTCGCCGGTAGAAGGTAAAGCAACA
ATAAATTAATGAGTGTTTTTGCAACCAGCCCCGCACCCTATACACCTGTACTACATACACCCACACTATA
TATGTATCCGAATCTGTATCTATATCGTCTCGTATCTCTCTACTTCTTGTGTATCGTTATTTGTGTGCCT
AGGCATTTCATAAAAGCTTGCTTTGTCCTCTCTGTGCTCTGTTTATTTTTTGGTCGATCGATCGACATTG
TAGGATCTGCTGCGCGAGAAGCAACAACATGTGGAGAAGCTGATGGTGGAGCGCGACCTGGACCGCGAGG
ATGCCCAGAACCAGGCGCTGCAGCTGCAGAAGAACATCAACGAGGTAATTATCTTCACCGACACCATGTA
CGCACCGCTGCCCTATGCGGACAGATCGCGACCCTATCGACCCTCCAAGAAGTCGAGGGTTAAGGTTCCA
CAGCAACAACACATTTGGGTGCCCACCACCCTGagcagcagcattaccaccagtaccagtactagaccaa
ccatagcagcagcaacagcagcagcagcagcagcagcagcatgcaacagacagcccctgcagcagcagca
acagccgcatctgcatcagaagcagcagcagGCCAAGCCAAAGAAAGTTTGGTTTTGCGATAAAAAGAAA
CTCTTTCCAGTTGGCTGACTTCGCGGCTGATTCGGGCCCAGTTTGCAATGTTTGCTCATTCATTTGTGCA
GTTTCGGTTTCGATTAattgtgttttgtttttgtttgattttatttttgCATTGCACCCGCGCTCTGGCT
GTTTACAAAACAAACACACACAACCATTTTCTCGTTTTCACACAGCTGtttttttttATGATTTTACCCA
TCATTGTCTTGTGTTTATTTGTTGTTGATTTAACTCAAACTCATCTAATTTTTGGCACGTTACGTTTTAT
GTTTTGTTCTTTAACCACAGTGAAGACACTCAACTCATTCCCAATTGCCAGTCATTTTCCCAAATTGATT
CATCAAATTAACTTGAAACGCTGAGAAGAGTACAAATTTCATAAGTTTAACTAGGCATGAAAGGTTTTGG
GGGTTCAACAGCATTTAACTCGGTCAAAATTTAAAATAACAATGGGTTAAGGTTATTGCTGATATGTATA
CAAACGAAATTGATTTTACATCAACTTGTATACATACATTTTATAATAATTATTATTCTTTTTGCATTTT
GTGCTGGCGCAAAATTTTTTATTTACCAATAACTTGCATCTGCGAAGATTGAATATGCATAAGATAACAA
AATAAGTTTTCATGGGTTCATCTCAGTTCAGATACACAGATTTCTACACTTAAATTATCATATACGCAAT
CTCGAAATTACAGCAAGAAAAACGCAAAAACAACCTATTGTTAATTATTTGCATTCTTAATTATATACGT
TAGCACTTTTGCACTGTTTGCTTATCCGCACAGTGCGTTTGGAGAAAAAAAGCTAGCGCACACAATTATA
CCCTATTATTGTTTCCTAAATTTGAAGAAGGGACTGTTTACTTTTATGTGCTTTTCGCAACCGCTTTGGG
GCTTTTCTAGAAGACGACTGTCTTGTGGGATGATTGAGTTTTGTGCTTTCTGTTTGGTTTTGTTTTCAAT
AAATTGTTTGTGTTTATATTTATATGCGGATACGTGTTAGACTTTTCTATTTGTCGAGCGCACTTTGAGT
TTGAGTGTTAAATTAGCAACAAAATTATCACCTGAAATTCCCGGTCCCAGCCACACCAAATCCATATTTT

CCTGCCTTTCTCATCCACTGATCAAGCTATCCATCCTTTTTAACGCAAAACCAAAAAGATAATCTGACTA
CCAATTTGAACAAAATTAGGCTGGTCCTTAAGAGTTGAAATTCTGTTTGCACAAGACTATTTTTGTATGA
TTCTTATAGGGTATGCTAACTCATCTACAAGATCCACAAATAAATTGTATGGTATTCGCTGTTGGTTTTT
CCTTTTCGCTCTTAAAAACTATTCGTTTGCCGAGCTGTAGTCGCTGTTAGCAAACCCAATCCATTAACGT
TAAAATTTTGCAAAATATTGTACGCAGAATTAATGGTTTGGCTATTAAATTAGGTTAAACAAAAAATGTA
TAAAGATTTTGACGTTAATGGATCTGTGCCCTCTTCTGTATAATTGTGGTTTAACCCTTAGTTTGTAAGC
ATTTAACTAACATAATacacactaacccacacaaaacacaacacaaaagacacaccccaaaaacaaaaaa
aaactcccaccaaaacaaaCTATGTAAATTAATAATAAATTTATGCCTATTAAGTCTCAATGCATTTTAT
TATGTCATGTGTTTGAGTTGGTTTATATATATCACAATGTACAATAAAAATATTTAAATACTTCCTGCAC
GCTTTCGCTGTTCAAGTGTTGATTGTTCAATGTTTTTGTTAAATTTGCTTCTATTTGCATGTGTGTGCGT
TCGCTGTCGCCGGTAGAAGGTAAAGCAACAATAAATTAATGAGTGTTTTTGCAACCAGCCCCGCACCCTA
TACACCTGTACTACATACACCCACACTATATATGTATCCGAATCTGTATCTATATCGTCTCGTATCTCTC
TACTTCTTGTGTATCGTTATTTGTGTGCCTAGGCATTTCATAAAAGCTTGCTTTGTCCTCTCTGTGCTCT
GTTTATTTTTTGGTCGATCGATCGACATTGTAGGATCTGCTGCGCGAGAAGCAACAACATGTGGAGAAGC
TGATGGTGGAGCGCGACCTGGACCGCGAGGATGCCCAGAACCAGGCGCTGCAGCTGCAGAAGAACATCAA
CGAG
>dme_circ_0000604_junction_seq
GCTGCAGCTGCAGAAGAACATCAACGAGGATACTTTGAGTGCAAGCCCAAGTACGG
>dme_circ_0000605
GCTGtttttttttATGATTTTACCCATCATTGTCTTGTGTTTATTTGTTGTTGATTTAACTCAAACTCAT
CTAATTTTTGGCACGTTACGTTTTATGTTTTGTTCTTTAACCACAGTGAAGACACTCAACTCATTCCCAA
TTGCCAGTCATTTTCCCAAATTGATTCATCAAATTAACTTGAAACGCTGAGAAGAGTACAAATTTCATAA
GTTTAACTAGGCATGAAAGGTTTTGGGGGTTCAACAGCATTTAACTCGGTCAAAATTTAAAATAACAATG
GGTTAAGGTTATTGCTGATATGTATACAAACGAAATTGATTTTACATCAACTTGTATACATACATTTTAT
AATAATTATTATTCTTTTTGCATTTTGTGCTGGCGCAAAATTTTTTATTTACCAATAACTTGCATCTGCG
AAGATTGAATATGCATAAGATAACAAAATAAGTTTTCATGGGTTCATCTCAGTTCAGATACACAGATTTC
TACACTTAAATTATCATATACGCAATCTCGAAATTACAGCAAGAAAAACGCAAAAACAACCTATTGTTAA
TTATTTGCATTCTTAATTATATACGTTAGCACTTTTGCACTGTTTGCTTATCCGCACAGTGCGTTTGGAG
AAAAAAAGCTAGCGCACACAATTATACCCTATTATTGTTTCCTAAATTTGAAGAAGGGACTGTTTACTTT
TATGTGCTTTTCGCAACCGCTTTGGGGCTTTTCTAGAAGACGACTGTCTTGTGGGATGATTGAGTTTTGT
GCTTTCTGTTTGGTTTTGTTTTCAATAAATTGTTT
>dme_circ_0000605_junction_seq
GTTTGGTTTTGTTTTCAATAAATTGTTTGCTGtttttttttATGATTTTACCCATC
>dme_circ_0000606
CAATAACCTTAACCCATTGTTATTTTAAATTTTGACCGAGTTAAATGCTGTTGAACCCCCAAAACCTTTC
ATGCCTAGTTAAACTTATGAAATTTGTACTCTTCTCAGCGTTTCAAGTTAATTTGATGAATCAATTTGGG
AAAATGACTGGCAATTGGGAATGAGTTGAGTGTCTTCACTGTGGTTAAAGAACAAAACATAAAACGTAAC
G
>dme_circ_0000606_junction_seq
GTTAAAGAACAAAACATAAAACGTAACGCAATAACCTTAACCCATTGTTATTTTAA
>dme_circ_0000607
GTTTAACTAGGCATGAAAGGTTTTGGGGGTTCAACAGCATTTAACTCGGTCAAAATTTAAAATAACAATG
GGTTAAGGTTATTGCTGATATGTATACAAACGAAATTGATTTTACATCAACTTGTATACATACATTTTAT
AATAATTATTATTCTTTTTGCATTTTGTGCTGGCGCAAAATTTTTTATTTACCAATAACTTGCATCTGCG

AAGATTGAATATGCATAAGATAACAAAATAAGTTTTCATGGGTTCATCTCAGTTCAGATACACAGATTTC
TACACTTAAATTATCATATACGCAATCTCGAAATTACAGCAAGAAAAACGCAAAAACAACCTATTGTTAA
TTATTTGCATTCTTAATTATATACGTTAGCACTTTTGCACTGTTTGCTTATCCGCACAGTGCGTTTGGAG
AAAAAAAGCTAGCGCACACAATTATACCCTATTATTGTTTCCTAAATTTGAAGAAGGGACT
>dme_circ_0000607_junction_seq
ATTGTTTCCTAAATTTGAAGAAGGGACTGTTTAACTAGGCATGAAAGGTTTTGGGG
>dme_circ_0000608
TAAACAGTCCCTTCTTCAAATTTAGGAAACAATAATAGGGTATAATTGTGTGCGCTAGCTTTTTTTCTCC
AAACGCACTGTGCGGATAAGCAAACAGTGCAAAAGTGCTAACGTATATAATTAAGAATGCAAATAATTAA
CAATAGGTTGTTTTTGCGTTTTTCTTGCTG
>dme_circ_0000608_junction_seq
ATAGGTTGTTTTTGCGTTTTTCTTGCTGTAAACAGTCCCTTCTTCAAATTTAGGAA
>dme_circ_0000609
GCGAAGATGCGCAGAAGATTGCCGACCTTAAGACCCTTGTGGAAGCCATCCAGGTGGCTAATGCCAATAT
ATCAGCTACAAATGCGGAGCTCTCCACTGTATTGGAAGTTCTTCAGGCGGAGAAGAGTGAAACAAATCAC
ATATTCGAGCTCTTTGAAATGGAAGCCGATATGAACTCAGAGCGGTTGATCGAAAAAGTTACTGGGATTA
AGGAGGAACTAAAGGAAACCCATCTGCAACTGGATGAGCGACAGAAAAAGTTCGAGGAGTTGGAGGAGAA
ATTGAAGCAAGCTCAGCAAAGTGAACAAAA
>dme_circ_0000609_junction_seq
TGAAGCAAGCTCAGCAAAGTGAACAAAAGCGAAGATGCGCAGAAGATTGCCGACCT
>dme_circ_0000610
GTTACTGGGATTAAGGAGGAACTAAAGGAAACCCATCTGCAACTGGATGAGCGACAGAAAAAGTTCGAGG
AGTTGGAGGAGAAATTGAAGCAAGCTCAGCAAAGTGAACAAAAGTTGCAACAGGAGTCTCAGACTTCCAA
GGAGAAACTTACGGAAATACAACAATCCTTGCAAGAACTCCAAGATTCT
>dme_circ_0000610_junction_seq
ACAATCCTTGCAAGAACTCCAAGATTCTGTTACTGGGATTAAGGAGGAACTAAAGG
>dme_circ_0000611
GCTCAGCAAAGTGAACAAAAGTTGCAACAGGAGTCTCAGACTTCCAAGGAGAAACTTACGGAAATACAAC
AATCCTTGCAAGAACTCCAAGATTCTGTAAAGCAAAAGGAAGAACTTGTCCAGAACTTGGAAGAAAAGGT
TAGGGAAAGCAGTTCCATCATAGAAGCGCAGAACACGAAACTAAATGAAAGCAAT
>dme_circ_0000611_junction_seq
GCAGAACACGAAACTAAATGAAAGCAATGCTCAGCAAAGTGAACAAAAGTTGCAAC
>dme_circ_0000612
GTTAGAACAGGCCCAGCAAAAGGAGAGGACTCTTCAAGAGGAAACATCCAAGTTGGCGGAGCAACTGAGC
CAATTAAAGCAGGCTAATGAAGAGCTCCAGAAATCCCTTCAGCAAAAGCAGTTACTTTTGGAAAAGGGTA
ATGAATTCGACACCCAGCTGGCAGAATATCAGAAAGTCATTGATGAGATGGATGATGCGGCTTCCGTTAA
ATCCGCGCTGCTGGAACAGCTACAAAATAGA
>dme_circ_0000612_junction_seq
CGCGCTGCTGGAACAGCTACAAAATAGAGTTAGAACAGGCCCAGCAAAAGGAGAGG
>dme_circ_0000613
GATACCCCTTCTGACTAGAAACATTGAGGATTCCGTGCAAACATGACCCTCTTGCCAGACATCAAGGCAT
CGGATGCCGCCTGCTGCGTGGGAGGTGCGAACAGCGATGCCAGCAGCAGTTCCGGCGTCTGCAGCCTCAA
GGGTCACCATGGTGACCCCGACAGCGGTTGCCTGGGCATCAACTGTTGCACCACGGCGGCCAGCTCGGAG
CTGTCCATCGACGAGACGTCATCGACCTCGTCCAGAGGCTCCTGCGAGTTGACCAGCAAGGTGACCAACG

ACCTGAACGGATTCCAGAGTCCGAACTACCACCAGGCGGTGGCGCACGCCAACTTCGACCACTGCGATCC
CGTGGACATCCAGTTCGCCGACGTGCGCTACACAGTGAAGAAGTTCTCCTTCCCGGAGCGGAAGTTCGGT
GGGTACCTCTGCCTCTGATCACTCTGAATGTTCTATATTAAAATATTTCCTTATTGTGCCGCAGTCACCA
AGGAGATCCTTCATGGCCTGAACGGCAGCTTCCGGTCTGGCGAACTCACAGCCATCATGGGTCCTTCGGG
CGCCGGCAAGAGCACGCTGCTGAATGTGATGTCCGGATTTTG
>dme_circ_0000613_junction_seq
CGCTGCTGAATGTGATGTCCGGATTTTGGATACCCCTTCTGACTAGAAACATTGAG
>dme_circ_0000614
GATACCCCTTCTGACTAGAAACATTGAGGATTCCGTGCAAACATGACCCTCTTGCCAGACATCAAGGCAT
CGGATGCCGCCTGCTGCGTGGGAGGTGCGAACAGCGATGCCAGCAGCAGTTCCGGCGTCTGCAGCCTCAA
GGGTCACCATGGTGACCCCGACAGCGGTTGCCTGGGCATCAACTGTTGCACCACGGCGGCCAGCTCGGAG
CTGTCCATCGACGAGACGTCATCGACCTCGTCCAGAGGCTCCTGCGAGTTGACCAGCAAGGTGACCAACG
ACCTGAACGGATTCCAGAGTCCGAACTACCACCAGGCGGTGGCGCACGCCAACTTCGACCACTGCGATCC
CGTGGACATCCAGTTCGCCGACGTGCGCTACACAGTGAAGAAGTTCTCCTTCCCGGAGCGGAAGTTCGGG
TGAGTCCTCCGCTCGGCTGAACTTGAAAGTCGACATGCACGTTGCGGGCAGCTTCGCTTTTAATTTATGG
TTATATCGCTGAATATGCAATCTGTTTCGTGCCATTTAATTACACATTGGACACATCGGCTGTGCATATA
AAGTGCAGACGTCGCAGTCGTTCAATCAATCAGCACATTGTTTGAGTGTCGGCAATACAGTAAGACAGCT
GCATTTGGCTAGCTATATAATTAAATTTTCACATGGCTGGGTTAGAGATCGAAGGGTCTTGTGGAATTTC
ACCTAGTTCTAGTATCTGCCCATGCAGCTTAGAAAGCATTTGATTGTAAGGTGTAGGAATTGCATTTAGA
TTTCCTCTGCACTCCGCTAAATGTCTAAACTGATAGCTGAACTAACTGAATGTGCGAAATACACATGAAT
CACAATCGCGCGTTTCGTTTGACGTCTGGAAGGCAGTGCACTTGCGATTGCCGTTCCATCCCGGACATAA
TCTCATCACTTCCTCCGGCAGCTCGGTTCAAGTTCTCAGCCATTGCATAATTAGGACTTCAGTCGCGAAT
GGGAGGCAATCTCTCTGAGTCACAAGCCCGCCAATTGACGTATCTCGCAGGAAATCGCCGTTTAATCGCA
GCCCTCTTTTGATGGCCAACTGACAGCTATTACAGCATTTTTAGTAAATAAACAGCAATTTATACATGCA
CCGAACGTGCAGAGGGATGAACTGTGAGTCTGGCCAGTGGAGCTATTAACTGCAGGCTGCTGCCTTAATT
ATCATGATTTTAGTAATGGACGTGGTGGTAAAGACGATGCCCCAGGCGGTACACGGGTTTTCATTGCTAA
ACGTTATCGGTCAGTAAACTATGAACTTGAAAGTGGCGCATGAGCATAATATACACACATATGTAGCTCG
ACTCACTTCCCCGAACTAGACGCTTGTGATTGAAGCTATTTTTCATTCAGTCCAGCCCTTGTTGAACCCA
TGTACGTTATTGTACCTAAATTAACCGCTTCGCCCGATTATATATTGTGATTTTTGTTCGACACATTTTC
ACTCGGTACTCGACCACGCCTCTTTTTCTGCAGGCTCCACTGGCGTGTCGGGCGATATCCGGGTTAATAG
AAAGCCCATGGCTCCCAGCTCCGAGAGGTTCCGCCAAATGCTGTGCTACATCCACCAGGACGATCTGCTG
CGTCCACAGCTGTTGGTCGGCGAGATAATGCTGCTGGCGGCACATCTGAAGCTGGGCTTCAAGGTCACCA
AGGCGTACAAGATGGATCTG
>dme_circ_0000614_junction_seq
GGTCACCAAGGCGTACAAGATGGATCTGGATACCCCTTCTGACTAGAAACATTGAG
>dme_circ_0000615
GATACCCCTTCTGACTAGAAACATTGAGGATTCCGTGCAAACATGACCCTCTTGCCAGACATCAAGGCAT
CGGATGCCGCCTGCTGCGTGGGAGGTGCGAACAGCGATGCCAGCAGCAGTTCCGGCGTCTGCAGCCTCAA
GGGTCACCATGGTGACCCCGACAGCGGTTGCCTGGGCATCAACTGTTGCACCACGGCGGCCAGCTCGGAG
CTGTCCATCGACGAGACGTCATCGACCTCGTCCAGAGGCTCCTGCGAGTTGACCAGCAAGGTGACCAACG
ACCTGAACGGATTCCAGAGTCCGAACTACCACCAGGCGGTGGCGCACGCCAACTTCGACCACTGCGATCC
CGTGGACATCCAGTTCGCCGACGTGCGCTACACAGTGAAGAAGTTCTCCTTCCCGGAGCGGAAGTTCGGG
TGAGTCCTCCGCTCGGCTGAACTTGAAAGTCGACATGCACGTTGCGGGCAGCTTCGCTTTTAATTTATGG
TTATATCGCTGAATATGCAATCTGTTTCGTGCCATTTAATTACACATTGGACACATCGGCTGTGCATATA

AAGTGCAGACGTCGCAGTCGTTCAATCAATCAGCACATTGTTTGAGTGTCGGCAATACAGTAAGACAGCT
GCATTTGGCTAGCTATATAATTAAATTTTCACATGGCTGGGTTAGAGATCGAAGGGTCTTGTGGAATTTC
ACCTAGTTCTAGTATCTGCCCATGCAGCTTAGAAAGCATTTGATTGTAAGGTGTAGGAATTGCATTTAGA
TTTCCTCTGCACTCCGCTAAATGTCTAAACTGATAGCTGAACTAACTGAATGTGCGAAATACACATGAAT
CACAATCGCGCGTTTCGTTTGACGTCTGGAAGGCAGTGCACTTGCGATTGCCGTTCCATCCCGGACATAA
TCTCATCACTTCCTCCGGCAGCTCGGTTCAAGTTCTCAGCCATTGCATAATTAGGACTTCAGTCGCGAAT
GGGAGGCAATCTCTCTGAGTCACAAGCCCGCCAATTGACGTATCTCGCAGGAAATCGCCGTTTAATCGCA
GCCCTCTTTTGATGGCCAACTGACAGCTATTACAGCATTTTTAGTAAATAAACAGCAATTTATACATGCA
CCGAACGTGCAGAGGGATGAACTGTGAGTCTGGCCAGTGGAGCTATTAACTGCAGGCTGCTGCCTTAATT
ATCATGATTTTAGTAATGGACGTGGTGGTAAAGACGATGCCCCAGGCGGTACACGGGTTTTCATTGCTAA
ACGTTATCGGTCAGTAAACTATGAACTTGAAAGTGGCGCATGAGCATAATATACACACATATGTAGCTCG
ACTCACTTCCCCGAACTAGACGCTTGTGATTGAAGCTATTTTTCATTCAGTCCAGCCCTTGTTGAACCCA
TGTACGTTATTGTACCTAAATTAACCGCTTCGCCCGATTATATATTGTGATTTTTGTTCGACACATTTTC
ACTCGGTACTCGACCACGCCTCTTTTTCTGCAGGCTCCACTGGCGTGTCGGGCGATATCCGGGTTAATAG
AAAGCCCATGGCTCCCAGCTCCGAGAGGTTCCGCCAAATGCTGTGCTACATCCACCAGGACGATCTGCTG
CGTCCACAGCTGTTGGTCGGCGAGATAATGCTGCTGGCGGCACATCTGAAGCTGGGCTTCAAGGTCACCA
AGGCGTACAAGATGGATCTGGATCAAGCACATCTTATCGCTGCTGGGTCTGGACCATCGCTACAATGTGC
CCACTGGGAAGCTTTCGGGTGGCCAGAAGAAGCGACTCGCAATCGCCCTGGAGCTGATAAGTAATCCTCC
CGTGCTATATCTGGATGAGCCGACGAC
>dme_circ_0000615_junction_seq
CCGTGCTATATCTGGATGAGCCGACGACGATACCCCTTCTGACTAGAAACATTGAG
>dme_circ_0000616
GATACCCCTTCTGACTAGAAACATTGAGGATTCCGTGCAAACATGACCCTCTTGCCAGACATCAAGGCAT
CGGATGCCGCCTGCTGCGTGGGAGGTGCGAACAGCGATGCCAGCAGCAGTTCCGGCGTCTGCAGCCTCAA
GGGTCACCATGGTGACCCCGACAGCGGTTGCCTGGGCATCAACTGTTGCACCACGGCGGCCAGCTCGGAG
CTGTCCATCGACGAGACGTCATCGACCTCGTCCAGAGGCTCCTGCGAGTTGACCAGCAAGGTGACCAACG
ACCTGAACGGATTCCAGAGTCCGAACTACCACCAGGCGGTGGCGCACGCCAACTTCGACCACTGCGATCC
CGTGGACATCCAGTTCGCCGACGTGCGCTACACAGTGAAGAAGTTCTCCTTCCCGGAGCGGAAGTTCGGG
TGAGTCCTCCGCTCGGCTGAACTTGAAAGTCGACATGCACGTTGCGGGCAGCTTCGCTTTTAATTTATGG
TTATATCGCTGAATATGCAATCTGTTTCGTGCCATTTAATTACACATTGGACACATCGGCTGTGCATATA
AAGTGCAGACGTCGCAGTCGTTCAATCAATCAGCACATTGTTTGAGTGTCGGCAATACAGTAAGACAGCT
GCATTTGGCTAGCTATATAATTAAATTTTCACATGGCTGGGTTAGAGATCGAAGGGTCTTGTGGAATTTC
ACCTAGTTCTAGTATCTGCCCATGCAGCTTAGAAAGCATTTGATTGTAAGGTGTAGGAATTGCATTTAGA
TTTCCTCTGCACTCCGCTAAATGTCTAAACTGATAGCTGAACTAACTGAATGTGCGAAATACACATGAAT
CACAATCGCGCGTTTCGTTTGACGTCTGGAAGGCAGTGCACTTGCGATTGCCGTTCCATCCCGGACATAA
TCTCATCACTTCCTCCGGCAGCTCGGTTCAAGTTCTCAGCCATTGCATAATTAGGACTTCAGTCGCGAAT
GGGAGGCAATCTCTCTGAGTCACAAGCCCGCCAATTGACGTATCTCGCAGGAAATCGCCGTTTAATCGCA
GCCCTCTTTTGATGGCCAACTGACAGCTATTACAGCATTTTTAGTAAATAAACAGCAATTTATACATGCA
CCGAACGTGCAGAGGGATGAACTGTGAGTCTGGCCAGTGGAGCTATTAACTGCAGGCTGCTGCCTTAATT
ATCATGATTTTAGTAATGGACGTGGTGGTAAAGACGATGCCCCAGGCGGTACACGGGTTTTCATTGCTAA
ACGTTATCGGTCAGTAAACTATGAACTTGAAAGTGGCGCATGAGCATAATATACACACATATGTAGCTCG
ACTCACTTCCCCGAACTAGACGCTTGTGATTGAAGCTATTTTTCATTCAGTCCAGCCCTTGTTGAACCCA
TGTACGTTATTGTACCTAAATTAACCGCTTCGCCCGATTATATATTGTGATTTTTGTTCGACACATTTTC
ACTCGGTACTCGACCACGCCTCTTTTTCTGCAGGCTCCACTGGCGTGTCGGGCGATATCCGGGTTAATAG

AAAGCCCATGGCTCCCAGCTCCGAGAGGTTCCGCCAAATGCTGTGCTACATCCACCAGGACGATCTGCTG
CGTCCACAGCTGTTGGTCGGCGAGATAATGCTGCTGGCGGCACATCTGAAGCTGGGCTTCAAGGTCACCA
AGGCGTACAAGATGGATCTGGATCAAGCACATCTTATCGCTGCTGGGTCTGGACCATCGCTACAATGTGC
CCACTGGGAAGCTTTCGGGTGGCCAGAAGAAGCGACTCGCAATCGCCCTGGAGCTGATAAGTAATCCTCC
CGTGCTATATCTGGATGAGCCGACGACCGTGAGTGACACTATATATTGTAAAAGCACACTTATGTATTTA
GAGTAGCCATGATAGTGATCATCTCCAGCATTCTGATGCTCATGGCGATGATGAGCAAACTTTGCCTGGC
GGAAGTTCAGCaaaaaaaaaaaaaaCACCAACTGACCCACAAGTCAAGTTTGTCCCCATCTATATGTCTA
TATGACTTAGAGCTTATCAATTGTCCAGATAGTCTGTTTAATAGTTTCGACTTGTTTTGATTAGTGGTGT
TCGGTGAGCTGTAAACAGTTTATTATTTAAAttttttttattgattattcatttaattaCTGCGCTATCT
GGCAGATAATCCCGTAATTAATTAAATTGTTCATTAGTTGTAGATTACTTTAATAGGGATTGATGAGTCT
GCTGTGGTTATATGAACCTACAAAATCGGATTTATGCAGCAATAAAGGGCCAGCTATTTTGAGTCGTCTG
GCTGGGGATTATGAGTATGAGTCCCTCCTTTTCATGTGGCATGACTAAGTATAAGTTGATTCAGACAACA
ATCTCAAAGTGAAAATCATGACTGGATACTTCGCAAACAAAGGGAGTTGCCAGTTCCAGAAATATCTCAA
GCTTTATTTTTAAGACTTGCTTTAAAATCATATGCTTTTCTTCTTAGACCTCACTTTTCATCATTCATTG
GCAATTAGTAAACTCGAAGTCGTGAGATTCATTTAAAGAAGGGTATTAGGCAAAGTGTATACTTGAATTT
GATATATATGGATACGAGTAATTCAAATAATACGTATGTCTCGATTTCAGGCAGTTAATAACACTTGAAA
GAACTCGTTTTTCAAAGTGTCAAATAAAATGCCGACTGCAAGTCGATCAAATCTTTGCAATGCCGCCCGA
CTTGCATCGGCAAATGGATTTTGGGAACAAACTTGTTTCTCGTTTTCATTTGACTTCGCTACAAGTACAA
GTTCAAGCCGCAATCTCTCACCTACCATTAAAGTTTCAGTTGCCCTGTTGCTTGATGTTCTTTGCTGTCA
GTTAGACAGCGGTTTAGTTTAATGGGGTTGTTATTCGTTAGATCTTCAAAGGTTAGGCAGCAATTAAACA
AAGTTGAGTCACACCGAAAGTTGTATTTCTGCTCGTTTGTTCGGATTCCAGGTGGCCTGGACAGCTCCTC
GTGCAGCTCCTGCGTGGCTCTGCTGAAGAAACTGGCGTCGCAGGGCCACACGATAGTCTGCACCATCCAT
CAGCCAAGTGCCCTCATCTTCGAGATGTTCGACAAGCTCTACACCGTCGTCGATGGCCACTGCATGTACC
AAGGACCTGTGCGCGAACTGGTGCCCTTCCTGGCCGACCAGCAGCTCGTCTGCCCGAGTTACCACAACCC
AGCTGACTATCGTACTGGAAGTGGCCGTGGGCGAGCATCAACGTGACCTGAATGAGCTAATCCATGCGGC
CAATAAAAAGTATTACGAGGATGTGGATCGCCATAGGTATATGAGCAGTGATGATATGGCACGCCTCGTG
GAAAGCATTAAAGGGTGAGTTCTATACTGATCAGATCTGTTGTCATGTTTCGGGTATTAAATTATTCTAA
ATATAAAAGTTTGAATGAAGTTTTATGCGAATATTGTTACGGTCCAGTGATAACGATCTTTATTTGTCCA
CGTTTTAAAGTTGAGTCGTTAAAGTAGAGGAGGTGTGCTACTAGTTTCGGTGTTATAAATGCGTTAGCTA
TTAAATCTGTCAAGCAATTTCGAACATACATGTATACACATGTATACAAGCAAAGCttttttttttttt
ttAAGGAAAACTGCCAATTATTGGCGCATGGTGCAGAAATTTCCCCCACTAGAGCTGCCACTTACTAACT
TGAACTTTCTCCAAATTGCAGGAAAACATGGGCGGCAAGGCAGTGGTAAAAACCAGTGAAGCGCTGGCAG
CATTTGCGGCGGCGCAATTCTCCAGCTTCGACTATGTAAAGCCCTCGCCGCAGGAGCTGGCTCTGGAGGA
GATCAAGGCACTGAGCGGCGGCCCCGAGAGCGCGGATCCCGATCTCCTCGAGAAAAATCTGAGGCCACAG
CCACAGCCGCTTGCCAAAGCCGGTGAGCTTGCCAGGCCGCCGAATGCCATTCGATCGGCCTCGTTCCTCA
TGCAGTATGTGCTCCTGATGCAGCGCATCTTGATTTGCGCCAAGCGAAACTAC

>dme_circ_0000616_junction_seq

CATCTTGATTTGCGCCAAGCGAAACTACGATACCCCTTCTGACTAGAAACATTGAG

>dme_circ_0000617

GATACCCCTTCTGACTAGAAACATTGAGGATTCCGTGCAAACATGACCCTCTTGCCAGACATCAAGGCAT
CGGATGCCGCCTGCTGCGTGGGAGGTGCGAACAGCGATGCCAGCAGCAGTTCCGGCGTCTGCAGCCTCAA
GGGTCACCATGGTGACCCCGACAGCGGTTGCCTGGGCATCAACTGTTGCACCACGGCGGCCAGCTCGGAG
CTGTCCATCGACGAGACGTCATCGACCTCGTCCAGAGGCTCCTGCGAGTTGACCAGCAAGGTGACCAACG
ACCTGAACGGATTCCAGAGTCCGAACTACCACCAGGCGGTGGCGCACGCCAACTTCGACCACTGCGATCC

CGTGGACATCCAGTTCGCCGACGTGCGCTACACAGTGAAGAAGTTCTCCTTCCCGGAGCGGAAGTTCGGG
TGAGTCCTCCGCTCGGCTGAACTTGAAAGTCGACATGCACGTTGCGGGCAGCTTCGCTTTTAATTTATGG
TTATATCGCTGAATATGCAATCTGTTTCGTGCCATTTAATTACACATTGGACACATCGGCTGTGCATATA
AAGTGCAGACGTCGCAGTCGTTCAATCAATCAGCACATTGTTTGAGTGTCGGCAATACAGTAAGACAGCT
GCATTTGGCTAGCTATATAATTAAATTTTCACATGGCTGGGTTAGAGATCGAAGGGTCTTGTGGAATTTC
ACCTAGTTCTAGTATCTGCCCATGCAGCTTAGAAAGCATTTGATTGTAAGGTGTAGGAATTGCATTTAGA
TTTCCTCTGCACTCCGCTAAATGTCTAAACTGATAGCTGAACTAACTGAATGTGCGAAATACACATGAAT
CACAATCGCGCGTTTCGTTTGACGTCTGGAAGGCAGTGCACTTGCGATTGCCGTTCCATCCCGGACATAA
TCTCATCACTTCCTCCGGCAGCTCGGTTCAAGTTCTCAGCCATTGCATAATTAGGACTTCAGTCGCGAAT
GGGAGGCAATCTCTCTGAGTCACAAGCCCGCCAATTGACGTATCTCGCAGGAAATCGCCGTTTAATCGCA
GCCCTCTTTTGATGGCCAACTGACAGCTATTACAGCATTTTTAGTAAATAAACAGCAATTTATACATGCA
CCGAACGTGCAGAGGGATGAACTGTGAGTCTGGCCAGTGGAGCTATTAACTGCAGGCTGCTGCCTTAATT
ATCATGATTTTAGTAATGGACGTGGTGGTAAAGACGATGCCCCAGGCGGTACACGGGTTTTCATTGCTAA
ACGTTATCGGTCAGTAAACTATGAACTTGAAAGTGGCGCATGAGCATAATATACACACATATGTAGCTCG
ACTCACTTCCCCGAACTAGACGCTTGTGATTGAAGCTATTTTTCATTCAGTCCAGCCCTTGTTGAACCCA
TGTACGTTATTGTACCTAAATTAACCGCTTCGCCCGATTATATATTGTGATTTTTGTTCGACACATTTTC
ACTCGGTACTCGACCACGCCTCTTTTTCTGCAGGCTCCACTGGCGTGTCGGGCGATATCCGGGTTAATAG
AAAGCCCATGGCTCCCAGCTCCGAGAGGTTCCGCCAAATGCTGTGCTACATCCACCAGGACGATCTGCTG
CGTCCACAGCTGTTGGTCGGCGAGATAATGCTGCTGGCGGCACATCTGAAGCTGGGCTTCAAGGTCACCA
AGGCGTACAAGATGGATCTGGATCAAGCACATCTTATCGCTGCTGGGTCTGGACCATCGCTACAATGTGC
CCACTGGGAAGCTTTCGGGTGGCCAGAAGAAGCGACTCGCAATCGCCCTGGAGCTGATAAGTAATCCTCC
CGTGCTATATCTGGATGAGCCGACGACCGTGAGTGACACTATATATTGTAAAAGCACACTTATGTATTTA
GAGTAGCCATGATAGTGATCATCTCCAGCATTCTGATGCTCATGGCGATGATGAGCAAACTTTGCCTGGC
GGAAGTTCAGCaaaaaaaaaaaaaaCACCAACTGACCCACAAGTCAAGTTTGTCCCCATCTATATGTCTA
TATGACTTAGAGCTTATCAATTGTCCAGATAGTCTGTTTAATAGTTTCGACTTGTTTTGATTAGTGGTGT
TCGGTGAGCTGTAAACAGTTTATTATTTAAAttttttttattgattattcatttaattaCTGCGCTATCT
GGCAGATAATCCCGTAATTAATTAAATTGTTCATTAGTTGTAGATTACTTTAATAGGGATTGATGAGTCT
GCTGTGGTTATATGAACCTACAAAATCGGATTTATGCAGCAATAAAGGGCCAGCTATTTTGAGTCGTCTG
GCTGGGGATTATGAGTATGAGTCCCTCCTTTTCATGTGGCATGACTAAGTATAAGTTGATTCAGACAACA
ATCTCAAAGTGAAAATCATGACTGGATACTTCGCAAACAAAGGGAGTTGCCAGTTCCAGAAATATCTCAA
GCTTTATTTTTAAGACTTGCTTTAAAATCATATGCTTTTCTTCTTAGACCTCACTTTTCATCATTCATTG
GCAATTAGTAAACTCGAAGTCGTGAGATTCATTTAAAGAAGGGTATTAGGCAAAGTGTATACTTGAATTT
GATATATATGGATACGAGTAATTCAAATAATACGTATGTCTCGATTTCAGGCAGTTAATAACACTTGAAA
GAACTCGTTTTTCAAAGTGTCAAATAAAATGCCGACTGCAAGTCGATCAAATCTTTGCAATGCCGCCCGA
CTTGCATCGGCAAATGGATTTTGGGAACAAACTTGTTTCTCGTTTTCATTTGACTTCGCTACAAGTACAA
GTTCAAGCCGCAATCTCTCACCTACCATTAAAGTTTCAGTTGCCCTGTTGCTTGATGTTCTTTGCTGTCA
GTTAGACAGCGGTTTAGTTTAATGGGGTTGTTATTCGTTAGATCTTCAAAGGTTAGGCAGCAATTAAACA
AAGTTGAGTCACACCGAAAGTTGTATTTCTGCTCGTTTGTTCGGATTCCAGGTGGCCTGGACAGCTCCTC
GTGCAGCTCCTGCGTGGCTCTGCTGAAGAAACTGGCGTCGCAGGGCCACACGATAGTCTGCACCATCCAT
CAGCCAAGTGCCCTCATCTTCGAGATGTTCGACAAGCTCTACACCGTCGTCGATGGCCACTGCATGTACC
AAGGACCTGTGCGCGAACTGGTGCCCTTCCTGGCCGACCAGCAGCTCGTCTGCCCGAGTTACCACAACCC
AGCTGACTATCGTACTGGAAGTGGCCGTGGGCGAGCATCAACGTGACCTGAATGAGCTAATCCATGCGGC
CAATAAAAAGTATTACGAGGATGTGGATCGCCATAGGTATATGAGCAGTGATGATATGGCACGCCTCGTG
GAAAGCATTAAAGGGTGAGTTCTATACTGATCAGATCTGTTGTCATGTTTCGGGTATTAAATTATTCTAA

ATATAAAAGTTTGAATGAAGTTTTATGCGAATATTGTTACGGTCCAGTGATAACGATCTTTATTTGTCCA
CGTTTTAAAGTTGAGTCGTTAAAGTAGAGGAGGTGTGCTACTAGTTTCGGTGTTATAAATGCGTTAGCTA
TTAAATCTGTCAAGCAATTTCGAACATACATGTATACACATGTATACAAGCAAAGCtttttttttttttt
ttAAGGAAAACTGCCAATTATTGGCGCATGGTGCAGAAATTTCCCCCACTAGAGCTGCCACTTACTAACT
TGAACTTTCTCCAAATTGCAGGAAAACATGGGCGGCAAGGCAGTGGTAAAAACCAGTGAAGCGCTGGCAG
CATTTGCGGCGGCGCAATTCTCCAGCTTCGACTATGTAAAGCCCTCGCCGCAGGAGCTGGCTCTGGAGGA
GATCAAGGCACTGAGCGGCGGCCCCGAGAGCGCGGATCCCGATCTCCTCGAGAAAAATCTGAGGCCACAG
CCACAGCCGCTTGCCAAAGCCGGTGAGCTTGCCAGGCCGCCGAATGCCATTCGATCGGCCTCGTTCCTCA
TGCAGTATGTGCTCCTGATGCAGCGCATCTTGATTTGCGCCAAGCGAAACTACCGTGAGTAGAAACCTCC
ATATCACGAACTAAATTCAACCAGCAAAGTTCAAGGCTTGAATTATGGCAAATATATTTGTTATATATAT
TTGCCAGCTTTTTAAAAATCTTAAGTTTAGCTTTTTAAGACTCGACTGCAATAACTTTAATGCACAAAGT
AACTTGGGTAACAATTAGTTGAAAGCCGGTTTAGGGCAGCGGCTTTAAGAGTTGATGGCCTGTCGAGTTG
ATTTGGTAATTGGTGCGGCGTCCTCGAAAAGTGAATCCAGTTTCGGCTAATCTCGTTTCTGCGTAATTCT
TAATGGCTAATCTTTTAAATAGCCGCTTTTTACTTTATTTTTATATTTCCCTCGGCAATTACAGCCCAGT
TGCACATTACCATTTAGTGGCCGTACATTAAGTACTATACGTTATACGtttttttttttGGGAAGGTTGA
ACACTTCGCCACGCTTTCGGATTTTGCTAAAAAATCATACGAAAATGGGACTTATGCATGCTCTTAATGG
TGATATAAGATCATGCATGCGGAATATAATAGGGCACTAGCTGAATTTTTTTATACATTATGCTTAAGCA
TATAATCAGGGCATTAAGCTACTTTTTAATAGAGCGACTTGTGGTGGGCTCATAAGTAAGTAATTCATGC
ACACAACTCAATTGTGCAATTGAATTTCTCATAGCTATGTAACATATATTCTACACTTAACAACGATTTT
CAGTTCGTTGGCACTGGGCACTACGTAAAAACAAATGACTCAGGGCAAAAGGCTCAAGCACTTACCATAA
AAAAGAGATAAATACAATAAATATGAAGCTGCGGAATCAGAAATGCCATCGAGATTGATGGCCGGCGTTT
ATCATCCGAATGTGTCCAATTAGCACTTGGCGTATGTTTGAATGTGAAATTGAACAGCTTGTGTGATGAC
CACTTAGCGAGCTAACTCACTTTTATGTGACTGCACACGATCAATCTGCGATGGATCTGCATACACACTG
CACTCGGCCACTCAAATAGTGGAGTGTGAACATAGCTTCGTTAGCACCTCCAGTTCCGCTGATATTGACT
TGTcaaccggccaagcggccaagcggccaaacggTGACTCCGAGAATCTCGGGCCCACTCGAGCGGCTCG
TAACTCAAAAGTAATGCCAGCCAGCACTCAGCCTTCACATAGATTTCGGCTTTAGACGGTTCTAATGACC
AACATCCACGGCACGATCATTTACTGGCTATTAGTTGCCAAAAGGTACAACTGCGGTCAATGCCATTAAA
TTGGGGAGTCTTGTCTTATTACCAGCTACCTGaaaaaaaaaaaaCGTTTTTTTAAGTTATGGTATACGCA
ATATGTCATAGTTTAGACCCACTTTCCCGCCAGATAGTTAAAAGCTTAGTGCTGTTATAGACATGACATA
TGAGAAAACTGCCCATAAATGTGTCACTTCGAGTGAAGAAACCAGCTGTGAACAAAAAAACTAACAACAA
ACTAGCAATGAGCATATTTGCAAGGACATTAAAGAGTTCTTTGCATTCGCATGAGAAAGTTAAGCAAAGT
GTGCTAACGTAAAGTATCTCAATTTCCAGTTTGCCTGAGTTGCTTGATTTGCATCTTTATCAACCTGTGT
GCCACATACATACCTAATACTCCGTATCTCTTTCAGGTTTCTGCTGCTGGCCCGCATCTTCTCGCACATT
TTCATCGGAGTCGTCTTCGGGTATCTGTACATGAACGTGGGCAACAATGCCCAGAGTGTGCTGGGAAACT
ACGTGTATCTGTACGGCTCCACGCTGCTCTTGGTCTACACCGGTAAAATGGCTGTGGTCTTGACAT
>dme_circ_0000617_junction_seq
ACCGGTAAAATGGCTGTGGTCTTGACATGATACCCCTTCTGACTAGAAACATTGAG
>dme_circ_0000618
GGATTCCGTGCAAACATGACCCTCTTGCCAGACATCAAGGCATCGGATGCCGCCTGCTGCGTGGGAGGTG
CGAACAGCGATGCCAGCAGCAGTTCCGGCGTCTGCAGCCTCAAGGGTCACCATGGTGACCCCGACAGCGG
TTGCCTGGGCATCAACTGTTGCACCACGGCGGCCAGCTCGGAGCTGTCCATCGACGAGACGTCATCGACC
TCGTCCAGAGGCTCCTGCGA
>dme_circ_0000618_junction_seq
CATCGACCTCGTCCAGAGGCTCCTGCGAGGATTCCGTGCAAACATGACCCTCTTGC

>dme_circ_0000619
GGATACGATGGGATCCAGGGGCACTTTTGAGTTGCCCACTGTGATGAAGGCCTGGTAGCAGAGGCGCAAC
TCATACCGATTGATCTTGTCGATCTGGTCCTTGTGATCGAATTTGGCTGAAAGGAAAGTATAGTTACTAA
AATAATAGTAGGAACTATTATAGTTTGAATTGCAGGAGGTATCATATAGTTTTAACATCCTTAAGTAAAG
TGTTAATAAGATGACTCACCATTGAAGGGATCGATGTTTCTCCTTTCCCTCTCCACCAGCGAGTCGCGCA
TCTCCAGCTTCTTGGCGCACT
>dme_circ_0000619_junction_seq
TCGCGCATCTCCAGCTTCTTGGCGCACTGGATACGATGGGATCCAGGGGCACTTTT
>dme_circ_0000620
GACGTCATCGACCTCGTCCAGAGGCTCCTGCGAGTTGACCAGCAAGGTGACCAACGACCTGAACGGATTC
CAGAGTCCGAACTACCACCAGGCGGTGGCGCACGCCAACTTCGACCACTGCGATCCCGTGGACATCCAGT
TCGCCGACGTGCGCTACACAGTGAAGAAGTTCTCCTTCCCGGAGCGGAAGTTCGGTGGGTACCTCTGCCT
CTGATCACTCTGAATGTTCTATATTAAAATATTTCCTTATTGTGCCGCAGTCACCAAGGAGATCCTTCAT
GGCCTGAACGGCAGCTTCCGGTCTGGCGAACTCACAGCCATCATGGGTCCTTCGGGCGCCGGCAAGAGCA
CGCTGCTGAATGTGATGTCCGGATTTTG
>dme_circ_0000620_junction_seq
CGCTGCTGAATGTGATGTCCGGATTTTGGACGTCATCGACCTCGTCCAGAGGCTCC
>dme_circ_0000621
GCTCCACTGGCGTGTCGGGCGATATCCGGGTTAATAGAAAGCCCATGGCTCCCAGCTCCGAGAGGTTCCG
CCAAATGCTGTGCTACATCCACCAGGACGATCTGCTGCGTCCACAGCTGTTGGTCGGCGAGATAATGCTG
CTGGCGGCACATCTGAAGCTGGGCTTCAAGGTCACCAAGGCGTACAAGATGGATCTGGATCAAGCACATC
TTATCGCTGCTGGGTCTGGACCATCGCTACAATGTGCCCACTGGGAAGCTTTCGGGTGGCCAGAAGAAGC
GACTCGCAATCGCCCTGGAGCTGATAAGTAATCCTCCCGTGCTATATCTGGATGAGCCGACGAC
>dme_circ_0000621_junction_seq
CCGTGCTATATCTGGATGAGCCGACGACGCTCCACTGGCGTGTCGGGCGATATCCG
>dme_circ_0000622
GCTCCACTGGCGTGTCGGGCGATATCCGGGTTAATAGAAAGCCCATGGCTCCCAGCTCCGAGAGGTTCCG
CCAAATGCTGTGCTACATCCACCAGGACGATCTGCTGCGTCCACAGCTGTTGGTCGGCGAGATAATGCTG
CTGGCGGCACATCTGAAGCTGGGCTTCAAGGTCACCAAGGCGTACAAGATGGATCTGGATCAAGCACATC
TTATCGCTGCTGGGTCTGGACCATCGCTACAATGTGCCCACTGGGAAGCTTTCGGGTGGCCAGAAGAAGC
GACTCGCAATCGCCCTGGAGCTGATAAGTAATCCTCCCGTGCTATATCTGGATGAGCCGACGACCGTGAG
TGACACTATATATTGTAAAAGCACACTTATGTATTTAGAGTAGCCATGATAGTGATCATCTCCAGCATTC
TGATGCTCATGGCGATGATGAGCAAACTTTGCCTGGCGGAAGTTCAGCaaaaaaaaaaaaaaCACCAACT
GACCCACAAGTCAAGTTTGTCCCCATCTATATGTCTATATGACTTAGAGCTTATCAATTGTCCAGATAGT
CTGTTTAATAGTTTCGACTTGTTTTGATTAGTGGTGTTCGGTGAGCTGTAAACAGTTTATTATTTAAAtt
ttttttattgattattcatttaattaCTGCGCTATCTGGCAGATAATCCCGTAATTAATTAAATTGTTCA
TTAGTTGTAGATTACTTTAATAGGGATTGATGAGTCTGCTGTGGTTATATGAACCTACAAAATCGGATTT
ATGCAGCAATAAAGGGCCAGCTATTTTGAGTCGTCTGGCTGGGGATTATGAGTATGAGTCCCTCCTTTTC
ATGTGGCATGACTAAGTATAAGTTGATTCAGACAACAATCTCAAAGTGAAAATCATGACTGGATACTTCG
CAAACAAAGGGAGTTGCCAGTTCCAGAAATATCTCAAGCTTTATTTTTAAGACTTGCTTTAAAATCATAT
GCTTTTCTTCTTAGACCTCACTTTTCATCATTCATTGGCAATTAGTAAACTCGAAGTCGTGAGATTCATT
TAAAGAAGGGTATTAGGCAAAGTGTATACTTGAATTTGATATATATGGATACGAGTAATTCAAATAATAC
GTATGTCTCGATTTCAGGCAGTTAATAACACTTGAAAGAACTCGTTTTTCAAAGTGTCAAATAAAATGCC
GACTGCAAGTCGATCAAATCTTTGCAATGCCGCCCGACTTGCATCGGCAAATGGATTTTGGGAACAAACT

TGTTTCTCGTTTTCATTTGACTTCGCTACAAGTACAAGTTCAAGCCGCAATCTCTCACCTACCATTAAAG
TTTCAGTTGCCCTGTTGCTTGATGTTCTTTGCTGTCAGTTAGACAGCGGTTTAGTTTAATGGGGTTGTTA
TTCGTTAGATCTTCAAAGGTTAGGCAGCAATTAAACAAAGTTGAGTCACACCGAAAGTTGTATTTCTGCT
CGTTTGTTCGGATTCCAGGTGGCCTGGACAGCTCCTCGTGCAGCTCCTGCGTGGCTCTGCTGAAGAAACT
GGCGTCGCAGGGCCACACGATAGTCTGCACCATCCATCAGCCAAGTGCCCTCATCTTCGAGATGTTCGAC
AAGCTCTACACCGTCGTCGATGGCCACTGCATGTACCAAGGACCTGTGCGCGAACTGGTGCCCTTCCTGG
CCGACCAGCAGCTCGTCTGCCCGAGTTACCACAACCCAGCTGACTATCGTACTGGAAGTGGCCGTGGGCG
AGCATCAACGTGACCTGAATGAGCTAATCCATGCGGCCAATAAAAAGTATTACGAGGATGTGGATCGCCA
TAGGTATATGAGCAGTGATGATATGGCACGCCTCGTGGAAAGCATTAAAG
>dme_circ_0000622_junction_seq
ATGGCACGCCTCGTGGAAAGCATTAAAGGCTCCACTGGCGTGTCGGGCGATATCCG
>dme_circ_0000623
GCTCCACTGGCGTGTCGGGCGATATCCGGGTTAATAGAAAGCCCATGGCTCCCAGCTCCGAGAGGTTCCG
CCAAATGCTGTGCTACATCCACCAGGACGATCTGCTGCGTCCACAGCTGTTGGTCGGCGAGATAATGCTG
CTGGCGGCACATCTGAAGCTGGGCTTCAAGGTCACCAAGGCGTACAAGATGGATCTGGATCAAGCACATC
TTATCGCTGCTGGGTCTGGACCATCGCTACAATGTGCCCACTGGGAAGCTTTCGGGTGGCCAGAAGAAGC
GACTCGCAATCGCCCTGGAGCTGATAAGTAATCCTCCCGTGCTATATCTGGATGAGCCGACGACCGTGAG
TGACACTATATATTGTAAAAGCACACTTATGTATTTAGAGTAGCCATGATAGTGATCATCTCCAGCATTC
TGATGCTCATGGCGATGATGAGCAAACTTTGCCTGGCGGAAGTTCAGCaaaaaaaaaaaaaaCACCAACT
GACCCACAAGTCAAGTTTGTCCCCATCTATATGTCTATATGACTTAGAGCTTATCAATTGTCCAGATAGT
CTGTTTAATAGTTTCGACTTGTTTTGATTAGTGGTGTTCGGTGAGCTGTAAACAGTTTATTATTTAAAtt
tttttattgattattcatttaattaCTGCGCTATCTGGCAGATAATCCCGTAATTAATTAAATTGTTCA
TTAGTTGTAGATTACTTTAATAGGGATTGATGAGTCTGCTGTGGTTATATGAACCTACAAAATCGGATTT
ATGCAGCAATAAAGGGCCAGCTATTTTGAGTCGTCTGGCTGGGGATTATGAGTATGAGTCCCTCCTTTTC
ATGTGGCATGACTAAGTATAAGTTGATTCAGACAACAATCTCAAAGTGAAAATCATGACTGGATACTTCG
CAAACAAAGGGAGTTGCCAGTTCCAGAAATATCTCAAGCTTTATTTTTAAGACTTGCTTTAAAATCATAT
GCTTTTCTTCTTAGACCTCACTTTTCATCATTCATTGGCAATTAGTAAACTCGAAGTCGTGAGATTCATT
TAAAGAAGGGTATTAGGCAAAGTGTATACTTGAATTTGATATATATGGATACGAGTAATTCAAATAATAC
GTATGTCTCGATTTCAGGCAGTTAATAACACTTGAAAGAACTCGTTTTTCAAAGTGTCAAATAAAATGCC
GACTGCAAGTCGATCAAATCTTTGCAATGCCGCCCGACTTGCATCGGCAAATGGATTTGGGAACAAACT
TGTTTCTCGTTTTCATTTGACTTCGCTACAAGTACAAGTTCAAGCCGCAATCTCTCACCTACCATTAAAG
TTTCAGTTGCCCTGTTGCTTGATGTTCTTTGCTGTCAGTTAGACAGCGGTTTAGTTTAATGGGGTTGTTA
TTCGTTAGATCTTCAAAGGTTAGGCAGCAATTAAACAAAGTTGAGTCACACCGAAAGTTGTATTTCTGCT
CGTTTGTTCGGATTCCAGGTGGCCTGGACAGCTCCTCGTGCAGCTCCTGCGTGGCTCTGCTGAAGAAACT
GGCGTCGCAGGGCCACACGATAGTCTGCACCATCCATCAGCCAAGTGCCCTCATCTTCGAGATGTTCGAC
AAGCTCTACACCGTCGTCGATGGCCACTGCATGTACCAAGGACCTGTGCGCGAACTGGTGCCCTTCCTGG
CCGACCAGCAGCTCGTCTGCCCGAGTTACCACAACCCAGCTGACTATCGTACTGGAAGTGGCCGTGGGCG
AGCATCAACGTGACCTGAATGAGCTAATCCATGCGGCCAATAAAAAGTATTACGAGGATGTGGATCGCCA
TAGGTATATGAGCAGTGATGATATGGCACGCCTCGTGGAAAGCATTAAAGGGTGAGTTCTATACTGATCA
GATCTGTTGTCATGTTTCGGGTATTAAATTATTCTAAATATAAAAGTTTGAATGAAGTTTTATGCGAATA
TTGTTACGGTCCAGTGATAACGATCTTTATTTGTCCACGTTTTAAAGTTGAGTCGTTAAAGTAGAGGAGG
TGTGCTACTAGTTTCGGTGTTATAAATGCGTTAGCTATTAAATCTGTCAAGCAATTTCGAACATACATGT
ATACACATGTATACAAGCAAAGCttttttttttttttAAGGAAAACTGCCAATTATTGGCGCATGGTG
CAGAAATTTCCCCCACTAGAGCTGCCACTTACTAACTTGAACTTTCTCCAAATTGCAGGAAAACATGGGC

GGCAAGGCAGTGGTAAAAACCAGTGAAGCGCTGGCAGCATTTGCGGCGGCGCAATTCTCCAGCTTCGACT
ATGTAAAGCCCTCGCCGCAGGAGCTGGCTCTGGAGGAGATCAAGGCACTGAGCGGCGGCCCCGAGAGCGC
GGATCCCGATCTCCTCGAGAAAAATCTGAGGCCACAGCCACAGCCGCTTGCCAAAGCCGGTGAGCTTGCC
AGGCCGCCGAATGCCATTCGATCGGCCTCGTTCCTCATGCAGTATGTGCTCCTGATGCAGCGCATCTTGA
TTTGCGCCAAGCGAAACTAC
>dme_circ_0000623_junction_seq
CATCTTGATTTGCGCCAAGCGAAACTACGCTCCACTGGCGTGTCGGGCGATATCCG
>dme_circ_0000624
GTACGAGAACTGGAGCAACTGGATTTGGGTCAGCAGTTGGAGCCACGTCCTTTGACGGCCAACGATAAGA
TCACCGAGTGGATGAAGTCCAGTGAGATTGAACAGCAAGTCCATGAGCCCAGTCCCACTGCTGAGGCAGA
TGTTTTGGACTCCGCCCTTGAGATATCCAAGGCAGATAAGACCCTTGACGAACTCCTTGAGACCGTGGCC
GAACTGGATGAGATATACACGGACTTCAAAGTTCAGAGGGATACG
>dme_circ_0000624_junction_seq
CACGGACTTCAAAGTTCAGAGGGATACGGTACGAGAACTGGAGCAACTGGATTTGG
>dme_circ_0000625
GTGGCCTGGACAGCTCCTCGTGCAGCTCCTGCGTGGCTCTGCTGAAGAAACTGGCGTCGCAGGGCCACAC
GATAGTCTGCACCATCCATCAGCCAAGTGCCCTCATCTTCGAGATGTTCGACAAGCTCTACACCGTCGTC
GATGGCCACTGCATGTACCAAGGACCTGTGCGCGAACTGGTGCCCTTCCTGGCCGACCAGCAGCTCGTCT
GCCCGAGTTACCACAACCCAGCTGACTATCGTACTGGAAGTGGCCGTGGGCGAGCATCAACGTGACCTGA
ATGAGCTAATCCATGCGGCCAATAAAAAGTATTACGAGGATGTGGATCGCCATAGGTATATGAGCAGTGA
TGATATGGCACGCCTCGTGGAAAGCATTAAAG
>dme_circ_0000625_junction_seq
ATGGCACGCCTCGTGGAAAGCATTAAAGGTGGCCTGGACAGCTCCTCGTGCAGCTC
>dme_circ_0000626
GTGGCCTGGACAGCTCCTCGTGCAGCTCCTGCGTGGCTCTGCTGAAGAAACTGGCGTCGCAGGGCCACAC
GATAGTCTGCACCATCCATCAGCCAAGTGCCCTCATCTTCGAGATGTTCGACAAGCTCTACACCGTCGTC
GATGGCCACTGCATGTACCAAGGACCTGTGCGCGAACTGGTGCCCTTCCTGGCCGACCAGCAGCTCGTCT
GCCCGAGTTACCACAACCCAGCTGACTATCGTACTGGAAGTGGCCGTGGGCGAGCATCAACGTGACCTGA
ATGAGCTAATCCATGCGGCCAATAAAAAGTATTACGAGGATGTGGATCGCCATAGGTATATGAGCAGTGA
TGATATGGCACGCCTCGTGGAAAGCATTAAAGGGTGAGTTCTATACTGATCAGATCTGTTGTCATGTTTC
GGGTATTAAATTATTCTAAATATAAAAGTTTGAATGAAGTTTTATGCGAATATTGTTACGGTCCAGTGAT
AACGATCTTTATTTGTCCACGTTTTAAAGTTGAGTCGTTAAAGTAGAGGAGGTGTGCTACTAGTTTCGGT
GTTATAAATGCGTTAGCTATTAAATCTGTCAAGCAATTTCGAACATACATGTATACACATGTATACAAGC
AAAGCttttttttttttttttAAGGAAAACTGCCAATTATTGGCGCATGGTGCAGAAATTTCCCCCACTA
GAGCTGCCACTTACTAACTTGAACTTTCTCCAAATTGCAGGAAAACATGGGCGGCAAGGCAGTGGTAAAA
ACCAGTGAAGCGCTGGCAGCATTTGCGGCGGCGCAATTCTCCAGCTTCGACTATGTAAAGCCCTCGCCGC
AGGAGCTGGCTCTGGAGGAGATCAAGGCACTGAGCGGCGGCCCCGAGAGCGCGGATCCCGATCTCCTCGA
GAAAAATCTGAGGCCACAGCCACAGCCGCTTGCCAAAGCCGGTGAGCTTGCCAGGCCGCCGAATGCCATT
CGATCGGCCTCGTTCCTCATGCAGTATGTGCTCCTGATGCAGCGCATCTTGATTTGCGCCAAGCGAAACT
AC
>dme_circ_0000626_junction_seq
CATCTTGATTTGCGCCAAGCGAAACTACGTGGCCTGGACAGCTCCTCGTGCAGCTC
>dme_circ_0000627
GTGGCCTGGACAGCTCCTCGTGCAGCTCCTGCGTGGCTCTGCTGAAGAAACTGGCGTCGCAGGGCCACAC

GATAGTCTGCACCATCCATCAGCCAAGTGCCCTCATCTTCGAGATGTTCGACAAGCTCTACACCGTCGTC
GATGGCCACTGCATGTACCAAGGACCTGTGCGCGAACTGGTGCCCTTCCTGGCCGACCAGCAGCTCGTCT
GCCCGAGTTACCACAACCCAGCTGACTATCGTACTGGAAGTGGCCGTGGGCGAGCATCAACGTGACCTGA
ATGAGCTAATCCATGCGGCCAATAAAAAGTATTACGAGGATGTGGATCGCCATAGGTATATGAGCAGTGA
TGATATGGCACGCCTCGTGGAAAGCATTAAAGGGTGAGTTCTATACTGATCAGATCTGTTGTCATGTTTC
GGGTATTAAATTATTCTAAATATAAAAGTTTGAATGAAGTTTTATGCGAATATTGTTACGGTCCAGTGAT
AACGATCTTTATTTGTCCACGTTTTAAAGTTGAGTCGTTAAAGTAGAGGAGGTGTGCTACTAGTTTCGGT
GTTATAAATGCGTTAGCTATTAAATCTGTCAAGCAATTTCGAACATACATGTATACACATGTATACAAGC
AAAGCttttttttttttttttAAGGAAAACTGCCAATTATTGGCGCATGGTGCAGAAATTTCCCCCACTA
GAGCTGCCACTTACTAACTTGAACTTTCTCCAAATTGCAGGAAAACATGGGCGGCAAGGCAGTGGTAAAA
ACCAGTGAAGCGCTGGCAGCATTTGCGGCGGCGCAATTCTCCAGCTTCGACTATGTAAAGCCCTCGCCGC
AGGAGCTGGCTCTGGAGGAGATCAAGGCACTGAGCGGCGGCCCCGAGAGCGCGGATCCCGATCTCCTCGA
GAAAAATCTGAGGCCACAGCCACAGCCGCTTGCCAAAGCCGGTGAGCTTGCCAGGCCGCCGAATGCCATT
CGATCGGCCTCGTTCCTCATGCAGTATGTGCTCCTGATGCAGCGCATCTTGATTTGCGCCAAGCGAAACT
ACCGTGAGTAGAAACCTCCATATCACGAACTAAATTCAACCAGCAAAGTTCAAGGCTTGAATTATGGCAA
ATATATTTGTTATATATATTTGCCAGCTTTTTAAAAATCTTAAGTTTAGCTTTTTAAGACTCGACTGCAA
TAACTTTAATGCACAAAGTAACTTGGGTAACAATTAGTTGAAAGCCGGTTTAGGGCAGCGGCTTTAAGAG
TTGATGGCCTGTCGAGTTGATTTGGTAATTGGTGCGGCGTCCTCGAAAAGTGAATCCAGTTTCGGCTAAT
CTCGTTTCTGCGTAATTCTTAATGGCTAATCTTTTAAATAGCCGCTTTTTACTTTATTTTTATATTTCCC
TCGGCAATTACAGCCCAGTTGCACATTACCATTTAGTGGCCGTACATTAAGTACTATACGTTATACGttt
ttttttttGGGAAGGTTGAACACTTCGCCACGCTTTCGGATTTTGCTAAAAAATCATACGAAAATGGGAC
TTATGCATGCTCTTAATGGTGATATAAGATCATGCATGCGGAATATAATAGGGCACTAGCTGAATTTTTT
TATACATTATGCTTAAGCATATAATCAGGGCATTAAGCTACTTTTTAATAGAGCGACTTGTGGTGGGCTC
ATAAGTAAGTAATTCATGCACACAACTCAATTGTGCAATTGAATTTCTCATAGCTATGTAACATATATTC
TACACTTAACAACGATTTTCAGTTCGTTGGCACTGGGCACTACGTAAAAACAAATGACTCAGGGCAAAAG
GCTCAAGCACTTACCATAAAAAAGAGATAAATACAATAAATATGAAGCTGCGGAATCAGAAATGCCATCG
AGATTGATGGCCGGCGTTTATCATCCGAATGTGTCCAATTAGCACTTGGCGTATGTTTGAATGTGAAATT
GAACAGCTTGTGTGATGACCACTTAGCGAGCTAACTCACTTTTATGTGACTGCACACGATCAATCTGCGA
TGGATCTGCATACACACTGCACTCGGCCACTCAAATAGTGGAGTGTGAACATAGCTTCGTTAGCACCTCC
AGTTCCGCTGATATTGACTTGTcaaccggccaagcggccaagcggccaaacggTGACTCCGAGAATCTCG
GGCCCACTCGAGCGGCTCGTAACTCAAAAGTAATGCCAGCCAGCACTCAGCCTTCACATAGATTTCGGCT
TTAGACGGTTCTAATGACCAACATCCACGGCACGATCATTTACTGGCTATTAGTTGCCAAAAGGTACAAC
TGCGGTCAATGCCATTAAATTGGGGAGTCTTGTCTTATTACCAGCTACCTGaaaaaaaaaaaaCGTTTTT
TTAAGTTATGGTATACGCAATATGTCATAGTTTAGACCCACTTTCCCGCCAGATAGTTAAAAGCTTAGTG
CTGTTATAGACATGACATATGAGAAAACTGCCCATAAATGTGTCACTTCGAGTGAAGAAACCAGCTGTGA
ACAAAAAAACTAACAACAAACTAGCAATGAGCATATTTGCAAGGACATTAAAGAGTTCTTTGCATTCGCA
TGAGAAAGTTAAGCAAAGTGTGCTAACGTAAAGTATCTCAATTTCCAGTTTGCCTGAGTTGCTTGATTTG
CATCTTTATCAACCTGTGTGCCACATACATACCTAATACTCCGTATCTCTTTCAGGTTTCTGCTGCTGGC
CCGCATCTTCTCGCACATTTTCATCGGAGTCGTCTTCGGGTATCTGTACATGAACGTGGGCAACAATGCC
CAGAGTGTGCTGGGAAACTACGTGTATCTGTACGGCTCCACGCTGCTCTTGGTCTACACCGGTAAAATGG
CTGTGGTCTTGACAT
>dme_circ_0000627_junction_seq
ACCGGTAAAATGGCTGTGGTCTTGACATGTGGCCTGGACAGCTCCTCGTGCAGCTC
>dme_circ_0000628

GAACGAGGCCGATCGAATGGCATTCGGCGGCCTGGCAAGCTCACCGGCTTTGGCAAGCGGCTGTGGCTGT
GGCCTCAGATTTTTCTCGAGGAGATCGGGATCCGCGCTCTCGGGGCCGCCGCTCAGTGCCTTGATCTCCT
CCAGAGCCAGCTCCTGCGGCGAGGGCTTTACATAGTCGAAGCTGGAGAATTGCGCCGCCGCAAATGCTGC
CAGCGCTTCACTGG
>dme_circ_0000628_junction_seq
GCCGCAAATGCTGCCAGCGCTTCACTGGGAACGAGGCCGATCGAATGGCATTCGGC
>dme_circ_0000629
TTACTTAAACGTCCATTTAATTTAATTaaaaacaaaaaagaaaaaCTTTAAGGAAGATTAAACAATAACG
TAAAGCATCGTATTTTTCTATACACCAATGGCGTACTTGTCTTAAAGACGCCTGATTGGTTTGATCTGAA
TTTTGTCGCGATGATTTTTTGATTTTTGTTAACAACGCTGTGCGCAAGCGAATAAATTGACAAACTAAAA
AGATACACAAATTGATGTTGAACAGATGGTATTGATATTGGCAACTGAAACATGCATTAATTGGCCAACC
CGATAATTAAAAGTATTTTTCCAAGGTCCAGTTTAAGCTAATTGGCCAACAAATATG
>dme_circ_0000629_junction_seq
AGTTTAAGCTAATTGGCCAACAAATATGTTACTTAAACGTCCATTTAATTTAATTa
>dme_circ_0000630
TTGAAGAGTTTGCTGTCCGTGAAGCGCGAGCAGATTGGAACTCTGCGCAACGTGCTCAAGTCAAACAAGC
AAACCGCTGAGGTGGCACTGACCAATCTCAAGTCCAAGTATGAGAACGAGAAGATCATTGTCAGCGACAC
CATGTCCAAGCTACGTAATGAGCTCAGGCTTCTTAAGGAGGATGCTGCCACATTCTCAAGGCCTGCGTGC
CATGTTCGCCGCTCGATGCGAGGAGTATGTGACCCAGGTGGACGATCTCAACCGCCAATTGGAGGCAGCA
GAGGAGGAGAAAAAGACTCTAAATCAGCTGTTGCGCTTGGCTGTCCAGCAGAAGCTGGCACTCACTCAGC
GACTCGAGGAGATGGAAATGGACCGGTGGGTGCACTTGGGTCGTTAAACGTCAGCTGCATCACCACTCGT
AGTGACAAGCAAAAACACATACATCCTTTTATTGATTAGGGCACCCAATATCCTTGTTTTCCCCTGAAAC
TTCCTTAAGAATCCAGCAATTGGGTGGGTGCACTTGGGTCGTTAAACGTCAGCTGCATCACCACTCGTAG
TGACAAGCAAAAACACATACATCCTTTTATTGATTAGGGCACCCAATATCCTTGTTTTCCCCTGAAACTT
CCTTAAGAATCCAGCAATTGGTAAGCCCCTGCAAGTGGCAGAAATTTTGTTGCATGCACGCTCGCCTGCT
AACATGCTGCTGGAATCCGCCTAACCCCAAGAGAAACCGTTGTGCGCTGATTAATATTTTAATTTGATCT
AGCTTAAATTATTAAATGTATGTTCCCAATTAACAACTAACAAATGTCCTGTATTCACTCCCAACAGCGA
AATGCGTCACGTCCGTCGGCCGATGCCAGCCCAGCGTGGCACGAGCGGCAAGTCCTCCTTCAGCACGAGA
CCTTCGAGCAGGAATCCAGCAAGCAG
>dme_circ_0000630_junction_seq
GACCTTCGAGCAGGAATCCAGCAAGCAGTTGAAGAGTTTGCTGTCCGTGAAGCGCG
>dme_circ_0000631
GTTGCTCTTGTACACCTCGTTGTCCGGTTCCAGCTCGATGGCCTTGGCGTAGGCCTGTTCCGCCTTCTCG
AAATTGCCCATGTTGGAGTACGCCACGCCCAGGCGGCAGTACGCCTTGCTGTAGTTGTTGTTGTACACCA
GGGCGGACTTGCAATCGGTTACGGCGCGTTCGTTCTCACCCAGCCGGATGTGGGCCGCAGCCCGATTGCA
GTAGAAGATCGGATTCTTGGGATCGAAGGCGATGGCACGATTGTACTGCAGCAGTGCTTCATTGTACTTG
TTCTCCTTCATCAGCCGG
>dme_circ_0000631_junction_seq
ATTGTACTTGTTCTCCTTCATCAGCCGGGTTGCTCTTGTACACCTCGTTGTCCGGT
>dme_circ_0000632
GTTTCTGCTGCTGGCCCGCATCTTCTCGCACATTTTCATCGGAGTCGTCTTCGGGTATCTGTACATGAAC
GTGGGCAACAATGCCCAGAGTGTGCTGGGAAACTACGTGTATCTGTACGGCTCCACGCTGCTCTTGGTCT
ACACCGGTAAAATGGCTGTGGTCTTGACATTGTAAGTAGCTGCAATCGCCGAGATAATCGCCTCACTTCA
CCTTGCCCACATCTAACCACCACCCAAATGCATTTGTCCAAGTGGTTCTCGAGTGCTATCGAGTGGACTT

GGCCTCTCCCAGTTTCCAGTCACTGGTTTCCTCGGATAGCCAATTTATGCAAAAATTTGTCGCCAGATTT
GACCAAAAGAAAAAAACTGCAGCTGTTCTTGCGCAATTTTGGCAACTTTGCTTTGTCTTTAGCTGCCGCA
TTCTATGATATTTTGTCAAGATACTTATTTATCGCGGAATGCGGATTAGCCAATAATATATTGGCATTTG
CACAGAAGTTTCGTGGTTCTTAATTGGCaaaaaaaaaGAACCAAGATTCTGAGCTTAGATAGTGGATCAT
TAGCCAAAGAATAAGAATCTAGTATAGCCACAAATAATAAATTATCAGCGTTCCAAATACGCCATAGTAA
ACTTGATTAAAATATATTGATTGGAACATTAACCCTCGAGGCGAGGCCAGATCTGATTAGCGATAAAAGA
TCTCTGTCGATCCACTTTGTCATCTATAATTTCCATGTAAATTGCCAAAAGTGCACTTCTCGCCCCGAAA
AATGCGATCCTGAAGCCCGCTAAGTAAATAATTATAAAATACAAGAGACACCAGCTGCGATTATTGTATA
ATAAAACCGGTTGGCCAGCGATTCTCGACTCTTTTTACAATAAATTGCTGCAGACAGGAGGCATtggagg
agctggtggagctgggggagctgggTATCCGATCTTTACCGGTTTCGGTTTACTGACCTGACCTACATAT
GTATAATGCCCTCACCTTCGTGCGCACACTTCGCATCGCATCGCATCACATTGAACCTAACGAATTTAAT
CGCAATCTCGTTTATCAATTAATTTTGTCCGCAGTTCCGCTGGAAATTGACATGTTGACACGGGAGCACT
TCAACCGCTGGTACAAACTGGGTCCCTACTTCCTCTCGTTGATCTCCTTCGAAATACCCTTCCAG
>dme_circ_0000632_junction_seq
GTTGATCTCCTTCGAAATACCCTTCCAGGTTTCTGCTGCTGGCCCGCATCTTCTCG
>dme_circ_0000633
GATGAACAAGCTGCCGCTCATCCTGACCTTTGTGGGCGCGGAGAACTGTAACACGGGACACATTCTCGCA
CTGGAGCACCAGGTCGATGGCTATTTGGAGGACATTAAACAGGCTGTCACCGAGGCCTAAAGTTAGTCTA
CCTTGTAATATGCATATTTTTAATATTTACTAAATGTTTATACATACATACTTTATTGAGAACAGAATGA
GTTTTTAAAAATCTCAACTCACCATTGTACTATTCCGGGAATTTATTTCATATCCTGCGAAGGGTGATCT
CAAAGATGAACAAAGTTCCGA
>dme_circ_0000633_junction_seq
GTGATCTCAAAGATGAACAAAGTTCCGAGATGAACAAGCTGCCGCTCATCCTGACC
>dme_circ_0000634
TTACCGAACTGATGGTCGACATGAATCTGGACAGAGATCGCAACGGCGTGGTAACTGTGGAGGAGGCCAA
GTACTTCCTGGACGAGCGCGAACGAGTGGATCTAGATGCTTTCGTAACGCTGGCCTGGCCGCGTATTAAG
TCTCTGCAAATGCTGGCCGAGGGTCTGTTCCAGCCGCCGCAGCCGGAGGAAGTTGCCCAGCAGCCAGAGG
TCACCACCGAGTCGATCCAGCCCACTCCACCAAAGCTAAGCGAAGG
>dme_circ_0000634_junction_seq
AGCCCACTCCACCAAAGCTAAGCGAAGGTTACCGAACTGATGGTCGACATGAATCT
>dme_circ_0000635
GTCGGAAAACGGAAAAACAAACAAGCCGGCGAGTTGCCCTTGACAGCAGGCAAAGAGCAACAATTCAATT
CGCACACAAATCACGCTAGAAATCATTTTGGCCTCCCAGTAAATTTCGGAAATTTTTTGGAAAAATGCTG
CTACAAAGGTTTTTCACACGCGCACTTCACTCCACGCGGCAGCTTTATGCCGGCTCTGGCGGATCCGGTG
GCCTGGAGAAGAGTGCCCTGGCGGCGCTGAGGAAGAAAACCGGATACACCTTCGCCAATTGCAAGAAGGC
GCTGGAGAAGCACAACAACGACG
>dme_circ_0000635_junction_seq
AAGGCGCTGGAGAAGCACAACAACGACGGTCGGAAAACGGAAAAACAAACAAGCCG
>dme_circ_0000636
GGCTTCAACATGCAGGGCAGACAGAATCCGAATCGGGAGTTCAGCATCCCCAGCTTCACGTCCATATGCG
GCGGTCGCCGCCTATGCGAGAAGTCGAAGATGCTGTACACGCGACCCAAGCCGGATGTGCTGCAGAGCAT
CCTGCAGTCAAAGGCCAATCCGGAGTACAAGTCGCGTCGCCTGTGGTACGAACTGAACATGCCGGAATGC
GCCAGCTCCCTGCTGGACGGCAAGATGAAATCCGACCTGCCGGACAACATACTCTGCGCGAAGAAACAGC
AGAAGAAACGCCAGAAGAAGCGTGAGAGCGGCGTGGCCAAGGCCAACTACATGACCATGCGACTGGAGCG

GGAGCACTTCCGCCGCAAGCTGGTGGAGCTCATCCTGCACATGGACGACGACCAGGATCCCGAGCTGGCC
GCCCAGTGCGCCGTACCCTTTCCCGACCAGGAGGAGCGCGAGATTCTGCGATATTACTATTACATCAAGC
ATGGCATTGACACCATTCACGTGTCGCCGTTGAGCAAGAAGATCCTCAAGAGGGATAACCGATCTTGTGC
CACCACTGCTCTACAAGTGGCAGACGGCACTCAATGAAAACATAGCCGAAGTCCGGGCGGACTACGTGTT
CGCCATGAAGAAGGCCGTTGTGGACTTCGTCCTGCGGCACACGGTCCTCGATCGCCTAACCAAGGAGGGC
GTGGGTGGCAAATACGACACCGCCGAGCGGCGGGAGGTGCAATCCATGACGAACTTTTGGCGCTATCG
>dme_circ_0000636_junction_seq
AATCCATGACGAACTTTTGGCGCTATCGGGCTTCAACATGCAGGGCAGACAGAATC
>dme_circ_0000637
TATTTGGATGTCATCCACGGCAGCCAGTTTGAATGTGCCGGAATCCCTGCGGGTGGCATTTCAATTATAG
GCCGTATCCCCCACCTGGCAGAAAGGTAAATGCTACTCACCTGTATGGCGAAATGGAGAACTCTACACCT
TCCCACTCGTTGACCATCTTGGCCATTGCCCTCTCCAAATTGTTCTCCTTGGTGGCGCTCTCCGAGATGG
CCTCGAACTTGGGCACATACTCGTCCAGCTGG
>dme_circ_0000637_junction_seq
GAACTTGGGCACATACTCGTCCAGCTGGTATTTGGATGTCATCCACGGCAGCCAGT
>dme_circ_0000638
GTTTCACTCAACGAAGTTCGGGGTAATTTGCATGTGAGTCTTGGCTACGATCCAGTTGGAGGATTGCTAA
ATGTTCGACTCCTGGAGGCCCAGAATCTGCAACCGAGGCAATTTAGTGGAACTGCCGACCCATATGCCAA
AGTCCGCTTGCTGCCGGATAAAAAGAACTTTTGGCAGACGCGTATACACAAGAGGACCCTGAATCCAGGT
TTTCGACGAGCAGTTTGTTTTTGAGGTAACAGCCGGAGTAATTGACAAGCGTACTGTGGAAATTTTACTA
TACGACTTTGATGCTTATTCCCGGCACGTTTGCATCGGAGGAAGTAAGCTACATTTGGCCAACTTGGATC
TCAGCGAGCAATTGAAGTTGTGGACTCCTCTTAGTTCTGCCTCGGCCCAAGATATGAAGGTGGATTTGGG
CGATATAATGGTGTCCCTAGCCTATTTGCCATCAGCGGAGCGTTTGATGGTCGTCCTTATCAAGGCACGA
AACCTGCGGATTGTCGACGATGCCCGGAACTCCTCGGACCCTTATGTAAAGGTAACCCTCCTCGGTCCCG
GTGGCAAAAAGATAAAGAAACGCAAGACGGGCGTTCAAAGGGGCACCCTGAACCCAGTCTATAATGAGGC
TCTTGCTTTCGATGTGGCCAAGGAGACTTTAAAAAACTGTGTACTAGAATTCACCGTAGTCCACGATGGC
CTATTGGGGTAAGCAGAGTTTTTACAATCGTTGTATgtttgttttttccatttacctggctgttaattgt
tttactttattgatttttttttaattatttcgttttactttatttttattgtctatttaGTTGCCTTTAA
TTTGGTTTAAGTTTCTTCGACTCTAAAATTGTTTCCTATCTTGTTGGATTGCTTATAGAATAATGTTATT
ATGTATCATATTTTATTATAATGATTTTTTCTTCAAAAAATGTTTGAAATCAGTTTACAGTTTCCTAAAT
TCAAACTTCATATCATTGTAGAATTGGCCTTAATATTTCAACACTGTATTTTTAAACAAAAATTAATAGA
ATATCTtttatggatatataatattataatatattgcaattttagttaagaataaatattttttttGCAG
GGATCAAGTGAAATATTAGGACGAACTCTCATTGGAAACTCTCCGGAAGTACGCACCGAGGAAAAAATAT
TTTTTGAAGAAGTTTTTCGTGCCAAAAATGCTACGGCTCAATGGGTTCCACTGCAAGAACCGGCAAACAA
TTTGGCCACATCGGCCAAAAGTTCAAAGAACTAGAGTTTT
>dme_circ_0000638_junction_seq
GGCCAAAAGTTCAAAGAACTAGAGTTTTGTTTCACTCAACGAAGTTCGGGGTAATT
>dme_circ_0000639
AGGGTGCCAACGGTGATGGCATGGCTGTGATATCGTCCCTAAGATCCTTCGATCGCGAGCAGCAAAAGGA
ATATATGATTCCTATTGTAATCAAAGACCATGGTTCCCCGGCAATGACTGGAACTAGTACTCTGACCGTC
ATAATTGGCGACGTGAACGACAACAAAATGCAGCCAGGATCTAAGGACATCTTCGTTTACAACTACCAAG
GACAATCGCCAGACACTCCGATTGGACGGGTCTATGTCTACGATTTGGATGATTGGGATCTGCCCGACAA
GAAGTTCTATTGGGAGGCCATGGAGCATCCAAGATTCAAGTTGGATGAAGATTCCGGCATGGTAACGATG
CGAGCAGGAACTCGTGAAGGACGCTATCATCTCAGGTTCAAGGTCTACGATAGGAAGCACACTCAAACCG

ATATACCAGCCAATGTTACGGTTACAGTGAGAGAAATTCCCCATGAAGCTGTTGTAAACTCGGGATCCGT
ACGTCTATCAGGCATTTCGGATGAGGACTTTATTCGAGTCTGGAACTACAGAACCCAAAGCATGAGCCGC
TCGAAGATGGATCGTTTTAGGGATAAGCTGGCTGACTTGCTAAACACTGAAAGGGAAAATGTGGATATAT
TCAGTGTGCAATTGAAGAGGAAACATCCGCCTCTAACGGACGTTAGGTTTTCCGCACATGGATCTCCTTA
CTATAAACCAGTTAGGCTAAATGGAATTGTACTGATGCACCGCGAGGAGATCGAAAAGGATGTGGGCATT
AATATCACCATGGTGGGCATCGATGAGTGTCTGTATGAGAACCAAATGTGCGAGGGCAGTTGCACAAACT
CGCTGGAAATCAGTCCTCTGCCCTATATGGTTAACGCCAACAAAACTGCCTTGGTCGGTGTTCGTGTGGA
TACCATTGCTGACTGTACTTGTGGTGCTCGCAATTTCACCAAACCAGAATCCTGTCGAACCACACCCTGT
CATAATGGTGGTCGCTGTGTGGACACCAGATTTGGTCCTCACTGCTCGTGTCCTGTGGGATACACGGGAC
CCAGGTGTCAGCAAACCACTCGCAGTTTCCGTGGAAACGGTTGGGCCTGGTATCCTCCCTTGGAAATGTG
CGATGAATCGCATTTAAGTCTGGAGTTTATTACCCGAAAACCCGACGGCTTAATCATCTACAACGGTCCA
ATTGTTCCTCCAGAACGAGATGAGACGCTCATCTCAGATTTCATTGCCCTGGAATTGGAGCGAGGTTATC
CTAGACTGCTTATCGACTTTGGTTCGGGAACTTTGGAGCTGAGGGTGAAGACGAAGAAGACTTTGGACGA
CGGTGAATGGCATAGAATCGATCTGTTTTGGGATACCGAAAGCATTCGTATGGTTGTCGACTTTTGCAAA
TCAGCAGAGATTGCCGAAATGGAGGATGGCACTCCGCCGGAATTCGATGACATGTCCTGCCAGGCGAGAG
GACAAATTCCGCCATTTAATGAGTACCTCAACGTTAATGCTCCTCTGCAAGTGGGTGGCCTATATCGGGA
ACAGTTCGATCAGAGTCTGTACTTCTGGCACTATATGCCCACGGCCAAGGGATTTGATGGCTGCATCAGA
AATTTGGTGCATAACTCAAAACTCTATGATCTGGCCCATCCTGGATTGTCGAGAAATAGCGTCGCGGGTT
GTCCTCAAACCGAGGAAGTTTGTGCTCAAACTGAAACCACAGCTCGCTGCTGGGAGCACGGAAATTGTGT
TGGATCCTTGTCCGAAGCTCGTTGCCATTGTCGACCAGGATGGACAGGTCCGGCCTGTAATATCCCTACA
ATACCCACTACCTTCAAGGCCCAGAGTTATGTTAAATATGCTCTGAGTTTCGAGCCCGATCGATTCAGCA
CGCAAGTCCAGCTAAGATTCCGGACAAGAGAGGAGTACGGCGAACTTTTCCGAGTTAGCGATCAGCACAA
TCGGGAATACGGAATCCTTGAGATCAAGGATGGTCATCTGCACTTCCGTTACAATCTGAACTCTCTGCGC
ACTGAAGAAAAGGATCTCTGGCTAAATGCCATAGTAGTAAACGATGGCCAATGGCATGTGGTGAAGGTAA
ATCGATATGGCTCAGCTGCTACCCTGGAATTGGATGGTGGAGAGGGTCGTCGTTATAACGAGACCTTTGA
GTTCGTGGGACATCAGTGGCTGTTGGTGGACAAGCAAGAGGGTGTCTATGCGGGCGGCAAAGCTGAATAC
ACCGGAGTGAGAACCTTTGAAGTGTACGCCGATTACCAAAAGAGCTGTCTCGATGATATACGGTCTCGAA
GGCAAACACCTGCCATTGCCACCGGCGATGAATGGTACTCAGTGGGGACAGGCTACCATGGCCAGGAATC
TGGAGAAGGGTTGTCCTTCGAATAAACCATGCTCCAACGTGATTTGCCCCGATCCGTTCGAGTGCGTAGA
CCTGTGGAATGTCTACGAGTGCACG
>dme_circ_0000639_junction_seq
AGACCTGTGGAATGTCTACGAGTGCACGAGGGTGCCAACGGTGATGGCATGGCTGT
>dme_circ_0000640
TTTGCGCCTCCCACAGGAAAACAAACAAAAATGTATTGATCAGCAGTGGAGAAGGTAGACTGGACAAAGG
AATTTACCCATGAATAAGTCAAGTGTTAATAGTAAAGTCAACAATTGAAGAGTGAGCACATTGAAAAGGG
AAAGTCGCAAAAAAAGGTGTTAATACCAAACAGGATCAGAAAGTGCCGCGGCAAAATGGCGGCACGACGT
TGCCTGAATCAGCTCAGGCAACGTTATATAACCAACCGATTTAATATATGCACCTGCGCGATATTCTTGA
TATCCCTTCCATTTATTTTGGCCATAGAGGAGACCACATTTGCTGGTCTTTCGGCGGAAAATGCGGCTCG
CATGCTGGCCGGCAGTCCCGGGGATGTGGAAAAGTCCTCGTTGTCGCATCACAGTGAAATGTCCTTGGTC
CTGCCACACGATACCTATCCTGGATTCAGTATCAAAAAGTTCAAAACCCATCCTGTAAAGATAAACGGTA
GCTCCCACTCTGGTGCAGCTGCCTATCACATGTTGGACACGGACTACTCCAAGTACTTCACCGTACTGGA
AGATGGTGTGGTGATGACCACGGCGGATATTTCCCCGCTTGTCAATCGTCCGGTTCAGTTGGTGGTTGTT
GAACAAACGCCCAATGCCACCAATACCCACAATCTGCAGCTGTTTGTGATGCACCGCAATGATATGCTGC
GATTTTCTGGATCCCTACTCGATGCCAGTGGTGAAGTTAGGGAGAATCAACCGGCGGGTACCAGGGTGAG

AGGAGTGCCCCTGATGCAGGCCTTCAGCGGTTCCATCCTGGACGAAGAACTGGCCACACCCAAAAAGGTT
CGGTACACCATTATTGACGGCAACGTGGATGATGCATTCGCCCTGCAGGAACGTAAGGCCAACAAGAACA
TTCAAATAAGTGCCAAGTCCTTGGTGATTAATGGCGATGACGAATCCGGTGTTTGGCTGGTCACCAATCG
TCCGCTGGATCGAGAGGAGAGGGCTCACTATGACCTGTCCGTGGAAGCATCAGATGTCGATGGTTTAGAC
AGGACAGTTTCCAAAATACAAATCACCGTGTTGGATGAAAACGACAATAGACCGATTTTCAAGTCCCTGG
ACTACAAGTTCGCCATCGCTGGACAGAAATCTGCAAGTATGGAAAGCAACAGTAGTGTGACATATCAGCG
ATTTGCGATCATGGGAAAAGTTGAGGCCACCGATGCTGATGGTGACAAGATTGCCTATCGGCTAAAGTCG
CCCAGTAATGTTGTCATTATTGTACCCCAAACTGGCGAAATAATGCTGGCTGGAGAGCCCACTTCAAATG
AACTTCTTATCGAGGTGATAGCCCACGATCTAAGGTACCCGAGTTTGGTGAGCGCAAAACCCGCGAAGGT
TCTATTGGAATTTTTGGCCGCCGAACCAGTTTCATTTATAATGCAACATCTGGAACACGATGACATCAAC
AATCACTCACATCATCGAGAGAAAAGACGTGTCACACGAGCTGTTCGACCCACCAAGAGGATTGAGTTCA
CCGAAGCTGATGGAGACACTGAGGGAAAATCGGTGTTCCAGTTGGAAAAGGAGACCGATAAGGAAACATT
CAAGATCCGGGATGATAATCCCTGGGTGACGGTGGAAACAAACGGAGCTGTGCGAGTTAAAAAGAAGTGG
GACTACGAGGAGTTGGGACCAGAGAAGACTATCGATTTTTGGGTCATCATCACAAATATGGGACACAATG
G
>dme_circ_0000640_junction_seq
TCATCATCACAAATATGGGACACAATGGTTTGCGCCTCCCACAGGAAAACAAACAA
>dme_circ_0000641
GAGGTGACAATGAAAATGGGGTGGCCATCATTTCATCCCTCCGTCCCTTTGATCGCGAGGCGCAAAAGAG
CTATGCCATTCCCATTGAGATCAAGGATAATGGAGCTCCTGCCATGACGGGCACCAGTACTCTAACCGTT
ACCATTGGCGATGTAAATGATAATAAAATGCAGCCCGGCAGCAAATCCGTATTGGTATATAACTACCAAG
GACAGTCGCAGGATACGCCGATTGGCAGGGTCTATGTCAATGATCCCGATGACTGGGATGTGCCGGATAA
GAAGTACTACTGGGAGGTTCAGGAGCACCAAAGATTTAAACTGGATACGGATACTGGTATTTTAACCATG
AGAGCGGGAACGAGAAGAGGTCGCTATCAGTTGCGGTTCAAGGTCTACGATCGTGAGCATGGTCAGGAGG
ATATTCCTGCCAATCTCAGCGTTACAGTGCGCGACATAACAGCAGAGGCCGTTCAGCAAGCTGGTTCAAT
GAGGTTGTCTCACATAACCGATGAGGATTTCGTTAGAACTTGGAACCCGGTTAAGAATCAAGTAGAGCCC
TCCAAGCTAGAGAGATTTCGCAACAAGCTGGCCGAATTATTGTACACCGATAGAGACAATGTCGACGTAT
TCAGTGTGCAACTTAAGGAAGGTTCTCCTTATCCCCTAACCGATGTCCACTTTGCAGCTCGATCTGCTAC
TCAGCAACCGTATTTCAAAGCAGTCCGCCTAAATGGTGTGGTCCAAATGCATCGTGAGGAGATTGAAAAA
GACGTGGGCCTGAACATCACCATGGTCAATATAAATGAGTGCTTGCATGAAGGTAAAGGGAAGTGTGGCT
CCAATTCCTGTACCTCCAAAGTTGAGTTGGGCAAAAAACCCTATACGGTTAGTGTAAATCGCACTGCTTT
GGTGGGCGTTCGTTTGGACATTAGTGCTCAATGTGTTTGCCGGGCGAGGAACTTCACCCACCAGGATCAC
AATTGCAGGACCCACCTTTGTTATAATGGCGGTCGGTGTGTGGAAACCCGAAATGGTCCGAAATGCGTGG
CCTGTCCGGTGGGGTACAATGGCCCGCGATGTCAGCAATCCACGAGAAGTTTTCGCGGCAATGGCTGGGC
CTGGTATCCTCCATTACAACTCTGCCAGGAATCCCATCTCAGCTTGGAATTCATCACTCGCGTTGCAGAC
GGCTTGATACTCTACAATGGACCCATTGTCCCACCAAAGCCAGAGGAAACCGTGATCAGCGACTTTATAG
CTCTCGAACTGGAACAGGGCTATCCGCGTCTACTCATCGATTTCGGCTCGGGAACTTTGGAGTTGAGGGT
AAAGACCAAGAAAACGCTGGATGATGGGGTCTGGCATCGATTGGATATTTTTTGGGATACCGAAAATGTC
CGGATGGTTGTGGACTTTTGTCGCACTGCCTTGGTCTCCGAAATGGAGGATGGCACTCCGCCGGAATTCG
ATGATAACGCCTGTCAGGCGAGAGGACAAATTCCGCCATTTGCTGAGTCCCTAAATCTAAATCAACCCCT
TCAGTTGGGCGGACTCTATAGACAGCACTTCGATCAAACCCTTTACAATTGGCAGTATGCCTTCAGCTCC
AAGGGCTTTGATGGTTGTATACGCAACGTTATCCACAACTCAGAGCACTATGATTTGGCTTTTCCGGCTT
TGGCCAGGAATAGTTTTCCCGCCTGTCCGCAGACCGACGAAGTTTGCCTGAAAACCGAGCACACAGCTCG
TTGTTGGGAGCATGGAAACTGTGTGGCCAGCTTGGTTCAGGCCAAGTGCCATTGCCAGCCGGGTTGGATG

GGTCCTGGCTGTAATGTACCCACCATTCCGACGACTTTCAAGGCCCAGAGCTATGTGAAATTCGCCCTCA
GCTTTGAGCCCGACAGATTTTCCACCCAGTTGCAGCTGAGATTTCGAACTCGAGAGCAGGGAGGCGAACT
TTTCCGGGTTAGTGATCAACACCACAGGGAGTACGCCATACTGGAACTTCGAAGAGGCCACCTGCAGTTC
CGCTATAATCTGAACTCCATGAGAAACGAGGAGCAGCTGCTCACCCTAACCGCAATCGCAGTGAACGATG
GCCAATGGCATGTGATTCGCATTAGTCGATATGGATCGGCAGCTCTAATGGAACTGGATGGCGGGGAGTC
GCGGCGCTACAACGAGTCCTTTCACTTCACAGGGCATCAGTGGCTGACCATCGACAAGCAGGAGGGTGTT
TATGCGGGCGGCAAGGCCGAGTATACGGGAATCAAGACCTTTGAGGTGCAGTCCGATTTCCAGAGAAGCT
GCTTGGACGACATCAGGTAAGCTCAAATGCTGATTAGAGCCAATTGCGTAGCAGAGATACACTCTCCAAA
GGGGTAATACAAAATATGTCTTTTCGatgaaaagaaaatgaattcaatgaaatgaattaaaaatgtttag
aaaaaaagccaatttaagagtagaaaacaagtaacaagaaaataaaaaatATACAGACTATTTCGAATTT
GAACACAACTTGTAAAGTAAAAGTGTTTAAACTAAGAACGAAGTGGTATGCACTTAAAGCAAGATAAAAA
GAAGCACACATGAATAGGAATCCAATAAATATATGGTACTCCTTAGCATCTTAACATCTGTTAACCAAAG
ATAAAGGAAAAGGACACAGTTTTATAGCCCATCGATTGCCGGCATTCATGCGTAGTTTTCCCATTTTTCC
CCTCTCCCGACCAACAACAGGCTGGATGGAAAGCACTTGCCCCTGCCGCCCGCCATGAATGGCACCCAAT
GGGGCCAGGCGACGATGGCCCGCAACCTGGAGCGCAACTGCCCCTCGAACAGGCCGTGCTCGAATGTCAT
CTGTCCCGATCCCTTTGACTGCGTCGACTTGTGGAACGAATACGAGTGCACG
>dme_circ_0000641_junction_seq
CGACTTGTGGAACGAATACGAGTGCACGGAGGTGACAATGAAAATGGGGTGGCCAT
>dme_circ_0000642
GGAGGCCTGAATTCACTGCTGTCGCCCAATGAGTTGGACGTCGATGTTAAAGTTATTATCATCCGTGGAC
CCAGGAATTCCATTACAATCGGTCCAGCGCTGCGGCAGTTCATGAAACTGGAGATACTCCGCATCACCGA
CTCCAATCTGCCGGCAATAGGCGCGGAGTCGTTTTGGGGCCTCAAATATCTGCGGATATTAGGGTGAGTA
ATCAATGCCTTTACGATCGCCGAGGCTCTTAAATTCAGCCTGAACGTATAATTCCAAAAGGGGCAGCGAT
TTCAATTTAAGCAGCTGCGCGCTCTTAAAACCCCACTCGCTGATATGCAAATAATACGTAGCAATCGGAA
GAAAAAGGAGCGGGTGGCATCACAGCGATGCGGTTGTCCTGCAGCCTCGTAAAATGCGAAACGCAAACGA
AAAGGCAAATAAAGGAGCGGACGCGTCGCAAAACGTTAAATGAGCAGGCCGAAAAAGGACAGCGCAGGAT
AACGCTGATTTCAAAAAATTTTAAGTTGGGTCTTCCAAATTTATCATCAGTTTATGTTTAATTCCAAAGG
TTGAAGAGCTTTTTTTGTTTATAAAATCATGAAGCAAATAAACTAAGATGCAAGGTTAAATAACAGCTTT
AACTCTGTTCCttttttttACTGATTAAAAGTTAATTTTCTTTAAAATACTTAATCAGCAGAATCCTTTG
TATAGAGTAATCGAGCTTAAAATCATTGTACGAAATCTACATAAAAGAGTAATGAAACTTCGCTTTCGGC
CTGCCAAACGCCTGTTGTCTTGTCAATAAAATTGAAATTGATGCGCCAAACAGCAGAAAGCGCCCGAAAA
ATAAAAGAGGATTCGACTGGCGGCGGCAGAAAGCAAGTGTAAAGCCAAAGAAAGCAATGCCCCGATATCG
TTGGGGCTTTAAACTTGTTTATTTGTGCTTTATGTGTGCCGTAGCGCCCCCTGCTGGCGGTTTACAGCCA
TGGTACTGCCCTCCAATTATTTACATGCTCGGATCTTATTTTTTTGGCTCGCCCCCCTCAGCCATTGGTG
TTTCATTCGCTTGTCAGGAGTTTCACAAGCTACAAAAACCGAGTTGCACTGCAGTTAAGGATTTTTTCCG
GTCTGCCAGCAAAGAGCAGCACGGAACAATTGGCGTTTCTTTTGGCTTGACTTTGCCTTTTCCTATTTCT
ATTTAAATTAATTTTAGCGAATGTTTAAGGGGCGTGGTCGCCTCTTGATTTTTGCTAGACAATGGGGCGT
ATGTGTAATGTGTTTTGGGTTTATTTAAGCTGCGTCAAAACACACGGAGAACTGAGCTGCTTTAATGCTT
TATTTCTATTGTAGTAAGTCGTGTTAAATTGATTTTTTCATTCATAGCTTATCATATTGTTCTTTTGGTA
GAGAACTGACAATAAAATCCAGGCCACCATGCATTTCTGTTGCAGATCTGTCCAAGAACAATATAACCAA
TATCACAGAGAACAACTTTCGAGGACAGGACAACCTACTGGAATTGGACTTATCGAAAAATAAAGTTTTA
CGCATGGCCAGTTCAACGTTTCGCCATTTGACG
>dme_circ_0000642_junction_seq
GGCCAGTTCAACGTTTCGCCATTTGACGGGAGGCCTGAATTCACTGCTGTCGCCCA

>dme_circ_0000643
GGAATACGCCATTATTCCATACTGCCATTTTCACAATTAACGAACTTTACCAGTGTTTCACTAAGATACT
TGTAACTTAATTTTAATGTTTTTATATGCGCATCTGTATATAAAATTTGTCTACATTTAGGTAAA
>dme_circ_0000643_junction_seq
ATATAAAATTTGTCTACATTTAGGTAAAGGAATACGCCATTATTCCATACTGCCAT
>dme_circ_0000644
TTATAAATATAATACACATACACACATACAATATATGGATATTGGATTTGCTATATAGGAAAAGTACGAC
GACTGCAGTTCAGAACATTTCAGCAACATCAGCATTTTTTAGGATCTACAAAAATCGGTTTGACTTGTAG
AAATACGCCATTTTTTTCGTTGGTTGAGG
>dme_circ_0000644_junction_seq
AATACGCCATTTTTTTCGTTGGTTGAGGTTATAAATATAATACACATACACACATA
>dme_circ_0000645
GTAACACATCATTTAGATTCCATACTTCAAATAAATACAATGAAATATTACAAGAATACCAGCGATTACG
AAAAGCTCGATTGGCCCTTCTTTTTGCAATACTTATGTGTGTAGGAGAGAGGGTTGCTTTAGATAATAAT
ACTTAGTTTATAGTACAATTAGCTAACTGCGATTTCGATTCTACATTCTACCAGCTCGCTTTCAAGAGTG
CCACAACTCAAAAATCCGTCCGAGCTGCATTCCAATAG
>dme_circ_0000645_junction_seq
AAAATCCGTCCGAGCTGCATTCCAATAGGTAACACATCATTTAGATTCCATACTTC
>dme_circ_0000646
TTTCCAGAAGCAATAATAGCAACAGTTGCTGCCGCAGTCGCAGCAGTAAAGCACTTTCAATGGGAGCAGC
AACATCCTCCGCCTCCGGATCCGCATCCACATCCGTGTCCACATCCACATCCTCGAAGCGACGATGCTGC
AAGTGCAAGTGCCGCGACGCCATCCGAGGATTAAGGGATATGCTGCCGAGCAGCAGCAGTTCCGTGAGCA
ATAAAAAGGATGCCGCTGCGGTGGTGGCGATGGTTCCGAGGAAATCCCGGACAGCTGACCGCCGCTCCAC
ACCGCCCACGCTGGGCACATCGAGTGGTGGCTCACAGCGCCGGATGCAGAGCCAAAGGATACGAAATTCC
GTCGCCGTTCCGGACGCCAGCCATCACCATCATCAGCTCATCATTCGGATCACATCGCCGGGCTTCGTGG
TCGAGTCGCCCAATGGCGGCCGCGTAACTGTGGTCACCGAgcccgccccccagtcgccccagactccgcc
ccggccacgtcccctggccacggtcgccccCGCCGGAGGGGCTGGCCATGCCGGAATCGGAGCCGCCCGC
AACATGACCGTGCACTCGCAAATCGACTTCATGCACTGCCTGGTTCCCGATCTCGAGAAGATCACGAACA
GCAGCTTCTACTGGGGCAAGATGGACCGCTACGAGGCGGAGCACCTGCTCGAGGGCAAGCCGGAGGGCAC
GTTCCTGCTGCGCGACTCCGCCCAGGAGGAGTTCCTCTTCTCGGTCACATTCCGGAAGTACGGCAGGTCA
CTGCACGCTCGCATCGAGCAGAGCGGTCACAAGTTCAGG
>dme_circ_0000646_junction_seq
CATCGAGCAGAGCGGTCACAAGTTCAGGTTTCCAGAAGCAATAATAGCAACAGTTG
>dme_circ_0000647
GATTCGATGCAGCCAAGCACTGGCTGGCAGTGAGCTCCATCCTCCTGATAATTGGCGCTGCCAGTGTGGC
CGTGCCACTGGCGCTACGTGTGGCAGCAAGTGAGTATCGCACCGAATCGAATCGAATCAAGTCCAAACGG
GGGCCCCCAGTCCACCTTCACATACCGCACATATCAAGACCTAGGCATGAACACTTCATAGACAATAAAT
CAATTACCATAACGCATTCGACACGTGGGCCGCCGCTGGGTGTGATTCTATGAAGCTATATTGTTGCAAA
GGGGCCTCTAAACTAGTTAGCAAGTGACTCTAGTGTGCACAGAGTGAGGTTTTAACTAGAGGATAGAACA
TAAGGATTGATTGATTAAAGCACAATGTTCTGCTAACGAATCAATGTTGTAATTATATCATATTAATTCG
GATGATTACATCGCCAATCCCATTCTCAGGTGCACCCTTTGAGGAGCGTTTGCGAGTGGCAGTTCAGCTT
CTGGATCAAGTGCCTTTAATCGACGGGCATAACGACCTACCATGGAATATTCGCAAGTTCCTTCACAACA
AACTCAACGACTTCAAGTGAGCATCCATCACATAGAAATGCAATTAAAACGTAATACCAATTTCTGTTAT
TATCCCCACCTGCCGCCGACCACCTCATCGTCCGCAGTTTTGATGAGGATTTGCGCAACGTAATGCCCTG

GGGTCGCAGCCATTGGAGTCACACGGACCTCACGCGACTGAAGAAGGGACGCATATCAGCACAGG
>dme_circ_0000647_junction_seq
CTGAAGAAGGGACGCATATCAGCACAGGGATTCGATGCAGCCAAGCACTGGCTGGC
>dme_circ_0000648
TTATTATAGACAAACAACCGGCAGCGACTAAGCCAAGTCTATTAACAGGGCAAACAAAGCCAGGCCGCCA
AAGAGAAACTGATTGTaaagaagcgaaaagaatgtaagcagtcaaaaaaggaagaaagtcgaaaaagCTG
GCAATGAGAGGGCCAAGAACGCAAGGGGCGCGGGCCAGAATTGAAACAGTTAGCTGGCAAATCGGATTGC
GAATCTGCTGTATTGACACAAGGAAACCGTTAGCCGGCTTAGCCAGCAAGAGGGCAAATTTAAAATTAAG
AATATAGCAAGCAAGACCGTTTCAAATCAAAGTGATATGCTAATGCCCCAGTCGCGAAGGACTTGAAGTC
GTGTGGCATGAATATTTCATGAGCGCAGGAGTGATGACAATAAGCCGGCTCCGGGAATAAAAAAGAAGCG
CAGAGTGAAATCAGGACGATGACGGAGCTAAGACGAACTAATGGCCTGCACAAGCAGCACTCCCTGAGAG
ATCGACAGGTGCATCCGATGCTGCACGCCCTGGCGGATCACGGCTGCAACCTCAGCAGCGGTGGCAACAG
TGCGGCCAGCACCACCAGCAGTAGTACGGCCACGGCCACCACAGCACTTGGTGGTGCCGGCGGTTTGGGC
GGTCTGGGTGGCCTGAACGGTGGTGTCGGCATAACAAGTCAGCGGAGCGTTGAGTTCGATGAGCACGACA
TCTTCTACGTCTCACCATCCAAGCGAAAAGGAGCCACTTCAGGTAGGTTCCTCTGGCAATGTGGTAACCT
TCTCGAATTTTGTAAGTGAACAACGCCTGACGCCCTCGTCGTCGAACCGCGGATCCCTGAAGCGGAGCAA
GGGCCGCTCCAATCAGTCCCTGTGCAGCTGCGATGCCGGCGACGAGGCCCAACTGGATTTGCAACCGAAT
GCGGCAAGGCCGCTGTACGAGTACAGTCTGGAACGAAAGCGTAAGACACACACCTACTCCTGCGAGCAGA
ATGCGCAGATACTTATGAGACTGGAGCGCGAGCGTAATCGTAAATTATCGCTGACtgggatggaaacggg
attgggaatcggactgggagtgggattgggaatgggatCTGGAGGAGGAACAGGAGGAGCCGGAGACCCC
CAGG
>dme_circ_0000648_junction_seq
GGAACAGGAGGAGCCGGAGACCCCCAGGTTATTATAGACAAACAACCGGCAGCGAC
>dme_circ_0000649
aataaattcGGCCACAATTAATATACTTATTTATTCATCAAAACCAAACCACCACAAGAGCATAACCAAT
CATGTTAAAGTTTATCAGAGGAAAAGGGCAGCAGCCCAGTGCTGACAGACAACGCTACATCGTAAGGTAA
AACAAACAATTTTCTCTTTCCGTTCTAttttttttttttAAGTATTTGTAGCCGATTGGTATATTTTGT
GGGGTCATTTATTTTGATCTTGGGTGGTAAATACATTTTTATGGAAGTTAGAAGAAAGCTTATAATTTAT
GAGGAATAAATAAATACCTCCTTTTGAATCTTTTGTTATGTCTTCTCCTATGTGCATACTTTTGGGCATT
AACATTTAGGTAAATTTTTTCCACCGCAACTACAAGTAAACCGCATACACACACAGTATTTTGTAGACTC
GCGCCACAAAGCACCGCATAAAAAAGGTTACGGTGCTTTTTTGCTCGTTTCTTTTGTGCACTCCTTCTAT
GCGGTGCCAAATATGAGGAGGTACCTGAAGCTTGGTTGATTTTGTGAGTGTCTTTTTCCACAGCATATTT
TTGTGTATTGGCTACTTTAAAAATTCTATTATATCGTTGTGCTCGTAATGTTGTCCATTCCTTTTAAATA
TTTTCGAATTAAATATACCATAGATGTTTCCCTATTACCATAATAGTTTAATAAATATACGTATATGAAA
ttttttttATCCTATGTTAGTATAAACATGTTATTCAACAAATAAAAGTAGGTCAGAGGTAATCGCCATC
CAGCTCCAGAAAGAGGGGGGAGCTGATAAGATTGCGTTTATGTACCTAATGGGTTGGGTAAGTGGCTTGG
TTTCACAAACACCCTTATTTCAGGTACAAAATAAGTATGTCATAGACGGCGTAAAATTGATAAGATTTTA
AAGTCAAAAGAAAATGAGTTTATTCGCCTGTAACTGCGTTAGAAATCCAAATACACCCGCAATATTATAT
GTATAGAAAGAAAAAGTTTGATGCAACAACAAATTTAAAGTTGTTTGGTATTTTGTAATGGAAAGTTTGT
GTCACTAAATATGGTTTTACTTGAACGCAGATTGGACATGGTCACACTGCCCACAAAATCACGACATTGC
GTTATAAATGAAGCACATCTAGTTTTGTGTTGAAAATGTGGTTCACAATTTATATAGCAGGTTATGGTAA
AACATCGATCGTTCCGAAAAATTCCGCTCGTTGGCACATTTCCTAAATTTTTCGTGCGACAGCGGTGATT
AATTACAAAAATTATTATTATAATTATATAAAAGTTGGCGACCATATCAAAAAGGTTGCTATAAAAACAA
ATGACAATTTATAGCGGAGTTTCACTCCCAAAGCGATGTTCTCCACCCATTCCAGTCAGTCATGGAAAAT

ATACCTTCTAAAAAATATATGATTTTTTCGATAGAGATACACGGGCACACTAGACCCACCGATCGCGACT
TTGTGTGTGTTAAAATGACGCGCATATCGTTTTGGGCTAATAATATTGTTCACGTTTTATAAAACAGATA
CACGGTTAAGCTCTGACCGGTCGTATTATTATCTGTGTGTTCTTGGTTGACACGTTTATTTCATTTATTC
TCATTTAAAGCAACGATGTGAAGAACAGTGCGGGCGTTTCTCAACATAAGAATACATACAAACTTATGCG
TGTTGGTGAGCAAATAAAATAGTTCAAAAAATTACTCGGCCACCGCCTTAACCGTACAAACGGAAATACA
TAAAGGTAAAGGGCTCAACAACAGTATTACTGATTAGGCAATTGATTTATATTTCAAACTTCTTAAATAA
AGTGTCCACAAATATGTATGTTCTTAGCATAGAGGCGTAGTCGGTGAGTCAGCCACGCCAACAGTTAACA
GTCGTCATTTTGCTGTCTGTCTAACACGCAATTTGTGTGTGTATATGTGAATAAACAGATACCGGTATGC
AATCGCAAAGCTGGTCAACTGTAAAGATCGGTATGTACTCAAAATTAGGTCATCACCTTGTTATGCAGAT
ACATTTGGGAAACAGTTGACTTACTTGGTATTGGTTATCAGCCGAAAAGCAAATGGTGATACGGTTTTGC
ACCTCAGCTTACTCACGGATACATACCTACGTACATAAATGTATGTATACGGTATACAGTCTTCGAAAAA
ATATTAGTACCGACCAGCTAGCGTTTGCCTAAAACGCTTTTACAAAAATAAACGTATATGTTTTAAGTTG
TTTTTTGTCATAATTAATACATTTATTATTATTTATTTATGAATTTCAGAGCTTTAATAGTAACTAAATT
GTTTAAACAAACTTaaaaaaagatttacaaataaaaaaatATTTCGTGTGCGTGACGGAATTGCTAGTTC
ATTAAAGGGAGGTACATATGTACATACATACACATTGCAAAGAGTACAGATCGCTCATCTTGCATCTCAT
TTTTCCTAATGTACATATACATATGTACAAACATGTTTATTAAACTGCCTACATGCAAATCTTTGAAGTT
TTAAGAAAATAAGCACAACTAGTTTGGAAAATCTTGGGAGCCAAGATCTATCTGTCCACTGAATTTCCTA
TGACCAAGAATGTAAAACCCTTTTTGTTAAACAAATTTTGATTAAAGTATACCAGAATAATATTAAACGT
ATCGTCCGTATCTATAAAAGGTATTAATAGTATTACCTAAATTTAATTTCTCGGTTTTATTCATAAGAGT
GAAAAAATATACAAGTTCCTTTTTAATTTTTTACTTATTGAAAATGTGATTAATTTTTATTTATTATTTT
CAGTGTAAGGTAAAACAAACAATTTTCTCTTTCCGTTCTAtttttttttttttAAGTATTTGTAGCCGAT
TGGTATATTTTGTGGGGTCATTTATTTTGATCTTGGGTGGTAAATACATTTTTATGGAAGTTAGAAGAAA
GCTTATAATTTATGAGGAATAAATAAATACCTCCTTTTGAATCTTTTGTTATGTCTTCTCCTATGTGCAT
ACTTTTGGGCATTAACATTTAGGTAAATTTTTTCCACCGCAACTACAAGTAAACCGCATACACACACAGT
ATTTTGTAGACTCGCGCCACAAAGCACCGCATAAAAAAGGTTACGGTGCTTTTTTGCTCGTTTCTTTTGT
GCACTCCTTCTATGCGGTGCCAAATATGAGGAGGTACCTGAAGCTTGGTTGATTTTGTGAGTGTCTTTTT
CCACAGCATATTTTTGTGTATTGGCTACTTTAAAAATTCTATTATATCGTTGTGCTCGTAATGTTGTCCA
TTCCTTTTAAATATTTTCGAATTAAATATACCATAGATGTTTCCCTATTACCATAATAGTTTAATAAATA
TACGTATATGAAAttttttttATCCTATGTTAGTATAAACATGTTATTCAACAAATAAAAGTAGGTCAGA
GGTAATCGCCATCCAGCTCCAGAAAGAGGGGGGAGCTGATAAGATTGCGTTTATGTACCTAATGGGTTGG
GTAAGTGGCTTGGTTTCACAAACACCCTTATTTCAGGTACAAAATAAGTATGTCATAGACGGCGTAAAAT
TGATAAGATTTTAAAGTCAAAAGAAAATGAGTTTATTCGCCTGTAACTGCGTTAGAAATCCAAATACACC
CGCAATATTATATGTATAGAAAGAAAAAGTTTGATGCAACAACAAATTTAAAGTTGTTTGGTATTTTGTA
ATGGAAAGTTTGTGTCACTAAATATGGTTTTACTTGAACGCAGATTGGACATGGTCACACTGCCCACAAA
ATCACGACATTGCGTTATAAATGAAGCACATCTAGTTTTGTGTTGAAAATGTGGTTCACAATTTATATAG
CAGGTTATGGTAAAACATCGATCGTTCCGAAAAATTCCGCTCGTTGGCACATTTCCTAAATTTTTCGTGC
GACAGCGGTGATTAATTACAAAAATTATTATTATAATTATATAAAAGTTGGCGACCATATCAAAAAGGTT
GCTATAAAAACAAATGACAATTTATAGCGGAGTTTCACTCCCAAAGCGATGTTCTCCACCCATTCCAGTC
AGTCATGGAAAATATACCTTCTAAAAAATATATGATTTTTTCGATAGAGATACACGGGCACACTAGACCC
ACCGATCGCGACTTTGTGTGTGTTAAAATGACGCGCATATCGTTTTGGGCTAATAATATTGTTCACGTTT
TATAAAACAGATACACGGTTAAGCTCTGACCGGTCGTATTATTATCTGTGTGTTCTTGGTTGACACGTTT
ATTTCATTTATTCTCATTTAAAGCAACGATGTGAAGAACAGTGCGGGCGTTTCTCAACATAAGAATACAT
ACAAACTTATGCGTGTTGGTGAGCAAATAAAATAGTTCAAAAAATTACTCGGCCACCGCCTTAACCGTAC
AAACGGAAATACATAAAGG

>dme_circ_0000649_junction_seq
TAACCGTACAAACGGAAATACATAAAGGaataaattcGGCCACAATTAATATACTT
>dme_circ_0000650
tttagaataaattcGGCCACAATTAATATACTTATTTATTCATCAAAACCAAACCACCACAAGAGCATAA
CCAATCATGTTAAAGTTTATCAGAGGAAAAGGGCAGCAGCCCAGTGCTGACAGACAACGCTACATCGTAA
GGTAAAACAAACAATTTTCTCTTTCCGTTCTAttttttttttttAAGTATTTGTAGCCGATTGGTATAT
TTTGTGGGGTCATTTATTTTGATCTTGGGTGGTAAATACATTTTTATGGAAGTTAGAAGAAAGCTTATAA
TTTATGAGGAATAAATAAATACCTCCTTTTGAATCTTTTGTTATGTCTTCTCCTATGTGCATACTTTTGG
GCATTAACATTTAGGTAAATTTTTTCCACCGCAACTACAAGTAAACCGCATACACACACAGTATTTTGTA
GACTCGCGCCACAAAGCACCGCATAAAAAAGGTTACGGTGCTTTTTTGCTCGTTTCTTTTGTGCACTCCT
TCTATGCGGTGCCAAATATGAGGAGGTACCTGAAGCTTGGTTGATTTTGTGAGTGTCTTTTTCCACAGCA
TATTTTTGTGTATTGGCTACTTTAAAAATTCTATTATATCGTTGTGCTCGTAATGTTGTCCATTCCTTTT
AAATATTTTCGAATTAAATATACCATAGATGTTTCCCTATTACCATAATAGTTTAATAAATATACGTATA
TGAAAttttttttATCCTATGTTAGTATAAACATGTTATTCAACAAATAAAAGTAGGTCAGAGGTAATCG
CCATCCAGCTCCAGAAAGAGGGGGGAGCTGATAAGATTGCGTTTATGTACCTAATGGGTTGGGTAAGTGG
CTTGGTTTCACAAACACCCTTATTTCAGGTACAAAATAAGTATGTCATAGACGGCGTAAAATTGATAAGA
TTTTAAAGTCAAAAGAAAATGAGTTTATTCGCCTGTAACTGCGTTAGAAATCCAAATACACCCGCAATAT
TATATGTATAGAAAGAAAAAGTTTGATGCAACAACAAATTTAAAGTTGTTTGGTATTTTGTAATGGAAAG
TTTGTGTCACTAAATATGGTTTTACTTGAACGCAGATTGGACATGGTCACACTGCCCACAAAATCACGAC
ATTGCGTTATAAATGAAGCACATCTAGTTTTGTGTTGAAAATGTGGTTCACAATTTATATAGCAGGTTAT
GGTAAAACATCGATCGTTCCGAAAAATTCCGCTCGTTGGCACATTTCCTAAATTTTTCGTGCGACAGCGG
TGATTAATTACAAAAATTATTATTATAATTATATAAAAGTTGGCGACCATATCAAAAAGGTTGCTATAAA
AACAAATGACAATTTATAGCGGAGTTTCACTCCCAAAGCGATGTTCTCCACCCATTCCAGTCAGTCATGG
AAAATATACCTTCTAAAAAATATATGATTTTTTCGATAGAGATACACGGGCACACTAGACCCACCGATCG
CGACTTTGTGTGTGTTAAAATGACGCGCATATCGTTTTGGGCTAATAATATTGTTCACGTTTTATAAAAC
AGATACACGGTTAAGCTCTGACCGGTCGTATTATTATCTGTGTGTTCTTGGTTGACACGTTTATTTCATT
TATTCTCATTTAAAGCAACGATGTGAAGAACAGTGCGGGCGTTTCTCAACATAAGAATACATACAAACTT
ATGCGTGTTGGTGAGCAAATAAAATAGTTCAAAAAATTACTCGGCCACCGCCTTAACCGTACAAACGGAA
ATACATAAAGGTAAAGGGCTCAACAACAGTATTACTGATTAGGCAATTGATTTATATTTCAAACTTCTTA
AATAAAGTGTCCACAAATATGTATGTTCTTAGCATAGAGGCGTAGTCGGTGAGTCAGCCACGCCAACAGT
TAACAGTCGTCATTTTGCTGTCTGTCTAACACGCAATTTGTGTGTGTATATGTGAATAAACAGATACCGG
TATGCAATCGCAAAGCTGGTCAACTGTAAAGATCGGTATGTACTCAAAATTAGGTCATCACCTTGTTATG
CAGATACATTTGGGAAACAGTTGACTTACTTGGTATTGGTTATCAGCCGAAAAGCAAATGGTGATACGGT
TTTGCACCTCAGCTTACTCACGGATACATACCTACGTACATAAATGTATGTATACGGTATACAGTCTTCG
AAAAAATATTAGTACCGACCAGCTAGCGTTTGCCTAAAACGCTTTTACAAAAATAAACGTATATGTTTTA
AGTTGTTTTTTGTCATAATTAATACATTTATTATTATTTATTTATGAATTTCAGAGCTTTAATAGTAACT
AAATTGTTTAAACAAACTTaaaaaaagatttacaaataaaaaaatATTTCGTGTGCGTGACGGAATTGCT
AGTTCATTAAAGGGAGGTACATATGTACATACATACACATTGCAAAGAGTACAGATCGCTCATCTTGCAT
CTCATTTTTCCTAATGTACATATACATATGTACAAACATGTTTATTAAACTGCCTACATGCAAATCTTTG
AAGTTTTAAGAAAATAAGCACAACTAGTTTGGAAAATCTTGGGAGCCAAGATCTATCTGTCCACTGAATT
TCCTATGACCAAGAATGTAAAACCCTTTTTGTTAAACAAATTTTGATTAAAGTATACCAGAATAATATTA
AACGTATCGTCCGTATCTATAAAAGGTATTAATAGTATTACCTAAATTTAATTTCTCGGTTTTATTCATA
AGAGTGAAAAAATATACAAGTTCCTTTTTAATTTTTTACTTATTGAAAATGTGATTAATTTTTATTTATT
ATTTTCAGTGTAAGGTAAAACAAACAATTTTCTCTTTCCGTTCTAttttttttttttAAGTATTTGTAG

CCGATTGGTATATTTTGTGGGGTCATTTATTTTGATCTTGGGTGGTAAATACATTTTTATGGAAGTTAGA
AGAAAGCTTATAATTTATGAGGAATAAATAAATACCTCCTTTTGAATCTTTTGTTATGTCTTCTCCTATG
TGCATACTTTTGGGCATTAACATTTAGGTAAATTTTTTCCACCGCAACTACAAGTAAACCGCATACACAC
ACAGTATTTTGTAGACTCGCGCCACAAAGCACCGCATAAAAAAGGTTACGGTGCTTTTTTGCTCGTTTCT
TTTGTGCACTCCTTCTATGCGGTGCCAAATATGAGGAGGTACCTGAAGCTTGGTTGATTTTGTGAGTGTC
TTTTTCCACAGCATATTTTTGTGTATTGGCTACTTTAAAAATTCTATTATATCGTTGTGCTCGTAATGTT
GTCCATTCCTTTTAAATATTTTCGAATTAAATATACCATAGATGTTTCCCTATTACCATAATAGTTTAAT
AAATATACGTATATGAAAttttttttATCCTATGTTAGTATAAACATGTTATTCAACAAATAAAAGTAGG
TCAGAGGTAATCGCCATCCAGCTCCAGAAAGAGGGGGGAGCTGATAAGATTGCGTTTATGTACCTAATGG
GTTGGGTAAGTGGCTTGGTTTCACAAACACCCTTATTTCAGGTACAAAATAAGTATGTCATAGACGGCGT
AAAATTGATAAGATTTTAAAGTCAAAAGAAAATGAGTTTATTCGCCTGTAACTGCGTTAGAAATCCAAAT
ACACCCGCAATATTATATGTATAGAAAGAAAAAGTTTGATGCAACAACAAATTTAAAGTTGTTTGGTATT
TTGTAATGGAAAGTTTGTGTCACTAAATATGGTTTTACTTGAACGCAGATTGGACATGGTCACACTGCCC
ACAAAATCACGACATTGCGTTATAAATGAAGCACATCTAGTTTTGTGTTGAAAATGTGGTTCACAATTTA
TATAGCAGGTTATGGTAAAACATCGATCGTTCCGAAAAATTCCGCTCGTTGGCACATTTCCTAAATTTTT
CGTGCGACAGCGGTGATTAATTACAAAAATTATTATTATAATTATATAAAAGTTGGCGACCATATCAAAA
AGGTTGCTATAAAAACAAATGACAATTTATAGCGGAGTTTCACTCCCAAAGCGATGTTCTCCACCCATTC
CAGTCAGTCATGGAAAATATACCTTCTAAAAAATATATGATTTTTTCGATAGAGATACACGGGCACACTA
GACCCACCGATCGCGACTTTGTGTGTGTTAAAATGACGCGCATATCGTTTTGGGCTAATAATATTGTTCA
CGTTTTATAAAACAGATACACGGTTAAGCTCTGACCGGTCGTATTATTATCTGTGTGTTCTTGGTTGACA
CGTTTATTTCATTTATTCTCATTTAAAGCAACGATGTGAAGAACAGTGCGGGCGTTTCTCAACATAAGAA
TACATACAAACTTATGCGTGTTGGTGAGCAAATAAAATAGTTCAAAAAATTACTCGGCCACCGCCTTAAC
CGTACAAACGGAAATACATAAAGG
>dme_circ_0000650_junction_seq
TAACCGTACAAACGGAAATACATAAAGGtttagaataaattcGGCCACAATTAATA
>dme_circ_0000651
CATAGAGGCGTAGTCGGTGAGTCAGCCACGCCAACAGTTAACAGTCGTCATTTTGCTGTCTGTCTAACAC
GCAATTTGTGTGTGTATATGTGAATAAACAGATACCGGTATGCAATCGCAAAGCTGGTCAACTGTAAAGA
TCGGTATGTACTCAAAATTAGGTCATCACCTTGTTATGCAGATACATTTGGGAAACAGTTGACTTACTTG
GTATTGGTTATCAGCCGAAAAGCAAATGGTGATACGGTTTTGCACCTCAGCTTACTCACGGATACATACC
TACGTACATAAATGTATGTATACGGTATACAGTCTTAGAAAAAAATATTAGTACCGACCAGCTAGCGTTTG
CCTAAAACGCTTTTACAAAAATAAACGTATATGTTTTAAGTTGTTTTTTGTCCTAATTAATACATTTATT
ATTATTTATTTATGAATTTTAGAGCTTTAATAGTAACtaaattgtttaaacaaactttaaaaaagattta
caaataaaaaaatatttCGTGTGCGTGACGGAATTGCTAGTTCATTAAAGGGAGGTACATATGTACATAC
ATACACATTGCAAAGAGTACAGATCGCTCATCATTTCATTTTTCCTAATGTACATATACATATGTACAAA
CATGTTTATTAAACTGCCTACATGCAAATCTTTGAAGTTTTAAGAAAATAAGCACAACTAGTTTGGAAAA
TCTTGGGAGCCAAGATCTATCTGTCCACTGATTTCCTATGACTTCAAGAATGTAAAACCCTTTTTGTTA
AACAAATTTTGATTAAAGTATACCAGAATAATATTAAACGTATCGTCCGTATCTATAAAAGGTATTAATA
GTATTACCTAAATTTAAATTCTCGGTTTTATTCATAAGAGTGAAAAAATATACAAGTTCCTTTTTAattt
tttacttattgaaaatttgattaatttttattattattttcagtttagaataaattcGGCCACAATTAAT
ATACTTATTTATTCATCAAAACCAAACCACCACAAGAGCATAACCAATCATGTTAAAGTTTATCAGAGGA
AAAGGGCAGCAGCCCAGTGCTGACAGACAACGCTACATCGTAAGGTAAAACAAACAATTTTCTCTTTCCG
TTCTAttttttttttttAAGTATTTGTAGCCGATTGGTATATTTTGTGGGGTCATTTATTTTGATCTTG
GGTGGTAAATACATTTTTATGGAAGTTAGAAGAAAGCTTATAATTTATGAGGAATAAATAAATACCTCCT

TTTGAATCTTTTGTTATGTCTTCTCCTATGTGCATACTTTTGGGCATTAACATTTAGGTAAATTTTTTCC
ACCGCAACTACAAGTAAACCGCATACACACACAGTATTTTGTAGACTCGCGCCACAAAGCACCGCATAAA
AAAGGTTACGGTGCTTTTTTGCTCGTTTCTTTTGTGCACTCCTTCTATGCGGTGCCAAATATGAGGAGGT
ACCTGAAGCTTGGTTGATTTTGTGAGTGTCTTTTTCCACAGCATATTTTTGTGTATTGGCTACTTTAAAA
ATTCTATTATATCGTTGTGCTCGTAATGTTGTCCATTCCTTTTAAATATTTTCGAATTAAATATACCATA
GATGTTTCCCTATTACCATAATAGTTTAATAAATATACGTATATGAAAttttttttATCCTATGTTAGTA
TAAACATGTTATTCAACAAATAAAAGTAGGTCAGAGGTAATCGCCATCCAGCTCCAGAAAGAGGGGGGAG
CTGATAAGATTGCGTTTATGTACCTAATGGGTTGGGTAAGTGGCTTGGTTTCACAAACACCCTTATTTCA
GGTACAAAATAAGTATGTCATAGACGGCGTAAAATTGATAAGATTTTAAAGTCAAAAGAAAATGAGTTTA
TTCGCCTGTAACTGCGTTAGAAATCCAAATACACCCGCAATATTATATGTATAGAAAGAAAAAGTTTGAT
GCAACAACAAATTTAAAGTTGTTTGGTATTTTGTAATGGAAAGTTTGTGTCACTAAATATGGTTTTACTT
GAACGCAGATTGGACATGGTCACACTGCCCACAAAATCACGACATTGCGTTATAAATGAAGCACATCTAG
TTTTGTGTTGAAAATGTGGTTCACAATTTATATAGCAGGTTATGGTAAAACATCGATCGTTCCGAAAAAT
TCCGCTCGTTGGCACATTTCCTAAATTTTTCGTGCGACAGCGGTGATTAATTACAAAAATTATTATTATA
ATTATATAAAAGTTGGCGACCATATCAAAAAGGTTGCTATAAAAACAAATGACAATTTATAGCGGAGTTT
CACTCCCAAAGCGATGTTCTCCACCCATTCCAGTCAGTCATGGAAAATATACCTTCTAAAAAATATATGA
TTTTTTCGATAGAGATACACGGGCACACTAGACCCACCGATCGCGACTTTGTGTGTGTTAAAATGACGCG
CATATCGTTTTGGGCTAATAATATTGTTCACGTTTTATAAAACAGATACACGGTTAAGCTCTGACCGGTC
GTATTATTATCTGTGTGTTCTTGGTTGACACGTTTATTTCATTTATTCTCATTTAAAGCAACGATGTGAA
GAACAGTGCGGGCGTTTCTCAACATAAGAATACATACAAACTTATGCGTGTTGGTGAGCAAATAAAATAG
TTCAAAAAATTACTCGGCCACCGCCTTAACCGTACAAACGGAAATACATAAAGGTAAAGGGCTCAACAAC
AGTATTACTGATTAGGCAATTGATTTATATTTCAAACTTCTTAAATAAAGTGTCCACAAATATGTATGTT
CTTAGCATAGAGGCGTAGTCGGTGAGTCAGCCACGCCAACAGTTAACAGTCGTCATTTTGCTGTCTGTCT
AACACGCAATTTGTGTGTGTATATGTGAATAAACAGATACCGGTATGCAATCGCAAAGCTGGTCAACTGT
AAAGATCGGTATGTACTCAAAATTAGGTCATCACCTTGTTATGCAGATACATTTGGGAAACAGTTGACTT
ACTTGGTATTGGTTATCAGCCGAAAAGCAAATGGTGATACGGTTTTGCACCTCAGCTTACTCACGGATAC
ATACCTACGTACATAAATGTATGTATACGGTATACAGTCTTCGAAAAAATATTAGTACCGACCAGCTAGC
GTTTGCCTAAAACGCTTTTACAAAAATAAACGTATATGTTTTAAGTTGTTTTTTGTCATAATTAATACAT
TTATTATTATTTATTTATGAATTTCAGAGCTTTAATAGTAACTAAATTGTTTAAACAAACTTaaaaaaag
atttacaaataaaaaaatATTTCGTGTGCGTGACGGAATTGCTAGTTCATTAAAGGGAGGTACATATGTA
CATACATACACATTGCAAAGAGTACAGATCGCTCATCTTGCATCTCATTTTTCCTAATGTACATATACAT
ATGTACAAACATGTTTATTAAACTGCCTACATGCAAATCTTTGAAGTTTTAAGAAAATAAGCACAACTAG
TTTGGAAAATCTTGGGAGCCAAGATCTATCTGTCCACTGAATTTCCTATGACCAAGAATGTAAAACCCTT
TTTGTTAAACAAATTTTGATTAAAGTATACCAGAATAATATTAAACGTATCGTCCGTATCTATAAAAGGT
ATTAATAGTATTACCTAAATTTAATTTCTCGGTTTTATTCATAAGAGTGAAAAAATATACAAGTTCCTTT
TTAATTTTTTACTTATTGAAAATGTGATTAATTTTTATTTATTATTTTCAGTGTAAGGTAAAACAAACAA
TTTTCTCTTTCCGTTCTAttttttttttttAAGTATTTGTAGCCGATTGGTATATTTTGTGGGGTCATT
TATTTTGATCTTGGGTGGTAAATACATTTTTATGGAAGTTAGAAGAAAGCTTATAATTTATGAGGAATAA
ATAAATACCTCCTTTTGAATCTTTTGTTATGTCTTCTCCTATGTGCATACTTTTGGGCATTAACATTTAG
GTAAATTTTTTCCACCGCAACTACAAGTAAACCGCATACACACACAGTATTTTGTAGACTCGCGCCACAA
AGCACCGCATAAAAAAGGTTACGGTGCTTTTTTGCTCGTTTCTTTTGTGCACTCCTTCTATGCGGTGCCA
AATATGAGGAGGTACCTGAAGCTTGGTTGATTTTGTGAGTGTCTTTTTCCACAGCATATTTTTGTGTATT
GGCTACTTTAAAAATTCTATTATATCGTTGTGCTCGTAATGTTGTCCATTCCTTTTAAATATTTTCGAAT
TAAATATACCATAGATGTTTCCCTATTACCATAATAGTTTAATAAATATACGTATATGAAAttttttttA

TCCTATGTTAGTATAAACATGTTATTCAACAAATAAAAGTAGGTCAGAGGTAATCGCCATCCAGCTCCAG
AAAGAGGGGGGAGCTGATAAGATTGCGTTTATGTACCTAATGGGTTGGGTAAGTGGCTTGGTTTCACAAA
CACCCTTATTTCAGGTACAAAATAAGTATGTCATAGACGGCGTAAAATTGATAAGATTTTAAAGTCAAAA
GAAAATGAGTTTATTCGCCTGTAACTGCGTTAGAAATCCAAATACACCCGCAATATTATATGTATAGAAA
GAAAAAGTTTGATGCAACAACAAATTTAAAGTTGTTTGGTATTTTGTAATGGAAAGTTTGTGTCACTAAA
TATGGTTTTACTTGAACGCAGATTGGACATGGTCACACTGCCCACAAAATCACGACATTGCGTTATAAAT
GAAGCACATCTAGTTTTGTGTTGAAAATGTGGTTCACAATTTATATAGCAGGTTATGGTAAAACATCGAT
CGTTCCGAAAAATTCCGCTCGTTGGCACATTTCCTAAATTTTTCGTGCGACAGCGGTGATTAATTACAAA
AATTATTATTATAATTATATAAAAGTTGGCGACCATATCAAAAAGGTTGCTATAAAAACAAATGACAATT
TATAGCGGAGTTTCACTCCCAAAGCGATGTTCTCCACCCATTCCAGTCAGTCATGGAAAATATACCTTCT
AAAAAATATATGATTTTTTCGATAGAGATACACGGGCACACTAGACCCACCGATCGCGACTTTGTGTGTG
TTAAAATGACGCGCATATCGTTTTGGGCTAATAATATTGTTCACGTTTTATAAAACAGATACACGGTTAA
GCTCTGACCGGTCGTATTATTATCTGTGTGTTCTTGGTTGACACGTTTATTTCATTTATTCTCATTTAAA
GCAACGATGTGAAGAACAGTGCGGGCGTTTCTCAACATAAGAATACATACAAACTTATGCGTGTTGGTGA
GCAAATAAAATAGTTCAAAAAATTACTCGGCCACCGCCTTAACCGTACAAACGGAAATACATAAAGG
>dme_circ_0000651_junction_seq
TAACCGTACAAACGGAAATACATAAAGGCATAGAGGCGTAGTCGGTGAGTCAGCCA
>dme_circ_0000652
TGTCCACAAATATGTATGTTCTTAGCATAGAGGCGTAGTCGGTGAGTCAGCCACGCCAACAGTTAACAGT
CGTCATTTTGCTGTCTGTCTAACACGCAATTTGTGTGTGTATATGTGAATAAACAGATACCGGTATGCAA
TCGCAAAGCTGGTCAACTGTAAAGATCGGTATGTACTCAAAATTAGGTCATCACCTTGTTATGCAGATAC
ATTTGGGAAACAGTTGACTTACTTGGTATTGGTTATCAGCCGAAAAGCAAATGGTGATACGGTTTTGCAC
CTCAGCTTACTCACGGATACATACCTACGTACATAAATGTATGTATACGGTATACAGTCTTAGAAAAAAT
ATTAGTACCGACCAGCTAGCGTTTGCCTAAAACGCTTTTACAAAAATAAACGTATATGTTTTAAGTTGTT
TTTTGTCCTAATTAATACATTTATTATTATTTATTTATGAATTTTAGAGCTTTAATAGTAACtaaattgt
ttaaacaaactttaaaaaagatttacaaataaaaaaatatttCGTGTGCGTGACGGAATTGCTAGTTCAT
TAAAGGGAGGTACATATGTACATACATACACATTGCAAAGAGTACAGATCGCTCATCATTTCATTTTTCC
TAATGTACATATACATATGTACAAACATGTTTATTAAACTGCCTACATGCAAATCTTTGAAGTTTTAAGA
AAATAAGCACAACTAGTTTGGAAAATCTTGGGAGCCAAGATCTATCTGTCCACTGATTTTCCTATGACTT
CAAGAATGTAAAACCCTTTTTGTTAAACAAATTTTGATTAAAGTATACCAGAATAATATTAAACGTATCG
TCCGTATCTATAAAAGGTATTAATAGTATTACCTAAATTTAAATTCTCGGTTTTATTCATAAGAGTGAAA
AAATATACAAGTTCCTTTTTAattttttacttattgaaaatttgattaatttttattattattttcagtt
tagaataaattcGGCCACAATTAATATACTTATTTATTCATCAAAACCAAACCACCACAAGAGCATAACC
AATCATGTTAAAGTTTATCAGAGGAAAAGGGCAGCAGCCCAGTGCTGACAGACAACGCTACATCGTAAGG
TAAAACAAACAATTTTCTCTTTCCGTTCTAtttttttttttttAAGTATTTGTAGCCGATTGGTATATTT
TGTGGGGTCATTTATTTTGATCTTGGGTGGTAAATACATTTTTATGGAAGTTAGAAGAAAGCTTATAATT
TATGAGGAATAAATAAATACCTCCTTTTGAATCTTTTGTTATGTCTTCTCCTATGTGCATACTTTTGGGC
ATTAACATTTAGGTAAATTTTTTCCACCGCAACTACAAGTAAACCGCATACACACACAGTATTTTGTAGA
CTCGCGCCACAAAGCACCGCATAAAAAAGGTTACGGTGCTTTTTTGCTCGTTTCTTTTGTGCACTCCTTC
TATGCGGTGCCAAATATGAGGAGGTACCTGAAGCTTGGTTGATTTTGTGAGTGTCTTTTTCCACAGCATA
TTTTTGTGTATTGGCTACTTTAAAAATTCTATTATATCGTTGTGCTCGTAATGTTGTCCATTCCTTTTAA
ATATTTTCGAATTAAATATACCATAGATGTTTCCCTATTACCATAATAGTTTAATAAATATACGTATATG
AAAttttttttATCCTATGTTAGTATAAACATGTTATTCAACAAATAAAAGTAGGTCAGAGGTAATCGCC
ATCCAGCTCCAGAAAGAGGGGGGAGCTGATAAGATTGCGTTTATGTACCTAATGGGTTGGGTAAGTGGCT

TGGTTTCACAAACACCCTTATTTCAGGTACAAAATAAGTATGTCATAGACGGCGTAAAATTGATAAGATT
TTAAAGTCAAAAGAAAATGAGTTTATTCGCCTGTAACTGCGTTAGAAATCCAAATACACCCGCAATATTA
TATGTATAGAAAGAAAAAGTTTGATGCAACAACAAATTTAAAGTTGTTTGGTATTTTGTAATGGAAAGTT
TGTGTCACTAAATATGGTTTTACTTGAACGCAGATTGGACATGGTCACACTGCCCACAAAATCACGACAT
TGCGTTATAAATGAAGCACATCTAGTTTTGTGTTGAAAATGTGGTTCACAATTTATATAGCAGGTTATGG
TAAAACATCGATCGTTCCGAAAAATTCCGCTCGTTGGCACATTTCCTAAATTTTTCGTGCGACAGCGGTG
ATTAATTACAAAAATTATTATTATAATTATATAAAAGTTGGCGACCATATCAAAAAGGTTGCTATAAAAA
CAAATGACAATTTATAGCGGAGTTTCACTCCCAAAGCGATGTTCTCCACCCATTCCAGTCAGTCATGGAA
AATATACCTTCTAAAAAATATATGATTTTTTCGATAGAGATACACGGGCACACTAGACCCACCGATCGCG
ACTTTGTGTGTGTTAAAATGACGCGCATATCGTTTTGGGCTAATAATATTGTTCACGTTTTATAAAACAG
ATACACGGTTAAGCTCTGACCGGTCGTATTATTATCTGTGTGTTCTTGGTTGACACGTTTATTTCATTTA
TTCTCATTTAAAGCAACGATGTGAAGAACAGTGCGGGCGTTTCTCAACATAAGAATACATACAAACTTAT
GCGTGTTGGTGAGCAAATAAAATAGTTCAAAAAATTACTCGGCCACCGCCTTAACCGTACAAACGGAAAT
ACATAAAGGTAAAGGGCTCAACAACAGTATTACTGATTAGGCAATTGATTTATATTTCAAACTTCTTAAA
TAAAGTGTCCACAAATATGTATGTTCTTAGCATAGAGGCGTAGTCGGTGAGTCAGCCACGCCAACAGTTA
ACAGTCGTCATTTTGCTGTCTGTCTAACACGCAATTTGTGTGTGTATATGTGAATAAACAGATACCGGTA
TGCAATCGCAAAGCTGGTCAACTGTAAAGATCGGTATGTACTCAAAATTAGGTCATCACCTTGTTATGCA
GATACATTTGGGAAACAGTTGACTTACTTGGTATTGGTTATCAGCCGAAAAGCAAATGGTGATACGGTTT
TGCACCTCAGCTTACTCACGGATACATACCTACGTACATAAATGTATGTATACGGTATACAGTCTTCGAA
AAAATATTAGTACCGACCAGCTAGCGTTTGCCTAAAACGCTTTTACAAAAATAAACGTATATGTTTTAAG
TTGTTTTTTGTCATAATTAATACATTTATTATTATTTATTTATGAATTTCAGAGCTTTAATAGTAACTAA
ATTGTTTAAACAAACTTaaaaaaagatttacaaataaaaaaatATTTCGTGTGCGTGACGGAATTGCTAG
TTCATTAAAGGGAGGTACATATGTACATACATACACATTGCAAAGAGTACAGATCGCTCATCTTGCATCT
CATTTTTCCTAATGTACATATACATATGTACAAACATGTTTATTAAACTGCCTACATGCAAATCTTTGAA
GTTTTAAGAAAATAAGCACAACTAGTTTGGAAAATCTTGGGAGCCAAGATCTATCTGTCCACTGAATTTC
CTATGACCAAGAATGTAAAACCCTTTTTGTTAAACAAATTTTGATTAAAGTATACCAGAATAATATTAAA
CGTATCGTCCGTATCTATAAAAGGTATTAATAGTATTACCTAAATTTAATTTCTCGGTTTTATTCATAAG
AGTGAAAAAATATACAAGTTCCTTTTTAATTTTTTACTTATTGAAAATGTGATTAATTTTTATTTATTAT
TTTCAGTGTAAGGTAAAACAAACAATTTTCTCTTTCCGTTCTAtttttttttttttAAGTATTTGTAGCC
GATTGGTATATTTTGTGGGGTCATTTATTTTGATCTTGGGTGGTAAATACATTTTTATGGAAGTTAGAAG
AAAGCTTATAATTTATGAGGAATAAATAAATACCTCCTTTTGAATCTTTTGTTATGTCTTCTCCTATGTG
CATACTTTTGGGCATTAACATTTAGGTAAATTTTTTCCACCGCAACTACAAGTAAACCGCATACACACAC
AGTATTTTGTAGACTCGCGCCACAAAGCACCGCATAAAAAAGGTTACGGTGCTTTTTTGCTCGTTTCTTT
TGTGCACTCCTTCTATGCGGTGCCAAATATGAGGAGGTACCTGAAGCTTGGTTGATTTTGTGAGTGTCTT
TTTCCACAGCATATTTTTGTGTATTGGCTACTTTAAAAATTCTATTATATCGTTGTGCTCGTAATGTTGT
CCATTCCTTTTAAATATTTTCGAATTAAATATACCATAGATGTTTCCCTATTACCATAATAGTTTAATAA
ATATACGTATATGAAAttttttttATCCTATGTTAGTATAAACATGTTATTCAACAAATAAAAGTAGGTC
AGAGGTAATCGCCATCCAGCTCCAGAAAGAGGGGGGAGCTGATAAGATTGCGTTTATGTACCTAATGGGT
TGGGTAAGTGGCTTGGTTTCACAAACACCCTTATTTCAGGTACAAAATAAGTATGTCATAGACGGCGTAA
AATTGATAAGATTTTAAAGTCAAAAGAAAATGAGTTTATTCGCCTGTAACTGCGTTAGAAATCCAAATAC
ACCCGCAATATTATATGTATAGAAAGAAAAAGTTTGATGCAACAACAAATTTAAAGTTGTTTGGTATTTT
GTAATGGAAAGTTTGTGTCACTAAATATGGTTTTACTTGAACGCAGATTGGACATGGTCACACTGCCCAC
AAAATCACGACATTGCGTTATAAATGAAGCACATCTAGTTTTGTGTTGAAAATGTGGTTCACAATTTATA
TAGCAGGTTATGGTAAAACATCGATCGTTCCGAAAAATTCCGCTCGTTGGCACATTTCCTAAATTTTTCG

TGCGACAGCGGTGATTAATTACAAAAATTATTATTATAATTATATAAAAGTTGGCGACCATATCAAAAAG
GTTGCTATAAAAACAAATGACAATTTATAGCGGAGTTTCACTCCCAAAGCGATGTTCTCCACCCATTCCA
GTCAGTCATGGAAAATATACCTTCTAAAAAATATATGATTTTTTCGATAGAGATACACGGGCACACTAGA
CCCACCGATCGCGACTTTGTGTGTGTTAAAATGACGCGCATATCGTTTTGGGCTAATAATATTGTTCACG
TTTTATAAAACAGATACACGGTTAAGCTCTGACCGGTCGTATTATTATCTGTGTGTTCTTGGTTGACACG
TTTATTTCATTTATTCTCATTTAAAGCAACGATGTGAAGAACAGTGCGGGCGTTTCTCAACATAAGAATA
CATACAAACTTATGCGTGTTGGTGAGCAAATAAAATAGTTCAAAAAATTACTCGGCCACCGCCTTAACCG
TACAAACGGAAATACATAAAGG
>dme_circ_0000652_junction_seq
TAACCGTACAAACGGAAATACATAAAGGTGTCCACAAATATGTATGTTCTTAGCAT
>dme_circ_0000653
GGTCTACAGAGGAACCACCGACAAACAGCTTCGAACGGAATTATTACGACCGCACCACCAGCCGTTTAGT
CACGCAGTATCAAGCGAACAACTCAACTTCCTTGGCCAATTCAAACTCCAGTCCCTCGTCCGTGAGCGCC
TCCGCGTCAGTCTTCGCTACGGCGGCAGGAGGGTCTTCCGAGAGATCACGCAACCGAGATCGCCCGTACA
GGAATGGTTCTGCCAGCGTTCAAGGCGGCGGCATCAACAGCAGCAACACGACGACGACGACAGCAGCCTG
CACAGCTGGAGGATCTGGATCGGGAGCAATTGGAACTGGGACTGGGGGTTTGGTAGGCTCAGGTCCTGGG
GGAGTACCGCAGGCGCTAGGCGACCGCAGCAGCACCCAGAATATCCACCAGAACCATCAGAGCGCGCGGG
TGGCTCCACCACAGTCATGGTACGAAGCCGCCACTGCTGCAACGACGGCCCAATTGAAAAGTTCTGGTGG
CAGTGGCAATGCCGGCGCCTCAGCAGCCGTGGGCTTCACAATGAGCAGCTCACCGATCAACCATCATCCG
CACCAGCATCCGCATCTTCAGAATCCTCAACACCCGCACTACACCAGCAGTCCAGTGGTAGGAGCCGGAT
CTTGTCCCTCCGCCGCTCAAGGACAGCCACAGATTCAGTCGCAATCGCAGACCACGGCCGTGCATCGAAG
CGTGGCCTATGCGGGAAGTGCTGCGGATGACTTACTGAACACCGCCACCTCACGAAATATGCTTCTGCAC
AGCAGCAAACTCAATAAGCTACTTAAGGGGGCAGGAGCAACGGGAAGCGGAGGAGAACGCTCTGGTTCGG
AATCACCAGGAAGAGCTGGAGGAGCTACTCCTTTGACTACGACAAGTACAATCACAAACAATAGTTTTAG
TAGTAATAGTTTAAATAATACAATAACCACGGCGACTCCAACAATGCCCACGATAGCTTCTGGAGCAGCA
GGATCGGTGGGGTTAGGATCTGGAGCAGAAGCGGGAGTATGTTCCAATTCCGGTACAGCCAGCGGTGACA
TTCTAAATGTGGCCGCTGTTTTGGCCGCCGCCGTGGACAATGGGGTGCCAACGCATCCGATCCGGACACG
ACACAACCTGCACGGACGCAGCACCACCTCCTCCAGTCGCTCGCACTCGCGCTCACCGAGTAGCTACAGC
AGTTCGCACAGctcctcctcctcatcgcactcctcatcccactcgcacGCGTCCAGTCCCGTCCAGTCTT
CGGGCAATTGTGCGATGGCAGAGGGGCGCAGCAGTCGCACCGTTAATTCTGTCACAGTAACCAGGCAACT
CTTCAAATCCCAGTGGCACAGCTGTTACCGTCAGCAGTGCAGGCGTCGGAGGGGGATGTGGGTCCAGTTC
ATCTTCAAGCAGTAGCTCTAGTTCCAGCGGATCCTCCTGTCTGACGGCCAATCCCGTCGTGCATTCCGAA
GACAACCGGCCGCTAGCGATACGAGTCCGCAACCTGCCTGCCCGTTCCTCGGACACATCCCTCAAGGATG
GTCTATTTCATGAGTACAAAAAGCACGGCAAGGTTACGTGGGTTAAGGTTGTGGGACAAAATTCTGAACG
CTATGCTTTGGTTTGCTTTAAAAAGCCAGATGACGTTGAAAAAGCGTTGGAGGTCTCTCATGACAAACAC
TTCTTCGGCTGCAAAATTGAAGTGGAACCGTACCAGGGTTATGATGTCGAGGACAACGAGTTTCGACCCT
ACGAAGCCGAGCTGGATGAGTATCATCCCAAGTCGACAAGGACACTCTTTATCGGGAACCTTGAGAAGGA
CATTACAGCCGGCGAACTGCGTAGTCATTTTGAAGCATTTGGCGAGATTATTGAGATCGATATCAAGAAG
CAGGGCCTGAATGCCTATGCATTCTGTCAGTATTCAGACATTGTCTCGGTGGTAAAGGCAATGCGCAAAA
TGGACGGTGAGCACTTGGGCAGCAACAGGATAAAATTGGGCTTTGGAAAATCCATGCCCACCAATTGCGT
CTGGATCGATGGTGTGGACGAAAAAGTCTCCGAGTCGTTTCTTCAGTCGCAGTTTACACGGTTTGGTGCA
GTTACTAAAGTGAGCATCGACAGAAACAGGCAGTTAGCTTTGGTTTTGTATGACCAGGTACAAAACGCCC
AAGCCGCGGTAAAGGATATGCGTGGAACGATCTTACGGAGAAAAAAGCTTCAAGTGGATTTTGCATCTAG
AGAGTGCCAGGACGCATTTTACGATAAACAAGAGAAACAGCAACAACAAAGTTCGGGGTTCTAACCCGCG

CTTTAGTCGCTATGAGTCAAGCGCCTCTTCTTTGCAATCTCGATCTCGTGCATCTTCCTTTAGCCGCCAT
CAAAACAACTCTAACGATGACTGCTCTCCCATTAACACGCCAGGAGGAGCAAGCAGTGGAATCTCCAGCG
CGAGCAATTTAATTAACCAAAGCACCAGCATCAACATTTCAAACATTGGTACTAATGCCTGCAGTGCGAT
GCCAGCCCCCAGCCTGGCGTCCGCTGTAGTCAGCTGTAATGTCAACGCCTCCGGAACGGTACCAGCGAGC
ACATCCATGCCCTCAGGGGTTAGCTCCTCTTCAAGTTCATTGCCTATGTCTCCGGCCGCTCTGGCCCAAA
GACATCGAATGGTTCGTAATGCGCGTCAAACCGTCGACTGTGACTTCAATGAAGTTGGTCGCCTCCGGTT
TCGTAGCTCCGAGGAGGTTTCTGGAGGTGCAGGAAATTCCACGCAGTTTGAAGATGTCCGCTGCGATTCT
CCAGTTACCGCTCGACAAGGTTCAGCTGTAAATTGTTTTACTGGACCTACAGCAGCCGTTGGAGAATCTA
TTGATGGAACGTTAAATAACAATCAAATTACGGGTGGAGCTGAAGGGTTTACTGGATCCGGAGGCAGCAT
TCTGAGCAGGAGACGTTGCG
>dme_circ_0000653_junction_seq
GGCAGCATTCTGAGCAGGAGACGTTGCGGGTCTACAGAGGAACCACCGACAAACAG
>dme_circ_0000654
GATGCACTCACCTGGGCCCAGGTGAATGTGGAACCCAACACAGCGCTCCTCAACGAAGGCGATCGCACCG
AGCTGCTCTGTCGGTACGGCAGATCCATCAACTACTGCCGCATTGAGATTCCCGGCGAGCAGAAGGTACT
CAACCTGTCGCCGGAGTGGAGCAAGACACCCGGATTCACGTACTTCGGAGCGGGTCTCACGGCCGGACAG
TGCGGCGTGAGCATCGAGAGGGTCAAGGCCAGCAACAATGGCCAGGTCAAGTGCAGTCTGGGCGTCGAGG
GAGAGGAGCTGTCGGGCACCATCGACCTGGTCGTGGCACGTGCGTCCCCAGCAGCCCATCATCGAGCTGT
TGTCGCGGCCAAATCGCGAGGGCTACTTCAACGAGGGCACTGAATTCAGGGCCCGCTGCAGCGTTCGCGA
TGGCCGTCCGCCGGCTAACATCTCGTGGTACATCGACAACATGCCGGCCAACAAGCGCACCACTCCCCTG
GAGGTCATGTCCTCGACCAACGACAACGTGGAGCTATCGACCTCCGTGCAGGAAATCCAGTGGCACCTGT
CGCCCGAGGACAGCAACCGGAAACTGGTCTGCCGCTCCCACCACCAAACCGATCGCGAGAGTGTGCCGCC
CCAGGAAGCTGCCTACATCATCAACGTGCGCTGATGCTCCCGTCCATCAGCCAGATGCCGCCGTGTATGG
ACTCTACCTCGAGCACACTGCCATTGTGAACATAACCATCCGCGCCAGTCCGCAGCCCAAGATCGAGTGG
ACCATCGATGGAGCTATTGTGGGGCAGGGACGCACCGATGGACGCTACTCGGCGTATGAGCCGCAGTATC
TGGGCAACGATGAGTACAACGTTACACTGGCCATTGCCGGACTGACCCTGGAAGATACCACCAAAATCTA
CAATCTGAGGGCCAGCAATGAACTGGGACTGACGGACTACCAGGTGCGAATCAGTTCGTCGAGCAAACCG
CCCAGCAGCAGCCTCGATGTGGCGGCAATCGTTGGCATTGTGGTCGCCGTTGCCGTTTTGGTCCTGGTCG
TTTTACTGATTGTGTTTGCACGGGCTACTGGCCGATGGTGTTTTGGCG
>dme_circ_0000654_junction_seq
CGGGCTACTGGCCGATGGTGTTTTGGCGGATGCACTCACCTGGGCCCAGGTGAATG
>dme_circ_0000655
GCAGCTTCCTGGGGCGGCACACTCTCGCGATCGGTTTGGTGGTGGGAGCGGCAGACCAGTTTCCGGTTGC
TGTCCTCGGGCGACAGGTGCCACTGGATTTCCTGCACGGAGGTCGATAGCTCCACGTTGTCGTTGGTCGA
GGACATGACCTCCAGGGGAGTGGTGCGCTTGTTGGCCGGCATGTTGTCGATG
>dme_circ_0000655_junction_seq
GCGCTTGTTGGCCGGCATGTTGTCGATGGCAGCTTCCTGGGGCGGCACACTCTCGC
>dme_circ_0000656
GGATCTGGATCGGGAGCAATTGGAACTGGGACTGGGGGTTTGGTAGGCTCAGGTCCTGGGGGAGTACCGC
AGGCGCTAGGCGACCGCAGCAGCACCCAGAATATCCACCAGAACCATCAGAGCGCGCGGGTGGCTCCACC
ACAGTCATGGTACGAAGCCGCCACTGCTGCAACGACGGCCCAATTGAAAA
>dme_circ_0000656_junction_seq
ACTGCTGCAACGACGGCCCAATTGAAAAGGATCTGGATCGGGAGCAATTGGAACTG
>dme_circ_0000657

GGTGTCCTTAAAAATCTCCTCGAAGGGCGGTATGTAGATCTTATTGATGTTGCAGAACCGCTGCGCCAGC
AGTCCGTAGAACTTCTCGTATGTTCTCTGTTCCGCACAGCAATCGAGAAACATGTGACAGAGCTCGATTT
CTTGTCCGGGTTTCAGTTGCATTTTCATCAGTTTATGAGCACACTCCTCATAATCCAAACTGGAGTTGAT
TGTTAA
>dme_circ_0000657_junction_seq
CATAATCCAAACTGGAGTTGATTGTTAAGGTGTCCTTAAAAATCTCCTCGAAGGGC
>dme_circ_0000658
GGATACGTCCTTAATGGCATTGGCCAAGACAACCATGGCCTTGTCGGAGTTGCCCTTGGTAGTCTGGATG
TCAATGAGGCAGGAGTACGCGTTTCCGATCGCGCCCTCGGAGATTTTGCTGTTCTTCAGCTGGGAAATGA
CTGTGGAGGCGAGCTTCTCGTCCTGCTGCTCGCGAGCCGTCTGCAAAACGCGCTGGAACATCAGCCTTGG
GGCATTGGACAGGTGCTTATCCCAAATCTCCTTAGAAGCCGCCTCCTGGCCAGTGGAAATCAAATGCATC
CACAGGACATTCATGGGTCCTAGTTGATTGTGAGCAGCAAACTTCTCAGCGAGGGATTGATCTGAAAATT
ATCAGAAAAATTTATAGTTTTAACACTTGTTTAATAGGCATTTCTGGGACCTACATTGCGGCACCAATTC
CGGGTGTTTTTCCAAAATGCCAACTGCTCCTCCGCGTGGGAACTTCTCAGCCAGCTTGGTTGACTCCTCG
GGAGTTTTGGCCGCATCGATTTCTGTGGTCAAGTGCTTCAGGAACTCCTCCGCCTTTCCGGCCACTATAT
TGGCATGACAAAACCGATTGTCAAAGGAAATCAGACGCTTCTGCTCGTCATTCAGCTGCTGGCCAATTCT
CTGCATTGTTTCCAGATCGCCTGCAGCGGCAATTTTGTTCAGGTAAAACTTGAAGATCTTGGGCTGCGGG
TGCAGTTTATCGCCCAGGAGCTCCTCGACATACTTGGTGGCCTCCGTTAAGCGATCAGCTCGAACCAGGA
GCTCGATCAGTCGAGAGGTGTCCAAAGCTCCAATTTTCTCGCCACTCAGAACACTTTGTTTATGCGAGAT
GGCTGCATCTGGATCATTGGCCTGGATTGCCTTGCGGAAGCCGGACAAATGGGAGGCGGTTTTGGGTGGC
TTTGCTGTTTCCGTTTTAGCTGGCTCCTTTTTCGCAGCCTTCTCCTTGGGCTGTTCTGCTACTTTAGCTG
TTTCTGTGGCTGCTTCTGTTGTCGTTGCATCTTTCGCTTTGCTTTTTTTGGCCTTGGCTTGTTCGTTTTG
GGTCTCTGGCACCACAAAAGGCACGTCAATATTTTTCTTACTCAGGATTTCTGCCAATTTCAAAAGGAAA
GCATCGTTTG
>dme_circ_0000658_junction_seq
GCCAATTTCAAAAGGAAAGCATCGTTTGGGATACGTCCTTAATGGCATTGGCCAAG
>dme_circ_0000659
GGATACGTCCTTAATGGCATTGGCCAAGACAACCATGGCCTTGTCGGAGTTGCCCTTGGTAGTCTGGATG
TCAATGAGGCAGGAGTACGCGTTTCCGATCGCGCCCTCGGAGATTTTGCTGTTCTTCAGCTGGGAAATGA
CTGTGGAGGCGAGCTTCTCGTCCTGCTGCTCGCGAGCCGTCTGCAAAACGCGCTGGAACATCAGCCTTGG
GGCATTGGACAGGTGCTTATCCCAAATCTCCTTAGAAGCCGCCTCCTGGCCAGTGGAAATCAAATGCATC
CACAGGACATTCATGGGTCCTAGTTGATTGTGAGCAGCAAACTTCTCAGCGAGGGATTGATCTGAAAATT
ATCAGAAAAATTTATAGTTTTAACACTTGTTTAATAGGCATTTCTGGGACCTACATTGCGGCACCAATTC
CGGGTGTTTTTCCAAAATGCCAACTGCTCCTCCGCGTGGGAACTTCTCAGCCAGCTTGGTTGACTCCTCG
GGAGTTTTGGCCGCATCGATTTCTGTGGTCAAGTGCTTCAGGAACTCCTCCGCCTTTCCGGCCACTATAT
TGGCATGACAAAACCGATTGTCAAAGGAAATCAGACGCTTCTGCTCGTCATTCAGCTGCTGGCCAATTCT
CTGCATTGTTTCCAGATCGCCTGCAGCGGCAATTTTGTTCAGGTAAAACTTGAAGATCTTGGGCTGCGGG
TGCAGTTTATCGCCCAGGAGCTCCTCGACATACTTGGTGGCCTCCGTTAAGCGATCAGCTCGAACCAGGA
GCTCGATCAGTCGAGAGGTGTCCAAAGCTCCAATTTTCTCGCCACTCAGAACACTTTGTTTATGCGAGAT
GGCTGCATCTGGATCATTGGCCTGGATTGCCTTGCGGAAGCCGGACAAATGGGAGGCGGTTTTGGGTGGC
TTTGCTGTTTCCGTTTTAGCTGGCTCCTTTTTCGCAGCCTTCTCCTTGGGCTGTTCTGCTACTTTAGCTG
TTTCTGTGGCTGCTTCTGTTGTCGTTGCATCTTTCGCTTTGCTTTTTTTGGCCTTGGCTTGTTCGTTTTG
GGTCTCTGGCACCACAAAAGGCACGTCAATATTTTTCTTACTCAGGATTTCTGCCAATTTCAAAAGGAAA
GCATCGTTTGGTGTCACATTCTCCTCCTGCATTTTAGTCCACAGTCCCAAAGCCTTCTCCGCCTCATCTG

CCTTGTCGTAGCTCAAGAGGAGCGTGTA
>dme_circ_0000659_junction_seq
CCTTGTCGTAGCTCAAGAGGAGCGTGTAGGATACGTCCTTAATGGCATTGGCCAAG
>dme_circ_0000660
GCTGGCTCCTTTTTCGCAGCCTTCTCCTTGGGCTGTTCTGCTACTTTAGCTGTTTCTGTGGCTGCTTCTG
TTGTCGTTGCATCTTTCGCTTTGCTTTTTTTGGCCTTGGCTTGTTCGTTTTGGGTCTCTGGCACCACAAA
AGGCACGTCAATATTTTTCTTACTCAGGATTTCTGCCAATTTCAAAAGGAAAGCATCGTTTGGTGTCACA
TTCTCCTCCTGCATTTTAGTCCACAGTCCCAAAGCCTTCTCCGCCTCATCTGCCTTGTCGTAGCTCAAGA
GGAGCGTGTAGTAAATCTTATTCCTGTCGATAT
>dme_circ_0000660_junction_seq
GTGTAGTAAATCTTATTCCTGTCGATATGCTGGCTCCTTTTTCGCAGCCTTCTCCT
>dme_circ_0000661
GGATTTCTGCCAATTTCAAAAGGAAAGCATCGTTTGGTGTCACATTCTCCTCCTGCATTTTAGTCCACAG
TCCCAAAGCCTTCTCCGCCTCATCTGCCTTGTCGTAGCTCAAGAGGAGCGTGTAGTAAATCTTATTCCT
>dme_circ_0000661_junction_seq
AGAGGAGCGTGTAGTAAATCTTATTCCTGGATTTCTGCCAATTTCAAAAGGAAAGC
>dme_circ_0000662
ATACTGGAGGAGAACAAGAAGGAAGTTCCCGTAGCCGAGGCCGAAGGAAGCTATAATTACATGAGCACCG
TGTGGAGAATTTTGAACTCACTAGCCGAGGCCGGTCAGCCGGAGAGGCTGCGCAAATTGTTCGACG
>dme_circ_0000662_junction_seq
CCGGAGAGGCTGCGCAAATTGTTCGACGATACTGGAGGAGAACAAGAAGGAAGTTC
>dme_circ_0000663
GCTGTGTGCGTGTTGTGGTGTACTCGTATTGGCAGCGCGTATGTGTGTGCGTAACTACACTGCAAATTGT
GACGGATTCTTGTGTTCCTCAGGGGGAAATAACTTGCGTTTGACAGGTGCGATCTAGTGTGCCGTCTCaa
aaatgaaaaaaaaCGATGTCTGGTTGCATTCTCTATTTGCGCCACTG
>dme_circ_0000663_junction_seq
CTGGTTGCATTCTCTATTTGCGCCACTGGCTGTGTGCGTGTTGTGGTGTACTCGTA
>dme_circ_0000664
AAATTGTCACCCGAGAAATACGATCAACTTGTGGGCAATTTCGGTTTgggatctgtatcgggattgggat
ctggatctgtatcgggatcgggatcgggatcggCCAAGGCAGCTGAGAGACACATGCAGCAACACGCCGA
TGATATTGAGCTCTATGTGGATCCGTGCGACTATGCGAGTCTGCGGGAGATCGGGCTGCCGCTGGAGGAG
ATTCAGGAGATAAGCAAGAAGATGAAGCAGGAGCAGAAAGATGAGGTACCAGGTAAGGTCTTCCTACATA
ATATAGAATTCTTATTCAAGCAGAAAATGTACACATATATTACTAGTCCTGATAAATATAATAGATTGTT
ATATCAGAGAAAGATAGGATACTAGGAAACAAATTTTAACTACTTTTAAACTAACATAAGTTTTCCCATT
ATTCAAAATTTAACTTCCATACTGGTGCTCAAAACTTAGATGAATTCCAAAAAATTGTTTTCCTGTAGGT
ACAACTACTTAGGATTCAGAAAGG
>dme_circ_0000664_junction_seq
AGGTACAACTACTTAGGATTCAGAAAGGAAATTGTCACCCGAGAAATACGATCAAC
>dme_circ_0000665
ATATATCCAAGGATCAGCCGCCGGCGCTACCGTGTCCACCAGACTTGACTTCTGTGAGTCCGCAACACCA
TCTGCACCATCATCATGGCCACCATTACGCAAGTTCCGAGGCGGATTCCGTGCCGGCCACAGGAAATTCA
ACACCGTTACCGGGTTTCAGCAATATTCCCCATTGCAAGATAATACCCGTGGAGTCGTACAAACATGAGT
ATCCCGACTACGATCCCGATGAGGAGGAGACGGACGATCAGTGCGATCCGCAGCAAATGATGCTGCCGCA
GAGATATAATCCTTATCACATGGAGCACGACGCCTGGAGTTCGTCCTGCGATATGGCGAATGATTTTGCT

GGCTATGCCTCTGTGCCACAAAGTATTTCCGGATCGGTCAGCTCACCGCCCTCGGCCCCGCCAACAAATC
CACTACTTAGGCGGAATCAGTATTGGGTGTAAGGTTTAGAAGGATG
>dme_circ_0000665_junction_seq
AGTATTGGGTGTAAGGTTTAGAAGGATGATATATCCAAGGATCAGCCGCCGGCGCT
>dme_circ_0000666
TTTATTGCCTCGTTCGGCTTTGTGACCACGCAAACTTTCGACTTCCGCATCACATTCACGCGCCTGCGCA
TAACGAGCACCAGACCCTCGCAGATATCGATCAATGGCAGCCGGGAGATTCTGCTGACCAGCGATGTACT
CTCCTACGAGACCACGCCCATCGGAAGCTTTGCGCGCAGCGTGATCTACAAAATAACGAAGCCAACTCGC
TACGGGGGCATCTATGTGGAGGGCTCGCGGAAGGCGGCCAAGAAGCTGGACTCCTTCACCCAACAGGATA
TAGAGAAACGGCGAATCCGATATCAAACTCATCACACCAGCTACTCCAGCTTCAGTGATCATCTGGAGTT
TGTTGTTTCCG
>dme_circ_0000666_junction_seq
AGTGATCATCTGGAGTTTGTTGTTTCCGTTTATTGCCTCGTTCGGCTTTGTGACCA
>dme_circ_0000667
GTGATCTACTTCAGCATGGGCTCCTATGTAAAGAGCACTGATTTACCACAGGAGAAAACGGCGCTGATTC
TCAAGGCATTCGGCCAACTCAAGCAGCAAGTGATTTGGAAGTTCGAGAACGATTCCATTGGTGATCTGCC
CTCGAATGTGATGATCAAGAAATGGATGCCCCAGAACGACATTTTGGCCCATCCGAATGTCAAGCTCTTC
ATCACGCACGGCG
>dme_circ_0000667_junction_seq
AATGTCAAGCTCTTCATCACGCACGGCGGTGATCTACTTCAGCATGGGCTCCTATG
>dme_circ_0000668
GTGGTGTCCGAGTTGTGGGGCGCGCAGTGTGTACGCCTGAAGGTGACCACCAAAACCGACAATTGCGGCG
AGAACGCCGATTGTTCCGGCAAGGGAGTGTGCTACTCCAACTCCGGAATGGAGGAGTACGAGTGCCAGTG
CTGCAGCGGATTCGCAGGACCACACTGCGAGGAGATAGACGCCTGCAATCCGAGTCCTTGCACCAACAAC
GGCATCTGCGTGGACCTCTCGCAGGGCCACGAGGGCAACTCGTACCAGTGCCTGTGTCCGTACGGGTGAG
TCCCAGGCAAACAATAGACGAAAAGAAGGCAAGAATGGCTGAAAGATGAGTTGCTGAGCTGCACTTTGAA
AAACAAACTCCGGCACTGCATACAGAAATTACCAAGAACTATAATTAATGATTCTTGAGTAACTAACATT
TATTTGCTCCAATTTATGCGATAATTTATGCAAATAAGTTTATTTTTAAATCATTTAAACTGCAAATTTC
TGAATGAAGCCATCGTTTTTTGCTCCGAGTGCAGGGTACACATTCAGCTGGCGGAGCACATTCTCCAGCA
GCAGGAGCAGCCTTCATTCAATTATCCTGGCCATTGTTTTGCGGTTCTGTCTAGCCGCCGCCGTCTGTGT
TTGCTCATAAATGTTGACTTTTGTCGCATGTTCAAAAATCCCCAGTTGTTCCACCTCCGCCTCTCACTCA
CCCTACCTCCCCTTCTTTTTTTGTTTTATTTCTGCGACTCCCCACTGTACGAGTATTAACCCCTCTTTGT
CTCGCCCCGCAGGGTGAGTAAATGGGACCAACAGTTCCAGCCAAATAAACGCAAATTGCCTTCATTCTTT
TGTTGATTCGTTGGCGAAATCGCTCCCATAATGCCAATATTTTGTGAGTTATCATCGTCGAAATTTCACA
CATCCCTTGTTGGCTTTGGCTGGGATCTATTATATATTGGATTTTTGGGCTGCGTTGCAGGGGCAAAGCT
AGTGCCAGCTTTGTCATGAATGATATTTGTTTTGCAGCAGAAAGAAGAAACTAACTTATATCTAGCTTAA
ACTTGTAGTAGCTTAAACTGGGTTCTGGGTAAATCCACATACTTTAAGGCAATCAAATTTCGCTTTTGAA
ATCGAATTGAAATCAGCGCAAATCAAAAAATCTACATCGGAGCTCATAAATTATTTCATTGGCACAAACA
CAATTTGCATAAATCGCACTCAATCACAATGAAAGTAAATGTATAAAAAATCACTGAGACAGAGACGAGG
GCAATTCCGGGAAGACGAGACAGTAGTCTGTCGCCCAGCCAGAGCATTTCCCATTTCCCATTGAAATTTC
CATTTCATTTACTTTGCACATTTACATTACCCGCAAGCTTTTCAACTGAACCCTTCTCTCACACCACCCG
CCCTGGCACCTTCTCACCTTTTATTTCATTTCTTCGAATCCTGTCTCGCAGGGATTCGCCGGCGAGCTGT
GCAACTTCGAGTATAATGAATGCGAATCGAATCCGTGTCAGAATGGCGGCGAGTGCATCGATCACATTGG
CAGCTACGAGTGTCGCTGCACGAAGGGATTCAGCGGCAACCGCTGCCAAATTAAGGGTGAGTGAACAGTG

TCGCATACGTTTAATACAAAATACAAGAAATCAATATCTGAATCATCAGATAATTCCATCTAAATTGCAA
GAATTGTAATAACTTCAAGCACTTCGCCTTCACAGTGCAGAAGATATATTGTTTGTAATTTATCTTAAGC
CTTAGCTTGTAAGTAAACTTGAAAATTTACGAAGCAATCCGAGATGCGCGAAAACATGACTTGTGAAGCT
GCGATGAAAATTGAGCCCCAAGAGTGCCCATCAGTTGGTTCTAGCTCCCCAGGATAATCAATATTTTGTA
TTGAAAACAAATCCCGCGACAATTCCCCGATGAGAAAGTGGAAGGACTCCAGCCAGCCGGCAGTTGTCTG
CGCGTCCTGAATGGGTATTGATTTAATTTGCTCCGACCCGAGCATCGCGTACAGGCATAGGAAATGGCAT
TGGATGGTtgtgtgtgtgtgtgtgtgtgtgtgGGAGTGTATCCTTCAGATACGAATACTGGGCAAAAACA
TTTTTGAATACGTAATGAAGCTACAGATCTCTGCCGCCACAATATGCTTTGATTTGTGCCCGTTTCTCTG
TTGTGTGTTTTCTAGGTTGATTTCTGCGCCAACAAGCCCTGCCCCGAAGGACATCGCTGTATTGATCATG
GAAATGATTTCAGCTGCGAATGCCCAGGAGGTCGCAACGGACCGGATTGTAATCAAATGCCACGAACG
>dme_circ_0000668_junction_seq
ACCGGATTGTAATCAAATGCCACGAACGGTGGTGTCCGAGTTGTGGGGCGCGCAGT
>dme_circ_0000669
GCTGCGACTGTGCTCCCGGGTTCACAGGACCTCTTTGCCAGCACAGCCTGAATGAGTGCGAGTCCTCGCC
GTGTGTTCACGGCATCTGCGTCGACCAGGAGGACGGATTCCGCTGCTTCTGCCAGCCAGGTGAGTAAATG
GGACCAACAGTTCCAGCCAAATAAACGCAAATTGCCTTCATTCTTTTGTTGATTCGTTGGCGAAATCGCT
CCCATAATGCCAATATTTTGTGAGTTATCATCGTCGAAATTTCACACATCCCTTGTTGGCTTTGGCTGGG
ATCTATTATATATTGGATTTTTGGGCTGCGTTGCAGGGGCAAAGCTAGTGCCAGCTTTGTCATGAATGAT
ATTTGTTTTGCAGCAGAAAGAAGAAACTAACTTATATCTAGCTTAAACTTGTAGTAGCTTAAACTGGGTT
CTGGGTAAATCCACATACTTTAAGGCAATCAAATTTCGCTTTTGAAATCGAATTGAAATCAGCGCAAATC
AAAAAATCTACATCGGAGCTCATAAATTATTTCATTGGCACAAACACAATTTGCATAAATCGCACTCAAT
CACAATGAAAGTAAATGTATAAAAAATCACTGAGACAGAGACGAGGGCAATTCCGGGAAGACGAGACAGT
AGTCTGTCGCCCAGCCAGAGCATTTCCCATTTCCCATTGAAATTTCCATTTCATTTACTTTGCACATTTA
CATTACCCGCAAGCTTTTCAACTGAACCCTTCTCTCACACCACCCGCCCTGGCACCTTCTCACCTTTTAT
TTCATTTCTTCGAATCCTGTCTCGCAGGGATTCGCCGGCGAGCTGTGCAACTTCGAGTATAATGAATGCG
AATCGAATCCGTGTCAGAATGGCGGCGAGTGCATCGATCACATTGGCAGCTACGAGTGTCGCTGCACGAA
GGGATTCAGCGGCAACCGCTGCCAAATTAAG
>dme_circ_0000669_junction_seq
ATTCAGCGGCAACCGCTGCCAAATTAAGGCTGCGACTGTGCTCCCGGGTTCACAGG
>dme_circ_0000670
ATGTCTCGTCTCGAAGCTGTTCGATGTGTGCCCCCGCAGCACCGTGGAGGAAATGGAGCAGCAGGACGAA
GTGCGGGTGGCATGGGGCACGGGAATCGCCGGCCATGTGGCCGAAAGCGGGGAGCCCGTCAACATACCAG
ATGCTTACCAGGGTCAGTATCCAGCAAGTACTATCTTGAGGCAAATTTGACAAATTTGAATGCCAACCGG
GTCATAAATCAAGCTCTGCTGCGAGAGTTGCAGATTATTCATTCGATTGTAAGATTTATGGTGGCAGATA
CCAATCCAATGTGTGTGTTTGTGTATACTCGTATTATGGTGGTGCTGCTCTTTAAGTTTTCAATATTGCG
CAGTGAACTCGGTGGCCATCACCAGCACTTTTCACATTTCCCAGCTTCCGCCCTGGCCACTACTGCCACA
TTTTCCCAttttttttttttttGGCAGTTTCTCTTCGGCCTGTCTGCCAATTGGAGCGTAATTAATCTTA
CGCCAAGACAAAGCCGTGTTAGGCATGTTATATCACCCAGATATATGTACTCCCAATGCTTTTATTCTTT
TTTCGACTTTAAGTCGCTCGGCTAACGATAACAATTGCGGCTGCTCGAAATGTTTTCTTCAAGTCACATT
AAGCCAATGGGTGTAGATAAACTTACCGCAAATACGTGACGCACAGACAGATCGTTTGTAGCCCGCAGAT
CTCCTGATTGTTAGCACAATTAATCAAGGCCTAAATTTGACACTTTTTGGTACCAGACAGCGCGACCAAA
GGTTTTCGGTGCGAATGAGGCGCATTATGGTTAGGCACACCAGTTACGCTCGGGTGCAGATCTTTTAGTC
GATCCCAGTGGACTTTTGACTCGGCTAAATGGCTGGCCAAACAATTGTCCAGTCGGCAGCGACAGTCGGA
GGCGTCTGGAATCCGATAACCTCCCAATTTTTGGAGCAAAGAAAGGCAGATAGAATAGATCATGGGGAAT

GTGTGCCAAGTGGGATAAAAATAGAAAGGCCAACAATGGAATGATTGATAGATAAAGAGCATTCTCAGTG
TTAGATGGTGTAAAAGGGAGGCACATATTTGGAttttttttttAAAGAATCTTATTTAATAAGAAACTTA
TCTAAACGGTAGATTGGACAATCAGCCAAAATTTGTTGTGACCTTATTGCTAAGACAATATTTTTACTTT
GATAATTAAAACCAATTGAATCTGATATGTAAGAATAatttgcttttgttgcttattattattattcttt
ttcattttatatgcctgtattgtatttttaCTTAACCATCACAATATAAGAGAAAGATTTTCATTAAGTA
AATTTAGTTCTTTGTTCATTGGTACTTGCTaaaaaacggaaatcgaaaaaaaatGAAGGGAAACCGTTTGT
AAAGGCTTACGGACGCAGAGCTTAAGTTAACAACGAAAACCCATTTAAATGAGATACTAATTCGTTCGCA
AATTAATTTCGTTTCCCAGGGACGAGCGATTCAATTGTGAAATTGACTCGCTGACCGGCTATCGGACGAA
GGCCTTGCTTTGCATGCCCATCAAGGATTCATCCGGGGATGTGATTGGCGTGGCGCAGGTCATCAACAAA
ATGAATGGCGAGTGTTTCTCCGAAATCGATGAAAAGGGTGAGTGGACTGTGGGCTGAAGGCTGAGGGGTA
AATGCGGAAATGGGGCGAATGGGGCCATGTGGAGATCGCATGACGGGGCAGTCTGATGGTTTCTTTTGTT
CCGAATAAAGCCGCATAAGAGCTGCACCCACTGCGGTGGCAAATTGCATTCATTATTCAACAAAGGGGAA
ACTTGTTAAGAAAGCTTAGCAGAATGTCAGGCAAAATGCCATAAGGGATTATCCGATGTGTAATGCCAGT
GGGACGCTCTCATAATTTTTATGGCATTTTGCAAGCTCTACTCTGCTATTTTCTGTTGTTTGAGCCATGG
GTTTAATCCTTTGCTGTAATCCCCATCAGGGTCTTTTCCTCGTATCTGCAATTCTGTGGGATTGGGCTGC
GGAATGCACAGCTGTATGAGAAATCTCAACTGGAGATCAAGCGGAATCAAGTGCTCCTGGATCTGGCACG
CATGATCTTCGAGGAGCAGAGCACCATAGAGCACATGGTCTTCCGCATTCTCACCCACATGCAGAGTCTC
ATCCAGTGCCAGCGCGTCCAGATCCTCCTGGTCCACGAGGCGGACAAGGGCAGCTTCTCGCGGGTCTTTG
ACTTCGAGGCGAATGACTTGAGCGAGGAGGAGGCCACTTCACGCACCAGCCCTTACGAGTCGCGGTTTCC
CATCAACATTGGTATCACTGGACATGTGGCCACCACCGGGGAAACGGTAAATGTGCCCAATGCCTATGAG
GACGATCGCTTCGATGCGAGTGTGGACGAGAACTCCTGCTTCAAGCACCGATCTATTCTATGCATGGCCA
TCAAGAACTCGCTGGGTCAGATCATTGGTGTGATCCAGCTGATCAACAAGTTCAATGAGTTGGGTAAGTA
AGCCCTGAATAAATTTCTTTACTCTTTGTCAAAAAATTCAAACTGCAATGCTTAGACAAGTTAAATCTTT
TATCTTTGTCTAGAGTCTAGACACAGAACTATAATTTAAGAATATTAAGCCCCAATCGCGAGCTGCATAT
TTTAAACTGCTCCTATCTGTTCCCCCAGGGACTTCACCAAGAACGACGAGAACTTTGTGGAGGCGTTCGC
CATCTTCTGCGGCATGGGCATCCACAACACGCACATGTACGAGAAGGCCATTGTGGCAATGGCCAAGCAG
AGTGTCACCCTGGAGGTGCTCAGCTACCACGCATCGGCCACCATGGACGAGGCTCATCGACTGCGGG
>dme_circ_0000670_junction_seq
ACCATGGACGAGGCTCATCGACTGCGGGATGTCTCGTCTCGAAGCTGTTCGATGTG
>dme_circ_0000671
GTGAAGGACATTTGCAACGAGCTCGAGGTGCGCACTTTGTGCCACAAGATACTCCAGAACGTGTCCATTC
TGCTGAATGCCGATCGAGGATCTCTGTTCCTGGTGCAAGGACGATGCAACGGACCGGATGGCCTAAAAAA
GGTAAGTGTTAGTTAGCCAGATAACGTTTATCAACTATAGGATAATTCCATATTTAGTCCCCACATCATC
CAAATATCTTTAAACTTAAATTTTGTAAACTTCCCAGTGCTTCGCCAGTTTATGAAGAGAGCTGCAATGA
AGACAGCCATCTATCATAAATCATTCGCATTCTAATAGCACAAAAGTGGACTGGTTCATGGCGCTTAGCA
ATCAGCCAGTTACTTTAGTCCTCCGCGTTAGCTATTTTCAACAATTTGTTGATGACTTCATATAGCTGCA
TTTTTGTGGACATTGTGGATAGAGCAGGAGCTCAGCTCCAACTTTGTCTTGGCAACATTTTTATGGCATT
TATTTTTGGCATTTTATGAGTGCATCGCGCGGATGCTGCGTCCCCTTTTCCGTTCCAAAAGCCGTGCCAT
AAATGCCATAACCCGGCAGCAACCTGCAACGAGGGGGTCAATGGGGCACGTCAGCCATTTTGTATGCATC
GTATGACGCCATTTTGCTGCCAACACTAATTGCAATTGTCTGCACGCGCATTTGGCAAATGCATTCTGTT
GGGTAAAAAGTTTTTAAAGCCAAAGAAAAGCAGCAGGAAAGCTGCAAAAGTAGGCGAGGCACTGAGAAAC
AAATGGCTCAAATGTAAATCAATAATACTTATCAGGCTCGGATTGTGACTGCATATTCGAATTAATAAAT
TATGACCCAATTAAAAGTTTGGGAATTTTCGCTTGTTTTCATTATCAACAAACGGAAATTGTAGTTTCGT
TAGTTTGCTTTCGGGTAATTTAGCAAGCTGATGCCATGCTGCTTATAATATTTTGGAAAACACTATTATT

ATTTTTCCTCTCAGTGGAATAGGAAAAGTTTGCATTGTGTCCGATGACAATGGACGATAGTTGCCGCCTC
CAACACAATTTATAATTTTCAACTTTGGATTCCATTTCTCCTGCAGAATGTCTCGTCTCGAAGCTGTTCG
ATGTGTGCCCCCGCAGCACCGTGGAGGAAATGGAGCAGCAGGACGAAGTGCGGGTGGCATGGGGCACGGG
AATCGCCGGCCATGTGGCCGAAAGCGGGGAGCCCGTCAACATACCAGATGCTTACCAGGGTCAGTATCCA
GCAAGTACTATCTTGAGGCAAATTTGACAAATTTGAATGCCAACCGGGTCATAAATCAAGCTCTGCTGCG
AGAGTTGCAGATTATTCATTCGATTGTAAGATTTATGGTGGCAGATACCAATCCAATGTGTGTGTTTGTG
TATACTCGTATTATGGTGGTGCTGCTCTTTAAGTTTTCAATATTGCGCAGTGAACTCGGTGGCCATCACC
AGCACTTTTCACATTTCCCAGCTTCCGCCCTGGCCACTACTGCCACATTTTCCCAtttttttttttttG
GCAGTTTCTCTTCGGCCTGTCTGCCAATTGGAGCGTAATTAATCTTACGCCAAGACAAAGCCGTGTTAGG
CATGTTATATCACCCAGATATATGTACTCCCAATGCTTTTATTCTTTTTTCGACTTTAAGTCGCTCGGCT
AACGATAACAATTGCGGCTGCTCGAAATGTTTTCTTCAAGTCACATTAAGCCAATGGGTGTAGATAAACT
TACCGCAAATACGTGACGCACAGACAGATCGTTTGTAGCCCGCAGATCTCCTGATTGTTAGCACAATTAA
TCAAGGCCTAAATTTGACACTTTTTGGTACCAGACAGCGCGACCAAAGGTTTTCGGTGCGAATGAGGCGC
ATTATGGTTAGGCACACCAGTTACGCTCGGGTGCAGATCTTTTAGTCGATCCCAGTGGACTTTTGACTCG
GCTAAATGGCTGGCCAAACAATTGTCCAGTCGGCAGCGACAGTCGGAGGCGTCTGGAATCCGATAACCTC
CCAATTTTTGGAGCAAAGAAAGGCAGATAGAATAGATCATGGGGAATGTGTGCCAAGTGGGATAAAAATA
GAAAGGCCAACAATGGAATGATTGATAGATAAAGAGCATTCTCAGTGTTAGATGGTGTAAAAGGGAGGCA
CATATTTGGAttttttttttAAAGAATCTTATTTAATAAGAAACTTATCTAAACGGTAGATTGGACAATC
AGCCAAAATTTGTTGTGACCTTATTGCTAAGACAATATTTTTACTTTGATAATTAAAACCAATTGAATCT
GATATGTAAGAATAatttgcttttgttgcttattattattattctttttcattttatatgcctgtattgt
atttttaCTTAACCATCACAATATAAGAGAAAGATTTTCATTAAGTAAATTTAGTTCTTTGTTCATTGGT
ACTTGCTaaaaaacggaaatcgaaaaaaatGAAGGGAAACCGTTTGTAAAGGCTTACGGACGCAGAGCTT
AAGTTAACAACGAAAACCCATTTAAATGAGATACTAATTCGTTCGCAAATTAATTTCGTTTCCCAGGGAC
GAGCGATTCAATTGTGAAATTGACTCGCTGACCGGCTATCGGACGAAGGCCTTGCTTTGCATGCCCATCA
AGGATTCATCCGGGGATGTGATTGGCGTGGCGCAGGTCATCAACAAAATGAATGGCGAGTGTTTCTCCGA
AATCGATGAAAAGG
>dme_circ_0000671_junction_seq
GAGTGTTTCTCCGAAATCGATGAAAAGGGTGAAGGACATTTGCAACGAGCTCGAGG
>dme_circ_0000672
GCCTGCATTTGCGGAGCATCGAGGAGCCAGCGACGACGCCGCTCCAGTTCCAGCCCACTGGCAGGATGAA
CACGGAGCAGGGGGGCACGGGGTACGGCGGCTACGGATCCAGCGAGCACTCGCTGCTCATCGCCACACGC
CACGCCGGTGTCCCCCTGCCCCTCGCCCAGCACCAGCCACTCCCCGCCCACTATCAGCCGCTGAACCACT
CCGGAGCAGCCCCGCCCTCCTCATCCAACGGATCATCCTCCTCCGGCGGCGGAGTCCAGACGTCGGCCAC
gccgcagcaacagcagcagtaccaggtgcaacagccgtatcagtaccagtaccagcatcactatcaccac
cagGCCAACTCGCCACAACACCACAGACCCTACGATCCAGAGCACGCGCGGATGGAGGCCTGGCTGGATG
AGAATCAGGAATTTGTACAGGATTATTTTATAAGGGCAAGTCGAAGTTTGATCCTTTATTTTCGTTTCCT
ACTATTTTCGTATCTGTGTAAAATTCGGATGTCGAATTAGGAACTATATTAGATGGTATTTATTATATTT
AAATGTTTGAGCCGAATTTATTTATTGGCTTGTTGTTAAACTGCCACTTGCCAAAAACCAATCGAACTCT
GCTTAAAACGTCCCAGTTGACCAATCGATAGCCCTTCTGTTAGCCAAGACAGACAGAGAGAACTAACCGA
ATCCTTGTAGAGACATAAATTGCATCTGCCTACAAGCGTTTTCGATGAACCCATCGAGCCAATAATCTAT
TTTGTCCATCGACGGTATAAATTCTTAAATTTGAAACTGCATTGTCAGTTGACACAGCCGTACGGCAAAC
AATTGGGTTTGCCGATGGGGATTTGGCCATGGGCGTGGATGGCTCGTTTCCGCCATAAACCGGAAATTGC
CTTTGCCACAGCAGGACATCTAGGGCAGCCCGGCAGCAAAGCGGGTTAAAGGACGTCCGTTGCCTATGAC
ACATAATAAAAATCACTAACAACTAAAAGCATAAATTGGGCTTTATTTTCATGGTCGAGAGGAGCCGGCC

AACGTAGTCGGCTCCACTGCTACTCCGGCTTTTTGGCCTGAGGGGCGGTCATTAAATGAGGCCGAAAAAG
GCTTCATTCTCATGTGTGTGTATTTTGCATAAGGATATGCCGCATGTCCTAAACAGTTCAACCATTTTGG
TAGTAGTTCTGCCCCTTGTTTATATTTAAGGGAGTATTAGCTTTTCTGCAGAAAAAATTAAGCATTAAGA
AAAATTACTAAACCATTTATTGCTGACTTAATCCATTTAGACTATAATTTGTTAAATTTCAATTTGGCTA
ATGTAAATTTTTGGTTTCGTTATATCTTATATATTAGATTTATATGGTTATAAAAAAATTTAAATATAAA
ACATTAAATATTTTCCTAATCAAAGAGTTGTGTGCTGTGATTACCCGACGTTTTGTGGCGTTTTGGTAGG
ATTTTATGTCAAAAGCGCGTGCCGAAAAGGACACGTTGTGGGCAGGAGCAGGAGTTGGAGCAGCTGATGC
TGTGTAGTCTCGCTGCCTGAATATTTATGAGTTGGCATTTCATTAGACACTTTTATGCGGCACAAGCATA
TGGAACTCGGCGCAAAATGCGTCGGCCAGCGCCTTTTCATTTGTGATTTTTAATAAGATTTTAACGTGTT
TACTAACTGAATTTCACCGTCTATTTCCCACAGGGAAAGCCACACGTCAGACCGTGGATGCCTGGCTGGT
TTCCCATGCCACATCGGCGGGCAACGATGTGGTCAGCAGCACCTCACCCACACACGCCAATGGACAGACG
AGTTCCTCGCGGGGTGGATCCGGTGCCACCACACCAGTGCGGGTAAGTGATTCAATTGGGGCTTCGTAAA
TTAAATACTGGAGCATTCCATGTGGAAAATGTAGAGGAGAGTACTTTTACAGCAAAATAAATTGATGAAG
TAACGTACTTACAAAAATGAATGCATTTTCCAAACATCCTTATTTTTTTATGCCAGCCGAAAAGCCTTTA
CTTGCCAGCTTTGTGAGCTTTAAAAGATACTTTTTTAAAGATACTAATGATTATTGAATTCTTTTCAGAA
AAAATATCTGCTCACGAGTTTGAGCGTGGCGGACTGCTGAAACCGATTGTCAACACAATCGACGGCACGC
CCACATTTTTGAGCATTGGTCCGCCGATGGACAACGGCAGCGTAGGCGGCAGCTGCAGCAATCTGCAGAA
TGTCGGTGGCGTTGTGGCCGGACAGTATCAGTATAACCATCAGCAGCACCACCACAATCATGCCCATCTG
CACCACAGCCAGCACAGTCATTACCAGGCGGGCGGGGCCGTTGGGAGCAGCAGCTTGGGCAGCACCGGTG
GCGCTTCGGGAGCGGGAGGAGCACCCAGTCTGGGTGGCAGCGGTGGAGCCGGAAATGGCCATCAATATCC
ATACTATCATTGCCACCAGCGACCACAGCGGTTGTCACGCAACGAGCTCAAGCAGCTGGACGAGAAGGAG
TTGATTTTCGAGCTGGGTGAGTGAAAACAATAATATTTTTGATTATAAGAAAAATATGTTTAAGTTGTCC
GATCACGTTCTGATCAAAATAATAATAGCCCAATATGATTCAAAAGTGCAATTGATTCGGAAATAACGTG
ACACACTCGCTTATATAAAAGTAATTGAATAAGTGAATGAGTAAAGTTTCCTCTAAATGCTTAACCATTA
GATATCAAAGTGAAGGTTCCGGCGAATCTCGATAGCTCGTCAATGATTATTTGTTGAGTTCCGCAAACGT
GTTTGTTTGTCGGCCCATTGACGCTGTTATCGGCAAACACTCGAAAGACGTCAGTCACACAGTCTGTATT
TCCCCATGGCTCCCTTTCTTTTTCAAATGCAACTGCTTATCAGTCGTGTAAATAACCGGACTGTGCTGTT
TATCGCCACCAGGGTGAAGGACATTTGCAACGAGCTCGAGGTGCGCACTTTGTGCCACAAGATACTCCAG
AACGTGTCCATTCTGCTGAATGCCGATCGAGGATCTCTGTTCCTGGTGCAAGGACGATGCAACGGACCGG
ATGGCCTAAAAAAGGTAAGTGTTAGTTAGCCAGATAACGTTTATCAACTATAGGATAATTCCATATTTAG
TCCCCACATCATCCAAATATCTTTAAACTTAAATTTTGTAAACTTCCCAGTGCTTCGCCAGTTTATGAAG
AGAGCTGCAATGAAGACAGCCATCTATCATAAATCATTCGCATTCTAATAGCACAAAAGTGGACTGGTTC
ATGGCGCTTAGCAATCAGCCAGTTACTTTAGTCCTCCGCGTTAGCTATTTTCAACAATTTGTTGATGACT
TCATATAGCTGCATTTTTGTGGACATTGTGGATAGAGCAGGAGCTCAGCTCCAACTTTGTCTTGGCAACA
TTTTTATGGCATTTATTTTTGGCATTTTATGAGTGCATCGCGCGGATGCTGCGTCCCCTTTTCCGTTCCA
AAAGCCGTGCCATAAATGCCATAACCCGGCAGCAACCTGCAACGAGGGGGTCAATGGGGCACGTCAGCCA
TTTTGTATGCATCGTATGACGCCATTTTGCTGCCAACACTAATTGCAATTGTCTGCACGCGCATTTGGCA
AATGCATTCTGTTGGGTAAAAAGTTTTTAAAGCCAAAGAAAAGCAGCAGGAAAGCTGCAAAAGTAGGCGA
GGCACTGAGAAACAAATGGCTCAAATGTAAATCAATAATACTTATCAGGCTCGGATTGTGACTGCATATT
CGAATTAATAAATTATGACCCAATTAAAAGTTTGGGAATTTTCGCTTGTTTTCATTATCAACAAACGGAA
ATTGTAGTTTCGTTAGTTTGCTTTCGGGTAATTTAGCAAGCTGATGCCATGCTGCTTATAATATTTTGGA
AAACACTATTATTATTTTTCCTCTCAGTGGAATAGGAAAAGTTTGCATTGTGTCCGATGACAATGGACGA
TAGTTGCCGCCTCCAACACAATTTATAATTTTCAACTTTGGATTCCATTTCTCCTGCAGAATGTCTCGTC
TCGAAGCTGTTCGATGTGTGCCCCCGCAGCACCGTGGAGGAAATGGAGCAGCAGGACGAAGTGCGGGTGG

CATGGGGCACGGGAATCGCCGGCCATGTGGCCGAAAGCGGGGAGCCCGTCAACATACCAGATGCTTACCA
GG
>dme_circ_0000672_junction_seq
CCCGTCAACATACCAGATGCTTACCAGGGCCTGCATTTGCGGAGCATCGAGGAGCC
>dme_circ_0000673
GCCTGCATTTGCGGAGCATCGAGGAGCCAGCGACGACGCCGCTCCAGTTCCAGCCCACTGGCAGGATGAA
CACGGAGCAGGGGGGCACGGGGTACGGCGGCTACGGATCCAGCGAGCACTCGCTGCTCATCGCCACACGC
CACGCCGGTGTCCCCCTGCCCCTCGCCCAGCACCAGCCACTCCCCGCCCACTATCAGCCGCTGAACCACT
CCGGAGCAGCCCCGCCCTCCTCATCCAACGGATCATCCTCCTCCGGCGGCGGAGTCCAGACGTCGGCCAC
gccgcagcaacagcagcagtaccaggtgcaacagccgtatcagtaccagtaccagcatcactatcaccac
cagGCCAACTCGCCACAACACCACAGACCCTACGATCCAGAGCACGCGCGGATGGAGGCCTGGCTGGATG
AGAATCAGGAATTTGTACAGGATTATTTTATAAGGGCAAGTCGAAGTTTGATCCTTTATTTTCGTTTCCT
ACTATTTTCGTATCTGTGTAAAATTCGGATGTCGAATTAGGAACTATATTAGATGGTATTTATTATATTT
AAATGTTTGAGCCGAATTTATTTATTGGCTTGTTGTTAAACTGCCACTTGCCAAAAACCAATCGAACTCT
GCTTAAAACGTCCCAGTTGACCAATCGATAGCCCTTCTGTTAGCCAAGACAGACAGAGAGAACTAACCGA
ATCCTTGTAGAGACATAAATTGCATCTGCCTACAAGCGTTTTCGATGAACCCATCGAGCCAATAATCTAT
TTTGTCCATCGACGGTATAAATTCTTAAATTTGAAACTGCATTGTCAGTTGACACAGCCGTACGGCAAAC
AATTGGGTTTGCCGATGGGGATTTGGCCATGGGCGTGGATGGCTCGTTTCCGCCATAAACCGGAAATTGC
CTTTGCCACAGCAGGACATCTAGGGCAGCCCGGCAGCAAAGCGGGTTAAAGGACGTCCGTTGCCTATGAC
ACATAATAAAAATCACTAACAACTAAAAGCATAAATTGGGCTTTATTTTCATGGTCGAGAGGAGCCGGCC
AACGTAGTCGGCTCCACTGCTACTCCGGCTTTTTGGCCTGAGGGGCGGTCATTAAATGAGGCCGAAAAAG
GCTTCATTCTCATGTGTGTGTATTTTGCATAAGGATATGCCGCATGTCCTAAACAGTTCAACCATTTTGG
TAGTAGTTCTGCCCCTTGTTTATATTTAAGGGAGTATTAGCTTTTCTGCAGAAAAAATTAAGCATTAAGA
AAAATTACTAAACCATTTATTGCTGACTTAATCCATTTAGACTATAATTTGTTAAATTTCAATTTGGCTA
ATGTAAATTTTTGGTTTCGTTATATCTTATATATTAGATTTATATGGTTATAAAAAAATTTAAATATAAA
ACATTAAATATTTTCCTAATCAAAGAGTTGTGTGCTGTGATTACCCGACGTTTTGTGGCGTTTTGGTAGG
ATTTTATGTCAAAAGCGCGTGCCGAAAAGGACACGTTGTGGGCAGGAGCAGGAGTTGGAGCAGCTGATGC
TGTGTAGTCTCGCTGCCTGAATATTTATGAGTTGGCATTTCATTAGACACTTTTATGCGGCACAAGCATA
TGGAACTCGGCGCAAAATGCGTCGGCCAGCGCCTTTTCATTTGTGATTTTTAATAAGATTTTAACGTGTT
TACTAACTGAATTTCACCGTCTATTTCCCACAGGGAAAGCCACACGTCAGACCGTGGATGCCTGGCTGGT
TTCCCATGCCACATCGGCGGGCAACGATGTGGTCAGCAGCACCTCACCCACACACGCCAATGGACAGACG
AGTTCCTCGCGGGGTGGATCCGGTGCCACCACACCAGTGCGGGTAAGTGATTCAATTGGGGCTTCGTAAA
TTAAATACTGGAGCATTCCATGTGGAAAATGTAGAGGAGAGTACTTTTACAGCAAAATAAATTGATGAAG
TAACGTACTTACAAAAATGAATGCATTTTCCAAACATCCTTATTTTTTTATGCCAGCCGAAAAGCCTTTA
CTTGCCAGCTTTGTGAGCTTTAAAAGATACTTTTTTAAAGATACTAATGATTATTGAATTCTTTTCAGAA
AAAATATCTGCTCACGAGTTTGAGCGTGGCGGACTGCTGAAACCGATTGTCAACACAATCGACGGCACGC
CCACATTTTTGAGCATTGGTCCGCCGATGGACAACGGCAGCGTAGGCGGCAGCTGCAGCAATCTGCAGAA
TGTCGGTGGCGTTGTGGCCGGACAGTATCAGTATAACCATCAGCAGCACCACCACAATCATGCCCATCTG
CACCACAGCCAGCACAGTCATTACCAGGCGGGCGGGGCCGTTGGGAGCAGCAGCTTGGGCAGCACCGGTG
GCGCTTCGGGAGCGGGAGGAGCACCCAGTCTGGGTGGCAGCGGTGGAGCCGGAAATGGCCATCAATATCC
ATACTATCATTGCCACCAGCGACCACAGCGGTTGTCACGCAACGAGCTCAAGCAGCTGGACGAGAAGGAG
TTGATTTTCGAGCTGGGTGAGTGAAAACAATAATATTTTTGATTATAAGAAAAATATGTTTAAGTTGTCC
GATCACGTTCTGATCAAAATAATAATAGCCCAATATGATTCAAAAGTGCAATTGATTCGGAAATAACGTG
ACACACTCGCTTATATAAAAGTAATTGAATAAGTGAATGAGTAAAGTTTCCTCTAAATGCTTAACCATTA

GATATCAAAGTGAAGGTTCCGGCGAATCTCGATAGCTCGTCAATGATTATTTGTTGAGTTCCGCAAACGT
GTTTGTTTGTCGGCCCATTGACGCTGTTATCGGCAAACACTCGAAAGACGTCAGTCACACAGTCTGTATT
TCCCCATGGCTCCCTTTCTTTTTCAAATGCAACTGCTTATCAGTCGTGTAAATAACCGGACTGTGCTGTT
TATCGCCACCAGGGTGAAGGACATTTGCAACGAGCTCGAGGTGCGCACTTTGTGCCACAAGATACTCCAG
AACGTGTCCATTCTGCTGAATGCCGATCGAGGATCTCTGTTCCTGGTGCAAGGACGATGCAACGGACCGG
ATGGCCTAAAAAAG
>dme_circ_0000673_junction_seq
ATGCAACGGACCGGATGGCCTAAAAAAGGCCTGCATTTGCGGAGCATCGAGGAGCC
>dme_circ_0000674
GCCTGCATTTGCGGAGCATCGAGGAGCCAGCGACGACGCCGCTCCAGTTCCAGCCCACTGGCAGGATGAA
CACGGAGCAGGGGGGCACGGGGTACGGCGGCTACGGATCCAGCGAGCACTCGCTGCTCATCGCCACACGC
CACGCCGGTGTCCCCCTGCCCCTCGCCCAGCACCAGCCACTCCCCGCCCACTATCAGCCGCTGAACCACT
CCGGAGCAGCCCCGCCCTCCTCATCCAACGGATCATCCTCCTCCGGCGGCGGAGTCCAGACGTCGGCCAC
gccgcagcaacagcagcagtaccaggtgcaacagccgtatcagtaccagtaccagcatcactatcaccac
cagGCCAACTCGCCACAACACCACAGACCCTACGATCCAGAGCACGCGCGGATGGAGGCCTGGCTGGATG
AGAATCAGGAATTTGTACAGGATTATTTTATAAGGGCAAGTCGAAGTTTGATCCTTTATTTTCGTTTCCT
ACTATTTTCGTATCTGTGTAAAATTCGGATGTCGAATTAGGAACTATATTAGATGGTATTTATTATATTT
AAATGTTTGAGCCGAATTTATTTATTGGCTTGTTGTTAAACTGCCACTTGCCAAAAACCAATCGAACTCT
GCTTAAAACGTCCCAGTTGACCAATCGATAGCCCTTCTGTTAGCCAAGACAGACAGAGAGAACTAACCGA
ATCCTTGTAGAGACATAAATTGCATCTGCCTACAAGCGTTTTCGATGAACCCATCGAGCCAATAATCTAT
TTTGTCCATCGACGGTATAAATTCTTAAATTTGAAACTGCATTGTCAGTTGACACAGCCGTACGGCAAAC
AATTGGGTTTGCCGATGGGGATTTGGCCATGGGCGTGGATGGCTCGTTTCCGCCATAAACCGGAAATTGC
CTTTGCCACAGCAGGACATCTAGGGCAGCCCGGCAGCAAAGCGGGTTAAAGGACGTCCGTTGCCTATGAC
ACATAATAAAAATCACTAACAACTAAAAGCATAAATTGGGCTTTATTTTCATGGTCGAGAGGAGCCGGCC
AACGTAGTCGGCTCCACTGCTACTCCGGCTTTTTGGCCTGAGGGGCGGTCATTAAATGAGGCCGAAAAAG
GCTTCATTCTCATGTGTGTGTATTTTGCATAAGGATATGCCGCATGTCCTAAACAGTTCAACCATTTTGG
TAGTAGTTCTGCCCCTTGTTTATATTTAAGGGAGTATTAGCTTTTCTGCAGAAAAAAATTAAGCATTAAGA
AAAATTACTAAACCATTTATTGCTGACTTAATCCATTTAGACTATAATTTGTTAAATTTCAATTTGGCTA
ATGTAAATTTTTGGTTTCGTTATATCTTATATATTAGATTTATATGGTTATAAAAAAATTTAAATATAAA
ACATTAAATATTTTCCTAATCAAAGAGTTGTGTGCTGTGATTACCCGACGTTTTGTGGCGTTTTGGTAGG
ATTTTATGTCAAAAGCGCGTGCCGAAAAGGACACGTTGTGGGCAGGAGCAGGAGTTGGAGCAGCTGATGC
TGTGTAGTCTCGCTGCCTGAATATTTATGAGTTGGCATTTCATTAGACACTTTTATGCGGCACAAGCATA
TGGAACTCGGCGCAAAATGCGTCGGCCAGCGCCTTTTCATTTGTGATTTTTAATAAGATTTTAACGTGTT
TACTAACTGAATTTCACCGTCTATTTCCCACAGGGAAAGCCACACGTCAGACCGTGGATGCCTGGCTGGT
TTCCCATGCCACATCGGCGGGCAACGATGTGGTCAGCAGCACCTCACCCACACACGCCAATGGACAGACG
AGTTCCTCGCGGGGTGGATCCGGTGCCACCACACCAGTGCGGGTAAGTGATTCAATTGGGGCTTCGTAAA
TTAAATACTGGAGCATTCCATGTGGAAAATGTAGAGGAGAGTACTTTTACAGCAAAATAAATTGATGAAG
TAACGTACTTACAAAAATGAATGCATTTTCCAAACATCCTTATTTTTTTATGCCAGCCGAAAAGCCTTTA
CTTGCCAGCTTTGTGAGCTTTAAAAGATACTTTTTTAAAGATACTAATGATTATTGAATTCTTTTCAGAA
AAAATATCTGCTCACGAGTTTGAGCGTGGCGGACTGCTGAAACCGATTGTCAACACAATCGACGGCACGC
CCACATTTTTGAGCATTGGTCCGCCGATGGACAACGGCAGCGTAGGCGGCAGCTGCAGCAATCTGCAGAA
TGTCGGTGGCGTTGTGGCCGGACAGTATCAGTATAACCATCAGCAGCACCACCACAATCATGCCCATCTG
CACCACAGCCAGCACAGTCATTACCAGGCGGGCGGGGCCGTTGGGAGCAGCAGCTTGGGCAGCACCGGTG
GCGCTTCGGGAGCGGGAGGAGCACCCAGTCTGGGTGGCAGCGGTGGAGCCGGAAATGGCCATCAATATCC

ATACTATCATTGCCACCAGCGACCACAGCGGTTGTCACGCAACGAGCTCAAGCAGCTGGACGAGAAGGAG
TTGATTTTCGAGCTGG
>dme_circ_0000674_junction_seq
GACGAGAAGGAGTTGATTTTCGAGCTGGGCCTGCATTTGCGGAGCATCGAGGAGCC
>dme_circ_0000675
GGTTTGTCTAAAAAGTTTGTCATTTTTAAGTAGTTGCAATAATATAATCGAGCCATATGTTTGCCCTTTC
GGCATCGGCAACATGTAGATCTTATGTTTTTGTGATTGCAACTTAATCGGCTCACCCTAATGTATAATAT
TACTCGATAAAGCTACGAGCACACAAAAACGCACACAAT
>dme_circ_0000675_junction_seq
GCTACGAGCACACAAAAACGCACACAATGGTTTGTCTAAAAAGTTTGTCATTTTTA
>dme_circ_0000676
GAACTTAACGTGATCCTTGCGGGGATCCAGCATTAACAGATACTTTCGCACCTGGTGGAACATCAAAGCC
TGGGAGATCGAGCCCCATTGCGCTGCCTGAGTGGCAGGCGAGAACAAATGTAAAGCAATCACCAGGATGA
GGCACAGTATAATGCACGAAGAGGCGTAGATGAAGTTAATCACAAACATCATGATCAGAGTGCCCAGTAA
TCCAACCAGACAGGTGTGCCAGGTGAAGAACTTAAATAGCGGCCGGAAGTTTGGAGCTCCCGTGAGTTCA
ATGCCCAAGCAAGCCAGGTTGGTGGCTAAATAGGAGAGCATGAAGAGCACCGAATTTATCTGGGCGATGA
TGTTGAAGGATCCAATAAGCAGAATGCACTCCACGAGACACCAGCTGACAGCAACCGCTGCAATGGGATT
GCCCTTCCAAGTTCCATGAATCACAAAGTTTAGAAGACTGCCAAACACTTGATCCTTAGACAATGCCTCC
AGAATCCTGCTGGATCCAATCAGATTGCTCAGACTTGTTGAAAAGGTGGCGGTCAAAATGCCGATCGCCG
TAAATGGAGGCCAAAGATTCACGGGCATCAGGAACAGGTAATTGTTCTGCATGGTAAAGTACGGAGTCGT
GCAGCTCATGAGGAAGGACAGGATGATGTAGGAGACGAAGGTAAACGCCACTGCCGAGAGAGTGCCATAG
GGAATACTCTTTGATGGGTTCTTCAGTTCTCCGGACATATTCGCTCCCGCCATTATTCCAGTGACTCCGG
AAAACAGCACACCGAAGGTGGTGGAGAAATCGACTTGTTTGCCATTCGACGTGTAATCGCGACCGTAGTG
GGATCCTAGATTATCCCGCAGCGTTGTGGCATTCAGCCCTGTATAGTCGAGAAATGTTGTG
>dme_circ_0000676_junction_seq
CAGCCCTGTATAGTCGAGAAATGTTGTGGAACTTAACGTGATCCTTGCGGGGATCC
>dme_circ_0000677
GTGCCCAGTAATCCAACCAGACAGGTGTGCCAGGTGAAGAACTTAAATAGCGGCCGGAAGTTTGGAGCTC
CCGTGAGTTCAATGCCCAAGCAAGCCAGGTTGGTGGCTAAATAGGAGAGCATGAAGAGCACCGAATTTAT
CTGGGCGATGATGTTGAAGGATCCAATAAGCAGAATGCACTCCACGAGACACCAGCTGACAGCAACCGCT
GCAATGGGATTGCCCTTCCAA
>dme_circ_0000677_junction_seq
AACCGCTGCAATGGGATTGCCCTTCCAAGTGCCCAGTAATCCAACCAGACAGGTGT
>dme_circ_0000678
GCACCGAATTTATCTGGGCGATGATGTTGAAGGATCCAATAAGCAGAATGCACTCCACGAGACACCAGCT
GACAGCAACCGCTGCAATGGGATTGCCCTTCCAAGTTCCATGAATCACAAAGTTTAGAAGACTGCCAAAC
ACTTGATCCTTAGACAATGCCTCCAGAATCCTGCTGGATCCAATCAGATTGCTCAGACTTGTTGAAAAG
>dme_circ_0000678_junction_seq
AATCAGATTGCTCAGACTTGTTGAAAAGGCACCGAATTTATCTGGGCGATGATGTT
>dme_circ_0000679
GGAAGGACAGGATGATGTAGGAGACGAAGGTAAACGCCACTGCCGAGAGAGTGCCATAGGGAATACTCTT
TGATGGGTTCTTCAGTTCTCCGGACATATTCGCTCCCGCCATTATTCCAGTGACTCCGGAAAACAGCACA
CCGAAGGTG
>dme_circ_0000679_junction_seq

GACTCCGGAAAACAGCACACCGAAGGTGGGAAGGACAGGATGATGTAGGAGACGAA
>dme_circ_0000680
GGACAGGATGATGTAGGAGACGAAGGTAAACGCCACTGCCGAGAGAGTGCCATAGGGAATACTCTTTGAT
GGGTTCTTCAGTTCTCCGGACATATTCGCTCCCGCCATTATTCCAGTGACTCCGGAAAACAGCACACCGA
AGGTGGTGGAGAAATCGACTTGTTTGCCATTCGACGTGTAATCGCGACCGTAGTGGGATCCTAGATTATC
CCGCAGCGTTGTGGCATTCAGCCCTGTATAGTCGAGAAAT
>dme_circ_0000680_junction_seq
GGCATTCAGCCCTGTATAGTCGAGAAATGGACAGGATGATGTAGGAGACGAAGGTA
>dme_circ_0000681
TTGTTAGCCCATTCACGACAAGATGCAAGGAGCTCCTTTCACGCCCGCCTGGCGGGATGCACTCAATACG
AGCTCTTTGAATCGCCCTACTCTCAAAGAGTTCTTCATCCATTTATCTTTCGGAGCGAAACGATGGCTCC
TCCATGGCTGAAGCTTATGTGCGAGCTGCAGCATAGAGTAAACGGATCTCATCCCACAAGATCTACCATC
GACTTCTGTTTTGTGAGACCGCAGCATATTCCGGCCGTAAACGCATTACTCCAAAGTACTTTTTGGCCAA
ACATTGATGGTAAGCGAGTGTTTGAGCTACCCGGACTACAGTGTAGTGGCGTTGTACAAGAAACTGGTCA
TCGGTTGCGGATTCCTAGTGCCCGACGTAGGTTACAACGAGGCCTACATCTCATTCATGGCGGTGCGGCC
GTGCTGGCAACGCAGTGGCATCGCCAGCTTCATGTTATATCACCTTATCCAGACATGTATGTCCAAGGAC
ATAACGTTGCACGTTTCCGCCACCAACGCGGCCGTCATGTTGTACCAGAAATTCGGATTTAAGATGGAGG
AAATCATCTTGGACTTTTATGACAAG
>dme_circ_0000681_junction_seq
GGAAATCATCTTGGACTTTTATGACAAGTTGTTAGCCCATTCACGACAAGATGCAA
>dme_circ_0000682
GGTTCTAACCCGCGCTTTAGTCGCTATGAGTCAAGCGCCTCTTCTTTGCAATCTCGATCTCGTGCATCTT
CCTTTAGCCGCCATCAAAACAACTCTAACGATGACTGCTCTCCCATTAACACGCCAGGAGGAGCAAGCAG
TGGAATCTCCAGCGCGAGCAATTTAATTAACCAAAGCACCAGCATCAACATTTCAAACATTGGTACTAAT
GCCTGCAGTGCGATGCCAGCCCCCAGCCTGGCGTCCGCTGTAGTCAGCTGTAATGTCAACGCCTCCGGAA
CGGTACCAGCGAGCACATCCATGCCCTCAGGGGTTAGCTCCTCTTCAAGTTCATTGCCTATGTCTCCGGC
CGCTCTGGCCCAAAGACATCGAATGGTTCGTAATGCGCGTCAAACCGTCGACTGTGACTTCAATGAAGTT
GGTCGCCTCCGGTTTCGTAGCTCCGAGGAGGTTTCTGGAGGTGCAGGAAATTCCACGCAGTTTGAAGATG
TCCGCTGCGATTCTCCAGTTACCGCTCGACAAGGTTCAGCTGTAAATTGTTTTACTGGACCTACAGCAGC
CGTTGGAGAATCTATTGATGGAACGTTAAATAACAATCAAATTACGGGTGGAGCTGAAGGGTTTACTGGA
TCCGGAGGCAGCATTCTGAGCAGGAGACGTTGCGGTAAGACACCTAAAGACTTGCACCCAGTGCACAATC
AACGTATCCAATTGGCAGAACAAGTTGAGGAATGCCCGTCCAGTGGTGATGAAGGCGTTGTCAGTCCCCG
TAAGCGCATCAAAATGGATTACCATCATCACCACCATCATTCCAATGCTAGCGGGGGTAAGTAAATGTTT
TAACAAGGACACGACTTGTTCCACGTTTTGAATGGATATGCTTTCAAGTTAGTTTACAAGaaaaaaaaTA
TTTTAATACAATATAAAATAATTAGAAAGtttaatcattaattaatcattaatcattaatcattaataat
taatGCTAGCGGGGTAAGTAAATGTTTTAACAAGGACACGACTTGTTCCACGTTTTGAATGGATATGCTT
TCAAGTTAGTTTACAAGaaaaaaaaTATTTTAATACAATATAAAATAATTAGAAAGTTTAATCATTAAGT
GTTATTATTTTTTTGGTGCATATCACTCTAATGTACCCAATGTACCTAAGCTTGGACTCGCCAAATCTAG
CttttttttttttCTTATTTCTCTATGCTAAAGCATTAAAACTGGAACAGTCTTTAAAATACAAAAAATA
ACTTCTTATTGGTAATATCGTTTTAATTTAGGGTGGAGTCCACTGGCGAGCACAGCAGCATCAATAAACC
AAGCCCTCTTTACTGTCCAACTGTGATGTGATACACGATCCCCTAAACCGCAAAAGCGAGATTCGAAGG
GTATCCGAGACGCCAAGTGGTTCACCAAGCATTAAGTTTCCTGGCCACCTGCCCTCGGCTCCCCAATCCC
TGATGCTCAGTTGCCGGCGCCCTTCCATCGATGTGGGAGCTTTGTCGGCGCTCTCTAGTTCCTCGGCTTT
CCGGCACGGAATCGTGGGAGCCAGCAGCATGGATCAACAGCACATGATGAATGCCTCGGCGGCAGCAAAG

AGGAGGCGAGTGACCACTACTATGCAACAACCCAGCTCATCCTCAACCACTAATAGTTCTAGTGGATCTG
GATTGGGTGGGATAAGTAGTTTAACTCCGGCGGATGAATATCATCATCATGTCTCCAGAGGTAGGGGACA
TCAACTACATTCCCATCACTCACACGAGGCTAGCGGTGGCGAAAGTGCGGATGGATCTCGCCCTGGTACG
CCGCTGTGTGATGAGCGACCTGAAGTGCTGCCCACAGAGCCACGTCGATTGCCTCCGCCCAGAGAAAGAG
TTCGGGAAAGAACCAGGGATGTGATGTGGCTCCCCCTTCCAAAGTTTGGAGTGCTTTTCTTCCAACAACA
ACAGTCCAGAAGCAGTGGAGGAGGAGGAGCAGGAAATAGCTATCTGCAGCAGCAACTGGGAGGAGGATCT
ACCGGAGGGTTGGGGTGCATAGGAGCTGCTTCCTCTTCGGCATGCTCCCTTAACAACTCTAGTCTTAACG
CCAGTCAGGGAATGGGCTCCTGTTCTGGATCCACATTCCTGCCCAGCCCATCCTCGCGATATTGGCGTTC
CTCTTCACACCACCAAAACCAACAAAATAATCACCAACAACAGTCGCAGCAATTGCACGGATCCTCATCT
TCTAACACTTGCTTGATGGCCAGTCCAGCACGTCCACGATCACTGAGTTCCAACTCCAGCGATTCGGATG
TACCTGGCCAAAATGCCGGAGGAAGTCCTTCACTAGATGAAAGACTGCGCAACTTTGAGGAAAACTATGA
ACGATGGTCTGGGGGAAGCAGTAGAGAACATATCTCCGGTCATACGCCCTCGAGTGCCACGCCTTCTTGG
CAGCTCTCTATGCACATGAATTTGAGTACTGGTCTAAATTCCCATCAGACAAGCTCGGCATCCGGAAATA
GCAACAGTTCCAGCGGAACAGTATCCAGTTCAGCGAGCAATTCACGGCACAAGTTTTTGGATATTGATGA
ACTGCAGCCCTCTGACATTGTCAAATCTGTGCTAGCCAAGAAAAGTGTTTTTGATGACGATTTTCAACGG
CTAAATAAAAACCAATGGTACGACCCATCCAGCTCTGATTTTGCATTGGGATCGAGTTCCAATATCGTTA
CAGGATCTTCGCTGGTCGCTAATGTGTCCCGACATCCTGGAGGCCCATGTAGCGGAAACACCTCACCTGC
TTTGCCTAATCTCGCGGCGACCAAGGCTACTCCAATTATTGGAAACTGTAGTGGTGGATTGGGTAACTCG
ACAGGTAGCAAATCTGCGGGATTGCTTCAAAGACTAAGTAGTTTATCACCCATGAATTCTCCTCAAGCTT
CGATGTCGCCCTACAACAGCCCATCACCCTCGCCTTCGGTGGGAGGAGTTACCGCATGCCTGGGACAACT
TACCAAACCTGCAGCACCAGGAACCGCAAGTGCAGGACTAAGTGGGGGAACTGCTGCCTCCTCATCCTCA
CCAGCTGCCAACTCGGGACCTACAAAAGGATTGCAGTACCCGTTTCCCAGTCACCCGCCTTTGCCGAACA
CCGCTGCTCCTCCTCCAGCTGTACAGCCTGCTCCTCCACCTCTTCCAGAAATGGGAAAACAATCTAGATT
AACTGGCCAGAGCTCTGGAAATAATCTAACGAAGAGCTTAAGCGTGCCAGATGGTCCTCAGTCATCGCCA
GCGAGAGTTCAGCTTCAGAAATCTGCTTCAGTGCCAGGTAGTACAAACGTAGGCGCCCCAAGCAGCCTTT
CCTTGGATTCAACCACAGCTTCTGTGGAAACATCTGCATCTATTTCATCATCAACGTCCAACGGTAACAG
TTCCTTAACCTCAGCAGCGATTCAC
>dme_circ_0000682_junction_seq
CAGTTCCTTAACCTCAGCAGCGATTCACGGTTCTAACCCGCGCTTTAGTCGCTATG
>dme_circ_0000683
ACGAACAGAGGCAGGATGGGCAGCTGGATGTTCGCGTGGAACCACAAGAGGATGTTCGTAAGGTACAGAA
GGAAACTCTGAAAAGGCAACCAGAGGATGCACCTAAGCACCTTCCGAAAGCGGTAGCCCCAAAGG
>dme_circ_0000683_junction_seq
CACCTTCCGAAAGCGGTAGCCCCAAAGGACGAACAGAGGCAGGATGGGCAGCTGGA
>dme_circ_0000684
GTCTGGTGGTCGCCGTGTCCGAGTCGTGAGTGTCCGGAGGAGCCGGAGCTGGAATTGGCCGTGCATCAGT
GCGTCGACTATTGTTTGATGCAGGAGCAAGTTAATCCCCAGCTTTACACGAATACCCCGGCGTATGATGT
GCGCCCGGCAGCCACCACGTCCACCACGCAGAGATCCCAGCTAACAGCAACCACAGCAAGGACGACCACA
ATGGAACCCTCCTCGGAATCGAGCTCATCGCCGGCCCCAACCACATCGACCACCACATCGCCGGCGTCAT
CATTGCCAGCATTATTCGCAACGCAAACGAGGAGCAGATACCAAGAAGTACTTAATATGTCATCGACCAT
GTTCCCGCCGATGTCCCAGTTGAAGGACGCGTTGGAGGAGGGTGTGGAGCCCGGGCAGTCATCGTCAGCC
GAACTGTCTGCCATGTCCATGCAGGGATTGAATTG
>dme_circ_0000684_junction_seq
CTGCCATGTCCATGCAGGGATTGAATTGGTCTGGTGGTCGCCGTGTCCGAGTCGTG

>dme_circ_0000685
GCAGACATTGTTTTCGTCCATGGATTGCTGGGAGGTGTTTTTATCACCTGGCGTCAGCGAGATAGAAAGC
CCACTGAGCTGGGTCTCTACGGAAAAAACGCCTTCTATACCAGCGAAACAGACGACGTATTTCTGGTGGG
TGAGCAGAAGAGACTCAGTGGAGGCAAACTAAAACCCGGCGAGGCGGTCAAAAATGTCAATGGCAACCAA
AAGACAGAAACAATCAAAGG
>dme_circ_0000685_junction_seq
GCAACCAAAAGACAGAAACAATCAAAGGGCAGACATTGTTTTCGTCCATGGATTGC
>dme_circ_0000686
GTTTGCCTCCACTGAGTCTCTTCTGCTCACCCACCAGAAATACGTCGTCTGTTTCGCTGGTATAGAAGGC
GTTTTTTCCGTAGAGACCCAGCTCAGTGGGCTTTCTATCTCGCTGACGCCAGGTGATAAAAACACCTCCC
AGCAATCCATGGACGAAAACAATGTCTGCCTTTGGCTTCCTGCGGGTGCGAACTCGTGGATGCAGGGGAT
AGACATTTGGCGGATACGTGCACTGGTTCGGATCGTCGTGATCCAGATTGGCCATAGTTTTAGCCGAGAT
CACCTGCAGGCGGAGATCTGGGCATTGTTGCCACTCGGCCAGGGCGCCCACCCATCCGGACGTAAAGAAA
TGTTCCACGGCATCGGGCACCAGGGACATATTGGCCAACACTTTGCACAAAGTCGATAAG
>dme_circ_0000686_junction_seq
GGCCAACACTTTGCACAAAGTCGATAAGGTTTGCCTCCACTGAGTCTCTTCTGCTC
>dme_circ_0000687
GATCACTCGGATGATGAAGAAGAAAGAGAAGCCAGGGCAGAGCAGCGAAAGCGCCGCCAAAGGGATCGCA
CAAACTCCACAGATTCCGTGGACACGGTAACCTCGGTGGCCACGACAGCCACTAAGGCTTCTTCAGTTGC
TCCTCCTCCTCGTCAACGCGGACGTCGTGGGCAACGAGAGA
>dme_circ_0000687_junction_seq
CAACGCGGACGTCGTGGGCAACGAGAGAGATCACTCGGATGATGAAGAAGAAAGAG
>dme_circ_0000688
GTTATCTTCTTCTGCATCTGCTGAGCGCAGTGCTTTTGACGATTTTCACCGTGTTGCTACCTATTTCGCT
GACCTTCATCCACGCCTCCTTGCGACTGCGAAACATCAAGAACAAGCTGACCAACAGCATCGAGAGCTTT
GCTCCCTCCACGCCCATGGGCTCCTTGCTGGATGCCTTAAATGTCCGGGCCGAAGGCGTCTTTAATTAGA
CTGACCAGTTTGACACAGTTGGAATGATCGTTTTTATCGCTAG
>dme_circ_0000688_junction_seq
CAGTTGGAATGATCGTTTTTATCGCTAGGTTATCTTCTTCTGCATCTGCTGAGCGC
>dme_circ_0000689
GGCGGCAGCGACAAAGAGAATCCCCCGCCGGTTGACGCAGAAATGAAACGCCGTCTGCGCTTGCTTTCGG
CCAGTCGAAACAGTACCCCGCACCATGCTAACATCACACCACAATCCTCAAATCCTCTCTTGAAGCCTCA
GCAGTTGCTGGCCAAATCGTCAAGCGGACTTTCCGCCCAAATGGATGCAGATTGGCGCAAGGAATTCAGC
GAGCTAAGAGATTTTGTAGACCAGCGCTGCGAGAAGGTGGAGCGCGAATTGGAGTACGTTACCTTCTCTA
ATCAGCGGCTATTGGCTAATAAGATGACCAACTACATGGACCAGCAG
>dme_circ_0000689_junction_seq
TAAGATGACCAACTACATGGACCAGCAGGGCGGCAGCGACAAAGAGAATCCCCCGC
>dme_circ_0000690
CGACCTCATCCCTGCTGCCTTACCTAACGATTCACATGCAGTCGATTGGACTCACGGTGGAGGAGATTGC
CATCATCTACCTGGCCCTGCCCTTCACCACCTTCCTTAGCCCGCCCATAACAGGTGAGTGGATCGGGATC
GGCGGTTAATTGTCTTAATGCACTTTCATTAGCCTGCTGCGTGGGTTTTAATTAAAAGTTTGCTTTGTTT
GATTGCCCGGGCATGAAAACGAGAGCAAGAACCAAGGTGAAGCCAAGCGAGATTAGCCGAGGAGTGCAGT
GCATTCGCTTTATGTTCGTTATTAAAGTCAGCACTTTAAAGGAAACACTAAACCTGCTAAACCGAGCATT
TGCCCAAGAAAATTGTGAAAATCAATCGATTGGGCCTATGTGATTTATTTTCAAATATCCGCGATGCGCA

AACATATACATATGCATATATTTATATTATTTATTCAACCGCACAGCGGACATTTGATTGGTTTTATCCA
CCCTCGGAGCAGAATTTTTTCCACGCCCAACATTTTTTAGGCCACTTTTCCGTACGAGTGCGAATAATTT
AATTCACTTGCAGTTTAAATGACAATTAAACAGAGTTCTGTTGCTCTCCTCTGAATGTCTGTATGTGAAG
TGCCCCCATCGTGTGGTTGGTTGGTTGGTTGCACTCTGCTCCTTTTGTGCAGCGCGATTCGTGCGTTCTT
ATTATTTGCTTTCGCATTTTAAAAAATGCATTTGCGGTATGAAATGTTGGCGACAGCAAAGGTTTTAAAC
ATTTTATGTGGACACTCGCTGCAGACAAATGTTGTGGCACAGAAAGTGGGGGTGTGTGGCAAGTGCATAA
AGCTGCTCGAATTGCCGCGCATAAAAACTGCGAAAAAATATAAAATGTGCGCTAAGTGAAAACGCTCTCG
TAAAGTTGCGACATCAAAATTTCGATCATCCAACTTGCATTATAATCGCGCTTGATAGTCGCATTTAATG
GCATTGGAGGCCATAAAAGTGAAACGAGTTCGAGATGAACGAGCGAATAAAATGGGCAGCAAGGAGTTTG
GAAAGTTTGGAAATGAAACATAAAATTTGAAGTACTTATAGTAGTAATAATCATTGAAAGTTTTTAAGCT
CTAGCAGGAGATTTTAATATATGTGTTTCCCGAATAAAGCGCGGCGGGTATTACTTTTTGTGTATTGACA
AAACGCTGGAATGTGACAGCTTACTCGACTTACTCGCCTCCTCGGTGTGGCAAATCCAACTTAAACCGCA
TTCGAGCTGCCAGCAACCAATCAAATGGCAGTCAGGTTGCTTTGAGCAGCGAAGTATTGGATTTACACGC
CCGACGATGCAGTTTCCAAACGTCTTACAGCAATCTCGCCTACGTTTTCCCGTACTGATGCCATTTTCCC
CGACTCCCACCCGCAGGGCTTCCTCGTTGACAAATTTGGCAAATACAAACCGGTCGTTGTGATGAGTCTG
CTCCTGAATGCGATTTTCCACCACTCGCTGCTCTTTATTCCGCAGCAGGAGATTCCGGGCGTGGTGCCAT
CGGCGTTCGTATTGAGACATCCAGACAGCGGCGACATCGAGG
>dme_circ_0000690_junction_seq
AGACATCCAGACAGCGGCGACATCGAGGCGACCTCATCCCTGCTGCCTTACCTAAC
>dme_circ_0000691
GCCCATAGTAGACACTATAATGGATGGCTAGAGCCCACAGAAGATGTGTCCACGTGGTTGAAAAAAATCC
GAAGCGTTCAGGACGATTGTATAGGCGAGGCTAATTTACAGAGTTACATTAATGCTGGTTATGTTAGTAT
ACAACCCCAACCCATTATAAAAAGCCCTTCAGCACGTGCCACTCGATTTTTTACAATTTTAACCATTATT
TTCCCTTGCCTTTTTATAGCTGTGGTACGAGACGAATCGGTACGTGAATGCTGGCAGTGAAATGGTCGTC
GACGGGCGACCCAAGAGTCCTGTTCAACTGAACGAGGACTTCATGAACGGCGGCAAGGTCCTGGCAGCAG
CCGCAGCAGCAGCCGCCGCAGTGGTGGCCGCCTCCTCCTCGGGAGCAAAAAGCGGCGGTGGGGGCAGCTC
CGCGCCCAGCGATGATCGCAGCGATCGTGATAATGGGTGAGTTTCGTTTAAATTATTTGGTTTGCCTAAG
AACTAATACCAAGAAGTTAAAAACATTTGCACAAATTTTGAAGTTATATCAAACATAAGCATAATTTTAG
TGTGTAATTGATATCCAAACAtttttatttattttttttCCACTATTGAGAAAAGAATATCATGGGTTTAT
CTTTTTAAATGAAGTAATATTCAGCCTGCTGCGTACTAATAAAGATATCAATGCCCATCGTAACGCTTAG
TTACCCTGCCCAAAATGGGGCAATTCCATCAGGATTTCACATTGAATAGGAGTGTAATAACGTATGTGTT
GTGTCCATGCTGGTGTGGCTAATAATTGCAGCTGTGGTGCCAGGCAATTAACGTGCAACGCAACCACTGT
GGGTCACATCAAATCGGGTGTACACAATCTAAAATCTAGAATCCAAAGTTCAGAATCCGGCGGCAATGCC
CGTGACAGAGTTCCTGGCACGAGCGATACGTCCTGCGATACAATCTCAATTTCGGGTGCAGCCACTGTTC
GCTTTATGAGGGCTCACAGAATCGGAAACAGAAGAGCAGGCATCCCTTCTCCGATATTGCCGTGTCACCC
TTTTTGCCTCCCCTAAATGCGATCAATTTGTGCGCTGCGCATGCGCCCATGCAATATTGCGGCAACCGCA
CATGTTTTCATTTGTTATTAAAATGTTTGATAATTTCATTAGAGATCCATCAGCAAACAAAGGGTCCGAG
ATTGTGTCAGCGATATACGGCATGTGCGATATGTTGCCGACAGTCAAAATGGCACACGCTCAATTTAAGC
CTAATCCTGCATCCCTCGAATCCCGAAAAACGGGAAGCCGGGGCGTGTTTGATTATAAACATGTGGGATT
TATGGACAGCTCATTTGGCGTTTCACACTCTCTTTTTTTCATGCCTATCACAAATTTAAATCTCCACGAA
TATGTTTTCATTGGCGAGTCGGCTTTAAATTTGAATATGTAATGAACATAATGGTTAATTCCATATGCTT
ATCTGCCGGATCATAGTGCCTTACAATGGTCTCGCATTTATATTCGAGTTTTTCAATTAAAGCCAAGCTG
TCAAAGTTTCCGCGTAAGTCCTTTTTTAAGCTTGATATTCAACCCGCTTTTCCGTTCAACACAAGGGACA
CCTGTGTCTTGACTTTTTAACGTATTTTCGGGCAACTTTTCTCGCAGTTCACGCGCTGCTGAGCAGGGAA

ATTTCCTCTACTCTCTGGCAGAAGACAAGGCCCAGATGTCCTGTGCGTCCAAGGACATGCAGCGAGTGGC
ACCAGTGCAGCACGGGGGTGGCACTGCGATCGAGGTTCCGTCGCCGAAGCCCGGAAAGGAAAAGAGGTGG
CAAGGAACGATCGAAAGTGTGGTTGCAACGGTAGCGAATGATCGCTGGATTTCCAGGAGAGGGAGTTCCA
AGTTCCGACCGTTACTTTTCTCGAGGAATACTTAATGAGCGCCTGGGCTGGGAATTTTTCGCGTTCGAGA
ATTTTCAGTAAGGAAAATGCTAATGCCAAGGGGTGCACTCTAGACTGGGAATGTTTTTAAAATTTGTAC
ATAACATTAAATGCACATTGTTGTTTTCCaaaaaaaaaaaaGAGAATTTAATAATGAGTTTTTGAATGAC
TGTATTCAGTTTTAAAACAACTTACTTCATAATACCGCAACTTTTAAACAATGACTTCTAACCATTCCTC
CAATCACTCCGAAATCAACCAACCCACAAAGTTGTCAACTCAAAGCGAGCTCCCTTCACCTTGACTCCAC
CTCCGCCGCCaaaaaaccaaaaaaCCATTCCCAACTTTAACGAAACGAAACCAAGACACTTTTGAGATGG
CTTTCGGCTTAAATCGCGGCAACTCTTCAATCGACCAATCAAACAATGGAACAAACTATACACGAAGCAC
ATTTAGATACGTTCGTATCGCCAACTGGCGACTGGCAGAACACACGCCACATAGACCAAGCCCATAAACG
AGATCCTAACTAACTCAGATACTCAGATACGAATCCAAAATCGAAAAACTCAACATACCTCCGTCGATTG
ACACTTTGTCGCCGGCAAAGCTGTTGGATGATTAATGAGCTGAAGAATAAAAAACGAAAGAGTAAAATTA
AATTGAATAGAAGGCTCGTGTCGCGTCTTCCGCCAAATGATCATCGTGTGTCAATTTGGGCATCATTAGG
AGTGCCACCTAACCCtttttttgttttttttattttattttttattttGTGCTGCTGCCAAATCGATTAGC
AGGCGGATCTAACTTTGATTTTTTCACATTTCGTTTGAGCAAGTTGTAAGGTGTAAATAATTCAATCAAT
CATTTGTCAACAGTTTCGGCTTCCGTTTAGCAATCTAGCCTCCGATTCAGGGTCAGCCGCTGGCTTTTGG
CAGCCAGCAGCATATTCTCTGATATGTAATTGGCATGTCAAAGAGTTTCCAGCCCGCCCGCCTCCTTCGG
CATCCATTGGCATCCCTGGTTAATCCACATCCTGAACCCTCACAGTGACTCTATTGAATTGTCAGTTTGC
TGTTGGTCGCTGTAGTGAATCAATCTGTCAGATGAAAGGGGAATCCAGCTGGCAGAGATCTGGTGAAAGG
CGTGAAAGGATTGTTCAGGATTTGGGGGAGATGCTGACAAGGGGGCTGGGCAATGACCAAATGATAAGGC
ATCGATGGGCAAACTGACAGCGGAGATGCCTTTGTGGAAAGACAAATAAATTAGGTACTTTCATTGGGTA
TTTGATTAATTGCTTTGTTAACGCGGTTTTTAAAAGAGTCAATATAATCTTCAATTTGAATTTAGCATTA
GAAAAAATAGTAGtttattgaaagttattatttatttatttatttaaaatttttcttaaactttttttaAG
TACTTCCAAGAGCAGCTGCTCTTAATATCTTCAAACTTTGTTCCCAGGATTCCGCCCTACTTAAAGCGAC
CCACCCAAACATAAGTTTTCCCAAGCATTCTCAGGCTGAAGGAACCTACAGCTACCCTACCATTTGCCCA
GGACACCATATGTCAACCCACTCCTACCTTGACTCCTATGTGTCATCTCCACCCCCTAGAAACGGCGCCT
ACCCTCCTACTATCCCACGCTTGCCCACCCAAAAATTAAGAACTGTAGAAACTCCGGGCAAAGTTCAAGT
GCAGCCCACAAGGAAAACCCTTGACGTGGGAATCTTCGCTGCCCTTTGCGGCCATATTGCACACGCTGAA
AATTAACTTTTGACCCAAACAGCAATTACCACTCCGTTTTTAGAGCGGGTATGGCTTGGATGTTTGGTAA
GCACCCCTCGTCGAGTGGGCAACCAAGGGGGGTGGGCTGCCTTGAGAGGCAACACCTGTTTAGAGGCCAG
AAGCAGCGATACGCTGCTGCAAGGTGCCACATCTACATATATGACTTTTCAAACGAAATCAGTCCAACGG
AACTAGAACTGCAGGGTGGCAGAGGTTGAGGGGTTGACTGCTGACGCTGACCCTTCGGATGTGCTTGCAT
GCATAATCTCTGGTCATCGCCATCATCCATTAGTCGCTGTGTTGGTGTCACCTGGTGGTTGTCTACTGCC
CACCACCCCTGCTAACTAACTAGTACCACTACCAATGCGAGCACCACTACCAATACCGGTGGCAGTAGCC
TACCCCACCCTCTACACCCCCTAGAGCACCACAGCTTTCTCCTCAACCTCTCTGCTCATATTCAACACTC
ATTTCCCCTAGGAATTGTTTAAAGATGCAGAGAGAGAAAAAATTGATATGCAATTATGCTTCAAAGCCGA
CCTAGTTCATGATCATGATCACTAGTTCATTAAACCTAAAGCATTGTATTGTGAAGAACTTCTGTGAAGA
CAATGAAAACATACAGAATATTCCAAGTAAAATTCATCCATTCATTCAAACTCTTATTATATTTTGTATT
CTTATTTTTCAAACTATGCTTACAATACACCATATCGTGTGTAAGCCTCATTCTAAATATATATAAAACT
TATTTAAGACATACTCAGAGTTTTCTCCCCCTGTACCTCTGCACTTGGCACTCGTCGGGAGTTGGCCAAG
TTAAGAGAGGGGTGAGGATTGCCTCTAGAGATGGTGGGGTGGGTAGGGGTGTCTTGCCTTTACCTTACTT
TCATTTGGGACAATTATCTATTCAACGTCAAGCTATGCGAGTGCCATATATGAGTGCACACGATTTCGTC
TCTGCTACCAATTTTGTGTTAAGTGTTTGCGTGTTTACTCCCGAGCAAGCGTACAtgtgtgtgtgtgtgt

gtgtgtgtgtgctggtgtgcaaaggtgtgtggtggTCATATGCCAGTGCGTCCTGGCCAGAAAAAGGACC
TGCCGCAGATTGTTAGCGGACAAACAGAACATTAGCTCGTCGGCATTATGCTCCGCAAGCGACACACTTG
TTCATCAAACGCGAACCCGAACGGTTGTGTGTCAAGTGAGCAGCTCAAGTGATAACTGAAGGATCGAAGT
GATATATAGTGAACATACGCAATTGAACTTGATGGTATCCTGATTCTCTGTGAGTGCTAAATAAGTGCAA
ATAAATCATAATAGTTTCTTGAGATCTGTAATATCTTACGATCACTGAATAAAGACCAACGCTTGAATAT
TTTGAATTGTTCATCTCCATATATCTGCAATATACAGCAATTATCATCTGATTTATCTTTAACTTTATTC
TAATCGAGTGCATTTTATTTATTCCAGGTTCTCTGTACAGTGGTGACGAATTCTCCAAAGATAAGAAAAA
CTCCTCGTTAATCAGAGAGGGCGACATCGATTTCACAGACGACGAAAACGGTTTCGATATACGCTGTGAG
GTCTGTGATAAAGTCTATCCGGATTTAGATCTG
>dme_circ_0000691_junction_seq
TGATAAAGTCTATCCGGATTTAGATCTGGCCCATAGTAGACACTATAATGGATGGC
>dme_circ_0000692
GCCCATAGTAGACACTATAATGGATGGCTAGAGCCCACAGAAGATGTGTCCACGTGGTTGAAAAAAATCC
GAAGCGTTCAGGACGATTGTATAGGCGAGGCTAATTTACAGAGTTACATTAATGCTGGTTATGTTAGTAT
ACAACCCCAACCCATTATAAAAAGCCCTTCAGCACGTGCCACTCGATTTTTTACAATTTTAACCATTATT
TTCCCTTGCCTTTTTATAGCTGTGGTACGAGACGAATCGGTACGTGAATGCTGGCAGTGAAATGGTCGTC
GACGGGCGACCCAAGAGTCCTGTTCAACTGAACGAGGACTTCATGAACGGCGGCAAGGTCCTGGCAGCAG
CCGCAGCAGCAGCCGCCGCAGTGGTGGCCGCCTCCTCCTCGGGAGCAAAAAGCGGCGGTGGGGGCAGCTC
CGCGCCCAGCGATGATCGCAGCGATCGTGATAATGG
>dme_circ_0000692_junction_seq
GCGATGATCGCAGCGATCGTGATAATGGGCCCATAGTAGACACTATAATGGATGGC
>dme_circ_0000693
GCTCCGGAGGCGACGACTTCGATCCAAGTGCAAGTGTAGCGCGCCACCATCATCACCTCCATCACCTGGA
TCTGAGTGCCAGGAGTCAGGGCTTCAATCCACTGTCTGAACAAATCGAGGATTCATTCGCCGCCCGCCAT
CACTTACCGCAGCAGGAGCCCGTGGAAGGATCTCCGCCGAGGCGGGATCATCTTGATATACAGGATCAGG
AGCTACTTCACCATCCCAATCATTTCCACCAGCAGAACCAGCGACAACAGCCACCGCCTCCCACGTTGCC
GCTGTCCGTGGAATCCTCCTCCGCCGCAGCCGCCTCCGTCGACGGCATGGATGCCTTCTTCAAGGACCGC
GCCCAGGCGGAGCACATCCTGCAGGAGTGGGTGCGTAGGCGCGAACCAGTCTGTGAGCTGGACATCCGTG
ATAGTGGTGGCGTCTACGCCAAGACGCCGCTCCAGAGGGGCACTCGCTACGGACCCTTTCCCATGAAGCT
CAGCCACCAACCCAACGATCCTCAGCTGGCCTGGAAGGGTAAGTAGTTTTGACAATCCTTGAAAAAGAGA
TAAATAATTTTTTAAGTTCTGAGAAGTAAATATAACTGTGGGGAAGGAGTAGAAAATACATTGAATATTT
AGAATCGAACGATAGATAGAAAACTTGTAGTCTAAAGTTGATTGTTGGATCTGATATTTTGTAATGCTTT
GCATAACTTATTAAACTACTTTCTAACGATGATTTAAATATATTTTAGTTGCTTGATGTTCACTAACCTA
ATTAATTCAAAAATCGGTAGAACTCAACAATGCCTACCCAAAATTGTCGTTAGTTGCAATTACAATTATT
TTCCCATGCAATCAGAAGCACAAATCCCATGCCTGAAAATCCCAAAAAACTTTTATAGCACACATTTATC
ACCAAACGGACCTTAAGCCTTTCACATGTGTGCGCATTTTGGCAATCTCAAATTTCCGATATTAGTTTTT
CGCTGGTCGGGAAATTTAATCCCATCAGAACCGTACCAATTTTGATGTGCGAAAATTGCAAGCGAATTGA
GTCATCCCAATTTTTGGGTGGTTTTCGCAGGGAAAATCAAACGATTGGAAAGAGATTTGATTAGAGCTCT
ACCTCGAGGGTATCAATTGTCAGTCAAATGTGCCTAATCCCCGGCCAGAGCTAATTTACCTTCCGCCGGA
GTGGAAAACCAGGACGAAAATCCACAAAGTGCACCCCAAATGAGGCTTAAGTCGGGGCCGGCTGATGATT
CCAATTAGGGTATACGGATCGTCCGATCCCAGTCTGAACCCTTCGATCGGTTgtcagtcagtcagtcagt
caACTGACACTTTTCCTCCTCCCTCCGCTACCACTTTTCTCTACCGAAACACAGAGACAATCCAATCAAA
TTATCGCGCCTCGTCCTGTTGACAATTCGCACAAGGAGATCCCTCTTTTTACCTACTCGGCCCAGGTATT
GGTACGAAAATATTGTTCCTGCCACTCGAGCGAAAATTGCAAAAATAACACAACTCGGATCGGATCGGAT

CCCTTCTGGCCTTTATTCCTGGATGATCCTCAAATCAAGCGCACTTTTATCGTTCACGTTTCCGTTTTGA
CAGATACATTGGCCGTATCTGTATCTGTATCTGCCACCGTTTCCCGTCCGATCCTTCTGGAGGATCTGGA
TCTGATCTGGCAATTCGAAGCGAGTTTTGCCAGCCGAACCTAACCGAACCGAACCCAACACTTTGAGATT
ATCTCTGGCGCGTAAATATTTTTCCTGCCCATTGACGTTTTCGTGTTTTTCTGCTGTGAGCGTGGTGGCC
TCCTTTTTAATAAAGTTATGAATAGGCAAGGACATGTGTGATGTCTGTCCGGGGATTATCGACAGACAAA
ACCAGCAACTGGAACTGTTTGCCAAACGGTTACGGATATTTCTGGCCGTGTATTTGTACATATCCAGATG
ATCTGATCATTTTTTTAGTGGCTGCATCGGATTGCAGTTTCCTTTGCTGTCTTGAATAACCAAGGAAAAC
TGGGGAAATGTCATATAAAGATATTCCGGTGATCAGTACTGATTGCCTGAATAATCTTGAATATGAAACC
ATAAGGAAACTTAAAATATCTTACACTGAATTGTCATAAATTTGCCACAGTACTGCTTCAGAAGTCTTTA
TGTGCTTAGATATTAATTTGCAAACAGATAACTTACTTATTTTCTGTATTATAAATTCTTTGGGTTTGAT
GCTTAATATTGATTTAATAAATTAACTATGCCTTCCCCTTTGAAATGAGAGATTGCTTTGAACCCACATG
TTCCTACTACTCCACCATCTTCTAATTATTGTAATTGCGAATTGCCCCGTGTTTCGCAAATCAAAGCCCT
TCCAATTATACATTTTTGCAACTATAACTTGCGTGGCCCTCGCCGAATGGAAATGCACAGACTGCAACCT
ACTTGTTTGGCAAGCCACTCGACGAAACCTCTCTTCCTCGTCCTGAAAACGCAAAGtttatttcatttat
gcgcgcgggtatatttttgtatttattttatttttttatttttGCGCTGCATATGCCGCGGGGCAGCAGAA
GCCACTTGAGGCGTTGTTAACTTAATTTTTGGAGCGTGCACACATGGCTCActctctctctctctttctc
CCTAGACGcacacacacccacatgagcacacgcacacattaacacacacacacacacacacacCCTTATA
CCACTTCATCGCCAACTGCAAAATGCCGTTTTTCCAAATGACTTCATAGACAAGAGTTCGACGGCGGCGA
CTTGAACGTTGCGCATACGCAAAGTCATTCAGTCAACCGCAGCATTTGAGttttttgattttttttctttt
ttggtttttttgattttAATTTGTGGTTGCGGCAGCGTCAAACTTTCGGCGTCGCAAAGTCTAAGGCAGTT
TTTCTCATTTCCAGCCGCAACTACAAAACAAGaaaaaaaaagaaaaaaaTCCACAGCCGCTGTTAACGAT
TTGTGTAAATTATGCGTAATTCAGCTTGGCGCCGCTGCTTTTATTTGTACACAAAAACTGTTGGCGGTTT
TTGTTTAGTTTTCTGTTGTTTTGCTCTTTTCATGTATTTTCGCACCGTGGCTGCAGTTTCCTCTTACCGT
TTAGTGACCTTGATTTTGCGGGCAGACTTGTGGGCGGCGTCTTGCTGGGCCTGCTTTTTAATGGAGTGTG
TGAAGACCCTTATTAATTGGCTAAGTAATTATGTTGACTCTCAATGAAAATTTGTTTGACACTAAAAATT
TATTCTAATATGCTCAAATTGTACTGTGAGTTGTTGCAAGCTTGCATTTGGGAATAATAATTATTTAAAT
TGCAAAGCACTTTCAGTTACCCAGATATTTCTTAGTTTTGGGTTATAATAGTAtttttttttatttattt
tatgtatatctttgtattctgtattgctatttttttataatttatatttaaccatatttTACATCTAAGAA
GTTGATATCTACTTACAATTATACACAAATATAACACTTTTGAGGGTTGTGTCTTGGCTTAGCAAAGACA
AACGTGTATAAATAGCACAGTTAGCAGGGGTGGGCCAACAGATGGCGCCAGCAGATATTTTTGAAAACTA
ATTACGATTTTAATTAACTTTTTTAATGAAGAGGAAAAAACTTGAAATAAATATATTTTAAATTGATTAC
CCTTATACTGTGCTTGCAGGCCCATAGTAGACACTATAATGGATGGCTAGAGCCCACAGAAGATGTGTCC
ACGTGGTTGAAAAAAAATCCGAAGCGTTCAGGACGATTGTATAGGCGAGGCTAATTTACAGAGTTACATTA
ATGCTGGTTATGTTAGTATACAACCCCAACCCATTATAAAAAGCCCTTCAGCACGTGCCACTCGATTTTT
TACAATTTTAACCATTATTTTCCCTTGCCTTTTTATAGCCTGTGGTACGAGACGAATCGGTACGTGAATG
CTGGCAGTGAAATGGTCGTCGACGGGCGACCCAAGAGTCCTGTTCAACTGAACGAGGACTTCATGAACGG
CGGCAAGGTCCTGGCAGCAGCCGCAGCAGCAGCCGCCGCAGTGGTGGCCGCCTCCTCCTCGGGAGCAAAA
AGCGGCGGTGGGGGCAGCTCCGCGCCCAGCGATGATCGCAGCGATCGTGATAATGG
>dme_circ_0000693_junction_seq
GCGATGATCGCAGCGATCGTGATAATGGGCTCCGGAGGCGACGACTTCGATCCAAG
>dme_circ_0000694
GAAATAACAACGTTATCAGCCCCCAGCTTCCCCGTTTCTCAAGGATCACCACATTCCTAGAGGAACATTT
CAATGGGATCTACTAAAGCATTTAGGAGAAATGCATCCCATTGAATTGCTGGCGATCGGTTTTCACCATG
TTGTT

>dme_circ_0000694_junction_seq
GCTGGCGATCGGTTTTCACCATGTTGTTGAAATAACAACGTTATCAGCCCCCAGCT
>dme_circ_0000695
CTATCTAGATCTAGAGACAAAATGGTCTACACCGAACGTACCGACAAGTGCCTGAGCAGCACCACAAAGG
ACAACCAGTCCACGAAGGGTCAGAACCACTCCAAGTCGAGCAGCGGCTCTggatcgggttcgggatcagg
atcgggatcTTGGAGCAGTCAAGCATCGAGTGCTGACAAGTGGAAGTACAACAACATGGTGAAAACCGAT
CGCCAGCAATTCAATGGGATGCATTTCTCCTAAATGCTTTAGTAGATCCCATTGAAATG
>dme_circ_0000695_junction_seq
AAATGCTTTAGTAGATCCCATTGAAATGCTATCTAGATCTAGAGACAAAATGGTCT
>dme_circ_0000696
CTATCTAGATCTAGAGACAAAATGGTCTACACCGAACGTACCGACAAGTGCCTGAGCAGCACCACAAAGG
ACAACCAGTCCACGAAGGGTCAGAACCACTCCAAGTCGAGCAGCGGCTCTggatcgggttcgggatcagg
atcgggatcTTGGAGCAGTCAAGCATCGAGTGCTGACAAGTGGAAG
>dme_circ_0000696_junction_seq
TCAAGCATCGAGTGCTGACAAGTGGAAGCTATCTAGATCTAGAGACAAAATGGTCT
>dme_circ_0000697
GCACTCGATGCTTGACTGCTCCAAgatcccgatcctgatcccgaacccgatccAGAGCCGCTGCTCGACT
TGGAGTGGTTCTGACCCTTCGTGGACTGGTTGTCCTTTGTGGTGCTGCTCAGGCACTTGTCGGTAC
>dme_circ_0000697_junction_seq
GTGGTGCTGCTCAGGCACTTGTCGGTACGCACTCGATGCTTGACTGCTCCAAgatc
>dme_circ_0000698
CTGTGCAGGGATGAGTCGAGCTAATCCTTCTTAGTCTTCTGGGCGGCACTGGATCCGCAACAGAGTCGAA
GGAGCCACACCCAACTGGCAATGATGCTGCCCAGGACGAGGGCCAACACGGCATAGACATCCAGGGAGTA
GTAAACGAATCGTGACATCTCGACGGCACTGGGCTTCAACAGAGGAGCTCCGCCAGTGTGGGCCACATGC
TCCACCCACCAAATGGCTG
>dme_circ_0000698_junction_seq
GCCACATGCTCCACCCACCAAATGGCTGCTGTGCAGGGATGAGTCGAGCTAATCCT
>dme_circ_0000699
GGTGGCCTCATCGTACAACAACCGCATCCAAGGTCCCTTGGAGACGGCCATTTGGTGGGTGGAACATGTG
GCGGAGACGAAAGGAGCTCCTCTGACCCAACCAAGTGCCGTGCATCTCTCCCGCTTTGTTTACTACTCCT
TGGATGTCTACTCGGTCGTGGCCCTAAGCCTGCTACTACCTGTGATCACGCTCTTGGGACTAATCCGAAT
GTTTAAAAGAAGGGAACCCAAAG
>dme_circ_0000699_junction_seq
CGAATGTTTAAAAGAAGGGAACCCAAAGGGTGGCCTCATCGTACAACAACCGCATC
>dme_circ_0000700
TAGACATCCAAGGAGTAGTAAACAAAGCGGGAGAGATGCACGGCACTTGGTTGGGTCAGAGGAGCTCCTT
TCGTCTCCGCCACATGTTCCACCCACCAAATGGCCGTCTCCAAGGGACCTTGGATGCGGTTGTTG
>dme_circ_0000700_junction_seq
CTCCAAGGGACCTTGGATGCGGTTGTTGTAGACATCCAAGGAGTAGTAAACAAAGC
>dme_circ_0000701
GGAGAACGAGTGTCTCTGGCCAATAGGTGCATGCCTTTTAAAAATACACACTATAACAGCTCAGTGTACC
AGCCTGCATTAAAAAACTTTCAATTGTTGTATCTAAAAAGTGCTTGCGGTGTGGTTGGTGGTGTAAATAA
ATTAAAAAACTACATTTCTATCAA
>dme_circ_0000701_junction_seq

ATAAATTAAAAAACTACATTTCTATCAAGGAGAACGAGTGTCTCTGGCCAATAGGT
>dme_circ_0000702
TTGCGCAGTCTTTCATCTAGTGAAGGACTTCCTCCGGCATTTTGGCCAGGTACATCCGAATCGCTGGAGT
TGGAACTCAGTGATCGTGGACGTGCTGGACTGGCCATCAAGCAAGTGTTAGAAGATGAGGATCCGTGCAA
TTGCTGCGACTGTTGTTGGTGATTATTTTGTTGG
>dme_circ_0000702_junction_seq
CGACTGTTGTTGGTGATTATTTTGTTGGTTGCGCAGTCTTTCATCTAGTGAAGGAC
>dme_circ_0000703
ACCGTCTTCTGCTTTGTGGTGGCTATGATGCTAGTGCCTTTGCTGATGTTATCATTCCTGTGTAAGTGCT
CGACATACATCCCCTGCAGTGATCGAAGATCGCCGATCCGCGACACGCGCTGCCCCGCTGATAAGCCGCA
TAAATCAATAACTTCAAAAGGGGCCGGCCACATAAATAAACGATCCATGGGTTGATTAACCGATTCTCAG
TCAGCCAAAGGATCATCCGCCGGTGGAAACCTAAGAACGAAAGATAGTTAGTTATAGAGGAGGCATAGGG
AGAGAGCAGAGCAGTAGGAAAGTGAGAGAGTCGTGGAGAAAGTGCTGCAGATAAGGTTTTTGTTTATAAA
TAAAACAGATTACCTGTTGCACCTTTGCGGAACCCAGACAAGCGACCAAGCGAACCTTGACCAACTGAAT
TACTCATAAAAATTTCACCATTGATAAGACCAGTCTTCGCTGCGCTTGATGATACCCGCTAATCGTCGGG
GATGATGTTGTATAGGAAAGTCTAGTGCTTGATTAGGTTTATCGGGCTGGGGGCTGAAACCATCCATTAA
ATTCAATTGATTTAATCTCATGGTATACGATTCTTGACCGCGCTGATTATCATTCTTCTTATTTACTTTT
TCCAAATAGGGTATCAAGAATGCGGGAATCAAATTGCTTCCCACTGCTTAAATATTTTGTTATTATTTAC
GTTATAGGCTTCTTTAATGTTATGACTTTGATCGCAGATTGGAATACAGCGCTAGAGGTGGCCAGTGAGC
TATTAAATTTCGGCACTTCTAATCCCAATCCATATTCCCAGGGTGCTCACTTTGGCAACGAACTATCCAA
CTATGTGCTGAGCATCAAGTACCCGAACCTGACCATCTCAAAGTCGAAGAAGATCCGCACCTTAATCGAC
ACGCCGAAGAATGCGGGCGTATTTCACTTCCTGCTGCAAG
>dme_circ_0000703_junction_seq
GCGGGCGTATTTCACTTCCTGCTGCAAGACCGTCTTCTGCTTTGTGGTGGCTATGA
>dme_circ_0000704
GACACTGGAGTTCTCCCCCAGCGGGCGGGAAACCAATTTGCCAATGGCAATGGCCACCAGATAAGCGGGA
ATGGGCACCTCCTGCTTGAATAGTGTCTTTCCCGGTTCCTTCTTATCGATAAGGGCGCTCATCAGGGCCG
TGAGCTCGCTGGGATGCTCCACTGTGGCATCATAG
>dme_circ_0000704_junction_seq
GCTGGGATGCTCCACTGTGGCATCATAGGACACTGGAGTTCTCCCCCAGCGGGCGG
>dme_circ_0000705
GTGTTTCTTGAATTTGAAAGTTGTTCATTGCTAGCTTTCAGAGTGGATGTGGAACTAGTTATATCCTCTC
TAGAACTTAGTCTGGAACTCATTTTAGTTCGTGTGGCCGTGCTTAAAATGGAACCTGGATTA
>dme_circ_0000705_junction_seq
GGCCGTGCTTAAAATGGAACCTGGATTAGTGTTTCTTGAATTTGAAAGTTGTTCAT
>dme_circ_0000706
GTACTAGTGAGTGGAAAATGGGGCAGCATCTGCCCGCAATGTTGGAAGACAATTCTCAAAAGGGGGTACA
AAATTATATCCGCAATTGTGAAACTGATGCCCTCGGCGAAGCGGTGCTCAATAAGCAGCTCTTCCTTAGG
TGTAGTGCACTTGATTTGCACTCGTTCTCTCCTCTTGACCTTGCCGCCATTCTCAGTCTGATTGGCCTGC
TCTCTGGCCTGTTTATAGATGTTATGCATCCTCACCGGTTCGTTCATGTGCTGCTCGAACCTCACTACTT
CTTGGGGGACGAATTCCAAAACTTGCCCACTATGAAGTTTACTGACAACAGCCACTAAATCCACCTCACA
GAACTTAGTCCAAATCGATGCTTCTGACGGAGCCAAGAGGCAGCTTCCCTTAAAGCCTAAAAG
>dme_circ_0000706_junction_seq
AGAGGCAGCTTCCCTTAAAGCCTAAAAGGTACTAGTGAGTGGAAAATGGGGCAGCA

>dme_circ_0000707
GGAAGCCATCTGATCTGGGTACATCTAGTACTCGCGTATATGTATTTAGCAAACTAAATAGGTCTAGGTC
TAGTCACACGAAACAGCTTTCGCATTAGCGACAAGGCTGCTATCAGTGACCGGAGTTCTCTCCCGATTAC
GGCTTTATGTTGAGGCCATCGACGTACTTGCGGATCGCCAACACGTCCGTCGGATAGACCCGGCAACAGC
CGCCAACGATGCGCACTCCCAGCTGGATCCACTCGGGCACAAACTTGACCACCTCCTCGCCGGTTCCAGT
CCAATCTCCCTGCTCGACGTC
>dme_circ_0000707_junction_seq
TTCCAGTCCAATCTCCCTGCTCGACGTCGGAAGCCATCTGATCTGGGTACATCTAG
>dme_circ_0000708
TGATTTGAACGTAGACGTAGACGTAGTCTAAAAACTTCCTAGTAACGCTATGCTCAACTCAGCAAAGTGC
CGCCAAGGCGATCTCCTCGATATCCTCCATCCAAGGAGCCGCTTCCAGACCACATCGGCAGCCGCCGTAA
GATTCGATCAGCTCCAGCAATAAATTAACCGTATACTAGCCGATCGCTGCTCGATCGAAAAATTTAGCGT
GCCATATAGATGGGGAATGACCCGCGAATACTACTTAAGTGCAATCAATCAATTTGTAAATACCCCATGC
ACCTACTAACCAACCAAAAATATCTCGACGAACCTTCTTATCTTTTGTCATATGAGTTTATTCGGTGATT
AATATG
>dme_circ_0000708_junction_seq
CATATGAGTTTATTCGGTGATTAATATGTGATTTGAACGTAGACGTAGACGTAGTC
>dme_circ_0000709
GTGGTTTCGCCATGGTCTTTTTGGCAAGGGGCAACGGCGGAGGCAGTTCCTCGGCGACCAAGTACGCCCT
GAAGCGCATGTACGTCAACAATGAGCACGATCTGAACGTAGCCAAGCGGGAGATACAGATTGCGGAGCAA
CCTGAGTGGGCACAAAAACATCATTGGTTATGTGGACTCCAGCATCACGCCCACGGGAAACGGGGTGTGC
GAGGTGCTACTCCTGATGCCCTATTGCAAGCATCACATGCTGGCCATGATGAATGCGAGGATTGCACGTT
GGCTTCACGGAGCCAGAGGTGCTCAACATCTTCTGTGATATTGCCGAGGCTGTCTCTCGATTGCACTATT
GTCAGACTCCGATTATCCATCGGGATCTTAAGGTGGAGAACATTCTGCAAACGGACGCGGGCAACTTCGT
ACTCTGCGACTTCGGATCGGCAACGGCCAAGACGTTAAATCCTCAACAGCACGGAGTCACCGTGGTGCAG
GAAGAGATCCAAAAGTACACCACTCTATCTTATCGGGCTCCCGAGATGATCGACCTGTACGGGGGCAAGA
GCATAACCACAAAGGCGGACATCTGGGCACTGGGTTGCATGCTCTATAAGCTGTGCTTCTTTTCCTTGCC
ATTTGGGGAGAGCACTTTGGCTATCCAGAATGGACAGTTTTCCATTCCAGACTCCTCAAAGTACTCGAAG
GGCATGCACCAACTGATTAAATATATGTTGGAACCCGATATGGAGAAGCGACCAAACATTTGGCAAGTTT
GCGAGGTGGCCTTTCGACTGGCGGGCAAGGATAATCCCGTTCAGAATCTCCATGAAAACTCCCATTCCAA
ACTTCGACCTACTGGTAGTGCCTCCGTTTGAATCGGAAGCCAAGAGGATCAGTGCAGCGGCCTCCAAGGC
GGCGAAGGCTCAGAATGTGTCAGTTGTGGAGGCGGGAACCAGTGTAGCTCCTCGGTCTCGTCCTAAGGGT
TCCTCATCGGTACATGGAGGAAATCCATTGGGTCTCGGTTTGCCTCCCAGTCCCTCGCCAAGACAGAATA
TCACTTCTCCACAACCCCAACCTCAACCTGTGGTCGAGCAGTTCCAGGCCAACTTCCCAGCTATGCCACC
TGCTGTTCCTCCACCTCCTCAAGCTGTTACTCCGGCAGCTCCTGCTGCCAGTAAACTCTTCGAGTCAAGT
GGCTATCCAGATCCATTCAATGAGCCAGCTGCAGCAGTAATTCCTACTGAATTTCTACCAGCAGCAGAAG
AACCTAACAAGGAGGAAGTGCCTCAGGACTTAAACGTCATTGCAGGCACGCCCACCAAGAGCATGTTGAC
ACCGCCCAAGCCAAGCGGAGGAGGTGGTCATAGGCGTAACGTAAGCGACACCTCGGCCTTCAATAAG
>dme_circ_0000709_junction_seq
CGTAAGCGACACCTCGGCCTTCAATAAGGTGGTTTCGCCATGGTCTTTTTGGCAAG
>dme_circ_0000710
GAAAATCAAGAAGTCTGACAAAAATGCATCGTCCTCTGACAAGCGAAAGAACTCGTCTACCTCCCAGTCC
TCTAAGTCAGCTACTCCCCGCATTGAAGACGACTCCAGCGAGGCCGATGATACGGCGGACAAAGCTGAAA
AGAACCAGCGCCACGAGAAAGAGAAAAAGGAGCGTCAGGAAAAGCGTGAGAAGGATCTCCGTAAACAG

>dme_circ_0000710_junction_seq
AAAGCGTGAGAAGGATCTCCGTAAACAGGAAAATCAAGAAGTCTGACAAAAATGCA
>dme_circ_0000711
GGAGCGTCAGGAAAAGCGTGAGAAGGATCTCCGTAAACAGGTTGAACGGGAGGAAAAGGATCGTAAGGCT
CAGCAGGAGgaaagagaaaaagaagaccgaaaagctaaagaagaggaaaaagaaagagaacgggagaaga
aggcccaagaggatcgtgaaaaaaaggaaagagaaGAGCGAGAGCTTAGAGAAAAGGAGCAGCGTGAtaa
ggaacaaaaagaaaaggaaatccgtgaaaaagatctgcgcgaaaaagaacaaagagagagaGATAACAGG
GAGAAGGAGCTGCGTGATAAAGACTTGAGAGAAAAGGAAATGCGTGAGAAGGAGCAAAGGGAAAAGGAGC
TTCACCGCGAAAAGGATCAGCGCGAAAGAGAGCATCGAGAGAAGGAACAATCTCGCCGAGCTATGGATGT
TGAGCAAGAAGGCCGTGGGGGAAGGATGCGTGAGCTATCTTCATACCAAAAAAGCAAAATGGATATTGCT
GGCGAGGCTAGTAGTCTCACCGCAATCGACTGTCAGCACAACAAGGAAAATGCCATGGACACCATTGCTC
AAGGAACTCCTGGTGCATCCCCATCAACTCCTTCAGACAACACCCCGAAGGAGCGCTCGAGAAAGTTGTC
TAGAAATTCCCCAGTGCGCCTGCATAAGCGACGACTTAGCTCGCAGGAAAGCAACCACAGTGCTGGAGGT
GGTGGATCATGCGGCGGATCGAGTCATCAAATTCATCATGAGGATTATGTAAAGCGCATAAGAATGGAGA
ACTCCCAGAACATCTCGGTTCACAGCAGTAATCAAAGATTAAATGATCGCCGGGACTCCAAGGAACACAA
GAGCAGCAGTTTCAAAGAAGATAAAAATA
>dme_circ_0000711_junction_seq
AGCAGCAGTTTCAAAGAAGATAAAAATAGGAGCGTCAGGAAAAGCGTGAGAAGGAT
>dme_circ_0000712
CTCTGGCCAAACATATTCTCGCTCGCTGTCCACCGGACAGTGAAGCTCCGCGCTCGCCCACGATTGTCCC
ATCACCGTGCAACAACTCCAAATCCCGCTCCAGGGCACACCGCTTGAGCACAGTTCGATAGCGCTGCTTG
TCCATGGGCAAGCCAAATAGAATGTTGTCGCGAACAGATGCATTGAAAAGCCAGGGCTCCTGGGAGGCGT
AGGAATACTTTCCCGAGACTTGTACCGATCCAGATTCAGGCGGCAGCTCTCCTAGAATAGCCTGAATGAG
GCTTGATTTGCCCGATCCCACGGGTCCAATCACAGCTACCAATTGGCCGCGCCGCAGCGACATATTGACG
TTGTTTAAGACAAGATCGTGTTGCTCCTGGCCCCAGCGTGCTCTCAAGGCTTTGATTTCCACTAAGGTAT
CTGG
>dme_circ_0000712_junction_seq
AGGCTTTGATTTCCACTAAGGTATCTGGCTCTGGCCAAACATATTCTCGCTCGCTG
>dme_circ_0000713
GGGAAAAGGAGCTTCACCGCGAAAAGGATCAGCGCGAAAGAGAGCATCGAGAGAAGGAACAATCTCGCCG
AGCTATGGATGTTGAGCAAGAAGGCCGTGGGGGAAGGATGCGTGAGCTATCTTCATACCAAAAAAGCAAA
ATGGATATTGCTGGCGAGGCTAGTAGTCTCACCGCAATCGACTGTCAGCACAACAAGGAAAATGCCATGG
ACACCATTGCTCAAGGAACTCCTGGTGCATCCCCATCAACTCCTTCAGACAACACCCCGAAGGAGCGCTC
GAGAAAGTTGTCTAGAAATTCCCCAGTGCGCCTGCATAAGCGACGACTTAGCTCGCAGGAAAGCAACCAC
AGTGCTGGAGGTGGTGGATCATGCGGCGGATCGAGTCATCAAATTCATCATGAGGATTAT
>dme_circ_0000713_junction_seq
GAGTCATCAAATTCATCATGAGGATTATGGGAAAAGGAGCTTCACCGCGAAAAGGA
>dme_circ_0000714
CGATGGAGATACTTAAGGAGTCCACTCTCAACCGAGATCTGCACAGTGCGGCCTGCAATTTCACCAAAAA
ACCCGGACACATCTATCATCTTTATCAAAGGCCATCGGGTCAAAACTATTTCAGCATGCTATCACCGGAA
G
>dme_circ_0000714_junction_seq
AACTATTTCAGCATGCTATCACCGGAAGCGATGGAGATACTTAAGGAGTCCACTCT
>dme_circ_0000715

GTAAAACTTGTGAATCTCAGGAGCCTCCTCTGGCTGAGGCTCCGCGGCCGACTGCAGATTGGACTTCTTA
ATGACGCACAAGTGCATCGCATTGTCGCCATCCTCATCCTTGGCATTCACGTCGCAGGAGAGGCGAACGA
GTCGCTCGATTACCCCGGCATGGCCCTGGGAGACGGCCAGTAGGAACGGCGTCTGTTGGCGATTATTGCG
>dme_circ_0000715_junction_seq
GGAACGGCGTCTGTTGGCGATTATTGCGGTAAAACTTGTGAATCTCAGGAGCCTCC
>dme_circ_0000716
GATCCTGCCGATGGTGCGCTTTGAGCCGTGTCAGCATCAGATCGCCTGCGAGGAGTGCGGCATCCGAATG
AAAAAGTGTCTCCGCTGTGCCGTGGCGATCGAGCGGCGATTGACGGTCAGCGGCCGAGTGGTGGCCCTGC
CCACGTCCACGTCGTCACCTAGTGACCCCACTCGCCTACC
>dme_circ_0000716_junction_seq
CGTCACCTAGTGACCCCACTCGCCTACCGATCCTGCCGATGGTGCGCTTTGAGCCG
>dme_circ_0000717
AAACCATCCTCATCATCCTCGGCCATCTCCTTGGCCAGCTCAAGGTCCTGCTGCTCCCGCTCGCGTCTCT
CCTCGGCGCTCTCCAAATCCTTGCCGTAGGACTTTGGGGGATAGCGCATGGCCTTAACGCTCTGGTTGTG
CAGGTTCAGGCAGAACGAGATGCGCTCGTGAAAGGCCAGCTGCGGTTCCCGGGTGCTGTAGATGTCCG
>dme_circ_0000717_junction_seq
TGCGGTTCCCGGGTGCTGTAGATGTCCGAAACCATCCTCATCATCCTCGGCCATCT
>dme_circ_0000718
ATGAACTATTTGCATGACTACTGAAGTGCTCCCTCTCCTTTTCTCTTTTGGGTTGATGAGACTGCAGCTC
CTCCTCACTTGTGTTTAAGTTGTGCTTGGTTTGGGAAATGGGATCTACCACCACTTCAATGGAGGAGTTC
GTTTCTATGGAGCTGGCACTGCCCTTTTGATGATG
>dme_circ_0000718_junction_seq
TGGAGCTGGCACTGCCCTTTTGATGATGATGAACTATTTGCATGACTACTGAAGTG
>dme_circ_0000719
GAATGCTGTCCACTTAGGCAAGAGCCAATTGCCACCTATGCTTCCGTCGTTAAACTAATAAAAATTCAAT
ACTTGCCATAAAATTATAAAGTATTCAAGTAAATCTACAAGAAACTGCCAAATTTTGAAGCCAAATGCAG
TCAGCCCTAAAGTATGCAGTGGATTTTGTAAGCTTCGAGTGCAACTCAAGGCTCATTTTCTGAGCTCAAC
TAAATGCTCAAAGCTACACTCAGCGGAGTCATCCACCGACCGTCGCTCGCGTATAAGTGATACGCGCTGG
TTCGAAACCCCGCTGGAGAGCGGTGAGCGACGCAATTTCTAAAAAGTAAAAGAGAAAACAAAGTAGCAAA
CGTTATCTTTTATTTAATTTTCTTTTATTATTGCGTTTGCAATCCACAGCAACCATGGACGCTCCAGATG
TGGGACGCATTATAAGGGCGCCGGCGAGGCGCAAACGGAACGATGAACAGTTAATGGATAGG
>dme_circ_0000719_junction_seq
ACGGAACGATGAACAGTTAATGGATAGGGAATGCTGTCCACTTAGGCAAGAGCCAA
>dme_circ_0000720
GATGAAGAGCATACACCACAGCAGCATAATCCTCACAGGAGAATCTCTGCTGCGGGAAGTGGGTCTGCTG
GAGAGCTCAGCTCAGCAGCCACTAATACTTCGAGCGGAAAACTCCATCATCAACACCACAGGAGAAGTGT
GGAACGGAAGTCATCACGGGGATCTGATGAAGGACATCATTCTTCATCTAAATCTCTTCGCGCCAAATTG
ATGATGTTGAGCTCCGCAGACTCTGACGACACAGATGATGCCTCCAAGAAGCATTCCATCTTTGATATCC
CGGATGATTGTCCAAAT
>dme_circ_0000720_junction_seq
CTTTGATATCCCGGATGATTGTCCAAATGATGAAGAGCATACACCACAGCAGCATA
>dme_circ_0000721
ATACATTTGGACAATCATCCGGGATATCAAAGATGGAATGCTTCTTGGAGGCATCATCTGTGTCGTCAGA
GTCTGCGGAGCTCAACATCATCAATTTGGCGCGAAGAGATTTAGATGAAGAATGATGTCCTTCATCAGAT

CCCCGTGATGACTTCCGTTCCACACTTCTCCTGTGGTGTTGATGATGGAGTTTTCCGCTCGAAGTATTAG
TGGCTGCTGAGCTGAGCTCTCCAGCAGACCCACTTCCCGCAGCAGAGATTCTCCTGTGAGGATTATGCTG
CTGTGGTG
>dme_circ_0000721_junction_seq
CTCCTGTGAGGATTATGCTGCTGTGGTGATACATTTGGACAATCATCCGGGATATC
>dme_circ_0000722
GGTCTCCAGATATAGCTGCGAGCTCTGGGTGAGGTAGGCCTCCTCGCCGAAGTACTTAAGCTTGAAGAGC
GTTGAGCCACCTTCCACCTGGGTCTGTACCAGAGTCGGGGGCGTCACCTCGTTATAGCCACGCGCCTCGT
AGTGGCCACGGAATGCTTGCGTCACAACGGATCGCATCTTGAGCACTTTCGAGGTGTTCTCTCCACGAAT
CATGATATGCCGGTTGTCGAGCTGCACGTCCGGTTGTGCTTCCTCGTTGAGAATGGCATCGGCACCGCCG
GGCGGAGCCAGACCAATCAGCTCCCAGTAGTCCACGTTCAGTTCATGCCCTCCCGGCGCCGTCTTTCCTT
CTGGAACTAGTTTCAGGGTACCGAACAGGACTACAGAGCTTTCCGTGCTCAGGGTGAGCGCATCGTAG
>dme_circ_0000722_junction_seq
TTCCGTGCTCAGGGTGAGCGCATCGTAGGGTCTCCAGATATAGCTGCGAGCTCTGG
>dme_circ_0000723
TTCTTAGTAAACTCTTAGTCACAAAGCAAGCAAGATGCCCGCCGATCAGTTGGCTGAGTTAACCTTGGGT
AAGCACGTTTTTAAGTGATAGAACTGGGCCAACTATTTGGTCCACGTTGCCAGCGAACCAGATGTATCGA
TTCATAATGTGCAATGTTGATATGTACGACCATATGCAGGTGCAATCTTTACGTCGGAAAAGAAGGGTAG
TGATGAGTCCGGCGATGGCAGTGAGTCCAAGCCCTTCAAGACCATCCTGCAGGCGATGCGTCATGCGGGC
AAGGAACCCTTTCCCACGATTTACGTAGACACCAAGGATCCGAATGCAGCGGAGCCCTACGAGCCGGCTG
CCAAGTCCCAGCTGAAGAAAATTCAGAAACTGTTCGTCAGGGAGTCGCACAAGAATGCCGATAAGCTGCA
ACGGGAGGCGGAGGATGCGGAGAAGCGCCAGCAGAATCTGGAGGATGCTCGAAAGGTGAAGATCTCGGAG
GATCCCAGTCTGCCGGTGGCCCGGAAAATTCGAATCTACGAGGGCACTGCCAACCGCGACTCGCGTGTCA
AGGTCTACGGTTGGGTGCATCGTCTGCGTCGTCAGGGCAAGTCCCTGATCTTCATAACACTGCGCGACGG
CACCGGTTTCCTGCAGTGCGTGCTAAACGATCAGCTCTGCCAGACCTACGATGCGCTCACCCTGAGCACG
GAAAGCTCTGTAGTCCTGTTCGGTACCCTGAAACTAG
>dme_circ_0000723_junction_seq
GTAGTCCTGTTCGGTACCCTGAAACTAGTTCTTAGTAAACTCTTAGTCACAAAGCA
>dme_circ_0000724
TTTGTTGTAGAATTTTCTATATATTCCAGTTCGTTTCCTGGTTTCCTGATTTCTGCTTGTTGTTGTTGTT
TCTCGCTGCTGGTTGCGCCGTGTTGCTGTCTCGCTCTTGCTTGCTACGCAGTCTATATGATAAATTGCGT
GGGATGGTTGCCGTTGTTTTGG
>dme_circ_0000724_junction_seq
TTGCGTGGGATGGTTGCCGTTGTTTTGGTTTGTTGTAGAATTTTCTATATATTCCA
>dme_circ_0000725
ATCCATTGGCGGATGTGTTGCTCGAGGAGCTGTTGCTGCTGCTGTTGCTTCCCTTGCCCGCACCCACGAC
CAGACTGCTGATGCCCGAGTTGCCGCCATTGTTTGAGGACTGAAGATTGCTGCCGATAACGCCTGG
>dme_circ_0000725_junction_seq
ACTGAAGATTGCTGCCGATAACGCCTGGATCCATTGGCGGATGTGTTGCTCGAGGA
>dme_circ_0000726
GTGTTGCGCACGCCGGCCTGAATCGACTGATAGTTGGACATAAATTCGATCTCGAATGGTAGCTGCATCT
CCTGAATGCCCGTCTGTAGTTGTTGCTCCAGGGATTTGCGCAGGAGAAGGGCCTCTGTCAGCAAGGATGA
TGGCGGAGCAGGACTCCGCTCGACCGCCTCGCAGGTGGCCAGTCCCTTGTCGATCAAGTCGCGGGAGCGT
TGCTGCCACGAATTGTTGCTGTTAACCTTGGCCGTGTACAGCGTCTCCAGTTCCTTGAGCAATTCGG

>dme_circ_0000726_junction_seq
AGCGTCTCCAGTTCCTTGAGCAATTCGGGTGTTGCGCACGCCGGCCTGAATCGACT
>dme_circ_0000727
GGACTTCTGCTGGGCGGTCGCGATCGGAACGCTCTGGCAGTGGAACAGCATTTCGGTGAGCTGATGCCCA
AGCGAGGAGGAGGTGGCTACACGGGCAGCAATGGATCGGCCACCAGTGCCGTGGCCCACTACAATCCCTA
CGAGAAGTGGAGCAACGGCG
>dme_circ_0000727_junction_seq
AATCCCTACGAGAAGTGGAGCAACGGCGGGACTTCTGCTGGGCGGTCGCGATCGGA
>dme_circ_0000728
GATGAAATAAGAAGGAATGTGATGAAGCATAGCCATTTTGGAAAAAGGAACTCCAACTCCACTAGGATTG
CATCTGATTCTGAGTCTCAATCCCAGCCGGCTCCTGATTTGACTATTAAGCAAGAGCATCCTATAGCTCC
TGCTCAGGAAATCAAGCGGGAACAGCTTTCCGACGAGGAACAGAAGTTCAAGTCTCGGCATGATTCAAAT
TCTTCAATAGAGGAGAGGAAACTGAAGACGGAAC
>dme_circ_0000728_junction_seq
ATAGAGGAGAGGAAACTGAAGACGGAACGATGAAATAAGAAGGAATGTGATGAAGC
>dme_circ_0000729
GCTTGGAGATTTCTATAATTCTTCTGAATACACCTATACTGGAAAGCTTAAAGAGTACTCTCCAGAAACT
CGCAAAAAACATAAAAAGAGCAAGCGACGTTTGAAATCCTCTTCCACAGCGGATACTTCAGCAGCTCAAA
CTCCTTTGGTGATGACCCCGTTAACTCCCTCCATATTTGATGTTCATTCATCCTCCGAGTGCAAGACGAA
GTTCGATAATTTTGATGATCTAAAAACTGAGTGTTCTTCAATACC
>dme_circ_0000729_junction_seq
ATCTAAAAACTGAGTGTTCTTCAATACCGCTTGGAGATTTCTATAATTCTTCTGAA
>dme_circ_0000730
GTATAATGTCTTTATTGGTTTCCGCTTGCTCGGGAAACTGTGATTCTTCTTCATCGGAAATAATGCCAAA
TATAAACTCCATTTTATCCTCTCCTCTTTTTGAAGTTTGGGCCGTCGAAGGCGCTGACGTTGGAGTGGCA
GGCAAAGAAGGTGAGGTAGAAGGATGGGCTCCACTTGAGTTGTATATTTTGGTATTGCAGGAATTATCCA
TGCTCTTCGATTTCTTTGACTTTTTTAGTCGATGCCGCTCTTCTTTCGATAACTTTTCGGAACTTG
>dme_circ_0000730_junction_seq
TCTTCTTTCGATAACTTTTCGGAACTTGGTATAATGTCTTTATTGGTTTCCGCTTG
>dme_circ_0000731
TCATATCCACTGAGTTGTTGTCTTCGTTATCAGAGAATCGGAATACATCTGTCATATCCGATCTATATGT
GGCGGCCGTTGAAGGAGTGGCTTCTTCTGAAATCGGCTTGGTATCAAAGTCACTCATTAGTTGAGCCTCT
AGAGCCCTCCCTGCTTCATCCATATCCACGCTGTTATCATCATCCACCTTAGTTTCAACCTTGGAAGATT
TCTGGTGAATTTGATTTTGCTGTTCTCGTTGAGATTTCTCGCGTCTCTTTTTCTCGCGCTTCTCTCTCCG
GTGTCTTTCCCTATCAGCATGAGAAGATGAGCTGCTGCCTCCCGATAGGCGACTTCTCTCCAGCTGCTGT
TGTTGCTCAGGCTCATGCACAGTTAATTGAATATGTGCTTCCTCATTTTTGCTGTTAGGTGTGGAAGGTT
CTTGTTTGTACGTTTGCAAAGCAGCGGAGACAATTGGACCAGTAGTAGACACGGAACTAGGTATAATG
>dme_circ_0000731_junction_seq
AGTAGTAGACACGGAACTAGGTATAATGTCATATCCACTGAGTTGTTGTCTTCGTT
>dme_circ_0000732
ATATGCTTGGCGTCCAATTGCAGGTCTGCATGTTTGCTAGCAGCTTGAACGTTGACAGTAAGCTTTCCAG
GAGTAGGAGGTGCGGATGAAGTGGAAGCCACATTTGGCAGGGATTCCCTTCGCTGTTGTTGCAACAACtt
ttcttgcttttcctccttagaccgcttcttctttttcttcttgtctttacttttatGCTGTTCCTGCTGC
TCCGATTTGACACCCTGTTTAGTCATATCCACTGAGTTGTTGTCTTCGTTATCAGAGAATCGGAATACAT

CTGTCATATCCGATCTATATGTGGCGGCCGTTGAAGGAGTGGCTTCTTCTGAAATCGGCTTGGTATCAAA
GTCACTCATTAGTTGAGCCTCTAGAGCCCTCCCTGCTTCATCCATATCCACGCTGTTATCATCATCCACC
TTAGTTTCAACCTTGGAAGATTTCTGGTGAATTTGATTTTGCTGTTCTCGTTGAGATTTCTCGCGTCTCT
TTTTCTCGCGCTTCTCTCTCCGGTGTCTTTCCCTATCAGCATGAGAAGATGAGCTGCTGCCTCCCGATAG
GCGACTTCTCTCCAGCTGCTGTTGTTGCTCAGGCTCATGCACAGTTAATTGAATATGTGCTTCCTCATTT
TTGCTGTTAGGTGTGGAAGGTTCTTGTTTG
>dme_circ_0000732_junction_seq
GCTGTTAGGTGTGGAAGGTTCTTGTTTGATATGCTTGGCGTCCAATTGCAGGTCTG
>dme_circ_0000733
ATTTCTGGTGAATTTGATTTTGCTGTTCTCGTTGAGATTTCTCGCGTCTCTTTTTCTCGCGCTTCTCTCT
CCGGTGTCTTTCCCTATCAGCATGAGAAGATGAGCTGCTGCCTCCCGATAGGCGACTTCTCTCCAGCTGC
TGTTGTTGCTCAGGCTCATGCACAGTTAATTGAATATGTGCTTCCTCATTTTTGCTGTTAGG
>dme_circ_0000733_junction_seq
TATGTGCTTCCTCATTTTTGCTGTTAGGATTTCTGGTGAATTTGATTTTGCTGTTC
>dme_circ_0000734
GACAATACCATCCGGTGGCGAAATAGGAGCCAAGGGTCGCGGCAATGGAGCATGCCCAGATCTGAGACGC
TGTTGCGACATCCTGGCCTGCATGGTCACTATCATATCGTGTGGCACTTGCCATATGAAAATGCAACCGT
CGCCACTTGCGGAAATTAAATGTCTGCAATCGTTGGTGAACTTAAGTCCCGTGACCAACTCGCTGTGACC
G
>dme_circ_0000734_junction_seq
AAGTCCCGTGACCAACTCGCTGTGACCGGACAATACCATCCGGTGGCGAAATAGGA
>dme_circ_0000735
GTTCCACGCTATCCACTCAGTCTGCAGAAACACCTGTAAGCAGTGGTACAGTTATTTCCTCTTCTGCTTT
GGCTACCACTCCTACATCTTCGACAGCAGCGGGAGTCAGTGCAGCCCCGGGATTAGATAATTCGCCTACC
AGTGCTTCTGCTCAATGCAAAAAGAAGGAAAGCTTCATTCCCGGTTTCGATGGTCAGCTTGACGATAGGA
TTA
>dme_circ_0000735_junction_seq
TTCGATGGTCAGCTTGACGATAGGATTAGTTCCACGCTATCCACTCAGTCTGCAGA
>dme_circ_0000736
CTGACCATCGAAACCGGGAATGAAGCTTTCCTTCTTTTTGCATTGAGCAGAAGCACTGGTAGGCGAATTA
TCTAATCCCGGGGCTGCACTGACTCCCGCTGCTGTCGAAGATGTAGGAGTGGTAGCCAAAGCAGAAGAGG
AAATAACTGTACCACTGCTTACAGGTGTTTCTGCAGACTGAGTGGATAGCG
>dme_circ_0000736_junction_seq
GGTGTTTCTGCAGACTGAGTGGATAGCGCTGACCATCGAAACCGGGAATGAAGCTT
>dme_circ_0000737
CTCCGCCTCCAACTTCGGCAGCACCGCCGCTCACCAGGTGGGCAGCAATGTGGAGTTTGCTGATAACGCC
AATGCCGGCAACAAGGTTGACTTTGGTGGCGTAAACGCCGTGGACTCCAGTGCTCAGCTGCTCAGCGGAG
TTCAAACCGTGCAGTCCGTCCCCACTTCCGAG
>dme_circ_0000737_junction_seq
AACCGTGCAGTCCGTCCCCACTTCCGAGCTCCGCCTCCAACTTCGGCAGCACCGCC
>dme_circ_0000738
TACAATTTAAATCGCGAGTGTCGAACGTATTTTTCGGTCAAGAGCTAAAACGAATTGCCAAAATGAATAA
GCTGCTGATTTTGGCCCTTTGCCTGGGTGTGGTCTGTGGAGGTAGTGCACTCGTTTCCCGAATATCAACT
AGTTCAGAAAATTAAACAAGTGGTCCTGGCAAGCCTGGCAAACAAACTATTTTCCCTTGATTAGTGTTGT

AATAAAATATAATAATTAAAAAGTGAACTCTTTGGAAATAAAAATATGTTCTAAAGTGAATGAGAAATAT
CTTTAAACGAAAAACTTAAAGTGATTAAATTTCTATCAATTTCGTATTTAGGTTTTGATTAAATGGGATT
TTGAAATCTTCTTGCGTACGTTAACTAATACATTTTCCTCTTTTTATCATTACAGAGTAATTTTAATTTG
AAGTGCCTCATTTAATATACATTCTAATGGTTTGTTTTTTAAATTTTTTAAATCTAAAAGTTTGGGGAGA
ACCAAACGTGACAGTTTCCACTCTCGGTAAGTGCCTTGTTGCAAATGTTAACGATCTTTCTTTTCTGAAT
CGAGAAAATTGAAATATTCCCATTGGCTGACATGGTACTTCAAAATTTTGAAATTTAAATTTTAGAAAAA
TATGTCTAGAATCAACAAAGGAATACAAATATATAGCTTAGTTTAGCACCGCAAAACTAAGATCAATGCT
GAATTAAATTTTTGTTGTTTATTTTGAGTTGCTTTTACAATTTAAAATTGTTGATTCTAAGATCTTTTAA
AAGACCCAATGACAATCTCTCTCTATTAATTGTCAACTGCCTTAATCCTCAGCCACTACAGTACCCACGG
ACAAGCGGTGGAGCAGGCTCGCAAGAACGCCGGCTACGGTGGAGG
>dme_circ_0000738_junction_seq
CTCGCAAGAACGCCGGCTACGGTGGAGGTACAATTTAAATCGCGAGTGTCGAACGT
>dme_circ_0000739
CAAAAACGGCTCCGACCCGCAGACGACGAGCATATGCCGGCGGTTACGCCGGAGGGTATGCCAGCAGTGG
AGGAGGAGCCGGTGGCTATACGGGAAGCTATAACAGTGGCGGTGGACACAGCAGCTATGTGGGTACCGGT
GGTGGTGGCGGTAGTGGCTATTACGATTACGATGACTATGGCGGCACTTCACCCAACTTTGGCATCATCG
ATCCGTATCTATTCCACCAGCAGCTGACGAATCATATTTTGGCCCAGAACTTCGCCAACCAACAGGTGAG
CATCGATCATATCCAGGGGATAGATTGTTTGGGATCTTTGGGTAGTGATACGGCGGGCTATGGGTAAACC
TAACGCTTGAATATATCTGTTCTTGTAGAGCTATAACTGGTCTGGCCACGGCTGGCAATGCTTATGCATC
CGCGGATGCATCTCTTGTCAACGACAACGATATGTAAATACAAGTAGCTAGTCCTTAACTAATCCCAATC
ACTAATACGGTATCACGGTATGTTTTTCCTTATTCTAATGTCATTTTGTGCGCAGTTCGCATCCAGCAAC
AGATCGCCGCTCAACAGGCCTACCACGACAATCTAGTGCGCCAGAATCGGTACAGAGGAGGCACCAGCTC
TAGCTTCAGTTCCAGCtctggatccgggtctggatctggatcgggatcCTACAACTCACATCGCTATGCA
CCCAGCTATGCATCAGCTTCCGGATCTATCGGTTCCAATGGCTATCGCCAGACGGCCTTCATTAGCCCAT
CCAATCCGG
>dme_circ_0000739_junction_seq
ACGGCCTTCATTAGCCCATCCAATCCGGCAAAAACGGCTCCGACCCGCAGACGACG
>dme_circ_0000740
GGAGTAGTCCGTCGTAGAGGAAGTGCCAAAGCTTTCCCCCAACAGTGCGGATACGGCACTCTCAGTCTCC
TCCTGAGAGATGGTCACGCGGGACTTGCTTTCCTCTAACTTATCCAACGGTTTAGTCGCACGTGGAGGGG
ATGCTACCGGAATTTTTGGCTCATCTGCAATG
>dme_circ_0000740_junction_seq
TACCGGAATTTTTGGCTCATCTGCAATGGGAGTAGTCCGTCGTAGAGGAAGTGCCA
>dme_circ_0000741
ATGCTCTATTAGCCGACCTGCAAAACAGTGTGCCAGGCCAGCCGCAGCAACCTCAGCCGCAATATGGAAC
TGTCCAGCCCAAACATCAAGCTCTGCAGCAACAGCAGTTTGTGGACAACACTCCAGGTTATGGAAGTCTC
AGGGGCAAAGCCCAGCCCCAGGTGGTATCAAGAGCACTACAGCGTAGAGACACGCTCCCCAACGGCTGGA
CATGATTTTAATGGCTCCTCAACGACTCCGGGATACGCTAACCAGGGATCGTTGCCACGTCAGGCGGCGG
GTGCAAGCACAGGACTCTCAGAACTGGACTCCTTGCTGCAGGATCTGCAGAGGTAAGGAAAAATCCAATC
TTATTGGGGATATAGTGATCGGATTACTGCATTTTAATTTTATATAACTATTCAGTTGAGCTAAGTTTAA
AAAGTTAATTAGTCTGTCTGTATATATGTACATAGCTACCGGTTTCTTTGTAAAATTGGTTTGTACATTA
GCTTGAGTAATTTATTGCACTCTTTAATTAGCCACAAAATCCTATACTACCTTTTAAACAGAATTATGAA
CACAGAAACGAACATACTTTGTTTTGCTTTAGTAACGACTTGTTAATCGAATTCGCTTTGTTCAAAATGT
TTCTGTATGGCATTAATTGGGTTTTTATTGACTCCTTAGAAAATCGATGTACCGGTCAACTACAGTACTC

CAGTCTCGAAATACAATACGATGAATAGTTATGCCACCGTGGAGGAGCGTCCTTCAGTGGACAGCCTGCT
GAAAGAACTGGACAATGCTCACATCTATGCTGTTCCTAACGGGTTGGCACTGTCGAGGAGCAAATTGTGC
AGCAGAAGGATTCTTATACGCCCAATCATGCTGTTCCGGGACAACAGGTGCATCAGGCCTATACGTCGCA
GGCCACCAAGGAACTGGATGATCTGATGGCTTCACTTTCCGACTTCAAGGGTAAGTCCTTTTCATCATTG
AGAACTGTTAGCTTAGATATTGATAAAATTCTGTATAAACTGTAATTCCTCCACAATTTCTCATATGAAA
GATATGTGATATATATCCCACAATATCTTCTGTATGACATGATCTACGAGGATATATCTCGTATTCAATG
AATGAACAAATATTGCACTAAAATTTGAACGAATATATGCTATATATTTATTTTACAAAACACAGAAATA
TGCTATACTTGCTGTATTGAGTTATTACAATGAAATAATTCAACTTGGTTTATGATATGACTATACATTG
TTAATCTGTAAAACATATCGCTTCTACAGCTTAAATTAAAAAAATTAAAATGGATTGTGCTTTTTGTTTT
CCAAAGCATTAGCAATATTATATTTTAAAAGTAAGAAAGAAAGTATTCCTCAACTATTATATAAATATCT
GCAAGCAGCAAACCGATTGCTTTAGCAAGCAGATAACGCCATTTGAAACATCTTTGGAACGTAGATAAAT
AAGCTAAAGAAACACCAACAGTAACAGTGATAGAGTGTGAtagagtctgtatctcagttttccagctctt
atagttccgaagatcttaaagttcatacggacggacagctagatcgtcttagctagtgatcataatcaag
aatatactttatactctatagggatcttccttctgataaataatttttaataaatatagtatatctcttG
GGCACTGGGTTAAGATGCTCGATTTTCAGTCTATTCAAACCTTTAACTTCTTTGTTTTTAAGACTATTTG
GAAGGTTTTGTAATATCGATATGTAAATATTTTATTATTGGTTTATAATTGTGTATTGGCAGAAAGATTT
TTATATTTGACTAGGCATCACCGTCAATTGTTTTCAAAGCGGTATTCTTTCAATTAAAAATGAAAACAGT
TTCCAAAAAAATTATAATTCCAATCTATAGTGTTTATTAACATTCGCATACCAAGCCTCTTTGAATGTAG
ATTTTTATGAAAAGTATTTTGTAAAGAGCGCAAATGTTTTCACTGTCTTCACCTGTCGTAGGGATACTAT
AAAAGCGCCTAAACAAATTGCATATGGTCGAAGTCTTTCGACCCCCGTGCCACCACCCACCCGATCCTTC
TGATTTTCTACTATGTATTTTGCTATTTTTAGCAAGAGACGCCCCAAATGCTCGGTTTGGTTGTGAATTG
TAATAAAGACCATAAAATGAATCTATTTGTCATCGCAAATCGTCTGACGCAAGGCAACCGAGCGAATCTA
ATGACACCGCCGATCCATAGATCCCCATGCTATCCATTGAGGACGATCCCTTCGAGAAGTCGAAGAGCAG
CGGAGAAAGATCTTGACTTTGACATCTGCCAACGCAATGCGAAGGTGGATATTTCGGGGCATACTTTAAT
GGGCCACATAAATTGATTGGGCTAATGAAGTCTGATTATGATGGCGAGGGACTGTCACACGATTTATTGC
ACACGGCGCCAAGACATTTTAAGTTTAAGTTTTTCCCCAGATTAGCATTTACACATGGCCACGTTTATTT
TATATTCTATATCGGTGCCTCATTAGGGCATCAATCAATTTTTGGGTGGCAATCGAGTAAACAAAAAAGG
TATTTTTTCATGCTTTCATGCACTGCACATCTTCTCAGGATATACAAGCAAGAAAATATTGTTTCCGTTC
AATTGGATTGGATTGGATTAGCCTGCTTTTCTTTTATGGCGATGTGGCCATTCGCCTTCCTTCATATGTC
CGACATTTGTTTTTAGAACTTTCTGACATCTCCGAACGAAACGCCTATACGGATGATGTATTTCAGCAGA
TGTCGTTCGCATTTATTTGGCCtttttacgccactttttcgttttgttttttATGATCTAGTTAAATATT
TTGCGGTGCGTCTGCTCCTTTCTTTCATACGAATGAATTTGGGTCAAGACTTAGTTCGTTAATGAAATGC
ATCGATCTTTGGAAAGTAACTGAAAAATGTCAGTAAAGGCAAATGAATGCTACTTATTCCTATATTTCTC
TGTTGATATACCACTTGAATAGTTACTATTATACAATAGCTAGATAGCATCTAAATTCTTGTGGCTTAAC
TCAAAGCAATTTATTGCTTTATTCCCATACTTAATGTGACAGTTAGTAGCGATTCTTTTCAAATGTTTTC
TAACATTTTCTAAATGTTTAGAAAATATCCCCATCTGAGATTTGACCCTTGAAATGCCTTGAAATTGCAA
GATGTCGGCAGGGAATGCCGCGAATTGTCAGTTTGTCGCCGAGGCATTCGGATTGCATTAATTATTAATT
AATTACTAAGTGAAAAATATTTTTGCACACTTCTTTCATTTTGTTGGCAATCGATTCGATGGCCATTGCA
GTCAAATTAGCAGACATTCGTGCGGGGCGGGGTTGAATGGATTCTTTAGACTTCGAGTAAATTGCAATTC
AATGTCATTGACGAATGGCAGGCGTGCATGCGAAAAGTTGAAAAGTAAAGTATACTCGGTCGGGCCTGTT
AATTATTTAATATCATTTTCAATTTTCTGCCAACTGCGAGGGTGGTCGGTGAAGGGGGCAGAGGGCGGGG
GGACAAGGCGTGTCCGTGACTTTTGTTGATATGATAAATATGAAAATAAAATAGAAAAGCCTCTTGGTCC
CTCGTTCTTTTTCTGTACGCGTGGAGAAGAGCGGGGGAAAAAATAAAATACAAATATATTCGGATATAAA
CAATATTGCACACGGCCCGTCGGATTTTCCCGGCGCTTTTCCTACCCACACAAACACACCCAAAACGCAC

AGTTAGTCGcccacccacccccatccacccccccaccccctcacccacTTGTCAACGCGTTTTCCACAGC
GCACACCGCTTTTGCTATAGTTTTTCATTGCAAATCATTTGCATGGCTATTTTCGAAAGCGCTTTTTTAT
TATATTTTCTTTCATTTCTATAACCCAAGCGATCCATTTAGCTGTGGGTGTTTCAATGTGTCTCTGGTAT
ATTCTGTATATTTCATTGCTTGCGCTTTCATTGCCACAATCATCTGGCGTTCAAAACCGCATTCGTATCC
CCATTTTCCGCCCATTTTCATTTCACCTCCTGCCCGTCCCATTCATTGATCGAGATTTCCTCAGTGCCAG
CTTCCTGTCCCCCATTTCATAATCCCTCAATTCCCCACCTTTTCCCAGATTTTCTCCCGTCCACTGGCCA
CGACTGGAAACGTGTGCCGAACGAACATTCGGCTGCGCTTTTTATGGGGAAATCCTGTTGACCAGCCGGT
CGATGTTCATTTTATACTCCATGCCAAACGACGATTTCGGTGGTTCGACTGCGCTGTGTACGATTTGGGT
CTTAAAATAGGCAATGAAAATATTAGAATAAGAAAACCTCGGCGACGCTGAAGCCAATGAAAATTTGTGC
GGCAGGCGAAACAGGAGCGGAGTTTCGTTCGAAAGGGGCGGGCGTGAATATAAATCTTGATAACATTGAG
GCTTCCTTGATTCAACCAAAATGGAATCTCTGACTATAACATTTCGGGAAGGGTCGATTTAATTTGGAAT
AATAACAATATTCTAATAAATGGTGCGATTCGTCACTACGCAGACTGCTGTCCACAGCGATAGAGTCATT
CCATGTGGATACAACATTTTAATAAGTAGTACGatggaaatggaattgtaaaaaaaaatggaatgtaaaat
gtaaaaaaaatggaatgtaaaaatATTACCCACCACATCAATTGCAAGTCTTTAATGCAATGCCTTAAATA
TTGATCGGCACTATTTAATAAATGCTATTTTTATAAAGGTTTAAGGCTTAAGAAGTTTTCACGAAATGAA
TCTTGAATATTTTATGTGTTTAGAGAAATTAACTGTCTTTCAATTTCGTTTGAGAATTCCTATCCATGTA
TACAAAATAAGAAAGGGTTAGTGATTATTCACGTAGTGACGAAACGCACAAAGAAAACGAGCCAGAGCGT
CTACTAATTATAAAGAAATTATAATCTGCTGTGCTTATCCAAACATATTGAGAAATATAAAACTAGGTAG
CAGTAGATAAACGGTTGCATTAAGTAAAGCTTGCTAATCATTAAAGTAGATACGTACCTTTTCAATGCGA
ATCCTCCCCTTTCCCAATTCAGAGGACGCTCAATTAGGCGTCTACTTAGTTAGGCCGAAGCAGGCAGAAC
CCGAGCACGCCCAATGAAAATGCAGAGTTTATAAAAGAAAAATTAAATATTGAAATGGAGACTCGACAGA
CAAGATCCGCACAGAGGCAGTAGAGAAACCCCCTTGGCCGCCCACTCCTTTCGTTGTTCGTTCATCGGAA
AACGTTCTTTCGTATTTTTGGCAATGAACGAAAACTACGTCAACGTAGAAGAAAAATGCTTTGCATATGA
GGAGAGCGTCGTGCGCAACGAGGTCcacatacacacacacacacatacaTATAGCTATATAGTTCAGTCA
GGAGGAGAATCTGTGCGAAGAGGAAGAGAAACATTAtttttgatattttgattcttttttttGTTCGTT
TTCGTCGGCAGTGGCGGCTAAAAGCAAAAGTTTTGTGCTAGCGCTCACACAACTTTTGATTCAGTCGAG
GCAAGCACATCGACGTAAGCCCACCGCAACAGAATCCGAAGGATATAGCATGCTAATTGGAATCGTTATC
CCTCATTCCAGGGTTAGCAACGGAACAAACGGCATCGGAAACGGCAGTCATCCGCAACAGCACTCGTCGA
CAGTGCAGCACCAGACGGTAACCGACTACGCCAGACCAAACAAGGGTAGCCAGCAGGCACATCTCACCCA
GACGATCGAGGAGACGACCATCGTCGAGGACAGTCGCGAGGATCAGTTGGATTCTATGCTAGGCAATCTA
CAGGCCAATATGAGTCGCCAAGGAGTCAATACCGTGCAGAAGGGCTGCTGCAATGCCTGCGAGAAGCCCA
TTGTGGGCCAGGTGATCACAGCCCTGGGCAAGACATGGCATCCGGAGCACTTCACGTGCAACCACTGCAG
CCAGGAGTTGGGCACCCGCAATTTCTTCGAGCGCGACGGCTTCCCGTACTGCGAGCCGGACTACCACAAT
CTGTTCAGCCCGCGCTGCGCCTACTGCAACGGCGCCATTCTGGACAAGTGCGTAACGGCGCTGGACAAGA
CCTGGCACACGGAGCACTTCTTCTGCGCCCAGTGCGGCCAGCAGTTCGGCGAGGAGGGATTCCATGAGCG
TGACGGCAAGCCGTACTGTCGCAACGACTACTTCGAGATGTTTGCCCCGAAGTGCAATGGCTGCAACCGC
GCCATTATGGAGAACTACATCTCGGCGCTGAACTCGCAGTGGCATCCGGATTGCTTCGTCTGCAGGG
>dme_circ_0000741_junction_seq
TGGCATCCGGATTGCTTCGTCTGCAGGGATGCTCTATTAGCCGACCTGCAAAACAG
>dme_circ_0000742
ATGCTCTATTAGCCGACCTGCAAAACAGTGTGCCAGGCCAGCCGCAGCAACCTCAGCCGCAATATGGAAC
TGTCCAGCCCAAACATCAAGCTCTGCAGCAACAGCAGTTTGTGGACAACACTCCAGGTTATGGAAGTCTC
AGGGGCAAAGCCCAGCCCCAGGTGGTATCAAGAGCACTACAGCGTAGAGACACGCTCCCCAACGGCTGGA
CATGATTTTAATGGCTCCTCAACGACTCCGGGATACGCTAACCAGGGATCGTTGCCACGTCAGGCGGCGG

GTGCAAGCACAGGACTCTCAGAACTGGACTCCTTGCTGCAGGATCTGCAGAGGTAAGGAAAAATCCAATC
TTATTGGGGATATAGTGATCGGATTACTGCATTTTAATTTTATATAACTATTCAGTTGAGCTAAGTTTAA
AAAGTTAATTAGTCTGTCTGTATATATGTACATAGCTACCGGTTTCTTTGTAAAATTGGTTTGTACATTA
GCTTGAGTAATTTATTGCACTCTTTAATTAGCCACAAAATCCTATACTACCTTTTAAACAGAATTATGAA
CACAGAAACGAACATACTTTGTTTTGCTTTAGTAACGACTTGTTAATCGAATTCGCTTTGTTCAAAATGT
TTCTGTATGGCATTAATTGGGTTTTTATTGACTCCTTAGAAAATCGATGTACCGGTCAACTACAGTACTC
CAGTCTCGAAATACAATACGATGAATAGTTATGCCACCGTGGAGGAGCGTCCTTCAGTGGACAGCCTGCT
GAAAGAACTGGACAATGCTCACATCTATGCTGTTCCTAACGGGTTGGCACTGTCGAGGAGCAAATTGTGC
AGCAGAAGGATTCTTATACGCCCAATCATGCTGTTCCGGGACAACAGGTGCATCAGGCCTATACGTCGCA
GGCCACCAAGGAACTGGATGATCTGATGGCTTCACTTTCCGACTTCAAGG
>dme_circ_0000742_junction_seq
CTGATGGCTTCACTTTCCGACTTCAAGGATGCTCTATTAGCCGACCTGCAAAACAG
>dme_circ_0000743
GAGGCAGCTTTAGCTGCAAAAGCGATTGAAACAGCTGGAGAGCCTGCGTCCATTCTGGAGGAACCAGAAA
TGGAACCCGAAAGAGAGGCTGAACCAGACCCCGATCCTGAGGCAGAAATTGAATCAGAACCTGTCGTTGA
GGTCTTGGATCCGGAAGAACTCAACAAGGCAGTGCAAA
>dme_circ_0000743_junction_seq
CCGGAAGAACTCAACAAGGCAGTGCAAAGAGGCAGCTTTAGCTGCAAAAGCGATTG
>dme_circ_0000744
GCTTTAGCTGCAAAAGCGATTGAAACAGCTGGAGAGCCTGCGTCCATTCTGGAGGAACCAGAAATGGAAC
CCGAAAGAGAGGCTGAACCAGACCCCGATCCTGAGGCAGAAATTGAATCAGAACCTGTCGTTGAGGTCTT
GGATCCGGAAGAACTCAACAAGGCAGTGCAAA
>dme_circ_0000744_junction_seq
CCGGAAGAACTCAACAAGGCAGTGCAAAGCTTTAGCTGCAAAAGCGATTGAAACAG
>dme_circ_0000745
ATTCCACAGCTCACGATTGTTTTGCACAACCGCATGTGACACTCCCTCATCCGCTTTCTTTAGCCTCCAA
TCTCGGATGAGATCTCGTCCATCCTTCGACTGGCCGGCCCAAGTGTCCAGGGGATCGGCTGCTGAACCGG
TTACATCTGAGACCAGCATCTGACGACCACCGCCCATGATTACG
>dme_circ_0000745_junction_seq
CATCTGACGACCACCGCCCATGATTACGATTCCACAGCTCACGATTGTTTTGCACA
>dme_circ_0000746
AACCTCCCAAATGGCCGACTCCTTGAGGCGACTTTTGCTGTTGCTGGGGACCATGCATTTGCTGCAGCTT
TTGTATTTGCTGCAGTTGCTGTTGCTGAGCCTGATGCTGTTGGTTCAACtgctgctgagattgatgttgc
tgttgggataggtgttgttgttgtgcctgattttgttgctggacctgatgttgctgttgggattggtgtt
gctgAATTTGTTGATTAAACTGCTGCTGAGCTTGATGTTGTTTCTGAGTCGGATGTTGTTGGGCATGAAG
TTGTTGATTTAAATGTTGCTGGTTGTGATGTTGTTGCTGCTGCTGAGCTTGATGTTGCTGATGCATTTGG
TGTTGCGGGGCTGACGTTATTTGTTGCGACTGACCGTGAAGTTGTTGCTGCTGCATGTGTTGCTGTCGTT
GTATCATTTGCTGATGCATGAACTGCTGCATGTGCTGCTGGTGCTGATGTGCAGTCATCTTGGCTATCAC
CTGTGGCTGCTGCTGATTCTGATGGGCATTTAAATGCACGTTCTTGTGCGGCGGAAGGCTCCCCAAGGGT
GGCGAGCTGGCCACTTGCGATTGGGTTGCCTGTTGAGGCTGTACGGTGACCACTTTACTTATGATCGTCG
GATGATGTGGCACCTGTATCGGTGTCATTTTTTGTAGCGGAATAGTCATTAACTGCGGTGTCGTCTGCAC
CTCAGCCACAGATTTCGGCGAGGGTTGTTGTGGTACCTGCTGGTTTCTGGGTTGGTAACTGTTTACTTTG
CTGGTAGTAGGCGAATTGCTGCTAGACAACACTCGAGTCGTGGGACTTGCCAATGGACTAATGTTGCTAC
TTTGCGGGCTGGTTATTAGGGCATGTTGCTGCTGTGTCTGCTGAACCTGTTGCTGGGGTGATGGTTGGCT

TACCATTGGACGCCCAACAGGACTCAGTTGACCCACCAGCCTACTTTGGGCTACACCACGACCTGGTGAA
TGAAGCGGACTGTGTGGGGATGGGGCTCGAATGCCAACCATATAAGTTCCAGGTTGCTGAGGATGATGTG
ACTGAGGAGACAACACCATTTGGGGGACCGCAAAGTGAGGTGTTTGTTCTGGTATAGATATCGTTGGCGG
TTGCATTATCAAAGATTGCGTCCGGATTGCTTGCTGAGGTGTAATCGGACCCTGCTTGGGAGTTGAAGCC
TGGGGCATGGCATTCTGACTTGCAGTTGGACTTCCAAATGATATGGATGCAGACGCTGAAGCTGTGGCAG
ATGCCGCGCTCGTGTTCGAGCAACTGCTTATCACGTTGGACAGATCCAAGTTGATCACGGAATTTGCACT
TATGCCGCTTCCCGTTGAATGCGGTGCAGGCAGGACGGTCTGCACAGGCTGCACCAAAGGCTGTTGGAGT
TTACTTATAGTTGGTGGTTCAATCTTAACAGGAGAAGGGGGTAGGCCTGCTGGTACTGTTTTTGGCGTAT
CCAAAAATGGCAACTTAGTGATCACGGAAGGTTGCGGTGTGGACTCGTTGCTGAGCTTCGGTTGAGACTC
TACGTTAGGTATGTCTATATTTGCTGTTTCCCTAGGAGTAGGACTGTTATTGCTAATTGATTTCTCCGGC
GACGATGAGCTCTGCACAGTTTCATCAATCTTGAGACTAGGCCCACTACTATCCGCTTCGTCTGGATTCT
CTTCTGTGTCAGTATCAATCTGCAGATCCCTTTCTGACTGCGGAGTATCAGCCTTGATGTCCATCATATC
CTCGTGTTTTAAACTTTGCACTGCCTTGTTGAG
>dme_circ_0000746_junction_seq
GTTTTAAACTTTGCACTGCCTTGTTGAGAACCTCCCAAATGGCCGACTCCTTGAGG
>dme_circ_0000747
GTTTAAAACACGAGGATATGATGGACATCAAGGCTGATACTCCGCAGTCAGAAAGGGATCTGCAGATTGA
TACTGACACAGAAGAGAATCCAGACGAAGCGGATAGTAGTGGGCCTAGTCTCAAGATTGATGAAACTGTG
CAGAGCTCATCGTCGCCGGAGAAATCAATTAGCAATAACAGTCCTACTCCTAGGGAAACAGCAAATATAG
ACATACCTAACGTAGAGTCTCAACCGAAGCTCAGCAACGAGTCCACACCGCAACCTTCCGTGATCACTAA
GTTGCCATTTTTGGATACGCCAAAAACAGTACCAGCAGGCCTACCCCCTTCTCCTGTTAAGATTGAACCA
CCAACTATAAGTAAACTCCAACAGCCTTTGGTGCAGCCTGTGCAGACCGTCCTGCCTGCACCGCATTCAA
CGGGAAGCGGCATAAGTGCAAATTCCGTGATCAACTTGGATCTGTCCAACGTGATAAGCAGTTGCTCGAA
CACGAGCGCGGCATCTGCCACAGCTTCAGCGTCTGCATCCATATCATTTGGAAGTCCAACTGCAAGTCAG
AATGCCATGCCCCAGGCTTCAACTCCCAAGCAGGGTCCGATTACACCTCAGCAAGCAATCCGGACGCAAT
CTTTGATAATGCAACCGCCAACGATATCTATACCAGAACAAACACCTCACTTTGCGGTCCCCCAAATGGT
GTTGTCTCCTCAGTCACATCATCCTCAGCAACCTGGAACTTATATGGTTGGCATTCGAGCCCCATCCCCA
CACAGTCCGCTTCATTCACCAGGTCGTGGTGTAGCCCAAAGTAGGCTGGTGGGTCAACTGAGTCCTGTTG
GGCGTCCAATGGTAAGCCAACCATCACCCCAGCAACAGGTTCAGCAGACACAGCAGCAACATGCCCTAAT
AACCAGCCCGCAAAGTAGCAACATTAGTCCATTGGCAAGTCCCACGACTCGAGTGTTGTCTAGCAGCAAT
TCGCCTACTACCAGCAAA
>dme_circ_0000747_junction_seq
TAGCAGCAATTCGCCTACTACCAGCAAAGTTTAAAACACGAGGATATGATGGACAT
>dme_circ_0000748
TAGGACTGTTATTGCTAATTGATTTCTCCGGCGACGATGAGCTCTGCACAGTTTCATCAATCTTGAGACT
AGGCCCACTACTATCCGCTTCGTCTGGATTCTCTTCTGTGTCAGTATCAATCTGCAGATCCCTTTCTGAC
TGCGGAGTATCAGCCTTGATG
>dme_circ_0000748_junction_seq
TTCTGACTGCGGAGTATCAGCCTTGATGTAGGACTGTTATTGCTAATTGATTTCTC
>dme_circ_0000749
GAGTAGGACTGTTATTGCTAATTGATTTCTCCGGCGACGATGAGCTCTGCACAGTTTCATCAATCTTGAG
ACTAGGCCCACTACTATCCGCTTCGTCTGGATTCTCTTCTGTGTCAGTATCAATCTGCAGATCCCTTTCT
GACTGCGGAG
>dme_circ_0000749_junction_seq

ATCTGCAGATCCCTTTCTGACTGCGGAGGAGTAGGACTGTTATTGCTAATTGATTT
>dme_circ_0000750
TGATCACGGAAGGTTGCGGTGTGGACTCGTTGCTGAGCTTCGGTTGAGACTCTACGTTAGGTATGTCTAT
ATTTGCTGTTTCCCTAGGAGTAGGACTGTTATTGCTAATTGATTTCTCCGGCGACGATGAGCTCTGCACA
GTTTCATCAATCTTGAGACTAGGCCCACTACTATCCGCTTCGTCTGGATTCTCTTCTGTGTCAGTATCAA
TCTGCAGATCCCTTTCTGACTGCGGAG
>dme_circ_0000750_junction_seq
ATCTGCAGATCCCTTTCTGACTGCGGAGTGATCACGGAAGGTTGCGGTGTGGACTC
>dme_circ_0000751
CGTTCTGTACTTCGACTTAGCTAATTGCACTTGGCTGCCCAACGAAATTCCGCCGGACTTTAAAGGCTGA
CGTAGCTCCGTGCTCGAAGGTGATCGTCTGACTGGAGGACGGAATGACCGAATGGCTAAATGGCGGAATG
GCGGTCCGACACGCAGAATGGGCTCAGGCAAGCGCAAGAAATGGCATGGAAATACATTTGGAAACGGCGA
GCAGTGAAATACTTAATTAATTGCTCGTGCTTGAAACTAAAAGCAAATGAGCTCGAATTACCCACAGCCC
CCGTCAGTCAGTTGATCACTTGATCGTGGAACGCGGACACGCCAGTCGAAAGAAGGTACATTAGCTAGTG
TCCGGCAGAAAAGCAGAAAGTTACTGTTGTGTCGAACGGGAGGAGTGATAGCCAAAGCAATAACTTACAC
ACTTACGATCCGTAAGAGGGATTACAAACTCGTTTTTCACTCAGTCACAGTGGCGAAGGCGATTAGTCTT
TAAACTACTCGTGTACGGAGCGTTCAAAAGTTCAAGTCTAATTGCATCCAAGTCGCGTAACCATCAGAAG
ATTAACCACGATACACTGTGCGAAACGCTCAGAACACGCACTTAatataaatatatatacatatataAAG
CTGTGCCGCGATTGCGAAAGTGCCCCGATGCTATATAGCTAACTTAAAAATTTCGTATTTTGCGACGAGA
AATGAAAATATTCCTTCGTTTACATTCTAGAACAAATATAGGAAGTGGACTTTTCGCAAGTGGCAGATGA
GAATTTCTCGAAAGTGCTAAACAGTACAAGTTACAAAGTGTATATAGTTACAAAATATAGCAAAGTGGAT
TTCCTGATAGAAAGTACGGATTTTTTCAATAATAATAATAATAACTATATTGTTTTAAGTCAAGAACATT
TCTAAAAGTGCACACAAAGTGAAGAAAGTTCTTGAATTTTGTAGTACTCACTTACTTTCAGTGTACCTTC
TGCAACATTATATTTAATAATGTTAATTGCTGTGGAAAATCGATTGAGTGCGCTGCACCGTGAGAAATAC
CGCACTTGTCTTGGCACTTTAACGTACTAAAAACAATGGAAATAACATAAAATTAATGCTGAGCTCAGTG
AACCATTTTCAGGTGAACTTATGCAAAACAAGCCaaaaaaaaaaaaGAAGATCCCATGCGGCGATTCGCG
ACGTGATCTTTAGAGCTCTTAACTCGAATCGGTCGCTTATAATGACCTATTAAGTCGCGCTGGCCTTACG
AGTGCCCGACTATTCCGACCGACCTATGTATATATCTTGATAACTATAACTTTGTCGTACAATACATATG
TGCAATGCACGCAGTGCATAAAAGGGGCGGCTATAAAACGATTTGACAAGCAGCTTTGTATTTTCAGTCT
TCAAACGCGACCCAAGCTCACTAGGTACATATATCGGAGTCGAACGGGAGTTCCGAAGGATTTCCACTGT
ACCAGCCAACTATTTGTGGATTAGCCGATTTTCAATTCAAACAAGG
>dme_circ_0000751_junction_seq
GATTAGCCGATTTTCAATTCAAACAAGGCGTTCTGTACTTCGACTTAGCTAATTGC
>dme_circ_0000752
GCATTCGTAGTGACATAGGCAGTCTCCTCCGCGACGTTGCTGTACtcctcgtctccctcgtcttcctcgt
cgtcctcgtcgccctcgctatcctcatctGCTTCTTCGGTAGTATCGTTGGCGTGCTCGTGTACTTCCTC
TTCCTCTTCGTAATCTGCGTCCTGCTTGTCAGCCTGG
>dme_circ_0000752_junction_seq
GTAATCTGCGTCCTGCTTGTCAGCCTGGGCATTCGTAGTGACATAGGCAGTCTCCT
>dme_circ_0000753
GGCGGCTCTAAGACCTCACCCAAGGCCAAGAAGGCGGTTGTCAAGAAGGAGCCGGGAGAAAAAAAGCCGC
GCCAAAAGAAGGAGAACGGCGGCGGGCTCAAGCAGAGCAAGATTGACTTCAGCAAGGCCAAGGGCGAAAA
AGAGCGACGATGACGTGGAGGAAGTAACTCCTCGTGCAGAGCGTCCTGGACGTCGACAGGCCAGTAAGAA
GATAGACTACAGCTCGCTCTTCTCGGATGAGGAGGAAGACGGTGGCAACG

>dme_circ_0000753_junction_seq
TCGGATGAGGAGGAAGACGGTGGCAACGGGCGGCTCTAAGACCTCACCCAAGGCCA
>dme_circ_0000754
AAAAACGAGCTACTCAAACAGCGCGACACTAAACTGTCCGAGTTGGAAAGTCTGCGCAAGAAGACGCCGG
AGATGCTTTGGCTGGATGATTTGGATGCCCTCGAATCGAAGTTAAATGAAGTAGAGGAGAAGGAGCGCGC
AGAAGAGCAGGGGATCAATCTCAAGACCGCGAAGGCTTTGAAGGGCCAGAAGTCGGCCTCAGCTAAGGGC
AGAAAGGTTAAGTCAATGGGAGGCGGAGCGGGTGCCGGAGACGTATTCCCAGATCCCGATGGAGAACCCG
TCGAGTTTAAGATCACCGAAGAAATCATTAAGAAAATGGCAGCGGCTGCCAAGGTAGCTCAGGCGGCTAA
GGAGCCGAAGAAACCCAAAGAGCCCAAGGAGCCAAAAGTGAAGAAGGAACCGAAGGGCAAGCAGATTAAA
GCCGAACCTGATGCCAGCGGCGATGAGGTGGACGAATTTGATGCAATGGTCGAGGGCGGCTCTAAGACCT
CACCCAAGGCCAAGAAGGCGGTTGTCAAGAAGGAGCCGGGAGAAAAAAAGCCGCGCCAAAAGAAGGAGAA
CGGCGGCGGGCTCAAGCAGAGCAAGATTGACTTCAGCAAGGCCAAGGTAAGTGTTTCAGTAATTGCCCAT
ATTTGTTTTTAAACCCAAATCTGTATTTAATTGTCAATTAATATTTTTCATCTTTAGGCGAAAAAGAGCG
ACGATGACGTGGAGGAAGTAACTCCTCGTGCAGAGCGTCCTGGACGTCGACAGGCCAGTAAGAAGATAGA
CTACAGCTCGCTCTTCTCGGATGAGGAGGAAGACGG
>dme_circ_0000754_junction_seq
CGCTCTTCTCGGATGAGGAGGAAGACGGAAAAACGAGCTACTCAAACAGCGCGACA
>dme_circ_0000755
GATGCCGAGCAAGCTGACGAAGAGGACGAAGAAGAAGAGGAGGCAGCTCCCAGTGTCAGCTCAAAGGCTA
AGAAGGAAAAGGAAGTCGATCCGGAAAAGGCCTTTAAGAAACTAACTGATGTCAAAAAGTTTGACTACCT
TCTGGGTATGTCCATGTGGATG
>dme_circ_0000755_junction_seq
CTACCTTCTGGGTATGTCCATGTGGATGGATGCCGAGCAAGCTGACGAAGAGGACG
>dme_circ_0000756
GTGGCTTGCTGTCCTTCAGATCGAGGCTTCCAACAGGTCTCGTTTGTCAACTCGATAGCTACCTACAAGG
GCGGTCGGCATGTGGACCATGTGGTAGACAATCTCATTAAGCAGCTGCTCGAGGTTCTGAAGAAAAAGAA
CAAAGG
>dme_circ_0000756_junction_seq
TCGAGGTTCTGAAGAAAAAGAACAAAGGGTGGCTTGCTGTCCTTCAGATCGAGGCT
>dme_circ_0000757
GTTGCTGAGGATGATGTGACTGAGGAGACAACACCATTTGGGGGACCGCAAAGTGAGGTGTTTGTTCTGG
TATAGATATCGTTGGCGGTTGCATTATCAAAGATTGCGTCCGGATTGCTTGCTGAGGTGTAATCGGACCC
TGCTTGGGAGTTGAAGCCTGGGGCATGGCATTCTGACTTGCAGTTGGACTTCCAAATGATATGGATGCAG
ACGCTGAAGCTGTGGCAGATGCCGCGCTCGTGTTCGAGCAACTGCTTATCACGTTGGACAGATCCAAGTT
GATCACGGAATTTGCACTTATGCCGCTTCCCGTTGAATGCGGTGCAGGCAGGACGGTCTGCACAGGCTGC
ACCAAAGGCTGTTGGAGTTTACTTATAGTTGGTGG
>dme_circ_0000757_junction_seq
GCTGTTGGAGTTTACTTATAGTTGGTGGGTTGCTGAGGATGATGTGACTGAGGAGA
>dme_circ_0000758
GCCTTTGGTGCAGCCTGTGCAGACCGTCCTGCCTGCACCGCATTCAACGGGAAGCGGCATAAGTGCAAAT
TCCGTGATCAACTTGGATCTGTCCAACGTGATAAGCAGTTGCTCGAACACGAGCGCGGCATCTGCCACAG
CTTCAGCGTCTGCATCCATATCATTTGGAAGTCCAACTGCAAGTCAGAATGCCATGCCCCAGGCTTCAAC
TCCCAAGCAGG
>dme_circ_0000758_junction_seq

ATGCCCCAGGCTTCAACTCCCAAGCAGGGCCTTTGGTGCAGCCTGTGCAGACCGTC
>dme_circ_0000759
CTCCTCCGTACCTTTGATCAGCTCCTCCACCTCACTGCGGATCTCATGGATGGAGTCCAGATCCTCAGCT
GCAGCCGCGCATAGGTAAATGCATATCGTTAGCAAAGTCAGTCGCACTCTCATGGTGTTTCTCTGATGGC
CGCTCCCCGCTGAG
>dme_circ_0000759_junction_seq
GTTTCTCTGATGGCCGCTCCCCGCTGAGCTCCTCCGTACCTTTGATCAGCTCCTCC
>dme_circ_0000760
TTTCGTTTCCGACTCCTTGGAGCTGGCAAATGGCACAGATCCCCATATCCGTATCCGAAACTATATATTA
AGCGCACTTTGGTCAGGGCTCAGGTTCTCCAGTCGGAGTTCTCCGATCCTGCTAGCCACCCACATTCTCT
CTCTATCTCCGATTTGGTTATTGATCTAGCGATACATACAAACAGATTG
>dme_circ_0000760_junction_seq
TGATCTAGCGATACATACAAACAGATTGTTTCGTTTCCGACTCCTTGGAGCTGGCA
>dme_circ_0000761
CAAGGAGGTGCACTTTAACACCTGGCACGGCCGAGTGGGTGAGTAAATAATTAGGTTAATTCAGGAAAAC
CGATTAAAGCTGACATTTTGCAGGGGGAGCTGCTTCCTTTTTCTGCGCGGCCAGCATTGTAGGCGGTTTT
GTCAACTATTTCCAGCCCAAGTTCGCCCTGAAAGTAATGCCTCCATCAGAGCTTCGCTTCCGCCATAATC
TATTTGGATTGGTCACCTTTAGTCTGGGAATGGGCGCCATTTACCTGGGCTACTATTCGAAATTCTTTAC
CAAG
>dme_circ_0000761_junction_seq
GGGCTACTATTCGAAATTCTTTACCAAGCAAGGAGGTGCACTTTAACACCTGGCAC
>dme_circ_0000762
GGTAGTAGGCTAAAGTAGCGGCCAGGCAAGTTTCAATCCGCAAACTCAATTTAACTTTGCAACTACAGAG
CAGCTAACTACTTGTTCTAGCCTTTAGCTTTCCAACTTCATTGCTACGGTTACGCATATTTATCCTTCTA
ATTAAAAACGCTCGCTTCCGCTGACCTTGAATAGCAATCAAT
>dme_circ_0000762_junction_seq
CTTCCGCTGACCTTGAATAGCAATCAATGGTAGTAGGCTAAAGTAGCGGCCAGGCA
>dme_circ_0000763
GAGGAGCCAAAGGCCACCAAGCAAGAGGGTTCTTCGGCCAACGCTGAAGCTCCTTCTGTCGACGATCCGA
GCAATTTCACCATCCACGACGCCGTTGAATGCGATGGCTGCGGCTTGGCTCCCTTGATTGGGTTCCGCTA
CAA
>dme_circ_0000763_junction_seq
TGGCTCCCTTGATTGGGTTCCGCTACAAGAGGAGCCAAAGGCCACCAAGCAAGAGG
>dme_circ_0000764
TTATTAAGGATTTCGATGCCAGCACTCATGTACTCGGGTGGCACAATCTGCGAAATGTTATCCAGATTAA
TCACTGGAGTAGTTGGCTGAGTGGAACGTGGAGTCGTCACTGGATCTTCGGTTCTAGGGACACTTTGATC
AGTGTTAACCTTCTTCGGCTCAGTTGGTTTGGCCTCGGATTCAGCTGCCTTTTCAGCGGTAACAGTGGGC
TCAGCTTGTGGAGGACTTTCAGTTTGTTCTGCAGCCTTCGGTTTCTGCGGTTCGGCTGGTGCCGTGGCCG
TTGTTGTGTTCAGCGGCAAGTTGGTCATCATCTCGACAAACTGAGTCAAGACTCCGGCATGGCGGCGGGC
CTGACGCCGTTCACGGCGACTATCCCTTGCGGGTTCGCCAGCAGGATCTGCCTGGTTCGTCTCCTGAAAC
GGGCAATGTCCCCTGGAGCGCCGGCCACTGCGGCGTCCCAATCCTGGACCTGTGAACCAGGCATCGACCA
TACCGGGTCCATTGTTGGTCGGCATGCGTAGCATCAAGTGCTCAGGATGCTTGTGAGCCAACTCGCATTT
CTGGCAAAGATCATAGTTGCTGCACTGAACGCACTTGTAGCGGAACCCAATCAAGGGAGCCAAGCCGCAG
CCATCGCATTCAACGGCGTCGTGGATGG

>dme_circ_0000764_junction_seq
CCATCGCATTCAACGGCGTCGTGGATGGTTATTAAGGATTTCGATGCCAGCACTCA
>dme_circ_0000765
TTGGCTGAGTGGAACGTGGAGTCGTCACTGGATCTTCGGTTCTAGGGACACTTTGATCAGTGTTAACCTT
CTTCGGCTCAGTTGGTTTGGCCTCGGATTCAGCTGCCTTTTCAGCGGTAACAGTGGGCTCAGCTTGTGGA
GGACTTTCAGTTTGTTCTGCAGCCTTCGGTTTCTGCGGTTCGGCTGGTGCCGTGGCCGTTGTTGTGTTCA
GCGGCAAGTTGGTCATCATCTCGACAAACTGAGTCAAGACTCCGGCATGGCGGCGGGCCTGACGCCGTTC
ACGGCGACTATCCCTTGCGGGTTCGCCAGCAGGATCTGCCTGGTTCGTCTCCTGAAACGGGCAATGTCCC
CTGGAGCGCCGGCCACTGCGGCGTCCCAATCCTGGACCTG
>dme_circ_0000765_junction_seq
CCACTGCGGCGTCCCAATCCTGGACCTGTTGGCTGAGTGGAACGTGGAGTCGTCAC
>dme_circ_0000766
TAGTTGGCTGAGTGGAACGTGGAGTCGTCACTGGATCTTCGGTTCTAGGGACACTTTGATCAGTGTTAAC
CTTCTTCGGCTCAGTTGGTTTGGCCTCGGATTCAGCTGCCTTTTCAGCGGTAACAGTGGGCTCAGCTTGT
GGAGGACTTTCAGTTTGTTCTGCAGCCTTCGGTTTCTGCGGTTCGGCTGGTGCCGTGGCCGTTGTTGTG
>dme_circ_0000766_junction_seq
TTCGGCTGGTGCCGTGGCCGTTGTTGTGTAGTTGGCTGAGTGGAACGTGGAGTCGT
>dme_circ_0000767
TTTGTGTTTTTCATGGGCGAAGCATTGCTGGTCCGGAATAGCGCATATTTGGAGTCCTCGTTGGGTCCAT
TGAACCTCAACCGCCTGCACGCCGTCCTGGGGCTGTTGGCATTCCTGGTCGGAGCCGGCGGAATCGGAAT
CAAGACATGGCAGAAGCTGGAGCGCAAACGGGAAGACCCCAATGCCACCGTTCGTCACTTTAAGAGCAAC
CATGCTTTTTACGGGTATAATCGGTTGTGCTCTTCTTCTGGGTAGCGTTCTGAGCGGTCTTCCGCTGTAT
TTCATCAACAGTGGCTTCGCCTTGAAAATGCTTCATCGATTTTTCGGATTGTCCGGATTCCTCGTTCTGA
TGGTATCTCAAATGTTCGGATATAACACGGGATTCGGTCGGCGACAGTGGAAGGCACACCACCAGAAGCT
CTTCAAATTTTTCACATTTATTGCCACCATTACGACTGCAAATTACGAACTCCGGAGATTTGTGCGAGAT
GTTGCCGGCTTGGCGACAGACTATTTTATTGGATCAGATGCAGCAGAAGCTAGAGAAGATCTTTGAGGCT
CTTTCGACAAGCGAGTAGTGTTCACACTGGAATATCAATACTACAGGAAACGAATCGAATTCCGAAACAC
AGTATTGTGGATTTGTGTATCACTCAAGAAATAATTCGAGTGCTTTGCGGATTTGAACTCTGAAAAACCA
GAATCGATTTATACCATACGTATAAACACATTATCTAAGTGGAGGAGGCTTTTGTTGTCTGTCAATCGTA
CTCTACTAAACAAAACCATATCAGG
>dme_circ_0000767_junction_seq
GTACTCTACTAAACAAAACCATATCAGGTTTGTGTTTTTCATGGGCGAAGCATTGC
>dme_circ_0000768
GCAGATCACCTTCAAACTGCCCCCATTGGAGGCGGGTGAATATAATCTGACGGCGGAGGGCGTCAAGGGA
CTGGAGTTCAAGAACTCCACCAAGCTGAACTGGGAGAATTTCAAGCCATACATTAAAATCCAGACCGATA
AGGGAAAGTACAAGCCCG
>dme_circ_0000768_junction_seq
CAGACCGATAAGGGAAAGTACAAGCCCGGCAGATCACCTTCAAACTGCCCCCATTG
>dme_circ_0000769
GAATTTCAAGCCATACATTAAAATCCAGACCGATAAGGGAAAGTACAAGCCCGGTGATACTATCAACTAT
CGTGTGATCTTCCTGGACGAGAACCTGCGGCCGGATACCGCTAAGGATGAAGTGGTGGTTTG
>dme_circ_0000769_junction_seq
ATACCGCTAAGGATGAAGTGGTGGTTTGGAATTTCAAGCCATACATTAAAATCCAG
>dme_circ_0000770

ATCCATGGTCAACTTGGCCTTGCCTCCGACCATGTCGGCGGTGCCAACAACAGAATGTCCGGGGTAGTCC
GTTTGCACTGTCTTGCCGTCCACATTCTCCCAACTGCTCGTCGAGTCCAGATGGACATTGACAAGAACTT
TACCATTCACGGGCTTACCATAGGTGTAGCTGGGGGATAATGCTGATATAAGAATGTCCTAAAGCATAAA
ACTTCCGGTAATACTTACTTGGCCTTCAGGACGACATTGAACTCGCCATCTCGCACTGAAACCTGCTGCG
TGGCATCCATTTTGACGGAGTACTTGGGCAGCACGTACTTCTCCACCTCAAAG
>dme_circ_0000770_junction_seq
GGGCAGCACGTACTTCTCCACCTCAAAGATCCATGGTCAACTTGGCCTTGCCTCCG
>dme_circ_0000771
GTTCTTGTCAATGTCCATCTGGACTCGACGAGCAGTTGGGAGAATGTGGACGGCAAGACAGTGCAAACGG
ACTACCCCGGACATTCTGTTGTTGGCACCGCCGACATGGTCGGAGGCAAGGCCAAGTTGACCATGGATCT
CAAGGACTTCGCCAGTTATCTGCCGCACAAGACCTCTTCTTCATATGCCCAGATTACGGCTACCGTGGAG
GAAGACTTCACCGGCGTGAAGCTCAATGAAACAGGCGGTGTGCAGCT
>dme_circ_0000771_junction_seq
AGCTCAATGAAACAGGCGGTGTGCAGCTGTTCTTGTCAATGTCCATCTGGACTCGA
>dme_circ_0000772
TAGAAGTACACCCGACCATAGGGAGTAGTGAGGAAAGTGGGCTTAATGGTTACGTTGACGGTCGTTTGGG
GTTCCGCGAGGTCCACACGTTTCGTTTCCAATATGTTTCCACGTCCCACAATGTTG
>dme_circ_0000772_junction_seq
CAATATGTTTCCACGTCCCACAATGTTGTAGAAGTACACCCGACCATAGGGAGTAG
>dme_circ_0000773
AGATCACCCACCAATCCGAATTCCACACGCTCGGAGTCGGGGACCACATCCTCCGGCACTTCCAGCTCGA
AGGTGTTCTTAAACTCACCCGTATCCTTAAGATTGATAAAGAATGCCCTGTTCTGGTACTGGGTAATTCC
TTCGGGCACTACCTTTAGCGGCTTGTGTATAGCATCTCCGGCCAGCGGTGAGATGGCCTTGAATTTCAGC
AAAATATTTCCAATGACCTTGGGTCGGATCAAAAATGAGGCTCCTGCCGCTTCGTTGGCGCCCACTCTGA
TGTTCTGCGTACGCTTCTGGTCACCAATAACCTCATTCGAGGCATCCACGAAGTCATACTCTTGATCCTC
GTTGTCCAGGGTCAGTTCCACGTCCAGTGTCTTGGGCAGGTAGTTAAAGACCAGTGCCGGTACATTGATC
ACCTCACCTCGCTTCACGGAGTATGGCAATCGAACGGAGACAAAGAACGGCTGGAATGTCTTGATATTTG
TCTGATCATTGGTGACACCTAATCCCTTCTGGGGATGCAGTGAAAAGCCGGTGACCACCCAGTTGGTGAT
CGTATCGGGTATGGTCTTTACCCACTTGAACACCTCCTCCTCGGTGCTCTCGATATCGGCAAAGATCCAG
GTCTCTGCGAAATTCTTTCGCACTACTGGTGCTGCAGATGCAGACGCACTTTCGGCGGAGAATCCCACGG
CCTGAGTTGGTCCGCCAGCACCGGAATGGAATACATGACTATCATGTGCCACTGTAGTCTTTCTCATGGC
GAAAGAGCTTCCACCAAATCCTTCTGTAACTTCCAATAATTGCAAATTAATGTGGTTTATTATTATGACA
AATTTATACGTACGAATATTGTAGTCTGGGGCGGTGCGATTATAGAAGAAATAGGCATTGGTCATAGTCA
CGACTCCAGTTCGCTCTCCGGGGTAGTAGGAGTATCCACCCTGCCAGGGAGTTGAGGTGTCATAGCCATT
TAGGCGCCAGTTGAAGGATTCCTTGTTCAAATCGTTGTTGCTGCCCAGCAGAAGGACACTCTGATCGACG
GCCAACAATCCAACGAATGACTTTGGCGACGTCTTGATCTCCAGTGCAACGTCGGCACCCGGTTTAACTT
CCGCGGGAGCCTTGATTTCGATCTGGTTCTGCAGCTCGACCTCCACACTGAATGTTTCCTCGGTATATCG
GAACTCTCCTGTCTCATCCACGTAGTAGAAGTACACCCGACCATAGGGAGTAGTGAGGAAAGTGGGCTTA
ATGG
>dme_circ_0000773_junction_seq
GGAGTAGTGAGGAAAGTGGGCTTAATGGAGATCACCCACCAATCCGAATTCCACAC
>dme_circ_0000774
TTGAAGGATTCCTTGTTCAAATCGTTGTTGCTGCCCAGCAGAAGGACACTCTGATCGACGGCCAACAATC
CAACGAATGACTTTGGCGACGTCTTGATCTCCAGTGCAACGTCGGCACCCGGTTTAACTTCCGCGGGAGC

CTTGATTTCGATCTGGTTCTGCAGCTCGACCTCCACACTGAATGTTTCCTCGG
>dme_circ_0000774_junction_seq
TCGACCTCCACACTGAATGTTTCCTCGGTTGAAGGATTCCTTGTTCAAATCGTTGT
>dme_circ_0000775
CATCTCCGGCCAGCGGTGAGATGGCCTTGAATTTCAGCAAAATATTTCCAATGACCTTGGGTCGGATCAA
AAATGAGGCTCCTGCCGCTTCGTTGGCGCCCACTCTGATGTTCTGCGTACGCTTCTGGTCACCAATAACC
TCATTCGAGGCATCCACGAAGTCATACTCTTGATCCTCGTTGTCCAGGGTCAGTTCCACGTCCAGTGTCT
TGGGCAGGTAGTTAAAGACCAGTGCCGGTACATTGATCACCTCACCTCGCTTCACGGAGTATGGCAATCG
AACGGAGACAAAGAACGGCTGGAATGTCTTGATATTTG
>dme_circ_0000775_junction_seq
AAGAACGGCTGGAATGTCTTGATATTTGCATCTCCGGCCAGCGGTGAGATGGCCTT
>dme_circ_0000776
GACATTCCAGCCGTTCTTTGTCTCCGTTCGATTGCCATACTCCGTGAAGCGAGGTGAGGTGATCAATGTA
CCGGCACTGGTCTTTAACTACCTGCCCAAGACACTGGACGTGGAACTGACCCTGGACAACGAGGATCAAG
AGTATGACTTCGTGGATGCCTCGAATGAGGTTATTGGTGACCAGAAGCGTACGCAGAACATCAGA
>dme_circ_0000776_junction_seq
TGACCAGAAGCGTACGCAGAACATCAGAGACATTCCAGCCGTTCTTTGTCTCCGTT
>dme_circ_0000777
AGATCACCCACCAATCCGAATTCCACACGCTCGGAGTCGGGGACCACATCCTCCGGCACTTCCAGCTCGA
AGGTGTTCTTAAACTCACCCGTATCCTTAAGATTGATAAAGAATGCCCTGTTCTGGTACTGGGTAATTCC
TTCGGGCACTACCTTTAGCGGCTTGTGTATAGCATCTCCGGCCAGCGGTGAGATGGCCTTGAATTTCAGC
AAAATATTTCCAATGACCTTGGGTCGGATCAAAAATGAGGCTCCTGCCGCTTCGTTGGCGCCCACTCTGA
TGTTCTGCGTACGCTTCTGGTCACCAATAACCTCATTCGAGGCATCCACGAAGTCATACTCTTGATCCTC
GTTGTCCAGGG
>dme_circ_0000777_junction_seq
TCATACTCTTGATCCTCGTTGTCCAGGGAGATCACCCACCAATCCGAATTCCACAC
>dme_circ_0000778
ATAGTTGGGCACCAATTTGGACATGGTCTGCTCACCGCAACCGCTGGGCAATCGCAACAGGTTCTCAAGG
TTCTTGACCACTGGTCCCAAGAGATCACCCACCAATCCGAATTCCACACGCTCGGAGTCGGGGACCACAT
CCTCCGGCACTTCCAGCTCGAAGGTGTTCTTAAACTCACCCG
>dme_circ_0000778_junction_seq
AGCTCGAAGGTGTTCTTAAACTCACCCGATAGTTGGGCACCAATTTGGACATGGTC
>dme_circ_0000779
CTACCATTTTCTGCCTGTCGAGTTAACAGGAATTCGTAGCCCTTGGCCAGGATCTGCTCATCCAAGTCAA
TTATATCCTTGATCTTGCTAAACGAGCGCAGCACATAGGCCGTCAGCCAGGTGGAACCATTGCGCACTGG
ATCCTCCTGCCTCCATTTGGTGGGCCCAAAGGAACTGAAGCTGCCGTCGTCGTGGCGATAGTGCAACATA
TG
>dme_circ_0000779_junction_seq
CGTCGTCGTGGCGATAGTGCAACATATGCTACCATTTTCTGCCTGTCGAGTTAACA
>dme_circ_0000780
CTTATCTGGACCATAGCAGCTCCAGTTCCCTTGGCTTCAAACTCCAGCGACTTGGTGCCCTGTGGGAACT
CGACTGTCTGGAGGAGCAGGTCATTTTCCTCCGATGTGTTGAGCTTCTCGGTCTTTTCACGCTTTCCCTT
GTTGGAAACGGTCACCTCCCACTTGGCCGCCTCGTAGCCCGACTTCTCAGCGAACTTAATGAGGGCGGTG
AGGCCGACAACCGTGTCCTGGCTGGAGGCAAATCCTCCGAAGCCATTACGCTGGGCGATCAACCAGCGAA

CGGTGTTCAGAACAGAGTCGGCTGTCTCCTGGTCTGAATCCAGAAGGGAAAGCAGCGCATATGATGTGAT
CTCCACATCCTGACTCCAGATCCAACACCACCAGCGTCCGCAGTTCTTGGAGGCACGCAGCTTATCCAGA
TCCTCGGTCCACCAGGTGCGATCGTCCTCGTGTTTGGCCAGGGACTTAAGACTGGCCACCTGCTTGGCAG
CTTCCGGCGCCTTGGCCTTTTGCAGGGCATAAATGGCAATGGATTTGGGCAGGAGCTCAATGGACTCGGC
AGTATTGGCGCTCAGATAGGCAACCGCCTTGTCAATGGCAGCCTGGTTCGGTTTCTCCTCCTCCAGCAGG
GCGAGCAAGGAATTTGCGGTCAATGTCAATAGAGATCGCTGGGAGCTATAGAAATACTCTCCGTGTTCCG
>dme_circ_0000780_junction_seq
GAGCTATAGAAATACTCTCCGTGTTCCGCTTATCTGGACCATAGCAGCTCCAGTTC
>dme_circ_0000781
CAGCGCATATGATGTGATCTCCACATCCTGACTCCAGATCCAACACCACCAGCGTCCGCAGTTCTTGGAG
GCACGCAGCTTATCCAGATCCTCGGTCCACCAGGTGCGATCGTCCTCGTGTTTGGCCAGGGACTTAAGAC
TGGCCACCTGCTTGGCAGCTTCCGGCGCCTTGGCCTTTTGCAGGGCATAAATGGCAATGGATTTGGGCAG
GAGCTCAATGGACTCGGCAGTATTGGCGCTCAGATAGGCAACCGCCTTGTCAATGGCAGCCTGGTTCGGT
TTCTCCTCCTCCAGCAGGGCGAGCAAGGAATTTGCGGTCAATG
>dme_circ_0000781_junction_seq
AGGGCGAGCAAGGAATTTGCGGTCAATGCAGCGCATATGATGTGATCTCCACATCC
>dme_circ_0000782
GGAAAGCAGCGCATATGATGTGATCTCCACATCCTGACTCCAGATCCAACACCACCAGCGTCCGCAGTTC
TTGGAGGCACGCAGCTTATCCAGATCCTCGGTCCACCAGGTGCGATCGTCCTCGTGTTTGGCCAGGGACT
TAAGACTGGCCACCTGCTTGGCAGCTTCCGGCGCCTTGGCCTTTTGCAGGGCATAAATGGCAATGGATTT
GGGCAGGAGCTCAATGGACTCGGCAGTATTGGCGCTCAGATAGGCAACCGCCTTGTCAATGGCAGCCTGG
TTCGGTTTCTCCTCCTCCAGCAGGGCGAGCAAGGAATTTGCGG
>dme_circ_0000782_junction_seq
TCCAGCAGGGCGAGCAAGGAATTTGCGGGGAAAGCAGCGCATATGATGTGATCTCC
>dme_circ_0000783
GATCACATCATATGCGCTGCTTTCCCTTCTGGATTCAGACCAGGAGACAGCCGACTCTGTTCTGAACACC
GTTCGCTGGTTGATCGCCCAGCGTAATGGCTTCGGAGGATTTGCCTCCAGCCAGGACACGGTTGTCGGCC
TCACCGCCCTCATTAAGTTCGCTGAGAAGTCGGGCTACGAGGCGGCCAAGTGGGAGGTGACC
>dme_circ_0000783_junction_seq
CTACGAGGCGGCCAAGTGGGAGGTGACCGATCACATCATATGCGCTGCTTTCCCTT
>dme_circ_0000784
TATTAATGATTCCTTTTGGTTTGGATACTCTAGCACTTGCTCTTGCAGTCGTCACCCTCGCAAATGTCGC
AGAGCTTCGACTTGATGGAGTAGTATTCGGTGGCCTTCTTGTTCGTGTCATAGTAATCGTAGAGGACCAC
CGATGATGGCTTCTGGTTGGCCACCGCATGTGTTCTGTACGCCTCTATGCGAATGCATTTTTCTTCGTTC
TTGGCCAGATTCTCGAAGTATATGACCACCACGGAGTCACCGTTCTTGGTTTCCACCAACTGCAAAGCGA
ATAATTTATAGGTAATTGAAGAGAAAAGTGTACAGGTATTAAGGATTCTGAAACTTACCCGCACACGTTC
GATGTTCCGGATGTCGGCGAAGCTATCCTCATCAGCAGTATAACCAGACGGCAGGGATACCTCCAGAATG
GCCATGTTGGATTCCTTGGACTCGCCCTCCTCCACGTAGTCCACGCAGACGCTTAGTTCCAAATTGGCTG
GCGACGATTCCGGCAAAACGG
>dme_circ_0000784_junction_seq
TTGGCTGGCGACGATTCCGGCAAAACGGTATTAATGATTCCTTTTGGTTTGGATAC
>dme_circ_0000785
AGCACCAGCTGCCCCGCAACCGCCTCCCAAAGTCAGGAGCACCCAGTGAATGGTGGTCTTCGTCTGGCGC
TTGTATCCATTTGTCAGCACATTGCCGCCGTAGTGGATCATGACGCCCTCGGCCATGAAGAAGGAGAACT

GTGGTGTGGAATGAAGATCTTCAGTGCCCGCTTAGACGGTGATGGTGATCCCCCAACTATTTACCCCGAT
GGTCACTAGCCAGGCATGCAGACCCGTGCCCGAGAGCCCCGTTCGCAGCGTGAGATAGGAAATATAGATG
GTGATAAATCCAATGCACATCTGGTTGAGAACGTTAAGGAAGTACTCCAGTCTCTGGAGGCGATTCTCGG
CCTCGTGGCCGGACAGGGAACCGTGGGCCATTTTCGAATAGCGACGCGGGGATTCGATCGGAATAAATG
>dme_circ_0000785_junction_seq
CGACGCGGGGATTCGATCGGAATAAATGAGCACCAGCTGCCCCGCAACCGCCTCCC
>dme_circ_0000786
gtgctgaacccgatgatccactgctgcgatcagtgcgacaagccgatcctcgtctacggccgcatgatac
cctgcaagcatgtattttgcctcaagtgcgcccgCGccgagcccatcaagagttgtccccgctgcacgga
caagg
>dme_circ_0000786_junction_seq
aagagttgtccccgctgcacggacaagggtgctgaacccgatgatccactgctgcg
>dme_circ_0000787
AACCTCCCAAATGGCCGACTCCTTGAGGCGACTTTTGCTGTTGCTGGGGACCATGCATTTGCTGCAGCTT
TTGTATTTGCTGCAGTTGCTGTTGCTGAGCCTGATGCTGTTGGTTCAACtgctgctgagattgatgttgc
tgttgggataggtgttgttgttgtgcctgattttgttgctggacctgatg
>dme_circ_0000787_junction_seq
gtgcctgattttgttgctggacctgatgAACCTCCCAAATGGCCGACTCCTTGAGG
>dme_circ_0000788
GGTGATGCAAACAAAACGAACATTTCTATAGAGCTAAGTGGTTGAAAAAGTAAAACAATGACTATGTTTC
AACACGTGAGTAAATTATATTAAATCATTCATTTATTCTGGTACTAATATTTAAAGAACTCTTATATAAA
TTGTAAGAATATCATGTTTAGAACTGAGATATGCGGTGCACTAAAAATATAAAAATACACCAGTTCAACA
AAATGAGTTAAAAGATTATATATAAAGTATATATAAAATAAAGGAACTGTAAAGGTTTGATTCTTTGACA
AAAAATTTGAATATTAATAAATGAAATTTGATATTATAATACGTATTATGCATTGCTGCAGTGAATTCAG
TGCGATTCATAGTAAGAGCGATGAACAAAAGTTATGCTCTATTTATGATGAAAATGAACTCTTCTTCATT
TTCGAAATAGTTTTCCCTTatatccttatccatatccatatccataatccttTTGTTACCCTATAAATAC
ATACTGTTTTAAAAAAATATCAATTTTTTTCTAGTAAGACGTACTTATATAAATATTATGTTTATTCAGT
TTCCTGTTATAAAAAAATAAATTTGATTTAAAAGAAATACTTCTCTCTGTTGTTGATAAACAGAATGTTCC
TTCGTTTTTCCCCTTATTTTGCGTTTTTATTTCTTGGAAGAAAAAAGAAAATTGGACAAAAATTCTTAGT
GGCTAGGTTTAACGAGCAAAAGGCGTAACCCTTCCAATAACTATTGAaggaacattatcatttttatgca
tttatttttatattacgcatttatgcgaaagtttttttgcttggctatcacattttcatctcttgctcatt
tgtcaggacttgtcaaatccggtcaacaacttagcacaccctgagctattcccaaggagtttatttctgc
cattgctccttttttttcgattatcaattcgcatacgtgccaaggcaagctggagaaagcttttcaagtgt
ctagacttctgacaactatggaatgaatgcagtaaacagttgcccaagaatatttgcctttcactcaaaa
tgtcctgggaatgcggggcgtagtgatttagatctgctcggagaaaaggaccatgtatcctggcttaatt
actgcgcctggtcagcagaggaagtggtgggcaggggacagggggcatggggcaaggccccaatcgaact
gatttgattgtggggcggctcgtttaatgcaatattcccttcataaagaagccctttgtgtgagggtgtg
tcgctcgtatgcgggtcctttcaaaaactttgtaaaccggtcagggatatccttgtcatacggctcacac
acacacaaaggcaacaaattaaactgttcctatttgcaaatgaattgtgcgtggcgcaatttcgcagaaa
actttctaagggataaGGCTTTCTAAGGGATAGTGGATGCGATTGGGACTGCGGGGATGGTTTGCACAAG
GCGCACACGTTCCTTCCGCTTGAAATAAAATAAGTAGTATAATACAGAAAGCAACTTTGCTGGCATCGGA
ATTGAAATGAAAGAAAAGAAAAGGATTCTTGGGCcacacacacacacacacacacaAAGAGGCAAAAAGA
AAAAAGAAGCTTGTATCTCTGCTGATATCTCTttgtgcgtgtctctgtcttgtgtccgtgtgtatgtgtg
tgtgtgtgagctctgggttctggtgtcctggctcgcagaaactttttgtgagggcccaggacctttctgg

cgcttcaacgctcaattgctggtggttagccgagcccgtctgcgtgttctggtcgccagtcaattgaaaa
TGAAATTGGTAGAAGTTAGCAGAAACGGGAACACGGCCAGGACCAAAACCGAAAACTCTGCTCCTGGCGT
GTCTCAgtgtgtgtgtgtgtgtgtTGGGGCATCTGTTCCTTGAAGTTGGACGATGTCGGGGGAGTGCAGG
GCTACGTGCGGACAAGCATGCATAATGAGGACCACGATCCTTTCACCTGGATGCGGGTTACCCCTACAAA
TTAATACTAAAGAGACGCGTAAAAGTATGCAACGCCAAGTGGCAGGGAACTACGTGCCTGCTGCATGGTC
CTTTATCCTCGAAACTcttgtcttatttttattaatactttccgctactttactctttgatcgtaaacaa
aaaaatacacaacttcgcgtttttttcgacgcatttttcgtccatggtcacgtccctttgtttggaatttt
tatgttttatggatcccagttttgagtgcatttgcttgttgcgttcacaacgcacatttccctccaattt
ggcttgctttgtcatttttttacgatttatcagccaacgatttggctcacacactttgtgacagattgttg
aagattagtttggctggagtggagactcaaggaattgcgtgtcagaagggattagcaagttgtgcggttt
agtgcgaaaaaatgtgtaaagttcgcataaGGGATAAGCTCAGTGAAAATACTTTTCATCAAGGTATAGT
GAATTAATGAAGAAAACATTGGACCTGTTGAAAGTGGCTTAAGTTGTTAAGACCCTTAATTTAaaccgat
tttatgacgttattatttagtttaatgcattcgaaaaatgcttattactatttgtgcatattcttaaaac
caaaaacctacatttaataattattattctaatatctgtgttttgattggctgaagctataaaacatcaa
ttaataactgattaaaattaataactgataatttttataacttatctttttgccaagttaactgcttttt
acactttttgtgacctttttattaaaggaatcgattttcaaattttaagaagcaacttaattgtagtaa
tataaaaatatgtaacagcaaatgcatttttaatgaattcttaatttattaataaatttattgaaattga
gtcaacgtgtacttacttttgtaacagaaagaggggtcaacaaatggataaatcagttttcattgcaaa
aagttaatttgtcaaaactttttcatcgcatatctaatatcacatatctgcaaaatatagaaaaaatatg
ttcggttaaaaaaataattttaaggtgtccatagtggtaaatataaaatatttctgctcggcacttgtat
ttatgttaaacctaatgctgtttgtttttaccagctgcctaattttgtttgcttttgataacgaacacgt
ttttttttgcattctcaattcaaactttttagacctgaaatgggattcgcatgcaattgtatttttatt
ctagtttgaccaattctgtgcacttatgcaaatatatttgatatcgaacttttcatctggaatttgattt
tagtttttgctgctttttttgccatgcgaatatatttaaatcCAACTCTCGAATTGACAGTCCTTGATCCC
AATAAATGGTTACATGGTAATTGTTTGCCAATAGCAAAAACAAGCAGTGTCCGAAATAATTAAAATCTTG
Tgcagttttgaaattttgtgtttcacttgtaaattgaaattttgttgtttctagcgagaatttctatttg
tcctcattctgttgtcatcataaagatgttttaaactatgcagcaagatatttccactttaactgaatac
ttttgtacatttaatttttaatacgatgggaaacacattaatatacatatgtgccttgtttatatggacc
taactgaatttaattatcttgatttttatgcttatcactcctgattgcacttattgtgtggatttttatt
gaattctccgtagcgcctttactttggtggcattatgggcgaacgcactcacagaaaaagaaatcgcaca
taaaattttgttatgacacgccaaccactccaattgtttcacgttaaagccgcataaaagtaatacataa
gtaaggaagtcggagaagtaacaaaacaaaaaaaaaaaagagataatttctttgaggcgggatactcaca
tgagcaacaaatgcagttcattgtcggatccttcgtttccttggctttttctgttggcgtttcccagttt
ttcccacggcgactgctgtgtcgtttacatatcgtttttttggccctcatctcctttttctgggccacgtc
gcgctgtctaaacgatttacgacactttggccaactgcctcacgcacacatttgcaatattgtttgtttg
taagtttgtgtgggttgtgcgcgggcgagtgtctgtgatattagcatgtaacgaacacacacacacacac
acacagatgcaggaacaggagttaacttggccaaaaggcaacacaaaaTGCCGCACACGTTCGTTTTTAT
TATGTTAAATGTCTCCTGGGGCAGGAACATCAGCATCTGCGAGTTGTTTCCATCTGTTCGGTGGCTTTCT
GCCTTTTAGctttcctcccttccttttgtcttctgacttttggtaactgtcacttggcttgttacatttc
acattcacttagactgcaggattaaatctggagcgcgccaaaaatctagctagccaggaaaattagcaca
ggccgtttctcgccttttcctgctcggcactgcgctggttcgcccttctgcgaaatatcctgccagctcg
tcctttcagccgatttcagcttactgcccgccgctccagttgaaactgacgactggcgaacgacggtcaa
agtcaatggcgggcttttcgtggacgcgattcgcttttcaccttcgtcgacgggctcggcgcctaaaagc
cgaacctttccgattcacccacgtcgaaagctcgtttagccatctcaccaggctctcttcgagcaagtcc

ttgctgagaaggattgagttgccttaatcgcagatttgcttctctttggtttcatgttctaagcctcgca
ggtctcgaatctcgaaattgagtgagtctgttttgttgcctttgagcatttaaaatgcgtttacatttct
catataagcctgtactctttgcgtctgcaattcgagtcctttttagttgagctataaatgttattaccca
gcaagcacttcactctaagagaaacattgctggttttgctataaaagcactcagtgattatctcaatgtt
aaataaattagctcgaatcgagcgagcagtgtaaactggatttaccattcccatacatttataagaatta
aataataatttttataatgctaaagaaaatcccaattttatattaaatataaaatatttTTTGAAACTCA
TGCTAAAAATGATGTTTCGTAATTCCTCTCAgtttattttacatttttttcaaatttattttatgttatt
ttatcaaatATTCCTTGCACTCAACTGTTCcataacattttaaactgagtgatttttgaatgctatttag
aatcactccgatttgtgtgctcttaacatttcccagaatggtttgtctattcgattgagttctgagtaca
aagtgagttccttttgagtagccatcctttggtgtttctcttccatcgctcgcagcttcggctggcgaat
ttctcggcttttccgctctccggaaaaatccaccacactcggcgctcggtcggcgcatcctcgggcacgc
tgaaggactcttcgggtcgagtggcgactcacaaacgcacacactgaacgcgaggaaaacacgagcattg
ctgctagcagccttagccaatcgtaaaagttttgtacgtatttagtacaatttcgggatttggcaggatg
taacgagcgtgtaagaaacagagcgcgtccttctcagccgtgtgtgtgcgataatcgggcaacaagcagc
gcgacagcaaattgtatattacacaaaattcgcaaaaaatccaacgaatcgtttgaatccaatttcgagt
gatataacataaccacagatcgtactcggttataatgcaatagcaaagagccgcagcatttcgaaaatcc
ggcaaagtgattttagccaagttaaccaaaaaccaaatcgtcgaactcctcggcttgcctcggttcaacc
cccttcaaccctttctccattaactcgttgttgccgctgtactcgtacatatgtgtgcacttgtttttgt
gaacgtgttttgccgcaagccggttaaaaaattattcccataatTACACCATCCGAGTTGCAGGCGAGGA
ACTTCCGCGAAACATAATTACCGAAGAACAACTGACGTAGCCGATAATACCTGGCGGATAAATCGGTAAA
ATCTAAAGAGAAAACAAACTCTGAAaattatcagaagcctggtgtgtggtgtgtgcgtgtgacctggaat
ttccttatgaatatcgatatatatatGgactttatccttatcgtcctggccctgctaataagaaacggca
atcctgtgccgctgctcgagatggagattgaaacgattgccccggggagaaggggattccttcggtctcc
ccatcctttcttccgtaatagtgtcgtttcctttatttgtccttttatgtttacctactttttcttttttg
tgctccgtcgtttcgtcgtggtttggtttggtttggtttagtttgctcgcacacaattgaaaattcaatt
gatttacaaaatgaagtttactggttgggacgacagatactttttatctATTTTCATTTGCAGTTTTACG
AGCGAGCAGCTCTTTTTTCGGCCCGCTCCTTTTGGCCTCAAAGCGAAACGGTTGGCAATTTTTCACAGTT
CTTTTTCGATTGAGGTCCTGGCGGGGTTTCCTGCAgtttggtgcgaaaataacgctgcgtcctgcaagga
ttgttgtggctgatgttttcgctgccttccgattcgggtgtgccgagagtgtgtgtttgtgattggagcg
gcctaaaatcgttttaggaccagctgcgaagtgattttcagctaaggcgaaaaaaggactgcagtgtgtg
tcctgtgtgagtgtgggtctgcagattgacatagttatgtcctcggcaagtaatgggaagttcttggcaa
gagctcaagaaaatttaagtttggcctggccttgcaaagctaaataagattctatatatatataggttgttt
taaAGCCAAAATGTTTACTAATGCTTAAAGCTGTTCTCAAAATGAAGCATTTTTCAATTGACATTATCTG
CTATTCAATATTTTCAAGCGAGTTCCTTATTCAgagccttctgctcaattctctagtataaaatattatt
tttgagtaaattctcaataaaacttataaaagtaaatattctgaaaaaaagcctttccaagtgttttgac
aagtatttaatattttcgttgtagaaactcgtacaacacaactcaacctaaaatgtctttcaccacttgc
ctcaaggaactttcctcgtttttggtgtcaagtgtttttattagaagcagaagcagaaaggagaaaaatt
gccgacgaaggggcgaagtaaaaatttttcctttcattcctttttcgatccaaacttgaaatgaggtttg
aaacaaaaacgtttttttctgcgccagtgtaaaataatacgcatacgccatgtataacgaatacccaaagt
aggcttcacaagtggtaagggctactttctttggcagaaacagaatcgaaaatcgtaggatatggttgtt
aaaaactataggctattctatggcttacattagcctaattgaaattatatcgcttgatacatcgttcgca
gggcttggggttaattaggtgctgaccaagttgaaattactatgtaacttgtttattatattaacatgtg
gttgctcctattgaatgaaaacagtagctagatatatatatatatatagatggatggattgatattgata
aatgagaggggggcttaatttgctctttgcatttcacagcgactcgaactatccccactcccattagccct

atgggcctcagttggagccaaagcaaaaggactagtacttcaatgggcacccccccccccccccaccttcc
attctcactgtTCAAACAGCGTTTTACCATTCGTTCGTCATGCCATCGTTGGCTATATAAAGGCGGCTGA
TCGGCCACGCCCACTCTTCTTTTTCGCCGCTTGTTGTTGACgtttacaatttggtgttgaaaacttcagc
tggaaattgggtaatactcgtgatgatggcgttggttggatgactgcagcaatcctgcattaagtaccct
cctgtgtctggatgtcggtgtgtgtgtgtgtgtgaacgcgaaggggtttgcaacaaagaaattttaattt
atatagaatgagtttgggggcggcaatacctccattttccaccacctctctctctccttttttttcggctg
cagtcgtcgacattccgatgttcgtgttctgtgctcacagtgctctcactcactcactcgttcgcacatg
gagaccgaaaaaaatccaattattttaaaacttttgacaactgcaacaacaaggcgttgcaaggctatga
aaacaataagaaactgactgaacagccagaggcaacaaaaacaatatatgtgtatgtatgtatgtagggg
aaagtcgaaagtccgcaaaaaatcaagcagaaaaatgagtgtgagtgctcgaaagggttgccagccatta
taacttcacttgaaaagtttaattgatttttacaagtgcatacatatacatacatacatacatatatata
tatatatacggagatggacgcaaaaacatgcatacgtacatatgccacggacctacctgcagtcgtgatt
ctcgtgatttcgctttctccgtgttggccaagtgggtctataacttggtgtaaacaaagtggttgcaggg
ggatgggggttactgaagggggggtagaaaagaaatcaaaagtagcattgccccatgaactgaggtgcaa
attcgttaggctcttctctTCCAATTGGCTTGGCTTTTATAGTTTGTTCAATTCTTGCCTGACTTTTTGG
TATTTTCCTTTCCTTATTTTCACTTGGCTGCTACACCTGACTTTTTAATTGCTGCTGAGTGTGCTCCTGT
TACTGTAATTATTGGGTTTGCCTTTGAGGGATTTGCTTTTAGGTGATTGCCTTAAtgagtcctctactca
cagtaggcaaaaagttaaaccgatgaagtctactgcgattaactttgttcgtatcacatctgtaatttag
ttttccaattttcattcaattcgatgacatcCTTAATTGGAATACCATTCTTACTTTTAAAGATATTTGC
TTGTGGCTCAATAATAGTGTTCCATATGCATTTTTAGTATTTCAATAATATTTCTTTAAAGGAAACGCTA
AACTCTTTAATTGTCCTTTAAAGAAGTCGAAAATTAAATATGCCTGTGTTTGGTTTATTGATCAAATTCG
TACGATTTGTTTATTTTTAGTTCAATCGTTTATTTGCAAAGACACGAATTTCCAATCAAAAATAATTTGC
AAAGTATTTGTGTTCGGACATTTCTGAGTATAGTATTGCGTTGAATGTTTTTAAATTTTTAAATCACCTA
AATACCTTTCCAATTTGCGTTGCCTTTGTTTTTGCGGGGACCGGAGTGTATTGTTAAAAACGTATTTATT
GTTGAATTTTGCAAGCTAAACGAGACCAACGGATTTTCACTTCTCCCCCCGGCCGCAAAACCACACACAA
AGGTCTCTGACAATTTAAGCCAGGTCGACTGACTTTTATCCTTAACAATTGTTTCTCCTCCTGCTCCCGT
CTTATGTGTATCCTTTTTTGCAGgaataccgaatgccgaatgccgaatACCAAACGGCACACAGCACTGA
ATCACAGCaaaaaaaaaaaaaaaTAAAACCAAATATAAATCAATAAGACAAAGAATAAACTGCATATCTC
AGATTATATTTAGATATATGTAAATGAAGTAGCCATAAGAGTTCATTAAGTATTAGcttcagacatagt
caggtaaacgtattctggtcagataagtaacaacagcttccctccacatggtaatcatatatctatcgat
gaatcgacaaccggcgcttgcaactgcgacgctcaaattcaaattgcgataatgtcgacaacaatcaatt
tgtgtgatctgcaacgacaacaccttctacaaatgaatgcgacgtgacgacaacaactgcaaatatttat
aacgcatacgacatgttgctgccatgccaataatgatgatgacatcgcatttactaacacatcttgtaat
atccgatgattttcggctttcgaaaatgtatttaacgaacgattgaaacacataataactgcgactataa
accaacataaaataatcaccaaaaaaacacaccaacgcacctccccccgaacacacacactatatataca
cacaaacttcaaaacaatcctacactgtaccgaaaacttgcaacacttgcaacacaaacgttgacgttgg
tttttcctaacctgcagGGTAAAATCTAAAGAGAAAACAAACTCTGAAaattatcagaagcctggtgtgt
ggtgtgtgcgtgtgacctggaatttccttatgaatatcgatatatatatGgactttatccttatcgtcct
ggccctgctaataagaaacggcaatcctgtgccgctgctcgagatggagattgaaacgattgccccgggg
agaaggggattccttcggtctccccatcctttcttccgtaatagtgtcgtttcctttatttgtcctttta
tgtttacctactttttctttttgtgctccgtcgtttcgtcgtggtttggtttggtttggtttagtttgct
cgcacacaattgaaaattcaattgatttacaaaatgaagtttactggttgggacgacagatactttttat
ctATTTTCATTTGCAGTTTTACGAGCGAGCAGCTCTTTTTTCGGCCCGCTCCTTTTGGCCTCAAAGCGAA
ACGGTTGGCAATTTTTCACAGTTCTTTTTCGATTGAGGTCCTGGCGGGGTTTCCTGCAgtttggtgcgaa

aataacgctgcgtcctgcaaggattgttgtggctgatgttttcgctgccttccgattcgggtgtgccgag
agtgtgtgtttgtgattggagcggcctaaaatcgttttaggaccagctgcgaagtgattttcagctaagg
cgaaaaaaggactgcagtgtgtgtcctgtgtgagtgtgggtctgcagattgacatagttatgtcctcggc
aagtaatgggaagttcttggcaagagctcaagaaaatttaagtttggcctggccttgcaaagctaaataa
gattctatatatatataggttgttttaaAGCCAAAATGTTTACTAATGCTTAAAGCTGTTCTCAAAATGAAG
CATTTTTCAATTGACATTATCTGCTATTCAATATTTTCAAGCGAGTTCCTTATTCAgagccttctgctca
attctctagtataaaatattatttttgagtaaattctcaataaaacttataaaagtaaatattctgaaaa
aaagcctttccaagtgttttgacaagtatttaatattttcgttgtagaaactcgtacaacacaactcaac
ctaaaatgtctttcaccacttgcctcaaggaactttcctcgtttttggtgtcaagtgtttttattagaag
cagaagcagaaaggagaaaaattgccgacgaaggggcgaagtaaaaatttttcctttcattcctttttcg
atccaaacttgaaatgaggtttgaaacaaaaacgtttttttctgcgccagtgtaaaataatacgcatacgc
catgtataacgaatacccaaagtaggcttcacaagtggtaagggctactttctttggcagaaacagaatc
gaaaatcgtaggatatggttgttaaaaactataggctattctatggcttacattagcctaattgaaatta
tatcgcttgatacatcgttcgcagggcttggggttaattaggtgctgaccaagttgaaattactatgtaa
cttgtttattatattaacatgtggttgctcctattgaatgaaaacagtagctagatatatatatatatat
agatggatggattgatattgataaatgagagggggcttaatttgctctttgcatttcacagcgactcgaa
ctatccccactcccattagccctatgggcctcagttggagccaaagcaaaaggactagtacttcaatggg
caccccccccccccccaccttccattctcactgtTCAAACAGCGTTTTACCATTCGTTCGTCATGCCATC
GTTGGCTATATAAAGGCGGCTGATCGGCCACGCCCACTCTTCTTTTTCGCCGCTTGTTGTTGACgtttac
aatttggtgttgaaaacttcagctggaaattgggtaatactcgtgatgatggcgttggttggatgactgc
agcaatcctgcattaagtaccctcctgtgtctggatgtcggtgtgtgtgtgtgtgaacgcgaaggggt
ttgcaacaaagaaattttaatttatatagaatgagtttgggggcggcaatacctccattttccaccacct
ctctctctcctttttttcggctgcagtcgtcgacattccgatgttcgtgttctgtgctcacagtgctctc
actcactcactcgttcgcacatggagaccgaaaaaaatccaattattttaaaacttttgacaactgcaac
aacaaggcgttgcaaggctatgaaaacaataagaaactgactgaacagccagaggcaacaaaaacaatat
atgtgtatgtatgtatgtaggggaaagtcgaaagtccgcaaaaaatcaagcagaaaaatgagtgtgagtg
ctcgaaagggttgccagccattataacttcacttgaaaagtttaattgatttttacaagtgcatacatat
acatacatacatacatatatatatatatatacggagatggacgcaaaaacatgcatacgtacatatgcca
cggacctacctgcagtcgtgattctcgtgatttcgctttctccgtgttggccaagtgggtctataacttg
gtgtaaacaaagtggttgcagggggatggggggttactgaagggggggtagaaaagaaatcaaaagtagca
ttgccccatgaactgaggtgcaaattcgttaggctcttctctTCCAATTGGCTTGGCTTTTATAGTTTGT
TCAATTCTTGCCTGACTTTTTGGTATTTTCCTTTCCTTATTTTCACTTGGCTGCTACACCTGACTTTTTA
ATTGCTGCTGAGTGTGCTCCTGTTACTGTAATTATTGGGTTTGCCTTTGAGGGATTTGCTTTTAGGTGAT
TGCCTTAAtgagtcctctactcacagtaggcaaaaagttaaaccgatgaagtctactgcgattaactttg
ttcgtatcacatctgtaatttagttttccaattttcattcaattcgatgacatcCTTAATTGGAATACCA
TTCTTACTTTTAAAGATATTTGCTTGTGGCTCAATAATAGTGTTCCATATGCATTTTTAGTATTTCAATA
ATATTTCTTTAAAGGAAACGCTAAACTCTTTAATTGTCCTTTAAAGAAGTCGAAAATTAAATATGCCTGT
GTTTGGTTTATTGATCAAATTCGTACGATTTGTTTATTTTTAGTTCAATCGTTTATTTGCAAAGACACGA
ATTTCCAATCAAAAATAATTTGCAAAGTATTTGTGTTCGGACATTTCTGAGTATAGTATTGCGTTGAATG
TTTTTAAATTTTTAAATCACCTAAATACCTTTCCAATTTGCGTTGCCTTTGTTTTTGCGGGGACCGGAGT
GTATTGTTAAAAACGTATTTATTGTTGAATTTTGCAAGCTAAACGAGACCAACGGATTTTCACTTCTCCC
CCCGGCCGCAAAACCACACACAAAGGTCTCTGACAATTTAAGCCAGGTCGACTGACTTTTATCCTTAACA
ATTGTTTCTCCTCCTGCTCCCGTCTTATGTGTATCCTTTTTTGCAGgaataccgaatgccgaatgccgaa
tACCAAACGGCACACAGCACTGAATCACAGCaaaaaaaaaaaaaaaTAAAACCAAATATAAATCAATAAG

ACAAAGAATAAACTGCATATCTCAGATTATATTTAGATATATGTAAATGAAGTAGCCATAAGAGTTCAT
TAAGTATTAGcttcagacatagtcaggtaaacgtattctggtcagataagtaacaacagcttccctccac
atggtaatcatatatctatcgatgaatcgacaaccggcgcttgcaactgcgacgctcaaattcaaattgc
gataatgtcgacaacaatcaatttgtgtgatctgcaacgacaacaccttctacaaatgaatgcgacgtga
cgacaacaactgcaaatatttataacgcatacgacatgttgctgccatgccaataatgatgatgacatcg
catttactaacacatcttgtaatatccgatgattttcggctttcgaaaatgtatttaacgaacgattgaa
acacataataactgcgactataaaccaacataaaataatcaccaaaaaaacacaccaacgcacctccccc
cgaacacacacactatatatacacacaaacttcaaaacaatcctacactgtaccgaaaacttgcaacact
tgcaacacaaacgttgacgttggtttttcctaacctgcagcaaaagtttaagtgccatttggcgagtgag
tgagtccgtcagccgacgagtaagtctgacagagacacggcctgtgtgtaaataataaataataaataat
aaaaaaaaagagaataaaaagagcatttaaagttattagtaaccatcgttgttgttgagttgttgttgtt
gttaccaactgtcgacatacccttgcaactgccggcgaaaacatagcgaaataatgggtctgcagatgac
agcagcccgtccaatcgcagctctcagcctactagtgttgtccttgctcacctggactCACCCCACAATC
GTGGATGCAGCCC
>dme_circ_0000788_junction_seq
actCACCCCACAATCGTGGATGCAGCCCGGTGATGCAAACAAAACGAACATTTCTA
>dme_circ_0000789
GGTTCTACGTCGATTTTTGCATCCCAACAACATAATTCCCAGCTTCCAGCGCGAGGTGTACCTCAGCAGC
AACATCCTCAACAATTGTCCCACAGTTCACCTTGCAAACCGAATACTTTGGTTTCTGTTAATCAGGGAGT
CCAGCCTCCCGCCATATTAACACGAGTTGGATCTCATTCGCAGCCAAACCAACAGCAACAATTACCACAC
CAGCAATCGTCGTCTGGTCATCCGCATCAGAAACAGCTGTCGTCGCCAGGAGCAAATCTTCCTCTTCAAA
CGCCTTTGAAT
>dme_circ_0000789_junction_seq
AAATCTTCCTCTTCAAACGCCTTTGAATGGTTCTACGTCGATTTTTGCATCCCAAC
>dme_circ_0000790
GCGAACCGCAAACGGTGCTCAGCATCGAGAGCCGGGGACGAAGGAGTAGCGAGGGACGCGGAGTCCTGG
TGGCCAAGGATGTGGCCAATGCCGCTGGCCAGTTAAATGCACCCAGTGTATTGTCAATG
>dme_circ_0000790_junction_seq
GTTAAATGCACCCAGTGTATTGTCAATGGCGAACCGCAAACGGTGCTCAGCATCGA
>dme_circ_0000791
TAGATTCCTTGGAGGACGTCCTTTCCTGCCTCTGGTTACAGGAGCTGTTACGGGTTTTCTTAGCCTGGTT
TGCACCCCATTGTCACCCGGTTGTACAACCAATGAATCCACTCCAGGCGGCGCAGCTGTTACCGAGTTAA
GCGGGAATCCTCCCACTTTCTTCGCCCCTCTTGGCTGGCGTCCTCTCTTAGCACTACCTCCCCTTCCTGG
AGGTCTCCCAACAGTGGGTATAGGTGGCTTTGCAGGGGGCAACTGCCTGGTCTCCGTTTCGTCCTCTGTA
TCATGATCGTTTGAGTTGTTCACACTCATTGAAGTTGTTGGAAGAGCAGTTTCTTGGGCCATAGACTGAT
TCCCAGGCTGCGGATTGGGAATAGGAGCCGGTGCAATTGCCTGCATTTCCACTGCTCGCTTCGCATCCTT
ACTAGCCAATGGAGTATCTAATTTCTTAATGACACTGTCTATATTTACCTCCTTTGCTGACCAGTAATCG
CTGTCAACTTCCTTTGAGTCCTGTTCGATCAACTCATCCTGCTTTGGCTGCGAAATCTTAATTAGATTCT
CCTCCGTTAACAGAGAGCCAACCGTATTTGCCGAGATTACTGCCAAACCTGTTGTAGGAGTGGGTGTGCG
AATCACCGAAACACTTTCCGATGTTTTCTGAGCGGACATAGCTGGTGTTGGTTCTACATGACCAGGTGGA
GTGCTATCCTTGCCCTGGTTTTGAGGATAGTGTATGGCATTACCTTGGG
>dme_circ_0000791_junction_seq
TGAGGATAGTGTATGGCATTACCTTGGGTAGATTCCTTGGAGGACGTCCTTTCCTG
>dme_circ_0000792

GCTATGTCCGCTCAGAAAACATCGGAAAGTGTTTCGGTGATTCGCACACCCACTCCTACAACAGGTTTGG
CAGTAATCTCGGCAAATACGGTTGGCTCTCTGTTAACGGAGGAGAATCTAATTAAGATTTCGCAGCCAAA
GCAGGATGAGTTGATCGAACAGGACTCAAAGGAA
>dme_circ_0000792_junction_seq
TGAGTTGATCGAACAGGACTCAAAGGAAGCTATGTCCGCTCAGAAAACATCGGAAA
>dme_circ_0000793
GATACTCCATTGGCTAGTAAGGATGCGAAGCGAGCAGTGGAAATGCAGGCAATTGCACCGGCTCCTATTC
CCAATCCGCAGCCTGGGAATCAGTCTATGGCCCAAGAAACTGCTCTTCCAACAACTTCAATGAGTGTGAA
CAACTCAAACGATCATGATACAGAGGACGAAACGGAGACCAGGCAGTTGCCCCCTGCAAAGCCACCTATA
CCCACTGTTGGGAGACCTCCAGGAAGGGGAGGTAGTGCTAAGAGAGGACGCCAGCCAAGAGGGGCGAAGA
AAGTGGGAGGATTCCCGCTTAACTCGGTAACAGCTGCGCCGCCTGGAGTGGATTCATTGGTTGTACAACC
GGGTGACAATGGGGTGCAAACCAGGCTAAGAAAACCCGTAACAGCTCCTGTAACCAGAGGCAGGAAAGGA
C
>dme_circ_0000793_junction_seq
GCTCCTGTAACCAGAGGCAGGAAAGGACGATACTCCATTGGCTAGTAAGGATGCGA
>dme_circ_0000794
GGACGAAACGGAGACCAGGCAGTTGCCCCCTGCAAAGCCACCTATACCCACTGTTGGGAGACCTCCAGGA
AGGGGAGGTAGTGCTAAGAGAGGACGCCAGCCAAGAGGGGCGAAGAAAGTGGGAGGATTCCCGCTTAACT
CGGTAACAGCTGCGCCGCCTGGAGTGGATTCATTG
>dme_circ_0000794_junction_seq
AGCTGCGCCGCCTGGAGTGGATTCATTGGGACGAAACGGAGACCAGGCAGTTGCCC
>dme_circ_0000795
GAAATGGAAAGTGCACCACGTAATGTCCAAGTGCGTACGCTGAGCTCGTCCACGATGGTTATTACTTGGG
AACCACCAGAGACGCCCAATGGACAAGTGACCGTAAGTACCTTGCATCAAGTGCGCATATTAAACGATAA
GATCCGGCGTCCAAAGTCCGGACTCCGAGCACCGGGCACCGGGTCCGGAACCCGTAGCCTAAAGCCACCC
GGCCAGCAACccgtagcccgaagtccgtagtccgtagtccgtagtccgACCAACGACCAACGCACTTTGT
CACGTCAGGCCGAATGGCTCGAATGGGTCGAATAGAGGGTCCCATCGAAGGGAAATTGGCAAAATGCCTT
TGGCCATGTTCCGGGACACGAATGGCTGGCGGACGTTCGTATGCAAATCCTAGACACCTGTCCACAGGTC
TCCATTCCGAGTCGGCAATCGCTGTGGTCCCTCTGGCCTCCGGATACCGGCGGGGCAGGCTTACTTGTCC
GGGCCTTTTAGGCTCACAAATCTCCCAGCAGCAGCAATATATCATTTTCGGTTTATCAGTTAATGAAAGG
TGAAAATCGGATTTGGAGTTTAGATTTCGAATGGCTTCAATTTCGTAGCCCAGCTGTTTTCCATAACCCT
CAAAATGTGGCTGTTAAGCTTAACTTGAAAGTTAACAAAAGTTTTAAACTTATTAAATTTCTTTTTGAAT
ATCAAATTTAACTAAGATCCTTCTTAATTCAAATTACCTGCTCTTCTTGCTGCTGTGGTTCGGGAACATT
TAAAATATTTACGATCCATTAGCGCTACATTCATAAAAGATGAGCGTATCTGTTCGGAATATTAAACCTC
TAACTCCTTTACACTTTCTCTTGGTAAAAACAGGGGCTACAAGGTGTACTACACGACCAATTCGAATCAG
CCGGAGGCGTCGTGGAACTCCCAGATGGTCGACAATAGCGAACTGACCACAGTCTCGGAGCTGACGCCCC
ACGCCATCTACACGGTCCGGGTTCAGGCCTACACATCGATGGGAGCCGGTCCAATGTCCACGCCGGTCCA
GGTGAAGGCCCAGCAAG
>dme_circ_0000795_junction_seq
ACGCCGGTCCAGGTGAAGGCCCAGCAAGGAAATGGAAAGTGCACCACGTAATGTCC
>dme_circ_0000796
GTACCAGGTGCTCCGCCTCGAAATATCACCGCCATAGCCACCAGCTCGACGACCATATCCCTCAGCTGGC
TGCCTCCGCCCGTCGAGCGATCGAACGGCCGGATCATATACTATAAGGTGTTCTTCGTGGAGGTGGGTCG
CGAAGACGACGAGGCCACCACCATGACCCTCAATATGACCAGCATTGTACTGGACGAGCTGAAGCGCTGG

ACAGAGTACAAGATCTGGGTGCTGGCCGGCACCTCCGTCGGGGATGGGCCGCGGTCGCATCCCATCATTT
TGCGCACCCAAGAGGATG
>dme_circ_0000796_junction_seq
CCCATCATTTTGCGCACCCAAGAGGATGGTACCAGGTGCTCCGCCTCGAAATATCA
>dme_circ_0000797
TTCCTACTTGACTTGCGACTTGGTTTTGTTCTTGAGCCTGAGTTAGGGCTTGATTCCTCGCCTTTTTCTC
TGCAGCTGTCAACACAGAGCTGGTAGGAATAGGAGTTGGCAGTGGAGTCGAAGATGTTGCCGACGTCACC
ATTTCCATTCCCTTTCGCTGAATATCGAGCTGCTGCTGCTGAAGTTGCTGTTGCTGCAATAG
>dme_circ_0000797_junction_seq
GCTGCTGAAGTTGCTGTTGCTGCAATAGTTCCTACTTGACTTGCGACTTGGTTTTG
>dme_circ_0000798
AATTTCCTCGCCATTAACAACGCCGTAGAAATCCGGCTTCACCATTGGCTGAGGTGCAGCCATTTGCGTT
GTGACCGTAATCTTTGTGGGCGGCCGGTAGGAATAATCCGAGGGAATGGCACTCACATTCACATTGTACG
TGGTAAACGGACTGAGTTCGTTGATAGTGTGGGTCTTCACATAG
>dme_circ_0000798_junction_seq
TTCGTTGATAGTGTGGGTCTTCACATAGAATTTCCTCGCCATTAACAACGCCGTAG
>dme_circ_0000799
GGTTGCGATTGGGAGTGGAGGATCCGGCTTTCTCTTCACTTCATCGGATGGTGCCTGGGGACTTAAGTGC
CTCGTGGGAATGGCGTTTCGCATGAGACGTCTTACGCCAATCAACTCGGGTTCAAAGTGTCGGCTGCAAA
TCAGGCGAGTGTTTC
>dme_circ_0000799_junction_seq
TGTCGGCTGCAAATCAGGCGAGTGTTTCGGTTGCGATTGGGAGTGGAGGATCCGGC
>dme_circ_0000800
ATAAAGGAGGAGGATGAAGATTCAATGCCGTCCCTGGAACCAGAACCGCAGCTTCACGAAGACCAAGAGG
TGGAGGACTCAGAAGCTATGCAAATACCCTTAAACCAGACAAATTGGAAGGGCCAACTGCGTTTGCTTGT
GAAGCAGGAAAAGGTGACCTACAACCAGGTGAAGTCAGGATACGATAAGTGCTCCTTAAGTCATTGCCAG
CGGCAGAGATCTAAGCATGGCG
>dme_circ_0000800_junction_seq
TGCCAGCGGCAGAGATCTAAGCATGGCGATAAAGGAGGAGGATGAAGATTCAATGC
>dme_circ_0000801
TTTTGTCAGATCGAGGGATGTATGAATCACCTGACCACTGAAAACATAACGTTGCACAAATTTCCCCATT
CGGAGGACATGTGCCTCAAGTGGCAGCACAACACGCAGGTGCCTTTCGATCCTTTCCACCGGTGGCGATA
CCGGATATGTAGTG
>dme_circ_0000801_junction_seq
CACCGGTGGCGATACCGGATATGTAGTGTTTTGTCAGATCGAGGGATGTATGAATC
>dme_circ_0000802
GGTAAGTTCAGAGTTGGAACGCTCCACGGATGCAGCCGCCTGTCGTCAAACGAACATCGGGACTCAAAGT
GATTGCGGCATACCCTAAGCATATATTGCTGCTGCGGCTCCGTAATCTCCAGTTGGCCATTCTCAACCCA
CATTCTCA
>dme_circ_0000802_junction_seq
AGTTGGCCATTCTCAACCCACATTCTCAGGTAAGTTCAGAGTTGGAACGCTCCACG
>dme_circ_0000803
GATTATGAATCCATTTCTTAGCCAACGCCAATGTTTTTGGAAATCCACTAAACTTAGGGTCTCCAGGCTG
CCATATTCGACCACATGCAGTGGCACAGCAAACCTCGGCTTTTTTCTGCCAGAGAATTTCTGGGTTCGGT

AACATCTCCACCTCTTCGTCGTGGCCTAGCATGAGAGTCGGCAGTGCGCCAGGGCGTAATCCTTTGTGGG
CACCGAAGCAAAAGGGCTCAAAGTGACGCTCACAGATGAGCTGCTCCCCCAATCCGAGGTCCTGGCAACT
GATCTTCAGGTTGTTGCTCCAAGCATTCAGTTGAGCAGTGGTTTTTGGCACCTT
>dme_circ_0000803_junction_seq
TCAGTTGAGCAGTGGTTTTTGGCACCTTGATTATGAATCCATTTCTTAGCCAACGC
>dme_circ_0000804
GACGAAGTTTTCCGCTGGGAGCGGACTTTGTTGCCGGATAGATAAAGATGCCGCCGTATTTAATGGTGCG
ATGCACATCCGCGACCATGGAACCCACGTACCGCGCTCCATAGGGCTTTCCCTTGGCGGGATCCTTCTTG
GCCGCAATGTAGTTGAAGACACCATCCTCCCAATCCGCTGCATATCCCTCGTTGATAGAGTATATCTTTC
CCTTCTCCGGCACCCGCAT
>dme_circ_0000804_junction_seq
ATATCTTTCCCTTCTCCGGCACCCGCATGACGAAGTTTTCCGCTGGGAGCGGACTT
>dme_circ_0000805
GGTTATATCAAATACTGATCCAATCGGAGGAGACAACTCCGAGTCTTGTAACACACGCAAATCTCGCCGT
TTACAAGAAAAGGAGGATCGCAGCACAGTAGACGATATCATTGAAGATGTGGTTCGAAATACCAACACGC
CAACTGGAACAGGACCACATTTGCCAAAAGGTGCACAAACTCCACCACGAC
>dme_circ_0000805_junction_seq
CCAAAAGGTGCACAAACTCCACCACGACGGTTATATCAAATACTGATCCAATCGGA
>dme_circ_0000806
GAACGGTAACCGGGGCAGCTACACTAACAGGCGCTGGTGTAGAAATTGGTGTCACAACTGATACCGGTTC
CACTTCTTTAGCATCTAATTGGGGGACATGCGACTCCACACCAGCTCCTTCTGGTGGTGCCAAATGACTG
GCGGCAACAGTTGCATTAGACGCAGTAACTTCTTCAATCAAGTTTGCTGTTTGTTCACCGATTGTTTTTC
TGTCCTTGGAACGCCTTGGACGACCCACTGCATTAATGATCTGTACCGCATCCGTCTTTTTGGCTTGAGC
ATTTCTGCCAGAACGTCGTGGTGGAGTTTGTGCACCTTTTGGCAAATGTGGTCCTGTTCCAGTTGGCGTG
TTGGTATTTCGAACCACATCTTCAATGATATCGTCTACTGTGCTGCGATCCTCCTTTTCTTGTAAACGGC
GAGATTTGCGTGTGTTACAAGACTCGGAGTTGTCTCCTCCGATTGGATCAG
>dme_circ_0000806_junction_seq
TCGGAGTTGTCTCCTCCGATTGGATCAGGAACGGTAACCGGGGCAGCTACACTAAC
>dme_circ_0000807
GGAGACAACTCCGAGTCTTGTAACACACGCAAATCTCGCCGTTTACAAGAAAAGGAGGATCGCAGCACAG
TAGACGATATCATTGAAGATGTGGTTCGAAATACCAACACGCCAACTGGAACAGGACCACATTTGCCAAA
AGGTGCACAAACTCCACCACGACGTTCTGGCAGAAATGCTCAAGCCAAAAAGACGGATGCGGTACAGATC
ATTAATGCAGTGGGTCGTCCAAGGC
>dme_circ_0000807_junction_seq
ATCATTAATGCAGTGGGTCGTCCAAGGCGGAGACAACTCCGAGTCTTGTAACACAC
>dme_circ_0000808
GAAGGCGATCAGTTTGCCGTGCTTACGCAGCACCGGCTTAGTGGGTGAAGCATCCAAGTGACCAACTAGC
GGTGCTGGCATTGGAAAATTCCTGGGCACCTTATCGCCGATAGTGGCCACACAGGATTGAGGGAAAAAAC
CCACTTCGTAGAGGGACTTCTGCAGATGCGACTTCTTGCCGCGCCACCAAATCGACTCCGCCGGACTGGG
CATGTCGATCACGGAAATCATGTCGCCCACCTCAATGTTGATCTCGTCCGGAGCTTGGGCCTGATAACGC
CTCACTCCGTAGGCGGCACCCACCGCAGGTGTGTTGATA
>dme_circ_0000808_junction_seq
GGCGGCACCCACCGCAGGTGTGTTGATAGAAGGCGATCAGTTTGCCGTGCTTACGC
>dme_circ_0000809

TGTGATTCCTGTGGGCGAGATTGTCCTAGAAGATTGCTACAACGACCAGGACGAGGACTACGACTCGGAG
GACTCGAACCAGGAGAACTACTTTACCAATGATTACCCGGACGACGACGAAGCGGGCGCCATGGGTTCGG
ATGATGAGTTGTGCCGGCAAATGAACAAGTTTATGCTCGGATGATGACGAGGATGAGTTTGCCAGCACCA
GCGATGATGATGACTATGCCACCTTTCGCGATCCCTACGTGCACACGATCGACACTGAGGAAGACTCGTT
TGTGGACGACGTGGACTTTTACAATGTCGACCGGGAGAGGGGCAGCGCCTATGAGCGCTACAAGCGAAGG
ATTCTGAAGGAACTGGAGGGTCAGGAAGAAGAGGATGAGGATGATGATGATGATGACGACTCCTTTGCGA
GCGCTGATGGCGAATGAGAGGTTGGAAAGCCAATTCATTCTTGCACCTTAAATTACTGGCTTTAAAAAAG
AATGTAGTAGTATAATTTAGCGTTTAATAACTAAAAAATTTAAGTTAATATAAAAATTCTGTTTGCATGC
TTTGCTATGTTTAAGCTAACCTTATCTTATCATCTATAATTTTTGCTGTCTTTCTTCTCAGTCTTGATGT
GCTGAAGTTGAGGTTTCGGCGCACAGCCCTCCATGTAAAGGCGAAGGGTTTCCTCGTCCATCTTCTCGTT
TTCCTTGGGAGGCTTGTAGTCGGCCACGTGATCGACCCTTAGTGTCCTATCGAGGATCTTGATGCCATTC
AGGTTGTCCACGGCCAGCACTGTGGATCTCTGGTCCTCGTAGCACAGGAAGCAGAAGCCCTGGACAAGTT
TAAACAATGATTTCAGGAACTTTCTTGAAAGGGTAACTAAGCATTACCTTGGACTTTCCCGTTTTTGAAT
CTCGAATTAGATTGATGTTGACCACTTCGCCGTACTGCGAGAATACGCAAACCAAGTCGCCCTCAGTTAG
GGTATACGGAAATCCTGCGACGAAAATCCAGGCTGAGTCCTTGTACATGTCGTGCCAGCTCTTCTTTCCG
CCATGTTGCAGTTCGTGTTCGCTCAGTTTGAGCACATTTTTCATATTTGTTAGCGGATTCATCTTCAAAT
CGTTTTATTTGTTTATGTTTTTGTGGCGATGTGACTGCTTGCTGTTAGGTAGAAAATACCATCGGTCTGT
TTTGAACTGTATTAACATAGCAAGCAAGCAGTGTAGGTAGTATTTCAGGCAATATATCGATAATTGGATA
CTTTCTTTAACTCATAGAGTTTCCTAAAACTAAAAGGCTAATATAATTAATATGTAGCATACTTATTTAA
TTTCCAACTAATTTAAATACCAAAGTAGTTTTTAATTGCTAGGTATTTGTATTTGGTATTTCTTGTATGT
GCAGTGGTATTAAATAGTGCGTTATAATAAGGCCGGTGGTATTTTAATCGTTATCGTCACATTTGTTGTC
GCGTTGTAAAAGGCTGCGGATCGGAAGCCAGTTAATTGTGCACTGGGACGGACAGTGAGAAATGTTGTAA
TATTGCCATTGCCACGGTGATCTATTCGAGAAAAGG
>dme_circ_0000809_junction_seq
TTGCCACGGTGATCTATTCGAGAAAAGGTGTGATTCCTGTGGGCGAGATTGTCCTA
>dme_circ_0000810
GAACGGTAACCGGGGCAGCTACACTAACAGGCGCTGGTGTAGAAATTGGTGTCACAACTGATACCGGTTC
CACTTCTTTAGCATCTAATTGGGGGACATGCGACTCCACACCAGCTCCTTCTGGTGGTGCCAAATGACTG
GCGGCAACAGTTGCATTAGACGCAG
>dme_circ_0000810_junction_seq
CTGGCGGCAACAGTTGCATTAGACGCAGGAACGGTAACCGGGGCAGCTACACTAAC
>dme_circ_0000811
GTGGCCTAACAGTGGTATGCTGCGCATGGTGCAGCACAGTGGGCAGGGGATTCTGTTGTAGGATTATGGG
TTGTTTTGCTGGAGCCACAATTTGCGGTTGTTGCTGCTGAATAACCTGCGAGGCGTTTCCTTTCACCAGA
ACACTTACAGGTTTGTTCAACAAAGCGGCTGCCGAGGTCTCAAG
>dme_circ_0000811_junction_seq
TCAACAAAGCGGCTGCCGAGGTCTCAAGGTGGCCTAACAGTGGTATGCTGCGCATG
>dme_circ_0000812
GGTTATTCAGCAGCAACAACCGCAAATTGTGGCTCCAGCAAAACAACCCATAATCCTACAACAGAATCCC
CTGCCCACTGTGCTGCACCATGCGCAGCATACCACTGTTAGGCCACCTCAACCACTGAAAGCTCAC
>dme_circ_0000812_junction_seq
TAGGCCACCTCAACCACTGAAAGCTCACGGTTATTCAGCAGCAACAACCGCAAATT
>dme_circ_0000813
AGTGCGGAGAGTGTGCTGTTGGTGGCGGCACGTTGACCTGAAGATGTTGTTGCTGCTGCAGGTGACGTGC

AATGATCTGGGGCTGCACTAGTTGTTGATTTCCTGCAGTATCCGTGAGCAACATGTGACCGGAATGCGGT
GCGTGCTGTGGTGGTTGAGCTACTGCTTGCTTTGTGGGCGTCAATTGCTGCTGGATATTCTTTTCCCTAT
TCAGGACGTGAGCTTTCAGTGGTTGAGGTGGCCTAACAGTGGTATGCTGCGCATGGTGCAGCACAGTGGG
CAGGGGATTCTGTTG
>dme_circ_0000813_junction_seq
GCAGCACAGTGGGCAGGGGATTCTGTTGAGTGCGGAGAGTGTGCTGTTGGTGGCGG
>dme_circ_0000814
aataaattcGGCCACAATTAATATACTTATTTATTCATCAAAACCAAACCACCACAAGAGCATAACCAAT
CATGTTAAAGTTTATCAGAGGAAAAGGGCAGCAGCCCAGTGCTGACAGACAACGCTACATCGTAAGG
>dme_circ_0000814_junction_seq
TGCTGACAGACAACGCTACATCGTAAGGaataaattcGGCCACAATTAATATACTT
>dme_circ_0000815
tttagaataaattcGGCCACAATTAATATACTTATTTATTCATCAAAACCAAACCACCACAAGAGCATAA
CCAATCATGTTAAAGTTTATCAGAGGAAAAGGGCAGCAGCCCAGTGCTGACAGACAACGCTACATCGTAA
GG
>dme_circ_0000815_junction_seq
TGCTGACAGACAACGCTACATCGTAAGGtttagaataaattcGGCCACAATTAATA
>dme_circ_0000816
AAGCTGACCGTGGTGTTCCTCTTGAGGATGCACTCCGCCTTGGTGGTGTCGCAGCCCTCGATGGCCACCC
GGGTGAACTTGCCCGTCTTCGATCCGCAATCGCTGAACTCGAGGGCTCCGGCGAAGACCACCAGTGCAGC
ACAGGCAATTACCGCG
>dme_circ_0000816_junction_seq
CACCAGTGCAGCACAGGCAATTACCGCGAAGCTGACCGTGGTGTTCCTCTTGAGGA
>dme_circ_0000817
GTTCACCCGGGTGGCCATCGAGGGCTGCGACACCACCAAGGCGGAGTGCATCCTCAAGAGGAACACCACG
GTCAGCTTCTCCATCGACTTCGCCCTGGCCGAGGAGGCAACGGCGGTGAAGACAGTC
>dme_circ_0000817_junction_seq
CGAGGAGGCAACGGCGGTGAAGACAGTCGTTCACCCGGGTGGCCATCGAGGGCTGC
>dme_circ_0000818
GGACGAGTCGTACCGCTACACGGCCACCCTGCCGGTGCTGAGATCCTACCCCAAAGTGTCCGTGCTGGTC
AAGTGGGAGCTGCAGGATCAGGACGGAGCGGACATCATCTGCGTCGAGATTCCCGCTAAAATTCAGTAGG
CA
>dme_circ_0000818_junction_seq
CGAGATTCCCGCTAAAATTCAGTAGGCAGGACGAGTCGTACCGCTACACGGCCACC
>dme_circ_0000819
TTTTTGCTGCAGTAGCATGCCAGGATGGGTAGGCATAATTATTCCGTGAGGAGGCATTTGTATCTGAGCT
CCCTTTTGCTGATTGTTTTGCTTATACAAATGCTGTTGTTGCTGCTGCTGAACGGCTGGCAAAGTGGGTG
TCAAAACAGTGGGTGCATAGCTATAG
>dme_circ_0000819_junction_seq
TGTCAAAACAGTGGGTGCATAGCTATAGTTTTTGCTGCAGTAGCATGCCAGGATGG
>dme_circ_0000820
TTTTGTCAAAATTCAATAGCCGGAAAGCACCCACAGGGCATCGGGAATGCGTCTGAGTTTTTCCCCCTTC
GAAATTTGACTTTTCTGGGTCATGTGGGCGTCTAATTGTTGGCATTTTTCCCCGCCGATTGGCTTTGTCT
ATTGAAATTTATGCAATTGCAGCTTGCATTGGCATTTAATGGTGCATTTGGTATCTATTG

>dme_circ_0000820_junction_seq
CATTTAATGGTGCATTTGGTATCTATTGTTTTGTCAAAATTCAATAGCCGGAAAGC
>dme_circ_0000821
TTACTCCTCAGGGAAATGGTCCTCTGCCTTCGGGGTTTCTGCAAGACCGGCGGGAGTTGGGCACCATTTT
CCGATTCCGCCCTTATCGATCGGGATCGGGTTAATCCGCGGGCTCGTATCGTGGCATAGCCATCG
>dme_circ_0000821_junction_seq
GCGGGCTCGTATCGTGGCATAGCCATCGTTACTCCTCAGGGAAATGGTCCTCTGCC
>dme_circ_0000822
GACCTCTCCAGCAGTTCCGTACTGTTGCTCCTTTGGGTCACAATTGGAAGCTCGTACAGATTAAGGCGTG
GACTGGTCGGCGGTCGCATGGGTGTGGCATAAATCTTTGGCTTGGCATAGAGACTTTCCCGACGAACTGG
CGCCGG
>dme_circ_0000822_junction_seq
AGAGACTTTCCCGACGAACTGGCGCCGGGACCTCTCCAGCAGTTCCGTACTGTTGC
>dme_circ_0000823
CGCACGGCGGGCAGAGGGTAGTCCACGCGATCCGCGTAGCAGGCGGTGCCGTTCCAGCCGTAGCCAACGA
TCTCCCGCTTGCCGATCTTGTCCAGTGTGTGGACGGCGGCCACGCTGCCCACCTGGGCACTCTTGGGCAG
CTGAGAGGCCAGCTGGCGGAGCACAGCACTGTTGAGTAG
>dme_circ_0000823_junction_seq
GCTGGCGGAGCACAGCACTGTTGAGTAGCGCACGGCGGGCAGAGGGTAGTCCACGC
>dme_circ_0000824
GAAGGCGCAGCTGCAGCGCATCATCGACCTGGAAATCAACCCCGTCACCGGATTGACCTCCAAGTGGGAC
TACGAGAACAAGAAGTGGAAGAACTAAGTGCGCTGTCGTTAAATAATTCCGCGATTGTGTGTGATTAAGT
TAGCCGACGTCTATGTGTATTAAGTAAAAGCAAGATAGTCGAAATTAAA
>dme_circ_0000824_junction_seq
AAGTAAAAGCAAGATAGTCGAAATTAAAGAAGGCGCAGCTGCAGCGCATCATCGAC
>dme_circ_0000825
GTCGCCGCCGCGCATAAACTCCAGCACCATATACACAGAATCCGGCTTGTCCACGATGTCGTGCATGCGA
ACCACGCACGGATGTGAAAGATTCTTCATAATCTTGGCTTCGTTCAGCACCCGGTCGGGATCACTGAAAT
TGGTACTGGG
>dme_circ_0000825_junction_seq
GGTCGGGATCACTGAAATTGGTACTGGGGTCGCCGCCGCGCATAAACTCCAGCACC
>dme_circ_0000826
GTTTATGCGCGGCGGCGACCTGCTTAACCGGATCATCAGCAACAAACTACTTAGCGAGGACATCTCGAAG
CTTTACTTTTACCAAATGTGCCATGCCGTCAAGTACCTACACGATCGGGGTAAAACAAATTAGTTTTATT
TGGGTTCGAATGTGTTGCTGATTAAGCAAGCCGTTCATTTCTGTTATGCAAATAAGTAAGTCTTCGGAAT
GGTCATTGAAAACGCCCTTAATTGCAAATCATTCCTGCGCCTTTACGAATTTGCTAGGTAAAACAAATTA
GTTTTATTTGGGTTCGAATGTGTTGCTGATTAAGCAAGCCGTTCATTTCTGTTATGCAAATAAGTAAGTC
TTCGGAATGGTCATTGAAAACGCCCTTAATTGCAAATCATTCCTGCGCCTTTACGAATTTGCTAGTACGA
CTACTGCGAACACATCAATCGAATTATTCAACTAACTAGTTTCTACCTTTCAGGCATCACCCATCGTGAC
CTTAAGCCTGACAATGTTTTGCTGGAGACCAATGACGAGGAGACCCTACTGAAGGTCTCCGATTTCGGGC
TGAGTAAGTTTGTGCAGAAGGACTCG
>dme_circ_0000826_junction_seq
GCTGAGTAAGTTTGTGCAGAAGGACTCGGTTTATGCGCGGCGGCGACCTGCTTAAC
>dme_circ_0000827

GTTTAGCAACTTCCTGTTCATCCTACTGCCGCCCGTCCTTATAATGCTCTTCAAGGAGTACGGACGCTTT
GTGACGCCCGGAATCCACGTCATCTGGGTGCTGCTCATCGTGGTTGGCCTGAGTTCGATGTACTTCCATG
CCACTTTGAGTCTGATTGGCCAGCTGCTGGACGAACTGGCCATACTCTGGGTCTTCATGGCGGCCTTTTC
GCTCTTCTATCCGAAGCGATACTATCCCAAGTTCGTGAAAAACGATCGGCAAAACCTTCAGTTGGCTCAT
GCTCTTGTCGGCGATTGCTGCGACGGGCTTGTCGTGGTGGAAGCCCATTGTTAACGCCTTTGTTCTCATG
TTCATGAGTGTGCCGACCATGGTAATGCTCTACACAGAGCTGCAGAG
>dme_circ_0000827_junction_seq
TGGTAATGCTCTACACAGAGCTGCAGAGGTTTAGCAACTTCCTGTTCATCCTACTG
>dme_circ_0000828
GAGGAGGCCCAAGGTTGAAGATTTCCACAAGAAAGGCAGAGGGAACACATGGTTATTAGCCTGCGGTACT
TTTAATATTTTTATAAACCAATATTTGGATGTTGACACAAAGTTGTTCGAAAACCGTGATAAACTTTCGA
AGAGG
>dme_circ_0000828_junction_seq
CGAAAACCGTGATAAACTTTCGAAGAGGGAGGAGGCCCAAGGTTGAAGATTTCCAC
>dme_circ_0000829
TGGTTTAGACAGTAGCGATGACAATCAGGAGGTGTGCAAGCTCAGCTACGCGATTCTTAAGATCCGGAGC
GGCGCGCTCTAGGTTTTCATTAGCAAAATCGCATGATGGAAATATGTGTACCACGTAGGCTGCTTGTTCA
CTTCCTGGAATGGGTATGTTCACTATGCCGGCTGCCATTTTCTGTTGCAAATAGG
>dme_circ_0000829_junction_seq
CCGGCTGCCATTTTCTGTTGCAAATAGGTGGTTTAGACAGTAGCGATGACAATCAG
>dme_circ_0000830
GTGAACAAGCAGCCTACGTGGTACACATATTTCCATCATGCGATTTTGCTAATGAAAACCTAGAGCGCGC
CGCTCCGGATCTTAAGAATCGCGTAGCTGAGCTTGCACACCTCCTGATTGTCATCGCTACT
>dme_circ_0000830_junction_seq
TGCACACCTCCTGATTGTCATCGCTACTGTGAACAAGCAGCCTACGTGGTACACAT
>dme_circ_0000831
GTTCATTGAGGCCAAGATCACATAAAAGTGTAAAGAGATCCGCAAGAGACTCAAACAAAACGAAAGAAAA
CCCTTTAAATTCCGACACTTTGAAAGCAAAATATTTGTCTAAATTAATTATATAACTCTCCGTAATTAAA
AACCTTAAAGAAAAAGCTTGCAAAGTATCGTTAAGTTTGATCTCAAGAAAGAAT
>dme_circ_0000831_junction_seq
ATCGTTAAGTTTGATCTCAAGAAAGAATGTTCATTGAGGCCAAGATCACATAAAAG
>dme_circ_0000832
TTGATGATCAACGATGTGGCCATCATCAAGCTGAGCTCTCCCGTTCGCCAGACCTCTAAGATCCGGGCCA
TCGAACTGGCCGACTCCGAGGCCGTTTCCGGAACCAATGCCGTGGTCTCCGGCTGGGGCACCACCTGCTT
CCTTTTCTGCTCCTCCCCCGACACCCTTCAGAAGGTGGAGGTTGATCTGCTGCACTACAAGGACTGTGCC
GCCGACACGTACAACTACGGCAGCGACTCGATTCTGGAGACCATGGTCTGTGCCACCGGCGAGAAGAAGG
ACGCCTGCCAGGGTGACTCCGGTGGTCCTTTGGTCGCGGACAACAAGCTTGTGGGCGTGGTTTCCTGGGG
CAGCGGATGTGCCTGGACCGGCTACCCCGGCGTCTATGCCGATGTCGCCAGCCTGAGGAGCTGGATCGTT
GACACCACTGACTCGTTG
>dme_circ_0000832_junction_seq
CTGGATCGTTGACACCACTGACTCGTTGTTGATGATCAACGATGTGGCCATCATCA
>dme_circ_0000833
GATTATCAGCGAACTGGCGGAAATCGTCTACACCGCCTTGGATTACAATCTGCCGGAGGACGAGGAGTGC
CAAGTGTCACAGGAGCTGGAGAATCTGTTCAACTTTATGACGGCAGATGGTGAGTATGGATTCTCGGGAA

TGAGCTATAGATTTGGCCCATAATCATCTTAGTATATTAAGGTCAAGCTTCAAGTATTATGAGATAAAAG
AACATTCCGTATTGACACTATTTTCTTCAAATGGTGAAATCGCGTTGTACTCGTTTCTTTGTGACTCAGA
TATACATATACTATGTAGCTTAGAACAGCAATTAAATCTCTCAATAAAGTTCCCAATCTGAGCTCAGTTT
TTTTATATTTAGGCAGGGACTTTCTTAAACCAAAAGCCCCGTTTATCGGGCTTCACAAGCGAAAACAAGA
TGAAGGTTAAATGAGGCGGCGTGCCATGAATCACGTATTCTTTGTGATACATTTAATAAAAATAATTATA
ATACAATAAACTCCGAACCTTATAGAGCTGATTGGATCTTGTTCATTGCAGAGACAGACGACGATTGCAT
TGACGAGGGCATCGACGAGGGCGACAAGCGCTGGGACGACGAATCCGAGGAGGAGCGCAACGATACCAAA
GAACTAGAGCACATAATCGAG
>dme_circ_0000833_junction_seq
TACCAAAGAACTAGAGCACATAATCGAGGATTATCAGCGAACTGGCGGAAATCGTC
>dme_circ_0000834
GATTATCAGCGAACTGGCGGAAATCGTCTACACCGCCTTGGATTACAATCTGCCGGAGGACGAGGAGTGC
CAAGTGTCACAGGAGCTGGAGAATCTGTTCAACTTTATGACGGCAGATGGTGAGTATGGATTCTCGGGAA
TGAGCTATAGATTTGGCCCATAATCATCTTAGTATATTAAGGTCAAGCTTCAAGTATTATGAGATAAAAG
AACATTCCGTATTGACACTATTTTCTTCAAATGGTGAAATCGCGTTGTACTCGTTTCTTTGTGACTCAGA
TATACATATACTATGTAGCTTAGAACAGCAATTAAATCTCTCAATAAAGTTCCCAATCTGAGCTCAGTTT
TTTTATATTTAGGCAGGGACTTTCTTAAACCAAAAGCCCCGTTTATCGGGCTTCACAAGCGAAAACAAGA
TGAAGGTTAAATGAGGCGGCGTGCCATGAATCACGTATTCTTTGTGATACATTTAATAAAAATAATTATA
ATACAATAAACTCCGAACCTTATAGAGCTGATTGGATCTTGTTCATTGCAGGGTGGGTTTCCAATTCGAG
TCGATCGTATCTGACATTTGCAATCGGTTCGTGGCATGATGTGCCAAAAACCAGGCACCCAAACCAAATC
GAACCAATGCAAATTCGAACTCCGATCCAAGGGTGAGTCAGACTAGTTTGGAGGTGCGCCAATGTCTGAC
CTTTTGACCAGGCGCTCAGTCGCTCTATTTGCCAGCTCTTTTAACATATTGTGAATAAATCTATATGGAG
CCAATGCATCGATATTCTTCGGTGGGAAAACTTGTTTGGCTCACTTTACAGTTAACCAAAGCACAGAAAG
AAATGTACAATCAAATCTCGGGAAATTAGTGTGCCAAGTCTAGAAAATAATAAAATAAAAATATTATTAA
TTAGGGTTCCTGGTTCAAATGCTAATTACCTCAGACTGTATTCCATTTTCAATAGTTAAACGTAAGATCA
AGGTCTCCAATTCTCAATGTCTATGCGAACATTTTTCTTTCTGTGCAGAAATCAAAGTATCTGGCGGATA
CTTGTTGTGCGCGTATCTGTATCGCGGCTAACCGCAAACAAGCCGATTTAGTCCGGGTTCTAGTCAGATA
CCTAGCTTATGATGCCCGGTGGAGCGACCGACTGGATTTCAACAATTTCAATTGCGGCTCGATTGCATCG
ATATTAAATAACCGAAATAACCATAAAACCAAACGCTTTCGTAATCGTAGGAAATCAGCCGCAGATCTGT
AAATCAGAAATCTAAATCTGAATTTGAATTCGAAACCGCCTACCTGCCGCAGTTGATTGCGCTTGGGGAA
TGCTTCCGCGTTCTCCGGCAGCCAGCGAATCATCTGCCGCGCCATTCGCTCCACCGCAGATCCATTGGAG
GCCCACACGAGGATCCCCGATTTGGGGCGAGTGGGGCGTGTGATGAACCTCACACCGTTGCTGATTGAGC
CGGTGTCTTCGGTTCCTCGGTTCTGTTTGAGTGCTCCGAGCGGGGCGGGCGGGGCTGGGTGGTTCTTGTC
TAATGAGCTTTATGGAATTGACACATTGCTGTCAGGCAACACTGAGCAGTGAGTGGATCCCTGATCTTTT
TCCACCTGCCTGACTTTTCTAGCACACCATCGTAAAGTGGAAAAGTATCCGCGACTGATACAGATAGATT
TGCATTTCTACAGTGTCTGTGCTTCTCGGGTTAGATAAGATTTGTTTGGGGGAATAAATTGAGTTGGTCT
ATATGCGCGATATGATTATCGTAATAAGTGAAAGGAAATTGCCGAAGGCGTGAAACGTAATTAGCTTTTG
GTACGCAGAACTTATGTATGGGTTTTGGGTTATTCCCAGAATTTGTTATGAATAAATGTAGGTGAGATGG
AACCGCAGAAAATAAATTAAAACCAATATAGTGCGGTTTAGGTGTGATATAAAATATATAGGCTACGAGT
ATGTGGCACACGGATAAGTTTGAATGTCTGATTAGCATCGAATGTACGGTTCTTGCATTGGTTTGTTATT
CCGCCGCTCATTTTATGTAGTTATTAGTGATTTTAATGAATTGCATTTTTTAATTCACTCCCCAATAGAC
CTGCAGAAATCACATAAAAACCACCCTGCCTGAGAACCACTATAGAGCTGTCTGCAGAGCCCTCGTGACG
GAGACCATAGAGCTCCGAGTATTTCTGCAGCAGGTCCTGAACAATGGTAAGTGATCCTACTGTTAGACTG
AGAACGCAGCTTGTTAACTAATTAATTATCTTAAGAAGCAGGTGCCGAGAAACTGATCAAGGCGTCGGAA

TCATCGGCCACCACGCAACAAGAACTGGCGAAGCTGGGCTTCAACGACTGG
>dme_circ_0000834_junction_seq
ACTGGCGAAGCTGGGCTTCAACGACTGGGATTATCAGCGAACTGGCGGAAATCGTC
>dme_circ_0000835
GAGACAGACGACGATTGCATTGACGAGGGCATCGACGAGGGCGACAAGCGCTGGGACGACGAATCCGAGG
AGGAGCGCAACGATACCAAAGAACTAGAGCACATAATCGAGGTGGGTTTCCAATTCGAGTCGATCGTATC
TGACATTTGCAATCGGTTCGTGGCATGATGTGCCAAAAACCAGGCACCCAAACCAAATCGAACCAATGCA
AATTCGAACTCCGATCCAAGGGTGAGTCAGACTAGTTTGGAGGTGCGCCAATGTCTGACCTTTTGACCAG
GCGCTCAGTCGCTCTATTTGCCAGCTCTTTTAACATATTGTGAATAAATCTATATGGAGCCAATGCATCG
ATATTCTTCGGTGGGAAAACTTGTTTGGCTCACTTTACAGTTAACCAAAGCACAGAAAGAAATGTACAAT
CAAATCTCGGGAAATTAGTGTGCCAAGTCTAGAAAATAATAAAATAAAAATATTATTAATTAGGGTTCCT
GGTTCAAATGCTAATTACCTCAGACTGTATTCCATTTTCAATAGTTAAACGTAAGATCAAGGTCTCCAAT
TCTCAATGTCTATGCGAACATTTTTCTTTCTGTGCAGAAATCAAAGTATCTGGCGGATACTTGTTGTGCG
CGTATCTGTATCGCGGCTAACCGCAAACAAGCCGATTTAGTCCGGGTTCTAGTCAGATACCTAGCTTATG
ATGCCCGGTGGAGCGACCGACTGGATTTCAACAATTTCAATTGCGGCTCGATTGCATCGATATTAAATAA
CCGAAATAACCATAAAACCAAACGCTTTCGTAATCGTAGGAAATCAGCCGCAGATCTGTAAATCAGAAAT
CTAAATCTGAATTTGAATTCGAAACCGCCTACCTGCCGCAGTTGATTGCGCTTGGGGAATGCTTCCGCGT
TCTCCGGCAGCCAGCGAATCATCTGCCGCGCCATTCGCTCCACCGCAGATCCATTGGAGGCCCACACGAG
GATCCCCGATTTGGGGCGAGTGGGGCGTGTGATGAACCTCACACCGTTGCTGATTGAGCCGGTGTCTTCG
GTTCCTCGGTTCTGTTTGAGTGCTCCGAGCGGGGCGGGCGGGGCTGGGTGGTTCTTGTCTAATGAGCTTT
ATGGAATTGACACATTGCTGTCAGGCAACACTGAGCAGTGAGTGGATCCCTGATCTTTTTCCACCTGCCT
GACTTTTCTAGCACACCATCGTAAAGTGGAAAAGTATCCGCGACTGATACAGATAGATTTGCATTTCTAC
AGTGTCTGTGCTTCTCGGGTTAGATAAGATTTGTTTGGGGGAATAAATTGAGTTGGTCTATATGCGCGAT
ATGATTATCGTAATAAGTGAAAGGAAATTGCCGAAGGCGTGAAACGTAATTAGCTTTTGGTACGCAGAAC
TTATGTATGGGTTTTGGGTTATTCCCAGAATTTGTTATGAATAAATGTAGGTGAGATGGAACCGCAGAAA
ATAAATTAAAACCAATATAGTGCGGTTTAGGTGTGATATAAAATATATAGGCTACGAGTATGTGGCACAC
GGATAAGTTTGAATGTCTGATTAGCATCGAATGTACGGTTCTTGCATTGGTTTGTTATTCCGCCGCTCAT
TTTATGTAGTTATTAGTGATTTTAATGAATTGCATTTTTTAATTCACTCCCCAATAGACCTGCAGAAATC
ACATAAAAACCACCCTGCCTGAGAACCACTATAGAGCTGTCTGCAGAGCCCTCGTGACGGAGACCATAGA
GCTCCGAGTATTTCTGCAGCAGGTCCTGAACAATGGTAAGTGATCCTACTGTTAGACTGAGAACGCAGCT
TGTTAACTAATTAATTATCTTAAGAAGCAGGTGCCGAGAAACTGATCAAGGCGTCGGAATCATCGGCCAC
CACGCAACAAGAACTGGCGAAGCTGGGCTTCAACGACTGG
>dme_circ_0000835_junction_seq
ACTGGCGAAGCTGGGCTTCAACGACTGGGAGACAGACGACGATTGCATTGACGAGG
>dme_circ_0000836
GACCTGCAGAAATCACATAAAAACCACCCTGCCTGAGAACCACTATAGAGCTGTCTGCAGAGCCCTCGTG
ACGGAGACCATAGAGCTCCGAGTATTTCTGCAGCAGGTCCTGAACAATGGTAAGTGATCCTACTGTTAGA
CTGAGAACGCAGCTTGTTAACTAATTAATTATCTTAAGAAGCAGGTGCCGAGAAACTGATCAAGGCGTCG
GAATCATCGGCCACCACGCAACAAGAACTGGCGAAGCTGGGCTTCAACGACTGGGTAAGATTCTTAAAGA
GTGCTGTCCCCTTAAAACACACAGATACTACAACACCATAGCGTACCATACCATAGTTATGTAATGGTGC
TCCACTAATGGCCCACAGTGGCGATAGCGTCTCACGATTTGTTTGGCGCACCTCTTCGAACGTAAACATA
CATATGCCATGAGCCATCATCATCATAATCATTATCGCAAAGATCACGAGCATTTGTGGCGGGGTTTTCC
GCTCTGGGCTGGAATAAAAAGTGAACTATTTCATGCATTGCCAAATGAAACTCACTTTTAATTTCAATAA
TTGGCCCGCGGTGGTCGCCTCCATTTTGGCCATCATCCTAATGAGTTCAACGGCGCACCATTCGTATACA

CTCGTATGTACTATGTGATTTCGTGAACTATTTAATGGACGCATATATCATCTATATATTTGGCGTTC
GCTGTGACCTTTGCCGAATATGTCGAGGCCGCCACAGGAGTTTGCCATCATCACCACATCGGCCCGCCAT
AGCCGTCATTCAACCTCCGAAACTCATTGAGACACACACGCAAATTGGCCAGGCACGCAAGCCAAAATTC
GCTCTTGTTTTTGCTTCAGCTTCTTGTTGACTTTTTGTGGCGTGAAAAGTTATACTATAGTAGTCGGCTT
TACCGTTCGAGGTGTGTTTGAttttttttAATCAGATGGAAACCCAAGCCTGTCAAAGAACAGTTGGCAA
AGTTTGTCCTCAAAAGTTGATAAGGCTGAAAAAGTATTAAATATTGAGTTCTTTAAGCTTCAATGTCTTT
AGACTTATTTGCATACTTCGGGAAATTCTACAGCTGGTAAACTATCGATGTTCCAGCCACGATAATAACC
CAGATTGAGTATGAATGTAAATCAAATGACTTATTATTTCAGTTGCTAATTTGTTTGTTCAAATTATTGC
CACCAACAAATTCTGAAATGACTTTATTTATATGAACTGCTAATTGCTTAAATTTAATAATGCCGCTTCT
TTGTTTTGAGGAGTCAAGAGAGCCCAATAAAAGACAAATTATCTGTAGTTTAGTGCAGTAATAGGTTTTA
TTGTATCCAAAAACGACCAAGAAAGTTCAATTTCAAAAGAGTTTAGGCTCGTCTTTTCCTCGCATGTCCG
ATGATTACAAATCGCAAAAAAATAAAGAAACCTTTGCGTGTGAAATAGACGACACATCATTATTTGCCTA
TACTCTGGAGTACAAAAGAAACACAACCAGATAGCGAAATAGACCATAAATATTTGCACTCCATGAATTT
GCATGTGCCGATAGGCGGGGATTAAAAAAGAGGGTGCAGCCGTGGAATTGGCCAATTAATTGGCCAGACA
TACACCATATATCATATGCGCAATGAAGACCTGGCAACGAATCCAGTCAAGTGTGTTTATTTTGTGTCTT
CTTGGTTTTGTTGGTATTGCGAAACGAGCGTTGATTAAACGCCATCTTATCTATTTCTACTTCTCCGATA
AGAGTTCTCTACCTTTGTTTGGTCTATTGTTTATAGGGAGTGTTTTTCTTTTATTTGCACTCGTTCTTTA
GCTACTGCCAAACGAAGTTCAAGAAGTGTGTCTTTAGTCAGCGTTTTATCGTGTTTTCTGGGAAGACAAC
AGCCTTCAGAATGTCACACAGCCAACTGATTGTTAAAACACTTTTTGACTGAATTTACATTGTTTTTGTT
TGGGCATTATAATGCTGGAATTGCGGAAATATAGCTGGTTCTGTTTTATTACTATTTTTTCTCTTTGGCT
TTGAACGTGTTTCGCTGGCAGGGGAAAAAAGGTTTTCCGAGGAAATCTCTCTTTTCGGCAGACATCGTGT
CTGTGTCTCGCCTAGCTGCCTTCGATGATGATGATGATTTGGTATGCTAATTTTCGATCCAGACGTTACC
AATCTTGTACGCACATGGTCCAAAAGTATTTCACAAGCAACAGCAGATACATCCCAACTTTTGCGGAAGC
TGCTGCCACTCAAAGCTGGCCTGATGTTGCTGCTGCAAGTTGATGTTGCAGCTGCTGCGTCTAATGCTGC
TGCGGTGAGCGTAGTTGAAGCGTGGCGCGGCATCTTAATCGTCGGTACATTTATGTATGTGCAACATGGT
GTCAAAACATGTGCTGCCATTGATGCCCTCAGCTGAAATCGGACCCTAGATACGCTCCACCCTCCTCGCC
TCGTTTTGCATGTTTAATGAGGTGTCTCAAGATGCATGGCCACCAATTGGCGGCGAACGAACAGGAAGGC
GATGGCGAATGGAAAGTGAAATTGCGGATCACTGTTACATATCAGGCCCCATTGTGTGCTAAACAGTCTT
CCTGCCATCGCGCAGCGTTCAACACTCATTACTCATACGCACAGTTAACTTGTCAGGCCGATGGATCGGA
TCGGTCCCCCTCGCAGCGGGAGTCTAAACTATTGGCTAATTAAATGAAAATAATTGCAAAAGTGAAATGC
ACCTCTCACCACTCTGTCTGCTTGTTTTTCTTCCTGTCTCAAGCACGAACTGCAGCTGTGGAGGGCACTG
CAAGTAGGTCCCTGGTTCCATTGCATGTTCTCCATtttgtttgttcgttgtttgtttgtttgCCTGCTTG
CGTTTGCCGTGTCCCACATCGCCAGCGATCCGTCGCAGCTATCTAGTATCGAATCTCTATTGATTAACCA
CACCTTCCCGAATCCATAGGGCACGCTTCTGGGTGCAGGTGATCGACGAACTGCGACGGGGAGTGCGGCT
GAAGAAGAGCAACCACGAGCGCACTCCCATCGAATATGAGCTGACACCCTACGAAATACTCATGGGCGAC
ATCCGAGCCAAAGTGAGTACAAACTAAACTAGTCGATAATCGCCAAGAAAGCTTACACAGTTGAGATACT
TCTTCATTAAATTACAATGATTGTAGATGATTTGATTTTCAGACCTTACATAGATAACATAATTAGCACC
GCTTATTTGTAGCCAACAAAAGAATGAAAGCAATACCCCATTTAGAAAACGCATCTAAGCCCGAATTTCT
CTTCACAGAAAGTATCAGTTGCGCAAGGTGATGGTCAACGGAGATATTCCGCCGCGCGTCAAGAAAGATG
CCCACGCCATGATCTTGGAGTTCATCAGGTCACGGCCGCCGCTGAAGAAG
>dme_circ_0000836_junction_seq
CATCAGGTCACGGCCGCCGCTGAAGAAGGACCTGCAGAAATCACATAAAAACCACC
>dme_circ_0000837
GGCACGCTTCTGGGTGCAGGTGATCGACGAACTGCGACGGGGAGTGCGGCTGAAGAAGAGCAACCACGAG

CGCACTCCCATCGAATATGAGCTGACACCCTACGAAATACTCATGGGCGACATCCGAGCCAAAGTGAGTA
CAAACTAAACTAGTCGATAATCGCCAAGAAAGCTTACACAGTTGAGATACTTCTTCATTAAATTACAATG
ATTGTAGATGATTTGATTTTCAGACCTTACATAGATAACATAATTAGCACCGCTTATTTGTAGCCAACAA
AAGAATGAAAGCAATACCCCATTTAGAAAACGCATCTAAGCCCGAATTTCTCTTCACAGAAAGTATCAGT
TGCGCAAGGTGATGGTCAACGGAGATATTCCGCCGCGCGTCAAGAAAGATGCCCACGCCATGATCTTGGA
GTTCATCAGGTCACGGCCGCCGCTGAAGAAG
>dme_circ_0000837_junction_seq
CATCAGGTCACGGCCGCCGCTGAAGAAGGGCACGCTTCTGGGTGCAGGTGATCGAC
>dme_circ_0000838
GGCACGCTTCTGGGTGCAGGTGATCGACGAACTGCGACGGGGAGTGCGGCTGAAGAAGAGCAACCACGAG
CGCACTCCCATCGAATATGAGCTGACACCCTACGAAATACTCATGGGCGACATCCGAGCCAAGAAAGTAT
CAGTTGCGCAAGGTGATGGTCAACGGAGATATTCCGCCGCGCGTCAAGAAAGATGCCCACGCCATGATCT
TGGAGTTCATCAGGTCACGGCCGCCGCTGAAGAAGGTGTGTAAAGCCCCCAATGCAAGTTCCCCAACGAA
TTCCCAATCCACTCGCATGGCCAGTACCCAAATCAACCGCAATCCCAACTGCAATCCCTCCTCATCAAAA
TCCCCATTTCACTTTGGCAAAGTGTGAAATATTAAACTACAGGTTTTCCCTTTTTCCGACTTCATTTCAT
TCGCCGTGATTCCTAATTTTTTTGTACCAAATTGTTGTTTTTCTTCGAAATAAAAATTATTAGTTTACTG
TGGCCAGCGATCGCCAATTGGGCCCGCCGCGCATGTGCGAGCCTTCTCCGCGGGAGCAGCTCATGGAGTC
CATACGGAAGGGGAAGGAGCTCAAGCAGATCACCCCGCCAGAAGCACCGACCCTCAGGGAAAGGG
>dme_circ_0000838_junction_seq
CCAGAAGCACCGACCCTCAGGGAAAGGGGGCACGCTTCTGGGTGCAGGTGATCGAC
>dme_circ_0000839
GAGCAACCACGAGCGCACTCCCATCGAATATGAGCTGACACCCTACGAAATACTCATGGGCGACATCCGA
GCCAAGTGAGTACAAACTAAACTAGTCGATAATCGCCAAGAAAGCTTACACAGTTGAGATACTTCTTCAT
TAAATTACAATGATTGTAGATGATTTGATTTTCAGACCTTACATAGATAACATAATTAGCACCGCTTATT
TGTAGCCAACAAAAGAATGAAAGCAATACCCCATTTAGAAAACGCATCTAAGCCCGAATTTCTCTTCACA
GAAAGTATCAGTTGCGCAAGGTGATGGTCAACGGAGATATTCCGCCGCGCGTCAAGAAAGATGCCCACGC
CATGATCTTGGAGTTCATCAGGTCACGGCCGCCGCTGAAGAAG
>dme_circ_0000839_junction_seq
CATCAGGTCACGGCCGCCGCTGAAGAAGGAGCAACCACGAGCGCACTCCCATCGAA
>dme_circ_0000840
GTGCTTCCAAGTGCAAACTCCACGCTGTCACGATCCAGACAGCGGCTCATCAAAGTGGACTTCTCCAAGT
TTCAGGTGGGTTTCGCGGAATCCAAAAAACCTACAAGGGGATCTGACCTTTTTTCCTGATTTTCTTATTT
TCCAGGACGACGATCTCTTCTACGATGAGAACAGCATCAGCAGCTCCCACTCCACAGCGGCCACCCACCA
GCATCATCCACATTTCGCCGAGATGCACCGCTGCTCGCAGCCCAAGATGCCGCCGTATCCATTCGGTGGC
TACATGGTGCCCAGTCAGGCACGACAAGATTGCCGGGAGACGGCGTCGCTGATGCGGCCCCGACGCACAA
>dme_circ_0000840_junction_seq
GCGTCGCTGATGCGGCCCCGACGCACAAGTGCTTCCAAGTGCAAACTCCACGCTGT
>dme_circ_0000841
GTGGACTACCTTAAGGAGAAGCAGGAGGAATACGCCCAGAAGAACGAGAAGCGCAAAAACAAAAAGGAGG
CCAAGCCTGAGCCTGCCTTCGAGCTGCCCAAGAACGCCATTGTTGTCTTCGAGGGCGCTCCGGAGACATC
TAGCCGCGAGGAAATTCGCGAAGCCTTCGAGAAGATCAAGGACTTCGAGGTGGCCTACATTGAGTTCGCC
AAGGGCGAAACCAAGGGCTCTGTGCGGCTGACGGAGGCCGATGCGGCCGAAAAGTACATTGCCAAGG
>dme_circ_0000841_junction_seq
GATGCGGCCGAAAAGTACATTGCCAAGGGTGGACTACCTTAAGGAGAAGCAGGAGG

>dme_circ_0000842
GATGTCTCCGGAGCGCCCTCGAAGACAACAATGGCGTTCTTGGGCAGCTCGAAGGCAGGCTCAGGCTTGG
CCTCCTTTTTGTTTTTGCGCTTCTCGTTCTTCTGGGCGTATTCCTCCTGCTTCTCCTTAAGGTAGTCCAC
CTGCCATTTACGCAGCAGCTCGCGCTCCTTATAAACGATCTTCTCCTGTTCGAGGAAGGCCTTTGCCTGG
TCCTTGGTCTCGAAAGTAAGGAAGATGCTGCCCTTGAACTTGTACGACTTGGTGGGCTTGTCATAGTGCT
TGCGCATGGTCAGGTTGACCACTTTGTCGTAGTTGGCAGCAAAGTCGAGCAGTTCGCTAATCTGCGAATC
TAGGGGGAAGCCCTTGGCGTATGCGGTCCGCTCCTGGATCTCCTTGCGCCGCTCTTCGTTGTGCTCTGGA
ATAGGACGCTCCGGATGGCGACGCAGGCTAA
>dme_circ_0000842_junction_seq
GGACGCTCCGGATGGCGACGCAGGCTAAGATGTCTCCGGAGCGCCCTCGAAGACAA
>dme_circ_0000843
GATGTCGCCGCCATCAAGCCACATACTCCTACCGCCCGGCGGCCGTCCGCCAGCCACTCCCAGCAGCATC
CCGGCCGGCAGCAGCAGCGGAGGGAGCCACAGCCACCAGGGGTCTCCGGCCACCAACTCGATGTCGCATC
ACAAACGGAGCCAGTCGTTCAACCACCATCTCAGCTACAATCAGTACACGCCCACTCAGCCTCCACCACA
TCATCTGCATCCACCACAACCACCAAGTCTGCCAAGTCATCTGGGGGCATCCGGCCCCAGATCAAGTTCA
GCCCAGACACCAAACAGCAAGACAGCAGCTCCACTCCAGGCGTCATCCATGGACGCCAGTCCAGCAATTC
AGGCAGCGGCAGCAGCAGCGGCGGCAGCGGTGGGAATGGGGGTGCCAAGCGCAAGGAAAGGAAGCACAAA
GGGCTAACTGGACCATCAGCTGCCTGCGACCCACGCCGCCGTTGCGACCCAGTTTATTGAATGCCACCAG
CGGATCGGGAAgtggcagcggtggcagcggtggcggcggCAGCAGCTCCAGCAATGCCCTGGCCAGCTAC
TGCAGCGGAAGGAAGAACCAGCCCACCTATGAGGAGGACGCCCTGGTGCTCCGGGTTTTCGAGGCCTACT
GTGCCGCCTATCAGAACAATGCCAGGAACACCATCCACTCGG
>dme_circ_0000843_junction_seq
AACAATGCCAGGAACACCATCCACTCGGGATGTCGCCGCCATCAAGCCACATACTC
>dme_circ_0000844
GTTATGATATTCTGTTGGTTTTCCATTTGGCCAAACAAAGAGTTTAATTTCTATTTGCGAGAGTAAAGTA
AATTAATCAACGTAAAAATGAATAATTACTCAATTACTGAAATATGTTTTGTTTGTAACTCACTCGTTGA
GGGCGTATGTGAATCCCTTGTTCGTTTGAAATTCCTCTCGGGACTTATGCAACCATATCTAAAATACTCA
AAGTGAATG
>dme_circ_0000844_junction_seq
ACCATATCTAAAATACTCAAAGTGAATGGTTATGATATTCTGTTGGTTTTCCATTT
>dme_circ_0000845
TTGTTCTTGTTCGCGTACTCAAACAAGCAGTACATGGGATTCAGAACCTCGTGGGAGAGCAAGAAGAACC
ACTCGCGAGATACACCACCATAATCCAGTCCCTCCTCGCCGCGGAATATGATATAGAGCCGCCTTCGCAG
TTCGTAGGCTGGCAGACGCATGATCTGGTGGTATGAATCCTCGAACAGGGTTTGTCGCGTCACCG
>dme_circ_0000845_junction_seq
TCCTCGAACAGGGTTTGTCGCGTCACCGTTGTTCTTGTTCGCGTACTCAAACAAGC
>dme_circ_0000846
GACTGAAGCCTGAATCGATAGACATACGAATAGCAGACTTCGACGGCGTCCTTTATCACATTTCCAATGT
GAATGGCGATAAAACAAAAGTTCGGGTAAGTGTACCGCGTACTATTCATTTAGGATAAATCCAACATTAA
CGATTTCTCACTTTTCTTGGGCTGCGAAACAGATCAGCATATCGCTGAAGTTCTACAAACAGCTGCAAGA
ACATGGAGCCGACGAGTTGCTGAAGCGTGAATATGGCAGTCTGCTTACAGACACGGAAGAAGGTACGTTC
GTGTTTAAGTTCGGCATTTAAAGAAGTTAATTACTCTCGTCTAATTGCAGGCTACAATGTTTCCGTACTT
ATCAACCTGGAGGAGATTCCCGAGGACTGCGAGCAAATCGCAAAGAGAATCGGACTGCTGAAGCGCAACT
GTTTCGCATCGGTTTTTGAAAAGTATTTTGACTATCAGGAGCAGGGCGAAGAGGGCCAAAAGCGTGCCGT

CATTAACTACCGCAACGATGAGACTTTGATATGTGGAAGCCAAGCCTGATCGTGTCACCGTCGTCTTTAG
TACCATTTTCCGGGACGAAGACGATGTCATTATCGGCAAAGTATTTATGCAGGAATTGAGGGAAGGTCGG
CGTGCCTCGCACACCGCACCACAAGTGCTCTTTTCGCACCGAGAACCACCATTGGAATTGGCCAACACGG
ACGCTAGGGTGGGCGACAACATTGGCTATGTCACATTCG
>dme_circ_0000846_junction_seq
GGCGACAACATTGGCTATGTCACATTCGGACTGAAGCCTGAATCGATAGACATACG
>dme_circ_0000847
GTTTGTTAATATTTGCAGTACGTTTTTAAATGCGCTTTCTACACATACAGGCTAATTGCGGGGATGAATC
CTCCATTGCGACTATATAACAAGTTGGTGGCATCCCTTAGTCGTGTTGCTCGCTTTGACATGGTAGTTTT
AGTTCTCATGTTGCACTTAAGTCTACGTTTATTTGTTTAAAATGCGGCTCAGCTGTGGCGCTGACTCGGC
CTATGACTGTAACAAATTGGCGGAGACACGGATTTGACGATGACTCGACACAAACACACACAATTCATTT
ACTAACGCGTAACTATTAATTCTTTGATCATTATTTTGCTGAGCTTACATTTAGCCTTACGGTTCTGTTT
GGAATACTTGGG
>dme_circ_0000847_junction_seq
CCTTACGGTTCTGTTTGGAATACTTGGGGTTTGTTAATATTTGCAGTACGTTTTTA
>dme_circ_0000848
GTTGCTGATAACGTATACAAGTGGCAATAACAGAATAAATTAAGCCGGAAAAAAGGTCGAAAGGCCAAAA
GTGAAATCCAATCCGGTGTGAAAACCAGTGCAAAAGTCAACAACTTGTGGGAGTCGAGCGTTCGTCCTCG
AGTCCTTGAATCCTGCCAGTGTGCTCAGTGCTTTAAGTGTGCCAAGTGTGCGTCTGCGTGTGCGTGAGCG
TGAGCCTGTGAGTGCGTCCGGGTAGCGGAGAAGCCACAACGTCGTCCACAAAACCATCGACGTCCACAgc
cgccgccgccgctgctgctgctgccgccgctgcAGGTTCGGGATCGGGACAAGGTTCGGGAGCGGGTGCG
GTATCAGGAACTGCTCCAGGCCACCAGAGGAGCGGCGGACGAAGCGGCCAGCGACGTCGCAGTAGCAGCG
GTAATCCACACGCCGGCACAACGAAAGCGACCAGTGGCAgcggtggagctggcagcgccggcggaggagg
aggcggcggcggcggCAGCGGTGGAGGCGGGGTCAAGCAGAGCGTCAGCCGGAACAGCAACATCCGGAGC
AGCAGCTCCAACAGCACCATGGCCTCTGCCAACCATCGCGGTGGCGGCGGACGCGGTGGAGGTGGCGCTG
TCGCCGGTGCCGGAGCGGATGGCAATGCAATCGTGGGCCTGCAGATCGGCTCCGCCTGGTTTCAGCGCAA
GATCAACCTGAGGCCGCAGCACCGCGGCGTCCACCTGGTCACCGAGGAGATACTCCGACAGATGCCGGAG
CTGGCGCACTTCTCAGTGGGATTATGTCACATGCAAA
>dme_circ_0000848_junction_seq
TTCTCAGTGGGATTATGTCACATGCAAAGTTGCTGATAACGTATACAAGTGGCAAT
>dme_circ_0000849
GCTGAAAAACACAAATGCAGCCATACAGAGCTACCGAAAGGCAGTGGAGGTCAACAAGCGGGACTATCGC
GCCTGGTATGGACTGGGCCAGGCCTACGAGATCATTAAAATGCACTACTACAGCCTGTACTACTTTAAAA
TCGCCCACCAACTGCGTCCCTACGACTCTCGAATGCTGGTTGCCTTGGGCGAGACCTATGAGAAGCTGGA
CAAGTGCGAAAATGCTGTGAAGTGCTACTGGAAGGCCATCGATGTGG
>dme_circ_0000849_junction_seq
AAGTGCTACTGGAAGGCCATCGATGTGGGCTGAAAAACACAAATGCAGCCATACAG
>dme_circ_0000850
AAAAATAAGGCGTCGCAGGGTCTTGCCATTGGCCTCCACTTCCTCGATGCGGAAATCTCCAGATATCTTA
GAGAACCCACAAATAAGAGTCTCCCTTTTGCCCACATCGGATCCAAGACTTAGATACGGTATCTGATCAG
TGAGTCCAGCTGGCGATAGGTTCTTTATGCTGTCGGCCAACTCTTGCTTCACTTCGTCTAAGGTACTGTA
AACCTGATCTCTATGAAGGGTAACCACAGCCAGTCGTTGGAAGTTGGCGGAGTCCTGGAGCTTTTTCCGG
CCCGCAGGCGTGGAAAAAATCCACTCCACCTCTCTTCCCTGAGGAACGATAAACGCCGCG
>dme_circ_0000850_junction_seq

TCTTCCCTGAGGAACGATAAACGCCGCGAAAAATAAGGCGTCGCAGGGTCTTGCCA
>dme_circ_0000851
TTTTCCCGAGATCATTACGGAGCTGCCAGCTGTCACAATAACAGAGCTCCCGCTGGATCGAGTTATGAAC
CGCCTGGATTCGGTTATACTTGACGGGTCCCCGGCCACAGCCGGAAACCACAGCGACGAGGAGCAGCATC
AGCAGCAGCAGCAACCTCATGAGGATCAGCCCATGCAAGTTTCGGAGGCTGACGAGGAAGTTCCTCAAAA
AGGACGAGCAAATGCCGAAGATAAACGATCCTGGCGGAGGCATTCAAGTGGAAGGAATGGTCACACCAGA
AGCTGCGACAGTGGGCGAAACCCAAGAGAGCAGCGAGGAGTTGCAGCCCGGATCAGCTGCATTCAATGGG
ACAGAGGGCACGGCTAACTTGACGAATGCCAACGAGGAAGTGCCCATGCCAGTGTTTTCCGAGTGGGCGC
AGAAGCAAATGGAGGCGGAGGCCAGCAGGGAGCAGGCCATGGAGTTGGAGCAGCAGGTGGTAAATAAGTC
CGCCCAACGCAAGAACAACACTGGATCTTCCAGTGGAAAGCCGCCGACGCTGAAGCTGCGCTCCAAGAAC
TACGCCTCGCCGGACTGCGGAGCCAAGATCATCGCACACAATTCCGAGTCCAAGCATACGGAGGCTGTGC
TAACCCAATCCACCGATGAGTACATGCTGAGTACTTGCGAAAGTCGGATTTGGTTCGTGGTGGAGCTGTG
TGAGGCCATTCAGGCGCAGAAGGTGGACGTGGCCAACTATGAGCTGTTCAGTTCGTCCCCCAAAAACTTT
ACGGTAGCCGTTTCTAAACGATTCCCCACACGAGACTGGAGCAATGTGGGACGCTTTGCGGCGGAGGACA
AGCGAACAATTCAGACTTTTGAGCTTCATCCACACTTATTCGGAAAATTCGTGCGCGTCGATATCACGTC
GCACTACGCCAACGAGCACTTTTGTCCGCTGTCGTTGTTCCGAGTGTTTGGCACCTCCGAGTACGAGGCT
TTCGAAACGGAAATCCGACCAAGCGATGACCTGGACGATTTCTACGACGACTACGGCGCTCAAGAACAGA
AGGCAGCCGTGGGCAGTGGTGGAAACATCTTTCAAAGCGCCTCGGATGCAGTGATGCAAATGGTCAAGAA
GGCCGCAGAGGTTCTCGTAAAGCCCACAAAGGCTCTAAAATGGTCAGAGGAATCGGTGCTCTGTCAAACG
CCAGCATTTGAAGCCTTCAGCTGTATCAATTGTAATACCACCCTGGTCGAGAGGATTAACAGTCTTTTAT
CCTGCCAGTTCCAGCAGCTGCAGGCACTCCTCAGTCTTAGTCACTTACGGTCAGATCTTCTAAACAGTCG
CGTGTGTCAGGAGGAGTTCGGCATAAGCCTTACGGGCTCGGAGTTCGCCAGTAAAATGGGTAAGGAGCAG
AGCTATTTCCTTAGTATGTTGCCAGCGGAGCACGTGGGAGCCATGTGTAAACTCATTCAGGCGGAGCAGA
ATGTGACTGACCATAACCATACAAAGGCGCCTTCTCTGAAGCAGCACGTAAGCTCACCAGAACCAGTGCA
GGATAACGCCACTGCTACGGGTGTTCGGCAGGATTGTGAGAACAGTAAAACGCCCACAAAGGAACCGCTT
ACACCAAGTCTTGAGGTTGTGGTGCCCGAGGTGAGCCAAGAAGTGCCTTCAATGGAAGACCAGTCGTCGA
CCTCCAGCGAAACAGTCTCCACAACGAACTCCACGCCTGCCGACGTCAATATATTCAATATGCCGTCGGA
ACCTGAGGAAGTGGTTGTCAAAGTTCAGCTTCCTCCGGAGCCAACACTTCCAACAACTTTGCAGCCCAGC
GATGTGGAATCGTTCACTGACGCTCCTAGCACAAATGCACTCCCTGGCTCTAGTGAAGCTGTAGCGAACG
GAGATCTGGGTATGGAGGAGGGTAATCCGGCTAACTGGGACGGCATTGACAACCTACTGACCACGACGGT
GGCTTCGATAACCGCTGGCGGAGGTGCAGCTGCCGCTGCAGCCGCCGTGGTGAACGGAAACGCCAACATC
GGAGGAGCCGGCATTGTCGGAGCAGGAGGACCAGCCAGTCTATCCAGTGTTAACATGCAGCAGAAGCTGA
CGAATGGAGCCCAGTCGGAGAGTGTGTTCATCAGGCTCTCCAATCGCATAAAGG
>dme_circ_0000851_junction_seq
TTCATCAGGCTCTCCAATCGCATAAAGGTTTTCCCGAGATCATTACGGAGCTGCCA
>dme_circ_0000852
TTAGACTTATCCTACGCCGAAGTTTTGCGCCGGGCCGATGCCGGCGAACATGGACCCACAAGATCACATT
CCCAGCGACAGCAACACAACGGAGCCAACATTCGCAAGAGTCATCATCATCAGTTTCGTCAAAAGTAAAC
TTGTTTAGAGAATGTAAATAAGCAATTAAACAG
>dme_circ_0000852_junction_seq
TAGAGAATGTAAATAAGCAATTAAACAGTTAGACTTATCCTACGCCGAAGTTTTGC
>dme_circ_0000853
GAGCTGGAAACCTTCATCAAGGAGTACAAGATTAAGGGGGATGAGCAGGAACTGGAGATCTTGCATCTGC
GCAAGCACATTAATGCGATGACCGAGGTGAATGATTCACGGCTTAAGGTCTACGAGCAGCTGGAAGTGGG

CATTCAGGATCTGGAGCGTGCCAATCAGCGGCTAAATCTGGAGAAGAATCGGGACAAGAAGCAGATTAAG
ACGCTGACCACCAACACGGAAG
>dme_circ_0000853_junction_seq
ATTAAGACGCTGACCACCAACACGGAAGGAGCTGGAAACCTTCATCAAGGAGTACA
>dme_circ_0000854
GCAAGGAACAGTGGAGTAACGAAGCGCGCAATAATTTTTAAGCAGTAAACAGCGAACGAGTTGGAGTTAA
AACGACTTGAAGCATCTTTTGTATAGCTCTTCGTTCTGTTAATCTTTAGGCGTAAAATTTAGCATTGTCC
ATTTCCCAGCTCTAGCAACGATTCCAAGAAAAGAAGAAATTATCAAGaaaaaaaaaaCTAAAATCCCTTT
AAATGTACTGAGCTGCATAACGAAGTACTTTAAACCCCGTTCTCTACTTGTAATATCAAAGCCTAACAAC
TGTAATTAGTGGCTCGTCTTAGCCGTAATTCGAGCAGGATTGAATTCACG
>dme_circ_0000854_junction_seq
CCGTAATTCGAGCAGGATTGAATTCACGGCAAGGAACAGTGGAGTAACGAAGCGCG
>dme_circ_0000855
ATTCCGGACCAATCGATCTACACACAGCCTATTCCTGGAATCGTTATGGGCGGCGTTCTACCCTTTGGCT
GCATCTTCATTCAGCTGTTCTTTATCCTGAGCTCGCTGTGGTCCAGCCAGATTTACTACATGTTCGGTTT
TCTG
>dme_circ_0000855_junction_seq
CCAGATTTACTACATGTTCGGTTTTCTGATTCCGGACCAATCGATCTACACACAGC
>dme_circ_0000856
GAAACGGAAAGTGAAGTAGAAATCATATAGAGAGGAAAAGAGCGAAGACATGACTTCCGATATCGCCATG
CAATTGATTGCCATTTTGGAAAAGACGGTGTCGCCAGGTGAGTTTCCCCACTGCTACAAACGTGTGTGTA
TAGATCCGAAAAATATGGATTTTTCTTCGATTGGCGGGAGGGACGCGCGTTATATGTTGCGCAGGGTTGC
AATAGGCGCTCGCATATGTTTGGTTGCTAGGGGCCTCCGCACACATGTGCATCCACGTACATACTCCAAG
GGAAACTTGGCGGCGCGTCTGTATGTATTCGCAATGCTTGCGCTCCTACAATTAAGGATGTACGAACCGC
CTGCGACATAACCTCACTAACTCTAGGAATCGATGAGGCGTCGCTTCGGAGTCACCACCGTATGCTTGGC
GGCCAGTTCTTCCGGTTGCTGATAAGGAATGCCTGCAAACTTGCCCCTTGCCTGAAGATTTTATGCTCCA
CCGCTACAATTCATTACTAGACCTTACTGCTTAGCTCTTCATATGCACACACTTCTTTCACCATCTATTG
TATTCTTATTAAACAATATTCTCACTATATACGCCTTGCCTAAAAGTTCCTATCTTGCCTTCACATTCCC
AAAAGTCAATTGTTGTAAAATTCAATGGCCTGATACCAAAGCTTTAAGCCGACCCAAAATGGTGGGCGTG
TGTAGGCCCACAAATAAAATCTTTCGTTGACATAATTGGAAATTAAAAATGTCTAACCAACCAAATGACG
CATTTTCAGGACAAAAATGAGCTGCTATCGGCGAAGAACTTTCTGGAGCAGGCGGCGGCCAGCAACCTGC
CCGAGTTTCTCAAGGCGCTGTCAGAGATACTGGTGAACACGGCCAACAGTGCGGTGGCGCGAATGGCAGC
CGGTCTCCAGCTGAAGAACCACCTGACCAGCAAGGACGAGAAGGTCAGCCAACAGTACCAGGATCGCTGG
CATCAGTTTCCCAGCGAGATCCGCGAGTTGATCAAGAATAACGATCCTGGCTGCTTTGGGTACCGAGAAC
ACCCGACCCTCCTGCGCCGCCCAGTGCGTGGCCTATGTGGCCGTGATTGAGCTGCCGATAAACCGCTGGC
CCATGCTCATCCAGACACTGGTGAACAAGGTGGTCAGCGAAGGATCCAGCGAGATGCATCGCGAGTCGGC
GCTGGAGGCCATTGGCTACATTTGCCAGGACATTCGTTTCGGCGTCATGGAGAACCAGTCGAACGATGTG
CTGACTGCTATTATTCACGGCATGAGAAAG
>dme_circ_0000856_junction_seq
GACTGCTATTATTCACGGCATGAGAAAGGAAACGGAAAGTGAAGTAGAAATCATAT
>dme_circ_0000857
GAATGTCTCGTCACGAGAAAACGAAATCCACGGGCGGCGGGCTCCTGGACAGTCTGTTCGGAAGACCCTC
GAAGTCCAAGGGAGGAACCATCAGCAGTGGCACCCTGGCCCATGGCGGACGACCCGTGTCCGCGGACAAC
TATGTGGTGCCGGGCGTGGAGGACTTTGAGCAGTACATCCAGCAGCTAAGCGTTGCGGAGCTGGATGCGA

AGTTTCTGGAGATCATCGAGGACATGAACATTCCGAAGGACAAGAGGGAGCCCCTGTTGGCCAAATCGAA
GGAGGAGCGACAGAAGATGATTATGTGGCACTTGAAAG
>dme_circ_0000857_junction_seq
CAGAAGATGATTATGTGGCACTTGAAAGGAATGTCTCGTCACGAGAAAACGAAATC
>dme_circ_0000858
GGAGTCGAAGCGGTCGGAGGAGTACGAGAAGAAGATCGAGCAGCTGGAGAGTGCCAAGCAAGAGGCGGAA
GCGAAGGCGGCTCATCTGGAGGAGAAAGTCAAGTTGATGGAGGCTAATGGTGTGGCGGCTCCGTCGCCCA
ATAAGCTACCCAAG
>dme_circ_0000858_junction_seq
GGCTCCGTCGCCCAATAAGCTACCCAAGGGAGTCGAAGCGGTCGGAGGAGTACGAG
>dme_circ_0000859
GTAGCGAAGTTGGAGTTTTCATCGATATGCATTGCTAATTTCTTGACATGAATTTAGTTTCAACTAGAAG
TTTTTTTATAGTATATTCGACTTGTAAAATTGGTACTTTAATATTGGGACAGTCATAGCAGTAAAATAAA
ATGTACAGCTGAAATTAAAAGGCCTGCCAATCATTTTAATTAAATAAAAGTGATCTTG
>dme_circ_0000859_junction_seq
TCATTTTAATTAAATAAAAGTGATCTTGGTAGCGAAGTTGGAGTTTTCATCGATAT
>dme_circ_0000860
GGCAAAACCCGCACCAAGGATAAGTACCGCGTGGTGTACACCGACTTCCAGCGCCTGGAGCTGGAGAAGG
AGTACTGCACCTCCCGCTACATCACCATCCGGCGGAAGAGCGAGCTCGCCCAGACGCTGTCGCTGTCGGA
GCGCCAGGTTAAGATCTGGTTCCAGAACCGCCGCGCCAAGGAGCGCAAGCAGAACAAGAAGGGTAGCGAT
CCGAACGTGATGGGCGTGGGAGTGCAGCATGCGGACTACAGCCAGCTGCTGGACGCCAAAGCGAAGCTGG
AGCCGGGCCTGCACCTGTCCCACTCGCTGGCCCACTCGATGAACCCCATGGCGGCGATGAACATTCCCGC
CATGCGCCTGCATCCCCACCTGGCCGCCCACAGCCACTCCTTGGCGGCGGTGGCGGCGCACTCACACCAG
CTGCAGCAGCAGCACAGTGCGCAGATGTCCGCTGCGGCGGCAGTGGGCACGCTCTCGATGTGACACGACC
ATTCCTGTTATGCGGCGAGCGGCGACAGTAACAACTACATGGAGCCCCATCATCCGCTGCTGGGTGGTAG
CGAGCGGGGTGGAgtaggaggcgtaggaggcgtaggaggAGGAGGCGGACAACCGTGCGATGTGTCCTTC
AGTGCTCCCGCTCTCGCCTTGGACTTGGCTTAACCCTTAGGTCGCGCAGTCAGACTGTTTCGACCGAACC
GAAAAGTTAATAGGCAGCCGGACGAATGGAGGACTTGGCGGCCGTTGCACCTGGAATATTGCACGTTGTT
AATTTTTGTGATTGTATATTCCTGGTTTCGACACGCGCCAGAGTCCTCACAGCTAAACAAGTCTTATATT
ATTCTTGTATTATGTTTGTTTTTTGTTCAACGTGTGTAGTAGCTTAAAGTAAAATGAATAGCTCGTAAGC
AGTAGTAAGTAAAGTTGCCCGAGAAAAACAAGAACAATTCAAACCAGCCGTCCCAGCCGATTAACGTTTA
AAAGTACTCGCTGCAGTTAAACATAATTTTAGTACAAGCAACTCATTTTAGAGCGCAACTTGATTCTTCA
GCCCGTGTAAATAGCCGCATATATAAAGAGTTTTAACGTTTATTACTTTTATGTATCGTAATAACAATTA
GCCGTAGTTAATATAGCCGTACTAAATGTTAAATGTAACGCACTCTTCGAGCACACAAGCAAAATTACCA
TTTAAATAAATAATTTTAACGTATTCACCCGCATCTTTACTTAGTTCAATTTGGCGGCAATTAATTTATA
ACTTGTGAATGTGTACGTGTGTATATGTATGTGCATAGCTATTTAAATAAAATATGAACACCATAGATAA
AGCAACTCCGATTATCTTGTCAATTATCGTCGATATCGAGTAGGCATCGACACAAGTACCAGCTCTAATT
CGCACGGAATTTGTTTGTCATTCAAGAAACAATTATAATTCGCCGCATTTTTATTCTAAAAGCGCTTACG
AATATGGTATCAGCCATCACTGAAAGTCCAGCCCGCCGAGGTGTCCGTGCTGAACGCCACCGGCCGTGTG
GGAATGCCCACCGAGGCCAACTGCCTGAACCAACTGGCCCACGTCCACCGGCTGCGCGACTCGGAGCTGA
ACTACAACGAGCACCAGTACAACCGCAACATGCAGATGCTGCGCAACCACGAGGGCCTCGGCGTGCCCCT
CAAGATGGGCATGGAGCGCTTCGCCGCACGTCAGGTGGGCCGCCTGCCCTTCCTTTCGTCCAGGGTGAGC
ATCTAGCCCGGTCTGGGAGCTTCTCAGTCTGGACAGGAATCTCTGCAAGGGCGGAAGCATTCAGAAATCT
TCTGTTTCCACATTATAAGAATCCCGGAGAAACCTGAGATATCCTACAATCTAGAAAATCTAAACTTCTT

GCCTAATAATGGATACCTATATACGTACATACATACATACGTGTATACCAAGTATACCTGTGATTCCCAT
AGTACCATTATTACCGAAAACCCAGCAATGACTACTTATCTTCTATATTAATTCTTTCCTTTATATTGCA
GGCAACTTCATGGACGATGTCCTGACTGGCCGCTGTGATTCCATTGGCTTCGAGGACTTCATGAACTTGC
CCGAGAACAGCGAGCATATGCGTCAGCCCCACGCTGTGGTGGAGAAATCTCTGGGAATTTACCAATAAGT
CCCTGCAAATGAAGTTGCTAAATGCCTGGCATTCAGCCTTTAAACTCTAAGACACTCTTAAGATCAGCAA
ATAAAATATATTTTGGTGCGACCAGCTAGAGGTAAACAAAATTCACCATTTCTTTGAGCCAAATTTATTC
GTATTTCGTTTGTTTAACTCCACATCAATACATTTAACTAGTTCGTTAAACTTACACATTTTATTCAAAC
AAATCTAAAAACTACACGAAAATATTGGCAGAAGGTTGTTGTACTGAGGAGATTCGTTAAAATAATAAAC
TGAAGAGATATGCGTTGACATCATTCATTATTTGTAAAAATAAAACAAGAGATGGTAAAATGGACGAATT
CGTTTCGTAAAATTAGGCTTATACTTAAGGAAAACTGAATTGTAAACGGAGGGCGCATTTCAACTTATAC
AAACATTGCATATATACATGATTTATATTTAAAGAGGAAAATGACAGTAATTGAGATAAACATAGTTAAT
AAAACGCATTACATTCCTCAAGCAAAACACGTCTAATCAACAATAAATATTGAAATCGCTAGCGTTTTAA
CATTCCGTTTTGAGCAGCGTGCGCGTACGGTGCTTTCCTCGCATGAAGAGTAAAAACAAATTAAATACCA
CTGTGGAATAACATCTCAGCGAACGAGTATACGTCGGAAACCGATTTGTCGCGCTAAGTAACACGAGATG
CTTTTGGGATTCAAAAAAACCCATATTTATGTGATGATATATTAATGTAAACCCACTGTTTCGTTTACAT
TTTCGTTTTTGAATGACTGGCTGCGGAAATAACTATAGATTCGCGAAAGTACACTAAATCAGGCAGTAAA
ATCGGATTGGCAATCTTGGGAAAAAATCGAAGGAAAAGAGACGCCAGCAGCTTCCTCGGATTGTTTTGTT
GTTTTCAGTTCGTTAGTGTGTGGCCGTTTTTCCGCCTGTGTTTGTGCAACAAAGTCAAAGTCGTCGAAGC
TGCTGCAAAAATACGTGAAATGCAAATTGTGAAAAAGTTGCAAAagcaacaacagcaactagcaacaacc
aacaacaacgcaacgacaacTATAACCGCAATCACACGGCCAACGCAACAATAACAACGGCCACAACAAA
ACGGCTTCGCGTGTTTTGAAAAATTGCATTGCAAATTAGTAATTAGCAACAACTAAAACAATAGCAATaa
aaaaaaggaaaattgcgcgccaaaatcgaaagaaacaaaaGGATTACACATAAACAAACCGAAGAAGCAG
AAGCAGCTGAGCGAAAGAGAGAGATAGCGAAAAATCAAAACAGAACAGGCAGAAATTCCTGTTTATCCAC
CTTCACCTCCGGCGACACCCCCCGTCGTG
>dme_circ_0000860_junction_seq
TTCACCTCCGGCGACACCCCCCGTCGTGGGCAAAACCCGCACCAAGGATAAGTACC
>dme_circ_0000861
AAATTCAGTTAAGTCCGGGCTAAGCACCGAAATTCCCGGCCAGTTCAACTTCAGCCTGAAGTCCCGCAGT
TAAGTCCGGGCCAGATCCTTTGGCTCATTTGCACAGCGGCAAGTGCCGGAAGGCTGCTGCCAGCAACAGG
GGCAGCATGAGGAGCAGGACCCGTGGAGTGGCGCCACTGGCTCCGTTGCAACCGTCCGTGGTGCAGGTGC
GGCAGTAGACGGTTTTTATGTAGGACGATGTCTGG
>dme_circ_0000861_junction_seq
GACGGTTTTTATGTAGGACGATGTCTGGAAATTCAGTTAAGTCCGGGCTAAGCACC
>dme_circ_0000862
GGATCCGACTGGCAGCCAGCTTGGATATTGTTGATGTCCCCGAAGTAGCAGCTCCTCACGATCTGCGGAT
GTCCGGCCACTGGAATGAGAGCAAATTGTTTGGTTTGGATGAGCAATTGGGCCAACAACTTTCTCTGGTT
GGGGTTGCTGAATGAACCCATTGTACCTTTGGCTTCGGATACTTGTTAATTGCTTATTTATTCAAGTCAA
AACACTGTAGTTTATTGTCAAGAAAAACAAAGATCAACAATTTGCTTCACACCTCGATGAAACATAATTC
GTTGCCCTGTTATCTCAAATGTCGTATTTTTCTACAAACTTTTTGTTTGATTCCTCTGAATAAATTGACT
AGATACGAAGCTGATTTGGCTTGGCGTTCAAAAACGATCAAAAACGATCACAATATTTGTGACGGATTAA
TTTATCGGGCGATCTGCCTTGTAAATAAATTCAGAAATGCTAAGTTTGATAAGTCGAATAGCTCGGAGCT
TGCGGCATTCTCTCTCGTCCCTGTCATTATTATTTTTACGAGAAAACGAATTAAGCTCTCAATTTGATGT
AATTGCTGGAAAATAAGACGCAAATAAAAGTGACTAGAAATCACGTCGAAAGGGAAAAAAGTAACGTCTG
AAAATCAGGTAGCGAAATATTCACGCATGACATCAGAGACTAATACAACCCGATTTTCTGCGATGATCTC

AGGGGCTTGTATTGCTTAATTGCGCTATTTCTAGCTTAATAATGGAGGAATCATAGAGTTTTCTTCTCAG
TTTTGCTGATTTATGAAATCTTAAGATTGTGTTTTCTTCCTATATGTTATAGTACTACAATATGATAGTC
CTAGGGACTGTGAACTCACCGCTTTCGAGGGTCTTCTTCATGCAACCAGTGGCGTTCCGCAGGGGGAAGA
AGTTCTGCAGGTAGCGTGGGGG
>dme_circ_0000862_junction_seq
GGAAGAAGTTCTGCAGGTAGCGTGGGGGGGATCCGACTGGCAGCCAGCTTGGATAT
>dme_circ_0000863
GTAATTTACTAGCTAGTTAAGATCTAGGCCAGCAGACGAGCCACGCCGAAGAAGAGCAGGATGGCTCCGG
CGATGGGGGCCAGGGAGGAGGATCCGTTGCACTCGTCCTTGGTGCAGACATCGCAGCCCAGCTGCTTAAC
GAACGGCATAGAGGGATCCGACTGGCAGCCAGCTTGGATATTG
>dme_circ_0000863_junction_seq
ATCCGACTGGCAGCCAGCTTGGATATTGGTAATTTACTAGCTAGTTAAGATCTAGG
>dme_circ_0000864
GCCCGTGCCATTACCTGCTACGAATGTGATTCCGTCAATAATCCAGGGTGTGGGGAGCGATTTGTGGGCG
ATGACATATCCACGACGGATTGCGATGTGGTGGCGAACATGCGATCCCTGGGAGCGGAGGCCACTTGCCT
TACCAAGTACCATGAGGGAAGTGAGTATACGAGTGCCAGGAGACACGCGCTTCGTGAGGCGCTCCTGTTA
CTTTGGCGATGCCTCCCCAATAGGCGTCAGTTGCGACGATGGACCAGATCCTGTGGTGCCCTTTATGAAC
TTCCTCGGCTGCACACTGTGCGATACGGATTTGTGCAACGCAGCCGCTGGATTATCCACACTACCCTTGG
TCATTGCCCTTTCGATACTCGGACTGCTTGTCCTACTGGCGACCTAAGGAAGATGAGTCGCGTGGAACGG
CGGCGGTCGTATTTCAATGTTTCCTATTAAAA
>dme_circ_0000864_junction_seq
GGTCGTATTTCAATGTTTCCTATTAAAAGCCCGTGCCATTACCTGCTACGAATGTG
>dme_circ_0000865
GCTTCATAGGTAGACATAAACAAAAACGGAGAACAATCAATAATTATTTTAAGATTCCATTCCATTTTCA
ATACCAACGAAATTCTTTGCTCACCTCCACCTAAAAACTGCACGTACACAAAATTTTTGCACACTTTTCT
AGTCAATTTTTATGTTTTACAAAAATAACATGGTGTGTCTCCTCCCATTTCCCTCTGGACGAACACTCAT
CTGCCTTTGAGTGTCTGCAAATTCCGATGGCTTATTGATCGTTGCACTACTCAAACGAATTGTTACATTT
AAATAAAAGAACTACAACGAATTGAGGGGAAGGGACAGGAGTGATCACAGATTGCAGAATGTAATGGGTA
ACACTCCACAACTATTTCATTGAGGAGCTGGTACTCCACAATTCCCGAACCCAAATCCCATACCCTAAAA
ATTATATTATAACCATCCAATATAACCCAGGTAACCTGCAATCAGTGAAAGGTGCAGCAGTGAAAAGCAA
ACTAAATATGAATAGGTTTTAAACAAACTCTGTGTGTTGTTGTTACTATGATTCAATGTATATGTGTACG
CACGCAGcattatataaacgaactacattatatacccaactacaAAACAAGTTGAACAAGTTGATCCATG
CAATGCAAATCTGTTTGTTATTTGTTTCTGACTGTGAATCGTGTTGAAACAATAAGTTTTACTATATTTG
GTTGACTAATGCTTACACGCTGATGATATGAATTGTTGATAGACTTTAAGACGCGGAAAGATGGACGACG
ATTGTTTGTGGCATTGACGCGGCAGTGGTCCTCGCTGGGATCGTAGAGATTCCGCCGGACAACGCCAGCA
TGGACAGTATTGGTGAAGCAAAAACAAACACAATCTTTTGTACATAAATTG
>dme_circ_0000865_junction_seq
ACAAACACAATCTTTTGTACATAAATTGGCTTCATAGGTAGACATAAACAAAAACG
>dme_circ_0000866
GTTATAGTTTATATGTGTAGTATCTGAGCATATCATTGGCGTCAGCAGCAGTACTAATCGAAAGAGGTTT
GGTTTTACTTTATCTCTATATTTTGCGACCAAATGGGACAACTTTCAGAAACATTCAGTTGTTTTCAATG
CAATTTACAAGTTACACTTTCTATACACGTAGCAAtaataatattaataacattaataacGAATTCAGTC
AAGTCAGTAAATTTGGTTTACAGAAAGAAAAATACAGTTTCATAAATTAAATGAAATAACGCTAACAAAC
AAAAATGATGTTGCAGCCCAAATTTTCCGGTGTGGTATTCTGCTTGAAATTTTTAAGGAATTTTTTCTTT

GCTCCGGGTACATGGCTATAGAAATACGTTTTACATATAAATATACACATTATAATAATTCATTTTATAT
ATACTTGTTTGTATAGATATCATGTATATACGTATGTAAAGGGGTTATAACAACTAGGGTATTGTAATTT
CAAAGCTTTGCGTTTTGAAACATGAACTCACAGA
>dme_circ_0000866_junction_seq
TTTGCGTTTTGAAACATGAACTCACAGAGTTATAGTTTATATGTGTAGTATCTGAG
>dme_circ_0000867
GCAAGGAACCGGCACTGCGCTCCTACACGGAAATCCAGCAGCTGCTGCAGCAGGGCAAGAAGCGCGACGT
GAAGAACATACTCAGGGAGAACTCGTGGCCCATTAACTCCCCGATCCGAGCCCAGCTATGGCCGATGTTG
TGTGGCCAGCACCAGACCAAACAGCAAATGCTAGATGGCTTCTACTGGGAGATGGTTCACCAGGGTCTTT
GGCACCACGGAGCTGTCGGAGAAGCCGATCATGCTGCCCGCCTTTGTGGATGCCACCCACTGTTTGCCCT
ACCACTTGACCAGCACGGGACGAGCCGTGGCCGATCGCATCGTGAATGTCCTGGGCTACGACTGCCCCGA
TATTACCTACAGTCCAGTATTGTATCCCATTACATCGATACTTTTGCATTTCATGTCGGGGTGAGTTATC
GCAGTGCCAGCACTCCAGCACACAACATTCCACACCTCTCCATCCGCAGCACACGTGAATGTCAATATTT
GATCGAGTCATTTTTATCGCCTCTACAAGGCACACAAAAATAAGTGGCACAATTGTAAGGCTCTACGCTC
CAGACCCTCGGTTAGCTCCCtatatatctataggtatatctatatttatatatctgtgtggtatatataC
GGGGGTTCTCGGCGCGTATCGCAGCTGGTTGCAAATTGATGTATCGTGATTTTCTACCTAATTGATTTGG
CCAACACGCAATGAGCCGCCAAATAATTTCTAATGTAAATCTTGTTATTCCTTTGTACTGTCTGAAGGTA
CAGTGAAAACCGGCATTGTAGGGCCGGCCGTTCAATCCTTGCCATTTACTTCAAGCAATTTCTGCTGCGA
TATGCAAATTCATCCTTGATTTGAGTCATTCTGTAATACATCCTGTAATATTTGTATAGCCTTGCGTCTG
CATTAGGTATACTTGTAGGTTGAACTGTATCGCACTGTACTTGACCATAGTCAAGTGATCAACGTCTAAC
TGTCTGCTTTAGTTCCGCATTTGTTGTTGCTTCTGAAAAATTGTCCGTACTACTTCATTATCAGTTAATC
ATTATTCATTGTTGTTATTGGCGACACAATAACAAAATCGAATATTTAACTTACAGTATAGATTGTTTAC
TAGGAATTTGGTTTCATCCAGTCAGGTGCGGAGTCGGATCGAGTACAAATGCGCTGAATTCCGCTCCATC
AATGATGGGAAAAATAATTGAATACGAAATGAGCAAAATTTAACCTAAAGGGATGTGAAAATTCGGAAGT
TTGGCCAAATAATCGCACACCGCGTATTTCGAGGTCTAGCTATATGAAATAGACAGCTTGTAATGCGAGT
GACCCATTGATAACCTCTTAATTGTGGCAATATGCATAATCTACAGGAATCTAATCAATTCCAATTGCTC
GGCCCTTAAATAGTTTTCATATTTTTAAAAAGTAAGACAATCCGTCAATCGTTCTATCAAACTTTTATTT
CTAAAAATATTTTCCGCTGCTCTCTGAAAATGATTTCAATTCGATTTCATATGTATACATATGTATGCAC
TTATATTCTGGAGATGCTGATAAGCTGATTTTTTGAGGAGGAGGTCAGACAGCCTGTCATTTTGAAGGTT
TATTTACACTGTGCGAAATACCTTCCCTCCTTTGTGTCTGTCTGGCAGACGAAACTGTTCGATTCCGGTC
TAGATTTCGAGGTAATTTCCCGCCCTCCAAAGAAATCTGATTTTTTCTAGGTCATACAAAAttttttttC
TTATATAAAACTGCTATTACttttttttttttGTGGTCATAAACATTGAAAGAAGTTGCAGTGAAATGAC
TCCaaaaaaaaaaCTGCCAAACCGAAGGATACTGGAATAAAGAAAATTGAAAAGTGAGCTGCGTTCAGCC
AACGCATTTAAATGTGGAACGTTTTTCTGCCTGCACTTTCAAGTTCAAGAGTCGCACATAAACGAATGTT
TAAACTTTAAAGTGGTATAGATAGCACATATGCATATGTCGGACTGTTGGAATTTCGCTGTTGACGTCAT
TGCATTCGACTCGGTTTGATTCGTCTTCTTTTGCAATTGGCGAATTTGCCTTTAGCCACTTAAAGTAAAC
AGAGCAAAAGTCGAAGCAGAACATTGAAATATTGGCATGTTTCAAGGCCTGGGGATACGTTTCTTAGTTT
ACTGACACACTTCTGTGTGTCTTTAATAACTTTTGCATATATACTCATGACGCTCCTCTAATTTACTTTT
GGAAAAGCATCACTCATCACACTGGTTTTTTGGGATTCGTGTGCTTTTAGTATTTCTTCTTTTTGTTCTA
GAATCTTGAAATGGTTCTTTGATTTGCGTTGTGCTTGGCTAATAATGCCAACACGTGAGCATAAAACTCG
CATAAAACGCAGACAGAAGACAAGGCAGCAGCAGATAGGCAAATGCGCACATATGTGCTCCCCATCGTTT
ATGGATTTGTTTGCCAGTTCTATTCTCCACCGTTCTGGCTCCCCATTGTTCTGTTTGTGTTTTTGCGCCC
TACCCAAACATTGTCGCTTTGGTATACCTGGCTTACCCGATTCCTATGCGGAGTGCTCATCATCACATCA
AATAGTTGGCAATGCCGGGCACGCACAATTCAGATTAAAGTATATGGAATATTTCGCAGTACTTGGCTTT

AATGGCCCAGCACTCTGACGTCAGACTTGACTCAGGTAGAGAGTGAAAATTGGAATATAGCCCTGCTTTA
ATTTGCATTTCTCTGCCCTTTCCCATGCATATGCATTAGCCAGCTAAATAAGTGAAATGGAGGCGGGTTT
TCAAGGTTCCCCAGGCGTAAAAGGGGGATTTGAATTGTATCTCTTTGTTTCGCGGCACTTTACGGGGCAA
AACACAGGTAAATAGTGTGTATCTTATCAGCTTCATTCAGAAAGCTCTAAAATCCATACCGTCTATTTCT
TATAGAGCTATTTCCCAGTTTCTTCATGCCTACTCAGATTTGCTTGTCTGCTGCTCTCCGCCGATTAGCA
ATTCAAAAATGTTATTTTTGCTTTTATCTAAGTCTATTAAGTGGGCCGTGTGTAAGTTCCGCTTTTGTTT
TTGTCTTGCCATATTTACATTGTTTATGCTTGAGTTTGTTGAGCTAATTAAAGTAGGAGAGCAGATGGAC
TGGACTCCTGGTTTCTGTTCGTGTATCTCGGCTGTGGGTCTTTTGGCTATCTTTGGCTATTTTTAGTTGG
CTTATATCTCATAAATAACTCGACACGTGCTCTCTGACGTCTACCGACTTAACATTCTGTCTCATTAGCC
AATCGACCTTTGTCTGTATTCAAAAGTAGCTTTGGTGATGCCTTTGGTTTCTGGTATTCTGTTTCTTTGG
CACGCTCGCGTTTACCACAGTTTTAAATCGTTATTATCAGTTACTTGATTAATTTCAATTGAAATTGTTT
ACTTTTGCTTTCGCGCCGACTGATAATGAGTATGTGAGGGGTCGTTAAAAGTTTGACCAGAATGCAAATG
TTGGCGAATTATTGCAATTGCAGTGTCTAACGCTCGTCATAGTCTATATATAGCGAGGCTCTTGAATATT
TAATGAACTTATCAAATGTTTGCCCGGACTCCAGCTGTCTTTCCGCAATAGATAAAGTTCAGCACACTAT
ATGTACTATACTCCAGACCCTGCCCTGCATCCGATGCCCTGGCAAATGAGAGCAATCCGGGGAAACTAAG
TCGAACCGCCTGGGGCCAGTGTAATCCACTGATAAGCCGGCGAAATCCGCTGATAAGACCTTTGTTTACC
CCGCGGCGTTTGAAACTAATTAAACTTTGTAATGCAGCAAATCAATCCGATGACTCCTTATCGCCCTGCG
AAGGTTCAAGTCGGTTCATTATCTAAATTAAATTTAAAGTCCTTTAAAGTCCACACCCCAAACTGCGCTC
CCTCCCGTTGCGCGCACAATTACTTTTTAAATGCCTGCCTAATATTTTCACGAACGCTAATGACTCTCTC
ACGATTGCCTGAAAAACAGCTCGAGCAAAACTCCCAGAGGGTATTTATAGCTCCAACACTGTGACATTGC
ATAATTTGATCTAATTTAGAAATCAAATAATCAGAGCGCACATACACAGGTTGCTGAAAAAATGGTGCCG
GCTAATTATGGAAAATTGAAGAGGGCGTGGTTGGGGCGGGGAAATGGCGGTGATGCTGGTCAAAGTGCAC
TCCACCGACGGATGCTCGACGCATTCTCAATTTAATTTGCGCTGCGTCATTGCGGTAATTCAGTGTGTAT
ACATACATACATATATGTACGTACGTATATGTAGATATGCACACCAAACCAAACCACCACGAATGTTATA
AGTCGAGCTGCTGCAGTTCCACAATTAAATTGGACGCGGATCAGGAAGGGAGGGGGTGCGGGGGTGGTAA
CTGCATAAATTGTGGCTAAGGTTAGGGCTGTTATTAAATAGATAATTGCTAGAAAAACTGCAATACGATC
TTGCGTACGAGTGCCAACGGGCGTATCAAATATCGAATATTACGTATACGACACGTTGGTATTGTAAGCA
GAAAATGAGCGGATAACTATTAAGGCAATTATATTCGAAATGAAATTATATTAGCAATATTTCAATTAAT
AATAAGAACTACATTTCAGTACATTTAAAATGAAGTCATTAGATTCTTTATTAACATTCGGTACTAATAA
TGCATTTTGATATAATTCATATTTTCCAGAAGGAGGAGGCGTACATATGTCTGGCCGGTCTGGTCGGTAG
CAAGGAGAAGGTATTCATCAATCAAACCAAACTACAGCACGAAGTCACTTGGAAGACGGTGATGCAGATA
GCCAAAAAGCACACAGAAAAGTGCTACTTCGTATTTCCAACGTATTTGCCCGGGCCTGAAGCTAGAACGC
ATCTTCATGGACTGGTGCTGGTGGATTCTAGCCGGTCTACCCTTCCAGCATCTGGTGCGGATCATGGACT
GCTACTTCCACGAGGGCATCAAGGTCCTGTACCGCGTGGCCCTCGTCATACTCAACCTGTTTCACAAGGA
GTGCCAGTCGAACAACGAATGGAGTCCGGACAACATTAAAAACGACATTGGCAACGCTTTGATCAAGTTC
TGCAAGAAGATACCAGTGTCGCCGGCGAAGTTGCTCCATGCCGCGTTTAGTATTAGGGGACTGAGGTACC
CAGTATATTTCTAGAATATTTATCAAGACTGAAATGCTACTAAAAAGCCGCTCCGTGCTCACCAGCGGTT
CGAAGCAATTAATCAAATCGCGTTCAAGTGATAATCTGCCCACTAGTCAGTCGCAGGTCAACATCCAAAT
GATGTCCCACACCCTGACCATCCGAGAGG
>dme_circ_0000867_junction_seq
ATGTCCCACACCCTGACCATCCGAGAGGGCAAGGAACCGGCACTGCGCTCCTACAC
>dme_circ_0000868
GCAAGGAACCGGCACTGCGCTCCTACACGGAAATCCAGCAGCTGCTGCAGCAGGGCAAGAAGCGCGACGT
GAAGAACATACTCAGGGAGAACTCGTGGCCCATTAACTCCCCGATCCGAGCCCAGCTATGGCCGATGTTG

TGTGGCCAGCACCAGACCAAACAGCAAATGCTAGATGGCTTCTACTGGGAGATGGTTCACCAGGGTCTTT
GGCACCACGGAGCTGTCGGAGAAGCCGATCATGCTGCCCGCCTTTGTGGATGCCACCCACTGTTTGCCCT
ACCACTTGACCAGCACGGGACGAGCCGTGGCCGATCGCATCGTGAATGTCCTGGGCTACGACTGCCCCGA
TATTACCTACAGTCCAGTATTGTATCCCATTACATCGATACTTTTGCATTTCATGTCGGGGTGAGTTATC
GCAGTGCCAGCACTCCAGCACACAACATTCCACACCTCTCCATCCGCAGCACACGTGAATGTCAATATTT
GATCGAGTCATTTTTATCGCCTCTACAAGGCACACAAAAATAAGTGGCACAATTGTAAGGCTCTACGCTC
CAGACCCTCGGTTAGCTCCCtatatatctatagg
>dme_circ_0000868_junction_seq
CTCGGTTAGCTCCCtatatatctataggGCAAGGAACCGGCACTGCGCTCCTACAC
>dme_circ_0000869
GCAAGGAACCGGCACTGCGCTCCTACACGGAAATCCAGCAGCTGCTGCAGCAGGGCAAGAAGCGCGACGT
GAAGAACATACTCAGGGAGAACTCGTGGCCCATTAACTCCCCGATCCGAGCCCAGCTATGGCCGATGTTG
TGTGGCCAGCACCAGACCAAACAGCAAATGCTAGATGGCTTCTACTGGGAGATGGTTCACCAGGGTCTTT
GGCACCACGGAGCTGTCGGAGAAGCCGATCATGCTGCCCGCCTTTGTGGATGCCACCCACTGTTTGCCCT
ACCACTTGACCAGCACGGGACGAGCCGTGGCCGATCGCATCGTGAATGTCCTGGGCTACGACTGCCCCGA
TATTACCTACAGTCCAGTATTGTATCCCATTACATCGATACTTTTGCATTTCATGTCGGG
>dme_circ_0000869_junction_seq
CATCGATACTTTTGCATTTCATGTCGGGGCAAGGAACCGGCACTGCGCTCCTACAC
>dme_circ_0000870
AACATACTCAGGGAGAACTCGTGGCCCATTAACTCCCCGATCCGAGCCCAGCTATGGCCGATGTTGTGTG
GCCAGCACCAGACCAAACAGCAAATGCTAGATGGCTTCTACTGGGAGATGGTTCACCAGGTGGGATCCAT
ATACCAGATTTCACCCTTTTGTCCCGTACTTAACTAATGTGTGTTTTCCTAAAATTCAGGTCTTTGGCAC
CACGGAGCTGTCGGAGAAGCCGATCATGCTGCCCGCCTTTGTGGATGCCACCCACTGTTTGCCCTACCAC
TTGACCAGCACGGGACGAGCCGTGGCCGATCGCATCGTGAATG
>dme_circ_0000870_junction_seq
CGAGCCGTGGCCGATCGCATCGTGAATGAACATACTCAGGGAGAACTCGTGGCCCA
>dme_circ_0000871
ACTCGAAAACGCCGACAATTGGGATTTGATTCACACACCGCTATGCTTTTGCAGTACTCGTGCTAAAACG
TTTACTATAATGAATTCCGCGCGGCACCATCGTATTGGAGTATTTCTGTCTCCATAACTTGGCCACCGTT
TACATAAAATTAAAACTTAAATATAATAGTAATATTATACTTAGCCCGACTACAAGCCCAAACAAGACCC
GCATATCGGTTGCCACACCGCGGACAGCAAAGCACTGCAAGGTAAAAAGCCATCTAGTCAAACAGAGACA
ACGAAGGAAAGCCGCCAACTGCCTGGAGGGCAGAACGGATCCGAGTGGACGCAAACCCAAATCAAAACTT
GACAGAGGACCCGGATCCAGAGACCGCAAACCTGGCTAAGAAGATGCCATACCATCGAGGCGGAGATGCC
TCTTCCCAGGCCGACAAGCTGAGCGGCATCGTGGAGGAGAGCGATCTGTACGAAGGGTTCGCCCCGCATG
TCGAGACGTCCGAGATCAAAACCCTCGACTTCTACAATTTACCAAAGCAAACTGG
>dme_circ_0000871_junction_seq
ACTTCTACAATTTACCAAAGCAAACTGGACTCGAAAACGCCGACAATTGGGATTTG
>dme_circ_0000872
GAAATATAGTTTTTATGCAAGGAATCTCCAATAATCTCTTGAGTAGAATCTACAAGTCCCTAAAAATGAG
GTTGCACATTTCGTTGTTTTTTTGGTTGAGGGCAAAAGTGCAGCCGGCAGTTTTGTGGGAGAGTGTAGAG
CGCACATGTTTGGCATAAGGACGATCAACAGACTGCAGGAAAACCGAACGAACGTTGGTTTGTCTGTGAA
GGAGGCTTTCAATTAGCATGTGAGTCATTAAAATAGTTCTAGCAAACTTACTTGAGAAGCTGTTCAGGTC
GCTGGAGTTGCATGCACAATTTAATGCCGCTGTAGAAGTCCAAGTCCGCATTCAGACTCAACTTTGGTTC
GTGGGTGATGCTGAAATCCTGCACTAATTTCGCATACGTAAAGCCAACGGCCAAATGACCAAGAACAGCG

CTGCCAGTGCTATAAGCCAAAAGTTAATCGTGAGTTAAATAACATTCCAATCAAATGCTTTGGCTTTTTC
ACTTACTTCTGCTGGATTTCCGTCTGGGCATTTCGATTCCACAGGCTGAATCCCACCTTGCCATTGAGAT
CCACACTCCTGGCGCCCAGAACTCGCCAGTGGAGCGTCGCCCCGGAGGTCAGGATTATGTAGTGCTCGTT
ATCCTGGGACAAAGTGGTTGCCTGGTAAGCGGGCGTTGAGTCCGAGGCACTTCCTCCCCACACGTGACCC
ATTAGCTCGGTCTGACCGCTGAAGAATACTAGGGGTCGCAATTGAGCACCCTGCACACTGATCTCCATGC
CCGCGGTGACCGCTTCGTCTTCGCTGAACTCATCATCCGCCGGGATTGCATCATTTCCATCGCCGGAGTC
ACCATCGCCTACCAGCGAGCCCAAGCCGGCAGTGTAGATGCCCAATTTAAAGCTGCTGGCTTGGGAACGG
CCTGCGTGGAGCAGAAATTCCACGGAGCCACGTTTCAGGATTCCCTGATACACTTCCTGG
>dme_circ_0000872_junction_seq
TTTCAGGATTCCCTGATACACTTCCTGGGAAATATAGTTTTTATGCAAGGAATCTC
>dme_circ_0000873
CTGAGGGAAAGCGTTATCCAAACCGTGGCCACCTTGACCCGCCAAAGCGGACTAGATGTTGAAGATCCTT
TGTTGAAGGAAGTGCGTTCTTATCTACTGCAAGGTCTCACCTCCAAGGAGCCTACCTTGTACATAAGGGC
TCTGCAAAATCTCCAGGATCCTGCTACCATTGAAGCTCTGCTGGAGCACGCTCAAACTGGCGAGGCTCCC
AATTTATCGG
>dme_circ_0000873_junction_seq
CAAACTGGCGAGGCTCCCAATTTATCGGCTGAGGGAAAGCGTTATCCAAACCGTGG
>dme_circ_0000874
GGTGAGACCTTGCAGTAGATAAGAACGCACTTCCTTCAACAAAGGATCTTCAACATCTAGTCCGCTTTGG
CGGGTCAAGGTGGCCACGGTTTGGATAACGCTTTCCCTCAGCTTTAAATGCTTTTTGATAGATTCCTGTT
CTAGCAATCCAAACAAGTGCTCAACTATTTTCCTGTCTGGATGGGTGGCGACCGCCAGGGACTGCAGGTA
TTTCTCTAGCAGATCCAATTGCTCGCTTGTGGTTTCCGACTCCTTGTAAAGGAATCCAAAGGTGGCATTA
TGAGCATCGAACGTTTGAACAGCACCCAAAAGATCCACCAACTGGGGCAGAACTTCTGCGTGTTCCGTAA
GTAGATCTTCAAATTGTTCTTGCCTTGTGATGCGAGCCAGGGGAATTAGTTTTACGTACGCAAGAGCCAG
CGAGGATTTGCCCACGTCGGCGGATTGCAATTCCGTCAGAGAGGCCTTTAATTG
>dme_circ_0000874_junction_seq
GCAATTCCGTCAGAGAGGCCTTTAATTGGGTGAGACCTTGCAGTAGATAAGAACGC
>dme_circ_0000875
TTACTCAGCAAGCCCAGGAGACGGTCTTCTACGAGCTCTCTTCAGAGGGCACACTCTTGCATGCAGAGTC
TCAGGAGAATCACCGCCTCAATCTAGCTGCCAAACCGGATGTGGGAAGCTTCGTGAAGAGTTCGTTGATT
CTGCAACACGTCTCGCAAGGATCGGAGGAAG
>dme_circ_0000875_junction_seq
CAACACGTCTCGCAAGGATCGGAGGAAGTTACTCAGCAAGCCCAGGAGACGGTCTT
>dme_circ_0000876
TGGCCTGGAACAAGATAACCGGAATCGGAGTGGACGTCCACGTCCACCGCCTTTCTAACAGGCTGGGCTG
GGTGCCGAAGCCCACCAAGGAGCCCGAGCAAACCCGTGTGGCGTTGGAGAAGTGGCTGCCATTCAGCCTC
TGGTCAGAGGTCAATCATTTGTTCGTGGGATTCGGACAAACCATTTGCACCCCAGTGAAGCCAAATTGTG
GGGAG
>dme_circ_0000876_junction_seq
CACCCCAGTGAAGCCAAATTGTGGGGAGTGGCCTGGAACAAGATAACCGGAATCGG
>dme_circ_0000877
GATCTTTACTACATTGCCTTCGGCTATCCCGAAAAGGAGCTCAACACAGACATTGCACTGATCACAGGTG
GTGGCAACGGACTTGGTCGCCTTCTTGCTGAGAGACTGGGAAAAATGGGAACCAAGGTCGTTATCTGGGA
CATCAACAAGAAGGGTGAGTCTTTGGATCACGCGACATTCAAGTGGGTCATCAAATAATTTCATCATAAT

CCCTTCCTGGCAGGCATTGCGGAAACCGTCCAAATTGTCGAAGAGGCGGGTGGCTATTGCAAGGGCTATG
TGGTGGATATATCCAAAAAGGAGGAGGTCTACAAGGCAGCCGATGTCATCAGAGACGAAGTTGGTGATG
>dme_circ_0000877_junction_seq
GATGTCATCAGAGACGAAGTTGGTGATGGATCTTTACTACATTGCCTTCGGCTATC
>dme_circ_0000878
GCCTTGTGAAGATCGGGTAGAACGTTCTGGTAGACAGCGATTTCCTTTTCGAACACTCCAAAATCCTCTA
GGTATTCCATTCGCGACTCATTACCCACGGGCGCCGACTTCGAGAAGAATGTGAGCTGCCGGACCTCCTC
GGAATTGTGAAGCTTCAGAGTGACGTGCAGGTAGAGGTGGCTGCCCAAG
>dme_circ_0000878_junction_seq
GACGTGCAGGTAGAGGTGGCTGCCCAAGGCCTTGTGAAGATCGGGTAGAACGTTCT
>dme_circ_0000879
GTTGTTCCAGCATCTCCAGCAAAGAGCTGTGATTCCACAAGCCACGAAGCTGAGGCTTCGGAAATTGGAG
TTCCGACTGCATCGCCTGCACACGCGACACCGCTACAAAGCTAAGCAGCGACTAAGTCATGTGAACAATC
GGCGGCAGAAATTCGAGAACCTAGTGCCACAAGATGTTATGAACCTGTTCAGTGTCCTAAAGCTGGATGA
GAGTAGTAAGCCGAAAAAGGAAAAAACAGGCAAATGTAAGCTGAATGGCAACAGTAAACCCAACACTAAA
GCCAAAAAAATCCCTGCTAGCGCGGCTCCCTTGCAGGGGCTGGCGCAGATTTCACATGTGCAATTTGGAG
CTGGGGATCAGGCTCATCTGGTGGCGGTCTGCACTGAATCTAGGGTGCTCATATGGAATTTGCTAACGTT
GCGATTGCAGGCGGGCTTAAAACTATCCGTTCGTCAGTTGGCCTTTGATCCGCTCACCAACTTAATGGCT
GTAATTACTAAAAACGATGAGTGTGCATGTGTTTCAGCCAAATGTTCCGCTGCCCCTCTATCAGCGCAGT
AATCTACCCAAAACACATGGCTTGGCCTGGTTGCCTAGGAGACAGCCCAGGCAGAGCTCTATTAATATCG
ACTGGCAGGCGCAGTCAACGCTGCTAATGCTGACCCACTCGCAGGAGATCGCTTACCTGGCGCTACCGGG
TCAGAGTCCGTCGGATGATGCGCCTACACCGATCAGTTTTTCTCAGCCCGCCGCAACAGACATTATTTTG
AAGCACGCGACTTTCGGAATCCATGTGATCAAGCCGCAGCAGGCTTCCGGTGAATCCTTGGAGAAAAACG
GACCACTGATCGTGGGACGGGAAGAACACAGTGCCGTTAACGCGGGTAAGTTTAAATTTTAAAATGCCTA
CAATGCCGTAGTTTAGTATACTTACATTTTTATTATCCTCATTGTTTGCTTCCATCTTCCCAGTCAAATT
TGCAATTTTATATATTTAGTCGTTTTATTGAAATCTTACTCAACTATGAAGTACAATTGGTTAGACTTAT
ACTAAGTTCCAGAAATTGTATATTTGGTAGTATATATTTGATTTAGCTTTGGTCTGGTTAGAAATCCTTT
AAAAAATGTTCTTTTAATTGATCTTCTGGTTCAGTCGTTTTGAGTATTGTGGACGATTGTCTGTGGGCCT
TATACTATTTCTTCTTTCGCTTGAGGACATCGCCCAGTCGTGGCGCTGTAGGGACTCGCAAGTGGCGACA
GCGGCGCATCATCAGGACATCATGCTTAAAGTAGCGCAGGCAGTGCCATTGAGGCACTTTCGGCATCAAT
TGTTGGTGCTGGAAGAGTCGTTATATGGGGGAAATATAGTTAATCTTGGCAAATATTGTGTTTTAAAGAA
CGGTATAATATTATGAAAACGCACCAACAACGGAAAGGGTTTCTATGGCATTTACATGTTGTTTTCCCTC
AATATGACGTCAAATTGTTTCGCCACGAGTCACCAAActtttgtgccttttgttttttagttgcggtttt
cttttgtgctttgttaTCTGCGCCCAAACAGTGTTCAACTTAATTTAAATAATTTCCATTGAGATTTATA
ACGTAGTTTTCTTAAAGCGCGTTTTATTTTTGGAACCGCCTTTCAATTGTGTGTTGTGTAAATCGTGTGA
GTTAAAGCGGCCTAGCAACTGAACAATATTAGATTTAAGCTCTTGCCGTTAGGGTTTTTGCAAAGATCTC
CCGGAGGTGATCTTCACATAAGACGTTCTATCACATGATACCCAGAATTCTAAAATAATGATTAGACAAA
AATATTGGTTAGTATTTAAACACTCAGCTGAACTTGACTCCTCGAAATTGGGGTAGTCCTTCCAAAGATC
ATTGCGGTAATCAGTTGTTATTTTTATAATACATTAATTACTTTGTTTATTCCATCAAGGGTCAAGTCTT
CATGGGAATTGATAAGGGATCGTTTTCATATGGAATATTATATAAACGGCTGTCTCGTTGAGTCTTAAAA
ATAAAAAGTCTTCTCTCCTATATGTATGTAGAACTTAAGATTAATTACACACGCTACAAACGTGCACGAA
AATCCTGGTTGTTATCCTTTACAACCAGGCACACTAATGACTAATATGCTGGCAAGTTTTCCAGATGGCA
TACAAGAATATTTAGTTAGATACATTGATGATAGGCCCATGATATTAGTTGTAGCAGAAAATATTCTAAT
CATTTTCCTCGGTTGCAGTTTCGGGTTTTTAATCGTGTAATAACTTTGAAATACAAGTGTTTAGTTTTGA

AGTAGGTGTTTCACCGAAATGTGTGGCAGATGTTACGTAAATTATTTATGTTGCTGAACCTTCACTTGCC
ATATAATGTTTTCTTGTTATGGATTTGTTCCCCACGCTGTTCTGAGTTCAAACGCAATCTTTTAATTAAG
AGTGCACTCTGCACTCTGTAGTTCTCGCGGTACTCGACGCCCACTTTTAATTAGCGAGTTTGCACTTCCT
GCAGAGTGCAGCTGGCATCCATTTTATTTTGGAATGCGAGTAAAGTCATTGCCCCAAATTCGTGCCCCCT
GCTGTACTACTCCGTTTTCGCCGTGCCCCGTCGGCCCCCTGTCTTCCGCTCACCTCATTCACAATTGGCA
CAGGTCCAGCTTTTGCCCAAGCGAAGAATGCAAGGCACAGTAGGGAAACGACAGCCAAAAAGTTGGGCCA
CATTTTCCGATATTCCCAATCCGTTTCCGGATGCGGGTGCGGAGGAGATGTGTTCTTAGAGTTCTGCAAT
GCCTTTGCAATGCACACTGCACCGCCCGAAATGtttttccttttttttttAGTCGCTCGTAAATCGTTTAA
TCTGGATTCTGGCACTTTGTCAGTTTCAGGCGAGGATGAACCTTTGTCCGCCTTTATTTCCGTAAACTTT
AGCGGCACGCGCTTCCGGCTCTCTTCTTTCCGGCTAACGGTTGTTGCTGCGGAGCACAACGGTAAAGTGT
TGAGCTGTCGCTGCGGCTCTCGGCAATTTATACTCGCAGCTCGGTACTTCGGCTCCGAACATCCACTTCG
GCTTCGGCACGGGCTTCGGCTTCGTCCGCTCTCCAAATTCTTAtttttttttttATTATTACTGTGAGTC
GAGTGAAGCTCGCTCTTTTGTGCTCTTTGCCACGATGACTTGTGCACTTGCCAGTGCTCCCATTGTGAAT
GGGCAACGAGGGGCTGGTGCAAACGAACCGCCACCCACCACTGCACAGTGGGACGGATACttaaatcatt
agattcaatattaaacagtagattaaaattaaTAGTTTTCCCACTTTATGTATTATAGAATAAGTTTTCC
TTTTGAATTCTGGTATATCAAAATATGGTTTTCAGTTaaaaaaaaaacaaaaTACATTATATACCTTACG
GAGAACATGAAAATAATATCTAACGCAGCTAAGTAAAACTAAGAAATTAAAACTGAATTTTTCTGTGCTT
AAATATACAATGTTATAACTGTGCACTTACCAAATAAGGATTATGGTTTCGTGTTTTCTAAATGTTTCGT
ACATTTATTTCATAATAAGTGTTCCTAATTCCTGTGCCATTGTGCCATGAATTAAGCCCACCGACTCTTC
CCCTTCTCTTATATTTACCATTGAAGCTTGTTGCGAGAATTTTCCATTGCGTGCTTGTGACACTTTAGTT
TTCCCCAATAGCACTCGATTGCGTTGGGCCAATAATTAAGATCGCTTTGTTCGGCGTCGTAAGTTTTCAC
ATCACTGTTACTACGAGCAATATTCTACTTGCCTTAATTACTTTTGCCAGTTACCGTATTTCTTCGACGA
TTTATACCAGTTTTAGTCGGTTTCTGAAGCTGAACTAAACTCAGGCTTATCGCTTTGAGTCATCAATAAG
TGTGTCTTCCTGGAAAAAACAATAATATTTTGCATCTTGCCACTTAACAAATTGACAGACATGTGTGCAC
TTGGATTGTTTTTGCCAATTTTGTTTGGTTTGCACTGAAATGGATCAACGATATCCACAGGCGACAAGCG
CTTGCGCGAAGGTTAATAGACACATATCACTCGGCACATTTTAAAGACTCAGCAATGACAAAACAGAAAG
CCGAACAACAATCACGTGAACTTGAACCGCAATCTCGACACCCAAAAAATCAAGTTTTACGTACACACTT
GATAGTGTTGGTTCTTCGCCCTATTAAAAGCTACGGACAGTCGTGATAGTACTCTACAAGAGGTAAACTA
ACATTGATTGAGTTCTCAACTACATGAGGTCAATAATTAATGCACAATCGTTAAAAGAGCACTGCATTAT
TATCATTTAACACTTTTTGAGCATGCTACCTAGTTTGATGTATAAAAAAACTTTATATTTTTTCTAGTTA
CTTTTACTCTCAAAAAAAATCGGGCAGCACAGCCAATAGTAACACAGTTTTTGAACTTCAGCAAGGTCAAT
GTCAATTAATGTTATATTTTGTGTTACAGTTTTGTCAATCTTTCCGCCCACACGATGCCTGCCATGAGCC
TGATTTGTGGCGAATTCGTGAAATCGCTGCTTATTCCGGCGGACTCCACCTCGAGGCACTCATCATCTGC
CGCGAGTGATGCCGCCTTGACCAATGGGGTAACCGTCAATGGCAATGGGGTGGGCACCAGGACAGTGAT
GGGGAGCACGAGGACGAGGAGCTGGGTGAGGAGGCAAGGAACACGCTGGACACCCGGAAAACGCTGCTGG
AGCAAAATGAAAAGCTGGTGGACCTGCCGCTTGAGGGAGAAACTGACCTGCAGGGCCTTGATAATCGATT
AAGGACCATCACGGCGTTGAGGACGAAACTGAGGATTTGAACGTAAACAAATCATTTAG
>dme_circ_0000879_junction_seq
AGGATTTGAACGTAAACAAATCATTTAGGTTGTTCCAGCATCTCCAGCAAAGAGCT
>dme_circ_0000880
GATTTGATAGTAGGTCCTCCTGCCAAAGCCAAGCTTGTGAGTGAACCAGACAATACCACCAAGGAGACGG
TATTTAAGTACATAATGAAAATGCCGGAACTCGGCTTGCATCAAAAATTGCAACTGCTGCGTCGCTTTGG
AGTTGAATGTTCTGTCCCATTGGCCCATCGGCAAAGG
>dme_circ_0000880_junction_seq

TTCTGTCCCATTGGCCCATCGGCAAAGGGATTTGATAGTAGGTCCTCCTGCCAAAG
>dme_circ_0000881
CGCGGAACGATGTGGAGTTGCCTGGCGCAGACTAGTTATCGAAACCTTCCGATTGGTTCCTTGTGTTTTT
CCCAGGATGGGTCTCTGTTGGCCGTGGGTTATGGCAACACTTTGGCACTTTATGATGCGAGGAATCCACG
GCTTCCTCTCCAAACCCTCAGCTGTCCTCCCGGCTTAGATGGTGTTATATCCAAAGCTCAACTGAGATTG
GCCCAAACTCCACTTAATGGTGCTAGGAAAGAATTTACCCAGCAAAGACAGCAGTTGTGGGCTCTGCTGA
AAACGCTGCTAAATAGTAATGATGAAAATTTAATACAGCAGGCAAAGGATTTGATAGTAGGTCCTCCTGC
CAAAGCCAAGCTTG
>dme_circ_0000881_junction_seq
GTAGGTCCTCCTGCCAAAGCCAAGCTTGCGCGGAACGATGTGGAGTTGCCTGGCGC
>dme_circ_0000882
GCACCGTCGTCTTAGTTATAGCCGACACGCAGTAGATGACAGAGATTTGTCAACCCAGGTGAGTACCTGT
CCACCCAAAACACCAGCAACTCGCTTCTGCCAATTGCATATCTGTTTGAAAAAAATTCTAACCCAAATGT
TGCTCTCTTGCAGGTCAATTACAAGATGCAAATGAACTTCAGGCGGATGTTTAATGATCGAAAACATCAC
AAACGCAGCAAACGTGGTGCCAGCAACAAGGTGAGCGCCTTCATTTATTTTGTATTCGAAGAATTCTATT
TTAATGGATACCGCCACTATCCACAGGAGGCCAAGGAGAACGTTAAGCAGAATCATGTCTCGTTCCATGA
CTTTCAACAGAACGGCACCACTAAGCAACTCACCAATG
>dme_circ_0000882_junction_seq
AACGGCACCACTAAGCAACTCACCAATGGCACCGTCGTCTTAGTTATAGCCGACAC
>dme_circ_0000883
GCACCGTCGTCTTAGTTATAGCCGACACGCAGTAGATGACAGAGATTTGTCAACCCAGGTGAGTACCTGT
CCACCCAAAACACCAGCAACTCGCTTCTGCCAATTGCATATCTGTTTGAAAAAAATTCTAACCCAAATGT
TGCTCTCTTGCAGGTCAATTACAAGATGCAAATGAACTTCAGGCGGATGTTTAATGATCGAAAACATCAC
AAACGCAGCAAACGTGGTGCCAGCAACAAGGTGAGCGCCTTCATTTATTTTGTATTCGAAGAATTCTATT
TTAATGGATACCGCCACTATCCACAGGAGGCCAAGGAGAACGTTAAGCAGAATCATGTCTCGTTCCATGA
CTTTCAACAGAACGGCACCACTAAGCAACTCACCAATGGTACGGAATCGAGTTCAAACCATTCACGAAAA
AAATCTTACAGAAAAAGACATACTCACAATTCACTTAACAAATATCGTAGATTAGAAATAGGGTACGGAA
TCGAGTTCAAACCATTCACGAAAAAAATCTTACAGAAAAAGACATACTCACAATTCACTTAACAAATATC
GTAGATTAGAAATAGGTAAATGACGCTAAAATCAGCAACAAATTTTTATACAATCGAACAGCATATTGTT
CTAGTCGAAAAAATGTCCGTAAAGTTTATCTCGAATCAGTTCGTAGAGTTAACGATCATAAAGTTCTTAT
TATTTTCTTTAAGTTTCTCATTGTACAATGATCAGACTGCGCTGTCTATTTTTCGTGCGATTCTTAGTTT
GTAGTGAAAAGTGAAGTAAATTTTAAGTTCCCAGTAAAGTTTCATGCGTCATACTAACCTAATTCACGAT
ACTTTTAGTTTCCTTGTAAAGCACACCGTTAAAACCAAGTTTTAAttttttttttATGTTCTTCATAAAG
GAGTTTGTTAGGTTTGGCAAAATCATGTCATTGCGTTACTGCATGTCTTCTAATTTCATTTCCGTGTACA
AATAATGAATACTTTTATGAGCTCCTTTAAACCTATTATCAAAATCGATTTCATAAGGaaaaaagtaaaa
aaaaaaTTGGTATTAATTTGGTAACTAATTTAATTTCATCCGAATCCTTCTCTTTCTCCCCTGCATCGCG
CTTCCTTCCAACTGATGCCAAAAACCACACCCATTTGGCCAAAAACCAAACTCGAAATTTGTTCACTAAT
CGAAATCAATAACCCAACCTGCAACACACACTCATAATTATCCTGACTTGCCCGATTGTTTGTTTACTCA
TTCCGAACCATACAAAGACTATATTAACAATGTGCTTAATGAAACAGCCGAGGAGTGCCAACAGAATCCC
AACGAGATCAATGTTGTTGGCCCGAGCGACGATTGGGATGATGGCCTGACCTTCACCGCCAAATCATCAC
>dme_circ_0000883_junction_seq
GGCCTGACCTTCACCGCCAAATCATCACGCACCGTCGTCTTAGTTATAGCCGACAC
>dme_circ_0000884
GTGGCCGAACATCCCATTCCCGAGGAGGATCGTAATCTGTCCCGCGAATCCGACGGAGAAAGGCGTGTGG

CCACACCCACCGCCACGGAATCCCAATTGCCGTGGAAAAGACAGGGTGACGAATGCACGGATGCAGTGCA
GCAGAACGAGTTTCCCGCTTGGGCCTCCAACAAGGAGTACTTGGCTTACAATTCCCCCAGTGCAACATTC
CTAGGGTAATGTATACATACCCACATTAACCAtttttttttttttGTGACAATAACTCGGTGGGAAAAGT
TGACATTCACACAAGAACTTGGAAATAGTTATGAGAGGGCTCTCCTAAGATTCTTCTTTTTGTGTTGAAT
ATTTATGTCCTCAGTTTATTACTCTTTGTGAATTGGCAAAAACCACTGGCTGGGTACACAGAAAAAAAGC
GGCGCTAGTTGCCTTTGTAAAATTTTGAGATCCCATGTTAAATGACGAATTTTTAAATTACTTAAATACA
TAACTGCAATCGCTCTAATAAATACATACTCGTCAAGGTTGCTTGTTTTCTGTGCAAGCCCAACCCACAT
TGTCTATATCAATGTGGCTTCTTACTTGTCTAAATGTAAATAGCTTAAATGTTTGTATTTTGACCACAAA
TGATATAATGATACAAATGCTTACTTATATCCGATTGCAAAATTGCACCCAGTTTTTATGTGGCTTTACT
GATTTGATGTTCATTTCGTTGGGATTTTCCTAAAATATTCGTTTTTCTATCTCTCTTTTTCCCGCATTGG
TATTCCCCGATAACTGTTGCCCACTTCTACACCCACCACCCACCCACAACGTGCCACACGATACACCACA
CGAACTCCCTGTAAAAACCACCGAAAACGCGTACAACGATCCCGCAACAAAGGGTGGTATAAACAAGCCT
AAACAGCCCAAGTCCGTCATAGGTCTCTTCCGCCGTGAGAGTTCCAGCTCGAAGGCCGGGAGCGTTGGCG
TCGGCAGCACAGGAGCCATGGACTCCGCCGCCAGTGGCTCCGAAACGATGGTGGTGTCCATGTCCAGCCA
ACCGCCCAACGCTCCATCGACGTCCATCCACAATCCGCGGCTGGACAAGCGCTCCCAGTCGATCTCCTCA
AGCTCGCTGGGCGCCGGAGCCCATCAGCTAGGTCCGGAGGGTCATTCCGGCCCATTTCCGGTTACGGCCA
GTCATCGGCGGAACGTGCGCAGGGGCTCCATGCTGGAGCTAAGCGG
>dme_circ_0000884_junction_seq
GCAGGGGCTCCATGCTGGAGCTAAGCGGGTGGCCGAACATCCCATTCCCGAGGAGG
>dme_circ_0000885
GAGAGTTCAGTATACAACATATTGGGGATGGAAGTGTGGAAAATGGTTTAGTTTGAGGAGAATACATGGT
GCCTTACTATATTAAAAACAGTTAAGCAATACGAGTCATTTTAAGATTATGAATTATGTACAGGTATTAT
ATATGTATAGAGACTGCAT
>dme_circ_0000885_junction_seq
AGGTATTATATATGTATAGAGACTGCATGAGAGTTCAGTATACAACATATTGGGGA
>dme_circ_0000886
CCTTTATCATCTTCACCACTTAGTGCCGCCTCGCTGTCCCCTTCCTTATGATTGTTATCTTCATCTTCCT
GCTCCTCCTCGCTACCTAGATCCTCCTCTTCGTCACTCGTTTGTAGCTGCGTCTGTTCGTCCTCTGAAG
>dme_circ_0000886_junction_seq
TGTAGCTGCGTCTGTTCGTCCTCTGAAGCCTTTATCATCTTCACCACTTAGTGCCG
>dme_circ_0000887
GAGGACGAACAGACGCAGCTACAAACGAGTGACGAAGAGGAGGATCTAGGTAGCGAGGAGGAGCAGGAAG
ATGAAGATAACAATCATAAGGAAGGGGACAGCGAGGCGGCACTAAGTGGTGAAGATGATAAAGGCTCCGA
GGACGACGCAGCCGAGGAACAGAAGCTAACCTGGAAAGATCTCGGGTCTCAATGAGGCTTTGTGTCAAGC
ATGTGACGAATTGAAGTGGAAGGCGCCTTCGAAGATCCAACGTGAGGCCATTCCGGTCGCCTTACAGGGC
AAGGATGTAATTGGCCTAGCGGAGACCGGTTCCGGCAAGACGGGCGCCTTCGCTCTGCCAATTTTACACG
CCCTTCTCGAGAATCCGCAGCGGTATTTTGCCCTCGTGCTAACCCCCACGAGGGAACTGGCATTTCAAAT
TGGCGAGCAGTTTGAAGCCCTTGGGTGAGTTTTCGCATAGTTTATGTGCTTTTAATTCGCAGTGATGTTA
AACCTGTTTTGATTTTGTGTATCAACGAAAATATGAAATTATAATTTTCTTATTCGTCACTACCTCTGAG
TAAAAAAACCTTCCCATTTATCGTATTCTTGAATTTCCTTCTGCCAAACTTATGCTAGCTCTGTTTTTTT
CAGGGTAGCGGGATTGGCATAAAGTGTTGCGTGGTAGTCGGCGGCATGGACATGGTAGCCCAGGGTCTTC
AACTGGCCAAGAAGCCGCATATTATAATTGCCACACCGGGTCGCTTGGTTGATCATCTAGAAAACATGAA
AGGTTTTAACCTTAAGGCTATTAAATACTTGGTCATGGACGAGGCGGATCGCATTCTTAACATGGATTTT
GAGGTGGAGCTGGACAAGATTCTGAAGGTATTGCCGCGTGAACGACGAACCTTTCTGTTCAGTGCGACCA

TGACCAAGAAAGTAAAGAAACTGCAGCGTGCCTCCCTTAAGGATCCCGTAAAGGTAGAGGTGTCTAACAA
>dme_circ_0000887_junction_seq
ATCCCGTAAAGGTAGAGGTGTCTAACAAGAGGACGAACAGACGCAGCTACAAACGA
>dme_circ_0000888
GGTGATGGCCCTTCAAGAACGAGTAGCCGAAGCACAGCGCACTGCAAAACTGGAGCTGAAGGATCTGGAA
GATACAAGGGGTGGTCACAAGAGGGGCGGGGACACCCACGATGATTCGGAGAACTTCACGGGCGCACGCA
AACGCATGAAACCGATGGGCGGCACTGGCG
>dme_circ_0000888_junction_seq
CGCATGAAACCGATGGGCGGCACTGGCGGGTGATGGCCCTTCAAGAACGAGTAGCC
>dme_circ_0000889
AAAACTTCCAGCTGTGCTATACATGCTCTGTAAACGATCAAGACTAGCACAGGCGCCTTCAAAGGGTTAA
TGCTTTTAATTTGAAACCTAAAAAAGACCCAATTTCATTTAAAAGTTGTCGCCAATATTATCGGACAATA
AACCTTAACGAATGGCTAAATATTATGGAGTACTAAAATAATTATTATTTATTCGTGATGATACATTTGA
AGACCAAG
>dme_circ_0000889_junction_seq
ATTCGTGATGATACATTTGAAGACCAAGAAAACTTCCAGCTGTGCTATACATGCTC
>dme_circ_0000890
GTCCACGATTCCTAAGCACTTACGAAGCTTGGCTGGAGTTATTGGAGAGCGCCACGACCTCACTAGACAT
AGCATCCTTCTATTGGACTCTGAAGGCCGAGGATACGCCCGGGGTGTCGGACAACAGCACTCGGCCGGGC
GAGGACGTCTTCGCAAGACTCTTAGCCAACGGAAACGGCGGTAGCAG
>dme_circ_0000890_junction_seq
TCTTAGCCAACGGAAACGGCGGTAGCAGGTCCACGATTCCTAAGCACTTACGAAGC
>dme_circ_0000891
GGTAGACATTGTGGCCGGTGGCGATCTGTAGCACGCACAGCTGATTGTCTCCGCACACGCTCTGCATCCA
TTCAGAATCCAGGTATATGACGCACTCCTGCTGCAGATGGTATAACATACGATCGAACTCATCAGCCTTG
TTCACAATGATAAGGCACTCGTCGGGCAGGTCCATGGTCAG
>dme_circ_0000891_junction_seq
GGCACTCGTCGGGCAGGTCCATGGTCAGGGTAGACATTGTGGCCGGTGGCGATCTG
>dme_circ_0000892
GTCACCCAATGGTCCATCAGTCTGCAGTTGACGCACGAGTTCGACATGCTGGAACTGGCTTTCCCGCTGA
TTGCAATTGAGAAACTACCACTGGCCGAGGAATATCTGGACCATGCCACACAGCAGAGG
>dme_circ_0000892_junction_seq
ATATCTGGACCATGCCACACAGCAGAGGGTCACCCAATGGTCCATCAGTCTGCAGT
>dme_circ_0000893
GGGCCAGGAGCAAAGGATCCTGATGGCCCATTAAAGCCCTTCCAAATTCCTGGCGCAGATGTGGCGACTT
TTTCTTGCTATACATGTTCCACTTCTCGCGGAACAGCTTAAAAAACTCCTCCGCCTTTGCGTCCAAGGTG
GCGTCAAAGTTACAGCCGTCGATTCCAGCACCTGTCGTGATGTCCTCCAAGCGCTTGATCTTCAG
>dme_circ_0000893_junction_seq
TGATGTCCTCCAAGCGCTTGATCTTCAGGGGCCAGGAGCAAAGGATCCTGATGGCC
>dme_circ_0000894
GCGGTTTACAACTGGCAATTTATTAACTCGCTGCGCTTGTGGGCAGATCTCCTGGGAGCAAGTGCGAACA
AACCACAGTTGCAGCCTCTGATTTATCCCCTTGTTACCATTGCCACCGGAGTGATTCGACTGATACCCAC
CGCTCAATACTTCCCATTGCGATTCCACT
>dme_circ_0000894_junction_seq

GCTCAATACTTCCCATTGCGATTCCACTGCGGTTTACAACTGGCAATTTATTAACT
>dme_circ_0000895
TGAACAGTGGAGGGCACAGCCACCTTTTTCAGTCCGGAGGAGATAAACTGAAGCAGAGCCATCTGTGCAG
TGGCATCCTGCATAGCCACACGATCGGGGCGAAGACGCAGGTACGAAGTACCGCGCACGATGTCCTGG
>dme_circ_0000895_junction_seq
GTACGAAGTACCGCGCACGATGTCCTGGTGAACAGTGGAGGGCACAGCCACCTTTT
>dme_circ_0000896
GCCACCCAAGCCACCACCGTTGGGGGTGTGTGAGTCAGTACCAATCATCAGCAGGCCGGGGAAAGCGTAG
TTCTCCAGAATGATCTGATGGATGATTCCGCTGCCTGGCTTCCAGAAGCCCAATCCGTACTTTGCACAGG
>dme_circ_0000896_junction_seq
CAGAAGCCCAATCCGTACTTTGCACAGGGCCACCCAAGCCACCACCGTTGGGGGTG
>dme_circ_0000897
GGTGTCTAGGTTGATTTCAATCAGCTCATCGTACTCGCAGTTCTTGTCAGCGGACAGAATCTTGGCCTGG
TACTTCTGGGCTTCGGAGGCAATGCCAGCACGGCCTGTCGATTTCAGGTAATCAGCCATGCGCTGGTTGA
AGGGGAACAGAGAGGTGGTAGCTCCGATCTCAGCACCCATGTTGCAGATGGTGGCCATGCCTG
>dme_circ_0000897_junction_seq
CCCATGTTGCAGATGGTGGCCATGCCTGGGTGTCTAGGTTGATTTCAATCAGCTCA
>dme_circ_0000898
TGAATGGTCCGTTAACATGGGGCTCCAGGGTGTCTAGGTTGATTTCAATCAGCTCATCGTACTCGCAGTT
CTTGTCAGCGGACAGAATCTTGGCCTGGTACTTCTGGGCTTCGGAGGCAATGCCAGCACGGCCTGTCGAT
TTCAGGTAATCAGCCATGCGCTGGTTGAAGGGGAACAGAGAGGTGGTAGCTCCGATCTCAGCACCCATG
>dme_circ_0000898_junction_seq
GGTGGTAGCTCCGATCTCAGCACCCATGTGAATGGTCCGTTAACATGGGGCTCCAG
>dme_circ_0000899
GAATCTTCGACTTGAGGCCATGGCTCATGGCATCCTTGGCGATGCTGGCGCAGCGACCCATGTCCTCATA
CGAAGAGTTGGTGCAAGAGCCAATAAGACCTACACGGATGTCCATAGGGTACCCGTTCTTCTTCGAGTTC
TCGCCTAGCTTGCTGATGGGATGGCCCAAATCGGGAGTGAATGGTCCGTTAACATGGGGCTCCAGGGTGT
CTAGGTTGATTTCAATCAGCTCATCGTACTCGCAGTTCTTGTCAGCGGACAGAATCTTGGCCTGGTACTT
CTGGGCTTCGGAGGCAATGCCAGCACGGCCTGTCGATTTCAGGTAATCAGCCATGCGCTGGTTGAAGGGG
AACAGAGAGGTGG
>dme_circ_0000899_junction_seq
CGCTGGTTGAAGGGGAACAGAGAGGTGGGAATCTTCGACTTGAGGCCATGGCTCAT
>dme_circ_0000900
GAATCTTCGACTTGAGGCCATGGCTCATGGCATCCTTGGCGATGCTGGCGCAGCGACCCATGTCCTCATA
CGAAGAGTTGGTGCAAGAGCCAATAAGACCTACACGGATGTCCATAGGGTACCCGTTCTTCTTCGAGTTC
TCGCCTAGCTTGCTGATGGGATGGCCCAAATCGGGAGTGAATGGTCCGTTAACATGGGGCTCCAGGG
>dme_circ_0000900_junction_seq
AATGGTCCGTTAACATGGGGCTCCAGGGGAATCTTCGACTTGAGGCCATGGCTCAT
>dme_circ_0000901
GAATCTTCGACTTGAGGCCATGGCTCATGGCATCCTTGGCGATGCTGGCGCAGCGACCCATGTCCTCATA
CGAAGAGTTGGTGCAAGAGCCAATAAGACCTACACGGATGTCCATAGGGTACCCGTTCTTCTTCGAGTTC
TCGCCTAGCTTGCTGATGGGATGGCCCAAATCGGGAGTGAATGG
>dme_circ_0000901_junction_seq
TGGGATGGCCCAAATCGGGAGTGAATGGGAATCTTCGACTTGAGGCCATGGCTCAT

>dme_circ_0000902
CTCGTCGGTCAGCGGGTTGAAGTCCAGACGACCGGCGATGGACAGAGCGGTGACCAACTCGGGACTAGTG
ACAAAGCAATGGGTGGCCGGGTTAGCGTCGTTCCTGCCAGTAAAGTTTCGGTTGTAGGAGGTGACAATGG
TGTTCTTGTCGCCCTTCTTAACG
>dme_circ_0000902_junction_seq
AATGGTGTTCTTGTCGCCCTTCTTAACGCTCGTCGGTCAGCGGGTTGAAGTCCAGA
>dme_circ_0000903
CACCAGTAAGCTCGTCGGTCAGCGGGTTGAAGTCCAGACGACCGGCGATGGACAGAGCGGTGACCAACTC
GGGACTAGTGACAAAGCAATGGGTGGCCGGGTTAGCGTCGTTCCTGCCAGTAAAGTTTCGGTTGTAGGAG
GTGACAATGGTGTTCTTGTCGCCCTTCTTAACG
>dme_circ_0000903_junction_seq
AATGGTGTTCTTGTCGCCCTTCTTAACGCACCAGTAAGCTCGTCGGTCAGCGGGTT
>dme_circ_0000904
GCTTACTGGTGCTGATGGCAAGAAGTTCAAGCTCAAGGCTCCCTTCGGCGACGAGCTGCCCGCCAAGGGT
TTCGATCCCGGCCAGGACACCTACACTGCTCCCCCACCCGTAAGTACATTTAAATTCCCAAACTGATTTA
TATTGTTCTTAAGAATCAATATGGAAATCCCACAGAGCGGCGAAAATGTCAAGGTGGCTGTCGACCCCAA
GTCGACGCGTCTGCAGCTGCTGGAGCCTTTTGACAAGTGGAACGGCCAGGATCTGACCGATCTGACTGTG
CTGATCAAG
>dme_circ_0000904_junction_seq
TCTGACCGATCTGACTGTGCTGATCAAGGCTTACTGGTGCTGATGGCAAGAAGTTC
>dme_circ_0000905
GGCTCCCTTCGGCGACGAGCTGCCCGCCAAGGGTTTCGATCCCGGCCAGGACACCTACACTGCTCCCCCA
CCCGTAAGTACATTTAAATTCCCAAACTGATTTATATTGTTCTTAAGAATCAATATGGAAATCCCACAGA
GCGGCGAAAATGTCAAGGTGGCTGTCGACCCCAAGTCGACGCGTCTGCAGCTGCTGGAGCCTTTTGACAA
GTGGAACGGCCAGGATCTGACCGATCTGACTGTGCTGATCAAG
>dme_circ_0000905_junction_seq
TCTGACCGATCTGACTGTGCTGATCAAGGGCTCCCTTCGGCGACGAGCTGCCCGCC
>dme_circ_0000906
GGGAAAGTGCACCACTGACCACATCTCCGCTGCCGGACCCTGGCTGAAGTACCGTGGCCATTTGGACAAC
ATCTCCAACAACATGTTTATCGGAGCCACCAACTACGAAAACAACGAGATGAACAACATCAAGAACCAAC
GCAACGGTAGCTGGGGAGGAGTTCCCGACGTAGCCCGCGACTACAAGGCCAACGGCATCAAGTGGGTGGC
CGTCGGCGATGAGAACTACGGCGAGGGTTCGTCCCGCGAGCATGCCGCTTTGGAGCCCCGTCACCTTGGT
GGTCGTGCCATTATC
>dme_circ_0000906_junction_seq
CCGTCACCTTGGTGGTCGTGCCATTATCGGGAAAGTGCACCACTGACCACATCTCC
>dme_circ_0000907
GTGAGAGGCAGAAGACCCTGTTTCTTCAGGTTGGTCTCGTGGATACGGGCGAAGGACTTGACGATAATGG
CACGACCACCAAGGTGACGGGGCTCCAAAGCGGCATGCTCGCGGGACGAACCCTCGCCGTAGTTCTCATC
GCCGACGGCCACCCACTTGATGCCGTTGGCCTTGTAGTCGCGGGCTACGTCGGGAACTCCTCCCCAGCTA
CCGTTGCGTTGGTTCTTGATGTTGTTCATCTCGTTGTTTTCGTAGTTGG
>dme_circ_0000907_junction_seq
TTGTTCATCTCGTTGTTTTCGTAGTTGGGTGAGAGGCAGAAGACCCTGTTTCTTCA
>dme_circ_0000908
TGCTGCAACGATAAAGAAATGAACTCAAACATTTTTCTGGGCACAGCAGAGAATGGCCTGCGGCATGATA

AGATTGTTATACTTGATGCGGGAGCACAGTACGGCAAGGTAAGCTTAAAACCAAGAGTAGTTTTAACCTC
TTCTTAAAATGGTCCTGGTTTCTCCGCAGGTTATCGACCGTAAGGTACGCGAACTCTTCGTTGAGACGGA
TATCCTTCCTCTGGATACGCCAGCTGCCACGATACGCAACAATGGCTATCGAGGCATCATCATCTCCGGC
GGACCCAACTCAGTCTACGCTGAGGATGCGCCCAGCTATGATCCCGATCTGTTCAAGCTAAAAATACCTA
TGCTGGGCATCTGCTACGGCATGCAGCTAATCAACAAAGAGTTCGGGGGCACAGTGCTCAAGAAGGATGT
TCGAGAGGATGGCCAACAAAATATCGAGATCGAGACCTCGTGCCCGCTCTTTAGGGTGAGCCAGGAAATA
TTGATTCCCAAAACAAGCAGCAACAAGAAAGTGCTTAGGCTTAAATGCGTTTACCTAAGCGCTGATACAA
TGCTGCCAGAACAATTTTAAAGCCAAGAACAGTTTAGTTTAGAGTAGTTTATAAACTGATCTACACGTGG
CCTAACATATTATCTATCATTACCATTAATTATTCTAATACATGCAATTAACAAACATAACATCTTCGTT
TCCCGCAGTTCGCCTCAGTCGCACACAGTCCGTGCTGTTAACCCACGGAGATAGCGTTGAGAGGGTAGGC
GAGAATCTGAAGATTGGTGGCTGGTCTACAAACCGCATTGTGACAGCCATTTACAATGAAGTACTACGCA
TCTACGGCGTACAGTTCCATCCTGAGGTGGACCTCACTATCAATGGCAAACAGATGCTATCGAACTTCCT
GTACGAAATCTGCGAACTGACACCTAACTTTACCATGGGTAGTCGAAAGGAGGAGTGCATACGCTATATC
CGTGAGAAAGTGGGCAACAATAAGGTGTTGGTGAGTTTGGGAAAATAAATTTCAATCAAGACATGAATA
TCTCAATACAATCTACATTTTACAGCTCCTGGTCAGCGGCGGCGTGGATTCGAGTGTCTGTGCAGCTTTG
CTCCGCCGTGCTTTGTACCCTCATCAGATAATTGCCGTGCATGTAGATAATGG
>dme_circ_0000908_junction_seq
AGATAATTGCCGTGCATGTAGATAATGGTGCTGCAACGATAAAGAAATGAACTCAA
>dme_circ_0000909
GGTAAAGAACTTCCTGGAACGAAAGTACAATGAGTACGGCAGTGATATAAACAAGAGACTGAAGCAGCAG
CAGGAGAACGCCTACGAGTCCAAGGTGGCGAGGGAGGTGGATCAGGAGCTTAAGAAGTCTATCCACGTGG
TTACATCCACCCCGCAACCCCTGAAAAATCCGCATAATCCTATTAAACGGCAATCGGCGGTGAGCACCAC
GTTTCAACGTCCTCCGCCAGTCGCTGCCGCCGTGGCATCTACATCCCAGTCAAGTGCTCCCGTATCTGCT
GTTTTTACGGATCCAGTCTTCGGACTGCGCATGATCAATCCGCTAGTCTCCAGCTCACTGCTGCAGGAGC
GCATGACGGGCAGGAAACCTGTGCCCTTCTCAGGCGTTGCGTATCACATCGAGCGAGGCGATTTGGCCAA
AGATTGGGTCATTGCTGGCGCGCTGGTTTCCAAAAATCCT
>dme_circ_0000909_junction_seq
TGCTGGCGCGCTGGTTTCCAAAAATCCTGGTAAAGAACTTCCTGGAACGAAAGTAC
>dme_circ_0000910
GGAGGAGCCACCGCACTATGAAACGGATCCTGGTTCGCCACTCTTCCGGTCCGATCACGAATTTTCACCA
GAATCGGAGCTTGAAGATGAGTCTCAAGTGGTGCCAATGAAAAGGGCGCGCACCGTACGGAAGGAGAACG
CCGATGACCTAGAGGAG
>dme_circ_0000910_junction_seq
GAAGGAGAACGCCGATGACCTAGAGGAGGGAGGAGCCACCGCACTATGAAACGGAT
>dme_circ_0000911
GTTTTACAACACAATGGAACTGCATCAACGACACCTCTCAAACTTTTACAAATTGCACAACTGAGAAATA
GTCTTTGATAAATAAATAAAATATAAGAAATCGCTACTGAAACAAGATGCCAAACATGTCCAGCATCAAA
GCGGAGCAGCAAAGCGGTCCTCTTGGAGGAAGTAGCGGCTATCAAGTACCGGTCAACATGTGCACCACCA
CAGTCGCGAATACGACGACCACTTTGGGAAGCTCCGCCGGGGGAGCCACTGGCTCCCGGCACAACGTCTC
CGTGACAAACATCAAGTGCGAACTAGACGAACTACCGTCACCGAACGGCAACATGGTGCCGGTTATCGCA
AACTACGTTCACGGTAGCTTGCGCATTCCACTCAGTGGACATTCAAATCATAGGGAGTCCGATTCGGAGG
AGGAGCTGGCAAGTATTGAGAACTTGAAGGTTCGGCGAAGGACGGCGGCGGACAAAAATGGTCCTCGTCC
AATGTCCTGGGAGGGCGAGCTGAGCGATACTGAGGTCAACGGGGGCGAAGAGCTGATGGAAATGGAGCCA
ACAATTAAGAGTGAGGTGGTCCCTGCTGTTGCACCCCCACAACCCGTCTGCGCACTACAACCGATAAAAA

CAGAGCTAGAGAACATTGCAGGCGAGATGCAGATTCAAGAGAAGTGTTACCCCCAGTCCAACACACAACA
TCACGCTGCCACAAAATTAAAAGTGGCCCCGACGCAAAGTGATCCGATCAATCTCAAGTTCGAACCGCCT
CTGGGAGACAATTCTCCGCTACTGGCTGCACGTAGCAAGTCCAGCAGTGGAGGCCACCTACCACTGCCAA
CGAATCCCAGTCCCGACTCCGCCATACATTCCGTCTACACGCACAGCTCCCCCTCGCAGTCGCCTCTGAC
GTCGCGCCACGCCCCCTACACTCCGTCTCTGAGCCGCAACAACAGCGACGCCTCGCACAGTAGCTGCTAC
AGCTATAGCTCCGAATTCAGTCCCACACACTCGCCCATTCAAGCGCGTCATGCCCCACCCGCCGGCACGC
TCTATGGCAACCACCATGGTATTTACCGCCAGATGAAGGTGGAAGCCTCATCCACTGTGCCGTCCAGTGG
GCAGGAGGCGCAGAACCTGAGTATGGACTCTGCCTCTAGCAATCTGGATACAGTGGGCTTAGGATCTTCG
CACCCCGCATCTCCGGCGGGCATATCACGTCAGCAGTTGATCAACTCGCCCTGCCCCATCTGCGGTGACA
AGATCAGCGGATTTCATTACGGGATTTTCTCCTGCGAGTCTTGCAAGGGCTTCTTCAAGCGCACCGTGCA
AAATCGCAAGAACTACGTGTGCGTGCGTGGTGGACCATGTCAGGTCAGCATTTCCACGCGCAAGAAATGT
CCAGCCTGCCGCTTCGAGAAGTGTCTGCAGAAGGGAATGAAACTAGAAGCGATTCGGGAGGACCGAACCC
GTGGCGGCCGCTCCACATACCAGTGCTCCTACACGCTGCCCAACTCAATGCTTAGTCCGCTGCTTAGTCC
TGATCAAGCGgcagcagctgccgccgcagcagcagtggcaagtcagcagcagccgcaccagcGactacat
caactaaatggatttggaggtgtacccattccctgctctacttctcTTCCAGCCAGCCCTAGTTTGGCAG
GAACTTCGGTCAAGTCGGAAGAGATGGCGGAGACGGGCAAGCAAAGCCTCCGAACGGGAAGCGTACCACC
ACTACTGCAGGGAAATCATGGATGTAGAGCATCTGTGGCAGTACACCGATGCAGAGCTGGCCCGCATCAA
CCAACCACTGTCCGCATTCGCCTCTGGCAGCTCTTCGTCGTCGTCATCGTCAGGTACATCCTCAGGCGCC
CATGCACAACTCACCAATCCACTACTGGCTAGTGCTGGTCTCTCGTCCAATGGCGAGAATGCCAATCCTG
ATCTTATCGCTCATCTCTGCAACGTGGCTGATCACCGTCTTTATAAAATCGTCAAATGGTGCAAGAGCTT
GCCGCTTTTTAAGAACATTTCGGATCGATGACCAAATCTGCTTGCTCATTAACTCGTGGTGCGAGCTGTT
GCTCTTCTCCTGCTGTTTTAGATCAATTGATACTCCTGGAGAGATTAAAATGTCACAAGGCAGGAAGATA
ACCCTATCGCAGGCCAAATCAAATGGCTTGCAG
>dme_circ_0000911_junction_seq
ATCGCAGGCCAAATCAAATGGCTTGCAGGTTTTACAACACAATGGAACTGCATCAA
>dme_circ_0000912
ATACAACTGATAAAGGAAAATTAAAGAAACAAATTGTAAATACAAAATCGAATGGAGAACTTATTATATA
GAAAGTTTAACAGAAAATGATTTTCAAACTGAGAGTGTTAGCACAATATCCATGAACGATGTGTTGAAAA
TTTGTCGATACTTTCAGCCAAATCTCTTGGACTTCCGGCAACATATTCCTATTCATCAATTTGTCTTTTA
ATCTTTCTTTG
>dme_circ_0000912_junction_seq
CATCAATTTGTCTTTTAATCTTTCTTTGATACAACTGATAAAGGAAAATTAAAGAA
>dme_circ_0000913
GGTTTGTAAATAACCGGCCCCAAACACAATTTACTGCTCAGAGACAAAGTCGACAATGAAAGCAATTGCT
AAGGAACCAGCCCaaaaaaaaaaggaaaaactaaaaaTTCTAGAAAGTGTATCACATCTATAACTACTAAA
GGTACCGTGATTCTATAACCGTCAATCAACTGATTCAAAGGCTAAGTTGAGGCGGTTTGGCACAACCGCT
GAGAGCTCTACATTCATTTTGTGGTAGGAGGGCGAAAGAACTTACGTTGTTGGACGGAAATTAATGACAC
TTCTTGGGCCTAGCGCCCCAGGCATGGCCTTTATGATGAAAAAGAAGAAGTACAAGTTCAACGTTGAAGT
GCAGCTGCAGGATTTGGTAGAGGTGGCCCTGGTCAATGAGGTGCTGTTCGCAAAGATACGGTTGCTCGAT
GGAGGTTCCTTTCAGGAGTACAGTAGTCG
>dme_circ_0000913_junction_seq
GAGGTTCCTTTCAGGAGTACAGTAGTCGGGTTTGTAAATAACCGGCCCCAAACACA
>dme_circ_0000914
GGTTTGTAAATAACCGGCCCCAAACACAATTTACTGCTCAGAGACAAAGTCGACAATGAAAGCAATTGCT

AAGGAACCAGCCCaaaaaaaaaggaaaaactaaaaaTTCTAGAAAGTGTATCACATCTATAACTACTAAA
GGTACCGTGATTCTATAACCGTCAATCAACTGATTCAAAGGCTAAGTTGAGGCGGTTTGGCACAACCGCT
GAGAGCTCTACATTCATTTTGTGGTAGGAGGGCGAAAGAACTTACGTTGTTGGACGGAAATTAATGACAC
TTCTTGGGCCTAGCGCCCCAGGCATGGCCTTTATGATGAAAAAGAAGAAGTACAAGTTCAACGTTGAAGT
GCAGCTGCAGGATTTGGTAGAGGTGGCCCTGGTCAATGAGGTGCTGTTCGCAAAGATACGGTTGCTCGAT
GGAGGTTCCTTTCAGGAGTACAGTAGTCGGCGAGGAAGTGCGCAACCATCGAGTAGAATGGAACCGCAGC
TTTGAGTTTCCTTGCAAGATGAGCGCGAATGCTTCAACCGGCGTTTTAGATCCTTGCCATCTGCGTATAT
CAATACGCAAAGAAATGAAGGGCGGACGAAGTTACTATAAGCTTGGTTTTATCGACCTCAATCTGGCCGA
ATTCGCCGGCGCCGGCCTCACCTCCCGTCGGTTTCTCCTGGAGGGCTATGACTCAAGACACCGCCTAGAC
AACTCGATGTTGCGTGTGAGCATTAAGATGCATATGCTTAGCGGTGACATTCTGTTCAAAGCGACCCACG
CCAAATCTAAAAAGCAAGCAGAGCAAGCCTTCAATAGATGATTTTTCAAATGCAGCCACTGGAGTCGGCC
TGCAAATTCCAACGGCTACCGCATTGCCCAGTATAGGTGGCGGAAGCGGTGGCGCATCCGTTTCCTTGGG
CGGTAGTGGAGTCACTTTGGGCGGCATCCCCGGTAATCAGTCGTTACCCGGAACCCGACCCGTTTCCACC
ACAAAGGAGGAGG
>dme_circ_0000914_junction_seq
CGACCCGTTTCCACCACAAAGGAGGAGGGGTTTGTAAATAACCGGCCCCAAACACA
>dme_circ_0000915
GGTTTGTAAATAACCGGCCCCAAACACAATTTACTGCTCAGAGACAAAGTCGACAATGAAAGCAATTGCT
AAGGAACCAGCCCaaaaaaaaaggaaaaactaaaaaTTCTAGAAAGTGTATCACATCTATAACTACTAAA
GGTACCGTGATTCTATAACCGTCAATCAACTGATTCAAAGGCTAAGTTGAGGCGGTTTGGCACAACCGCT
GAGAGCTCTACATTCATTTTGTGGTAGGAGGGCGAAAGAACTTACGTTGTTGGACGGAAATTAATGACAC
TTCTTGGGCCTAGCGCCCCAGGCATGGCCTTTATGATGAAAAAGAAGAAGTACAAGTTCAACGTTGAAGT
GCAGCTGCAGGATTTGGTAGAGGTGGCCCTGGTCAATGAGGTGCTGTTCGCAAAGATACGGTTGCTCGAT
GGAGGTTCCTTTCAGGAGTACAGTAGTCGGCGAGGAAGTGCGCAACCATCGAGTAGAATGGAACCGCAGC
TTTGAGTTTCCTTGCAAGATGAGCGCGAATGCTTCAACCGGCGTTTTAGATCCTTGCCATCTGCGTATAT
CAATACGCAAAGAAATGAAGGGCGGACGAAGTTACTATAAGCTTGGTTTTATCGACCTCAATCTGGCCGA
ATTCGCCGGCGCCGGCCTCACCTCCCGTCGGTTTCTCCTGGAGGGCTATGACTCAAGACACCGCCTAGAC
AACTCGATGTTGCGTGTGAGCATTAAGATGCATATGCTTAGCGGTGACATTCTGTTCAAAGCGACCCACG
CCAAATCTAAAAAGCAAGCAGAGCAAGCCTTCAATAGATGATTTTTCAAATGCAGCCACTGGAGTCGGCC
TGCAAATTCCAACGGCTACCGCATTGCCCAGTATAGGTGGCGGAAGCGGTGGCGCATCCGTTTCCTTGGG
CGGTAGTGGAGTCACTTTGGGCGGCATCCCCGGTAATCAGTCGTTACCCGGAACCCGACCCGTTTCCACC
ACAAAGGAGGAGGGGTAAGTAGTAGTTACAAATCAGGATTTTGTTTATTGTAGAACAAAGTTAACATCTG
GATGAGAAACTTTGAAATAGTGATGTTAATTTATAGGGTCGGAAGCGGTTCttttttttGTTCTTTATAC
GCATATACGCATCATAACGCATCTAGTATACCATTTTCCCCTTAAAGTAATAACAATACAATATTACAAA
AAATTTTATTTTCAATGTATTGTAAATAATCGGTTTCGATAGGTTATTTCCTTATTATTATAGTTGGTAA
AATGGTATACTAGATTCGGTGAGAAGTGTGTATTAATCAGACGTCACACCTACGTAACGTTTTTATCTCT
GTTTTACATGAACTATTTAAATTATTTTGTCATGAAATGGTTACGTTTCCATCCATGTAAATTATGTATA
TTCTTATATCAatatccgtctgtccgtgtgaacgtctggatcttaggaactatacgaaagattaggcatg
caggtttcagagatttcagagactttcgctgtgcttgtttgtatcgaaattttgccactcccactctgac
gcccacaacccgctcaaaacacacactttgaatatttgttgtacatttattcatttgaatgtttccaaaa
atctagcgatattgcagaaaattatacagtttctcattcaactgaaattagctgaataacgggtatctgc
tatttcgaactcaactaaaagcaatctcctttgATTTTGATTGAAATGTTCGTTTTATCGTATATCTATT
GTAGTCGCTTTCGTGTTCTTTCCTCGTGCATTTCGTCATTAGATGCACAGCTATAAGAGGCGCTAGCCTG
TAAAAACGGATGTTTTGTTCACCTATACCGCATACTATAAAGCCCACAAATAAGCATATACTTTATCGCT

AAAATTTAGAAGTGTTTAATCGGTACAGAATTTTACTACTGCAATACTTACAGGATATCGATAACCAGGC
TTTGATAGCAGCTATTGTGACAGACTCCGGCTTGTCGGAGTCCTCGGAATCGGGGGTAACGTTGACTACG
GATAATCTCCAGCAGCATGGCTCAGTCGTCGTAGCTAATCCTATTAGTCCTGTGACGCAGGCGCTACCGC
AACTGGGAATCATTGGCAGCAACAATTACGGAACAACAGGAGGGGCTATCGGATTTACTGGCGCCGGCAA
CGCCACCCTCATAGAAATGGGTCATTCAAGAAATTCCAGTAATACTAGTCAA

>dme_circ_0000915_junction_seq

TTCAAGAAATTCCAGTAATACTAGTCAAGGTTTGTAAATAACCGGCCCCAAACACA

>dme_circ_0000916

GGTTTGTAAATAACCGGCCCCAAACACAATTTACTGCTCAGAGACAAAGTCGACAATGAAAGCAATTGCT
AAGGAACCAGCCCaaaaaaaaaaggaaaaactaaaaaTTCTAGAAAGTGTATCACATCTATAACTACTAAA
GGTACCGTGATTCTATAACCGTCAATCAACTGATTCAAAGGCTAAGTTGAGGCGGTTTGGCACAACCGCT
GAGAGCTCTACATTCATTTTGTGGTAGGAGGGCGAAAGAACTTACGTTGTTGGACGGAAATTAATGACAC
TTCTTGGGCCTAGCGCCCCAGGCATGGCCTTTATGATGAAAAAGAAGAAGTACAAGTTCAACGTTGAAGT
GCAGCTGCAGGATTTGGTAGAGGTGGCCCTGGTCAATGAGGTGCTGTTCGCAAAGATACGGTTGCTCGAT
GGAGGTTCCTTTCAGGAGTACAGTAGTCGGCGAGGAAGTGCGCAACCATCGAGTAGAATGGAACCGCAGC
TTTGAGTTTCCTTGCAAGATGAGCGCGAATGCTTCAACCGGCGTTTTAGATCCTTGCCATCTGCGTATAT
CAATACGCAAAGAAATGAAGGGCGGACGAAGTTACTATAAGCTTGGTTTTATCGACCTCAATCTGGCCGA
ATTCGCCGGCGCCGGCCTCACCTCCCGTCGGTTTCTCCTGGAGGGCTATGACTCAAGACACCGCCTAGAC
AACTCGATGTTGCGTGTGAGCATTAAGATGCATATGCTTAGCGGTGACATTCTGTTCAAAGCGACCCACG
CCAAATCTAAAAAGCAAGCAGAGCAAGCCTTCAATAGATGATTTTTCAAATGCAGCCACTGGAGTCGGCC
TGCAAATTCCAACGGCTACCGCATTGCCCAGTATAGGTGGCGGAAGCGGTGGCGCATCCGTTTCCTTGGG
CGGTAGTGGAGTCACTTTGGGCGGCATCCCCGGTAATCAGTCGTTACCCGGAACCCGACCCGTTTCCACC
ACAAAGGAGGAGGGGTAAGTAGTAGTTACAAATCAGGATTTTGTTTATTGTAGAACAAAGTTAACATCTG
GATGAGAAACTTTGAAATAGTGATGTTAATTTATAGGGTCGGAAGCGGTTCttttttttGTTCTTTATAC
GCATATACGCATCATAACGCATCTAGTATACCATTTTCCCCTTAAAGTAATAACAATACAATATTACAAA
AAATTTTATTTTCAATGTATTGTAAATAATCGGTTTCGATAGGTTATTTCCTTATTATTATAGTTGGTAA
AATGGTATACTAGATTCGGTGAGAAGTGTGTATTAATCAGACGTCACACCTACGTAACGTTTTTATCTCT
GTTTTACATGAACTATTTAAATTATTTTGTCATGAAATGGTTACGTTTCCATCCATGTAAATTATGTATA
TTCTTATATCAatatccgtctgtccgtgtgaacgtctggatcttaggaactatacgaaagattaggcatg
caggtttcagagatttcagagactttcgctgtgcttgtttgtatcgaaattttgccactcccactctgac
gcccacaacccgctcaaaacacacactttgaatatttgttgtacatttattcatttgaatgtttccaaaa
atctagcgatattgcagaaaattatacagtttctcattcaactgaaattagctgaataacgggtatctgc
tatttcgaactcaactaaaagcaatctcctttgATTTTGATTGAAATGTTCGTTTTATCGTATATCTATT
GTAGTCGCTTTCGTGTTCTTTCCTCGTGCATTTCGTCATTAGATGCACAGCTATAAGAGGCGCTAGCCTG
TAAAAACGGATGTTTTGTTCACCTATACCGCATACTATAAAGCCCACAAATAAGCATATACTTTATCGCT
AAAATTTAGAAGTGTTTAATCGGTACAGAATTTTACTACTGCAATACTTACAGGATATCGATAACCAGGC
TTTGATAGCAGCTATTGTGACAGACTCCGGCTTGTCGGAGTCCTCGGAATCGGGGGTAACGTTGACTACG
GATAATCTCCAGCAGCATGGCTCAGTCGTCGTAGCTAATCCTATTAGTCCTGTGACGCAGGCGCTACCGC
AACTGGGAATCATTGGCAGCAACAATTACGGAACAACAGGAGGGGCTATCGGATTTACTGGCGCCGGCAA
CGCCACCCTCATAGAAATGGGTCATTCAAGAAATTCCAGTAATACTAGTCAAGTGAGTATAGGGTTGAAT
CACTTGACTTTACATATACGTGATGTTCTGTTAATGTTTTCTGAAATAGATGTCAAAAGGTTCGGGTTAC
AGTAGTTTTTCGCACAGTCAACATTCAAGGCAAAGTTCTGAGGGTGATTCGGGACATGCTAG

>dme_circ_0000916_junction_seq

GTTCTGAGGGTGATTCGGGACATGCTAGGGTTTGTAAATAACCGGCCCCAAACACA

>dme_circ_0000917
GCTTATCGGCGTAACATACAACAAGGCAGCTCTTCGACGCCTTTGGCTACTCAGGATTTCTTCGCCGTGC
GACCACATTCCACAAATGCTGCCTATGAGCTAATGAGGTGCGTCGCGTTAAAATATCAAAACTTCATGCA
GTAAATTCTATTAAAAACTACTCTAATTTAGTCTAGAAACCAGAGcgttactcgttactcgttactcttt
aAAATGTATCTGTTACTGTCGAACTGTTTTCTAAATGTTTAGTCTAAGACTTCGTTTTCGGGACCTAGTC
CTAGTCTAGGAATTGCCATTGGTCTCCTGGTATTGGTCCACTACCCCAAAATGCCGATATCATTTTCGCT
TACTATTTTCACTGGTTTTTTCGATAGATGATTGTTTTACTTCTGTTTTGTTTAAATTCGCACTAATTAT
TTTTTGCTTTTGTTGAGTGGATTTTTACATTTTAAAACAGTTCTATTGCAAAGAAGGTTGTACAGTATTT
CAGTTGCCACTGACGGTTTTAGTTTAATCACCATATTAATCACCTAATCAAGGCATCCTAATTATGTATT
ACATGATATATAAGCTATTATAAACATTCACAGGTCTCAAAAATGAATTGATTCCATTTTTATCACCACC
AAATAACATTTCTGCAACATATTTCGCTCAACGAAATCCATAGTATTAGTCACTTAAGGAGATATTTGGG
AACAACAGAAACGTGCTTTCAAAATCAAAAACTGAAGATAAAAATATAAGTAAACACAACAGATCAAGAA
TTATGTAGCAAGTAGAAAAATATGACACTGACACTGATTTCATAGGCCATTTGATTCTCTTTTTAACATT
AATAAACTAGTATAAATAATTAATTGATTTGCCGAAAATATTAATTCGGTAGCGGTTTTGCTTTAAAATT
ACATGGGCGTTGAAAAATATGCTACGTTCATAAGACAAAAGCACGTAGAACGTGTGCTACGTTTCATAAG
AGAAAAGAAAACAAAGATGTTCAGATGCATTTCCCGATCATGAATATGTATACTGTATATAGGATTGATC
ACACTTGAAAACCATATTTTATTACTATTATTTTTCTGTCTAAATTTTTGTTTCTATTACGCAGTTATTG
AGATCGCGAACAAGATTTTGCCCCGTTTCTTTAGAATACCGATAGAAGTTAGCaaaaaaaaaaatatttaa
aaaaaaatatttGCAAGAAAATCACGTCCACAATTTATTTAATATTAATACTTGTTCTGCTAATTTCTAT
CAATTTATTAATTTTTCATTAAATCAGCAAGTATACATACATATATGTATATACATATTAAATAGTAGCC
CCTGCCAATCTCTTATGTTACGAAGAACGCTTTTGCTAAGCTTATTCGCTTTATTTAAGAGGAGTTACAC
TATGAGTAAAATACTATTTGTTTGTTCGATTTCTTTTATAATTTTACTCTATTGTTTTTTAAATGTTAA
CCGCTTTAATTATTTATACGTGTTTTTATAATATGGCCGTAATGTTCTGTGTCTAAAACAAATATTCGCA
TTTATGGACGCGTTCATTCAGAAACTTCAGTGGAGTTCCTCGACGACTCTTTGATGGCAATATCACTACT
GCAGGCCAGAACAGCAACATGGGTGCTGTCAGCACCAATAGACAGCAGCAACCCTGTGCTCCAGGAACTA
ACAGTTTTATTGGATCCAATTCATCACCTTCTGGAACTTTGGCCAGTATACACTCAAGCCATTCTGCCTC
TTCTTCAAATGGCAGCAACAGCAGCAGCAGTGGAGCCGTCATTACCTTCCAGTGTACCGCAGGCAGTGGA
CAGGATACAGTAGACTCGGCAAACCATCAACAGCAAATGACAGTGGGATCGCCCAACGGTCATGGAAGTA
ACCCCCGGTTGGGCAATGTCATGTCAGGCGATGTCATTAGCTCAATGGCCGCCAGTCCCACAGACGCTTG
TAGCATCCGTTCTAATCCGTCGGCTGCCTCACTTCTTCTTTCCACGTCGGCAGCTGCAGCAGAGGCTTCT
GCATTGGCCTCGGCAACTACTATGTCCGTTTCGGGAGCAACACTATCACCTCTAGCCACCACCCAAATCA
TTCGCCGCCCTAGCATCACAATGATGTGGGTTCATTTGCAGTTTCTACTTTTTATATATTCACCTCATCC
TCATACTTTGAGCTTTTAAGTTGTAATGTCATACTTCAGACAACTTCTGGGTTCTGCAAAAATATTTTTC
TTTTTCAAATTGTGAACTATTTAAGCAGAAAGCAGATAGTTTGAGCTGTATTCTCTAGTACCATACCGAA
CTAAAAGtttttttttAATGTAAAAATCAGTTTTTTCAACTTTTGTAAACAAACCACTCTAAAGAAATAA
TTCTCAAGAGTTTCAGAATATGTAGCTGCGTGTAAAATGTGAATTTTATTTTTATAGCTTGTTTTTATCC
TCTTTTTAGAAAATTTGTTTAGACAAATAAAAATCTTTATGGTTTTTAAAAAAATTAATCAATCTGTTAA
TTGATCTTATTGATTTAATACCATTTAAAGATTTTTCACAAGCCCTGCATAAGCGTAATTATTCATTTAT
TAGGAGGTCCGTGTTTAATTGGCATGGTACAACCAAAAAGTGTGCAAACGTTTTATTGTTTTGCCAAGAT
ATTGCCTGTGCTTTTGTTGCCTTGTTTATCTCGCAATTATTATTAATTTCTTATTTACAATTTAAATTAA
TTTGATTGTTGTTTAAATAATTTCATGTATTTACTCATTTCTATCAACGAAATTGATTATTCTCATTTTG
TTCCTTCAGGGAATCCCAGCTCCGGTTCGTTAGTTATTAGTGAGACGGGATCTCTAGATCGGACGAAAAG
CAGCGGTGAGAAGCGCAAAAAGGGCGCGCTCGATGACGGGCCACGTGTTTCCGACCGAGTGGAGGAGACA
CGGGTCAATCCAGACTCATTAATTGATGAGATTTTAAAGGACACTAAACTTGATCAGCTGGAGGAATCCG

CAGAAAGCCTCAGGCCTTCAACTGTACATCGCTCGCGATGGAACCGCCTCCCTGGGCGGACATGAGGTTA
AATCAAGCGTACGAGCGGGCGCCCTAAAGCAGGTGGTAATGCAGGATAGAAGATAGTAGTATCCGAGACT
CACAACTCCTTTTTAAAATTAATTAGAAGACTTATTGATCAATTAAAATGGAGTATTGAACTAAAAGAAA
TAGTTTAGGTAATACAAACATGTTTACTCTCAAACAAAAGTGAGAATGCAAATACGCTATATAAGAGTAT
ATAAAATGTTTGTCGGAACTACACTGCTATGAAT
>dme_circ_0000917_junction_seq
TGTTTGTCGGAACTACACTGCTATGAATGCTTATCGGCGTAACATACAACAAGGCA
>dme_circ_0000918
CGGACCACATAAAGCAACTGCGACAGGAGCGCAATGTTTTAAAGGATAAAATTGAAGCTTTGCGCATGGA
GCGAGACGAGCTAAACAATTCGCTGACGTGAGCTTGTCCCAACACAACCATACCCTTTAACAAATAAAGA
TATACTTTTTTCTTAATTAGGCACCTTCACTCAATTTTGCCAGCTAATGGAGCGCCTGTAACGAGGCAGG
GTACAGAACATGTTCGGCAGCTTTACGACATTTATGTACGATACAATACAATGAACGACTGGAAATTTTG
GATTGTAAGTAGAACAAGATAAAACGTTATAGTCCCCTTATAGACCATAGTCGCAACATAGCGGCGGTTA
CGTTTATTCCTAACGACATTTTAACATTATTTACCGTGGTTAATACTTAAAATCTTATTTTATTACATTT
TAATGTAATGGAGTGCCTTTGACTATTTTCAAGGTTTTGGTAAATGTTGTCTGTAGAAAAAGTGATTTGG
GCGAATAAAAGTGATTCCTTTTGTATACGCTAGTGAGCGCGCCAAATTTTTGGCATATCGATAATAATGA
ACAAGACAAAAATTAATTCAAAACATTGTTGGAGTGACAGATTTAGGCGTTGTTTTGCCGTTAAAGTGAC
GAGGAAAATAACAACTTTCAAATAACAAATTGTAATAAAAAATTTTGTGACGATTAATAATATGTGTTTA
ATAGTTTGGGGGATCCAGTAATGTTTAGAGACCTATTCTTGATTTTTTTGGAGTCTTAAGCTTTGAAGAA
AATGCCATAAACTTATATAACATCATTTTTAAAGTTacgttacgtttaaacgttacgtttaaaattacgt
ttTATCAATAATTCATTGCTGTTTTTTAGTTTGGGTCTCATTCTGGAACCTTTGCTTGCCTCTTACACGT
CAACAGTCTCGAGTGCTAGTCTTGATGAGCTACGTCGGACCGCTTTTCTTTGGGTTGATCAGCACTGTTC
CCTTATTGATCTTCGTCCAGG
>dme_circ_0000918_junction_seq
ACTGTTCCCTTATTGATCTTCGTCCAGGCGGACCACATAAAGCAACTGCGACAGGA
>dme_circ_0000919
AAAAGCGAATTAGACTTTTTGGAATGGTCGCCTCTGAACGATAGGAACTTAATGCCAGACGATTGGACCA
CAGACACCTTATTCTCGGCTATAAACGTTCCATTCCCATTTCCAGATTCTAGGGAAATCGGACTTGATTC
CAACCACACGCTTGCCTCCAGTTCCAGAAGAGGGAACCGACGCTGAAATGCTCAAAAACGACGAATATTG
CCTGTCAGCGATTGTCGGTGCTCCTCATACGCTGTATTGCAACGATAGCATAATGGATGTGGTAAACAGC
AGTAACAATGGCGCTGTAAATGTAGATGCTAGCATGCTTGAGCTTAACACGCCCATAAATAACGTATCGT
ACGGTGAGGTAGAGCAACGATTTTCTGTTGCACGGCAGGTGCAGCAATCCAACAATGATTCTCAGCTTCT
CGGAGACTCGGGTTCAATGTCCGGACGAATACACGGAGGCAAGCATTTTGGACCTGCTAGCGCAAACGAC
AACAACACCAATAGGTGTGTTATAAACGGTCGCGTGGCCAAGAGCACTCAGCGGCCATTCCGACGCGAGC
CCCACAATTATGACAAGGCCCAGCCCACAATGCACCAGACGAACCTGTATCAGCAAATGTTGGCCGAACA
ACAGAAGAACCAGCAGCAGCAACACGTGGTAGTGTCTTCATTTCAGGCTTCCCAACAACAGCAGCTCGCT
CAACAGCAACCCTTTTCGGCCCAGGCAACAAACGCCTTCAATAATCAGAGTTTTATTAGCAGTTACGCAG
ATAACTATCAGCATCAGCAGCAACTCAACGTCAAGGTAGAACCTCTACATGCCGATGTGCTGTCATTACT
AAACGACAATGCATACAACTCTATTGGCTACAAGCCCTATCCGCAATTTAAGAAGTCCGCCTCGACGGGA
ACTTTTCTTAATGTCCCCAGGCAGACGCAGCAGCAGCAAAGTTATCTGCAGCAAGAGCCCCCGCAAATGC
AACAGTCCCTTCCCGTAGGCATGGGAAATCAACAAATTTTGCAGCTTACGCGTCAGCAGCAGGTTAACAA
TACACAGCAACAGCAGCACCAATCCACAGTGGCTAGCTTGTTGCAGCAGCAGCACCAGCAGCAAAACCAT
CAGCAACAGCAACTACAGACTGCTGTCAGCTCAGCCACATGGGTTTCGCCAAACACCATTTTGCCTAAAG
AAATCTACCGCTCCAACAGCTTACCACTGAATGTTTCCCTTAATAAACTACCCGATGGTCGCCAGCTGCA

TCATGAGCAGCCCTTCGCAGTGCCCAAGTATCACTCCAAAAGCAAGTCGAGGGTGCGGAGTAATTCGATG
CACCAGCAACACACTTCAGTGGTGTCTGCTGCTGGAGCGGGAAGTTCTTCATTAGGCGCACATAGTTGCA
GCAACCTGCTGGTCACTCAGCAAATGCAGCAGGCAACCAGCGATCCCATGCTAAACAGCACCCTGGCTCA
ACTGCTCACTTCGAGATTCACGCCTACAAACAGCAGTGGCGAACAGTTCGTCATCAAATTCACCTTCCGT
CAGTGCCATAGCCACCACTAACAGCAACAGCAATGCGCCTCCGGTTCATTACGCTAACGTCACATCGCCG
TTGTCGGTTCCGGTGCCAACACATCAACACTCACCAAAAAAGTCCGCCTCCTTGCCTATGGCTGTCAACT
CTGCATTGCACAGTCCCCCTCTAGGAAGTAAAATACACGGTATTGGGCTGGCCACAGCTAACGTGGGCAG
TAATAGCTTGAGTTTGTCACCGGAATCGACGTTCCACGAGTCACAGGATTCCCCACTGTCTCCTACAACC
AGCCTAAAATTCCAGCCGCGAGACACGCAGCGTCGTGCTGGCCATATCCACGCCGAGCAGAAGCGGCGTT
ACAACATAAAGAACGGATTTGACACTCTGCACGCGCTCATACCACAGCTTCAGCTAAATCCCAACGCCAA
GTTAAGTAAGGCAGCCATGCTACAAAAGGGAGCGGACCACATAAAGCAACTGCGACAGGAGCGCAATGTT
TTAAAGGATAAAATTGAAGCTTTGCGCATGGAGCGAGACGAGCTAAACAATTCGCTGACGTGAGCTTGTC
CCAACACAACCATACCCTTTAACAAATAAAGATATACTTTTTTCTTAATTAGGCACCTTCACTCAATTTT
GCCAGCTAATGGAGCGCCTGTAACGAGGCAGGGTACAGAACATGTTCGGCAGCTTTACGACATTTATGTA
CGATACAATACAATGAACGACTGGAAATTTTGGATTG
>dme_circ_0000919_junction_seq
ACAATGAACGACTGGAAATTTTGGATTGAAAAGCGAATTAGACTTTTTGGAATGGT
>dme_circ_0000920
GCTGAGAATCATTGTTGGATTGCTGCACCTGCCGTGCAACAGAAAATCGTTGCTCTACCTCACCGTACGA
TACGTTATTTATGGGCGTGTTAAGCTCAAGCATGCTAGCATCTACATTTACAGCGCCATTGTTACTGCTG
TTTACCACATCCATTATGCTATCGTTGCAATACAGC
>dme_circ_0000920_junction_seq
ATCCATTATGCTATCGTTGCAATACAGCGCTGAGAATCATTGTTGGATTGCTGCAC
>dme_circ_0000921
TCATACAAAAGCGCAGAACCCCGGTGTGTCAATTTGCGTCGCCGCTGGACGTTGATATACATAGTAATCC
TCAAGTAAGTACCTGTAGATTTGGGTATAGCCAACTGGCATCCTTTTAAATAATTTGGCCACGAACAAAG
TGTCGTAAGAAGTTCTGAGAAAAATTATTTGACGTAAACAGTATTTCCTGCGATTCCAATTAAAATTGTT
TATGTTAGATTAATTTAATGAATTGATCTTATTACTACATTTATGTTTTATTATAGTGGGAAAAGACGAA
ATATTTTCTTTAAGAATAATATAAAATGGGATCACAGTTGTACATCGTATTTGTAGAACCTCAAAACTAA
TTGATAAATGGGATTAACACGAGCGTAGCAAAATTCTTTACATTAATTGCAAAACCTTGGCGTAATTAGA
AACtgcataatttcaacgttctattatttaacatagttcccgaatcaaagcgtttatatgtatgtacagc
cggacggaaattaccatatcgacctggctggtgatcttaagcaagaataCACACACTTTATACGTATTAA
CTAGAAGGTTTATTCTGTTGCGCCTAAAACGTAAACTCCCAAACTTAACACTGTTTATTTTTGCATTCTT
GCCAATTTCTACCGTTGGAACAAATTGTTCGGACTTCCTTTACCTGAATAATATCTGTGTGTCGTGTTCT
TTCTTGTTGGTTGTGAAGATTTCTTCTTGATGATACTTTTGTTGATGATACATGTTAGGTAATGCTGTAT
ttaaacccttcatttgaacaataaaaagttatactagatgggctgataaatataaatgaggtagatgaat
gcgtttccggtcctatgtgtattcaggataactacccaagtcaatttgctcatgatctgtaacagtatga
acAGAAAAATAAAAGCTAACAAGTAAGCAGTAGTAACGCTATCACTGTCCATAAGTTCTAAAATTATATT
GAACAAATatctgttagttcttatattatccccaaaacctcgacgttcatacggacaaacggatatggcc
atatcgacacggctagtgatcctgatcaagaCAAAGCACGTACATCTTTATAACTGTTACGTACTTTGCA
ACAAATCTATCGGTGGTCTAGTTAATCTAGTAGGCATTTTATAGTATAATAAAAATAAACTTCATTTTAA
ATACTCGTAGAAGCAAAAGTCATTAGAGGATTCAAATGTGTATGATGGAACTTAATCTTTAACAGATAAA
AGCCAGACATTTGTAACACATTCTTCAATGAACACAATGTACCCTTCCAATTTTAAATGAGTAAAATAAA
ATcaggagaaaatgctatagtcgagtttcccgatcatctcatctgaccgttactcggctagtgtgaatgc

gaacgcgaaattttataatttttctgggatatcgatagatattgggaaataatatgagaaaaaatttgaa
aattgttcaaatgtgtagacttgTATATATTTATATATGTATGTACATGTAATATTGTCAGCAACAGTCA
TTGCTTCCGTAATTTTACTACTTTAAATGCCATGGATCAGTTCATTTCGTACCATCGATGCGCATCATAA
ACGTAAAATTCCTTGTGAAATGTGGCGCACGCCTGGATTGATATCGACAAAACACTACTTTCCGAGGGTA
TAAAGCACACCCTCTCTAAAAATCACTGACCGGTAGGTGGCTTACGCTGCTGGAAAAAAGGCATCACTCG
ATTGCGGCGTGATCGTCGACGCCATTAAATACCAGGGTTAGATACAGCTTGGACCCCATGGATTGCCGTG
AGTCTACCGTCTACTTTCCGGCGTATTCTCTCTTGCGACAAGTGATAAGAGCAGTGGGACTTTTTGCAGT
GTTATTTTTAGCCGTCATGAACCTTTTGGAGGGGGGCATTTTTATTTATAAACTGAGATTGAGCATAAGT
ACCAGAATGTATAAACATTTTTGTTGTTATCGTTCGACTATTTTCCCTGAAATTTTAATAATGATCTTAT
TTTTTGTATTTCAATAAATGTTTGTATTATCAACCATaacaagagaaaccactagaatcgagttcgacga
ctatcagatacccgttcctaagaaagtgggagtgcgagggagaaTTTGGCCAGGCAAATAATGAGATATA
TAATCAAATAATATTCAAAACATCTTTTTAAAGAATAGGCAGTGTTGGTCGGTTAGGGGTTATGGGGTAT
TCAGAAACAAGAAACAAATTAGCAAGACAAATTATGATAATATGAAAAATATTGAGAAATGTGCAAGGTT
TGTGTTCGCTCTTTTTTCTCAAAAGTAGCTTTTTTGTGTGCAAATGcaaacatagccagatcgaccctga
tattgatcctgattaataataaatatacttcataggtccggaaacgcttcgctccaccttttgcataatt
tcaaggaatcttgtTATGGCATGAGAAAATATTGtagaatcagcatttttttttaacctcaaccttctata
attccagagattttgacggacagacggacggatagagacacatgtccagatcgactctgctagtgaactt
gatcaaaaatataaatatgcttatggggttgaaactctttcttTCTTATACCTGCAAGTACCGGGTATGA
AAACTTCTTAATGAGGTGACCGATATTAAAGTAAAGATGTAACTTTTAAACATTAACattaataaaaaga
caatacaaaaaattaaattatattaaaaatGAATTGTACGTTTTTGGTAGATTTTCTACAGTTTAGGCAC
CAAATCGAGACTAAGAATATTATTTTCAGTTTTTACATAACGTAAACATATTACTTTAAAATTGAATACT
AATGCAAAACATTTTTAATCGCAAATTATGGACTTAAAGGTCCCAAAGTCTTATTTGTGTTTCCTTGTTT
GATTTTATAAATACCTTGACAACAGGAAGTTTTTCTTAGAGATTGTTTTTTATTaaaaaaaaaTTTTATA
CGAATTGAATTTTTTATATGGTAGTAGACAGTAGATTGTTACGAAACAAATTTTTAAAATTAAAACTAAT
GTTCTAAAGCAATAGACTGATTAGTTTTGTTTTGATTAGTTTTATTCTAGCTGAAAAAATTAATACCCAT
GATTCGTTAATTTTAAGGGCTTCAAGGGATGTACAAAATAACTTAATTTGCTTAGTTATAATTTTTTGAA
AAATTCTTTTGCACGGCATACATACATATATAGAAATTAAACGCGGAATATCAATCTTTTCTGTCCCTTT
ATAAAGATATAAATCAATaaaaaaaaaGGACGAGCTTATAAAATAAAAACGAGAACGATTGATCGTAACAG
ATTTTCTATTCTTTATAGTCGCATACACGCTCATCGTCATTGCGTGCATTGCTATCTGCTCTTTGTATTG
TTTTTAGTTCTTGGGCTTTGCGAAAAACttttttttACAAGATGTGCGTGGGTATATTATTTAAGGATGT
ACAATAAGGTTTTTGAATGTTCACGGTTACATTACATTACAGATCTGAGCGTGGGCTTAGCAATTTAAAT
GGGTTTTGCAAAGTGAGTCATATATTATGCAATTCGCTAATTAAGTTAGGATACTTTTGAAAATATATTT
GTGGATGGAAAATTAATTCGAATTTGCCGTGTTTCTTTTCTCATCACGCTTTTTCTTTCTGTTGTAAACC
ACGCAGACTGTTGTTCTTGAAGGCAAATACTGGAAACGGCACTCAGCTGTTATAAAAGCCGAATACAGGA
AATGGCGCAGGAACTATAGGTCGAAGGCTACGGGCTGCTTAACATATGATTCGG
>dme_circ_0000921_junction_seq
GCTACGGGCTGCTTAACATATGATTCGGTCATACAAAAGCGCAGAACCCCGGTGTG
>dme_circ_0000922
TCAAAAACTAACCTCACCCAAATGGAATCATTTCAAAGGAGTACGACTTCGGTGGAAGGACAAAATTCGC
CTGAACAATGTCATCTGGCGTTGCTGGCACATGCAGTGTGAGTAATATACTAAGCCCTTCATAGTATACT
TGAACTGCAAGCAACATCATACCCAAATAAATGATTTAATTAAAATAAAATTTGGCTAGGGTTTCCCAAA
TGCCTAATAAAAATATGGAAGTAGTTGTCGTTACCTTGTGATAAGGAGATGATAAGGCTCCAAACTATTT
GCTTGTTACGGCATGTTGGGCACTCGGCATTGGTAAAATATTCCAAACAAATAAACTAATATTATGATCA
TTTCTTTGAAAAGTGCTACACGTGCAAAAGCCTCATGAACaaaaaaaaaTCTGAGTGAATCAGCGAGCCAG

AGACCTCCATAAGCTTTAAAAGCACAAGTTATCTTCTCATCCGCTCTCGCTTTTGAAGCTGCTGGCTAAC
TTTTGGCAATTTTCGGCCAATAGTTGTCAGCTTGTTCGGGGTAGATTCTAGGCATGATCCCCGTCTAGCT
GGCTGGACGGAAGACTGGATGAATTAGCGTTGCCAAGCGGGCATCGCCGACTCCTCAGTCGCCTTCGCCA
CTTCGGGCTCTTATCATAGTCTGGTCGGCTGTGCCGCTGGCCCTTATCATAGTCTGGACCCTAGCCGATT
GTCATTTCATTTTTGTTCTCTGCTGACTTTTGTTTTTGCCTTGCGTGTAATTAAAAGCTAAACATATGAT
TTCGGCGCTGCTAGGCTTCACTGACCACCCGCTCGATCGTCTAGACAGCCTCGGAGTTTCCCGCGCACTT
CAGCCGCGGTAGGAGTGAGTCGCAGTTTCAAGTATCCCATTGATTAGCACTTTCAAAAGCAGAAGTAAAG
ACATAAAGCATAAGTCAGACAAACATCGAATCGGTTTGACTATGCATTTAGCTTGACAGCTAGTCGACCG
ACAGTCTGACACAGTTTTCTGCTGATAGTATTTCCAAAATTTTCCGTTACATCATTTGTGCGAGTGGGAG
GCCGGCAGCCCTGTGTTACAAAGCCATCGTGAGGGCAGTGATTATCTCTTCGCTCTTCTAGATGTCGGCG
ACAAAGGCAAATGGAAATTTTTTGCTTATTTATAGGTAGGCACTTCAACCGCCTCATCAGCAAATATAAT
TAATTTTTGTTATTATCTAAGTTCATGTATACCATTCGTAAATACCTCATATGTGAAGGTCCTGGAGAGA
TAATGGAAGCATATAATCTGTTGCAATTCCATATTTTTTTACCTGCGCGTTTTTGGGTTGTGTTAATCGT
GACTAAACCATGTTTTAAATGAATACACACGAATTTCGGTAGAGATCTATTAATTCACAAAATTAGTCTT
CCGGATTTTCGATGTGTCAAAAGAAATCTAAGTCCACACTTTTTATATAAGTTTATATAACTGTCTGTGG
GAAAATTCCCCATTTATTTCTATAAACAAACCAAATTTATTTACCATATACTTGCaaaaaaaaaTTAGTA
ACCCAAAAAAATTACCTGTATTTCCGAATAAAATTCTGTAGTCTACAGGTTATAACCTGAGATACCGGAAA
TCAAGGttttttttGGGGTCTTTTAATGAGAAAGAATAGTTCGAATTCGTTTAAAAAATAGCATGGTTTT
AGTTTGTTTTCACCCTTCTAAAGTACTATATAAAATGTTACCCAAAAGGTTCAAATTCGTAAACCCTCaa
aaaaaaTAAAGATaaataagaaagaatgctatagttgagttctccgactatgagatacccgttaacgcta
gtgtgaatgcaaaggcgaaaacacataatttttctgggatgttgggtaataaaatgagaaaaaaattatt
ccaaagtgagtgggcgtggtaaaaagttgtttggcaaatcgatagaagtttacaagactaattaaattat
gaaaaaatattgaaaaatggttcaaaaatgtgggcgtggcggttttgtgcgcgttagagtgggtaacatg
ggtcaacaaacttgcgctgcgtctatgtctctagaatctgtatgctgaatcttaacgttctagcttttat
agttcctgagatcttgaagttcatacggatagtcgaacagacggacatggctagatcgattcggctatgg
atcctgatcaagaatatatatactttatatagtgttcaagtatagttttcaacgaatctagtatacctct
tagtctacgcggaacgggtataaTTATCTCTTAACAAGGTAAGATTCAAGGGACCTTGGGACCTTTAACA
CCAAAAATGTTGTATATTTTGTATGAattcgttgaaaagtatgtaacaggcagaaggaagcgtttccgac
catagtatatatatttttgatcaggattaatagccgaatcgatttggcgatgtccgtctgtcgaactgtt
cgactgtccgtatgaactcacagatctcaggaactgtaaaagattcatcatacagattctagagacatag
acgcagcgcaagtttgttgacccatgctgccacgcccactcttacgacataaaaccgaccaaaactgcca
cgcccactttttttgaaaaattgttggatactttttcataatttcattattttaatataacataatttcat
tagtctcttaaatttctattgatttgccaaacaactttttgccatgcccactttaacgccctaaagccgc
caaagcggtcacgccacacttctgaacaatttttaaatttttttcattttattcgccaatatctatcgata
ttccagaaaaattatgaaatttcgtgttcgcattcacactagccgagtaacgggtatctgacagtcgggg
aattcgactatagtattctctcttgttATGTTTAAATTCAAAATTCGCTAACAAAGAGAAAAAGATACTT
TTTTAAAATTAAAGAAATCTGTTGTTATATCTGAAAATAGCAGAACAGTCACATACTTTTTTATTCTTTA
TTTATTTCAAAAAGATTTTTCTCAGCCTTATTACCATTTAGAACCAATTTTTTAAAAACCATTTTTTTAT
TCCACTTTATTCAACATGTTAGTGTTAACGAAGTGTTCTCAATGTTTCCTTTGCTGATTCAAATACACTA
GGGTATTCTTTGTATCCATATGCCTAAGGCCTAAAGTAAGAATTAAGAGCAGCTTGCTCTTTTAGGCAAT
TGTTGGCTACGGCACAGCTTTCCAGATAGTTGTTAGGAAGAACCACTGGTAAAACGACACGTGTTTCTGA
CACATTTTGTCATCTCTGGGGAACTCACTATCTCTATCTATCCCCAGAATCTTATCACGTCGTTTTTTAT
GTTTTCCATACTAAACGGTGTTTGTGTGTTTTCCATTTTTGCAGTCATACAAAAGCGCAGAACCCCGGTG
TGTCAATTTGCGTCGCCGCTGGACGTTGATATACATAGTAATCCTCAAG

>dme_circ_0000922_junction_seq
GACGTTGATATACATAGTAATCCTCAAGTCAAAAACTAACCTCACCCAAATGGAAT
>dme_circ_0000923
GGTCTCAGATAATAATATGTTGTAAGCAAATTGCATTTCATTTTGCAAAATTAATTCATTCTGATATTGC
TTTGGCAATACTTTAATATTTTCTCTATGATTGCGCTGTCGTTTGCAGAAATGGGAGAAGTAACAGAAAG
AGGGGTATAAAGGGTGGCAGAGGAGCGATTGAGGCAATGTGGGGCCAAAGTAACCTGAGCAGCGACGTCA
CGTCTCAACATGTGCCGCGATTATGGGACCGAAGCGGTCCAAGCTTTTTCGTTTGCTGGATTTTCGTTGG
CCAGTTGAGCGTTATCACGCTCGAAACGCGTGAACAAGCGGGAAACAACATTTCATAAATCTTCAGGGCC
CCGGTGTTCGGTTGTG
>dme_circ_0000923_junction_seq
ATCTTCAGGGCCCCGGTGTTCGGTTGTGGGTCTCAGATAATAATATGTTGTAAGCA
>dme_circ_0000924
GGATTGCCATAACGCAGCCAATCATCGGGCTCCTCCACCTGCTCGCCGTTCTTGATCTTCTGGGCGAAGA
TACCGTACTCATAACGGATGCCATAGCCATAGGCGGCCAGACCCAGAGTGGCCATCGAGTCGAGGAAACA
GGCGGCCAAACGACCCAGACCACCATTGCCCAGACCGGCGTCCTCCTCCATCTCCTCCAGGTTCTCGATA
TCCAGACCCAACTGGTACATGGCCTCCTCGCACTCGCTCTGGATTCCCAGGTTGATCATGGTGTTGGTCA
GTGAGCGACCCATGTAGTACTCCAGCGACAGATAGTAGACGCGCTAAAAAAATACAGGGGTCGTTATTAG
ATCTATTGTATCCATGATTAATTAGCAACTATATTGGAAGATCGAAATGTCATAAGATTATTTCATGAGT
TGGATTCAGATTTCGTGCATTTCCGCAGTGCATGGGTCACTCACACCAGGTTCAGCTGTACAGTGCATAC
AATAACTATAATACAACGAAAGCCTGATAAGAGGGGCGCCGACTGCGGCTGAAAGACCTAGTTATGTTTG
GCCAGAAGGTCAACCAGTTTCCAACTTTTACGGTTCCCAACGGAATACTCGCTTATCAGCGAAAAATCGC
ACGGTAATTTCAGGGCAACCCAACTGGAAACTATAAATAGAGGAAGGCATTCACTATCACCGGTTCGATT
CAGTGCGAATCTATCGATCGATGTTACGAGGGCATTAATTGGGTTTATAGCAACGCTATCAAGCGATTGC
AGGCTGCACTTTGCATGCTGGGCAAACATCATGGCTAAGCATGAATCTATACAGCTTGGAATGGCGATTA
TTATCACCAGCCAATGCTGGGCTAATCATGGATCGATCACGGATCAGCAATCACCAATCAGCATTGACGA
CAACATCGCCCACAGTGTCGCCAGTCAGTAGCTAATGGCTCCCAGGGTATGCTAATCCTATCCCTTATCA
GTCAACCTACCTATGTAATCTATGCCCTAAAACGCATACCTATCGCACATGATGCTTTCAGATGTCTGAT
GCTATGCGATCCGGGAAAAAAACTATAGCTAATTAGTTCGGAATGTTTGGAGAGCTTGTTTTTGTGTGCA
TATTTGTAGGTGAAAGGGGTTAAGGCGCCTATATAGAATCGGGGACTTTCGATTAGGAATATTTTGCAGT
CCATTTAGCAGATAAGATATACAGAATTAGGTGAACATGGAGTATGAACTGATTCGGGCAATATGTGCTG
GGCAATGGAAATGCTGTGATAAAACACATTATTGTTTATATCTATATAGGTATACCAAAATATGAACTAT
AAAAGTAGGTATTTTCTTAGTTATGAAATATGAGGTGCACTCAGTAAATACTGAGTTGGAAAAAATCCAA
ATATTAAAACCAATTCTAAGCATGATCTTCTTTGCTGCAGaaaaaaaaaGAAGCTAATAACCTATTTAGC
TTATCTAATCGAAGATAAGAGCTTCACAACAAAACGTGAGTAATCTTCCAGATCCATAAAAAGCTTTTGG
AGTCATTCAACTGCCGGCGCTAATGTTTTCCACAGCAGTTTGTTGTGTAGGGTCTTTGATATTCGTTCTG
GCCAGTTCTACAGACGAATTAGATGCCAGACATATGTTTTTTGCGGTTTTGATAAAGAGCGCCTCACCAA
CAAACGACCAGTGTTTGTTTGCCTTTGTTTATTGGttttttttCCCTATATGCAATAAATAAATGACAGA
TGTGCATAGGAAGTTGGAAATGCAATAGGTAGTGGCGCCCTGGCATTGAATTTTTACATGATCGGTGATA
GCTAAACGCTTTTAATTTGGTGATAAGTGGAACAGTATTTTCGAACTGCTTGTGGAAAACTACAGGGAAA
TAAACTCTTTTGTGTGACTATTCACCCCGCTGAACTTTCTTCAATTTGCATAAATTGGTTTAGAGACCGA
TGGACGCCAAGTTGTCTTCAATTAACTCTAAGTACCAATCCACGCACAACAACAGCTGTTTAAGTATCCA
TAAGATACAATCGTCGTCAGTTCATATATTTCTATTTAGATGGTTGCGTGAGCTAATTGCATGTGGGGGG
ACAACTAAACAAAATCGGGCCTATAAAAGTTCCACAACCTGAAAGAACTtatatatacatacgtacatat
atatatatTTGAGAACGAACAAAAACCGAAATGGTAGCCAAAATAAAAATGAGAGAGGAAAAATGGGGGA

AATTTTCCTTGATATTTTTGTCAGTTGGTTGTTGTTAATAACCTTTGATTGACCTTGAAAAAATGTGTGC
CGACGCCGTGACGGTATCCACAATTTAGTTGAATTGAAAATATTTATCACCCAAAAGTGTATATAGCTCG
AACTTCTAGTCGCCAAAATTGCTCGAGTCAATGGAAAATTAATAGATACATTTTGGTGGGCTTGAAATTG
AAAGTGATTTAAGCTGACATTCAGCAAGGACAAAAATGTTTAGCAGCCGATGCTGACAAATTTGCAAATG
GAAAGCTCACGCTTTGGGCACGTGTTTGGATACATGTACATATGTATGTATATACATATATTGTGTATCT
GATGGATGTGGCTAAAAGGGGGCACTTATTATGCACCCTTGCCCTACTTAAACTGCATCGCCATGGCCCC
AGGAAAACTTATTACTAAAAACTCATCAATATGTTTGGATAGAGCTACTTCTATAGTAGCTCGGCGACAG
CTGCTAAATGCAGAGCTATTTTTAAGCTTTTGTTGCTTCGTCAGCTGGCGGGGAAAATTGTCAAGTGGGC
GCGTAAAAAGGGGGTGGCTGATAAATTCGCCGGCTTTTTGCAGCACCTCTGCAATTTGTTGCAGTGAAAG
TGTCACACCTTTTTTTGGCGCTACTTCTTTGGCATTGATCCCATTGCAGTCTTTGCTTTTTGGCAATTTC
GCGAATAATTAAATGAAACGAAACAATTATGTAGTAGCAAAACAACGAAAGCGTGGGAAATGCAGTAAAA
GCCGAAATTTCAAATAAACAGTGAGCATTCGGAAATTTTTAGAATTTTAGAAAATAAGGATTAAAATAGG
ATTATGAAATGTTGCATGGAATATTCATTTATAATAATAAAAGACATAGTGGAAATACTTGGGATAAAAG
CAAAACTGCTTCAGTTATTTATGAATACGTTTATCGTTTCCCATTTAAtttttagttttttttttGGCTG
AGTGTTCACTGTAAATTCGTTGGCCAAAGCGCATCGGGGAATAAATCAGCAGCGAATAAAAGAGAATGGA
GCAGGCCGAAAAAAATGTCGTAATAGCCAAAGAAATTTAGGAAAAAATATTTCAAAGGAAATTATTTCAA
AAGTTTTGGTTGCCGCAGGAAGGGATAATCAGGCCTAAGAACTCCAGAGCCCGGATAAAGTTAGGTTAGG
TGGTCCGACCACTTCTTTTTGGAAAATTCCACGGACTAAATCAATAAGTGGGTGGCCTGGATGGGCGCGG
AACTCACCTTGGGATCCTTCTCGTAGTAGTGCTGCTGCGTGCGAATCCAGCGGCCCACCATGTTGTCCTT
GACGGTGTTGGCCAGGGCGAAGTAGTAGTCCCTCAGGGTGGACACATTGCGATCCTTGACCAGGGTGTAG
TGCAGGTGGCGATTGAAGTTCTTCTTCACTTCGGTGACATTGCCCACCTCGGCGATCCCGCGCACCGAGA
TCTGTTTGCGCCGATCGGCGTCGGATTGGGGCTTCGACATGGTGTCCTAG
>dme_circ_0000924_junction_seq
GGATTGGGGCTTCGACATGGTGTCCTAGGGATTGCCATAACGCAGCCAATCATCGG
>dme_circ_0000925
GCGCCATCATGGCATTTTCACAACAACAAAACACACCGCGAAAACAACCATTCTCATTCTTCGGACCGGA
TTTCATACTCTCAAAACAAAACATATCTTTGACCCCTGATTTGAACTCTGGATATGGACGCGACATTTCA
CCAAACCATGGAGAGCTTGCAGTTGCCCAAGGAGCTGTACTGGGACCTCAAGATGGAGCCACAATCCCCA
ACATCGGTTTTGGGCTCCGATCTGTTTCCGCTCACTGACTCAAACGATACGGAGTGGCTGTATGACGATA
ACTTTGCAAATGGTTTGTGGTGCCTAAAAGTTTTTTTGAAAGGATTTTTAAGGATTTTAATTTAAGGCAT
TACTCTTATTGGCGATGATGAAGCCTTGACCCTGGAAGAGGTAGCTTCTCTCCAGTTGCTTAGTGATGAG
GAGATGGTTGTGGAGATCTTTGACCTGAAAGATGAAG
>dme_circ_0000925_junction_seq
GTGGAGATCTTTGACCTGAAAGATGAAGGCGCCATCATGGCATTTTCACAACAACA
>dme_circ_0000926
TTATCGTCATACAGCCACTCCGTATCGTTTGAGTCAGTGAGCGGAAACAGATCGGAGCCCAAAACCGATG
TTGGGGATTGTGGCTCCATCTTGAGGTCCCAGTACAGCTCCTTGGGCAACTGCAAGCTCTCCATGGTTTG
GTGAAATGTCGCGTCCATATCCAGAGTTCAAATCAGGGGTCAAAGATATGTTTTGTTTTGAGAG
>dme_circ_0000926_junction_seq
GGGTCAAAGATATGTTTTGTTTTGAGAGTTATCGTCATACAGCCACTCCGTATCGT
>dme_circ_0000927
GAGAATTCCTTATATTATGAAGATAGAAAAAATATTAGCAGTAATGTATAATCAAATTTCCAAATGAAAG
TAAGAAATCACAGAATCAAATTAGAAAGTACAAATAAAAAGCTGGAAAATGGGTAGGTTAGGCTGGTATA
AGTTCACCACATTTTCTTATTaaataaaaaacaaaaacggtacgtggtaaaaacggtaaaaacg

>dme_circ_0000927_junction_seq
acggtacgtggtaaaaacggtaaaaacgGAGAATTCCTTATATTATGAAGATAGAA
>dme_circ_0000928
TTGGTTCTCTCGGGCAATTTCGCTGCCGCCTACCCAGTTGGTAAAGTTAAAGGGCGTGGCAGATGCTGCG
TGTGTAACATCAGGAGTATAGCATTGCTGCAATATTTGAACGTATATTAAGAACACATTTATATCCAATG
CACACTCATATCATGACTTACCACCTTTTGCTCGGCCTTTACGAGCACGGGTTTTGGCTGTGCATCGCCC
GCCTGTTTGTAAGCATCGAATTGAGGCGGAGACTGCGGCGGCGTTAATTGCTG
>dme_circ_0000928_junction_seq
GCGGAGACTGCGGCGGCGTTAATTGCTGTTGGTTCTCTCGGGCAATTTCGCTGCCG
>dme_circ_0000929
TTGGTTCTCTCGGGCAATTTCGCTGCCGCCTACCCAGTTGGTAAAGTTAAAGGGCGTGGCAGATGCTGCG
TGTGTAACATCAGGAGTATAGCATTGCTGCAATATTTGAACGTATATTAAGAACACATTTATATCCAATG
CACACTCATATCATGACTTACCACCTTTTGCTCGGCCTTTACGAGCACGGGTTTTGGCTGTGCATCGCCC
GCCTGTTTGTAAGCATCGAATTGAGGCGGAGACTGCGGCGGCG
>dme_circ_0000929_junction_seq
TCGAATTGAGGCGGAGACTGCGGCGGCGTTGGTTCTCTCGGGCAATTTCGCTGCCG
>dme_circ_0000930
TCCTCATTGAGCTGCTGCCAGTTCGTGGACAATTCGAGCTCCTTAGCACGCATATTGACGATATCATCGA
CCAGTTGGTTCTCTCGGGCAATTTCGCTGCCGCCTACCCAGTTGGTAAAGTTAAAGGGCGTGGCAGATGC
TGCGTGTGTAACATCAGGAG
>dme_circ_0000930_junction_seq
GCAGATGCTGCGTGTGTAACATCAGGAGTCCTCATTGAGCTGCTGCCAGTTCGTGG
>dme_circ_0000931
ATGAAGACGCCTGAGACTCGCAGTCCTCATTGAGCTGCTGCCAGTTCGTGGACAATTCGAGCTCCTTAGC
ACGCATATTGACGATATCATCGACCAGTTGGTTCTCTCGGGCAATTTCGCTGCCGCCTACCCAGTTGGTA
AAGTTAAAGGGCGTGGCAGATGCTGCGTG
>dme_circ_0000931_junction_seq
AGTTAAAGGGCGTGGCAGATGCTGCGTGATGAAGACGCCTGAGACTCGCAGTCCTC
>dme_circ_0000932
GGAGCTCGAATTGTCCACGAACTGGCAGCAGCTCAATGAGGACTGCGAGTCTCAGGCGTCTTCATCTTTA
GACAGCAGAAGCACAGGAAGCGGAGTGTGCAGCAGCATCGCCGACGCCGACGAGGACTGGGTTCCAGAGC
TTATCAGCAGCTCCTCCTCGCCAGCTCCTACAACCATCGAGCAGTCTGCGTCCCAGCCAAAAAAGCGTAC
TCGCACTTACGGGCGCGGTGTAGAGGATCGAAAGATCAGGAAAAAGGAGCAGAACAAGAACGCTGCCACA
CGATATCGCCAGAAAAAGAAACTTGAAATGGAGAATGTGCTGGGCGAGGAGCATGTGCTGTCTAAAGAAA
ACGAGCAGCTTCGTCGCACTCTGCAGGAGCGGCACAACGAAATGCGCTATCTGCGCCAGCTGATTCGAGA
GTTCTACCATGAACGCAAGCGCTAGGCTTTATTTTCTTTGAAATCAATTTTATCGTCGTTTGCAAAGTTA
TT
>dme_circ_0000932_junction_seq
CAATTTTATCGTCGTTTGCAAAGTTATTGGAGCTCGAATTGTCCACGAACTGGCAG
>dme_circ_0000933
GGAGCGGCACAACGAAATGCGCTATCTGCGCCAGCTGATTCGAGAGTTCTACCATGAACGCAAGCGCTAG
GCTTTATTTTCTTTGAAATCAATTTTATCGTCGTTTGCAAAGTTATTGTTATAGTTAAACCCAAGCCTCT
TCCATAAACTAATTGTCATCATAATGTTTCAATGTAATACTATCAATAACAATCTCCGG
>dme_circ_0000933_junction_seq

ATGTAATACTATCAATAACAATCTCCGGGGAGCGGCACAACGAAATGCGCTATCTG
>dme_circ_0000934
ATTAAAAATATAAGTGTTTTTGAAATAGCACAGCAGCCGCAACACTGTCCGATTTGCAGAAAAACAAATC
AATGAGCTTTTCAATTAATGTGTTTTTACATTGGACCGTGCTGCTTGGGATATGGGGTCGCCTTTAGGCC
CCACTGGCAACAAATATTTCCGAATTCAGAGACTCGTCATTAGTGCCAAAAATGTAGGACAAAGGACAGG
CTGCGTACTTTGGTGATTTGTAGATTTTGGGCAACCCGGAGATTGTTATTGATAG
>dme_circ_0000934_junction_seq
TGGGCAACCCGGAGATTGTTATTGATAGATTAAAAATATAAGTGTTTTTGAAATAG
>dme_circ_0000935
ATTAAAAATATAAGTGTTTTTGAAATAGCACAGCAGCCGCAACACTGTCCGATTTGCAGAAAAACAAATC
AATGAGCTTTTCAATTAATGTGTTTTTACATTGGACCGTGCTGCTTGGGATATGGGGTCGCCTTTAGGCC
CCACTGGCAACAAATATTTCCGAATTCAGAGACTCG
>dme_circ_0000935_junction_seq
AACAAATATTTCCGAATTCAGAGACTCGATTAAAAATATAAGTGTTTTTGAAATAG
>dme_circ_0000936
GCTAATGAACCTGGACCACTAAGAGGAAGGGATCCCCCCGCAGCCGCCATTGCCTGTGCAGCCATTTCAG
CTAAAAAGGCCTGTGGGAAATCGCTGGACATGCCAGGAATTTGTGGTAATTGAGCTGCCGCTGCTGTCGC
AGCTGCTTTCGAGCTCTTACTAGAGGAAGTGGATGTTCCC
>dme_circ_0000936_junction_seq
GCTCTTACTAGAGGAAGTGGATGTTCCCGCTAATGAACCTGGACCACTAAGAGGAA
>dme_circ_0000937
GATGGAGGAGGACGCCGGTCTGGGCAATGGTGGTCTGGGTCGTTTGGCCGCCTGTTTCCTCGACTCGATG
GCCACTCTGGGTCTGGCCGCCTATGGCTATGGCATCCGTTATGAGTACGGTATCTTCGCCCAGAAGATCA
AGAACGGCGAGCAG
>dme_circ_0000937_junction_seq
CGCCCAGAAGATCAAGAACGGCGAGCAGGATGGAGGAGGACGCCGGTCTGGGCAAT
>dme_circ_0000938
GGTGGCCATTCAGCTGAACGATACCCATCCATCGCTGGCCATCCCTGAGCTGATGCGCATCCTGGTCGAT
GAGGAGCATCTGACCTGGGAGAAGGCATGGGACATCACCGTCAGGAGTTGCGCGTACACCAACCACACCG
TGCTCCCAGAGGCCCTGGAGCGCTGGCCCGTCTCCCTGCTGGAGTCGATCCTGCCCCGCCATCTGCAAAT
CATCTATCACATCAACTTCCTGCACATGGAGAATGTGAAGAAGAAGTTCCCCGACGATTTGGACCGCATG
CGCCGCATGTCGATGGTGGAGGAGGATGGCGAGAAGCGCATCAACATGGCTCATCTGTCCATCGTCGGCT
CCCACGCCGTCAACGGTGTGGCCGCCATCCACTCGCAGATCCTAAAGGACTCGCTGTTCCATGACTTCTA
CGAAATGGAGCCCCAGAAGTTCCAGAACAAGACGAACGGTATTACCCCGCGTCGTTGGTTGCTGCTCTGC
AATCCCGGACTCTCCGACCTGATCGCCGAGAAGATCGGCGACGA
>dme_circ_0000938_junction_seq
ACCTGATCGCCGAGAAGATCGGCGACGAGGTGGCCATTCAGCTGAACGATACCCAT
>dme_circ_0000939
GTTCAAATAAAGCAGCCAGCGAGTAACGAAGCAATACTTTTACTCATTGAAGTATAATGGCATCCGGCGG
CTTAACATGTGTAAAATACTTGACATTCTTTTGCAATCTTCTTTTTGCGGTAAGTCAATATCACTGACTA
TTTAATATAGTTGATTGCAATTTCAGCTTAAGTGGAGCTTTAATTTATATTTAAAAATCCTAATTTGttt
ttttttATAGTTAAGACACGGGGACCTCTGTGTACTTATCTTATTTTATATCCCTTGTATGATAAAGAGT
GACGATTTTGAAATTTTTCTCATCTAAATCGGCAGTTTTTATCCTTTTTAATAATGTAATATTTTGGTGC
TACATAGTGTTACCTTCGATTACAAAAACTTCAAAGCTTTAATTTAGATCACATTACTTGCCATTTATTC

TTTTCATTAAGAGGACCTCTtaaaaaaggtttctaaaataaaaaaaTTGAATTTTAGAAAGAATACTTTT
ACGATAATTTTATTTTATATTGTAATGTTTTATCTCATATaaaaaaaaTATTTAGTTTAAATATTTTAAC
ATTTATAAATATGTTAATAaacaatttatgtgtgttatctaacattatatttctcttatctaacaaAGGC
TCACTGGGCTTCTTATTTTTCTGGTGGGAGGCATGGTCCAACTAAACTATGCACATTATTCGAACTTCGT
TAGCGATCACGTCTGGACCGCTCCTATCATACTTATGATTGTCGGTGCCGCTGTGGCAGTCATTTGCTTC
TTGGGATGTTGTGGAGCCTTAAAAGAGAGCAGCTGTATGATACTTAGCTTTGCCCTTCTTGCCGTCGTGA
TATTTTTGTTCGAGATCGGACTGGGCCTTGCTGGCTATGTGAAGCATACCGGTCTTCATCAGATCATGGA
GAGTCAGTTTAACTCGACCATGCAGCACTACAAGGAACGGGCCGACTATCGCGATGCTTGGACTTTGCTG
CAAACCGAGCTAGATTGTTGCGGCATCAATGGGCCCAACGACTGGGAAACCGTTTACCGTAATAGTACCC
TACCTGCAGCCTGCTGCTCTGTAATTAATTTAAGCGAGGCCAAGGAATGTACAAATACTCACGCAACTCA
GCATGGCTGCTTGCAGAAGCTCTTAGAGATTTTGGATTCAAAGACCTTAATTTTGGCCTCCGTTGTTTTG
GGAGTGGCGGGTATACAGGATGCTCACTATACTGTTCGCTTGCTGCCTCTATCGTTCGTTTCGCAGGAGC
TACGATCATGTTTAAAATGCGAAGTAGCCGATTTTGAAAGGAAACAACAGTTTTTGGCATACCATGCTTC
ACAAGAACTATTACAAAAATGAAATCACAAAATATTCTATTTTACATGCCCATTGAAGGCTGATtatctt
atttataactagatatattatattaatttaatttattatattaattcttcaaattattgtagttcttcaa
aAAATATGGGCGCATCCGATCCTACTACAGTTTCTTTGTTCTTTGTAATCTGAATACCTCAGAATCATCT
GATGGTTAATGGGTTTAATGAAGAAACCTTTGTTGTACTTTTTAATTAATAATTTTTTAATGTTTGCTTG
AATTTGTATGCCATATACAAATTTAAGTACGTTTTATTAAGGCTGTCACTTTGGTTCGATTTCCATTCAT
TCGTACTTCAATATTTCATATTATAATAAAGTGACCTTGTAGCGTTTATCTATGTACTACCTTTATTCAC
ATAAAGAATGTTCGAAGGTATGTAGAGTGACGATGTGCATCTCTTTACAGTaaaaaaaaaaCAATTGTTT
GATAATCATACTACCGATTGACCAACAAAATATGTGTTCAGTCAGTTCCAGGTGTAAAAGAAAAAATACA
AACTTCTCTTTTTACGTTTGTTCTTTAAGTTTTAAAACATTTCTTTAGAATTCGAGACTGAATGACATAT
TTAGTGTTATTCGAAATGTATTCAAAAATGTGACGAGAGGATACAGAAAATTAAATTTGATTTACGGAAC
CAAAACATTATCAAATAAAACTAATGATCTGTAAAAATATTTGTATTTTTTCGGTGTTTTTCGGAAATGT
TGTTATTATTAAGCTCGGTTGTGGAATTTCCTGTTAGAATATCCCCAAAAGAATGTCAATTGCATTTCAA
TTAATTTTTGCCACATATTTGTTGTACGATATTGGGACAATCCAGATAGTTTGGTTCTGCCAGTAAAAAT
AATTATTCAAGTGAGGAATGAAATGAATTAACGATTGCAAGGAAACTGCTAAAAGTCTGAGAACTGTATA
TCATGATGAAAAATTGATATTCTGTGAACATTTACATATGTATGTGTATGTAGCTATTTAAATACAATAT
TTAAATATATGGTTTTCATGTATTGCATTTTCTTCAGATAAGTAGCAATATTTTCAACATAAAACATTTT
ATCACAGTACAGACAAATAAAGACAACTAATaaaaaaacataaaaaaaaaTATACCTAAATATATCGAAT
TTACAATTTTCATATCGACTTATATGTAGATTAACTTCGAGGCTTTACAATAAAACAACCACTATAAAAC
TATTTACATTTGTGTTTTACAAAATAGTACTTAATTGTCTACAAGTCTAGTCAACTTCCGAATATTATGT
TGGTAACTTTTTAATTTTTTGTTTGGTTTGAAACTGTCATTaaaaaaaacttagcacatcaataaaaaat
attttcaatttGGTTTTCAGTGCATAGAAAGCTTGGAAGAGTATACAGAGCTTAAGTGTTTGTGGTTAGA
ATGCAATGCCATTTCCGAAATTCAGGGCTTAGAGAAACTTAGCAAACTGAAGTGTCTCTTTCTCCAAAAC
AACCTTATAACGAAAATTGAAAATTTAGACCCATGTCGTGAGCTAGATACTTTAAATTTGAGCTCAAATC
ATATTCGGAAAATACAAAACATTGGGACCAATGTCTTGCCAGTTTTAAATACTCTTACCATTTCATCAAA
CTATCTTAAGGACAGTGAGAGCCTTTCAGACTTGATTCAATGTAAAACACTATCGGTGCTAGATTTATCA
AACAACAGAATAGACGATATTTTGATTGTTAAAATATTTGAGCAGATGCTTAATCTAAAGGTCTTGGTTC
TACAAGGAAACCCGGTTGTTTCCCGACTCCCCCAGTATCGGAAGACATTGATACTAGCCTGTGGTACCAT
ACGTATGCGCAATAGTCACCGTCCACCTGATCAGCAGGATCCTTTGCTGAGGTCTAGCGATTCGGAAGAC
GATACATGTGCCGAAACTGCCCGTAAGAAAGTTGCGCTTGAGAACGGTTGTGTCGATGATTTATGGGAGG
AGGTGTCGGGTGAACAGCCTATCTCAGAGGACGGTACCAACTCATCTAGTTCTCTGGAAGACAATGATGG
CACAAGCTCTCAGGACGATCTTATAGCAGAGAAATTGAGTAATCGTAGAACACTCGAGGGGCGCCCAACA

GTCCTTTATGAAACTGAAGTGAGCAACGTAAAGAGTGCCAATAACGATATTAACATATTTGAGGAGGGTG
TTGGCAAGGCATCGCAGATTATTATAGAAGACAAACCCGTTACGAAAATAAAGCTTATAAACGAACCAAG
CTGTATGAATGATAAAAGTGCGATGAATTGTACTTCCATGGGACCAGTGGTAAAGGAAAACGATTTTGAC
GAAGACGCAGAAATAAAAAATGTGGATGATATGGTCCCATGCCAGAATTTAATTAAATCAAACGAAGATA
TAAACTCTGAATTCGGTTTCAGCTCAAAGCTGAAACAAGATTTTGATCAAACTTGTACTGCCCTAAGAAA
TGATGCTGAATGTAAGGAATTTGATGAAGAACTAACCATCAAAGAAAGTGAAAGCAAGCCTTTTAACGAA
ATGTACGACATTTTTGCAGCTGAAGACGATAAGCTCAACGAAACACTCGATTTAAATCTAAATGAAACCA
CATGTCCACACCATGTGCACGACAAttttttttCCGAGCAAAACAAAATGCCAAGCTTTTATGAAGAAGA
TATCATGATCAACCTTTTACCCAAAACCAAGGAGAAAACACTGTTGGAAAAAGACAGTATCATTGAAAAT
GAAAAGTGCGCTCATGACTTAGATGAAATGGGTCGCCAAATGGAAGAGGATCTGGCAGAACTTAGGCAGT
CCACTCAGAATTTGATCGGAATTTCAATAGATGAGACTGCTCGTACAGACAGTGAAACAGATGAAGAGGA
TCTGATAGCACAGCAGGACCCATACTCTCCACTTCTCAAGCAGCAATTCAAAGACCGTCGTATGAAAATT
ATGTTAACAGAAGAAACCAAAGCGCAAGAGGAATCACTAAGTGATAGAAATATTGCACTCTCAAACGAAT
CTGACCAAGATGACAAGCAAGACCAGTTATTTGCCAAAATATTAGATGATGCAACAGAAAATATACCCAA
GCGCATCTTTGGCACTGGGTGTGATGCACTTAGTTCCGTTTGGCCTCAAGAAGAGTGCTTGCTTCAATTG
ACTCTACCCGAAAACAAAGAATCTCCAGCTCAAGAAGACACCTTCAAGAAATCTATAACAAATAGTACAA
GTTTCGAGAAGGCGAATGAAATTTGCGTTCGTATGGATCAGAAAATGGCCGAGGAAGAGGAAGCTTTAGG
TAAGCTGTTGCATGACCTTGAAGGCGAGATCAACAACGTGTATGATATAAACACGAAGGTGAAGTACGAA
GAAAACACTTCTACTAATGAGAGTGACGCTGTTAGTATTTGTACATCCTTGCTAGACGATATTATAGTTG
GTCTTACGTTCGACGAGGTACTGTGTCATGAGAAACCCAAAAGTTTCGAATTCGGTCTTATCGAATCTGA
CGAGGAGTTCAGTTACTCATTTGAACCGAAATTGGAAAAGTTGGTTCCCCCTGCACTAGAGGATCCGGCC
CGAGGAAAAAGCCTTCGAGAATGTTTGGACACTTTTTCTGACTTTGTCTCTTCCATGGCAGATCCGAAGC
TGCCCGTAATGTTGGGTCGTAACCCCACATCGGGTGTGGAGAAAATCCGTGCCGCTCAAGAGTTACTAAA
GACTAAAAATCTTTCGGACCTATACAAGGATACGGCAGAAAGCTTAAACTCCCAGGTAGCCAAAGAAATT
GAAAAGCGCAAACGCCGCGTGGCAGCATCTGCTACGCGTTGTTTTAATCAACGCGATAAATATGATGATA
CCTTGGAGTTAGTCGAAAATCGGTTGATGATTGTCAAGAAAGATTCCGGGGATCTGGAAGAACTGCCACC
ACCACCCCCCTTAATAAGTGACACAGAGTCCGAAGACGACAACCCAACTGAGGATAATTATACACCTGGC
AATGAGGTTCATAGACATGGTACAAAAACTCAGGACTCGAAAGAACATCTAATGAGCAATTTGTTGAATC
AAAATCAGGACGATATTATAGAAGGTaaaaaaaaTGACAGTGCTGAAGATGAATTTTATTCGTTGGAAGC
AATGACAACCTTTGGCAACCTAGACGCGGAATTTTTTCAGAAGCTGGATCTTCAGAAAGTTAACGCTTCC
GAAGACTCAGAACCGGCCATCAACTGCATGCGAAGTTATAATGAACTTCAAGCTTACATGAAATCCGGAT
CATTAAACACCCAGCTGAATAGTGAGGAAGCCGAGATGTTGCAGACAATGTGTTCCAGGGTTGCCTCAGA
TGTAGACAAGCCGAAGTCACAAAATTCCAAAGAAGAAGAGGAGGATGACCTTCTTAAAAAAATGGTTCTT
CGCATGAAAGAGTACGAGGAACGGGAGCATCAGCTTCAGCTAATATCGCATGAAACCTCCAGTGAGTTAG
GTCCCATTAAGCTATCCATAGGTGGGTCTAAGCTTTTCGAGCAAAATACTAAAATTCCAGAATCTGTCCT
TGTACATTCAGAAAATGAGCCCACTAGAAACAACAAACCTATAAATACCAAGAACACAAACGATAAAGAG
AGCTCAGATATAATGGAAAAAAATGGGGATCCAAGTGTCACGACCTCCTTTAAAACATCACACAAAAGTA
TAGACGACGATATACAGTCCGATGTTTCAACTGACTACGAATCTGGCGAAGAAGTAGTTGTTGTAGAGCC
GCCTACACTTTCCGAGGCAGTTCTCAAATCGTTCTACTCTGATGAATTTGAAGCAGACTCAAAAATGGTT
CATGAACTTGAAGAAGCTACTCGGCGTAATCTCCGCCGCTATAATCCAAACATAATGCATATTTCAACAA
GCAATCATCCATTTTCAACAAATAATATACTGCCTACTAAAACCTCTACTTCGGAGCTGTCTGAAGGCGC
TAAGGCCAAGTGGGCAAAAATAGCGGAACGACTACACGAATTTCTTGATCCGGAGACGATTGCGAATCTA
AATAAGGAACAGTTCGGGGAAAGCGATGAATGTGAAGATAGTCAAGATGATAATATTACAGATATCACAG
TTGAGGAAACCAATTTAAAAAAAGATACAAAATTAGTAGTTTTGGAAGAATATAACAATACACACACTTG

CGATGGAAACTCATGTCCATCTAAGCTTGATGACAGCGATCAATTCGCAAGCCTCAAAGGATTTGAAAAA
ATTAAAGAAACGCCAACACATAATATGGGATTAAGTAGTGTTATGAGCGCATCGCAAAATAAAACTTCCG
AAACTTTGCCAAACGAAATTGGAAATAAAATCACAGATTTCTCTTCTTTTAAAACTTCGTCAACGGATCT
CATTAATTCAGAAGGTGTAAAAACGGAACAAATTCAGTGCAATCTTCAGATTTTGAGCGACGAAGGAGAC
GTTGTGGTCGAAGAGCTCAGTGTCAATGCTCAGGTGTCGTCTTTTAAGTAAAAATCGATCCCTATTAATT
TGTTAAAATGTAATCATTTTCCAATTGTTTTTTATTTATAATAATTAAATGGATGATAATTCAAAAGCGC
aaaaaaaaaGTATTTTAATTGCCCCACTATGATAACGTATAGAAACTTAAATTTAAAATCCAGCTCGTAC
CGCTCAAGTAGCAGTAATCTTaaaaattgtaaaaaaaaaGTTGAAAGTACTTATAAAAAGATTAGATGGA
CTTCGGCTATAACTTCTTTCCCCCGTGCCCAACGAAACTGGCACGAATTGAACTACTTGAAACCGGATCG
CGGTTTAATGGTGTATCACAGAAACCGGTTTGTTTTGTTGACTGCTTGGTGGTTTCTAACGCAGGTCAAT
CGTTTGGGTTATCACTTGGCTGACATTGATAACAGTTTTCTGTGATTAGCAGTATTACCAAGCGAAAAAT
AAAATTTTTATTTATGATCAGCCCCAAAATTTTAGGATCGAAACTTATTCAGTCTATAGGTTTTGATATT
TTTTCCTATCAGGATTCTTTTTTGGACCCCCTGCACTTTGTCGATGGTCATACTAGATCAACAATAAAAT
ATATATGGCGGCGAGGAACTAAATCCCGTTTAGGAATAAAATACAGTGTGATAATTTTATGAAGTGATAC
ATTTTGTTTCATATATTTTCGTAGTCCTTTTTAAATTAGAGAAAAGGGTTAAGCAATTACTAGACTGAAC
TAAATTGCATAAGCACGATTAAATAGGTAACTTTCGCAGCCAAAATCTGAAAATGTAAAAATATATTTAA
ATAGCGCTTAGTTTGGAAACGAAATCATACAAACCGACATTAGAAGCGAATTATCAGTGCTCATAAAGCC
CTCAGCATTGATAGCCAATACATTTTGTTCCACTTGCAAAATAAACTCCATCAGCAGATCTTTATACGAA
GTTTCTCTTCCAATCGAGGGATGGCAGGACAAGTTCCTTAAATAACTTCCAATCAAGACACTCTGAATGG
GGTGGAAATAACTTTATTTCAACATGGAAGGCAGCTGTTTATAACATCACATTTTTATGATTACGCAATT
TACAATATGCATGTGTGTATGTGAATGGATTTTATATTTTGATATTAAAAAAAGTTCAGTTGTATACCCT
TGCATATGGTACATTGATTTCAGTCAGATTATTTTCAACCGTTCGACCGTTTCGACACATCCGTTCGATT
CTATCAGTTTAACTGCAGGTATAAATCAGAAGTAGACGATTTTCCTCTTGGAAAATATTAGAAACAAAAT
TATAATTCGATATACACCAGTATTCGAAAATATATAATTTTGATAAACATCTCAGGAACGAATATACAAT
AGAATATACCTAGAGGAAGAGTCGAAAATAGTTCAGCTAATTAATGGAAAATAAAATCCTTCCTTAATTT
GATGGTTTGATAGTTTTTAGACAAAAGATATTTTTGATTTGAATTTGATCAAAATCGGACGACAATATCA
TAAAGCTACCTTGGGAATatttgattttgtttttgatcataaattgtttctttttttttcataaattAAAT
TGTTGCAAGGGTATATAAGCTTCGGCTTGCCGAAGCTAGCTTCCTTTCTTGTTTTTTTATAACCTGTCGT
TGGCAAAACTCTCCACAACGGCAAAACACTAAGATGTTGAAGAATGAGGGTACAAAAACGGAAAATGTTG
CGTAAAGGTATTTGTATTCATAAACCTCTACCAGAACCTGCTGGGTCCAGTAAATATTGACGGTACTATC
TGAAAACAGAACCACGTAGGTGCCAAGTATGGACCAGCCGAAAAGATCGTTGAAGTGGGTAAACAGATAA
TGCAGTTGCATATGGCTCTGCTTAAGTGCTAACAGAAACTCTAGGGAGCAAAGCCGATTGGCATTGCCGT
CCAATGTGCAGTTGGCGCCCATCAGATGACACTCTAGTACGTTTTGAAGTCGTATGCCTAACAGACTCAC
GTGGTGACCCAGCAATTCTACGTTCAAAAGAACCAATACGAATTGCATCCTTATAATAAGGATTGACAGG
AGGGAGGACCAAAAGTAGAGAAGGCTACCGCTTAGAGCAACATAGATGGATGGCAATACCGTCACCAAAA
ATCCAATACCGAGTGCTAGAATTTTAGCATACGACTGCCACTTTAACCGCCCTACATGTATTTTAAGTTC
GCACTGCAATCGAAGTCCCAAGTACTTCATCTCTCTGCAGAACCTGAAGTAGTCATCGTAGTGAACCATC
ACCTGAAGTATAATCACAGTAACACACACAATCCTAGCTACGAAAACAATCACGTTAACCATAAGAGAAA
GAAATAGTTCTGCACTCTGAGGAAAATAAATTGAAATCCCGAAATGAACTGCATTTAAAATTATTAGGAT
CGCTGAGTACACTTTCTGTACGAACTTAGATTGGGCACAACTCTCTGACCAAGGCACAAGCCCCAAAAGG
GCAAAGTATTTGAGGTACGGATGAAAGGAATAGAGCATGCCGTACGGTTGAAAATATACTGGCTCTATGC
TGTAAGTAAATGATATTGCCTGAATATTTAAATTCTACCACTTGGCGTTATGAGCTACTAATTTTCTAAT
TGATCGGATAACTTGCCTTCTCAGTCAATATTGTTCCGTTATTGCTAACCACCTAGGAATCCACCTTTTT
GGATTACCTGCCTCATATATATATATGCCAATGCAAACAATCTTTTCAGGAGAAAGAGTAATTTAATAAA

TGTGTTGTTGAATTTGTAATGGAATTGATATACCTTCGTTGTACACTGGAACAATATTAATTCCAATAAA
AATTTTGTCCAGCCTTCTGGTCGTAAACGGGAGAAAGCGGTCCGAGGGCCGCGACTGTCAGATACCCATT
ATGGAGATAATAGAAGTGCGAAGGTACGTTATAGATCTATGTAAGCATAAATTTCCTTAGTAAACTGAAA
TTTCAGCATGCAGTGATGTGATGGCACTTTCCAAAAAACTTGGAACAAACTTTTAACTATTATCGCCAAT
TTTCTAAAAACGAATTATTATTTAAATATTGATGACCCaaaaaaaaaaaaaaaTCagataatgctatagt
tccccgactatcagatactcagctagtgtgaatgcgaacgcgaaatttcattattttttccatataaaat
cgaaaatttttttcaaaaatttgggcgttgcagttttggcggttttagggcgttaacacatttataaaaag
tgtgggcttggcagttttgtgcggtttgtgggagtggcaacatgggtcagcaaactttgcgctgcgtctt
tgtttctagatctgtacgctgaatctcaaccttctagcttaaatagttcctgagatcttgacgttcatac
ggatagacggatgtggctggaacttagtatagtcggaaacgcttccttcagcctgttacatacttttcaa
cgaatctagtatacccttttactctacgagtaacgggtataacaatttaatcACCTACGAATACCTTGAG
CACGACGTGGCCGCCAGAGAGACTTTAGTGTTGTTGGCGATTTTGTACGGTTTACGTTAGGATTCACTAA
AAATTGTATGCGGATGAGTTAAATatataccttaaagcggcgaaaacggtccattgtacctgttacatac
tttttaacgaatccaccatactccattctcaacgagtaacaggtataCATTTATTAGTACGCTCATCATA
TTCGCTTTCCGAAGTTATCGCCCGCTGTATTATCAGCTCGTTTCTATAATTTAATTTGTGAGCTATTGTC
AATAGGCTGCATACGTTTTTGTATACCCTTGTAGAGAGtatatattcttgatttgtataaacagctgagt
cgatatagctgtccgtccgtTGTTATTGCAAGGGTATAACAGCTTCGGCTTGTCGAATTGAGCTTCCACT
TTAGGGGTTTTTGTTTTTAATAGTTGATAACGATAATGAAGTGCTCAAGTGCTGTCAAGGTATAAATACG
GCCCGAATTGTAAACACGAAGTCAGTTGATAGTTTGCAGGCTCTGAGAAAATACCCAAGTGAAAACGGAG
TGTAGAGTACAATATTGAAAGCTACCTGATTAATATTGTTTTTTCTAAAGTGCTGAAGAGTATTCACCAG
TAATGGCT
>dme_circ_0000939_junction_seq
TGCTGAAGAGTATTCACCAGTAATGGCTGTTCAAATAAAGCAGCCAGCGAGTAACG
>dme_circ_0000940
ATAACCTTGAGCTTATCGCCCACAATGGGATCGTTGTTCACAACGTTGCCCACGGCGCAGATGAGCTTGA
TGATCTGCTTGGCCACATAGTAGCCCGGAGCAGCCTTGCCTCCGATCATGATTGTCCTCGGGGTGAAGTT
GGCTGTGGGATCCTTCTTGATCCTGTTGTACAGGGTGATGATGTGCAGGCAGTTCAGCAGCTGGCGCTTG
TACTCGTGAATACGTTTCACCTGAATG
>dme_circ_0000940_junction_seq
GTACTCGTGAATACGTTTCACCTGAATGATAACCTTGAGCTTATCGCCCACAATGG
>dme_circ_0000941
GAAGATAACCTTGAGCTTATCGCCCACAATGGGATCGTTGTTCACAACGTTGCCCACGGCGCAGATGAGC
TTGATGATCTGCTTGGCCACATAGTAGCCCGGAGCAGCCTTGCCTCCGATCATGATTGTCCTCGGGGTGA
AGTTGGCTGTGGGATCCTTCTTGATCCTGTTG
>dme_circ_0000941_junction_seq
GGCTGTGGGATCCTTCTTGATCCTGTTGGAAGATAACCTTGAGCTTATCGCCCACA
>dme_circ_0000942
GATAAACAATTCGCCTAGTTCTCTCATCACTGTGgaatcgaatcgaatcgaatcAACAACGGTACCTACC
TAAAAAGACCTCAGGAACACTTTCAAAGTAAAAGAAAAGTCTATTTAAAATAATGGAAAGAGTTGTAAAA
AACGTTAGTGGCAAGCTTGTTTAGATATCTGATATGCGTGTTTTAAATCGAGATTAAACCTCAAGTTCAA
CT
>dme_circ_0000942_junction_seq
AAATCGAGATTAAACCTCAAGTTCAACTGATAAACAATTCGCCTAGTTCTCTCATC
>dme_circ_0000943

GGTGGCCGGCTTCAGCATCTTTTGGGCAGATGACGGTCTTGACACAGGACCCCTACTGCTAACCCGACAA
ACAAACGTGGAATCCACGGACACTCTGGACACCATATACAAGCGCTTTTTGTACCCCGAAGGTGTGAAGG
CGATGGGCGTCGCCGTGGACATGGTGGCTAGTGGTACAGCTCCCAAGATAATTCAGCCGGAAGTGGGGGC
AACCTACGACCCGGCTATGTTTAAAACAGAAAATCAGGTTATCAATCTTCAGGCTACAGCAGAAAAAATT
TGGAACTTTGTTCGTGGTCTGGACTCAGTTCCTGGAGCCATAGCAACGGTTCTGCACGGGAATGGCACAG
AGGAGCAAATTCGACTCTTTGGTGCTCATTTGTACTCTGCTGGCCCCGTGTCGAATGGAAAGGTTCTTCG
TCTAAGAGGCTTAACAAAGACCGCCTGGATACACAGCGGTGGTCTTCTTATAGAGGGAACAGACGGTGCC
TTTGTCAACATTCGCCGCATTAAGCGTGGCACAAAGGTAATCAGTGCCAGCGAGTGGTTTAAGCAAGCTG
AGGACCAGTCTATCACTGACTTCAGCGAGGACGAATTGGTAAAGCGATCGTTGCTGGCTGGAATTTGGAA
GGCAATACTTAAGGAAGATGTCGAGTCATCGACTGATTTCTTTGCATCCGGAGCTGGTTCAATGGACGTG
>dme_circ_0000943_junction_seq
TGCATCCGGAGCTGGTTCAATGGACGTGGGTGGCCGGCTTCAGCATCTTTTGGGCA
>dme_circ_0000944
GTTCCTGGAGCCATAGCAACGGTTCTGCACGGGAATGGCACAGAGGAGCAAATTCGACTCTTTGGTGCTC
ATTTGTACTCTGCTGGCCCCGTGTCGAATGGAAAGGTTCTTCGTCTAAGAGGCTTAACAAAGACCGCCTG
GATACACAGCGGTGGTCTTCTTATAGAGGGAACAGACGGTGCCTTTGTCAACATTCGCCGCATTAAGCGT
GGCACAAAGGTAATCAGTGCCAGCGAGTGGTTTAAGCAAGCTGAGGACCAGTCTATCACTGACTTCAGCG
AGGACGAATTGGTAAAGCGATCGTTGCTGGCTGGAATTTGGAAGGCAATACTTAAGGAAGATGTCGAGTC
ATCGACTGATTTCTTTGCATCCGGAGCTGGTTCAATGGACGTGGTGCGCTTAGTCGAGGAAGTTAAAGAA
GCCTTTGACGTGCCCCTGCAGAACGAGCAAGTTTTCATGGCGCCTGTGTTTGAAGAGTTCTTTAGCCTGC
TGGTTAGGTTCCTTCGACAGGGAAGTGCCGGTTTCGGTAACCAGCAGCTCGATTTTGAGGCATTCACGCT
GAAAGCCAACAAAAGAGAGATTCGAGTGCCCACACAACTTTTTATCAACGGAGAGTTTGTTGACGCCGAG
GCTCAACGCACTTTGGAAATT
>dme_circ_0000944_junction_seq
CGCCGAGGCTCAACGCACTTTGGAAATTGTTCCTGGAGCCATAGCAACGGTTCTGC
>dme_circ_0000945
GCGTTATTAAACCGCATACACCAATTGGCTCCTTACGGGTGAAGGTCAGTACGTTGTTGGGTCTCGCAGG
GTTAACAGGGATTGTGTTGCCCTGGATCTTGTCGCACCATCCGGCAAAGTAGCGCCACGCTTCGATGGAC
ATTCCAACATGGGTTTTAAGGGCTAGCGTGTAAACCGCTCCCGAATCGACTGACTCAATTGTGGCCAGCT
CCTCCTTATTTCGTTCCATCAGATCAGCCAAGTTCAGCATCAACTGGCCTCGTTGTCTGGG
>dme_circ_0000945_junction_seq
TCAGCATCAACTGGCCTCGTTGTCTGGGGCGTTATTAAACCGCATACACCAATTGG
>dme_circ_0000946
GTATTCCCGGCGGCAATGCAGGCGGCCATTTTCCAGGAAAGCATCATCAGGGGGTAGTTCCAAGGCGTTA
TTAAACCGCATACACCAATTGGCTCCTTACGGGTGAAGGTCAGTACGTTGTTGGGTCTCGCAGGGTTAAC
AGGGATTG
>dme_circ_0000946_junction_seq
TTGGGTCTCGCAGGGTTAACAGGGATTGGTATTCCCGGCGGCAATGCAGGCGGCCA
>dme_circ_0000947
GTAACGATTCACATTAAATGCAAGTCACAACAATACTATGGTTACAGACGTTTaaaaatatataaaaaaa
aTTTTGCACCAGAACAGTTGATAAAAGAAACTTAAAATAACGACTTAAAACAAAAATAAATTCGAAATAA
TTCAACTTGAAAGACAATACGCCCATCAACACACTACAAAGTTAAAATGGCCGATAAAAAAATTGGTGAA
TACTATGCACCACCTGTGAAAATGGGCAAATGGGAGGGTTTCAAAAAATTCCTATGGAACAGTGAAACTA
GCCAATGCCTTGGACGCACCGGATCCAGTTGGGGGTAAGTACATTGAATCCCTTCATTTTTATTTAATAT

TATGTCTAATAACAACAATTTACTGAGTGGATTTTGGTATGTCCAGACCTAAATATACATTGCTACACAA
AATTAAAAACTATTAAAACAGCTTATCAATAAAATCGATATCGGCGTGTGATCCCAATATATGTATATAT
TTTGTAGGACGTATAAAGTTTTTCTACTTCTCATCGACGTAATATAGATTGATGGTTATCGGCATATAAA
TACAGATATTTTATTAAATTATATTACCAACCAAAAATCAATTATTGATCAAGTAGTTCAAGCTATTCGG
TTTCGGAAATTGAAGTAGCTAATAAAACAAATTCTAGCCCCATTGAATAGATTTCTATAATTGTACCAGC
TTTGTAATAGGTCATGTAAGAAACGTCAGAATTGTGTCACCTACTTACTGGCAAACGGGAGTATGTCACT
ACAGTTAAATTTAAGGTAAACCTCTATTCTCTTTTTGTAATGCAAACAATGGCTGTGTTGTCCTAATTCT
TACAAGGGTTAGTTAGATTATGGGTATGGCTAACTGTTTTATTTATTACGGTCTTATTAATTTATCTTAA
AATATACATACACATACATACATACAATACCTATTATGATATTAACATTAAATAATTTAAACTTTGCAGT
CAAACGAAGTATGTGTACATTTTTAGATGAAAACACATTTGTTAGCAAGGTATCAAAATCTCGGTCCATT
TATCAATTGCCACAATTATCGTAAAGGCAATTTAAAAAATAGATTCATCTGGGGCTCAATTATTGAAATC
AACTTGCGGCGCAGCAATCTGTGCTACCAGTAAAGCAATTAAATGAATATCGTTTCAATTTACAGTGATA
CTCGAAAGTTAGCTAACAACATGCTAAATAAAACGCTGCGATTTTTAACGAAACCGTAATAATTCAACTA
AATACTATAACCTTTACACAAGTGACACCGTAATTAGCTTGCGAATCATAAAAAATTTGAATGAATttcc
ccgactaacagatacccgttactcagctagtggaaatgcgagcgcgaaatttcaccatttttctggaata
tcgatagatattggAAACTCTCTTGATTTTTATTAGTCATGTAAATTTATATCGCTGTTTACATTTTtta
tacccgctactcgtagagtaaaagcaaagacgctagaataattattctagtgtctttggtgaaagggtat
tctagattcgatgaaaagtatgtaacaggcaggaggaaacgttttcgactatataaagtatatatattct
tgatcaggatcaatagccgagtcgatttagccatgtccgtataaacgtcgagattccaggaactataaaa
gcatacagattctatagacaaagtcgcagcgcaagtttgttgatccatgttgttaagcccactctaactc
taaaatgtgttgatatattttcatatttttattagtattgtacatttttattgatttgcaaaaaaaaaaa
ttgccacgcccactctacctcctaaaaccggccaaaactgccacgcccacatttttttttaacaattttt
cgatatttttttaataattttattagtcttgtaaaatctatctatttgccaaaaaacttgttgccacgccc
actctagcgccataaagaacaattcctctttggaacaattttgataatttttcttatttttcttctccaat
atctatcgatatcccagaaaaattatgaaatttcgcattcacacttgctgaatagcgggtatctgatagt
cggggaTAATAATCAAGATCAATTTATTTCAAATTATCTAATACTGGCAATGTTTTATTGTAGATAAGCA
GATTCTTACATGCATATTTCCTTTTCATTTTCTTTGACCACCTTATAGTTAGTTCAGAAGATTGGTCTCA
CTTGTTGGTATCTCGTTCGCCCTCtcttattcactaactattaactaactattcactaactaatcactaa
ctatttaacgggtatctgatagtcgggaaactctgctatgcgaactcgtttgttATTTATTAAGCTAAGA
ATTATACCTATGAGTACTTTATAACTGTTGACTCTTTCCGTGTTTCACATCTTTCCAGGCGAAAATTCTC
CTATTTTACATAATTTTTTATGCGGCGTTAACTGGTTTTTTTGCTGCAATTTTCACTGTATTTTATCAAA
CTTTGGACAATGAAAAGCCAAAATGGATGCTTGACAATGGTTTGATAGGGTCCAACCCAGGGTAAGTAAC
GAAAAAACATACTACCTTTAAATCCCCATGAATCTTGTTAAAATGAATATACTGTGTTGGGGAATACAGT
TAAGATTaaaaaaaaaaaattaaaaaattaggttaatttaacatacaaatttaatatttacttaatttta
catattaTTTGAAAAAAGGGTTCTGGATTCTATATTCTTTTTCATTGTGATGTTCTCTGTACTAAGTGTT
TCGCACAATATTCCAGATAGAAAGAAATACAATATTTCATTTTAAATTTCTGTAACCACAGTTTTTTCAA
ACAGGAAACCAAAGTTCTGAGCCAAAGAGTAAGAAATCAGATTAAGAATGCATCAGTAAAATTCATTACT
TTACGTTTCATTAAAATTATGAAAAGGAAAACTAAACGTTTACAAATTAAAGTTTATTTACTTTGAAATT
TCAAGATGTCGACCACATCTTTTTTATTTTAAATACCAATAGCAAATTTACTGATAATTTATGCTTATTT
ACGGAGATAAAACACAAGTCTCTTTGGCCGGAATTCTAAAAAAGACAATTACGAATGTAACCAGAGAGAT
GAATGTTTAAAAAATGTATATACAAGTAACCTGCCTTAAAGAGAAAATAGTTACAATAATTTATATTTTC
TCAACTCAACTCAACGACGATAATATTTTTCCCTCTACATCAGGTCTAGGCTTTCGACCAATGCCACCGG
AAGCGAATGTTGAGAGCACATTAGTTTGGTACGAGTCATCAAAGAAGGATAACTATAAGTACTGGGTGGA
CGAGACTTCACGTTTTCTGAAAT

>dme_circ_0000947_junction_seq
GTGGACGAGACTTCACGTTTTCTGAAATGTAACGATTCACATTAAATGCAAGTCAC
>dme_circ_0000948
GCGAAAATTCTCCTATTTTACATAATTTTTTATGCGGCGTTAACTGGTTTTTTTGCTGCAATTTTCACTG
TATTTTATCAAACTTTGGACAATGAAAAGCCAAAATGGATGCTTGACAATGGTTTGATAGGGTCCAACCC
AGGGTAAGTAACGAAAAAACATACTACCTTTAAATCCCCATGAATCTTGTTAAAATGAATATACTGTGTT
GGGGAATACAGTTAAGATTaaaaaaaaaaaattaaaaaattaggttaatttaacatacaaatttaatattt
acttaatttttacatattaTTTGAAAAAAGGGTTCTGGATTCTATATTCTTTTTCATTGTGATGTTCTCT
GTACTAAGTGTTTCGCACAATATTCCAGATAGAAAGAAATACAATATTTCATTTTAAATTTCTGTAACCA
CAGTTTTTTCAAACAGGAAACCAAAGTTCTGAGCCAAAGAGTAAGAAATCAGATTAAGAATGCATCAGTA
AAATTCATTACTTTACGTTTCATTAAAATTATGAAAAGGAAAACTAAACGTTTACAAATTAAAGTTTATT
TACTTTGAAATTTCAAGATGTCGACCACATCTTTTTTATTTTAAATACCAATAGCAAATTTACTGATAAT
TTATGCTTATTTACGGAGATAAAACACAAGTCTCTTTGGCCGGAATTCTAAAAAAGACAATTACGAATGT
AACCAGAGAGATGAATGTTTAAAAAATGTATATACAAGTAACCTGCCTTAAAGAGAAAATAGTTACAATA
ATTTATATTTTCTCAACTCAACTCAACGACGATAATATTTTTCCCTCTACATCAGGTCTAGGCTTTCGAC
CAATGCCACCGGAAGCGAATGTTGAGAGCACATTAGTTTGGTACGAGTCATCAAAGAAGGATAACTATAA
GTACTGGGTGGACGAGACTTCACGTTTTCTGAAAT
>dme_circ_0000948_junction_seq
GTGGACGAGACTTCACGTTTTCTGAAATGCGAAAATTCTCCTATTTTACATAATTT
>dme_circ_0000949
GATTTACAATTGGATACCAGAAATTTACAACGATTCTAAGACTTTGCCAGATCATATGCCAGAGGAACTA
AAACAGCACATCAAGGAAAAGCAAAGTCTTAGGCCCAATGAAGTAAGCTTAGAAAGAATATTTTAATAAT
ATTAAATTTTAATAAAAAAATGGTTATTTAAATACTACTTCCAGACAAATGTAGTTTGGGTCTCGTGCGAG
GGAGAAAATCCCGCTGATGTCGAGAACATAAAAGCACGCGACTACTATCCCCGAATGGGATTTCCTC
>dme_circ_0000949_junction_seq
GACTACTATCCCCGAATGGGATTTCCTCGATTTACAATTGGATACCAGAAATTTAC
>dme_circ_0000950
TAACGAGGAAATCCCATTCGGGGATAGTAGTCGCGTGCTTTTATGTTCTCGACATCAGCGGGATTTTCTC
CCTCGCACGAGACCCAAACTACATTTGTCTGGAAGTAGTATTTAAATAACCATTTTTTTATTAAAATTTAA
TATTATTAAAATATTCTTTCTAAGCTTACTTCATTGGGCCTAAGACTTTGCTTTTCCTTGATGTGCTGTT
TTAGTTCCTCTGGCATATGATCTGGCAAAGTCTTAGAATCGTTGTAAATTTCTGGTATCCAATTG
>dme_circ_0000950_junction_seq
ATCGTTGTAAATTTCTGGTATCCAATTGTAACGAGGAAATCCCATTCGGGGATAGT
>dme_circ_0000951
CAAGACTTTATCATTTATATGGCTCAATACCCTATACGATCAAAGAAATGTATCTTGTTAAGTGGATGGA
AAAGGGGTGACTCAAATTTCTCTTATGATTTCCACAATCTGGTCAACGGGTTGCCTGTGAGTAAATAACA
AAATTTTATATAATATACATATAGTATATTGCAGTTCATATGCCACTTTCTGCATCCCGAAGGATGATTG
ACTTATCAGGAACTTAACTATGGGAACGGATGGACTAAGAAATTCTGAACTCTCCTGGATTTCGCTGTAA
AACACACATATGATGGACACGGGTAAATTGGG
>dme_circ_0000951_junction_seq
CACATATGATGGACACGGGTAAATTGGGCAAGACTTTATCATTTATATGGCTCAAT
>dme_circ_0000952
AGTCGTAATAGAGATGTGGAACGAGACCGACATGATCAGCAAGACCAACGAAGCATTAGTAGCCATCGCA
GTTCCAGAGACATTGTGCGGGTCAAGGAGCGCACAGAAGCCGAGCTGCATCAGAGG

>dme_circ_0000952_junction_seq
GCGCACAGAAGCCGAGCTGCATCAGAGGAGTCGTAATAGAGATGTGGAACGAGACC
>dme_circ_0000953
GATTTGGCGCATGAACTCCAGTGAGCGAAAGTATGGCCAGTGGGACATCTCGATTCGTTTCTGCTGTATC
TTGCGCACCTCTGCCCGGTAGCTGTCCCGCATTCCCTTGAAACGTTTCTTGCAGATCGAGACTGCAAAAT
GACCAGGTGTTATTTATAGGTTTCCTAGGCATATAAAACCCTCCTCGTTTATCCGCCACTCACCATCCTG
CTGCATTATCTGCGCGACACTCGCCCACTGGAGAGCTGCGTCCTGGTTGCGGTACGAAATGGACATGTTT
>dme_circ_0000953_junction_seq
CTGGTTGCGGTACGAAATGGACATGTTTGATTTGGCGCATGAACTCCAGTGAGCGA
>dme_circ_0000954
GTTTCCTGGATATACTCATTGGCTCCGGAACTGTAGAATCGGTTCTACACGATGAAGCAAATCCCTTTGC
AGAGGATATCTTTAGGCTGCTCAACCTGGAGGTTAAGGGCTACAAGAAGCTGTACAAGACTGCGACCAGC
ATCAGTGCTTTTTGCCAGTTGCTCCAG
>dme_circ_0000954_junction_seq
CATCAGTGCTTTTTGCCAGTTGCTCCAGGTTTCCTGGATATACTCATTGGCTCCGG
>dme_circ_0000955
GCCTTCCGTTGAGCGGGGGGCAGGAACTGCATCATCTTTTGCTGTTGTTTGAGGAACGTCTGAGGGTCGG
TGGCCATTGTCTGTGCCAACAGGGCATTGAGACTGTCTTTGGATGACAGACCTCCCATTGCCATATTCTG
CAGGGCGCTCAAgccgctgccgccggctcctccgccgctggccgacgagcttcctccTGTGCCGGATCCG
CTGTTGGACGAGCCGCTCTTGGATTCTGAAAGACAGATACATTCGATTAGTGAAAAATCCTATTAGATGG
ATGCCAATCTTCGGCTGGTGAAAAGGTAAAAGCGCAGAGACTCAAACTCACTTCCAGGCACTGTGGTAAC
AGTGATAGTAAAGGGTTTCTGATTTGACTGCTGGTTGGACGAGGATTGAAGGTTTGCTGGCGTCACCGAT
ACGCTTGGAGGGATCTGCGGTTTCTTTGATGATGGCAGAGATGGGTCAGGGAAGGTCTTTTGCTCTTTAG
CAGCCGATAGGCCGAGAACGATCTCGTCCAACTTCTTCCTCTTTTTCTCCTTACCAATAACAGGCTCTTC
AATGGTCTACAATAAAACAAAACAAAGGTGTAAGTTGCCTTAAATAATTTCAATTTAATTTAATTTAAAA
CTTACGCAATTGTTTTTCTTCATCAATTTATTAAGCGTGTCGTCCAATTTGCTCTTGTTTCCCATCGAGG
GAAAAGCGGAACTTAAAGCAGCATGTACACTGGATTTCTTAGAAGGCATAGAGAAGTCCTGAACCTCACT
CAAATCGATGGCTCCCGAACTGGAAGCCGACTTAAGCATCTCAGTCATATTCATTTTCGGCAGGCTGTTA
GACACAGTTTTAAAAAAATATTTGTACAATGGGAAACGTAATGACCTATTTTAAAGAACACACCTTGATG
AAAGGTCCATTGGTGCACCCATTGAGGGAGGAGGAGTCTTCATTGCGGGGACAGCTGGCTTGCTGAACTG
TCCAGATGATTGTCGCGAGAGTGAAGCATGTTGATGAGCTGGAGGCGGCTGCATGCCACTCGAAGCAGAA
GAATTGCCACCTCGACCACGACCAGAGGAGCCACTCAAGGCCTCCATGTTTGCAATCTCACTCTCCCAGT
CTTTCGGCGCCACTAAAGTTGAGATAAGGTTAATTGGGGTAAACAGAATTTTAGTTTAAAATACACTTAC
TTGTAGAACTGTTAAGCAGAGCGTGGAGCTTAGCCCTTTCCGTCTCCACGTCAATGGCGATGTGTCGCTT
GCGCTTCTTTCCCACTCCAGACCCGGCACTCGGAACGCTCAATCCGCTCACTCCACCGGCCGAAGAGCCA
GTTGCTGCACTCAGTGACATTCCGGGAAGCAGAGACGATAGGGAGGTGGCATTTCCACCTGCTGCAGCTG
CCGCAACGGCCGCTGCCACAGCAGCATTGATGCTTTTAGCATCCAAGCCAGGTAAACTGTTGGAAGAAGA
GGGCTTTGATTGGGTGACCTGGGGAACTCCCGAGGAAGTGGATGCAGCGGACGCCGAGCTGGCAACTTGC
TGCATGTGGGAGGTCTGCAAGTGCTTTGTGACACCTTGGTCCGTTGTTATGGTAATGACATCGGGTGTTG
TGGAGCGACGTTCCAAACTGCTCAAGTGAGGAATGGATTCGTGAAGAGCAATGCTTAGATCAGTGGCCCC
CTTGCAGGTTGCAAAGGCACTGTATTTACTATCCACAGGCCAATTTCCTGACTCAACGCAAGCGACAATG
TGCGCAATACGGCGTTCTAACGCAAAATCCTTAAACCATCGGATCATGCACATTTCAAGCTTTGGCTTCT
CCAACTTTTGCTGCTTAACAGTTGCCGGTTGAATCACTTCCAAGTCCGGAAAGCGGGCTTTAAGTTCTGC
TGCCTGCTTCTTGCATTCTTCTTCTACTGCTTTCTCTTTTTCCTTCATCACCTCGTCATCATCGGGATCT

GAAGGACCAGCCACCAGGTCGAGTATGTCTTGGTCGCCAGTGGATTTTTTGGGCACAGCTACTTCGGGTT
CGAGGACAGTTTCAGATTTTTCAGAGGGCTCTGATTCTATACTCGATTTTTCCAGTTTTTCC
>dme_circ_0000955_junction_seq
TTCTATACTCGATTTTTCCAGTTTTTCCGCCTTCCGTTGAGCGGGGGGCAGGAACT
>dme_circ_0000956
AATCCAAGAGCGGCTCGTCCAACAGCGGATCCGGCACAggaggaagctcgtcggccagcggcggaggagc
cggcggcagcggcTTGAGCGCCCTGCAGAATATGGCAATGGGAGGTCTGTCATCCAAAGACAGTCTCAAT
GCCCTGTTGGCACAGACAATGGCCACCGACCCTCAGACGTTCCTCAAACAACAGCAAAAGATGATGCAG
>dme_circ_0000956_junction_seq
CCTCAAACAACAGCAAAAGATGATGCAGAATCCAAGAGCGGCTCGTCCAACAGCGG
>dme_circ_0000957
GTGTAGTAGATTAACGTATAGAAAAACTGCAACACAACTTAAGTGAGATTGCCATTCAATATGTTCGCCA
ACAAATTGTTGACCTGCTCGGCATTACTAATAATGTTATTTTTACTAAGCAGTACAG
>dme_circ_0000957_junction_seq
ATAATGTTATTTTTACTAAGCAGTACAGGTGTAGTAGATTAACGTATAGAAAAACT
>dme_circ_0000958
CCCCAATGGATGGTTGCGGATATGGTTGCGGGGCAATGGTCGTGCTTATCACTGGTGCGGTGGTCGATGG
TGATTCCGTTGAAGGTGTGGTAGATCTCGGAGTTATTGTAGAAGTTGATCTGGTTGTGATAGGTTTCTCA
GTTGTTGTCGAAGACGTTGAGATTGTTGAGGAGCTGCTGG
>dme_circ_0000958_junction_seq
GACGTTGAGATTGTTGAGGAGCTGCTGGCCCCAATGGATGGTTGCGGATATGGTTG
>dme_circ_0000959
GTAATGACACCACTGTGCTTTCCTTTATGCAAATTCATCTGGCTTTGCTGCCTGAGGATTTTCCAGATGC
AAAGGGTAAGAAAATATAAAAAATAGTAGACAGATCTGTGTCGAATAATCCCCAATTCCAGAAAATCAAA
CGGTTCAGCTTATTACCAGGAGCGACGGAGCGTTTAAGACACCAGAAAATATGTCGTATCACTGCACTCG
TGTTCAGAAGATAAATATGACTGAGACACTCGATGCTGAGCAGTTAATTG
>dme_circ_0000959_junction_seq
GAGACACTCGATGCTGAGCAGTTAATTGGTAATGACACCACTGTGCTTTCCTTTAT
>dme_circ_0000960
GCTTATTACCAGGAGCGACGGAGCGTTTAAGACACCAGAAAATATGTCGTATCACTGCACTCGTGTTCAG
AAGATAAATATGACTGAGACACTCGATGCTGAGCAGTTAATTGGTTGGATCAGCGTCAGTCATGTTCAG
>dme_circ_0000960_junction_seq
TGGTTGGATCAGCGTCAGTCATGTTCAGGCTTATTACCAGGAGCGACGGAGCGTTT
>dme_circ_0000961
GATAAATATGACTGAGACACTCGATGCTGAGCAGTTAATTGGTTGGATCAGCGTCAGTCATGTTCAGGTG
GAGGCTTTCCGGAGGGCCAACGACACTGGTTTCTCAGTTGGACACGATTGCGATAGCTCGGAAACTTCGG
ATGTGGTTCCCATTGCCGTTGGAATTGCTTTAGCTGCATTAATTTTGGTGGTATTAATATCATATCTGTG
TGCTAGGCGTCGTTCCACCTCCCGCG
>dme_circ_0000961_junction_seq
TGTGCTAGGCGTCGTTCCACCTCCCGCGGATAAATATGACTGAGACACTCGATGCT
>dme_circ_0000962
GTATGGACAGCAAATTGTTAGAGAGAAATTTAAGATTGATTTTTATTATTGGAAGCCAAAATTGATTAGT
TGAAGTTGAATTAGtttttttttCAAGAGGGTCATCGAAATCATCAAGCGCAATTGGGTTTAGAATAATA
AAACGGTTCTTTAAAAACTTTTTTACGTCTCTGTCTTTGTAAGTGTCCGATATTGGTTCTTGTATGCTGA

TATTTGTGTTTTATTTAATTGGTTTGAGCAATGTAAATGTaaaataaaaaaaaaaTTTCTAAATAAATTA
ATCTCTATAATGTAGCTACCGT
>dme_circ_0000962_junction_seq
AAATTAATCTCTATAATGTAGCTACCGTGTATGGACAGCAAATTGTTAGAGAGAAA
>dme_circ_0000963
GtttttttttCAAGAGGGTCATCGAAATCATCAAGCGCAATTGGGTTTAGAATAATAAAACGGTTCTTTA
AAAACTTTTTTACGTCTCTGTCTTTGTAAGTGTCCGATATTGGTTCTTGTATGCTGATATTTGT
>dme_circ_0000963_junction_seq
ATATTGGTTCTTGTATGCTGATATTTGTGtttttttttCAAGAGGGTCATCGAAAT
>dme_circ_0000964
GAATATGGTATTGCTCGAATTTTGGAATATCAAAAGCTAAAGGCAGCTTATCAAGCTAATTAAGACAAAT
ATCTGAACCTTTATATAAATCGAATCAGAACCATAGTTAACTAGCCTAGACTTAATTGACCTTGCAATAC
CTGTCCGTCTTTGTCCGTGTCTAGAAATATTTTTAACTAAAAATGTTTGAATAG
>dme_circ_0000964_junction_seq
ATATTTTTAACTAAAAATGTTTGAATAGGAATATGGTATTGCTCGAATTTTGGAAT
>dme_circ_0000965
ATCCAACTTCGACGAAACTTTGGGTGATAGCCTTGATGGAAATAGCAATATATCGGATACGGAGACACAG
TAAGTATACCACGATCGTTCAAGAGCGAAGTCAACTGATAACCCGTTAAATTTTATTCAAAAAATCTTCG
TGTCGAATAGCAAATCACGTAGCGTGTCGCCTTCACCATGCCCGGAGTTGGACACTACGAATGACTCAGC
CCTGGATTCCAGCTTCATGGCCGATAATGAGCCGGATCTTATTATCATCGACTTTGAGTATTGTGCCTAC
AACTACCGTGGCTATGATCTTGCCAATCATTTTATTGAGTGGACTTTCGACTACACTAACCCACAGTTCC
CTTACTTCTACCACAACTCCAGCAACTGCGCAACTGTTCAGCAACGGCGCGACTTCATTGTAAACTATTT
AAAGAAATTTCACGATGACGAGAACTATAATATCACTGGCCAGGAGTTGATGAAGGTAGACGCTGAGATT
CAGTTCTTTACAATGCTGTCTCATCTGTTCTGGAGCCTTTGGTCCGTAATTAATGTCACATCGGCGATTG
AGTTTGGTTATTGGG
>dme_circ_0000965_junction_seq
ACATCGGCGATTGAGTTTGGTTATTGGGATCCAACTTCGACGAAACTTTGGGTGAT
>dme_circ_0000966
ATCCACAGTCTTAATATACCCATGTCTAAGGAGCCCGATTGGATTTGGAATTGTATGCAGCGTTGGGTTT
CCGGTTTAGAGAGCATTGTTAACGGCAGTGTCCAAACGAATCAAAAATCTTCGGTACTGAAGAAGCAAAT
GGAATTGATGCGGACCTTTGACTACGTTCAAGAGATGGCCTGGATTAGGTCGATTATCGATGAAGGAGAT
TATCCAGTTGTGTTCTGTCATAATGATCTGCAAGAAGGTAATATTCTGATGCGACAACCGTCAGCTGGTC
AAAACGAACGGACCCCCCGGGAGTCAATCAGTAGCCTGAGG
>dme_circ_0000966_junction_seq
CCCCCGGGAGTCAATCAGTAGCCTGAGGATCCACAGTCTTAATATACCCATGTCTA
>dme_circ_0000967
GCACGCGCTTTGACCACAGCAGAATTGGGAGAGCAACGAATATTAAAGAGAGTGGCTGAGAAAATGGGTG
AGATCCACAGTCTTAATATACCCATGTCTAAGGAGCCCGATTGGATTTGGAATTGTATGCAGCGTTGGGT
TTCCGGTTTAGAGAGCATTGTTAACGGCAGTGTCCAAACGAATCAAAAATCTTCGGTACTGAAGAAGCAA
ATGGAATTGATGCGGACCTTTGACTACGTTCAAGAGATGGCCTGGATTAGGTCGATTATCGATGAAGGAG
ATTATCCAGTTGTGTTCTGTCATAATGATCTGCAAGAAGGTAATATTCTGATGCGACAACCGTCAGCTGG
TCAAAACGAACGGACCCCCCGGGAGTCAATCAGTAGCCTGAGG
>dme_circ_0000967_junction_seq
CCCCCGGGAGTCAATCAGTAGCCTGAGGGCACGCGCTTTGACCACAGCAGAATTGG

>dme_circ_0000968
GCCACCCTTGAGGAAATTCGACATGCGGCCGCGCGTATTTGCCGAGACTATCTAACTGGGCCCTGGAAAG
TGGTTACTCCGGAGAGTTTGGTAGTGAAGCGCATAAGGTAAGCCCTCTAGTTCATTAAATATAAATGTAT
TGCCTTTATTCTTGTGTAGTTGTTCCAATAGGATAGTTAATCATAAGGCTCCTTCATGTATCTAATGAAT
TTAAAAGGGAAATGTAAATTGTTAGAAGACGGAAAAGACTCTGCTGCATCACAATATTTTCAAAACTCCT
TTCTTGCAGTGGCGGGCTGTCAAATTTCTTGTATTACGTAAGCCTTCCCGATTTAAATGACTACGATGAG
CTGGACGAACAGAAACAGCAGCAGGAAGACATCATAGGGGCCACCGCAACTGTGACCAATAGAAGCGGAG
TTTTCATACACGACGATGAGTCGGCTGCCAAGGCGGTGGGCGTAACATTGATGGCGAGGGAATTAATAAA
TAACGATGACGTCGCCGACGCAGCTCCTGGCGCCACACAAGCACACAAACGTCAGCGATGCGATAGTAAT
AGTCGCGAATGCAGTTCTTCGAAACGATTGCACGCCCCCAAGCAGCAACCCCGTGAGGGTCCTTCTGCGA
ATATATGGTCAGACCCATGGTGATCACGCCCTGGAGAGCATGATTACCGAGTCCGTGGTTTTCGCCCTTC
TCAGTGAACGGAACTATGGGCCCAAACTGCACGGAATCTTTCCGGGCGGACGTATTGAACAATATATTCC
GGTATAGTCAATAAGCCaaaaaaaaaaaTGAGCAAAGCTTAAGCAAGATGTATTTTTCTGCGCATTTCTG
TTCTTAACAAGAGAGAACGCAATTATCGAGTCTCTCGCCTATCAGAAATTTTCAATTTTGGCAAATCGAT
AGAAGCATGCAAGAAAGATAATATGGAAATAACTTTAAATTATGGTCGAGATTAGAAAGCTGCGTTTCCA
ATAAGTTTTTGATATTCTTATAGAATTTGGCAAGACGTgtttttgagcgttagagtgggcgcggcagcat
cagtaaagaaacttgcgctgcttctatgtctcggaatcaaccttctaggctttgtagttactgagatctc
gacgttgacaaaacaatagacgatggatatagccagaacacctcgtattttgttcctggtttagaatata
catatatactttatatgttcgtaaaagcctccttctttctgttacatattacatctagtatacccttgaa
cgctacgagtaacggAAAACAGAATATACGTTTCAAAAAGATCTCTTTTCAAAATAGTTTTAATTATTGT
GTATCTATTCGTGGCTTCAAACGAATATTAAAACCAAACTACATTCGGCGTTCCTtaaagagtaaagagt
aaaagagtaataagaTCCAAATATTCAATAAATTTAAATAAACAGATATTAAATAAAATTTGCTTTATTT
CCAATAGGCACGCGCTTTGACCACAGCAGAATTGGGAGAGCAACGAATATTAAAGAGAGTGGCTGAGAAA
ATGGGTGAGATCCACAGTCTTAATATACCCATGTCTAAGGAGCCCGATTGGATTTGGAATTGTATGCAGC
GTTGGGTTTCCGGTTTAGAGAGCATTGTTAACGGCAGTGTCCAAACGAATCAAAAATCTTCGGTACTGAA
GAAGCAAATGGAATTGATGCGGACCTTTGACTACGTTCAAGAGATGGCCTGGATTAGGTCGATTATCGAT
GAAGGAGATTATCCAGTTGTGTTCTGTCATAATGATCTGCAAGAAGGTAATATTCTGATGCGACAACCGT
CAGCTGGTCAAAACGAACGGACCCCCCGGGAGTCAATCAGTAGCCTGAGG
>dme_circ_0000968_junction_seq
CCCCCGGGAGTCAATCAGTAGCCTGAGGGCCACCCTTGAGGAAATTCGACATGCGG
>dme_circ_0000969
GATTTTTGATTCGTTTGGACACTGCCGTTAACAATGCTCTCTAAACCGGAAACCCAACGCTGCATACAAT
TCCAAATCCAATCGGGCTCCTTAGACATGGGTATATTAAGACTGTGGATCTCACCCATTTTCTCAGCCAC
TCTCTTTAATATTCGTTGCTCTCCCAATTCTGCTGTGGTCAAAGCGCGTGCCTATTGGAAATAAAGCAAA
TTTTATTTAATATCTGTTTATTTAAATTTATTGAATATTTGGAtcttattactcttttactctttactct
ttaAGGAACGCCGAATGTAGTTTGGTTTTAATATTCGTTTGAAGCCACGAATAGATACACAATAATTAAA
ACTATTTTGAAAAGAGATCTTTTTGAAACGTATATTCTGTTTTccgttactcgtagcgttcaagggtata
ctagatgtaatatgtaacagaaagaaggaggcttttacgaacatataaagtatatatgtatattctaaac
caggaacaaaatacgaggtgttctggctatatccatcgtctattgttttgtcaacgtcgagatctcagta
actacaaagcctagaaggttgattccgagacatagaagcagcgcaagtttctttactgatgctgccgcgc
ccactctaacgctcaaaaacACGTCTTGCCAAATTCTATAAGAATATCAAAAACTTATTGGAAACGCAGC
TTTCTAATCTCGACCATAATTTAAAGTTATTTCCATATTATCTTTCTTGCATGCTTCTATCGATTTGCCA
AAATTGAAAATTTCTGATAGGCGAGAGACTCGATAATTGCGTTCTCTCTTGTTAAGAACAGAAATGCGCA
GAAAAATACATCTTGCTTAAGCTTTGCTCAtttttttttttGGCTTATTGACTATACCGGAATATATTGT

TCAATACGTCCGCCCGGAAAGATTCCGTGCAGTTTGGGCCCATAGTTCCGTTCACTGAGAAGGGCGAAAA
CCACGGACTCGGTAATCATGCTCTCCAGGGCGTGATCACCATGGGTCTGACCATATATTCGCAGAAGGAC
CTGTGGGTGTATATCAAGGATTAGCTTTCAAAGAGGGTAACAGGTACTGTGGATGACCTACCTCACGGGG
TTGCTGCTTGGGGGCGTGCAATCGTTTCGAAGAACTGCATTCGCGACTATTACTATCGCATCGCTGACGT
TTGTGTGCTTGTGTGGCGCCAGGAGCTGCGTCGGCGACGTCATCGTTATTTATTAATTCCCTCGCCATCA
ATGTTACGCCCACCGCCTTGGCAGCCGACTCATCGTCGTGTATGAAAACTCCGCTTCTATTGGTCACAGT
TGCGGTGGCCCCTATGAT
>dme_circ_0000969_junction_seq
TGGTCACAGTTGCGGTGGCCCCTATGATGATTTTTGATTCGTTTGGACACTGCCGT
>dme_circ_0000970
CTGGACGAACAGAAACAGCAGCAGGAAGACATCATAGGGGCCACCGCAACTGTGACCAATAGAAGCGGAG
TTTTCATACACGACGATGAGTCGGCTGCCAAGGCGGTGGGCGTAACATTGATGGCGAGGGAATTAATAAA
TAACGATGACG
>dme_circ_0000970_junction_seq
GCGAGGGAATTAATAAATAACGATGACGCTGGACGAACAGAAACAGCAGCAGGAAG
>dme_circ_0000971
TGAGACCCCTGTTGTCGAGAAGGTCGCAGCTGAGGAAGTTGATGCTGTGAAGAAAGATGCAGTTGCCGCC
GAGGAGGTAGCAGCTGAGAAGGCGAGCATCACAGAAAACGGTGGCGCCGAAGAGGAGAGCGTAGCCAAGG
AGAACGGAGCCGCCGACAGCAGCGCGACCGAGCCCACCGACGCAGTCGATGGTGAAAAGGCGTCTGAGCC
CACTGTTTCTTTTGCCGCCGATAAGGATGAGAAGAAGGACGAGGATAAAAAAGAGGATTCCGCTGCCGAT
GGGGAGGACACCAAAAAAGAGAGCAGCGAAGCTGTTCTACCTGCTGTCGAGAATGGCTCCGAGGAGGTCA
CCAACGGTGACTCAACAGGATGCTCCCGCCATTGAGGCTGTAAAGCGGAAGGTGGATGAGGCTGCAGCCA
AGGCAGATGAGGCCGTCGCCACGCCGGAGAAAAAGGCTAAGCTTGATGAGGCCAGCACAAAGGATGAGGT
TCAGAATGGGGCCGAGGCTAGCGAAGTGGCCGCCTAAGGGAATCGCCCGCGGAGCAGCAAACTCAGTAAT
CTACTTATTAAATACTTACTAACACAAAAAAAGCAAATCACAGAAATTACGGTTCTACCGCCAGGAACCC
GGACTGTATCCATTTTCCGCTCTTGTGAGCGGGTGATACGTGGGTGGCTGCTTGGGTGGCGGGCGCAATT
CTCCAATAACAATCCGGCAATGGGAGCGTAGTTTTATAGCGTACCGGTTCTCTCTCTCTCTACTAACAAA
CAACCTACCAACCAACAATACCGGCAACAAACGAAAACAAAAAATCCATTATTATTTAAAAACTTACTTA
AAATCTTAACTTTAAAGCAAAGtatatatatatataAAGCGAAGGATTAAGAGAACAATATTAAGCGAAA
TTAAGATGCGAGACCCTCTATGTTTTAAGAATAAAAGTTGTAAACAAAAGCGTCAAATTTAATAATTGTa
aaaaaaaaTACGAAGCGAATGAAACAAATTGCAAACAAAATCTTTAAGTAAGAAAAACATCAAAACAATT
ACGGCAAAATAACATATGTAATCCTTTACAATTTTTAATTTTACAACCGTATCTTATTTTTGAGTATGAA
TATTTATATCGAACAAAGTAAAACTAAAAATTCAGTAAAATGTAACAAAAAGCTGCCAGCAACAACCCCA
GAAAAATGTATGCCAACCGGCTAAAAAAATACTACCACATAGAAACAATAAAAAGTACGAAAAACAACTT
TTACAGCAATACTCTGAAATGTTTATTTTTAAAATGCTTTCGAAATCGTACCTTTTTTATTTGTAATCAA
GGTACCACCTATTTCACATTCCCAATTACATTTTATTTACTTGTCTAAGCCTACGAAATAGTCTGCGACG
AAACTCTCATTAATGTTCAATGTTTAACAACGATTTAATGGGCAGGAAAGGTAATGGCCAGTAAATCCAG
CATTCAGCAAAATCAAGATTCGAGATTATtaaaaaataaaatgaaacgtaaaaaattaaaatcaaactgta
aGCGTAACTTTTATTAATTTGAGCATGTTTGTATTCGCACAAATAACAGCTGTCGCCAATAGTAGAAATT
ATTATTTTCTTGGTAGGGTGCGGTCCAAGTACATATTAGTGGAAAGTAAACTATTTTAGGTAATATGATT
TCGACACAGAACGTTACAAATAGTATTACTATTTTGCCTACGGTAAGTTGTtttttttttttgtatttttG
GACTTTCGTTTAATTTTAATCAGAAAATTTAATATTTGATAATCTCTTGACTCATGACATTATATCCCAC
TAAGTAGTCGAATCTGAATCGTTCTTGTGAGTAGGCGCTTTGAAACCGTTAACGGAGACTGCGTATATAC
TCAATGTTATTTTATATTGCACTATAATAAAAACCACGTGACGCCCAATTCACCGCAAAAATCTGTTTTT

GAAGTGCTGCTGTCAGACACCGCTTATTTGTCGGTGCCTGGCTTccaaaattaaattaccaaaattaaaa
taccataaataaatAAGAAAGCGAAGGACAATGGCCACCAACCTGCAAAAGG
>dme_circ_0000971_junction_seq
AGGACAATGGCCACCAACCTGCAAAAGGTGAGACCCCTGTTGTCGAGAAGGTCGCA
>dme_circ_0000972
GATAAAAAAGAGGATTCCGCTGCCGATGGGGAGGACACCAAAAAAGAGAGCAGCGAAGCTGTTCTACCTG
CTGTCGAGAATGGCTCCGAGGAGGTCACCAACGGTGACTCAACAGGTATGatgatatcaatgatatcaat
gCCAAACAAATTTTCCACGTTCATGTTCTTATTGTTCAATTTTTATTATCGCCTTACAGATGCTCCCGCC
ATTGAGGCTGTAAAGCGGAAGGTGGATGAGGCTGCAGCCAAGGCAGATGAGGCCGTCGCCACGCCGGAGA
AAAAGGCTAAGCTTGATGAGGCCAGCACAAAGGATGAGG
>dme_circ_0000972_junction_seq
CTTGATGAGGCCAGCACAAAGGATGAGGGATAAAAAAGAGGATTCCGCTGCCGATG
>dme_circ_0000973
GTAGCAGCTGAGAAGGCGAGCATCACAGAAAACGGTGGCGCCGAAGAGGAGAGCGTAGCCAAGGAGAACG
GAGCCGCCGACAGCAGCGCGACCGAGCCCACCGACGCAGTCGATGGTGAAAAGGCGTCTGAGCCCACTGT
TTCTTTTGCCGCCGATAAGGATGAGAAGAAGGACGAGGATAAAAAAGAGGATTCCGCTGCCGATGGGGAG
GACACCAAAAAAGAGAGCAGCGAAGCTGTTCTACCTGCTGTCGAGAATGGCTCCGAGGAGGTCACCAACG
GTGACTCAACAGGTATGatgatatcaatgatatcaatgCCAAACAAATTTTCCACGTTCATGTTCTTATT
GTTCAATTTTTATTATCGCCTTACAGATGCTCCCGCCATTGAGGCTGTAAAGCGGAAGGTGGATGAGGCT
GCAGCCAAGGCAGATGAGGCCGTCGCCACGCCGGAGAAAAAGGCTAAGCTTGATGAGGCCAGCACAAAGG
ATGAGG
>dme_circ_0000973_junction_seq
CTTGATGAGGCCAGCACAAAGGATGAGGGTAGCAGCTGAGAAGGCGAGCATCACAG
>dme_circ_0000974
GAAGTTGATGCTGTGAAGAAAGATGCAGTTGCCGCCGAGGAGGTAGCAGCTGAGAAGGCGAGCATCACAG
AAAACGGTGGCGCCGAAGAGGAGAGCGTAGCCAAGGAGAACGGAGCCGCCGACAGCAGCGCGACCGAGCC
CACCGACGCAGTCGATGGTGAAAAGGCGTCTGAGCCCACTGTTTCTTTTGCCGCCGATAAGGATGAGAAG
AAGGACGAGGATAAAAAAGAGGATTCCGCTGCCGATGGGGAGGACACCAAAAAAGAGAGCAGCGAAGCTG
TTCTACCTGCTGTCGAGAATGGCTCCGAGGAGGTCACCAACGGTGACTCAACAGGTATGatgatatcaat
gatatcaatgCCAAACAAATTTTCCACGTTCATGTTCTTATTGTTCAATTTTTATTATCGCCTTACAGAT
GCTCCCGCCATTGAGGCTGTAAAGCGGAAGGTGGATGAGGCTGCAGCCAAGGCAGATGAGGCCGTCGCCA
CGCCGGAGAAAAAGGCTAAGCTTGATGAGGCCAGCACAAAGGATGAGG
>dme_circ_0000974_junction_seq
CTTGATGAGGCCAGCACAAAGGATGAGGGAAGTTGATGCTGTGAAGAAAGATGCAG
>dme_circ_0000975
GAGAGCGTAGCCAAGGAGAACGGAGCCGCCGACAGCAGCGCGACCGAGCCCACCGACGCAGTCGATGGTG
AAAAGGCGTCTGAGCCCACTGTTTCTTTTGCCGCCGATAAGGATGAGAAGAAGGACGAGGATAAAAAAGA
GGATTCCGCTGCCGATGGGGAGGACACCAAAAAAGAGAGCAGCGAAGCTGTTCTACCTGCTGTCGAGAAT
GGCTCCGAGGAGG
>dme_circ_0000975_junction_seq
CCTGCTGTCGAGAATGGCTCCGAGGAGGGAGAGCGTAGCCAAGGAGAACGGAGCCG
>dme_circ_0000976
GTAGCAGCTGAGAAGGCGAGCATCACAGAAAACGGTGGCGCCGAAGAGGAGAGCGTAGCCAAGGAGAACG
GAGCCGCCGACAGCAGCGCGACCGAGCCCACCGACGCAGTCGATGGTGAAAAGGCGTCTGAGCCCACTGT

TTCTTTTGCCGCCGATAAGGATGAGAAGAAGGACGAGGATAAAAAAGAGGATTCCGCTGCCGATGGGGAG
GACACCAAAAAAGAGAGCAGCGAAGCTGTTCTACCTGCTGTCGAGAATGGCTCCGAGGAGG
>dme_circ_0000976_junction_seq
CCTGCTGTCGAGAATGGCTCCGAGGAGGGTAGCAGCTGAGAAGGCGAGCATCACAG
>dme_circ_0000977
GAAAACCTTAAGATGACGAATTTAAGGACCTAGAAATAAAACTGCGAGGAAGGTCGAGCAAAACCTCCAA
TCACTTTCACTCATTGCATACATGAACATTAAAACTGCCGCCGATAAAGCAGCGTCGCTGACTGGGAAAT
TGTAGTTATCAAAATATCCGACATCAACAAAGAAACCACTGCCGACGAGCTCTACCGCCGATGGTTTAAT
GAACATCCCATAATGGCAGCGGCACATGAATCGCCTAAAGCACGCGATTCGACCAACGTCGAGACTGAGC
GGACGTTTCAGCTGGAGGCAGGGCAATCCTATGACACAATGGCAGAATCTCATCGCGCATTCCATTCCCT
GGAATTGGAGGCGGGAGCGGAGGTAGCCGTAGATGCCGCTGTTGAAGCTGACTCAAAAAGACAAAAGGTA
TGTGAAACAAATTTTATACGACCTTGATGAGCAATTATGATTTCAAAACTTTATCAATGAAGTGAGCTAG
TTTATGAAGTAATCCGAGTCAATAAAATTAAATTGATAAGGCTTAATGTTGTTGCTTTTAACCTCAGTAA
CCTAAAAGCTTACCGCCAGAGGCGTCAGAACCTGGTGAATTTCCAAGTCCGTATGTCCGTGCATCTGCCA
GATTTTGTGAACTATTAGAGCTACGCAGCTAGTACTAGCATGCTTTTTCTTGTGATAGCTGCACAGCATT
AGGTTTAAAACATTGTCTATATTTACCCTAGGGGCCCACAAGTCAGTCAAAAATAAACAATAAAGCCGTT
ATAAGCGTGAAATTAAATAACTATTTCTATGCAGCCGATAATCAACTGCAGTCGTTGGAACCCATTTCCT
TGGATGACATACCCGAACCAATAAAAGTCCTCGACGAGATTATTTCCGAATTTGAGGAGGCAGCAACGAA
ACCAGTGGCCTTGAACTGCAATAGTGGCGAGAATCAGTCCGAGGACGATGGCTACATGAGCCTAAGTCGC
AAGAAGGTAAGTAACAGACTAGTTCATTAGTGCCAACATAAAAACACATGCAACATAGTTAATAGTTAAT
ATTATAATAACAATAATTACCATACAATGCCATTTATTTAAAATTTTCAAGTGTGCCACGGAAAAATGTA
TCGTTTATATCTAAGATCTGTGCTGAAGTAAAATAAAACGGACATGACTTCGGACAGCTCAGAATCATCA
TAGTGATGATTATTAATTAATTAATTAAATTATCGAtaaacgggtatactagattcgataaaaagtacat
atataacaggtagaaagaagcttttccaatatatacatatatatatattcctgatcaggattaatagtcg
gatttgatctggccctgtctgtccgtatgaacgtcgagatctcaggaactataaaagctagaactagaac
gtcatacagcatacagatttcgaaaaaatagacgccattgttgttgacacatgttaccacgctcacaagc
cgccaaaaaatgcaacgcccaTTTTTCTCATTATATTCTTCAATTTAAATAAAATTAAATTTTTCGTTCT
CACTTCtatttgatagtcggcgaaatcgactatagcgttattttttgtttTATAACTAGGATCATTAATC
ATTAATCATAATCTGGCCATGTATGTCGGCCTGTCCGTCGCTGAGATCTCGGAAATTAAGCATGTATATC
AGGAAGATTGCTGTGATTTGTCATACGAATTTTGTAATGCTTTGATCCTGTTAAAATTTGTCAATAAAAT
CTGTTGTGCCTATTTTAATGCAAAAAATCCCTAGTCTGTGACACCAAATATTTCTTTTCAAAAAATATTA
TTATTATGATTATTTAAGTACTCTTCGTCCGTTTTCTAAAAAAACCCTTAACTTTAACATAACAGCTTTTA
ATGCAAAAAATCCCTAGTCTGTGACACCAAATATTTCTTTTCAAAAAATATTATTATTATGATTATTTAA
GTACTCTTCGTCCGTTTTCTAAAAAAACCCTTAACTTTAACATAACAGCATGTCCAAAAAGGATTCCGAAG
ATGTGCCCCAAACACCCAGCACAGACACTGTGCCAGAGGGAAGCTTCAACGAAGTGGATGGTACGGCTGA
GAATCTAACTAAATTATCTGAGGTTGTACACGGACAGGAAACTACATCGCCTCTCATTCAGACAACGCTG
GCCAAAGGCTAGAGGATCTAGCTCAACTCCTGCCGTAAGCTCAAACTACTCTAGTTTACCGTGCTGTGGT
GTCAGGGCTTCGAATTTGGGTGGAGTTTCTGCTGCCAATTCATCAAGTGGATTGTCAGCCATCCGGCATC
CAGGCAGCTGCAACGGGGGTCGCAACGCACTGGGCATCGACATTAGCGCTGTTCATAACGCTGTTCTGCG
TGGTAATTTGGCGAAGCTGCCGGAACCATTCGTCAACGAACATCCAGTGACGATATACCCAGGTCCCTCT
TCTAAGACAGCCGTGGGGGAGGGAGCTCGCTGCAAGCGACTACTCAGCTCTGTCGAAAAGCACAAGGAAG
TGTGCCACATGCTAAATCCACTATCCAGCGGCTCAAACTCTTCGTCCGGGGAGGCTTCGCCTGCTGCGTC
TCACGTCAATGTGGCGAAATCAAAAGCTGCTGCCAGGGATGACGAATATTCGGAAGATTCGCTGGAGGAG
TCAACCATCTCTACTGAACTTACACCCGTTAAACAGAACGGAGTCGCCTGGGAGATTCACTTTAGAAACA

GCAAGAAGAAATCCAGTGGCTTCAAAAATACGTCTGCATCTAAAACGG
>dme_circ_0000977_junction_seq
TTCAAAAATACGTCTGCATCTAAAACGGGAAAACCTTAAGATGACGAATTTAAGGA
>dme_circ_0000978
ATCCAGTGCCCCGAGGTTGCTGTAGGAGGAATATACGGTGTGCTCAACAGGCGACGTGGCCATGTATTCG
AGGAAAACCAGGTTGTGGGCACTCCCATGTTCGTTGTGAAGGCATACCTGCCCGTCAACGAGTCATTCGG
TTTTACCGCCGATCTGCGCTCTAATACCGGAGGACAGGCCTTCCCTCAATGTGTCTTCGATCATTGGCAG
GTGTTGCCTGGCGATCCGAGCGAGCCCTCAAGCAAGCCCTATGCCATCGTCCAGGATACTCGTAAGCGCA
AGGGTCTTAAGGAAGGATTGCCCGATCTATCCCAGTACTTGGACAAGTTGTAAAGCACCACCACCCGATC
CCCCACAACATCCAAGTGCAGCTGGCTGAGCGGAAAAGCATAGAAAAATATGGAGTGACCGAATTTAACA
TCTTTATTCGGCAAGGCACTTGAAACATCGCCTTTGGTCAATG
>dme_circ_0000978_junction_seq
GCACTTGAAACATCGCCTTTGGTCAATGATCCAGTGCCCCGAGGTTGCTGTAGGAG
>dme_circ_0000979
GGCGATGTTTCAAGTGCCTTGCCGAATAAAGATGTTAAATTCGGTCACTCCATATTTTTCTATGCTTTTC
CGCTCAGCCAGCTGCACTTGGATGTTGTGGGGGATCGGGTGGTGGTGCTTTACAACTTGTCCAA
>dme_circ_0000979_junction_seq
GGGTGGTGGTGCTTTACAACTTGTCCAAGGCGATGTTTCAAGTGCCTTGCCGAATA
>dme_circ_0000980
GGCGATGTTTCAAGTGCCTTGCCGAATAAAGATGTTAAATTCGGTCACTCCATATTTTTCTATGCTTTTC
CGCTCAGCCAGCTGCACTTGGATGTTGTGGGGGATCGGGTGGTGGTGCTTTACAACTTGTCCAAGTACTG
GGATAGATCGGGCAATCCTTCCTTAAGACCCTTGCGCTTACGAGTATCCTGGACGATGGCATAGGGCTTG
CTTGAGGGCTCGCTCGGATCGCCAGGCAACACCTGCCAATGATCGAAGACACATTGAGGGAAGGCCTGTC
CTCCGGTATTAGAGCGCAGATCGGCGGTAAAACCGAATGACTCGTTGACGGGCAGGTATGCCTTCACAAC
GAACATGGGAGTGCCCACAACCTGGTTTTCCTCGAATACATGGCCACGTCGCCTGTTGAGCACACCGTAT
ATTCCTCCTACAGCAACCTCGGGGCACTGGATCTCGCACAGGTAAACAGGCTCCATCAGGCGCGGCTTGG
CGGTTATAGCGGCAGCGTACAGACAACGACGAGTGGTTGGAATGATCTGGCCACCACCACGATGGATGGC
ATCAGCGTGAAGTGTCACATCGTAGATGTTGAAGCGGACACCGCGCAAGTTTTCGTCGGCTAAAATACCC
TCCTTTGAGGCCCATTGGAATCCAGCGACAACAGAGTCCTTGATTTCGTTAAGGTACTGCACGGACTTGG
TACAGTCGAGGATGAAATTCGGACCGGTTCCGTCGGGGCCGAAGCACCAGATCTTACGGGCCTCAGTCAC
ATCGTAGTCGTACTTCTCAGACAAATAACGGGCACGAGCCTTGAACTCATCCTTGGCACTCACATCGCCG
TTATCAATGTCCTCGGGAAGACCGTCTGGCATAGGTAGGGCTTTCATCAGCAGACGGTTGTGCTTGTTGG
GCGACTTGGACAGACACATCTGGTCGGACTCCTCCGACACAGTCTCACGGTAGGAGACGACGGGATCAGA
CTTCTTCAGTGGGATACAAGCGTGGTCTTCCTCTAGATCCTTAAGGCAGATCTCCAAATGCAGCTCACCG
GCACCAGCAATGATATGCTCGCCGGACTCTTCGATGATACACTGCACCATAGGATCGGACTTAGCCAAAC
GCTTCAAGCCCTCCACCAGCTTAGGCAAGTCCGCGGGGTTCTTTGGCTCAACAGCCACGCGCACCACAGG
GGACACGGAGAACTTCATGAcctaaaaacgaaaaaattttaaaagtcaataataaatttgtatgctgctt
aaaaaaagaagaaaatgaaaaaCGCTGCAAACCTTCATGTTGTGGGCGTCCTTGAAGGTGGTAATCGTAC
CGGTCTTAACCAAGAACTGATCAACACCGACCAGACCGCAAATGTTGCCAGAGGGCACATCCTCGATGGC
CTCGACGTAACGACCCATCATGAGAATTGTGCGCTGAATAGCCTTCTCATATAAGTCCTCCTTCTTGCCG
GGAGTGTAGTTGGGGCCCATGATTCGGCACTTTTGCCCGGTGGCCACCTTACCGGCGAACACACGACCGA
AAGCATAGAAGCGACCCTTGTCAGAGGTCGGTACCATCTTAGAAATGTACATCATTAGAGGACCATCAGG
ATCGCAAGACTTAACGGCGATAGCGGCCTCATCGTCATGGGGACCTTCGTACAGCATCTCCATGCGGTAC
TTCTGGGCAACCACTGGCGACGGCAAATGAATAGCAATCATCTGAAGTAAAGCCTCGCCGGCTGGCAGCC

AGGTGCGCATCACAGTCTTCAGCAGAGCTTTGCCATCTTTGTCCTTGTCTTCATGCTTCAGAGTCACACC
GATTTTCTCGAGCAAGGTACCTATCTCCTCCTTCTTGTAATTCATAATGGCATCGAACACCTTGTAGATG
GGGTCCAGGATGTACATGCAGAACGAGCGTTTGTTGTCAGCCTCTTTCTGTTTCTGCCATTTCTTGGTCT
TGGCGTTAAAGAAGTTTTCACCCCACAATCTGTGAAGAAGTTGAACGATTAATTGTGCCTTCAGATATTT
CTTATTAACTACTTATCACTTAATGAAATGCTTAATGACCAGTTCAAATCGCTTATTTAAAAAAATAATT
AATATTATTTTTCTGGAAATTTTGTATAATACAAAATGATTCATTTCAGCCAATAAATTTGTTGCAAATT
TGATTTATAAATTTTAAGTCTTAGATATCAAAAATGACGTCCGAATATATCGATTTGTATAATATGAGGC
CAAACAACAATTGATTTCAGATATATGAAGAAATTATGTGAACAATAAAAGATTTCTGTATACAATTTAA
AAATAATTAGTGTAAGGTAACTGCCTTAACATGACTCGTTTGAACATTAGGAAAGCGGTCCCGAGTCAGC
GTTTAAACAGCATCCGAAAGTTTATTCTTCTTCTATTTAAGGTCCACTTGTGGCGCCGTCAGCACGAGAG
ACTCTAAAAGGACTTTCCTTGCGGAGATGTCTTTCAAGAAGCCGCCCGCTAAGCGCCACACGTTTTGTGG
TAGAAGTTTGAAAACATTTACAAACAAACTTGAGACATGACTCACCGGTTCATTAGCTTGACAACATCAA
TCTTGAATTTCTCGGAGTACATCTCAGAGAACTGCTTGAGGGTGAACGCCCAGCCGTGAAGCCCAGAACC
GAAGCCCACTGATCCCTTAGAGGGATCCACGCGAACCTCACCCATCGGTCCTCCGTCATCGTTATAGGTG
GCAATGATAACGTTTACGTTCTCGACGATGCGCTGGAAGGTCTGGTACAGCTCCTCGGCATCTAGTTGCA
GCTCAAGCAGAGCGCGGTCCATTTTGTTCATGAATAGGATGGGCTTAATACGCTCAGCGATGGCCTGACG
CAGCACGGTTTCGGTCTGGACACAAACTCCGGAGACGCAGTCTACAACAACGA
>dme_circ_0000980_junction_seq
ACTCCGGAGACGCAGTCTACAACAACGAGGCGATGTTTCAAGTGCCTTGCCGAATA
>dme_circ_0000981
CAAGCCCTATGCCATCGTCCAGGATACTCGTAAGCGCAAGGGTCTTAAGGAAGGATTGCCCGATCTATCC
CAGTACTTGGACAAGTTGTAAAGCACCACCACCCGATCCCCCACAACATCCAAGTGCAGCTGGCTGAGCG
GAAAAGCATAGAAAAATATGGAG
>dme_circ_0000981_junction_seq
GAGCGGAAAAGCATAGAAAAATATGGAGCAAGCCCTATGCCATCGTCCAGGATACT
>dme_circ_0000982
CGAGCCCTCAAGCAAGCCCTATGCCATCGTCCAGGATACTCGTAAGCGCAAGGGTCTTAAGGAAGGATTG
CCCGATCTATCCCAGTACTTGGACAAGTTGTAAAGCACCACCACCCGATCCCCCACAACATCCAAGTGCA
GCTGGCTGAGCGGAAAAGCATAGAAAAATATGGAG
>dme_circ_0000982_junction_seq
GAGCGGAAAAGCATAGAAAAATATGGAGCGAGCCCTCAAGCAAGCCCTATGCCATC
>dme_circ_0000983
GTACTGGGATAGATCGGGCAATCCTTCCTTAAGACCCTTGCGCTTACGAGTATCCTGGACGATGGCATAG
GGCTTGCTTGAGGGCTCGCTCGGATCGCCAGGCAACACCTGCCAATGATCGAAGACACATTGAGGGAAGG
CCTGTCCTCCGGTATTAGAGCGCAGATCGGCG
>dme_circ_0000983_junction_seq
TCCTCCGGTATTAGAGCGCAGATCGGCGGTACTGGGATAGATCGGGCAATCCTTCC
>dme_circ_0000984
GTACTGGGATAGATCGGGCAATCCTTCCTTAAGACCCTTGCGCTTACGAGTATCCTGGACGATGGCATAG
GGCTTGCTTGAGGGCTCGCTCGGATCGCCAGGCAACACCTGCCAATGATCGAAGACACATTGAGGGAAGG
CCTGTCCTCCGGTATTAGAGCGCAGATCGGCGGTAAAACCGAATGACTCGTTGACGGGCAGGTATGCCTT
CACAACGAACATGGGA
>dme_circ_0000984_junction_seq
CAGGTATGCCTTCACAACGAACATGGGAGTACTGGGATAGATCGGGCAATCCTTCC

>dme_circ_0000985
GTACTGGGATAGATCGGGCAATCCTTCCTTAAGACCCTTGCGCTTACGAGTATCCTGGACGATGGCATAG
GGCTTGCTTGAGGGCTCGCTCGGATCGCCAGGCAACACCTGCCAATGATCGAAGACACATTGAGGGAAGG
CCTGTCCTCCGGTATTAGAGCGCAGATCGGCGGTAAAACCGAATGACTCGTTGACGGGCAGGTATGCCTT
CACAACGAACATGGGAGTGCCCACAACCTG
>dme_circ_0000985_junction_seq
CAACGAACATGGGAGTGCCCACAACCTGGTACTGGGATAGATCGGGCAATCCTTCC
>dme_circ_0000986
GGGCTCGCTCGGATCGCCAGGCAACACCTGCCAATGATCGAAGACACATTGAGGGAAGGCCTGTCCTCCG
GTATTAGAGCGCAGATCGGCGGTAAAACCGAATGACTCGTTGACGGGCAGGTATGCCTTCACAACGAACA
TGGGAGTGCCCACAACCTGGTTTTCCTCGAATACATGGCCACGTCGCCTGTTGAGCACACCGTATATTCC
TCCTACAGCAACCTCGGGGCACTGGATCTCGCACAGGTAAACAGGCTCCATCAGGCGCGGCTTGGCGGTT
ATAGCGGCAGCGTACAGACAACGACGAGTGGTTGGAATGATCTGGCCACCACCACGATGGATGGCATCAG
CGTGAAGTGTCACATCGTAGATGTTGAAGCGGACACCGCGCAAGTTTTC
>dme_circ_0000986_junction_seq
TGTTGAAGCGGACACCGCGCAAGTTTTCGGGCTCGCTCGGATCGCCAGGCAACACC
>dme_circ_0000987
GAGGAATATACGGTGTGCTCAACAGGCGACGTGGCCATGTATTCGAGGAAAACCAGGTTGTGGGCACTCC
CATGTTCGTTGTGAAGGCATACCTGCCCGTCAACGAGTCATTCGGTTTTACCGCCGATCTGCGCTCTAAT
ACCGGAGGACAGGCCTTCCCTCAATGTGTCTTCGATCATTGGCAGG
>dme_circ_0000987_junction_seq
CCTCAATGTGTCTTCGATCATTGGCAGGGAGGAATATACGGTGTGCTCAACAGGCG
>dme_circ_0000988
GACACATTGAGGGAAGGCCTGTCCTCCGGTATTAGAGCGCAGATCGGCGGTAAAACCGAATGACTCGTTG
ACGGGCAGGTATGCCTTCACAACGAACATGGGAGTGCCCACAACCTGGTTTTCCTCGAATACATGGCCAC
GTCGCCTGTTGAGCACACC
>dme_circ_0000988_junction_seq
CATGGCCACGTCGCCTGTTGAGCACACCGACACATTGAGGGAAGGCCTGTCCTCCG
>dme_circ_0000989
GTGCCCACAACCTGGTTTTCCTCGAATACATGGCCACGTCGCCTGTTGAGCACACCGTATATTCCTCCTA
CAGCAACCTCGGGGCACTGGATCTCGCACAGGTAAACAGGCTCCATCAGGCGCGGCTTGGCGGTTATAGC
GGCAGCGTACAGACAACGACGAGTGGTTGGAATGATCTGGCCACCACCACGATGGATGGCATCAGCGTGA
AGTGTCACATCGTAGAT
>dme_circ_0000989_junction_seq
CATCAGCGTGAAGTGTCACATCGTAGATGTGCCCACAACCTGGTTTTCCTCGAATA
>dme_circ_0000990
ATCATTCCAACCACTCGTCGTTGTCTGTACGCTGCCGCTATAACCGCCAAGCCGCGCCTGATGGAGCCTG
TTTACCTGTGCGAGATCCAGTGCCCCGAGGTTGCTGTAGGAGGAATATACGGTGTGCTCAACAGGCGACG
TGGCCATGTATTCGAGGAAAACCAGG
>dme_circ_0000990_junction_seq
CGTGGCCATGTATTCGAGGAAAACCAGGATCATTCCAACCACTCGTCGTTGTCTGT
>dme_circ_0000991
ATCATTCCAACCACTCGTCGTTGTCTGTACGCTGCCGCTATAACCGCCAAGCCGCGCCTGATGGAGCCTG
TTTACCTGTGCGAGATCCAGTGCCCCGAGGTTGCTGTAGGAGGAATATACGGTGTGCTCAACAGGCGACG

TGGCCATG
>dme_circ_0000991_junction_seq
GGTGTGCTCAACAGGCGACGTGGCCATGATCATTCCAACCACTCGTCGTTGTCTGT
>dme_circ_0000992
GAGGGTATTTTAGCCGACGAAAACTTGCGCGGTGTCCGCTTCAACATCTACGATGTGACACTTCACGCTG
ATGCCATCCATCGTGGTGGTGGCCAGATCATTCCAACCACTCGTCGTTGTCTGTACGCTGCCGCTATAAC
CGCCAAGCCGCGCCTGATGGAGCCTGTTTACCTGTGCGAGATCCAGTGCCCCGAGGTTGCTGTAGGAGGA
ATATACGGTG
>dme_circ_0000992_junction_seq
GAGGTTGCTGTAGGAGGAATATACGGTGGAGGGTATTTTAGCCGACGAAAACTTGC
>dme_circ_0000993
CCGACGAAAACTTGCGCGGTGTCCGCTTCAACATCTACGATGTGACACTTCACGCTGATGCCATCCATCG
TGGTGGTGGCCAGATCATTCCAACCACTCGTCGTTGTCTGTACGCTGCCGCTATAACCGCCAAGCCGCGC
CTGATGGAGCCTGTTTACCTGTGCGAGATCCAGTGCCCCGAGGTTGCTGTAGGAGGAATATACGG
>dme_circ_0000993_junction_seq
CCGAGGTTGCTGTAGGAGGAATATACGGCCGACGAAAACTTGCGCGGTGTCCGCTT
>dme_circ_0000994
GGTATTTTAGCCGACGAAAACTTGCGCGGTGTCCGCTTCAACATCTACGATGTGACACTTCACGCTGATG
CCATCCATCGTGGTGGTGGCCAGATCATTCCAACCACTCGTCGTTGTCTGTACGCTGCCGCTATAACCGC
CAAGCCGCGCCTGATGGAGCCTGTTTACCTGTGCGAGATCCAG
>dme_circ_0000994_junction_seq
GGAGCCTGTTTACCTGTGCGAGATCCAGGGTATTTTAGCCGACGAAAACTTGCGCG
>dme_circ_0000995
CACAACCGTCTGCTGATGAAAGCCCTACCTATGCCAGACGGTCTTCCCGAGGACATTGATAACGGCGATG
TGAGTGCCAAGGATGAGTTCAAGGCTCGTGCCCGTTATTTGTCTGAGAAGTACGACTACGATG
>dme_circ_0000995_junction_seq
TATTTGTCTGAGAAGTACGACTACGATGCACAACCGTCTGCTGATGAAAGCCCTAC
>dme_circ_0000996
TGTATCATCGAAGAGTCCGGCGAGCATATCATTGCTGGTGCCGGTGAGCTGCATTTGGAGATCTGCCTTA
AGGATCTAGAGGAAGACCACGCTTGTATCCCACTGAAGAAGTCTGATCCCGTCGTCTCCTACCGTGAGAC
TGTGTCGGAGGAGTCCGACCAGATGTGTCTGTCCAAGTCGCCCAACAAGCACAACCGTCTGCTGATGAAA
GCCCTACCTATGCCAGACGGTCTTCCCGAGGACATTGATAACGGCGATGTGAGTGCCAAGGATGAGTTCA
AGGCTCGTGCCCG
>dme_circ_0000996_junction_seq
CCAAGGATGAGTTCAAGGCTCGTGCCCGTGTATCATCGAAGAGTCCGGCGAGCATA
>dme_circ_0000997
TTCTTGGTTAAGACCGGTACGATTACCACCTTCAAGGACGCCCACAACATGAAGGTTTGCAGCGtttttc
attttcttcttttttttaagcagcatacaaatttattattgacttttaaaattttttcgtttttaggTCAT
GAAGTTCTCCGTGTCCCCTGTGGTGCGCGTGGCTGTTGAGCCAAAGAACCCCGCGGACTTGCCTAAGCTG
GTGGAGGGCTTGAAGCGTTTGGCTAAGTCCGATCCTATGGTGCAGTGTATCATCGAAGAGTCCGGCGAGC
ATATCATTGCTGGTGCCGGTGAGCTGCATTTGGAGATCTGCCTTAAGGATCTAGAGGAAGACCACGCTTG
TATCCCACTGAAGAAGTCTGATCCCGTCGTCTCCTACCGTGAGACTGTGTCGGAGGAGTCCGACCAGATG
TGTCTGTCCAAGTCGCCCAACAAGCACAACCGTCTGCTGATGAAAGCCCTACCTATGCCAGACGGTCTTC
CCGAGGACATTGATAACGGCGATGTGAGTGCCAAGGATGAG

>dme_circ_0000997_junction_seq

TAACGGCGATGTGAGTGCCAAGGATGAGTTCTTGGTTAAGACCGGTACGATTACCA

>dme_circ_0000998

CATATCATTGCTGGTGCCGGTGAGCTGCATTTGGAGATCTGCCTTAAGGATCTAGAGGAAGACCACGCTT
GTATCCCACTGAAGAAGTCTGATCCCGTCGTCTCCTACCGTGAGACTGTGTCGGAGGAGTCCGACCAGAT
GTGTCTGTCCAAGTCGCCCAACAAGCACAACCGTCTGCTGATGAAAGCCCTACCTATGCCAGACGGTCTT
CCCGAGGACATTGATAACGGCGATG

>dme_circ_0000998_junction_seq

CTTCCCGAGGACATTGATAACGGCGATGCATATCATTGCTGGTGCCGGTGAGCTGC

>dme_circ_0000999

TGCCATTTCCATGTACTTTGAGGTGGAGGAAAAGGATCTGGTGTTCATTACCCACCCCGATCAGCGCGAG
AAGGAGTGCAAGGGTTTCCTGATCAACTTGATCGACTCGCCCGGTCACGTTGACTTCTCCTCGGAGGTGA
CCGCCGCCCTTCGTGTAACCGACGGTGCACTCGTTGTTGTAGACTGCGTCTCCGGAGTTTGTGTCCAGAC
CGAAACCGTGCTGCGTCAGGCCATCGCTGAGCGTATTAAGCCCATCCTATTCATGAACAAAATGGACCGC
GCTCTGCTTGAGCTGCAACTAGATGCCGAGGAGCTGTACCAGACCTTCCAGCGCATCGTCGAGAACGTAA
ACGTTATCATTGCCACCTATAACGATGACGGAGGACCGATGGGTGAGGTTCGCGTGGATCCCTCTAAGGG
ATCAGTGGGCTTCGGTTCTGGGCTTCACGGCTGGGCGTTCACCCTCAAGCAGTTCTCTGAGATGTACTCC
GAGAAATTCAAGATTGATGTTGTCAAGCTAATGAACCGGGTGAGTCATGTCTCAAGTTTGTTTGTAAATG
TTTTCAAACTTCTACCACAAAACGTGTGGCGCTTAGCGGGCGGCTTCTTGAAAGACATCTCCGCAAGGAA
AGTCCTTTTAGAGTCTCTCGTGCTGACGGCGCCACAAGTGGACCTTAAATAGAAGAAGAATAAACTTTCG
GATGCTGTTTAAACGCTGACTCGGGACCGCTTTCCTAATGTTCAAACGAGTCATGTTAAGGCAGTTACCT
TACACTAATTATTTTTAAATTGTATACAGAAATCTTTTATTGTTCACATAATTTCTTCATATATCTGAAA
TCAATTGTTGTTTGGCCTCATATTATACAAATCGATATATTCGGACGTCATTTTTGATATCTAAGACTTA
AAATTTATAAATCAAATTTGCAACAAATTTATTGGCTGAAATGAATCATTTTGTATTATACAAAATTTCC
AGAAAAATAATATTAATTATTTTTTTAAATAAGCGATTTGAACTGGTCATTAAGCATTTCATTAAGTGAT
AAGTAGTTAATAAGAAATATCTGAAGGCACAATTAATCGTTCAACTTCTTCACAGAATTGTGGGGTGAAA
ACTTCTTTAACGCCAAGACCAAGAAATGGCAGAAACAGAAAGAGGCTGACAACAAACGCTCGTTCTGCAT
GTACATCCTGGACCCCATCTACAAGGTGTTCGATGCCATTATGAATTACAAGAAGGAGGAGATAGGTACC
TTGCTCGAGAAAATCGGTGTGACTCTGAAGCATGAAGACAAGGACAAAGATGGCAAAGCTCTGCTGAAGA
CTGTGATGCGCACCTGGCTGCCAGCCGGCGAGGCTTTACTTCAGATGATTGCTATTCATTTGCCGTCGCC
AGTGGTTGCCCAGAAGTACCGCATGGAGATGCTGTACGAAGGTCCCCATGACGATGAGGCCGCTATCGCC
GTTAAGTCTTGCGATCCTGATGGTCCTCTAATGATGTACATTTCTAAGATGGTACCGACCTCTGACAAGG
GTCGCTTCTATGCTTTCGGTCGTGTGTTCGCCGGTAAGGTGGCCACCGGGCAAAAGTGCCGAATCATGGG
CCCCAACTACACTCCCGGCAAGAAGGAGGACTTATATGAGAAGGCTATTCAGCGCACAATTCTCATGATG
GGTCGTTACGTCGAGGCCATCGAGGATGTGCCCTCTGGCAACATTTGCGGTCTGGTCGGTGTTGATCAGT
TCTTGGTTAAGACCGGTACGATTACCACCTTCAAGGACGCCCACAACATGAAGGgTCATGAAGTTCTCCG
TGTCCCCTGTGGTGCGCGTGGCTGTTGAGCCAAAGAACCCCGCGGACTTGCCTAAGCTGGTGGAGGGCTT
GAAGCGTTTGGCTAAGTCCGATCCTATGGTGCAGTGTATCATCGAAGAGTCCGGCGAGCATATCATTGCT
GGTGCCGGTGAGCTGCATTTGGAGATCTGCCTTAAGGATCTAGAGGAAGACCACGCTTGTATCCCACTGA
AGAAGTCTGATCCCGTCGTCTCCTACCGTGAGACTGTGTCGGAGGAGTCCGACCAGATGTGTCTGTCCAA
GTCGCCCAACAAGCACAACCGTCTGCTGATGAAAGCCCTACCTATGCCAGACGGTCTTCCCGAGGACATT
GATAACGGCGATG

>dme_circ_0000999_junction_seq

CTTCCCGAGGACATTGATAACGGCGATGTGCCATTTCCATGTACTTTGAGGTGGAG

>dme_circ_0001000
GTCAACTTCACCGTCGACGAGATCCGTGGCCTCATGGACAAGAAGCGGAACATCCGCAACATGTCTGTGA
TTGCCCACGTAGACCACGGCAAGTCCACTCTGACCGATTCCCTTGTGTCGAAGGCTGGTATCATTGCCGG
AGCCAAGGCTGGTGAGACTCGTTTCACTGACACCCGCAAGGACGAGCAGGAGCGCTGCATTACCATCAAG
TCGACGTGCCATTTCCATGTACTTTGAGGTGGAGGAAAAGGATCTGGTGTTCATTACCCACCCCGATCAG
CGCGAGAAGGAGTGCAAGGGTTTCCTGATCAACTTGATCGACTCGCCCGGTCACGTTGACTTCTCCTCGG
AGGTGACCGCCGCCCTTCGTGTAACCGACGGTGCACTCGTTGTTGTAGACTGCGTCTCCGGAGTTTGTGT
CCAGACCGAAACCGTGCTGCGTCAGGCCATCGCTGAGCGTATTAAGCCCATCCTATTCATGAACAAAATG
GACCGCGCTCTGCTTGAGCTGCAACTAGATGCCGAGGAGCTGTACCAGACCTTCCAGCGCATCGTCGAGA
ACGTAAACGTTATCATTGCCACCTATAACGATGACGGAGGACCGATGGGTGAGGTTCGCGTGGATCCCTC
TAAGGGATCAGTGGGCTTCGGTTCTGGGCTTCACGGCTGGGCGTTCACCCTCAAGCAGTTCTCTGAGATG
TACTCCGAGAAATTCAAGATTGATGTTGTCAAGCTAATGAACCGGGTGAGTCATGTCTCAAGTTTGTTTG
TAAATGTTTTCAAACTTCTACCACAAAACGTGTGGCGCTTAGCGGGCGGCTTCTTGAAAGACATCTCCGC
AAGGAAAGTCCTTTTAGAGTCTCTCGTGCTGACGGCGCCACAAGTGGACCTTAAATAGAAGAAGAATAAA
CTTTCGGATGCTGTTTAAACGCTGACTCGGGACCGCTTTCCTAATGTTCAAACGAGTCATGTTAAGGCAG
TTACCTTACACTAATTATTTTTAAATTGTATACAGAAATCTTTTATTGTTCACATAATTTCTTCATATAT
CTGAAATCAATTGTTGTTTGGCCTCATATTATACAAATCGATATATTCGGACGTCATTTTTGATATCTAA
GACTTAAAATTTATAAATCAAATTTGCAACAAATTTATTGGCTGAAATGAATCATTTTGTATTATACAAA
ATTTCCAGAAAAATAATATTAATTATTTTTTTAAATAAGCGATTTGAACTGGTCATTAAGCATTTCATTA
AGTGATAAGTAGTTAATAAGAAATATCTGAAGGCACAATTAATCGTTCAACTTCTTCACAGAATTGTGGG
GTGAAAACTTCTTTAACGCCAAGACCAAGAAATGGCAGAAACAGAAAGAGGCTGACAACAAACGCTCGTT
CTGCATGTACATCCTGGACCCCATCTACAAGGTGTTCGATGCCATTATGAATTACAAGAAGGAGGAGATA
GGTACCTTGCTCGAGAAAATCGGTGTGACTCTGAAGCATGAAGACAAGGACAAAGATGGCAAAGCTCTGC
TGAAGACTGTGATGCGCACCTGGCTGCCAGCCGGCGAGGCTTTACTTCAGATGATTGCTATTCATTTGCC
GTCGCCAGTGGTTGCCCAGAAGTACCGCATGGAGATGCTGTACGAAGGTCCCCATGACGATGAGGCCGCT
ATCGCCGTTAAGTCTTGCGATCCTGATGGTCCTCTAATGATGTACATTTCTAAGATGGTACCGACCTCTG
ACAAGGGTCGCTTCTATGCTTTCGGTCGTGTGTTCGCCGGTAAGGTGGCCACCGGGCAAAAGTGCCGAAT
CATGGGCCCCAACTACACTCCCGGCAAGAAGGAGGACTTATATGAGAAGGCTATTCAGCGCACAATTCTC
ATGATGGGTCGTTACGTCGAGGCCATCGAGGATGTGCCCTCTGGCAACATTTGCGGTCTGGTCGGTGTTG
ATCAGTTCTTGGTTAAGACCGGTACGATTACCACCTTCAAGGACGCCCACAACATGAAGGgTCATGAAGT
TCTCCGTGTCCCCTGTGGTGCGCGTGGCTGTTGAGCCAAAGAACCCCGCGGACTTGCCTAAGCTGGTGGA
GGGCTTGAAGCGTTTGGCTAAGTCCGATCCTATGGTGCAGTGTATCATCGAAGAGTCCGGCGAGCATATC
ATTGCTGGTGCCGGTGAGCTGCATTTGGAGATCTGCCTTAAGGATCTAGAGGAAGACCACGCTTGTATCC
CACTGAAGAAGTCTGATCCCGTCGTCTCCTACCGTGAGACTGTGTCGGAGGAGTCCGACCAGATGTGTCT
GTCCAAGTCGCCCAACAAGCACAACCGTCTGCTGATGAAAGCCCTACCTATGCCAGACGGTCTTCCCGAG
GACATTGATAACGGCGATG
>dme_circ_0001000_junction_seq
CTTCCCGAGGACATTGATAACGGCGATGGTCAACTTCACCGTCGACGAGATCCGTG
>dme_circ_0001001
TGTATCATCGAAGAGTCCGGCGAGCATATCATTGCTGGTGCCGGTGAGCTGCATTTGGAGATCTGCCTTA
AGGATCTAGAGGAAGACCACGCTTGTATCCCACTGAAGAAGTCTGATCCCGTCGTCTCCTACCGTGAGAC
TGTGTCGGAGGAGTCCGACCAGATGTGTCTG
>dme_circ_0001001_junction_seq
GTCGGAGGAGTCCGACCAGATGTGTCTGTGTATCATCGAAGAGTCCGGCGAGCATA

>dme_circ_0001002
TTCTCCGTGTCCCCTGTGGTGCGCGTGGCTGTTGAGCCAAAGAACCCCGCGGACTTGCCTAAGCTGGTGG
AGGGCTTGAAGCGTTTGGCTAAGTCCGATCCTATGGTGCAGTGTATCATCGAAGAGTCCGGCGAGCATAT
CATTGCTGGTGCCGGTGAGCTGCATTTGGAGATCTGCCTTAAGGATCTAGAGGAAGACCACGCTTGTATC
CCACTGAAGAAGTCTGATCCCGTCGTCTCCTACCGTGAGACTGTGTCGGAGGAGTCCGACCAGATGTGTC
TG
>dme_circ_0001002_junction_seq
GTCGGAGGAGTCCGACCAGATGTGTCTGTTCTCCGTGTCCCCTGTGGTGCGCGTGG
>dme_circ_0001003
TTCTTGGTTAAGACCGGTACGATTACCACCTTCAAGGACGCCCACAACATGAAGGTTTGCAGCGtttttc
attttcttctttttttaagcagcatacaaatttattattgacttttaaaatttttttcgtttttaggTCAT
GAAGTTCTCCGTGTCCCCTGTGGTGCGCGTGGCTGTTGAGCCAAAGAACCCCGCGGACTTGCCTAAGCTG
GTGGAGGGCTTGAAGCGTTTGGCTAAGTCCGATCCTATGGTGCAGTGTATCATCGAAGAGTCCGGCGAGC
ATATCATTGCTGGTGCCGGTGAGCTGCATTTGGAGATCTGCCTTAAGGATCTAGAGGAAGACCACGCTTG
TATCCCACTGAAGAAGTCTGATCCCGTCGTCTCCTACCGTGAGACTGTGTCGGAGGAGTCCGACCAGATG
TGTCTG
>dme_circ_0001003_junction_seq
GTCGGAGGAGTCCGACCAGATGTGTCTGTTCTTGGTTAAGACCGGTACGATTACCA
>dme_circ_0001004
GACACATCTGGTCGGACTCCTCCGACACAGTCTCACGGTAGGAGACGACGGGATCAGACTTCTTCAGTGG
GATACAAGCGTGGTCTTCCTCTAGATCCTTAAGGCAGATCTCCAAATGCAGCTCACCGGCACCAGCAATG
ATATGCTCGCCGGACTCTTCGATGATACACTGCACCATAGGATCGGACTTAGCCAAACGCTTCAAGCCCT
CCACCAGCTTAGGCAAGTCCGCGGGGTTCTTTGGCTCAACAGCCACGCGCACCACAGGGGACACGGAGAA
CTTCATGAcctaaaaacgaaaaaattttaaaagtcaataataaatttgtatgctgcttaaaaaaagaaga
aaatgaaaaaCGCTGCAAACCTTCATGTTGTGGGCGTCCTTGAAGGTGGTAATCGTACCGGTCTTAACCA
AGAACTGATCAACACCGACCAGACCGCAAATGTTGCCAGAGGGCACATCCTCGATGGCCTCGACGTAACG
ACCCATCATGAGAATTGTGCGCTGAATAGCCTTCTCATATAA
>dme_circ_0001004_junction_seq
TTGTGCGCTGAATAGCCTTCTCATATAAGACACATCTGGTCGGACTCCTCCGACAC
>dme_circ_0001005
gTCATGAAGTTCTCCGTGTCCCCTGTGGTGCGCGTGGCTGTTGAGCCAAAGAACCCCGCGGACTTGCCTA
AGCTGGTGGAGGGCTTGAAGCGTTTGGCTAAGTCCGATCCTATGGTGCAGTGTATCATCGAAGAGTCCGG
CGAGCATATCATTGCTGGTGCCGGTGAGCTGCATTTGGAGATCTGCCTTAAGGATCTAGAGGAAGACCAC
GCTTG
>dme_circ_0001005_junction_seq
TTAAGGATCTAGAGGAAGACCACGCTTGgTCATGAAGTTCTCCGTGTCCCCTGTGG
>dme_circ_0001006
ACCGGTACGATTACCACCTTCAAGGACGCCCACAACATGAAGGTTTGCAGCGtttttcattttcttcttt
ttttaagcagcatacaaatttattattgacttttaaaatttttttcgtttttaggTCATGAAGTTCTCCGT
GTCCCCTGTGGTGCGCGTGGCTGTTGAGCCAAAGAACCCCGCGGACTTGCCTAAGCTGGTGGAGGGCTTG
AAGCGTTTGGCTAAGTCCGATCCTATGGTGCAG
>dme_circ_0001006_junction_seq
TTTGGCTAAGTCCGATCCTATGGTGCAGACCGGTACGATTACCACCTTCAAGGACG
>dme_circ_0001007

GGATCGGACTTAGCCAAACGCTTCAAGCCCTCCACCAGCTTAGGCAAGTCCGCGGGGTTCTTTGGCTCAA
CAGCCACGCGCACCACAGGGGACACGGAGAACTTCATGAcctaaaaacgaaaaaaattttaaaagtcaata
ataaatttgtatgctgcttaaaaaaagaagaaaatgaaaaaCGCTGCAAACCTTCATGTTGTGGGCGTCC
TTGAAGGTGGTAATCGTACCGGTCTTAACCAAGAACTGATCAACACCGACCAGACCGCAAATGTTGCCAG
AGGGCACATCCTCGATGGCCTCGAC
>dme_circ_0001007_junction_seq
CAGAGGGCACATCCTCGATGGCCTCGACGGATCGGACTTAGCCAAACGCTTCAAGC
>dme_circ_0001008
GGATCGGACTTAGCCAAACGCTTCAAGCCCTCCACCAGCTTAGGCAAGTCCGCGGGGTTCTTTGGCTCAA
CAGCCACGCGCACCACAGGGGACACGGAGAACTTCATGAcctaaaaacgaaaaaaattttaaaagtcaata
ataaatttgtatgctgcttaaaaaaagaagaaaatgaaaaaCGCTGCAAACCTTCATGTTGTGGGCGTCC
TTGAAGGTGGTAATCGTACCGGTCTTAACCAAGAACTGATCAACACCGACCAGACCGCAAATGTTGCCAG
AGGGCACATCCTCGATGGCCTCGACGTAACGACCCATCATGAGAATTGTGCGCTGAATAGCCTTCTCATA
TAAGTCCTCCTTCTTGCCGGGAGTGTAGTTGGGGCCCATGATTCGGCACTTTTGCCCGGTGGCCACCTTA
CCGGCGAACACACGACCGAAAGCATAGAAGCGACCCTTGTCAGAGGTCGGTACCATCTTAGAAAT
>dme_circ_0001008_junction_seq
TGTCAGAGGTCGGTACCATCTTAGAAATGGATCGGACTTAGCCAAACGCTTCAAGC
>dme_circ_0001009
GGATCGGACTTAGCCAAACGCTTCAAGCCCTCCACCAGCTTAGGCAAGTCCGCGGGGTTCTTTGGCTCAA
CAGCCACGCGCACCACAGGGGACACGGAGAACTTCATGAcctaaaaacgaaaaaaattttaaaagtcaata
ataaatttgtatgctgcttaaaaaaagaagaaaatgaaaaaCGCTGCAAACCTTCATGTTGTGGGCGTCC
TTGAAGGTGGTAATCGTACCGGTCTTAACCAAGAACTGATCAACACCGACCAGACCGCAAATGTTGCCAG
AGGGCACATCCTCGATGGCCTCGACGTAACGACCCATCATGAGAATTGTGCGCTGAATAGCCTTCTCATA
TAAGTCCTCCTTCTTGCCGGGAGTGTAGTTGGGGCCCATGATTCGGCACTTTTGCCCGGTGGCCACCTTA
CCGGCGAACACACGACCGAAAGCATAGAAGCGACCCTTGTCAGAGGTCGGTACCATCTTAGAAATGTACA
TCATTAGAGGACCATCAGGATCGCAAGACTTAACGGCGATAGCGGCCTCATCGTCATGGGGACCTTCGTA
CAGCATCTCCATGCGGTACTTCTGGGCAACCACTGGCGACGGCAAATGAATAGCAATCATCTGAAGTAAA
GCCTCGCCGGCTGGCAGCCAGGTGCGCATCACAGTCTTCAGCAGAGCTTTGCCATCTTTGTCCTTGTCTT
CATGCTTCAGAGTCACACCGATTTTCTCGAGCAAG
>dme_circ_0001009_junction_seq
CAGAGTCACACCGATTTTCTCGAGCAAGGGATCGGACTTAGCCAAACGCTTCAAGC
>dme_circ_0001010
GGCAAGTCCGCGGGGTTCTTTGGCTCAACAGCCACGCGCACCACAGGGGACACGGAGAACTTCATGAcct
aaaaacgaaaaaaattttaaaagtcaataataaatttgtatgctgcttaaaaaaagaagaaaatgaaaaaC
GCTGCAAACCTTCATGTTGTGGGCGTCCTTGAAGGTGGTAATCGTACCGGTCTTAACCAAGAACTGATCA
ACACCGACCAGACCGCAAATGTTGCCAGAGGGCACATCCTCGATGGCCTCGACGTAACGACCCATCATGA
GAATTGTGCGCTGAATAGCCTTCTCATATAAGTCCTCCTTCTTGCCGGGAGTGTAGTTGGGGCCCATGAT
TCGGCACTTTTGCCCGGTGGCCACCTTACCGGCGAACACACGACCGAAAGCATAGAAGCGACCCTTGTCA
GAGGTCGGTACCATCTTAGAAAT
>dme_circ_0001010_junction_seq
TGTCAGAGGTCGGTACCATCTTAGAAATGGCAAGTCCGCGGGGTTCTTTGGCTCAA
>dme_circ_0001011
ACAAGGACAAAGATGGCAAAGCTCTGCTGAAGACTGTGATGCGCACCTGGCTGCCAGCCGGCGAGGCTTT
ACTTCAGATGATTGCTATTCATTTGCCGTCGCCAGTGGTTGCCCAGAAGTACCGCATGGAGATGCTGTAC

GAAGGTCCCCATGACGATGAGGCCGCTATCGCCGTTAAGTCTTGCGATCCTGATGGTCCTCTAATGATGT
ACATTTCTAAGATGGTACCGACCTCTGACAAGGGTCGCTTCTATGCTTTCGGTCGTGTGTTCGCCGGTAA
GGTGGCCACCGGGCAAAAGTGCCGAATCATGGGCCCCAACTACACTCCCGGCAAGAAGGAGGACTTATAT
GAGAAGGCTATTCAGCGCACAATTCTCATGATGGGTCGTTACGTCGAGGCCATCGAGGATGTGCCCTCTG
GCAACATTTGCGGTCTGGTCGGTGTTGATCAGTTCTTGGTTAAGACCGGTACGATTACCACCTTCAAGGA
CGCCCACAACATGAAGG
>dme_circ_0001011_junction_seq
ACCTTCAAGGACGCCCACAACATGAAGGACAAGGACAAAGATGGCAAAGCTCTGCT
>dme_circ_0001012
GGTGGTAATCGTACCGGTCTTAACCAAGAACTGATCAACACCGACCAGACCGCAAATGTTGCCAGAGGGC
ACATCCTCGATGGCCTCGACGTAACGACCCATCATGAGAATTGTGCGCTGAATAGCCTTCTCATATAAGT
CCTCCTTCTTGCCGGGAGTGTAGTTGGGGCCCATGATTCGGCACTTTTGCCCGGTGGCCACCTTACCGGC
GAACACACGACCGAAAGCATAGAAGCGACCCTTGTCAGAGGTCGGTACCATCTTAGAAAT
>dme_circ_0001012_junction_seq
TGTCAGAGGTCGGTACCATCTTAGAAATGGTGGTAATCGTACCGGTCTTAACCAAG
>dme_circ_0001013
GGTCGCTTCTATGCTTTCGGTCGTGTGTTCGCCGGTAAGGTGGCCACCGGGCAAAAGTGCCGAATCATGG
GCCCCAACTACACTCCCGGCAAGAAGGAGGACTTATATGAGAAGGCTATTCAGCGCACAATTCTCATGAT
GGGTCGTTACGTCGAGGCCATCGAGGATGTGCCCTCTGGCAACATTTGCGGTCTGGTCGGTGTTGATCAG
TTCTTGG
>dme_circ_0001013_junction_seq
GGTCTGGTCGGTGTTGATCAGTTCTTGGGGTCGCTTCTATGCTTTCGGTCGTGTGT
>dme_circ_0001014
GACCGCAAATGTTGCCAGAGGGCACATCCTCGATGGCCTCGACGTAACGACCCATCATGAGAATTGTGCG
CTGAATAGCCTTCTCATATAAGTCCTCCTTCTTGCCGGGAGTGTAGTTGGGGCCCATGATTCGGCACTTT
TGCCCGGTGGCCACCTTACCGGCGAACACACGACCGAAAGCATAGAAGCGACCCTTGTCAGAGGTCGGTA
CCATCTTAGAAAT
>dme_circ_0001014_junction_seq
TGTCAGAGGTCGGTACCATCTTAGAAATGACCGCAAATGTTGCCAGAGGGCACATC
>dme_circ_0001015
ATGATTGCTATTCATTTGCCGTCGCCAGTGGTTGCCCAGAAGTACCGCATGGAGATGCTGTACGAAGGTC
CCCATGACGATGAGGCCGCTATCGCCGTTAAGTCTTGCGATCCTGATGGTCCTCTAATGATGTACATTTC
TAAGATGGTACCGACCTCTGACAAGGGTCGCTTCTATGCTTTCGGTCGTGTGTTCGCCGGTAAGGTGGCC
ACCGGGCAAAAGTGCCGAATCATGGGCCCCAACTACACTCCCGGCAAGAAGGAGGACTTATATGAGAAGG
CTATTCAGCGCACAATTCTCATGATGGGTCGTTACGTCGAGGCCATCGAGGATGTGCCCTCTGGCAACAT
TTGCGG
>dme_circ_0001015_junction_seq
AGGATGTGCCCTCTGGCAACATTTGCGGATGATTGCTATTCATTTGCCGTCGCCAG
>dme_circ_0001016
GAAATGTACATCATTAGAGGACCATCAGGATCGCAAGACTTAACGGCGATAGCGGCCTCATCGTCATGGG
GACCTTCGTACAGCATCTCCATGCGGTACTTCTGGGCAACCACTGGCGACGGCAAATGAATAGCAATCAT
CTGAAGTAAAGCCTCGCCGGCTGGCAGCCAGGTGCGCATCACAGTCTTCAGCAGAGCTTTGCCATCTTTG
TCCTTGTCTTCATGCTTCAGA
>dme_circ_0001016_junction_seq

ATCTTTGTCCTTGTCTTCATGCTTCAGAGAAATGTACATCATTAGAGGACCATCAG
>dme_circ_0001017
GAAATGTACATCATTAGAGGACCATCAGGATCGCAAGACTTAACGGCGATAGCGGCCTCATCGTCATGGG
GACCTTCGTACAGCATCTCCATGCGGTACTTCTGGGCAACCACTGGCGACGGCAAATGAATAGCAATCAT
CTGAAGTAAAGCCTCGCCGGCTGGCAGCCAGGTGCGCATCACAGTCTTCAGCAGAGCTTTGCCATCTTTG
TCCTTGTCTTCATGCTTCAGAGTCACACCGATTTTCTCGAGCAAG
>dme_circ_0001017_junction_seq
CAGAGTCACACCGATTTTCTCGAGCAAGGAAATGTACATCATTAGAGGACCATCAG
>dme_circ_0001018
GAAATGTACATCATTAGAGGACCATCAGGATCGCAAGACTTAACGGCGATAGCGGCCTCATCGTCATGGG
GACCTTCGTACAGCATCTCCATGCGGTACTTCTGGGCAACCACTGGCGACGGCAAATGAATAGCAATCAT
CTGAAGTAAAGCCTCGCCGGCTGGCAGCCAGGTGCGCATCACAGTCTTCAGCAGAGCTTTGCCATCTTTG
TCCTTGTCTTCATGCTTCAGAGTCACACCGATTTTCTCGAGCAAGGTACCTATCTCCTCCTTCTT
>dme_circ_0001018_junction_seq
CGAGCAAGGTACCTATCTCCTCCTTCTTGAAATGTACATCATTAGAGGACCATCAG
>dme_circ_0001019
CTCTGCTGAAGACTGTGATGCGCACCTGGCTGCCAGCCGGCGAGGCTTTACTTCAGATGATTGCTATTCA
TTTGCCGTCGCCAGTGGTTGCCCAGAAGTACCGCATGGAGATGCTGTACGAAGGTCCCCATGACGATGAG
GCCGCTATCGCCGTTAAGTCTTGCGATCCTGATGGTCCTCTAATGATG
>dme_circ_0001019_junction_seq
TTGCGATCCTGATGGTCCTCTAATGATGCTCTGCTGAAGACTGTGATGCGCACCTG
>dme_circ_0001020
GTACCTTGCTCGAGAAAATCGGTGTGACTCTGAAGCATGAAGACAAGGACAAAGATGGCAAAGCTCTGCT
GAAGACTGTGATGCGCACCTGGCTGCCAGCCGGCGAGGCTTTACTTCAGATGATTGCTATTCATTTGCCG
TCGCCAGTGGTTGCCCAGAAGTACCGCATGGAGATGCTGTACGAAGGTCCCCATGACGATGAGGCCGCTA
TCGCCGTTAAGTCTTGCGATCCTGATGGTCCTCTAATGATG
>dme_circ_0001020_junction_seq
TTGCGATCCTGATGGTCCTCTAATGATGGTACCTTGCTCGAGAAAATCGGTGTGAC
>dme_circ_0001021
GGACCATCAGGATCGCAAGACTTAACGGCGATAGCGGCCTCATCGTCATGGGGACCTTCGTACAGCATCT
CCATGCGGTACTTCTGGGCAACCACTGGCGACGGCAAATGAATAGCAATCATCTGAAGTAAAGCCTCGCC
GGCTGGCAGCCAGGTGCGCATCACAGTCTTCAGCAGAGCTTTGCCATCTTTGTCCTTGTCTTCATGCTTC
AGA
>dme_circ_0001021_junction_seq
ATCTTTGTCCTTGTCTTCATGCTTCAGAGGACCATCAGGATCGCAAGACTTAACGG
>dme_circ_0001022
GGACCATCAGGATCGCAAGACTTAACGGCGATAGCGGCCTCATCGTCATGGGGACCTTCGTACAGCATCT
CCATGCGGTACTTCTGGGCAACCACTGGCGACGGCAAATGAATAGCAATCATCTGAAGTAAAGCCTCGCC
GGCTGGCAGCCAGGTGCGCATCACAGTCTTCAGCAGAGCTTTGCCATCTTTGTCCTTGTCTTCATGCTTC
AGAGTCACACCGATTTTCTCGAGCAAG
>dme_circ_0001022_junction_seq
CAGAGTCACACCGATTTTCTCGAGCAAGGGACCATCAGGATCGCAAGACTTAACGG
>dme_circ_0001023
CATGAAGACAAGGACAAAGATGGCAAAGCTCTGCTGAAGACTGTGATGCGCACCTGGCTGCCAGCCGGCG

AGGCTTTACTTCAGATGATTGCTATTCATTTGCCGTCGCCAGTGGTTGCCCAGAAGTACCGCATGGAGAT
GCTGTACGAAGGTCCCCATGACGATGAGGCCGCTATCGCCGTTAAGTCTTGCGATCCTGATGG
>dme_circ_0001023_junction_seq
TCGCCGTTAAGTCTTGCGATCCTGATGGCATGAAGACAAGGACAAAGATGGCAAAG
>dme_circ_0001024
GACTTAACGGCGATAGCGGCCTCATCGTCATGGGGACCTTCGTACAGCATCTCCATGCGGTACTTCTGGG
CAACCACTGGCGACGGCAAATGAATAGCAATCATCTGAAGTAAAGCCTCGCCGGCTGGCAGCCAGGTGCG
CATCACAGTCTTCAGCAGAGCTTTGCCATCTTTGTCCTTGTCTTCATGCTTCAGAGTCACACCGATTTTC
TCGAGCAAG
>dme_circ_0001024_junction_seq
CAGAGTCACACCGATTTTCTCGAGCAAGGACTTAACGGCGATAGCGGCCTCATCGT
>dme_circ_0001025
AAGGAGGAGATAGGTACCTTGCTCGAGAAAATCGGTGTGACTCTGAAGCATGAAGACAAGGACAAAGATG
GCAAAGCTCTGCTGAAGACTGTGATGCGCACCTGGCTGCCAGCCGGCGAGGCTTTACTTCAGATGATTGC
TATTCATTTGCCGTCGCCAGTGGTTGCCCAGAAGTACCGCATGGAGATGCTGTACGAAGG
>dme_circ_0001025_junction_seq
AGTACCGCATGGAGATGCTGTACGAAGGAAGGAGGAGATAGGTACCTTGCTCGAGA
>dme_circ_0001026
GAGATAGGTACCTTGCTCGAGAAAATCGGTGTGACTCTGAAGCATGAAGACAAGGACAAAGATGGCAAAG
CTCTGCTGAAGACTGTGATGCGCACCTGGCTGCCAGCCGGCGAGGCTTTACTTCAGATGATTGCTATTCA
TTTGCCGTCGCCAGTGGTTGCCCAGAAGTACCGCATGGAGATGCTG
>dme_circ_0001026_junction_seq
TGCCCAGAAGTACCGCATGGAGATGCTGGAGATAGGTACCTTGCTCGAGAAAATCG
>dme_circ_0001027
GCATCTCCATGCGGTACTTCTGGGCAACCACTGGCGACGGCAAATGAATAGCAATCATCTGAAGTAAAGC
CTCGCCGGCTGGCAGCCAGGTGCGCATCACAGTCTTCAGCAGAGCTTTGCCATCTTTGTCCTTGTCTTCA
TGCTTCAGAGTCACACCGATTTTCTCGAGCAAG
>dme_circ_0001027_junction_seq
CAGAGTCACACCGATTTTCTCGAGCAAGGCATCTCCATGCGGTACTTCTGGGCAAC
>dme_circ_0001028
GCAATCATCTGAAGTAAAGCCTCGCCGGCTGGCAGCCAGGTGCGCATCACAGTCTTCAGCAGAGCTTTGC
CATCTTTGTCCTTGTCTTCATGCTTCAGAGTCACACCGATTTTCTCGAGCAAGGTACCTATCTCCTCCTT
CTTGTAATTCATAATGGCATCGAACACCTTGTAGATGGGGTCCAGGATGTACATGCAGAACGAGCGTTTG
TTGTCAGCCTCTTTCT
>dme_circ_0001028_junction_seq
AACGAGCGTTTGTTGTCAGCCTCTTTCTGCAATCATCTGAAGTAAAGCCTCGCCGG
>dme_circ_0001029
GTAAAGCCTCGCCGGCTGGCAGCCAGGTGCGCATCACAGTCTTCAGCAGAGCTTTGCCATCTTTGTCCTT
GTCTTCATGCTTCAGAGTCACACCGATTTTCTCGAGCAAGGTACCTATCTCCTCCTTCTTGTAATTCATA
ATGGCATCGAACACCTTGTAGATGGGGTCCAGGATGTACATGCAGAACGAGCGTTTGTTGTCAGCCTCTT
TCTGTTTCTGCCATTTCTTGGTCTTGGCGTTAAAGAAGTTTTCACCCCACAATCTGTGAAGAAGTTGAAC
GATTAATTGTGCCTTCAGATATTTCTTATTAACTACTTATCACTTAATGAAATGCTTAATGACCAGTTCA
AATCGCTTATTTAAAAAAATAATTAATATTATTTTTCTGGAAATTTTGTATAATACAAAATGATTCATTT
CAGCCAATAAATTTGTTGCAAATTTGATTTATAAATTTTAAGTCTTAGATATCAAAAATGACGTCCGAAT

ATATCGATTTGTATAATATGAGGCCAAACAACAATTGATTTCAGATATATGAAGAAATTATGTGAACAAT
AAAAGATTTCTGTATACAATTTAAAAATAATTAGTGTAAGGTAACTGCCTTAACATGACTCGTTTGAACA
TTAGGAAAGCGGTCCCGAGTCAGCGTTTAAACAGCATCCGAAAGTTTATTCTTCTTCTATTTAAGGTCCA
CTTGTGGCGCCGTCAGCACGAGAGACTCTAAAAGGACTTTCCTTGCGGAGATGTCTTTCAAGAAGCCGCC
CGCTAAGCGCCACACGTTTTGTGGTAGAAGTTTGAAAACATTTACAAACAAACTTGAGACATGACTCACC
GGTTCATTAGCTTGACAACATCAATCTTGAATTTCTCGGAGTACATCTCAGAGAACTGCTTGAGGGTGAA
CGCCCAGCCGTGAAGCCCAGAACCGAAGCCCACTGATCCCTTAGAGGGATCCACGCGAACCTCACCCATC
GGTCCTCCGTCATC
>dme_circ_0001029_junction_seq
GAACCTCACCCATCGGTCCTCCGTCATCGTAAAGCCTCGCCGGCTGGCAGCCAGGT
>dme_circ_0001030
GGTGCGCATCACAGTCTTCAGCAGAGCTTTGCCATCTTTGTCCTTGTCTTCATGCTTCAGAGTCACACCG
ATTTTCTCGAGCAAGGTACCTATCTCCTCCTTCTTGTAATTCATAATGGCATCGAACACCTTGTAGATGG
GGTCCAGGATGTACATGCAGAACGAGCGTTTGTTGTCAGCCTCTTTCTGTTTCTGCCATTTCTTGGTCTT
GGC
>dme_circ_0001030_junction_seq
TCTGTTTCTGCCATTTCTTGGTCTTGGCGGTGCGCATCACAGTCTTCAGCAGAGCT
>dme_circ_0001031
GGTGCGCATCACAGTCTTCAGCAGAGCTTTGCCATCTTTGTCCTTGTCTTCATGCTTCAGAGTCACACCG
ATTTTCTCGAGCAAGGTACCTATCTCCTCCTTCTTGTAATTCATAATGGCATCGAACACCTTGTAGATGG
GGTCCAGGATGTACATGCAGAACGAGCGTTTGTTGTCAGCCTCTTTCTGTTTCTGCCATTTCTTGGTCTT
GGCGTTAAAGAAGTTTTCACCCCACAATCTGTGAAGAAGTTGAACGATTAATTGTGCCTTCAGATATTTC
TTATTAACTACTTATCACTTAATGAAATGCTTAATGACCAGTTCAAATCGCTTATTTAAAAAAATAATTA
ATATTATTTTTCTGGAAATTTTGTATAATACAAAATGATTCATTTCAGCCAATAAATTTGTTGCAAATTT
GATTTATAAATTTTAAGTCTTAGATATCAAAAATGACGTCCGAATATATCGATTTGTATAATATGAGGCC
AAACAACAATTGATTTCAGATATATGAAGAAATTATGTGAACAATAAAAGATTTCTGTATACAATTTAAA
AATAATTAGTGTAAGGTAACTGCCTTAACATGACTCGTTTGAACATTAGGAAAGCGGTCCCGAGTCAGCG
TTTAAACAGCATCCGAAAGTTTATTCTTCTTCTATTTAAGGTCCACTTGTGGCGCCGTCAGCACGAGAGA
CTCTAAAAGGACTTTCCTTGCGGAGATGTCTTTCAAGAAGCCGCCCGCTAAGCGCCACACGTTTTGTGGT
AGAAGTTTGAAAACATTTACAAACAAACTTGAGACATGACTCACCGGTTCATTAGCTTGACAACATCAAT
CTTGAATTTCTCGGAGTACATCTCAGAGAACTGCTTGAGGGTGAACGCCCAGCCGTGAAGCCCAGAACCG
AAGCCCACTGATCCCTTAGAGGGATCCACGCGAACCTCACCCATCGGTCCTCCGTCATCGTTATAGGTGG
CAATGATAACGTTTACGTTCTCGACGATGCGCTGGAAGGTCTGGTACAGCTCCTCGGCATCTAGTTGCAG
CTCAAGCAGAGCGCGGTCCATTTTGTTCATGAATAGGATGGGCTTAATACGCTCAGCGATGGCCTGACGC
AGCACG
>dme_circ_0001031_junction_seq
ACGCTCAGCGATGGCCTGACGCAGCACGGGTGCGCATCACAGTCTTCAGCAGAGCT
>dme_circ_0001032
GGTACCTATCTCCTCCTTCTTGTAATTCATAATGGCATCGAACACCTTGTAGATGGGGTCCAGGATGTAC
ATGCAGAACGAGCGTTTGTTGTCAGCCTCTTTCTGTTTCTGCCATTTCTTGGTCTTGGCGTTAAAGAAGT
TTTCACCCCACAATCT
>dme_circ_0001032_junction_seq
CGTTAAAGAAGTTTTCACCCCACAATCTGGTACCTATCTCCTCCTTCTTGTAATTC
>dme_circ_0001033

GCTTGACAACATCAATCTTGAATTTCTCGGAGTACATCTCAGAGAACTGCTTGAGGGTGAACGCCCAGCC
GTGAAGCCCAGAACCGAAGCCCACTGATCCCTTAGAGGGATCCACGCGAACCTCACCCATCGGTCCTCCG
TCATCGTTATAGGTGGCAATGATAAC
>dme_circ_0001033_junction_seq
CGTCATCGTTATAGGTGGCAATGATAACGCTTGACAACATCAATCTTGAATTTCTC
>dme_circ_0001034
GCTTGACAACATCAATCTTGAATTTCTCGGAGTACATCTCAGAGAACTGCTTGAGGGTGAACGCCCAGCC
GTGAAGCCCAGAACCGAAGCCCACTGATCCCTTAGAGGGATCCACGCGAACCTCACCCATCGGTCCTCCG
TCATCGTTATAGGTGGCAATGATAACGTTTAC
>dme_circ_0001034_junction_seq
CGTTATAGGTGGCAATGATAACGTTTACGCTTGACAACATCAATCTTGAATTTCTC
>dme_circ_0001035
GTACATCTCAGAGAACTGCTTGAGGGTGAACGCCCAGCCGTGAAGCCCAGAACCGAAGCCCACTGATCCC
TTAGAGGGATCCACGCGAACCTCACCCATCGGTCCTCCGTCATCGTTATAGGTGGCAATGATAACGTTTA
CGTTCTCGACGATGCGCTGGAAGGTCTGGTACAGCTCCTCGGCATCTAGTTGCAGCTCAAGCAGAGCGCG
GTCCATTTT
>dme_circ_0001035_junction_seq
GCAGCTCAAGCAGAGCGCGGTCCATTTTGTACATCTCAGAGAACTGCTTGAGGGTG
>dme_circ_0001036
GAACTGCTTGAGGGTGAACGCCCAGCCGTGAAGCCCAGAACCGAAGCCCACTGATCCCTTAGAGGGATCC
ACGCGAACCTCACCCATCGGTCCTCCGTCATCGTTATAGGTGGCAATGATAACGTTTACGTTCTCGACGA
TGCGCTGGAAGGTCTGGTACAGCTCCTCGGCATCTAGTTGCAGCTCAAGCAGAGCGCGGTCCATTTT
>dme_circ_0001036_junction_seq
GCAGCTCAAGCAGAGCGCGGTCCATTTTGAACTGCTTGAGGGTGAACGCCCAGCCG
>dme_circ_0001037
GAAAAGGATCTGGTGTTCATTACCCACCCCGATCAGCGCGAGAAGGAGTGCAAGGGTTTCCTGATCAACT
TGATCGACTCGCCCGGTCACGTTGACTTCTCCTCGGAGGTGACCGCCGCCCTTCGTGTAACCGACGGTGC
ACTCGTTGTTGTAGACTGCGTCTCCGGAGTTTGTG
>dme_circ_0001037_junction_seq
GTTGTAGACTGCGTCTCCGGAGTTTGTGGAAAAGGATCTGGTGTTCATTACCCACC
>dme_circ_0001038
TCCACTCTGACCGATTCCCTTGTGTCGAAGGCTGGTATCATTGCCGGAGCCAAGGCTGGTGAGACTCGTT
TCACTGACACCCGCAAGGACGAGCAGGAGCGCTGCATTACCATCAAGTCGACGTAAGACCAGTCATGTTC
CAGCACCCACGGCTTTTTAATCCGCCTTTCATTATTGCAGTGCCATTTCCATGTACTTTGAGGTGGAGG
AAAAGGATCTGGTGTTCATTACCCACCCCGATCAGCGCGAGAAGGAGTGCAAGGGTTTCCTGATCAACTT
GATCGACTCGCCCGGTCACGTTGACTTCTCCTCGGAGGTGACCGCCGCCCTTCGTGTAACCGACGGTGCA
CTCGTTGTTG
>dme_circ_0001038_junction_seq
CGTGTAACCGACGGTGCACTCGTTGTTGTCCACTCTGACCGATTCCCTTGTGTCGA
>dme_circ_0001039
GTCAACTTCACCGTCGACGAGATCCGTGGCCTCATGGACAAGAAGCGGAACATCCGCAACATGTCTGTGA
TTGCCCACGTAGACCACGGCAAGTCCACTCTGACCGATTCCCTTGTGTCGAAGGCTGGTATCATTGCCGG
AGCCAAGGCTGGTGAGACTCGTTTCACTGACACCCGCAAGGACGAGCAGGAGCGCTGCATTACCATCAAG
>dme_circ_0001039_junction_seq

CGAGCAGGAGCGCTGCATTACCATCAAGGTCAACTTCACCGTCGACGAGATCCGTG
>dme_circ_0001040
GTGTCGTTCCGGGTGGTTAACTTACATACTCCAGTGACCTAGTCAATGATGGCTCTGACCGGCTGTAATT
ACGATGTGTATGTTTTACACATTTTGTGTAAACTTTATTTTGGTTTTTAACATTTAAGTGACAAATGTTA
ACTGCAGTGCACTGTTGAGTATATGGAGTCCTTAGGGGATTTTCAGCGCTTGGTCCACCCTTTAATTAAG
GGTCACAGTGTGGTTCAACTCAGGCTAAAAGGGAGGAGTTGCTAAGAACATCGAAATGCTTTTACTCATG
TTTGGCGCTGGGTACCCCTCTTGCACATTTCATGAATTCAATTGGGCTCAATTATCCATTTCTGTTTGGA
CAACTGCAattatttttttattctattattttattttatACGTGACAGGTTATCAAATGTCGGTACGACT
TTATTTCATTCTTTTCGAAGAATTTCCGCTTCTAACTAGAGGGGCTCAAAGCTGGGATATCTTTCATCTT
ATTGCCAACT
>dme_circ_0001040_junction_seq
TGGGATATCTTTCATCTTATTGCCAACTGTGTCGTTCCGGGTGGTTAACTTACATA
>dme_circ_0001041
GGTTACAAAATGTCCCGTCCAATATGCAGGAATCGCCTAACCAACGCCGATGTCGATGATACGCTCTGCA
GCGTTACTAATCGTCCGGAGGCGTCGGTGGAACAATGTAATACACACAGTTGTCCTCCCCGGGTGAGTGT
TAGGAATAGCTTCCATAAAGGTCATGTGGTGTTGACAAAGTCACCGATGATTTTGCGTACGGCAATGGTG
GGGAGCGTCAACACGTTATTGTCGTGTTATTTATACTCTGTCTTTTCCAACAGGTGGCCGATATCATGTG
CCGCACACCCAAGCCACCAACACAAGAGACCTGCATTATCGAGGAGTGTCCGCACTGGGAGGTCGAAGAC
TGGACCGGT
>dme_circ_0001041_junction_seq
GCACTGGGAGGTCGAAGACTGGACCGGTGGTTACAAAATGTCCCGTCCAATATGCA
>dme_circ_0001042
GGTTACAAAATGTCCCGTCCAATATGCAGGAATCGCCTAACCAACGCCGATGTCGATGATACGCTCTGCA
GCGTTACTAATCGTCCGGAGGCGTCGGTGGAACAATGTAATACACACAGTTGTCCTCCCCGTGTAAGTAC
TTTGGATCTCAGCCTATTTCCCACCAAGTTGTCTAACAACCACACGCCTTGAGCGCTGCACAAGAAACTG
TAGGAGCCAAACCTAGGCGCAGGACCGCAGTACTATACCTTGCACCTTGACTATGTCAGAATAAGCGTAC
AGAGGTCTCTCATTTTATGCACCGGATATGTGTCTATGATTGGAAGATGCTGTTTGCAAGCTGATCTAGC
AAAttatacccgttactcgtagagtaaaagggtatgttagattcgttgaaaagtatgtaatacgcagaat
gaagcgtttccgactatataaagtatatatattctatcaggatcaatagccgcgtcgactagcaatgtcc
gtctgtccgtatgaacgtcgagatctcaggaactataaaagctagaaggttgagatGCAGATGAAAGGAG
TAAATCTTGAATTTAATCTTTAATTAATTTTACCAATTCTAAATTGTTCAGACTTCCTGGGCAGACATTT
TTTCAAAATTAAAAATTACTATATATATGTAATATTCCCATAGTATTTGAAATTCGAGTAAGCTTTTCTT
ACCTTCTTCCGCACTGGATATTACTTTGCTTTGTGACTCGTTATAACCTGCTCGCCTCTAGACAGAATGG
CCTGAGTATGAAACAGCGGCAGTTTCGTTGCCACCGGGTGTCTGCTTAAGTTTGTTCCTTAGTTCCCATT
CTCACCTCACGTGCTTCCTTCTCCTGTTACCATGCATTTTCAATGTGttttttttgtttttttGTACTTCT
ACTTTATTAGGCTCAACAAGCCAAAACCTCTTTTACGTAGGGGAACTTATGAGGACCGCTTGGGGTGTTA
AGTTTTTACGTTACTCAATTGATTTGTTTAAAGACTTTTTCTGAATGGTATTTAACGCCTGCTACTGTTA
GATTTCTTTAAATTTGGGACAACCTTAGTGCAATTAAGAGTAAAAGGTATAACAAGTTAACGTTTATATC
CAGTAAAACGGGCAACGAAAGAAGAATTATATTGAATTTATAACAATGAAGCTGCGGATGCATCGTTACG
CATACGCCCCGTGGCACGACGCAACGCAGACAATATGGGAATTGGCATTTGTATGCTTGTAACGGGCACG
TGGCACTGCGATGTGGTTAAATGACTGTGTGTGAAGAAGGGGAATGTAGGGAGTATAGGGGACTTTAGTT
GGATTTGGCTTTTCGTTTGTGAGCCACGTCACTGAAAACATTTGCTAAGGAGAAGCAAATTTTGTCATGT
TGCGGCTCATCCATTGTGATGGCGAAAAGAGGCGTATTGAAATAATTTAAAATGCTCACGGAAGCCACAC
CTCCCTCTTTTAAATCACAGGTGTTCGGTTTCCTGCGGACAGGGTATACAAATGCGAGGCGTCGAGTGCA

AATCGACAGACGGCAGCCTGAGCGCGAAGTGTGATCCACTTACAAAGCCGGGCAGCATGCAGCAGTGCTC
CACCGGAATTCATTGCGGCGGATCGTTGAACAAAGTTGGTGGCACCATCATCGTAGGCAGCAGCCGGTCA
CTTAACGAAGCGATCGGAGCGACAGTTGGACAGCTCGGATGCAGATGAAGACAACGAGGACGAAAATGAT
GAGGGCGATGATGTGGACGACCTGGAATCGGGACAGGATACAGATGATGGCGAAGGATTGTCATATGCTG
ATCAACCTTTGCTTTACGCTCATCGCACACAGAGTCGACTGAATCAGGAAGCCCCAGATGAGCCGCGCAC
CATGCATCTAATGAACGGAAATTCAAACAATAATTTCAATCGGGGAGAGGACGAGTCAGAGGGACCATCA
CTTGATCCCACCGTGAGTATGGGGTGATTTATTATTTTAAGGTCGATTTTCTTGAGTGCCAGGAGTGCTA
GTAAGATTTTTCCAAATTTTGTTGGCATAGCGATAGAAATTGGAAATATTTTGTTTAAACTTGCCGAAGT
TAGATTTTTCTAATATACAACTAAAAATTTGTGACCTTTAGTATCATTCATTGATTGTAGTATCAATATT
GTACATGCTTAACTGGTGGCACGCCTTGGTGTGATTACTTGTGTCCTTTGGGACATGAGATACTTTGCCT
ACTTGGGGTTTTAGATTGGAGCATTATCTTAAAGGCACCACTTGACATATTCCAACTGTAAATTTGGCTT
TGAGCATTATTGACACCTTAAATTAAGTTAAAAGAGTTCTGAACCAGAACTGGTGTAGATTGTACATAAG
TTCGGCGAGTGTCCCTAATGACAACTAACGTGCACATTGTATTTATTCGAAAACGATACAAATTCCAGAG
TTAAGCCTTTCCAGACATATTGGAGACTCACCTCAACAGGAAAGTACATATTGTAGTATACACAGTATAC
ATATAATTATTTAGCAAAACAAAGGAAATCATTTGTATTAAATGGTCAAAGTCAAGTGCTTTTATCCGAT
TATTGCCGCGTGCACATTTCTCAGGCGATGTATTAAATGACAGCTCATACTATAGATCCCTGTTATACCA
CTTGATTACGTACCAAAGTGGGGTACTAGGTCCATGAAAATTGCGTTGGCGATTTCAAATCTTGAATCTT
GCCTTGATTATTATTATTATTACTAGTATTTCTGGCGATTTGAATATTTTTaaaaatgaaaaaacaaaat
aaaaaTTTTCTAAGCAGCGAGTTTAATCGAttttttttCTAATTTCTTCGGAAATTAGTAGTATTATTAA
TATAAATGCAGAATGTTGTACGGAAAGTAGTATGTATTACTTTTCTCCGATAAAATGATACAATATAATA
ATTTCATAATATCTTAAACACCGACGTGACCTTTTCCAAAGAGTAACAAAAATGCGGTGTACTTATAGTT
TTCCAAAATTTTCGCTTAGCTTAAACTGTGCCAATCTGGCGCCGCCAAAAAGGCGCGACATTGTGCCGCC
ACCAAAACCTTGGCCTCTGATGCCGGTTTTGCaaaaaaaaGGCCGTAAAAAGCTTAGAGATGGCAGCGCG
CCAGTCAGCCAAACAAAGAAAAACTAGAGAAAGAATATCCTAGCCTACGCGCCGCGACGATTACGAAATC
AGCACTGAGAAAAAATAAGAAACAAAGATCTCTAAGTAAATGTAATAAAATAAATCAATTTAGATTTTGA
GTAGCACGGCCTATAATAACAAAAATGTCATAAATGTAACAGGCGCTGAAAATTCAGTAACGCCGAGTGA
TAAGGAAAATATGAAAATAAAATAAATTGATGCGTTTTTTTGTACACACTCTCTCagagagaacactata
ttcgcattcctcgactatcagatacccctatatctaactagcaggagtgtgaacaagaagtttcaacattt
tATTATTTTTTGTCCATGGTCCATGGTATTATTTTTTGTCTGCGCAGCCTGGCCCACTTAGGGCATCATG
CTAAGATGTCTACATCCAGATGGCACCGCATCGCCATTCTGTCCAGCGGATTTGTTAGCCGTATTTTGTA
ACCATTAGGGCGGTGGAACCGAACggaagcttttccgactataaatatgtatatatttctgatcaagttc
aaccgccgagtcaatctgacgatgtcagtccgtatcaacgcttcacggataacggaaactaataaagcta
gagatattatatctctagattagatattagatatgaagattccagacgcatatacgcagggcaagtatat
tatacccacacccaacggcttgagccagagccggattctaaagcgtgcacccgcaagtacaggttccctt
cccttccatcaatttatttccttacacgttacaaacggcatcatgtcttttaggtgccgacgggcttcag
cgaggttgttgtcgctaagcgcaacgatttggcgttgtaatttttttatttactatattatttgttggttt
ttatttattaaagtttttagagtttaCGAGAGTTTTCAGGGTATAATCTCTTAGATAAACGAAtatttaa
aattttttaaattgaattttgtagtagttttattttattttaacttgttttAACTTCTTGTAGTTTTTAT
TTCGAATAATAAGCCTCAAtgcttaaagaatattgtattgtaaacttggatccttaagtttagaagattc
aaagagagcaggcaaagctgcttaggtcaggctttaggatttgtttacaagaacggctaagtgctgttta
ttagaatgtttattggaacgtatactgcgcatacgcatacTTCATTATTCGAAATAATGAATTGGCCGGT
GTGGGTGGCGCAAAAATACGTCACTATATACTATATATATGTttatacccgttactcgtggagtaaaagg
gtacaatagattcgttgaaaagtatgtaacatgcaaagggaagcgtttccgaccatataaagtatatata
ttcttgatctggatcaatagccgcgtcgatctagccatgtccgtctgtccttttcacagttttgttagtc

gtgtaaatttctatcggtttagcaaaaaaactttgaaacgctcactctaacgaccacaaaaaactgtcgc
gcccacacttttaaaatatgatttgatgtttttcttgttagtctcgtaaatttctaacggtttgccaaaa
aactttttgtcacgcccactctaacTTCTAACGTTTTTATAGTCTACTATAGCGCGCTCTGTTGTATAAA
AATGTACTATTGGAAGGCCGATTCATTAATACTGTATATATTTTATTTATTTGATGATTAACGGGTATCT
CATTCTTCCCAGCGGCATCCTTTTTTGATGGTATAATGTGAAATTACCTAAATatttatttttgattttc
tttttttATTATACACAGGCTACATCAAGGACAATGAGTGGTCCCCATGTAGCGTTACCTGTGGGGAAGG
AATCCGCCGGCGCACCTACAACTGCAAGATTTTCCTTGAATACTCTCGAACGGTGGCCACCGTTAATGAT
TCGTTGTGCGAGGGAAAAAAGCCACATGACGAAGTAGAGCGGTGTGTGGAGGATCCGTGCATGCTACCCT
CCCACGGCTTTGACGACCAGTTCCCCCGTGATTCCATTAAAGTGGGTGTGTCGGAACCGGGTAAGACGTA
TGTCTGGCGGGAACAGGGATACACCTCCTGCAGTGCCTCCTGTCTCGGCGGTGTCGAGGAACTGATAATC
AACTGCGTTAGAGAAGATAATGGTAGGGTGGTGTCGCCCTTTCTCTGTTCCCCGGAAACCAAGCCGGAGG
CCAGGGTTCGTACCTGCAACGACCGTCCCTGCCCACCCAGATGGAACTACTCAGACTACACACCCTGCTC
CAAGAGCTGTGGCATTGGCATCAAGACCCGCGAGGTGCAGTGCATCCACGAGGTTACGCGCGGAGGGGAC
AACACAATGGTGGTGCCGAACAGTATGTGCCCACAGCCACCACCAGCAGATCGGCAGTACTGCAACGTTC
TCGATTGCCCCGTACGTTGGGAAGTCGGTGAGTGGAGCAAGTGCTCCCATACGTGTGGCTATGGGTTTAA
GGATCGCAAGGTGGAGTGCAAGCAGATAATGGCGCAAGAGCACAAGATCGAACGACCCGAATCGATGTGT
CCGAGTGCTAAGCCGGCGGACAAAAAGCCCTGCAATGTGAAGCCCTGCCCGCCGGAAGATCCCAAGCCAG
TCATCCAGATCAACAACTCAACACACATCCAGCACGACCCCAAGAAGACCAAGATCACCCTTAAAGTGGG
CGGCGCTGGACTTGTTTTCTTCGGCACCCAGGTAAAGATCAAGTGTCCCGTGAAGCGGTACAACCGCACC
AAGATCAAGTGGAGCAAGGATCACAAGCCGCTGCAGCGTTCGCGAAAGTACAAAGTGTCGAAGAAGGGAG
CCCTGCGTATACTTGATATTACGTTTCGCGATGCAGGGGTGTACTCCTGCCACGCCGGTCTCAGCTCCGC
AGAAATAAGCATCGAAGTGAAGGCCAAGCCAGGACAGCAGATTGAGGAGCTGGAACAACAGGAAACTGGT
TAGTTATCCTATCTGGTTTTGATATACTCTTTTGGCTAAGTAACGCGTTTATATCGCTTTTTCAGAACGA
TTGGTGCGTGAGAAAAGTGGCACAGAGGCTCTCACCTCTGCGGATATGAAGTCCGCTGATGGAACTGGGA
CTTCCACTGCGGTGGGACCTAATAGCCACGTTAACGG
>dme_circ_0001042_junction_seq
CGGTGGGACCTAATAGCCACGTTAACGGGGTTACAAAATGTCCCGTCCAATATGCA
>dme_circ_0001043
GGTGGATAGCAGACGATTGGAGCACTTGCAGCCGCCTTTGTGGTCACGGCTACCGTGAGCGGATGGTTGT
CTGTGCCGAGGAGTCGAACGGCATCAAGACGAGGGTGAGTGTTAGGAATAGCTTCCATAAAGGTCATGTG
GTGTTGACAAAGTCACCGATGATTTTGCGTACGGCAATGGTGGGGAGCGTCAACACGTTATTGTCGTGTT
ATTTATACTCTGTCTTTTCCAACAGGTGGCCGATATCATGTGCCGCACACCCAAGCCACCAACACAAGAG
ACCTGCATTATCGAGGAGTGTCCGCACTGGGAGGTCGAAGACTGGACCGGTGTAAGTACTTTGGATCTCA
GCCTATTTCCCACCAAGTTGTCTAACAACCACACGCCTTGAGCGCTGCACAAGAAACTGTAGGAGCCAAA
CCTAGGCGCAGGACCGCAGTACTATACCTTGCACCTTGACTATGTCAGAATAAGCGTACAGAGGTCTCTC
ATTTTATGCACCGGATATGTGTCTATGATTGGAAGATGCTGTTTGCAAGCTGATCTAGCAAAttataccc
gttactcgtagagtaaaagggtatgttagattcgttgaaaagtatgtaatacgcagaatgaagcgtttcc
gactatataaagtatatatattctatcaggatcaatagccgcgtcgactagcaatgtccgtctgtccgta
tgaacgtcgagatctcaggaactataaaagctagaaggttgagatGCAGATGAAAGGAGTAAATCTTGAA
TTTAATCTTTAATTAATTTTACCAATTCTAAATTGTTCAGACTTCCTGGGCAGACATTTTTTCAAAATTA
AAAATTACTATATATATGTAATATTCCCATAGTATTTGAAATTCGAGTAAGCTTTTCTTACCTTCTTCCG
CACTGGATATTACTTTGCTTTGTGACTCGTTATAACCTGCTCGCCTCTAGACAGAATGGCCTGAGTATGA
AACAGCGGCAGTTTCGTTGCCACCGGGTGTCTGCTTAAGTTTGTTCCTTAGTTCCCATTCTCACCTCACG
TGCTTCCTTCTCCTGTTACCATGCATTTTCAATGTGtttttttgtttttttGTACTTCTACTTTATTAGG

CTCAACAAGCCAAAACCTCTTTTACGTAGGGGAACTTATGAGGACCGCTTGGGGTGTTAAGTTTTTACGT
TACTCAATTGATTTGTTTAAAGACTTTTTCTGAATGGTATTTAACGCCTGCTACTGTTAGATTTCTTTAA
ATTTGGGACAACCTTAGTGCAATTAAGAGTAAAAGGTATAACAAGTTAACGTTTATATCCAGTAAAACGG
GCAACGAAAGAAGAATTATATTGAATTTATAACAATGAAGCTGCGGATGCATCGTTACGCATACGCCCCG
TGGCACGACGCAACGCAGACAATATGGGAATTGGCATTTGTATGCTTGTAACGGGCACGTGGCACTGCGA
TGTGGTTAAATGACTGTGTGTGAAGAAGGGGAATGTAGGGAGTATAGGGGACTTTAGTTGGATTTGGCTT
TTCGTTTGTGAGCCACGTCACTGAAAACATTTGCTAAGGAGAAGCAAATTTTGTCATGTTGCGGCTCATC
CATTGTGATGGCGAAAAGAGGCGTATTGAAATAATTTAAAATGCTCACGGAAGCCACACCTCCCTCTTTT
AAATCACAGGTGTTCGGTTTCCTGCGGACAGGGTATACAAATGCGAGGCGTCGAGTGCAAATCGACAGAC
GGCAGCCTGAGCGCGAAGTGTGATCCACTTACAAAGCCGGGCAGCATGCAGCAGTGCTCCACCGGAATTC
ATTGCGGCGGATCGTTGAACAAAGTTGGTGGCACCATCATCGTAGGCAGCAGCCGGTCACTTAACGAAGC
GATCGGAGCGACAGTTGGACAGCTCGGATGCAGATGAAGACAACGAGGACGAAAATGATGAGGGCGATGA
TGTGGACGACCTGGAATCGGGACAGGATACAGATGATGGCGAAGGATTGTCATATGCTGATCAACCTTTG
CTTTACGCTCATCGCACACAGAGTCGACTGAATCAGGAAGCCCCAGATGAGCCGCGCACCATGCATCTAA
TGAACGGAAATTCAAACAATAATTTCAATCGGGGAGAGGACGAGTCAGAGGGACCATCACTTGATCCCAC
CGTGAGTATGGGGTGATTTATTATTTTAAGGTCGATTTTCTTGAGTGCCAGGAGTGCTAGTAAGATTTTT
CCAAATTTTGTTGGCATAGCGATAGAAATTGGAAATATTTTGTTTAAACTTGCCGAAGTTAGATTTTTCT
AATATACAACTAAAAATTTGTGACCTTTAGTATCATTCATTGATTGTAGTATCAATATTGTACATGCTTA
ACTGGTGGCACGCCTTGGTGTGATTACTTGTGTCCTTTGGGACATGAGATACTTTGCCTACTTGGGGTTT
TAGATTGGAGCATTATCTTAAAGGCACCACTTGACATATTCCAACTGTAAATTTGGCTTTGAGCATTATT
GACACCTTAAATTAAGTTAAAAGAGTTCTGAACCAGAACTGGTGTAGATTGTACATAAGTTCGGCGAGTG
TCCCTAATGACAACTAACGTGCACATTGTATTTATTCGAAAACGATACAAATTCCAGAGTTAAGCCTTTC
CAGACATATTGGAGACTCACCTCAACAGGAAAGTACATATTGTAGTATACACAGTATACATATAATTATT
TAGCAAAACAAAGGAAATCATTTGTATTAAATGGTCAAAGTCAAGTGCTTTTATCCGATTATTGCCGCGT
GCACATTTCTCAGGCGATGTATTAAATGACAGCTCATACTATAGATCCCTGTTATACCACTTGATTACGT
ACCAAAGTGGGGTACTAGGTCCATGAAAATTGCGTTGGCGATTTCAAATCTTGAATCTTGCCTTGATTAT
TATTATTATTACTAGTATTTCTGGCGATTTGAATATTTTTaaaaatgaaaaaacaaaataaaaaTTTTCT
AAGCAGCGAGTTTAATCGAttttttttCTAATTTCTTCGGAAATTAGTAGTATTATTAATATAAATGCAG
AATGTTGTACGGAAAGTAGTATGTATTACTTTTCTCCGATAAAATGATACAATATAATAATTTCATAATA
TCTTAAACACCGACGTGACCTTTTCCAAAGAGTAACAAAAATGCGGTGTACTTATAGTTTTCCAAAATTT
TCGCTTAGCTTAAACTGTGCCAATCTGGCGCCGCCAAAAAGGCGCGACATTGTGCCGCCACCAAAACCTT
GGCCTCTGATGCCGGTTTTGCaaaaaaaaGGCCGTAAAAAGCTTAGAGATGGCAGCGCGCCAGTCAGCCA
AACAAAGAAAAACTAGAGAAAGAATATCCTAGCCTACGCGCCGCGACGATTACGAAATCAGCACTGAGAA
AAAATAAGAAACAAAGATCTCTAAGTAAATGTAATAAAATAAATCAATTTAGATTTTGAGTAGCACGGCC
TATAATAACAAAAATGTCATAAATGTAACAGGCGCTGAAAATTCAGTAACGCCGAGTGATAAGGAAAATA
TGAAAATAAAATAAATTGATGCGTTTTTTTGTACACACTCTCTCagagagaacactatattcgcattcct
cgactatcagataccctatatctaactagcaggagtgtgaacaagaagtttcaacattttATTATTTTTT
GTCCATGGTCCATGGTATTATTTTTTGTCTGCGCAGCCTGGCCCACTTAGGGCATCATGCTAAGATGTCT
ACATCCAGATGGCACCGCATCGCCATTCTGTCCAGCGGATTTGTTAGCCGTATTTTGTAACCATTAGGGC
GGTGGAACCGAACggaagcttttccgactataaatatgtatatatttctgatcaagttcaaccgccgagt
caatctgacgatgtcagtccgtatcaacgcttcacggataacggaaactaataaagctagagatattata
tctctagattagatattagatatgaagattccagacgcatatacgcagggcaagtatattatacccacac
ccaacggcttgagccagagccggattctaaagcgtgcacccgcaagtacaggttcccttcccttccatca
atttatttccttacacgttacaaacggcatcatgtcttttaggtgccgacgggcttcagcgaggttgttg

tcgctaagcgcaacgatttggcgttgtaatttttatttactatattatttgttggtttttatttattaa
agtttttagagtttaCGAGAGTTTTCAGGGTATAATCTCTTAGATAAACGAAtatttaaaatttttaaa
ttgaattttgtagtagttttattttattttaacttgttttAACTTCTTGTAGTTTTTATTTCGAATAATA
AGCCTCAAtgcttaaagaatattgtattgtaaacttggatccttaagtttagaagattcaaagagagcag
gcaaagctgcttaggtcaggctttaggatttgtttacaagaacggctaagtgctgtttattagaatgttt
attggaacgtatactgcgcatacgcatacTTCATTATTCGAAATAATGAATTGGCCGGTGTGGGTGGCGC
AAAAATACGTCACTATATACTATATATATGTttatacccgttactcgtggagtaaaagggtacaatagat
tcgttgaaaagtatgtaacatgcaaagggaagcgtttccgaccatataaagtatatatattcttgatctg
gatcaatagccgcgtcgatctagccatgtccgtctgtccttttcacagttttgttagtcgtgtaaatttc
tatcggtttagcaaaaaaactttgaaacgctcactctaacgaccacaaaaaactgtcgcgcccacacttt
taaaatatgatttgatgtttttcttgttagtctcgtaaatttctaacggtttgccaaaaaactttttgtc
acgcccactctaacTTCTAACGTTTTTATAGTCTACTATAGCGCGCTCTGTTGTATAAAAATGTACTATT
GGAAGGCCGATTCATTAATACTGTATATATTTTATTTATTTGATGATTAACGGGTATCTCATTCTTCCCA
GCGGCATCCTTTTTTGATGGTATAATGTGAAATTACCTAAATatttatttttgatttttctttttttATTA
TACACAGGCTACATCAAGGACAATGAGTGGTCCCCATGTAGCGTTACCTGTGGGGAAGGAATCCGCCGGC
GCACCTACAACTGCAAGATTTTCCTTGAATACTCTCGAACGGTGGCCACCGTTAATGATTCGTTGTGCGA
GGGAAAAAAGCCACATGACGAAGTAGAGCGGTGTGTGGAGGATCCGTGCATGCTACCCTCCCACGGCTTT
GACGACCAGTTCCCCCGTGATTCCATTAAAGTGGGTGTGTCGGAACCGGGTAAGACGTATGTCTGGCGGG
AACAGGGATACACCTCCTGCAGTGCCTCCTGTCTCGGCGGTGTCGAGGAACTGATAATCAACTGCGTTAG
AGAAGATAATGGTAGGGTGGTGTCGCCCTTTCTCTGTTCCCCGGAAACCAAGCCGGAGGCCAGGGTTCGT
ACCTGCAACGACCGTCCCTGCCCACCCAGATGGAACTACTCAGACTACACACCCTGCTCCAAGAGCTGTG
GCATTGGCATCAAGACCCGCGAGGTGCAGTGCATCCACGAGGTTACGCGCGGAGGGGACAACACAATGGT
GGTGCCGAACAGTATGTGCCCACAGCCACCACCAGCAGATCGGCAGTACTGCAACGTTCTCGATTGCCCC
GTACGTTGGGAAGTCGGTGAGTGGAGCAAGTGCTCCCATACGTGTGGCTATGGGTTTAAGGATCGCAAGG
TGGAGTGCAAGCAGATAATGGCGCAAGAGCACAAGATCGAACGACCCGAATCGATGTGTCCGAGTGCTAA
GCCGGCGGACAAAAAGCCCTGCAATGTGAAGCCCTGCCCGCCGGAAGATCCCAAGCCAGTCATCCAGATC
AACAACTCAACACACATCCAGCACGACCCCAAGAAGACCAAGATCACCCTTAAAGTGGGCGGCGCTGGAC
TTGTTTTCTTCGGCACCCAGGTAAAGATCAAGTGTCCCGTGAAGCGGTACAACCGCACCAAGATCAAGTG
GAGCAAGGATCACAAGCCGCTGCAGCGTTCGCGAAAGTACAAAGTGTCGAAGAAGGGAGCCCTGCGTATA
CTTGATATTACGTTTCGCGATGCAGGGGTGTACTCCTGCCACGCCGGTCTCAGCTCCGCAGAAATAAGCA
TCGAAGTGAAGGCCAAGCCAGGACAGCAGATTGAGGAGCTGGAACAACAGGAAACTGGTTAGTTATCCTA
TCTGGTTTTGATATACTCTTTTGGCTAAGTAACGCGTTTATATCGCTTTTTCAGAACGATTGGTGCGTGA
GAAAAGTGGCACAGAGGCTCTCACCTCTGCGGATATGAAGTCCGCTGATGGAACTGGGACTTCCACTGCG
GTGGGACCTAATAGCCACGTTAACGG
>dme_circ_0001043_junction_seq
CGGTGGGACCTAATAGCCACGTTAACGGGGTGGATAGCAGACGATTGGAGCACTTG
>dme_circ_0001044
GGTGGATAGCAGACGATTGGAGCACTTGCAGCCGCCTTTGTGGTCACGGCTACCGTGAGCGGATGGTTGT
CTGTGCCGAGGAGTCGAACGGCATCAAGACGAGGGTGAGTGTTAGGAATAGCTTCCATAAAGGTCATGTG
GTGTTGACAAAGTCACCGATGATTTTGCGTACGGCAATGGTGGGGAGCGTCAACACGTTATTGTCGTGTT
ATTTATACTCTGTCTTTTCCAACAGGTGGCCGATATCATGTGCCGCACACCCAAGCCACCAACACAAGAG
ACCTGCATTATCGAGGAGTGTCCGCACTGGGAGGTCGAAGACTGGACCGGTGTAAGTACTTTGGATCTCA
GCCTATTTCCCACCAAGTTGTCTAACAACCACACGCCTTGAGCGCTGCACAAGAAACTGTAGGAGCCAAA
CCTAGGCGCAGGACCGCAGTACTATACCTTGCACCTTGACTATGTCAGAATAAGCGTACAGAGGTCTCTC

ATTTTATGCACCGGATATGTGTCTATGATTGGAAGATGCTGTTTGCAAGCTGATCTAGCAAAttataccc
gttactcgtagagtaaaagggtatgttagattcgttgaaaagtatgtaatacgcagaatgaagcgtttcc
gactatataaagtatatatattctatcaggatcaatagccgcgtcgactagcaatgtccgtctgtccgta
tgaacgtcgagatctcaggaactataaaagctagaaggttgagatGCAGATGAAAGGAGTAAATCTTGAA
TTTAATCTTTAATTAATTTTACCAATTCTAAATTGTTCAGACTTCCTGGGCAGACATTTTTTCAAAATTA
AAAATTACTATATATATGTAATATTCCCATAGTATTTGAAATTCGAGTAAGCTTTTCTTACCTTCTTCCG
CACTGGATATTACTTTGCTTTGTGACTCGTTATAACCTGCTCGCCTCTAGACAGAATGGCCTGAGTATGA
AACAGCGGCAGTTTCGTTGCCACCGGGTGTCTGCTTAAGTTTGTTCCTTAGTTCCCATTCTCACCTCACG
TGCTTCCTTCTCCTGTTACCATGCATTTTCAATGTGtttttttgtttttttGTACTTCTACTTTATTAGG
CTCAACAAGCCAAAACCTCTTTTACGTAGGGGAACTTATGAGGACCGCTTGGGGTGTTAAGTTTTTACGT
TACTCAATTGATTTGTTTAAAGACTTTTTCTGAATGGTATTTAACGCCTGCTACTGTTAGATTTCTTTAA
ATTTGGGACAACCTTAGTGCAATTAAGAGTAAAAGGTATAACAAGTTAACGTTTATATCCAGTAAAACGG
GCAACGAAAGAAGAATTATATTGAATTTATAACAATGAAGCTGCGGATGCATCGTTACGCATACGCCCCG
TGGCACGACGCAACGCAGACAATATGGGAATTGGCATTTGTATGCTTGTAACGGGCACGTGGCACTGCGA
TGTGGTTAAATGACTGTGTGTGAAGAAGGGGAATGTAGGGAGTATAGGGGACTTTAGTTGGATTTGGCTT
TTCGTTTGTGAGCCACGTCACTGAAAACATTTGCTAAGGAGAAGCAAATTTTGTCATGTTGCGGCTCATC
CATTGTGATGGCGAAAAGAGGCGTATTGAAATAATTTAAAATGCTCACGGAAGCCACACCTCCCTCTTTT
AAATCACAGGTGTTCGGTTTCCTGCGGACAGGGTATACAAATGCGAGGCGTCGAGTGCAAATCGACAGAC
GGCAGCCTGAGCGCGAAGTGTGATCCACTTACAAAGCCGGGCAGCATGCAGCAGTGCTCCACCGGAATTC
ATTGCGGCGGATCGTTGAACAAAGTTGGTGGCACCATCATCGTAGGCAGCAGCCGGTCACTTAACGAAGC
GATCGGAGCGACAGTTGGACAGCTCGGATGCAGATGAAGACAACGAGGACGAAAATGATGAGGGCGATGA
TGTGGACGACCTGGAATCGGGACAGGATACAGATGATGGCGAAGGATTGTCATATGCTGATCAACCTTTG
CTTTACGCTCATCGCACACAGAGTCGACTGAATCAGGAAGCCCCAGATGAGCCGCGCACCATGCATCTAA
TGAACGGAAATTCAAACAATAATTTCAATCGGGGAGAGGACGAGTCAGAGGGACCATCACTTGATCCCAC
CGTGAGTATGGGGTGATTTATTATTTTAAGGTCGATTTTCTTGAGTGCCAGGAGTGCTAGTAAGATTTTT
CCAAATTTTGTTGGCATAGCGATAGAAATTGGAAATATTTTGTTTAAACTTGCCGAAGTTAGATTTTTCT
AATATACAACTAAAAATTTGTGACCTTTAGTATCATTCATTGATTGTAGTATCAATATTGTACATGCTTA
ACTGGTGGCACGCCTTGGTGTGATTACTTGTGTCCTTTGGGACATGAGATACTTTGCCTACTTGGGGTTT
TAGATTGGAGCATTATCTTAAAGGCACCACTTGACATATTCCAACTGTAAATTTGGCTTTGAGCATTATT
GACACCTTAAATTAAGTTAAAAGAGTTCTGAACCAGAACTGGTGTAGATTGTACATAAGTTCGGCGAGTG
TCCCTAATGACAACTAACGTGCACATTGTATTTATTCGAAAACGATACAAATTCCAGAGTTAAGCCTTTC
CAGACATATTGGAGACTCACCTCAACAGGAAAGTACATATTGTAGTATACACAGTATACATATAATTATT
TAGCAAAACAAAGGAAATCATTTGTATTAAATGGTCAAAGTCAAGTGCTTTTATCCGATTATTGCCGCGT
GCACATTTCTCAGGCGATGTATTAAATGACAGCTCATACTATAGATCCCTGTTATACCACTTGATTACGT
ACCAAAGTGGGGTACTAGGTCCATGAAAATTGCGTTGGCGATTTCAAATCTTGAATCTTGCCTTGATTAT
TATTATTATTACTAGTATTTCTGGCGATTTGAATATTTTTaaaaatgaaaaaacaaaataaaaaTTTTCT
AAGCAGCGAGTTTAATCGAttttttttCTAATTTCTTCGGAAATTAGTAGTATTATTAATATAAATGCAG
AATGTTGTACGGAAAGTAGTATGTATTACTTTTCTCCGATAAAATGATACAATATAATAATTTCATAATA
TCTTAAACACCGACGTGACCTTTTCCAAAGAGTAACAAAAATGCGGTGTACTTATAGTTTTCCAAAATTT
TCGCTTAGCTTAAACTGTGCCAATCTGGCGCCGCCAAAAAGGCGCGACATTGTGCCGCCACCAAAACCTT
GGCCTCTGATGCCGGTTTTGCaaaaaaaaGGCCGTAAAAAGCTTAGAGATGGCAGCGCGCCAGTCAGCCA
AACAAAGAAAAACTAGAGAAAGAATATCCTAGCCTACGCGCCGCGACGATTACGAAATCAGCACTGAGAA
AAAATAAGAAACAAAGATCTCTAAGTAAATGTAATAAAATAAATCAATTTAGATTTTGAGTAGCACGGCC
TATAATAACAAAAATGTCATAAATGTAACAGGCGCTGAAAATTCAGTAACGCCGAGTGATAAGGAAAATA

TGAAAATAAAATAAATTGATGCGTTTTTTTGTACACACTCTCTCagagagaacactatattcgcattcct
cgactatcagataccctatatctaactagcaggagtgtgaacaagaagtttcaacattttATTATTTTTT
GTCCATGGTCCATGGTATTATTTTTTGTCTGCGCAGCCTGGCCCACTTAGGGCATCATGCTAAGATGTCT
ACATCCAGATGGCACCGCATCGCCATTCTGTCCAGCGGATTTGTTAGCCGTATTTTGTAACCATTAGGGC
GGTGGAACCGAACggaagcttttccgactataaatatgtatatatttctgatcaagttcaaccgccgagt
caatctgacgatgtcagtccgtatcaacgcttcacggataacggaaactaataaagctagagatattata
tctctagattagatattagatatgaagattccagacgcatatacgcagggcaagtatattatacccacac
ccaacggcttgagccagagccggattctaaagcgtgcacccgcaagtacaggttcccttcccttccatca
atttatttccttacacgttacaaacggcatcatgtcttttaggtgccgacgggcttcagcgaggttgttg
tcgctaagcgcaacgatttggcgttgtaatttttttatttactatattatttgttggtttttatttattaa
agtttttagagtttaCGAGAGTTTTCAGGGTATAATCTCTTAGATAAACGAAtatttaaaatttttttaaa
ttgaattttgtagtagttttattttattttaacttgttttAACTTCTTGTAGTTTTTATTTCGAATAATA
AGCCTCAAtgcttaaagaatattgtattgtaaacttggatccttaagtttagaagattcaaagagagcag
gcaaagctgcttaggtcaggctttaggatttgtttacaagaacggctaagtgctgtttattagaatgttt
attggaacgtatactgcgcatacgcatacTTCATTATTCGAAATAATGAATTGGCCGGTGTGGGTGGCGC
AAAAATACGTCACTATATACTATATATATGTttatacccgttactcgtggagtaaaagggtacaatagat
tcgttgaaaagtatgtaacatgcaaagggaagcgtttccgaccatataaagtatatatattcttgatctg
gatcaatagccgcgtcgatctagccatgtccgtctgtccttttcacagttttgttagtcgtgtaaatttc
tatcggtttagcaaaaaaactttgaaacgctcactctaacgaccacaaaaaactgtcgcgcccacacttt
taaaatatgatttgatgtttttcttgttagtctcgtaaatttctaacggtttgccaaaaaactttttgtc
acgcccactctaacTTCTAACGTTTTTATAGTCTACTATAGCGCGCTCTGTTGTATAAAAATGTACTATT
GGAAGGCCGATTCATTAATACTGTATATATTTTATTTATTTGATGATTAACGGGTATCTCATTCTTCCCA
GCGGCATCCTTTTTTGATGGTATAATGTGAAATTACCTAAATatttatttttgattttcttttttttATTA
TACACAGGCTACATCAAGGACAATGAGTGGTCCCCATGTAGCGTTACCTGTGGGGAAGGAATCCGCCGGC
GCACCTACAACTGCAAGATTTTCCTTGAATACTCTCGAACGGTGGCCACCGTTAATGATTCGTTGTGCGA
GGGAAAAAAGCCACATGACGAAGTAGAGCGGTGTGTGGAGGATCCGTGCATGCTACCCTCCCACGGCTTT
GACGACCAGTTCCCCCGTGATTCCATTAAAGTGGGTGTGTCGGAACCGGGTAAGACGTATGTCTGGCGGG
AACAGGGATACACCTCCTGCAGTGCCTCCTGTCTCGGCGGTGTCGAGGAACTGATAATCAACTGCGTTAG
AGAAGATAATGGTAGGGTGGTGTCGCCCTTTCTCTGTTCCCCGGAAACCAAGCCGGAGGCCAGGGTTCGT
ACCTGCAACGACCGTCCCTGCCCACCCAGATGGAACTACTCAGACTACACACCCTGCTCCAAGAGCTGTG
GCATTGGCATCAAGACCCGCGAGGTGCAGTGCATCCACGAGGTTACGCGCGGAGGGGACAACACAATGGT
GGTGCCGAACAGTATGTGCCCACAGCCACCACCAGCAGATCGGCAGTACTGCAACGTTCTCGATTGCCCC
GTACGTTGGGAAGTCGGTGAGTGGAGCAAGTGCTCCCATACGTGTGGCTATGGGTTTAAGGATCGCAAGG
TGGAGTGCAAGCAGATAATGGCGCAAGAGCACAAGATCGAACGACCCGAATCGATGTGTCCGAGTGCTAA
GCCGGCGGACAAAAAGCCCTGCAATGTGAAGCCCTGCCCGCCGGAAGATCCCAAGCCAGTCATCCAGATC
AACAACTCAACACACATCCAGCACGACCCCAAGAAGACCAAGATCACCCTTAAAGTGGGCGGCGCTGGAC
TTGTTTTCTTCGGCACCCAGGTAAAGATCAAGTGTCCCGTGAAGCGGTACAACCGCACCAAGATCAAGTG
GAGCAAGGATCACAAGCCGCTGCAGCGTTCGCGAAAGTACAAAGTGTCGAAGAAGGGAGCCCTGCGTATA
CTTGATATTACGTTTCGCGATGCAGGGGTGTACTCCTGCCACGCCGGTCTCAGCTCCGCAGAAATAAGCA
TCGAAGTGAAGGCCAAGCCAGGACAGCAGATTGAGGAGCTGGAACAACAGGAAACTGGGTAAGCAAGCAA
TCTGTCATGGATCAGTCGGCAAAGCttttttttttATAAttatgccctacttgtagagtaaaaggatata
ctcgttttgttaaacagtaacttacagatagaaggaagcctttccaagcatataaaatatatctggtcat
gtccctcagcctgtgtatcacaggcactataaaagctagaacgttgtgactaggcaggcagattccagaT
TGGCTGGTTGGTTTTGCCaatttcttcgtacttccactagataagtaatgggtatctaatggtcaggaaa

ctcgaAGAGAACACGATGTGGTTGGTAGATAGTGCAAGAAATGAATAGATGTCATTCGACACAATTTAAT
TTAATAAAAAGAACACATAAAGGGTtcagctagtggaaatgtgaacgcgaaatttcatagtttctctggg
atatcgatagatattgggcaataaaatacgaaagttcaaaagtgtgggcgtgcccgttttgggcgttttg
taggtgtaaggaggggcgtggccaaattgtttttagtataccgattgaaattcaaacaatataactgaga
aaaatcaaaacacttttcaaaagtgtgagcgtggcagttttggtctgtttgtggacgtttttgcccaatc
gatagaaatgtacaagactaataaaaatatgaaaaactataaaatgtcaaatgtgtgggcgtggcagtAT
TCCTGATCaaaaaaaaaaaaaaaaaCgttatatacttttcaacgaattcagtacagaactttacgagtaa
aggatataGCTAGGGAAAAAAGttttttttttGTGGATTTCCCGATATCAAGTCTAACGACATGGCTTAA
AGACACTAGAATAATTCTAGAATTCTAGTGTCTTTGCATTTACCAAGTAAATATTTTCAGAAAGCGTACC
AATCGATTAAATTCTTTATATTGTATTTATATGTAGACAAGTAAAAAAACATTTTTATTGCATCAACAC
TTTTTTGATATTAAAACTTTTGGATGTTGTTTTTACTTAGTCAAAAGGGTTTGACTATAAAATATACAC
AGGGTCGGCGGTAACGGAAGCAAACGTAACTATTACATACATTTCaaaaaaaaaaGTTTTGCAGTAGTTA
TACAAAATCTGTAAAAAAGTATTTACATACAAACAGATTTTCGAGAAAATATGGTTACTTAAATGGTTTG
CCAAATGGTTTGTACCTCTTAGGTCAGGGTCACAAGAAAAGTTGGCTTATCGTCTAGGTTCACGTTACTT
AATGAATGCGTCATCCAAGTGTTGTTGACTGGACTTTATGCAataaaaattgtaataaccttttataaccg
aaaataaaaaaaataaataacctaaaaatGTTGTTTGTAAACAACAACAAGACATATTAAAGCCATACGGC
TTGAAGGGTTTAAACATATTGGTTATATTACATATTACATACCTAATGAATGTAAATTGGTAAGTAGTAA
AGGAAATTCATTTTATTTTCGACGCAATCATTTACTTTTCGTTCCGGTCTTGTTTGCTTTCTACAATATA
AAGAAATAATTACCATTTTAAATTAATATTAATATATTTAATATGTTGACTTACGTTATTTTTTATTATT
TCTTGTTATACCTTTCAAGCTGTATTGCTTTTAATATACCTTCTATGCTCGAAAACTATAGTTCAGAACT
GTTTTAAATATTGCGATCTCGTATATACCCACTTGGCCTTGTGGGTTGTGCACGGAAATTCCCATTTGAC
ATCTTTTACGTCAAAGACTGAGCTGTAAGCCTCGTCTAATAGCGTACATCTTATGCCGCTTTCCTCCACA
TTTTTCCAGGTTCGGCACAACAGCATGGTTCGCGTCGCAGGCAGCAGCAGTCGGAGCGATTGCAGAACGG
GAGGGAACGGAGCCGGAGACCCAAGTCCGATGGAGTTCAGCATGCGGATAGCAGCATTATGGAAGACGAT
>dme_circ_0001044_junction_seq
TGCGGATAGCAGCATTATGGAAGACGATGGTGGATAGCAGACGATTGGAGCACTTG
>dme_circ_0001045
GTGTTCGGTTTCCTGCGGACAGGGTATACAAATGCGAGGCGTCGAGTGCAAATCGACAGACGGCAGCCTG
AGCGCGAAGTGTGATCCACTTACAAAGCCGGGCAGCATGCAGCAGTGCTCCACCGGAATTCATTGCGGCG
GATCGTTGAACAAAGTTGGTGGCACCATCATCGTAGGCAGCAGCCGGTCACTTAACGAAGCGATCGGAGC
GACAGTTGGACAGCTCGGATGCAGATGAAGACAACGAGGACGAAAATGATGAGGGCGATGATGTGGACGA
CCTGGAATCGGGACAGGATACAGATGATGGCGAAGGATTGTCATATGCTGATCAACCTTTGCTTTACGCT
CATCGCACACAGAGTCGACTGAATCAGGAAGCCCCAGATGAGCCGCGCACCATGCATCTAATGAACGGAA
ATTCAAACAATAATTTCAATCGGGGAGAGGACGAGTCAGAGGGACCATCACTTGATCCCACCGTGAGTAT
GGGGTGATTTATTATTTTAAGGTCGATTTTCTTGAGTGCCAGGAGTGCTAGTAAGATTTTTCCAAATTTT
GTTGGCATAGCGATAGAAATTGGAAATATTTTGTTTAAACTTGCCGAAGTTAGATTTTTCTAATATACAA
CTAAAAATTTGTGACCTTTAGTATCATTCATTGATTGTAGTATCAATATTGTACATGCTTAACTGGTGGC
ACGCCTTGGTGTGATTACTTGTGTCCTTTGGGACATGAGATACTTTGCCTACTTGGGGTTTTAGATTGGA
GCATTATCTTAAAGGCACCACTTGACATATTCCAACTGTAAATTTGGCTTTGAGCATTATTGACACCTTA
AATTAAGTTAAAAGAGTTCTGAACCAGAACTGGTGTAGATTGTACATAAGTTCGGCGAGTGTCCCTAATG
ACAACTAACGTGCACATTGTATTTATTCGAAAACGATACAAATTCCAGAGTTAAGCCTTTCCAGACATAT
TGGAGACTCACCTCAACAGGAAAGTACATATTGTAGTATACACAGTATACATATAATTATTTAGCAAAAC
AAAGGAAATCATTTGTATTAAATGGTCAAAGTCAAGTGCTTTTATCCGATTATTGCCGCGTGCACATTTC
TCAGGCGATGTATTAAATGACAGCTCATACTATAGATCCCTGTTATACCACTTGATTACGTACCAAAGTG

GGGTACTAGGTCCATGAAAATTGCGTTGGCGATTTCAAATCTTGAATCTTGCCTTGATTATTATTATTAT
TACTAGTATTTCTGGCGATTTGAATATTTTTaaaaatgaaaaaacaaaataaaaaTTTTCTAAGCAGCGA
GTTTAATCGAttttttttCTAATTTCTTCGGAAATTAGTAGTATTATTAATATAAATGCAGAATGTTGTA
CGGAAAGTAGTATGTATTACTTTTCTCCGATAAAATGATACAATATAATAATTTCATAATATCTTAAACA
CCGACGTGACCTTTTCCAAAGAGTAACAAAAATGCGGTGTACTTATAGTTTTCCAAAATTTTCGCTTAGC
TTAAACTGTGCCAATCTGGCGCCGCCAAAAAGGCGCGACATTGTGCCGCCACCAAAACCTTGGCCTCTGA
TGCCGGTTTTGCaaaaaaaaGGCCGTAAAAAGCTTAGAGATGGCAGCGCGCCAGTCAGCCAAACAAAGAA
AAACTAGAGAAAGAATATCCTAGCCTACGCGCCGCGACGATTACGAAATCAGCACTGAGAAAAAATAAGA
AACAAAGATCTCTAAGTAAATGTAATAAAATAAATCAATTTAGATTTTGAGTAGCACGGCCTATAATAAC
AAAAATGTCATAAATGTAACAGGCGCTGAAAATTCAGTAACGCCGAGTGATAAGGAAAATATGAAAATAA
AATAAATTGATGCGTTTTTTTGTACACACTCTCTCagagagaacactatattcgcattcctcgactatca
gataccctatatctaactagcaggagtgtgaacaagaagtttcaacattttATTATTTTTTGTCCATGGT
CCATGGTATTATTTTTTGTCTGCGCAGCCTGGCCCACTTAGGGCATCATGCTAAGATGTCTACATCCAGA
TGGCACCGCATCGCCATTCTGTCCAGCGGATTTGTTAGCCGTATTTTGTAACCATTAGGGCGGTGGAACC
GAACggaagcttttccgactataaatatgtatatatttctgatcaagttcaaccgccgagtcaatctgac
gatgtcagtccgtatcaacgcttcacggataacggaaactaataaagctagagatattatatctctagat
tagatattagatatgaagattccagacgcatatacgcagggcaagtatattatacccacacccaacggct
tgagccagagccggattctaaagcgtgcacccgcaagtacaggttcccttcccttccatcaatttatttc
cttacacgttacaaacggcatcatgtcttttaggtgccgacgggcttcagcgaggttgttgtcgctaagc
gcaacgatttggcgttgtaatttttatttactatattatttgttggttttatttattaaagtttttag
agtttaCGAGAGTTTTCAGGGTATAATCTCTTAGATAAACGAAtatttaaaatttttttaaattgaatttt
gtagtagttttattttattttaacttgttttAACTTCTTGTAGTTTTTATTTCGAATAATAAGCCTCAAt
gcttaaagaatattgtattgtaaacttggatccttaagtttagaagattcaaagagagcaggcaaagctg
cttaggtcaggctttaggatttgtttacaagaacggctaagtgctgtttattagaatgtttattggaacg
tatactgcgcatacgcatacTTCATTATTCGAAATAATGAATTGGCCGGTGTGGGTGGCGCAAAAATACG
TCACTATATACTATATATATGTttatacccgttactcgtggagtaaaagggtacaatagattcgttgaaa
agtatgtaacatgcaaagggaagcgtttccgaccatataaagtatatatattcttgatctggatcaatag
ccgcgtcgatctagccatgtccgtctgtccttttcacagttttgttagtcgtgtaaatttctatcggttt
agcaaaaaaactttgaaacgctcactctaacgaccacaaaaaactgtcgcgcccacacttttaaaatatg
atttgatgtttttcttgttagtctcgtaaatttctaacggtttgccaaaaaactttttgtcacgcccact
ctaacTTCTAACGTTTTTATAGTCTACTATAGCGCGCTCTGTTGTATAAAAATGTACTATTGGAAGGCCG
ATTCATTAATACTGTATATATTTTATTTATTTGATGATTAACGGGTATCTCATTCTTCCCAGCGGCATCC
TTTTTTGATGGTATAATGTGAAATTACCTAAATatttatttttgattttcttttttttATTATACACAGGC
TACATCAAGGACAATGAGTGGTCCCCATGTAGCGTTACCTGTGGGGAAGGAATCCGCCGGCGCACCTACA
ACTGCAAGATTTTCCTTGAATACTCTCGAACGGTGGCCACCGTTAATGATTCGTTGTGCGAGGGAAAAAA
GCCACATGACGAAGTAGAGCGGTGTGTGGAGGATCCGTGCATGCTACCCTCCCACGGCTTTGACGACCAG
TTCCCCCGTGATTCCATTAAAGTGGGTGTGTCGGAACCGGGTAAGACGTATGTCTGGCGGGAACAGGGAT
ACACCTCCTGCAGTGCCTCCTGTCTCGGCGGTGTCGAGGAACTGATAATCAACTGCGTTAGAGAAGATAA
TGGTAGGGTGGTGTCGCCCTTTCTCTGTTCCCCGGAAACCAAGCCGGAGGCCAGGGTTCGTACCTGCAAC
GACCGTCCCTGCCCACCCAGATGGAACTACTCAGACTACACACCCTGCTCCAAGAGCTGTGGCATTGGCA
TCAAGACCCGCGAGGTGCAGTGCATCCACGAGGTTACGCGCGGAGGGGACAACACAATGGTGGTGCCGAA
CAGTATGTGCCCACAGCCACCACCAGCAGATCGGCAGTACTGCAACGTTCTCGATTGCCCCGTACGTTGG
GAAGTCGGTGAGTGGAGCAAGTGCTCCCATACGTGTGGCTATGGGTTTAAGGATCGCAAGGTGGAGTGCA
AGCAGATAATGGCGCAAGAGCACAAGATCGAACGACCCGAATCGATGTGTCCGAGTGCTAAGCCGGCGGA

CAAAAAGCCCTGCAATGTGAAGCCCTGCCCGCCGGAAGATCCCAAGCCAGTCATCCAGATCAACAACTCA
ACACACATCCAGCACGACCCCAAGAAGACCAAGATCACCCTTAAAGTGGGCGGCGCTGGACTTGTTTTCT
TCGGCACCCAGGTAAAGATCAAGTGTCCCGTGAAGCGGTACAACCGCACCAAGATCAAGTGGAGCAAGGA
TCACAAGCCGCTGCAGCGTTCGCGAAAGTACAAAGTGTCGAAGAAGGGAGCCCTGCGTATACTTGATATT
ACGTTTCGCGATGCAGGGGTGTACTCCTGCCACGCCGGTCTCAGCTCCGCAGAAATAAGCATCGAAGTGA
AGGCCAAGCCAGGACAGCAGATTGAGGAGCTGGAACAACAGGAAACTGGTTAGTTATCCTATCTGGTTTT
GATATACTCTTTTGGCTAAGTAACGCGTTTATATCGCTTTTTCAGAACGATTGGTGCGTGAGAAAAGTGG
CACAGAGGCTCTCACCTCTGCGGATATGAAGTCCGCTGATGGAACTGGGACTTCCACTGCGGTGGGACCT
AATAGCCACGTTAACGG
>dme_circ_0001045_junction_seq
CGGTGGGACCTAATAGCCACGTTAACGGGTGTTCGGTTTCCTGCGGACAGGGTATA
>dme_circ_0001046
GCTACATCAAGGACAATGAGTGGTCCCCATGTAGCGTTACCTGTGGGGAAGGAATCCGCCGGCGCACCTA
CAACTGCAAGATTTTCCTTGAATACTCTCGAACGGTGGCCACCGTTAATGATTCGTTGTGCGAGGGAAAA
AAGCCACATGACGAAGTAGAGCGGTGTGTGGAGGATCCGTGCATGCTACCCTCCCACGGCTTTGACGACC
AGTTCCCCCGTGATTCCATTAAAGTGGGTGTGTCGGAACCGGGTAAGACGTATGTCTGGCGGGAACAGGG
ATACACCTCCTGCAGTGCCTCCTGTCTCGGCGGTGTCGAGGAACTGATAATCAACTGCGTTAGAGAAGAT
AATGGTAGGGTGGTGTCGCCCTTTCTCTGTTCCCCGGAAACCAAGCCGGAGGCCAGGGTTCGTACCTGCA
ACGACCGTCCCTGCCCACCCAGATGGAACTACTCAGACTACACACCCTGCTCCAAGAGCTGTGGCATTGG
CATCAAGACCCGCGAGGTGCAGTGCATCCACGAGGTTACGCGCGGAGGGGACAACACAATGGTGGTGCCG
AACAGTATGTGCCCACAGCCACCACCAGCAGATCGGCAGTACTGCAACGTTCTCGATTGCCCCGTACGTT
GGGAAGTCGGTGAGTGGAGCAAGTGCTCCCATACGTGTGGCTATGGGTTTAAGGATCGCAAGGTGGAGTG
CAAGCAGATAATGGCGCAAGAGCACAAGATCGAACGACCCGAATCGATGTGTCCGAGTGCTAAGCCGGCG
GACAAAAAGCCCTGCAATGTGAAGCCCTGCCCGCCGGAAGATCCCAAGCCAGTCATCCAGATCAACAACT
CAACACACATCCAGCACGACCCCAAGAAGACCAAGATCACCCTTAAAGTGGGCGGCGCTGGACTTGTTTT
CTTCGGCACCCAGGTAAAGATCAAGTGTCCCGTGAAGCGGTACAACCGCACCAAGATCAAGTGGAGCAAG
GATCACAAGCCGCTGCAGCGTTCGCGAAAGTACAAAGTGTCGAAGAAGGGAGCCCTGCGTATACTTGATA
TTACGTTTCGCGATGCAGGGGTGTACTCCTGCCACGCCGGTCTCAGCTCCGCAGAAATAAGCATCGAAGT
GAAGGCCAAGCCAGGACAGCAGATTGAGGAGCTGGAACAACAGGAAACTGGTTAGTTATCCTATCTGGTT
TTGATATACTCTTTTGGCTAAGTAACGCGTTTATATCGCTTTTTCAGAACGATTGGTGCGTGAGAAAAGT
GGCACAGAGGCTCTCACCTCTGCGGATATGAAGTCCGCTGATGGAACTGGGACTTCCACTGCGGTGGGAC
CTAATAGCCACGTTAACGG
>dme_circ_0001046_junction_seq
CGGTGGGACCTAATAGCCACGTTAACGGGCTACATCAAGGACAATGAGTGGTCCCC
>dme_circ_0001047
ATACCAAAATGTGTGTGCACTGCAGAATTCATTCAATCAAAGCAAACAAACCAAATGTACCGAAAACATC
GAATGAACGAAATGACCCTAAACAAAAACAGTTGCCATCAAAATGCGAGATTGGTCAAATAAAAACGAAA
TATGGCGCACAGAGTATTGCCGGATACTGAAGTAATCGAATCAAGCAAGGCTGGGGGTGCAAAGGGATGC
GTCTCACCTGGAGATTGCTAAGCAGAGCCTGGAAATATGGCATAGCCAGCAGCGTCCTGGTGCGACTACC
GCCGCTACTGGCACTCGCCGGCTGTGGTATTCTGTCCTCGGATTGCACTAATTGCGAAAGCTGCAGATGC
CACCCTCACAGGTAGATATATATGTATTGGACGGGGATTGGGTTGGGTTAAGAAAGAAATATGGCGAGAA
TAGTTATTAAGTGCGGCGTTATTTATTAGCAATATGTTTCGACTTAAAGTTATGGATATGAGCGTAATAT
ATTATGGGAGATTGTGCTGGGCGAGATGAAGTCAGGTTTGTTAATGGGAACGGGGGAATACTGTGGAGCT
GTTAAAACGGTTCAACCTGAAAGCGAATGCATTAAGTTCGGAAACAAATGTATTATAGATATATTTACAT

AAACGTCTGCATAAATATCTTTAACATTTATACAACAATATACGAGTATGAAGTAGTAAAGCAGACAAAC
AGAGATTTGACCAGGTTGACACAGCAAGAAAGTTTTGCCAATTAATACCTCAGCTTTTGTTGTAGTCTAG
TTTACGCTCACGCAAACGTTATCTCTATATGGTCATATATATAGTCATACAGATACAGAGACAGCCACAT
AAAAATCTGACAGTTGGCAAAGATATTTCTTTCGTTTATATGAGGAAAACTATGAAATCACGAAGATGTT
GACACGGGACAAAAGAAACCGACTTGAGTGCATACACGACGAAATTGTGTAAACATTAAAGGGGGCAACG
TTCCAACATATTTAAAACGTAAGCCCGGCAATGACGTTGATCGAGTGGGATAATCATAACGTTTCCAATA
CTCACATCGTCTTCCATAATGCTGCTATCCGCATGCTGAACTCCATCGGACTTGGGTCTCCGGCTCCGTT
CCCTCCCGTTCTGCAATCGCTCCGACTGCTGCTGCCTGCGACGCGAACCATGCTGTTGTGCCGAACTGGA
AAAATGTGGAGGAAAGCGGCATAAGATGTACGCTATTAGACGAGGCTTACAGCTCAGTCTTTGACGTAAA
AGATGTCAAATGGGAATTTCCGTGCACAACCCACAAGGCCAAGTGGGTATATACGAGATCGCAATATTTA
AAACAGTTCTGAACTATAGTTTTCGAGCATAGAAGGTATATTAAAAGCAATACAGCTTGAAAGGTATAAC
AAGAAATAATAAAAAATAACGTAAGTCAACATATTAAATATATTAATATTAATTTAAAATGGTAATTATT
TCTTTATATTGTAGAAAGCAAACAAGACCGGAACGAAAAGTAAATGATTGCGTCGAAAATAAAATGAATT
TCCTTTACTACTTACCAATTTACATTCATTAGG
>dme_circ_0001047_junction_seq
TACTACTTACCAATTTACATTCATTAGGATACCAAAATGTGTGTGCACTGCAGAAT
>dme_circ_0001048
GTTCGGCACAACAGCATGGTTCGCGTCGCAGGCAGCAGCAGTCGGAGCGATTGCAGAACGGGAGGGAACG
GAGCCGGAGACCCAAGTCCGATGGAGTTCAGCATGCGGATAGCAGCATTATGGAAGACGATGTGAGTATT
GGAAACGTTATGATTATCCCACTCGATCAACGTCATTGCCGGGCTTACGTTTTAAATATGTTGGAACGTT
GCCCCCTTTAATGTTTACACAATTTCGTCGTGTATGCACTCAAGTCGGTTTCTTTTGTCCCGTGTCAACA
TCTTCGTGATTTCATAGTTTTCCTCATATAAACGAAAGAAATATCTTTGCCAACTGTCAGATTTTTATGT
GGCTGTCTCTGTATCTGTATGACTATATATATGACCATATAGAGATAACGTTTGCGTGAGCGTAAACTAG
ACTACAACAAAAGCTGAGGTATTAATTGGCAAAACTTTCTTGCTGTGTCAACCTGGTCAAATCTCTGTTT
GTCTGCTTTACTACTTCATACTCGTATATTGTTGTATAAATGTTAAAGATATTTATGCAGACGTTTATGT
AAATATATCTATAATACATTTGTTTCCGAACTTAATGCATTCGCTTTCAGGTTGAACCGTTTTAACAGCT
CCACAGTATTCCCCCGTTCCCATTAACAAACCTGACTTCATCTCGCCCAGCACAATCTCCCATAATATAT
TACGCTCATATCCATAACTTTAAGTCGAAACATATTGCTAATAAATAACGCCGCACTTAATAACTATTCT
CGCCATATTTCTTTCTTAACCCAACCCAATCCCCGTCCAATACATATATATCTACCTGTGAGGGTGGCAT
CTGCAGCTTTCGCAATTAGTGCAATCCGAGGACAGAATACCACAGCCGGCGAGTGCCAGTAGCGGCGGTA
GTCGCACCAGGACGCTGCTGGCTATGCCATATTTCCAGGCTCTGCTTAGCAATCTCCAGGTGAGACGCAT
CCCTTTGCACCCCCAGCCTTGCTTGATTCGATTACTTCAGTATCCGGCAATACTCTGTGCGCCATATTTC
GTTTTTATTTGACCAATCTCGCATTTTGATGGCAACTGTTTTTGTTTAGGGTCATTTCGTTCATTCGATG
TTTTCGGTACATTTGGTTTGTTTGCTTTGATTGAATGAATTCTGCAGTGCACACACATTTTGGTATCTGT
AAATCACATTAAATACATCTCTGTAAACCCACCGCAAAAAAATTATAGCACGTCAAATTTTAAATACACC
ATTCAATGCACTACGTTGTACATTTTTATTTATTGATCGACAGATGGTGCGACTGTTTAATCAAAATGTA
TTTATTTGTATGTGAAATGCGATAAAAAATAACTTTAATAAAATCTAACGTTCAGCAATGAAAAAACAGT
GAATGCTTTCTAACGAAATAAAACAAATTCAAATTTTACCGGGTACAAGAGAGTTAACCGCCAATTCGAC
CAAATAAAATTATTAAGCCTTAAGTGATATATATATTATAAACACGATATGTTTTATCAAAGGCTTGGCG
TGCTTTCAAATTATTTTTACGATTCGTTCTGTGCTTTTTAGTGATGTTTTTATGTTTCCCACTCTTAACA
ATAACTTACCAACTTTGTCTTTCTATTTTGAAAATCGTAACATAAATATATTTCTTTACTGTTCCGTTAT
TCGGGGCCCTGTGAAAATATTGTACAACACATACAACATCTAAAAATAACACTACGGGAACCATTTTCGA
ATATACCGTAAACATAACAAAAAAACGTAGGTAAACTTTATACAATTACCACCACAATGCCTGATTCCAC
TCGAAAACGAGCTTTTTACTCCACTCCCAGCGCGTAAACAGTTTATCTGAAACTTTATATTTTCTTAGCG

TTGAGGAATTTAATTGGTACCaaataattttaattacgaatttttttataataattttctcttctttcta
ataatCTGAAAAGAGAGAACTTTTTGCGCGCTTGTCATTTAAGGAATGCTTATCttttgtttttttgcatt
ttttataccctttttttATAATTCCCCTGTCGTTATTATATATAATTAATGCCACTACTTCATTTCAGTA
ATTAATGATTGTAGAATTTGTTTATCTTTATCGATATGTGTAAAAACATATGTTTTATGGGAAATGCATT
TTATGTTCTATGTATTTGTTATTTTTTAAACAAACAAAAAACAACCCTTAAAAATTGTAATTCTGTTCTA
ATTAAGTGAAGTTCGGTACTTACAATGATCAGAATATATATTATTTATCAAATATTTAGCCACGTCATGA
AAAACATCATACCCAGCTTCAGTCTTAAGAATAAATAACATTTTTATCGGAAAGTTTGTTTTGGTTTTTC
ATCCTTGCACGATATAAATGCATAAGTCAACATGAATATATTGATACTTtttaaacacataatttacaaa
aataatttaaaaaaaaaaCTTATATTTATACCCAAGCTGATCGTTAGCTTGCTATAGTTTTATATGCCGG
CAACGTACATTAGTGTAATCGGCAACTGCCCGTAAATGTTTCTGAGCTGGCTGTGCCCCAAACTCTCTTT
TGTTGGTAAAGCCCCATCACTGTGCAACATTTTCCGCTTTACGCTTTCCGGGTCTGAATGCCAATTACAA
TATTATTTCTCATATATTCTCACAATATGTTTAGTAGCGCCTTGGTAATTGTGCAATTAAAAATCACGAC
AAAATATTACCACATAACGAAAGATGATCGCTTAAAAAGTCAAAATGGCGATAACTTTAATGAAAATCAA
GAGACAAAGTTGTACTCGAAGTCCCCGACTCAGGTACTGCTAGTGCAAATGAACAAATGCATGACAGCAT
TGGTAAACCGATAGAAATTGGCATAATAAATAAGAAACCGGaaaaataaaaaaaaaaTTCATACTTTAGA
AAACAGGCAACAAAAATCTGCTATCTTTATGGGTCGGAGTACATCATACTAACTTATCATACGTACCTAC
TTATAATACTTATATAATGCTCATACGAATACTACGAATTACAACAATACTAATTATTACCAAAAGATAT
CGCAGAGAGAAATATAAAATTCAATAGAAAATTTAGCTTCATTTATAAGCTATTCAATGTAATGTTAGGC
AATTACTTTTTCATTGAGAACTGTCGCTTGCAGCGTGTCGAACTTTAAAGCTTATGAATATTATACTAAT
ACTAAGGCGCCTAGAAATCAAGTCTGTTCATTAAGTCTAGACAAAAATTATATATTTAACATAGCAAGGG
ATCACCTAAAAGAGTCACATTACCACTTTAATTAGTGTGTCATTGTTCTGGGTGTGATAAACGGAATATG
CTGAGTCTAGTATTTACAAACTGATGGCGCCATTAAAACCAATTTGCCAGCATATTTTATGAGAGTTTTC
GCGGCGAACCACCAACCGTATACCACAAAAAATGTTCAGTGCTTGAAATCCAACAGATACGCACCCATTG
TGCCACATATGCCAGCTAATGGGTGACAATAAATCAATTTGCGGGCAAGAATATGCGGCGTTTTGCGAAC
TGGTTAATTGTAATATTTATGAGCTTATATCTTCAGTGGGCTAGGCATGTCAAATCTTGCTCTGACCTCT
TAAATATGAAGCGTGTAAGTCTTGGCTATTTGCACTTTTACATGGTCTAGCAATAAAAATAATTGTGAAC
GAAGCGCGCCCATAGTGACGAAGCGCGCCTAGTTGGTGCACTTATATAGCTTGCCTATTAAGTAAGCTTG
ATTGTGGTTTGTTGAGGTCTTTTATTGAATAAAGCGCAATAAAATTAGTAGGatacataaataaaaaatc
ataacaaaataatataaaaaaatcggctaaataaGATATTTTCGGTTCTGTCAATAGATTTGACTTCCTG
TTATTGTATTGTTATCCATGGCATCCGTGACCCACCTGTTACAACTATGAATGCAAATTCTATGCATTTA
ATTAAGAGCTCATCCTGTGACGAATGGATTAATGGAAACCTGAAACATGGCACTTGTTCATTTCTCTGAT
AAGCTTTGGAATACAATTTATTGCCTGGGCAAAATATTTAATTTTCCTTCTATTCATTATTAAACCGGGA
CTATTGGTCCTGCTAGGAATGCTCAACGACCTGTATTACCTATATTGGCCCCAGAAATTTAGAGGACGTA
GGGTCTGCTTGGAAAGGCGCTAGACCGCGATATTACATTTGCATAAACCGGAGTCCTTTTAAGAACTCCA
GCATCCTCTTGATTTTGTTTATTTGCAGACAGTGCGCTCAGCACGACACATTTGAAGTGAAATTGTATGT
AATCCTACATATTTTGACATCATAGAGTATTGTTGCCCATGGCATCCGTGGTCAGCCAGAGGGTACTTCA
GTAAAAAAGTGACAGGTTTCGCATATCAGGCGTAAGAATTTATATGTAACAATGTGCGCAGTCCGATGCT
AGCTTTTCATATATTAAAAGTAAGTTAATTCCTGTGACCGAATAGGCAACGAAGAACatttttagtataa
ttctatatattattaatatttatatattaataCCCAAATTCAAAGCTTCAGTGTATTTCCGAAAATTGAT
ATGGTAATCCCACAGAATGTAGTATGTAATATTGGCAAGATCCAAACACCAGCAGCAGGAATAACACCAA
CAATTACGACTGCTGTGACAACAATAAACATTAACTGGGGCGTCGGTGAGGGCCTACTTCTTTGGGGCAG
CAAAAGATGAAGTGGTGCAGCAAGTTATAGCGCTGTATATGCAGACAACAATGCAGCAACGACAGAAGCA
ATGTACAATAGGTGCTTTTTTCGCTTGCAAATGACGCTTGGCAAATCATCTTTGTGCGGCCCTGTCCCAG
CAAAACCCTTTAATTCCCACCCAACTCTGCTGCGACACCGACTCCTGGCAACTGAAAGTGCCAACGGCCA

GAGATATGACATGTTGTTTGACAAATGTTTCACTGGGAACTGCAAAAGACAATTTATGATAAGGGTTTCA
GGCACTTAAAAATGGAATTTACTGTGCCGTTCAATAGTTCAATGGTTTTTCAGTTCGATAATTAACAAAA
GTAAGAGAACTCTTATCAAGAAATCCTGAACATTATACAGTAAAAATTTTAGAGGAGTTGGCATGACGGC
AAAGTACTCACAGAAAGGGGTAATCAGAGAGGGGATATAGGATATGCTGACTTTGTTCGCAAGCTCAGTG
GATAATCGGACTTTGCAAATCAGTGAACGTGAACCAATTGATGATGTTAATTTAAGTGAGCATGAATGAA
ACCATACCCATTCTCCCCGACCCCTACATCCAAAAACATTTAAGCATTTACGTACCTCCTCATAGCGCCT
CATGCATAGCGCTGCAGTAGATTCAGTAAACCGGAAACGGAAACGCGGTGGCTGTCGAGCACTGAAGTTT
AGCCTGGCATATGGAACAAATAAAATACGATTGGCTTGGGCCCGTGAACTACAATACGTTTTCCAATGTC
AAAACCAATGATATTTCGCCCACAGGGTGAGACGCATCCCTTTGCACCCCCAGCCTTGCTTGATTCGATT
ACTTCAGTATCCGGCAATACTCTGTGCGCCATATTTCGTTTTTATTTGACCAATCTCGCATTTTGATGGC
AACTGTTTTTGTTTAGGGTCATTTCGTTCATTCGATGTTTTCGGTACATTTGGTTTGTTTGCTTTGATTG
AATGAATTCTGCAGTGCACACACATTTTGGTATCTGTAAATCACATTAAATACATCTCTGTAAACCCACC
GCAAAAAAATTATAGCACGTCAAATTTTAAATACACCATTCAATGCACTACGTTGTACATTTTTATTTAT
TGATCGACAGATGGTGCGACTGTTTAATCAAAATGTATTTATTTGTATGTGAAATGCGATAAAAAATAAC
TTTAATAAAATCTAACGTTCAGCAATGAAAAAACAGTGAATGCTTTCTAACGAAATAAAACAAATTCAAA
TTTTACCGGGTACAAGAGAGTTAACCGCCAATTCGACCAAATAAAATTATTAAGCCTTAAGTGATATATA
TATTATAAACACGATATGTTTTATCAAAGGCTTGGCGTGCTTTCAAATTATTTTTACGATTCGTTCTGTG
CTTTTTAGTGATGTTTTTATGTTTCCCACTCTTAACAATAACTTACCAACTTTGTCTTTCTATTTTGAAA
ATCGTAACATAAATATATTTCTTTACTGTTCCGTTATTCGGGGCCCTGTGAAAATATTGTACAACACATA
CAACATCTAAAAATAACACTACGGGAACCATTTTCGAATATACCGTAAACATAACAAAAAAACGTAGGTA
AACTTTATACAATTACCACCACAATGCCTGATTCCACTCGAAAACGAGCTTTTTACTCCACTCCCAGCGC
GTAAACAGTTTATCTGAAACTTTATATTTTCTTAGCGTTGAGGAATTTAATTGGTACCaaataattttaa
ttacgaatttttttataataattttctcttctttctaataatCTGAAAAGAGAGAACTTTTTGCGCGCTT
GTCATTTAAGGAATGCTTATCttttgtttttttgcatttttttataccctttttttATAATTCCCCTGTCGT
TATTATATATAATTAATGCCACTACTTCATTTCAGTAATTAATGATTGTAGAATTTGTTTATCTTTATCG
ATATGTGTAAAAACATATGTTTTATGGGAAATGCATTTTATGTTCTATGTATTTGTTATTTTTTAAACAA
ACAAAAAACAACCCTTAAAAATTGTAATTCTGTTCTAATTAAGTGAAGTTCGGTACTTACAATGATCAGA
ATATATATTATTTATCAAATATTTAGCCACGTCATGAAAAACATCATACCCAGCTTCAGTCTTAAGAATA
AATAACATTTTTATCGGAAAGTTTGTTTTGGTTTTTCATCCTTGCACGATATAAATGCATAAGTCAACAT
GAATATATTGATACTTtttaaacacataatttacaaaaataatttaaaaaaaaaaCTTATATTTATACCC
AAGCTGATCGTTAGCTTGCTATAGTTTTATATGCCGGCAACGTACATTAGTGTAATCGGCAACTGCCCGT
AAATGTTTCTGAGCTGGCTGTGCCCCAAACTCTCTTTTGTTGGTAAAGCCCCATCACTGTGCAACATTTT
CCGCTTTACGCTTTCCGGGTCTGAATGCCAATTACAATATTATTTCTCATATATTCTCACAATATGTTTA
GTAGCGCCTTGGTAATTGTGCAATTAAAAATCACGACAAAATATTACCACATAACGAAAGATGATCGCTT
AAAAAGTCAAAATGGCGATAACTTTAATGAAAATCAAGAGACAAAGTTGTACTCGAAGTCCCCGACTCAG
GTACTGCTAGTGCAAATGAACAAATGCATGACAGCATTGGTAAACCGATAGAAATTGGCATAATAAATAA
GAAACCGGaaaaataaaaaaaaaaTTCATACTTTAGAAAACAGGCAACAAAAATCTGCTATCTTTATGGG
TCGGAGTACATCATACTAACTTATCATACGTACCTACTTATAATACTTATATAATGCTCATACGAATACT
ACGAATTACAACAATACTAATTATTACCAAAAGATATCGCAGAGAGAAATATAAAATTCAATAGAAAATT
TAGCTTCATTTATAAGCTATTCAATGTAATGTTAGGCAATTACTTTTTCATTGAGAACTGTCGCTTGCAG
CGTGTCGAACTTTAAAGCTTATGAATATTATACTAATACTAAGGCGCCTAGAAATCAAGTCTGTTCATTA
AGTCTAGACAAAAATTATATATTTAACATAGCAAGGATCACCTAAAAGAGTCACATTACCACTTTAATT
AGTGTGTCATTGTTCTGGGTGTGATAAACGGAATATGCTGAGTCTAGTATTTACAAACTGATGGCGCCAT
TAAAACCAATTTGCCAGCATATTTTATGAGAGTTTTCGCGGCGAACCACCAACCGTATACCACAAAAAAT

GTTCAGTGCTTGAAATCCAACAGATACGCACCCATTGTGCCACATATGCCAGCTAATGGGTGACAATAAA
TCAATTTGCGGGCAAGAATATGCGGCGTTTTGCGAACTGGTTAATTGTAATATTTATGAGCTTATATCTT
CAGTGGGCTAGGCATGTCAAATCTTGCTCTGACCTCTTAAATATGAAGCGTGTAAGTCTTGGCTATTTGC
ACTTTTACATGGTCTAGCAATAAAAATAATTGTGAACGAAGCGCGCCCATAGTGACGAAGCGCGCCTAGT
TGGTGCACTTATATAGCTTGCCTATTAAGTAAGCTTGATTGTGGTTTGTTGAGGTCTTTTATTGAATAAA
GCGCAATAAAATTAGTAGGatacataaataaaaaatcataacaaaataatataaaaaaatcggctaaata
aGATATTTTCGGTTCTGTCAATAGATTTGACTTCCTGTTATTGTATTGTTATCCATGGCATCCGTGACCC
ACCTGTTACAACTATGAATGCAAATTCTATGCATTTAATTAAGAGCTCATCCTGTGACGAATGGATTAAT
GGAAACCTGAAACATGGCACTTGTTCATTTCTCTGATAAGCTTTGGAATACAATTTATTGCCTGGGCAAA
ATATTTAATTTTCCTTCTATTCATTATTAAACCGGGACTATTGGTCCTGCTAGGAATGCTCAACGACCTG
TATTACCTATATTGGCCCCAGAAATTTAGAGGACGTAGGGTCTGCTTGGAAAGGCGCTAGACCGCGATAT
TACATTTGCATAAACCGGAGTCCTTTTAAGAACTCCAGCATCCTCTTGATTTTGTTTATTTGCAGACAGT
GCGCTCAGCACGACACATTTGAAGTGAAATTGTATGTAATCCTACATATTTTGACATCATAGAGTATTGT
TGCCCATGGCATCCGTGGTCAGCCAGAGGGTACTTCAGTAAAAAAGTGACAGGTTTCGCATATCAGGCGT
AAGAATTTATATGTAACAATGTGCGCAGTCCGATGCTAGCTTTTCATATATTAAAAGTAAGTTAATTCCT
GTGACCGAATAGGCAACGAAGAACatttttagtataattctatatattattaatatttatatattaataC
CCAAATTCAAAGCTTCAGTGTATTTCCGAAAATTGATATGGTAATCCCACAGAATGTAGTATGTAATATT
GGCAAGATCCAAACACCAGCAGCAGGAATAACACCAACAATTACGACTGCTGTGACAACAATAAACATTA
ACTGGGGCGTCGGTGAGGGCCTACTTCTTTGGGGCAGCAAAAGATGAAGTGGTGCAGCAAGTTATAGCGC
TGTATATGCAGACAACAATGCAGCAACGACAGAAGCAATGTACAATAGGTGCTTTTTTCGCTTGCAAATG
ACGCTTGGCAAATCATCTTTGTGCGGCCCTGTCCCAGCAAAACCCTTTAATTCCCACCCAACTCTGCTGC
GACACCGACTCCTGGCAACTGAAAGTGCCAACGGCCAGAGATATGACATGTTGTTTGACAAATGTTTCAC
TGGGAACTGCAAAAGACAATTTATGATAAGGGTTTCAGGCACTTAAAAATGGAATTTACTGTGCCGTTCA
ATAGTTCAATGGTTTTTCAGTTCGATAATTAACAAAAGTAAGAGAACTCTTATCAAGAAATCCTGAACAT
TATACAGTAAAAATTTTAGAGGAGTTGGCATGACGGCAAAGTACTCACAGAAAGGGGTAATCAGAGAGGG
GATATAGGATATGCTGACTTTGTTCGCAAGCTCAGTGGATAATCGGACTTTGCAAATCAGTGAACGTGAA
CCAATTGATGATGTTAATTTAAGTGAGCATGAATGAAACCATACCCATTCTCCCCGACCCCTACATCCAA
AAACATTTAAGCATTTACGTACCTCCTCATAGCGCCTCATGCATAGCGCTGCAGTAGATTCAGTAAACCG
GAAACGGAAACGCGGTGGCTGTCGAGCACTGAAGTTTAGCCTGGCATATGGAACAAATAAAATACGATTG
GCTTGGGCCCGTGAACTACAATACGTTTTCCAATGTCAAAACCAATGATATTTCGCCCACAGTTGCTATG
GCCGCTGCAGGTTGCTATGGCCGCTGCAGCGCTTTAAAGACTCTCGTGGTCAGCACTTGCTTCAAGGCGA
GGCGCTTAAGTTCGGCATTGATTTAAGCGGCAAAGACTTTGACCAGGACGAGTCGGAGCGTGTTCCGGCA
GAACAGCTTCCCCCAGAAGTATTGCATTCGCCATCGGACCCAGAGTACCATGAGCCGTCGAAGTCCGGTC
CGGAAGCGGATGTTCCGCATGTTCCGCCACACATGCCACACGCTCGATGGGAAACGGGAGCTGCAGCTTC
GACTACGGCGGTGCCAAGCCTTCCGAACGACGGTTTCGTCTACAGATGGATGTTGGGCGAATGGTCCCAG
TGTTCGCAGGAGTGTGGAGCAGCCGGCAGTGGGTTGCAGGGTAAGTGAAATGGGTGCAGTGGCTGTCAGG
ACTGTTAAATACCCAGCACTAGAATACCAatacaagaataagaagatgcgtaacggccatacattgtttt
tgcactatgcagccactttttttggtgacggccaaaattgcgctctgtccgctggcgtacgctgagagcg
taaggaatctataaatcgaatttgcttgattgtgtgagtaaaaagaatagactgaaacaatacgtgtgat
atatgtgatttcaagtctaagaacttctaaggtgaattgcatattttgtcaaatttacaatgcatgagca
tacgtgtgaacacatagagttgtcggcttttcgctgtagagctctccgctctctcgctcttgaacaaaaa
ttcgagagagccaggggagctacctctagagccaccccgaaaaaatcgtgtgcaaaaaaatcgtgtggcg
ttacacatcttgttattctagtgtctttgTTAATACCCTTGCAACTAGTCAGCCAGTAACATGGCTATGC
ATGAAACTTTTCCCTAACGTTTCTCGCTTTTGCAGGTAGATATGTAGGTCGCCGATGGCTGAATCAGACC

AATAAATAATATAGCTCTTATAAGAATAATCGAAAAATaacacgagagaatgctgtagtcgcgttctccg
actttcagatacccgttactcagcttgtgtgaatgcgaacgcgaaatttcgtcatttttctgggatatca
atagatattggggaataaaatgaccaaaaatttaaaaatggtttaaaatcgttaaaattgtttaaaggtg
tgggcgtgaccgttttggtcgttttgtacgtggaaagaaattacaagactaataaaattatgaaaaaata
gcaacacattttcaaatgtgcgggcgtggcagttttggtcggtttgtgggcgttagaatgggcgtggctt
catgggtcaacaaactttcgctgcgtctatgtctctagaatcgggtttccttaaatcttgacgttaatac
ggacagacggatggacagtgccagatcgacttggctattgatcctcatcaggagtatatatactttatac
ggtcggcttcaacgaaactagtatactcttttactctacgagtaacgggtataaAAATAACAGTCGTCAA
AAAAGAAAAACACGTCTTTAGTAAGCGGCATTAAAAAAGGGAAACAGCTATATAGTCTGATGTATATCAA
ATAAAAATTTAAATTATTATTATTACTGCTTCTGCTTCTGTATATAAATATGTCGAACATAAAATATTAT
TGGATATTTAGCCGACTAATTTCAAGCAAAACTATTGTATATACCACTGCAGTAAAGATATCATAAAACA
GGAACGCGTCCCAAGATCGCTTAAGCAATAATTATCACATTGCACAGTACTCTGAAATCCACCGCCAAAG
GCCTCCTAAATTAAGCAAAATTATATACAGCAAAAGTCACAAGACCCCATCCCGTGCCAAAATGCTTTTT
ATTTCATTGTCGGGAGGAAACAATAAAGATATAAAAGTCATTCTTCCTCTACTCCCAAAGGCGGCGCACG
GTGAGCTGCCAGAGAGCTGCAAAAGCCACGAGCTCCGGCAGTACTAGCGACACCGAAAACAATGTCGTGG
ATAATTCAGAATGCTTCGCGCACGGCCTGGAGCTGCCCGAATTTTTCCAGAGCTGCGGCAACGAGGCATG
CCCGCAGTGGAGCAAGGGCGACTGGACTCCCTGCCAGCGATCCCACTGCCACGGAAGGAATACGGCCATG
CAGCGCCGCGAAGTGACGTGCCGCTACCCCAACGGTACGGAAGGAAGCATCTGCGACGAGTACGAGCGAC
CTGCGATGCGACAGGAATGCTACAATGAGCGATGCGAGGGAGTGTGGCGCGTGGAGCCTTGGTCCGAGGT
GGGTCCTGGATCACCTATTAGCCGTGTTAAATATTAATGCCTCCTTTACTCCTACCAGTGCAATGCGCCG
TGCGGCCGTAAGGGCATCAAGTACCGCATCCTGCAGTGCGTCTGGTACGGAAGTCGCCGCCCAGCCGGCA
ATGTGTGCAAGCACCAGCCACGCCCGGCGGTTATGAAGGTCTGCAAAAGTCCGGCATGCCAGGCGCAGC
>dme_circ_0001048_junction_seq
TGCAAAAGTCCGGCATGCCAGGCGCAGCGTTCGGCACAACAGCATGGTTCGCGTCG
>dme_circ_0001049
GCGGCGCACGGTGAGCTGCCAGAGAGCTGCAAAAGCCACGAGCTCCGGCAGTACTAGCGACACCGAAAAC
AATGTCGTGGATAATTCAGAATGCTTCGCGCACGGCCTGGAGCTGCCCGAATTTTTCCAGAGCTGCGGCA
ACGAGGCATGCCCGCAGTGGAGCAAGGGCGACTGGACTCCCTGCCAGCGATCCCACTGCCACGGAAGGAA
TACGGCCATGCAGCGCCGCGAAGTGACGTGCCGCTACCCCAACGGTACGGAAGGAAGCATCTGCGACGAG
TACGAGCGACCTGCGATGCGACAGGAATGCTACAATGAGCGATGCGAGGGAGTGTGGCGCGTGGAGCCTT
GGTCCGAGGTGCAATGCGCCGTGCGGCCGTAAGGGCATCAAGTACCGCATCCTGCAGTGCGTCTGGTACG
GAAGTCGCCGCCCAGCCGGCAATGTGTGCAAGCACCAGCCACGCCCGGCGGTTATGAAGGTCTGCAAAAG
TCCGGCATGCCAGGCGCAGC
>dme_circ_0001049_junction_seq
TGCAAAAGTCCGGCATGCCAGGCGCAGCGCGGCGCACGGTGAGCTGCCAGAGAGCT
>dme_circ_0001050
CTTGCTGTTCTTTAGGAGCTTTCCAGACCACTTGAGCACCAGCTCCCACTCGTAGAAAAATATGAGCTTG
CCCTTGCGATTGTTCACGGTGGCCTCCCCGGAGCATTTGTCCACGGAGTCCACGGCACACTCAATGTCGC
TTTGCCCTGCGTTTTGAGAAGTTTACTTTTAGGTTAGGATATACCTAAAATGTATGTTCTACACTACTAC
AAATTCTTATATACTCGCCTATTTTGAAATCCTGAAAAAGCTGATGCAGGCGGTCCTTGGACCATGGCGT
GGCGTTCTTTTCCGTCCAGTGCCAGTTGTTCACG
>dme_circ_0001050_junction_seq
CTTTTCCGTCCAGTGCCAGTTGTTCACGCTTGCTGTTCTTTAGGAGCTTTCCAGAC
>dme_circ_0001051

GGAGGAGTACTCCAAGAACCTTATCCTACCAAAGAAGGGCGACGAGGCCGGAGCGGGAAATACTGTGGCG
AATTTTAAGGATGCCAACAATACCCGTAATGCAGCCCAAAATATCGCTTTAAACTCCTCGGTGGCGGCTC
CTAGGCTGAAGAACTCTGGCATCGGCTGTAAACTGGACGTTCGCACTCTCTCGATGACCGAAGAATTTCA
CTGCAGCGCCAATGACCTTTACAACGCTCTCACAAAGCCAGAAATGGTCACTGCCTTTACGAGGGCACCT
GCCAAAGTAGATGCCGTGCGCG
>dme_circ_0001051_junction_seq
GCACCTGCCAAAGTAGATGCCGTGCGCGGGAGGAGTACTCCAAGAACCTTATCCTA
>dme_circ_0001052
GCTTTCAATGCTGAAAAGGCAGAGAAGTTAGCGGCAGctgaaaaagatgctgcaactgaaaaagctgcgg
cgactgaaaaGGCGGCAGCAGAAAAGGCAGCTTCCGTGAAGAATGAGGTCATAGACCTTGATGATGAGCT
CATGACTAATGAGAGTGTCATAAAGAAAGAATCGCCGGTTACTCCCATAAAAGAAGAAATTAAATCTGAA
GAGAGTCCTGAAAAGCATGATACTGCTGATAATCTCGAAGATAAAAATTCAGATGCTGAAGAGAGTACAA
AGATAAAGGGTAAAGAGGATTTTAATCCAGAAACCACGGAAAAGGAAGCACTGGACGAATCTACAAATTT
GAATAAGGATAAGTCTAGCACTGAAACATTGGTTCCAGAAATTGAAGATAGCAAACCTAGCGAAGATAAA
ATGGAAGTTGAGGAATTGCCCGTTGCCGAAAATGGTGAGAAGGAAGGTTCGCCTGAGAAATGCAGTGATG
CTGAGGATAAGAAATTTTCAGAAGAAAAGCCAAGTTCCCCTGCAGTTCCTGATGGTAAAGTGTCAGAAAT
GGAGGCAGATGAAGCTGCCCCTAATACAGAAGAAAACTGTTCTGGGGATTCGGAATCGCCCGAAAAGGAA
TGTGAAGACAAGGTTG
>dme_circ_0001052_junction_seq
CCCGAAAAGGAATGTGAAGACAAGGTTGGCTTTCAATGCTGAAAAGGCAGAGAAGT
>dme_circ_0001053
GAATACCTATGAGCGTGAAATGTTCGACAAAGCTTCAATGAAACTTGGATTGGACAAGGCTGTATTGCAG
TCGATGAACACCCAAGGGTCTAAAGATGGCAATAACAAGCAGCTGTCCAAAAAAGAAATTGAAGATCTTT
TAAAAAAGGGTGCTTACGGGGCCGTCATGGATGACGACAATGCTGGTGACAAATTTTGCGAAGAGGACAT
CGATTCAATCCTTAAGCGACGCACTCAAGTCATCACAATGGAATCGGAGAAGGGTTCAACCTTCTCAAAG
GCTTCATTTGCTGCATCCGGTAACCGGTCGGATATTACGATAGATGATCCTGATTTTTGGACCAAGTGGG
CCAAAAAGGTTGACATTGATCCCGATGCTTGCGAAAGGGATGAAACGGAGGACTTGGTTTTGTCCGAACC
CAGAAGACGCACCCAAATCAAACGCTACGGCCACGAGGACGTAATGGAGCTCAATTCCGAGGAATCTTCC
AATGAGAATAGCGACGAGGAGGGAGGAATAGGGGCTCCGTTCCCGTCGCCGCAAGGAGAAGCGAGATCGT
GCTCGTGAAAAGAAGGGCAACGATGAATACATTCCACGAGAACGGGATGCATTAGCTGCTTTGGGCTTGG
AGGAAATTCAATATGGTAACTGGGCCAAGTCTGAGTGCTTTAAAGTGGAAAAGGGACTGCTTTCCTTTGG
GGTAAGTTTATAACGAGCTAAAATGCCACAATTTAATTTTCTTCGAGCCGATTTCGCCGACGTATGGCAT
TGTTGAGCTTCATATTGAGGATGTAGATCTTTCTCTGCAGCATTTTTTCTTCCTTTTCAAGGACGTGGGC
CTCTTCCTTTCGCTCGATATACTCGCTGACACTGGGGGCCTCATAGTCCTGGAGCTGTGCTCTCAGCTCA
TCCAGAGTCACGTAGTCTTTGGTATTGTGCATTTGAACTGCAGCCACCTCCTTTTCCAGTTTCTGAATGG
CTCGCGTGACTTCATCCGTTCTATTCAGAACGGTACGCATTTCTGTTTCCAAGTTCATCATGGCCTGTTT
TTCCTCAGTCATGGCCAAGGAAGTTTTTCCTGTGACTCCTTTAAGTCCCGCCATGTGTATGTTTTTTTCT
TCCAGTTGATTCACCAGCTCGGTTCTCTTTATAATAAGTTTTTCAAAATCTACAGCCGTCAGGATACCAC
TCAGGTCTGATTTGGTTATCAAATCCGCCCCGATGCTGCTGACATGTTTCTTGAAGCGATACTATGGTTAA
CTTCATAGTTCCCAGTAAAGCGCGTGCATTTTTAAACCAATTTGAAGTGAACTTGCGCAATTTCCGTTCA
ATCTGTCGCTCAGTTGCAGACTCCAAAAAGGCCACATCGTTCTCATCCCGTAAAAAGTGGAGCATAAAGT
TGTTCTCCGTCTCAATGGTTTCCTCACTTCGAAAGCGGAGCTCTTCTATCTTGGCTCTTAACTGTTTCAC
TTCGGTCATGGCTAAAAAGTGCATTGTGAATGAATGAGTTTTGAAAACGAGATCTAAAATGCAATTAACC
CACCATTTCTTTCAATATCCGCGACCCGCTTCTCCACCTCAGCTGCTCTTTTCTCTGCCATTTCGGCTTT

GGCCTTAAAATTTAGTTTGTAGTCCATTCCACGTCGCTTTTCTGGAGCAGATATGCCACTTATGGATACT
AGCTGATTCACTGACATGGGTATGGAGCGTTTGGAGCTGGAGTGCCGGCTATTCACGCCATGACTTCCCT
GTGCCGATCCTCGCTTTGTTTGCAACTTTCTAAGAATGGAAGTACGGCGCTGTCTCAATCCCAATAACAA
CTTTGGATCGTTTTTCTCAAGGAATTCGACCATTATGTGGTTCTCCAGCCTTAAAGTAGCCACATCATTA
TGCACTTGCTCTATGGTGTGGAGCATGTCGATGGCGTCCAAACTGGACAATCGCAGTTGCCGCTCTTCTT
CCGTAAAGTTGCGAAAGTCCTCCAGATAGAACTTGTAGATAATAGCCACGACGTCCAAGTCCTGCGGAGC
AGCATATTCGAATACGTGAAGCTTGCTTGGATGAGATTTCACCGAGCGTCTGCTCTGCGTGTCGGTTTTA
AGGCTGTGTCCTGACGTGCTCATCATGAGACCAGTAACGTTAAGGAATAATTTATGGAATTTGTTTGAAA
TATATGTATTTTGACCGGTTTGTTGGTGATAAAGGGAAAGTATACAATGACTAGATTGGTAACATACAAC
AAAGTCTATCTAGTCTATCCCTTTGCCCAGGCATATTACAAACAAACGTGATCTCATTGGTAAATACttt
tttttCAGTGTAAGTTAAATGAAACTCAAACCAAAAATAAACGTGTGCAAAAAAATGATGACCAACCATG
CTAATATTGAGAAAACTCAAGGTTTTCGCTGAGTAAAACTATCTTCTCAACCCGCTGATATGAATGTCAG
AAAGTTTATAGAATGCACTTTACAAAAGCTTTAACATTAATATTGTTCTATTCTCGCAGCCTTCTTTACT
GCCTGCAGGTGTACAAGGGTGATGAGAAAATCAAGACGTTCATCTGGGACTTGATAACACCGACGGAGGA
CGGGGAAGTGCAGAAGATTAGCAGGGATCATAGTGGCCTACACAACTTGGTGCCACGAGGTAGGAATGCC
AAAGGGTAGGAATGCCAAAGGTATGGTAAAAGATACATTTACGCACACTCCAACTGCTGCACGGACGTAG
AGAGTTTTAAAAAACCAATACAAAAGTAATATAAACGAAACGGTCTACCAGAAAAAAATAGCAAACACGT
TTTGTAAAAAAATTTCTTTATGCTATCTGTACTGGGCTCTGGACTTTCTACATCATTTTAATTGCGTTCT
TCTACTTATTTGTATCCGTAAATGTCAGATACTTAAAGCAAGAATATAATTTAATTTGTGTGTCTAATGG
CCTCTCCTCCGTAAAAACTTCCTTCAATATTAAATCGAATCACCCAATAAAGACTGAACCAGAACAGAGA
CATAAATTATTGACTCATATACGCGACGCGACGAAAAGAAGTGTGTGTATGTTTTGTTGCCATGCAAATG
TATGCCTTTTACTATATCAAATCTGTTACTTTTATGTACATAATAAATAACATTTTACTTACATACCCTT
TAAAATTCATATTAACTCTTTATATAGAGTAGCCTAAACCATTTTCTAATTTACTACTAATTTATTTTTT
ATTTGCTGTTGTTGAGTCGAACAATGTGCTAGCTTAAAAGGTAACATTGTAGGAAAGATACATAATATCT
CGAAATTTTAAACTATTTAATTTTTAATATGTATTTAATTTTTAAAATCATATATCCCTTGTGTATCATT
TTCATGTATAGGCAATATTTTAAATTATAGCCAATTATATTAAGTACCCTTCAAAAACACCTCTTTTAAA
CGAAAACGATCAGAGATCCGGTGGTGCTGTAAATTCATTTTGATTTTACATTTTGATACCTAGAGCTTTG
TTGTCCTTCAGATTAGTGTATATATCAGTCTGAAGGCAGCTAGAGAAATATGAATATACTAATGTTTGTG
TAAACACTACTTTTGCTTTACAGGGTCGCAACGGTGGAAAGTCAAACAAAGAATCCACAGGAGTGTCTAC
TCCTGCTCCGGGCTCTGCATCCGGAAGCAATAGTGGAAACACGACTCCGGCTCACAAATCTAGTGCGCTG
GATGGGTCCGATAAGGACAGCGGCGGCATAAATGCCAGCGCAGTAGCCGCGGCGGTCACAAGCATTCACG
ATCCAAATCACTGGAGCAAGAAGGAGAAGTACGACGCTGATGCGTACTTGGAAGGCGCCTACAAGAAGCA
TTTAGGCAGACACGCCAATAAGGTAGGCGTGGCACTCGGAGTCATCTGCTAAACACTTGGCATTCATTAA
TCATTTTGTTTGTTGTTGTTGTTGATTGAATGATTGACTGGCTGATTATCAAAGAGTTTTCATTAATTTT
GTATTTAATTTAACCTTCTGTTATAGATACTTTATAGCCTCTTTACTAATGCCAATTTGTGTCttttttt
ttaattattattaaccctttttttaTAGAACTTTGTGTCTGCTGATTGAAGCAATGATCAGTGGGCGTT
TCAAGGTTCAGGTGGAATGTGATTCTCTTTTATTCGCTATGTATCTCTATACTGCAACTCTCTTTGTATT
TTCGATTGCTTGTCCATTAAAATGCTAACACTTGTACATCTTATACACGATTCTGCTCATTTGTAACCAC
AACATTTAAATTCTATATTTTGCCAACTAATCTTCGGCGTATCGAATCTTCTTCTATCGCTGTGTTTTGT
ATCTTTTCGATTCGTTTAACAAACCTTAAAAATTCCTAATTGACCTCATTGCGATATCTATACGTATATT
ATATAACCACCAAAAACTGCAGCAGTACGAGGGCCAAGCAGCAGTTGGCACTGAATCTGAATTTGGAAAG
GTAATATCCCCATATATTCCTCAACCACCGATATCGATCGCCATCGCTCGTCGCCCTCGCTTGCACGCAC
GGTGTTTTCCACTTTTAAGAAAAAAGCTGGCGAATAGGGAAATTCACGCTACTCAACATCTTTATTAACC
TCCATACGTGCAGTCGTCCTCATAATACAAAAACCGCCAAGTTATACATGCAATTCAATAATTAATTTAT

ATTTTTTAATACGAATACCAAATTCaaaaaaaaaaaGCCATCAAGAGGCTCAACTAACCTTTTAAATTTT
GCTTTGTATGATTCTGCATTTTGTAGTGGTAAATCTGTAGTACAAATTGGCAATTAACAATGTTACAatt
tttactttatttccctttatggtctcatttttgatttttgttttatttaaCATCATACAAAAAATTGTTG
AACCGAGAAAATTGAATTTTTACTAAACAACGCATTTCTTTTTTCGCGCATCTCTCAACTTCCCCAATCC
ACCAAACACATTTGAATGTTATCGAATGTACTCATCGAACGATACGATCATATGTAGCGAACTCCCCATT
CGCCCGCCGCCAACGCCAGATCAAGTGCCATCTTCATGGTGGAATCCCATTTGTTGTGATAAGAGCCTGT
TGGTGGGAACCTACAAGCATGGCTGTGAAATGTATCGTCAAATGCGCGCCGATCCCAATCTGTGCTTTGT
CACCCATGTGGGCGCCGGTGCGGCCGGTGACGATCTGGCTGTCACGAGTTTGCCGATGTAAGTCTTCAAA
ACGAAAACAATCAATTCTAAATTAATTCGGTGTGTTCTAAGGAATCTCTATTTCAACGTGTACCTTCTGC
ACACACGTAACATTAATATTGATGGTAATTGATAACCGTTTTAACCGCTACTGTGGTGAAAGAATTTGAG
AAATGATATAGAGGTTTTTTTGAACACGCCGGATAGAAAAAACAACAGATGCAAAGAGATATAAACCATA
CATAAACGCCATTGAGTAATTCATATACATCACACTAAAGTATtaaaattaaaaaaataaataaataCGC
TTCCAATTAGACCTGAATGGAAGGAACGTGCAGGATTTGCTTAAACTCATTTTCATTCCCATTCCCCACG
TGCACATGCATTCATCTGAAAGTCTTGGCATTAAAATGTGCTTCAAACATTTTTTCAGCTATTAGCGCTT
AATATAATATTACATCAAAGATTACAGTTTGGTAGACAGTGCTATTTATCCATCAAATGTTAAGGTCATA
AATCATAATAATTGTTTTGTTGACTTTTAATATAGACTAGATTTAATTTAAATTATTCAAATCAAATTAA
AAAAGTACTTTCAATTCACATTAATAATTTTAATAATCATTGTGCTTGTTCATCGAATTCTCGCCGTATA
ATTTCTTGATAAGGTATTCAACGAAATATTCTCAGATACAACAGCGCAACCTACGGGGCTTCCATTAACG
CTCACATTGAATGCATTGATCCATTGACATTGCGCTGAAtgtgtgtgtgtgcgtgtgttttgtgAATTTT
CTCTCAGTGTTTTAACTGCGTTGGGCATGGCCTGCATCTGATATATGATCAGTAAATTCCATTAAATTGC
TACTAACATTCAAACCCTTTTTTCTGGAACCACCACAGAAAACGAAGACGATGCTAATTCGAAGCACGAT
GATGGTGACGAGGTGGACGATGACGGAACTACAACGACCAAAGACTCTGACTCTACTAAACTCACCGGGG
GCGATAACAAGGACTCGCTTCTCGACCCGGAGCGTCCCAGTTCGTCTGGTAAGAGTAGCAGTGAAAATGC
TAGCGGCGGCAACGACAAAACGGAAAGCGAAAATGATGCCACAACCGTTTCCGAATCCTCATACAAATCC
GAAGTCTTGCCGGGCAAAGGCAGCGATCTGGATGTGTCGGTCGCCCAGCAGGACGCATTATCAGCAACTC
CTGAATTGGCAAATACATCAGATCCTAAAATGAATAAAAATGACGCACAAGTCTCAGCCTCTGTTTCCTG
CGATCCGGCCGACTCTAGTTCACCCGAGGCGGCAGGAAAAGCTCAGGGGACGGGGGTCCAGACTGGCGAT
GCCGGAGAATTGGACATCGAATCGGAGAAAAAAGATAACGAAAAGGAAACCAAGGAGTCAACTGGTGATG
CTCCTGCTGTGGAAGCCAAAAAAGATAGCGCTGCCATTGCCCGAGCTGAAGGCGATAAAACGGAATCATG
CACTAACAATGCTACCACAAACATCTccaccaccactactactactaccattaccaataccaccaccacc
accaataATCCATCATCCAAAGCGGCTAACTCTAACAACACCACCGACCATGCTAACTCAAACTCACATT
TAGGCTTGGACGAGGAGGAGAGTGTCGCCGGCAGCTATCCGCCAACCGTGGCAGCCGTCGAGGACGCCAC
TACTATGTGGCCATCTATGCAGGACCTTAACACGCGTCTCCGGCGGGTGATCACTGCCTATCAAAGGAAT
TACAAGAAAGAGGAATTAAAACTGCAGCAGAAGGCCAAGGGTAGGTGCAATATCCTTTATCCACATACCA
ACCCTTTTCATATTATCTTCTCCATTCGTTGCCACTTTAATGAGAGTACATATATATTTTAtattccctt
tactctcagagtataaaggtatactaaattcgttgaaaagtatctaacagtcaaaattgaacgtttctgT
CTGTCCGTATTAACATCGAGATCTCGATGCTGCAAGTTTGCTTGAGTACGTTGCTTGAGAACTGTCACGC
TCACGATGTTTTCATTTTCTTTTATTTAGTTTTGCCAATTTCTAGTGATACTTTAAAATTAGCGTTTTCT
TCAAATTAAATTGTTATCTTTGAGATTTATATTCACCCACCCTTATTAACGACTATGAAAAATCTAAAAT
TCAAATAGTTTCCGAAACTGTCTGTTGCCTAACCCAAATGACTATTGAATCATTTTACATTTTTTGTTCT
ACGTTTCATGATTATGTTGTAGTGTCCAATGTTAGAAATATGTAGTTTTGTTGCTTAGCTGTTGGCTGTA
GTTAGTTTCGAACCGTACACCGTAGTTAGCGTACAGAACGGACAGTGGACATTGACTTGGACAAAGAAGC
GGTTCCAAAAATGCAACCGAATTCGATTTACCCAAAGCATCAAAAGCTGTAAATAAGAAAAAATCATTAA
CTAATGCAGCCAACAGCTGATAAACTAAACTATGTAAAACGCTGACAGAGGATTGCACTTAATTTCTTTA

CCATCAAGACCATCTTAATAAAATCATCCTCAGCCCACCCTGAATCTGTGACAGTTCCTGACCTTTTTGC
CGAACTTGCAAAGTTACCAACTCCACCAACTCTTTCTCTGTGATTGTCACATTTGTCCCACAGCTCCAGG
CATTGGTTTCATCGACACCGCCGCTGTCGGTTCCCACCACTCCCACCCTTCAGTCTATGCAAATGCAGCT
GCAGAGCGGCTCCGGCAGTGGTGGGTCCGGAATAGGATCCTCTGCTCAGCACGATGCCAGTGGCCGGAGT
CTTCAGGCGTTGATGCAATCGCAGGGGGCCAGCACATCCGCAGCAGCAGCCGCCGCCGCATCAAGTGCTG
GAGGATCCTGTAGTGGATCCGGCCAGGTGGGCATGGGCGGAGCGGGAGTCATGCCAGCTATGCCAACCGC
CGCCGACATAAGCCTAATGCTCAGCATACTCAACACCACAGATGTCAACCAGCTGGCCAACTTGGACATC
AATAAATTGGCCATGTACCTGGTCAGCATGAACAATGTACGTTAGTGATGAGTACCTCCTCTCTATATAA
AACATTTTTATATTATAACCACTTACACTGCCACTTCGAACAATCATCCCGTTGCCCTTATAAGCAACCC
CCACTCAAATAAATCCCCAACCTCCACTCGTTGGCTAAAGCAAAAACAGAAAAGCCACGCTTTGAGGCTC
TAAAAGACTATGAACACTTTAATAAACTCCTTCGAACCGATACCTAAATaaaaaaaaaaaaaaaaCACTG
ACAAAAAAGCACGCAACTACACAAAACACTATTAACCTTACTAACCTGCATGTATTCGTAAACTATCATA
ATATAAACAAAAACCTAACATTTTGTCTAACCCATATGTTTAAAGGGTGACGTTTAAAGTCGTATACTAT
TATAAGTAAAGCAATTGGTTTTTCGATTTGACTATTCAGAAAACTATTCGCGATATGGGTTCCTAGCAGT
AGTTCTGATACCCATTTTAACCTTTGTCCACATACTCACTTTGCGATGGTCTTATGTAGAAGGCACACGA
AAATAATGCAGGGAATCAGCCTAAATAAAGGACGAAGTCAATACCCATTCCCACAGTGGGGTTTTGAAGT
TTTAGAGGAGAACATTTTGTTAACTCGCCTTGCGACTTTGTATCCTCCCTTTTGTGTGTGTGTGCGTTCG
TACTGAACGAAGAATCAAAAGCGAGCGACGACAAAACGAACAGGAGGCAGCGACAGAGCTGAGAACCGAC
AGCATACACTTATGCCTAATTTATGCAAAATAAAAGAAATACATACACTCCAATTTGAACCCGAATGCGG
TGCACACGATGTTCAGATGGTCCTCTCGTTGGATTCCCCGTCCTCTTTTCTCATCGCATTTTATCTCATC
TCGCGAATGGGAATGGGCATGCGAATGAACGGCCAAAACGGAGGAAATGTCAAACCACTTTGCACACCCG
GCGGCAGAAACAAAAACAGCAACACACCGAACTCCTTCTCAAATCCACTGTCTAGTGCAAAACCGTTGCT
TTCGATTCCTTGCCTCCGCTTCGCCTTTTGCTGCCCCTTGATGTGGCAGCAAAAGCAGATACACCGCAAA
ACAGTGGAACCAGCAACGTGTTGGTCACAAGGCATGGGGCGACATATGGGAAAAGGCAAAACAGCGAAGT
CAATTGTACAAGCTGCCCTTTTCGATGCGCAGCAATCCCACTGTGTTGGCGGGCTAGAGATGCGCCAATT
GCAAGCCAAATGAGAACGATTTTTTAATATTTCATATTCACATATTTATAAATATCCGAGTAGTCCGGAA
TCGATTTGAATAAGGGTGCCGATACTAAAGCGATACTGCGCTCCAATACATCTGATTATATATGCCATGG
CTATAGAAATGTAGAAATTTTAGAAGGCCTAATTCGGGAAATTCCCCCTACAACTGGTCATCGCTGAATG
GGCGACGTTGCCATTATCAAAAGGCAGAGAGGCAGCGGTAAAGGCAGCGCACCATCAATCAAACATACAA
CAACAACAATAACAGAAGTAAAAGGAGCGAAACAATCCGGTAATGTCGGGGGGCCGGAAGAAACGGCGGC
AGCAAGAAGAGCAAAGCCAAGTGGTTGTTGCTGCCGTTGCCGATGTTGTTGTATCGACGAACTGGAGTTC
TTTGGTGACGATGGTTGCTGCGGCTGCCGCGACGTGAGCAGTAGCGTTGGTGGACGACTTGGTTTTATTG
CTGTAGTTGTTGTTGGCCATTCGATATCATCGGGTGGTGGTGCTGCCTAGCTGCGTTGGCTGTACTTACG
CTGCCGCCGCCTCGCTGTATGTATTAGTTTTCCATGCGAGAGTGCCTCACTCTCTATCTCTGTCCCGCTC
CCTAGATGTCTGCAATGTGTGTTTGTAATTTTACATTTTCTGGATTTATCCAATGTATAATTTTTTCCTT
TCGTATGCGCCCACCCAAATGTCTGGGGGGTGGTTGGTGCTGCTTCTGCTTCTGACACCACTGTTTCGGC
TACTGCTCTCACGAACTGAATTATTGAGCGCAAAAAGGAGACGGGGATAATGGTGACCTGTTCCTCTTAT
GGTTTCCTGTTCTCTTCTTTCGCATACCGCGAGAATTCGATAAAGACGCCTAATTGTGCTCATTTGGACT
GGGAATGGGAATTCCTTTGCTCCCCCAGACCTCCGGGGCGGCCGTCCCATTGCACGCTGCGCTGCTGCTT
TTGCTTAAACCTACTTTTTGCTTTATTCTGTTGATTCGTTTTTGAAGTTGCAACGTAACGTGCATTTGAA
ACACCTAACATAGAGAAGTTGATGGCTATCCTCCTGTGCGTGTGATTCGACTGGTGCGGGTTGCCATTCT
TTTTGTTTCCATCTTCGGTCCCACCTTCGACTTGCTTGGAAGCTTTTGCTTTTGTTGCTGACCGCTGTCG
TTGCTCTTTTTTAGTGACGTTGTTGTTGGTGCTGCCATTCGATTTATTATTTTATTAGCATTTCGCTGCC
ACTTAAGTGTGCACAGTGGAGCGAATCGAAACTTTTCTGATGTGTAAATACATAAAATTTTGAAAGGGAA

AAGTTGtttttgccatgctgactttttgagtaatattttttttgttgaagaattttttATATTCTTTTATGC
TCTCGGACACTCCACTGTGCGTGTGCTCGTAACAGTCGCTGCTAGATCCCATTGCCGCTCTCTCACTCTC
TCTGTAGTGACTGCATTCAAAGGATGCAGCCGCCACGCCTGCGCATTTTTCACTTTACACCTGTCACTCA
TAAACCATTTGAAAAAGTATATGTCTAACCGATATGCTGAACGGCTTGTCAAGTTTAAATCCGACTTGTC
TACCTGCCATCACCCTTCTTACTTTCTTTCCCTTTTTCGATATAGTTTAGTCCAATTTAGGATAGAGAAG
AGTGGCCTCCTGCCCTCTTCGGTGATCTTGCGCAAGATTTCTGTTGGTGTGAGTGAGTGCATTGTCGAAG
ATATATATATGTCTCTGTCGCATATCGATTGTGCGATTGTAGCCGCCCTGCTCACTCATGCATGCATTTC
GGTGTGTGCAATATCCACTCACGGACTCGCTCACTCATGTGGTATCTGCATCTGTATCTTGCGGATTGAT
TAATAATCTCGTTTCGTTTTTGCTCTGGCTACGTGCTTATCGCACTTTGGCACTGCTTCCATCTTTCATG
CTTTCTGACTGCCTCGTGCTCTCTCTCCCTGTCTCCTGTCTGGATAGTGTGTTGCTGTCGATGTAAAGCC
CAAAGGTCGAAATAATAAAACAATGCGAATTTCTAATCATACGATAAAAATTGTTAATTGATATTGATAT
CTTAAAATATGGATTAGGAGACGAGGGTGAACTCCATACCGATAAGGTATTAGGCGTCATTTCTAAAAGA
TTTTCGAATTAAACCATTTCATGGTTTTACTTCTCTTTGCTTTGCGCTGTTGTTGCGCCTGCTGTTATTA
TTTTATTTTATCCCTTTTTTCCGTTGTCCTGCGTCCGTCCGTCATTACCCCGTTCATCTTCCCCCATTTG
GCTGCCTCGCTCTTTCGCTCTTTCGTGCCGTTCTCTATGTGTCGTCAACAGTTTCTGTGGTACATTTGTT
TCATTGCCTTAGCTTTTTTTGCCTGCGATTCCGCCGTGTGTTGTTCTCTCCCCGCTCCAGGGTTTGTGTT
TAGGTGCGAGTAAGTGTGCGGCAGGCTCTTGTCTCGCTTCTTTGCTTACATTACACAGTTGACACAATGT
GCCCGCTGCGGCCGCTCTTCTTCAACTCGTCCATCCACTGCACACTCACAGTCACAATCGCATCTAGGTT
GCTTCTGCCCGCGCCCTCCCTTGAATTGTACTCTCGCGAAGCCGAAGTCGATGCTCTCTCTGTCGCTCTC
CCTTATTGCAAGTGACGCTCATTCACCTCCGAGTTGGATGTCGGTCTCAGTTTTAGCCCACCTCATTCAT
ATTCACATTCACCCAAAACGGCGTTTGACTTAATCTCACGATTGTTTGGTTTCCTACTCTCCCGCGCTCC
CTCTCTTCTGAGTCTCTCTTTCTGGCTGATTCGCATTCGATTTTAGCCGCTGCCATCGCCGTTGTTTGCC
TACCTATGTGTGTGTGTGAGGAGTGTGTCTTGTATTTTAGTCCGCAATGCGCTCCGCTCATTATTTGTTT
GAGCGCCGCGGTGTAAAGTTGTAAAAAGTCCAAGTGCTCGTGGAAACTCGATGCAAGACGGGGAAAAACGA
AACGCGATAAATCGTGAGAAAGAGAGTGCGCTAAAGGAAGAGGGAGTGATAATCAAACGAAATGGAATAA
TGTATTTGCAGAGGCAACAACAACAATGCAAATAGTTGTCATTGAGGCGCAATGAATGATAATTAGTGCT
TAATTGAAATCAATAATCATGACGAAAGCGTAAAGCTTCGATTTTGCCAATATATTCTTGATTACCAGTG
AGTCTGTGATATTGCCGTGTGTTCGAAAATGGAGGTTATTAAACCCATGGACTTCAGCAGCTTCTCGGCG
TTCTGCGAGCATCTTAACAAATCTTCACAAATTGCAGCAACAACTGCAACGACGATACCGCCAACAATAA
ACAATGGAAAAGGCATTTATTTGGAGGTAAAAGCAAAAAATACAAATATTTCGATGCGAAATTGCAAGCA
TGGACATGATGCGTCTCCTAATTAAAATGAGAATGCAAAAATGCAGAGAGAATATGCAAAGAATGTGTGC
ATGCAGTTAGTCAAGATAAAGTGAACGAGACCCAAAAAGAGTGGGACAGACGACGAGGAGGGGGATCATT
TGGCCCTCTCGCTCGTAATTCGGTCTGTAGCGAGTGGAAATACAAAACAATAAATTTAAGCCTATAAATT
TCACATGAACAAAAAGCGTTCACGTTGCCCCTTTTAGGGTTTAATAGGGTTTAGGTTGGGAAGTTGTGTG
CGCTTGTGAGGGGTAGAGAGAGAGAGAGAATTCCATGTTGCAAGTAGGTGTTATTGGTAAGGCAGGTCCACA
CTAAACCGTTTCaaaataaaattactgataatataaacataaagaaaattaaaaataatttttaattcaC
GACTGAATTGTTGTACGATAGAATCTTAATGTGATCTTTTAAATGAATTTGTAGGCCAATGTGGTTTCAA
GTATTATTATTATTTAATTGATCTACTCTTAATCAATTTCTTAAAAACCACTTTGTGCAACGGTTATATC
TATTTATTGCCTTTTAAGTGCACTCACTCAAATTCTTTTTTGCGCGGCTGTTTATTGCTCCGCGTTCTCT
ATTTTATTTACCCTATTGCTATTTACCTACCGCCGCCTCCAAGCCTGTAGCCATGTTCATAGCCAGCGGT
TCTACTAATATTCATAATCAATCGCTCAAGTCGCCTCGCACTTGTGTAGTGTAAATGCGATGGTGGAACG
GTGCGGAGAAAACGAGTGATAGAGAGGCGGAGTGAGTGCGAGCGAGAGCCAAATGTATAGTACGCGAGAA
GGGAAGAAAGAAGAGGCCTCGAGTCGCTGTCGTGTTATCTTCTTTTGTCGTTTGTTTTTGTTTGTCCCCT
TTTCGGATTTCGTTATACCATCGTTCGTAGCCTGCAAGCAATGGTTGGTTAGGAGGGGAGGGAGGCCTAG

CAGTACAAACAGAGGCATGGAATCACAAGGATACACCGTATACAGAGTTGCCAACCTGGGCGCCACTTGG
CATCATTTTAATTTTTGGATTCGCTCCAGGAAGGATTCCATAACGTTTTCAGTTAGGTTACGCATTTATA
CATACATGTATGCCAAttttttttATTATATGTACATATTTAGAAAAGAAGACAAATTCGTTGTGCCCAT
TTACCTCGATAAAGATAGAAACTAATGTAAATTCAATCAAAGGATAGAATAACTATTTACTCCTTATATT
TTTCAAATTATAACATTAAGACTTTGGAATGAACCGCATATTTGGCAGCCGTAAATCCAATGGCTGTTAC
TATGTTGACCCCTAGGACGGCTTACAATTTTGTGTATTTGCGTGACGTTTGCTTCTGTTTCTAGCCCTTG
CAGCAGTGTGTGCCACCTATTATTTTTAGTTTAAATCGACGCACCAAACAGCGCATAAAGTAAGTGTGGG
TGAGTGGGAGTGTATGAACTTCCTTGGCTGTGGCGCGTGCAATTGGTCTCACAGGATTCATTTGTTTGTC
TCTATTTATTTTTGAAATCACCTTCGCATGCCCCTTACTTTTGAGCGCCATTGCTTGGTGCGGCTTGCTG
GTTGTTCCGTGTTCCGATTCCACGTCGTTCTCTGTTGCTGTCGCCTTTTGGCCTCTGTCCTCCGGCCGAA
GCGTTATTAGGGGTGTAAAATGGatttttttacttttattattatgattttattgtttttCCAGTTTCGGT
CTCCTCTCTCTTTTTTTGAGCCTTTGTCTCGCTCTTATTTTCTATATGCGCGTGTCATTCGCATTATGTC
TTTCCCTTTTTGCTTTTCGCAAAAATTTGTGTGTAATTCGAAAGCCAGGAGCCTGCTATGTGCTGATGAT
TATAGTGATGAGGGAAGGTGTCGTAACGTGGCCACCCCACATCACCACCAACACAGTCGCGCTGTGTGAT
CCCAACCCCTGGGGACGCAGTGATGGGTGGTGCAATGCAAAGGGTCGTTTAAATATTTTTTCAAGCTAAT
TTGGAAAATATTCGTAATGAATTTGCATTGCAAAATGTGCACATCACATATGGGGTGTTCTATTTCGCTG
AGCTGATATGTAGAAATATGCTTCTCTGTTGTAAAAATTTTTTGAAATATACTATTATTGAAAAGAATTA
TTTaaaaaaaaTATAATGCTTACTTTATTTTTAATTAAATAGTAGTTAAGTTTTGATAACATTCGAATGG
CCTGATTTAAACATTTTTTATACTTGCATAGATGGTTATTAATTCGATATATTTATTTATTACCAATATA
TTTTGTGGACATTGACGAACACCTCAAATACTTGGGGTCAGACAGACTATGTTTCGATACGTGCAGTACC
TTTGTACTTTGGGGTTCTATGCTTTTTATAAAAAAATTTACTTTTGGTTATTGCAAAATCAAACTAAAAAT
CTAACTTAAAAATATGTTAATTCTATGACTCTAAGACTTGCACCATGTTTTTGCAAGATAAAATTTATTA
TTGTTTTAACGAAAATATTTATAATAAATATTTTATATTTTCGGTAATTTTTACAGAAAAATGGAGCGAC
AGGGAAAGATGGAACTGGCGGCGAAGGAGCGCGAGTCCCAACGGCTGCAGCTCATTCCCAAAAAATGGAA
CCGCCGGGAAGAATACGAATTTCTTAGAGTTCTTACCGGGTATGGTGTGGACCTACACGTCTCAACACCT
ATGGCTTCCTCTAATGGAAGTTCCCTTTCGCCCGACTGGACCAAGTTCAAACAGATGGCTCACCTAGAGA
GAAAAAGCGATGAAACACTGACGGACTATTATAAGG
>dme_circ_0001053_junction_seq
GATGAAACACTGACGGACTATTATAAGGGAATACCTATGAGCGTGAAATGTTCGAC
>dme_circ_0001054
ATTCGCGAGCAGCAGGAGAGCCTGGCCAAATTGCATTTCGAACTGGCTGCCGCCGAGGAGGATTCGCTGG
AGCACGGCAACGAGTTCAACACCAACAAGGCGCAGGAGAACATGCTGCAGCTGATGCAGCGCCTGGAACA
GCTATCCATCTCCATCGAGCAGCTGCAGACGAGCCACACGGGTCTCTGAGATTTGGCCCAGCCGCAGGCC
TGGGATCAACGCCTGCTCTGGCTCTGAGCACACCTATGGGGAAACGAGGAATGCCAAAGTGGAGCTGGAT
TGGGATTTCACACAAGACTAGCGACTTTTCGACGGTGCGGACAGTTGGGATACTGTG
>dme_circ_0001054_junction_seq
CGACGGTGCGGACAGTTGGGATACTGTGATTCGCGAGCAGCAGGAGAGCCTGGCCA
>dme_circ_0001055
GCTGCAGCATGTTCTCCTGCGCCTTGTTGGTGTTGAACTCGTTGCCGTGCTCCAGCGAATCCTCCTCGGC
GGCAGCCAGTTCGAAATGCAATTTGGCCAGGCTCTCCTGCTGCTCGCGAATCTTGGTCATCTGGTCCATT
>dme_circ_0001055_junction_seq
CTCGCGAATCTTGGTCATCTGGTCCATTGCTGCAGCATGTTCTCCTGCGCCTTGTT
>dme_circ_0001056
GGCTCATTCCTTGCCCAACACATTGCACACAGGCTCAAATTTTTCGCGCAGCACTAGTGTCCCCAGCGAA

CACCCAGAGCCTAAGCTGACTGGTGCTAAGCCAAAGCGTTTTTTGCACACCTCGTCTGTGATGACTAAAA
GCTCCTCATATAAGTCGCGGCGCTCTCATACATCAGCTAGCTTTGATGAACGTTGCTACAGCTGCACTGA
TTTCCGGGCAACAACATGTCAGCAGCAACAGTCAGGGTCGAAGTCAAAGCAAGATCAAGTGCCTCCTATA
CTGGTGGCTCCTGCGACTGTTGGGGCTTTGACCACGTCTTCTTCGTGCACGGGCCAATTTGACTTGGGAA
TAACCGAGGAGAACTGCTTAGACTGCCTAGAAAAGGAGTTCTTGGCCAATACCATAGGCAAAGTCGTAGA
CCCGGGAACATGTCCTAAGTGTCGTAGATCAGCTGGCAGTCGGGGCAGTAGTATTTCGCTTTACTCACGG
TCGGACCAGGTTCGCTGTCGAAGCGGGTCCCCAGTATACTGCTATGGAGGTCCGTTCGCCGGCTGGCACA
GCTTAATAGCAACCGGAATTGTTTCTGGTTCCAATTTGGGTCCACCCTGCAACCAAAG
>dme_circ_0001056_junction_seq
CCAATTTGGGTCCACCCTGCAACCAAAGGGCTCATTCCTTGCCCAACACATTGCAC
>dme_circ_0001057
ATTGAATTTTAAATTTAAAACAGCAGAAATTCTTGATTTGTTTTTTAGTAGCCGGCTTACTAACtatacg
cgtgactcgtatagagagagtatactagattcgttgtaaagtaACAGTGACATTCATACGTGCTAGAAAC
TAACTTGTGAGTTTTAAAGCAACACAAAAATTTGGACCAGCAAGAAATATGTCGTTCTATACGCAGGGAA
CTCTTAG
>dme_circ_0001057_junction_seq
TGTCGTTCTATACGCAGGGAACTCTTAGATTGAATTTTAAATTTAAAACAGCAGAA
>dme_circ_0001058
TAACAGCAGCTCCTCCTCCTCTTCGCAAAGTTTCTCGAAATTCTTTGGTATGGATTTGGACTTTTCCCGC
TTTAGGGTTCTTGGCTCGCAGTAGCCGGAGTCCTCGGACAGGTTCTCAGCTGAGTTGGGGGGAATTCGAC
GAGAATTCGTCCCGTCCTGTTTCCCCCTTTCGGTTGCTACTGCATCTCCGGGAACAAGGCAAGAGCTTAG
AAGGCTGTGACCACTGCTATCGCTGGACGAGCTGGAGCTAGAGGAGCTCTCTAGGTCAGATTTTAGCTCC
GCCTCTCGCAGGCTTCTGGAGAGAAGTTCCTGCTGCTGCTTATATTCAAAATTGAGATTGTTCAGCTCAT
AGTATGTCTGGTGCAGATGACTACAGTCGCTGGATTCATAACGCTTTAGCAGGGACAGCGTCGGGGCTGA
TCCCTTCCTACTCTCTGGACTGCTGTCCCTGGTCCTGGCCCGCGTTGAAGCACCAGACTCGCAGGTGAGC
GAGGGCGAACTGTTGCCCAGACTGTTCATACTCTGATGCTGCTCCTGGAGCGTGAGCTGGCGTTCCAAGG
ACTCGAACTGTACCAAGGAGGAGCTGCGCGATAGCGAGTGGGAGGAGTCCCTGTCCGAATAAAAGTCCTC
GAAGGTGCCCCCTCGCCTACTGCCTATACTGCTGCGTTGGCCCTGGCTTCCGAAGCTATCGCGAGCACAT
GCCTCCAAAGCCAGAAAGCGTTCAAAGCTATTGTGGTACTCGGGATCGTAAGGCATAGGGCGGCGAAGTT
GCACGCAATCCTCTGAAATCTGGCCAGAATTTAAGTCATGGCTGCGCCTTGGTCGCTTGGGTGGGCAGAC
AAAGTCCCTCTCAGAAACAGCATCGATATCTGCTTTTAAAGTGCTAGTATAGTCGATTGTTCAGAATGAC
AGAACCAACGCTCTATTAGCTTACCCTTAATCGTTTCTATGCGTAAGACTGATCCAGTTTCAGACTCCGT
AGAGCTCCAGAGGGTGGAGCGGCGATCTAGGCTTACAATCAAGCTACTTCGGGCACGTTCCTCATCCTCG
CTAG
>dme_circ_0001058_junction_seq
CTTCGGGCACGTTCCTCATCCTCGCTAGTAACAGCAGCTCCTCCTCCTCTTCGCAA
>dme_circ_0001059
GCCCCCTGAAAACGAACTGCAGAAATCACATGACCACAATCCAGGCGCACTAAGCGAGCATCAACTGCGT
GTCCAAGCTTCGCTTCAGCGGCTAAATATTCCCGACTGGTTCCGTCATTACAACAAAGGCCCCGAAAAGG
CAGCAGATGGCGAAAACGCATGCACAAGTGCGACTGGCGGTGGTTACCGACCTGGCAACTTCACCCGCAA
ACGCGCACAGGAATCGGGTCGCTGGCAAGGGCTCAACTCAAAGACGACTTCCCTCAGCTCGCTGGGATCT
CAACGCTCCGACCGAAGTCCTTTGTTGATGAGTCCCTCAGCGCACAGTCATCACGGCGGCCAAAGCTCCT
ATTCATGTGGGTCGACGACAATTCACGGTCCGGCACCTGTACAGATAGGCATGGGTGCAACTCGCTGGTC
AACCTCTCATCTGAATTCCACTCAGATGTCTCCCGGTGCCTCGCAGCGCGGAAGCTTTACGCGCGGCGCC

CCCATTAATAGCAGCTTTATATCTGTGGCCAGTGGTAGTGGCTTGTTGCGCAATTCCTACCGACAGCCGT
ATTTGGGTTGGCGCAGCACAGAGAAGCTCTCCCAACGAACTCCCCACGAACGGTAGGTTCCAATAACTCA
TTACCATAAGGTTTGGAGGTCAAAACTCTTGCTGCTTGCTAATATTGAGTGGATCGAGCTCGATTCTTAA
AAACAAGCACGATGAAATTGAGACCTAAAAAACGAAAGGAGAACAATATTTTAGTTTAATTTTAGAGCAG
CCATCCAAAGTTCTAGAGAACACTAAAGCTAAGTGCCTCGATTAGCAGGTACCTATCAATCAGCTAAGGG
GATTGTGAAGTATATTAAGATATGCAGCAAAGCGACTACGTGTTGCACATAAGCTGTTACAATAATTGCT
ATGTTTCTCTAGCTTTTCTTTAGCGTATAAATGGCTATTGCTAACCAAACCACAATCTTGTTAACACAAT
TATGTCAGTGTTGTGTGCGTATGTGAGCTATAACGGCCAAAGTGGAGAGTAAAAGTTCTTGGGGCTTGTC
GGGGTTTTATTGTAGTAAGTGAGTCACGGCTTAATAATACTAGAGTTACGCAAAAATCTGTTTAACTGAT
TCCTAGCTGTGCTCTAGCTCCGATCTAGTTCTTAACATGAGGGAAAGCTATTGTCGACTTTTGCCTGTTA
CATACAATACTTTTCAACTAATCCATTAGATAATTTTCAATTTACTACTCGTACTACTCCTAGGTTGAGT
TCAAATTTGGTAAGCTAGGTAATAGTTGAGTATAAGAACTATTTTGTTCTTAAATCGTTGAGTTTAAAAA
AACTATGTAGCAGGTAGAAGGAGGAGTTTCTGATAAagttgagtagacttgtccgtctgtctctcttttt
gaacgctgttatccattaaaagccagaaagttgagattaggcattcggattcgagTAACGATTTTGGTGT
TCATATGGTCGTATCTCTCCATATAAACGCTGTGATCTCAGGAGATAGAAAAAAGTAGATTCTAGTAATG
CTTTCTTATTGTAATATTTGTCTTGTcaatttctatcgttatgccacacaatttaaaaaatatcttcgca
gaactttcactagctgagtaacgggtatttgatggtcgagcaaatcagctatagcgttctctATATTAAT
AATTCTTTTGAGCGgtattaagtgttatcgtattaactattattgtataaagtatATTATTGTTTCGTCC
TAAACCCAATAACGTTCATAACTAAAAATCATGTAAAATATTCACGTTCGACTTTTTATATTTTGATCTT
GCAGGTCTGGCCAATTCGCTGCTAACCCAACGGACAGCTCCGCCCTCCACACGGGCGGGCAATGAAACCC
GCACCTTACATTCTGTATCGACAGAGATTCAAAGCTCCATTAAGGAAGTCACATCCGCCATCGTTCACTT
CGTGAACGACCAGCAGCAGTCCCAACATCCGCGGAGCCGTTCTACCAGTCCAAATTCAAG
>dme_circ_0001059_junction_seq
GGAGCCGTTCTACCAGTCCAAATTCAAGGCCCCCTGAAAACGAACTGCAGAAATCA
>dme_circ_0001060
GTTTATGCTTTTTGTTTGGTGCACGGCATTTTTGTAGCCTATCTGCAAACAGTAAATTAATTGTGTGAAC
ACGTAGGCATCCACACCTTTATTCCGTATAAAAATCATCAGCCTATATGAACGCaaaaaaaaTATTTTTA
TTTATCTTCTATAGTGGATCTTAATTGTTTTACATTTAATTATTAATTTATTAAATTCAAAGGAGCAGAA
ACTTTTGCTCTTCTAATTATTTATCGAATACTCATGATTTGCCTTTGCCTCGGGTATAGAAAATTGTAGA
TTTGGGCAGAAAACCatatccgttacttctgcagtaaaatgtcaaatagatccgcggaaaaaaat
>dme_circ_0001060_junction_seq
aatgtcaaatagatccgcggaaaaaaatGTTTATGCTTTTTGTTTGGTGCACGGCA
>dme_circ_0001061
CATGCAATGAACTACATAACGGATACCGAGCCAAGTTTTTGACCATAGTGTATTGGATAGCAGCGACCTA
TGTTTTGGCCGATGTATATTCAGCTCAACTGACCAGCCAATTTGCACGTCCAGCTCGCGAGCCACCAATC
AATACTCTTCAGCGCCTGCAAGCAGCGATGATTCATGACGGTTACCGGCTATATGTGGAGAAGGAAAGCA
GTTCATTGGAGATGTTGGAGAATGGGACAGAACTGTTTCGTCAGCTTTATGCTCTGATGAGGCAGCAGGT
GATCAATGACCCTCAAGGATTTTTTATTGACTCTGTGGAAGCGGGAATTAAACTAATTGCAGAGGGCGGC
GAGGACAAGGCAGTACTCGGAGGGCGTGAAACACTGTTTTCAACGTTCAGCAATACGGATCAAACAACT
TTCAGCTCAGTCAAAAACTTTACACTCGTTATTCGGCTGTGGCTGTTCAAATCGGATGTCCCTTTCTAGG
TAGCCTCAATAATGTG
>dme_circ_0001061_junction_seq
TCCCTTTCTAGGTAGCCTCAATAATGTGCATGCAATGAACTACATAACGGATACCG
>dme_circ_0001062

GTTTTGATGGATGTCGATCAACTTCAGAGCTTCAAAACAACCTATGGGAGAGCATGACGGAGATCACGAT
ATGGTTGGCGTATGTGGCACTGAAAGGCCACAAGCCTGATGCAAAATGTAGAAAAGGCCAAACCACAGCA
GCAATTGTCAAGACTTGGCTCGTCTGGAAATGCCCTCACGTATATATTACTCCAGCACTGTCCGCAGACA
TAAATGTAATCAAATATTCGCCGTAGCCTTGAGGACGGATTTGATTTTATCTTTCTTAGTCTAGCAGCAA
TTTCGCAACTGCGAGAAACAATCTGTGAGCTACTGTGGTTTGAGTGAATAAAT
>dme_circ_0001062_junction_seq
TGAGCTACTGTGGTTTGAGTGAATAAATGTTTTGATGGATGTCGATCAACTTCAGA
>dme_circ_0001063
GAAAATGAGCGATTGCTGAAGGAGGCGAAGGAAAAGGGACAATGGGTCAGTCTGAAGCGCCAGCCGGAAC
AGCCGAAGAAGGCGCACTTTGTTAAGAAACTTGAAGAACCGATTGCTCTGGCCCCGATTCCATACGAATT
CATTGCCTAAAGGCATTTTACTGCTCGGAAATTG
>dme_circ_0001063_junction_seq
CTAAAGGCATTTTACTGCTCGGAAATTGGAAAATGAGCGATTGCTGAAGGAGGCGA
>dme_circ_0001064
GAGCAATCGGTTCTTCAAGTTTCTTAACAAAGTGCGCCTTCTTCGGCTGTTCCGGCTGGCGCTTCAGACT
GACCCATTGTCCCTTTTCCTTCGCCTCCTTCAGCAATCGCTCATTTTCCTTTACGCGGCGCAAAAAATCT
TCGCGGCACTTGGAGTGGTGGATGTGCTCAATGCGCACATTGACCCGCTTAGCAAGGATTTTGCCTCTTA
CGCGTTTGTTTACTATTACACCAACGGCGTGCTGAGTCACATTGAATATGCGCCCGGTTTTGCCATGATA
TGCCTTATAGGGCAAACCCTTCT
>dme_circ_0001064_junction_seq
TGATATGCCTTATAGGGCAAACCCTTCTGAGCAATCGGTTCTTCAAGTTTCTTAAC
>dme_circ_0001065
AGGCAAAATCCTTGCTAAGCGGGTCAATGTGCGCATTGAGCACATCCACCACTCCAAGTGCCGCGAAGAT
TTTTTGCGCCGCGTAAAGGAAAATGAGCGATTGCTGAAGGAGGCGAAGGAAAAGGGACAATGGGTCAGTC
TGAAGCGCCAGCCGGAACAGCCGAAGAAGGCGCACTTTG
>dme_circ_0001065_junction_seq
CCGGAACAGCCGAAGAAGGCGCACTTTGAGGCAAAATCCTTGCTAAGCGGGTCAAT
>dme_circ_0001066
ATACCCTGCCGTCACATGGTACGGCTTTTTGAAATAGGGGAATTTGGCGGATATGGCATCTATGATAATA
TCGAAAGCCATTGGCACGGCAGTTAAGATGCGATCCAGTACTTTATGGATGGGTTTCAACTCATTTCGCA
CGTGCTCCGAAGGGCATCCGTGGACCCAATCGGATTCATCCATATCTCCAGGAATCCAGTGGACAATTAG
TTTAGAGACTCCAATCGGTAAATATCTATTATGGG
>dme_circ_0001066_junction_seq
ACTCCAATCGGTAAATATCTATTATGGGATACCCTGCCGTCACATGGTACGGCTTT
>dme_circ_0001067
GATATGGATGAATCCGATTGGGTCCACGGATGCCCTTCGGAGCACGTGCGAAATGAGTTGAAACCCATCC
ATAAAGTACTGGATCGCATCTTAACTGCCGTGCCAATGGCTTTCGATATTATCATAGATGCCATATCCGC
CAAATTCCCCTATTTCAAAAAGCCGTACCATGTGACGGCAGGGTATCT
>dme_circ_0001067_junction_seq
AGCCGTACCATGTGACGGCAGGGTATCTGATATGGATGAATCCGATTGGGTCCACG
>dme_circ_0001068
GTTGAAACCCATCCATAAAGTACTGGATCGCATCTTAACTGCCGTGCCAATGGCTTTCGATATTATCATA
GATGCCATATCCGCCAAATTCCCCTATTTCAAAAAGCCGTACCATGTGACGGCAGGGTATCTGTATAACG
TGCTTTGGTTATTAGAATACAAGCCTATTTTCGAAGAACTTGTTTTTCAACTA

>dme_circ_0001068_junction_seq
TATTTTCGAAGAACTTGTTTTTCAACTAGTTGAAACCCATCCATAAAGTACTGGAT
>dme_circ_0001069
GATTTGCTCTTCCTCCAATCCCTCCAACTATCGCGCCTTGCTATGTGTCATTTAAATCCCCTGCGGTATT
GTTTAGCCCCAGTGGCCACAGCATTTGCTGGTGTTACAAGAACTTACCAGCTGGCCTATTGTCATACTGT
TTTGGAGCGCAACGCTCGCCGTAAACTGGCTACAGTTTACGGTCATGAAAAATGCATGCCCGATGAGACG
CTGGAATCGTTTTTTCCCTTCGATCCTTATGTACTTAAGTT
>dme_circ_0001069_junction_seq
TTCCCTTCGATCCTTATGTACTTAAGTTGATTTGCTCTTCCTCCAATCCCTCCAAC
>dme_circ_0001070
GATTTGCTCTTCCTCCAATCCCTCCAACTATCGCGCCTTGCTATGTGTCATTTAAATCCCCTGCGGTATT
GTTTAGCCCCAGTGGCCACAGCATTTGCTGGTGTTACAAGAACTTACCAGCTGGCCTATTGTCATACTGT
TTTGGAGCGCAACGCTCGCCGTAAACTGGCTACAGTTTACGGTCATGAAAAATGCATGCCCGATGAGACG
CTGGAATCGTTTTTTCCCTTCGATCCTTATGTACTTAAGTTGATCAAAAAAATATATTGAGACTAATTAT
ATGGTTTACCAGAGCAATGACACGGATGAGTATGTCTATGGGTCTAACAAATACGGGCATTCTAGGAAAC
GTGGTGACTCTGAAATGCTAGAGGAAGACGAATTCCTAATTGTCGACAAACGTCCAAAGAATTTTGATCT
GTCTAAAAGTCAGGAATTTGACAAACAATTTCATTTCGGATCTTCGCCATGATTTTACGAATAAGTCGTA
TTTGTTCAAAATTACTTATAAAAAAGAAATTATAAATGCAATAAATTTTTTACTCTGGGTTGCAGTTAAG
TTTTGTTTGGGATGTCAACATTTTGTTCTTCACTGTAGCTGCCATAGAATCGATGGTTATTTTATAGCAT
ATCGATTGGAATACATTATTTTGGTGAGACTGAAACTTATTGCAATACATAATACTTTAATAATATATAC
TGTCGGGTCTCGAATTGGCTGCCAACCAACAATTAGGCACCTCAGCGCTGGTACCTTTGGCTTAGCGATC
TACATTTGCTCTACAATTAAAGTCGCACATCACTCGCTTCCGCATCAATGCAGAATCTCGTTTGAACAAA
CAAGAACATAATAAtttttttttAGCTCAACATTTAGTGACCCCGACGTGAGCAATACATAGTGCATGCC
ATACAACATTAAATAAACAAATTTAATTAAATCCCTGTATTCACCAGCATGCtttttttttttttttttt
tttttttttgtagcgtgcgtttgtttatttaaaatctgtcctagtggacaataattaaatggttttgcgg
gggaacattagcttaagggatactattgtacatgtatttgtggcgggttttatatgtgttgtctgttgtt
gtggaagatcgagatggtgtctcctcatgagacgtctgcttgtttggctatagtctaacaggttggtggc
gaggtggttagggtggttttcgagccttttcaggtatctctcgctgagcctggagatttcatcagcgact
tgtgggattccaagttccctatgagtgggatctgggggagaagtgggtttcggctacgagtaggatatcg
atctggtgggtcttcagatggtgctcgactatggccttgctgttctgcaggccgttcgcattccagacga
ctaggtggagcttttattgcatgacttgctgttgagaatggtggccaccagctgtgtcatctgggtgaac
atgcgctccatcatcctttccatcattccttccatgcgagcgaacatcgcttctatctggccggagtgtg
gggtttctgtttgggtttggactggggttgtgggattctgcggtgctgcgtgcactccgttgagggcgtc
tcggtagctcctgccagaggtgacgtttggtgatgccacgggcggcgccttgttagcattagcgacgggc
cttgttttcggagcagcgagtgctttggcccttttgtatgcagggcatcccttgaaagacgcaggatggt
ccgcctggcagtggaagcagcacgctttgtcgtcctccctcttctcacatgccctcgattcgtgagggcc
tccacactttacacatcggtattccaggaagcagtaacgctgtgtatgaccgaatccttggcacctgaag
cattgaggcacgtcgttgaacttctgcggcggttcaatggttactaccgacctgcatatccgcttgactt
ggtagacgtctttgttgttgctggaaggctccaggttggcgaagaatattcccagcggttctttagttct
tctgccaatgggattgtgcagatctctaacagcgtgcccttgtctgcgaaggtcttcaatgatttcctca
cgatcggtggagtggtggagtcctttgatgacaaccttgtatgcacgttcatctttcggctggaaagtcc
gatgcctcttgttttgggccacaagttggagacgcagagctgtataggactctttgtccggcatcatgac
acgtacgttgttgccattcacggtcttgtaggtaaaattgttcttcgggcctacaagagtcgtgatcatc
ttgacgagtgccgagatgttggtcacatcggggataaaaatcggaggtggcttgatggtcttcggggtgg

gctttggtttctcgggggtcttgggcttggaatcgtctttaggtccgggttcgtcatcggagctctcttg
gtcgtcctcgtcttcttcgctcgagagtaaagcgaaggcgttgttggcgagcttcttgcgcagagctgcg
ctagtgcttggcttctcttccagctcctctccctgtgctttctttgccggttttcgctttatctcgtccg
gctctgtatgcgaggtgcttccctcgctgctggggctgctcttgcgtgttctcagagcctttttgcgtgg
tgttgttccaagcttgtgggtgcgcggggggtctgccagtccatcttctccaaagttgataaggagtga
ggagggggggaagctgggcaccgtaagccggtgagccaggcagcacgtggctgctgctttgcaagtata
ccgctgcttcgagcactgggtttttgcgctggtaacacgcacactcgggtgtgcgatggtgttagtgcg
aactaccgttaggtacgttggctgggccaatcaaaacgcgcgacaagatcacgcgacgcgaagtggctgg
gcacaagtaacgggacttaaaactgtgaacaatttgctgtcgatccgcaggcacttctgcactcgtcgag
tgttcgatcgcgactgAGCATGCTTTTTTGAACAACAATTCAATATGCAATATTCAGACATGATTGAAAG
TAAATCCGTTGTTTACGCAAGAGTGCATATACATTAGGAATCATATTTTTGGGTGGACATAGCCAAACAA
CGGATACTGttttttttATATTCCTTTATTTACACATATACTATTCTGCCGGCTGGAACGGGCTTAGGAT
GTTATGGGGCAGGGTGTATCGTCCACGGGCGAGGCAACGCTCGGCCTGAGACCACCTTTGCTGACCACTG
ACCCTGTCAGACACGCCAGGTTCTTCTTCTCCACTCCAGGATCACTCTTCACTTTCAGTGTTCGTCCTTC
CGAGTCctgccgtctgctcctctccaccagttcggtatttgaaaactcccgcctTCCGTTCCAACGCTCA
TCATCttttcccgcctggcaacaccgccgaactcaccatgatcgtgtcatcgatatcggtgagcggcgtt
gccactaccgacattccgacgctgccatcttgtgtcgatcgccgcccatagtgtggccgcgtgttgagaa
tccgtgctattccagtattttttttCTGCAAGTTACCCCCCCTCGTTAGGCGACCGTACCACgtttctt
gctgttcggcgttttgttcggccatttcgaccctcacttccccgcctcgccgcaccttgaggcgatagcg
ctgcccgcaaagctcgaacagcttggcccagtgcacctttcgcttccgcgccccttggcgcgtggcctAC
TCGGCCACCCGGAGCAGCCACTTCTCCAACGGCACTTCCCTCCACCTCTGGTCGTCCAGCGGACCCTGAC
TGCGGGAGCAGCGGTGTCTTTCGCGCCTTTGGGCAGACGCGgcagctttggtggcggcagcgcctcacct
agcgcgggccaattgcactccccccttctccgggttctcttcttccgaactcacccccctttgggtgGTTGA
GTCGTCGGCGGCGTCAGTGGCAACGGCGGCGcgggtggctgtgttggcggcggcgttttcggcaatggcg
actcttttggcgtctgcgccggcatcggctggcccgccgttggtgcccacagtgcttcctcttcctcctg
cagccgagccaaccgctcctgccatgcggccttggcggcagcgaccttccgcgcatccactatctgttgg
aaccgccgcagggtggcgactcgtgcctccatgaaccgggtcctcggtcggcggtgcgacccggggccta
tccgcccctgacctgcgtagcccgccgcacgattcggtacgccatccgagccttggccatccgcacgttc
tcgtcctcgtccgacgacacctccggggtgaccgtctcgcccccgtcgtcgtcctcctcactttcagggt
gacgacctcgaccgcgcatttcgtatccattaccggtgctcgtctttcagttcgacatccggaatctctt
aagggattatagtggcgcgcctgatcctgcggcactctcttcatgtttctccgaagatttccttcaattt
ctccgcgttctccgccggcatctgtgtgcacttgccagtcccggtcgtcgtcgaataactcgttgggcag
cctcgagtcggcgagtatcctgtggtctccgccaaacttgtgctctaccgccagctgcagctccggccag
tgttcgtcccatgtcctctgatcggcacctgtgaactgcgcgatcattgtttttacggtcctgttggccc
tttccgtagggttctcttgaggagtatggagccgtgagctggtgttgcaccccccgcttctccagaaacc
tcatgaacgccctgcttttgaagtgcacaccgtggtctgtaatcataactttgggcaccccgtacctggc
cactatctgctctcgACcttctcatagaaacgatctcggtccacttttagaatatgtccaccaggaccaa
cggcattgaattcccgtgtttcgaccgcggcaagggCGTTGACGTTGGGCATCAGATGTAACGAACTGAT
TTTCTATTTCTGCTCGCTGCGGATTGTAACGGCTAGCTCAGAGAGTTTGTGCGTTCGTCCCACCAAATTT
ATGCTTTTTGTGACTGCCGaaacaaaaatctccacgtgctcttcagtccttgcttgctcaccgggactgg
cagtcaaggtatgacttcggtgagcaggaagtgaggccaggtcgcactccctcccttaagtgtagtccat
gagggctcctcctccaccctttagtcgccttttacgacgagccgggaacgctgaggtagaattcttcgcc
cgccgtcacccaggacgcctgttttgacttagacacccactctgttggccatctcatctgatcggacgcg
cttcatcatattaatgataaggctgtgtcccagcaccaaagacactagaataacaagatgcgtaacggcc

atacattggtttggcactatgcagccactttttggtgacggccaaaattactctctttccgctcactcc
cgctgagagcgtaagaaatctaaaaatataatttgcttgcttgtgtgagtaaaaacaaaagtcgagaccg
cgtatatgtgtgcgtgtcgtgctagaagacgattttcgggccgaaattaattctgatcgaagaaacgaat
ttacattgtacatattagggtagttttatagcaatatgataaaataaaataatttttaaaaattcgcgcc
ctgactattataattttaaagctttttaattttgtttgctaaaatcgccgaattagctaccgtttacaca
tttatatttttgtttaattctaatttgtctttcatctgaccatttttttaaaagcgaaatattttttttta
acacttttaatgttaatgttacatcatattaagtcaaatgatttaataaatatactaaataattaaatat
gcaattaaattaaattgcaaaagtaatatcaaagacaatacaattattctagtgtctttgctttgttcat
atcttgaggcacgaagtgcggacacaagcactcaacaatcattgccttattaatttttcacacgccgcaa
gatgaatactctaatgacaaatattcttatatagtcattttgaaatttatttttgtgataatatgtaca
tagatttgactatttctaatctattttcaaataataataacgttaaggcaatgcaaaacaagaatttttc
gcatggtgccaattgatcaaaaataatatagatttaaagccaaagaacttctaaggtgaagggcatattt
tgtcaaatttacaatgcatgtgaatacgtgtgcacacatacagatgtctgctatcactttgtgcgttgaa
aagagctgttcgctgtagcgctcttcgctctctcgctctctaacaaaaattcgagagagcctggagccac
ctctagagccacggccaaaaaatcgtgtgccaaaaaatcgtatggcgttacgcatcttgttattctaggg
tctttgccagcacccatgccgcgctgttcgccagcatagccgcgaacaagccactagggctgaacggggt
acctgccagagactttagctgctccctctactccgtaaacctggagcagtgcattagcacatgttcagct
gtttcttcacgatcaacacagaacaagcattgggctgactgggcggaacctaattagataggcccgaaag
cagccatggtccgtggggaactgggttagatggtagtccaggcatttgtgttgacactctgcccagaccg
tcaactcagggatgagctggtgtgaccaccttccccgccgcgacgtttgccatctgttctgccactcacc
agatcaattggcggcaggcctgccagcgctagcgccgcgtcttcggatatggccctgaatcctctaatga
gcctgagggccattgaccgtagcaccgaacgagctcctttcaggtatcagcccctgcaagtggcattgct
ccagattggtgcagcgtatagcagcgaagcctttgctaccgccaccagcagtttcctggccggatatcta
gggcccccgacgttcggcatgagtctagccagcgaggtggctgccttcttgctggcgtaactcgcgtgat
ccttgaacgatattctgtggtctatcattacacccagatactttcacctgtgtcacctgtgtgccattga
cggagacatattcatgtcatattctccaccatcttcctgctgcttagcaggactgcttcggccttgtggg
ccgctgttgctagcccggctttctcgagccagatgatggcagcaccaatcgtagtgttacatttatcttg
caacccagcgattgatagctgctctgcctgactcgtctaggatcatggaggatggtcccgtctcagtgat
ttaaggttcgcttaggagcatgattatcattgagctgtattacgctaatacgggcgtgcattatggtggc
tcttggcttcttttaggctcgctctgtaggtcgggcacgcaaagcttcccgtcgggtgctttctgtccac
gtcgttactccaaatcaggctttttggtgctgaaacacacccatttgccttgtgaccacgctcaccgcat
ctaagacaacagtccgcaccttggtagcgttcagcagcactgaggctatctgcgtcccgtctcgcagggg
cccctgacatcctctgggttcaactgtatgccctggaattgggcgaccatgcaAATTAACTTTTTGCGGT
ATAAATTATTTAAAtttttttttatttttttaatatttttATTGGTGTTAAACTTTTCAACTTATCACTTTA
TTACAAGACACTTATTCTTATCTATCGATACATTTGTAACCCGTTCTTTTAATCCTCCCGAATCAGACTG
ATCTTCAAATTCTGAGTCATAATCCAATCAACTTCGGCCAGAAGCTTTTCTTTGAATCTTTCTGAACATT
CGATAAAAGCTTTATGCTTAAATTTTTCCACTGCATTGTGAAATTCAATTATGCTGATCGATGCACTTTT
TGATTGAAGCGCAATAAGTTGGCCGTGGTTTGGGTTGGGTAAGTTTGGGTTGTTTATTTTAGCGGGTAGC
TAAGCGCAGGCAAAGGCGATGACAAAAACAAAGACAAAGGAAATGCCGACGAGATTGAGAATTGAAATTT
GTTTATAAACTGTGAAAGGGTGCTATGTTTATGGGTTGGATAACTCTCCCCCTTCGAAAATGACGCGTCC
CGCTTCATTATTAATTTCGTTTAAACGCTCATTCAATTCTGTTAAAAATTCTACTTCATTATTTGGTTTT
TCTAGTTCTGGCCGTTTTTGTTTGGTTACAAATATATAATTTCTGAATTTTAATATTAACATAGTAATGA
AGTACAAAATGATTAAAATTAGTAATATAAATGAAATTGAAATTTTCCAATTGTAAATTTTTAATAAATG
GGTTTAAAATATTAATGTTGTCAAGAGAAAGTTCTGAATTGTTGGATTTTATATATTCTGATATTTCCAG

GTTTTTAAAATGGTTGGTAGTGATGTACTCAAGTATTTGTTTTCTGAGTTGGTGTATGAAATATTATTT
ATTGTAGTTGTGCTGTTAAATGTTATTAAATATGACCCGTTTAAATGAGAATCGTTCACAATATTATTTC
CATTTATTAGAATGGCACCTTCTTGAATGATATCTATATTCTTATTTTTTTCTAGAATTTTAGTGCAAAT
TGATTTTTCTCCATTTAGTAAACGGGTAAAACAGGTCTTATCTTTACTTATAATACAATAGTTGTTAAAA
AGTTAGTTCTTAAAATTAGATATTTCATAAAAGGTATTTTCGCATTTTGCGACCTGATTACTTATTAGTA
ATTTTCCATCATTATGTGAAATGGATCTGGCATAGTAAATTTCGCATCTGTTTGTTATAACGGGGTATTT
TATATAAATAATTAAGAGTTCTTTATGCAATACAATTTTGAATATGGAGATGTCCATTAGGTCTGCAATA
ATTATAGGTTGTTGTTCGTGATCTAATATTTCTTTAATGTCTTCATTATTTAAAATTTGTGTGTTGAATA
AATCAATTTTGGCTATTCTTGATCAATAAAAATGTTTTTAAAGTGATCGGAAAGTGATTTTATCTCTTTA
AACGATTTGGAATTAATAATAAATTGTTGGTTATTGTTTTCAATCAAATCATTTATTTTATTCTGTATTT
TAATGAAATCATCGTGATCCGGAGTTCCAGCTAACCAATTCCAAACAGTGCCTAACTCATTTATTCCCCT
TTTTGATCTAGTTGGTATCAATTGTGACAGAATTAATTCCAGTATCTGTATTTCGTAAGTAATTTCCCAT
TTTGTCCGAATATTTGTATCCTTTCTAACATATCCTGATTGTAGATTGATTATTTATTTATAAAAACTTA
AGTTAGTTACGTGGAATAAGTCGGCATATGATTCATAGGTAAGGACATCCTTTTTGTCCATGAACAGGAA
GAATTCGTGATTGGAGTAATCAATTATTTCTGAAGTGGTTATCATTACGAATGGTATTAGAAGTAATAAG
AAATGCATTTTCTGTAATTTAATaaaataaaaaaaaaaaTGGTATTTGTTAAAACTTAATGTTGTCTTTG
TGGATAGTTCTGTTTCTATTATTGATTATAACTTTGTTAGGTAAGTTTTCCTTAACAACCTGTTTTTTGT
ATCTAGTTTTTAGCTTATTTCTTTCTCCGTGTTTCTTTTCATAAACAACTTGACCTACATGGTATTCTTT
TTCTTTGCAACCTTCATTATGTGTTACTAACATTTTTTCTTGTGTGTTCTTTAGAATTCTTGAAATATTT
TCGTGATTAATTTTATTATAAAGAATATCAAAAGGTTTTTGGTTTGTTGTTGAATGGATGCTCTGATTGA
ATTCTTTTGCGGCTCTAATAATTATTTCAGAATAGTCAGTAAGATTAAATTCTTCCtttatacaacgagc
taattctgttaatgttgaatgtgccctttctacttgtccgttcgaggtactgtgTCTGGGGTCTGCGAAA
TGTAGAGTTAAACCACATCTTTGTGCAAAGGATTTAAATTGGACAGAGGTAAAGCTCGGTTCATTATCAG
TTATAATTACTTTGGCGCGGGGAAATTGTTGGAGTAGTTCCATTACTTTATTCTCGATGTTTAGTTTGTT
TTGTATTTCTTTTACAACCAGGaattttgaatacgcgtcaatgcaagttacgaaaataagactttgcgcg
taataaatgtcgatgtgtaaattatctccttctttagttggaataggggcttccccaataggaattttaa
taggatgcctgttgtatttattttcgttgcaaattgtacaatttTTTATATATTCTTTTAATTTGATAAA
TAAATTTGGCCAATAATATAATCTATTGATTTGTTTGTAATTTTCATCTAAACCTCTATGAGCTCGACTA
TGTGTGTCTTCTAGCTTTATCTTCATTATTTTCCACGTCTTGTAGAAATATTTTAGTATAAAGAAATTTG
TTGGTAAAGTTATTCGTTAATGGTAATTGAATATGATAAAGATCTTCTAAAGTGCAATGAATTCCTACCG
TTATGTTCGGTTTTAGATACTCCCCTAGTATTTatattaaattttctggcgtgtcatattcaattatatg
tcgtgtcttatcaaaAACATTCAATGACTCATGTATTGTATACCTCCCCTTTGTTAATAACAGTTGTTGT
TGAAACTGATTTAACGGTTTTCGGGTCTCTTGTATTACATTTTCAAAACTACTTTCGGCAGAATGGTGTG
TATTTTGATCTGAGCTTGATTGTTCTAAATCGCTGTTAGTGATATTATTTATTTTTATTcgagataatgc
gtcagctacgacattagttgttccgggtttatatattattttcggggtgaaactttctatgaaagagtac
catcttttcatttccacgttcgggtttttatctgagattgtgaatgataaagattggtggtctgtttgta
tttccataccaacaacaccatataagtaattcctgagatttttaagagcccaaactattgccaacaattc
ttttttatttgaggcataaagttgttcggttttatttaatgttttggatatgaaagtaattggtttatta
tcctgtgataaaactgctccgatagctatatctgatgcgtctgtcgttaaagtgaatttttttgttataat
caggttgaacaaattccactcgtgctattaaattatctttaagttcgttaaaagcttcaacagctgggtc
atctaactgtattgtaatttttgtagacattctcctagaaatttttttcattatttcctcctaagtatttg
gttacaggtttggcaatttttatataatttcggacaaatttacggtaatagccggtgaggcctagaaaac
tttttgtgctttttgtaaaaattgaatttgaattgaatcagtgtaatgttattcaattgtattagaaaat
tattatattattattattatattattattaaatcatccatataaacatgacaagtttttccgacttgttc

tctcaatatgtcatccattgctctttgaaatatggcatacgtaagaattcgaattttccgtaatttatac
agaaagcggtttttttcaatatctgaatttttcattaaaatttagtggaatcctgatttcagatcaattgt
taagaaatatcatatcatatcaaggtgtctggagtagtcaaaatattaaacattggatagggagaagaaa
gtaaatttgtataattaaagataagaaaagtaaaaaaaaaaaaaaatgtaacaaatagcggctaagaacaa
tgtaatagttataagttgggaatttcgacaagatcaggtgatcttaaatttcaggatggtcgaatTGTAG
TAGGCTAGAGGTACATGTCGCAGCGTTGACATTTTCTTGTGCCTGATCTCCCATGTTGGCAATTAAACTA
TTTGAGTGAAGCGAATTCCAACCGGCGTGCGCGATCGTCTGTATGCAACTGATCGTATTTTCTACGCATC
GCATTCTACACATGCTAGCTGGACTACACTAGGCGACAGCGCGACAACGCCGTCGCCTCGACACTCGCCT
CACTCGTAACGGTACTTTGGCGCCAGTCATATTCTTCGTGGTAGTCAAATTCTAAGAATGACCTCTCAAT
ATACCCAACTCATTGCGCAGCTGCGTCTTGCAAGCACAGTGGTCAAACCATGTTAATAATATGACAGACA
CGTACAGTGGTCAGAATACATCCGTAATGCGACAcggccttcctgacataatacacgtcctcgtgtgttG
TCTTAAATCTACCACATAAGTGATAATACTTCAGGATTCCAAGGCTCTCTGGATATTTCTAAGAATGTTT
TCCATTTATTCTATTTCTATTTCTTTTTAGCCTCCTTTCTCAAATTTTCTTTGTCTACCTCATCATCGTT
ATCTTCGTTTAGCggcaacaagccaaaaggtcgagcttctttaccaatcaataattccaaagggctaaac
ttagtcacacgattaatcgtgcaattcattcctagctgcacttcagccaacgcatcctgccatgatctct
gactagactcgacagccgttagcatacctttcagggtgctcatgacacgtcctacctgaccgtttgctct
actttcacctgtagcaattaaatgtagattaatttttttgagaggagcaaaaatcgcgaaagcttttactt
gcgaaactacgtccttgatctgctataattcgagtgggtaaaccaaacaaggacacggctgattttacag
ccttaatgcagctatcagaatatacatttagtgtgtgatgaaaataaacaaacttggtgaaggcatctaa
aggacgatgaaatattccttcaaatcatttttgccactcaatttgcctgtgatgtcaatatgtatcgtat
gccacggtatttctattttaggaataggattcagctcagcctgcacttttcccgctagtggcttagaaaa
tttacatgtaatgcaattttctacgaatctacacacatatttaggcatattttcgaaccagtaataacaa
tagactttgtcCTTTATGTTCTTCAAgcactggatttcgagagagaaaatccacatgcgccaattcgctc
gccttttatatactctatttcgaaatcaaatgcctgtaaaaacgcccaccatctatgcaccctcggtgtt
aaatctagttttgtacggctggcctttaaagaattacagtcagtatgtactacaaatttacgtccctgca
aataatgccggaagtgttttatagcatttaccactgctaacgtctccagttcgtatgaatggtatttaga
ttcggcgggagacgtacacttactgtaatactctacaacatgaggtttattgtcaattttatgtaacagg
atcgctccatacccaatacaacttgcgtcagtatgcagctctatttggtattgcggatcgaatatgatta
aaactggcgtctgggtcaatatagttattatttttattcgcacctgttcatgcactggtaaccaaacaaa
gtcagtgtttttcttagatgtgagaagatacagaggtttaagtgtttgggagaaatttaacacaaattga
cgaaaatatgtggctaaaccaatgaattgtcttagttgtgagacattttggggtgatggtaatgcttta
aagcttgaatttttcgtgtatttggcctgacttctcccttgcttatttcaaatccgagatactctatttt
tgtggtgagaaaggaacacttagtgacattaaatgaaaatccagccttacttaatgtctccaacactcta
tgcaatctaactagcgcatcttctttactttctgctattatgagaacatcatcaatatacacaattgcat
aagtgtaggctaaatctcctagggccttcattatagcccgctggaaaacagatggtgcgtttttcaaacc
aaacggcattgtcaagaactcgtattgaccctctggcgttacaaacgctgttcgttcaattgagtcctca
tggactggaatttgataaaagccgcttgccatatctatgcacgaaaaatatttagcaccccgaagcctag
caatttggtctgagatgaggggcaatggatacttgtcagccactgtattttcatttagcatgcgatagtc
cacacccagtcgatcggtgccattcttttttttttgactagcatcatgggactagcgaaagttgaacagct
tggccgaataagcttttagtaattaatcaactttaccccgcactttgcttttctcttcttcactaagagt
tctttaaattgtggccatgtgatgccagcaaagcatatttgggacagccattgtgatgcacttccctcta
gcgccttacttaaagttatcatcaggctgctaccttcgggcattttatcagcaaaaatgagatccactgt
tgtacaccaagctgaggcatctgcaccagctccctcaggatgaaactttgggagaattatatattttccc
ttttcatcagtttccgatgcctttggtgtctgcatatttttgatgatctccattaggttgttgttttgca

cttgtaaagctgccaggtatgcatcggtgatatttttaggcaaagcagatcccacttctgaTGTCGCAGC
GTTGACATTTTCTTGTGCCTGATCTCCCATGTTGGCAATTAAACTATTTGAGTGAAGCGAATTCCAACCG
GCGTGCGCGATCGTCTGTATGCAACTGATCGTATTTTCTACGCATCGATCTATCCTTTACACACGCTAGC
TGGACTACACTAGGCGACAGCGCGACAACGCCGTCGCCTCACTCGTAACGGTACTTTGGCGCCAGTCATA
TTCTTCGTGGTAGTCAAATTCTAAGAATGACCTCTCAATATACCCAACTCATTGCGCAGCTGCGTCTTGC
AAGCACAGTGGTCAAACCATGTTAATAATATGACAGACACGTACAgtacacatacataagttcagttcat
aatagcaacatagtatataaacattaagtcattgttcgttttcgggtgcattctctctgtttcgcaacat
cggctctgtagcaacaagcatagaggcagatcgcaaacggagagggagtcagtcgaatgctaaagctgaa
cacgggaagtaacaaataaaccaaagagtccgactcctgcttttgtgttttagttaactaagaggatata
tagtacccctacaCATCTTTGTTTCGACATTTTTGGACACTACTAAGACTGGTGAATTGTAAGGACTTC
TACTTGGTCTTATAATTTGTTCTTTTAGCATTCTATCGATTTCATAATTAACGAAATCTGAGACCGACAT
AGGATATTGTTTGCTATAATTTGCCCTGTCATTTTCAGTATGTATTTCTCCTCGAATATCGGTACGAAAA
GGAATTTGTATATTTATTTTAGAATGCTCCTTTTCAATTTTATTCATAACTTTATTTTCAAGATCTTTTA
TTTGATTTGAAATTATATTTAGGTTATGAATTTTTTCTTTGTcggataactcaacgacggcgtgctcagg
atttgtggatcctaaactatttgatttaaaaatgactgaattaacatcgtcggcttgttcaggattgctc
gatcccaaactgatatattgtttattagcggataaatctacgctggcgtgctcaggattttcagatcccg
aaatattagttttattaatgacggataattcaacgtcggcgtgttcaggattttcagatcccgaactata
taAACTGCCTTCAGCAATAACCGTTTCGGTTTTAAATAACTTTTGCTCATCAAAGTATTTTTTGATAATA
TATTCTTTCAATGGAAGAATATTTTTTTCCTCAATTAAACATAAATCGTTTTTTTCCTCGTCAAAATATT
CGAGTTTCTCTTTATTTCCCTTATACTCCATGATTCCTTTCTCGATATCTAATTTAGCTCCAATCTTTCT
TAACAGATTGAATTCAACTAATAGATCAGATTCTAGACCCTCAATTTCATAAAATAAATGTTGTTTCCCA
AATATATTTATCAAATGATAATATTTTATGATTGAATATCCATGAACAGTAGATACTCTTTTGTGTATTG
ATAATTCTCTTTTATTCTCATAAATACCCTTTTTATATAGCACGGTGGCTCCTGTATCTATTAATATTTT
AAATTGTCTTTTAGTTTTTCTGTCTACCCTTGTTATATGAGGCATTCCTCTGTAGGGTGCTGATCTAAAA
AATGGCCCTCGTTGATATTATTTACCCCCATCGGGAGGTGTGTTTCGGACTGATTAACCTGTCGTGCGTT
TGTGATGGGTTTTTGCGCTGGGTATTGGTTATAATTATTTCCTTGGAAATTATTATTAAAATTTTCTCTG
TTGTTATTATAATTCCACTTGCTGTTATTATTCCTAGGACGGTTATAATTTTTATACGGACGATTTTGAA
CTCTTATGGAGTTGTCTAATTCCATAGGTTCTGGTGGTGGTTGAACTTTGGAATTATTGTTAAAGTTCCA
ATTGTTATTCGTTCCCAAGTTCTCGTTTTGTGGCCAATTATTATTTATTCTATTTGGCGTAATATTATTC
TGTCTAGAGGCAAATCTAAGGTTGTTTCCTTGATTTTTATTTTCATTCCTAAATCCATTGTACCTATTTG
CAAATTGAGCTCTAAAgttgttagactctagttcctgacactttgccaaagcatttggcaagtctggtgg
gctaagagagaagagtgtttctgagattgaactgtttaatccagaaataaaaattctaatagcattgctt
ctaattgttttatttgtttccttggttataacgttgtcctttccataagtcattatagttttatttatga
gtaaagtcattttcttgttaacttcgttgtaaaattctgtaatggtcatacgtccctgtctaaggatgct
taattcttattcgagtatatggattggtcttttatcgctataaataaaatccaatcctgaaagaattgct
tgaaagtttaaaacggtaccatggttggttagagcatcgtgtgctgctcccatgatttGATTACGTAGTA
TTGTTAAGGCGATACAATATTGTTCACTTTCAATTTTGTATAGACTCATTGCGGTTTCTGCCACTTCTCT
CCAGCCTACGTATTGTGTTATTTCACCCGTGAACTTAGGTAAGGATTTAATTACCTCTAATGTAGTTTCG
AATTTTATGGTTCGGTCAATTGTTTGGATCTTATAGTCTTCCACTTCATTTCTGTTAGCGGTTAACTGCC
CTTCTATCCCTTCAATTTTTCTGGTCACTTCACTCAACTGTACATTATTAATCTATTTATTAATTCCATT
GTCAGGTTACTGGCGCTGGATATCCCGCCCGCAGAAAGGGTTGGTACTGAACCTCGGGTTGCCATTGTTA
AATTTAAATTTTCAAAACTATTTATAAAATCTTCCAGATTGTTTTAAAACTGATAATGTTTAATTTTAAA
GCTTGTAGTACTCTCGGTATATTGATCTTAATTTTTTAGATTCTATTTAAAGACTTAGTGTTTTATTTTT
TATCTGCCTTCAGCTCTTCCTTCCTTCTAGGTGTTGatttttttacgtttttgtttttttttttaatttt

tttggttttaattgggtttgtagttaagttctgtttttttATTATTGTGTTCTCGTAATTAGTGTTAAGT
ATTCAGTCCACCTTTTATATTAAATTCCTAATTGGATTATTGCATTTATTGGTTTCCAATTAAGTTTATT
CAGGATCACTGTCACAGTAGTAAGGTGTCTTTGGATTGTTGTGGATGTTTTTAATTCTTCCACTAAAAAT
ATGGGTTGTTGGCGGCGCGAGTTCCGTTTTGTATTTAACTTATTTTCGTTAATATTTATTTAGAACTTTT
AAGTACGCCGCCCACGTTGGGCGCCAATTAACTTTTTGCGGTATAAATTCTTGaaaaaaaatattttcta
atttttattatttttatTGGTGTTAAACTTTTCAACATATCACATTATTACAAGACACTTATTCTTATC
TATCGATACATTTGTAACCCGTTCTTTTAATCCTCCCGAATCAGACTGATCTTCAAATTCTGAGTCCTAA
TCTAATCAACCTCGGCCAGAAGCTTTTCTTTGAAACTTTCTGAACTTTCGATAAAAGCTTTATGCTGAAA
TTTTTCCAATGCATTGTGAAATTCAATTATGCTGATCGATGCACTTTTTGATTGAAGCGCAATAAGTTGG
CCGTGGTTTGGGTTGGGTAAGTTTGGGTTGTTTATTTTAGCGGGTAGCTAAGCGCAGGCAAAGGCAATGA
CAAAAACAAAGACAAAGGAAATGCCGACGAGATTGAGAATTGAAATTTGTTTATAAACTGTGGAAGGGTG
CTATGTTTATGGGTTGGATAACGTGATgctgtcgagctcctctgctgacgtagcctggtccagtccgctg
cagtatagcgctattctttgcgcttccgtgcgcacaggggacaagtcgttgagcgccgctacaagatcgc
tcttaaacttgggaacgctttccgaggcttttccctctacatcgagcagcagctcaccttgctaagttct
ccggattttgcgtacgtggatgcccagttcgctaaggctcgggtccgatgttagcttcctaagcatctct
gcgtacgaagcctcgcccgtcttcttaacgatcaaggcatccggtttttttgcgcaggctcataggcttga
ccttggtccactctgaacccgtgcATCGATTGCTTCAAAAGGCAGCGCTAATACTGGGTCTCTGACGAGA
GGCCCAAAACAAGCCATCCGGAAAAGTTTCTGACGGGCGTTTTAACATCCCACCTGCTCTACGCAGCTCA
AGTGTGGGCCGAAGCTGTTTGCACCTGTTTCGTACATGCGCGTACACACAATATAAGATGATGTCGCTAC
ATAGTCCTTGCTGGGCAGTTTCCCATTTGAGAACTAGTGCGGGCACAGGCCCAGCGCAGAGTCAATAATC
GACTTTACGTTCGTTAACAGCCCACTGGCACGCCTCTCCCGTTGGAGCAGCAAGGAGGTGTACACAGGTC
GAAATCGACATTTTTTGTGTGTATCAACAATATAGGGAAtttttatttgatcaacgttacctatgtggc
aactacatttgatgtgtttatcgagttctagtttcagtgcgtgtttgttacAGAAGTTTTATCCTATTAT
AACTATGGAGAATTATTATTAATGTTTAGTAAATCCTCCGATgtcaagtcccgatcggggaggtcaagag
ggtggagtagttagagccttcttctgactccagcccataaagccattccatgtacttttcagcgatgctc
tgagctttggtgccttgagatcgcgtttgatgtccctggttattatctaccaaggagctccagaaaccct
tcttagggtcttctcctgtacaacccggatcttatttaggttcttggcagcaacgccgtatacttgaaga
ccgtagaacaaactgggggacaatgtggacttgtaaatattcaccttgcagtcaagcgatagtttgttga
gtgttttaatgagccaagacatcctagctatcgtggtcctgaacgcttgttaatgtgcctgctgagggtt
attccaacttatttctagacctgaaggtggttgattcttcttccattcttgaacgtcgcattgacacaat
tctcagggttaactcacacgttccagttctcagcccgttgctgggatgcttccaagtattcctgtatact
ataagtggcagccaaaatattttagctcttcgcacagccgtatcgtccgtaagtggctaagagcacactt
tcttcctctacttctgtcactggtttccgaagcaaatacccttcctacgctaggatgcttccttgcagaa
ctccagctgcaattgtcttctttgacgatttgaattcatcagcagagacgtgtgcgttcgatctttcaga
aaacttttaataatcaaatacagctgaagtggaaacaacctgtttgctttgtacagaagcccaggatacc
atactctgcccgttgtatgtaagaaaaagttaaggcagctactgcatacactttctttccaatagcctct
acagcaaaACACCGTGCACAAAACTAAGGACTACCGTCACGACACCATGTGTCTATAGGAGTTCGCCGAC
CTGAAGGTCGACAAAGCGGACCCGCGAACGCATCAGCGGAGAAAATGGCAGTGAGggtctacagaaatac
ttgaaagcataccagcaacgggctgagaactggaacgtttgagttaaccctgagaaccaaccgagcaggt
gttgtcctggtgttagtcttaatggaagaagaatcaaccaacttcaggtctataaataacttggtattac
cctcagcaggcccattaccaatattcaacaagcgttcaggaTTACCCgtgcaaaaaaatcgtatggcgtt
attcatcttgttattctaatgtctttgGCCAAACCAAAGCTTTTCCCAGAACCACCTTCACAAACACCAC
TCCTGTCACAAGACACCTACCAAAGACTTTTGACCTAAAGTCACTTAAAAGTGACTTAAAGTCACAGTGG
ACCTAAATTGAccactgtaaggccctaaaaccacggaaaactgtcacgtccacacttttgaaaaacttgt

agatatttttttcataattttagtagtcgagtaaattactatcgatttgactaaaaacttatttccacgcc
cactctaacgaccATATCCAATGAGACCCAAAAACTTTCGATCTTCGGTGATATTATTTGGCTTTTCAAA
GTTTGCAATGACATTAGTTTTGACTTCTCCGGGCATGATTCCATCTGGGTGTATTTGATGACCTAGAAAT
ACGATTGATAACAACAACTCACATTTCTCACATTTCGCTTCTGTAACTCTGCAATGCATCATATGAATGC
AGCACGTGCATTCTTAAGTCCGAAAGGAAGGCGTTTATACTCAAACAATCCATCCGTGGTGATAAACGCA
TTGAACTGGCGGTAATTGATGCAAAGTCGATCACTACCGATTAGCTTCTTGATAGTCACCACCGGGCTGG
CTTGTTCTGACGTGGATGGAAAAATATTGTCGCGCTTTAAAAGTTCATCCACAATCTTGGCAACAACTTC
CTTCTGCGGCTCGGTATGGCGCTTAAGAAAGTACTTGGCCACTTTCAACAACTATTTTTGCCGAGACGAC
GTTCGTCTTGCCGATGCCTACCAATTCTGTGGAAAATACGTCCGTAAACTTGTCTAGAAAACTACATACT
TTGTTCCGTTCGTATTCTTTCTGAAAATGAACACGGTTTTTAAACGGTTTACTTGACTTCTATCTCGCTT
GGCTCGAATTTCCAAACTCAGCATATGTAATGACATCTTGTCCCAAACAACAAAACTTTGGCTGTAACCA
TTTTTTCCGCGATTTCAAAACGCACAACCATAGCTTCGGTAAGGATACGTGAACCTCCACATATGCCCAC
TATTTTTATGAGGCACTCCTGTCGATCTCGTCCGCAATCGATTTCCGAATTTTACTGATTATACTGATTT
GCGAATTATACCTTCCAGTATCAATGAAAGCGTTATAGCCGTTATCGTTGATTTAGATCATCCGTGTGAA
CAGCTTATCCTGATTGGATGTGCCCAAACGCATCACCTGTGCGGCTTGGCATATTTCATCGGTGGCCAGA
AACCTAGCTTCTCTTGGCTTTGATTGGTTCATATAATTTTTTAGCCTTACCTCAAAAGTATTGAGTTTGG
GTAAAAACTCCTTATTTGTATTGTGCCTCCCTCTATTTATAAAATATGACTCGGTAGCGTCGCAAAACTC
CTTCATCGACGAAAAATTAATGGCTGCAATATTGTTTTGCAACTCTCTGTGCTGTATACCTGTAGACATA
CGGAAACAATACTCCTTTACGGTTTCCTTCGATCTTCTGGCATTTGCAATGGACTTATACCTCGTCAGGC
TCTGATCCGAATTTCTTATGTAAAAGAATCAAGACACACTTTAAAAGGACCTTTCAGCTTTGCGTATATA
TAGCAAAAAGTATAAATTTTTCGTAGCATTAATACGCATTGATCTCCGGGGTCATAGTCTGGTGAGGTAT
ATGCAATTTTCTTAACCGACGCTTTTTTACCATGGCTGATCGCCGCTTGATCTCCTGGGTTACGTTTGAC
AAACTCGTCTATATCAATCTCATCTTCCTTTGCTGCTGCCGATTAGCTGTTGGCATATTGCTGACAAACT
GCGCTAACATATTTGTCATCTACTGTTGTAGTTGTGAAATCGCAACCGCCTGGCTGCGAATGATTCCTCG
TGTTGGCTCATAATTCAAACTGCTTCTGGCAAGTCCACTTTTTCAGAACTCACTTGTTCCACTAGTCTCT
GCAATAGTTCTGCTCTGAAGCCTGCAACTTCCAAGTTCCTGTCTCTTAGCAACTACTGCCTTGAGCTCCG
CTATCTTAACCATCTTCTCCAAAAGTGATCGCTCCTCTCCGATAATGTTGACAGTTTTCCTTCCTATGTA
CGCACAACCAGAATCGTTTGCACACACAATCTTGCACCCACtgtaacgtgtaacaaaataaagggatgga
agactagagtacctgtactcgcgggtgcacgctttagaggccggctctagctcatggcgttgggtgtgtt
ggtcttgcttgtatagatatcttttcgttacacacaataatgaataagaccaagaaattgagcgtgctta
caacaacaaaatgaaggagctagttaatacctcctgaaggggacagttctagaaaaggaggaaaagcttg
agagttttgtcgcctgcgaagcacaggtggaatcgtgttcgcgtattgccctaccctacctaagaccact
ttccttaagagcatggctctagtggccccttaagtacccaacgtccggagaaacatcagccgagtcagta
tttaagtttttagaaattcatattttcaatattcaacgttgctcaataattaataataatatttatagct
acaaataagtacataataaaagaaacaagtaaatatgtaaacacaaactaaagtaagtcgagggaatcta
aatcaaggtttttttgttgcgaaatcagaaaagaaaggtttaagccgttattcaagaacgttataattaa
agagttaaaggtactttcagtgtggtttttctctataatcttgttccatgaattattttttttccattat
tataatgaattattaaagagaagaggaaaaacatttttttgtttgagagtgggccgcgagtaaatcgggt
tagatttttcttttttttttctttttataattcaatcggctactttcgccactccattaggaacaataatt
tatgagcagtgtgtataaaaataaatgaaattttgtacgccatcaaaatgatgacctcccgtaaaatttt
atcgtcgtactttaccgaatatttccaataataaaaaaagttttataaatgaaactcactcaacttttca
ctcctcggcgaaagatcgatctcccggcgcagatgagctgaaacgaaagtgtggcattttccgcaaatac
tttgagaagctataatttcggattacacgactgcgaagaaacttttataaatccttactcgaaaattcaa
acgaggactctcagaaaatgaactcttcttaaaaaaaaaaatgattagcttaggttagcttacatactatt

ttaagtactaatgggaattcaatttaaatgcaagaaaaattagaaaatgacatgactgagtctcctgctc
gagtaaacatttttttttatagcttattgttaaacttaaaatacatcataaaaaaaaatttaacttcttact
ctctacgctcaaaatggcggttcggggattaatacatttgagtaaaggttaggtcagacatttagcgttt
ttacgcataattaaaatttttttaatcaatttaaactcgctttaatttaattttaatgtccattctggact
accaatcacgctataattctccgtcttttttttattaatttaaataaccctcctgggggtcccggcttat
tgccggttgcgatcccgctgctgctgcttgtccttctctgcccctcgctgggaaggcgctccactacacg
gcctggcttagttatcgcctctccttctgcccgccggtgacggcccccgtcgaaccgtggggtcaggata
gtgaagcggatgcgtaaaagctgccaagcgaagactctcctgccttcaatttcagctacaataatattta
ccgcatcaaaccacagttcacttttttccgaagtttactgacctgatattgccccagtcgaaacttgggg
tctggcgacaaggctgcggcgcgatggctgtgccaagcgacgatctttccggcttaaaaaaatttttat
tgccgggaaacacacgacggacttatctcgcgcaccaatattgtcgaagaaacgcagtacgataatattt
cccacgacaagaaaatacttatgaccgcaggttacgacttttcgaaaggctctttcacaactagactcgt
cgactgttctattcgaaaaaaaaacaaacaggaaacacaagccgaggtgtaggccatcggcggaaatcac
gatcagctgaaacgcacatttttttcgataaccgaattatcccaaactttccgggtgcagcttatatggg
ttgcattccgtctggtatattccagctacttcatactgcatctggtatgaccttccattggtgtgtcaca
gctgaccgcaagcacactatcgattattcatcgagactttaatacccactgcttcagtcgcctcttcaCC
ACGAGCACCTCGGTATGATCGCTATTTCCCAACAGATCTCGTGAATTTCAACTCTTGTGAATTTCACCAC
GACACTACTCATACACACAAGCTTTTTGCATATATACCCTTAGGTTCACCAAGATTCCTGAGTACTCCGC
TTTGAATCCATCAGCTACGTAGGAAAGGATGCCTTATCCGTAATTTGTAAACTCTTTGGGTGtttacaag
ctttacaagcttCCAACGGTGATGACTCTGCAGATTCTGCTTTTCCGTGTCTCTTACATTGTTACTGCCA
AATTGTTAACACAAAAATCCTTCCGTTATGCAGGTAGAGGAAGGTGTTTCCGACCATataaaaaaacatg
ttaaggtaaaaaagttttgttttcgcttcgggaataaaatcaaaaaaTTGTTTTTCTTTGGTAAGATTCT
TTTTAGATTCCCAATGTAAAACTGATTTTACAAAGTGCATTATTCTTATAACTGTGGCTTTTGTCGGTGA
CAGCGACGAGGGCAACGAAAGTGGACTTTGGTCAGAATATTTTGATTGGTTTAACCTGTGGCCTATATAC
TACTCTATACATCGCCAGGCTGCATCTTTTGTTAAATTAATTTGGATTATTAAAATTTTTTATGATGATC
AAAGGGTTTACAGGCTGGACCCTTTGTTTAAGAGTTTGAAAAGTGTTTATGTAaattaacagacaacaat
tttagttattgtggtttattctgcgactatgcgcgttcagatgagttcttaaaactgaagacagaaaact
taagcttaatataaagtagcccatcttctgaagcagcgctcacggcagaacgctgacattgcgcttcaca
cgctccgggcagtagaatgttggaatgcaggagtcatatttcgcgacttagttgcagtcgcgctgacgtt
actgcggccgcgtccggtcttatcgtggagttagcgattttggccctgcgcgtgttaactcctccccgcg
ttaggttaaagtcgtcctcggcttttcgggaaggtatcgtcatctcgatgctggagtcggggtgtcgtct
tttgactcgacagtaacggatgctttggatgatcccataaacaattaggaccacggcgatggtgatgcca
ctgcttaaaatgggtctcgaaatgatgtcagatttcagactaccaatatgttggaggttctgcagcgaca
gctcgtgcaagaacgacaagcttagttgatctcgatggctagtcacattgatctgagaggccactgccgc
agcgggtttcttctttaggacgccctggtggttttcgtagtgggtgccgtttagagatatacgatcgtca
tatgtggctaagtaggttcccgagatcatcttactgctgcctaggctatcttcgaccctgaaggttgcgt
cgttgataatcaggacaccttcttcaacaagtgtcaccggatccagatggccaaggagagtggtgcactg
tgcgacggttccagacaccaattgttgagcacaagtcgggttctgcaatctcttgcagaaggtggtactc
atggctgcgttacattccttgacggcgtagattcgtgcaccgcattcggctactacattattattctcaa
agtctaatattatcttattatgttgaactgggaaaacgttaatttttttacaaactgtttctggtttagg
atattttattaagaaatacaataaatcgttgttctgaaaggctttaacgctagatactcttaaaatatct
gctaagctcgtatttgtgagaagttcattggccagctcatgaatgtcaacactatccaaaataagtgggc
ttattaaatttattttagctagagttaccgacagtatcaagtcttctaggtttgtaataattattctatt
tttagcaagtatactttcaaataggtgttctgagtccgcgttgtctgttttacttattgcattcattgac

cttgtaatttcgtttagtcgagattgaagtttcgtatttatttcgatctgtctatttgctgaatctatta
attgcccttgtttaaatttaatctgttcccagtcgtcgaagtctggggttccggctattaccttgagtgc
tgtacctagtagatttatactgcgagcatgtctatggtgtaccctaatcgtagctattagtgattctatg
tgccccaagtcagtagttatcaaatttcgcatatggtcttttccaaagtagttcagttgccgtctggtat
cttcaacataagtttcataagaagttatgttagaagtgtgtccgagatatccgtatctgtcccatatcgt
taagtcaccgtctattataggtatgtagtctgcgtgtgaatagtcattaagtttcatagtcgttattgtc
aaggtgattgcggaagccagcacacacaaatatcctaacctgtaataaaaatattaaaattaaaagtact
ataagcgatgaaatttttatttatttatatttgtttttaattttttttttcttgggtttacttaaggttg
tccttatggaccaccccccttcctttcattagaaccgtcgtacctaagtcagcatcaatggcttcttccgt
gcacagtggtgacagtttattacctaatcgtttgattgatttagccatactttttcacctactttatac
tttttatggattctatctcggttcatgtacccaagttgtttttcttgcgcaagtacaattttatccctag
tttctgttaataaatccgaaggggcggagcaaattaattctgtcggttttctgtttgttactgagtgaat
tgacctattatactcaatggtcgccaaaaggattatgtccttggtttcgttaaggtgtttatctattttt
agacaacgggctatttctgtaagggtactgtggaatctttccacttgaccattagaggtgctgtggagtg
gtggggcattacaaatctgaatatcaaaattgtttagtatatttttttattgtttcggaatttaaagattt
ttcgttgtcgcaataaatgatccttattttgggaaacaagtttaccagctgtaaaatcggtgactttacg
tgcacgattgcgcgtgaagtaatatattggacgaccgcaaactttgagagtttatcgacgcatgttaaaa
aaaatgttttgtcggtactaaaaatgtcaacatgcaatatttccccaacatatgatgggattggggtctc
acccatcgcttgcttgaccggatgtcgattatatttacccattgaacaaaccttacagtttacgacaatc
tctgttgccagcgaattcatctttgggaaaaagtagtcccggagaatttgcttcacattttcctgcgctg
ctcggtgcgctcggttatgctctgcaataattatttctctctgttcggttttatcgacaatgtctatcac
gatatttttggagtgtcggaatgtggttgacggaaacgcttttactagactgtgttgtatgaatgccaaa
atgggtaggtcgcaatgtattgcgttaactaccttggcatttacgacgtcttgtactgtttgcaaaagtg
tgttacggtcaacaaatcttataacgtgcctggtttttttccctgaacaaaatagtagatctaactgatgg
ggcgttagattcttcaattacgatctggttcctaaagcaatttacaggcttttcggtcgtttcgatagta
taggtaagagattcttcgctatgtattgtggcaatatcagagtttgggcaatcttcgagtacatttacat
tctggcgagatagggcgtctgccacgaaattctctttgccaggcttgtaaaaaatatttgcgttgtgctc
gtcaataaaagctttccagcgttttatcttcgcatttgggttcctatccgacacggcaaaagtcaacggt
tgatggtccgtgaagatatttaaatttttttacgccatataaataatttcgaagatttttttagggcccaaa
caatggccaagagttccctctcatttgttgcaaagtttacttcgttgtctcgtaatgttcgagaaatcaa
ggttattggacgtccgtcctgagacaacacagctccaagaccgtgccctgaggtatccgtagttaaatcg
aatggttttttaaagtttgggtatgctaaaataacgtcctcagaggctaatacgtcttttagtctattaa
aagcctttagttgatcttgtgttaattcaaggtttactttccttgaattgttggcacttattttaccatt
ttcgccttttaagacgtccgttaggggtcgtgcgatgttggcaaagccttttctaaagcacctataatag
cttgctagccccaaaaatgatctgagactaaacaaggtttttggcggtttgaagtcttttatggcctgaa
ccttacttggcgaagtttgaatcccgccgcgggataccgtgaatcccagatattccacgctctttttgaa
gaacttggatttttcttgagacaccctcatgccagccgtctgaagactgtttaacacccaatcgatgtgt
ttaacgtggtcttctttgttgttagagaaaataattacgtcgtcgacgtagacataacaggttttcccta
tctgctctcttaagacatcatcaatggctctttgaaatatgcttggcgcatttttcaaaccaaaagggag
tctacagaattcatattttccgttgtttacggagaaagcggtttttttcccggtccctttccgctaattcg
atttgatgaaatcccgatttgaggtcaagagtagtaaagtactgtgcctcgcccatatttgataatatgg
ttgaaatacttgggatggggtacttatcatcagtcgtttttttggttaagtttacgaaaatcgatgaccat
acgctttagcttgtggtccgattggtctacccccttttttatcgacaacccagatcgggttgttgtaaggt
gatttggacggtcttattatgccattcgctagaagttgtttaacctctgcgttgacaaactcggatacgc

ccattggctaagggtacagtttagaatatactggctccccgtcagttcgtattgtggcaactgtattgat
attgtagggcagggactcattaacatctgcaaatgaccctcttcttttattaatcattttgttaaagtca
tctttaatgacttcaggaacatcgccatcgtctatttttatgaagtttacgttctggcattttgtaaact
tgattggctctgaaccgcgatcataagtgatacaatttttttcgaaatcaacattggcgttaatcttttt
tagtaagtctaggccaataatgccgtgaaaatcacttagattttctaaaataaaaaaatatgttttagtt
ttaaaaagttgaatttggcacttccgttcaatttttgtatgaccatgtagtgattttacctggaatggct
tgtcgactggcacaaagtgtggcagccctagaagtggcttaatgtaatttttcgaagtccctgtatcgat
aagaagttttatggtctgccccgctactgtacgtgtgatgtacggaagcagggagtgacccctaaaaaat
tacagctttcgacctcagacaaatcgtcttcagccgcatttgcctcggattctgcctgttcttcgtattc
ggtttttggtcttgggcatgtggttcaatgattggcgacttttcgctgaatgaccacctttaaaatttg
ttgcttgagtgggctgtctaaagcgtgatgacgtgttgggttcgtggacgttccatgggttcggaagttg
attcttgtggcttctgctaatatctctgtctagcgctacctgaagtgttcggcttttgaccgttaccttt
ctggtaatggggatttttcctaaagatagtgggagcgtcgctctcttgctgggagttttgacgcgaacct
tgggacctctgattcccagattttttgcgctttttcctctatgtgccgagcaaagtttgcggcaaaaatgc
ttctctcgttacttgattctgcctcttgtgctaatgccagggcagtctgtaagtctcgaggttgggctgg
aaaaactgcccatttagagatttcttcaggcctgagataaatgtgtgaagtgcatcgtttctatatttg
tcatttaacacagctgccgaagctgcgtcgtgggtcatgagagttttgttagttaaaagcgtcagctttc
tttctatttcatagaacttccgtcataagcttcgaagactttatatgcggcgttggccgcttggcgccaa
gagatataatgttcttgtttgccgtcgaactcaggaatagatttaactatgtctaggggctcttcacatc
taacttctggtatgatttctataggcttataagctgtcacctctggcgcagtagcctttaaggcgttctg
ttaaagttgctatttttggtcaaatatttgtgcttgctctgccagacaatttcgtatgtgaaggtcaat
ctgtactgattcttgttgcgtcattgtgcggtttttgtattctcggtaagaggggatatagtcttctaaa
ctactgtcgctaaatcttttctgcatttataatggcacaatgaacaaatcacttacatgtatttaatttc
tgggtaactgcttaaactgccgatgcgtgcacttgttgatttttttctttactctctcgacacttcgtgtt
ggggttctatttttttaacttgtatgttgttttagccgtttttttctttttaatttgtatgttgttttaa
ccgtttgtttagctttttttattttattcttgttttcgctttgtttgctttgttcttggtcggaaaaaat
acttcactttgcactttttcgtttatttgtcacagctttggctggtgaacaaatattctgagttctataa
ttagctttgcacttgtaatttgcttgtcacagttcacttcgcactttttcgtttatttgtcacagctttg
gctggtgaacaaatattgtgagttctataagagtgccgtgggcgccaattaacagacaacaattttagtt
attgtggtttattctgcgactatgcgcgttcagatgagttcttaaaactgaagacagaaaacttaagctt
aatataaagtagcccgtcttctgaagcagcgctcacggcagaacgctgacattgcgcttcacacgctccg
ggcagtagaatgttggaatgcaggagtcatatttcgcgacttagttgcagtcgcgctgacgttactgcgg
ccgcgtccgatcttatcgtggagttagcgattttggccctgcgcgtgttaactTGTATGCTGCCGACGTT
ACCGTCGCGATCGATCGTGGGAAAGATATATGTCagagacagtccagctctttaaggcttttgaaggcac
tttgtttttattgctgtcatatgggctgagttcgatggtggtggactcccagataacagtgggagttgac
gttgcctatcatcccactattgtaaatttggccagtgaaggtatcggccgaaacgcgaaggtcacacaat
cggacttagcaccgtttatagctgtgttccacttggctgacttcaatgtcgtccagccattactgtactg
cgttcatggccgtttgcagtgacatgttcgatgtaaccatagctgtgtcgtcagcgtatgtagccactag
taccttggccgagggaatactgggggacggaactggtatatctgctgtgcaaaaggtaCAAGTACACTTC
CTTGTCGAACGCCTACgaccacgaacgggggccttaattttgtacaaaagtccctcatgccatattttgt
caaaggctttcttaacctcagaaTaggttggaaggagccttattgggcgatatgagtctgtctgcgttgg
gggtttgccggctttgggtatttcttaggaaagtgatctagtctcagcatggcgctataaatttttacta
gctaactcctttgcgtaggaggaatttggctgcacgattgtcaatgtcatcaggtcctcgtgtctttttc
agttgaagggcctttagctggagactgacttcttgcggagtgtcatatctcatggggtgggcagattgag

taggcacatccaaaaagtttacgacttcttctcggtgggagtcgtccgtcagttggaagggggtgaagat
ggagaacaaatggtcagcacgtgcattcgctttagaaccatctgtttgagccaatgccattgtgtccatg
tactggcgattggtagagcggctgcctttttattcctatcgttaacttccagactgagaattcgctgttg
ttgtcaaggtcagattctacgagcagggtgtccGAGGATTTCGTACCGGTTTCTGAGAGGGTctgcttag
ttgcaggatcccctgatactatgaacttctctctgaggctccttttgaggttaaaaaaagctcatggctc
agaattaagtatgcgtgttctaacagtttttgggcctacttggcgcggtgatggcgtagcccgtcgtcca
gtcttgacgacaatagcagtgaagcagtgaacataagcatccagTTCGTCGCTAGATGATTGGCAAATGC
AGCACGACGTCCTTGACGTGTGTTTTAAAAAGAGTAACAACATTTTCACTAGAGCGCAGTTCTGATTTAC
TTGGTGATGGATTCATGTTCTTTGCATAGCAGGTTCAGAGACCGTCTAAACTTGTAGACCAGTTTAAAGC
TTGTAATATTGAAACTTAAACTTATCTTTTAAACGCAGTGGACCAGCAGCACGTAATATTATAAcacaac
atattcaactgaggaattcatcccaattcctttttttcgaggcttagatatgccgttattgcagcccttat
tgaagcgagtgaacatcggtcaacgtgtgcttaacgccgtactgctccatcagtttgtggaacgttctag
cttgaaattgagatccgttgtctgatagtatctctactaattttgtgtactcatcagactgaaaaaattc
tgaatctaaatcaataatcttgcaaatccacagcggccacaatctcttcgtttacacgagacaatgtatc
cgggacaacatttaaagaacccttgcggtgttcaatctcaaaattgaatctttgcaaagctagtgccaat
cgctctagtcgtgaatttaagtcatgattggacattaaccacttgagcgacattaagcatgatcggttat
tatcttgaattggagtccttccacataagttctaaagttcttaggagctactacagcggccaaacattcc
tgctatgcaactgtgtaattgcgttgagctttgttcagtttctttgagacgaaggcgataggacgctcgt
caccctcttcagatacttgcaccgcccctacgtctgacttactggcgtcgcaatgtattgcaaacggctt
cgctaaatccggactacacaagtccggagctttactgagacactctttgagtttgctgaacgcttttgaa
cgcaattgcctcctttgttaagccaaacttcggcttcataatcattatgacggtcaagggtgcagaaagc
attgcgaagtttgcgacgattttccggtaccatccacacaatcccatcaaactccgcaaacttttcatac
cctttggtaacggaaaatcggtgatcgcggctaccttttcagggccgtctgtattttcccgtcaccgata
atctgggccaaatagcgtactcgtcgcatacaaaagtgacttttagctctgtttaacgctaagcccgctc
gctttatAACTaacttaactttcaaaaatttaaacgcccactctctgaaaatgtattcatcttggtaaaa
taattagattaggtaagattaattaatataaaaaacattaaatactaaaatttaaataaaattaaaaata
caaaagtaatgaaaactaagtctcctcctactatacccgacagtatataatataaaaatataattgttca
gacaatttttcttagcgtagattcgcagttcggtaattttttttttgccgatgtagggttaggctctacc
tagttcagcaagcattttatatatatttttgatttttatatgttcacttcaacagttgtgcttttttttat
taatacatttcctagcaattaaattaagtacacatcacagggtctcaatttctctgcggctgcggtcccg
ctCCACACAACGGCATGCGCGGTTCATTTTTCTCTTTCTGCCCGCCTGTTACTGCCCCAGTCGACCCTTT
CAGATTAAATTTGATTAAAATACGTTTGCTAATGATTTTGGCGAATTTTCGAAATAAAATACTTTTTATT
GAAATTAATTATTCTTAATCAAGTCAATTTTTATAATTCTGTATATTTGTGTTTCTTTAAATCAAGTGGT
TATATGAGCTAGAATAAAAGTgtctttagtctttagacttttgtctttGGTGTCTTTTAGTCATTTATGT
TTTCTATTTCTTCAAGTTTTAATATTTTGGGATTAAATATTCCTACTCTTGTTGATTGTAAACGCATTTA
TTTGATGTTAAATATTAAAGGCTTATTGAATAGATGCAATAACAACACTAATGTTGGTCGATTAGACGTT
CGAGATCGCATTGAAAGGTGACGAACCAGTGACACAAGCTGCCGATTTTGTCCCTTAAGTTGAAGCTGAA
TTGAATGGGATTGTTATTAGAGGCAGTTGAGATCTAGAGTCGATACGTCTAGTGGAGCACAACATCTCTA
CCATGATCCTGTGCAATAAGAGCAACAGACGTTCCAGGCAATGCAGAAGGTTCTTCGGTATCATGCGACT
TCGAGGCAAGCGTTTCTACAGCCTGTGATCTTTCATGTTGTGAAGGCGAAGCCAAGTTTCCCAGATGACG
TAGGGCGTGGTGCACTCAATTGATGCATGTCAGCAAAAGTTGTTGTCTCCGTGCGATCCGAGGATTTAAG
AGTCACGAGAATGTCATCAAATTTCTTATTCAAGTTAATTTCTTCAAAATTAAGTTCTTCGAGCTCGTTA
AGAATTTTCCTAAACGTTTGCTGAAGCTCATCAATCAGCTTCAACCTCACAGTAATAACGGACTCATCTA
ACTAAAATATACCAGAGCTGATAGGGTGGCAGCTCGATTTTAAATTGTAGCCACCTTGCTGTTTAAAAAT

GTCACCCTGTTTGCATCAGCAGTAACGTTTCCAGAATTTCCAGCAGCCATTGCAACAACAACTAAACGGC
AGAAACAGCGACTCGAATTCAGTTCAAGCGGGAGGATGAGCGCAAAGAAGAATATATGTATATGCAATCT
TGCGTAACCAACGGAGTTTCTTTTTTCTTTTCACTGCCTTTATTTTGTCTGAATATTGCAGATACAAACT
GTTGTTGAAAAGCACACGCGAATCAACAATAGCGAATTAATTTAAGTTTAGGACTTAAATAGCTTTGTAT
GAATTGCACTATATATTGTTTATGCATAAACCAGCTGACGTCGGGGTCACCATATGTTCAGCTAATAATT
TTTTTATAAGCTGTTATGGAGCACAAGGTTTATTTAGCGATTTCGAACGATATGTGTGATAATGATATGC
ACAAGATAATAAAAGCGAATGCTGGTTCTGCGTGAGCCGACAGATAACAAATATTAAATTTATAAAACTT
AATTTTTTTATATATTTCTAAAATAGAAATAATGTTGTCCCAATGTTATTGACCTTTACTTTATATTACA
TGTATTCTAGTGAACCGCTACTTAAATGCTTTCAACCGGAAAAATTTTTTATAAGGACCTTCATCATTTA
TATGACAACCGCTGAGAGCAGTTTCCTGCAGTTCAAGTATCAACCTTAGAATGTTTGGTCTGCAAAAAAA
GCACATCATAATTAGAAATATTATAACTAAATAAAATCTCTTTAGAACACTTTTCAAATTTTTTAACTGT
TGGGCTAATAAGCTTTTTTATTAGCTATTTGTAGAGCTATTTATCGCAACCGCCAGCAATTCCTAAGTTA
ATACAATTTATTTGTGTAGCGATTTCAAATGATTATGTTACATAATCGCGCAGAACGCGAGGCCAAAAAT
GTAGGTGCGAGAGTATAAGAGAGAGAGCGCGAAAGATGGAAGCTTAGATAACGAAAATATTTCTGCACAG
CTGAGGTGCCGTTAAGTTTAATTGTCTCCAAAGATGGCATGTTTTTTAAATAAGAAATTTGCTATGGCAT
CCGAAAATAACTCATACCCTAATTGAATTATATTATAGTTTCGTCAAGACTTTTTAAGCTAGCCACAATA
ATAATATTTATAATTAATTTATGTCTCAAGTAAAGAAACAATAAAATAAAAATTGCAACATATATAAACA
CATTTATTACTTTTATGTAAGAATTATTTTAAATTTATTTTCAATACCAAAGAAAGTAGCGCGTTGTGTG
TTTGTGTATTTATATTCTTTAAGGCTTATTATGTAAACACTTAAGCCAGaaacaagacagaatgatgcaa
tcgagttccccgtctattatataccctttactcagctaatggaaatgcgaaatgcatcatttttctggaa
tatccataggtCCGTTTCGGACGTTTTCTAGGTGTAATTAGGGGtcttgttaatttgacgatagtcaact
agaaatcgccatatcttttccgccgaattaggcagtgatttctcaggtaccaggagaagtgggctgttat
CTATATCTTTTCTATTATAtttgtcatttcatctgtaattttgcctaaaaagttattagctgatatggtt
tctgtttcgagactaaaattgcacagttttgttaaatcgtttttaaataccgacggaattttaagaacta
ctaatacttttgaagtgctttgtatatttgcgctgttaacaatagcgtctttaacggatgtatttataaa
gagagtcaacacgttctaagtgattacaagtagacttgctattataaagccaggaatgttatggagataa
gaaaatgtgtgtcagtagacattagttcaatctttctaattacttctgatctagctggcaaagctatttg
ctattttattgcctagggagtgtattattggaatatttatatacgcgacggtttcgggcctcgaagtaaa
cgaatAAATATTAAATAATAAAAAACTATTTTTTTAAATAAATTAATAGTAACACTTTTAATTGGAGCCC
AACGTGGGGTGGTGTACttaaaaagttataaaaaaatattaacgataataagttaaatattaaatGGAAC
TTACGACGCCAACAACTCACATTTTTAGTGGATAAACTAAAACATCCACATTAATTCTACGACATCTAAC
TACTGTGGCAGTGATCGTCGAAAAACTTAATTGGAAATTAATTAATGCAATAATCCATAAAAATTTAATA
TTTATAAAGATGGACTGAATACTTAAAACAAAGTACAAGGCACTCTTAATAACAAAAATCAGACTCAACA
AGAAACAAAAATAAAACCAAACACAAATCTTTAACCACAAATAATGAAAAAAATCAACACTTAGAAGGAA
GGAAGTGCTCAGGGCAATCCCTCGCTACAACTGAATAGaaaaaaaaGAGGAAAGACCCAAATAGATAATA
ATCAGGACTATTAAACTGAAGGAAGGCCCCAACAGAAAGTAATCAGCAAAACTAAACGAAAGGAAGACTC
CAACGAAAAGTATCCTGAGCAGAAAATAACATTGAGTCTTaaaaaaaaCAATAATTTCATAAAATAAGAG
CAGCTATAGGGAATGCTATAAAGATTAAAATTAAACATTACAAAATCAATTAAAGAAAACTAGAAGCTGG
AAAAGTTAATAAATGGTTTTGAATGGTTTTGGTTCAGCACCAACCTTTTCTGCGGGCAGGATATCCACCG
CCACTGAGCTGAAAATGGAATTTATTTACCGTAAAGTGACCAGAAAAATTAAAAGGATAGAAGGGCAGTT
AAACGCTTGCAGAAATAAGGTGGAGGATTATAAAATTAAAACAATTGACCCAACTATAAAATGCGAAACT
ACCCTAGAAGTAGTTAAATCCTTACCAAAGTTCACAGGTGAAATAACATAATATGGAAAGTAATGGCAGA
AACTGCCATGAGCCTATTTAAAATTCAAAGCGAACAAtattttattattatttattattttattgcttaa
tattatataatataatatattatattatataaatattttatatttttatTGCTTTAACAATTCAACGTAA

TAAAATCATGGGAGTAGAAAATGATGCTCTAACTAACCATGGTACAATTTTAAACATTCAAACCATTTTT
TCTAGATTGAatttcatttataacgataaaagaacaatccatatcctcgaatcagaattaagcatcctta
gacagggacgcttaagcattacagaattctacaacgaagtaaacaagaaaatgtctactcataaataaaa
ttatggaaatcatgacatatgGAAAGAACAGTGAAATAACCAAAGAAACGAATAAAACAATTAGGAGCAA
CGCTCTTAGAATTTTTATTTCTGGATTGAATGGCTTAATCTCAGAAACACTCTTTTCCCTAAATTCCACC
AGACTTGCCAAATGCTCTGGCAAAGTGTCACGAACTAGAGTCTAAAAACTTTCAAGCCCAAATTTCCAAA
TAGGTTTTATGGATTTAGGAATGAAAATAAAAATCAAGGAAAAAACGATATATTTAATCCAGTAGATTCC
AGACAGAATAATAATACATCAAATAGAAACAATAATAATTGGAACAATAATCAGAATAATAATTGGCCGC
AAAATAATAATTGGAATTTAAATAAGAATTCTAAGGTACAAACCGCGCCAGAACCATGCAGGTAGATAGT
CCCACAAGGTAACTCCTATCGTCGGAGtaataattacaaacaaaggtataataataacagacagaatcat
aataattTTTCGACAAGACATAATTTTagatttgtcgcacatattcccttgaggttgcagctgagtctgt
tattttatttgggatttgtattcgaatctaagctttcttcctttaacaatctaaaaaggtaatgttatta
tcaatagccgtgcatgattatttatagtgtattgttgcacctctctattttaccttatacatttgtacga
cttgtcgtaagtcggtttaatatcgttaaattcttccatttataatattaatataagttaatctgtgcaa
gagaagtttaaaatCATAGAATtatatatattcttgatcaggatcgatttgccaaaaaaaatttggccgcg
cccactctaacgccctaaagccggtcaatatcccccaatatctgtcgatatcccggaaaaatgataaaat
ttcgcgtAATTGTTATTTAATAATTAGAATTTAAATAGCAATTCTAACGTACAACCCCGACCAGAACCAA
TGCACGTAGATAGTCCCATAAGGTATGAAAATCGTCCGAATAATAACTACTATCGTCAGAGTAATAATTA
TAAACGAAGGTataataataacggacataatcataataatttttaataatttttattaaAAAAAGGCAAC
TTCATTTAACCAGAAAAGACAGaaaaacaaaaaaaaaaaagcaaaacaaaaaaTATTAATTGAGACAGGAG
CCACATCCAGCTATATCAAAAATCGATTTTATGAAAATAAAAATGAATTGCCAATTTTCattactattaa
gtgggtttgtatgtgtactgcgacccagtcggatctattgtactaccccttcatgactcaaggatccccc
tgaagtccaatgtactgtataaatgccagaattttattgggggccagggctgcaattgtttcccgtggga
ctacatacatgcccaggagtctcttcctcctgatggaaagcgccatgcatatcatatcatcacatataat
atgtgcagagctctcctcctccatgcagcagaagcgacagagttcactgtctgcaatgccaatcttgtga
aaatggctgtgagacggcaatgccccgtaagaaggtcagtcaaaatgcgcacggtgtttttctcgtgtgt
cataaaggtcttgaaccttttatggttatattcacggagaagaatcttagactCGGTAGTGGAGTGGGTA
GAGCTCCCCAGTTAGTAGAGCTCTACCGCCAGCCAAGGTTAAAGCCCAGGTATTCTGAAGCCAAAAATTA
CTGACGAATTTAGTTTTAATCTAATCAAAAACAGACATAGTATTATGAAGAATTTAGTTAAGCACAAACA
CACATAAATACAAAATATAACAAAAATATACATTAATCAAAACTGGCCAATCGCATTCACGTCTGAAAGT
TCAGCCAAATGAACCATATAATGAAACAGATATTGTCCCAACATGAAGTTGAACCCACGTGATAAATCTA
TGTTGATTGCCGATCTTAATTCAAAGTCACGTCATTTTAAAAAACGGGAATCGAAAAGTTCTGAGTCGGA
CGTGTACCATATATTTTGATTAAAACCGCATTcggtatgtttgttatacccgttactcgtagagtaaaag
ggtatactagagtcgttgaaaagtatgtaacaggcagaaggaagtgtggccatatCCGTTTTTCCGACGg
tccgcctgtctgtatgaacgtcgagatctcaggaaatataaaaatagaaggttattcaggctcgacgacc
tgatcgctcacgtcagtaaagtcaagggtttcattattcatttgttgtaccagccaccgtttggccaatt
aagactgtTTTATTAACAGATCCgcagagatccgcagtaaccggtgaggctaaccagaccgggcgaacgc
cacttgcccggactctccgttagcgactggcctagttgtttcccgggtccccagcctggcagactctcgg
cactggtcgaatgacaccttagtccggcccggagatgtggaaatttttgagagttaacagttccagtcca
actatgattagccagcacccttagcagaggcatgacctaactaagagcctgtttccagccgccctcgcgt
gtagagtgtagttgcatttccagtaatgtgtcaccataacggcgcgtaccaccgttggtggggcgttgat
gcccttgtgatacctactggtatgCCAACACACAAGAGAGTATTTACAGTTCTTGGATACTCAATTATAA
AATATTACCATATATTAACAATATTTGAAACAAAACATCAATTTTATGAAGTAAAAGGTTTAGAATCCGG
AGCTAAACTAGATGCAGGGTATCATGAGCGAAGAAAGTTTGTATAGCTTGGAATCAAAGAATACTGAGCA

CGTCGACGTTGAATTATCCGTCAACAAAAAAAGTAATCCACAAATCCTttttttaaacatctcattctat
ttattttggttccaaaaggaatcttttagtcaattcctctgaactaatgtgtgtataaaggtatatgaga
ccaagtggtttcttaattatcaagtacagaagtgtagtaggctacaggtacacctacataagttcagttc
ataatagcaacatagtatataaacattaagttattgttcgttttcgggtgcattctctctgtttcgcaac
atcggctctgtagcaacaagcatagaggcagatcgcaaacggagagggagtcagtcgaatgctaaagctg
aacacgtgaagtatcaaataaaccaaagaggccaactcctgcgtttgtgttttatttagataagaggata
tatagtacccctacaTtatcagaagtgggccgatcacgagccaaaaaagaaaaaaaaaatgaatataaac
aatgcatcgacggcgcaattaaaaggatggttgagtgaagtaaaagaaccaaccacgggaacaaaaagag
aattgattttgcgtttgaataatttaacaagcgaaaagcgaaatcaattgaaattattatcaaaaagcga
ggaagaagatttggacggaagcagcagtaataatgttcaaaaaggaaaaacaaataacggaaaagacgga
gagcataaagacgacgaaagtgacagcgaagctggagatagcaacgaagacggcgatgagaagagcactg
tgcgcaacgacaaaagtaacggcgaacatgacgacaaaagcggggtacgcagttacaagaacaacagtgc
agacggcgcccgcaacaacaacagcggcgacgggaatagcgacgaaaacaaagtttgcgacagcgaagac
ggcggcggtgagaaaagcaacaaagaatgtggcaagaataaacgaaatacaatgggacaagctgtgcaaa
atatggatttattattgcgtatggcaaccgaggcagtaaatgaattttcgggagatacatgtgcgcgcaa
atggatcttacaggcgaaaaacatagccagcgtttatggaataaaggaaccgtatataaagatgttgatt
gtcagtaagataaaaggaaaagcatgcatgtggttgcatgcagatccagaacgtgtattgctaccaacgg
agcagttggcggctgagctaatatcaatgtttggtgagaggaagtcgaagttagaaacaaggcgaaaatt
tgaggaacgaaagtggacagcgagcgaaagctttgttgcttatgcagacgataaggtgatgcttgcacat
ggaataaacatggataaggaagaattggtggcgctcctcattgaaggtattccgaatcaaatgctacgta
accaagcccgtatccaatgctttgaagatatccagcacataaagaaggcatttgcggaagtgaaactacc
gaaaatggatgaagcagacaagaaggtggcatcggtgaacaataatagcagcacactcttgcgttgtttc
aattgtaattcgaagggacactgggccaaagagtgcaggaagccaaagcgcgaaaaagggtcatgctatg
cctgtggtgagatggggcactctgcagcaaggtgcctgaaaaacaagaatggggatgaaaacaattacgt
aagatattttgaaattaattttatgaacaatgctaaatcaaaatttattacagcatgcctcatagacacg
ggaagccccatttcgttcataaaaaatagtaaagtacctaaagatgttattggagtaccagttttaagtt
cattttacggattaaacaaaagtcccttgaaaacatatggaaaaatattgttttacattataaaaaattt
gaattaaatacactttaatttaataatggtggcaaacgagtctatgaatcatgatgtagtattcatgatg
tacttgaatctggacaccttgaaaatgattacagtggataaaattgaaagtcgatgtaacaagaaaagcg
ataaaatgttaaaggaagaaaatatgtacaacaataataaaacagttagcgatagtgttagtcaaggtag
tagtaaaagtcaaattagtgctgtcaatgctgaaataagcacagcggagagggaaatgtttgaaattaat
gtaatagatgactcaattgattacaaaaaaggtgatcaggtggataataatatgaaatgtcaatttattg
agttggtagaaaactattatgttaataaagaaagaccgaatgaaccggagattcggtgtgaaatacaatt
aagattcaatgacctgaaaccgtttagttgttctcctagaagattagcatgtactgaaaaagaaaagctg
cagattatcttagatgaatatctaaaaaacggaattattaaaccgaggtattcagagtatgcatctccga
tggtgttagtgaaaaagaaaacaggagacctaaggttatgtgttgattacaggaaacttaacaaaacaat
ggtcaaagataatcctttaccattgattgatgatctgttggatagattagttaataaaattctattattc
tcgaagttagatcttaagcatggctactttttaagtttttgttaacaaggaatcaatgaaatatacattt
tttaagacgtgccatcctttttaaggatgccaatggggctgaaaaatgcaccggcggtctttcagagatt
tattaatagaattttcgaggatatgataaggcaggataaagttattatatatatggatgacattatgata
gctagcaaaggaattaaggaacaaatggaagtacttagggaagtttttgacaggttgacgaggaacaaat
tagagctgagaatggataaatgtgaatttctgcaatcgagtgtacaatatttaggatttttaataacaag
taacggaatacaggccaattacaatggaatagaagcaattaaagattttcaagtaccagataaggagcat
ggtgtcagaagcttcttaggattgtgttcatattttagaagatttattaagggtttttcaacgattgcaa

aacctttatatgacttattaaagaaagacaaagaattttcgtttgaagaaaaagagttagaatgttttga
gacacttaagaaaaagttagtggaagctccagtgttagggttatattgttataaggatgaaatagagtta
catacagatgcaagtgcgcaaggttttggtgcagtgttattgcaaaagaaagaggatatgaaatggcacc
ctatattttattactcgaaagccacaacaaaggatgaggccaaatatcatagttttgaacttgaaacatt
ggccatcctgtatgcattaagaagatttagaatctatgtacagggaaagagatttaaaattgttacggat
tgcaacgcattaagtctagcgttaaataaagtagaattcaaccctagatttgcgaggtgggcattagaac
tattagagtacgatttcgaggtagttcataaagcaggaaagcatatgcaacatgtggatgcattgagcag
aaatacgaacatattagtgatagaaactaatacttttgaagataatttaattatttgtcaagcaagagat
gaaaagttgaaagatattaggaaaaagttagagaaaacggaagataaaatttttgaaatgagaaatggtg
tactttataggaaggcaaacggtggaagattgttgttttgtgtaccagaagaaatggaaaaaaaatttt
atataaatatcacaatgaattaggtcacttagggagagataaggttatagatgctattgttaagacttat
tggttttcgaatatgaaagaaaaagttgttaggcatatagaaaattgtttgagatgtgtggcactttcgc
cgaagtcgggaaaagaagaaggaatgctgcatagcatcccaaaggtaaatgtgccgtttgagataataca
catcgatcattacgaaccggtagataacggtagaataaagaaacatctgtttgtaataatatatagataa
cggtagaataaagaaacatctgtttgtaatcatatagcaggtcgaaatttatagtatcagatagggagac
ctgctttacatccgaagattttgcaaaatttatgggagagtataatattaaacacataaagatagcgaca
ggttcaccccaggccaatggacaggtagaaagagtcaatagaatagtagggcctatgatagctaaattAA
CAGACATtgtgttttatttagataagaggatatatagtacccctacagaagggaaaatttattaagttat
taattacttaatgtgtaatatattttactatcctccgggtagggagatcgctagggtgtaccctcttcag
ccttcgatgggagatgggattaactaggatatttgccagtcggttcgggtaactttcaggtctctttcga
ttaccttgttcgtcacgtaccaggggagccacaaatCCTGAACACGCTGTCGTTAAATTTTCCGACaaag
aaaaaatgtataatctaataataatttcaaatcaaaaaaaaaTATATAAATATGCAACTTCCTTTTCGAA
CTTCCCTCCTTCCTTTGGAACTCTTAAACATCGACTAGTAatatggatatcattcataataatcttttca
aatacaactgaacaacagtataccaatctaattcaaataattcatattttacaacaagccaatatgaaaa
tctaaattctttaaataagatacagcatttcttggttatattgtttctcacaaagtaTGCGACTCCACAA
AACCTTAGAGAGCTTCGAAGTTTTTTAAGCCTCAAtggatatttccgaaaatttgtccgaaattacgcaa
aaattgcaaaacctttagccaaatatttaggaggagagaatggtaaaatttctaggagaatgtctacaaa
aataagatgatgatccagctgtaaaagcccttaacgaactcaaaaagataatttaaaagcacaagttgaa
ttagtgcaaccagattataataaaattttactttgacaacagacgcatcagatgtagccatcggagcggt
tttatcacaggataataaagcaattactttcatatctaaaaccttaagtaaattcgaacaactttatgtt
agccatagtttgggctcttaaaaaTTATTTATATGGACTAATCGGTATGAGATACAAACAGACCATCAAT
CTATATCTTTTACAAAAACTTTTTCTTTTATAAAAACCCGAACGTAGAAATGAAAAGATGGTATTCTTTC
ATAGAAAGTTTCACCCCAAAAATAATATAAAAATCCAGAACAAGAAATGTCGTAGCTGACGCGTTATCTC
CAATAAAAATAATTAATATTACAAACAACGATATAGAACAATCAAGCTCAGATCAAAATACACAGCATTT
TGCTGAAAGTAGTTTTGTAAGATGTAATACAAGAGACCCGTAAACCGTTAAATGAGTTTAAGCAACAACA
GTTTTTAACAACGGGGAAGTATACAATACATGATTCATGGAGAGTTTTTGATAAGACACGACATATAATC
GAATTTAAAAAATAATTTTCATACAAAATTTATAAACCACAAAAACACATAATAAACCACTTAATAGAAA
ACAATAACCAACAATTTATTATCAATTCCAAATTGTTTAACGAGATAAAATCACTTAAAAACATTTTTAT
TAATCAAGATTTGCCGTAAAGAAAACATCGTTTACGATTGATAACATTCAACTTGCAAAACATAATTAAT
ACTACTACACTGGCAAAAATTGATGTATTTAACACAAAAATATTAACTAATTAAGACATTAAAGAAATAT
TAAAACACCAACAAACGCTTGTAATTATTGGAGATCTAATGGATATCTCCGTATTCAAAATTTTACTACA
TAACGAACTCCTTTTATTATTTATATCAAATACCCTGCGATAACAAACAGATGCGAAATTTATTATGCCA
AATCTATTTCACATAATGATGGAAAATTACTAATAAGCAATCAGGTCGCAAAATGTGAAAATACTTTTTA
TGAAATTCGTTCTTGAAAAAGAACGAACTTCTTAATAACAATGGTATTTTAAGTAAAGATAAAACTTGTT

TTACCCGCTTACTAAATGGAGAGCAAATACGAGAAAAAAATAAGATAGATATCTTTCAAGAAGGTGCTAT
TCTAATAAATAGAAATAATAATTCAAACAATTCTCATTTAGACGGGTCATATTTAATAACATTCAACGGG
ACAACTACAATAAATAATATTTCATAGTATATAACCACTAACAGAATATTTAAAATCCAACAACTCAGAA
CATTAATATTTTAACCCCATTTATTGAAATTTACAATTGGAAAATATCAATTTCATTTATATTAAAATTT
TTAATTATTTTGTATTTGATTACTACATTAATAATAAAATTCAGAAAGATTATATTTGTAAACAAACAAA
AACGGCCAGAACTAGAAAAACCATATACTGAGAAAGAATTTTTAACAGAATTAAGAGAGCGCTTAAGCGA
AATTCATTATGAAGCATGGCGCGTCATTTTAGAAGGGAGGAGAGTTATCCAACTCCATAAATTTGAAACT
CCAATCTCAGCTGCATTGCCTTTGCCCTTGTTTTTGTCGCCGATAAATAACCAAACTTAAAGTGAACATA
ACTTCCACTTGGAGACTTATGATCAACTTATTGCGGTTCAATCAAACTGCGACAGTCAGCTAATTTGAAT
TTCAGAATGCTTTGAAATATTGTAATTTTAGTGCAAAGCTTTTATCCTAAACATATTTGAAAGCAGaaaa
aaaaCCCTAAAGTAAAGCTTCAGGCCGAGGTGGAGTGGGTTAGGATTAAGATTTAGAAGATCAGTCTGAA
AAGGGACGAGATCGTGAACAGCTTTAAATTAAAGATAAGCTagtaataaattgtactgtaatagtataag
tgaataaaccgaataaatattaaataataaaaaataaaaactatttttttaaataattaaaTGGTAACAAT
TTTAGTTTGCAGGGGTCGATGGAAGGGAGAGGCACGATCGCCTCTGCCGGCGTACTTGATCCAGAGCAGT
TTCCCCTCTCCAACTGGTAGACAGGATGCATGGCGCAGGTGAAATGAGATACGTCAACCAAAAGATATAA
GCCAGAGTATGTCAAAATGCTGGGAGGGAGGGAGGATAAGATACAGAAGACACAgagtcgaactaatgtc
ccatcagttgactaaggacaaatctaaggaataaaaaattattgaagaatagtgttcacgtatttcatct
tttgatcttagcttaagactaaatattaGTGACAAAAATTGTTTCTATACTGCCCTCTTTCTGGAGCGAG
AGATAgtaagtattgtatttggttcaacgtattttcctttgagggagtcggcatgaacttacaaattaaa
ttcttacgcttaattatatttttaatgagtattattattattatttttttttttttgtattgattata
cattttattctcatcttcaaaatatgtttgttgatctgaatagtctaactgatacccattatggccagga
tacggaattatattaatggtgttgatggtttttttggttaatagagatatgtgaaatgagtaataattcga
tatttttgtggtcaatccaagttgatactttgattaaaagtatttaaatctttcagtagtttattgtata
agtatttcattctgtgattttgttaccaaaagaattaatgatagaattgatttccgtaaaatttattctt
agacaatattgtttatataccactggtattttaattggattgctcgaaaagagtatgtagtcaaggtcgg
aatcgatttttgttatttgaatttgtcgagtgtggcgcgtggttaataatagaagcaacgtcgttatact
caagctactcgtgaaagtttaatggtattattatcttttgtttctttaattgtgttttgtagtatttggt
tagtgagtttgtaatggtcttcgtttatgttttctacagttttataacttataaatggattttgtaatgg
atttttctgtatttgatgtcaatattaaattcctgtcggtcttcatttagcttttctacaatatattcct
ttctttactttttgagtatctcatattggatgcccagcgtataagaaaatttgagcaggtagtgtcactt
tacttaattttacttcttcatcatcagatgaattgattatacgaatttttttaatttattgttttgtgtaa
tctttcgacgtctgataccctgtttttttgctttattaaactgtaattcgatttttttcttcatcaagccat
cgcttcaaagctaaactggaaaaatttgggcatacctaactgaataaaaatttgcattagtgcgtttttg
cattctatccagttctttgttttaatttcaagtgtatctagaataactagaataaatgtcaatgcaactg
atgtaatgtttccctcagatgaataaatatctactacaaatttgtctcggcaatgttccgggttaggtgc
cattttaaaaggcattttggtgtttctatgttctgttttggccaaattgcatatgttgcattcgtttatt
atattctgaattagtagctggctatttggaataaatcaagtaaataaataaaatttaataaaataaataa
ttttgtcattttctgtataacagggtgtaacagtttctcatgtgattgaagtatgatttctttgaattcg
gcgtatgaacgaaccaacgttgttcaataggaaaggactgcgaattacttttgggtaagtggtattaata
atttctatgtgtgctctttgaataattttaagatctacatcgatctcaatataaatggtaatgtttctat
gagtaaaatgatcaagtaaaatttgtttgggcttttcaagtgttattatatcatattgaattgtggtaat
ggagttaccgaatattattgaatgctctactttatttttatgggatttaatgaagattaattgtttttta
aataattaattaaaataattttagttcttgataatgcatcggaaacagaattttccttccctttaataga
atcgattttaagttggtattcgaataatctaactcctcatctttctaacttagcattcggttcctttaat

ttatgaagctatctaagagcttgatgtcgaaaagtttttcttgcctaaactatgacgagtaattcttttt
cgatagctctgtaatgtaattcatgttcattgattgatctcctaataaaggatattggatttgagaacga
gaaatttattaatttaactggcatttgtggttaaatttatttatttttcgaaatcgggttataggttgt
cttattattaaacctatatgtttttttgaatgactctatgtacttaaggttttgtgcatctatcgttgca
cccatttttaaacatttggtcattggttttgctatgtctgcgcaatttgggatacatttacgatgataac
cgggtaatccaaggaaagctctgcattctttcacctttttttggaattgtatatgaatctatggctttatt
ttaagagggttcggttttataccatcatggatTTCGATACATTTTGTTGtttttttggtactacccaggta
ggattcattattccctgatttagcaattcctgtactgggtttactacttcaatttggtgtgtttccggaa
gggggtattgttttgaataaattggggagttatgtgttgcatttagtacctatttaatggtatttgtaaa
tgtcagtttatctcctcctttatattgaagatttttaaatttatttaataaacttctcaatttaaaatat
tcctccttatttagatgatgtaattgatattgtgaaaaatcaatttctttttgattcctgatataaatca
ggattgtaattggattggtgtttttttggatataaaaattctgggttctttccgattctgaggtaattaat
tatttattaatttctaactgattgcgcatttgtcaacaattttctaccaattacgatatgctaattatcg
taactgtgtacatgaaatgtttgatttatttttaaaatactattgctgggtaagataatcaagcttttcg
aagtagtaggcatttgatgttaaaacttcacatatactattttgatatgtattttccattgatcatatta
tttgttgatcctgtatctagtaggcatttgtatgtgcaccctttgtatactatttttatgtattcgtgtt
gtcggggccgttgaccgaaaattttcgtttagttcagaaccattttgatatatatatattttgatatata
atatttcgttattaatttgtacgggttggatcggttgttgcccttcatttatgaacccatgattttgata
tggattgtattgattttggtcataatactgttgttcgtaataattaggttggacttgttcgaattcatgt
gcgtcttggaatctcatttcgtcaatagacattttgaagttagaattcttagttgtggaggcttgttgta
ttgtagttgagggtatgtatttcttataaaatgtgctgtttgaggattttggatttgttggaaatgactt
ttgggaatacttggttgaatgctgatatatgggctggacttgaaataattgggtggtattgattaaaatt
aggatggaagggttgagaatattgtgggtaacttggtggatattgatacaaatacccaattgtattgaag
ttgtgtgattttgaattcagataaggatttgagttttattacggatttcgagaaatttgttcaatccaaa
attattatgttctttataaattccctcactctgtgcaatagtaaagtctgtaatatcacgatgagccaaa
atattgtagtaaataattgtattggtagttATAAGAgtcactcgtggagtcaaaaaggcataccagattc
gtagaaaagtatgtaacagtcgggagaaagcgtttccgactgtatagagcttacatatgtataaatatat
ttgatagggatcaatagccggagtcgatctgtccgtgtcaacgtcgagatctcaggaaatataagaacta
gacggttgagattaagcatacagattctagagacatagacgcagcgcaagtttgttgacgcatattgcca
cgcccactctaacggccacaagccgcccaaaactgtcagttattagtcgatttgacaaaaactttttgcc
acgcccactgtaacgccaagaagccgccgaagtggtcacgcccacacatttgaacaatttttaattactt
ctcattttattccccaatatctatcgatatcccacaaaaatgataaaaatttcgcgtttgcattcatact
agctgagtaaagggtatctgatagttggggaaaacgactatagcattctctcttgttGTATATCTGTAAG
TTCATTGGATGAATTGAATTTCCTCCCAAtttttcctttttttatttttttttttGTTTGTTGAACAAGGT
GCTTGTTAAAGAAACTACAATTGAGCCAAGACaattttataaaactacaaaaagaaaaattatttaatca
aaataataaaaatttaattaaCCCCAAACTTTTCAATCACCTTCCAGTGCCTATGCCCGCCACCTTCCAG
CCTCCACACAAGTCATCAATGTTTTCTACACACGTAGCCTCCTCAGTTGTTTAAGGGATCACAAAGTCTA
AGCTTCGCATGTGCCGTGTTGTTTTAAGTGAATTTTGAGTTTTATTGTCGTAAATTATATAATTAGTTCA
TATACAATCAT
>dme_circ_0001070_junction_seq
ATTATATAATTAGTTCATATACAATCATGATTTGCTCTTCCTCCAATCCCTCCAAC
>dme_circ_0001071
GAGTCAGTgaaaacaacaacacacaaaaacacaagacacaagtacacagagaaaaacgcaaacaCCCGCA
CTCGCGCAAACAGacacacacacacacTGTGAAACTCCCAACGTCGAGTCAGCAAATT

>dme_circ_0001071_junction_seq
GAAACTCCCAACGTCGAGTCAGCAAATTGAGTCAGTgaaaacaacaacacacaaaa
>dme_circ_0001072
ATAACTTGGTTGCGGCAGAACGACAAACTGGAGAGCGTGTTCCGACACTTCCTCGTGGAGGACCGCGGTA
CAGACGGTTCCGCCAGCTACGTGGATTTCCTGTGTCACATGCACAAGGAGATTAAGGATCTGCTGAGTTA
GCACGCCGGCTACTCCGGCACATACAAGCCCGAGCAGCGATGGGCGGACAG
>dme_circ_0001072_junction_seq
ACAAGCCCGAGCAGCGATGGGCGGACAGATAACTTGGTTGCGGCAGAACGACAAAC
>dme_circ_0001073
ATCTGCCGACTCCCGTTCGCTGCACGCACGAAAAGACGCAAGAGGATGGCGCCTACATCCTGGAGAATGG
AGTCCACCTCTTCGTCTGGCTGGGCCAGGCATTGTCGCCTGATTTTGTGCAGTCCGTG
>dme_circ_0001073_junction_seq
ATTGTCGCCTGATTTTGTGCAGTCCGTGATCTGCCGACTCCCGTTCGCTGCACGCA
>dme_circ_0001074
GTCTCTTGCCATCCACGTCCGCCTGGAAGTAGGTGTACTTGAACACCTCTCCACCAGTCAGCCGAGAAAC
CTGCCCAATCGTGGCCAAGTCGATGTAGGCGTTGTTAAACACGAATAAGTCCACGGAGCAACCCTGCTGG
ACGCACTCCTGGCCCAGTGTGTTATATGCA
>dme_circ_0001074_junction_seq
GCACTCCTGGCCCAGTGTGTTATATGCAGTCTCTTGCCATCCACGTCCGCCTGGAA
>dme_circ_0001075
GCTTCCGATCATCGCGATTCTTTAGCTTGCCAGGCGCCTCCGCGATGGGTAGCGTTGAGTTAAACACCAG
CAGTTTTCCAGCTGCATTCGATGCCTTCAGTGCCTCCAAACCCGCTTGAATGGCAGGGTACAGAATCGTT
TCGGTTTCCTTGGTGTCGGCAAACATACGAGGGATCTCTTCCATCAGAGCATCGATTACAGCAGCCGACT
CTTCTGGGTGGCACAGGAATCCATCCAGCAGGGGCATGAACATCTCCTGGACATCGCCCACAACCATCAT
TTGCGGCTGGGCCAGACTGCTCTTGATATTGTAGAAATGCACGGTGCTGTTGTACGTGATGAAGCCCACC
CGCACCTTGGATTTGTCTTGACCCTGGTCGACAGGCAAATGCTTGAGTAT
>dme_circ_0001075_junction_seq
CCTGGTCGACAGGCAAATGCTTGAGTATGCTTCCGATCATCGCGATTCTTTAGCTT
>dme_circ_0001076
ATGAGCTTGAACAGCGCCACTTTGGCGGGATTGCCGCACATGCCGCCACCGAAAGCGGCAACGCCGGGAG
CAGCACCTGGTCAGCCTCCGATTCCAGCAGCTGGATCTACTTCTCAGCCGCCTCTACCCGGACAACCGCC
ACTTCCCGGACAGCCACCCTTTTCCGGTCAGATTCCCACATCCCAGCCTGCCCCAAGTCCGTATGGAGTA
CCGAGCTCACGACCGGGTCAACCCCAGCTACCTCCTGGAGCCACTCCTCCAACTTACACGCAACCCGGAT
TGCCACCTcagcagcagcagggaatcccgccattgcagcagccagggataccacagcagcagccgggatt
tcccccacagcagcctggactgccaccacTATCCCAACCCGGGTTGCCTCCGCAACCAGGAGCGCCTTAC
GGAGCTCCCCAACAAGGCGGATATTCAGGTGGCTTTCCAGGTCAGGCTCCCGGAGGCTTCCCTGGAGCAC
CTCCACCGCTTCCAGGTCAGCAAGCAGCTGCTCCGCCGCAATTCGGAGCTCCACAACCTGGTTATCCGGG
CCAGCAACCGGGCTATCCCCCACAACCCGGTCAGCAGCCGATGCCAGGATATCCGCCACAACCGGGACAG
CAACTAGGAGGACCAGGATATCCACCTCAGCCAGGTGCCGGTTTTCCAGGTCAGCCAGGACGTCCTGGCT
TTAATGCAACCTCCCATGCCAGGAGCAGGAAATATGTATCAACAGGCTCCGCAGGCACGACGCCTTGATC
CCGATCAAATGCCCAATCCCATCCAGGTGATGATTGAGAACCAGCGATTGTCTGGAGGACCGTTTGTGAC
CAACCAGCCAGGATTGCTGCCTCCATTGGTGACCACCAAATTCGTGGTTCACGACCAAGGCAACTCTTCA
CCCCGCTTCCTGCGCTCCTCGCTCTATTGCATCCCCAACACGGGTGATCTTTTGAAGACCACAGCTCTAC
CCCTGACATTGAACATCTCGCCGCTGGCCAAAGTTGGCGAGGGGGAAATGGAGCCACCGATTGTGAACTT

CGGCGAAATGGGACCAATTCGTTGCAACAGGTGTAAGGCGTACATGTCGCCCAACATGCAGTTCGTGGAT
GCCGGCCGCCGATTCCAGTGCCTGATGTGCAAAGTAACTTCGGAAGGGTAAGTTATACATATATTGGAAA
TACTACTAATAATATTTCTAGACACCAAAATGTGGTGCTTTTAAAAATAGCTATTTTTGAAAATGGAACA
GCTTTTCTGAACATATTCCAATAAATATGTTGAACATATGGCATGTTTGTTAATAGATCATGTATTTCCA
CAGAATGAAAATTTGTCTCGGTTGTTTTTAATGATAATCATTTTTAGAGTAGATACACACTAAACTGATT
TTTAATTATTTCCCTTTGCCGCAGAAACAATACTCCCCCGGAGGTTCCCGCCTTTATCTTCATCATCGAC
GTCTCCTACAACACCGTGAAGTCGGGACTGGTTCATCTGCTCTGCTCCCAGATCAAGAACATACTCAAGC
ATTTGCCTGTCGACCAGGGTCAAGACAAATCCAAGGTGCGGGTGGGCTTCATCACGTACAACAGCACCGT
GCATTTCTACAATATCAAGAGCAGTCTGGCCCAGCCGCAAATGATGGTTGTGGGCGATGTCCAGGAGATG
TTCATGCCCCTGCTGGATGGATTCCTGTGCCACCCAGAAGAGTCGGCTGCTGTAATCGATGCTCTGATGG
AAGAGATCCCTCGTATGTTTGCCGACACCAAGGAAACCGAAACGATTCTGTACCCTGCCATTCAAGCGGG
TTTGGAGGCACTGAAGGCATCGAATGCAGCTGGAAAACTGCTGG
>dme_circ_0001076_junction_seq
GCATCGAATGCAGCTGGAAAACTGCTGGATGAGCTTGAACAGCGCCACTTTGGCGG
>dme_circ_0001077
CTCTACCCCTGACATTGAACATCTCGCCGCTGGCCAAAGTTGGCGAGGGGGAAATGGAGCCACCGATTGT
GAACTTCGGCGAAATGGGACCAATTCGTTGCAACAGGTGTAAGGCGTACATGTCGCCCAACATGCAG
>dme_circ_0001077_junction_seq
TAAGGCGTACATGTCGCCCAACATGCAGCTCTACCCCTGACATTGAACATCTCGCC
>dme_circ_0001078
ATGAGCTTGAACAGCGCCACTTTGGCGGGATTGCCGCACATGCCGCCACCGAAAGCGGCAACGCCGGGAG
CAGCACCTGGTCAGCCTCCGATTCCAGCAGCTGGATCTACTTCTCAGCCGCCTCTACCCGGACAACCGCC
ACTTCCCGGACAGCCACCCTTTTCCGG
>dme_circ_0001078_junction_seq
CACTTCCCGGACAGCCACCCTTTTCCGGATGAGCTTGAACAGCGCCACTTTGGCGG
>dme_circ_0001079
GATGCTGGCCGTTTGATAGAAACGGAAAAGTCGAAAACATATATGTAATGGAACATTCCGATCCCGTGCC
TTATCAACAGCTATCGACATTATCGCCGACCACCGCACTATACTCGAACTTTAACAACTCTTTGGAAGCA
GGAACCGCAGAAAGACTACGCCTTAATAAAACTGGGTCACAGGTCCACAGCCTATATGTCGGCGACGGTC
AAGTGCAAAATGCTTCTGAACCCTTATCTTTTCCCAGCCACCTTAATCTGTACCAATATGTTCGTACCCC
GGCAAACTTCACTATTAACAGCCGCAACCGATATATTCCGTACTATAACAATCGTTCCTACTCTGCTTCA
ACAACTGGTTTCCCTATAGCCAGTACTCCGCTGATCAGCAGCCATGCGCACATCCAAGACCAACAAGGTG
CAGCGTCTTATCAAACGATTGTTCCGCTTTATCAGACCGCAAGCCACTGTGCGCAGCAACATACAACATT
GGCATCGGTAGCTCCAATCGTGGACAATTCATTCGTTGAAAGCACCACTTGCCCCATGGCCGGCATCACC
TCTCACAACATCAAAATGAAAATGATTCCTCCCAATGTATTGTTGCCTTGCTCCAGGCCTACGACAACCG
CGGTGACCATCCAAAGGTCAACCGGAGAAGACGGTGGAGATAAAGAAGGTCATACTTTTGCTAAGATTAC
GGAGATGAGCGATGCTGTTACTTTGCAGATCACCAACTTGGACTACTCGCTGGACGAATCACATATACGC
AGCTTTCTACTTAATCAGCTTAAGCCCATTACGCCAGTTGTGTCACTTGTCTTCGAGGGAAGCTCCTATG
CCAAAGTCACTGTTCCAGATCTTTATTGTAAGGGGCAATCCTAATTCGAATTAAATATTTTAAAGTAATA
CCGGCAAATAATCAAGTAGAATAGTATTGGTGGTAAAAATATAAAAATATTCGAATCGTAAGTGTCAGTT
TCTTATAGAGGAACTCTTGACTTTTTTCTGTTCTGTTTGCTTTTTTAAATAACGGGTACCATCTTAAAAA
ACAACAGGATAACTTTCATTTCGATCTTTATGTACCTAAAATCAGGTAACTAAAACGCTATCCAGACAGT
CCATAAAATTTAAACTTGTTTACCTCTTATTATTTTAGAGTTTCTATATTTTTACGGACAAAAACAAAAA
CTTTATTTTCTGAAGTATTTAGTTCTCTTTTTATTATTAAAGTTTATTAATATTGTTTAATGGTGTGGGA

TACGTATTTTTTTCTACAAATTTCAAACAGTCCGATTTTCTTTCTGTTTTCTCTTGTGTTAGGAACACAC
CTTAGGTACACTTAAAATAGTTTTAGTTTAAAAAAAgattattattaaataatataataatacaatatta
ttatactattatattattatattattatattattatattattatattattatattattatattattatat
tattatattattatattattatattattatattattatattattatattattatattattatattattat
attattatattattatattattatattattatattattatattattatattattatattattatattatt
atattattatattattatattattatattattatattattatattattatattattatattattatatta
ttatattatattatattattatattattatattccctattatattCCCATTAAGCACTTTTCGACTGATC
ATATTTTTTCGTCAGGTTCGCAAAGCAGGTCGTCTCCAATTTGCACCGCAAGAAGATTGGACACAAACGC
ATGCTGGTCAGTTATACTCGAGATTCCTCCCTCACCGAGGTCAACACACTCCGCTGCCAGGTCGCAGGAC
TTCTAAAG
>dme_circ_0001079_junction_seq
CCGCTGCCAGGTCGCAGGACTTCTAAAGGATGCTGGCCGTTTGATAGAAACGGAAA
>dme_circ_0001080
GATAAAGAAGGTCATACTTTTGCTAAGATTACGGAGATGAGCGATGCTGTTACTTTGCAGATCACCAACT
TGGACTACTCGCTGGACGAATCACATATACGCAGCTTTCTACTTAATCAGCTTAAGCCCATTACGCCAGT
TGTGTCACTTGTCTTCGAGGGAAGCTCCTATGCCAAAGTCACTGTTCCAGATCTTTATTGTAAGGGGCAA
TCCTAATTCGAATTAAATATTTTAAAGTAATACCGGCAAATAATCAAGTAGAATAGTATTGGTGGTAAAA
ATATAAAAATATTCGAATCGTAAGTGTCAGTTTCTTATAGAGGAACTCTTGACTTTTTTCTGTTCTGTTT
GCTTTTTTAAATAACGGGTACCATCTTAAAAAACAACAGGATAACTTTCATTTCGATCTTTATGTACCTA
AAATCAGGTAACTAAAACGCTATCCAGACAGTCCATAAAATTTAAACTTGTTTACCTCTTATTATTTTAG
AGTTTCTATATTTTTACGGACAAAAACAAAAACTTTATTTTCTGAAGTATTTAGTTCTCTTTTTATTATT
AAAGTTTATTAATATTGTTTAATGGTGTGGGATACGTATTTTTTTCTACAAATTTCAAACAGTCCGATTT
TCTTTCTGTTTTCTCTTGTGTTAGGAACACACCTTAGGTACACTTAAAATAGTTTTAGTTTAAAAAAAga
ttattattaaataatataataatacaatattattatactattatattattatattattatattattatat
tattatattattatattattatattattatattattatattattatattattatattattatattattat
attattatattattatattattatattattatattattatattattatattattatattattatattatt
atattattatattattatattattatattattatattattatattattatattattatattattatatta
ttatattattatattattatattattatattattatattatattatattattatattattatattcccta
ttatattCCCATTAAGCACTTTTCGACTGATCATATTTTTTCGTCAGGTTCGCAAAGCAGGTCGTCTCCA
ATTTGCACCGCAAGAAGATTGGACACAAACGCATGCTGGTCAGTTATACTCGAGATTCCTCCCTCACCGA
GGTCAACACACTCCGCTGCCAGGTCGCAGGACTTCTAAAGGGATGTCCCATTCAACACCCTCCCCATGTA
TAAGTTTAGAGAACTATTTCAGTCCCGCTTCAAAACCTCTATCAGTGTTTTAGATTTGTACAAAATGCAA
GACATATGTACCATTAACTCGGATAACAATGAAGAAAAGTTTATCAGCTTAAATCCGGAATTA
>dme_circ_0001080_junction_seq
AAAGTTTATCAGCTTAAATCCGGAATTAGATAAAGAAGGTCATACTTTTGCTAAGA
>dme_circ_0001081
TTTTTGAAATAGCTTCCACAATTTGTGCGATCACTTGCAGTCAGGCTACATGTATTAGAACTGGCATTAA
AAGTTCCTGATTGCATTTCTTTGTTTATTTCTAACCATCCGAGAATTTTAATTCCAAAATTGTTTGCCCG
AACTTGAATTCCCTGAACACAGCAGATCAAATGCTCCAAGTTAACGCCATTTTCGTTCGCCATTATTGAC
ACATTAAGCTCTTCTTTAACGCAGTGAAGAAGGGTAGCCACCGGAATATCCCCCGTGTGTACCTTGAGAA
GTGGATAAATTAGTTTCTGAATTTCCGAAATGCTCATGAAGACGTTTGGTAGTGGCTCGATTTCCTGCTC
GGCCCATCCCTTGTGCTGCTCCTTTTTAAAATGAATACTACAGTAAGGAACACTATGTTGGAGTCCTTCC
ATTAAGGGGGATATATCGAGGGTGTTTACTAATTCCGGATTTAAGCTGATAAACTTTTCTTCATTGTTAT
CCGAGTTAATGGTACATATGTCTTGCATTTTGTACAAATCTAAAACACTGATAGAGG

>dme_circ_0001081_junction_seq
TTGTACAAATCTAAAACACTGATAGAGGTTTTTGAAATAGCTTCCACAATTTGTGC
>dme_circ_0001082
GGGACCCTTCTGCATCAGTTTGGACAAACGTTAGAGGTGAAGTTAAGTTAACTAAATCTCAACAATTCGT
AAAACCAATTGAAAAAAGCATATTGGTTAGGGATTTCAATGCTAAGTATAAAGACCATCTTTCAGAGTCA
GCTCTTTTAGCCATGCGACAAGCGATTGAG
>dme_circ_0001082_junction_seq
TCTTTTAGCCATGCGACAAGCGATTGAGGGGACCCTTCTGCATCAGTTTGGACAAA
>dme_circ_0001083
TTTTCCTTGTTGCTGTAACTATTGGAAAAACTTCCACTTTCCAGAGATGATCCGAAACTATTAGGCAATG
ATAAGTTGGTTCTTGGCGGTAAACAATTCGCTGGCACATTAAATATGTCGGTATCAGCATGATATGTTTG
GAATGTTGAACAATTATTGATTGTTGCGCTTCG
>dme_circ_0001083_junction_seq
TTGAACAATTATTGATTGTTGCGCTTCGTTTTCCTTGTTGCTGTAACTATTGGAAA
>dme_circ_0001084
GCGAATTGTTTACCGCCAAGAACCAACTTATCATTGCCTAATAGTTTCGGATCATCTCTGGAAAGTGGAA
GTTTTTCCAATAGTTACAGCAACAAGGAAAACTCTATGAAATCTCTTCACTTGTTCAACAGTTCATTTAA
TTCGTCTGGCGAGCTAAATGCCAGTCTAGGCGCAGGAGATTTAACAAATATATCTTTAGGGACAG
>dme_circ_0001084_junction_seq
GATTTAACAAATATATCTTTAGGGACAGGCGAATTGTTTACCGCCAAGAACCAACT
>dme_circ_0001085
TGAATTTATAAATGAGCACATTAAATAATTATTATTGTTATTACATTAAGTTATTTTGACAATGGAAGTA
TGAGTTGTGATCAAGCTATTTTACGATCGTGATTATCAAAACTAAACAACTGTCGCTTCAAATTTTCATT
TTTCGAAATGGTGTAAGGCGAAAGTATATTGGAAAACGTTTTAGTTGTTCTGGCCTTAGGAAGTCGAATG
TTAAGTATGTCATGCCTGAGCTGTTCCGATCCTTTGCTCCAAATGGGATCAATCCAAAAGGGTAATTTCT
AATTGAAAAAACAACATAATAAATAATATATTTAAAAAATATATTTTGGATTTTTTGTAGCTTTTACCTC
TTTGTCTTGACCATTATACTTTAGTGGCTCGTAAATGGATCCTATTGGTGGTGCGATGTAATTTCCAATG
GTGTAAGTACCGTCAGTTCCATTATCTGATTTACGAAAATTTGATGAATTAGGGCGATTAAAGTTTTGTT
GACGTCTACGCCAAAGTTCGGATGGTTCATTTGATGTATCAACTGCGATACCTGTCCCTAAAGATATATT
TGTTAAATCTCCTGCGCCTAGACTGGCATTTAGCTCGCCAGACGAATTAAATGAACTGTTGAACAAGTGA
AGAGATTTCATAGAGTTTTCCTTGTTGCTGTAACTATTGGAAAAACTTCCACTTTCCAGAGATGATCCGA
AACTATTAGGCAATGATAAG
>dme_circ_0001085_junction_seq
TGATCCGAAACTATTAGGCAATGATAAGTGAATTTATAAATGAGCACATTAAATAA
>dme_circ_0001086
GCGAAAGTATATTGGAAAACGTTTTAGTTGTTCTGGCCTTAGGAAGTCGAATGTTAAGTATGTCATGCCT
GAGCTGTTCCGATCCTTTGCTCCAAATGGGATCAATCCAAAAGGGTAATTTCTAATTGAAAAAACAACAT
AATAAATAATATATTTAAAAAATATATTTTGGATTTTTTGTAGCTTTTACCTCTTTGTCTTGACCATTAT
ACTTTAGTGGCTCGTAAATGGATCCTATTGGTGGTGCGATGTAATTTCCAATGG
>dme_circ_0001086_junction_seq
ATTGGTGGTGCGATGTAATTTCCAATGGGCGAAAGTATATTGGAAAACGTTTTAGT
>dme_circ_0001087
GCAATTCGTAACGACCCTACACACAAGGTCACCGCTAAGAAGTCTTCTGCCGTTACGAAGAAAAGGTGGA
ATGCTAAGAAACTCACAAACGAGCAACGGAAGACTAAGATTGCAGCTCATAAGGCAGCATATGTTGCCAA

GCTGCAGTCTGAAACTGAGGCTTAAAAGTGTTCGTCACCAGCTTCAGTTTATATTGGTGTAAAAATAAAT
AAACAGTTCTAATGACACCAGCGACAATTGGTAG
>dme_circ_0001087_junction_seq
TTCTAATGACACCAGCGACAATTGGTAGGCAATTCGTAACGACCCTACACACAAGG
>dme_circ_0001088
GTGGAATGCTAAGAAACTCACAAACGAGCAACGGAAGACTAAGATTGCAGCTCATAAGGCAGCATATGTT
GCCAAGCTGCAGTCTGAAACTGAGGCTTAAAAGTGTTCGTCACCAGCTTCAGTTTATATTGG
>dme_circ_0001088_junction_seq
GTTCGTCACCAGCTTCAGTTTATATTGGGTGGAATGCTAAGAAACTCACAAACGAG
>dme_circ_0001089
GTCACCGCTAAGAAGTCTTCTGCCGTTACGAAGAAAAGGTGGAATGCTAAGAAACTCACAAACGAGCAAC
GGAAGACTAAGATTGCAGCTCATAAGGCAGCATATGTTGCCAAGCTGCAGTCTGAAACTGAGGCTTAAAA
GTGTTCG
>dme_circ_0001089_junction_seq
GTCTGAAACTGAGGCTTAAAAGTGTTCGGTCACCGCTAAGAAGTCTTCTGCCGTTA
>dme_circ_0001090
CCCACCAGGCAATTCGTAACGACCCTACACACAAGGTCACCGCTAAGAAGTCTTCTGCCGTTACGAAGAA
AAGGTGGAATGCTAAGAAACTCACAAACGAGCAACGGAAGACTAAGATTGCAGCTCATAAGGCAGCATAT
GTTGCCAAGCTGCAGTCTGAAACTGAGGCTTAAAAGTGTTCG
>dme_circ_0001090_junction_seq
GTCTGAAACTGAGGCTTAAAAGTGTTCGCCCACCAGGCAATTCGTAACGACCCTAC
>dme_circ_0001091
GGTTTCGTTAAGGTAGTCAAGAACAAGCAGTACTTTAAGAGGTACCAAGTCAAGTTCCGAAGGCGTCGCG
AAGGAAAGACCGATTACTATGCCAGGAAACGCCTAACATTTCAGGACAAGAACAAGTACAACACTCCCAA
GTACCGTTTGATCGTACGTTTGTCCAACAAGGACATCACAGTACAGATCGCCTATGCTCGCATCGAGGGT
GATCGCGTGGTGTGCGCTGCTTATTCCCATGAGCTTCCCAAATACGGGATCCAGGGTTGGATTGACCAAC
TACGCTGCTGCTTACTGCACAGGCCTGCTGGTCGCCCGTCGTGTCCTTAACAAGTTGGGACTGGACTCCC
TATATGCAGGATGCACTGAAGTGACTGGTGAGGAGTTCAACGTCGAGCCTGTTGATGACGGCCCAGGGGC
ATTCCGTTGCTTCTTGGATGTTGGACTCGCTCGTACTACAACTGGTGCCCGTGTGTTTGGCGCTATGAAG
GGAGCAGTTGATGGTGGTCTAAACATACCTCACTCTGTGAAACGCTTTCCTGGATACTCTGCTGAAACCA
AGAGCTTTAATGCCGATGTGCATCGCGCTCATATATTTGGCCAGCACGTTGCAGACTATATGCGTTCTTT
GGAGGAGGAGGATGAGGAGAGCTTCAAAAGGCAGTTTAGCCGATACATCAAGTTGGGCATTCGTGCTGAT
GATG
>dme_circ_0001091_junction_seq
ATCAAGTTGGGCATTCGTGCTGATGATGGGTTTCGTTAAGGTAGTCAAGAACAAGC
>dme_circ_0001092
TTGATGGTGGTCTAAACATACCTCACTCTGTGAAACGCTTTCCTGGATACTCTGCTGAAACCAAGAGCTT
TAATGCCGATGTGCATCGCGCTCATATATTTGGCCAGCACGTTGCAGACTATATGCGTTCTTTGGAGGAG
GAGGATGAGGAGAGCTTCAAAAGGCAGTTTAGCCGATACATCAAG
>dme_circ_0001092_junction_seq
CAAAAGGCAGTTTAGCCGATACATCAAGTTGATGGTGGTCTAAACATACCTCACTC
>dme_circ_0001093
GCTCTCCTCATCCTCCTCCTCCAAAGAACGCATATAGTCTGCAACGTGCTGGCCAAATATATGAGCGCGA
TGCACATCGGCATTAAAGCTCTTGGTTTCAGCAGAGTATCCAGGAAAGCGTTTCACAGAGTGAGGTAT

>dme_circ_0001093_junction_seq
CAGGAAAGCGTTTCACAGAGTGAGGTATGCTCTCCTCATCCTCCTCCTCCAAAGAA
>dme_circ_0001094
GGGCATTCCGTTGCTTCTTGGATGTTGGACTCGCTCGTACTACAACTGGTGCCCGTGTGTTTGGCGCTAT
GAAGGGAGCAGTTGATGGTGGTCTAAACATACCTCACTCTGTGAAACGCTTTCCTGGATACTCTGCTGAA
ACCAAGAGCTTTAATGCCGATG
>dme_circ_0001094_junction_seq
GCTGAAACCAAGAGCTTTAATGCCGATGGGGCATTCCGTTGCTTCTTGGATGTTGG
>dme_circ_0001095
GGAAAGCGTTTCACAGAGTGAGGTATGTTTAGACCACCATCAACTGCTCCCTTCATAGCGCCAAACACAC
GGGCACCAGTTGTAGTACGAGCGAGTCCAACATCCAAGAAGCAACGGAATGCCCCTGGGCCGTCATCAAC
AGGCTCGACGTTGAACTCCTCACCA
>dme_circ_0001095_junction_seq
AACAGGCTCGACGTTGAACTCCTCACCAGGAAAGCGTTTCACAGAGTGAGGTATGT
>dme_circ_0001096
GGAAAGCGTTTCACAGAGTGAGGTATGTTTAGACCACCATCAACTGCTCCCTTCATAGCGCCAAACACAC
GGGCACCAGTTGTAGTACGAGCGAGTCCAACATCCAAGAAGCAACGGAATGCCCCTGGGCCGTCATCAAC
AGGCTCGACGTTGAACTCCTCACCAGTCACTTCAGTGCATCCTGCATATAGGGAGTCCAGTCCCAACTT
>dme_circ_0001096_junction_seq
CTGCATATAGGGAGTCCAGTCCCAACTTGGAAAGCGTTTCACAGAGTGAGGTATGT
>dme_circ_0001097
GTTGTAGTACGAGCGAGTCCAACATCCAAGAAGCAACGGAATGCCCCTGGGCCGTCATCAACAGGCTCGA
CGTTGAACTCCTCACCAGTCACTTCAGTGCATCCTGCATATAGGGAGTCCAGTCCCAACTTGTTAAGGAC
ACGACGGGCGACCAGCAGGCCT
>dme_circ_0001097_junction_seq
AAGGACACGACGGGCGACCAGCAGGCCTGTTGTAGTACGAGCGAGTCCAACATCCA
>dme_circ_0001098
GTTGTAGTACGAGCGAGTCCAACATCCAAGAAGCAACGGAATGCCCCTGGGCCGTCATCAACAGGCTCGA
CGTTGAACTCCTCACCAGTCACTTCAGTGCATCCTGCATATAGGGAGTCCAGTCCCAACTTGTTAAGGAC
ACGACGGGCGACCAGCAGGCCTGTGCAGTAAGCAGCAGCGTAGTTGGTCAATCCAACCTTAAATAAATAT
AATGGGCCATCAAGTTGAGTACGCCAGAACGCAGTGGGAAAACTCACCTGGATCCCGTATTTGGGAAGCT
CATGGGAATAAGCAGCGCACACCACGCGATCACCCTCGATGCGAGCATAGGCGATCTGTACTGTGATGTC
CTTGTTGGACAAACGTACGATCAAACGGTACTTGGGA
>dme_circ_0001098_junction_seq
CAAACGTACGATCAAACGGTACTTGGGAGTTGTAGTACGAGCGAGTCCAACATCCA
>dme_circ_0001099
GTTGGATTGACCAACTACGCTGCTGCTTACTGCACAGGCCTGCTGGTCGCCCGTCGTGTCCTTAACAAGT
TGGGACTGGACTCCCTATATGCAGGATGCACTGAAGTGACTGGTGAGGAGTTCAACGTCGAGCCTGTTGA
TGACGGCCCAGGGGCATTCCGTTGCTTCTTGGATG
>dme_circ_0001099_junction_seq
CCAGGGGCATTCCGTTGCTTCTTGGATGGTTGGATTGACCAACTACGCTGCTGCTT
>dme_circ_0001100
GAAAGACCGATTACTATGCCAGGAAACGCCTAACATTTCAGGACAAGAACAAGTACAACACTCCCAAGTA
CCGTTTGATCGTACGTTTGTCCAACAAGGACATCACAGTACAGATCGCCTATGCTCGCATCGAGGGTGAT

CGCGTGGTGTGCGCTGCTTATTCCCATGAGCTTCCCAAATACGGGATCCAGGTGAG
>dme_circ_0001100_junction_seq
AGCTTCCCAAATACGGGATCCAGGTGAGGAAAGACCGATTACTATGCCAGGAAACG
>dme_circ_0001101
GCTCATGGGAATAAGCAGCGCACACCACGCGATCACCCTCGATGCGAGCATAGGCGATCTGTACTGTGAT
GTCCTTGTTGGACAAACGTACGATCAAACGGTACTTGGGAGTGTTGTACTTGTTCTTGTCCTGAAATGTT
AGGC
>dme_circ_0001101_junction_seq
TACTTGTTCTTGTCCTGAAATGTTAGGCGCTCATGGGAATAAGCAGCGCACACCAC
>dme_circ_0001102
GCTCATGGGAATAAGCAGCGCACACCACGCGATCACCCTCGATGCGAGCATAGGCGATCTGTACTGTGAT
GTCCTTGTTGGACAAACGTACGATCAAACGGTACTTGGGAGTGTTGTACTTGTTCTTGTCCTGAAATGTT
AGGCGTTTCCTGGCATAGTAATCG
>dme_circ_0001102_junction_seq
TGTTAGGCGTTTCCTGGCATAGTAATCGGCTCATGGGAATAAGCAGCGCACACCAC
>dme_circ_0001103
GCGTCGCGAAGGAAAGACCGATTACTATGCCAGGAAACGCCTAACATTTCAGGACAAGAACAAGTACAAC
ACTCCCAAGTACCGTTTGATCGTACGTTTGTCCAACAAGGACATCACAGTACAGATCGCCTATGCTCGCA
TCGAGGG
>dme_circ_0001103_junction_seq
TACAGATCGCCTATGCTCGCATCGAGGGGCGTCGCGAAGGAAAGACCGATTACTAT
>dme_circ_0001104
GAGAAGGTGGGAAACTGACCGGAAAACGTAAAGCCGACGCTGTCGATGTACTAGGCCAAGTTGACAGGTT
GCTTGTGAGGCTCGAGTGCAGGGAAATAGAAGGAGAGAGTGGAGGGGAGAGCCGTGCACTGCAAACTGCA
AGCGCGGGAGACACCCGCTGACTATATGAGGGACTTAATCGAGAGAGTCTCGAAAACTTGGAAAACCAGC
CAGAGGAGGTAGGACACGTAACAGACCAAATGGAGACCACAATGGGTGAAGCAGCCGGAGGAATAGAACT
ACCACCACTTGGCTATACTAGGTGTCCCTCCAGTGTGCTTCCGCGGTACCAGTCAAGTTCAGAAGACCGG
TCGGATGCTACGGACAGGCGTGAACGAGAGCTAATGAAAATGAGCGTACAAATAAACGCTCTGAAGAAGA
AGGTGGATGAATTACACGCCGCAGAGACCTTTACGGAGCAAGACCAATACCACCACATGCACTACCTAGA
ATTCGTACGGATAGCTTTCTAAGGCTTCAAGACACACCGACACCAGTAAAAGTGTCGCAAGAGTGCTGTA
ACGACCAGATGCATCGATTGAGGGAGCCCAGGCGAACGGCGAAACCACAGTAGATACCTTAAGAATGCGT
TGCCGAGGAACCCAGTCACCAGTTTTTCTCCAAGTAATGACCATCACCTCCGGTTTTCGAATTCGTAAAA
TTTTTTGATTTGTGAAATGGGTACTTTTGTCTGTCCGAAATGCCTGTAAAAGTAAAGTAAATGCCTGTTT
TCATATCAAATCAGATCTTATTTTTAAAGGGAAAATTAGTTCACTTAGCCTATTGATGTTTATTCCATAT
CCCCAAAATGTCATTCTAGCCACTAAGGTATTTTATAATAAATTTATTTGCGACTCTACCACAGATTTGA
CATgccacggccaaaaaaattgtgtgccaaaaaaatcgtatggcgttacgcatcttgttattctagtgtctt
tgCTAGTACGATAGATTGCTGGTTCATAATTGTACTCAATTTTAAGTGTCTTTACTGCTTTTACTTTGCT
TTTGTGCAAATATATGTGTAGATGTTTCACAAGTGTGAACAGGCCTTAAACATTGTGTTTTTCTGGCATT
CTAGTCGGCTTTATTTGAGCTGTTTCACAAACGAAAGATATGGCTTTCTTGTAATCGTGAACCGTAAACT
TGTGTGTTGCGCTAGGAATAGGGTGGGCTGTGTAGAGCTCTTCATGTTTATATATATTATAACAAAAATA
GGAGCATCCGACAAATCAACCCTCACTCGTATAGTCTATGCGACGTATATCAAGAACTTATACCAACATC
TGAAATTACGCCAATCGATTAATGGAGCAAAACTCAACTGCGGCTGGCAACGCCAACCCTGCTACAGTTC
CTGCCAAAAGGGGTACGCAACAAGGTCGCAAGAAGTCTGGCCCCAAGTTCGAACTGTCTGAGGCCCAAAA
ATGCGACATTAAGGAGGCCTTTGATCTATTTGACAACGAAGGCACGGGTTACATTGAGGTAAAGGAACTT

AAGGTGGCCATTAGGGCTCTGGGATTCGAGCCAAAAAAGGAGGAGATTAAGAGGATGATTTCCGACATTG
ACAAGGACTGCAGTGGGCGCATTGCGTTTAACGTCTTTCTGCAACTGATGACTATAAAGATGGCCGAAAA
GGACACTAAGGAAGAGATTCTTAAGGCTTTTCGGCTTTTTGATGATGATGACACTGGAAAAATTTCATTC
AGAAATCTAAAGAGGGTGGCTCGTGAACTTGGTGAAACACTTACTGACGAGGAACTGCGAGAGATGATAG
ACGAAGCCGATTTGGACAATGACGGGGAG
>dme_circ_0001104_junction_seq
CGAAGCCGATTTGGACAATGACGGGGAGGAGAAGGTGGGAAACTGACCGGAAAACG
>dme_circ_0001105
GAGAAGGTGGGAAACTGACCGGAAAACGTAAAGCCGACGCTGTCGATGTACTAGGCCAAGTTGACAGGTT
GCTTGTGAGGCTCGAGTGCAGGGAAATAGAAGGAGAGAGTGGAGGGGAGAGCCGTGCACTGCAAACTGCA
AGCGCGGGAGACACCCGCTGACTATATGAGGGACTTAATCGAGAGAGTCTCGAAAACTTGGAAAACCAGC
CAGAGGAGGTAGGACACGTAACAGACCAAATGGAGACCACAATGGGTGAAGCAGCCGGAGGAATAGAACT
ACCACCACTTGGCTATACTAGGTGTCCCTCCAGTGTGCTTCCGCGGTACCAGTCAAGTTCAGAAGACCGG
TCGGATGCTACGGACAGGCGTGAACGAGAGCTAATGAAAATGAGCGTACAAATAAACGCTCTGAAGAAGA
AGGTGGATGAATTACACGCCGCAGAGACCTTTACGGAGCAAGACCAATACCACCACATGCACTACCTAGA
ATTCGTACGGATAGCTTTCTAAGGCTTCAAGACACACCGACACCAGTAAAAGTGTCGCAAGAGTGCTGTA
ACGACCAGATGCATCGATTGAGGGAGCCCAGGCGAACGGCGAAACCACAGTAGATACCTTAAGAATGCGT
TGCCGAGGAACCCAGTCACCAGTTTTTCTCCAAGTAATGACCATCACCTCCGGTTTTCGAATTCGTAAAA
TTTTTTGATTTGTGAAATGGGTACTTTTGTCTGTCCGAAATGCCTGTAAAAGTAAAGTAAATGCCTGTTT
TCATATCAAATCAGATCTTATTTTTAAAGGGAAAATTAGTTCACTTAGCCTATTGATGTTTATTCCATAT
CCCCAAAATGTCATTCTAGCCACTAAGGTATTTTATAATAAATTTATTTGCGACTCTACCACAGATTTGA
CATgccacggccaaaaaaattgtgtgccaaaaaaatcgtatggcgttacgcatcttgttattctagtgtctt
tgCTAGTACGATAGATTGCTGGTTCATAATTGTACTCAATTTTAAGTGTCTTTACTGCTTTTACTTTGCT
TTTGTGCAAATATATGTGTAGATGTTTCACAAGTGTGAACAGGCCTTAAACATTGTGTTTTTCTGGCATT
CTAGTCGGCTTTATTTGAGCTGTTTCACAAACGAAAGATATGGCTTTCTTGTAATCGTGAACCGTAAACT
TGTGTGTTGCGCTAGGAATAGGGTGGGCTGTGTAGAGCTCTTCATGTTTATATATATTATAACAAAAATA
GGAGCATCCGACAAATCAACCCTCACTCGTATAGTCTATGCGACGTATATCAAGAACTTATACCAACATC
TGAAATTACGCCAATCGATTAATGGAGCAAAACTCAACTGCGGCTGGCAACGCCAACCCTGCTACAGTTC
CTGCCAAAAGGGGTACGCAACAAGGTCGCAAGAAGTCTGGCCCCAAGTTCGAACTGTCTGAGGCCCAAAA
ATGCGACATTAAGGAGGCCTTTGATCTATTTGACAACGAAGGCACGGGTTACATTGAGGTAAAGGAACTT
AAGGTGGCCATTAGGGCTCTGGGATTCGAGCCAAAAAAGGAGGAGATTAAGAGGATGATTTCCGACATTG
ACAAGGACTGCAGTGGGCGCATTGCGTTTAACGTCTTTCTGCAACTGATGACTATAAAGATGGCCGAAAA
GGACACTAAGGAAGAGATTCTTAAGGCTTTTCGGCTTTTTGATGATGATGACACTGGAAAAATTTCATTC
AGAAATCTAAAGAGGGTGGCTCGTGAACTTGGTGAAACACTTACTGACGAGGAACTGCGAGAGATGATAG
ACGAAGCCGATTTGGACAATGACGGGGAGGTAAATCAAGAAGAATTTCTGCGAATCATGAAGAAGACTAG
TTTGTATTAAAGAGCAGCGCTTAATCTTCATTTTAAAACACTTCGTGCGTTCCTGGTGCACTTAATGAAA
CATTCTCAAAATAATTTGCCGTTTTAAGTCATCAATTATAAAT
>dme_circ_0001105_junction_seq
TTGCCGTTTTAAGTCATCAATTATAAATGAGAAGGTGGGAAACTGACCGGAAAACG
>dme_circ_0001106
GGTAAATCAAGAAGAATTTCTGCGAATCATGAAGAAGACTAGTTTGTATTAAAGAGCAGCGCTTAATCTT
CATTTTAAAACACTTCGTGCGTTCCTGGTGCACTTAATGAAACATTCTCAAAATAATTTGCCGTTTTAAG
TCATCAATTATAAATGTAAGTTTTAGATATTTTAAAGTTTTACGTATGAGTATTACATCAAGAAATAATT
GTTCAGCTCGTTTTGAATTTTAAACAAAAATACTTTTTCCCGATTTGCGATAAAAGGGCGTAGTAACATC

AACTGCAAACATTTATAATATCAAATTTCATGATGGTGAATAGTAAAATTTAAAACAAAATGTAAGGGAA
CTAATTATTGGTATATGTTACTTGAATTAGGTCACAACGCTACTTTTTACAGCTTATTGTCTAAATCTAA
GCAAG
>dme_circ_0001106_junction_seq
TACAGCTTATTGTCTAAATCTAAGCAAGGGTAAATCAAGAAGAATTTCTGCGAATC
>dme_circ_0001107
GCAGTCGGATCAACGGCATTAAACAGAACTGGCACAACCGACACGGCCACGGAATTACCTCAGACGACCA
GATATCCATCCGGGGAAGAACCTGCTGTGAAGCCAGAGGAGGCGCAATACGCTTTAAACATGGAAACTGA
AAAAAATTTGGAGCAGGACAATTTCTTTCATATAATGGACATAAAAGAAGATATGGCTGATAGTAACAAC
GAGGCGTCCACGAAGAACCAGGAAAATAATAATATGGAACTTTGTGATTCCAACACAGTATGTATTGACC
GACGTCCGGCTGAAGTTTCCCAGCATGCTATGAATATGGCCATGAACTCTGTGCTGAATGCAAATAACAC
CGGGGTTGGAGTTCGAGTAACTACTGCCTGCAGTCTGTTTAGCCCACTAGGAGTACTAAACAATAGTGCT
TTACAAAGGGAG
>dme_circ_0001107_junction_seq
ACTAAACAATAGTGCTTTACAAAGGGAGGCAGTCGGATCAACGGCATTAAACAGAA
>dme_circ_0001108
TTCATGGCCATATTCATAGCATGCTGGGAAACTTCAGCCGGACGTCGGTCAATACATACTGTGTTGGAAT
CACAAAGTTCCATATTATTATTTTCCTGGTTCTTCGTGGACGCCTCGTTGTTACTATCAGCCATATCTTC
TTTTATG
>dme_circ_0001108_junction_seq
GTTACTATCAGCCATATCTTCTTTTATGTTCATGGCCATATTCATAGCATGCTGGG
>dme_circ_0001109
GAAGATATGGCTGATAGTAACAACGAGGCGTCCACGAAGAACCAGGAAAATAATAATATGGAACTTTGTG
ATTCCAACACAGTATGTATTGACCGACGTCCGGCTGAAGTTTCCCAGCATGCTATGAATATGGCCATGAA
CTCTGTGCTGAATGCAAATAACACCGGGGTTGGAGTTCGAGTAACTACTGCCTGCAGTCTGTTTAGCCCA
CTAGGAGTACTAAACAATAGTGCTTTACAAAGGGAGGTGGCATCGGAATGCGGCACTTTACAAGTCTCTT
CAGTGCCCCCAACTTCTGGCGTAGATCTAGACATAGGATTGGAGTCTGCCCCAACGCCATGTAAGGGTGC
AGCAAAAATCGTACTCCTATGTGAACCAAAGTCGCACAAGTTTGAAACGAGATCTATATTGCTGCAGCCC
AACCAGGATTGCAAG
>dme_circ_0001109_junction_seq
ATTGCTGCAGCCCAACCAGGATTGCAAGGAAGATATGGCTGATAGTAACAACGAGG
>dme_circ_0001110
GAATCCGGCCTGCCGTTGTCACTCTCTGTAAAAGTGCAAATTAACATGCACTTTAAGGACCTGAGCAATT
TTCCAGTAGTATCTGTATTTAATCATCTAACAGTGCCAATGTTGTGGTTTGAAATCGTAAGTACCACATA
CACGTCCGCTCCGATGTCATCATTGTGAATTGTATATTTCAGATGATGTCAAAGCTGCCCGACAGCTTGG
ACTCGCGGTTCAACTTTTACCTGAACATTTTGCCGTTAGTTAATCCCTTAGGCTTTTGGAGTGGCATACT
ATTGGGAATAAGTCTTTTGGGGTATGCAATAACCAAGGCCACGCTTCACATGTCTACCTTTTCTCGACGA
GCTGCTAATATAACAGATGTTAAATATGTACGAGCAAATTTAATTCAAAACATCGCAGGTGAAGCAGCGA
ATCATGCTTACAGTCCGTGCGATATTAAGTTACTAGATGATATTTCGGTTGTAAGGCATCCTGTTAATAA
GCAAACAGTCCAGGATTCTGGATTCAGTTCAAAAACAACATACGCCTTGGATATGGAACCTGCTTTATTA
GACACGAGCGAGAGCAGCGACGAGGGGCGAGATTGTGATATTGTTACCATATCCCGTAATTCTACAAGCA
CCCGCTTTTGCGCAGATGaaaaaaaaTGCTGGCTCGGCGACGATATACGAGTCGAGTGTTTCACCGAGAG
TCGAGCTCCTCAAACGATTTTGAAAAGGGAAGAACAAATACATTAAGGCAACAGG
>dme_circ_0001110_junction_seq

GGAAGAACAAATACATTAAGGCAACAGGGAATCCGGCCTGCCGTTGTCACTCTCTG
>dme_circ_0001111
GCAATATCACACCTTACTGCCAACCAGCCGTTCTTAGTTAGAATGGCTCTGAAGACACTACTACTGAGTA
CAAAGTCGGAGCCAATAGTTCGCACCACTGCCAAGGAATTCATGTTTGGCTATCCCTCCGCCCTGGCCAC
TCTCGGCAACACCTTCCTTCCAAATTGGATTTCCTTTGAAAAGGTTGGGCTTATCGATCGCATGTACGAC
TTCTCAACGGATTTCGAGACGTTCTACACAGGCGTGCCCAATCCAGCGCTTTCGGGATTATATGCATCGT
ATCGCGGAGAGACAACGCTGCCACAGTGGGACGGGGACCATTGCAGCAACATAGAGTTTGCATCTGATGG
TACAAAGTTCAAAAGCTTCATTCAGCCTAACGAGACCGTTAAGTTCTTCCGAAAAAGCATGTGTAGACCG
ATAAACTTGGTATGTTTTGAACAAGAATTCCGGGATAAGCCCCAACTAATTTATATTGTCTTTTAGTACC
GGGTGGGAAACGAAAAAACCTATGGCAGCCTTAAAGGATATAATTATGTATTTGAGGATAACGCCTTTGA
TAATGGCGCAACTAATGAAGCTAACAAATGTTTTTGTAGAAAAGG
>dme_circ_0001111_junction_seq
AAGCTAACAAATGTTTTTGTAGAAAAGGGCAATATCACACCTTACTGCCAACCAGC
>dme_circ_0001112
GTTTATTTATACTTCTAGTCATCGGATTTTTGGCTATTATAACATCTACACTGATTAAAGTTTTGGAACC
ATACGATTTGATATTTAAATGGGTAAGACACAAACGACTTTTTATGATTTTTTTAAATGATTACGCAAGA
GTACTAAGTGGACGGAAATTTGCTCGGCACATGTAAATGATTAATCACAAATTTGATAGTATTCACCACC
CAAAAAAATACACATTTACTCTAACGTTCTTCTTGCAGAAATTAATCATGACAGAGGGAGGTGAGATCTT
TAATCTGTGGGCCCAGCCTCCAGTCGATCTGTATATTAAGATATATTTGTTCAACATAACCAATGCCAAC
GCGTTTTTAGCGGGTCGCGAGCAGCTTAGAGTCGAACAAGTTGGTCCCTATGTTTATAAGGGAGATAATG
ACACATGAGAATGTAACGTTCAATTCGAATAACACCATGTCCAGCACTCCCAGTCATCCCTTAGTCTGGC
AGGAGGAAATGTCTGGGAATTGTCGTGAGGACGATGAGGTTGTAATGCTCAATATTGCCATGCTGG
>dme_circ_0001112_junction_seq
GTTGTAATGCTCAATATTGCCATGCTGGGTTTATTTATACTTCTAGTCATCGGATT
>dme_circ_0001113
GTCGCAACGGTGGAAAGTCAAACAAAGAATCCACAGGAGTGTCTACTCCTGCTCCGGGCTCTGCATCCGG
AAGCAATAGTGGAAACACGACTCCGGCTCACAAATCTAGTGCGCTGGATGGGTCCGATAAGGACAGCGGC
GGCATAAATGCCAGCGCAGTAGCCGCGGCGGTCACAAGCATTCACGATCCAAATCACTGGAGCAAGAAGG
AGAAGTACGACGCTGATGCGTACTTGGAAGGCGCCTACAAGAAGCATTTAGGCAGACACGCCAATAAGGT
AGGCGTGGCACTCGGAGTCATCTGCTAAACACTTGGCATTCATTAATCATTTTGTTTGTTGTTGTTGTTG
ATTGAATGATTGACTGGCTGATTATCAAAGAGTTTTCATTAATTTTGTATTTAATTTAACCTTCTGTTAT
AGATACTTTATAGCCTCTTTACTAATGCCAATTTGTGTCttttttttaattattattaacccttttttt
taTAGAACTTTGTGTCTGCTGATTGAAGCAATGATCAGTGGGCGTTTCAAGGTTCAGGTGGAATGTGATT
CTCTTTTATTCGCTATGTATCTCTATACTGCAACTCTCTTTGTATTTTCGATTGCTTGTCCATTAAAATG
CTAACACTTGTACATCTTATACACGATTCTGCTCATTTGTAACCACAACATTTAAATTCTATATTTTGCC
AACTAATCTTCGGCGTATCGAATCTTCTTCTATCGCTGTGTTTTGTATCTTTTCGATTCGTTTAACAAAC
CTTAAAAATTCCTAATTGACCTCATTGCGATATCTATACGTATATTATATAACCACCAAAAACTGCAGCA
GTACGAGGGCCAAGCAGCAGTTGGCACTGAATCTGAATTTGGAAAGGTAATATCCCCATATATTCCTCAA
CCACCGATATCGATCGCCATCGCTCGTCGCCCTCGCTTGCACGCACGGTGTTTTCCACTTTTAAGAAAAA
AGCTGGCGAATAGGGAAATTCACGCTACTCAACATCTTTATTAACCTCCATACGTGCAGTCGTCCTCATA
ATACAAAAACCGCCAAGTTATACATGCAATTCAATAATTAATTTATATTTTTTAATACGAATACCAAATT
CaaaaaaaaaaaGCCATCAAGAGGCTCAACTAACCTTTTAAATTTTGCTTTGTATGATTCTGCATTTTGT
AGTGGTAAATCTGTAGTACAAATTGGCAATTAACAATGTTACAatttttactttatttccctttatggtc
tcatttttgatttttgttttatttaaCATCATACAAAAAATTGTTGAACCGAGAAAATTGAATTTTTACT

AAACAACGCATTTCTTTTTTCGCGCATCTCTCAACTTCCCCAATCCACCAAACACATTTGAATGTTATCG
AATGTACTCATCGAACGATACGATCATATGTAGCGAACTCCCCATTCGCCCGCCGCCAACGCCAGATCAA
GTGCCATCTTCATGGTGGAATCCCATTTGTTGTGATAAGAGCCTGTTGGTGGGAACCTACAAGCATGGCT
GTGAAATGTATCGTCAAATGCGCGCCGATCCCAATCTGTGCTTTGTCACCCATGTGGGCGCCGGTGCGGC
CGGTGACGATCTGGCTGTCACGAGTTTGCCGATGTAAGTCTTCAAAACGAAAACAATCAATTCTAAATTA
ATTCGGTGTGTTCTAAGGAATCTCTATTTCAACGTGTACCTTCTGCACACACGTAACATTAATATTGATG
GTAATTGATAACCGTTTTAACCGCTACTGTGGTGAAAGAATTTGAGAAATGATATAGAGGTTTTTTTGAA
CACGCCGGATAGAAAAAACAACAGATGCAAAGAGATATAAACCATACATAAACGCCATTGAGTAATTCAT
ATACATCACACTAAAGTATtaaaattaaaaaaataaataaataCGCTTCCAATTAGACCTGAATGGAAGG
AACGTGCAGGATTTGCTTAAACTCATTTTCATTCCCATTCCCCACGTGCACATGCATTCATCTGAAAGTC
TTGGCATTAAAATGTGCTTCAAACATTTTTTCAGCTATTAGCGCTTAATATAATATTACATCAAAGATTA
CAGTTTGGTAGACAGTGCTATTTATCCATCAAATGTTAAGGTCATAAATCATAATAATTGTTTTGTTGAC
TTTTAATATAGACTAGATTTAATTTAAATTATTCAAATCAAATTAAAAAAGTACTTTCAATTCACATTAA
TAATTTTAATAATCATTGTGCTTGTTCATCGAATTCTCGCCGTATAATTTCTTGATAAGGTATTCAACGA
AATATTCTCAGATACAACAGCGCAACCTACGGGGCTTCCATTAACGCTCACATTGAATGCATTGATCCAT
TGACATTGCGCTGAAtgtgtgtgtgtgcgtgtgttttgtgAATTTTCTCTCAGTGTTTTAACTGCGTTGG
GCATGGCCTGCATCTGATATATGATCAGTAAATTCCATTAAATTGCTACTAACATTCAAACCCTTTTTTC
TGGAACCACCACAGAAAACGAAGACGATGCTAATTCGAAGCACGATGATGGTGACGAGGTGGACGATGAC
GGAACTACAACGACCAAAGACTCTGACTCTACTAAACTCACCGGGGGCGATAACAAGGACTCGCTTCTCG
ACCCGGAGCGTCCCAGTTCGTCTGGTAAGAGTAGCAGTGAAAATGCTAGCGGCGGCAACGACAAAACGGA
AAGCGAAAATGATGCCACAACCGTTTCCGAATCCTCATACAAATCCGAAGTCTTGCCGGGCAAAGGCAGC
GATCTGGATGTGTCGGTCGCCCAGCAGGACGCATTATCAGCAACTCCTGAATTGGCAAATACATCAGATC
CTAAAATGAATAAAAATGACGCACAAGTCTCAGCCTCTGTTTCCTGCGATCCGGCCGACTCTAGTTCACC
CGAGGCGGCAGGAAAAGCTCAGGGGACGGGGGTCCAGACTGGCGATGCCGGAGAATTGGACATCGAATCG
GAGAAAAAAGATAACGAAAAGGAAACCAAGGAGTCAACTGGTGATGCTCCTGCTGTGGAAGCCAAAAAAG
ATAGCGCTGCCATTGCCCGAGCTGAAGGCGATAAAACGGAATCATGCACTAACAATGCTACCACAAACAT
CTccaccaccactactactactaccattaccaataccaccaccaccaccaataATCCATCATCCAAAGCG
GCTAACTCTAACAACACCACCGACCATGCTAACTCAAACTCACATTTAGGCTTGGACGAGGAGGAGAGTG
TCGCCGGCAGCTATCCGCCAACCGTGGCAGCCGTCGAGGACGCCACTACTATGTGGCCATCTATGCAGGA
CCTTAACACGCGTCTCCGGCGGGTGATCACTGCCTATCAAAGGAATTACAAGAAAGAGGAATTAAAACTG
CAGCAGAAGGCCAAGGGTAGGTGCAATATCCTTTATCCACATACCAACCCTTTTCATATTATCTTCTCCA
TTCGTTGCCACTTTAATGAGAGTACATATATATTTTAtattccctttactctcagagtataaaggtatac
taaattcgttgaaaagtatctaacagtcaaaattgaacgtttctgTCTGTCCGTATTAACATCGAGATCT
CGATGCTGCAAGTTTGCTTGAGTACGTTGCTTGAGAACTGTCACGCTCACGATGTTTTCATTTTCTTTTA
TTTAGTTTTGCCAATTTCTAGTGATACTTTAAAATTAGCGTTTTCTTCAAATTAAATTGTTATCTTTGAG
ATTTATATTCACCCACCCTTATTAACGACTATGAAAAATCTAAAATTCAAATAGTTTCCGAAACTGTCTG
TTGCCTAACCCAAATGACTATTGAATCATTTTACATTTTTTGTTCTACGTTTCATGATTATGTTGTAGTG
TCCAATGTTAGAAATATGTAGTTTTGTTGCTTAGCTGTTGGCTGTAGTTAGTTTCGAACCGTACACCGTA
GTTAGCGTACAGAACGGACAGTGGACATTGACTTGGACAAAGAAGCGGTTCCAAAAATGCAACCGAATTC
GATTTACCCAAAGCATCAAAAGCTGTAAATAAGAAAAAATCATTAACTAATGCAGCCAACAGCTGATAAA
CTAAACTATGTAAAACGCTGACAGAGGATTGCACTTAATTTCTTTACCATCAAGACCATCTTAATAAAAT
CATCCTCAGCCCACCCTGAATCTGTGACAGTTCCTGACCTTTTTGCCGAACTTGCAAAGTTACCAACTCC
ACCAACTCTTTCTCTGTGATTGTCACATTTGTCCCACAGCTCCAGGCATTGGTTTCATCGACACCGCCGC
TGTCGGTTCCCACCACTCCCACCCTTCAGTCTATGCAAATGCAGCTGCAGAGCGGCTCCGGCAGTGGTGG

GTCCGGAATAGGATCCTCTGCTCAGCACGATGCCAGTGGCCGGAGTCTTCAGGCGTTGATGCAATCGCAG
GGGGCCAGCACATCCGCAGCAGCAGCCGCCGCCGCATCAAGTGCTGGAGGATCCTGTAGTGGATCCGGCC
AGGTGGGCATGGGCGGAGCGGGAGTCATGCCAGCTATGCCAACCGCCGCCGACATAAGCCTAATGCTCAG
CATACTCAACACCACAGATGTCAACCAGCTGGCCAACTTGGACATCAATAAATTGGCCATGTACCTGGTC
AGCATGAACAATG
>dme_circ_0001113_junction_seq
GCCATGTACCTGGTCAGCATGAACAATGGTCGCAACGGTGGAAAGTCAAACAAAGA
>dme_circ_0001114
GATGGCTTCTTAACTCAGAATAGCGTGCTGATAGCACAAGATGTGCATGCCGGCTCATTTCAGGGTTAAT
ACACGAAAATTGAGTTAGTTTTTCGCATTTTACTAAAATATCCTTGTAAAACTTTGACACGATTCGTATG
ACCAACAGAAAGTCGTCTGAATTTTGAAATCCATACTTGT
>dme_circ_0001114_junction_seq
TCGTCTGAATTTTGAAATCCATACTTGTGATGGCTTCTTAACTCAGAATAGCGTGC
>dme_circ_0001115
AAACGAAGACGATGCTAATTCGAAGCACGATGATGGTGACGAGGTGGACGATGACGGAACTACAACGACC
AAAGACTCTGACTCTACTAAACTCACCGGGGGCGATAACAAGGACTCGCTTCTCGACCCGGAGCGTCCCA
GTTCGTCTGGTAAGAGTAGCAGTGAAAATGCTAGCGGCGGCAACGACAAAACGGAAAGCGAAAATGATGC
CACAACCGTTTCCGAATCCTCATACAAATCCGAAGTCTTGCCGGGCAAAGGCAGCGATCTGGATGTGTCG
GTCGCCCAGCAGGACGCATTATCAGCAACTCCTGAATTGGCAAATACATCAGATCCTAAAATGAATAAAA
ATGACGCACAAGTCTCAGCCTCTGTTTCCTGCGATCCGGCCGACTCTAGTTCACCCGAGGCGGCAGGAAA
AGCTCAGGGGACGGGGGTCCAGACTGGCGATGCCGGAGAATTGGACATCGAATCGGAGAAAAAAGATAAC
GAAAAGGAAACCAAGGAGTCAACTGGTGATGCTCCTGCTGTGGAAGCCAAAAAAGATAGCGCTGCCATTG
CCCGAGCTGAAGGCGATAAAACGGAATCATGCACTAACAATGCTACCACAAACATCTccaccaccactac
tactactaccattaccaataccaccaccaccaccaataATCCATCATCCAAAGCGGCTAACTCTAACAAC
ACCACCGACCATGCTAACTCAAACTCACATTTAGGCTTGGACGAGGAGGAGAGTGTCGCCGGCAGCTATC
CGCCAACCGTGGCAGCCGTCGAGGACGCCACTACTATGTGGCCATCTATGCAGGACCTTAACACGCGTCT
CCGGCGGGTGATCACTGCCTATCAAAGGAATTACAAGAAAGAGGAATTAAAACTGCAGCAGAAGGCCAAG
G
>dme_circ_0001115_junction_seq
GAATTAAAACTGCAGCAGAAGGCCAAGGAAACGAAGACGATGCTAATTCGAAGCAC
>dme_circ_0001116
GTCGCAACGGTGGAAAGTCAAACAAAGAATCCACAGGAGTGTCTACTCCTGCTCCGGGCTCTGCATCCGG
AAGCAATAGTGGAAACACGACTCCGGCTCACAAATCTAGTGCGCTGGATGGGTCCGATAAGGACAGCGGC
GGCATAAATGCCAGCGCAGTAGCCGCGGCGGTCACAAGCATTCACGATCCAAATCACTGGAGCAAGAAGG
AGAAGTACGACGCTGATGCGTACTTGGAAGGCGCCTACAAGAAGCATTTAGGCAGACACGCCAATAAGGT
AGGCGTGGCACTCGGAGTCATCTGCTAAACACTTGGCATTCATTAATCATTTTGTTTGTTGTTGTTGTTG
ATTGAATGATTGACTGGCTGATTATCAAAGAGTTTTCATTAATTTTGTATTTAATTTAACCTTCTGTTAT
AGATACTTTATAGCCTCTTTACTAATGCCAATTTGTGTCttttttttttaattattattaacccttttttt
taTAGAACTTTGTGTCTGCTGATTGAAGCAATGATCAGTGGGCGTTTCAAGGTTCAGGTGGAATGTGATT
CTCTTTTATTCGCTATGTATCTCTATACTGCAACTCTCTTTGTATTTTCGATTGCTTGTCCATTAAAATG
CTAACACTTGTACATCTTATACACGATTCTGCTCATTTGTAACCACAACATTTAAATTCTATATTTTGCC
AACTAATCTTCGGCGTATCGAATCTTCTTCTATCGCTGTGTTTTGTATCTTTTCGATTCGTTTAACAAAC
CTTAAAAATTCCTAATTGACCTCATTGCGATATCTATACGTATATTATATAACCACCAAAAACTGCAGCA
GTACGAGGGCCAAGCAGCAGTTGGCACTGAATCTGAATTTGGAAAGGTAATATCCCCATATATTCCTCAA

CCACCGATATCGATCGCCATCGCTCGTCGCCCTCGCTTGCACGCACGGTGTTTCCACTTTTAAGAAAAA
AGCTGGCGAATAGGGAAATTCACGCTACTCAACATCTTTATTAACCTCCATACGTGCAGTCGTCCTCATA
ATACAAAAACCGCCAAGTTATACATGCAATTCAATAATTAATTTATATTTTTTAATACGAATACCAAATT
CaaaaaaaaaaaGCCATCAAGAGGCTCAACTAACCTTTTAAATTTTGCTTTGTATGATTCTGCATTTTGT
AGTGGTAAATCTGTAGTACAAATTGGCAATTAACAATGTTACAatttttactttatttccctttatggtc
tcatttttgatttttgttttatttaaCATCATACAAAAAATTGTTGAACCGAGAAAATTGAATTTTTACT
AAACAACGCATTTCTTTTTTCGCGCATCTCTCAACTTCCCCAATCCACCAAACACATTTGAATGTTATCG
AATGTACTCATCGAACGATACGATCATATGTAGCGAACTCCCCATTCGCCCGCCGCCAACGCCAGATCAA
GTGCCATCTTCATGGTGGAATCCCATTTGTTGTGATAAGAGCCTGTTGGTGGGAACCTACAAGCATGGCT
GTGAAATGTATCGTCAAATGCGCGCCGATCCCAATCTGTGCTTTGTCACCCATGTGGGCGCCGGTGCGGC
CGGTGACGATCTGGCTGTCACGAGTTTGCCGATGTAAGTCTTCAAAACGAAAACAATCAATTCTAAATTA
ATTCGGTGTGTTCTAAGGAATCTCTATTTCAACGTGTACCTTCTGCACACACGTAACATTAATATTGATG
GTAATTGATAACCGTTTTAACCGCTACTGTGGTGAAAGAATTTGAGAAATGATATAGAGGTTTTTTTGAA
CACGCCGGATAGAAAAAACAACAGATGCAAAGAGATATAAACCATACATAAACGCCATTGAGTAATTCAT
ATACATCACACTAAAGTATtaaaattaaaaaaataaataaataCGCTTCCAATTAGACCTGAATGGAAGG
AACGTGCAGGATTTGCTTAAACTCATTTTCATTCCCATTCCCCACGTGCACATGCATTCATCTGAAAGTC
TTGGCATTAAAATGTGCTTCAAACATTTTTTCAGCTATTAGCGCTTAATATAATATTACATCAAAGATTA
CAGTTTGGTAGACAGTGCTATTTATCCATCAAATGTTAAGGTCATAAATCATAATAATTGTTTTGTTGAC
TTTTAATATAGACTAGATTTAATTTAAATTATTCAAATCAAATTAAAAAAGTACTTTCAATTCACATTAA
TAATTTTAATAATCATTGTGCTTGTTCATCGAATTCTCGCCGTATAATTTCTTGATAAGGTATTCAACGA
AATATTCTCAGATACAACAGCGCAACCTACGGGGCTTCCATTAACGCTCACATTGAATGCATTGATCCAT
TGACATTGCGCTGAAtgtgtgtgtgtgcgtgtgttttgtgAATTTTCTCTCAGTGTTTTAACTGCGTTGG
GCATGGCCTGCATCTGATATATGATCAGTAAATTCCATTAAATTGCTACTAACATTCAAACCCTTTTTTC
TGGAACCACCACAGAAAACGAAGACGATGCTAATTCGAAGCACGATGATGGTGACGAGGTGGACGATGAC
GGAACTACAACGACCAAAGACTCTGACTCTACTAAACTCACCGGGGGCGATAACAAGGACTCGCTTCTCG
ACCCGGAGCGTCCCAGTTCGTCTGGTAAGAGTAGCAGTGAAAATGCTAGCGGCGGCAACGACAAAACGGA
AAGCGAAAATGATGCCACAACCGTTTCCGAATCCTCATACAAATCCGAAGTCTTGCCGGGCAAAGGCAGC
GATCTGGATGTGTCGGTCGCCCAGCAGGACGCATTATCAGCAACTCCTGAATTGGCAAATACATCAGATC
CTAAAATGAATAAAAATGACGCACAAGTCTCAGCCTCTGTTTCCTGCGATCCGGCCGACTCTAGTTCACC
CGAGGCGGCAGGAAAAGCTCAGGGGACGGGGGTCCAGACTGGCGATGCCGGAGAATTGGACATCGAATCG
GAGAAAAAAGATAACGAAAAGGAAACCAAGGAGTCAACTGGTGATGCTCCTGCTGTGGAAGCCAAAAAAG
ATAGCGCTGCCATTGCCCGAGCTGAAGGCGATAAAACGGAATCATGCACTAACAATGCTACCACAAACAT
CTccaccaccactactactactaccattaccaataccaccaccaccaccaataATCCATCATCCAAAGCG
GCTAACTCTAACAACACCACCGACCATGCTAACTCAAACTCACATTTAGGCTTGGACGAGGAGGAGAGTG
TCGCCGGCAGCTATCCGCCAACCGTGGCAGCCGTCGAGGACGCCACTACTATGTGGCCATCTATGCAGGA
CCTTAACACGCGTCTCCGGCGGGTGATCACTGCCTATCAAAGGAATTACAAGAAAGAGGAATTAAAACTG
CAGCAGAAGGCCAAGG

>dme_circ_0001116_junction_seq

GAATTAAAACTGCAGCAGAAGGCCAAGGGTCGCAACGGTGGAAAGTCAAACAAAGA

>dme_circ_0001117

GGTCAACTGGCTTTCGGTAGTTGGCGCACTCGCTTCGGACGACTTTCTAATAAGACGGAATTGGGCCAAG
TTATTGCGGGAACTTTTTGCGCAGTGCCCGATGATTGGCTGCGAAAAAAATTGGGTAAATAACGATATCA
GCCACTAACACAAGCTGTGCCAGTAAAAATATTACAGCAGCCGGACCATCATCGATCTCTTGTCGCAATG
AAGGTATCATACTTTCATTTTTATTTTGCTTCCAGGGTCAACTGGCTTTCGGTAGTTGGCGCACTCGCTT

CGGACGACTTTCTAATAAGACGGAATTGGGCCAAGTTATTGCGGGAACTTTTTGCGCAGTGCCCGATGAT
TGGCTGCGAAAAAAATTGGGTAAATAACGATATCAGCCACTAACACAAGCTGTGCCAGTAAAAATATTAC
AGCAGCCGGACCATCATCGATCTCTTGTCGCAATGAAGGTATCATACTTTCATTTTTATTTTGCTTCCAG
TTGAGACGGCAGCATCCCCCACGGCAGGTCAATTGTACAGCAGTAAGTAAATGTCAAAACTTGTTGGCTT
ATAAGTCATCGTATATTTGCAGTTTTCTTTGTAAATGCAGTGCCGTTTATCGCTTTCGGATTCCTTGACA
ATTTCATAATGATCATGGCG
>dme_circ_0001117_junction_seq
CCTTGACAATTTCATAATGATCATGGCGGGTCAACTGGCTTTCGGTAGTTGGCGCA
>dme_circ_0001118
GGTCAACTGGCTTTCGGTAGTTGGCGCACTCGCTTCGGACGACTTTCTAATAAGACGGAATTGGGCCAAG
TTATTGCGGGAACTTTTTGCGCAGTGCCCGATGATTGGCTGCGAAAAAAATTGGGTAAATAACGATATCA
GCCACTAACACAAGCTGTGCCAGTAAAAATATTACAGCAGCCGGACCATCATCGATCTCTTGTCGCAATG
AAGGTATCATACTTTCATTTTTATTTTGCTTCCAGGGTCAACTGGCTTTCGGTAGTTGGCGCACTCGCTT
CGGACGACTTTCTAATAAGACGGAATTGGGCCAAGTTATTGCGGGAACTTTTTGCGCAGTGCCCGATGAT
TGGCTGCGAAAAAAATTGGGTAAATAACGATATCAGCCACTAACACAAGCTGTGCCAGTAAAAATATTAC
AGCAGCCGGACCATCATCGATCTCTTGTCGCAATGAAGGTATCATACTTTCATTTTTATTTTGCTTCCAG
TTGAGACGGCAGCATCCCCCACGGCAGGTCAATTGTACAGCAGGGTGAATACATTGAATATTATTTGGGC
CACTTTATAACGCTGTCTACAATGGCAGCCGCAGGACTAGGAAACACCATAAGCGATATTTTAGGCATCA
CTATGGCCACCTACGTTGAGAATGGGTGCCAGATATTGGGTCTAAAGCAGCCCAATTTGACTCCAGCGCA
ATTTGAATTAAAGTCTAGCAAGCGGTCTTCAAGTTAC
>dme_circ_0001118_junction_seq
AAAGTCTAGCAAGCGGTCTTCAAGTTACGGTCAACTGGCTTTCGGTAGTTGGCGCA
>dme_circ_0001119
GGTCAACTGGCTTTCGGTAGTTGGCGCACTCGCTTCGGACGACTTTCTAATAAGACGGAATTGGGCCAAG
TTATTGCGGGAACTTTTTGCGCAGTGCCCGATGATTGGCTGCGAAAAAAATTGGGTAAATAACGATATCA
GCCACTAACACAAGCTGTGCCAGTAAAAATATTACAGCAGCCGGACCATCATCGATCTCTTGTCGCAATG
AAGGTATCATACTTTCATTTTTATTTTGCTTCCAGGGTCAACTGGCTTTCGGTAGTTGGCGCACTCGCTT
CGGACGACTTTCTAATAAGACGGAATTGGGCCAAGTTATTGCGGGAACTTTTTGCGCAGTGCCCGATGAT
TGGCTGCGAAAAAAATTGGGTAAATAACGATATCAGCCACTAACACAAGCTGTGCCAGTAAAAATATTAC
AGCAGCCGGACCATCATCGATCTCTTGTCGCAATGAAGGTATCATACTTTCATTTTTATTTTGCTTCCAG
TTGAGACGGCAGCATCCCCCACGGCAGGTCAATTGTACAGCAGGGTGAATACATTGAATATTATTTGGGC
CACTTTATAACGCTGTCTACAATGGCAGCCGCAGGACTAGGAAACACCATAAGCGATATTTTAGGCATCA
CTATGGCCACCTACGTTGAGAATGGGTGCCAGATATTGGGTCTAAAGCAGCCCAATTTGACTCCAGCGCA
ATTTGAATTAAAGTCTAGCAAGCGGTCTTCAAGTTACGTAAGTTCCTCAAAGAACGTATAAAAGGGATTT
TTTTCATAGAAATATCTATTATTTTAGGGACGGATTGTCGGGATCACTGTAGGCTGTTTGCTTGGAATGT
GTCCTTTATGGTTTATGGATGAAAAAGCAGATAAG
>dme_circ_0001119_junction_seq
ATGGTTTATGGATGAAAAAGCAGATAAGGGTCAACTGGCTTTCGGTAGTTGGCGCA
>dme_circ_0001120
GGACTCTTGGGCCGATAGCATAAATGAGGAAGATGTGGAGCCCAAATTTAAGTATCACCGTCTCGCAAAT
GATTTAAAATACATGCTCAACGCTGATGTCATCACTTGTAGCGCTGTACACTTAAAGGTGTGAGCCAACC
ACATATTATATAAGTATTTTAAAAAACGTACATATTTAGTTTCTTATATTTGGAACTTTCCGAGGACGTG
TTTGTATATTTGACCATCAAGGAAACTCAGTCTACTCAAATCTTAGCGCTAGCGAACGTCACACTCACCA
AGTGGCAGTAAACAATATAGATGTAGATCATAAGGGCGAGTATGTGGCCACATGCTCCGACGACGGAAAA

GGCGACGACAAACTTATCTTGTACGAGCGAAACTTGCTAAAGAAGCTTAAACCAGTTGAACTGTGCTCAG
TCGAAGGCAGTGTTTTGTCAATCTGCTGGCATGGCAACTTTATTGCCTGGGCAAGTCACATAGGAGTTCG
AGTCTACGATTTAAACGAAAGATGCTCACTTGGATTAATAAAGTGGGAGGTTCCTCCTCAGGAGCGATTA
GAAAATTTTCGATGCCACCTTCGTTGGTCCAACAAGCATACATTATTAATTGGCTGGGTAGACACTATTC
GAGTTTGCGTAATTCGAAAGAGAAATTCAATCGAAGCTTCAACTGGCAATCTGCCTGTCTACATTGTGGA
TCCAAAGTATGTAAAGTTTATTAAAAAATCAACAGTGATTTACCGTAACcaaagacattagaataacaag
atgcgtaacgctattattattgagttttattattactagtttttgttcaagagcgtgagagtggagagct
ctactgccatcagctcttttctacgtatacagagacagcttgtatgcacacgtatgcttattcattgtaa
atttgacaaaatgtgcccttcactttacaagttcttagactttaaatctatattatatatgattaattgg
taccatataaaatattcatgttttgcattgcttaaggcaatgatattattatttgaataacacttagaaa
taataaataacaaaataaatttcaagtatgactatattagaatatttatcattcaagtattcagcttgcg
acgtgttaaaaattagagtagacattggttattaggtgcttataaccgcacgtcgtgcctcaagaattca
actttgcattaaacaggtagcatatttgtatttattttataaatattaacttttaactatgtatgtttgt
attatttttataaaatatatttttaaatatgtatttccttttttgaaaattattgttttaaattacaaa
agtaataataacaattccggatcggaacttaattttatttataagttgaaatattgattaacttaatgtt
aatgctgtaaaaggtattgctcacagtaacctgttgaaattttgcaacgaaattgaccaaaacttcaaat
atgtaaattcgattcttatatcaaaattgatttcagtttgaaaatcgtcttctagcccaagtaggcacac
atatacacgttttcgtttattgtttttactcacaaaagcaagcaaatattatatttatatttagatttat
taggctctcagttattttttctaaagtccccaaaaaaattgccggcataggacaaaatcaatgagtggccg
ttacgcatcttgttattctaatgtatttgCCGTTACTAAGTGTTCTGTTGACCGTTTTGCAGGTGTAAGG
AGGGGCGTGGCCAAAGTGTTTTTGGTATACCGATGGCAAGAGAAATAATAAAACAAAGAATAATTGAAac
agttcaggttcggtttatgggcgttagagtaggcgtgacaaaatttttttttttaaatttgcaagataca
aaatatcaaaaggggttgtggcaacatttgaacacacaacaaacttgacctgcgtctatgcctttagaac
ctgtatgcttaatctcaactttctggcgtatgtagttcctgagatctcgacgttcatacggacggacagg
ccagatcgactgatcaaaaaaaaatgtatatatatccttcagatggttggacaaacttctctctatgtgtt
aaatacttttcaacgaatctagtatattctttcattctacgagtaacggttataaTGAAATATTTTGGGC
TCACGCAGTGAAATTGAAAATCCTCACATTTATAATTTCACTTTACCATTGCAGGTCTCGACATTTCAAA
CAACTTTCTACGTATGTGGGCTTGCACCATTATCTGCAAAGCAATTGGTTGTTCTTGGCTTTCGCAAGGA
AAAAAGCTCTTGTTTTAAAGCCCTACGACCTGTATTATGCGTTATTGAATACAAAATGAATAATAGCGAG
GAAATCTGCACAGACAGTTTAACTTTACGCGGCTTTGAGGAGTACACCGTGAATGACTATAGTCTGGGTG
GCATAATCGAGGAGAATCGCTTTTATATTGTGGCCCCAAAGGATATTGTGGTAGCTAGTCTTATTGAAAC
AGATGATCGTATAGAATGGTTGATTAAACACAG
>dme_circ_0001120_junction_seq
ATCGTATAGAATGGTTGATTAAACACAGGGACTCTTGGGCCGATAGCATAAATGAG
>dme_circ_0001121
GGACTCTTGGGCCGATAGCATAAATGAGGAAGATGTGGAGCCCAAATTTAAGTATCACCGTCTCGCAAAT
GATTTAAAATACATGCTCAACGCTGATGTCATCACTTGTAGCGCTGTACACTTAAAGGTGTGAGCCAACC
ACATATTATATAAGTATTTTAAAAAACGTACATATTTAGTTTCTTATATTTGGAACTTTCCGAGGACGTG
TTTGTATATTTGACCATCAAGGAAACTCAGTCTACTCAAATCTTAGCGCTAGCGAACGTCACACTCACCA
AGTGGCAGTAAACAATATAGATGTAGATCATAAGGGCGAGTATGTGGCCACATGCTCCGACGACGGAAAA
GGCGACGACAAACTTATCTTGTACGAGCGAAACTTGCTAAAGAAGCTTAAACCAGTTGAACTGTGCTCAG
TCGAAGGCAGTGTTTTGTCAATCTGCTGGCATGGCAACTTTATTGCCTGGGCAAGTCACATAGGAGTTCG
AGTCTACGATTTAAACGAAAGATGCTCACTTGGATTAATAAAGTGGGAGGTTCCTCCTCAGGAGCGATTA
GAAAATTTTCGATGCCACCTTCGTTGGTCCAACAAGCATACATTATTAATTGGCTGGGTAGACACTATTC

GAGTTTGCGTAATTCGAAAGAGAAATTCAATCGAAGCTTCAACTGGCAATCTGCCTGTCTACATTGTGGA
TCCAAAGTATGTAAAGTTTATTAAAAAATCAACAGTGATTTACCGTAACcaaagacattagaataacaag
atgcgtaacgctattattattgagttttattattactagtttttgttcaagagcgtgagagtggagagct
ctactgccatcagctcttttctacgtatacagagacagcttgtatgcacacgtatgcttattcattgtaa
atttgacaaaatgtgcccttcactttacaagttcttagactttaaatctatattatatatgattaattgg
taccatataaaatattcatgttttgcattgcttaaggcaatgatattattatttgaataacacttagaaa
taataaataacaaaataaatttcaagtatgactatattagaatatttatcattcaagtattcagcttgcg
acgtgttaaaaattagagtagacattggttattaggtgcttataaccgcacgtcgtgcctcaagaattca
actttgcattaaacaggtagcatatttgtatttattttataaatattaacttttaactatgtatgtttgt
attatttttataaaatatatttttaaatatgtatttcctttttttgaaaattattgttttaaattacaaa
agtaataataacaattccggatcggaacttaattttatttataagttgaaatattgattaacttaatgtt
aatgctgtaaaaggtattgctcacagtaacctgttgaaattttgcaacgaaattgaccaaaacttcaaat
atgtaaattcgattcttatatcaaaattgatttcagtttgaaaatcgtcttctagcccaagtaggcacac
atatacacgttttcgtttattgtttttactcacaaaagcaagcaaatattatatttatatttagatttat
taggctctcagttattttttctaaagtccccaaaaaattgccggcataggacaaaatcaatgagtggccg
ttacgcatcttgttattctaatgtatttgCCGTTACTAAGTGTTCTGTTGACCGTTTTGCAGGTGTAAGG
AGGGGCGTGGCCAAAGTGTTTTTGGTATACCGATGGCAAGAGAAATAATAAAACAAAGAATAATTGAAac
agttcaggttcggtttatgggcgttagagtaggcgtgacaaaatttttttttttaaatttgcaagataca
aaatatcaaaaggggttgtggcaacatttgaacacacaacaaacttgacctgcgtctatgcctttagaac
ctgtatgcttaatctcaactttctggcgtatgtagttcctgagatctcgacgttcatacggacggacagg
ccagatcgactgatcaaaaaaaatgtatatatatccttcagatggttggacaaacttctctctatgtgtt
aaatacttttcaacgaatctagtatattctttcattctacgagtaacggttataaTGAAATATTTTGGGC
TCACGCAGTGAAATTGAAAATCCTCACATTTATAATTTCACTTTACCATTGCAGGTCTCGACATTTCAAA
CAACTTTCTACGTATGTGGGCTTGCACCATTATCTGCAAAGCAATTGGTTGTTCTTGGCTTTCGCAAGGA
AAAAAGCTCTTGTTTTAAAGCCCTACGACCTGTATTATGCGTTATTGAATACAAAATGAATAATAGCGAG
GAAATCTGCACAGACAGTTTAACTTTACGCGGCTTTGAGGAGTACACCGTGAATGACTATAGTCTGGGTG
GCATAATCGAGGAGAATCGCTTTTATATTGTGGCCCCAAAGGATATTGTGGTAGCTAGTCTTATTGAAAC
AGATGATCGTATAGAATGGTTGATTAAACACAGGGTAAGCCAATAGTCCTATATTTTCATGACATTAATA
CACTAATAATCTACACTACTACTTTTTCATCGGAGAATTACTGTTAGGTATTCATATTTTGGCTTAATTA
CTGCTCAATTTGTTCTACGGACTTATTGCTAGGTAAACCATCACAAAAACAGCACTCAAACTGCGCAGTA
TCCTCAGTAGTAATCATTTCTATTTATGCACATATTCATTCATCACCGTACTCCGTGGAAATATGCTCAT
ACCTTGAATAATGAGAACATAATTTGAAGTCTTTAAATGATCCTGCCTACCTTGATTTTCCGTATGTTGA
TTTATGGTAATTTACCTGTTTTTACCTTTTTGCTTGGCTACCTCGAAGGCATTCCTTACGGATTTTGTAC
TGTTAGATAGCCAAAAAACTTTAtgtcacgccaacactttttaaaaatattttcatatttttgttattct
tgtacatttttttcgacttgataaaaaacgtttggccacgcccactctaacgcccacaatccgcccttttt
tttttatttttttaccttactttacctttattttcactctcccagaactatcgatatctcagaaaaatta
tgaaatttcgcgttttcacttccacaaccagtatctgttagtctggatctcgactatagcattctctctt
TATTTACACGATACACGACGTTACTCGTAGAAGTATGAAAAGTTGAAAAGTGTAACACGTAGAAGGcagg
atcaataaccgagtccttcaggccctgtctgtctgtccgtctgtatgaacgtcgagatatcaaagaagta
taacagctagaaggttgagattaagcataATACATACAAAATTGTAACtttataattatttaacaaaata
atttttattttgattattttgtatttggatttttttttttgtattttgatttCGGTGATAGACCTTTTCA
CTTATGCTATTACAAGACACTTTATTAATAGCCTAGCTGTTCACGATCTCGTCCCGATTCAGACTGATCT
TCTTAATCTGAATCCTAATCCACTCAACCTCGACCAGAAGCTTTTCTTTAAGATTTTCTTGAATTTTCAA
ATAAAATAAAAGCTTTACACTGAAATTATTTCACAGCATTGTGAAATTCAAATTTGCTGAGTGATGCAGT

TTCATTGAAACGCAATAAGTTGATCATGGCTTGGTCTCCAAGTGGAAGTTGTGTTTACTTTAAGTTTGGT
TATTTATCAGTTTGGCGAAAACGGATGACTAAGTGTTGGCGACAAAAACAAAAGCAAAGGAAATGCTGAT
GAGATTAAAGTTTGAAATTTGTTTATTAACTGTGGCTGAGTGCTTTATTTACGGGTTGGATTacttagac
gaaacgcaagtttgttgacccatgtatacgggcccacaaaccgcccaaaactgccgcTTATAAAAATGtt
ttttttttCTGGATATGCCAAAAAACACAttaaaaaatatatttcataaaaataatacaaacatacatag
ttaaatgttaatatttataaaaTGAGTAAAATTATGCGACCTacgacgtgcggtcataatcacctaataa
tcgatgcctactagacaatgtttactctactttttagcacgtcgcaagctgaatattcaaattattataa
gtcctttttgaaatttattttataatattatgtacatagattcggctattactatttctaagctatattt
aaataataataaagtaaaggcaatgcaaaaaaagaatttttgacactaaaactaaacggcatattttgtc
aaatttacaatgcatacgtgtgcacacatacagttgtcggctgttactgtatgcgtagaagagagctgct
ggctgtagagctctccgctctcttgctcttcaacaaacatccgagagagcctcaaaccagctctagagcc
agccaaaaaaaaatcgagtgcaaaaaatcgtattccgttacttatcttgttattctagtctctttgCCCA
CACTTTAGAAAAATGGTACATAAAGGGTTGTTCCACATAAACTAAGGAATTTAGAATAAAAATATATTTT
GACCCGTTTAGACAGTAATTACAACGAAATTTTTTGTGAAAATCCCGTAGCGTGTTTGACAAGGGTTACT
TATTTAATTTTGAAAACATTTTAGTTCATCGACCACCTCAAGTAAGAAAATATTTGGTTTGTAGATTTCT
GACTAAAATTGTGGTCTACATTCATCTACGATGAATAACAAAATCCTTGAAAATGTTGTAAAATTTCAAT
AATATAAATAATTTTTATAGGGATTTGAAAAGAACTGGCTTTATTATCCTACAAATCGCACGCAAAAAAA
CATTTTTTCCATttttaaagttactgacatgtcgacgttcatacggacggacatggccagatcgactcgg
ctaattttcctcaacgagtatatatcttttatatgatcagaaacgcttccttctatttactacatacctt
tctgcaaatctggttaactctttaactctacgagtaacgagaataaATTCTTTAGAGCTAAATTATTCAT
TAAAGAGAGAAACCCCTATTAGGTAAAGAGTGTATAGGAGTGCAAACAGAGCTAGAAATAAGTACGGTGA
TCTACTGAAGTAGTAACTAACATAGCAAAATTTACATAAGTAAAGTATTGTTGTTTCACGAGCAAGAAGT
AGACTTAAAGATATACATCCAAATGACAATTTCAAATGGATTTGAATTGTCTGTTTGTCCAGTTTCTCTC
CATATTCGTTATCTTTATCTGTAGCTAGTACATGAAATTACTGTTTGGGCCATCAACTGAAGAAATTTTG
GTTGAGAAGAATACCAATAAATCATTATTTTTATACACACACAGACATCGTGGCGGATGAATAATTGTTT
TCTACGTTGAAGATGGGATCGTCTCTAATTCTTACCACCATTTGGAGGTTGTTTTTGAtagtaaatctcc
tcggaacaaagaagtttagccgactaaccaagtcggggatatcaactttaccacgaacaagcttttaaat
aaagcaagcatcgtactacggttagctggctaaaggtaaattaattaaaagcagtctattgtaataagga
ggtaataaggtttgcatactaatttaggcaccgtaaagcaaacagtaaggagtttttttggaccgattcaa
tgcggtccgagtgaactacgtattgaggactccaaaaccaagcacacccctggccttatggacaattgtc
gaaatatggggtgaaaaaattagtttaaagaccagaagaacaccaagctcatcaactcgtgctattctct
tcaaagagcaccccctaagagt
>dme_circ_0001121_junction_seq
ttctcttcaaagagcaccccctaagagtGGACTCTTGGGCCGATAGCATAAATGAG
>dme_circ_0001122
GTCTCGACATTTCAAACAACTTTCTACGTATGTGGGCTTGCACCATTATCTGCAAAGCAATTGGTTGTTC
TTGGCTTTCGCAAGGAAAAAAGCTCTTGTTTTAAAGCCCTACGACCTGTATTATGCGTTATTGAATACAA
AATGAATAATAGCGAGGAAATCTGCACAGACAGTTTAACTTTACGCGGCTTTGAGGAGTACACCGTGAAT
GACTATAGTCTGGGTGGCATAATCGAGGAGAATCGCTTTTATATTGTGGCCCCAAAGGATATTGTGGTAG
CTAGTCTTATTGAAACAGATGATCGTATAGAATGGTTGATTAAACACAG
>dme_circ_0001122_junction_seq
ATCGTATAGAATGGTTGATTAAACACAGGTCTCGACATTTCAAACAACTTTCTACG
>dme_circ_0001123
GTCTCGACATTTCAAACAACTTTCTACGTATGTGGGCTTGCACCATTATCTGCAAAGCAATTGGTTGTTC

TTGGCTTTCGCAAGGAAAAAAGCTCTTGTTTTAAAGCCCTACGACCTGTATTATGCGTTATTGAATACAA
AATGAATAATAGCGAGGAAATCTGCACAGACAGTTTAACTTTACGCGGCTTTGAGGAGTACACCGTGAAT
GACTATAGTCTGGGTGGCATAATCGAGGAGAATCGCTTTTATATTGTGGCCCCAAAGGATATTGTGGTAG
CTAGTCTTATTGAAACAGATGATCGTATAGAATGGTTGATTAAACACAGGGTAAGCCAATAGTCCTATAT
TTTCATGACATTAATACACTAATAATCTACACTACTACTTTTTCATCGGAGAATTACTGTTAGGTATTCA
TATTTTGGCTTAATTACTGCTCAATTTGTTCTACGGACTTATTGCTAG
>dme_circ_0001123_junction_seq
TCAATTTGTTCTACGGACTTATTGCTAGGTCTCGACATTTCAAACAACTTTCTACG
>dme_circ_0001124
GTCTCGACATTTCAAACAACTTTCTACGTATGTGGGCTTGCACCATTATCTGCAAAGCAATTGGTTGTTC
TTGGCTTTCGCAAGGAAAAAAGCTCTTGTTTTAAAGCCCTACGACCTGTATTATGCGTTATTGAATACAA
AATGAATAATAGCGAGGAAATCTGCACAGACAGTTTAACTTTACGCGGCTTTGAGGAGTACACCGTGAAT
GACTATAGTCTGGGTGGCATAATCGAGGAGAATCGCTTTTATATTGTGGCCCCAAAGGATATTGTGGTAG
CTAGTCTTATTGAAACAGATGATCGTATAGAATGGTTGATTAAACACAGGGTAAGCCAATAGTCCTATAT
TTTCATGACATTAATACACTAATAATCTACACTACTACTTTTTCATCGGAGAATTACTGTTAGGTATTCA
TATTTTGGCTTAATTACTGCTCAATTTGTTCTACGGACTTATTGCTAGGTAAACCATCACAAAAACAGCA
CTCAAACTGCGCAGTATCCTCAGTAGTAATCATTTCTATTTATGCACATATTCATTCATCACCGTACTCC
GTGGAAATATGCTCATACCTTGAATAATGAGAACATAATTTGAAGTCTTTAAATGATCCTGCCTACCTTG
ATTTTCC
>dme_circ_0001124_junction_seq
TAAATGATCCTGCCTACCTTGATTTTCCGTCTCGACATTTCAAACAACTTTCTACG
>dme_circ_0001125
GTCTCGACATTTCAAACAACTTTCTACGTATGTGGGCTTGCACCATTATCTGCAAAGCAATTGGTTGTTC
TTGGCTTTCGCAAGGAAAAAAGCTCTTGTTTTAAAGCCCTACGACCTGTATTATGCGTTATTGAATACAA
AATGAATAATAGCGAGGAAATCTGCACAGACAGTTTAACTTTACGCGGCTTTGAGGAGTACACCGTGAAT
GACTATAGTCTGGGTGGCATAATCGAGGAGAATCGCTTTTATATTGTGGCCCCAAAGGATATTGTGGTAG
CTAGTCTTATTGAAACAGATGATCGTATAGAATGGTTGATTAAACACAGGGTAAGCCAATAGTCCTATAT
TTTCATGACATTAATACACTAATAATCTACACTACTACTTTTTCATCGGAGAATTACTGTTAGGTATTCA
TATTTTGGCTTAATTACTGCTCAATTTGTTCTACGGACTTATTGCTAGGTAAACCATCACAAAAACAGCA
CTCAAACTGCGCAGTATCCTCAGTAGTAATCATTTCTATTTATGCACATATTCATTCATCACCGTACTCC
GTGGAAATATGCTCATACCTTGAATAATGAGAACATAATTTGAAGTCTTTAAATGATCCTGCCTACCTTG
ATTTTCCGTATGTTGATTTATG
>dme_circ_0001125_junction_seq
ACCTTGATTTTCCGTATGTTGATTTATGGTCTCGACATTTCAAACAACTTTCTACG
>dme_circ_0001126
TTTTCCTGTTTGCATATGACCAACGCCTCCTGAATGTCATAGTCCTTTGAACGCTTTAGAAAGGGCAATA
GTTTGTTTCGATCGAAAATGGCATATAAGGAGATAAGttttttttGAAACACATTACTGGGATCCTTTTT
AAGTAAAGAATCTAAGTACTGGAAAAAACAAAAATTTTAATTTTTATTCTTATTATTACATACGCCACTT
ACCCAGTACAGGTACTCTTGATTGTGCTCTAGCTGGTGGACAACTATCTCAGTCTTTATTTTTTTCTTGT
CAAGTAGTATTTCAAAGGCGCAGTCACGATCCAATTGAATAAGTGGAATAATCAGTTTTGAAATGACATC
GTAAAGCTCATACCGTCGAATTAATTGGAAGACATCCTTGTTCTGCAACCTACGAACATTGTTTAATTAA
GTTATTTTAATTGACTTTGCTACATACTTTAAATACATGCGAAGAGCACTTTCGAAGTCGCCTTGATAGG
AATACAAAAGAGCCAATGATTCCAGCAACTGATTGGCATAGTGCTTTCGAAAGTTATCGTGAATAGCGTT
AATAACAGCCAGGCCGTCATAAAGATGAGATGGCCATTCCTTGATAAGATTCAGGAAACCACATACATCA

AATTTGAGAAACTCGTATAAAACCATTTCGTATACATGGGGATCAAGTTTGCATTCGTCTGAAGTCGGCA
GATAGGCGCTAACTGATCGTAGCTGCTGACACTTTACAAACTTAAAAACTTCCTCTTCCCAAAGGACTTT
GTCATTGCCTAGCATGCGGAGGCAAAGTTTTGCTGCG
>dme_circ_0001126_junction_seq
TAGCATGCGGAGGCAAAGTTTTGCTGCGTTTTCCTGTTTGCATATGACCAACGCCT
>dme_circ_0001127
GACGCCAGGATTTGTACCACAACCGTCTCCCCAGCTAACCATGCCTATGAGTTGCCTATGGCCGCCTTTC
GTTGTTTCAATCAAAGGACCACCGGAATCACCTTGACAAGTATCCCATCTTTGTCC
>dme_circ_0001127_junction_seq
CACCTTGACAAGTATCCCATCTTTGTCCGACGCCAGGATTTGTACCACAACCGTCT
>dme_circ_0001128
GGATTTGTACCACAACCGTCTCCCCAGCTAACCATGCCTATGAGTTGCCTATGGCCGCCTTTCGTTGTTT
CAATCAAAGGACCACCGGAATCACCTTGACAAGTATCCCATCTTTGTCCGTCGGCACAGATCATGTCGAT
AGTTATATTAACGTGCTT
>dme_circ_0001128_junction_seq
TCATGTCGATAGTTATATTAACGTGCTTGGATTTGTACCACAACCGTCTCCCCAGC
>dme_circ_0001129
GGATTTGTACCACAACCGTCTCCCCAGCTAACCATGCCTATGAGTTGCCTATGGCCGCCTTTCGTTGTTT
CAATCAAAGGACCACCGGAATCACCTTGACAAGTATCCCATCTTTGTCCGTCGGCACAGATCATGTCGAT
AGTTATATTAACGTGCTTGTAAGCTTTCAGACAATCCGTTCTATTCAGAATGGCTACATGTACGCCTTGC
A
>dme_circ_0001129_junction_seq
TTCAGAATGGCTACATGTACGCCTTGCAGGATTTGTACCACAACCGTCTCCCCAGC
>dme_circ_0001130
GCTACAGTGAAATCCTTCGGTAACTATGATAAATAACTATATATAATGATAAAAACCGAGACTACAGCAT
TTTAATATGCATATAGACTTATTTAAACCCATCGGCAAACTTAGTTATCGTGGTATTTGTGATATAATAA
TATTAATAACAACTTTTTAACATATGCATTTTATATGGTAGCATATGTTGATTTATATTAATATTATCGA
TATATTAAAACGGATGTTGTCAATATCAAAAATATTGTTTTATATGTATTTTGCGTGCATCTTCGTTTTG
AAAAGCCATTAACCAATTTATGTGAGATTTTAAAAGCAGCTGAAATATTTTTAAATGGTCGTTAAAA
>dme_circ_0001130_junction_seq
CTGAAATATTTTTAAATGGTCGTTAAAAGCTACAGTGAAATCCTTCGGTAACTATG
>dme_circ_0001131
GTGAGCATTGGGCGGACGACGGCCAGTCACATCCTTTAGCTTCGTTGTTCCAGTGAAGACCGCTTGGACA
AAACTGTTGCTGTAACTCTCCCGCAAAAAAACAGTGATAGTAAGCGTTACAATTATTTGA
>dme_circ_0001131_junction_seq
AGTGATAGTAAGCGTTACAATTATTTGAGTGAGCATTGGGCGGACGACGGCCAGTC
>dme_circ_0001132
GTCGCAACGGTGGAAAGTCAAACAAAGAATCCACAGGAGTGTCTACTCCTGCTCCGGGCTCTGCATCCGG
AAGCAATAGTGGAAACACGACTCCGGCTCACAAATCTAGTGCGCTGGATGGGTCCGATAAGGACAGCGGC
GGCATAAATGCCAGCGCAGTAGCCGCGGCGGTCACAAGCATTCACGATCCAAATCACTGGAGCAAGAAGG
AGAAGTACGACGCTGATGCGTACTTGGAAGGCGCCTACAAGAAGCATTTAGGCAGACACGCCAATAAGGT
AGGCGTGGCACTCGGAGTCATCTGCTAAACACTTGGCATTCATTAATCATTTTGTTTGTTGTTGTTGTTG
ATTGAATGATTGACTGGCTGATTATCAAAGAGTTTTCATTAATTTTGTATTTAATTTAACCTTCTGTTAT
AGATACTTTATAGCCTCTTTACTAATGCCAATTTGTGTCttttttttaattattattaaccctttttt

taTAGAACTTTGTGTCTGCTGATTGAAGCAATGATCAGTGGGCGTTTCAAGGTTCAGGTGGAATGTGATT
CTCTTTTATTCGCTATGTATCTCTATACTGCAACTCTCTTTGTATTTTCGATTGCTTGTCCATTAAAATG
CTAACACTTGTACATCTTATACACGATTCTGCTCATTTGTAACCACAACATTTAAATTCTATATTTTGCC
AACTAATCTTCGGCGTATCGAATCTTCTTCTATCGCTGTGTTTTGTATCTTTTCGATTCGTTTAACAAAC
CTTAAAAATTCCTAATTGACCTCATTGCGATATCTATACGTATATTATATAACCACCAAAAACTGCAGCA
GTACGAGGGCCAAGCAGCAGTTGGCACTGAATCTGAATTTGGAAAGGTAATATCCCCATATATTCCTCAA
CCACCGATATCGATCGCCATCGCTCGTCGCCCTCGCTTGCACGCACGGTGTTTTCCACTTTTAAGAAAAA
AGCTGGCGAATAGGGAAATTCACGCTACTCAACATCTTTATTAACCTCCATACGTGCAGTCGTCCTCATA
ATACAAAAACCGCCAAGTTATACATGCAATTCAATAATTAATTTATATTTTTTAATACGAATACCAAATT
CaaaaaaaaaaaGCCATCAAGAGGCTCAACTAACCTTTTAAATTTTGCTTTGTATGATTCTGCATTTTGT
AGTGGTAAATCTGTAGTACAAATTGGCAATTAACAATGTTACAatttttactttatttccctttatggtc
tcatttttgatttttgttttatttaaCATCATACAAAAAATTGTTGAACCGAGAAAATTGAATTTTTACT
AAACAACGCATTTCTTTTTTCGCGCATCTCTCAACTTCCCCAATCCACCAAACACATTTGAATGTTATCG
AATGTACTCATCGAACGATACGATCATATGTAGCGAACTCCCCATTCGCCCGCCGCCAACGCCAGATCAA
GTGCCATCTTCATGGTGGAATCCCATTTGTTGTGATAAGAGCCTGTTGGTGGGAACCTACAAGCATGGCT
GTGAAATGTATCGTCAAATGCGCGCCGATCCCAATCTGTGCTTTGTCACCCATGTGGGCGCCGGTGCGGC
CGGTGACGATCTGGCTGTCACGAGTTTGCCGATG
>dme_circ_0001132_junction_seq
CGATCTGGCTGTCACGAGTTTGCCGATGGTCGCAACGGTGGAAAGTCAAACAAAGA
>dme_circ_0001133
atttgacagaaataaagcaaactgTTGTtcgcgcatataattattcctatgcatttttgcgaaataatcc
gtgagaccaggacggagagtttgaagatcaaggtgatcataaagtgccacaggtcgagccagtgaggtct
gcgactttgaaatcgaataataagaaaatttAAACTTATAGATCATTTTCGATCATACggctggtgg
>dme_circ_0001133_junction_seq
AGATCATTTTCGATCATACggctggtggatttgacagaaataaagcaaactgTTGT
>dme_circ_0001134
GTCGCAACGGTGGAAAGTCAAACAAAGAATCCACAGGAGTGTCTACTCCTGCTCCGGGCTCTGCATCCGG
AAGCAATAGTGGAAACACGACTCCGGCTCACAAATCTAGTGCGCTGGATGGGTCCGATAAGGACAGCGGC
GGCATAAATGCCAGCGCAGTAGCCGCGGCGGTCACAAGCATTCACGATCCAAATCACTGGAGCAAGAAGG
AGAAGTACGACGCTGATGCGTACTTGGAAGGCGCCTACAAGAAGCATTTAGGCAGACACGCCAATAAGTG
AGTCGTGTGGAATCGAACTCGTTATAAGATATTTCCAAATTTGAGATGGACTGTTGCTAACAATTTTTTT
CGCTCTCTTTTCCCTTTATTTTCATACATTTTGTTTATTTAATGTAAACATTTACAGGGTTTTGCTGCGC
GTGAGGATGCTCTATTATATTCAACATGAGGTTATTGGCGACTTTGTTCAGCAGATAAAGGACAACACGC
CTATCAGG
>dme_circ_0001134_junction_seq
GCAGATAAAGGACAACACGCCTATCAGGGTCGCAACGGTGGAAAGTCAAACAAAGA
>dme_circ_0001135
GATTGCAAATTGAAATGGCAGCAACAATGTGAACGACCAGAAGAGCAACAATACACCGCTGAAGAGCTCT
GAAAATAAGCAGCAGAAAAAAAGAGGAAATATCAGATAGAAGCGGTCGAAATCATAAACAAGAATGCCGG
CCACTTGCGgaggactgtgaggactgcaaggactgccaggaTGCTGATGCGATGCGAAGGCGACGCAGAT
TGCATTCAAGGACCGCTGGGCCAGCAAACAATCAGTGGGGCAGACAATGAGAACTAATAAAGCACTTAAG
CGGATAGAAGACCAGAAACAAGGACACCCAAGTGGAAGCGACCCTACAGCAGCAGCAGCCGCAATAGCAT
CAATCAAAATAAGATTCACCCTGCCACGGGGCGTATGAGCAACGTGCATCAGTTTGATACGCAGACGATG
GCGGAATCGCCTCAAATTAGACGCGACATGGGTCGCCTATGTGCCACGTGGCCTTCGAAGGACTCTGAGG

ACGGGGCCGGCACAGCTTTGAGGGCAGCCACACCTCTGACAGCCAACGGAGCCACCACCACCGGGCTGAG
TGTTACCCTTGCCCCGAAGGACATGCAACGAAACCACCTGCTAAAGATGCCAACAGCAACAATAGAGAAA
CCAACGATAACAGCCACCATCGCCAGCAGCAGCAGCACCAGCACTAGCACCACTAGAAAATCAGTTACAG
CCACACGGAGCCTGAAGTTGAACCCGAACATTCTCCTTCCGACACTAAGGATATTAGCAAGGGGTCTGCT
GCTACCCGCTCTGATCCTGGCCATCCTCGTGGGCAGCAGCCAGGCGGGATTTGCCTGCCTCAGCAATCCC
TGTGTGTTCGGCGTGTGCATCGATGGCCTAAACAGGGTGAGTTCAGGTGCAAAGCCCTCTAACCAATTAC
TAGAGATGGGAACGGTTGTATCTTGTGCAAATTTACTTTATTCACAAAATATCAGAACAGGATTTTAACA
TTATTACCCCGACTTGATGCAGACCAATTGGGATAGGTACTCTTGATTAAATTCGACATATCTGGTGGCA
TTTTTTCGAACATCACTAAATGTGTAAATGCACTCTGTAACCGTTTTACTTTTTTACTTTCTGATTATCG
GCCAGGCAGCGACCGACCAATAAACCCGTCCAACACATTTCGAGTGGCAATAAACTTGTGCCACTTGAAC
ATCAGGAGGGGGCATCCAGCTTCAGTTTCAGTAATCCAGATATATGAACCTTTCCATACCAACCCATGCC
ATATGCGGAACCCTCGATGTTGATGCCGCCACTTAGCAGGGAAATGAATTTGCCACAGCTGTCCTCCTCC
CTTCGCTTGTATTTTTGTGCTCCTGTTTCAGGGCAGGAATACCCATACCCACCGGTCTCACATCCTTCAC
ACTTTTTAATGCCTGCCCGTTTAAATGGGGTTAAGTTGCGGCCACTGCAAGTGTGTTGGTGTGGGAAGGG
GAAGGCGGAAAAGCCAGAGGGCGGACCAAAGGGTTAAGTGGGTTTCAATTGCAAGCAGCTGGAAAATTAT
TAACTTGGACGAGCAACCGAAGTGCATTCTTAATGCAGAAAATTTATAGCCAAGATATGCAAAATATTTC
AAGTTTTATTAAAAATGCGGCACCCACAGCTGGGGGAATTTAATTAAATGTGATGCTTAAAGATAATCAA
GCTTAAAGAATATTGCGGTCATTTGAATCTCTCTTAGTTACACTTTTCAAATGAACTCTGGCGGTTTGAA
GCCAAAAATACGTTTTTCTTGCACAAATTCCGATAAGAAAGCTATGCAAATCCATTGCAAATATCGCCAC
AGTCTGCACTTCATCTCAAATTCAAATCTAAGTAGCTCGCCAAATTGCTGCACTTTCAGCCATTCAGTGG
GCTAAACTTTGAGGTTTAACTGTCAGAGATTCCACCATCCGACTTTTCCCAGACGTACACACGTATGTAC
GTATGTGTAACTCAAAACCGATTGCACAAACAAGTTAGAAGACGAGGCAATCCTTCTCCTTGACGCCATT
TATACGACTAAGTAACTGCCATTTTATTTTCCTATGCTCACTTCGCGATATATGCAAGCGCTAATCCGAC
CCAGAAAGCATCCAGATCCAGAACCAGAACCACCCAACCACCGACGGGAAATGCGGTTAAGATATTTTTT
ACGATAGCTCCATGGGAAAGCTGCAAGTCAAATGCGAAATGCGAAAACCAGAAGCTTGAAATCGCTTCCC
GCCTGCATTTCCCCTCCCCATCAACCAATAGGAGTGGAAAAACTCAGGCTGGCCAAGGCGGATGGATGGG
AAAGTTTATATGCTGGCAGGCGAAATTTATAGCTGCAATTAAATTTAACACGATGCCATGAGATTTAAAC
GGATTAACTGAATAGTTTTCGACCAGCTCAAACTTTGGGGTCGACTGCGTCGAGGGGGAAATATCGCGAG
GAGTTTACTGGCTTCGTAGAACATTGATATTTTATTGGTTTATCCTGGCTTATTCGAGCGATAAGATGGC
TCTTAACCGTTTTATGACTTCGCTCTGCACGCTCCAATAAAAGTTGGCTATTCAAGATGAGCAAAAGGAT
ATGATTTGCTTGGCAAATTGCCAAAGCCATTAATCGTGCACTTCATTTAGTCATGATAAAATCGGAATGA
GAAAATTTCTAAATTATGTCTTTAAATGCTTTCGATTAGAACTCGGCGCAGAGTAATGGTTAAATTATTA
TGCAATACATTATTGATTACCCCCAATATTTATAAATAGAGTAAATGCTTCGCAGGACCACAAAAAATTA
GCCACTCCAGCGAGCGTCCAATATCCTGATGGCCAGGCACTCTGATATTCCGTCCACAAGGAGCCAGTCC
AATGCCATTACGTAGTCATTTCCCAATTGCTCCTGGCTGAGCACAACAAATCAAATAGTCTCCCGGGTGA
AAATGTCCAACCGTAGCCCATAATCAGCCGAACTGGAATCTCAGGGGCTTCACTGGTGAACTGAAAACAA
GGGCCGGGAACAACAGGCGAGTGTATCTGGGTGCTTATGCCGTGCCAAGACCATAACAGACATGTGCACC
GGGAGCCATCAGCTGCCTCCTGCATCCTAATGTAATCCATTATAAGGGACAACAGTAAGCCAAAGCTACC
TACATACCCCCGCACCCCAGCCCCACTGGCCCATTTACACGGCAAAGAAACTTCTTTAATTAAGCCGGTT
ACCTGGCAGCAGTTCCTGTTTGCCATGTTTGCAGTGCTCCTCGGATAGCACTCGTCCACAGTTCCCCTGA
CACTTTTTTCGCTCTTTGCATTTCCGTTTCCGTTTCGATCCGAACTCGGCTTGGCCaaaatataaataga
aatggaaatagaaataggaatgaaaatggaaaACCCAATCCCAAATGTCTACCAAATATTTATTTGCACA
ACTCCTCAAGTCCAATGTTGCGCTCTTTGAATGTTTATAGCACTCAACTGGCCGGCGATTTAACTGCAGG
GCATGTGCATTATGTCTGAATGTAGGGGATCTGAGCCTGAAAAGTCGGGGAAATTTATTCAGGAAAATAC

TGGACTGCAGGCCAAAACAAATGCGAGACTCGAGTGCCACCAGCTCAGTTCCATTTTCATAATTGTGAAA
TATGCATATGCAGAATATTAAGATAGAAGCAAAAGTAGTATAAAATATAGATGTATGATTGACTAATATT
GTTTAAAGAATTGATCTAGATATATATCTCAGGGAGATGGCGAATTTATACGTGTCCGCCCATCTGCACT
TCAATTACTGCCTACTGCCATTATAGTCCGGctgtatctgtatctgtgtatctgtatctgtTCCTGCCCT
CCCGAAACAATTTGTATCCAAAGCATTTGGTTTGTTCTGCCACTAAACCTTCGTTTTCTCTGTTTTCTTT
TGGTGGTGCTGCAATCCGGCTGTTTTTTACCACCGTTTGATGCATCATTGACACACCGTTCCGCACACCC
ACCGCACCCGGATGGAAACAACTAAAACAACAGCTCGTACTCGTGTTACTGCATTGATGGCTACACAGGG
ATCCAGTGCCAAACGAATTGGGACGAGTGCTGGTCGAGTCCTTGCCAGAACGGCGGGACATGTGTGGACG
GTGTGGCCTACTACAATTGTACGTGTCCGGAAGGATTTTCTG
>dme_circ_0001135_junction_seq
AATTGTACGTGTCCGGAAGGATTTTCTGGATTGCAAATTGAAATGGCAGCAACAAT
>dme_circ_0001136
ATCAATATCCTTTTATATGCATCTTACTCGGACTTAAATTAAAGCTCTCTAAAAATACGCTCAAAACCGA
AATGTGCACTACTCCTCGTTGAAAAATTACCGTTTTTCGTGAAAAACTTAAAAAAGTTGATTAGTACTAA
CAATAACTTATTCGAAATCTTTCTGAAATGTTCTTTTTCAGCCTGTGAGCGAATGTGTTTCACTGCGAAT
ATGTCAAAGGTCGGCCATGAAGTGCGCGAAACACGAAACACAAATGACTTTTACCCTATAAAAGCAACAA
CAAGACGTTAAATTGCACACCAGTTGTAATTAAGCTTAGTAGTTAGAACCAACAACCAACTAATATATAA
TCATAATCGATAAGGCGAAGGTGGATTGAGACACCAGAGACCGACCTGATCCACGGGGTAAAGTGAGAAG
TGAGGAACAAACATCTTATCCCCTGAGAAGTGAAACTTCTCCGCAATTGTCTGGCTCTCAATTATCGACT
ACATTTAGGCTTCCGACATTTTGACAACGGCCACGCGCAGAAAGCAGCATCAAAATTAAGCCAATTGACG
ACCAAATCTGAAACGCAGCGGCGTCGCGTCTTTCGACGCGTAACGGACATTTCTGTGTGCGCTGCGTTTA
AAAAATCTGATGGATGGAAATGCACAAAATATGTTTCACGGAAATCATCAAGGGAGATCCCTATTACCGT
TATGAtgcggcagctgccgcagcagccgcagcCGCGGCGAACCCGCGTGCAATGGGACCGCCTGGCGGCT
ATCCGCCGCGCCACCGCCCCCCACATCCGCTACAGCAACAGCCCCAATACCCATCGTACCAGCCAACGGC
GGAGAATCTCTACGGGCTGGGGGCCGCCGATCACCAGGCGGCTATGGGCCTCGGCGTTGGAGTAGGAGTT
GGTGCCGGAATGGGCGATCTAAGCGGTTGGGGAGCGGCGCCCTCGGTTCCGGGCCAGGCAGCGGCTTATC
CCGGAGCCGGACTGGGGGCCTATGGGCATCCGCAAGCGGCTCCACCGTCGCAGCAACAGCGTCAGAGCAA
CGCTAGCGCATATCGTCAACACATGCCCGCCTATGGACAACAGGACCAGCAGAAACTTGCTTCATACGGC
GCGCAGCAGAGCATGATGGCGGGGTACGGATCACTGGCGCCCcagcagcaacagcagcagcaaccgccca
cgccccagcagcaacagcaagcacagcaagtgcaacagcaggcgcagcaacaacaacaacagcagcagca
gcagagtcagcagcaggctcgactgccgcagcaTTATCCGCAAGCTCCTGGTCAGCATCCGTTGTATCCT
GGAGTGGGAGCACCCACCGTCCAGGCGGGACAACCTCCCACGCCTCAAGCTTATGCTCACAGTCCGTACG
GAAGTCCCATGCAACACCAACCAAGTAGAGGAGCTCCCCAGCATGTGGGCTACGGTCATACAGGACTAGA
TCAGGCTTCTGCTTACGGACAGCATGTGCCGGGAGGAGCGCCACCGGGTCACCACTTGTCAGCGCAGCAG
CAGCAGGCTGCGCAGCAATTGGGTGCCCACCAGCAGCAGCAGCTCCAATCGCAGCAGCAACAGCAGGCCC
AGCAGCAGCAACCTCACGTCTCGCTAATGCAGCAGCAACAGTTGCCGGGTGCTGGATTGCCGCCGCCGCA
CATGGGAATGCCTCCTAGTTACGGAAGCCACCATcaacagcaacagcagcaacaacagacccagcaacaa
cagcagcagtcagcgCCGCCTTACATGTCCAGCAGCAAGCATCAACAGGCGGCTGCCCAGCTAAATTCCT
CGCCCCAGTATCGTGCACCTTTCCCTCAGCTTTCCCCACAAATGTCGCCACGTCCTCCGACAATGTCGCC
GCACCCACAAATGTCCCCGCGACCAGTTGGCGTGTCGCCGGCGAAACCACCGCCCCCCCAACAGCAGCAA
CAACAAACGCAAGCGCAGCAGCCGCAACAGGTTGTAAGCAATCAGCCAAGCCAGCACAGCATTCAAGGCA
TGGTAGGCCTGCCATCGCCACGCCCACAGCCGCCCACAAGTGGAGCAGGCGGAGCGGGAGCGAAGGGTCC
TGCGCCAGTTGCTGCTCCCGTTAACACATTACAGGCCCTCGAACAAATGGTGATGCCATCTCAGGCTCTC
GGACTACCACCCATGGACTACCCATCGGCTTACAGAAGTGCCGTGGGTCCTAGGATGCCAGCTTCCCCTc

aacagcagcagcaacaacatcaacaatggggggcggctcatggagcacagcacttggcggccctgcagca
gcagcaggcccaacaacaacaacagcaggtacaacagcagcaacaagcacctccgcagcagcagcaacag
gcggcgatgttgcaggctcagcagcaacatcaccagcagcagTTAAGCATGTATGGTCAGCCGATGGGAC
AGCAACCACAAACCGCGCAGCAACAAGTTGTACCACCGGTGACTTCACAACCGCCAGTAGTGGCCGCCTC
TCAGCAGCAGCAACAACAGCAACACCAGCAGCAGTCACTCAACGATCAATTGCAGCACTCGATCAACGAT
ATTGTTGGGGGTGGTAACAGTAGTGGAGCAACAGGGGGTGCTCAGAATCAGCACCAGCAGCAACCCCAAC
TGGCGGCCATGTCTTCGCCGTTGCATCAGCAAAATCTGCCTAACATGCACCATTTAATTCAACCTACCAT
GGAGacaacacagccgacgcagcagcaacagcaaacgtcacaacaacagcagcaaccacagcaaccgcag
GCGACTTTAACATCGCCGCACCACCAGCAAATGCAGCACCAGTCTCCAATACACCAGCAGCAACAGTCGC
CGCACCACCAGCAACATCTACCCAGCTATAATCAGTCACAGCAGCAATTTGATCCCTTCGCTAACATACT
AGGAGACAATCAACCGGCTGCATCCCAACAGACAATGTCGCCCGCTCATGTAAGTTCGCCAGTACCCGCG
CAAATGCAACAGCAGCAACATCAAACCCAACAACAACCATCCCAACAAGTACCACAAAGCCAGCAACAAC
AGGTGTCGCAGCCGCAACAGCAAACGCAGCAGCAGCAACCCTCGACCCCATCACATCACCTGAGCAGCAT
TCTCAATGAAACAGCCAACTCGAATGATTCCTCCACTCTGGCGGTGGCTGCTAATGAcagcaacaacagc
agtagcaatagtagcaccaatagcattgtcaacaaccagcccacgtcggtgcacgacagtaattcgcaaa
gcagcattaacagcagcagcaacaaccagcaacagTTGCTCATGGACAACACGAATTCGCACGGAGGAAA
TTCGGAATCGGCCCAGAATGCTGGAGTAGATGTTGGTCTTTTTGATAACAACTCGATGTCTTCAAATTCA
GCGGCAGTTGCTGCCGTGGCCTCCAATGCAGCTTCTGCAGCAGCAGCACTTTTAGATAGCAATTCTCAGT
CCTCGAATGCGGAGTTTGAGAAGAATCAAAGCGAAGGTGAGGGACTCGGTGTTGTGGAAGGAGTTATTGC
CAATGAAACTGATTTGCCACAGGATGAGAACAGTGTTTCTAAAACTCAAGACATAACTCTAAGCACGGAA
TTGCCAACAATACAAGACGATCTGCAAGAAAAGCCCACCGCTGAGATGGAACAAACTCAATTGGGACAAT
CTTTAGGGGATCTATCTAAATCTTTGTTAGAGGAACCCAAAACAGTGGAGGAATCCGGAGAGGATAAATC
TCAATCGGCCCCTGGAGTTGCACCCCCTGCAGATCCTATTATCCCCGTTACACAACCGCCTCAGCAACAG
ATGCCTCCTATAGGGATGCCTATGCATCCTATCGCCCCCGGATATGAGGCCCAGGCTCAGGGACAAGGGC
ACATGCCTCCCATGATGCCTCCGTATGGAGGAGCGCCAGGAATGTATCCACCTTATCCGATGCTGCATCA
ACAAGAAATTGCTGCACTGCAGCAGCAGATACAGGAACTGTATTGCATGCCTCCAGGACATGAGCTTCAA
CACCAAGATAAGTTAATGCGTATGCAGGAGCGATTAAATCTTCTGACGCAGCACGAGGTAAACGATCAGT
GTGCTGGCGGTCCACAGTGCTTATTGTTCCAAAACGTTCCGCCAATGTATGGACCACCAGGAAACCCCCT
GCTTAACCAGCAAATGGTAGAGAGCCCTCAAGTATCTAGCACCACGGGAAGAGGTCGAGGAAAGAGTGCC
AATAAGCCGCGTAAGCCTCGCGCCAAGAAGGGTGAGAAAGCCCAGGTGGGTCAGCAGCAGGATTTGATGG
ACATCAGCGGCAATGTGGCAATGGGAGCAGCCAACGCCGTTTCAAGTATTCCAACGACACAACTACCCGT
CTCTGAGGATTGCGTGACTCAAGGAGCGGGAGATACCAGTGTGGTCGGTATGCTGGAGTATAGTGAAGGG
ATGGGTGATCTCTCTCAAGATGTGCACTCAGCCAACGAACTGGATACCTCGACTGATGGCAGTGGCAAAA
AGAAGAAGCCCCGCAAGCCTAGAACACCTAAGGATCCAAATAAACCCCCACGAGATAGAACGCCGAAGGC
GAAGAAGCCCAAGGATCCAAACGATCCCAACTCTACCGAGACACCAGCAGCGGTAAAAAAGCGAGCTGGT
GCTAGTAAACGCAGGAAACAGTACGGAGAAGATGGGGCTGAGCAAACGGGAGAGGGTAGCGAAGTGGAAG
ACAACAAGCCATTGGTACCAAAAGCAGGATCTGCTGATGGTGAAGCTGAAACCACTTCGGGAGGAACTGG
AGTGGATGGAGAATCTCCAGACTACGATGACATTCCCGTTTCAAAAATTCCGCGTGGCTCCAATGAAGAT
GAAAAGGAAGCGGAGGCCGGCGATGAAACAGTAGACTCTGTTCCAGATTCGGCAGGAGATCCTGCTTCTA
CACCAAGCAGAAGAGATCGTAAAAGATCCACTGCAAGGAGTCGCCGCAATGCAAATTCTGAGGAGGGTGG
CAGTGCTCGCAAGAACCGTGGATCTCTTTCCGCCAAGGCACTAAAGAAGCGAAGGAATAGGGGTCGCATT
GTACCTGAATCCGATGGCGAAGATGACACACTGGACAGAACTCCACCTCCATCACCGCCACCAGACTCTG
AAATGGATTCCAACAAGCGAAGATCGTCAAGGAATACTCAAAGAAAGAAATACATCGATGATGTTATGCT
AAGGTTCTCCGATGATGAGAACTCTCTCTTAGTGGCATCTCCCGTAAAGAAGGATAAGAAACCATCTGCG

AATGCGAGTAATTCAAATGCAGGAAGTGATGTGGAAAAAACGGAACCTCAATCTGGTGCCGAGGGAGACG
CTGCCCAGGAGGTGGGTGAAGAAAAGTCAAATCTACCACTGGATGAAAGCAGTCAACTGGAAGCCAGTTC
GAGTACTTCTGCAGTAGCGGAGAAGGAGCGACAAATCTCTACTGATGCTGCAAATGCAGCCATGTCTTCC
AAACCCAATTACGTGTACATAAACACTGGCGATGAGGACAGCATGGTGGTTCAATTAGTTTTAGCCATGC
GAATGGGAAAGCGCGAACTTATTCTGGACAAACCCAAAGAAAAAGCACCCGAACCTAAACAAGATGAAGA
GAAATCTGAATTAGATGAAGCCACTACTGATAAACCTGAGGGAGATGAAAAATTCAAAACTGAGGGTGAA
TCCAAGAAAGATTTAACCGATTCCGAGGAAACGAAATTAGAATCCTCCGCAATGGAAGTAGACAGTAAGG
AAGAATCTGAGCCTGATGATTCTAAGAAATCGGATGAAGACAATAAGGACAAGGACAAAATGGAAGTAGA
TGATGAAGTTGGAAAATCAGATAAGGAAAGTAAACCAGAAGAACAATCAGAAACAGTGAAAACTGAAGAA
AATTCTAAAGCCATCGAGGAAGACAAGTCCTCTACCGTTTTAACAGCAGACCACGCCAAGGAACCTGAGA
CTGTCTTGGAGAAAATGGAGGTTGATGAAAAGGCAAATGATGACCAATCGGCTGTATCGAAAGCAGAGGG
ATCTGACGAAAAATCTACTGATGACTCCAACCCCGAAGAGGCTACTACAGAAAAGAATAAGGAAAGTTTA
GAAATTGAAGGGGAAAAGGAGCGGGTCAAGGAGGGAGAAGAATCCGTTAAGAAGGAGAATGACGAGAAAA
CAGAAGCTGATATGGAAAATAAGCCAGAGCCCGTCTTTATCGATGTGGAGGAGTACTTTGTGAAATATCG
CAATTTCAGTTATCTACATTGCGAGTGGCGAACAGAAGAGGAACTATTAAAGGGTGATCGCCGCGTAGCT
GCTAAGATTCGTCGCTTTCAGCAAAAGCAGTCTCAGCAATTGAATATATTTGAGAATATCGAAGACGAGC
CCTTCAACCAGGACTTTACTGAAGTGGATAGAGTGTTAGACATGTCTGTGCATACAGACGAAACCAGTGG
AGAGACTACGAAGCACTACCTGGTCAAGTGGAAGTCGCTTCCCTATGAGGATTGCACTTGGGAGCTAGAA
GAAGATGTAGATAACGACAAAATTGAGCAGTACCTGCGCTTTAACAAAATCCCTCAACGGAGCGAGTGGA
AGTCTAAAAAACGACCGCATCCAGAATTGTGGAAAAAACTGGAAAAGACGCCCGTCTATAAGGGAGGAAA
TAGCCTTAGACCCTATCAACTTGAGGGTCTTAATTGGTTGAAGTTTTCTTGGTACAACACCCACAACTGC
ATACTCGCTGACGAAATGGGCCTTGGAAAAACTATTCAAAGTTTGACATTTGTGCACTCTGTATATGAGT
ACGGCATTAGAGGACCTTTCTTAGTTATAGCTCCTCTATCTACAATTCCAAACTGGCAGCGAGAGTTCGA
GGGCTGGACGGATATGAACGTAGTTGTTTATCACGGCTCGGTGACAAGTAAACAAATGATACAGGACTAT
GAATATTACTATAAGACAGAAAGTGGAAAGGTATTGAAGGAGCCCATCAAGTTTAACGTTTTGATCACCA
CTTTTGAAATGATCGTGACAGACTACATGGACTTAAAAGCCTTTAACTGGCGCCTTTGTGTGATTGATGA
GGCACATCGTCTTAAGAATAGGAATTGCAAACTCCTTGAGGGTCTGCGACAGTTAAATTTGGAGCACAGA
GTATTGCTCTCCGGAACTCCCCTACAAAACAACATCAGCGAGCTGTTCTCGCTGTTAAACTTTCTGGAAC
CCTCGCAGTTCTCCTCACAGGAAGAGTTCATGTCTGAGTTTGGAAGTCTTCGCACTGAAGAAGAAGTAAA
TAAGCTGCAAGCTCTACTGAAACCAATGATGTTACGTCGTCTAAAAGACGACGTAGAGAAAAGTTTGGCG
CCCAAGGAAGAAACCATTATCGAAGTGGAGCTAACTAACATACAAAAGAAATATTATCGAGGTATACTGG
AACAGAACTTTAGTTTCCTGAAAAAGGGGACCACATCTGCTAATATCCCAAACCTTATGAACACCATGAT
GGAGTTGAGAAAGTGCTGCATACACCCTTATCTCTTGAATGGAGCGGAGGAACAAATCCAATATGATTTC
AAGTCCCAGCATGGCGAAGATCCTGAGTCCTATTATAAAAATCTGATTCTTTCCGCTGGTAAAATGGTTT
TAATTGATAAATTGCTACCTAAACTAAAAGCAAACGGCCATCGCGTTCTAATATTCAGTCAGATGGTGCG
TTGCTTGGATATCCTTGAAGATTATCTAGTGTACAGAAAATACCCCTTTGAGCGAATCGATGGGCGCATT
CGCGGTAATCTCCGCCAGGAGGCCATCGATCGTTACTCCAAGCCAGGTTCCGATCGCTTTGTATTTCTCC
TTTGCACCAAAGCTGGTGGATTAGGTATTAATTTAACAGCTGCTGATACTGTTATTATTTACGATTCGGA
TTGGAATCCACAAAACGATTTACAGGCTCAGGCCCGATGCCATCGTATTGGCCAGAGAAAGATGGTAAAG
ATTTATAGATTGCTTTGCAGGAATACCTATGAGCGTGAAATGTTCGACAAAGCTTCAATGAAACTTGGAT
TGGACAAGGCTGTATTGCAGTCGATGAACACCCAAGGGTCTAAAGATGGCAATAACAAGCAGCTGTCCAA
AAAAGAAATTGAAGATCTTTTAAAAAAGGGTGCTTACGGGGCCGTCATGGATGACGACAATGCTGGTGAC
AAATTTTGCGAAGAGGACATCGATTCAATCCTTAAGCGACGCACTCAAGTCATCACAATGGAATCGGAGA
AGGGTTCAACCTTCTCAAAGGCTTCATTTGCTGCATCCGGTAACCGGTCGGATATTACGATAGATGATCC

TGATTTTTGGACCAAGTGGGCCAAAAAGGTTGACATTGATCCCGATGCTTGCGAAAGGGATGAAACGGAG
GACTTGGTTTTGTCCGAACCCAGAAGACGCACCCAAATCAAACGCTACGGCCACGAGGACGTAATGGAGC
TCAATTCCGAGGAATCTTCCAATGAGAATAGCGACGAGGAGGGAGGAATAGGGGCTCCGTTCCCGTCGCC
GCAAGGAGAAGCGAGATCGTGCTCGTGAAAAGAAGGGCAACGATGAATACATTCCACGAGAACGGGATGC
ATTAGCTGCTTTGGGCTTGGAGGAAATTCAATATGGTAACTGGGCCAAGTCTGAGTGCTTTAAAGTGGAA
AAGGGACTGCTTTCCTTTGGG
>dme_circ_0001136_junction_seq
AGTGGAAAAGGGACTGCTTTCCTTTGGGATCAATATCCTTTTATATGCATCTTACT
>dme_circ_0001137
ATTATCTAGTGTACAGAAAATACCCCTTTGAGCGAATCGATGGGCGCATTCGCGGTAATCTCCGCCAGGA
GGCCATCGATCGTTACTCCAAGCCAGGTTCCGATCGCTTTGTATTTCTCCTTTGCACCAAAGCTGGTGGA
TTAGGTATTAATTTAACAGCTGCTGATACTGTTATTATTTACGATTCGGATTGGAATCCACAAAACGATT
TACAGGCTCAGGCCCGATGCCATCGTATTGGCCAGAGAAAGATGGTAAAGATTTATAGATTGCTTTGCAG
GAATACCTATGAGCGTGAAATG
>dme_circ_0001137_junction_seq
TTGCAGGAATACCTATGAGCGTGAAATGATTATCTAGTGTACAGAAAATACCCCTT
>dme_circ_0001138
AACTTTAGTTTCCTGAAAAAGGGGACCACATCTGCTAATATCCCAAACCTTATGAACACCATGATGGAGT
TGAGAAAGTGCTGCATACACCCTTATCTCTTGAATGGAGCGGAGGAACAAATCCAATATGATTTCAAGTC
CCAGCATGGCGAAGATCCTGAGTCCTATTATAAAAATCTGATTCTTTCCGCTGGTAAAATGG
>dme_circ_0001138_junction_seq
AATCTGATTCTTTCCGCTGGTAAAATGGAACTTTAGTTTCCTGAAAAAGGGGACCA
>dme_circ_0001139
GGATCTTCGCCATGCTGGGACTTGAAATCATATTGGATTTGTTCCTCCGCTCCATTCAAGAGATAAGGGT
GTATGCAGCACTTTCTCAACTCCATCATGGTGTTCATAAGGTTTGGGATATTAGCAGATGTGGTCCCCTT
TTTCAGGAAACTAAAGTTCTGTTCCAGTATACCTCGATAATATTTCTTTT
>dme_circ_0001139_junction_seq
TCCAGTATACCTCGATAATATTTCTTTTGGATCTTCGCCATGCTGGGACTTGAAAT
>dme_circ_0001140
GTTAGCTCCACTTCGATAATGGTTTCTTCCTTGGGCGCCAAACTTTTCTCTACGTCGTCTTTTAGACGAC
GTAACATCATTGGTTTCAGTAGAGCTTGCAGCTTATTTACTTCTTCTTCAGTGCGAAGACTTCCAAACTC
AGACATGAACTCTTCCTGTGAGGAGAACTGCGAGGGTTCCAGAAA
>dme_circ_0001140_junction_seq
GTGAGGAGAACTGCGAGGGTTCCAGAAAGTTAGCTCCACTTCGATAATGGTTTCTT
>dme_circ_0001141
TTTTCTTGGTACAACACCCACAACTGCATACTCGCTGACGAAATGGGCCTTGGAAAAACTATTCAAAGTT
TGACATTTGTGCACTCTGTATATGAGTACGGCATTAGAGGACCTTTCTTAGTTATAGCTCCTCTATCTAC
AATTCCAAACTGGCAGCGAGAGTTCGAGGGCTGGACGGATATGAACGTAGTTGTTTATCACGGCTCGGTG
ACAAGTAAACAAATGATACAGGACTATGAATATTACTATAAGACAGAAAGTGGAAAGGTATTGAAGGAGC
CCATCAAGTTTAACGTTTTGATCACCACTTTTGAAATGATCGTGACAGACTACATGGACTTAAAAGCCTT
TAACTGGCGCCTTTGTGTGATTGATGAGGCACATCGTCTTAAGAATAGGAATTGCAAACTCCTTGAGGGT
CTGCGACAGTTAAATTTGGAGCACAGAGTATTGCTCTCCGGAACTCCCCTACAAAACAACATCAGCGAGC
TGTTCTCGCTGTTAAACTTTCTGGAACCCTCGCAGTTCTCCTCACAGGAAGAGTTCATGTCTGAGTTTGG
AAGTCTTCGCACTGAAGAAGAAG

>dme_circ_0001141_junction_seq
TTTGGAAGTCTTCGCACTGAAGAAGAAGTTTTCTTGGTACAACACCCACAACTGCA
>dme_circ_0001142
TTTGACATTTGTGCACTCTGTATATGAGTACGGCATTAGAGGACCTTTCTTAGTTATAGCTCCTCTATCT
ACAATTCCAAACTGGCAGCGAGAGTTCGAGGGCTGGACGGATATGAACGTAGTTGTTTATCACGGCTCGG
TGACAAGTAAACAAATGATACAGGACTATGAATATTACTATAAGACAGAAAGTGGAAAGGTATTGAAGGA
GCCCATCAAGTTTAACGTTTTGATCACCACTTTTGAAATGATCGTGACAGACTACATGGACTTAAAAGCC
TTTAACTGGCGCCTTTGTGTGATTGATGAGGCACATCGTCTTAAGAATAGGAATTGCAAACTCCTTGAGG
GTCTGCGACAG
>dme_circ_0001142_junction_seq
TTGCAAACTCCTTGAGGGTCTGCGACAGTTTGACATTTGTGCACTCTGTATATGAG
>dme_circ_0001143
TTTTCTTGGTACAACACCCACAACTGCATACTCGCTGACGAAATGGGCCTTGGAAAAACTATTCAAAGTT
TGACATTTGTGCACTCTGTATATGAGTACGGCATTAGAGGACCTTTCTTAGTTATAGCTCCTCTATCTAC
AATTCCAAACTGGCAGCGAGAGTTCGAGGGCTGGACGGATATGAACGTAGTTGTTTATCACGGCTCGG
>dme_circ_0001143_junction_seq
ATGAACGTAGTTGTTTATCACGGCTCGGTTTTCTTGGTACAACACCCACAACTGCA
>dme_circ_0001144
GAAAACTTCAACCAATTAAGACCCTCAAGTTGATAGGGTCTAAGGCTATTTCCTCCCTTATAGACGGGCG
TCTTTTCCAGTTTTTTCCACAATTCTGGATGCGGTCGTTTTTTAGACTTCCACTCGCTCCGTTGAGGGAT
TTTGTTAAAGCGCAGGTACTGCTCAATTTTGTCGTTATCTACATCTTCTTCTAGCTCCCAA
>dme_circ_0001144_junction_seq
GTTATCTACATCTTCTTCTAGCTCCCAAGAAAACTTCAACCAATTAAGACCCTCAA
>dme_circ_0001145
GAAAACTTCAACCAATTAAGACCCTCAAGTTGATAGGGTCTAAGGCTATTTCCTCCCTTATAGACGGGCG
TCTTTTCCAGTTTTTTCCACAATTCTGGATGCGGTCGTTTTTTAGACTTCCACTCGCTCCGTTGAGGGAT
TTTGTTAAAGCGCAGGTACTGCTCAATTTTGTCGTTATCTACATCTTCTTCTAGCTCCCAAGTGCAATCC
TCATAGGGAAGCGACTTCCACTTGACCAGGTAGTGCTTCGTAGTCTCTCCACTGGTTTCGTCTGTATGCA
CAGACATGTCTAACACTCTATCCACTTCA
>dme_circ_0001145_junction_seq
AGACATGTCTAACACTCTATCCACTTCAGAAAACTTCAACCAATTAAGACCCTCAA
>dme_circ_0001146
GAGGGAGAAGAATCCGTTAAGAAGGAGAATGACGAGAAAACAGAAGCTGATATGGAAAATAAGCCAGAGC
CCGTCTTTATCGATGTGGAGGAGTACTTTGTGAAATATCGCAATTTCAGTTATCTACATTGCGAGTGGCG
AACAGAAGAGGAACTATTAAAGGG
>dme_circ_0001146_junction_seq
GGCGAACAGAAGAGGAACTATTAAAGGGGAGGGAGAAGAATCCGTTAAGAAGGAGA
>dme_circ_0001147
GCAAATGATGACCAATCGGCTGTATCGAAAGCAGAGGGATCTGACGAAAAATCTACTGATGACTCCAACC
CCGAAGAGGCTACTACAGAAAAGAATAAGGAAAGTTTAGAAATTGAAGGGGAAAAGGAGCGGGTCAAGGA
GGGAGAAGAATCCGTTAAGAAGGAGAATGACGAGAAAACAGAAGCTGATATGGAAAATAAGCCAGAGCCC
GTCTTTATCGATGTGGAGGAGTACTTTGTGAAATATCGCAATTTCAGTTATCTACATTGCGAGTGGCGAA
CAGAAGAGGAACTATTAAAGGG
>dme_circ_0001147_junction_seq

GGCGAACAGAAGAGGAACTATTAAAGGGGCAAATGATGACCAATCGGCTGTATCGA
>dme_circ_0001148
GAATAAGTTCGCGCTTTCCCATTCGCATGGCTAAAACTAATTGAACCACCATGCTGTCCTCATCGCCAGT
GTTTATGTACACGTAATTGGGTTTGGAAGACATGGCTGCATTTGCAGCATCAGTAGAGATTTGTCGCTCC
TTCTCCGCTACTGCAGAAGTACTCGAACTGGCTTCCAGTTGACTGCTTTCATCCAGTG
>dme_circ_0001148_junction_seq
GCTTCCAGTTGACTGCTTTCATCCAGTGGAATAAGTTCGCGCTTTCCCATTCGCAT
>dme_circ_0001149
GATTACAGACACTTCCGAGAAGAGTTTCGCCACAAAGATTTGCCACGTCACCCTGCCGGAATACGAAGCC
TTCAAGGATCTCTACGCCAAGGAACTGGAGCAAAAGTCCAGAAGAAAGGAGGAGGATGAGCTTAGTGATG
AAGATGACTAATGTTAC
>dme_circ_0001149_junction_seq
GCTTAGTGATGAAGATGACTAATGTTACGATTACAGACACTTCCGAGAAGAGTTTC
>dme_circ_0001150
CCCCGCAAGCCTAGAACACCTAAGGATCCAAATAAACCCCCACGAGATAGAACGCCGAAGGCGAAGAAGC
CCAAGGATCCAAACGATCCCAACTCTACCGAGACACCAGCAGCGGTAAAAAAGCGAGCTGGTGCTAGTAA
ACGCAGGAAACAGTACGGAGAAGATGGGGCTGAGCAAACGGGAGAGGG
>dme_circ_0001150_junction_seq
AAGATGGGGCTGAGCAAACGGGAGAGGGCCCCGCAAGCCTAGAACACCTAAGGATC
>dme_circ_0001151
GAGTTGGGATCGTTTGGATCCTTGGGCTTCTTCGCCTTCGGCGTTCTATCTCGTGGGGGTTTATTTGGAT
CCTTAGGTGTTCTAGGCTTGCGGGGCTTCTTCTTTTTGCCACTGCCATCAGTCGAGGTATCCAGTTCGTT
GGCTGAGTGCACATCTTGAGAGAGATCACCCATCCCTTCACTATACTCCAGCATACCGACCACACTGGTA
TCTCCCGCTCCTTGAGTCACGCAATCCTCAGAGACGGGTAGTTGTGTCGTTGGAATACTTGAAACGGCGT
TGGCTGCTCCCATTGCCACATTGCCGCTGATGTCCATCAAATCCTGCTGCTGACCCACCTGGGCTTTCTC
ACCCTTCTTGGCGCGAGGCTTACGCGGCTTATTGGCACTCTTTCCTCGACCTCTTCCCGTGGTGCTAGAT
ACTTGAGGGCTCTCTACCATTTGCTGGTTAAGCAGGGGGTTTCCTGGTGGTCCATACATTGGCGGAACGT
TTTGGAACAATAAGCACTGTGGACCGCCAGCACACTGATCGTTTACCTCGTGCTGCGTCAGAAGATTTAA
TCGCTCCTGCATACGCATTAACTTATCTTGGTGTTGAAGCTCATGTCCTGGAGGCATGCAATACAGTTCC
TGTATCTGCTGCTGCAGTGCAGCAATTTCTTGTTGATGCAGCATCGGATAAGGTGGATACATTCCTGGCG
CTCCTCCATACGGAGGCATCATGGGAGGCATGTGCCCTTGTCCCTGAGCCTGGGCCTCATATCCGGGGGC
GATAGGATGCATAGGCATCCCTATAGGAGGCATCTGTTGCTGAGGCGGTTGTGTAACGGGGATAATAGGA
TCTGCAGGGGGGTGCAACTCCAGGGGCCGATTGAGATTTATCCTCTCCGGATTCCTCCACTGTTTTGGGTT
CCTCTAACAAAGATTTAGATAGATCCCCTAAAGATTGTCCCAATTGAGTTTGTTCCATCTCAGCGGTGGG
CTTTTCTTGCAGATCGTCTTGTATTGTTGGCAATTCCGTGCTTAGAGTTATGTCTTGAGTTTTAGAAACA
CTGTTCTCATCCTGTGGCAAATCAGTTTCATTGGCAATAACTCCTTCCACAACACCGAGTCCCTCACCTT
CGCTTTGATTCTTCTCAAACTCCGCATTCGAGGACTGAGAATTGCTATCTAAAAGTGCTGCTGCTGCAGA
AGCTGCATTGGAGGCCACGGCAGCAACTGCCGCTGAATTTGAAGACATCGAGTTGTTATCAAAAAGACCA
ACATCTACTCCAGCATTCTGGGCCGATTCCGAATTTCCTCCGTGCGAATTCGTGTTGTCCATGAGCAAct
gttgctggttgttgctgctgctgttaatgctgctttgcgaattactgtcgtgcaccgacgtgggctggtt
gttgacaatgctattggtgctactattgctactgctgttgttgctgTCATTAGCAGCCACCGCCAGAGTG
GAGGAATCATTCGAGTTGGCTGTTTCATTGAGAATGCTGCTCAGGTGATGTGATGGGGTCGAGGGTTGCT
GCTGCTGCGTTTGCTGTTGCGGCTGCGACACCTGTTGTTGCTGGCTTTGTGGTACTTGTTGGGATGGTTG
TTGTTGGGTTTGATGTTGCTGCTGTTGCATTTGCGCGGGTACTGGCGAACTTACATGAGCGGGCGACATT

GTCTGTTGGGATGCAGCCGGTTGATTGTCTCCTAGTATGTTAGCGAAGGGATCAAATTGCTGCTGTGACT
GATTATAGCTGGGTAGATGTTGCTGGTGGTGCGGCGACTGTTGCTGCTGGTGTATTGGAGACTGGTGCTG
CATTTGCTGGTGGTGCGGCGATGTTAAAGTCGCctgcggttgctgtggttgctgctgttgttgtgac
>dme_circ_0001151_junction_seq
ttgctgtggttgctgctgttgttgtgacGAGTTGGGATCGTTTGGATCCTTGGGCT
>dme_circ_0001152
GAGTTGCAAGCGACCATGCGCGCATGGCTTCTACTCCTCGCAGTGCTGGCGACTTTTCAAACGATTGTTC
GAGTTGCTAGCACCGAGGATATATCCCAGAGATTCATCGCCGCCATAGCGCCCGTTGCCGCTCATATTCC
GCTGGCatcagcatcaggatcaggatcaggaCGATCTGGATCTAGATCGGTAGGAGCCTCGACCAGCACA
GCATTAGCAAAAGCATTTAATCCATTCAGCGAGCCCGCCTCGTTCAGTGATAGTGATAAAAGCCATCGGA
GTAAAACAAACAAAAAACCTAGCAAAAGTGACGCGAACCGACAGTTCAACGAAGTGCATAAGCCAAGAAC
AGACCAATTAGAAAATTCCAAAAATAAGTCTAAACAATTAGTTAATAAACCCAACCACAACAAAATGGCT
GTCAAGGAGCAGAGGAGCCACCACAAGAAGAGCCACCACCATCGCAGCCACCAGCCAAAGCAGGCCAGTG
CATCCACAGAATCTCATCAATCCTCGTCGATTGAATCAATCTTCGTGGAGGAGCCGACGCTGGTGCTCGA
CCGCGAGGTGGCCTCCATCAACGTGCCCGCCAACGCCAAGGCCATCATCGCCGAGCAGGGCCCGTCCACC
TACAGCAAGGAGGCGCTCATCAAGGACAAGCTGAAGCCAGACCCCTCCACTCTAGTCGAGATCGAGAAGA
GCCTGCTCTCGCTGTTCAACATGAAGCGGCCGCCCAAGATCGACCGCTCCAAGATCATCATCCCCGAGCC
GATGAAGAAGCTCTACGCCGAGATCATGGGCCACGAGCTCGACTCGGTCAACATCCCCAAGCCGGGTCTG
CTGACCAAGTCGGCCAACACAGTGCGAAGTTTTACACACAAAG
>dme_circ_0001152_junction_seq
AACACAGTGCGAAGTTTTACACACAAAGGAGTTGCAAGCGACCATGCGCGCATGGC
>dme_circ_0001153
GGGCACTTCCCGTGGCAGTAATATGCATCGTAGCCCAGAGGCGCCACAATCCAGTCGTCCCAGCCCACGT
CCGAGAAGTCCACGTACAGCGAGTGCCGCCGGCAGGTGTCGTCGTGGTTCTTGCGCCTCGTAGGCCGTCT
CGGCTGCCGCTTGTTCCGGCCGCCCTTGCCACCGCCCTCTCCGCCAGACACGTCCCGAATGGAGCGCGCC
TTGTGCCGCCCGTCGTCCGTGTAGGTGAACAGGAGCGGCTGCTTGTGCTGCCACCGCTCGTGCGCCTCGT
CCGCGCTGCGGCGCAGGCGTACATGGTGGTGTGGGGCCGGCTTCAGGGAGCGGACCGTCCGCACCTCCAC
CAGCAGTCCGTAGTTGCGCTGCGGACTCGCCAGCCACCGGTCCACGGCCGGCTGGACATCGAGGCTCACC
GTGTCCGTGCTGTTAAGCCGGACCGTCTTGGTGTCCAACAGCAGATAGCTCGGCTCCCGCTGACCACGCA
CCCCGACGCGCGTGATGTCGTAGACAAGCACCTGGTAGCGCGTCCGATTCGCCGACGATCTGCTGGCCAC
CACCTGTTGACTGAGTGCGTCCCGGGTCAGCTGCAGCTCCGCCGCCTTCAGCTTCTCGTCGGCGGGAATG
CTCTTCACGTCGAAGTGCAGCCGAAACCGGTGGTGGTGCGGAAATCGATCGTCGATTTTACTATCTGCAA
GGAAACGGAAAAGAAGGATATTGCGGGTGAGCTTGGAGCAAAGAAATCATAGGAACTTAGCTATTGAATC
TaaaaaaaaGGACTACCTCTAGCACACCCCATCTTTTTAAAGGTGATTCTAAAAAAACAGCCCACTTCCA
GCTGTCTGCTTTATCCACCTGAAATCGGAATCAGCGAATAAAACCGTACAATCCGGAGGATCAAGTGCAT
GAGCCTCTATCTGTGGGGACATAACGCGGGCTCGGATCTCCGGCTAGATAATTAGACACACCTTCCTTCA
TCTACCTGGTTTCCTGGACCAGTAAGTACCCCCTGCTTAAATACACCCACTACTCTAGAGCGTGGAAAGT
GCAGGGAAATCGGGGACGACAAAGGTAGGTCGAAAATCGCACGTTCCAGTTGTCCAGCCGCCTTTTTACG
ACTGGCGCCTGTGCTAAAGACCCTGGCCCACACCGGCCTACCTTTTCGGATCTGTCTATATACACATATA
GGTAAAGTATATGTATTTGTGCGGCAAGGTGCAAACGCTGCACGCTTGCCGAAAAATCTTAATTTGAATC
GACGCCGCCGCTGCCGTTTTCAAGTACCTTGCACAAAGAGCCAAGCGAGTCGTAAATTCTTTTGGGAAGC
GGGAATCAACCGGAGGGTTGGGTTCCGAGTCCGAAAACCAGAAACCAACAACGGCACAGATGCCACAGCC
TAAAGACGTGGCCAGAACTGAACCGTAACCAAGACAAACTCGACTCGCCAGTCGGCTTAGACACTTGGCT
TTAGACACTTGGTTTGTGCGGCTTGCTTACCTGCACCTAACTGCTTTATTGTTCGACCCTATAATCCTGC

GGAAAATAATGCGAAATACGCTTTAATAATAATTGTTATTCCTACAAATACAATATAAAAGCCATTTAGC
GCATGCGCTAAACGGTCCTGGTGTAATTCTTAGGAAAAATTTCAAGGTCACTGCCGAGCTGTCGTCGCGG
AAGAGCAGGTAAAAGGACTCTCGTGTCCTTTTAGCTGTTCATTTTGGAAAGCCGTGGAATTTCCGCGTGA
TTTCACAGCGCTTGCGTGAATGATATGAGGGGTGGATCGATGGATCGAGGACAGAAGGATCTAGGGATCG
GCAGGTATGCAAATCGCTTAGATGAAACAGAAATTTCAGGAAAATCCGACTGGCTCTTCATTCGCATTTT
GGTAGCGGAAAGTACGCATTGCGATGAGTGGGAATATAAATTACGGCTAATACACATCGAAGCCTTTCGC
GGTGCACAAAAGCCTAGGCGGATGGCAGGTGGAAAATTCAGGTATAGGAAGTAATTGAAAATGGATTGGA
TTACCTTTATGTCCTAATAAAAGGGGCAAGCTACTCTGATTTTTTTGCATATATAAGATTCGTTAGTTTT
AATTTTAAGCAAACCTAAGATGTACCATGCGATCTAGATAAACCAGATTGAAATCAATAAAAATGGAAAT
TATTCTTCTTATAAAAAGCAAATATATATCTGAAAACGTTGGATGAAATGTAAATATATTCGAAAGTTGC
CCATAACAACATTTTGTCTGTTTTAGTTTGTGACCATTGCATTAGTTTTAATTGACTAACATTCCAAAAG
GAATCATACTCGCTGTATTAAAAGTAATACCATTAGAATGCAAAGTTTTTCTATATCCGCTTCCGGTTTT
CTAGTTCTGGTTTTAAACATTTGAAAAGGAGACTCACCTTTGTGTGTAAAACTTCGCACTGTGTTGGCCG
ACTTGGTCAGCAGACCCGGCTTGGGGATGTTGACCGAGTCGAGCTCGTGGCCCATGATCTCGGCGTAGAG
CTTCTTCATCGGCTCGGGGATGATGATCTTGGAGCGGTCGATCTTGGGCGGCCGCTTCATGTTGAACAGC
GAGAGCAGGCTCTTCTCGATCTCGACTAGAGTGGAGGGGTCTGGCTTCAGCTTGTCCTTGATGAGCGCCT
CCTTGCTGTAGGTGGACGGGCCCTGCTCGGCGATGATGGCCTTGGCGTTGGCGGGCACGTTGATGGAGGC
CACCTCGCGGTCGAGCACCAGCGTCGGCTCCTCCACGAAGATTGATTCAATCGACGAGGATTGATGAGAT
TCTGTGGATGCACTGGCCTGCTTTGGCTGGTGGCTGCGATGGTGGTGGCTCTTCTTGTGGTGGCTCCTCT
GCTCCTTGACAGCCATTTTGTTGTGGTTGGG
>dme_circ_0001153_junction_seq
CCTTGACAGCCATTTTGTTGTGGTTGGGGGGCACTTCCCGTGGCAGTAATATGCAT
>dme_circ_0001154
GGGCGGCGAGGTGATCCAGGAGAGTATTACCTCCAACATTGGCGAGGACCTGATAACGCTGGAGTTTCAG
AAGACCGACGGAACGCTCATCACCCAGGTCATCGACTTTCGCAATGTGAGTGAAGAATCCGAGGCTCTAG
AGAGCAGGGCGAGAGACCCAATTGAAATTGAAAACCTCTTATCCATCTTCCCCTCAGGAGGTTCAAATCC
TCAAGGCTCTGGTTCTCGGCGAGGAGGAGCGTGGCCAGAGCCAGTACCAGGTCATGTGCTTCGCAACCAA
GTTCAACAAGGGCGACTTCATCTCCTCGGCGGCAATGGCCAAGCTGCGCCAGAAGAATCCGCACACCATC
CGCACTCCCGAGGAGGACAAGGGCCGTGAGACCTTCACCATGAGCAGCTGGGTACAGCTCAACCGCTCGC
TGCCCATCACCAGACATCTGCAGGGACTCTGCGCCGAGGCCATGGACGCCACCTATGTCCGGGATGTGGA
CCTTAAAGCTTGGGCGGAGCTACCAG
>dme_circ_0001154_junction_seq
GACCTTAAAGCTTGGGCGGAGCTACCAGGGGCGGCGAGGTGATCCAGGAGAGTATT
>dme_circ_0001155
GATCGGTAAAACTGCCATTACGGGCTTTGACTTGGGGTTTGGCTGGTAGTTTTATGTTCTGCCGCGGGAT
TTCTTCCTGCAACTGAATCTCCTGCAGCTTGCGGCGTTTGATGAACTGTAGTGGAGT
>dme_circ_0001155_junction_seq
TGCGGCGTTTGATGAACTGTAGTGGAGTGATCGGTAAAACTGCCATTACGGGCTTT
>dme_circ_0001156
GGCGCGCCATCTTGGCGAATGGCGACGTGACAGGAGCTCGCCCATTGGCCAATCTTTTGCGGAACGACTT
CTGGGGCACTCCACTGGTGGACAGTGGATCCCACGGTTCTTGGCGACCGCTGTGCGTGCTCAGCTTCCGG
CTGAACTACCTCGCCGGCGGAATGACACCCTTGGGCTATCACCTGGTCAACGTAATGCTCCACTGTGTGG
CCACGTGGCTGGTGTTCCTGGTGGCGAGAACGCTCCTGCCATCTCGAATGGGCGTCCTGGCCGCTGGCGC
TCTTTTCGCCGTTCATCCCGCCCACACGGAGGCGGTGGCCGGCTTGGTGGGCAGGGCGGATTTGGCCTCG

TGTGTTTGTTACCTGCTCGCCTACCTCTCCTACCGCCGCCACATGCTGAACCGGGAGTGGGGATCCCTCA
TCCTCACCATTATGCTGGCGTTGGCAGCACTTCTTTGCAAGGAGACTGCCATAACGGCGCTGCTGCTGTG
CGGATTGTGCGATGTCCTATCGCCTGTCGGTCGGGAAAATAGCGATAAGGTGTGTGATGGATCCATCTCA
GGATTAGCTTCTTTCAACTTTCAGCGCCGCTTCCGGTCGCTTAGCATTTTGGGATTTACCCTACTGTGCG
GCCTCTACTGTCGACTGAGCCTCCTGCCACGCCCCTCAACCGCCTTTTCGGCCGCCGACAATCCGACGGC
CCATGAATCCTGTTTTTGGACTAGGACTCTAACTTTTCTGTACCTGCCAGTGGCCAACTTTGGGATTCTC
CTCTGGCCACAGGAGCTAAGCTTCGACTGGGGAATGGAGGCTGTGTCGCGGATCAGAACGCTTTGGGATG
CACGAAATATTCTGACGGCTGGCTTCTACGGATCTCTAGTTGCCATACTGTGGAAGGGATCTGGACTGCG
TAGTGCAGCCTCACCGATGGATTTTGCGGAGGTGGCGAACATTTCGCTGCCCCTACTGAGGCGATTGGGC
GGCAATAGCTGTCACACCTGGCTGGGCCTCACCTGCGATTGCCACCACCAACTCTCTGCACCCAGCTATC
GCTCTGCGTCAGCAATCTACTCCACCTCCTCCAAATCGAAAAGCGCCTCATGGACAGCAGCGCCCATCTT
GGGCACCGCCTTCCTGGTGTTGCCATTCCTGCCCGCCAGCAATCTGCTCTTCTACGTGGGATTCGTAATG
GCCGAAAGGGTGCTCTATTTGCCAAGCGTTGGCTATTGTCTACTCTTTGGCCTCGGTTTCGGACATCTCT
GGCAGCGGGTCAATAGCTCCTGGCGATCCCGCCTGATGCTCCTATGCGGCCTGGCCCTGCTCCTTGGCGT
CCACGGAGTGCGCACCTTCAGGCGCAACTTGGATTGGCGGGATGAGGAGCAGCTATTCCGCAGTGCCATC
TCTATAAATCCTCCAAAGGGTGAGTGCATACTTTCTTAGATATTCATATGTACAAAATACAAGAAATATA
TTGAATCTATCTACAGCATTTGGAAAAATCAATATATTATTCTATATATTTCAGCTCTGGGCAACCTGGG
CAGCGTGTTGAGTGCCCAAGGAAGATACGAGGAAGCGGAACTCACTCTGAGAATGACCCTTGGACACCGG
CCCACCATGGCTGATGCTCACTTTAATCT
>dme_circ_0001156_junction_seq
CCACCATGGCTGATGCTCACTTTAATCTGGCGCGCCATCTTGGCGAATGGCGACGT
>dme_circ_0001157
GCTGTGACTGTGCCAGCAGGAAATCCAAATCCTTGCTCGCAATGAAAGGCAGTCATTTGCTGCATGGCAA
ACTCCACAAACGACTGGATGGCAATATTTAGATTGGGCGGGTTTGGACAGTTTTTGAGACCATGATAACT
ACCGGAGCAATTACGACATATCATCTGACACTGGAAGGAG
>dme_circ_0001157_junction_seq
ACGACATATCATCTGACACTGGAAGGAGGCTGTGACTGTGCCAGCAGGAAATCCAA
>dme_circ_0001158
GAGAAGAAGTCCGTGCTGAAGGGCATCAAGGGTACGTTCAAGTCGGGCGAACTGACCGCCATAATGGGCC
CCTCGGGGGCGGGCAAATCTAGTCTTATGAACATCCTCACCGGTCTGACCAAATCCGGCGTCAGCGGGAA
GATCGAGATCGGGAAGGCGCGCAAACTGTGCGGCTACATTATGCAGGACGATCACTTCTTTCCCTACTTC
ACCGTCGAGGAGACCATGCTGATGGCGGCCACACTTAAAATCTCCAATCAGTGCGTCAGTCTGAAGGAAA
AGCGAACTCTGGATCGACTATCTGCTGAACTCGCTGAAGCTGACGAAGACGCGGCAGACGAAGTGCTCCA
ACCTGAGTGGCGGCCAGAAGAAGCGCCTATCCATCGCCCTGGAACTGATAGACAATCCAGCTGTGCTATT
TTTAGACGAGCCCACAACGCGGATTGGACAGCTCCTCCTCCTTCGACACCATCCAGCTGCTGCGCGGCCT
GGCCAACGAGGGACGTACCATCGTGTGCACCATCCACCAGCCGTCGACGAACATCTACAATCTCTTTAAC
CTGGTCTACGTGCTAAGCGCGGGTCGATGCACCTACCAGGGCACGCCCCAGAACACGGTCATGTTTCTCA
GCAGCGTGGGCCTGGAGTGCCCGCCCTACCACAATCCCGCCGACTTCC
>dme_circ_0001158_junction_seq
CCGCCCTACCACAATCCCGCCGACTTCCGAGAAGAAGTCCGTGCTGAAGGGCATCA
>dme_circ_0001159
GATACGATCAGCAGTTGATGCAGGGCGAGGATGCCGATGCGCCCAGCGAGACGCAGGTGGCCAAGTTCAA
TGAATCTCAGTCACCGGGGCAGGTCCAGGTGCAGGTGCAGAAGATCGAGATCCAGAACATGGAGTCGTCG
AAGGATCTGACCAAGCACACCTATCCGCCCACGGAATACATGCGACTGTGGCTGCTCATCGGCCG

>dme_circ_0001159_junction_seq
ACATGCGACTGTGGCTGCTCATCGGCCGGATACGATCAGCAGTTGATGCAGGGCGA
>dme_circ_0001160
ttttttATAAAAAAAGTACACAtaaaatacaattaaaataaggtaagaaataaaaaattatttcttaaac
ataaaaatTATATACTTTTGCGAtttttttgtggtttttttCTCCTCCTCCTCCTCATTTTCTCCTTGTT
GTGGTTTATTTTCTGCTTGTTGGTTTATTATTATAttttttttgttttgtttttgcttgtaacttttatt
tattattaatttaaatttttgtttgtttttgtttg
>dme_circ_0001160_junction_seq
aatttaaatttttgtttgtttttgtttgttttttATAAAAAAAGTACACAtaaaat
>dme_circ_0001161
TATACGAAATTCATGTGAGACTAGCGAGCCGCGGCCTCACTAGCCAAAGCCAGTCACAAAAAACCAAAAA
CATAAATCAGGCAGCAGCAGCAGCACACATACATCGACAAGCGCCAGGACCCGCATCACCATATCCTTCC
TGCCAAATAGTCGCCACTGCTGCCGACCCTCCCCGCTCCCCTCGCAGGAGTCACGCTTCGTTAAACGCTG
CTCAAAACCCATCATCCTAACTAATAAAGCGGACGATAAACAAGCGAATTAAGTACCAAAAACGACCGAG
CTATTAACGCTAGTCTGGATCCCAATTGCTGATTGGAAAGGAGAAACATTGGCAGCATTTCTGATTAGGG
GGTGAGTGTTGGTATTTACAAGAACGTAGCACACTTTTTGGGGCAGTCCATAGAGTGAATGGAGAAACAT
ACATAACTAAAGGGTTGCTGCAGCTAAAGAAAAACTAATCTCTAAGCTATCAGAGAAAATGTGATTGCTT
ATTATTCTAAGATACAAACATTTCCCATGGTTAACATTCATCTTGAATTATATTAATATCTAAATAATCA
AGTTAATTAATAGATTCACAAAAATTTGCAGGAATTTCCTTTACCAATTAAACCCAAAAGGTCTGAAATA
CTTTGGGTTTATTAACTTGATTAATTGCCAGACTGCCAAATTCTCTGCCCAGTGCCACACATCACTTGTT
CATCGTGTCCTTTCTTATCTTCGTCTAATAAGAACTAAACCTGATTTAGTCCTGCTCTTCGCTAATTAGC
TTTGGACAGCCCGAAGTTGGGCTGGAATTCCTCGGAGGGCAGGCTATTAAGGGCATTGCCGAAGCATAGC
AATAAAAATGCTGCAAACAAGCTCGGCAGGCCGCACATAATTGCCAATAATCAAGTTGGCCCGAACGGCA
GCGTAATTTTGACCCTTCGCGTCGCCCGAAAATCCTTTTGTGTGAAATTCGAGAATATGAATGAATTGTT
TGCACGGAGTCTATATTTGATTTTGAAAACATCGAGACGGATTGTTCAATGAAAGCGAAAAGCATAATGA
TGTGGAAAATTGGTTTTCTCCGCCAGGCATTCTTCTGGCCTGAGGCAATGTCGGGCTCCAACGTCTAGCT
TGGATTTTCCTTCATGTTCATTTCtttttttttttATTTCCTTTGCTATTTTCCGAGTTTTATTTTGTT
GCCTTCTCTGGGTTATGTGCGCAGTAAAGAGAGCAGAAACAGAAACAGAAagagagagagagagagagag
CTGTGGGCATGTGTGGCTTTTCCGCTTTTCCATCCTTCCGCTCTCATAGTTTTCCACTCTCCGGCTCGCC
AAAAAGTTTAACCGTTTCCCCATTCGTGAGCTTCGAGAGCCTCCTGGCCATTTTCTACGCGATTCGCCCG
TATTACGTGCGTGGGTTCCACAGATGTTGCCATAGATCCGAATCCGAACCCGAATCATATTGCGAGCTCC
ATTTGCTCGCTGGCGGGTTTGTTTGGACCCGGTAATGGGTAATGACTGCCTGTCCAATCCATCCCATGTT
AATTTGTTTTGACAGCTTGATGGTAAGACCGGTAGAAAACTCTATTTTTACTCGGGTTCTTCGTTGCTTA
CTCGCAGTATCCTTTTACCATTTATCTTATCGCCATGCGAACCCCGCGCTAATCTAAAATGCCCAGCCAA
TTGCCGTTATCTGTGCTCCACTTTTTGTCACTGCATTGCACCACCCACCGGGCCACCAACCCAGCAGCCC
ACGCACCCTTATCAGCTTTTTCTGCACCACCGCCCACAATTTATGAAAAAGCAGCAGACAGCTGCCTATT
TATAGGTCCTTCGCCAAAGGTCAAATCGTAGCAATTATTACTAGGAAAGCACACATTCCTCTTATCGTCC
CTAAATTGGAGCTCCGAACTGAGCTTTGATTAATTTAGTAGGGTCTTCGACACGAAAGTTCTGTTCATAT
TGAGTAAGTTCATCAAAAGTGGTAATTGAATTTTACAACGTTAAGTGGAATGCTGATTTTTGGAACTTAT
TAGGAGATTTCAGTGTACCTTGCTTTATTTGCTCATTTCAATTATAATACTTTTCCGAGACTGGCTTTCA
AAAGCAGTACTTCCTTCATGTTTATGGCACTTTTGACAGTTACTGGAAATGTTTTGCCATCAATCGAGTG
CTCAACAATGAACTCACTGACCACTCAAGCGTCTTACTACCAATAACAAATACTGGAAGCAGCTGTTTTG
GCCCTAAACATAATTTACACTTTAGCACGAAACGAAGCCGTTTTGAACTGCGTAGTTTGTTATTTAGCTG
CCACTTCTTGGTGCCCAGACAAGTGGGCACCGCAAAATAACCAGCACACCCATACGCCCCGTTGACCCGT

GGGCaaaaaaaaaaaaaaaaaCAGTGCCACGAAATGCTGGCAGAATGACAAAAGTGTGGCAACTGGCGCA
TACAATATTTGTGACCTTCTTGTGCCTTCCTGGAAATCACAGTAGCTGCACATCTTGTTCCTATTAATGT
TAAGTTCTTTTTCTTGCGGGTTTTTCGGCCCCTTATGCCAGGAAACATAATTCAGCATGAGATTTTTTGG
TGCATAAATAAAATATGAATAACAAAGTGGTCCACCTTTGGGTGATTGCCATTTTCCGGGCAACTTTCGG
TAGCTAGGTGTTTCTGATTGTTGCGTCCACATTTATTATGGATGAGTCTTTCGGAGGGTAGAAAGCCCTT
GTTTATCCATTTTATTTATCTGCCACTGGGCTGCAACGCAATTTGTCAGGGAATCACACGGAAATCGTGA
CATCTCCCAGCTTTCCACTTTTTGCTGCCACATGACTGGTTTTCCCCTTCCTCCATCTCAAGGAGTCATT
TCCAGGCAGCTTCTGTGCCGCACAACTTGTCCTAGATGCATTTAAATTTATTTATTTCTCGGTAATTTTC
CTTTTTGTCCTCAAGTGGCCAAGAGTCGTGGTCGCTCATTCTAAAAAGTAAAATAAGTTAGTGCATTTAA
AAGTAGGACAACATTTAAAATTTATATTGATCATGTTAAAGTTATGATGTTTATCAATTGTGCACTCTTA
AATAATTTACAGAGAAATTGTCTGTCTTAAGTTTAATCTTCGCGAAATACGAGATGAGCTGGCAAAAACT
CATTTAAGAAAGCGATAAGCGGAGAAAAGAGACAGCTGTTTATTATTCATATGCAAAATTGGTGAAAAGT
AAACAATTTCATTAAATTCGTGATAAGAACATGTATGTATATTAGTCCAGCAGCAGTACTTTTATTTAGT
CGCTCTAGTTTAACTGTGAGGTATGGACATCAAATAAGTATGTGATTAAGTTATACAGGTAAAAATTATA
TATAAGAAATATTTCTATAATTAGTTTTCATTACTTAAGCCATGATGCATGCCTTTTTATTTGAAAAATA
TGTTTAGCTTAATTTTATCGATTTTAAAGATGCCTTTTTCGACCCAAAACTGAATTAAATCGCAAATTGC
GTTTGCCTAGAAGGCAATTAACCCGCATAACTTAATTTCGCACATTATTCATGCAAATTGTATTGTATGT
TGAGCAAACACAGAGTTTGCATCTAAATTTTCCACCCGCGTGGCATCCACAACTTTTGAATTTTAAGCAT
ATTTATGGGGCCAAATAAACAAAGCTGCCTTCAGGCTTTTCATTTAAGCGGCTTGccgagtttttttcgag
ttttccgagttcttttccattttccgTTGGTGTGTGTTAGCGTGTTTCAGGGTATGGATGTCCTTTGACG
TCATTTGCAAAGTAAACAAGAGAAAAGCCATAGGAGAAGCATCGCTTTCCAGTCATTTTGGCTTTAAATT
GCTCTACACACACAGTTTGCTTTTCTTTCTTTCTGGCTGAGAGGATAAGTGGTTTTGGTGACATATTAAG
TTATCTTTGGCTCACGAGATGCTTTATGGAAACAATGAGATGCAAACAAAAGGTGAACAAACAATGGATA
GCGGACTTGGAATATCATTTAACATAAAATCAATTTGACAATAGGATTCCTCGTGAATAAATTTCATATT
CTGCTGTTCAGATTTAAGTGAACCAATAATATTCCAATGAAATTCGACTTTTAGTATTCTTTCGGCTGAC
ATGTGTCGTTCAACTTTTGATGGATTACCCAAATTGCAGTCTTCAAATGAGAGAAGAAATTGCCAATCAG
CGAGACATAAGTACCCGCGTATATTTTCCACTGGGGCAACCCCCAAGCTCCCAAAGTCCCAGGAGTCCGT
CTCTGCAACAATGGCAGTTCAGGGTCAAAGGATGTCTGACAAAAAGGAAAAGAGGAGCCAAGCACCCAAC
TCAAGCAGCCATCCGCCGTCCATAATTAATACGACTCAAGTTGTTAAAGTAAACGAAATGAAGTAAAATG
CGGAAAATTCAAAACGAGCGACTACAAAAAGCAGCTGGAGAACAGAACCAGCAGCTCGGTGTTAATATTA
ATGAATTCAATTAAAGGAGAAAGTGAGCGAAATGGGAAAAAAACTGAGCAAAATCGTAAGGGGGGATCCCA
GCCGTTCAAAGCGTTTTGCGTGGGTGCCATTGTAGAAACACatatatatgtatatatatatgtatatgtG
GATGGGTGGCTGGTTGGGTGGCTTTGGGAGTGCGTGCCCTTGGTGGCTAAGGATAAGCACTTTTACTTGG
TGCGTTTGGTGCCACTGCCACCACCACCCAACAGCCCGTTTTAGATGACGCCGTCAGCGCATTTCTAGTG
TTTCAAGGATGCGAGTGTAGGAGAAATTGCTAACGACTTTTGTGGGATTTCTTTGTAGCCACACTCGAGT
TTAGCGCTATTTTTAAGCCGGCGACAGCCCGGAAATTAAACACACGCATCATTAATATCCATTTACGTTC
AGCTACTCATTAAATTTTCACTTTGCAGCCAACAACACAGCAAAACAAAATGCCGCCAAATGCAAAATCG
GAAACGGACGCCAAACCGGAAGCGGAACCAGCGCCAGCGTCTGAGCCGGCAGCGGATCTGGAGTCCGTGG
ACCAGAAGCTGGAAGAAACACATCACTCCAAATTCCGTGAAGTGGACAGGCAGGAGCAGGAGGTGCTCGC
CGAGAAGGCGGCGGAAGCGGCCAGTCAAAGAATCGCACAGGTGGAATCAACAACACGGAGTGCTACCACA
GAGGCTCAGGGAATCCACCACCACCGCAGTGCCGGTGATCAAAAAGATCGAGCATGTCGGCGAAGTGGTC
ACCGAGGTGATCGCGGAGCGCACGGGGCTGCCCACATGGGGCGTGGTGGCCATCATCATACTCGTGTTCC
TCGTCGTCTTTGGTATAATCTTCTTCTGTGTGCGGAGATTCCTGAAGAAGCGAAGAACCAAAGATGGAAA
GGGTAAGAAGGGTGTCGACATGAAGTCGGTACAGTTGTTGGGATCGGCGTACAAGGAGAAAGTAAGTGTC

TGACGCGCCATTAAATCTTAGTAGTTATTGGGTTCTGGTATGTCATAGGTTCAGCCTGATATGGAGGAAC
TCACCGAAAATGCCGAGGAGGGTGACGAGGAGGACAAGCAGAGCGAGCAGAAGCTGGGGCGCCTCAACTT
CAAGG
>dme_circ_0001161_junction_seq
CAGAAGCTGGGGCGCCTCAACTTCAAGGTATACGAAATTCATGTGAGACTAGCGAG
>dme_circ_0001162
GACGAAACTTAACGAAAAGTTTAAAATATTGAACATGCATCATTCAAGCGTATAAGTCGTTGTTATATAA
AATCATAAAAAACGCATAAACGTTTTAAAAGTGCTTAAACAAGGCTATAAAACGGCAACAATAAGTGAAA
CCAGCTGCAATTCTATGAGTTTTGTAAACCTGAAAGCCGGAAAAAATTGATGTTTTAATGCTGCCAAACAG
ATAGCAATCAGCCCCAGCCGACAAAACAAGAGTGAAACCAAAGGCGATCCAGAAGATCATAAGATACATA
TAAAATATCTCACAGTTaaaaaaaaaGAGAAGTCATAAGTACATATTCAGAAATGGCCAAGTACTTTATA
GCCCTGGTGTTGCTGGTGTGTCTGGCCCTGGAGAACAGGAACGTATACAATCGCCTGCACGCCCGCTCCC
ATCCGAGTTCCCGTGGCGACATCCTTCGTCCGCATCCTAAGCACCAGCCGCATCCCCATGTGCAGCACAC
CTCGCAACAGCTGCAGCAGCAACACCATATCCGGCAGCAACGATCCCGTCAGCTGAAACACCTGGAACCG
GATCCGACCAGCGAGGAGGACGATGTACCGATCACCAAGCGGGAGTACTATGCCCGCCTGAGGCGCCACG
TCATCCACAAGAGGCAGCACCTCCTGCAGCAGGAGCTCAGCTACAACCATCCCGGCTCCCATTCGGAGCA
GCTAAAGGTGCCCCGCCTGTGGCAACACCTCATGGAGCAGGAGGAGAAGGCACACCGACATGTGAGCCCG
CCCGTGGAGTTGCCTATCATGTACGGAGATGCCCCCGAGGAGTCATCCCTCTCCTCCGATGATCACTTTG
ATGATCTGTCGCCGCTGGACTTTGTGCGCAACGAACTGATGGCCGAGGACCAGCAGAATCAGGAGAAGGA
TCTCGATCTCGATCTGGACCTAGATCAGCAGCCGGAAACGCCCATAGAGCCAGAGCTCCCATTGGGTGAG
AGGAACATCACCATTTCGGTGAAGTCCAGCGCCGGAGGATGCCCCAAGTGCGAGAGCAATCGCCAAGTGG
AGCACATCACCGAGGAGCAGCTGACCCACTTACGCATCGAGTTCGTTAAGCAGCAGATCCTGGAGAAGCT
GCGCCTCAAGGAGAGCCCCAAGGTGTCGGCCGTGGAGCTGCCCAAGCCCATCTTCGACGGGATGACACTC
TCCCATCCGGACGATAGCACCAAGAACAAGGAGCTGGACGATTACTACGCTCGCACTAGCAAGAAGTTCA
TACTGCTCAATAGAG
>dme_circ_0001162_junction_seq
AGCAAGAAGTTCATACTGCTCAATAGAGGACGAAACTTAACGAAAAGTTTAAAATA
>dme_circ_0001163
GACGAAACTTAACGAAAAGTTTAAAATATTGAACATGCATCATTCAAGCGTATAAGTCGTTGTTATATAA
AATCATAAAAAACGCATAAACGTTTTAAAAGTGCTTAAACAAGGCTATAAAACGGCAACAATAAGTGAAA
CCAGCTGCAATTCTATGAGTTTTGTAAACCTGAAAGCCGGAAAAAATTGATGTTTTAATGCTGCCAAACAG
ATAGCAATCAGCCCCAGCCGACAAAACAAGAGTGAAACCAAAGGCGATCCAGAAGATCATAAGATACATA
TAAAATATCTCACAGTTaaaaaaaaaGAGAAGTCATAAGTACATATTCAGAAATGGCCAAGTACTTTATA
GCCCTGGTGTTGCTGGTGTGTCTGGCCCTGGAGAACAGGAACGTATACAATCGCCTGCACGCCCGCTCCC
ATCCGAGTTCCCGTGGCGACATCCTTCGTCCGCATCCTAAGCACCAGCCGCATCCCCATGTGCAGCACAC
CTCGCAACAGCTGCAGCAGCAACACCATATCCGGCAGCAACGATCCCGTCAGCTGAAACACCTGGAACCG
GATCCGACCAGCGAGGAGGACGATGTACCGATCACCAAGCGGGAGTACTATGCCCGCCTGAGGCGCCACG
TCATCCACAAGAGGCAGCACCTCCTGCAGCAGGAGCTCAGCTACAACCATCCCGGCTCCCATTCGGAGCA
GCTAAAGGTGCCCCGCCTGTGGCAACACCTCATGGAGCAGGAGGAGAAGGCACACCGACATGTGAGCCCG
CCCGTGGAGTTGCCTATCATGTACGGAGATGCCCCCGAGGAGTCATCCCTCTCCTCCGATGATCACTTTG
ATGATCTGTCGCCGCTGGACTTTGTGCGCAACGAACTGATGGCCGAGGACCAGCAGAATCAGGAGAAGGA
TCTCGATCTCGATCTGGACCTAGATCAGCAGCCGGAAACGCCCATAGAGCCAGAGCTCCCATTGGGTGAG
AGGAACATCACCATTTCGGTGAAGTCCAGCGCCGGAGGATGCCCCAAGTGCGAGAGCAATCGCCAAGTGG
AGCACATCACCGAGGAGCAGCTGACCCACTTACGCATCGAGTTCGTTAAGCAGCAGATCCTGGAGAAGCT

GCGCCTCAAGGAGAGCCCCAAGGTGTCGGCCGTGGAGCTGCCCAAGCCCATCTTCGACGGGATGACACTC
TCCCATCCGGACGATAGCACCAAGAACAAGGAGCTGGACGATTACTACGCTCGCACTAGCAAGAAGTTCA
TACTGCTCAATAGAGGAGGAGGTCGAGTGCAACCGAGCGAGAGATGGAAAATCCAATCCGTCCATGTGCT
TCACCTTCAAGATAGACGACGCAGATGCCGAGGGCTTCGACGTGAGCACCGCCGTCCTGTGGCTCTTCAA
GAACAAACAGAACCGCACCGACACGGCATCGGTGAACAGCACCAGTGCCCAGCAGACGATCGTAGTCTCC
GAGGTGGAGGTGGATCAGCAGAAGGACTCCAAGTATTTGTCGGCGGCCAAGACCATAGCCATCCAGTCGG
TGAATGTGCAAG
>dme_circ_0001163_junction_seq
ATAGCCATCCAGTCGGTGAATGTGCAAGGACGAAACTTAACGAAAAGTTTAAAATA
>dme_circ_0001164
GAGATGACTTTGATGGAGGTCAACAGGGACATAATGTTGGCGCGGGCGGAGACACGGGAGCGGCGCCAAA
AGCGACTGGAGTACTTCTTCCGTGAGGCTTACGCCCTGACCTTTGGCCTGAGACCCGGAGAACGACGAAG
GCGTTCAGATGCCTCCAGTGATGGCGAGG
>dme_circ_0001164_junction_seq
CGTTCAGATGCCTCCAGTGATGGCGAGGGAGATGACTTTGATGGAGGTCAACAGGG
>dme_circ_0001165
TGCTCTTGAGATTCGGAAACTGGTCGCTTGGAATTAAACTTGGAATCGCACTTGGACAATGATGCCGCAT
CTGATGTTGTCCGCTGTTGCTGGTGCGGCTGCTGGTTCTCATCTGATTTGATTTGGTGCCACTTGCCAAG
CGATCTCGTTGCTATATAGCAGCTATATAGTTGGCCAGAAGAAGTCGTTGTTGCTGCTGCTGCTGCTGGC
GGAGGGGCAGCGTTACAGTTCGGAAAATCGGTCAATGGAAACGGGCTTGTAAGCAGGTGCAATTGCTGCG
AGAGCCACGGAATAGACCAAACAGCCGGGGATCCAGAGGCCGGGCGAGCTTGGAGGCGCAGGCATGTCGC
TTAAGGGTGAGTAAATGCTCAGGTATTGACCCATATAACACTTAATTGACTCAACCGGATCGCGTTTGAT
GGCTCTTTTAATGGGAAAAACTCAAATGAATAGCTTCGGCAACTGCGAACTATGATTTAAGCGCACTGTC
ATGGATTTGAAATTGAGAAAAATTGAAAGAGTTCAATGGATTGAAATCAGATCTTGATGCCCAGATTATG
TCTGAAGCTTTAAAAGTGTTTCTCACCTGCTGCTACTCACAAACATATATCTTGGTCGTCGTCATCAGGA
ACGTGATTATCACGATGGCATCACCGTCTTCATTGTCATTATGTCACTTCAAAGTGCAGCAGCATTTGCA
TTGTTACCCTCGCAACTTGAGTCACGTCATTTTCATGCAAGGGTTTTCACTTGTCTTTGCCTTTCAACAG
GTGCTTCAAACCACTTCATCAATGGCAGAAACCTGGCTAAAAAGCAAACACACAGACAAACACGGGCACA
TCTGGGCCACTTAATTCGCTTCATTAACGCAATGTTGACATTTCAAACACACTTAGCTGGAAAAAATATT
TGTTGCATTTCCTCCTTCAGCTGAAATTGGCATGCCAGTTAACCCTCaaaaaaaaaaCAAATGCATCATA
TTTAGATTTCGACACATATTACTCATACCACCCGTAAGCCGCCCTCTGCATTTCATTTCACTGTACAGCC
CTGTCCATCTGAGGGTTAGGTTTCATTTTGAAAAAATGCATTCAATGTGGCACAACATCCGCTGCGAAAT
AACtttttttttAGTCACTGCTCTGACCCTAATTACTGCAAACAGGAAGAGTAATATTCCCACGTGGCGCC
CAAATGCGGAATgggggggggCTATGAAGTGGGAACTTTGGCCAGAAAGCTCGGGGCACGGGTATCACTT
CCCCGAAAATCTCTCTATTTGGACAGCTGCTGCGGGGCAAGTTGATAATTGATTTGATTCCGTTTTCAAT
TTCATTTTTGCGACTGCAGAAGGAGACGCCCTCGGATGTGCAACTGCACATGGCAGCGATGAGGGGAGAT
GAGGTGGCTCTTCTTCGCGTTCTGGACAGTGGCAAAGTGCATGTCGACTGCAAGGATGAGG
>dme_circ_0001165_junction_seq
AAAGTGCATGTCGACTGCAAGGATGAGGTGCTCTTGAGATTCGGAAACTGGTCGCT
>dme_circ_0001166
GCCGCACCAATGACTCCAATTCTGAGGACGAGTCCTTTGACAATGTGAGCGTTTACTCTCATATGTCCGA
AGTCGCATCCTCGGAGGCCACCGACGAGCTGGCCAATGAGCGTTTCGAGGAGAAGTTCGAGAAGGCCCTG
GAACAGGCCACCGAGAAGTCGGCCCAGACACGTGTGCAGGCTCTGCAAGCGATTTGCGAACTGCTCATGC
ACCGCTACATGCCGGACTTCGTGGAGGACCGCAAGATGACCCTGATGGATTTCGTGGAGAAGAGCATCCG

TCGAGGCAAGGGCCAGGAGCAGGTGTGGGGCGCACGACTCGCGCCCCTGCTGGTGCTCCAAATGGGCGGG
GATGAAGGCATATCCAAGGCGATGAATCAATTCCTGTTGAACACCGTTCAGGACAAGTCCGTGGGCTTCG
ATGCCCGTGCCAAGTGCTGCACAGCTGTGGGATTGTTGAGCTTCCTGGGCTGCGAGGATGTTGGGGAGTT
GGTACACCTGATGCAGAGCTTCGAGGCCATCTTCGCAGGCAGTTATTTGCGTGGCGATGACAAGACACCG
GTATCCGTCACCGCCGAAGCTGGGACTTTCCATGCGGAGGCCTTGAACGCCTGGGGGCTTCTACTAACAC
TTATACCTTCGGGGGATTTTGTTTCGTTGATGACCACTGGCCAAAACATGTTTCCGATCTATTAAGAAGT
TCTTGGGTCTCTTGCAATCTACACATTTGGACGTCCGCATGGCCGCTGGCGAGACCATTGCTTTAATTCT
GGAGTCGGGACGTGCTCACGAAGAGGATTTCCTAGAAGATGACATCGCTGAGTTATCGGAGGCCGTCAAG
CAGCTGGCCACGGATTCGCATAAGTACCGCGCCAAGCGCGATCGCAAGGCCCAGCGTGCCACCTTCCGTG
ATGTGCTGCGATACCTGGAGG
>dme_circ_0001166_junction_seq
TTCCGTGATGTGCTGCGATACCTGGAGGGCCGCACCAATGACTCCAATTCTGAGGA
>dme_circ_0001167
ATCTATTAAGAAGTTCTTGGGTCTCTTGCAATCTACACATTTGGACGTCCGCATGGCCGCTGGCGAGACC
ATTGCTTTAATTCTGGAGTCGGGACGTGCTCACGAAGAGGATTTCCTAGAAGATGACATCGCTGAGTTAT
CGGAGGCCGTCAAGCAGCTGGCCACGGATTCGCATAAGTACCGCGCCAAGCGCGATCGCAAGGCCCAGCG
TGCCACCTTCCG
>dme_circ_0001167_junction_seq
ATCGCAAGGCCCAGCGTGCCACCTTCCGATCTATTAAGAAGTTCTTGGGTCTCTTG
>dme_circ_0001168
AGCATCAAAATCAATAAATCGGAACCAACACGTGCGTTTCAGTAGTTGCCGCCAAAGCAACGAAAGTGTA
GACATTTTCGCCGTTGTGTAGACTTCGACGCCTGTTCTCCACCTTTTGAGGACCCAGAACAGGAAAAGGA
CACTCGGCTACAAAGCATCTGttttttttGTGTTTCGTACCTTGTGTTTGAAACAGTAGGCTAAACAATA
GAAACCAAAACGTCGTCG
>dme_circ_0001168_junction_seq
CTAAACAATAGAAACCAAAACGTCGTCGAGCATCAAAATCAATAAATCGGAACCAA
>dme_circ_0001169
GCCCGCGCCTTCATACCCAAGGAGCACTGGTCTTCCACACCGCTGGTTCTGAAAGCGACCGCCGGCCTGA
GACTCCTGCCCGCCAGCAAGGCGGAAAACATTCTAAATGCCGTCCGAGATCTCTTCGCCAAGTCCGAGTT
TAGTGTGGATATGGATGCCGTGGAGATCATGGACGGCACGGACGAGGGCATCTTCAGCTGGTTTACCGTG
AACTTCCTGCTGGGCCGCCTGTCCAAGACGAACCAGGCAGCTGCCTTGGACTTGGGCGGCGGTAGCACGC
AGGTAACCTTCTCGCCAACGGATCCGGACCAGGTGCCCGTGTACGATAAGTACATGCACGAGG
>dme_circ_0001169_junction_seq
CCCGTGTACGATAAGTACATGCACGAGGGCCCGCGCCTTCATACCCAAGGAGCACT
>dme_circ_0001170
GCTGGCAACGCCGTGCAAAATACGACCAGACGCTGCTTTTTTCGGTTTCCGAAAATAATTGAAAATTATC
ACTAAATAAACAGGCGGCTAACATAATTGCAGTTTGACCGCGTTGCAGAGACAAGCGGGTAGATAAGTTA
CACATTTTGGCCACAGCAACACCACAG
>dme_circ_0001170_junction_seq
ACACATTTTGGCCACAGCAACACCACAGGCTGGCAACGCCGTGCAAAATACGACCA
>dme_circ_0001171
CTGAATGGTCTTCTCCGCATCTTCTAGCGCCTCCTGGAACTTTCCAGCCTTGGCGAATGCCGCCGAACGG
TTGCTGTAGAGCACATGGTTCTGGTCGTCCAGAGCGATGGCCTCTGTGTACGCCGCCACCGCCTCGTCGA
ACTTCTCGGCACTGAGCGCCTGG

>dme_circ_0001171_junction_seq
GTCGAACTTCTCGGCACTGAGCGCCTGGCTGAATGGTCTTCTCCGCATCTTCTAGC
>dme_circ_0001172
TTTAAATTTCTGTTAATGGTAGTTATCGTCGTTCTGCGAATAGGCGATGTGATTTCTTAGGTAATTACGT
TTAGCATTAATTGTGGCCCGTTTCATCAAAAACGATATATTGGTTAACCAGCTTAGAGACGCCATAATAA
TGCGTAATGGTTCTCTCCTGAATG
>dme_circ_0001172_junction_seq
ATAATGCGTAATGGTTCTCTCCTGAATGTTTAAATTTCTGTTAATGGTAGTTATCG
>dme_circ_0001173
GACGCCACCCAGCTTAACGGAGAAGGTGGCGCGTGCGCGGCGCTGCTTGGCGCCCGGCGTCTCATCCGTC
GCCGCAGTCTTACTCTCTTCCTCCTTGTGCTCGTGCAGGAACTGAACGCAGCGGTCATGCAGCATCTCTC
CGAGGCGCTTCAGTTCCGCCAGTGGCTTTTCCTGCAGCTCAGCATCACATGCGATGGCCTCCATGCGGTG
GAGAGGAGCGGGAAACTTTTTGTAACTGCGAATGAAGCGACGCAACTCCGCATCAGTGAATCCGGTGATC
TTCTCCTTCATGGTCGGCCGCCCGCGCTTGCGAGGTCGTCCATCGTCACCACTGCCGTCGGAGCCCAGCT
CGTAATCCGAGTCGCCACCGGAGTCGTCTGCCTGCT
>dme_circ_0001173_junction_seq
GAGTCGCCACCGGAGTCGTCTGCCTGCTGACGCCACCCAGCTTAACGGAGAAGGTG
>dme_circ_0001174
GTAGCTTATCGAGCAGCACAAGCTTTCCGGATCCCTTAAGAAGAGTTTGCAATGCCTCATCCTGCTGCAG
ACCCATAAGCTCAAACTCGGATGGTCGGATGAGAGCCGCGTGGTTGCAGCACTTCTTTAGCTCGATGACT
ATGTTGAGGAAAGTGGAGGTACTGCCCCGCTTTCCCTTGCGCAGTGCATCAAA
>dme_circ_0001174_junction_seq
CCCGCTTTCCCTTGCGCAGTGCATCAAAGTAGCTTATCGAGCAGCACAAGCTTTCC
>dme_circ_0001175
GAAACAATAAACTGCAAACAATTGCTAGAATCCACGGAAGTCCTCATCGGCACTGTCATCACTGGCGGGC
GTGGATGATCGTTTGCGGCTTTCTGGTTTTTCCTCTGGCTGATCTGTTGCTTCCTCCTCAGCACTCGGCA
CAATTTCCTGAGCCTGCGATCGGTGGTGGAAGACAAAGGACACGCACTTGCAATCCATCCAGGCCTGGGC
GAGAATATTGTCAGGCAGGCCTCTGTTGTTGGAGCAATGGTACCATTGTGACAAATGCGAG
>dme_circ_0001175_junction_seq
GCAATGGTACCATTGTGACAAATGCGAGGAAACAATAAACTGCAAACAATTGCTAG
>dme_circ_0001176
GTCCTGATGAATTCGTTGCTCAGCAGACAAAGATCCCCGTGCGGCTGCGACTCCTCATCCTCGCGGGCTC
GCACAATCGGATTACTCAGTGTTATGTCCAGCGTCTTGTTGTACATGCTCAGAGAGTTCTCCTTGTAGTA
GTTGATCATCTCCACCACCGAATTGAACAGG
>dme_circ_0001176_junction_seq
GATCATCTCCACCACCGAATTGAACAGGGTCCTGATGAATTCGTTGCTCAGCAGAC
>dme_circ_0001177
GCTTCTAAATATTTAAAAAGCGAGCAACATTGAAATACTCACCACTCACACAGTCGGACAAAAGCACAAA
ACGAGAGATAACAGAGCACCAGCACCACCAATTCATGCCAAACGAAACGCACACGGAGCAGTTATCAGCG
GACCAGCGCCACTAAAAGCCACCGAATCGCACACACACCGCACCGACAACGATGTGGCTGCGCCAGAGCA
GCGGTGGAGGCGTTGCCTCCGTCGGACACGTCGGCCCACTGCGACAGCGACCCATCGACGCTGCCACAGA
CTGCGATCCGCGCGCTTGCTACGACAGCTTCTGTAAGCACTGGCAGCAGGCCTTCGAGATCATCCAGCAC
AGTGCACCGCCCTCGCACGACGACGTGCTGGGCGTGGTCTCGCACTTGGACTACATGGTCACCCTGCTGC
TCGTGGAGCTGCATCACTGCAACAAAGTCTCGCTGCCGGCGGCCGAGGCTAGTGGTCCGCCTGCGGCGCC

CTGCCTGGAATTCCTGCTCAGCGAGAATCTGCTGGACAAGCTGTACGAGTGGGCCTGCACCACGGGACGG
TATGCCAACGCTGTGAGGCTGGAGCAGCTGAAGCTGTACGAACTGCTCGTCAGCCACTCGCGCCACCAGC
TGCTCTGCCACGAGCCCTTCCTGCGACCCCTGCTCAAGATACTGGCATCCAGCCAGGGCGAGATCTTTCC
GCCCGATCTCGAGAAGCGCCTGGTGATCCTGCTGAACCAGCTGTGCGTGGTTCTTATGCAGAATGTCCAC
CTGCTGGACCTCTTTTTCTTCTCCGCCCAGACGCAGGTGCAGGAGCAGATATTAAATGGCAACGTGGCGC
CACCCAAAAGCGGAACCACTACCAAGCTTCATAATATTTTCGCTGCTTATCCCGTACGTGCATCGCGAAG
GCAGTCTTGGTCACCAAGCGCGCGATGCTCTGCTGCTGTGCATGGCGCTTTCGCAGAAGAACTCCAACAT
TGGAACGTACATAGCCCAGTACTCCTCGATCTGCCCGCTGTTGGTGACCGGTCTGGGTGGGCTTTACTCG
CGTCTGCCCAACAGCATCGAGATTAGCTCCATCGACTGGCACCGGATAACGCCAGACGATGTGACAGAGA
TCCCGGAGCTGACGCTGTTCATGAACGCCCTCGAGTTTTGCAATGCGGTGGTGCAGGTGGCCCATGAGAT
GATCAAGCAGCAGTTGCTGGACTTCATGTACCAGGGCTTTATTGTTCCCGTGCTGGGACCTGCGATCCTT
CAG
>dme_circ_0001177_junction_seq
TCCCGTGCTGGGACCTGCGATCCTTCAGGCTTCTAAATATTTAAAAAGCGAGCAAC
>dme_circ_0001178
GACACTGAAGGGCAAACATTTTCAGACGAACATCGACTCGCAAATCTCGGCAATGTCGTACCTGGACCTG
ATCCTGCGCTCCATCACGGAGCCCGGACTGCTGAGGGCCTTCGTTCGGTTTCTGCTCGATACGGAAAAGT
TTGACGGCGAACGGATACTTGATGCCCTGGTCGAGCGCCTGAACTCGCCGGATGCCAATCTGTGCATGGT
CACGATGGCATTGTTTGACACCCTGCTGGGTCTGCACTGCGAGGACCTGATGCTGGAGCTGCTGCTCAAG
TTCATGCTGCCGGGCAAGCATGTGCCCATCTCACATCGCCACAAGATCAACAAAATCGATCCGTATTTGA
ATAGCAGTGAGTTCTTCCTCGAGCTCTCGCCCGATGTGATGAAGAGAGCCAGAGATCTGGCCAGGCCGAA
GAGCGTCCACGAGCCTGTGGTGAGTGAACTGACGCCTCTGCCGAGTCTCCCCTCTCCGGTCATGAGCAAG
ACGATTGGAGCCAACTGGAACTATTACGGAGTGCACACCGGTGATAGTTTGTATGCGAATATACAGGCAT
ATCTCTTCGAGGCTCATTGGCGAATCGCCCAGTGTCAAAAGGATTGCCTAAAATGGGCCAACAGCTATCG
CTACCAAAAGTGGCCTCGTCACGGGCAAGGAAGAGTCCATGCCCATGCCTTGGAGCTGGCGCGACAGTTC
TTTAGTGAGTTCGGTGGCGGTCCTATCGCCGCCAACGAGACGGGTGAAAAGCAGCTGGACAGCTTGCAGT
CAATTGGCGAGTCAAGCGGCTACGAATCCTTCAAGTGGCGGCCAGCGGACGAGGAAAGCGAAGCCACGGA
TACAACTGTGGCCACGACAGCCAGCGAGGCGGATATGGATCACAATAGCAGCAGCCTTAGCAGCGTTCTT
GGTGCATCCGGCAAACGCGAGTCATGGCGGACTTCGAACAGCAATCGCAATGAATTAATATTGACGGACC
TTGACTTCTCGGAAGATTTGTTTGCGCAGGGCACCGTAAGCTTAGGGTCCCTTTCTCAATGCCATTTGGG
GCAAACTGCAAACCTTCACGAGCAATTCGCTGTACGTCAATCTCCACTTGACCGGGCTGATCACTCGCTT
GGCCTGGTATCCCCTGCCGTTGATTCACTCGCTGCTGCTGCGCTCTGACATAGCCATCACATCGGATACG
CCCTCTTTTCACCAGGTGCTGCGCATTCTGAAGCAGCAAATAGATGCCGAGCTGCCAGTGACGGAGGATT
CGCTGGAGATCATTGATGTGGCGCGTTCGTCGCTGATTGATCGAGAGTTCCGCTTGGCAAACGCGCGCAA
GGGCAACGAGGGCTCTCCAATGCATCATAGCCAACAGCAACAGATGGTAACGAACTCCGGCCAGCAGCAG
GGTCAACTGCGATCAGCCTACGCGACCCTCTCGGCCGCCACGCCCGTGCAGGCCACGCCCACCAGTGCCT
ACGATCCGTTCAAGCGCAGTGATAACAAGCGTCGCAGCATCAGCAAATCCATTACCAGCATGTTTAGCAG
AAAGTCCGCCTCCACGTCCACAGCGCCACCCAATGGATCCTCCGGGTGAGTAACAGAATCGGTTGCCTGC
TTTTGTCACACTCTTGCACAACTTCTATATTTTTCAAAAAAGAAAAACCAAGCAAACAAACTAGATATTA
CCAACAGTTTACAAATCTGCAGCGATGGATGTTGGAAGAAAGTTGGTTTTGGGtcacattacacacacac
ttcactcacaaaaacacacattcacacacacGTACTTGAGCCCTGCATTAGAGCCTGGTTAATTGCCAGC
TAATGCAAAGTAAACCAAAGAAATCATTTTAACATTTAAATGAACTATTAATGTTATAAGAGTAAACAGG
TCTCATTCAGGGCTAAATTTGGTTGACACACATCTCTGTAAAATGCCGCCGGCATCGTTTATAATAAATT
AATTTCTTTTATTTCTTTATTTTTATCGAATCGATCAAAAAATCAAACAAATCTTAGCTTCATCTGGCTT

ATCACAAATTTACGCATTCTTCACCG
>dme_circ_0001178_junction_seq
TTATCACAAATTTACGCATTCTTCACCGGACACTGAAGGGCAAACATTTTCAGACG
>dme_circ_0001179
GACGAACATCGACTCGCAAATCTCGGCAATGTCGTACCTGGACCTGATCCTGCGCTCCATCACGGAGCCC
GGACTGCTGAGGGCCTTCGTTCGGTTTCTGCTCGATACGGAAAAGTTTGACGGCGAACGGATACTTGATG
CCCTGGTCGAGCGCCTGAACTCGCCGGATGCCAATCTGTGCATGGTCACGATGGCATTGTTTGACACCCT
GCTGGGTCTGCACTGCGAGGACCTGATGCTGGAGCTGCTGCTCAAGTTCATGCTGCCGGGCAAGCATGTG
CCCATCTCACATCGCCACAAGATCAACAAAATCGATCCGTATTTGAATAGCAGTGAGTTCTTCCTCGAGC
TCTCGCCCGATGTGATGAAGAGAGCCAGAGATCTGGCCAGGCCGAAGAGCGTCCACGAGCCTGTGGTGAG
TGAACTGACGCCTCTGCCGAGTCTCCCCTCTCCGGTCATGAGCAAGACGATTGGAGCCAACTGGAACTAT
TACGGAGTGCACACCGGTGATAGTTTGTATGCGAATATACAGGCATATCTCTTCGAGGCTCATTGGCGAA
TCGCCCAGTGTCAAAAGGATTGCCTAAAATGGGCCAACAGCTATCGCTACCAAAAGTGGCCTCGTCACGG
GCAAGGAAGAGTCCATGCCCATGCCTTGGAGCTGGCGCGACAGTTCTTTAGTGAGTTCGGTGGCGGTCCT
ATCGCCGCCAACGAGACGGGTGAAAAGCAGCTGGACAGCTTGCAGTCAATTGGCGAGTCAAGCGGCTACG
AATCCTTCAAGTGGCGGCCAGCGGACGAGGAAAGCGAAGCCACGGATACAACTGTGGCCACGACAGCCAG
CGAGGCGGATATGGATCACAATAGCAGCAGCCTTAGCAGCGTTCTTGGTGCATCCGGCAAACGCGAGTCA
TGGCGGACTTCGAACAGCAATCGCAATGAATTAATATTGACGGACCTTGACTTCTCGGAAGATTTGTTTG
CGCAGGGCACCGTAAGCTTAGGGTCCCTTTCTCAATGCCATTTGGGGCAAACTGCAAACCTTCACGAGCA
ATTCGCTGTACGTCAATCTCCACTTGACCGGGCTGATCACTCGCTTGGCCTGGTATCCCCTGCCGTTGAT
TCACTCGCTGCTGCTGCGCTCTGACATAGCCATCACATCGGATACGCCCTCTTTTCACCAGGTGCTGCGC
ATTCTGAAGCAGCAAATAGATGCCGAGCTGCCAGTGACGGAGGATTCGCTGGAGATCATTGATGTGGCGC
GTTCGTCGCTGATTGATCGAGAGTTCCGCTTGGCAAACGCGCGCAAGGGCAACGAGGGCTCTCCAATGCA
TCATAGCCAACAGCAACAGATGGTAACGAACTCCGGCCAGCAGCAGGGTCAACTGCGATCAGCCTACGCG
ACCCTCTCGGCCGCCACGCCCGTGCAGGCCACGCCCACCAGTGCCTACGATCCGTTCAAGCGCAGTGATA
ACAAGCGTCGCAGCATCAGCAAATCCATTACCAGCATGTTTAGCAGAAAGTCCGCCTCCACGTCCACAGC
GCCACCCAATGGATCCTCCG
>dme_circ_0001179_junction_seq
TCCACAGCGCCACCCAATGGATCCTCCGGACGAACATCGACTCGCAAATCTCGGCA
>dme_circ_0001180
GTCCCTCCGTGGTGGGACGACTCCTGAAGAAGAGAAACCCACCGTCGTCATCCTCATCATCGCCTACCTC
ACCACCCGATCCCTCCACATCATCGCTGGCTGGCGAAGATGCAGGTGCTGCCGAGGTGGTAGATGCCTCT
GTACTGGATCGGAGCGGTGTGGGAG
>dme_circ_0001180_junction_seq
TCTGTACTGGATCGGAGCGGTGTGGGAGGTCCCTCCGTGGTGGGACGACTCCTGAA
>dme_circ_0001181
GACGAGCACCGGCTTTGGATTTGCTGCCCCTTACAATATATATGCCACGCTGAAGAGTCTGGTGGAGCAC
TATGCCAACAATTCGCTGGAGGAGCATAACGACACGCTGACCACGACATTGCGCTGGCCTGTCTTGTACT
GGAAGAACAACCCGCTGCAAGTTCAGATGATCCAATTGCAGGAGGAAATGGATCTGGAGTACGAGCAGGC
AGCCACGTTGAGGCCGCCGCCAATGATGGGCAGCAGTGCGCCGATTCCCACGAGTCGGTCCCGTGAACAT
GACGTC
>dme_circ_0001181_junction_seq
CACGAGTCGGTCCCGTGAACATGACGTCGACGAGCACCGGCTTTGGATTTGCTGCC
>dme_circ_0001182

GCTTCTCCTTGTCTTCCTTGCGCTCTTCGTCATCATCGCCGTCATCGTCCTCGTCCTCGAGCTGTTTGTC
TGTAGCCTTTTTGTCCCTGGGATGGGACTCAGAATCGTGCGAAGATTTGTCCTTCTCCACAGGCTTTG
>dme_circ_0001182_junction_seq
GAAGATTTGTCCTTCTCCACAGGCTTTGGCTTCTCCTTGTCTTCCTTGCGCTCTTC
>dme_circ_0001183
CTGTGATGATCGCGCCTCTTCACGCAGGTTCCGCAGACGGGACAGTGGTATGCCCGCCTTGGAGTAACCT
TCCGGCAGATCTCGCAGATATTGGGCTGACCGTGCATCAAGCCAGATCGCTCAGCAGTG
>dme_circ_0001183_junction_seq
GTGCATCAAGCCAGATCGCTCAGCAGTGCTGTGATGATCGCGCCTCTTCACGCAGG
>dme_circ_0001184
GGTCCACATGCGAGTACTTGCCGCGCTTTTCGCAGTCGCCAAAGGTGCGCTCCACGCGGTTCTCGTCCTC
GTCGTTAGACACAGGCACGGACTCGTGCAGGTGGTTGCCCACCTCCCGCAGTGAGGTGTTCCTCGTCTGC
TCGGCAAGCTCTAGCGACTTCTGGTTCTCGGTCATTGC
>dme_circ_0001184_junction_seq
CTAGCGACTTCTGGTTCTCGGTCATTGCGGTCCACATGCGAGTACTTGCCGCGCTT
>dme_circ_0001185
GAGCCGGTTGGTGCCATGAGCGAGGACCTGCCCGCAGACGTGACCAAGGACCTCACAGAGATTGTAGCCG
AGACACTGCAGCCGTTGACCGTCAACCAGATCAAGCAGCTACGCGTTCTCATCGACGACGCAATGACCGA
GAACCAGAAGTCGCTAGAGCTTGCCGAGCAGACGAGGAACACCTCACTGCGGGAGGTGGGCAACCACCTG
CACGAGTCCGTGCCTGTG
>dme_circ_0001185_junction_seq
CAACCACCTGCACGAGTCCGTGCCTGTGGAGCCGGTTGGTGCCATGAGCGAGGACC
>dme_circ_0001186
GGTCCTTGGTCACGTCTGCGGGCAGGTCCTCGCTCATGGCACCAACCGGCTCCTTCTTCTTCATCTTCTC
GCCGATCACCTTGCTGCAGACGTTCTTGACCTTGTTCAGGTTGTCGGCACGGTGGCGGCACTGACGCCAC
TCCGTATCCTTGGCGATCACAGTCTCCACCAGCGCCACATCCTTGAAGCGCTTCTTTTGATTTTCGCGCA
CGAG
>dme_circ_0001186_junction_seq
AGCGCTTCTTTTGATTTTCGCGCACGAGGGTCCTTGGTCACGTCTGCGGGCAGGTC
>dme_circ_0001187
TCGTCGATTGCTGCCGCTGCCCTAGGCCCTAGCACCTCAACCTATCAGCCACCAGTGTCAGCGGGTAAAA
CCCAAAAGTTTCAAATCGGCTTCGCTACCAACAATAAAAATCACCAACTCCAAAAGAGCAGCATCAACCA
TAATGGGCCCGCAGCCTCAGCCTCTGTGAAGTGCTCCGATAGCACACTAAACGCCAAATGCAACGAAATC
TCTTCAAGG
>dme_circ_0001187_junction_seq
CGCCAAATGCAACGAAATCTCTTCAAGGTCGTCGATTGCTGCCGCTGCCCTAGGCC
>dme_circ_0001188
AACAATTTCTTAATTAAAAACAAATCAAACGCGAAATGCTGCAAATTTTGTAAATAGCATAGGAAAACAT
CGTATTTTTAAGTGAAAAGCGAATTTATAGTGCAATAATGTTTCTGCCCGAAACAGCCACCTTCTGTATC
ACCCTGTTGG
>dme_circ_0001188_junction_seq
ACAGCCACCTTCTGTATCACCCTGTTGGAACAATTTCTTAATTAAAAACAAATCAA
>dme_circ_0001189
GGAAAAGCCAAACAAATACTCTTTCCCAAAACGCGGTTAGGGGTTCAATAATTACATACTCGAAAACAAT

AGGTTACTAAATGTAAGCTTTTATGAATCAAATTTTTACAAAGCTGATGAATAATTATTTGGCTGGGAAA
GAGTATTTCGTACAAG
>dme_circ_0001189_junction_seq
TTGGCTGGGAAAGAGTATTTCGTACAAGGGAAAAGCCAAACAAATACTCTTTCCCA
>dme_circ_0001190
GATCCTTGTATATTTGGGCCCTCATACAGACTGAGCCAGAACAAAGAAAAAACGCCGAAATGCCGAGCAG
GAAGAAAGCCCAGGGGGAATCGGCCGCTGGTGAGGATGCGGACATTGTGATCTCGGGACTTTCCGGGAAG
CTGCCGGAGAGCAGCAACATCGAGGAGTTCAAGTACAACCTGCTCAACGGCATCGACATGGTCACAGATG
AGCCGCGCCGTTGGGAGGCAGGGCAAGTACCAAGCATATATCACATTAATTTATGAAATTTTTGGGGGAG
TAGACacaaaatgcaaacttatggcaaaaataccatatattcaaagggtctcttcatcaaaatctggcca
aaaaaggtcgcaaagctgagggaattactgttttgtacagctaattaactaatctaaccacccatatcac
ttttttgttaattttcgaaaatccatttttttgactaattttcgcatttttgataaggggtaccatcatca
aattttacgaaaaatgtcaaaattttaaaatttcaaggcttgaatacagatcgatagtaaatttcattac
gaattcagggagctatgatactccactcgttcatcatttttttggatagttatagacaaaatattgattat
ttatatacaaaaatcggtttaacatcttttccgagctgttttctaagcataacttgatcaaaaaacccaga
tcgcacagctaaatgatggattgtttcgtaaatctaataaacagagctaattatACTTAATCCCACGACA
ATATTTGATAATAGATTAATAGTGGGCTGATTTGTACAGTTTTTCAATAGAACTAGTTATCCTAATGCCT
ATAAGAAATTGAAAGAGAACAATATATTTTCGATCGACTGCTCAATTGGCCAAGCAAGGAATAACACTAT
CGCTTGGGTCTccccccccccTCGTTTGAAAGCAAGCCGTTCGAGCGGCGCAAGTGGCTGACTCATAGC
AAACATCCACCCCTCTTGGTTCGCTTTCGCGATCATCTTTCAGAGCGCCCTCGTTTTCCTTCAGGCGCAC
AAGTAATTCTCAACTGGTTATGGCAAGCCAACGTAGAATCCCCATCGAGTGTCGGGGATCATCATGTCTC
GATCGCCGCTTCAGTTGTGGAGCGAGAGCTTACGCAATGGAGCGGAGTGATGAGCACATTATCCGAGGCA
ATTTTTTTAGTGCCTGGCCGCGAAATGCCGCCGGGCCGTTAGAAATGAATATGAAACCATCTACTTTAAA
TATGATTGTAATGTAAAAACTTGCATCAACACTAAAAGGGCATCTATGGACTCCCCGAGCGTATGGCCAA
AATGAAGGACTCCGATCTGGAGAAGTTCGACGACAAGTTCTTTAGCGTCCACCAGAAGCAGGCGGAGCTG
ATGGACCCCTGCATGCGGATGCTGCTGGAGCTGACCCACGAGGCGATCATCGATGCGGGCATCAATCCCG
TACAGCTGCGCGGCAGTCGGACGGGCGTCTACATAGGCCTGTCCTTTGTGGAGACGGAGCACGAGATCCC
CAACATGGAGCCGAGCAGTATCAACGGCTACTGCCTGACGGGCTGTGCGCGTGCCATGTTCGCCAATCGC
ATCTCCTACACGTTCGACTTCAAGGGGCCCAGCTTTATCGTGGACACCGCATGCTCCAGCTCGCTGGTGG
CCCTTAACCACGCCTTCGCGGACATGCGGGCGGGTCGCTGTGATTACGCCCTGGTTGCGGGCGTGAATCT
CATCCTAAAGCCCATCTTTGCCCTCCAGTTCCTCAGGCTGGGCATCGTGAGCCACGACGGCTCCTGCAAG
ACCTTCGATGCGGCGGCCAACGGCTATGCCCGGGCGGACACCTGCGCCGTTGTGCTGCTCCAGAGGCGTA
AGGAGGCCAAGCGGGTGTACGCCAGCATCCTAAATGTTCGGACCAATACGGACGGATTCAAGGAGCAGGG
CGTAACCTTCCCAGACGGCCGCATGCAGCAGGCCCTGCTGGAGGAGACCTACAGCGAGATCGGCCTCAAT
CCGGACGAGGTGGTCTACGTGGAGGCACACGGCAGCGGCACACCGGTGGGCGATGACCAGGAAGCCAACA
TGCTGAGCAACTTCTTCTGTCGCCCCTCGCGATCCACTCCGCTGCTTATTGGCTCAGTGAAGTCGAACAT
GGGTCACGCGGAGCCAGCCTCGGGAGTGAGTGCCCTGGCCAAGATGATCATCGCCATGGAGGAGGGCATT
ATTCCGAAGAATCTGCACTATCGCACGCCCAATCCTTCGGTTCCGGCTCTGGTCGAGGGTAAGCTTAAGG
TGGTGGATCGCAATCTTCCCTGGCAGGGTGGCATTGTGGGTCTGAACTCCTTCGGCTTTGGAGGAGCCAA
TGCCCATGTGGTACTAAAGTCGCACACTAAAGCCAAAGAGCCTAAGGCATGCCCGAAGGCTGGGGAGGCC
GAACAGTCCCTGAGTCTAGTTACCTGTTCTGGCCGCACGGAGGATGCAGTGAATCAGTTGCTGAAAGTTG
CCACCGAGCAGAGGCACGATCAGGAGCTGCTGACCCTCATCAATGACATCCACTCACATCCTATTCCCCT
GCATCCGTTCAGGGGATACGCTGTTCTCGGTACCAGCGGATGCGTTAAGGAGGAGGTGCTGCCCTACGAG
GAGGAGGAGCGACCCATCTGGTTCGTCTACGCCGGCATGGGCAGTCAATGGGCCAGGATGGCTAAGGATC

TCATGCAGCTGGAGGTTTTCCGGAACTCCATACAGCACTGCGCCGAG
>dme_circ_0001190_junction_seq
CCGGAACTCCATACAGCACTGCGCCGAGGATCCTTGTATATTTGGGCCCTCATACA
>dme_circ_0001191
TTATTGGCATGACCTCGCTGCATCAGACTCACATAGCTAATCTGCGGACTTAAGGATCGGAGAATCGCCC
TGAAGAGTCCATGGGGTGCGATCTCCACGATGAGAGCGTTTTGGGGAATCCGCTTGACAGCTTGATGAAA
GAGCACGGGGGATATGAGGTTGTTTATAAAGTAGCCAGCCGATGCCAGGTGGTTTTTCTCCGAGTCCCAC
TCCGCTTCTTTCACACTAG
>dme_circ_0001191_junction_seq
GAGTCCCACTCCGCTTCTTTCACACTAGTTATTGGCATGACCTCGCTGCATCAGAC
>dme_circ_0001192
GATACACAAGATGAAGACCGCCCAGACCCAAAGGCGTTCTGGTGGCCAATCTCCACCCAGCTTGGAGCGC
TATAGATTCTGGCCTCATTCCCAGCACTTGGGTCAGCGGGAGGAGCAGGAAAAGTCAAGGAGCACAGCTC
TAGCGGTGGCCATTCAATTGATCATCGAGAACAGCGGTGGAGCTGTAAAGCTCAAGGGCGTGGAACTGGC
TGGATCCCGGTCCACTTTGGATGCGCTGAGTGCCGTCCAGCTTCTCGAGCTGATCGAAAGGGAACCGATT
CTGGTGGGCGACATGGCGGTGGTTACCAGCTCCTCCAATGAATCCGAACTGGGTGAGCAGCTCAAGGAGC
ACGGTATTCGGGTGATAGTCAAGGATATCAAAGCTGGTGCGGTGGAGCAGCAATGCCATTTCGT
>dme_circ_0001192_junction_seq
GTGCGGTGGAGCAGCAATGCCATTTCGTGATACACAAGATGAAGACCGCCCAGACC
>dme_circ_0001193
GATCCGCCAGCTAACCTTTAGCGCCCTGCGCCAGATAGACGCCCAGGAACCGCCCTCCGCTTCAGTCTCA
TCGCCCTCAGCCTCTTCCACCCTATCAAGGATCAAGGAGGAGGCCTGCGTGGAGGTCCAGCCCCAGAGCG
ATGAGACGCGCAAGGTATTCGCCAAGGAAGTATTACCCCAGGAG
>dme_circ_0001193_junction_seq
ATTCGCCAAGGAAGTATTACCCCAGGAGGATCCGCCAGCTAACCTTTAGCGCCCTG
>dme_circ_0001194
GTGCTGCTGACGGGGCACATAGTGACCACCACCCAGGTCCAATTGGGGGGCGGCACGTTGTGCGGGCTCA
GCGGTGATGACTTCCTCGGCGAATCGGGCGGGCATATTGATGGATTCGGGATTGAATTGGGAGTTTAGGT
GGTCCTGTCTATCGTTGGGTGCAGAGTGCGGGGTGGACGAGGAGG
>dme_circ_0001194_junction_seq
GGTGCAGAGTGCGGGGTGGACGAGGAGGGTGCTGCTGACGGGGCACATAGTGACCA
>dme_circ_0001195
GTGCTGCTGACGGGGCACATAGTGACCACCACCCAGGTCCAATTGGGGGGCGGCACGTTGTGCGGGCTCA
GCGGTGATGACTTCCTCGGCGAATCGGGCGGGCATATTGATGGATTCGGGATTGAATTGGGAGTTTAGGT
GGTCCTGTCTATCGTTGGG
>dme_circ_0001195_junction_seq
AGTTTAGGTGGTCCTGTCTATCGTTGGGGTGCTGCTGACGGGGCACATAGTGACCA
>dme_circ_0001196
GTGCTGCTGACGGGGCACATAGTGACCACCACCCAGGTCCAATTGGGGGGCGGCACGTTGTGCGGGCTCA
GCGGTGATGACTTCCTCGGCGAATCGGGCGGGCATATTGATGGATTCGGGATTGAATTGGGAGTTTAGG
>dme_circ_0001196_junction_seq
GGATTCGGGATTGAATTGGGAGTTTAGGGTGCTGCTGACGGGGCACATAGTGACCA
>dme_circ_0001197
GGTGAACTCTATCACTGACTTCTTCTGCAAGGACCGTACGACCCCTCTGTTGATCGGATCGGTCAAGTCC

AACATGGGTCACTCGGAACCCGCTTCCGGTGTCTGCTCTGTGGCCAAGATTCTGATCGCCATGGAGGAGG
GCGTCATTCCCGGTAACTTGCACTACAACAAGCCGAACCCAGATCTTTATGGACTCGTTGATGGACGTCT
CAAG
>dme_circ_0001197_junction_seq
TTATGGACTCGTTGATGGACGTCTCAAGGGTGAACTCTATCACTGACTTCTTCTGC
>dme_circ_0001198
ATCTGGGTTCGGCTTGTTGTAGTGCAAGTTACCGGGAATGACGCCCTCCTCCATGGCGATCAGAATCTTG
GCCACAGAGCAGACACCGGAAGCGGGTTCCGAGTGACCCATGTTGGACTTGACCGATCCGATCAACAGAG
GGGTCGTACGGTCCTTGCAGAAGAAGTCAGTGATAGAG
>dme_circ_0001198_junction_seq
GTCCTTGCAGAAGAAGTCAGTGATAGAGATCTGGGTTCGGCTTGTTGTAGTGCAAG
>dme_circ_0001199
GTTGCTCGAGTCCGCCTCTACTAATGCCGATGATGATGAGTACCTGCAACTGATCAACGAGATCCACTCA
AAGGCTATTCCGAATCACTTCTTCCGTGGCTACGGAGTGGTGAGCAGCAAGGGCACCCACCAACGTGAG
>dme_circ_0001199_junction_seq
GAGCAGCAAGGGCACCCACCAACGTGAGGTTGCTCGAGTCCGCCTCTACTAATGCC
>dme_circ_0001200
TTTAGCGACCAGAGCCTCGATGGAAGCCTCCGGGCCAGAGATGGTGCAGTTGTCCTCGCTGTTGTGGCAC
ACCGGGAAGCAATCGCTGGGCACACGGCTGTGCGCATCTTCCCAGCTCAATCCCACAGCGGCCATCTTGC
CCTTGGCCAGTTGAGTGTCCAGAATGCTCTTTCCTCGCCAGTAAGCAGCCAGCACGGTCTGCTCCGGGGT
GAAGCAGCCATCGGCGTAGGCGCATCCAAGCTCTCCCACCGAGTGTCCCACAATTCCGTCGGGGTGGATG
CCCAGCGAGCTGAGCAAATCGGTAAGAGCCACTTGCATGGCAGCAATCGAGATGAAAGAGTTCAGAATGT
TTTCGAAGCTCTTGTCCG
>dme_circ_0001200_junction_seq
TTCAGAATGTTTTCGAAGCTCTTGTCCGTTTAGCGACCAGAGCCTCGATGGAAGCC
>dme_circ_0001201
CGAATGTCTGCCAAGCCAGAGTCATGTAGCCAGTGGCCGGGAAGAGGATACGTCCGTCGATCGTGTGTCC
AGCCAGGAAAGCATCATCCTCCTTGGAGAGATCGACTTCCACGATGGTCTCACCAGAAGAAGTTTCCTTG
CCGAACTTGGCCACCAGCCACTTCTGGGTGTGGTCCCATCCGACCTTGGAGTTCAACATGGGGGTTCCAC
GACCCACCGGGTAGCTGATAGGACGCACTAGAGTAAG
>dme_circ_0001201_junction_seq
GGTAGCTGATAGGACGCACTAGAGTAAGCGAATGTCTGCCAAGCCAGAGTCATGTA
>dme_circ_0001202
CGCCAGTTCCATCGAAGAAGTTAATGCCAAACTTGACCACTGCGTTCTTGTTAAGAATAGTGGCACGGTG
GAACACGAGGTTCTCCATTACCACCGGGGTCTTGTGGAACTCACTTCCCTGCATCTTAGCGAATGTCTGC
CAAGCCAGAGTCATGTAGCCAGTGGCCGGGAAGAGGATACGTCCGTCGATCGTGTGTCCAGCCAGGAAAG
CATCATCCTCCTTGGAGAGATCGACTTCCACGATGGTCTCACCAGAAGAAGTTTCCTTGCCGAACTTGGC
CACCAGCCACTTCTGGGTGTGGTCCCATCCGACCTTGGAGTTCAACATGGGGGTTCCACGACCCACCGGG
TAGCTGATAGGACGCACTAGAGTAAG
>dme_circ_0001202_junction_seq
GGTAGCTGATAGGACGCACTAGAGTAAGCGCCAGTTCCATCGAAGAAGTTAATGCC
>dme_circ_0001203
GATACGTCCGTCGATCGTGTGTCCAGCCAGGAAAGCATCATCCTCCTTGGAGAGATCGACTTCCACGATG
GTCTCACCAGAAGAAGTTTCCTTGCCGAACTTGGCCACCAGCCACTTCTGGGTGTGGTCCCATCCGACCT

TGGAGTTCAACATGGGGGTTCCACGACCCACCGGGTAGCTGATAGGACGCACTAGAG
>dme_circ_0001203_junction_seq
ACCGGGTAGCTGATAGGACGCACTAGAGGATACGTCCGTCGATCGTGTGTCCAGCC
>dme_circ_0001204
ACTCTGGAATGGTTATTTTTCCAGAAACCGCCAAGCTACCGCTCTCACAAATCTCAAAAGCGCCAGTTCC
ATCGAAGAAGTTAATGCCAAACTTGACCACTGCGTTCTTGTTAAGAATAGTGGCACGGTGGAACACGAGG
TTCTCCATTACCACCGGGGTCTTGTGGAACTCACTTCCCTGCATCTTAGCGAATGTCTGCCAAGCCAGAG
TCATGTAGCCAGTGGCCGGGAAGAGGATACGTCCGTCGATCGTGTGTCCAGCCAGGAAAGCATCATCCTC
CTTGGAGAGATCGACTTCCACGATGGTCTCACCAGAAGAAG
>dme_circ_0001204_junction_seq
ACTTCCACGATGGTCTCACCAGAAGAAGACTCTGGAATGGTTATTTTTCCAGAAAC
>dme_circ_0001205
GTTTCATGGACACCATGTTGCAGTTCAGTATCCTTAGCAAGAACCTTCGTGAGCTGTACCTGCCCACTCG
CATCGAGCGAGCGGTGATTAATCCCGCCAAGCACTTTGAATTGCTGAGCGCCCTCACCAAGGAGGAACAG
GTTGAGACTGGACTACCTGTGCAGTGGTACAGCGACATTAACGTGATCAAGAGTGCGGGCGTGGAGCTGC
GCGGTCTAAAGGCCAATCTCGCCCAGCGTCGTCCTGGCACACAGGCACCTCCCACTCTGGAGCGCTACCA
GTTTGTGCCCAACATCAACACCACGGATCTAAATGAAAACTCTGAGAAGGCTCGCCTGCACGCCTTGGAT
GTGGCCATCCAGGTGATCATCGAGAACTCCAGCGGAGCCGTCAAGCTAAAGGGCGTGGAATTGGCCAATG
GACGCAACCCCGACGTTTTGGTTGCCAACCGCCTGCTGCAGATTATCGAGGGTGAGCCCGTGCTTACTGG
TGATGTGGCTGTGGTCACCTCGAACAACAATGAAGAGACCATAACCGCCGCCCTAGGAGATTCAGGA
>dme_circ_0001205_junction_seq
CATAACCGCCGCCCTAGGAGATTCAGGAGTTTCATGGACACCATGTTGCAGTTCAG
>dme_circ_0001206
GGTTGAGACTGGACTACCTGTGCAGTGGTACAGCGACATTAACGTGATCAAGAGTGCGGGCGTGGAGCTG
CGCGGTCTAAAGGCCAATCTCGCCCAGCGTCGTCCTGGCACACAGGCACCTCCCACTCTGGAGCGCTACC
AGTTTGTGCCCAACATCAACACCACGGATCTAAATGAAAACTCTGAGAAGGCTCGCCTGCACGCCTTGGA
TGTGGCCATCCAGGTGATCATCGAGAACTCCAGCGGAGCCGTCAAGCTAAAGGGCGTGGAATTGGCCAAT
GGACGCAACCCCGACGTTTTGGTTGCCAACCGCCTGCTGCAGATTATCGAGGGTGAGCCCGTGCTTACTG
GTGATGTGGCTGTGGTCACCTCGAACAACAATGAAGAGACCATAACCGCCGCCCTAGGAGATTCAGGAGT
TCGAGTGGTGTCCAAGGACGTGCTGAAGGAACCG
>dme_circ_0001206_junction_seq
GGTGTCCAAGGACGTGCTGAAGGAACCGGGTTGAGACTGGACTACCTGTGCAGTGG
>dme_circ_0001207
TGCCCCAAGCGCCGTTCTTCAGCACGTTCGAGATGAGGTCCTTCTCCAGCTGCTGGCGGTACAGGGTCGA
TGTAAGGGAGAACTTCTCAGCCTTGGCATCTTGCACGAAGACCAGGCGAGCCAGTTTGCCGCCATTCTCG
TTCTTGATGCACGTCATCAGACCCACAGCTCCAAACAGCTCCTCTCCCTGGCAAACCACGTACACATACT
GCTCCTCCGTAGCAGCAGTAGCCAGAGCGGCCTTCAAGTCATCCACCCAGTTAAAG
>dme_circ_0001207_junction_seq
GGCCTTCAAGTCATCCACCCAGTTAAAGTGCCCCAAGCGCCGTTCTTCAGCACGTT
>dme_circ_0001208
GATCTTCTCACCCTTCTTCATTTGTCCTCGCACAACCAGAGCGTAGTAGACGGTGGAGTACACGCAAGGA
ACGGTCGAAGCCTCTTCCATGGTCCACTTCTCAGGGATTTGCCACATCATGCGCTTGCTTGCCACGCAGG
TGGTAGCAAGTGATTTAGCCGGCACCATGGCCATTACACGACGACCCTGGG
>dme_circ_0001208_junction_seq

ACCATGGCCATTACACGACGACCCTGGGGATCTTCTCACCCTTCTTCATTTGTCCT
>dme_circ_0001209
GGAGGAGATGCAAAACCAGGTTGTCTCCCTGGTCGCTGAGGGCATCAAGACCGGAGCCGTTGTACCCCTT
CCGACCTCGGTGTTTAACGACCAGCAGGTGGAGCAGGCCTTCCGTTTCATGGCCTCTGGCAAGCACATCG
GCAAGGTAGTGATCAAG
>dme_circ_0001209_junction_seq
CAAGCACATCGGCAAGGTAGTGATCAAGGGAGGAGATGCAAAACCAGGTTGTCTCC
>dme_circ_0001210
GTACTTGGTGGCCGTCACCTTGGGATCAGCCACTGTCTTGAAGTCCTTGGCGGTCTGATCCTCGATCAGA
GCATCGCGGAGAACAGCCGCCAGGTTGAAGATGCCTCCGACCAAAGCCAGCTTGTTACTATTCTCCAGCA
GCTTTTTAGCTCCAGCGGCGGTGGTCACATCACTGGTGTCAATCACCACCTTTACGCCACGTTCCTGCCA
GCGACGGATCATCAGACCCTGGTAGCCGGTCTTAACTCCGGAACGAGAGGTCAGCACAATGTAGCGGGCT
CCACGCGTCACCAACCAGTTGGTCAGCTCCAAACCGAATCCGCCAAGACCTCCAACCAGGATGTAACTCT
TCTCCGGG
>dme_circ_0001210_junction_seq
TCCAACCAGGATGTAACTCTTCTCCGGGGTACTTGGTGGCCGTCACCTTGGGATCA
>dme_circ_0001211
GGTGACGGCCACCAAGTACCTGGATCAGTTCTCTCGCGATATCTGCACTGAGCTGGACTACTTCATCTGC
TTCTCCAGTGTTTCCTGCGGTCGTGGTAACATTGGTCAGACCAACTACGGATTGGCCAACTCTGCAATGG
AGCGAATTTGCGAGCAGCGCCAGGTGAGCGGATTCCCAGGCACCGCCATCCAATGGGGAGCCATCGGAGA
CACCGGTCTGGTGCTGGAGAACTTGG
>dme_circ_0001211_junction_seq
GACACCGGTCTGGTGCTGGAGAACTTGGGGTGACGGCCACCAAGTACCTGGATCAG
>dme_circ_0001212
AATTTGGCTGCTGATTCCAGAGAATCGGAGGGCACGGCTTCCGTGTACTGCAGTCCATAGGCGGGCACCT
CCAATCGCTTGGCCAGCGGCTCCAGTGCGGAGGCGAACCCCTCGATCGGGGAAATGAAGAAGATGGGGCT
CTGTTTGCTGTTTGCAGGTGCCTTTGTGTCCAGTTG
>dme_circ_0001212_junction_seq
TGTTTGCAGGTGCCTTTGTGTCCAGTTGAATTTGGCTGCTGATTCCAGAGAATCGG
>dme_circ_0001213
CATGATCACATTGGCCACCTCGTTGGTCTCTTCCAGAATACCGGCCATCACGTAGGTGAGCAGGCAACCG
AAGGAGTATCCGGCTAGTTTGTAGGGACCCTTTGGCTGGACGGTGCGCAGCTGCTTGATGAAGAATTTGG
CTGCTGATTCCAGAGAATCGGAGGGCACGGCTTCCGTGTACTGCAGTCCATAGGCGGGCACCTCCAATCG
CTTGGCCAGCGGCTCCAGTGCGGAGGCGAACCCCTCGATCGGGGAAATGAAGAAGATGGGGCTCTGTTTG
CTGTTTGCAGGTGCCTTTGTGTCCAGTTG
>dme_circ_0001213_junction_seq
TGTTTGCAGGTGCCTTTGTGTCCAGTTGCATGATCACATTGGCCACCTCGTTGGTC
>dme_circ_0001214
CTGCTTGATGAAGAATTTGGCTGCTGATTCCAGAGAATCGGAGGGCACGGCTTCCGTGTACTGCAGTCCA
TAGGCGGGCACCTCCAATCGCTTGGCCAGCGGCTCCAGTGCGGAGGCGAACCCCTCGATCGGGGAAATGA
AGAAGATGGGGCTCTGTTTGCTGTTTGCAGG
>dme_circ_0001214_junction_seq
AGATGGGGCTCTGTTTGCTGTTTGCAGGCTGCTTGATGAAGAATTTGGCTGCTGAT
>dme_circ_0001215

GATCACCCAGCCCGTGGAAACGGTAAGAGCACAGAGACTTCTGCAAAGATAGATCACAGCTTACTAAATG
ATTTGTACGCCAACTTACAGATCAAGAAATCCGCCACTCTGTTCTACAAGAAACTGGAGCTGGCCGATGG
CTACCAGCCCACTCTGAAGCTCAAGACGAATGTGACCTTGGTCAAGCCCACAGACAACTCTGCCAAGCTG
GACGAGGACTATCGCCTTAAGGAG
>dme_circ_0001215_junction_seq
GCTGGACGAGGACTATCGCCTTAAGGAGGATCACCCAGCCCGTGGAAACGGTAAGA
>dme_circ_0001216
GGTCTGCACAAAGCCCGTGGAAGTACACACTGTTGAGGGCAACCACCGCACCTTCCTCATCGAGGATCAG
TCCCTGAAGACCATCCAGAGCATACTGAAGCGTCTGTTCAACTAAGCTAGAAAGAGGATTAGAATAAGAG
TTGTGAATGTGGCATAGGCCCACTACCCACACGATCATCACCCTCTCACCGTCACTCACCCAATACTCGG
GTTCTATGCACCATGTGGAACCACGGAACACATCGACACCTAGTTAGCATACTTA
>dme_circ_0001216_junction_seq
ACACATCGACACCTAGTTAGCATACTTAGGTCTGCACAAAGCCCGTGGAAGTACAC
>dme_circ_0001217
GAATGTCGGGTACTAAATTGTACGGTGCACAAAGCGTTAAGTGTTATGGACTGAGCCTCTCATCTTGCAT
CATTTCAACTGCCCGGAATCGCAGAGACTGTAGACTTAATTTTGGACCGAAGCCTACAGCCCTGCAAGAC
AGCAGTTAGGCAGATTCAATCCGATCGACGCAAATCCAATCTGTTAACTTATCTCTTCTAGCTGTGCGTA
ACTGGGATGGGGCGAGCCATTCCTTGCAGCTCAACAACGTAAATTCATTCACTAAGTATGCTAACTAGGT
GTCGATGTGTTCCGTGGTTCCACATGG
>dme_circ_0001217_junction_seq
TGTCGATGTGTTCCGTGGTTCCACATGGGAATGTCGGGTACTAAATTGTACGGTGC
>dme_circ_0001218
GAATGTCGGGTACTAAATTGTACGGTGCACAAAGCGTTAAGTGTTATGGACTGAGCCTCTCATCTTGCAT
CATTTCAACTGCCCGGAATCGCAGAGACTGTAGACTTAATTTTGGACCGAAGCCTACAGCCCTGCAAGAC
AGCAGTTAGGCAGATTCAATCCGATCGACGCAAATCCAATCTGTTAACTTATCTCTTCTAGCTGTGCGTA
ACTGGGATGGGGCGAGCCATTCCTTGCAGCTCAACAACGTAAATTCATTCACTAAG
>dme_circ_0001218_junction_seq
GCTCAACAACGTAAATTCATTCACTAAGGAATGTCGGGTACTAAATTGTACGGTGC
>dme_circ_0001219
ATATATTTGGTAAGTTGGGACGTCTGGTGCCGTCTAGCTCTCAAGGAATGTCGGGTACTAAATTGTACGG
TGCACAAAGCGTTAAGTGTTATGGACTGAGCCTCTCATCTTGCATCATTTCAACTGCCCGGAATCGCAGA
GACTGTAGACTTAATTTTGGACCGAAGCCTACAGCCCTGCAAGACAGCAGTTAGGCAGATTCAATCCGAT
CGACGCAAATCCAATCTGTTAACTTATCTCTTCTAGCTGTGCGTAACTGGGATGGGGCGAGCCATTCCTT
GCAGCTCAACAACGTAAATTCATTCACTAAG
>dme_circ_0001219_junction_seq
GCTCAACAACGTAAATTCATTCACTAAGATATATTTGGTAAGTTGGGACGTCTGGT
>dme_circ_0001220
GAATGAAATTCATTACATGCGCGACTACAAACTGTAGGCTTACAACTAGGCAATAAAACTAGAATATGCG
ACATACATTATAGATAGAGCATAAAAGATATATTTGGTAAGTTGGGACGTCTGGTGCCGTCTAGCTCTCA
AGGAATGTCGGGTACTAAATTGTACGGTGCACAAAGCGTTAAGTGTTATGGACTGAGCCTCTCATCTTGC
ATCATTTCAACTGCCCGGAATCGCAGAGACTGTAGACTTAATTTTGGACCGAAGCCTACAGCCCTGCAAG
ACAGCAGTTAGGCAGATTCAATCCGATCGACGCAAATCCAATCTGTTAACTTATCTCTTCTAGCTGTGCG
TAACTGGGATGGGGCGAGCCATTCCTTGCAGCTCAACAACGTAAATTCATTCACTAAG
>dme_circ_0001220_junction_seq

GCTCAACAACGTAAATTCATTCACTAAGGAATGAAATTCATTACATGCGCGACTAC
>dme_circ_0001221
GCAATAAAACTAGAATATGCGACATACATTATAGATAGAGCATAAAAGATATATTTGGTAAGTTGGGACG
TCTGGTGCCGTCTAGCTCTCAAGGAATGTCGGGTACTAAATTGTACGGTGCACAAAGCGTTAAGTG
>dme_circ_0001221_junction_seq
AATTGTACGGTGCACAAAGCGTTAAGTGGCAATAAAACTAGAATATGCGACATACA
>dme_circ_0001222
AATCCCTGCGCTCTGGCGTGGATCAAACCGAACTTCAGGAGCTGAACAATGGGCAATCTGCTACCGGTTG
TCCGACGCCGCCGATGGCGCGGGACTCAAAGCGTACGTCCATTGGAAATGAGTTACCCTGTGAGACGGAG
GCCAAGTTGAAGAGATGCGAGGACCAGCAGGATCCACAAGCATCCACGACGGCATCACCCCTACCGATGA
TCAATCCACAGGAACAAGAAGCCAGTGGCCAGGTCAAAGAAGCAACAATCACCACTGCAAATGAGGCCAA
ACGCCTTGTCCTTGCCCGACGAAATCAGCAATTCGGTCAGCTCGACGACGATGCTGGCCACGCCCCGCGT
AGACATAGCCGCCCAGTATCCCGTCCTGAAACCATTCACAGAGAACGGAGAGCTGGACTGTGCCCGACTG
GCCAAGTTCTTCCAGGACACCCAGTTCGAGAGGAAGGAGGAGCAACGCCAGATTCTGGGCCTCTCCGTAA
TGGTGCAGTTCATGTCCAAAGAGG
>dme_circ_0001222_junction_seq
GTAATGGTGCAGTTCATGTCCAAAGAGGAATCCCTGCGCTCTGGCGTGGATCAAAC
>dme_circ_0001223
ATAAACGTTTAAATGGCACTTTGGCCAGCACGGCAGCAATTGCAACACCCATCAAAGGAGTGGCCACCAA
ATCCGGAGCCACAAACAAGCTGCAACAATCGGCAAAAACTAAATCAACCGCGAGTATCAAAAGTCCAGCA
ATTGTCGGCGTTTCCGAGGG
>dme_circ_0001223_junction_seq
GTCCAGCAATTGTCGGCGTTTCCGAGGGATAAACGTTTAAATGGCACTTTGGCCAG
>dme_circ_0001224
GAGCTGCCAAAAAGCGCGATGTGCGCAATCTGAGCATATCGCCAACCGATCTGGGTCCGCCACCAAAAAC
CCAACAGACAACCAAGGGACAATCGACGCGCAGCAAATCATCGGCCACGCCCACGCCAACGCCCACATCC
ATACGGTAAGTGCAGAGATTTACATATCACTAGGATAAGTTGAAATCTTAATGGGTAAAGCCTTAAATAC
ATATTTTATTTATAAGAGTTGTCAACTTGCTGAGCTTGGAGCAAAGTATAGCTTTGAATATTTTAGAATT
ATATTCCAATTAAACTTTTGTGACTTGAGCTATGCCACTTGAAATCGCATAAAATCTCCAACATCGTTCT
TACTCTGTTCACAGACGGAAGACACTTTTTACAGCTTCTTGGAAATCCCCTTGAAAGGTGTCAACTGTTG
TCAAGAGTCCGCCCTGCAACGCCCCACCTTTTTCGCTCACTAGAGTTGTTATAGCCTGGCTAAATGCGAA
ATCATCACTCAGGTGAAATGATGCACAACTTGTTGCCCTCAATGAATTGCTAAATCTGTCCACCTGACCG
TCGGTCTGTTCCCTTATTTCACTCACCTTGCTCCGCCGTAATGGTGTCGTATCTTGAAGTCACTGTAATT
CAATTGAGGGCGTGTGTGCTGGCTTCCAGTTGGTGTTGGAAAAATAATTTAGCGCTCCATTCCCACAGTT
TGATGTTTGGATCTGAAGGGGAAAACTGGGAAAAATCTGTGCCAGCTGCAGTTGGGCAACAGCTGCTGAG
GTGACACTTTGTCTGCAGTCTCCTTCGACGCAGCTTGCCCCCAAACTCCTGGCAGAGTACATGTTGCCAC
ATGTGCCTGCGAATTCTGCGCAAATTTGGCTCCGTTGACTCATTCATTCCCTGTGAGTCATCTTAAATCC
CCCATCAAGTTGAAATGAATGTGGTAATTGCTTCGCCTGGAGTCACTGATTGCTTCATTGATTTTGTTGA
AGAGGCAAGAGATACATTTTCTCGAGAGCCTCTCAAATTATTTGGTATTATCCTAACATAAATCTCGAGG
AATTTTAAAGTACTGTGTTGATTGAAATCATCACGTCTGCGGAAAACCTAATTAAATCATTTTTGCCACT
TGCTCAATTAACGCATTTCGTTCATTGTATTCGAATTTCGATGACCCCATAAATTTGTATATTTATGCTG
ATACAAAACTGGAGCAACCAAAACTGAAACTTAATAAATCATATTACTGCTATAATTGTTTTAAAGAATT
AGAAAGGATGGAAGAGTTAACTTCCCGGAGTTTTAGTGGTAGTTTTAAACTCCAATTATTTTCTTTAGTT
TTTTAAGTTTCTGCCTGACTTTGTGCGGGTACCATTATTTTGTTTACGATCGTGTACGGGTCATGCAAAT

TTTTGCAGGCCTGCATAAATTAGCCTTAATAGGGAAATGCATTGTGTTCGCCTGCCGAGATGTTGCTAAA
TTGCTTGAAATGTCGCAGGGCTGGAAATGAAATCGCTAAGAGAATGCTTACTCCTCCGTTGGATTCTATT
TATAAAGTCGGTCTAATTTAAAATGACTTCTTTTGTCCCGATCTCCGATCATCGTCATTGTCCAGTAGGA
ATTTCCAGAGTGAACATGCTTATCGCACCATTTTCACAAGCAGAAAAGCATGAAAAACGATGTAGAAACG
TGTTTTGCATACCCGATGGGGAAAAACGAGCTTTTTGCTTTCGGCTGGAATTTCTTGTACTAATTAAGAT
TTATTTTGCCAACATGCAATTTCATGCAATTAATGCCTCTAGAGATTAATCAAAAAGAAAACATATTTAC
AAATTCTAAGCCAAGACCATATATAATAGCTACTCTATTTTTAATGGTTTATCAACCAAAACGTGCCGTG
AGTTCTTTCACCTCGCCACTATCTCCGCTGATAAGCAGAAGTTCGATTAACACACCCATTCCAGCTAATT
GGATGGCCCATAATCGAATAACTATTCAGTATTCACTGGACAAGCAGCGCGATGGTGCAAACGTGATGGT
TTTCAAGCCCACACTGCACTCCATTAACCCACGTCTGCCGGTTTTGGCCAAAAGAAAACCACTAAAGAAA
TGATTATTGGCATAATTGCGCAAGATCGATTGAGTCGTCGGCACTTTCGCACCTCTTTGTAACGTTCAGA
TTAGCTGCCCACGCATCTGATTTTCGTAAGAGATCCGGTTAGGTTTTATACCTTATGGGTTTAATTCACC
AATTGAAAGATGTGGTGGTCATAATAGTTCTTCATGGGTGGTAGCTCCAATAAAGTTCAGAACTCTCGTC
TGACATTGACATTCGTGGCATTGTAAACTCTTTTTTCGGCCTGCACAATTCCATTGTTTGTACTTGTGCC
CGGtttttttttCTAGACTTGTACTAGCCATTTATGGCTTCTGGCGCTGGtttgtttagcgttttgtttt
gcgttttttttttttccttttttttgcctttttgtttttgCGGTTAGCATTGAAACCGAAGCGGTGACAA
CCGGGCTAAAAAGATCTCAGCACGAGCTGGAGAATTGCAAAGACAATATGGGAGATACATACGTATTCCC
AAGTGGAAGTGGTCGTGAGGTCAGTTGGTGGACAGTACTATTCGTACAGGAAAATAAACAGAACAAAAGC
AGCAAATTATGCTAATTATTCAAGGAAACCTGTATTGTGTGCAAGTTGCAAGAGAAACTTTAGAAACTAA
AGAGTTAGGTTAAGAGTTGGAAACTTAAGAGAGCTGCCATATGGCATTCGATTTAAGAGAGATCCTTAGC
AACATTTAACTGCTTGCCTATGATGCTTTAGCAACATTTCAATGCATTAGAGAAAATGAACAACTAGATT
AATTGGGATAACATATTTAAAATGCCCATGCTGTTACCTATTTTAGTTCTCAAATGAAATAAAACTCAGC
AACATGTTGCGCTCTCTCGACCAGCAACATGTGAACTGCTTTCTGGCACTGCTTGCTGCACGCACCTGCC
TTTTTCATGCCACTCTAAAAAAACAGGTGTTTCTGTTTTGGCATTCTTTCCCTGTTTTGCACCTTGTGTT
GCAGCACACaaaaaaaaaaaaCGCATGAGCACTGCAACCTGGAAATGCAAACTGGTTTTCTTTTGATTTC
ATTAATTTGGTCAAATTATGACATGCTGGGCGGCCAGCGACTCGGGATCGCTGTTTCCCCAGCCGAATTC
AGACTCGGATTCAGTTGCAGATGCAGATTCTGGCGTTTTCTGCGCTCCCTGCCACTTGAGCGTTCAGGTG
CATTGATAGCCGGAAAGCGGGATTTTTATCATAATAGTTGAACCAAGTAGGCTCAACGAATGTGACAATT
GCTGTAGAAATATTTCGTACAAAAATTATAAACTTTGCCTTATTCCAATTTGAACAAATAAATATTGCAT
CCGTATTTTCATTCTTTATATCATATTTCGAATGAGACCTGATCCTCGGAGTGGTATAGATAATAGATAA
TGAAAATATGGGCCTACGTAGGTTTATTCGCAAGCATTTTGGCAGTAAAAATTCATTGGGCAGTGCAGTT
AAAATTATAAAAAGCATAGAGAACGACGAGTTGAAAAGTGAGTTTTTTCCGGTTGCCTGAAAGTATGCCG
CATTATTTACATACGCCCTAATCATATTACCATTCGACACATTCATTGCAGAATAAACGTTTAAATGGCA
CTTTGGCCAGCACGGCAGCAATTGCAACACCCATCAAAGGAGTGGCCACCAAATCCGGAGCCACAAACAA
GCTGCAACAATCGGCAAAAACTAAATCAACCGCGAGTATCAAAAGTCCAGCAATTGTCGGCGTTTCCGAG
GG
>dme_circ_0001224_junction_seq
GTCCAGCAATTGTCGGCGTTTCCGAGGGGAGCTGCCAAAAAGCGCGATGTGCGCAA
>dme_circ_0001225
GCAGCCGAGTGCGTGTGTGTTTCTCCGTATCTGTGCGTTGTGACTAAGCCGACAGTTAAAAACTATAGCT
AAACTAAAATAGGAGAGCGAATCCAATAAAAAATCCCATTACCACCGGAGAGGTTATCTTCGTTTGCCAG
CGG
>dme_circ_0001225_junction_seq
CGGAGAGGTTATCTTCGTTTGCCAGCGGGCAGCCGAGTGCGTGTGTGTTTCTCCGT

>dme_circ_0001226
GAAAACAGCCAGAACTTTTAATCAGTCCCAGAATGCAGTCCAAAGAGCTCGCATGCGGTGCAAAGTCAAT
ACCGCTCTAACTGTGCCAGGACCATGTCGATGATTCGGATACCAACCAGGACTATGGCGTCATTTGTATG
CGGTGTGGCTATTCTGCGACAACTAAGGGAAAGGAAATGAGGAGCCGCGGCTAATCCAAGAATCGCACAG
AAAGTAAACAGAAAGCGTTCATCAGTGGGTCAGTAGCAGCCCGTAAACGAGATTTTAAACCCAAAAATTT
GTGAGGATGATGAGCGCCGGAGGAACCTACATCTCCACCGCCAGCGTGGAGGTGCCACAAGCTCTGCAAG
ATGGCGAGAAGTTCATTCGATGGGATGAT
>dme_circ_0001226_junction_seq
TGGCGAGAAGTTCATTCGATGGGATGATGAAAACAGCCAGAACTTTTAATCAGTCC
>dme_circ_0001227
GAAAACAGCCAGAACTTTTAATCAGTCCCAGAATGCAGTCCAAAGAGCTCGCATGCGGTGCAAAGTCAAT
ACCGCTCTAACTGTGCCAGGACCATGTCGATGATTCGGATACCAACCAGGACTATGGCGTCATTTGTATG
CGGTGTGGCTATTCTGCGACAACTAAGGGAAAGGAAATGAGGAGCCGCGGCTAATCCAAGAATCGCACAG
AAAGTAAACAGAAAGCGTTCATCAGTGGGTCAGTAGCAGCCCGTAAACGAGATTTTAAACCCAAAAATTT
GTGAGGATGATGAGCGCCGGAGGAACCTACATCTCCACCGCCAGCGTGGAGGTGCCACAAGCTCTGCAAG
ATGGCGAGAAGTTCATTCGATGGGATGATTGTGAGTAGTGAGCTGTGTACTTAGACGATTTTGTCCGGGG
ATATTGATTATTAATTACTTATACTTGTAACTGGTACATTCCATTATTTCCAATTCGTTTGCAAAAGGTC
AAAAGACTTTTTAGGCTTGTTCAAAGTATTTGTTTTGCCTAAAAAAGTCTTTAACACTAATCAATTTAAT
TTTGATAGTTGAGTTATCAGGTATTTTTTGGAAAAAAATATCCACTAACCCAATGTGTCATTGTTTGAGTA
CAGAATGTTCGTTTATTAAGTGGTTATATAAGAGAATAGGTTTTTGTCTATTTCGCCTTACGGTTCTCAT
CGATCCAAGGCAAAAACGAAGACACTGATGTGTATACGGAAGGATAGGTTGTGGACAAACAGGGTGTGTA
TCCCCAGGAGACGATGCCAATCAGCTCGGCTCCTCGACTGCTGGATTGCGAGACCAGGGGGCCGCCACTG
TCTCCGTTGCAGGATCCGTCGGCTTTTCCGGGGGTGTGGGTGCATACATTGGTTTCTGCCAGCGAATTGT
TGGATGGCAACGCCGCCTTGCATTCGTTGTAGTCAACAATGATAGCGTCCAGTTTCTGAAGGTTCAGCGG
CAGCAATCCTGAGTTGTCGCGGCCCCAGCCGTACAGATATCCATCGCTAGTGCCTTGGAATGTCTTTGAA
GGAAGACTTACTGCGGAAACCGGATTGCTGCCATCACGGGCAACGGCATTCAGGTCAAAGGCATCCTCTT
CCTTGAGGAGAATTAGGCCGATGTCGTTGGGTCCCACACCGCCACCGTAGTTCTCGTGGATCACGTACTG
GGCATTGGTGAACTTCCTAATCTGAACGTTCTCCTGGTCAGTGCGGCTGTGTGCACCGGCAACTGCCTGA
CCCTGATTGTAGGTCAGGCAGTGTGCGGCGGTCACAATGGTCACCTCATCGAGCAAGCTTCCTGCACAGA
AGTGCGAGTTGGATGTGGTCTGTAAGGACACAATATAGGGCGCCTCTCCCTTCGCCGCTTCATAGCCGTT
GATGATGCGACCTTCGGGGAATCCCGGCTGAGGGACAGAAATGGCCGAAACCGAAGCCAGAGCCAGTGCG
AATATAACCGCGAATTGTTTCATTTTGAGTTCTCTGTCGAGTTTGTTCCACTGCCAAATAGCCTAGCGCC
TTATATAGGTCTCTTCAATCTCCAATTTGAACTGTAAGCACTCCAAGCACCAGTTATCGTTATCACCGAA
GAGATTTACGATCATCACCGGCATATTCTTGGCCCCGATATGATTTCGCTTTGTCCTGCTCTTGCGCCTT
TGACTGATACGAGTTCTAATATCTCAACACATGCATGCACTTAAGGCACAGAAGAAGCTTCCTTGCAGCC
AACATGTCTGGCTTTTGGCCCCGATCTGGATGGCAAGTTTCTATTTGTTCACATACAGGTTACTTTGTCT
GAGTGTGTTAATTGTATTTGTCTTAAAACAATAGTAGAGACTTTGTGTATTTGGCAAACAATGATTAGAA
GCTAGCATGCCCTGATAATAAATGATGAAATATGAAAACATAAGTGTAAATATTATTGAGTCTTCTAAAA
ACAACCTTAAATGTATTTGGGAATTATTTTTAGTAGCGCCAAAAAGCATCTAGAAAAATTTAACTTTTAA
AGTTGTAAATTTTCATGCGTCATCCTTGTATTCTTTGTTGTATTAAACGTTGTTTTAGCAACATGTTGAT
TCTTAGAATTCGAACTTTGTAAAGCTTTTCCAATCTCTTTGTTCAATATTATTTAGCACTTTTTTAATAG
CAATGATAAGGAGCTTTCAGTCCGGTTAGATTTGATTGGTCGAGTGCATGACTTTCGATACTGGATCGTC
TATCAATGGCACACGCCTCCAAAAGTCTGGGCTTTTCCAGAGCTTATATTAGAAACCATAAATCAAGAGA
CAAATGGTGTGTTCGGGACCGTCTTTAATCCAGTTCCGATTACAGTTCGATTCAAGGGTAATCAGACACA

ATAAATTGTAGAAAAGCAAACAGAGACTGGACTTTCATTGCCTATAATTATTTCACAATCGATCAATCGG
AGGGGTGTACAAAACTAGTTTCTTTTTGAACGTTTTTGCTTTGCGTTGAAGAGGATGGTATTTTAGAAAG
AATAAACGCAAAAAAGTTACAAGACAAGTTTTAGTTGGATGCTTTTATTCAACAATCGATCATGACTCTG
ATCAATAGCGATTTCGATCCTGGCTAATAGATCTGACTAATAGTTTCTTCCTTTTGTTTAGAAGAGCTTG
TAGTAGGCACTCTGAATATTGGTTATCCAATCAATGTAGGCCGACACCTTGGTGTAAATGGAAGGCATGT
TGGCCAGTCCGCAGGGGATGTAGCCCCAGGATACGATGCCAATCAGCTCGGAGGGGGCGTTTGTGAACTC
AACAACCAGGGGGCCGCCGGAGTCACCGTTGCATGCAGACTTGCTCTGTTGCAGGGAAGAGGAGCAGATG
TTGGACTCAGCGATAGGAGCGCTTTCCGGCAGCTCCTCCTTGCACTCCTCGTAGTTCAAAATTTGGGTGG
TCACAGTCTGCAGGGTCTTGGCGCCCGAGAAGATGTAGGACTTTGGCTGTCCCCAACCGTACAGGTGGGT
CTCTCCCTCATGGACTTGTTCACGGCTGGGCAAGGTGGCGGGCTGCACCCACTCGTTGAAGATGAAAGAC
TCGTTCACGTGCAGCAGGGCAATGTCGTAGGGCCCAACGCCACCGGTGTACTTCTCGTGGACGCGGCCAA
AGTCAACTTGGCGCTGCTGGGTCAGCTCATCCACCTCGGCACGGGTGTGGAGTCCGGCAATGATGCTCAT
GCCTACTGGCTCGGAAATGCAATGAGCAGCAGTCACAATCCAGTCCTTGTTAATCAAGGTGCCTCCGCAG
ATGTGCGAGTGCTTCAAGTAGTTGGTCGCCAGGGAAACAATGTACGGGGCGGAGTGGGGTTCTGCCTCCG
TGCCGTTGATCACGAAGCCGGTCGCAAAGCTAGGCACGAGTTTGGCCAGCTTGTCGGAGAGCTTGGCTCC
CTGGGCGGCGGCCACAAGGGCAATAACCAAAGTCACAGTGATCAGTTTCATTCTGCAGATTTACGGAGAA
TGTATACTGACTACCAACTAATGGCACCTTTTATACTTGCCCACCACTAGAGATAAGCAGATAGATTTAT
GAATAGTATTTGACGATTAGTTAATTTCGTCTGAATTGATTACGATTGTCCGATATGAGTACGCGTATTT
AATAGTAACTTGTTCTTGAATGGGAAAAGCCCTGGAAATCTGCGCCCACGTGGGTGTGCGTAATGTGCTA
ACCGAGGTGTTGACACGGCAAGGTCGACCACGGCCCAGTCTCAAAGGATGCTATAAATACTGACCCCTCT
CGATGGGGTTCTCCCAGCTGGCGTTCAAAGCCAATTAAGAGAGGAGGAAGAGATTTCATACCCCTGAAGT
GACCGCGTGTTTGCGTAAACATTTTCAGGACAACATCCACACCATGGGACATGTGGCCCTAATGACACAT
GGTTGCAGCTTGTTCCGGACAGTCGGCAATTAGTGCATGCAAGGATGCACTCCGCGATCTTCATTGTGTC
CTCTTACGCGCTTCAAAGGTTAATTGCAAAATTTAAATGCCACAGCACCAGGAGGTTAAAATAGTACATG
TAGCCTAAGACGGAGCTTAAACTTAAATTATATTTTACATGCTACATACATACATACAATATATGCATAT
ATGTTAGGTAATGTGGAGAAGTTGAATGTCCCACTCCATGTAATTTTTTAAAGCCACATTAAAAACGTAA
TCTCTTAATTCCCGGTATCAGAGGTTAGTCCAATATTTCTATAATCGAACTAGTTCTTTGAGTTACTGCT
GCACTCCAGGGGTTTCTCTCTTTCCTCTGCATGGCAAAGCTTTTTGGTCTTGTACTATAAAAGGCCAGT
TGCTTTTCGGCGTCCGGTTTAGACGATTCAAATTCTCAACCGTATCAGTGATGTTATCGCCGCTTTTCCT
GCTGCCCCTCTTGGCTTTGGCCGGAGCGGCTAAACTTCCGCACATTCAACACCTGACATTGAGGGATACA
GAACAAGTTCACGCAGAGATCCAGCCCCTGATTATAGATGGCTACGATGTGCAGGGGGTGGACAATGTGC
CCTACCTGGTGTCCCTGTCCTTGACAAGGGCCACCTATACCCACTTGTGCGGGGCATCCATCATTGGCAA
ACGATGGCTCCTGACGGCTGCTCATTGTGTCGATGAACTGCGCACCTTCAACGGAGATGCCGTAGGAACA
CCCGTTTACGCTGGAATCATCAACCGGTCGAACGTGACGGCCGCCCAAGTGCGCTACGTCGACTTCGCCT
CAACGCATCGGTCTTTCAATGGAAACGCCGGAAGTGACAATATAGCCTTGCTCCACGTCTCTGAGAGCTT
CGAGTACAACGCTCGAGTTCAGCAGATCGCCCTGCCCGACATCAATGATGATTACAGCAATAAGACAGCT
GCTGCTTATGGCTGGGGACTCACCGACCCCGATGGCGACGAGTACTCCAAAGAGTTGCAGTATGCCTTCG
CTCCCCTACTCAATAGCACGGGTTGCAAGGAGTTGCTGCCCGCAGACGCTCCTCTGACGGCGCAGCAGGT
GTGCTCTCAGGTAAAGACCTGCTACGGAGACGGAGGCACTCCCCTGATCTACTGGCCCATCACTGGACCG
GCGGAGTTGGTGGGCCTCGGATCCTGGAGCTATATGCCGTGCGGATATGCCAATCGGCCAACGGTGTACA
CCTCTGTACCCCCTTACATTGGATGGATCCACCAAACGATCGGCGCCTACTACCAGCTTAACTGAGAGTC
GTGGTGTCGAAGGGGAGATCTTTGCGTAATACTCTAGCAGTACATTAAAGTAAACACCGCGCGTTTTCGT
ATCTTATCACAGAGCTCGTTAACGTGAATAGAAAACCTTGTTTTATTGCTCGATAAGTGGACGGCTGATT
GCCTGAAATCGAACTACTATTTATAATTGCCGATCAGCTGGCGATTTATGATTTATGATAGAGTCTTTGT

ATTTGCTGAGTAAGCGGTAAGGTGTAAGCTGACTTTCTAGTCAGATGGGTAAATTTGGTAAGATCATAGT
TACTGCAAATTTGGTGACAGACCCTGACGAGAGCGAGACTCATTCCTATCACGACTCTGCGGTAGTTTGT
CAGGTTATCCTGTCAACAGCAGTGGCAGGCAAAGTTTTCTAGTTCCATGAAAACTTTATCAGGTAATTTG
AGGGGGATGGAAATGGGTTAGGTGGAATATGTATGTAAAGAGAACTAAGCAGGTACATATTTTATACTAT
GATTTGCATGTAGCACAGCCAGCAGCGGTTTAAACGTATTTCTATAGTTCGAAGTGTACTCTTCAAGGGA
GATGAGCTGACTGTTGGGTGTTCCTCAGCAGCATTAGCATATCCTAACCTGCCAATCGTCCTCCCAATGC
TTCCTAATGCACTGAAGCACTCCACCTTTCGCTTTGGACTCCCGACAGCCCTTTTAGAACTCAATTCCCT
AAGCATGATTGACAGCGAATCGAGAACAGCGTCTGACAATTGTCACTAACAGATGTGTTTCATAGATACA
GATGTGCATGTATTTTTAAAGGAAATCGCACTCAAAAAGCCCTACATACGTAGGTATAGACATTTTTCAC
AGTGCACGCATCTGAGACACTTTTGGCTGGCTGCAGACATTCCCTTTTGCTGTGCTCGGTGTGCGGTCTG
CTTTAAGCGTTTTCTACAGTCGAGTGACATATGTACGAGGAGCAGATCCAACAGCTAAACCAATTCAATT
ATGCATGTAATCGTTCTCATCCTAAAGGCACACCCAAATACAAGAATATACATTAGCAGAAATATACACT
GATGTAGTAGATGCTGAAACGCGTGCCAGCGTTGGCTGTTTGTATTTGAGGAGCGTGTCAAAAGGCAGTA
ACTTCGAACACAGTCTTTCAGGAGCAGTCACGAAGTAGAAACTTAAATTCGCACACAATTTCTGCAGTTA
TTGGATTTCCACTGAATTTTGCATATTGAAATTGAATTAACGAACTTTTTACCGCCCATCCGCGCGAGCT
CAACTCAATTAGTTTACAGTAATTAGACAACAGGAAAAGCAACCAAGACAACTCGAGTGGGGATTGTGGC
TACCAATTAGCGATGGTAGAACCCTTGTCCTCGCTCCGGACTTTCCTAAACAACCAACAGACCAGCCCAT
CTGAAAATCCCCAGAAAATATGCGCCTAATTGAATATCACAAGTGGTGGGGGTGAATCCGAGTTGATGTT
TTCAATTTCAATTATTAAGGAACCTCTCGGCCTCGTCTGTGAAAAGGGGAAAGTGTTTGCCAGGCAGGCC
ACTTTGGCACCTAGTGGGTTCATTAAACTCAAACCCAAAATTCGATCCCAAAAGGATAATGAACTTGTGG
CTGCGCGAATTAAATATGCTCGTAACGAGTATTTGAAAGCTCATCAATTTTGGTCGTAACAACAAGCGTT
CCCTAATCTTAATGGACCGATGACGGGGTCAAGGAATTTCACCCTCTAATTGGAACTAAACTCTGGAGTC
AAGGAATTTTTTCAGCGAACAGATAGGTGGTACCAAACAAACAAAGTAAACAAAACAAACTTCGAGTAAC
GGGCTTTAATATAGGTAGTTATACCAGAAGCTCGCATTTTTATTTTACTCGTAATTCGTTGCCGGTGATG
CATTGAGTGAGGTACCTAATACCGATATGAGAGTCAATAAATATTTCAGTGAAATCGCCCCCTTAAAATA
ACAACGCCTGCACCCCAAGGTGGATCCACTTTGTTGCCACATTCCCTGAAGCAAAACTGGCGTGACCGTC
GTATTTTGCCCCACTTAGTTGCACACACATTCATGGCTTCTTTGTTGCACGTTGTCTGCGTTGACTTCAC
GCCCAGATGAAGGGCCCTCTACCCCCATTGTCACCCACCGGGAAAATCTTTCTAGAAGTGGAGCCGAATT
TTCGGGACATCTTGTTAGCTGGATACCTTGTCATCTTGTTAGCTGGATACCAGATTTGGCTGCCACTTAT
TTGCATGCATATTTGAGAAATACAAAATATATTAATTTCGGCTGGGGGTTACTCGGAATCCGCATCTGTT
TCGCATTGCCAGCTGCGGTCGGAGAATTTTCACTTTTCGCCAGCTTTTTCCTTTATGAATAGACGGCCGT
GGAATTAAATATTTATGGCTGCCGTCAGATGCCCATTTAATTTGACTTAAATAATAAGCAGAAATAACAG
ACGACTGCACTCATGCGGCAGTTCAGCTGAAATATTGAATGGGATAAATTCATTAGGGGCAGTTCAATAA
ACTCTGAAATTACGACTGTATGTATCATGTGTTTACAATTTATTGGACCCGAATCGAAAGAATACGCGCT
TTGATTATCATTCGCGCTATTAATAACGACTAGGGTTCGTCTTCATTTCAGCCTGCTTCATTTTTGGTTC
TCATGCGGTTTCTTCATCTGATTTCAGGGACTCGGGCACTGGAACACCGGTTACAATGCGCGTCGATGCC
AAGGGCTTCTTCCTCTACTGGGTCGACCAGAACAACGAGCTGGATATCCTTGATATAGCCACCATACGCG
ACGTTCGCACAGGACAGTACGCTAAGCGACCAAAG
>dme_circ_0001227_junction_seq
CACAGGACAGTACGCTAAGCGACCAAAGGAAAACAGCCAGAACTTTTAATCAGTCC
>dme_circ_0001228
GAATTTGTATTCTCGTTTTTATTATCCTGCCGCTTGTTCCTGCGACCCCACGAATTGCTGGGCAGACTGC
TGGACTCGGTACCTGAAAGCGAATGCTTGGAGTCCCTGGTGTCCCTGCTGGCCGAGTGGACGATGAAGTT
CCCCTACGACTATCGGGATGAGCGGATGATGAGCCATGTCAAACATATCGTTGCCAGGTGAGGAGAAACT

GCTTTCCAAATGGCACCCAATCATCAACTCAAGTCTTCTACACGATTCCCGCCACACAGATGTTCCAATT
CGCATTTGGAAGCCGCTGTCTCCCAAACACTGAGTGCCTTACTAAAACGATTGACTGATTTAGAGAGACA
TGAGGCGGACCTACGAGCCTGCCAGACCAACGACAAGGTGAGTGGATGTTTGGCCAGTGCTGTCATCAGT
GAAAAGCATGTTCCAGAAAAATATCTGAAAGTTTTTAGGTGCACATACTTCCTTTTAAAACTGTATTATG
CTTAAAACCATCTCAGACTTAATTTCTGTAGGCTATAATATAACCCTATTGAAAATAGCTTGGTTTCCCA
GGCAAGCTATCCAAGTTGACAGCACTGACCGGCCTGAAAGCAATCTGCCTAATCTCCGGCAATTGTCCTG
TTCTCCCTTCTTTTTCCATTATGTCTTTCTGTGGCGATTTTCCCCGCAGTGTGAGTGGACGAGCTCATAA
CCATTTGGGGGTCAGCACGAACACTGCTCCGCACTCCGTACTCCAACCAACTCCGGTGCTGAAGTTAAAC
GGAGCGCATGAGTCCTTAGACCCAGTGGATTGAATTAAAATTCCCATGCATGTTGAATATGCTGGGAGAG
CACGTCCATCCAAGTGCTGCTCCCTACAGCCGCGGTCGCAAGAATAGGAATCACTTAAGCTATCTATAAG
TGTGTGTCTTACAAATAGGGATATCTTCTATCTGAACTCATCGCTTACTTCTAGTTGACATTGAACAGTA
GCTCATTTATGGGCTAGATATTTTAATATCGTTCCTATTCTTGTGACCGCTGCTGCTCGAGGTCTGTATG
CAAACAGAACGACTGGTAAGATGGCTTATAATTTATGCCGTTCGTAACTGTCTCATTGACAGTTTGCCAG
TTGGCCGTGTAAGCGACTCGTTAAAACTTTGCCCAAGTCACGTGGGTCCCGGCATGTCCAATGTCCTTTT
CCCTCCCTGCCCTTGTCCTTGCTGCTGGCCCCTGCCCCTTAACGTCTAACTAACTTCCGTAATCAAATAA
ATCTTTCATTTTGCCCGTGCCCGCTGCAACATCATTGTTTTTGCCACCGCTTTTCCAGAGTGAGTTTGCC
AAATAATATCTATTTAATTTGTGCAATCCTGTAATTAATGGCGACGACCGAAAAAATAGGGCTGATATAT
ATGCAATCATGTATCAGCGAGAGATAATGTCTAATTTATATTGGTTTTATTTATCGCAAACAAAAACTAA
TTTGATGAACAATTTATTGCTGCGGGATATAATTTGTTTTCTGTAATTAACCAGCCGCTGGGGATAACAT
TCAAAAGGAATTGATACAATTTGGTTCGATCGAATTATCCTGATGACGGAAATGACTAGTTTTTAAATCA
ATTTAAATAGAAATTTAAAAATAAATTGATAGCACAAACAAAACGTTCATTTAACGATACTAAAATTTTT
AATGTTACAAAATCACAGACATTCTTTAACTTTCGCAGAAGAAAGTTAAACTTTATTTTCTCCAATTCCG
AATTTAACTATAGCGAATATCACGTAGTACGTGCTGCTTCATAAATTTCAGAAATAAGCACTCGAAATTA
AATAGACAGCAGTTTTATAGGCAAATTACGTGGCAAGGCGGCGAAATGGGTTCCTGGTTCATAATTTCCC
ATGGCAAAGTGCGCAGTGAAACTTTAATTGGACTGAAACAACACAACGGCGTTCTTTGCATAATGTGATG
CGTAAACCAAATGGAAAATCATTTGGCCCCCGACATCAATTCCTGCCTGACATTTTCGCATGACGTACAG
CCAACATTCGAGGAGCAAATTTCCATAAACTCGGGGATCTGAAACTTAAGCTTAGCTAAAGTTAGTCGGT
ACAGTCAAGGAAACGAGCAAGGATAATAAAATTGTGCCCTTTGCCATGTGAGAGTGCGACTGGATCCTTA
CACTCGTATATGCACTGTAATTGTACGCTGTTAAAAGTTAGAAAGCAGCGCCTTGGGGCCATTTTCATTC
ATGGATTGAGCTAAAAGCGCTGCAGTCTATTTGGTCGATGGCACAAATACACTATTTTTTATTAAATTAA
GATCTTCAAAGCTGCTAAGCTATTCGCAGTAAGGTAGTAATTTGAGATATTCACTTTTGAGAACAGAGAA
TGGCCATTGGTTCGGCCAAGGAAATGTACATGAAACTAGAAATGTGATATTAATGTTACTTTCCAAGGAA
GGACCATAGAGCAAGTCGAAGATGTTACTACCTCCTTGTTTCTCCCCAACCATCATATCCTTTTTCTTGC
ACTTTTCTGTTTGTGAACTCGAAGCAAATCCTTTTGCCTCGAAGCTGGACGCCTAGTGCCTAAGATATTT
GTTCGTGTCAGCAGATATTTATGCAGCCTAATGCATTTTGGAGAAAAGTAGCTTAAGAGATTTAGGCCGA
GTTCCTAGAATTAAAGTCAAATATTGAAAGAAGTAATGGAGAAGCTTAGAAAAATGCTAGTTGTTTGGCC
TGTAAATTACAGTATTTAAATTTGTAGATACTCTTGAGAAATACGACTTTCTGTTATTGCCGTTGGTTCT
TACCAAGGAAAATATTTAAATAATATTTTTGCGCCACTTTGTCACAACAACAGAAGTTTTCTCCAGCGAT
AAAAAGATTTTTCTCCGGCTGCCaaaaaaaaGAAATGAATTGCGGGGGCGAACTGAAAGAGAAATGACCA
CGCCCAAGCAACCACATTTTCGCTGCAGTTGGCCCAATTTCAACAAGTTTATATATTTACTCACTTATTT
GGCACATTGCCCACAccttcgtccttcatccttcatccttcgtcctgcgtTCTGTTTGCCGTGTCCTGTG
TCCTTCCGCCCACTCCTTCAATAGACCGCAGCGTGTTGCCGTGCATTCAGCAGCAAATGAAGCTAGTTTT
CATTAAAAAACTTTTTAATTAGTTAATGGATTCCGCATTTCAGCCCTCCTTTGTTGCCACCACAATTTAA
CACTAGGCTTGGCCTTCTTTTCACCCACTCATAATGAAAAAATCAAGTTAAAAGTAACTTAATGTTACTT

TTTGGTGATTTAAATGCCTTCGGCTTCTGCTTTATACATTATGAATGACTCTTCTTTTCCCCACTGCTAT
GTTGTGTGTGTTCTTTGCTCTCAGTACAAATGTGCAGGTATTTAGAAGGCAAACAGCACTGAAATATTCC
AGTCAGAAAGAAAACTTATATTTTAAACCAAATTAACTTGTCCAGAAGCCGTCTGTTAGTTAGTTTAATG
AGCGTATATATTCAGTGCACTTATTGAGATATAATTACTTTTCATAATACATACATACTCCAAACACTTT
GTGCACCTTTTCTCTGCAATATTTAATTCCTAGCGCTGCTGCACTTTTTCCTCTATAAATTCTGTGGACA
ATGGGTGCTGAAAAGTATGCAGCGAGAAATGAAAAAAGGAATCCTCTTGCCAAATAATAAATGAAAGGGA
ATTGCATAAATTTACAGAACTATGCAAAATTCGAGCAGCAAAAAGTGGGCGTGCATTGTATTTTCGAGAG
ATATATGTATACAAATATATTTCTATAGCTGCTGTTGAAATGTTTATGTGTTCGCATTGGATGATGGTAA
TAAATGTGGTCGGAAAACTGAAAACTTTAAATTATTCGCCATGGTGAGAGGTGTAAAAAATGTTATTATA
AATGGGTTTCTTAACGAGCAGCCAACTTTCGGATTCCGTTTTTACAACAAAACGAATGTTTTCTAATGTA
AAAGCACAATAAATTCGCTGAGATCCAGATTAAGATGCCGTCCCTTTTTTCCAGTTTAATGCGTGTGAAA
TCCCGAAAAAGCATTTCCCTGATGCCTCGGGAAATCCTAATTAATATGTTATCACGCGAAGGCCAAATGT
AGCCCATAAAATTCGATCTAAAGCTTTGCGGGGAAAATTACAAGCATAAAACTCGCACAGACCGACAGAA
CaaaaaaaaCCATAAACTTTTACAGCCCCaaaaaagcgaaacaaaaaccaaaaaaaaaaaaaaaaaacaG
CAATCTTCTCCACCCAAGTGGGCGGAAATCGGGCGAGCGTATCAAGATTGCCACTACAAACGATGCTCAT
TTATGGATTTCGATTTGATTACAGTGTAAGTTCAATTTGCCTGCCCTGATTGATGGCCAAATCCAGGGCA
AATATATGTTATGTGACCGCGGCCAAGGGGTCGTATGATTAATATACATATATGGCCCATCCGCCATGGC
CAACTATCGCATTTCCAGCGGACTTTCGTTTCACTTAGCGCCCAATCGTATTTACATTCACGTTTGTATT
TATTTACGCTTTAAATTAATTAACCACAGAGTGGTAAAATAAATGATTATGTGCCAAACGACAGGGCGGA
AAATCAATTTAATGGATGTTCATAAAAAATAACATAAATATAAAGCTCTTTAGCCGTTGAATTTATTTGT
TTTTATGCCGAGCGTTGAAGAGTAAAATCGCTGGAAATGCGTATTTTTTCGCATTTCTAAAGCATTTATA
TTGAATTGACTTCAACAAGTGACCGAACAATCAGAAAATAATTAAAAGAGATTTTGTTTTTAAAGCCTTT
GTTCATGAGTAGTGTTTAATGTTCTTAACAATAAAACGGGATTGAAGTTTTTGACCATTAATTGCTGTTC
TTATCCCATAATTGGGATGTGGTTAAAATCaaaaaaaaaaaaaaGGTCAATAACATAAATCCTTTGGCAGC
ACAAAAAAAGTCAAATCATTTGAATTACTACCCGAAAATAATACATGTAATTGAAATGAATATATTTCAA
TAATTAATACTTTTCCCATTGCCCAATGGCAGATGCCTTCTGATTATTTGCGGCTGCCACTATTTTCCCA
TCCAATGGCAAATCTGAACCATATCCTTCCCTTAATGGGCCCCAATCCTCCCATATGCATTGCTCCCCTT
TGGACCAAGCGATACGGAGCTCAAATAAACAATAAAGTATGCACCACCGACCACTGGTGGTCACAATTGA
CCTCATTAGGCTGGCCACTGCGAATATACTCGACATTCCGGCGTTGCAATTTATTCCGTGAAATATAATC
GAAACGCAGTAGTTGCCACAGAAATAGGCGAAATAAATCCGCATTGATTCAGACAATTTCAATGTCGCAT
TCAGTTTCGCCATGAAATGTAGCGTGTAAGCGTGAATCGAGTTCTAAGAGCGCTAATTAAGCGATTCGCT
TTTGCATTAATTGCGAGGAATCCACTGGAAATCGAAAAAAATACCGCTTCGATTCAAATTTAGTATGCTT
AAATATTTGAGGTCTTTGATAGAATACAGATGGTGATAAATAGCAGCAGCACTTTTCACTGTAACTCCGC
CCTTTTAAGCCCTTTTGCTGCGCTTTTTGCAttgccttttgcattttccattttgcatGGCAAACACCAG
CCATGGCTTTTTATCAGCCAGCAAAACATACTTTTCCCACAACTCGCACTTATGTTATCCGCTTTTCCAC
CCTTTTTCCGTTCTTCCAGCTTAGCAAATGGCCACATCAATATGGCTGCCTTCGATCGAATGGCCGCCAT
TGTTGGTGCATTCACCAAACACATGCAGGCGATGAAGGCGGCCCAGGCGCCATCTAGCGGCGAGTACGAG
CACTTCTGCCGCCACATGCAGCAAACAACGAAACTTGGCGAGGCGGG
>dme_circ_0001228_junction_seq
AGCAAACAACGAAACTTGGCGAGGCGGGGAATTTGTATTCTCGTTTTTATTATCCT
>dme_circ_0001229
GGCATGTTGGCCAGTCCGCAGGGGATGTAGCCCCAGGATACGATGCCAATCAGCTCGGAGGGGGCGTTTG
TGAACTCAACAACCAGGGGGCCGCCGGAGTCACCGTTGCATGCAGACTTGCTCTGTTGCAGGGAAGAGGA
GCAGATGTTGGACTCAGCGATAGGAGCGCTTTCCGGCAGCTCCTCCTTGCACTCCTC

>dme_circ_0001229_junction_seq
TTTCCGGCAGCTCCTCCTTGCACTCCTCGGCATGTTGGCCAGTCCGCAGGGGATGT
>dme_circ_0001230
GGATGAACATGGTCTGCAGCGAGGTCTGCACCACTCCAAAGACCTCCGCTATAATGCCCTCCACCCGGTC
GGGCACTGTGTCCCATTTGGCGAAGTAACTGCCCAGGATGCTGAACATTCCGTACAGGTACACCCCGGTC
TGGGCCACAATCAGCAGGAGATTGTCCAGATCCATCGA
>dme_circ_0001230_junction_seq
TCAGCAGGAGATTGTCCAGATCCATCGAGGATGAACATGGTCTGCAGCGAGGTCTG
>dme_circ_0001231
ACGATATTATCGACATACTGCAGCTACAATCCTGCCGCAGGACCCTGGTGAAGAATTTATCCGGAGGCGA
GCACAAGCGCTTGTCCATTGGCATTGAGTTGGTAACGAATCCGCCCATTATGTTCTTCGATGAGCCCACC
AGCGGATTGGATTGTGTGGGCAGCTACCAGG
>dme_circ_0001231_junction_seq
GGATTGGATTGTGTGGGCAGCTACCAGGACGATATTATCGACATACTGCAGCTACA
>dme_circ_0001232
GATCCATATTCCCCCACGAACAGTACAAAACCATCAAGAAGATCAACATTGGCTTCGAGAATATACGCTA
CACTACCAAATTCGGAGTCTTCCAGAGGGGTAAGTGCTTCCAATCGGTCCTTATTCGGAGGTGATCTTTT
TGAATTTGGGATAGTCTTTGGATTAGCCATATCTAACTATGGAATTAGGGTGGAATATCTTAAATAACTG
CTGGTTTGATAGAAAAATGAACTTACCATACTATAACAAACCCTATTGACTGTTTAGTTTGGTCTTTTAA
ACAATCTTGACATTGAAAAACAAACCCATAAAGTTTGTGTTTGTTTTGCTCAATTGGAAATAGGACACGC
GCCACTTTGAAATAAAAATTGTATAGTACTCAGAAAAAAACAATAATATTGATCCATTTATAAAGTGTGT
ATTTGGAATGCTAAGAAATGTTCGAAACTAAGTTAAGTGGAAAGTGTATGTTAATACTTTGAGAGCTCTT
ATGTAAACAACAAAGATTTTGATATTTGCCTGACAAGACATAGTCTATCTATATGGATATCGACACTCTG
AGAAGCGATCGTTTCATGTATACTAAATATTTTCGGTTCAAAGTTTGGAGTCCTACTTGAACAAAAAGCT
GTCATCTGGAGAGCATTCCCCCCAAGGTCATTAAATGGCTTTTTTGCTCCCAGTCACGCATTGTCGGCAA
TTATAATTAAGGGCTGGCTATATGCTACATATATGTACATGTATGTGCATACTAGCGTGGCAATGTCCAG
TAAACCCAATTAAAATCAGCCAGTTCTCTGACACTCGAATGAGTCTTTTAAATGAATGGTCAAATAAATG
GCTTTAATGCCAAAACAAAAATAGTGCTACAATTATTTAAATCGTCAGCACTTAATGCTGTGATATCTGA
TAATGTTTCGGTTCATCAATCTCGACATAAAGCCAGATAAAGCTGCACTTAGGTAACTTTGAGATTAGGA
CACTTGCGTAGTATCAACTTCTTGGGGCAAAGTCCGGCTTGCAGGAAACAAGCTGGGCAAAGTGGGGAAT
ATCGGTTGCCTTTATTGGATATATGTAACCATCTGGGACTCTTCAATTCAGTATATTGACAAGCTGCTGA
TTGGGCGAATCACTGGGTTCAAAGAGCCTCTTAAATTAAATTGAACATATCTTGGGCACACGCAGAAAAT
AAAAAAGTGTAGTTGCAGGGTTGGGGTCAGGTGATTTTATTTAAGCAGAGCCGATGGTCCTATATGCACA
TCGTGTAGTGTGGCGGCTTTACTGTACTGTTTGTTTACGATTCGCCCATCCCACAGTTTTAATGGCGATA
AGAAAGCCACTTACAACATGAATACAGATAGTTGATACTTCTCTGGAATACCATTGGTTATCGTGCATAC
AAGAAGGGACATTTAACATGTGACTCACGCTAGTGCACCACTGATAGATAGGCATTCCCGAAAAGATATT
TCGTCGAGCAGTGGCCCAATAGAGTATCCCAGGTGACTAACGTTCTGAGTCATTCGTGTATCCGATTATG
ATCCTGGTGGAAATTATGACACGTGAATTTTGCTGATTATATGCACGATAAATCATAGGCAGTCATTATG
GGGGTGATTACCATGAGTTCGTGTTTTGGATAACCAGTGCTTTGTGCTGCGTACAAGACTAATTGAGGTG
GATAGCTCCATGTGTCCTATCCTGCGTAATTGCAAGTCACATTAAGTTAGTACACTCAAAAAACTTTCAA
CACAGCCCATTTACCGATTCGAAGGCGAAGCATAACGCAATCCAAAGTGTCCCCAATGTTACTCGTGTTT
CATATCTACAAACCGCTTTATCGCCCACACAACAATTGATAATAAAATGTACAGCGTGTATAACATACAT
CAGTTCTTCACCATTAATGTATTTTAGATTATTCAAAGTCGGTAGCCGGTGAAAGCAAATAGATAGTTGG
GAACAAATGATATGAGAACTGAACGACATGGTTATCATTACAATTCCCAGGCATGTTATTATGACGGCGA

ATAAAATAATACACCATATATGTATGCCCCAGTTAGATATCTGATATGAAAACATTTTATAGTTGTTTCT
TCGCGCTATGAAAGAAGGCGCAAGCGTTTTAATTGAAGTCACTTAAATTGCTTCTTAGATTTTCCGTTGC
TCTTCGCGTGTCGTCTTCATTGACTCACTAATTAAGTGAATTTTCTCATTTTCCCTCTTAAAGCGGTGGA
GTCTGTTAAATATATTTACAAAAGATTCCGACTTTGGAACAGACTTTGGATATGAGTCAGGTGATTTGAG
ATTGGAGCTTCCTATTTCAATTAAGATTTTAGCACGCTTTGAATGTAATTGCTAGGTTCAGTTTCCTGAC
TAATACAATGTAGGCCTTTTAAAATTACCGGAGATCTCTGGTTGTGCGTGCTGAGAATTTTAGGAGGCTT
AAATAGATACTTTCGCTTTCTTGCAAACAATTAGATACTTCTTAATAAAGTAATAAGTTTAAAAGTAAAT
TTAACCAGAAACTTCCATATAAGGCTTATTGCATTCTCAAAATTGTATTTAATTACCAGCGCAATTAGCA
ATCAATTTGATAAGAAAGCAGATCCAATAACTCGATGTGACGAGTTGGTAGTCCAAATAATATGTAATAT
GTTGTGTAAATATGGGACGTGGTCAGATGCAGGTGGTGCTCATAATCCCCCCTTACCAAATGCACTTCCA
AGTACAAGGCCATAGAAAACACTGATAAGGAGGCCAACTCCAGTGGAAGTGCAATAAACATTGCTCGTTG
TACACCTGTTTGCCCAGCGATGAGTCTAGTCTAACATCTGGCCCACGGCATTCAAAAAAAGGCGTAAAGG
CAGTTTAACGACATTTTTGGTCATAGAACTTGAGTGCGATAAGATTCGACTGCGGAGTGATTGATAAGTG
GGCGCGCAGACCCCTCAAAATCTAAATTGGACAGGAGCCATGGCTAATCGACAGATTAACCGGGCACATT
CGACATTTAGAATGGGCGCCTACAATGTATTTGGCCCAGCGAAACGAACCCCTCACAAAATTGAACATAT
TGAACTTAAGGTTTATAAGGCATTATCATCCAGCCACTGGGGATTCAAGAAAGCGTGCAAACCGAGATTA
GACAACATTGAACGCCAGTAATCGCGGGCATTGGGATTTCCTTATCACTCGGGATGTACGAGTTAAGTTA
ATGTGGGGCACTCTACATATGTGGGAATTAAATAAAGCAAAGTCAATCGGACTTCGTTTGCAACTCATTA
ATACCAGATATTGCTCAAAGTCATTCATGTTCTTATGTAAAGCAGTCACCCTCAATATTTTATGAAATAA
TCTTTAAAACAACGTATGAAAATCGTGAACTAATCTAGAAATTCAAATAGTGACACTTGGAGTTTGATTT
AACACTTTTAGTGAGTAATCAAATTAAGCTGAAATTCTTGAAAACTGAGGTCTGTCAATGATTAAGTAAG
TTTATAGAACTGACTTACTAATCAGCCATAAACCAGAACAATAAATGTAGTGCGACTTCTTGAGATTAAA
TGATTAAAAAGTACTTTTTTAATTTGTAGTCTTAAAGACATAAGTTTCGCTTTAATTTTCATTTCTTTAA
TTTTCCGATTCTTACAGAAACATATGGCTTTACGGGGGATTTCCGGGTCAATGGCAACAGGCGGGATTTA
AAGGCCTTCAAGTCGAACGTGGCGTTCATCCGCCAGGATACGAGTCTTCAGGCCTTTCTCTCCGTCAAGG
AGGCGATGCATTTTGCAGCCAATTTGAAAATCGGCACTCACATGACACATAGCGAGAAAAGAGAGCGGGT
AAAGTGCATTCTGGAGGCCATCGGTATGTACGAAAACCGGCATACCAGGACGGGGCAGCTAAGTGGAGG
>dme_circ_0001232_junction_seq
ATACCAGGACGGGGCAGCTAAGTGGAGGGATCCATATTCCCCCACGAACAGTACAA
>dme_circ_0001233
GGACAACAAGCTGCGCCAGATTGTGACGCTGGGCCCCCAGGACACGCTCGAGGAGAAGACAGTGACGGTA
TGCCACGGCTCGGACTTCGTCAACATGACGTTCGTCAACTTCTGCTGCACCCGGCGGGACATAGCCCAG
>dme_circ_0001233_junction_seq
CTGCTGCACCCGGCGGGACATAGCCCAGGGACAACAAGCTGCGCCAGATTGTGACG
>dme_circ_0001234
GCTCTGGACCGACGGACTCATCAAGTTGGCCTACAGCTTGGCCCAGCTCAATGGCTCGGCGATTATGTTC
CTGCAGAAGGCCCATACCAAACTGTGTCTCCAGGTGGACAAAAGCGGCCGCATACCAGTGAAAAAAGTAA
TTGACAACAGGCACCCACCAAAACAAATACCAATCCCAATACCCACTCCCAAGCCCTCTATACCCACTAC
ACTGCCTCACCTCAACGCGCGATGTCCTGAGTCCTGGATCCCGGCCAACTttcgggttcgggttcgggtt
cgggttcCTAGCTCTTGTGCATTGTGCGTTGAAGCACTTGACAAATATTTGTTAAGAGTAGCCATCGACG
TTGAGCTGAAAATTGAAATTTACGCCAATTTCTATTTCGCAATTTCCCTTTTTTATGCTCCCTGTCCTGC
TGCTGGTGCCGCTGCTTCTGAAACGTGGCTCATGCATCACTGGCGACCCACGATGATGATGACGACGACA
ACGACAACAACCTCATCAACACGAACCACACCGCATTGTCTGCCATCCAACCGTGCTACCGGCTACTCTC
TGCTCCACTCCGCCGCTGGTCAAATTCTGGTCGTGACCACTTTAGGCATCATAAAACTTTTTGCCCAAAA

CAAGGAGGATCGCAAACGTGTCGAGAAGGCGCTGGACGTGACCGGTTTGCCGTCGGGAAAGGTCGATAGC
ATATCGGTGTCCAAATTTCAATTCGAGGACTTTTACAATTTGTACAAATATCTCACGCAGCGTTCGGAGG
TGGAACGCCTCTTTGACAGCATGCGTTGGCAACTCGAAGCGCAAGTGCATGTCCATCGCTCAACTGGTGG
AGTTCCTGAACAAGACACAGCGTGATCCCCGCCTGAACGAGATCCTCTACCCTTATGCGAATCCCGCCCG
CGCCAAGGAGCTCATCCAGCAATATGAGCCCAACAAGTTCAATGCGCAAAAGGGCCAACTCAGCCTGGAT
GGCTTTTTGAGGGTGAGCTGACTAGTTGCTGCTGGAAACTGGTTGTGGAAAGAGGTGCGTGCCAGAGGCA
CTGCATCCATTCACTTGGGGGAACCATCCTGGGCGTGACAGCTTATCGGACGCAGTGCAAGCTGTTGGGG
CTGTTAGCGTCACTCGAAGGTCCTCTTGAGTGCAAGTGCGAGTGCATGTGAATATACATATGCAAGTGAA
CACGAATATGACGTTGCGCTGTTTTTCGCACTCGGGAAAATTGAAATGGTATGGCTGTCAGGAGAGTTTA
GTCTACCCTGAGTGGCTTAGAAGCCGTGAAACCCCGCATTTTACTGTTACAACCAGCAGCCAAAGATCTT
AGTAACCGTGGGCGACGCAAAAGCTGGCGACATCTTTAGCTGCTTAAACACTTCAGCGAACTGTGTGCCA
CGTTGAGTGTCTCCATGTTGGCGACTGAGTAATCCCTACGTGTACCGATTTCATCCCACAGGTACTTAAT
GGGCGACGACAATCCCATCATGGCGCCCAGCAAGCTCGATCTCTGCGATGACATGGACCAGCCGATGTCG
CACTACTTCATCAACTCCTCGCATAACACCTACTTGACGGGACACCAGCTGACCGGCAAGTCGTCCGTCG
AGATTTACAGGCAGTGTCTCCTGGCGGGATGCAGGTGAGTGTATGCGGATGCGGGTCACTCAGTGGAATG
CAGATGACGCAATTGATGTGGTCAGCTGCCGTTGCTCGAGTGTTGGCCGTGACCTTAATCCCGAGGTGTG
TGTGTTTGCGTGTGTGGTGGCCTTCGGATGAGAAACACACATATAAAAAGCAACTCTCGATGGCATTCAC
TTAAAACGTCAGGTGCAGCTTGCATCGCCAgaaaacagcattcactgaaaacaaaaatcattcaaaaaaa
gtatataaaaCGCAACGGTAGAACATAAAATTCGATACCCCCACTTTTTACGCAAGGCATGTGTTCTGTG
CACAGTTTAACCTTTTGTTAGCACATACAAGAGGAATAGAGTATGAAATGCAAAAACTATATTATATCCT
TTTTATTATCCGAGAGGAGAGATATCCAAAATAAAATGGGTTTGAACAAATTTTTTCTTGAGTCTGCTCT
TTTTTCGTCTGTAACTGACAACGATGATCATGACGATGCACACATCACAAAATTTATTGACACAGAAGAT
GTTACTTTACTTTTATTCTCCGCTTGTACGACTGTCACATTGCATTCGACACTTTGCGTGCGATTTATAG
AGGAAAATGGGAAatggctatgggtatgggtatggttatgggAAGGGGAGTGGAGTGGAAAAATAAAAGT
GAGAAGTAGGAAATTGTCATTTGCTTTTACCACTTTCAACTTGTGTTCTTCTGTCTTAACCACTTTATTG
CAGGATGCGTTGAGCTCGACTTCTGGAACGGACGCACCGAGGAGCCAGTCATTGTCCACGGCTATACGTT
CGTGCCCGAGATATTTGCCAAGGACGTGCTGGAGGCCATCGCAGAGAGTGCATTTAAAACATCCGAATAC
CCTGTGATATTGAGCTTTGAGAATCACTGCAACCCTAGGCAACAG
>dme_circ_0001234_junction_seq
TGAGAATCACTGCAACCCTAGGCAACAGGCTCTGGACCGACGGACTCATCAAGTTG
>dme_circ_0001235
GCTCTGGACCGACGGACTCATCAAGTTGGCCTACAGCTTGGCCCAGCTCAATGGCTCGGCGATTATGTTC
CTGCAGAAGGCCCATACCAAACTGTGTCTCCAGGTGGACAAAAGCGGCCGCATACCAGTGAAAAAAGTAA
TTGACAACAGGCACCCACCAAAACAAATACCAATCCCAATACCCACTCCCAAGCCCTCTATACCCACTAC
ACTGCCTCACCTCAACGCGCGATGTCCTGAGTCCTGGATCCCGGCCAACTttcgggttcgggttcgggtt
cgggttcCTAGCTCTTGTGCATTGTGCGTTGAAGCACTTGACAAATATTTGTTAAGAGTAGCCATCGACG
TTGAGCTGAAAATTGAAATTTACGCCAATTTCTATTTCGCAATTTCCCTTTTTTATGCTCCCTGTCCTGC
TGCTGGTGCCGCTGCTTCTGAAACGTGGCTCATGCATCACTGGCGACCCACGATGATGATGACGACGACA
ACGACAACAACCTCATCAACACGAACCACACCGCATTGTCTGCCATCCAACCGTGCTACCGGCTACTCTC
TGCTCCACTCCGCCGCTGGTCAAATTCTGGTCGTGACCACTTTAGGCATCATAAAACTTTTTGCCCAAAA
CAAGGAGGATCGCAAACGTGTCGAGAAGGCGCTGGACGTGACCGGTTTGCCGTCGGGAAAGGTCGATAGC
ATATCGGTGTCCAAATTTCAATTCGAGGACTTTTACAATTTGTACAAATATCTCACGCAGCGTTCGGAGG
TGGAACGCCTCTTTGACAGCATGCGTTGGCAACTCGAAGCGCAAGTGCATGTCCATCGCTCAACTGGTGG
AGTTCCTGAACAAGACACAGCGTGATCCCCGCCTGAACGAGATCCTCTACCCTTATGCGAATCCCGCCCG

CGCCAAGGAGCTCATCCAGCAATATGAGCCCAACAAGTTCAATGCGCAAAAGGGCCAACTCAGCCTGGAT
GGCTTTTTGAGGGTGAGCTGACTAGTTGCTGCTGGAAACTGGTTGTGGAAAGAGGTGCGTGCCAGAGGCA
CTGCATCCATTCACTTGGGGGAACCATCCTGGGCGTGACAGCTTATCGGACGCAGTGCAAGCTGTTGGGG
CTGTTAGCGTCACTCGAAGGTCCTCTTGAGTGCAAGTGCGAGTGCATGTGAATATACATATGCAAGTGAA
CACGAATATGACGTTGCGCTGTTTTTCGCACTCGGGAAAATTGAAATGGTATGGCTGTCAGGAGAGTTTA
GTCTACCCTGAGTGGCTTAGAAGCCGTGAAACCCCGCATTTTACTGTTACAACCAGCAGCCAAAGATCTT
AGTAACCGTGGGCGACGCAAAAGCTGGCGACATCTTTAGCTGCTTAAACACTTCAGCGAACTGTGTGCCA
CGTTGAGTGTCTCCATGTTGGCGACTGAGTAATCCCTACGTGTACCGATTTCATCCCACAGGTACTTAAT
GGGCGACGACAATCCCATCATGGCGCCCAGCAAGCTCGATCTCTGCGATGACATGGACCAGCCGATGTCG
CACTACTTCATCAACTCCTCGCATAACACCTACTTGACGGGACACCAGCTGACCGGCAAGTCGTCCGTCG
AGATTTACAGGCAGTGTCTCCTGGCGGGATGCAGGTGAGTGTATGCGGATGCGGGTCACTCAGTGGAATG
CAGATGACGCAATTGATGTGGTCAGCTGCCGTTGCTCGAGTGTTGGCCGTGACCTTAATCCCGAGGTGTG
TGTGTTTGCGTGTGTGGTGGCCTTCGGATGAGAAACACACATATAAAAAGCAACTCTCGATGGCATTCAC
TTAAAACGTCAGGTGCAGCTTGCATCGCCAgaaaacagcattcactgaaaacaaaaatcattcaaaaaaa
gtatataaaaCGCAACGGTAGAACATAAAATTCGATACCCCCACTTTTTACGCAAGGCATGTGTTCTGTG
CACAGTTTAACCTTTTGTTAGCACATACAAGAGGAATAGAGTATGAAATGCAAAAACTATATTATATCCT
TTTTATTATCCGAGAGGAGAGATATCCAAAATAAAATGGGTTTGAACAAATTTTTTCTTGAGTCTGCTCT
TTTTTCGTCTGTAACTGACAACGATGATCATGACGATGCACACATCACAAAATTTATTGACACAGAAGAT
GTTACTTTACTTTTATTCTCCGCTTGTACGACTGTCACATTGCATTCGACACTTTGCGTGCGATTTATAG
AGGAAAATGGGAAatggctatgggtatgggtatggttatgggAAGGGGAGTGGAGTGGAAAAATAAAAGT
GAGAAGTAGGAAATTGTCATTTGCTTTTACCACTTTCAACTTGTGTTCTTCTGTCTTAACCACTTTATTG
CAGGATGCGTTGAGCTCGACTTCTGGAACGGACGCACCGAGGAGCCAGTCATTGTCCACGGCTATACGTT
CGTGCCCGAGATATTTGCCAAGGACGTGCTGGAGGCCATCGCAGAGAGTGCATTTAAAACATCCGAATAC
CCTGTGATATTGAGCTTTGAGAATCACTGCAACCCTAGGCAACAGGGTGAGAACGAGAACCAATTAACGG
AATGGGCTTGGGAAGACCAAAATTAACTGGGAGAACTGTCTTAAAAAATTTTAATATTTTTGATAACGGC
AAAGCCGAAGGATAGGCGAGCAGTTTTTACAAAGTATGTTCTCCTTATCTGACGCAAGCTAGGAGACTTG
ATATGCATCTATCTTTGAAAACCCCTGGTATATAAATATAAATATATTAAGGCAAAAGTTCCACTTCCAG
GAGGCAAAAACTGAATAAACTACCAAAGTAAATATAGGGCAATAAACTCTCCTTCGGACACGCTAAATTG
AAGAACGCAGAAGGCCACAATGTTACTTGTCCTAAAACAACAGTTGAGTCCCAACTCATCAGCCTAATCC
ATCATTCGGGGAAAGACAAACAAATGAATTGGCGGTGGCACACATGGGGGCTCCTTGAGCCTGAAAACAT
TACTTAAGGAGCATGAAGGGAGTCAGGAGTCCCGGAACTTGGCGAATCTCAGAAGCCACTCGAACGGTAA
TTGCCGGCTGTTTAAGCTTCACGAGCGGGATAATAACAAAGGCAAAGTTTTTACGGCCTGAAAGGTAAAA
GCAGCAAAAGGAGGAGACCAGGTACCGTCAAAGAGTCAGAAGTTTTGCCATTAAAAGTAGTAATATAAAC
TAAATTTCCCGCTGGGAGTCCAGGCAGGTAACAAAGTCTGCTAGCAAACGTGCCACGTGCCAAAAACCAA
ACTCATGCCAGTAAAGCTCAACATATGAAGTCTTCACATTTATACATATAATTGTTTAAATTGAATGTCA
GTGTAGCCCTTTAAAATAATAATAATACCTAATACTTATTCCTATCTAATATATGGTTAGTTTTGAGTGT
CATGAAAATACCGGTTAAAGCGGTAAGACACAGGACGATTCGAGCAGTTATGTAAATTATGGACTATATG
TAGAATATTTGAAAGTAGCATCCTTTTCTCTCTGTGTACAAGGAAACCCCTTTGCGTGGAGCTAATGCAA
TTGCCAATTAGCAGTTAGTTAACAAGACCCCTGCGCACTACCTTATGTCACATCACTTATTTCCTCACTT
GGCACCTTCAACAGGGCGAAAATTGCCAACTATTGTCGCGAAATATTTGGCGATATGCTGCTCGATAAGC
CTCTGGACTCTCATCCGCTGGAGCCGAACATGGACTTGCCACCGCCGGCGATGCTGCGCCGCAAGATTAT
AATCAAGAACAAGAAGAAGcatcaccaccaccatcaccatcatcaccaCAAAAAGCCAGCGCAGGTGGGC
ACGCCGGCAGCCAACAACAAACTGACTACAGCGAATTCAGGGTGTGTTATTTAATCCTCCACTAATCTAA
TCTATAATGCAACTCCTTTTGCGCACCAAGCACATGCACCAGGTTCATGCTTAATGTGGTCTGAGCCAGG

TTCGGTTCGCTCCTTGTTTTTTCTGGATTTTAAGTCTGACTCACATATATTTCCGCATCGAAGCTTCCTG
CCTTTACCCCGCTCTTCTATTCACCCCTTCCGACCCCATTGGCATATGAAAAATGCAAATGAGTTCCGCA
CACGCAGCAGCTGCTCGGTGTTTTCTGCAAAAGTTCAGCAGCCAGAAAGGCAGGCTGGGAATATCAAATT
TGGAACACAGTCGAAGTGTGGGAATGCGGGGGTGGCTCTTAATGAACAGAATGTTTGAGTTTGCTAGCAC
ACGGGAAGCACTTGGGACTTAAAAAGCTAGAAAGCAGGGCTACCTACTGCTTGACATCACAAATGGATGG
TCCCTTCCTGCGTTTATTATAGAGTTTCAATAACAAGCGTTTAATAGGTAACATGGCCACATTTTTGAAG
TGCAATATAAATAGTACTTACCAATTTAGTGCTTACTGTCTAGATGTCAAATATTTAGCCGAAGTTACCA
TGTCCTACTTTCAGTTAGTAAAACTTTACGTAGAGCATTCGCGTTGCTTCCATTGAAAGTTTTGAAGCGA
AAATGTTGTTGATAAATTTGCCGCATGGccacttccacttccacttccTGTTTGCGAGCTTTGAGTTCCT
GCAGGACCCATTTGCATGCGCAGCTGCACAGTCGTCCGGGGGCCCAACATTTGTCGCTCCTCCGCCCCCC
TAGGAGCCACATCCTTCTGGCATTGGCCGGATGACATTTAGCTCATGAATTGCAAAAAGCCAATTTATAA
ATGCAAATTATTTTTCCCCTCCCAGCCAGCGGCTGCAGATCCCAGCTGTCATCTCTCCATCCCCGGGATC
CCAGCACGGCAATTGCATCCTATTTAGAGGACCCCTTCTGGTTTATGTAATTTGATTTATACATATTTTT
ATTACATTCCATTAGGGCGAAACTGTAATTTAAAAGCCTTGCGCTTTTTGGATTCTTCCATAATCCAGtt
ttttttttGTCAGGAAAGGGGCGCTGGCAAATTTAAATTTATATTCACACTTATATTAGTAAGATATTTG
TTTGGTTTTAAGTTTCTTTCTTATTGTAGATTAAGCCTAAATGGTTCATTTTTCATTTCTTTCAATTAT
AAATATTTTTTCTGTCTGTAGTACATTTACATTTACATACATTTTTCAGGTGGATGCCAAGGCGGCGCAA
CAAGTCGGTTTGTCGGCCTCGCATGAGGATGGGGGCGTGACCAGGAGCACCGCCAACGGTGACGTTGCCA
CTGGAACAGGAACCGGAAGTGCTGCTGGGACAGCAGGACACGCGCCGCCGTTGCAA
>dme_circ_0001235_junction_seq
GACAGCAGGACACGCGCCGCCGTTGCAAGCTCTGGACCGACGGACTCATCAAGTTG
>dme_circ_0001236
GGTTCCTGCTTTTTTCAAGCTTTTGCTTTGTTTATTTTTGCTCTGGTACATTAAAATGCAACTACTACAG
ACTAACAATACTTGGCCatatatacggattatatatatggtgtatatataGAGTTACGCAGTTACTGGCC
ATAGCCTTGACTTGCGATTTGGATTAAATAGTTTGAAGCATCTTGTTTCTGATTCTTGTTGTGGTATTGG
TATCG
>dme_circ_0001236_junction_seq
TCTGATTCTTGTTGTGGTATTGGTATCGGGTTCCTGCTTTTTTCAAGCTTTTGCTT
>dme_circ_0001237
GTGTATTGGGTTTGTGACTAAATGTTAGTTGAATTTTAAATTCATTTTCAGATTTTTTGGTGGCTTTGCT
TTTGGCCCAAAAAAATTGTTTCGGGTGTTTTGCTAACATGTCGAAGACAATTGGATTGGATTCATTTTACT
TGCACATTTTGTTAAATAATTTTACTGCTAGCGTACTACGATTTCAGCCAAAGTTCTTTCAGTCTGAAAA
ACAAAAGGATTTTGAGTAAAACAGTTTAAAAGCAAACTTATGTTCTCCACACAAAAAGCGTTTTACAGGG
AAGCATCCAGGATACAAATTAGTCAAACATAAAAAGGATTAGTTGAGAGTGAATGAGTACTTTCTACAAG
GGTAAATACAAAATTAAAAATTACTTACAAAAATACAAGGTTTTGACAGTGAGTTTGTGTGGTGCCATGG
GAATCTCTTCTCCATTCTTCATTCAGAATATGAAAATTTTTAAAGGTTTTTCATTACAATTGCAATC
>dme_circ_0001237_junction_seq
TTAAAGGTTTTTCATTACAATTGCAATCGTGTATTGGGTTTGTGACTAAATGTTAG
>dme_circ_0001238
GCGCGATTCGATTACCGAGGAGATCACGATAACGAGCACTACAAATAGCAGTCAAAGCACCAAACTCCTG
ACCATTCAGGAAGCTGCCGGCAGTTTTGAGCAGAGCAGCGTCTCTGCCGTATCGACGTCCTCCAAACAGA
TCGCTTCCACCACAGCCGGCGGTTCGCCACCAGCTGCTGCAGCTCCAGGTGCCAATGAGTTCAACGGCAG
CGTCCTCCGATTAACACAGGCCGCAACTTCCACTAGTCCCGGCTCCATCTCGAGTGCCGGCAACACAGCT
ACACTAGGAGCCGCTCCGAGCATTAACATCGTGTCCAGCGAGTCCACGCGGGATCAGACACAACAGCCGC

AGAGCGTTGACCCCAACGGCGTTGCCGCTCCAGGCGACAGTCCCAGCAGCCGGAAGAGCTCCACCAGCTC
CAAGGGGAAGGCCAGCCAGGCCAAGCTATCCACATCCAGCAGTGGACGCGATGAGGGTGAGTTCGAATGG
CCTGCGTGTGGGATTTAAGGCAGCATTAGGGGACCATGTCGCTGGTTCAGAAAAGGTTTATCATGTGCAT
ATAATTTCTACAGATATTTCTGCATTACGTTACTGTGCTATGAGTCATGTCGAGTTGGTCAGTTTCTAGT
TTTCTCAGCGATAACAAATTTATTACGCACCACTCGAATTTTCTAAGAATGCTTCACTACTTGGCACTAA
TGGCATTAAGTCATTTTTTACCAGTGAGCATGATAGTTCAAGGCAATCACATTTGCTTGGTTTAGCTTTA
TATAGTACAAATTTGAATAGCAACGTCACTTCGTTGCTTTTTGATTAGACCGATTTCTATGGGCTTTAAA
ACTGTGCTGGGCTGGGCATCATAAAAAGTAGGTTTGAACCATGTAGGGAGTGACTTCCGAAATTATATTT
TTAAAATTGATAAAACATTTACTTTTGGCGTCGTGGTTTTATCGACGTTCTATGCAGTTTGAGGGTGCGA
GTTTTTGTCATAATTTGAATGCTTAACCCTAGGAGTACACATAGAATTTGTTTTTGTTTGATGAACTAAT
GATGATAATGTTTAGAGGCTTTTTTGTCACATCTTTTCAAGTTCGAATGAACCAATGTCAACACTTTTGC
GTATCTATATGACTAGCAAGGGCTAGTCGAGAAACCCTGACTATTTCGATGAGCTTTCAAAAACTAGCCG
TTTGTTTATTTTGCCCATTAGCCAGTCATACACGCCCCGAAAAAGGTGTGAAATTGGTGGAGCCAAGTGT
TCGAATCAAATTAAAGGCACTCGAAGCGACGATCAATCGATCAGGCGATTGACAGATAGAAAACTGCACA
GATAGCCAGTTGGGCCGACAGATAGACAAGGGCGATTTGGATTAATGGCCCCATATTGGCCATCTAGCAG
CGCAATCTACTTGCTGTCCGATAAGGAGCATGTGTCACCATGTGTGTAGCTGCCCTCTCACCCCATCGAG
AGGCAGCTGATTAGCTGATTAGAATCACCCTTCGATGCGAAAGCGAAAGCTCGCTAAATAAATGTTTTGG
GCCCAAGCTTTTTCCGGCCAACTGGGCGGTGATAACGATTCGTGTGCGGCGTAGCTCTTCCATTGACGTT
TGCCTGGCTGGCAATGCCAAATTATAATTCGACAAGCGACGGAGCGTCTCAGCGCGAATATAGAGACACC
aaaaaaaaaaaaaaaaaaaaaagaagaaaaaaaCCAAAATAACAAACTAATATCGAGCATTGCAGCGAAC
TTTGTGTAAATAATTGGGGGTCGTAAACGAACGACAAGCGTGTGGGGTGATAAGAAGTAAAACCAAACAC
CTGGCCGAAGAGACTTTCCTACGAACGGGCGTGAATCACCCGAAAAAAAGAGCTAACAAACAGAGTGTTG
GTGCAAACAGGTGGCCAAAAAGCCAAAGGCAACGATACCCAACTGCCTGGGGCGATCCATTTAGAGTTAA
AATGTTTGTACACGCAAACACAGTCCGCAATAAATGGAAATTCTCATGGTGTTTGATCAGCTCTCCCTTT
CGCTTGTCTCCCAGCAGGCAGGACATCTCGCAGCATCGGCTGAGCGATCTCACCCAGCACGAGCTGAGCC
TGCTGCGCGTCGACCGGGAGCAACAGGTGACCAGCAGCAGCTCCACCTCCAACGAGAAGCCGGCGAAGAC
GTCGAGGCTCTCAGAGCGGGCGAAGAAGAAGTCCTGGTACAACGTCATCTATCCCAACTACAAGTCGCGG
GCCGAGGACTTCAAGAAGCTCTTCAAGGACGTGCCCAACGACGAGCGTCTGATTGTGGGGTGAGTTTTCA
TGTGGCCCGGTAGCAGCCCGCAATGATTACCTTTGTTTTTCATTTAATGAAATCAGTCACATGCAGATTC
AGTCCGATAAAGGTAATTATGCATACATGAGCTCCTTTAAAGGCAAACTTTGCAAAAGCGTATGTGATTT
CATATATATTTATATTCCATTTAATTGTTAAGGGAATCTATATAATATAGTTATCAATGAGTTACCCTTT
AAACACAAAAATAGTTTCTATTTGAGCAATCAATTTTTCATATTATTTTTAAATTAATATGTAGTACGGA
CAAATGCAATTGTGATAATTGACGACTGATGCAATTGAGCATTAGAAAATCAGCATGATCATTTGAAATT
TTAATGCAGTTCGGTGATCTTTTGCTCTGATAAATGAACTGAGAAGTGTAATCATCAATGAATTCCCGTT
AATGAAACACGCCTTAATTGAAGCAATTTACTCAAGTGATTATCTTCTTCCGCAGAACTACTCGTGTGCC
CTACAACGCGACATTCTGGTCCAGGGCCGTCTGTATGTGTCACAGAACTACGTCTGCTTCCACGCGAACA
TCTTCAGTTGGGAGACCCACGTGAGCATCAAGTGGAAGGATGTGACGGCCATAACCAAGGAGAAGACAGC
ACTGGTCATACCGAATGCCATTTCGATATCCAGCGGCAAGGACAAGTACTTCTTTGCCACGTTCACCTCG
CGCGACAAGAGCTTCTTGATGCTCTTCCGCGTCTGGCAGAACACGCTGATGAACAAGCAGTTTTCGCCGC
AGGAAATTTGGCAACATGTGCACACCTGTTACGGCGACGAGCTGGGCCTGACCACCGACGACGAGGACTA
CATTGATCCCACGCTGAACAAGACCAACGAGCCAGACTTCGACTTTCAGACGGCCATCGATGACGATAGC
TACTCGCAGCGCCAGTCGCAGCAGTCGAATCAATCCAACCAGTCGAATCCCCTGCTTCCCCAGAGCGGAA
ACAGCAGCGCCAGCAGCGGTGGCGGCGTCCGCGCCTCTGCCCCCCGCAAGTCTAAAACGAAATATTTCTT
CAATTCGTCCAAGTCATCGGCCAACGCCTCGGCCAGCGGATCGGATAACAATAAGACCAGGGAGAGCTCA

CGCAAATTGAACAAGAAGATGAAACAGAATGCCAAGGAGCTGACTCTCAGCTCGGTGAAGCCGACGGAGC
TATCGGTGAGCGTCACCATGAGCGCTCCCAACGTCAGCAGCAGCAGCACAGCGGGCTCCACCACTGCCTC
CTCCTCCACGATTGCCAACCACACGGGCTCTGGCTCCGGATCGGGATCTCTGTCGGGCTCAAATACGGCG
GCAGCCAGCGGCAGTGGCAGCGACAAGATGAGTGCGGAGAAGAAGGTTAGCACGGCTTCTAACTCGGCCG
CCGTAGCAGCGGCAGAAGCCGCTCAATCGCTGGCCAGTGCCAGTTCCGGGTCAGGATCCTTGGAGTCCAA
GATGGAAATGAAGCGAAAGCTAGGGAAAATGCATCGGCAACGCGAAGAGGGCAAAAATGCAGCCGAGAGT
GTGCCGACAGATGTCTCCGACTCGTCTGATTCCGAGGAGAACAATTTGCCGATTTGTGCCCACCACAGAG
TGCACCTCGACGCACGAGGGCCGCCAAATCGTGCATACCATTCTGCCAATTAACGTGGACACCTTGTTTA
ATCTTTTGTTCAGCAAGTCGAAATTCATAACTGACTTCCATGCCATGCGCAAGTCAACGGACCTCGTGTT
AGGCGAGTGGACCAAGAACGAGGAGGGTCTGCAAGTGCGCACAGTGAACGTCACTGTTCAATTGGCCGCC
TCTGTGGGGCCCAAGACCTCAAAGGGTAACCGAGTATCAGACGCAGCGGGAGTGTAGCAAGCCGGGCGAA
CTGTATTCCATAGACGTAAATAGTGTTAATGCAGGCATTCCATATGCGGAAAGTTTCAGTGTGCTCATAC
ACTTCTGCCTGGCCAGGAACTGTCGATGATCACACTATGCTCTCAATTCACACCCAGATCAAGTACAAGA
AGTCGATCTGGGGCGTGGTCAAGGGCTTCATTGAGAAGAACACGTGGGCGGGCCTCGAGGACTTCTTTGG
CGCTCAATTGCATGCGCTCCAGAGCGAGACGTGCATTCCACCTGCCAAGGGAAAGGGTCGACGGCCGAGG
AGAGGGTAAGCATTGCGACTCTAGACGCAGTAACCAATTAACTACAATGcaacaaccacaacaacaagaa
caacaaatacagcaaatacagcaacaaagcaaagcaatTTCTTTCTAATATTTTGTAATGAACATATCAA
ATCAGAGTTCGAATTCGATAACTGCCAATGCCAATTGCCATTGCTTCTTCTAATCACACCAAAACGCACG
CATGCTCACAATAAACATGAAACCACTCGTTACGAACCAAAAGCAACATCCAGCTACAATCAACATACAT
CTCAGGCATGAATCAGCAATCCGCCACCTCCACGTCGACCACCTCCATCACGACAGGTGCGCTCCATACG
GCCACCAGCACGGACAATGACAGCATTCAAGAGTCGCGAcatccccatcagcaggatcaacatcagcacc
agcacttgcacatgcaccatcatcaccaccatcaccatggtagtcagcatcatcatcacgatgccaggca
ctcccatcatcatcatcatcagcctctgcttccccatcatcagcagcaacatccccatcactggcatcac
cgtAAGGCTCTACTTCAGTCCGGCAGAGGTACAGCTTAAGTATTTGGATCCTTTCTGACTAGCAATTGCC
ACAGGGGAATTGTGTATGTCTATCAGTACTAAAATATAGTTAGTTTATAATCTACTCAGAATTGACCACT
TCATCTTTAAAGAAAAGATGTCAAAAATAATAGTTTCAAATGTTGTCGGCTTATTGTCattagctattaa
ctattaactattTCTTTTCTATTTGAGAAGCGAATGTTTAGTAATGAGGTTTTGAAACGTAATTTAGAGG
GATTTCGGGATTTCGCTCTTTTAATGCGCTTAACTTCAAATATACATTTCAAAGTAAATTCGATAAGAAG
CACAGCTGGCACTGCCAAAGATTTATGAGCACACGTGATACAGGTTGCCGATCATAAAACGATTTCACCA
ATTTTAACCTTCCTTTTGAATGCTTCTTTTTATTAATCTAATAATTTTTCTTGGTAATGATTACTTTTAA
TGGCTTTATCAAATTTCGATAAGGCTTCATTCATATTTTGCTTAAAGTTTTGTCCCCTTCTAGTTCATAA
TGGATTTACCTTCAACTAAGTCACAAGAACTTGACGAAGCTTTCGAATCAATTCGATTTAGTTCGCGCAT
TAATTTCGTTAAAGAAACCTAATATTATGTCGATCATTAAATTTTTTGCAAGGATATTTTTCACCGAAAG
GCTTGGTTCATTTCATAAACAGTTATCTACTGGTAATGCAAACTTTTCTGGAAATGTCTTGAATGATATG
TCTTTTATCTTAAATACAGTCTCTCGTGACGATTGCTAGCCCATCATGTACCTCCTAATCACTTGCTTTT
TATTGCAGCTTAATTTTTTTACTAGTGATTTATATTTTGTTGTACTCTTAATGTTGTGATTTTGTTGCCG
GCCTATGTTCATTTCGATTCTTCCGTTCGAATGGTTTGATTTCTAACCGTTCCATATGCCATTCCTTCGC
TCGTCCCCGTTTCTCCGCAGGGTACCGCCACCCAAATTCGTCCTCTGGAGGAGGCAGTTGGTCCGGCCCC
AGCCGTTGATCCCCTGGAGACCCAAATGCTGGCCGCGGGAGGTATTCCCGGGGCGGCAGTCGGCGCCGCC
GCCGGTCATCCGCTGCTGCTCGAGGGCGGCAagcagcaacagctccaccaccagcagcaaatgcaccatc
accaccttcagccgcacaagcagcacctacagcagcaGCAGTTGCACCTGGGCGGCGGCGACGGTCAGCC
TCTGGCATCgggtcaagggtcacagggtcacagACTTAGACACAAGGGCTTGTGGTTGTTGGTTATACTC
TTGCTGTGCTTGATGCTGGCATTAAATGTGATATTATTACTTAAGCTCTGGAGGCTGGAGGAACGCATTG
ACGTGGATCTCAATCGGCGGGCCCGCATGCCCAGTTTGGCGGCCTTGAGGTGAGTCCAAATCCATACTGA

CAACCAAATTCGCTATAATGTATGCAAATGAACCTACGCTTTTCAGTGAGCTGCCGAGCACGAATCAGGA
ATGGCTGGAATTGCTGCGAGACCAGGAGATGGCGCACGAGAACGAGCTGCACAAATGGCAGCAGGTGCTC
CAAACTGCCATCGAGCTGCTGAAAAAGG
>dme_circ_0001238_junction_seq
CAAACTGCCATCGAGCTGCTGAAAAAGGGCGCGATTCGATTACCGAGGAGATCACG
>dme_circ_0001239
GATTAAAGATAGTGATAAAATCAACCCAACAACATCAAGTGTAAACATAACCGTTACGACTGCCGAAAGT
TCGAGCAGTTGTAAAAACCCTGCCAATGCCAATATGATACCCGAGACCACCCAGCCCGATCAGCTCCAGT
CACAAACACAGACACAAGCCCAGCTCCAGCCGCAGTCTCAATCGCCGTCACAGTGCCAGCAGGCGCAGGA
GAATCCCGAGCATCTCCAGCAGCAATCGCAGCCGGAGGAGGAGTTGCAGGCGGAGGCGCCTGGTAGCAAT
GCTGGCGACAGGTAGGTGGGATCAGTGCACCGGAAACTCTCCAGCCAGGCCTTGGTCCAATTCCGTAAGA
GAACTCATCCATTCCTTCCAAATTTGCATGCCTAGTTCAAGAGTTTTTCCATTTAATCGAAGTAGCAGTA
GTTCGGGGTGGCCAGTTGAATCCGTGCTAACCTCGTTTCCTTGTACCATTCACAGGGCGCGATTCGATTA
CCGAGGAGATCACGATAACGAGCACTACAAATAGCAGTCAAAGCACCAAACTCCTGACCATTCAGGAAGC
TGCCGGCAGTTTTGAGCAGAGCAGCGTCTCTGCCGTATCGACGTCCTCCAAACAGATCGCTTCCACCACA
GCCGGCGGTTCGCCACCAGCTGCTGCAGCTCCAGGTGCCAATGAGTTCAACGGCAGCGTCCTCCGATTAA
CACAGGCCGCAACTTCCACTAGTCCCGGCTCCATCTCGAGTGCCGGCAACACAGCTACACTAGGAGCCGC
TCCGAGCATTAACATCGTGTCCAGCGAGTCCACGCGGGATCAGACACAACAGCCGCAGAGCGTTGACCCC
AACGGCGTTGCCGCTCCAGGCGACAGTCCCAGCAGCCGGAAGAGCTCCACCAGCTCCAAGGGGAAGGCCA
GCCAGGCCAAGCTATCCACATCCAGCAGTGGACGCGATGAGGGTGAGTTCGAATGGCCTGCGTGTGGGAT
TTAAGGCAGCATTAGGGGACCATGTCGCTGGTTCAGAAAAGGTTTATCATGTGCATATAATTTCTACAGA
TATTTCTGCATTACGTTACTGTGCTATGAGTCATGTCGAGTTGGTCAGTTTCTAGTTTTCTCAGCGATAA
CAAATTTATTACGCACCACTCGAATTTTCTAAGAATGCTTCACTACTTGGCACTAATGGCATTAAGTCAT
TTTTTACCAGTGAGCATGATAGTTCAAGGCAATCACATTTGCTTGGTTTAGCTTTATATAGTACAAATTT
GAATAGCAACGTCACTTCGTTGCTTTTTGATTAGACCGATTTCTATGGGCTTTAAAACTGTGCTGGGCTG
GGCATCATAAAAAGTAGGTTTGAACCATGTAGGGAGTGACTTCCGAAATTATATTTTTAAAATTGATAAA
ACATTTACTTTTGGCGTCGTGGTTTTATCGACGTTCTATGCAGTTTGAGGGTGCGAGTTTTTGTCATAAT
TTGAATGCTTAACCCTAGGAGTACACATAGAATTTGTTTTTGTTTGATGAACTAATGATGATAATGTTTA
GAGGCTTTTTTGTCACATCTTTTCAAGTTCGAATGAACCAATGTCAACACTTTTGCGTATCTATATGACT
AGCAAGGGCTAGTCGAGAAACCCTGACTATTTCGATGAGCTTTCAAAAACTAGCCGTTTGTTTATTTTGC
CCATTAGCCAGTCATACACGCCCCGAAAAAGGTGTGAAATTGGTGGAGCCAAGTGTTCGAATCAAATTAA
AGGCACTCGAAGCGACGATCAATCGATCAGGCGATTGACAGATAGAAAACTGCACAGATAGCCAGTTGGG
CCGACAGATAGACAAGGGCGATTTGGATTAATGGCCCCATATTGGCCATCTAGCAGCGCAATCTACTTGC
TGTCCGATAAGGAGCATGTGTCACCATGTGTGTAGCTGCCCTCTCACCCCATCGAGAGGCAGCTGATTAG
CTGATTAGAATCACCCTTCGATGCGAAAGCGAAAGCTCGCTAAATAAATGTTTTGGGCCCAAGCTTTTTC
CGGCCAACTGGGCGGTGATAACGATTCGTGTGCGGCGTAGCTCTTCCATTGACGTTTGCCTGGCTGGCAA
TGCCAAATTATAATTCGACAAGCGACGGAGCGTCTCAGCGCGAATATAGAGACACCaaaaaaaaaaaaaa
aaaaaaaagaagaaaaaaaCCAAAATAACAAACTAATATCGAGCATTGCAGCGAACTTTGTGTAAATAAT
TGGGGGTCGTAAACGAACGACAAGCGTGTGGGGTGATAAGAAGTAAAACCAAACACCTGGCCGAAGAGAC
TTTCCTACGAACGGGCGTGAATCACCCGAAAAAAAGAGCTAACAAACAGAGTGTTGGTGCAAACAGGTGG
CCAAAAAGCCAAAGGCAACGATACCCAACTGCCTGGGGCGATCCATTTAGAGTTAAAATGTTTGTACACG
CAAACACAGTCCGCAATAAATGGAAATTCTCATGGTGTTTGATCAGCTCTCCCTTTCGCTTGTCTCCCAG
CAGGCAGGACATCTCGCAGCATCGGCTGAGCGATCTCACCCAGCACGAGCTGAGCCTGCTGCGCGTCGAC
CGGGAGCAACAGGTGACCAGCAGCAGCTCCACCTCCAACGAGAAGCCGGCGAAGACGTCGAGGCTCTCAG

AGCGGGCGAAGAAGAAGTCCTGGTACAACGTCATCTATCCCAACTACAAGTCGCGGGCCGAGGACTTCAA
GAAGCTCTTCAAGGACGTGCCCAACGACGAGCGTCTGATTGTGGGGTGAGTTTTCATGTGGCCCGGTAGC
AGCCCGCAATGATTACCTTTGTTTTTCATTTAATGAAATCAGTCACATGCAGATTCAGTCCGATAAAGGT
AATTATGCATACATGAGCTCCTTTAAAGGCAAACTTTGCAAAAGCGTATGTGATTTCATATATATTTATA
TTCCATTTAATTGTTAAGGGAATCTATATAATATAGTTATCAATGAGTTACCCTTTAAACACAAAAATAG
TTTCTATTTGAGCAATCAATTTTTCATATTATTTTTAAATTAATATGTAGTACGGACAAATGCAATTGTG
ATAATTGACGACTGATGCAATTGAGCATTAGAAAATCAGCATGATCATTTGAAATTTTAATGCAGTTCGG
TGATCTTTTGCTCTGATAAATGAACTGAGAAGTGTAATCATCAATGAATTCCCGTTAATGAAACACGCCT
TAATTGAAGCAATTTACTCAAGTGATTATCTTCTTCCGCAGAACTACTCGTGTGCCCTACAACGCGACAT
TCTGGTCCAGGGCCGTCTGTATGTGTCACAGAACTACGTCTGCTTCCACGCGAACATCTTCAGTTGGGAG
ACCCACGTGAGCATCAAGTGGAAGGATGTGACGGCCATAACCAAGGAGAAGACAGCACTGGTCATACCGA
ATGCCATTTCGATATCCAGCGGCAAGGACAAGTACTTCTTTGCCACGTTCACCTCGCGCGACAAGAGCTT
CTTGATGCTCTTCCGCGTCTGGCAGAACACGCTGATGAACAAGCAGTTTTCGCCGCAGGAAATTTGGCAA
CATGTGCACACCTGTTACGGCGACGAGCTGGGCCTGACCACCGACGACGAGGACTACATTGATCCCACGC
TGAACAAGACCAACGAGCCAGACTTCGACTTTCAGACGGCCATCGATGACGATAGCTACTCGCAGCGCCA
GTCGCAGCAGTCGAATCAATCCAACCAGTCGAATCCCCTGCTTCCCCAGAGCGGAAACAGCAGCGCCAGC
AGCGGTGGCGGCGTCCGCGCCTCTGCCCCCCGCAAGTCTAAAACGAAATATTTCTTCAATTCGTCCAAGT
CATCGGCCAACGCCTCGGCCAGCGGATCGGATAACAATAAGACCAGGGAGAGCTCACGCAAATTGAACAA
GAAGATGAAACAGAATGCCAAGGAGCTGACTCTCAGCTCGGTGAAGCCGACGGAGCTATCGGTGAGCGTC
ACCATGAGCGCTCCCAACGTCAGCAGCAGCAGCACAGCGGGCTCCACCACTGCCTCCTCCTCCACGATTG
CCAACCACACGGGCTCTGGCTCCGGATCGGGATCTCTGTCGGGCTCAAATACGGCGGCAGCCAGCGGCAG
TGGCAGCGACAAGATGAGTGCGGAGAAGAAGGTTAGCACGGCTTCTAACTCGGCCGCCGTAGCAGCGGCA
GAAGCCGCTCAATCGCTGGCCAGTGCCAGTTCCGGGTCAGGATCCTTGGAGTCCAAGATGGAAATGAAGC
GAAAGCTAGGGAAAATGCATCGGCAACGCGAAGAGGGCAAAAATGCAGCCGAGAGTGTGCCGACAGATGT
CTCCGACTCGTCTGATTCCGAGGAGAACAATTTGCCGATTTGTGCCCACCACAGAGTGCACCTCGACGCA
CGAGGGCCGCCAAATCGTGCATACCATTCTGCCAATTAACGTGGACACCTTGTTTAATCTTTTGTTCAGC
AAGTCGAAATTCATAACTGACTTCCATGCCATGCGCAAGTCAACGGACCTCGTGTTAGGCGAGTGGACCA
AGAACGAGGAGGGTCTGCAAGTGCGCACAGTGAACGTCACTGTTCAATTGGCCGCCTCTGTGGGGCCCAA
GACCTCAAAGGGTAACCGAGTATCAGACGCAGCGGGAGTGTAGCAAGCCGGGCGAACTGTATTCCATAGA
CGTAAATAGTGTTAATGCAGGCATTCCATATGCGGAAAGTTTCAGTGTGCTCATACACTTCTGCCTGGCC
AGGAACTGTCGATGATCACACTATGCTCTCAATTCACACCCAGATCAAGTACAAGAAGTCGATCTGGGGC
GTGGTCAAGGGCTTCATTGAGAAGAACACGTGGGCGGGCCTCGAGGACTTCTTTGGCGCTCAATTGCATG
CGCTCCAGAGCGAGACGTGCATTCCACCTGCCAAGGGAAAGGGTCGACGGCCGAGGAGAGGGTAAGCATT
GCGACTCTAGACGCAGTAACCAATTAACTACAATGcaacaaccacaacaacaagaacaacaaatacagca
aatacagcaacaaagcaaagcaatTTCTTTCTAATATTTTGTAATGAACATATCAAATCAGAGTTCGAAT
TCGATAACTGCCAATGCCAATTGCCATTGCTTCTTCTAATCACACCAAAACGCACGCATGCTCACAATAA
ACATGAAACCACTCGTTACGAACCAAAAGCAACATCCAGCTACAATCAACATACATCTCAGGCATGAATC
AGCAATCCGCCACCTCCACGTCGACCACCTCCATCACGACAGGTGCGCTCCATACGGCCACCAGCACGGA
CAATGACAGCATTCAAGAGTCGCGAcatccccatcagcaggatcaacatcagcaccagcacttgcacatg
caccatcatcaccaccatcaccatggtagtcagcatcatcatcacgatgccaggcactcccatcatcatc
atcatcagcctctgcttccccatcatcagcagcaacatccccatcactggcatcaccgtAAGGCTCTACT
TCAGTCCGGCAGAGGTACAGCTTAAGTATTTGGATCCTTTCTGACTAGCAATTGCCACAGGGGAATTGTG
TATGTCTATCAGTACTAAAATATAGTTAGTTTATAATCTACTCAGAATTGACCACTTCATCTTTAAAGAA
AAGATGTCAAAAATAATAGTTTCAAATGTTGTCGGCTTATTGTCattagctattaactattaactattTC

TTTTCTATTTGAGAAGCGAATGTTTAGTAATGAGGTTTTGAAACGTAATTTAGAGGGATTTCGGGATTTC
GCTCTTTTAATGCGCTTAACTTCAAATATACATTTCAAAGTAAATTCGATAAGAAGCACAGCTGGCACTG
CCAAAGATTTATGAGCACACGTGATACAGGTTGCCGATCATAAAACGATTTCACCAATTTTAACCTTCCT
TTTGAATGCTTCTTTTTATTAATCTAATAATTTTTCTTGGTAATGATTACTTTTAATGGCTTTATCAAAT
TTCGATAAGGCTTCATTCATATTTTGCTTAAAGTTTTGTCCCCTTCTAGTTCATAATGGATTTACCTTCA
ACTAAGTCACAAGAACTTGACGAAGCTTTCGAATCAATTCGATTTAGTTCGCGCATTAATTTCGTTAAAG
AAACCTAATATTATGTCGATCATTAAATTTTTTGCAAGGATATTTTTCACCGAAAGGCTTGGTTCATTTC
ATAAACAGTTATCTACTGGTAATGCAAACTTTTCTGGAAATGTCTTGAATGATATGTCTTTTATCTTAAA
TACAGTCTCTCGTGACGATTGCTAGCCCATCATGTACCTCCTAATCACTTGCTTTTTATTGCAGCTTAAT
TTTTTTACTAGTGATTTATATTTTGTTGTACTCTTAATGTTGTGATTTTGTTGCCGGCCTATGTTCATTT
CGATTCTTCCGTTCGAATGGTTTGATTTCTAACCGTTCCATATGCCATTCCTTCGCTCGTCCCCGTTTCT
CCGCAGGGTACCGCCACCCAAATTCGTCCTCTGGAGGAGGCAGTTGGTCCGGCCCCAGCCGTTGATCCCC
TGGAGACCCAAATGCTGGCCGCGGGAGGTATTCCCGGGGCGGCAGTCGGCGCCGCCGCCGGTCATCCGCT
GCTGCTCGAGGGCGGCAagcagcaacagctccaccaccagcagcaaatgcaccatcaccaccttcagccg
cacaagcagcacctacagcagcaGCAGTTGCACCTGGGCGGCGGCGACGGTCAGCCTCTGGCATCgggtc
aagggtcacagggtcacagACTTAGACACAAGGGCTTGTGGTTGTTGGTTATACTCTTGCTGTGCTTGAT
GCTGGCATTAAATGTGATATTATTACTTAAGCTCTGGAGGCTGGAGGAACGCATTGACGTGGATCTCAAT
CGGCGGGCCCGCATGCCCAGTTTGGCGGCCTTGAGGTGAGTCCAAATCCATACTGACAACCAAATTCGCT
ATAATGTATGCAAATGAACCTACGCTTTTCAGTGAGCTGCCGAGCACGAATCAGGAATGGCTGGAATTGC
TGCGAGACCAGGAGATGGCGCACGAGAACGAGCTGCACAAATGGCAGCAGGTGCTCCAAACTGCCATCGA
GCTGCTGAAAAAGG
>dme_circ_0001239_junction_seq
CAAACTGCCATCGAGCTGCTGAAAAAGGGATTAAAGATAGTGATAAAATCAACCCA
>dme_circ_0001240
GCGCGATTCGATTACCGAGGAGATCACGATAACGAGCACTACAAATAGCAGTCAAAGCACCAAACTCCTG
ACCATTCAGGAAGCTGCCGGCAGTTTTGAGCAGAGCAGCGTCTCTGCCGTATCGACGTCCTCCAAACAGA
TCGCTTCCACCACAGCCGGCGGTTCGCCACCAGCTGCTGCAGCTCCAGGTGCCAATGAGTTCAACGGCAG
CGTCCTCCGATTAACACAGGCCGCAACTTCCACTAGTCCCGGCTCCATCTCGAGTGCCGGCAACACAGCT
ACACTAGGAGCCGCTCCGAGCATTAACATCGTGTCCAGCGAGTCCACGCGGGATCAGACACAACAGCCGC
AGAGCGTTGACCCCAACGGCGTTGCCGCTCCAGGCGACAGTCCCAGCAGCCGGAAGAGCTCCACCAGCTC
CAAGGGGAAGGCCAGCCAGGCCAAGCTATCCACATCCAGCAGTGGACGCGATGAGGGTGAGTTCGAATGG
CCTGCGTGTGGGATTTAAGGCAGCATTAGGGGACCATGTCGCTGGTTCAGAAAAGGTTTATCATGTGCAT
ATAATTTCTACAGATATTTCTGCATTACGTTACTGTGCTATGAGTCATGTCGAGTTGGTCAGTTTCTAGT
TTTCTCAGCGATAACAAATTTATTACGCACCACTCGAATTTTCTAAGAATGCTTCACTACTTGGCACTAA
TGGCATTAAGTCATTTTTTACCAGTGAGCATGATAGTTCAAGGCAATCACATTTGCTTGGTTTAGCTTTA
TATAGTACAAATTTGAATAGCAACGTCACTTCGTTGCTTTTTGATTAGACCGATTTCTATGGGCTTTAAA
ACTGTGCTGGGCTGGGCATCATAAAAAGTAGGTTTGAACCATGTAGGGAGTGACTTCCGAAATTATATTT
TTAAAATTGATAAAACATTTACTTTTGGCGTCGTGGTTTTATCGACGTTCTATGCAGTTTGAGGGTGCGA
GTTTTTGTCATAATTTGAATGCTTAACCCTAGGAGTACACATAGAATTTGTTTTTGTTTGATGAACTAAT
GATGATAATGTTTAGAGGCTTTTTTGTCACATCTTTTCAAGTTCGAATGAACCAATGTCAACACTTTTGC
GTATCTATATGACTAGCAAGGGCTAGTCGAGAAACCCTGACTATTTCGATGAGCTTTCAAAAACTAGCCG
TTTGTTTATTTTGCCCATTAGCCAGTCATACACGCCCCGAAAAAGGTGTGAAATTGGTGGAGCCAAGTGT
TCGAATCAAATTAAAGGCACTCGAAGCGACGATCAATCGATCAGGCGATTGACAGATAGAAAACTGCACA
GATAGCCAGTTGGGCCGACAGATAGACAAGGGCGATTTGGATTAATGGCCCCATATTGGCCATCTAGCAG

CGCAATCTACTTGCTGTCCGATAAGGAGCATGTGTCACCATGTGTGTAGCTGCCCTCTCACCCCATCGAG
AGGCAGCTGATTAGCTGATTAGAATCACCCTTCGATGCGAAAGCGAAAGCTCGCTAAATAAATGTTTTGG
GCCCAAGCTTTTTCCGGCCAACTGGGCGGTGATAACGATTCGTGTGCGGCGTAGCTCTTCCATTGACGTT
TGCCTGGCTGGCAATGCCAAATTATAATTCGACAAGCGACGGAGCGTCTCAGCGCGAATATAGAGACACC
aaaaaaaaaaaaaaaaaaaaaagaagaaaaaaaCCAAAATAACAAACTAATATCGAGCATTGCAGCGAAC
TTTGTGTAAATAATTGGGGGTCGTAAACGAACGACAAGCGTGTGGGGTGATAAGAAGTAAAACCAAACAC
CTGGCCGAAGAGACTTTCCTACGAACGGGCGTGAATCACCCGAAAAAAAGAGCTAACAAACAGAGTGTTG
GTGCAAACAGGTGGCCAAAAAGCCAAAGGCAACGATACCCAACTGCCTGGGGCGATCCATTTAGAGTTAA
AATGTTTGTACACGCAAACACAGTCCGCAATAAATGGAAATTCTCATGGTGTTTGATCAGCTCTCCCTTT
CGCTTGTCTCCCAGCAGGCAGGACATCTCGCAGCATCGGCTGAGCGATCTCACCCAGCACGAGCTGAGCC
TGCTGCGCGTCGACCGGGAGCAACAGGTGACCAGCAGCAGCTCCACCTCCAACGAGAAGCCGGCGAAGAC
GTCGAGGCTCTCAGAGCGGGCGAAGAAGAAGTCCTGGTACAACGTCATCTATCCCAACTACAAGTCGCGG
GCCGAGGACTTCAAGAAGCTCTTCAAGGACGTGCCCAACGACGAGCGTCTGATTGTGGGGTGAGTTTTCA
TGTGGCCCGGTAGCAGCCCGCAATGATTACCTTTGTTTTTCATTTAATGAAATCAGTCACATGCAGATTC
AGTCCGATAAAGGTAATTATGCATACATGAGCTCCTTTAAAGGCAAACTTTGCAAAAGCGTATGTGATTT
CATATATATTTATATTCCATTTAATTGTTAAGGGAATCTATATAATATAGTTATCAATGAGTTACCCTTT
AAACACAAAAATAGTTTCTATTTGAGCAATCAATTTTTCATATTATTTTTAAATTAATATGTAGTACGGA
CAAATGCAATTGTGATAATTGACGACTGATGCAATTGAGCATTAGAAAATCAGCATGATCATTTGAAATT
TTAATGCAGTTCGGTGATCTTTTGCTCTGATAAATGAACTGAGAAGTGTAATCATCAATGAATTCCCGTT
AATGAAACACGCCTTAATTGAAGCAATTTACTCAAGTGATTATCTTCTTCCGCAGAACTACTCGTGTGCC
CTACAACGCGACATTCTGGTCCAGGGCCGTCTGTATGTGTCACAGAACTACGTCTGCTTCCACGCGAACA
TCTTCAGTTGGGAGACCCACGTGAGCATCAAGTGGAAGGATGTGACGGCCATAACCAAGGAGAAGACAGC
ACTGGTCATACCGAATGCCATTTCGATATCCAGCGGCAAGGACAAGTACTTCTTTGCCACGTTCACCTCG
CGCGACAAGAGCTTCTTGATGCTCTTCCGCGTCTGGCAGAACACGCTGATGAACAAGCAGTTTTCGCCGC
AGGAAATTTGGCAACATGTGCACACCTGTTACGGCGACGAGCTGGGCCTGACCACCGACGACGAGGACTA
CATTGATCCCACGCTGAACAAGACCAACGAGCCAGACTTCGACTTTCAGACGGCCATCGATGACGATAGC
TACTCGCAGCGCCAGTCGCAGCAGTCGAATCAATCCAACCAGTCGAATCCCCTGCTTCCCCAGAGCGGAA
ACAGCAGCGCCAGCAGCGGTGGCGGCGTCCGCGCCTCTGCCCCCCGCAAGTCTAAAACGAAATATTTCTT
CAATTCGTCCAAGTCATCGGCCAACGCCTCGGCCAGCGGATCGGATAACAATAAGACCAGGGAGAGCTCA
CGCAAATTGAACAAGAAGATGAAACAGAATGCCAAGGAGCTGACTCTCAGCTCGGTGAAGCCGACGGAGC
TATCGGTGAGCGTCACCATGAGCGCTCCCAACGTCAGCAGCAGCAGCACAGCGGGCTCCACCACTGCCTC
CTCCTCCACGATTGCCAACCACACGGGCTCTGGCTCCGGATCGGGATCTCTGTCGGGCTCAAATACGGCG
GCAGCCAGCGGCAGTGGCAGCGACAAGATGAGTGCGGAGAAGAAGGTTAGCACGGCTTCTAACTCGGCCG
CCGTAGCAGCGGCAGAAGCCGCTCAATCGCTGGCCAGTGCCAGTTCCGGGTCAGGATCCTTGGAGTCCAA
GATGGAAATGAAGCGAAAGCTAGGGAAAATGCATCGGCAACGCGAAGAGGGCAAAAATGCAGCCGAGAGT
GTGCCGACAGATGTCTCCGACTCGTCTGATTCCGAGGAGAACAATTTGCCGATTTGTGCCCACCACAGAG
TGCACCTCGACGCACGAGGGCCGCCAAATCGTGCATACCATTCTGCCAATTAACGTGGACACCTTGTTTA
ATCTTTTGTTCAGCAAGTCGAAATTCATAACTGACTTCCATGCCATGCGCAAGTCAACGGACCTCGTGTT
AGGCGAGTGGACCAAGAACGAGGAGGGTCTGCAAGTGCGCACAGTGAACGTCACTGTTCAATTGGCCGCC
TCTGTGGGGCCCAAGACCTCAAAGGGTAACCGAGTATCAGACGCAGCGGGAGTGTAGCAAGCCGGGCGAA
CTGTATTCCATAGACGTAAATAGTGTTAATGCAGGCATTCCATATGCGGAAAGTTTCAGTGTGCTCATAC
ACTTCTGCCTGGCCAGG
>dme_circ_0001240_junction_seq
TGTGCTCATACACTTCTGCCTGGCCAGGGCGCGATTCGATTACCGAGGAGATCACG

>dme_circ_0001241
GATTAAAGATAGTGATAAAATCAACCCAACAACATCAAGTGTAAACATAACCGTTACGACTGCCGAAAGT
TCGAGCAGTTGTAAAAACCCTGCCAATGCCAATATGATACCCGAGACCACCCAGCCCGATCAGCTCCAGT
CACAAACACAGACACAAGCCCAGCTCCAGCCGCAGTCTCAATCGCCGTCACAGTGCCAGCAGGCGCAGGA
GAATCCCGAGCATCTCCAGCAGCAATCGCAGCCGGAGGAGGAGTTGCAGGCGGAGGCGCCTGGTAGCAAT
GCTGGCGACAGGTAGGTGGGATCAGTGCACCGGAAACTCTCCAGCCAGGCCTTGGTCCAATTCCGTAAGA
GAACTCATCCATTCCTTCCAAATTTGCATGCCTAGTTCAAGAGTTTTTCCATTTAATCGAAGTAGCAGTA
GTTCGGGGTGGCCAGTTGAATCCGTGCTAACCTCGTTTCCTTGTACCATTCACAGGGCGCGATTCGATTA
CCGAGGAGATCACGATAACGAGCACTACAAATAGCAGTCAAAGCACCAAACTCCTGACCATTCAGGAAGC
TGCCGGCAGTTTTGAGCAGAGCAGCGTCTCTGCCGTATCGACGTCCTCCAAACAGATCGCTTCCACCACA
GCCGGCGGTTCGCCACCAGCTGCTGCAGCTCCAGGTGCCAATGAGTTCAACGGCAGCGTCCTCCGATTAA
CACAGGCCGCAACTTCCACTAGTCCCGGCTCCATCTCGAGTGCCGGCAACACAGCTACACTAGGAGCCGC
TCCGAGCATTAACATCGTGTCCAGCGAGTCCACGCGGGATCAGACACAACAGCCGCAGAGCGTTGACCCC
AACGGCGTTGCCGCTCCAGGCGACAGTCCCAGCAGCCGGAAGAGCTCCACCAGCTCCAAGGGGAAGGCCA
GCCAGGCCAAGCTATCCACATCCAGCAGTGGACGCGATGAGG
>dme_circ_0001241_junction_seq
TCCACATCCAGCAGTGGACGCGATGAGGGATTAAAGATAGTGATAAAATCAACCCA
>dme_circ_0001242
GATGCGTTGAGCTCGACTTCTGGAACGGACGCACCGAGGAGCCAGTCATTGTCCACGGCTATACGTTCGT
GCCCGAGATATTTGCCAAGGACGTGCTGGAGGCCATCGCAGAGAGTGCATTTAAAACATCCGAATACCCT
GTGATATTGAGCTTTGAGAATCACTGCAACCCTAGGCAACAGGGTGAGAACGAGAACCAATTAACGGAAT
GGGCTTGGGAAGACCAAAATTAACTGGGAGAACTGTCTTAAAAAAATTTTAATATTTTTGATAACGGCAAA
GCCGAAGGATAGGCGAGCAGTTTTTACAAAGTATGTTCTCCTTATCTGACGCAAGCTAGGAGACTTGATA
TGCATCTATCTTTGAAAACCCCTGGTATATAAATATAAATATATTAAGGCAAAAGTTCCACTTCCAGGAG
GCAAAAACTGAATAAACTACCAAAGTAAATATAGGGCAATAAACTCTCCTTCGGACACGCTAAATTGAAG
AACGCAGAAGGCCACAATGTTACTTGTCCTAAAACAACAGTTGAGTCCCAACTCATCAGCCTAATCCATC
ATTCGGGGAAAGACAAACAAATGAATTGGCGGTGGCACACATGGGGGCTCCTTGAGCCTGAAAACATTAC
TTAAGGAGCATGAAGGGAGTCAGGAGTCCCGGAACTTGGCGAATCTCAGAAGCCACTCGAACGGTAATTG
CCGGCTGTTTAAGCTTCACGAGCGGGATAATAACAAAGGCAAAGTTTTTACGGCCTGAAAGGTAAAAGCA
GCAAAAGGAGGAGACCAGGTACCGTCAAAGAGTCAGAAGTTTTGCCATTAAAAGTAGTAATATAAACTAA
ATTTCCCGCTGGGAGTCCAGGCAGGTAACAAAGTCTGCTAGCAAACGTGCCACGTGCCAAAAACCAAACT
CATGCCAGTAAAGCTCAACATATGAAGTCTTCACATTTATACATATAATTGTTTAAATTGAATGTCAGTG
TAGCCCTTTAAAATAATAATAATACCTAATACTTATTCCTATCTAATATATGGTTAGTTTTGAGTGTCAT
GAAAATACCGGTTAAAGCGGTAAGACACAGGACGATTCGAGCAGTTATGTAAATTATGGACTATATGTAG
AATATTTGAAAGTAGCATCCTTTTCTCTCTGTGTACAAGGAAACCCCTTTGCGTGGAGCTAATGCAATTG
CCAATTAGCAGTTAGTTAACAAGACCCCTGCGCACTACCTTATGTCACATCACTTATTTCCTCACTTGGC
ACCTTCAACAGGGCGAAAATTGCCAACTATTGTCGCGAAATATTTGGCGATATGCTGCTCGATAAGCCTC
TGGACTCTCATCCGCTGGAGCCGAACATGGACTTGCCACCGCCGGCGATGCTGCGCCGCAAGATTATAAT
CAAGAACAAGAAGAAGcatcaccaccaccatcaccatcatcaccaCAAAAAGCCAGCGCAGGTGGGCACG
CCGGCAGCCAACAACAAACTGACTACAGCGAATTCAG
>dme_circ_0001242_junction_seq
AACAACAAACTGACTACAGCGAATTCAGGATGCGTTGAGCTCGACTTCTGGAACGG
>dme_circ_0001243
GATGCGTTGAGCTCGACTTCTGGAACGGACGCACCGAGGAGCCAGTCATTGTCCACGGCTATACGTTCGT

GCCCGAGATATTTGCCAAGGACGTGCTGGAGGCCATCGCAGAGAGTGCATTTAAAACATCCGAATACCCT
GTGATATTGAGCTTTGAGAATCACTGCAACCCTAGGCAACAGGGTGAGAACGAGAACCAATTAACGGAAT
GGGCTTGGGAAGACCAAAATTAACTGGGAGAACTGTCTTAAAAAATTTTAATATTTTTGATAACGGCAAA
GCCGAAGGATAGGCGAGCAGTTTTTACAAAGTATGTTCTCCTTATCTGACGCAAGCTAGGAGACTTGATA
TGCATCTATCTTTGAAAACCCCTGGTATATAAATATAAATATATTAAGGCAAAAGTTCCACTTCCAGGAG
GCAAAAACTGAATAAACTACCAAAGTAAATATAGGGCAATAAACTCTCCTTCGGACACGCTAAATTGAAG
AACGCAGAAGGCCACAATGTTACTTGTCCTAAAACAACAGTTGAGTCCCAACTCATCAGCCTAATCCATC
ATTCGGGGAAAGACAAACAAATGAATTGGCGGTGGCACACATGGGGGCTCCTTGAGCCTGAAAACATTAC
TTAAGGAGCATGAAGGGAGTCAGGAGTCCCGGAACTTGGCGAATCTCAGAAGCCACTCGAACGGTAATTG
CCGGCTGTTTAAGCTTCACGAGCGGGATAATAACAAAGGCAAAGTTTTTACGGCCTGAAAGGTAAAAGCA
GCAAAAGGAGGAGACCAGGTACCGTCAAAGAGTCAGAAGTTTTGCCATTAAAAGTAGTAATATAAACTAA
ATTTCCCGCTGGGAGTCCAGGCAGGTAACAAAGTCTGCTAGCAAACGTGCCACGTGCCAAAAACCAAACT
CATGCCAGTAAAGCTCAACATATGAAGTCTTCACATTTATACATATAATTGTTTAAATTGAATGTCAGTG
TAGCCCTTTAAAATAATAATAATACCTAATACTTATTCCTATCTAATATATGGTTAGTTTTGAGTGTCAT
GAAAATACCGGTTAAAGCGGTAAGACACAGGACGATTCGAGCAGTTATGTAAATTATGGACTATATGTAG
AATATTTGAAAGTAGCATCCTTTTCTCTCTGTGTACAAGGAAACCCCTTTGCGTGGAGCTAATGCAATTG
CCAATTAGCAGTTAGTTAACAAGACCCCTGCGCACTACCTTATGTCACATCACTTATTTCCTCACTTGGC
ACCTTCAACAGGGCGAAAATTGCCAACTATTGTCGCGAAATATTTGGCGATATGCTGCTCGATAAGCCTC
TGGACTCTCATCCGCTGGAGCCGAACATGGACTTGCCACCGCCGGCGATGCTGCGCCGCAAGATTATAAT
CAAGAACAAGAAGAAGcatcaccaccaccatcaccatcatcaccaCAAAAAGCCAGCGCAGGTGGGCACG
CCGGCAGCCAACAACAAACTGACTACAGCGAATTCAGGGTGTGTTATTTAATCCTCCACTAATCTAATCT
ATAATGCAACTCCTTTTGCGCACCAAGCACATGCACCAGGTTCATGCTTAATGTGGTCTGAGCCAGGTTC
GGTTCGCTCCTTGTTTTTTCTGGATTTTAAGTCTGACTCACATATATTTCCGCATCGAAGCTTCCTGCCT
TTACCCCGCTCTTCTATTCACCCCTTCCGACCCCATTGGCATATGAAAAATGCAAATGAGTTCCGCACAC
GCAGCAGCTGCTCGGTGTTTTCTGCAAAAGTTCAGCAGCCAGAAAGGCAGGCTGGGAATATCAAATTTGG
AACACAGTCGAAGTGTGGGAATGCGGGGGTGGCTCTTAATGAACAGAATGTTTGAGTTTGCTAGCACACG
GGAAGCACTTGGGACTTAAAAAGCTAGAAAGCAGGGCTACCTACTGCTTGACATCACAAATGGATGGTCC
CTTCCTGCGTTTATTATAGAGTTTCAATAACAAGCGTTTAATAGGTAACATGGCCACATTTTTGAAGTGC
AATATAAATAGTACTTACCAATTTAGTGCTTACTGTCTAGATGTCAAATATTTAGCCGAAGTTACCATGT
CCTACTTTCAGTTAGTAAAACTTTACGTAGAGCATTCGCGTTGCTTCCATTGAAAGTTTTGAAGCGAAAA
TGTTGTTGATAAATTTGCCGCATGGccacttccacttccacttccTGTTTGCGAGCTTTGAGTTCCTGCA
GGACCCATTTGCATGCGCAGCTGCACAGTCGTCCGGGGGCCCAACATTTGTCGCTCCTCCGCCCCCCTAG
GAGCCACATCCTTCTGGCATTGGCCGGATGACATTTAGCTCATGAATTGCAAAAAGCCAATTTATAAATG
CAAATTATTTTTCCCCTCCCAGCCAGCGGCTGCAGATCCCAGCTGTCATCTCTCCATCCCCGGGATCCCA
GCACGGCAATTGCATCCTATTTAGAGGACCCCTTCTGGTTTATGTAATTTGATTTATACATATTTTTATT
ACATTCCATTAGGGCGAAACTGTAATTTAAAAGCCTTGCGCTTTTTGGATTCTTCCATAATCCAGttttt
tttttGTCAGGAAAGGGGCGCTGGCAAATTTAAATTTATATTCACACTTATATTAGTAAGATATTTGTTT
GGTTTTAAGTTTTCTTTCTTATTGTAGATTAAGCCTAAATGGTTCATTTTTCATTTCTTTCAATTATAAA
TATTTTTTCTGTCTGTAGTACATTTACATTTACATACATTTTTCAGGTGGATGCCAAGGCGGCGCAACAA
GTCGGTTTGTCGGCCTCGCATGAGGATGGGGGCGTGACCAGGAGCACCGCCAACGGTGACGTTGCCACTG
GAACAGGAACCGGAAGTGCTGCTGGGACAGCAGGACACGCGCCGCCGTTGCAA
>dme_circ_0001243_junction_seq
GACAGCAGGACACGCGCCGCCGTTGCAAGATGCGTTGAGCTCGACTTCTGGAACGG
>dme_circ_0001244

GATGCGTTGAGCTCGACTTCTGGAACGGACGCACCGAGGAGCCAGTCATTGTCCACGGCTATACGTTCGT
GCCCGAGATATTTGCCAAGGACGTGCTGGAGGCCATCGCAGAGAGTGCATTTAAAACATCCGAATACCCT
GTGATATTGAGCTTTGAGAATCACTGCAACCCTAGGCAACAGGGTGAGAACGAGAACCAATTAACGGAAT
GGGCTTGGGAAGACCAAAATTAACTGGGAGAACTGTCTTAAAAAATTTTAATATTTTTGATAACGGCAAA
GCCGAAGGATAGGCGAGCAGTTTTTACAAAGTATGTTCTCCTTATCTGACGCAAGCTAGGAGACTTGATA
TGCATCTATCTTTGAAAACCCCTGGTATATAAATATAAATATATTAAGGCAAAAGTTCCACTTCCAGGAG
GCAAAAACTGAATAAACTACCAAAGTAAATATAGGGCAATAAACTCTCCTTCGGACACGCTAAATTGAAG
AACGCAGAAGGCCACAATGTTACTTGTCCTAAAACAACAGTTGAGTCCCAACTCATCAGCCTAATCCATC
ATTCGGGGAAAGACAAACAAATGAATTGGCGGTGGCACACATGGGGGCTCCTTGAGCCTGAAAACATTAC
TTAAGGAGCATGAAGGGAGTCAGGAGTCCCGGAACTTGGCGAATCTCAGAAGCCACTCGAACGGTAATTG
CCGGCTGTTTAAGCTTCACGAGCGGGATAATAACAAAGGCAAAGTTTTTACGGCCTGAAAGGTAAAAGCA
GCAAAAGGAGGAGACCAGGTACCGTCAAAGAGTCAGAAGTTTTGCCATTAAAAGTAGTAATATAAACTAA
ATTTCCCGCTGGGAGTCCAGGCAGGTAACAAAGTCTGCTAGCAAACGTGCCACGTGCCAAAAACCAAACT
CATGCCAGTAAAGCTCAACATATGAAGTCTTCACATTTATACATATAATTGTTTAAATTGAATGTCAGTG
TAGCCCTTTAAAATAATAATAATACCTAATACTTATTCCTATCTAATATATGGTTAGTTTTGAGTGTCAT
GAAAATACCGGTTAAAGCGGTAAGACACAGGACGATTCGAGCAGTTATGTAAATTATGGACTATATGTAG
AATATTTGAAAGTAGCATCCTTTTCTCTCTGTGTACAAGGAAACCCCTTTGCGTGGAGCTAATGCAATTG
CCAATTAGCAGTTAGTTAACAAGACCCCTGCGCACTACCTTATGTCACATCACTTATTTCCTCACTTGGC
ACCTTCAACAGGGCGAAAATTGCCAACTATTGTCGCGAAATATTTGGCGATATGCTGCTCGATAAGCCTC
TGGACTCTCATCCGCTGGAGCCGAACATGGACTTGCCACCGCCGGCGATGCTGCGCCGCAAGATTATAAT
CAAGAACAAGAAGAAGcatcaccaccaccatcaccatcatcaccaCAAAAAGCCAGCGCAGGTGGGCACG
CCGGCAGCCAACAACAAACTGACTACAGCGAATTCAGGGTGTGTTATTTAATCCTCCACTAATCTAATCT
ATAATGCAACTCCTTTTGCGCACCAAGCACATGCACCAGGTTCATGCTTAATGTGGTCTGAGCCAGGTTC
GGTTCGCTCCTTGTTTTTTCTGGATTTTAAGTCTGACTCACATATATTTCCGCATCGAAGCTTCCTGCCT
TTACCCCGCTCTTCTATTCACCCCTTCCGACCCCATTGGCATATGAAAAATGCAAATGAGTTCCGCACAC
GCAGCAGCTGCTCGGTGTTTTCTGCAAAAGTTCAGCAGCCAGAAAGGCAGGCTGGGAATATCAAATTTGG
AACACAGTCGAAGTGTGGGAATGCGGGGGTGGCTCTTAATGAACAGAATGTTTGAGTTTGCTAGCACACG
GGAAGCACTTGGGACTTAAAAAGCTAGAAAGCAGGGCTACCTACTGCTTGACATCACAAATGGATGGTCC
CTTCCTGCGTTTATTATAGAGTTTCAATAACAAGCGTTTAATAGGTAACATGGCCACATTTTTGAAGTGC
AATATAAATAGTACTTACCAATTTAGTGCTTACTGTCTAGATGTCAAATATTTAGCCGAAGTTACCATGT
CCTACTTTCAGTTAGTAAAACTTTACGTAGAGCATTCGCGTTGCTTCCATTGAAAGTTTTGAAGCGAAAA
TGTTGTTGATAAATTTGCCGCATGGccacttccacttccacttccTGTTTGCGAGCTTTGAGTTCCTGCA
GGACCCATTTGCATGCGCAGCTGCACAGTCGTCCGGGGGCCCAACATTTGTCGCTCCTCCGCCCCCCTAG
GAGCCACATCCTTCTGGCATTGGCCGGATGACATTTAGCTCATGAATTGCAAAAAGCCAATTTATAAATG
CAAATTATTTTTCCCCTCCCAGCCAGCGGCTGCAGATCCCAGCTGTCATCTCTCCATCCCCGGGATCCCA
GCACGGCAATTGCATCCTATTTAGAGGACCCCTTCTGGTTTATGTAATTTGATTTATACATATTTTTATT
ACATTCCATTAGGGCGAAACTGTAATTTAAAAGCCTTGCGCTTTTTGGATTCTTCCATAATCCAGttttt
tttttGTCAGGAAAGGGGCGCTGGCAAATTTAAATTTATATTCACACTTATATTAGTAAGATATTTGTTT
GGTTTTAAGTTTTCTTTCTTATTGTAGATTAAGCCTAAATGGTTCATTTTTCATTTCTTTCAATTATAAA
TATTTTTTCTGTCTGTAGTACATTTACATTTACATACATTTTTCAGGTGGATGCCAAGGCGGCGCAACAA
GTCGGTTTGTCGGCCTCGCATGAGGATGGGGGCGTGACCAGGAGCACCGCCAACGGTGACGTTGCCACTG
GAACAGGAACCGGAAGTGCTGCTGGGACAGCAGGACACGCGCCGCCGTTGCAAAGTGAGTATTGTTCCAT
GCTATTGGGCACGGTAAGAAAAGGTCAAAGGACACTGCGTATCTATCCATTATTGTTATTGTGGGAGTGT
CACTGGCATGCCCATAATTGCATTTAATAGCACACTCCACATCACATACACTGATGAACGGATATGGCGA

TAGCGAGGAGTACTTTTCGGTTCCTCTGTTGACTCCGGCTGATTTGTTTTTCTGTTTGCTGTTGGCCGTT
GTTCTCCCtgatgatgatgatgatgatTGTCATCGGGCTGCTCTCTTTACGTCCGGCGTTTGCATACGCA
ATAATCATGATGACAGAACGGGATAGAGGGAATAAGCCATTGATGCACCGTCAAAAAGTAGGGCTTCGAA
GTGAAAAACAGTTCATGTTAAGAAAACTACTGAAAACTATTAAATCTATTCTATGCAACTGGATTTTTAC
AATAGTTTGACACGAAGTGCCAACCAACCAGCCACAGCACATCTCAGTTTAAGAAATCCAAGTTCTCTTC
ttttttttttGAGTACATGTGTGTTCTACAATATGTTCTAAATGAATGTGGGCATGGAAATGAATGAAA
TAGAGTGGATGTGGCTGGCAATGGGAATGTATGCGGTTGGCTGCCTGATGACCAGTGACAGTCAACGAAA
TGCGAACTGTGGATCCTCGACGTGGGCATGGACGGATATGCCCACTAATCAAAAGTTACCCAAACCATCA
GTATTGCAGGCAGTAATCAAAACTGGAGCCACGTGGGATCCCCCGAAACTTGAGCGTAGTCGAAACCCAT
ACTAATTTGGCTAAATAGTTGCACACTTATGCATTTTAAATTAATTTGCCTGTCTGCTATTAGTGCAGTG
TTATAATTAATGATTTTTTGGTGTGGTCCCGGCCTAAGGGTAATGACCGTATTTCATAATAAATGCCCTA
TGGGAGGCATCAGCTAATGGAAGTCAAGTTACCATTGTTCACATGGTGGAATTTACATTTTGGGAAGCGC
GGTATGCTGTGCATTTTGCTCTCATTAGCTGGTAGGGACTCGATATGGTCCTGGTCCTCGAGCAGGCGCA
CATTATGCGCCTTCCGGATTGGGGCTTTTATAAATCGATTTCGTAGTGACGTGGTTCCGGATGGACAATG
CGTAAAAGGAGGATTAAACCGCAAACCGCAGACCGCCGTGTTCTAGTTGGTCTCATGTCGTAAACTTTAT
GCAATGCACAGAAATGCATTGTGTACTTCCCCTTTTATCCATTTCTCGCCACGAAGGGAACACAATCTGT
GCAATTTTCTTCGCTTTATCTGCACACAGAGAAAAATTATTTATATATTTATTTATATAAAGATGTGTGA
TCAAATGATTTTTTATAGTATAGTTTGTATAGTTCACTTTACGCTTAAATAAATAAAATATATCTAAAAT
GAATAAGTGTACTATACTGAATACAAATTTATTTTCACTGTGGAAAAAAACACATGGAGTAAGCAGGATC
TCTGGCAGCTACAACAGCACAACATATAATAATGGGTTTCATATTCCGTCTTTCATTGCTCGTATGTAGC
TGGCCCTTGTGAACAGCAATGCAATGACATGCTATTTAATAGTTCCACTATGGGGTCCCACCCTGCGTAT
ACGTAATATGTGATTGGGGACTGCTGTTGGTGACCTCAGGAGGACGAAAAAACCGCATAACTAACCATCT
TTTTATCTATTTAATCTCCCACCACAGGCAAATCCGCCAAAGCTCCAAAGACAGCACTGGATCCTCCGAT
TCGGACAGCTCTTCGGAGGATGAATCCCTGCCTAACACCACGCCCAATTTGCCCAGCGGCAACGAACCTC
CGCCAGAAAAGGCTCAAAAGGAAACGGAGGCGGGAGCAGAGATTTCAGCTTTGGTCAACTATGTTCAGCC
CATCCATTTTAGCTCTTTTGAGAATGCGGAAAGAAAAGAATCGATGCTACGAAATGTCTTCGTTTGATGA
AAAACAGGCGACAACATTATTGAAGGAGCGACCGATTGAATTTGTGAACTACAACAAACACCAATTATCT
CGTGTCTATCCGGCGGGCACTCGCTTTGATAGCTCCAACTTTATGCCGCAGTTGTTCTGGAATGCCGGAT
GTCAGCTCGTGGCACTTAACTTCCAAACCCTCGATCTGGCCATGCAACTCAACTTGGGGATCTTTGAGTA
TAACGCTCGGTCCGGTTATCTACTTAAGCCGGAGTTTATGCGCCGATCAGATCGCAGATTGGATCCCTTC
GCGGAAAGCACTGTGGATGGCATCATAGCAGGCACAGTATCAATCACGGTTCTATCTGGTCAGTTTCTCA
CGGATAAGAGGGCGAACACTTTTGTGGAAGTTGATATGTACGGTTTGCCAGCGGACACGGTGAGAAAGAA
GTTTCGCACCAAGACAGTGCGGGATAATGGAATGAATCCCCTTTATGACGAGGAGCCGTTCGTGTTTAAG
AAGGTGGTTCTCCCCGAGTTGGCGAGCATTCGAATTGCAGCATATGAAGAGGGTGGCAAACTCATTGGTC
ACCGCGTTTTGCCCGTAATCGGTCTTTGCCCTGGTTACCGACATGTGAATCTCCGATCTGAAGTGGGTCA
ACCAATAGCCCTTGCCTCTCTTTTCCTGTGCGTGGTGGTCAAGGATTATGTACCCGATGATCTTTCCAAT
TTCGCAGAGGCGCTAGCTAACCCTATTAAATACCAAAGTGAGCTAGAGAAGCGCGATATTCAACTATCTG
TTTTGACCGACGAAGCCGAAGCGCTGGGCAGTGCGGACGAGGATCTCTCCAAGTCGT
>dme_circ_0001244_junction_seq
AGTGCGGACGAGGATCTCTCCAAGTCGTGATGCGTTGAGCTCGACTTCTGGAACGG
>dme_circ_0001245
GGCGAAAATTGCCAACTATTGTCGCGAAATATTTGGCGATATGCTGCTCGATAAGCCTCTGGACTCTCAT
CCGCTGGAGCCGAACATGGACTTGCCACCGCCGGCGATGCTGCGCCGCAAGATTATAATCAAGAACAAGA
AGAAGcatcaccaccaccatcaccatcatcaccaCAAAAAGCCAGCGCAGGTGGGCACGCCGGCAGCCAA

CAACAAACTGACTACAGCGAATTCAG
>dme_circ_0001245_junction_seq
AACAACAAACTGACTACAGCGAATTCAGGGCGAAAATTGCCAACTATTGTCGCGAA
>dme_circ_0001246
GACTCCACCTGTCGGATGGAGGTGCTTACGGACTGGTCTAGAGAGTGCTGATGCTGTATTGAGGGTGCTA
CAATAGAGATGCTATCTTCTCCCCTTCCGCCATCGGTCCGATCCACGGTAACGTCAACACTCATTGCTGC
GGCGGCCGAGATGCTCGGCCTATGCTTCGGCGAAGTCGCAAGCGATTCAACCGGACGCAGCTCCTTTTTT
TGGCCACCTACTTGGACGAAAACGACTGGAGGATCAGACAGAATCGAGCAGATCGAGTGGGCAGGGGAAG
AGGTTTATAGGTTGTGCAGTTTATGTGTCCGTCAACCGTTTTGagtgtgggagtgtgggtgggtagtgga
ttgtgggttgggtagtgtttgtggtgttggttTAACCAAATCCGAAAATCCAAAAGTACAACCGAAAGTT
ATATGATACGTTACCAAAAAGAAAAGTAGAAAGAAAAGTAGGTAAATTAAAATCGCAATTCGAAGTGGGT
AAGTGGAGCTACGCTTAACAGCTAATGCAGTTTACTCACACGACTTGGAGAGATCCTCGTCCGCACTGCC
CAGCGCTTCGGCTTCGTCGGTCAAAACAGATAGTTGAATATCGCGCTTCTCTAGCTCACTTTGG
>dme_circ_0001246_junction_seq
AATATCGCGCTTCTCTAGCTCACTTTGGGACTCCACCTGTCGGATGGAGGTGCTTA
>dme_circ_0001247
GAGCAAGAAGATCTACACCAATCGCTGCAATGCCAAGGGCTGCAAGCGAAAGGAGCTCATCCCGGTGACC
TGCTCGCAGTGTCGCTTGAACTTTTGCCTGCGTCATCGACACACCAGCGACCACGACTGCAAGCCAGTGC
CTGCATCTTCGACAACGTCCAGCCGTGGGGGATTTCAGTCCATATTCAAGACCTCCTCGGACTCACGATC
CATGGCTGCCCAGGCGGCCGAGAGAAGGCAGAACAGAAGGCCCGCGAGCAATAGCATCAGCAATACCAAT
TCCAGACCTCGACCTGTGCAGGCCACACAAGTACAAAACATACAGGGCAATATG
>dme_circ_0001247_junction_seq
ACAAGTACAAAACATACAGGGCAATATGGAGCAAGAAGATCTACACCAATCGCTGC
>dme_circ_0001248
ATTTTGAGGACGCAAAGGCAGCGGCTGAAGAGGCGAAGGAGAAGGAGAAGGACCTGCACGCAATCAGTGC
TGAGCTGCAGAAGGAGGACAGTGACGAGCCAACACTTAAGCGAAAGAGAACCCGTACTCTCAAAGCGTCA
CAAGCTGCCAAAATTGAGCCTGTACCGTCTGAGGTCAAGACTAAAGTGCAATCTGGAAAGGGCGCTCTTC
GCAGGATTCGGGGCAAGGACAAGGAGGAAAAGGTGAAGCCTCCGAAGCCGAAGTGCCGCTGCGG
>dme_circ_0001248_junction_seq
AGCCTCCGAAGCCGAAGTGCCGCTGCGGATTTTGAGGACGCAAAGGCAGCGGCTGA
>dme_circ_0001249
CTCCACATCCAAACGGTGGCATTCGGCGGTCCCTCGTGCACATCCAGGCACACTGAATCGAAGCCCTTCA
TCCGGAGGTCTCGGAATATGGAGAGCTCCCAAAACTGATTCGGTTGCGGATGGGTCCTATTGTCCGCACA
GCTGAACATACCCACCGCCTCCTCGGTGGACTTGCCCATGTTGTCCAGGCAATAGACCGGGTTGGCCACG
TTCTGAATGATGCCGGCGGCGTAGCTAGGTGGTTCCACGGGCGGGAATTTGACGAGGAAATCAGGCGCCA
CCTCCG
>dme_circ_0001249_junction_seq
TTGACGAGGAAATCAGGCGCCACCTCCGCTCCACATCCAAACGGTGGCATTCGGCG
>dme_circ_0001250
GGAACTGCCCATTTACATGTATGTGTGTGTATGTGGTAGGTAGGTATACCGAATACGATTGGAAACTAAG
GTTAACTTAAGAGTATCTAGTGGGCTAGCATAGGCAAACAGTCTGCAGAGCCTCACATTCATTAACCAAA
TAATGCACTTTTCTTCACTTTCTGCTTGGACTTTTTTTGTTTATCCGCGTAATTTACGTTATGCGTAGCT
AATTAATCAGGTATACTTTTGCATTTAGAGGTCATAAAAT
>dme_circ_0001250_junction_seq

ATACTTTTGCATTTAGAGGTCATAAAATGGAACTGCCCATTTACATGTATGTGTGT
>dme_circ_0001251
CATTGGCTCCTCGTCGACAACCGTGGACGATGAGGGATTTGGCAAGTCCATCAGTGCAGCCACTTCGCAG
GCGGCGAGCACCTCCATGAGCACGGTTAATCCCACAACCACTTTGAGCCTGAACATGCTAAACACCTTCA
TGGGAAGCCACAACGAGAACAGCAGCAGTTCTGGTTGCGGGGGCACCGTCTCCTCCCTGTCCATGGTGGC
TCTGATGAGCACCGGCGCGGCAGGCGGAGGAGGTAACACCTCCGGGCTGGAAATGGATGTGGACGCCTCC
ATGAAGTCCTCGTTCGAGCGGCTGGAGGTAAACGGATCGCACTTCTCGCGGGCCAACAACTTGGACCAGG
AGTACAGTGCCATGGTGGCATCTGTGTACGAAAAGGAGAAGGAATTAAAG
>dme_circ_0001251_junction_seq
TGTGTACGAAAAGGAGAAGGAATTAAAGCATTGGCTCCTCGTCGACAACCGTGGAC
>dme_circ_0001252
TTACAGATACCGCGCAAATGGCTAAGAAGTACCTCAATGTTCGCAGTCGACAATGAGCAGAGTTAGGCAG
CTCCACAATCACATCTGGAATAATCAGAACTTTGATAAAGTGAAATCGGTTATGGACTGGTTACTAGCAA
CTCCGCAGTTGTACAGCGCGTTCTCCTCCTTGGGTTGCTTGGAGGGCGATACCTATGTGGTCAACCCGAA
TGCATTGGGCCATTCTGGAGGAGATCAACTACAAGCTCACCTATGAGGACCAAACACTGCGCACCTTTCG
ACGGGCCATTGGATTTGGCCAGAATGTGAGGTCAGACCTGATACCGCTGCTGGAGAATGCCAAGGATGAT
GCGGTCCTGGAGTCGGTCATCCGGATACTCGTCAATCTGACGGTGCCGGTGGAGTGCCTCTTCTCCGTGG
ACGTGATGTACCGCACGGATGTGGGTCGCCACACCATCTTCGAGCTGAACAAGCTGCTGTACACCAGCAA
GGAAGCATTTACCGAGGCCAGGAGCACCAAGAGCGTGGTGGAGTACATGAAACACATACTGGAGTCGGAC
CCTAAGCTGTCGCCGCACAAATGCGATCAAATCAACAACTGTCTGCTGCTGCTGAGAAATATCCTGCACA
TTCCAGAGACGCATGCCCATTGCGTGATGCCCATGATGCAGTCGATGCCGCATGGCATCTCCATGCAGAA
CACGATCTTGTGGAATCTCTTCATCCAGAGCATCGACAAGTTACTCCTGTATCTGATGACCTGTCCGCAG
AGAGCCTTCTGGGGAGTGACCATGGTGCAACTGATTGCTTTGATCTACAAGGATCAGCATGTGAGCACTC
TGCAAAAACTACTTAGCCTCTGGTTCGAAGCCTCTCTGTCGGAGAGCTCTGAGGATAATGAGAGTAATAC
CTCGCCCCCCAAACAGGGCAGTGGCGATTCCAGCCCCATGCTGACCTCTGATCCTACCTCTGATTCCTCG
GACAATGGCAGCAATGGCCGTGGCATGGGCGGTGGCATGCGGGAAGGAACAGCGGCCACTTTGCAGGAGG
TGAGCCGCAAGGGTCAGGAGTATCAGAACGCCATGGCCAGAGTGCCAGCGGATAAGCCCGATGGCTCCGA
AGAGGCCAGCGATATGACGGGGAACGACAGCGAGCAGCCTGGATCGCCGGAGCAATCGCAGCCCGCCGGC
GAGTCCATGGACGATGGAGATTACGAGGACCAGAGACACAGGCAACTGAACGAGCATGGCGAGGAGGATG
AAGATGAGGGACGAAGTGGAGGAGGAAGAGTACCTACAATTGGGCCCAGCCTCGGAGCCCCTAAACTTAA
CACAACAACCAGCTGACAAGGTCAACAACACTACCAACCCAACGTCCAGTGCGCCACAAGGCTGCCTGGG
CAATGAGCCATTCAAGCCACCACCTCCTCTGCCAGTCAGAGCCTCCACCTCGGCACACGCTCAAATGCAG
AAGTTCAACGAATCGTCCTACGCGTCCCACGTATCTGCGGTCAAATTGGGCCAAAAGTCCCCACATGCCG
GCCAGCTCCAGCTGACCAAGGGCAAGTGTTGCCCACAGAAGCGGGAATGCCCCTCCTCGCAGTCGGAGCT
ATCGGATTGCGGTTATGGCACCCAGGTGGAAAATCAGGAATCCATTTCCACCTCCAGCAACGACGATGAT
GGGCCGCAGGGCAAGCCGCAGCACCAGAAGCCTCCGTGTAACACGAAGCCACGGAATAAACCACGGACGA
TTATGTCGCCAATGGACAAAAAGGAGCTTAGACGCAAAAAACTGGTCAAGCGCAGCAAAAGCAGCCTGCA
TCAACATGAAGGGTCTGGTACAGCACACCCCCACCGATGATGACATCTCCAATCTGCTGAAGGAATTCAC
CGTGGATTTCCTCCTCAAGGGTTACAGCTATCTGGTGGAGGAACTGCACATGCAACTGCTTTCCAATGCG
GGTATGTCGATATTGATATAGAAATTATTATGAGGACTATAATTTCGATTTTCTTAAAAACAAATTTACA
CACACTAACTAACTAATTGAGACAGAACAAGGTGAATTTTTGTCATTTATGATGCGCTCATCGCTTTTCA
TATGTTTTTCGGAGGCTTAAAACTTAACAGGCTTAAAGTAGATTAGGTTCATTATCTTTTGAAATACCTC
ACATCTTGCtattattattttaattataatattatatattatCATAAAAACTAAAGAGAACTTCTCTGAC
TTTCCTCCAGAAGGAAGGTGCCCATTGACACATCGCACTTCTTCTGGCTGGTCACCTACTTCCTGAAGTT

TGCCGCCCAACTGGAGCTGGATATGGAGCACATCGACACTATTCTCACCTACGATGTTTTGAGCTACTTG
ACCTATGAGGGTGTGTCCCTATGTGAGCAACTGGAACTGAATGCCCGACAGGAGGGCAGTGACCTGAAGC
CCTATCTAAGGCGAATGCACTTGGTGGTGACGGCCATCCGGGAGTTCCTCCAGGCCATTGATACGTACAA
CAAAGTGACTCATCTGAACGAGGACGACAAAGCCCATTTGAGGCAGCTTCAGCTGCAGATTAGCGAAATG
TCCGATCTGAGGTGCCTTTTTGTGCTTCTGCTGAGGCGTTTCAATCCCAGCATTCATTCCAAGCAGTATC
TTCAGGATCTGGTGGTTACCAATCACATCCTCCTACTCATTCTGGACAGTTCGGCCAAACTTGGTGGATG
TCAAACCATTCGCCTGTCGGAGCACATAACACAGGTTTGCCACGCTGGAGGTGATGCACTACTATGGCAT
TCTGTTGGAGGACTTCAACAACAACGGAGAGTTTGTCAATGACTGCATCTTCACCATGATGCATCACATC
GGTGGCGATCTGGGCCAGATTGGGGTTCTATTTCAACCAATTATTTGAAAACCTATTCAAGAATTTGGG
AAGCGGACTATGAACTGTGCGATGGACTGGTCTGATCTTATCGAGTATGTGATTCACAAGTTCATGAATA
CTCCTCCGAAGTCGCCACTCACCATTCCTACAACTTCCTTGACGGAAATGACCAAGGAACACAACCAGGA
GCATACCGTTTGGTAAGGATTTCAGAAGATTTCCTTGAAGCAATATATTTAATAAGGGTATAAGCAATAT
AATCTTTGATCTAAGATACAACTAACATAAGCAATAAACATAACATTTTTAACATTTGTGGACCATAGTA
AGGATTTCAGAAGATTTCCTTGAAGCAATATATTTAATAAGGGTATAAGCAATATAATCTTTGATCTAAG
ATACAACTAACATAAGCAATAAACATAACATTTTTAACATTTGTGGACCATAAGAACAAGGTGTCTAGCG
TTTTCTTTTAGACATATTTAGTCGTCCTAAACAAAACTTCATCTGTGGATGAGCTACAAGAGATTTTTAA
AATTCAATCGTTTGTAGGGATCCAAGTATTTATGGGATTCTTTAGTTGATTTACAAGGCTTCCTAACATG
ACTTTCCCTAAGAATTTGCTTACTAATCACAACTATATTAGGTTCACTTGAATTAGACCTTTGGTATATG
TTATTAACCTATAACTATATTCTTTAGCCTCTTGGTCGCAGGAGGAAATGGACACACTTTATTGGTATTA
TGTGCAGAGCAAGAAGAACAACGATATTGTGGGAAAGATAGTTAAGCTCTTCAGCAACAACGGCAACAAG
CTGAAAACCAGGATTTCTATTATCCAACAACTTTTGCAACAAGGACATTATCACCCTGTTGGAATACGAT
GACCTGATGAAGTTCGAGGATGCGGAGTATCAGAGAACTTTGCTGACAACTCCCACTTCCGCAACAACAG
AGTCTGGAATTGAGATTAAGGAGTGCGCCTACGGCAAACCCTCAGATGATGTTCAGGATCCTGCTGGACC
TGATCATTAAGGAAAACAAGGCGCAGCATTTGTTATGGCTGCAAAGGATCCTCATTGAGTGCTGCTTCGT
TAAACTGACCCTGCGGAGTGGTCTCAAGGTTCCGGAAGGCGATCACATCATGGAGCCGGTGGCCTACCAC
TGCATCTGCAAGCAGAAGTCCATTCCGGTGGTGCAGTGGAACAACGAGCAATCCACTACGATGCTGTACC
AGCCTTTTGTTCTCCTGCTCCACAAGCTGGGCATTCAGCTGCCGGCGGACGCGGGCTCGATCTTCGCCAG
AATTCCGGACTACTGGACACCGGAGACAATGTACGGACTCGCCAAAAAGCTGGGACCGCTGGACAAACGT
GAGTTAAAGTCAACCACAGAAAAAAACAACCCATTTGTCATTCCACAAAGGTGATGTATATACCGTTATC
AACAATTTTTGCTCTCTCTCTGTGTGAAATTTTGATCATGGGAATCTCGCCCGAAACCGAATCCAAATCC
GTGCTTCCATCCATTCCCATTCTCATTGTGTGTCCGTCGGTGTATCTCGATATGCTGTGTGCCTCTCTCC
TTCTCCTCCTTCTGGGCTCTTATAGTCAACCTCAAGTTCGACGCCAGTGAACTGGAGGATGCGACGGCGT
CGAGTCCGTCGCGTTACCACCACACCGGACCCCGCAACTCGCTCAGCTCGGTAAGCAGCCTGGACGTGGA
TCTCGGCGATACCGAGGAGCTGGCCCTTATACCCGAGGTGGATGCGGCCGTGGAGAAGGCACACGCCATG
GCATCCACGCCATCGCCCAGCGAGATTTTCGCGGTTCCCAAGACGAAGCACTGCAACTCGATCATCAGGT
GAGCATCTATCGGAATACAAGAAGGACTGTCCTCTCTCCTGACCTTCCAATTGCTAATTTCGCCTTATAT
TATCGCTATCCCACAGATACACACCAGATCCCACGCCTCCAGTGCCCAACTGGCTGCAGTTGGTCATGCG
CAGCAAATGCAATCATCGCACAGGTCCGTCTGGTGATCCCAGCGATTGG
>dme_circ_0001252_junction_seq
AGGTCCGTCTGGTGATCCCAGCGATTGGTTACAGATACCGCGCAAATGGCTAAGAA
>dme_circ_0001253
AAGGTGCCCATTGACACATCGCACTTCTTCTGGCTGGTCACCTACTTCCTGAAGTTTGCCGCCCAACTGG
AGCTGGATATGGAGCACATCGACACTATTCTCACCTACGATGTTTTGAGCTACTTGACCTATGAGGGTGT
GTCCCTATGTGAGCAACTGGAACTGAATGCCCGACAGGAGGGCAGTGACCTGAAGCCCTATCTAAGGCGA

ATGCACTTGGTGGTGACGGCCATCCGGGAGTTCCTCCAGGCCATTGATACGTACAACAAAGTGACTCATC
TGAACGAGGACGACAAAGCCCATTTGAGGCAGCTTCAGCTGCAGATTAGCGAAATGTCCGATCTGAGGTG
CCTTTTTGTGCTTCTGCTGAGGCGTTTCAATCCCAGCATTCATTCCAAGCAGTATCTTCAGGATCTGGTG
GTTACCAATCACATCCTCCTACTCATTCTGGACAGTTCGGCCAAACTTGGTGGATGTCAAACCATTCGCC
TGTCGGAGCACATAACACAGGTTTGCCACGCTGGAGGTGATGCACTACTATGGCATTCTGTTGGAGGACT
TCAACAACAACGGAGAGTTTGTCAATGACTGCATCTTCACCATGATGCATCACATCGGTGGCGATCTGGG
CCAGATTGGGGTTCTATTTCAACCAATTATTTTGAAAACCTATTCAAGAATTTGGGAAGCGGACTATGAA
CTGTGCGATGGACTGGTCTGATCTTATCGAGTATGTGATTCACAAGTTCATGAATACTCCTCCGAAGTCG
CCACTCACCATTCCTACAACTTCCTTGACGGAAATGACCAAGGAACACAACCAGGAGCATACCGTTTGGT
AAGGATTTCAGAAGATTTCCTTGAAGCAATATATTTAATAAGGGTATAAGCAATATAATCTTTGATCTAA
GATACAACTAACATAAGCAATAAACATAACATTTTTAACATTTGTGGACCATAGTAAGGATTTCAGAAGA
TTTCCTTGAAGCAATATATTTAATAAGGGTATAAGCAATATAATCTTTGATCTAAGATACAACTAACATA
AGCAATAAACATAACATTTTTAACATTTGTGGACCATAAGAACAAGGTGTCTAGCGTTTTCTTTTAGACA
TATTTAGTCGTCCTAAACAAAACTTCATCTGTGGATGAGCTACAAGAGATTTTTAAAATTCAATCGTTTG
TAGGGATCCAAGTATTTATGGGATTCTTTAGTTGATTTACAAGGCTTCCTAACATGACTTTCCCTAAGAA
TTTGCTTACTAATCACAACTATATTAGGTTCACTTGAATTAGACCTTTGGTATATGTTATTAACCTATAA
CTATATTCTTTAGCCTCTTGGTCGCAGGAGGAAATGGACACACTTTATTGGTATTATGTGCAGAGCAAGA
AGAACAACGATATTGTGGGAAAGATAGTTAAGCTCTTCAGCAACAACGGCAACAAGCTGAAAACCAGGAT
TTCTATTATCCAACAACTTTTGCAACAAGGACATTATCACCCTGTTGGAATACGATGACCTGATGAAGTT
CGAGGATGCGGAGTATCAGAGAACTTTGCTGACAACTCCCACTTCCGCAACAACAGAGTCTGGAATTGAG
ATTAAGGAGTGCGCCTACGGCAAACCCTCAGATGATGTTCAGGATCCTGCTGGACCTGATCATTAAGGAA
AACAAGGCGCAGCATTTGTTATGGCTGCAAAGGATCCTCATTGAGTGCTGCTTCGTTAAACTGACCCTGC
GGAGTGGTCTCAAGGTTCCGGAAGGCGATCACATCATGGAGCCGGTGGCCTACCACTGCATCTGCAAGCA
GAAGTCCATTCCGGTGGTGCAGTGGAACAACGAGCAATCCACTACGATGCTGTACCAGCCTTTTGTTCTC
CTGCTCCACAAGCTGGGCATTCAGCTGCCGGCGGACGCGGGCTCGATCTTCGCCAGAATTCCGGACTACT
GGACACCGGAGACAATGTACGGACTCGCCAAAAAGCTGGGACCGCTGGACAAACG
>dme_circ_0001253_junction_seq
CCAAAAAGCTGGGACCGCTGGACAAACGAAGGTGCCCATTGACACATCGCACTTCT
>dme_circ_0001254
AAGGTGCCCATTGACACATCGCACTTCTTCTGGCTGGTCACCTACTTCCTGAAGTTTGCCGCCCAACTGG
AGCTGGATATGGAGCACATCGACACTATTCTCACCTACGATGTTTTGAGCTACTTGACCTATGAGGGTGT
GTCCCTATGTGAGCAACTGGAACTGAATGCCCGACAGGAGGGCAGTGACCTGAAGCCCTATCTAAGGCGA
ATGCACTTGGTGGTGACGGCCATCCGGGAGTTCCTCCAGGCCATTGATACGTACAACAAAGTGACTCATC
TGAACGAGGACGACAAAGCCCATTTGAGGCAGCTTCAGCTGCAGATTAGCGAAATGTCCGATCTGAGGTG
CCTTTTTGTGCTTCTGCTGAGGCGTTTCAATCCCAGCATTCATTCCAAGCAGTATCTTCAGGATCTGGTG
GTTACCAATCACATCCTCCTACTCATTCTGGACAGTTCGGCCAAACTTGGTGGATGTCAAACCATTCGCC
TGTCGGAGCACATAACACAGGTTTGCCACGCTGGAGGTGATGCACTACTATGGCATTCTGTTGGAGGACT
TCAACAACAACGGAGAGTTTGTCAATGACTGCATCTTCACCATGATGCATCACATCGGTGGCGATCTGGG
CCAGATTGGGGTTCTATTTCAACCAATTATTTTGAAAACCTATTCAAGAATTTGGGAAGCGGACTATGAA
CTGTGCGATGGACTGGTCTGATCTTATCGAGTATGTGATTCACAAGTTCATGAATACTCCTCCGAAGTCG
CCACTCACCATTCCTACAACTTCCTTGACGGAAATGACCAAGGAACACAACCAGGAGCATACCGTTTGG
>dme_circ_0001254_junction_seq
GGAACACAACCAGGAGCATACCGTTTGGAAGGTGCCCATTGACACATCGCACTTCT
>dme_circ_0001255

TTACAGATACCGCGCAAATGGCTAAGAAGTACCTCAATGTTCGCAGTCGACAATGAGCAGAGTTAGGCAG
CTCCACAATCACATCTGGAATAATCAGAACTTTGATAAAGTGAAATCGGTTATGGACTGGTTACTAGCAA
CTCCGCAGTTGTACAGCGCGTTCTCCTCCTTGGGTTGCTTGGAGGGCGATACCTATGTGGTCAACCCGAA
TGCATTGGGCCATTCTGGAGGAGATCAACTACAAGCTCACCTATGAGGACCAAACACTGCGCACCTTTCG
ACGGGCCATTGGATTTGGCCAGAATGTGAGGTCAGACCTGATACCGCTGCTGGAGAATGCCAAGGATGAT
GCGGTCCTGGAGTCGGTCATCCGGATACTCGTCAATCTGACGGTGCCGGTGGAGTGCCTCTTCTCCGTGG
ACGTGATGTACCGCACGGATGTGGGTCGCCACACCATCTTCGAGCTGAACAAGCTGCTGTACACCAGCAA
GGAAGCATTTACCGAGGCCAGGAGCACCAAGAGCGTGGTGGAGTACATGAAACACATACTGGAGTCGGAC
CCTAAGCTGTCGCCGCACAAATGCGATCAAATCAACAACTGTCTGCTGCTGCTGAGAAATATCCTGCACA
TTCCAGAGACGCATGCCCATTGCGTGATGCCCATGATGCAGTCGATGCCGCATGGCATCTCCATGCAGAA
CACGATCTTGTGGAATCTCTTCATCCAGAGCATCGACAAGTTACTCCTGTATCTGATGACCTGTCCGCAG
AGAGCCTTCTGGGGAGTGACCATGGTGCAACTGATTGCTTTGATCTACAAGGATCAGCATGTGAGCACTC
TGCAAAAACTACTTAGCCTCTGGTTCGAAGCCTCTCTGTCGGAGAGCTCTGAGGATAATGAGAGTAATAC
CTCGCCCCCCAAACAGGGCAGTGGCGATTCCAGCCCCATGCTGACCTCTGATCCTACCTCTGATTCCTCG
GACAATGGCAGCAATGGCCGTGGCATGGGCGGTGGCATGCGGGAAGGAACAGCGGCCACTTTGCAGGAGG
TGAGCCGCAAGGGTCAGGAGTATCAGAACGCCATGGCCAGAGTGCCAGCGGATAAGCCCGATGGCTCCGA
AGAGGCCAGCGATATGACGGGGAACGACAGCGAGCAGCCTGGATCGCCGGAGCAATCGCAGCCCGCCGGC
GAGTCCATGGACGATGGAGATTACGAGGACCAGAGACACAGGCAACTGAACGAGCATGGCGAGGAGGATG
AAGATGAGGGACGAAGTGGAGGAGGAAGAGTACCTACAATTGGGCCCAGCCTCGGAGCCCCTAAACTTAA
CACAACAACCAGCTGACAAGGTCAACAACACTACCAACCCAACGTCCAGTGCGCCACAAGGCTGCCTGGG
CAATGAGCCATTCAAGCCACCACCTCCTCTGCCAGTCAGAGCCTCCACCTCGGCACACGCTCAAATGCAG
AAGTTCAACGAATCGTCCTACGCGTCCCACGTATCTGCGGTCAAATTGGGCCAAAAGTCCCCACATGCCG
GCCAGCTCCAGCTGACCAAGGGCAAGTGTTGCCCACAGAAGCGGGAATGCCCCTCCTCGCAGTCGGAGCT
ATCGGATTGCGGTTATGGCACCCAGGTGGAAAATCAGGAATCCATTTCCACCTCCAGCAACGACGATGAT
GGGCCGCAGGGCAAGCCGCAGCACCAGAAGCCTCCGTGTAACACGAAGCCACGGAATAAACCACGGACGA
TTATGTCGCCAATGGACAAAAAGGAGCTTAGACGCAAAAAACTGGTCAAGCGCAGCAAAAGCAGCCTGCA
TCAACATGAAGGGTCTGGTACAGCACACCCCCACCGATGATGACATCTCCAATCTGCTGAAGGAATTCAC
CGTGGATTTCCTCCTCAAGGGTTACAGCTATCTGGTGGAGGAACTGCACATGCAACTGCTTTCCAATGCG
G
>dme_circ_0001255_junction_seq
CTGCACATGCAACTGCTTTCCAATGCGGTTACAGATACCGCGCAAATGGCTAAGAA
>dme_circ_0001256
GTTTTTTGCGTCTAAGCTCCTTTTTGTCCATTGGCGACATAATCGTCCGTGGTTTATTCCGTGGCTTCGT
GTTACACGGAGGCTTCTGGTGCTGCGGCTTGCCCTGCGGCCCATCATCGTCGTTGCTGGAGGTGGAAATG
GATTCCTGATTTTCCACCTGGGTGCCATAACCGCAATCCGATAGCTCCGACTGCGAGGAGGGGCATTCCC
GCTTCTGTGGGCAACACTTGCCCTTGGTCAGCTGGAGCTGGCCGGCATGTGGGGACTTTTGGCCCAATTT
GACCGCAGATACGTGGGACGCGTAGGACGATTCGTTGAACTTCTGCATTTGAGCGTGTGCCGAGGTGGAG
GCTCTGACTGGCAGAGGAGGTGGTGGCTTGAATGGCTCATTGCCCAGGCAGCCTTGTGGCGCACTGGACG
TTGGGTTGGTAGTGTTGTTGACCTTGTCAGCTGGTTGTTGTGTTAAGTTTAGGGGCTCCGAGGCTGGGCC
CAATTGTAGGTACTCTTCCTCCTCCACTTCGTCCTGGAAGAGTACGATATTTTGTTATATATTTCAATGG
CACTGGTCATAGAAACTCACCTCATCTTCATCCTCCTCGCCATGCTCGTTCAGTTGCCTGTGTCTCTGGT
CCTCGTAATCTCCATCGTCCATGGACTCGCCGGCGGGCTGCGATTGCTCCGGCGATCCAGGCTGCTCGCT
GTCGTTCCCCGTCATATCGCTGGCCTCTTCGGAGCCATCGGGCTTATCCGCTGGCACTCTGGCCATGGCG
TTCTGATACTCCTGACCCTTGCGGCTCACCTCCTGCAAAGTGGCCGCTGTTCCTTCCCGCATGCCACCGC

CCATGCCACGGCCATTGCTGCCATTGTCCGAGGAATCAGAGGTAGGATCAGAGGTCAGCATGGGGCTGGA
ATCGCCACTGCCCTGTTTGGGGGGCGAGGTATTACTCTCATTATCCTCAGAGCTCTCCGACAGAGAGGCT
TCGAACCAGAGGCTAAGTAGTTTTTGCAGAGTGCTCACATGCTGATCCTTGTAGATCAAAGCAATCAGTT
GCACCATGGTCACTCCCCAGAAGGCTCTCTGCGGACAGGTCATCAGATACAGGAGTAACTTGTCGATGCT
CTGGATGAAGAGATTCCACAAGATCGTGTTCTGCATGGAGATGCCATGCGGCATCGACTGCATCATGGGC
ATCACGCAATGGGCATGCGTCTCTGGAATGTGCAGGATATTTCTCAGCAGCAGCAGACAGTTGTTGATTT
GATCGCATTTGTGCGGCGACAGCTTAGGGTCCGACTCCAGTATGT
>dme_circ_0001256_junction_seq
GACAGCTTAGGGTCCGACTCCAGTATGTGTTTTTTGCGTCTAAGCTCCTTTTTGTC
>dme_circ_0001257
GGTATTACTCTCATTATCCTCAGAGCTCTCCGACAGAGAGGCTTCGAACCAGAGGCTAAGTAGTTTTTGC
AGAGTGCTCACATGCTGATCCTTGTAGATCAAAGCAATCAGTTGCACCATGGTCACTCCCCAGAAGGCTC
TCTGCGGACAGGTCATCAGATACAGGAGTAACTTGTCGATGCTCTGGATGAAGAGATTCCACAAGATCGT
GTTCTGCATGGAGATGCCATGCGGCATCGACTGCATCATGGGCATCACGCAATGGGCATGCGTCTCTGGA
ATGTGCAGGATATTTCTCAGCAGCAGCAGACAGTTGTTGATTTGATCGCATTTGTGCGGCGACAGCTTAG
GGTCCGACTCCAGTATGT
>dme_circ_0001257_junction_seq
GACAGCTTAGGGTCCGACTCCAGTATGTGGTATTACTCTCATTATCCTCAGAGCTC
>dme_circ_0001258
GAGGCTAAGTAGTTTTTGCAGAGTGCTCACATGCTGATCCTTGTAGATCAAAGCAATCAGTTGCACCATG
GTCACTCCCCAGAAGGCTCTCTGCGGACAGGTCATCAGATACAGGAGTAACTTGTCGATGCTCTGGATGA
AGAGATTCCACAAGATCGTGTTCTGCATGGAGATGCCATGCGGCATCGACTGCATCATGGGCATCACGCA
ATGGGCATGCGTCTCTGGAATGTGCAGGATATTTCTCAGCAGCAGCAGACAGTTGTTGATTTGATCGCAT
TTGTGCGGCGACAGCTTAGGGTCCGACTCCA
>dme_circ_0001258_junction_seq
TGCGGCGACAGCTTAGGGTCCGACTCCAGAGGCTAAGTAGTTTTTGCAGAGTGCTC
>dme_circ_0001259
CAACTCCGCAGTTGTACAGCGCGTTCTCCTCCTTGGGTTGCTTGGAGGGCGATACCTATGTGGTCAACCC
GAATGCATTGGGTGAGTAATCAATATAGGACTACTACATTAATTAGGTATTTAACTTATTTTCAATCCCC
AGCCATTCTGGAGGAGATCAACTACAAGCTCACCTATGAGGACCAAACACTGCGCACCTTTCGACGGGCC
ATTGGATTTGGCCAGAATGTGAGGTCAGACCTGATACCGCTGCTGGAGAATGCCAAGGATGATGCGGTCC
TGGAGTCGG
>dme_circ_0001259_junction_seq
GCCAAGGATGATGCGGTCCTGGAGTCGGCAACTCCGCAGTTGTACAGCGCGTTCTC
>dme_circ_0001260
TTTTCCTGAGTGAACTCAATTTGAAAACGCAGCAAACCGATGAGAATTACCGAAAGATGTTGTTCAAGGA
GGCTCTAAGGAGTGGCTTCTACGAACTGCAGTTGGCCAGGGACAAGTACAGGGAGCTGTGTGGCGCAAAT
GGAATGCATGAAGATCTGGTTCTGGAGTTCATTCGCAGACAAGCTCTTTTGGTATCCCCTATTTGCCCCC
ATATGGCGGAGCATGTCTGGGGACTGCTGGGTAACAAGGAGTCCATTGTTCATGCTCGGTGGCCAGAAGT
GGGAGCCATCAACGAAGTCGATATCCTGTGCTCGGAGTACCTCATGGAAGCCGCTCACTCG
>dme_circ_0001260_junction_seq
GGAGTACCTCATGGAAGCCGCTCACTCGTTTTCCTGAGTGAACTCAATTTGAAAAC
>dme_circ_0001261
GCCGTCCTCCTCGTCGCTCTCCTGGTTTTGTGCTTGCTTCCGCGGCTCCTTGGCAGGCGGATCGTCGCGG

GACTCGCCGTGGCTGGATCCTGCTCCTCCGGAGAACGAATCTGCATTCTTGGCCGCTGGCTCAGCGTAAA
AATTGCCGTCGTCGAATTCGGCAAAATCATCACTGTCG
>dme_circ_0001261_junction_seq
GTCGAATTCGGCAAAATCATCACTGTCGGCCGTCCTCCTCGTCGCTCTCCTGGTTT
>dme_circ_0001262
GTAGTATGTTCGTAAAATATCCGTTGGACAAGGCAAACATTACCATCATCGCTATGAAAGTATAGTCGTG
TTTTACCAAAACTGGCAGAAAGCTGTGTTCGCTGGAGTTTGAACATAGAAATAACGGAACAAACGCCATG
CGCACTACAATAAATAGTAGCGAAGTGTTCTGGTTTAATGGTCGCTCCATCCAGCCAGCAAACAGACGAC
CAAAATAGTCACCGCAGTTAAATATCAG
>dme_circ_0001262_junction_seq
CAAAATAGTCACCGCAGTTAAATATCAGGTAGTATGTTCGTAAAATATCCGTTGGA
>dme_circ_0001263
GGTAGAACACGAGGTAGGTGAAGAGTCGTCCACTCTCGGGTTCGTTGGACACCAAAACTACCTCGTCCTG
CTGGCGTTCCATGAGACATTGCTCATCCGCATGGTGGTCCTGCGGACGAAAAGCGGGAACGAAGCTGCCC
TCCGTGCCCAGAATCTCGTCATCCTCGTCCAGGGCGTCCACCAGTCGTCGTGTGTCACTGGTGCCTGGCA
TTGCCACTGAATTGCACTACTGTTTTGTACGCAGCCCTTG
>dme_circ_0001263_junction_seq
TGCACTACTGTTTTGTACGCAGCCCTTGGGTAGAACACGAGGTAGGTGAAGAGTCG
>dme_circ_0001264
GTTTGTTGTATCCACAACGCTTTTCACGGCGGGCTTAATGGGCGGTTCCAGTGTGCAGTTCTGCAGGCCC
CACCAATAGAAGCCATCGAACCATCTGTGCGGGAAATAATAATCGAATAAGTATCTGCTTCACATACTAT
TTGAAATGGATCTTACTTGTGCTTTTGGATCTCGCTGATTCCCCCACGCTGGTAGCCCAAACGCTCGGCT
GGATTGTCGCGACAGAGCTTCTTGATCAGGTTGCTGGCATTGCGGGTGATATTCCTTGGGAATTCGATGG
CGTCGATGCCCTTAAGTATAAT
>dme_circ_0001264_junction_seq
CGATGGCGTCGATGCCCTTAAGTATAATGTTTGTTGTATCCACAACGCTTTTCACG
>dme_circ_0001265
TGTGCCCATCTTCAAAGACCTGGCGGAAGACACGCTCATCAAAATCTCCGATGTTTTGGAGGAGACGCAC
TACCAGCGTGGCGACTACATAGTGCGCCAAGGCGCCCGAGGCGATACCTTCTTCATCATATCCAAGGGAA
AAGTGCGAGTGACGATCAAGCAGCAGGACACGCAGGAGGAGAAGTTCATTCGCATGCTGGGCAAAGGGGA
TTTCTTTGGAGAGAAGGCTCTCCAGGGGCGATGATCTGCGCACGGCGAATATTATTTGCGAGTCCGCCGA
TGGCGTCAGTTGTCTGGTCATCGATCGCGAGACCTTCAATCAGCTAATTTCCAATCTAGACGAGATCAAG
CATCGCTACGACGACGAGGGCGCCATGGAACGCAGAAAG
>dme_circ_0001265_junction_seq
CGACGAGGGCGCCATGGAACGCAGAAAGTGTGCCCATCTTCAAAGACCTGGCGGAA
>dme_circ_0001266
GCTGCCATATTGGATAATGATTTCATGAAGAATCTGGATCTGACGCAGATCCGCGAGATCGTTGACTGCA
TGTATCCGGTTAAGTATCCAGCCAAGAATCTGATCATCAAGGAGGGAGATGTCGGAAGCATCGTTTATGT
CATGGAAGG
>dme_circ_0001266_junction_seq
TCGGAAGCATCGTTTATGTCATGGAAGGGCTGCCATATTGGATAATGATTTCATGA
>dme_circ_0001267
GGCTCCCAACGCCTGGCTGATTGAGTTTTCCGTGGCGGCTGTTGATCAGGAAAGCCGCCTGGATGCGTTT
CTGCTTTGATCGGCTGTGCTTCGCAACCAAGCGGCCAGCCCAAAACTCCAACAGCAATGCACCACACAGC

AGCACCACAGTAGATGCACCACCACGTCCAGCGGATGTAGATGTAGCCACTGTACCAGTAGCAACACCAG
CACCACCACCACAACAGCCAGTTAGTAATCTGTTCTATGCCGACTACCAGAAGCTGCAGCCGGCGATAAT
CGATCGGGACTGGGAGCGGGACAGAGATACAGATACAGATACCAGGAGCGAGGCCAAGCCACCGGACATT
GTGGAGCACATAGAACCCGTAGAGGAGCAGAGACAGATACACACACAGATACAGTCGCCGGCAGAGATAC
AGATACAGATACCCCCGACGCCCCCTGCCCCATCGATACAGATACAGATACAGCAGCGCTATCGCCGGCA
CAGCAGTGCCGAAGATCGCAATCTGAACACGAGGCGAAACGATTCGAATATTACGGAGGCGTTACGAAAA
GCAGCCAGTATGCAGCAGGAGCCGAACGCCAACTATCAGTTCCCAACCGACTTGGGTCTGGTGAGCATCG
TCAACAATAATAATAACACAAACACCCATCCGAGTGGATCCAATAGCGGCAGCAACAACAACAGCAATAT
CAACAATAATCTGGTGGGTGGCATTGTGACCTTGCCGGCGGCTGGTGGTCTCATAGGTCTGGAGCATACG
GCGTCGGGTCTGCGACTGATACCAGCACCGCCTACCCACTCGGATGTGCTGACCCACACGCTGATCTATG
GCACACCGCCCTCGGGCGCCCAGCAATTGAACCAGGATCCCCGAAGCCTTCTGCACCAACAGGAGCTGCA
GCTACAGCAGCGCTATCAGCAATTGCAGCAGCTTCAGGCGCAGACGCAGGGCCTGTACACCAGCCAAGGA
AGTCCGGTGCTGTATCACCAACCCAGTCCGGGATCGAGTCAACCGGTGGCCATACCCGGAGCCACTTGCC
ATTCGCCCACGCAACTGCAGCCGCCCAACACGCTGAACCTGCAGCAGCAGATGCAGAGTCTGCGGATCTC
GGGATGCACGCCCAGCGGTACGGGTGGCTCGGCCACGCCCTCGCCGGTGGGCCTGGTGGATCCGAATTTC
ATAGTCAGCAACTATGTGGCCGCCTCGCCGCAGGAGGAGCGCTTCATTCAGATCATTCAGGCCAAGGAGC
TCAAGATACAGGAAATGCAAAGGGCCCTCCAGTTCAAGGACAACGAAATAGCCGAGCTAAAGTCGCACTT
GGACAAATTCCAGAGTGTCTTTCCCTTCAGCCGTGGCAGTGCGGCCGGTTGTGCGGGCACGGGCGGAGCG
TCGGGATCTGGAGCTGGCGGAAGTGGTGGCAGTGGTCCCGGCACCGCCACAGGTGCCACACGCAAGTCGG
GTCAGAATTTCCAGAGGCAGAGGGCATTGGGTATCTCCGCCGAGCCACAGAGCGAGTCCTCGCTGCTCCT
CGAGCACGTCAGCTTTCCCAAATACGACAAGGATGAGCGGGTGAGTGCTTTGACTCATTTATCCATTTAT
GTTTTTATTTTATGTAGCTCATCATATTATTCAAGCTCACATTGTACATATTGATATTTGTTTACATTAT
GATGCTCAACATGGCGCGTGATCAGTGATACTATAAATGTAATCATTACTTTCACTTTGTGTGCTTAGTA
ATTCTCCGGCTTTTATCGTACATTATTATAAAGGCTAAAGTAAATAAGTTTGCTTTACTCTAAGAGAGAT
TTTTGAGTGTTTGGCTTGAGGAAATCGTTGGCTTGCCTTGCACAGTAGCATCAGGTTAAACCTAATCGCG
CCGCCCAGATGAGCCCATCATTATAGCGATCCCAATACGGATGTACAAAGTTTAGACTAGATGCTATATA
GCCGAAACGATAACGATCGGAATCGGCGTCATTGCGCCGATCTTAATCATATATTGGGAACAGCGCCCTG
TCGCCTGTCGTCGGTTGCTTGTGTCGCCTGCCACAAAATGCGTTTATTTAAAATTGAGTTGGGGTGACAG
GCGCGGTTGCACCAGCCACCCACCACCCCTTGCCAATCCGCCCAATGGCCTTGGGCAGTGTCAGTACTCG
GATATAAAAGTCCGTATGAAAATATAAGCCACATATATGATTTGCAACCCACAAAGAGGCTGCCAATTGG
CAGCCAACTCACGCTACGTTGCGTCACATTCGTTTCGTTCTATTGGGAAttttttaagacacatttttttc
gtgtgtttttttttCGATCATGCAAAATTCGTTGCAGTCACTCCGAAATACATACCTATATACATATATA
CCCATATAATTAACAGAGTATAAAATTAAATCTGCGAAAATGCGTGGGCGGATCTGTCGGCTCCGCGATT
TTCACACTTTGGCTGGCCACTGGAAGTCGTTGCTCTTGGCCAGATTTCCCCACTTTGTCTGCTGCAAAAC
ATAAGAAAACATAAATATTTATTCTTTGGCTATGTGGCGCTGTTTCCTGAATGCCTTTCAATAATACACG
GGGGCATGTGTGATCACAGATCATTTAAATGGCGACACGCGTTGTCCAAAAACCGCTGATCCAAATTTCT
GACTAATTTTTATGAGCTATAACTTAATTTTATAGTGTTTTGTGTGCTATCAGAGATATTTAATTTTTAT
ACGCAAAAAATTCCATTTAAAATATTTTCCACGATTTTCCCATTGTTGCGGATAAGCAGAAATAAATATA
TTTGAATTTTCATTGCGTTTGGCATAAACAAACAAATGTATATTTCCTATTTTAAATGGGtttttttttt
AAGAATGGAAATCTTTGTGTTAAGAATAGGCGAGCATTTGGAGGTTTTCTAAAAATAACCATGTCTTCTT
TTGCGAtttttttttAAACAAACAATTAATTACAGTTAGTTCAAGGCTATGCCATAAAATAGGAATATTA
AAAAAATAAAATAATGTGAATAAGATACTTGCTATGATCATAGCGCTACTATTGCTGTAATCTCAAAGAA
CCTGAATGACAAAATGATATGCGATATACATTTATTTCTTATATTTATCTTCAATTTTAACATGGAGCTG
GTTTAGATTAATCTGAATCGGACGCTAAGTAATGCGGCAAATTTGTTTAAATCGGCCATTTTTATGACCC

CAAGCTGTTGACACAATCACAAAAAATCACCGGCAAATTCGCGAAATCCAATTGCATTCAAACATTTTAC
TGGCCAAAATGATTGCGAGGGGGGCGAGAAGGGGTCCCATAGACTTTGCATTTCGGAGCCGGCCAATTGA
GGTCCATTTTATCTCTGGCCAAAATCTGTGCCGACGGCGACGCTTGTGTGTGCAACAGGACATTTTTTGA
CACTTGCATGGCATAAATCAAAAGCGCCGTAGGCCTCCACCTCGGCTTTACAGatatatatatatatatc
gtgtatatatCTGTGCGATCGGGATTGAGACTGAGGTTGGTCCGAGTACTTTTGTGGCCATTTACACCAC
GCAACTGTCGCTTGTTGGCTTTCAACGGGGCCATCTAATAAATAATGCCAGAAAATTGTGCGAAATCGCC
TTCACGATGGAGTTTCCAGGAGGGGTGGCGTCATGTGGGCAGTGGCGTTCCTGGCAGGGGGTATTTTGTC
AGAACTTGCTACATGTTGCACATACATGTGTCCACGGTCAAGATGGCAAGCAAACAGTTTGTGGGTCATG
CAATTATAATGCTAATTAGAGCAGTGCTAGATTGAGAGAATAATAAAGTGCTATTGAAGTATTCAGATGA
TCGTGTACATATGAAATGAGCTATTTTGACTATGCTTATTAATTAATATAGCTAATATAATATATAATTG
CAGTTTTAGTAGCTGTTGGCAAGCAATAAATCAAAAGAAATTACGAAATTTGGACACTATATTTTTTAGA
TAAGGAAAACCCAAACTGAAGTCAGTCATTATGCACTTAGCAGTTAGCTACTAACTATTATTTAATTTGT
ATAACAACATAAAATTGGCGTGCAGAGATTTTCTTTTCGCAGCCGAATCCCGATAAGTCTCCACCCCCTT
GACAAACATGCCTTGATTGCCATCTCACAGATAGTTAACCGTCTCACACATGGATATACACATGTGTACA
TAGATATAGATTGAGCCAAAAATCAAGCCCCACCCAAAATAGCTCACTTTGCAGTTGGTTTGTCGCTTCC
ATTGGCGCTGACTTCTATGCGCAATTTCCATTTTCAATTCCCCCGCCGAGACGCCAGTTGGCTTTTATTG
TCAACTGCTCTTAGCCGCTAAGTATTTGCACTTTCAATTCGGGTTTGCCAGTCAGCCGCCAACTGAACGC
ACCCGCATCCGAAGGAGATCTCTCTGGGGACAGACTCCGTGTATTTCTGAGGTGCAGCCAATTTCATTCC
ATGCCTTGGCACACATTTCAAATTCAATTTACGCACGAATATAGCCATATGAATGGTTATAGCTTCCGAC
AACTGGAACGCTCGAGCGATCCGGCCCGTAGGTCTAATTGAAGCTATTAATTGTTTTGGCTCGTACACGC
AGtggcaccactggcaccacttgcatcacCGCCATTGTGCCACGCATGTGCGTGGTGCTGCAGCCAAAGA
AAATGTTAATTTAGGCATATAAAAAGTGCTCCGCTGGCAAAACCCCACGGGGCTGACACCATCGAGCGAC
CCACCGCCTCGGGCGACTCGTCTACACTCGAAAAATATATTATAAAAAAGAATTTAAAGCTAATCTTAAG
TTACCAAATCAACAGTCATTATACGATTTTTGCCAAAGATATTTGCTCAAGATTGGGATTGTGTAACTAT
TGGTATTGTTTTATGAATGTCCTAAAACAAGGATATTCTTATACTGTGATATGTCTCAATTTCTCTGAGT
GTATGCGCCTCCTTCTTCTTTGGTTTCAACTCCATTATTAAAACTAGTTTCGATTTGTGGGGCGCTGAAA
TCTAATACTTCACTATGCCAGAAAACTATCCATGTTAGATTGTCGCTTCCCAGACTCACTGGTCGTGACT
AGGGTATCGATTAGACATGATTCGGtttttttttcgcattttattatttttcattaaattttaattgTGAG
TGTTATGCGGCGTAGCCGAAAATACCACAAAGATACATGACTACCCGCCAAACGGGCGCCACAGGTGTTG
GAGTCTGGGTGTAGCTGTCGGCTTTGGCCAAATGTCCCCACTCCATCGAACCGAACCAAGCCAACTCATC
ACCACATTTTGGGGCAATCAGGTAGCTGGTGGTTTCTCCCAACTCAGACTGCTGCCAAAAACGAGGCTTA
CAAATTGGCCCCCAACCCCATTGACGACAGCCTGGGCAACGCCCTCGTGATTAACTTGATCATTGATCAG
AGATCGGTGGGACCAATTATGAAAAATGAATTTTAAACCATAAAACGTTTTTTAAGCCAGAAAACATAAT
TAACACATTTAACTAACATTAATCCCTTTCATCGTAGTTTCTCCCTAGTATTTTAAGTATTTTAAATCAT
ACAAAATGTTGGCCACACTGGCTGGCAGTGCTCTGGTTGGCTTTAATTAATCACCCAGGTCAGGTATCAG
CATTGGCTTCCAATTTTATCCGATCCACTGCTTCTATTCCCATTACTGTTTCTCTGGCCCGGGCGAGTTG
TCAAGTTCGCTGACAGATAAAGTCATTTGACATTTACAGGCATTGCCGACATGGCATGGTACGGATCCCC
ACTCCCCAGCCGAAGCATACAATTTTCAAATTTGTATTAATTTCATATCGCTCGATTGCGCGCATGCCAA
CATCCATCGACATTGGGCTCCAAAAACAACCGAAAAAATTGAGTTAAAATGCCGCCAAATTTTTGGTGGC
ACAACTTGTTGTGCGGCTTGTAAGCCAAACTGGCCAACCAGATAAAACGATTTATATGTGTCAGTCGGAA
AATTAATTGGACTCTTGCCTTTGCTCATTTCATTGGAAGTCTCTTGCCATTTTCAATATTCACATCTAAT
TGCATTTATCTATCGTTTTGCAGCCTCCCGTGAACTTATCAAGGCTGCCATATTGGATAATGATTTCATG
AAGAATCTGGATCTGACGCAGATCCGCGAGATCGTTGACTGCATGTATCCGGTTAAGTATCCAGCCAAGA
ATCTGATCATCAAGGAGGGAGATGTCGGAAGCATCGTTTATGTCATGGAAGG

>dme_circ_0001267_junction_seq

TCGGAAGCATCGTTTATGTCATGGAAGGGGCTCCCAACGCCTGGCTGATTGAGTTT

>dme_circ_0001268

GGCTCCCAACGCCTGGCTGATTGAGTTTTCCGTGGCGGCTGTTGATCAGGAAAGCCGCCTGGATGCGTTT
CTGCTTTGATCGGCTGTGCTTCGCAACCAAGCGGCCAGCCCAAAACTCCAACAGCAATGCACCACACAGC
AGCACCACAGTAGATGCACCACCACGTCCAGCGGATGTAGATGTAGCCACTGTACCAGTAGCAACACCAG
CACCACCACCACAACAGCCAGTTAGTAATCTGTTCTATGCCGACTACCAGAAGCTGCAGCCGGCGATAAT
CGATCGGGACTGGGAGCGGGACAGAGATACAGATACAGATACCAGGAGCGAGGCCAAGCCACCGGACATT
GTGGAGCACATAGAACCCGTAGAGGAGCAGAGACAGATACACACACAGATACAGTCGCCGGCAGAGATAC
AGATACAGATACCCCCGACGCCCCCTGCCCCATCGATACAGATACAGATACAGCAGCGCTATCGCCGGCA
CAGCAGTGCCGAAGATCGCAATCTGAACACGAGGCGAAACGATTCGAATATTACGGAGGCGTTACGAAAA
GCAGCCAGTATGCAGCAGGAGCCGAACGCCAACTATCAGTTCCCAACCGACTTGGGTCTGGTGAGCATCG
TCAACAATAATAATAACACAAACACCCATCCGAGTGGATCCAATAGCGGCAGCAACAACAACAGCAATAT
CAACAATAATCTGGTGGGTGGCATTGTGACCTTGCCGGCGGCTGGTGGTCTCATAGGTCTGGAGCATACG
GCGTCGGGTCTGCGACTGATACCAGCACCGCCTACCCACTCGGATGTGCTGACCCACACGCTGATCTATG
GCACACCGCCCTCGGGCGCCCAGCAATTGAACCAGGATCCCCGAAGCCTTCTGCACCAACAGGAGCTGCA
GCTACAGCAGCGCTATCAGCAATTGCAGCAGCTTCAGGCGCAGACGCAGGGCCTGTACACCAGCCAAGGA
AGTCCGGTGCTGTATCACCAACCCAGTCCGGGATCGAGTCAACCGGTGGCCATACCCGGAGCCACTTGCC
ATTCGCCCACGCAACTGCAGCCGCCCAACACGCTGAACCTGCAGCAGCAGATGCAGAGTCTGCGGATCTC
GGGATGCACGCCCAGCGGTACGGGTGGCTCGGCCACGCCCTCGCCGGTGGGCCTGGTGGATCCGAATTTC
ATAGTCAGCAACTATGTGGCCGCCTCGCCGCAGGAGGAGCGCTTCATTCAGATCATTCAGGCCAAGGAGC
TCAAGATACAGGAAATGCAAAGGGCCCTCCAGTTCAAGGACAACGAAATAGCCGAGCTAAAGTCGCACTT
GGACAAATTCCAGAGTGTCTTTCCCTTCAGCCGTGGCAGTGCGGCCGGTTGTGCGGGCACGGGCGGAGCG
TCGGGATCTGGAGCTGGCGGAAGTGGTGGCAGTGGTCCCGGCACCGCCACAGGTGCCACACGCAAGTCGG
GTCAGAATTTCCAGAGGCAGAGGGCATTGGGTATCTCCGCCGAGCCACAGAGCGAGTCCTCGCTGCTCCT
CGAGCACGTCAGCTTTCCCAAATACGACAAGGATGAGCGG

>dme_circ_0001268_junction_seq

CTTTCCCAAATACGACAAGGATGAGCGGGGCTCCCAACGCCTGGCTGATTGAGTTT

>dme_circ_0001269

GTTTCCACCCAGAGCTCGCAGTTGGAAAAGACGCACGCCGGCAGTTCCTTCGAAAGCGCCAGTTCCCTTT
ACTCCATGAGGGAGGATCTCCTCCAGCACGACGAGAAGGAGCGGGATAAGCAGACCACTCTTACCAAGGC
TCAACTAAAATCGCCCATTGGCTCAGTGGCTGAGCTGACCAGGAAATCGCCATCGCACTCCATCAGCAGT
ACCACCTCTTCAGGCAGCTGTCCTGTCTTGGGGGCGGCCATCAAATCTCCTGCCAAGGAAAGTCTGCCCC
AAACAGTGGCCAGTTCGGTAACTTCACAGATGAAAGTCAGCTCCGTAGAATGCATACCGCCTGGAGTTAA
GCCCAAGCCGGATTCTATATCGGAGGATGAGCGCAGCGAAGTGCGGTACTCCTCGTCCGGTTATTACGAG
AGTCCCCACGAGGAGGATGACGAAGAGCAGGGCACCAGGAGCAAGGCGCGCCGCCTGCGACAGGAAGACG
AAAGGAAAAGGCGCAAAACAAGCATGAAGCTGGACATTGAGAAGGAGAACATGCGTGCCCTAACCAGTCC
CATTAAAAAGCCCACGGGATCCAGCAAGATCACATCGCCAGAGCAATCGATTTCTGGAACCATGGACAAT
GGCAGCAGTCCCAGCAAAATGAAACGCTTCCGTCCCAAGATACGCAGGCAGCTGAGAAAAAGTTCACGGG
AAGATGTCCTGGCGGCGGCAGCGGTACGCAGGAGTCGTGCCACGCCCACTATTTTCGGCCTGAGCAGTGG
CAGTGGAGACACAGAGTTGCTGCTAGATGCCAGCATGTCAACTGGCGCCGTGGCAGGAACCACCACTTCT
GCCGTGACATCGGTATCCGCACCTTCATCCGCATCCAAGTTACCAACATCGGAATCCTCAGTGACCACAC
AACCCGAGGCAGTAGTGGCTCCATTGCCGGCTAAGAAACCACACAGTTGCGCCACGCCCACATCCCTGCT
GTCGCCCAAGCTGCCCACAACTAGCGGCACCCAGTCGAAATCCACATCGGACACCTTTCAGCTCAAGGCC

AAGTCAATTGAATCCTTGCGCTCTGTGTCGCCTGGTTCCGATTCCGTGTTCTACAGCGAAGCCGATGGCA
ATGCGGCCAGTGGCGAGCAGAGTCACTGTCACCATTGCGGCAAGGAGATGGAGGGCAAGCAGCAGAGCAA
CACCATCAGCGAACTGGCTGGTGACTCGGTCGAGTCGATACCCTACATCGAGCAGGACATCGTCAAGCCT
CCGTCGGACTTTGCCGACTCGCCGGTGACCACCAAGACCACCCAGCGGTTGTACAAAAAGATGGACAAGC
GGTTCCGATCCGAGGAGCGATACCACGGCGAAAGGGGCAGGCACTACAAAACCAGGCAGGAGAATATTAG
GGCAAAGGGTGAGTAAAAATAGGATTAACAATCGATAAGGAAATGGGAGGTGTAAATTACATTTTGCAAA
ACTAATGGCATTCGACACAGTTGACTGGGAAAATGTTCCTACTCAGGTCTTATAACCGCATTTCAATGGA
AAAGCTTAGCTATAGCCCATTCCAAGAATGAAAATCTATCTTTTTATCCTCTCACTCGCATTGCGTCTTC
TGAATGTCTTGGCCCTTAAGGATCGTAAGCATTGGGTGTCTATAACGTGTTCTTAGCTATAATTATTATT
TTTTAGCCATTTGCGGTCTGAAGCCTAGGGTTAATAGATGCATATCGGCAAAAGAATCTCTAGTATTGTA
TGGTTATAGTGAATCACGAAATATTTGTTACAAAATCAAAAATCCCTGTCAAACCTTAGTCAGAAGATTT
GCAACAAAAGAGATCTGCCAGAAGTTGTGCCAGGAGGAGCTGGCAGAAGTATGAACATATTTTGTTAGTA
ACATAATCACATGAACTGTAATAAAATAACCTTCAAGAGTAAAGGCAACAATATTCTTTCTCTACAGATC
GTTCCAGGGAGCGCAGAGCTTCAACGGAGTCCGAGAAGGGCTTCCATGCCAAGTATCAAGTGATCCTGCA
TCGTCTCGTCCAGCGACGCTGCACCCTGGAGATGTACCACCGCCAGAAGCACAACAGCTTCC
>dme_circ_0001269_junction_seq
TACCACCGCCAGAAGCACAACAGCTTCCGTTTCCACCCAGAGCTCGCAGTTGGAAA
>dme_circ_0001270
GTATACCTGAAGTCTTCCCTCATTGGCACCCGCTCCTCCGACCAAGCGAACTGGAACTCGAGGTACACCA
TCCGCGGGTATTACTTCCGTTTCCATATCTATGGCCTCAGTTTCGCTATCCAAATCGATGGTTTCCGTAT
CATTTGATTCGCCCATAATTG
>dme_circ_0001270_junction_seq
TCCGTATCATTTGATTCGCCCATAATTGGTATACCTGAAGTCTTCCCTCATTGGCA
>dme_circ_0001271
GGAGTAACCCAATTGGTGGCAAACCAGAGCTGCGTTCAGGACATTCCAGCCATAATCACATACAGTGCCC
CATCGTCCCTTTAGGTATACCTGAAGTCTTCCCTCATTGGCACCCGCTCCTCCGACCAAGCGAACTGGAA
CTCGAGGTACACCATCCGCGGG
>dme_circ_0001271_junction_seq
CTGGAACTCGAGGTACACCATCCGCGGGGGAGTAACCCAATTGGTGGCAAACCAGA
>dme_circ_0001272
GTATGTTGGCCGAAAGAGCGGATCTACAGTATGTAACTGTGGAAAAGGCCGGACTTTTCGATTATACCAC
CAATGCATTTAAGCCCGCTGTTCAGATGGATCATGCGCGTCACAATCTGGAAAACGTAAGAATCGTGAAT
AATCTACAAGACGGTTTGG
>dme_circ_0001272_junction_seq
ATCGTGAATAATCTACAAGACGGTTTGGGTATGTTGGCCGAAAGAGCGGATCTACA
>dme_circ_0001273
GATTTCAATTCTTTCGATACTGACTACGATCCTTATCTCTTGCCCCGTGAAATAAGCAACATCGACTTGG
GAACCTTCGAACACAAGTACATAAGAACTGAAGAGCTATTGGGACAAAATATTAATCGCAAAATAGTGGT
TCAATGTCCGGCAGGCTAT
>dme_circ_0001273_junction_seq
AATAGTGGTTCAATGTCCGGCAGGCTATGATTTCAATTCTTTCGATACTGACTACG
>dme_circ_0001274
GATTTCAATTCTTTCGATACTGACTACGATCCTTATCTCTTGCCCCGTGAAATAAGCAACATCGACTTGG
GAACCTTCGAACACAAGTACATAAGAACTGAAGAGCTATTGGGACAAAATATTAATCGCAAAATAGTGGT

TCAATGTCCGGCAGGCTATGTTATTGGAATACAGCTCTTGAATCCCATACACAATCTGTCCACAGAGAGC
ATAAACATCCTGAATGCCAGGACAGAGAATATTAGAAGTGATCTGTGGCAAGTTAAGCGAGATTTAAACG
TATTCCCA
>dme_circ_0001274_junction_seq
AGTTAAGCGAGATTTAAACGTATTCCCAGATTTCAATTCTTTCGATACTGACTACG
>dme_circ_0001275
GGTGGTCTTCGGATTAGAGCTGATTCTCGAGGTTCAGCCACCTCACTTCGAGGCTTTGTTCATCACAATC
TCTTCATGAGGAATCGCAATCGTCCAGCTCTGTACGTCGAAGGCAGACAATCCTCGCCGTATCAAGAAGT
GGAACTTTATCGCAACTACTTTGCCCAGAATATGGCCGGTTATGAGGATGTAATAAGATTGTGCCAA
>dme_circ_0001275_junction_seq
TTATGAGGATGTAATAAGATTGTGCCAAGGTGGTCTTCGGATTAGAGCTGATTCTC
>dme_circ_0001276
ATCCCGTGGACGAACTGATCATATCGAAGCACTATCTAAATACAGCAGCGAAGGAACATAATAATCCGAA
AGCCAAACGCGATCGTCCACTGAGCGAAATCAGTGTGACGTCGGTGGACACCCTATCACCGAATACCGCA
AAAGCCGTGCTCGAGCTGCAGAAACGCTGCCGAGCTGATAAGTTGGCCTGCCTGAGCCTAACTCGCGTCA
CCTACCTCAGCATAGCCAATGATCTGCAGGAAGTCGAGGAGCCGGATCGGGCCAGCATATTGCACGATAC
GGATCTTTTGGTAGCACCCAATAGCCCGGCGGAAAGTTCGCCGGACGAGGAACTCATGGCCTTGCAGCTA
GGCAAGAAGTTGGCCCAGGTTTTGGGCAGTGGAGCGGGCTCACCTTTGACCCCCGGCACAATGGAGCCTT
GTGCAGCGGGTTCGGGCTCACCGCTGGCAAATGGAGAGCTCTTCAACGTCTCCAAGGCCAAAAAGGTAGA
GCTACAGAACCTTTCGTCTCGATTCACAGCCGCCGTCACCCAAACACCGCCAGGTGTCACGTCATCCACT
CCCAATGAATCAGGTAAGGATTGGTCAAGTCAACCTATGCTCAAGAGGAACTATTAATCTATTAAAATAG
CATGCTACTGCTATAGCAATAGCATTTTTGATTAGTaaaaaaaaaaaaCCGAAAATGAAATTATTCAAAT
TTAATATTTCTATTGAAACAAAACAGTGACGATGAGTAAACAACAAGTTAAACTAATTGCTTTAAACTAA
GCTCTTATTAAAGCTTCTTGTTTGTCATATGTTTTTTGGCCCGAGGACATCAGTTAATTAAATTTTAACG
ACCACGCAGCAGGCAAATAAAAACTATTTTAGACAAAAGATGTCATTTTAAACTATTGATAGAATATGCA
AAGTGTTTAAAACCATTTAAAGATTTCGGATAATGCCCATTGTTTTGTGAAATCTGATAAAACAATAGTG
CATCGAGTTGGCATAAAGCACTTATGCGTTAATGAACAAAATGCAAAAATCGCATTGCCAAGGGAATAGA
AGTGCAAAAATTAAGTTTAAATAAAGTCCACCACATTTACCCTTTAAAATTGCCAATCTACACGTTTTGT
TGGCCCCCACTTGTAGCCATATGTGTGCTCCAGAAAGTGGTCCTTCAGCCCaaaaaaaaaaaaaaggaaaa
aaaCGGAACAGGGAGACCTGTCTAAAAGCTTGTCCCTTTTTTTGGTGGAGATTGTTGCTTATTCCGGCTC
TGCGCCAATTTCAAAGGTAAACACGAGGCGCACATAAGTGCTTGTCAAACTGTAGTTTTGAGTGTTTGTT
GCTCCTCTCGAAATTGAAGTACCCTTTGCCCCTTGGGAGGTGATAGCAAATGAAAAAGGACCTACGCCTA
AACACTTCTTTaaaataaaaaaaaTTTCAAATTTAAACAAAGTGTTTGATTTGTACAATTAAACAAAATA
TAAAGTAATTGTATGAAATGCGACTCTTACTTCTTTAGAACAAACTCATTTGAGCAAAGACAGCCGCTAG
GAGAAAAGGTGTTGCCCTTGGACATGAATGCAAAGTTTTTTAATTAACTCATGTAGCAGCCATTCCATTT
TCCCCTTTTCCGCAAGCAACTCTTTGTTTTCCGAACTGATCCGATCAGCGACGCTTTTGAAAACACaaaa
aaaaaaacgcgagaaaatgcttcgaaaaaaaaaaGATTGAATAAAAAAGAAGCAGGGACCCGAAAGTAATT
ATTGGCACATTTATGCAAGGACTCGTTTTCCTCTGCTCCCACATGATATTTTAAATACATAATTGCCCCC
GAAACTCCCAGTACTTTCCGTCCCTGCCCAACAAAATCACCTAATTGCGTTGGCCCAACTCGCTCAGCTC
GATAAACAAAAAATAAACAAAAGCGAGCATGGTTCGCTGCTTTTTCCGGCCTTCTGGCCCTCTCTGCCAC
ATCGTACCCATGTACTCCATATAGCCCGGaaaaaaaacactacgtaccaacacgaaaaaacatttgtaaa
aacttaaaaatttcaaacaCGTCTTCATTGTTTACAAAATGTTGGCCGGAAGCTGGCGCTTCAGGCTCAG
CTTCAGTTTCCTTTTCCCCGACTTCTTTACttttttttgtttttttttttttttGATTCCCCATTGTG
GCTCCGCAGGGAGGCACCGAAAATCCCaaaaaaaaGCAAGTTTAAATTTTAATTAAATCATGTTATATAG

AATGATGCACGCGGGACTTATCCGCCAAATCGAAGAGCGGGCGGCAAATGGAGGCAGTCGGCCCAACCCA
CTTGGGTGCACAGTCAATGATGATGGGCCGTGATAAAGGCTCCACAGGGTCTACTCAATCCGAGTGGAGG
GACCATAAAAATTTGCTACACAAAATCAAGGATACCGGCGAAGATTTAATGCACTAGAAAAATTATATGC
TTACTTTTTGGGGGGGTGTACGCAATTTTTTATGAAGCTACGAACATGAATTTCGATTTAATGAATGTAA
ATCATTTGTATATTGGCATGATGCTGGGGATATTTCAGAGCTGGATGATTCTTAATGAAAGCAAGAAACA
GAAAACCCTTATATACATAACTCATTTGAAAAGGCTTAATTAGTAATTAGGTATTAAGTATACCAGATTA
AATTTAATTTTCAATGGGTTGAGTCTGCCTAGCACAGCTTACATAAATTTCATTACAATAAGTGTTGAGC
ACTTAAATTTAAAATGTGAATACAGGCACTCTTTAAGCTTTGCATTCTTGTTTTTTAATACCAAAACCAC
TCGGAGTTTCCATCGATTTACCCGCACCGCCAGCGAGAATGATAAATGTTTGGCAAATGTTTGCCGAACA
TTCTATAATTTAACCAGTAATTATGGGAAGTGACTTATGCGCTGGCAGGAATACATTTATCCTCAATTTT
TCACTGATTTAAGCAGCACGGGAATTTTCTATACAAATTTATCCAGCTAATGCCATTGCCTCGTCATTCA
TTCATTCATTCTTTCGTTCGTTTTGCAGGGAGTCACAGGACCTGCAGGACCTTTGGGGGCTACAACATCC
TCGCCGTCGCTGGAAACGCAATCAACTGTTATAATTTCGTTTAAATCATCTCAAACACCTGTGCAGTCTC
AAACGAATTCTGCAGCCTCCGAAAATGTTGAGGATGACACAGCGCCCCTGCCACTTCCACCGCCGCCCCC
CGGCTTCGGTACGCCCACCACGCCCCTTTTGTCAAGCAATGTGCTGAAGAAGGTCGCCAGCTTCACGGTC
GAGAAGTCTTCGGCGGGCAATAATAGCTCGAATCCTCCGAATTTGTGCCCCACCAGTGACGAGACCACCC
TCTTGGCCACACCATGTTCTTCATCGCTGACGGTGGCAACCCTGCCGCCCGAAATCGCCGTGGGCGCAGC
GGCGGGGGGCGTGGCCGGGGGTGCTGGTTCGCGACGCGGCTCATCTTATGTACCGGAAAAGTTAAGCTTC
GCTGCATATGAAAAGTTCGAAGG
>dme_circ_0001276_junction_seq
GCTTCGCTGCATATGAAAAGTTCGAAGGATCCCGTGGACGAACTGATCATATCGAA
>dme_circ_0001277
GTGGACGCCGACCGCCGATGTTGAACTCGATGAAGGGAGCTCTTGGTGGCACCACATCCACCTCGATAAT
GGGTCGTGGTGGTGGCGGCTGGTAGACGGCAGCTGGTGTGATGACCTCCACCCTGGGCGG
>dme_circ_0001277_junction_seq
CTGGTGTGATGACCTCCACCCTGGGCGGGTGGACGCCGACCGCCGATGTTGAACTC
>dme_circ_0001278
GGTCATCACACCAGCTGCCGTCTACCAGCCGCCACCACCACGACCCATTATCGAGGTGGATGTGGTGCCA
CCAAGAGCTCCCTTCATCGAGTTCAACATCGGCGGTCGGCGTCCACCTCCCAGGGAAGAG
>dme_circ_0001278_junction_seq
CGGTCGGCGTCCACCTCCCAGGGAAGAGGGTCATCACACCAGCTGCCGTCTACCAG
>dme_circ_0001279
CTGTTCCAGTGTTCCGATGTACTCCACCCTTTTCCACTTTAGGTTCCAAACGGGCCTCATCATCCTGGCA
GGACCCGTTGCAATGTAAAGCCCTGGAAACTGACCGTTCTTCTTGAAGGGTATAAAGCCGATTTCCATCA
TCTGGGGAAGGGTGCCGAATATCTTCCCGTAGCGCAATTCGTCAACTATTTTTTCGGCTTCTGATTGATG
AATG
>dme_circ_0001279_junction_seq
TATTTTTTCGGCTTCTGATTGATGAATGCTGTTCCAGTGTTCCGATGTACTCCACC
>dme_circ_0001280
GCACTCGTAGCTCTGAGTTCCACGACTCTGGCCGATGAATCCATTGACACACGTGAGAATGCTGACTTAA
CCCTCAAGTGCCGGTTTAACGACAAGTATGAGGCGAACGATTTCTCTTTCTTCTGGACCCGCTGGACAGC
GAATCCCGCCCAATTCGATAACGTAGCCATCGGTGAAGTGCAGTTGAGTTCCGGCTATAGGGTAAGTAAT
CTGTAATCTGCAAAAATATCATAAGCATAGTTTTTATTTTTTATGGGTCTGAAATACTTTTAAAGTTAAA
GTTTGACAACAATGTACAATAGTATCGATACTATCGATGTTTTTTCGACCCTAGTTGGTAATGGTTATCG

AAGCACGACCTAGCAACAAGGATGATAATTGCGGAAACGACTCGCTTTGAGGGATGTTAAATGCTGGCCA
TAAAGCTGATGTCCTTAATGGGAGTGTGTGTGTGGGGTCATTAAGTGAAACGTGGAGGGAAATCTCGCAG
CCACTGTCCTTCTGTGGCATTAACGGTTATCCTTTTGCCGACCGGCAAGTACAGTTTATCCCGGCAGCAG
CAGTTGGCAGCCATTTGTTAAGTTAATTAAATGTTTCACACTCCTCTCTGCGCCCACCCGACCTGGATAA
TGCGATTTATGCATGTGACTGACTGGGCTGGCAACTTAATTAAAATCAAGTTCTTGGCAGTTTAAACAAA
TGCCAGGCGACTGAAACAGTTAAGTCCGAACGAGGCTACGGCTTTGCATTTGTATTGGATTATGATGATT
TATTAATATGAAAACCGTCGTTAAAATGTTGTGAATAACGTAAACGAATGAAAGTCACAGAATCTAAGCG
TAGAGCAATCCTTTCATCAAATTTATTAATATATAGGAGTGGTTCTATGTTTTCAGTACTATTTTTGGGT
AGGAAATAAAACATTTTGCTTCTTACCAATTTTTGTATCCTATAATTTTCCTTATCCAGAATATTTTTAC
GATTTGTCGCGATCCGTTAGGTCATCACTATTGCCCGTGTCCTTAATGTCAAAGCCGCGTGTTGCATGTT
GAGACTTGCAGAATACACACATGGCCTTCCGCCTCTTGGTGTTTGTGTTTATGTTTCATTTTCATTGCCC
CCTTTCATTCCCAGATTTCAATTGTATTTTTATTTGCCTGGTGCCTGCGACTTCCGTTTCCCGTTGGCCG
CCTGCTTTTTAATGTTCGGTTTGGTTTCTTTATGTGGAAACGCATAAATTTTAAGTGCCAACTGTGCCAC
ACGAGCCCCCAACTTCTTCCTTCTCGTCTTCCTCTTCTTCGCGAAAAATGCAACTCTCCATCCGttccat
tccgtgtcgttgcgtttccgtttcattccattccgtttcaACCTTCATGCAGCTTGCCAAATAAAATGGC
CACATGCCAGCGTTACATTTTATGGGTGCACTGGGGAAAATAGCTATAAAAAGAATAAACCAAAAATCAT
ATAAATAAATGTTTTCTAACCCAAAGAGTGCTGGCACACAAACACATATAAGAATTTGGTTGACAATATC
TTTTCAAAAGTTCTGTGAAATGATTAAATGTAACACCGAATTTTCTATCTGTACGCATTGCTATGAGTAT
GGAGTGTGGGCATAATAAAAGCAAATCAACAAATAGAAAAACAGCGTAAAGAGGCGTTGAGGGGCCAAAG
AAATCGCGCTGCGGGCCCAGGCAAATTGTAAAATATATTTTTACTGCAATTTGTGCCGAAagctaacaag
gagccgaaaacaagcagccaacaaggacgagaaacagcagccaGGACACAAGAACACGGCTCAAGGAGGC
TAAAACGAAACTGGCCACATAATGAAGGCGAAGATAGTACCCAACCCGAAAAAACCCCCTGGAAATTTCT
ATCTGTGCAAAAGTGAAATTACTGCAAATTTGCTGGCAATGCCGAACAACACACACACACaaaaaaaaaa
GAAATAAAGGGAATTACAGATTTTTTCTCTGGGCCAAAATGTCGCGACAGGTCGAAGGGAAAAAGAAAAG
CTCTGGGCTAATATTTCGCTGAACATTTAATTTAACGAGACATCAGGGGAATGCTCCTCTCACAGAGATT
GAGAAACAATGAGAGCGGCATTTAGAAGGGAAAACGATTCCCTTGGATGCGAAATTGTACATGTAAAGAA
TTACTTCTGAGCAAAGTGTACGATTGCATACTTGAGAGAAAATATGTTGGAATATCCATTTTCAGTATCC
AAAATCTGGTCAAATGGTTTGACGACTAATGCAATTTGTATAAAATTTAAAATTAAAACTTTCTATATAT
TTGTCTGCCTTTCGTTTAGGCGAATAATTGGCGGCCGTTTAAAAATGTCCTCTGTCCTGGTACGAAAATT
ATAAAAAAATTTTACTTTTTGCCCAGAGGGAGCTACAAAATACGCGAATCGAGGGCAACACTGTGGTAGC
AACAACAAATCACAAATTGCAATGCACGAGGAGAATTTATCTGGGTAGGGGAAGGGATGCTATAAATAAA
GGGACTGTTGGGTATAAACTTGAAAATTGCAACAAGAATAAGGCAGCCGGGGGCCTTTTGCTTATCCCTC
TAGAGTCCGCTTTTTTGTTGTTACGCTCAAGGACACGCGTCCTTTcccccccccACATTCCGCGGCTCGT
TTAAAGCGCTTTTCATTTGCCGATAGGAGGTGGAAAATTGAGGGGATTCCCCAGAGCGACGGCAATGACG
ATGACGATGACTGCGCTTTGCCAGCAGTTTTTATAATTTGCACtttttttttttATTTCCACCGAGCACG
TGTCCTGAGAATTCTATCTATCCCAGCTGCAAATGAGCAAATCGCCAGAGACTTGGCCCCAAAAACTGTG
CTGAAAGTGGAGGAAGGCAGGGGAGCAGCTGCAAATAGAAAAATGCTCGTAGTAATaaaaaaaaaaaaaa
aTCAACTAGACAAGGACACGCGGTTTTGGGTGGCCAAAGGAGGTAAGCGGGCATGTCTGTGGAGAGTTCA
GTCAATTCTGCTGTCTGTTGCAGAGGCAAAAATGAGAGTACAGACAATTATTAACATTTAGTTTAGATAG
AGCAACGATATGAAAGTAAAAGTATTTAAGAGGCAGTGCTTATATTTTATAAATGGATCTATAAGTTTTA
TAACTAACTACGATTTATTGTGATAAATAACATTTAGGTTTGCATTTTTTTGAGTGCACTTTACGTTCCC
TTTTGGATTTCGTTTGCGAGTGTTTTTCTTCTTTACCCCGAATTTTTTCGCCTGTCTGCGCGTTTATTCT
GTTTAGGGATTTCAAGACCATGAGACCTGGGCAGTTTCGGCAttttttttttCGTCCTGCTGCGGTTTGC
TGCTTGGCAAATTAAATTTTCACACAAACGCGTGttttattttcttttttttCATGGGACTGCGGTTTTT

GGCAGCAATTAGCACAAGAGTAAAATGAGTGAGTAAATAAAACGAACTTGCGACCGAGCTAATCAGTGTG
AGCCCTAAGCCAGTCCAATTAGAATCGCAAAATTGTCTAGACCAAAAGTGCACAAAGCTAAGATGTCTCA
GAAAATCGGATGGCAAAAATACCTCGCTAAAAATCCTCAAGATGAGAAGCAATTTCAGTGTCAGTCAAAA
GTCCACTTGCAGCTTGACAAATGAACTCGGAAAAGTCTCTGCAAATGGAAAATGAAAATTTTATCTCCCA
GCTGTGGGCAGTAGGCAGTGTGAGGAGGGGCATCTTGTTTGCCAATCTAATAAACTGCTGACTGGCCAGC
GAGGAAAAGCCGAGAATCCAGCGAAGCGCATTCGGGCGGCATCAATTTTCAGTAGTTGTCCACGACAACG
ACGATGACGACCAAATAATCATCATCATCCTATTGAGAAATTGTGAAAAATGGCAGCACCAAACaaaaaa
aaaaaaaaaTCGCAAATGGAGGACACTGAAGTGGCCTTGAAATATTAAGACATTGGCAAAATGAAGTCAA
GATAAATGATGGAGGGGGAATGAATGTCTGACAGAAGCCAAAAGCAGAAAATTGACAAACTTGTCCCTTT
TTGCTTTGGATAGATAAAAGGGAGCGAATTTTCAATATATTTCATATTGAATTTAAGGCTTTAAGATTAC
GGGTATTCCGAATTAGTTATTCATTAATTTAGAATGTTATaaaaaaaaaGAACACTTGCAACTTTCCAAA
AACGAAAAATAAATGATATTTAGTAAGAAACAAAGAACGTTTGCTTAGCTCAATATGCAACATGATAATA
AATGAAATAGATTTTCCAATTATACAttttttttGCAATTAATTTGGTTTACGAGGCTGCAAAGCAATTA
CGCCATAAGAACCCTCAGAGCTTAATAGTCCGGCACATAAAAATAACAATAAGGTAAACATTGGTAAAAT
TCCAAAGACTGTGTGAAAATTACTTTCCTTCGTTCCCTGTTTACATTTCACTCGAGTTGCCAGTGTCTGG
GACGCAGGTTCTTATTGAAAATTGTCTAGGAAAAATATGCAAACATTTATTCGAACGTTGATTTTATTTA
TATTGGTCCTTTGTGCATACCCCTGAAAACGCCTTGTCGTGTCGccccccccccccccTCGTCAAATATT
ACCATTTCGTATTTATTTATATTACGAGCGTTTAAAGTTTGGCCACCTGCGGGCAGAAAACAGGAAGCGA
ATCAAGGACAAGAGTGGGAAAATCCAATATTAGCACAATTAGTGACGGCCACTGAGGAGTCCTGGCATTG
GCGCACAGAAGTCGCCTGGAAGTTTTCAAATAAAAATAAATTGATTTCGAGGCAAATGGAAATTGATTGA
CTGAATTTTGACATTTAACTTTTGAAGCTTGTTTCCTTTTTCGCTTTTCCGAAAAATACACGCAATACGC
AGTTTAATTTATATATTTAAGTATTTTAATGCACAGATCTTGGTATTAATTACCTATAATTCATTTTATT
TTATTTCCTTCCTTCCTTCCAGGACTCGACTTTCAACCGGAGCGTGGCATTTACGATCTGCAGATCAAGA
ATGTGTCCTATAACCGTGACAATGGACGCTTCGAGTGCCGCATCAAGGCCAAGGGAACGGGCGCAGACGT
CCATCAGGAGTTCCACAATCTCACGGTGCTCACGCCACCTCATCCACCCGTGATCTCACCCGGAAACATA
GCCGTGGCCACCGAGGACAAGCCCATGGAGCTGACATGCAGCAGCATCGGTGGTTCCCCCGATCCCACCA
TAACGTTGGTATCGCGAGGGTTCGAATACGCCCCTGCCAGCGACGGTGTTAAAGGGCGGTACCAAGGATC
AGCCCACCAATGCCACGCTATCCATAATTCCTCGCAGAGAAGATGACGGTGCCAAGTACAAGTGTGTAGT
CAGAAATCGTGCCATGAACGAGGGAAAGCGATTGGAGGCCACTGCCACATTGAATGTTAACT
>dme_circ_0001280_junction_seq
GAGGCCACTGCCACATTGAATGTTAACTGCACTCGTAGCTCTGAGTTCCACGACTC
>dme_circ_0001281
GACTCGACTTTCAACCGGAGCGTGGCATTTACGATCTGCAGATCAAGAATGTGTCCTATAACCGTGACAA
TGGACGCTTCGAGTGCCGCATCAAGGCCAAGGGAACGGGCGCAGACGTCCATCAGGAGTTCCACAATCTC
ACGGTGCTCACGCCACCTCATCCACCCGTGATCTCACCCGGAAACATAGCCGTGGCCACCGAGGACAAGC
CCATGGAGCTGACATGCAGCAGCATCGGTGGTTCCCCCGATCCCACCATAACGTTGGTATCGCGAGGGTT
CGAATACGCCCCTGCCAGCGACGGTGTTAAAGGGCGGTACCAAGGATCAGCCCACCAATGCCACGCTATC
CATAATTCCTCGCAGAGAAGATGACGGTGCCAAGTACAAGTGTGTAGTCAGAAATCGTGCCATGAACGAG
GGAAAGCGATTGGAGGCCACTGCCACATTGAATGTTAACT
>dme_circ_0001281_junction_seq
GAGGCCACTGCCACATTGAATGTTAACTGACTCGACTTTCAACCGGAGCGTGGCAT
>dme_circ_0001282
TTAACATTCAATGTGGCAGTGGCCTCCAATCGCTTTCCCTCGTTCATGGCACGATTTCTGACTACACACT
TGTACTTGGCACCGTCATCTTCTCTGCGAGGAATTATGGATAGCGTGGCATTGGTGGGCTGATCCTTGG

>dme_circ_0001282_junction_seq
AGCGTGGCATTGGTGGGCTGATCCTTGGTTAACATTCAATGTGGCAGTGGCCTCCA
>dme_circ_0001283
GTTATCCATGGTGGTCACCACCTCCCAGGCGGCCATCGGAGAATCTGCATTCACCATGGACTCCACAACG
GCCACCAGGTTCAAGCACGTGGCCCCCACATCGATGCCGAGAGTTCGAGTATTCGGCTCATAGGTCACCT
GCAATGGTGGCGGAATCCTGCTGACCTTTGTCGTCACCATGATTTCACTCGAGTACGGTGAATCGCTCTT
GGGATTCAGAGCCTTGACCTTAAAGGCATAACTCTGGTGTTGCTCCAGGGCTGTCACCTTGCAGGGAATA
TCCCGCTGGCAATCGACTTCCACCCAAGCCGAATTCGAGTTGGCCAAAGTGGCGCAATCCGGTATCAATT
GTTCTTCCCTTGGCATGGCCACACGGCGATAGCTAACAAAGAACTTGGTCTTGCTCAGACCTCCATCGAA
TCCGGGATCCCAGCTCAGTGATACGTAATTGTGACCCACCTCGGTGGCTCGCAGATTTGTTGGTTTCTCT
GGGGGTCCTTTCTGCTGCAGTCTAATGGGAGCTCGAATCGTATCCAGGGTGTTGGCCACCCGGCAGGTGT
ATTCTCCGTAATCCGAATGAGTCAGCGAGTTGATCTTGAGCACGGACGTATAGATGTCGTTG
>dme_circ_0001283_junction_seq
CTTGAGCACGGACGTATAGATGTCGTTGGTTATCCATGGTGGTCACCACCTCCCAG
>dme_circ_0001284
TGATACGTAATTGTGACCCACCTCGGTGGCTCGCAGATTTGTTGGTTTCTCTGGGGGTCCTTTCTGCTGC
AGTCTAATGGGAGCTCGAATCGTATCCAGGGTGTTGGCCACCCGGCAGGTGTATTCTCCGTAATCCGAAT
GAGTCAGCGAGTTGATCTTGAGCACGGACG
>dme_circ_0001284_junction_seq
GTCAGCGAGTTGATCTTGAGCACGGACGTGATACGTAATTGTGACCCACCTCGGTG
>dme_circ_0001285
AACTTGGTCTTGCTCAGACCTCCATCGAATCCGGGATCCCAGCTCAGTGATACGTAATTGTGACCCACCT
CGGTGGCTCGCAGATTTGTTGGTTTCTCTGGGGGTCCTTTCTGCTGCAGTCTAATGGGAGCTCGAATCGT
ATCCAGGGTGTTGGCCACCCGGCAGGTGTATTCTCCGTAATCCGAATGAG
>dme_circ_0001285_junction_seq
CAGGTGTATTCTCCGTAATCCGAATGAGAACTTGGTCTTGCTCAGACCTCCATCGA
>dme_circ_0001286
GTATTCCATTGACGCCGACGCCACTGCCTCCGATTCCGCCCACTCCGCCGCCCATTCCGCTGACCACGCT
GCCGCCAACACCCACTCCCACAACGCCGCCGCCCATGCCAACGCCCACCACCCCACTTCCCGGATGATAG
CTATATCCATTCTGGGTGGCGTATAGTGCCGTTTTCTGATCCTCCATGCTGAGGGCGAGATCCATTGAG
>dme_circ_0001286_junction_seq
CTCCATGCTGAGGGCGAGATCCATTGAGGTATTCCATTGACGCCGACGCCACTGCC
>dme_circ_0001287
GATAGCGCAAGTTGGTCTCCCGATTGCTAAGGAACTGTCCCAACTGGTTGCAAGCGCGGACAAGAAGGTT
CGGCTCGCTGTCGCTGTGAATGATCAGGTTGATGGCCTCGAAGAGCACGGCGTTCTTTGCGTTAGAGTGC
TGCACCTTCTTACTCTTTGGCGGCTCTTGCGCCTTGTTCAAAATCGTCTCCAAA
>dme_circ_0001287_junction_seq
TTGCGCCTTGTTCAAAATCGTCTCCAAAGATAGCGCAAGTTGGTCTCCCGATTGCT
>dme_circ_0001288
AGTACCCATCATTAAGATCGAGGGACCTTTGATCATCGTCCAGCTGCTGGAGACAACACTACTCACCTTG
GTAAATTATGCCAGGTAGGCATCGCTTATACACTATCTGGAAATTTCCTAATAATACCACCTCCACTTGC
CACACAACACAACAGTTTGATGGCCACCAATGCAGCCCGTTATCGCATGGTAGCTGGTAAACATGTCAAA
CTATTGGAATTTGGACTGCGTCGTGCCCAGGGACCAGATGGAGGTCTATCCGCATCGAAATACTCCTACA
CAGGTAACTACACCGCCATCAAATATGAACTCTAGCAGGCCCCAAACTACGAGTCTCTTGGAGTTCTTTA

AAATCGTGACAAGAAAGGTCAGCCGTGTGCGATATGAATATTTTTTCGGCATTGCCAAATTGCATGCAGA
CAGCTCATTATCAATTAAAAATAATAAGGGGAAAGGGAAACATCAGGATTCGGGCACAGAGTTTATTTAC
AGTCACCTTCTGTTGGAAAGCTTACATTGTGTGATTCCGATCTACAAATTCTTAGATGTTATATATGAAC
TATATACTTTTAATCACAAATACGAGCCTAAACGAACATTTTGTTTGTCAGCGCTTAAGTAATTATCCAT
AATAAGTTTACAGGGCATTTAAGTAGTCCATAGGACAGCATATCTGAGGCATTGATCCTTTCAAAGGGCC
TGCAGGAAGTCGGTGCTTTTCATATTGCCTCTGGCCGACTTTTATGATGTGCATGCATGCAAAGCACGTG
TCACTGTCAGGAGCTGAGGCTTCGCTGACTCTGATTTCCGATTTTGCCATTTCTCAAGGCCCTCCCTCCG
AACTATGAGTAATTCTCCGCCTTCATCAAGGCTTCCGGCTCGTCTCCACCTGCTCCTCTCGCGACAAAGT
TCAAGAACTCTAGTTTATGTGTTAAACTAAATGCGCCCGGGCGGCAGTAAAATGCCCAAAGTGGGTGGCA
CACGCAACGCCTACGTGGGGTGTCCATGTATATGGAGTATATCGGTGCCAGGCACCCATCGAATCCGGAT
TCGAACATGGCAAACCCTGCTCCTGCTCCGGAGCCTCTCGATTGGCGCTCACACATCGTAACTTTTTCGG
TTTCGTTTGTTTGGTAAGCAACGCTCCAAGTTTTTAATGCGTTCCACATTGTGCTTGGACTATACATATA
TCATCTATATAGTATCACCCCAAAATCGCGTGGCGTCAAGGTTCCCGTCAAGGttttttttttGTGATAA
AGTGAAGGGAAATTGAAATGTTTATAGCCGGAATTTTCGCTCTTAATGGAGGAAAATAATGTAACGAAAC
CATTAAGCGAGTATTTAAAAGTTTGGAGGCTTTGTATCCTTGTGTAAATGTATAAACTAAATTATACGAT
CATTCCTTATTCTTTTACCAGGGCGGTTTTGATGGCACCTCCAATGTGCTGGCTGGAAAATTGTTCAACA
TACCCGTAAAGGGAACGCATGCCCATGCCTATATCACTAGTTTCAGTAGTATTGGAGAGCTGAAGACCCG
TCTAATTAAGCACAAGCAGACAGGGCATCCTGGAAGATTTGCTGGAGCATGCTGTCAGGCACCGGGCATT
GCTGTCGCATCTCCTTGACGTTTCTACGGAGGAATCTAGTGAGGGGGAACTGGCTGCGATGGTCTCCTAT
GCCATCGCATTCCCTGATGGATTCATGGCACTTGTCGATACGTACGATGTTAAGAGGGTATTTCGCTTTT
AGGCAGATTCTCTCGGAACGGTTTTAGTTTTCGGTTTATTCCTACGTTTGTGATATTATCCCATAACTAT
CCCGGTAACCCTCTTCTTGCCCTCGTTTATTTTGAGTATGGATTTCTTTGTTATATTTCGTATTGTATTT
GAGACTCTCGAGACGTGCACCAGCCAGTTACAAAAACCCAAAAACCAGAGTAACTATTACATACATACAT
AGCTAACAAGGCTCCGCCAATGAAGAGTTTTTAATTTTCCCTGCCTCATCCCAATCATTTCTTGAGCTTT
TCATTTCGTTCTTTCGTTTGAGTAATAATCAAAAGTATTATCCAACAGCCGAGAAACAGAACAAGCCAAC
ACAGAACCAAAGCATATCAATGATATCAAGTCTGAGAACAGTCCGCCAAAGCCAAAGGCAAGTCCCTCGA
CCAATGGCCATCAAAATGGTCAGGGCGTTGGCCACGTAAACGGCCACAAGACCACCAATGGTCATCAGAA
TGGCTCTGCTCAGAATAATGGCAGTAGAACGCCAAAAGATACAGATATACAAGATACTAGTACCAGCACA
ACAACTACGAAAACAACTGCAACAGCAACAGGAACCTTAAAGACAACAACCACAGTGTCCAATGGGACGG
CATACCTACCAAAATATGTCGAGAAGTCATTCAGG
>dme_circ_0001288_junction_seq
ACCAAAATATGTCGAGAAGTCATTCAGGAGTACCCATCATTAAGATCGAGGGACCT
>dme_circ_0001289
GGAGGCGTAGACGCAGGCGAGCTCGGCTTTGGTGGACATGTCGAAGTGCTGTGGCTAATCACTGATTGTG
CAAGTTGCCAAAAATTAATTGTTTATAACAAATGGCCTTAGCTCGGGACGAGAACAAGCACGCTGG
>dme_circ_0001289_junction_seq
TAGCTCGGGACGAGAACAAGCACGCTGGGGAGGCGTAGACGCAGGCGAGCTCGGCT
>dme_circ_0001290
GATGGTGTTGATCTTCTCACCCTGGTGAATCCAAAAAATAAGATCAGCACAAGTTGCAGCAGTGCGAAAG
AAACCATAGGACCCGCAAATTGTCCAATACTTCCGGCCGATGGCCGCGGCTTTGGAGGATTTGCTTCGTC
CCCGATTGCAATATCGAAAAACTTACGGTGACGGCGACATCGTCATCGACGAGGATGAGGGAGGCGTAGA
CGCAGGCGAGCTCGGCTTTGGTGGACATGTCGAAGTGCTGTGGCTAATCACTGATTGTGCAAGTTGCCAA
AAATTAATTGTTTATAACAAATGGCCTTAGCTCGGGACGAGAACAAGCACGCTGG
>dme_circ_0001290_junction_seq

TAGCTCGGGACGAGAACAAGCACGCTGGGATGGTGTTGATCTTCTCACCCTGGTGA
>dme_circ_0001291
CACAAACACAACGGATGTCCGGTTGTCGAGAAGTTCTCTAGAGGATTTAGTCGAACAGACCGAAGCCCAT
GTCGTCGTCAGACTGGTCGGACTCCTCCTCCTTCTTCTTCTCCTCCTTCTTGGACTCGGCGGCTGGAGCA
GCGGCGGCGGCAGCAGGGGCAGCACCACCGGCGGGAGCGGCACCAACTCCGGATCCGATGTTGGTGATCA
GG
>dme_circ_0001291_junction_seq
AACTCCGGATCCGATGTTGGTGATCAGGCACAAACACAACGGATGTCCGGTTGTCG
>dme_circ_0001292
GTCGGGAAACGGGTACACGTCCACCTCTCGAGGATTACAGCACAAACACAACGGATGTCCGGTTGTCGAG
AAGTTCTCTAGAGGATTTAGTCGAACAGACCGAAGCCCATGTCGTCGTCAGACTGG
>dme_circ_0001292_junction_seq
ACCGAAGCCCATGTCGTCGTCAGACTGGGTCGGGAAACGGGTACACGTCCACCTCT
>dme_circ_0001293
ATTCCCGGCATCTACAACGTGCACTTCGATGTCAAAAAGGACGAAAGAGTGGTTCCCTATTTGCACGGAC
CCGTGGACAATCCGGAGTTCCACGTATCGGTGCCAATCACGATGAACTGCTGCTTGGGCTTGGACACCGA
CGAGACTCGCAGCGCCATACAG
>dme_circ_0001293_junction_seq
CACCGACGAGACTCGCAGCGCCATACAGATTCCCGGCATCTACAACGTGCACTTCG
>dme_circ_0001294
GATCTTCCAACGCTGCGAGAAGCGCGGAGGGCAGGGGAAAACAAAAACACAAACATACGTACATACAATT
AGCCCTAAGAATGATTCTATCGGGATTACTTGACTATGTATACTGGATAGTGATTACATACATATTGGGG
GAGTGGAGTATATGGTATTGC
>dme_circ_0001294_junction_seq
ATTGGGGGAGTGGAGTATATGGTATTGCGATCTTCCAACGCTGCGAGAAGCGCGGA
>dme_circ_0001295
GTATGGAAACGGCCACTGTGATGCCCAGCAGGTAGATATTGGTGTCCAGCAGAGTCTCCTTCAGCGAGTC
CTGATCCTCGTCACTCTCCTCCGGCTGGCTGGCGGCCATCAGCTCACCGAGCATATTGCCAGCCCACTTG
TTCTTCATCTGTTTGGCGGCATACAGCTGCCACTTGAACATGCCCAGCGGCTGGAAAGTGAGGTGCAGCT
CCAGTTCGGGAGTGGTCTCATTAATGGGTTGATACTCCCGCTGAAGATTCCAGTAGTCGTTGACGAACAG
GATGGGCAGATAGGTCTTACCGCCGTCTACGAACTTTACGTAGTCATCCAGTGGCGGCGGCACAGATCCC
TCGGCCCAGTTAGTTTG
>dme_circ_0001295_junction_seq
GCACAGATCCCTCGGCCCAGTTAGTTTGGTATGGAAACGGCCACTGTGATGCCCAG
>dme_circ_0001296
GTCTCCTTCAGCGAGTCCTGATCCTCGTCACTCTCCTCCGGCTGGCTGGCGGCCATCAGCTCACCGAGCA
TATTGCCAGCCCACTTGTTCTTCATCTGTTTGGCGGCATACAGCTGCCACTTGAACATGCCCAGCGGCTG
GAAAGTGAGGTGCAGCTCCAGTTCGGGAGTGGTCTCATTAATGGGTTGATACTCCCGCTGAAGATTCCAG
TAGTCGTTGACGAACAGGATGGGCAGATAG
>dme_circ_0001296_junction_seq
GTCGTTGACGAACAGGATGGGCAGATAGGTCTCCTTCAGCGAGTCCTGATCCTCGT
>dme_circ_0001297
ATGTGGTTTCACAAAATGTACATATATTTACTTAGATGGTTAACCATGGTTTTGTCGATAGAAAGACCAG
CCTGGACCGTCGACGGATGATCATAGTCTACTTCAACTTCTTCTTGGGGCGCAGGTTGTTGG

>dme_circ_0001297_junction_seq
AACTTCTTCTTGGGGCGCAGGTTGTTGGATGTGGTTTCACAAAATGTACATATATT
>dme_circ_0001298
GGGCTCCGATCTGCCCATCTATATCTGGTATGAGAGCTATCCGGAGCATATCGAGGAGGGCTACAAGGGA
CGCGTGTCCCGCGTATCGCAGGACTCGCCCTTCGGCTCCGCCTCGCTCAACCTGACCAACATCCGGGAAT
CGGACCAAGGATGGTACGAGTGCAAGGTCGTCTTCCTCAATCGGGACCCCAAGCAGCATAAGAACGGTAC
ATGGTTCCACTTAGACGTACATGCACCGCCGCGCTTTAGTGTTACGCCAGAGGATATTATATACGTTAAT
TTAGGGTGAGTATGAGCTAATAATGTTTTAATTTAAGAGGAAACCATTAAAATATCGAAATAATCGAAGG
AAAGTTATCGGCAAAGTTGTGGTAATGTAAGTTATCGTATTTATCGAAGGGTTATCGATACGAAACGTTA
AGTACTCTACTCTCTACTTTCACTGATATCCATCGACACACTTATCGGATTTTTGTACCATCGCTAGTGC
TCAAAACCTGGCGCTTTGCTGCCTGACAAATAGCAAGGTCATAAATAAGCGCATATTCAGCTGGCCAACT
TATTTGGGGCCTGTCATCTCACAGAATTAAGTGGAGTGCGACCCCCGTACGCTTTTGGCCCACTTTTCGA
GTGTGTCAAATGGCCGGCCATTTGTTGCAGCGCCCGAATGAGTTTGTCACCTTGAGAACTCGTATTCCCA
GGAGTCGTAAAAAGTGCCACCAGCCACACGAAACGAGTTATGGCCGCCCATTTGTCATCTTCCGATGGAA
GACGCTGCCAAAGTGGAAGAGTTTCACGAAAATCGATAGGCTCTTTATGCTTGCAATCGTATCAAATCAA
ATTTCCGTCGCTTAATCAAAGTTAAAATGTTTACAAGTCCAAGTGGCAGGCGGCGAAACAAAAGGCAGTT
GAAAGGGCCGGAAATGCGTTGAGCGAAAATGGCGCTGCAGCGCATAAATCAGACAAACACAACGGCTGAT
TCAGAAAACAACGGGGCCAGTGAGGCGAGTAGGGAACTGCAACAAAGAGCCCGGTTTCATTGTCTGCCGC
AAATGGTGGCAGGACGAAGGACAATAGATAAGGGACTGCCGAGCCGGAAGGAGGCGAGCTGTGGGCTTAC
CCGCCCATGCTCCTACTCCTGCTCGTCGTCTCCTCGTTGAAACCAAAAAGCGTAACAATTGCCACCACTT
ACCTTATTAATTCCTCGCTCTCGTTCTTGTGCCCATTCTCATTCTCATTTCGTTCTGGCATCACATTTCC
TTGCAGGGTGATTCAATTATACTCAATTGCCAGGCAGATGGCACGCCCACGCCGGAAATACTCTGGTACA
AGGATGCCAATCCCGTCGATCCCTCGCCCACGGTGGGCATCTTCAACGACGGCACCGAGTTGCGGATCTC
TACCATTCGGCACGAGGACATCGGAGAGTACACTTGCATTGCACGCAATGGTGAGGGACAAGTCTCCCAT
ACGGCCCGTGTTATAATCGCGGGCGGCGCTGTAATCATGGGTAAAAACCTAAATACAAATCACTTAATTT
TGAGATACATAAGGGGCTGTCCCAGAAATCTCGTCCTGACATAATTCCATCCGATGTTTCGCAGCAATCT
CCTTTAAATTTGTCGTATAAGTGGTGAAATCAGAATCACAACGTGTCTATGAGTATGCAGCATAAATACT
TTATACTATGCATGGTTTATAGTGATTTATGCGGCACCATCTATGAAAATATTTGAAATATTCAACAAAT
TCTGTGATACCAATGAATTTTTCGCATGAATTCCAACCTACTCTACCGAAAACCATCAGAGATTTCTGGA
ACGCCCCCGATAGAAAACCCAACCGATTCAAAAGTAGTATTAGGAGCAAAGTTCATGCTGGGATTATGGG
AGGAGGTGATGAGCATTGGCAGATAAAAACCAGGGTTCGATTAGGTTACTAATATGTAGTTTTATTCTCT
AATCCCCATCCACGTTAAATACCCCCAAAACCAATTCCCCAAATAAAAAACCCGTAGGATAAGGCGGCGC
CCGACAAACACACACGCACCAAGTCCAAATCGAATGTTGTGAATGAGAGGCTGACCATACGTGTAAGTCC
CAACTGAAAATCGCTCCTTGCATTTTGCATTCCGGACTCCACATTCCGCATACTGCATAAACTGTTCAAC
GCTTTATCATTAACTAAGCCCTCAGCTGGCTACCGAATCCCATTCACAGCAGACCCAATCACTCCGAAAC
TCCGGGCTTTGGGATTTGGGCTTTGGAGAGCAGCAGCCAAATGGCTTCAGAAATCACCAGACCAAACCAT
GTCCAGATTCCGATTCCGGATCCGTGGGACTGCAGAGCAGGCCGAAAAACGGATaaaaaaaaaaaaGCCG
TAACAAGCGTGAAACTTTTTGTGCCGCCATGTGGCCTCCATCCGGCCCGTATTTTTGCATGCCAGAAAGG
GCAGACCGCCACACCCCCCACAAAACAGCTAAACAGAACAGAACAGAACAACCAGCAGAAGGAGCACTCG
GTACACTTGACATGGTCATTCGCCTAGTCTCAGGCAGATCCCCCTATTTACAACCTGCATACAAGCATGC
TCACATGCCCCGCTCACACCCACCACCACGCCCACTTTTCCGACCATTCTGGGAAGCTTTCGTGCATGTG
TGGGTGGAAACATGGCcgtcgtcgtcgtcgtcgtcTGTATAGCATACTTTTGTGCGCCCCGCTAATGCAT
TTGCATTTCGGTTATTTACAGTTAGCTGCAACTGCAAGAGGGCGGGATTAGGGGGCGGCTAAGCCTGCAC
AGTGAGAAAAATTCAAGCTTAAAAACTATCACAGAAGAAGACTATGAAATGTTTGATCTTTTGTGATAGT

TGTATACATTTATTAATCTTTATTAATTCCAAATATGAACTTAGATTGATCCTTGAAACTATCGCAAATC
TTTGTCACTGCACCTCACAAGCAATCCTGCCTTTTGGTCTATCCTTGCTGGCTTAGCGTTTTCCGCCCTA
CATGCACAAGCGGCTTGCCGTCTTGGAGCGCTTGAACTTTGACCTTTTGCTGGCTTTTCAGGCCAGCCAA
GGAAAGCATGCAGCAGCAGGGTGGGAAGTGTACATATAGTACATAGCTTGTGCTTCAATTACAGCTCAAC
CGTTTTGCAGCAATCTGCGCATAAATATTGCAATAACATTTATCTGACCGCAGGCAAAGTGGACATTTTA
TAGATGACCCCAAGGGAAATCGAAGCCAAACCGAAAGGCAAATCCAGCCAATTCAATTTACGAGCCCATT
TCGGCCACCAGCAGTCGGAGTCAACCCACATCAATTTTGGCCATAACTAAAACTAATTCAAATGAAACAG
TCTGCCAGCAAATTGCCCGAAAGCAAAGCGCCAGCAAATGTGCCCATTAAATAAACCAAGCCGTACACAA
CGGCCCAGAATGCACTCATGCAATTACCGGGAAGGACGAAAAGGGGTTTCGGATCGTAATGTTTGGCTCC
TGACATTTGACTGACAAGTTGTCAATGGCAGCGAGCAGGGAAGCGGGGGTAGCGGGGATTTTGGGGATAC
TGAGGGACTTGAGGAGGCGGAAAGCGGGGCACATTTCATAGCTGTGACAGCAGAAATGTTATTTCTCGGG
AGCGAGTGCACACAAAGGATATCGATGACGGCGACAGCCAAAAATTAGCTGTCAAGCTCCAGTCTCCAAA
AAGGCAACGGCCAGGGGTGGTTCCTTTTCACGATTTGAATTAGTCAACAGTCCACAGTCAACCGTCATAA
GTTTGCAGTTCGTTACCCGATCGTTGAACCCTCTTTTCTAAATATACCATCCCATCCCATTTTCCCATAC
CCAAATCAGGGTGCCTCCGACCAACCAAACGAAGCTCGAGGGCGAAAAAGTGATATTCTCCTGCGAGGCT
AAGGCCATGCCCGGCAACGTCACGGTGCGCTGGTACCGCGAGGGTTCACCCGTCCGGGAAGTGGCCGCCC
TGGAGACCAGGGTGACCATCCGCAAGGACGGCTCGCTCATCATCAATCCCATCAAGCCGGACGACTCGGG
CCAGTATCTCTGCGAGGTGACCAACGGCATCGGCGATCCGCAGAGCGCCTCCGCCTACCTCAGCGTCGAA
TGATCCCGCCAAGGTCACCTTCACGCCCACGGTGCAATACCTGCCGTTTCGCCTAGCCGGCGTTGTTCAG
TGCTACATCAAGTCGAGTCCGCAGCTGCAGTACGTGACCTGGACGAAGGACAAGCGCCTGCTGGAGCCGT
ACCAGATGAAGGACATCGTGGTGATGGCCAACGGGTCGCTGCTGTTCACGCGGGTCAATGAGGAACACCA
GGGCCAGTACGCCTGCACGCCGTACAATGCACAGGGCACGGCTGGAGCTTCCGGGGTGATGGACGTGCTC
GTCCGGAAGCCGCCCGCCTTCACCGTAGAGCCGGAGACGCTCTACCAGCGCAAGGTGGGCGACAGCGTAG
AGATGCACTGCGACGCCTTGGAGGCGGAGGGCACGGAGCGACCCACCATTAAGTGGCAGCGTCAAGAGGG
CGAGCAGCTGACCGAGTCACAGCGCAATCGCATCAAGATCTCCGGGGGCAATATCACCATTGAGAATCTG
AGGAGGGAGGACTTTGGCTACTATCAGTGCGTGGTCTCCAATGAAGTGGCCACCCTGATGGCCGTTACCC
AGCTGGTAATCGAGGGCACCCAGCCGCATGCGCCCTACAATATCACGGGCAAGGCCACCGAGTCCTCGAT
AACGCTGCAATGGCTGCCAGGATACAGTGGCGGATCGGAATACAAGCAGGACTATACGATCTGGTTCCGG
GAGGCGGGCGTCAACGACTGGCAAACGATTTCCGTGACGCCCTCGGGCAGCACCCAGGTGACCATCAATG
GCTTGGCCTCCGGAACCACCTACGAGTTCCAGGTGGTGGGCCGTAATGTCCTGGGCGACGGAATGATGAG
CAAAGTGATGACTGTGCGCACACTGG
>dme_circ_0001298_junction_seq
AGCAAAGTGATGACTGTGCGCACACTGGGGGCTCCGATCTGCCCATCTATATCTGG
>dme_circ_0001299
GAGCTGAAAGCTGAGTGGTACAATCAACATATAACGTTTACTTCCAATGCAGATGCCAATCGCCAGCGTA
TTCAATTAAATAACAGCATTCAAGATCCGCAAATACGTATCACAAAAAAATATAAAGCATTAATCAATTG
AGCCAAATGCTGATGCCATATGCCAAAAGCGATTGCAACAAAAACTAAACGCATTCTCAACTAGAACTAC
ACAGAAACTAGCGTCATGAATGAGCTGCAGGCCTCCAGTGTCCCGCTGGTGGACAAGTGCTCCTCCCACG
CGGTCATCCTGCCGGCGGCCAAGAGTCCCAACAGAGTCTCCGATTCGGCAGCCACTACGGCACCTAATGC
CGTTCTCTACACAAGCGGGAACATGTCCCTATCAACGCTCTCCACGGGCAGCGGCAATCCAAATCCAAAT
CCGAATGCGAGTACCAATTCCAGACAGGAGCCAGTGCCACTGCTCAGCCAACTGAAGCTGACCAACGATC
TGAAGCAGAACAGTCACGG
>dme_circ_0001299_junction_seq
CCAACGATCTGAAGCAGAACAGTCACGGGAGCTGAAAGCTGAGTGGTACAATCAAC

>dme_circ_0001300
TAAATGTCTACGGTACTAATGGTCCTCCTTAGACGGCCACTATCTTGCTGGGATCCACTTTCACGCGCAG
AAACACGGTATCGCCCTTGATGAAGGGTCGGCTGTGCAGCAGTTCGTGGGAGATGAAGCGTGGGAAGCCG
AATCCTAGCTGATCTGG
>dme_circ_0001300_junction_seq
GTGGGAAGCCGAATCCTAGCTGATCTGGTAAATGTCTACGGTACTAATGGTCCTCC
>dme_circ_0001301
GACGCTCTAATTAAGAAGAAGGTTATCGAGGAAAACCACATACATAATGAGGAGATCATAGGAGCGAGCA
ACATAGTAGACGTAGAGGttcatgagaatcatcgcgcaaataacgacgtgatcctcatacaggattcctt
cgctgaggagg
>dme_circ_0001301_junction_seq
ctcatacaggattccttcgctgaggaggGACGCTCTAATTAAGAAGAAGGTTATCG
>dme_circ_0001302
GGGGAAGCGCTTTTTCAGTAGGTTTTCCAGCTTTCTGATGCCCGTCTGGGAACTTCCATCGGCATTTTTA
TCTGCGCTTCCCGTAAAGCTGCCCACGCTGAAGATAAAGGCTCGTTTGGGGGTTGAGGAAGTTGCCGTTG
TTGTTTCAGTTGGTACTGCTGAGGTTTCAGCATCGTCTGTGGTCCCCGAAGTTGCGACTACTGTTTCTGC
ACCTTGGGTCTCCTCCTCTCCAGTAGTTGGCTCTATTAAGCCACTCACAGCCGCTGCAGATGCCGTGTGC
TTGAAAAGATCAATCAGTTGCAGTTGATCCACACGTTTGACCGCCTTCATATCCAGAATCTTTTGGTACT
CCGCAATG
>dme_circ_0001302_junction_seq
ATCCAGAATCTTTTGGTACTCCGCAATGGGGGAAGCGCTTTTTCAGTAGGTTTTCC
>dme_circ_0001303
GTGCAGTGGCAACACATTAACAACGTGGGCGACCAGAGCGCCTTCATCAGCAGCCTGTGCGGTAACTTTA
AGCAGACAGTGCCTACCATCAGGGACACGCTAGCCAGTTCGCGGAAGTATTTCACCCAGTTCTGCCATCG
CTTCGTCGCCGCCTTCATACCGAAGTTCATCAATGTGCTCTATCGCTGCAAACTAACCCTCTCGGATGGT
TCCAACAATGTTCTGGGCTGCGAGCAACTCTTGTTGGACACCCATTCGCTAAAGACGGCTTTATTAGAGC
TACCTTCCGTGGGATCGAGTGTGAACCGCAAGGCGCCCACCAGTTACACCAAAGTTGTGGTCAAGGACAT
GACTCGGGCCGAGATGATCATCAAGGTGGTGATGACGCCGGTACAGCCGCCGGCGCACTTCACTCAGCAA
GTGCTTAAGCTGCTGCCAGACATTACCATTGCGGAGTACCAAAAGATTCTGGATATGAAGGCGGTCAAAC
GTGTGGATCAACTGCAACTGATTGATCTTTTCAAGCACACGGCATCTGCAGCGGCTGTGAGTGGCTTAAT
AGAGCCAACTACTGGAGAGGAGGAGACCCAAGG
>dme_circ_0001303_junction_seq
CAACTACTGGAGAGGAGGAGACCCAAGGGTGCAGTGGCAACACATTAACAACGTGG
>dme_circ_0001304
GCAACGTATTTCGAACGCGCGCGCTGTATACGGAAAATGATGCAGCATGCGAGCATTTTGCACCGTCAGC
GCGCCTTTGGAAGTATGCGCGTCCTCAGCCGAGCAACTATCACCAGGCTCGCGATTCCTGGCCCTGAGGA
CTTCTGGGGCAGCAGCAGCCGAAAACCTTGTACTTCCTAGTCGATGCCAAGAGTCGAGTTCGAGAGGTTT
ACACGCAGACATGTCTGCACTTTGCCACGCAAGGGATGCTGGACACCGAACTCTTCGGTCTGGCTGTGCT
CATAG
>dme_circ_0001304_junction_seq
GAACTCTTCGGTCTGGCTGTGCTCATAGGCAACGTATTTCGAACGCGCGCGCTGTA
>dme_circ_0001305
ATGTCCCATGGCGGGAATGGGATGTGCAGGAAGAAAGCCAGTCGGCAGGGCAGGTTCTTCTCCTCGGCGT
GCTCGCGCACCCAATTGGCGGCCAGCATGAGATGGTAGTCGTGGATCCAGACAATCGGTGGACTCTTCTC

GCTGCCCTGGTTTTTGGCCAGGCACTTCTCCAGAGCCTCGATGGTCCGCACGGCGAAGTGCTTGTTGACA
GTGACATAGTCGTGCCAGTGCTCGCCTCCGAAGTTGGCTCTTCCCGGCATCGAG
>dme_circ_0001305_junction_seq
TCCGAAGTTGGCTCTTCCCGGCATCGAGATGTCCCATGGCGGGAATGGGATGTGCA
>dme_circ_0001306
GAAACAACGCATCCAGTGGCTCACATCGCACTCGGCTTCACGACGGCGCAGCCGAGCCATACGTAGGACG
CGCTCATCCTCGGGCATGGTCAGTGCTCGGTGGATCACCTCGGCGGCCTCGTTCACCTCGTACGGATTGC
AGAGCAGCGCCTCGTGCATCATCTCGCCGGCTCCTGCGAACGGTGAGATGACCAGCACGCCGGGCACCTC
GTTGATCTGGCAGGCCACGAACTCCTTGGCCACCAGGTTCATGCCATCGCGAAGTGGAGTTACCAGGCAA
ACAGCCGCATCCCTGTACAAAGCAGCCAACTCGTCTTGGCTGACATAGTCGTAGATGTAGCGTATGGGCG
CCCAGTTGGCTGTGG
>dme_circ_0001306_junction_seq
TAGCGTATGGGCGCCCAGTTGGCTGTGGGAAACAACGCATCCAGTGGCTCACATCG
>dme_circ_0001307
TAGTCATCGAAATCGTCCACGGACACTGGCTGCATAATGGTGGTGCCCACATCATCCATCTCCAGAGCGC
CCACCGCCTTTAGGAAACAACGCATCCAGTGGCTCACATCGCACTCGGCTTCACGACGGCGCAGCCGAGC
CATACGTAGGACGCGCTCATCCTCGGGCATGGTCAGTGCTCGGTGGATCACCTCGGCGGCCTCGTTCACC
TCGTACGGATTGCAGAGCAGCGCCTCGTGCATCATCTCGCCGGCTCCTGCGAACGGTGAGATGACCAGCA
CGCCGGGCACCTCGTTGATCTGGCAGGCCACGAACTCCTTGGCCACCAGGTTCATGCCATCGCGAAGTGG
AGTTACCAGGCAAACAGCCGCATCCCTG
>dme_circ_0001307_junction_seq
AGTTACCAGGCAAACAGCCGCATCCCTGTAGTCATCGAAATCGTCCACGGACACTG
>dme_circ_0001308
TTATCTGAGTTACGAGGCATGCATAGATAATGCATGGATATCTGGGCTCACACCTCCAGATTGTTCGCTG
AAGCGGCCACCTCCAGGGACATCTGCATCTGGACACCGTCGCCCTTGGTGGGCCTGTAGGTCAGCGAATT
GGCGCGCGCCTTGCGTCCCATGAAGTGTCGTTCCACCCATTTCAGCAGGGTG
>dme_circ_0001308_junction_seq
GTGTCGTTCCACCCATTTCAGCAGGGTGTTATCTGAGTTACGAGGCATGCATAGAT
>dme_circ_0001309
GACGCTGCCTTTGCGAACTGGCCAAACAGATTGGATTCACCGACCATGCCACTGATCGGTTCGCGCTGGA
GGGTCAATTGGCTAGCTATCGCCACTTGGTAAGACTCGGTGACGTTGTAAAATAGTATTCTAGTTATTAT
TTAAAATATCTCCATTGCAGCAACCTGATGTTGTGCGAAGGGACATTCGTTTCGCCCGGCAGCTCCAGCT
GTCCTCCAAGGTGAAGGTCCCATTCCCGCACTCTTTGTCTGTGGTCATGCGCGAGAATCCAGGCGGCTAT
TTATCACTTTTCACCCAGGGAACAGCAGACATTATACTGGACTGCTGCGATGACTTTTGGGACGGCATTG
ATCTGCGACCATTGTCCGCACAGGATCGCAAGCGTGCACTGGACTTTTATCAGCGCAGCGCGCTGACGTC
CTATTGTACAGCGTTTGCCTATCGACCTCTAAGGCACGGCATCCATGGAGCCCTTAGCGGTCCTCAACGG
AGTGGAGGTGACTGTTTGTATCTGGAGCTACCGCCGGAGTCAAAGCTGCGCATGCAGCACGTGGACAAGA
CCCATTGCGACTTCCAGGGCGGACATCACGTCAAGCTGAGCCACAGCATTAGCACGGACTCATTGCTATT
TTCGGAGAACAAGGACGAGGAGTGTAACGATGTGGAGGGCTGCTTCGAGATGCAGTGCCATCAGGTATTC
ATCGGCATGGTGACAATGCAGTACCAGGCGCAAAACGATATCGTACGACTCGTTGAGCATCTCGAACAGG
CCTGCATACGCTTTGTTCACTTTAGCAAAGAAAACGAACTGCGCTCGCGTGTCTTCTCTGAGAAAATGGG
TCTAGAGTCCGGATGGAACTGCCACATCTCGCTACTGAGCGAACCTGAGCCGGATAGTGAAAAGGAGCGG
ATAAGTGCCAAGCCAAAGGAGTTCCCGGAAAATATTG
>dme_circ_0001309_junction_seq

AAGCCAAAGGAGTTCCCGGAAAATATTGGACGCTGCCTTTGCGAACTGGCCAAACA
>dme_circ_0001310
ATACAAACAGTCACCTCCACTCCGTTGAGGACCGCTAAGGGCTCCATGGATGCCGTGCCTTAGAGGTCGA
TAGGCAAACGCTGTACAATAGGACGTCAGCGCGCTGCGCTGATAAAAGTCCAGTGCACGCTTGCGATCCT
GTGCGGACAATGGTCGCAGATCAATGCCGTCCCAAAAGTCATCGCAGCAGTCCAGTATAATGTCTGCTGT
TCCCTGGG
>dme_circ_0001310_junction_seq
TCCAGTATAATGTCTGCTGTTCCCTGGGATACAAACAGTCACCTCCACTCCGTTGA
>dme_circ_0001311
GCTAACTGGACAATCATCGGAGATCGCCGAGTCGGAAAAGAGTTCGCACTACGGGATGTTCCAGCCCCAA
AAACTAGAAGAAACCCACGTGCAACATTCTGATTCCGTAGATGGAAAAAAGAAAGAAGACTTCAGACCGC
GATCGGATTCTAACGTCAGCACAGGGAGTTCCTTTAGAGGCGATGGCAGCGATCCAACAGACAACAAACA
TTCCTTGCTCTCCGCAGAAGAGTTAACCAATTTGATTGTGGGCAGGGGGACCTACCCCAGTCGGAAAACA
>dme_circ_0001311_junction_seq
CAGGGGGACCTACCCCAGTCGGAAAACAGCTAACTGGACAATCATCGGAGATCGCC
>dme_circ_0001312
CATCCCGGAGGTCGCGGTAACGCTGGTGGCATGCACCATCACCGCATCAACTTCGACAAATACCATCCTG
GTTACTTCGGCAAGGTGGGCATGAGGAACTTCCATCTGCGTCGCCAGCACAAGTTCAGGCCCGAGATCAA
CCTGGACAAGCTCTGGTCCCTGGTCGGAGCCGAGAAGTTCGCCGAGCTGGAGAAGGAGAAGAGCACCAAG
GCTCCCGTCATCGACTTGGTTAAATTCGGTAAGTTTCGCCCGAATTCCCCATCCAGCTACGCGAACTCTG
CTGCCGCTTTCAACATGAAGATACACCCAAGGCGATGGGCGGACAGAATGCTACCGTACAATTGCTCTCA
CCGTGTAGAGGACAGCCTTTCAGATCAAAGCCAGCAGTTCTTGAAGAGGATGTGCACCCGTTCTCACCCC
GTTGTAACCCATTGTATTGACTCTGGTGTCCTTCTCATGTCTCCACAGGGGCTACTACAAGCTGCTGGGC
CGTGGTCACCTGCCCGCCCGCCCCGTCATCGTGAAGGCCAAGTACTTCTCTAAGAAGGCTGAGGACAAGA
TCAAGAAGGCCGGCGGTGTGTGCTTGCTGAGCGCCTAAGTTAAGG
>dme_circ_0001312_junction_seq
GTGTGCTTGCTGAGCGCCTAAGTTAAGGCATCCCGGAGGTCGCGGTAACGCTGGTG
>dme_circ_0001313
TCGAATATCAAGCGGAAGAAGACCAGGAAGCTGCGTGGTCATGTGAGCCACGGTCACGGCCGTATCGGCA
AGCACCGCAAGCATCCCGGAGGTCGCGGTAACGCTGGTGGCATGCACCATCACCGCATCAACTTCGACAA
ATACCATCCTGGTTACTTCGGCAAGGTGGGCATGAGGAACTTCCATCTGCGTCGCCAGCACAAGTTCAGG
CCCGAGATCAACCTGGACAAGCTCTGGTCCCTGGTCGGAGCCGAGAAGTTCGCCGAGCTGGAGAAGGAGA
AGAGCACCAAGGCTCCCGTCATCGACTTGGTTAAATTCGGTAAGTTTCGCCCGAATTCCCCATCCAGCTA
CGCGAACTCTGCTGCCGCTTTCAACATGAAGATACACCCAAGGCGATGGGCGGACAGAATGCTACCGTAC
AATTGCTCTCACCGTGTAGAGGACAGCCTTTCAGATCAAAGCCAGCAGTTCTTGAAGAGGATGTGCACCC
GTTCTCACCCCGTTGTAACCCATTGTATTGACTCTGGTGTCCTTCTCATGTCTCCACAGGGGCTACTACA
AGCTGCTGGGCCGTGGTCACCTGCCCGCCCGCCCCGTCATCGTGAAGGCCAAGTACTTCTCTAAGAAGGC
TGAGGACAAGATCAAGAAGGCCGGCGGTGTGTGCTTGCTGAGCGCCTAAGTTAAGG
>dme_circ_0001313_junction_seq
GTGTGCTTGCTGAGCGCCTAAGTTAAGGTCGAATATCAAGCGGAAGAAGACCAGGA
>dme_circ_0001314
GTCGATGACGGGAGCCTTGGTGCTCTTCTCCTTCTCCAGCTCGGCGAACTTCTCGGCTCCGACCAGGGAC
CAGAGCTTGTCCAGGTTGATCTCGGGCCTGAACTTGTGCTGGCGACGCAGATGGAAGTTCCTCATGCCCA
CCTTGCCGAAGTAACCAGGATG

>dme_circ_0001314_junction_seq
TGCCCACCTTGCCGAAGTAACCAGGATGGTCGATGACGGGAGCCTTGGTGCTCTTC
>dme_circ_0001315
ACCCCTGGGGGTGGATGACGATGACAGCTACCTGGACATGTCCGATGAGACGGCCCAGCTCAAGCCGCGC
CAGCAGCACTGGGCCAATAAGATGCAATTCGTCCTGGCCTGCATAGGATACTCGGTGGGTCTGGGCAACG
TCTGGCGTTTTCCGTACATGTGCTACAAGAGTGGCGGTGGGTAAGTTCTTATGGTTATAAATTGAATAAC
ATTAAGGTAGCATTTACGATAAGATTATGTTGGTGATTTCTATAGATATATTATGAGATTTTATTCTTTA
TTTAATTAATAATTTGACTGAGCTATACTTGCACAATACTATTTATACGCTTATCTACTGCCTCCGAAAA
AGATCTGCGGTTCAATTGGGAATTAAGAAAGGGGTCAGTCGTAGGCAATTCAGAGATTTTCCGGGGAACG
CTGGTGGGCGGAAGTGCCTCGTGAGAGTCCAGCAAGTGACTAAGAGTGCCGCCaaaaaaaaaaCCTGCAC
ATGCGATAAGGCAGATATAGAGATAGTTACTTTGATTTTGTGCGACGGCTCCGGGAAGATATGGGGGTTT
AGTTGTTTATTTTAGTTGCTCTAAAAAACATTTGACAATTTTGCACTTTAAGCGGCATTTCCGCTGAAGC
GTTTAAAAGCCATAAATTAAAATTTGTTTAAGCGCAGAGCAGCGGGAACAGAGAATAGAGCGGTCACTGT
CATCATTTGGCCGGTGTTTCTCGATCACGCGAGCTCGGAGGCTCGCGTCCATCTTCATTTGCACTTGGAG
ACGAGAAGCTGGGCCCCTACTTAGCTACCCCGTTTCTCGAGGGGATCGGGCATGAAAATGGGGGCGTAAT
GAGCTGGTGTGACAGTAGAGAAACGGGCACACGACCTTCTGCTAATGACGCGTATAACGATAGACAGTAG
TTTTATGCCATTTGCATTTGCCTATGTCAAATATTTATAATTAATTTAAATTATTTGCTTAGCTGCTCTC
GTCCATGGCTAAACGTACATATGTAAATGTTTTGTTATTCGTTGCTTACAGGGTGTTTTTCTAGTACCTT
ATTGCATAATACTGTTCATATGCAGCATTCCGCTGCTCTTCATGGAGCTGTCCGTTGGCCAATACACCGG
TCGAGGACCCATTGGAGCACTGGGTCAACTGTGTCCTCTGTTCAAGGG
>dme_circ_0001315_junction_seq
TGGGTCAACTGTGTCCTCTGTTCAAGGGACCCCTGGGGGTGGATGACGATGACAGC
>dme_circ_0001316
GCTAATTGGAAACCGGAACGGAAGTGGACATTGGGGGTCCAATACGACACTCTTCTCTCTAGTTCGATTG
CTGTTCGAAGTGCTGATGTATGAGTGTGCCCAGGTGAAGAGCTAATTCTGCGTTCGGAAATTTGCATAGA
TCGGGTGGTGTAGC
>dme_circ_0001316_junction_seq
GAAATTTGCATAGATCGGGTGGTGTAGCGCTAATTGGAAACCGGAACGGAAGTGGA
>dme_circ_0001317
GGCCTGTGGTTGCATGTAGAGCGGATTGGGGGCCTGTAATCCGGAAGTGGCCATCGATACGCCTCCAACC
AGATTGCCGCTACTGTCGCTCAGGCCTGGAAGATAACCTGCAGGGGATTTGGAATGATAAGCGCATTATA
ATAGTAACAGGTAAAATATATTCTATGGAACTTCATTGCCTATCAATATCATAATCAATAATTCTAGAAA
CTGCTTTCATTAAAATCATACTATGGTTAATTTAGACTAAATTTGCTAGCTATGCCATTTATATAGCTTA
TTATGAAGCAATTATAGGCAGACATTTAATTATTAGTGGGACAACATTATCAACAGCCTGTTCAAGTATT
TCTTGTAATTAAAAGATTAAGAGGCGTTACCAATCAAAGCAATTGAGTACAAGCAATTACATACGAGCAC
TTGGAAATGCAATATGGGGGCGAGGGTTTGAGGGTCACCAACCTGAGACTACGGAGCGCATGCTGCTGTG
ATGGCTGCCTAACCGCGACTTCAGCTCCATGCTGAGCGGCGAGACGGCCACCGAATCACCGGCCATGGTG
>dme_circ_0001317_junction_seq
GACGGCCACCGAATCACCGGCCATGGTGGGCCTGTGGTTGCATGTAGAGCGGATTG
>dme_circ_0001318
GAACACCGCCGGGAAGCGGTGCGGTAGCAGGACCACTTCCCTTTCGCCAGACCAGCTCAACCACATCGCT
GGCGGCCATGGCAGCTGCGCTGCACAAACAGTCTCCACTGCGCCATACATCGGTGCGAACCAGGCCCAGT
TCGGAGGTATTGCAACCGGGCCAGGTGCTCGCCAATCAGCCAACGGCAGCTGTATCCACCTCCACGGTGA
CCTTCACCATTGACGACTGCAATGAAGAAGGCGATGCCATTCCTGCTGCAGCTTCAGTTCCAGCTCCCGT

GACCATGCCCGCCATCTCACCCACGCCGGCCACCTTGACCTGCACCACCACCACCACCATGGCCGGTGAT
TCGGTGGCCGTCTCGCCGCTCAGCATGGAGCTGAAGTCGCGGTTAGGCAGCCATCACAGCAGCATGCGCT
CCGTAGTCTCAGG
>dme_circ_0001318_junction_seq
ACAGCAGCATGCGCTCCGTAGTCTCAGGGAACACCGCCGGGAAGCGGTGCGGTAGC
>dme_circ_0001319
GACTACGGAGCGCATGCTGCTGTGATGGCTGCCTAACCGCGACTTCAGCTCCATGCTGAGCGGCGAGACG
GCCACCGAATCACCGGCCATGGTGGTGGTGGTGGTGCAGGTCAAGGTGGCCGGCGTGGGTGAGATGGCGG
GCATGGTCACGGGAGCTGGAACTGAAGCTGCAGCAGGAATGGCATCGCCTTCTTCATTGCAGTCGTCAAT
GGTGAAGGTCACCGTGGAGGTGGATACAGCTGCCGTTGGCTGATTGGCGAGCACCTGGCCCGGTTGCAAT
ACCTCCGAACTGGGCCTGGTTCGCACCGATGTATGGCGCAGTGGAGACTGTTTGTGCAGCGCAGCTGCCA
TGGCCGCCAGCGATGTGGTTGAGCTGGTCTGGCGAAAGGGAAGTGGTCCTGCTACCGCACCGCTTCCCGG
CGGTGTTCCTGGC
>dme_circ_0001319_junction_seq
CGCACCGCTTCCCGGCGGTGTTCCTGGCGACTACGGAGCGCATGCTGCTGTGATGG
>dme_circ_0001320
ACGTCAGCCAGGTGCTAAACACCACACCCGACCAGGTGGTCATCAATCGTGCCCCGCCCACCGGACTAGC
GCCCTTGAGGCACAGCCAACTCTCTGATAGCGGAGCGGAGAGCTGCTACGAGGGCGATGAGCAGGCACGG
CTCATCCGGTCCTCATCGTCGCGGGCGCACAAGTTCATCATAATACCGGCCACGCCAGGAACACCGCCGG
GAAGCGGTGCGGTAGCAGGACCACTTCCCTTTCGCCAGACCAGCTCAACCACATCGCTGGCGGCCATGGC
AGCTGCGCTGCACAAACAGTCTCCACTGCGCCATACATCGG
>dme_circ_0001320_junction_seq
AAACAGTCTCCACTGCGCCATACATCGGACGTCAGCCAGGTGCTAAACACCACACC
>dme_circ_0001321
TTATAAATTTAGTATAAGTTTAAATAAAAATCAATCACAGTTGTTTGGGCGGAAGTAGTTAGTTTTAAGT
ATTCGCATTTCTGGCCTAACTAAGTATTTGCAGCTAAGACTAAAGATCTACGATGTAGTTTAGTAAATTA
GGTAGTGGTCGTATTTTATACTTGAGTATTGTTGCTTCCCATTTTGATAAGCTTCCTCTCTATTTCCGAT
GTTGTGTCATGTTGTGGTCTGTGCTGTGGTTACGTTACCAATTCCCTTAAGTGACTAGACTGGGTTCTGA
TTATTTGAATGTGTATTTTGAGGTTACGTTGCTCACGTCCTGTAGGCTTAAGTATTGACTCTGTGATATT
CAGTTGTTTCAGTGGCTCGTGTATTTTACCATCTGAAGTTGGTGGACCTGCTAGTTTGTGTTTTTCTAAA
AAACTGTCTTCGCTAGTTAAATGCATGTTAGTTCCTACTAAATAGCTATTGTTTCTGCTGTTCTGACAAA
TGG
>dme_circ_0001321_junction_seq
CTATTGTTTCTGCTGTTCTGACAAATGGTTATAAATTTAGTATAAGTTTAAATAAA
>dme_circ_0001322
CTAAGACTAAAGATCTACGATGTAGTTTAGTAAATTAGGTAGTGGTCGTATTTTATACTTGAGTATTGTT
GCTTCCCATTTTGATAAGCTTCCTCTCTATTTCCGATGTTGTGTCATGTTGTGGTCTGTGCTGTGGTTAC
GTTACCAATTCCCTTAAGTGACTAGACTGGGTTCTGATTATTTGAATG
>dme_circ_0001322_junction_seq
ACTAGACTGGGTTCTGATTATTTGAATGCTAAGACTAAAGATCTACGATGTAGTTT
>dme_circ_0001323
GCTTGGGATGTTTCTGGATGACCAGCACGAAGCTTGCCACATCCTTTACAGCCTGCATGCCGCAGCTACC
GAAGTGGACACGGAAGATCTCGGTACGCGGAACAGTCTCGCCAGCCAGGTTAACCACCCGACGGCACTCC
TCGTCCTTGCTGTGTCCCTTAACGTACAACACTCCATTGAATCCTGGTTCGGTGAT

>dme_circ_0001323_junction_seq
ACACTCCATTGAATCCTGGTTCGGTGATGCTTGGGATGTTTCTGGATGACCAGCAC
>dme_circ_0001324
GATCATCGTCCTTACGGGCAAGGTATCACGGACACTGCAGAAGGCATTGACTCCGCATGGTTGGGTGGCA
TTGCACGGATTCACACACTCTCCGCGCAGACAGGCTTTCTGGCTGGGACAGTCCGCATCGCTTATGCAGA
TGGGAAT
>dme_circ_0001324_junction_seq
AGTCCGCATCGCTTATGCAGATGGGAATGATCATCGTCCTTACGGGCAAGGTATCA
>dme_circ_0001325
CTTGCCGTAAGATGATGCCGCCTAGGCTTCCTGGCTGCGAGAGCGACCAGGACTGTCCCGACCAAGAGGC
CTGCATACACGCTCAGTGCCGCAATCCATGCAACTGTGGAACTAACGCCGTCTGCCAAGTGACGCAACAT
CGCGCCGTATGCAGTTGCCAAGATGGATTTGAGGGTAATCCCTATGCCTCCTGTCGCTCAATTGGATG
>dme_circ_0001325_junction_seq
CCTATGCCTCCTGTCGCTCAATTGGATGCTTGCCGTAAGATGATGCCGCCTAGGCT
>dme_circ_0001326
GTTTCCGCCACGACAGGCTTCCGTATCCTGGCATTGATCATGTGACGTACAGCCCTGATCACGAGGGGGT
TCTGAGCAAAAAAATTAGAGTGGATTAGAGTTTTTCCAAACTAAATATTTAGTTGCTAAGTGTTTATGTG
CAACAGTTAAGAGTTATAGAGTGCAGTCAAAATATATATAGGTGCAACTGCTCAGGATAATGGTTTTGCA
TCACAATAAATGGGATTGTAATTCAAGATAAATATTTGTAAAAGCACTTTATAAAAAACCTGCTGCTGCA
ATGAGAAAAGCAAACTCAAAAGTAAAGCGTATTTAAATAAGTTGGAATAAGGAGTACCAAAAGCTGGGGT
GGGTTGAGCTATTGGACAGCTGTCTAACAAATCAACCCACTCTAAGGCTTCTTTTGGTCTTTCCACCAGC
CATATCTTACCTTTGTGACATCCAATGTCGGCTTCACCCACATAGCCAGGTAGACAACTGCACATCATGG
TGCGCAGAGGCAGTGTGTCGACCACTTCGCAGATGGCATTGGCGCCACACGGATGCGTGACATCGCACGG
GTTCTTGCATTCGCCGCCGAAACAGGCCAGTTTGCTGGGACACTCGGCATCCATGGTGCAGCCCTCCGGT
TGTGTTGGCACAAG
>dme_circ_0001326_junction_seq
TGCAGCCCTCCGGTTGTGTTGGCACAAGGTTTCCGCCACGACAGGCTTCCGTATCC
>dme_circ_0001327
CCCCTGCACCGGAATACGTGAACCCCTGTCAGCCGTCGCCTTGCGGTGCCAATTCTCAGTGTCGCGAGTC
CCAGGGACAGGCTATCTGCTCCTGTCTGCCGGAGTTTGTGGGCACACCGCCCTCGTGTCGTCCGGAATGT
GTGATCAGCGCCGAGTGTCCAGCGGATAGGGCGTGCATTAACCAGAAGTGCCAGGACCCTTGTCCAGGAG
CATGTGGCTTGAATGCCCAGTGCCATGTGCGCAATCACAGTCCGCTGTGCTCGTGTCAGCCCGGATTCAC
CGGAGATGCTCTGACCCGCTGTCTACCAGTACCGCCTCCACAACCGCCGAAATCGAATGATATTCGCGAT
CCTTGTGTGCCATCTCCTTGTGGACCTTACTCCCAATGCCGGGTGGTTAATGGCGGAGCTAGTTGCAGCT
GCCTTCCCAATTATGTGGGAGCGGCGCCCAACTGCCGTCCGGAATGTACCATCAATGCGGAGTGTCCCAG
TAATCTGGCCTGCATCAACGAGAAGTGTCGCGATCCTTGTCCTGGAGCCTGTGGCTTTGCTGCCCAGTGC
AGTGTCATCAATCATACGCCCAGTTGTTCGTGTCCCGCTGGTTACACGGGAGATCCGTTTACCAGCTGCC
GAGTTCTGCCACCACCACCGCCTCCGAAGAGTATGCCCGACCTTTTACCTAAAAGCCAAGAAGATTTCCC
TAAGCTAGGCCCCATTTCTCTTGCAGCTCCTTCCGATCCCTGCCAGCCTTCGCCCTGCGGAGCCAATGCT
CTGTGCAACAATGGCCAGTGCTCTTGCCTGCCAGAGTATCATGGTGATCCTTACACTGGATGCCGTCCCG
AATGCGTTTTGAATTCTGATTGTCCCAGGAATAGAGCGTGTGTCAACCAGAAGTGCGTTGATCCTTGCCC
AGGTCACTGTGGTCTCAATGCTCTCTGCGATGCCGTTAACCACATTGCCATGTGCCACTGCCCCGAGCGG
ATGACGGGCAATGCGTTCGTTTCCTGCCAGCCAATTCGCGATGATCCACCACCACCAACGACACCCAATC
CATGCCAGCCCTCGCCGTGCGGTGCAAATGCCCAGTGCCTAGAGAGGAATGGCAACGCCATCTGTTCTTG

CCTAGCCGGATACTTTGGTCAGCCGCCCAATTGCCGTTTGGAGTGCTACTCCAGCTCTGATTGCTCCCAA
GTGCACAGCTGCATTAACAACAAGTGTGTGGACCCGTGTCCAGGCAAATGTGGTCTAAATGCTGTGTGCC
AGGCGATCCAGCACAGAGCCCATTGCGAGTGCATCCCGAGATACACAGGAAATGCCTTTGTGCAGTGCAA
CCCCATTCCAGTTCCCAGAGTACCAGAACCAGTGCGCGATCCTTGCCAACCGTCACCCTGTGGTCCCAAC
TCGCAGTGCACGAATGTAAATGGCCAAGCGGAGTGTCGCTGTCTGCAGGAGTTCCAGGGTACCCCGCCAA
ACTGCCGACCAGAGTGTGTAAGCCACGATGAGTGTGCCAATACCCTGGCTTGCATGAACCAAAAGTGCCG
CGATCCCTGTCCCGGCAGCTGTGGCCAGAGTGCCCAGTGTACGGTCAGCCTACACATTCCGAACTGTCAA
TGCCCTGTGGGCATGACGGGTGATCCATTCCGAATTTGCCTGCCCAAACCACGTGG
>dme_circ_0001327_junction_seq
TCCGAATTTGCCTGCCCAAACCACGTGGCCCCTGCACCGGAATACGTGAACCCCTG
>dme_circ_0001328
GGTAAAAGTGAGGGAAGGAGGAGTAAATGGGTGCTTTGTCTCGGCACGCCTTGCACTCCACGACACCGGT
CTTGGGACATTCATCAAATCGCGCCTCATCACAGAAGCACGCCTGGTTAGGGTAGTTTTCTCCGGAGTCC
AGAGTCTCCTCGGTGCCTACCCACTTAGTGGCGGTCAGGCCGTGATTCTCGTATGTGCCCCGGGGCCGGA
GGTTCATAAATCTGCAAGCATCGGTGGCAAAAATC
>dme_circ_0001328_junction_seq
AAATCTGCAAGCATCGGTGGCAAAAATCGGTAAAAGTGAGGGAAGGAGGAGTAAAT
>dme_circ_0001329
GCACACATTCGGGTCGACATCCTTCATACGGGTTACCGTAATATTCCGGTAGACACTGGCACGAAGCAGC
CTCTCCACGCTCTCTGCAGACCGCATTGCTTCCGCACGGGTTCTGGTAGCATGGATTGATTTGCTCTCGC
TGAATGGGCGGCCGCGCATAGCATCCAGTGTAAGGGTCTCCAATATA
>dme_circ_0001329_junction_seq
AGCATCCAGTGTAAGGGTCTCCAATATAGCACACATTCGGGTCGACATCCTTCATA
>dme_circ_0001330
AACGCATTGTCAATGAGCAGGTGACTCCCTGCGAGCCCAATCCCTGTGGCTCGAATGCTGTGTGCCGCGA
AAGAAATGGCATCGGTTCCTGCCAGTGCCTGCCGGATCATTTTGGTGACCCGTATCAGTCGTGTCGTCCG
GAGTGCGTACGCCACTCGGACTGCGCCTCGAATAAGGCGTGCCAACAGCAGAAGTGTCGCGATCCCTGTC
CAGGCACCTGCGGCAGCAATGCGGACTGCAGTGTAACAAACCATCTGCCCACCTGTACATGCCGCATTGG
ATATACCGGTGATCCGTACCGCTACTGCCACGTGGAACCACCCCAGCGTAAGATAACCCACTTGAACGCA
CCTCGTTTTCGACCCTGACCTTCGACCCTTTCTAGTCCCTGCCCGAGTGACCGAACCATCTCAACCCTGC
CGCCCTTCACCATGTGGTCCCAACTCTCAGTGCCGCGAACTGAACGGTCAGGCCGTGTGCTCCTGCCTGG
AGCTTTACATCGGTCTGCCACCCAACTGCCGACCGGAGTGTGTGCTGAGCACAGAGTGTCCCACCGATAA
GGCTTGCATCAGTCAACGTTGTCAGGACCCGTGTCCGGGCACTTGTGGCATCAATGCTGAGTGCCGGGTG
CGTAATCATAGTCCTCTGTGTCAGTGTCGTCAAGGTTTTACCGGTGATTCCTTTACCCGTTGTTATCCCC
TGCCACGTAAGAGATTGCTTCTTTTTCGCATCAAACTCCATCCTGTAAATGCTAATATCCAACCTAGCCC
CGCCTCCTGTAATCGAACGAGTTGAGCGTGATCCTTGCCTGCCATCGCCCTGCGGATTGAATAGCCAATG
CCGCAATGTCCAAGGTGTGCCGTCCTGTACCTGCCTGCCAGACTTTTTGGGGGCACCGCCCAACTGTAGG
CCGGAGTGCACAATCAGTGCAGAGTGTCCCTCAAATCTCGCTTGCATACGGGAGAGGTGTATAGATCCCT
GTCCTGGTTCTTGTGGCTATGCCGCCGAGTGTAGTGTGGTAAACCATACGCCAATTTGCGTCTGTCCCGC
TGGCTTTACGGGTGATCCGTTCTCCAGTTGTCGTCCAGCGCCGCCGCCAGAGCCCACACAAAGTAAGAAA
TTGGAAAAATATCCAAACATGTTGCTGCAGCTAAACCAAACAACTCAATTAATAGGCGAATATGTGGATC
CTTGCAACCCATCACCTTGTGGACCGAATGCCCAATGCAACGCAGGAATCTGTACTTGTCTGGCCGAATT
CCATGGCGACCCCTACTCAGGTTGCCGTCCCGAGTGCGTTCTCAATTCGGACTGTCCCAGGGACAAGGCT
TGCCATAGCAGCAAGTGCGTAAACCCTTGTCCAGGAACTTGTGGCGAGAATGCCATTTGTGATGTCATCA

ATCACATACCCATGTGCAGATGTCCCGAACGCACCGCTGGCAGCGCATTCATCCGCTGCTCTCCTGTGCA
GAGTACGTCAATGTGGTTACCCTCCTCAAAAGCTCCTCTAACACTTGCCCCCATAGTTACAGTATCTAAT
CCCTGCCGACCATCACCGTGTGGTCCAAACTCGCAGTGCCGAGAGGTTAATCAGCAAGCAGTGTGTTCGT
GCCTGCCAAGCTTCATTGGAGCTCCGCCATCTTGCCGACCCGAGTGCACCTCGAATTCGGAGTGTGCACC
CACTCAAGCTTGCCTGAATCAACGATGTGGAGATCCCTGCCCCGGTACATGTGGAGTGGGTGCTAATTGC
GCTGTTGTCAGTCATAGCCCCTTCTGCACATGTCCTGAGAGGTTTACGGGTAACCCATTCATTCGTTGCC
AGCCCCAGAGTAAGACCACTATGCCGGAAAAAGAGTCTTCTTTACAAGAGTTACCATTCCCAGTTGAACC
CCCCGTTCGTGATGTTGCGCCCGTTGATCCCTGTCGCCCATCTCCCTGTGGACCTTATTCGCAGTGTCGC
CCAGTTGGTGAGGCCCCGGCCTGTTCCTGTGTGGAGACCTATATTGGTCGCCCTCCAAACTGCCGCCCAG
AGTGTGTGACCAGCTCGGACTGCTCCAGTCAGCTGGCCTGTGTGAACCAGAAGTGTGTCGACCCATGTCC
AGGACGCTGTGGTCTTAATGCCGAGTGCTTTGTGGTTAGCCACGCCGTGCAATGTATTTGCCAGCAGGGC
TTCAACGGAGATCCGTTCGTGCAATGCAAACCGGAGATCGCTTATGAAAATGAAATCCGCACTCCGTGCA
GCCCGAGTCCTTGTGGCCCGAATGCTGTGTGCCGCGATCGCAACGGAGTAGGCTCCTGCCAGTGCCTGCC
GCAATACTTTGGAGATCCTTACGAAGGCTGTCGCCCGGAGTGCATGCTTGACTCGGACTGCCCGTCAAAC
CGGGCTTGCCAACAGCTGAGATGCCAAGACCCTTGTCCAGGAACCTGTGGACTGAATGCCAACTGTCAAG
TGGTGAACCATTTGCCAACCTGTACTTGCCTGACCGGATATGTGGGTGACCCATACCGTCAATGTAATCG
ATTGCCAGAACGTAAGCCGGAATTAACAACAAATACTTTCCCCCTACTCAAGCCCCCCATTTTAGCACCC
CAAAACGAGTATGTAAACCCATGTCAGCCGACTCCTTGTGGTCCCAACTCCCAGTGCCGCGTTTCGAATG
AACAGGCGGTTTGCTCTTGCCTGCCCCTGTTCGTAGGAACTCCACCATCCTGCCGGCCCGAGTGTACCAT
ATCCTCGGAGTGTTCTGCTGATAGGGCCTGTGTGAATCAGAAATGTGTGGATCCTTGTGCAGCAGATACA
TGCGGAAATAATGCCATATGTAGGGTCCGCAACCACAGCCCTATTTGCAGTTGCATTAGCGGCTATACAG
GCGACGCCTTTACTAGATGTTTCCTTATTCCACGTAAGCCCATCTCCTATTACCCAGGGGTGAAATATAG
TAAACCCTTCCGTACCACAGCGCCGATTATTGAGACCAAGGACGAACCTTTAAGGGATCCCTGTATACCA
ACACCCTGTGGTCCCAACTCCGAGTGCCGTAATATCAATGGTGTGCCTGCCTGTTCCTGTCTGGTTAATT
TCATTGGCCAGGCACCAAATTGTCGTCCCGAGTGCACAATCAACTCCGAGTGTCCCAGTCAGCTGGCGTG
TATCAACCAAAAGTGTCGCGATCCCTGCCCGGGAGCCTGCGGACAAAACGCTGTTTGCAGTGTCATCAAT
CATACGCCACTGTGCGCCTGCATCGATGGTTACATAGGAAATCCATTTACCAACTGCAACCCCAAACCTC
CAGAACGTAGGTGTCTCAAGAGATCTTACAGATCCGGTGATTGTTTACCCCCTTCGATAAATCTTCCCTT
TGTAGCCCCAGCGCCACCAGTTGCGGACGATCCTTGCAATCCGTCGCCTTGCGGAGCTAATGCTCAGTGC
CGAAATGGCCAATGCTCTTGTATACCTGAATACAAAGGGGATCCATACGTCTCCTGTCGTCCAGAGTGTG
TGCTTAACACCGATTGCCCAAGGGATCGAGCCTGTGTGCGCAACAAGTGCATCGATCCCTGTTCTGGAAC
TTGTGGGGTAAATGCCTTGTGCGAGGTCAACAACCATATCCCAATTTGCCGTTGTCCAGAGCAAATGTCT
GGCAATGCCTTTTTCGAGTGTCGACCCGTGCCGCCTGCCAAGATTCAAAATCCCTGCCAGCCGTCGCCGT
GTGGTCCGAACAGCCAGTGCCGCGTGGTGCAACAAACTGCCGTCTGCTCCTGTCTGGCTAACTACGTAGG
CAGTCCGCCGCAATGTCGGCCGGAATGTGTAACTAACTCAGACTGTCCTGCCGATCAGGACTGCCAGAAT
ATGAAGTGTCGCGACCCGTGTCCTGGTACATGTGGCTTTAATGCTCTATGCAACGTCGTCAATCACCGTC
CCTTCTGCAGCTGCCCCACTGGCATGTCCGGAAATCCTTTCGTGAGCTGTCAGCAGCTAAGTAAGACATC
ATAGTTTTTATATACGGCCCATGCTCAAATATGAAGTTCTTCCAGTTATACGCGATGAAAGACCTCAAAA
TCCGTGCCAGCCTTCTCCTTGTGGTCCCAACTCCGAATGTCGTGTATCTGGAGATTCACCGTCTTGTTCA
TGTCTGCCTGAATTTGTAGGAGCACCGCCGAACTGCCGACCCGAGTGTATATCCAACTCTGAGTGCCCCA
CAAACCAGGCTTGCATCAACCAGAAATGTGTGGACCCCTGTCCAGGACTATGTGGTCAAAATGCCATTTG
TCGGGTGTTCAGTCATAGTGCCATGTGCTTGTGTGACGGTGGCTTCACCGGAGATCCGTTTAGTCAGTGT
AGTCCAATAAGAGATAGTCCTCCTGAAGTTCTGCAGCCCTGCAACCCCAGTCCTTGCGGCGTGAATGCCA
AGTGCGAGGAACGTGGTGGGGCCGGATCCTGCCAGTGTCTGCCTGATTACTTCGGAAATCCATATGATGG

CTGTCGTCCCGAGTGCGTTTTGAACTCTGACTGCCCTTCGAATCAGGCATGTGTCAACCAGAAATGCCGT
GACCCATGTCCCGGCACTTGCGGCCAAAACGCCGAGTGTCAAGTGGTCAATCACTTGGCCACCTGTAATT
GTTTGGTTGGATACACCGGTGACCCGTACAGCATATGCCGCATCACAGTCAATGAGCCGCGTAAGATAGA
TTTTCTTCTGCATAAGAACACCGTTACCATAGATGGTCTTCTTCTAGCTGAACGCGTCTATGTGAACCCT
TGTCAGCCGTCTCCATGCGGTCCAAACTCGCAATGCCGTGAAGTAAACGAGCAAGGAGTTTGTTCCTGCT
TGCCAGAGTTCATCGGCAGTCCTCCTGCCTGTCGTCCGGAATGCACCAGTAGCTCGGAGTGCGCCGCCGA
CAAGGCTTGTGTGAACCGAAAATGTGTGGACCCTTGTCCAAATGTTTGTGGTCAGCAAGCCGAGTGTCGC
GTGCGTAACCACAATCCCATTTGCACTTGCCTAAGTGGATTCACTGGTGACCCATTCACCCGTTGCTACC
GTCAGCCGCGTAAGAAACCAGTCACGAATAGAATTGGTCCTTTCGATACCATAAAATTAAAATTAATTTC
AGCTCCGCCTCCAGTTGTCGAACGTGAACCCCTTGACCCTTGTGTTCCTTCACCCTGCGGTGCAAATTCC
CAGTGCCGCGAAATTCACGGCACACCATCCTGCTCTTGCCTTCCGCAATATTTGGGTACACCACCCAATT
GCCGCCCGGAATGTTCCATCAATGCCGAGTGTCCCAGTCATCAGGCCTGTATTAATCAAAAATGCAGAGA
CCCCTGTCCTGGCTCTTGTGGTCTAAACACTCAGTGCAGTGTCATCAACCACACGCCAATCTGCAGTTGT
CTCGCGGGTTATATAGGTGACCCATTCAGCGTTTGCAACCCCGAGCCAATACCCGAGAAAAGTAAGATGC
ATTATCTACCAAGGCTAAAGCTCAAAAATCATAACATACATTTTAATAAATCAGTTCGAGACCCACTGCC
ACCGGAGGATCCTTGCAACCCATCACCATGTGGCTCTAATACACAGTGCAACAATGGAGTTTGCTCTTGC
CTGCCCGAATACCATGGTGACCCATACACGGGATGTCGTCCAGAGTGCGTTCTGCACACGGATTGTGACC
GTAGTAGAGCTTGTGTTCGTCACAAGTGTGTTGATCCCTGTCCCGGTACTTGTGGAACCAATGCGATATG
CGAGGTTCTAAACCATATACCCAACTGCCGTTGTTTAGAAGGAATGCAAGGCAATGCATTTATCCAGTGC
AGCCCAGTTCCAAGTAAGAAGATTTTGACCGAAGTCCCTATTCCAAGTTACCATAAACTCACCAACCTTA
CAGAGCTCGACGTCGTACAGAATCCGTGCCAACCCTCGCCCTGCGGACCAAACTCACAGTGTCGCGTTGT
TAATCAGCAAGCAATCTGCTCCTGCATCACGTCCTTCATTGGAAGTCCGCCATTCTGCCGACCTGAATGC
ACTACAAACTCGGAGTGCCCCTTGAACCTGGCGTGTCGCAACCAGAAGTGCAGTGATCCCTGTCCCGGCG
TCTGTGGTCGCGGTGCCCAATGTCATGTGACAAACCACAGTCCATTCTGTCGCTGCTTAGAGCGCTACAC
GGGTAATCCGTTTGTGAGTTGTCAGCAGATCATCGAACCTCCGGTTCCACCACCACGGCAAACATGCCTG
CCATCACCCTGCGGACCGTATTCCCAATGTCGTGAAGTTAACGAATCACCTTCTTGCACTTGCCTGCCAG
AGTACATTGGAGCTCCACCTAACTGTCGGCCGGAGTGCGTGACTAGCTCGGAGTGTCCCACGAATCAGGC
GTGCATTCAGCAGAAGTGTCGCGATCCTTGCCCAGGACTATGTGGACAATCTGCGGAATGCCGAGTCCTT
TCGCATACGCCAAGCTGCGTTTGCCCCGAAGGCATGGAAGGTGATCCGTTCACTTTATGTAAGGAAAAGA
GGATTCAGGAACTAGACCAGCTAGACCCCTGCAGTCCCAGTCCTTGTGGAATCAATGCCCGTTGTACTTC
CCGCCAAGATGCCGGCTCATGCCAGTGCCTGCCAGATTACTTCGGAAATCCTTACGAGGGATGTCGCCCT
GAGTGCGTTCTTAATTCGGACTGCCCATCGAACAAGGCTTGCCAACAGCAAAAGTGTCAGGATCCCTGTC
CAGGAACCTGCGGTCAGAATGCTCTCTGCAATGTCCTGAATCATATACCCAGTTGTAGCTGTATATCAGG
ATATTCCGGTGATCCTTACCGCTCGTGTGTCCCAGAACGTAAGACTAATTTTCAGATTAAAACGAATTTT
GTAATCTTTCACGGATTACCTACTCTTATAGCGGTCAAGGAATATGTCAATCCCTGCCAGCCATCACCGT
GTGGTCCAAATTCTCAGTGCCGTGAAGTGAATGAACAAGCCATATGCTCGTGTCTTCCGGAATATGTAGG
AGCTCCGCCTGTGTGTCGCCCTGAGTGTACGATATCTTCAGAGTGCCCGGCTGACAAGGCCTGCGTGAAC
CAGAAATGTGTTGACCCTTGTCCAAACACTTGTGGAGATCAAGCGATCTGTCGAGTGGTGAACCACAGTC
CGATTTGCTCATGTCGTGCCGGTTACACGGGCGACGCCTTCTTCCGCTGTTTCCCCAAGCCCCGTAAGTC
CTGTCATTCCCAAATGGTAGGTTCCAGTCCTAACTGTTTTCATATTTCAAAAGCGGTACCACCAACACCA
GTACAAAAGACACCGGTTGATCCTTGTGTTCCCACACCTTGTGGCCCCTACTCCCAGTGCCGATCCCAGG
GAGATGCCCCAGCCTGTTCCTGTTTGGTCGGCTACATAGGAGCCCCTCCAAATTGCCGACCCGAATGTCG
CATCAACGCTGAATGTCCTAGCAGCCAAGCGTGTATTAATGAGAAATGTAGAGACCCTTGCCCCGGCTCT
TGCGGCTATGGTGCCATTTGCAATGTGATCAATCACACGCCTAGCTGCACCTGTCCACCAGGATACTCGG

GTGATCCATTTAGTCAGTGTCAGCCCGTGCCACCTCCGCCTCCAACACGTAAGACTCAGTACGAACCGAA
AAGTGTAACATGGACTCAAACCATTCCTCCTTCCTCAGCCGTTAAGCTTGATGATCCATGCAATCCCTCA
CCATGTGGTCCGAACGCTCAGTGCAATAATGGAGTATGTACTTGCATTCCCGAATACCATGGCGATCCCT
ACAGCGGTTGCCGACCCGAGTGCATCACCAGTGCCGATTGCTCACGAGAACTAGCCTGTTCGAGAAACAA
GTGCTTCGATCCTTGCCCCGGTACCTGTGCCCCCAATGCCATTTGTACTGTCCTTAACCACGTGCCCATG
TGCACCTGTCCGGAGGGATATAATGGCAATGCATTCGTGCAGTGTAAACCCACACCACGTAAATATTTCC
CCTCAAGGCCGTGTTCAAAGCCCAACGATACCCAACTCAACGATTTCCTTCCAGCCCCTGCCCTTGTCCA
GCCTTGCCAACCATCTCCGTGTGGACCCAACTCTCAGTGCCGCGAAGTCAACCAGCAAGCTGTTTGCTCC
TGTGTGCCAGGATACATCGGAACCCCGCCACTCTGTCGACCGGAGTGCACGTCAAACTCAGAGTGTTTGT
CACACCTAGCATGTGTTAACCAAAAGTGCAATGATCCCTGTCCCGGCTCATGTGGCCGTAATGCCCAGTG
CAGTGTGGTTAACCACAATCCCTTCTGTACGTGCTTGCCAAGGTTCACTGGCAATCCTTTCGTGGGTTGT
CAGCAGATTATTGAGCCACCACGTCAAGATATTGTGCCACAGGATCCGTGCCGACCGTCACCTTGTGGTC
CGAACTCTGAATGTCGCGCCGCTGGTGAGACAGCAACATGTACATGCCTTGGTGATTTTGTCGGCTCCCC
GCCATATTGCAAACCCGAATGCGTGGCCAATTCAGAGTGTCCCTCGAACCTGGCGTGTATCAATCAAAAG
TGTCGCGATCCCTGTCCTGGATTGTGCGGTTCAAGTGCTACTTGCCGCGTCGTCTCTCACACAGCAATGT
GCATTTGTGATGCTGGTTTGACCGGAGATCCATTTACACAATGTCAACCCATTGTACAGGATGTTGAGAT
CATTAATCCTTGCCAGCCGTCACCTTGCGGAGCCAATGCCGAGTGCATACAACGGAATGGAGCCGGAGCC
TGCCAGTGTCTCACAGACTATTTCGGAAATCCCTACGAAGGCTGTCGACCGGAGTGTGTTTTGAACTCGG
ACTGCCCATCGAACCGAGCTTGTCAGCAGCAGAAGTGCCGTGATCCTTGTCCTGGAAGCTGTGGACAGAA
TGCCGAGTGTAATGTGGTGAACCATACACCCATGTGTAACTGCTTCGCTGGATTCATCGGCGATCCGTAT
CGCTATTGTAGCCAACCGCCAGAACG
>dme_circ_0001330_junction_seq
ATCGCTATTGTAGCCAACCGCCAGAACGAACGCATTGTCAATGAGCAGGTGACTCC
>dme_circ_0001331
GCACGTACAGAAGGGATTGTGGTTAACCACACTGCACTGGGCATTACGGCCACATGAGCCGGGACAGGGA
TCATTGCACTTTTGGTTAACACATGCTAGGTGTGACAAACACTCTGAGTTTGACGTGCACTCCGGTCGAC
AGAGTGGCGGGGTTCCGATGTATCCTGGCACACAGGAGCAAACAGCTTGCTGGTTGACTTCGCGGCACTG
AGAGTTGGGTCCACACGGAGATG
>dme_circ_0001331_junction_seq
CACTGAGAGTTGGGTCCACACGGAGATGGCACGTACAGAAGGGATTGTGGTTAACC
>dme_circ_0001332
TTAACGAATCACCTTCTTGCACTTGCCTGCCAGAGTACATTGGAGCTCCACCTAACTGTCGGCCGGAGTG
CGTGACTAGCTCGGAGTGTCCCACGAATCAGGCGTGCATTCAGCAGAAGTGTCGCGATCCTTGCCCAGGA
CTATGTGGACAATCTGCGGAATGCCGAGTCCTTTCGCATACGCCAAGCTGCGTTTGCCCCGAAGGCATGG
AAGGTGATCCGTTCACTTTATGTAAGGAAAAGAGGATTCAGGAACTAGACCAGCTAGACCCCTGCAGTCC
CAGTCCTTGTGGAATCAATGCCCGTTGTACTTCCCGCCAAGATGCCGGCTCATGCCAGTGCCTGCCAGAT
TACTTCGGAAATCCTTACGAGGGATGTCGCCCTGAGTGCG
>dme_circ_0001332_junction_seq
CCTTACGAGGGATGTCGCCCTGAGTGCGTTAACGAATCACCTTCTTGCACTTGCCT
>dme_circ_0001333
GTTCCTGAATCCTCTTTTCCTTACATAAAGTGAACGGATCACCTTCCATGCCTTCGGGGCAAACGCAGCT
TGGCGTATGCGAAAGGACTCGGCATTCCGCAGATTGTCCACATAGTCCTGGGCAAGGATCGCGACACTTC
TGCTGAATGCACGCCTGATTCGTGGGACACTCCGAGCTAGTCACGCACTCCGGCCGACAGTTAGGTGGAG
CTCCAAT

>dme_circ_0001333_junction_seq
CCGGCCGACAGTTAGGTGGAGCTCCAATGTTCCTGAATCCTCTTTTCCTTACATAA
>dme_circ_0001334
TTGTCAGCAGATCATCGAACCTCCGGTTCCACCACCACGGCAAACATGCCTGCCATCACCCTGCGGACCG
TATTCCCAATGTCGTGAAGTTAACGAATCACCTTCTTGCACTTGCCTGCCAGAGTACATTGGAGCTCCAC
CTAACTGTCGGCCGGAGTGCGTGACTAGCTCGGAGTG
>dme_circ_0001334_junction_seq
GGCCGGAGTGCGTGACTAGCTCGGAGTGTTGTCAGCAGATCATCGAACCTCCGGTT
>dme_circ_0001335
CAAGCAATCTGCTCCTGCATCACGTCCTTCATTGGAAGTCCGCCATTCTGCCGACCTGAATGCACTACAA
ACTCGGAGTGCCCCTTGAACCTGGCGTGTCGCAACCAGAAGTGCAGTGATCCCTGTCCCGGCGTCTGTGG
TCGCGGTGCCCAATGTCATGTGACAAACCACAGTCCATTCTGTCGCTGCTTAGAGCGCTACACGGGTAAT
CCGTTTGTGAGTTGTCAGCAGATCATCGAACCTCCGG
>dme_circ_0001335_junction_seq
AGTTGTCAGCAGATCATCGAACCTCCGGCAAGCAATCTGCTCCTGCATCACGTCCT
>dme_circ_0001336
TGCCGAAATGGCCAATGCTCTTGTATACCTGAATACAAAGGGGATCCATACGTCTCCTGTCGTCCAGAGT
GTGTGCTTAACACCGATTGCCCAAGGGATCGAGCCTGTGTGCGCAACAAGTGCATCGATCCCTGTTCTGG
AACTTGTGGGGTAAATGCCTTGTGCGAGGTCAACAACCATATCCCAATTTGCCGTTGTCCAGAGCAAATG
TCTGGCAATGCCTTTTTCGAGTGTCGACCCGTGCCGCCTGCCAAGATTCAAAATCCCTGCCAGCCGTCGC
CGTGTGG
>dme_circ_0001336_junction_seq
AAAATCCCTGCCAGCCGTCGCCGTGTGGTGCCGAAATGGCCAATGCTCTTGTATAC
>dme_circ_0001337
GCGTGTATCAACAACAAATGCAGCAATCCATGCCCAGAATCATGCGGAACCAATGCGGAGTGTCGTGTTA
TTGGACACGCTGTGTCCTGCTCTTGTCCAACAGGATACGCCGGCAACGCTTTCGTACAG
>dme_circ_0001337_junction_seq
AGGATACGCCGGCAACGCTTTCGTACAGGCGTGTATCAACAACAAATGCAGCAATC
>dme_circ_0001338
GGGATCTCCGGTGTAACCTGGCACACACTCGCATGCTGGTCGGTGCTTGATGACCGTGCACACGGAGTGC
AGGCCGCAAGCGCCGATACATGGGTTCGAGCAGCGCTGGTTCACACAGGCCAGGTTAGCCGGACAGTCGG
CACTACTGAGGCACTCGGGCCGGCAGTTTGGCGGAC
>dme_circ_0001338_junction_seq
AGGCACTCGGGCCGGCAGTTTGGCGGACGGGATCTCCGGTGTAACCTGGCACACAC
>dme_circ_0001339
GGACATGGGTCCGCACAGCGCTGGTTCACACATGCTCTGTCCTGAGCGCATTCGGAGCTAACCGAGCACT
CCGGACGACAAGCGGGCGGTGTGCCAATATAATTGGTAACGCAGGAGCAGACCGCATGTCCGTTAACCTC
ACGGCACTGGCTGTAGGGTCCGCAGGGCGAAGGACGACATGGTTCAACGGGAGCGGGCACTAAAATCAGG
GTGATCGTTATTAAGCCTGTTTGAGCAATGGGGAACACTAAGAGTGGGCCAATTTTTAAGGAAACTCCAC
ATTTTTAGGGCTATTTGCACGTTATGATCAAAATAATCTGCTTAGATACTTGTGTGGAAACTACAATTCG
AAAGTTCCCTTAAAATTCGACCCACCCAACTCGAAAGTAGTTCCTACATCTTGGTATCTCCCGACAAAAT
TGCGAGGGATTTCCAGTGAAACCGGAAGGACAACTGCAGCTGGGGGCGTGGTTGATCACGTGGCACTCGG
CCGAGACGCCACAGACTCCCGGACAGGGATCGCGGCACTTGTTGTTGAGGCAGGCGCGGTTCTTCGAGCA
>dme_circ_0001339_junction_seq

TGTTGAGGCAGGCGCGGTTCTTCGAGCAGGACATGGGTCCGCACAGCGCTGGTTCA
>dme_circ_0001340
AGATTGGATGCGCATCGAGTGATGAGTGTCCGTCCCAGCAGGCGTGCATTAATGCTCTTTGTGTGGATCC
TTGCACCTTTAACAATCCCTGCAGCCGTAATGAAGATTGCCGAGTTTTTAACCACCAGCCATTGTGCTCA
GCCGGTGAGTATTAATaaaaaaaaaCTGAATAACTGGCTCAAACTATTAATGATCTAATCCCGTAGAGCA
TGGTCGCACGCCTGGATGCGAGCATTGTCCACCAGGCGCGAACTGCGATCCAACAACCGGTGCTTGTATA
AAGGGTAAGTTCTACTGTCAGTCCTTCTCCTTAATCAACCTAAAACTCTTAGCTTTAAGTGTAAATACAA
TGTGTACATATTCTAATTTAAAATTAACCTCCCGTTACACCCGTGTAAATACATCTCTCTCCTTAGCTAA
TGTAACAATAACAACTATTACTACCAAGAACTCCACATCTACCAAGATACCAACGAAACCAAGAACAACA
GCTAACCCAAACACAGGCGTCAAAACGACGCCGACAACCACAAGGGTCACCACAAGAAATACCACCACGA
CCACAACCACTACTACCACTTCCTCCACTAGCACTGAATCGAGCACAATCACAAGCGCTACTAACCAGAC
CAGTAAGAACCAAAAACCAGACACTGAAAGCACCACCTCCCACACCGATGCCACGAGGCGGTATCGCGAT
GGTGAGAACAATATCACCGATACTCCGACACCGAGGCCCACGATCCAGACGACCACGCTGCGGGGCGAAG
GTGTCATGGGTGACAGTCAGAGGCGGTCCACTACGACGCCCAAAATGAAGACCACCCGGCTGGATACTTC
CAACGAGGTACCAGATACCACATCGCCGTGGCCAATAGAGCTGCCCACAACGGAGGGCACCACCACGGAA
GTCTACAACACCATGTTTGCCCCGGTGGTCAACACCACTGACACCTCATTAATTAACCCATGTACAGTAG
ATACTAATTGTGCCCCTAACGAGCACTGCAAACTGGGCCATTGCCGTAAAAAAGGTACATTAAGTTTATA
TGTGAAATGCACGCCACTTTGGGATTTCATCCTATTAATTGACTAAATAACAAGTCATTTTAATTGGAAA
ATATAATTTTCCCAAAGTGGCACTAATACTATTTATTTGTACATAAACTAGTATGTATAGGGCTCGTATT
CTGGCATTACAAACTAATTTTGTTGTAAAAATTGTACATATTTAGGATAGCCCTTGTACATATTTGGGAT
AAAATTTATCTAAAAATGAAATCACCATCATTTAATTAACTATTTAACTCACTAAATCCTTTCTAACAAG
TAGAACCGCCGGGTTCACCGAAAACACCCGAACCCTGCCAATCTAACAACGACTGTATTGAGAGCGAGGC
CTGTTACATGGGTTTGTGCCAGGATCCTTGTGAGTTTGCCAAGATCTGTGCCGCGACTGCCAAGTGCACG
GCCAAGAGTCATCGACCAGTGTGCACCTGTCCGCAGGGCCATGAGGGCAATCCGATGGTCAAATGTGTAA
CCACTCAAACCTCAAGTAAGGCACTAATCAATTAATCAACAACTTTCATTTAATGCATCGGATGCACTAC
CAATAATTGACCATAAGGACTCTTCTCTTTCTATCTGAATAACAATATTTAATCAACTTACAGTTTGAAT
GCACTGACGATTCGGACTGCGGTGTCACGGAAGCGTGCATCAACCAACTTTGCCAGCATCCTTGCGATGT
TCACGATCCTTGTGCCACGAATGCTGTTTGCATCAACAGCAACCACGCAGCTGACTGCAGTTGCGCGGAT
GGCTTCCAGGGCAATGGATTTGTTGGCTGTCAGCCAGGGTTAGTATACCCTTAATCAACACGATGACGCA
CCTCTGTTGTCCTTCAAGAAATCTCCTGCTAATGTGTCACCATGTCTTGTTTCTAAAAATCTCTCTCTTT
CATACACTGTACAAATCCCCGTACACCACGACACATGTATAAAACTTGTACCGCCCCGACACCTACTCAA
CTCTAAAAACCAACCAACAAAAAAATATAAATACACCCACCGATCCAACCGAATCGCACCGTTATCCTAAC
CAAAACCACAGCCACGTTCCCATGTCTGCCAATACAACGAGGACTGTCCACCGACGAAGCTCTGCGATCG
CCTAAACCGACGCTGCATCAATCCCTGCCAAGAGGACTCTTGCGGCGAGAATGCCGAGTGCATACCCGTG
AACCATGGCACTGAATGTCGCTGCCTGCCCGGATTTCTGGGCAACGCCTACGTGCAGTGTCTCCCTAGTC
AGGGATGCCGCTCCGATTCCGAATGTGATTCGAGTCAGGCCTGCATCAACGGAAAGTGCTCATCGCCCTG
CCAGTGTGGAGCCTATGCCCTCTGCGATGTGGTTAATCATCGTGGTGTTTGCAAATGCCCTCCCGGCTAC
AACGGAAATCCCAAGGTGGGCTGCAGTCCGCCTCAAGATCCATGCGACCCCAATCCATGTGGCTTAAATG
CCTTGTGCGAACTGGACAATGGCAACCCCATCTGCTACTGCCCGAAGGGTCTTACCGGAAATCCGTTCAA
AAATTGCAGTTCCTGAGGGAGATGAGTGCACACCGAATCCCTGTGGTCCCAACTCTGGATGTCGTCGCGT
AGGTGGAAACCCGGTGTGCTTCTGTCTGCCGGAATATGAGGGTCAACCACCGTCGATTCCTTGTGAGCTA
CCTTCCAATCCCTGCGACCCATCGCCTTGCGGACCGAATACTCAGTGCTCTGTGCTCAGCAATGGGTTCT
CCAAGTGCACTTGTCTGCCGAACTATGTGGAGAGTCCGAACACCATCCGTGGATGTGTTGAACCTATTAA
CCCGTGTGACCCGAACCCTTGTGGAACTGGTGCAATCTGCGATTCATCACGTCACCCCGTGTGTTACTGC

CCGGACAACAAGATTGGCAATCCTTTCAGACTGTGCGACAAACCTGCAGTTACCATTGAACTTTGCCAGC
CAGGACCATGTGGTCGAAATGCCGAGTGCTATGTGGCTGGAAATCGCGAGGAGTGCTACTGCCGCTCGGG
ATATGTGGGAGATGCTTACCAGGGATGCCGTGAACCGAGTCGCACCGTTTGCGACCCCAATCCTTGTGGA
CCCAATGCCAACTGCGTTGTGGCTGGCGACGGACAAACGGCATGCGTTTGTCCTGACGGTTTGTCAGGAG
ATCCCACGTCCGTAATTGGCTGTCATGGCTACGAATGTCAGGTGGATGCCGATTGTCCAAATAGCAAGGC
CTGCATGGGCTACCGCTGCTACGATCCCTGTCCAGGAGCATGTGGCCAAGGAGCTCACTGCCAAGTGGAG
GAACATCACCCAGTTTGCTCTTGTAACTCCGGCTTGACTGGTAACCCTGGAATTCGCTGCTATGCTTTGG
ACCATCCCAAAAAGAATCCTTGCGTTCCTAGCCCTTGTGGCCGAAATAGCGAGTGCAAGTTGCTAAATAA
CCGCGCTGTTTGTTCGTGCATCCCAGGTTACTTGGGCGATCCTCAATCGGGATGCCAACCTGAGTGCGAC
ATTAACTCCGATTGCGGTGATACCTTGTCGTGCATTAACCACAAGTGCGTGGATCCATGTGCCGGAGCGA
TCTGTGGAATTAATGCCATCTGTAATGTGCGCCAGCATACGCCAGTATGTCTTTGCCTGGACGGCTTCGT
CGGCGACGCCTTTTTGCAGTGCGTTCCCATTGGAATACTGAAGAACGTTTCTCGGGATCCGTGTGCTCCG
TCGCCCTGTGGACCTCATGACGTTTGTTCGGTGTACGGCGATGGAGTGGCCCTTTGTGATCCCTGCTTTG
GGCCAAATGCCCAGCAGAATCCACGCTGCCGACCCGAGTGCGTCGGCAATTCGGATTGTCCGTTTGATCG
CGCCTGTTTGGGTCAGCGTTGTCTGGATCCCTGTCCCGGATCCTGTGGCCGGAATGCTATCTGTAATGTC
TACGAACATAATCCCGTATGCGCTTGTCCCACAGGACTATTTGGCAATCCGTACGAACAGTGCACAACTA
AGTCGGTGGTCGAGACGCCACCTCAACCCAGCTGTGCTAAACTCCATTGCGGTGCCAATGCGGAGTGCAA
GCGTCAGCACAGCGGTCTGGCCTGCGTCTGTCGTAAGGGTTACTTCGGAGACCCTCATATTGGTTGTCGG
CCAGAATGTGTTCTTAACAGTGACTGTCCCGCTGAAAAGGCTTGCTTGAACTCCAAGTGCGTGGAAGCGT
GTACCGGTGTGTGTGGCGTTAATGCAGTTTGCCGTGTGGTTAACCATGCGCCTGTTTGCATATGCGCCGA
GGGATATAGTGGAGATGCCTCTATTGCCTGCAATCCGTTCTACCTGCCACCTCCAGAGCGACCTCATCCG
TGTGAGCCATCACCATGCGGACCAAACTCCCGATGCAAGGCCACCCCTGATGGCTATGCAGCCTGTTCCT
GCCTGCCGAACTTCAAAGGAGCTCCACCAGTCTGTCAGCCAGAGTGTGTGGTTAGCTCGGAGTGTGCTCC
CAACCAGGCATGTCTGAATCAGAGGTGCACAGATCCATGCCCAGGTATTTGCGGTGGTGGAGCTCGCTGC
GAAGTGCTGAACCATAACCCCATCTGCTCCTGTGAGGCCAACTTCGAGGGTGATCCGTTTGTGGCGTGCT
CACCCATTCAGGATCCAGGTCGCGACATCCCTGTGCCAAAGAACCCGTGCGTGCCATCTCCTTGCGGACC
CAACTCCATTTGCCAGATTAAACAAAACCGACCAGTCTGCTCCTGTGTGGCCAACTATATCGGCAGCCCA
CCGTATTGCCGACCGGAGTGCACGCTGAGCAGTGAGTGCCCATCGGACAAGGCCTGCATCAACGAAAAGT
GCCAGAATCCGTGTGCCAACGTATGTGGACACAATGCCCGTTGTACGGTCATCGCTCACTCCGCTCACTG
CAGTTGTGACGAGGACTACGAGGGAGATGCCTTCATTGGTTGCTCTAAGAAGATTACAGAAAGGGCCCGG
TGATCACATTGATCCATGCTACCCTAACCCATGTGCCGAGAACGCTGTTTGCACGCCATACAATAATGCC
GCACGCTGTACCTGCATTGAGCCCTATAATGGCGATCCTTACAGTACCGGATGTCGACCGGAATGTATCT
ACAGCTCGGAGTGCCCCTCATCGCTGGCCTGCATCAAGCAGCACTGTCGCGATCCATGCACGGCTGCTTG
TGGAGCGAATGCCGAATGTACGGTGGTCAACCACTTACCCTCTTGCAGTTGCACCAGAGGATTTGAGGGT
AATCCGTTTGACGGATGCAAACGGGTGGTCGTTGTGCGACCCGAAACCGTGTGTGAACCCAATCCCTGTG
GACCGAACAGCATTTGTCGCTCTGTAGAGGGACATCCAACGTGCAGTTGCCAGGTCGGTTACTTCGGAGC
TCCGCCACAGTGCAGACCCGAATGTGTGGTCAGCTCGGAGTGTGCTCAGCATTTGTCCTGCATAAATCAG
AAGTGTATGGATCCGTGTGTGGGCACTTGTGGATTCAACGCCAAATGTCAAGTCAACAACCACAATCCTA
TTTGCTCCTGTCCAGCCAACTATGAGGGAAATCCATTTGAGCAATGCATGCCCAAACGCCGCAGAACCAA
CTCGCAACGTAGATCCATGCCTTCCCAGCCCATGCGGTTCCAATTCCATTTGCCGAAATGTGAACAATAG
GGCAGAGTGCTCTTGTGCGCCAGGAATGTTTGGAGCACCACCGAACTGTCGACCTGAATGTGTGATTAAC
CAGGATTGCCCATCAAATCGAGCTTGTATACGGCAGCGGTGTGAGGATCCTTGCATTGGCATTTGTGGCT
TCAATGCCGTGTGCTCCACGCAGAACCACCAGCCCAAGTGCAGTTGCATTGAAAGCTTTGAGGGTGATCC
GTACACCGCCTGCAAAATGCGTGAGAG

>dme_circ_0001340_junction_seq
CGTACACCGCCTGCAAAATGCGTGAGAGAGATTGGATGCGCATCGAGTGATGAGTG
>dme_circ_0001341
GGTAGCTCACAAGGAATCGACGGTGGTTGACCCTCATATTCCGGCAGACAGAAGCACACCGGGTTTCCAC
CTACGCGACGACATCCAGAGTTGGGACCACAGGGATTCGGTGTGCACTCATCTCCCTCAGGAACTAAATC
ATGGTTAAAGCAAGTTTTAACTTTTAGAAATCTTTTGTAATATAGACAATACTCACTGCAATTTTTGAAC
GGATTTCCGGTAAGACCCTTCGGGCAGTAGCAGATGGGGTTGCCATTGTCCAGTTCGCACAAGGCATTTA
AGCCACATGGATTGGGGTCGCATGGATCTTGAGGCGGACTGCAGCCCACCTTGGGATTTCCGTTGTAGCC
GGGAGGGCATTTGCAAACACCACGATGATTAACCACATCGCAGAGGGCATAGGCTCCACACTGGCAGGGC
GATGAGCACTTTCCGTTGATGCAGGCCTGACTCGAATCACATTCGGAATCGGAGCGGCATCCCTGACTAG
GGAGACACTGCACGTAGGCGTTGCCCAGAAATCCGGGCAGGCAGCGACATTCAGTGCCATGGTTCACGGG
TATGCACTCGGCATTCTCGCCGCAAGAGTCCTCTTGGCAGGGATTGATGCAGCGTCGGTTTAGGCGATCG
CAGAGCTTCGTCGGTGGACAGTCCTCGTT
>dme_circ_0001341_junction_seq
AGAGCTTCGTCGGTGGACAGTCCTCGTTGGTAGCTCACAAGGAATCGACGGTGGTT
>dme_circ_0001342
GGAATCGACGGTGGTTGACCCTCATATTCCGGCAGACAGAAGCACACCGGGTTTCCACCTACGCGACGAC
ATCCAGAGTTGGGACCACAGGGATTCGGTGTGCACTCATCTCCCTCAGGAACTAAATCATGGTTAAAGCA
AGTTTTAACTTTTAGAAATCTTTTGTAATATAGACAATACTCACTGCAATTTTTGAACGGATTTCCGGTA
AGACCCTTCGGGCAGTAGCAGATGGGGTTGCCATTGTCCAGTTCGCACAAGGCATTTAAGCCACATGGAT
TGGGGTCGCATGGATCTTGAGGCGGACTGCAGCCCACCTTGGGATTTCCGTTGTAGCCGGGAGGGCATTT
GCAAACACCACGATGATTAACCACATCGCAGAGGGCATAGGCTCCACACTGGCAGGGCGATGAGCACTTT
CCGTTGATGCAGGCCTGACTCGAATCACATTCGGAATCGGAGCGGCATCCCTGACTAGGGAGACACTGCA
CGTAGGCGTTGCCCAGAAATCCGGGCAGGCAGCGACATTCAGTGCCATGGTTCACGGGTATGCACTCGGC
ATTCTCGCCGCAAGAGTCCTCTTGGCAGGGATTGATGCAGCGTCGGTTTAGGCGATCGCAGAGCTTCGTC
GGTGGACAGTCCTCGTT
>dme_circ_0001342_junction_seq
AGAGCTTCGTCGGTGGACAGTCCTCGTTGGAATCGACGGTGGTTGACCCTCATATT
>dme_circ_0001343
GCTGGTTAAAAAGAATGAATTGCTGAATCAAACAGGATCAAAAACGAAATGCAGCTTATCAAGTGCTTGG
TTATAATTCAGCTGTCCCTCTTGCTGGTGGAGGAGTCATTTGCCGGCCGGGACTTCTACAAAATACTGAA
C
>dme_circ_0001343_junction_seq
CGGCCGGGACTTCTACAAAATACTGAACGCTGGTTAAAAAGAATGAATTGCTGAAT
>dme_circ_0001344
GCCTGTAACGAAACCCGCCTCAGGCACCAGGAAATGCAACTGCCGCCAGGAGATGGTCACCCGGAACCTT
GGACCCGGGCGCTTCCAGATGATCCAACAGACGGTGTGCGACGAGTGTCCCAACGTGAAGCTAGTCAACG
AGGAGCGCACATTGGAGATCGAGGTGGAACAGGGCATG
>dme_circ_0001344_junction_seq
ATTGGAGATCGAGGTGGAACAGGGCATGGCCTGTAACGAAACCCGCCTCAGGCACC
>dme_circ_0001345
TTGTCGGTCGCAAGGCCCAATGTTCATGTCCGCCGGACTTCTTTGGCAATCCGACTAGTGAGTGCCGACC
GCTGGAAGGTGGATGCTCTAGCAAACCCTGCGGTGAGAACTCCAAGTGTACTGAAGTGCCCGGAGGCTAT
GAATGCGCTTGCATGGACGGCTGCATTGGAGATGCCCACCAGGGATGCCTTTGCGGCGGACCTCTAGTGA

ATGCCTGTCGCGATCAACCGTGCGGTCTGAATGCCGCCTGCCACG

>dme_circ_0001345_junction_seq

CCGTGCGGTCTGAATGCCGCCTGCCACGTTGTCGGTCGCAAGGCCCAATGTTCATG

>dme_circ_0001346

AAACGACCAGAACTGCTTGGCCGATGAGCGTTGCATGAGAGGAACATGTAGAACCGTATGCAACACCGAT
GAGGCATGTGCTCAGGGACAGATTTGCGAGAATCGAATGTGCCAGACTGGATGTCGTACCGATTTGAGTT
GTGCCACCGACGAGGCCTGCGTGAACAAGAAGTGCCAGAATCCATG

>dme_circ_0001346_junction_seq

GCGTGAACAAGAAGTGCCAGAATCCATGAAACGACCAGAACTGCTTGGCCGATGAG

>dme_circ_0001347

GTACATTCACAATATTCACAACAACCGCAACCATTCAAGACGAACTTAAGGGCGAATAGTCGGTTTCGCG
GCGAGGTCTTCTACCTGAACCTCGAGAATGGCTACTTTGGCTGCCAAGTCAACGAGTCCACCGAATATTT
GCAGCTATTCAACCTATCGAAACTATGCGACGGTACCCAAGATTGCTTCCTGGGTGCCGATGAGTTGAGC
AAAGAGCTCAAATGCACAAGTGAGTATCTAAGCGCGAATCGCTTAAAAGCCTAATGGGTTTTGCCTAACC
TCGGGTTCGTGTACTTTTACGCAGATGACTGTGATAAGGATGGCACCAAGTGCACACATGGCGCGTGTTT
GAACGGCGTCTGCCATTGCAATGACGGCTACGGCGGCTGCAATTGCGTGGACAAAGGTGAGTCTGATTGT
GTCTGTTCTGGTCTTAACTTACATAACCGTTAAAATCCCCTGCTAACTCCCCAATTTCTGCTGCCCCCAG
ATGAGAACGAATGCAAACAGCGGCCGTGCGACGTGTTCGCCCATTGCACAAACACACTGGGCAGCTTCAC
GTGTACCTGCTTTCCGGGCTATCGAGGCAATGGTTTTCATTGTGAAGG

>dme_circ_0001347_junction_seq

ATCGAGGCAATGGTTTTCATTGTGAAGGGTACATTCACAATATTCACAACAACCGC

>dme_circ_0001348

GTCTTATCTTTGTAGCTATACGCACTCTTGATGACCGTCTCCCGATCTGTTCCACTCAATATACTGCTTC
TGAATTCGGAAGAATTCAAGGCCGAGACACTCAGTCCTTTGAGACTAGGGGGATTACGCAGATCCGGGGT
CGCATCGTATTTCTTCATCAGCCTCTTGATCAGAGGCAGCCGTTTCTCTTTTAAAAT

>dme_circ_0001348_junction_seq

TCAGAGGCAGCCGTTTCTCTTTTAAAATGTCTTATCTTTGTAGCTATACGCACTCT

>dme_circ_0001349

AGCCACGAAAGGAAAGATGGCAACGTAGTAGGCCACACATACCACGGACACCATCCAGAAGTCCAGCTTG
AAGGTGACTATATCGCTTAGTTTGGCCAATTCTCCACCAGGATTGTTATTTCGCTTAAGGATTCTCTCCG
CGCGCTTGTCCATCCAACCTAAG

>dme_circ_0001349_junction_seq

CTCCGCGCGCTTGTCCATCCAACCTAAGAGCCACGAAAGGAAAGATGGCAACGTAG

>dme_circ_0001350

TCAAGTGTAGTGGAAATGGCATTAGCATCGCAATTGGTTATTGGTTAGTAACAAACAGAATCGATCTATT
GCACATAGTTGCCCAATAAAGGCCGCCTGTTCATGTGGCGGCACTGCATCGCGCTTTTGGCATG

>dme_circ_0001350_junction_seq

GGCGGCACTGCATCGCGCTTTTGGCATGTCAAGTGTAGTGGAAATGGCATTAGCAT

>dme_circ_0001351

GACTAGAGAAAACAAAATACAATTTCAAATCTTGAAAAGTGACATTCAGCGCAAGTCACAAGAGAATTAC
AAAATAATAATAATAAAACACATAGATGAATTAATACATTTCCATTTCCGTTTTGAGAATTATTTGGTTG
GGAGAAACAAAGTTTGCGTAAGGATGAGAAACAAATTTCCAACTAAAAACGTGTCATTCTCTATTGA

>dme_circ_0001351_junction_seq

CAACTAAAAACGTGTCATTCTCTATTGAGACTAGAGAAAACAAAATACAATTTCAA

>dme_circ_0001352
TCAACCCGGGTGCCAAGGAGCGTTTGGTGCAGATTACTGGTCCAGCTGAGGATAAAATTAAGTGAGTAAA
GTAAATAAATTAAAGCTAATTTACCTTTATGTCAAAATGTTTTAATTCTACGACAGCTATGCCAAACAAC
TTATGGAGGACACCATTCGTCGGAACGCCTCGCCGGTGCGCCTGGAACCCGCTCCTGCCGTTGGAGGATC
CTGCTCCTCGCTTAACTCGTCCAACTCGGACGACGCCATTGTGCAACCAAGAACGCCAACCGGAAGCAGT
TTGGCCAACCGGCTAAGCTTTAACTCGGCCCAAAACTTCATGACCgccacagctgctgcacagcagatct
cgcagcagatgcaccaccaaacgcatcacctgcagcaccaacagcagcagcaggtcgccgcagtagccgc
cgcagcagcagcggcggcaCATGCTCAGGCGACAGCCGCTGCCGGTAAAGTCTTGCGTCCCAATCAACAG
CTGCTAATGCACTCATATTCCACAAACGATGCTTCCGTAGGTGAATACAAGTTCACGGTCAACGTGGGCC
AGCACCTGATCAAGATCACCGGCGACTGCTGCGAACTAGTGCGCGGTGGCCAAACTCGTGCTCGATGACT
ATTTTAGCAGCTCGGAGTTTCTGGCATCCATTGAGGCTGGCGCCGCCTTCGACGGCACTTCGCTGGTGAC
CACGCCATCGACTCCGCTTCCCGGAGCCGGGCCGCCGCAATTTTGGCTGCCCGGCACGGACAGTGGCATT
GGACTGAATTGCGTTGTTTCCTCGTCAGCGAACAACAATGGTGAAGGCGACGACGAGGTGTTCGCTGAGC
CCAGCAATGGAGGATCATCGACTAGCAATCAGAATGGACTGGCCAGATCGCGTCGCAGCCACTTTTCGCG
CAAGGAGTCCACGCCGGAGACCAAGGGCGCTCGGGAAAAGGGTGACTTGGACGATTTGGCAGGCACCAAT
TCCTTGAAAAGCAATGCATGTAAGATTACAATCCTTTTATGCTTAACCAAGTCTTTGATGCTTAAACTTA
AATTACAGCTCGCGTTTCTTACGATATTGAACACTTGCTCTACTACTCCATGAGTCCGCACTCGTGGACC
CTGCCCACCGACTGGCAAAAGATGCAGGAGACAGCGCCCTCGATTCTGCGCAACAAGG
>dme_circ_0001352_junction_seq
ACAGCGCCCTCGATTCTGCGCAACAAGGTCAACCCGGGTGCCAAGGAGCGTTTGGT
>dme_circ_0001353
GTTTTCCGGATTCAATTCGCCAACCACTCGCCATTCAGATGAAACCCCGAAGCGAAATTCAATCTAAACC
AAATCAATACTGAAAATCAACAAATGAAAATATCAAAAACCCTGTGCATAAAAACCGCGTAAAGCCAAAC
TGCAACCAACCAACCAACAAAATCAACAACAATTTCTGGAATTAATTTCATTTGCCGCGCGCGTCCATAA
AAATCTACATAGAAAGCGTCACAAAATAAATACACCAGCCAGCCAGCCAGCGAGGAAAGTGAATTGGTTA
GCCATGGAGAAGGCGTAATGGCCATCTGCTGACCCAGGACCCATAACTTTAGGGTTAGTATGTCGTCGCC
GTCTTACTGACTTTGCACTCCACGAAATGGGGGAACAACCCGATTTCCATTGCCTTCGAGCTTCCTTTTC
CTCGACGTTTTTCCATTTTACTTCTAGCCGCTTTTTTATCTACTTAGTACTAGTGGGTTTCttttttttt
tGGGGCATGCTAGACTCATTAGAATCCAATAAATGTGGCAATGATAACTTTGTTACAACAGCATATTGCA
CTAAGTTTGTATAACAACTAGAGTAATGAATTTACACAGAGTAATGAATTTATATCATCTATCTTAAATT
CGATTGTATACAAATGAAAGTAAATACAGGGTATCTTGCATTTGCAACGCTTTATTGCCGCTTTCCTTTG
TTGAGATGCGTTGTTCGCTCGTTTTGTTTATCCTTTGGCCCCGATCAAATAGGCATCATTTGCCTGGCGG
CATCTCGGATTGGTAGGGAAAAAGGTTCGCGACCGCATCAGACGCTTCGCCGTGAATCCgcatgctccgc
atccttggcatcctccgcgcccttggcatccCCATCCGCTTCCATGTCCTTCGCAGTGACAGCAATTTGA
GCGACAGACAGTTGATGACTCTGTTGACAGGAGCGGGGCATCTTCGGTGTGGAGCCGGAAGGTGGGGCCA
TCGCTTCCCTATATAGTATATAACCCATCGTCTTGGGCGATATTAACCCTATTTTTGTCGTAAATTAATT
ACGACCGGCAGATGGAGGGTACGGAAATAGGCACGCACATTTTTTCTATGAAATTTAATATTGAGCGGTC
AAAGCGAAAGCGAAAAGTTGGTAGATCCTTTAGAAGATATAATCTTAATAGTTTGGGTCTATGAAAATAC
GCTGGGTATCCTTAATCGCGAATTTAGTATGCATCAAATCTTATGCTTGTTAATATTTAAGCGGAATTAT
AATATCTGCTGAAATTGTTACATTTCTTTGATAAAAATGATTATTTTATGTTTTCGATGATTCACTCAAC
TGTCATAACTTGAATATATGTATATGGATGCAACATATTTTAATGCGGGTAATATTTCAATAGCCAACGA
AAAGGTTGTCCATTTATTTTATTTTGTTTGTTTTCAATTCGCCGCCTCATTCAGCAGTTGAATCCTTGGG
CAGCCACCCTCAATTCCCATGACTCCAGACCATATATCATCGGCTCCTGCGACTTCCGGTTGTCGCTTTT
GGCACCCGCCATTCCCACTGTCTATTTATAGGCGCCGCTCACGCAGACAACCGCACATGAAAACCGCCGC

CACTCCCCCCATCCTTCTGCGCCGCCCCACAATGCAAAGAAAATTGAAAAGCAAGTTATGTGTATTTTCC
TGCCACTCGGACTTAAGGTGGCTAAGTCTGTCAGCATGTGTGAAAATGGGAAAAGAACAGGCAGGCTTTC
TTCATGCGCAATTCCTCAACTCAACCACGCACCCACTCAAAACGATACTCCATTAGTTTTAATACTTTTT
GCCCTTAAACGAAAGATACAATTGCGGTAGCAATAATAGAAACACTCAAGATATCAAGAAACATGGACAA
AGATAGTGTATCTTAAAGCTAACATTTCTTCAAGACATTCAGATAAAGTAGTTAAAAGGAATAATAATAA
ACTGGTATTTTATTTTAAACTTTATATATTCACTTCATGATAGCTAGTATTTTAACCGCAGCTGTGGCTA
GCATTGTGTGTGGAATTTATTGAATTTTTATTGGACTTCTCTGGCGAGCCACGCTGGCTGCTGTGTACTC
GTTAGGCGTGGCATAATTCATTATGTTGACTCACCGACACCACGCATCCCACTCCGTGGCGGCCCCACCA
CCAACAGCACCCACATCCTGGCGAATGTCCTGTCCGTGAAGCCACCACCCATATCGTCAGCAGGCTCCAC
TAATGACATGCCGCGTGCTTTGCATGCTAATGCATGGAGCATAGAACGAGCTGCATTCCACATTGGCAGC
TCAATTTGTGAGTTAAAGAGCCTAGCGTAGGTTTAAATATGCCTGCTTTCTGTGTGAGACGGACAGCAAG
AGAGGTCCTTTTCAAGGACACGCTTCGTGATTAGCAGAGAAGCGAGGCGACTGGCAAACGGGCCATTGTT
ACGCTGCAAAGATCAAAACCGGATTGCCAGGGCAGCGAGCTGCCAATCTCACCTTTTCATATCAAAGCAA
ACCAATTTTTGGGCAGTCAAATGTGAGAGGATtgtgtatatgtatccatgatatatgtatatatatgtat
gtatgtatgtatgtatatgtgtaAAGGAATGAACCACCCTGCAATGTTGTCCTTGGCGCTGTATTTTTAG
AAGAAGTTCCCAGAAAATACACGATTTGTCTGCTGAAAGCAAAAAGGCGTATGAAAGCTTTCATTTAACT
TTCAATGATTTGTGTTTCAGTGAATTGAATAATAGAAGGTATTATTAAGCACATAAATAGGGTTATTTTT
AAAGAATTAGAGCTTAGAATATATTCTAGCAAGGATTACAATCCTTTTTGGTTAGATGTATGTTTCTTGC
TTTATATACTGCCACTTCACTTATATATAGATATTTAATTACTATTTTACTTTCAAGGGTCTGAAATATA
TTCGACCATATCAACAAGCTCGAGTTAATCCCAATCTGGAAATGATTTCGCAGTGAACTGGTCTAATTGC
GAGGCCGTGATCTTCCAGCTGGGTTGCTTAACCATACCGATGATATCTGGCAACTTCCGGCTTGCATGCA
TGGGCTATTTACATATCTGCTCTCCATTTCCATTCCCATAGCCGAATTCATGGAGCCTGGCAGCAAACTT
TCGCCTTTGCCCAGAACTTTGGCCCGGCGAAAAGTTTGCCCAAAACACTGAGACCAGACCATGTCGCCTG
GACaaaaaaaaaaacaaaaaaCCAAAGTTTCGGGGGATGAAATTGGGCAATATCGCAATTccagttccca
gttcccagttcccagttTACCAGTTTTCCTCGCATTTCTAAGCAAAACGTTTAATATTCACGCTTGAGTG
CCTTTTCTCCTTGGCAGTGGCTATGAAAATGTGTGAAACTGTTTGACGCTTATCTCTGCGTCCTGCTCGG
CGTTTTGACCAAATTTTATTGCCTGTTTCCAGGCCAAACGCTTCGTTTCTGCTCAAGTGAACCCGGTGTT
CCTTAGTGTTCCGCTATTCCGCTGTTATGCAATTAGCCACCGCCCATGTGTCTGCCGTTCCAGCAcggat
gcggatgcggatgtggatgTCCATTGCTGTGTGTGATGATGATGATTAGGAAACGCTCGCATATTAATCA
TACGCCATGTTGTGCGCACTTATGTGCTAATGGAGCCCCCGTTCAACTTCTCGGCCAATGTCGAGCGTTC
AAATGCAGTTTCGAGTTACTTGTTAACTGTCACTTGTTTACATACTCGAAAATCGGACTGTCCTGAATCC
AAAGTGAATCCGTGAAAGCACGGACTGCAATTCGGACGTGGTCTGCAATTGCGATTCCTGATTTTCAGTT
TGGTTCAGCTTGCAGCTATCATTCAACTTTCATGCGAAcatttgttttgcccattctgtttttccaacgc
ttttttcttatttttcattttcttttcctttttGGATTGGATTGTGGCTGAAAAAAAGGATAGAGGCAGTG
GTCCACAGGCTACTTAGTTGCTAGTCCACAGTGGTTTGGTACTCTTTTTCACTACCATTTTCAATTTATG
TTGAGCTAATGCAATAGAAATGTGAGTAGATAATACATATTATTAAAAAAATACTGCAGTAAGAAATCTA
AACCATTTTTCTAGACATACCATTGAATAGTAATATCCAAAGTTAACTTAAACCCACCTAAGTATTCCAA
GTAAGTTTTACTAAGATAACATTGAGACACCTTGCCAGTCATCGATATTAGATGCCTTGTTTACAGACAC
TTTTTATTGATGCCAAGAAGTTGGCCAGGACTTTTCTACGCAGTCCAAGTTCGACTGAAAGTCGTAGTCA
GTGTGCCGGAGACCAGAACAGTGCTCATCTAGGACTCGACGAACCGAACGGCGGAAGTGCTCGTCGCCAT
TTTTAGTGCACTTCTCGAAAGTGCGCACATTTCCGTTTGGCATCAATGATTCGAACCGAAGCCATCATCC
CACCCACTTTACTGCCCACATTTCGTGTCCATTCTCCTTCGTGttttttttttcttttttttAGCCTCTCG
CTTTTGGCCACTGGCTGTTTGTTGGCCTCTCACTTTTGCTGATTTTAATTTTGGTAAAAGTTGTCACATT
TGATTTTTGGCAATTGTTCGGTTTTATGCGCTCTAGTCTCCGCCTTATTCAGCTTTTTTTATGGCGCTTT

TTATGTGGTGCCACGTCGCTGTTGCTTTGTTTGGACGCGCGGACAGCATTTGGCTTTATTGTCTTGGCCA
CCCCGATACATTCCGGCTTTATGGTGGAGTGGCAAGGATACACAGAGTGGCATGGAGTGCCTGTTGCATG
GCATGGAAGTGGATTTCTTAAAAGAAAAGACGGGGAAAAAGAGATTACGAGGGCGGAAAAAAGTTTGGCA
GAACTCGAAGGAATCTCTGTACGTTTGGCTACaaaaaaaaaaaTCTAATTAGCCAGAGAATTAATTTGGA
CTTGGTAGTTAGCAATGAATGTTGGGGATTTGGCGGAAAAGAAAATGTTGAGATAGGTTACAAAAGAAAG
TGCAATTAGCTTAAGCAGTGGATTGCATTTTTATGAGCCTGAAATAGTTAGAAGGCAGTTTATTTCAGTT
TAAAACATATAATATACTAAATACATTATCCGGTAAATTTGCAAATGAATTAATTAAGTTCATAACGTGA
ACATTTAAATTTCAAAGCCATTGAATAGAATTTGGCTAATAAATCCGATTATAACTCATTCATGTCTGCC
AAAATTGTTTACCAAACTATTATCGCACCGAAGCAAAGCCATTTAGCTTTAAGCCCCTCAAACAAAAGTA
AATGCTTGGCTAACACTTAAAGTATGGACAACTTGTTTGCCCATTCACCTCGGTAATCTGGGTGCATTGT
CATCGATATCAGATCATCTGGCACCCCCATTGTCCTGCATGATAATGCTCGTTTggggggggggggggTC
CTTTTGGGCCAGATGGGCGTTCTATCTGCGAGCTGGAAGGATAATAACCACTAAGATATTTATGAACTGC
TATTTCTTATGCAATTAAATGTGGCCTTTAAGTCGGCCCCTCTACATCCACATTCACATCAGCATCCCCA
GCCGCATCCTCGTTCACATCCACATCCACCCACTTGACATTGTCATGGCTACGGCTGAGCCTCAAATGAC
ATCGGCTGAGAATGGGAATTTTCCCGTGCTTTTCCGGCAACCAAAGCCGCTCCTCTTGCTCCAATCCCCC
GCCATCAATCGTTTTCCATGTTCTGTTGCCACTTGACTGCACTTTGGATACACATATTGGATGCATGTTG
TACACAGCTCCGATCCGCATGGGAAAATGGGACTGGATGAGGTGAATTGGGGCAACTGGAGCAGGGAACA
GGCGGCACGAGTGGAATGAATCTGAAATAAAATCAAGTGAAATTTGAACTTTTGTGTGAAGAGCGAAAAT
GGCAAAGGCGTTGTTTGTAAATGGCAATCGAAACGGCAATCGGAGCGACACTTTTTCACGATCCTCCAGG
CGAAAATTGTACGCCAACTATATACTATATACTAAGTGTCCTGTACAGTAAAGATGGGCTAGGCCAAATA
AATATGATAATGATAATGATAAACATAACAAAAGCGTGATTTGATAAGTAACATCACATCAAGTAGTAAT
ACAATCTTAATCTCTTGTAATTGAATATGTGCATGATGAATTTTAAGGAGGCTTCACTGTTTGAGACCCT
TCCCCCCACCCCCTTGTCGCAACCTTCATGCTAAGGCCAACAACAAAGCTAATAAAAAGCTATGACCTCG
AAAAGTTCAATTTTGTTGCTGTACATAAACGTTGAGTGTGCTTGACAATTTTTCACTTCAAGCAACAAAT
TATGCCGTAGCATAACATTAGGAGCACCCATCcacacacacacacacaGCCGCAATCACATGGTTGGTCC
TTCGACATTTTGAAGTGACCccgcccaccgcccaccgcccacctcccCTTTCCTTTTCCTTTTCCGCTTG
TGCGTGCCAAGCCGACTCGGCAGCGACTTTATCTAAAAGCGCACTTAGTCCAAGTAGGCCAAGTAGATTA
CGGCCCAAGAACCGCTTGCCTTTGGAGGAGGCATCCATAGAAGAAGATACACAGATACATAGATAGATTA
TAAATACATCCTATATCCTGTGCCATCAGTCTGCCCACGCGCAGCCCATCCAGTGAATGAGTGATGAATA
ATCGTAATTTGAAGGATGAATATACCGCATATGGTTATATTTATTAAATTTGTGGGTTATTTATTACGGT
GAAAAAGGATCTAACTTCAAAGTACGTTTGATTGAAACAAATCTTTTGCTTTCTTTCTCGGAAAGTTACT
TACTAAAACGAGCAATGAAATCTAACAGTCTATTCAAAAATGAGGATCTAAGGTCGTGCTTTTAATGATA
TAAACACTACGTGGCAGCACTTGAGTAAATTTTGCATGCAAACACGGGCTCCCaaaaaaatacaaaaaaa
aaaaaGGGGAGAAGCAAGTTGGCGGCGGTGGGGGATCACAGCAAAGTGCGGCGCCAGACCCTGAAAAGTG
ATTAAAACGATTGTTGTACAACAATGTGCGCGTCCTTGTAAGGACTTTGCAGGCCACCCACTCCGCCCTC
AAGTTGCCaaaaaaaaaaaaGTGGCGCAGCCATTCTCCACTTTTGTAATTTTTATATTTTCACCCCCCCT
CTTCCATTATACAGTCTGCACTGGGTAACGTGAATTTGCATCTCCCCAAGTTTAAGTACAATAAAAAATA
CATTAATACATACACAAAAAACTTGAACTAAGCTACTCAACATTATTCTTTGGGTTTTTTCATTTTAAAT
CATCTTGCGTTGGTCAAACGGACATGCCATGCTAGACTCCACTGTATCTGCATATTGTATCTATATAAAT
GGCTGAATTTATCTAGAAAAGCTTAATTGGAAAAGTGTTTTGGGACTGGCACTCGAAGCTTTTAGCCAAA
AATGTTTCGACAAAGGCACAAGGGAAGCGATTTCAAGCGATGTTGCCTGATTTGCTAGCTTTTCGGGGGT
TTTCTGGGTTAGGTGTGTGCCCAAGTGGGTGGTGTTGTCCGCCTTTTTGGGGCGTCTTACACAATGGAAT
GCTGTGGGTGGTATGGTGATTCTTGGAGACCGGCGAACAATTTGTCCTCGAGCCTGTCAGCGGCACTCCT
TATTATTATTAtgctgctgttgctgctgcttctgcaattgctttgcttgttgctCTCTCATTGTTTAATT

GTTAACCATTTTGTTTGCTAgtgtgcgtgtgtgtgtgtgctggtgtgtgtgtgtgCGGCTTTGTTGCATT
GTTTGATTGTTTCACACGCCCCAGCTTTGTCTCTCATTCTGTGCGTGTGTGCGAAATAACATTTGTCTAA
TGAAAAGCGCCCTTGTCTCTTGTCTCGCTTGCACCCGGCTCATTGTCTCGCTCGCACCACGTCTGGGCAT
CTTAAGCTATTTTTAGCTCATTATGTTGCCATGGCCCAAAGGACATTGAGTCCTGGGACACGCCTCAAGA
ATTCCGGCTGCCTCTTCACTCAAACTGAATGATTCGTGGTTATTTCATATTGTTATTCCTGTTCTACAAC
ATAATGATTGAATTTATATTGCTGGTTATTTTGGCCATATATTTTCATTTGCTCTCAACTCAATTGAATG
AAATATTGATGTTTTATTTCAAATTTCAATTTGCACACCAGCATTTGCGTGCTTCTATGCCAAAACTCTG
CAAACTCAATTAACTTTAATATTTAAGTCAACTAGTGCGTCAAGTGTATACACAGTTGATAATAAATTTG
CAAAATGCTCCAAGGAGTCGCATCTCCGGGCAGCTTTTTCATGTTTTGCCACAAATTACAAAAGCCGGTT
TGGTCACAAAATATGGTTTTGATTGCTCTGCAAACACGAATAAAATAATTGCCAGAAAAGCATGAATATT
CAACAGATGGAATGACAAAATATCCATCTCTTGTTGTTTGCCCATCAAATTTGCATGCCGAAGTTATCAA
ACAAAAGCAGCAACCTCAACAAATCCAATTGGTTACCAAAAGTGCGGAGGTTGCTTGTTTTCGATTTGTG
TTTCGCCATTTTTGATTCGAGTTTGCAGTTGGCCGAAAGCGACAAGACAAGACAGTTGGCAAACAAGGCG
GCCAATTGGGTTGTGAATGCAAACGTGCAACACACCAGGTGACAGACTGTGGAACCTGAACACGACACGG
CAGCAGCTACAACCGAAATCCGGTTAATTGAAAAACCGAATGGTCCACAGAGACATTCGCCAGgatacag
acgcagatacagatgcagatatagttagagatacagatacagatacagatgcagatacagttacagatac
agttagagatacagatacagatacagGCAGTGGGTTGGCTTTCTCGCATCCTTACCGCATTAGCTGCAGT
TGAGCGAGTGTCAAGGTGAGGCCAGGTGAAAGCAGCCAGGCGGAAAACAGGCAGCAAAAACAGGCCAAAG
TGGTAAAAGAAACTTGGCAACACACGAGTGTAGTGGTCACGCACACTGCAAGAAAAAAATGTACGCTCAAC
GTGCTGTTTTTGCCAATGGTTTAGCGTTAAGACTTCATTTATCTAACCAAAGCATGCAGCTTAAGGATGC
TTCATACTAGATTTATACTGATTATGCATTATGAACTACAAATAGCTGAGGGATGTACTACATTTTGAGG
AATTCGCAGGATTCAGGAAATGCAAAATTTGGGAGTCCCACCTTAAATGGACATGGGCGACTGTTTTTTG
CCCCTTGGGCTGCTGGCATCTGGCCAAATCACGTAATCACAACTTGTTTATGGCGCATGTGCCAACTATT
CCCGACTTTCACCTTCGCCTACACCCCCTTCCCCCCATCCCTTTTTACAAATCCGTGGGCCCTGACCCTC
CGAAAAAGAGACATACCACACATACCGCCGTGCAGTTGCAGCCATGAAAATTAATTTCAATTAGCGGAAA
TTGTTTTTACACCTGTTTGCCTTCCAGCAAAACTGCTGCTACTGTGTAGAAGCCGTCCAAATCGTTGGGC
CAATTTGCGGCCTGTCATCGCATTGATGTGCTAATGCCGCACCATATAATACCCTGCCCATTGCTTGTGC
CACCTTCACGCCCTGTGCAAATTCATAAATCAGaattgcaaattgcaaattgccaagtgcaGCAACAATG
GGGAATGTGAATGTGCAACaaaaagaagaagaaaaaaagtggaaaaTTATGGCATACCGCTGGGCGACAC
TTGAGTGCAGCAAGTGCCACGCCCACTCCCACGGGAGCTCGTAACTGGCCAAGCAAATTCTTTTTAGCAC
TGCTTTCAGGGGTTTcatggctccatggctccatggctccatggctGTGGGATTGGGTGTGGGCATGGGC
ATGGGAGTGCCGGAATGTGGCTCCAAATTGCGCACAAGTTTGTCTGAACCGAGCAATTTGATGCGATTTA
ATTGCAGCTTCATCCGGCTGAGGAAGATGCATCCACAAACTATGGTTGTTATTCAGATTTATTCTTCAAA
AAAGAACTAGAACAAACTTTTGTGGTCTTTTATGTCATTCTGTTTATATGTAATTGAAAGTCACATTGAA
AATTTGTATATTCAGTACAGAGTACTTCCAAAGTCTATCATGCTCCATTTGATTTTCATGCACTTTGGTT
TTGCACATTTAGCGGACGTCATCAGCAATTATTTGTGCTGGATTCTCAATTAAATTGCAATTGGCGCAGC
TATATAAATTTTCATTGACGTGGGCGTTGTGGATTTTGAATTTGACGTTCGGATTCTGATTTCTTGTGGG
CTTTTTTTAATTGATCAGCATGCATTTGTTAAAATTAATTGAACATAATTGTAAAAATATTCAACCACAA
GCATTTAGTGAGTAATCGAAACGGTCCGCTCAATCGTTTGTAGCCAATTTAATTGTGTTTACTTCGTAAT
TGCTGGTAATTGTTAAATTTAGGGATTTAATATATAATAACATATGATTTAATACGTGACTTTAATATGT
GTTTATGTGTAATGGAAAGTACTTCCAAATAATTCGAATTGTTTCATATGTTTAGTGTTCATTTTTGCTG
CATGTGCATGCCACATTTGATAACAAAGCCATGCTATGTGGCAGCAACTTTAAATTTATTCACCGACTAA
GCCGCAACTAAACAATAGCCAAAACTTTTCTAATTTTTGCAGGATGAGCGCGCCACTGTGCCACAATTTC
GGTTTACACCTGTCGAAGCTGCCACACCCACGAGCAACTTTTTCTACCACCAAACCGGCAACAGCGGCAA

CAACAACTGGAGGCGCCACCAGCATAGGAATAAGCAAAAGAGCAACAACATCAGTGATATGGAACTGTGC
ATAGCAACAAAGTCAAAAATTGAAACCGCTCAGAGTTcaatgccgcaaagcagccaatgcagcagcagca
gcaactgccgAAACTATCAAAAGAAATGCCACCAAAATCAAACAAACACTCAAGTACAGAGCACTCAGCG
AAGCAATTGCAGCAGCCACAGCGATCGAGACCACCTGACAAACcaacaaaagcaacagaaccaacgaaca
aatagtgcaacagcagcaagtgcagcagctgcaacattatccgaatccactaggtgcaacatcatcatca
gcagcagcagcagcagcagcagcagcactaaaagcagtaaaaacatcagggagccgagcagcagcagcag
caCACAATTAAACACCCTCTACTCGCTGCTGGTGCTTTTGATAGTCGGCCTTGCCACAGCCACCGCATCG
CCCAGCCCCAGCCCCAGCCACGCCCCCTTTGGCCGCGAGAGCAGGCGCGATGCTCTTTTGGCCTACTACA
AAAGGGCACACCTGCCGCAGAGCAGCAGCAGCGGCATTTCCCTGGGGCACAAGTATCACTTGCTGCACGA
TGCCAATGGCCTTGACTTTGACGAGCTAACGCCCACCGCCAGCAGTGTGTCCGCTCTCAGTCGGGCGGAG
CGTTTTCGATTGCGTAAGCGCAGCGCCACGCCCACAACGCCCGCCACTGCAGCAGGAGTCACTTCACTAG
ATGTTGCAACTGCAGCAGCAGCGGCAACAGATGCAGCAGCAAAGGATGCGGGCAGCGGGAAGAAGCCGGA
GGAGAAGTGCGAGCCAAAAGTACTCGAAACGCTGCCAGATGAGCCGGGTAGGTTAATAAGTTATATTTAT
ACTATTATTATTAAATTATAATATAATGATTACATATGCAATGATAGACTGAAATGGTGCTAGCCAGATA
ATAGGAATATATAAAACAGCTTACTCTTAGGTATTAACTTCTTTCCATAACTTATTCCTAATCACTACTG
CTTCACTCAACATTATTTACTTGTAACTTTCCATAGTTATGTAAATATCAACTTAATAACTACTATCCTA
AAGTAAATTGAGATTTACGCAGCATTAACACAAAAGTTCTTCAATCGAGTGCCAGCAGTTTGAGAAGTGT
GCACATTTTGTTTAACTTTGCAACTGCCACTGTTAGAGTGCCACCTTATAATTTGTTGCATAAGACGGTT
GGGTGAAAGTTCCAGCTGTGCTATGCACTTGGTCACGAGAACTACTTAGAAGCAATGCAATTGCGAGAAG
CAATAAGCCAGCGTACAACTTGGTGCCCCTCAACTTTTGAGGCAATcacacacacacacacaAATGCCAA
CGCAAATTCAAAGGATTGCCGTAAGGATTGGTGGAAGGTGGAGCGGGGGGATTCACCTTCCGGTTGCCTT
CATAACTTTTGACGGACATTTGTGAGAACGAGTGCAGCCTGCATACGGCGCATCGATCCAAGGCAGAGAC
AATTAACGACAATTTGCGAAATTGCCAACGCGGCGCAATGGCGAATAATCAATAAAATTGCTGAGATAGC
AGCCCATATAATGTGCTCCTTCACTTTTCGGTGTGCTTCGTCCTTTCGATGttttttttttCTATCTCCT
GCTTCCTGCTGACACAAATCTCTGCGCAAATCTGTGTCCGCCCCTCCTCACCTTGTTGATTGTCGTGGAA
ATATTACGCATACGACATGGCTGACGCAAGTGAATGTGGGAGTCGTCAAGTTCTTTTCACTTTCGAGGGC
GCTGTGCTGCACAGAAATCGCGCGATTTTGGAAAGGCGGCGGCTCATATGTGGCGCCTCCAGCCAAGACT
CTAGTTTGCTCACTCTGTGCAGATGCCTCTTGTACAAGTTAATTAAGAGATGCTAATGAAGTCGCAGCTC
CAGGGCACTGGAAAAAAAGGCTAGATATACAGTAGACCTGGTGAACTAATTAAAATGGAAAATGTCCTTTT
TAAATGCTCAAAAACAAGTTGGAACACTTTTTATGTAAATATTAAAACCTTTTGTAATTGAAATTAGAAT
TGTCTAACCAGAACATTTTCGCGCCGTGTGCGCATCACAATATTTTAATTGAATTTGCATGACAATTGCG
GACGAGGACTTTAAGCAGCCGGAGGACATGTTAGGCGAGGATTTGCTCCCACTGTCTGCCCGCCAGTTGG
CTCCTTGGGGATGCTATTTTTAGTGCGCCAAGCGAATCGAGTATAAATATTCAATGAGTCGGCGGTGTAA
ACAGAGTCCAATACATGACGTGCACGAATCGAATCGATTGCGCTGCCAACTTTCCTCTGACGCAATCGGG
ACAGGACCTTTGTGTAATTAGAAGGCGGGTAGTGAGCGCGGTGCGTGGTTGGAGATGAATGAAAATAAGG
ACCATCCTTGGTGCGATTTGAATCTCGAGTGTGGTCAGATAAAATGATAAATAAATGTGCTGCGCAGCAA
AGGCAGCAATAGCAGCAGCATCGGGAGCAACACCGGCTGACAGCTGCCAACTTGGGCATTATCCTGGGCC
ATCCTGCTCGTCCCCCTCGCAGGAAAGCGCCCTGTCTCGCTCCTGTGGCCTGCGGTCAACTGCGAGCGCC
TTGTGTCGAATGGCTGTCAAGGATTATTgttgctcctgttgccgctgcggctgctcttgttgctgctgtt
gttgcctgcggctgcttttgttTCGCTCCCAGCTGGAGTGTGTCTGCCTGTCCTTGTCTCACCGTCCTTC
CCTTCTctttcttttcgtccttttttttgtgtttttttcttcttttttttGCTTCGTGTTCGTGTTGTTT
TGCTAACAATGCTGAAGCTGCAAAGTTATTGATAATTGTTTTATACACACTCTACTTCGTGATGCTGGAC
ACGCATATTGTTGCTCTGCCTTCTTTAAAAGCCCTATTTACTTCTGAATGGGAAAATATATGAGTTAAGC
AGCATAGCTTTTATCTCGAAAATATCATATAATCAATATGTTATTGAGCCCATTTAATTTAATTATTAAA

ATAGCCAGCAATTAGTGCAATCGATGCATTTAACTCTGTTTTCTCCTATTAAGTTGTCAACTAATTAGTT
TAGTACTAAAGCTGGCCAACTGTGGTTTCATTATTTATAGGGACATAAATTTAAAAACCATTGCCACTCA
ACAGAAGCATTTATGTTAATACGATAAATAAAACAGTcacacacacacacacacacacTCAACCGTGCTT
CCGTAGCAATCAGCTTATGTCATTCGCCTTGTCGTCCTGAATAATGTAATGAATGCAGAAGTACAGTGAA
GCCTGTCAGTAATCGACCGGGATTTGCAGAGTGTCAACAGCAGTTGAGCCAAAGATgagaaagaaagaga
gagagagagagagtgagacaacaaaacaaaggaaaacgaaataaatacgagagcaaaaagcaaaaggaaa
aggaaaaCAATGTCTGACATATTTTATTCATTAAGCGCAGAGCATAGAAAATAAAAGGAAAAACATGCAA
ATGACAATGAGGAAGCATACAGTTACGCCATGTAAATATTTTTATGGTCATAAGTAGGAATCCTGTACAT
ATGTATGTACATGCCGGAAAATGCAGATGGAAATGCGGGGAAAGGCGCGCACGGACATTAAAAAGCCAGT
AAAACGCGCTTTACTGATTTCATTTGGCAGCCAGGATTCAGTGCTCgtgtgtgtgtgtgtgtgtgtgtgt
gGGCCAGACTCTTGGCATAGTTGCTCAAATacacacacacacacacGCGCGCGGGCACATTTGTTTCGGC
CATGTTTGCATAAGTATGCATGTTTTATTGCACACACACTCGGTGTGTGTTTTGTGGCTGCTGCCGCTAT
TTACAAGTGTTTTCCCCATACATTTATACCCTGAAAAAACGAGGGGCGCTAGTGCGAGGGTATGTGTGTA
ATTGCGGACATTTGGGAAAAAGGATATCAAAAGGATTTGGCGGTAATGAAGACATTCTACTGCTTCCAAG
AACTGTGGTGGGATTACTTAAAGTTCAGATAACTCCTGAAGAAAAAACAATGAATAATGGTGTAAAAATA
GAAATATTTCCTTACTTAGTTTAATATCTGAAAACACTTACCCAACTACAATATTTCTCTAGCATGCAAG
AGTATACCAAATGTTTATGATTATGGCTGCGCGCGAGTGCTAGGAGCACTACATAATGACTTTATCTGGG
AGCAGGTCTCGTGCCATTTCTCCAGCTGCCTTATACTGATTGCTTTTGCCTCGATGTGTAAATTTTGGCT
CACGTTTGGCAGGaaaaaaaaTGAAGAAACACACTCACACACACATGGTAGAGGAGCATCCAGCAAACGT
TTGTATATCGATGTTACTTGTTGGCAATTTATTTGGTGGCTTCGGTGGCGTATACGCAATGTGGTCGCTC
ATCACCGAAAGCCCAGCTAATGGGAAATATTTGTTTTAAAAGCCAAAAGGTGCCGCCAGCCCAACTACAT
GACTCACGCCCAAACAACGTGTCCTTTTTCCCCGCTACCAGCAACCCCCTCCCTGCTCCCTTCTGTGGTT
ACTTTTGTTTCCCAAAGCCATAATGATGTCATCAAAGTGGCCCTACCATTTCCTACACTTTGGTGCGTAG
ATCTCGAGAGTTTCTTGTAACGTGTAAATTATGTATAAACATTTGGTCGGTTAGGAGCTGAACGAACTTG
GATTAAAAACCCTTCCCGCAGTCCCACAGCACGGCGAGGGTATTAAATCCTATTATTTCACAGTCGAAAC
GAAGCAAAACTTTTCGCACTCTTGCCGCAAGGTGTTGCTGTGAAACGTGTCCTGTGCGCCAGGTGAATAA
TTAATGGTCCCAAGTCGGGGGAAGTGGCCGTGCCGCATGGTCACTTAAATCACGTGGCGACCAATATACG
TGGCATATACGTAATTTTTTCGCAATTTCCGAAAATATCTTCAATTAGAAAGCACACGTTTTCCGTTTCC
TATATAGTGTGGTATAAATAAATTGAGTTTAGCAAAGCGTAAATTTATGCTGTGTTAATTTTATTGAACT
TTTTCTTTGTGTATTTAAATCGAATTCGATTTAAATGATATTTATATGCCCATTTCATATACATTGAAAT
GATTATTTATGACACAACGCAATTAATTGTGATCTGGTTTTATGAATTCAACAGGTTTTACGACTACACC
GACATCGCCGAGGATGCCGCACGCCAGTTTATTGAATTTTTATCGGGCAAATTCCCAAATGCCAACACAC
CGATTGCCATCGATGAGCCGACACGTGCGGAGGTGAGTCGCCGGGCCAATGGAATCGCCAGCTACGCCCT
CAACGAGGACGACAATCTGCTGGCATTCGCCATTGCCGCGCCCAGCATTCACACGGTGGTCGTTAAATTC
AGGGATAACGTTACGGTTGCGATCGGAATACGACCTTCTGCCTGCTCACCGAGAACAAACCCGCCGCCAC
CCGGGATGTGTACACCTGTCTGTGCCGGGAATCCTACTACCTGCCCAATTCCACGCTCCAGGGATTCCGT
GGCGATCGAGTGGAACTGTCCGAGGGCTACGACAACTACTCGTGCATTCCGTGTCCCGGCGGATGCACTA
ACTGCGATAGCAACGGCGTCTGTCTGACCTTTCAGGAGGAGGAGGTGCTCAATGTGGACGCCTGTCTGCG
CCTCCTGGTGGCCATCGTACTGGGCGCCTGCATCCTGTGCTGCATTGTCCTCGGGGTGATTGTCTTCCGG
CAGCGAAAGTGCAAGGGTTGCCGTCCATTTCTTTCCCGCATCCACCGAGCGCTGCCTTCTGGAGCCCTGG
CTTCGGGAGCTGGGCTTCATCACCTGCTACGGCGCCATCATACTGAAGTTGTACCGCCACCTGGTGGACT
TTCGCACCCGGAAAGCGCATCGCTGGGTGCTGCGCGACGTGGACCTGCTCAAGTATCTGGGCACCATGGT
CTTTGCCGTCATCTGCTACATGGCCGCCTTTACGGCCTCGTCGTTGGATCTTCTCGAAAGTGCGCAGCTG
GAGAGCCTCAGGGAGGCGGACACGAACACCTGCCATCCGCTCAAGTGGGAACTGGTCACGCAGACCAGCG

AGATGCTCATCCTGTGCTTTGGACTGCACCTGTCCATCGCCAGTCGGAATGCCAACACCCAGTTCCGGGG
TAAGATGTACAGTCAAATCGATTGCAGATCTTTCTTTATCAATTAAGCGCAAGAGATTGGCTTAGATGAT
TAACTAAGTAGAAGGGGAATTTAAGTCAAGTCAAATGAATTAATCGGTAATTTCCTTTTGTCCCATTGAA
GGGTAACCGATTTGAGTTCGAAACGATATGATTTACTTACTTTGCTTTGGttttttttttttttttttG
GTTTGAGTACGCTTTTGTGTTCTACACATTGAGATATATGATGTAATTAGGAAGTGTTTTGTCCTAGTCG
GGCACCAAACGAATATAATTCATATGCTTAGAGTATTCACACCCGTTCATTTTGTGCCTTTCGTCTGTCG
TGGGCGTGGTCGTCATTATAGTCATCGAAATGCGTACTTGGTATTGACATTGGTTGAGGAAACGATTTTT
GTTAAATAACTAGCCAAAACACAAGAAGTATTAAAATGTGGACATCCACTCAAAAGCTTGGTTGCTTTTG
CTAATTAAAATACAATTTGAATATTTCATTTGTTTATGGTTATATTTTGAGTTCTTGTTCTGGTTTTCAC
CGAGAAAATTAACATAATTTTAGTAATAAGCTAACATTGAATTGAAACTTTAGTCAAGCTATGAAGTTGC
TGCAAGTGTGTCTCGTTTAAACAGAGAATGTTCGACTGTTTTTAGCTCTTATTATTTATGCCTGAATGTA
TTGCACTTTAACTTGAACTCAATAAGCACGCCATTTAATTTGCATGTTTAGTGTTGATTTTTGCATGAAT
TTAACAAGAAAACACAACATCTAACTACTAACAAACAAATGACAAAAATCGGTTTCCTTTCAAAATTTCC
AATATGCATACCAGaaaaaaaaaTTCCAAACTAGTGTATCCAAGACTCTGCCTCACTGTAACTTTCTCTG
TCTTTCTGTGCCTCAACTGAATAATCTTAAACTGAAGTTCTCTGTCTAAGTAGTCTTCTTATAGCTATCG
TCCATCTTGTTTACCTTTTGAGTTACTTTTTAATTTGATTTGTTTCTTTTCGTTCATTTCCAGCACCCC
CAAAGATACAATCACGACCAATAAGCACAGACCACCCATACACATACTATATACATATAACTATACATAA
CTCTTCAGCACAGATCTATACCATGCGATTTTGCTCTCTCTTACCTTTGAgttccgtttccatttccgtt
tccgttccgttctccattttccgtttGACTTCCTGAGTGTGGTTTGCGGTGTTTGTTGCATCTTCATAGC
TTGCAGGCACCCCCAAAGATACAATCACGACCAATAAGCACAGACCACCCATACACATACTATATACATA
TAACTATACATAACTCTTCAGCACAGATCTATACCATGCGATTTTGCTCTCTCTTACCTTTGAgttccgt
ttccatttccgtttccgttccgttctccattttccgtttGACTTCCTGAGTGTGGTTTGCGGTGTTTGTT
GCATCTTCATAGCTTGCAGGTTCGTTCGCTAGCGTATGATCTATCAATACGTTTACCTGTGGATGCTTTC
AAGGGTACGTCACACGACGCCGGCCAGCGGCTGGGCGGAGGATATGCCGGGCTCTGTCTGGGCGATCCGG
ACATTGGTGAGCTGACCATATCCGAAATGAGTCCCGAGGACATACGCGCCGAACTCAAAAGGTACGAAGC
AtatctatatgttctattatctctattttctatACATTTTCGATTCCAGACTGTATACCCAATTGGAGAT
AATGAAGAACAAGACTCTGCGGCAGGACAATCCGCACATCAGTAAGCGACGTGGCGGACGCAAGGCGGGT
CACCGTCGCTTCTCCCTGCAG
>dme_circ_0001353_junction_seq
GGCGGGTCACCGTCGCTTCTCCCTGCAGGTTTTCCGGATTCAATTCGCCAACCACT
>dme_circ_0001354
GTTTCAACGCTCTTCCAACGAGCACGCAGCAACAGCAGAGCAACAACAGCAGCAACTGTCCACGGCAGCA
ACAGCGTTTTGAGTTTCGATTTCGAAACAGTTCTAGAATTGCCTGCGCTTGCGCTAGAAAACTCGTCGCT
TGACGTTTTCAAAAATAATCAACAGCTGTCCCATCCGGCATTGTTGAAACC
>dme_circ_0001354_junction_seq
AGCTGTCCCATCCGGCATTGTTGAAACCGTTTCAACGCTCTTCCAACGAGCACGCA
>dme_circ_0001355
GAGATGCCAGTGTGGTCACGGATCCAGTCCAAGTGGTAGGTGACGCGCTGGAATCCAGCAGGAGCACCCG
ACTGGCAGCCGTTGGAGGAGACGAAGTTGCTAACTCCCACCAGCTTGGAGCCGTCGTGTGTGACAAGGGG
ACCGCCAGAGTCACCTCCGCAAATGGACTTGCCGTCGACGGTGCGGGTGCAGATGACGTT
>dme_circ_0001355_junction_seq
CGTCGACGGTGCGGGTGCAGATGACGTTGAGATGCCAGTGTGGTCACGGATCCAGT
>dme_circ_0001356
GAGATGCCAGTGTGGTCACGGATCCAGTCCAAGTGGTAGGTGACGCGCTGGAATCCAGCAGGAGCACCCG

ACTGGCAGCCGTTGGAGGAGACGAAGTTGCTAACTCCCACCAGCTTGGAGCCGTCGTGTGTGACAAGGGG
ACCGCCAGAGTCACCTCCGCAAATGGACTTGCCGTCGACGGTGCGGGTGCAGATGACGTTGTCGCCAACG
CTGCCGTAGGTCCAGCCGCACTCTTCGTTGTGGACGATCTGGAGATCGACGCACTGCAGCCAGTCGGGCA
GTGGGCTGCCGTCGTAGGTGCCACCCCATCCGCAAGCGACGGCCCACCATTCGTTGTAGTTGTTGTAACG
>dme_circ_0001356_junction_seq
CCCACCATTCGTTGTAGTTGTTGTAACGGAGATGCCAGTGTGGTCACGGATCCAGT
>dme_circ_0001357
GAGATGCCAGTGTGGTCACGGATCCAGTCCAAGTGGTAGGTGACGCGCTGGAATCCAGCAGGAGCACCCG
ACTGGCAGCCGTTGGAGGAGACGAAGTTGCTAACTCCCACCAGCTTGGAGCCGTCGTGTGTGACAAGGGG
ACCGCCAGAGTCACCTCCGCAAATGGACTTGCCGTCGACGGTGCGGGTGCAGATGACGTTGTCGCCAACG
CTGCCGTAGGTCCAGCCGCACTCTTCGTTGTGGACGATCTGGAGATCGACGCACTGCAGCCAGTCGGGCA
GTGGGCTGCCGTCGTAGGTGCCACCCCATCCGCAAGCGACGGCCCACCATTCGTTGTAGTTGTTGTAACG
GTCGTTGTAGCTGGGGAGCTCCACCTTGTTCACCATGTGCCAGAA
>dme_circ_0001357_junction_seq
GCTCCACCTTGTTCACCATGTGCCAGAAGAGATGCCAGTGTGGTCACGGATCCAGT
>dme_circ_0001358
GATGCCAGTGTGGTCACGGATCCAGTCCAAGTGGTAGGTGACGCGCTGGAATCCAGCAGGAGCACCCGAC
TGGCAGCCGTTGGAGGAGACGAAGTTGCTAACTCCCACCAGCTTGGAGCCGTCGTGTGTGACAAGGGGAC
CGCCAGAGTCACCTCCGCAAATGGACTTGCC
>dme_circ_0001358_junction_seq
CAGAGTCACCTCCGCAAATGGACTTGCCGATGCCAGTGTGGTCACGGATCCAGTCC
>dme_circ_0001359
GGGGACCGCCAGAGTCACCTCCGCAAATGGACTTGCCGTCGACGGTGCGGGTGCAGATGACGTTGTCGCC
AACGCTGCCGTAGGTCCAGCCGCACTCTTCGTTGTGGACGATCTGGAGATCGACGCACTGCAGCCAGTCG
GGCAGTGGGCTGCCGTCGTAGGTGCCACCCCATCCGCAAGCGACGGCCCACCATTCGTT
>dme_circ_0001359_junction_seq
ATCCGCAAGCGACGGCCCACCATTCGTTGGGGACCGCCAGAGTCACCTCCGCAAAT
>dme_circ_0001360
GGTGCCACCCCATCCGCAAGCGACGGCCCACCATTCGTTGTAGTTGTTGTAACGGTCGTTGTAGCTGGGG
AGCTCCACCTTGTTCACCATGTGCCAGAAGTCGACGTGGGGGATGCGGATCAGGGCGATATCAGCGTTGG
AATGCTTGATGAAGTTGCCGTTGCCAACGGTGTGGGTGAACTGGGCGTTG
>dme_circ_0001360_junction_seq
GCCAACGGTGTGGGTGAACTGGGCGTTGGGTGCCACCCCATCCGCAAGCGACGGCC
>dme_circ_0001361
ATGCCGCTTCCGTGATCGTTTACTTCGGTGCCACCTGGCGCACCAACGCCCAGTTCACCCACACCGTTGG
CAACGGCAACTTCATCAAGCATTCCAACGCTGATATCGCCCTGATCCGCATCCCCCACGTCGACTTCTGG
CACATGGTGAACAAGGTGGAGCTCCCCAGCTACAACGACCG
>dme_circ_0001361_junction_seq
AGGTGGAGCTCCCCAGCTACAACGACCGATGCCGCTTCCGTGATCGTTTACTTCGG
>dme_circ_0001362
CTATCCTGGCTCTGGCCGTGGCTTCGGCCTCCGCCTTCGACGAGAAGGTCTTCGTAAAGGACCTGCCCAA
GGCCACCAAGATTGAGGGTCGTATCACCAACGGATACGCCGCCCCCGAGGGTAAGGCTCCCTACACTGTG
GGTCTTGGCTTCAGCGGAGGCTGGTGGTGCGGTGGCTCCATCATCGCCCACGACTGGGTCCTGACTGCCG
AGCACTGCATCGGAGATGCCGCTTCCGTGATCGTTTACTTCGGTGCCACCTGGCGCACCAACGCCCAGTT

CACCCACACCGTTGGCAACGGCAACTTCATCAAGCATTCCAACGCTGATATCGCCCTGATCCGCATCCCC
CACGTCGACTTCTGGCACATGGTGAACAAGGTGGAGCTCCCCAGCTACAACGACCG
>dme_circ_0001362_junction_seq
AGGTGGAGCTCCCCAGCTACAACGACCGCTATCCTGGCTCTGGCCGTGGCTTCGGC
>dme_circ_0001363
GGTGGCACCGAAGTAAACGATCACGGAAGCGGCATCTCCGATGCAGTGCTCGGCAGTCAGGACCCAGTCG
TGGGCGATGATGGAGCCACCGCACCACCAGCCTCCGCTGAAGCCAAGACCCACAGTGTAGGGAGCCTTAC
CCTCGGGGGCGGCGTATCCGTTGGTGATACGACCCTCAATCTTGGTGGCCTTGGGCAGGTCCTTTACGAA
GACCTTCTCGTCGAAGGCGGAGGCCGAAGCCACGGCCAGAGCCAGGATAGCTAGGAACACCTTCATGCTG
ATGTTTATACTGGATTCTACTCTCGGATGGCAGCCTTTTATACACATTTTCAGCTAAAACCATATCTGCA
ACTCGAAAAGGGCTCGTAAATAATCCTCAAGACTCCCATAAACGATGTCATCGATAAGAATGGAGGAACC
ACTCTACCAAGCCAGTAGTAGAAATCAAAGAAACTCTAGGGGGGGACTAGAccccccccACTAGACtatat
atatttatatatattatattattttattatttaccatatattaAGTATGCAGCAATAAACAGCCAAATTA
TTATCTATCTAAATGGAATAATATTTTGTGGCTAATCAAATATGTTTGTAGTTTTATTCATAATTATAAA
ATATATATTTGTCAACCTTTGCGCAGACCATTCTATCTTATCATCATTTTTCAGAACTTAAAATTTACAA
TGATCTTTGGACAGGAATGATTTTCAATAAAGTTGTTTGTTAACTTTTACATAAAATGTATGTCTGCTTT
TTGTCCTGATAAGGATAATCTGTAATAATGGCTGGAGGTTTTCCGCTTGTTTTCAAATCTATTACTAACT
GTTGCCAAGTTAGGCTGAAGGATTCTCGACTATTATTAATTTAATAGACAATTAATAAGAACAACAACAA
ACCAAAACTTGATGCACAAGTGTATGATAACCCTATATACTCACAGGTTTTTGATAACAAGAATTACAAG
TGCTTAAAAATAAAATAGTTTACAATTATTGTTCAAGTGTAAACAAATGTTTGAAAGTGATAATAAAATA
GGTGTTGGAAAAATGTTAAAAACTTGTGTGTTATTTTCATAAAAATGCTGATTGAACTTCTTTACAGCAA
AATTGTCAAAATTACTAAAAAAGTTAGCTGTCATTGAAGATAAGATTAAATCTAGTACAAATCTGTAAAT
TATTATTTGCTTGCTATTATATATAGCTTTTGCTTAAAGTTTCTAAACTATCCAATCGTTTTAATACTTA
CAAAAATATATCCAAAATAAAACAGAAACAAAATATACATCTGTATTTAATATTCTAAGAAAAACTGAAA
TATAAATTATCATTATTTAAATGAAGGAAAATTTTATTTATTATAAACAAGTTAAACAAAATGGAGATTT
TAGCCACATGCGTAAACCCTTTAATCTTATCGAAGGTAGGTCTGAAAAATAAACAAATCCCTCCAAATCA
ATTGATAGAAAACTGAGATAGTAAGAGAAATAAACTAGACTGGCACTAACAACAAATGAAATACGCAATT
CTTTATAACTTATAACACTACTTATCTTTCTGATTTAATTTAAATATGAGGCTTAACATTTAATCATAAA
TAATAATAAATTGGAAACCATATCATTCGTATTCTATGAAAACATTCTATGAAGATATCGAAAATCAATC
TAAACCATACAAAATCAAGTATCTTGATCCTTAAGATAATCCATTTTAGTTTCCCCATTCCTTAAATTTA
AACTATCGAATTAAAAAACAATTATCTATTAAATGCAGAAGGGTTTATTTCGAGAACGAAATTAGTAGGA
GATGCCAGTGTGGTCGCGGATCCAGTCCAGGTAGCTGGTGACGCGGGTGAAGCCATCGGGCAGGCCCGAG
GTGCAGCCGCTGGCGGCCACAAAGGAGGTGACACCGACCAGCTTGGAGCGGTCGTGGAGGACCAGTGGGC
CGCCAGAGTCGCCGGCGCAGGTGGACTTGCCGCCGGGGGTGGAGGTGCAGATGACGTTGTCGGTGATCAC
ATCGGTTCCATAGACGCTGGAGCACTCGTCGCGGGTGATGATCTGGGAGTCCACGCAGTTCAGGTAGTCG
GAGACGCCACAGCTATCGCAAGGCCTGCCCCATCCGGAGGCTAGAGCCCACCAGCCGGCAAAGCTGTCGT
GGCGCTCGTTGCCATCGGGCAGCTCCACCTTGTTGATCAGGTGCCAGAAGTCCACGTGCGGGGTGTTGAT
CAGCGAGATGTCGTTGTTCAGGTTGTTGGTGTTGTAGTCCGAGTGCTGGCGGAAGTGGCCGCTGCCCACG
GTGTGGGTGTACTGAGCCTGGAGGCGCCACAGAGCACCGTAGTAGATGGTGACGGAGTGAGCTCCGTGGG
TGCAGTGGGCAGCCGTGATCACCCAGGTGTGACCGATGATCGAGCCACCGCACCACCAGCCACCGCTGTC
GCTGCTGAAGCCCAGACCCACGATGTAGGGCACCTTGCCCTCGTAGGCGGGATAGCCGTTGGTAATGCGA
CCCTCGATCGAGCCGGATCCGGATCCCAGGACGGCGTCCTTTAGGGGCACCGGCTTCACCTTCTCAGGCT
GGGCGGCGGCGCAGGCAATGGCCACGGCCAGGACAGTTAGGAACAGCTTCATGTTGATTACTTGAGTGCA
AGTGGATACTCCGCCGACGGGCAGTGATTTATATACCCCCCAAATCCGACGTATTCATATCGCTAGAGAT

CTTCACCATTCGCCACTCGAAAGTTCTAAACACTCCGGTCGGACTAATCTTATCAAATTAATTTCGCACG
CCTCCAAAATGCGGCACTATCATTACAGGGTACTCCAATGATCTACCCATCCGATTTGGACACTTTCCGG
CTCATGACTTGCCACCACTCAATCTGACGATTGCCCACTGCCACTCATTCATTAGAAGATTGTCCGACCC
TATCAACATGGACGTGCGGCCACTCGGAGCTGATCTTAAGATAGGTGCCAATAAGCCCCCATTCATGCCC
AGAGCCACAACTCAATGGAACACTCGAACAGGAGCGCACAGAGATTAGGGCACACGTCCTGTGGTCATTC
AAAAGTAGTATTAcagtataattcgcttagctgcatcgatagttagctgcatcggcaagatatctgcatt
atttttccatttttttgtgtgaatagaaaatagaaaatagaaaaaaaaaaattaagttagctgcattttt
aagttacctgcatcgaggcattgtgcaaagtactcgaggcagctaagcgaattatactgTACTTAACGTT
CGTAAGTGCGCTACATTGTTTAGGTTTTAATATTAAACGCACTCCCATTAAAGTTATTAAAATTAAAATT
TAAAACAATAAACTAAACATTTACAAGTAAAAAGTGAAATATTTTAAAGACGAAAGTTGTATTATTTTTT
AGAAATATATTTCATATATTTAATAAATATATCACTTCGCTTTTGTGATTTGTCAGTATAataatccttt
ggaatatataatcttttggtaaatataTATATTATTTCATTATCTGATTATTGACTAGTATAGACCACAG
TTGCTAGTATTCCTACTTATGTCTATCATGGCTACATATATAGTGTTTAATCATGTTTTCCAGATAACAA
GTGTTCGATTGCCAAGAAACCATCCCATTTCTTAGCCATTCATGGCTTCTTATCTATAAGACGTTTATTA
TAGATTCGGATTCTGCATGACTAGACTTTTGAAACTTAAAAATATCCATGTGATTTTTTAAATGTAATAA
AACCAGAAACATAAAGGCGTCAAATTAATTGTTATGCATTTCGTTTATTTAAAAATAAAGATAATCAAGC
TTAGTAGTAAGCAACGCCGGAGTTGTCGCGGATCCAGTCCAGCTGGTTGGTAACACGGGTGAATCCGGAG
GGCAGGCCAGCGGTGCAACCGTTGCCGGAAACCCAGGAGGTGACACCCACCAGGCGGCCACCGTCGTGGA
GAACCAGGGGGCCACCGGAGTCACCGGAGCAGGTGGACTTTCCGCCGGAGGTGGACACACAGAGGATGCC
ATCGGGCTGGGTGCCGTAGGTGCGGGAGCATTCCGAGTTGCTAATGATCTGCAGGTCGACGCACTCCATC
CAATCGGGCTGGGATCCGGCGGTGGTCAGTCCCCAACCGCAGGCGACGGCCCAATAGTTGTCGTACATGT
TGTAGCGATCGTTGAAGCTGGGCAGCTCCACCTTGTTAACCATGTGCCAGAAGTCCACGTGGGGGGTGCG
GATCAAGGCGATGTCGTTGCCGTTCTGGTTGGGCCAGTTGTGGTTCTGGATGAAGTCGCCGCTGCCGACG
GTGTGGGTGAACTGGGCGTTGGTGCGCCAG
>dme_circ_0001363_junction_seq
GTGGGTGAACTGGGCGTTGGTGCGCCAGGGTGGCACCGAAGTAAACGATCACGGAA
>dme_circ_0001364
GTAAACGATCACGGAAGCGGCATCTCCGATGCAGTGCTCGGCAGTCAGGACCCAGTCGTGGGCGATGATG
GAGCCACCGCACCACCAGCCTCCGCTGAAGCCAAGACCCACAGTGTAGGGAGCCTTACCCTCGGGGGCGG
CGTATCCGTTGGTGATACGACCCTCAATCTTG
>dme_circ_0001364_junction_seq
TCCGTTGGTGATACGACCCTCAATCTTGGTAAACGATCACGGAAGCGGCATCTCCG
>dme_circ_0001365
GGTGGACTTGCCGCCGGGGGTGGAGGTGCAGATGACGTTGTCGGTGATCACATCGGTTCCATAGACGCTG
GAGCACTCGTCGCGGGTGATGATCTGGGAGTCCACGCAGTTCAGGTAGTCGGAGACGCCACAGCTATCGC
AAGGCCTGCCCCATCCGGAGGCTAGAGCCCACCAGCCGGCAAAGCTGTCGTGGCGCTCGTTGCCATCGGG
CAGCTCCACCTT
>dme_circ_0001365_junction_seq
GCTCGTTGCCATCGGGCAGCTCCACCTTGGTGGACTTGCCGCCGGGGGTGGAGGTG
>dme_circ_0001366
GGTAGTCGGAGACGCCACAGCTATCGCAAGGCCTGCCCCATCCGGAGGCTAGAGCCCACCAGCCGGCAAA
GCTGTCGTGGCGCTCGTTGCCATCGGGCAGCTCCACCTTGTTGATCAGGTGCCAGAAGTCCACGTGCGGG
GTGTTGATCAGCGAGATGTCGTTGTTCAG
>dme_circ_0001366_junction_seq

TGTTGATCAGCGAGATGTCGTTGTTCAGGGTAGTCGGAGACGCCACAGCTATCGCA
>dme_circ_0001367
GGTAGTCGGAGACGCCACAGCTATCGCAAGGCCTGCCCCATCCGGAGGCTAGAGCCCACCAGCCGGCAAA
GCTGTCGTGGCGCTCGTTGCCATCGGGCAGCTCCACCTTGTTGATCAGGTGCCAGAAGTCCACGTGCGGG
GTGTTGATCAGCGAGATGTCGTTGTTCAGGTTGTTGGTGTT
>dme_circ_0001367_junction_seq
AGATGTCGTTGTTCAGGTTGTTGGTGTTGGTAGTCGGAGACGCCACAGCTATCGCA
>dme_circ_0001368
GATAATCAAGCTTAGTAGTAAGCAACGCCGGAGTTGTCGCGGATCCAGTCCAGCTGGTTGGTAACACGGG
TGAATCCGGAGGGCAGGCCAGCGGTGCAACCGTTGCCGGAAACCCAGGAGGTGACACCCACCAGGCGGCC
ACCGTCGTGGAGAACCAGGGGGCCACCGGAGTCACCGGAGCAGGTGGACTTTCCGCCGGAGGTGGACACA
CAGAGGATGCCATCGGGCTGGGTGCCGTAGGTGCGGGAGCATTCCGAGTTGCTAATGATCTGCAGGTCGA
CGCACTCCATCCAATCGGGCTGGGATCCGGCGGTGGTCAGTCCCCAACCGCAGGCGACGGCCCAATAGTT
GTCGTACATGTTGTAGCGATC
>dme_circ_0001368_junction_seq
AATAGTTGTCGTACATGTTGTAGCGATCGATAATCAAGCTTAGTAGTAAGCAACGC
>dme_circ_0001369
GGTGGACACACAGAGGATGCCATCGGGCTGGGTGCCGTAGGTGCGGGAGCATTCCGAGTTGCTAATGATC
TGCAGGTCGACGCACTCCATCCAATCGGGCTGGGATCCGGCGGTGGTCAGTCCCCAACCGCAGGCGACGG
CCCAATAGTTGTCGTACATGTT
>dme_circ_0001369_junction_seq
CGACGGCCCAATAGTTGTCGTACATGTTGGTGGACACACAGAGGATGCCATCGGGC
>dme_circ_0001370
CCGTAAGCTCATCACCGGCGCCGGTGATATGACCGCTAAGATCTGGGACGTCGAGTACGGCACCGTTATC
GCCTCCATTCCGACCAAGTCATCGGTGCGCACCAGCAACTTCAGCTTCTCGGGCAACCAGGCAGCCTACT
CAACGGACAAAGCGATGGGACAGAGCTG
>dme_circ_0001370_junction_seq
CAACGGACAAAGCGATGGGACAGAGCTGCCGTAAGCTCATCACCGGCGCCGGTGAT
>dme_circ_0001371
GCTGAAGTTGCTGGTGCGCACCGATGACTTGGTCGGAATGGAGGCGATAACGGTGCCGTACTCGACGTCC
CAGATCTTAGCGGTCATATCACCGGCGCCGGTGATGAGCTTACGGCTCTCCCAGTCCACATCCAGGCACC
AGACCGCACCCTGGTGACCATCGTAGGTGCCCAAACGTTCTCC
>dme_circ_0001371_junction_seq
GACCATCGTAGGTGCCCAAACGTTCTCCGCTGAAGTTGCTGGTGCGCACCGATGAC
>dme_circ_0001372
GGCGGCAACATCACCTGGCCAACACCACCGCCTTTGGACCAGCTAAAGAGCAATACTTTCGGCAGCGTGG
AAACACCATCTCCGGTGACCAATGAACCGCCCTTCGACAATGGCATACACATTTCCAATCCTAACACGCA
TGGGCTATAATGTTCAGATACAGAGTACACCCACGATGGCGTCTTCTACGGCCAGCCAAGGATCTCGTCC
GGATACGGGTATATCCACGCAATCCCAGTCCCAAACGCAATCGATCTCGTCCGCCTCGAAGAGTGCCAAG
AGTGCAGCTGCTCGATCGGGGGCAACTGACACCGTGCCCAGTCTCACCAAGGAGATCAACTCTGGTCTGC
TGTCCATGAAGAAGGGCTTCTCCAGCTTCATGACTTCCATTGACTCGGCCATCAAGTCGGGCACGCCCAA
TGACGATGCCAGTGATACCTTCTCCATACAGAGCGACATTAGCTCCGATTCGGATAATTTTGCCAATGTT
CTTGGCGATGACAAGACCATGGATTGCATGGATGTTATGTTCCGACTTAATCCCTTCACCACGGACAATA
TGAAGGCTTCTCCCGTGGAGGTGGCCAGTGAAGTGTATGAGGAGCAACCGAGTAGCTACAAAACAAACAT

GTCTTCCCCATCCGAGCCATCGGAAGGCAGCACCTGGCGGCGACGCGATCTGGTGTCCATGGCCACCTTC
AGGGTAGACTTTATCGAGGGTTTACCTTTTCTTTTGCTGGAATCTTTTCAGTTATTTGTCATTCAttttt
tttttGTGTTCGATTTGTAAATACGAAAACGGACATTAATTCTATCGAGATTCTTTAGTTCCTCTAAAGA
TTTTGCTCAATTCAATGTTTGCGCTTAACTTTTTGCATGTTTCGATATATTTTATCTGAACATTTTCCAG
TAAAATCACTTTTGAAGTTTATATATTTTTCAATCTGTTGGGGAATCCCCCGAAATGTTTATTTAAAGCC
AAAGAAGTCTTTCTGTAAGATAACTTATCATAAAATCATATTCTCATTATATTTTCTCTCTCTCTCTTAA
AACAAATGTtaaatttactcaaaatcaatctcaatgtgtgctaatcaaaaccaaaaacaaactcaaaatc
aatTGTTGTGCTCAACTCTTTATAATCAACATGAAACGAACACTTGTAAATTAATTTATGACAATCCTTA
CATGACACAACTTTCAACAATTACAACAACCAACAACACTCCAACAATAAAAAGATCGCGCGTCAAGCAA
ACAAG
>dme_circ_0001372_junction_seq
TAAAAAGATCGCGCGTCAAGCAAACAAGGGCGGCAACATCACCTGGCCAACACCAC
>dme_circ_0001373
GGCGGCAACATCACCTGGCCAACACCACCGCCTTTGGACCAGCTAAAGAGCAATACTTTCGGCAGCGTGG
AAACACCATCTCCGGTGACCAATGAACCGCCCTTCGACAATGGCATACACATTTCCAATCCTAACACGCA
TGGGCTATAATGTTCAGATACAGAGTACACCCACGATGGCGTCTTCTACGGCCAGCCAAGGATCTCGTCC
GGATACGGGTATATCCACGCAATCCCAGTCCCAAACGCAATCGATCTCGTCCGCCTCGAAGAGTGCCAAG
AGTGCAGCTGCTCGATCGGGGGCAACTGACACCGTGCCCAGTCTCACCAAGGAGATCAACTCTGGTCTGC
TGTCCATGAAGAAGGGCTTCTCCAGCTTCATGACTTCCATTGACTCGGCCATCAAGTCGGGCACGCCCAA
TGACGATGCCAGTGATACCTTCTCCATACAGAGCGACATTAGCTCCGATTCGGATAATTTTGCCAATGTT
CTTGGCGATGACAAGACCATGGATTGCATGGATGTTATGTTCCGACTTAATCCCTTCACCACGGACAATA
TGAAGGCTTCTCCCGTGGAGGTGGCCAGTGAAGTGTATGAGGAGCAACCGAGTAGCTACAAAACAAACAT
GTCTTCCCCATCCGAGCCATCGGAAGGCAGCACCTGGCGGCGACGCGATCTGGTGTCCATGGCCACCTTC
AGGGTAGACTTTATCGAGGGTTTACCTTTTCTTTTGCTGGAATCTTTTCAGTTATTTGTCATTCAttttt
tttttGTGTTCGATTTGTAAATACGAAAACGGACATTAATTCTATCGAGATTCTTTAGTTCCTCTAAAGA
TTTTGCTCAATTCAATGTTTGCGCTTAACTTTTTGCATGTTTCGATATATTTTATCTGAACATTTTCCAG
TAAAATCACTTTTGAAGTTTATATATTTTTCAATCTGTTGGGGAATCCCCCGAAATGTTTATTTAAAGCC
AAAGAAGTCTTTCTGTAAGATAACTTATCATAAAATCATATTCTCATTATATTTTCTCTCTCTCTCTTAA
AACAAATGTtaaatttactcaaaatcaatctcaatgtgtgctaatcaaaaccaaaaacaaactcaaaatc
aatTGTTGTGCTCAACTCTTTATAATCAACATGAAACGAACACTTGTAAATTAATTTATGACAATCCTTA
CATGACACAACTTTCAACAATTACAACAACCAACAACACTCCAACAATAAAAAGATCGCGCGTCAAGCAA
ACAAGGTATAGAATATACAAAATTTGAAATTTTTAAATTAGTTAATTATTAAGTATTTTATTTTGGCGCT
TTGTATATACTACCCAAATTATTATTTATTAACATAATTTAATGGTTTTTGGTGCGACATTTTTCCTTTT
AACGTAGTGAACTTTTGGCTTGCTCGCAAACGCATTTTGGCGTTACTGGCCTCTAGTTGAGTAGTTATGA
GTTTATCGTGGGTTCATTCATAGCTGGTAGCCACTGGGAATACCTCTGAAAGTACGTTGATCAAACATTC
TGCCCTCATAGCAATCGAGATGCTCTTGCGGAACTTGTTTGAAAATGGTTTACTCATTCGAATGTTATTT
TTGAGCATGATGCCGGTTAGAGAACAGCTATCTCCAACTATTTTCTAGACCTACCCACACAATAAGTACA
TACCTAGATCCCCACCTGAATTCCATTTGATTTTATAATTTCATTGTTATTGGTATTGCTTTGTTGATGA
CTTATGATTTGCAACGAATTGGTTTCTTCAGAAATATACGtttttttttCGAAAGCGTACACATACTGCA
TAGGCAAATACGGCCAAAAGAAGTACAGTAGAACAAAAAATAGAGCACCCACTGACTAAAACATTGGCGT
CCTACACGACTAACAACTGTACTGGTAAAACTAACTAAGTAACTAAAAACACCCGCTGCTTCAACAAAAA
TAAACCCATTTTCCATCTCTCCCATAACACCTACATCAACACCATCGAAACCCACACAATCAAAAAACCC
ACATTGCATGATGTTGCCTCTAGCTTAGCATGTTGTTCCTTAGCTGTTTCCATTCCAAAGTAGCTGATCA
CGAGCACAGATTTATGAGACTTAAATCTAACGCACGATATGCTTTCTAACCCCTTCACTTAGGACGAAGT

TCGGAGCACGCTGCAAGGCTTGGAATCTGGCGCCATATAATCCAGAGGCTCCACCGTGCATCCGGATGCG
CTTGGAGGAGACTTTGAATATGCCAAAGGAAATTGAGGGCATCATCGATCGCAAACGCATCCAGAGGGTA
AATCTGGAAAATGTGCGGATCAACTTGCTTGAGGACCGCCCACCCGTAAATATTACCTCACCGGGTCCCA
TCCCCATAAATCTGTGCATTGGTCGCATGCGTTTGGAGCGTGACCAGAGCGGATTGCTCAACATTCAGCC
TATAGGATACCAACATGAGTGACGCACAGCATCAGGCGTTGGGCAGTGCCTTATTTGGAGCACCTCGTGA
ACGCGATCGGGAGCTGTTGTCCATGCAGCTGGTGATGCAGCAGATGAAGCTAGACAACGATCAACTGCGC
CGCCAGCTTGTGGACTCCAAAGTGAACACGGATAATTATAGGTGAACGTTTATAGTTGTATTGATCTCAT
GCATTTACTCATGATTGTCGGAATTCTCTATAGGCACAAGACCAAACAGGAGGCGGATGTGCTGCGATCG
TATTTGAAAGCCGCCCAGGACGATATAAGCATATTGTTGGAGGAGAAGAAGGCTTTGCTGGACACCATCC
GCTCCTTGCAG
>dme_circ_0001373_junction_seq
TTTGCTGGACACCATCCGCTCCTTGCAGGGCGGCAACATCACCTGGCCAACACCAC
>dme_circ_0001374
GGAATGGCTCCACATTCGTCACAGGAAACGGCGGCCACCTGCAAGCGAACAGAAGATTTGGGGCCCTCCT
GCTGACGAATAAGCTCCACGGTGGTCAATCTGAATGTGGATAAGCAAATTGTTAACTGATGTGTAAAGTA
ATTCAATGCACTGAATCAACCTGAAGGTGGCCATGGACACCAGATCGCGTCGCCGCCAGGTGCTGCCTTC
CGATGGCTCGGATGGGGAAGACATGTTTGTTTTGTAGCTACTCGGTTGCTCCTCATACACTTCACTGGCC
ACCTCCACGGGAGAAGCCTTCATATTGTCCGTGGTGAAGGGATTAAGTCGGAACATAACATCCATGCAAT
CCATGGTCTTGTCATCGCCAAGAACATTGGCAAAATTATCCGAATCGGAGCTAATGTCGCTCTGTATGGA
GAAGG
>dme_circ_0001374_junction_seq
GAGCTAATGTCGCTCTGTATGGAGAAGGGGAATGGCTCCACATTCGTCACAGGAAA
>dme_circ_0001375
GACGAAGTTCGGAGCACGCTGCAAGGCTTGGAATCTGGCGCCATATAATCCAGAGGCTCCACCGTGCATC
CGGATGCGCTTGGAGGAGACTTTGAATATGCCAAAGGAAATTGAGGGCATCATCGATCGCAAACGCATCC
AGAGGGTAAATCTGGAAAATGTGCGGATCAACTTGCTTGAGGACCGCCCACCCGTAAATATTACCTCACC
GGGTCCCATCCCCATAAATCTGTGCATTGGTCGCATGCGTTTGGAGCGTGACCAGAGCGGATTGCTCAAC
ATTCAGCCTATAGGATACCAACATGAGTGACGCACAGCATCAGGCGTTGGGCAGTGCCTTATTTGGAGCA
CCTCGTGAACGCGATCGGGAGCTGTTGTCCATGCAGCTGGTGATGCAGCAGATGAAGCTAGACAACGATC
AACTGCGCCGCCAGCTTGTGGACTCCAAAGTGAACACGGATAATTATAGGTGAACGTTTATAGTTGTATT
GATCTCATGCATTTACTCATGATTGTCGGAATTCTCTATAGGCACAAGACCAAACAGGAGGCGGATGTGC
TGCGATCGTATTTGAAAGCCGCCCAGGACGATATAAGCATATTGTTGGAGGAGAAGAAGGCTTTGCTGGA
CACCATCCGCTCCTTGCAG
>dme_circ_0001375_junction_seq
TTTGCTGGACACCATCCGCTCCTTGCAGGACGAAGTTCGGAGCACGCTGCAAGGCT
>dme_circ_0001376
GATAATCAAagagagagagagagCTCGAACTATGTACAGCCAGCCGTGTCATTGATATTGCTCCTACTCC
ACATATTGTATATTAAAACCAATTCCACTAAGATTGGATAGGACATATTGTTGTGATTCATGATCATATC
AAAACTGAATTCTAGAAGCTCTATGCATTGCTGTT
>dme_circ_0001376_junction_seq
AATTCTAGAAGCTCTATGCATTGCTGTTGATAATCAAagagagagagagagCTCGA
>dme_circ_0001377
CTTCTCGGTGATCAGCGTGTTCGCCTACTTGTCGCTCCTAACCCTCTTCGGCACCGTCGCCTTCAGAATC
TACAAATCTGTGACACAGGCCGTGCAAAAGACAAACGAGGGTCACCCCTTTAAGTGAGTATTGTAGACTG

ATGTCTCCCAACaaaaaaaaaaGTTTAACTAACGTAACTCCTTACGAAATCAGGGATTACCTGGAGCTGG
ATCTGACGCTGTCGCACGAAAAGGTACAGAACATTGCCGGCGTGGCTGTGGCACATATCAATGGCTTCAT
CTCCGAGCTGAGGCGTCTG
>dme_circ_0001377_junction_seq
TGGCTTCATCTCCGAGCTGAGGCGTCTGCTTCTCGGTGATCAGCGTGTTCGCCTAC
>dme_circ_0001378
TACCAGTTGCCAAACCCCAGCCGGTTCCAGTGGCTGTACCCGTGACAACCGCTTTGATAACCGACCTGGA
TGACGAGGAGGTGGTGTTCAAACCCACTGTCCAGGAGGCGCCGAAGGCACCCACCATCGCCGCACCCGTA
GTCGAGCCGAAACCAgagccacccaaacccaaggtggagccggccaagccgaaggtTGTGCCAGTGGCGC
CAG
>dme_circ_0001378_junction_seq
aagccgaaggtTGTGCCAGTGGCGCCAGTACCAGTTGCCAAACCCCAGCCGGTTCC
>dme_circ_0001379
CCGGTTCCAGTGGCTGTACCCGTGACAACCGCTTTGATAACCGACCTGGATGACGAGGAGGTGGTGTTCA
AACCCACTGTCCAGGAGGCGCCGAAGGCACCCACCATCGCCGCACCCGTAGTCGAGCCGAAACCAgagcc
acccaaacccaagg
>dme_circ_0001379_junction_seq
CCGAAACCAgagccacccaaacccaaggCCGGTTCCAGTGGCTGTACCCGTGACAA
>dme_circ_0001380
GGCAAACTGGCAATCCGGCCGGTCTTCCAGTCCTGGCTCTCAGTCCAGTCCTGGTTCCAGCTCCGGATCC
TGGTCATctggatccaatgccggagcttatcctggctccaatgccggagcttattctggctccaatgccg
gagcttatcctggctccaatgccggagcttatcctggctccaacgccgCCTGGCAATCGAGGACCAATGC
TGGATCCAGCTGGCATTCCGGACCCGTAGCAGGATCGGTCTCCAATCCGGGATCGTACTCTAGCTGGTCC
ACGGGCTCCAGTCCTGG
>dme_circ_0001380_junction_seq
CTAGCTGGTCCACGGGCTCCAGTCCTGGGGCAAACTGGCAATCCGGCCGGTCTTCC
>dme_circ_0001381
ATATCGGCTTCCAGGGCGAACCTGGCTGGGATGGCGCCAAGGGCGAGCAAGGATATCCCGGACTTCCGGG
TAAGAACGGAAGGGTGGGTGCCCCAGGACCACGGGGACCCACTGGCGACGCCGGCTGGGGTGGAATCGAT
GGCATGGACGGACTCGTTGGCCCCAAGGGTCAACCAGGTG
>dme_circ_0001381_junction_seq
CTCGTTGGCCCCAAGGGTCAACCAGGTGATATCGGCTTCCAGGGCGAACCTGGCTG
>dme_circ_0001382
GACCACGCGGATTGATTggtgagcagggagagcagggtgagcagggtgaCGAAGGTGAAGTCGGTATTCC
GGGTCGCTTGGAGAACCTGCGGGATCGGAGCTTCTATCGCGGCTTCACCGGAGATCAAGG
>dme_circ_0001382_junction_seq
TCTATCGCGGCTTCACCGGAGATCAAGGGACCACGCGGATTGATTggtgagcaggg
>dme_circ_0001383
GGAAGTCACCGCGCTGGCCCTTGTTGCCAGGCAGTCCTGTTTGTCCAACTGCGCCATCGGGTCCTTGCTC
GCCCTTCACACCCTTCGGTCCTGGGCGTCCGGCGAGTCCGTATTGGCCGGGTGCGCCGACATCACCGGGG
AAGCCCTTAATGCCTGATCGACCCGGTGGCCCAATCTCGCCCTGAATCACCTCCTGACCAGGTACACCGC
GTTGTCCCTTGTATCCAATGCCTCCATCCAAACCAGGTTCACCATCGCGACCAGGCAGTCCGGGAGCTCC
CTGCTGTCCGTAATCGCCAGTTGGGCCGGGATAACCCTTGGCACCCACGGGTCCGGGAATAACTTCGCCA
GCCGGGCCACGGTCACCAATTTCGCCACGAAGTCCAATAGCTCCTTGCTCGCCCTTGGCTCCCTTAACGC

CCTTCGGTCCGGTATAACCATCCTCGCCAGGCTCACCAGTGTTGTCGTAGATCTGACCGGGATTCGGCTC
ACCCTTGACGCCCTTGAAACCG
>dme_circ_0001383_junction_seq
CGGCTCACCCTTGACGCCCTTGAAACCGGGAAGTCACCGCGCTGGCCCTTGTTGCC
>dme_circ_0001384
CTATTGGACTTCGTGGCGAAATTGGTGACCGTGGCCCGGCTGGCGAAGTTATTCCCGGACCCGTGGGTGC
CAAGGGTTATCCCGGCCCAACTGGCGATTACGGACAGCAGGGAGCTCCCGGACTGCCTGGTCGCGATGGT
GAACCTGG
>dme_circ_0001384_junction_seq
GACTGCCTGGTCGCGATGGTGAACCTGGCTATTGGACTTCGTGGCGAAATTGGTGA
>dme_circ_0001385
GGTTCACCATCGCGACCAGGCAGTCCGGGAGCTCCCTGCTGTCCGTAATCGCCAGTTGGGCCGGGATAAC
CCTTGGCACCCACGGGTCCGGGAATAACTTCGCCAGCCGGGCCACGGTCACCAATTTCGCCACGAAGTCC
AATAGCTCCTTGCTCGCCCTTGGCTCCCTTAACGCCCTTCGGTCCGGTATAACCATCCTCGCCAGGCTCA
CCAGTGTTGTCGTAGATCTGACCGGGATTCGGCTCACCCTTGACGCCCTTGAAACCG
>dme_circ_0001385_junction_seq
CGGCTCACCCTTGACGCCCTTGAAACCGGGTTCACCATCGCGACCAGGCAGTCCGG
>dme_circ_0001386
GTAACCCCGGACGGGCTGGTCACAAAGGTCTGCCCGGTCCAGCGGGTATTCCCGGCGAGCCAGGCAAAGT
TGGAGCAGCCGGACCCGATGGAAAGGCGATCGAAGTGGGCTCCTTAAGGAAAGGCGAAATCGGTGACACC
GGCGACAGTGGACATCGCGGAGATACGGGTGACGACGGTGAAAAGGGTCGGGATGGATCCGATGGTAGCA
AAGG
>dme_circ_0001386_junction_seq
GTCGGGATGGATCCGATGGTAGCAAAGGGTAACCCCGGACGGGCTGGTCACAAAGG
>dme_circ_0001387
GCCCACTTCGATCGCCTTTCCATCGGGTCCGGCTGCTCCAACTTTGCCTGGCTCGCCGGGAATACCCGCT
GGACCGGGCAGACCTTTGTGACCAGCCCGTCCGGGGTTACCTTGCAAGCCCTGTACGCCACGCGGTCCAG
TGAGGCCGATAGCTCCACGGTTTCCATGGCTACCCGGGTAGCCGGTGTCGCCCTCCTGGccacggggtcc
acggggtccGGCATTGCACACGGGGCAGTCGTCACCGCGCAATCCCTTCTCACCGGGCAGTCCACGTA
>dme_circ_0001387_junction_seq
AATCCCTTCTCACCGGGCAGTCCACGTAGCCCACTTCGATCGCCTTTCCATCGGGT
>dme_circ_0001388
ACTATGGATTCATTGGACCACCGGGACCGCAGGGTCCCCCTGGCGAAGCTGGTCTACCCGGCCGATACGG
ACTGCACGGAGAACCTGGACAAAATGTGGTCGGACCGAAGGGCGAGCCTGGCCTAAATGGACAACCTGGT
TTGGAGGGCTACCGCGGTGATCGCGGAGAGGTCGGTTTGCCCGGTGACAAGGGTCTGCCCGGCGAGGGCT
ACAACATCGTTGGTCCTCCTGGTTCCCAGGGACCGCCCGGTTTCCGTGGACTTCCCGGTGACGATGG
>dme_circ_0001388_junction_seq
GTTTCCGTGGACTTCCCGGTGACGATGGACTATGGATTCATTGGACCACCGGGACC
>dme_circ_0001389
ATGGTCCCTCCATGCCAGGGAAACCCATTTCGCCCTTCAATCCCGTGGGTCCAAGAGGGCCCGGCAGACC
ACGGTTGCCCTTCTCCGCTATGCACTTGGGCACACAGCCCGCATATCCGGCCGTACAATTCTTGGGCGGC
AGATCGCCGCGCGCCACGCCGGCAGAGTCTACAATG
>dme_circ_0001389_junction_seq
GCGCGCCACGCCGGCAGAGTCTACAATGATGGTCCCTCCATGCCAGGGAAACCCAT

>dme_circ_0001390
GGGCAACCGTGGTCTGCCGGGCCCTCTTGGACCCACGGGATTGAAGGGCGAAATGGGTTTCCCTGGCATG
GAGGGACCATCTGGTGACAAGGGTCAGAAGGGTGATCCCGGCCCATACGGACAGCGTGGTGATAAGGGTG
AGCGCGGATCGCCTGGTCTTCATGGTCAGGCTGGTGTACCCGGAGTCCAGGGACCCGCCGGCAATCCCGG
AGCCCCTG
>dme_circ_0001390_junction_seq
GGACCCGCCGGCAATCCCGGAGCCCCTGGGGCAACCGTGGTCTGCCGGGCCCTCTT
>dme_circ_0001391
GGATGGTATTCCTGGCTTGGAGGGTCTATCCGGAATGCCCGGACCTCGCGGATATGCCGGCCAGCTTGGC
AGCAAGGGCGAGAAGGGTGAACCGGCAAAGGAGAACGGTGATTACGCGAAGGGCGAGAAGGGTGAGCCCG
GTTGGAGGGGGACTGCCGGTTTGGCTGGACCACAAGGATTTCCTGGAGAAAAGGGCGAGCGCGGCGACAG
TGGACCTTACGGAGCCAAAGGACCCCGGGGTGAGCACGGTCTGAAGGGAGAGAAGGGTGCCTCCTGCTAC
GGACCCATGAAGCCTGGTGCACCGG
>dme_circ_0001391_junction_seq
TACGGACCCATGAAGCCTGGTGCACCGGGGATGGTATTCCTGGCTTGGAGGGTCTA
>dme_circ_0001392
GTGCGCCAACATAGCCGCGGGGTCCTTTGGGTCCCGGGGGTCCTGGTGTTCCACGGCCACCGCCTGGCGG
TCCCGGAGGACCGAGCAGACCAGGCTTACCTGTCGCTCCAGCACGTCCTGGCTCACCCTTCTGTCCGGGA
TTACCGGGCAGACCATTAAGGCCCGGCTCGCCACGATCTCCCTTTTGTCCTG
>dme_circ_0001392_junction_seq
GGCTCGCCACGATCTCCCTTTTGTCCTGGTGCGCCAACATAGCCGCGGGGTCCTTT
>dme_circ_0001393
GTATGCCAGCATCTCCCTTAGATCCTTGAATGCCATAACCGGGCAAACCGGCGTCACCCTTCTGTCCCTC
TGGTCCCGGTGGTCCTGGGTGTCCAATGGGTCCGATGGATCCCTTTGGTCCATTAAG
>dme_circ_0001393_junction_seq
GTCCGATGGATCCCTTTGGTCCATTAAGGTATGCCAGCATCTCCCTTAGATCCTTG
>dme_circ_0001394
ATACTCCGGGCAGACCGGCATCGCCTCTATCTCCAGGATATCCCACACTGCCATCGGCACCGGGAAAACC
AATGGGTCCGGTTGGACCACGGTCGCCCTTCTCACCTTGCAGACCATTGAGACCGGGTGGGCCCATAGGG
CCGGGTCTCGAAATACCAGGCTCTCCCCTCGGTCCAGGCTGTCCATTGCGTCCGGGAATGCCGCGAAGGC
CGTCCTTGCCAGGATTACCAGGTACTCCGACAGCGCCCTGGTTGCCCTTTTCAccgcgaggtccattcaa
gccgcggggtccttcaacgccaggaggtccCTGTGGTCCAGTGTTGCCCTTCAATCCGG
>dme_circ_0001394_junction_seq
TGTGGTCCAGTGTTGCCCTTCAATCCGGATACTCCGGGCAGACCGGCATCGCCTCT
>dme_circ_0001395
GAGGTCCGGCAACACCAGCGGGTCCGATCGGGCCCACGTCTCCCTTCTCACCAACAATTCCGGGACGTCC
AGATACTCCGGGCAGACCGGCATCGCCTCTATCTCCAGGATATCCCACACTGCCATCGGCACCGGGAAAA
CCAATGGGTCCGGTTGGACCACGGTCGCCCTTCTCACCTTGCAGACCATTGAGACCGGGTGGGCCCATAG
GGCCGGGTCTCGAAATACCAGGCTCTCCCCTCGGTCCAGGCTGTCCATTGCGTCCGGGAATGCCGCGAAG
GCCGTCCTTGCCAGGATTACCAGGTACTCCGACAGCGCCCTGGTTGCCCTTTTCAccgcgaggtccattc
aagccgcggggtccttcaacgccaggaggtccCTGTGGTCCAGTGTTGCCCTTCAATCCGG
>dme_circ_0001395_junction_seq
TGTGGTCCAGTGTTGCCCTTCAATCCGGGAGGTCCGGCAACACCAGCGGGTCCGAT
>dme_circ_0001396

GGGCGCTGTCGGAGTACCTGGTAATCCTGGCAAGGACGGCCTTCGCGGCATTCCCGGACGCAATGGACAG
CCTGGACCGAGGGGAGAGCCTGGTATTTCGAGACCCGGCCCTATGGGCCCACCCGGTCTCAATGGTCTGC
AAGGTGAGAAGGGCGACCGTGGTCCAACCGGACCCATTGGTTTTCCCG
>dme_circ_0001396_junction_seq
GGTCCAACCGGACCCATTGGTTTTCCCGGGGCGCTGTCGGAGTACCTGGTAATCCT
>dme_circ_0001397
GATATCCCACACTGCCATCGGCACCGGGAAAACCAATGGGTCCGGTTGGACCACGGTCGCCCTTCTCACC
TTGCAGACCATTGAGACCGGGTGGGCCCATAGGGCCGGGTCTCGAAATACCAGGCTCTCCCCTCGGTCCA
GGCTGTCCATTGCGTCCGGGAATGCCGCGAAGGCCGTCCTTGCCAGGATTACCAGG
>dme_circ_0001397_junction_seq
GAAGGCCGTCCTTGCCAGGATTACCAGGGATATCCCACACTGCCATCGGCACCGGG
>dme_circ_0001398
GGTGAGAAGGGCGACCGTGGTCCAACCGGACCCATTGGTTTTCCCGGTGCCGATGGCAGTGTGGGATATC
CTGGAGATAGAGGCGATGCCGGTCTGCCCGGAGTATCTGGACGTCCCGGAATTGTTG
>dme_circ_0001398_junction_seq
GGAGTATCTGGACGTCCCGGAATTGTTGGGTGAGAAGGGCGACCGTGGTCCAACCG
>dme_circ_0001399
GGGTGACCAAGGATTGGCAGGCGCCCCTGGACAACAAGGACTGGACGGTATGCCTGGCGAAAAGGGTAAC
CAAGGATTCCCCGGTCTGGATGGACCTCCTGGTTTGCCTGGAGATGCCTCCGAGAAAGGACAAAAGGGTG
AACCCGGTCCATCCGGACTCCGCGGCGATACAGGTCCGGCCGGAACGCCCGGTTGGCCAGGAGAGAAGGG
TTTGCCCGGTCTGGCTGTTCACGGTCGTGCTGGTCCGCCAGGCGAGAAGG
>dme_circ_0001399_junction_seq
GGTCGTGCTGGTCCGCCAGGCGAGAAGGGGGTGACCAAGGATTGGCAGGCGCCCCT
>dme_circ_0001400
GGACTGGACGGTATGCCTGGCGAAAAGGGTAACCAAGGATTCCCCGGTCTGGATGGACCTCCTGGTTTGC
CTGGAGATGCCTCCGAGAAAGGACAAAAGGGTGAACCCGGTCCATCCGGACTCCGCGGCGATACAGGTCC
GGCCGGAACGCCCGGTTGGCCAGGAGAGAAGGGTTTGCCCG
>dme_circ_0001400_junction_seq
GGTTGGCCAGGAGAGAAGGGTTTGCCCGGGACTGGACGGTATGCCTGGCGAAAAGG
>dme_circ_0001401
CGTGGCACACAGGATCCGGGGGATCCAAGGTCCTGGTTGTGGGCATAGTCATTGCCATCGACGTACAACA
GGGAGTAACCCGTCCACAGTTCCGTGTGTCCAGCCGAGCAAGCGGGCACCGTTTCCGATTGACTGTGTCG
CGTAATCAGGATACCGGTGAGATAG
>dme_circ_0001401_junction_seq
TCGCGTAATCAGGATACCGGTGAGATAGCGTGGCACACAGGATCCGGGGGATCCAA
>dme_circ_0001402
AAGCAGGCGAAAGGTGAACTGAGTTCATTGTGCTGTTGGGCATTACGTGTGCGTCCATCCGGTCTCCCTC
CTCCGCCGGAACATCATCCCCCCCATCCACTCGAGAACTCGGGAAGCTGCCaaaaaaaaaGAGAAAACAA
AGACCAAAGAACACGCCGCTTGGCATAATCAATTAAAATGCCTAGGAATCTGCCGCTCCTCCCGCTGACC
CTCTGTGCCGTCGCCTTGCTGGCCGCCGTGGGTCCCATGCCCGCCCATGGGGCGGTGGCCAACAGTCACA
AGCACGAGAAGCACCTTAGCAAGGAGCGGGTCAAGGACGGCATCTACGCTCCCCGGGATGCCCACCACCA
CGGCGAGGATGGGGAGCACAACGTGGAGTTCGATCACGAGGCCATCATCGGGTGAGTCAGCGCTAGTTAG
CAGAGAAAGCAGATCATATTCAGTTTGAGAAAATCACTTTGAAAAGAGCGAAAAAGACTCCTTACAATGA
AGGTCATAAAAGTAGTGCTCATTTCCTGAAAAGCAAATAAAATATAATCACGTTTTTTTGCCAGAACAAC

AATAGAATTTCCAGTTTCAGTTGTTAATTTGTAGAAATATAAAAGTTTATTTTAAGGATACTTTCCAACC
CAATGAAATTATGAGTATTGACCATAATTTTGTATTATTTTAGTTATGAAAAGGACTCATACtttttttt
tGTGTATGCTATGGTGTATGCCATATGTTTGTTTACTTGCCTGAGGTTTATTTTTGTGGGTCTGCACACG
GAAAGACTCATTCAAGCATCAAGAGAATACGGATCAGTCAATACACATACCCATTTATGCTCAAAAATAT
TGCTTGAAGGTCGAAATATAGTTTACTAATGAATTGGAATATGCTGTAAGAAAATGTATTATTGAAGTAT
ATAAGTTTGTATAGTTTTATAGTGAAATCATTAAAATTGTTTTCTAAAAGCAATACGTCAATGGCATTGC
TGCTGTTTTATGTTCCATTCAGTTTTAAATGCTAATGTATGTGTGTGCTATTATTACTCTTTAACCACAA
AAAAGGGCAACACGAAAGAGGCTCAGGAATTCGACTCACTTTCGCCAGACGAATCGAAGCGACGACTGTT
GATATTGATCAAAATGATGGATCTGAATAAGGACGAATTTATTGACCGGCATGAGTTGAAAGCCTGGATA
TTAAGGTCCTTTAAG
>dme_circ_0001402_junction_seq
GAAAGCCTGGATATTAAGGTCCTTTAAGAAGCAGGCGAAAGGTGAACTGAGTTCAT
>dme_circ_0001403
ATTCCCATTGTGATCAAGAACTCGCCGCTGACCAACATTATGATGAGCGAGCTGAACGAGCTGCTGCCCG
AGGACAAGGGGCACAACTTCCTGGACCTGGGCACCGCCACCGTATTGGAGAACCAAATGCGTAGCCTGAT
CGAGCGCGTCGACGAGTTG
>dme_circ_0001403_junction_seq
TAGCCTGATCGAGCGCGTCGACGAGTTGATTCCCATTGTGATCAAGAACTCGCCGC
>dme_circ_0001404
GGAATCCGCGGGATGACTTCTGCGTATCGTAGACGACAACGACGGACTCTTCAATGCTGGTCTGGTAGTG
GTACTGCGATTCCAACAGAGCCATTGACAGGCTGTTGCCCACGTCGGAGCTCTGGTACCAGCCCACATGG
AGATGATCTACGTTGACGCGACGCAGGCGACGCATCACGGTCAGCTG
>dme_circ_0001404_junction_seq
GACGCAGGCGACGCATCACGGTCAGCTGGGAATCCGCGGGATGACTTCTGCGTATC
>dme_circ_0001405
TTCCTTGAACTCCTTCTTCTTGGTCCTTCGAGTGGTTCTTAGGGTCACATTGAATGGCGCCAGATGTTCC
TCGACATTTCCGTGCTCGGCACCGGGACTCATGCGACTGGTGGTGCGCAGGGTCACATTGAAGGGACTCG
CCACCTGG
>dme_circ_0001405_junction_seq
GGTCACATTGAAGGGACTCGCCACCTGGTTCCTTGAACTCCTTCTTCTTGGTCCTT
>dme_circ_0001406
GATCGAGGAGAACAGCCGGAACCTAGAGTATTTGGGTCATGGTATTGGCGGTTCGGTTAGTTCCCTCGAC
AGTGTTGGCAACCAGAAGCACGGCGATCTGAAGGCTGAGAAGTGGAAGCAGGGTGCCAGGAAGACCCTGC
TCAACTGGGTGACTAATGCCTTGCCAAAGGGACAGTGGTGTGGAGGTAAAGGATTTCGGCGCCAGCTGGA
GAGATGGCGTCGCTTTCCTGGCCCTCATCGATGCCATCAAGGCGAACCTGGTCAATTTGGCTGAGCTGAA
GAAGACCAGCAACCGTCAGCGTTTGGAGACCGCTTTCGATGTGGCCGAAAGCAAATTGGGCATTGCCAAG
CTCCTGGACGCTGAGGATGTGGACGTACCCAAGCCGGACGAGAAGAGTATCATGACATATGTGGCGCAAT
TTTTGCACAAGTATCCCGAACCAAAGGGGTGCTTCCCGCGACCAGTCGCATGTGCAACAGGAGGCGGACG
AACTGCGTCGCTTCCTGGTGGAGAAGACCACCGAGTACGAGCCCATGGTCATGATGAGCTCGTTCCCACG
CGATTTTGGTGAATATTTACTGGCACGTTCTGAGGTCGATGCACATTTGGCGGCCTACAATCGCTTGAAA
CAGCTCATTGAGAGCCAGAGCGGATTCCTGCAGGTCTCCCGCCAATCCTGGGAGGAGATTAACGAGCTGT
GGCAGCGCCTCCAGTACCAGATGATGTACTGGCTCTGGTTGCTGGATTCGGAGCTGCCCGGCGACTTCGG
CACCGTTGGCAAATGGTTAGCCGAAGCAGAGAAACTATTAATGGACAACGATATACCCAATGCAATGAAT
GAGGAGACGGCCGCAGTGATCAGCAGGAAGCTGGAGGAGCACAAGCTCTTCTTTGCCGACCTGCCGCGCA

TTTTGGCCATGTTTGATAATGCCAAGCGGTCACCGGTGGCTCAACAGATTCCCCTGGAGCAGTTGCGCAA
CATGGAGCGTAGACTTCAGGAGGTCGGACCGAAGGCAGCGGAGCGAAGGATACGTTTGAAGTTCCTGGAG
CACAAGTGCTGTTTGATTGCCTTCCTCAATCTGGTGGAGAACAAGATGCGCGGATGGACTGGAAAGTATG
GTCACGAGGAGAAGGTGGCCCAACAGCTGGAGCAGTACAAGAACTTTGTGTCGCGCAACAAGATCTTCCA
GGAGTTCCAAAAGGCCTTTGTCGATATGCAACAGGTGGTGGAGGAGTACAAGCGCGATGGCAATGTGCCG
CGCAAGGAGATCAACGACATCGATCGATTCATGTATGAAACCGAGGAGCGCTGGAAGCGCGTCTCCATGG
AACTGAAGTGCTGTCAGAACTCCCTGGAGGAAGTGGTCAACTGCTGGCGCAGCTGGAACCAATTGGCCCC
CACCTGCGAGGAGTGGCTGCAGCTGGCCGAGCAAAAGGTCAACCAAAGCGAGGATGAGCGTTTGGACTTC
TTTCAGGATATACCCGTTTGGAAGGACAAGTTTGATGCCCTCGCTAGCTCGGCTAACTATCTGATTGCCT
CCTGCGAGGAGCCCATTGCCCAGCAATTGCGTCAGCGACATGGAGCTCTCTCTGAGCGATTCGAGCGTCT
GTTTGCCAACACCAAGCAGTACATGCATGCTGGTGACATTATCCGATCCCGACAGGAGTACAAGTCGGGC
ATAGAACAACTGTCCAGATGGCTGCGAGGAGCCGAAAGCGTTCTAGACCAGCGACAAGTTCTGGGCAACA
GTGAACAAGTCAAGGAGTACGGCCAGCAGTTGCAGCAATTGGCCAGCGAAATCGATGACAACGAAGAGCT
GTTCAAAACCATCAGCAGGAACTTCCAATCGCTGATCCAAGACCTTTCCCGCGATGAGGTGGATAAGATG
ATGAAGCTACTCAAGCAAGAGAAGGAATCTCTGGTGCGCATACGTGCACAATTGCCTGCCAAGCTGCATC
TGTTCCACCAGCTTCAGATCCAACAGGAATCCCTGGAGGCTGGACAAAAAGAGATTCACCAGTGGCTGAG
TGAGGCTGAGCAGCTTCTGGGAACCCACAATCTCTCCGGAGGCCGTGATGCCATCAATGAGCAATTGCAC
AAACACAAGACCTACTTCTCGCGCACTGTCTACTACCGATCCATGCTGGAGAGCAAGAACAAGGTGTTCC
AGAATCTCTTGAAGGCTGTATCCTCCGATGATAAGATCGATACTGCTCCTGCTTCCCAGCAAATGCAGCA
GCTGAACGAGCGTTTCAACTATGTGATTCAAAATGCCCAGCAATGGGAACAGCGCTTGGATTCGGCAGCC
GGTGGATGGAGTAACTTCAAGGACAACGAACGTGTGGTCAGCGAATGGCTAACCCAAGCTGAATCCATGT
TGGTGGAGAAGCACATCGAATCGAAGACGACAATCGAGACACAGAAGTACTTCTTTGAGCAGGTCAACGA
TCGCTGGATGAATGACCTGGTGCAGTCGGCTCAACAACTTCTGACCACATTGCCCGCCCAGGAGCAACCT
GCTGTGGTGCACAGTGTGGAGCAGTTGCAGTCCCGCTGGAAGAATGTATTGTCCCAGGCTCCTTTGCATC
TATTGAAACTGGAATTTAGACTGGATGAGAACGCATTCTATCAATCTCTGAAGGATGTAGAGAAGGAGTT
GCAGCTGGAGCAGCAGGCCCTCAATCGCAATGAGGATGTGGACTCCATACTCCAGCGTAACCAGCAGTTC
CTTCTGCAACAGGATGTCGTTCCTCGCCTGGAGCGATGCCTCCAGAACATGCAACGTTTGGCTCAGGCCC
ACAGGCAACAGCAGCCCGGTGACATTAGTCTGGATCAGGCCTACGACAATGCCAAGTCGCAGTGGCAACT
GTTGTCCAACAAATTGGGCGACATGCGTCAGACTCTCCAGCAAATCCCGGCCCAATGGCAGGGCTATCAT
CTGAAGTTCAACGACATGGTCGATTGGATGAACGGCGTGGATCAGAGCCTTAAGAATATTGTGAACGAAG
TGAATACCATGGAGGAATTCGAAAAGGAGAAGGTGGTCTTCCAG
>dme_circ_0001406_junction_seq
ATTCGAAAAGGAGAAGGTGGTCTTCCAGGATCGAGGAGAACAGCCGGAACCTAGAG
>dme_circ_0001407
TCTAAATTCCAGTTTCAATAGATGCAAAGGAGCCTGGGACAATACATTCTTCCAGCGGGACTGCAACTGC
TCCACACTGTGCACCACAGCAGGTTGCTCCTGGGCGGGCAATGTGGTCAGAAGTTGTTGAGCCGACTGCA
CCAGGTCATTCATCCAGCGATCGTTGACCTGCTCAAAGAAGTACTTCTGTGTCTCGATTGTCGTCTTCGA
TTCGATGTGCTTCTCCACCAACATGGATTCAGCTTGGGTTAGCCATTCGCTGACCACACGTTCGTTGTCC
TTGAAGTTACTCCATCCACCGGCTGCCGAATCCAAGCGCTGTTCCCATTGCTGGGCATTTTGAATCACAT
AGTTGAAACGCTCGTTCAGCTGCTGCATTTGCTGGGAAGCAGGAGCAGTATCGATCTTATCATCGGAGGA
TACAGCCTTCAAGAGATTCTGGAACACCTTGTTCTTGCTCTCCAGCATGGATCGGTAGTAGACAGTGCGC
GAGAAG
>dme_circ_0001407_junction_seq
GGATCGGTAGTAGACAGTGCGCGAGAAGTCTAAATTCCAGTTTCAATAGATGCAAA

>dme_circ_0001408
GAAAATCTGCCAAGATGCTGACAACAAACGCGAGGACATGAAATGGTTGGTCAAGACCTTGGACTCTCTG
CTTAGTTACGCCACCGAAGATGAGGCCAATCTGGAGCAGAAGAAGCTGGAGGATCTGATCGCCCGCTACA
AAAACCTTATCCCAACGATTGAAATCACGATGGTCAAGACGGAAGTCTTCTCAAAGTGCTACACTTACCG
CCGAGAAGTGCACGAGGTTGTGTGTCTGCTGAGCAAGGTCAAGGATCAGACTGCCAATATACCAGCTCCC
GATAGTCTGGATCGTGTGAATCGTCTGATTGAGGAGCAGCAGTATGCCATCAACCAATTGGATCACCAGC
GTCCGCATATCATGTCCATGCTGCAAAGAGGACGTGATCTCATCAAGGATGTCCATGCCCCGGCCTTTGT
AAATGCCGAGGTCAAGAATCTGGAGACGGGATGGAATCAGGCGTACACAGAGACCTCCGACAAACTGCAG
GCCCTCAAGGGCACCCAGGCCGTTTGGAGCGAGTTTGTGGACCAGAAGAACGATATTTTCTCAATGCTTC
AAACTGCGGAGACGGAGCTGCGTTCCCTGACACCATTGCAAACCGATCCCAAGAACGTCAGTCAGGATCT
GAAGTCCAAGAGAGATCTTAACGTACAACTGCAGCAGGCTTCCCATCAACTGTTGCCTAAGTTGCATGCT
CTCAAATCGGAACTGGCTCCTCTGGCCGCACCCGATAAGCGTCCCATCCTCGAGAAGGAAGTGACCGAGG
TGGAGAAGATGTTCTTCAACACCATGGAGCACGTAAAGGATCGCGTCGGCTATCTGGAAGACTACAGTGC
CAAGTGGAATAACTACAAGACCCGTCTCGCTGAACTGCAGGAATGGGCCAACAAGGTCGCGCCCAAGAAT
ATCGAGGCCCTTCAATCGGAGGACCTCACACCGGAGGAGCGTGTGGTCAAGGTGCAGGCGTTCAAGCGTA
TCCTTGGGGATCGCATGAAGCAACTGGATCTTCTGGCCGCCGATGCTTCCGAGTTGGCGCCCAAGGAGGG
CAACATTGCCGAGGCCAAGCGACTGAAGGGAGAGATTACCAAGCTGCAGGAGGTCCTCAGTGCCATCAAT
CGCAACGTGGACCATCAGGCACAGGCTGTGCAGGAGGATCTTGTCAACTGGCAGCAGTTCCAGGCCGGTC
TGCAGCAAATCAAGCCAGCCGTGGAGCAAAGCGAAGTCAAGGTAAATAATGTAGTTTCTAAGCCCATCTC
CCTCGAAGAAGCCGTTGCCATGCAACAGAATGCCCAACAATTCGAGACACAGTGCCAGGAGCAATTGGAC
AAGCTCCATGGCATATCAAACATCAGTCATAAAATGTTATGTAAGACCAATGCCCCCGACGAATTGGACG
CCATGCATTCGCGTTGGACTGCCGTCCATGAGAACGCGAAACAGGCGAGCGCCAAGCTCGAAAAGTTGGT
GGCCAATTGGAAGTCATTCGACGCCGATGCCGCCAAGCTCGAGGATTGGGTTGGACAGGGTGAGCAGCAA
ATGTCCCGCCGTCCAGCTGTCCTCAACACCCCGCACATTGACAAGCTCGAAAAGGAGCTGGTCAAGCTGA
AGTCGTTCAACAACGAGATCTCGCAGCAACAGGCCAAACTGGTGACTCTGGGTCAAAATGCCGATCAAAT
CAGTTTGCACTTGGCTCCTGAAGGAGCAGCCGCTTTGAAGGATCGTGTTAACCAAATGAAGGGCAAGCTA
CAGAAGCTTTCGGAAGCAACTCGAGGTCACATCAACGAGGTGTCCGACGCCATTATCTCCAGGCAGGATT
TCAATGCCAAGTTGGTGAACTTCTCCAACTGGATGGAGCAGCTGCGTAACCAGGTCACTCAGGTGGAGGA
GATCAACCCGGAGCGGGTGGAAACCAGTCTCCATGTCATACACGCGCTACTGCAGGAGCATGCCGATAAG
AAGCCAAGTTTCAATGCCATATACGATGAAGTTAAGCAATTGGCTTTGGGAGCAACTCCCGAAGAGTCGA
ATGCCCTGAACGATGCCTACACCGCTTTGGTGGTGAACTATCAGAACCTCGAGACCAACATGCTGCAAAA
GAAGGCGGCTCTGGAGAAATGGACAGAGCTGCTGGGCTGGAAGAATGACACCGAATCGCATTTGAACTAC
CTCAAGCATCAGCTGGACAAGCCGGAAGGACCTGCTGCCGAGGAGCTGTCCAAGGTCATTGATGAGATCG
ATAATTTGGGTCAGGGTATTGGATACTGGAAGGGTCAGGCTAAGGAGATTGATGAAAACCCGGCCATTCA
GTTGAGGGATGCTCTGTCTCGTCGTCCATTGATTGCCACCCAGATTGTGAACGATGTTGAGAATAAACTA
GAGAATCTGAAACTGCGCTCGCAGAGCCAGCAACAGCAAATCCAACAGATGACCGTGCGCAAAGATAAGT
TCCATGCCCTGGAGCATAACTTTGGACAGGCACTTCAAGAAAATCGGGCTAAGCTAGACGAGATTCTCAG
ACAGCATCCGACTTTAAATAATATCGATCAAATCATTGCCGATTTGGTCGCTCTGAATGATGCCCTGAAA
TACCAAGCCGATCTGAAGAATCGCATTCATGACGAAGGTTCATTGCTGATGCGTGAGGATATTGCTAGCA
TGCCCGCCATTCAGGAGAGCCTACTTATCATGGACAAGAACTACGATTCTCTGCAGAATGAAATAGCTGA
TCGCATTCAGAAGTACAACCTGATTAGCCAGGCCCTGAGAGAATATGCCGATTCCAAGGACAAGTTCTCC
AAGGAACTGAAGAAGGCAGAAGATCTATACAATGCTATACCTCAACAGCCACGCGATGAAACCGAGCTCC
ATCAAGCTTCCGAGAAGACCAGAAAGACCATGGAGCAGTTGCGTAAGTCGAAGCTCAGTTTGGATGAACT
GGAGCGTCGGGGCAACAATGTGGGCAAACTTTTCAGTGCCATTGGAGAACCCATTCCGCAGGAAGTGCCA

CAGGAGGTCACCGCAGCCAAACAACACTGGCAGGATTTGCACGACAAAACCGCTAAGAATGCGCATGTGT
ACGAGACGGAAGCTGTTATTTGGTCACAGATCGAGGATGCCAAGAAGGATTTGCTTCCCTGGTTGTCCGA
AACGAATCAGGGACTCTGCGATGCTGCGGATAACTCAATTGAAATTGAGTTTGGACCCATGCGTCTGAGC
AAGTACCGCACGGAGCTACCTTCGTATCAGGCTCTGAAGGATTCGATAGTGGAGAAAACCAATGATTTGG
TTAAGATCAACAAAGGTGCGGAAATTCCCGCTCTATCTGCACTCAACAAACTATTGTCTGAGCAGTTTGC
TGAGGTTAACAACAATGCAGATCGCCTAAGTGCCATAACCACCTCGTTCAATGACCAGGAACAAGAGTTG
CGCAGGCGTTCGAAGGAAGCTGGCGAACGGGTGAGCAAACTTCGTGAGCAGCTGATCAAATGTGATGACA
TGTCCGGTGATAATAACAAGATCATGGAGCGTCTTCAGCAATGCCGTGCCCTGCGTGGTGAGCTCGATAA
TAGTGGAAACGAAATCGACAACATCAAACAAAAGGTTGACGAGCTGCGTAATTTGTACCCAACTTTCAGT
GAGTCCATCATACCCAAAGAACTGAACAATGTGCAAAAACGCTATGAGAACGTTGATCTTTACGCCAAGA
AGATTGAAAGTTCTCTGCTGCAGTTCCTCAAGAAGTTCCACGCCGATAAGGTGGGCATGCTGAAGCGTAT
CATTGCCACTCAACGCGAAAAGGTGGCCTGGTGTCAGCCGGAGTCCTCCAGTGACAAATACAATCTGGAT
GTCAAGAAGTCCTCTCTGCAGGAAGTCAGCAAGAGCATTGATGACTGCAAGGCACGCCATGCCGAGACCC
TGAAGTCCTTGGAGATGTTGAAGGCGGTAGAGTCGCCACAGAACCTGGCTGAACTCACCAGCGATGCCGA
GTTACTGCGCAAGGATATGCAGGCTCTGCAGGACAGCTTTGACCAAATCAAGGGAATCCTTGACGAGAAC
GTCGATTTGTGGTCGCAGTATGAGCAATCGAATGAGCAGATTTCCAACTGGCTGCGTGACGTTGAAGGAC
GCGTTAAGGCAGAAACTAGCAGCCAAGTAAATCTGTCCGAGGTTCCACAGAAGCTTCAAGAATTGTCTAT
CCTCCAACAGGATGTGTTGGCCCATGAGCCCATTATTAATAACCTCGAGCAGACATCCCAACAGCTAATT
GAAAAGAATCCGGAGGCAAGAATTGGCCAGTTTGTCACCCATTTGGTGCAGCGATATCAAGCTGTGTCCA
AGGCTTTAACCAGCTACATCGATAAGATCCGTGGTGCTCAGTTATCAAACGCTAACTTTGCCAAGGCCGC
CAAGGATTTCAACGAGTGGTTTGGTGATGCCAAGATCGAGTTCCAGGAACTTGCTCGCATGGGATCTCCC
GGATCATCCTCTGCCACAGCTCAGCAACTGCAGACCGTCAAGAATTACATCAAGACCTTCGACAATGGTC
AGATACTACTGAATAATGCCGTCGATATTGGTGAGGCTTTGTACCCGGTGGTATCGCCCGATAATCGCGA
ACGTATCCGTGCTGATCTTCGCCAGATGCGTGAGAAGTTCGACTACTTGCGAGATGAGGCCAATGCTTTT
ATGCAGCAAGTCGAGGGTGTTTTGATTCAAAAGACTTCAATCGAAGAAAGCTACACTCAGGTTTCTCACT
ATCTTAACGAATCGAAAGCTAAGGTTCCCACCACTGACGAATTGTATCCCACTTTGGCCACCAAAAAAGC
TGCCCTCCAGAACTACAAGACCCAGCTGCAGGAGATCACTCTGCACAAGAATGCTTTGAAGCAGTTGCAC
GACAAGGCTGTCACCCTTTGCGATGATGAGTCCGAGAGAAAGACCGACGAGTCTATCCAGGAGTATAACA
CGCTATCCAAGAAGATTTCCGATAGGATTACAACAGTGGGAAATCATGTGGTCAAGCATGAAGCGTATGA
TCAGGTCCTGGAAAAGGCACAAGACTGGCTGAATACCATCAAATCCGAGGCCATTGATATCCTCAACGAG
ACGACATTTGAAAAGGAAGGCGCCGAGGAGAAACTGCTGGTTGTGGAAAATCTTCTGCAACATAAGCCTG
AGGGTGATTCCATTTTCGATACCTGTCACAAGTTATTGGAAACGGTGCTCACACAAACCCATCCCAGTGG
CCATCCAGCTCTGCTGAAGGGTTTCGAGGAACCGAAGCAATCTTGGGAGGACTTTATGACTCTTTGTCAG
GATTCGTTGGTAAAACTGAAGCAGCTTTGCTCTAAATGGGATGAGTTTGATACAATCATTGAGGAGCTAG
ACAACTGGATGAAGAATGTCGAGGCGGTGGTTAAGAACCAGAACCTTAAGAGTACGGCGGAAGCCAAGAA
CGCCCATTTGAAGCAGCTTCAGGATATTTCAAAGGACATCGAACGGCGCGGTGCGGCCATCAATGAGCTA
ATGGATCAAGGCCGTGAGATTGAGGGTGAAACCGATTTGAACCTTAAGTTGTCGCGCCTGAATACTCGCT
ATCAGACCCTGAAGAATCTGTGCAAGGAATCGATAGCCAAGTATGTGAACTATGTAAAGGATCACGAAAG
CTTCGACAAGGACTTTGATTCATTCAAGCAGAATCTTCAATCTTCAGTCGACGAGCTGGCCAAGACCAAT
GAAATTGTCGGCGATCAAAGCGTGCTGCAGGATCAGCAAAATAAACTCCGTGAGATGTCCGACAAGCGCA
TTTTGGATTCCACTCTATTCGAGGGACTCATCGATCGTGGCGAGAAACTCTATGGTCACACCAGCCCCGA
AGGCCGTGAAATCATTCGCCAGCAATTGCGTGCCCTGCGAACCCTTTGGGACAACTATACCGATGATTTG
AACTCGGCGACGCAAAAGATCGATCAGTGTTTGCTGCAGTTTAACGAGTTCAGCATTGCCCAGGATCAGC
TTACCAAGTGGCTGAAGGATGTGGACAAGGCCATGCAGAGTCACACTGAACCCAAGACGACGCTGCAGGA

GAAGCGTGCTCAACTGCAGAACCACAAGCTGCTCCATCAGGAGATCACCACACACAATGTTCTGGTTGAT
AATGTCTGCGACAAGGCACAAATACTGGTCGACCAGATCAAGGATAACTCGTTGAATGTGTACTTGACCT
CGATCAAACAGCTTTTCCAGAGCATAGTCCAGAAGTCGGATGAAATTTTGCACAATCTGGATGATTGTGT
TCAGAAGCACAATGAATTAAACAACGCTCTGTCCTCGGCCAAAACTTGGATTTCGAATGAGAAGGCCAAA
TTGCTGGAGTGCGACGATGCTTATGGTGAGAAGGCAGACATTAAGCGAAAGATTGAAACTTTGGGACAAT
TGGCTCAGAACAAGCCACAGGCAATGAAAATAATCAGCGACATCCGTGATCTCTTTGAGAAAGTGAAGGC
AACGACCTCCGAAAAGGGTAACGAAGTGCTGGACAAGGAGATCGAGGAATTGGAGACCACCATGAAGAGT
CACTTTGATGACATCGAGGGCATTGAAGGCAAACAGAAGGATGTGCTTGCCCAGTGGGACAAGTTTGAAA
AGGCGTTGGAGGAACTTACCAAGTGGTGCCGCAGTGCAGAGGCAGTGTTCCGCGAACAGCAGCTGCAGTC
CACGCTCCATGAGAAAGTGGAGCAGCTGGAGAAGTACAAGATTCAAAGGGAGTTGATACTCCAGAAGGAG
AAGGAGATCGATGCCTTCGGAGATGCTGCCCACGCTCTGCTTAACAACTGTGGAGCCGATCGTCTAAAGA
CTTTAACCACTCAGATCACCAATCGGTATCAGCTGCTTCAAGTCCTTAGCAAGGAGGTTGTTAACCGCTG
GTCAAATCTGGTCGATGATCACCAGTTCTACCAGGATAAGTACAACGAGGTGGATTTGTGGCTGCAACCC
ATCGAATCGCAGATGGCGAAGGTCCTTTTGGATGAGCCAACCCAGAGTTCTAACATTTTGCAAGTGCTGT
TGTCTGAAAAGGAGCAAGCGGAGAGCTTATTTGCTGCTCTAAATGCAGCTGGAGAAAAGGCTCTTCCAGA
GACATCCACTCAGGGTAGGGAGAAGATCCGTAAGGACCTGCGCGATATCCGCGATCGTTGGGACAAACTG
GACGAGGGCATTCGCAACCTGGAAAAACGCCAGGAAGCGCAGGGAGTTCAGCTCTCCAGCTACCAGGATA
TTCTTAATCAAACCGTCAACTGGTTGGATCAAGTGGAGAAACTGATCCACAATGAGAACCCTGCTAGCTG
GACCAGTGCCCAAGAAATTCGCTCCAAGTTGTACAAGTACAAGGCCACCAACCAAGACATCAACTCGCAT
AAACGCATAGTCGAGGCTGTGAATGAAAAGGCAGCAGCTCTTCTGGGCAGCGCCGCTCCAGCCAATGCGG
ATGAGATCTCCAAGGCGGTGGCTGAGGTGAACAAGCGATATGATCAAGTGGGCCAGGACTGTGCCAAGTT
AGTTGCCGATCTGGATGGAGCTTTCGATGTGTACCAGCAGTTCAGTGAGCTGCAGAAGGCTCAACAGGAC
TACCAGAAGAATCTTTGGGATCGTCTTACCGGCTACTCGGACTACTCAGGCAATAAGGCAGCTCTGCAAG
CTCGCCTTCAAAAGATCAACGAGATCCAAGATGCTTTGCCGGAGGGTGTGGCCAAACTCAAGTCCCTGGA
GGATCACATTGAGCAACAGGCCAGTAATATACCAGCCCGATCCAAGGAGGTAATGGCACGTGATCTGGCC
AACTTGCATGCCGATTTCGAGAAGTTTGGAGCTTCGTTGAGCGACGTAAAGAGTGGACTAGAGAATCGTC
TACAACAATGGAATGATTACGAAATCAACTTAGATCGACTGATCACTTGGTTGGGCGAGGCTGAGAATTC
CCTCAAGAACTACAATCTGAAATCCTCGTTTGAGGAGAAGGAAGAACAGCTGAACGGTTTCCAATCCTTG
GCTCAGAATTTGCGTCAAAACGAAGCTGACTTTGACAAGGTGAAGGACGACACCTCAGAGTTGGTCCAGA
GTTCTGGTGAAACAAGGATTGCTGTCAATGTGCAACAGGTTTCCTCCCGCTTCCAATCGATACAGGCTAC
AGCTAAGGAGATTCTCAAGAAGTGCGAGCAAGCTGTCCAGGATCATGGTCATTTCAATGACAAGTATAAG
CAGTGCGCCGACTGGTTGGCAAATGCTCAGGCCCGTTACGATGATTGCTGTGACTTGTCCACGGTGGCAT
CTCGAGACGATCTTCTCAAGAAACAAGTGGTCATCCAGGAGCTTCTGGCCCAGCAGCCAACAGCTACTCA
GCTTCTAAATAGCACCGTTGAGCTGGGCGAGAAGTGCTATGGGTCCACAGCAACCGAAGGACGCGAGGCC
ATTCGTAGTCAACTGGATGATCTGACATTCGATCAGTTGTTCGACAACATTGCCATCACTGCAAGAAAGA
TTCAGGATAAGATCGCCAAGTGGTCTGGCTTTGATGAGATCGCTGACAGCTTGAAGAGCTGGTTGGATGA
AACGGAGAATGCCCTTCCAGCTGATATTGAGCTGAAAACGACTCTGGATGAGAAACGCAATAAACTGCAA
ACCTATCGTGACATCTTGAACGACATCAACAATCATCAGGTGGAGTTGGGTAATCTTCAGGAGATTGCCG
CCAATCTGCCAGAGAAAACAGAGCTTGTGGACCAAATCATAAAGGATATCTCTGACCGCTTTGGAAAGCT
GCAAAAACGTGCCCAGAACTACGTGGAGCGTTACGAGGGTATTGTGAGTGCACATCAACAGTACTCCAAG
GCTGTTATGGATGCTCAAGAGTTTATCGATGCTACCCTGAATACGGTTCACTATTGGGGCGATCTGGATC
TGGAACAAATCTCACTGCACACGAACCTCGACCGCCTAAAGAATCTGAAGGCCAGTTTGGCCGATGAGTT
CCCACGCGTTGATCAAGTTAGGGCTTTGGGCGAGAAGGTTATTCCGGGTACGGTGGATGTGGGACAGGTA
AACATTAAGTCCCAGATCGACACCACCCAGCAAGAATGGGAAAGCCTACTGACCACAATCAGCTCCACAA

TTGAAGCTATCGAAGCTCGCCTACAGCATTGGTCGGAGTACGAGCAATTACGTGACCAGTGTTTGGCTTG
GATTCGAGATACGGATAACAACCTTCATGCTATCGATCTGAAGGAGGATCTGCCGAAAAAGCGTGCACAG
TTGGATGCACTGAAGGCCCTCCAAGGAGATGTACGCGCCAAGGAATTAGAGGTGGATAATGTCACGGAGA
AGGCCCAAACCCTTCTGAAGGGTCCCAGCAGCAATCGTGCCTCGGGTCCTGAGTTGGTAACCAAGTACCA
GCAGATCTTCCACAAGGTCAAGGAGCTCAACAACCGTTGGCAACAGTATGTTACCTCACACGAAGATTTT
GATAACGCTATCTCTGATTGCTCCTCTTGGATCAATGAGATCAAAGAAAAATTGGACTATTGCTCCGACA
TGTCATCTATGAGTCCAAAAGAACTGGATAAGAAACTGGCCACTATTCAGGATGTTATCTTGCTAAAGGA
CGAAGGTTCTGCTCGTGTTTTGAAGATCTTAGAGCAGGCCCAGCATGTTTTGGCTAACACAGCGCCCGGA
GGTCACGAAGCCATCAATAAGGAACTCACAGATCTCCAGGATCTCTGGTCCGGAATCGCTCTGCGCATCA
TGGATGTTAAGTCAAATCTGGATGACTCAATTACTCAATGGTCTGGTTTCCTGGATCAAGTGCAAAATGT
TCGCAAGTTTAATGAGTGGTTGGATGGTCAGGTTAAGGAGTTGAGTGAGCATCAAACAACCATGACAGAG
AAACGAGCACAATTGGATCGGGTAAAATCTACGGAGGAGAAGGTGCGCGTCGAGAAGATTGATGTGGATG
CACTCAAGATCCAAGCCAAGGAAATGATTGCCAGTGGTCAACAGAGTCAGGCGGCCTTCCAGGCGCAAAA
AGTTCTGGATACATTCGACGAGCTCTTTGCCAAGACCCAGAAATTGTTGTCGCATCGTCAAGATCAGTAC
AGGGATCATCGATTGTTCAAGGAGGCCTACGATGATTTGGTTAGCTGGATTGGACGAGCCCGCGAGAAAT
TCCCTTCGTTGAAGCAGAGCAGCTTAAGCGATAAATTGGCCATTGAAAACGCTGTCCAAGCTACGGAAGC
TCTTCTCAACAAGCAGGCACAGGGTGAACTCCTCGTGGAGCACTTGGTCCACACTGGAGAGGTGGTGCTG
GCCAGTACATCTGCTCAAGGTCAGGAAATCATTAGGAATGATATTCGTGCCCTGCGCGACAGCTTCGAGG
GCTTGTTCCGTGAAATCAACCAGCAGAAGGAGAACCTTGAGGTGACCATGGTTCAGTGGCGAGCCTATAA
GGAGGAATATGAACGTCTGATGGAATGGCTTCAACAGATCGACATCCTTGTGAAGAACCACAAACTGAAC
CTGTGCCCCAACCTACCCGAAAAGGAAAAACAGGTGGCTGATATGAAGGAAGTAATGTCCCGTCTCGAGA
AAGGCAAAGATGACATCGATAAGTTTAATGCTTCGGCTGCCAGTCTGCTAAAGTCCCATTTGGATACTTA
TGTGAACAATCAGCTAAGGCACTTGAGCTCCGTGTATCAAGTACAGGTAAATCTGGCCAAGGATGTTCTT
AAGAAGGTGGAAACCAACCGCGATCAGCATCGTGAATACGATGCCAATATGAAATCTGCCAAGGATTGGA
TAGCAAATGCCAAGGCTACAATTCAGTCGGCGGGTGAAGGAGCAGGTTCCAAGGAGGCTCTTCAGCGTCG
ACTGGAGCAAATTCAGGATCTGATTCGCAACCGTGAGCTTGGCCAGAATTTGGTGCACACCGCTATTAAT
AATGGCGAAAAGATAATAAGGAACACTCGCTCTGACGGACGTGACGCCATTAATACCGAGATGAAGGAAC
TGCAAACCGAATGGGATCGACTGGTGAAGAAAATGTCCACAGCCAAGGTGCAGTTGGAGACAAATCTATT
GCAATGGGCTGACTACAGTTCCAGCTACTCTCAGCTGCAGCAATGGATTACTGACCGGGAGGCCAAGTTG
CAGCAGGCTTGTGAACAGAAGATCGTTAAGTCCAAGAGGGGTCAACCTGGTCTCAGCAGTGGATTGAGCG
AACGCAAGGCCAATCTTCGCCAGACGAACAACATTGTCCAGGATATTGTGTCATTTGAGCCCATGATTCA
GTCGGTTACGTCCAAGGCTTCGGTTCTTCAGCAGGGTGCTCCGGGCACTGAAATCTCCGATAAGTACGAG
AATCTTACCAAGCAAGCTAAGGACTTGTACGAGAAACAGAAGAACACCATCGAAAGCTATCAGTCCTTGA
TTGACGCGGGCAATGAATTTGCCACATGGCTGAGAAATGCCAAGGAACGATTGAGCAAATGCTCTGAACC
CACGGGCGATAAACAAGCTCTGGCCGAGAAGACCCACCAGCTGAAGATTCTGCAGGGTGAGCTGCCGGAA
GGCGCCCAAAAACTTAAGAATGCTCTGGAACAGGGCGAGATCGCTTGTCGCAGTGCCGAGCCCGAGGATT
GTGAGATTATCGAGCAGGAAGTGGCTCTGCTTCAAGAAGAATTCGATGCCTACAGGGAAGCTCTTAATAA
GGCCAAGGATTATCTGGAAGTGGGTATTGTCAAGTGGTCCGACTACCAGGATCAGTACACCGAAGCTTTG
GAGTGGTTAAGTAAGACCGAAGCTTTGGTGCAATCCTACAACAAACTGCAGGATAGTTTGATCCAGAAGA
AGGTTGTTCTCGAGCAATTCCAGGGTCATCTGCAGACCCTATTCGACTGGCAGAAGACTCTGGATGATCT
GAACATGAAGGCACAGGTTCTACTGGAGACTTGCTCCGATACAAGAATCAGCAATGCTATTATGCAACTG
ACCACCAAATACAATGCTCTACTAACTTTGGCCAAGGAGGTTATGCGTCGCCTGGAGATGCACTACCAGG
AGCATCAGCAGCATCACAGTCTGTACGAGGAGTGCCAATCATGGATTGAGAAGACTCGTGAGAAGCTTTC
CGAGTGCGAACAAATTCCCGGAACTCTTAACGAGGTTCAAATCAAATTGAATACCGTAAAGAATCTTCGC

CAAGGTTTTGAAACCGGTCAGAATAAACTGCGTTATCTTTTGGAGCTCAAGGAGAAGGTGATAATGAACA
CCGAACAAAATGGTGCAGCCAAGATTCAGGAAGATACCGAAGCCTTAAAGCAGGACTTTGACAAACTGTT
GGTGGATCTGAACGATGTTCGCCAGAAATTGGCCAATCGTCTTGCCCAGCTGGAGGAGATCTTTAAGCTG
TACAAGATCCTGATTGAATGGCTGGAGGATGTGGAACCGAGTGTCAAGACCAGTGATGAGTTCCTGAATG
ACTTGAGTGAGAAGCGAGCGGCGCTGGAGAAGTTCCGCGTCATTCAGCGTGATATCAATGGCCACAATGA
TATTGTGGAAAAGATCAATCAGCGTCTTAAGGAGGACAACAGTTTGGATCTTAAGGACTTCCAGCCCGGC
TTGACCAAATTCGATGACCTGCAGACCCAGGTGAATAAAATCATCGAGTCGCTGGAGAACCAGGTAAACA
GCCACGAGAAATACAAGCAGGCCTACAATGAGCTGCAAGATTGGCTGCGACGCACTCGCATTGAGGTGGA
GCAGTGTGCCGACTGCCATGGCGAGAAGGATCAAGTGGAGAGTCGTCTGAATCGATTGGGAGATATACAA
TCATCATCTCTGGAGGGCAAAGCCCTTCTTGAAGCTTGTGAAGAGCTGAGTCAGGCTGTGATTGCCACCA
GCGGAAGTGAAGGCCAGGACAATGTGGCCCAGGAGATTAAGCATCTCACCTCGGAGTGGGAGACCCTCCA
AACGATATCCCGCGATGCTCGCAGCAGCTTAGAATCCTGCTTGGCCGCCTGGCAGACCTTCCTGCAGAAG
TTCAACAAGATCAACCTTTGGATCGAAACGATGAACAAGCGCGTTACCAAATCCCAGGAAGGCGAGAACA
AGACACCCGAAGATTTGGTTAATGCCAAGGGTAAATAATGTAGTTTCTAAGCCCATCTCCCTCGAAGAAG
CCGTTGCCATGCAACAGAATGCCCAACAATTCGAGACACAGTGCCAGGAGCAATTGGACAAGCTCCATGG
CATATCAAACATCAGTCATAAAATGTTATGTAAGACCAATGCCCCCGACGAATTGGACGCCATGCATTCG
CGTTGGACTGCCGTCCATGAGAACGCGAAACAGGCGAGCGCCAAGCTCGAAAAGTTGGTGGCCAATTGGA
AGTCATTCGACGCCGATGCCGCCAAGCTCGAGGATTGGGTTGGACAGGGTGAGCAGCAAATGTCCCGCCG
TCCAGCTGTCCTCAACACCCCGCACATTGACAAGCTCGAAAAGGAGCTGGTCAAGCTGAAGTCGTTCAAC
AACGAGATCTCGCAGCAACAGGCCAAACTGGTGACTCTGGGTCAAAATGCCGATCAAATCAGTTTGCACT
TGGCTCCTGAAGGAGCAGCCGCTTTGAAGGATCGTGTTAACCAAATGAAGGGCAAGCTACAGAAGCTTTC
GGAAGCAACTCGAGGTCACATCAACGAGGTGTCCGACGCCATTATCTCCAGGCAGGATTTCAATGCCAAG
TTGGTGAACTTCTCCAACTGGATGGAGCAGCTGCGTAACCAGGTCACTCAGGTGGAGGAGATCAACCCGG
AGCGGGTGGAAACCAGTCTCCATGTCATACACGCGCTACTGCAGGAGCATGCCGATAAGAAGCCAAGTTT
CAATGCCATATACGATGAAGTTAAGCAATTGGCTTTGGGAGCAACTCCCGAAGAGTCGAATGCCCTGAAC
GATGCCTACACCGCTTTGGTGGTGAACTATCAGAACCTCGAGACCAACATGCTGCAAAAGAAGGCGGCTC
TGGAGAAATGGACAGAGCTGCTGGGCTGGAAGAATGACACCGAATCGCATTTGAACTACCTCAAGCATCA
GCTGGACAAGCCGGAAGGACCTGCTGCCGAGGAGCTGTCCAAGGTCATTGATGAGATCGATAATTTGGGT
CAGGGTATTGGATACTGGAAGGGTCAGGCTAAGGAGATTGATGAAAACCCGGCCATTCAGTTGAGGGATG
CTCTGTCTCGTCGTCCATTGATTGCCACCCAGATTGTGAACGATGTTGAGAATAAACTAGAGAATCTGAA
ACTGCGCTCGCAGAGCCAGCAACAGCAAATCCAACAGATGACCGTGCGCAAAGATAAGTTCCATGCCCTG
GAGCATAACTTTGGACAGGCACTTCAAGAAAATCGGGCTAAGCTAGACGAGATTCTCAGACAGCATCCGA
CTTTAAATAATATCGATCAAATCATTGCCGATTTGGTCGCTCTGAATGATGCCCTGAAATACCAAGCCGA
TCTGAAGAATCGCATTCATGACGAAGGTTCATTGCTGATGCGTGAGGATATTGCTAGCATGCCCGCCATT
CAGGAGAGCCTACTTATCATGGACAAGAACTACGATTCTCTGCAGAATGAAATAGCTGATCGCATTCAGA
AGTACAACCTGATTAGCCAGGCCCTGAGAGAATATGCCGATTCCAAGGACAAGTTCTCCAAGGAACTGAA
GAAGGCAGAAGATCTATACAATGCTATACCTCAACAGCCACGCGATGAAACCGAGCTCCATCAAGCTTCC
GAGAAGACCAGAAAGACCATGGAGCAGTTGCGTAAGTCGAAGCTCAGTTTGGATGAACTGGAGCGTCGGG
GCAACAATGTGGGCAAACTTTTCAGTGCCATTGGAGAACCCATTCCGCAGGAAGTGCCACAGGAGGTCAC
CGCAGCCAAACAACACTGGCAGGATTTGCACGACAAAACCGCTAAGAATGCGCATGTGTACGAGACGGAA
GCTGTTATTTGGTCACAGATCGAGGATGCCAAGAAGGATTTGCTTCCCTGGTTGTCCGAAACGAATCAGG
GACTCTGCGATGCTGCGGATAACTCAATTGAAATTGAGTTTGGACCCATGCGTCTGAGCAAGTACCGCAC
GGAGCTACCTTCGTATCAGGCTCTGAAGGATTCGATAGTGGAGAAAACCAATGATTTGGTTAAGATCAAC
AAAGGTGCGGAAATTCCCGCTCTATCTGCACTCAACAAACTATTGTCTGAGCAGTTTGCTGAGGTTAACA

ACAATGCAGATCGCCTAAGTGCCATAACCACCTCGTTCAATGACCAGGAACAAGAGTTGCGCAGGCGTTC
GAAGGAAGCTGGCGAACGGGTGAGCAAACTTCGTGAGCAGCTGATCAAATGTGATGACATGTCCGGTGAT
AATAACAAGATCATGGAGCGTCTTCAGCAATGCCGTGCCCTGCGTGGTGAGCTCGATAATAGTGGAAACG
AAATCGACAACATCAAACAAAAGGTTGACGAGCTGCGTAATTTGTACCCAACTTTCAGTGAGTCCATCAT
ACCCAAAGAACTGAACAATGTGCAAAAACGCTATGAGAACGTTGATCTTTACGCCAAGAAGATTGAAAGT
TCTCTGCTGCAGTTCCTCAAGAAGTTCCACGCCGATAAGGTGGGCATGCTGAAGCGTATCATTGCCACTC
AACGCGAAAAGGTGGCCTGGTGTCAGCCGGAGTCCTCCAGTGACAAATACAATCTGGATGTCAAGAAGTC
CTCTCTGCAGGAAGTCAGCAAGAGCATTGATGACTGCAAGGCACGCCATGCCGAGACCCTGAAGTCCTTG
GAGATGTTGAAGGCGGTAGAGTCGCCACAGAACCTGGCTGAACTCACCAGCGATGCCGAGTTACTGCGCA
AGGATATGCAGGCTCTGCAGGACAGCTTTGACCAAATCAAGGGAATCCTTGACGAGAACGTCGATTTGTG
GTCGCAGTATGAGCAATCGAATGAGCAGATTTCCAACTGGCTGCGTGACGTTGAAGGACGCGTTAAGGCA
GAAACTAGCAGCCAAGTAAATCTGTCCGAGGTTCCACAGAAGCTTCAAGAATTGTCTATCCTCCAACAGG
ATGTGTTGGCCCATGAGCCCATTATTAATAACCTCGAGCAGACATCCCAACAGCTAATTGAAAAGAATCC
GGAGGCAAGAATTGGCCAGTTTGTCACCCATTTGGTGCAGCGATATCAAGCTGTGTCCAAGGCTTTAACC
AGCTACATCGATAAGATCCGTGGTGCTCAGTTATCAAACGCTAACTTTGCCAAGGCCGCCAAGGATTTCA
ACGAGTGGTTTGGTGATGCCAAGATCGAGTTCCAGGAACTTGCTCGCATGGGATCTCCCGGATCATCCTC
TGCCACAGCTCAGCAACTGCAGACCGTCAAGAATTACATCAAGACCTTCGACAATGGTCAGATACTACTG
AATAATGCCGTCGATATTGGTGAGGCTTTGTACCCGGTGGTATCGCCCGATAATCGCGAACGTATCCGTG
CTGATCTTCGCCAGATGCGTGAGAAGTTCGACTACTTGCGAGATGAGGCCAATGCTTTTATGCAGCAAGT
CGAGGGTGTTTTGATTCAAAAGACTTCAATCGAAGAAAGCTACACTCAGGTTTCTCACTATCTTAACGAA
TCGAAAGCTAAGGTTCCCACCACTGACGAATTGTATCCCACTTTGGCCACCAAAAAAGCTGCCCTCCAGA
ACTACAAGACCCAGCTGCAGGAGATCACTCTGCACAAGAATGCTTTGAAGCAGTTGCACGACAAGGCTGT
CACCCTTTGCGATGATGAGTCCGAGAGAAAGACCGACGAGTCTATCCAGGAGTATAACACGCTATCCAAG
AAGATTTCCGATAGGATTACAACAGTGGGAAATCATGTGGTCAAGCATGAAGCGTATGATCAGGTCCTGG
AAAAGGCACAAGACTGGCTGAATACCATCAAATCCGAGGCCATTGATATCCTCAACGAGACGACATTTGA
AAAGGAAGGCGCCGAGGAGAAACTGCTGGTTGTGGAAAATCTTCTGCAACATAAGCCTGAGGGTGATTCC
ATTTTCGATACCTGTCACAAGTTATTGGAAACGGTGCTCACACAAACCCATCCCAGTGGCCATCCAGCTC
TGCTGAAGGGTTTCGAGGAACCGAAGCAATCTTGGGAGGACTTTATGACTCTTTGTCAGGATTCGTTGGT
AAAACTGAAGCAGCTTTGCTCTAAATGGGATGAGTTTGATACAATCATTGAGGAGCTAGACAACTGGATG
AAGAATGTCGAGGCGGTGGTTAAGAACCAGAACCTTAAGAGTACGGCGGAAGCCAAGAACGCCCATTTGA
AGCAGCTTCAGGATATTTCAAAGGACATCGAACGGCGCGGTGCGGCCATCAATGAGCTAATGGATCAAGG
CCGTGAGATTGAGGGTGAAACCGATTTGAACCTTAAGTTGTCGCGCCTGAATACTCGCTATCAGACCCTG
AAGAATCTGTGCAAGGAATCGATAGCCAAGTATGTGAACTATGTAAAGGATCACGAAAGCTTCGACAAGG
ACTTTGATTCATTCAAGCAGAATCTTCAATCTTCAGTCGACGAGCTGGCCAAGACCAATGAAATTGTCGG
CGATCAAAGCGTGCTGCAGGATCAGCAAAATAAACTCCGTGAGATGTCCGACAAGCGCATTTTGGATTCC
ACTCTATTCGAGGGACTCATCGATCGTGGCGAGAAACTCTATGGTCACACCAGCCCCGAAGGCCGTGAAA
TCATTCGCCAGCAATTGCGTGCCCTGCGAACCCTTTGGGACAACTATACCGATGATTTGAACTCGGCGAC
GCAAAAGATCGATCAGTGTTTGCTGCAGTTTAACGAGTTCAGCATTGCCCAGGATCAGCTTACCAAGTGG
CTGAAGGATGTGGACAAGGCCATGCAGAGTCACACTGAACCCAAGACGACGCTGCAGGAGAAGCGTGCTC
AACTGCAGAACCACAAGCTGCTCCATCAGGAGATCACCACACACAATGTTCTGGTTGATAATGTCTGCGA
CAAGGCACAAATACTGGTCGACCAGATCAAGGATAACTCGTTGAATGTGTACTTGACCTCGATCAAACAG
CTTTTCCAGAGCATAGTCCAGAAGTCGGATGAAATTTTGCACAATCTGGATGATTGTGTTCAGAAGCACA
ATGAATTAAACAACGCTCTGTCCTCGGCCAAAACTTGGATTTCGAATGAGAAGGCCAAATTGCTGGAGTG
CGACGATGCTTATGGTGAGAAGGCAGACATTAAGCGAAAGATTGAAACTTTGGGACAATTGGCTCAGAAC

AAGCCACAGGCAATGAAAATAATCAGCGACATCCGTGATCTCTTTGAGAAAGTGAAGGCAACGACCTCCG
AAAAGGGTAACGAAGTGCTGGACAAGGAGATCGAGGAATTGGAGACCACCATGAAGAGTCACTTTGATGA
CATCGAGGGCATTGAAGGCAAACAGAAGGATGTGCTTGCCCAGTGGGACAAGTTTGAAAAGGCGTTGGAG
GAACTTACCAAGTGGTGCCGCAGTGCAGAGGCAGTGTTCCGCGAACAGCAGCTGCAGTCCACGCTCCATG
AGAAAGTGGAGCAGCTGGAGAAGTACAAGATTCAAAGGGAGTTGATACTCCAGAAGGAGAAGGAGATCGA
TGCCTTCGGAGATGCTGCCCACGCTCTGCTTAACAACTGTGGAGCCGATCGTCTAAAGACTTTAACCACT
CAGATCACCAATCGGTATCAGCTGCTTCAAGTCCTTAGCAAGGAGGTTGTTAACCGCTGGTCAAATCTGG
TCGATGATCACCAGTTCTACCAGGATAAGTACAACGAGGTGGATTTGTGGCTGCAACCCATCGAATCGCA
GATGGCGAAGGTCCTTTTGGATGAGCCAACCCAGAGTTCTAACATTTTGCAAGTGCTGTTGTCTGAAAAG
GAGCAAGCGGAGAGCTTATTTGCTGCTCTAAATGCAGCTGGAGAAAAGGCTCTTCCAGAGACATCCACTC
AGGGTAGGGAGAAGATCCGTAAGGACCTGCGCGATATCCGCGATCGTTGGGACAAACTGGACGAGGGCAT
TCGCAACCTGGAAAAACGCCAGGAAGCGCAGGGAGTTCAGCTCTCCAGCTACCAGGATATTCTTAATCAA
ACCGTCAACTGGTTGGATCAAGTGGAGAAACTGATCCACAATGAGAACCCTGCTAGCTGGACCAGTGCCC
AAGAAATTCGCTCCAAGTTGTACAAGTACAAGGCCACCAACCAAGACATCAACTCGCATAAACGCATAGT
CGAGGCTGTGAATGAAAAGGCAGCAGCTCTTCTGGGCAGCGCCGCTCCAGCCAATGCGGATGAGATCTCC
AAGGCGGTGGCTGAGGTGAACAAGCGATATGATCAAGTGGGCCAGGACTGTGCCAAGTTAGTTGCCGATC
TGGATGGAGCTTTCGATGTGTACCAGCAGTTCAGTGAGCTGCAGAAGGCTCAACAGGACTACCAGAAGAA
TCTTTGGGATCGTCTTACCGGCTACTCGGACTACTCAGGCAATAAGGCAGCTCTGCAAGCTCGCCTTCAA
AAGATCAACGAGATCCAAGATGCTTTGCCGGAGGGTGTGGCCAAACTCAAGTCCCTGGAGGATCACATTG
AGCAACAGGCCAGTAATATACCAGCCCGATCCAAGGAGGTAATGGCACGTGATCTGGCCAACTTGCATGC
CGATTTCGAGAAGTTTGGAGCTTCGTTGAGCGACGTAAAGAGTGGACTAGAGAATCGTCTACAACAATGG
AATGATTACGAAATCAACTTAGATCGACTGATCACTTGGTTGGGCGAGGCTGAGAATTCCCTCAAGAACT
ACAATCTGAAATCCTCGTTTGAGGAGAAGGAAGAACAGCTGAACGGTTTCCAATCCTTGGCTCAGAATTT
GCGTCAAAACGAAGCTGACTTTGACAAGGTGAAGGACGACACCTCAGAGTTGGTCCAGAGTTCTGGTGAA
ACAAGGATTGCTGTCAATGTGCAACAGGTTTCCTCCCGCTTCCAATCGATACAGGCTACAGCTAAGGAGA
TTCTCAAGAAGTGCGAGCAAGCTGTCCAGGATCATGGTCATTTCAATGACAAGTATAAGCAGTGCGCCGA
CTGGTTGGCAAATGCTCAGGCCCGTTACGATGATTGCTGTGACTTGTCCACGGTGGCATCTCGAGACGAT
CTTCTCAAGAAACAAGTGGTCATCCAGGAGCTTCTGGCCCAGCAGCCAACAGCTACTCAGCTTCTAAATA
GCACCGTTGAGCTGGGCGAGAAGTGCTATGGGTCCACAGCAACCGAAGGACGCGAGGCCATTCGTAGTCA
ACTGGATGATCTGACATTCGATCAGTTGTTCGACAACATTGCCATCACTGCAAGAAAGATTCAGGATAAG
ATCGCCAAGTGGTCTGGCTTTGATGAGATCGCTGACAGCTTGAAGAGCTGGTTGGATGAAACGGAGAATG
CCCTTCCAGCTGATATTGAGCTGAAAACGACTCTGGATGAGAAACGCAATAAACTGCAAACCTATCGTGA
CATCTTGAACGACATCAACAATCATCAGGTGGAGTTGGGTAATCTTCAGGAGATTGCCGCCAATCTGCCA
GAGAAAACAGAGCTTGTGGACCAAATCATAAAGGATATCTCTGACCGCTTTGGAAAGCTGCAAAAACGTG
CCCAGAACTACGTGGAGCGTTACGAGGGTATTGTGAGTGCACATCAACAGTACTCCAAGGCTGTTATGGA
TGCTCAAGAGTTTATCGATGCTACCCTGAATACGGTTCACTATTGGGGCGATCTGGATCTGGAACAAATC
TCACTGCACACGAACCTCGACCGCCTAAAGAATCTGAAGGCCAGTTTGGCCGATGAGTTCCCACGCGTTG
ATCAAGTTAGGGCTTTGGGCGAGAAGGTTATTCCGGGTACGGTGGATGTGGGACAGGTAAACATTAAGTC
CCAGATCGACACCACCCAGCAAGAATGGGAAAGCCTACTGACCACAATCAGCTCCACAATTGAAGCTATC
GAAGCTCGCCTACAGCATTGGTCGGAGTACGAGCAATTACGTGACCAGTGTTTGGCTTGGATTCGAGATA
CGGATAACAACCTTCATGCTATCGATCTGAAGGAGGATCTGCCGAAAAAGCGTGCACAGTTGGATGCACT
GAAGGCCCTCCAAGGAGATGTACGCGCCAAGGAATTAGAGGTGGATAATGTCACGGAGAAGGCCCAAACC
CTTCTGAAGGGTCCCAGCAGCAATCGTGCCTCGGGTCCTGAGTTGGTAACCAAGTACCAGCAGATCTTCC
ACAAGGTCAAGGAGCTCAACAACCGTTGGCAACAGTATGTTACCTCACACGAAGATTTTGATAACGCTAT

CTCTGATTGCTCCTCTTGGATCAATGAGATCAAAGAAAAATTGGACTATTGCTCCGACATGTCATCTATG
AGTCCAAAAGAACTGGATAAGAAACTGGCCACTATTCAGGATGTTATCTTGCTAAAGGACGAAGGTTCTG
CTCGTGTTTTGAAGATCTTAGAGCAGGCCCAGCATGTTTTGGCTAACACAGCGCCCGGAGGTCACGAAGC
CATCAATAAGGAACTCACAGATCTCCAGGATCTCTGGTCCGGAATCGCTCTGCGCATCATGGATGTTAAG
TCAAATCTGGATGACTCAATTACTCAATGGTCTGGTTTCCTGGATCAAGTGCAAAATGTTCGCAAGTTTA
ATGAGTGGTTGGATGGTCAGGTTAAGGAGTTGAGTGAGCATCAAACAACCATGACAGAGAAACGAGCACA
ATTGGATCGGGTAAAATCTACGGAGGAGAAGGTGCGCGTCGAGAAGATTGATGTGGATGCACTCAAGATC
CAAGCCAAGGAAATGATTGCCAGTGGTCAACAGAGTCAGGCGGCCTTCCAGGCGCAAAAAGTTCTGGATA
CATTCGACGAGCTCTTTGCCAAGACCCAGAAATTGTTGTCGCATCGTCAAGATCAGTACAGGGATCATCG
ATTGTTCAAGGAGGCCTACGATGATTTGGTTAGCTGGATTGGACGAGCCCGCGAGAAATTCCCTTCGTTG
AAGCAGAGCAGCTTAAGCGATAAATTGGCCATTGAAAACGCTGTCCAAGCTACGGAAGCTCTTCTCAACA
AGCAGGCACAGGGTGAACTCCTCGTGGAGCACTTGGTCCACACTGGAGAGGTGGTGCTGGCCAGTACATC
TGCTCAAGGTCAGGAAATCATTAGGAATGATATTCGTGCCCTGCGCGACAGCTTCGAGGGCTTGTTCCGT
GAAATCAACCAGCAGAAGGAGAACCTTGAGGTGACCATGGTTCAGTGGCGAGCCTATAAGGAGGAATATG
AACGTCTGATGGAATGGCTTCAACAGATCGACATCCTTGTGAAGAACCACAAACTGAACCTGTGCCCCAA
CCTACCCGAAAAGGAAAAACAGGTGGCTGATATGAAGGAAGTAATGTCCCGTCTCGAGAAAGGCAAAGAT
GACATCGATAAGTTTAATGCTTCGGCTGCCAGTCTGCTAAAGTCCCATTTGGATACTTATGTGAACAATC
AGCTAAGGCACTTGAGCTCCGTGTATCAAGTACAGGTAAATCTGGCCAAGGATGTTCTTAAGAAGGTGGA
AACCAACCGCGATCAGCATCGTGAATACGATGCCAATATGAAATCTGCCAAGGATTGGATAGCAAATGCC
AAGGCTACAATTCAGTCGGCGGGTGAAGGAGCAGGTTCCAAGGAGGCTCTTCAGCGTCGACTGGAGCAAA
TTCAGGATCTGATTCGCAACCGTGAGCTTGGCCAGAATTTGGTGCACACCGCTATTAATAATGGCGAAAA
GATAATAAGGAACACTCGCTCTGACGGACGTGACGCCATTAATACCGAGATGAAGGAACTGCAAACCGAA
TGGGATCGACTGGTGAAGAAAATGTCCACAGCCAAGGTGCAGTTGGAGACAAATCTATTGCAATGGGCTG
ACTACAGTTCCAGCTACTCTCAGCTGCAGCAATGGATTACTGACCGGGAGGCCAAGTTGCAGCAGGCTTG
TGAACAGAAGATCGTTAAGTCCAAGAGGGGTCAACCTGGTCTCAGCAGTGGATTGAGCGAACGCAAGGCC
AATCTTCGCCAGACGAACAACATTGTCCAGGATATTGTGTCATTTGAGCCCATGATTCAGTCGGTTACGT
CCAAGGCTTCGGTTCTTCAGCAGGGTGCTCCGGGCACTGAAATCTCCGATAAGTACGAGAATCTTACCAA
GCAAGCTAAGGACTTGTACGAGAAACAGAAGAACACCATCGAAAGCTATCAGTCCTTGATTGACGCGGGC
AATGAATTTGCCACATGGCTGAGAAATGCCAAGGAACGATTGAGCAAATGCTCTGAACCCACGGGCGATA
AACAAGCTCTGGCCGAGAAGACCCACCAGCTGAAGATTCTGCAGGGTGAGCTGCCGGAAGGCGCCCAAAA
ACTTAAGAATGCTCTGGAACAGGGCGAGATCGCTTGTCGCAGTGCCGAGCCCGAGGATTGTGAGATTATC
GAGCAGGAAGTGGCTCTGCTTCAAGAAGAATTCGATGCCTACAGGGAAGCTCTTAATAAGGCCAAGGATT
ATCTGGAAGTGGGTATTGTCAAGTGGTCCGACTACCAGGATCAGTACACCGAAGCTTTGGAGTGGTTAAG
TAAGACCGAAGCTTTGGTGCAATCCTACAACAAACTGCAGGATAGTTTGATCCAGAAGAAGGTTGTTCTC
GAGCAATTCCAGGGTCATCTGCAGACCCTATTCGACTGGCAGAAGACTCTGGATGATCTGAACATGAAGG
CACAGGTTCTACTGGAGACTTGCTCCGATACAAGAATCAGCAATGCTATTATGCAACTGACCACCAAATA
CAATGCTCTACTAACTTTGGCCAAGGAGGTTATGCGTCGCCTGGAGATGCACTACCAGGAGCATCAGCAG
CATCACAGTCTGTACGAGGAGTGCCAATCATGGATTGAGAAGACTCGTGAGAAGCTTTCCGAGTGCGAAC
AAATTCCCGGAACTCTTAACGAGGTTCAAATCAAATTGAATACCGTAAAGAATCTTCGCCAAGGTTTTGA
AACCGGTCAGAATAAACTGCGTTATCTTTTGGAGCTCAAGGAGAAGGTGATAATGAACACCGAACAAAAT
GGTGCAGCCAAGATTCAGGAAGATACCGAAGCCTTAAAGCAGGACTTTGACAAACTGTTGGTGGATCTGA
ACGATGTTCGCCAGAAATTGGCCAATCGTCTTGCCCAGCTGGAGGAGATCTTTAAGCTGTACAAGATCCT
GATTGAATGGCTGGAGGATGTGGAACCGAGTGTCAAGACCAGTGATGAGTTCCTGAATGACTTGAGTGAG
AAGCGAGCGGCGCTGGAGAAGTTCCGCGTCATTCAGCGTGATATCAATGGCCACAATGATATTGTGGAAA

AGATCAATCAGCGTCTTAAGGAGGACAACAGTTTGGATCTTAAGGACTTCCAGCCCGGCTTGACCAAATT
CGATGACCTGCAGACCCAGGTGAATAAAATCATCGAGTCGCTGGAGAACCAGGTAAACAGCCACGAGAAA
TACAAGCAGGCCTACAATGAGCTGCAAGATTGGCTGCGACGCACTCGCATTGAGGTGGAGCAGTGTGCCG
ACTGCCATGGCGAGAAGGATCAAGTGGAGAGTCGTCTGAATCGATTGGGAGATATACAATCATCATCTCT
GGAGGGCAAAGCCCTTCTTGAAGCTTGTGAAGAGCTGAGTCAGGCTGTGATTGCCACCAGCGGAAGTGAA
GGCCAGGACAATGTGGCCCAGGAGATTAAGCATCTCACCTCGGAGTGGGAGACCCTCCAAACGATATCCC
GCGATGCTCGCAGCAGCTTAGAATCCTGCTTGGCCGCCTGGCAGACCTTCCTGCAGAAGTTCAACAAGAT
CAACCTTTGGATCGAAACGATGAACAAGCGCGTTACCAAATCCCAGGAAGGCGAGAACAAGACACCCGAA
GATTTGGTTAATGCCAAGGTAAGAACTCTTCGTCAAGTCGAGATCTCGCAACTGTCGCACGCACCTCTAA
CCAAAAGAAAAGTAGTAAAAATGTAATCAAATATAAACATAGCCAAAGCGTGCACTTTAAGTAGAACTCA
CCGACAACAAAATTTGTATCTAAATATCTCGTACCGTGAGAAATTAAATCATGTACTAAACCCTCACTAA
TCACTACTGCTAACTTTTCAGCTAATCACCTATTTGTATAATCAATTGCAGCTAATCATTTACACAGACA
CCTGTCTAATGCCTAATTGCTTTATGTGCTATAATTAGCCAATATTAGATCTTGTAACTAAATATTATTT
CTTTCTATTAGGAAACTGCTTGAGGAAGTGCTGGCCGAGAAGGATAATGTGGAGGATTTGAACGACAACT
GTGAGCTTCTCATGGAGCAGAGTGCCTGCACTCGCATTCGTGACCAGACCATCGAAACTCAGGCCAACTA
CACCAAGCTCCTGACTTCCGCTCAGGGATTGGTTGCCAAGATCGAGAAGAATCTGTCCGATCACACCGAG
TTCCTTAACTATAAAAAGGAAATGGACGCCTGGATCGAAAAGGCTCAACAGGTTCTCGACGATTGCTCCA
CCGATGGCGATGCTGCCATCATTGCCCAAAAACTGGACACGGTCAATGTCCTTGGCCTCTCGTCTTCCCG
AGGGTCAGCATCTGTTGGCCTTGGTGCAGGATGCCTACTCGAAGGCATCGAACATCACTCCAGAGGATAA
GCAAGAGAAGCTGCGGGAGCTGATGACCAAGGTTCGCGAGGATTGGGATGCCCTGGGACTGGCCGTTAAG
CAAAAGTTAAGTGACCTGAAGCAGGCGCAGAATCGTTGGAACGATTTTGCTGCCAACAAGGATAAGCTTG
AGAAGTGGCTCAATGAAACCGAAACGACACTCAAGGTGGCACCGGAAACCAAGGGTGAACTGAGCGAAAT
GAAGACCCTGCTGGAACGATACAAGACCCTTTCCAATGAACTGAAACTGAAGGGTAACGAGCTGGAGCAG
CTGCAGTCGGAGGCTCGCGACCTGGGCACCGAAGTGGATGCAGTGAATCGCTTGCAATCGCGATGCGACA
AGCTCAAGAACGATTGTTCCGCTCACATTACGGCACTGGAACAGGAGATGTTCGATTATAATGCCTACCA
CCAGAGTCTGCAGGATGTGGAGAAGTGGCTGCTGCAGATATCCTTCCAGTTGATGGCCCACAATTCGCTG
TTCATCTCGAATCGTGAACAGACTCAGGAGCAGATTAAACAGCACGAAGCTTTGTTGGTTGAGATTCAAA
AGTACCAGACCAATTTGGATGACTTGAATGCCAAGGGTCAGGCGCAGATCAAGCGTTATGAATCCTCAAC
ACCAGCCATTCGTCCTACGGTTGAGTCACAGCTGAAGAATATCCAAGATAGCTATAACTCACTGCTGCAA
ACGTCAGTGCAGATTAAGAACCGCCTGTTGGAATCGCTGGCCAAGTTCCAGGAGTACGAGGATACTTTGG
ATAGCATTATGCGTAATCTGGAGACATATGAGCCCATTATTCAGACCGAACTTGATGCTCCGGCTACCAG
CTTGGAGTTGGCTCAAAACCAATTGAGGTGTGCCCAGGAAATGCAGAACAAACTGAACAACGAGAAGTCC
CGACTGGCAGCTGCCGTTCAGGCCTGTGAGGCAGCTACTGCCTCCATCAGTCGACCAAGTTCTCCACTGG
AAACAGCCATGCAGGCTATTCCCGAAAGGGAGCTCATTGTGCGCGCCAAGCTCGAGGATCTGCTGGATCA
GGGTAGGATAATGTTTAATGCCTTTGCGATGCATTTGTAGAGTTGCGACCACTTTCAATAGTTCAAAGTC
AAGCAATTAAAATGAATGGAAGAATCATTTTGTAGATCTATGTTGTTCTATCATATTTTCTTAAAACCTT
TTTAGATGTAATCAGTAGAGATTATGCTTGATTTCAAAACCTATTTTCTTTTTTAGATAGCTATTCTTCC
ATTCTATTATTCTTTTATCACTATTATTTGCTAACCAATGACTTGTTCTTTTTATTAAACCCTTAACCCC
ACAAACCCAACACTGCCCTAACCCTTGTTGATTTGCCACTCTGCCCGTAAATGTTGTAAATTGCACTACC
CACCAAAAAAATGCTATGCCTTGCGCTTTGCTGCCACAAAGAAACCGCCTCCAAAGACTCGGAGCTCAAC
CGGAGGTGTCAGCACAGATGATGATAAAGACGAGGCTGATGTTGAGATTCAGGTTGAGCTCAGCGATGTG
AATGAGGCGCTTTTGGATCCCATTGCCCACGAGCGTGTCAAGAACTATCGTCGCATAGTTCGCTTGAATA
GCGCCCATGTGGGCAAGCTGAATGAATTAGTTGCTAAGGTAAGAGTAAGGTCTATTCCTACACAGGATCA
AACAGGTGGCGGTCTGCATGGCTATGATGTTTTAAATTGCTTGTTATGCTTAGAATATAACTTGTAACTC

AATTTAATTTAATTGCGTGTTTGTTTTTACTAGGGTGCAATCCCACTTGGGTGGTTTGACTGCATCCGTT
AGCGAACTGGAGCAACAGCAGAAGCAGCGCGCCGAGCTGCAGGATTGGGTGAAGAAGCAGCAGAGCTCCG
TCTCCGACTGGATGATGCGTCCTTGCAAACTGCGCCCGGAGGCAGCTCAACAGGAGCTGGTGTCCATGAA
CGATTTGCTAAACTCCATCGGTGACAAGCGGTCGCAGTTGATGCTGGAAATGACAGGATCAT
>dme_circ_0001408_junction_seq
CAGTTGATGCTGGAAATGACAGGATCATGAAAATCTGCCAAGATGCTGACAACAAA
>dme_circ_0001409
GCTCGAAAAGTTGGTGGCCAATTGGAAGTCATTCGACGCCGATGCCGCCAAGCTCGAGGATTGGGTTGGA
CAGGGTGAGCAGCAAATGTCCCGCCGTCCAGCTGTCCTCAACACCCCGCACATTGACAAGCTCGAAAAGG
AGCTGGTCAAGCTGAA
>dme_circ_0001409_junction_seq
AGCTCGAAAAGGAGCTGGTCAAGCTGAAGCTCGAAAAGTTGGTGGCCAATTGGAAG
>dme_circ_0001410
GTATAGCATTGTATAGATCTTCTGCCTTCTTCAGTTCCTTGGAGAACTTGTCCTTGGAATCGGCATATTC
TCTCAGGGCCTGGCTAATCAGGTTGTACTTCTGAATGCGATCAGCTATTTCATTCTGCAGAGAATCGTAG
TTCTTGTCCATGATAAGTAGGCTCTCCTGAATGGCGGGCATGCTAGCAATATCCTCACGCATCAGCAATG
AACCTTCGTCATGAATGCGATTCTTCAGATCGGCTTGG
>dme_circ_0001410_junction_seq
ATGAATGCGATTCTTCAGATCGGCTTGGGTATAGCATTGTATAGATCTTCTGCCTT
>dme_circ_0001411
GATCTATACAATGCTATACCTCAACAGCCACGCGATGAAACCGAGCTCCATCAAGCTTCCGAGAAGACCA
GAAAGACCATGGAGCAGTTGCGTAAGTCGAAGCTCAGTTTGGATGAACTGGAGCGTCGGGGCAACAATGT
GGGCAAACTTTTCAGTGCCATTGGAGAACCCATTCCGCAGGAAGTGCCACAGGAG
>dme_circ_0001411_junction_seq
ACCCATTCCGCAGGAAGTGCCACAGGAGGATCTATACAATGCTATACCTCAACAGC
>dme_circ_0001412
GTGCCATTGGAGAACCCATTCCGCAGGAAGTGCCACAGGAGGTCACCGCAGCCAAACAACACTGGCAGGA
TTTGCACGACAAAACCGCTAAGAATGCGCATGTGTACGAGACGGAAGCTGTTATTTG
>dme_circ_0001412_junction_seq
ATGTGTACGAGACGGAAGCTGTTATTTGGTGCCATTGGAGAACCCATTCCGCAGGA
>dme_circ_0001413
GTTCCACGCCGATAAGGTGGGCATGCTGAAGCGTATCATTGCCACTCAACGCGAAAAGGTGGCCTGGTGT
CAGCCGGAGTCCTCCAGTGACAAATACAATCTGGATGTCAAGAAGTCCTCTCTGCAGGAAGTCAGCAAGA
GCATTGATGACTGCAAGGCACGCCATGCCGAGACCCTGAAGTCCTTGGAGAT
>dme_circ_0001413_junction_seq
ATGCCGAGACCCTGAAGTCCTTGGAGATGTTCCACGCCGATAAGGTGGGCATGCTG
>dme_circ_0001414
GAACCTGGCTGAACTCACCAGCGATGCCGAGTTACTGCGCAAGGATATGCAGGCTCTGCAGGACAGCTTT
GACCAAATCAAGGGAATCCTTGACGAGAACGTCGATTTGTGGTCGCAGTATGAGCAATCGAATGAGCAGA
TTTCCAACTGGCTGCGTGACGTTGAAGGACGCGTTAAGGCAGAAACTAGCAGCCAA
>dme_circ_0001414_junction_seq
ACGCGTTAAGGCAGAAACTAGCAGCCAAGAACCTGGCTGAACTCACCAGCGATGCC
>dme_circ_0001415
GCTAATTGAAAAGAATCCGGAGGCAAGAATTGGCCAGTTTGTCACCCATTTGGTGCAGCGATATCAAGCT

GTGTCCAAGGCTTTAACCAGCTACATCGATAAGATCCGTGGTGCTCAGTTATCAAACGCTAACTTTGCCA
AGGCCGCCAAGGATTTCAACGAGTGGTTTGGTGATGCCAAGATCGA
>dme_circ_0001415_junction_seq
ACGAGTGGTTTGGTGATGCCAAGATCGAGCTAATTGAAAAGAATCCGGAGGCAAGA
>dme_circ_0001416
GGTTTCTCACTATCTTAACGAATCGAAAGCTAAGGTTCCCACCACTGACGAATTGTATCCCACTTTGGCC
ACCAAAAAAAGCTGCCCTCCAGAACTACAAGACCCAGCTGCAGGAGATCACTCTGCACAAGAATGCTTTGA
AGCAGTTGCACGACAAGGCTGTCACCCTTTGCGATGATGAGTCCGAGAGAAAGACCGACGAGTCTATCCA
GGAGTATAACACGCTATCCAAGAAGATTTCCGATAGGATTACAACAGTGGGAAATCATGTGGTCAAGCAT
GAAGC
>dme_circ_0001416_junction_seq
TGGGAAATCATGTGGTCAAGCATGAAGCGGTTTCTCACTATCTTAACGAATCGAAA
>dme_circ_0001417
GATATCAATGGCCTCGGATTTGATGGTATTCAGCCAGTCTTGTGCCTTTTCCAGGACCTGATCATACGCT
TCATGCTTGACCACATGATTTCCCACTGTTGTAATCCTATCGGAAATCTTCTTGGATAGCGTG
>dme_circ_0001417_junction_seq
CCTATCGGAAATCTTCTTGGATAGCGTGGATATCAATGGCCTCGGATTTGATGGTA
>dme_circ_0001418
CAAAGCTGCTTCAGTTTTACCAACGAATCCTGACAAAGAGTCATAAAGTCCTCCCAAGATTGCTTCGGTT
CCTCGAAACCCTTCAGCAGAGCTGGATGGCCACTGGGATGGGTTTGTGTGAGCACCGTTTCCAATAACTT
GTGACAGGTATCGAAAATGGAATCACCCTCAGGCTTATGTTGCAGAAGATTTTCCACAACCAGCAGTTTC
TCCTCGGCGCCTTCCTTTTCAAATG
>dme_circ_0001418_junction_seq
TTCTCCTCGGCGCCTTCCTTTTCAAATGCAAAGCTGCTTCAGTTTTACCAACGAAT
>dme_circ_0001419
GTTTAACGAGTTCAGCATTGCCCAGGATCAGCTTACCAAGTGGCTGAAGGATGTGGACAAGGCCATGCAG
AGTCACACTGAACCCAAGACGACGCTGCAGGAGAAGCGTGCTCAACTGCAGAACCACAAGCTGCTCCATC
AGGAGATCACCACACACAAT
>dme_circ_0001419_junction_seq
GCTCCATCAGGAGATCACCACACACAATGTTTAACGAGTTCAGCATTGCCCAGGAT
>dme_circ_0001420
CTCCATCCAGATCGGCAACTAACTTGGCACAGTCCTGGCCCACTTGATCATATCGCTTGTTCACCTCAGC
CACCGCCTTGGAGATCTCATCCGCATTGGCTGGAGCGGCGCTGCCCAGAAGAGCTGCTGCCTTTTCATTC
ACAGCCTCGACTATGCGTTTATGCGAGTTGATGTCTTGGTTGGTGGCCTTGTACTTGTACAACTTGGAGC
GAATTTCTTGGGCACTGGTCCAGCTAGCAGGGTTCTCATTGTGGATCAGTTTCTCCACTTGATCCAACCA
GTTGACGGTTTGATTAAGAATATCCTGGTAGCTGGAGAGCTGAACTCCCTGCGCTTCCTGGCGTTTTTCC
AGGTTGCGAATGCCCTCGTCCAGTTTGTCCCAACGATCGCGGATATCGCGCAGGTCCTTACGGATCTTCT
CCCTACCCTGAGTGGATGTCTCTGGAAGAGCCTTTTCTCCAGCTGCATTTAGAGCAGCAAATAAGCTCTC
CGCTTGCTCCTTTTCAGACAACAGCACTTGCAAAATGTTAGAACTCTGGGTTGGCTCATCCAAAAGGACC
TTCGCCATCTGCGATTCGATGGGTTGCAGCCACAAATCCACCTCGTTGTACTTATCCTGGTAGAACTGGT
GATCATCGACCAGATTTGACCAGCGGTTAACAACCTCCTTGCTAAGGACTTGAAGCAGCTGATACCGATT
GGTGATCTGAGTGGTTAAAGTCTTTAGACGATCGGCTCCACAGTTGTTAAGCAGAGCGTGGGCAGCATCT
CCGAAGGCATCGATCTCCTTCTCCTTCTGGAGTATCAACTCCCTTTGAATCTTGTACTTCTCCAGCTGCT
CCACTTTCTCATGGAGCGTGGACTGCAGCTGCTGTTCGCGGAACACTGCCTCTGCACTGCGGCACCACTT

GGTAAGTTCCTCCAACGCCTTTTCAAACTTGTCCCACTGGGCAAGCACATCCTTCTGTTTGCCTTCAATG
CCCTCGATGTCATCAAAGTGACTCTTCATGG
>dme_circ_0001420_junction_seq
TCGATGTCATCAAAGTGACTCTTCATGGCTCCATCCAGATCGGCAACTAACTTGGC
>dme_circ_0001421
CTCCATCCAGATCGGCAACTAACTTGGCACAGTCCTGGCCCACTTGATCATATCGCTTGTTCACCTCAGC
CACCGCCTTGGAGATCTCATCCGCATTGGCTGGAGCGGCGCTGCCCAGAAGAGCTGCTGCCTTTTCATTC
ACAGCCTCGACTATGCGTTTATGCGAGTTGATGTCTTGGTTGGTGGCCTTGTACTTGTACAACTTGGAGC
GAATTTCTTGGGCACTGGTCCAGCTAGCAGGGTTCTCATTGTGGATCAGTTTCTCCACTTGATCCAACCA
GTTGACGG
>dme_circ_0001421_junction_seq
TTCTCCACTTGATCCAACCAGTTGACGGCTCCATCCAGATCGGCAACTAACTTGGC
>dme_circ_0001422
GTTGTACAAGTACAAGGCCACCAACCAAGACATCAACTCGCATAAACGCATAGTCGAGGCTGTGAATGAA
AAGGCAGCAGCTCTTCTGGGCAGCGCCGCTCCAGCCAATGCGGATGAGATCTCCAAGGCGGTGGCTGAG
>dme_circ_0001422_junction_seq
GGATGAGATCTCCAAGGCGGTGGCTGAGGTTGTACAAGTACAAGGCCACCAACCAA
>dme_circ_0001423
CTCCATCCAGATCGGCAACTAACTTGGCACAGTCCTGGCCCACTTGATCATATCGCTTGTTCACCTCAGC
CACCGCCTTGGAGATCTCATCCGCATTGGCTGGAGCGGCGCTGCCCAGAAGAGCTGCTGCCTTTTCATTC
ACAGCCTCGACTATGCGTTTATGCGAGTTGATGTCTTGGTTGG
>dme_circ_0001423_junction_seq
CGTTTATGCGAGTTGATGTCTTGGTTGGCTCCATCCAGATCGGCAACTAACTTGGC
>dme_circ_0001424
CTTCGTTTTGACGCAAATTCTGAGCCAAGGATTGGAAACCGTTCAGCTGTTCTTCCTTCTCCTCAAACGA
GGATTTCAGATTGTAGTTCTTGAGGGAATTCTCAGCCTCGCCCAACCAAGTGATCAGTCGATCTAAGTTG
ATTTCGTAATCATTCCATTGTTGTAGACGATTCTCTAGTCCACTCTTTACGTCGCTCAACGAAGCTCCAA
ACTTCTCGAAATCGGCATGCAAG
>dme_circ_0001424_junction_seq
TCCAAACTTCTCGAAATCGGCATGCAAGCTTCGTTTTGACGCAAATTCTGAGCCAA
>dme_circ_0001425
TGATGGCAATGTTGTCGAACAACTGATCGAATGTCAGATCATCCAGTTGACTACGAATGGCCTCGCGTCC
TTCGGTTGCTGTGGACCCATAGCACTTCTCGCCCAGCTCAACGGTGCTATTTAGAAGCTGAGTAGCTGTT
GGCTGCTGGGCCAGAAGCTCCTGGATGACCACTTGTTTCTTGAGAAGATCGTCTCGAGATGCCACCGTGG
ACAAGTCACAGCAATCATCG
>dme_circ_0001425_junction_seq
CACCGTGGACAAGTCACAGCAATCATCGTGATGGCAATGTTGTCGAACAACTGATC
>dme_circ_0001426
TTTATTGCGTTTCTCATCCAGAGTCGTTTTCAGCTCAATATCAGCTGGAAGGGCATTCTCCGTTTCATCC
AACCAGCTCTTCAAGCTGTCAGCGATCTCATCAAAGCCAGACCACTTGGCGATCTTATCCTGAATCTTTC
TTGCAGTGATGGCAATGTTGTCGAACAACTGATCGAATGTCAGATCATCCAGTTGACTACGAATGGCCTC
GCGTCCTTCGGTTGCTGTGGACCCATAGCACTTCTCGCCCAGCTCAACGGTGCTATTTAGAAGCTGAGTA
GCTGTTGGCTGCTGGGCCAGAAGCTCCTGGATGACCACTTGTTTCTTGAGAAGATCGTCTCGAGATGCCA
CCGTGGACAAGTCACAGCAATCATCG

>dme_circ_0001426_junction_seq
CACCGTGGACAAGTCACAGCAATCATCGTTTATTGCGTTTCTCATCCAGAGTCGTT
>dme_circ_0001427
GGTTAAGGAGTTGAGTGAGCATCAAACAACCATGACAGAGAAACGAGCACAATTGGATCGGGTAAAATCT
ACGGAGGAGAAGGTGCGCGTCGAGAAGATTGATGTGGATGCACTCAAGATCCAAGCCAAGGAAATGATTG
CCAGTGGTCAACAGAGTCAGGCGGCCTTCCAGGCGCAAAAA
>dme_circ_0001427_junction_seq
GAGTCAGGCGGCCTTCCAGGCGCAAAAAGGTTAAGGAGTTGAGTGAGCATCAAACA
>dme_circ_0001428
GATAATAAGGAACACTCGCTCTGACGGACGTGACGCCATTAATACCGAGATGAAGGAACTGCAAACCGAA
TGGGATCGACTGGTGAAGAAAATGTCCACAGCCAAGGTGCAGTTGGAGACAAATCTATTGCAATGGGCTG
ACTACAGTTCCAGCTACTCTCAGCTGCAGCAATGGATTACTGACCGGGAGGCCAAGTTGCAGCAGGCTT
>dme_circ_0001428_junction_seq
GACCGGGAGGCCAAGTTGCAGCAGGCTTGATAATAAGGAACACTCGCTCTGACGGA
>dme_circ_0001429
GGAACACTCGCTCTGACGGACGTGACGCCATTAATACCGAGATGAAGGAACTGCAAACCGAATGGGATCG
ACTGGTGAAGAAAATGTCCACAGCCAAGGTGCAGTTGGAGACAAATCTATTGCAATGGGCTGACTACAGT
TCCAGCTACTCTCAGCTGCAGCAATGGATTACTGACCGGGAGGCCAAGTTGCAGCAGGCTTGTGAACAGA
AGATCGTTAAGTCCAAGAGGGGTCAACCTGGTCTCAGCAGTGGATTGAGCGAACGCAAGGCCAATCTTCG
CCAGACGAACAACATTGTCCAGGATATT
>dme_circ_0001429_junction_seq
CCAGACGAACAACATTGTCCAGGATATTGGAACACTCGCTCTGACGGACGTGACGC
>dme_circ_0001430
GATTATCGAGCAGGAAGTGGCTCTGCTTCAAGAAGAATTCGATGCCTACAGGGAAGCTCTTAATAAGGCC
AAGGATTATCTGGAAGTGGGTATTGTCAAGTGGTCCGACTACCAGGATCAGTACACCGAAGCTTTGGAGT
GGTTAAGTAAGACCGAAGCTTTGGTGCAATCCTACAACAAACTGCAGGATAGTTTGATCCAGAAGAAGGT
T
>dme_circ_0001430_junction_seq
GCAGGATAGTTTGATCCAGAAGAAGGTTGATTATCGAGCAGGAAGTGGCTCTGCTT
>dme_circ_0001431
ATAACGCAGTTTATTCTGACCGGTTTCAAAACCTTGGCGAAGATTCTTTACGGTATTCAATTTGATTTGA
ACCTCGTTAAGAGTTCCGGGAATTTGTTCGCACTCGGAAAGCTTCTCACGAGTCTTCTCAATCCATGATT
GGCACTCCTCGTACAGACTGTGATGCTGCTGATGCTCCTGGTAGTGCATCTCCAGGCGACGCATAACCTC
CTTGGCCAAAGTTAG
>dme_circ_0001431_junction_seq
GACGCATAACCTCCTTGGCCAAAGTTAGATAACGCAGTTTATTCTGACCGGTTTCA
>dme_circ_0001432
GAACTCATCACTGGTCTTGACACTCGGTTCCACATCCTCCAGCCATTCAATCAGGATCTTGTACAGCTTA
AAGATCTCCTCCAGCTGGGCAAGACGATTGGCCAATTTCTGGCGAACATCGTTCAGATCCACCAACAGTT
TGTCAAAGTCCTGCTTTAAGGCTTCGG
>dme_circ_0001432_junction_seq
TTGTCAAAGTCCTGCTTTAAGGCTTCGGGAACTCATCACTGGTCTTGACACTCGGT
>dme_circ_0001433
GGTGAATAAAATCATCGAGTCGCTGGAGAACCAGGTAAACAGCCACGAGAAATACAAGCAGGCCTACAAT

GAGCTGCAAGATTGGCTGCGACGCACTCGCATTGAGGTGGAGCAGTGTGCCGACTGCCATGGCGAGAAGG
ATCAAGTGGAGAGTCGTCTGAATCGATTGGGAGATATACAATCATCATCTCTGGAGGGCAAAGCCCTTCT
TGAAGCTTGTGAAGAGCTGAGTCAGGCTGTGATTGCCACCAGCGGAAGTGAAGGCCAGGACAATGTGGCC
CAGGAGATTAAGCATCTCACCTCGGAGTGGGAGACCCTCCAAACGATATCCCGCGATGCTCGCAGCAGCT
TAGAATCCTGCTTGGCCGCCTGGCAGACCTTCCTGCAGAAGTTCAACAAGATCAACCTTTGGATCGAAAC
GATGAACAAGCGCGTTACCAAATCCCAGGAAGGCGAGAACAAGACACCCGAAGATTTG
>dme_circ_0001433_junction_seq
AGGCGAGAACAAGACACCCGAAGATTTGGGTGAATAAAATCATCGAGTCGCTGGAG
>dme_circ_0001434
GGATCTCGACGAACACAAGATCTTCTTTGGCAATGAGGCACCCATCCGTAACCTGGTGCACAAACAGATC
CAGGAGGCCGCCGATAAGATCTGGTCCTCGCTGAACAACTACGAACAGTCGGAACTCTCGGCGGAGTTGG
CTCAGTTCCAAACCAA
>dme_circ_0001434_junction_seq
CGGCGGAGTTGGCTCAGTTCCAAACCAAGGATCTCGACGAACACAAGATCTTCTTT
>dme_circ_0001435
GAGCCAAAACAGCGACTTAACGCTAGTCAACCAGCAGGCCCAATCGTTGATCCGCCAGGCGGACGCTCGC
AATCGCCAGCTGATCGAGCAGGATAACGCCGGACTGAATAGATCTTGGCAGGATCTGGTGCGCAGCCTGG
AACAGCGACGCGACAACCTGCAACAGTTGGCCGAACATTGGGACGGCTTCGAGAACAGCCTGCACGCCTG
GGAAAAGGCACTTGGTCGACTGGAGGACAAGTTCCGCAATGTCGATCCGACTGTAAGATCCCGACGTCAC
TTGGAAGATACGAAGAATGCCATTCAGGGTGAGTTGCGACATTTTCGGGTGACACTGGGAAAAAGTCACC
TGAGGAGTGTATTATTTTAAAAGGGGCTTGGATTGGATTCAACATATGAAAGAGTAGATTTCTTTATTAT
CATTTCTTTATTTTATTTAAAAATATCCCTCTCTTTCATACATTTGATAACAAATCATGTTCATATCACA
GTGTGTTTGCACTTGGGGGGCACCCATTCATTCAGCTTCGTTGTGGCAAAACCGCCCTCCCTTGGGGGCT
TTTATGTTTGTCAAATATTTTTACCAATATCCATGCATAAACACATTCCGACAGATATGTAAGTGTTAGA
TATATATGTATGTAGTATATTCGTTTATTGTATAGCCGTGCCGACATCAAGTTCATGGTCACGTTGTGGA
CCGGAGCTTGCGCTCTTGTAGTTTATCACGCTCTTCGGACTCTCTTAAAACGACTCGAGCTTCTCGAAAG
CCATGGAGCAGCTGAGTTTTCGAGGCAGCTCCAAAATATACATAGCCAACGATGTGTTTTGAATGATTAA
CACGGAAAGCCACTAAATCACAGCAATCAAGAGTGCGAATAGCGAATAACAACAAAACAACATTCTAACT
TTCGGTTTCGGTAGACTCTTGATTTTAGCTAACCTACAGAACCAGAGATACTCCGATGATTAATGAGCCA
TAAATAACTTGAAACTGTTTGCATACACCTGATTTTATTTATATTTACATATTAATTGCATTATTATATT
GTAACTTCACGAACTCGAGTTAGTTAACTAAACCAGCAATTATCATTTGTAGTACTTATCAGCAATGATT
TGAGTGATTTGTAGGCGAGTGAAATCACTGTTTGGAGCGATAACAACTTTGGACGCTTGTTTTCAATCGG
TCAACTAAAAATAGTACGAATACACAAATTAAGTAACTGAAAAAGGAAGTCCCACCTCAACGTCAATAGC
AGGCTTTCAAATCACATTTCAATTGAAGCAATTTTCAGCCATAAAAGCTAGGCCCGTCCAAATCAAACTA
ACGATGTTGTCATACACGTCCCATATAAAGCAGCATTCAGCGATAACGAAACaaaaaaaatgaaaccatc
aaacttgccaatgataaacaaagacaaacaaacaCTTAGTTAAGGTTTTAGTTTGTCTTTTCGAAACTAT
AAATTGTATTTCTTTTTTGTTTCATGCTTAAACCACATATTTCATCTTCTCGATCAAATAACCTCAAGCT
CCAAAAGCTGCCAAGTCAAGTCAACAAATTTATTTACTTTTATGCGGCGGTGACCTTCCAGAAAAACTGG
AAATAAAACCGAAATTGAGGGAGATACACAAAAAAGAGATCTAGAATGTATAGCATCACATAACCACACC
AAATGAATCATAATTGAACAAACATTGTAATTAGTAGTAATAATTTCTTATATTTTATAGGGAACTGCGT
GAAGAATCAAATCAACTTAAATCATCACATAAAGAAATCGAGGCGCTCTCTAAATCCATCCTCACATTCC
TTGGGGAAGTACACAAACCCTCGGCGGAGGCCATACAGGCCAAAGTGGATAAGTTAGTTGAACAGCAGGC
CAA
>dme_circ_0001435_junction_seq

TGGATAAGTTAGTTGAACAGCAGGCCAAGAGCCAAAACAGCGACTTAACGCTAGTC

>dme_circ_0001436

GAGCCAAAACAGCGACTTAACGCTAGTCAACCAGCAGGCCCAATCGTTGATCCGCCAGGCGGACGCTCGC
AATCGCCAGCTGATCGAGCAGGATAACGCCGGACTGAATAGATCTTGGCAGGATCTGGTGCGCAGCCTGG
AACAGCGACGCGACAACCTGCAACAGTTGGCCGAACATTGGGACGGCTTCGAGAACAGCCTGCACGCCTG
GGAAAAGGCACTTGGTCGACTGGAGGACAAGTTCCGCAATGTCGATCCGACTGTAAGATCCCGACGTCAC
TTGGAAGATACGAAGAATGCCATTCAGGGTGAGTTGCGACATTTTCGGGTGACACTGGGAAAAAGTCACC
TGAGGAGTGTATTATTTTAAAAGGGGCTTGGATTGGATTCAACATATGAAAGAGTAGATTTCTTTATTAT
CATTTCTTTATTTATTTAAAAATATCCCTCTCTTTCATACATTTGATAACAAATCATGTTCATATCACA
GTGTGTTTGCACTTGGGGGGCACCCATTCATTCAGCTTCGTTGTGGCAAAACCGCCCTCCCTTGGGGGCT
TTTATGTTTGTCAAATATTTTTACCAATATCCATGCATAAACACATTCCGACAGATATGTAAGTGTTAGA
TATATATGTATGTAGTATATTCGTTTATTGTATAGCCGTGCCGACATCAAGTTCATGGTCACGTTGTGGA
CCGGAGCTTGCGCTCTTGTAGTTTATCACGCTCTTCGGACTCTCTTAAAACGACTCGAGCTTCTCGAAAG
CCATGGAGCAGCTGAGTTTTCGAGGCAGCTCCAAAATATACATAGCCAACGATGTGTTTTGAATGATTAA
CACGGAAAGCCACTAAATCACAGCAATCAAGAGTGCGAATAGCGAATAACAACAAAACAACATTCTAACT
TTCGGTTTCGGTAGACTCTTGATTTTAGCTAACCTACAGAACCAGAGATACTCCGATGATTAATGAGCCA
TAAATAACTTGAAACTGTTTGCATACACCTGATTTTATTTATATTTACATATTAATTGCATTATTATATT
GTAACTTCACGAACTCGAGTTAGTTAACTAAACCAGCAATTATCATTTGTAGTACTTATCAGCAATGATT
TGAGTGATTTGTAGGCGAGTGAAATCACTGTTTGGAGCGATAACAACTTTGGACGCTTGTTTTCAATCGG
TCAACTAAAAATAGTACGAATACACAAATTAAGTAACTGAAAAAGGAAGTCCCACCTCAACGTCAATAGC
AGGCTTTCAAATCACATTTCAATTGAAGCAATTTTCAGCCATAAAAGCTAGGCCCGTCCAAATCAAACTA
ACGATGTTGTCATACACGTCCCATATAAAGCAGCATTCAGCGATAACGAAACaaaaaaaatgaaaccatc
aaacttgccaatgataaacaaagacaaacaaacaCTTAGTTAAGGTTTTAGTTTGTCTTTTCGAAACTAT
AAATTGTATTTCTTTTTTGTTTCATGCTTAAACCACATATTTCATCTTCTCGATCAAATAACCTCAAGCT
CCAAAAGCTGCCAAGTCAAGTCAACAAATTTATTTACTTTTATGCGGCGGTGACCTTCCAGAAAAACTGG
AAATAAAACCGAAATTGAGGGAGATACACAAAAAAGAGATCTAGAATGTATAGCATCACATAACCACACC
AAATGAATCATAATTGAACAAACATTGTAATTAGTAGTAATAATTTCTTATATTTTATAGGGAACTGCGT
GAAGAATCAAATCAACTTAAATCATCACATAAAGAAATCGAGGCGCTCTCTAAATCCATCCTCACATTCC
TTGGGGAAGTACACAAACCCTCGGCGGAGGCCATACAGGCCAAAGTGGATAAGTTAGTTGAACAGCAGGC
CAAAGTGAGTACCCACCGAACTTACCAATTACCAGAGCCATATACCTTATACTACTACATACCAATGTGC
CTAACTACGCGTGACTGGGGACCcaaaaagtgaaaaaaaaaatgccaaaaacaaaacacaaacaaaGCTCC
CAATCGAAGCGTTTAATTCCGGAAATTGTATTGGCTTGCATACATTATATCCAGCTGCCAAACTTGTTGC
CTAATGAAAATAAATGGCAATTTAGAGAACTTAACTGGTTACAGATGAATATCGCAGCGGCTTTCATGTT
TATATCACACTCATATTTAAAGTGCTATGAGGCAATATTCTAACAGAACTTTTTACAGCTAAACTACATA
CTTATAAGGTTTAGTTAGTTAGAGCTCTAAAAAGAAATGCTTCTCGCCTTTTCAAACCTTTTACTTGTTG
CTTGGCCCTCGAAATGCCATCTTATAGTTATAGTTGAGTCGAAATCTGACGTCAGACCAAGTGTTACGCT
TCGTTGTCGGCGTTGTTTTGTGTGCATTACGTAAGCCGAGGCTTATAGttttttttttGTACCCCTCTCA
GTCTGCTATTTCCCGAAATAATTGTTATTTTCATTGTTCTGGGAATGAGCCGTTTATGACGGAAAACTCT
TTATTTTTAATCACTTGTTTTCTCTTTGtttttttttttttttGGGCTATCCCGGTCTTCAAAGAACTGT
GCCAAAGCTTCGGTCTTATCAAACGCGATAGGGTTCTTAGGAGCTATTAGatatatatatatatatatTA
CGTGTAGTATGCTTTTTGCTTTTGCGATAAGCTACACAGCGAAAGAAGAATTCTTACTTGTTTTTATCGG
AAATGCCTAATATTTATTTATATATGTGTTGTTGCCTGGCGCTAGAAAAACACAGAAACTGTGCCAATAT
GCCAATTCATTTTATTGCTCTTGTTGTTCATTGTATCTTTGAAATTCTTGTGGATTTTCTTTCGCTAATT
AATCACGTGCCAAAAGTTTGGCAACATGTTGCCAGCAACATTTGTATATGTTGATAAGCCACGATATTCG

GATCGCGTAAACACTTTTCCGCTGACAGCGCCCCACATTTCTCCCATTTGGCCTTTCACACTGACACAAT
TTTTGGGAAGCTCACCGGCCCCTCGTTTTCCCCCCAAAACTATTTTGTTTTATTTTTACACACAATTTGT
TGTTTTCTTGACAGCAGTCTGCTTGAAAAACGAGAGCAGCAGCTGTGTCTATAattttcctcaaatttc
cccgcattttccctcaaaaactcaccgcaATGTGCGTAAGTTTTCAGTAGGCTTAAAGCGAGTGAAATGA
CAAATGTTTTGACTGCTGCCAGACGATGACGGGTTCTATACATCATGTACAAATTCGATGGATAATCACG
TTTCACTGTTGATTGATTAAATAACAATCGAATATCATTTCGATGTACATCACTTGAATGATATAATAAT
AATATAATGTCATTCCATTGATTACCCAGAGGCTTCCCCTTTAATTCACTGAAGTATTTCTTCCTTTAAT
TGTGATCACATTCAAATGTGGCTGTCCCAAACTGAACAAATAAACTATGCCATCATCGCGCCAAAAAGAG
TGATCTAATATACCTCCAAGCGGCATGTCATCGATAATTATATAGACACAACTGAGATATATTGTATATC
CCAAAATGCGTCGCAAAATTTCAATGCATTTACACACTTGAGATATTAAACACATTGTTTCGGCATGCAA
AAATCTAATCAGCAAAAATACATTAAGTCTCTTGCGTTTTGTGGGGATTTAATAATTTATTTTTAACAAT
TCGCGGCGTTTGTGAGCGTGTTTATCTTGAAGGCGACAATTAACTCCATTAGCGAGTAAATTTGATTTTT
GCACAAATACTATATAGAGATTTTAAAGCAGAatataggtatatatatataggtacatatatGCCATTGA
TGAAATGCCTGAGAGGAAACAAGCTAGGCAATGTGAGCAGAATGTgtaaacaaaaagccgaaaaaatcat
aaagaataagtaaataaaaagcacaagtgaaaaTTGAATTTTTTGCTTGAGTTTTGGGTGACTTCATCAG
TAAGAAATGGTAAAAGTATGAAGAAAAGGTGAACTAAAAACCATATGTGCAACTTATTAAAGTGAATGAC
AAGCGAACCACTGCCGACTTGCAGATTCACTAACCTTATCAGGTGTATAAGCCATCATTAGGCACCCAAA
GAAATCCAAGTTTAATCTGAGATTTGCATATGCATTTGCATGAACAATTTGTCATTCGGGTAAGGCCGAA
ATAAAAAAGAGAAAATCATAAATAACAGACAACAACAGAAACGAATAATAATAAAAACTTTGACCAAATG
AGGTAATCGGGTAATAATAAGTATATTTTCGCCACTGTTAGTCGTTAACAGAGTGCTGTTAGCTTTTTCG
TTCCATTCAACTGCTTTGCTCTCTCCTTTTCTTTTTCCGGTTCTCTCGAGTGGTAGGTTTATTGCCGGCT
TTTCTTATCTAAACCGGTGAATTGGCATTATGGTCTTATCAATCAGCCCTGCATTTGGTGTACTATGTTG
CCCTGGAAAAGGGTGAGAGTTTCCTGCTAAGTTCGTTGATTTTAGCCGGTTTATTCAATGTCAAGCGATT
TGGCCATAAAAATAATAGCTTTCAGTTCCCACTTTTGCTCAGATATTTCGGTGTGGTGAGTTTGGGCTCT
TAGCAGTTTTCGTTCTTTTATTTCCGCAGCGCCTTCGTGCGCCATTAACGATCGACAGCCGACGAAAGCC
GAAGCAAAGTTTTTGGCAATTTTTAGCGTTGAGAATTCTATTCAAATCAGTTTTTATTTAGAGTTAGTTG
CAAGCGGACGTGTTTCTCAAACTGGATGGCTGGAAACTAAATGAAAGAGCAAACGAACGAAGCGACCGCA
AATACATATACGCATATTTTTAAATCAAATTAATTAACTAAGAAGTAGTAAAGACCAGAGAAGCAGCAAA
GCaaaaaaaaaaaaCACAATCAAACCGGCGCCCCAAGAATACAAGAGAGAGATGTATGACAATGGATATA
TATTCTTTTGGGCCAACGGCTGCTAATCGGAATTTACATTTCTTCTCCCCTTCGATTCTTTCTATTTTCA
TGTTGAAGTTTCGAACGCCGCTTTGTGGATTACCAGGCGTATACGTAACGAGCGAACAGCAACGAGTCCG
GGAATTAATTTTCCATTTGCAGCGTCTGCTTGGGTTTCACCGCAATTCCGCTTACGGAATTGAGGCACTG
GAAGTCAAGCAGAGTGCTCGCAAGTTCCAAAAGTTGAGCACACTTCTGCCTCCAAAAGTTACAGGAGGTC
CGAAAGTTGTCGCCAACGAGCCAAAATCGCAGACCGAAGACTGAGCAGCAATTAAAAATTGTGTAGCTAA
TTGCCGGCCACTTACGCACACGTAGTTCAAGTCGTGAAAAGCATAAGGTGATTAATTTCCCACACATGaa
aaaaaaaaaTAGAAGAAGAAGATAGGAATCGCGTACTTAAACGAGAGCTGTTAGAAATTTTGCAAGACTT
GATTAAGTCATCGTAGATATGCAAAACATCACCGGTCTCTGGTACTCGTATTGTCATATACTCGAATACC
TGCCAGGGTCGTCAGGTGTGTTTGTTTTTGTTATTTTCGCTTCATTCGATTCGCTAGAGAAACGGAGAGA
AAGAGCATATTGAATCGCAGGCGTTTCTACTTTAAACGATGACGTAGCCAAGGCGACAGAAATAGCCAGA
CCCAAATGCTTATCGTCGTCGCTGTGGGAATGATTACATTCCTACTACCGCCACAATTGATCAAACTATT
TTTCGTAAATGTTTTGCTATATTTTGCGATTTGTTTTTATGCACACTCTCGTTTCGTTTTGGAACAACAC
GCGCCAATTTATACCAATACCATTTGCCTTTCACTTTCACTCGTGCTCGCCACGGCATTTCTAGCATATT
TTGCCATTTAAATTAGAGCCGCGTACTAAATTTAAATCGTTAATTTGTACAAAATGGCAGCCGACTGAAA
ATAGCGTAGAGGAAAAATAATTTTCGTAtttttttttttttGCTCTTAGATTTATTTTGCTTGCCTGTCGG

CGGCGGCGGCAATTCAAACCCAAACAAAATTGAGCAATTCACTAGTTCGATATTTGTTGGCTGCGAAAAA
TGACGCCGAAATAATTCTGGCTAGTTTATAGACGAGTATCGTTTATTTCCGCTGCCCGATTTTCTCAGTT
TCGGCGCAGCGCATTTAACAATTAAATTTGAAAATGTGTTCAGCCGACTTTTCGGTCAATTGGCCAAGAG
CCAGAATGTGAGTTGACTTGGCCTCATCTTGGCCAGCATTTCGCTACGCCACTCGGTGTCTGTAAAGGGC
AACAAACGTTCCATTAACGAATCCGTCATTCCATGCCAAGTGCAATGGTTGGACATGAAAATAACGAACA
AGTATGAACAACGTGATGCTTGAAGTATACCTCAAGCGCTTTTGCTTTGGGCCACCAGGGCGTATGAGTG
ATATGCGTCATATGCTGGAAATACGGAGCGGAAATCAATGCGAATTTCTTTATATACGATCCATGTTACT
CATTGATTTAATGCGTTCACTGCAACGCCCCAATGACGTCAGCTTGTCTACGGCCGAAATGCAAATCGaa
aaagcaaaaaaaaaaaaaCATAAATAAATGAGGGGAATATAAATTTAAACATGCGCCGCTTTTAAAAATAC
ACCCAAGCTAAAACTATAACAAAAACAAGACATTTTGAAAGTGGTGCGTGTCCAAGAAGCGTTAGGGAAT
ACATGTATTTAttttttttctttctttggcggctttttAATGAAGAAAAGTTTCGCTCTTTGGCTCTCAA
TGGGATCAAGGCGATTTCGAATTATGAGAAATGTACGAAAAACTTTTTCGAAAAATTGCCGAGGCCCAAG
CAAATGTGCTCGAGGCATTTCATTTAGAAAACTCTCTTGTAGCCAAACGGCAGCATTCATTTTTCACGGA
AATCTTCGCAGCCTCGAGGCCAAAGCCCAGTGGATCTTACACACAAACACACatatatatatatatatat
agcgatttatatatatatGCGCAGCAAAAGAACATTGAAAACTTCAGAATATTTCAGCCTTTTGTGCCAG
CAGAATAACTTTTAGCACCACCAAATATCTCTATAGATAGAGGTCTTTCAGATCTCTACGCGTGTACGGA
ATAGAACTTGGTCTATGCCATCAGGAACCGCGTGGTCTTCACACTGTCTCCCCATCCACAGTGCATGCCA
TTGAATAACGTGCCCCTGAAACTTAAAAAACCGCTCTGTTTCCTTTCAGGATTGAACGACACTCTGCGCG
ATAAGGAGCAACAGGTTAGCAAGGATCTCGAGGAGATCGAGCAAGTCTTCCGTCGCATCTCGCAGCTGCA
GGACAAGCTAAACGCGCTTCACGAGCAACTACAATCGGTTCACGTCTACGACGAGCACATAGCCCAAACG
GAACAGCTCCTCATCACATTGAACAGCCAGGTGCAGCAGGCCGCCGAGGAGAGCAAATTACTGGTGGCCC
AGACAACGGCCCACTACCAGGCCAAACAGAATCAGCTGCCCAGCGATATTGCCCAGGAGTTTACGGCTCT
TGAGCTACTTGCCGAACGCGTCCAAGTGACCATGGAGACCAAAGAGAAGGATTTCAAGCGGGCCAAGACC
GTGCGCACCGAATATGTGGACGGTGTGGACGAGGTGCAGCGTTGGCTGCTCCAGGCAGAGGTGCAGGTGC
AGGAGAGAAGCCTCACACCCACACAGATGAAGGAGCTGCTGCAGCGCATTAACCACGAGATCACCGCCAT
CTACGAACGCTTCACCCTGGTCAAGACCAACGGTCAGCTGATCATCGAGAACTGTCGCAACAGCGAGGAG
AAGACGCTGGTGCAGACGACCATCGATCAGTTGGCCGCATCGCTGGCCCAGGTACGCGGTTGGCTGGATG
AGAAGAAGCAGGCGGTGGGCGATAGTCTGGACGCGTGGACGAGGTTCATGAACCTCTACCAGATCGTGAT
GTCGTGGGCATCCGAGAAGCGCAACTTCATTGACCAGACCATCGAGCTGCGCACCCTGCCCGAGGCACGC
AACAAATTGAACGACTATGTGACGTCTGTGAAGAGTATTAAACCGATTGTGAAGCATCTGAGCGAAATGG
ACAAGGAACTGGAGCACATTGGCCAAGTGACGACTGTGGGCGATCTCAAGGACAAACTGCAGGAGGCCGA
GGATGCGAAGATATCCGTGGAAGCGGTGCTGCTAGAGAGG
>dme_circ_0001436_junction_seq
ATCCGTGGAAGCGGTGCTGCTAGAGAGGGAGCCAAAACAGCGACTTAACGCTAGTC
>dme_circ_0001437
GATTAAGGATATACGCTCCTGGCACGAAAAGACGAAGCAGGGACTGGACTCCTCGCAGCAGCAGAAGAAA
CCGCTGCGCGATCAGCTCGGTTTCTGTGAGAAGACCCTGGCCGATATCAATGTGCAGAAAACGAAACTGA
GACTGTCCATCGAAAAACTGGAGGGTTCATTTCCGCAATGGCATGGGCGGTGATCCGCGCCTGAGCGAGA
ACGTCGATGATCTGGTGCGCGTGCTCGACGGCCTCGGCGAATTGGTCAAGGCCAAGTCGCAGAGCCTCGA
GCAGACGCTGGCCCAGATCGACGTTTACCAACAGCAGATGCAGTCCCTGCGCCAGCGTATCATCCAGGAG
GAGCAACAGCTCCGCCTGGTGATGGCGCCCACGTACTTGCCGCACGATCGCGAGCGCGCATTAGCCGAGC
AACAGGGTACGTACCGTCCAGACTTTGCTAGATCTACAATAGTTAGACCCGTTTAGCAAACAGCTAGGCA
GCTAGGAGTTAAATTCAGTTGTAGCAGATATATGACCAGGATACTCGTTAGTAAAGTCGGTTATCCAATG
TTGAAGCCCCACCCCTTTTGATATCTATCTCATTTcttcattgctgtgaacgaaccttcatcgctatgaa

caaacAGTTGACAGAACAACCAAACAAAGTTATTAACCCCTTACCCGAATATATAATATAAACTTGATTA
ATCTTTGAAATCGTTTTGCCCCAAACGCTTTACTGTGTGCGGGTAGGTCTGAAGAAAAACAGTTGCACGG
TGGCTGCGTATATTTAAGTTATTTTATTTAGTTTGTTTAAAAACAAATGAAATCAAGGCGCCTTATTATT
TTCCTATTTTCATTACATTCTATTTTGTTCGGCTAAATTCTATTACGTTTTTTTATTTTCGAATTCGTTT
TGCGCCATTATATCTCACACAAATGCTCACACCAATCAGCATTCATCATTTTCATTTCATCTTTGTGTTT
CCTTATGTTCAAACATCATTCATCGCTCATTTACCAACCGTGCAGCTCACAATCTCAAAACTCATTGCTT
TCACATCGCTCTCATTGGGAAAACTACCCACAAAATTATACCACATCAATATCGgatctttaagatcttt
aagaACTCATCATTACCATTTGTCACGTGCTGCGGCCCAAAAGCATTTTGAATGCCAGAAAAAAACAAAT
AAGTTCTTGCTGTCAGGGTTAGGCCACCCAGATGAGAAAAGCGAATTCCTCCTCCAGATAATCACACGTA
CTATTTTTAGAGCCACGCTGCTTGCCATTGtttttttttttGTTGTTCATAAACAAAGTGATTATATTTA
GAACCAGGTGGCCTAGCAGCAATAGGTTAAAATCAAGATTTCCCTAGTTTTTTAAATAGCTCGTAAATTT
ATTACTAAAATTTAACTTTACATTGCGAACTGCAATGTTGGCGGTAAGTGGCAATCTTAGAATTCAATTC
AATTTGGGGCAGTAGCTAATTTTGATGATATTTGTTGGGGCAGCTCTTGCTATGAGAACAATTTTTGATT
TCCCTTCACCTGACCTTTcacacacacacacacacacacacaAGAGAAAGTACAGTCATCACATCGCATT
TAAATCGTCACATCTTATGGCACATTTATGACACATTAAGCTTTGGCCTAGACATTTAAACAACAATact
tttggtttaatttcatttactttttattttagttttttttttttttttGATTTATTCATTTAGCCAGCTT
AGCCAAGCAATCATAATATTCTTCACCAAGCAGCTCTTTCTCTTTCTCTCTATAAATCTTTCTATATCTT
TCTGGTATCTTGTAATAGCGAAAGTCGGTTAATATTTAATTATGGTTACGGttataatttgatttggtat
tttatttttgacaattgtatttctttttatcttttCTCTACGTAACTTTTGTTGAATATAAACAAAAAAC
CTGGTGTCGTTGCGCTGAAAACAATCGAAAAACAAACATGCAAATTATTAAACACACTTCATAAACATTG
TTCTTACTAATCGTATATTAAACCAATTACAACTTATCTTGGGCTTTGCTCGCTTGCTTGTTATGCTACT
TACAAAACGGTGACTAATTCAAATCTGCCCCATTCCGATTTAACCTTTAACCCCATCGAATAACAACCCC
TTATcacacacaaacacacacacGTATATAAACATACATGGGTACTGCTTACTTCTTCTAATAAATCGAA
ATATCGAACATGGTGAATGGTCGCTTTTCCTGTATCTATCCCACATGGCGTATGATGTGCTCGCTGTCTA
TGAAATACCATAAACGATACTCAATATATATGCTGACATCTCTTGTATTTCTTCTCtatatatatatata
aatatatatCTCTGCTCTCACGCCAAACATTCGATCCATGCACCATCTATACCCTATATACGAACAATAG
GCATGCCGGGAACGTGTCAAAAATCTGCATTCGAAAATAACTGCACGTAATGAGCGCATCAAGTTGTTAA
TACATCGCGGCACGCCGGACGATGCCAAGCTAGAGATTTAGGATGCCACAACCTTAACCATAAGATAAAG
CCAATCTAGGACTAGATCAAAGCCAAAGCAGCCACCCCGGCGTCTTCCGTTTGCCCCTGCCACCGATTTC
CTTCTCCCAGGAACGGTGGCTACGGTACAACTGTCAACTGCCGTCATTACTCTAATCAATGAAACATGAC
CCGTGTTTTAGAATTTACTATtatctatatatatatatatatatatatatacatacatatataCTATTTT
ACAGAATAAATTAAATTACCGAATTATATTTATAAAACGCAAGCCCCAATCGAGTATAATTTATGAGGGA
GTTAGGATTTGCAACTTTTTACTCTTAGGTAGCTTAGCCTTGAGTCCCCATTTTTCAAAACCCCTGCAAT
CATATCAAAGCTAACGTTTTTGGCAAATCTTCCCCCCATCTCTCCCACTTTCTCTCACTCTGATACGTGA
ACCGTGATGATGAACAATTAGGCAATGCGGGAATCAATCGATGGCATGCAGCAAGTCTACGATGCGACCG
CTCGAAAGTCCTATGCGCTGGATCCCCATTCAGTGGTGACCACTTCATCGACTGTCTCGTTGCTTATGAA
GCCAACGGCGGCAGTAGGTCTAACCTATGCTGAAGTGCTGTCGGGACAAGCGAGTCCGGTAAGCAGCGGT
AATGAATCGGTAACCAATTCCCGACCAGAACGAGACCCCTCCACGACGACAACAACAACGACAACACGGC
AGCAACAATCGAACGATACTCAGAACACGACCACTATTACAACCATTAAGACTACCACAACTACGACAAC
GACTTCGAGAAGTACGCACGGCGATGATGATCAAAGCCAGGAGAGACAAGAGCTCAATGGCGGTGGCAGC
ACCACAGCCACAGCAAACTATAAGCGCCCCCAACAGGTCACACCACCCAGCGAGAATACCTCAAACACGG
TTCCTAACACAGCTACGACAACGATAACCAATCCCAATATCCAGTTCATCGAAAGCGAGCGGAGGCATCA
GCATCACCACGCGAAAACGGGTAAGAAGCTTCATGCGCCGCGGCCAGAACGACGTGGTCGATCGCCCAGA
CGACGACCACCTCAACAGCAAAAGCCAGTGGAATCATCTCAACAACCCTCGATCGAAATTCAATTGGAGC

GCCAGCAACCACAAGAACAGGAGCAACTATTCTTGCCAGCTGATGACCGAACTAGATCTCGCAGCCCCAT
GTGGGTACCAGGATCGACTTCCTACGCTGAGGTTCTTCGGGGACTAGAGCTGGAGCGGATCAGGGCCGAA
CAGCAGGCCGCCCAGGCAGTTCAGGGACGTTCCGATCTGACCATTCAGGAT
>dme_circ_0001437_junction_seq
GGGACGTTCCGATCTGACCATTCAGGATGATTAAGGATATACGCTCCTGGCACGAA
>dme_circ_0001438
GGCAATGCGGGAATCAATCGATGGCATGCAGCAAGTCTACGATGCGACCGCTCGAAAGTCCTATGCGCTG
GATCCCCATTCAGTGGTGACCACTTCATCGACTGTCTCGTTGCTTATGAAGCCAACGGCGGCAGTAGGTC
TAACCTATGCTGAAGTGCTGTCGGGACAAGCGAGTCCGGTAAGCAGCGGTAATGAATCG
>dme_circ_0001438_junction_seq
GAGTCCGGTAAGCAGCGGTAATGAATCGGGCAATGCGGGAATCAATCGATGGCATG
>dme_circ_0001439
GATACACCAGAGATCCCCACAGAAGAGCCCACAAATGTTGTGGTGCACCCCAAGCCTCAGAGGCGAAGCA
ACCAACAGGTCCGGCCCCAGGACATTAGCACAGGCAGCACTTACGCCGAAGTCCTTTTCGGTCTCCACCA
CAAAGAACATCCAGTTTTCCAGCCATCCAGTCAGCAACCGACCCCAGTGCGCCTCACGTCCTCGCCGCCT
TTGCAACGCAAGACAGAACAGGCTGGTCCTATCA
>dme_circ_0001439_junction_seq
CGCAAGACAGAACAGGCTGGTCCTATCAGATACACCAGAGATCCCCACAGAAGAGC
>dme_circ_0001440
GCAATACCATTTGTATCCGCAGACTGGGGCTTTCTAACCGTCCTACCGTCTTTCCTGGTACTCTGTTTAA
TCGGTAGTGGTTCCGGTTGCGCTTCGTGCCTCACTCTCTTACTTCGCGAGTTTCGTGAGTCTACATTATC
ATTCTTGTGTTCCCATGTCGTCGATTTCTCGACTTGACGGCCTGGTTGGG
>dme_circ_0001440_junction_seq
GATTTCTCGACTTGACGGCCTGGTTGGGGCAATACCATTTGTATCCGCAGACTGGG
>dme_circ_0001441
GAAGTCTAAGAAGACTACTACTGATAATGTGGTTGAATCAGCCGTCGATATTTCCGTGCCATCTAAGAAA
GATACGCAATCGGTCCTTCCCTCTGTAGGGAAAAAGGAAACTGTGGTCGAAAAATCTGTGATAAAGAAAA
CCGTTGAACCATCTGTTGAGCCAGTAGAACCAGTTCCAGTTCCCTCGAAGAGAGAAAAGAAACACAAGCC
GGTCAAGGAAAAGGAAACTCCTCAGTCAGATGAACCAGTTCTTGAAGTTTCACCAGTTGCTCCAACCAGA
AAAGAGAAGAAACAAGTCAAGACAGTTGTTAAGAATGTTGAAACTACCATCAGGAACGAACCATTAATTG
AAAAGGAGACTTCTAAGCCAGCTGATGTTCCAAAGACACAAGTAAAGCAAACTAAATCTGTGGTCGAAAC
GGTAGTTGAAAAGAAG
>dme_circ_0001441_junction_seq
TGTGGTCGAAACGGTAGTTGAAAAGAAGGAAGTCTAAGAAGACTACTACTGATAAT
>dme_circ_0001442
GGATACAATCGTACAGCAGGGATCGATAACTAAGACCGTTATTACCAAGACAAAGCGCATCCTGAAACGC
ATCACCGAAGACGGAGAGGAAATAGTTGAGGAGATCTTCGAGGATGAACCCGAGGTGGAGCGCGACGTTA
CACTACTGCCAACAACAGTGGTGACTTCGTTGGATGTCATTAAGGATGCTGCCCATAATGTGGTTGCGGA
AGTTATTAAACAGGCTGAGGAAATAACGCAACCTGAATTGCAAGAATTGCCTGCTAAGGTAGAAAGTCCC
ACTGAACCAGAA
>dme_circ_0001442_junction_seq
TAAGGTAGAAAGTCCCACTGAACCAGAAGGATACAATCGTACAGCAGGGATCGATA
>dme_circ_0001443
GTTATTAAACAGGCTGAGGAAATAACGCAACCTGAATTGCAAGAATTGCCTGCTAAGGTAGAAAGTCCCA

CTGAACCAGAAGTTATCAGCGAAGTTGTTGTTGAGCAGGAGCAAAATAAGCCACAAAAGCCTTCGAGAAA
GGACAAGAAGAAGCAAAA
>dme_circ_0001443_junction_seq
CTTCGAGAAAGGACAAGAAGAAGCAAAAGTTATTAAACAGGCTGAGGAAATAACGC
>dme_circ_0001444
GTCCCACTGAACCAGAAGTTATCAGCGAAGTTGTTGTTGAGCAGGAGCAAAATAAGCCACAAAAGCCTTC
GAGAAAGGACAAGAAGAAGCAAAAGTTATTGGAGAACCAACCAGAAACTGTAATCGAAACGAAGGTT
>dme_circ_0001444_junction_seq
ACCAGAAACTGTAATCGAAACGAAGGTTGTCCCACTGAACCAGAAGTTATCAGCGA
>dme_circ_0001445
GATTTCCAATGAGACCGAAAATATCAGCGAGACAAGCAGTGAACCCGAAGTATCTCTACCAATTGTGGAA
ACAGTCATTCAAGATGCACCTGTAGTAGCTGAATCGGTTACAACCGAATCAAATAGCGATGGTATAACCA
AGACCACAACGACGCGGACTACAAAGGTTGTGGCCAAGACTGTCACCGAAAATGAACAAGAAATCCTTAA
TCTACCGGATGTTCCTGTTGAACCAGAGAATACAACCGTTTTGGGTGTAATTGAAACCGTCGAACTGCCA
GAAGTTCCGGAGGCCCCTGTTGGAGTCGAGGACAAGCTCCTAGTCACGGCAACATCTACTAAACTTTCCA
AGGCTAATCAAGAAAAGTCCAAAATATTGGACTTTATTAGCGCAGAACAAAATGCGACTGATAAATATAT
AGCGCCTGGAAAAACTGTACCGCAGATTCCA
>dme_circ_0001445_junction_seq
GCCTGGAAAAACTGTACCGCAGATTCCAGATTTCCAATGAGACCGAAAATATCAGC
>dme_circ_0001446
GAATACAACCGTTTTGGGTGTAATTGAAACCGTCGAACTGCCAGAAGTTCCGGAGGCCCCTGTTGGAGTC
GAGGACAAGCTCCTAGTCACGGCAACATCTACTAAACTTTCCAAGGCTAATCAAGAAAAGTCCAAAATAT
TGGACTTTATTAGCGCAGAACAAAATGCGACTGATAAATATATAGCGCCTGGAAAAACTGTACCGCAGAT
TCCA
>dme_circ_0001446_junction_seq
GCCTGGAAAAACTGTACCGCAGATTCCAGAATACAACCGTTTTGGGTGTAATTGAA
>dme_circ_0001447
GGCTAATCAAGAAAAGTCCAAAATATTGGACTTTATTAGCGCAGAACAAAATGCGACTGATAAATATATA
GCGCCTGGAAAAACTGTACCGCAGATTCCAGTTATCGAGGAGCTTGAGAACAGTTATCCAATTGAAGAAA
GTACGGCACCTGAGCCAGAATCTTCAGGTGTTGGGGATGTTGTCAAGACCACAATTGTTCGAACAACGAA
AATTGTTAAAAAG
>dme_circ_0001447_junction_seq
TGTTCGAACAACGAAAATTGTTAAAAAGGGCTAATCAAGAAAAGTCCAAAATATTG
>dme_circ_0001448
TCACAGCAGGAGAGGTTGACCTCGACGATATAAGTGGACTAATTGGTCGTTGATCGGGCATTTCGATGCT
AGAAGTTACGATTACATCACTTGGTGTCTCGCTTTCCGTAAGGTGAATACGTCCGTCATTGGAGACCCTC
TTTGTAATGCGCATCGTGCGAATCGTCGTAGTTTTGATAATTGCCCCATCGATTTGGCTGGATGTGGTTG
AAGTATCGCTCGGAACTTCCGAAAAAGCTGGCGAAGGTTCGCGCACTGTCACTGGGTCACTGGCAGTTCT
GGTCTCCTCCGTGGTTG
>dme_circ_0001448_junction_seq
CTGGCAGTTCTGGTCTCCTCCGTGGTTGTCACAGCAGGAGAGGTTGACCTCGACGA
>dme_circ_0001449
GAACAATTTCATTAGCGATATTAGTAGGAGCAGGGCTTGATTGAGTTTCACATGGATTTTCTACTTGCAC
TTCTTCTTGGGTAGAAACAGGGACAACTTCTACTATAGATTCGTGGTAAAGAATTTCTGAATTGCTTTGC

TCGATTAACTTAGAGGTTGAGATTAGGGACGAGTCTTCAAAGATGTTGAGCAGTTTCTCTGACTCTGGAA
CAGGGATCAGCTTAG
>dme_circ_0001449_junction_seq
TCTGACTCTGGAACAGGGATCAGCTTAGGAACAATTTCATTAGCGATATTAGTAGG
>dme_circ_0001450
GTTTTTTGGTTGCCTTCTGTTTCTTGGGCTTTTGTGGCTTTTTGTCCTTTTTGATTGGTTCTTTTACATC
AGGCAATGGGGTGACAGCGGTTTTAATGTGAGTAACAGTGTTGTCAATCCATTCGCCTGGTCTCTTTACA
AGTGAGGCCCAGGAGAATGCGTTGCTACTCTCTAGCTTCTGAACAACTTCAGGCTCCGGTTTTGACTCTA
TTGGTGCAGGCATTGCCTCCACTGGTTCGGGCACAGTCTCGGGCTGTTCGTCCTCAGATGTAGTCGAGAC
GGGATCTACAACGGGTTTCTTGGACTTCTTTGTCTTCTTCGCCTTTGTGGTCTTTTTGGGCTCCTCCTTT
TCTTTCTGGCGATGTGTTTCTTTAGTTGTGACAGCCGTATGAGAAGTCTGCTTTTTATTTACGGTTGAAT
CTGACCACTCGCCGGGTCTTTGCACAAGGGCTGACCATGTCAACTCGGATGTGCTGGAAACATCCCTTTC
ATCAGAAGACGGCTCTTTGGAactttctgtcgttggtttggataattcactttctgtcgtaggcttagat
tgctcactGTCGCTGTTTGGCTTCGATTTTTCGCTTTCGATGCTTGCTGAGGACAGTTCAATAACCTTTT
CTGCCTGCCGCTTCTCTGGCTTCGATTTCTTCTTTGGCTCTTGGGCTTTAGTTTTGACTATGTCTGTTTT
GTGAGGGATTTCCTTATGTAACTCAGTAGATGGGACGTCGTCACCTGTAACTCCAGAGTTGAGTAGCACG
TCAGCATACGATTTACCATCCCGGTTCATGTTCCAAACATTCTCCACTTCTTCGACAATTTGTACCGGTT
CTACAATATTTATTTCTTGAGCAGGTTGGACATCAATAATTTTTTCTCGGATTTCCACCTTCTTGTTCTG
TCGCTTTGATGGCTTTGCCTTTTTAGGCTTTTGGTGTGGCCCAATTGTTTGTTCTGGCCTATTCCTGGGA
ATAAATTCGTTGTATATCCTTTCATTGGCAAGGAATGTATGCGTTTGATCGGAGAAGGTATGTCTCAAAT
AAGATGGAGTAAACTCAGGTGCGTCCGCCGATAAAGTTGAGGTATAACCAATGTTTCCAGTATTAGAAAT
GGTGTCCTCCAAAACGCTGGACCATGTATCAATGGGTCCCTCAAGAGGCTTCTCCAATTGACTGACATCA
TCTACCACATTACTAATAATTTGCGGTTCAGCTGGCGCGGGAAGAGAGTTCTCCTCTTCCAAAGTATTAG
TTTGATTGGTAGATGTAACAATTGTGGTCGTAACAGTTGTTATTGTTTTGCTTTCATCCACAACAGAAGA
TTCAACAATTGGCTCGACTACTTCTTCCACAAGAGTTTCTTCTTTCAGTGGCTCAGGAACTGGTTCCTCT
TCGTCGTCAGAGAAGTTCATAGGTTCGTCGAATGACGAAGGttcagctttcttctccggttctactacct
tcttgggttcctcgggaataggaagttcactaatggtttcggtagttgttgttgtggtggtaactgttgt
aatagtcttggaatcgtcgacaacagaaacttcaacaattggcttttcctcgactacttctttcacaaca
gtttcttccttcactggctcaggaactggttcctcttcgtcgtcagagaagttcataggttcgtccaaga
catccatccacgagctagaagactcatcgacgggaagaccggcatatgctggctttttgggttcttcagg
tatcttctccggttcttctaccttcttgggttcttcggaaataggcagttcactaatggtttcggtagtt
gttgttgtggtggtaactgttgtaatagtcttggaatcgtcgacaacagaaacttcaacaattggctttt
cctcgactacttctttcacaacagtttcttccttcagtggctcaggaactggttcctcttcgtcgtcaga
gaagttcatgggctcatccaagacatccatccacgagctagaagactcatcgacgggaagaccggcatat
gctggctttttgggttcttcaggtttcttctccggttcgacaaccttcttggtttcctcgggaataggaa
gttcgctaatggtttcggtagttgttgttgtggtggtaactgttgtaatggtcttggaatcgtcgacaac
agaaacttcaacaattggcttttcctcgactattttttccacaacagtttcttcctttagtggttcagga
actggttcctcttcgtcgtcagagaagttcatgggctcatccaagacatccatccacgagctagaagact
catcgacgggaagaccggcatatgctggctttttgggttcttcaggtatcttctccggttctactacctt
cttgggttcttcggaaataggaagttcactaatggtttcggtagttgttgttgtggtggtaactgttgta
atggtcttggaatcgtccacaatagaaacttcgacaatgggcttatcctcgactacttctttcacaacag
tttcttccttcagtggctcaggaactggttcctcttcgtcgtcagagaagttcatgggctcatccaagac
atccatccacgagctagaagactcatcgacgggaagaccggcatatgctggctttttgggttcttcaggt
atcttctccggttctactaccttcttgggttcttcggaaataggaagttcactaatggtttcggtagttg

ttgttgtagtggtaactgttgtaatagtcttagaatcgtcgacaacagaaacttcaacaattggcttttc
ctcgactatttcttccacaacagtttcttcctttagtggttcaggaactggttcctcttcgtcgtcagag
aagttcatgggctcatccaagacatccatccacgagctagaagactcatcgacgggaagaccggcatatg
ctggctttttgggttcttcaggtatcttctccggttctactaccttcttgggttcttcggaaataggaag
ttcactaatggtttcggtagttgttgttgtagtggtaactgttgtaatagtcttggaatcgtccacaacT
GGTTTTTTGGGCTCTTCAGGTATCGTTTCAGGAATAGAAGTTTTGCTCAAAGACTCGGTCGTTATTGTTG
TTGTGGTTTCAGTTATAATGCTTCCATCTTCAAGTGGAGCTTTAATTATTGGCTTAACGAATTCTTCTGT
TACAGATTCCTCATGTTGTGGCTGAGGAACTGGTTCAGGTTTTTGGGGATCCTCAGAAATATAAGTTTCG
TTGGCTGTTGTGTGATCTTCTTCAGTTCTGGATGCTTCCTGAAGCTCGTAAGCTTGAAGAATTCTCTGTC
TGCTTTCCGCCTTACTAAGGGACAATATGTCAAATTTGGGAATCTCGAAGTGATATATAGCTGGAATATT
ATCTTTTTGTTGGTCTACATTGGAAACCTGTGTTGTTGTTGTTGAACCCACGTCGGTGACGGTAGATTCC
ATTTTTGTTTCGGTGTCAATGTCGACGCTTACCGACTTTGGTGGCGATACGGGAACAGTTTCGGTAGGAT
CCAACTTCTCAATTGGTTTAG
>dme_circ_0001450_junction_seq
GTAGGATCCAACTTCTCAATTGGTTTAGGTTTTTTGGTTGCCTTCTGTTTCTTGGG
>dme_circ_0001451
ggtagaagaaccggagaagatacctgaagaacccaaaaagccagcatatgccggtcttcccgtcgatgag
tcttctagctcgtggatggatgtcttggacgaacctatgaacttctctgacgacgaagaggaaccagttc
ctgagccagtgaaggaagaaactgttgtgaaagaagtagtcgaggaaaagccaattgttgaagtttctgt
tgtcgacgattccaagactattacaacagttaccaccacaacaacaactaccgaaaccattagtgaactt
cctattcccgaggaacccaagaaggtagtagaaccggagaagaaagctgaaCCTTCGTCATTCGACGAAC
CTATGAACTTCTCTGACGACGAAGAGGAACCAGTTCCTGAGCCACTGAAAGAAGAAACTCTTGTGGAAGA
AGTAGTCGAGCCAATTGTTGAATCTTCTGTTGTGGATGAAAGCAAAACAATAACAACTGTTACGACCACA
ATTGTTACATCTACCAATCAAACTAATACTTTGGAAGAGGAGAACTCTCTTCCCGCGCCAGCTGAACCGC
AAATTATTAGTAATGTGGTAGATGATGTCAGTCAATTGGAGAAGCCTCTTGAGGGACCCATTGATACATG
GTCCAGCGTTTTGGAGGACACCATTTCTAATACTGGAAACATTGGTTATACCTCAACTTTATCGGCGGAC
GCACCTGAGTTTACTCCATCTTATTTGAGACATACCTTCTCCGATCAAACGCATACATTCCTTGCCAATG
AAAGGATATACAACGAATTTATTCCCAGGAATAGGCCAGAACAAACAATTGGGCCACACCAAAAGCCTAA
AAAGGCAAAGCCATCAAAGCGACAGAACAAGAAGGTGGAAATCCGAGAAAAAATTATTGATGTCCAACCT
GCTCAAGAAATAAATATTGTAGAACCGGTACAAATTGTCGAAGAAGTGGAGAATGTTTGGAACATGAACC
GGGATGGTAAATCGTATGCTGACGTGCTACTCAACTCTGGAGTTACAGGTGACGACGTCCCATCTACTGA
GTTACATAAGGAAATCCCTCACAAAACAGACATAGTCAAAACTAAAGCCCAAGAGCCAAAGAAGAAATCG
AAGCCAGAGAAGCGGCAGGCAGAAAAGGTTATTGAACTGTCCTCAGCAAGCATCGAAAGCGAAAAATCGA
AGCCAAACAGCGACagtgagcaatctaagcctacgacagaaagtgaattatccaaaccaacgacagaaag
tTCCAAAGAGCCGTCTTCTGATGAAAGGGATGTTTCCAGCACATCCGAGTTGACATGGTCAGCCCTTGTG
CAAAGACCCGGCGAGTGGTCAGATTCAACCGTAAATAAAAAGCAGACTTCTCATACGGCTGTCACAACTA
AAGAAACACATCGCCAGAAAGAAAAGGAGGAGCCCAAAAAGACCACAAAGGCGAAGAAGACAAAGAAGTC
CAAGAAACCCGTTGTAGATCCCGTCTCGACTACATCTGAGGACGAACAGCCCGAGACTGTGCCCGAACCA
GTGGAGGCAATGCCTGCACCAATAGAGTCAAAACCGGAGCCTGAAGTTGTTCAGAAGCTAGAGAGTAGCA
ACGCATTCTCCTGGGCCTCACTTGTAAAGAGACCAGGCGAATGGATTGACAACACTGTTACTCACATTAA
AACCGCTGTCACCCCATTGCCTGATGTAAAAGAACCAATCAAAAAGGACAAAAAGCCACAAAAGCCCAAG
AAACAGAAGGCAACCAAAAAACCTACTCCCGTTGCTCTGCCCGAAGATGAAGTAAGCAGTGAACCCTCTG
AGGAAATAGTTGTGGAGTGTGAGGTGGAAGAGAAAGAAACTAAATCTATCAAAAATACAGAAAACGCCTT
CTCTTGGGCGTCCCTGGTAAAGAAGCCCGGCGAATGGGTTGATGACATTGCTTCTCGTAAAAAGCCAGTT

GAAATCATTCACGAGGAGCCTAAGGAGGTGCATCCACGGAAAGAACGTAAAATTTCAAAACCTCAGCCCA
AACCTCAGTCTGAAAACACCACCAAGCGTAATAACAAAAAGGAAACTCGTGAGCCCGTAGTTGTTCATTC
GACTTCAGAGGAAAGTGATGTGGTTGAGGTATTAGAGCCTACAAATGAAAATACAATTACTTGGGCTAAA
ATAGCCAGTCAAATTGATCATATGACTGACTTgaaaccaaaggctaaacaagaaacaaagccaaaacaag
GAGAAAGGGTAAAAGAGCAAATAAGATTCGATCGAATTGAGAAACATCCAAAAGATCATGCTATATCATC
AGATCTAAGTCAACAAAAGGGAGATTCTGGTCATTCCTGGTCATCTGTGGTAAGTCATCAAGTCACTGAT
TTTATTGAAGCTGAAAGTATAAAACCAGTTCCTAAGAAGTCTGTGAAAAAGTCGAGTGGCAATACCCAAC
CAAAAATTGCACCAAAACTGGAGGAACCTTTGCAAGAACCCGTTCAAAAGGACGTAGAAAGTACACCCCT
AATGCCATGGTCTGAAATCATTGACGATGGGTTGGATGACGTGGTCGTTGAACGAAAATCGTGGAAAGAA
CTTATAGAAGACGAAATAGAAATTCCTCTTCCTCATGAGAATGGAGAGAAAGCCAACATTGACAAGATTA
TTGAAACCATCCAGGAAATACAATCAACCAGCGAAGAAAAACCAAATAACGAATCCAAAACAATTACAAC
AGTAACCACCACAACACTATCAACTGAGTCTATCAGCGAATCTGTTACTCCAATGGAACCCAAGAAAGTT
GCCgaaccagtgcctgagccactgaaggaagaaactcttgtggaagaagtagtcgaggaaaagccaattg
ttgaagtttctgttgtggacgattccaagaccattacaacagttaccaccacaacaacaactaccgaaac
cattagcgaacttccaattcccgaggaacccaagaaggttgtcgaactggagaagaaacctgaagaaccc
aaaaagccagcatatgccggtcttcctgtcgatgactctacgaactcgtggatggatgtcttggacgaac
ctatgaacttctctgacgacgaagaggaaccagttcctgagccagtaaaagaagaaactgttgtgcaaga
agtagtcgaggaaaagccaattgttgaagtttctgttgtggacgattccaagactattacaacagttacc
acaacaacaacaaataccgaaaccattagtgaacttcctattcccgaggaacccaagaag
>dme_circ_0001451_junction_seq
acttcctattcccgaggaacccaagaagggtagaagaaccggagaagatacctgaa
>dme_circ_0001452
ggtagaagaaccggagaagatacctgaagaacccaaaaagccagcatatgccggtcttcccgtcgatgag
tcttctagctcgtggatggatgtcttggacgaacctatgaacttctctgacgacgaagaggaaccagttc
ctgagccagtgaaggaagaaactgttgtgaaagaagtagtcgaggaaaagccaattgttgaagtttctgt
tgtcgacgattccaagactattacaacagttaccaccacaacaacaactaccgaaaccattagtgaactt
cctattcccgaggaacccaagaaggtagtagaaccggagaagaaagctgaaCCTTCGTCATTCGACGAAC
CTATGAACTTCTCTGACGACGAAGAGGAACCAGTTCCTGAGCCACTGAAAGAAGAAACTCTTGTGGAAGA
AGTAGTCGAGCCAATTGTTGAATCTTCTGTTGTGGATGAAAGCAAAACAATAACAACTGTTACGACCACA
ATTGTTACATCTACCAATCAAACTAATACTTTGGAAGAGGAGAACTCTCTTCCCGCGCCAGCTGAACCGC
AAATTATTAGTAATGTGGTAGATGATGTCAGTCAATTGGAGAAGCCTCTTGAGGGACCCATTGATACATG
GTCCAGCGTTTTGGAGGACACCATTTCTAATACTGGAAACATTGGTTATACCTCAACTTTATCGGCGGAC
GCACCTGAGTTTACTCCATCTTATTTGAGACATACCTTCTCCGATCAAACGCATACATTCCTTGCCAATG
AAAGGATATACAACGAATTTATTCCCAGGAATAGGCCAGAACAAACAATTGGGCCACACCAAAAGCCTAA
AAAGGCAAAGCCATCAAAGCGACAGAACAAGAAGGTGGAAATCCGAGAAAAAATTATTGATGTCCAACCT
GCTCAAGAAATAAATATTGTAGAACCGGTACAAATTGTCGAAGAAGTGGAGAATGTTTGGAACATGAACC
GGGATGGTAAATCGTATGCTGACGTGCTACTCAACTCTGGAGTTACAGGTGACGACGTCCCATCTACTGA
GTTACATAAGGAAATCCCTCACAAAACAGACATAGTCAAAACTAAAGCCCAAGAGCCAAAGAAGAAATCG
AAGCCAGAGAAGCGGCAGGCAGAAAAGGTTATTGAACTGTCCTCAGCAAGCATCGAAAGCGAAAAAATCGA
AGCCAAACAGCGACagtgagcaatctaagcctacgacagaaagtgaattatccaaaccaacgacagaaag
tTCCAAAGAGCCGTCTTCTGATGAAAGGGATGTTTCCAGCACATCCGAGTTGACATGGTCAGCCCTTGTG
CAAAGACCCGGCGAGTGGTCAGATTCAACCGTAAATAAAAAGCAGACTTCTCATACGGCTGTCACAACTA
AAGAAACACATCGCCAGAAAGAAAAGGAGGAGCCCAAAAAGACCACAAAGGCGAAGAAGACAAAGAAGTC
CAAGAAACCCGTTGTAGATCCCGTCTCGACTACATCTGAGGACGAACAGCCCGAGACTGTGCCCGAACCA

GTGGAGGCAATGCCTGCACCAATAGAGTCAAAACCGGAGCCTGAAGTTGTTCAGAAGCTAGAGAGTAGCA
ACGCATTCTCCTGGGCCTCACTTGTAAAGAGACCAGGCGAATGGATTGACAACACTGTTACTCACATTAA
AACCGCTGTCACCCCATTGCCTGATGTAAAAGAACCAATCAAAAAGGACAAAAAGCCACAAAAGCCCAAG
AAACAGAAGGCAACCAAAAAACCTACTCCCGTTGCTCTGCCCGAAGATGAAGTAAGCAGTGAACCCTCTG
AGGAAATAGTTGTGGAGTGTGAGGTGGAAGAGAAAGAAACTAAATCTATCAAAAATACAGAAAACGCCTT
CTCTTGGGCGTCCCTGGTAAAGAAGCCCGGCGAATGGGTTGATGACATTGCTTCTCGTAAAAAGCCAGTT
GAAATCATTCACGAGGAGCCTAAGGAGGTGCATCCACGGAAAGAACGTAAAATTTCAAAACCTCAGCCCA
AACCTCAGTCTGAAAACACCACCAAGCGTAATAACAAAAAGGAAACTCGTGAGCCCGTAGTTGTTCATTC
GACTTCAGAGGAAAGTGATGTGGTTGAGGTATTAGAGCCTACAAATGAAAATACAATTACTTGGGCTAAA
ATAGCCAGTCAAATTGATCATATGACTGACTTgaaaccaaaggctaaacaagaaacaaagccaaaacaag
GAGAAAGGGTAAAAGAGCAAATAAGATTCGATCGAATTGAGAAACATCCAAAAGATCATGCTATATCATC
AGATCTAAGTCAACAAAAGGGAGATTCTGGTCATTCCTGGTCATCTGTGGTAAGTCATCAAGTCACTGAT
TTTATTGAAGCTGAAAGTATAAAACCAGTTCCTAAGAAGTCTGTGAAAAAGTCGAGTGGCAATACCCAAC
CAAAAATTGCACCAAAACTGGAGGAACCTTTGCAAGAACCCGTTCAAAAGGACGTAGAAAGTACACCCCT
AATGCCATGGTCTGAAATCATTGACGATGGGTTGGATGACGTGGTCGTTGAACGAAAATCGTGGAAAGAA
CTTATAGAAGACGAAATAGAAATTCCTCTTCCTCATGAGAATGGAGAGAAAGCCAACATTGACAAGATTA
TTGAAACCATCCAGGAAATACAATCAACCAGCGAAGAAAAACCAAATAACGAATCCAAAACAATTACAAC
AGTAACCACCACAACACTATCAACTGAGTCTATCAGCGAATCTGTTACTCCAATGGAACCCAAGAAAGTT
GCCgaaccagtgcctgagccactgaaggaagaaactcttgtggaagaagtagtcgaggaaaagccaattg
ttgaagtttctgttgtggacgattccaagaccattacaacagttaccaccacaacaacaactaccgaaac
cattagcgaacttccaattcccgaggaacccaagaaggttgtcgaactggagaagaaacctgaagaaccc
aaaaagccagcatatgccggtcttcctgtcgatgactctacgaactcgtggatggatgtcttggacgaac
ctatgaacttctctgacgacgaagaggaaccagttcctgagccagtaaaagaagaaactgttgtgcaaga
agtagtcgaggaaaagccaattgttgaagtttctgttgtggacgattccaagactattacaacagttacc
acaacaacaacaaataccgaaaccattagtgaacttcctattcccgaggaacccaagaaggttgtcgaac
tggagaagaaacctgaagaacccaaaaagccagcatatgccggtcttcctgtcgatgactctacgaactc
gtggatggatgtcttggacgaacctatgaacttctctgacgacgaagaggaaccaattcctgagccacag
aaggaagaaagtcttgtgaaagaagtagtcgaggaaaagccaattgttgaagtttctgttgtggacgatt
ccaagtccattacaacagttaccaccacaacaacaactaccgaaaccattagtgaaattgctattcccga
ggaacccacgaaggttgtcgaaccggagaagaaacctgaagaacccaaaaagccagcatatgccggtctt
cccgtcgatgactctacgaactcgtggatggatgtcttggacgaacctatgaacttctctgacgacgaag
aggaaccagttcctgaaccactgaaggaagaaactgttgtggaagaagtagtcgaggaaaagccaattgt
tgaagtttctgttgtggacgattccaagaccattacaacagttaccaccacaacaacaactaccgaaacc
attagcgaacttcctattcccgaggaacccaagaag
>dme_circ_0001452_junction_seq
acttcctattcccgaggaacccaagaagggtagaagaaccggagaagatacctgaa
>dme_circ_0001453
ggtagaagaaccggagaagatacctgaagaacccaaaaagccagcatatgccggtcttcccgtcgatgag
tcttctagctcgtggatggatgtcttggacgaacctatgaacttctctgacgacgaagaggaaccagttc
ctgagccagtgaaggaagaaactgttgtgaaagaagtagtcgaggaaaagccaattgttgaagtttctgt
tgtcgacgattccaagactattacaacagttaccaccacaacaacaactaccgaaaccattagtgaactt
cctattcccgaggaacccaagaaggtagtagaaccggagaagaaagctgaaCCTTCGTCATTCGACGAAC
CTATGAACTTCTCTGACGACGAAGAGGAACCAGTTCCTGAGCCACTGAAAGAAGAAACTCTTGTGGAAGA
AGTAGTCGAGCCAATTGTTGAATCTTCTGTTGTGGATGAAAGCAAAACAATAACAACTGTTACGACCACA

ATTGTTACATCTACCAATCAAACTAATACTTTGGAAGAGGAGAACTCTCTTCCCGCGCCAGCTGAACCGC
AAATTATTAGTAATGTGGTAGATGATGTCAGTCAATTGGAGAAGCCTCTTGAGGGACCCATTGATACATG
GTCCAGCGTTTTGGAGGACACCATTTCTAATACTGGAAACATTGGTTATACCTCAACTTTATCGGCGGAC
GCACCTGAGTTTACTCCATCTTATTTGAGACATACCTTCTCCGATCAAACGCATACATTCCTTGCCAATG
AAAGGATATACAACGAATTTATTCCCAGGAATAGGCCAGAACAAACAATTGGGCCACACCAAAAGCCTAA
AAAGGCAAAGCCATCAAAGCGACAGAACAAGAAGGTGGAAATCCGAGAAAAAATTATTGATGTCCAACCT
GCTCAAGAAATAAATATTGTAGAACCGGTACAAATTGTCGAAGAAGTGGAGAATGTTTGGAACATGAACC
GGGATGGTAAATCGTATGCTGACGTGCTACTCAACTCTGGAGTTACAGGTGACGACGTCCCATCTACTGA
GTTACATAAGGAAATCCCTCACAAAACAGACATAGTCAAAACTAAAGCCCAAGAGCCAAAGAAGAAATCG
AAGCCAGAGAAGCGGCAGGCAGAAAAGGTTATTGAACTGTCCTCAGCAAGCATCGAAAGCGAAAAATCGA
AGCCAAACAGCGACagtgagcaatctaagcctacgacagaaagtgaattatccaaaccaacgacagaaag
tTCCAAAGAGCCGTCTTCTGATGAAAGGGATGTTTCCAGCACATCCGAGTTGACATGGTCAGCCCTTGTG
CAAAGACCCGGCGAGTGGTCAGATTCAACCGTAAATAAAAAGCAGACTTCTCATACGGCTGTCACAACTA
AAGAAACACATCGCCAGAAAGAAAAGGAGGAGCCCAAAAAGACCACAAAGGCGAAGAAGACAAAGAAGTC
CAAGAAACCCGTTGTAGATCCCGTCTCGACTACATCTGAGGACGAACAGCCCGAGACTGTGCCCGAACCA
GTGGAGGCAATGCCTGCACCAATAGAGTCAAAACCGGAGCCTGAAGTTGTTCAGAAGCTAGAGAGTAGCA
ACGCATTCTCCTGGGCCTCACTTGTAAAGAGACCAGGCGAATGGATTGACAACACTGTTACTCACATTAA
AACCGCTGTCACCCCATTGCCTGATGTAAAAGAACCAATCAAAAAGGACAAAAAGCCACAAAGCCCAAG
AAACAGAAGGCAACCAAAAAACCTACTCCCGTTGCTCTGCCCGAAGATGAAGTAAGCAGTGAACCCTCTG
AGGAAATAGTTGTGGAGTGTGAGGTGGAAGAGAAAGAAACTAAATCTATCAAAAATACAGAAAACGCCTT
CTCTTGGGCGTCCCTGGTAAAGAAGCCCGGCGAATGGGTTGATGACATTGCTTCTCGTAAAAAGCCAGTT
GAAATCATTCACGAGGAGCCTAAGGAGGTGCATCCACGGAAAGAACGTAAAATTTCAAAACCTCAGCCCA
AACCTCAGTCTGAAAACACCACCAAGCGTAATAACAAAAAGGAAACTCGTGAGCCCGTAGTTGTTCATTC
GACTTCAGAGGAAAGTGATGTGGTTGAGGTATTAGAGCCTACAAATGAAAATACAATTACTTGGGCTAAA
ATAGCCAGTCAAATTGATCATATGACTGACTTgaaaccaaaggctaaacaagaaacaaagccaaaacaag
GAGAAAGGGTAAAAGAGCAAATAAGATTCGATCGAATTGAGAAACATCCAAAAGATCATGCTATATCATC
AGATCTAAGTCAACAAAAGGGAGATTCTGGTCATTCCTGGTCATCTGTGGTAAGTCATCAAGTCACTGAT
TTTATTGAAGCTGAAAGTATAAAACCAGTTCCTAAGAAGTCTGTGAAAAAGTCGAGTGGCAATACCCAAC
CAAAAATTGCACCAAAACTGGAGGAACCTTTGCAAGAACCCGTTCAAAAGGACGTAGAAAGTACACCCCT
AATGCCATGGTCTGAAATCATTGACGATGGGTTGGATGACGTGGTCGTTGAACGAAAATCGTGGAAAGAA
CTTATAGAAGACGAAATAGAAATTCCTCTTCCTCATGAGAATGGAGAGAAAGCCAACATTGACAAGATTA
TTGAAACCATCCAGGAAATACAATCAACCAGCGAAGAAAAACCAAATAACGAATCCAAAACAATTACAAC
AGTAACCACCACAACACTATCAACTGAGTCTATCAGCGAATCTGTTACTCCAATGGAACCCAAGAAAGTT
GCCgaaccagtgcctgagccactgaaggaagaaactcttgtggaagaagtagtcgaggaaaagccaattg
ttgaagtttctgttgtggacgattccaagaccattacaacagttaccaccacaacaacaactaccgaaac
cattagcgaacttccaattcccgaggaacccaagaaggttgtcgaactggagaagaaacctgaagaaccc
aaaaagccagcatatgccggtcttcctgtcgatgactctacgaactcgtggatggatgtcttggacgaac
ctatgaacttctctgacgacgaagaggaaccagttcctgagccagtaaaagaagaaactgttgtgcaaga
agtagtcgaggaaaagccaattgttgaagtttctgttgtggacgattccaagactattacaacagttacc
acaacaacaacaaataccgaaaccattagtgaacttcctattcccgaggaacccaagaaggttgtcgaac
tggagaagaaacctgaagaacccaaaaagccagcatatgccggtcttcctgtcgatgactctacgaactc
gtggatggatgtcttggacgaacctatgaacttctctgacgacgaagaggaaccaattcctgagccacag
aaggaagaaagtcttgtgaaagaagtagtcgaggaaaagccaattgttgaagtttctgttgtggacgatt
ccaagtccattacaacagttaccaccacaacaacaactaccgaaaccattagtgaaattgctattcccga

ggaacccacgaaggttgtcgaaccggagaagaaacctgaagaacccaaaaagccagcatatgccggtctt
cccgtcgatgactctacgaactcgtggatggatgtcttggacgaacctatgaacttctctgacgacgaag
aggaaccagttcctgaaccactgaaggaagaaactgttgtggaagaagtagtcgaggaaaagccaattgt
tgaagtttctgttgtggacgattccaagaccattacaacagttaccaccacaacaacaactaccgaaacc
attagcgaacttcctattcccgaggaacccaagaaggttgtcgaaccggagaagaaacctgaagaaccca
aaaagccagcatatgccggtcttcctgtcgatgactctacgaactcgtggatggatgtcttggacgaacc
tatgaacttctctgacgacgaagaggaaccagttcctgaaccactgaaggaagaaactgttgtggaagaa
gtagtcgaggaaaagccaattgttgaagtttcttttgtggacgattccaagtccattacaacagttacca
ccacaacaacgactaccgaaaccattagtgaacttcctattcccgaggaacccaagaaggtagtagaacc
ggagaagaagcctgaagaacccaaaaagccagcatatgccggtcttcccgtcgatgagtcttctagctct
ttgatggatgtcttggacgaacctatgaacttctctgacgacgaagaggaaccagttcctgagccagtga
aggaagaaactcttgtgaaagaagtagtcgaggaaaagccaattgttgaagtttctgttgtggacgattc
caagtccattacaacagttaccaccacaacaacaactaccgaaaccattagtgaacttcctattcccgag
gaacccaagaag
>dme_circ_0001453_junction_seq
acttcctattcccgaggaacccaagaagggtagaagaaccggagaagatacctgaa
>dme_circ_0001454
ggtagaagaaccggagaagatacctgaagaacccaaaaagccagcatatgccggtcttcccgtcgatgag
tcttctagctcgtggatggatgtcttggacgaacctatgaacttctctgacgacgaagaggaaccagttc
ctgagccagtgaaggaagaaactgttgtgaaagaagtagtcgaggaaaagccaattgttgaagtttctgt
tgtcgacgattccaagactattacaacagttaccaccacaacaacaactaccgaaaccattagtgaactt
cctattcccgaggaacccaagaaggtagtagaaccggagaagaaagctgaaCCTTCGTCATTCGACGAAC
CTATGAACTTCTCTGACGACGAAGAGGAACCAGTTCCTGAGCCACTGAAAGAAGAAACTCTTGTGGAAGA
AGTAGTCGAGCCAATTGTTGAATCTTCTGTTGTGGATGAAAGCAAAACAATAACAACTGTTACGACCACA
ATTGTTACATCTACCAATCAAACTAATACTTTGGAAGAGGAGAACTCTCTTCCCGCGCCAGCTGAACCGC
AAATTATTAGTAATGTGGTAGATGATGTCAGTCAATTGGAGAAGCCTCTTGAGGGACCCATTGATACATG
GTCCAGCGTTTTGGAGGACACCATTTCTAATACTGGAAACATTGGTTATACCTCAACTTTATCGGCGGAC
GCACCTGAGTTTACTCCATCTTATTTGAGACATACCTTCTCCGATCAAACGCATACATTCCTTGCCAATG
AAAGGATATACAACGAATTTATTCCCAGGAATAGGCCAGAACAAACAATTGGGCCACACCAAAAGCCTAA
AAAGGCAAAGCCATCAAAGCGACAGAACAAGAAGGTGGAAATCCGAGAAAAAATTATTGATGTCCAACCT
GCTCAAGAAATAAATATTGTAGAACCGGTACAAATTGTCGAAGAAGTGGAGAATGTTTGGAACATGAACC
GGGATGGTAAATCGTATGCTGACGTGCTACTCAACTCTGGAGTTACAGGTGACGACGTCCCATCTACTGA
GTTACATAAGGAAATCCCTCACAAAACAGACATAGTCAAAACTAAAGCCCAAGAGCCAAAGAAGAAATCG
AAGCCAGAGAAGCGGCAGGCAGAAAAGGTTATTGAACTGTCCTCAGCAAGCATCGAAAGCGAAAAATCGA
AGCCAAACAGCGACagtgagcaatctaagcctacgacagaaagtgaattatccaaaccaacgacagaaag
tTCCAAAGAGCCGTCTTCTGATGAAAGGGATGTTTCCAGCACATCCGAGTTGACATGGTCAGCCCTTGTG
CAAAGACCCGGCGAGTGGTCAGATTCAACCGTAAATAAAAAGCAGACTTCTCATACGGCTGTCACAACTA
AAGAAACACATCGCCAGAAAGAAAAGGAGGAGCCCAAAAAGACCACAAAGGCGAAGAAGACAAAGAAGTC
CAAGAAACCCGTTGTAGATCCCGTCTCGACTACATCTGAGGACGAACAGCCCGAGACTGTGCCCGAACCA
GTGGAGGCAATGCCTGCACCAATAGAGTCAAAACCGGAGCCTGAAGTTGTTCAGAAGCTAGAGAGTAGCA
ACGCATTCTCCTGGGCCTCACTTGTAAAGAGACCAGGCGAATGGATTGACAACACTGTTACTCACATTAA
AACCGCTGTCACCCCATTGCCTGATGTAAAAGAACCAATCAAAAAGGACAAAAAGCCACAAAAGCCCAAG
AAACAGAAGGCAACCAAAAAACCTACTCCCGTTGCTCTGCCCGAAGATGAAGTAAGCAGTGAACCCTCTG
AGGAAATAGTTGTGGAGTGTGAGGTGGAAGAGAAAGAAACTAAATCTATCAAAAATACAGAAAACGCCTT

CTCTTGGGCGTCCCTGGTAAAGAAGCCCGGCGAATGGGTTGATGACATTGCTTCTCGTAAAAAGCCAGTT
GAAATCATTCACGAGGAGCCTAAGGAGGTGCATCCACGGAAAGAACGTAAAATTTCAAAACCTCAGCCCA
AACCTCAGTCTGAAAACACCACCAAGCGTAATAACAAAAAGGAAACTCGTGAGCCCGTAGTTGTTCATTC
GACTTCAGAGGAAAGTGATGTGGTTGAGGTATTAGAGCCTACAAATGAAAATACAATTACTTGGGCTAAA
ATAGCCAGTCAAATTGATCATATGACTGACTTgaaaccaaaggctaaacaagaaacaaagccaaaacaag
GAGAAAGGGTAAAAGAGCAAATAAGATTCGATCGAATTGAGAAACATCCAAAAGATCATGCTATATCATC
AGATCTAAGTCAACAAAAGGGAGATTCTGGTCATTCCTGGTCATCTGTGGTAAGTCATCAAGTCACTGAT
TTTATTGAAGCTGAAAGTATAAAACCAGTTCCTAAGAAGTCTGTGAAAAAGTCGAGTGGCAATACCCAAC
CAAAAATTGCACCAAAACTGGAGGAACCTTTGCAAGAACCCGTTCAAAAGGACGTAGAAAGTACACCCCT
AATGCCATGGTCTGAAATCATTGACGATGGGTTGGATGACGTGGTCGTTGAACGAAAATCGTGGAAAGAA
CTTATAGAAGACGAAATAGAAATTCCTCTTCCTCATGAGAATGGAGAGAAAGCCAACATTGACAAGATTA
TTGAAACCATCCAGGAAATACAATCAACCAGCGAAGAAAAACCAAATAACGAATCCAAAACAATTACAAC
AGTAACCACCACAACACTATCAACTGAGTCTATCAGCGAATCTGTTACTCCAATGGAACCCAAGAAAGTT
GCCgaaccagtgcctgagccactgaaggaagaaactcttgtggaagaagtagtcgaggaaaagccaattg
ttgaagtttctgttgtggacgattccaagaccattacaacagttaccaccacaacaacaactaccgaaac
cattagcgaacttccaattcccgaggaacccaagaaggttgtcgaactggagaagaaacctgaagaaccc
aaaaagccagcatatgccggtcttcctgtcgatgactctacgaactcgtggatggatgtcttggacgaac
ctatgaacttctctgacgacgaagaggaaccagttcctgagccagtaaaagaagaaactgttgtgcaaga
agtagtcgaggaaaagccaattgttgaagtttctgttgtggacgattccaagactattacaacagttacc
acaacaacaacaaataccgaaaccattagtgaacttcctattcccgaggaacccaagaaggttgtcgaac
tggagaagaaacctgaagaacccaaaaagccagcatatgccggtcttcctgtcgatgactctacgaactc
gtggatggatgtcttggacgaacctatgaacttctctgacgacgaagaggaaccaattcctgagccacag
aaggaagaaagtcttgtgaaagaagtagtcgaggaaaagccaattgttgaagtttctgttgtggacgatt
ccaagtccattacaacagttaccaccacaacaacaactaccgaaaccattagtgaaattgctattcccga
ggaacccacgaaggttgtcgaaccggagaagaaacctgaagaacccaaaaagccagcatatgccggtctt
cccgtcgatgactctacgaactcgtggatggatgtcttggacgaacctatgaacttctctgacgacgaag
aggaaccagttcctgaaccactgaaggaagaaactgttgtggaagaagtagtcgaggaaaagccaattgt
tgaagtttctgttgtggacgattccaagaccattacaacagttaccaccacaacaacaactaccgaaacc
attagcgaacttcctattcccgaggaacccaagaaggttgtcgaaccggagaagaaacctgaagaaccca
aaaagccagcatatgccggtcttcctgtcgatgactctacgaactcgtggatggatgtcttggacgaacc
tatgaacttctctgacgacgaagaggaaccagttcctgaaccactgaaggaagaaactgttgtggaagaa
gtagtcgaggaaaagccaattgttgaagtttcttttgtggacgattccaagtccattacaacagttacca
ccacaacaacgactaccgaaaccattagtgaacttcctattcccgaggaacccaagaaggtagtagaacc
ggagaagaagcctgaagaacccaaaaagccagcatatgccggtcttcccgtcgatgagtcttctagctct
ttgatggatgtcttggacgaacctatgaacttctctgacgacgaagaggaaccagttcctgagccagtga
aggaagaaactcttgtgaaagaagtagtcgaggaaaagccaattgttgaagtttctgttgtggacgattc
caagtccattacaacagttaccaccacaacaacaactaccgaaaccattagtgaacttcctattcccgag
gaacccaagaaggtagtagaaccggagaagaagcctgaagaacccaaaaagccagcatatgccggtcttc
ctgtcgatgactcttcgaactcgtggatggatgtcttggacgaacctatgaacttctctgacgacgaaga
ggaaccaattcctgagccactgaaggaagaaactgttgtggaagaagtagtcgagataaagccaattgtt
gaagtttctgttgtggacgattccaagactattacaacagttaccaccacaacaataactaccgaaacta
ttagtgaacttcctatttccgaggaacccaagaaggtagtagaaccggagaagaaacctgaagaacccaa
aaagccagcatatgccggtcttcccgtcgatgactctacgaactcgtggatggatgtcttggacgaacct
atgaacttctctgacgacgaagaggaaccagttcctgagccagtgaaggaagaaactcttgtggaagaag

tagtcgaggaaaagccaattgttgaagtttctgttgtggacgattccaagactattacaacagttaccac
cacaacaacaactaccgaaaccattagtgaaccagttatatccgagcaacccaagaaaacagtagaacca
gtgaagaaacctgaagaatccaCAGAAGTCGAGCCAAAACCATTGTCTTCTAATATATGGGAACTGCATT
CGTCATATGCTGATGTCTTGCGTCATACTGTGGACTTCATAAGTTTAGAAAAGAATAATGTTGAAGTCCC
AACTGAATCAAATTTAGGTAAAGTGCCAACAGTAAAAGAGCCTGCTAAACTTCCAGAACCTTTGATTAAG
ACAAAGGCTAAGAAAGAAAAACCGTTAAAGAAGGAAAAGGGAGCTAAGCTTTTGACGCCTAAAGATGAAG
TTCTACCAATCGAAAGAGAATCAGCAAAGGATGAAACAAAGACGTCGTGGTCAACAATGGTCGAGAACGA
TTTTGATGATAACGTTCAAACAAACATACTCAGTGCCGATAGCACTTTGTGGTCTTCAATTGTCCGACAA
GGTGCCCAGGAACCTATTACCAAAATGACACAATCTGCCGACCATGATGGTAAAGTTCTAGAACCTGCAT
TCCAGAATTCACAAGAAATAAACAAATTCATTTCCCAAGAAAGAAGCTCCACAGTGATTGAAAAGACTAA
GAAACAAAAATCAAAGAAGGAAAAGAAACACAATAAGATAGAATCAGTAGTTGAAGTGGCTTTAGAACCA
TACGTTGATAGCACTTTCATGCGCGATGATCCTAAAACACAGCAGAATGCAGAAATTCCGTCTGTTTTGG
AAAGTTTACCTATTCAACCCAAGTCTTGGTCATCTATCGTCTCACAAACCGAGGTTCTGCCTCCAACAAG
TATAGAAAGTAAAATATCCCAGAAAGCCGTTGATCCTCATAGGTCAAAAGAAGTGCAGGAGTTCATTAGT
AAAGAACAAATCTTTTCAACGACTGTAAATGCCGATTCTAAGAaacctgaagataagaaacccttagaag
aaaaaccgaaaaaaacaaaaatcaaagaaggaaaagaaaactaaggtggtggaagctccagaacccatcgt
tgagtctgcagttcctgaggccgttgttgaacccactgttgaagtgacatttgttgaggaaccagtggtt
gaggtgactacagttgtagaagaagtaacgcccgagacacaaccggaacccactccagtaagtgaggtgg
ccccatcgcaaccactatcatggtcgtctatcgtttctcaaaccacggaagttacgacaaatgtaacaga
gactcgcttcacggaagagcctgaagataagaaacccttagaagaaaaaccgaaaaaaacaaaaaccaaag
aaggaaaagaaaactaaggtggtggaagctccagagcccatcgttgagtctgcagttcctgaggccgttg
ttgaaccaactgttgaagtgacatttgttgaggaaccagtggttgaggtgactacagttgtagaagaagt
aacgcccgagacacaaccggaacccactccagtaagtgaggtggccccatcgcaaccactatcatggtcg
tctatcgtttctcaaaccacggaaattacgacaaatgtaacagagactcgcttcacggaagagcctgaag
ataagaaacccttagaagaaaaaccgaaaaaaacaaaaatcaaagaaggaaaagaaaactaaggtggtgga
agctccagaacccatcgttgagtctgcagttcctgaggccgttgttgaacccactgttgaagtgacattt
gttgaggaaccagtggttgaggtgactacagttgtagaagaagtaacgcccgagacacaaccggaaccca
ctccagtaagtgaggtggccccatcgcaaccactatcatggtcgtctatcgtttctcaaaccacggaagt
tacgacaaatgtaacagagactcgcttcacggaagagcctgaagataagaaacccttagaagaaaaaccg
aaaaaaacaaaaaccaaagaaggaaaagaaaactaaggtggtggaagctccagagcccatcgttgagtctg
cagttcctgaggccgtggttgaacccactgttgaagtgacatttgttgaggaaccagtggttgaggtgac
tacagttgtagaagaagtaacgcccgagacacaaccggaacccactccagttagtgaggtggccccttcg
caaccactatcatggtcgtccatcgtttctcaaaccacggaagttacgaccaatgttacagagactcgct
tcacggaagaacctgaagataagaaaccctcggaagaaaaaccgaaaaaaacaaaaagcaaagaaggaaaa
gaaaactaaggtggtggaagctccagagcccatcgttgagtctgcagttcctgaggccgttgttgaaccc
actgttgaagtgacatttgttgaggaaccagtggttgaggtgactacagttgtagaagaagtaacgcccg
agacacaaccggaacccactccagtaagtgaggtggccccatcgcaaccactatcatggtcgtctatcgt
ttctcaaaccacggaagttacgacaaatgtaacagagactcgcttcacggaagagcctgaagataagaaa
cccttagaagaaaaaccgaaaaaaacaaaaaccaaagaaggaaaagaaaactaaggtggtggaagctccag
agcccatcgttgagtctgcagttcctgaggccgttgttgaaccaactgttgaagtgacatttgttgagga
accagtggttgaggtgactacagttgtagaagaagtaacgcccgagacacaaccggaacccactccagta
agtgaggtggccccatcgcaaccactatcatggtcgtctatcgtttctcaaaccacggaagttacgacaa
atgtaacagagactcgcttcacggaagagcctgaagataagaaacccttagaagaaaaaccgaaaaaaca
aaaagcaaagaaggaaaagaaaactaaggtggtggaagctccagagcccatcgttgagtctgcagttcct

gaggccgttgttgaaccaactgttgaagtgacatttgttgaggaaccagtggttgaggtgactacagttg
tagaagaagtaacgcccgagacacaaccggaacccactccagtaagtgaggtggccccatcgcaaccact
atcatggtcgtctatcgtttctcaaaccacggaaattacgacaaatgtaacagagactcgcttcacggaa
gagcctgaagataagaaacccttagaagaaaaaccgaaaaaacaaaaatcaaagaaggaaaagaaaacta
aggtggtggaagctccagaacccatcgttgagtctgcagttcctgaggccgttgttgaacccactgttga
agtgacatttgttgaggaaccagtggttgaggtgactacagttgtagaagaagtaacgcccgagacacaa
ccggaacccactccagtaagtgaggtggccccatcgcaaccactatcatggtcgtctatcgtttctcaaa
ccacggaagttacgacaaatgtaacagagactcgcttcacggaagagcctgaagataagaaacccttaga
agaaaaaccgaaaaaacaaaaaccaaagaaggaaaagaaaactaaggtggtggaagctccagagcccatc
gttgagtctgcagttcctgaggccgttgttgaaccaactgttgaagtgacatttgttgaggaaccagtgg
ttgaggtgactacagttgtagaagaagtaacgcccgagacacaaccggaacccactccagtaagtgaggt
ggccccatcgcaaccactatcatggtcgtctatcgtttctcaaaccacggaagttacgacaaatgtaaca
gagactcgcttcacggaagagcctgaagataagaaacccttagaagaaaaaccgaaaaaacaaaaatcaa
agaaggaaaagaaaactaaggtggtggaagctccagaacccatcgttgagtctgcagttcctgaggccgt
tgttgaacccactgttgaagtgacatttgttgaggaaccagtggttgaggtgactacagttgtagaagaa
gtaacgcccgagacacaaccggaacccactccagtaagtgaggtggccccatcgcaaccactatcatggt
cgtctatcgtttctcaaaccacggaagttacgacaaatgtaacagagactcgcttcacggaagaacctga
agataagaaacccttagaagaaaaaccgaaaaaaccaaaggcaaagaaggaaaagaaaactaaggtggtg
gaagctccagagcccatcgttgagtcttcagttcctgaggccgttgttgaacccactgttgaagtgacat
ttgttgaggaaccagtggttgaggtgactacagttgtagaagaagtaaagcccgagacacaaccggaacc
cacttcagttagtgaggtggccccatcgcaaccactatcatggtcgtccatcgtttctcaaaccacggaa
gttacgacaaatgtaacagagactcgcttcacggaagaacctgaagataagaaacccttagaagaaaaac
cgaaaaaaccaaaggcaaagaaggaaaagaaaactaaggtggtggaagctccagagcccatcgttgagtc
ttcagttcctgaggccgtggttgaacccactgttgaagtgacttttgttgaggaaccagtggttgaggtg
actacagttgtagaagaagtaacccccgagacacaaccggaaccaactccagtaagtgaggtggccccat
cgcaaccactatcatggtcgtccatcgtttctcaagccacggaagtaacgacaaatgtaacagagactcg
cttcacggaagaacccgaagataagaaacccttagaagaaaaaccgaaaaaaccaaaggcaaagaaggaa
aagaaaactaaggtggtggaagctccagagcccatcgttgagtctgcagttcctgaggacgttgttgaac
ccactgttgaagtgacttttgttgaggaaccagtggttgaggtgactacagtcgtagaagaagtaacgcc
cgagacacaacaggaacccactccagtaagtgaggtggccccatcgcaaccactatcatggtcgtccatc
gtttctcaaaccacggaagttacgacaaatgtaacagagactcgcttcacggaagaacctgaagataaga
aacccttagaagaaaaaccgaaaaaaccaaaggcaaagaaggaaaagaaaactaaggtggtggaagctcc
agagcccatcgttgagtcttcagttcctgaggccgtggttgaacccactgttgaagtgacttttgttgag
gaaccagtggttgaggtgactacagttgtagaagaagtaacccccgagacacaaccggaaccaactccag
taagtgaggtggccccatcgcaaccactatcatggtcgtccatcgtttctcaaaccacggaagtaacgac
aaatgtaacagagactcgcttcacggaagaacctgaagataagaaacccttagaagaaaaaccgaaaaaa
caaaaaccaaagaaggaaaagaaaactaaggtggtggaagctccagagcccatcgttgagtctgcagttc
ctgaggacgttgttgaacccactgttgaagtgacttttgttgaggaaccagtggttgaggtgactacagt
cgtagaagaagtaaagcccgagacacaaccggaacccactccagtaagtgaggtggccccatcgcaacca
ctatcatggtcgtccatcgtttctcaaaccacggaagtaacgacaaatgtaacagagactcgcttcacgg
aagaacctgaagataagaaacccttagaagaaaaaccgaaaaaacaaaaaccaaagaaggaaaagaaaac
taaggtggtggaagctccagagcccatcgttgagtctgcagttcctgaggacgttgttgaaccaactgtt
gaagtgacttttgttgaggaaccagtggttgaggtgactacagttgtagaagaagtaaagcccgagacac
aaccggaacccactccagtaagtgaggtggccccatcgcaaccactatcatggtcgtccatcgtttctca

aaccacggaagtaacgacaaatgtaacagagactcgcttcacggaagaacctgaagataagaaacccctta
gaagaaaaaccgaaaaaacaaaaaccaaagaaggaaaagaaaactaaggtggtggaagctccagagccca
tcgttgagtctgcagttcctgaggacgttgttgaaccaactgttgaagtgacttttgttgaggaaccagt
ggttgaggtgactacagttgtagaagaagtaaagcccgagacacaaccggaacccactccagtaagtgag
gtggccccatcgcaaccactatcatggtcgtccatcgtttctcaaaccacggaagtaacgacaaatgtaa
cagagactcgcttcacggaagaacctgaagataagaaacccttagaagaaaaaccgaaaaaacaaaaacc
aaagaaggaaaagaaaactaaggtggtggaagctccagagcccatcgttgagtctgcagttcctgaggac
gttgttgaaccaactgttgaagtgacttttgttgaggaaccagtggttgaggtgactacagttgtagaag
aagtaaagcccgagacacaaccggaacccactccagtaagtgaggtggccccatcgcaaccactatcatg
gtcgtccatcgtttctcaaaccacggaagtaacgacaaatgtaacagagactcgcttcacggaagaacct
gaagataagaaacccttagaagaaaaaccgaaaaaacaaaaaccaaagaaggaaaagaaaactaaggtgg
tggaagctccagagcccatcgttgagtctgcagttcctgaggacgttgttgaaccaactgttgaagtgac
ttttgttgaggaaccagtggttgaggtgactacagttgtacaagaagtaaagcccgagacacaaccggaa
cccactccagtaagtgaggtggccccatcgcaaccactatcatggtcgtccatcgtttctcaaaccacgg
aagtaacgacaaatgtaacagagactcgcttcacggaagaacctgaagataagaaacccttagaagaaaa
accgaaaaaacaaaaaccaaagaaggaaaagaaaactaaggtggtggaagctccagagcccatcgttgag
tctgcagttcctgaggacgttgttgaacccactgttgaagtgacttttgttgaggaaccagtggttgagg
tgactacagtcgtagaagaagtaacgcccgagacacaaccggaacccacttcagttagtgaggtggcccc
atcgcaaccactattatggtcgtctattgtctctcaaactactgaagATGTAGCAGAGGCTCTTATCACT
GATGTAGCTGAAGATCAGAAACCTTTAATGGAAAAACCGAAAAAACTTAAGGCAGGGAAGGAAAAGAAAT
CCAAGGTTCAGAATGTTGAAGAGTCAATTTTGGAGTCTGATGTTCCATTGTCCGTTGTTGAACCCATTAT
TGGATTTACCACTGTTCAAAAGGATGTACCACCAAAAACGGAATCGCATACGTTTTCATGGTCGTCTATT
GTTGCTCAGTCTGTTGAGGTTCCACGAACAATTCCAAAACATGTAGAATCAAAAACGGGCGGAATTCAAA
ACTTTATCGCACAGGAGCAAAATTACTCTACGGTTGTGAATGATTCAGTCAAAAAGCCGAAACCTAAAAA
GGAAAAGAAAATAGTTCAGCCTATTCAACAACCAACCGGTGAAAGCGTTCCTGTCCGAAAAGATCACATG
CGAGAAGGAGTAACTAAAGCAGTTCCTGAGCCACAAACTTTGTCTTGGTCGTCAATCGTTTCGCATAACA
TCGATTTGACGCCAACGTTTACTGAAAACCGTGTCATTGAAGCCGTCGATGAGATCAATCCCGATGACAG
CAGTAGTAATCAAGAAGGTGAGCTTCCCATTCATGTTGAtgaaaggaaacgaaaacccaaaaaggacaaa
aaacgagatcaaaatattgaaacTTCTGTTGTCTACGAAGAGATTCCAGAAACTGAACGCGTAGCTGTGT
CTTCCACGCCTGAATTCAGCATTCAGTTGCCGGCACCAACAAAACCATCAGTGTGGTCATCATCAGACAC
CTATGCCGAAGTTGTTAAGAAGTCTGGTTACAACAACAATACGAACATTAAATATCAGAAAATTGAGGCA
AGGGAACCAATCAAGAGTGAACCAAAACACACATTTGTCGAAGTGCAGACACATGAAGATCTTCTGGAGT
TCCCGGACCCTATCATTCCTGCAATTGAAAGAGATGAAGCACTTGAGAAACCCTCTCAGCTATCATGGAA
GGACCTTGTAGATGAGGATGATGTGGATCCGCTGGAGTCGTGGCATAAGGGAGAAACAGATTCTGTAGAG
ATAAAGACACAAAAACCAATACAGGAAAGACATTCCCGAGTGAGCGTAAAGCCGGAAATTCAAATAACCG
AGGTAGTTACTGAAACTCAATCGCAATCCGAGACTGACCAAGAATTCTTGGAAATTACTTCAAAGAAACG
AAATCGCTCACGCTCACGTTCGCAACAGTCACAGGCTTCGAACATTGATgaaaagcatcaaaagaaaaaa
tcaaagaagccaaaaaaTACGGTACCTAAAGCACCAGCTCAACAGCCTACGGAAGCTCCTGAGGATGTCC
AGCCTCCTAAGGAATCTGTACCATCTGTAGAAGAACCGTCTCCACCACCAGCTCCTTCTCCTAATACCTC
AGGTATGTCATGGGCTGCTATAGCCGCCCATAATGTGCCAGAACCAGTTCAACATATTCGACCCCTCCAA
GCTGAGGCCAAACCAACTGTCGTTGAAGATGTAAGGAAATCAACAAATGAGCACACAGTAAACATTCCAA
TCACTTTCGAAACTACTGAGCCAAAGACTAAAAAGCCAAAGCAAAAGTCTAAGCGAAAGTTGCCAACCGA
TGTGGTTGATTTCATTAATGCAGAAAAATCGATACATCAAGTGATTGCTTCCAAACCTGAAATAGTTGAA
GAACCTGGATCTATAATAGTTGAAGACGTCAACAATACTTTAACATCATGGGAAATAAAAACTCCACTTG

CTCCCTTTGAATTTGTTGCCGAGTCTGAAGTTGAACCAGTATCTGAGTCACATATGGAAGACCTCCCTGT
CGAAAAAGTTTTGAAGACGGagccaattgttgaatctacggcggtagacgaatctaagactactacaaca
gtaaccacaacaacaacaactaccgaaaccactagcaatgtttctattcccgaggaacccaagaaggttg
tcgaaccagagaagaaacctgaagaacccaaaaagccagcatatgccggtcttcctgtcgatgactcttc
gaactcgtggatggatgtcttggacgaacctatgaacttctctgacgacgaagaggaaccagttcctgaa
ccactgaaggaagaaactcttgtggaagaagtagtcgaggaaaagccaattgttgaagtttctgttgtgg
acgattccaagtccattacaacagttaccaccacaacaacaactaccgaaaccattagtgaacttcctat
tcccgaggaacccaagaag
>dme_circ_0001454_junction_seq
acttcctattcccgaggaacccaagaagggtagaagaaccggagaagatacctgaa
>dme_circ_0001455
ggtagaagaaccggagaagatacctgaagaacccaaaaagccagcatatgccggtcttcccgtcgatgag
tcttctagctcgtggatggatgtcttggacgaacctatgaacttctctgacgacgaagaggaaccagttc
ctgagccagtgaaggaagaaactgttgtgaaagaagtagtcgaggaaaagccaattgttgaagtttctgt
tgtcgacgattccaagactattacaacagttaccaccacaacaacaactaccgaaaccattagtgaactt
cctattcccgaggaacccaagaaggtagtagaaccggagaagaaagctgaaCCTTCGTCATTCGACGAAC
CTATGAACTTCTCTGACGACGAAGAGGAACCAGTTCCTGAGCCACTGAAAGAAGAAACTCTTGTGGAAGA
AGTAGTCGAGCCAATTGTTGAATCTTCTGTTGTGGATGAAAGCAAAACAATAACAACTGTTACGACCACA
ATTGTTACATCTACCAATCAAACTAATACTTTGGAAGAGGAGAACTCTCTTCCCGCGCCAGCTGAACCGC
AAATTATTAGTAATGTGGTAGATGATGTCAGTCAATTGGAGAAGCCTCTTGAGGGACCCATTGATACATG
GTCCAGCGTTTTGGAGGACACCATTTCTAATACTGGAAACATTGGTTATACCTCAACTTTATCGGCGGAC
GCACCTGAGTTTACTCCATCTTATTTGAGACATACCTTCTCCGATCAAACGCATACATTCCTTGCCAATG
AAAGGATATACAACGAATTTATTCCCAGGAATAGGCCAGAACAAACAATTGGGCCACACCAAAAGCCTAA
AAAGGCAAAGCCATCAAAGCGACAGAACAAGAAGGTGGAAATCCGAGAAAAAATTATTGATGTCCAACCT
GCTCAAGAAATAAATATTGTAGAACCGGTACAAATTGTCGAAGAAGTGGAGAATGTTTGGAACATGAACC
GGGATGGTAAATCGTATGCTGACGTGCTACTCAACTCTGGAGTTACAGGTGACGACGTCCCATCTACTGA
GTTACATAAGGAAATCCCTCACAAAACAGACATAGTCAAAACTAAAGCCCAAGAGCCAAAGAAGAAATCG
AAGCCAGAGAAGCGGCAGGCAGAAAAGGTTATTGAACTGTCCTCAGCAAGCATCGAAAGCGAAAAAATCGA
AGCCAAACAGCGACagtgagcaatctaagcctacgacagaaagtgaattatccaaaccaacgacagaaag
tTCCAAAGAGCCGTCTTCTGATGAAAGGGATGTTTCCAGCACATCCGAGTTGACATGGTCAGCCCTTGTG
CAAAGACCCGGCGAGTGGTCAGATTCAACCGTAAATAAAAAGCAGACTTCTCATACGGCTGTCACAACTA
AAGAAACACATCGCCAGAAAGAAAAGGAGGAGCCCAAAAAGACCACAAAGGCGAAGAAGACAAAGAAGTC
CAAGAAACCCGTTGTAGATCCCGTCTCGACTACATCTGAGGACGAACAGCCCGAGACTGTGCCCGAACCA
GTGGAGGCAATGCCTGCACCAATAGAGTCAAAACCGGAGCCTGAAGTTGTTCAGAAGCTAGAGAGTAGCA
ACGCATTCTCCTGGGCCTCACTTGTAAAGAGACCAGGCGAATGGATTGACAACACTGTTACTCACATTAA
AACCGCTGTCACCCCATTGCCTGATGTAAAAGAACCAATCAAAAAGGACAAAAAGCCACAAAAGCCCAAG
AAACAGAAGGCAACCAAAAAACCTACTCCCGTTGCTCTGCCCGAAGATGAAGTAAGCAGTGAACCCTCTG
AGGAAATAGTTGTGGAGTGTGAGGTGGAAGAGAAAGAAACTAAATCTATCAAAAATACAGAAAACGCCTT
CTCTTGGGCGTCCCTGGTAAAGAAGCCCGGCGAATGGGTTGATGACATTGCTTCTCGTAAAAAGCCAGTT
GAAATCATTCACGAGGAGCCTAAGGAGGTGCATCCACGGAAAGAACGTAAAATTTCAAAACCTCAGCCCA
AACCTCAGTCTGAAAACACCACCAAGCGTAATAACAAAAAGGAAACTCGTGAGCCCGTAGTTGTTCATTC
GACTTCAGAGGAAAGTGATGTGGTTGAGGTATTAGAGCCTACAAATGAAAATACAATTACTTGGGCTAAA
ATAGCCAGTCAAATTGATCATATGACTGACTTgaaaccaaaggctaaacaagaaacaaagccaaaacaag
GAGAAAGGGTAAAAGAGCAAATAAGATTCGATCGAATTGAGAAACATCCAAAAGATCATGCTATATCATC

AGATCTAAGTCAACAAAAGGGAGATTCTGGTCATTCCTGGTCATCTGTGGTAAGTCATCAAGTCACTGAT
TTTATTGAAGCTGAAAGTATAAAACCAGTTCCTAAGAAGTCTGTGAAAAAGTCGAGTGGCAATACCCAAC
CAAAAATTGCACCAAAACTGGAGGAACCTTTGCAAGAACCCGTTCAAAAGGACGTAGAAAGTACACCCCT
AATGCCATGGTCTGAAATCATTGACGATGGGTTGGATGACGTGGTCGTTGAACGAAAATCGTGGAAAGAA
CTTATAGAAGACGAAATAGAAATTCCTCTTCCTCATGAGAATGGAGAGAAAGCCAACATTGACAAGATTA
TTGAAACCATCCAGGAAATACAATCAACCAGCGAAGAAAAACCAAATAACGAATCCAAAACAATTACAAC
AGTAACCACCACAACACTATCAACTGAGTCTATCAGCGAATCTGTTACTCCAATGGAACCCAAGAAAGTT
GCCgaaccagtgcctgagccactgaaggaagaaactcttgtggaagaagtagtcgaggaaaagccaattg
ttgaagtttctgttgtggacgattccaagaccattacaacagttaccaccacaacaacaactaccgaaac
cattagcgaacttccaattcccgaggaacccaagaaggttgtcgaactggagaagaaacctgaagaaccc
aaaaagccagcatatgccggtcttcctgtcgatgactctacgaactcgtggatggatgtcttggacgaac
ctatgaacttctctgacgacgaagaggaaccagttcctgagccagtaaaagaagaaactgttgtgcaaga
agtagtcgaggaaaagccaattgttgaagtttctgttgtggacgattccaagactattacaacagttacc
acaacaacaacaaataccgaaaccattagtgaacttcctattcccgaggaacccaagaaggttgtcgaac
tggagaagaaacctgaagaacccaaaaagccagcatatgccggtcttcctgtcgatgactctacgaactc
gtggatggatgtcttggacgaacctatgaacttctctgacgacgaagaggaaccaattcctgagccacag
aaggaagaaagtcttgtgaaagaagtagtcgaggaaaagccaattgttgaagtttctgttgtggacgatt
ccaagtccattacaacagttaccaccacaacaacaactaccgaaaccattagtgaaattgctattcccga
ggaacccacgaaggttgtcgaaccggagaagaaacctgaagaacccaaaaagccagcatatgccggtctt
cccgtcgatgactctacgaactcgtggatggatgtcttggacgaacctatgaacttctctgacgacgaag
aggaaccagttcctgaaccactgaaggaagaaactgttgtggaagaagtagtcgaggaaaagccaattgt
tgaagtttctgttgtggacgattccaagaccattacaacagttaccaccacaacaacaactaccgaaacc
attagcgaacttcctattcccgaggaacccaagaaggttgtcgaaccggagaagaaacctgaagaaccca
aaaagccagcatatgccggtcttcctgtcgatgactctacgaactcgtggatggatgtcttggacgaacc
tatgaacttctctgacgacgaagaggaaccagttcctgaaccactgaaggaagaaactgttgtggaagaa
gtagtcgaggaaaagccaattgttgaagtttcttttgtggacgattccaagtccattacaacagttacca
ccacaacaacgactaccgaaaccattagtgaacttcctattcccgaggaacccaagaaggtagtagaacc
ggagaagaagcctgaagaacccaaaaagccagcatatgccggtcttcccgtcgatgagtcttctagctct
ttgatggatgtcttggacgaacctatgaacttctctgacgacgaagaggaaccagttcctgagccagtga
aggaagaaactcttgtgaaagaagtagtcgaggaaaagccaattgttgaagtttctgttgtggacgattc
caagtccattacaacagttaccaccacaacaacaactaccgaaaccattagtgaacttcctattcccgag
gaacccaagaaggtagtagaaccggagaagaagcctgaagaacccaaaaagccagcatatgccggtcttc
ctgtcgatgactcttcgaactcgtggatggatgtcttggacgaacctatgaacttctctgacgacgaaga
ggaaccaattcctgagccactgaaggaagaaactgttgtggaagaagtagtcgagataaagccaattgtt
gaagtttctgttgtggacgattccaagactattacaacagttaccaccacaacaataactaccgaaacta
ttagtgaacttcctatttccgaggaacccaagaaggtagtagaaccggagaagaaacctgaagaacccaa
aaagccagcatatgccggtcttcccgtcgatgactctacgaactcgtggatggatgtcttggacgaacct
atgaacttctctgacgacgaagaggaaccagttcctgagccagtgaaggaagaaactcttgtggaagaag
tagtcgaggaaaagccaattgttgaagtttctgttgtggacgattccaagactattacaacagttaccac
cacaacaacaactaccgaaaccattagtgaaccagttatatccgagcaacccaagaaaacagtagaacca
gtgaagaaacctgaagaatccaCAGAAGTCGAGCCAAAACCATTGTCTTCTAATATATGGGAACTGCATT
CGTCATATGCTGATGTCTTGCGTCATACTGTGGACTTCATAAGTTTAGAAAAGAATAATGTTGAAGTCCC
AACTGAATCAAATTTAGGTAAAGTGCCAACAGTAAAAGAGCCTGCTAAACTTCCAGAACCTTTGATTAAG
ACAAAGGCTAAGAAAGAAAAACCGTTAAAGAAGGAAAAGGGAGCTAAGCTTTTGACGCCTAAAGATGAAG

TTCTACCAATCGAAAGAGAATCAGCAAAGGATGAAACAAAGACGTCGTGGTCAACAATGGTCGAGAACGA
TTTTGATGATAACGTTCAAACAAACATACTCAGTGCCGATAGCACTTTGTGGTCTTCAATTGTCCGACAA
GGTGCCCAGGAACCTATTACCAAAATGACACAATCTGCCGACCATGATGGTAAAGTTCTAGAACCTGCAT
TCCAGAATTCACAAGAAATAAACAAATTCATTTCCCAAGAAAGAAGCTCCACAGTGATTGAAAAGACTAA
GAAACAAAAATCAAAGAAGGAAAAGAAACACAATAAGATAGAATCAGTAGTTGAAGTGGCTTTAGAACCA
TACGTTGATAGCACTTTCATGCGCGATGATCCTAAAACACAGCAGAATGCAGAAATTCCGTCTGTTTTGG
AAAGTTTACCTATTCAACCCAAGTCTTGGTCATCTATCGTCTCACAAACCGAGGTTCTGCCTCCAACAAG
TATAGAAAGTAAAATATCCCAGAAAGCCGTTGATCCTCATAGGTCAAAAGAAGTGCAGGAGTTCATTAGT
AAAGAACAAATCTTTTCAACGACTGTAAATGCCGATTCTAAGAaacctgaagataagaaacccttagaag
aaaaaccgaaaaaacaaaaatcaaagaaggaaaagaaaactaaggtggtggaagctccagaacccatcgt
tgagtctgcagttcctgaggccgttgttgaacccactgttgaagtgacatttgttgaggaaccagtggtt
gaggtgactacagttgtagaagaagtaacgcccgagacacaaccggaacccactccagtaagtgaggtgg
ccccatcgcaaccactatcatggtcgtctatcgtttctcaaaccacggaagttacgacaaatgtaacaga
gactcgcttcacggaagagcctgaagataagaaacccttagaagaaaaaccgaaaaaacaaaaaccaaag
aaggaaaagaaaactaaggtggtggaagctccagagcccatcgttgagtctgcagttcctgaggccgttg
ttgaaccaactgttgaagtgacatttgttgaggaaccagtggttgaggtgactacagttgtagaagaagt
aacgcccgagacacaaccggaacccactccagtaagtgaggtggccccatcgcaaccactatcatggtcg
tctatcgtttctcaaaccacggaaattacgacaaatgtaacagagactcgcttcacggaagagcctgaag
ataagaaacccttagaagaaaaaccgaaaaaacaaaaatcaaagaaggaaaagaaaactaaggtggtgga
agctccagaacccatcgttgagtctgcagttcctgaggccgttgttgaacccactgttgaagtgacattt
gttgaggaaccagtggttgaggtgactacagttgtagaagaagtaacgcccgagacacaaccggaaccca
ctccagtaagtgaggtggccccatcgcaaccactatcatggtcgtctatcgtttctcaaaccacggaagt
tacgacaaatgtaacagagactcgcttcacggaagagcctgaagataagaaacccttagaagaaaaaccg
aaaaaacaaaaaccaaagaaggaaaagaaaactaaggtggtggaagctccagagcccatcgttgagtctg
cagttcctgaggccgtggttgaacccactgttgaagtgacatttgttgaggaaccagtggttgaggtgac
tacagttgtagaagaagtaacgcccgagacacaaccggaacccactccagttagtgaggtggccccttcg
caaccactatcatggtcgtccatcgtttctcaaaccacggaagttacgaccaatgttacagagactcgct
tcacggaagaacctgaagataagaaaccctcggaagaaaaaccgaaaaaacaaaaagcaaagaaggaaaa
gaaaactaaggtggtggaagctccagagcccatcgttgagtctgcagttcctgaggccgttgttgaaccc
actgttgaagtgacatttgttgaggaaccagtggttgaggtgactacagttgtagaagaagtaacgcccg
agacacaaccggaacccactccagtaagtgaggtggccccatcgcaaccactatcatggtcgtctatcgt
ttctcaaaccacggaagttacgacaaatgtaacagagactcgcttcacggaagagcctgaagataagaaa
cccttagaagaaaaaccgaaaaaacaaaaaccaaagaaggaaaagaaaactaaggtggtggaagctccag
agcccatcgttgagtctgcagttcctgaggccgttgttgaaccaactgttgaagtgacatttgttgagga
accagtggttgaggtgactacagttgtagaagaagtaacgcccgagacacaaccggaacccactccagta
agtgaggtggccccatcgcaaccactatcatggtcgtctatcgtttctcaaaccacggaagttacgacaa
atgtaacagagactcgcttcacggaagagcctgaagataagaaacccttagaagaaaaaccgaaaaaaca
aaaagcaaagaaggaaaagaaaactaaggtggtggaagctccagagcccatcgttgagtctgcagttcct
gaggccgttgttgaaccaactgttgaagtgacatttgttgaggaaccagtggttgaggtgactacagttg
tagaagaagtaacgcccgagacacaaccggaacccactccagtaagtgaggtggccccatcgcaaccact
atcatggtcgtctatcgtttctcaaaccacggaaattacgacaaatgtaacagagactcgcttcacggaa
gagcctgaagataagaaacccttagaagaaaaaccgaaaaaacaaaaatcaaagaaggaaaagaaaacta
aggtggtggaagctccagaacccatcgttgagtctgcagttcctgaggccgttgttgaacccactgttga
agtgacatttgttgaggaaccagtggttgaggtgactacagttgtagaagaagtaacgcccgagacacaa

ccggaacccactccagtaagtgaggtggccccatcgcaaccactatcatggtcgtctatcgtttctcaaa
ccacggaagttacgacaaatgtaacagagactcgcttcacggaagagcctgaagataagaaacccttaga
agaaaaaccgaaaaaacaaaaaccaaagaaggaaaagaaaactaaggtggtggaagctccagagcccatc
gttgagtctgcagttcctgaggccgttgttgaaccaactgttgaagtgacatttgttgaggaaccagtgg
ttgaggtgactacagttgtagaagaagtaacgcccgagacacaaccggaacccactccagtaagtgaggt
ggccccatcgcaaccactatcatggtcgtctatcgtttctcaaaccacggaagttacgacaaatgtaaca
gagactcgcttcacggaagagcctgaagataagaaacccttagaagaaaaaccgaaaaaacaaaaatcaa
agaaggaaaagaaaactaaggtggtggaagctccagaacccatcgttgagtctgcagttcctgaggccgt
tgttgaacccactgttgaagtgacatttgttgaggaaccagtggttgaggtgactacagttgtagaagaa
gtaacgcccgagacacaaccggaacccactccagtaagtgaggtggccccatcgcaaccactatcatggt
cgtctatcgtttctcaaaccacggaagttacgacaaatgtaacagagactcgcttcacggaagaacctga
agataagaaacccttagaagaaaaaccgaaaaaaccaaaggcaaagaaggaaaagaaaactaaggtggtg
gaagctccagagcccatcgttgagtcttcagttcctgaggccgttgttgaacccactgttgaagtgacat
ttgttgaggaaccagtggttgaggtgactacagttgtagaagaagtaaagcccgagacacaaccggaacc
cacttcagttagtgaggtggccccatcgcaaccactatcatggtcgtccatcgtttctcaaaccacggaa
gttacgacaaatgtaacagagactcgcttcacggaagaacctgaagataagaaacccttagaagaaaaac
cgaaaaaaccaaaggcaaagaaggaaaagaaaactaaggtggtggaagctccagagcccatcgttgagtc
ttcagttcctgaggccgtggttgaacccactgttgaagtgacttttgttgaggaaccagtggttgaggtg
actacagttgtagaagaagtaacccccgagacacaaccggaaccaactccagtaagtgaggtggccccat
cgcaaccactatcatggtcgtccatcgtttctcaagccacggaagtaacgacaaatgtaacagagactcg
cttcacggaagaacccgaagataagaaacccttagaagaaaaaccgaaaaaaccaaaggcaaagaaggaa
aagaaaactaaggtggtggaagctccagagcccatcgttgagtctgcagttcctgaggacgttgttgaac
ccactgttgaagtgacttttgttgaggaaccagtggttgaggtgactacagtcgtagaagaagtaacgcc
cgagacacaacaggaacccactccagtaagtgaggtggccccatcgcaaccactatcatggtcgtccatc
gtttctcaaaccacggaagttacgacaaatgtaacagagactcgcttcacggaagaacctgaagataaga
aacccttagaagaaaaaccgaaaaaaccaaaggcaaagaaggaaaagaaaactaaggtggtggaagctcc
agagcccatcgttgagtcttcagttcctgaggccgtggttgaacccactgttgaagtgacttttgttgag
gaaccagtggttgaggtgactacagttgtagaagaagtaacccccgagacacaaccggaaccaactccag
taagtgaggtggccccatcgcaaccactatcatggtcgtccatcgtttctcaaaccacggaagtaacgac
aaatgtaacagagactcgcttcacggaagaacctgaagataagaaacccttagaagaaaaaccgaaaaaa
caaaaaccaaagaaggaaaagaaaactaaggtggtggaagctccagagcccatcgttgagtctgcagttc
ctgaggacgttgttgaacccactgttgaagtgacttttgttgaggaaccagtggttgaggtgactacagt
cgtagaagaagtaaagcccgagacacaaccggaacccactccagtaagtgaggtggccccatcgcaacca
ctatcatggtcgtccatcgtttctcaaaccacggaagtaacgacaaatgtaacagagactcgcttcacgg
aagaacctgaagataagaaacccttagaagaaaaaccgaaaaaacaaaaaccaaagaaggaaaagaaaac
taaggtggtggaagctccagagcccatcgttgagtctgcagttcctgaggacgttgttgaaccaactgtt
gaagtgacttttgttgaggaaccagtggttgaggtgactacagttgtagaagaagtaaagcccgagacac
aaccggaacccactccagtaagtgaggtggccccatcgcaaccactatcatggtcgtccatcgtttctca
aaccacggaagtaacgacaaatgtaacagagactcgcttcacggaagaacctgaagataagaaacccttta
gaagaaaaaccgaaaaaacaaaaaccaaagaaggaaaagaaaactaaggtggtggaagctccagagccca
tcgttgagtctgcagttcctgaggacgttgttgaaccaactgttgaagtgacttttgttgaggaaccagt
ggttgaggtgactacagttgtagaagaagtaaagcccgagacacaaccggaacccactccagtaagtgag
gtggccccatcgcaaccactatcatggtcgtccatcgtttctcaaaccacggaagtaacgacaaatgtaa
cagagactcgcttcacggaagaacctgaagataagaaacccttagaagaaaaaccgaaaaaacaaaaacc

aaagaaggaaaagaaaactaaggtggtggaagctccagagcccatcgttgagtctgcagttcctgaggac
gttgttgaaccaactgttgaagtgacttttgttgaggaaccagtggttgaggtgactacagttgtagaag
aagtaaagcccgagacacaaccggaacccactccagtaagtgaggtggccccatcgcaaccactatcatg
gtcgtccatcgtttctcaaaccacggaagtaacgacaaatgtaacagagactcgcttcacggaagaacct
gaagataagaaacccttagaagaaaaaccgaaaaaacaaaaaccaaagaaggaaaagaaaactaaggtgg
tggaagctccagagcccatcgttgagtctgcagttcctgaggacgttgttgaaccaactgttgaagtgac
ttttgttgaggaaccagtggttgaggtgactacagttgtacaagaagtaaagcccgagacacaaccggaa
cccactccagtaagtgaggtggccccatcgcaaccactatcatggtcgtccatcgtttctcaaaccacgg
aagtaacgacaaatgtaacagagactcgcttcacggaagaacctgaagataagaaacccttagaagaaaa
accgaaaaaacaaaaaccaaagaaggaaaagaaaactaaggtggtggaagctccagagcccatcgttgag
tctgcagttcctgaggacgttgttgaacccactgttgaagtgacttttgttgaggaaccagtggttgagg
tgactacagtcgtagaagaagtaacgcccgagacacaaccggaacccacttcagttagtgaggtggcccc
atcgcaaccactattatggtcgtctattgtctctcaaactactgaagATGTAGCAGAGGCTCTTATCACT
GATGTAGCTGAAGATCAGAAACCTTTAATGGAAAAACCGAAAAAACTTAAGGCAGGGAAGGAAAAGAAAT
CCAAGGTTCAGAATGTTGAAGAGTCAATTTTGGAGTCTGATGTTCCATTGTCCGTTGTTGAACCCATTAT
TGGATTTACCACTGTTCAAAAGGATGTACCACCAAAAACGGAATCGCATACGTTTTCATGGTCGTCTATT
GTTGCTCAGTCTGTTGAGGTTCCACGAACAATTCCAAAACATGTAGAATCAAAAACGGGCGGAATTCAAA
ACTTTATCGCACAGGAGCAAAATTACTCTACGGTTGTGAATGATTCAGTCAAAAAGCCGAAACCTAAAAA
GGAAAAGAAAATAGTTCAGCCTATTCAACAACCAACCGGTGAAAGCGTTCCTGTCCGAAAAGATCACATG
CGAGAAGGAGTAACTAAAGCAGTTCCTGAGCCACAAACTTTGTCTTGGTCGTCAATCGTTTCGCATAACA
TCGATTTGACGCCAACGTTTACTGAAAACCGTGTCATTGAAGCCGTCGATGAGATCAATCCCGATGACAG
CAGTAGTAATCAAGAAGGTGAGCTTCCCATTCATGTTGAtgaaaggaaacgaaaacccaaaaaggacaaa
aaacgagatcaaaatattgaaacTTCTGTTGTCTACGAAGAGATTCCAGAAACTGAACGCGTAGCTGTGT
CTTCCACGCCTGAATTCAGCATTCAGTTGCCGGCACCAACAAAACCATCAGTGTGGTCATCATCAGACAC
CTATGCCGAAGTTGTTAAGAAGTCTGGTTACAACAACAATACGAACATTAAATATCAGAAAATTGAGGCA
AGGGAACCAATCAAGAGTGAACCAAAACACACATTTGTCGAAGTGCAGACACATGAAGATCTTCTGGAGT
TCCCGGACCCTATCATTCCTGCAATTGAAAGAGATGAAGCACTTGAGAAACCCTCTCAGCTATCATGGAA
GGACCTTGTAGATGAGGATGATGTGGATCCGCTGGAGTCGTGGCATAAGGGAGAAACAGATTCTGTAGAG
ATAAAGACACAAAAACCAATACAGGAAAGACATTCCCGAGTGAGCGTAAAGCCGGAAATTCAAATAACCG
AGGTAGTTACTGAAACTCAATCGCAATCCGAGACTGACCAAGAATTCTTGGAAATTACTTCAAAGAAACG
AAATCGCTCACGCTCACGTTCGCAACAGTCACAGGCTTCGAACATTGATgaaaagcatcaaaagaaaaaa
tcaaagaagccaaaaaaTACGGTACCTAAAGCACCAGCTCAACAGCCTACGGAAGCTCCTGAGGATGTCC
AGCCTCCTAAGGAATCTGTACCATCTGTAGAAGAACCGTCTCCACCACCAGCTCCTTCTCCTAATACCTC
AGGTATGTCATGGGCTGCTATAGCCGCCCATAATGTGCCAGAACCAGTTCAACATATTCGACCCCTCCAA
GCTGAGGCCAAACCAACTGTCGTTGAAGATGTAAGGAAATCAACAAATGAGCACACAGTAAACATTCCAA
TCACTTTCGAAACTACTGAGCCAAAGACTAAAAAGCCAAAGCAAAAGTCTAAGCGAAAGTTGCCAACCGA
TGTGGTTGATTTCATTAATGCAGAAAAATCGATACATCAAGTGATTGCTTCCAAACCTGAAATAGTTGAA
GAACCTGGATCTATAATAGTTGAAGACGTCAACAATACTTTAACATCATGGGAAATAAAAACTCCACTTG
CTCCCTTTGAATTTGTTGCCGAGTCTGAAGTTGAACCAGTATCTGAGTCACATATGGAAGACCTCCCTGT
CGAAAAAGTTTTGAAGACGGagccaattgttgaatctacggcggtagacgaatctaagactactacaaca
gtaaccacaacaacaacaactaccgaaaccactagcaatgtttctattcccgaggaacccaagaaggttg
tcgaaccagagaagaaacctgaagaacccaaaaagccagcatatgccggtcttcctgtcgatgactcttc
gaactcgtggatggatgtcttggacgaacctatgaacttctctgacgacgaagaggaaccagttcctgaa
ccactgaaggaagaaactcttgtggaagaagtagtcgaggaaaagccaattgttgaagtttctgttgtgg

acgattccaagtccattacaacagttaccaccacaacaacaactaccgaaaccattagtgaacttcctat
tcccgaggaacccaagaaggtagtagaaccggagaagaaacctgaagaacccaaaaagccagcatatgcc
ggtcttcccgtcgatgagtcttctagctcgtggatggatgtcttggacgaacctatgaacttctctgacg
acgaagaggaaccagttcctgagccagtgaaggaagaaactcttgtggaagaagtagtcgaggaaaagcc
aattgttgaagtttctgttgtggacgattccaagtccattacaacagttaccaccacaacaacaactacc
gaaaccattagcaaacttcctatttccgaggaacccaagaaggtagtagaaccggagaagaaacctgaag
aacccaaaaagccagcatatgccggtcttcctgtcgatgactcttcgaactcgtggatggatgtcttgga
cgaacctatgaacttctctgacgacgaagaggaaccagttcctgagccagtgaaggaagaaactcttgtg
gaagaagtagtcgaggaaaagccaattgttgaagtttctgttgtggacgattccaagtccattacaacag
ttaccaccacaacaacaactaccgaaaccattagtgaacttcctattcccgaggaacccaagaaggtagt
agaaccggagaagaaacctgaagaacccaaaaagccagcatatgccggtcttcctgtcgatgactcttcg
aactcgtggatggatgtcttggacgaacctatgaacttctctgacgacgaagaggaaccagttcctgagc
cagtgaaggaagaaactcttgtggaagaagtagtcgaggaaaagccaattgttgaagtttctgttgtgga
cgattccaagtccattacaacagttaccaccacaacaacaactaccgaaaccattagtgaacttcctatt
cccgaggaacccaagaag
>dme_circ_0001455_junction_seq
acttcctattcccgaggaacccaagaagggtagaagaaccggagaagatacctgaa
>dme_circ_0001456
gttcgtccaagacatccatccacgagttcgaagagtcatcgacaggaagaccggcatatgctggcttttt
gggttcttcaggcttcttctccggttctactaccttcttgggttcctcgggaataggaagttcactaatg
gtttcggtagttgttgttgtggtggtaactgttgtaatggacttggaatcgtccacaacagaaacttcaa
caattggcttttcctcgactacttctttcacaagagtttcttccttcactggctcaggaactggttcctc
ttcgtcgtcagagaagttcataggttcgtccaagacatccatcaaagagctagaagactcatcgacggga
agaccggcatatgctggctttttgggttcttcaggcttcttctccggttctactaccttcttgggttcct
cgggaataggaagttcactaatggtttcggtagtcgttgttgtggtggtaactgttgtaatggacttgga
atcgtccacaaaagaaacttcaacaattggcttttcctcgactacttcttccacaacagtttcttccttc
agtggttcaggaactggttcctcttcgtcgtcagagaagttcataggttcgtccaagacatccatccacg
agttcgtagagtcatcgacaggaagaccggcatatgctggctttttgggttcttcaggtttcttctccgg
ttcgacaaccttcttgggttcctcgggaataggaagttcgctaatggtttcggtagttgttgttgtggtg
gtaactgttgtaatggtcttggaatcgtccacaacagaaacttcaacaattggcttttcctcgactactt
cttccacaacagtttcttccttcagtggttcaggaactggttcctcttcgtcgtcagagaagttcatagg
ttcgtccaagacatccatccacgagttcgtagagtcatcgacgggaagaccggcatatgctggcttttttg
ggttcttcaggtttcttctccggttcgacaaccttcgtgggttcctcgggaatagcaatttcactaatgg
tttcggtagttgttgttgtggtggtaactgttgtaatggacttggaatcgtccacaacagaaacttcaac
aattggcttttcctcgactacttctttcacaagactttcttccttctgtggctcaggaattggttcctct
tcgtcgtcagagaagttcataggttcgtccaagacatccatccacgagttcgtagagtcatcgacaggaa
gaccggcatatgctggctttttgggttcttcaggtttcttctccagttcgacaaccttcttgggttcctc
gggaataggaagttcactaatggtttcggtatttgttgttgttgtggtaactgttgtaatagtcttggaa
tcgtccacaacagaaacttcaacaattggcttttcctcgactacttcttgcacaacagtttcttcttta
ctggctcaggaactggttcctcttcgtcgtcagagaagttcataggttcgtccaagacatccatccacga
gttcgtagagtcatcgacaggaagaccggcatatgctggctttttgggttcttcaggtttcttctccagt
tcgacaaccttcttgggttcctcgggaattggaagttcgctaatggtttcggtagttgttgttgtggtgg
taactgttgtaatggtcttggaatcgtccacaacagaaacttcaacaattggcttttcctcgactacttc
ttccacaagagtttcttccttcagtggctcaggcactggttcGGCAACTTTCTTGGGTTCCATTGGAGTA

ACAGATTCGCTGATAGACTCAGTTGATAGTGTTGTGGTGGTTACTGTTGTAATTGTTTTGGATTCGTTAT
TTGGTTTTTCTTCGCTGGTTGATTGTATTTCCTGGATGGTTTCAATAATCTTGTCAATGTTGGCTTTCTC
TCCATTCTCATGAGGAAGAGGAATTTCTATTTCGTCTTCTATAAGTTCTTTCCACGATTTTCGTTCAACG
ACCACGTCATCCAACCCATCGTCAATGATTTCAGACCATGGCATTAGGGGTGTACTTTCTACGTCCTTTT
GAACGGGTTCTTGCAAAGGTTCCTCCAGTTTTGGTGCAATTTTTGGTTGGGTATTGCCACTCGACTTTTT
CACAGACTTCTTAGGAACTGGTTTTATACTTTCAGCTTCAATAAAATCAGTGACTTGATGACTTACCACA
GATGACCAGGAATGACCAGAATCTCCCTTTTGTTGACTTAGATCTGATGATATAGCATGATCTTTTGGAT
GTTTCTCAATTCGATCGAATCTTATTTGCTCTTTTACCCTTTCTCcttgttttggctttgtttcttgttt
agcctttggtttcAAGTCAGTCATATGATCAATTTGACTGGCTATTTTAGCCCAAGTAATTGTATTTTCA
TTTGTAGGCTCTAATACCTCAACCACATCACTTTCCTCTGAAGTCGAATGAACAACTACGGGCTCACGAG
TTTCCTTTTTGTTATTACGCTTGGTGGTGTTTTCAGACTGAGGTTTGGGCTGAGGTTTTGAAATTTTACG
TTCTTTCCGTGGATGCACCTCCTTAGGCTCCTCGTGAATGATTTCAACTGGCTTTTTACGAGAAGCAATG
TCATCAACCCATTCGCCGGGCTTCTTTACCAGGGACGCCCAAGAGAAGGCGTTTTCTGTATTTTTGATAG
ATTTAGTTTCTTTCTCTTCCACCTCACACTCCACAACTATTTCCTCAGAGGGTTCACTGCTTACTTCATC
TTCGGGCAGAGCAACGGGAGTAGGTTTTTTGGTTGCCTTCTGTTTCTTGGGCTTTTGTGGCTTTTTGTCC
TTTTTGATTGGTTCTTTTACATCAGGCAATGGGGTGACAGCGGTTTTAATGTGAGTAACAGTGTTGTCAA
TCCATTCGCCTGGTCTCTTTACAAGTGAGGCCCAGGAGAATGCGTTGCTACTCTCTAGCTTCTGAACAAC
TTCAGGCTCCGGTTTTGACTCTATTGGTGCAGGCATTGCCTCCACTGGTTCGGGCACAGTCTCGGGCTGT
TCGTCCTCAGATGTAGTCGAGACGGGATCTACAACGGGTTTCTTGGACTTCTTTGTCTTCTTCGCCTTTG
TGGTCTTTTTGGGCTCCTCCTTTTCTTTCTGGCGATGTGTTTCTTTAGTTGTGACAGCCGTATGAGAAGT
CTGCTTTTTATTTACGGTTGAATCTGACCACTCGCCGGGTCTTTGCACAAGGGCTGACCATGTCAACTCG
GATGTGCTGGAAACATCCCTTTCATCAGAAGACGGCTCTTTGGAactttctgtcgttggtttggataatt
cactttctgtcgtaggcttagattgctcactGTCGCTGTTTGGCTTCGATTTTTCGCTTTCGATGCTTGC
TGAGGACAGTTCAATAACCTTTTCTGCCTGCCGCTTCTCTGGCTTCGATTTCTTCTTTGGCTCTTGGGCT
TTAGTTTTGACTATGTCTGTTTTGTGAGGGATTTCCTTATGTAACTCAGTAGATGGGACGTCGTCACCTG
TAACTCCAGAGTTGAGTAGCACGTCAGCATACGATTTACCATCCCGGTTCATGTTCCAAACATTCTCCAC
TTCTTCGACAATTTGTACCGGTTCTACAATATTTATTTCTTGAGCAGGTTGGACATCAATAATTTTTTCT
CGGATTTCCACCTTCTTGTTCTGTCGCTTTGATGGCTTTGCCTTTTTAGGCTTTTGGTGTGGCCCAATTG
TTTGTTCTGGCCTATTCCTGGGAATAAATTCGTTGTATATCCTTTCATTGGCAAGGAATGTATGCGTTTG
ATCGGAGAAGGTATGTCTCAAATAAGATGGAGTAAACTCAGGTGCGTCCGCCGATAAAGTTGAGGTATAA
CCAATGTTTCCAGTATTAGAAATGGTGTCCTCCAAAACGCTGGACCATGTATCAATGGGTCCCTCAAGAG
GCTTCTCCAATTGACTGACATCATCTACCACATTACTAATAATTTGCGGTTCAGCTGGCGCGGGAAGAGA
GTTCTCCTCTTCCAAAGTATTAGTTTGATTGGTAGATGTAACAATTGTGGTCGTAACAGTTGTTATTGTT
TTGCTTTCATCCACAACAGAAGATTCAACAATTGGCTCGACTACTTCTTCCACAAGAGTTTCTTCTTTCA
GTGGCTCAGGAACTGGTTCCTCTTCGTCGTCAGAGAAGTTCATAGG
>dme_circ_0001456_junction_seq
CCTCTTCGTCGTCAGAGAAGTTCATAGGgttcgtccaagacatccatccacgagtt
>dme_circ_0001457
gttcgtccaagacatccatccacgagttcgtagagtcatcgacgggaagaccggcatatgctggctttttt
gggttcttcaggtttcttctccggttctactaccttcttgggttcctcggaaataggaagttcactaata
gtttcggtagttattgttgtggtggtaactgttgtaatagtcttggaatcgtccacaacagaaacttcaa
caattggctttatctcgactacttcttccacaacagtttcttccttcagtggctcaggaattggttcctc
ttcgtcgtcagagaagttcataggttcgtccaagacatccatccacgagttcgaagagtcatcgacagga
agaccggcatatgctggctttttgggttcttcaggcttcttctccggttctactaccttcttgggttcct

cgggaataggaagttcactaatggtttcggtagttgttgttgtggtggtaactgttgtaatggacttgga
atcgtccacaacagaaacttcaacaattggcttttcctcgactacttctttcacaagagtttcttccttc
actggctcaggaactggttcctcttcgtcgtcagagaagttcataggttcgtccaagacatccatcaaag
agctagaagactcatcgacgggaagaccggcatatgctggctttttgggttcttcaggcttcttctccgg
ttctactaccttcttgggttcctcgggaataggaagttcactaatggtttcggtagtcgttgttgtggtg
gtaactgttgtaatggacttggaatcgtccacaaaagaaacttcaacaattggcttttcctcgactactt
cttccacaacagtttcttccttcagtggttcaggaactggttcctcttcgtcgtcagagaagttcatagg
ttcgtccaagacatccatccacgagttcgtagagtcatcgacaggaagaccggcatatgctggcttttttg
ggttcttcaggtttcttctccggttcgacaaccttcttgggttcctcgggaataggaagttcgctaatgg
tttcggtagttgttgttgtggtggtaactgttgtaatggtcttggaatcgtccacaacagaaacttcaac
aattggcttttcctcgactacttcttccacaacagtttcttccttcagtggttcaggaactggttcctct
tcgtcgtcagagaagttcataggttcgtccaagacatccatccacgagttcgtagagtcatcgacgggaa
gaccggcatatgctggctttttgggttcttcaggtttcttctccggttcgacaaccttcgtgggttcctc
gggaatagcaatttcactaatggtttcggtagttgttgttgtggtggtaactgttgtaatggacttggaa
tcgtccacaacagaaacttcaacaattggcttttcctcgactacttctttcacaagactttcttccttct
gtggctcaggaattggttcctcttcgtcgtcagagaagttcataggttcgtccaagacatccatccacga
gttcgtagagtcatcgacaggaagaccggcatatgctggctttttgggttcttcaggtttcttctccagt
tcgacaaccttcttgggttcctcgggaataggaagttcactaatggtttcggtatttgttgttgttgtgg
taactgttgtaatagtcttggaatcgtccacaacagaaacttcaacaattggcttttcctcgactacttc
ttgcacaacagtttcttcttttactggctcaggaactggttcctcttcgtcgtcagagaagttcataggt
tcgtccaagacatccatccacgagttcgtagagtcatcgacaggaagaccggcatatgctggctttttgg
gttcttcaggtttcttctccagttcgacaaccttcttgggttcctcgggaattggaagttcgctaatggt
ttcggtagttgttgttgtggtggtaactgttgtaatggtcttggaatcgtccacaacagaaacttcaaca
attggcttttcctcgactacttcttccacaagagtttcttccttcagtggctcaggcactggttcGGCAA
CTTTCTTGGGTTCCATTGGAGTAACAGATTCGCTGATAGACTCAGTTGATAGTGTTGTGGTGGTTACTGT
TGTAATTGTTTTGGATTCGTTATTTGGTTTTTCTTCGCTGGTTGATTGTATTTCCTGGATGGTTTCAATA
ATCTTGTCAATGTTGGCTTTCTCTCCATTCTCATGAGGAAGAGGAATTTCTATTTCGTCTTCTATAAGTT
CTTTCCACGATTTTCGTTCAACGACCACGTCATCCAACCCATCGTCAATGATTTCAGACCATGGCATTAG
GGGTGTACTTTCTACGTCCTTTTGAACGGGTTCTTGCAAAGGTTCCTCCAGTTTTGGTGCAATTTTTGGT
TGGGTATTGCCACTCGACTTTTTCACAGACTTCTTAGGAACTGGTTTTATACTTTCAGCTTCAATAAAAT
CAGTGACTTGATGACTTACCACAGATGACCAGGAATGACCAGAATCTCCCTTTTGTTGACTTAGATCTGA
TGATATAGCATGATCTTTTGGATGTTTCTCAATTCGATCGAATCTTATTTGCTCTTTTACCCTTTCTCct
tgttttggctttgtttcttgtttagcctttggtttcAAGTCAGTCATATGATCAATTTGACTGGCTATTT
TAGCCCAAGTAATTGTATTTTCATTTGTAGGCTCTAATACCTCAACCACATCACTTTCCTCTGAAGTCGA
ATGAACAACTACGGGCTCACGAGTTTCCTTTTTGTTATTACGCTTGGTGGTGTTTTCAGACTGAGGTTTG
GGCTGAGGTTTTGAAATTTTACGTTCTTTCCGTGGATGCACCTCCTTAGGCTCCTCGTGAATGATTTCAA
CTGGCTTTTTACGAGAAGCAATGTCATCAACCCATTCGCCGGGCTTCTTTACCAGGGACGCCCAAGAGAA
GGCGTTTTCTGTATTTTTGATAGATTTAGTTTCTTTCTCTTCCACCTCACACTCCACAACTATTTCCTCA
GAGGGTTCACTGCTTACTTCATCTTCGGGCAGAGCAACGGGAGTAGGTTTTTTGGTTGCCTTCTGTTTCT
TGGGCTTTTGTGGCTTTTTGTCCTTTTTGATTGGTTCTTTTACATCAGGCAATGGGGTGACAGCGGTTTT
AATGTGAGTAACAGTGTTGTCAATCCATTCGCCTGGTCTCTTTACAAGTGAGGCCCAGGAGAATGCGTTG
CTACTCTCTAGCTTCTGAACAACTTCAGGCTCCGGTTTTGACTCTATTGGTGCAGGCATTGCCTCCACTG
GTTCGGGCACAGTCTCGGGCTGTTCGTCCTCAGATGTAGTCGAGACGGGATCTACAACGGGTTTCTTGGA
CTTCTTTGTCTTCTTCGCCTTTGTGGTCTTTTTGGGCTCCTCCTTTTCTTTCTGGCGATGTGTTTCTTTA

GTTGTGACAGCCGTATGAGAAGTCTGCTTTTTATTTACGGTTGAATCTGACCACTCGCCGGGTCTTTGCA
CAAGGGCTGACCATGTCAACTCGGATGTGCTGGAAACATCCCTTTCATCAGAAGACGGCTCTTTGGAact
ttctgtcgttggtttggataattcactttctgtcgtaggcttagattgctcactGTCGCTGTTTGGCTTC
GATTTTTCGCTTTCGATGCTTGCTGAGGACAGTTCAATAACCTTTTCTGCCTGCCGCTTCTCTGGCTTCG
ATTTCTTCTTTGGCTCTTGGGCTTTAGTTTTGACTATGTCTGTTTTGTGAGGGATTTCCTTATGTAACTC
AGTAGATGGGACGTCGTCACCTGTAACTCCAGAGTTGAGTAGCACGTCAGCATACGATTTACCATCCCGG
TTCATGTTCCAAACATTCTCCACTTCTTCGACAATTTGTACCGGTTCTACAATATTTATTTCTTGAGCAG
GTTGGACATCAATAATTTTTTCTCGGATTTCCACCTTCTTGTTCTGTCGCTTTGATGGCTTTGCCTTTTT
AGGCTTTTGGTGTGGCCCAATTGTTTGTTCTGGCCTATTCCTGGGAATAAATTCGTTGTATATCCTTTCA
TTGGCAAGGAATGTATGCGTTTGATCGGAGAAGGTATGTCTCAAATAAGATGGAGTAAACTCAGGTGCGT
CCGCCGATAAAGTTGAGGTATAACCAATGTTTCCAGTATTAGAAATGGTGTCCTCCAAAACGCTGGACCA
TGTATCAATGGGTCCCTCAAGAGGCTTCTCCAATTGACTGACATCATCTACCACATTACTAATAATTTGC
GGTTCAGCTGGCGCGGGAAGAGAGTTCTCCTCTTCCAAAGTATTAGTTTGATTGGTAGATGTAACAATTG
TGGTCGTAACAGTTGTTATTGTTTTGCTTTCATCCACAACAGAAGATTCAACAATTGGCTCGACTACTTC
TTCCACAAGAGTTTCTTCTTTCAGTGGCTCAGGAACTGGTTCCTCTTCGTCGTCAGAGAAGTTCATAGG
>dme_circ_0001457_junction_seq
CCTCTTCGTCGTCAGAGAAGTTCATAGGgttcgtccaagacatccatccacgagtt
>dme_circ_0001458
gttcgtccaagacatccatccacgagttcgaagagtcatcgacaggaagaccggcatatgctggcttttt
gggttcttcaggtttcttctctggttcgacaaccttcttgggttcctcgggaatagaaacattgctagtg
gtttcggtagttgttgttgttgtggttactgttgtagtagtcttagattcgtctaccgccgtagattcaa
caattggctCCGTCTTCAAAACTTTTTCGACAGGGAGGTCTTCCATATGTGACTCAGATACTGGTTCAAC
TTCAGACTCGGCAACAAATTCAAAGGGAGCAAGTGGAGTTTTTATTTCCCATGATGTTAAAGTATTGTTG
ACGTCTTCAACTATTATAGATCCAGGTTCTTCAACTATTTCAGGTTTGGAAGCAATCACTTGATGTATCG
ATTTTTCTGCATTAATGAAATCAACCACATCGGTTGGCAACTTTCGCTTAGACTTTTGCTTTGGCTTTTT
AGTCTTTGGCTCAGTAGTTTCGAAAGTGATTGGAATGTTTACTGTGTGCTCATTTGTTGATTTCCTTACA
TCTTCAACGACAGTTGGTTTGGCCTCAGCTTGGAGGGGTCGAATATGTTGAACTGGTTCTGGCACATTAT
GGGCGGCTATAGCAGCCCATGACATACCTGAGGTATTAGGAGAAGGAGCTGGTGGTGGAGACGGTTCTTC
TACAGATGGTACAGATTCCTTAGGAGGCTGGACATCCTCAGGAGCTTCCGTAGGCTGTTGAGCTGGTGCT
TTAGGTACCGTAtttttttggcttctttgatttttttcttttgatgcttttcATCAATGTTCGAAGCCTGTG
ACTGTTGCGAACGTGAGCGTGAGCGATTTCGTTTCTTTGAAGTAATTTCCAAGAATTCTTGGTCAGTCTC
GGATTGCGATTGAGTTTCAGTAACTACCTCGGTTATTTGAATTTCCGGCTTTACGCTCACTCGGGAATGT
CTTTCCTGTATTGGTTTTTGTGTCTTTATCTCTACAGAATCTGTTTCTCCCTTATGCCACGACTCCAGCG
GATCCACATCATCCTCATCTACAAGGTCCTTCCATGATAGCTGAGAGGGTTTCTCAAGTGCTTCATCTCT
TTCAATTGCAGGAATGATAGGGTCCGGGAACTCCAGAAGATCTTCATGTGTCTGCACTTCGACAAATGTG
TGTTTTGGTTCACTCTTGATTGGTTCCCTTGCCTCAATTTTCTGATATTTAATGTTCGTATTGTTGTTGT
AACCAGACTTCTTAACAACTTCGGCATAGGTGTCTGATGATGACCACACTGATGGTTTTGTTGGTGCCGG
CAACTGAATGCTGAATTCAGGCGTGGAAGACACAGCTACGCGTTCAGTTTCTGGAATCTCTTCGTAGACA
ACAGAAgtttcaatattttgatctcgtttttttgtcctttttgggttttcgtttcctttcaTCAACATGAA
TGGGAAGCTCACCTTCTTGATTACTACTGCTGTCATCGGGATTGATCTCATCGACGGCTTCAATGACACG
GTTTTCAGTAAACGTTGGCGTCAAATCGATGTTATGCGAAACGATTGACGACCAAGACAAAGTTTGTGGC
TCAGGAACTGCTTTAGTTACTCCTTCTCGCATGTGATCTTTTCGGACAGGAACGCTTTCACCGGTTGGTT
GTTGAATAGGCTGAACTATTTTCTTTTCCTTTTTAGGTTTCGGCTTTTTGACTGAATCATTCACAACCGT
AGAGTAATTTTGCTCCTGTGCGATAAAGTTTTGAATTCCGCCCGTTTTTGATTCTACATGTTTTGGAATT

GTTCGTGGAACCTCAACAGACTGAGCAACAATAGACGACCATGAAAACGTATGCGATTCCGTTTTTGGTG
GTACATCCTTTTGAACAGTGGTAAATCCAATAATGGGTTCAACAACGGACAATGGAACATCAGACTCCAA
AATTGACTCTTCAACATTCTGAACCTTGGATTTCTTTTCCTTCCCTGCCTTAAGTTTTTTCGGTTTTTCC
ATTAAAGGTTTCTGATCTTCAGCTACATCAGTGATAAGAGCCTCTGCTACATcttcagtagtttgagaga
caatagacgaccataatagtggttgcgatggggccacctcactaactgaagtgggttccggttgtgtctc
gggcgttacttcttctacgactgtagtcacctcaaccactggttcctcaacaaaagtcacttcaacagtg
ggttcaacaacgtcctcaggaactgcagactcaacgatgggctctggagcttccaccaccttagttttct
tttccttctttggtttttgtttttttcggtttttcttctaagggtttcttatcttcaggttcttccgtgaa
gcgagtctctgttacatttgtcgttacttccgtggtttgagaaacgatggacgaccatgatagtggttgc
gatggggccacctcacttactggagtgggttccggttgtgtctcgggctttacttcttgtacaactgtag
tcacctcaaccactggttcctcaacaaaagtcacttcaacagttggttcaacaacgtcctcaggaactgc
agactcaacgatgggctctggagcttccaccaccttagttttcttttccttctttggtttttgtttttttc
ggtttttcttctaagggtttcttatcttcaggttcttccgtgaagcgagtctctgttacatttgtcgtta
cttccgtggtttgagaaacgatggacgaccatgatagtggttgcgatggggccacctcacttactggagt
gggttccggttgtgtctcgggctttacttcttctacaactgtagtcacctcaaccactggttcctcaaca
aaagtcacttcaacagttggttcaacaacgtcctcaggaactgcagactcaacgatgggctctggagctt
ccaccaccttagttttcttttccttctttggtttttgtttttttcggtttttcttctaagggtttcttatc
ttcaggttcttccgtgaagcgagtctctgttacatttgtcgttacttccgtggtttgagaaacgatggac
gaccatgatagtggttgcgatggggccacctcacttactggagtgggttccggttgtgtctcgggcttta
cttcttctacaactgtagtcacctcaaccactggttcctcaacaaaagtcacttcaacagttggttcaac
aacgtcctcaggaactgcagactcaacgatgggctctggagcttccaccaccttagttttcttttccttc
tttggtttttgtttttttcggtttttcttctaagggtttcttatcttcaggttcttccgtgaagcgagtct
ctgttacatttgtcgttacttccgtggtttgagaaacgatggacgaccatgatagtggttgcgatggggc
cacctcacttactggagtgggttccggttgtgtctcgggctttacttcttctacaactgtagtcacctca
accactggttcctcaacaaaagtcacttcaacagttggttcaacaacgtcctcaggaactgcagactcaa
cgatgggctctggagcttccaccaccttagttttcttttccttctttggtttttgtttttttcggtttttc
ttctaagggtttcttatcttcaggttcttccgtgaagcgagtctctgttacatttgtcgttacttccgtg
gtttgagaaacgatggacgaccatgatagtggttgcgatggggccacctcacttactggagtgggttccg
gttgtgtctcgggctttacttcttctacgactgtagtcacctcaaccactggttcctcaacaaaagtcac
ttcaacagtgggttcaacaacgtcctcaggaactgcagactcaacgatgggctctggagcttccaccacc
ttagttttcttttccttctttggtttttgtttttttcggtttttcttctaagggtttcttatcttcaggtt
cttccgtgaagcgagtctctgttacatttgtcgttacttccgtggtttgagaaacgatggacgaccatga
tagtggttgcgatggggccacctcacttactggagttggttccggttgtgtctcgggggttacttcttct
acaactgtagtcacctcaaccactggttcctcaacaaaagtcacttcaacagtgggttcaaccacggcct
caggaactgaagactcaacgatgggctctggagcttccaccaccttagttttcttttccttctttgcctt
tggtttttttcggtttttcttctaagggtttcttatcttcaggttcttccgtgaagcgagtctctgttaca
tttgtcgtaacttccgtggtttgagaaacgatggacgaccatgatagtggttgcgatggggccacctcac
ttactggagtgggttcctgttgtgtctcgggcgttacttcttctacgactgtagtcacctcaaccactgg
ttcctcaacaaaagtcacttcaacagtgggttcaacaacgtcctcaggaactgcagactcaacgatgggc
tctggagcttccaccaccttagttttcttttccttctttgcctttggtttttttcggtttttcttctaagg
gtttcttatcttcgggttcttccgtgaagcgagtctctgttacatttgtcgttacttccgtggcttgaga
aacgatggacgaccatgatagtggttgcgatggggccacctcacttactggagttggttccggttgtgtc
tcgggggttacttcttctacaactgtagtcacctcaaccactggttcctcaacaaaagtcacttcaacag
tgggttcaaccacggcctcaggaactgaagactcaacgatgggctctggagcttccaccaccttagtttt

cttttccttctttgcctttggttttttcggtttttcttctaagggtttcttatcttcaggttcttccgtg
aagcgagtctctgttacatttgtcgtaacttccgtggtttgagaaacgatggacgaccatgatagtggtt
gcgatggggccacctcactaactgaagtgggttccggttgtgtctcgggctttacttcttctacaactgt
agtcacctcaaccactggttcctcaacaaatgtcacttcaacagtgggttcaacaacggcctcaggaact
gaagactcaacgatgggctctggagcttccaccaccttagttttcttttccttctttgcctttggttttt
tcggtttttcttctaagggtttcttatcttcaggttcttccgtgaagcgagtctctgttacatttgtcgt
aacttccgtggtttgagaaacgatagacgaccatgatagtggttgcgatggggccacctcacttactgga
gtgggttccggttgtgtctcgggcgttacttcttctacaactgtagtcacctcaaccactggttcctcaa
caaatgtcacttcaacagtgggttcaacaacggcctcaggaactgcagactcaacgatgggttctggagc
ttccaccaccttagttttcttttccttctttgattttttgttttttcggtttttcttctaagggtttctta
tcttcaggctcttccgtgaagcgagtctctgttacatttgtcgtaacttccgtggtttgagaaacgatag
acgaccatgatagtggttgcgatggggccacctcacttactggagtgggttccggttgtgtctcgggcgt
tacttcttctacaactgtagtcacctcaaccactggttcctcaacaaatgtcacttcaacagttggttca
acaacggcctcaggaactgcagactcaacgatgggctctggagcttccaccaccttagttttcttttcct
tctttggtttttgttttttcggtttttcttctaagggtttcttatcttcaggctcttccgtgaagcgagt
ctctgttacatttgtcgtaacttccgtggtttgagaaacgatagacgaccatgatagtggttgcgatggg
gccacctcacttactggagtgggttccggttgtgtctcgggcgttacttcttctacaactgtagtcacct
caaccactggttcctcaacaaatgtcacttcaacagtgggttcaacaacggcctcaggaactgcagactc
aacgatgggttctggagcttccaccaccttagttttcttttccttctttgattttttgttttttcggtttt
tcttctaagggtttcttatcttcaggctcttccgtgaagcgagtctctgttacatttgtcgtaatttccg
tggtttgagaaacgatagacgaccatgatagtggttgcgatggggccacctcacttactggagtgggttc
cggttgtgtctcgggcgttacttcttctacaactgtagtcacctcaaccactggttcctcaacaaatgtc
acttcaacagttggttcaacaacggcctcaggaactgcagactcaacgatgggctctggagcttccacca
ccttagttttcttttccttctttgctttttgttttttcggtttttcttctaagggtttcttatcttcagg
ctcttccgtgaagcgagtctctgttacatttgtcgtaacttccgtggtttgagaaacgatagacgaccat
gatagtggttgcgatggggccacctcacttactggagtgggttccggttgtgtctcgggcgttacttctt
ctacaactgtagtcacctcaaccactggttcctcaacaaatgtcacttcaacagttggttcaacaacggc
ctcaggaactgcagactcaacgatgggctctggagcttccaccaccttagttttcttttccttctttggt
ttttgttttttcggtttttcttctaagggtttcttatcttcaggctcttccgtgaagcgagtctctgtta
catttgtcgtaacttccgtggtttgagaaacgatagacgaccatgatagtggttgcgatggggccacctc
acttactggagtgggttccggttgtgtctcgggcgttacttcttctacaactgtagtcacctcaaccact
ggttcctcaacaaatgtcacttcaacagtgggttcaacaacggcctcaggaactgcagactcaacgatgg
gctctggagcttccaccaccttagttttcttttccttctttgctttttgttttttcggtttttcttccga
gggtttcttatcttcaggttcttccgtgaagcgagtctctgtaacattggtcgtaacttccgtggtttga
gaaacgatggacgaccatgatagtggttgcgaaggggccacctcactaactggagtgggttccggttgtg
tctcgggcgttacttcttctacaactgtagtcacctcaaccactggttcctcaacaaatgtcacttcaac
agtgggttcaaccacggcctcaggaactgcagactcaacgatgggctctggagcttccaccaccttagtt
ttcttttccttctttggtttttgttttttcggtttttcttctaagggtttcttatcttcaggctcttccg
tgaagcgagtctctgttacatttgtcgtaacttccgtggtttgagaaacgatagacgaccatgatagtgg
ttgcgatggggccacctcacttactggagtgggttccggttgtgtctcgggcgttacttcttctacaact
gtagtcacctcaaccactggttcctcaacaaatgtcacttcaacagtgggttcaacaacggcctcaggaa
ctgcagactcaacgatgggttctggagcttccaccaccttagttttcttttccttctttgattttttgttt
tttcggtttttcttctaagggtttcttatcttcaggctcttccgtgaagcgagtctctgttacatttgtc
gtaatttccgtggtttgagaaacgatagacgaccatgatagtggttgcgatggggccacctcacttactg

gagtgggttccggttgtgtctcgggcgttacttcttctacaactgtagtcacctcaaccactggttcctc
aacaaatgtcacttcaacagttggttcaacaacggcctcaggaactgcagactcaacgatgggctctgga
gcttccaccaccttagttttcttttccttctttggtttttgtttttttcggtttttcttctaagggtttct
tatcttcaggctcttccgtgaagcgagtctctgttacatttgtcgtaacttccgtggtttgagaaacgat
agacgaccatgatagtggttgcgatggggccacctcacttactggagtgggttccggttgtgtctcgggc
gttacttcttctacaactgtagtcacctcaaccactggttcctcaacaaatgtcacttcaacagtgggtt
caacaacggcctcaggaactgcagactcaacgatgggttctggagcttccaccaccttagttttcttttc
cttctttgatttttgtttttttcggtttttcttctaagggtttcttatcttcaggttTCTTAGAATCGGCA
TTTACAGTCGTTGAAAAGATTTGTTCTTTACTAATGAACTCCTGCACTTCTTTTGACCTATGAGGATCAA
CGGCTTTCTGGGATATTTTACTTTCTATACTTGTTGGAGGCAGAACCTCGGTTTGTGAGACGATAGATGA
CCAAGACTTGGGTTGAATAGGTAAACTTTCCAAAACAGACGGAATTTCTGCATTCTGCTGTGTTTTAGGA
TCATCGCGCATGAAAGTGCTATCAACGTATGGTTCTAAAGCCACTTCAACTACTGATTCTATCTTATTGT
GTTTCTTTTCCTTCTTTGATTTTTGTTTCTTAGTCTTTTCAATCACTGTGGAGCTTCTTTCTTGGGAAAT
GAATTTGTTTATTTCTTGTGAATTCTGGAATGCAGGTTCTAGAACTTTACCATCATGGTCGGCAGATTGT
GTCATTTTGGTAATAGGTTCCTGGGCACCTTGTCGGACAATTGAAGACCACAAAGTGCTATCGGCACTGA
GTATGTTTGTTTGAACGTTATCATCAAAATCGTTCTCGACCATTGTTGACCACGACGTCTTTGTTTCATC
CTTTGCTGATTCTCTTTCGATTGGTAGAACTTCATCTTTAGGCGTCAAAAGCTTAGCTCCCTTTTCCTTC
TTTAACGGTTTTTCTTTCTTAGCCTTTGTCTTAATCAAAGGTTCTGGAAGTTTAGCAGGCTCTTTTACTG
TTGGCACTTTACCTAAATTTGATTCAGTTGGGACTTCAACATTATTCTTTTCTAAACTTATGAAGTCCAC
AGTATGACGCAAGACATCAGCATATGACGAATGCAGTTCCCATATATTAGAAGACAATGGTTTTGGCTCG
ACTTCTGtggattcttcaggtttcttcactggttctactgttttcttgggttgctcggatataactggtt
cactaatggtttcggtagttgttgttgtggtggtaactgttgtaatagtcttggaatcgtccacaacaga
aacttcaacaattggcttttcctcgactacttcttccacaagagtttcttccttcactggctcaggaact
ggttcctcttcgtcgtcagagaagttcataggttcgtccaagacatccatccacgagttcgtagagtcat
cgacgggaagaccggcatatgctggctttttgggttcttcaggtttcttctccggttctactaccttctt
gggttcctcggaaataggaagttcactaatagtttcggtagttattgttgtggtggtaactgttgtaata
gtcttggaatcgtccacaacagaaacttcaacaattggctttatctcgactacttcttccacaacagttt
cttccttcagtggctcaggaattggttcctcttcgtcgtcagagaagttcataggttcgtccaagacatc
catccacgagttcgaagagtcatcgacaggaagaccggcatatgctggctttttgggttcttcaggcttc
ttctccggttctactaccttcttgggttcctcgggaataggaagttcactaatggtttcggtagttgttg
ttgtggtggtaactgttgtaatggacttggaatcgtccacaacagaaacttcaacaattggcttttcctc
gactacttctttcacaagagtttcttccttcactggctcaggaactggttcctcttcgtcgtcagagaag
ttcataggttcgtccaagacatccatcaaagagctagaagactcatcgacgggaagaccggcatatgctg
gctttttgggttcttcaggcttcttctccggttctactaccttcttgggttcctcgggaataggaagttc
actaatggtttcggtagtcgttgttgtggtggtaactgttgtaatggacttggaatcgtccacaaaagaa
acttcaacaattggcttttcctcgactacttcttccacaacagtttcttccttcagtggttcaggaactg
gttcctcttcgtcgtcagagaagttcataggttcgtccaagacatccatccacgagttcgtagagtcatc
gacaggaagaccggcatatgctggctttttgggttcttcaggtttcttctccggttcgacaaccttcttg
ggttcctcgggaataggaagttcgctaatggtttcggtagttgttgttgtggtggtaactgttgtaatgg
tcttggaatcgtccacaacagaaacttcaacaattggcttttcctcgactacttcttccacaacagtttc
ttccttcagtggttcaggaactggttcctcttcgtcgtcagagaagttcataggttcgtccaagacatcc
atccacgagttcgtagagtcatcgacgggaagaccggcatatgctggctttttgggttcttcaggtttct
tctccggttcgacaaccttcgtgggttcctcgggaatagcaatttcactaatggtttcggtagttgttgt
tgtggtggtaactgttgtaatggacttggaatcgtccacaacagaaacttcaacaattggcttttcctcg

actacttctttcacaagactttcttccttctgtggctcaggaattggttcctcttcgtcgtcagagaagt
tcataggttcgtccaagacatccatccacgagttcgtagagtcatcgacaggaagaccggcatatgctgg
ctttttgggttcttcaggtttcttctccagttcgacaaccttcttgggttcctcgggaataggaagttca
ctaatggtttcggtatttgttgttgttgtggtaactgttgtaatagtcttggaatcgtccacaacagaaa
cttcaacaattggcttttcctcgactacttcttgcacaacagtttcttcttttactggctcaggaactgg
ttcctcttcgtcgtcagagaagttcataggttcgtccaagacatccatccacgagttcgtagagtcatcg
acaggaagaccggcatatgctggctttttgggttcttcaggtttcttctccagttcgacaaccttcttgg
gttcctcgggaattggaagttcgctaatggtttcggtagttgttgttgtggtggtaactgttgtaatggt
cttggaatcgtccacaacagaaacttcaacaattggcttttcctcgactacttcttccacaagagtttct
tccttcagtggctcaggcactggttcGGCAACTTTCTTGGGTTCCATTGGAGTAACAGATTCGCTGATAG
ACTCAGTTGATAGTGTTGTGGTGGTTACTGTTGTAATTGTTTTGGATTCGTTATTTGGTTTTTCTTCGCT
GGTTGATTGTATTTCCTGGATGGTTTCAATAATCTTGTCAATGTTGGCTTTCTCTCCATTCTCATGAGGA
AGAGGAATTTCTATTTCGTCTTCTATAAGTTCTTTCCACGATTTTCGTTCAACGACCACGTCATCCAACC
CATCGTCAATGATTTCAGACCATGGCATTAGGGGTGTACTTTCTACGTCCTTTTGAACGGGTTCTTGCAA
AGGTTCCTCCAGTTTTGGTGCAATTTTTGGTTGGGTATTGCCACTCGACTTTTTCACAGACTTCTTAGGA
ACTGGTTTTATACTTTCAGCTTCAATAAAATCAGTGACTTGATGACTTACCACAGATGACCAGGAATGAC
CAGAATCTCCCTTTTGTTGACTTAGATCTGATGATATAGCATGATCTTTTGGATGTTTCTCAATTCGATC
GAATCTTATTTGCTCTTTTACCCTTTCTCcttgttttggctttgtttcttgtttagcctttggtttcAAG
TCAGTCATATGATCAATTTGACTGGCTATTTTAGCCCAAGTAATTGTATTTTCATTTGTAGGCTCTAATA
CCTCAACCACATCACTTTCCTCTGAAGTCGAATGAACAACTACGGGCTCACGAGTTTCCTTTTTGTTATT
ACGCTTGGTGGTGTTTTCAGACTGAGGTTTGGGCTGAGGTTTTGAAATTTTACGTTCTTTCCGTGGATGC
ACCTCCTTAGGCTCCTCGTGAATGATTTCAACTGGCTTTTTACGAGAAGCAATGTCATCAACCCATTCGC
CGGGCTTCTTTACCAGGGACGCCCAAGAGAAGGCGTTTTCTGTATTTTTGATAGATTTAGTTTCTTTCTC
TTCCACCTCACACTCCACAACTATTTCCTCAGAGGGTTCACTGCTTACTTCATCTTCGGGCAGAGCAACG
GGAGTAGGTTTTTTGGTTGCCTTCTGTTTCTTGGGCTTTTGTGGCTTTTTGTCCTTTTTGATTGGTTCTT
TTACATCAGGCAATGGGGTGACAGCGGTTTTAATGTGAGTAACAGTGTTGTCAATCCATTCGCCTGGTCT
CTTTACAAGTGAGGCCCAGGAGAATGCGTTGCTACTCTCTAGCTTCTGAACAACTTCAGGCTCCGGTTTT
GACTCTATTGGTGCAGGCATTGCCTCCACTGGTTCGGGCACAGTCTCGGGCTGTTCGTCCTCAGATGTAG
TCGAGACGGGATCTACAACGGGTTTCTTGGACTTCTTTGTCTTCTTCGCCTTTGTGGTCTTTTTGGGCTC
CTCCTTTTCTTTCTGGCGATGTGTTTCTTTAGTTGTGACAGCCGTATGAGAAGTCTGCTTTTTATTTACG
GTTGAATCTGACCACTCGCCGGGTCTTTGCACAAGGGCTGACCATGTCAACTCGGATGTGCTGGAAACAT
CCCTTTCATCAGAAGACGGCTCTTTGGAactttctgtcgttggtttggataattcactttctgtcgtagg
cttagattgctcactGTCGCTGTTTGGCTTCGATTTTTCGCTTTCGATGCTTGCTGAGGACAGTTCAATA
ACCTTTTCTGCCTGCCGCTTCTCTGGCTTCGATTTCTTCTTTGGCTCTTGGGCTTTAGTTTTGACTATGT
CTGTTTTGTGAGGGATTTCCTTATGTAACTCAGTAGATGGGACGTCGTCACCTGTAACTCCAGAGTTGAG
TAGCACGTCAGCATACGATTTACCATCCCGGTTCATGTTCCAAACATTCTCCACTTCTTCGACAATTTGT
ACCGGTTCTACAATATTTATTTCTTGAGCAGGTTGGACATCAATAATTTTTTCTCGGATTTCCACCTTCT
TGTTCTGTCGCTTTGATGGCTTTGCCTTTTTAGGCTTTTGGTGTGGCCCAATTGTTTGTTCTGGCCTATT
CCTGGGAATAAATTCGTTGTATATCCTTTCATTGGCAAGGAATGTATGCGTTTGATCGGAGAAGGTATGT
CTCAAATAAGATGGAGTAAACTCAGGTGCGTCCGCCGATAAAGTTGAGGTATAACCAATGTTTCCAGTAT
TAGAAATGGTGTCCTCCAAAACGCTGGACCATGTATCAATGGGTCCCTCAAGAGGCTTCTCCAATTGACT
GACATCATCTACCACATTACTAATAATTTGCGGTTCAGCTGGCGCGGGAAGAGAGTTCTCCTCTTCCAAA
GTATTAGTTTGATTGGTAGATGTAACAATTGTGGTCGTAACAGTTGTTATTGTTTTGCTTTCATCCACAA
CAGAAGATTCAACAATTGGCTCGACTACTTCTTCCACAAGAGTTTCTTCTTTCAGTGGCTCAGGAACTGG

TTCCTCTTCGTCGTCAGAGAAGTTCATAGG
>dme_circ_0001458_junction_seq
CCTCTTCGTCGTCAGAGAAGTTCATAGGgttcgtccaagacatccatccacgagtt
>dme_circ_0001459
gttcgtccaagacatccatccacgagttcgaagagtcatcgacaggaagaccggcatatgctggcttttt
gggttcttcaggtttcttctccggttctactaccttcttgggttcctcggaaataggaagtttgctaatg
gtttcggtagttgttgttgtggtggtaactgttgtaatggacttggaatcgtccacaacagaaacttcaa
caattggcttttcctcgactacttcttccacaagagtttcttccttcactggctcaggaactggttcctc
ttcgtcgtcagagaagttcataggttcgtccaagacatccatccacgagctagaagactcatcgacggga
agaccggcatatgctggctttttgggttcttcaggtttcttctccggttctactaccttcttgggttcct
cgggaataggaagttcactaatggtttcggtagttgttgttgtggtggtaactgttgtaatggacttgga
atcgtccacaacagaaacttcaacaattggcttttcctcgactacttcttccacaagagtttcttccttc
agtggttcaggaactggttcctcttcgtcgtcagagaagttcataggttcgtccaagacatccatccacg
agttcgaagagtcatcgacaggaagaccggcatatgctggctttttgggttcttcaggtttcttctctgg
ttcgacaaccttcttgggttcctcgggaatagaaacattgctagtggtttcggtagttgttgttgttgtg
gttactgttgtagtagtcttagattcgtctaccgccgtagattcaacaattggctCCGTCTTCAAAACTT
TTTCGACAGGGAGGTCTTCCATATGTGACTCAGATACTGGTTCAACTTCAGACTCGGCAACAAATTCAAA
GGGAGCAAGTGGAGTTTTTATTTCCCATGATGTTAAAGTATTGTTGACGTCTTCAACTATTATAGATCCA
GGTTCTTCAACTATTTCAGGTTTGGAAGCAATCACTTGATGTATCGATTTTTCTGCATTAATGAAATCAA
CCACATCGGTTGGCAACTTTCGCTTAGACTTTTGCTTTGGCTTTTTAGTCTTTGGCTCAGTAGTTTCGAA
AGTGATTGGAATGTTTACTGTGTGCTCATTTGTTGATTTCCTTACATCTTCAACGACAGTTGGTTTGGCC
TCAGCTTGGAGGGGTCGAATATGTTGAACTGGTTCTGGCACATTATGGGCGGCTATAGCAGCCCATGACA
TACCTGAGGTATTAGGAGAAGGAGCTGGTGGTGGAGACGGTTCTTCTACAGATGGTACAGATTCCTTAGG
AGGCTGGACATCCTCAGGAGCTTCCGTAGGCTGTTGAGCTGGTGCTTTAGGTACCGTAtttttttggcttc
tttgattttttcttttgatgcttttcATCAATGTTCGAAGCCTGTGACTGTTGCGAACGTGAGCGTGAGC
GATTTCGTTTCTTTGAAGTAATTTCCAAGAATTCTTGGTCAGTCTCGGATTGCGATTGAGTTTCAGTAAC
TACCTCGGTTATTTGAATTTCCGGCTTTACGCTCACTCGGGAATGTCTTTCCTGTATTGGTTTTTGTGTC
TTTATCTCTACAGAATCTGTTTCTCCCTTATGCCACGACTCCAGCGGATCCACATCATCCTCATCTACAA
GGTCCTTCCATGATAGCTGAGAGGGTTTCTCAAGTGCTTCATCTCTTTCAATTGCAGGAATGATAGGGTC
CGGGAACTCCAGAAGATCTTCATGTGTCTGCACTTCGACAAATGTGTGTTTTGGTTCACTCTTGATTGGT
TCCCTTGCCTCAATTTTCTGATATTTAATGTTCGTATTGTTGTTGTAACCAGACTTCTTAACAACTTCGG
CATAGGTGTCTGATGATGACCACACTGATGGTTTTGTTGGTGCCGGCAACTGAATGCTGAATTCAGGCGT
GGAAGACACAGCTACGCGTTCAGTTTCTGGAATCTCTTCGTAGACAACAGAAgtttcaatattttgatct
cgttttttgtccttttttgggttttcgtttcctttcaTCAACATGAATGGGAAGCTCACCTTCTTGATTAC
TACTGCTGTCATCGGGATTGATCTCATCGACGGCTTCAATGACACGGTTTTCAGTAAACGTTGGCGTCAA
ATCGATGTTATGCGAAACGATTGACGACCAAGACAAAGTTTGTGGCTCAGGAACTGCTTTAGTTACTCCT
TCTCGCATGTGATCTTTTCGGACAGGAACGCTTTCACCGGTTGGTTGTTGAATAGGCTGAACTATTTTCT
TTTCCTTTTTAGGTTTCGGCTTTTTGACTGAATCATTCACAACCGTAGAGTAATTTTGCTCCTGTGCGAT
AAAGTTTTGAATTCCGCCCGTTTTTGATTCTACATGTTTTGGAATTGTTCGTGGAACCTCAACAGACTGA
GCAACAATAGACGACCATGAAAACGTATGCGATTCCGTTTTTGGTGGTACATCCTTTTGAACAGTGGTAA
ATCCAATAATGGGTTCAACAACGGACAATGGAACATCAGACTCCAAAATTGACTCTTCAACATTCTGAAC
CTTGGATTTCTTTTCCTTCCCTGCCTTAAGTTTTTTCGGTTTTTCCATTAAAGGTTTCTGATCTTCAGCT
ACATCAGTGATAAGAGCCTCTGCTACATcttcagtagtttgagagacaatagacgaccataatagtggtt
gcgatggggccacctcactaactgaagtgggttccggttgtgtctcgggcgttacttcttctacgactgt

agtcacctcaaccactggttcctcaacaaaagtcacttcaacagtgggttcaacaacgtcctcaggaact
gcagactcaacgatgggctctggagcttccaccaccttagttttcttttccttctttggtttttgttttt
tcggtttttcttctaagggtttcttatcttcaggttcttccgtgaagcgagtctctgttacatttgtcgt
tacttccgtggtttgagaaacgatggacgaccatgatagtggttgcgatggggccacctcacttactgga
gtgggttccggttgtgtctcgggctttacttcttgtacaactgtagtcacctcaaccactggttcctcaa
caaaagtcacttcaacagttggttcaacaacgtcctcaggaactgcagactcaacgatgggctctggagc
ttccaccaccttagttttcttttccttctttggtttttgtttttttcggtttttcttctaagggtttctta
tcttcaggttcttccgtgaagcgagtctctgttacatttgtcgttacttccgtggtttgagaaacgatgg
acgaccatgatagtggttgcgatggggccacctcacttactggagtgggttccggttgtgtctcgggctt
tacttcttctacaactgtagtcacctcaaccactggttcctcaacaaaagtcacttcaacagttggttca
acaacgtcctcaggaactgcagactcaacgatgggctctggagcttccaccaccttagttttcttttcct
tctttggtttttgtttttttcggtttttcttctaagggtttcttatcttcaggttcttccgtgaagcgagt
ctctgttacatttgtcgttacttccgtggtttgagaaacgatggacgaccatgatagtggttgcgatggg
gccacctcacttactggagtgggttccggttgtgtctcgggctttacttcttctacaactgtagtcacct
caaccactggttcctcaacaaaagtcacttcaacagttggttcaacaacgtcctcaggaactgcagactc
aacgatgggctctggagcttccaccaccttagttttcttttccttctttggtttttgtttttttcggtttt
tcttctaagggtttcttatcttcaggttcttccgtgaagcgagtctctgttacatttgtcgttacttccg
tggtttgagaaacgatggacgaccatgatagtggttgcgatggggccacctcacttactggagtgggttc
cggttgtgtctcgggctttacttcttctacaactgtagtcacctcaaccactggttcctcaacaaaagtc
acttcaacagttggttcaacaacgtcctcaggaactgcagactcaacgatgggctctggagcttccacca
ccttagttttcttttccttctttggtttttgtttttttcggtttttcttctaagggtttcttatcttcagg
ttcttccgtgaagcgagtctctgttacatttgtcgttacttccgtggtttgagaaacgatggacgaccat
gatagtggttgcgatggggccacctcacttactggagtgggttccggttgtgtctcgggctttacttctt
ctacgactgtagtcacctcaaccactggttcctcaacaaaagtcacttcaacagtgggttcaacaacgtc
ctcaggaactgcagactcaacgatgggctctggagcttccaccaccttagttttcttttccttctttggt
ttttgtttttttcggtttttcttctaagggtttcttatcttcaggttcttccgtgaagcgagtctctgtta
catttgtcgttacttccgtggtttgagaaacgatggacgaccatgatagtggttgcgatggggccacctc
acttactggagttggttccggttgtgtctcgggggttacttcttctacaactgtagtcacctcaaccact
ggttcctcaacaaaagtcacttcaacagtgggttcaaccacggcctcaggaactgaagactcaacgatgg
gctctggagcttccaccaccttagttttcttttccttctttgcctttggtttttttcggtttttcttctaa
gggtttcttatcttcaggttcttccgtgaagcgagtctctgttacatttgtcgtaacttccgtggtttga
gaaacgatggacgaccatgatagtggttgcgatggggccacctcacttactggagtgggttcctgttgtg
tctcgggcgttacttcttctacgactgtagtcacctcaaccactggttcctcaacaaaagtcacttcaac
agtgggttcaacaacgtcctcaggaactgcagactcaacgatgggctctggagcttccaccaccttagtt
ttcttttccttctttgcctttggtttttttcggtttttcttctaagggtttcttatcttcgggttcttccg
tgaagcgagtctctgttacatttgtcgttacttccgtggcttgagaaacgatggacgaccatgatagtgg
ttgcgatggggccacctcacttactggagttggttccggttgtgtctcgggggttacttcttctacaact
gtagtcacctcaaccactggttcctcaacaaaagtcacttcaacagtgggttcaaccacggcctcaggaa
ctgaagactcaacgatgggctctggagcttccaccaccttagttttcttttccttctttgcctttggttt
tttcggtttttcttctaagggtttcttatcttcaggttcttccgtgaagcgagtctctgttacatttgtc
gtaacttccgtggtttgagaaacgatggacgaccatgatagtggttgcgatggggccacctcactaactg
aagtgggttccggttgtgtctcgggctttacttcttctacaactgtagtcacctcaaccactggttcctc
aacaaatgtcacttcaacagtgggttcaacaacggcctcaggaactgaagactcaacgatgggctctgga
gcttccaccaccttagttttcttttccttctttgcctttggtttttttcggtttttcttctaagggtttct

tatcttcaggttcttccgtgaagcgagtctctgttacatttgtcgtaacttccgtggtttgagaaacgat
agacgaccatgatagtggttgcgatggggccacctcacttactggagtgggttccggttgtgtctcgggc
gttacttcttctacaactgtagtcacctcaaccactggttcctcaacaaatgtcacttcaacagtgggtt
caacaacggcctcaggaactgcagactcaacgatgggttctggagcttccaccaccttagttttcttttc
cttctttgatttttgtttttttcggtttttcttctaagggtttcttatcttcaggctcttccgtgaagcga
gtctctgttacatttgtcgtaacttccgtggtttgagaaacgatagacgaccatgatagtggttgcgatg
gggccacctcacttactggagtgggttccggttgtgtctcgggcgttacttcttctacaactgtagtcac
ctcaaccactggttcctcaacaaatgtcacttcaacagttggttcaacaacggcctcaggaactgcagac
tcaacgatgggctctggagcttccaccaccttagttttcttttccttctttggtttttgtttttttcggtt
tttcttctaagggtttcttatcttcaggctcttccgtgaagcgagtctctgttacatttgtcgtaacttc
cgtggtttgagaaacgatagacgaccatgatagtggttgcgatggggccacctcacttactggagtgggt
tccggttgtgtctcgggcgttacttcttctacaactgtagtcacctcaaccactggttcctcaacaaatg
tcacttcaacagtgggttcaacaacggcctcaggaactgcagactcaacgatgggttctggagcttccac
caccttagttttcttttccttctttgatttttgtttttttcggtttttcttctaagggtttcttatcttca
ggctcttccgtgaagcgagtctctgttacatttgtcgtaatttccgtggtttgagaaacgatagacgacc
atgatagtggttgcgatggggccacctcacttactggagtgggttccggttgtgtctcgggcgttacttc
ttctacaactgtagtcacctcaaccactggttcctcaacaaatgtcacttcaacagttggttcaacaacg
gcctcaggaactgcagactcaacgatgggctctggagcttccaccaccttagttttcttttccttctttg
ctttttgtttttttcggtttttcttctaagggtttcttatcttcaggctcttccgtgaagcgagtctctgt
tacatttgtcgtaacttccgtggtttgagaaacgatagacgaccatgatagtggttgcgatggggccacc
tcacttactggagtgggttccggttgtgtctcgggcgttacttcttctacaactgtagtcacctcaacca
ctggttcctcaacaaatgtcacttcaacagttggttcaacaacggcctcaggaactgcagactcaacgat
gggctctggagcttccaccaccttagttttcttttccttctttggtttttgtttttttcggtttttcttct
aagggtttcttatcttcaggctcttccgtgaagcgagtctctgttacatttgtcgtaacttccgtggttt
gagaaacgatagacgaccatgatagtggttgcgatggggccacctcacttactggagtgggttccggttg
tgtctcgggcgttacttcttctacaactgtagtcacctcaaccactggttcctcaacaaatgtcacttca
acagtgggttcaacaacggcctcaggaactgcagactcaacgatgggctctggagcttccaccaccttag
ttttcttttccttctttgctttttgtttttttcggtttttcttccgagggtttcttatcttcaggttcttc
cgtgaagcgagtctctgtaacattggtcgtaacttccgtggtttgagaaacgatggacgaccatgatagt
ggttgcgaaggggccacctcactaactggagtgggttccggttgtgtctcgggcgttacttcttctacaa
ctgtagtcacctcaaccactggttcctcaacaaatgtcacttcaacagtgggttcaaccacggcctcagg
aactgcagactcaacgatgggctctggagcttccaccaccttagttttcttttccttctttggtttttgt
ttttttcggtttttcttctaagggtttcttatcttcaggctcttccgtgaagcgagtctctgttacatttg
tcgtaacttccgtggtttgagaaacgatagacgaccatgatagtggttgcgatggggccacctcacttac
tggagtgggttccggttgtgtctcgggcgttacttcttctacaactgtagtcacctcaaccactggttcc
tcaacaaatgtcacttcaacagtgggttcaacaacggcctcaggaactgcagactcaacgatgggttctg
gagcttccaccaccttagttttcttttccttctttgatttttgtttttttcggtttttcttctaagggttt
cttatcttcaggctcttccgtgaagcgagtctctgttacatttgtcgtaatttccgtggtttgagaaacg
atagacgaccatgatagtggttgcgatggggccacctcacttactggagtgggttccggttgtgtctcgg
gcgttacttcttctacaactgtagtcacctcaaccactggttcctcaacaaatgtcacttcaacagttgg
ttcaacaacggcctcaggaactgcagactcaacgatgggctctggagcttccaccaccttagttttcttt
tccttctttggtttttgtttttttcggtttttcttctaagggtttcttatcttcaggctcttccgtgaagc
gagtctctgttacatttgtcgtaacttccgtggtttgagaaacgatagacgaccatgatagtggttgcga
tggggccacctcacttactggagtgggttccggttgtgtctcgggcgttacttcttctacaactgtagtc

acctcaaccactggttcctcaacaaatgtcacttcaacagtgggttcaacaacggcctcaggaactgcag
actcaacgatgggttctggagcttccaccaccttagttttcttttccttctttgatttttgtttttttcgg
tttttcttctaagggtttcttatcttcaggttTCTTAGAATCGGCATTTACAGTCGTTGAAAAGATTTGT
TCTTTACTAATGAACTCCTGCACTTCTTTTGACCTATGAGGATCAACGGCTTTCTGGGATATTTTACTTT
CTATACTTGTTGGAGGCAGAACCTCGGTTTGTGAGACGATAGATGACCAAGACTTGGGTTGAATAGGTAA
ACTTTCCAAAACAGACGGAATTTCTGCATTCTGCTGTGTTTTAGGATCATCGCGCATGAAAGTGCTATCA
ACGTATGGTTCTAAAGCCACTTCAACTACTGATTCTATCTTATTGTGTTTCTTTTCCTTCTTTGATTTTT
GTTTCTTAGTCTTTTCAATCACTGTGGAGCTTCTTTCTTGGGAAATGAATTTGTTTATTTCTTGTGAATT
CTGGAATGCAGGTTCTAGAACTTTACCATCATGGTCGGCAGATTGTGTCATTTTGGTAATAGGTTCCTGG
GCACCTTGTCGGACAATTGAAGACCACAAAGTGCTATCGGCACTGAGTATGTTTGTTTGAACGTTATCAT
CAAAATCGTTCTCGACCATTGTTGACCACGACGTCTTTGTTTCATCCTTTGCTGATTCTCTTTCGATTGG
TAGAACTTCATCTTTAGGCGTCAAAAGCTTAGCTCCCTTTTCCTTCTTTAACGGTTTTTCTTTCTTAGCC
TTTGTCTTAATCAAAGGTTCTGGAAGTTTAGCAGGCTCTTTTACTGTTGGCACTTTACCTAAATTTGATT
CAGTTGGGACTTCAACATTATTCTTTTCTAAACTTATGAAGTCCACAGTATGACGCAAGACATCAGCATA
TGACGAATGCAGTTCCCATATATTAGAAGACAATGGTTTTGGCTCGACTTCTGtggattcttcaggtttc
ttcactggttctactgttttcttgggttgctcggatataactggttcactaatggtttcggtagttgttg
ttgtggtggtaactgttgtaatagtcttggaatcgtccacaacagaaacttcaacaattggcttttcctc
gactacttcttccacaagagtttcttccttcactggctcaggaactggttcctcttcgtcgtcagagaag
ttcataggttcgtccaagacatccatccacgagttcgtagagtcatcgacgggaagaccggcatatgctg
gctttttgggttcttcaggtttcttctccggttctactaccttcttgggttcctcggaaataggaagttc
actaatagtttcggtagttattgttgtggtggtaactgttgtaatagtcttggaatcgtccacaacagaa
acttcaacaattggctttatctcgactacttcttccacaacagtttcttccttcagtggctcaggaattg
gttcctcttcgtcgtcagagaagttcataggttcgtccaagacatccatccacgagttcgaagagtcatc
gacaggaagaccggcatatgctggctttttgggttcttcaggcttcttctccggttctactaccttcttg
ggttcctcgggaataggaagttcactaatggtttcggtagttgttgttgtggtggtaactgttgtaatgg
acttggaatcgtccacaacagaaacttcaacaattggcttttcctcgactacttctttcacaagagtttc
ttccttcactggctcaggaactggttcctcttcgtcgtcagagaagttcataggttcgtccaagacatcc
atcaaagagctagaagactcatcgacgggaagaccggcatatgctggctttttgggttcttcaggcttct
tctccggttctactaccttcttgggttcctcgggaataggaagttcactaatggtttcggtagtcgttgt
tgtggtggtaactgttgtaatggacttggaatcgtccacaaaagaaacttcaacaattggcttttcctcg
actacttcttccacaacagtttcttccttcagtggttcaggaactggttcctcttcgtcgtcagagaagt
tcataggttcgtccaagacatccatccacgagttcgtagagtcatcgacaggaagaccggcatatgctgg
ctttttgggttcttcaggtttcttctccggttcgacaaccttcttgggttcctcgggaataggaagttcg
ctaatggtttcggtagttgttgttgtggtggtaactgttgtaatggtcttggaatcgtccacaacagaaa
cttcaacaattggcttttcctcgactacttcttccacaacagtttcttccttcagtggttcaggaactgg
ttcctcttcgtcgtcagagaagttcataggttcgtccaagacatccatccacgagttcgtagagtcatcg
acgggaagaccggcatatgctggctttttgggttcttcaggtttcttctccggttcgacaaccttcgtgg
gttcctcgggaatagcaatttcactaatggtttcggtagttgttgttgtggtggtaactgttgtaatgga
cttggaatcgtccacaacagaaacttcaacaattggcttttcctcgactacttctttcacaagactttct
tccttctgtggctcaggaattggttcctcttcgtcgtcagagaagttcataggttcgtccaagacatcca
tccacgagttcgtagagtcatcgacaggaagaccggcatatgctggctttttgggttcttcaggtttctt
ctccagttcgacaaccttcttgggttcctcgggaataggaagttcactaatggtttcggtatttgttgtt
gttgtggtaactgttgtaatagtcttggaatcgtccacaacagaaacttcaacaattggcttttcctcga
ctacttcttgcacaacagtttcttcttttactggctcaggaactggttcctcttcgtcgtcagagaagtt

cataggttcgtccaagacatccatccacgagttcgtagagtcatcgacaggaagaccggcatatgctggc
tttttgggttcttcaggtttcttctccagttcgacaaccttcttgggttcctcgggaattggaagttcgc
taatggtttcggtagttgttgttgtggtggtaactgttgtaatggtcttggaatcgtccacaacagaaac
ttcaacaattggcttttcctcgactacttcttccacaagagtttcttccttcagtggctcaggcactggt
tcGGCAACTTTCTTGGGTTCCATTGGAGTAACAGATTCGCTGATAGACTCAGTTGATAGTGTTGTGGTGG
TTACTGTTGTAATTGTTTTGGATTCGTTATTTGGTTTTTCTTCGCTGGTTGATTGTATTTCCTGGATGGT
TTCAATAATCTTGTCAATGTTGGCTTTCTCTCCATTCTCATGAGGAAGAGGAATTTCTATTTCGTCTTCT
ATAAGTTCTTTCCACGATTTTCGTTCAACGACCACGTCATCCAACCCATCGTCAATGATTTCAGACCATG
GCATTAGGGGTGTACTTTCTACGTCCTTTTGAACGGGTTCTTGCAAAGGTTCCTCCAGTTTTGGTGCAAT
TTTTGGTTGGGTATTGCCACTCGACTTTTTCACAGACTTCTTAGGAACTGGTTTTATACTTTCAGCTTCA
ATAAAATCAGTGACTTGATGACTTACCACAGATGACCAGGAATGACCAGAATCTCCCTTTTGTTGACTTA
GATCTGATGATATAGCATGATCTTTTGGATGTTTCTCAATTCGATCGAATCTTATTTGCTCTTTTACCCT
TTCTCcttgttttggctttgtttcttgtttagcctttggtttcAAGTCAGTCATATGATCAATTTGACTG
GCTATTTTAGCCCAAGTAATTGTATTTTCATTTGTAGGCTCTAATACCTCAACCACATCACTTTCCTCTG
AAGTCGAATGAACAACTACGGGCTCACGAGTTTCCTTTTTGTTATTACGCTTGGTGGTGTTTTCAGACTG
AGGTTTGGGCTGAGGTTTTGAAATTTTACGTTCTTTCCGTGGATGCACCTCCTTAGGCTCCTCGTGAATG
ATTTCAACTGGCTTTTTACGAGAAGCAATGTCATCAACCCATTCGCCGGGCTTCTTTACCAGGGACGCCC
AAGAGAAGGCGTTTTCTGTATTTTTGATAGATTTAGTTTCTTTCTCTTCCACCTCACACTCCACAACTAT
TTCCTCAGAGGGTTCACTGCTTACTTCATCTTCGGGCAGAGCAACGGGAGTAGGTTTTTTGGTTGCCTTC
TGTTTCTTGGGCTTTTGTGGCTTTTTGTCCTTTTTGATTGGTTCTTTTACATCAGGCAATGGGGTGACAG
CGGTTTTAATGTGAGTAACAGTGTTGTCAATCCATTCGCCTGGTCTCTTTACAAGTGAGGCCCAGGAGAA
TGCGTTGCTACTCTCTAGCTTCTGAACAACTTCAGGCTCCGGTTTTGACTCTATTGGTGCAGGCATTGCC
TCCACTGGTTCGGGCACAGTCTCGGGCTGTTCGTCCTCAGATGTAGTCGAGACGGGATCTACAACGGGTT
TCTTGGACTTCTTTGTCTTCTTCGCCTTTGTGGTCTTTTTGGGCTCCTCCTTTTCTTTCTGGCGATGTGT
TTCTTTAGTTGTGACAGCCGTATGAGAAGTCTGCTTTTTATTTACGGTTGAATCTGACCACTCGCCGGGT
CTTTGCACAAGGGCTGACCATGTCAACTCGGATGTGCTGGAAACATCCCTTTCATCAGAAGACGGCTCTT
TGGAactttctgtcgttggtttggataattcactttctgtcgtaggcttagattgctcactGTCGCTGTT
TGGCTTCGATTTTTCGCTTTCGATGCTTGCTGAGGACAGTTCAATAACCTTTTCTGCCTGCCGCTTCTCT
GGCTTCGATTTCTTCTTTGGCTCTTGGGCTTTAGTTTTGACTATGTCTGTTTTGTGAGGGATTTCCTTAT
GTAACTCAGTAGATGGGACGTCGTCACCTGTAACTCCAGAGTTGAGTAGCACGTCAGCATACGATTTACC
ATCCCGGTTCATGTTCCAAACATTCTCCACTTCTTCGACAATTTGTACCGGTTCTACAATATTTATTTCT
TGAGCAGGTTGGACATCAATAATTTTTTCTCGGATTTCCACCTTCTTGTTCTGTCGCTTTGATGGCTTTG
CCTTTTTAGGCTTTTGGTGTGGCCCAATTGTTTGTTCTGGCCTATTCCTGGGAATAAATTCGTTGTATAT
CCTTTCATTGGCAAGGAATGTATGCGTTTGATCGGAGAAGGTATGTCTCAAATAAGATGGAGTAAACTCA
GGTGCGTCCGCCGATAAAGTTGAGGTATAACCAATGTTTCCAGTATTAGAAATGGTGTCCTCCAAAACGC
TGGACCATGTATCAATGGGTCCCTCAAGAGGCTTCTCCAATTGACTGACATCATCTACCACATTACTAAT
AATTTGCGGTTCAGCTGGCGCGGGAAGAGAGTTCTCCTCTTCCAAAGTATTAGTTTGATTGGTAGATGTA
ACAATTGTGGTCGTAACAGTTGTTATTGTTTTGCTTTCATCCACAACAGAAGATTCAACAATTGGCTCGA
CTACTTCTTCCACAAGAGTTTCTTCTTTCAGTGGCTCAGGAACTGGTTCCTCTTCGTCGTCAGAGAAGTT
CATAGG
>dme_circ_0001459_junction_seq
CCTCTTCGTCGTCAGAGAAGTTCATAGGgttcgtccaagacatccatccacgagtt
>dme_circ_0001460
gttcgtccaagacatccatccacgagttcgaagagtcatcgacaggaagaccggcatatgctggcttttt

gggttcttcaggtttcttctccggttctactaccttcttgggttcctcgggaataggaagttcactaatg
gtttcggtagttgttgttgtggtggtaactgttgtaatggacttggaatcgtccacaacagaaacttcaa
caattggcttttcctcgactacttcttccacaagagtttcttccttcactggctcaggaactggttcctc
ttcgtcgtcagagaagttcataggttcgtccaagacatccatccacgagttcgaagagtcatcgacagga
agaccggcatatgctggctttttgggttcttcaggtttcttctccggttctactaccttcttgggttcct
cggaaataggaagtttgctaatggtttcggtagttgttgttgtggtggtaactgttgtaatggacttgga
atcgtccacaacagaaacttcaacaattggcttttcctcgactacttcttccacaagagtttcttccttc
actggctcaggaactggttcctcttcgtcgtcagagaagttcataggttcgtccaagacatccatccacg
agctagaagactcatcgacgggaagaccggcatatgctggctttttgggttcttcaggtttcttctccgg
ttctactaccttcttgggttcctcgggaataggaagttcactaatggtttcggtagttgttgttgtggtg
gtaactgttgtaatggacttggaatcgtccacaacagaaacttcaacaattggcttttcctcgactactt
cttccacaagagtttcttccttcagtggttcaggaactggttcctcttcgtcgtcagagaagttcatagg
ttcgtccaagacatccatccacgagttcgaagagtcatcgacaggaagaccggcatatgctggctttttg
ggttcttcaggtttcttctctggttcgacaaccttcttgggttcctcgggaatagaaacattgctagtgg
tttcggtagttgttgttgttgtggttactgttgtagtagtcttagattcgtctaccgccgtagattcaac
aattggctCCGTCTTCAAAACTTTTTCGACAGGGAGGTCTTCCATATGTGACTCAGATACTGGTTCAACT
TCAGACTCGGCAACAAATTCAAAGGGAGCAAGTGGAGTTTTTATTTCCCATGATGTTAAAGTATTGTTGA
CGTCTTCAACTATTATAGATCCAGGTTCTTCAACTATTTCAGGTTTGGAAGCAATCACTTGATGTATCGA
TTTTTCTGCATTAATGAAATCAACCACATCGGTTGGCAACTTTCGCTTAGACTTTTGCTTTGGCTTTTTA
GTCTTTGGCTCAGTAGTTTCGAAAGTGATTGGAATGTTTACTGTGTGCTCATTTGTTGATTTCCTTACAT
CTTCAACGACAGTTGGTTTGGCCTCAGCTTGGAGGGGTCGAATATGTTGAACTGGTTCTGGCACATTATG
GGCGGCTATAGCAGCCCATGACATACCTGAGGTATTAGGAGAAGGAGCTGGTGGTGGAGACGGTTCTTCT
ACAGATGGTACAGATTCCTTAGGAGGCTGGACATCCTCAGGAGCTTCCGTAGGCTGTTGAGCTGGTGCTT
TAGGTACCGTAtttttggcttctttgatttttttcttttgatgcttttcATCAATGTTCGAAGCCTGTGA
CTGTTGCGAACGTGAGCGTGAGCGATTTCGTTTCTTTGAAGTAATTTCCAAGAATTCTTGGTCAGTCTCG
GATTGCGATTGAGTTTCAGTAACTACCTCGGTTATTTGAATTTCCGGCTTTACGCTCACTCGGGAATGTC
TTTCCTGTATTGGTTTTTGTGTCTTTATCTCTACAGAATCTGTTTCTCCCTTATGCCACGACTCCAGCGG
ATCCACATCATCCTCATCTACAAGGTCCTTCCATGATAGCTGAGAGGGTTTCTCAAGTGCTTCATCTCTT
TCAATTGCAGGAATGATAGGGTCCGGGAACTCCAGAAGATCTTCATGTGTCTGCACTTCGACAAATGTGT
GTTTTGGTTCACTCTTGATTGGTTCCCTTGCCTCAATTTTCTGATATTTAATGTTCGTATTGTTGTTGTA
ACCAGACTTCTTAACAACTTCGGCATAGGTGTCTGATGATGACCACACTGATGGTTTTGTTGGTGCCGGC
AACTGAATGCTGAATTCAGGCGTGGAAGACACAGCTACGCGTTCAGTTTCTGGAATCTCTTCGTAGACAA
CAGAAgtttcaatattttgatctcgtttttttgtccttttttgggttttcgtttcctttcaTCAACATGAAT
GGGAAGCTCACCTTCTTGATTACTACTGCTGTCATCGGGATTGATCTCATCGACGGCTTCAATGACACGG
TTTTCAGTAAACGTTGGCGTCAAATCGATGTTATGCGAAACGATTGACGACCAAGACAAAGTTTGTGGCT
CAGGAACTGCTTTAGTTACTCCTTCTCGCATGTGATCTTTTCGGACAGGAACGCTTTCACCGGTTGGTTG
TTGAATAGGCTGAACTATTTTCTTTTCCTTTTTAGGTTTCGGCTTTTTGACTGAATCATTCACAACCGTA
GAGTAATTTTGCTCCTGTGCGATAAAGTTTTGAATTCCGCCCGTTTTTGATTCTACATGTTTTGGAATTG
TTCGTGGAACCTCAACAGACTGAGCAACAATAGACGACCATGAAAACGTATGCGATTCCGTTTTTGGTGG
TACATCCTTTTGAACAGTGGTAAATCCAATAATGGGTTCAACAACGGACAATGGAACATCAGACTCCAAA
ATTGACTCTTCAACATTCTGAACCTTGGATTTCTTTTCCTTCCCTGCCTTAAGTTTTTTCGGTTTTTCCA
TTAAAGGTTTCTGATCTTCAGCTACATCAGTGATAAGAGCCTCTGCTACATcttcagtagtttgagagac
aatagacgaccataatagtggttgcgatggggccacctcactaactgaagtgggttccggttgtgtctcg
ggcgttacttcttctacgactgtagtcacctcaaccactggttcctcaacaaaagtcacttcaacagtgg

gttcaacaacgtcctcaggaactgcagactcaacgatgggctctggagcttccaccaccttagttttctt
ttccttctttggttttgtttttcggtttttcttctaagggtttcttatcttcaggttcttccgtgaag
cgagtctctgttacatttgtcgttacttccgtggtttgagaaacgatggacgaccatgatagtggttgcg
atggggccacctcacttactggagtgggttccggttgtgtctcgggctttacttcttgtacaactgtagt
cacctcaaccactggttcctcaacaaaagtcacttcaacagttggttcaacaacgtcctcaggaactgca
gactcaacgatgggctctggagcttccaccaccttagttttcttttccttctttggttttgtttttcg
gtttttcttctaagggtttcttatcttcaggttcttccgtgaagcgagtctctgttacatttgtcgttac
ttccgtggtttgagaaacgatggacgaccatgatagtggttgcgatggggccacctcacttactggagtg
ggttccggttgtgtctcgggctttacttcttctacaactgtagtcacctcaaccactggttcctcaacaa
aagtcacttcaacagttggttcaacaacgtcctcaggaactgcagactcaacgatgggctctggagcttc
caccaccttagttttcttttccttctttggttttgtttttcggtttttcttctaagggtttcttatct
tcaggttcttccgtgaagcgagtctctgttacatttgtcgttacttccgtggtttgagaaacgatggacg
accatgatagtggttgcgatggggccacctcacttactggagtgggttccggttgtgtctcgggctttac
ttcttctacaactgtagtcacctcaaccactggttcctcaacaaaagtcacttcaacagttggttcaaca
acgtcctcaggaactgcagactcaacgatgggctctggagcttccaccaccttagttttcttttccttct
ttggttttgtttttcggtttttcttctaagggtttcttatcttcaggttcttccgtgaagcgagtctc
tgttacatttgtcgttacttccgtggtttgagaaacgatggacgaccatgatagtggttgcgatggggcc
acctcacttactggagtgggttccggttgtgtctcgggctttacttcttctacaactgtagtcacctcaa
ccactggttcctcaacaaaagtcacttcaacagttggttcaacaacgtcctcaggaactgcagactcaac
gatgggctctggagcttccaccaccttagttttcttttccttctttggttttgtttttcggtttttct
tctaagggtttcttatcttcaggttcttccgtgaagcgagtctctgttacatttgtcgttacttccgtgg
tttgagaaacgatggacgaccatgatagtggttgcgatggggccacctcacttactggagtgggttccgg
ttgtgtctcgggctttacttcttctacgactgtagtcacctcaaccactggttcctcaacaaaagtcact
tcaacagtgggttcaacaacgtcctcaggaactgcagactcaacgatgggctctggagcttccaccacct
tagttttcttttccttctttggttttgtttttcggtttttcttctaagggtttcttatcttcaggttc
ttccgtgaagcgagtctctgttacatttgtcgttacttccgtggtttgagaaacgatggacgaccatgat
agtggttgcgatggggccacctcacttactggagttggttccggttgtgtctcgggggttacttcttcta
caactgtagtcacctcaaccactggttcctcaacaaaagtcacttcaacagtgggttcaaccacggcctc
aggaactgaagactcaacgatgggctctggagcttccaccaccttagttttcttttccttctttgccttt
ggtttttcggtttttcttctaagggtttcttatcttcaggttcttccgtgaagcgagtctctgttacat
ttgtcgtaacttccgtggtttgagaaacgatggacgaccatgatagtggttgcgatggggccacctcact
tactggagtgggttcctgttgtgtctcgggcgttacttcttctacgactgtagtcacctcaaccactggt
tcctcaacaaaagtcacttcaacagtgggttcaacaacgtcctcaggaactgcagactcaacgatgggct
ctggagcttccaccaccttagttttcttttccttctttgcctttggtttttcggtttttcttctaaggg
tttcttatcttcgggttcttccgtgaagcgagtctctgttacatttgtcgttacttccgtggcttgagaa
acgatggacgaccatgatagtggttgcgatggggccacctcacttactggagttggttccggttgtgtct
cgggggttacttcttctacaactgtagtcacctcaaccactggttcctcaacaaaagtcacttcaacagt
gggttcaaccacggcctcaggaactgaagactcaacgatgggctctggagcttccaccaccttagttttc
ttttccttctttgcctttggtttttcggtttttcttctaagggtttcttatcttcaggttcttccgtga
agcgagtctctgttacatttgtcgtaacttccgtggtttgagaaacgatggacgaccatgatagtggttg
cgatggggccacctcactaactgaagtgggttccggttgtgtctcgggctttacttcttctacaactgta
gtcacctcaaccactggttcctcaacaaatgtcacttcaacagtgggttcaacaacggcctcaggaactg
aagactcaacgatgggctctggagcttccaccaccttagttttcttttccttctttgcctttggtttttt
cggtttttcttctaagggtttcttatcttcaggttcttccgtgaagcgagtctctgttacatttgtcgta

acttccgtggtttgagaaacgatagacgaccatgatagtggttgcgatggggccacctcacttactggag
tgggttccggttgtgtctcgggcgttacttcttctacaactgtagtcacctcaaccactggttcctcaac
aaatgtcacttcaacagtgggttcaacaacggcctcaggaactgcagactcaacgatgggttctggagct
tccaccaccttagttttcttttccttctttgatttttgtttttttcggtttttcttctaagggtttcttat
cttcaggctcttccgtgaagcgagtctctgttacatttgtcgtaacttccgtggtttgagaaacgataga
cgaccatgatagtggttgcgatggggccacctcacttactggagtgggttccggttgtgtctcgggcgtt
acttcttctacaactgtagtcacctcaaccactggttcctcaacaaatgtcacttcaacagttggttcaa
caacggcctcaggaactgcagactcaacgatgggctctggagcttccaccaccttagttttcttttcctt
ctttggtttttgtttttttcggtttttcttctaagggtttcttatcttcaggctcttccgtgaagcgagtc
tctgttacatttgtcgtaacttccgtggtttgagaaacgatagacgaccatgatagtggttgcgatgggg
ccacctcacttactggagtgggttccggttgtgtctcgggcgttacttcttctacaactgtagtcacctc
aaccactggttcctcaacaaatgtcacttcaacagtgggttcaacaacggcctcaggaactgcagactca
acgatgggttctggagcttccaccaccttagttttcttttccttctttgatttttgtttttttcggttttt
cttctaagggtttcttatcttcaggctcttccgtgaagcgagtctctgttacatttgtcgtaatttccgt
ggtttgagaaacgatagacgaccatgatagtggttgcgatggggccacctcacttactggagtgggttcc
ggttgtgtctcgggcgttacttcttctacaactgtagtcacctcaaccactggttcctcaacaaatgtca
cttcaacagttggttcaacaacggcctcaggaactgcagactcaacgatgggctctggagcttccaccac
cttagttttcttttccttctttgctttttgtttttttcggtttttcttctaagggtttcttatcttcaggc
tcttccgtgaagcgagtctctgttacatttgtcgtaacttccgtggtttgagaaacgatagacgaccatg
atagtggttgcgatggggccacctcacttactggagtgggttccggttgtgtctcgggcgttacttcttc
tacaactgtagtcacctcaaccactggttcctcaacaaatgtcacttcaacagttggttcaacaacggcc
tcaggaactgcagactcaacgatgggctctggagcttccaccaccttagttttcttttccttctttggtt
tttgtttttttcggtttttcttctaagggtttcttatcttcaggctcttccgtgaagcgagtctctgttac
atttgtcgtaacttccgtggtttgagaaacgatagacgaccatgatagtggttgcgatggggccacctca
cttactggagtgggttccggttgtgtctcgggcgttacttcttctacaactgtagtcacctcaaccactg
gttcctcaacaaatgtcacttcaacagtgggttcaacaacggcctcaggaactgcagactcaacgatggg
ctctggagcttccaccaccttagttttcttttccttctttgctttttgtttttttcggtttttcttccgag
ggtttcttatcttcaggttcttccgtgaagcgagtctctgtaacattggtcgtaacttccgtggtttgag
aaacgatggacgaccatgatagtggttgcgaaggggccacctcactaactggagtgggttccggttgtgt
ctcgggcgttacttcttctacaactgtagtcacctcaaccactggttcctcaacaaatgtcacttcaaca
gtgggttcaaccacggcctcaggaactgcagactcaacgatgggctctggagcttccaccaccttagttt
tcttttccttctttggtttttgtttttttcggtttttcttctaagggtttcttatcttcaggctcttccgt
gaagcgagtctctgttacatttgtcgtaacttccgtggtttgagaaacgatagacgaccatgatagtggt
tgcgatggggccacctcacttactggagtgggttccggttgtgtctcgggcgttacttcttctacaactg
tagtcacctcaaccactggttcctcaacaaatgtcacttcaacagtgggttcaacaacggcctcaggaac
tgcagactcaacgatgggttctggagcttccaccaccttagttttcttttccttctttgatttttgtttt
ttcggtttttcttctaagggtttcttatcttcaggctcttccgtgaagcgagtctctgttacatttgtcg
taatttccgtggtttgagaaacgatagacgaccatgatagtggttgcgatggggccacctcacttactgg
agtgggttccggttgtgtctcgggcgttacttcttctacaactgtagtcacctcaaccactggttcctca
acaaatgtcacttcaacagttggttcaacaacggcctcaggaactgcagactcaacgatgggctctggag
cttccaccaccttagttttcttttccttctttggtttttgtttttttcggtttttcttctaagggtttctt
atcttcaggctcttccgtgaagcgagtctctgttacatttgtcgtaacttccgtggtttgagaaacgata
gacgaccatgatagtggttgcgatggggccacctcacttactggagtgggttccggttgtgtctcgggcg
ttacttcttctacaactgtagtcacctcaaccactggttcctcaacaaatgtcacttcaacagtgggttc

aacaacggcctcaggaactgcagactcaacgatgggttctggagcttccaccaccttagttttcttttcc
ttctttgatttttgtttttcggtttttcttctaagggtttcttatcttcaggttTCTTAGAATCGGCAT
TTACAGTCGTTGAAAAGATTTGTTCTTTACTAATGAACTCCTGCACTTCTTTTGACCTATGAGGATCAAC
GGCTTTCTGGGATATTTTACTTTCTATACTTGTTGGAGGCAGAACCTCGGTTTGTGAGACGATAGATGAC
CAAGACTTGGGTTGAATAGGTAAACTTTCCAAAACAGACGGAATTTCTGCATTCTGCTGTGTTTTAGGAT
CATCGCGCATGAAAGTGCTATCAACGTATGGTTCTAAAGCCACTTCAACTACTGATTCTATCTTATTGTG
TTTCTTTTCCTTCTTTGATTTTTGTTTCTTAGTCTTTTCAATCACTGTGGAGCTTCTTTCTTGGGAAATG
AATTTGTTTATTTCTTGTGAATTCTGGAATGCAGGTTCTAGAACTTTACCATCATGGTCGGCAGATTGTG
TCATTTTGGTAATAGGTTCCTGGGCACCTTGTCGGACAATTGAAGACCACAAAGTGCTATCGGCACTGAG
TATGTTTGTTTGAACGTTATCATCAAAATCGTTCTCGACCATTGTTGACCACGACGTCTTTGTTTCATCC
TTTGCTGATTCTCTTTCGATTGGTAGAACTTCATCTTTAGGCGTCAAAAGCTTAGCTCCCTTTTCCTTCT
TTAACGGTTTTTCTTTCTTAGCCTTTGTCTTAATCAAAGGTTCTGGAAGTTTAGCAGGCTCTTTTACTGT
TGGCACTTTACCTAAATTTGATTCAGTTGGGACTTCAACATTATTCTTTTCTAAACTTATGAAGTCCACA
GTATGACGCAAGACATCAGCATATGACGAATGCAGTTCCCATATATTAGAAGACAATGGTTTTGGCTCGA
CTTCTGtggattcttcaggtttcttcactggttctactgttttcttgggttgctcggatataactggttc
actaatggtttcggtagttgttgttgtggtggtaactgttgtaatagtcttggaatcgtccacaacagaa
acttcaacaattggcttttcctcgactacttcttccacaagagtttcttccttcactggctcaggaactg
gttcctcttcgtcgtcagagaagttcataggttcgtccaagacatccatccacgagttcgtagagtcatc
gacgggaagaccggcatatgctggctttttgggttcttcaggtttcttctccggttctactaccttcttg
ggttcctcggaaataggaagttcactaatagtttcggtagttattgttgtggtggtaactgttgtaatag
tcttggaatcgtccacaacagaaacttcaacaattggctttatctcgactacttcttccacaacagtttc
ttccttcagtggctcaggaattggttcctcttcgtcgtcagagaagttcataggttcgtccaagacatcc
atccacgagttcgaagagtcatcgacaggaagaccggcatatgctggctttttgggttcttcaggcttct
tctccggttctactaccttcttgggttcctcgggaataggaagttcactaatggtttcggtagttgttgt
tgtggtggtaactgttgtaatggacttggaatcgtccacaacagaaacttcaacaattggcttttcctcg
actacttctttcacaagagtttcttccttcactggctcaggaactggttcctcttcgtcgtcagagaagt
tcataggttcgtccaagacatccatcaaagagctagaagactcatcgacgggaagaccggcatatgctgg
ctttttgggttcttcaggcttcttctccggttctactaccttcttgggttcctcgggaataggaagttca
ctaatggtttcggtagtcgttgttgtggtggtaactgttgtaatggacttggaatcgtccacaaaagaaa
cttcaacaattggcttttcctcgactacttcttccacaacagtttcttccttcagtggttcaggaactgg
ttcctcttcgtcgtcagagaagttcataggttcgtccaagacatccatccacgagttcgtagagtcatcg
acaggaagaccggcatatgctggctttttgggttcttcaggtttcttctccggttcgacaaccttcttgg
gttcctcgggaataggaagttcgctaatggtttcggtagttgttgttgtggtggtaactgttgtaatggt
cttggaatcgtccacaacagaaacttcaacaattggcttttcctcgactacttcttccacaacagtttct
tccttcagtggttcaggaactggttcctcttcgtcgtcagagaagttcataggttcgtccaagacatcca
tccacgagttcgtagagtcatcgacgggaagaccggcatatgctggctttttgggttcttcaggtttctt
ctccggttcgacaaccttcgtgggttcctcgggaatagcaatttcactaatggtttcggtagttgttgtt
gtggtggtaactgttgtaatggacttggaatcgtccacaacagaaacttcaacaattggcttttcctcga
ctacttctttcacaagactttcttccttctgtggctcaggaattggttcctcttcgtcgtcagagaagtt
cataggttcgtccaagacatccatccacgagttcgtagagtcatcgacaggaagaccggcatatgctggc
tttttgggttcttcaggtttcttctccagttcgacaaccttcttgggttcctcgggaataggaagttcac
taatggtttcggtatttgttgttgttgtggtaactgttgtaatagtcttggaatcgtccacaacagaaac
ttcaacaattggcttttcctcgactacttcttgcacaacagtttcttcttttactggctcaggaactggt
tcctcttcgtcgtcagagaagttcataggttcgtccaagacatccatccacgagttcgtagagtcatcga

caggaagaccggcatatgctggctttttgggttcttcaggtttcttctccagttcgacaaccttcttggg
ttcctcgggaattggaagttcgctaatggtttcggtagttgttgttgtggtggtaactgttgtaatggtc
ttggaatcgtccacaacagaaacttcaacaattggcttttcctcgactacttcttccacaagagtttctt
ccttcagtggctcaggcactggttcGGCAACTTTCTTGGGTTCCATTGGAGTAACAGATTCGCTGATAGA
CTCAGTTGATAGTGTTGTGGTGGTTACTGTTGTAATTGTTTTGGATTCGTTATTTGGTTTTTCTTCGCTG
GTTGATTGTATTTCCTGGATGGTTTCAATAATCTTGTCAATGTTGGCTTTCTCTCCATTCTCATGAGGAA
GAGGAATTTCTATTTCGTCTTCTATAAGTTCTTTCCACGATTTTCGTTCAACGACCACGTCATCCAACCC
ATCGTCAATGATTTCAGACCATGGCATTAGGGGTGTACTTTCTACGTCCTTTTGAACGGGTTCTTGCAAA
GGTTCCTCCAGTTTTGGTGCAATTTTTGGTTGGGTATTGCCACTCGACTTTTTCACAGACTTCTTAGGAA
CTGGTTTTATACTTTCAGCTTCAATAAAATCAGTGACTTGATGACTTACCACAGATGACCAGGAATGACC
AGAATCTCCCTTTTGTTGACTTAGATCTGATGATATAGCATGATCTTTTGGATGTTTCTCAATTCGATCG
AATCTTATTTGCTCTTTTACCCTTTCTCcttgttttggctttgtttcttgtttagcctttggtttcAAGT
CAGTCATATGATCAATTTGACTGGCTATTTTAGCCCAAGTAATTGTATTTTCATTTGTAGGCTCTAATAC
CTCAACCACATCACTTTCCTCTGAAGTCGAATGAACAACTACGGGCTCACGAGTTTCCTTTTTGTTATTA
CGCTTGGTGGTGTTTTCAGACTGAGGTTTGGGCTGAGGTTTTGAAATTTTACGTTCTTTCCGTGGATGCA
CCTCCTTAGGCTCCTCGTGAATGATTTCAACTGGCTTTTTACGAGAAGCAATGTCATCAACCCATTCGCC
GGGCTTCTTTACCAGGGACGCCCAAGAGAAGGCGTTTTCTGTATTTTTGATAGATTTAGTTTCTTTCTCT
TCCACCTCACACTCCACAACTATTTCCTCAGAGGGTTCACTGCTTACTTCATCTTCGGGCAGAGCAACGG
GAGTAGGTTTTTTGGTTGCCTTCTGTTTCTTGGGCTTTTGTGGCTTTTTGTCCTTTTTGATTGGTTCTTT
TACATCAGGCAATGGGGTGACAGCGGTTTTAATGTGAGTAACAGTGTTGTCAATCCATTCGCCTGGTCTC
TTTACAAGTGAGGCCCAGGAGAATGCGTTGCTACTCTCTAGCTTCTGAACAACTTCAGGCTCCGGTTTTG
ACTCTATTGGTGCAGGCATTGCCTCCACTGGTTCGGGCACAGTCTCGGGCTGTTCGTCCTCAGATGTAGT
CGAGACGGGATCTACAACGGGTTTCTTGGACTTCTTTGTCTTCTTCGCCTTTGTGGTCTTTTTGGGCTCC
TCCTTTTCTTTCTGGCGATGTGTTTCTTTAGTTGTGACAGCCGTATGAGAAGTCTGCTTTTTATTTACGG
TTGAATCTGACCACTCGCCGGGTCTTTGCACAAGGGCTGACCATGTCAACTCGGATGTGCTGGAAACATC
CCTTTCATCAGAAGACGGCTCTTTGGAactttctgtcgttggtttggataattcactttctgtcgtaggc
ttagattgctcactGTCGCTGTTTGGCTTCGATTTTTCGCTTTCGATGCTTGCTGAGGACAGTTCAATAA
CCTTTTCTGCCTGCCGCTTCTCTGGCTTCGATTTCTTCTTTGGCTCTTGGGCTTTAGTTTTGACTATGTC
TGTTTTGTGAGGGATTTCCTTATGTAACTCAGTAGATGGGACGTCGTCACCTGTAACTCCAGAGTTGAGT
AGCACGTCAGCATACGATTTACCATCCCGGTTCATGTTCCAAACATTCTCCACTTCTTCGACAATTTGTA
CCGGTTCTACAATATTTATTTCTTGAGCAGGTTGGACATCAATAATTTTTTCTCGGATTTCCACCTTCTT
GTTCTGTCGCTTTGATGGCTTTGCCTTTTTAGGCTTTTGGTGTGGCCCAATTGTTTGTTCTGGCCTATTC
CTGGGAATAAATTCGTTGTATATCCTTTCATTGGCAAGGAATGTATGCGTTTGATCGGAGAAGGTATGTC
TCAAATAAGATGGAGTAAACTCAGGTGCGTCCGCCGATAAAGTTGAGGTATAACCAATGTTTCCAGTATT
AGAAATGGTGTCCTCCAAAACGCTGGACCATGTATCAATGGGTCCCTCAAGAGGCTTCTCCAATTGACTG
ACATCATCTACCACATTACTAATAATTTGCGGTTCAGCTGGCGCGGGAAGAGAGTTCTCCTCTTCCAAAG
TATTAGTTTGATTGGTAGATGTAACAATTGTGGTCGTAACAGTTGTTATTGTTTTGCTTTCATCCACAAC
AGAAGATTCAACAATTGGCTCGACTACTTCTTCCACAAGAGTTTCTTCTTTCAGTGGCTCAGGAACTGGT
TCCTCTTCGTCGTCAGAGAAGTTCATAGG
>dme_circ_0001460_junction_seq
CCTCTTCGTCGTCAGAGAAGTTCATAGGgttcgtccaagacatccatccacgagtt
>dme_circ_0001461
GAATGACCAGAATCTCCCTTTTGTTGACTTAGATCTGATGATATAGCATGATCTTTTGGATGTTTCTCAA
TTCGATCGAATCTTATTTGCTCTTTTACCCTTTCTCcttgttttggctttgtttcttgtttagcctttgg

tttcAAGTCAGTCATATGATCAATTTGACTGGCTATTTTAGCCCAAGTAATTGTATTTTCATTTGTAGGC
TCTAATACCTCAACCACATCACTTTCCTCTGAAGTCGAATGAACAACTACGGGCTCACGAGTTTCCTTTT
TGTTATTACGCTTGGTGGTGTTTTCAGACTGAGGTTTGGGCTGAGGTTTTGAAATTTTACGTTCTTTCCG
TGGATGCACCTCCTTAGGCTCCTCGTGAATGATTTCAACTGGCTTTTTACGAGAAGCAATGTCATCAACC
CATTCGCCGGGCTTCTTTACCAGGGACGCCCAAGAGAAGGCGTTTTCTGTATTTTTGATAGATTTAGTTT
CTTTCTCTTCCACCTCACACTCCACAACTATTTCCTCAGAGGGTTCACTGCTTACTTCATCTTCGGGCAG
AGCAACGGGAGTAGGTTTTTTGGTTGCCTTCTGTTTCTTGGGCTTTTGTGGCTTTTTGTCCTTTTTGATT
GGTTCTTTTACATCAGGCAATGGGGTGACAGCGGTTTTAATGTGAGTAACAGTGTTGTCAATCCATTCGC
CTGGTCTCTTTACAAGTGAGGCCCAGGAGAATGCGTTGCTACTCTCTAGCTTCTGAACAACTTCAGGCTC
CGGTTTTGACTCTATTGGTGCAGGCATTGCCTCCACTGGTTCGGGCACAGTCTCGGGCTGTTCGTCCTCA
GATGTAGTCGAGACGGGATCTACAACGGGTTTCTTGGACTTCTTTGTCTTCTTCGCCTTTGTGGTCTTTT
TGGGCTCCTCCTTTTCTTTCTGGCGATGTGTTTCTTTAGTTGTGACAGCCGTATGAGAAGTCTGCTTTTT
ATTTACGGTTGAATCTGACCACTCGCCGGGTCTTTGCACAAGGGCTGACCATGTCAACTCGGATGTGCTG
GAAACATCCCTTTCATCAGAAGACGGCTCTTTGGAactttctgtcgttggtttggataattcactttctg
tcgtaggcttagattgctcactGTCGCTGTTTGGCTTCGATTTTTCGCTTTCGATGCTTGCTGAGGACAG
TTCAATAACCTTTTCTGCCTGCCGCTTCTCTGGCTTCGATTTCTTCTTTGGCTCTTGGGCTTTAGTTTTG
ACTATGTCTGTTTTGTGAGGGATTTCCTTATGTAACTCAGTAGATGGGACGTCGTCACCTGTAACTCCAG
AGTTGAGTAGCACGTCAGCATACGATTTACCATCCCGGTTCATGTTCCAAACATTCTCCACTTCTTCGAC
AATTTGTACCGGTTCTACAATATTTATTTCTTGAGCAGGTTGGACATCAATAATTTTTTCTCGGATTTCC
ACCTTCTTGTTCTGTCGCTTTGATGGCTTTGCCTTTTTAGGCTTTTGGTGTGGCCCAATTGTTTGTTCTG
GCCTATTCCTGGGAATAAATTCGTTG
>dme_circ_0001461_junction_seq
TGGCCTATTCCTGGGAATAAATTCGTTGGAATGACCAGAATCTCCCTTTTGTTGAC
>dme_circ_0001462
CCGTATGAGAAGTCTGCTTTTTATTTACGGTTGAATCTGACCACTCGCCGGGTCTTTGCACAAGGGCTGA
CCATGTCAACTCGGATGTGCTGGAAACATCCCTTTCATCAGAAGACGGCTCTTTGGAactttctgtcgtt
ggtttggataattcactttctgtcgtaggcttagattgctcactGTCGCTGTTTGGCTTCGATTTTTCGC
TTTCGATGCTTGCTGAGGACAGTTCAATAACCTTTTCTGCCTGCCGCTTCTCTGGCTTCGATTTCTTCTT
TGGCTCTTGGGCTTTAGTTTTGACTATGTCTGTTTTGTGAGGGATTTCCTTATGTAACTCAGTAGATGGG
ACGTCGTCACCTGTAACTCCAGAGTTGAGTAGCACGTCAGCATACGATTTACCATCCCGGTTCATGTTCC
AAACATTCTCCACTTCTTCGACAATTTGTACCGGTTCTACAATATTTATTTCTTGAGCAGG
>dme_circ_0001462_junction_seq
GTTCTACAATATTTATTTCTTGAGCAGGCCGTATGAGAAGTCTGCTTTTTATTTAC
>dme_circ_0001463
GATTCAACCGTAAATAAAAAGCAGACTTCTCATACGGCTGTCACAACTAAAGAAACACATCGCCAGAAAG
AAAAGGAGGAGCCCAAAAAGACCACAAAGGCGAAGAAGACAAAGAAGTCCAAGAAACCCGTTGTAGATCC
CGTCTCGACTACATCTGAGGACGAACAGCCCGAGACTGTGCCCGAACCAGTGGAGGCAATGCCTGCACCA
ATAGAGTCAAAACCGGAGCCTGAAGTTGTTCAGAAGCTAGAGAGTAGCAACGCATTCTCCTGGGCCTCAC
TTGTAAAGAGACCAGGCGAATGGATTGACAACACT
>dme_circ_0001463_junction_seq
GAGACCAGGCGAATGGATTGACAACACTGATTCAACCGTAAATAAAAAGCAGACTT
>dme_circ_0001464
ATTTAGTTTCTTTCTCTTCCACCTCACACTCCACAACTATTTCCTCAGAGGGTTCACTGCTTACTTCATC
TTCGGGCAGAGCAACGGGAGTAGGTTTTTTGGTTGCCTTCTGTTTCTTGGGCTTTTGTGGCTTTTTGTCC

TTTTTGATTGGTTCTTTTACATCAGGCAATGGGGTGACAGCGGTTTTAATGTGAGTAACAGTGTTGTCAA
TCCATTCGCCTGGTCTCTTTACAAGTGAGGCCCAGGAGAATGCGTTGCTACTCTCTAGCTTCTGAACAAC
TTCAGGCTCCGGTTTTGACTCTATTGGTGCAGGCATTGCCTCCACTGGTTCGGGCACAGTCTCGGGCTGT
TCGTCCTCAGATGTAGTCGAGACGGGATCTACAACGGG
>dme_circ_0001464_junction_seq
ATGTAGTCGAGACGGGATCTACAACGGGATTTAGTTTCTTTCTCTTCCACCTCACA
>dme_circ_0001465
AAGCAATGTCATCAACCCATTCGCCGGGCTTCTTTACCAGGGACGCCCAAGAGAAGGCGTTTTCTGTATT
TTTGATAGATTTAGTTTCTTTCTCTTCCACCTCACACTCCACAACTATTTCCTCAGAGGGTTCACTGCTT
ACTTCATCTTCGGGCAGAGCAACGGGAGTAGGTTTTTTGGTTGCCTTCTGTTTCTTGGGCTTTTGTGGCT
TTTTGTCCTTTTTGATTGGTTCTTTTACATCAGGCAATGGGGTGACAGCGGTTTTAATGTGAG
>dme_circ_0001465_junction_seq
AATGGGGTGACAGCGGTTTTAATGTGAGAAGCAATGTCATCAACCCATTCGCCGGG
>dme_circ_0001466
GAACCAATCAAAAAGGACAAAAAGCCACAAAAGCCCAAGAAACAGAAGGCAACCAAAAAACCTACTCCCG
TTGCTCTGCCCGAAGATGAAGTAAGCAGTGAACCCTCTGAGGAAATAGTTGTGGAGTGTGAGGTGGAAGA
GAAAGAAACTAAATCTATCAAAAATACAGAAAACGCCTTCTCTTGGGCGTCCCTGGTAAAGAAGCCCGGC
GAATGGGTTGATGACATTGCTTCTCGTAAAAAGCCAGTTGAAATCATTCACGAGGAGCCTAAGGAGGTGC
ATCCACGGAAAGAACGTAAAATTTCAAAACCTCAGCCCAAACCTCAGTCTGAAAACACCACCAAGCGTAA
TAACAAAAAGGAAACTCGTGAGCCCGTAGTTGTTCATTCGACTTCAGAGGAAAGTGATGTGGTTGAGGTA
TTAGAGCCTACAAATGAAAATACAATTACTTGGGCTAAAATAGCCAGTCAAATTGATCATATGACTGACT
TgaaaccaaaggctaaacaagaaacaaagccaaaacaagGAGAAAGG
>dme_circ_0001466_junction_seq
agaaacaaagccaaaacaagGAGAAAGGGAACCAATCAAAAAGGACAAAAAGCCAC
>dme_circ_0001467
TTTCTTTCTCTTCCACCTCACACTCCACAACTATTTCCTCAGAGGGTTCACTGCTTACTTCATCTTCGGG
CAGAGCAACGGGAGTAGGTTTTTTGGTTGCCTTCTGTTTCTTGGGCTTTTGTGGCTTTTTGTCCTTTTTG
ATTGG
>dme_circ_0001467_junction_seq
TTTGTGGCTTTTTGTCCTTTTTGATTGGTTTCTTTCTCTTCCACCTCACACTCCAC
>dme_circ_0001468
GGACGCCCAAGAGAAGGCGTTTTCTGTATTTTTGATAGATTTAGTTTCTTTCTCTTCCACCTCACACTCC
ACAACTATTTCCTCAGAGGGTTCACTGCTTACTTCATCTTCGGGCAGAGCAACGGGAGTAGGTTTTTTGG
TTGCCTTCTGTTTCTTGGGCTTTTGTGGCTTTTTG
>dme_circ_0001468_junction_seq
CTGTTTCTTGGGCTTTTGTGGCTTTTTGGGACGCCCAAGAGAAGGCGTTTTCTGTA
>dme_circ_0001469
AATCTCCCTTTTGTTGACTTAGATCTGATGATATAGCATGATCTTTTGGATGTTTCTCAATTCGATCGAA
TCTTATTTGCTCTTTTACCCTTTCTCcttgttttggctttgtttcttgtttagcctttggtttcAAGTCA
GTCATATGATCAATTTGACTGGCTATTTTAGCCCAAGTAATTG
>dme_circ_0001469_junction_seq
TGACTGGCTATTTTAGCCCAAGTAATTGAATCTCCCTTTTGTTGACTTAGATCTGA
>dme_circ_0001470
ATGACCAGGAATGACCAGAATCTCCCTTTTGTTGACTTAGATCTGATGATATAGCATGATCTTTTGGATG

TTTCTCAATTCGATCGAATCTTATTTGCTCTTTTACCCTTTCTCcttgttttggctttgtttcttgttta
gcctttggtttcAAGTCAGTCATATGATCAATTTGACTGGCTATTTTAGCCCAAG
>dme_circ_0001470_junction_seq
TCAATTTGACTGGCTATTTTAGCCCAAGATGACCAGGAATGACCAGAATCTCCCTT
>dme_circ_0001471
TATGACGCAAGACATCAGCATATGACGAATGCAGTTCCCATATATTAGAAGACAATGGTTTTGGCTCGAC
TTCTGtggattcttcaggtttcttcactggttctactgttttcttgggttgctcggatataactgg
>dme_circ_0001471_junction_seq
ttttcttgggttgctcggatataactggTATGACGCAAGACATCAGCATATGACGA
>dme_circ_0001472
GCTCTTTTACTGTTGGCACTTTACCTAAATTTGATTCAGTTGGGACTTCAACATTATTCTTTTCTAAACT
TATGAAGTCCACAGTATGACGCAAGACATCAGCATATGACGAATGCAGTTCCCATATATTAGAAGACAAT
GGTTTTGGCTCGACTTCTGtggattcttcaggtttcttcactggttctactgttttcttgggttgctcgg
atataactgg
>dme_circ_0001472_junction_seq
ttttcttgggttgctcggatataactggGCTCTTTTACTGTTGGCACTTTACCTAA
>dme_circ_0001473
gttatatccgagcaacccaagaaaacagtagaaccagtgaagaaacctgaagaatccaCAGAAGTCGAGC
CAAAACCATTGTCTTCTAATATATGGGAACTGCATTCGTCATATGCTGATGTCTTGCGTCATACTGTGGA
CTTCATAAGTTTAGAAAAGAATAATGTTGAAGTCCCAACTGAATCAAATTTAGGTAAAGTGCCAACAGTA
AAAGAGCCTGCTAAACTTCCAGAACCTTTGATTAAGACAAAGGCTAAGAAAGAAAAACCGTTAAAGAAGG
AAAAGGGAGCTAAGCTTTTGACGCCTAAAGATGAAGTTCTACCAATCGAAAGAGAATCAGCAAAGGATGA
AACAAAGACGTCGTGGTCAACAATGGTCGAGAACGATTTTGATGATAACGTTCAAACAAACATACTCAGT
GCCGATAGCACTTTGTGGTCTTCAATTGTCCGACAAGGTGCCCAGGAACCTATTACCAAAATGACACAAT
CTGCCGACCATGATGGTAAAGTTCTAGAACCTGCATTCCAGAATTCACAAGAAATAAACAAATTCATTTC
CCAAGAAAGAAGCTCCACAGTGATTGAAAAGACTAAGAAACAAAAATCAAAGAAGGAAAAGAAACACAAT
AAGATAGAATCAGTAGTTGAAGTGGCTTTAGAACCATACGTTGATAGCACTTTCATGCGCGATGATCCTA
AAACACAGCAGAATGCAGAAATTCCGTCTGTTTTGGAAAGTTTACCTATTCAACCCAA
>dme_circ_0001473_junction_seq
TTTTGGAAAGTTTACCTATTCAACCCAAgttatatccgagcaacccaagaaaacag
>dme_circ_0001474
GTTCTGGAAGTTTAGCAGGCTCTTTTACTGTTGGCACTTTACCTAAATTTGATTCAGTTGGGACTTCAAC
ATTATTCTTTTCTAAACTTATGAAGTCCACAGTATGACGCAAGACATCAGCATATGACGAATGCAGTTCC
CATATATTAGAAGACAATGG
>dme_circ_0001474_junction_seq
GCAGTTCCCATATATTAGAAGACAATGGGTTCTGGAAGTTTAGCAGGCTCTTTTAC
>dme_circ_0001475
AACTTTACCATCATGGTCGGCAGATTGTGTCATTTTGGTAATAGGTTCCTGGGCACCTTGTCGGACAATT
GAAGACCACAAAGTGCTATCGGCACTGAGTATGTTTGTTTGAACGTTATCATCAAAATCGTTCTCGACCA
TTGTTGACCACGACGTCTTTGTTTCATCCTTTGCTGATTCTCTTTCGATTGG
>dme_circ_0001475_junction_seq
CATCCTTTGCTGATTCTCTTTCGATTGGAACTTTACCATCATGGTCGGCAGATTGT
>dme_circ_0001476
GTAAACTTTCCAAAACAGACGGAATTTCTGCATTCTGCTGTGTTTTAGGATCATCGCGCATGAAAGTGCT

ATCAACGTATGGTTCTAAAGCCACTTCAACTACTGATTCTATCTTATTGTGTTTCTTTTCCTTCTTTGAT
TTTTGTTTCTTAGTCTTTTCAATCACTGTGGAGCTTCTTTCTTGGGAAATGAATTTGTTTATTTCTTGTG
AATTCTGGAATGCAGGTTCTAGAACTTTACCATCATGGTCGGCAGATTGTGTCATTTTGGTAATAGGTTC
CTGGGCACCTTGTCGGACAATTGAAGACCACAAAGTGCTATCGGCACTGAGTATGTTTGTTTGAACGTTA
TCATCAAAATCG
>dme_circ_0001476_junction_seq
GTTTGTTTGAACGTTATCATCAAAATCGGTAAACTTTCCAAAACAGACGGAATTTC
>dme_circ_0001477
ATGACCAAGACTTGGGTTGAATAGGTAAACTTTCCAAAACAGACGGAATTTCTGCATTCTGCTGTGTTTT
AGGATCATCGCGCATGAAAGTGCTATCAACGTATGGTTCTAAAGCCACTTCAACTACTGATTCTATCTTA
TTGTGTTTCTTTTCCTTCTTTGATTTTTGTTTCTTAGTCTTTTCAATCACTGTGGAGCTTCTTTCTTGGG
AAATGAATTTGTTTATTTCTTGTGAATTCTGGAATGCAGGTTCTAGAACTTTACCATCATGGTCGGCAGA
TTGTGTCATTTTGGTAATAGGTTCCTGGGCACCTTGTCGGACAATTGAAGACCACAAAGTGCTATCGGCA
CTGAGTATG
>dme_circ_0001477_junction_seq
CCACAAAGTGCTATCGGCACTGAGTATGATGACCAAGACTTGGGTTGAATAGGTAA
>dme_circ_0001478
GTTGAAGTGGCTTTAGAACCATACGTTGATAGCACTTTCATGCGCGATGATCCTAAAACACAGCAGAATG
CAGAAATTCCGTCTGTTTTGGAAAGTTTACCTATTCAACCCAAGTCTTGGTCATCTATCGTCTCACAAAC
CGAGGTTCTGCCTCCAACAA
>dme_circ_0001478_junction_seq
TCACAAACCGAGGTTCTGCCTCCAACAAGTTGAAGTGGCTTTAGAACCATACGTTG
>dme_circ_0001479
GTTGAAGTGGCTTTAGAACCATACGTTGATAGCACTTTCATGCGCGATGATCCTAAAACACAGCAGAATG
CAGAAATTCCGTCTGTTTTGGAAAGTTTACCTATTCAACCCAAGTCTTGGTCATCTATCGTCTCACAAAC
CGAGGTTCTGCCTCCAACAAGTATAGAAA
>dme_circ_0001479_junction_seq
GAGGTTCTGCCTCCAACAAGTATAGAAAGTTGAAGTGGCTTTAGAACCATACGTTG
>dme_circ_0001480
AATCGGCATTTACAGTCGTTGAAAAGATTTGTTCTTTACTAATGAACTCCTGCACTTCTTTTGACCTATG
AGGATCAACGGCTTTCTGGGATATTTTACTTTCTATACTTGTTGGAGGCAGAACCTCGGTTTGTGAGACG
ATAGATGACCAAGACTTGGGTTGAATAGG
>dme_circ_0001480_junction_seq
TAGATGACCAAGACTTGGGTTGAATAGGAATCGGCATTTACAGTCGTTGAAAAGAT
>dme_circ_0001481
AATCGGCATTTACAGTCGTTGAAAAGATTTGTTCTTTACTAATGAACTCCTGCACTTCTTTTGACCTATG
AGGATCAACGGCTTTCTGGGATATTTTACTTTCTATACTTGTTGGAGGCAGAACCTCGGTTTGTGAGACG
ATAGATGACCAAGACTTGGG
>dme_circ_0001481_junction_seq
GTGAGACGATAGATGACCAAGACTTGGGAATCGGCATTTACAGTCGTTGAAAAGAT
>dme_circ_0001482
GATCAGAAACCTTTAATGGAAAAACCGAAAAAACTTAAGGCAGGGAAGGAAAAGAAATCCAAGGTTCAGA
ATGTTGAAGAGTCAATTTTGGAGTCTGATGTTCCATTGTCCGTTGTTGAACCCATTATTGGATTTACCAC
TGTTCAAAAGGAT

>dme_circ_0001482_junction_seq
TATTGGATTTACCACTGTTCAAAAGGATGATCAGAAACCTTTAATGGAAAAACCGA
>dme_circ_0001483
GCTGAACTATTTTCTTTTCCTTTTTAGGTTTCGGCTTTTTGACTGAATCATTCACAACCGTAGAGTAATT
TTGCTCCTGTGCGATAAAGTTTTGAATTCCGCCCGTTTTTGATTCTACATGTTTTGGAATTGTTCGTGGA
ACCTCAACAGACTGAGCAACAATAGACGACCATGAAAACGTATGCGATTCCGTTTTTGGTGGTACATCCT
TTTGAACAGTGGTAAATCCAATAATGGG
>dme_circ_0001483_junction_seq
TTTGAACAGTGGTAAATCCAATAATGGGGCTGAACTATTTTCTTTTCCTTTTTAGG
>dme_circ_0001484
GTTTCGGCTTTTTGACTGAATCATTCACAACCGTAGAGTAATTTTGCTCCTGTGCGATAAAGTTTTGAAT
TCCGCCCGTTTTTGATTCTACATGTTTTGGAATTGTTCGTGGAACCTCAACAGACTGAGCAACAATAGAC
GACCATGAAAACGTATGCGATTCCGTTTTTGGTGG
>dme_circ_0001484_junction_seq
AAAACGTATGCGATTCCGTTTTTGGTGGGTTTCGGCTTTTTGACTGAATCATTCAC
>dme_circ_0001485
ggacaaaaaaacgagatcaaaatattgaaacTTCTGTTGTCTACGAAGAGATTCCAGAAACTGAACGCGTA
GCTGTGTCTTCCACGCCTGAATTCAGCATTCAGTTGCCGGCACCAACAAAACCATCAGTGTGGTCATCAT
CAGACACCTATGCCGAAGTTGTTAAGAAGTCTG
>dme_circ_0001485_junction_seq
ACCTATGCCGAAGTTGTTAAGAAGTCTGggacaaaaaaacgagatcaaaatattgaa
>dme_circ_0001486
GATTCCAGAAACTGAACGCGTAGCTGTGTCTTCCACGCCTGAATTCAGCATTCAGTTGCCGGCACCAACA
AAACCATCAGTGTGGTCATCATCAGACACCTATGCCGAAGTTGTTAAGAAGTCTGGTTACAACAACAATA
CGAACATTAAATATCAGAAAATTGAGGCAAGGGAACCAATCAAGAGTGAACCAAAACACACATTTGTCGA
AGTGCAGACACATGAAGATCTTCTGGAGTTCCCGGACCCTATCATTCCTGCAATTGAAAGAGATGAAGCA
CTTGAGAAACCCTCTCAGCTATCATGGAAGGACCTTGTAGATGAGGATGATGTGGATCCGCTGGAGTCGT
GGCATAAGGGAGAAACAGATTCTGTAGAGATAAAGACACAAAAACCAATACAGGAAAGACATTCCCGAGT
GAGCGTAAAGCCGGAAATTCAAATAACCGAGGTAGTTACTGAAACTCAATCGCAATCCGAGACTGACCAA
GAATTCTTGGAAATTACTTCAAAGAAACGAAATCGCTCACGCTCACGTTCGCAACAGTCACAGGCTTCGA
ACATTGATgaaaagcatcaaaagaaaaaatcaaagaagccaaaaaaTACGGTACCTAAAGCACCAGCTCA
ACAGCCTACGGAAGCTCCTGAGGATGTCCAGCCTCCTAAGGAATCT
>dme_circ_0001486_junction_seq
TGAGGATGTCCAGCCTCCTAAGGAATCTGATTCCAGAAACTGAACGCGTAGCTGTG
>dme_circ_0001487
AATCTGTTTCTCCCTTATGCCACGACTCCAGCGGATCCACATCATCCTCATCTACAAGGTCCTTCCATGA
TAGCTGAGAGGGTTTCTCAAGTGCTTCATCTCTTTCAATTGCAGGAATGATAGGGTCCGGGAACTCCAGA
AGATCTTCATG
>dme_circ_0001487_junction_seq
GGTCCGGGAACTCCAGAAGATCTTCATGAATCTGTTTCTCCCTTATGCCACGACTC
>dme_circ_0001488
GACACAAAAACCAATACAGGAAAGACATTCCCGAGTGAGCGTAAAGCCGGAAATTCAAATAACCGAGGTA
GTTACTGAAACTCAATCGCAATCCGAGACTGACCAAGAATTCTTGGAAATTACTTCAAAGAAACGAAATC
GCTCACGCTCACGTTCGCAACA

>dme_circ_0001488_junction_seq
GAAATCGCTCACGCTCACGTTCGCAACAGACACAAAAACCAATACAGGAAAGACAT
>dme_circ_0001489
TGATTGGAATGTTTACTGTGTGCTCATTTGTTGATTTCCTTACATCTTCAACGACAGTTGGTTTGGCCTC
AGCTTGGAGGGGTCGAATATGTTGAACTGGTTCTGGCACATTATGGGCGGCTATAGCAGCCCATGACATA
CCTGAGGTATTAGGAGAAGGAGCTGGTGGTGGAGACGGTTCTTCTACAGATGGTACAGATTCCTTAGGAG
GCTGGACATCCTCAGGAGCTTCCGTAGGCTGTTGAGCTGGTGCTTTAGGTACCGTAtttttttggcttctt
tgattttttcttttgatgcttttcATCAATGTTCGAAGCCTGTGACTGTTGCGAACGTGAGCGTGAGCGA
TTTCGTTTCTTTGAAGTAATTTCCAAGAATTCTTGGTCAGTCTCGGATTGCGATTGAGTTTCAGTAACTA
CCTCGG
>dme_circ_0001489_junction_seq
TGCGATTGAGTTTCAGTAACTACCTCGGTGATTGGAATGTTTACTGTGTGCTCATT
>dme_circ_0001490
GGTAGTTACTGAAACTCAATCGCAATCCGAGACTGACCAAGAATTCTTGGAAATTACTTCAAAGAAACGA
AATCGCTCACGCTCACGTTCGCAACAGTCACAGGCTTCGAACATTGATgaaaagcatcaaaagaaaaaat
caaagaagccaaaaaaTACG
>dme_circ_0001490_junction_seq
gaaaaaatcaaagaagccaaaaaaTACGGGTAGTTACTGAAACTCAATCGCAATCC
>dme_circ_0001491
GTTACTGAAACTCAATCGCAATCCGAGACTGACCAAGAATTCTTGGAAATTACTTCAAAGAAACGAAATC
GCTCACGCTCACGTTCGCAACAGTCACAGGCTTCGAACATTGATgaaaagcatcaaaagaaaaaatcaaa
gaagccaaaaaaTACGGTACCTAAAGCACCAGCTCAACAGCCTACGGAAGCTCCTGAGGATGTCCAGCCT
CCTAAGGAATCT
>dme_circ_0001491_junction_seq
TGAGGATGTCCAGCCTCCTAAGGAATCTGTTACTGAAACTCAATCGCAATCCGAGA
>dme_circ_0001492
attcgtctaccgccgtagattcaacaattggctCCGTCTTCAAAACTTTTTCGACAGGGAGGTCTTCCAT
ATGTGACTCAGATACTGGTTCAACTTCAGACTCGGCAACAAATTCAAAGGGAGCAAGTGGAGTTTTTATT
TCCCATGATGTTAAAGTATTGTTGACGTCTTCAACTATTATAGATCCAGG
>dme_circ_0001492_junction_seq
TGACGTCTTCAACTATTATAGATCCAGGattcgtctaccgccgtagattcaacaat
>dme_circ_0001493
ATGTATCCTTATGTGTAGTCACCGTTGTGGTCACATATGTTGTTGTCGTTGTTGTTATATTCTCCGAATC
AACCACGTTCTTCAGAGGCTTCGGCTCCTGGAAGTCAGTTGTTTTGCTCACAACATGAGTAGACCAATCA
GGAGTCTTTTCATTGTCCTTTTGTACATCTGGTGTTGGGAACACAGTTGTCTCGGTTTGGACAATTGCTG
ACCATGTTGTTGGCTGATCAGTGGGTGACTTTACCGCCACTTCGTCAATGCCTTCATCGTCTGGAATAGT
TACTTTAATGGGTTGGCTTTCCTCTTGTTTTGTCTCGGTTGTTTCAACTGTGGATGCCGGTTTTTGACCT
GGCTTGAGAGTTGCTGACCAGTAGGAAGTCTCACATATAAACGCATCGTCTAAAGGAGAGTCAGGAATTT
CTTCGAAGGGCGCCAGGGGAGTCTTCTCGGCTTCAGTATTTCCAGAGTCAATAGATATATTAGCTGTTTC
GACTGGTTTTTGAACCTTATCAACTGCAACATCCTTAAATGGTGCCAAGGGTGTTACTTCAGTTTGTGAC
TCTGGTACGggttcttcttcactgtctgagaagttcatgggctcatccaagacatccatccaagagcttg
aagaggcatcaaccggcaggccggcatatgctggtttctttggttcttcaactttgacgactgcttgttc
gaccttctcggg
>dme_circ_0001493_junction_seq

tgacgactgcttgttcgaccttctcgggATGTATCCTTATGTGTAGTCACCGTTGT
>dme_circ_0001494
TTAGTTCAAAAGGTTCTGGTTCCAAATCCAGTTCATTTTTATGGCTGGTTACCACAGTGGACACATAAGA
TGTTGCTGGCGATTCATTCTCCACGATAGATGTATCCTTATGTGTAGTCACCGTTGTGGTCACATATGTT
GTTGTCGTTGTTG
>dme_circ_0001494_junction_seq
GTGGTCACATATGTTGTTGTCGTTGTTGTTAGTTCAAAAGGTTCTGGTTCCAAATC
>dme_circ_0001495
GTTATCCGGTTTAGTTAGTTCAAAAGGTTCTGGTTCCAAATCCAGTTCATTTTTATGGCTGGTTACCACA
GTGGACACATAAGATGTTGCTGGCGATTCATTCTCCACGATAGATGTATCCTTATGTGTAG
>dme_circ_0001495_junction_seq
TCCACGATAGATGTATCCTTATGTGTAGGTTATCCGGTTTAGTTAGTTCAAAAGGT
>dme_circ_0001496
ATTAAGAGCAAGGATTTCGGAATTGATTTCAGGTATCGCTTCGGGTTCAATTATCACATAATCTTCTACC
AAAGGAACATCTTCTTTGGGTTCCTCCAAATACGGTTCTTTCGTTATATTTACCACCTTCAGTTGTTGGT
ATTGTCCCTGCAAGAATTCGTATTGGGGATGCACTTTGAATGCCTCGCGATTAAGATTCTCGATAAGGTT
ATCCGG
>dme_circ_0001496_junction_seq
GATTAAGATTCTCGATAAGGTTATCCGGATTAAGAGCAAGGATTTCGGAATTGATT
>dme_circ_0001497
GAATGATTGGTAATGACGATGCATCTGGTTCAGTCTTTTCTACAACGCGATCGGTTATCGTGGTCGTCGT
GGTTGTCACTACAGTTTCTGTAATGCCGTCAGTCTTTTGCTTATCGTCTTGAGTTTCCTGAGCTATCGTC
GTGTCTGTGGTTGTCCTTGGCAACAATGCGAATGCATCGCCTGCCGTTTGTTTATAG
>dme_circ_0001497_junction_seq
GAATGCATCGCCTGCCGTTTGTTTATAGGAATGATTGGTAATGACGATGCATCTGG
>dme_circ_0001498
ATACAGATTCAGACGCAACTGATCGAGGTTACCAAGCGTTGGTGCCGTCAATGGCTGGGCGGTAGGAATG
ATTGGTAATGACGATGCATCTGGTTCAGTCTTTTCTACAACGCGATCGGTTATCGTGG
>dme_circ_0001498_junction_seq
TTTTCTACAACGCGATCGGTTATCGTGGATACAGATTCAGACGCAACTGATCGAGG
>dme_circ_0001499
TTTAACCTGAGGCTGTTCGGCTTCTAGAGCCAGATAAGTACTGTCGTCACGCCAATGACCCACACCACCC
GGTAGATCGGTGGACCTATACTCCGATGAACAGCTACGGTTCTGACCGTACTGGGCATCGTGCTCGGGTG
TGGGGGTCACATTACCACTACTACCGCTACCAGGATCCTTGTCGTTCGGTTTATCATCATCGTCGTTCGG
CTTGCGAATTTCTGGCACAGTCGGCTTACCTCCCtcgtcgtcgtcatcgtcgtcgtcgtcgtcatcatcg
tcactgtcatgatcgtccttgtcTGGTATTCCAGTAACACCATCTGGCTTTTTGGCATCACCGTCAGGTT
GTTGCTCCTGG
>dme_circ_0001499_junction_seq
GGCATCACCGTCAGGTTGTTGCTCCTGGTTTAACCTGAGGCTGTTCGGCTTCTAGA
>dme_circ_0001500
GATCCTTGTCGTTCGGTTTATCATCATCGTCGTTCGGCTTGCGAATTTCTGGCACAGTCGGCTTACCTCC
CtcgtcgtcgtcatcgtcgtcgtcgtcgtcatcatcgtcactgtcatgatcgtccttgtcTGG
>dme_circ_0001500_junction_seq
cgtcactgtcatgatcgtccttgtcTGGGATCCTTGTCGTTCGGTTTATCATCATC

>dme_circ_0001501
GATCCTTGTCGTTCGGTTTATCATCATCGTCGTTCGGCTTGCGAATTTCTGGCACAGTCGGCTTACCTCC
Ctcgtcgtcgtcatcgtcgtcgtcgtcgtcatcatcgtcactgtcatgatcgtccttg
>dme_circ_0001501_junction_seq
atcatcgtcactgtcatgatcgtccttgGATCCTTGTCGTTCGGTTTATCATCATC
>dme_circ_0001502
GGATCCTGGTAGCGGTAGTAGTGGTAATGTGACCCCCACACCCGAGCACGATGCCCAGTACGGTCAGAAC
CGTAGCTGTTCATCGGAGTATAGGTCCACCGATCTACCGGGTGGTGTGGGTCATTGGCGTGACGACAGTA
CTTATCTGGCTCTAGAAGCCGAACAGCCTCAG
>dme_circ_0001502_junction_seq
TCTGGCTCTAGAAGCCGAACAGCCTCAGGGATCCTGGTAGCGGTAGTAGTGGTAAT
>dme_circ_0001503
TTTCGGTTAGTACAGATTCAACACCCGATATAGTGGGTGCCAGTGGTACACTTTCTAGTTTAACCTGAGG
CTGTTCGGCTTCTAGAGCCAGATAAGTACTGTCGTCACGCCAATGACCCACACCACCCGGTAGATCGG
>dme_circ_0001503_junction_seq
CAATGACCCACACCACCCGGTAGATCGGTTTCGGTTAGTACAGATTCAACACCCGA
>dme_circ_0001504
GGATCTAATTACGCAAGAGCTCGACGAACTGCTCCAATCGCTTTCGAGCGTCGAGGATGGCATCGCCAAC
ATGAACCAGAGCAGCCTGGATGGCATGCTACATGGATTGAAGGTAATAATATTATTAATCTAAGGCCGAA
ATAACACCACCTTTTTGGATGCTTGATTTGGCATGTACTCAAAGTAGTTAGATTAATTTGAATTAAAATA
TCTACTCACACCTTGTACATAACTTTTGTAAACGATTATTGGATGCTGGCGTGTGTCAGAGAACTTTAAT
AACTCAATCGCATCACTCCATAAAGAATCGTACATATTTTGTTAGGCTGTTATTTTTGTATCAACAGAAT
ACCGTTTAGTTGTTAGTTTTGTCCAGTTGTAGTGGTCGAACCTTTGTCTGTCTAAGTAATACACTTTTCT
AGGCCCAAAATCCAATTGTATTGTTACGTAGATTTGTATATTTCGAAACAACATATATCTGAAACACATA
TTTTACACGCCCGATGTACAGGCTAATCCAGAGCAATCTCGAAGTACACGAAAGGGACGCCATCGAGTTA
AAGAACCAGGCGAAGAAATTACCCACGGATCCGGCTACCGAACGGTTGCTCAACGACACCGTGGACCGTA
TTGACTTACTACTACGCCGCACTCAGCAAGGCATCACCATGATAGCGGAATGCTATGCATGGTCAGAAGA
AGCGCCAGCAGGAGATCGACGAGTACCAGCAACATCTGCTGGAGCTGGAGCAATGGATTATCGAGGTATC
CGCCGAGCTGGCTTCCTTTGAACCCACCTCGGACAGCAGCACGGATGAGCAGGTGCTCAAGTCGCAGGTG
GAGAGGAGCCAGCAATTGCTGCGCACCCTCAAGGATCGCCAGCAGTCCATGGAGGATTTGGTGGAGCAGA
CGCGCCAACTGCAATCCCATCCCGATGTTTCTCCCTTGGCTGACACGCTGATGGAGCAGCTGCAGAGCAT
CATTACCATTCTGCGGGAACAGGTCACAGTGGCCACCAAGCGCATCTTTACCATTGAAAAGCGCATTGTG
GATTTGCGAAAGGCCAAGAGCGAGGAGGCGCAACGCCAGCGTGTGCTTGCCGATTCACTCATCAAGCCAC
CAACCGAAGCTCCAGCGAGTCCAGAGGCGCATGAATCCATCGAGTCCAACGAGAACACCATCGACTCGAG
TTCCATGCCCGAAGAGGAGATCAAACCGACTGGAGTGTATGTGGAGACGCAGACATCGCTCAGCCTGCAG
CAGCCGCCAGTTCAGGTGGTGACCACCACGACTGTGGAGGCCCAGACCAGCTTCAAGGAGCCAGCGGTAG
AAACCGCAGAGGTGGCTCTACAAACCCAAAAGGAACGTTCGCCCACCGAAAACATTATGGTAACACAAAC
GGTGCATCATGGCCAGGAAACGATCCAGATCGACACGACTCGCAACAAGGATGTGCCCGATGAACCCGAA
GATGTCCAGATTGAGGCTCGCTACCATCAACGACCCAAGGGCGATGTGGACCGCGCCACCGAGCTGATCC
TGAAAAATGTACCTCAGGCATTCGAAACCACTTTCGTGGAGCCAGATGAGACGACCACCGAAGTGATTGT
GGGACCTGATGGCACCAAGCACATAGTACTCAAGAAAGTAACTCGTACCCGCCAGCAAGTTGTACAGCAG
CAGCAGATTAGCTCAATTGAGACTATTAGCGATTCAGATGGTAACATCGAGGTTCACTCCACGGGACAGA
TTAATCTGGAGAATGTGCACACCACGGACACCAAAGCTGATCCGGAGGAGGGCAGTGTCCACACAGTAAT
CACACAGCAAACGCGTGGTGCTGTTGTGGATTCAACGCAGCCCGAGGGTGTGATCTTGCAGGAATTCGAA

ACCGAGCCAACTATTGAGACGTACGAAGAAGTGATTGCCCCAGGCTCGCAGGCACAACTGATTCCCATGC
AGCCGGGAGATGTGCAAACCCAAGGCACCATCCGAGCAGTAGTTCAGCAGGTGACCCGTAAGGTGATCCG
CAAGACGCGCAAGATAATCAAGCGAGTGGTTATCATCGACGGAAAGGAGCACATCACTGAGGAAGTGGTC
GAGGAGCCAGAGGAAGTGGAGATCACCGAGGAGGAGACAGCACCGCATATCAACGTAAATATTGTGCGAA
CCGTTGACGGAAAGGTGGTGAGCGAGGAGGAGTTCCAGCGAATGATGCAGGAGCCCGGAGTTCTAATCGA
AGAAGTTGCCACGGATCTGCAAAAGCCGACAGCAGAGCCGCAGCAAGAGGTATTTGATATTGAGTCTACC
CAAGTCACCACCACCACAAGaactacaaccgcaacccacacaagaacaagaacagccagaacaacagacac
aaccaacaacaacaGAAACGACTAAAGAAGCACCAGTAGAGTTGCCAGCACCTCAAGTAGATGTTGAACA
ACCCGTAGTTGTTGCCACTACCAGTCCCGTTCATGTTCCAACAGCGGATGTAGTCGAGCCCAAGGACTCC
TCACCGACATCCACTACCGCAGCTGTTGTAGATGTCGAAGCAGTGGTTGAAGATATCAACGAGATTTGGC
CACTGGAACATCACCTGAAACCCACCAACATTGACTTCTCCCAGCACGTCGAAGAATTGGCAGCACCAGC
AGCGGTCACAGCAGAGACTGAAGCATCCATGCCAGTAGAGGAAATTTGGCCGACAAGTCCAGAAACCGGA
AACTCATTGACCCTGGAGCAGTACGAGTTTGAGCCACAATCACCACATGAAGAGAGCACAAAATCTGATC
TAGTTAAGCCCCAAGAGACTGAACCGCAAGTTGTTGCTGAAACTAAGCCAGAAGGCATTACCACTGGAAG
TATTACCATTACCAAGACTACAACCACCATCACAAGCTCCACAGAAGTACCGGAGGAAACTCTTGTCCAA
AATGTGCCTGCCGATGAACAACAGCCTCCTGCAAACAAAATCAAGACTGACATTCAAAGTTTCCTTGAAG
CCGAACAGACTTTGGCCGCAGCTCTAAAGGAGCAGTCTTCAACTCCGACTGGAGCTTCGGTTGCTGAAGA
TGTTCAGACTCAGCCTGAGGAAATTGTTCTTGAGGAAAGGACTGTTGAAATTTCAACCATCAAGACTGAA
GAGAATCAACAAGAGCCAGTTATAGTGGAGGAAGTGAAATCACTCCCTGTAGAGCCTGAACCAGTAGAAC
CAGAGCTGGAGGAAGTGGCCATTGCAATTGTTGAGCAGACTGAGGAAAAGCCAGAAGAGCCCGTTATTGA
GAAACAACCAGCTTCTGGCCCAATCGACCTGCGTGCCGCCACTCAACTCTTCATCTCTGGAGAGGCCGCA
GCTTCAACGGCTCCACAGAAAACCTTCCAAATCTCAGCACCCTCTCTGGAGGACAATGGCGCTGGTGTGC
TGAAGGTTGTCCTGGGCAAGGAAAGTACCAACGAAGAGGATACCGCAGCTCCAACTACCGGCAAGGTTAG
CATGACCATCATTGAGACCGCTGCTGCTCCCGCCGCCGACGCCAAGCGGCGTCGAAAGAAAAAGAAGAGA
AGAGATACAAAGCACGAGGAGGAGCTTGAGCAAGAACAAGAGACTGAGCCGGAACCAGTTGCCGCCGTTA
AGGAGCCTGAGGTGAGCTCTGATGTGCCTGTTTCTCCGGAGGATTCACCACGCGATACCGTTCGTCATGA
GTCCATTGTTGAGATTAGTCCCGATAGCGATTTATCCAGCATTGAGATTGACACGAAAGTGAAGATTGTG
GAGGATGCCGTTGTATCTTCACCATCGGAGTCGCCACGAACGCCAATGGTCGAGCTGGTCATTCCCACTG
AGGTTGTTGAGTTAGCTTTGGTCGAGGATGAGGAACAACAGACCACACCGCGTATTCCATCGCCCACTGA
AAAATCGGAAGTCGAGCAGGATATTAAATCCGTGCAGACTTCACCACAGCATCAACCCAAATTAGATGAG
ACTGCAGTGCAGACGAGTCTAGAGGTGCAGCCGGATAACCAGGAGAATGAATCGCAAACACTCATTGTCG
AGATAACGGAGACTGAAGCACAAACCACTCCGCGAAGCGAGGAGCAGTCGGTGGCTGTTGAGATTTCCAC
CACTGAGATTCAAACCGATGTGAGTGGCCAGCCAGCTGAAACTGTAGAGATTTCCAGCCAAACTACCGTG
ACCACCACTATCGAAAAGGAACTGCAGACCACGCCAAAGGATTCTCCTCGAGCACCAGAAGCAGGCAGCA
GTGATGTGGTGGAATCTCTGGTGCAGGATTTGGTTAAGGATATGACTACCGATCTGCCAGTGCGTACAAG
CGAACAATCTACCGTGACTGAAACTACCACAACCACTGAGACCCATGTGCAGACAACCACTCCGGAGCCA
AGGGAACAAACTGAAGTCATTAAACCGGAAACTGCACATGAAGAAACCTCAACCGTTGAACTTGTGCAGT
TCGCGGATGGTGAGATGCAAACCACGCCTCCTGGAGATCAGCAACCCGCATCTTTAGATGATAGTTCTCT
GACAGCCACTTCGATATCCGTTTCGGAACCCTATGAACTGGAGGTCAAGACTACGGTGGCCATTCCCGCT
GATTCGGATACATCTGTGGCTGAGCCAACGGTCTATGAATACACACAGACCATGCAGCTGCCGAAGCAGG
AGAAGAAATCCAAGAAGGACAAGAAAAAGAAGCAGAAGAATGTGCCTGAAGTGGAGCAGCAGCTGCCAGA
GGATCAACAGATCAGTGTGACCGTTGAAATCGCACCAGAGTTACTATCTGAGTCCGGCATTGTGGTATCC
ACCAACCAGCAGATTGAAGATGTGCCTCATGTAACACCCGTGGTAGACACTCCCATCGAATCCGAAGAAG
TGGAAACGCCCAAAGCTCAAAGAGTGCAACTTCAAATCACCAAGACCACTGTCTACGATGAGTATCCCGA

TCTGCCAGTGCATATAACTGAGCAGAACAAGGTGCTCATTGCATCCCAGCAGAGCAAACGATCCGGAGCA
GGACCCACATCCTCAGCGGTGACCATCGAAGAGGTGGGCTCACCCACGGAGGAGTTGGTAGTGCCCATCA
CCCCGGGACCAGACAATCTGAGTGGCGAGCCCCACAACATCTGGTTCAGTGCCACCACCAGTGTGGATAA
GACACCCATTGAGCTCTCCCAGGCTTTAATTATGAGCGAGAGTTTGCAGCATTATCCAGGACAACAGAAA
CTGACCCAGGAGCCAATCCTGATTTCCACGAAGGAGGCCATTGGTGACCGCATCAAGCAGCTCAAGCAGG
CGTCGCCCCAACAAGCCACTCCACTGAGCAATGTCCTCCACTTGGCCACCTTATCTGAACAGATTAAGGA
ACTGCCCACCGAGCAACGCATCTTGGAGGTCAACGAAGGTCTCAAGGATCTCGATGTTGCCATTAAGAAT
GGTGATAAGACTGTGATTCAAACCACTGTGATCACGGTTATCGAGAAGGTATCAACTTGGTTGGAAACCA
TCGAGTATCGCGTGTACCTCATCAGGCAGAACTCTAATGAGGGACCCTCCGAAGAGAAGCTGGATAACTA
TAACCAACTAAATGACGAGCTTAGCACCATCAAGCAAAATGTCGTCCAACTTGAGCGACAATTGTCCAAG
GCTGAGCCGGAACCACAGTTGCTGCAATGCGTGGATAGCCTGAAGGAGCATGTGGATGCAGTGGAGCAGG
TTACACAACAGAACCAGGTGCAGGACAGCAATGATCTGGACAAGTGGCACAACTTCGAGGTACTTTTGTA
CAACGTCTCCTCCGTTTTGGCCGACCTGCAGCAGAGCTACGATCTTTTGATCAACCAGGAGTATCCTTTG
TCCGCTAAACTGGCCCAGTTGGACGAGTTGGAGCAGCAGCATGAGGCGGCGCAGCAGCAACTTGCGCATC
TCTGTCAAAACGCTCGAGCTTTCCAACGCGATTTTCCAGGCAAGAAGATGCCGCAGGATGTGCACAATGC
GTTCGAGACCAGCAAGAATATTGCCAACAACATTCAGGCAGAGCGCGAGCGTGTGCTTCAACTTCAATCC
CTAGCGGAGGAGTACGAGCAGACCCTTAAAGAGTTCACCAAAATCACGGTTCTTGCCGACAAGCTGGTGG
AGAGTCCAATTGTATCCAGTTCCCTCGAGCAGCTAAACAACGAGGTGCAGAAACAGAGGAAGTTCTTCGT
AAATCTCAGCCACTGTCGCGCCATGTTGGAATCCCTGGAGGAGAACATCGATAGTGAGACCCGCGAGAAG
CACTCGGAGTTGCACAAGGAGCTCTACAACAGAGCTACCAGTCTGCTGGACAAGGCATCCGAAAGGTCCT
CCAAATTGGTGCAAGCTGCCTCCCGTTGGACTGTGCTGGAGAAGGGCATGCGAGATGAACTGCAATGGCT
TCAGGTGGCTCAGCAACGAGTTCCCGACTTATCTGCCGTAACTTCCGCAGACTACGATCAATACACCACT
CTGTACCAATCCCTGAGCAATGATATCTCACATCACTATGTGAAAATGACACAGCTTTCGGGAATAGCCA
ACAAGTTGCAGCTTTTGGTGCAAGCCCCCAATCTCGTAGAGGAAACCAATGAGGCGTTGATAGTGCTACT
CAAACTCCGCGAGGAGGTTGCTCTCTACCTTCACCGCCTTCTGGTCTTCAAGGAAATCTGGGTGCAGTAC
GAACAGCAAACGGATAAGCTGGAGGCCTTTGTACGCGAAGCAGAGCAGGAATTGAGGAACATCCAGATAC
CCTCGCAGCCCACTCACCAGCCCATCGAGCACATGCGTCAATTCTGGGAGATTAAGGCCCGTTTCGAGCT
CCACAATAATGTGAGAACCGATACGGGTCTGAGCTTTGAGAAATCCCTGCAGGTTATCCCCTTGGCGGAC
GAGATGCTACAGCGGCAGTTCCATGCCCAACTGGAGGATCGCTGGCAGGCAGTGGCCCAGGCCATTGAAC
TCATTCAGCACAACATTGTGGAATGCCTGTCGTCGGAGGATGTGCCCGCTGACGAGAAGCTCAAAATGGT
AGAGCGAGAGCTGCAGGAGATCTACCTGACCATGACCAGCATGAAGGGAGTCATCAAGAACGAGGAGGAG
CTCTGTCTGTACATCGAACGAGTCCAAGTGCTACGCACTCGCGTCGGATTCATTGGCAACGAGCTTGGCA
GGATCGGACTGCAAGAGCCCGCCATTGAGCCGGAAAAAGTTGGTGAGCTATTCTCGCTCTCGCACAAGAT
AAGTACCCAGATTGCCGAGGAGCTAGAGGGTGCATCTGTGCTGCGCGACCAACTGCAGGCCATCCAGGAG
GGCATTAGCAATCAACGCAAACACCAGGCCAAGATCTCGGTCATTTTGGATGAATGTGAAGCCGCCGAGC
GCCAGGGAGCAGATGTTCTCGAAAAGGCCGTCGCCGATTGCCAGGCCGCCGGCGAGGAGCTGGTGATCAG
CTGGCAGGAGATTATGCGAATCCGCCAGATGCTCCACACCCTGCCCATGCGCCTCAAGATGTCCGTTTCA
CCGGTTAAGTTGGAGAGGGACATCTCCCAATTGCAGGACGACCACGCCTTCCTCGAGAGCAAGTGCACCA
ACATAATGGCCATCCTGAGGAGCCGCCTAGCCGTCTGGCTGCGCTATGAGCGTCAGCTGGAGCTGGTCCA
CGGATCCGTCCAGGAAACCGACTTCATGATGGAACTCATCCGGGTGCACGGTCAAGTGGACTACGAGCGC
CTGCGCAAAGCAACTGAACGTTTGGAGGGTAAGTTTTTAGCTCACAATTTTATGACTTTGTTTACGCGCG
CGTTTAGGGATCGATTTGTATCTCAGATATATTTGTATCTTTCAAAATGCGCTACCTGCTGAGCGGAATT
TTTGCGAACAGCTTATCAGCTTAGTTTTCCCAGAGGAAAATTAGAAATAAAATCCGCTACATATTTGTAA
CGCGCCTCATTCATTTTCCGATACTCGAAACAGAAACATCATCTATACGTTTTGTTCTAAATAATAATTT

AATCATTTAAATTTTAAAATTTATTCAATACTATTTGCGAAGCAAAGACTTTCATGTAGAATGTAAATTT
AAAAAGCTATCGGTCGTCAAACTTTCGGCAAGCTTTTTCGTAAGCTCCCGCCGCATGTGGAGCTCTTTAC
GTAGCAAGTTTATCGCGGCCCCAGTTGACGTTAACTTCGAACCGAGCGCGTATCTCCCGTCGCGGTCGGA
ATCCCATATACAACTAGACTAATCCATCTTTATACGTGCAAATTAACCCATCGCAGGGGACTGGCTGGCG
ATCTGCATAACCGCGAGCAACTGATCGATGAGCTGAAGGGTGCCGCCAAACCGCTGATCGAGAGCTGCGA
TGTCCAGATTGTCGAACAGATCGAGTCCGCTGTCCAGGAGGCGGTAGTCGCCTGGAATGACACCAGCGAG
AATCTGCAGCAGCTGCGAACCCGCTACCAAAGAGCCGTCGAACTGTGGGATAAGTATCGCAACGCCTCAG
CCGCCGTCAAGAACTCCATTGACCAGCAGATGGATGCGGTCAAGTCGCTGGAGCAGCCCCTGGATGCCCT
GCAGCACGCCAAGGGTGAGTGCCCACAAATGCCGTGACCCGTCCGGCACACGGGTCGTATGAGTGATTTA
TATCGCGTGTGGGCGACAGTGATGCCAACGACCTGTTTGTCACATAGGTAAAATCGAACCGGAAATATAT
AATAATTGAACAAACAACAGAGACAGAAAATCGGTTAATAATTTGTGCATTCTAAGTACAAAGATATATC
GATTGGTTTGTTATTTAAAGTCAATTAATCTAATTTAGTTTGAGCATATTTCAAGTGTAGTGTAACAtaa
ggtaaggtaaggtaaggtTTTTTGAGATCTTTTGGGAACATAAGTTATCTAGATAAGAGGCTCTTCTAAA
ACTGATAAATCCGCCTTAGGTGCACATATCtttttttttatttttGGCGGCTAACTAGGACAGAAATGGGG
CTCTTTAATTGGCCACTGGGGACGCGGGTGTTCCAACTAGTAATTACATTGATTTTCTCCGTAGTGCTCG
CCCTATCAATTTTTTTGGGCGCGTCAATGTAATCGGTTCGTAATAGTTCCGCCGTGAATGTACATATATG
AGCTAACTTGGCCGGGCTGTGTTTGTGTCATAAATTATGTTTTGGGTAAATATGACCGACATCGTACGTA
CGATTTCCTTTTCGGCATTTACTTAAAAAATTAACGCATACTCGGATCGCCGTTTTGTGTTTATTGATCA
TTAGGGTCATTATATTATGGTAGGGGGCCCAACTCGATGGTGTAATTATATTTTCGGACAGGCGACCAAC
TGTCATTTCACACAGGAGAGTTTATCAACCCGCTGCTGTTGGGTGGCTATATTTTCGTATTATTATAGTA
TTTTATGTATACATAATCAGCTTTATCAACAGCAGCAGTAGGAGCAGCAGCTGAAAAACAGCGGGAACAG
GTTCGCAAATGCCTTGAGCTCCGAAAGAAGATAAATGTGGAAAAAGATAGACAGACACAGAGCGAACAAT
ACCTAATCTATGTTGGTATTTTCAATGCCAGTTTTCTAAGCGTTTGTTTGCTTATGTAGCTCCGTGATAA
AGCATCGCCAAATACTTGCCAGATGATATCATAGGGCGGACATTACAAAGGGCATTAGAGAACAACTTAT
GCGTCGGCAAGTGGTGGTGACCCGTTTTCCCCTCGAATTGAAATCGAAAGTGCGCCGGGCACTCACAGCC
CTAATTACCATTGGCGAGCAGGGAAATGCACTCGACAACACAATTGATAAGGGTCTGAGGCACGAAATGG
AACACCAGCCCGGCCGGCTTGGGCTAAATACATTCGTCGTTGTGGTCGTCGTCGAGTTAAATATAAACCA
GTGACATTTTGCCAGGCATCCAATATAATTGGGGTATGTTTAGTTCAGAAAGAGCTTTTCGGTGGCGAGA
AAGAACAGAAAGTATTAGAAATAGTTAGGCAAAAGGCAAAGGGAATGTTAAATGAATACAATTGATAGAT
GACTTCTATTGAGATGGTTTTAAGTTGATCATTTAAGCTAATCACTTGTTTAGAGGAACAAAAACGAGTG
ATTATACCCTGGACTTCATTAACTAAATTGGCCTCCATATCTCTACATTTACACAGGGTTTGCCAAGACA
ACCTGACGACCCAGAATGACAGAATCTTGGAGTTGCGCGACATTGTGGCCAAGATCGCCGCCGATGTGGG
CCTGGATGCCTCAGCGCTCATGCAAGGTGAATTGGATGCACTGGGTCAGCGATTGGCCGAGTGCAAGGAT
GCCATCACCACGCTGGCCAACGTGGCCGAGACGCAGGACAAGGAGCGCAAGGAGCTCGACAAGGAGGTGA
CTCTGGCCAAGGCCTACTTCAACAATGTGCAGCAGGGTGAGTGAGAGGCGAATGAATGAAATTGTGTTAG
TACACGGAAGTAGGACGTGTATCCCACTGGGATTAGACCCATCTATCCTGACCAGCTATCGATTGTGGCG
TCGAATAGATGGATATTTTGCATATGCTAGCTTGGGCTTGGCGTGTGGAAATTCCCACACTAACAAATAC
CGATTAGCCGGCAAATGTTTTTGCTGTTGTGCTTGAAGCTTGTAGTTGTTAATCCAATACGAAGGACATG
CTATTTTAAGTGGGTTAAAAGTTGAAAAAGTTTTTGTGTATCCTGGCTGGTAGCCCACTCACCGTGGTCC
CAGTGCTCCTGCCCATCATTATTGATTGATCAGAGGCGTGAATATAATTTTTGAGAGTACGATTCCCCAG
TGAAAAACTTATAAAATCGTAATTGACACTTGCTGGCCCGTGGGAAGGTTGATAAATTTTTAATGACAAT
ATGTGCATATATGTGTTCCATGTTTTGGTCTTTGGGGCTAGGAAACTTATTGCGGACTATTTCTGTTTTG
CACTTCATTTCATTTATCAAACAACTAATTCACTTCCCTGGGGAACGGAAGTTGAGCATTTTATGAAATT
ACAAATAAATTAGTTCTGTGATAAGCGCATTCTTTAAGAGCTCTAAAGAGCTTTCCCCTTTTAAATCAAT

GCCACCATAAATATAAACGGAAGCTTGGCTGATTAACGATCGTACAGTTTTACGATTCATTCAAAAGAGG
GGGCATAAACAAGTGATTTCATTGATAGGTGGTGGCATACAAGTGCCTTTACAAATTAACGTACCAAATT
GGGCATTTTTTGGTAGAACTTTCCAAGCACCTTGCACCACGGTGGGCTCATTTGCATGGCACATGCCAAC
CAGTTTGGATTTCCCCCGGCTCCCTTTTCCGCTCACAGTCCAACTCCGCCGCCCACTTTTGGCCCCCTCA
TCGACGCCTCAGACAATTGAAGTTGGTCACTGGGAGCGCGTGTCGTTGGCATGAGGTCATTGCCAGTTGG
CGGAAAGAGTTGCAATGTGCCACAGCCACAGCGATTCCCATTGTCTTGGAAGCCATGCAGTAGTGGACAG
ACGGACGAATGGTAACCGGAGACCTGCGAGCAGCCATATTAGGGTCAGGCCAATTAGCGATGCACGAAGC
ATATTCAAAGCTGACCACTGAATGAATGAATAAATAATAATTGTTTTCGTTTGCCTCCTTGCAGGGACAT
ATCCCGAGAGGCACCGCAAAATCCCAAAGAAAGCGAGGAGCAGCTGGCCGCCTTGCGAGCCCATCTCCAG
ACTTTGGCCCGAACGGAAGAGCAACTGCGTCAGCTGAAGGAACGCCATCAGAATAGCGAAGTAGCACCAT
CGGTGGCCAGCAGCGATGATGACGGCATCCTGGAGGTTCTAGCCCTGTGGCAGAAGATTTTCCAGGACAC
CTTCCAGGAGTATCATCGCCTGTCCACGCGACTCGCCCGCAGCCAAAACAGTTCGGAGGCTCTGCGATTG
TGGAGGCAGTACCTGCAGCACGTCCAGTCCTTCCTGTCCTGCGCCATTCCCGAGGATTACAGCAGCCTGC
GAGAGCAGCAGCAGCTGTGTGCCATCCACCAGAACTTGCTCATCTCGCAGCAGAGTGTGCTTTCCGAGAC
ACCACTGGAATCGGAGCTATCGGAGCAGTACAAGGCTCTGACCAACCTGCACAATGAAACCCTCTCGAGG
ATCATGCAGCGCAATGGTGAGCTGGAACGGCGAGTTAGTGGCTGGAATGCCTACCGCCAGCAGCTGGCAG
CCCTTTTGGACTGGCTGCGTCAACGGGAAGCGGAACGCAATGCCCTTCAACTGCGCTACATTCACCTGAA
GCGAGTGCCCCACCTGAAGCACCGCCTGGATGCCATGATCCAGCAATTGGATCAGGGAGAACAACAGAGC
AAGGCTTTGCAGGAGCAGCAGCAGGAGCTGGCCAGGCATTGTGACGACGCGCTGGCCACCGCCATGCGAA
TGGAGCAGGCCAGCATTGGTCAGCGGATAAGCAATCTGCGCGCAGCCCTAAAGACCTGGCAAGGATTCTT
GCAGCGCGTTACCCAGCTTTCGGAAAGCTACGAGCAGCGAGTTAACCAGTTGCAGCAGGAATTCGGAGCA
GCACAGAAGCTTCTGGATGCCAACAGCGAATCTCTGCCCACTCAGCCAGCGGCAATTGAGCAACTACTGG
GATCCCTGCGTGCGCAAAGGGTCCAACTTGGTGCCCAAGTCTCCGCTCTGGAGAGTCTCACTGTGACCCA
GGAGGAGCTCAAGGAGTGCATCAGTCCCCACGACATGAAGACCATTCGCCAAAGGAACTGGTTGCTCTGG
CAGCAGCACGCCGATCTGGACTACCAGCTGGCCAACCTGATCAACTCGATTGAGGAGCGGCTTTCCCTAC
TGTCCAACTATCAAATCAGATACGATCGCATCTCCCAGTGGCTGCAACGTTTGGAACAGCGTGTGGAAAA
GGATGCTGATGTCACTGCCATGACAAATCCCGAACAGGCAGCCAAGCAGCTGGAGCAGCAGGTGAATTCC
GAGCTGCAATTGAGGGACAAGGAACGGGAGTGGCTTCTATCCACCAGCCGGGAACTGCTCACCCTCTACT
CCGAGCCAGAAGTGCGATCCCAGGTGCAACAACAGAGTGACTCCCTCATCGATCGCTGGCAGCGTCTCAA
GTATTTGGCCAAGCAGAAAGCCACCAAGATAGGAGAGCTCAAGATGACACTTCTCCGTTTGGAGGAGCGA
ATCGCTCTGATCCGCGCCTGGCTTTTCGAAGTCGAATCCCAACTGGACAAACCCTTGAATTTCGAAAGCT
ACACACCGAATGTGATCGAGGCCAAGCTAAAGGAGCATGAGCAAATCCAACGCTCCATTGAGCACCACAG
CAGCAATGTGGGCGAGGTCCTTAATCTCGTAGAGATGCTCCTCAACGATGCGGACAGCTGGCGCACGCAG
GTGAACACTTCTGGATTAGCTGCCTCAGCTCAGAATCTGGAGCAGCGCTGGAAGAATGTGTGCAGCCAGT
CGGCGGAACGAAAGGCCCGCATCCTGACCATTTGGAATCTTCTCCAGCAGCTCATCAAACTTACTGCAGA
GCATAAAAATTGGCTGGGAAAACAAGAAAGCCAAATTGCCGGCTTCGAAAGAGATCAGAAGTCCCACAGC
AAGCATAAGCTGGAGGAGCGCCAGATGGAGCTGAGAGCCAAGCTGGAGGAGCTGGAAAGTCAGTCGGTGA
ATCTTCGCCAGCTGGAGCAGATCTATGCTAAGCTGGCCATGAGTGCTGGCGTCGAGCCGGAGAACATCCA
GAAACTGACACTGCCCACCAAGGTGATGGTGAGCATGTGGCGCCAGTTGACGCCACGCTGCCATGCCCTG
TTGGATGCCATCGACAAGGATGCCAAGCTAATGCGAGAGTTTAACAACGCACAATTGGAAGCCACCAACT
CGCTGAATGCCATACAAAAGGCTTTGGAACAATTGCCCAGCGCAGAGAATCAACAGACCTCCAAAGCGGA
GCCGAAAGCGGTGCTCCAACGTCTGGAGAGTCTAGAGAAGAAGTTGCAGGACGCACAACAACATGTCCAG
CAGGCGGATAACCTGGCCCAGGAGGCCAAGACGAGGACCAAGCAGCAACCCCAACTGAAGCAATTGTTGG
AACTTGTGTCCGCCTATACCACCCTTTGGCAGACGGTCCAGACCCGCATTGTCACCCTCAAGACCACCTG

GCTGACCAGAGCAGCTCAGGCAGCCGCCTCTCTGCCGGTCAGTGAGGCAGCCAACGCCGCCGTCCAGGTA
AACACCCTATCCCAGCGGAAACTCCGCCAGGCCCAACAGATGCAGCGGGAAACCTCCATCACGGCCAAGG
ATGCCTACATCATGGAGCTGCAGACGGCCATCACGGAATGCCAGAACAATCTGGACGAGCTGCAGCGCAC
GGTGGTGGACAAGACCCGCAAGCCGGGACCCCAGAAGATCGCCAAGCTGCTGGGCAACGCTCAGTCCTCC
ACCGAGTTAGTGAAGCATCTCAGCCATCTACTGCTCACCGAGTGCAAGGCAGATGACCAGGCTGCCGAGG
TGGACACCGTGGCAGAGCTCACGTTGCGCTTCGATACACTGCAGTCGCAGTGGAAGGCGCGACAGCAGCA
CGATCAAAATGCAAG
>dme_circ_0001504_junction_seq
CGCGACAGCAGCACGATCAAAATGCAAGGGATCTAATTACGCAAGAGCTCGACGAA
>dme_circ_0001505
GGATCTAATTACGCAAGAGCTCGACGAACTGCTCCAATCGCTTTCGAGCGTCGAGGATGGCATCGCCAAC
ATGAACCAGAGCAGCCTGGATGGCATGCTACATGGATTGAAGGTAATAATATTATTAATCTAAGGCCGAA
ATAACACCACCTTTTTGGATGCTTGATTTGGCATGTACTCAAAGTAGTTAGATTAATTTGAATTAAAATA
TCTACTCACACCTTGTACATAACTTTTGTAAACGATTATTGGATGCTGGCGTGTGTCAGAGAACTTTAAT
AACTCAATCGCATCACTCCATAAAGAATCGTACATATTTTGTTAGGCTGTTATTTTTGTATCAACAGAAT
ACCGTTTAGTTGTTAGTTTTGTCCAGTTGTAGTGGTCGAACCTTTGTCTGTCTAAGTAATACACTTTTCT
AGGCCCAAAATCCAATTGTATTGTTACGTAGATTTGTATATTTCGAAACAACATATATCTGAAACACATA
TTTTACACGCCCGATGTACAGGCTAATCCAGAGCAATCTCGAAGTACACGAAAGGGACGCCATCGAGTTA
AAGAACCAGGCGAAGAAATTACCCACGGATCCGGCTACCGAACGGTTGCTCAACGACACCGTGGACCGTA
TTGACTTACTACTACGCCGCACTCAGCAAGGCATCACCATGATAGCGGAATGCTATGCATGGTCAGAAGA
AGCGCCAGCAGGAGATCGACGAGTACCAGCAACATCTGCTGGAGCTGGAGCAATGGATTATCGAGGTATC
CGCCGAGCTGGCTTCCTTTGAACCCACCTCGGACAGCAGCACGGATGAGCAGGTGCTCAAGTCGCAGGTG
GAGAGGAGCCAGCAATTGCTGCGCACCCTCAAGGATCGCCAGCAGTCCATGGAGGATTTGGTGGAGCAGA
CGCGCCAACTGCAATCCCATCCCGATGTTTCTCCCTTGGCTGACACGCTGATGGAGCAGCTGCAGAGCAT
CATTACCATTCTGCGGGAACAGGTCACAGTGGCCACCAAGCGCATCTTTACCATTGAAAAGCGCATTGTG
GATTTGCGAAAGGCCAAGAGCGAGGAGGCGCAACGCCAGCGTGTGCTTGCCGATTCACTCATCAAGCCAC
CAACCGAAGCTCCAGCGAGTCCAGAGGCGCATGAATCCATCGAGTCCAACGAGAACACCATCGACTCGAG
TTCCATGCCCGAAGAGGAGATCAAACCGACTGGAGTGTATGTGGAGACGCAGACATCGCTCAGCCTGCAG
CAGCCGCCAGTTCAGGTGGTGACCACCACGACTGTGGAGGCCCAGACCAGCTTCAAGGAGCCAGCGGTAG
AAACCGCAGAGGTGGCTCTACAAACCCAAAAGGAACGTTCGCCCACCGAAAACATTATGGTAACACAAAC
GGTGCATCATGGCCAGGAAACGATCCAGATCGACACGACTCGCAACAAGGATGTGCCCGATGAACCCGAA
GATGTCCAGATTGAGGCTCGCTACCATCAACGACCCAAGGGCGATGTGGACCGCGCCACCGAGCTGATCC
TGAAAAATGTACCTCAGGCATTCGAAACCACTTTCGTGGAGCCAGATGAGACGACCACCGAAGTGATTGT
GGGACCTGATGGCACCAAGCACATAGTACTCAAGAAAGTAACTCGTACCCGCCAGCAAGTTGTACAGCAG
CAGCAGATTAGCTCAATTGAGACTATTAGCGATTCAGATGGTAACATCGAGGTTCACTCCACGGGACAGA
TTAATCTGGAGAATGTGCACACCACGGACACCAAAGCTGATCCGGAGGAGGGCAGTGTCCACACAGTAAT
CACACAGCAAACGCGTGGTGCTGTTGTGGATTCAACGCAGCCCGAGGGTGTGATCTTGCAGGAATTCGAA
ACCGAGCCAACTATTGAGACGTACGAAGAAGTGATTGCCCCAGGCTCGCAGGCACAACTGATTCCCATGC
AGCCGGGAGATGTGCAAACCCAAGGCACCATCCGAGCAGTAGTTCAGCAGGTGACCCGTAAGGTGATCCG
CAAGACGCGCAAGATAATCAAGCGAGTGGTTATCATCGACGGAAAGGAGCACATCACTGAGGAAGTGGTC
GAGGAGCCAGAGGAAGTGGAGATCACCGAGGAGGAGACAGCACCGCATATCAACGTAAATATTGTGCGAA
CCGTTGACGGAAAGGTGGTGAGCGAGGAGGAGTTCCAGCGAATGATGCAGGAGCCCGGAGTTCTAATCGA
AGAAGTTGCCACGGATCTGCAAAAGCCGACAGCAGAGCCGCAGCAAGAGGTATTTGATATTGAGTCTACC
CAAGTCACCACCACCACAAGaactacaaccgcaaccacacaagaacaagaacagccagaacaacagacac

aaccaacaacaacaGAAACGACTAAAGAAGCACCAGTAGAGTTGCCAGCACCTCAAGTAGATGTTGAACA
ACCCGTAGTTGTTGCCACTACCAGTCCCGTTCATGTTCCAACAGCGGATGTAGTCGAGCCCAAGGACTCC
TCACCGACATCCACTACCGCAGCTGTTGTAGATGTCGAAGCAGTGGTTGAAGATATCAACGAGATTTGGC
CACTGGAACATCACCTGAAACCCACCAACATTGACTTCTCCCAGCACGTCGAAGAATTGGCAGCACCAGC
AGCGGTCACAGCAGAGACTGAAGCATCCATGCCAGTAGAGGAAATTTGGCCGACAAGTCCAGAAACCGGA
AACTCATTGACCCTGGAGCAGTACGAGTTTGAGCCACAATCACCACATGAAGAGAGCACAAAATCTGATC
TAGTTAAGCCCCAAGAGACTGAACCGCAAGTTGTTGCTGAAACTAAGCCAGAAGGCATTACCACTGGAAG
TATTACCATTACCAAGACTACAACCACCATCACAAGCTCCACAGAAGTACCGGAGGAAACTCTTGTCCAA
AATGTGCCTGCCGATGAACAACAGCCTCCTGCAAACAAAATCAAGACTGACATTCAAAGTTTCCTTGAAG
CCGAACAGACTTTGGCCGCAGCTCTAAAGGAGCAGTCTTCAACTCCGACTGGAGCTTCGGTTGCTGAAGA
TGTTCAGACTCAGCCTGAGGAAATTGTTCTTGAGGAAAGGACTGTTGAAATTTCAACCATCAAGACTGAA
GAGAATCAACAAGAGCCAGTTATAGTGGAGGAAGTGAAATCACTCCCTGTAGAGCCTGAACCAGTAGAAC
CAGAGCTGGAGGAAGTGGCCATTGCAATTGTTGAGCAGACTGAGGAAAAGCCAGAAGAGCCCGTTATTGA
GAAACAACCAGCTTCTGGCCCAATCGACCTGCGTGCCGCCACTCAACTCTTCATCTCTGGAGAGGCCGCA
GCTTCAACGGCTCCACAGAAAACCTTCCAAATCTCAGCACCCTCTCTGGAGGACAATGGCGCTGGTGTGC
TGAAGGTTGTCCTGGGCAAGGAAAGTACCAACGAAGAGGATACCGCAGCTCCAACTACCGGCAAGGTTAG
CATGACCATCATTGAGACCGCTGCTGCTCCCGCCGCCGACGCCAAGCGGCGTCGAAAGAAAAAGAAGAGA
AGAGATACAAAGCACGAGGAGGAGCTTGAGCAAGAACAAGAGACTGAGCCGGAACCAGTTGCCGCCGTTA
AGGAGCCTGAGGTGAGCTCTGATGTGCCTGTTTCTCCGGAGGATTCACCACGCGATACCGTTCGTCATGA
GTCCATTGTTGAGATTAGTCCCGATAGCGATTTATCCAGCATTGAGATTGACACGAAAGTGAAGATTGTG
GAGGATGCCGTTGTATCTTCACCATCGGAGTCGCCACGAACGCCAATGGTCGAGCTGGTCATTCCCACTG
AGGTTGTTGAGTTAGCTTTGGTCGAGGATGAGGAACAACAGACCACACCGCGTATTCCATCGCCCACTGA
AAAATCGGAAGTCGAGCAGGATATTAAATCCGTGCAGACTTCACCACAGCATCAACCCAAATTAGATGAG
ACTGCAGTGCAGACGAGTCTAGAGGTGCAGCCGGATAACCAGGAGAATGAATCGCAAACACTCATTGTCG
AGATAACGGAGACTGAAGCACAAACCACTCCGCGAAGCGAGGAGCAGTCGGTGGCTGTTGAGATTTCCAC
CACTGAGATTCAAACCGATGTGAGTGGCCAGCCAGCTGAAACTGTAGAGATTTCCAGCCAAACTACCGTG
ACCACCACTATCGAAAAGGAACTGCAGACCACGCCAAAGGATTCTCCTCGAGCACCAGAAGCAGGCAGCA
GTGATGTGGTGGAATCTCTGGTGCAGGATTTGGTTAAGGATATGACTACCGATCTGCCAGTGCGTACAAG
CGAACAATCTACCGTGACTGAAACTACCACAACCACTGAGACCCATGTGCAGACAACCACTCCGGAGCCA
AGGGAACAAACTGAAGTCATTAAACCGGAAACTGCACATGAAGAAACCTCAACCGTTGAACTTGTGCAGT
TCGCGGATGGTGAGATGCAAACCACGCCTCCTGGAGATCAGCAACCCGCATCTTTAGATGATAGTTCTCT
GACAGCCACTTCGATATCCGTTTCGGAACCCTATGAACTGGAGGTCAAGACTACGGTGGCCATTCCCGCT
GATTCGGATACATCTGTGGCTGAGCCAACGGTCTATGAATACACACAGACCATGCAGCTGCCGAAGCAGG
AGAAGAAATCCAAGAAGGACAAGAAAAAGAAGCAGAAGAATGTGCCTGAAGTGGAGCAGCAGCTGCCAGA
GGATCAACAGATCAGTGTGACCGTTGAAATCGCACCAGAGTTACTATCTGAGTCCGGCATTGTGGTATCC
ACCAACCAGCAGATTGAAGATGTGCCTCATGTAACACCCGTGGTAGACACTCCCATCGAATCCGAAGAAG
TGGAAACGCCCAAAGCTCAAAGAGTGCAACTTCAAATCACCAAGACCACTGTCTACGATGAGTATCCCGA
TCTGCCAGTGCATATAACTGAGCAGAACAAGGTGCTCATTGCATCCCAGCAGAGCAAACGATCCGGAGCA
GGACCCACATCCTCAGCGGTGACCATCGAAGAGGTGGGCTCACCCACGGAGGAGTTGGTAGTGCCCATCA
CCCCGGGACCAGACAATCTGAGTGGCGAGCCCCACAACATCTGGTTCAGTGCCACCACCAGTGTGGATAA
GACACCCATTGAGCTCTCCCAGGCTTTAATTATGAGCGAGAGTTTGCAGCATTATCCAGGACAACAGAAA
CTGACCCAGGAGCCAATCCTGATTTCCACGAAGGAGGCCATTGGTGACCGCATCAAGCAGCTCAAGCAGG
CGTCGCCCCAACAAGCCACTCCACTGAGCAATGTCCTCCACTTGGCCACCTTATCTGAACAGATTAAGGA
ACTGCCCACCGAGCAACGCATCTTGGAGGTCAACGAAGGTCTCAAGGATCTCGATGTTGCCATTAAGAAT

GGTGATAAGACTGTGATTCAAACCACTGTGATCACGGTTATCGAGAAGGTATCAACTTGGTTGGAAACCA
TCGAGTATCGCGTGTACCTCATCAGGCAGAACTCTAATGAGGGACCCTCCGAAGAGAAGCTGGATAACTA
TAACCAACTAAATGACGAGCTTAGCACCATCAAGCAAAATGTCGTCCAACTTGAGCGACAATTGTCCAAG
GCTGAGCCGGAACCACAGTTGCTGCAATGCGTGGATAGCCTGAAGGAGCATGTGGATGCAGTGGAGCAGG
TTACACAACAGAACCAGGTGCAGGACAGCAATGATCTGGACAAGTGGCACAACTTCGAGGTACTTTTGTA
CAACGTCTCCTCCGTTTTGGCCGACCTGCAGCAGAGCTACGATCTTTTGATCAACCAGGAGTATCCTTTG
TCCGCTAAACTGGCCCAGTTGGACGAGTTGGAGCAGCAGCATGAGGCGGCGCAGCAGCAACTTGCGCATC
TCTGTCAAAACGCTCGAGCTTTCCAACGCGATTTTCCAGGCAAGAAGATGCCGCAGGATGTGCACAATGC
GTTCGAGACCAGCAAGAATATTGCCAACAACATTCAGGCAGAGCGCGAGCGTGTGCTTCAACTTCAATCC
CTAGCGGAGGAGTACGAGCAGACCCTTAAAGAGTTCACCAAAATCACGGTTCTTGCCGACAAGCTGGTGG
AGAGTCCAATTGTATCCAGTTCCCTCGAGCAGCTAAACAACGAGGTGCAGAAACAGAGGAAGTTCTTCGT
AAATCTCAGCCACTGTCGCGCCATGTTGGAATCCCTGGAGGAGAACATCGATAGTGAGACCCGCGAGAAG
CACTCGGAGTTGCACAAGGAGCTCTACAACAGAGCTACCAGTCTGCTGGACAAGGCATCCGAAAGGTCCT
CCAAATTGGTGCAAGCTGCCTCCCGTTGGACTGTGCTGGAGAAGGGCATGCGAGATGAACTGCAATGGCT
TCAGGTGGCTCAGCAACGAGTTCCCGACTTATCTGCCGTAACTTCCGCAGACTACGATCAATACACCACT
CTGTACCAATCCCTGAGCAATGATATCTCACATCACTATGTGAAAATGACACAGCTTTCGGGAATAGCCA
ACAAGTTGCAGCTTTTGGTGCAAGCCCCCAATCTCGTAGAGGAAACCAATGAGGCGTTGATAGTGCTACT
CAAACTCCGCGAGGAGGTTGCTCTCTACCTTCACCGCCTTCTGGTCTTCAAGGAAATCTGGGTGCAGTAC
GAACAGCAAACGGATAAGCTGGAGGCCTTTGTACGCGAAGCAGAGCAGGAATTGAGGAACATCCAGATAC
CCTCGCAGCCCACTCACCAGCCCATCGAGCACATGCGTCAATTCTGGGAGATTAAGGCCCGTTTCGAGCT
CCACAATAATGTGAGAACCGATACGGGTCTGAGCTTTGAGAAATCCCTGCAGGTTATCCCCTTGGCGGAC
GAGATGCTACAGCGGCAGTTCCATGCCCAACTGGAGGATCGCTGGCAGGCAGTGGCCCAGGCCATTGAAC
TCATTCAGCACAACATTGTGGAATGCCTGTCGTCGGAGGATGTGCCCGCTGACGAGAAGCTCAAAATGGT
AGAGCGAGAGCTGCAGGAGATCTACCTGACCATGACCAGCATGAAGGGAGTCATCAAGAACGAGGAGGAG
CTCTGTCTGTACATCGAACGAGTCCAAGTGCTACGCACTCGCGTCGGATTCATTGGCAACGAGCTTGGCA
GGATCGGACTGCAAGAGCCCGCCATTGAGCCGGAAAAAGTTGGTGAGCTATTCTCGCTCTCGCACAAGAT
AAGTACCCAGATTGCCGAGGAGCTAGAGGGTGCATCTGTGCTGCGCGACCAACTGCAGGCCATCCAGGAG
GGCATTAGCAATCAACGCAAACACCAGGCCAAGATCTCGGTCATTTTGGATGAATGTGAAGCCGCCGAGC
GCCAGGGAGCAGATGTTCTCGAAAAGGCCGTCGCCGATTGCCAGGCCGCCGGCGAGGAGCTGGTGATCAG
CTGGCAGGAGATTATGCGAATCCGCCAGATGCTCCACACCCTGCCCATGCGCCTCAAGATGTCCGTTTCA
CCGGTTAAGTTGGAGAGGGACATCTCCCAATTGCAGGACGACCACGCCTTCCTCGAGAGCAAGTGCACCA
ACATAATGGCCATCCTGAGGAGCCGCCTAGCCGTCTGGCTGCGCTATGAGCGTCAGCTGGAGCTGGTCCA
CGGATCCGTCCAGGAAACCGACTTCATGATGGAACTCATCCGGGTGCACGGTCAAGTGGACTACGAGCGC
CTGCGCAAAGCAACTGAACGTTTGGAGGGTAAGTTTTTAGCTCACAATTTTATGACTTTGTTTACGCGCG
CGTTTAGGGATCGATTTGTATCTCAGATATATTTGTATCTTTCAAAATGCGCTACCTGCTGAGCGGAATT
TTTGCGAACAGCTTATCAGCTTAGTTTTCCCAGAGGAAAATTAGAAATAAAATCCGCTACATATTTGTAA
CGCGCCTCATTCATTTTCCGATACTCGAAACAGAAACATCATCTATACGTTTTGTTCTAAATAATAATTT
AATCATTTAAATTTTAAAATTTATTCAATACTATTTGCGAAGCAAAGACTTTCATGTAGAATGTAAATTT
AAAAAGCTATCGGTCGTCAAACTTTCGGCAAGCTTTTTCGTAAGCTCCCGCCGCATGTGGAGCTCTTTAC
GTAGCAAGTTTATCGCGGCCCCAGTTGACGTTAACTTCGAACCGAGCGCGTATCTCCCGTCGCGGTCGGA
ATCCCATATACAACTAGACTAATCCATCTTTATACGTGCAAATTAACCCATCGCAGGGGACTGGCTGGCG
ATCTGCATAACCGCGAGCAACTGATCGATGAGCTGAAGGGTGCCGCCAAACCGCTGATCGAGAGCTGCGA
TGTCCAGATTGTCGAACAGATCGAGTCCGCTGTCCAGGAGGCGGTAGTCGCCTGGAATGACACCAGCGAG
AATCTGCAGCAGCTGCGAACCCGCTACCAAAGAGCCGTCGAACTGTGGGATAAGTATCGCAACGCCTCAG

CCGCCGTCAAGAACTCCATTGACCAGCAGATGGATGCGGTCAAGTCGCTGGAGCAGCCCCTGGATGCCCT
GCAGCACGCCAAGGGTGAGTGCCCACAAATGCCGTGACCCGTCCGGCACACGGGTCGTATGAGTGATTTA
TATCGCGTGTGGGCGACAGTGATGCCAACGACCTGTTTGTCACATAGGTAAAATCGAACCGGAAATATAT
AATAATTGAACAAACAACAGAGACAGAAAATCGGTTAATAATTTGTGCATTCTAAGTACAAAGATATATC
GATTGGTTTGTTATTTAAAGTCAATTAATCTAATTTAGTTTGAGCATATTTCAAGTGTAGTGTAACAtaa
ggtaaggtaaggtaaggtTTTTTGAGATCTTTTGGGAACATAAGTTATCTAGATAAGAGGCTCTTCTAAA
ACTGATAAATCCGCCTTAGGTGCACATATCttttttttatttttGGCGGCTAACTAGGACAGAAATGGGG
CTCTTTAATTGGCCACTGGGGACGCGGGTGTTCCAACTAGTAATTACATTGATTTTCTCCGTAGTGCTCG
CCCTATCAATTTTTTTGGGCGCGTCAATGTAATCGGTTCGTAATAGTTCCGCCGTGAATGTACATATATG
AGCTAACTTGGCCGGGCTGTGTTTGTGTCATAAATTATGTTTGGGTAAATATGACCGACATCGTACGTA
CGATTTCCTTTTCGGCATTTACTTAAAAAATTAACGCATACTCGGATCGCCGTTTTGTGTTTATTGATCA
TTAGGGTCATTATATTATGGTAGGGGGCCCAACTCGATGGTGTAATTATATTTTCGGACAGGCGACCAAC
TGTCATTTCACACAGGAGAGTTTATCAACCCGCTGCTGTTGGGTGGCTATATTTTCGTATTATTATAGTA
TTTTATGTATACATAATCAGCTTTATCAACAGCAGCAGTAGGAGCAGCAGCTGAAAAACAGCGGGAACAG
GTTCGCAAATGCCTTGAGCTCCGAAAGAAGATAAATGTGGAAAAAGATAGACAGACACAGAGCGAACAAT
ACCTAATCTATGTTGGTATTTTCAATGCCAGTTTTCTAAGCGTTTGTTTGCTTATGTAGCTCCGTGATAA
AGCATCGCCAAATACTTGCCAGATGATATCATAGGGCGGACATTACAAAGGGCATTAGAGAACAACTTAT
GCGTCGGCAAGTGGTGGTGACCCGTTTTCCCCTCGAATTGAAATCGAAAGTGCGCCGGGCACTCACAGCC
CTAATTACCATTGGCGAGCAGGGAAATGCACTCGACAACACAATTGATAAGGGTCTGAGGCACGAAATGG
AACACCAGCCCGGCCGGCTTGGGCTAAATACATTCGTCGTTGTGGTCGTCGTCGAGTTAAATATAAACCA
GTGACATTTTGCCAGGCATCCAATATAATTGGGGTATGTTTAGTTCAGAAAGAGCTTTTCGGTGGCGAGA
AAGAACAGAAAGTATTAGAAATAGTTAGGCAAAAGGCAAAGGGAATGTTAAATGAATACAATTGATAGAT
GACTTCTATTGAGATGGTTTTAAGTTGATCATTTAAGCTAATCACTTGTTTAGAGGAACAAAAACGAGTG
ATTATACCCTGGACTTCATTAACTAAATTGGCCTCCATATCTCTACATTTACACAGGGTTTGCCAAGACA
ACCTGACGACCCAGAATGACAGAATCTTGGAGTTGCGCGACATTGTGGCCAAGATCGCCGCCGATGTGGG
CCTGGATGCCTCAGCGCTCATGCAAGGTGAATTGGATGCACTGGGTCAGCGATTGGCCGAGTGCAAGGAT
GCCATCACCACGCTGGCCAACGTGGCCGAGACGCAGGACAAGGAGCGCAAGGAGCTCGACAAGGAGGTGA
CTCTGGCCAAGGCCTACTTCAACAATGTGCAGCAGGGTGAGTGAGAGGCGAATGAATGAAATTGTGTTAG
TACACGGAAGTAGGACGTGTATCCCACTGGGATTAGACCCATCTATCCTGACCAGCTATCGATTGTGGCG
TCGAATAGATGGATATTTTGCATATGCTAGCTTGGGCTTGGCGTGTGGAAATTCCCACACTAACAAATAC
CGATTAGCCGGCAAATGTTTTTGCTGTTGTGCTTGAAGCTTGTAGTTGTTAATCCAATACGAAGGACATG
CTATTTTAAGTGGGTTAAAAGTTGAAAAAGTTTTGTGTATCCTGGCTGGTAGCCCACTCACCGTGGTCC
CAGTGCTCCTGCCCATCATTATTGATTGATCAGAGGCGTGAATATAATTTTTGAGAGTACGATTCCCCAG
TGAAAAACTTATAAAATCGTAATTGACACTTGCTGGCCCGTGGGAAGGTTGATAAATTTTTAATGACAAT
ATGTGCATATATGTGTTCCATGTTTTGGTCTTTGGGGCTAGGAAACTTATTGCGGACTATTTCTGTTTTG
CACTTCATTTCATTTATCAAACAACTAATTCACTTCCCTGGGGAACGGAAGTTGAGCATTTTATGAAATT
ACAAATAAATTAGTTCTGTGATAAGCGCATTCTTTAAGAGCTCTAAAGAGCTTTCCCCTTTTAAATCAAT
GCCACCATAAATATAAACGGAAGCTTGGCTGATTAACGATCGTACAGTTTTACGATTCATTCAAAAGAGG
GGGCATAAACAAGTGATTTCATTGATAGGTGGTGGCATACAAGTGCCTTTACAAATTAACGTACCAAATT
GGGCATTTTTTGGTAGAACTTTCCAAGCACCTTGCACCACGGTGGGCTCATTTGCATGGCACATGCCAAC
CAGTTTGGATTTCCCCCGGCTCCCTTTTCCGCTCACAGTCCAACTCCGCCGCCCACTTTTGGCCCCCCTCA
TCGACGCCTCAGACAATTGAAGTTGGTCACTGGGAGCGCGTGTCGTTGGCATGAGGTCATTGCCAGTTGG
CGGAAAGAGTTGCAATGTGCCACAGCCACAGCGATTCCCATTGTCTTGGAAGCCATGCAGTAGTGGACAG
ACGGACGAATGGTAACCGGAGACCTGCGAGCAGCCATATTAGGGTCAGGCCAATTAGCGATGCACGAAGC

ATATTCAAAGCTGACCACTGAATGAATGAATAAATAATAATTGTTTTCGTTTGCCTCCTTGCAGGGACAT
ATCCCGAGAGGCACCGCAAAATCCCAAAGAAAGCGAGGAGCAGCTGGCCGCCTTGCGAGCCCATCTCCAG
ACTTTGGCCCGAACGGAAGAGCAACTGCGTCAGCTGAAGGAACGCCATCAGAATAGCGAAGTAGCACCAT
CGGTGGCCAGCAGCGATGATGACGGCATCCTGGAGGTTCTAGCCCTGTGGCAGAAGATTTTCCAGGACAC
CTTCCAGGAGTATCATCGCCTGTCCACGCGACTCGCCCGCAGCCAAAACAGTTCGGAGGCTCTGCGATTG
TGGAGGCAGTACCTGCAGCACGTCCAGTCCTTCCTGTCCTGCGCCATTCCCGAGGATTACAGCAGCCTGC
GAGAGCAGCAGCAGCTGTGTGCCATCCACCAGAACTTGCTCATCTCGCAGCAGAGTGTGCTTTCCGAGAC
ACCACTGGAATCGGAGCTATCGGAGCAGTACAAGGCTCTGACCAACCTGCACAATGAAACCCTCTCGAGG
ATCATGCAGCGCAATGGTGAGCTGGAACGGCGAGTTAGTGGCTGGAATGCCTACCGCCAGCAGCTGGCAG
CCCTTTTGGACTGGCTGCGTCAACGGGAAGCGGAACGCAATGCCCTTCAACTGCGCTACATTCACCTGAA
GCGAGTGCCCCACCTGAAGCACCGCCTGGATGCCATGATCCAGCAATTGGATCAGGGAGAACAACAGAGC
AAGGCTTTGCAGGAGCAGCAGCAGGAGCTGGCCAGGCATTGTGACGACGCGCTGGCCACCGCCATGCGAA
TGGAGCAGGCCAGCATTGGTCAGCGGATAAGCAATCTGCGCGCAGCCCTAAAGACCTGGCAAGGATTCTT
GCAGCGCGTTACCCAGCTTTCGGAAAGCTACGAGCAGCGAGTTAACCAGTTGCAGCAGGAATTCGGAGCA
GCACAGAAGCTTCTGGATGCCAACAGCGAATCTCTGCCCACTCAGCCAGCGGCAATTGAGCAACTACTGG
GATCCCTGCGTGCGCAAAGGGTCCAACTTGGTGCCCAAGTCTCCGCTCTGGAGAGTCTCACTGTGACCCA
GGAGGAGCTCAAGGAGTGCATCAGTCCCCACGACATGAAGACCATTCGCCAAAGGAACTGGTTGCTCTGG
CAGCAGCACGCCGATCTGGACTACCAGCTGGCCAACCTGATCAACTCGATTGAGGAGCGGCTTTCCCTAC
TGTCCAACTATCAAATCAGATACGATCGCATCTCCCAGTGGCTGCAACGTTTGGAACAGCGTGTGGAAAA
GGATGCTGATGTCACTGCCATGACAAATCCCGAACAGGCAGCCAAGCAGCTGGAGCAGCAGGTGAATTCC
GAGCTGCAATTGAGGGACAAGGAACGGGAGTGGCTTCTATCCACCAGCCGGGAACTGCTCACCCTCTACT
CCGAGCCAGAAGTGCGATCCCAGGTGCAACAACAGAGTGACTCCCTCATCGATCGCTGGCAGCGTCTCAA
GTATTTGGCCAAGCAGAAAGCCACCAAGATAGGAGAGCTCAAGATGACACTTCTCCGTTTGGAGGAGCGA
ATCGCTCTGATCCGCGCCTGGCTTTTCGAAGTCGAATCCCAACTGGACAAACCCTTGAATTTCGAAAGCT
ACACACCGAATGTGATCGAGGCCAAGCTAAAGGAGCATGAGCAAATCCAACGCTCCATTGAGCACCACAG
CAGCAATGTGGGCGAGGTCCTTAATCTCGTAGAGATGCTCCTCAACGATGCGGACAGCTGGCGCACGCAG
GTGAACACTTCTGGATTAGCTGCCTCAGCTCAGAATCTGGAGCAGCGCTGGAAGAATGTGTGCAGCCAGT
CGGCGGAACGAAAGGCCCGCATCCTGACCATTTGGAATCTTCTCCAGCAGCTCATCAAACTTACTGCAGA
GCATAAAAATTGGCTGGGAAAACAAGAAAGCCAAATTGCCGGCTTCGAAAGAGATCAGAAGTCCCACAGC
AAGCATAAGCTGGAGGAGCGCCAGATGGAGCTGAGAGCCAAGCTGGAGGAGCTGGAAAGTCAGTCGGTGA
ATCTTCGCCAGCTGGAGCAGATCTATGCTAAGCTGGCCATGAGTGCTGGCGTCGAGCCGGAGAACATCCA
GAAACTGACACTGCCCACCAAGGTGATGGTGAGCATGTGGCGCCAGTTGACGCCACGCTGCCATGCCCTG
TTGGATGCCATCGACAAGGATGCCAAGCTAATGCGAGAGTTTAACAACGCACAATTGGAAGCCACCAACT
CGCTGAATGCCATACAAAAGGCTTTGGAACAATTGCCCAGCGCAGAGAATCAACAGACCTCCAAAGCGGA
GCCGAAAGCGGTGCTCCAACGTCTGGAGAGTCTAGAGAAGAAGTTGCAGGACGCACAACAACATGTCCAG
CAGGCGGATAACCTGGCCCAGGAGGCCAAGACGAGGACCAAGCAGCAACCCCAACTGAAGCAATTGTTGG
AACTTGTGTCCGCCTATACCACCCTTTGGCAGACGGTCCAGACCCGCATTGTCACCCTCAAGACCACCTG
GCTGACCAGAGCAGCTCAGGCAGCCGCCTCTCTGCCGGTCAGTGAGGCAGCCAACGCCGCCGTCCAGGTA
AACACCCTATCCCAGCGGAAACTCCGCCAGGCCCAACAGATGCAGCGGGAAACCTCCATCACGGCCAAGG
ATGCCTACATCATGGAGCTGCAGACGGCCATCACGGAATGCCAGAACAATCTGGACGAGCTGCAGCGCAC
GGTGGTGGACAAGACCCGCAAGCCGGGACCCCAGAAGATCGCCAAGCTGCTGGGCAACGCTCAGTCCTCC
ACCGAGTTAGTGAAGCATCTCAGCCATCTACTGCTCACCGAGTGCAAGGCAGATGACCAGGCTGCCGAGG
TGGACACCGTGGCAGAGCTCACGTTGCGCTTCGATACACTGCAGTCGCAGTGGAAGGCGCGACAGCAGCA
CGATCAAAATGCAAGGGTAAGATTTTATATTAATTATCTATATATATTTGTTGGGATATTTTTCAAGATA

CTTGCCAAACCAGAAAACCATTTTGATAGGAGGCAAAGTGGCATATTTGGGAATATACCTTCCCAATTGT
GTCTCAAATACTCCAAAAACAACTATGTAATAGGGACGAACGGCATCCTGAATAGTGCATCATACTATAG
CAGATGACTTGTGTTCGTGCACATGTTAAAAGTTTTCTAAATTTTTATATATATAATAATAGAAGTAACC
CTTTTCGGTTTGGCAAGTTCTGCAACATTTTCCGCTGGTGATTTGGGACCTGTTCTTGACTAACCAATTG
TTTTATTGCTTTCCATTTCGCTGTTGCCTACTTTCTCCTTATGTCAAAACACGCACACGCATATTCACAC
TCAAATATATAAACTTACCCATGCTTAACTCGAACCTGCGCATTCATGATGACACCAGGTGCCACCTACC
TGCATCCTACAAGTACAACTGCATCCAGTAAGTGTATCTACCTGTCGGTGGGCAGTTCCGACCTCCGACC
TCCTGTTGTTTGATCCTTTGTTCCATGTTCTACGTGGTTTTCCACTAGACAAAAGCTGGCAGTGGACTAC
AAATGGCTGCCAGTCTCCAAGGTGGCGAACAGCAAGAGGTCGCCGAGCAAACGCCCTAACTATCTAAGTT
AACCTTAAAGCCAAATCAATGGACTTTGGAAAGGCGAGCTTAAGCTAACATTTAATCGATTACTACCATA
ATCACTATCTGCTATGATTATTCAATTACTAATTTCTCCACTTTTTTGACCAATTCTAGGCGAGGTCGGC
CGGCTAACGTGTCCGCTCTGCACACAGCGAAATTGGCAGCAGATCGACAACGATCTGTGGCGCCTGGAGC
AGTGGCTGCAGTTCGCCGAGAGCACCCAGAAGGCCCAGTCAGCTCCACCCTCAAACATCGAGCTCCTCGA
GGACGTCACGCAGGACCATCGCGAGTTCCTCCTGGATTTGGAGAGTCACAAGTCCATCATATCGTCGTTG
AATGTGGTCGGTGACCACTTGGCTACGCACACTTTGGATACCGAAAAGGCGCGACAGCTGCGATCTCGTC
TAGAGGCGGACAATGAGCGCTGGAACAATGTGTGCATCAATGCCACCAAATGGCAAGGTCTTCTCCAGAC
CGCCCTGATGGGCAACAGCGAGTTCCATCAGACCATCGGTGAGCTGGTCGAGTGGCTGCAGCGCACCGAG
CAGAACATCAAGGCCTCCGAGCCCGTTGATCTCACCGAGGAGCGCTCCGTTCTGGAGACCAAGTTCAAGA
AGTTCAAGGATCTGCGAGCCGAACTAGAGAGATGCGAACCTCGGGTGGTCAGCCTGCAGGATGCCGCCGA
TCAGCTGCTCCGCTCCGTCGAGGGCTCCGAGCAGCAATCGCAACACACGTACGAGAGGGTAAGGTGTCCA
GTGGGCCAGTTCCGAGGGCGGAACGAAACGATTCGTGGGTGCCGCTTGAGCTTAGGTCCTGGAGAGGTTC
CAGTGTTAGTTGGGCGGAAAACAATTAAATTCTATATATTTTATATGGTTTCAGTTGGTATTCTTAGTTC
ATTTGACTAGCTTTAGCAATGATCACTTGGAAACTGTAGATAATTTGTGCTTTTTTGATATGTGCCCTTT
CTATAAACTCCTTGTAGAATCCAAACATTAGTTTTTATCCTTTACCCAAGAGCTTTTTCCTATTTCTCT
TATTCTATTAGCAAGTAATTCGGCCTTTTAACTATCCAAATATCCCAAAACTTCCACTGTGCACTATCCA
CCAAAATCCTTTTCGGTAGTTTCCGCTTCAAAAATTTGAATTCACATTCTTTCAAAATTATACTAAAAAA
ATATCGCCAAGGATAATTAAGCCTTTTGGAACCTTCCACAACTAAACATTTTATAGCCATTTAAACTAGT
AGAACGTATTTCAGCTATATACAAAATCAATCGTATCGATCGATCATTTATTTTTTGCCTCTACACATAT
TTTGTGTTGCCGTTTTTGTTGCGTATATTATTTTGTGTTGTATTTTTGGTACTCAAAATTCTTTCGTCCG
CCCATTCTAACCCTCCATCCAAACATACACCGCCAAGAGCACATGCAAATGTTTTAAATGCTCCAGGAAC
CCTTTCCAGATTGACCGATCTCCGGCTGCGTCTCCAATCCCTGCGTCGTCTATCCGGCATCTATATCGTC
AAGCTGGGTGCCGTCCTCGGCTACGAGGGCGACAATCTCGGCGTGCCATTGCACATGTTGTCTAGTGAGA
GTAAATATCTATAGAAATTGATTTTAAGTTTTTGAGTTTTCTCACATGCATCGTAGTTGAAAAGTATTTA
TTGTTTGAACAACTTGTGCACTTTATAGTTGAAACTGAAAAGAGCACGTAGTTTGTACTCAATTAGTTGC
ATGATAGTGCTTTAAAACATATTGAACATAAACTAATGGACTTATAATTCGAATCTTTAGGCAACACCGA
TGGCGGCGATGCTGTCGATGGCGATGTCATCAATACCACGGTTCTGGCGCGCGGTGCACGATTCCTAGGC
CGCGTTGCACGTGCCTCGCTGCCCATTCAGGCGCTCATGCTGCTGCTTTTAGGTGTGGCCACCCTGGTGC
CCCACGGTGAGGACTACACCTGTATGTTCTCCAACACCTTTGCGCGCAGCCTGGAGCCCATGTTGTCGTA
TCCCCACGGACCGCCACCCACGTAAAGCTGTCGAGTTGCAATTGGATTAGTGCAATGAAACACGATCaaa
aaaaaaGTCAAGAAACCCTATAccctaagaagaaccccctatgaaaaacgccctatgaaaaaaacccAGTT
TAAAGATGCCTTTTGAAAGCTAAATTTCAA
>dme_circ_0001505_junction_seq
AAGATGCCTTTTGAAAGCTAAATTTCAAGGATCTAATTACGCAAGAGCTCGACGAA
>dme_circ_0001506

GCTAATCCAGAGCAATCTCGAAGTACACGAAAGGGACGCCATCGAGTTAAAGAACCAGGCGAAGAAATTA
CCCACGGATCCGGCTACCGAACGGTTGCTCAACGACACCGTGGACCGTATTGACTTACTACTACGCCGCA
CTCAGCAAGGCATCACCATGATAGCGGAATGCTATGCATGGTCAGAAGAAGCGCCAGCAGGAGATCGACG
AGTACCAGCAACATCTGCTGGAGCTGGAGCAATGGATTATCGAGGTATCCGCCGAGCTGGCTTCCTTTGA
ACCCACCTCGGACAGCAGCACGGATGAGCAGGTGCTCAAGTCGCAGGTGGAGAGGAGCCAGCAATTGCTG
CGCACCCTCAAGGATCGCCAGCAGTCCATGGAGGATTTGGTGGAGCAGACGCGCCAACTGCAATCCCATC
CCGATGTTTCTCCCTTGGCTGACACGCTGATGGAGCAGCTGCAGAGCATCATTACCATTCTGCGGGAACA
GGTCACAGTGGCCACCAAGCGCATCTTTACCATTGAAAAGCGCATTGTGGATTTGCGAAAGGCCAAGAGC
GAGGAGGCGCAACGCCAGCGTGTGCTTGCCGATTCACTCATCAAGCCACCAACCGAAGCTCCAGCGAGTC
CAGAGGCGCATGAATCCATCGAGTCCAACGAGAACACCATCGACTCGAGTTCCATGCCCGAAGAGGAGAT
CAAACCGACTGGAGTGTATGTGGAGACGCAGACATCGCTCAGCCTGCAGCAGCCGCCAGTTCAGGTGGTG
ACCACCACGACTGTGGAGGCCCAGACCAGCTTCAAGGAGCCAGCGGTAGAAACCGCAGAGGTGGCTCTAC
AAACCCAAAAGGAACGTTCGCCCACCGAAAACATTATGGTAACACAAACGGTGCATCATGGCCAGGAAAC
GATCCAGATCGACACGACTCGCAACAAGGATGTGCCCGATGAACCCGAAGATGTCCAGATTGAGGCTCGC
TACCATCAACGACCCAAGGGCGATGTGGACCGCGCCACCGAGCTGATCCTGAAAAATGTACCTCAGGCAT
TCGAAACCACTTTCGTGGAGCCAGATGAGACGACCACCGAAGTGATTGTGGGACCTGATGGCACCAAGCA
CATAGTACTCAAGAAAGTAACTCGTACCCGCCAGCAAGTTGTACAGCAGCAGCAGATTAGCTCAATTGAG
ACTATTAGCGATTCAGATGGTAACATCGAGGTTCACTCCACGGGACAGATTAATCTGGAGAATGTGCACA
CCACGGACACCAAAGCTGATCCGGAGGAGGGCAGTGTCCACACAGTAATCACACAGCAAACGCGTGGTGC
TGTTGTGGATTCAACGCAGCCCGAGGGTGTGATCTTGCAGGAATTCGAAACCGAGCCAACTATTGAGACG
TACGAAGAAGTGATTGCCCCAGGCTCGCAGGCACAACTGATTCCCATGCAGCCGGGAGATGTGCAAACCC
AAGGCACCATCCGAGCAGTAGTTCAGCAGGTGACCCGTAAGGTGATCCGCAAGACGCGCAAGATAATCAA
GCGAGTGGTTATCATCGACGGAAAGGAGCACATCACTGAGGAAGTGGTCGAGGAGCCAGAGGAAGTGGAG
ATCACCGAGGAGGAGACAGCACCGCATATCAACGTAAATATTGTGCGAACCGTTGACGGAAAGGTGGTGA
GCGAGGAGGAGTTCCAGCGAATGATGCAGGAGCCCGGAGTTCTAATCGAAGAAGTTGCCACGGATCTGCA
AAAGCCGACAGCAGAGCCGCAGCAAGAGGTATTTGATATTGAGTCTACCCAAGTCACCACCACCACAAGa
actacaaccgcaaccacacaagaacaagaacagccagaacaacagacacaaccaacaacaacaGAAACGA
CTAAAGAAGCACCAGTAGAGTTGCCAGCACCTCAAGTAGATGTTGAACAACCCGTAGTTGTTGCCACTAC
CAGTCCCGTTCATGTTCCAACAGCGGATGTAGTCGAGCCCAAGGACTCCTCACCGACATCCACTACCGCA
GCTGTTGTAGATGTCGAAGCAGTGGTTGAAGATATCAACGAGATTTGGCCACTGGAACATCACCTGAAAC
CCACCAACATTGACTTCTCCCAGCACGTCGAAGAATTGGCAGCACCAGCAGCGGTCACAGCAGAGACTGA
AGCATCCATGCCAGTAGAGGAAATTTGGCCGACAAGTCCAGAAACCGGAAACTCATTGACCCTGGAGCAG
TACGAGTTTGAGCCACAATCACCACATGAAGAGAGCACAAAATCTGATCTAGTTAAGCCCCAAGAGACTG
AACCGCAAGTTGTTGCTGAAACTAAGCCAGAAGGCATTACCACTGGAAGTATTACCATTACCAAGACTAC
AACCACCATCACAAGCTCCACAGAAGTACCGGAGGAAACTCTTGTCCAAAATGTGCCTGCCGATGAACAA
CAGCCTCCTGCAAACAAAATCAAGACTGACATTCAAAGTTTCCTTGAAGCCGAACAGACTTTGGCCGCAG
CTCTAAAGGAGCAGTCTTCAACTCCGACTGGAGCTTCGGTTGCTGAAGATGTTCAGACTCAGCCTGAGGA
AATTGTTCTTGAGGAAAGGACTGTTGAAATTTCAACCATCAAGACTGAAGAGAATCAACAAGAGCCAGTT
ATAGTGGAGGAAGTGAAATCACTCCCTGTAGAGCCTGAACCAGTAGAACCAGAGCTGGAGGAAGTGGCCA
TTGCAATTGTTGAGCAGACTGAGGAAAAGCCAGAAGAGCCCGTTATTGAGAAACAACCAGCTTCTGGCCC
AATCGACCTGCGTGCCGCCACTCAACTCTTCATCTCTGGAGAGGCCGCAGCTTCAACGGCTCCACAGAAA
ACCTTCCAAATCTCAGCACCCTCTCTGGAGGACAATGGCGCTGGTGTGCTGAAGGTTGTCCTGGGCAAGG
AAAGTACCAACGAAGAGGATACCGCAGCTCCAACTACCGGCAAGGTTAGCATGACCATCATTGAGACCGC
TGCTGCTCCCGCCGCCGACGCCAAGCGGCGTCGAAAGAAAAAGAAGAGAAGAGATACAAAGCACGAGGAG

GAGCTTGAGCAAGAACAAGAGACTGAGCCGGAACCAGTTGCCGCCGTTAAGGAGCCTGAGGTGAGCTCTG
ATGTGCCTGTTTCTCCGGAGGATTCACCACGCGATACCGTTCGTCATGAGTCCATTGTTGAGATTAGTCC
CGATAGCGATTTATCCAGCATTGAGATTGACACGAAAGTGAAGATTGTGGAGGATGCCGTTGTATCTTCA
CCATCGGAGTCGCCACGAACGCCAATGGTCGAGCTGGTCATTCCCACTGAGGTTGTTGAGTTAGCTTTGG
TCGAGGATGAGGAACAACAGACCACACCGCGTATTCCATCGCCCACTGAAAAATCGGAAGTCGAGCAGGA
TATTAAATCCGTGCAGACTTCACCACAGCATCAACCCAAATTAGATGAGACTGCAGTGCAGACGAGTCTA
GAGGTGCAGCCGGATAACCAGGAGAATGAATCGCAAACACTCATTGTCGAGATAACGGAGACTGAAGCAC
AAACCACTCCGCGAAGCGAGGAGCAGTCGGTGGCTGTTGAGATTTCCACCACTGAGATTCAAACCGATGT
GAGTGGCCAGCCAGCTGAAACTGTAGAGATTTCCAGCCAAACTACCGTGACCACCACTATCGAAAAGGAA
CTGCAGACCACGCCAAAGGATTCTCCTCGAGCACCAGAAGCAGGCAGCAGTGATGTGGTGGAATCTCTGG
TGCAGGATTTGGTTAAGGATATGACTACCGATCTGCCAGTGCGTACAAGCGAACAATCTACCGTGACTGA
AACTACCACAACCACTGAGACCCATGTGCAGACAACCACTCCGGAGCCAAGGGAACAAACTGAAGTCATT
AAACCGGAAACTGCACATGAAGAAACCTCAACCGTTGAACTTGTGCAGTTCGCGGATGGTGAGATGCAAA
CCACGCCTCCTGGAGATCAGCAACCCGCATCTTTAGATGATAGTTCTCTGACAGCCACTTCGATATCCGT
TTCGGAACCCTATGAACTGGAGGTCAAGACTACGGTGGCCATTCCCGCTGATTCGGATACATCTGTGGCT
GAGCCAACGGTCTATGAATACACACAGACCATGCAGCTGCCGAAGCAGGAGAAGAAATCCAAGAAGGACA
AGAAAAAGAAGCAGAAGAATGTGCCTGAAGTGGAGCAGCAGCTGCCAGAGGATCAACAGATCAGTGTGAC
CGTTGAAATCGCACCAGAGTTACTATCTGAGTCCGGCATTGTGGTATCCACCAACCAGCAGATTGAAGAT
GTGCCTCATGTAACACCCGTGGTAGACACTCCCATCGAATCCGAAGAAGTGGAAACGCCCAAAGCTCAAA
GAGTGCAACTTCAAATCACCAAGACCACTGTCTACGATGAGTATCCCGATCTGCCAGTGCATATAACTGA
GCAGAACAAGGTGCTCATTGCATCCCAGCAGAGCAAACGATCCGGAGCAGGACCCACATCCTCAGCGGTG
ACCATCGAAGAGGTGGGCTCACCCACGGAGGAGTTGGTAGTGCCCATCACCCCGGGACCAGACAATCTGA
GTGGCGAGCCCCACAACATCTGGTTCAGTGCCACCACCAGTGTGGATAAGACACCCATTGAGCTCTCCCA
GGCTTTAATTATGAGCGAGAGTTTGCAGCATTATCCAGGACAACAGAAACTGACCCAGGAGCCAATCCTG
ATTTCCACGAAGGAGGCCATTGGTGACCGCATCAAGCAGCTCAAGCAGGCGTCGCCCCAACAAGCCACTC
CACTGAGCAATGTCCTCCACTTGGCCACCTTATCTGAACAGATTAAGGAACTGCCCACCGAGCAACGCAT
CTTGGAGGTCAACGAAGGTCTCAAGGATCTCGATGTTGCCATTAAGAATGGTGATAAGACTGTGATTCAA
ACCACTGTGATCACGGTTATCGAGAAGGTATCAACTTGGTTGGAAACCATCGAGTATCGCGTGTACCTCA
TCAGGCAGAACTCTAATGAGGGACCCTCCGAAGAGAAGCTGGATAACTATAACCAACTAAATGACGAGCT
TAGCACCATCAAGCAAAATGTCGTCCAACTTGAGCGACAATTGTCCAAGGCTGAGCCGGAACCACAGTTG
CTGCAATGCGTGGATAGCCTGAAGGAGCATGTGGATGCAGTGGAGCAGGTTACACAACAGAACCAGGTGC
AGGACAGCAATGATCTGGACAAGTGGCACAACTTCGAGGTACTTTTGTACAACGTCTCCTCCGTTTTGGC
CGACCTGCAGCAGAGCTACGATCTTTTGATCAACCAGGAGTATCCTTTGTCCGCTAAACTGGCCCAGTTG
GACGAGTTGGAGCAGCAGCATGAGGCGGCGCAGCAGCAACTTGCGCATCTCTGTCAAAACGCTCGAGCTT
TCCAACGCGATTTTCCAGGCAAGAAGATGCCGCAGGATGTGCACAATGCGTTCGAGACCAGCAAGAATAT
TGCCAACAACATTCAGGCAGAGCGCGAGCGTGTGCTTCAACTTCAATCCCTAGCGGAGGAGTACGAGCAG
ACCCTTAAAGAGTTCACCAAAATCACGGTTCTTGCCGACAAGCTGGTGGAGAGTCCAATTGTATCCAGTT
CCCTCGAGCAGCTAAACAACGAGGTGCAGAAACAGAGGAAGTTCTTCGTAAATCTCAGCCACTGTCGCGC
CATGTTGGAATCCCTGGAGGAGAACATCGATAGTGAGACCCGCGAGAAGCACTCGGAGTTGCACAAGGAG
CTCTACAACAGAGCTACCAGTCTGCTGGACAAGGCATCCGAAAGGTCCTCCAAATTGGTGCAAGCTGCCT
CCCGTTGGACTGTGCTGGAGAAGGGCATGCGAGATGAACTGCAATGGCTTCAGGTGGCTCAGCAACGAGT
TCCCGACTTATCTGCCGTAACTTCCGCAGACTACGATCAATACACCACTCTGTACCAATCCCTGAGCAAT
GATATCTCACATCACTATGTGAAAATGACACAGCTTTCGGGAATAGCCAACAAGTTGCAGCTTTTGGTGC
AAGCCCCCAATCTCGTAGAGGAAACCAATGAGGCGTTGATAGTGCTACTCAAACTCCGCGAGGAGGTTGC

TCTCTACCTTCACCGCCTTCTGGTCTTCAAGGAAATCTGGGTGCAGTACGAACAGCAAACGGATAAGCTG
GAGGCCTTTGTACGCGAAGCAGAGCAGGAATTGAGGAACATCCAGATACCCTCGCAGCCCACTCACCAGC
CCATCGAGCACATGCGTCAATTCTGGGAGATTAAGGCCCGTTTCGAGCTCCACAATAATGTGAGAACCGA
TACGGGTCTGAGCTTTGAGAAATCCCTGCAGGTTATCCCCTTGGCGGACGAGATGCTACAGCGGCAGTTC
CATGCCCAACTGGAGGATCGCTGGCAGGCAGTGGCCCAGGCCATTGAACTCATTCAGCACAACATTGTGG
AATGCCTGTCGTCGGAGGATGTGCCCGCTGACGAGAAGCTCAAAATGGTAGAGCGAGAGCTGCAGGAGAT
CTACCTGACCATGACCAGCATGAAGGGAGTCATCAAGAACGAGGAGGAGCTCTGTCTGTACATCGAACGA
GTCCAAGTGCTACGCACTCGCGTCGGATTCATTGGCAACGAGCTTGGCAGGATCGGACTGCAAGAGCCCG
CCATTGAGCCGGAAAAAGTTGGTGAGCTATTCTCGCTCTCGCACAAGATAAGTACCCAGATTGCCGAGGA
GCTAGAGGGTGCATCTGTGCTGCGCGACCAACTGCAGGCCATCCAGGAGGGCATTAGCAATCAACGCAAA
CACCAGGCCAAGATCTCGGTCATTTTGGATGAATGTGAAGCCGCCGAGCGCCAGGGAGCAGATGTTCTCG
AAAAGGCCGTCGCCGATTGCCAGGCCGCCGGCGAGGAGCTGGTGATCAGCTGGCAGGAGATTATGCGAAT
CCGCCAGATGCTCCACACCCTGCCCATGCGCCTCAAGATGTCCGTTTCACCGGTTAAGTTGGAGAGGGAC
ATCTCCCAATTGCAGGACGACCACGCCTTCCTCGAGAGCAAGTGCACCAACATAATGGCCATCCTGAGGA
GCCGCCTAGCCGTCTGGCTGCGCTATGAGCGTCAGCTGGAGCTGGTCCACGGATCCGTCCAGGAAACCGA
CTTCATGATGGAACTCATCCGGGTGCACGGTCAAGTGGACTACGAGCGCCTGCGCAAAGCAACTGAACGT
TTGGAGGGTAAGTTTTTAGCTCACAATTTTATGACTTTGTTTACGCGCGCGTTTAGGGATCGATTTGTAT
CTCAGATATATTTGTATCTTTCAAAATGCGCTACCTGCTGAGCGGAATTTTTGCGAACAGCTTATCAGCT
TAGTTTTCCCAGAGGAAAATTAGAAATAAAATCCGCTACATATTTGTAACGCGCCTCATTCATTTTCCGA
TACTCGAAACAGAAACATCATCTATACGTTTTGTTCTAAATAATAATTTAATCATTTAAATTTTAAAATT
TATTCAATACTATTTGCGAAGCAAAGACTTTCATGTAGAATGTAAATTTAAAAAGCTATCGGTCGTCAAA
CTTTCGGCAAGCTTTTTCGTAAGCTCCCGCCGCATGTGGAGCTCTTTACGTAGCAAGTTTATCGCGGCCC
CAGTTGACGTTAACTTCGAACCGAGCGCGTATCTCCCGTCGCGGTCGGAATCCCATATACAACTAGACTA
ATCCATCTTTATACGTGCAAATTAACCCATCGCAGGGGACTGGCTGGCGATCTGCATAACCGCGAGCAAC
TGATCGATGAGCTGAAGGGTGCCGCCAAACCGCTGATCGAGAGCTGCGATGTCCAGATTGTCGAACAGAT
CGAGTCCGCTGTCCAGGAGGCGGTAGTCGCCTGGAATGACACCAGCGAGAATCTGCAGCAGCTGCGAACC
CGCTACCAAAGAGCCGTCGAACTGTGGGATAAGTATCGCAACGCCTCAGCCGCCGTCAAGAACTCCATTG
ACCAGCAGATGGATGCGGTCAAGTCGCTGGAGCAGCCCCTGGATGCCCTGCAGCACGCCAAGGGTGAGTG
CCCACAAATGCCGTGACCCGTCCGGCACACGGGTCGTATGAGTGATTTATATCGCGTGTGGGCGACAGTG
ATGCCAACGACCTGTTTGTCACATAGGTAAAATCGAACCGGAAATATATAATAATTGAACAAACAACAGA
GACAGAAAATCGGTTAATAATTTGTGCATTCTAAGTACAAAGATATATCGATTGGTTTGTTATTTAAAGT
CAATTAATCTAATTTAGTTTGAGCATATTTCAAGTGTAGTGTAACAtaaggtaaggtaaggtaaggtTTT
TTGAGATCTTTTGGGAACATAAGTTATCTAGATAAGAGGCTCTTCTAAAACTGATAAATCCGCCTTAGGT
GCACATATCtttttttttatttttGGCGGCTAACTAGGACAGAAATGGGGCTCTTTAATTGGCCACTGGGG
ACGCGGGTGTTCCAACTAGTAATTACATTGATTTTCTCCGTAGTGCTCGCCCTATCAATTTTTTTGGGCG
CGTCAATGTAATCGGTTCGTAATAGTTCCGCCGTGAATGTACATATATGAGCTAACTTGGCCGGGCTGTG
TTTGTGTCATAAATTATGTTTTGGGTAAATATGACCGACATCGTACGTACGATTTCCTTTTCGGCATTTA
CTTAAAAAATTAACGCATACTCGGATCGCCGTTTTGTGTTTATTGATCATTAGGGTCATTATATTATGGT
AGGGGGCCCAACTCGATGGTGTAATTATATTTTCGGACAGGCGACCAACTGTCATTTCACACAGGAGAGT
TTATCAACCCGCTGCTGTTGGGTGGCTATATTTTCGTATTATTATAGTATTTTATGTATACATAATCAGC
TTTATCAACAGCAGCAGTAGGAGCAGCAGCTGAAAAACAGCGGGAACAGGTTCGCAAATGCCTTGAGCTC
CGAAAGAAGATAAATGTGGAAAAAGATAGACAGACACAGAGCGAACAATACCTAATCTATGTTGGTATTT
TCAATGCCAGTTTTCTAAGCGTTTGTTTGCTTATGTAGCTCCGTGATAAAGCATCGCCAAATACTTGCCA
GATGATATCATAGGGCGGACATTACAAAGGGCATTAGAGAACAACTTATGCGTCGGCAAGTGGTGGTGAC

CCGTTTTCCCCTCGAATTGAAATCGAAAGTGCGCCGGGCACTCACAGCCCTAATTACCATTGGCGAGCAG
GGAAATGCACTCGACAACACAATTGATAAGGGTCTGAGGCACGAAATGGAACACCAGCCCGGCCGGCTTG
GGCTAAATACATTCGTCGTTGTGGTCGTCGTCGAGTTAAATATAAACCAGTGACATTTTGCCAGGCATCC
AATATAATTGGGGTATGTTTAGTTCAGAAAGAGCTTTTCGGTGGCGAGAAAGAACAGAAAGTATTAGAAA
TAGTTAGGCAAAAGGCAAAGGGAATGTTAAATGAATACAATTGATAGATGACTTCTATTGAGATGGTTTT
AAGTTGATCATTTAAGCTAATCACTTGTTTAGAGGAACAAAAACGAGTGATTATACCCTGGACTTCATTA
ACTAAATTGGCCTCCATATCTCTACATTTACACAGGGTTTGCCAAGACAACCTGACGACCCAGAATGACA
GAATCTTGGAGTTGCGCGACATTGTGGCCAAGATCGCCGCCGATGTGGGCCTGGATGCCTCAGCGCTCAT
GCAAGGTGAATTGGATGCACTGGGTCAGCGATTGGCCGAGTGCAAGGATGCCATCACCACGCTGGCCAAC
GTGGCCGAGACGCAGGACAAGGAGCGCAAGGAGCTCGACAAGGAGGTGACTCTGGCCAAGGCCTACTTCA
ACAATGTGCAGCAGGGTGAGTGAGAGGCGAATGAATGAAATTGTGTTAGTACACGGAAGTAGGACGTGTA
TCCCACTGGGATTAGACCCATCTATCCTGACCAGCTATCGATTGTGGCGTCGAATAGATGGATATTTTGC
ATATGCTAGCTTGGGCTTGGCGTGTGGAAATTCCCACACTAACAAATACCGATTAGCCGGCAAATGTTTT
TGCTGTTGTGCTTGAAGCTTGTAGTTGTTAATCCAATACGAAGGACATGCTATTTTAAGTGGGTTAAAAG
TTGAAAAAGTTTTTGTGTATCCTGGCTGGTAGCCCACTCACCGTGGTCCCAGTGCTCCTGCCCATCATTA
TTGATTGATCAGAGGCGTGAATATAATTTTTGAGAGTACGATTCCCCAGTGAAAAACTTATAAAATCGTA
ATTGACACTTGCTGGCCCGTGGGAAGGTTGATAAATTTTTAATGACAATATGTGCATATATGTGTTCCAT
GTTTTGGTCTTTGGGGCTAGGAAACTTATTGCGGACTATTTCTGTTTTGCACTTCATTTCATTTATCAAA
CAACTAATTCACTTCCCTGGGGAACGGAAGTTGAGCATTTTATGAAATTACAAATAAATTAGTTCTGTGA
TAAGCGCATTCTTTAAGAGCTCTAAAGAGCTTTCCCCTTTTAAATCAATGCCACCATAAATATAAACGGA
AGCTTGGCTGATTAACGATCGTACAGTTTTACGATTCATTCAAAAGAGGGGGCATAAACAAGTGATTTCA
TTGATAGGTGGTGGCATACAAGTGCCTTTACAAATTAACGTACCAAATTGGGCATTTTTTGGTAGAACTT
TCCAAGCACCTTGCACCACGGTGGGCTCATTTGCATGGCACATGCCAACCAGTTTGGATTTCCCCCGGCT
CCCTTTTCCGCTCACAGTCCAACTCCGCCGCCCACTTTTGGCCCCCTCATCGACGCCTCAGACAATTGAA
GTTGGTCACTGGGAGCGCGTGTCGTTGGCATGAGGTCATTGCCAGTTGGCGGAAAGAGTTGCAATGTGCC
ACAGCCACAGCGATTCCCATTGTCTTGGAAGCCATGCAGTAGTGGACAGACGGACGAATGGTAACCGGAG
ACCTGCGAGCAGCCATATTAGGGTCAGGCCAATTAGCGATGCACGAAGCATATTCAAAGCTGACCACTGA
ATGAATGAATAAATAATAATTGTTTTCGTTTGCCTCCTTGCAGGGACATATCCCGAGAGGCACCGCAAAA
TCCCAAAGAAAGCGAGGAGCAGCTGGCCGCCTTGCGAGCCCATCTCCAGACTTTGGCCCGAACGGAAGAG
CAACTGCGTCAGCTGAAGGAACGCCATCAGAATAGCGAAGTAGCACCATCGGTGGCCAGCAGCGATGATG
ACGGCATCCTGGAGGTTCTAGCCCTGTGGCAGAAGATTTTCCAGGACACCTTCCAGGAGTATCATCGCCT
GTCCACGCGACTCGCCCGCAGCCAAAACAGTTCGGAGGCTCTGCGATTGTGGAGGCAGTACCTGCAGCAC
GTCCAGTCCTTCCTGTCCTGCGCCATTCCCGAGGATTACAGCAGCCTGCGAGAGCAGCAGCAGCTGTGTG
CCATCCACCAGAACTTGCTCATCTCGCAGCAGAGTGTGCTTTCCGAGACACCACTGGAATCGGAGCTATC
GGAGCAGTACAAGGCTCTGACCAACCTGCACAATGAAACCCTCTCGAGGATCATGCAGCGCAATGGTGAG
CTGGAACGGCGAGTTAGTGGCTGGAATGCCTACCGCCAGCAGCTGGCAGCCCTTTTGGACTGGCTGCGTC
AACGGGAAGCGGAACGCAATGCCCTTCAACTGCGCTACATTCACCTGAAGCGAGTGCCCCACCTGAAGCA
CCGCCTGGATGCCATGATCCAGCAATTGGATCAGGGAGAACAACAGAGCAAGGCTTTGCAGGAGCAGCAG
CAGGAGCTGGCCAGGCATTGTGACGACGCGCTGGCCACCGCCATGCGAATGGAGCAGGCCAGCATTGGTC
AGCGGATAAGCAATCTGCGCGCAGCCCTAAAGACCTGGCAAGGATTCTTGCAGCGCGTTACCCAGCTTTC
GGAAAGCTACGAGCAGCGAGTTAACCAGTTGCAGCAGGAATTCGGAGCAGCACAGAAGCTTCTGGATGCC
AACAGCGAATCTCTGCCCACTCAGCCAGCGGCAATTGAGCAACTACTGGGATCCCTGCGTGCGCAAAGGG
TCCAACTTGGTGCCCAAGTCTCCGCTCTGGAGAGTCTCACTGTGACCCAGGAGGAGCTCAAGGAGTGCAT
CAGTCCCCACGACATGAAGACCATTCGCCAAAGGAACTGGTTGCTCTGGCAGCAGCACGCCGATCTGGAC

TACCAGCTGGCCAACCTGATCAACTCGATTGAGGAGCGGCTTTCCCTACTGTCCAACTATCAAATCAGAT
ACGATCGCATCTCCCAGTGGCTGCAACGTTTGGAACAGCGTGTGGAAAAGGATGCTGATGTCACTGCCAT
GACAAATCCCGAACAGGCAGCCAAGCAGCTGGAGCAGCAGGTGAATTCCGAGCTGCAATTGAGGGACAAG
GAACGGGAGTGGCTTCTATCCACCAGCCGGGAACTGCTCACCCTCTACTCCGAGCCAGAAGTGCGATCCC
AGGTGCAACAACAGAGTGACTCCCTCATCGATCGCTGGCAGCGTCTCAAGTATTTGGCCAAGCAGAAAGC
CACCAAGATAGGAGAGCTCAAGATGACACTTCTCCGTTTGGAGGAGCGAATCGCTCTGATCCGCGCCTGG
CTTTTCGAAGTCGAATCCCAACTGGACAAACCCCTTGAATTTCGAAAGCTACACACCGAATGTGATCGAGG
CCAAGCTAAAGGAGCATGAGCAAATCCAACGCTCCATTGAGCACCACAGCAGCAATGTGGGCGAGGTCCT
TAATCTCGTAGAGATGCTCCTCAACGATGCGGACAGCTGGCGCACGCAGGTGAACACTTCTGGATTAGCT
GCCTCAGCTCAGAATCTGGAGCAGCGCTGGAAGAATGTGTGCAGCCAGTCGGCGGAACGAAAGGCCCGCA
TCCTGACCATTTGGAATCTTCTCCAGCAGCTCATCAAACTTACTGCAGAGCATAAAAATTGGCTGGGAAA
ACAAGAAAGCCAAATTGCCGGCTTCGAAAGAGATCAGAAGTCCCACAGCAAGCATAAGCTGGAGGAGCGC
CAGATGGAGCTGAGAGCCAAGCTGGAGGAGCTGGAAAGTCAGTCGGTGAATCTTCGCCAGCTGGAGCAGA
TCTATGCTAAGCTGGCCATGAGTGCTGGCGTCGAGCCGGAGAACATCCAGAAACTGACACTGCCCACCAA
GGTGATGGTGAGCATGTGGCGCCAGTTGACGCCACGCTGCCATGCCCTGTTGGATGCCATCGACAAGGAT
GCCAAGCTAATGCGAGAGTTTAACAACGCACAATTGGAAGCCACCAACTCGCTGAATGCCATACAAAAGG
CTTTGGAACAATTGCCCAGCGCAGAGAATCAACAGACCTCCAAAGCGGAGCCGAAAGCGGTGCTCCAACG
TCTGGAGAGTCTAGAGAAGAAGTTGCAGGACGCACAACAACATGTCCAGCAGGCGGATAACCTGGCCCAG
GAGGCCAAGACGAGGACCAAGCAGCAACCCCAACTGAAGCAATTGTTGGAACTTGTGTCCGCCTATACCA
CCCTTTGGCAGACGGTCCAGACCCGCATTGTCACCCTCAAGACCACCTGGCTGACCAGAGCAGCTCAGGC
AGCCGCCTCTCTGCCGGTCAGTGAGGCAGCCAACGCCGCCGTCCAGGTAAACACCCTATCCCAGCGGAAA
CTCCGCCAGGCCCAACAGATGCAGCGGGAAACCTCCATCACGGCCAAGGATGCCTACATCATGGAGCTGC
AGACGGCCATCACGGAATGCCAGAACAATCTGGACGAGCTGCAGCGCACGGTGGTGGACAAGACCCGCAA
GCCGGGACCCCAGAAGATCGCCAAGCTGCTGGGCAACGCTCAGTCCTCCACCGAGTTAGTGAAGCATCTC
AGCCATCTACTGCTCACCGAGTGCAAGGCAGATGACCAGGCTGCCGAGGTGGACACCGTGGCAGAGCTCA
CGTTGCGCTTCGATACACTGCAGTCGCAGTGGAAGGCGCGACAGCAGCACGATCAAAATGCAAGGGTAAG
ATTTTATATTAATTATCTATATATATTTGTTGGGATATTTTTCAAGATACTTGCCAAACCAGAAAACCAT
TTTGATAGGAGGCAAAGTGGCATATTTGGGAATATACCTTCCCAATTGTGTCTCAAATACTCCAAAAACA
ACTATGTAATAGGGACGAACGGCATCCTGAATAGTGCATCATACTATAGCAGATGACTTGTGTTCGTGCA
CATGTTAAAAGTTTTCTAAATTTTTATATATATATAATAATAGAAGTAACCCTTTTCGGTTTGGCAAGTTCT
GCAACATTTTCCGCTGGTGATTTGGGACCTGTTCTTGACTAACCAATTGTTTTATTGCTTTCCATTTCGC
TGTTGCCTACTTTCTCCTTATGTCAAAACACGCACACGCATATTCACACTCAAATATATAAACTTACCCA
TGCTTAACTCGAACCTGCGCATTCATGATGACACCAGGTGCCACCTACCTGCATCCTACAAGTACAACTG
CATCCAGTAAGTGTATCTACCTGTCGGTGGGCAGTTCCGACCTCCGACCTCCTGTTGTTTGATCCTTTGT
TCCATGTTCTACGTGGTTTTCCACTAGACAAAAGCTGGCAGTGGACTACAAATGGCTGCCAGTCTCCAAG
GTGGCGAACAGCAAGAGGTCGCCGAGCAAACGCCCTAACTATCTAAGTTAACCTTAAAGCCAAATCAATG
GACTTTGGAAAGGCGAGCTTAAGCTAACATTTAATCGATTACTACCATAATCACTATCTGCTATGATTAT
TCAATTACTAATTTCTCCACTTTTTTGACCAATTCTAGGCGAGGTCGGCCGGCTAACGTGTCCGCTCTGC
ACACAGCGAAATTGGCAGCAGATCGACAACGATCTGTGGCGCCTGGAGCAGTGGCTGCAGTTCGCCGAGA
GCACCCAGAAGGCCCAGTCAGCTCCACCCTCAAACATCGAGCTCCTCGAGGACGTCACGCAGGACCATCG
CGAGTTCCTCCTGGATTTGGAGAGTCACAAGTCCATCATATCGTCGTTGAATGTGGTCGGTGACCACTTG
GCTACGCACACTTTGGATACCGAAAAGGCGCGACAGCTGCGATCTCGTCTAGAGGCGGACAATGAGCGCT
GGAACAATGTGTGCATCAATGCCACCAAATGGCAAGGTCTTCTCCAGACCGCCCTGATGGGCAACAGCGA
GTTCCATCAGACCATCGGTGAGCTGGTCGAGTGGCTGCAGCGCACCGAGCAGAACATCAAGGCCTCCGAG

CCCGTTGATCTCACCGAGGAGCGCTCCGTTCTGGAGACCAAGTTCAAGAAGTTCAAGGATCTGCGAGCCG
AACTAGAGAGATGCGAACCTCGGGTGGTCAGCCTGCAGGATGCCGCCGATCAGCTGCTCCGCTCCGTCGA
GGGCTCCGAGCAGCAATCGCAACACACGTACGAGAG
>dme_circ_0001506_junction_seq
AGCAGCAATCGCAACACACGTACGAGAGGCTAATCCAGAGCAATCTCGAAGTACAC
>dme_circ_0001507
GGATTTGGTGGAGCAGACGCGCCAACTGCAATCCCATCCCGATGTTTCTCCCTTGGCTGACACGCTGATG
GAGCAGCTGCAGAGCATCATTACCATTCTGCGGGAACAGGTCACAGTGGCCACCAAGCGCATCTTTACCA
TTGAAAAGCGCATTGTGGATTTGCGAAAGGCCAAGAGCGAGGAGGCGCAACGCCAGCGTGTGCTTGCCGA
TTCACTCATCAAGCCACCAACCGAAGCTCCAGCGAGTCCAGAGGCGCATGAATCCATCGAGTCCAACGAG
AACACCATCGACTCGAGTTCCATGCCCGAAGAGGAGATCAAACCGACTGGAGTGTATGTGGAGACGCAGA
CATCGCTCAGCCTGCAGCAGCCGCCAGTTCAGGTGGTGACCACCACGACTGTGGAGGCCCAGACCAGCTT
CAAGGAGCCAGCGGTAGAAACCGCAGAGGTGGCTCTACAAACCCAAAAGGAAC
>dme_circ_0001507_junction_seq
GAGGTGGCTCTACAAACCCAAAAGGAACGGATTTGGTGGAGCAGACGCGCCAACTG
>dme_circ_0001508
TACTATGTGCTTGGTGCCATCAGGTCCCACAATCACTTCGGTGGTCGTCTCATCTGGCTCCACGAAAGTG
GTTTCGAATGCCTGAGGTACATTTTTCAGGATCAGCTCGGTGGCGCGGTCCACATCGCCCTTGGGTCGTT
GATGGTAGCGAGCCTCAATCTGGACATCTTCGGGTTCATCGGGCACATCCTTGTTGCGAGTCGTGTCGAT
CTGGATCGTTTCCTGGCCATGATGCACCGTTTG
>dme_circ_0001508_junction_seq
TCGTTTCCTGGCCATGATGCACCGTTTGTACTATGTGCTTGGTGCCATCAGGTCCC
>dme_circ_0001509
TACTATGTGCTTGGTGCCATCAGGTCCCACAATCACTTCGGTGGTCGTCTCATCTGGCTCCACGAAAGTG
GTTTCGAATGCCTGAGGTACATTTTTCAGGATCAGCTCGGTGGCGCGGTCCACATCGCCCTTGGGTCGTT
GATGGTAGCGAGCCTCAATCTGGACATCTTCGGGTTCATCGGGCACATCCTTGTTGCGAGTCGTG
>dme_circ_0001509_junction_seq
ATCGGGCACATCCTTGTTGCGAGTCGTGTACTATGTGCTTGGTGCCATCAGGTCCC
>dme_circ_0001510
TCCTTGGGCTCGACTACATCCGCTGTTGGAACATGAACGGGACTGGTAGTGGCAACAACTACGGGTTGTT
CAACATCTACTTGAGGTGCTGGCAACTCTACTGGTGCTTCTTTAGTCGTTTCtgttgttgttggttgtgt
ctgttgttctggctgttcttgttcttgtgtggttgcggttgtagttCTTGTGGTGGTGGTGACTTGGG
>dme_circ_0001510_junction_seq
gtagttCTTGTGGTGGTGGTGACTTGGGTCCTTGGGCTCGACTACATCCGCTGTTG
>dme_circ_0001511
CCGTTGAAGCTGCGGCCTCTCCAGAGATGAAGAGTTGAGTGGCGGCACGCAGGTCGATTGGGCCAGAAGC
TGGTTGTTTCTCAATAACGGGCTCTTCTGGCTTTTCCTCAGTCTGCTCAACAATTGCAATGGCCACTTCC
TCCAGCTCTGGTTCTACTGGTTCAGGCTCTACAGGGAGTGATTTCACTTCCTCCACTATAACTGGCTCTT
GTTGATTCTCTTCAGTCTTGATGGTTGAAATTTCAACAGTCCTTTCCTCAAGAACAATTTCCTCAGGCTG
AGTCTGAACATCTTCAGCAACCGAAGCTCCAGTCGGAGTTGAAGACTGCTCCTTTAGAGCTGCGGCCAAA
GTCTGTTCGGCTTCAAGGAAACTTTGAATG
>dme_circ_0001511_junction_seq
CTGTTCGGCTTCAAGGAAACTTTGAATGCCGTTGAAGCTGCGGCCTCTCCAGAGAT
>dme_circ_0001512

GTTTCCTTGAAGCCGAACAGACTTTGGCCGCAGCTCTAAAGGAGCAGTCTTCAACTCCGACTGGAGCTTC
GGTTGCTGAAGATGTTCAGACTCAGCCTGAGGAAATTGTTCTTGAGGAAAGGACTGTTGAAATTTCAACC
ATCAAGACTGAAGAGAATCAACAAGAGCCAGTTATAGTGGAGGAAGTGAAATCACTCCCTGTAGAGCCTG
AACCAGTAGAACCAGAGCTGGAGGAAGTGGCCATTGCAATTGTTGAGCAGACTGAGGAAAAGCCAGAAGA
GCCCGTTATTGAGAAACAACCAGCTTCTGGCCCAATCGACCTGCGTGCCGCCACTCAACTCTTCATCTCT
GGAGAGGCCGCAGCTTCAACGGCTCCACAGAAAACCTTCCAAATCTCAGCACCCTCTCTGGAGGACAATG
GCGCTGGTGTGCTGAAGGTTGTCCTGGGCAAGGAAAGTACCAACGAAGAGGATACCGCAGCTCCAACTAC
CGGCAAGGTTAGCATGACCATCATTGAGACCGCTGCTGCTCCCGCCGCCGACGCCAAGCGGCGTCGAAAG
AAAAAGAAGAGAAGAGATACAAAGCACGAGGAGGAGCTTGAGCAAGAACAAGAGACTGAGCCGGAACCAG
TTGCCGCCGTTAAGGAGCCTGAGGTGAGCTCTGATGTGCCT
>dme_circ_0001512_junction_seq
GGAGCCTGAGGTGAGCTCTGATGTGCCTGTTTCCTTGAAGCCGAACAGACTTTGGC
>dme_circ_0001513
GATTCAAACCGATGTGAGTGGCCAGCCAGCTGAAACTGTAGAGATTTCCAGCCAAACTACCGTGACCACC
ACTATCGAAAAGGAACTGCAGACCACGCCAAAGGATTCTCCTCGAGCACCAGAAGCAGGCAGCAGTGATG
TGGTGGAATCTCTGGTGCAGGATTTGGTTAAGGATATGACTACCGATCTGCCAGTGCGTACAAGCGAACA
ATCTACCGTGACTGAAACTACCACAACCACTGAGACCCATGTGCAGACAACCACTCCGGAGCCAAGGGAA
CAAACTGAAGTCATTAAACCGGAAACTGCACATGAAGAAACCTCAACCGTTGAACTTGTGCAGTTCGCGG
ATGGTGAGATGCAAACCACGCCTCCTGGAGATCAGCAACCCGCATCTTTAGATGATAGTTCTCTGACAGC
CACTTCGATATCCGTTTCGGAACCCTATGAACTGGAGGTCAAGACTACGGTGGCCATTCCCGCTGATTCG
GATACATCTGTGGCTGAGCCAACGGTCTATGAATACACACAGACCATGCAGCTGCCGAAGCAGGAGAAGA
AATCCAAGAAGGACAAGAAAAAGAAGCAGAAGAATGTGCCTGAAGTGGAGCAGCAGCTGCCAGAGGATCA
ACAGATCAGTGTGACCGTTGAAATCGCACCAGAGTTACTATCTGAGTCCGGCATTGTGGTATCCACCAAC
CAGCAGATTGAAGATGTGCCTCATGTAACACCCGTGGTAGACACTCCCATCGAATCCGAAGAAGTGGAAA
CGCCCAAAGCTCAAAGAGTGCAACTTCAAATCACCAAGACCACTGTCTACGATGAGTATCCCGATCTGCC
AGTGCATATAACTGAGCAGAACAAGGTGCTCATTGCATCCCAGCAGAGCAAACGATCCGGAGCAGGACCC
ACATCCTCAGCGGTGACCATCGAAGAGGTGGGCTCACCCACGGAGGAGTTGGTAGTGCCCATCACCCCGG
GACCAGACAATCTGAGTGGCGAGCCCCACAACATCTGGTTCAGTGCCACCACCAGTGTGGATAAGACACC
CATTGAGCTCTCCCAGGCTTTAATTATGAGCGAGAGTTTGCAGCATTATCCAGGACAACAGAAACTGACC
CAGGAGCCAATCCTGATTTCCACGAAGGAGGCCATTGGTGACCGCATCAAGCAGCTCAAGCAGGCGTCGC
CCCAACAAGCCACTCCACTGAGCAATGTCCTCCACTTGGCCACCTTATCTGAACAGATTAAGGAACTGCC
CACCGAGCAACGCATCTTGGAGGTCAACGAAGGTCTCAAGGATCTCGATGTTGCCATTAAGAATGGTGAT
AAGACTGTGATTCAAACCACTGTGATCACGGTTATCGAGAAGGTATCAACTTGGTTGGAAACCATCGAGT
ATCGCGTGTACCTCATCAGGCAGAACTCTAATGAGGGACCCTCCGAAGAGAAGCTGGATAACTATAACCA
ACTAAATGACGAGCTTAGCACCATCAAGCAAAATGTCGTCCAACTTGAGCGACAATTGTCCAAGGCTGAG
CCGGAACCACAGTTGCTGCAATGCGTGGATAGCCTGAAGGAGCATGTGGATGCAGTGGAGCAGGTTACAC
AACAGAACCAGGTGCAGGACAGCAATGATCTGGACAAGTGGCACAACTTCGAGGTACTTTTGTACAACGT
CTCCTCCGTTTTGGCCGACCTGCAGCAGAGCTACGATCTTTTGATCAACCAGGAGTATCCTTTGTCCGCT
AAACTGGCCCAGTTGGACGAGTTGGAGCAGCAGCATGAGGCGGCGCAGCAGCAACTTGCGCATCTCTGTC
AAAACGCTCGAGCTTTCCAACGCGATTTTCCAGGCAAGAAGATGCCGCAGGATGTGCACAATGCGTTCGA
GACCAGCAAGAATATTGCCAACAACATTCAGGCAGAGCGCGAGCGTGTGCTTCAACTTCAATCCCTAGCG
GAGGA
>dme_circ_0001513_junction_seq
TGCTTCAACTTCAATCCCTAGCGGAGGAGATTCAAACCGATGTGAGTGGCCAGCCA

>dme_circ_0001514
ATTTACGAAGAACTTCCTCTGTTTCTGCACCTCGTTGTTTAGCTGCTCGAGGGAACTGGATACAATTGGA
CTCTCCACCAGCTTGTCGGCAAGAACCGTGATTTTGGTGAACTCTTTAAGGGTCTGCTCGTACTCCTCCG
CTAGGGATTGAAGTTGAAGCACACGCTCGCGCTCTGCCTGAATGTTGTTGGCAATATTCTTGCTGGTCTC
GAACGCATTGTGCACATCCTGCGGCATCTTCTTGCCTGGAAAATCGCGTTGGAAAGCTCGAGCGTTTTGA
CAGAGATGCGCAAGTTGCTGCTGCGCCGCCTCATGCTGCTGCTCCAACTCGTCCAACTGGGCCAGTTTAG
CGGACAAAGGATACTCCTGGTTGATCAAAAGATCGTAGCTCTGCTGCAGGTCGGCCAAAACGGAGGAGAC
GTTGTACAAAAGTACCTCGAAGTTGTGCCACTTGTCCAGATCATTGCTGTCCTGCACCTGGTTCTGTTGT
GTAACCTGCTCCACTGCATCCACATGCTCCTTCAGGCTATCCACGCATTGCAGCAACTGTGGTTCCGGCT
CAGCCTTGGACAATTGTCGCTCAAGTTGGACGACATTTTGCTTGATGGTGCTAAGCTCGTCATTTAGTTG
GTTATAGTTATCCAGCTTCTCTTCGGAGGGTCCCTCATTAGAGTTCTGCCTGATGAGGTACACGCGATAC
TCGATGGTTTCCAACCAAGTTGATACCTTCTCGATAACCGTGATCACAGTGGTTTGAATCACAGTCTTAT
CACCATTCTTAATGGCAACATCGAGATCCTTGAGACCTTCGTTGACCTCCAAGATGCGTTGCTCGGTGGG
CAGTTCCTTAATCTGTTCAGATAAGGTGGCCAAGTGGAGGACATTGCTCAGTGGAGTGGCTTGTTGGGGC
GACGCCTGCTTGAGCTGCTTGATGCGGTCACCAATGGCCTCCTTCGTGGAAATCAGGATTGGCTCCTGGG
TCAGTTTCTGTTGTCCTGGATAATGCTGCAAACTCTCGCTCATAATTAAAGCCTGGGAGAGCTCAATGGG
TGTCTTATCCACACTGGTGGTGGCACTGAACCAGATGTTGTGGGGCTCGCCACTCAGATTGTCTGGTCCC
GGGGTGATGGGCACTACCAACTCCTCCGTGGGTGAGCCCACCTCTTCGATGGTCACCGCTGAGGATGTGG
GTCCTGCTCCGGATCGTTTGCTCTGCTGGGATGCAATGAGCACCTTGTTCTGCTCAGTTATATGCACTGG
CAGATCGGGATACTCATCGTAGACAGTGGTCTTGGTGATTTGAAGTTGCACTCTTTGAGCTTTGGGCGTT
TCCACTTCTTCGGATTCGATGGGAGTGTCTACCACGGGTGTTACATGAGGCACATCTTCAATCTGCTGGT
TGGTGGATACCACAATGCCGGACTCAGATAGTAACTCTGGTGCGATTTCAACGGTCACACTGATCTGTTG
ATCCTCTGGCAGCTGCTGCTCCACTTCAGGCACATTCTTCTGCTTCTTTTTCTTGTCCTTCTTGGATTTC
TTCTCCTGCTTCGGCAGCTGCATGGTCTGTGTGTATTCATAGACCGTTGGCTCAGCCACAGATGTATCCG
AATCAGCGGGAATGGCCACCGTAGTCTTGACCTCCAGTTCATAGGGTTCCGAAACGGATATCGAAGTGGC
TGTCAGAGAACTATCATCTAAAGATGCGGGTTGCTGATCTCCAGGAGGCGTGGTTTGCATCTCACCATCC
GCGAACTGCACAAGTTCAACGGTTGAGGTTTCTTCATGTGCAGTTTCCGG
>dme_circ_0001514_junction_seq
TTGAGGTTTCTTCATGTGCAGTTTCCGGATTTACGAAGAACTTCCTCTGTTTCTGC
>dme_circ_0001515
GTCGGCCAAAACGGAGGAGACGTTGTACAAAAGTACCTCGAAGTTGTGCCACTTGTCCAGATCATTGCTG
TCCTGCACCTGGTTCTGTTGTGTAACCTGCTCCACTGCATCCACATGCTCCTTCAGGCTATCCACGCATT
GCAGCAACTGTGGTTCCGGCTCAGCCTTGGACAATTGTCGCTCAAGTTGGACGACATTTTGCTTGATGGT
GCTAAGCTCGTCATTTAGTTGGTTATAG
>dme_circ_0001515_junction_seq
GCTAAGCTCGTCATTTAGTTGGTTATAGGTCGGCCAAAACGGAGGAGACGTTGTAC
>dme_circ_0001516
AGATGCGCAAGTTGCTGCTGCGCCGCCTCATGCTGCTGCTCCAACTCGTCCAACTGGGCCAGTTTAGCGG
ACAAAGGATACTCCTGGTTGATCAAAAGATCGTAGCTCTGCTGCAGGTCGGCCAAAACGGAGGAGACGTT
GTACAAAAGTACCTCGAAGTTGTGCCACTTGTCCAGATCATTGCTGTCCTGCACCTGGTTCTGTTGTGTA
ACCTGCTCCACTGCATCCACATGCTCCTTCAGGCTATCCACGCATTGCAGCAACTGTGGTTCCGGCTCAG
CCTTGGACAATTGTCGCTCAAGTTGGACGACATTTTGCTTGATGGTGCTAAGCTCG
>dme_circ_0001516_junction_seq
GACATTTTGCTTGATGGTGCTAAGCTCGAGATGCGCAAGTTGCTGCTGCGCCGCCT

>dme_circ_0001517
GCTTAGCACCATCAAGCAAAATGTCGTCCAACTTGAGCGACAATTGTCCAAGGCTGAGCCGGAACCACAG
TTGCTGCAATGCGTGGATAGCCTGAAGGAGCATGTGGATGCAGTGGAGCAGGTTACACAACAGAACCAGG
TGCAGGACAGCAATGATCTGGACAAGTGGCACAACTTCGAG
>dme_circ_0001517_junction_seq
TGATCTGGACAAGTGGCACAACTTCGAGGCTTAGCACCATCAAGCAAAATGTCGTC
>dme_circ_0001518
GTATCCTTTGTCCGCTAAACTGGCCCAGTTGGACGAGTTGGAGCAGCAGCATGAGGCGGCGCAGCAGCAA
CTTGCGCATCTCTGTCAAAACGCTCGAGCTTTCCAACGCGATTTTCCAGGCAAGAAGATGCCGCAGGATG
TGCACAATGCGTTCGAGACCAGCAAGAATATTGCCAACAACATTCAGGCAGAGCGCGAGCGTGTGCTTCA
ACTTCAATCCCTAGCGGAGGAGTACGAGCAGACCCTTAAAGAGTTCACCAAAATCACGGTTCTTGCCGAC
AAGCTGGTGGAGAGTCCAATTGTATCCA
>dme_circ_0001518_junction_seq
AAGCTGGTGGAGAGTCCAATTGTATCCAGTATCCTTTGTCCGCTAAACTGGCCCAG
>dme_circ_0001519
TTGAAGCACACGCTCGCGCTCTGCCTGAATGTTGTTGGCAATATTCTTGCTGGTCTCGAACGCATTGTGC
ACATCCTGCGGCATCTTCTTGCCTGGAAAATCGCGTTGGAAAGCTCGAGCGTTTTGACAGAGATGCGCAA
GTTGCTGCTGCGCCGCCTCATGCTGCTGCTCCAACTCGTCCAACTGGGCCAG
>dme_circ_0001519_junction_seq
GCTGCTCCAACTCGTCCAACTGGGCCAGTTGAAGCACACGCTCGCGCTCTGCCTGA
>dme_circ_0001520
GGAACTGGATACAATTGGACTCTCCACCAGCTTGTCGGCAAGAACCGTGATTTTGGTGAACTCTTTAAGG
GTCTGCTCGTACTCCTCCGCTAGGGATTGAAGTTGAAGCACACGCTCGCGCTCTGCCTGAATGTTGTTGG
CAATATTCTTGCTGGTCTCGAACGCATTGTGCACATCCTGCGGCATCTTCTTGCCTGGAAAATCGCGTTG
GAAAGCTCGAGCGTTTTGACAGAGATGCGCAAGTTGCTGCTGCGCCGCCTCATGCTGCTGCTCCAACTCG
TCCAACTGGGCCAG
>dme_circ_0001520_junction_seq
GCTGCTCCAACTCGTCCAACTGGGCCAGGGAACTGGATACAATTGGACTCTCCACC
>dme_circ_0001521
GGATTCCAACATGGCGCGACAGTGGCTGAGATTTACGAAGAACTTCCTCTGTTTCTGCACCTCGTTGTTT
AGCTGCTCGAGGGAACTGGATACAATTGGACTCTCCACCAGCTTGTCGGCAAGAACCGTGATTTTGGTGA
ACTCTTTAAGGGTCTGCTCGTACTCCTCCGCTAGGGATTGAAGTTGAAGCACACGCTCGCGCTCTGCCTG
AATGTTGTTGGCAATATTCTTGCTGGTCTCGAACGCATTGTGCACATCCTGCGGCATCTTCTTGCCTGGA
AAATCGCGTTGGAAAGCTCGAGCGTTTTGACAGAGATGCGCAAGTTGCTGCTGCGCCGCCTCATGCTGCT
GCTCCAACTCG
>dme_circ_0001521_junction_seq
CGCCGCCTCATGCTGCTGCTCCAACTCGGGATTCCAACATGGCGCGACAGTGGCTG
>dme_circ_0001522
GGTCCTCCAAATTGGTGCAAGCTGCCTCCCGTTGGACTGTGCTGGAGAAGGGCATGCGAGATGAACTGCA
ATGGCTTCAGGTGGCTCAGCAACGAGTTCCCGACTTATCTGCCGTAACTTCCGCAGACTACGATCAATAC
ACCACTCTGTACCAATCCCTGAGCAATGATATCTCACATCACTAT
>dme_circ_0001522_junction_seq
CCTGAGCAATGATATCTCACATCACTATGGTCCTCCAAATTGGTGCAAGCTGCCTC
>dme_circ_0001523

AAGGCGGTGAAGGTAGAGAGCAACCTCCTCGCGGAGTTTGAGTAGCACTATCAACGCCTCATTGGTTTCC
TCTACGAGATTGGGGGCTTGCACCAAAAGCTGCAACTTGTTGGCTATTCCCGAAAGCTGTGTCATTTTCA
CATAGTGATGTGAGATATCATTGCTCAGGGATTGGTACAGAGTGGTG
>dme_circ_0001523_junction_seq
ATTGCTCAGGGATTGGTACAGAGTGGTGAAGGCGGTGAAGGTAGAGAGCAACCTCC
>dme_circ_0001524
AAGGCGGTGAAGGTAGAGAGCAACCTCCTCGCGGAGTTTGAGTAGCACTATCAACGCCTCATTGGTTTCC
TCTACGAGATTGGGGGCTTGCACCAAAAGCTGCAACTTGTTGGCTATTCCCGAAAGCTGTGTCATTTTCA
CATAGTGATGTGAGATATCATTGCTCAGGGATTGGTACAGAGTGG
>dme_circ_0001524_junction_seq
TCATTGCTCAGGGATTGGTACAGAGTGGAAGGCGGTGAAGGTAGAGAGCAACCTCC
>dme_circ_0001525
ATTTCCTTGAAGACCAGAAGGCGGTGAAGGTAGAGAGCAACCTCCTCGCGGAGTTTGAGTAGCACTATCA
ACGCCTCATTGGTTTCCTCTACGAGATTGGGGGCTTGCACCAAAAGCTGCAACTTGTTGGCTATTCCCGA
AAGCTGTGTCATTTTCACATAGTGATGTGAGATATCATTGCTCAGGGATTGG
>dme_circ_0001525_junction_seq
ATGTGAGATATCATTGCTCAGGGATTGGATTTCCTTGAAGACCAGAAGGCGGTGAA
>dme_circ_0001526
GCTTTCGGGAATAGCCAACAAGTTGCAGCTTTTGGTGCAAGCCCCCAATCTCGTAGAGGAAACCAATGAG
GCGTTGATAGTGCTACTCAAACTCCGCGAGGAGGTTGCTCTCTACCTTCACCGCCTTCTGGTCTTCAAGG
AAATCTGGGTGCA
>dme_circ_0001526_junction_seq
TTCTGGTCTTCAAGGAAATCTGGGTGCAGCTTTCGGGAATAGCCAACAAGTTGCAG
>dme_circ_0001527
GGGATAACCTGCAGGGATTTCTCAAAGCTCAGACCCGTATCGGTTCTCACATTATTGTGGAGCTCGAAAC
GGGCCTTAATCTCCCAGAATTGACGCATGTGCTCGATGGGCTGGTGAGTGGGCTGCGAGGGTATCTGGAT
GTTCCTCAATTCCTGCTCTGCTTCGCGTACAAAGGCCTCCAGCTTATCCGTTTGCTGTTCGTACTGCACC
CAGATTTCCTTGAAGACCAGAAGGCGGTGAAGGTAGAGAGCAACCTCCTCGCGGAGTTTGAG
>dme_circ_0001527_junction_seq
AGAGAGCAACCTCCTCGCGGAGTTTGAGGGGATAACCTGCAGGGATTTCTCAAAGC
>dme_circ_0001528
GGAGATCTACCTGACCATGACCAGCATGAAGGGAGTCATCAAGAACGAGGAGGAGCTCTGTCTGTACATC
GAACGAGTCCAAGTGCTACGCACTCGCGTCGGATTCATTGGCAACGAGCTTGGCAGGATCGGACTGCAAG
AGCCCGCCATTGAGCCGGAAAAAGTTG
>dme_circ_0001528_junction_seq
GAGCCCGCCATTGAGCCGGAAAAAGTTGGGAGATCTACCTGACCATGACCAGCATG
>dme_circ_0001529
GTACCCAGATTGCCGAGGAGCTAGAGGGTGCATCTGTGCTGCGCGACCAACTGCAGGCCATCCAGGAGGG
CATTAGCAATCAACGCAAACACCAGGCCAAGATCTCGGTCATTTTGGATGAATGTGAAGCCGCCGAGCGC
CAGGGAGCAGATGTTCTCGAAAAGGCCGTCGCCGATTGCCAGGCCGCCGGCGAGGAGCTGGTGATCAGCT
GGCAGGAGATTATGCGAATCCGCCAGATGCTCCACACCCTGCCCATGCGCCTCAAGATGTCCGTTTCACC
GGTTAAGTTGGAGAGGGACATCTCCCAATTGCAGGACGACCACGCCTTCCTCGAGAGCAAGTGCACCAAC
ATAATGGCCATCCTGAGGAGCCGCCTAGCCGTCTGGCTGCGCTATGAGCGTCAGCTGGAGCTGGTCCACG
GATCC

>dme_circ_0001529_junction_seq
GCGTCAGCTGGAGCTGGTCCACGGATCCGTACCCAGATTGCCGAGGAGCTAGAGGG
>dme_circ_0001530
GCATCCAGGCCCACATCGGCGGCGATCTTGGCCACAATGTCGCGCAACTCCAAGATTCTGTCATTCTGGG
TCGTCAGGTTGTCTTGGCAAACCTGTGTAAATGTAGAGATATGGAGGCCAATTTAGTTAATGAAGTCCAG
GGTATAATCACTCGTTTTTGTTCCTCTAAACAAGTGATTAGCTTAAATGATCAACTTAAAACCATCTCAA
TAGAAGTCATCTATCAATTGTATTCATTTAACATTCCCTTTGCCTTTTGCCTAACTATTTCTAATACTTT
CTGTTCTTTCTCGCCACCGAAAAGCTCTTTCTGAACTAAACATACCCCAATTATATTGGATGCCTGGCAA
AATGTCACTGGTTTATATTTAACTCGACGACGACCACAACGACGAATGTATTTAGCCCAAGCCGGCCGGG
CTGGTGTTCCATTTCGTGCCTCAGACCCTTATCAATTGTGTTGTCGAGTGCATTTCCCTGCTCGCCAATG
GTAATTAGGGCTGTGAGTGCCCGGCGCACTTTCGATTTCAATTCGAGGGGAAAACGGGTCACCACCACTT
GCCGACGCATAAGTTGTTCTCTAATGCCCTTTGTAATGTCCGCCCTATGATATCATCTGGCAAGTATTTG
GCGATGCTTTATCACGGAGCTACATAAGCAAACAAACGCTTAGAAAACTGGCATTGAAAATACCAACATA
GATTAGGTATTGTTCGCTCTGTGTCTGTCTATCTTTTTCCACATTTATCTTCTTTCGGAGCTCAAGGCAT
TTGCGAACCTGTTCCCGCTGTTTTTCAGCTGCTGCTCCTACTGCTGCTGTTGATAAAGCTGATTATGTAT
ACATAAAATACTATAATAATACGAAAATATAGCCACCCAACAGCAGCGGGTTGATAAACTCTCCTGTGTG
AAATGACAGTTGGTCGCCTGTCCGAAAATATAATTACACCATCGAGTTGGGCCCCCTACCATAATATAAT
GACCCTAATGATCAATAAACACAAAACGGCGATCCGAGTATGCGTTAATTTTTTAAGTAAATGCCGAAAA
GGAAATCGTACGTACGATGTCGGTCATATTTACCCAAAACATAATTTATGACACAAACACAGCCCGGCCA
AGTTAGCTCATATATGTACATTCACGGCGGAACTATTACGAACCGATTACATTGACGCGCCCAAAAAAAT
TGATAGGGCGAGCACTACGGAGAAAATCAATGTAATTACTAGTTGGAACACCCGCGTCCCCAGTGGCCAA
TTAAAGAGCCCCATTTCTGTCCTAGTTAGCCGCCaaaaataaaaaaaaGATATGTGCACCTAAGGCGGAT
TTATCAGTTTTAGAAGAGCCTCTTATCTAGATAACTTATGTTCCCAAAAGATCTCAAAAAaccttacctt
accttaccttaTGTTACACTACACTTGAAATATGCTCAAACTAAATTAGATTAATTGACTTTAAATAACA
AACCAATCGATATATCTTTGTACTTAGAATGCACAAATTATTAACCGATTTTCTGTCTCTGTTGTTTGTT
CAATTATTATATATTTCCGGTTCGATTTTACCTATGTGACAAACAGGTCGTTGGCATCACTGTCGCCCAC
ACGCGATATAAATCACTCATACGACCCGTGTGCCGGACGGGTCACGGCATTTGTGGGCACTCACCTTGGC
GTGCTGCAGGGCATCCAGGGGCTGCTCCAGCGACTTGACCGCATCCATCTGCTGGTCAATGGAGTTCTTG
ACGGCGGCTGAGGCGTTGCGATACTTATCCCACAGTTCGACGGCTCTTTGGTAGCGGGTTCGCAGCTGCT
GCAGATTCTCGCTGG
>dme_circ_0001530_junction_seq
GTTCGCAGCTGCTGCAGATTCTCGCTGGGCATCCAGGCCCACATCGGCGGCGATCT
>dme_circ_0001531
GAACTCCATTGACCAGCAGATGGATGCGGTCAAGTCGCTGGAGCAGCCCCTGGATGCCCTGCAGCACGCC
AAGGTGAGTGCCCACAAATGCCGTGACCCGTCCGGCACACGGGTCGTATGAGTGATTTATATCGCGTGTG
GGCGACAGTGATGCCAACGACCTGTTTGTCACATAGGTAAAATCGAACCGGAAATATATAATAATTGAAC
AAACAACAGAGACAGAAAATCGGTTAATAATTTGTGCATTCTAAGTACAAAGATATATCGATTGGTTTGT
TATTTAAAGTCAATTAATCTAATTTAGTTTGAGCATATTTCAAGTGTAGTGTAACAtaaggtaaggtaag
gtaaggtTTTTTGAGATCTTTTGGGAACATAAGTTATCTAGATAAGAGGCTCTTCTAAAACTGATAAATC
CGCCTTAGGTGCACATATCttttttttatttttGGCGGCTAACTAGGACAGAAATGGGGCTCTTTAATTG
GCCACTGGGGACGCGGGTGTTCCAACTAGTAATTACATTGATTTTCTCCGTAGTGCTCGCCCTATCAATT
TTTTTGGGCGCGTCAATGTAATCGGTTCGTAATAGTTCCGCCGTGAATGTACATATATGAGCTAACTTGG
CCGGGCTGTGTTTGTGTCATAAATTATGTTTTGGGTAAATATGACCGACATCGTACGTACGATTTCCTTT
TCGGCATTTACTTAAAAAATTAACGCATACTCGGATCGCCGTTTTGTGTTTATTGATCATTAGGGTCATT

ATATTATGGTAGGGGGCCCAACTCGATGGTGTAATTATATTTTCGGACAGGCGACCAACTGTCATTTCAC
ACAGGAGAGTTTATCAACCCGCTGCTGTTGGGTGGCTATATTTTCGTATTATTATAGTATTTTATGTATA
CATAATCAGCTTTATCAACAGCAGCAGTAGGAGCAGCAGCTGAAAAACAGCGGGAACAGGTTCGCAAATG
CCTTGAGCTCCGAAAGAAGATAAATGTGGAAAAAGATAGACAGACACAGAGCGAACAATACCTAATCTAT
GTTGGTATTTTCAATGCCAGTTTTCTAAGCGTTTGTTTGCTTATGTAGCTCCGTGATAAAGCATCGCCAA
ATACTTGCCAGATGATATCATAGGGCGGACATTACAAAGGGCATTAGAGAACAACTTATGCGTCGGCAAG
TGGTGGTGACCCGTTTTCCCCTCGAATTGAAATCGAAAGTGCGCCGGGCACTCACAGCCCTAATTACCAT
TGGCGAGCAGGGAAATGCACTCGACAACACAATTGATAAGGGTCTGAGGCACGAAATGGAACACCAGCCC
GGCCGGCTTGGGCTAAATACATTCGTCGTTGTGGTCGTCGTCGAGTTAAATATAAACCAGTGACATTTTG
CCAGGCATCCAATATAATTGGGGTATGTTTAGTTCAGAAAGAGCTTTTCGGTGGCGAGAAAGAACAGAAA
GTATTAGAAATAGTTAGGCAAAAGGCAAAGGGAATGTTAAATGAATACAATTGATAGATGACTTCTATTG
AGATGGTTTTAAGTTGATCATTTAAGCTAATCACTTGTTTAGAGGAACAAAAACGAGTGATTATACCCTG
GACTTCATTAACTAAATTGGCCTCCATATCTCTACATTTACACAGGTTTGCCAAGACAACCTGACGACCC
AGAATGACAGAATCTTGGAGTTGCGCGACATTGTGGCCAAGATCGCCGCCGATGTGGGCCTGGATGCCTC
AGCGCTCATGCAAG
>dme_circ_0001531_junction_seq
GGCCTGGATGCCTCAGCGCTCATGCAAGGAACTCCATTGACCAGCAGATGGATGCG
>dme_circ_0001532
GTAGCACCATCGGTGGCCAGCAGCGATGATGACGGCATCCTGGAGGTTCTAGCCCTGTGGCAGAAGATTT
TCCAGGACACCTTCCAGGAGTATCATCGCCTGTCCACGCGACTCGCCCGCAGCCAAAACAGTTCGGAGGC
TCTGCGATTGTGGAGGCAGTACCTGCAGCACGTCCA
>dme_circ_0001532_junction_seq
TGTGGAGGCAGTACCTGCAGCACGTCCAGTAGCACCATCGGTGGCCAGCAGCGATG
>dme_circ_0001533
TTCCTTTGGCGAATGGTCTTCATGTCGTGGGGACTGATGCACTCCTTGAGCTCCTCCTGGGTCACAGTGA
GACTCTCCAGAGCGGAGACTTGGGCACCAAGTTGGACCCTTTGCGCACGCAGGGATCCCAGTAGTTGCTC
AATTGCCGCTGGCTGAGTGGGCAGAGATTCGCTGTTGGCATCCAGAAGCTTCTGTGCTGCTCCGAATTCC
TGCTGCAACTGG
>dme_circ_0001533_junction_seq
TGCTGCTCCGAATTCCTGCTGCAACTGGTTCCTTTGGCGAATGGTCTTCATGTCGT
>dme_circ_0001534
GGAAAGCCGCTCCTCAATCGAGTTGATCAGGTTGGCCAGCTGGTAGTCCAGATCGGCGTGCTGCTGCCAG
AGCAACCAGTTCCTTTGGCGAATGGTCTTCATGTCGTGGGGACTGATGCACTCCTTGAGCTCCTCCTGGG
TCACAGTGAGACTCTCCAGAGCGGAGACTTGGGCACCAAGTTGGACCCTTTGCGCACGCAGGGATCCCAG
TAGTTGCTCAATTGCCGCTGGCTGAGTGGGCAGAGATTCGCTGTTGGCATCCAGAAGCTTCTGTGCTGCT
CCGAATTCCTGCTGCAACTGG
>dme_circ_0001534_junction_seq
TGCTGCTCCGAATTCCTGCTGCAACTGGGGAAAGCCGCTCCTCAATCGAGTTGATC
>dme_circ_0001535
GTGGCTGCAACGTTTGGAACAGCGTGTGGAAAAGGATGCTGATGTCACTGCCATGACAAATCCCGAACAG
GCAGCCAAGCAGCTGGAGCAGCAGGTGAATTCCGAGCTGCAATTGAGGGACAAGGAACGGGAGTGGCTTC
TATCCACCAGCCGGGAACTGCTCACCCTCTACTCCGAGCCAGAAGTGCGATCCCAG
>dme_circ_0001535_junction_seq
CTACTCCGAGCCAGAAGTGCGATCCCAGGTGGCTGCAACGTTTGGAACAGCGTGTG

>dme_circ_0001536
ACGCTGCCAGCGATCGATGAGGGAGTCACTCTGTTGTTGCACCTGGGATCGCACTTCTGGCTCGGAGTAG
AGGGTGAGCAGTTCCCGGCTGGTGGATAGAAGCCACTCCCGTTCCTTGTCCCTCAATTGCAGCTCGGAAT
TCACCTGCTGCTCCAGCTGCTTGGCTGCCTGTTCGGGATTTGTCATGGCAGTGACATCAGCATCCTTTTC
CACACGCTGTTCCAAACG
>dme_circ_0001536_junction_seq
CATCCTTTTCCACACGCTGTTCCAAACGACGCTGCCAGCGATCGATGAGGGAGTCA
>dme_circ_0001537
AAGTGTTCACCTGCGTGCGCCAGCTGTCCGCATCGTTGAGGAGCATCTCTACGAGATTAAGGACCTCGCC
CACATTGCTGCTGTGGTGCTCAATGGAGCGTTGGATTTGCTCATGCTCCTTTAGCTTGGCCTCGATCACA
TTCGG
>dme_circ_0001537_junction_seq
CCTTTAGCTTGGCCTCGATCACATTCGGAAGTGTTCACCTGCGTGCGCCAGCTGTC
>dme_circ_0001538
GTTATCCGCCTGCTGGACATGTTGTTGTGCGTCCTGCAACTTCTTCTCTAGACTCTCCAGACGTTGGAGC
ACCGCTTTCGGCTCCGCTTTGGAGGTCTGTTGATTCTCTGCGCTGGGCAATTGTTCCAAAGCCTTTTGTA
TGGCATTCAGCGAGTTGGTGGCTTCCAATTGTGCGTTG
>dme_circ_0001538_junction_seq
CGAGTTGGTGGCTTCCAATTGTGCGTTGGTTATCCGCCTGCTGGACATGTTGTTGT
>dme_circ_0001539
GAACCCTTTCCAGATTGACCGATCTCCGGCTGCGTCTCCAATCCCTGCGTCGTCTATCCGGCATCTATAT
CGTCAAGCTGGGTGCCGTCCTCGGCTACGAGGGCGACAATCTCGGCGTGCCATTGCACATGTTGTCTAGT
GAGGGTAAGTCCAAGTGTCCCATATTCATTGCCCAACACCTTGGTTTCCTGTTCaaaaaacatgatttgt
ccaaaaaaaatggaaaataattaaaTTTTTGATTTGTTGATTTGTGAGCGCACCAGAAGTAAGTGAAACC
CATTGGCAGTCTAACGAAAAAATATGAAAAGAATTGAAACTTATGatttctattatttttaatttattta
tttttctttctttctttttttcTGTATGGCAAACATAGCTTTTGGATAATACTACATTATCAACCTCTTCC
ATGCAGGCCGCCGCACCCAACACAGAAAATGCAAA
>dme_circ_0001539_junction_seq
CCGCCGCACCCAACACAGAAAATGCAAAGAACCCTTTCCAGATTGACCGATCTCCG
>dme_circ_0001540
GATTGACCGATCTCCGGCTGCGTCTCCAATCCCTGCGTCGTCTATCCGGCATCTATATCGTCAAGCTGGG
TGCCGTCCTCGGCTACGAGGGCGACAATCTCGGCGTGCCATTGCACATGTTGTCTAGTGAGGTAAGTCCA
AGTGTCCCATATTCATTGCCCAACACCTTGGTTTCCTGTTCaaaaaacatgatttgtccaaaaaaaatgg
aaaataattaaaTTTTTGATTTGTTGATTTGTGAGCGCACCAGAAGTAAGTGAAACCCATTGGCAGTCTA
ACGAAAAAATATGAAAAGAATTGAAACTTATGatttctattatttttaatttatttatttttctttcttt
ctttttttcTGTATGGCAAACATAGCTTTTGGATAATACTACATTATCAACCTCTTCCATGCAGGCCGCCG
CACCCAACACAGAAAATGCAAA
>dme_circ_0001540_junction_seq
CCGCCGCACCCAACACAGAAAATGCAAAGATTGACCGATCTCCGGCTGCGTCTCCA
>dme_circ_0001541
AAGCAGCAAAGTACACAAATTTAACATTTAAATCAAACTTAATTTTATACATAGTAAATCGTTCATAGGC
CTAATAAATAAATGTATGTATTTTCTCAATAATAATTGGCCAAGCATTAAATAATAGAAAATAGTTGTAA
TAAACAAGTTGGATAAGTATAAGGCAAATATAATTAGGATTATCAAAAAATATTAACAACAAAATAGGTA
TCTTTTACTGGATGGACTTACGTCACTTGTCAATATGCATATTGCATTTTTACGAttttttttttCAAA

GAATTAATCTCTTCttttatgaattcataatatttttgtttttttgtatacgcattttacattttttttcttt
atacaGAAAATTTGTAGGTTGGTTTAGCACCGGCATAGGACACTTGCTTCGTTTTGTTCAATGCTCGTGG
CACGCACGTTTTCCATTTTCTTTTCTTTGAGAGCTGGCTTCGATGGTCAATGTCTATTGGTAATTTGGAA
ATGAGTATCAGGAATGCTTGAAATG
>dme_circ_0001541_junction_seq
GAAATGAGTATCAGGAATGCTTGAAATGAAGCAGCAAAGTACACAAATTTAACATT
>dme_circ_0001542
GTCAGCTCGGCGGTACCGATCTCCTCCCTCAACTTCATCTGCTCCTTGGGATTGCGTGCCAGGAAAAGGA
GTGTGTGGGTAAGCACTTGTGCGGTGGTCTCGAATCCGTCCGTTAAAAATGTCATTGTGTGCGAGG
>dme_circ_0001542_junction_seq
TCCGTTAAAAATGTCATTGTGTGCGAGGGTCAGCTCGGCGGTACCGATCTCCTCCC
>dme_circ_0001543
TTTTGGGATTAACCTTGATGTCGAAGTTTCGCACGATTTCCACCAGGGCAGCTTTGATTTGGGTAAGTGA
AAACCGCATACCTACAAATTATGCATAGAttttttttAGTAACTTTAATTGTTTCAAAAATGATGTTACC
CACCGGGGCAAATACGTGGTCCATCGCCAAACCCAAAGAATAGACCCTGATCTCTGTACTTTTTGGCTCC
GCCATTGATGTTCAGGAATCGCTCGGGCTTGAACGATTGAGGTTCCTCATAGTATTGGGGATCGTGG
>dme_circ_0001543_junction_seq
AGGTTCCTCATAGTATTGGGGATCGTGGTTTTGGGATTAACCTTGATGTCGAAGTT
>dme_circ_0001544
GGTGAAACATAAGCTAGCCACGATCAGCATAAACACGGACAGGGAGATGATGATGGAGCCAAAGACGGCC
AGCGTGTGTAGGGACTCGCTGCTCACGGGCAAATCCGGCGCTGGTGTGGTCAGCTTGGGTGTGTAG
>dme_circ_0001544_junction_seq
CGCTGGTGTGGTCAGCTTGGGTGTGTAGGGTGAAACATAAGCTAGCCACGATCAGC
>dme_circ_0001545
GTGAATTCAGTGCGAATGTTCAGCCAGACGTCGAAGCTATGGCCGCCGCCGCTTTGAGTGGCATGGAAAT
GGGATCTGGACCAGGATCAGAGGGATATGAGGATGCCGACAACGAGAAAAGCAAGACCGTGGAAAATGG
>dme_circ_0001545_junction_seq
ACGAGAAAAGCAAGACCGTGGAAAATGGGTGAATTCAGTGCGAATGTTCAGCCAGA
>dme_circ_0001546
ACACCGTGACGCCGCAGCAATTGGAGACGATCTCAGTGCATAGCATTATGGAAGCCTGGGAGCTGGCCAG
CATTCCCAACACTCGCAACCTACTTCACGTCCTGGGATTCGATGAGGAGGAGGAGGTGAACCTGCAGCAG
CTAACTAAGGCATTGGAGGAGGAGCTGCGGGGCATCGATGGGGATCACGAGCAATCGAATATGTTGCGCG
CTCTGGCTGCTCTGCAGGCCACCGAGTTGGGCAACTACAGACTTGCCTATAGGCAGCAGCATGAGGAGAA
CCTCAAGCTGAGGGCCGATAATAAGGCGGCCAACCAAAGGGTGGCTTTGCTTGCCGTGGAAGTGGATGAG
CGGCATGCGTCGCTGGAGGATAACTCCAAGAAGCAGGTGCAGCAGCTGGAGCAAAGACACGCCAGCATGG
TGCGTGAAATAACGCTGCGGATGACTAATGACCGCGATCACTGGACCAGCATGACGGGAAAGCTGGAGGC
ACAGCTTAAATCGCTTGAGCAGGAGGAGATCCGTCTGAGAACGGAACTTGAACTGGTGCGCACTGAGAAC
ACGGAGCTTGAGTCGGAGCAGCAAAAGGCTCACATCCAAATCACAGAGCTTCTCGAACAGAACATTAAGC
TCAACCAGGAACTGGCCCAAACGTCGAGCAGCATTGGTGGCACCCCGGAGCACAGTCCATTGCGACCGAG
AAGGCATAGCGAGGACAAGGAGGAGGAGATGCTCCAGCTAATGGAGAAGCTGGCTGCTCTTCAAATGGAG
AACGCCCAGCTGCGTGACAAGACTGACGAACTGACCATCGAAATCGAGAGCTTAAATGTGGAACTAATTC
GCTCGAAAACCAAGGCTAAAAAGCAAGAAAAACAGGAGAAACAAGAGGACCAGGAGTCGGCGGCCACGGC
TACCAAAAGGCGTGGGGATTCGCCGAGCAAAACACATCTAACAGAGGAGAGCCCTCGCTTGGGGAAACAG
CGCAAGTGCACCGAAGGAGAGCAGAGCGATGCCAGCAACAGCGGAGATTGGTTGGCTCTAAACTCCGAGC

TGCAAAGAAGTCAAAGCCAGGATGAGGAGCTAACAAGCCTTAGACAGCGGGTTGCTGAGCTAGAGGAGGA
ACTCAAGGCTGCAAAGGAAGGCAGATCTCTCACCCCGGAAAGCCGTTCGAAGGAACTGGAGACCAGTCTA
GAGCAAATGCAGCGTGCCTATGAGGATTGCGAGGACTACTGGCAAACGAAACTTAGCGAGGAGCGGCAGC
TGTTTGAGAAGGAGCGACAGATCTACGAAGATGAGCAGCACGAGAGCGACAAGAAGTTCACCGAGCTGAT
GGAAAAGGTGCGCGAGTACGAGGAGCAGTTCAGCAAGGATGGCCGCCTCTCGCCCATTGATGAGCGCGAT
ATGCTGGAACAGCAGTACTCGGAATTGGAGGCAGAGGCAGCCCAGCTGCGCTCGAGTTCCATTCAAATGC
TCGAGGAGAAGGCTCAGGAAATCAGTTCACTGCAATCAGAGATCGAGGATTTGCGACAGAGATTGGGTGA
GAGCGTTGAGATCCTCACAGGCGCCTGTGAACTCACCTCGGAGTCGGTAGCCCAACTGAGTGCCGAGGCG
GGAAAAAGTCCAGCCAGCTCACCCATCAGCTACCTCTGGCTGCAGAGCACCATCCAAGAGCCAGCGAAAT
CGCTTGCCGATTCCAAGGATGAAGCCACCGCCAGTGCCATCGAATTGCTCGGAGGCTCACCATCGCACAA
GACAGCCAGCCGGTGAGTATGAGAAGCCTCTCGGTGTGTCCTTGGTGTGAGCATCCCTGTGTCTTCCTCA
TAATTTGCACTGTATGTCCTGTATATATGTTTCAGTTTTGTCCCTCACATCTAACCATGTCTAATATAAG
CTAATTTAATCCTTTTAATTGTATGTTTGTGCTTGTTTAATAAATATAATTTATATTCATATAGACAATT
GGAGACGATCTCAGTGCATAGCATTATGGAAGCCTGGGAGCTGGCCAGCATTCCCAACACTCGCAACCTA
CTTCACGTCCTGGGATTCGATGAGGAGGAGGAGGTGAACCTGCAGCAGCTAACTAAGGCATTGGAGGAGG
AGCTGCGGGGCATCGATGGGGATCACGAGCAATCGAATATGTTGCGCGCTCTGGCTGCTCTGCAGGCCAC
CGAGTTGGGCAACTACAGACTTGCCTATAGGCAGCAGCATGAGGAGAACCTCAAGCTGAGGGCCGATAAT
AAGGCGGCCAACCAAAGGGTGGCTTTGCTTGCCGTGGAAGTGGATGAGCGGCATGCGTCGCTGGAGGATA
ACTCCAAGAAGCAGGTGCAGCAGCTGGAGCAAAGACACGCCAGCATGGTGCGTGAAATAACGCTGCGGAT
GACTAATGACCGCGATCACTGGACCAGCATGACGGGAAAGCTGGAGGCACAGCTTAAATCGCTTGAGCAG
GAGGAGATCCGTCTGAGAACGGAACTTGAACTGGTGCGCACTGAGAACACGGAGCTTGAGTCGGAGCAGC
AAAAGGCTCACATCCAAATCACAGAGCTTCTCGAACAGAACATTAAGCTCAACCAGGAACTGGCCCAAAC
GTCGAGCAGCATTGGTGGCACCCCGGAGCACAGTCCATTGCGACCGAGAAGGCATAGCGAGGACAAGGAG
GAGGAGATGCTCCAGCTAATGGAGAAGCTGGCTGCTCTTCAAATGGAGAACGCCCAGCTGCGTGACAAGA
CTGACGAACTGACCATCGAAATCGAGAGCTTAAATGTGGAACTAATTCGCTCGAAAACCAAGGCTAAAAA
GCAAGAAAAACAGGAGAAACAAGAGGACCAGGAGTCGGCGGCCACGGCTACCAAAAGGCGTGGGGATTCG
CCGAGCAAAACACATCTAACAGAGGAGAGCCCTCGCTTGGGGAAACAGCGCAAGTGCACCGAAGGAGAGC
AGAGCGATGCCAGCAACAGCGGAGATTGGTTGGCTCTAAACTCCGAGCTGCAAAGAAGTCAAAGCCAGGA
TGAGGAGCTAACAAGCCTTAGACAGCGGGTTGCTGAGCTAGAGGAGGAACTCAAGGCTGCAAAGGAAGGC
AGATCTCTCACCCCGGAAAGCCGTTCGAAGGAACTGGAGACCAGTCTAGAGCAAATGCAGCGTGCCTATG
AGGATTGCGAGGACTACTGGCAAACGAAACTTAGCGAGGAGCGGCAGCTGTTTGAGAAGGAGCGACAGAT
CTACGAAGATGAGCAGCACGAGAGCGACAAGAAGTTCACCGAGCTGATGGAAAAGGTGCGCGAGTACGAG
GAGCAGTTCAGCAAGGATGGCCGCCTCTCGCCCATTGATGAGCGCGATATGCTGGAACAGCAGTACTCGG
AATTGGAGGCAGAGGCAGCCCAGCTGCGCTCGAGTTCCATTCAAATGCTCGAGGAGAAGGCTCAGGAAAT
CAGTTCACTGCAATCAGAGATCGAGGATTTGCGACAGAGATTGGGTGAGAGCGTTGAGATCCTCACAGGC
GCCTGTGAACTCACCTCGGAGTCGGTAGCCCAACTGAGTGCCGAGGCGGGAAAAAGTCCAGCCAGCTCAC
CCATCAGCTACCTCTGGCTGCAGAGCACCATCCAAGAGCCAGCGAAATCGCTTGCCGATTCCAAGGATGA
AGCCACCGCCAGTGCCATCGAATTGCTCGGAGGCTCACCATCGCACAAGACAGCCAGCCGG
>dme_circ_0001546_junction_seq
CTCACCATCGCACAAGACAGCCAGCCGGACACCGTGACGCCGCAGCAATTGGAGAC
>dme_circ_0001547
GAGCGACAGATCTACGAAGATGAGCAGCACGAGAGCGACAAGAAGTTCACCGAGCTGATGGAAAAGGTGC
GCGAGTACGAGGAGCAGTTCAGCAAGGATGGCCGCCTCTCGCCCATTGATGAGCGCGATATGCTGGAACA
GCAGTACTCGGAATTGGAGGCAGAGGCAGCCCAGCTGCGCTCGAG

>dme_circ_0001547_junction_seq
AGGCAGAGGCAGCCCAGCTGCGCTCGAGGAGCGACAGATCTACGAAGATGAGCAGC
>dme_circ_0001548
GGTTCTCCTCATGCTGCTGCCTATAGGCAAGTCTGTAGTTGCCCAACTCGGTGGCCTGCAGAGCAGCCAG
AGCGCGCAACATATTCGATTGCTCGTGATCCCCATCGATGCCCCGCAGCTCCTCCTCCAATGCCTTAGTT
AGCTGCTGCAG
>dme_circ_0001548_junction_seq
CCTCCAATGCCTTAGTTAGCTGCTGCAGGGTTCTCCTCATGCTGCTGCCTATAGGC
>dme_circ_0001549
GTGAAGCCATCCGTTTGCACCACCTTCCGGAACTTGGTCTTCTCACCGTCCGGGAACAGGGTCATATCGA
TAGCACAGCCGAAGTCAATCAAACGTAGCGATGGCAACGGACTGTCCACATTGGGCACACGCATTAGCAG
GAAGTTGTCCGGTTTAATGTCCGCATGGATGATGTGCTGGCGATGCAGG
>dme_circ_0001549_junction_seq
CGCATGGATGATGTGCTGGCGATGCAGGGTGAAGCCATCCGTTTGCACCACCTTCC
>dme_circ_0001550
GCTCATCGGTGTCCGGCAGTGCGGCCAGTGTGCTGAATCTATCGCGTCGGGCCTGCTCACCCAGCTACGA
GCACATGCTCTCCTCCACCACCTCCTCCACTCTTTCCTCGGCCTCCTCATCGGGAGCTGTTTCCGGCGAT
GATGAGCAGGAGCACGAACCAGCGCACATGGCTCCACTGCAGCTGCAGCGAAGCAGTCCGCAGCAGGGCA
GCGATGCCAACAACTGTCTGCCCCTCAAGCTGCGCCACAAGTCCCATCTCGGCGACAAGGATGCGGCGGC
CACGGCGCTACTCTCACTGCAGCACATCAAGCAGGAGCCCAACTGCAGTCGGGCATCGCCGCCGGCTTGG
AACGATGGCGGCGACAATTCCAGCGACGAAAGGGACTCCGGCATCTCCATCGCCAGTGCGGAGTGGACGG
CGCAGCTGCAGAGGAAGCTACTGGCGCCCAAGGAGGCCAATGTG
>dme_circ_0001550_junction_seq
GCTACTGGCGCCCAAGGAGGCCAATGTGGCTCATCGGTGTCCGGCAGTGCGGCCAG
>dme_circ_0001551
AATTTTGTAGGCGATGGTCACCACGGCGGTCAGGCCCAGGGAAACGGCCAGGTTGCGCTTGATGGTGGCA
TTGTGGAGGCCACGGAGCACGGGTCCGGCAGAGGAGGTGGCTGGAGTGTTGGCCATG
>dme_circ_0001551_junction_seq
AGAGGAGGTGGCTGGAGTGTTGGCCATGAATTTTGTAGGCGATGGTCACCACGGCG
>dme_circ_0001552
GACATAGAAACGCTAGCCATGCGCCTGGAATCCGAATTCCTAAACGATCTGCACGGCGGCATGTGGTTTC
TCGATGAGGCTACTCTGTGCCTCCTTGTCCTTCTCCGCATTCCGGCCGATTGAGACGTGCTCCGGTTTTA
GATA
>dme_circ_0001552_junction_seq
CGATTGAGACGTGCTCCGGTTTTAGATAGACATAGAAACGCTAGCCATGCGCCTGG
>dme_circ_0001553
GTGCCATTGTATCGCCGCAGCTGATCAAGGATGTGAGACAGGTTCTCAACGTGGAAGCGGTGCATAGTGT
CTATGGCCTGACGGAAACCACAGCTGTGATCTTCCAATCCTTGCCCGGCGATAGCAGTGATGTTGTCCTC
AACTCAGTTGGCCATCTGACGGATCACATCGAAGCCAAGGTGGTGGATGCCGAGGGCAGGTGTG
>dme_circ_0001553_junction_seq
AAGGTGGTGGATGCCGAGGGCAGGTGTGGTGCCATTGTATCGCCGCAGCTGATCAA
>dme_circ_0001554
GGATAATTATAATGTGCTACATATTGCCGCAATGTATTCGCGTGAGGATGTCGTCAAATTGTTGCTAACA
AAACGCGGCGTGGATCCCTTCTCCACCGGTGGCGTGAGTATTCCAATAGCTTTATATACTACATATATAC

GTATGCGCCCCAAGAAAGTGTTACCCCAATAGTTGAGGTAGCGACACGTCAGGCGACACACTCAATACTC
GAGTTCCTACTTTCGAGTCAATGAAATAGCTGCATACCTTGGGGCTGCTGTCAGCCCGATTCGCAGGCAA
TTTGCGGCTATTAGACGCATACTTCACCTGGCTACGAAAGAGAAGaaaaaaaaaaaaaCCTATCCAAAGG
TCAGAGCCATGCGAAGATGCAACTTTGAGGCTCGCATGTTGCATGTTACTTTGGCGGGACCAGCAATTAA
CTGGCGACAAGGTTAAGATGGTAATGTCTAGGGCCCGCTTAAGAACACTTTAAGACCTGAAAACAAATTT
AAAGTAACCCTAGGTTTCACGAAAAACTTTACTCATCAGATTAAACAGAAATTTAAGCTTAGATACCGTC
ATTAAAATTAAAATTTAACATTTTGCATGATTTCCAAGTCTGACTTCTGTTTAAATACTACAATGTATAA
ATATTAAAGTCTGAGCAAGATTAGTGACACCATCTTTATATTGTCTAAAATCATAAAGCGTTAACCATTT
AATACAATGCATTTTCTCATAGGTAACATTTTTAACAAAATATATGATGATCACATCGTCAAGCATTTTG
GCAATTATTTCTCCAAGTTTATTTCTCGTGTCGGCATTAATTTGCTTTTCTTTATTTTTTTCTCGGCCGC
ATTGGGTTTTCGAGACTTGGTTATTTAGGGGGCGTGCGCCTTGCCCAAATTACTGATGGTTATCAGAAGA
GAGCTCTAAGCACGTGTGGGAGCGAGAGAAGTGGAGCTGCGGAAGCGAGACAGACAGATGCAAACTTTTG
TTTTAGCAACAGCCAAGTTTGAAGTGTTCCGTTAGCGTGTGTGCGTGGCAAAAAGGACTCCCACATCCAC
AACCGACACCTGCCCCCCATGTTGCCTACACCTGCTGCTCGACCACCCCTCCCCCACCATCACCTATATA
CACCTCTCTCGCTCACTCCCGCAGCGGTTGTCGGTGGGAGTTCTTTATTATGCTTTTTTCGGGCTGTCAA
TCTGTGATATGAGCGGGAGAGGCCAAAAAAGAAAAATGACACGAAATGTGCTTATAAACGCAAAAACGAG
CCACTTGCCTATTCAGTAGCAAATGGAATTTTGAAGCGAATAGGGAAACAGTTTGCCAGTTTTTTAGGTG
CCAACATTAACCACACAGTAGTGCACATAGCTGCATATTAATTTTGGCTAGAAAAAAAGTGTAACCCCAG
CAATAAGTGCGTTTGCAGTGTGTGCATAGTTTAATCGAAGACTTAATTGGATTTTTTTCCCTTTTCAGCG
TAAGTGTTACCAtgtgtgcttgtgattgagtgtgccagtgtggctgtgtgtgtgCGACGGAGAGCCACAA
GTGTTGGCCGCCCAATTGATGCCGCTTTATCTCCACTAGTTTATGATAGCTAAGCCACCCAAATGCAAGC
CGATGTGAAGTCAAGTACTCTCGACAGCGGTGCCAGGCGGTGCCGACGTAAACAAAGACTTAATAAAAAT
CACCAAAAAATATATACATTACAATAATGGCACCAACAAAATCGAGAGGAGTTAGTAACATAAAGCAAAC
AAAATTGTGTGGAAAAATCGATATGCAAAACTGCTCGCGGTAAATGCATTTCGACTGGCTGTAAATCAGA
AAAGGCCCAAAAAAGTTAATGCGGCTATTACACAGCGAGGAATTGAATAGGTAATTTTTGAGTCAATTTT
AGCTTATAATTTGTGGTActtttatgaatttttttaaaatttttatttCAAATTATTAGAGAGCTAATAT
ATTTGAATTATGCTTATATAACTTAAAATACTCAAAATTTATAGACAGCAATAAAGTATGGGATCTGCAA
CACATCTTTTTCTACACTGTATCAATAAGTAGCTCTCACCACAGTGGGTAGGCTCCAGCGAGCTTTGAAT
TACCATCGAAGCAGTTGTCTCCGCCTGATGAACTTGCTGGGGCTAACCGAGCTCCAGATCCCTTTTTCGA
GCTCCCCCCTTGGAAATCTGAACAGAAATGCGGAACTATTTGTCGCATCACGTGCCCCGGGTGAAAATGC
ACAGGCGATATTTCCATTACGCACGCGAAGAAAGCGCATAAATTTCCAACGAATTGCTATCAAGCGATTG
TAAGGATTTGGGGTATATGGGGGCTGATTGAGGGAATCCCGGGTGCCACCGATTGATTGTCTAGACAAAA
TGGGTAACCCACCTCGATTTGTGCCTCGAGGGCTGCGGCAAATGGCAAACAGCAACTTGATTTAAATCAA
TTAGAGAGAGGTGGAATGGCACTGTCAGGCGAAATTAGTCGGATGAAGTATTTAGCTTTCGATGGCATTC
AGTTCGATTCGTTTCGATTCGCttttcttttttttttCTACACGCATTTCCGGTGTGCATATACATGCAA
ATATATATATTGTATGTGTGTGGATAGTACTGTAGTTTTCCCCCGCGAGGGCGCTCAACTCGTTGCCAAC
AACAAACAAATATAACAAAGCGAGGAAAACTCTACCGAAAAAAGGGGGTCAAGTCGCTGTACAACTTGAT
TTACTCGCCTTTCCTGGCAGATAGGGATAATGGCTCCCCGTCACGCCCCCCTCTTACGACTCGCCCCCAA
AAGGTAGTTGGTTGCAAGTTGGAGCGCCAAAGTTGCGAACTTGGCTAAAAATAGCGAAACATGTTGCCGT
TAACACTTGAGGCTCGAATTGGCTAATTGGATATTTATGATTATATGTTCGCGAGTGTGAATGGATGTGT
GTTCGCTGTCCTTATCTTAATTATATTTTATACTATATATAACCTATCTCTAACCTAGGATCGCACTCCG
ATGCACTTGGCCGCCGAGAATGGGCACGCGCACGTCATCGAGATACTGGCCGACAAGTTCAAGGCGAGCA
TCTTCGAGCGCACCAAGGATGGCAGCACGCTGATGCACATTGCGTCACTCAACGGTCATGCTGAGTGCGC
CACGATGCTCTTCAAGAAGGGCGTCTACCTCCATATGCCCAACAAGGATGGAGCCCGGAGTATTCACACC

GCCGCCGCCTATGGTCACACGGGAATCATCAACACCCTGCTACAGAAGGGCGAGAAAGTGGATGTGACCA
CCAATGGATAACTATACAGCACTGCACATAGCCGTGGAATCGGCTAAGCCCGCCGTTGTGGAAACCCTGC
TGGGATTTGGAGCAGATGTCCATGTCCGTGGCGGAAAACTACGTGAGACCCCGCTGCACATTGCGGCACG
AGTGAAGGATGGAGATAGGTGTGCCCTCATGTTGCTGAAGTCGGGAGCCAGTCCAAATTTGACCACGGAT
GACTGTCTGACCCCCGTGCATGTGGCGGCTCGTCATGGCAATCTGGCCACGTTGATGCAACTCCTCGAGG
ACGAAGGAGATCCGCTGTACAAATCGAATGACTGGAGAGACACCGCTGCACATGGCCTGTCGTGCTTGCC
ACCCGGATATTGTGCGTCATCTCATCGAGACGGTGAAGGAGAAACACGGTCCGGATAAGGCCACCACCTA
TATAAACTCGGTAAACGAGGACGGCGCCACGGCGTTGCATTACACCTGCCAAATCACCAAGGAGGAGGTT
AAGATTCCCGAATCCGACAAGCAGATCGTTCGGATGCTCCTCGAAAATGGTGCGGATGTCACGTTGCAAA
CGAAAACTGCCTTGGAGACCGCTTTCCACTACTGCGCCGTGGCCGGCAACAATGATGTGCTGATGGAGAT
GATCTCACATATGAATCCCACAGACATCCAAAAGGCCATGAACCGGCAATCATCGGTGGGCTGGACTCCA
CTGCTGATTGCTTGCCATCGAGGGCACATGGAGCTGGTCAATAATCTACTGGCGAATCACGCTCGAGTGG
ATGTCTTCGATACGGAAGGACGATCTGCCTTGCATTTGGCTGCTGAGCGAGGATACCTGCATGTGTGTGA
TGCCCTGCTGACCAATAAGGCTTTTATTAACTCCAAGTCCCGCGTGGACGCACTGCACTACATCTGGCA
GCCATGAATGGATTTACGCATCTGGTGAAATTCCTGATCAAGGATCACAATGCAGTTATCGATATTCTAA
CGTTGAGAAAGCAAACGCCGCTCCATTTGGCGGCAGCCAGCGGGCAGATGGAAGTCTGTCAGCTGCTCCT
CGAGCTGGGCGCCAATATCGATGCGACGGACGATCTGGGCCAGAAGCCAATCCACGTCGCCGCCCAGAAC
AACTACTCTGAAGTGGCCAAACTCTTCCTGCAGCAGCATCCATCCCTGGTGAATGCCACCAGCAAGGATG
GAAACACATGTGCCCACATTGCCGCCATGCAGGGATCCGTCAAGGTGATCGAGGAGCTGATGAAGTTCGA
TCGATCGGGTGTGATTTCGGCGCGGAATAAACTTACGGATGCCACGCCCCTTCAGCTGGCCGCCGAGGGC
GGACATGCGGATGTGGTGAAGGCTCTTGTGAGAGCTGGTGCCTCCTGCACCGAAGAGAACAAGGCGGGAT
TCACCGCCGTTCATCTGGCGGCACAGAATGGACATGGTCAGGTCTTGGATGTGCTGAAAAGCACAAACTC
ACTAAGGATCAATAGCAAAAAGTTGGGTCTGACGCCGCTTCATGTGGCTGCCTATTACGGACAGGCGGAT
ACCGTGCGGGAATTGCTGACCAGTGTTCCCGCCACCGTCAAGTCGGAAACTCCAACGGGACAAAGTTTAT
TTGGGGATCTGGGCACGGAGTCCGGAATGACACCACTACACTTGGCGGCCTTTTCCGGCAACGAGAACGT
GGTGCGACTGCTCCTCAACTCTGCGGGTGTTCAAGTGGATGCGGCGACCATCGAGAACGGGCTATAATCC
ACTCCATTTGGCTTGCTTCGGTGGTCACATGTCAGTGGTCGGTTTGCTCCTAAGTCGGTCGGCGGAACTC
CTCCAATCGCAGGATCGTAACGGCAGGACGGGCCTGCATATCGCCGCCATGCATGGCCACATCCAGATGG
TGGAGATTCTGCTCGGCCAGGGCGCGGAGATCAACGCAACCGATCGGAACGGTTGGACGCCACTGCATTG
TGCTGCCAAAGCTGGCCACTTGGAGGTGGTGAAGTTGCTGTGCGAGGCGGGTGCCTCGCCAAAATCGGAG
ACCAACTACGGTTGCGCCGCCATTTGGTTCGCCGCCTCCGAGGGACACAACGAGGTCCTGCGGTATCTGA
TGAACAAGGAGCACGACACCTACGGCCTGATGGAGGACAAGCGATTCGTGTACAACCTGATGGTGGTGTC
CAAGAACCACAACAACAAGCCCATTCAGGAGTTTGTCCTGGTATCACCAGCACCCGTGGATACAGCCGCC
AAACTGTCCAACATCTACATAGTACTCTCGACAAAGGGAAAAAGAGCGCGCCAAGGATCTGGTAGCAGCT
GGCAAACAGTGCGAGGCAATGGCCACGGAGCTCTTGGCCCTGGCAGCTGGGTCAGATTCCGCCGGAAAGA
TCCTTCAAGCCACCGATAAGCGAAACGTGGAGTTTCTCGACGTTCTCATTGAAAATGAGCAGAAGGAAGT
GATTGCCCACACGGTAGTTCAGCGATACTTGCAAGGAACTCTGGCATGGCTCCCTGACGTGGGCATCCTG
GAAAATCCTTCTGCTGCTCGTGGCCTTCATAGTCTGCCCACCAGTGTGGATTGGATTCACATTCCCGATG
GGTCACAAGTTCAACAAGGTGCCCATCATCAAGTTCATGTCGTACCTAACCTCTCACATTTACCTCATGA
TCCACCTGAGCATCGTGGGCATAACGCCCATTTACCCAGTGCTCCGATTGAGTTTGGTGCCCTACTGGTA
CGAGGTGGGTCTTCTCATCTGGCTGAGTGGATTGCTCCTTTTCGAGCTGACGAATCCGTCAGATAAATCG
GGACTGGGATCGATAAAGGTGCTCGTGCTGCTGCTCGGCATGGCCGGAGTGGGTGTCCATGTCTCAGCAT
TTCTATTCGTCTCCAAGGAGTACTGGCCAACTTTGGTGTATTGTCGAAATCAGTGCTTCGCGTTGGCCTT
CCTGCTGGCCTGTGTGCAGATCCTCGACTTTTTGTCCTTCCACCACCTATTCGGTCCCTGGGCCATCATC

ATTGGGGATCTGCTGAAGGATCTGGCTCGGTTTTTGGCCGTCCTGGCCATCTTTGTGTTTGGCTTTTCCA
TGCACATTGTGGCCCTGAATCAGAGCTTTGCCAATTTCTCACCGGAGGATCTGCGCAGCTTCGAGAAGAA
GAACCGAAATAGAGGCTACTTCAGTGACG
>dme_circ_0001554_junction_seq
AACCGAAATAGAGGCTACTTCAGTGACGGGATAATTATAATGTGCTACATATTGCC
>dme_circ_0001555
GGTCCCGCATCTGTGGAGCGCTATTCAGGCCAGGATGCGGACGATGATGTTGACGTTGATGTGGTCGACT
GCAGTGACTCGGAAATGCCGTcggcaactgcggcagcagcagcggcggcaacaacagcagcggctgcagc
ggcagcattgcaggcgcagcaacaagcgcgtcaggcattgcgtgttgcacagcaacaacagcagcagcag
caacaacaaaggcagcagacacatcaccacgcaacaactggcaagcaacaacgccaacatcacaatcatc
acagcagcaacaccaacaacagcagcaactcaggcaacagcaacaccaacagcaaatcaagcaGCCAGCG
AGGGCGTTCTGCTGCCGCAGTGGGAGCCGCTGCCACGCCCTCGCCACCGCCGCCCCCGCCCTCGCAAAGT
CCGGAGGAACTCGAACGCCTGTCGCCAGAGGAATCACCAGCCCAGGTAAAGCTGCACAGAGACATAAATC
CTTTTTGCTTCTAGAAAAAGTGGCTAGCAAAAAGTATTATTAGCAAAATTTATAAAATAAGATATAGGTA
CAAAAAATTTGatataggattatttcctatcttatatattatattatcttatataatagattttatatat
agtagatatGATTTTCTTTGTGTGTATGCATCACCAACTAAGTGTGGCGAAAGAAATATGCATCTCCTCG
AGCGTCCTACTAAAATCGCCTCCTAATTCTCTACTCCTTCTTTTTCATCTCTCTTGGCAATGCCACGCCC
ACAGCAGCCCACGCCCAAGATAGTGGGCTCGTGCAACTGCGACGATCTCACGCCCGTCCAGTGCCACCTG
GAGACCAAGGAGCTGTGGGATAAGTTCCATGAATTGGGCACTGAAATGATCATTACCAAATCGGGCAG
>dme_circ_0001555_junction_seq
CTGAAATGATCATTACCAAATCGGGCAGGGTCCCGCATCTGTGGAGCGCTATTCAG
>dme_circ_0001556
CGCCGCTGAGCCGTCAGAGTGTACTCCATGTAGCCGTAGTAGTCCTGACTGACAAAGTGGGCTCGCGAGT
CCACGATTTCCCACTCCGGTGACTGAACCAGGTCGTCGTGGTCAAGGGACTCTCCTCTCGCCGTGCCGTT
CTCCGGCAGGACGACCTTCAGGCCCCAGGAGCCGATCTTCAACTTGCAGCTCTGCTTGTCGTGGGGCCAG
TTGAGCATGTTGAGTTCGCAGTAGGCCGTGTACACGGCTGGAGGCATCCACCGGAAGTGGCCATCGTGGC
TGAGGGTCACCTGGGTTTCGGCCATCAGGCCACCTTCGTCGCCGTTGAAGAGTGTGATTTGGGGGGTCCA
GACCTCGCTGGACTTCAAAG
>dme_circ_0001556_junction_seq
GGGGTCCAGACCTCGCTGGACTTCAAAGCGCCGCTGAGCCGTCAGAGTGTACTCCA
>dme_circ_0001557
GAAATGCGGTCCACGGCGGTGGCCAGCAGCGCCCAGTCGAACTGGTTGGCCTTGGCGCCCGGCACCTCGG
TGTGGTTGATGCCCAGCGGACTGGACTCCTGCTCGTCCGCCTCCTCGTACGGGTGCTCGTCCATCTCCTT
GGTCTTCTCCGCCTGCGACTCGCCGGTGGTGCTGAACACCGAGAGCAGCAGCCACGTGCCCAGGTGCCCG
TGCAACAGCTTTCTCAGCGCCTCCGGCAGGCGCCTCTTGTGCTTGCCTGTGGCCAGG
>dme_circ_0001557_junction_seq
GCGCCTCTTGTGCTTGCCTGTGGCCAGGGAAATGCGGTCCACGGCGGTGGCCAGCA
>dme_circ_0001558
GAAATGCGGTCCACGGCGGTGGCCAGCAGCGCCCAGTCGAACTGGTTGGCCTTGGCGCCCGGCACCTCGG
TGTGGTTGATGCCCAGCGGACTGGACTCCTGCTCGTCCGCCTCCTCGTACGGGTGCTCG
>dme_circ_0001558_junction_seq
CTCGTCCGCCTCCTCGTACGGGTGCTCGGAAATGCGGTCCACGGCGGTGGCCAGCA
>dme_circ_0001559
GCTCACTGATGTTCACATCGAAACGATCCTGTACTTCGTTAAGTATCTTGGCGTCGTTCTCGTCCGAAAC

GAATGTGATTGCCAGTCCCTTGGTGCCGAAGCGACCGGCACGGGCCACGCGATGCAAGTAGGTGTCGGAA
TCCTCGGGCAT
>dme_circ_0001559_junction_seq
GCAAGTAGGTGTCGGAATCCTCGGGCATGCTCACTGATGTTCACATCGAAACGATC
>dme_circ_0001560
GAGGCCCCCAAGAAGGATGTCAAGGGCACCTATGTGTCCATTCACAGTTCCGGCTTCCGCGATTTCCTCC
TGAAACCGGAGATCCTGCGCGCCATCGTGGACTGCGGCTTCGAGCATCCCTCGGAGGGTTCAGCACGAGT
GCATTCCGCAGGCCGTACTGGGCATGGACATCCTCTGTCAGGCCAAGTCCGGCATGGGTAAGACCGCCGT
CTTCGTTCTGGCCACGCTGCAGCAGCTGGAGCCGTCGGACAACAACACCTGCCACGTCCTGGTCATGTGC
CACACCCGCGAGCTGGCCTTCCAGATCAGCAAGGAGTATGAGCGATTCTCCAAGTACATGCCCACAGTCA
AGGGTGGCTGTCTTCTTTGGCGGAATGGCTATTCAAAAGGACGAGGAGACCCTCAAGAGCGGCACCCCGC
ATATTGTGGTGGGCACCCCTGGCCGAATTCTCGCCCTCATTCGCAACAAGAAACTTAATCTGAAGCTTTT
GAAGCACTTTGTGCTCGACGAGTGCGACAAGATGCTGGAGCAGCTGGGATATGCGTCGTGACGTTCAAGA
GATTTTCCGTAGCACGCCGCACGGCAAACAAGTGATGATGTTCTCTGCCACATTGAGCAAGGACATTCGT
CCCGTTTGCAAAAAGTTCATGCAAGATGTAAATATCAACGTGTTCATAACCAAAATGCTCGTACTTCTCC
ACCTCGCATCCACCGCTATCACGACAATTAGTTATTATTTTGTTAATGCCACGCGATATCGAACATTCGA
TCTTGATCCCCTCCGTATAAGCCAACCACCATCTTGCAAATGGTTAAACGCGTACAACCAAACGTCCAGC
GCTCTGTGAGCATAACTCGTATCGAAAAACTTAGTTAATGACTATCGGACACCATTTTCTAAATGGTTAA
TCGTGGAAATTGGCAACCGTTGCAAAAGCATAAACAAGCAGTAACCGCTGAATGAATCAATTGAAATCGC
AAAAGATTTAACAATACCAACGCTAGCAAAAACCAATACCTAAATAAAACTACAAGCAAACAGCAAAGAT
CCCGCCGACTGGCCGATGAGACGAGTTGAATCAATCCAGAGCGAATGCGATGCGATAGGCCCGCTCATCT
GTAATCCGCACACTTCTCAGAGCGATCATCGATCCTCGGGAGCAGTTCAGAGACTAGTTGATGAGATGTG
GTGGTAGCGTGGCCGTATGTAGAATATTCTATGCATATTGTTGTATGTTCCGAGGCGAGCCTAGTCTAAG
TTCGATTTCTCTGTACCCCTCCTAATTGTGTCCTTTAAAATGCGCCACATTCTATTTCCCAAATCCAGCC
CATGGAGGTCTACGTCGACGATGAGGCCAAGCTGACGCTGCACGGACTGCAGCAGCACTACGTCAATCTG
AAGGAGAACGAGAAGAACAAGAAACTGTTCGAACTGCTCGACGTGCTCGAGTTCAATCAGGGTGGTCATC
TTTGTGAAGTCTGTGCAACGTTGCGTGGCTCTGTCGCAGCTGCTGACGGAGCAGAACTTCCCCGCCATCG
GCATCCATCGTGGGATGACCCAGGAGGAGCGTCTGAATCGCTACCAGCAGTTCAAGGACTTCCAGAAGCG
CATTCTGG
>dme_circ_0001560_junction_seq
TTCAAGGACTTCCAGAAGCGCATTCTGGGAGGCCCCCAAGAAGGATGTCAAGGGCA
>dme_circ_0001561
GTTATTTTTTGTGGCCGCAGCGCCAATTATTTAATCAAAAACATTTGTGTAGAAGACATCCTGAATCTTC
GTTTCGTTTGTACACTTTCGTTTTCCCTTTCTTAACAAATTCTATAGTTTATTGTTTCGATGTTTTTGTA
TTTCCGC
>dme_circ_0001561_junction_seq
TATTGTTTCGATGTTTTTGTATTTCCGCGTTATTTTTTGTGGCCGCAGCGCCAATT
>dme_circ_0001562
GTCTTTGCTGCCGGAAACAATCAAGCGGGAGTCCGCGGACCAGGCAACCGTGTAAACAGCCTGCACATGA
CCCCGGAAGGTGGCCATGTACTGACCATCGCTGGCTCGCCACAGACGCACTGACTTGTCAAATGAAGCAG
ACGCAATTAGCTTTACATCCGGCGAATATTTCACGTCGTTGACCACGTTCTGGTGCCCTGTCATGCGCTC
AACGCACTTGTTCTGGTTGTTCCGCCACAGATAGAGGGTGTTGTCATCCGAACAGGAAACCAGCGACTCC
ACCTCGTCAGGGCACACGGCCTGGTAGCGCTTCAAGGCAGATTCCTGCAATTCCTCAGCTGTAAAACAAT
CTCTTTTAGGATATACAGCATATATAGAGAGCATATGAATTCTTACTGCTCAAACTGAGGTGGCTCTTGG

AGCGATCCTTCACCGGATGGAATGGACCAGTGCGCAGGACGTAGTCGGTGCTCAGCGCAATGTTGTTTAC
CCAGTGAGCGTGGCCAGAGAACGTCCGGCACAAGATTCCATCAGCTGCTCGCCACATCTTCACTGTGCGA
TCTTTGGAGGATGTATAAATAAGGCCCGCTCCACCCCATCTCACTGCTGTCACAGCATTTGTGTGTCCGG
CAATGTTCATAAGGCACTGGCCCAATTTCACGTCCCAAATCCGGCAGTCCCCGTCTCCACTGGCGGAAGC
AAGTTTCCTGCACTCCGGATCGCGATGATACGGTTCCCAGGCGAGGCAGTTGATGTGTTTCTTGTGCCCA
CTCAAGGGTCGCCCCTTCTGCTGACCCGTCTCCGGGTCCCAGATGATTATAGAGCCCGCTTTGCAACCGC
TGGCCAACCGTTTGCCATCCGGAGCCCAGGATACGCACAGAACCCACTGCTTATGACCTGTGCAGGTGAA
GTGCGGTGTCTCTGTGTTAAGATCCCACAATCGCACTGTG
>dme_circ_0001562_junction_seq
TGTGTTAAGATCCCACAATCGCACTGTGGTCTTTGCTGCCGGAAACAATCAAGCGG
>dme_circ_0001563
GATTGTACTAAAGGTGGTGAAGCAATGTTGCGCCACCGATGGTGTGGAACCGCAGTACATCAAAGAGGAG
ATTCTTCCGCACTTTTTCAAGTTCTTCTGGAATCACCGCATGGCCCTGGACAGGCGTAACTACCGCCAGC
TGGTGGACACCACCGTGGAGATTGCCAACAAGGTGGGCGCCTCTGAGATTATCAATCGTGTGGTGGATGA
CCTGAAGGATGAGAACGAGCAGTACAGGAAGATG
>dme_circ_0001563_junction_seq
GGATGAGAACGAGCAGTACAGGAAGATGGATTGTACTAAAGGTGGTGAAGCAATGT
>dme_circ_0001564
GCAATGTTGCGCCACCGATGGTGTGGAACCGCAGTACATCAAAGAGGAGATTCTTCCGCACTTTTTCAAG
TTCTTCTGGAATCACCGCATGGCCCTGGACAGGCGTAACTACCGCCAGCTGGTGGACACCACCGTGGAGA
TTGCCAACAAGGTGGGCGCCTCTGAGATTATCAATCGTGTGGTGGATGACCTGAAGGATGAGAACGAGCA
GTACAGGAAGATG
>dme_circ_0001564_junction_seq
GGATGAGAACGAGCAGTACAGGAAGATGGCAATGTTGCGCCACCGATGGTGTGGAA
>dme_circ_0001565
GACCACAAGGTAGCAGCAAAATGGATCGCTATGAGAAGCTCAGTCGGCTGGGCGAGGGCTCCTACGGTGT
GGTCTACAAGTGCCGGGATCGGGAAACGGGTGCTCTGGTGGCGGTCAAGAGGTTTGTGGAGTCCGAGGAT
GATCCAGCGATTCGTAAAATTGCACTGCGAGAAATTAGGCTACTGAAGGTAAGCCTTaataacttaaatc
acttgaatcacttaatcactcgaaATTTCCTGCAGAACCTGAAGCATCCGAACCTTGTCTCCCTGCTAGA
AGTGTTCCGACGGAAGCGACGCCTCCATCTGGTCTTCGAGTTCTGCGAGCTGACCGTGCTGCACGAACTG
GAGCGGCATCCACAGGGCTGCCCGGAGCACCTGACCAAACAGATCTGCTACCAGACCCTGCTGGGCGTGG
CCTACTGCCACAAGCAGGGCTGCCTACACCGCGACATCAAGCCGGAGAACATTCTGCTGACGGCCCAGGG
TCAGGTGAAGCTGTGTGACTTCGGCTTCGCCCGGATGCTCAGTCCGGGCGAGAATTACACGGACTACGTG
GCCACCAGATGGTACCGGGCGCCGGAGCTGCTGGTGGGCGACACTCAGTACGGCACTCCGGTGGACGTCT
GGGCCATCGGTTGCCTCTTCGCCGAGCTGGTCCGGGGCGAGGCCCTCTGGCCAGGACGTAGCGATGTGGA
TCAGCTCTATCTGATCCGCAAGACGCTCGGCGACCTGCTGCCGCGCCACATCCAGATCTTTGGACAGAAC
GAGTACTTTAAGGGCATCACGCTGCCAGTGCCGCCGACGCTGGAGCCGCTGGAGGACAAGATGCCGGCCA
AGTCGCAGCAGAATCCCCTGACCATTGACTTTCTCAAGAAGTGCCTGGACAAGGATCCGACCAAGCGCTG
GTCCTGCGAGAAGCTCACAAAGCACTCCTACTTCGATGACTACATCGCTAAGCAGCGCGAACTGGAGCAC
GTCAACAGCCTGGAGGCGGCCAATCTCCGCCAGCAGCAGCTCGCCTCCCAGCAGTTCATGCTGGCCACGG
CGGCCCAGCAGCTCCAGACGGGTCCTGCTCAGGCGGCGGCCATCGCGGCGGCCCGGGATAAATCAAAGGA
CTTCAAACACATCACTACCGCTGCTGCCTAGCACGCAGCATCATCATCACCCGCATCAAGATTACGTGAA
GCTGCAGCCCCTGAACAAGAATGCAAACCTGCTACATCGCACCGAGCATCATCTGCCCACAATTTGAGTT
TGAGCAGAGAATTTGTAACGAAGTAATGTCCTTGAGCGAGAATTGAGCCCGAAATTTAACTTATTTACTG

CTGGGAATAAATGTGTAAAAGTGTGCTTGTGGCCTACGACACATTGGCGTTGATTGCCTTCGTGCAAATA
ATGTTCCTAGTGCGGAGTCAACGTTATATACGACTTTACGGTCCAGATCCTTTGAATCCCGTAACAAACG
GAGTGCAATCCAAGCAAATGGCATCTATTAATCCGATCTCACAACAGCCAATAATTATTTACGTAATGCC
AAAAGCCGGGCAAAAATTGTGGGATTTGCCACAATCGAAGTGGTGGCAGGATGAAAAAACTGATGACAAC
GTTCCACAAAATATAAATTATGAAGAGTCCTCTGAATTTTTTCAGCGGCAGGAGCTTTTGTCAAAAGTTT
TACTACGCAATGCCATCAAAGAAGACTATCTACACAAGGAAAGAGTTGCAATCTAAGCCATCCCTATTGT
CAATGGTGCGTTTGTTTACATTCACAAAAGTTTCAGTTTCTGCTGTAAGTAATATATACACGTCTAGCCA
GGATGTCGGACAACGATTACGAGCGTTTCGAGATAACGGACTACGATCTGGACAATGAGTTCAACATCAA
TCGGCCCCGAGGCCGCCAGAGCAGGCACCAACAGATCTACGGGTATTTGGGCGGATGACAGCGAGGAGGA
GAGTGGTGGCGAGGGCGGAACTAAGAGAAGGGGGCGCGCCGCCCGTAAACCCAAAGACTACACCATGCCC
GTCAATTTTGTGGCCGGCGGCATTCAGCAGGCGGGCAAAAAGAAGAAGAAGGCGCTCCAAGCCGATGATG
AGAAAGGATCGCAAAAAGAGGGAGCAGAGGCTGACCAGGGCGAGGAGAGCGACGATTCCGCCGCCAGCGG
ACGGCCAGCCTTCGGCCAGAACGATCCGGGCAGCTCCAACAGCTCATCAGAAGAGGAGAGGCCAACGCTG
AGCCGGAAACAGCCAAGCACCACATTTCAGCATCGCTCCCACATCGCCAGCGAGCGCAACGTGGGCGCCT
GGGAGCAGCATACACGCGGCATTGGAGCTAAGCTACTCCTTCAAATGGGCTATGAGCCTGGCAAGGGTTT
GGGCAAAGATCTGCAGGGCATTTCGCATCCTGTGCAAGCCCATGTCCGCAAGGGCAGAGGTGCTATCGGC
GCCTATGGACCCGAAACTGCGGCAAGCATCGGAGGAAAGACGAACAAGAGCATCAAAGTGGATGAGGACG
TGCGCGAGGCGAAAGAGTTCAAAGACCAGCTGAACAAGTGGCGAAAGGGCTCAGCCGGTGGCGCCGAACC
AATGGAGCGGCAGGGTAAGCGCTACTACTACAAGTCCGTGGAGGAGGTGATTGCCAAGGGTCACACGTCC
GGCCACCTACTGTCGGAGAAGCTAAGCAAGAAACTGGGCAATGTGCGGGTGATCGATATGACAGGCCCAG
AGAAACGAGTGCTGAGTGGCTATCATGCCTTGGGACAGGCAAAAATTACGCCAGAGGAGACGCTCTACGA
CACGGAGGCGACGGAAAAGGGATCCGCTCCCGCCTGCGTTTTTGCCATGCCCGAGCTGACGCACAACCTC
CAGCTCTTGGTTAGCCAGTGCGAGCAGCAGATCATCGCCATCGACAACCAGGAACGCGAGTGTTCCAGCC
AGCAGGCAGCGCTGGAAAGCGAGCACCGGAAACTGGAGGAGATAGTCCAGCTAGAACGAAACCACATACG
CACGCTGGAGGAATCGTTGGAGCGCGTGGAGCGGTTAATCGATAATCCAGACTTGAGTTTACCTCAGGCT
GAACGACTCTTTCGGGAGCTGCTCGTGGACTACGCTGCCGAATTCCATGAGTTCGGACTTGCAGATCTGG
CAGCCGGTGTAATAGCGCCGCTCCTGAAGCGAGAACTAGTTCAGTGGCAGCCACTGGAAAACCCAACGGA
ACCGCTGCCACTCATCAAGAAATGGCGCGGCATGCTCCAGCAAGGAGATGCAGCAGAGCAGCAGCCCCGC
AACGTCTTCGATCCGTACTCCTCGCTCATCTGGGCCGGCGTGATGCCTTCGTTTCGGTCCAGCGCTGCCG
CATGGCAGCCGAAGGAGCATCCGCCAATGGCTTCACTCCTAGATGCCTGGGCACCCCTGCTACCTAGCTG
GGTCTTGGATTCGGTTCTCGAGCAACTGGTTCTGCCACGACTGGTGGCTGGTGTCCAGGAGTGGGATCCG
CTCACCGACACCGTTCCGATCGACAGTTGGGTGCTGCCGTGGCATGCCATCTTGGGCAGCAAGCTGGAGG
AGGCCGTCTATCCTCAGATCCGCAGCAAATTGGGTATAGCATTGCGCGCCTGGTCACCGCATGACCGATC
AGCCAGAGCCATGCTTACGCCCTGGCAGAAGGCCTTCCCCGAGGAAGAGATGCAAGAGTTCCTGCAGCGG
TACATAGTGCCCAAACTGCAGGCGACTCTAGGCGAGCTCATCATCAATCCAATGCACCAGGACCTGGAGC
TGTGGCAGCAGGTATGGGAGTGGCACGAGCTCATCGATCCCATGTACATGGCCCAACTACTGGACAGACA
CTTCTTCCCGCGCTGGATGCAAGTGCTCGTCGTCTGGCTGAATCAGTCGCCGGACTACGCGGAGATCTCG
CGATGGTACACCGGCTGGAAGAGCATGTTGAGCGAACCGCTTCTGCGCGAGCCCAGCGTTAAAGAGCATC
TGCGGCGAGCACTGGAAATTATGCACAGAGCCAGCGATACCCTGCTGCAGCCCACGGTGACGCCCACGCC
CCCGCCTCCAGTTCCTCCGGCTCCAGTCATAATGATGGACCTGATTCATCCGCCCGCTCAGCTGGAGTTC
AAAGAGTTGGTATCGCAGCAATGCGCCGACTTGGGAATCATCTTCGCTCCTCTGCCAGGGCGACGGGAGA
TGGGCAAACAGGATTTATCGTGTGGGAAAGCTGTTTTGCTACATCGATCGTCACGTCTGCATGGTCAGCG
ATGGCAGCTTCAGCAACTGGAAGCCGGTGTCGCTAAATCATCTGCTAGAACGCTCTCAAACGGGAATTCT
TTAAATAAATAGCTGCgaaaatgtttCACCTCTGGCCATCGCAGCTGACTCTAGAACGCGTCGTGGAAAT

AGGAATAAACTGACTGAGTTCGCGTTCGGTTCGTTTGGCGCACGCAAAGCAAGCAAGTAGTGAGCCAGAT
GCGGTTCGAAACGGTGCAGTCAACTGAAGCTCGCGTAGTGCAATACAATTAATTGTAAAACCAAGACACA
AGCACTCCTATCGCGATAGGCACTGTTAGCAAATTGAAACCGCGTGTCTTTCGTTTCGCTTCTCCCGCTC
TTTCGTTGCACTTCGCCCACACTATTCGCCAATGCAGTCGTGAGTGCGTGTGTGCGTGCGTATTCCCCCC
CAAATTCAATGAATTCGACAGGCGGTGCAATAAAAGCGCAACTAAAACAAAAGTGATTCCACCACGTGCG
CCACTCTCTCGCGTCTTAAGTTCAGTTGTTGTAGCTGGTTTCGTTTGTTTTGCTGGCGTCTCGCTCTAAC
AGCTCCCACTGCTAAATTTACCGCTCGCCGAACAACCGGCACGGAAATATCCGTTGGATTAGGAGGACTC
ACTAACGGGACACCCGCAAAAGGACATCAAAATGGTGCAG
>dme_circ_0001565_junction_seq
CCCGCAAAAGGACATCAAAATGGTGCAGGACCACAAGGTAGCAGCAAAATGGATCG
>dme_circ_0001566
GTGTCTCGGCCACCGAAATGCTGTGGGCTCATCTTCTAACCCAGCTCATCATCATGGCCCTGCAGTCGTT
CGAGGTTATTATGTATATTGGCCTAGTGTTCGATACTTACAATAATGGGGATACCACAACGCTCATTGGA
TTGCTAACACTGACCGCATTTTGTGGCATGTTG
>dme_circ_0001566_junction_seq
AACACTGACCGCATTTTGTGGCATGTTGGTGTCTCGGCCACCGAAATGCTGTGGGC
>dme_circ_0001567
TCTACAAGGATAGCGACAACGAGAGCTGTCTATTCAACAAGTTATCCTGCAGATACATACGCGTGTTGGG
TGACGATGTGGCCACGCGGAAATACTATGCCAGCGAAGCAGATGCTCTGAACGATGCAAAGCGGGCCACA
ACCGTTGGCTATTTGCACTTTGCCCAGAACTTCAGCGATTCGATTCTCTCGGTAATGGAGGATGGCATCC
ACTCCAGTGACGGAGCTGTGGACCACGCCGAGCTTAGTATCCACATCGATATGACGGG
>dme_circ_0001567_junction_seq
AGCTTAGTATCCACATCGATATGACGGGTCTACAAGGATAGCGACAACGAGAGCTG
>dme_circ_0001568
GTTCGAGATGATCGCGTGGCCGCGCTGGCTAAAATGGAAGCAGTCGTGAGAGAAGAACCTCCAGTCCGTG
CTCCCATCGGGCAAAGTGGGAGCAGTTAGTTTGGTCAGCATCGGATGGGCGACGATGGCGAAGTCCTGGC
GACGGAACTCCGGCAGCCGGGCGATCTCCATGTCCAGCTGCTGCCAACGCGTCAGGGTGTCCATGCGCTC
ATTGAACTCGGTGCGG
>dme_circ_0001568_junction_seq
GTCCATGCGCTCATTGAACTCGGTGCGGGTTCGAGATGATCGCGTGGCCGCGCTGG
>dme_circ_0001569
GGCACTGGACTACGATTGCGAGGCACAGGGCCAGGACATCCCGCAGGCGATTCAGTTGCTCGGGGAGATG
AACAGCAATGTGAAACAGGTGACTGACTTGGTGGAGGGCATGCTGCAGCGAGTCAAGCGAGGCGAGCTAA
CCACAGAGTACGGCCTGAGCTTTCTGGAAGTGAAGTACCACATGCTACTGGACTACTTGATCAATCTGAC
ATATGTGGTGCTGCGCAAATGCTCCGGCGAGACCATTGAGGGTGATCCCTCCATCGAACGTCTGATCGAG
ATTCGCACTGTGCTGGAGAAGATTCGGCCCATAGACCACAAGCTACGCTATCAGATTGACAAACTGGTAA
AGACAGCCACCACTGGCGTCTCAAGCAGTACGGATCCCATACTTTACAAACCCAACCCAGATGATATGAT
GAGCAGCGCAGGCGGAGCAGGCCGAGATGAGGATGATGGAGAAGATGACAGcgatgaggaggacgaggac
gacgatgaggaggatgaggacgaGGCCGGCGCAGCGAAAATGCCCCGGAAAGCGGCCACAGCCGGAAAGT
CCGGAGTGTACGTGCCACCGCGCATCAAGCCCGTTTATTACGATGGCGATGAACGCGATGCCGACAAGGA
GAAAAAGGCACTGGATCGGGCCAAGAAGCGGGCCATTACGGTCTTCCATGCTGCAGGACCTCAAAGAGGA
GTACTTGGATGCTCCTACAGAGATTTCATCTGGTTCGCGTGCTCAGCAGATGCTTTCTCAAGCTCAGAAG
GAGAAGCAGGAGTACGAGGAGACCTACTTGATGCGTCTGCCGGTGACCAAGGCGGAGAAGCATCGCCAGC
GAAAACTGACCACCTTGGGGCACACTTGGCGATGAAATTCTTGGGGAGATCAGTCGGGAATCAGCCCTGC

GTGGAGATGGAAGCAAAAAGCGCAAACTGGCCAAAAAGGGGAAGGGAAAGAAACGTGGCGGTAAAAAGCG
AAAATTCCATTAATTGGTGGCACAAAATATCCTATTTAA
>dme_circ_0001569_junction_seq
AATTGGTGGCACAAAATATCCTATTTAAGGCACTGGACTACGATTGCGAGGCACAG
>dme_circ_0001570
GGTCTGGCCTGGAATCCGGCCACCTACAAGTGCGATTGGCCCGATCAGGTGGAGGACTGTGACGCCGAGG
CCTTCCTGGGCTTCCGCTGCCCAGCGCCGGCACCCAGATCTGAGCTGCTCGGCGAGCAGGAGGCTGACTA
CACCTTCCACCCGTCGCAGGACAATTGCCAGG
>dme_circ_0001570_junction_seq
TTCCACCCGTCGCAGGACAATTGCCAGGGGTCTGGCCTGGAATCCGGCCACCTACA
>dme_circ_0001571
GGTGTAGTCAGCCTCCTGCTCGCCGAGCAGCTCAGATCTGGGTGCCGGCGCTGGGCAGCGGAAGCCCAGG
AAGGCCTCGGCGTCACAGTCCTCCACCTGATCGGGCCAATCGCACTTGTAGGTGGCCGGATTCCAGGCCA
GACCCTCGGGGCAGTCGAACACGAAGCCACGGCCCGCGGCGCAGTTCATGAACTGTCCACAATGGCTGGC
ATCACCCATGCGATAGTATCCGAACTGGTGGGGGCACTCATCCGTTGGCTGAGCGGCCTGCAGGCGGGCC
TGGCCCTGGGTGCACTCCACGTCGATCGGATAGCCGCAGGGGTAACCC
>dme_circ_0001571_junction_seq
GTCGATCGGATAGCCGCAGGGGTAACCCGGTGTAGTCAGCCTCCTGCTCGCCGAGC
>dme_circ_0001572
AAAGTGGTGGCGCCGTCGCCGTAAAGGAAGTAGGTGAAAGTCTCCACCTCGGTGTCGTTAAAGAAGCCCA
CGGCCAGCCTGCTTCCATCCGATGACCACCAGATCGCGCTGCCGCTGCTCAGTACCTCCTCTTCGTAGAC
CCAGTCGGGTACTCCGTTGTAGACCACCCCGTCCACGCCGTCGTCCGTGATGCTGATCTCCAAGCTCTCG
CTGAAGTGGATGAACACATTGTTCAGGTAGACG
>dme_circ_0001572_junction_seq
GTGGATGAACACATTGTTCAGGTAGACGAAAGTGGTGGCGCCGTCGCCGTAAAGGA
>dme_circ_0001573
CAGGTGCGTCGAGTTCGACCAAACAACGCTCTGCAGGATGTGATCGTCTCCGACGATATCCACGGGCGCC
ACGATGGTGTGCATGGTGGGGTCATTGTCGGACACATCGTAGACGCGTAGGCTCACCACGGGGTTCTTGG
AGCCTGACTTCGGATACTTGAGCTGCTCCTCGTGCGGATACTGATAGAAAG
>dme_circ_0001573_junction_seq
TGCTCCTCGTGCGGATACTGATAGAAAGCAGGTGCGTCGAGTTCGACCAAACAACG
>dme_circ_0001574
TTGCCGAACACGCTGCTCTCGTTGTATTTCTTCAAGTTGTCATCGTCAGTGGGAAGTCCCATATAGCGCT
CGGTATAAATCGTATCTATAATAGAACATTGAACATGGTAAAACCCCAATAAAATCAAAAGGCAAGGTAG
ACAAACCATAGTACAGCCATGAGGTAACTGGAGCCACCGAAACTCCGCACTGAAAGACCCGATCGTCATC
CTTCTCAATGGTCTTGGCTGTCATATAGCCACCATAGCTCCATCCCCAAATGCCGCAACGCTCGGCATCC
ACGAAGGCCAGATTTTGTTGCATCCAACGAGTGACGAACAGTTGATCCTCGACCTCGTGATCACCCAGAT
CATTGTTGACCGAGAAGAGCAGATCCTTGCCCTTATTACCCGTACCTCGGCCATCAATATACGCATAAAT
CGTATCCCGCGAGGTCGTGACAAACGCCTCGTAGCCCAGGGTGAACCCGTTGGTCACTCGCACCGAGTTG
GGTCCCTGGTACACAACCACGATCAGTGGGTACTTCTTCGTCTCCACGAAATTGGGAGGAAGGGCTAGCT
TGGCGTAACCAATACTTCCATCGGCCAGGGTGACGTTCATGAAGCGATAGCTGGGACGCAGCTTTTCTTC
TATTTGCTTGCGG
>dme_circ_0001574_junction_seq
ACGCAGCTTTTCTTCTATTTGCTTGCGGTTGCCGAACACGCTGCTCTCGTTGTATT

>dme_circ_0001575
GCGACGTCCTTTATTGGCGGCCCAAGGAGTCGTCGCCACACGCACGAAATGATTATGACGATGATGCAGA
CGGTAAGGGCATGGCAACAGGAATCGGATGTGGAGCACCGAAAACAACATAAGCAGCGATGGCGACCTGA
TGGGGCACACATATCAGCGGCTTACGACCTCAACAGTGATAATGACGACGGCCACCACAGGGTGGTGCAT
AATCAAAACAACGGCAGCCCAAACAGCTCTCCGAATCAGAGCACTTCCGCCTTCCGACAGCGGCAACCGC
ACCACCCACCCACCGGACAACAACCGCCCCGGCTGCCCTGCACCGTAACCCACTTCTCCGCACACTGGAA
AACGctgctcatcctgctcaccctgctcTCCGCTTCCACGCTGACAGCCTCCGCCAATGTCACCAGCACC
ATCAGTCCGCCCATCAACGGCAGCAGCACCGATTACATCCTGCTCTATGGCGAATCCACCACATCCCTTG
TGCCCGCCCTGACCACCGGACTCTCGGGGGACGGAAGTGGTGCGGTTATCGAGGACGAGGAGGATGCCGA
GAAGGCCAGCGAGTACATCTTTGATCGCACCGATGTGCGCATAATATTCATCACACTCTACACACTGGTG
TTTTGCTGCTGTTTCTTTG
>dme_circ_0001575_junction_seq
ACACTGGTGTTTTGCTGCTGTTTCTTTGGCGACGTCCTTTATTGGCGGCCCAAGGA
>dme_circ_0001576
GCGACGTCCTTTATTGGCGGCCCAAGGAGTCGTCGCCACACGCACGAAATGATTATGACGATGATGCAGA
CGGTAAGGGCATGGCAACAGGAATCGGATGTGGAGCACCGAAAACAACATAAGCAGCGATGGCGACCTGA
TGGGGCACACATATCAGCGGCTTACGACCTCAACAGTGATAATGACGACGGCCACCACAGGGTGGTGCAT
AATCAAAACAACGGCAGCCCAAACAGCTCTCCGAATCAGAGCACTTCCGCCTTCCGACAGCGGCAACCGC
ACCACCCACCCACCGGACAACAACCGCCCCGGCTGCCCTGCACCGTAACCCACTTCTCCGCACACTGGAA
AACGctgctcatcctgctcaccctgctcTCCGCTTCCACGCTGACAGCCTCCGCCAATGTCACCAGCACC
ATCAGTCCGCCCATCAACGGCAGCAGCACCGATTACATCCTGCTCTATGGCGAATCCACCACATCCCTTG
TGCCCGCCCTGACCACCGGACTCTCGGGGGACGGAAGTGGTGCGGTTATCGAGGACGAGGAGGATGCCGA
GAAGGCCAGCGAGTACATCTTTGATCGCACCGATGTGCGCATAATATTCATCACACTCTACACACTGGTG
TTTTGCTGCTGTTTCTTTGGGTGAGTAGTAGACTTGGATTTATCTCATCCTAATAGCACAAATGATTTAA
TTACGAAATTATTGATTTAGAAACATTAGCCCGCCAGCTATGGCCTTTGGTCATAATTTTAGTCTAATTA
AGAGATAATAATATTGTAGTTAATGTTTTCAGACAACATTTTCTCTGTGTGCAGGACTTGCCGTACACTT
ACAACGTTTTTCCTGTGTATATAGTGCTATGCTATATACACAAGATTTATAGTTCTCATACGCATTTATC
AGAGCAGAGCTCGATGTTATGTTCGATTGTGCAGCATGTAGCATGATGGGGCACAAAGGCCTGCCGAAAC
ATACATGAAAGTAAAGAAAAGACGGCCTGCCGGCCGTTTGCGGTGCCCCAGGGACATCTTTCTGGGTCGA
CCAGGATGTGCAAACTTACTGCTGACTTGCCAATTACTCAATGCCCTGCTGTTCGGTTTATGCTGTGTTT
TTGTCCAGTCTGCCTAGTATTTAAGTACTCTCAGGGCTTTTCCACTCTGCTCTTGGCGCAGCAGGAAAAT
AGGCAAAGTACtaatagttatattgcgagcataatacttaacttgctagcaATTTTAATGTGTTATTTCA
GTGTTGCTGTTTCTAACTTTTAAGCGGATTGAAGAATCTAAAATACTAGAAGAGTTTGTTACAACACACT
CACTATCAAAATATCGATCTATTTACGAACCCATTTTCCATTTCATGCGGAATAAATTAGGCAGTCTTCC
TTCTCAACAACAGACTTTTTTTCATATACATTCGCACAACACTCATACGCCCCATGTGGCGCATTATAAT
GAAAACATTTTATTGATAGCGCCCATTAGGCCATTTGCTATTTGTTTCTGCAATGCTTAATTGCAGacac
acacacacacacacacGAATACGGAATGTCCTTTAACTTTGCGTCTTATCTGGGTGCGCGTGCGAGTACG
GATGGTTAGAATCAATTACGTTAGTCGTTCGATTGGGCTCTTTGCGGGTGTTGGCCAATCTCAGCCATCG
TCTGTTGTAGGCTTTATTACCCGCAGTGACACCAGCATGCACAATAAAGCAATGGAACATGTGTAGTCGC
CATTCCGAATGGCATCTAAACGCCGCCCCCTCCGCACAGACATGGGCGTGGCCGTTCATTGTGCGGACAA
TTGCAAAGTGTAAGCAATTTGGTTGGTTTCACTGGCGCCCGGAATGTGGGCGTAAACGATGGGGTGGTGG
GCGCAAGGACGACCAGCGACACACTCGCCCACTAAATTTACATCGGGCGGACACGTTTAAAGCTTTCGTT
TGGTTACTTCTTTGTTGCCGTCACTTGAACAAGATACCTTATTGTCATATTCAAATGGGAATGCTTGGGT
GACGGTAACCTTGGCGATAGAACAATATCATAACAGTTGAGTGGGGCGAACGCAACCTATAAATATAATC

GAAATTTGAGGTGTACTTCAAGAAGGAGCAATCAATAGTTTAGCTGTTCTAAGAATGATAACTAATTCAA
TAAAACTACAGATTTCAATAACTAAAGCATACATGTAtatgtatctatgtatctatgactatgtatctCG
AATCCTCCGCTGAAACTCTAAACTATTCCCATGCAGTTTCTTTTCCTTCGTATCGAGTTTCAGAGCACGG
CTTGTAGTGTTGCACgttttttcgtattttttggcagctctggctggttttttttcattttcctttttttttt
tGTACTCCCATTTCCTGTCACCCTGCCCGGTGTAATTAAAAAAGTTTTTCTCGACATTTGCTTGCCATGT
GAGGCTCCAGGAACAAATGCGTTACATTGTGGACCATCCGATTGTAAAATAAATTTTAAGTGCAATACGA
AGTGCAAACTCAGCCGTAACAATAAGATTTTTGCAATAATATTTAACTTTTAATTGTAACTTTGGTAAAG
TCAATGTTTTCTTTTTCCAAGTTGGCCAATAAAAAGAGCATAAAGCAAAGCGAAAACTTTAGTGCGTACT
TTTATTACAACATTAACTTCATGCTATATATTTTATTTAAATATTTTGGCTTTTCTATCCATTGTTTTTC
AGTTAATATCTTAAACAAACAATTTTTGATGCACTACAAATGCTGTTGATTTTAATAAACGATTTATAGC
TTACATGGATTATTAGCTATGTACATATAATTACGAATATTTTTAATAAAGGGCATACTTAATAAATTGA
AATTCTGGAAAACACTCATTTATTTAACACTAGCAAATTAGCGCACAAATAATGTGCAAAGTTCGTTAAT
CTTTAAAGTGTATTTGTGACCACAGCACAATTAAACGGTGGTCCGAAATTCTATATCTTCTAATTCCGCA
TTGtttttttttttttGTGAATTTTTCAGGGTGAGTGACAATTGGCAAAAACAACATTATTTCAGTATTA
AATTAACGTTTCATCATGCACGAGCGGAGCTGCAAAATTCTCGGAAAGCATATACGAAGAATACGTGAAC
TAACTTCACTGTAAAGTTGCCAATAGTTTTTAATGTCCGTTTTCATACGCAAAAAatttaagtgaagcat
ttaagtgaagcatttaTAGAAAAGTTTTTAGTTATAGTAGGTTAAAAATTAACACAAAAGTTAAAGTAGC
GTTTCAGCTAGTACAGATAGTAGAATGTTATGTGGTCGATTAAATTTATTTATTTATCAGCTGCAGAATG
TGGCTGAAACGGTATCTGAGTGCATACGTATGTATGCATGTGCCTCGAAAACAAAGCCCCaaaaaaaaGT
TTCAACTTTCAACCGTGAAACATTTACGCCGGCTGTCACATAAAGGAAACTAAAACTCCGCAGGCGTCAC
TTTCACttcaggcttttcaggcttttATCCCCACGCTCCGCTTTCACTCCAATCATGTTTATTTGCATTG
TGTTTCCTTAACTTTCCCAAAAACACGTCGCAGTGACGTGTTCACTTAAAATAAAGCGTGGAGTGCTGCT
TGAAAATTCAAACAAAAAGAAGCCTTTTTCAGATTTGCAGGGCAAATTAAAAAAGTATTTTGACAAATGA
GTTTGGCTTACAGTTTCTATAAGTTATTTAATTTAAAATTTTAGTCGCAGTGTCAGAAATAAATAATGCT
TCATTAATTTAAATTTGTGAAATGCATGTGTTTAATATGTGTTTAATTAAAAGCTATGTTCTATACATTT
AGaaaaaaaaGTAATTACCTATCTCATTAGAATTCTTGAAATGACCATTTATTTATGCACTTAATAATAA
ACAGACATAATGATTTATTTGCATTCCCGATGTTAAATCCTGAAATTGGTTTGCATATTTTCCATTTCAT
TTTCTAATATGCTTTTTGTGGCCAAAGAACTAAATTATTGAGATGACCCATCTGTGTTTGGCATAATTTT
AGCTAAATTATGCGCACAACTTGACGCCCACCAGAAATCATATGCAAAATGAGATGAGATTACGAAAAAT
CATAATTTATGCATTTATAATTTATATGTGCGGCATACGGCTGCCAGTGACAATTTAATTTAATGATTAT
AAACATCAAGTCGTTTTAAGAAAGTTTTTAGTTCATTATGTTAAGGAATGATTTGTCCCTTTTAAAGAGA
GAAATTGAATCACCAGGCAAATGGGGAATCTTTTTAAGAAACGCATTATCCAAAATCTTGGCGACAACCT
TCAGGATTATTATGTTTATTATAGTATTATTGGGCACATTTAAACGCATGAGTGCGAAATTACGTGGCAT
TGCTAATAACGAGGCACAAATGCGGTTTTCGAATGTAATTTCAGCCGAAATAGTAATAAAAAGGAAAAAG
CTTTGCGCCTCATAAAATACTAAAGACACCTTAATGTCGCATTAGTTTTGGCGGGCGTGTCATAACATTT
TATGTGGCTTGTGCTAAATTTGGCTCTGCCACCAATATTGCCAAGTATATGCTCTTTAAAGAGCGAAACT
TTTGCCCATAAATAATTATTTATGTCCTTTCAGGATGGTCATTGTCACAGTTTGGATTACATCGGCAGTT
TACTCCACGCCCAAGTTTGTTTTCAGCAAGACCATTAAGAACATCCACACGCAGGACGGGCAGGAGGAGG
AGATATGTGTCCTGGACCGTGAGATGTTCAACTCCAAGTTGCTGGACATGATTAACTTTGTGCTTCTCTA
CGTTATGCCGCTCTTGGTGATGACGGGTGCTGTACAGCAAAATCGCCATCGCCTTGTGGCGCAGTTCGCG
CGGCCTCACGCCGCATGTGGTGCAGCATCAGCATCAGCAGCCGCAGCAGCCATCCTGCCAGGACATTGGC
ATGGGTATGCACAACAGCATGtaccatcaccatccgcatcatcatcaccaccaccaccagcatcaccaGC
TGCAATCGGCGGCTTCGTCGGCAGGAgtggtgggagtgggtttgggtggtggtggtgggggCGGTCCTGG
TCCGTCACTCGCTTCCGGCGGCAGCAGCACCACGTCCTTGTCCCGCAAACAGAGCAGTAAATATGAGAAG

CGCGGCGTCAGCATCACGGAGAGCCAGGGTGAGCTGCCAGGAGAACACTGCATCAGAAAAATCGCCGGTT
GAGCACGGAGGAGTAGCCTTAAAAGCCTAACAATAGCAATAAACCGAAAGTGGAGTCGAACCTTTGTTCC
CTAATTGGCCAGAACGGCTTTAACTTCGATTGAGTCCGCACGTGCTGTGTGGTAAATAATGTTTTTTGAA
AATCACATCGCTATCGGAGCACTAATTTATTAGATACACCATTTGTCGGCCTGGCCAAGTTGGCAGTCGT
AAAAACTTTACCCGTAGCTGTTACTCCCGCACTTTCGCCGGGCCAAGTGGCACGGTGCTCGGAGCAGGTG
TCAATTCACATTAAGGCCATCGCAGGACCATGCCACAAGCTCGGCCACCGATGTGACACCTGGAGTGTGG
AGTGGTGGAGCGAAGTGACCACCGGCACAGGGCGAAACTGCAAGTGCGAGGCCAAAGTTATGGCAAAAGT
GtttttttttttCCATTATCGGTGGCCGGACAGCAGGTTCAATTGGTCGGGTGCACCATGGCGtatttct
tatttcttatttcGAGCTCGACAGCAGCTTTGCCACACAAACTGGCACACACATCATTGTTCTTGATCAG
CATCAGCAGCTCACTTTGTCAATAAAAGCCGGCGGATAAAAATCGAGAATTTGTGACACGATGACATTCG
GAAAAGTTGTGCAACTGCCGCAGATTGAAAATTGGTAAACACCAAAGGCAAGGCCCTTGAATTTTTTAAT
ATTCCTCGAAGTATCAAATGTCAGGGGAGCACTGGTTCGATTTCGGTATGTTTTCCGTTTTAAAAGTCAC
AAAAATGGAAGTTTGGTAGGAAAAACAATGGCGGGAACACCTTTATAAAACTTTTACATAACTTTAGTTT
ACGCTGTTCCTTAACTTATGACCCCGATATACACAGATAACAACCTAACTATCTTTCTGTTGAACTCTTT
TCTCTCTCTCTACCTTTCGTTTGATCAATAAAACTCTGTGCTCTCCCCCTCTACTAACTCCCCAATTATA
ACAACAACCACACACAATCCCACACCACAAATTAGCTCGATAATTGCAAGGTAGGTTGTAATCGAATATC
AAAATCGATATGATAATGTTCAACAACTGTTGCGGCCAGCGACCTAATGCCATTACACCTGTTAATCCGC
ACAGGGTGTCCCTGGAGGCCGATCGTCCCATTGTCAGTGCCTGCCGCAAGACGAGCTTCTACCACCACGG
CCACGCCCATCACCAGCGGGCGGGCAACGCCTCAGTGGGCGGTGGCAGCGGCGGAGCCGGGGCCGGAGCC
ACCCACATGTCCCACTCGTCCAGCAACGTCCTACGCGCCCGGCGCGGCGTTGTCCGCATGCTGATAATAT
TCGTGCTGACATTCGCCCTCTGCAATCTGCCGTATCATGCCCGCAAAATGTGGCAGTACTG
>dme_circ_0001576_junction_seq
ATCATGCCCGCAAAATGTGGCAGTACTGGCGACGTCCTTTATTGGCGGCCCAAGGA
>dme_circ_0001577
GATGGTCATTGTCACAGTTTGGATTACATCGGCAGTTTACTCCACGCCCAAGTTTGTTTTCAGCAAGACC
ATTAAGAACATCCACACGCAGGACGGGCAGGAGGAGGAGATATGTGTCCTGGACCGTGAGATGTTCAACT
CCAAGTTGCTGGACATGATTAACTTTGTGCTTCTCTACGTTATGCCGCTCTTGGTGATGACGGGTAAGTG
TAACAAATGATACGTAGCTTGAAGTTAATAATTATGTTTAACCAATCCTTGGATAGGATGTTCATAAATT
CATTTTCAATCCAAAAGTGTTTCCTAGTCACGTTACCTAGCATTGAGCAGACTATAATTAATAATTAAAT
GTATTTTTTTGAATTGCTTATTAAAGTGATTAATTATATCAATAACAACTATCTCTCTAATGAGATAGAA
TATAAATAATTTTGGGTACATAATAAATAATCTATGAAATGATTTTTCTTCGTCTGTTAAAAAAGTGATT
AATGGAAGTGTAGATTTCCCTTTATTTTTTTAACGACTCCATTTTTCAATTTCTTCTGAGAAATTTGATA
AGAATTGAGAAGCTTTTTGCATAAAATCGCCCCTTGTTCCGCAATGAAATTTGAATAATGAATTTAACGA
ACCCATTTGCATTGCAGGGTGCTGTACAGCAAAATCGCCATCGCCTTGTGGCGCAGTTCGCGCGGCCTCA
CGCCGCATGTGGTGCAGCATCAGCATCAGCAGCCGCAGCAGCCATCCTGCCAGGACATTGGCATGGGTAT
GCACAACAGCATGtaccatcaccatccgcatcatcatcaccaccaccaccagcatcaccaGCTGCAATCG
GCGGCTTCGTCGGCAGGAgtggtgggagtgggtttgggtggtggtggtgggggCGGTCCTGGTCCGTCAC
TCGCTTCCGGCGGCAGCAGCACCACGTCCTTGTCCCGCAAACAGAGCAGTAAATATGAGAAGCGCGGCGT
CAGCATCACGGAGAGCCAGGGTGAGCTGCCAGGAGAACACTGCATCAGAAAAATCGCCGGTTGAGCACGG
AGGAGTAGCCTTAAAAGCCTAACAATAGCAATAAACCGAAAGTGGAGTCGAACCTTTGTTCCCTAATTGG
CCAGAACGGCTTTAACTTCGATTGAGTCCGCACGTGCTGTGTGGTAAATAATGTTTTTTGAAAATCACAT
CGCTATCGGAGCACTAATTTATTAGATACACCATTTGTCGGCCTGGCCAAGTTGGCAGTCGTAAAAACTT
TACCCGTAGCTGTTACTCCCGCACTTTCGCCGGGCCAAGTGGCACGGTGCTCGGAGCAGGTGTCAATTCA
CATTAAGGCCATCGCAGGACCATGCCACAAGCTCGGCCACCGATGTGACACCTGGAGTGTGGAGTGGTGG

AGCGAAGTGACCACCGGCACAGGGCGAAACTGCAAGTGCGAGGCCAAAGTTATGGCAAAAGTGttttttt
ttttCCATTATCGGTGGCCGGACAGCAGGTTCAATTGGTCGGGTGCACCATGGCGtatttcttatttctt
atttcGAGCTCGACAGCAGCTTTGCCACACAAACTGGCACACACATCATTGTTCTTGATCAGCATCAGCA
GCTCACTTTGTCAATAAAAGCCGGCGGATAAAAATCGAGAATTTGTGACACGATGACATTCGGAAAAGTT
GTGCAACTGCCGCAGATTGAAAATTGGTAAACACCAAAGGCAAGGCCCTTGAATTTTTTAATATTCCTCG
AAGTATCAAATGTCAGGGGAGCACTGGTTCGATTTCGGTATGTTTTCCGTTTTAAAAGTCACAAAAATGG
AAGTTTGGTAGGAAAAACAATGGCGGGAACACCTTTATAAAACTTTTACATAACTTTAGTTTACGCTGTT
CCTTAACTTATGACCCCGATATACACAGATAACAACCTAACTATCTTTCTGTTGAACTCTTTTCTCTCTC
TCTACCTTTCGTTTGATCAATAAAACTCTGTGCTCTCCCCCTCTACTAACTCCCCAATTATAACAACAAC
CACACACAATCCCACACCACAAATTAGCTCGATAATTGCAAGGTAGGTTGTAATCGAATATCAAAATCGA
TATGATAATGTTCAACAACTGTTGCGGCCAGCGACCTAATGCCATTACACCTGTTAATCCGCACAGGGTG
TCCCTGGAGGCCGATCGTCCCATTGTCAGTGCCTGCCGCAAGACGAGCTTCTACCACCACGGCCACGCCC
ATCACCAGCGGGCGGGCAACGCCTCAGTGGGCGGTGGCAGCGGCGGAGCCGGGGCCGGAGCCACCCACAT
GTCCCACTCGTCCAGCAACGTCCTACGCGCCCGGCGCGGCGTTGTCCGCATGCTGATAATATTCGTGCTG
ACATTCGCCCTCTGCAATCTGCCGTATCATGCCCGCAAAATGTGGCAGTACTG
>dme_circ_0001577_junction_seq
ATCATGCCCGCAAAATGTGGCAGTACTGGATGGTCATTGTCACAGTTTGGATTACA
>dme_circ_0001578
CTGCTTTGGAATCTGACCAAAATTGTTAATGAACCCAATTGTCGCATTCTTTTTGAGCGGATCATCAATG
CTGTCAAATAACAAGGGAAATATAGTTAGCTAATATGCAAGCAGAGAATAATGAATGTGCTTATTACTTG
TAGATATCCACGTTTCCCTCGTAGAAAAGATGATGAAACACATTAACTGCGTCAACGGCGGCTGGTCCCT
GTTGCTTACAGCCAAAGATTAAG
>dme_circ_0001578_junction_seq
TCCCTGTTGCTTACAGCCAAAGATTAAGCTGCTTTGGAATCTGACCAAAATTGTTA
>dme_circ_0001579
GTACAATTACAATTGATTAATCGCGCCAAATCGCGGAGCCAAGTAGTACTTAGCAAGTAGCAAGAGGAAG
CACATCAGCGAGTCCATCGACAACAATAACAAAATATGGCGGATAAAGTAAATGTGTGCATTGTGGGCTC
CGGCAACTGGGGGTTCGGCCATAGCGAAAATCGTGGGCGCCAATGCCGCCGCTCTGCCGGAGTTCGAGGA
GCGTGTGACGATGTTCGTCTACGAGGAGCTCATCGATGGCAAGAAGCTGACGGAGATTATCAACGAGACG
CACGAGAACGTCAAGTACCTGAAAGGACACAAGCTGCCCCCGAATGTGGTGAGTGTTGTTCGCTGGTTGC
CAATCAATGGGGTGGACTTATGTAACGCCGGAGCGGATTGTTAACAAACAGCCGTGCGCTTACATAAAGG
CTGCATACCTTGATCTTGGCCGTGCGGACCCAAATTATCTAATCGGTCGAATCCGCGTCTATTTTTACAC
CGCGCTCGctctctctctctctctGCGCTGCGCTCTGGCTGCAGTCAGATTGCACTCTTCTTGCACTTTT
CAGGGCCAGCATCGCACTCTCGCTCTCCGCGAAAGAGCGCGTCCGGCCCCAGTGCCTTTGACGTAGGTCA
AGGCCGGTGGGCGTGCAATGGGCCACCCCCTCGCAGGGCCGCCCAGAATGCGTCAACATTCTATTAGCCG
ATAATTGGACAGGCAGGCGATAAGCAGGCGATCCACTTGAAACGGGGTGAGTGTTGTTCGCTGGTTGCCA
ATCAATGGGGTGGACTTATGTAACGCCGGAGCGGATTGTTAACAAACAGCCGTGCGCTTACATAAAGGCT
GCATACCTTGATCTTGGCCGTGCGGACCCAAATTATCTAATCGGTCGAATCCGCGTCTATTTTTACACCG
CGCTCGctctctctctctctctGCGCTGCGCTCTGGCTGCAGTCAGATTGCACTCTTCTTGCACTTTTCA
GGGCCAGCATCGCACTCTCGCTCTCCGCGAAAGAGCGCGTCCGGCCCCAGTGCCTTTGACGTAGGTCAAG
GCCGGTGGGCGTGCAATGGGCCACCCCCTCGCAGGGCCGCCCAGAATGCGTCAACATTCTATTAGCCGAT
AATTGGACAGGCAGGCGATAAGCAGGCGATCCACTTGAAACGGGTGCGTTGCTACACATGTACATGCTCC
ACAGGCACTCGAGCTGTTCTGTTTGCCGATTATTGATAAGTCCAACTGGTCCGGGTCGGTTGATAAAGTC
CATCCATAAAGGAGAGTCAGAAGTTCATCCGCGACAAAGTTTGCCCCATGCCTTGGAAAAAACCTAGCCA

TGCGTTGGCAGCATGGGGATTGATAAACGTGAAAAACAGAAACCAAACAAAATGTGGGCGACACTTTTCT
ATGCCGCACCGTTTGTTTGCCTATATAATTTACACACATATATAAATTTGGCAGACTTTCAGCGACTTTC
GGCTTTTGTGGGAGAGGGGCGAATCAAAACAACAGATGAGCCAAAAGAAAATAGATAAACGAGGCTGAAA
TTCTATAAGCAATGATCGCCACCAATGTCAGATATGCAAAAAGTATGCAAAAGGCAATGAATATGTGTCA
GCAAGCTAATGAGCTTAATGCAACAAAGAACTAATGAAAATATTTGGAAATTCTGTTTATTTGCCCGTAT
GGAATTACTGTTTGTCCATATGACGCAGTGAGGAGCGAAATACGTGTCGAATAGCTTAGAAGTCTGAATT
ACCTGAATTGCTAATTGGCAGCTGGTTTTTCTAGTTAAATTTACTTTTACCGATGTCAAATCATAGATTG
TTACGTTATTGCAAAATATATAACAATTATTTAAATGTCATTGCCCTAGTAAGATCATTAAAATGCAAGT
AGCGCAACATCACACTATACACACTTTATAACTTAACCTTTTAAAATTTCCATTTGCTTGCCATTTTTGT
CTTTTTTGTGAACCCATAATCACTATCGTCGCGCGTAGAAAAAAACAAATACATTAGCAATATGAACTCA
TTTCAAATGCTACAAACAGGTTGGCGAGCCAGCATGGCGATATGGAGAAATAATACTCTGATGTACAAAG
TCACCCATATCAAGCTCAGCCGATCGATCTGACCAGCCAGTCCGCGATTTAAATTGTCTAGACAGCTGAG
GAACGAGCCTCCAAAGGTTGGAGTGGAATACACGTGCTGGGAAAATGATTTCCGTGATCCGTCTGCAGTT
CCCTTCATTTAACCTTCAGCCCGCTTATCGGAGTTATATAttttttttgtttttttCCTTATCACAGTAAT
TAGAAAAGTTTACTTTGTGGCCCCTATTCCACAGGGTTGCCGTGCCCGACCTGGTTGAGGCCGCCAAGAA
CGCTGACATCCTGATCTTCGTGGTGCCCCACCAGTTCATTCCCAACTTCTGCAAACAGCTACTGGGCAAG
ATTAAGCCAAATGCCATCGCTATCTCCCTGATCAAGGGCTTCGACAAGGCCGAGGGCGGCGGCATCGATC
TGATCTCGCATATAATCACGCGTCACCTGAAGGATCCCATGCGCCGTGTTGATGGGCGCAAACCTGGCCA
ACGAGGTGGCTGAGGGCAACTTCTGCGAAACGACAATCGGCTGCACGGACAAGAAGTATGGCAAGGTGCT
GCGCGATCTCTTCCAGGCGAACCACTTCCGCGTGGTGGTCGTGGACGATGCTGATGCCGTGGAGGTTTGC
GGTGCCCTCAAGAACATCGTGGCCTGCGGCGCTGGCTTTGTTGATGGCCTCAAGCTGGGCGACAACACCA
AGGCGGCTGTCATCCGGCTGGGCCTCATGGAGATGATTCGCTTCGTGGACGTCTTCTATCCCGGAAGCAA
GCTGTCCACCTTCTTCGAGAGCTGCGGCGTGGCCGATCTGATCACGACGTGTTACGGGTAAGTGCAAAAG
TTCGCCAGGGCATGTGAGTTTGGAGCTCGGTTAAATTGAGAAAGTTATCGGGAACGGGCAACAACGAGCG
TAATAAGTCAACAAACTGCTAGTCAAATGCTATTATTATTTTAAGGCAAAAACAACTCTGCCAGTGACTC
ACATTCAATAGTAATTGCGAATTGCTTTATTAGCCTTTACTAATAGCTGTGTAACAAATTAGTTCTGTTT
ATGACTGTAAGCTTGTTACTTTAAGTTGTTTGTTTTATGTAAGGTACACTTAGGTAGCAATTACAATTTT
AGAATGTTTGATATAATAACTATGCATCTCTTCCAGGGTGGCCGTAACCGCCGCGTCTCTGAGGCGTTTG
TGACTTCTGGAAAAACCATTGAGGAGCTGGAGAAGGAAATGCTCAACGGCCAGAAGCTGCAGGGCCCACC
CACTGCCGAGGAGGTCAACTACATGCTGAAGAACAAGGGTCTGGAGGACAAGTACGACTGAGCTTATTAT
CCAATCCAATTCAAATAGTTAACTTAACACTGATCTTCTTTGCTTGCAGATTCCCCCTGTTCACGGCTAT
TCACAAAATATGCACAAATCAGCTCAAGCCTAATGATTTAATTGATTGCATACGCAATCACCCTGAGCAT
ATGTAAGTGTTTC
>dme_circ_0001579_junction_seq
AATCACCCTGAGCATATGTAAGTGTTTCGTACAATTACAATTGATTAATCGCGCCA
>dme_circ_0001580
GGTTGCCGTGCCCGACCTGGTTGAGGCCGCCAAGAACGCTGACATCCTGATCTTCGTGGTGCCCCACCAG
TTCATTCCCAACTTCTGCAAACAGCTACTGGGCAAGATTAAGCCAAATGCCATCGCTATCTCCCTGATCA
AGGGCTTCGACAAGGCCGAGGGCGGCGGCATCGATCTGATCTCGCATATAATCACGCGTCACCTGAAGGA
TCCCATGCGCCGTGTTGATGGGCGCAAACCTGGCCAACGAGGTGGCTGAGGGCAACTTCTGCGAAACGAC
AATCGGCTGCACGGACAAGAAGTATGGCAAGGTGCTGCGCGATCTCTTCCAGGCGAACCACTTCCGCGTG
GTGGTCGTGGACGATGCTGATGCCGTGGAGGTTTGCGGTGCCCTCAAGAACATCGTGGCCTGCGGCGCTG
GCTTTGTTGATGGCCTCAAGCTGGGCGACAACACCAAGGCGGCTGTCATCCGGCTGGGCCTCATGGAGAT
GATTCGCTTCGTGGACGTCTTCTATCCCGGAAGCAAGCTGTCCACCTTCTTCGAGAGCTGCGGCGTGGCC

GATCTGATCACGACGTGTTACG
>dme_circ_0001580_junction_seq
GTGGCCGATCTGATCACGACGTGTTACGGGTTGCCGTGCCCGACCTGGTTGAGGCC
>dme_circ_0001581
GGTGGCCGTAACCGCCGCGTCTCTGAGGCGTTTGTGACTTCTGGAAAAACCATTGAGGAGCTGGAGAAGG
AAATGCTCAACGGCCAGAAGCTGCAGGGCCCACCCACTGCCGAGGAGGTCAACTACATGCTGAAGAACAA
GGGTCTGGAGGACAAGTACGACTGAGCTTATTATCCAATCCAATTCAAATAGTTAACTTAACACTGATCT
TCTTTGCTTGCAGATTCCCCCTGTTCACGGCTATTCACAAAATATGCACAAATCAGCTCAAGCCTAATGA
TTTAATTGATTGCATACGCAATCACCCTGAGCATAT
>dme_circ_0001581_junction_seq
ATTGCATACGCAATCACCCTGAGCATATGGTGGCCGTAACCGCCGCGTCTCTGAGG
>dme_circ_0001582
GTCAGAAAAATCGTTCCACGTCAACTGCATTACTTTTTCCTCTAATTCTCGCAAGTGTTTTTCAATGTGT
ACAGTGCTTAAGTGTTAGATAATCGAACCAAAACAAAGAGTGTCATAATTGTTGAACCTAATTGGAATTG
TTAAAATAAATATAAATTAAATACAA
>dme_circ_0001582_junction_seq
TGTTAAAATAAATATAAATTAAATACAAGTCAGAAAAATCGTTCCACGTCAACTGC
>dme_circ_0001583
GGGATGACCGAAATGAGATACCTCAGGATGGCCCCGCCAGCATGGAACCCGAGGGCGTCATCGAGTCCAC
CTGGCACGAGGTGTACGACAACTTCGATGACATGAACTTGCGCGAGGAGTTGCTGCGCGGCATCTACGGT
TATGGTTTCGAGAAGCCGTCGGCCATCCAGCAGCGCGCCATCATTCCCTGTGTGAGGGGTCGCGATGTCA
TTGCCCAGGCCCAGTCGGGGTGAGTAAATGGCTCCGAGTCCCCCATCATCATCTTCACCATCATCCCCCT
CGATCAGTCACAAAGAAAAACTTTCGAGAATGTGCAAAATCGGCGAAAAAACAGCACGATGACTCAGCCG
CTGCGCCTGTATTTCTTTGTTGAAATTCCGCTTTGCAGcactctctttcttctccttatactctctctct
tccgtccgctctctTCGATTGTCGGCGTCGTTGCGCTTGCGCGCCACTTTTCGCTGACCAGCTGCGTTTC
GGTTTCTCGGCCGCCAGGCCAGAAGAAAGTTCCCAGAAAGAAGGTCGCTCTTCGAGCGCTCTTGGCGAAT
TCCAGCTGCTATGCGTTTTTAAGGTCATCTGTTCCAAATGCGATCTACCTACATTGCTCATTCGGCACTA
ATCCATTTTGCAGGCTCTGCAAAAGAGGCTCTATATACATATTAATATAGTTCATTCTCAACTCCTGTTT
TGTGTTCTGGCACTTGCACCTGCCTATCGATGTATAGGCAGGGATTAGTGGCCAGCTCGACCCTGAAATG
GCACATCAGCTACCTGATGAAACGATGGACAACTGTCTTTATTCCATTGATCTTTCCGTCATTCAAGTTT
ACGCCTAACAGAATTGCATAACAAATGGCGTACTGGTTAGTCGAAAATTGGAATTTTGCTGAATTTCCAT
AAGAATATCACATAGTTAGAAAAGTAATTGTGTATAGTCCGTAGCACACACTATTCTGCATACTAACCAT
TTTACCTTTAATCTCCTCCGTAGGGAACTGGCAAGACTGCCACCTTCTCGATTGCTATCCTTCAGCAAAT
CGACACGAGCATTCGCGAGTGCCAGGCGCTGATCCTGGCCCCCACTCGCGAGTTGGCCACGCAGATCCAG
CGCGTGGTGATGGCGCTCGGCGAGTACATGAAGGTGCACTCGCACGCCTGCATTGGCGGCACTAACGTGC
GCGAAGACGCCCGCATCTTGGAATCCGGTTGCCATGTGGTGGTGGGCACTCCTGGCCGCGTCTACGACAT
GATCAACCGCAAGGTGCTGCGCACCCAGTACATCAAGCTGTTCGTGCTGGATGAGGCCGATGAGATGTTG
TCCCGCGGTTTCAAGGATCAGATCCAGGATGTCTTCAAGATGCTGCCCCCAGATGTGCAGGTCATCCTGC
TGTCCGCCACCATGCCGCCGGATGTGCTCGAAGTGAGCCGTTGCTTCATGCGTGATCCCGTCAGCATCCT
GGTTAAGAAGGAGGAACTGACCCTTGAGGGTATCAAGCAGTTTTACGTCAACGTGAAGCAGGAGAACTGG
AAACTGGGCACCCTTTGCGATCTGTACGATACGCTGTCCATCACCCAGTCGGTAATCTTCTGCAACACCC
GTCGCAAGGGTGGACCAACTGACCCAGGAGATGTCTATCCACAACTTCACCGTCTCGGCCATGCACGGCG
ACATGGAGCAGCGTGATCGCGAGGTCATCATGAAACAATTCCGTTCGGGCTCGTCTCGTGTTCTGATTAC
CACTGATTTACTGGCGCGCGGTATTGATGTGCAGCAGGTGTCGTTGGTCATCAACTATGATCTGCCCTCG

AACCGCGAGAACTACATTCATAGGAATTGGTCGCGGTGGTCGTTTCGGTCGCAAGGGTGTTGCGATCAAC
TTTATTACAGATGATGATCGACGAATCCTTAAGGATATTGAACAGTTCTACCACACAacaattgaggaaa
tgcctgctaatattgccgatttgatttaaattttTTCTGCTGAGCCGCAAAACAACTAAGAAAGAATTCT
AAACAAAAA
>dme_circ_0001583_junction_seq
ACAACTAAGAAAGAATTCTAAACAAAAAGGGATGACCGAAATGAGATACCTCAGGA
>dme_circ_0001584
GGGATGACCGAAATGAGATACCTCAGGATGGCCCCGCCAGCATGGAACCCGAGGGCGTCATCGAGTCCAC
CTGGCACGAGGTGTACGACAACTTCGATGACATGAACTTGCGCGAGGAGTTGCTGCGCGGCATCTACGGT
TATGGTTTCGAGAAGCCGTCGGCCATCCAGCAGCGCGCCATCATTCCCTGTGTGAGGGGTCGCGATGTCA
TTGCCCAGGCCCAGTCGGGGTGAGTAAATGGCTCCGAGTCCCCCATCATCATCTTCACCATCATCCCCCT
CGATCAGTCACAAAGAAAAACTTTCGAGAATGTGCAAAATCGGCGAAAAAACAGCACGATGACTCAGCCG
CTGCGCCTGTATTTCTTTGTTGAAATTCCGCTTTGCAGcactctctttcttctccttatactctctctct
tccgtccgctctctTCGATTGTCGGCGTCGTTGCGCTTGCGCGCCACTTTTCGCTGACCAGCTGCGTTTC
GGTTTCTCGGCCGCCAGGCCAGAAGAAAGTTCCCAGAAAGAAGGTCGCTCTTCGAGCGCTCTTGGCGAAT
TCCAGCTGCTATGCGTTTTTAAGGTCATCTGTTCCAAATGCGATCTACCTACATTGCTCATTCGGCACTA
ATCCATTTTGCAGGCTCTGCAAAAGAGGCTCTATATACATATTAATATAGTTCATTCTCAACTCCTGTTT
TGTGTTCTGGCACTTGCACCTGCCTATCGATGTATAGGCAGGGATTAGTGGCCAGCTCGACCCTGAAATG
GCACATCAGCTACCTGATGAAACGATGGACAACTGTCTTTATTCCATTGATCTTTCCGTCATTCAAGTTT
ACGCCTAACAGAATTGCATAACAAATGGCGTACTGGTTAGTCGAAAATTGGAATTTTGCTGAATTTCCAT
AAGAATATCACATAGTTAGAAAAGTAATTGTGTATAGTCCGTAGCACACACTATTCTGCATACTAACCAT
TTTACCTTTAATCTCCTCCGTAGGGAACTGGCAAGACTGCCACCTTCTCGATTGCTATCCTTCAGCAAAT
CGACACGAGCATTCGCGAGTGCCAGGCGCTGATCCTGGCCCCCACTCGCGAGTTGGCCACGCAGATCCAG
CGCGTGGTGATGGCGCTCGGCGAGTACATGAAGGTGCACTCGCACGCCTGCATTGGCGGCACTAACGTGC
GCGAAGACGCCCGCATCTTGGAATCCGGTTGCCATGTGGTGGTGGGCACTCCTGGCCGCGTCTACGACAT
GATCAACCGCAAGGTGCTGCGCACCCAGTACATCAAGCTGTTCGTGCTGGATGAGGCCGATGAGATGTTG
TCCCGCGGTTTCAAGGATCAGATCCAGGATGTCTTCAAGATGCTGCCCCCAGATGTGCAGGTCATCCTGC
TGTCCGCCACCATGCCGCCGGATGTGCTCGAAGTGAGCCGTTGCTTCATGCGTGATCCCGTCAGCATCCT
GGTTAAGAAGGAGGAACTGACCCTTGAGGGTATCAAGCAGTTTTACGTCAACGTGAAGCAGGAGAACTGG
AAACTGGGCACCCTTTGCGATCTGTACGATACGCTGTCCATCACCCAGTCGGTAATCTTCTGCAACACCC
GTCGCAAGGGTGGACCAACTGACCCAGGAGATGTCTATCCACAACTTCACCGTCTCGGCCATGCACGGCG
ACATGGAGCAGCGTGATCGCGAGGTCATCATGAAACAATTCCGTTCGGGCTCGTCTCGTGTTCTGATTAC
CACTGATTTACTGGCGCGCGGTATTGATGTGCAGCAGGTGTCGTTGGTCATCAACTATGATCTGCCCTCG
AACCGCGAGAACTACATTCATAGGAATTGGTCGCGGTGGTCGTTTCGGTCGCAAGGGTGTTGCGATCAAC
TTTATTACAGATGATGATCGACGAATCCTTAAGGATATTGAACAGTTCTACCACACAacaattgaggaaa
tgcctgctaatattgccgatttgatttaaattttTTCTGCTGAGCCGCAAAACAACTAAGAAAGAATTCT
AAACAAAAAGTGAGTGAGCAAAACAGCAAACAAGAACTGCGAAATCGATAACCGAGCGCAACTATAAATT
ATTCAAAGATCAACAAGTCAAGTTAAATATTTATTATTCAAATATTAATTCGAAAAGGCAGCCGACCGAC
CACCCAAAAAAAAGGACTAAACCAGGACAGagcagcaacatcttcagcagcggcgcagcagcagtaacatc
agcagcagcggaagcggcagcaacaccaccaccaATTGCCGATTAGCCGGAACCCCTCTGTATCCATTTA
GTTCTCTGTGTATCATCAATTTTAAAACTGCGTTGTCAACCAGCAACAACAAATATATACAAATATATaa
aaaaaaaaaaaCAAAACTTAAAACAACATTTGCATCTATCTAAAAAAAGTAATATCTGAAACCAGGTACA
AGATACCACTTGCTAAGAAGAGCGAGGAACTAAACGCAAAAGCCACAATATCAGAACACCTTTTTGCCGC
ACCTTTGTTTTAGCTTTTTCTAGAACTATTGATAAAAATATATTAAAACAAAACAAAATACATATGTACA

CATTATGTTTAAATATACCACTGTCAGGTCATATTAATTTTTCAATCCGCCCAGTTGGATGTTTGGACAG
TTTCCTTCCTCGCGCCGGTCGATAATGTTGGCCAACTAACACCGACCTGTAGCACGTCACCCGTAATAAC
CCATAACCGCAACCAGTCCAAGCATCCAAGATGGGACAGAAAGTTTCGCGCACCGACTTCGAGTGGGTGT
ACACGGAGGAGCCGCACGCCTCGCGGCGCAAGATCATACTGGAGAAGTACCCGCAGATCAAGAAGCTCTT
TGGCCACGACCCGAATTTCAAGTGGGTGGCCGGCGCAATGGTGCTCACACAGATCCTGGCGCTGTTCGTC
GTGAAGGACCTGTCGTGGTCGTGGCTCATCGTGGCCGCCTATTGCTTCGGCGGCATCATCAACCACTCGT
TGATGCTGGCCGTTCACGAGATCTCGCACAACCTGGCCTTTGGACACAGCAGACCTATGCACAACCGGAT
CTTGGGCTTCATCTGCAACCTGCCCATCGGGTTGCCCATGAGCATTTCCTTCAAAAAGTACCATCTGGAA
CATCACCG
>dme_circ_0001584_junction_seq
TCAAAAAGTACCATCTGGAACATCACCGGGGATGACCGAAATGAGATACCTCAGGA
>dme_circ_0001585
GAAGCCGTCGGCCATCCAGCAGCGCGCCATCATTCCCTGTGTGAGGGGTCGCGATGTCATTGCCCAGGCC
CAGTCGGGTGAGTAAATGGCTCCGAGTCCCCCATCATCATCTTCACCATCATCCCCCTCGATCAGTCACA
AAGAAAAACTTTCGAGAATGTGCAAAATCGGCGAAAAAACAGCACGATGACTCAGCCGCTGCGCCTGTAT
TTCTTTGTTGAAATTCCGCTTTGCAGcactctctttcttctccttatactctctctcttccgtccgctct
ctTCGATTGTCGGCGTCGTTGCGCTTGCGCGCCACTTTTCGCTGACCAGCTGCGTTTCGGTTTCTCGGCC
GCCAGGCCAGAAGAAAGTTCCCAGAAAGAAGGTCGCTCTTCGAGCGCTCTTGGCGAATTCCAGCTGCTAT
GCGTTTTTAAGGTCATCTGTTCCAAATGCGATCTACCTACATTGCTCATTCGGCACTAATCCATTTTGCA
GGCTCTGCAAAAGAGGCTCTATATACATATTAATATAGTTCATTCTCAACTCCTGTTTTGTGTTCTGGCA
CTTGCACCTGCCTATCGATGTATAGGCAGGGATTAGTGGCCAGCTCGACCCTGAAATGGCACATCAGCTA
CCTGATGAAACGATGGACAACTGTCTTTATTCCATTGATCTTTCCGTCATTCAAGTTTACGCCTAACAGA
ATTGCATAACAAATGGCGTACTGGTTAGTCGAAAATTGGAATTTTGCTGAATTTCCATAAGAATATCACA
TAGTTAGAAAAGTAATTGTGTATAGTCCGTAGCACACACTATTCTGCATACTAACCATTTTACCTTTAAT
CTCCTCCGTAGGGAACTGGCAAGACTGCCACCTTCTCGATTGCTATCCTTCAGCAAATCGACACGAGCAT
TCGCGAGTGCCAGGCGCTGATCCTGGCCCCCACTCGCGAGTTGGCCACGCAGATCCAGCGCGTGGTGATG
GCGCTCGGCGAGTACATGAAGGTGCACTCGCACGCCTGCATTGGCGGCACTAACGTGCGCGAAGACGCCC
GCATCTTGGAATCCGGTTGCCATGTGGTGGTGGGCACTCCTGGCCGCGTCTACGACATGATCAACCGCAA
GGTGCTGCGCACCCAGTACATCAAGCTGTTCGTGCTGGATGAGGCCGATGAGATGTTGTCCCGCG
>dme_circ_0001585_junction_seq
GATGAGGCCGATGAGATGTTGTCCCGCGGAAGCCGTCGGCCATCCAGCAGCGCGCC
>dme_circ_0001586
GACTGCCACCTTCTCGATTGCTATCCTTCAGCAAATCGACACGAGCATTCGCGAGTGCCAGGCGCTGATC
CTGGCCCCCACTCGCGAGTTGGCCACGCAGATCCAGCGCGTGGTGATGGCGCTCGGCGAGTACATGAAGG
TGCACTCGCACGCCTGCATTGGCGGCACTAACGTGCGCGAAGACGCCCGCATCTTGGAATCCG
>dme_circ_0001586_junction_seq
CGCGAAGACGCCCGCATCTTGGAATCCGGACTGCCACCTTCTCGATTGCTATCCTT
>dme_circ_0001587
GCGCGTGGTGATGGCGCTCGGCGAGTACATGAAGGTGCACTCGCACGCCTGCATTGGCGGCACTAACGTG
CGCGAAGACGCCCGCATCTTGGAATCCGGTTGCCATGTGGTGGTGGGCACTCCTGGCCGCGTCTACGACA
TGATCAACCGCAAGGTGCTGCGCACCCA
>dme_circ_0001587_junction_seq
TGATCAACCGCAAGGTGCTGCGCACCCAGCGCGTGGTGATGGCGCTCGGCGAGTAC
>dme_circ_0001588

GCTGTTCGTGCTGGATGAGGCCGATGAGATGTTGTCCCGCGGTTTCAAGGATCAGATCCAGGATGTCTTC
AAGATGCTGCCCCCAGATGTGCAGGTCATCCTGCTGTCCGCCACCATGCCGCCGGATGTGCTCGAAGTGA
GCCGTTGCTTCATGCGTGATCCCGTCAGCATCCTGGTTAAGAAGGAGGAACTGACCCTTGAGGGTATCAA
GCAGTTTTACGTCAAC
>dme_circ_0001588_junction_seq
TGAGGGTATCAAGCAGTTTTACGTCAACGCTGTTCGTGCTGGATGAGGCCGATGAG
>dme_circ_0001589
GAAGGAGGAACTGACCCTTGAGGGTATCAAGCAGTTTTACGTCAACGTGAAGCAGGAGAACTGGAAACTG
GGCACCCTTTGCGATCTGTACGATACGCTGTCCATCACCCAGTCGGTAATCTTCTGCAACACCCGTCGCA
AGGTAAATTCACTCATTCTTATAATTGTGTGAAGCTGGATTAGTTTCAATCGCAAAGCCGATAAGTTGCT
AACAAAAATGTCTACCCATTCTTCAGGTGGACCAACTGACCCAGGAGATGTCTATCCACAACTTCACCGT
CTCGGCCATGCACGGCGACATGGAGCAGCGTGATCGCGAGGTCATCATGAAACAATTCC
>dme_circ_0001589_junction_seq
GATCGCGAGGTCATCATGAAACAATTCCGAAGGAGGAACTGACCCTTGAGGGTATC
>dme_circ_0001590
GGAGGAACTGACCCTTGAGGGTATCAAGCAGTTTTACGTCAACGTGAAGCAGGAGAACTGGAAACTGGGC
ACCCTTTGCGATCTGTACGATACGCTGTCCATCACCCAGTCGGTAATCTTCTGCAACACCCGTCGCAAGG
TAAATTCACTCATTCTTATAATTGTGTGAAGCTGGATTAGTTTCAATCGCAAAGCCGATAAGTTGCTAAC
AAAAATGTCTACCCATTCTTCAGGTGGACCAACTGACCCAGGAGAT
>dme_circ_0001590_junction_seq
TTCAGGTGGACCAACTGACCCAGGAGATGGAGGAACTGACCCTTGAGGGTATCAAG
>dme_circ_0001591
GGAACTGACCCTTGAGGGTATCAAGCAGTTTTACGTCAACGTGAAGCAGGAGAACTGGAAACTGGGCACC
CTTTGCGATCTGTACGATACGCTGTCCATCACCCAGTCGGTAATCTTCTGCAACACCCGTCGCAAGGTAA
ATTCACTCATTCTTATAATTGTGTGAAGCTGGATTAGTTTCAATCGCAAAGCCGATAAGTTGCTAACAAA
AATGTCTACCCATTCTTCAGGTGGACCAACTGACCCAGGAGAT
>dme_circ_0001591_junction_seq
TTCAGGTGGACCAACTGACCCAGGAGATGGAACTGACCCTTGAGGGTATCAAGCAG
>dme_circ_0001592
GGGTATCAAGCAGTTTTACGTCAACGTGAAGCAGGAGAACTGGAAACTGGGCACCCTTTGCGATCTGTAC
GATACGCTGTCCATCACCCAGTCGGTAATCTTCTGCAACACCCGTCGCAAGGTAAATTCACTCATTCTTA
TAATTGTGTGAAGCTGGATTAGTTTCAATCGCAAAGCCGATAAGTTGCTAACAAAAATGTCTACCCATTC
TTCAGGTGGACCAACTGACCCAGGAGAT
>dme_circ_0001592_junction_seq
TTCAGGTGGACCAACTGACCCAGGAGATGGGTATCAAGCAGTTTTACGTCAACGTG
>dme_circ_0001593
ACGGTGAAGTTGTGGATAGACATCTCCTGGGTCAGTTGGTCCACCTGAAGAATGGGTAGACATTTTTGTT
AGCAACTTATCGGCTTTGCGATTGAAACTAATCCAGCTTCACACAATTATAAGAATGAGTGAATTTACCT
TGCGACGGGTGTTGCAGAAGATTACCGACTGGGTGATGGACAGCGTATCGTACAGATCGCAAAGGGTGCC
CAGTTTCCAGTTCTCCTGCTTCACGTTGACG
>dme_circ_0001593_junction_seq
TTTCCAGTTCTCCTGCTTCACGTTGACGACGGTGAAGTTGTGGATAGACATCTCCT
>dme_circ_0001594
GGCAGATCATAGTTGATGACCAACGACACCTGCTGCACATCAATACCGCGCGCCAGTAAATCAGTGGTAA

TCAGAACACGAGACGAGCCCGAACGGAATTGTTTCATGATGACCTCGCGATCACGCTGCTCCATGTCGCC
GTGCATGGCCGAGACGGTGAAGTTGTGGATAGACATCTCCTGGGTCAG
>dme_circ_0001594_junction_seq
AGTTGTGGATAGACATCTCCTGGGTCAGGGCAGATCATAGTTGATGACCAACGACA
>dme_circ_0001595
GGCAGATCATAGTTGATGACCAACGACACCTGCTGCACATCAATACCGCGCGCCAGTAAATCAGTGGTAA
TCAGAACACGAGACGAGCCCGAACGGAATTGTTTCATGATGACCTCGCGATCACGCTGCTCCATGTCGCC
GTGCATGGCCGAGACGGTGAAGTTGTGGATAGACATCTCCTGGG
>dme_circ_0001595_junction_seq
GTGAAGTTGTGGATAGACATCTCCTGGGGGCAGATCATAGTTGATGACCAACGACA
>dme_circ_0001596
GATGTCTATCCACAACTTCACCGTCTCGGCCATGCACGGCGACATGGAGCAGCGTGATCGCGAGGTCATC
ATGAAACAATTCCGTTCGGGCTCGTCTCGTGTTCTGATTACCACTGATTTACTGGCGCGCGGTATTGATG
TGCAGCAG
>dme_circ_0001596_junction_seq
ACTGGCGCGCGGTATTGATGTGCAGCAGGATGTCTATCCACAACTTCACCGTCTCG
>dme_circ_0001597
CGCACCTCAACCACAGCCACcaataataacaacaacaataataataacagcacgaacaatagcaacagca
GTACCAATTTGAACAGCAACACCACCAGCGGCCAGGGAGCAGCTACTGGAGGCAATAATGCGACTGGCGG
TCAGAAGAAGCACGCGCAACGGTCTCAACAGGCGGCGCAGGAGGACGACGAGGAGGAG
>dme_circ_0001597_junction_seq
GGCGGCGCAGGAGGACGACGAGGAGGAGCGCACCTCAACCACAGCCACcaataata
>dme_circ_0001598
GAGCACACGGAGTGGCTGGTCCAGCAGCTCTGACTTGCCGTTCTTGAGTACCACTCTGGAATCCAGTCCG
AATCCCTCGGTCACGCCCAAATAGTAGTTTGGTACTCCTGCCTTTTCGTAGGTAGATCGAACTCGTTCCA
GGTCTGTGTCCAGGACTTCCACCACCCATCCACACTCCTCGGCGAAGAGAACAGCTAGTTCGGGACGATT
AA
>dme_circ_0001598_junction_seq
AAGAGAACAGCTAGTTCGGGACGATTAAGAGCACACGGAGTGGCTGGTCCAGCAGC
>dme_circ_0001599
GAGTTCCAACTCGGCGATCCCACGATCACTGCCCTCGAACTGTGGGGTGCCGAGTACCAGGAAAACAATG
CCATTCTTTGCAACGCTGATCAACGCGAACTGCTGGAAAAGATCTGCCGCCGTGAACGCTGTCCCATCAG
CTTCGTGGGAGTGGTGACCGGAGATGGGCGAG
>dme_circ_0001599_junction_seq
GTGGGAGTGGTGACCGGAGATGGGCGAGGAGTTCCAACTCGGCGATCCCACGATCA
>dme_circ_0001600
GAATGGCATTGTTTTCCTGGTACTCGGCACCCCACAGTTCGAGGGCAGTGATCGTGGGATCGCCGAGTTG
GAACTCCTTGGAGAAGATAACAGCTCCGGCAAAGCCTGGTTCCACTAGCTCCTTGAGGACATTGCCGTTG
CCGCCAGCCCCCTGGTCATGGATGGCTAATATGGGGTTTTGCTCGCCCAGGTCTAGACAGGCTCGCACCA
CACG
>dme_circ_0001600_junction_seq
CCAGGTCTAGACAGGCTCGCACCACACGGAATGGCATTGTTTTCCTGGTACTCGGC
>dme_circ_0001601
ATACAAGGATCCGGAGATGCTGAGCTAGATTTTAATGCCGTACAGCGAGGAGACGCCGAAATGGAGAATA

AATTGAACCGTGTGGTGCGAGCCTGTCTAGACCTGGGCGAGCAAAACCCCATATTAGCCATCCATGACCA
GGGGGCTGGCGGCAACGGCAATGTCCTCAAGGAGCTAGTGGAACCAGGCTTTGCCGGAGCTGTTATCTTC
TCCAAGGAGTTCCAACTCGGCGATCCCACGATCACTGCCCTCGAACTGTGGGGTGCCGAG
>dme_circ_0001601_junction_seq
CACTGCCCTCGAACTGTGGGGTGCCGAGATACAAGGATCCGGAGATGCTGAGCTAG
>dme_circ_0001602
GAGCTGGGCTTAGCCTTCAACGACTACGATTTGGACTACTACCACGACTTGTTTGCCAAGGAGCTGGGCC
GCAATCCCACCACTGTGGAGCTCTTCGATTGCGCCCAGAGCAACAGTGAGCACTCGCGCCACTGGTTTTT
CCGCGGACGTATGGTGATCGACGGCG
>dme_circ_0001602_junction_seq
TTCCGCGGACGTATGGTGATCGACGGCGGAGCTGGGCTTAGCCTTCAACGACTACG
>dme_circ_0001603
GCCACCGTGACCGTCTTTGGTGTGCCGCTGTCCCACTACATGGAGGACCTGCTCACCTCGACCATTAGCA
CAAATGCCGGCAAGGGACGCCAGGGCCTGGAGTGGGTGATCGGGCTGATCAACACGGAGG
>dme_circ_0001603_junction_seq
TGGGTGATCGGGCTGATCAACACGGAGGGCCACCGTGACCGTCTTTGGTGTGCCGC
>dme_circ_0001604
GAGCTTGGTGCCCGAGTTACGGGTGCGACCGAAGCTGGCGAACCATACACCGCCACGACGGCAAATGGTT
GGTGCAACGGCCACGGTCTCCACATTGGTAACTGTGGTTGGGCAACCGAACACACCCACATCAGCTGGGA
ATGGCGGCTTCAAGCGTGGCTTTCCCTGCTTTCCTTCCAGCGATTCGATCAGAGCGGTCTCCTCGCCGCA
AAT
>dme_circ_0001604_junction_seq
CGATCAGAGCGGTCTCCTCGCCGCAAATGAGCTTGGTGCCCGAGTTACGGGTGCGA
>dme_circ_0001605
GCTACTTCATCATGAGCGCACCCACCGACCAACCACCGCGCTCCGAGGGCGCTCAGACCAACAGCTCCGA
GAGATCCTCACAGCAGCAGGAGCAACCGCAGCAATCTCAATCACAGAACGTACCGGCCAAGCTCCTGCAG
CATTTTCAGACAAATCGCATAGATTCCGCGCTATGGGCACTACGTCTGCTGGTCATCTTCTTCACCGTCA
GCTACGTGCTGCCCATCTTTACGCTCACAACAGAGCGCATTCAGCAAGGTTATGCTGGCTAATGCCGCCA
TCTCTGCCTTGCGTTTGCACCAGAGATTGCCCGCGTTCGCGTTCAGTCGGGAGTTCCTTGCTCGACTGTT
TGCCGAGGACTCGTGTCATTACATGAT
>dme_circ_0001605_junction_seq
TTGCCGAGGACTCGTGTCATTACATGATGCTACTTCATCATGAGCGCACCCACCGA
>dme_circ_0001606
GGATGAGATCACCAATGAGTTTATTACTCAGACCAAGGAGCAGCTGGAGTCGAAAATGGAGCTGCATGTG
GAGAAGCGAGAGGCCATCATCAGTGACATGAAGGAGAAGCTAAAGGTAGGCTACCAAACCTATTGCATCC
CATAGTATATTCCCAACTAACCAAGTATTTTCTTCCGTTTAACGAAAGATCCATGCCCAGGATATCGAAA
AGACGCGTGAGACTCTTGAGCagcagaaggccaacgagcagaaggcaatcgagGAAAAGTTGAAGATTGC
TCAGTCTCTTCGCGATGAGAATATCAAGAAAAT
>dme_circ_0001606_junction_seq
CTCTTCGCGATGAGAATATCAAGAAAATGGATGAGATCACCAATGAGTTTATTACT
>dme_circ_0001607
GGCAGCAGACAACAACCCGACACCCAGACACAATGGCAGCCACATTGAAGCCCTACCTGACCGCCGTGCG
ACACTCGCTGACGGCGGCGATGTGCCTGCAGGACTTCCCCTCGCAGGTGGTGGAGCGGCACAACAAGCCG
GAG

>dme_circ_0001607_junction_seq
GGTGGTGGAGCGGCACAACAAGCCGGAGGGCAGCAGACAACAACCCGACACCCAGA
>dme_circ_0001608
GACTGAAAACCGATAATGACGGAGGAGCCTGGTGTCCCAAGCACATGGTGTCGAATGCGTTGAAGGAGTA
CCTGCAGGTAGATCTGCTGCAGACGCACGTCATCACGGCGATCCGCACCCAGGGTCGCTATGGCAAGGGT
CAGGGTCAGGAGTACACCGAGGCCTATGTCCTGGAGTACTGGCGACCGGGCTTCGACAAGTGGCAGCGCT
GGAAGAACATCCAGGGCAAGGAGGATTCTGCCCGGCAATATCAACACATACAGCGAGGTGGAGAATGCCC
TGCAGCCGATCATCTTCGCCTCCAAGATTCGCATATATCCGTATGGCCAGTACGATAGAACCGTCTGTCT
GCGCGCCGAGATCGTCGGTTGTCCATGGGAAG
>dme_circ_0001608_junction_seq
GCCGAGATCGTCGGTTGTCCATGGGAAGGACTGAAAACCGATAATGACGGAGGAGC
>dme_circ_0001609
GGTTATGAATGGATTGGCTGGCGTAACGATACGCCCGGACTGCTGGGAAAACCGGTTGAAATAGTCTTTG
AGTTTGATACGGTTCGCAATTTCAGTGCCATCGTCCTGCACACAAACAATATGTTCACTAAGGATGTTCA
GGGTATTTGTGCATGCCAAGGTGTTCTTCAGCATCGGAGGTCGCTACTTTTCGGGTGAACCTGTGCAATT
TTCCTATATGCCCGATACGATAATGGATCATGCAAGGGATGTTACCATCAAACTGCACCACCGGGTGGGC
AAATATTTGAAAATCAATTTGTACTTTGCCGTCAAATGGATTATGCTCTCCGAGATCAGCTTTATATCCG
>dme_circ_0001609_junction_seq
ATGCTCTCCGAGATCAGCTTTATATCCGGGTTATGAATGGATTGGCTGGCGTAACG
>dme_circ_0001610
GCTAATGAAACATGTTAAGAACACCCTGTAAATATACGAGTTTAGGAATCGATAGTGAAGAATCTGCATC
TGATGGCAAGGAGCTGAGTTGCACGGAGGAATCCAGGACATCATGCaaaagaaaaaaaaaaaataaaaca
aaaaaCTCACGACAAGATTATACACGAATCATGAGAATATTGAGACGAATTTAAGGTCTAGCATTTAGCG
ACACAATTTGTTTATAAAACGAAGCAAGAAATAATATTATATTGAATAACACCTATACGACTAACTAGCT
TATAATAATGTTTAACTGATTTGTAGTACTCTTTCGGGAAGAACACTGCGATCTGCGAACTAACTGTACA
TGCCTGTGAACCTACCCCGACTAAACCtgaactatatgaactatctgaattatatAGTATGAACTATCTG
ATGAACCCTATAAGCGCCTACACTCACACTACTTAAGCCCCACTGTACATTTTCTGTGTATAAACTTATA
GACATAGATTGTTTGCTATAGATGGAAACACGATGACGCGTTGAGACAAAATTACGTAGGAAAATCCAGG
AGATGAAAATCTCGGGAAATGATGATGATGATAAAATTGTAACGGAATTTAACGGACCAAGAGAGCGTGC
AGAGCGACTTCGCCTAAACCAAGAAAAAATGAAGTTTTCATCAATGATTTGTGATTCAATATTCAACTAT
TTATATTTATAGACACGCGGATGCGAGTATTCAGTTCTTAATGCTAACTCTAATTTATCGAAACGTACTA
CCCGCAAACAAATGACTCTGACTATAAAGAAAGCACTATGAATATAAAACAAGAAACAAATCGTATTTAT
AATTAATATTAATGGATAATCATGTATATGTTCCTTTTTAGTTTAATGCGCATTAGTATCACCCGAAACT
TACTAAGAGTTAAACGTAGTAGCTTCGTAGTAGTTTAATAAAGCAGCAGCAAAGATAAAATAATTTTAAA
CAACCGGATTACATAGGCAAACAATAGGCGTGTTGGAAATAGCATAAAGTATAAACGAAACGAGACACTC
GATACTCGACACCCATTAATAACACTTAAAAATTAGTTTAAATAGTAAAACCGAAAGAAAAGAAACCCAA
AACCGGCACACGCTCCACACAAACAACGTTTAAAATGTTTGTGCAACAATTTGCGAAGTTTTTAACAAGA
AAAGATATACAACTTAAAATTAAGCAAGCGGTTACAACAATATTTAAAAGTATTTAATAACATTAGGCTT
CGAGATATGTCCGTAT
>dme_circ_0001610_junction_seq
AACATTAGGCTTCGAGATATGTCCGTATGCTAATGAAACATGTTAAGAACACCCTG
>dme_circ_0001611
GTCATACACGTACCACCATGGCCAGCGAGCAAACGATTGATGGAGCAGCCGCAATTCCCAGCGGCGGCGG
CGACGAACCCTTCCTGGGACTGCTGGACGTGGCCCTATTGGCGGTGCTAATCGGCGGCGCAGCCTTCTAC

TTCCTGCGTAGCCGCAAGAAGGAGGAGGAGCCAACCAGGAGCTATTCCATACAAGTAAGTATAGTTAAAT
TTGAGCAATCTAAAGCAATCTAATCCGAAGTCCAAAACCATGGCAATTCTTGAATTGAATAGTCAGTTTC
TTGATAAACCTTTCAAAAAACATGCATATCACGCGTACAATTCAATATTTAAAACTGCTTCTTGAATGAA
ATGCCCTCTAGATCTATTCGGTTCATTCCGTCACAGATCCATATGTTCCGCCGATTTGTTGGAATCGATC
GGCCATTATTTGACGCTGAATGATTCACACATCGCAAATGAATGTGTGAATACCATTGTGTGATTCATTC
GTTGATCTGAGTGTTTACCTGCGTGTTAAGATAAGATTCAAATTTGGCTGAGATTTTAACGCATATCTTT
ACCTTCTTCAGGACCCACCACAGTGTGCACCACCAGTGCGTCGGACAATTCGTTCATCAAGAAGCTCAAG
GCATCCGGACGCAGTCTGGTCGTCTTCTATGGCTCACAGACCGGAACCGGCGAGGAGTTCGCCGGCCGTC
TGGCCAAGGAGGGCATTCGTTACCGTCTGAAGGGCATGGTTGCCGATCCCGAGGAATGCGACATGGAGGA
GCTGCTGCAGCTGAAGGACATCGACAACTCGCTGGCGGTCTTTTGCCTGGCCACCTACGGCGAGGGAGAT
CCCACTGACAACGCCATGGAGTTCTACGAGTGGATCACCAGCGGCGATGTCGATCTGAGCGGGCTAAATT
ATGCG
>dme_circ_0001611_junction_seq
TGTCGATCTGAGCGGGCTAAATTATGCGGTCATACACGTACCACCATGGCCAGCGA
>dme_circ_0001612
CAAACGGTACTGGCGGATGAGCACTTCCTCGCCGCCGCCCTCGATGCCGAAGTGATCGCACACGGCGGGC
CAGAAGCGATCCTTCCACGTGATGAAGTCATCCTCGATGCTTTTGGGGAAGTATCACTTGAGTATGGTTC
ACATTGTTGGCTACACCTTCATCACTTACTTGGCATCATCGTCGCCCAGTCCCAGTTCAAAGACCCGGTT
GGCGCCCAGCTCCTCCAGTCGCTTG
>dme_circ_0001612_junction_seq
GTTGGCGCCCAGCTCCTCCAGTCGCTTGCAAACGGTACTGGCGGATGAGCACTTCC
>dme_circ_0001613
GTGAAAATTTTGTCCACTAAGGGTAACATGAGCGAGGCTGACGCCGTGCAGTATATCAAGAAGATGGAGG
CTCAGAAGCGCTACTCGGCCGATGTCTGGAGCTAATCTGATCCAAAAGCCAACCGATGATAAGCGTAAAG
TTTTCTAGTTCACTAAGCTATGTAAGCGTAATTTTTG
>dme_circ_0001613_junction_seq
TCACTAAGCTATGTAAGCGTAATTTTTGGTGAAAATTTTGTCCACTAAGGGTAACA
>dme_circ_0001614
GTGAAAATTTTGTCCACTAAGGGTAACATGAGCGAGGCTGACGCCGTGCAGTATATCAAGAAGATGGAGG
CTCAGAAGCGCTACTCGGCCGATGTCTGGAGCTAATCTGATCCAAAAGCCAACCGATGATAAGCGTAAAG
TTTTCTAGTTCACTAAGCTATGTAAGCGTAATTTTTGGTTTGGGAACTTCATGCTGCAGTTCAACAAACG
AATGAGTAACGATTAA
>dme_circ_0001614_junction_seq
GTTCAACAAACGAATGAGTAACGATTAAGTGAAAATTTTGTCCACTAAGGGTAACA
>dme_circ_0001615
ATTCCATCTCAGTTTGTCCCATCAAACAGTCCAGGGTCAAGCTAGAGTCAATTACACTAGACATACACTT
AATCGTTACTCATTCGTTTGTTGAACTGCAGCATGAAGTTCCCAAACCAAAAATTACGCTTACATAGCTT
AGTGAACTAGAAAACTTTACGCTTATCATCGG
>dme_circ_0001615_junction_seq
AACTAGAAAACTTTACGCTTATCATCGGATTCCATCTCAGTTTGTCCCATCAAACA
>dme_circ_0001616
GTTCACTAAGCTATGTAAGCGTAATTTTTGGTTTGGGAACTTCATGCTGCAGTTCAACAAACGAATGAGT
AACGATTAAGTGTATGTCTAGTGTAATTGACTCTAGCTTGACCCTGGACTGTTTGATGGGACAAACTGAG
ATGGAATCTAAGTAATGAAATGAGCGGAGTAACAAGTTAACGATTAAGTAGCTGTGTACGAGTGCAGATT

AGTTAAAGTCCTTTATACGAGCAATTTACTTCGTATTCTCTTG
>dme_circ_0001616_junction_seq
TACGAGCAATTTACTTCGTATTCTCTTGGTTCACTAAGCTATGTAAGCGTAATTTT
>dme_circ_0001617
AGAATACGAAGTAAATTGCTCGTATAAAGGACTTTAACTAATCTGCACTCGTACACAGCTACTTAATCGT
TAACTTGTTACTCCGCTCATTTCATTACTTAGATTCCATCTCAGTTTGTCCCATCAAACAGTCCAGGGTC
AAGCTAGAGTCAATTACACTAGACATACACTTAATCG
>dme_circ_0001617_junction_seq
TCAATTACACTAGACATACACTTAATCGAGAATACGAAGTAAATTGCTCGTATAAA
>dme_circ_0001618
GGATTTGCTCGTAGCTGGGCACCGGGATGCCCATGTCCAGGTCGAGCAGCGTTTTGGCCAGCCGCGCGTG
GTATGTGCGCAGCAGCTGGGTCCGCCGATGACGCAGCACTTCCAATGTCAGGCTAGTGTAGAAGAAATAG
TTGAGGTCGATGCCCGGACTACCCCACACGCTCAACTGGAAGTCGATGAGGATGAGGTCCTGCGGACGCT
GTGCTCCGTC
>dme_circ_0001618_junction_seq
TGAGGTCCTGCGGACGCTGTGCTCCGTCGGATTTGCTCGTAGCTGGGCACCGGGAT
>dme_circ_0001619
ATTTTATTTATTCATCACTGGATCCTGCCATGTCCTGTGGGAGAGATGGAAAGAGAACTATTACCAGATG
CGGCACCGCTGGCTGGCCTGCATCTTGGCCTTCTTATCGCACCCGAACTCCCTAGAGAACTCTTCAAAAT
TGGCCAGCGTCTGCATCACACGCAACTCATCCGCGGCGTGCTTCAAATGAGCCTCCGACTGAGATCGCGG
ACTGAGCACGGGCGTCCGGCCGCAAAAGAACTGGGCGAAGGTGATGAAGAATAGCTGCCGCTGGCTGAAC
TGCGGCAGCAGAGGATCCCTGGGCTCCCGCTTGTCCCGCACCCCGAAAAAGGTCTCATACGCTAGACGCA
GAGCCACAAAGTCGGCCAGCTTTTCGTCGATCCACTTGGAACCGGTGGCCATCTGTTGCTGAAGACACTG
GAGTCCTTGTCGGAACTGACGGCTAGCGCCGATCTCCTCCACGGGCCCCATGATGTTTCCCATGGCGTCG
TAGTCCATTCCCAACATATCGAATCCGTGTGCCAGCTCGTGGGCCAGGACAAATCCCATCACGGCGTGCT
GCTGCAGGGCGCTAATGTTTCGATGCCAGATGGGCAGCTGGAGCAGACCGTGCGGCACCAGGACCAGATT
GCGTGGTCTCACATACAGGGGCGACGAGTTGCTGGCCTCCCAGCTTTCCGACAGGTAACTCAAGTTGCTC
CCCGTGGAATTTGCGAAGTTTGTCACATTTATGTAAAGCAGATCGTGGTTCGCCTCAACGCGTTGTCGCA
GCAAGACCAGCTGGTTGCCATAGAAGGAGTGGCCGCTCAGGTCGATGCGGACGTGGTACTGCTCCACATC
CTCAATTTCCTCCGTCTGAAAGAAGCCCATCTGCAGTCGAACTGCGCGCAGTTTCTGCTGCAGGATGTGC
ACCAGTGGTGGCGATAGTCTCATTCGGTTCTCAGCCAAGATTTGGCCAAATCGCACCTTCAAACGCTGGA
ACAGTGAGCTTACGTAGGATTCGGATTCCGGATCGGAGAACTGCTGACCCACCAGCCAGCTACTGGCCAG
AGGCATGGCTCTTCTGAGGGCGGCCACGCACTCACCTCGTGAGATGTCCGCGGGTCCCTGTTGCTTGAGG
AAGGCCAACCACTTGAGCATGATGTAGCTGCAAACGGTGTCCGGGCTCTCCGATTCCATCAGATCGCTCA
GGGCCCTGAGGTACGGAATATTCTCGATGATTAATGTGGAGCGCAGATCCAGCTCGGAGTCAATGCGTAC
CTGGCGCATGAAGGATAACCATCGTAGCTGTGGCAGGTACGAGTCCAAGTAACCAAGCTGCATTTCTCGT
GGTCCTTCGTCGTCCTCCAGCTCCTGCAGGCGGTGCAATTTTCGTTCGAAGTCGTCCACCTGGCGAGCCA
AGGCATTGGCCGTTCGCTTTGTCTGCCCAATGTCCAGAAGCAGCTCGATCATGGCACCTTCTCCCATGGG
CTGCGTCTCCTGGCGACTCGGCTTGTTCACATAGATACTGTAGTTGCGAGAGTCATCCCATCGCGGCAGT
ACCTCCTCCCGCAGCAGCACCCCGTTCAGTCCGTGCAGACGCAGTCGCCCCAAGGTCGTTATCCAGTCGA
ACCGCTCCGGACGCCAGCCCCGACCCACCGATGGCCACTGCGTGGAGTTGCTGGGGGGCAGGAGCTGCAG
GTACTTCTTGAGGTTGTACGGCTTAAGGCGTTTGCACGAACGGTAGTAGAGTCGCATCTGCTCCAGAATC
GAGGAGTCCGACTCGAAGGAGCTCTCATTCCTTCGCCGCAGCAGCAGCTCCAGCTTCCGATTCACGGAGT
GGTCTATCAGTCCCAGGGTGTCGGTGGGCAGCATCTGACCCTGGTACCGCTGCCGATCCCTCGTCCAATC

TCTGTCGCGCAGGGAGTGCTGCTCCTGCTGCTGGATCTGCCAGTTGCCGCAGGCGTACTGGTAGAAGTTC
TCGCAGGGACGCGCCTTCGTATCCATGTAGCTTTGCAGCTGCTCGGCCAGCATTTCCTGAACGCTCTGGG
GTGCGTTGGTCTGGCGACAGGCGGTGGGCGTGGTGCGCCAAGCCAGCGAAAATATGAGCCAGGCCACTAG
GCTTAGCTTCAGCATGATGGTTTCGACTGTGACAAATTGCAACTGGCCGGACTACGATTTGCCCGGATCG
CGGAGACTGCGGACTAGAGCAGGCCCATCGCCTGTCAGTCATCATCGTGGGAGGAGCAGTGGATCCTAGC
TATATATACACACCCAGTACTTGACCACTTGACCTTTCTATTTTCAGGCAAAGGTGATAATTAACAAAAA
TGCTTTAATAGGTTCAAAGTTCGAAGAACATGATCCTAGAACGTACTTTATACCCCATTTGGTGGCATAT
TCTTCAAAGCATTAAACACTCGTAAGATATTATAATATTTGACTTTAAATTGGATCTTCAGTACCTGGAG
CCACTGTACTTTGTAGGCTAGGTCGCATTTTGATTAGACATAAGTGCGCGCTTATCAGTTTCACCTTGGC
CTTGATACCCGATTGTGGGCTGTTGTGGAACTGACCCACAATGAGCACAATGGCTGTCATTGAACTTGCC
TCACGCTGATGCCTCCGTCTCTCCACTTTCAGAAACCGAAACCATTCCCGGCCATCCGTCGTACAAGGCG
ACCATGCGGCTCACGATTACCAACGTTCAGAGCAGCGATTATGGCAACTACAAGTGCGTCGCCAAAAATC
CTCGGGGCGACATGGACGGCAACATAAAGCTGTACAGGTAAGTGACTAGGCTATATGTGTTATTTATACC
CTTTGATATTGCGCCACTGTCATAGGCAAATGTATATGTACACCCCTTTTTAAATGGGCGCCAGTATTTT
GGGGGGTCTTTAAACTTGAAACTCCGACAGTTCCTTGATTCCAAAGGAAGCAGATTGTACTGTTCATTTT
ATCAGGATTTTTGGTGCCCAAATACTTTTTAATATACAGCAAGATTAGATTTTGATATTTAATAAGGAAA
TGACGCGTTTAATTTGTCTCACCGACGTTTTTCTTCATATCCTTGCCGCCGTCAACGGGGCGTATGCGCA
ATCCTCACAACCGGCAATTAGAGCCACTGtagtgtcaataaagtgtcaatattgtgtcaatTTGCTAAAT
TACTCCCCAGTCACTGTTTGGGCCATTAAAGCTCAGCTCGGCAGCAGCGATTCCGATTACAAAAGTTCAT
AAAACAAAATAAAATGCCCGCTTGTTggtggtgaggttgtgaggtggtgaCAAGAGCCCAACGCCACCAA
GAATGGCGAATGTGAAAAGACAACTTAATTTATATATACACGGTTTTCATTCTGCTGCCACAACAATGGC
CTGTGTAGCCCATCGAAAAGTGGGCGTCATTCTACCAATTAAAGGTAACAATGGCCGCATCGATAATAGA
AGGGGCAGCTCCGACAATGAGAACAATGAACCTGTGACAATTTTATTGAGTGGTCAGGAGGCGATACAGT
CGCAGAAAGGCGACATCAACCAGGCATTGGAGTGGGCTATAGTTTCGTGATTCCCAGCCGAGGCAAGGCC
AAGTGTCAAGCCCAATAAATTTCCTCTACAAATTGTCTTCAATAATGCCAACGGAGGGCAGCCAGCCGAG
AAAAGGAACCGAGAGTTGTTAATCCTCCCCGGGGAGATGGCCAAGAAATTGAAAAGAATCGGGGGGGAAG
GGCGGGCAAAAGGGATTGCACTGAATTTACGATGGCTGATAAGTGAAGACAATGCGGCTGGGTAACCGAA
AATATCTGTTTAGAAATCGAGCAAAGGAGTTTCGGCACTGATGGGGCGCACATTTTTGGGGCAGGACTGG
CTTATAACTCACAGTTAATTTAAGTTGTTTATGGATTGTTTCCGATTGAGCAATCCACTTTTGTTCGGTA
CCGTTTTTTGGCGCAGACATCACGCTCTTACCCCTTATTTTAATTACTACACTGACAGAAAGTAGATAAA
ATAATGCaaaaaaaatgtataaaagaaaaaGGATTCATTTCTGACATGTAGCGTTACACTTCCTTTAAAG
TTCCACTTTGCGATAGCTTCTATTTATTGAATTATTTCTACCACTCATGTACGCCAATATTTCTCTACGT
GTAGTTGTCGAGTCCGCCGACAAcacaaccgccgcccacaaccacaacgctgcggcggacgacaacaacc
gcggcggaAATCGCACTGGACGGCTACATCAACACGCCCCTGAATGGCAATGGCATTGGCATTGTGGGCG
AAGGACCCACAAATTCGGTGATTGCGTCCGGCAAATGCGAGCATTAAATACCTATCGAATCTGAACGAGA
TCGACAAGTCGAAGCAGAAGCTGACGGGCAGCAGTCCCAAGGGATTCGATTGGTCCAAGGGCAAGAGCAG
CGGGAGTCATGGCAACCTAATGGCGTCCAGTTGGCCCCTCATCTGCTGCATCCTCGCACTGCTGACGACA
CACTCGTGTGGTTGAGAACTATGTGCGATTCCATTTTAATAAAGTGCCATTACAAACAGTCGAAATGAAA
GGTGTTCGAAGCCGATGCATACGATTAGCCATAACAACTGTGTAATTAATTAAGCGAATTGCAATTTGCA
AACGTTAACGAAGTCAAGCTTTGGTTTTCCCCGAAACTCCCTATGCAGCCCATTAATATTTGATTACGTT
TGGTGGCGAAAACCACAGCGTGTTTTTAGGCAAACAAAGTTACCAAATTAATCAACCTCGCATATTTGTT
TACCATTCGTTCGGAAGCACTCTAAAATATTTTCTAAAAATTCGTTTGGTTTTAAAACCACTATACCATC
GGTGAGGGTATGCGAAGAATGTTGTAAAAAGCATTTCGGCAGGGCCAAAGCAAACATGCGATGCAAACCA
AAGCACATAAATCCTGGTTAG

>dme_circ_0001619_junction_seq

CAAACCAAAGCACATAAATCCTGGTTAGATTTTATTTATTCATCACTGGATCCTGC

>dme_circ_0001620

TCATCAGCGAAGATCCCGAGTTCACGGATGTGATCGAAAATATTACAGTGCCTGCGGGGCGTAATGTAAA
ACTGGCTTGTTCAGTCAAGAACCTGGGATCCTATAAGGTAAGACTAAACAAAGAATTTTCGAGATTTATA
ATTTATATAAACTTAAGTAAAAAACGTAGTATTTGGCTTTGCAAACCAAAATCCAAAAGCTGAAGTTTCT
GGCCGAGCAGGAATGAAGTCAACTTTCGGAACGACTTTTGGTTTGCGCTTAAATAAATGCTCCACCGTCA
ATCGATTGAAGTGGTTGATGCGGGTGTGGGGTTAATGCCAACTGGACACTACTCCAACCAACTGATCAAT
ATTCATTGTGCGGTGCACACTTTTGGGCGGCGCTCGGCGTGCCTAGAAGAATCcatcgcatcgcatcgca
tcgAAACGCTCTGTCTTGTGGCTCAGGCGACAATTTAAATTTGCATCCAATTGAATTTCCATAATGTGCG
TGCGACCAAAACGTGCATAAATTTCATTCTCGACTTTCGCCTCGACACTCGGCTTGTAATAATTTTTATG
TCTCTTAAGTTCGGCATGTGCGACCCGCGACCATCATCATccatagccacccttagccacccatagccac
ctataaccacccataaccaaccatTACCGCCCAAACTACGCCCGCCTCCGCCCAGCCATTCATCTCCAGG
TGCTATCCTCATTGGCCTCAGGTAAGCTCAAAAGTTAACCAGCGAGGACCGCTTGTAGCTGCAATAAAAC
GTGACTTTGCATGCAAAATAGTTGCCTAGCGAGTGGGTGGCTGGGCCACCCATTTCGCCCATCGCAGCCG
CACATTGGCTTTAGCACAAACAGACAGGTTGCCGGGAATTGATTTTAAAGCCCTCGGCGGGTATAACCAC
TTTTCCAACAAACTGGCCACTCGACAAAAGAACATGTATTAAAAGCAAACAAGCAGTTTAATGTTCCAAT
GAAAGTTGTAAATCGATGTACTGAAAAAATAGCTGGGGTACAAGTGGTTTTGGAAATAATTCAGATTTAC
AGCAAAGGAAATACTTATATCAAGTTATAGTTATAAATATTTATAATTTTTTTGGGCTACTAACTGTGCA
CTATGAAAATATGCTGTCTTGTAATTAAACTTTATATAAATACACTGAAATATTTCATATATTTTAATTA
ATATTTGGCAAGCATATCACATAGGGCATACGCAATTCGTTATGCTTTGTGTTTCAAAACTTTCAGTACT
TCTTGTGCTTCCAAATATTTGAGCTGCGTGGAAATTAACTCGCGTAGTTCATAAGCGCTTTTTTCACACG
TTTACTCGCAAATTGTTAATGTCGAGCGAGTAGGTTTTTATTAGCTGAAGCCCTTTAGCTACCCGTTTTG
CCCATTTTCCACATTTTCCCGCAGCACTCCGTATTGTCCTTGTTTGCATTTCAATTTTAAACGCCTACAC
AAACAGTCGAAAAACTCGCACACATAGCCGAACGAACTAACAACAATGCCAGTAACATGAGCATAAGTTT
ACTTTGTTTATGTTGTTGCTTGGCCCCACTTCCAACCCGATACGCCCACTCCGCCCCCACAGAACATAAA
CATTTTCAGGCATAATAAAGtttgtttatttttgcttcggctacaagtttttcaacttgttttctttttt
agtttttacttaaaattttatgacttcatttagtatttTCTATGGCAGGCGTCGTCGGCTAGCGGCGAGG
AATTTTGTGCGGGGGATTGCGTTGGTTCTGGCAAGAGTTGTGCCATTACGCAAATTGCAAATTGGTCATT
TTGTTTGTCGGCTTCTATCGAATCGAGCCATCAGCATGTGTCTGAGACAAGTGAAAGCTTCTGCTCCATG
AAAATGAAAATGAAGATAAACACAAAAGAGCTTGCAGCCTTTTCCAAATAAAAAAACTGCTTTAATGGAG
CTCGTGGACATTCGGCGGCTTTTCTTTAAGTGAAATATGTGGTGGCACAGTGGCAGGGAAGATTCACTTA
AGATGGGATTGTTTAGTAAACCGAATATGCCACTACCAAACTATTACCTTTGCAATTACCAtgtccgttt
gtcagttcgtttgtctgtccgtaagtctgtccgtaagtctgttagtatgtttgtccgttGTATATTCAAG
TGATGCAAACACACCCAAGTTCCTTTAATCTTTTACCTCTAATCTTTAATCTTACTTATACCATTTTGCA
TACATATGGCAATGACCGTTTAACTCAAATTAGCAAAACTTTCAAATTGGGACCAGCATAAGTGCTGGTG
CCACTAATAGTAATCTTCTGGGCACGGCTCGTGCCATTTGGTGGTAGCCATAAATGGACTAAAATATGCG
TGAAATTTTAATGGCAGCTGGTGCTTGGCCTGTGGAGAAGACTTTTAAAAGTCGCACTCTTACATAACCG
CCCAAAAGTCTACATTGCCTTGACTTTTACTCGAAAATCATAAAATAATTTGGTCAAGAGTCGCACTGTG
GCAGCGCCTTCCTTTAAGAGGATGGAAGTCAACTTTTTTTGATGGCCTAACATGGCGTCTAATTAAATTT
GTTGAAATGGCCTTTAAGCATAAGCCAAATTTGAAATGAACTTGCAAACTCAAGCAGATTTAGCTGGCTA
CAAGTTTGAGCAAACTTTCTAATAACTTAAATTCGCTCAGCAATTCATTTCTATTGATTTTAAGTGCACT
ATCTTACCGCCTGCCTGATAAACGTATCTACATTAATTCTTCCATTCGATTCAACTTTGAGCAACAGCAA
CGCAAAACTAGCATTTGGCTGACTTCTCCTGACTTTTAATATGCCAAAGTCAACGGCGCCGAAATGTAAA

ATAAGCAAATTTGATGGCGACTTCCTCGTCGCCGTGTCTCCGATTCCCGCTCATCGTCTATCGGGCTGCA
CGTTTCCCGAGCAGACGTTGAAAAATTAACCGATATCAGTCCACGTTTGCCAGTCACAATCCTTCTTGGC
TCCTCCTGCCTGCGGCGTCCTTCGGGGGATGTTGATAGTGCAGCGTTTCGGGATATTTCATTCAATCCCC
GTTTCGCCGACGCCACTGGGCGAGCAGCGCCCACCATTACCATGGATTCCCTGACACTTCTACGGAATTT
ATCATTGAAATGGCATCGACCTTCTTTGTTGCCCGCGCTCGCCATGTCTCCTCAGCCAATGCGTTGGGTG
CTTATATTTGCATAAATTGCTTGTTGTTCTCGACTCCTGCCCCGCCATCTGGTGGGCGGAAAAAGAAAAC
TATTGGTGGTGTTGCATAAGCGTGTGGATGAATGGGGGAGTATGATGCTTAGCCTATAGCGCTGGAGGAG
GAGAAGGTTCACGATGGTCTGCGCATCGGTTGTGACATGTGGAAAAAGTTTAGTACCAAATGCGGTCGTT
AATCCTTCATTGAAATTGATGATGATGGCCCCAAGTTTGGCCAATAAAGGCGTTGGCAGCTTGTCGTCAA
ATGACCCACACATATGTACATGTTGCCGCATTTGGTTGACATCCATCATAAGTTGGTCAATAGCAACGCC
TTGAATAATTAACGTATTCTGTCTTTCAGGGTCGCGTGGATGCACTTCGAGCAGTCGGCCATTCTCACAG
TTCACAACCACGTGATAACGCGTAATCCAAGGATTAGTGTCACCCACGATAAGCATGACAAGCACCGCAC
CTGGTTCCTGCATATCAACAATGTCCAGGAGGAGGACAGGGGCCGATACATGTGCCAAATCAATACGGTT
ACGGCCAAGACCCAGTATGGATTTGTCAAAGTAGTGGGGTAAGTCTTCGCTATGGTCCTACCATGTCCTT
TGCATTTTTCATTTCATTTGCCAAATCTGTCGGCGGATATTAGtttttttttAGTCCCTTTTCGACCCTA
CTCTACTTTTGAGAACTACATGGATATGCGTGATCCATCATGTTCGAATCCAAAACTCTGGAAATAACTT
AACAAACAATATTAAAAACCAATATAATTATAAAGATCCTTAAAAAGAATGGACAAACTACAAGGAAGTG
CTGGAATTTGGGAACAAGTTCCGCCTTTCAAGTTGACAAATCAACATCATCCGGCTACCGAACGGCACTG
GCACACATCCACATCCACATCCTATTCCACAGAAGCCTTCATTGCCTCCTGCAGGGTCGaatgggcatgg
gcaatgggcaatgggaaatgggaaatgggcGTGCCTCGGTCCATTCGCATAAATCAGTTTTTGTGGCAAA
ACTTGAAAAATATGTGCGCAAACAAATGGCAACGGACGATGAAGTGAAATGGACGGGGAGAATCAGCAGG
CAGTCCGTGCAACAGTTTGGGCCCAAAGTACCACAGTGGCAacacacacacatacagacacaagggacac
atgcagacacagaaacatacacaggcacagacaTCGCATGGAAATGCAAGGACACATATGCTGCACATAG
TAGGATTTGGGCTTAGGTTATTTTTCGAAGGAGAAGACTCGGCAACGCCGTCTGTCGTGACTGGTAACAC
CCATTACTGAAACTTAATGAATTCTTCTGATTCCTCTTTGTATTATTCCCTTAACGTAAAGTAAGGTAAC
TTATTTTTTAATCATATGAGTCAAGCTGTTCCTTACTTCAAGTTTTTAAGGCTAAGTGTAAGGACTCTCT
TTTAAATTTGTTAAATATTCACAAATATACTATACTATAATCTTATCACTCATGACACAAAATACACTTC
TATAATGGGTTTCTTTAAGAACTATAAAAAATTGTATCAAAGTTTCGTGACTACGCAGAGCAATTTTTCA
AAAATCACAAAAGACAGTAATCCCCATTTAAATTCGATTAAATCTCGTTGACAAATTAAAAGTGCAGCAT
CGAGTGTAAGCCAGATTCCGGTTGATCACACTTTTTGCCTGGGCCAAGTAATGAAACTTTGTTAAGCCTA
ATTTCCCAGGCCAATTTAAATCGCATATTGGGGTTCATGCGGCGTACGCCCTTAAAACTTTTGCAGGTAT
GAAAATGGCTGCGGCATATTTTAACCATTTTTTGTTGCCTGCCTTACACATTTGTGGCTCGTTTTGTTGC
CTCTGTCCAGAGCGACCAGAGCTGTCATAGTTGCAttttttgtgtttttcttttttttttttGGTTCTGGT
AAATTTGGCTTGCATTTTTGCCGTCCCAGAACTTTTGCCACATCAGCGTGTGGCAAGGATACATACATAT
GTGTGTGTGTATCTGTGTGGATGATAACGTGCGTGAGTATGAGTGCCGCAAAAAACACACAAAAAATGGC
GACAACGACAGCCAGAGTTGGCCAGAAAGGGTTAACCGGAAAAAAATTGTTAACAGTTACAGTCGCTCTGG
TCACGCCGCATCAAATGGCCAAGACGCGTGCTGCGTGTTATATGAGCATTTTACAGCACCGACGCCTGCT
TAACGACTTTTTAAAAGGCATAAGGCATAAAACCGCAGGAATAGCAGAAACAATTGTCTCTTCGAAGCTT
TTCAGGCCGGTTTAATGGACAGATTTTGGGCCTTTTGCCTTGTAAGTCGTACTTCCTAAATCCGGCTTAG
TTGGGATCAAAATTGGGCCGTCAATTGCCTGAAAACGGATCCATAAAAGATCGCATATTGCATCATTCAA
ATAAAATCAAACAATTTTAATTTACATAACAAATCGAATTAATTGCTATAAAACTAAATGGTGCGCACTG
GTCTAAATAAAAATCGGAAACGCCAAACTGGCGCACCTCGATTTAACAAAACTAAACAAGAGGAACGATT
TAGTTAGATCGCGTCGAATAACAGATACCTGCTACTCTGTAAACTGAATGCGTGTGAATTTCAAGCAAAT
TTCTGTATTTTTCAGTGATTTTGGACACAAACTTTATTGCATTGGTTTTGGAAAGCTTAATCTATCTATT

TATGTATGTATAGTTATTTTCAAAATAGCCAACATACTCCAACTAGTAACAGGTATGCTGTATGCGAAAG
AAAAACGCAATAAAAACATAACATACTCTTAATTACATTTAGCGACAAACATAAAACGAAATTAAAATGA
TGCATTTGTCGTGCGTATATTGAGGGGGGGAAAACAAACGTGAAAACGTTGTCGGGCAAATGAGTAAATA
AATAAATTTATTTTTGGTAGTGCAACATTTGTGAATTTAATTTGCTGACTCGAATGCGATGCGCGGAATA
AAATCCACAGACATCACGGAGGCTACGCAGAAATTTGTGGCTTGAGCATGCAAAAACAAACATTAAATGC
GCCCGAATTTATGGCCCATGCACACAGAATGCATTTAAATGCGCCAGCGATGGCGATGGCGTTGGTGATG
CCGATGGCAGCCTGCTGTTGTCCATCCTCTGATGCTGTGCCGCCTGATGCAAACTGGGCACggagcatca
ggagcagcaggagcagcagCAGGACCCCCATAGTTGCTCCCCTTTTTTCCCGCCAAATTACCGCACATTG
CGAAAGTAGCAGCAAATGCAGCTTGTAACCGAAGCAAAATAACAAACACCAACAATTACGAGCTGGACGG
GAATGGGCACGAGGATGTGGATGTGGATACGGGGTGTTCTTTGCGCGTCTGCCGCATACATATATTCTCA
TTTATGTTCACATAATGAGGCCATTTATTAAATTCAATTATTTTGTTGACATCGGTGCGGTACGAAAGCA
ATATTACAATGTGCTAAGGTAACAAATAACAGAAATAGCCGCTAAACTGGGAGCCACAAAATAGGCAACA
GTATCCAATGGACATTACCTTAACCTGGTCATAAGCTACTGTATAGAACTGTCaaaatttatattaaatt
taaactgaaaagtactgaaaataaaacaagatactaaatattaaaaaACGGCAAATTAAAATGAATTATT
CTTGTCTAATGCCATTATCGTTTTAAGCTAGCAAAAAAAGATAAATAACTAAAAACTTTCCAAACACATC
AATTAAAAAGTTTATAAGGATTTTGCAGCGCATGATGGTCTTGCTAAGCGAAGTGCTAAGCCAAAAACAC
CAATTGACGCATTATGTAATAAGCGATAGAAATTCGTGTCCAGCACTTGCACCACTAATTAAATTTCACA
TGCACATATGTATGTCGAGAAAGAAAGTTTCTTGGTTTAGGGTCCAAAGTTTGCGCAATGTCGGCAGCTG
CTAAAAATATCAATAAGAAACTAATATGTCAAAAGCAAAGGGAACAACATCTGCTGGGTCACCAAGTTGG
GCCACCACttttttttttCATCCTGGTGCTGTCCTGGATCTGTCCTGGTTTGTCCGGCGATTAGCACATC
AAGCGTTTATTAATAGCATGAAATAAAGCTTTGGCAAAAAGTTTTTTGGTCATTATTGGGCATGAGAAAA
GAAAAGGTAAAAGGGCGAAACTTTTGAAAAGTTTTTTCTTTTAATTACGCTTTTTTCCCCCTACGGTTAC
CATAGTGCTCGGATGGGAAAAGTTGAAACAGGGGTTTTTCCTTATGAAAGGCAACTTTCTACGACGACAA
GATAACCTGGCCATTAATGTTAATCCCCACGATGCAATGATACGCGAACAatatatgtatatatatattt
ataGCCCCGTGGCCTCCAGTTCCATCAATTAAAAACGTGTGCCGAAAACCAGACATTTGCTGACCCGACT
GCCATTGATTAGCTGTCCAATTCAGAATGAATGGCGACAGGTCACTGCAAATACGTGACCTGTCATGGAA
GACCTGGCCTTGACCCCCGGAAAATTGCAGGAGCAAATAAAATTTATGATTTTTTGTTGGAATGGAAAAC
TGCATTTCAGGCAACTTTAGAAATTTATaaaaaaaaaCAACTGTCAAAGCTAACAAATGTACACAACTAA
CACAGCTCCAACTACCAACTCCAAGTTCTGGTAAACACTCCATTTAAATAAGATACATTTGAAAAAGAAT
GGTAATTTATCGCAGCTAACTTCGGGTTGGAACCAGTGTGCATTGCCAGATATTAAGCCAGTGCGGTATG
CTGTCCATTCCATGACCTTTTCCATGCTTCCCTGTGATTTGCATAGATTTTTCATTAAAGATTTTCTGGG
ATTTTCGACCAAATACAAAAGAGTTTCCCGGAAGATTGAAAAAATCCCTCAAACAAAGTGAATGGCAAGG
GAAGTGAAACCTGATGACAACTTGATGAAGGTTGCCGCATCGATTTTTGAATTAACAGAATGATTCCTTA
TTTCGCCAGGAGCAACGGATTTCAAACGGACTTCAAATCTGAATCTGGATGCAAAGCTAATTAGGCTTAA
AGTTACGCAGTGACTGTGAGTAGAACAAGAACTCACTGGGGAGTGAGCGCATTGGAGTCAACAATGCGTG
AAATGTTCCttttttttCCACTTCCTAACTGCCTTGGGGAAGTCCAACTTGTCTGGGGGTAGCTTTGATA
AGCCAGTTATTAGCATTACAAGTGCTTCTCTTTGAAAGGGTATTCCCATGTCTCTGTAGTCCTAGGAGTG
TTTTTCACCGGTTTCCGCTTTCTTGGCAACGACATTTACATGAAAGTGGAACTTTTCACTTCTGTCCTTT
GCATTTAAATCGAGCTTTGCACGGTTCCGTGTAGTTTTTGGGTAAAATACTCTCCTAATGTCCGCCTCTC
GCTCTCTCCATCCAATAGTTGCCGCCGAATATAGACGACGCTCTGACCTCCAGTGACATCATCGTGCGCG
AGGGCGATAACGTTACGCTACGCTGCAAGGCGAAAGGTAGTCCGGAGCCGACCATCAAATGGAAACGCGA
TGACGGCAACAAAATCGTTATTAACAAGACGCTCGAGGGGTAAGTTGAAGTCATCGTGCTATTGTCTTTT
CACCTCTCGATTGAGGATGTTTTGGGTTATCTCTTGGCTGGATTATCAGCTGTGGGCACATTATATTTCG
CTGATATGTCGACGTTCCGCGGCCAGTCGCTTCCAGCAATCTGGGATCTCGGCTCAGTTTGCTGTCTGCG

ATGTGGTTGATTGCCTGCCTGTTTATTGTTCCTATTTGCGCTGCTCCAACAACCCAGAATGTCGAGGCGG
ATCCCAATTCACGGCTAATGAACAACATCCTCAGCTACGTGGCGGAGGATTCCAGTGCGTGCGGCACCTA
CTTTCAATATGCCTGCGGCAAGTACGCAGTACGTCACCGCGACGATCCCTTCACGGAGATCATCCAGATG
CTGGACCACAAGGTGAACGGAAACCTAGTCCAACTGATGCACGAGCTGCACCAGCGCTCCCAGTCACCCG
GCTTCAACGAGTCCAGTGTGGAGGCCAAGGTCTTGCACTTCTACCGCACCTGCCGTGAAGCTCCGCCGAG
TACACGCAAGATGGAGCACTACCTTAGACTAGCCCCTCCTGGCCAGGCACTTACATGGCCCCAGTTTATG
CCCCGCGGCGAGCCATGGCCAAAGGCTCAATTCAAGTGGATGAAGTCCTTGGGACACCTATACCGCTATG
GGTTTACCAATGTTTTGGTGAACGTGTTGGTCTTACGGAGCTCTGAGAACAGTAACCGCTTCCGACTGGA
CATCAGTATGCCTAGTCTCGAAGGGGACGCGCAGCACCTGGGCGGTTTTCTAAGTACCCTCGCCACTCTC
CAGATCATTGGTGTGCCGACGAAAAGCATATTACCCCTTGTGCGGAAGATCAGAAGGCTAGAGTCCGCCA
TACGCGTCCTAACCGAAGTGGACGACGATGATGAAAGCGAGACTTGGACTTTGCGCCAGTGGGAGTACAA
AACCGGCTACGCATGGCAGGGTTTCGTCGAGTCAATAGTTGGCCATAGTGTCTCACATCACTACGTGATG
CAGGCGCAGAATGCAAAGTACTTCACGGGCCTTAAGCGGCTGATGGACTCCACGGACGCCGAGGTGGTGG
CCAACTACATCATGACCCGATTCGTGCTCTACCTCATAGACGACTTTATTGACAGCAATGAGCACATTGA
TTGCGTCACGGATATGCGCCGCAACATGAATCTGGCATCGAACCTGCTCTACAAGGAGCGCTTCCTGGAC
CCTGCGACACTACAGCAGTACACCCAGGAAGTTACGAAGGTCTTTGAACAGCTGCGCCGGCAGTTTCTAC
TGCAAATCAATGAGAATCGCCTTGGGCTAACCAGTGAGCAGAACAGTATGGTTGCCACAAAAGCTCAGCA
TGTCGTTCTCAATATTGGGAATTTGCCAAGAGGTCGTGACCATCGTAGCTTTGTCAGCCGGCACTATGAG
GATCTTGTCTTTCCGCTCCCTGATTTCGACTACGCACGTGAACATCTTAACCTGCTGGAGTTCCGCACCA
GAAAGCAGGTTTTACAACTTAACCAAAGTGCTCCGACACCCGATGAGTTCTTCTACTTGTCGGATCCTAA
AACGGCCATGAGCTCCGGACCCTACTACCTAATGCGACTAAACATCATCGTTGTGCCACACGGACTTCTA
CAGGAGCCATTCTTTGCAGCGGATAGCCACGACATATTCAAGTACAGCCTGTTGGGATTCGTACTTGCCC
ATGAGTTGATGCACTCAGTCGACACCGTGGGTCTTTATTATGACAGATATGGGAACATACACGAAATCGG
ACCGCAGATATCCAGTTCGCCACGCTTTGTGGCGGGTTTACAGTGCCTGAACCGGAACAGTACACAGTAT
CTGGACGAGCGTATTGCAGACATTGCTGGACTGGATCTGGCCTATTCCACCTACTTCCAGAACAGCTCGC
AAATGAGTCTAACCGATTTTACGACCATTCCGCCGCAACAAATCTTCTTCCTCAATCTGGCACAGTTCTT
CTGCGGCGATGGTGATCCAATAAACTTCGTTGGTCACGACAACGACGAGACGCGTCTGCAGCAAATGCTC
AATGGGTTCGCGCCGTTTCACGAAGCTTTTGGTTGTCCAGCAAAGAGCAATCAGCCTGAAAAGTGCCAAC
TGTGGTAACTGGATTCAAGTAGTGCTTGAAATCTACTAGTGTATAAAACTATATTAAACAATAAACTAGA
TGGATCTGAAAAGAGTTCACTACCCCATCGAGTTACTCACTCACATTTAAAAAACTCTTCGTTAGGTGAG
ATTTTATTTATTCATCACTGGATCCTGCCATGTCCTGTGGGAGAGATGGAAAGAGAACTATTACCAGATG
CGGCACCGCTGGCTGGCCTGCATCTTGGCCTTCTTATCGCACCCGAACTCCCTAGAGAACTCTTCAAAAT
TGGCCAGCGTCTGCATCACACGCAACTCATCCGCGGCGTGCTTCAAATGAGCCTCCGACTGAGATCGCGG
ACTGAGCACGGGCGTCCGGCCGCAAAAGAACTGGGCGAAGGTGATGAAGAATAGCTGCCGCTGGCTGAAC
TGCGGCAGCAGAGGATCCCTGGGCTCCCGCTTGTCCCGCACCCCGAAAAAGGTCTCATACGCTAGACGCA
GAGCCACAAAGTCGGCCAGCTTTTCGTCGATCCACTTGGAACCGGTGGCCATCTGTTGCTGAAGACACTG
GAGTCCTTGTCGGAACTGACGGCTAGCGCCGATCTCCTCCACGGGCCCCATGATGTTTCCCATGGCGTCG
TAGTCCATTCCCAACATATCGAATCCGTGTGCCAGCTCGTGGGCCAGGACAAATCCCATCACGGCGTGCT
GCTGCAGGGCGCTAATGTTTCGATGCCAGATGGGCAGCTGGAGCAGACCGTGCGGCACCAGGACCAGATT
GCGTGGTCTCACATACAGGGGCGACGAGTTGCTGGCCTCCCAGCTTTCCGACAGGTAACTCAAGTTGCTC
CCCGTGGAATTTGCGAAGTTTGTCACATTTATGTAAAGCAGATCGTGGTTCGCCTCAACGCGTTGTCGCA
GCAAGACCAGCTGGTTGCCATAGAAGGAGTGGCCGCTCAGGTCGATGCGGACGTGGTACTGCTCCACATC
CTCAATTTCCTCCGTCTGAAAGAAGCCCATCTGCAGTCGAACTGCGCGCAGTTTCTGCTGCAGGATGTGC
ACCAGTGGTGGCGATAGTCTCATTCGGTTCTCAGCCAAGATTTGGCCAAATCGCACCTTCAAACGCTGGA

ACAGTGAGCTTACGTAGGATTCGGATTCCGGATCGGAGAACTGCTGACCCACCAGCCAGCTACTGGCCAG
AGGCATGGCTCTTCTGAGGGCGGCCACGCACTCACCTCGTGAGATGTCCGCGGGTCCCTGTTGCTTGAGG
AAGGCCAACCACTTGAGCATGATGTAGCTGCAAACGGTGTCCGGGCTCTCCGATTCCATCAGATCGCTCA
GGGCCCTGAGGTACGGAATATTCTCGATGATTAATGTGGAGCGCAGATCCAGCTCGGAGTCAATGCGTAC
CTGGCGCATGAAGGATAACCATCGTAGCTGTGGCAGGTACGAGTCCAAGTAACCAAGCTGCATTTCTCGT
GGTCCTTCGTCGTCCTCCAGCTCCTGCAGGCGGTGCAATTTTCGTTCGAAGTCGTCCACCTGGCGAGCCA
AGGCATTGGCCGTTCGCTTTGTCTGCCCAATGTCCAGAAGCAGCTCGATCATGGCACCTTCTCCCATGGG
CTGCGTCTCCTGGCGACTCGGCTTGTTCACATAGATACTGTAGTTGCGAGAGTCATCCCATCGCGGCAGT
ACCTCCTCCCGCAGCAGCACCCCGTTCAGTCCGTGCAGACGCAGTCGCCCCAAGGTCGTTATCCAGTCGA
ACCGCTCCGGACGCCAGCCCCGACCCACCGATGGCCACTGCGTGGAGTTGCTGGGGGGCAGGAGCTGCAG
GTACTTCTTGAGGTTGTACGGCTTAAGGCGTTTGCACGAACGGTAGTAGAGTCGCATCTGCTCCAGAATC
GAGGAGTCCGACTCGAAGGAGCTCTCATTCCTTCGCCGCAGCAGCAGCTCCAGCTTCCGATTCACGGAGT
GGTCTATCAGTCCCAGGGTGTCGGTGGGCAGCATCTGACCCTGGTACCGCTGCCGATCCCTCGTCCAATC
TCTGTCGCGCAGGGAGTGCTGCTCCTGCTGCTGGATCTGCCAGTTGCCGCAGGCGTACTGGTAGAAGTTC
TCGCAGGGACGCGCCTTCGTATCCATGTAGCTTTGCAGCTGCTCGGCCAGCATTTCCTGAACGCTCTGGG
GTGCGTTGGTCTGGCGACAGGCGGTGGGCGTGGTGCGCCAAGCCAGCGAAAATATGAGCCAGGCCACTAG
GCTTAGCTTCAGCATGATGGTTTCGACTGTGACAAATTGCAACTGGCCGGACTACGATTTGCCCGGATCG
CGGAGACTGCGGACTAGAGCAGGCCCATCGCCTGTCAGTCATCATCGTGGGAGGAGCAGTGGATCCTAGC
TATATATACACACCCAGTACTTGACCACTTGACCTTTCTATTTTCAGGCAAAGGTGATAATTAACAAAAA
TGCTTTAATAGGTTCAAAGTTCGAAGAACATGATCCTAGAACGTACTTTATACCCCATTTGGTGGCATAT
TCTTCAAAGCATTAAACACTCGTAAGATATTATAATATTTGACTTTAAATTGGATCTTCAGTACCTGGAG
CCACTGTACTTTGTAGGCTAGGTCGCATTTTGATTAGACATAAGTGCGCGCTTATCAGTTTCACCTTGGC
CTTGATACCCGATTGTGGGCTGTTGTGGAACTGACCCACAATGAGCACAATGGCTGTCATTGAACTTGCC
TCACGCTGATGCCTCCGTCTCTCCACTTTCAGAACCGAAACCATTCCCGGCCATCCGTCGTACAAGGCGA
CCATGCGGCTCACGATTACCAACGTTCAGAGCAGCGATTATGGCAACTACAAGTGCGTCGCCAAAAATCC
TCGGGGCGACATGGACGGCAACATAAAGCTGTACAG
>dme_circ_0001620_junction_seq
ACATGGACGGCAACATAAAGCTGTACAGTCATCAGCGAAGATCCCGAGTTCACGGA
>dme_circ_0001621
TCATCAGCGAAGATCCCGAGTTCACGGATGTGATCGAAAATATTACAGTGCCTGCGGGGCGTAATGTAAA
ACTGGCTTGTTCAGTCAAGAACCTGGGATCCTATAAGGTAAGACTAAACAAAGAATTTTCGAGATTTATA
ATTTATATAAACTTAAGTAAAAAACGTAGTATTTGGCTTTGCAAACCAAAATCCAAAAGCTGAAGTTTCT
GGCCGAGCAGGAATGAAGTCAACTTTCGGAACGACTTTTGGTTTGCGCTTAAATAAATGCTCCACCGTCA
ATCGATTGAAGTGGTTGATGCGGGTGTGGGGTTAATGCCAACTGGACACTACTCCAACCAACTGATCAAT
ATTCATTGTGCGGTGCACACTTTTGGGCGGCGCTCGGCGTGCCTAGAAGAATCcatcgcatcgcatcgca
tcgAAACGCTCTGTCTTGTGGCTCAGGCGACAATTTAAATTTGCATCCAATTGAATTTCCATAATGTGCG
TGCGACCAAAACGTGCATAAATTTCATTCTCGACTTTCGCCTCGACACTCGGCTTGTAATAATTTTTATG
TCTCTTAAGTTCGGCATGTGCGACCCGCGACCATCATCATccatagccacccttagccacccatagccac
ctataaccacccataaccaaccatTACCGCCCAAACTACGCCCGCCTCCGCCCAGCCATTCATCTCCAGG
TGCTATCCTCATTGGCCTCAGGTAAGCTCAAAAGTTAACCAGCGAGGACCGCTTGTAGCTGCAATAAAAC
GTGACTTTGCATGCAAAATAGTTGCCTAGCGAGTGGGTGGCTGGGCCACCCATTTCGCCCATCGCAGCCG
CACATTGGCTTTAGCACAAACAGACAGGTTGCCGGGAATTGATTTTAAAGCCCTCGGCGGGTATAACCAC
TTTTCCAACAAACTGGCCACTCGACAAAAGAACATGTATTAAAAGCAAACAAGCAGTTTAATGTTCCAAT
GAAAGTTGTAAATCGATGTACTGAAAAAATAGCTGGGGTACAAGTGGTTTTGGAAATAATTCAGATTTAC

AGCAAAGGAAATACTTATATCAAGTTATAGTTATAAATATTTATAATTTTTTTGGGCTACTAACTGTGCA
CTATGAAAATATGCTGTCTTGTAATTAAACTTTATATAAATACACTGAAATATTTCATATATTTTAATTA
ATATTTGGCAAGCATATCACATAGGGCATACGCAATTCGTTATGCTTTGTGTTTCAAAACTTTCAGTACT
TCTTGTGCTTCCAAATATTTGAGCTGCGTGGAAATTAACTCGCGTAGTTCATAAGCGCTTTTTTCACACG
TTTACTCGCAAATTGTTAATGTCGAGCGAGTAGGTTTTTATTAGCTGAAGCCCTTTAGCTACCCGTTTTG
CCCATTTTCCACATTTTCCCGCAGCACTCCGTATTGTCCTTGTTTGCATTTCAATTTTAAACGCCTACAC
AAACAGTCGAAAAACTCGCACACATAGCCGAACGAACTAACAACAATGCCAGTAACATGAGCATAAGTTT
ACTTTGTTTATGTTGTTGCTTGGCCCCACTTCCAACCCGATACGCCCACTCCGCCCCCACAGAACATAAA
CATTTTCAGGCATAATAAAGtttgtttatttttgcttcggctacaagtttttcaacttgttttctttttt
agtttttacttaaaattttatgacttcatttagtatttTCTATGGCAGGCGTCGTCGGCTAGCGGCGAGG
AATTTTGTGCGGGGGATTGCGTTGGTTCTGGCAAGAGTTGTGCCATTACGCAAATTGCAAATTGGTCATT
TTGTTTGTCGGCTTCTATCGAATCGAGCCATCAGCATGTGTCTGAGACAAGTGAAAGCTTCTGCTCCATG
AAAATGAAAATGAAGATAAACACAAAAGAGCTTGCAGCCTTTTCCAAATAAAAAAACTGCTTTAATGGAG
CTCGTGGACATTCGGCGGCTTTTCTTTAAGTGAAATATGTGGTGGCACAGTGGCAGGGAAGATTCACTTA
AGATGGGATTGTTTAGTAAACCGAATATGCCACTACCAAACTATTACCTTTGCAATTACCAtgtccgttt
gtcagttcgtttgtctgtccgtaagtctgtccgtaagtctgttagtatgtttgtccgttGTATATTCAAG
TGATGCAAACACACCCAAGTTCCTTTAATCTTTTACCTCTAATCTTTAATCTTACTTATACCATTTTGCA
TACATATGGCAATGACCGTTTAACTCAAATTAGCAAAACTTTCAAATTGGGACCAGCATAAGTGCTGGTG
CCACTAATAGTAATCTTCTGGGCACGGCTCGTGCCATTTGGTGGTAGCCATAAATGGACTAAAATATGCG
TGAAATTTTAATGGCAGCTGGTGCTTGGCCTGTGGAGAAGACTTTTAAAAGTCGCACTCTTACATAACCG
CCCAAAAGTCTACATTGCCTTGACTTTTACTCGAAAATCATAAAATAATTTGGTCAAGAGTCGCACTGTG
GCAGCGCCTTCCTTTAAGAGGATGGAAGTCAACTTTTTTTGATGGCCTAACATGGCGTCTAATTAAATTT
GTTGAAATGGCCTTTAAGCATAAGCCAAATTTGAAATGAACTTGCAAACTCAAGCAGATTTAGCTGGCTA
CAAGTTTGAGCAAACTTTCTAATAACTTAAATTCGCTCAGCAATTCATTTCTATTGATTTTAAGTGCACT
ATCTTACCGCCTGCCTGATAAACGTATCTACATTAATTCTTCCATTCGATTCAACTTTGAGCAACAGCAA
CGCAAAACTAGCATTTGGCTGACTTCTCCTGACTTTTAATATGCCAAAGTCAACGGCGCCGAAATGTAAA
ATAAGCAAATTTGATGGCGACTTCCTCGTCGCCGTGTCTCCGATTCCCGCTCATCGTCTATCGGGCTGCA
CGTTTCCCGAGCAGACGTTGAAAAATTAACCGATATCAGTCCACGTTTGCCAGTCACAATCCTTCTTGGC
TCCTCCTGCCTGCGGCGTCCTTCGGGGGATGTTGATAGTGCAGCGTTTCGGGATATTTCATTCAATCCCC
GTTTCGCCGACGCCACTGGGCGAGCAGCGCCCACCATTACCATGGATTCCCTGACACTTCTACGGAATTT
ATCATTGAAATGGCATCGACCTTCTTTGTTGCCCGCGCTCGCCATGTCTCCTCAGCCAATGCGTTGGGTG
CTTATATTTGCATAAATTGCTTGTTGTTCTCGACTCCTGCCCCGCCATCTGGTGGGCGGAAAAAGAAAAC
TATTGGTGGTGTTGCATAAGCGTGTGGATGAATGGGGGAGTATGATGCTTAGCCTATAGCGCTGGAGGAG
GAGAAGGTTCACGATGGTCTGCGCATCGGTTGTGACATGTGGAAAAAGTTTAGTACCAAATGCGGTCGTT
AATCCTTCATTGAAATTGATGATGATGGCCCCAAGTTTGGCCAATAAAGGCGTTGGCAGCTTGTCGTCAA
ATGACCCACACATATGTACATGTTGCCGCATTTGGTTGACATCCATCATAAGTTGGTCAATAGCAACGCC
TTGAATAATTAACGTATTCTGTCTTTCAGGGTCGCGTGGATGCACTTCGAGCAGTCGGCCATTCTCACAG
TTCACAACCACGTGATAACGCGTAATCCAAGGATTAGTGTCACCCACGATAAGCATGACAAGCACCGCAC
CTGGTTCCTGCATATCAACAATGTCCAGGAGGAGGACAGGGGCCGATACATGTGCCAAATCAATACGGTT
ACGGCCAAGACCCAGTATGGATTTGTCAAAGTAGTGGGGTAAGTCTTCGCTATGGTCCTACCATGTCCTT
TGCATTTTTCATTTCATTTGCCAAATCTGTCGGCGGATATTAGtttttttttAGTCCCTTTTCGACCCTA
CTCTACTTTTGAGAACTACATGGATATGCGTGATCCATCATGTTCGAATCCAAAACTCTGGAAATAACTT
AACAAACAATATTAAAAACCAATATAATTATAAAGATCCTTAAAAAGAATGGACAAACTACAAGGAAGTG
CTGGAATTTGGGAACAAGTTCCGCCTTTCAAGTTGACAAATCAACATCATCCGGCTACCGAACGGCACTG

GCACACATCCACATCCACATCCTATTCCACAGAAGCCTTCATTGCCTCCTGCAGGGTCGaatgggcatgg
gcaatgggcaatgggaaatgggaaatgggcGTGCCTCGGTCCATTCGCATAAATCAGTTTTTGTGGCAAA
ACTTGAAAAATATGTGCGCAAACAAATGGCAACGGACGATGAAGTGAAATGGACGGGGAGAATCAGCAGG
CAGTCCGTGCAACAGTTTGGGCCCAAAGTACCACAGTGGCAacacacacacatacagacacaagggacac
atgcagacacagaaacatacacaggcacagacaTCGCATGGAAATGCAAGGACACATATGCTGCACATAG
TAGGATTTGGGCTTAGGTTATTTTTCGAAGGAGAAGACTCGGCAACGCCGTCTGTCGTGACTGGTAACAC
CCATTACTGAAACTTAATGAATTCTTCTGATTCCTCTTTGTATTATTCCCTTAACGTAAAGTAAGGTAAC
TTATTTTTTAATCATATGAGTCAAGCTGTTCCTTACTTCAAGTTTTTAAGGCTAAGTGTAAGGACTCTCT
TTTAAATTTGTTAAATATTCACAAATATACTATACTATAATCTTATCACTCATGACACAAAATACACTTC
TATAATGGGTTTCTTTAAGAACTATAAAAAAATTGTATCAAAGTTTCGTGACTACGCAGAGCAATTTTTCA
AAAATCACAAAAGACAGTAATCCCCATTTAAATTCGATTAAATCTCGTTGACAAATTAAAAGTGCAGCAT
CGAGTGTAAGCCAGATTCCGGTTGATCACACTTTTTGCCTGGGCCAAGTAATGAAACTTTGTTAAGCCTA
ATTTCCCAGGCCAATTTAAATCGCATATTGGGGTTCATGCGGCGTACGCCCTTAAAACTTTTGCAGGTAT
GAAAATGGCTGCGGCATATTTTAACCATTTTTTGTTGCCTGCCTTACACATTTGTGGCTCGTTTTGTTGC
CTCTGTCCAGAGCGACCAGAGCTGTCATAGTTGCAttttttgtgtttttcttttttttttttGGTTCTGGT
AAATTTGGCTTGCATTTTTGCCGTCCCAGAACTTTTGCCACATCAGCGTGTGGCAAGGATACATACATAT
GTGTGTGTGTATCTGTGTGGATGATAACGTGCGTGAGTATGAGTGCCGCAAAAAACACACAAAAAATGGC
GACAACGACAGCCAGAGTTGGCCAGAAAGGGTTAACCGGAAAAAATTGTTAACAGTTACAGTCGCTCTGG
TCACGCCGCATCAAATGGCCAAGACGCGTGCTGCGTGTTATATGAGCATTTTACAGCACCGACGCCTGCT
TAACGACTTTTTAAAAGGCATAAGGCATAAAACCGCAGGAATAGCAGAAACAATTGTCTCTTCGAAGCTT
TTCAGGCCGGTTTAATGGACAGATTTTGGGCCTTTTGCCTTGTAAGTCGTACTTCCTAAATCCGGCTTAG
TTGGGATCAAAATTGGGCCGTCAATTGCCTGAAAACGGATCCATAAAAGATCGCATATTGCATCATTCAA
ATAAAATCAAACAATTTTAATTTACATAACAAATCGAATTAATTGCTATAAAACTAAATGGTGCGCACTG
GTCTAAATAAAAATCGGAAACGCCAAACTGGCGCACCTCGATTTAACAAAACTAAACAAGAGGAACGATT
TAGTTAGATCGCGTCGAATAACAGATACCTGCTACTCTGTAAACTGAATGCGTGTGAATTTCAAGCAAAT
TTCTGTATTTTTCAGTGATTTTGGACACAAACTTTATTGCATTGGTTTTGGAAAGCTTAATCTATCTATT
TATGTATGTATAGTTATTTTCAAAATAGCCAACATACTCCAACTAGTAACAGGTATGCTGTATGCGAAAG
AAAAACGCAATAAAAACATAACATACTCTTAATTACATTTAGCGACAAACATAAAACGAAATTAAAATGA
TGCATTTGTCGTGCGTATATTGAGGGGGGGAAAACAAACGTGAAAACGTTGTCGGGCAAATGAGTAAATA
AATAAATTTATTTTTGGTAGTGCAACATTTGTGAATTTAATTTGCTGACTCGAATGCGATGCGCGGAATA
AAATCCACAGACATCACGGAGGCTACGCAGAAATTTGTGGCTTGAGCATGCAAAAACAAACATTAAATGC
GCCCGAATTTATGGCCCATGCACACAGAATGCATTTAAATGCGCCAGCGATGGCGATGGCGTTGGTGATG
CCGATGGCAGCCTGCTGTTGTCCATCCTCTGATGCTGTGCCGCCTGATGCAAACTGGGCACggagcatca
ggagcagcaggagcagcagCAGGACCCCCATAGTTGCTCCCCTTTTTTCCCGCCAAATTACCGCACATTG
CGAAAGTAGCAGCAAATGCAGCTTGTAACCGAAGCAAAATAACAAACACCAACAATTACGAGCTGGACGG
GAATGGGCACGAGGATGTGGATGTGGATACGGGGTGTTCTTTGCGCGTCTGCCGCATACATATATTCTCA
TTTATGTTCACATAATGAGGCCATTTATTAAATTCAATTATTTTGTTGACATCGGTGCGGTACGAAAGCA
ATATTACAATGTGCTAAGGTAACAAATAACAGAAATAGCCGCTAAACTGGGAGCCACAAAATAGGCAACA
GTATCCAATGGACATTACCTTAACCTGGTCATAAGCTACTGTATAGAACTGTCaaaatttatattaaatt
taaactgaaaagtactgaaaataaaacaagatactaaatattaaaaaACGGCAAATTAAAATGAATTATT
CTTGTCTAATGCCATTATCGTTTTAAGCTAGCAAAAAAAGATAAATAACTAAAAACTTTCCAAACACATC
AATTAAAAAGTTTATAAGGATTTTGCAGCGCATGATGGTCTTGCTAAGCGAAGTGCTAAGCCAAAAACAC
CAATTGACGCATTATGTAATAAGCGATAGAAATTCGTGTCCAGCACTTGCACCACTAATTAAATTTCACA
TGCACATATGTATGTCGAGAAAGAAAGTTTCTTGGTTTAGGGTCCAAAGTTTGCGCAATGTCGGCAGCTG

CTAAAAATATCAATAAGAAACTAATATGTCAAAAGCAAAGGGAACAACATCTGCTGGGTCACCAAGTTGG
GCCACCACttttttttttCATCCTGGTGCTGTCCTGGATCTGTCCTGGTTTGTCCGGCGATTAGCACATC
AAGCGTTTATTAATAGCATGAAATAAAGCTTTGGCAAAAAGTTTTTTGGTCATTATTGGGCATGAGAAAA
GAAAAGGTAAAAGGGCGAAACTTTTGAAAAGTTTTTCTTTTAATTACGCTTTTTTCCCCCTACGGTTAC
CATAGTGCTCGGATGGGAAAAGTTGAAACAGGGGTTTTTCCTTATGAAAGGCAACTTTCTACGACGACAA
GATAACCTGGCCATTAATGTTAATCCCCACGATGCAATGATACGCGAACAatatatgtatatatatattt
ataGCCCCGTGGCCTCCAGTTCCATCAATTAAAAACGTGTGCCGAAAACCAGACATTTGCTGACCCGACT
GCCATTGATTAGCTGTCCAATTCAGAATGAATGGCGACAGGTCACTGCAAATACGTGACCTGTCATGGAA
GACCTGGCCTTGACCCCCGGAAAATTGCAGGAGCAAATAAAATTTATGATTTTTTGTTGGAATGGAAAAC
TGCATTTCAGGCAACTTTAGAAATTTATaaaaaaaaaCAACTGTCAAAGCTAACAAATGTACACAACTAA
CACAGCTCCAACTACCAACTCCAAGTTCTGGTAAACACTCCATTTAAATAAGATACATTTGAAAAAGAAT
GGTAATTTATCGCAGCTAACTTCGGGTTGGAACCAGTGTGCATTGCCAGATATTAAGCCAGTGCGGTATG
CTGTCCATTCCATGACCTTTTCCATGCTTCCCTGTGATTTGCATAGATTTTTCATTAAAGATTTTCTGGG
ATTTTCGACCAAATACAAAAGAGTTTCCCGGAAGATTGAAAAAATCCCTCAAACAAAGTGAATGGCAAGG
GAAGTGAAACCTGATGACAACTTGATGAAGGTTGCCGCATCGATTTTTGAATTAACAGAATGATTCCTTA
TTTCGCCAGGAGCAACGGATTTCAAACGGACTTCAAATCTGAATCTGGATGCAAAGCTAATTAGGCTTAA
AGTTACGCAGTGACTGTGAGTAGAACAAGAACTCACTGGGGAGTGAGCGCATTGGAGTCAACAATGCGTG
AAATGTTCCttttttttCCACTTCCTAACTGCCTTGGGGAAGTCCAACTTGTCTGGGGGTAGCTTTGATA
AGCCAGTTATTAGCATTACAAGTGCTTCTCTTTGAAAGGGTATTCCCATGTCTCTGTAGTCCTAGGAGTG
TTTTTCACCGGTTTCCGCTTTCTTGGCAACGACATTTACATGAAAGTGGAACTTTTCACTTCTGTCCTTT
GCATTTAAATCGAGCTTTGCACGGTTCCGTGTAGTTTTTGGGTAAAATACTCTCCTAATGTCCGCCTCTC
GCTCTCTCCATCCAATAGTTGCCGCCGAATATAGACGACGCTCTGACCTCCAGTGACATCATCGTGCGCG
AGGGCGATAACGTTACGCTACGCTGCAAGGCGAAAGGTAGTCCGGAGCCGACCATCAAATGGAAACGCGA
TGACGGCAACAAAATCGTTATTAACAAGACGCTCGAGGGTTTCGCCCATGGTCTGGATACCGCATCAGCT
GGTGGGTATTCCCATTGGATTCAACATCACCCTGGAATGCTTCATCGAGGCGAATCCGACCTCACTTAAC
TATTGGACGCGCGAAAACGATCAAATGATCACTGAGTCATCAAAGTACAAG
>dme_circ_0001621_junction_seq
AATGATCACTGAGTCATCAAAGTACAAGTCATCAGCGAAGATCCCGAGTTCACGGA
>dme_circ_0001622
GTTTGGTTTTATTATAAATGTCATCTCATCTTAGCTCATTGCACTTGGCCCGGATCATGTTGAAGGTAAC
CGTGCCGACGCTTTGTGCCGCGTTGTGGAGCAGCTCAAAGATGTGCCGGTTGCGCTGACCCTGCTCCACG
GTCCGCTCGTGCAAGCGACGCAGTGCGTCCGAGTAGGCCGTGTACTTGGCCTCGCACTCCGTGTCCAGCT
TGAAGACCTGATACAGCTGCTGCAGATCGTACAATTGGCGTGCGGTCTCGTCGTATAGCAAACTGGGTGA
CTGTGGCAACAGCTGCTCCCAGTGCTCGTGCACCAGGTCTAGGAATTCAAAATTGCTAAAAAGAAAGATG
ACGAACAG
>dme_circ_0001622_junction_seq
AATTGCTAAAAAGAAAGATGACGAACAGGTTTGGTTTTATTATAAATGTCATCTCA
>dme_circ_0001623
GGTCTAGGAATTCAAAATTGCTAAAAAGAAAGATGACGAACAGGTCCAGTCCCTTGGTCTTCAGCTGGGC
GAGAAGATGTTCGGTGATGTGCTCGCTGCTCGATGTGTCCCGCATCTCCTCGTAATCGGTGCGCAAGTGG
TGCTCCAG
>dme_circ_0001623_junction_seq
CGTAATCGGTGCGCAAGTGGTGCTCCAGGGTCTAGGAATTCAAAATTGCTAAAAAG
>dme_circ_0001624

GGTACGCATACACCCGTTGACGTTTCTCGAAGAAGTCCTCCGTGGCAAGCATGACGTCCAGCTGCTGCTG
CATCTTGTCCAGCTTGGCACTGCTCCCGGCCAGATCAGCATGGTCACCGTCGATGATGTCCTGCAGCAGG
GAACGCA
>dme_circ_0001624_junction_seq
TCGATGATGTCCTGCAGCAGGGAACGCAGGTACGCATACACCCGTTGACGTTTCTC
>dme_circ_0001625
GAAGTCCTCCGTGGCAAGCATGACGTCCAGCTGCTGCTGCATCTTGTCCAGCTTGGCACTGCTCCCGGCC
AGATCAGCATGGTCACCGTCGATGATGTCCTGCAGCAGGGAACGCAGTATGTGCAGGTAGTTGTCGATGA
TTTCGGCGCCGGATAACTTGACCCGCTCGTACAGCTGCGCCCCGAACTCATTGAGATCCAAGCCGGTGGA
GTTCACCTCCTTTTTGGTCAGATTGAGTTGCCCCAATGCCGCCCCGACTGTCT
>dme_circ_0001625_junction_seq
AGTTGCCCCAATGCCGCCCCGACTGTCTGAAGTCCTCCGTGGCAAGCATGACGTCC
>dme_circ_0001626
GCTACGGAAACAGATTCGAGTGTATTCGGTTCGAGTTCGGTTGTTATTCTAGGCTCTAAATGCTTACAAC
GTATGGCTGTTCCGGCTCCTGCTGCTCCTCGCATTTCAGAGAGTCTTTGTTGGCTTTGTTGTCGTTACTG
CGATTGCAGTGCATCCTTGCCCCACAGGAACTGGGTTAGGTTGGTTAGTTGGATTTTTAAAGCCAAATTA
AAAGTGATTGGTTTTGTAAT
>dme_circ_0001626_junction_seq
CCAAATTAAAAGTGATTGGTTTTGTAATGCTACGGAAACAGATTCGAGTGTATTCG
>dme_circ_0001627
GGTAATCCCAGTTGTAGCTGCCAGTCAATTCCTCCTCACTGTGAACTATGGAGCTCAGGGAACCACCCAC
ATTACTACCGCCAACCACAACCGGTACTGGAACAGGAACTTCTCCGTAACTGCTCATCACCCCATGGCCA
TGGGTTATAGCCGTTCCGATGGTTCCGATGGAGCCCGCAGTGGTTCCCGCGTCGTCTGCACTGCTTCCTA
TCTCTGAACCAGCTCCTGTCACATCATTAAGTTTGGCATAGATGAGGTTC
>dme_circ_0001627_junction_seq
ATCATTAAGTTTGGCATAGATGAGGTTCGGTAATCCCAGTTGTAGCTGCCAGTCAA
>dme_circ_0001628
AAAAAGGAGCTCGCCCGTATCAACCAGTTGGCCAGGTACTACGGCATGTCGCAGAGCGATCTGCAGCAAT
TCCGTGAGGGTGAGCTTCGCCAGCTGGCGTACATCAATCACCGCCAGCTACTGTCCGCCGCTGAGGCCCA
GAAGTGGGAGAAGCGTCATCAGTGGACTCTGAGTCGCCTCCAGAGTCGCTATGGCAAG
>dme_circ_0001628_junction_seq
GAGTCGCCTCCAGAGTCGCTATGGCAAGAAAAAGGAGCTCGCCCGTATCAACCAGT
>dme_circ_0001629
GTGGAGGAGATCGAGCAGTGGATCAAGAGCGAATTGAAAAAGTTCCAGTCGGAGGGTCTGCTCAAGGGTG
TGGAGCAGGAGTTGATCCAGTGGCAGCAGAAGGAACGGGAACGTCTCCAGGCCATTGTGCAACAGAACTC
GTTGACGGTGGAGCAGCTGGAGGTGAGGATCAAGAACGATCAGGACCACTTCTTCAAGCTGGCCGACAAG
TACAAGATCAATGTCGAGGACATTCAGGACTGGCTGAAGAAGGAGCTGCTGCGCCTCCAAAGCGAGGGTC
TGGTCAAGGCGGAAACCCTTAAGGAGTGGCAACAGCAGGAGCGCGCCCAGATTTCGTTGCTGGTGCAGCA
GAACAAGTACTCGCTGGACGAGTTCGAGCGCAAGATGCTGGCCGACCGGGCACGTCTCCAGGAGCTCTCC
AATACCTACAACGTAAAGG
>dme_circ_0001629_junction_seq
GAGCTCTCCAATACCTACAACGTAAAGGGTGGAGGAGATCGAGCAGTGGATCAAGA
>dme_circ_0001630
GTAAAGGTGGAGGAGATCGAGCAGTGGATCAAGAGCGAATTGAAAAAGTTCCAGTCGGAGGGTCTGCTCA

AGGGTGTGGAGCAGGAGTTGATCCAGTGGCAGCAGAAGGAACGGGAACGTCTCCAGGCCATTGTGCAACA
GAACTCGTTGACGGTGGAGCAGCTGGAGGTGAGGATCAAGAACGATCAGGACCACTTCTTCAAGCTGGCC
GACAAGTACAAGATCAATGTCGAGGACATTCAGGACTGGCTGAAGAAGGAGCTGCTGCGCCTCCAAAGCG
AGGGTCTGGTCAAGGCGGAAACCCTTAAGGAGTGGCAACAGCAGGAGCGCGCCCAGATTTCGTTGCTGGT
GCAGCAGAACAAGTACTCGCTGGACGAGTTCGAGCGCAAGATGCTGGCCGACCGGGCACGTCTCCAGGAG
CTCTCCAATACCTACAACG
>dme_circ_0001630_junction_seq
CTCCAGGAGCTCTCCAATACCTACAACGGTAAAGGTGGAGGAGATCGAGCAGTGGA
>dme_circ_0001631
CAGCCAGACCAGTCGCTAGAATCGAACTCCAACATGGTTGCATTGAGCACAAAGTAAGTACCCCTGCCCC
ACAGCCCCATCGCCTCGACTGACGACCACAACGTCCACCAATCCAACATTCCAACAAGAGCCAGTTCTCT
GGGCAATGGCGAGCGGAGTAGGCGATGGCGATGGTGGTGACCAAAACGGTCTGAACTCATTACTAACCGC
ACTGGGCGACTATTACAGCAGTTCTCTGGGCAATGGCGAGCGGAGTAGGCGATGGCGATGGTGGTGACCA
AAACGGTCTGAACTCATTACTAACCGCACTGGGCGACTATTACAGCGGCCGCCGGGAGGAGAAGGCTGCA
GCCAACATCTACGATGACAAACGGATCTTTCCCGACTTCCGTAGCGGACCTTTGGAAGAGTACCGTGAGC
GGGCTAGCTTCTGCTACAAGCGCATGAACG
>dme_circ_0001631_junction_seq
GCTAGCTTCTGCTACAAGCGCATGAACGCAGCCAGACCAGTCGCTAGAATCGAACT
>dme_circ_0001632
GCCTATGAGCGGCGGTTTCCCACATGCCCGCAGATGCCCATCGTCCTGGACTGTGAGGTCATTAAGGACG
AGTCCCTGGAGGACGGGGCCAAGCGAAACACGAGCCGACGCTGCAAGCTGGCGGTCGACGCGCCGTACAT
CTTCAAAAAGCTTATTGGCGTGGACCATGTGTACTTCCTGCAGCACAACTTCCTGGACCTGGCCAACCGC
ACGCTGAGCATCGAGGCGGTCAACGAGAGCTTCTCGTCGCGCATCGAAATCTTCGAGCGGTGTCGCTACT
ACGCCCATCCGGACAACTCCGAATGGACGTGCTTTGACCAGTCGGCCACGCTGGACATTAAGAACTTCTT
CGGCTTCGAGCACTCGATGGAAAAGATGGGCATGAAGCAGTACACCCAGACGACGCTGAAGGGCAAAGAA
ATCATTGAGTTCTTTATCGGGCAGCTGCGCGAGGAAGGCATCACGCATGTGGAGCGTTGGACATCGCCCA
GTGATGCAACCAAATCGCCAACCCTCGATCAGGCTTCGGATCAGCAGCATAGCATCTTGCTGGACGGCGA
CTTTATCGCACGCAGCCTGGGCCAGCTGTCGCCCATGCAGGAGTCCAAGCTGCTGGAGCTGCGAAAAATG
CTGGATGGTGTCGATGACTTGGAGCGTGTGCCCAGTTATCAGACCATCCTGCGTTTCTTAGCAGCCAGGG
ACTGGCACGTTAGCCAGGCCTACGCCATGCTCTGCGACTCGCTTCGCTGGCGTCGGGAACATCGCATCGA
CGCACTGTTGGCGGAGTACTCCAAGCCAGCCGTGGTAGTGGAACACTTTCCGGGCGGCTGGCATCATTTG
GACAAGGACGGTCGACCCGTTTACATTCTGCGCCTGGGACACATGGACGTCAAGGGCCTGCTAAAGTCAC
TGGGAATGGATGGACTACTGCGATTGGGCTTTGCACATCTGCGAGGAAGGCATTCAGAAGATCAATGAAT
CCGCCGAACGCCTGGAGAAGCCAGTTCTCAACTGGTCACTGCTTGTCGATCTGGAGGGTCTTTCTATGCG
CCACCTCTGGCGTCCCGGCATCAAAGCCCTGCTCAACATCATCGAGACTGTGGAGCGCAATTACCCAGAG
ACCATGGGCCGTGTCCTAGTGGTGCGAGCGCCCAGAGTGTTTCCCATTGCATGGACAATCGTTAGTGCTT
TTATTGGTGAGTCTGAACAAGCCGTCATATGGAATTGCCTGTTAACAACGCTTTGAAATCCTGCAGATGA
GCACACCCGATCCAAGTTCCTGTTCTACGGCCCCGACTGTGCGCACATGAAGGATGGCCTGGCCCAGTAT
CTCGACGAGGAGATTGTGCCTGACTTCCTGGGCGGACCGTGCAAGG
>dme_circ_0001632_junction_seq
CCTGACTTCCTGGGCGGACCGTGCAAGGGCCTATGAGCGGCGGTTTCCCACATGCC
>dme_circ_0001633
ATGACATCGATTACCCTGCGGCTGGTGCCGGTGGCACTCTTGCGCACCAGCTTATCCCACATGTCCTCCA
GGGAGACGTAGAAGGGAGCGCCCATGATGCAGAAGTAGTGTCCCGGCCGGAATCCGTGCCAGTAGGCAGA

GCACAGCAAAGTGGCGCCAGTCCTAGGAAGATCACAAATGCTAATTAGTAGGGGCAACATAAGTCAAGCT
TATAGTTTAGGTAACTACTAGGAATACCTGCCAACCCTGCGATCAGTAGCAAAGGGAAAGAACAACAACG
acaagagtaatacgagagtaatacaagcagtaaaacaaCAAAAACTACGATTCTATGAAACAAGTTAACA
TTTGAACTTCGATGTGGAGTAATCGAACTATTTAGTTTCATAGAACATACTTGTATTCAAACGCACCTGT
ACTTTTTGCTGGGGAAGAGTTTGTACACATTGACAGCCAACCAATACTGGACGCACACATTCCAGTGCTT
CATGCCCTCGCGGAAGGTCCAGCATCTTTCCACCTCCAGAACCCTGGTGTTCACGATCGTTGTAAAGTTG
TAGGTGTGCTTATCGGCGTCGCGCTTTAGGTGCTGATAGCGTTTCCGAGGGCCCTCGCCATTGTTGGGAT
CGGATTCATCCGGATAGGCTCCGAAGCCGGCCATTGTGCAGACACATTCGCTGAGGGTGAGTCCGGTGTA
GATGCGAGCGCGGAATGTGAAGAACGTGGGCCACACGTACAGCAAACGGTACACAAAGGAGCGATCGTTG
AAGAACTCATCACTCAACGCATACTGAAATGTATTCAATTTAGTAATGTTTTGCGGTGTGCTCAAGCACT
CACATCCAGTGGCCACATGTAGTTGGTTGCCAGGTATAATGCGCAGTAGAAGACGGCGTACTTCAGCTTC
TCCAGGGTGGCTTCAACAGTTGGAGCGTAGGTCTTGAAAGGCATCTCAAAGTAGTCCCTGTATGTGCGAT
ACCGATAATAGGGACCTGCAATCAATGGACATGAGTGGTTTCGTTTTAAATGCAGATTTACAAATTATTA
TCTACTTGATACTGATGATTATATTGATGCGGTTGAAATCGATTATAAGCATGTGTGTGTAATGTTTTAC
AATCAGCAATTGAATTGGACTTTGGTATCTGGTTGTATTGTGACTACATTTTTAGTGCTTGTGGATAGGA
AACTAGGTTGAATCTGGTCCAATCTTCACCTGTAAGAACTCCGATGTAGTTGAAGCTGTAGTGCAGGATC
TCAGCGGCACTCAGGCTCTGCAATTCCACATCGTAGTCGGTGATCTCGATTGGACTCTCCTGGTGCACAT
CCCGCTGGTCGTTCTTCTTTTGCTCGTCGTGCGCTTGCAGTCGCTTCCAGGCGGCAGTCTTCTCGAAGGC
AATGCCCGAAACCTGCCGATGCAAGGATTACGAGCTAAGTAATGGCAATGAATGGTTATGCAACCCACCT
TTAGTGTTAGTATCATTTGTATCATGTTCGTGTGGCCGGGAATGCCGAAGTAGAAGTCGAAAATGCGGAA
GAACACCAGGTAGCCAAACATGACCGCAAACGTGACCAGGTGACCCTTGCTGGAAGAGGGAGCAGTGTGG
TTATTGGTAATTTTCCACAGGTTCAGGATCACCAACTAACCTCGGGTGCACGAGAAGCACGGCGGCCGTG
CCCAGTGCCAGGGAAACAAAGCAGTGCAGACTGTGGAGCCCCGAGACAATGACAACCACAAGAACGCCTA
GCCCAGTGGACACCAGCTTGCGCTGGCCCTCGTCCGCGATCTTCTTCACATAGCTGCCGGCTCCGATGCA
GCCCAGCAGGCAGATCACATAGATGACGTCGTCGATGCTCATCCTGCAG
>dme_circ_0001633_junction_seq
GATGACGTCGTCGATGCTCATCCTGCAGATGACATCGATTACCCTGCGGCTGGTGC
>dme_circ_0001634
GGTGGAAAGATGCTGGACCTTCCGCGAGGGCATGAAGCACTGGAATGTGTGCGTCCAGTATTGGTTGGCT
GTCAATGTGTACAAACTCTTCCCCAGCAAAAAGTACAGGTGCGTTTGAATACAAGTATGTTCTATGAAAC
TAAATAGTTCGATTACTCCACATCGAAGTTCAAATGTTAACTTGTTTCATAGAATCGTAGTTTTTGttgt
tttactgcttgtattactctcgtattactcttgtCGTTGTTGTTCTTTCCCTTTGCTACTGATCGCAGGG
TTGGCAGGTATTCCTAGTAGTTACCTAAACTATAAGCTTGACTTATGTTGCCCCTACTAATTAGCATTTG
TGATCTTCCTAGGGACTGGCGCCACTTTGCTGTGCTCTGCCTACTGGCACGGATTCCGGCCGGGACACTA
CTTCTGCATCATGGGCGCTCCCTTCTACGTCTCCCTGGAGGACATGTGGGATAAGCTGGTGCGCAAGAGT
GCCACCGGCACCAGCCGCAGGGTAATCGAT
>dme_circ_0001634_junction_seq
CACCGGCACCAGCCGCAGGGTAATCGATGGTGGAAAGATGCTGGACCTTCCGCGAG
>dme_circ_0001635
GTTCATCAACGGTATACGAAAGATTTACAAATAATCGAAACGACTACTATCCGTCCATCAGTCTATGCGT
CCATGCAGATCCCCTACATCTTAAAGGTGTCCGTGCAAGGGATCGCGGCTGTCCCGCAGGTAGACGGGAC
TGGTGAACGACTTGCCCTCCACATGCGAGGCGAGATTCTCGCCGGCGTAGGTGACGAAGGGCGAGATCTC
GCAGACTTCGCCATGCACAATGCCACCGGCGCCTTCGATATACTTCTTGTGCAGGCGATGGAGGTCCGAG
CGGGCAGTACTGGGGCAATCCTTGCCAGCTGCATCGGAGTTCTTTAGCGCGCTGAACTCTTCGTCGCGTG

GCACTTCCATGGCCACGAACTTCTGGGCGAACTCGAAGACGTCAAAGACAAACTTCTCTATCTTGATGCC
GTTCGGCTTGTCGGGCGTTAGACGCTTGCCGGCGTTGTCCACGAAGGGAATCTTCTTCTTGGCCACGTGC
AGCTTCAGCTCCTGCTCGTATGTGCTGCCAATCTTCTGCAGGAAGTTCGAGCTGAAGAAGTGATTGCAAA
TGTTGCCAGCACTGAAGGTCAGCCTGCCGTCTGAATTGCGCATCTCGGCTGTTTTGGCCGAGATTTCGCT
GTACTCCACCACCTGGTATTTGCCATCGACAATGGCCACCACGCCCACAGCCTCGTTGGGAGCTGCCTTC
TCCACCACCTTAGCGGCGCAGTCGGCCTTCTCCTGCACGCAGTAGCCGATAAAAACGGGATCGGCCACCT
TGATCAGGATGTTGTCCACGCTATGGGCATGCAGATATAAGACACCACGCTTCTGCATATCGTCCAGAAT
CCCCTGGCGCTTCATAGCCCGATAGATTCCTCCGTTGCCGTCCGGCGCACGCGCCACCCGATGCTTTTCG
TCCAGAATGATGCGTCCATCGTATTCAAAGCACGGCAGTGATCCCTGCTCGAACAACAGTACGTTCTCCG
CCTTCAGGCCAAAGAAGTTATTGGCCACAAAGTAGTCGTATGTGGGTTGCACCGTGTGCTCCGACGTCAT
GATGTACCAA
>dme_circ_0001635_junction_seq
CGTGTGCTCCGACGTCATGATGTACCAAGTTCATCAACGGTATACGAAAGATTTAC
>dme_circ_0001636
GTGATGTGTCCACGTTTTCCATTCGCCTCTTGGGCGAGCTCCTCCAGCTTCAGGATTCGCTCGGCCTGAA
TGCGGAACAGGGTCTTTCGAGACTGCAGTCCCACATCGTACATTCCCTTGGGATGATCGAAGCCAAGTCG
TGTGCCTGCAAAGAGAAAGGACAACATTACGAAGGTTTCCACCAATACAGTTCCCCAATATAATAATTAT
TGCAGTATGTATATAGATAACAATTCCTGAATGTATTAATCACTTGTTAACGACAAGAACTGCTGCATTT
TTTATATCTCACCCGGCTGACCTTAAAAACAAGTTTATTTTCGACCATCGTTTCGCTCTTTAGGTTGTCT
TTTGTCTGCGCCTTTTTTAACATATATGTATATAAGATACAAGCTGGCTTGTTTAAGGTCAGTTCCACTA
CCGGTTTCAAAATGATCTGACTACATTTAATCTACGGCTTATCGGTACAGCAAACGGAACCTCCACTCCT
GACCAGAAATTCTCAGAAATCAGAAACCGTTATCTAGGTTCACTACTTATGACTCTTCTCGAATTTGAAC
GTGCAAGAAATAATGCTATGAGCAATCGGTGTTCAACTCCGTTTCTTGGTTTCTCAAAAGACAATTACGT
GATTGGACTCCAGCGTTTGCCCAAGAAACTGGTAAGGAAATGTCAAATGCCGCCCACATTTCAATGTTTC
GATTCGAAAGAGCACTGGCCCCGCCTGAAATCAAAATGAGAAAGGCAAGTCTGGGGCTGTAAGAATGCCT
TCCACATTGCTGCTGGCCTGGCGTGTTTCTTAGTTTTATACGCTTTTTGTCTTGCAAACGGAATTACAAT
TGCAATTATTACAATGCAGCTGCATGAGTGGGCAGCTGATTGGGATTGCGTGTATTTGTGAGCACTTTTA
TATCGAGTTAATTAGCTTTTATAAAGCACACACACTCTGCGAATGCTCAAGTTTCTGTTTTAGTTCGGCT
TACACTATGTTTTTTGCTCGCAAAGGCGCAAttttttttttGATGGGTAGCCGATTGGGGCATGATAAA
AATATCTCAACTTGTACGGTATCTAAGGAAGTATCTTTCAGAGATACAAGCAAATGTGAACTCTGAAACG
AACGAACTATGAGTGCAATGGAAAATTTCTTTACTTGTAGCCTTTGATTTGTATATATTTTGATTTTTAA
ACTGACTACCATATCGTGCTCGCGCATTGGAAACGTTAAAAACCATTATGGTAAGTCTCAAAACGAAAAT
CGTTCATTTTCGAATAACCTAATCGTTTTTGCTATCAATGATTGACGACTTAAAATCCATTGCTATCTGA
AGTTTATATAAACGCCATACAACACTACCAACTGGACCCACAAAGGTCTGTCCCACTTCCAGTGCGTCAA
ATTGCTCCATAATCACACTATTGACTTAAATATGCTACCAACTAAACACAGGTGTACTATTTCCCTAGCT
ATTATTCTGTTTTTTCTGTTAAATTAGAGAAACTCCAGCTCACCTTGTCCGCCTGCCATAAGCAGCACAG
CGACATGTCCGTTGCTGATCTGGAGGAGACCCTCGTCTCGGTAGGCGTCCAATTTCTCCAGCGGCGCCCT
GGCAATGGAGATTAGCTTGCCCTCGGGCAGCGGCTGCAGGCGATCGTCCAGCTTGATGCCATTCTCGTTC
ATTGAGACCGTGGCGCGGTCGAAATACAGCTTGATCTCGTCCAGATTCAGCTCCTCGATGTCCCGCACCA
GGTCGATGCGTTCGTCATTCGTTA
>dme_circ_0001636_junction_seq
ACCAGGTCGATGCGTTCGTCATTCGTTAGTGATGTGTCCACGTTTTCCATTCGCCT
>dme_circ_0001637
GATTCAGCTCCTCGATGTCCCGCACCAGGTCGATGCGTTCGTCATTCGTTAGTTCCGGCCAGAACTTGAG

CAGGTGCTCCTGGCCCACCTGGGCGAGTCGCGAGTGCAGCGACAGGTAGTCCGTCATT
>dme_circ_0001637_junction_seq
CGAGTGCAGCGACAGGTAGTCCGTCATTGATTCAGCTCCTCGATGTCCCGCACCAG
>dme_circ_0001638
GTTCCGGCCAGAACTTGAGCAGGTGCTCCTGGCCCACCTGGGCGAGTCGCGAGTGCAGCGACAGGTAGTC
CGTCATTGTTGGCGAAGTTTTGGCTGCGTTTGGTGATTTTGATGTGGCACCGCCAGCTGCAAGAATCCAG
AGACAAGATATTTGACTAACAAATTATAAACAATAGAGACAAAATATTATTACGCGGTATCCTACTTAAT
ACGTTATATTCTTCACCATAATTTTGGAACATTTTAAATGCGAACACACAGTTAAAAACAAGGTTTTATC
TTTAGAGATCACATTGGAATCATATATTAATTGCTGCTATCTGTTTGTTAATTCAGCTAATGGAACGGGC
TCTCCCGAAGGTGTGTATTTACGTTTCTCTCCGCTGGCTGACTGATTATTTGGCGAATGCGGTAAAAAGA
CAGTGGTCGGGTTGTCCGGGCTTTGTTGCGTTCCGCTATACGTGGTAACCAAAGCGCAACTGAGCGCCAA
GCGATCGCCCTCAGTGGTTTTTCTTGCTCAAAGTGGAGCCGTCAGACAACCACCATCGCCTTCGCCTCCA
TCTGGTTGTGTCTCTGTGGGAACGCCTGAATATCTGAATGGCAGTAGCTCGTGTGCAGGTTATATTGGTA
TCTGGTATTTGGGCGCGAATGTGTGCGAACGTGTGTCTCTTAACGTATTTGCAGCCGCGTTTTAACATGG
CCACGCCAGTTGTTTTGGCCATACCCTGTAAAAACCTGAAGGGCGGGATCGCGATCATGGCGGAAAATGT
GCCCAATCAATCACAGTCTCTTTTTGGTATTAGATACTACATTGTTTAATTATAATCTGTTTCAGAAAGC
GAAAGAGAAACATATTCCTTATAGGTGAAACGTAAAAGTTATAATTATAATTTAACAGCTTTGCTTCGTA
TTTCTACATATGTAGATATAAATATTTGTATGTATGTACATCTGTTAAGTGATCTTAATGAGCTCAACCT
TGTTTTTATTAAAATTGAATAAGAGGAATGCGGGAATTCCCAACGACTTAAGACTTAAGAACATGTAACT
TGTGAGTGAGGGTATCAATTAGTCGTGTCTGCGGTCGGCTCTCTTGATTACACGGCTGATACGGCTACTG
GGACTGCAGCAATTTCAATCGGATTTTCGCTGCTTGGCCAATTCGCCAAGCAGCAGCGTGTTGCAAATGC
AAAGCGAAATATCAGAAACCGAAATCGAATTCGAAACCAAATGGCCCTGAACACGGGCTTTAGTTTTAGT
GAAAGTAAGAGGTGCCACAGTCCAAGAAACGCCAGCCACAAGGAAACTCCTAAGGGCGACACATATTTAT
ATATAGTTCATACAGTTCAGTTCCTGATAAACGGGTTACAAACTAGTTGAGTGGGTTATCGGGATTTGTT
GGAAAGGTTATGGCATTAAGGCGAGCCAAGAACTGACAAAAACATGGCAAAACTAAGTCGGTGCTCAGCC
GGTTTATTCGGTTTTAATACCCATTAACTTAAGCGCAATGGTTTATATTAAATATTATGGAATGGAAAAG
TAAGCGCAATAATAAGGTATCAACAAGAATAACAGTAATTGAAAAATATCACCATTATATGCATCTTtat
aagataagataagatgtcatataaataaaaatgtataaatgtataacaaatcaatatatatCTATATCGA
TTTAAGGAAAACGGGGTAAAAAATACATTTAAGGAGTGATAAGAGGGTATTTTACATTCGCTACACTGAC
CATTATTGTTGTTTTTGCCACGAAGCAAAACACTTGTCTGCATGTATGAGTGCGCATaaaaaaaaaGATA
AAGCTGCAGCAACAAAAACACTCCCCGcacacacacacacacacacaAGTACCCAAAAACGGAACAAAAC
AGCGATAGCAAGTAAGTTGTTTACATACACCCACTGCtgtgtgtgtgtgtgtgATTGTGGGCTTTCCGCT
TTTTTCTTATCGCAGCTCTGGGCTCCAACTATTGCGCTACCATTATCAGTTTGCTTTTTCGAGTGGGTTT
TTACCAACTAACCATTGCGGTGGCGCCTCACATGAGTCTGCTGGTGATGCGAACTGAAGTTGCTGCGACC
CATTTTGTCTGGGAACACCCT
>dme_circ_0001638_junction_seq
TGCGACCCATTTTGTCTGGGAACACCCTGTTCCGGCCAGAACTTGAGCAGGTGCTC
>dme_circ_0001639
GGGTCTCTCATCCGCTTTTATGTACGCTTCTATTGGTTGGGAAGATGAAAAATAGCAGCCAAAGGCCTTA
CAATTTACGCTTACTGTGGTTGTTTTGACAGACGACGAAAAAAACAAAGCAGGTTATTAAGTTTGTTTTA
ATGGGAGCGAGCGTATGGGAAGTATGATGAAGTATTAGTTGTTGCTGATTGGATCGCAGGTGATCTTCTT
GACTGCTTAGAACAGCAGCGTGCCCATGCCGCCCATTTCGGACTGCTCCTTAACGAAAATCTCAAAGTAT
TG
>dme_circ_0001639_junction_seq

GCTCCTTAACGAAAATCTCAAAGTATTGGGGTCTCTCATCCGCTTTTATGTACGCT
>dme_circ_0001640
GGCAACTTCTTCATTAACCTTCTGGGTGTGTGGGCTGATGTTGGAGGCGGCGGCCCGGCTCGCTCCTATC
CCATCGGTGGTCTGTGCTACTATCTTTCGCCCCCAGAGAGCGTGGGCCACATCCTGACCGACCCCATTCA
CGCGTTGCTCTACATTGTCTTCATG
>dme_circ_0001640_junction_seq
TCACGCGTTGCTCTACATTGTCTTCATGGGCAACTTCTTCATTAACCTTCTGGGTG
>dme_circ_0001641
GCCAGCATCTGGGAGATGACGTACAAGTTGGACACGAGCGCAGACTGCAGGATGATGGGAATGTTGGAGG
TGTAGAACAGCTTGATGGGATAGCTGCTGTACTGGCCGCGGTAACGGGCGCTCTTGATGG
>dme_circ_0001641_junction_seq
TGGCCGCGGTAACGGGCGCTCTTGATGGGCCAGCATCTGGGAGATGACGTACAAGT
>dme_circ_0001642
tttgccgttgcagctgccccactaaaggcgtctgttgctgctgtgattgctgttgcagctgccccactga
aggcttctgttgctgctgtgattgctgttgaagttgccccactgaaatcttctgttgctgttgtggttgc
tgttgcagctgcgccactgaagtcttctgttgctgttgtgg
>dme_circ_0001642_junction_seq
ccactgaagtcttctgttgctgttgtggtttgccgttgcagctgccccactaaagg
>dme_circ_0001643
AAACGGTCCCGACATTCACCAATGTCACCTCTTCTACTTCATCATCCTGCTCTCTTCCCGCCATCACAGT
CTGCTGCCCCGGCGCTTTTACTATCGCTGGCTGCTTGGGTGTGGTCAATGTGggtggaagtgggaactgg
ctagctggtggaagtgggaactggctagctggcggaaTTGCTCGTACTGTGGGAAACATTTTGGTCAGCC
TCCGCGATGCTAACTCATCTAACACGTACTTTAGGTGCACGTACACCAGGTACGATTCGCTTCGGATCCT
CGCCATAGCCATTGTAACCAGGTTGCGAAAGCCACATGACATCACGAAGCTTTCGGGCGCACTGTAG
>dme_circ_0001643_junction_seq
CATCACGAAGCTTTCGGGCGCACTGTAGAAACGGTCCCGACATTCACCAATGTCAC
>dme_circ_0001644
GTGCTGCTCACGCCCCCCGATGAGGGACCGCGGGTCAAAGGGCAGCGCATCAAGCTGCAGGACATCGTCG
ATGGCCTCTTCGTGCCGCAGCACTCCAACGGCAGCTGGATCGATGGTGAGTGCTACACTTTGGTGAAAAT
CCACAGCAAAGGTGCTTAAAAGTATGCAACAGCAAGCTGTAAAAGAACGTTACCATTTCATAGCATTCTT
TTGGGCACAAGTGGTTTTAGTATCGAATTCATATATGTCTTTCGTTTTCTCTATCAGATTATTACAAACC
tttttgtttttttttGCGTGTATGCCATGAGTGCCCCTTAGTGGCAGTTCTATGTGGCAAATCGCTTTTA
ATTTCCGCTTTCATTTCAttttttttttGCAGGCGAGGAGTTCCTGTACCAGGACCATTTGGGTCGCATC
TGTCTGCTGAATGCAGCCAATCGCTCGGAACGTGTCCTCATGTCCAATGTGACATTTGTAAGTACATTTC
GTTGAATCCCCTTGCCCCCTTGCCGGACTGTTTGTTTGCTTATTGCTTATCTTTCTGAGCTGAATAAATA
TTTGCCCCATCTTATTTACGACTAGCGAAGTCCTAGTCCGCAGTCCTAGAAAGTGTAGTTAGTTGGCAAG
GGGCGGACCCAAGGCAGATATCTCAAGTGTACACACTCGACTTGGCAATGTTAACTGATATTATGGCGCG
CACAAATGTGGGCGAAACAAATGGGCCAAGTTATCGTTAACGGAGTGCGGCGACTCTCGCCCCAATACAG
CGATAGCGATAAAAGGCAAAAAGATTTTTATGTCAATGACTGGGTAAACATACACATGTGGAAAACGTGC
GGGGAAATCGCGGGACATATAACGCTTTCTAATGAATAATGCGCGAATGGAATTGAAAAACAAACAAATA
AGACAAAACTGAAAGGGAGGAAAATGAATGGAAATGAATGGAGAGGGGAGGGGGAGGGGGTCAAAGGGCG
GTAACCAGAAGCTTTTCCTTGCCAACTTGGCAAACGAACATTCCGAGTGCCTCGAGGGCCCAAAAATACA
GACAGGGAGTGCAACAAGTGCAGCCACTGCAAGTCAACAGTTGCCAGTTGAAACAGTTGAGTGGGAAATA
ATTAATGTGCAGCCGAATCCAAAGAAACTCGAGCGTACAGTCGCAACGGCATAATGGCAATACATATTCA

TAGTTGGCTAAGGATCTGGCTTTAAAGTTACTTCGCCGTCAAACTCAAACACGAAATGAGCAATGAATAG
CAAAATACTAGACATCAACTTGGCTCTCGGTTACATTGGTCTGCCGAGAATAATTTTCAGATTGTTTTCC
CAGGCAAATTAAGGCGGCAACTCGTTGGCAAACAATTTTCGACATCCAGAGCCGTAAACAATGCCGCAAA
TTTCAAAAATTTCCAACCAGTCACCACCAAACTGGTGGCAAACTTCCGCCATTTTGTGTGCGAATCAGAA
AAGACCATTAAAATTTTAATTAAAATTGCGTATACGCAGTGTGCTCGCACTGTTACAGCCGCTCAACAGT
ATTGCGCATAATTTGCAACCAGCATTTAATACCACTCAATGGCTCCACTTGCCCCACACAGACGAGAGCA
TCAAGCTGCGCCACAGTCCGCACCAGGATGAATGGCCATACCTGCACTACGCACGATTTACGCCAGCTGG
AAACGCACTGGTCTGGGTCCAGAGCTACGATATCTATTACCGAGAGGAGGTCCGCAGTGCGTCCGTGCAT
CGGATTACCCACGATGCGGTGCCCGGAGTGGTGTACAACGGCATTCCCGATTGGCTGTACGAAGGGTTAG
TGGCATTTGCAAGAAAATATCGCATGTGTGGATGAGGATTTATTTGTAGAGATAATCAGGAAGAATCAAA
GAAGAATCACCTGAATGCGTGCTCAAAGCAAATCTTATCTGTTGTTTAATCTTCTAAAGCTGCTGATATT
GGTAGCATATAATTTAAGTTTTATAATATATATGTTCTTAGCATAAGTCTTATGTGAAATGAAGATATTC
CTCGAGTTGCAAACTGCATTACTTGTCAACCGGCACTTATTTCTATTTTCCGTTCTCTTTGGCACTTCGC
AGAAGGAGATCCTTCATGCAAATAATGCAATTTGGATGTCCGACAATGGCCAACTGATGCTGTATGCCAC
CTTCAACGACACGCACGTCCAGGAGCAGCACTTCGCCTGGTACGGCACGACGGGTCCGTCCGCCGGTGGA
GCAGCAGCTGCAGCGGCAGTCGGCGCTGGAGGAGCCGGAACTGGGAGCCCAGGAGCTGGCGGCAGTAATC
CGCATGCCAGCCTGTATCCGGAAATCAGGTCCTTACGGGTAAGTCGTGACCCCATTAGGTCCAAAGCTCT
GGCTAAATCGCTTTTCGATTATGTGTTAATGATGCCTCATCGTCGTCGAGCCGCTCGTTCCCTCGTAACA
TCTAATGGGCTCCTAGTGCCATGCTTAAAATTCATGAAGTGACAGTGGCGAAACGTTTTCACAGGTGGAT
TTGGGCACTGGCTTCATCCTTTTTCGGGTCGCCATACCCTTGCCAACTCCGGATTCGGTTGTGCTCATAG
TTGGGGGCGTTAGAATCGGTTGCTATGCGTAAGACAAAACAATTTTTGGGGTGTCTAAACAAGCTGTGAT
CCCCTGTCAATTGGGTTTGATAAATTGCTAGAACGGATCTGGAAAGGTTAATGCGAGATGTCAGTGTTGT
CTCATTTGAGGGTTTGGGTCGTCTTCTAGAAATAACAAATGAGATTAAAGCTTTAACTCACCATTCATAT
TGTTCTCATGTTTTCCTCATTGTGATAGCTTACTTTCTGGGAACTCCGGAGCGTCTTCCTTCGCAGCAGC
ATCTGTATCGCGTATCCGCCCTGCCGGCCCGCCAAGGTCAGGCGCTTCGCTCACCCGACTGTCTGACTTG
TCCGGCAGTGTCGCAGTGGTCCGAGGGCTACGACGAAGGTCACACCAAGTCGCCGCCCAAGCTGGTGACC
GCCTGGGACGATGACTGGGAGGATTCCGAGGAAGCGGAGGCTCAACCGCCGCAGCCAGCTCTGCCGGTGG
AGCAACAGCCTCCGGGGCGGGGTCAAAGTGCTCCACTGCCACCGCCTCCCAGTGATTGCCTGTACCACGA
GGCGAAGTTTCCAATTTCGCGGCAGGCGAAGTACGTACTAATCGATTGCCTGGGACCAGTGGTACCCACC
TCCATACTATACGGCTTGAAATCCGCCGCTGCGGACAGTGCCAAGACGAAACGACACAGCACCCAGCAGG
AAGAACCACCCACGGAGGGTGGTGATGAGAAACCAGGGGAGGAGCCGAAGTCTCAGTTCCTGGAACTCCT
GGTCATCGTGCAGAATAACACGCGCTTGAAGGAGAAGATGGCCAAGACGGCTATGCCGCAAATCAAGACC
TTCCCCGTGATGATCTCCGGCGGTTATCATGCCCAGGTTCGACTATATCTGCCGCCTGTGCTGCGCGAGG
ACGAGATAACACGGTATCCCACCATTCTGCATGTTTACTCCGGACCCGGCAGTCAGCTGGTCACCGATCA
CTGGCACGTCGATTGGAACACCTATTTGTCCGGCAGCAAGGATTACATTGTCGTTGAGATCGACGGACGG
GGATCTGCTGGACAGGGCTACCAGCTGCTCCACGAGGTCTACAAGCGTCTGGGCAGCGTGGAGGTCTCCG
ATCAACTGGAGGTGAGCGAGTACCTCAGGGATAACCTGCACTTCATCGACTCACGCAGAATGGGCGTCTG
GGGCTGGAGTTACGGAGGATACACAGCCGCATTGGCCCTCGCCGGACAGCAGTCCATTTTCCAGTGCGGC
ATCAGTGTGTCACCGGTTACCAATTGGAAGTTATATGGATTCCACGTATGCGGAACGCTATCTGAGTTTC
CCCAACGTGACGGACAACTACAAGGGCTACGAGGAGAGTGACCTGTCCAAGTACGTGGACAACCTGCGGG
ATCGCCAGTTCCTGCTGGTCCACGGAACCGCCGACGATAATGTCCATGTGCAGCAATCTATGGTCCTGGC
CCGTTCTCTGACCAGCAAGGGAGTGCTCTATAAGCAGCAGATATATCCGGATGAGGGGCACAGCCTTTCG
GGGGTTAAGCGGCACTTGTACCGCTCGATGACCGCCTTCTTTGAGGATTGTTTCAAGAAACTGGGTAAGT
TAAGTGTGCACACCCCAAATCTTACTTTGTAATATATTCCACTCTCCTACCACAAatatatattatatat

ataACTCTCTCTATTTCTCCTGCTCTCCAAATTAAAAACAAACCCACCAAATCACCGCCAAAATAAAACC
ACACCCACTGTCATTTCCGTTTTGTTAAATTATTGGTTTTTAATGCCCATCGTCCCTGGTGTAGATCGAT
CAGCAACAACAAAATCAAACAGATTCCATGGATTTTCTGGACTTTCATAGTTTTTATGGTCGCTAAGTTT
GATTCCATATCCCACATCCTTCAGATACTAGGTACAGTCGCGGTTGTCAGATTTATTTTATTGGTTCCTC
ATCGTTGTCAAGTTGTTAAGTGTCGTTCTCATTGTCGATGTTGTCATTAGTTGCAGGATTTTCCAATTTT
ATATCAATTATTCGCTGTCTAATCGCCAACTCTTAATGCGGACCAAAATATTTACATGCTTTCATATCGT
TTAAAGTTAAGATAGATATTGATGTTTGATGTTTGAATATGCATAAGCCTATTATCCTCTTTCTATATCA
TTTTATTGTGAAAGACAGTGTATTTTTTCGCCAGAAAATAATATGTGAACAAATCAAATCTAAttttttt
tATAATTATTAAGCCATATAGTTTTTAAATGTGTTTTGATTTCGAGCATAAGCCACACAGTCTCTTTGGC
ATCCCGCATAAGCAATGGTGTTTTCCTTTTCCTTTATCTTCCCAGAAACTTTTCCGGTTCGGTTGCTTCT
TAATTTAATATTGAACGCGTTGAGTCTGCATTACACTTGTTACACTTTGGGCTTAATATGGTTTACCAGC
CGAACTTTATTTTGGGCCTAAGTCCCAGTCATAATCGTATGTGTTTGCTGTTGGTTTGTTGCCCTTTTTC
TGGGCCTTGGATCTTGGCGAAATATTGTGgtttttcttttggtttagtttgtgtttgctttagtttttgg
CTGCTACGTTCTTTGGATTATTTGTTTCCCTTGTTTGATGCTAGTTTTCCCTATGAGTTCCATCACATTT
CTGttttttttttttgaagctttttttCCATTATTATTCCGAAAGTTTCGCCTTGAATACTTTTTGCGTTTG
CCCCGAATTTTCGTTGATCCCTCGTTTGATTCGAACAAATTGTTTTGGCTGTCCAACATTTGTATCCTTT
GGCGTATGTTTGTGTGTGGGACTTGTTGTTGCTCGATGTGTTTTCCTTTTACGCAGGCTAGATacacaca
ctctcacacacacgcacacacacatccagagacactagaaacacatacacCCAAACTGGCTATTTTACCT
AAATGTTTGCTAATGCCTTGATTGTGCTCCTTTCTCCTTTCCACTTTAATTTTCTCTGATGCTGTACTGT
CACTTAATGTAGAAAATAATTGATATTGATATTTTTGAGTAATGGCTCACTTTCTGTCCAACAACAGGGT
GCCGCCGGAGTCGAAAGCTGGTCTTGGCAATGGCGGCGATATGCAACAGCAATAAACTTAAATTATGGCA
GCAGAAAATTCTGATAACAAAAGTATGGAACACAACAAACTTCAACCGGAACGTGCGAACGAAATGTTTT
TCTGTAAATTGCATTCAATGTGATTTATCATGAACACACCCACGACACTCACATACACAGCAAGCGGCTA
AATGGCAACTCAAATAGCCAAACGGCGGCGGCGACGGCAAAAAAAGCAACAACTAAGTTAAATGGCATTT
TGTGTAAAATTAATAAGCAAagcagcagcagcagcagcagcgaaatgcgaaatgcgaaatgcCAAG
>dme_circ_0001644_junction_seq
agcgaaatgcgaaatgcgaaatgcCAAGGTGCTGCTCACGCCCCCCGATGAGGGAC
>dme_circ_0001645
CTACCCCAAGCCGGGCACTCAGAATCCCACGGTAACCCTACGAGTGGCTGACCTAAAGGATCCGCTGAAA
GTTCACATCACCGACCTGCATCCTCCTCAGATTATAGCCAATGAGTAAGTTCGTTTTCTCTTTGTGTATA
TATTAGTTTCGGTATAATAACATGCATAATATTACCTTATTCCGAATCATTCTAACCCACAGAGATCACT
ACTTCAGCAGTGCCAGTTGGGTGAGTCACTCGAAGATCGCGGTGGTGTGGCTAAATCGTCCGCAAAACAT
CTCGGTGGTCAGTGTGTGTAAGGCTCCATTGTTCCAGTGCATCGAGGTGAGTTTGTTGGTGGCAGGTCCG
GCCAGCTGGCTAGCAATCTTCTAATGAACCCCTTCCGCCGTTCGCCATTGCAGACCCACCGGGTGAGCGG
AGATGGACGTGGTTGGGTGGACACTGTCGCAGTGCCGCTGTTTGCCGCCAATGCCAGCATCTACGTGGCC
ATATCCCCGCTACGAGATGGACTGTTTGG
>dme_circ_0001645_junction_seq
TATCCCCGCTACGAGATGGACTGTTTGGCTACCCCAAGCCGGGCACTCAGAATCCC
>dme_circ_0001646
GACGCCACTTGCACATCACGTGTTTGACTCAGGCTACAAATTTGTGGCCCACGATCTGAATTTGATCTCA
TCTGTGGTCGCGATGTTCTCACTACAACTACAGCAGTTGCTCCGAGCGACAGCAAGCGGCGTGACGTCGG
GAGCCACAAAGACACTGGTGCACAGCCACAGCGTCTGCCGCCACAAAGCACTCTAGTCAAGCGAAGTTCG
GCGACAACAGCAGTCACGGTTCGCCATCGCACACGGACGGTCGCAGTCACGGTGAATCAGAGTCGGATTT
GGAGTCGGCGCACGGCCAGAATTTCACATCACACCACTTAGCCAGACGGATACTTCAGCAGCGCAGCCAG

GACATCCAGCGCCACATCCACACGCAGTCAGCCAAGGGACAGACCAGTGCCAAGTCCTCGCAGCAACAGC
AGCATCAGCAGACCGCCACCAAGTTCGCCATGTCATCGCTAGCAGCTGAGCCTCTGCAGTCGCAGAAATC
GGATGAAATCACGGCCAGCACGGGCGGCGGAGATGTGGAGACGCCCGGACTGGGCGCATCCTGCCACGAG
GTGGCCGCCGCATCGGCCAGTGCCGGCAAGAAGCCCTGCTTCAGCTCGGGCCTCGGCGAGATACTTCAGT
TCATGGAGCTCATCGGCAACCTGAAG
>dme_circ_0001646_junction_seq
GTTCATGGAGCTCATCGGCAACCTGAAGGACGCCACTTGCACATCACGTGTTTGAC
>dme_circ_0001647
GTGTGTTAATATTTAAACATTAATATTTAGCTACTTCCCATCGGTTGCATCTGTTCCGTTTCTTTCGCAA
TAAATTAGTGAGTTAAGAATTAGATCTAGCTATGAGCGTGTCGAGGTAGGCTTAACAGTTTGTTAGTTGG
ATTGGCTTACAAATTAAGAGCGTGTTGGTTTGTATGTGAGGatgtgttcatg
>dme_circ_0001647_junction_seq
TTGGTTTGTATGTGAGGatgtgttcatgGTGTGTTAATATTTAAACATTAATATTT
>dme_circ_0001648
GTACAGTACATTTTTTAAGTAATTCCACTTAACTGATGAAGATACGTTGAAACATTTTGGAAAATATTTG
AAAAATTTGTTTGCTTTTGACTACTTAAATACGTACATAATATATAGATATATACACATATCCATATT
>dme_circ_0001648_junction_seq
TATATAGATATATACACATATCCATATTGTACAGTACATTTTTTAAGTAATTCCAC
>dme_circ_0001649
GGCGCTGGACTGAGAGACGGATAGGAACTGGGACTAAGTTCTTTATCCTTCAGGAGGCACTCCTCGGCAA
TGGTTACTTTTGGGGGCGGGAATGCATTTTCAAGTTTTTCTAGCGCTGGGACAACTGGCGTAG
>dme_circ_0001649_junction_seq
TTTTCTAGCGCTGGGACAACTGGCGTAGGGCGCTGGACTGAGAGACGGATAGGAAC
>dme_circ_0001650
GGTGAGCGAGCTGTCCGATGATTTTCGCTGCAGGAGGCTGGGCGCCGTCGACAGGATGATGCCCCTGGGG
GGCAGGCCCAGGGCTCCAGTGACTCCTCCGCCAGCGACAGTGCCACCGACACCACCTGCAACACCTCCTG
ATCCCGAACTGACGAAATCCGGGCAGATGGTCGAGCTGGGATTGGAGGCCAGCGAATCGGTGCGCCACAC
GGCCGTCTGCTTGGCATCGTG
>dme_circ_0001650_junction_seq
GCCACACGGCCGTCTGCTTGGCATCGTGGGTGAGCGAGCTGTCCGATGATTTTCGC
>dme_circ_0001651
gcgtttacaagaggaagcggatcagcagaagaggttgcaagaggaagctgcgctgaataaaaggttactc
gaagaagccgagcaacagaaaaggttacaagaggaagcggagcagcagaagagattgcaagaggaagctg
agctgaataaaag
>dme_circ_0001651_junction_seq
tgcaagaggaagctgagctgaataaaaggcgtttacaagaggaagcggatcagcag
>dme_circ_0001652
ATCTCCGTCACGAAATTGTACTTTTCGGCCGTCAAACGCTCCTCGCCCTCTTCGCTGGTGAGCAGGCAAG
TCTCGGCGTGGGCCCTTAGCATACTAACGCCCTTCGCCTGCCGATCTTTGTTGGGAACTGCACAATATGT
AGGGTCCACCAGGAGAACCTGCAGCGAATATTTAAAAAGTTATTACTAGATAAGGGGGCATGGCTAGTTC
AGAAGTGTATTTACCTTTAACGAGGGCATACATAGGGGAGCACAGAGACGAACTAGAACTGCAGTCAGGT
TGGTCATGAAGGCATCGCTTGACGTGCTGTGAGCTGTTTGCTCCAGGCTGGCATTGATGCTGCTCCATAG
GTGACCACGCGGCACATTTGCATCCAGGCAATTGCCTACCCATTGAAGCG
>dme_circ_0001652_junction_seq

TCCAGGCAATTGCCTACCCATTGAAGCGATCTCCGTCACGAAATTGTACTTTTCGG
>dme_circ_0001653
GGAGCGTTTGACGGCCGAAAAGTACAATTTCGTGACGGAGATCTTCTACATGACACACAAATGCTTCGAG
CTCTCCAATATTCCGTGCATTGAGCGCTTCGTTCGGGTGCTGCGCGAGCTGCAAAACACGCAGATGGCGT
ACGGAGAGATCGTGAACAGCGATCCGAATAGCGAG
>dme_circ_0001653_junction_seq
GATCGTGAACAGCGATCCGAATAGCGAGGGAGCGTTTGACGGCCGAAAAGTACAAT
>dme_circ_0001654
GGATCCGGCCATTGTTAACCAAAGACGCATTGGCAGCGGTCGCTTAAACCATTCCAAGTGGGGTTACAAC
GACGAAGACTACCACTCGTACCACAATGGCAAGTCGCAGCATATGGAGGAGGTCAACTCGAAGAACAGCA
AAAACATGACGGTGCTGCAGTTCTTCGACAACGGCGAGATCAGCAGTCAGCCTCAAAGACGGCCGAACAC
TCCTGTGATGGGGATGTCTATCAATCGATCCGAGAACGACACCCTGCACTCGAACGAGTCCAGTGAAGAT
TTGAGTCGCGCCAACGAGAACTATGTAAAGCGCGTAATGTCTGGATTCCTGGTTGTATCGAAGCCCAAGT
CACGAGATGTCGAGGATAGACATCACCGGCGGTACAGGAACCAGAACGAGGAGCCTGAGTGGTTTAGCTG
TGGACCCACGTCTAGACTGGACACTATAGAGTTGTGTGGGTTCGATGAGGACGAGGAGAAAATGCTAAAG
GAGGGCAACAAAAACCACGGATTAGGCGAAACAGAGCGAGAAACGTCAAAACAAAAGGATGGACCATAAG
TACAAATGGACGCATGCGGAGCCGATGGGTCGAAGCAAATACATGCCCAAACACGACACTAACAATAATC
ATAACGTGGAGAACATGAATAATGTTATGGCTACCGAGCATCAGCAACAAAAGGAAGAGAAACGCCCAGG
CAGTGGCCGTTCGTTTCAGTTTGATAAGTTCAATCAAAGCCAACAGAATTACGAGAGTAGCAGTTATGTT
AATCACCAAcagccgccgcaaacgcagcctcagcaaatgcagcagcagTCGAACACAAACACGAACAACT
CTAAATTCATGTCATTCTTTGCCAATGAGGGGAACTCCTCGTCTTCTTCGCTGAACGAGTTTTTCAAGCA
GGCCATTAACCAGGGTCATGGCAACAATCCGGAGCAGCCCAAATCGTTGGGCCACATCGGACAGATGCCT
TCGGTGGATCAGCTGGAGGCCAAATGGCGTCGAAACTCACTGAACAATGTTGGCGAGACTGCCAACAAGC
AGACTGATAACTTCCAGAAGCTAATTGGGTCTCTTTCTTCGGCTAAGCCACAGTCACAGGCGGTTGGCTA
TGATGCCATCTCCAACTTTATAATgcagcaacagcagtaccagcagcagcaacagaagcagcacctcatc
atacagcagcagcagcaacaTACCGCCTTTTTGGCGAGCCTGCAGCTGAAGGCGATCCTCGGCCGGGCCG
ACACCCAATTGCTACTGCTCCGTCTGACTAAAGGGGGAAATATCCAAGCACGGATTGCTGGTGCAGTTGG
CCAACCCACGCTTGACGGACATGGATCGCGAGGCCATCACTGCTGTATTGCAGTTCACCAACACCcagca
gcagcagcagcagcacaagcagcagcTGGACATGCTCTCAAGCACGGTCATCGCCAGCCAGTTGCAGAAT
CTCCACAACTTGGCCATTGTCCAGCAGACTCTTGCAGCCAGGCAGCAGCCGCAGCATAATCCACAGACGC
AGGCGCCGCACCAACTGTCGCAGGAGGACTTGCAAGCGCATGCCAATGTGATAATGCGAAACGCCGTAAT
GAAGCGAAAAATTGAGGAGCAGACCTCGAAGCTTATCAATGGCGGTGCCAAGCATCAGGCACAGCAGCAA
TATCTAAACCGTGGCCAGCAACGCCAGGCTCGACCAGATGCCAACTCGAATGCCTTGCTCCATGCCTTGA
TCTCTGGCGGTGGCAATAACCACGCCTCTGGTTATCCTATGAATGGACAGCCGCAGAAGCATCATTCCAA
TTTGAGATTCGGTGATAACCAAAACTTCCAATCTTTTGAGTCCAACCAGCCGCACTTCGCCACGCAGTAC
AAGCAGCAATACCAACAGTCTCAGCAGCAACATCCCCATCAGCAGCCCCAACAGCTGAATTCGCTTCATC
AGAACAACGCTGGAGCTGTCAATAGCTTCAATAAGGCCCAAATGCAAGCTCAATCAGCTATTTCCATGCT
GCCCAACAGCGGGG
>dme_circ_0001654_junction_seq
GCTATTTCCATGCTGCCCAACAGCGGGGGGATCCGGCCATTGTTAACCAAAGACGC
>dme_circ_0001655
CTGATCCACCGAAGGCATCTGTCCGATGTGGCCCAACGATTTGGGCTGCTCCGGATTGTTGCCATGACCC
TGGTTAATGGCCTGCTTGAAAAACTCGTTCAGCGAAGAAGACGAGGAGTTCCCCTCATTGGCAAAGAATG
ACATGAATTTAGAGTTGTTCGTGTTTGTGTTCGActgctgctgcatttgctgaggctgcgtttgcggcgg

ctgTTGGTGATTAACATAACTGCTACTCTCG
>dme_circ_0001655_junction_seq
TTGGTGATTAACATAACTGCTACTCTCGCTGATCCACCGAAGGCATCTGTCCGATG
>dme_circ_0001656
AACCTGGAAAACGACGAGCTAAAGAGCGCTCAGTGGGCCATGGAGGAGGTGAAAGTGAAGGTGACGCTGC
CCAAGTTCCATTTCGACTACCAGCAGAACCTTAAGGAGACGTTGCGCAGCCTGGGTGTGCGCGAAATCTT
TGAGGACAGCGCCTCGCTGCCGGGATTAACCCGCGGCGCTGATGTCGCCGGCAAAG
>dme_circ_0001656_junction_seq
ACCCGCGGCGCTGATGTCGCCGGCAAAGAACCTGGAAAACGACGAGCTAAAGAGCG
>dme_circ_0001657
GCGTCACCTTCACTTTCACCTCCTCCATGGCCCACTGAGCGCTCTTTAGCTCGTCGTTTTCCAGGTTCTT
GACCAAGTCATGGATGCCATTCAGGGCGTAGGGCAGCAGGACGAACAGGGAGTTCTTGCCCTTGTAGGGC
AGACGCAGAATCTGCGCCTTCAGCTTCTCCGAGGTGGTGTAGTAGAAGTAGTCGGTCTGCTCCATGAACT
CCGCCCGCGACTGGTCGTCCTTGCTGCGGAAGAAGGATCCCTGGAAT
>dme_circ_0001657_junction_seq
CTTGCTGCGGAAGAAGGATCCCTGGAATGCGTCACCTTCACTTTCACCTCCTCCAT
>dme_circ_0001658
GTCGGTCTGCTCCATGAACTCCGCCCGCGACTGGTCGTCCTTGCTGCGGAAGAAGGATCCCTGGAATGTG
GTGGCAAACTGCCGCCGCCACAGACCGTTGAAGTAGATCAGATTGGTCAGCAACATTACGCTGCTGCGCA
CGTTGTCCGGGGCTACTAGCTGCTGAAGTCTGCCCTGCGTGATGTTGGCGGCCCAGGCGTTGATGGCATC
TGCCGCCGCCTCGGGATTGGTGAAGTCTAGCGCCTCCACCTCGCTGTCGTAGAAGTGTTTCAGCGTGGCC
GTGAACTTCTGCTGTGTCTCGATGAAGCTGTCCGTGAAGAGCTTGGTGCGCACACTGAGCGTCTCGTGGA
GCTGGTTCTCCTTCTTGAAGGAATTAA
>dme_circ_0001658_junction_seq
AGCTGGTTCTCCTTCTTGAAGGAATTAAGTCGGTCTGCTCCATGAACTCCGCCCGC
>dme_circ_0001659
ATAATATCATTGAAAAGCTCTTGTAGAATAGATAACTAAATATGTATATGTACGTCCCAATCAAATGCCC
AGAAAGTGGAGTCTATAAACGTGTAACAAAATTTGCTTGCCCCGCCTGAAATCTCCCAATTATTG
>dme_circ_0001659_junction_seq
TGCCCCGCCTGAAATCTCCCAATTATTGATAATATCATTGAAAAGCTCTTGTAGAA
>dme_circ_0001660
TTGCTTCTGCTGAACTGGAGCAGTTTATACGCAAAGGAGATGTGGTCATATCAACGCGACACATTAGGTA
ATTAACGAACGATAACACTATTTATTCTAAAACTATGAATTCCCATCAATTAGACCTCGTCGAAAGTTGC
ACATATCCATAGAAGCACTCTTCATGATCGACTTCCCGATGCTGAAGCACAAGATGTCCTTCTTCCTGGA
CCGCAAACAGCAGCGGGTAACGTTGGACATTAGTGCGAACGGAGCCACCGAGTCGCGCAACTTCGAGATA
CCCAACATCAATGAGACAAGCACCATCCGATCCCTGGCACTGCAGTTCTCCAAGAATCGCATAACGCTCC
ATGTGGACTGCAAGGCGAGCACGCATCACGACATCGACATGAACCTGGCCAAGCTGTACACCCAGATGGA
CGATCCGGTGATCAAGCTGGAGAAGAACAAGAAGCGCGATGTGCGCGGCTGGTATGAGCCAACGATCGCC
CGGGAAGGAGTCGTGGATCACAGACACCAGGAGGTGCCAACGGACGTGGAGCGCGGTGATATTCCCGTTC
TGAATGGCGATTGCGAAGG
>dme_circ_0001660_junction_seq
TTCCCGTTCTGAATGGCGATTGCGAAGGTTGCTTCTGCTGAACTGGAGCAGTTTAT
>dme_circ_0001661
CGGACGGTAAAGATTTTGGAGTGAGGCGCTCATACCCCACGGTCCATCACTCCCGCACCCCCATCCATTG

CTTCCGAAGATCACAGAAGACACCAGCAACAACGAACACGATGAATTGGACGCGCGTGCTGTTAATCGGG
TTGACCGCCTTGGCGCTGACATTTGTGGATGTTGCATCACTCTCACTCGATCCAGGGTAAGTGCATCCGA
AGCACCTTTATCGCGCCGCTCTGCATTCCAGCGAACTCTTTCCTCAGCTGCACTGGGAGAAAAGCCAAGG
TTGTATCAATAAGTCAGCTGAAGAATTCGCGTGGTTTATGAACATAAAGGTAGCAGAACTCAAACGATAT
CTATACAAAGCAAGTCAGTTTATTCGCTTTAAAATACTTAGATAGATAGATAGATTCATTGATAATTTTT
AACTTTTAACCAATTGTATATTACAAAAATATATCCAAATTGGTTCCTAAGTGTTGATCAGTGTATGTAC
ACATATGGCTTTACGTTTCCTATGGCCAGCAAAGCAACTAGCTGCAATGCCAATATATGCTAAAATCCGC
CCGAAAGCCATACTTTTGGTTAACAACTTGGCGATTTATGTAAATATTGTACTCAAATCCCAATGCAAAC
GTCGCCAGCTGACAGCCGGGTCACCAGTTTCTGAGTTGGCGACCTCCCTGCTCCGTAGATGGTTATATTA
CAGCCCGATCACGAGCCAGAGGCTTTTATGCCCGTGTCCCCGGTCCCAAAACTATGTCCATTTCCATACC
TATGCCCAAGTCCACATCCATGTCGTGAAGGTCACCTGGCATGAGCCTGTCAAAAGTCGGTCGCTGTCAG
CATGACAAATAACCGCACAAATGCGTGAAATTCTATATAGAGTCGTCTTGATGGATTCTTTGACCCACAG
TTGTTCGTTCACTTACATACACACGGATTGTGCAAATAGCGATCGTGTATTTATTTGGGTTAACACGGTC
CGCGGGTATTAAAGACAATCTCCCTGGGTGACCAAAATAAATAGTTCCATGTTCCGTATTCGAGGTGCTG
TAATCTGTGGCAGGCGAAATAACCGATTGCCAATGTTTGTGGGCAAACAGAGCAAGTGGCTAAGACGGGG
TTTACTTGAAGAGATTAACAAACATGGACCAATGATGATTTGAGCATTATCTCTGTTTATTTTGTAATCA
ATATGTGTGGTTAAGGTAGAACGGAAGTTATTCTTAACAGTTTCGAAACTGATCAACCAATGCCAAACGA
GATAGGGCACGAAAATCAGAAAGGAATGTAGAATGAATTTGATTTATTTATATGTTATCATTGCAGTACC
TCGTCGAAAGTTGCACATATCCATAGAAGCACTCTTCATGATCGACTTCCCGATGCTGAAGCACAAGATG
TCCTTCTTCCTGGACCGCAAACAGCAGCGGGTAACGTTGGACATTAGTGCGAACGGAGCCACCGAGTCGC
GCAACTTCGAGATACCCAACATCAATGAGACAAGCACCATCCGATCCCTGGCACTGCAGTTCTCCAAGAA
TCGCATAACGCTCCATGTGGACTGCAAGGCGAGCACGCATCACGACATCGACATGAACCTGGCCAAGCTG
TACACCCAGATGGACGATCCGGTGATCAAGCTGGTTCCGTGAGCGCAAGTATCCCCTTCACTTCGACGGG
GACATGGAGCACTCCCTGCAGCGGGCCAATTGCCAGAAGGGCAACCATCGTCGTGGCAATCGTCGCATGC
TGCGCAACAAGATCACCGAGCGTGGAGAAGAACAAGAAGCGCGATGTGCGCGGCTGGTATGAGCCAACGA
TCGCCCGGGAAGGAGTCGTGGATCACAGACACCAGGAGGTGCCAACGGACGTGGAGCGCGGTGATATTCC
CGTTCTGAATGGCGATTGCGAAGG
>dme_circ_0001661_junction_seq
TTCCCGTTCTGAATGGCGATTGCGAAGGCGGACGGTAAAGATTTTGGAGTGAGGCG
>dme_circ_0001662
GGCCAGATGTTCAGAGTTCAATTGACGATCGGTCGGATCATTCCTTCGTATAAATATATACAATACACTT
GAAAACCTAAGGTCGCTAGCTCATTCCatatatatatatatatatctatatCTGCATATGTGTATATCTC
TTTCAGTAAGGTAGGTGGTAGTTGGTCTCCGACGCGGTTTGCCATATCCTCGCCCAGAATACGGCTA
>dme_circ_0001662_junction_seq
TGCCATATCCTCGCCCAGAATACGGCTAGGCCAGATGTTCAGAGTTCAATTGACGA
>dme_circ_0001663
GGTGGTAGTTGGTCTCCGACGCGGTTTGCCATATCCTCGCCCAGAATACGGCTAGTATTTGCTGCTGCGA
TCACAAGTTGGCGACATGATTCCCATTCCCCGCACTTAGTCCAGGAGGATCTGGAAGGTCACGGAGCCCT
TGCGATCGCGGACACTGCCACTGTACTGAATATTCTTGGCCCAGGCACGGCACTCCACGTCCAGCATTTG
ACCCTTGGGCGGGGAGTTGAACTGCACGGCCACCAGCGGACTCAGGTAACCCGGCTGGTTCAGGAACGGG
TAGTA
>dme_circ_0001663_junction_seq
AACCCGGCTGGTTCAGGAACGGGTAGTAGGTGGTAGTTGGTCTCCGACGCGGTTTG
>dme_circ_0001664

GAATACGGCTAGTATTTGCTGCTGCGATCACAAGTTGGCGACATGATTCCCATTCCCCGCACTTAGTCCA
GGAGGATCTGGAAGGTCACGGAGCCCTTGCGATCGCGGACACTGCCACTGTACTGAATATTCTTGGCCCA
GGCACGGCACTCCACGTCCAGCATTTGACCCTTGGGCGGGGAGTTGAACTGCACGGCCACCAGCGGACTC
AGGTAACCCGGCTGGTTCAGGAACGGGTAGTAGTAGCTTGGGAACCCTTGGCTGGGGAAGTAGCGGATGT
TCTGGAAGTTCTCCTTGTCCTTGCCCAAGTGACCGTTGCAGCTCACCCAGACTTGCTGCCGCTGGAGTAC
AGGAAGGCAACTTGAGTACAGATCCAGATACAAAACATACATTGTACTTACCTCCTCCGTTTTCGTCTCA
TTGATCACCTTTTTCAGATCATCAGGCATATCCTTCTCCTCCTTATCGTACACTTCCGGAATCCAGCCAA
AGATCTTATTCAGCTTCAAGAATATGCAGGGCTGGTTCGTCTTGTAGCCATA
>dme_circ_0001664_junction_seq
TGCAGGGCTGGTTCGTCTTGTAGCCATAGAATACGGCTAGTATTTGCTGCTGCGAT
>dme_circ_0001665
GAGAACTTCCAGAACATCCGCTACTTCCCCAGCCAAGGGTTCCCAAGCTACTACTACCCGTTCCTGAACC
AGCCGGGTTACCTGAGTCCGCTGGTGGCCGTGCAGTTCAACTCCCCGCCCAAGGGTCAAATGCTGGACGT
GGAGTGCCGTGCCTGGGCCAAGAATATTCAGTACAGTGGCAGTGTCCGCGATCGCAAGGGCTCCG
>dme_circ_0001665_junction_seq
GGCAGTGTCCGCGATCGCAAGGGCTCCGGAGAACTTCCAGAACATCCGCTACTTCC
>dme_circ_0001666
GTAGTAGCTTGGGAACCCTTGGCTGGGGAAGTAGCGGATGTTCTGGAAGTTCTCCTTGTCCTTGCCCAAG
TGACCGTTGCAGCTCACCCAGACTTGCTGCCGCTGGAGTACAGGAAGGCAACTTGAGTACAGATCCAGAT
ACAAAACATACATTGTACTTACCTCCTCCGTTTTCGTCTCATTGATCACCTTTTTCAGATCATCAGGCAT
ATCCTTCTCCTCCTTATC
>dme_circ_0001666_junction_seq
CATCAGGCATATCCTTCTCCTCCTTATCGTAGTAGCTTGGGAACCCTTGGCTGGGG
>dme_circ_0001667
GATCATTTCCGAGAAGGTTTGCTTCTTCGCTGGCTGCGGGAATTCGAATTCGCCTTTGGCGCCCTTTCCG
TTATTCTTTGACATTGCGGCGCTGTTCTTTCGCACTTGTTTACGGTTAGCTTTTCA
>dme_circ_0001667_junction_seq
TTCGCACTTGTTTACGGTTAGCTTTTCAGATCATTTCCGAGAAGGTTTGCTTCTTC
>dme_circ_0001668
GATACACGGCGCACTCTTGTGGTAGCTGTAGTTATTCTCCTTGGTGCACGGCGACCACGTCTTGATGTCC
ACGTCGCACACCTGACCCTTTGGCGGCGGCTGGTTGTAGTCGCAGTTGTAGATGTTCTGACCACGGCCGG
GTGTTAGACCAGGTACTTT
>dme_circ_0001668_junction_seq
CACGGCCGGGTGTTAGACCAGGTACTTTGATACACGGCGCACTCTTGTGGTAGCTG
>dme_circ_0001669
TAATCCAATAGACGGATGCACGTCTGAATTGCGACCGTCGGATCTTCGAACATCAACGTCAGTTCACGGG
CAAAGTCCAGCCGCGACTTGTCTGTGGACACTTTTTGTGGAACTTGCTCCAGAAGCACTTGAGACTCAAA
GATAATGCACAAGAACTGCCAAAGGTGCTCCTTGGGCTCCAGAACTCGGAACAGGG
>dme_circ_0001669_junction_seq
TCCTTGGGCTCCAGAACTCGGAACAGGGTAATCCAATAGACGGATGCACGTCTGAA
>dme_circ_0001670
GGGCAGTTCCAACAGTGCGCAGCAGGCGAAGCTTCAACAGACTGCGTTGCATGCCCTACAGTTGTTGGCC
CTCCGTCACGGACGCGACTACATCGAGGAGTGTCGCTCTCTTCTGGCCACCTTGACCAAGATCACCAAAC
GACGTGCGAACGTGCCCAAGGCGGTAGTGGGCAATGTGGTGCTGACCCTCGTCGAAATTTGCGCAAGTCT

TAAGGCCCATGCCTTGGCCCAATTGCCCAAGTTCGCTCCCCAACTTACTGAGTTACTGAAGGAACAG
>dme_circ_0001670_junction_seq
CCAACTTACTGAGTTACTGAAGGAACAGGGGCAGTTCCAACAGTGCGCAGCAGGCG
>dme_circ_0001671
TTTCTAGCTCCTGCACTCCAGTCCGGGTGTTCTTCTCCACACGTTGATGCTCGTCCTCCAGGAGCTCAGC
GATGAATGGAACTGTCTCTGGCAACAGGGCGGCATAGCTTTCACCCAGTTTGCGCGCGATAGCCACACAA
CTGTTGAATGCCAGAATCCGCACTTCGGGATTGGATGTGCGTGTCTTTAGGAGCACTTGGCTGTTGAGCT
GCTTCCACATCACATCGTTGGTGGCCACCGCAAACTGGGCAATGCAGTTACTGAGAACTTGCTGAAGCGA
CTCATTTCCCAATACCAGG
>dme_circ_0001671_junction_seq
CTGAAGCGACTCATTTCCCAATACCAGGTTTCTAGCTCCTGCACTCCAGTCCGGGT
>dme_circ_0001672
GATGCATGGTGGGATCGTCTGCGCGTAGACCCGCCGTTTGACCGAGCACCTTGATGTTCTTGATTCCGAG
GACGATCCCGTCGAGCCCCGAGTCATAGAATCCCACCTTGGTACGGATAAGGAGCTCGTGGAGCGCGTGC
TTGAAATTGGCCATGCCGTACGGGCCCATTGCTAG
>dme_circ_0001672_junction_seq
TGGCCATGCCGTACGGGCCCATTGCTAGGATGCATGGTGGGATCGTCTGCGCGTAG
>dme_circ_0001673
GTTTTTAGTTGTCCAAAACCATATTAGTTAAGGGTTTTAATTTATAGTGGAACTCGAGTTTGCATATTTA
GGTATTCTAATGTTCCAACACAACAACAAATACAAGCACACGCAGAACAAAGGAGAGCGCTTCGGAAAAT
CATTTGACGGAAGGAAATTTAAGATATAGAAATGTATCGGCAGCCGCAGATTAACGATCTAGTCAGGATT
GCTATG
>dme_circ_0001673_junction_seq
GATTAACGATCTAGTCAGGATTGCTATGGTTTTTAGTTGTCCAAAACCATATTAGT
>dme_circ_0001674
GATGTGCTGAACATTCAGTGTCTGTGCGACGAGATCATATCGATCAACGATTACCGCACCCACTTGTACG
ACTACTTGAAGGCCAGAATGATGGCCATGGCTCCGAATTTGACAGTGCTCGTGGGCGACACCGTCGGCGC
TCGACTGATTGCCCATGCTGGCTCGCTGATTAACCTGGCCAAGCATCCCTCATCTACTGTACAAATTCTG
GGCGCCGAGAAAGCACTCTTCCGTGCGCTGAAGACCAAGAAGGATACTCCAAAGTACGGTTTGATCTATC
ACGCCCAGTTGGTGGGACAGGCAAGCCAGAAGAACAAGGGCAAAATGTCGCGTTCACTGGCCGCCAAAGC
GTCGCTTGCCACGCGTGTTGATGCCTTTGGCGAAGAGGCTACCTTTGAGCTAGGAGCGGCTCACAAGGTT
AAACTGGAGTCTCGGCTGCGACTCCTGGAGGAGGGCAACCTGCGCAAACTCTCCGGCACCGGCAAGGCCA
AGGCCAAGTTCGAGAAGTACCAGGCCAAGAGGTATGTTTTAGCTGTCAATTATATAAGAGTAAACAGATT
TCCAGTGATGATACCCAAAAGGTTTCGCATGTATCCGATTAAGATGACGATAAACCTTGGCTGTCTGAGC
GAAATTGGGTTTACAATCAGGCTATCTCTCGATTGTTCAGTGTATTTAATTTATTTCTTAACTTTTTAGC
GAGGTGTTCACCTACCAACCAGAGGCCGACAACACCTTGAACGTGAAGAAGAAGCGCAAGCACTCCGAGT
CCGAACAGCAGACGCCTGTTAAGAAGGAGGAACCTGCTGAGGAGGAGGCGGAGGTCAAGTCCGagaagaa
gaagaaaaagaagaagaagcagaaggacgaagaGG
>dme_circ_0001674_junction_seq
aagaagaagaagcagaaggacgaagaGGGATGTGCTGAACATTCAGTGTCTGTGCG
>dme_circ_0001675
GATTACGTCAATTATTGAGAACCTACCCGGCGCCATAGCTCACAACCACAACCAAAACAACATCATGGAG
GAAGACGAGAGGGGCAAACTTTTTGTGGGCGGTCTATCCTGGGAGACGACGCAGGAGAACCTGTCGCGCT
ACTTCTGCCGCTTCGGGGACATCATTGACTGTGTGGTGATGAAGAACAACGAGAGCGGCAGGTCGCGCGG

CTTTGGCTTCGTTACCTTCGCCGATCCCACCAACGTCAACCACGTGCTGCAGAACGGACCGCACACGCTC
GACGGTCGCACCATCGACCCCAAGCCGTGCAATCCGCGCACTCTGCAGAAACCGAAGAAGGGCGGCGGCT
ACAAGGTCTTCCTGGGTGGCCTGCCCTCGAACGTCACCGAGACCGATCTGCGGACCTTCTTTAACCGCTA
CGGCAAGGTCACCGAGGTGGTTATCATGTACGACCAGGAGAAGAAGAAGTCCCGCGGCTTCGGCTTTCTC
TCCTTCGAGGAGGAGTCCTCCGTTGAGCACGTGACCAACGAGCGGTACATCAATCTGAATGGCAAGCAGG
GTATGTGATCGCTGGAAAGTTGTGCTATACTTACAAAACATAATTACCTATTTCTGCGAAGCATCTAGTC
TTGCAAAACTATAAATTGTGAATATGGAATATTCATAATTTTTTTCGATTTACTAAACTTTGTTTCATAT
AATACAAGAGTGCTATTTGTAACAAAACATATTGGAAATAATTTTTACTTAAAAACGCTCAAAAGAATAC
ACTAACAATTAATTGTACAAAGAATATAAGTTAACATTTTTGGCAACAGCACTAATAATCATTATTATCC
CCAGGGTCGAAATCAAGAAGGCCGAGCCTCGTGATGGATCTGGCGGCCAAAACTCCAACAACAGTACCGT
GGGAGGCGCCTATGGCAAGCTTGGTAACGAGTGCAGCCACTGGGGACCGCACCATGCTCCCATCAACATG
ATGCAGGGCCAGAATGGCCAGATGGGTGGACCGCCGCTGAATATGCCCATTGGAGCGCCGAATATGATGC
CTGGCTATCAGGGTTGGGGCACCTCGCCGCAGCAGCAACAATACGGCTACGGCAACAGTGGCCCAGGATC
GTACCAGGGATGGGGAGCTCCACCAGGACCCCAGGGACCACCACCGCAGTGGTCGAACTACGCTGGACCT
CAGCAGACGCAGGGCTACGGCGGATACGACATGTATAACTCGACGTCGACCGGAGCTCCTTCGGGACCAT
CGGGCGGCGGCAGCTGGAACTCGTGGAACATGCCACCTAACTCTGCCGGACCCACTGGGGCACCAGGAGC
CGGAGCGGGCACCGCCACTGACATGTACTCGCGTGCTCAAGCCTGGGCGACGGGCGGTCCCTCGACCACT
GGACCAGTGGGCGGCATGCCCCGGACCGGACCCGGTAATTCGGCCTCCAAGTCTGGCTCTGAGTACGACT
ACGGCGGCTATGGATCCGGGTACGACTACGACTACAGCAACTATGTGAAGCAGGAGGGCGCCTCGAACTA
CGGAGCAGGGCCGCGATCAGCGTACGGCAACGACAGCTCCACGCAGCCACCCTATGCAACCTCGCAGGCT
GTCTAAAGAGAGAAGGATCGTGTGGAGGAGAAGTAGGAGTAGCGGAGCAGAGAGGAGCGGAGTAGCATGT
CGTCCTCGGCGAATGATGAAGAAGACAGAAAAGATGGTG
>dme_circ_0001675_junction_seq
AATGATGAAGAAGACAGAAAAGATGGTGGATTACGTCAATTATTGAGAACCTACCC
>dme_circ_0001676
CCGCGGGACTTCTTCTTCTCCTGGTCGTACATGATAACCACCTCGGTGACCTTGCCGTAGCGGTTAAAGA
AGGTCCGCAGATCGGTCTCGGTGACGTTCGAGGGCAGGCCACCCAGGAAGACCTTGTAGCCGCCGCCCTT
CTTCGGTTTCTGCAGAG
>dme_circ_0001676_junction_seq
CCGCCGCCCTTCTTCGGTTTCTGCAGAGCCGCGGGACTTCTTCTTCTCCTGGTCGT
>dme_circ_0001677
GTGGCATGTTCCACGAGTTCCAGCTGCCGCCGCCCGATGGTCCCGAAGGAGCTCCGGTCGACGTCGAGTT
ATACATGTCGTATCCGCCGTAGCCCTGCGTCTGCTGAGGTCCAGCGTAGTTCGACCACTGCGGTGGTGGT
CCCTGGGGTCCTGG
>dme_circ_0001677_junction_seq
ACTGCGGTGGTGGTCCCTGGGGTCCTGGGTGGCATGTTCCACGAGTTCCAGCTGCC
>dme_circ_0001678
TTTAACTTTTAGCTAGTTTTTGTTTTGTTTACTTTAAATGGTAAATTTTTAATTTTGTAACGTATCTTTC
CCAAGTCAGTACCGCCCTATTTCGCCACCACACCACTCCCATCCAACGACCTATTGCCGATAAAACCAAG
TAAAACATTTGCCGATTGGGTTCAACCATTGTCTGAAAACAAAACTAACTACTACTAACCTCCCATATCT
GTGAACCGCGCGGCCGGTCAATGAGTCATAAAAGCTAAGGCAAGCAAAGTTCTCAAAATCGACCGATTCG
ATTCGACTCGATTCCTTTTCAAACCCTTACTAAACCTTGTCGCCTGATTCAGAAAGCGGGTGGTGGCGAA
AGTTGATCGGCTAACGACGTGCTTTTGCTTTAAAATGTACAATTTCGAATTTGTATTTACTAATTTTAGT
AATTAATGGGGATCTTTACTTGTTTACTCTTTAAATAAAATTATAAGTTCTTATGTTCTTTGTGTTGGTA

TTTCGCGCTCTATTCTTGACCTTCTTGTGTCTAATTAGCTAAACTTTATATTTGTAAATCATTACGTTTA
TATCTTCACATTTCTATTTTGTTTCTTG
>dme_circ_0001678_junction_seq
TATCTTCACATTTCTATTTTGTTTCTTGTTTAACTTTTAGCTAGTTTTTGTTTTGT
>dme_circ_0001679
GAAACAAAATAGAAATGTGAAGATATAAACGTAATGATTTACAAATATAAAGTTTAGCTAATTAGACACA
AGAAGGTCAAGAATAGAGCGCGAAATACCAACACAAAGAACATAAGAACTTATAATTTTATTTAAAGAGT
AAACAAGTAAAGATCCCCATTAATTACTAAAATTAGTAAATACAAATTCGAAATTGTACATTTTAAAGCA
AAAGCACGTCGTTAGCCGATCAACTTTCGCCACCACCCGCTTTCTGAATCAGGCGACAAGGTTTAGTAAG
GGTTTGAAAAGGAATCGAGTCGAATCGAATCGGTCGATTTTGAGAACTTTGCTTGCCTTAGCTTTTATGA
CTCATTGACCGGCCGCGCGGTTCACAGATATGGGAGGTTAGTAGTAGTTAGTTTTGTTTTCAGACAATGG
TTGAACCCAATCGGCAAATGTTTTACTTG
>dme_circ_0001679_junction_seq
TGAACCCAATCGGCAAATGTTTTACTTGGAAACAAAATAGAAATGTGAAGATATAA
>dme_circ_0001680
TTTAACTTTTAGCTAGTTTTTGTTTTGTTTACTTTAAATGGTAAATTTTTAATTTTGTAACGTATCTTTC
CCAAGTCAGTACCGCCCTATTTCGCCACCACACCACTCCCATCCAACGACCTATTGCCGATAAAACCAAG
TAAAACATTTGCCGATTGGGTTCAACCATTGTCTGAAAACAAAACTAACTACTACTAACCTCCCATATCT
GTGAACCGCGCGGCCGGTCAATGAGTCATAAAAGCTAAGGCAAGCAAAGTTCTCAAAATCGACCGATTCG
ATTCGACTCGATTCCTTTTCAAACCCTTACTAAACCTTGTCGCCTGATTCAGAAAGCGGGTGGTGGCGAA
AGTTGATCGGCTAACGACGTGCTTTTGCTTTAAAATGTACAATTTCGAATTTGTATTTACTAATTTTAGT
AATTAATGGGGATCTTTACTTG
>dme_circ_0001680_junction_seq
TTTAGTAATTAATGGGGATCTTTACTTGTTTAACTTTTAGCTAGTTTTTGTTTTGT
>dme_circ_0001681
taattaaagatatatgtataTATGTTATGTTCATAATCAATTAAAtttcttctcttcctttcctttcctt
tccttttcatctcttctcctttctttttctctactcattctcttcctttaccctcctctttGCTCTCTGC
GCTTCGCTCCTGCTGTTCTTGTTGTTGGTACTAAATGGGGTTAAGTGTGAGTGGGTGTGCGTGTTGATGG
>dme_circ_0001681_junction_seq
AAGTGTGAGTGGGTGTGCGTGTTGATGGtaattaaagatatatgtataTATGTTAT
>dme_circ_0001682
CATATAATATGTATGtttctcattttgttttttgttgtttttccatcattctttttgTAATAATAAATTG
TAAAATCGCAATCATTAATCATCATTAAAATAGTTTACGTTCATCTTGTTTTACACACAATGCACGCTTG
TTTAGTGAGCGTGCAGagtagttaagagtaaatatgtataagtaattaaagatatatgtataTATGTTAT
G
>dme_circ_0001682_junction_seq
aattaaagatatatgtataTATGTTATGCATATAATATGTATGtttctcattttgt
>dme_circ_0001683
GCTTATGTCAGCAACGAACTGTCGAATATCAATTCTGTGCTTTTTTCGCAGTTCGAATCAAATCCGGCCG
ACGCTATTAATGAAGCGCTTATCTCACTCCTTAAGCAGCAACAACAAGAAACAAAAGACCAGCGAAAAAC
TCGTCAGAGTTGTATTGGCTCTGACAGTGCAGTTAATTTAGAAGACATTGTACTGATCGATCCCCAACCG
CCTCTTGCAGTCGATCCGCATAATGTGAAGTACCTTGAGGATGACTACGTCTCTCCGCAGTCCCAGCCCG
GATCCCAATACAATTTTAAAAGGCGCAAAACAGACATTCGGTCATCAGAGCCGTCTGAAGTCGTTGTGCT
CGATAACAGCGGATCCCCAAATGAAAAGAATTCAAAAGAAGTATTTCCAAGTACGAAACAGACTTTATCC

AGAGAAACAAAGAAAAGTGCG
>dme_circ_0001683_junction_seq
TTTATCCAGAGAAACAAAGAAAAGTGCGGCTTATGTCAGCAACGAACTGTCGAATA
>dme_circ_0001684
GTGACTTGGAGCACTTGGACGATGGCGATGATAGCTTCAGTGCCTTTGAGCGCATGTGCGACAAAACCGG
TTCCGATCCGGATAGCACTCTTTTCCAGTACCTGCACAGCGAGGACCGGAGTCAGG
>dme_circ_0001684_junction_seq
TACCTGCACAGCGAGGACCGGAGTCAGGGTGACTTGGAGCACTTGGACGATGGCGA
>dme_circ_0001685
GGTGCCCCATAGCAATGTAGTTCCAGATCGGTGTCCATTTCGCTCGTAGATCTTGGTCACGCCAATGTTG
TCGCTGAAGTGCGGCTCATCCCAGGAGACCACCGCGGAACCGTTCTTGGCAATCACCCACAGATCTCCGG
GGCA
>dme_circ_0001685_junction_seq
TGGCAATCACCCACAGATCTCCGGGGCAGGTGCCCCATAGCAATGTAGTTCCAGAT
>dme_circ_0001686
CTGGCCTACTCGGATTCCGGATTCCAGGGCACAATAACCAAGGCTCAAGTGTGGGCCAGAGCCCTAGACA
TCACGTCAGAGATCCAGAAGCAGGTGCGCGACTGCCGTTCTGAACCGGTTCTCTATCCCGGCCTCATCCT
CAACTGGGCGGGATACGAGG
>dme_circ_0001686_junction_seq
CTCATCCTCAACTGGGCGGGATACGAGGCTGGCCTACTCGGATTCCGGATTCCAGG
>dme_circ_0001687
GGGTGAAGAAGATGCCGCGATCGTCCTTCTGGGCAAACTGCACCCACATGGCGACAGTCAGGCTGTTCGC
CTCTCCCGTGGGGAAGGGCACCACCTGGGCGGCGGTGGATCGCGATGGATCGCTGAA
>dme_circ_0001687_junction_seq
CGGCGGTGGATCGCGATGGATCGCTGAAGGGTGAAGAAGATGCCGCGATCGTCCTT
>dme_circ_0001688
GGATCACCGATGCAGCGTTCCGGAGCGGTCTCGCAGTTCTTGCCATCGGTGCCGCTGGGACACACGCAGA
AGTAGTCCTGGAACAGGTCGATGCAGCTGGCATCGTTCTGGCACGGCGCCTGAGCTCCGCACGTGGTCAC
CTGCTGCTCGCAGCGCATGCCACTCCATCCTGGCTCGCAGCCGCACTCAAAGCCACCGACGCGATCCCGA
CAGATGCCGTTGTTCAGACACGGCTGCGCCTCGCAGTCATCGATGTCCGTGGCGCACAGGG
>dme_circ_0001688_junction_seq
CAGTCATCGATGTCCGTGGCGCACAGGGGGATCACCGATGCAGCGTTCCGGAGCGG
>dme_circ_0001689
ATGTGAGGTTGCAGTTCAGCCCCGAGAGTGCCAACATCAAGATCGGAAACTTCGAGAACGTGACCGTCAC
GGCAACGGATAAGTACAACAACCGCGCCGCCTGCCACTTCCAGGTCTCTGTGAAGGCTTCACCCTGCGTG
GACTGGGAGCTCCAGCCGCCGGCGAATGGTGCCATCAATTGCCTGCCTGGTGATCGTGGTATCGAATGCA
TTGCCACGTGCAAGCCAGGATTCCG
>dme_circ_0001689_junction_seq
GCATTGCCACGTGCAAGCCAGGATTCCGATGTGAGGTTGCAGTTCAGCCCCGAGAG
>dme_circ_0001690
GGTTTAAGATGGAGAGCGTGGGCTCCAGGATGCGGAATTCGACGGCAGTGCCGTCGGGATTGGCATAATC
CTTGAACACATCCGTCAGATCGTTGGTGTACTGCAGACGCACCGCCGAGCTGAAGGCCACATTGCCATCG
GGACGCTGCACGGACATGGTGCGGAAGCCACGCAGAATGGTGGGTGCCTTCAGGTCGATGAGTATCCAGT
TGGCACCAGGCTCCGAAGAGCCACCGCACCAGCCATTCGTGTTCAAGCGCAGCTGCTCCTTGCTGTAGCC

CGGCTCACTGACCGAGGTGGTGATGCTGTCATCCGGTATGCCTCCATTACCCAAGCCCAAATCACCGACG
GGCCGACACTCGGTGGTAGCCGTGAATCCCGTCCTGCACTGGCAGTGGAAGGAGCCGTTCGTGTTCTGGC
ACTCGGTGGTGGTTAGGTCGCACTGCGAGGAGCTGCACTCGTTGATGTCCTCGCACGTCGGCGGCTGCTC
GAACTTCTGGGCCTCGCTGCAGCGCATGATGTTGCTGCCGATCAGTTTGTAGCCCTTGAAGCACTGCACC
CTGGCCTCATCGCCGAAGAGGTAGGCTCGCGTCGAGTCCACCACAAAGCCGTTGGCAATGGTCGGGAATT
CGAAGCACCGCTTGCGCGTGCATCTTG
>dme_circ_0001690_junction_seq
TCGAAGCACCGCTTGCGCGTGCATCTTGGGTTTAAGATGGAGAGCGTGGGCTCCAG
>dme_circ_0001691
GTTGAGCAACTAACCACCAGACGCGGCACATAGGAGGAGTTCCCAACCCAAAACGGTGTCCACGCCGCGA
ATCCCCAAAATCAAAGAGTAACAATTCCAAGACAATACTGCTAACCAGAAGCGATCACCATTTAATGCTA
TCCGCAACTTTGGGGGCCAATGGCGGCTCTCAGCCGCTCAGTCCCTCCGCCCAGGCGACGCTCAGCAAGC
ACCTGCCGCGCCTCCAGTCGAAGCGTCTTCTCCAGCAGACGCAGTTGCAGTCGCCGGCTGCCGCCAGACC
ACAGACCTGCGATGCCAGCTGCCTGACGGGGTGAGCAAATCATTTCGATCTCAACTACAGTCCAACTATC
ACAAGCTAAGCTTATCCATCCATTCATCGTTTTTCTGTGGTAAATCTTAGCATGGTAGTTGAATTGCAGG
TGCACTCTAAACAAAATTTCACACAAACGTGCTAATATATTATGTTGTAATATTCACAGGAGCTGTGCAG
CAGCGAGAATTTGCCCGAGGTGGAGATTTTCTCACTGCTGGAGGAGCAGATACCCAAGTACAAGGTGCGC
AACGATTTCCTCACAAACTTCTCGGGCTATGCGAATGAGGACTGGTTCGTTCCGGCACCAGCGCTGCCCA
TTCCGCCGGAGGGTCTGGGCTTGACCAAGGAGCAGACAAGAGAGTGCCTAAACTATTTCC
>dme_circ_0001691_junction_seq
CAGACAAGAGAGTGCCTAAACTATTTCCGTTGAGCAACTAACCACCAGACGCGGCA
>dme_circ_0001692
GTTAAAACTCTGTCAAGCGAGAGTCTCAACTCCCAGTCGGATGACGGTTATCCAGCCCAGCCCTCTGGTG
TGCCAGGAGCGCCCGGCGCCAAGGAGCTGGAGGCGGCTCTCAAGAGGCTGACGCCGGCTGAGGTCTTGGC
TCGCCGTGCTATGTT
>dme_circ_0001692_junction_seq
CTGAGGTCTTGGCTCGCCGTGCTATGTTGTTAAAACTCTGTCAAGCGAGAGTCTCA
>dme_circ_0001693
GCTAAAGAACGGAATGGCCAACCAGAGGCCAGATCTGGGCACAATTTCTGGCGCCGGAGGAGGCGGACGA
GTGCGCCCGGATCTGGGAAAGGTAGCCGACAGCGGCAGCAGCAGCAAGCTCAGCACCAAGCGAAGTGAGG
CTAAGCCTGCGGAGGAGGAAGAGGCGACACCACAGACCATCACACAGGCGTTTGTGGGTTCAGTTAGTTC
CTTGCTTTTTGGCCGCAAGGGCGGTTGGCT
>dme_circ_0001693_junction_seq
TGCTTTTTGGCCGCAAGGGCGGTTGGCTGCTAAAGAACGGAATGGCCAACCAGAGG
>dme_circ_0001694
GTTTAATTTTTCTGTTTAATACCCCGCATAATGCCTGTCATCGCCACCTTAAGCAACACTGGAAAGAAGG
TTTTAGTGTTTGTAGCTGTGATTTGTAAATACATATATAGAGAAATGTTTAAATTTAGTAGTTATTAATC
GAGTACTTCGCATATTATTATTATTATTTTTGCTTCAACCGACGACGGCAATATAAGAACAAAACGATGG
CCAGCTCGATAGAGAAACCGAGTTCCAAAGCAGCTATTTTTAATTTAAACTACATTTTGCCAATTTTGTA
TTTATTTTCCCTGTGTTTATTACCACTTAAACGAAAACGAAATCGAGTATACAtttttttttATTGATTT
CGTTGT
>dme_circ_0001694_junction_seq
ATACAtttttttttATTGATTTCGTTGTGTTTAATTTTTCTGTTTAATACCCCGCA
>dme_circ_0001695

gaaaacaaaaacaaaaCGCGCAGCCGAAAATCGGCAACAACAAAAGCGAGTGAGAGAAATGTGCGAGAAA
TTCACCTGTCCCCGTGGGCCGATTAATTGATGACGTGGAATCGGACGAAGGACGCAGGGCCACAGGATAT
ATACATATTTTCCAGTGCACatgcacatgcatgctgcacacgcacacacacatgcaaacacacacaGTAG
CGCCCATGCAGAAAACCAGTTGTTTGGCCgttgctgttgttgttgctgttactgctgctgctgcaaatga
gaagtgcAAGTGAAGCAGCAACAAAAATCCTGCTCTTAAA
>dme_circ_0001695_junction_seq
AAGCAGCAACAAAAATCCTGCTCTTAAAgaaaacaaaaacaaaaCGCGCAGCCGAA
>dme_circ_0001696
CTTCCTTTGCTTcctgttccgccctcgccagttccgccttcgcctgctccgcctCCAGATCCTCGGGCAA
CTTGATGACCACCTGGGACCTAATGTCGTAATCGAACTTGGTGATATTATTCACCAGTGGTTTGGCCCAA
CCAATGTGTAGAATGGGAGTCAAGCTATCCTCCTCCCATTGGCAGATGCCGTCGTAGAATCGCAGCAAAT
TGGCGAAAACCTCAGAATCTCGTAGAATACTTCTCGCCGAATGACCAGAACTTTCTGTTTTCATCACGTA
CGGTATGAGCTCGTTGGCAATGTCCAATAGCTCCTTGTAGATCTCCTCGTCATCACGACACTGATACGTA
TACAAACGAATAACGTCCGCCGCATTTGCCCATGCGGCAAAGGCGTCGCGGTATTTGAGCAATCGATTGT
AGTAGTTCCCCTGGTAGGTATACGGATATACG
>dme_circ_0001696_junction_seq
GTTCCCCTGGTAGGTATACGGATATACGCTTCCTTTGCTTcctgttccgccctcgc
>dme_circ_0001697
AAACGGGCAACTGGACAGAGGTGCCGCTGCCATTGCTTGTGGTTGTTGCGTTAGTTGTGGTCGCTGCGGC
CTCCTTTTTGTCCGCAttgtggttgttgttgttgttattgctgttgctgttattgttgttgttgtgGAGT
TCTGGATTATCGGATTCGCCGCCAGAAGGCTGTTGCTTCTGATCGGCGAACGGCTGACCGCCGCCCTGGA
GCAGGAAATCCGGATTGAGAATCTTCTCGCCACAGGCAGCAGTTAAATCGGCCAAGGTAGTCGGCAGCTC
CGAGTTCTTGGATTTCTGGCAaatataataataataataatatgaataaCTTGTAACGAGCACGAAACCC
ACAATCGTAGTAGGCTGTGACCCTGTTTTCTTTCTTCGGGGAATTCCATTTAGAATGTACCTCAGTCAAC
ACAGAATCGGTAAGAATAGCTGAACTTTAAAAGTAGACTTAACTATAAATTAAAATTTTTGTTTCCTTTT
AAGACCTGTACTCCTTACCCAAGTTAATATAACTGGAGTCAACAGACTTACATAAAGTACCATGGCACTT
TCCGCAACGAAGGACACTAGTCCATGGACCAACATGGTGAAGAGGGTAACAGCGAGCAAATACGATGTAG
TCTCGGTCAAGACGCTGCTGATGTTTATCAACAGCAAACTGGATTGCGAATTGCGAGGCTTTGAGGATCT
GAAAACAGGAGCAGTATATTGCCAACTGATGCACAGACTCTTTCCCAATTCGATACAAATTCATAAGGTC
AAGTTTTATACGAACGACATCAGCGACTTTCAGTTAAATTTCCGGTTACTAAACAATTGCTTTCAAAAAC
TAAGAGTAACCGTTTATATGCCAGTGCATGAGTTAACTCTGGGACACAATCAAGTGGTCTTCTGCAATTG
GATATACAAGTTCTATGAAGCAAACGACAAGGGGAACGAATACGATGCCAGAAAGGTGAGAAAAGGCTCG
CCAATCGGTTTAGACAATAGTTATAAGGTAGCCTCCATCTCAACTGGCAGCACCTGTTCAGTGCATAAAT
GCCAGTCGATGGTCTTTAATTATGCAAAAAAGCCCGTCCGATTCGAGAGGCAAAACAGCCTCGATGCTCT
ATCATTCAGACCGGGCATCTTTAAGAGTATTCGTAGGGAGAAGCCAACCGAACAGGCGAATCCAGAACCA
CAGCAGACAAAGAATATCAAAAAGTCCTCACAATTCCTTGCCGAGAGCAAGCACGTTTCTCTGCCATCGG
ATAGCGAAGATGAGTTGGAACTTAAAGCCCAAAAACGTTATTTGCAAGATCAGTTTGTGAAAAAGCTCAA
TAGCAGTAAGAATGATGAGAAGACTGGCACAGACGCAACGTCAAAAATCTCGGAAATTCCAAGACCAAAT
CCAGAAAACGAGCAACTAAACACTGTATGCGAAAAGTATGAGCAGGAAACGAGTGATCTGCGATCCAATC
TTAATTTGATCGACAAACTAGAATTACAGCTACAAAACTTACAGATTGATAAGGAAAAGCTGACCAACAA
GTTGTCACGCGTGGAGTCAATATTAAACAAACACGATTTAAATCCAGAAAAAGCTGTTCTAAAATTAAGA
AAATTGCTATTGAACCAAGCACAAACGGCAAGAGTGCATCCGCGTAAAGCGGATACACTTATTGATGAAG
ATGAATTAACCAAGAAAGAAGAATTCAAGTCTTGCACGGAATTTTCTCGTCAGGAAGACGTTAAGTTGAC
GCCAACTCAAATAAAAAATCAAAAATCAAATACTAGGTGTTCGCAAACTAAGGTCAGAAAGAGCCTAAAG

GTTTGATGCAAATGTTGCGTCCAATTTAGAATCCACGGAATATACAAACTACGTGAATGTAGGCATCTAA
AGAAGAATTACTCCATCGGATACGATTTTGATTTTCATACATAACACCTTATGaaaaaaaaaTGTCTGCA
AAATTTCGTTCCAAATGAATTTCAAGAATTATATATTTTCTATTTTCAACTGGACTGAAGTGTTAAATAA
AATTTATAATGTGAAGTAATGAGTATGCTTACCTCCTCCTTTGTGGCCATCCTATTGTTATTGCCTTCCA
TTGCCTCTGATCCTGCAGCTTCCTTCGACTCCTTAGCTTCCTTTGCTTcctgttccgccctcgccagttc
cgccttcgcctgctccgcctCCAGATCCTCGGGCAACTTGATGACCACCTGGGACCTAATGTCGTAATCG
AACTTGGTGATATTATTCACCAGTGGTTTGGCCCAACCAATGTGTAGAATGGGAGTCAAGCTATCCTCCT
CCCATTGGCAGATGCCGTCGTAGAATCGCAGCAAATTGGCGAAAACCTCAGAATCTCGTAGAATACTTCT
CGCCGAATGACCAGAACTTTCTGTTTTCATCACGTACGG
>dme_circ_0001697_junction_seq
CAGAACTTTCTGTTTTCATCACGTACGGAAACGGGCAACTGGACAGAGGTGCCGCT
>dme_circ_0001698
GATGTGGAGAACATTTACGCACGCAATTATGTTGCGGCGAAAGTTGGCCAGAAGAAGGAGGACCAGTTGG
GAACCCCCGAAGCGGTGATCAAAGCGAGACGCCTCCATGCTCAGAAGCAGAAGAGTGAACGAGGTAATCC
AATCCGGTGGGACTGCCTGTGGTGAAATCTATTTAACTTAATATTTCATCCATTTACAGCTCTTGCCCAC
GCCCATATGAACCAGGTGCTGAAGGAGGCCACCTGCCGCATTCCCCAGAAACGTTGTCAGCTGGTCCAGC
AGGATCCTTCCAAGATCTATACGCCTCACTGCACCATCCTGCATCGCTGCAGCGAGGACAGTGGTTGTTG
CCCCAGCAGAAGCCAAATTTGTGCCGCCAAGAGCACCCACAACGTTGAGCTGCACTTCTTCG
>dme_circ_0001698_junction_seq
ACCCACAACGTTGAGCTGCACTTCTTCGGATGTGGAGAACATTTACGCACGCAATT
>dme_circ_0001699
AAGTGTACAACTTACAGAAGTTCCAGATTGTGTGGGAGATTGCTGCTGACATTTTATTTTATGACGCTAC
ATGCACAATGTCCGACTGAAATTATATTCTGCCTCTTAAACTGGTTAGACTAACGAATTTCCAGGCTGAT
CAGCCGGATTGGTAATAGATTTTGTCGTAGTTTTCAGTTTTCCCGCTGTCGCCTCATGTTGCTGGCATCA
TGTTGCGACTAGATGCTCTTTTGTGGATCGCAACATGTGTTGCCAATGCGCATCTGCCGCGACTGAAATC
CATACGGAATCCGTGTGTCAGTCGTTATCTGAACGTCGGGAACTGTGGATTTGTCCGCCCCTACGTGGAG
CCACGCGATCTGAACTTACCATTTACCGGAGCCAAACGGAAGTGCCATATCTCCATCTATTCCTATGGAA
CTCCGCCATCTGGCACTGGAAATCAAAACAAACAAATCATTGCACAAACAAGCGCTAAAGCATCGCACAT
ACAGATATATACTCCGGAGATATATACTCGGATGTGCGTGAGTGGATTGGTGGGTGTGTTTTTCTTGCCT
CCTGGCATCGCGTCAAAAGTCGCGCGCGGATTCTGCGGCGGATTTATGGCCGCGGTAATTGGTTAAAGCT
AAGAATTTAATAACCATTAATCACTGAGTGTGTGAACAAATTGGCAAAAGATCAAAGGAAAAAGAATAAA
GAATATTATaaaaaaaaaTGAATGATTTGTGGTGATTAGTGTTGAAGTGCTAATATTTGGaaatacaatt
gaaaacgacaataaaaaagagataaataaaaaataaattgaCGTCGCGGTATATGGAATACCTATAAATT
ACAATAAAGAATAAACATATTGAAATGaaaaaaaaataaaaTGTTATCTTAATACTTAAATGAAGTTTCT
AGGAATCTCACACAACGCATAAATAAAGCTATAAATGCAATGGTATAATTAACAAAGACCCCGAGTAATA
TTTTAATGACATATAAAAATTAACATCCAGTAAAACATTTTTTACGAGAACAATTAAAATTCCATTTACG
AAAAAATAAACGACTTAATTCCAACTCTGAACAATTAATATTCCATTTTAAGCCGGTAATAACGAATTAT
TTTAACTTTCAACAAGTGTCAAAACAAAAAGTGACAATATTAATTGCGGATTGTGGGACAAAGGGAAATG
TTTTAAACAAAACAAACGACAAAGGCAAGTGCCAACAAACGCATGTGAAAAATATCGACACAAAGAGCAG
AGTATTCCTAGAAAAAAAGGCCAATATAAAACAATAAAATCCCCGCGGTGAAACGCAACGATCTTCGAAA
TGCATTGGCTAGTTCTGATACTAGTGCCCCTCGTCTGGGCCAATAGCAGTGATCTAAAGGGAGCACCTGT
GATGACCACAGTAGCCCCGACTGGCGGTCATCGAAGTCCCCATCAGCAGCAGAGATTGCAGCAATTGCAG
GAGGCGCAACAACTGGAGGCCCATCAGCAGCACCATCATCATTTGCGGCACGAACAGCATCTGCGGGCCG
AACTGGAGGGCAACCATCAGCCGCCGATCGAGGATGTCGAGAAGAAGGCCTTCCTCGATCCCCAATTGCC

CATGCAGCAGCATCACTTGCGTCATCACAATCGTCATCATGCGAGCTGGGAGCAGCGGGTATTTCCCTCG
CTGCGTCATCAGCAGCACCGCTTGTCCATCGGACCGAGCACGGCCAAGAACCTAATGACCACCGAGGCAC
CCACAACCAGGCACCATGGCCTGATCTCCAATCACAGTCGCTATTTGACAGGGACGGCATCTTTCCATC
CTGGGCTGAGCCTCGAACCACCAGAGGTCCCAATTGGCGACAGGAGGTGGACTCGAGTGATTCCGACGAG
GACAGCGATGAGGATGACGAAGATGACTACGATGAGTATGATGAGGATGGCGATACCTCGAATGGCGTTG
ATGAGGAACTGGCCCGACAAATGCCCAGATATTCGCTGTTCACCCAGAAGTTGCCACACATCGATTTAAA
GGATACCGATTACAATGCGTACGACCAGTCGGATGAACTGGACATAGTTTCGAGTAGCAATCGCAACAGC
AACAAGTATCCCAGCGTGGCCAATCCGCACGACAAGTCTGCTGGCAAGCGGAATATATTCGATTGGCTGT
TCAAACATGATAGGGAAAAAGAGGCTGTTAAAAAGCCACACATCACAACTACTTCAACGACAACAACAAC
CTCAACAACCAGCACAGTAAAGCCCACAATAATGCGAACCGAAAGGCCAAAACTGCAATTAATTGATGCG
AATCTAAAGAATGGCGGTGGCGAAGAGGACGACTCCAATGAGGATTCAGACTCCGAGTCGGAGGAGGGCT
TCTCCAATGAACAGTGGAACAAAATCGAGCACCAACACCATCTCAGGCAGCAGAAACACCAGAAAGAACT
ACTGGCGATGCGAGAGAAGAGCCGGAATACCCCTCTCATAAGGAACATTGAGAATGAGG
>dme_circ_0001699_junction_seq
CCTCTCATAAGGAACATTGAGAATGAGGAAGTGTACAACTTACAGAAGTTCCAGAT
>dme_circ_0001700
GATCTTGTCACTCGAATTCGAAAGAAAAGAACCGAATGATGAGGGGAATATAACAGAAGCGGATTTTGCA
GAACTCCTACTGGCCTACGCTGGATATCCGTTAAAAAAGAAACAAAAGAAGTTGAAGAGGGTTAAGAGGA
G
>dme_circ_0001700_junction_seq
AAAAGAAGTTGAAGAGGGTTAAGAGGAGGATCTTGTCACTCGAATTCGAAAGAAAA
>dme_circ_0001701
GAGAACTTCATATCCATTGATGATGTGAAAATCAAGAGTTTCCTGTGGCAAATCGGCCAGCAGCAGAGGG
AAAAGGAGTTGGAAATCAAAAGGATCATGGAACAAGAGAAAAGGGAGAAAAGAGAGAAGGAAAGGCGGGA
GGCCGAAAAGCGAAGAGATCAAGAGG
>dme_circ_0001701_junction_seq
GAGGCCGAAAAGCGAAGAGATCAAGAGGGAGAACTTCATATCCATTGATGATGTGA
>dme_circ_0001702
GTGAACCTTAGGCTAACATATTAAAACCGATTGATTCTAGGGGTGAAGGGAAGTGCGCAGCGTTAAATGA
TATGCAAGATGGTTTAGAACTTCAGACAAACAACTTAACTATGTACAACAAAGCTACGATCTAAAGTTAA
GCAAGTCGGAGGATCTTTTGAAACTCTCAACTTAAGCGTGTGTGTGTAATTGGTTATTTCGGGTCGATTT
TCTTAAACAAAAATCAAA
>dme_circ_0001702_junction_seq
GGGTCGATTTTCTTAAACAAAAATCAAAGTGAACCTTAGGCTAACATATTAAAACC
>dme_circ_0001703
TAAACAGAGATAGCTCACAATGGATTGGATCATCAATGATGAGATGGGCCTGACGGTCGGTCACGTGGTG
GGCTGGGCCGCCGCCTCCGCAATGGTTATTGGGGGCGTGATCCCCTACGTGCCCCAATACATTGAGATCA
AGAAGACGCAGGACGCGGAGGGATTCTCTCTGTACGTGTGCCTGGCGCTGCTGGTGGCCAACTCGCTGAG
AATACTCTTCTGGGTAAGACTTAACTTGTACGCTAATCAAAAATAACTAAATTTGTAATCGAAAATAGAT
ACTGGAATACTAGATACATAATAGATACCTAGGAAACTAAAACATTACTAGCATCCGTACGAAACTAATT
AAATTCATTACCTAATGCTCCATAATGTTAACAAAAGTGCTTAAATGTGAAAGCTCAAAATTTTGCAAGT
AATTATGACGATTTCATATCCAAATATGCCATTTTTGAGCAATAAATCAATCATGAAAGGCAAAACGAAC
TTTTATTGCACGCGCTTATTTTTCTTTTTGACTGTACAGCACTCGAAAATAACACAAATCACTTTTGAAC
GCTGCGTATTCAAATGAAAATAAACACCTGCAAATTCTCTCTCGttttttttcttttGATTTGGGTTGAG

AATACAATTGAAATACATCATAATTCTTTaaaaaaaaaaTCTCTAGAAAATTACTGATATGGCACTTCCT
TGTCTTTAAAATCTACCTAAAAACTTACTTTAGAAACATGCTATTATATCTACATATATATACAGAAAGA
TCTCTTTCGCAGTAGTTTCGAATCCTCTGGCACCCCTGCGGTGAAAACGTCAGGTACCTGAACCTCTCTA
AGTAAACCCCTGTCCACTTTTATCTCACCCACATTGGGATGACTAAACCCACTCGGCAAATACCTGGTAT
GGTATTACGGCTTATCTGGGTGGGCAATTCAAACCGCTTATCACGCGAAATTCTCTCATGGTCCGACGCG
ACAATTTACGGTTTGGAGCACTTGAGACCAAAACCCCTTCCTATCTGTTAGCTATGTCATGATTTCTGGT
CGATTTTGCTCACCATAGTCGATAGACGACTTCCACAATTTGGACGAAGGACCAGCGTACTGGTTTTATC
TTTATTGTACTCCCAGCGGTATTTAGGTATTCGGCACAATGCACAATGTCGTCGATAATCGCTATTTTCG
CTGGGGTTTAGGACACTTGATGGAAGATTTATCAGCTTTCATGCTGAACTAATGTTTTGTGGGATGTTTG
ATAAGATTGTTACACTCAACTCAATACAAGCGCAACTATAGTTTATAAGAAGTCaaaaaaaaCTATTTCG
TAATATACGAAATTCAGTCGTTTTTGGGAATATTTAGGTGACTTAAAAATGTTGACAGTCTGAACTTTCG
TTTCTGGAATGGTTTCATCCAGCTGCCCTGGATTGTTGATTCATCTTTTTGCAGAGCGAAACATATAAAT
CAAAATTAATAAAGTAAATTTTTAATTCAATTTGAATGAGTGTGCAAAGTAGCTTTCAAAATGTGACAAT
GCCATTGAAACCAGTTGAAACTGACACAGAGCCAACAAACAAAAAGATATAATTGAATTTGCGCTATTGT
TTATTCAGAACGTATTTTCATATTTAGTTCAAAATATATATTTGCCATATggaataaacggaataaacgg
aTTTTTAGGCCAGCTACTTCTCGACTGGCCTTTAACAACAGGGTCAACTACTTTTGTCCAGCTGGTTTCT
GTGTATTTTTCAGGTTGTTTAGCCCGTTTAAAGCTGCTGCTATTGTGGTTTTTCGAGACCCATCGCTTCC
TACTAATAATTTATTAACAAGCCAAGTGAAACGTGAACGCaaaaacaaaaaaaGAATAGGAGCAACTGGT
GGCCCTCATCGACGCCGTCAATTTGTCTGGCCTGGGTGGCAATCAATAAATAAATTATTTAGCTGGAAAC
TGTTGCCGGCGAGCAAAGTTGAATCAGCTGGGCAACGAGGCTTTGAGCCGAATAATCCCAATACAATATG
TATACGTTATATATAGATGTTTACGATTCCTTGTGGCCAGACAttttttttCCAGACACGCGATCAGTTT
CAGTTGGGTTTTATGAATAAAGTCAGTCAGTCTGGAGGCTAAGTAATAGTGCAATAGGAATGTTTTGGGG
GCTACAACAATGATAGGAAGTAGCTTTTAATTTATGCACGACTTTTTGCTTTCCGTACGCCGTAATTATT
ATTATTCCGACTGAATAAACCCCCGCaaaaaaaaaaaaaaTTTAGTTGAGTGCAACTGGCAATTTGTGTA
CAACAAAATGCACGAAACACTTCAACTATTAAAAGTTATTTCGTGAATTTACCATAAATTGCACGCAATT
ACAATTAATTAGTTCACGTGATGGAAATATATTTCACATATGTGATATTCAGAGaaaaaaaaaCACTCCC
AACAAAGGGAGGCAGATTATGGATAGGAACAAGAAGGAATGACACATCCCGTTGACTTTATTGTAAAAAT
TGACAACGTGGCTGCCTTTGTTGTTGATCGTAAATATGCATATTAAACACGGAATTAATCATAAAACTCA
CACCGTGGCACGACTAAAAGTAAATATAATAATTGGAAAAATTTTCTGTGCTTTTTGCATGAGCTCATTG
CTTGAAATCATCGCTGAATAATTATTGTAAAATCAAGATTAGAATTGTGGTTTAGATAATGCGAATAGAG
TTTACACTTGAGATAAGATATTTGGAAtttttttttCAGCTTTTGGTGTAGGAAGTAAAGTAAAGTTGTT
TTTATCTTATCTGTaaaaaaaaaaGTGCAGTCCATTACATACTTCATATACTAAATAAAAATTCTTTGTT
TGGCTATATTTTATGAAGTAGTTATTCATTCAACTTTCAGTTAATAAAATATTTTGCGATGTGGGCGTTT
TTTCCAGTTTACTAACGAAGCAGATTGCGtatatatatatatagaatatatacatatTTAACTAACTAAA
GTTTGCTCTATGGCAAAAGTCAACAAAGCGGCGGAGTTCGATTTCGTAAAAAGGTTTTCTCGCGCaaaaa
aaaaaaaCGCTACCATTATGACTGAATAAGTTGGTCAGGTCAGTGGGGCGTGACTACACAAGTCTGCAAT
ATAATTTACACTTTCATTCCAGGCATATAACAATAGATGTAAATATAGTATGCGAACGGTTAGCTCGGCA
CATGCAAACAGTCATTTCTATATTTGTAATTTTCGTGTATTTGCTTAAATATTTACACATTTTGTCAGCC
CGCCTGAAAACGCAAGCTGTTCACACTTAACAAGCTATCAAATTTGGCTGCAATTAAAAATTCTATAGTA
TGTACATCCGAAAATCCAAATATTCACATTTAAAATTCAATCTCAAATGTGAGGCAACGTTCGACAGGAA
TTTGCATAGTATAGTCGAACATTTAAATATTCATATTTGAAATTGACTCTAAAAATGCAGCTATAAGCTA
ACTTAAAATGTTGCAAAACTTGAATGACCTTTAAAAAATTTTCCAGCTATATATATATTATACAACTCAC
AGCTCAATCTGGCGAAAAAGTGAAGGGTTAAACCTGAGAACTTCGGAGTTCATTGAACCTATTTCAGCAA
ATTTTACGATCAACATGTTTAAAACTTGAGCAATCAAGAAAATTTGCTGACTAATTGGGCTCTACAGCTT

TGTTTTCTAAGGGAATTTTTGAATGGGGATTAAATTAGACATAAATAGCGTATATAAATAAGTACTTGCT
TGATTTAGATATTGTAAGAATATCTAGTTACTTCAAAACATACGCTTTTGATAAGAACTCTTTTCTAGGT
GGAAACGTTCAGTTTATAAAATGCACCATTCACACTGCATGCTTATGATATTTGTGTAAAGCAAGCTAAA
CTTCGCCTCTTAATTGAGGTTCCTCAAAGAGGTTCCTCCTTCTTATCACTATTGTACAAAGGAAAATGTG
TGTACGATGATAAGCCCAGTCTCGACATCAGGCGATAATTGTGTCTACTCCAACCAATTAATTTGTTTAC
GTAAACACTTATCGAAATAAGGCGCTTTCCTATACGTAAGCTAATGATATGCCTGCAAGATAATGCCATA
GAATAGATATTATTGTGTTTATCTACGAATGTCACACGATTGTGCCGAATTTATTTATGAtttttttttt
tATATAATTTGCTTATCAACTTTCGATTGAACGAAATGAAGACACTTTAAATTTAATCTAGCATTTTTAA
TAATTTACTAAGCACACCCAATTTTCATTCACCTTTATTTATACAAGCTTTGGCCCACTTTCCTTGTTTA
TTTATTATTAATGCATACGACTCACTGAATGGAAAAAAATGAAGGAACTTTTTTTCCAAAGGCAAACAAC
TTTATTGGACATTTGGTCGCGGAAGGTCAAGGTTTGTGGGTGGTTCttttttttttttGTGGTAAAGTCC
TTCAAACCAATTAATTTTTTCGGGGAAAAAATTTGCTGACCAGGAAACCTTGCTTATGGTCCATAAATGT
TTACAGCTTGTTGGTCTGTCGCTTTTTGTTACGCATCCCATGCAAATTGGAATTTAAATTTCTAAACCGG
TCCGTTGACGTAGTAGTTAATAATAATGTTGTTCGAGGGGTTCTGCTTATTTATCAGCTATAAAAACCCA
ATACCATTTTTGCAAACGAAGCACTTGTTTTCTGCGTGTGGAAATGTTGACTGGTTAGTTGGTAACTTAA
TTGTGGTTTTGGCATCTGTGAAATATTTATAATGTTGATGTTGAAGTTGCTACGGCACGAGATTCTTTAT
CAAGtttttttttAGGCGGCACTTGAAATTCAAGTTGAATTTGCTGTGCACTTGATGAGCAGCTATGAAG
TTTATGCAATCACTTGCTGAGGGGGTTTAGGTAATAATTCTGGCAAAAACATTGAAATGTACTCTATATA
TTTTCTGTTTTTCCAAAACGTTTTTAATACAAAGACTAACGTTTGCCTTGGATTTAGCGTGCTGCAAATC
CGTTAATTGCCTCGCCTAAGCACTCAAGTTTAAATAGTTACCAAGCGGAGATAATTTAAGTGTAGAGCCT
TTTGTTTTGCACAACTATTCGTATACCAGCAGTTCTAATTGTGAGCTCATCAAGTAGGCACCGAAAAACG
AGCTACCTAATTGTGTCAATTATCAGTTTCTGCCAGCCCATCCCATTCGAATGGATGAACCCAGGGGTTA
TCTCTGGCACATTACATATAATAGTTTTGAGTGCTCATTTTCCTCATGTAGAGACTCCATCTTGAGCCGA
TTGGCATTATCTCAATCGCATTTGTTTCGTTTGGAAAGCACGAACGTTTTGCTTTTAATGCTGATAACCA
GGGTGCGGTTACAGCCCAAAGGTCAAGCAGCGCAGTCTTATCTGCCAACCATGCCCCGCGAAATTAATTG
TATTTGTGTTGCAAGATTTTTAACGTAATTAAAAGTAGAGCTGCGCCGAAAGCGGTTCACGTCACATTCC
GTGCCGTGGACAATCCCTTAAATCCTACACGTCACTGCTGCCGAAAGGACGCACTCCACTCCAACGTTAA
TCTTTGTTCTTGGTTGTGTTTTATGGCCGAGACGTTGGCCTAATTGAATTTTAATGCCAAGCCAATGTCA
GGTTGTTACTTTTGTGTAAATCTTGGCACTAGCAAGACTCGAAATTCAACGGCATTGATGGAAAATTCAG
ATGGCAAAGCACAACTAATCGAAATCGGTGGCCATAGAATGTTTACGGCTTTGACTTTGGTCCGCTGCCG
GAATGAACAATCAGAATCATGGATGGGGCTTCTTTGCATGCGAGAAATGTGCGACGTTGGCTGACGCAAA
TGTTTGCACGAAGCGAACGGACAGAGATTTAAATTTCTCAATTAGAATAGATTTTTGACACCACCCTCAA
AATTTTTGGAAGCAGAAGCAGAGATAATCTGCGTGGGCAAACTTACGGAATATATATGTATATAACGCAG
AGTCACAGCTTTAATGTTTAATGGCGCTTGAAAGCTACGTTTTCTGGCTATTGAGTTATTAAGTTAACTT
TTTAAAAATACAGCCCAAGGTCTTTGACAACTTTGTATCGATTATTTGTACGCATAAGTTGATTCAGTTa
aaaaaaaaaCAGCATTATCACGCTTGATTTGTTTTCGAGAATTGAGAAGCCTTTAAGGCGCATGACTTTT
AAAGTTCTGATAGGCCAGCAGAAATAAAATTGGGGTAGGTCTCAAGACGAAATGATATGGAAATCAGAAT
GTAACTTAGTCAGCTCTCTATTTTCTATCTGCAGGCGAATATACATATTTGCTGGTATTCATGCCTATCT
GCCATCGAGTTGGCTACAAATTTAATGCTCGGCTTCCttttatttttcctttttttcaattgcggcattgt
ctgtttcccttttttccttcttttgtcctaagacttttttttgTGCCATCTGTGTTCCAGACTCCATTTC
CAATTCCACCAGCACGCCTACGAATTTTCCGGGGTGGGCGTAAATTGAAGTGTTTTCCTGAACAAAAGTC
ACACACATCTGGGTGACTGGAATCTTTTGTTTGTGCTTCGGTTTGGGGTTTTATGGGCTTAGTGCCATGT
GGGTGGGCTCAGCTCGCATTAATTTTCATTGAGCAAATCAAGTGATGTAATAAAATATTTATTTTTCCAT
TTATTTTGTTATTTTCGGAGAACATATCAGCTATTGTTGCTCttttttttATTCTAGATAAGTAAATATT

GCGTGCTGTTTTGTTTTTTGAATGAAATGCTTAAATTTCGTTTTCCAACACTTAACTCACAAACTGTTCA
GTTCATTGGCAATCGCTATCCATCTGTGCTCTAGTTCATCGTCACTACATCCTTTATCCCGCCGACAAGG
TCATTTCGAGCAGCTTAGCTCGGCACACCAGTAAATCCATCTAGGCTGTAGATCGACCCAGTTATGGCCA
TTCATCATCATCATCAGCGGCTGGCAATCCTTTCTATACATTCATTTACTCAATGGTTGGTGGAAAAATT
GTGCTTTGGCTGTAGGCTCTCATTTCCCACTGCTCATCATCAGGCGGCAGCTTGTGTGTTTACACAAATC
TCGAAGCAGGCAATCTACTAACGTGGCCAACACTCTTGTTCAGTGGTTCCGAAATAGCCGGAATGCATGG
CCTTAAGTGCTTTCGGTTCCGTTAGTTAGGAGCCCTTCAGCGGTGAGACAATGATGAGGACTGCGGCCCA
CGACAAGTGTAGCCTACTTACAGGCGACCACACCAGTTGGGCCAAATGAAATTTGTGTTAAGCGCACTGT
GCCAGCCTTGGCTTAAACAAAACGGCTTACAGCTGTGGTCGAAATTATAGGCCAATAGCTGGTAGAGATT
TCTAGAGAAATAACTTTAACTTTAACAAACATTTAGTCGCATTAAATAGAAGATATATGTTTAATAATCC
TTATACTGACATATTAAGATCCTTTTTGGCAAGATTGAAGTTATATAGTTATAGTTAGTTATAGCCAAGT
GACAACAGCTGTAATTCAGGCAGACAAAACGCACCCACACTTGAGTAAACATCGCGCCGAAGGCAACGCG
AAATTATAATTATGGCTAAAACACACACTAAAGGTTGGTTGCTGCTTAGCAAAGCCCCACACACACGTGT
GCTTGCCAAATTGCTTGGCTCCTCCATTTTGGTGACTTAATTTCTAACAAATTCTAGCAATAAAACTTTC
ATCAAGCCTAAGTGACATTTTCGGTACTCGCCTATTTAGTCAAATTGTTGCGGCTATTCGAGCAAATTGC
CAGTGAAAGTGAGCCAGAAAATGCAGGTGAAATGAGGGTGGGGATGGTGTTGGAAAATTGTTTACATTGC
AGGCTTAGGCACGCCGCCAAATGCGGCATGCTACATACATATATTGTATATGTACATGTATTCATGCCTG
TTTGTAATGCACGTATACGATTTTATCGATTTTTTACGACATTTTTTACACTTTGAAGCGTGCAAATGCC
ATGCCCGTGGGTGGAAGTAGGTGCCGGTGTGTTTTTTCTGTTTTTTGCCAATTTAAAAGGCAAAGCCAAC
CAATTGGACTAGTGACGAAAAAATAGCGATGTTTTCGGCTGTGAGTTCTCTAGTCCAAATTGGAGTGTTT
GCACGGAAAATATTAGCATTTGAGAAAATTGCACTTGTTATACAAACTCGAAGAGTGTGCTCTTTATTCC
TCTGATTTTCTGGTTTGTCGGGTGAGAACTTAGCATAGAATTTCCTTTTCATTACTCACTGAAGCTAGAG
TCCTTTAAGATGGAAAGAAATTCAAACGTAAACGTTTGCGAACTAAAAATTCAAATCAATTCATACTAGA
GCCAGTAAATTTCTTGAAATGTTCTAGTTCATTCGGAAAGTAAAATGTATTTTGTGGTGATTCAGTCGGA
AGCCGCGTGGAATGGCATTGGCGTGGCCCAACTGGTAATTGAATTTCGATGCCCCGCCAAAGCGACTTGC
CAATGAACATGGGCGTAAATAGCCACCGTGGCACAATTTCCGCGTTTTCCGCATTTTACTATCTGGTAAT
CGGAAGAAGAAACGCGGAAATGTATGCTATCTGCTGTTGTGATTGCGAGCGACAGCTGTGGCTTAAGTAA
AACAGTTGGCTCCACTAATCTACGCCAGCGAAATGCCGACAAATTGTCGGATGTTTGTTTGttttttttA
ATAACATTTGGGAATGACTTTTGTTTGGACACCAAAAGGACTTTCATCACAAAAAAATCCCCAGCAAATC
TGAGGACTTGCCTTTGGTTTGCTCGGCATTTGTTTGGGGCCTTGCCAAAATGCCCAACACATTTCAACCA
TTGTTTGTTGTTTGTGCTCGGGGACCCTTTTTCACCCACTTGGCATCTGTCAGATTTATGACAGTGCCGC
TTGACACAGTGCTGGGATTTTTCCAGCACACCCGCCACCTTTTACTTGTGGGAAATATGTGCACATTAAG
CTCTATAATCGATTTGATTCGGGAGTAAATTATGCTCTTGGGCGAACATCAATCACTTGGCGCCACCTAA
GCACCTGTCAATTAGCTGTAATCAACACTACATTGTGCAGCATACACTTGTCAACTGAGGTGTGATAAAT
AATAGTTATACTTAATTATTTAGGGTCCGGTGGAATGTCTTTCTGTCAGCGTATGACCATTGCGACTTAT
AATTAACCGACAAACTGTTTAAATAAATGCCACTCCGAATGCACTTTCCATTTCTGTTTTAAAGTTTACA
AAGTTTGAAATAAATTTCTTGAAGGACACATGCTAATTATATATGTCCCTGGAAATGAAATACCGTCAAG
GACATAACCGCATCTCGATAGGGGGCAGTCAATTAAAATGTCATTTTGAGTTGACCTTTTTTCAATTGAC
CTTTACCCAATTTCCCTCTGGCTTGCAGGGTTCTCCAGCCGCTATGAGCTTCCACTGCTGGTGCAGAGTG
TCGTGATGAACGTGACCATGTTCCTGATGATCCATCTGTGCGTGAAGGTGAAGCGGGTTAACGCCAATAA
CCGCGAGCACGCCCTGCGAGGGTGAGTCAGCTGATTCCAGGGTGTAGTAATGGGTCTGGACTTAACAAGT
CGACATGTCTGCGTTTATTGATTGACAAAGAAAGGAGAGTCTGCATACTAGCACTAGCATTGATTTACGG
ATCAAGATTTTAGCATAACAACTTTTCACTCAATTGACAGTTTGCTGAACAGATTGAAATTGAATCAAAT
GTGTGAGGATGGGAAGTTAGGTTATTGTAATTCCATTAGCAAAGTACATTCTGCTGTTTGATATCAAACC

CATATCTTTATGGCTGTTCGCATTTATTTCTTCTCCTTCGTTACTTCTTATCAGAGAAACTAGAAAAACA
TGAAACTGGGTTAGGAGGTACGTACGGTTGGTTCCAGGCGGCAAATTGATCAACGAACCAGAGAGCACAG
AACCAatatatatatatatataCACACACACCGACATGAGCTAATTATATTGGCACGCCTTGGGCTTCTT
TGTACATGGCTCGTCGTAAATTGTGCAGCGCCTAAAAGCTGGCAGAGATAATGGAAATTGATTTCCAACT
GGCTTCCCATCTGCAGCCTGGTAAAGGCTTCCTTCTTGGTTGTGAACAATTTATAGAGTAGTAAACTGTT
GGGGTGAGGTCATGACCTTTTGCTTTCCTACTGTAGCTAAATAAAAGTGCATGCAATGCCTGTAAAATAA
GTGGGTTAGCAAGTAGTCTTAAGTTGCTGGGTAAACTAATTCATTGAGCTGGTTATTACTAATAATATTC
AATCATTCACTCTTACAGAACCTGGACTTCAAATACTTTTGGAACTGGACAGACTTCCAATCGTATCTGG
ACTTCATGCTAGTCGTGTGGGCCGTTGGTGCTGCCATAACATATCTAATGTTGTCCGTACTTTGGTTTAT
GGAATCCATGGGTTTTGTTGCCGTCTTCACAGAAGCCATGTTGGGGGGCGCCACAATTTCTGCGCAACTT
TAAGAATAAATCAACATATGGAATGAGCATACACATGGTGATCATGTGGACGCTCGGTGATATGTTTAAG
ACTGGTTACTTTATTGTTAGGAAAGCACCATCGCAATTCTGGATCTGTGGCACGCTGCAGGGTCAGCTTG
GACATAGCGATCCTGTCACAGGTGTGGTTCTACCGTAAGAATTCCAAGCCGCGCGATCTGCGCCGCGGCG
ATTAGCAGTTTGCCCCCGAAGAACAGCCACAGCACATTCATCACCACGACATACATACATCCCATTCCGA
GGAGCATCAGCTATCGCTGCCCGTCACAGTGAGCAGCAGTGGAGACGATCATCTGCTGACAGCTCGAGCG
GATGACGAGCACTTTAAGGTGCTGGACTTTGGTCAGTGCCACGATAATCGTGCGTTTCAATATCACAATA
ATAACAAGATGTTGCCCAGACCAGGAGGCAGTGGCAAGAGTAAGGATGATAGCAGTATGGCAGCTGCCTT
GGAGGTCGTCATTGTCCATTCCCCGGCATCCAAAGAGCCGAGACTGATTGGCGCTCAGGAGGAGAGTGGG
CGCTGTCGCAGGAAGCAAAATAATGCCACCCAAACTAAATTGGAGGGTGGCAGCTGCTGCTGTGTGATCA
CCACCCATCATCACCACTGCCACTGCAATCACCATAGGCATCATCACCATCATCATCATTGTCACTACAG
GAGCACAAAGAGACGAGCTCTGCTGCATCAGAAAAGGAGTCACCACCATCAGAAGCATATGCCAATTAAG
CAGGCGAATCACAAACAAATGCTTCCATCTaacaacaacaacaacaacaaGTCTTCCTTGGACTCTAGTG
ATGAGGCAATGCATCACCAGGCCATTGCCATTGAAATCGATGCAGCAACGATGAGCGAGGACGGAGATGA
AAATAACAGCAGCACGGCCACTTCGAATTTTCCGTATAAAGTGGCCAGTGAGGGAGTAAAACCCATTATA
ATGGCTCCTCTTCGAGAACAACATCGTCACAGGAGAGGTTCGTTGAATTCCAATACCACAATTGAAACCG
ATGAATTGTCTCGCGATGAAGATGACGACGATGATGTGCGGATGGGATCCCACCACTTACCAGAAGATGA
GACCCAACTAACTCAGCCAGCAGTTACGAATAGTTCCAGCAGTTCCACAAAGAGGGGAAATCCTGCCTCT
GCACCAGCGCGAAATGAGTGTGTTCGGATCAAGAGGAAAAGGTTTATAGCTGAGGCTGGACAGGATTACT
GCAGCAGTTGCTCAAGTTCCTGCTCCTGCAGCAGTTTGGATGAGGATCTAGAGGCACATCATGAAATGAC
GGTGGCTGCAGAGTGTCACCAATGCATTCCCGGTTCTCGGAGAGCAAGTCTCTGCTCTTGCCAGAATTCC
AGCTGCTGTTGTGGTTCGTGTTCCCaggaagatgatgaggaggaggaagcggatgatgaggaCGACGAGA
CCACGGGACAAAGGGAAAGTTTTTGTACAGCTGCAGATCATACCATAACTCCCTCGCAAGAGGGTGAGGA
CATGCACAGCAGTACACTGACACCGCTTAGCACCCACAGGTCTTCGTTTATGGACCAGGATCATGGCGAT
CACACCATGAGGAGTGATATCGGCGGTGGCAACATAACCGATGCGGATGCCTCTTCATTAAGCCAATCAG
CGGAGTACTTCTCGCTGTCATCCACCGCTGGAGGCGGTACTTCTTTTCCCACCAAAGAAGAAGATAAAAT
GGATAAGCTGGAGATCAAGGAGACGAAGGATTTGCGAGAGAAGGAAGTGGAGCAACCGTCCTGCAAGCAC
TGGAATCGCAATCCCCATGGCAAGCATAAACGCGGATCTTCACCAGTGGACAACTCCTTATGACGAAGTG
TCTCGCTGGTCGCCTGGCCAGCAATCGATGTGAATGCCTTGCTGCAGCAGACCATAAGGAAATCAGCTGT
GCTGCAGGAGACACCAGTGCGCAGGAAAACCACTGCCAAAAGGGGTTCGGGAACAACGGGAAGCAATGAC
TCCTCCACGGCCACATTAACAGCCAGTGTTGTGACGGTAGCCAAATTTCCAGGAACTGGACTAGTCAGCA
CTGTCACCAGTTTGAATGGATGTGGAACCACCTATAGCTACACCACCACCACGGCTGCACCCTTGAATCT
TGCAGCTTCCAGCAAGAAGAACACCAAGTACTCGCCGGTACCAAACTCCGAGGCCTATCAGTGCGATCCA
CTGCAGCAAAGCTCGACGGAGATCATCTTATAACTCATGCAAACATACCTCTGCACCTTACACGATTATA
CAGTGCGAAACCGTATAAAAGTCAAGGTTAAACTTCATTAGTAAAATGGGATAATACATAATAATACATA

ACACCAAAACTCGTGGATATCTAAGGAGTGGTATAACGAAAACCAAAATTCATAAAGCTGTTCAATTTGT
TGTTATTCGGGGGTAACAAAAACCAAGATGAGGATGTAGAAAAGGCAGACCTAGTTTATACACACTAGAA
CATAAAACCAATGTACATTACTTCTTATGTGAGATGCAGCTACAAAAATTAGCTTACAATGTAAACTAGT
TTTAAACATTTAGTTTCGGACAATGTGAATTTAAAGAAAAGGGTTTCAGCTGGGGAAGGGACCAAGCCAG
AGTTGACACAGCCTTCAAGTTTTCCAAAATCCACGCAGAGACATATCCACATTGTGCAAATTTCTCGCCA
AGAAAACTTCCCTCAGCTCGGGCCTTGAACCTTGTTTAGATTTAACATTATAAATCGTCATTGTTTTTCG
TACGTCGTTAGTCGAATTAATTTTGTAGCCCTGTAAGTTCAATTTGTATTAAAATCAACAAAATCAAACT
TTAATCTCGATATTATTCTTTAAAGAATTTTTTAATTGTATAATTTCCTAGCAGAATTTATTAGCTAAGA
TCTATAAAAAATTATACGAAAATAAACGAAACCCAAGCAAATTTATGAttttcttttcttttgattttcc
taattttcatttttcattttctttcaAATGGCACACGTCCTAATCTTTAGCTATATTATTCCTAGATATA
ACGTATAAAAACTACTAAGCAAACAAACATCTTCCTTGCAATTTTGAGATTCCGTTGAAAACGTACACAC
AAGATAAATATGATAAAATTGAATTATTTTTAAGCCACGTAAATATTTATTCAAAGTGCTTAAGAATATT
TAAGAAATATTTAAAAACTACAATTACCCAATGGATGGGAAGATAAAATTTGTTGATTAGTATGTAGCAG
CTATTTTTGATTGTTTTCCTATGATATGTTGGCTATGTAGCAAACAAATAATAACAAATCTGTTGCAGAT
GCGCAGTATTATATTCATTGTAAAGCCATTATTAAAACTGTAACGAATTACTTACTAACAATGAAATCGA
GGCATTGTTATTTTGCATCTCTACCGCTGTCGCGGGCGAAAAAAGTGCAGTGCAACGAAAAGTTGGTGAT
ACAGTGTCCAGACCAGGAAATAATTCAAAAGTCGCGTGGAAACGCCGAACCTTATTGCTTTTTGCTAGCG
CTCAATAAAAGATATAATTGTTGGGAAGAACTGCATTCGAGATTGCTTCTCCCGTTGCTATTATTTGTTT
CTCTGTGCGAGTGGCGAAGGAAGCGAAAGAAACGAGAATCGGGAATAAAGCGGATTGCTGATTAGCACGA
ATCGTGGAAAGAAAACAGAAGAACAGCGAATTGCATTTTACACTTGGCAAG
>dme_circ_0001703_junction_seq
CAGCGAATTGCATTTTACACTTGGCAAGTAAACAGAGATAGCTCACAATGGATTGG
>dme_circ_0001704
ATGCAGCTACAAAAATTAGCTTACAATGTAAACTAGTTTTAAACATTTAGTTTCGGACAATGTGAATTTA
AAGAAAAGGGTTTCAGCTGGGGAAGGGACCAAGCCAGAGTTGACACAGCCTTCAAGTTTTCCAAAATCCA
CGCAGAGACATATCCACATTGTGCAAATTTCTCGCCAAGAAAACTTCCCTCAGCTCGGGCCTTGAACCTT
GTTTAGATTTAACATTATAAATCGTCATTG
>dme_circ_0001704_junction_seq
TTAGATTTAACATTATAAATCGTCATTGATGCAGCTACAAAAATTAGCTTACAATG
>dme_circ_0001705
TGGACAACTCCTTATGACGAAGTGTCTCGCTGGTCGCCTGGCCAGCAATCGATGTGAATGCCTTGCTGCA
GCAGACCATAAGGAAATCAGCTGTGCTGCAGGAGACACCAGTGCGCAGGAAAACCACTGCCAAAAGGGGT
TCGGGAACAACGGGAAGCAATGACTCCTCCACGGCCACATTAACAGCCAGTGTTGTGACGGTAGCCAAAT
TTCCAGGAACTGGACTAGTCAGCACTGTCACCAGTTTGAATGGATGTGGAACCACCTATAGCTACACCAC
CACCACGGCTGCACCCTTGAATCTTGCAGCTTCCAGCAAGAAGAACACCAAG
>dme_circ_0001705_junction_seq
TGCAGCTTCCAGCAAGAAGAACACCAAGTGGACAACTCCTTATGACGAAGTGTCTC
>dme_circ_0001706
GTTCTCCAGCCGCTATGAGCTTCCACTGCTGGTGCAGAGTGTCGTGATGAACGTGACCATGTTCCTGATG
ATCCATCTGTGCGTGAAGGTGAAGCGGGTTAACGCCAATAACCGCGAGCACGCCCTGCGAGGTAAGTCCC
ATCAGGCGTTTAAAAACCTGTCACCCGAAGGATAAACATGTCAACTTCTAGGCGATGAACTGCATTTGCC
CAAAGTGATGACGGATACGGATACGGGCGCCTCTATATCCACAGAAGCGGGCGGAAGCGTCCTGAAGCGA
GTTCGTTCCCGGCACTATCTCAATGG
>dme_circ_0001706_junction_seq

GAGTTCGTTCCCGGCACTATCTCAATGGGTTCTCCAGCCGCTATGAGCTTCCACTG

>dme_circ_0001707

GGACTGGACCGGTGGACCCGAGAATTGTCAGCGGCTGACCCGAAAAGAAGCCATAGCCCATGCCACAAAC

GAAACCAGAGACCAAGGACTCCATCGCAGCCATGTGCTTGCCAGTGGCCTCCGCCAGGAGTCCTCCGAAC

GTGATGATCGGCGACAGGCAGGCGAAGTACAGAAAGATCCACGATGCCACGCACTGCATGGATAGGGCGT

CCTTGTAATCGCTTATGTACCACGGCGCCTTCCTCTTGATGTCGTTGATGAGGCCTCCGAAAAGCCTTCC

CGTCCTGGACAGTCCGTTCTCCTCGCGAAGACGCGCTtcctcctcctcctcgtccacctcctcctTGGGC

AACTCTGGCGGCCGCTTCCTGACTTCCTGCGAGGGAATCGCCGCTGGTGGCTCAATTC

>dme_circ_0001707_junction_seq

GAGGGAATCGCCGCTGGTGGCTCAATTCGGACTGGACCGGTGGACCCGAGAATTGT

>dme_circ_0001708

GAGACCAAGGACTCCATCGCAGCCATGTGCTTGCCAGTGGCCTCCGCCAGGAGTCCTCCGAACGTGATGA

TCGGCGACAGGCAGGCGAAGTACAGAAAGATCCACGATGCCACGCACTGCATGGATAGGGCGTCCTTGTA

ATCGCTTAT

>dme_circ_0001708_junction_seq

TGGATAGGGCGTCCTTGTAATCGCTTATGAGACCAAGGACTCCATCGCAGCCATGT

>dme_circ_0001709

TGACTCCGCCGGCACAGCGAGTTCAATTCATACTTGGCGAGGATGTGGACGATGGCACACACGTATCGCA

TCCCCTGTTTTCGGAAATGGGGATGCTGGTCAAGGAGGGCGACGAGATCGAGTGGAAGGAGACAGCGCGA

TGGATCAAATTCGAGGAGGATGTGGAGGAGGGTGGCAATCGATGGTCGAAGCCGCACGTGGCCACGCTCT

CCCTGCACTCCCTGTTCGAGCTGCGTCGCCTGCTGGTCAATGGCAGCGTGATGCTGGACATGGAGGCCCA

AAATCTGGAGGTGATGGCCGATCTCGTGTGCGATCACATGGTCAGTGCGGGAACATTGCCGCCCGGTGTA

AAGGATAAGGTCAAGGACGCGCTCCTGCGACGACATCGGCATCAGCACGAGTATGCCAAAAAGACGCGAC

TGCCAATTATTCGATCCCTGGCCGATATGCGCAATCACTCGTCATCAAAAAGGTGAGTATGGGGGTGTTC

ACGATCGCTAGGTAACTCTAAGCTCATCTCGTTCGTTTGGTGTGAATCTATGATCCATCTGCGAGCTCAA

CGCCATCAATCAGTTCAGAACTCATTTAGGATCATTTTGTGAATTGTGTCTAAAACTCTCATCCGATCCG

ATACGAAACAATACTAACAACTCTCAGACCTGGGCAAGAGCACTCCACTGCACTCACTGCATGGCTCAGC

AGCTGGCCTGGATCTGACCGGAACCAGACCGGACCAGACACCCCTCTATGTGTCGAGTAAGGATCAGCGT

GGTTGTTATCTCGCTGTTCCCACAGGTATAGCAAAATCCTGTCACCACTCTACCTTATGCAATCTCTGTA

CAGCTGAGAAGCCAAGTTGAAATAATTTTTAGTTGCCTTGCACTTAACAAAAAGCACTCAGAATCAATCA

AATGTAGGTGTGCTAAGTAAACTTTAAACTACGGATACTTGAAAACTTAGAAGCTCGTAGAACCGACAAA

GGGTTTACAATTTACATTTTGACTTTAAGTGGTAAATAATCAGGTTGAAATAGTCAAAAGCTCCAAAACC

TTATTAGGAATCTTGTAAATTTTTTTGaaaaaaaaaaCCCACATTTGCTCACTCATAAATCACTCATATC

CATTAGCAATTGAATTGCGGAAGACGCTAAAGAATCATGAAATAAACTCGCGTTATTCGTTTACCCAATA

AGCTGACACTTCAACGCATCTGGGGGCCATCGGGGTCACCACCTGGTTCCATGCTGGGGCATCGCCCCAA

CATCAGGCACAGAATCATGCTCAGAATCAAGGACGACCGCAGTCACAAGCAATACCAAGTAATAGCCCTT

CAATGATATTCACTGTACTTTGCCGTACCTATCAAGAGCACGCTGTGTTTTTCGATGATTATCTCTTGCC

ATAGACTCTTGTATTTTCTCTACTCCTATTCTACTCACTATATATGCAACTTAAACCTTCAAAAGTTCGT

TAGTCCTTACTATGATGAAATATCCAATTTGTTGTGCTGTCTACCCAAACAATTTAGCAATTTATCTTTA

TATTTAAGCAAACAGTCTAACCAAAATATGAAAACCCTCTATTTTACTCTCAAAATCTTCGTTATATCTC

TAATTCTTCATTGATTTGTTTCCCATTCTTTGAAATACGTTTTCATTTCTCATTTTAACTTTCCCATTTT

TATAATAAGTTAAAATACCAATACTTTTATACACTGAGTAAAAATGTACTTACTTGAATGTTTATAAATA

TTGCGATCAATTTTACTATAGaacaagagggaatgctatagtcgaattccccgactaacagatacccgtt

acgcagctggtggaagtgagaacgcgaaattcatttttctggcataccgatagatattggagaatataat

gagaaaaagggcggtttgtgggcgttagagtgggcgaggggacatgggtctacaaacttgagctgggtct
aagtctctggaatctatatgcttaatcttaaccttctagcttttatagttcttgacgttcatacggactg
acagacaggcggacagggccagatcgactcggctattgatcctgatcaaatatatatatactttatatgc
tcggaaacgcttctttctaatctagtatgccttttactctacgaataaGAGAAAATTTTGAACGGAGAGG
CAGTTGTGTTAAGTGTCAAGTCCAAATAGACCCTACAGTCAGAAAACTTATATAGTGCGGGAAGTGAAGT
TCGATCTTAACATAAAAAAAGATTTTTCTGATTTTAAACAAAATAAGATTCTTGGTACGTATGAACACAG
AATGAACTGACCATTGTTATATCTAAGCTCATATAATTGCCAGACAACAAGGTGCTGTCACCATCACCAA
CaaaaaaaaTGGAACCCTCGTTTTCCAATACTAACAAATCGGAACTTCAGAACCAACTACGAGTAGAGAG
AGCTCTGCAAGATAAATATTTAAAAGAATTTTCTTCAATGGCGGATGAGCTAAATCACTTAATTGATGGC
ACTACTCCCAGCCATATTGAGACTTATGAATATTTGGACACTGTCGACACTGAAAGCGGTCGTTCTCTCG
AAGAAGAGAAAAAGTTAACTAACTTTTTCTATAAATTGAAGAGCGATCTACAGAAATTATTTAGAGAAAT
CGTAGAGAACCCAAAATATTTAAACGAAATCAGGGATTCGACTATTATCAGGGACAATATAGATTTACTG
GATGAAGCAGACGCTTTTGAAGGTTCGATGAATATGCTTTGACGGACAGGTGGGATTTGAAAAATGTAAA
AAAATATTGTTACTTCAATAAAAAATGGTTAAATAAAAAACAGGTACCCGTTACTCAGCTTGTGGGGAAA
GCTAAATTTTCAAAAGGGAAATTTTGGCAACATTTGGAAAAACAACTATATTTACTTCCAGTGAAGTTTA
ATAGAACTTGACAAGTTCGAGTTACTCAGAGAATACCCTCGACATAATGATAACTTGAAAAACAACCCGC
ATTTACAGTTGAAGAGCACTCTGCGAGCATTTTGGGCGCCACAACGCCAATTTCCCTAACGGCCAGCGAA
CCGGGACCGCCCGGATCCAATGGAAATTCAAACCTGAGTACCGCTGCCGGCGCCATGGGGCGTTTTCTAA
CGGTGCCCAGTGGAAAACCCAGCAACAGAACGCTCGG
>dme_circ_0001709_junction_seq
GTGGAAAACCCAGCAACAGAACGCTCGGTGACTCCGCCGGCACAGCGAGTTCAATT
>dme_circ_0001710
TGACTCCGCCGGCACAGCGAGTTCAATTCATACTTGGCGAGGATGTGGACGATGGCACACACGTATCGCA
TCCCCTGTTTTCGGAAATGGGGATGCTGGTCAAGGAGGGCGACGAGATCGAGTGGAAGGAGACAGCGCGA
TGGATCAAATTCGAGGAGGATGTGGAGGAGGGTGGCAATCGATGGTCGAAGCCGCACGTGGCCACGCTCT
CCCTGCACTCCCTGTTCGAGCTGCGTCGCCTGCTGGTCAATGGCAGCGTGATGCTGGACATGGAGGCCCA
AAATCTGGAGGTGATGGCCGATCTCGTGTGCGATCACATGGTCAGTGCGGGAACATTGCCGCCCGGTGTA
AAGGATAAGGTCAAGGACGCGCTCCTGCGACGACATCGGCATCAGCACGAGTATGCCAAAAAGACGCGAC
TGCCAATTATTCGATCCCTGGCCGATATGCGCAATCACTCGTCATCAAAAAG
>dme_circ_0001710_junction_seq
ATATGCGCAATCACTCGTCATCAAAAAGTGACTCCGCCGGCACAGCGAGTTCAATT
>dme_circ_0001711
TGTTTTGTAGGCAGTTAAATTCATTGCAAAACGTCTATACATACCGTAAACATCCTTAGTAGCACTCAAA
ACAATATCACGCAAACCCAACATAAACAGCAACAAACTCAAATGGGTCTAAACAGGACATATGGTGGACA
ACGGTCAGGGACCAATTCCCAGCATTCCCAGATCAGGGCCAAATTATTTTCGCCAATTAACTTTAGTACG
CATTCGCCGGAAGCCCAAGGATGCGGTGCAATCATTGTTATAAACGGGGTGGCAGTGGTAGGATGATGTT
GCTCCCAAGTCATAAATATTGATTAAGCTCATAAAGGTTTGCACAGGAAGAAAAGGCTGCACATAAATTA
AATGTTGTAAATGTTTATTTTATTTTGTAGTTTAGTTGATCATTTTATGCTTTCGATATATTTAGATATC
TTGCATTATTTTTTATCCATGTATAGACCTTCTGTTTCTCATAAAGTATCCTTGAACTTATAGTTGACTC
CGCCGGCACAGCGAGTTCAATTCATACTTGGCGAGGATGTGGACGATGGCACACACGTATCGCATCCCCT
GTTTTCGGAAATGGGGATGCTGGTCAAGGAGGGCGACGAGATCGAGTGGAAGGAGACAGCGCGATGGATC
AAATTCGAGGAGGATGTGGAGGAGGGTGGCAATCGATGGTCGAAGCCGCACGTGGCCACGCTCTCCCTGC
ACTCCCTGTTCGAGCTGCGTCGCCTGCTGGTCAATGGCAGCGTGATGCTGGACATGGAGGCCCAAAATCT
GGAGGTGATGGCCGATCTCGTGTGCGATCACATGGTCAGTGCGGGAACATTGCCGCCCGGTGTAAAGGAT

AAGGTCAAGGACGCGCTCCTGCGACGACATCGGCATCAGCACGAGTATGCCAAAAAGACGCGACTGCCAA
TTATTCGATCCCTGGCCGATATGCGCAATCACTCGTCATCAAAAAG
>dme_circ_0001711_junction_seq
ATATGCGCAATCACTCGTCATCAAAAAGTGTTTTGTAGGCAGTTAAATTCATTGCA
>dme_circ_0001712
ACCTTGGACCATGAACTCCTCCAGCGGCGATGACGAGGCGCCGAAGGATCCGCGCACTGGCGGCGAGGAT
TTTACGCAACAATTTACAGAGAATGATTTCGAGGGTGAGTCGAGCTTCACTTTTTAAAGCCCCATTTTTT
AATGCTTACCGAAAAGTGATAGAAAAAACATGAATATATAAAAGTTCACTCCACGAATGTACCAAAATTA
AAGTTGACTTGTAAAACCAGCCAAGTGACAAGTCGATAGACAAACAAGAAATTTGCGTTGTGTTAATTGC
AGGTTGACAACTTTAGGCGCGTAACCAAAATTAATACCGAAATGGACTATAAATTCTTCATAGAAATCAA
AGATATGAAAAACCAGATTTAACTAGAAATTTAAACTGTATATGGGGGTCATACAAATTAAGCTGTGCGT
TTAAATAATTAATTTAAGTCTCATCTTACATATCTTCCGGTTGTGGTCTTCTATGTGCTTACATCACTGA
AAATAACATCTTTACGCTTAATTAATTGTACATTTTAAAGGCAAAACAACATCTAGGAATAATTTATTAT
ATACTGTATAATAAAAGTGCATTTACCAACCAACTTTTGTGTCCCTAATTGCAACGCCCACAATTTCAAT
GCTTATGAGACCTAATCGCGTGACTCGGCGAAAGTGGAAACAAATTAAAAGATGTGCTCTGTATAATTAA
TTTAAATTTATGAAAAACTACTACTACTACTACTTGGCAGATGATATCACAATTAGTCAACTGACAGAGC
GCAAGTTCAACAAAGGCTTTGAAAATATAGACTAGGTCTCTCCTTCAAAGCGGACATCAAAATTCAGGGA
AATAGTAATTAACATAAATTTATTTGTAGTCACTCAAAAAACAACGAACAAAGCCGGGAATTTGATTAAG
ACGGCACTGCATCTAAGATTAAATTGGATTTGGGTTCGTAGATGATTCAACGTTGCGTATACGTGATTGT
CTGATGGCAGACAAAAGTTATGGACGAATCTTATCACTAGAGTAAACTAAATTTAAGCTGAACTCTGAGC
TGCTGATTGTTAGATTGAAAATGATGTCGGGCCCAAATACGTTTTAATAAAATGCCATTGAGTGTGTTTC
TGAACGTTCAGAGGTTTCAAAAGCAAACTACAAAAGTTACATCTATTGTTTGTCTAGATGAAATTATATT
GCAGCGTCCTTCAGAAAAGAAATACATTTAGAATTTAATTGAAAACGAGAAGAAAGAAAGAAATTTCAGG
AACACATTCAATGTGGCAAGTAATAGCACAAATTGAAACGAATATAAAGCACTTTAAGCGAATAGAATTA
TGTAACTCAATTGCAATTAGTAAACAACGTCGCACTCAATTTGGACCAACATCAATTGGACCCTCCCCAA
CCGAATCCAGCTGAAATTTTACCCGCATTTTTCCTGCAGGACATCGGGCCCACACCGTTTATGTGGGCGT
CCATGTGCCAGGAGGACGACGCCACTCGCAGCGCCGCCGCAAGCATCACCACAGTGGCCCCGGAGGTGGT
GGCGGTGGCGGAGGTGGAGGCGGCTCCATCGGCGGATCCGGAAGTGTGGGCGGTGGTGCGGGCAAGGACA
ACGTCAGCGAGAAGCAACAGGAAGTGGAGCGACCAGGTGCGTCAGGTGTCTCGCATTATTGCAGCCTCGG
GTTTCCCTGTGCAGATATAATTGCAATGTGGTGGCTGCGCAGTGTGGAATGCCAGTAAATGTGGCCAATC
AGTTCTGGCGAGGTCGAGAATCGAGGCAAATTATAGGAACCTAAAGTGCGAGGAGAATTTCTGAAATAAC
AGTCATCTTCTGAGACATTTCGCTCGTCAGACGGCTAATCAAAACGATAGATGATTGGCTTTCAGCTAAC
CGCCAACAAGAAGTTCTGATTGTAATGGGGGGTTGCGCACTAATGGGTGACATTGAATTAGGATAATCGA
ATAACCTTCAGTCGACTGAAAGCAGAGGAAGCTTCTTGAACTTGGACTTCAAATGCGTTTCttttttttt
ttttGCAAACAAAAGCAATCACAATGCTTTCATTAGTAGGATTTAGTTTAAAAAGTTATATATTAATGCG
CCTATTTCCACGATTATTCTGCTTAATACTTGCGTTATTGCTATTCATATATCCTTGCATAAACCTATCC
CAACATTTGTTTTATTGTAACTACATACTACTATAATCGTAGTAAGCAACATTCTCCATCTTCATCAATT
AATTTTTTAGCAAATTTAAGCTCTATACGTCATATAACCCAATTTAGATCGTAACAAATTCCTTCTTTAG
TGTTTTGTAGGCAGTTAAATTCATTGCAAAACGTCTATACATACCGTAAACATCCTTAGTAGCACTCAAA
ACAATATCACGCAAACCCAACATAAACAGCAACAAACTCAAATGGGTCTAAACAGGACATATGGTGGACA
ACGGTCAGGGACCAATTCCCAGCATTCCCAGATCAGGGCCAAATTATTTTCGCCAATTAACTTTAGTACG
CATTCGCCGGAAGCCCAAGGATGCGGTGCAATCATTGTTATAAACGGGGTGGCAGTGGTAGGATGATGTT
GCTCCCAAGTCATAAATATTGATTAAGCTCATAAAGGTTTGCACAGGAAGAAAAGGCTGCACATAAATTA
AATGTTGTAAATGTTTATTTTATTTTGTAGTTTAGTTGATCATTTTATGCTTTCGATATATTTAGATATC

TTGCATTATTTTTTATCCATGTATAGACCTTCTGTTTCTCATAAAGTATCCTTGAACTTATAGTTGACTC
CGCCGGCACAGCGAGTTCAATTCATACTTGGCGAGGATGTGGACGATGGCACACACGTATCGCATCCCCT
GTTTTCGGAAATGGGGATGCTGGTCAAGGAGGGCGACGAGATCGAGTGGAAGGAGACAGCGCGATGGATC
AAATTCGAGGAGGATGTGGAGGAGGGTGGCAATCGATGGTCGAAGCCGCACGTGGCCACGCTCTCCCTGC
ACTCCCTGTTCGAGCTGCGTCGCCTGCTGGTCAATGGCAGCGTGATGCTGGACATGGAGGCCCAAAATCT
GGAGGTGATGGCCGATCTCGTGTGCGATCACATGGTCAGTGCGGGAACATTGCCGCCCGGTGTAAAGGAT
AAGGTCAAGGACGCGCTCCTGCGACGACATCGGCATCAGCACGAGTATGCCAAAAAGACGCGACTGCCAA
TTATTCGATCCCTGGCCGATATGCGCAATCACTCGTCATCAAAAAG
>dme_circ_0001712_junction_seq
ATATGCGCAATCACTCGTCATCAAAAAGACCTTGGACCATGAACTCCTCCAGCGGC
>dme_circ_0001713
TGCTACCGGATGGATAAGCCTGAGGAGAAAAGTGTCTTACAGATTGAAAGACTGCCTGATGGATGGACTG
GGCAAGCAAACATGGAAAAACCTCGGCAAACCAAACTCCGGTTTATCTGGGTAAAGAAGATAAACGGAGT
GGCCGGAAAATGCGCGGTAGCTGGTACAAGTGACTAACCCAGAGACACTTGCCTGCCAATTAGCCACGGA
GCAATTACGGCTGACAAGTTGTCGCCATAATTAATGAAGTGATGTCCACATATTGTGACTGCGATCAATT
GGTAAGCAACATGTTGCAAGTTTGTCACTGGCTGCGCACGCACACGCATTTGCCTATCAGAACGGCCACC
AAAGACGGCCAGGCAcggctgcaacaccaccagcaataggaacagcagcagcaacagcacaagcagcaac
accaacagcagaagcagcagcaacaccatcggcaacggcaacaCGACGGTCGGCTGTCGATGATTCAGAT
TCAGTCGAGTGCCGAACGAGCTGATGGACGCACGTGTGGCGCGGTGGTTGAAGAAATTCCAAGCTAAAAT
CCCGAAATCAGATTCAAGCAAACAGACAGTACCGTCGAAAGCGAAACTCGCACGCATCAAAAGTGTTGGG
AAAGCGTGAAAACTTCTGGCATCCGGaaaaagaaaacaaaaaaaaaGAGAAGAAAACTTCGAAATTCTAG
TGGATCCCCGGTGTGTGATAACTTCTGTGATCTCTTAGCTGCTGTGCGTATGGCGAGTGGCCGGCCGGCC
GGCCCCAAGAAAATTCTCCAAATGCGCGGCGGCGACTTTTCATGTGCTGCGGAAAACCCTCAAGCCCAAG
CTGCCCAGATGGATGGATCGGCGGTATAAAGAAGCACTGGCGACGGCGTCGCTGGCGGTCAGCAACACTC
ATTGCAACTGCAGCAGCAGCAGGAGCAGCAACATTGCAGCACCAGCAACACCTTCGAAACGGGTTTCCCC
GCGTTTTGATACGCCCGATCGTCTTGGGTGGGGGGAAAAGTGCGCCTTTTGATCTAGCGCTCTGCGATCG
GAGAGATTTCGATTTCCCAGGAAGCGCAAAGGAAAATACCACTCGGGAAAAACGCACATAGCTTAAGCAA
ATAAATCATCTTTTGCCGTTCGAAATAACAAGAGAAAAACGTTTGAAGTGCCAATGTGAAAAGTTAAAAG
TTTATACGGTCCGCTAATAAGCAGGAAAAACAAAAATGTAGCTATCTGAAACAAGCAAGTGGAAATCAAA
CGAAAAGTATTAAGCAGTCCAAAATAAGTTACACAAAAATAAAATAAAATTCACGCAACTATCAAAAGCA
CATAATCTCAAAACAAAATCGCATCGAATAGAATATTAAACAATTTTTATACGGCCACTTAACAATTTTA
TATTAATCGAAATCATTTTGACAAACATACAAGAAGTAAATTTAATTATAACGCTAAAGAAATAAATATT
AAATAACGTAGAACCCTAGCATCTAGTAATTTATTAACTAAAGTACTTACCACAAAAGCATCCACAATAT
ATCACGCCAAAAAGTAGACTATAGAAACCGCATTTAGCAAAATCCACAATTCACCAACAACCGCTTCGAT
AATCAACAAGAAATATTAAAAAGATCCAATAAATCAGTAAAATGCCATCGCCAACGGGAGTCTTTGTGCT
AATGATCCTCACCCACCTGAGTTTTGGTTTGGGCCAGGTGCGAAACGAGGACCAGCTGCTGATGGTTGGC
CAGAATGGCGATCTCGACAGCAGCAATCCTGCGATTcaccaccagcaacaccaacagcaccaacaacacc
agcagcatcagcagcaccagagcaatcacaATCTGAACAACGGGAACATGAACAGCACGATACTGAATAC
ACTGATGGGAAACAATGCTGGTCAGGTGGTGAACAGTTCCCCGGGTGGCGGCGGATCCATGATCAACCAA
TTGGGCAGCAGTACATCGAGTGTACCCTCGGTGATTGGAGGCGGCGTGGGGTCCGTGGGAAACCCCTGGC
ATTCGGCAGTCGGACTCGGAGTTCCTGGCAACGGAATGGGGCTACCATCGAGCCACGGTCTCGGCGGCAA
CATGGGCTCCCATCCTCATGGACACGCTCTGGCTGGACTGGCCAAATTGGGCATCATAGTGCCGGGCGGC
CAAGGATTACCTGGGAATCTGGGATACGGCGGGACCATGCTGAACGGCGGAGGAGTTGGAGGTGCTGCCG
GAATGGGCCTAGGAATCGGCTCCAACACTAATAACATGGATATGCAGCAAGGCCTTTACAAG

>dme_circ_0001713_junction_seq
CATGGATATGCAGCAAGGCCTTTACAAGTGCTACCGGATGGATAAGCCTGAGGAGA
>dme_circ_0001714
GTTAAGGAGAAATTCGTGAAGGGCAGTGTGATCAATGTGCTCAAGAACTGCGACCAGAAGGGTCGTCGTG
TGCTCATCGTGAACTGTGGCAAACTCTGGGATCCCAGCGATATTACCAGCGATGAGATGTTCCGCATGCT
CTACATGGTACATCTCGCCGCCCAGCTGGAGGAGGAGACCCAGG
>dme_circ_0001714_junction_seq
GCCGCCCAGCTGGAGGAGGAGACCCAGGGTTAAGGAGAAATTCGTGAAGGGCAGTG
>dme_circ_0001715
GTTCTTGAGCACATTGATCACACTGCCCTTCACGAATTTCTCCTTAACCTGCTCGACCAGGAGTCCGCGG
ACCAGAGAGGCGTACTCCTTGCGGAAGCTGGCTGTGGTTTTCATCTGAAAGTCATTTTCAAATTAGTCAA
GAGCTTAGCAAACTTAAATTTATGTATTTTATGTGAGGATAAAAAATTTTAGTCTAGGCAACCAACTTAA
TTTAGCTTATTATGCTACTCGTGTAGCTAATTGAAAACTTTATATTTTTAATATTTTTATAGGGTACACA
TTATAAACAGAGGAATTGCCTCTATAAATCTTTGAACCGATGACGCTAACTTCTTACGGAACTAGTCATT
GTAACTTCATCTGTTGGCAGGTGTGACATGGCTAGATTAGAGTCCATAATTGATAAGGTATTGACCATTG
GCCAGAACTATAAAATGCCCATATAAGTTATGCGGCTTCCTATGATGATAAATTTAAAAATGGCGAACAT
TCGATTTTTATGACCCGATTCAGTCATTCTGCTAAAAAGTCATTGAACACTCTACATGCATAAAGTGAAT
CCGAATCGGATCGAAATCGACCACGCTTATCAGGGTTTTCTTTGCCGGTCAGCGAGTTCAGAAGCAATAA
GTTATCAGCAGACGGGAACTGTAAATGATCCTGTTGTTGGGTTTCATTTCAGATCGTATGTTGCTTCTTT
TACAATATCCCATATATTACTTTAGCTAGACTATAAATGAGGTGGCCATTTTCTGGGCAAGTTTTTATGC
AACCGCTTAATCATATATTATTATGTAAGAGATATGTAGATAAGGCCATGGTTATAAGCTGATGCAACTT
TCTAAACTTGGGGCAAAAATGCTTTTCTTGTAGCTCTTATGCATATACAATATTATTCCCCCACTATCTA
GTGCACTTGTTTGAACTTCGGTTATAGACCCCATTTCAATAGCTCTGGTTGACCGATGTTTGCTGGCGAT
AAGGCGTGTTCGAGAAGTTCTATACATTTGATAATCCCTCAAATGTTGAAACAAATAGACGGAGACACTT
GTGTTGCCGGCGTTGATAAGCAAAAAGCCATTGAATTGTGTTTCTAAGGCGGTCAATTCATAATATACAT
ATATATACTATGTATGCTTGCGATTTGATTGAAGCTTCAGATGATTTTCAGAGTGATGTGTAATGTTAAG
CGATTATTAGTATTATGAATTGAATATTGCTGGAACAATTTAGATATTCCTTAAACTATGTTCCTTTTTT
TATCATCATTGGATTCTGGACTATGTTTAAATAACTTTAAATTTGTAATTTATATATATTAAGAACTTAA
CGTTTGGTTATATTGCATTATGTATTAGGGAAGTTAAATGATTCAAAACATGACATTTTATGATATTTAT
TAATGGTGAAGTTAAAATGAAAAGTGAAGGGCACACGAAACTGAGACACTTGCATTGCTGACTTAACATT
GCATTATCAGTGGCTCGCGATATGCTTTCATGGTACGCACCTTTTCCAGAGCTCCTTCGGGATAAAAGTG
GCAGGCGCGCAGGAAAACTGTGAGGAAAGCGTCGTCATCCTTGTAGTTCAGCTCGGGCGTGGCCTTGAGC
AGTTCCCGTAGCTTGATGATGGCCTCCGCCTTGACTTCTTCGGTTTCGCGGAGTTCCACACGGGCGATTT
CCACGGTCTCCGGCTTCAGATAGCCCAGGCGCAAGTGAAAGGCCTCCTCGGCGATATCGGTGGCAGCCAT
TATGTCGCTTGGTTTTT
>dme_circ_0001715_junction_seq
GTGGCAGCCATTATGTCGCTTGGTTTTTGTTCTTGAGCACATTGATCACACTGCCC
>dme_circ_0001716
AGACTATTACTTAACGTACGACAATATTTGCAATATCACAAATGACCCAACTTAAGTGACTAAAATACGA
ATCGAAAGGGGGAAGGGAGGATTGTGCGGATTTGGGGGGTTAGTTTTGGACTATTCCTTG
>dme_circ_0001716_junction_seq
TGGGGGGTTAGTTTTGGACTATTCCTTGAGACTATTACTTAACGTACGACAATATT
>dme_circ_0001717
TGACACTCTAAAACACCCAACTGAAAATTGCAAGCATTCCCGTGCCTATGCCAAAGATTAGATAGCCTTC

GAAGTCGAAACGTCTGGTGGACTTCGCTTAGCTAACGAATACTCTTTTTACATTCCTTAGTTATTAGATA
TGTTTTG
>dme_circ_0001717_junction_seq
ACATTCCTTAGTTATTAGATATGTTTTGTGACACTCTAAAACACCCAACTGAAAAT
>dme_circ_0001718
AACAAGCCACTCGTGGTGGAGCCGATTGATTTTGAAGCCTTTATAGCCAAAAATAAAACTGTTATCCAAA
ATGATCCGCAGAGGGAGCTACTCATCTATCCCACAGATGATGTATCGGTGAGTAAAAGAATATACTATAC
AACTGACAAATTTTACAAATGTACGTGATACAATCTAATTTTCTTTTCTGTTCCGATAGGAAATTATCAT
GCCCCGCAAGCAGCGAACAAATGCCAAATCAGTGGCGGACCGATTCGATCCTCCCAATGAGGCGATTGTG
TGTCCCCTTCATGGTCCTTCCATGATAACGAATGGAAATGGACACAGTCAAGTGAGTCGGCAGGGTAGCA
TACAATCGAATGGTAGCCACCACAATGGAAATGGTAATGGGCACACAAGCAGCAGCAGTAGCAGCAGCTT
GAACAATTCCAATGGACATGGTCAACTCTCCAGAAAGAGTTCACAGTGCTCGAATGGCTCCTCCAGTCAA
AAGGATTCCTCCTATGAGTCAGCCCTGTCGTCGATAACACTCCGATCGAATTTGGCCCAGCCGGAGGAGG
TGGATGAGTTTGCGGACGATGGGAACGCCGAGGACTTAGTCGATGGACAGCCTCACGGATCCAGGGCTGA
GTGCACCAGATTCACACGGCAGGCTCTCTACACATACAGGGCTAAGAATCACTTGATCCATTATAAATAC
AATGCCTATGGTGGAAACTGTCACGATCTTCCTAGGCATATCGCCTGCAGAGGAACTGGTGGAAGAGATC
TATGAAATCGATGCGGATCAGGATCGCATTGACGAGCAGATGACTCGCTCACAGGCGGACACCATTACCA
AGCAGGGATATCTACTAAAAGGACCCGATTCGGCTTCAGATCGAATGTTTGCCAATATTGGCAATAAATC
GTTTAAACGACGCTACTGCTATCTGCGACAGGAGATAGATGGAACATATATATTAGAACTGCACAAGGAC
GAGAAACAAGGCGAAGCCAAAGCCACAATAGTAATGGACTTTTGCACAGATGTGGTTCAGGAATCCCAAA
CGTGGCCGCTTTTGTTTCGAGCTCCGCATGACGACGGGTCATAAATCCTTCACACTGGCTGCCGAAAACG
AACAGGATTTTAAGGACTGGCTCAGCAAGATTTCATCGGTTTTGGCCCAAAATCGCGCACAAGAGGAGAA
GCGAAATGCTTCACTGGAGCGGCACCCATCTATTGGTTCAAATCCAAGTCCACAGCTCCAGCCACCAGCA
ATGGATCCACCTACCTTTGGCACTTTGAAGGGCTTAGATCAATCACTGCATCCACAATTAATGAAGTATG
GACGAGAAACGGATCACTCGATTGCTTTGGCAAGAAGGGAACAGCGCCGCCGCCTTTTTGCTTGCTATCA
GTCGCCAGTCAAGGGGAGCGGTAGCGATAATGTTGAGCAATATCGAGAGCACTTTGGCACACGGTTACTG
CTCACCTGCCATAACCTGCGCTTCCGGCTGCAGTGCATTCCACAGGATGAGAGCTCCGCGGGTGGAGTCG
AACAGCAGGTGGAACCCTATATCACCAGCCTGGCCTTGTTCGATGCCAAGGCAAATCGAAAGTTAAGCGA
GAACTTTTACTTCAATGTAAATGAGCAGTGGGCAGCCCAGCTATTGCCGAATACTCCGGTACCTTCGTCA
GTCGCTGGTTGTGGAGTCCCTAGAAAGTCAGCGGAGGGCGATGAAAGGAGCACTGCCTGTCAAGCACCGC
ATTCCCTTTTTGATGGAGTGTCTGCCGAGCTGTTGCGATCGAATCGACAGCAATTTCAGCAACTGAGACA
ATGTCTACTTTCCGTGACAGCTCCACATGCCGATATATATTTGGGTTGTGCGCATAGAGAAAATACTGCA
ATCAGGAATAGCCCAGGTTACCGAACCGTATCTGAAAGCTGGCAAGGATCCAAAGCTAGGCCAAAAAGTC
TACAAAGCGGCAAAGAGCTATGCTCAACATATTGGACATTACCGGCAGCCTTTCGCCTGGGCTGCAAGAC
CGCTCTTCAAGCAATACAGTCACGAATTGGATGTAGATCCCAAAAGAGAGTTTGAGTTCAGTCCCATTTA
TCGCCAGGAGATACCCAAACTGAAGGATGAGGAACTTCTTAAGCTTTTGGTCGATTATCGCAAGCCGGAA
AAACTAAGCAAACTTACAATTATTCCTGGCAGTCTTAAGATGCAGATGCAGTTCCTAGACCAAACCACAC
CTTGCGGCTTAAGCAAATCGCTGGCACCCTTGTCCACCTTTAGCCCATCTTCCAAGCAATCGCCCACTGT
AGAAGTGGCAGAATTCCAAAGCCAAAGTGAACGGGATGCCCACCCTTATACCAGTTTCTGTAACCACCTC
TATGTGTACCCACTGAGTTTGCAATTCGACAGTCAGAAACTGTTCTCAAGAGCCAGGAATATTACGGTCG
TGGTGGAGCTACGTGATGGAGATGGGGAGTACAGCAAACCCTTAAAGGTGCATATATGGTCGCCCTGGTC
AGGATCTTTTAGTGTCCCAGATCGCCTGCCCGGTGCTGCATCACAATGTAACGCCCACATGGTACGAGGA
AATCAAGCTACGTCTACCGCTGGGACTATTTCCGGAACACCACCTGCTCTTTTCCTTCTACCACGTGTCA
TGTAATCTCAGCAAGAAAAGGGATGCAAATGCGGCGTTCGAAACACCTATTGGCTATGCCTGGTTGCCAT

TGCTTCAGAAGAATCGGATATGCCTGGAGGAACAACAGCTTCCGGTGGCTGCCACATTGCCAGTCGGCTA
TCTATCTATCCAACCCTTGGGATGGGGCAAAGGGGCAGAACTGCGGTCCGGATATCCAGTGGATCGACAA
TCAGAGGAATTTATATACGGTGGGACTGCGTCTGGATTCCACGGTTCTGACTGCCGATCAGCACTTGCAC
AACTTTTTTGGACACTGTGAAAGGTTGCTGGAGGGGGGCAAAACTGGAGCAGTGCCAGCGGAAACGGAAA
CCTGCAAGATCTTGAAAGCTGCCCACGCCATTGATATGAAATCGTTGATTAACTATTTACCCACGGTCCT
TAATGAGCTGTTTACTCTGCTAGTTCACACTCAGTCGGAAGAAATCGGCTTGAATGTAATCCGCTTGATT
ACAAACATCATCCATTTGATCAGTGATCAGGCGAAAAGATCCGATCTCTTGGGAGCCTATGTGAAATACG
TATTTCACGCACCCTACTATAGCCAACAAACTGCTAGGCAAAGGACGGTTCATGGAGAACTCTGCAGGCA
TTTACCCTACCTCCTTAATCCTAGCAATCCGGATTTCCTCATCGTCAACAAGTTCATGAGATACTCGTCG
ATATTCTTTGATCTGATTATAAAAAGCATGGCTCAGCACTTGTTGGCCACGGGTAGGATTCGAATGCTCC
GCAACGAAAGGTTTCCCAAGGAATATGGTGATCGTGTGGAACAGTTGATTAAGGCACTAATGCCCTACAT
AACCACACGATTTGAGGATTTAAGTGAGGAAACTCATTTGTTGAATCGATCTTTGGCTAAGTTTGTTCGC
CAGTGTTTGAGCTACATGGACAGAGGCTTTGTCTTTCGTTTGATCCGCTGTTATATGGGAGAATTTTCGC
CTGGTAATCCGCGTATACTTCACGAATACAAGTTCAACTTTCTGCAGGAGATCTGCCAGCATGAGCATTA
TGTGCCACTGAATCTGCCATTTGTTTTGAATCCAAAGAACAGACCTCCTGAAATGATGCAGCACTTCACT
CTTTCCGAACAATTCTGTAGACAACATTTTCTATCCGGGCTGCTACTCCAGGAATTGAAAAGTAGCCTCA
ATGAAGTGGGTCATGTGAGAAGACATGCGCTTGGCATTTTCAAGGATCTCTTGGCCAAACACGAGTTGGA
TAATCGATATCAGCAGAAAGGTCAGCTATCAAGGATTGCCCTGCTCTATGTTCCGTGGTTGGGCGTCGTG
ATGGACAACATCCATCGGATTGATGATTTATCGGAGTCTGGAGCTTGTACGCCCAATGGACACGTTTATG
CGGACTCCGCTTCCTATACTAAGCGCTTGAGTTGTTCAAGTAGCTATGTGTTCAGCAAGGACTCTTCCAC
TTTTGGCTCCTTGACGTCCACGCCGCGTTCAAAGAATCGTCTGACTATGCACTGTGATCAAGCGAGTCCA
TACCGCACCTCGGTTCACATGAAGGAGCACAACTATCTGGCCGCCATCGCTGGACAACCCATTAGCAATG
GGATCTCCAATCTCTCACTAAATTCAAATACGGACTCGGGGGCACTCTCAGGACACCACCACAATAGGAG
CCTATACGAATGGAGACACAGATGTGGCCCTACGAAATGGTCACAATCGCTCAGTGAGCGTTACCCATGC
ACAGATCCTGTCCCGCTGCGATAAGTTCAGTTCTGTGGAAAGCAAGGATCTTCTCCTGGGCTTTCTTTTC
ATCATCAAACACCTTTCACAAGAGCAAATGATTGGTTGGTGGCAAAATTGCAATGAATCCGAGACGCTGC
AGTTTCTCTCCATTTTGGATCTCTGCCTGCTGCAATTTCGATATGTGGGCAAGAAAAGTGTAGTGATAAC
TACAGAAACCCGTCAGGGACGTTTGGCCAAGGCTAACACTCTTCCAGCTAGGACTCAACCACCCACGGGT
TTGGAAAACGGCAGTCAGGAACAGCAGCCATCAAGTGGAACCCTGAATCAAACCCGGGAACACCTATTAG
AGGACATCGATACCCTAGCTAGAAATCAGTTGGCTCTCTATGAATCGAATTTGGCCACCGAAGTGGGCAT
GATTATTTTGGACTGCTTAGGTCTGTATGTTCTGCAGTTCAGACAACTTTTGGCCGATAGTTTAGTCCTG
CCCAAGGTGGCCAGAGTTTACCTAAGATTCCTGCAGTTGGGTCAATCAGAAAGGCTCTCCAAACACGTCT
TTGCTGCTCTGCGAGCATTTATTAACAACTATGCAGTGGCTCTTTTCAAAGGAAATGCCATGTTGTGTGG
TCAGATGGTATATGAGCTCCTTAAGGCCTGCGATAGTCGCCTAGTAGAGATTCGCCACGAATCTTGTGCT
GTTTTATATCTTCTCATGCGCAGTAACTTCGAATTCAGTGGCCGAAAGGCTCTAACTCGCGTACACTTGC
AAGTTATTATCTCAGTATCGCAGATGATTGGCAACGTAATTGGATTGAACAATGCTAGATTCCAAGAAAG
CTTGTCGATCATCAATAGCTATGCGAATAGCGATAAGGCGATGAAGGGCACAGGCTTTCCTATGGAGGTT
AAAGACCTAACGCGTCGAGTGCGTACTGTACTTATGGCCACTGCCCAAATGCAGGCTCATCATATGGATC
CTGAAAGATTGCTGGAACTGCAGTATTCCCTGGCCAATTCGTATGCCTCCACACCAGAGCTTCGTCATAC
TTGGTTAGTGACAATGGCCAGAAATCACGAGCAGAATGGGAATCTGTCGGAGGCTGCCTGTTGCCATCTC
CACATTGCTGCTTTAATGTGCGAATATCTCCGATTAAAAGGTGGTTGCACTCTTAGTTGGTCATCCACTG
CATTCGGAAAGATATCTACAAATATACCCCTAGATGAGCAAGGTCTCAAACTGGATGCCGGTGCTCAGGA
TTCCCAGTACACCGAACAAATGCTACTGGAGCAGCTTAAGCTATGTGCTGACTTCTTGGATCGCGCTGAA
AGATTTGAGTGCCTTGGAGAGCTCTACAAACTCATCCTGCCCATGTACGAAAGAGATCGTAGTTACCAGG

ACCTGGCACACTGCTATGAGCATCTCACGCAAGCCTATAATAAAATAGTTGAAGTGAATCGGTCAGGAAA
GAGAATGTTGGGTCGCTTTTACCGTGTTGTATTTTATGGATTAGATGTACTTTGAAGAAGATCATGCCAT
CGAGTTTGTGTACAAGGAGCCCAAGCTAACATCACTCAGCGAAATCTCTGAGCGACTTGCCAAACAATAC
AAAGAAAAGTTTGGAGCCGATGTAGTTAAGATGATCATGGACTCATCACCGG
>dme_circ_0001718_junction_seq
GTTAAGATGATCATGGACTCATCACCGGAACAAGCCACTCGTGGTGGAGCCGATTG
>dme_circ_0001719
GATATCTTTCCGAATGCAGTGGATGACCAACTAAGAGTGCAACCACCTTTTAATCGGAGATATTCGCACA
TTAAAGCAGCAATGTGGAGATGGCAACAGGCAGCCTCCGACAGATTCCCATTCTGCTCGTGATTTCTGGC
CATTGTCACTAACCAAGTATGACGAAGCTCTGGTGTGGAGGCATACGAATTGGCCAGGGAATACTGCAGT
TCCAGCAATCTTTCAGGATCCATATGATGAGCCTGCATTTGGGCAGTGGCCATAA
>dme_circ_0001719_junction_seq
TGAGCCTGCATTTGGGCAGTGGCCATAAGATATCTTTCCGAATGCAGTGGATGACC
>dme_circ_0001720
GGCCTCATTTTACGAATATGCAGATGCTGTGCCAAAATATTTTAACGACCAAATAGGGAAATTGAGCGAA
GGGATAAGTTTCACGGTGACTGGGAATCTCTCTGTAAACTGCGAGAGGTATGTTAAGTGGCCAAGTTTAC
GTGATTAATTTTTAAACTGTTCCTCGTCCTTTTTAGGTTTTCCATCAATCTGGTTTATAACAATGACTCT
CGAGATGTGGCGCTCCACATTAATCCACGGTTGCCACAAAACTATATTGTCCGCAACACCAAAGTACAGG
ACATTTGGGGAAACGAGGAAGTGTCCTCGGCATTGCCGTTTCTACTAAGTCGAGGCGAGGAGTTTTCCAT
TCAAGTGCTAGTTACTGAGGCATGCTATATGATATCGGTAAACGGACAACACTTTGCAGCGTACACTCAT
CGAATCCCTTATAGGGATGTTCGCATTCTGGAAGTGAAGGGAGAT
>dme_circ_0001720_junction_seq
TGTTCGCATTCTGGAAGTGAAGGGAGATGGCCTCATTTTACGAATATGCAGATGCT
>dme_circ_0001721
CTCCAACTCATCGATCGTTTCGGCCATTCCCGCCTCGCAGAGATTGCTGGAGGAGGCGCTGGCCAAACCA
GCACCGTACCCCATGATCTTGCTGCCACTGCACGGTGGTTACTGGATGGATGGCACCGAACATGAGTGCG
GCTACGATGCGAGAGGAAATCCTCTGCTACCACAGACTACCTGGATGGCAAAG
>dme_circ_0001721_junction_seq
GCTACCACAGACTACCTGGATGGCAAAGCTCCAACTCATCGATCGTTTCGGCCATT
>dme_circ_0001722
GTGACTGAAAGTTAGCGAATGGCGTGTAGTTTTTTCCCGTGTTACCCAATCCTGTCAATCGGACCTATCG
AGAAAGTCCTTTGCATTGCATACTTTTATGGTCGACTGCTGTGCAGAGTGAATGTGCAAAAAGATACAAA
ATGCGAGCAAAAGTGAGGCCAATAAAAAGGCGATAAAATTGACGCCGATACAAGAGGAGGGAGCAGAAGG
AAGAGCCGCTCGTCGGAAGAGAAACGATAAGCGTATCCTGTCGGCAGAGTGCATAAAAAGGCGATATATA
TTCCGGTGTCCTGTGCTGTGTGGCTCGTGTTTGTTTGTTGCTGCAGCCTCTGATAGCCGACGATTCAAAT
GCCTTGGTGATTAATGGGCCATTGGCCATTGCCGACGGGATGCGCATAATTCAACCGGTCCAAGGGACCA
GATACGGTCCATGGCCGGCCGTGGGACTGAGGCTAGTCCTGGCCCTTGCCTGGGCAACGTCGGCAGCGGC
TGCCATGGAGTCATCAGCCGAGCTGCAGGCCCTGGGCTACGAGGCAATTAGGCCAGGTGCTGCCTCAATT
AGCACATCCAGCCCATCCAGCTCGCCACCCGGAGAATCGGCATCGACTGTGACTGCAGGGGGGGACTCCGA
TTCCACCGCGCTCCGATTGGAAGTACAAACGGACGAAAGTCAAACGCCGGCAGCAGCGCCTCAATTCGCA
CAGCAATCTGCCCGGAAGCACCAATGCCTCCCACGCCCACCACCTCCTCAATCTGCCCCCCAGGCAGCGA
TACTTGAAGGTCAACCAGGTGTTCGAAAGCGAACGCCGCATGTCGCCGGCCGAAATGCAGCGCAATCATG
GCAAAATCGTGCTGCTCGGACTCTTTGAGCTGTCCACATCGCGGGGACCACGTCCGGATGGTCTGAGCGA
ATTGGGAGCTGCCACCATGGCCGTGGAGCACATCAACCGCAAGCGCCTGCTGCCGGGCTACACCCTCGAG

CTCGTGACCAACGATACTCAGGGTGAGAATGTCATTTCTACGTGGCGTAACCGTATGCCCCACGTTGGGC
GCCACTTCTAGGCGTCGCTGTTCCATTACGAAATTGAATTTTCATTCAGTAGCGCGTGCAACATTAAAAC
TTAATAAGAAAATCTGCAACAACAAGAACAGTGCGGATAGCAGCTGCGGCTGTGGCCCTAAAAGAGAGGC
AAATATGTCATCAACCTTCCGTCTTTCGCGCTTTTATTGCGATGAGTCGTTGCCGGTTTTGATGGGGCAC
GCAAATGTACAGAACACTGTCACTGAAGAAAAGCAATGCCACTGACTAAGTTACCAACAATGTAAACTAT
TTACCTGCAATCATTTTTAACTTGGTGCAGTAGTGTATTAAAGCTACAATTAATTGATGGTaaaaaaaag
aaaaTGCATAATAGTAGTTTAGTTTCTGATAAATTAAACCGTCAATACGTAATACCTCGACTGTGATGTC
AGCACTTAGTGAACTAGGTTTTCAAACGATTTTTTTAAGTGTATGCATATGCACATCGACCTTAAACAGA
CTGTCATATCTGGAGCCGCATCTCCGGCTGTGCCTCTCTAAAAGTCGACATTTTAAAAATGTTTATTGCG
CCATCATGTTGCCTTTTTATTGCGTCGAAACGAGTCAGCGAGCAACGGCCTCTGCCACTGCTATTTTCCC
TGCCCCCTCTATTACTCATCTCTGATGTGGTGTCCTTTGCTCCTCCGTTTCTTCACACATTTCTTCTCCG
ACAATCAGGGTGAGTCATCTGCGACTGATTAAAATGCAAATAATCCAGCTCGCTCGGTTCATCCAACCAA
CTCCTCGTATCGGGCAATTTCTCAAAAATCTAATCAACTCACGTGCGTAGCTCGATTAGCATAAATGCTG
CGGAATCCAGCCGATTCCAGCACCCCATAAACCTTTGAGCCATAAACATTTCCACAGCCCACAAATTATG
AGGTCCACAAATTCACTTAGAGCATTTGCATTTGGCCTTTGAAGTTTCATTTCGGCGGTGCGTGTGTTAA
ATGAGTGGGGAAACTGGGAAGACACATGACCTAGAGGGCTGGATTTCTTGATGCGTGTCTTGCATTTTAA
GCGACCCCATGTACGGTAGAGATAGTCTAACGATTTCGTTCGATGAAAGccttctcgttgtcctgtcttg
tccgtcttgCTGTGCGTGTCCGAAATGAAATGATTTATTTGATTAGACAATCCCGAGGCTGCGATTGCGT
TTCGCGATGGTTTCAGCAATGGCGATGGCGATGGTGATTGCCAAGTGCGAAGCCCCCTAAGTCCGGCCTG
TCATCGCATTCGCATTCACCATCGCAGACGCGTTTTTATGGCTCTTTTAGTGCTGGTCATGAAATTGGGG
CAGGAGTGGTAGCTGCCATAGGCAATCTCATTACTGGCGACATTTCTGCGGCATTCGAGGCTTGTTTAAT
CGCGTTTCCACTTCGCAGAACAATCACATGTTCAACTCCCAGCTGCGCAAGCAATCCGCCCAGTTCCACG
GCCAGGATGGATTTGGCTCCGGTTATGGTTCCAGGATCAGTATCGCTGCAACGCAATCTGACTCTCGTCG
GCGGAGGAGAAGGGGCGTGGGAGGCACCAGCGGAGGGCACCTCTTTCCGGAGGCGATCTCGCAGTACGCG
CCGCAAACCTACGACGCCGTGTGGGCCATCGCCCTGGCCTTGAGAGCCGCTGAGGAGCACTGGCGGCGGA
ACGAGGAGCAGTCGAAGCTGGACGGATTCGATTACACCCGCAGCGACATGGCCTGGGAGTTCCTGCAGCA
AATGGGCAAGCTCCACTTCCTGGGAGTGTCGGGGCCCCGTTTCCTTCAGCGGCCCAGATCGCGTTGGCAC
CACTGCCTTCTATCAAATCCAGCGCGGTTTGCTGGAACCGGTGGCCCTCTACTATCCGGCCACGGATGCC
CTGGACTTCCGGTGTCCCCGCTGCCGGCCGGTGAAGTGGCACAGCGGGCAGGTACCCATCGCCAAGCGGG
TGTTCAAGCTGCGGGTGGCGACCATCGCTCCACTGGCCTTCTACACCATCGCCACCCTCTCCAGCGTGGG
AATCGCTCTGGCCATCGCCTTCCTGGCGTTCAATCTGCACTTTCGGAAGCTGAAGTAAGGCGATAAGTAC
ATAAATTATTGGCTTACTCTTTTTATGGGCCCTGATTGTTTGAATATATTATTTTTCATTAAACGACTAT
AAAATAGTAAGGCGATAAGTACATAAATTATTGGCTTACTCTTTTTATGGGCCCTGATTGTTTGAATATA
TTATTTTTCATTAAACGACTATAAAATATTTAGTGGCCATATAAAAGAGAATTTGTGCGGGCTGTTTCTT
TATATTCCATGATGAGTTAACTGCTTTCGGTGCTCAACCCATTGAACCCATTACACATGTGACTTGTTTC
GGTTTTTGAAGTGAAGTAATTGCTTGGCTTTCCAGCTGTATTTCTGACCCATCGTTAGTTGAGTTCGTAT
ATATGTTTCGCATTCTACGGTTACTCTGCACTTTCTCTGCAAAGGATTGCGGCGCCTCAGCTTATAACCA
TGACCATCCCCCTTCTCACTCCACAGGGTGAGTTGTTATCCTTCGGCTCCGACATCCCCATCCCTATCGC
CATTCCTATCCGAATCCGCATTCTCATCCGCCTTTCCTCTCAGCCGTCTTTGTTTGACTTTGACGGGCTT
CTGTGTGTAAGCAAGCATGTGGGGTCCTCATCAAATATGACTTTCAGTTTCAGGCTGGTAACTTTCGAGT
CGCGCTCATGTGTTTGCTTTATTTATTCACTGAAAGAAATATTGCTTGAACTAATTGTCGTATTCGTATA
CACGAAGATGGCGATAAAATAGCCTGCGAAGTATAAAAATTACTGATAATTGTGGCAATTTTTTGTTAAT
TTCTATTATTTTAAAGTGGATTATATAAACTGAATATTGATTATTCCGCGTGTCTTTTCAAGATACACGA
CTGCCCCTGCTGGCTATAAATAGCCGTTCGGTTTCGCTGCAAATCTGCTGCTCTTTAAGCCAGAAGTCAA

GTCAAGTTTGACGAGCTTTCTTCTATCCCGATCTGCGCCTTTCCACTTTGACCCCCTTAACAACTGCCTC
GAACGCCACTCCTGGGAAATTTGCTGGTCGCTGTTGTTTCTGTTTCTGTAACCACTTCTACTGTCGTTCC
GCTTTTCCAGTTGCCTGCTTGTTTTTGTTTTGTTGAGGCTTGTGGCAGGGAAATGCAAACACATGACACG
GAAATTTACAACTTTTGTGATATTCCTTTATTTGCATAAATTTGAAACGCACAACAAAGTCTCAAGCACT
CTGAGAATTGTGGTCCATTGATGACTTGTTTCCCCCATTGTTGCCGCCGGCTTGTTTTCTTTGGGTCTGC
CTGACATGACACTTAGTGTGTTACTTTGCTGCTGCTACTACAGTACTTTCTCCCTAACCTGAACTTCCCC
GTCATCCGCTTATCATGCTCACCAGGGCCCGCGTCTATCTGCTCTCCGCCGGATTCTCGTTGGCCTTTGG
ATCGATGTTTGCCAAGACCTACAGAGTGCATCGGATATTCACTCGTACCGGCAGCGTTTTCAAGGACAAG
ATGCTGCAGGACATTCAACTGATCTTGCTCGTCGGCGGATTGCTTCTGGTGGATGCGCTGCTCGTAACCC
TTTGGGTGGTCACCGATCCAATGGAGCGCCATCTTCACAACCTGACGCTCGAGATCAGTGCGACTGATAG
AAGTGTCGTTTACCAGCCTCAGGGTTGAAGTTTGCCGTTCGCAGCACACGCAAACGTGGTTGAGTGTCCT
GTACGCCTACAAAGGCCTTCTTCTTGTGGTGGGTGTCTATATGGCCTGGGAGACGCGCCACGTAAAAATA
CCTGCTCTCAATGACTCGCAGTACATCGGAGTGTCTGTATACAGTGTGGTCATCACCAGCGCCATCGTCG
TGGTGCTGGCCAACTTGATTTCGGAGCGAGTCACCCTGGCCTTCATCACAATCACAGCTCTGATTTTAAC
CAGCACCACTGCAACCCTTTGTCTGCTTTTCATCCCAAAACTCCATGATATTTGGGCAAGAAGACGATAT
TATCGATCCGGTTATCCACAGTATGGGCCTTAAGATGGAGTGCAACACACGCCGATTCGTGGTCGATGAT
CGCCGAGAACTGCAGTATCGAGTGGAGGTGCAAAACAGGGTCTATAAGAAGGAAATCCAGGCTCTGGACG
CCGAGATTCGAAAGCTGGAGAGGCTACTCGAGTCGGGACTAACCACCACCTCCACCACAACTTCGTCGTC
CACATCACTCTTAACTGGGGGAGGTCATCTAAAGCCAGAACTGACGGTAACCAGTGGCATCTCGCAGACT
CCGGCTGCAAGTAAAAACAGAACTCCAAGTATCTCGGGAATACTGCCCAATCTCCTGCTTTCCGTGCTGC
CTCCTGTGATTCCACGGGCCAGTTGGCCGTCAGCAGAGTACATGCAGATCCCGATGAGGCGTTCTGTTAC
CTTTGCCTCCCAGCCCCAATTAGAGGAGGCCTGCCTGCCTGCACAGGACTTGATTAACCTCCGTTTAGCC
CACCAGCAGGCCACGGAGGCTAAGACGGGCTTGATAAACCGATTACGAGGGATATTTTCTCGCACCACTT
CGAGCAACAAGGGATCCACCGCCAGCTTGGCGGACCAAAAGGGTCTGAAGGCGGCCTTTAAATCGCACAT
GGGACTGTTCACCCGCCTGATTCCCTCCTCTCAAACGGCGTCCTGCAATGCCATATACAATAATCCAAAT
CAGGATTCCATTCCCTCAGAGGCGTCCTCCCACCCGAATGGTAACCACCTAAAGCCCATCCATAGGGGTT
CATTGACCAAAAGCGGTACTCACCTGGATCACCTTACCAAGGATCCGAATTTCCTGCCTATCCCCACTAT
TTCTGGCGGTGAACAGGGGGACCAAACGTTGGGTGGAAAGTATGTGAAACTGCTGGAGACCAAG
>dme_circ_0001722_junction_seq
AAAGTATGTGAAACTGCTGGAGACCAAGGTGACTGAAAGTTAGCGAATGGCGTGTA
>dme_circ_0001723
GCGTTCGGAGTTTGACACAAACTTTCGCCCTGGTCGGTCTTTTTGTTTAGCCATTGTTTGTACCAAAACA
TCCTTTGAGGTGTATGTCAATCGGCAGTTTATGACTGATTTTAAATATAAGGTTAGCCCTGAGGTGGTGG
ACACTGTCTACATACAAGGAGAT
>dme_circ_0001723_junction_seq
GGTGGACACTGTCTACATACAAGGAGATGCGTTCGGAGTTTGACACAAACTTTCGC
>dme_circ_0001724
TATCAGCCGAACAATGGACTCATTTGACATATCAAGCGATTCGGAGGAAGGTCTCCTGGAAGAGATTGTC
GTCGGTGGAAGCTGCCATCGCCAGCACTTGCTTCGCCTGCAGCAGTGGCACTCCGCCCACTCATATGTCC
ACTCGTATGCCTCGGAGTCGCCGATTCATTATTGTGACCCTGCGGCCGTCTATTCGGATGATAATAGCCA
GGAGCCGGGTCAGGATCCCCAGCCGCCGACCATTCGATGTAGCACCAGTAGTCAGAGCAATCTGGATGTC
CTTCTGGCTGAGGGAGCCGCGGACGAGGTATACGAGGAGCAGGAGGAGGAGGGTTTTGAGCAGGAAGAGG
AGGCAAGTGGCTCCTCGGGAACGCAGAGGCTTCAGGCTCCAGCCTGCACTTCGTCGGCTGCTGGGGCCTC
CTTCAACCATCAACTCACTGCCGATATGTGCAACTACAAGAACTCGAAGAGCAGGAGGTTGGAGTACTCC

CTCAAGTGTCTCAAGTATGGCCGCAGTAATCCCTCCCAGGACTCAGCCTTTGGCTCTCTGACTGACAACG
ACCTGTCCATTGGATCGTCCAGCATTAGGTTGAGCAGCTTTCAGTCCATTTCATCGCCCATTGACGAGGG
CGTCGAGGATGTCATCATAGCCACCACCACAGGTGGTAATGAAACCTGCCTGGAACTAGCCACCATTCCT
AGGAGTATGGTCTCCCAGGACTCGGCGATTTCATCGCCCTGTCGCGAGAGTTCCCCAAACAACTTCCTGC
ACATTGACGATGCGATGTCGGGAACGGGTTCAACTTCCTCTGGCTCTAATTGTGGCTCCTTCGGAAATAT
ACGTCCCCAACAGTTTCCCGTTTCGCTTTCACCAAAGATTTTGGTAACGGCCACCAGCAATTCGAGCAAC
TCGTCCTTAGCATCCAGCAGCAATGCGGGTCAGGGTCAGGCCCAGCTTCATGGACAAGGGCATCCCCAGC
AGCAATTTGATCCGCCCAAGATTCTAGTCAACGATCAGAACTCCATCTACACCACATCGCCCTCGAAGCG
ATCGGGGATGATAGAGGTGTTCTCACAAAAATATCGGGTGTCCCTTCCAGGTGAGCTCCTTCGAGGACAT
GTCCCCGAAGCGAACCTCAGACAAACAGCACCTGAAGCATGTGAATCGGCTGGCCTTTCGCTCCTTGGAG
GAGGAGCGTCGGATCGATAATGCCTTTCTGCCGATGGCAGATCGCACCCACTCGGACAATCGCGTGAATA
TGCAGAGCGAGAAGTACAAAAAGCTCACGCACGCCTCGTTCCGCATTAGCAAAGGTCAAGGCCAGGACTC
GCCCACGGCCACACCCACCAGCCAAACGTCCCCCGGTCAGCCTGGCAATTGCATTGAAATGCAACGAACC
GGAAGGCAGACCCTCTGGCGAGACAGCAAGTTCTACCGGAAGAACAGAATCGCCAAAAGCAACGACTCCC
TAATGGAAAACAATCCCTCGCCCAGGATATCGCCATGCTCCTTGCGGGATAATCAGTACGAGAGCAGAAG
CAGCTCACAGGCCAGCAGGAGATCGCTCAGTGGTAGTCAGCAAATGCTGAGTGTCACCACCAGTTTCTGC
GGCAACGGCAGTGCCTCCAGAAATTCGGCCAGATACTGCAGCTCGAAGAGCGAGGACTTGGGCGAGTCCT
CCGACTATCTGAGAGTTATGGATGCACGAAAATCGTACTCCGAGCGGCACCTGGTGCGACTCAAGCAGAC
GGCCATAAACAACACACACAGCGAGGACGATATGAGCTTCAACGATGACAATAATCAAACGGTATCATCG
CAGGGTCAGGGGTTACGGCAATGCGGCATCCAGTACAATCAGAAGGAGCACCACCAGAAGCACTATCAGG
GTGGCAGGTGGAGCAGCAGCTGCTCCATTGGCGGCCACCTGAAGACGACCAATATTAAGACCACCTCGCT
GTCAGCTCAGTCCAGCCAATCGAATTTAGTGGCCACCTCGGCAACTGCCAGCTATCGCACACCATCCAAG
TCCCTCGACCAGAGCATCGCCACCATCGCCATCCCGAGCATCCATGCCACCAACCAGAACAACACCGCCC
AGAATagcaccaccaagggcagcagcaccaccagcagtgacagcagtagcagcaccaccagCTCGGTGGA
TGAGTCCCCGTCGAGACATACCCTCAGCGGAGCGGACCTCTCCAGGATCTTCGTGATGGACATGGACGAT
GCACCGGGCAGTACATGCAGCGAAGAGAAGACTCCGCTTCTGGACAGTGTGGAAATGTCCCCGATGAGTC
CAACCGATCCAGAGGAACCCGACCTGGACAAGCTCTAATGGCCATCCCGTTAACACTATGTACACGCACT
TACAGATACGTTTTGAGCAGCCGAAATGCAAAGCATGTGATAAAGAAACCAAATATAGATGAAATCGAAC
GGGGAACCATCAAGTTATAGAACTACTAGGCTAGG
>dme_circ_0001724_junction_seq
CATCAAGTTATAGAACTACTAGGCTAGGTATCAGCCGAACAATGGACTCATTTGAC
>dme_circ_0001725
GACACTTGCAGCGAGGTCAACGTCCCGGCAGCGTCGCATCCGGAGCCGGAGCTCATCATCAGCTGCCACC
GGGATCAGCGGGATCAGTGCCATCGGAATAATCTCCAGAGGGAAAGCAAAGTTATCACCCGATCACAATC
ATCGGCGTCGACCGCGCGGCATGCTCCAGTGCATCAACTGAAGCAGCAGCAATCTCTGCCCACACCTCTA
ACACAGCAGCCCAGCCACAATTCGCACCACAATCCCCAGACCCCCAAGTCCGTCTCCATTCTCGTCTACA
AGACCTTGAACACGGTCTTCAAGAGCAGTCGCAAGATCATCGTTCGTGTGTGTGAGGTGGCCAGCGCCAA
GAAGTCCTTCACCATGTTCAAGTTCAACAAGGCCGCTCAGCAGCAGCGGATCGACAATCGGAACAGTGCC
GTCACAGGACACGATCCCTTCGTCCGACCACCAGTGCCCGAAAAGGTGAGTACCCAACCGATCGGCGAGA
TATATATGCGTGTGTATATAGGACTTTTGCAAtgtgtgtgcgggtgtgtgtgtgtgAGCCCCACTCACAC
ACTCAAGCAAAATCGAGCACAGTGCACAGGTACCTCGATTTTTGATTTGGTTTATCACACGTTTTGGTTT
CGTTAAGTGAAGCTTCTTTGACGAATCAAGTTATGAAGAGAACACACACTTATCGCAGTAttttcttttg
tatattttcttttttttttGCCAAATACCCACCAAACCAACCAAAAAACACCCACCGCAACCACCACTGT
GTACCCTTCTATTTGCAGCAATCGGTCGGCGAGCTCGGAGCAAGATAACTCAGATCTATCCGAGCACTCG

GAGAAATCGCCGCTGGTTAGCGCGAGGTTAGACAATCTAGCTAGGCTCTTCTTTAGCAAGTCGATGCCAG
CGGAAACCGGAAGTAGAGATACCATAGATTCAGTACTGACAACAAGG
>dme_circ_0001725_junction_seq
TACCATAGATTCAGTACTGACAACAAGGGACACTTGCAGCGAGGTCAACGTCCCGG
>dme_circ_0001726
GACACTTGCAGCGAGGTCAACGTCCCGGCAGCGTCGCATCCGGAGCCGGAGCTCATCATCAGCTGCCACC
GGGATCAGCGGGATCAGTGCCATCGGAATAATCTCCAGAGGGAAAGCAAAGTTATCACCCGATCACAATC
ATCGGCGTCGACCGCGCGGCATGCTCCAGTGCATCAACTGAAGCAGCAGCAATCTCTGCCCACACCTCTA
ACACAGCAGCCCAGCCACAATTCGCACCACAATCCCCAGACCCCCAAGTCCGTCTCCATTCTCGTCTACA
AGACCTTGAACACGGTCTTCAAGAGCAGTCGCAAGATCATCGTTCGTGTGTGTGAGGTGGCCAGCGCCAA
GAAGTCCTTCACCATGTTCAAGTTCAACAAGGCCGCTCAGCAGCAGCGGATCGACAATCGGAACAGTGCC
GTCACAGGACACGATCCCTTCGTCCGACCACCAGTGCCCGAAAAGGTGAGTGCTCCGTGCCCCAAGAATT
GAGGAACTCCAAACTGTTTGAAAAGCTTTGGAAACGAACATTTTGCAGATTGAGGCATTTAGTTAATACA
AAAAATTGCATTTCAAAGATTTGTCAGCATTTCTAAAATTCAATATTTTTCACTTCCAATTCCTTAGTAG
TAAAGaaaaaaaaaaaaCGTTAATCTTCGGAAACTTAATGATACCTCTGAAAGCAAAGTAAATCCTTTTC
AACCGCCTAATTAGAAAAAAGTTTGAAATTCCGAAATGTCAATGTCAATGTTTGTCTCTCAATACTAAAT
GCACCCGATAACCGATTTCGAACGCGATAGGAAAGTGAGGAACATTATGAAGAAGCTGCACAAGGCGAAC
GGCCTGAAACGCTCCAACTCGGCGATCGAGTTCGATGTCTCGGCCCTGACAGCTGCCAATCGCCGACAGA
TCTATAGTAGG
>dme_circ_0001726_junction_seq
TGCCAATCGCCGACAGATCTATAGTAGGGACACTTGCAGCGAGGTCAACGTCCCGG
>dme_circ_0001727
TAACTCCTCCTTCTTCAGCTGGTTCTGCTTTCGCCGCTCCAACAACTTTTTCTGGAAGTCCTTGCGTACC
AGATACCCGCGACTCAAGGCCTGCAGTTTTATGGTTCGACACCTGACCATCGTGAACTTGGTGGTCAACT
GCTGCGCAGCTATACAGGCTCCCAGGCGATGGAATCCCTGCCGCATAACCTGGTACTTACGACGCTGCAG
GCGACCTCGCCAATATCTCTGAACCGTAATAATGGCCTCGCGGTATCTCTTCATG
>dme_circ_0001727_junction_seq
AATAATGGCCTCGCGGTATCTCTTCATGTAACTCCTCCTTCTTCAGCTGGTTCTGC
>dme_circ_0001728
GAGATATTGGCGAGGTCGCCTGCAGCGTCGTAAGTACCAGGTTATGCGGCAGGGATTCCATCGCCTGGGA
GCCTGTATAGCTGCGCAGCAGTTGACCACCAAGTTCACGATGGTCAGGTGTCGAACCATAAAACTGCAGG
CCTTGAGTCGCGGGTATCTGGTACGCAAGGACTTCCAGAAAAAGTTGTTGGAGCGGCGAAAGCAGAACCA
GCTGAAGAAGGAGGA
>dme_circ_0001728_junction_seq
GAAAGCAGAACCAGCTGAAGAAGGAGGAGAGATATTGGCGAGGTCGCCTGCAGCGT
>dme_circ_0001729
TAACTCCTCCTTCTTCAGCTGGTTCTGCTTTCGCCGCTCCAACAACTTTTTCTGGAAGTCCTTGCGTACC
AGATACCCGCGACTCAAGGCCTGCAGTTTTATGGTTCGACACCTGACCATCGTGAACTTGGTGGTCAACT
GCTGCGCAGCTATACAGGCTCCCAGGCGATGGAATCCCTGCCGCATAACCTGG
>dme_circ_0001729_junction_seq
GCGATGGAATCCCTGCCGCATAACCTGGTAACTCCTCCTTCTTCAGCTGGTTCTGC
>dme_circ_0001730
GTAGACGACCACCGTGGAGTCGGAGTTCTTGGTCTCTACGCGCTTCACCCGATCCACTGCCTGGATTTTG
CCCAAGCTTGTGGAGTCACCAACGAACCCTGAGGGCAGGGCGATTTCCATCACTGCCATGTTGGAGTCCT

TGTCCTTGTCGGCGTCTTCTAAGGGCACGTACTCGGCACAGACGTCCACTATTAGTAGCTGGTTGGGAGT
GTCCTTGACCGTGGGTGTTACCTTGAAACTGGGCTTCTTCTCTTTCTCCGCTAGATTGTAGCGATAGGAG
AGCTGAACCATGGCAGATCCTGTTCCCTTGGCTGTAAAATCGACCTTGCGAGTGCTCTTGGGCAGGTCAT
GGGTCTGCAGAACCAGGGAATTCTCTGGGTTAACCTTAATTGTATTCTTGGATTCACCCGCAGATGAGAA
CTCAATATCCATGGTGCCTGAGCCAGAGCCCGTCTTGTATGCGAATTTTGTGAGCGCCTGAAGACCAATC
ACAGTATCCTGAGTGGAGGAGAATCCCCCGTTGCTGTTGCGCTGCGATATCAGCCACTTGATAATGGGCA
GGGCCTTCTCAGCCGGATCCTTTTCAAGAAGCGCAAGCAGAACATAGGAGGTGATCTCGACGTCGTTACT
GCGAGGTTTCCAGTGGAAAACTCGTCCATCCTCACCAGTCGATTCCGTGGCCTTGGACCACCACATTCGA
TCGTTTTCCTTTCGGGCCACGCTCTCCAGCTTAGCAATGACCTTCTCCGACTGCGGGTGCTTGGCCAGTT
GGAGGGCCACGGCGGCAATGGCCAGGGAATACTGGTCGTCCGTCTTGTCAGCCTCTTCGGCCACATAGCG
TACTGCTTTCTTAATGGCACTTTGGTACTTCGGGATCAGTTCCTAATGCAATTAAGGATAAATCTCTGGT
CAAAATACATAATCGGTATGATAATAGGCAGCTAACTAGGTTACTTACATGATTCTCGAAGAAAGCCAGC
AGAACGAACGAAGTTAGAGCCAATGGGTTTTGGTTCGCGTTGTCGAAGAGTTTGCCGACCTCCGGGAACT
CGCCACTCTCCTTCTGTTTCGATACAAGGAAGTCAAGACCAGCAGTAATGACTTTAGGATCGATATCC
>dme_circ_0001730_junction_seq
AGCAGTAATGACTTTAGGATCGATATCCGTAGACGACCACCGTGGAGTCGGAGTTC
>dme_circ_0001731
GACGACCACCGTGGAGTCGGAGTTCTTGGTCTCTACGCGCTTCACCCGATCCACTGCCTGGATTTTGCCC
AAGCTTGTGGAGTCACCAACGAACCCTGAGGGCAGGGCGATTTCCATCACTGCCATGTTGGAGTCCTTGT
CCTTGTCGGCGTCTTCTAAGGGCACGTACTCGGCACAGACGTCCACTATTA
>dme_circ_0001731_junction_seq
ACGTACTCGGCACAGACGTCCACTATTAGACGACCACCGTGGAGTCGGAGTTCTTG
>dme_circ_0001732
GTTAACCCAGAGAATTCCCTGGTTCTGCAGACCCATGACCTGCCCAAGAGCACTCGCAAGGTCGATTTTA
CAGCCAAGGGAACAGGATCTGCCATGGTTCAGCTCTCCTATCGCTACAATCTAGCGGAGAAAGAGAAGAA
GCCCAGTTTCAAGG
>dme_circ_0001732_junction_seq
GAGAAAGAGAAGAAGCCCAGTTTCAAGGGTTAACCCAGAGAATTCCCTGGTTCTGC
>dme_circ_0001733
TCCAAAGCTAGAAAGTTCCTGGAGATCGGCTACCAGAGGGAACTGACCTACAAGCACGACGATGGCTCAT
ATAGTGCATTTGGAAAATCGGACGCTTCGGGCAGCACTTGGCTTACCGCCTATGTCATGCGTTCCTTCCA
TCAAGCTGGAACATACACGGATATCGATCCTAAAGTCATTACTGCTGGTCTTGACTTCCTTGTATCGAAA
CAGAAGGAGAGTGGCGAGTTCCCGGAGGTCGGCAAACTCTTCGACAACGCGAACCAAAACCCATTGGCTC
TAACTTCGTTCGTTCTGCTGGCTTTCTTCGAGAATCATGTAAGTAACCTAGTTAGCTGCCTATTATCATA
CCGATTATGTATTTTGACCAGAGATTTATCCTTAATTGCATTAGGAACTGATCCCGAAGTACCAAAGTGC
CATTAAGAAAGCAGTACGCTATGTGGCCGAAGAGGCTGACAAGACGGACGACCAG
>dme_circ_0001733_junction_seq
CGAAGAGGCTGACAAGACGGACGACCAGTCCAAAGCTAGAAAGTTCCTGGAGATCG
>dme_circ_0001734
GAATTCACCCTGACCAAAAAGATACCGGATACGATCACCTCATGGGTGGTTACTGGTTTCTCCCTTAACC
CAACATCCGGAATTGCTTTAACTAAGAATCCGAGCAAGATTCGAGTATTCCAGCCCTTCTTTGTGTCCAC
AAATCTGCCGTATTCCGTAAAGCGAGGGTGAGGTAATTGCTATTCCCGTGGTCATCTTCAACTACCTGGA
TAAGACCCTTGATGCAGATGTAGTAATGGACAACTCTGATCAGGAATATGAATTCACCGAGGCCACCAAT
GAGGTGTTGGAAAAGGCAATCGATGAGGTGCGCCGGGTCAAACGGGTCACGATACCGGCCAATAGTGGCA

AGAGTGTTTCATTTATGATCCGACCGAAGAACGTAGGATTCACGACCCTGAAAATCACAGCCACCTCTGC
CTTGGCCGGCGATGCTATTCACCAGAAGTTAAAGGTTGAGCCAGAGGGCGTAACTTTGTTCGAGAACCGG
GCTGTCTTTATCAACCTCAAGGATCAGCCAGAGATGTCTCAGTCTCTGGATGCTGATATTCCCAACGAGG
TCGTGCCCCAGTCTGAATTTATAGAGTTTTCTGTGGTTGGTGATCTTCTGGGTCCGACGCTCCAGAATCT
CGACAATCTGGTGCGTATGCCGTACGGCTGCGGAGAGCAAAACATGGTAAACTTTGTGCCCAACATTCTG
GTGCTGAAATATCTGGAGGTTACTGGTCGAAAGCTTCCGTCCGTTGAGTCCAAAGCTAGAAAGTTCCTGG
AGATCGGCTACCAGAGGGAACTGACCTACAAGCACGACGATGGCTCATATAGTGCATTTGGAAAATCGGA
CGCTTCGGGCAGCACTTGGCTTACCGCCTATG
>dme_circ_0001734_junction_seq
TCGGGCAGCACTTGGCTTACCGCCTATGGAATTCACCCTGACCAAAAAGATACCGG
>dme_circ_0001735
GTTGAGCCAGAGGGCGTAACTTTGTTCGAGAACCGGGCTGTCTTTATCAACCTCAAGGATCAGCCAGAGA
TGTCTCAGTCTCTGGATGCTGATATTCCCAACGAGGTCGTGCCCCAGTCTGAATTTATAGAGTTTTCTGT
GGTTGGTGATCTTCTGGGTCCGACGCTCCAGAATCTCGACAATCTGGTGCGTATGCCGTACGGCTGCGGA
GAGCAAAACATGGTAAACTTTGTGCCCAACATTCTGGTGCTGAAATATCTGGAGGTTACTGG
>dme_circ_0001735_junction_seq
TGGTGCTGAAATATCTGGAGGTTACTGGGTTGAGCCAGAGGGCGTAACTTTGTTCG
>dme_circ_0001736
TTAAAGGTTGAGCCAGAGGGCGTAACTTTGTTCGAGAACCGGGCTGTCTTTATCAACCTCAAGGATCAGC
CAGAGATGTCTCAGTCTCTGGATGCTGATATTCCCAACGAGGTCGTGCCCCAGTCTGAATTTATAGAGTT
TTCTGTGGTTGGTGATCTTCTGGGTCCGACGCTCCAGAATCTCGACAATCTGGTGCGTATGCCGTACGGC
TGCGGAGAGCAAAACATGG
>dme_circ_0001736_junction_seq
CCGTACGGCTGCGGAGAGCAAAACATGGTTAAAGGTTGAGCCAGAGGGCGTAACTT
>dme_circ_0001737
TGTTTCATTTATGATCCGACCGAAGAACGTAGGATTCACGACCCTGAAAATCACAGCCACCTCTGCCTTG
GCCGGCGATGCTATTCACCAGAAGTTAAAGGTTGAGCCAGAGGGCGTAACTTTGTTCGAGAACCGGGCTG
TCTTTATCAACCTCAAGGATCAGCCAGAGATGTCTCAGTCTCTGGATGCTGATATTCCCAACGAGGTCGT
GCCCCAGTCTGAATTTATAGAG
>dme_circ_0001737_junction_seq
GGTCGTGCCCCAGTCTGAATTTATAGAGTGTTTCATTTATGATCCGACCGAAGAAC
>dme_circ_0001738
GGTGGCTGTGATTTTCAGGGTCGTGAATCCTACGTTCTTCGGTCGGATCATAAATGAAACACTCTTGCCA
CTATTGGCCGGTATCGTGACCCGTTTGACCCGGCGCACCTCATCGATTGCCTTTTCCAACACCTCATTG
>dme_circ_0001738_junction_seq
ATCGATTGCCTTTTCCAACACCTCATTGGGTGGCTGTGATTTTCAGGGTCGTGAAT
>dme_circ_0001739
GGTGCGTTTGAAGCGTTCGCAAAAGGATCCAGTGCTACGGTATATGTAGTAGATTCCATAACCAGGAATA
GCCAGTATGGGCAAAATCACTAGTATGATGGACATTCCGAGGACGATTGCTGAGGAATAAATGTGCTCCA
TGTAGCACACTAATATTCCTACTACCTAATATAGCGaaaaaaaaGAAAGACATTATAGAATACAAATCAC
CAGAACTACCTACCAGACAAATAATTAAAATTATTGGAGCTATAAAACGGAGTATGTAAACCTTCCAGGA
GGCAAAGGGCTGGCCCAACATGAACTCAATATCTCGCTGGAATCGTTCGCGACCATAGATCCACAAAACC
ACCAAAAGGAGCAGTAAGTGCAGCAAACTATGTGAGAAAATGGCATCAAGCGCCAAGGCCGAAAAGAAAG
TAATTCCATGCTAAAAAATATGAAGATAATTATATTTAAGTCTTTAAAAAATATTACTGAGAACTGTCTC

TTAATTTGTTGCAAACCTAATCCAGATAACTTACATTTGTGCAAAAGTAAAAGGAGCAGACTGCAATACC
TCCGATTAGCCCAAAGGTTACCTCCTGTTTTCTCACTCTAAGTACTTCAAACTCATCAAACAAACTTTGC
AAAACAGTAAAGATTTGCGTGGTCTGaaaaaaaaagaaaaaCACATAATACGTTTAAATCGATCAAAAAG
TGTTTGTTAATGTACAAAATTAAGAGTGTGTTGTAAATTTTTTTCACCGGAAAATCTAAACCAAAATAAA
CACATAGTCTTTGTTCTTTGTTCAGaaaaaaaaaaCTTACTATCACAATAAGAGCAGCCATCAACAACAT
TGTATAGTAAATAAAGGTCCACAGGTTCGGCCAACCCAGAGAAGACAATGCGGTAGCACTGGACAAATAC
AGAGCCCAATGAGTCAATACTTGTTTGTCGAAAGAACCTTCAATGCCTTCTGAAAGGCAAACGATACAAT
TAAGTTGTGATATTAGATATGAGTTAGTGGAATCGTAAACCTTTAGATTAGAAAACTCTCTTGTACATTT
CGTTCCTGCACCAAAACAATTTTTTATTATATTAAGGGAACTGGTCTTACCATTGTAGTAGTGATCCAGC
ATATAGGAAACCAATCCGAACATTGTAAAAATAAAAATCTGTCCGAAGGAAATGATCCAACTATACGACA
TGATGTCAGTCTTGAAACCATTGAAACTGGACAGGGCTATTACACTGCCCCAACCAGCTCCGAACGCATG
AAGAACCATTACAAATGTGGAGCCAATTCCCATGGCTATTTCGTCTGGACTCGGCGTCACATATTTCCTT
AGTCCCAAAAGGGCTCCTGGAAGGAAGAGAAAACGCCCAAAGCACACCAAGAGAAGAATCAGTGTCCCAA
TAACCATGTAACGTATAAATTTCCCAAACATCGCCGTCTCTGAGAATTTATAAAAAATGAAGGCAACCAT
TGCCCAAACTAGTACAAAGAGGCTCACAAATCGCCAGGATAGGTCATAATCTTGACGCAAATCTGAATAA
TTTTCCTCTTGGACGCTTTCAAAGTGTTTTCTGAAATAAATAGAAAATCAATCGATGATCATCTTTATAA
CACATCCTTACAAATTGCTTATGTGTTAGAAATAGCAACTCAAAAGTGATGATTGCGCTAATTAAATAAA
ACCGCATTGAAAATTCAAAAATTACGATAATGATTTAAACATATATGATACAAATAACAATATAGAAAAA
TACTTACAGAAAGTAGAGTACGGAAGGTACATGAAAGTGTGGTCTGTGAAAATCCGTGTTGTTATCAGAG
TTATCCGTAAGATCCTCACTGATTGTCATATTGCATGTCTAGAAGGCAAAGCAAGTTGCCTTTATTACTT
TAGTATATGTTGTTATTTTCTTACTGTCATTTTTCCATCCGATTCATTGTACCACGACTTAAAACCTTCA
CAACTCCATGGCAAGGTAGGTCGGAAGGAGTTGATCATGAAGAGCAGAGGAACTGCAGCAAAGATGCTGT
AGTAAATCAACATGCTGATGGTCAGGAAAACGCTAACATATCCCATTCCTACAATACAAAATAAACTTTA
AGCCAATTATTTAGTTTTTTCCTTTTAACATACCTTTAAAAAACGGTGACAGCCGGAAGGCGGAGATGAA
CCCACTGCTTGAGAACTGGCCCATGAAGGAATGAATGACCATGATTGGAACCAAATATATCACCATGTAA
ACTAGGTACGGCAGCATCCCAAAAACTTAGGAAGGCAAGAATCCAGAAAATTTCAGTGTTGCTACTATTT
TTAACAGGCATTTACATTAATTAAATTATATTTATTACTCTTATCTGATCAGAGCGTAGGAAATTTACAT
TTTACAATCAACGCCCCTAAAATGAATAGTCTAACCTTGGAGCATATATGCCCTATACCTATAGATACCT
CTGTTTTTAATACTTACTGCCCATATCAAAGAAGAACCAATACGAGGCCACAAATATATCCAGTTTAAGG
GCTAATCCAAAGCAGGCAAATATATAGTCGGTGGGCTTATCCCACTTTCCTCTCTTCAAGTCGGGGGAAA
AGGGTTTGTGCCCACTATCATACGATGTTTCATATATCATTTTGTGACGTTAGTATAGCCCGGTAAATTG
TGTGAGCAAAGGAATTTGAAAAACTTTTGTATAAAAAGAAATATATCCAATTTCACACGCCGATGGAGGT
ACTTATTATTAAAAGCTTTGGCAAACAACAAACATTTCGCGAGCGTTTTAAATTGACGAACTAATTAGTA
GCAGCGATGCGGACGAACTAAATCCGATTCCGCTGATAAACCGCAGTGTAGCTGATTCCACTGGACTGAG
ATGCTCATAGCCTATCCCGGACCATTCACACTAGATCGGCCATCAAATACACTTTTAAGAAATAATGTTA
AGCATAATTTTTTGAATAAATAGTCGGTATTACTTTGTAATACTATGTAATGTGAACAAGTTCAAACAGA
CAGTAAGAAGATCCCTAATAATTTAATGTAATATGATGTATATTAATTTGGATATGTTAAAACAAACATC
TCAATGAAACTAATACCGTTCCTAAAAGTAGTGAGTCACCGAAGTGGCATTCTGAAATTAAAAGGCATTT
CTATACACTAACATGACGTCAATAAGAGTACTTTGCTTATAACCATAATTAATCAATAACTACATTAATA
ATTACACGTTTTTAAAAGctctctctctctctcGATAGACAAACTATGTAAGCATTATAATTGTGGACGT
CACAGATTTTTTAAGCTCAACTTTATTAACTTTTCCGAGATATCTACGTTTCTCTGGACAGTCACACGTG
TGAACCTGATCGATTCAGGTGAGATAAACGCTGTAAAACCCATTGCTGTAAGCATAGTGCAATAAGTGTA
GCATTTCCACCTTTCACAATATGATTGTATATATATGCATACCTTTTTTTAAATACGCATTAAAATTACT
CATATATTTATATTTAGTACATTTCTAAGTGCATCAGTAACTAGTTGACCTCCTCAGTAACCTCGAAGAG

TTGGTGGGTCATCTCCGTGTTGTCCACAGCTTCCTCGTACTGCTGGCGGTGCTCCATCTCAACCGGATAC
CAATCGGTGGGTCGGCAG
>dme_circ_0001739_junction_seq
AACCGGATACCAATCGGTGGGTCGGCAGGGTGCGTTTGAAGCGTTCGCAAAAGGAT
>dme_circ_0001740
TTGTTAACCTATGATAATGCAAAGGCTATGTTCACTGTACCGCCCATCGTATTGTAATTTTCTGTAGATC
ACGAAACAGACTGTCGCCGCTGAGGAAGAGCTGCAGGATGTTGTTGAGGACGCCAAACCTACGGAAAACG
TCGAAGAAGTCTAGCTATGGCCGCTTCTTTTGCAATTGTGTTG
>dme_circ_0001740_junction_seq
TATGGCCGCTTCTTTTGCAATTGTGTTGTTGTTAACCTATGATAATGCAAAGGCTA
>dme_circ_0001741
GTGTATGCCGGAGACCTATGGTCTTTCGGAATCTCCGGAGGGTTGCTCTCTGTGTAACTGCGATGCTGGT
GGATCCTACGATAATTACTGCGATGTCATAAGTGGTCAGTGTCGCTGCAGGCCCCACATGACGGGTAGGT
CATGTTCGCAGCCGAAGCAGAATTACTTCATTCCCCTGCTGCCCGAG
>dme_circ_0001741_junction_seq
GAATTACTTCATTCCCCTGCTGCCCGAGGTGTATGCCGGAGACCTATGGTCTTTCG
>dme_circ_0001742
GAATATCTCCGACAGTGAAAACCAGTTCCGAGTTCTCAGGAACCCTGGTAAAACCAATGCCCGTGAAACT
GCCATCAGGAGTTTCGGCCACCAGGCTGCAGTTACCGTTGGCGCCATACGAGATGCACTCGTCCACCACC
TCTGCTTCATGAACCTCGGGCAGCAGGGGAATGAAGTAATTCTGCTTCGGCTGCGAACATGACCTACCCG
TCATGTGGGGCCTGCAGCGACACTGACCACTTATGACATCGCAG
>dme_circ_0001742_junction_seq
GCGACACTGACCACTTATGACATCGCAGGAATATCTCCGACAGTGAAAACCAGTTC
>dme_circ_0001743
AGAATCCACCAAGATCGTCGCTGTGGGACTGTCCACATCGTGTCGCTTTCGCTCAAAGTAAATCCGGAAT
TTATAAACCTTTCCAGCTTCCAAGCATACTTCATTCAAAGCAACCACCTGACGGCTGCGATCAGGAAGGG
AGAACGGAATCCTGGTTTCCGAAGAAGTTGCTGCAGCTAGTTCACCGCATCCTCCTTCAGGATCCACTTG
ATCGGGTCTGACAAGAGTGATAAAGGCGTTCTCCCAATCTCCTCGTGAAGTGCTCTGG
>dme_circ_0001743_junction_seq
CTCCCAATCTCCTCGTGAAGTGCTCTGGAGAATCCACCAAGATCGTCGCTGTGGGA
>dme_circ_0001744
GGCCAGGGACAGGCACTGGGGCTGATCGGCCTTCGACGTGGGCGAAACGACCTCGGGGCGCAGCTCCTCA
ACGGTCTTCTTGCATGCCAGATCCCAGATCTTGATCGAGGGTCCGTAGGCCACGCACAGCCAGTAGCGGT
TGGGCGAGAAGCACAGGGCGTTGATGATGTCGTTGTGCTCCAGAGTGTACAGGTTCTTGCCGTCATTGAG
>dme_circ_0001744_junction_seq
GAGTGTACAGGTTCTTGCCGTCATTGAGGGCCAGGGACAGGCACTGGGGCTGATCG
>dme_circ_0001745
GGTAGCCGTTGTGGCCGTGGTGGTTGTTCTTCAACTTGCAGTTAGCCAAGTTCCAGACCTTGACGGTGCG
ATCCCAGCCGCAGGACACGATGATCGGGTTGGAGTGGTTGGGCGAGAAGCGCACGCACGACACCCAGTCG
GTGTGGCCATCCTCCTGGATGGTGAACTTGCACTCAGCCAGGGTGTTCCACAGCTTGATGGTCTTGTCCC
GAGAGCCGGACACGATCTGACGGTTATCGGCCGAGAAGGCAACCGACAAAACGTCCTAGAAAGCGAACAA
GCATTTGACGATTAGCTAATGGTATTGGTATGGTTTTCATAAAAATAATATATTTACTGGATATTTCACT
GACCAACTGAATGGGCATAGTCCCAACTCAATTAGTCAGTGAACGGAATCCAGATGGGTTTCTTGCAACT
AATTAGGAATCAATGGGTATCAGAGATTGTTTGCCTACTTACGATGGAGCTTCAAGCAATTTATAGCCGA

GACTCGAGCGAAGGGTGAAGCGTACGTCGTACAGATGTCAACCAGTAATTCTGCCTCGCATAGGCGCAAT
ATATATTTTTTCGATTTGTACGCGGCAAAGGCGGGCCTAACCTCACTCCTAACCTTACATTTAGCCTTTT
GCCGGTACAGATCGGTTCAAAATCTCAGTTCCCTAAACGAACTAAATGCAATTGGAATCTTATCCCACCT
TAGTGTGTCCCTCGAAGCGACGGGTGGTCTTGCCGGCGGCCAAATCCCACAGGCGAAGGGTCTGATCCCA
GGATCCGGACAGGGCGTAGTTGCCATCGGAGGAGAGCACCACGTCGCTGATGAAGTGCGAGTGTCCGTAG
AGACGCTTCTGGGGGTAGCCGTAGTTGGTGTCCTCGTCGCGGGTCAGCTTCCACACGATCAGGGTCTT
>dme_circ_0001745_junction_seq
GGGTCAGCTTCCACACGATCAGGGTCTTGGTAGCCGTTGTGGCCGTGGTGGTTGTT
>dme_circ_0001746
GTTAGCCAAGTTCCAGACCTTGACGGTGCGATCCCAGCCGCAGGACACGATGATCGGGTTGGAGTGGTTG
GGCGAGAAGCGCACGCACGACACCCAGTCGGTGTGGCCATCCTCCTGGATGGTGAACTTGCACTCAGCCA
GGGTGTTCCACAGCTTGATG
>dme_circ_0001746_junction_seq
CTCAGCCAGGGTGTTCCACAGCTTGATGGTTAGCCAAGTTCCAGACCTTGACGGTG
>dme_circ_0001747
GACCTTGACGGTGCGATCCCAGCCGCAGGACACGATGATCGGGTTGGAGTGGTTGGGCGAGAAGCGCACG
CACGACACCCAGTCGGTGTGGCCATCCTCCTGGATGGTGAACTTGCACTCAGCCAGGGTGTTCCACAGCT
TGATG
>dme_circ_0001747_junction_seq
CTCAGCCAGGGTGTTCCACAGCTTGATGGACCTTGACGGTGCGATCCCAGCCGCAG
>dme_circ_0001748
CTGTGGAACACCCTGGCTGAGTGCAAGTTCACCATCCAGGAGGATGGCCACACCGACTGGGTGTCGTGCG
TGCGCTTCTCGCCCAACCACTCCAACCCGATCATCGTGTCCTGCGGCTGGGATCGCACCGTCAAGG
>dme_circ_0001748_junction_seq
TCCTGCGGCTGGGATCGCACCGTCAAGGCTGTGGAACACCCTGGCTGAGTGCAAGT
>dme_circ_0001749
ACCATCAAGCTGTGGAACACCCTGGCTGAGTGCAAGTTCACCATCCAGGAGGATGGCCACACCGACTGGG
TGTCGTGCGTGCGCTTCTCGCCCAACCACTCCAACCCGATCATCGTGTCCTGCGGCTGGGATCGCACCGT
CAAGG
>dme_circ_0001749_junction_seq
TCCTGCGGCTGGGATCGCACCGTCAAGGACCATCAAGCTGTGGAACACCCTGGCTG
>dme_circ_0001750
AAGCGTCTCTACGGACACTCGCACTTCATCAGCGACGTGGTGCTCTCCTCCGATGGCAACTACGCCCTGT
CCGGATCCTGGGATCAGACCCTTCGCCTGTGGGATTTGGCCGCCGGCAAGACCACCCGTCGCTTCGAGGG
ACACACTAAGG
>dme_circ_0001750_junction_seq
ACCCGTCGCTTCGAGGGACACACTAAGGAAGCGTCTCTACGGACACTCGCACTTCA
>dme_circ_0001751
GCCGTAGTTGGTGTCCTCGTCGCGGGTCAGCTTCCACACGATCAGGGTCTTGTCTGCGAATCGAGATGGT
GGCCATTAATATACGCACGTGCTACCAGATAAATCCGAAGAATTTCAATGGCGATTACTTACCACGGGAG
GCCGAAATTATGGTGTCGGGATCCTTGGGGTTG
>dme_circ_0001751_junction_seq
AATTATGGTGTCGGGATCCTTGGGGTTGGCCGTAGTTGGTGTCCTCGTCGCGGGTC
>dme_circ_0001752

GTGTAACCGGCGCCACGAGGCGAGATTCCCCAGCCACCCCTGTCATCGGGATCGGACCAGAGCAGATCGC
ACATGGGACCCTCGTGCGGAACCTCCTGCAAGCGATCGAGGGCCCGAATGTGATCCAGACTGTCGATCGA
GGGACTGAGGCCTCCGTGCAGGCAGAAGATCTGCCCGTCGACGAGTGCCGTCAGTGGCAAGTAG
>dme_circ_0001752_junction_seq
GTCGACGAGTGCCGTCAGTGGCAAGTAGGTGTAACCGGCGCCACGAGGCGAGATTC
>dme_circ_0001753
TCCATCCATCCATCACTCCTGCTCCCTGGCTTTCTGCTGCGACGCTTAGTTCGTTATTTTTATAAATTAA
CAAATTTAGCTAAGTAGTTGTAGTTCTTCTTGTTGCTTGCGGTTTTGTTGCTTGGTTTCTGGGTTGGTTG
GTAGG
>dme_circ_0001753_junction_seq
TTGCTTGGTTTCTGGGTTGGTTGGTAGGTCCATCCATCCATCACTCCTGCTCCCTG
>dme_circ_0001754
GGGAGCAGGAGTGATGGATGGATGGACTCATGGATTATGACGTGATGTGTGTGTGGGCGGAAGGGGCTGA
CAAAGGACTAAACGGGACGAAAGGACGAACGCCAGGACgtagcagcataagcaagcagcagcg
>dme_circ_0001754_junction_seq
GACgtagcagcataagcaagcagcagcgGGGAGCAGGAGTGATGGATGGATGGACT
>dme_circ_0001755
GGGAGCAGGAGTGATGGATGGATGGACTCATGGATTATGACGTGATGTGTGTGTGGGCGGAAGGGGCTGA
CAAAGGACTAAACGGGACGAAAGGACGAACGCCAGGACgtagcagcataagcaagcagcagcggtagcag
cagcagaagcagaagcagaCGACCCACGACCGACAAAGCAGGAAGATAACGATGAAGCAGCAGCAAAAAA
GTAGGAGCAGTAT
>dme_circ_0001755_junction_seq
AGCAGCAGCAAAAAAGTAGGAGCAGTATGGGAGCAGGAGTGATGGATGGATGGACT
>dme_circ_0001756
GTGATGGATGGATGGACTCATGGATTATGACGTGATGTGTGTGTGGGCGGAAGGGGCTGACAAAGGACTA
AACGGGACGAAAGGACGAACGCCAGGACgtagcagcataagcaagcagcagcggtagcagcagcagaagc
agaagcagaCGACCCACGACCGACAAAGCAGGAAGATAACGATGAAGCAGCAGCAAAAAAGTAGGAGCAG
TAT
>dme_circ_0001756_junction_seq
AGCAGCAGCAAAAAAGTAGGAGCAGTATGTGATGGATGGATGGACTCATGGATTAT
>dme_circ_0001757
GTTATTATTGTTCGTGGGGCGGTTGTGGAAGTTTTCGGGGTAAAATATTTACAATTTTATCGCACtttta
atttttttttatttCTTGTTTGACCGCGCAAAAAGAAGTTGAAAATCCGCACTTAGTCTACACCATTAAG
GGtttttttttcagagtttttctttttCAGAGGGACGGGGTTGCTCtacaacacataggtacatacatata
g
>dme_circ_0001757_junction_seq
TCtacaacacataggtacatacatatagGTTATTATTGTTCGTGGGGCGGTTGTGG
>dme_circ_0001758
GGACTCACGGAATAGATGAGGAGCATTACGAATCTTGaaaaaaaatttaataaaaaaaaTGTCAACTGAA
ATGTAGCTTTTAAAACAGTTTGCTTTGAATGCCCATATTTAAATAAGCAAATTATTAATATACATATGCT
TTGAAGTTTACAGTGCCGCTCCTTTACATTGAACTAATGATTGAACAATTTATAAGCAACTTTTTGCCAC
CTAGATTTCATTCCTTTCTGGCTATGAAGCATTTTACTTTCACTAAAATACCCCTATAATGAAAAACATG
TGCGTTCAGGCTTTGTGCTTGAATATCGGCTGCACTTTCTCCTACAAAAGAAATGGGATTTTAACTAAGT
TCTAACTGTTCTGTCgtgtgtgtgtgtgtgAAAGAGCTTTTCTTTGAGAGGTTTTCGGGGCTTCTATCTC

TAGGCTATTTCCTTTTTGATTT
>dme_circ_0001758_junction_seq
TATCTCTAGGCTATTTCCTTTTTGATTTGGACTCACGGAATAGATGAGGAGCATTA
>dme_circ_0001759
GTAAGCTGAGTGTGGCTCTCTGAAGCGAAGGTCATCGCGAAGTGGCATCATCATCTGTTTGTAAGGCCGT
ATCTAATTATCACCTAGGTTGCCTGCCATTTTCCTTGGGTGTGGGTGTGACCCACGCGTCACCTGGAAGT
CTGTCTATGTCTGGGCCACTGCAATTGAAATCGAAACTGAATAGGAGACTGATTGCTCGCAACCAGTCTT
CTAGTCAAACGACTTGCGGCATCGTGCCGCGAGCAATTATATTCCATGTTGCCTTTTGCTATAAATTAAT
TAAATGGAAAATAATCGTGTGCAGCGCCAGTCAGGCAACAAACAGCGTTCTTCAGAGACACCGATGCCGA
CAAACGAAGCCGAGTTCTAAATGCTTTTGTAATTTTAATTGCTCCGCGCCAAATGTTGTGCTGCCAAATA
TGGCAGCGGTAACTGAAACTCGCAAGTGCGGCACATCCACATTCAGATACAGGTGCAACTGCAGAAGTGG
CAGCTTTTGTTGCTAATGTCTCTGTTTAGGTAACTTCCTTCTCGTGTTCAGGCCAAAAGCGAATTTAATT
CATCACGAAAGGCTTTTACCCACAGCACGGTATTCATTGCCACCCCCTTTTGTGTATCTGCATCTTTGGC
TGTCGCCGAAGTTGTATCTGTATCTTTATCTGATGAATTTGAATCTGCATCTGGCTGTTGGCGGCTCAGC
GTCTAATCGCTGGCTTTGAATTATTTATGTGACTGGTGCTGCCTCAAGTGCGATGTGCAATCTTGACGAT
GAATGCTAATCGATTCGGTAATGCCCCTGATTGAGAAGTCATGGAATTGGAAAACACCTTTAAATTGGAG
ACAAATATTTCTTTGATTTATATAACTGAGAGGCATTGGGCAGACTTACTAATTACTAATTAAGATTAAT
TTCATCTGTTTGCTTATGTTACTCTTTCTTTAAATCCTAATTCGACAGAATAATGTATGCATTGATTTTT
AATTGGCATGCTCTTTTCCAAAAGTTATCGAACTCTGTCGCTTTCAATTCAGTTAAGGTAAATTATTCAA
CTGAGTTTGCACTCTCCTCGAGTTCTTTTGAACGAAAACGGATTTTTAAATGCATTCATTAACCTGTGGC
GACCGCTGTAAAAAGGGCTCAAAAGGCCTTCTGCCGTTGGTCAGAGTTGGCCCTTTGTTTTGGCCACAGC
AGCAAAGGCAGGGCAGCGTGTGGGAGCGTCGCTCATATTGAATTACCAAAGTACAGTGAACACTCCACTC
CGGGGCGCCTTTGTTTGCCTTTTGCGCTTTGTGCATGCCAAAATAAAAAGGCGTAGTCACTTTGTTGGCC
AGAGGAATTGCggggggggggggCCTGCAAATGCGGGGGCGGGGGCGTTCGCAGAGCGGTGGGCGTGGGC
CCGCTCAATAGGCACTGGCCGCTCCAAGCGAGCGAACGAATGTTGAGTGAGTGACGGACTGGTCGAGTCT
GACATGCACTGAGCAAAAAAATCTGCAATTGCCCCaaaaaaaaGTATGGATTAAAAGAAGATAACAAG
>dme_circ_0001759_junction_seq
aaaGTATGGATTAAAAGAAGATAACAAGGTAAGCTGAGTGTGGCTCTCTGAAGCGA
>dme_circ_0001760
GTATTTCCTTTTTGATCAGCGCCACAACTGGCGGAGATGCTTGGCACTTGCCATTGAGGCCAGGACTCTC
GCTCCTGCCATTGGTGACTATGTCGAGGACACCGGCGGTGGTGAGGGCGTGTCCTGGTCCCTGGAGCGAT
TTGGTGGCACTCGTCTGGTATTTCTTCAAGACGGTGCTAATCATTGGC
>dme_circ_0001760_junction_seq
TTTCTTCAAGACGGTGCTAATCATTGGCGTATTTCCTTTTTGATCAGCGCCACAAC
>dme_circ_0001761
GTTTGCACAGTGCTGGAATTTCAGCGGTGTTCTGACCACCATTGCCAGGAGTCTGGGAATACCGTCTAGG
ATCATCACTTGCTACTCCTCCGCCCACGATACACAGGCCTCACTCACCGTGGACGTGTTCATCGACGCGA
ACAACAAGAAGTTGGATGCAGAGACCACGGACTCGATCTGGAACTATCACGTGTGGAATGAGCTCTGGAT
GCAGAGACCGGATTTGGGCGTGGGTGAGCACGGGACCTTCGATGGCTGGCAGGTGGTGGACGCCACTCCG
CAGGAGGCCTCCGATAATATGTATCGCGTGGGACCCGCCTCCGTTGCGGCGGTGAAAAACGGTGACATCT
TGCGACCATTCGATGGTGGTTTCGTCTTCGCGGAGGTGAATGCGGATAAGCTCTATTGGCGCTACAATGG
ACCCAGCCAGCCGCTTAAGCTGCTCAGGAAGGATACGCTGGCCATAGGGCATCTCATCAGCACGAAAGCG
>dme_circ_0001761_junction_seq
CATAGGGCATCTCATCAGCACGAAAGCGGTTTGCACAGTGCTGGAATTTCAGCGGT

>dme_circ_0001762
GCAAACTACAAACATGTCGGGCACATCGCAGAAACACAGGAACTTCGTTGCGGAGCCAATGGGCAACAAG
TCGGTGACGGAACTGGCCGGAATTGGGGAAACCCTCGGTGGACGCTTGAAGGACGCTGGATTCGATATGG
TGCGTGTGTAAATACGATGACGCCATGTGCGCAAATAAACAATGCCAGTCAAGTGCTAACGATCATTTCC
ATTCGTTTTAGGCCTACACCGTTTTGGGACAGTATCTGGTGCTGAAAAAGGACGAGGAGCTGTTCAAGGA
CTGGATGAAGGAG
>dme_circ_0001762_junction_seq
GGAGCTGTTCAAGGACTGGATGAAGGAGGCAAACTACAAACATGTCGGGCACATCG
>dme_circ_0001763
GGATATAGCGTAGGACCAGTGAACAAACAAGTCCGAGAGAGCGACAAACAAAGGACCGTGCACTGGGCAC
CTGAAGGCAGACAGCTGGCAACGAATCAGGAGAGGATCAGGAGAGCAGGATCAGCGGGCACAAGGATCAC
CGGAGCAGGCAAAGGAAACACCTGGCACACAGCACAGCCACTGAGAGCGACGTGAACCAATGGGCACCAA
TTCGGGAGCCACCGCTGGCATAAACAACAAGCCGGTTGGCGGTGCAACAGGAGCCGGCGTCCTTGTAGGC
GGCGGTGTGGGCGGTGCCAATTCCTCGATCGGCGGTGTCCTGTCGAACAGCCTGGGCGGTGGCGGCAGCG
GCGGTCTGAGCATCAGCGGCCTCAACGCTGGTGGACAGAACGCCAATGTGGGCGGAATGGGCAACGTTGG
CGGCGACGACGGCGGAAACGGGATGGTGGGCGGCGGTGTAAATAACCAGCAGGCCACAACGCCCCAATAC
ACAATACCGGGCATCTTGCACTTCATCCAGCACGAGTGGTCGCGCTTCGAGCTGGAGCGATCACAGTGGG
ACGTGGACAGGGCCGAATTGCAGGGTGAGTGATCCTTAAATACTGAATTGCTAATAGCTCCTCTTTAATA
TTCGATTAATCTCGTACTTTGAGATAGGTGAATCAGAAAATCAGCTTAAGATCTTAACAATGTCCTTATT
ATTAGTTTGAACATTGATTTAGTTCTCATCCTCCACTATTTTATTCACTATTGATTAAGTAGGTACTGGA
CTCACGGACTACAGTAAACTTTATAGCGTGAACTTTGTGCAGATAGTGACAGTATAGTGCTTAGTATAAA
AGAACCAATGAAACTGCAAATTGTTTGTCCTAGAAAGAATACTATGCAACCAAAAGCAAGGCAATTTGGC
AAGGCCCAGGGAGTGCTTAAAAGGTCCTTGCTTATCGACAGCGGTGGCTCACTTTCCATTTCAATGATTT
TCCGAGGCGCTAATGCATGAACATGCAAGGAAAACGGCCAGGAGGAGCCACCACACCGCCTTGCCTCCGC
CATGTCCGCATGCAAGCGAATCGAGTTGAGCAATTGCCGTCATCGAGTGTTTATGCTCTGTTTGTCGCAG
ACGAATGGCCAAAAAGCCTGAAAGCCCCCAAAAAATGTAAAATGAGTGTAACCGCGGGTTGGATAACATT
ATCGTAATTTCGAAGGAGATTACTCCACCTCCAACCTGCGCCACCCTGCGCCTCCATGGCCATCGTCAAT
GTCAATGTCGACCCACGCTCTATCGCTTTCACCTGCCAAGTGGCCAGATAACGAGCGGTATCGATAACGA
GGCACAGTCCGTTACGCTCTGAATGCAACGTGACATTCAAATCCGTCTCTTCCCCTGCTTTGCATCATCA
TAATGTGGAAAAGTGGCCACaaaaaaaaaGGAACCAAGTTGACAACGCAAATTTGTCGCCGCGATTTATG
AACCTGTTCGCCTTGTTTGGCGTTTTTGGCCAAAATAAATCTGGTTCGGGTGGGCAGCAGAGGCAGAGGC
GACGCCATCCGAGATGGCTCTGTTTCAGCAGTCGTAGCATCCGTTCCCTGCCCGGCCGCCTGGATCTATT
TATATACTTCCATATGCACATCGCAAATCTCTTTGTGTGTCAGTGCCGACTGCTGTGAATGCAGCAACGT
CCTTGTCGCAACGAATCTCAGTTTTGCCGGTCTTCCCATGAATGCATCCACTTCCACTTCCTACTCGGTC
GGCTTTTCCTACTGTCGCGTTTGTCAGGCAATTAACTTGCACTGTGGTCGGAAACTCTTTATTTAAAGAA
CATTTACGTTTCTATGTTGAATTTCAATCAGATATGAGGTAACAAAAACAAAAAAGCGAACATTTAGGTT
CATCTTATACTATCATTTATAACTCTTTAATGTTTAATGGCATATTTACCTTTAAATTATTATCAAATTC
TTGAGAAATGATTATTTGTGCTTCGAAAGCACTGTAAGCAAGAATGACGCGGCAAATGCTAATATATTTT
CAATCATTTCGCCATTGCAAAAGCATTTGTTTGCAAATGCATTTACAGATTAGAGAAACCAAACGCCGCT
GGCGCTCTTGTCATATTAATAATTCTATTTATATGTCCTTGTGTACAATATGGTCACTTCCCCAACAGCA
TTATGCAATGGACAATTCCAAGAGCAGCCACGTAACCCCTTTGGTTAGCCAGCATTTTTCAAAAATTGTA
AAGCGAAAAATCTTGGAGCTTCTCCGCTCGGGCTCATTAAGTTACAAATGCGCATTTGTCATGGAATTCG
TAATTTCTAATTGCGAATGAAAATAAAAAGTAGTTGCATGTCGCGGAGCGATTTGTGTAGCAGCTCCATA
AGGCGTTGGCGTGTGTGTTTTGTTACCTTTCTGGCCCTTTTGCCTTCGAATATCCGGAATATTAGCGCGC

TGCTTTTAATTAGCCAGCTTAAGGAGCCAAATGAAATGCCATTGGGCGATAATTTAAATGCACAACATGT
TTCCATTAACAGCAACAGCACAAAGAGTACCCCACTCActcctcctcctcctcctcctcctcctcctcct
cctcctcctcctcctcctcctcctcctcctcctcctcctcctcctcctcctcctcctACCGCCCTTT
TCGTTACTATTAGCATGGGCAAATTGTTATTGTTGCTGGTTCTGGTTCTGCGCTTGCCTCATTCCATACA
AACATGCGTTCGCTTGCCACAGTCGCGGCCACAACTATAGGGACTGTTATACGTAAATGATACATTTAGG
AAGTAACATAGTTTAATATACGGATAGTTGTTTGATAAGCTAATGTTAGTTAAATAACGCAGACTTCTTT
CCAAATGAAAGAAATTCTGCTGTAATAGAATTAAATAATATGTATATCAATAGCTAAGCACACAGCTATT
CAGCTGACCTGCAACGTTGGAGCGTGGGTGCTGACCACTTCCCCTCCATTGTTGGttttatgttttttat
tttttattttttGCAGGGGCGTCATAGTTATACTTCTGGCACAAAGTTAGCCAGTTAGAGTATAGAGTTG
TGAAAAATAGGTAAAAGCAAAAGTTTCCTTCTCTTGCACACCGCATCTGCTTCGATGTCAGGTGCAGTGT
TTGCCTGAGATTTTCCCTCCTGAATTTTCATTCTCTCCCCTCCTCAAAATGCTCGGGGAAATGCGCAGTC
ATAACTGCACGCTGTTGCTACGCTTGCTGAATGAACACGAAATGGGTAGGAACGAAGGGGCGGAACGGAA
AAGTGCTGGCTACATTGCAGTTTTTACGCATTTCGATTAGTTGAACCGAAAACTAGCTTATTAGATACAT
ACCCCCACCAGCTGCCCCCAACCCTCTCACCTCTCAGCTAATTGTGCCAGTTGTAGTGTTTCTTTTTGTA
TTACTATTAATTGAATTTAATTGTTTTTCGTTGGCCCGACGCAGCGGAGCGTCAATTATGCTCCGcaaca
aacaaacaaacaaacaaacGCAATTGGATTGCAACGGCAGGCAAAATGGGGACAAAGCCAGCCATTAAGC
AGCAGTGTATTAATTAATTTTCTATAACTCATAGCTTGTACATATATGCATTTATTGCGCACATCGGGTG
TACTTTCTCTTTCGAAAATCGAAAATAATATCTATGAATAATTCTATTAATATTATAAATCTTAGCTAAA
TCTTTTTGTGCTACAAATTGAAATTAATCAAATGGCTTTCTATATCCTCGCAGGGCTCGCATTGCTATGC
TGCTTGGAGAGCGCAAGTGCTTGGAAAGCCTCAAATCGGATTTGACGCGCCGCATCAAGATGCTGGAGTA
CGCACTGCGCCAGGAGCGTGCGAAATTCTATCGTTTAAAATACGGCACCGATCCGCCGCAGCTCAATGAG
TTCAAGCCATCGAACGAGGATGCCGGACTGGCCGGTGAGGTGGCCACTGACTCGGAGGTGCCCTACAGCA
GTGTCTCGAACACTACGTGGCGGCAGGGGCGTCAGATGCTGCGACAGTATCTGGCCGAGATTGGCTACAC
AGACAACATCATCGATGTGCGTTCAAATCGGGTGCGTTCGATCCTTGGCCTAAACAATAATGCCGAGCAC
GACGGCAGCGGCGGAGGACTGGGTGGTGGACTTGGCGGCGGCACCGGCGGCGAGAACCTTAGCCCAAACA
TCAATGGAAACGAGAGCAACAAACGCGCATCGGAGACGGAAGGGCCGTCATACTCCCGCTAAAAAAGTGC
AACAATCGATCGATGAAATAATCGTGGACACCGAGGCAGCTGTGATGGCGAACTTTGAGTTCCTGGGCGC
CACCGAGATGTCCGACGACGACGAGATATCTGACGATCTGGAAATGGTGGCCACCGACAATGACGACACA
GACGTAAAGCTGGCCAAGCGCGCCAAGTCTGGCAAGGATATGCTCACCGAGG
>dme_circ_0001763_junction_seq
AAGTCTGGCAAGGATATGCTCACCGAGGGGATATAGCGTAGGACCAGTGAACAAAC
>dme_circ_0001764
GGCTCGCATTGCTATGCTGCTTGGAGAGCGCAAGTGCTTGGAAAGCCTCAAATCGGATTTGACGCGCCGC
ATCAAGATGCTGGAGTACGCACTGCGCCAGGAGCGTGCGAAATTCTATCGTTTAAAATACGGCACCGATC
CGCCGCAGCTCAATGAGTTCAAGCCATCGAACGAGGATGCCGGACTGGCCGGTGAGGTGGCCACTGACTC
GGAGGTGCCCTACAGCAGTGTCTCGAACACTACGTGGCGGCAGGGGCGTCAGATGCTGCGACAGTATCTG
GCCGAGATTGGCTACACAGACAACATCATCGATGTGCGTTCAAATCGGGTGCGTTCGATCCTTGGCCTAA
ACAATAATGCCGAGCACGACGGCAGCGGCGGAGGACTGGGTGGTGGACTTGGCGGCGGCACCGGCGGCGA
GAACCTTAGCCCAAACATCAATGGAAACGAGAGCAACAAACGCGCATCGGAGACGGAAGGGCCGTCATAC
TCCCGCTAAAAAAGTGCAACAATCGATCGATGAAATAATCGTGGACACCGAGGCAGCTGTGATGGCGAAC
TTTGAGTTCCTGGGCGCCACCGAGATGTCCGACGACGACGAGATATCTGACGATCTGGAAATGGTGGCCA
CCGACAATGACGACACAGACGTAAAGCTGGCCAAGCGCGCCAAGTCTGGCAAGGATATGCTCACCGAGG
>dme_circ_0001764_junction_seq
AAGTCTGGCAAGGATATGCTCACCGAGGGGCTCGCATTGCTATGCTGCTTGGAGAG

>dme_circ_0001765

GCTTACGTCTACAAGTTTATTTTGTTTTATTTGCCGCCGTGCAGCGACGCGAAAACAACATAAAACCACT

TCGTTAGCGAAAAAAGGATTTTTAATGGTGTAAAGAAGGTGCCTAGAACTGGATCATCTGTCCCTTGCGC

TTCTTGTCACCGCCCAGCTCGAAGTGCTTGCAACGCTTCAGGGGAGTCTGCTTGCGGTATTTGCACTCGG

TGCACTCCATACGCAGCACAATCTTCTTG

>dme_circ_0001765_junction_seq

GCACTCCATACGCAGCACAATCTTCTTGGCTTACGTCTACAAGTTTATTTTGTTTT

>dme_circ_0001766

GGTGCCTAGAACTGGATCATCTGTCCCTTGCGCTTCTTGTCACCGCCCAGCTCGAAGTGCTTGCAACGCT

TCAGGGGAGTCTGCTTGCGGTATTTGCACTCGGTGCACTCCATACGCAGCACAATCTTCTTG

>dme_circ_0001766_junction_seq

GCACTCCATACGCAGCACAATCTTCTTGGGTGCCTAGAACTGGATCATCTGTCCCT

>dme_circ_0001767

GTGACGCAGTACAAGAAGTCCAAGGAGCGCAAGGGAGCTCAGGGCAGGCGTCGTTACGACAGGAAACAGC

AGGGTTTCGGAGGTCAGACCAAGCCCATCTTCAGGAAGAAGGTAAGTGCCCCCATTCGTCTAATGCTACC

GGGATCGCTTGCCGCCGGATTGCCATCGACACTAATGGCGGCTAGATTGGAATCCCTGGAATGGCGCTTC

GTAGAACTAAATCGCTGCGCTCGTTGAGCATTATGCTTTTTGGTGGGAAAAAACTGTGCCAGATCAAAAC

TGTTGTTGCACTGGTGCGGCCGTGAAGGCAACGTTGTGTGGCATCCATGCCCACAACGCCCCACCGGCAA

CTTCAGACAATCCAAATGAATTCATGACCACCATTTGTATGCCGCTTGCTAACTGGAGTTCTAATTCCAT

TTTAGGCCAAGACCACCAAGAAGATTGTGCTGCGTATGGAGTGCACCGAGTGCAAATACCGCAAGCAGAC

TCCCCTGAAGCGTTGCAAGCACTTCGAGCTGGGCGG

>dme_circ_0001767_junction_seq

AGCGTTGCAAGCACTTCGAGCTGGGCGGGTGACGCAGTACAAGAAGTCCAAGGAGC

>dme_circ_0001768

GTCCACAAGCTGCACAAGGTGACGCAGTACAAGAAGTCCAAGGAGCGCAAGGGAGCTCAGGGCAGGCGTC

GTTACGACAGGAAACAGCAGGGTTTCGGAGGTCAGACCAAGCCCATCTTCAGGAAGAAGGGTAAGTGCCC

CCATTCGTCTAATGCTACCGGGATCGCTTGCCGCCGGATTGCCATCGACACTAATGGCGGCTAGATTGGA

ATCCCTGGAATGGCGCTTCGTAGAACTAAATCGCTGCGCTCGTTGAGCATTATGCTTTTTGGTGGGAAAA

AACTGTGCCAGATCAAAACTGTTGTTGCACTGGTGCGGCCGTGAAGGCAACGTTGTGTGGCATCCATGCC

CACAACGCCCCACCGGCAACTTCAGACAATCCAAATGAATTCATGACCACCATTTGTATGCCGCTTGCTA

ACTGGAGTTCTAATTCCATTTTAGGCCAAGACCACCAAGAAGATTGTGCTGCGTATGGAGTGCACCGAGT

GCAAATACCGCAAGCAGACTCCCCTGAAGCG

>dme_circ_0001768_junction_seq

AATACCGCAAGCAGACTCCCCTGAAGCGGTCCACAAGCTGCACAAGGTGACGCAGT

>dme_circ_0001769

TGCAAGGTCCACAAGCTGCACAAGGTGACGCAGTACAAGAAGTCCAAGGAGCGCAAGGGAGCTCAGGGCA

GGCGTCGTTACGACAGGAAACAGCAGGGTTTCGGAGGTCAGACCAAGCCCATCTTCAGGAAGAAGG

>dme_circ_0001769_junction_seq

CAGACCAAGCCCATCTTCAGGAAGAAGGTGCAAGGTCCACAAGCTGCACAAGGTGA

>dme_circ_0001770

GATGGGCTTGGTCTGACCTCCGAAACCCTGCTGTTTCCTGTCGTAACGACGCCTGCCCTGAGCTCCCTTG

CGCTCCTTGGACTTCTTGTACTGCGTCACCTTGTGCAGCTTGTGGACCTTGCACTTCTTGCAGAAG

>dme_circ_0001770_junction_seq

CTTGTGGACCTTGCACTTCTTGCAGAAGGATGGGCTTGGTCTGACCTCCGAAACCC

>dme_circ_0001771
GTATGACCAGTGAAAGGTATCCAGAGTCTCCATCTTGCACACTTCAGTCTCGTTGACGGATCGCAAATGG
CAGATATTGGTGAGAGGACACCAAGCACAGGAGGCGTCAGTGAGGCAGTGCAGACAGTTGGAGTACTGAG
TACACTTGCCAGAGTAGTATGGTTCCAAATACTCGAATGTGAAGGCGTTTAGCTGGCCGTTATGCAGG
>dme_circ_0001771_junction_seq
GAAGGCGTTTAGCTGGCCGTTATGCAGGGTATGACCAGTGAAAGGTATCCAGAGTC
>dme_circ_0001772
GTACCAATGTCGGCACTATATGGGCTGCAGCTACTGTACTATTCAGAACACTTATAGCTACAGTAGCCAC
TGCTTTAGTCATGGACGAACACCGTGTGCCAATCACAATGGCACCCTGGTGGTCAACAATGGCGCTGCAT
GCGATGATGACTGGATGGCGAGTAGAAACTGCTCGAGCTTTGCAACTTGTGGAGCCTGTTTGGCGGCCTG
GCCAACACATCAAGAAGTGGCTCCTGTATGCCACTGGTGTGACGACTGTGGCATTCGAGGCAGGTGCGTT
CCAGCTGGAGTTGATTGCGGAAGGAGGAGTGCCTGGTGCAACAAGGAACTGAGTGTTGGA
>dme_circ_0001772_junction_seq
CTGGTGCAACAAGGAACTGAGTGTTGGAGTACCAATGTCGGCACTATATGGGCTGC
>dme_circ_0001773
GTGGTCCGCAATCGAACATGCACTTCGTACCCTACCTGCTCTTCTACTCCGTCTATCTGTTGCTTTCGTC
GCGATCAGCGGCTAGAGACAGCAAGACTCTGCTGACGTATTTGCAGGCGCCGCCCAGCGAGAAGTGGTTG
GAATGCGGATATGAGG
>dme_circ_0001773_junction_seq
GAGAAGTGGTTGGAATGCGGATATGAGGGTGGTCCGCAATCGAACATGCACTTCGT
>dme_circ_0001774
GTGCTTTGCCAACCCGAGATCGGAGCCATCAACACGCTGCTTAAGGTGCTACAAATGTGTCTCCAGTCGG
AGAACGATTCGATTCAATCGGCGGTTACCGAGCAGTTATTGGAGATCATGGAAACAATACTATCGAAGGC
GGCCAGCGATACGTTGGACTCGTTCCTGCAGTTCTCCTTGACATTCGGTGGACCGGAGTATG
>dme_circ_0001774_junction_seq
TCCTTGACATTCGGTGGACCGGAGTATGGTGCTTTGCCAACCCGAGATCGGAGCCA
>dme_circ_0001775
GCTTATTATTTTGTTGTTGACCAGCAATTCCATGCCGTTATCATCTTCCAACAGGGCGATTAGTTCACAA
TCCGTACAAATCTTATTCTTCACATCCCGCATCAATGGACCCAATCCCATTTCACTCGAGGGGTAAGGAT
TACCCAGCATGCGACCTTGCAGAAAGTCCTCCTGCTGGGGATCTTTCTCTAAT
>dme_circ_0001775_junction_seq
GTCCTCCTGCTGGGGATCTTTCTCTAATGCTTATTATTTTGTTGTTGACCAGCAAT
>dme_circ_0001776
TATTTGGTCCTGAAAGGAGTATTAAGTCAAATCTCACAGCTTCTGGCTATTGAAATCAGCAAGGTGCACC
GCATGGAGGAGTACTCGCTTTCTTCAGATTTATCCTTAGGATATGCACTGCGCCAGTATGTGGAATTGTT
GTGGCTACTCCTAGAGTGTCCAAATATCCGCAGAACCTACAAGACTCGCATGCTAGGACCAG
>dme_circ_0001776_junction_seq
ACCTACAAGACTCGCATGCTAGGACCAGTATTTGGTCCTGAAAGGAGTATTAAGTC
>dme_circ_0001777
GTGGCCACTACAGATTTGTGTAGCATGAAGATGTTTGAGGGCAACACGGTGGACTATAGGGCCTGGCTGA
ACTCAGACCGTAATCATGAATACGCAGCGTGGAGCAAGCGGATGCCGTCAAACAACCAGGCTAAGCTAAA
GAATGCCAAGGATCAGAATG
>dme_circ_0001777_junction_seq
AAGCTAAAGAATGCCAAGGATCAGAATGGTGGCCACTACAGATTTGTGTAGCATGA

>dme_circ_0001778
GAAGTGCGCAACCTGTTGGTCGTACTGACTAAGGATAATGCCGAGGCATGCATGCATCTGTTGCAGTTGG
TGACCACAAGGGTGAAGAATGCTCTGATGGGATCGATTCCTCTGATAAGTTTAGAGGCAGCTGTGCACCA
AGAAATGACACTTTTGGAGG
>dme_circ_0001778_junction_seq
GTGCACCAAGAAATGACACTTTTGGAGGGAAGTGCGCAACCTGTTGGTCGTACTGA
>dme_circ_0001779
CTAGTCCAATGCCATACTATTTCCAAGTTGATCGTGCGGATTGCCGACTTAAAGCGGACAAAGATGGTGA
GAACCATTAATGTTTACTACAACAACCGCAGTGTCCAGGCGGTAGTGGAGCTAAAAAATCGCCCAGCTCT
GTGGCACAAAGCTCGCTCAGTATCCCTGCAATCGGCACAGACCGAACTGAAGATCGATTTTCCACTACCA
ATCACCGCATGCAATTTGATGATCGAGTTTGCTGATTTCTTTGAAACTGTGAGTGGATCCAGTGAGAATT
TACAATG
>dme_circ_0001779_junction_seq
TGAGTGGATCCAGTGAGAATTTACAATGCTAGTCCAATGCCATACTATTTCCAAGT
>dme_circ_0001780
AACGACTGCCAGAGTTCGTTTCCCGCGCTGTGGATGTTCTCAGGCAGCAGAACGAACTGCTTTGCAAGCA
TCCAAATGCCCCAATATACACCACCTTGGAGTCTATACTTCAAGTAAATGGTTACTACCTAGAATCGGAA
CCTTGTTTAGTGTGCAACAATCCAGAGGTGCCGATGGCTAATATAAAGTTGCCATCAGTTAAATCAGACT
CTAAGTACACCACAACCACAATGATTTATAAGCTAGTCCAATGCCATACTATTTCCAAG
>dme_circ_0001780_junction_seq
GCTAGTCCAATGCCATACTATTTCCAAGAACGACTGCCAGAGTTCGTTTCCCGCGC
>dme_circ_0001781
GTTCCAATGTGGCAACCGATGGATCAACATTGCGTACTTCTCCAGCAGAGCCTGCTGGCAGCGGTGGATC
AGAGAGTGGAGGCAGTGGAGTAGAGAGTATTGGTGGAACATCCGCCAGGAGCAGCAACTTTGGCGACCAC
GCGAATGCCTCGCCGCCGCGTCAGGGATCCACCAAGGACGATCAAGAGCAGCCAGGTCCCAGTGGCGTTG
CTGGCTCCGGTGGTGTTGCTG
>dme_circ_0001781_junction_seq
GGCGTTGCTGGCTCCGGTGGTGTTGCTGGTTCCAATGTGGCAACCGATGGATCAAC
>dme_circ_0001782
GTGGATTACCCGAACCGAATGCGGACGCGGAAGGTGCTGCAGTTGGAGTAGGAGGAGTCGGTCAGCAGCA
AATGCTCAATCTGGAGGCATTCATGGGTGGCGGTTTCCCTCGCTTACTCGGCCTACCCGAAGATGCCGAC
GATGAGGCCATCATGGATATTGCCATCGCATTGAGTCTCCAACAGCACGGTGGGGATGCAAACGCCTTGC
AGTCTCTTCAGCAAGGATTGGCCAATCTTCAGGGCATCCGTCAAGCTACTGCCATGGCAGCAGCTGTGAA
CGCAGCTGCCAATGTCTCCCTAGGTGGATCCGATGATGATGAAGGTTCCAATGTGGCAACCGATGGATCA
ACATTGCGTACTTCTCCAGCAGAGCCTGCTGGCAGCGG
>dme_circ_0001782_junction_seq
CTTCTCCAGCAGAGCCTGCTGGCAGCGGGTGGATTACCCGAACCGAATGCGGACGC
>dme_circ_0001783
TTGCATAAGGTTACACCGACGCTAGACGAACCCGTGAATATTGTGAAGCGAGGCTTGTGCCAGCCGGAGA
CCATTGTCCATTGTCTGGTAGAGATAATGTACGGATTTGCCTTGGCCGATCCCGGACAGGTTGGTCGCAT
GACCAAGTACTTCATTGATTTGCTGAAGCATGATGCTAGCGTGATTAGCCACAGCGCCAAAGAGGCCCTG
ATCCTTTTGCTTAGTCCAAGAATGAAGCGCAGAAAGGTGGCCATAGTTACACCCCCAGCATG
>dme_circ_0001783_junction_seq
AGGTGGCCATAGTTACACCCCCAGCATGTTGCATAAGGTTACACCGACGCTAGACG

>dme_circ_0001784
GTTGTTCTCCACGCAGATCTTCGCTAGCGATTGGGGTCGTGCGTTGGCAATGCCACGGACCATCAGAACT
AGGCGGTAGAAAGCCTCAGGGTCGATCTCCCGCAGAGTTTCAAGTTTTTCAAGCTTCGGCTGCATTGACT
GTAGTTCTCTATTTACAAACTGAAGAAGCACTCCATCCTTGTA
>dme_circ_0001784_junction_seq
CAAACTGAAGAAGCACTCCATCCTTGTAGTTGTTCTCCACGCAGATCTTCGCTAGC
>dme_circ_0001785
GTATATGGAAAATCGAAAGAGCTGGTGGGCTGGCCCGACGAAAGCGAGGATGTTACTGTCCCAGGATCCA
GTGCGCCAGCAGTATCTTCCTCCCAGGCAAGTAGCGCTAACTTTGGAGAAGGATTCAATTGCATTACTCA
GCTGGATCGAATGGCTAATCACCTTTTGGAAGTAATGGATTGTGCACTGCATTTGTTGGGATCTGGAGTA
CCTGCTTCTATGCGTCAAAAGGCAGTTAAAACAGCGAGTGCCCTATTGCTACTGCCAACGCCCAATCCTG
TTCAGACCCAGGCGCGCTACGTGCTGGCCACTCTATACGGAACTAGAGCATTG
>dme_circ_0001785_junction_seq
GGCCACTCTATACGGAACTAGAGCATTGGTATATGGAAAATCGAAAGAGCTGGTGG
>dme_circ_0001786
GTATATGGAAAATCGAAAGAGCTGGTGGGCTGGCCCGACGAAAGCGAGGATGTTACTGTCCCAGGATCCA
GTGCGCCAGCAGTATCTTCCTCCCAGGCAAGTAGCGCTAACTTTGGAGAAGGATTCAATTGCATTACTCA
GCTGGATCGAATGGCTAATCACCTTTTGGAAGTAATGGATTGTGCACTGCATTTGTTGGGATCTGGAGTA
CCTGCTTCTATGCGTCAAAAGGCAGTTAAAACAGCGAGTGCCCTATTGCTACTGCCAACGCCCAATCCTG
>dme_circ_0001786_junction_seq
CTATTGCTACTGCCAACGCCCAATCCTGGTATATGGAAAATCGAAAGAGCTGGTGG
>dme_circ_0001787
GAAGCAGGTACTCCAGATCCCAACAAATGCAGTGCACAATCCATTACTTCCAAAAGGTGATTAGCCATTC
GATCCAGCTGAGTAATGCAATTGAATCCTTCTCCAAAGTTAGCGCTACTTGCCTGGGAGGAAGATACTGC
TGGCGCACTGGATCCTGGGACA
>dme_circ_0001787_junction_seq
TACTGCTGGCGCACTGGATCCTGGGACAGAAGCAGGTACTCCAGATCCCAACAAAT
>dme_circ_0001788
GTATATGGAAAATCGAAAGAGCTGGTGGGCTGGCCCGACGAAAGCGAGGATGTTACTGTCCCAGGATCCA
GTGCGCCAGCAGTATCTTCCTCCCAGGCAAGTAGCGCTAACTTTGGAGAAGGATTCAATTGCATTACTCA
GCTGGATCGAATGGCTAATCACCTTTTGGAAG
>dme_circ_0001788_junction_seq
GATCGAATGGCTAATCACCTTTTGGAAGGTATATGGAAAATCGAAAGAGCTGGTGG
>dme_circ_0001789
ATTTCTATGTAACCAACACTTTGGAGCTGAGCCACCAACATATCAAGGATATTGCCGGTCATATCGGAGG
CGGTGGAGTATCCATCTACTATTCGCATACCCTACAGCTGCTCTTCTACAGTTATTCTTGCGGAAGGAGC
TTCTTTTCACCACTCACCAATGTAAGTGAAGGCGTGAAGGGAATATATCACCTGGACACAAATTCGGCTA
GTAAGTCAGCTTCGAAGGGACCACTACAGCCTCTCGTGCAGTGGACAGAGG
>dme_circ_0001789_junction_seq
CTACAGCCTCTCGTGCAGTGGACAGAGGATTTCTATGTAACCAACACTTTGGAGCT
>dme_circ_0001790
GGTGATATATTCCCTTCACGCCTTCACTTACATTGGTGAGTGGTGAAAAGAAGCTCCTTCCGCAAGAATA
ACTGTAGAAGAGCAGCTGTAGGGTATGCGAATAGTAGATGGATACTCCACCGCCTCCGATATGACCGGCA
ATATCCTTGATATGTTGGTGGCTCAGCTCCAAAGTGTTGGTTACATAGAAATCTCCATGAACCGCTAGAC

TCTGTTGATCCAACTGCTGAGTATAGATGTATCCGCTGCTTGCAAAGGTCAGCATATTGTAGTTGCCATC
CTGGTACACAAACGTGCAATCTCTAATTTTGCCAACGGCCACTAG
>dme_circ_0001790_junction_seq
AATCTCTAATTTTGCCAACGGCCACTAGGGTGATATATTCCCTTCACGCCTTCACT
>dme_circ_0001791
GGGTATGCGAATAGTAGATGGATACTCCACCGCCTCCGATATGACCGGCAATATCCTTGATATGTTGGTG
GCTCAGCTCCAAAGTGTTGGTTACATAGAAATCTCCATGAACCGCTAGACTCTGTTGATCCAACTGCTGA
GTATAGATGTATCCGCTGCTTGCAAAGGTCAGCATATTGTAGTTGCCATCCTGGTACACAAACGTGCAAT
CTCTAATTTTGCCAACGGCCACTAG
>dme_circ_0001791_junction_seq
AATCTCTAATTTTGCCAACGGCCACTAGGGGTATGCGAATAGTAGATGGATACTCC
>dme_circ_0001792
GGTAATAGTACTTTGGGCTATATGTATCCACTGCCAAATCGTAAATCTTTACATAATCCGAAGTTACAAC
GGCAAGAAGAGTCTGTGAGCCAGGCAGCCACACAGCTTTCTTGATGTAGTTTCCATTTTCCAGTTGGGGG
TTCACTACTATGTGTTCGTTAGTACTGCCACTGCTTGAGAAGGTGAGAATGTGACACTCTTTTAGGCCAC
AAACTGCAAGGCAATCCTCATTGCACGGATTAGCAGCTAGGGAGATAACCGTGCAGGCAATGGGTGCAGA
ACTCAATTGCGTTAGA
>dme_circ_0001792_junction_seq
AATGGGTGCAGAACTCAATTGCGTTAGAGGTAATAGTACTTTGGGCTATATGTATC
>dme_circ_0001793
GTGTTTGTCCGGTAATTGCAATTTGGCCAGTTCCAGCCAAAGGAGGGTGGTATTGAACAAGGCGACATGG
CCGCGTGCTGGACTGGCGTCAGCTAGAGTAATCATTATCTGTAGTACATCTGCAAAATCGCAGCCTTCCT
GGGGTGGTGAAATTAATGTTTGTCCGAGCTGAAGCAAAGCTTGGAACAGTGGTGGAGCTATCTGCGATGC
TTTGCTTTGATCTTTCACCAATGACTTCACGAAGCTTAAGAGCAACGCGCCATTTCGTTCGCTATCACTA
TGATCGCAAGCCTGTGAAGATTCTTCCGACGGATTTGGCTGCGCAGCCTGCCACAAACTCAGAATCTGCG
AGGTATTGGCTGCAGCTCCTCCGGTTCCTGGGTTACTTTCTCCGCCTCCCGCTGCAGTTCCGCAATCGTA
TTCAATTTCCATGGGATCCAACTGGTCTGAGATACTTGGCATAGCCGAAACGGTGGCCATATTTGTTGGC
GTCTGAGCATTGCTGCTGGAGCTACTCGACGGCTGCAAAATGCTGGTGAGCATCCGGCAGGTGGTGTCTA
ATGCCTCGGAGGTGCTTACATCG
>dme_circ_0001793_junction_seq
GTCTAATGCCTCGGAGGTGCTTACATCGGTGTTTGTCCGGTAATTGCAATTTGGCC
>dme_circ_0001794
ATTCTGAGTTTGTGGCAGGCTGCGCAGCCAAATCCGTCGGAAGAATCTTCACAGGCTTGCGATCATAGTG
ATAGCGAACGAAATGGCGCGTTGCTCTTAAGCTTCGTGAAGTCATTGGTGAAAGATCAAAGCAAAGCATC
GCAGATAGCTCCACCACTGTTCCAAGCTTTGCTTCAGCTCGGACAAACATTAATTTCACCACCCCAGGAA
GGCTGCGATTTTGCAGATG
>dme_circ_0001794_junction_seq
CCCCAGGAAGGCTGCGATTTTGCAGATGATTCTGAGTTTGTGGCAGGCTGCGCAGC
>dme_circ_0001795
TTCAGGCCATGCGCCAGTTTTTCAAAGATCAGCGCTCTGGGAGTCTTACCACACTTTTGCTATCGTTCAC
TGGCACCAGTCTGCCTGTTAGCTATGCGCGCAAGATGCTGCAATTTGTTAACCGACTTTTCCAGCTATCC
TTACAAGCGGATTCACAGTTCCAGCATGAAGATCTGGTTGAGTGTTTCTCGGAACTGGCCACAGTTGACG
TAGCTCGTTTAAAGCAATGGTTGGGCCACATTATCTACGGCCCTAATGTGAGTACCGATG
>dme_circ_0001795_junction_seq

ATCTACGGCCCTAATGTGAGTACCGATGTTCAGGCCATGCGCCAGTTTTTCAAAGA
>dme_circ_0001796
GGAGATAAGCAATTGCTTTAGCTGAGTCTCTACTTCTGCGTTCGCGGTGCGATGTGGTAGCAGCAACTCA
AACAATTGCAGGATAGACTTTAAGCAGTGTTTCAGAGTGTGCAGGGTAGAGTAATTGGATCCTCGTGTCA
GCTCAATAAT
>dme_circ_0001796_junction_seq
AATTGGATCCTCGTGTCAGCTCAATAATGGAGATAAGCAATTGCTTTAGCTGAGTC
>dme_circ_0001797
GTTTTAGAAAAGCTGCATGGAGTTCTAACATAATTGCTGGCTTATCGCTTACAATGGGCACAATCAAATT
CAGTGCCTTCTCCGGTGTTGAACTTGACAACTGCTTTAGCATTTGGCCACGCACGATCACCGAAAGCAAT
TGGTAAGAATTCATGAGCTTTACCATTAGCTC
>dme_circ_0001797_junction_seq
AAGAATTCATGAGCTTTACCATTAGCTCGTTTTAGAAAAGCTGCATGGAGTTCTAA
>dme_circ_0001798
ATTCTACGACTTATTTCAACGATTCTCTCTCCTGTTCGGACAACTCCGAGGAGGACGATAGTGAGAGTTA
CCTGGGCCACTGGTTTAAGGAAACTCTTTCTCCAGAAACCCATGATGATAATGCAAACACATCCACACAA
GAGCGTGCTGAACAGAAGAGCGCCCTTGTCCCCAAGCTAGATGAGCCACATGAGTATTTAGACTTGGCCG
CAGACATATTCTG
>dme_circ_0001798_junction_seq
ATTTAGACTTGGCCGCAGACATATTCTGATTCTACGACTTATTTCAACGATTCTCT
>dme_circ_0001799
GGCTCCGGGAGCTCTGTGGATTCGAGACTCTGTCCGCATGCGGCTTCAACCTTTAGATCTTCTATTAGCT
CTGAGAGCAATTTAATGCTGTGGTTAGCAATAGCTGCATTGAGTTCTCCAAATCCTTGCTGTACCTTGAA
AAGGTTGACTCTGGCAATAGGTGTAGTAGGTGCG
>dme_circ_0001799_junction_seq
GACTCTGGCAATAGGTGTAGTAGGTGCGGGCTCCGGGAGCTCTGTGGATTCGAGAC
>dme_circ_0001800
GCTGGAACCGCACCTACTACACCTATTGCCAGAGTCAACCTTTTCAAGGTACAGCAAGGATTTGGAGAAC
TCAATGCAGCTATTGCTAACCACAGCATTAAATTGCTCTCAGAGCTAATAGAAGATCTAAAGG
>dme_circ_0001800_junction_seq
CTCTCAGAGCTAATAGAAGATCTAAAGGGCTGGAACCGCACCTACTACACCTATTG
>dme_circ_0001801
AACCATCTCTGCTATGGCAGCTGGCTGCTAATTAGCGGTATACAAGGAGCAATGGGCGCCTCTGGAAGCG
GTGGATCTAGCTCGGATTCGGCATCGAAGTCGGCGTCAAAAGCGACCAAGTCTGGTAGTGAGGCTGGAAC
CGCACCTACTACACCTATTGCCAGAGTCAACCTTTTCAAGG
>dme_circ_0001801_junction_seq
CCTATTGCCAGAGTCAACCTTTTCAAGGAACCATCTCTGCTATGGCAGCTGGCTGC
>dme_circ_0001802
GAACCTCCTACTCCGCTTCCTTGACTAGTGGATGCAGTCTGTTTCTGAGAGCTGCTGGCCTGGCTCATAC
CGAGAACACTGGTGGCTATTGAGGTCAACACAGCATTGTATAGCGCAGCAATACCACACATTGATAGTCG
TTCAATTCTACTGGGA
>dme_circ_0001802_junction_seq
CATTGATAGTCGTTCAATTCTACTGGGAGAACCTCCTACTCCGCTTCCTTGACTAG
>dme_circ_0001803

GGTTTCGTTTATTACAAAAGAAAACACAAGACACTTCATAGAGATTCGATTCGATTCTTATGGTACGGAA
AGGAGCGACATTCTTAAGTTGTGTACAAAAATTTCCTTGTTTCTCGCTTAGTAGCTTCATGTACATGGCT
ATGTGAGATTTGTGTTGGGAATTTATAAACTAATTGTGGTTCATATGAAGGCGTATATACGTATAAAACT
TACAAACTATGCAGATTCATTATTTGCATAAATTAATTGGACG
>dme_circ_0001803_junction_seq
TTCATTATTTGCATAAATTAATTGGACGGGTTTCGTTTATTACAAAAGAAAACACA
>dme_circ_0001804
GATAAATTTCCATTCGATATGCTTGCAAACTTTTGTCAAGATACATTCGCTAATGATTTCTAAAATCTAT
TAAAGGCATTTTGTGAAAGACACATTGTTTGATTTTTTCATTCCGCACTTGGAGCCCGCTCTATGATAAG
GCATAGTACGGTTCGTTACGACTTACAAGATACATTAG
>dme_circ_0001804_junction_seq
GTTCGTTACGACTTACAAGATACATTAGGATAAATTTCCATTCGATATGCTTGCAA
>dme_circ_0001805
GATAAGCTGGTGCCAAACTATGATAATGCCGAGCATCAAATGAAATTTTACGACATCAGATCGCCGCTCG
TTCTCAGCTGCAACGTGAAAGACGGTACGCCGGGCGGCGTGCTCATATGGTAAGTCATTCCGACGTTCCC
CCATTTGGGGATACGGTGTCATAAAATATCCGGGAGTTGTTCCAAGCCAGAAAAGGGATGAGGATGGGTC
GGTCCCTGTGTTTGTGTCTGTGTCCGTGTCCCACATAGTTAGTAGTTATAAGAGCAATCATTTTAATACG
GCACAGCTAAGCACTTTTGCTTAATTATGTTGGCctgactgcctaactgactgcctgactgacGTAACCT
GGCAACCGAAACGTGAACGAATCGAGAAACACACTGCACTTGGAACTTACATACATGAACTGCATTAAGG
TACAAATCAAATCAAAGAAAAACTGCTATAGTGGATTCTATAGTATTATTTTTATTAGTTATTACATTTT
ATAAATGCATAGTTTTAGAGCTGCACATTCATCCTTAATTGGTCCAGACTGGATCCTCTACCTTATTCCA
ACCCTTGCACAAATTATGTGAATAATTAGCATACCCTCCTGCAAGGGTACAATAAGGTGCCATTTCTGCG
AGTGTGCACAGTTGAGTGGGTCTGATGGGTGGCACTTCTAGGGAAGGCGGTTACTGAAAAGTGCCTTCCT
GTCTGGGACCACCAGTAAGTGTTGGTGGCCAAAGCTCGGAGCTCTGTGGCAGGGTCTGGTAATGGTAATG
ACACTTCTGTTCATTCCGGGACCTGTCCGGGTCtctggttctcgttctcgttcttgttcttgttctGGGG
CCAGGGCCAAACTTTGCGTGTGCCTCGGTGTGGAATTAGTCTTTGAATAATTGGCAAAAGGCGTAGAAAA
CGGCGGAGGGCTTAGAGAAAGTTTGGCCCTCGAGTAGGTGCAGCACGGATACTCGGCGATAACTGCGGAA
TTCATTTAGATAAATGAGTATTTTCTGTAACCATATATCTACACAGAGAAAAAAGCAGAAAATAAACCAT
GAAGTAAGGAATTTTTCGGGTTCCATTATAAGTGGGAAGTGTCTATCATACTGCCTTTaaaaaaaaCTAA
TTCCGTATCGGGTATTCTCACGTGAATAGGGAAAAAGAACGGCACCGCTGTGACGGATGTACCTTCTCTC
AGAGGTCGCTTTAAGTTAATCGCAGACGAGAACAAGTTCATCATCGATAAGACGGATACGAACGACGATG
GCAAATACAGTTGCGAGTTTGACGGCGTGTCCAAGGAAATCGAAGTAATTGGCCAATGTAACGCTGACAA
ATGCCACAGATCGCTTCATTCTCAAACCAGACGATAATGGTGTTCCTAACGCCATTCTGACACTGGATAA
TGTGACATTGGACGACAGAGGCGAATACAAATGCATTGGACGAAATGCGGCCAATGTCTACGGTGGGAAC
ACCACCACTCCTGCCAGCGACGTCACAACTGTGCGTGTTAAGGGGTAAGTTTGCATCGGATGGGTGTGTC
GCCCGAGCTTTATTAAATATCCGCGGAAAAGTCATGGTCGCCATTCGCAATTGAGATTTATTTGATATTA
CATTTATCTCCAAAACGTGCTGTCCGTCACCAAATTGATAAATCTAAATTGAATTTGTATGTATATAAGC
TTTAAAAACTAGCATTTTAGAGTACAAATCTTTGAATTTATCATATCTCATCATATTGGCTAATGTAACT
GAAAATAAATTTGTGAGCCTGTTTAACTAGTATTTGTAATTGATAATAATATATAATTTTCTTTTATTAA
CAGGGCAAATTTGCCGCCTTGTGGCCTTTCCTGGGCATCTGTGCTGAGGTGCTGATTCTGTGCATCATCA
TTCTCATTTATGAGAAGCGACGCAACAAGAGCGAACTGGAGGAGAGTGATACTGATCCCCAAGAACA
>dme_circ_0001805_junction_seq
AGGAGAGTGATACTGATCCCCAAGAACAGATAAGCTGGTGCCAAACTATGATAATG
>dme_circ_0001806

GGTACCCAAAATCCCACCATTGTTGTGCGCTAGGAAAAGAATAGCTCGCATACACTGTAGATTCTTGTTA
GTCAGCATCACGGGCGCTTGCATCACGCTGTGGGGCAGCGATGCCGATGGCAGTGGT
>dme_circ_0001806_junction_seq
GTGGGGCAGCGATGCCGATGGCAGTGGTGGTACCCAAAATCCCACCATTGTTGTGC
>dme_circ_0001807
AACAGCAAATCCTGCAGTTCTTCGTTCAGCGGCCCAACGAAAACTTGCAGGCTATGGACGTCGTGAAGGT
GGTCTACAAGGAGACACCAGAAAACCTCTGGCCCGCCGCCGCCTATAACGTGAACCACCACCTAAGCAAG
CTGGAGAAGGAGGGAAAGCTGCGGG
>dme_circ_0001807_junction_seq
AAGCTGGAGAAGGAGGGAAAGCTGCGGGAACAGCAAATCCTGCAGTTCTTCGTTCA
>dme_circ_0001808
GTTCCATCGCATTCGTTGTCGGAATCGCAAGCGTGAGGGATCGTTATCATAATACTCACAAATTGAAGAC
GAGAAGCGGAGTCCAGACTGAAACTGAGACCTAGACTGACTGAGATTTGAGAGATTTTGCAAGTGCCACC
ATGGCGGCTATGGCCAATCTTCACAACACGCGCATGCTACGCTTCTTTTTAGTCCTAATCGTGGTGATCC
TAACCATTTTCATCTACGCCTCGTACTCAACGACTAGCACCCTCACAGCGAACCATGTGCACCCTGCGGC
ATCACCGCCACAGCAGCTGATTGGCAGTCATCCTGGGCGACAGCCCATGGACCAAGTGCCGCCAGTCAAT
AGCACGGATAAGGAGTACAATTCGCCACCTGGTGAAAGTTCGTCTAAGCAGCAAAGCGTATCCAGCAGAC
ACTCGCAACAATTACCGCCGCAACAGGCAGAGATACACAAGAGGCATCCGCAGCACCGCCTGCCCACCAT
CGACGAGGATGCTCTGCTCGACGGTGGCTCCGCGGGCGCCAGTCCCCAGGTTGGTGGCCCTGACCAAACG
CCCAATGTTATGGCGGATGAGCTGCTGGAGGAGCAGCAGTATGGTCAGAGTGTTGATGGCATCACAGGGC
AACATCAACAGTAGCACCAACAACAACAGCTCTGCCGACACGTTGGCTGTTAAACAATCGATTCCCGCCC
AGTCACCACAACAACAACTGCCCCAGTCACAGTCGCAGGTGCAGCCAGGCCTTTCCGATGCCGACCTCCT
CATACCCACTTCCAATTTGCAAAAGTTTATTGAGAACGCCGATAGGATACTGAAGAACATCACTTCGAAC
AGTAGCACGG
>dme_circ_0001808_junction_seq
AAGAACATCACTTCGAACAGTAGCACGGGTTCCATCGCATTCGTTGTCGGAATCGC
>dme_circ_0001809
GGCAATCCATCGGATATGGCACAACTCAACGCAGTGGACGGTAAACTGGAAGTCTCGGGCGTTGAGTCCG
AAAAGCAGGAACCGGAATCCAAGGTGATACAGGAAATTAAGGAGGCCAAGAAAGAGGCGGCAGTCCCTCT
GCCAAAATCCGCAAAACCACCAGTGCCACCTGTGAAAACGACGAAGAGCAAGGTGCCAAGTGCTCCTGTA
GATCCCTCCAAGGGAGTGGCCACCGAGACACTCTACGAGCCAGGCCACGTGGATGAGGAGATCGATGCGG
AGCGCATATGTCCGAAAGGCGGAGAGTTTATTAAGCTCCTGGTTCTTATTAGCTCAGCGATGTCTCACGA
TGCAGCCCGCATGTCCATCCGGCAGACTTGGATGCATTACGGGACGAGAAGGGACGTGGGCATGGCATTT
GTCCTAGGACGTGGCACCAATGAAACCATAAACAAAGCGCTTACCCAGGAGAACTTCATCTATGGAGATC
TGATACGGGGAAACTTCATAGACTCGTACAACAACCTCACGCTCAAGACAATATCGACGCTGGAATGGGC
AGATGTGCATTGCCCGAAGGCAAAGTACATTCTCAAGACGGACGACGACATGTTCATCAATGTGCCCAAG
CTGCTGACCTTCCTGGACAAGCACAAGGACAAGCGTACCATATACGGTCGTTTGGCCAAGAAGTGGAAAC
CGATTCGCAATAAGAAATCCAAATACTATGTGTCCGTCGATCAGTTCGCGGCGGGAGTATTCCCGTCCTT
CACCACTGGACCCGCCTACGTACTCACCGGAGACATTGTGCACGAACTCTACGTGCGATCTCTGAAGACC
GTTTATCTGAAGCTGGAGGACGTGTTCACCACGGGCATAGTAGCCAAGAGCCTTAATGTTAAGCGGGTGC
AGGCGAACGAGTTCGTTAACCGACGCATCTCATTTAACCCGTGCAACATCCGCAACGCAATCAGCGTGCA
CATGATCAAGTCGAACGAACAATTCGATTTGTGGAAAAAGCTGCTGGATCAGACCACCAAGTGTAAATAG
TATTTTAAGTTTAGGGTGTAGTTTTTCCGGTATGAAGAACACAGTGTGGTCTTTCGAGAAAAAAGGGGCG
AACATATATGTAATATGGTAGGAGTAGAGAATGATTAGCAAAGGCAGCTATATAGGATTATAAAATTAGA

CTAGTCTCTAAGTCCTAGAAATATCACAAATTCTGTTGTCGATCTCTGCATCCGCCTATATGCATGCGAT
CCAACAACGATTCGAGTACATTTTAAAACATCTGTAGTTTAATTTTAGCATTCTGAATGCCATTATTTAA
ATGATAATTACTGAGCGATAACTGTGGTTATATATTAACACTAAATACATTAATGTATATTGAAAGAATC
ACATAGTGCCTTTTCATTTCGAAGCGCTTCCCAAATAATACCTCTTCTGGAAGTCTAATGGTTTTGGAT
ATTTGAAGCCTTCGGTATTATTCAAAAAATAAATAATTCTTTTTCATCGTTCCATATTGTTGATTTCTGA
ATGTTGGTATTTTTCCAGGCCTTAGACCGCAACAAAAAATACCGCAACGGAGCAGTTATCGACTATTCGA
TAAGCTGAAACAGTAATTCGATTTATGAAAACCCGCCAAACTTGAAAACAATGGTAGTTATTTATTTTAA
AATTTGTTAGACATACACTTCGTTTAACATAAATGTTACAAAGTTAGTTGTTACACAGGCGATATTACAA
AAATAACAAATAGGTCTCATGGGTGCCAATCCGATCAATACATAGGTAATTTTGTAAAGCTCAAGAACTT
GCTACGGTTTTGTCACTCAAACGATAAAGACTTCTTATTTTGATAATACCTTTTACCATGTTGGAGTTGC
ACATATGGAAAATTCTTTGCATGATTCGGCTAACAAATAACATATCTAAATAAATTGGTACTAAATTTAT
ATATAGTTATGGTGTATACGGTACGTGGGATCGTTGGATCCAACAGAGCTCTCGTGTGGTTTTGTGGACT
CACATCGGCAACAGCGAGTGGCTAACACTGCTCTCATGCGAAACGGGTCGCTCGATGGTCATTGAGGACT
GCGGCACCTGGCTGTCTGTGCTGATTCCCAGGCTGGCATAAATCTCCTTCATCACGAAAAGGATCGTATC
TTCGGAGCCCCTGCGTTTGAGAAGTCATAATTG
>dme_circ_0001809_junction_seq
AGCCCCTGCGTTTGAGAAGTCATAATTGGGCAATCCATCGGATATGGCACAACTCA
>dme_circ_0001810
GACCACCTGTTCGCCATTGGCCAGCATAATCTTCTGATGCCATTCACCAGTCTCCGCAGATCGCTTGTAT
TCCGCTTCAAGTAGTGCAGCCGTATCCTCGGTGTATGGAACATACTTGGAGTCAACTCCCTTGTAGAACC
AGGAGCAGCGTCGCACTTCGATGGCCTTTCCCTCCCAGTAAACGGGAGTCTTGGTAC
>dme_circ_0001810_junction_seq
CCCTCCCAGTAAACGGGAGTCTTGGTACGACCACCTGTTCGCCATTGGCCAGCATA
>dme_circ_0001811
GATGCTGTTGGAGTAGATCGTCGCCCGATTGGCGATCGGTTTGTGTTGGTGCTGGAGCAAGGGTCGAGAA
TTCCGAACAGGCAGACGAAGGTGGCTCGACAAGATTTGAAACGGGCGTCTGTGAGCGCTCTTGCAGCTGC
TTAACAGCCACTCCGCTGTGGACAATGTCGTATTTGAGTTCACCTG
>dme_circ_0001811_junction_seq
TGGACAATGTCGTATTTGAGTTCACCTGGATGCTGTTGGAGTAGATCGTCGCCCGA
>dme_circ_0001812
GTTACCCCGGATCTGGACATGTATCTAAAGATCATCAATAGCTTGAACGAAGGGGACACAATCTTCACGT
TGGCGGATGCAGGTGCTTTGCGCGGCAAGATTGGACAAGTGGCAGAGGCCATGGACATGAGGAGCAAGCG
CATTCTAGCCATATTTT
>dme_circ_0001812_junction_seq
AGGAGCAAGCGCATTCTAGCCATATTTTGTTACCCCGGATCTGGACATGTATCTAA
>dme_circ_0001813
GCGCATTCTAGCCATATTTTGTGAACCAGGCAGCCGGGAGGAGGCATTAAAGAAGGCCATTCGGTTGGGC
TGCATCCAAGCCATCAAGGAGCGAATGTTATCGCTGCCCCCACTGCCAGAGGAATCGCACATACGCCAGA
TGCAGGAGCGTAGACGCATGGAAACGGAGCAGC
>dme_circ_0001813_junction_seq
GAGCGTAGACGCATGGAAACGGAGCAGCGCGCATTCTAGCCATATTTTGTGAACCA
>dme_circ_0001814
GTTGGTGCGGCAAAGCACGCGAAAGGCACGTACACCCCAACCGCTGGAAAGTGGATCCTCGGTGGCGCCA
AGTGTCACTCAGCGGGGCAACGACATGCAGCTGGAATCGATGGAGCAGCGATCGGAACAGGAGAACACCA

CCACATCCATACTGGATCAAATATCGTATGGATTCGTCAGCGTGGGAGGATTCATATATCAGAATAGCTA
TATATTCACCAATATTCTTATGATGGGCCTGGTCCATAGTGTATCACAGTTGGCTGACTTTTGTCCTCTT
GCTGTCGGCCAACGTACTGTGGATGATTCCCAACCAGAGGAAGGCCATGATGCGGTCCAGTCCCTTTATA
GTTCTGTATGCTGAGGCCCTTTTGATTGCCCAATACATATACGGCATGGATCTCAACAACGAAGAGCTGC
CCACGAGCGTTCCCGACTGCGGGCATTAACCTGCAACAAATTGGCTTCGAACGACCCATCGAGAACCAAA
TGCGGCCATGTGTGCCGCTGATCGTGAAGACAGCGTTTGTGCTAATGTTTTGGGTGACATCACGACAGTT
CTTCAAGGAGAAGCGCGATCGCCGACGGGACAGCACTCTGGCGGACTTCATTGCCCCACTGCAGATTACT
GTGGGATCGGCTGGCTCCAGCTACCTCATCAACGATGGCAAGAAGACCTCAAAGTTCCTAAAGAAGGCCG
GCGATGTGATCAAAAATCTATTGGTGCGTCTGTGGATCTGGCTACTCGTGCTGGTTATCTTCCTTTGCGC
CATCACTGGCGAGAACATGACCGGCTTCCGCATCTGCTACATGGCCCTGTTTCTATTCTTCTTGCTAGTC
TTTCAATCTTCGTCCAAGGCGTGGGTTAAGATCATGTACGGCTTCTGGCTGTTTCTGATCTTCTATGCCA
TGTCCATACTTATATTGATCTACACATATCAATTCGACAAGTTCGACACGTACTGGAGCGACTATCTTAA
TGTGTCCGCGACTTTGGCAAAAGGACATCGGCCTTAAGCGCTATCAGACCAAGGATCTGTTCCTCCATTT
GGTCTCACCGACGATAATTGTGATCCTGACCGTCATACAAGTGCACTACTTCCACAAGCGCTTTATTGCA
TCATTGCAGCAGCAGCCGTTGGCTGGCGGATCGGCACAGCAGAAACCCACGGAGACAACTGCCTTGGAAC
CGGCGCCATCGAAGCGACGTGGCAGCGCCGGTTCACTGCGTAAATCTCAGGGTCCATCGGCGGAGGCTGC
TCCAGGAGCCACCACCGATTTCGAGACATCTGTGCGAGACTTGGTGCGCATATCGTTCCGCAAGATCAAG
AACAAGTCGGAGTACATCTTCAAGAACTTCAAGGATGTCTTCTGGCGCTTCCTGGAGCTGCACATCATGA
AGGCTGTGTATATCGCAGCCTTCGTGTGCAGTGTCAGCGAAGTCTGCGTACTGCACATTATCTTTGTGGG
TTTCTGTGTGCTGGGCGCCACCTCGCGGAAGGCCGTTCAGGTGGTGATCAGCCGCCTCATCTCGTTCATT
GTCACCGTCATAGTTCTGTCCAAGATGATCTACCAGATCGAGTACTTGAGTCACTCGCAGCACAATGTGG
TTTGTGTCTGACAACCGGACTGCCAACAATGCGGAGTGGATTGGTCTCACCAAGGCTGACAAGGTAACTG
GCGGACTGATGAGCCTGTTGCGCACCTACATCATCTACATGGTTATTGTGACCATGCACGCAGTGATCAG
TTTGCGGCAGCTTCAAATGCGCGTCAAGATCGGAGCACTGAATGCTCCACCCACCAAGCTGCTGTTCCCC
AATATTATTCGAGCTGATGCTGAGAAGGATCTGGTGGGACTGGTCAAGTATCTCCTCAACTTTGGCTTCT
ACAAATTCGGCATTGAGATATCGCTAATCGCGCTGGTCTCTACCATCACATATCGCCAGGATATTGTTGC
CGTAGTCTATGCTCTGTGGCTAGTGGTGCTTTTGCTTCTACGGAGATCGCAGTGCGCCAAAATATGGGGC
GTGTTTCAGGCATTCTTTGCCATCTCCATACTGACACAGTATATAGTGCTGGTAGGATTGCCGCCGAGCT
CATGCCTGGGTGTTTCCCTGGGATGAAGGTCCCTTCGGCGAGGGCATACAACGCTGGGCGATGCTGCCAG
GAGCCCTGCACTTCAACCACGTACCCAAGCTGATCTTCGACTTTATTGTCTTGGTCATTCTGAATCGACA
GAAGAGTATCTTCTGCATCGAACAGCGTTATGCCAGTAACGATGACTACCCGGGTGGCAGCAATCGCAGT
GTGATCGCGGATATTGCTCAGCTAGGTCGCGTTCCCTTCGACAATCCCACCCACGACTTTTGTTCGTACA
TACGCAACTACTCGGACATCCTTAAGAACGGAGTGCTGTGCGGCTTCTACTGGTTTACTCTGGCAGTTGT
TTTCTTGGCCGGCACCAATATTGCTGATCTGCTGGCACTGGGTTATCTGATCGGAGCGTTTATCTTCCTG
TGGCAGGGATCGGATTTCTATCTGCGTCCCATACACACCATCATCTTTCGCTGGAAGTGGCTGCTGGCAT
TCAATGTGGCCAACATACTTATCAAGACGTCCTTCCAAATGGCCGGCTGTTTGTTCATGACCCAACTGAC
GAAAGACTGCTGCTGGCTGGTGCACATGCTCGGCATCACATGTACGAGCAATGTACTTACAGAGCAAATA
ATGCTGCCCGAGGAGGCTGAATTGGCGCTTAAGCCAGGCGAATGTCCTAAGATCACCCACCAGGTGGTCC
TTCTGTGGGACACGATTTGCTTTGCCTTCATCATCTTCCAGCTGCGCATCTTCAAGTCGCACTACTTCTG
TCACATCATAACGGACACCAAAGCAAATAACATCCTGGCCTCAAGGAGGAGCCGACATCATTGAGAGCCT
ACGACATAAGCAGATTGCACATCGACACGACCATGAAAAGCAGGTGCTGCATAAGATCAAGCGAAAGATG
GAGCGCATCCGTGCCACGCAGCAGAAGATGCTACGACCCTTGGACAAACAAACCCACTTCGACGGGTAAG
TGCTTGCACCATCATCACTTCCAGGGGTTTTTTTCTTCCCCTATCTGTAATTTTTTGGAGCTCGAACTTC
TGGTCGCGTTTTTTGCAATTTCCCCCTTTTGTATTTTGATCGTTGGAAATTGTCGCGTGTGTGTCTTACA

TTAATCTTTGGCTTCGTGAAGGGCGACGCACGCCCTCAGAGCACGCCATGTCCGATGTGGCGCCTTTGGT
CCACAATAGCAGCTTCACCTCTTGTCAAGGATCTGGCTATCTGACCCCAGATGAGCATAGCTCCGCCACC
TCAGGCACTTCATCGCCAGCCAGGGCATCGATCCTCTCCAGCAGTGGAGGCAGCGTGGAAAGCGTGGACA
GCGCAGACGCTGCCGTGATTATTGAACCGGAAATCAATGTGATGCCGTGTGATGAGATTACGGACTATGT
GGATGTTCAGTCTGTCAGCGAGGAGGAGACAACTGTGGCAATGCCTAAAAAGGATCTTAGCTGCCAATCA
AAGGAATGTGTTTTCAAAAGCCCAGAGCCTTCGAAGCCAACCACGCCGAGTACGGATGCCTTGTCTACTC
TCCTGACCGAGGGATTTTTGGAACCGAAAAGGATCTCGTTGGGATTGGCTCCTCCTGAGCTAAGTCCTCC
TGTTGGGATGCAGTGCCTGGCACCTCCGTTGGCTGCTTATGCCACAGCTATGGCTTCTAGTGCAGCCAGC
TCTGTGCTGTCCACCAATCTGACTCCAAATCTTCGCCGAGAACATCGGCGTCAGTCGTCAACCAATGCCG
CTGTTTCCTGGAACGAAACTGTATCCATCAAACGATCGCCCATGAAAAAGCAAATGGTAAAAAGTGGAAA
GTGCGGGATGCAAACCGTTCACATTGTGTTATTAAGTTTAAAGTGTTGATTTGATTAAAGAATTTAATTA
ATTTAGACAGAATAATGTTATAAATATTTATAAGTGGTAAGGCTGAAAAGTGTGTTCGGTTTGCATCCCA
GGAGAAAACAATCGCTCACTCATTTATCCCACTTGCCAAAACAGTCGGCGGATAAGGAGGAGGTGGTTAC
CATGCGGCACAAGTCGCTACACCGCCGACACCAGAGCATGGGAAACGCCAGTTACTCTTTGGACGAAGCC
AGTCAGTCGCAGCGCAAGCGGTTGTCCAGCAACTTATCCGGAGCTCGGGACGAGGCCGCTCTGCGATCCG
CACAGAGACCACATAGCTGGGGACCCGTGGGTACGAGCTCCTATATGGAGTCGCTGATGTGCGCCTTCTA
CGAGCCTGCTCTGCTGCATGGACCATGTATTGGATGAATCATTACTCTCAAATTCTAATGATAATTTTGC
TCAATTTTAACGAAACTCTGTACAGAAAAAACCATTTGTTCAATGTGCGTGATCTTTAATCTTCAATAAA
ACCGATTGGGGTCCTGTGTAGCAGACCCCAAGAAACTGCCTCCATGAGTCTGGCCTGTAAATAACACTAA
ACTAGATAACTTAGCTCCCAATTACTTCCATTTGGGTTAATGGTTAAGTGCGCAATCAGAACTCCTGAGC
GCTGCTACAAGTGTATTATAAATCTCTTCATCGATCTAAATGTGATTGCTAGAATCAGTTTCTTAAGCTT
CAGGGGATTCACTTGTGTTGTAAATTAAGATTTTTGATCTGCATGGATGAAACtcttctaaatttttcta
aaagctttttataatataatactttttctaaatatttattttatttACCTCAAAACGGATATAAAATCTG
ATTATATATTTCCACTGTCTATAAGCATATTGAATGCCATGGGTATCGGTTCAGAAATATACAATTTACA
ACATATGAAGCATCGGTTAGCCGTTGTTAGATTATGTTCCATCTTGATTTCAATTAAAGCCAAATGTCTA
AATTCTGTTCgtttttcaacgttttatttttgattattttgtatttgattatttttttAGAACATGGTTAT
CCACTTCCTGCACCAACAGTACGCAGAAGGAAGGAAATTAAATTACATCCACATG
>dme_circ_0001814_junction_seq
AGGAAGGAAATTAAATTACATCCACATGGTTGGTGCGGCAAAGCACGCGAAAGGCA
>dme_circ_0001815
GTTGGTGCGGCAAAGCACGCGAAAGGCACGTACACCCCAACCGCTGGAAAGTGGATCCTCGGTGGCGCCA
AGTGTCACTCAGCGGGGCAACGACATGCAGCTGGAATCGATGGAGCAGCGATCGGAACAGGAGAACACCA
CCACATCCATACTGGATCAAATATCGTATGGATTCGTCAGCGTGGGAGGATTCATATATCAGAATAGCTA
TATATTCACCAATATTCTTATGATGGGCCTGGTCCATAGTGTATCACAGTTGGCTGACTTTTGTCCTCTT
GCTGTCGGCCAACGTACTGTGGATGATTCCCAACCAGAGGAAGGCCATGATGCGGTCCAGTCCCTTTATA
GTTCTGTATGCTGAGGCCCTTTTGATTGCCCAATACATATACGGCATGGATCTCAACAACGAAGAGCTGC
CCACGAGCGTTCCCGACTGCGGGCATTAACCTGCAACAAATTGGCTTCGAACGACCCATCGAGAACCAAA
TGCGGCCATGTGTGCCGCTGATCGTGAAGACAGCGTTTGTGCTAATGTTTTGGGTGACATCACGACAGTT
CTTCAAGGAGAAGCGCGATCGCCGACGGGACAGCACTCTGGCGGACTTCATTGCCCCACTGCAGATTACT
GTGGGATCGGCTGGCTCCAGCTACCTCATCAACGATGGCAAGAAGACCTCAAAGTTCCTAAAGAAGGCCG
GCGATGTGATCAAAAATCTATTGGTGCGTCTGTGGATCTGGCTACTCGTGCTGGTTATCTTCCTTTGCGC
CATCACTGGCGAGAACATGACCGGCTTCCGCATCTGCTACATGGCCCTGTTTCTATTCTTCTTGCTAGTC
TTTCAATCTTCGTCCAAGGCGTGGGTTAAGATCATGTACGGCTTCTGGCTGTTTCTGATCTTCTATGCCA
TGTCCATACTTATATTGATCTACACATATCAATTCGACAAGTTCGACACGTACTGGAGCGACTATCTTAA

TGTGTCCGCGACTTTGGCAAAAGGACATCGGCCTTAAGCGCTATCAGACCAAGGATCTGTTCCTCCATTT
GGTCTCACCGACGATAATTGTGATCCTGACCGTCATACAAGTGCACTACTTCCACAAGCGCTTTATTGCA
TCATTGCAGCAGCAGCCGTTGGCTGGCGGATCGGCACAGCAGAAACCCACGGAGACAACTGCCTTGGAAC
CGGCGCCATCGAAGCGACGTGGCAGCGCCGGTTCACTGCGTAAATCTCAGGGTCCATCGGCGGAGGCTGC
TCCAGGAGCCACCACCGATTTCGAGACATCTGTGCGAGACTTGGTGCGCATATCGTTCCGCAAGATCAAG
AACAAGTCGGAGTACATCTTCAAGAACTTCAAGGATGTCTTCTGGCGCTTCCTGGAGCTGCACATCATGA
AGGCTGTGTATATCGCAGCCTTCGTGTGCAGTGTCAGCGAAGTCTGCGTACTGCACATTATCTTTGTGGG
TTTCTGTGTGCTGGGCGCCACCTCGCGGAAGGCCGTTCAGGTGGTGATCAGCCGCCTCATCTCGTTCATT
GTCACCGTCATAGTTCTGTCCAAGATGATCTACCAGATCGAGTACTTGAGTCACTCGCAGCACAATGTGG
TTTGTGTCTGACAACCGGACTGCCAACAATGCGGAGTGGATTGGTCTCACCAAGGCTGACAAGGTAACTG
GCGGACTGATGAGCCTGTTGCGCACCTACATCATCTACATGGTTATTGTGACCATGCACGCAGTGATCAG
TTTGCGGCAGCTTCAAATGCGCGTCAAGATCGGAGCACTGAATGCTCCACCCACCAAGCTGCTGTTCCCC
AATATTATTCGAGCTGATGCTGAGAAGGATCTGGTGGGACTGGTCAAGTATCTCCTCAACTTTGGCTTCT
ACAAATTCGGCATTGAGATATCGCTAATCGCGCTGGTCTCTACCATCACATATCGCCAGGATATTGTTGC
CGTAGTCTATGCTCTGTGGCTAGTGGTGCTTTTGCTTCTACGGAGATCGCAGTGCGCCAAAATATGGGGC
GTGTTTCAGGCATTCTTTGCCATCTCCATACTGACACAGTATATAGTGCTGGTAGGATTGCCGCCGAGCT
CATGCCTGGGTGTTTCCCTGGGATGAAGGTCCCTTCGGCGAGGGCATACAACGCTGGGCGATGCTGCCAG
GAGCCCTGCACTTCAACCACGTACCCAAGCTGATCTTCGACTTTATTGTCTTGGTCATTCTGAATCGACA
GAAGAGTATCTTCTGCATCGAACAGCGTTATGCCAGTAACGATGACTACCCGGGTGGCAGCAATCGCAGT
GTGATCGCGGATATTGCTCAGCTAGGTCGCGTTCCCTTCGACAATCCCACCCACGACTTTTGTTCGTACA
TACGCAACTACTCGGACATCCTTAAGAACGGAGTGCTGTGCGGCTTCTACTGGTTTACTCTGGCAGTTGT
TTTCTTGGCCGGCACCAATATTGCTGATCTGCTGGCACTGGGTTATCTGATCGGAGCGTTTATCTTCCTG
TGGCAGGGATCGGATTTCTATCTGCGTCCCATACACACCATCATCTTTCGCTGGAAGTGGCTGCTGGCAT
TCAATGTGGCCAACATACTTATCAAGACGTCCTTCCAAATGGCCGGCTGTTTGTTCATGACCCAACTGAC
GAAAGACTGCTGCTGGCTGGTGCACATGCTCGGCATCACATGTACGAGCAATGTACTTACAGAGCAAATA
ATGCTGCCCGAGGAGGCTGAATTGGCGCTTAAGCCAGGCGAATGTCCTAAGATCACCCACCAGGTGGTCC
TTCTGTGGGACACGATTTGCTTTGCCTTCATCATCTTCCAGCTGCGCATCTTCAAGTCGCACTACTTCTG
TCACATCATAACGGACACCAAAGCAAATAACATCCTGGCCTCAAGGAGGAGCCGACATCATTGAGAGCCT
ACGACATAAGCAGATTGCACATCGACACGACCATGAAAAGCAGGTGCTGCATAAGATCAAGCGAAAGATG
GAGCGCATCCGTGCCACGCAGCAGAAGATGCTACGACCCTTGGACAAACAAACCCACTTCGACGGGTAAG
TGCTTGCACCATCATCACTTCCAGGGGTTTTTTCTTCCCCTATCTGTAATTTTTTGGAGCTCGAACTTC
TGGTCGCGTTTTTTGCAATTTCCCCCTTTTGTATTTTGATCGTTGGAAATTGTCGCGTGTGTGTCTTACA
TTAATCTTTGGCTTCGTGAAGGGCGACGCACGCCCTCAGAGCACGCCATGTCCGATGTGGCGCCTTTGGT
CCACAATAGCAGCTTCACCTCTTGTCAAGGATCTGGCTATCTGACCCCAGATGAGCATAGCTCCGCCACC
TCAGGCACTTCATCGCCAGCCAGGGCATCGATCCTCTCCAGCAGTGGAGGCAGCGTGGAAAGCGTGGACA
GCGCAGACGCTGCCGTGATTATTGAACCGGAAATCAATGTGATGCCGTGTGATGAGATTACGGACTATGT
GGATGTTCAGTCTGTCAGCGAGGAGGAGACAACTGTGGCAATGCCTAAAAAGGATCTTAGCTGCCAATCA
AAGGAATGTGTTTTCAAAAGCCCAGAGCCTTCGAAGCCAACCACGCCGAGTACGGATGCCTTGTCTACTC
TCCTGACCGAGGGATTTTTGGAACCGAAAAGGATCTCGTTGGGATTGGCTCCTCCTGAGCTAAGTCCTCC
TGTTGGGATGCAGTGCCTGGCACCTCCGTTGGCTGCTTATGCCACAGCTATGGCTTCTAGTGCAGCCAGC
TCTGTGCTGTCCACCAATCTGACTCCAAATCTTCGCCGAGAACATCGGCGTCAGTCGTCAACCAATGCCG
CTGTTTCCTGGAACGAAACTGTATCCATCAAACGATCGCCCATGAAAAAGCAAATGGTAAAAAGTGGAAA
GTGCGGGATGCAAACCGTTCACATTGTGTTATTAAGTTTAAAGTGTTGATTTGATTAAAGAATTTAATTA
ATTTAGACAGAATAATGTTATAAATATTTATAAGTGGTAAGGCTGAAAAGTGTGTTCGGTTTGCATCCCA

GGAGAAAACAATCGCTCACTCATTTATCCCACTTGCCAAAACAGTCGGCGGATAAGGAGGAGGTGGTTAC
CATGCGGCACAAGTCGCTACACCGCCGACACCAGAGCATGGGAAACGCCAGTTACTCTTTGGACGAAGCC
AGTCAGTCGCAGCGCAAGCGGTTGTCCAGCAACTTATCCGGAGCTCGGGACGAGGCCGCTCTGCGATCCG
CACAGAGACCACATAGCTGGGGACCCGTGGGTACGAGCTCCTATATGGAGTCGCTGATGTGCGCCTTCTA
CGAGCCTGCTCTGCTGCATGGACCATGTATTGGATGAATCATTACTCTCAAATTCTAATGATAATTTTGC
TCAATTTTAACGAAACTCTGTACAGAAAAAACCATTTGTTCAATGTGCGTGATCTTTAATCTTCAATAAA
ACCGATTGGGGTCCTGTGTAGCAGACCCCAAGAAACTGCCTCCATGAGTCTGGCCTGTAAATAACACTAA
ACTAGATAACTTAGCTCCCAATTACTTCCATTTGGGTTAATGGTTAAGTGCGCAATCAGAACTCCTGAGC
GCTGCTACAAGTGTATTATAAATCTCTTCATCGATCTAAATGTGATTGCTAGAATCAGTTTCTTAAGCTT
CAGGGGATTCACTTGTGTTGTAAATTAAGATTTTTGATCTGCATGGATGAAACtcttctaaatttttcta
aaagctttttataatataatactttttctaaatatttattttatttACCTCAAAACGGATATAAAATCTG
ATTATATATTTCCACTGTCTATAAGCATATTGAATGCCATGGGTATCGGTTCAGAAATATACAATTTACA
ACATATGAAGCATCGGTTAGCCGTTGTTAGATTATGTTCCATCTTGATTTCAATTAAAGCCAAATGTCTA
AATTCTGTTCgtttttcaacgttttatttttgattattttgtatttgattatttttttAGGGTAAAGTTTT
TGTTTGTTGTGCCTCATTTGTTCGCTAACTAAGCCGCCCCCATGCCATGtatatatatatataATCTCTA
CCCGATTAACTGCATGAAATTCGGTGTGCTGCTATGTAAATTGAATGCATAAAGAAAACCCTACTACTCC
TAGCAACTATTTACTAATATCTATCTAAATGTTGTTAAACACCATAAATGCAACACTTCGCTGGAGCCGT
AATAATGTTGTTTTCGATGTTCCCTACTACCCTAGGCTACCCGTGCTGGTGACTACTATATGTTCGAGGA
GATGGACGATAAGTTTGAGCTCGACTTGATACACGACGAGATCGACTTCCTCGAGGAGGAGAACATCACC
GAGAGCGAGATGAAGATGCAGCGACGCAAGACCCTCTACGATTGTGAGTTTCCACATATTTCTAGTTGGG
ATTCCTCTGTGTTTAGGCCATGCTGCATGCAATACTTGCCCGCATGACTTTCGAACCGTACTTAAATTCT
TGTAAATTCCACCCCCTCCcaaaaaaaaaatccaaaaaccaaaaccaaaCCAACCAATTGCCCCGACTTA
TGATCGAAGGTGTGGAAGGATCTGAATGATGCCGAGTATCGTCGCCACTTCTATATGCGTGAGCGTTCCT
ACGCCTCAGAGCCCGGCTCACGCATTCTCCTGAAGTTGGACGACGAGAAGGAGAAGATTGGGCCCGATCT
CGGCCTGGCCAGCGACCACGATGAGGACGACGATACCATTGAGTGCGACCTTGGCATGCGCACCTCGACG
CTGTCCGAACCGCTCGACTTTGGTCGTCGCAGCCAGACGCTGCTCGAGGTTGTGAGTCTATGGAGCAGAG
ATACCTGCATATCTTGTCTTAAATTGGCGCACCTCCCGTTGATTTCACATATTTTGCTCACGAGCTCAAG
TTTTTCAACTGCGCCCGCAATATCAAGCTGCCGACCAGCAGTTGGGTGCACTCACCTTAAAATACTGTAC
aaaaaaaacaaaaaaTCATATAAAAACTACAAACGATTTCTTGTAACTGCATATTGTATAAGCTTGGTTT
TTCGTTAATATCGTGTAAATGTCTTTCTTACAGCTATTACCAGCCAGTGGTCTAACTCGCTATATTTATT
TGAACCCACAGGAAGTCGAAGGATGCACCCACAGGCGAGTTTCCCTCCACCAGTAAGGGTATTTCGAAGG
AGCGCGATGCGGCAACGGCTTCCAGTTCGGCTAGTCCAGCGCCCACTAGGGATGTGGGTGATCTGCCTGT
GATTCCACCACCCTCCACTGGCCTGGGACGTGAGCAAACCTCCAAGGAGACCTCCGATAGCAAGTCCAAA
ATGGAAGTGGACAGCGGAGAGGTGACGGCCAAGGATTCGGATGAGGACTTCGATACCAATCCCATTATCA
GACTGCTCGAGGGCTTCTTGGTCACGCTGACCATAAGACTGAACCGCTTTTCGCGCAACTATCGCTTTGT
GAATCGCATCCTGGCCGGCGAAAAGAAGACCCTAAAGGGTATTTAGAAAGCGATTCTGGAAACCAGCTCC
AGTGTGCGCCGCTTGCGTTAAAATTTTGAATTAAGTTTTGATTTTTGGAAACTGAAAACCCCCAAACGTC
GTAGAGTAGCGTGTTCTGATCGCGTTACGAACCGTTCAATATCTTTGTATTTAAGTTTGTTAATAGTTAT
GTTCGGtttttattcaattatttatttcgtattctttttcttctttttttatttgcatatccgttttact
tttgtgtttcgtgtgtttGAAACTCTGTACCTGCTTCCACAACATACTACTACTCTCGAAATCAACGACA
CACCTGCCAAAATGTTTCACCATCTCACCTCCATCATTTGTGCCACCATGTTGTTGTTGATACCCGATCC
GATCGATTGATGTTGCTGTTGCTGCTGTTGCGATGAAGCGATGAAAGTGAACCGCCCGCCTCCTCATCCA
CCCCGCGGCGGGTGGTGATCGCACCACCGAATGCCACCGAGCACTCAGACCCCACCAGCACCACACTGAA
CACGAACACGACAACCACACCGCTATCACCACCAGaaccactgcaaccactgcaaccactgcaaccaAAT

ACAACCAGTACACCACAGCAACAGCATCAGCATATTCGCGCTGCCGAAGAAATCATCGAACTGCCTGTAG
ATACCGTGGATGGAGTCGCCCATAG
>dme_circ_0001815_junction_seq
TAGATACCGTGGATGGAGTCGCCCATAGGTTGGTGCGGCAAAGCACGCGAAAGGCA
>dme_circ_0001816
GTTGGTGCGGCAAAGCACGCGAAAGGCACGTACACCCCAACCGCTGGAAAGTGGATCCTCGGTGGCGCCA
AGTGTCACTCAGCGGGGCAACGACATGCAGCTGGAATCGATGGAGCAGCGATCGGAACAGGAGAACACCA
CCACATCCATACTGGATCAAATATCGTATGGATTCGTCAGCGTGGGAGGATTCATATATCAGAATAGCTA
TATATTCACCAATATTCTTATGATGGGCCTGGTCCATAGTGTATCACAGTTGGCTGACTTTTGTCCTCTT
GCTGTCGGCCAACGTACTGTGGATGATTCCCAACCAGAGGAAGGCCATGATGCGGTCCAGTCCCTTTATA
GTTCTGTATGCTGAGGCCCTTTTGATTGCCCAATACATATACGGCATGGATCTCAACAACGAAGAGCTGC
CCACGAGCGTTCCCGACTGCGGGCATTAACCTGCAACAAATTGGCTTCGAACGACCCATCGAGAACCAAA
TGCGGCCATGTGTGCCGCTGATCGTGAAGACAGCGTTTGTGCTAATGTTTTGGGTGACATCACGACAGTT
CTTCAAGGAGAAGCGCGATCGCCGACGGGACAGCACTCTGGCGGACTTCATTGCCCCACTGCAGATTACT
GTGGGATCGGCTGGCTCCAGCTACCTCATCAACGATGGCAAGAAGACCTCAAAGTTCCTAAAGAAGGCCG
GCGATGTGATCAAAAATCTATTGGTGCGTCTGTGGATCTGGCTACTCGTGCTGGTTATCTTCCTTTGCGC
CATCACTGGCGAGAACATGACCGGCTTCCGCATCTGCTACATGGCCCTGTTTCTATTCTTCTTGCTAGTC
TTTCAATCTTCGTCCAAGGCGTGGGTTAAGATCATGTACGGCTTCTGGCTGTTTCTGATCTTCTATGCCA
TGTCCATACTTATATTGATCTACACATATCAATTCGACAAGTTCGACACGTACTGGAGCGACTATCTTAA
TGTGTCCGCGACTTTGGCAAAAGGACATCGGCCTTAAGCGCTATCAGACCAAGGATCTGTTCCTCCATTT
GGTCTCACCGACGATAATTGTGATCCTGACCGTCATACAAGTGCACTACTTCCACAAGCGCTTTATTGCA
TCATTGCAGCAGCAGCCGTTGGCTGGCGGATCGGCACAGCAGAAACCCACGGAGACAACTGCCTTGGAAC
CGGCGCCATCGAAGCGACGTGGCAGCGCCGGTTCACTGCGTAAATCTCAGGGTCCATCGGCGGAGGCTGC
TCCAGGAGCCACCACCGATTTCGAGACATCTGTGCGAGACTTGGTGCGCATATCGTTCCGCAAGATCAAG
AACAAGTCGGAGTACATCTTCAAGAACTTCAAGGATGTCTTCTGGCGCTTCCTGGAGCTGCACATCATGA
AGGCTGTGTATATCGCAGCCTTCGTGTGCAGTGTCAGCGAAGTCTGCGTACTGCACATTATCTTTGTGGG
TTTCTGTGTGCTGGGCGCCACCTCGCGGAAGGCCGTTCAGGTGGTGATCAGCCGCCTCATCTCGTTCATT
GTCACCGTCATAGTTCTGTCCAAGATGATCTACCAGATCGAGTACTTGAGTCACTCGCAGCACAATGTGG
TTTGTGTCTGACAACCGGACTGCCAACAATGCGGAGTGGATTGGTCTCACCAAGGCTGACAAGGTAACTG
GCGGACTGATGAGCCTGTTGCGCACCTACATCATCTACATGGTTATTGTGACCATGCACGCAGTGATCAG
TTTGCGGCAGCTTCAAATGCGCGTCAAGATCGGAGCACTGAATGCTCCACCCACCAAGCTGCTGTTCCCC
AATATTATTCGAGCTGATGCTGAGAAGGATCTGGTGGGACTGGTCAAGTATCTCCTCAACTTTGGCTTCT
ACAAATTCGGCATTGAGATATCGCTAATCGCGCTGGTCTCTACCATCACATATCGCCAGGATATTGTTGC
CGTAGTCTATGCTCTGTGGCTAGTGGTGCTTTTGCTTCTACGGAGATCGCAGTGCGCCAAAATATGGGGC
GTGTTTCAGGCATTCTTTGCCATCTCCATACTGACACAGTATATAGTGCTGGTAGGATTGCCGCCGAGCT
CATGCCTGGGTGTTTCCCTGGGATGAAGGTCCCTTCGGCGAGGGCATACAACGCTGGGCGATGCTGCCAG
GAGCCCTGCACTTCAACCACGTACCCAAGCTGATCTTCGACTTTATTGTCTTGGTCATTCTGAATCGACA
GAAGAGTATCTTCTGCATCGAACAGCGTTATGCCAGTAACGATGACTACCCGGGTGGCAGCAATCGCAGT
GTGATCGCGGATATTGCTCAGCTAGGTCGCGTTCCCTTCGACAATCCCACCCACGACTTTTGTTCGTACA
TACGCAACTACTCGGACATCCTTAAGAACGGAGTGCTGTGCGGCTTCTACTGGTTTACTCTGGCAGTTGT
TTTCTTGGCCGGCACCAATATTGCTGATCTGCTGGCACTGGGTTATCTGATCGGAGCGTTTATCTTCCTG
TGGCAGGGATCGGATTTCTATCTGCGTCCCATACACACCATCATCTTTCGCTGGAAGTGGCTGCTGGCAT
TCAATGTGGCCAACATACTTATCAAGACGTCCTTCCAAATGGCCGGCTGTTTGTTCATGACCCAACTGAC
GAAAGACTGCTGCTGGCTGGTGCACATGCTCGGCATCACATGTACGAGCAATGTACTTACAGAGCAAATA

ATGCTGCCCGAGGAGGCTGAATTGGCGCTTAAGCCAGGCGAATGTCCTAAGATCACCCACCAGGTGGTCC
TTCTGTGGGACACGATTTGCTTTGCCTTCATCATCTTCCAGCTGCGCATCTTCAAGTCGCACTACTTCTG
TCACATCATAACGGACACCAAAGCAAATAACATCCTGGCCTCAAGGAGGAGCCGACATCATTGAGAGCCT
ACGACATAAGCAGATTGCACATCGACACGACCATGAAAAGCAGGTGCTGCATAAGATCAAGCGAAAGATG
GAGCGCATCCGTGCCACGCAGCAGAAGATGCTACGACCCTTGGACAAACAAACCCACTTCGACGGGTAAG
TGCTTGCACCATCATCACTTCCAGGGGTTTTTTCTTCCCCTATCTGTAATTTTTTGGAGCTCGAACTTC
TGGTCGCGTTTTTTGCAATTTCCCCCTTTTGTATTTTGATCGTTGGAAATTGTCGCGTGTGTGTCTTACA
TTAATCTTTGGCTTCGTGAAGGGCGACGCACGCCCTCAGAGCACGCCATGTCCGATGTGGCGCCTTTGGT
CCACAATAGCAGCTTCACCTCTTGTCAAGGATCTGGCTATCTGACCCCAGATGAGCATAGCTCCGCCACC
TCAGGCACTTCATCGCCAGCCAGGGCATCGATCCTCTCCAGCAGTGGAGGCAGCGTGGAAAGCGTGGACA
GCGCAGACGCTGCCGTGATTATTGAACCGGAAATCAATGTGATGCCGTGTGATGAGATTACGGACTATGT
GGATGTTCAGTCTGTCAGCGAGGAGGAGACAACTGTGGCAATGCCTAAAAAGGATCTTAGCTGCCAATCA
AAGGAATGTGTTTTCAAAAGCCCAGAGCCTTCGAAGCCAACCACGCCGAGTACGGATGCCTTGTCTACTC
TCCTGACCGAGGGATTTTTGGAACCGAAAAGGATCTCGTTGGGATTGGCTCCTCCTGAGCTAAGTCCTCC
TGTTGGGATGCAGTGCCTGGCACCTCCGTTGGCTGCTTATGCCACAGCTATGGCTTCTAGTGCAGCCAGC
TCTGTGCTGTCCACCAATCTGACTCCAAATCTTCGCCGAGAACATCGGCGTCAGTCGTCAACCAATGCCG
CTGTTTCCTGGAACGAAACTGTATCCATCAAACGATCGCCCATGAAAAAGCAAATGGTAAAAAGTGGAAA
GTGCGGGATGCAAACCGTTCACATTGTGTTATTAAGTTTAAAGTGTTGATTTGATTAAAGAATTTAATTA
ATTTAGACAGAATAATGTTATAAATATTTATAAGTGGTAAGGCTGAAAAGTGTGTTCGGTTTGCATCCCA
GGAGAAAACAATCGCTCACTCATTTATCCCACTTGCCAAAACAGTCGGCGGATAAGGAGGAGGTGGTTAC
CATGCGGCACAAGTCGCTACACCGCCGACACCAGAGCATGGGAAACGCCAGTTACTCTTTGGACGAAGCC
AGTCAGTCGCAGCGCAAGCGGTTGTCCAGCAACTTATCCGGAGCTCGGGACGAGGCCGCTCTGCGATCCG
CACAGAGACCACATAGCTGGGGACCCGTGGGTACGAGCTCCTATATGGAGTCGCTGATGTGCGCCTTCTA
CGAGCCTGCTCTGCTGCATGGACCATGTATTGGATGAATCATTACTCTCAAATTCTAATGATAATTTTGC
TCAATTTTAACGAAACTCTGTACAGAAAAAACCATTTGTTCAATGTGCGTGATCTTTAATCTTCAATAAA
ACCGATTGGGGTCCTGTGTAGCAGACCCCAAGAAACTGCCTCCATGAGTCTGGCCTGTAAATAACACTAA
ACTAGATAACTTAGCTCCCAATTACTTCCATTTGGGTTAATGGTTAAGTGCGCAATCAGAACTCCTGAGC
GCTGCTACAAGTGTATTATAAATCTCTTCATCGATCTAAATGTGATTGCTAGAATCAGTTTCTTAAGCTT
CAGGGGATTCACTTGTGTTGTAAATTAAGATTTTTGATCTGCATGGATGAAACtcttctaaatttttcta
aaagctttttataatataatactttttctaaatatttattttatttACCTCAAAACGGATATAAAATCTG
ATTATATATTTCCACTGTCTATAAGCATATTGAATGCCATGGGTATCGGTTCAGAAATATACAATTTACA
ACATATGAAGCATCGGTTAGCCGTTGTTAGATTATGTTCCATCTTGATTTCAATTAAAGCCAAATGTCTA
AATTCTGTTCgtttttcaacgttttatttttgattattttgtatttgattatttttttAGGGTAAAGTTTT
TGTTTGTTGTGCCTCATTTGTTCGCTAACTAAGCCGCCCCCATGCCATGtatatatatatataATCTCTA
CCCGATTAACTGCATGAAATTCGGTGTGCTGCTATGTAAATTGAATGCATAAAGAAAACCCTACTACTCC
TAGCAACTATTTACTAATATCTATCTAAATGTTGTTAAACACCATAAATGCAACACTTCGCTGGAGCCGT
AATAATGTTGTTTTCGATGTTCCCTACTACCCTAGGCTACCCGTGCTGGTGACTACTATATGTTCGAGGA
GATGGACGATAAGTTTGAGCTCGACTTGATACACGACGAGATCGACTTCCTCGAGGAGGAGAACATCACC
GAGAGCGAGATGAAGATGCAGCGACGCAAGACCCTCTACGATTGTGAGTTTCCACATATTTCTAGTTGGG
ATTCCTCTGTGTTTAGGCCATGCTGCATGCAATACTTGCCCGCATGACTTTCGAACCGTACTTAAATTCT
TGTAAATTCCACCCCCTCCcaaaaaaaaaatccaaaaaccaaaaccaaaCCAACCAATTGCCCCGACTTA
TGATCGAAGGTGTGGAAGGATCTGAATGATGCCGAGTATCGTCGCCACTTCTATATGCGTGAGCGTTCCT
ACGCCTCAGAGCCCGGCTCACGCATTCTCCTGAAGTTGGACGACGAGAAGGAGAAGATTGGGCCCGATCT
CGGCCTGGCCAGCGACCACGATGAGGACGACGATACCATTGAGTGCGACCTTGGCATGCGCACCTCGACG

CTGTCCGAACCGCTCGACTTTGGTCGTCGCAGCCAGACGCTGCTCGAGGTTGTGAGTCTATGGAGCAGAG
ATACCTGCATATCTTGTCTTAAATTGGCGCACCTCCCGTTGATTTCACATATTTTGCTCACGAGCTCAAG
TTTTTCAACTGCGCCCGCAATATCAAGCTGCCGACCAGCAGTTGGGTGCACTCACCTTAAAATACTGTAC
aaaaaaaacaaaaaaTCATATAAAAACTACAAACGATTTCTTGTAACTGCATATTGTATAAGCTTGGTTT
TTCGTTAATATCGTGTAAATGTCTTTCTTACAGCTATTACCAGCCAGTGGTCTAACTCGCTATATTTATT
TGAACCCACAGGAAGTCGAAGGATGCACCCACAGGCGAGTTTCCCTCCACCAGTAAGGGTATTTCGAAGG
AGCGCGATGCGGCAACGGCTTCCAGTTCGGCTAGTCCAGCGCCCACTAGGGATGTGGGTGATCTGCCTGT
GATTCCACCACCCTCCACTGGCCTGGGACGTGAGCAAACCTCCAAGGAGACCTCCGATAGCAAGTCCAAA
ATGGAAGTGGACAGCGGAGAGGTGACGGCCAAGGATTCGGATGAGGACTTCGATACCAATCCCATTATCA
GACTGCTCGAGGGCTTCTTGGTCACGCTGACCATAAGACTGAACCGCTTTTCGCGCAACTATCGCTTTGT
GAATCGCATCCTGGCCGGCGAAAAGAAGACCCTAAAGGGTATTTAGAAAGCGATTCTGGAAACCAGCTCC
AGTGTGCGCCGCTTGCGTTAAAATTTTGAATTAAGTTTTGATTTTTGGAAACTGAAAACCCCCAAACGTC
GTAGAGTAGCGTGTTCTGATCGCGTTACGAACCGTTCAATATCTTTGTATTTAAGTTTGTTAATAGTTAT
GTTCGGtttttattcaattatttatttcgtattctttttcttcttttttttatttgcatatccgttttact
tttgtgtttcgtgtgtttGAAACTCTGTACCTGCTTCCACAACATACTACTACTCTCGAAATCAACGACA
CACCTGCCAAAATGTTTCACCATCTCACCTCCATCATTTGTGCCACCATGTTGTTGTTGATACCCGATCC
GATCGATTGATGTTGCTGTTGCTGCTGTTGCGATGAAGCGATGAAAGTGAACCGCCCGCCTCCTCATCCA
CCCCGCGGCGGGTGGTGATCGCACCACCGAATGCCACCGAGCACTCAGACCCCACCAGCACCACACTGAA
CACGAACACGACAACCACACCGCTATCACCACCAGaaccactgcaaccactgcaaccactgcaaccaAAT
ACAACCAGTACACCACAGCAACAGCATCAGCATATTCGCGCTGCCGAAGAAATCATCGAACTGCCTGTAG
ATACCGTGGATGGAGTCGCCCATAGGTAACCCAAAACCACATGAAAATCGTTGTTTTAAATGCGGGGATT
CCAAAAGCGTAATGATCATTATGACCAGTTGCAAAATTCGATTTTTCAAGTAAAATGCAAATTTTTAGCT
TAATAATACAACTTAAACACAATCAAAATGAAGTTAGCAAATATACTTAATCAATCTTTAGTTTTTTTCT
GAATCCAATCCGCACATTTTGTTTTACACGCACTTACATGTTCTTTTCGAAATTAGAAAATAATGAATTG
TCCCTCATTTCATTGGAGTAGAGATTTTAATTCCATGTGATTATGTATATGTCAAAGACAAAATTTCCAG
AATGAACAAAAACAGATCCTTACTAATTTATTTTTCTTACATTTTACATTTTTCTGCGCGCATTTCGATC
ATCCGAATCAATTTTTGTTTTGTTAGAAAACAATCAATCAATTCATCGCCGCCAGCCAAGGG
>dme_circ_0001816_junction_seq
CAATCAATTCATCGCCGCCAGCCAAGGGGTTGGTGCGGCAAAGCACGCGAAAGGCA
>dme_circ_0001817
GCATGAGCTCGGCGGCAATCCTACCAGCACTATATACTGTGTCAGTATGGAGATGGCAAAGAATGCCTGA
AACACGCCCCATATTTTGGCGCACTGCGATCTCCGTAGAAGCAAAAGCACCACTAGCCACAGAGCATAGA
CTACGGCAACAATATCCTGGCGATATGTGATGGTAGAGACCAGCGCGATTAGCGATATCTCAATGCCGAA
TTTG
>dme_circ_0001817_junction_seq
GATTAGCGATATCTCAATGCCGAATTTGGCATGAGCTCGGCGGCAATCCTACCAGC
>dme_circ_0001818
GATGTTATTTGCTTTGGTGTCCGTTATGATGTGACAGAAGTAGTGCGACTTGAAGATGCGCAGCTGGAAG
ATGATGAAGGCAAAGCAAATCGTGTCCCACAGAAGGACCACCTGGTGGGTGATCTTAGGACATTCGCCTG
GCTTAAGCGCCAATTCAGCCTCCTCGGGCAGCATTATTTGCTCTGTAAGTACATTGCTCGTACATGTGAT
GCCGAGCATGTGCACCAGCCAGCAGCAGTCTTTCGTCAG
>dme_circ_0001818_junction_seq
GCACCAGCCAGCAGCAGTCTTTCGTCAGGATGTTATTTGCTTTGGTGTCCGTTATG
>dme_circ_0001819

GTTTAAGCTGATCTGGTCCAATTACCACCAACTGCCCAATCAGCCGCTAACACCGGCCAAAATATTCGGC
GTTGAGAATAAGGCCCATTATGCGATTTACGACCTGATACTATTGCTGGTTCTATTCCTGCATCGCTATT
TGCTTAAATCACAAGGCCTGTGGAAATCGGGCTACAAGGACACGGATAACCAGTTCACCAAGCCCACCGC
TAGCATGTGATGAACGCGACGATAGCGACAACTTATCCCAACCGGATTCCCGCCAGCTAAACGATGATGC
TGCTCAGAAGCTGAGTCTCCAAGTGAGCCAGGCTTCCTTGCCAGGATCGCCCGAGTTTAGCAAGACAGGC
ATCAATCAGCTAGAGGCGCACCAAGTACACTTCATCGCTGTACAAATTCTTCTTCAGTTTGGTCCACAAA
TCCCGTCTAGCCACAGATGTTTACGCTCTGATGTTCCTCTGCGATTTTGTGAACTTCTTCGTGCTTCTTT
TCGGCTTCACTGCTTTTGGAGACTCAGCAAACGGAAAGTGATGAAGGTGTGCAGACATATCTTGCGGAGA
ACAAAGTGCCCATACCATTCCTGATTATGCTACTTGTTCAGTTCCTGCTCATTGTCATTGATCGAGCTTT
GTATCTGCGCAAAGCCCTGGTGAACAAGATCATCTTCCACTTCTTTTCGGTTATCGGAATACACATCTGG
ATGTTCTTCGTTGTGCCTGCAGTTACGGAGCGCACTTTCAACTCCCTCGCACCTCCAATAATATTCTACG
TTATCAAGTGCTTTTACATGCTGCTCAGCTCTTATCAAATCAAATCCGGTTACCCTAAGCGCATTCTAGG
CAACTTCTTCACCAAGGGTTTCTCGATGGTCAATATGATTGCCTTTAAGGTGTACATGCAGATTCCATTC
CTGTACGAGCTGCGAACCATATTAGATTGGGTGTGCATTGACAGCACCATGACCATTTTCGATTGGCTAA
AGATGGAGGACATTTTCTCGAATATATATCTTATCCGCTGCACCCGGCAGTCAGAGACTGATTTTCCGGC
CATGAGAGCTCAGAAGAAGGCCTCACTTTCAAAGCTGATAATGGGTGGCACCATCGTCCTGCTGATAGTG
ATTTGCATTTGGGGACCATTGTGTTTGTTTGCACTTGGCAACGCGGTGGGCACCTCGAATGTGCCGTTCC
ATGTGTCGCTATCAATACGAATTGGACCCTATGATCCCATCTACACAACGAACAACTACGACAGTATCTT
CGAGATCAATCCTGAGATGTACTCTCAGATGACTAATGCATACATAAAGGAGAAACAGGCTTTGACGTTT
ATTGCCGGCTATGATGCAACGGATGTAGCGGCGGTTAGACTAGCTGGCAACTCGCCATCTCTGTGGAACA
TAGCACCGCCGGATAGGCAGCGACTACTAAATGATTTAAGAAACAGATCACACGCTGAAGGCCCGGTTCT
CTTATTCCCTCACCCGAAAGGCTCCTGCCAAGGGGCTAAAGGAAAATGTGGGCGACGAGCATGCCATATC
GCTGGACGAGTCCTTTGAGGGACGAGCAGCACTCATTCACATGCTAAGCGAAACCCATGATGTGGAGCCC
ATTTACAGCAATGGCACCACAAATGGAACCACTCCCGAAGTTGAAGAAGTGGTGGTGATACCCGGAATGA
TACCCAAGTTTATCAAGGTTCTCAATTCGGGCGACGCTGCGGTG
>dme_circ_0001819_junction_seq
GGTTCTCAATTCGGGCGACGCTGCGGTGGTTTAAGCTGATCTGGTCCAATTACCAC
>dme_circ_0001820
GGCACAGCCACTGATCTTGTGGTGCAGATTGTCCTGATTGCCGAGTCCATGCGACTGCAGGCTATGATGG
CCACCTATGGCATTCAAACACAAACCCCCCATGAGGTAAGTCCGGTCACAGATCTCATATTTTTAAAGTT
CTACTTACACATTATTTTTTATTAGGTGGAACCTGTGCAAATCTGGAGCTCTACAGAGCTCATTAAAGTA
TATCAACATCTCGGAGTTAACAACAAGGTTGGCCTGTCCGGTCGTCCATGCAGACCGGTGGGTTCTTTGG
GCACCAGCAAGGTGTATCGCATCTGCGGCATGACCGTCCTCTGCTATCCACTAATCTTTGAGGTCTCCGA
CTTTTATCTCTACCGGGACATGGCTCTACTTATCGATGACATCAAGACAGAGCTACAGTTTGTGGGCAAG
TACTGGCGGCTATCGGGCAGACCTACAGTTTGTCTGCTTATTCGCGAGGAGCACATGCGTGATCCGCAGT
TTAAGGAGATGCTCGACCTGCTGGCCATGCTCAAAAAGGGCTACTGCGATGGCATGAAAGTTCGCATCGG
TCGTCTACAGAATCTGATTAGCAGCTCGTGCATCGAGCATCTTGACTTTATGAACCAAAGTGATCTGACC
GACAACGAGAATGCGTTCTCGCAGATAAACCATGAGTATATTGGCTACCAATCGCTAACAGATGTGCCCA
AAGCTCTGACATATGTGGAGGAAAAGATTTCTGTTGCGGCACTTCGATACCAAACCCACGCCGGATATCA
TCAACGCACTACGTAGCACGGATTCCATTTACTGTTTGTGCCAATTGTGGGGTATTATTCTCAACCGCGA
GGGTCCTCATTTTGAGGTAAACGGTCTGAACGTAAACACGGCCTTGACGCAGCTATACCATCGCGCTGGT
TCCTTGCGCTACTGGCGTGCCGTGCGATACTGTTCCTCTCTTCTTCACCACATTGTGGACTCCATTAGTC
CATTTATCACCACTGTGTTGGTGAATGGCAAGGAGCTCACAGTGGGCATCATTGGCCAGAAGGAGACGGT
ATTCGACAAGCCAATGACGCCGGCGGAGATTCAAAATGTCATGTACACGAGTGTCCAACCCTACGACGTC

ATCCAGGCAGTGTTGCAGCAGGAAGTGGTTCTGTATTGTGGCCGTTTAATAGCCACCAATCCATCAATGT
TTCGTGGCATATTAAAAATTCGCATTGGTTGGGTACTGGAGGCCATGCGCATCTATTTACAAATCTCAGG
ACAACAGAGCATTGATGTTGACAACCTTTCGCCCTTCCAAGTCCGCATACTTCTTCAGAAAGTCTTAACC
GTCAGCGAATGGGCTGTAGAAGAAAAGGCTCACTACCCTACAACGTCGTCAACTGGAGGGATGCTTGTGC
CGTGTGCCAAAACATTTTTATAACAAAATCTGGGAAATCCTACAGCGAACCCCTCAAGGCATACTGACCC
AGGGGCATCATTTGCCAGCCACGCCCACACTAACCAACATGAGCCGCGGCGAGTTGACCTTCAATCTGCT
GGTCGAGGAAACTCTGATTTGCATTGACCGTCCGGAAAGGCGTCAGATAACCGTGGAGCTTTTGTGCATT
GTGGCCACCATTCTTAATCGGTAACCCCGAGCTGCACTTTAAGCAAGCTCTAGACTTGGACGGCATCTTG
GCGGAAGCGTTCGCAATGTACTGCAAGGACAACAACATACAGCATCAACCAAAACCAGAGCATGAGCAGA
CCAAAAATGAGGATCTCAAGGCTTTCTATTCGTTGCCATATTCCGAGACCACGGGCTATTTGGCTCGCGC
CGCGGTCAACAAGGTTTTGCAGGGTGGCATCTTCTCCACCAATGAAGAGGATGTTCAGCTCGATGGCGAT
CGTTTGCACGACGACAACTGTAAGG
>dme_circ_0001820_junction_seq
GATCGTTTGCACGACGACAACTGTAAGGGGCACAGCCACTGATCTTGTGGTGCAGA
>dme_circ_0001821
GATGTCCAGCTGCTTCACCGTGTCCTCGTAGTTGGACGTTTTCAGGAACTGGTCCAGATTGATCTCCTCC
AAGCTGTTGTGGCGACCACTGTCCGGAGCACCCGAACTGCCGCCTGGAGTTGTGACCGAGAGACCGAGAC
TGGAAGTGGAAATATCCTCGCCATTAATATGGGATTATTTTCGATGTTTTCATACTTACGATTTCGGCAC
ATCGCGCATTGTTTCTTCAGCAATTGCAAATTATATTGCAATTGCGCTTAAGAAAATGTATCAAAGTCaa
aaaaaa
>dme_circ_0001821_junction_seq
TAAGAAAATGTATCAAAGTCaaaaaaaaGATGTCCAGCTGCTTCACCGTGTCCTCG
>dme_circ_0001822
GAGGAGTACGCCGTGGAAAAGATCATCGACAGGCGGGTGCGCAAGGGAAAGGTGAGTTTTCCGAACCAGA
CGTCCCATTATATTTGGTCCTAAAACGCCATTTTCCACCTGCAGGTGGAGTACTATCTGAAATGGAAGGG
CTATCCCGAAACTGAGAACACGTGGGAGCCGGAGAACAATCTCGACTGCCAGGATCTTATCCAGCAGTAC
GAGGCGAGCCGCAAGGATGAGG
>dme_circ_0001822_junction_seq
CAGTACGAGGCGAGCCGCAAGGATGAGGGAGGAGTACGCCGTGGAAAAGATCATCG
>dme_circ_0001823
GTGAGAACTGGTGCTGTGTTTATTGGCTGAGCTGCTACTTCTATTGTGAAATATTCATTAACAATCGCTT
GGCCATGACACAGGTGTTTATCAATGGTGCCAGAGACGATCTCAATGTACGGGAATTCCAGAGCCTGG
>dme_circ_0001823_junction_seq
CTCAATGTACGGGAATTCCAGAGCCTGGGTGAGAACTGGTGCTGTGTTTATTGGCT
>dme_circ_0001824
AAATTCGGACAATGATCCCCGATCGGGCACCCGAGCCGGACGAACCGGATCCGGAACAGCCACCTACAGC
TACGACCGTCAGATGGATACCTGTGCCACCGTAATGACCACCGTCCCAGAAGGTAGCTTCACCCCTAGAA
GATTCTTACAATATGTTTGTGCTTATTATATCGTAATCAACAGACGATCAAATAATGTCCAATAACTCCG
TACAACCCGCCTGGCGTTCCAATTACAAAACCGACTCCACGGAGGCGATGACAGTGACCACTGTGGATTT
GATCAGTTGGGCATTCCAAGTGGCAAGGGGCATGGATTACTTGTCCTCCAAGAAGGTGTTGCACGGCGAT
CTGGCCGCTAGAAATATTCTCCTCTGCGAGGACAATGTGGTAAAGATTTGTGACTTTGGTCTGGCTCGAT
CCATGTATCGAGGTGATAACTACAAGAAGTCAGGAGAATGGCAAATTGCCCATCAAGTGGCTGGCGCTGG
AATCGCTGAGCGATCATGTGTTCAGCACATACAGCGATGTTTGGTCCTACGGAATTGTTCTATGGGAGAT
GTTCTCGCTGGCCAAGGTGCCGTATCCGGGCATCGATCCCAACCAAGAGCTATTTAACAAACTGAACGAT

GGCTACCGCATGGAGAAGCCGAAATTTGCCAACCAGGAGCTCTACGAGATTATGCTAGAGTGCTGGCGAA
AGAAGCACTACCTGGACCTAAACAATCCGTACATGCAGAGCAACATTGAGTACATGAAGAAGCAGTCTAC
GGATTACCTGGCACTGATGGGATCACCCGACGAGCTGGCGCCTGCAGCTCCGCGCTACGTGAACGGGCAC
ATAGTGCCCGATATACG
>dme_circ_0001824_junction_seq
TGAACGGGCACATAGTGCCCGATATACGAAATTCGGACAATGATCCCCGATCGGGC
>dme_circ_0001825
ACGGCAAACCGCTGCCAAAGCCCGTGATCAGGTCCTCCGTGGAGCATCACGTCTTCACGGACACCAACTT
CACCCTGGATTGCGAGCAGTCCGCCTACGTTGAATCAGTATACGGAATGGAATGGTTCACTCCGTCCCGG
GATGAGGAATCGCATATTTGCCTCCCAATCAAGAACCGATCCCAAGACCAGGAACAGCACCCATCAGACG
GGCAGGAGCACCTTGACAGTGCTAAATGCACAACCCTCGGACACTGGTCTATACAAGTGTGTGACAACAG
ATAATTCTAACCAGAACGTACAACGTGCCACCTACAGGATTAAGGTGCTAAGAGCAAAACGAAAGTTACC
TGAACGTGGGCGAACCATCGGGTCATTACAACGTTCAGGAATATGCCAATCGCACGATCCAAATGACCGC
GAACTTTGAGGGATTTCCGACGCCCTCCTTCAGTTGGTTCAAACCCGATGGCACCGAGGTGCGACAATCG
GAGAATAACTTCAAGATTCTCTCCACGGAATTGAGCACAATGCTCCAGGTGCTGAACGCCCAATTGCAGG
ACAGCGGCACGTATGTCCTCCGTGGATCCAATTCCTTCGGCGTCGTTCAGCGGGAGTACAACGTCAGTGT
GATGGACGCACCGGCGCTGAAGATGTCGGACGCCTATGTCCAGGTGGGATCCGTGGCGCGACTGGAGTGC
ACAGTACGCTCCTATCCGCCGGCTATCGTGACCTTCTTCTTCCGCCCCTGCAGCCTGGAACCACAGTGGC
CCACTTGCTCTGTGCTCAATCAGAACTTTAGCGGTAAGTAATACGATCGAACTGGAATATATATCCAACT
ATAATTATATTTGTATGTAATGGATTCTATATGATGGGTTCTTAAGTTAATGTCTTGTCCTTATAATTAT
ATTTGTATTGCTTTCTTGTGTCGTCCCATGCTTTGGTGTTGTCGTTCTGCAGTTGCCGAGTGAACAGGAG
AAATATCAGGTATTTGTTTAAAATCATCTTCCCCAAACTTTTTGCGCACAATTACCAACTCTTTGTATAC
CATCTCCAGTTTCCAGACCCGGCCCAGACCCGGAAAGCTGAGTGTGGAACGCATATACGAGGTATCCTTC
CTGCCCACGGAGCCGGGAATCCTCACATGCATTGCCCAAAATATCATTGATGGAAAGGAACGAAGAACCC
TGACGAAGGCGCACGTTCTGCTGGGCAACATTTCCGAGAACATGACCATATATGGCTTCGATAAGGATCA
CAAAATCGCCAAGGAGGACAATGTGAACTTCACCTGCGAGGCGCTGGCCTATCACTTCGATGGAAATCTT
AAATGGTTCATCAATGGAGAGGATTTGAAGGAGTCGGATTGCGGTTCACATTGAGACCAGCCATACCAAG
TACTCCTACAAGAGCACTGTACACATCACAACGATATCCGACAGGGATCGTGGAACCTATGAGTGCCGGG
CCTACCACAACGACAAGGATGCCGTTTACAGCAGCCGGGAGATAGACTTGTACGTCCACGATCCCTCTGC
TCCTCAGTGGACAAACGGCGGACAGGAGGGTCACTCGAAAATAAAGCGCAAACTAAGCCAAACGCTGGAG
CTGGAGTGTGCCTCCACAGCGGTTCCCGTGGCAATTGTGCGTTGGTTTAAGGACGACAAGGAAGTGACCG
AATCAAAGCTCAGGCACATCATTGAAAAGGAATCCAAGCTGCTGATCACTCACCTGTATCCCGGAGATGA
AGGCGTCTACAAGTGTGTGGTGGAGAACCGATTGGACAGAATCGAACGCTCCTTCACGGTAGTGATATCA
GGATCTGCCCGGCATTAGCATGGCCTGGGTGTGGTTCGGTGTGATACTATTCCTCATCCTGATCGGTCTG
TGCGTCTTCCTCGCCGTGCGCTACCAGAAGGAGCACAAGCGGCATCTGGCCCTTAAGGCAGCCGGATTGG
CCAACTTCGAGGAGGGCGCCGTGGGACACATCAATCCCGATCTGACCCTGGACGAGCAGGCGGAACTGCT
GCCCTACAATCGGGAATTCGAGTTCCCACGGGAAAACCTGAAACTGGGCAAGCAACTCGGAGCCGGAGCA
TTTGGCGTTGTGCTCAAGGGCGAGGCCAAGGGCATCCGGCGAGAGGAGCCCACCACCACGGTGGCCGTCA
AAATGGTCAAGGCGACGGCTGACAACGAGGTGGTGCGGGCACTGGTCTCCGAGCTCAAGATCATGGTACA
TCTGGGACAGCACTTGAATGTGGTCAATCTCCTGGGTGCAGTTACCAAAAATATTGCGAAGCGGCGAACT
AATGGTCATTGTGGAATACTGTCGCTTTGGCAACATACAGAACTTCCTTCTGAGGAACAGAAAGTGCTTT
ATCAATCAAATCAATCCAGACACCGATCACATTGACCCCAGCATCATGACCCAGCGCATGTCCGACAACT
ACGAACTGCACCGG
>dme_circ_0001825_junction_seq

CATGTCCGACAACTACGAACTGCACCGGACGGCAAACCGCTGCCAAAGCCCGTGAT
>dme_circ_0001826
AAGACGGCAAACCGCTGCCAAAGCCCGTGATCAGGTCCTCCGTGGAGCATCACGTCTTCACGGACACCAA
CTTCACCCTGGATTGCGAGCAGTCCGCCTACGTTGAATCAGTATACGGAATGGAATGGTTCACTCCGTCC
CGGGATGAGGAATCGCATATTTGCCTCCCAATCAAGAACCGATCCCAAGACCAGGAACAGCACCCATCAG
ACGGGCAGGAGCACCTTGACAGTGCTAAATGCACAACCCTCGGACACTGGTCTATACAAGTGTGTGACAA
CAGATAATTCTAACCAGAACGTACAACGTGCCACCTACAGGATTAAGGTGCTAAGAGCAAAACGAAAGTT
ACCTGAACGTGGGCGAACCATCGGGTCATTACAACGTTCAGGAATATGCCAATCGCACGATCCAAATGAC
CGCGAACTTTGAGGGATTTCCGACGCCCTCCTTCAGTTGGTTCAAACCCGATGGCACCGAGGTGCGACAA
TCGGAGAATAACTTCAAGATTCTCTCCACGGAATTGAGCACAATGCTCCAGGTGCTGAACGCCCAATTGC
AGGACAGCGGCACGTATGTCCTCCGTGGATCCAATTCCTTCGGCGTCGTTCAGCGGGAGTACAACGTCAG
TGTGATGGACGCACCGGCGCTGAAGATGTCGGACGCCTATGTCCAGGTGGGATCCGTGGCGCGACTGGAG
TGCACAGTACGCTCCTATCCGCCGGCTATCGTGACCTTCTTCTTCCGCCCCTGCAGCCTGGAACCACAGT
GGCCCACTTGCTCTGTGCTCAATCAGAACTTTAGCGGTAAGTAATACGATCGAACTGGAATATATATCCA
ACTATAATTATATTTGTATGTAATGGATTCTATATGATGGGTTCTTAAGTTAATGTCTTGTCCTTATAAT
TATATTTGTATTGCTTTCTTGTGTCGTCCCATGCTTTGGTGTTGTCGTTCTGCAGTTGCCGAGTGAACAG
GAGAAATATCAGGTATTTGTTTAAAATCATCTTCCCCAAACTTTTTGCGCACAATTACCAACTCTTTGTA
TACCATCTCCAGTTTCCAGACCCGGCCCAGACCCGGAAAGCTGAGTGTGGAACGCATATACGAGGTATCC
TTCCTGCCCACGGAGCCGGGAATCCTCACATGCATTGCCCAAAATATCATTGATGGAAAGGAACGAAGAA
CCCTGACGAAGGCGCACGTTCTGCTGGGCAACATTTCCGAGAACATGACCATATATGGCTTCGATAAGGA
TCACAAAATCGCCAAGGAGGACAATGTGAACTTCACCTGCGAGGCGCTGGCCTATCACTTCGATGGAAAT
CTTAAATGGTTCATCAATGGAGAGGATTTGAAGGAGTCGGATTGCGGTTCACATTGAGACCAGCCATACC
AAGTACTCCTACAAGAGCACTGTACACATCACAACGATATCCGACAGGGATCGTGGAACCTATGAGTGCC
GGGCCTACCACAACGACAAGGATGCCGTTTACAGCAGCCGGGAGATAGACTTGTACGTCCACGATCCCTC
TGCTCCTCAGTGGACAAACGGCGGACAGGAGGGTCACTCGAAAATAAAGCGCAAACTAAGCCAAACGCTG
GAGCTGGAGTGTGCCTCCACAGCGGTTCCCGTGGCAATTGTGCGTTGGTTTAAGGACGACAAGGAAGTGA
CCGAATCAAAGCTCAGGCACATCATTGAAAAGGAATCCAAGCTGCTGATCACTCACCTGTATCCCGGAGA
TGAAGGCGTCTACAAGTGTGTGGTGGAGAACCGATTGGACAGAATCGAACGCTCCTTCACGGTAGTGATA
TCAGGATCTGCCCGGCATTAGCATGGCCTGGGTGTGGTTCGGTGTGATACTATTCCTCATCCTGATCGGT
CTGTGCGTCTTCCTCGCCGTGCGCTACCAGAAGGAGCACAAGCGGCATCTGGCCCTTAAGGCAGCCGGAT
TGGCCAACTTCGAGGAGGGCGCCGTGGGACACATCAATCCCGATCTGACCCTGGACGAGCAGGCGGAACT
GCTGCCCTACAATCGGGAATTCGAGTTCCCACGGGAAAACCTGAAACTGGGCAAGCAACTCGGAGCCGGA
GCATTTGGCGTTGTGCTCAAGGGCGAGGCCAAGGGCATCCGGCGAGAGGAGCCCACCACCACGGTGGCCG
TCAAAATGGTCAAGGCGACGGCTGACAACGAGGTGGTGCGGGCACTGGTCTCCGAGCTCAAGATCATGGT
ACATCTGGGACAGCACTTGAATGTGGTCAATCTCCTGGGTGCAGTTACCAAAAATATTGCGAAGCGGCGA
ACTAATGGTCATTGTGGAATACTGTCGCTTTGGCAACATACAGAACTTCCTTCTGAGGAACAGAAAGTGC
TTTATCAATCAAATCAATCCAGACACCGATCACATTGACCCCAGCATCATGACCCAGCGCATGTCCGACA
ACTACGAACTGCACCGG
>dme_circ_0001826_junction_seq
CATGTCCGACAACTACGAACTGCACCGGAAGACGGCAAACCGCTGCCAAAGCCCGT
>dme_circ_0001827
GATTTCCATCGAAGTGATAGGCCAGCGCCTCGCAGGTGAAGTTCACATTGTCCTCCTTGGCGATTTTGTG
ATCCTTATCGAAGCCATATATGGTCATGTTCTCGGAAATGTTGCCCAGCAGAACGTGCGCCTTCGTCAGG
GTTCTTCGTTCCTTTCCATCAATGATATTTTGGGCAATGCATGTGAGGATTCCCGGCTCCGTGGGCAGGA

AGGATACCTCGTATATGCGTTCCACACTCAGCTTTCCGGGTCTGGGCCGGGTCTGGAACTGGAGATGGTA
TACAAAGAGTTGGTAATTGTGCGCAAAAAGTTTGGGGAAGATGATTTTAAACAAATACCTGATATTTCTC
CTGTTCACTCGGCAACTGCAGAACGACAACACCAAAGCATGGGACGACACAAGAAAGCAATACAAATATA
ATTATAAGGACAAGACATTAACTTAAGAACCCATCATATAGAATCCATTACATACAAATATAATTATAGT
TGGATATATATTCCAGTTCGATCGTATTACTTACGCTAAAGTTCTGATTGAGCACAGAGCAAGTGGGCCA
CTGTGGTTCCAGGCTGCAGGGGCGGAAGAAGAAGGTCACGATAGCCGGCGGATAGGAGCGTACTGTGCAC
TCCAGTCGCGCCACGGATCCCACCTGGACATAGGCGTCCGACATCTTCAGCGCCGGTGCGTCCATCACAC
TGACGTTGTACTCCCGCTGAACGACGCCGAAGGAATTGGATCCACGGAGGACATACGTGCCGCTGTCCTG
CAATTGGGCGTTCAGCACCTGGAGCATTGTGCTCAATTCCGTGGAGAGAATCTTGAAGTTATTCTCCGAT
TGTCGCACCTCGGTGCCATCGGGTTTGAACCAACTGAAGGAGGGCGTCGGAAATCCCTCAAAGTTCGCGG
TCATTTGGATCGTGCGATTGGCATATTCCTGAACGTTGTAATGACCCGATGGTTCGCCCACGTTCAGGTA
ACTTTCGTTTTGCTCTGTGGATTTTGGAATGCAAATAAGTAGATATCTTCATAATAAAATTCATCATATA
TTTAATACTCACTTAGCACCTTAATCCTGTAGGTGGCACGTTGTACGTTCTGGTTAGAATTATCTGTTGT
CACACACTTGTATAGACCAGTGTCCGAGGGTTGTGCATTTAGCACTGTCAAGGTGCTCCTGCCCGTCTGA
TGGGTGCTGTTCCTGGTCTTGGGATCGGTTCTTGATTGGGAGGCAAATATGCGATTCTGCGGTAAATCAT
TATCTTAGTACTGCTCTCTTAAAAATGCATGTAGACTGAGGTTTACTGACCTCATCCCGGGACGGAGTGA
ACCATTCCATTCCGTATACTGATTCAACGTAGGCGGACTGCTCGCAATCCAGGGTGAAGTTGGTGTCCGT
GAAGACGTGATGCTCCACGGAGGACCTGATCACGGGCTTTGGCAGCGGTTTGCCGTCTTCTGTGGCGTTG
GTAGAAAGTTTAGCTTTATTAGATACCCTTCGCAATGGACTGGAACTGGCCAGCAATGCTACAGCTTCAT
GCAGATGGTTGGGTTAGACATAGACGTGGACACAGATGGGTTAACGTTTCTACAGTACTTAGGCGGTAGG
TGTTAGCATTGATTAGTAACGAATAAGTTTGCTAAAGCAGTTAAATCAGTCCATAACCAACATAAATCGA
ACACAAAAGGATGCAAGAAATTAAAACGCACAACCAGGACAAAACAACATGAACAGAATGAACACAGGAA
CAGATCACAAAACAGCCAGGACAACACTTACGCCCTGGTCTGGGTGAGGGCGACGCATTCATCGGAGCCA
GGCGGGCTGGACTTCGCCGTGCCCGCTCCCTGGATAAAGTTCCATCACCACCACGGATTAGTGCCAAATT
ACCCGTCGATTGGTTAGTAACAGTGAGGACCTCGTCATCGCCATCGGTGACACCTGGTGCATATGTGTAC
ACCATGTTAGTCGTTTGGGTACGAGGGATATCCAATCCGGTTGCTGGAGGAGTTAGCAGATTAACATGAC
ATTGGGTACGGCAGGTCAGTCAATTGTTTTTGAGGGCGGGAAGACAAAGGAGGCAAAAATACCATTGGTT
AGCACACTTACATATGTTACTTGCAAGCCGTGATTTTCAGTAGTGTGACACTTTTAAAGTTCATTTTTTA
AGCATAATTTTAAGAAATTAAGGGAAGTTTAAACCGAAAACtttttttttAACTTTGTGCGAAAGTCTAC
AACTAAATGCGTGCAGTGTAGTTTCGTCTATTGGCTGTAGACTGTGATTAAAACCAAAGAATTTGGAAGA
GAAAGTATTGTAGTTTGCATAGATTAAATCCTTGGAATCAGGACCAAAACCAAAACCATAACCGATCCCG
AAACCGTAACCGCTTCCGATGATCGATTCCAAATATAATTGAATAGTTGAATAGTGGAAAGCAATATTTA
CGACATGTATAGACGGAAATCACAAATCACAAACTTTGGGGTGCAAAAACTCACTATCAATGTGACCGTT
ACCAATAAAGCGCACTTCTATGCTGGTCATCTCCTCTTCGTTATGCGGGAATGGCGGATTGGGTCGGCAG
TAGTAGTCTCCGCCATCCACGATGCTTCGGATTTCGATGGTGAATCCCCGTTGCGGATCGTATCGACCGA
CAGATTTGCTGGAATGCATCTGCACAGAGGCATATTGGTCAGATATCAATCAAATGGGTATTGAATGCAA
ATGATGGCCTACATATGGACTGGCTATCTGCTGCCTATCAAGACTATAGCGAATTTGGAGCTGATTGGTT
AATACTACGCTACAATAATATATTATTATGTAGACTTATATAGATTGTCTGTACAGTAGAGTTCGAGTAT
ATATACATATATGGATCATAGGGGTTTAACTACGATCAATCAATAAAAGACAGACTGTGACATACTTTCT
GGATAGGTAACGTCAATGATCAATTCATAGTCCAACGTTTTGCAATACAAGTAACCGGATACACAGTCAA
TGACTCGAAAGGTAAAGCCCCATCTGGGGTGGTACCTCACACTATCGAAGAACTTGGGGTTACCCTGAAT
CTGACCTTTCGAGCTGATTTTCCACGTGCTCTGCGCGCCGAGAAAAGAAGATCATGAGTAATACCAGATA
TATGTACACAAGACCTGCATGAATCTCATACTGGTCTTAtttttttttAAATAGTTTTGTAATTTTAAAA
GTAACCCACTATAATCTTATATCCCCATCTCTCATTTCCTGTCTTGGATTCATGCAGGCCCTTTTTTGGC

CCATTAGACTAGATGTTTTCGAAAGTGTTTTCAAGCTGTAGATCCTGCCCACTTACTTCTCCATTACTGG
TCTCTAGCAGCACCTCTGTGTCCGGCATGGCTGGTTTACAGGGTATGACTACGTCGGTATACTGGCGTGC
CGTCACCACGTTATGACTATCGACCAGCTTAGTGTCCGGATCATTCACGTACACGTAGATGGAGCTGGCG
TATCCTTGATTAACCAATTCGATCATCGCCTCGTCCGACTGCTCCTCCTTGGCGATCTGACTGAATTTCG
AAGTCTTCACGCAATAGTAGGCTGCCACATAGTCAGCCGTCACTTCGATGAGATGCAGGCTAGTTCCGAA
TGGTCGAGCCGGATCCTCCGTATTATCGAAGGACTTCACATGCACATATTGCGATTGACTGGTCCACCAG
CTCATCGGCTCGTCGTCCTCGCACTTAAGCGTGGTGCTCGTCTGGGCATCCAGGATAATGGCGCTCTTGC
AGGGCGTCATCAGGGGAGCTCCATTCTCGCCGCCGCAGTTCTCGATGCTGTCATCGGGATCCGGTGAGAA
CTGCTGCAAAGGCACTGAGATCGTAGAAGAGAACATGCATATTAATATTATAATAAACCATAAAAATGTA
TATATATTAGTACATCTGTCCATTTGTCCGTATTTCTGTCTGGTAAATAGAAGGTCAGCTGCACTCGTAT
ATTGTTGTTCATTGATCGCTCGTATGCCCTACAACGGAGCACTCCACATGCCGCTTTATCTATTGCCTTA
GGCCTCCACCTCCTGCGGTCAACAATATCCCCATCCACTGGGTGGCGCTACAATAATAATGACTTCCAGT
GATTTGCGGTCGAGCGAGCGCCTCTCGGTGGAGGCGTCATGGGCGCAGATGCACAATCCTTTAGCCAATC
CCATGTTTCATATTTAATTGATCGTGGTATCCCAGAAGGGTGCGCGTAGCTACGCCTCTGCGATGGCCGC
TCAACTAGTTTGTTTTGCCTTCTAGGCTCGGGAATCAATTGTTTGTGTTTACTGCGATAACAATAGAGTT
GATAGCCATTATAATATGCTCTCCGCTCAGCCTCGTACTCTAACGAAAGTCTTTATGGATGCACAACAAT
ATCAAACTCGTAACTAATGCAAACGACACTTCTTGAATCGACATTAGTCCAAAATTCGGGCATACATATA
TCAACCAGAGTTCAAAGTTCAGAGCGGTGTCTATAAACTGTGGCTGTCGGATCTGTCTGGAGTGGCTGAA
GCGTAGAGATAGATAGATTATGGCATCACACCGAGAAAAATTTAATGCAAGTCAACAAAAACTTGTACTT
GAATTAAATCTCATCTATTCTTCAAATAGTTCTTCTATTTTGAGAAATGGAATTGTTATTGTACAAAATA
AAAAACCACCTTGATTCAGCATTTAGAATCGGATTACTCCGTTTTTTGTCTCCGTGCACTGAATAAAGAA
TGTGCGATTGTTGACAGGAAGCAAGCCATACATAAATATCAATGCATATTCGCAGATATGTATTGttttt
tttAATTGTGCAAATTGAAAGCCTTAGCCGTCGTGTATTTTCAGAGCTTATCAGTTCCGTAAAATTCTTC
GGAATTAAGTAAATGGTAGGAAAATATGTTCttttttgtgttttttttttAATGAGATTTGTGCAGGGCC
AACTTACCAGCATCGCTCCACGAGATCCGCAAAATCAGGAGCAGTGGCAGCAGAATCAACCGCGGAAGCA
TCGCCATCTGGCTTGGTTAGTTTTGTTTCCTCTCCGATTATATATTGTGCTAATTTTACACTCTTAAATA
TTCACAATTTTAACACATTTGGCCCTTTAATGCCATGGTCACGCTGAAAGACACTTTGCTCACTTAGCGT
TGCACTGaaaaaaaaaaaCGAAGATGTTTTTGTTGATTAGAGTTGAAGTTGTGAATTAAACCGATTATAG
ATATTGTTTGTTTGCTTTATGTTATATTAAGATAATTATTTTTTAAGTCGCATTTGCTACTTTTTATTTG
CCTCTTTTTATATGCAGTACATTTGGCTAATCAGTAAATATATACATATATATTTGTATTATGCACTTTG
TGTTCCATTCTTCGtttttttttGGTCCACACTTTACTTTAGTTACACACACATTTCTTTTATAAAATAT
TCGTATATACTTGATTATACTCCGCTTTTCGAGCAGAAGTGAACAGCATAGCCATCCGAGGAAGAATAAA
GCTTTTGCTGGATGTTCGGAGGAACGATGCGATAAGCGAATTGTGTATATTTTTTCCACACTTCATTCAA
GACCAATAAATTCGATAAGGCGGAAATAAAAGAAAATGAATCTTGTAAGTCTTTGCTCACACATTGTGCT
TGCGCATAAATAACAAGAACAACACAATGGGCTACACAGTTTTTCCCAttttttttttGGTGTCATATCC
GCATTTTGAAATGCAAGTTTCCGGGAGATAAACATATCtttttttttGGGTGCAACGGAGGGCGGTGGGC
CACACCCACCGACGGATGAGGCAACAGGTCATCCACGTGGCCATAACTCACTGTCACACAAGCTGAGCTA
GTCTAAGTCTCTAAATGACAACGGGACATGCACAATTAAACAAGCTGACTAACTATTGCCTACATGGCCA
CAAAAAAATACATATAATACATATATGAAATTAAATGAAGCAGCATTTTTATGTCCACATGGCATACTTT
TGTATAGGCTATTATAGCTTCAAATGTTTTGCAAAACAGAAATAAGATAACGATGTTTCTATATACCATA
TTGTATATATTATCAGATTCAAGCAACAGTACGTTAAATTTGAAGCTTATGCTTTCACTGGCGAGCTTTT
TATGTTTTTTGTGCTCACTATCAACAGTTTCTTATCATGTCAGCTTAATaaaaaaaaaaaaaCAAACAGC
AAGTGTGGATGAAAAATAAGCCGTTGCTTTTAATTTCAGTATGATATATTTTGTTAAATGCGATTTTCAA
TTGAATTGCCTTTTTTTCAGTGTGTGCAAGCGGCGCCTCCGTTTGCCGACAATTAGAGCAAACGAGCTGG

CTGCAATGTCGTATAAATGTAATCCCTCCGAGCCACAGACAACAAGTGTCCCGGCTGCTCGCCTGACACA
CCAATAAACTGGCATTTAATTTGCTGGCCAACTTTGAGGGCACTCCTCCAGCGCCTTCGTGATGACATTT
TGGCCTATGCCAAAACTCCCACTACTGGCTAATTGCCAGGTGGTCAGCCAACACAGAGCCATTCATCTTT
GTCACTCCCAGGACTCGGGGTCAGTGCTCAGTCCGCGTTTGCAGCGTTTGTGTTGGCCAATTGAATTTGC
TCTGGTTTCTCCTGCCCCACTTATTCGCAACGGTAGCATTGCGGATTAAACGCAATCAGCCGGCAAATAT
TGACTTGGTCGCTAATCACCTGAGAGGTGGAGCAGCTAATGGAATGATTAAAAGGCGAAAAACTAAGGGG
CATGAAGTTTAACCTCTACTGAATACACTTTCTTTTTAGAGCTAAACTAGAGTTAACTTAAACTTTGTAC
ATATTTCAAAACTCTGAATATTTCATTTGAATATTTAACTGTCCGTTTGGCATAAGCTATATATACGGAA
AAAGATACAATATATAGAAAAATGGAATATATAGAATTGGTATTATATATTTTACTCAGCAATATACCCT
CAGAATTTAAAGAAATACAAATTCGACAGATCTTATATTTATCGGCGAATTTGTTGGTCATTTGAATCAT
GCCGGGTTTTTCATTAGTCGATAGTAAGCACTTTTGCATAAGTGCCAAATCAAATGCGAATAGAGTATTA
CTTCCGGAACTGATACTTTGTTCCGAATTCATATTTTCCAAATGTATTATCGCATCAGGAATGTAGGAGA
GTAAAGCCCATGACTAGTAGCAGACAGGCTATCAAATGTGTCTGGCTGCTTGTGGCAAATTGCAAGTTGC
TCGCGCCGCCAAGGAAATCTTGGACTTTGACAAGGAGAACTTAAGACTTGAAACTCCGTTTTCTGCCAGT
CACTCGTGACTACACAGTGAGAAAGATCAAATCACTTTCGCAGGAGGCCAACTCTTAAATGGAAAGCATT
TCAATACCATTCGCAAGTAAGGTTTTTTTCAAAAAATTTTCAAAATGAAGTTATCAAAGCTGATTTTACT
TTTAAACTTCGATTTAAAAGTATAGCTGATTTCTTTCGGTGCATTGGTTTGTGTTGTCGCTGGAATGCAT
GTTGTCATGACGACACGCCCACTTTGGCCATTTTCAAGGGCGACTCCGGACTGTGGCATTTTCCTTTAGC
CCCCATTGCGTCTATTTATATCTGGGCAACAGTTTTTCTGCTGCTCTTTTAAGTGCCTCACGTGCCCGTG
ACGCAAATTACGGTGCTCCCTAATTGCAGTCCCAGCTGCAGGCGAATGAATCCCTTTTCTTCCAAGCTCT
GATTTTTCTCTCTGATTGTCTTCTTCAACAGTCCTCGGATCGCAATTAGCCATGGGCATAATGAGGCCAG
TCGGTTGGTAAGAGGGAATCTCTCCCACCTCATTCGCCCTATCTCGGAGTACACAATTCTGCACTTAGTC
ATTTTCAGACGTGGGCAAACAGAACGCTCAAAATATCTGCTCCAATTCGAGCATTTTCATTGTCAGCAAT
GGAATTATAGCTTATCACTTCTTATTATTGGCTTATAATCCTGTTTACAATGTCATCGCAAAATAATGGC
AATCGATTATCTTTTAAGAAGAAAATACACAAAAATCAGAAAATATGTTATGATCATTTTGTTTAAAACA
TTTTTTAATCCGAAGAGAATAATGACTTTCAATTGTGAATGCTAAAATGAATCTTGTTGAATGAAGGAAA
ACACAGCCAGATGAATAATTTGCTCGGCATTTTTCAGCTGTCTTGTTTTTGTGCTAAGAAAATACAATTT
CCCAAAGTCGTCACTCTACTTCGGTTTTTGCTGTTGCATTCTTTTCTGCGTAATCTATTAGATTTCTAAA
AGTTTAGAGTGTTTTTATGCGATACGAAATGGATATATGTAATTAATCAAGAGCTGCCCGAAACTTTCAT
ATCAATTCGCGACTATCAAAATTCACATCGATATACAACATATGTTTAACACACAATTGTTTACCACCAT
CGAGTCGTATAAATAATAAAGAATGACGTCATTCCACGGTCGAGTGGCGCCGCCTTAATTCGTTCATTCA
TTCACCAATGTCCCCCACATTTAATGAccccccccccccccTTCCACCTTTTTGCTTATTCATGTGTGAC
GTTTGCATTCACGTTTGCGTTTCATGTAAATCTGTTTATATAAGTGACGATATTTGCGTACTAATTAAAC
TGCCTTACGCTCACATTTATATCTGTTTATTGTTCAAATATGCTTTTTACGTATATATTATAATAAAACT
GTATAGCACGCAAACCGGTTGACCGAATGCATTTGTAAAAGGGGAGAACTTTCCTTGAGAGCAAGTGTCT
CCTTGCATTCATCATCATCATCATTATCATCGCATTGATTGCAAAATCTTTTGTTTCGAAGAGTGATCTT
CCAGCAGAATGTATAATGTATAAACAGTCATTGAAAATCGTTTAAGGAACTTACAGGGCTATAATAATCA
GAAATCAGGTAACAACAGACCATTTGTACTAGTTCTACCAATCACAAGAGCTTAAAAATAAAACACAACC
ACATTGTAGATAAACTGCAGCATTATTCACTTGAGTTTTTTTGAATTTCCCAAGCCTTTTTTATGCGATT
ATCTAATAAGCTTACCAGGGGAAGCAAGAAATAAACTTAGGCAAACTTTTGAAGTAAATTTATGCAATGA
TACAATGTGTATTTATGCGAAGACAAGCGAACTCTTTCATGAGTTTTTGCCATAAAAAACGGATGGTCAT
TTCATGTTTCGGCTACATCAGCGGCCAACATTCTTATTAGTTCCATGTTTTTCCAACaaaaaaaaaGAGA
AATGAAATAGCATTCCATTCCACCACTGTTCTTTACGCCGAAACTTTTGACAGTTTAACGCTTTGCCGGG
AAAATCTTTCAACGTTATTTGATTCTTTATGGCCAATGAACGGAAAACTTGCACAGCTCGTGCTTTTCAC

GCGCTGCATGTGCAACAGATTCATTTCGAGGCTTGAGTGCGGTTTTTGTGGCTTTCCCTCACGTGTTCCT
GaaaaaaaaaaTGTATTTCACTCTGAAAATTTAGCCGCCTACGTGCTACGTGCAACGGCTGACCCTCGTC
ACGGACGAATTTTCCTGCTGCTTTCCACCGTTGTCCATTTTAATTTGCAACTCCCTTGTACAAATTGAAT
TTATATGGGCCACAAGCTATGTGTTTATTTTTGCAATTATCCCGGATTCGAGAAAAGTAAGTGTGTCTCC
ATTGTTCCGACTACCTTTTGTTTGGGCCAACGAAAATTTACGACTCTTTCGTCGGAGTTTTTCCGTGCTT
TTATTTGATTTTCCCGCTGACCTTTGGCGTTGCGTGTTTCTGTGTTGGTGCACAAGTCAAGTGTTTTCCT
TTGTTTTTTCCATTACAATTGAAATTTCGCGCACCACATCTTAATGGAAAGTTGAATCGTTCACGAATTC
TGTGATTGCAATTGCAAACATGCCGCTGGCTTCTGGCCAAAATAATGAGCAGTCGCCATGGAGAAATACG
AAGAAACTACTTGTATCGGAAAATATTTTTATCCATCTGCATTGCCTGGAAGCGGACAAAGGGGTGTGGC
CTTTCATTTGCTTCATTAAAAATTCAGAAAAGCGCCACGCAGTCAAATTGAAATTTGCAATTTGGGCCAA
GCTGAGATTTGTTGGCTCGTAAATCAAACTATTTGTCAATCGGTTAACAGCGGTATCGAGAGATCTGGGG
CCAATCTGCCCACCCAGGCCAAAAAACCGCGTGTGGGCCTTTTGGCCAACTCTCGTTTCGTTGGCCCAAA
ACACCGTTCAACCAACGTTCTGCATAATGACGCTACTTTTGTCCCAGTTTGCGTGCCTCTTAATGATGGC
GGGTATGTGGATGGTGACATGGTAATGGTTCCTTACGCGGAATGCCAGCTAATTCCATTAACCAATAGGC
TTTCGTCAGGACAACAAATGTTCGCAAGTGGGCGTTAGCTAACCAAAAACGCTAATGAACGCAAGGATGT
CATAGGGATAAGCATTTAGTTAGCTTCAAATGCCAAAAAGATAATGGAATTGCTTTTGCCATCCAACTTA
TTTCGCAAAGAAAGCCAGCAAAGTTTCAAATAAAGTTTTTGGTGTCACACAAAATTTATTATCGCCTAAT
TaaaaaaaaTTGAATTGTTTGCGACTCGTAGCAGCAAATTATTGATTTAAATTGTCAACAAAAACAGAAT
CTTTAAACTGGCAATATATGAATCCATTTAAAGCCAAGTGTGCTATATAAAATATTTTTCGCTTTAATAA
AAGCTGACTAATTAATTTTTGGCGAGCTAGAAAGCTAATGACAATTCCAAGGTAATGCGTCTGCAGAATT
AATAGATCTTGATACTTGATTAATTGAGCAGACATTTTAGCACCCTTCGATTGAGGTCACTTGACTTGTG
GGTAATCTGCTATCAGCGGGAACATCTGGTTTAATGTCTATTGTCCGACTCTAATTAATTGATATATGGT
TCCGTTTCAATTGACATTTTTGTTCCtttttttttttGGGCAAGTGCACAGCGCCCGCTGTAAATTCCGC
CCACAAACAAGAGCTAGTTTTGCAGTTTTTGCGTTGGCTCCACTGGTTGTTGGCCACGCCCCCCCACCAA
CTACGCCAACGCCCACGACTATATCCCCGCCCATTTGGCATTCCACATACCAAATTCCCAGCTGAGAATA
GAAATACGGGCTTCTGTGCGCAGATCTAATGCATTTTGAAGCGATAGCTGCAAGACAAATGCCCAAACAA
CACGAGCTGTGAGCAAAAAGCAAAAGTGACGCCTTGATGGATTGGCAACTGGAAGCGAAGAATAATGGGT
TACAGAAATCAGTCGCAGCCTCAGATCCCCGAGAAGCTGCAAGATTAAGGGAAACTTCTAGCTAAACTGC
GGGCGCACTGCAAGATAAACTATCTGGCAAATAGCGAGTTCTTAACACCATTTCGCAACGGCCTATCTTT
TCAACCATTTGGCTGCGATAAGGTCAGGAAAGAGATACGGGGCTTGATTATGTTAAGTAATTGCCTAGCA
GAAGCGGCGAAAATAATTTAATGTCttttttttttGGCAACCTTTGCATAATGCGCAAAGAAAGTCAAAT
GGCATAAAAGTGAAATTACCGGAGTAATGTATCTTTCAGTTGAGTCATTTGGGAAACTTTATTTTTCGGT
TGTATAACTCTTCAGTTGTCACTCGAGAGATTTGGAAAAGTTGTTTCTTTTCTTTTCCATAAACTGGGAA
CTTAATAAAAATAATAGAAAGACCATTGCCGGTGAAATTGAATCGTTGGCATTCTGCCCACATATTATAC
AACTACAACGCGTGGCGACAACAAGTTGGTTGGTTGGCCAGCTGCTGGCGAATAAATAGAAATGTTATAT
GCGTTCACTTAATAGCCGACAAGTGTTTAATTTGTTTGTTTTGGGTTTCGCCCGATTAATAAATAAATAT
GGCCAATGTATATAGCCCATTTAGTGCCTCGCCGTGACCTCAAAACATGGCTCAATTTTATGCGATTTGT
TTACTCAATGCATTTTTTGTTTTGACTGGCTGGCTGCCTTCTGCCATTTTATTCCCGACGGAAATAAGAT
TGCTTTtatatatatatatacatatatTTTCTTATTTCCCTGATTCTCCGAAGTGCACTTGATTACGTGC
AGTTTATGGGGCAGATAAAATTTTCCCAACGAATGGGCTTTAGTCTAAAGTTTCCAGCGCTTATGAATGG
GCTTTAGTCTAGGGCTCAGGTGAAAGCCAATTGCCTGCTTAGCCtttttttttGCAGATTTATGGCCAAG
TTATTTCCACCTCTCCATGCAGACAGTTACAATGGCCTGGCCAAAACATTTGTTGTCCGCTGTCAattta
ttatttgttatttattatttaCGTTCTGACTCAGACCACGGCGGATGTAAAGAGGCGAAGGTGTCCATTC
ATAGAATGccaaaccaaccaaccaaccagccagccaaccaacaaAGTGGTGTGAAAACGTGTTCAATGCT

GGCCGAGATTGTCCAACGAATCGGTTTTATTTATGGCTTATTGGTAACCGGCAAATTCAATTAAACAGAT
ATTATCTTAAATGGAAATTTATGAGCCAATTCGCCCAAAGAGTTAAGGGGTTTCTCAAAAATGTTTCAGC
TTATCCGAACTGCCTTAGAGAAACGCAAAATGATTTTTCTCTCTACAGATGTACAAAATGTACCTAAACT
CAATTCTAGTGTTTTTATCTACAACAGGAAGTCAATCTTCTGTGCATAAGCAATCCTCTTGAGATTAAAA
AGATCTCTTGTGCAATAATACATATCAGCATTCTAAATATCTCTAGAGATATTACAATCGCTTGAAATGC
ACACAAATTCGTTGTCACACACATTCTTTGATTATTTTGAAGTCTATTTTCGGCGAGTTCTGCTTAAATA
TTTTGTGTGTATTTATAATTAGTTTCTATTAATGCGAAAGAACTACTTTGTTCATTTTTTCCAGTGCATA
CAAAGTAATTTATTCGTAATGGAGCTCTCTACTTCAGCACAATCAACTACAGTAAAGTATCTCTAATTTG
ACACCCACTGCGGGGGGCGGGGCAGGTGTGCGTATCTGGTGGATACTTGGCCATAAGCCCCACCTGATTA
ATGTGCCATGTGCATAATGGGTAGGTGTGAAAAAGAGGTGCAAGTGGCCAGTAGCAATGTGATGGGTGCA
CATTTTCCAAGAACCACTAGTATCCATTGATTATTCTGGCTTATCACAACAGTTGTTCAACGCTTGATGC
CAGGTGAATTGCGAATTTGGCCAGGGGAATTACAATAAATATTTCATCAACACCATTCGCGGACATTTTT
ATGACTCCAAACTTTGACGATTCGTAGCAGTTTTGTACCACATTTTCCGGACATTGAGAACGATGGCAAT
TAACAACAGCCTGGGCTGGGCAGCCTTTTGAAGTGGCCAGAGATAAATGGAGCAGTCGGCAAACTGAAGG
AGGTCTGTATTCAATAAACAAATTGGGCACAGTGTGCAATTGGAGGCAATGAATTATGGGCATCTGATTT
GATCAGAACTTCTCGATCCTGAGTCACGTATTGGGGGAAAATGTATCAACAGTTCGTTTGGTCAAGAAAC
GGATGGCGTGGAACCGGTTTAAGAAAGTCGCTATAGACACATTCAGTTGACCAGGCAAACAAAAGAAAGG
TGAACCCTTTCTTCtatatatatatgtacatatatatgtatatataCCAACTTTATTTGGGTGTTTCTGG
AAAAGCAGTATGAGTAAATTCGAAATCCATCGTAGTGTTATTCATATTTTCATAGTCTTACGCAGTCTAC
CGTTTGATCGCAGCAGCTTGGCAacaacaacaactattgaagatacaacaacaacttttgaacgtacaac
aGTCACGATCGGCGATGAATCACGATTTGAATACCTGGCACCGGTGGATGTTTGCAGTCTTGATTACTTG
GAATGCCGCTGACGTATTCACCATAGACACACGCACACTTTTCGTAGACCCAACACTTATATGCACTTAT
CAATGAATAATACCAGGCAGTGATAATAGcacacacacacacacacGCACTTAGAAAAAGGTCACTGATA
ATAGTTCATTATGTACACCATCTATAGGAGCATAGTGACCATGACTATTTTGGGTGttttttttttGTAT
AATTGCATCGAATTGAGCAACATCACGAGCGCCGAATTCGATTTCGATTTTCGAGCTTACGATTGACAAT
CACTTAGAACTCTTGTCAACCACCTGTGGCTGAGTTTGCACTTGTATTCGAATTCCGATTCGTATCTGTG
TTTGTTTTTTACTTTTGCGAAGGTGTGGGGGTTCGCTCACATCGATATCTAAACTTTTGAGTGCGGATTC
ATTTGCTTAGCACCTTTGATGCTTCACTTATACTTATATACGACACGATTTGGTTTCGTTTCCACACGAT
TCTATATCTATATTATCGGACGTATCTTCGAGCCTTTCAAAAATTAACGCTTACGTTGTGGTTTTCTGTG
CTTAACACTTTCTGCGTGGGGCACTTCAGCAGTATCGGTTGCTCTTGAAAGACAATAAATACTGCGTCGT
CGGCAAAGTTTGAACAGTTTGCACCGAACACAAGTCAAAAGCCCGCGAAATTTCCTTCCGCTTTGACGTC
CGAACAGCTCGAAAA
>dme_circ_0001827_junction_seq
CCGCTTTGACGTCCGAACAGCTCGAAAAGATTTCCATCGAAGTGATAGGCCAGCGC
>dme_circ_0001828
ATGCATTCCAGCAAATCTGTCGGTCGATACGATCCGCAACGGGGATTCACCATCGAAATCCGAAGCATCG
TGGATGGCGGAGACTACTACTGCCGACCCAATCCGCCATTCCCGCATAACGAAGAGGAGATGACCAGCAT
AGAAGTGCGCTTTATTGGTAACGGTCACATTGATAGTGAGTTTTTGCACCCCAAAGTTTGTGATTTGTGA
TTTCCGTCTATACATGTCGTAAATATTGCTTTCCACTATTCAACTATTCAATTATATTTGGAATCGATCA
TCGGAAGCGGTTACGGTTTCGGGATCGGTTATGGTTTTGGTTTTGGTCCTGATTCCAAGGATTTAATCTA
TGCAAACTACAATACTTTCTCTTCCAAATTCTTTGGTTTTAATCACAGTCTACAGCCAATAGACGAAACT
ACACTGCACGCATTTAGTTGTAGACTTTCGCACAAAGTTaaaaaaaaaGTTTTCGGTTTAAACTTCCCTT
AATTTCTTAAAATTATGCTTAAAAAATGAACTTTAAAAGTGTCACACTACTGAAAATCACGGCTTGCAAG
TAACATATGTAAGTGTGCTAACCAATGGTATTTTTGCCTCCTTTGTCTTCCCGCCCTCAAAAACAATTGA

CTGACCTGCCGTACCCAATGTCATGTTAATCTGCTAACTCCTCCAGCAACCGGATTGGATATCCCTCGTA
CCCAAACGACTAACATGGTGTACACATATGCACCAGGTGTCACCGATGGCGATGACGAGGTCCTCACTGT
TACTAACCAATCGACGGGTAATTTGGCACTAATCCGTGGTGGTGATGGAACTTTATCCAGGGAGCGGGCA
CGGCGAAGTCCAGCCCGCCTGGCTCCGATGAATGCGTCGCCCTCACCCAGACCAGGGCG
>dme_circ_0001828_junction_seq
ATGCGTCGCCCTCACCCAGACCAGGGCGATGCATTCCAGCAAATCTGTCGGTCGAT
>dme_circ_0001829
TGCAACGCTAAGTGAGCAAAGTGTCTTTCAGCGTGACCATGGCATTAAAGGGCCAAATGTGTTAAAATTG
TGAATATTTAAGAGTGTAAAATTAGCACAATATATAATCGGAGAGGAAACAAAACTAACCAAGCCAGATG
GCGATGCTTCCGCGGTTGATTCTGCTGCCACTGCTCCTGATTTTGCGGATCTCGTGGAGCGATGCTGGGT
AAGTTGGCCCTGCACAAATCTCATTaaaaaaaaaacacaaaaaaGAACATATTTTCCTACCATTTACTTA
ATTCCGAAGAATTTTACGGAACTGATAAGCTCTGAAAATACACGACGGCTAAGGCTTTCAATTTGCACAA
TTaaaaaaaaCAATACATATCTGCGAATATGCATTGATATTTATGTATGGCTTGCTTCCTGTCAACAATC
GCACATTCTTTATTCAGTGCACGGAGACAAAAAACGGAGTAATCCGATTCTAAATGCTGAATCAAGGTGG
TTTTTTATTTTGTACAATAACAATTCCATTTCTCAAAATAGAAGAACTATTTGAAGAATAGATGAGATTT
AATTCAAGTACAAGTTTTTGTTGACTTGCATTAAATTTTTCTCGGTGTGATGCCATAATCTATCTATCTC
TACGCTTCAGCCACTCCAGACAGATCCGACAGCCACAGTTTATAGACACCGCTCTGAACTTTGAACTCTG
GTTGATATATGTATGCCCGAATTTTGGACTAATGTCGATTCAAGAAGTGTCGTTTGCATTAGTTACGAGT
TTGATATTGTTGTGCATCCATAAAGACTTTCGTTAGAGTACGAGGCTGAGCGGAGAGCATATTATAATGG
CTATCAACTCTATTGTTATCGCAGTAAACACAAACAATTGATTCCCGAGCCTAGAAGGCAAAACAAACTA
GTTGAGCGGCCATCGCAGAGGCGTAGCTACGCGCACCCTTCTGGGATACCACGATCAATTAAATATGAAA
CATGGGATTGGCTAAAGGATTGTGCATCTGCGCCCATGACGCCTCCACCGAGAGGCGCTCGCTCGACCGC
AAATCACTGGAAGTCATTATTATTGTAGCGCCACCCAGTGGATGGGGATATTGTTGACCGCAGGAGGTGG
AGGCCTAAGGCAATAGATAAAGCGGCATGTGGAGTGCTCCGTTGTAGGGCATACGAGCGATCAATGAACA
ACAATATACGAGTGCAGCTGACCTTCTATTTACCAGACAGAAATACGGACAAATGGACAGATGTACTAAT
ATATATACATTTTTATGGTTTATTATAATATTAATATGCATGTTCTCTTCTACGATCTCAGTTGCCTTTG
CAGCAGTTCTCACCGGATCCCGATGACAGCATCGAGAACTGCGGCGGCGAGAATGGAGCTCCCCTGATGA
CGCCCTGCAAGAGCGCCATTATCCTGGATGCCCAGACGAGCACCACGCTTAAGTGCGAGGACGACGAGCC
GATGAGCTGGTGGACCAGTCAATCGCAATATGTGCATGTGAAGTCCTTCGATAATACGGAGGATCCGGCT
CGACCATTCGGAACTAGCCTGCATCTCATCGAAGTGACGGCTGACTATGTGGCAGCCTACTATTGCGTGA
AGACTTCGAAATTCAGTCAGATCGCCAAGGAGGAGCAGTCGGACGAGGCGATGATCGAATTGGTTAATCA
AGGATACGCCAGCTCCATCTACGTGTACGTGAATGATCCGGACACTAAGCTGGTCGATAGTCATAACGTG
GTGACGGCACGCCAGTATACCGACGTAGTCATACCCTGTAAACCAGCCATGCCGGACACAGAGGTGCTGC
TAGAGACCAGTAATGGAGAAG
>dme_circ_0001829_junction_seq
GTGCTGCTAGAGACCAGTAATGGAGAAGTGCAACGCTAAGTGAGCAAAGTGTCTTT
>dme_circ_0001830
GTTGTGTTATCATATGGGTGTTGTAGCTTAAAAGATCCTCCTCCTCCTCCAAAGCTCAGGCCTGTTGCCT
AGCCTCGTTGGCCTCGTAGGCCTCCACCTCGGCGATGTTGGCCAGGTCGTACTCGATGACGCCCTTGCCC
ATGAACTCGGCGTACTTGGCTGACTCCGGCAGCGGATCAGCCGATCTCTTCGAGACTGTAATGGGTCGAG
GAATGGGAATGGATCAGTAAGTTGCTGGCACCTGTAAGACATTGCTACTCACCGCCTGAGACGAACTTCT
CCATCATGTAGACCTTCATATCGAAGCACTCGTTGGCAATCCGCTCCGAGATGAGCGAGACATCGGTGAT
GCGGGGCATGACCACACGCATCTGGCGCCGCACGCGCTCCGTATCTATG
>dme_circ_0001830_junction_seq

CTGGCGCCGCACGCGCTCCGTATCTATGGTTGTGTTATCATATGGGTGTTGTAGCT
>dme_circ_0001831
GGAGGATCTTTTAAGCTACAACACCCATATGATAACACAACCTAAACAAATTATTACTTAGAATCGATTT
ATACATACACAGAGCCATGCCTACAAGTCTAGATCTGGTTACCACTGGTTAGCAGGACACACACCCACTC
CGGGTGGATATATCATCATTGGCCAAGCGA
>dme_circ_0001831_junction_seq
GGTGGATATATCATCATTGGCCAAGCGAGGAGGATCTTTTAAGCTACAACACCCAT
>dme_circ_0001832
CTCCGAATAGCTATTGCCCTGAAAAGGTCACGCAATCAAAAAAGAATGCGCCACGCTACACTTTCGGTAG
ACGAACTAAAATCGAACACGATCAGGGCACACCAGGTAATAAATCCCAGCCTCCCCCCCAAGTAACTGTA
TCTGCATCTGTAATTGTATCCGTATCTACCCCAATCGAGGCACCACTTGCTCAAGCGTACCCAATTGGAC
AGTATCTATAGCGCTTTCCTTTTCTATTCCCGTGACTTTTGGCCAACTTAATACTGATATCCCATCCCAT
CCGCAGCTCCTGGCGCATATTGTCCAGAGAAGGTGAAGCTCAACAAGACGCCAGAGTTCAGTTTCGGCAT
CAAGCACCATGAACAGCGACCAGATTACACTCCAGGTTTGTAAACAGTATGCCTTGTAGGTTTGAGCCAC
GTTAATATAGCTGCTTGATGCTTTCACCACGACGACACTTTCATTAGCGCCAGGCACCTACAAGCCCGAG
CAGGTTGTCCTCGAACACATTCCCGCCTACAGCTTTGGCTTGAAGACCAAACACATCCAGGTCAGCGATA
CTCCAGGTAAGGCTTCCCGCTCAGCTGGGCTGTAGTAACTTGGCTTATAGCGCACACTTTTATGTCCTTA
TGACCAGCACCCGGTGCCTACAGCCCCGAAAAGTCAAGGTTGCACTCCGCACCCGCTTACTCAATCGCCG
GAAAGGCTTCGCAGGACGTGGTCGATTGCACACCTGGTGAGTTCTAGAATCGCATTTCAATGGATCTTAG
TCTTAGCGTGCGACCCGCACACCAAAACCCAACTCTTTTTACACCTTATCTTCACCCAATCTCTTGCAGC
ACCCGGAGCATACGAACCCGAGAAGTGCGTCCTGAGCAGGACGCCTGCCTTCAGCTTTGGCCACCGTGCC
GAGCTGGCCAAGTCGAGTGACACGCCAGCGCCGGGCACCTACAATCCGGAGAAGGTGCGGCTGGACCACA
CGCCCGCATTCACCCTGTCCGGACGCCCAGAGACGAGGTCCGTGAGCGAGACTCCGGCGCCCGGTTCGTA
TGCCCCGGAAAAGTATCGCAACGATCGAACGCCGGCCTTCACCTTCGGCGGTAAGCACGAGCAGCGACTC
GAGAGCTCcactccggcaccgggcgactactgtcccgaaaaagtgcggcacgatcacaatcccgctttct
cattcgccggtcgccacgatctgcacaagccaagcgacacaccagcaccgggagcctacgacaccgagaa
agtccggcaagatcacaatccggccttctcctttgccgggcgccacgatctgcacaaacccagtgaaaca
cccgctccgggcgcctactccccggagaaggtgcgacaagatcacaatcctgccttctcGATGGCCGGGA
AACACAGTCAAAAGGTGACCAGCGATAGCCCGGCTCCTGGGGACTATTGTCCCGAGAAGGTCCGCTTGGA
TCACACTCCCGCCTACAGTTTTGGCGTTAAGAGTGATCCTAAAGTAGAGAATAACACTCCGGCCCCGGGT
GATTATCATCCCGAAAAAGTTAGGCAGGATCACAATCCCGCATATTCGTTTGCCGGTCGTCACGACCTTC
AGAAACCAGACAACACTCCCGCTCCTGGAGCCTACTTCCCGGAGAAAGTGAGACTGGACCACAATCCAGC
TTTCTCAATGGCCGGCAAGTATGATGCCAAGGTTACCaacgatacacctgctcctggtgattatagcccc
gagaaagttaggcaagatactaatccagcgtactcctttgctggccgccatgatctccacaagccaagcg
agactcccgctccaggtgcttactcgccggagaaagtgagattagatcacaatccagctttcacgatggc
tggcaagcacgatcagaagattttaaatggcactccggcaccaggagactatagtcccgaaaagtgcaga
ttagaccacacacctgcttacagttttgggggaaagaatgatcctaaagtagagaataacactccggcac
caggagattaccatccggagaaggtgaggttggaccacaatccagcgtactcctttgccggtcgtcacga
ccttcaaaagccaagtgatactcctgctccaggagcctactctcccgagaaggtacgtcaagatcataac
ccagcattttcaatggcaggcaagccagttgaaaagctaactaatggtactcccgctcccggagactaca
gcccggagaaagtacgcttaaatcatactccagcctacactttcggtggcaaaaacgatccgaaagtaga
gaacaatactcccgctcctggggattatcatcccgaaaaggtcaggcaagatcataatccagctttctcc
tttgctggtcgtcatgatcttcacaaaccgaatgatactcccgcccctggtgcctattgtccggaaaagg
tccgtcaggatcataatcctgccttctcaatggcgggtaagtacacccaaaaggtagccaacgacactcc

agctccgggcgattacagtccggaaaaggtgcgtctagatcacacacctgcctatacttttgggggaaag
aacgatcccaaagttgagagcaacaccccagctccaggtgattatcatcccgagaaagttaggcaagatc
ataacccagcattctctttcgctggccgtcataatcttcaaaaacctagcgacactccagcacctggagc
ctattctcctgaaaaagtcagacaggatcataatcctgccttcacaatggccggaaagcatgaccctaag
gtttccaatgatactcctgcccctggcgactacagtcccgaaaaggtccgtctagattacacaccatcct
ttacctttgctggtaaaaacaatcctaaagtagaaaacgataccccagctccaggtgactatcatcccga
gaaagttagacaagatcacaatcccgccttctccttcgccggtcgtcacgatcttcataagcccagcgac
actcctgctccaggagcatactcaccggagaaggtgaggcaagatcacaatcccgcattctcaatggctg
gtaaatacattgagaagattaccaatggcaccccagctcctggtgactactgtcccgaaaaggttcgctt
agatcacactccagcttttacatttgggggcaagaatgatcccaaggtggagaacaatacaccggcacca
ggagattatcaccctgaaaaggttaggcaagatcatgttcccgcattttcctttgccggccgtcatgatc
tccacaagcccaatgagactcctgccccccggagcttactcccccgagaaagttaggcaggaccataatcc
tgcttttacaatggccggcaagcatgatccaaaggtaacccatgacactccagctcccggggattaccat
cccgagaaagttaggctagatcacaATCCCGCCTTCTCCTTCGCCGGTCGCCATGACCTGCATAAGCCCA
GCGAAACGCCCGCACCCGGAGACTACTTCCCGGAGAAGGTCAGGCAGGACTTCAATCCGGCGTTCACCTT
CGCCGGCAAACACGATCCGAGATCACTGAGTGAATCCCCCGCTCCCGGCGACTACAGCCCCGAAAAGGTG
CGACTGGACCATGCACCCGCTTTCAGCTTTGGCGGCAAGCACGACCCCAAGACGGAGCACCTTCATCCCG
CGCCATGCGACTACGCCCCCGAGAAAGTTCGTCTCGACCATACACCCGCCTACACCATTGCAGGTCGTCC
AGCCGCCGATCACGTGAGCCAGACGCCGGCACCCTGCGATTACCATCCGGAGCAGTGCCAGGTGGACAGC
ACACCAGCGTTCACCTTCGGCATGCGACTGGGAAGGGAGCGCATATCCGATACGCCAGGCAAGTAACTTC
CAAAAGGAAGCCGCATTAGGATTAGTTCCTTAAAGGATGTCTAGGGTTATATCCTCAATTATCCTAACGA
GCATCAGTACCATTGCAGCACCCTCCGCCTATGAGCCGGAGAAGCACTCACTACACTCCACACCCGCCTA
CAGCTTTGGTACCAAGTCCGATATCCGCGTGACCACCGATGCTCCAGG
>dme_circ_0001832_junction_seq
ATATCCGCGTGACCACCGATGCTCCAGGCTCCGAATAGCTATTGCCCTGAAAAGGT
>dme_circ_0001833
gtatcattcggtttgtgaagatcatgacgaccagcaaaggagaaagctggattatgatcttgcctgacct
tttcgggatgataatccccaggagcgggagtattgttctctactttcggatcgtttttgccaccgaaagt
gtaggctgga
>dme_circ_0001833_junction_seq
gtttttgccaccgaaagtgtaggctggagtatcattcggtttgtgaagatcatgac
>dme_circ_0001834
actatagtcccgaaaagtgcagattagaccacacacctgcttacagttttgggggaaagaatgatcctaa
agtagagaataacactccggcaccaggagattaccatccggagaaggtgaggttggaccacaatccagcg
tactcctttgccggtcgtcacgaccttcaaaagccaagtgatactcctgctccaggagcctactctcccg
agaaggtacg
>dme_circ_0001834_junction_seq
caggagcctactctcccgagaaggtacgactatagtcccgaaaagtgcagattaga
>dme_circ_0001835
GTCGAGTTCAAAAACAAGAACAAGGATCAGGGACCCTGCGCCCGCGTGCGTAAGATTCGTCCAATTAACA
TGGGCGGCAACGTGGAGCAGCGTCCTTGGACTCCAACTGTTAGGATCGGCAAGATCGCCGCCGAGACCAC
CAATCCGGGTCCAGCCACTGTCCAACTTCCAACCTTGATAGGGCAGCAAAGTTCCCGACTCCAAGAAGAA
GGCCGCACCCTCGTACTCCTTTGGCCACAAGTTGGGCGGAAAGTATGACACCTCCGGACCGGGTCCGGCG
CAGTACAATGTTACGGGTATGCGAGCCAAGGGTCGGGATTATCCTCGAGCAGCCACTCTGCAGAGCAGAC

CCAAGGAACTAACACGCTTCTCGAATCCCGGACCTGGGGAATACGATGTGGTGCCGGCGGCCAAGGCGGT
GATCGATGCCACGCCCAAATACACTTTTGGCCAACGGCCGGTCGCGTTGAAAACCTTCCAGATACCAGG
>dme_circ_0001835_junction_seq
TCGCGTTGAAAACCTTCCAGATACCAGGGTCGAGTTCAAAAACAAGAACAAGGATC
>dme_circ_0001836
GAGGAGAAGCGCGTGTAGTTGAGCACTTCGGGCGGGGCCCACTTGATGGGGAACTTGGTTCCGCCAGAGC
TGGTATACTGATCGTCGAGAACGTATCGGGCCAATCCAAAGTCGGCCACTTTAACGACATTCTCGGAACC
CACGAGACAGTTGCGGGCAGCCAGATCCCG
>dme_circ_0001836_junction_seq
CGAGACAGTTGCGGGCAGCCAGATCCCGGAGGAGAAGCGCGTGTAGTTGAGCACTT
>dme_circ_0001837
GACCCACGCCACTTGCCGCGCCGCACAACACCAAACTGTCCCGATCCAAGCTCCTCCATCAGCATCAGTT
CCATCGGATGGATCTCCCATTTGTCGTGGGACAATCCCGCCGTCGGTGGAACTGGGCGATCGCAGGGCGA
GGATTTGAGTCGGCAAGCCAAACCCCCAGAGTTGTGGCGATG
>dme_circ_0001837_junction_seq
AAGCCAAACCCCCAGAGTTGTGGCGATGGACCCACGCCACTTGCCGCGCCGCACAA
>dme_circ_0001838
CGGAGACGCGAACGTGGTCGCATTCATTTGAAGGGCGTTCGCCTGGTGGAGGAGGCGACGGTCAGTGGCG
AGGGCGGCGATCCCTTTGCACCGGATGTGAGTAGCCAGAAATGGTTTGGGAAAATATGTTTCATTAACAA
ACGTTCAAATCACCAGGGTTATCCGTTCCAAGTGGGCTACTGTGAGATCTCTGCGTCGGCCAATTCGCAT
CAGCTGGAGAACGGCAACGGCGGTGGTAGTGGCGTTGGAATCGAGGGTCAGCAGAGTGGACGAGCGGTGC
CACAGTACACACTCTACGTGATCGCCAACAGCGAAAAGGAGCGCAGCGAGTGGATCCGCGCCATCCGCCA
AGGTGAGCCCCCATTGACTCGATTGATGATGACCCAGTAAACACAATGGCAAGGGAGCCAGCCAAACTGC
AGCTGTTTTTGGCCAGATCAATCGAAATCGATAAGAAGTAGTCGTGTTCAGTTCAAGAAGCGCCTGTAAA
TTGGGAAAAAGTAACTTAAAGCAATCAAGAAGGTTTAAGTGGTTAAAGGCAGATGGATTTATCAGTTAGG
GAGACATGGATATAACATCATGGAAATAGTGTATTCATCAAAAATTGACAATAAATAATATTATTCATTC
CCTAATCTTTATTACTTATATTCATTGATTTTCAGTTTTGTGAGGATAGTAATACTCCAAAATCGTATCG
CTATCATCCGGGCTTGTGGTCTGGAAAGAAGTGGAGCTGCTGCAAGGGATTATCTCGAACCACATTTGGT
TGCAGAGCAGCCGCCCATTGGCGTGAGGCCAACAATAATCCAAG
>dme_circ_0001838_junction_seq
ATTGGCGTGAGGCCAACAATAATCCAAGCGGAGACGCGAACGTGGTCGCATTCATT
>dme_circ_0001839
ACACTTTTTCTTGTCAAGATGATGGGCACTAAGCATCGAAACTCGCATGTCAATGGCAGCATTAAGTCAT
CATCATCGCTGCGATCGAGCAGCAAATCCTTTCAAGCAAAAATGGATCTGATGTCCGAAAGGCTGTACGA
TGTCGTGAAAAGTGGATCGATGGTGAAAAGGGCTCAAAACAAGAAGCGTTTCACCCCCGTGAATTACAAA
CATCGATGGTTTGAGTTGACCAAGCGAACTTTCAGCTACTTCGATGTGGAAAATGTGGAGGGTGAGTCAT
GCAAATTGCTGTACGCATTTCGGCCAACAGGTTTTATTTGGCCAAAACTAATTCAGCCAAAATAATGAAT
CGACATAATTTAAGCTAAATTAGCGAACACAAAATGAGAATGTAGATAACAGCGaaaaaaaacaaaaaTAT
TATGAGAAGCTGCTACGTATTTTTATTCGATTCTGATCGGTAGAATCAATAAATAGAGCTTATCTATTGG
TAGATAATATGAAAATAATTCATGGATAATCTGCAAATGGTGttatacatactatagcaattatacacca
tactatagcaattaAGTAATTGCTAACTAATACTTCAAAAGATTCATATCTGTATGCTACATTGTATTTT
GTAAAAATAATATGAATACGCTACTCAAAATGCACATCGGAGTTAGTCAGTTCCGGCTGACAATGAAAGA
TCTCAGGATTCGAAGGACCGGCAACTGGGCCCCGCTTTGGAATTGGCCAAAATGGTGAATTGAGGAGGGTC
CTTTCATTATGCATGTTCAAGTGGGCGTCTCATCTTGTCGTCGTGGCTACGTCATTTATTGCGTCGCTAT

TACGGCACCTAACGGGCTCGCAAAATTTTCACCATCCGGCGCCCGGGAAACTCAGCTTACTCTGCTCCGG
CGACCGGCTGCATTCGTTTCAAAACAAATGTGGTTTTCGGAGGAAAAAAGTCTCCAGTGCGCTCGGCTTG
GAACGTATATGTACCACCGTTCAATCCAGTCTCCGGCTGGAAAGTAAGCACTGTGGGGGTTGGAAAATAC
ACACACAAAAGCAGCTGTTAGCCGGCACAATGTGTGTGGAAACCCACCCTCGACAGTCCTTGGGTGTAGA
CTACGGTGTCGTCCTATCCCGTTATGCCGTTCCAATTGGAAGCGGAGTTTCCCTTGAGTTTCCCTCTGAA
TATCCTGGATGAAGTTCAGTAACCGTGGCAGGAGTTTCTATCCTGGCCTCGTCGCGTGTGGACGTTCGTT
AGATTTTCGCATTTAATATTTTATTAAGCCCAAAGACTGTGTTTTAGTTGCTTaaaaaaaaTATATCTGA
TTTGCATGACATTTACGAATAAACACAAGTGTGCGTTAAAGTTTCGAGTAACGTGTTTATTTGCAATACT
TTTCAAATTACAAACATTTTTCTAATGCGCCGGCCGCTACCCTTGAAAATTCGGTTGGTTTTCAGCGGTG
AAGTGAAACCACCCTTATTTACCCTCTTACTAACTGTATTTAAACAAACTTCTATCTCTACTAAATCATA
TAAATGATTCGAAAAGTGCAGTGCATCCTACAAAGATAGCCGAAAATAGGAAAACACCCAAATAAAACAT
TTAAATCGCAAATCCTTTCCATGGCGTCGTTGGAAAATTTTTTGCGAATCTAACCGaaaaaaaaGAGAGA
AGAAAACGCCCCACCCAAGGCAAAAGTTTTTCAAGTGTATCCAAGCGGCCTTtgtgtgcgtgcgtggtgc
gtttgtgtgtgtgcgtgagtcctagtgttgttgtcgCTGTTGTTGTTGCTTTTGCTACTTTTGCCGCACC
CCGAATTTCGAATTTCGTGTGCCTGTGCCTAAAATTAAAAGGATTATCCTGACGCCCAAAAGGACGCATA
TTTAGTCGTAGGTAAGCATTGAACTGAAAGGGAGAAATGCCCACGGGAGTTACTAGTTTTATGTGCCAAG
TTTGAATATCGTAATTGAATTAGATGGGGTTTTCCTTGTAGGGAATACGCCTAAAATTATGCAATAAATA
TATACATTTTATGCCTGATTGAAATTTATGGAAATTGaaaaaaaaaGTTGATTACATTATATAAACACAA
CACGAAGAAAAACTTCTCTGATAAAACATCATTACTTCATTAAAATCAAATGATAGATATATCATTTTAA
GTATTTTCCATCTACTTGAACGTCAAAGGGACCTTCCTAAGCTCCTTTCAAATTTCAGGAGACACTGTCG
GTTGTCAATCACTCCTGTCACTCGATCCCATCATTTTCCTATACATTTCGCTGGTTTTCCTTTGGCACTT
TTCTTTTGTCCCTATTTTAAGGGCTGCTGCGGAGTTCGGGGGTTCGATGAACTCGTGTAGAAAATATGTG
CAATTAATTGATTGATTCGCGGTGGCTTCTTTGTGTGAGCTCCCACCCACCCCATTTCAACTAAAGTCGA
GTATGAATCCAGCTGCCTGGCACTCTTCAGTCTGTCCGTCTTCCTGCCTGGTTGTAATCCAATGTCCGAG
CCTGTCATGTTTATTGGGGTCAGACCCTTTGGACGCAGGGCGGGCTGATTAATGGCTAGCCAGCAACAAG
CTGAGCCGAACAACTCATCCGAGTAGCCAACCAGTCATTGCACCTATACACTAGCAAAAAACCGAGCAAG
TAAGTTATTTGTTTTAAAGTGGATCAATATCTAAGTCAAAAAGGCAAATCTGTAAATGATTTCATTTTCA
TCACACAAATCCCTGAGCTTTAGTTTTAGATCACTTTCTTGCTACTTTCGTTGTTTGTTTAATGTTCAAT
TCATTGCTTAATTAAAAAGTAATGTACTAGAATTTTTGTTTGTATTTTTCCTGATTGGTGGGTATTGCAC
TCCCGCCTGGTCTTCATCATCATCAGCAGCACCTTCATTGTCGTAGTTGTGTCATTAACCTGTCATAAAA
ATCGCAGCTTGGGCCAGGTCCCAGTTACAGTCGCAGTCCCACGAGTGAAGAGTTGGGCAGGCTGGATACG
GGGGGTCTGGTGGTCTGGGGGCTGTAGAGCCTAACCATCCTCTATGGCTCACCGCCGTGGTATGCTAATT
GAAGCTGGACTTTTTGTTTGCCAACACTTTGCAGTTCTCTACACTCGCCGCTCCATGGCAATGCGTTCGT
TCTGCTCTCAAATACATTTCGCACAGATTAAGGCTGTAAATAACAATTTTCCAAAGGAGGCGGGTCGCCA
CGCAATCCGCGTTAAATGGGGAAATGTGCTTGGGGGCGAAGTGTGAATAACGAATGTGGGAATGCACTTT
CCATCAGAGATGTCAAGAAAATTAGAAGCAAGCTAAGCAATAATATAGTTAACAAACTAGCAATATATAA
ATCATATGACAACGGAGTTATTAAAAACAAACCTTACAATGATATGTTCCATTTAACACTTTTTATCTGA
ACTTAAAGACATTTTTCCTGGCTACCAATGGCTGGATGTATTTATAGCATCGAAAGATGAAAGATATAAT
GTGCATATAATATACCCTTTCCCCATATCTATTTTAGAGCCCATGGCCACTGCCCTTTGGCTAATGGAGG
TGCCATGTGGCATATGGTTGTTGGGCTGGAGTTCCTTGCCACCTTGGCTCgtaaatggaaatgctgtaaa
tgtaaatgctTTCACCATTATTGATGTTTGCCTGTGAGAATGTGCGAGGACTAATCCTCCTGTCCGGTAG
CCGGCAACGCAGCGAAAAACAttttttttttacgacttttcggggagtttttatctttttACGGCCTGCAC
TCAAAAGTATCTTTTGCAGTATGAGAACGCGGCCTAGAGCTGCTGATCAATCTACCACAATTGTGAGCCA
TGCCCAATTAGACGTTCAAATTTATCACTTTACGGGCGGGCAACttttttttttttgtttttttttttC

ATATTCTACCCCGTTTTTTAAGCCGCTTCGGTGAAAGAAAAATATGTGTCTGTCGTTGGGGATTATTACT
CATCGCATGCATTTTAAAATTCTTCACGAATTGACACCTTTGGGAGGCCAGCGCCGAATGGCTTAAAGCT
GATTTCGTAGAATTACAAAACCCGCATCGCTCGTCTTCCCCCTTCTCTGGCTTCACCCCCTCCACCATCT
CTACCAATGCTGACAAATTAGCGAGCGAAGCTGAATATTTTTAAATATGTTGGGTGGTCCCGTTTCCAAT
CGCCGTCTTTATGCCTTTATAAATCTTCGCGCTGCATTTTATTTAACACGAAATTGGCGCCAAAGCCAAA
ATGTTTCTGATAAGCCACGTCTTCCGTTGGCTTGGGGTTGGGGGGTTTGCAACTTGACTCTGACATATGT
TAGCCTTCATTTCAGATCCGAAAATGATCGGATGGCATGACTCCTGGTTGCTCCTGGACCTTGGAACACA
ACCTGACTGCTCTTGCGGTTGCCAGTTGCAGTGGCACATGACCGCAGGCTAAATGACCGAGAAGTTTAAG
CCTGATTACAGTTACTTGTTTAAGGATGCTACCGCTTGATGTGCAGGGTATATAAGGATAACAACTCTTT
TTATAACCTTGAACGTAATGTGATATTTACTCAAGTTTTTGTACAAGCAATCAATTTAGGAGGGTATTAA
AAATTCTTGGAAACAATAATCTCAAATTTGATTCCCTCTTTCCAAGTGACAGCCCGACATATGCTGACTA
ATTACAAGCACTTCTCGAACCATTTACATATGCACATATGCTCAATACATTTCAATTGTGCAGAGACCAA
GCCCTTTTCGTAAGCACTTGTAACAATTATGAATCACAATCGAAAAGGTAACACTCACTCCAGCTACGGC
GGACTCGGATTCGGATTCGAACTGGAACTCGAACCGAGCATGATGGTAATAAAATTATAAGGAAAACCTG
TTGGACAATTGGTGGTCGGTTGGTACATGGTTTGCCTATTAACGGATTTAAGACCGCGAGATGATGATTC
ATTCGATGAATGGTGGAGttttttttttttttGGTGTTATCCGTCGTGTCTTGAATGCCAAATGAAGGTT
TTGCAGCGTCTAATGAGTTGTAAATCCCAGAAGGAAGAGCCAGCAGCGGACCTAGGAATATATGTATATA
TATTTCATACCTTTGTGCCTGGGACTCCAGACGTCGCATTGTCTGTTGAAGCTTAAGCTTTTCATGCTTC
ATTATGCTGCCTTGAATGAATCTCTCCTATTCGTCCTCCGGTGGCTGCCTTTGTTTTCCTTCGCCCACGA
GCTGACATTGAGTTATTACATCCAGACAGGGCAGACAACCAAGTTGGCCGTAAACATAAAGCCCAGTTCC
AATCAGTTTGCTCCCACAGCAAGTGGGCCTAAGCCTAAATACCTTTTACGCCTTATTATCGGCCAGAGAC
AATAAACCATCGTTTAATTGGAATTAAGTTCGAAAATAAACGCCTTTCTATGGAAAAACTAAAAGTCTTA
AGCGTTTTTATTGGCATTATCGCTTTAGCTATTTCCGCTGTTAATTTAAAGCTCACGAGCCATCGAATCA
AATATCAAAAATAAGAAAAAACACCGCAGACCCCGAAAAAAACAAACCGATCCCCAATAATTAGAAATCA
AAGCCGGTTTCATCCAAATGTGTGAGCATGTGTGGGACAGTGCGGTTCGTTATCATAAATACTAAATAAG
CTGGAAAATAATAAAATAAATAAGTTAAGCTTAGACAAGTTTTATAGTTGTTTCAATGAATTAAGTCAAG
ATATTAATTATGCAGtttttttttGTATTCTAAACCAAGTTGCGTACATTTTCGAAACGCACTGTATCTG
AAAATGATTCCGAAAACTTGTCTACTTTCGTATTCAATAGTGTTATCATGAGAGATGGTCTGAATGGCCA
GACGCAGACCGCAAACGCGACTCCGGTCGCCGAGTTCCAGATTGTCGAGAGCTCGCTCGTAACTTCCCCC
ATTTCCCCTCCTTTGTGGTTGTTGATGATTGATCTTATCGCGGCCCGCTCGCCCCGTGAGTCATCATAAA
AAACACATATTTCTCTTCTTCAGCAGCCGCAGCAGCATCATCTGTAGCCGCCTCAGCAGCGATCGCCATA
AGTTGTTAGCTGTTTTGTACTTTATGGCCTTTTTGGCGGCGAAATAGGGGCGGTGGATCGCAATACGGAG
AGGGGGATGGGGTTAGGGGGGCAAGGATGGGTTAGCTTCATAACTCAATGTGGGTTGTCGTTTCGGTTCT
GTTTAAGCTGCGTGTGATACTTACTTTCCTCCGGtttttttttttttttttGGCCCACACAAAGGCCGTT
GCTAGAGTACTATGGAACGAAACGTAAACTCGGACAGTaaaaaaaaaaTATGCTCTCGGACATGTGGGAA
AAAATGTAATTACAGCGAATGTTCAAAATTACTTTTTCAACTCGGTGAACATTTACGACTCGGCATAAAT
CATTAACACAAGGAGCTTACGTTTTCAATAGCTTTGTGCCATTTTGGCAAAGTAGGAAAGCTATTAAATG
TACTTATTAACTTACTTTCAACACAAAAATCGATTGCTATCTTAGGCAATTAGGATTGTAACAAACAAAA
ATATACAAATTTGTTTTCTTACTTTTGTTGCAATTGAAATTAGCCATCTTTACCACTGTTCATCAAGCCA
TTCTTATTAAATGCGTAACTGTAAAATCATGCTAGCAATTGTCCAAACAACAACAAAATGGGCAACAAAA
TATTCACACACCGCCTGAAAAATGTCCAAAATGATAAATGACTTTCGCCGTTTTGCCGCTTTTGACTCCA
ACTAATTTCCGCTTCCAGTGCAATTTCGCCAAATCCAAATTCCCTCGAATTTGTCGTGTAAAAGATCCCG
ATATGAAAATGCAGCCGGCAACTTAACACttttttttGTACCGACTTTTACCGACTGCAGCAGCATAGGA
TCACATGCAGGCATGTCCAGTGATTCAATCTGCTTGGAATCCACCCCATTTCCGGTTTCCATTACAGTTT

CCCAGACAGCTCTTTCACCCGCCATTGCGGAGTGGTCAACCGTCGTGGCTCTTTCTTTTTCCATTTTTAG
TTTTGGGAGGCGTTTCGCAGTCATCGGTCAGGAATTGGCGCAGACACTGCGTTTCTGTCAGAAGGAGGAG
GATTGCAGAGTTTCGACCCAGACAAATTGCCCCGGCGGCCGTTGCTTGTGTTCACTGCTCCAGTATGCGG
TTGCGCCAGTCCAAGCTACAGTGTTACCCAGATAAAAGGACCTTTATGGCAAAACGTAGCCAAACAACTT
TGGTCTTGTTGACTCTTACTCAGTGCATTTATTATTGGGAGTTCTTTCTTTCACCATTTGTATCTTTAAT
TATTCGTTTTATCGAAGCTTCACGTTAAATCAAAGCTTAACGCCCTCTTCCTGGCCTAATTATGTTGTTG
CACATGCACATTTTTATGAATGAATCACAGCCTCCAGCTCTTTCCCACACGAATGAAAAATATTTATAAA
AATGAATTAGTCTGCGCTGaaaaaagttaaaaagtggaaaaaaaTGATGTTTCGCCCGAGCAGGGACTTG
AACCCTGGACCCTTGGATTAAAAGTCCAATGCTCTACCGACTGAGCTACCCGGGCAGATGGCAACAGGCT
CGCGAAAAATGCAATAAGAGCAAAGCTTGCCAAAATCAAGAGAACAATTCTGGCGCTCTGCCATTCTGTA
CTCTCTCTTTAAGTTGTTTGTTCTATCATTAATTGTAAAAAGTATTACTTTTCTCTACATGTATTATATT
TAAAACTTATTTAAATACTTTAGTATGTCCTTTATAAAGCAGGAATTTGATCATTACTTTATTTCGTTTC
TATTTAAAGAATTGATGTTTTTACACTTGTATGCATATGCATAATTGCACTGCTGTATTTTTAGAAAAAT
TCAAACAAGCGAATATTATAACCGAATAAAAATCTAATTACCTGCATCGAAATTCCGGCATTGCTTAATC
AGTAATATTATTCTAGGATTTTATGAAAAGCCTAGACCATGAAATTTTTAAATGAGCTCCGCTCAAAATT
GAATTCGAAAATTAATCCGATTTCATATTGATATCCCAATTAGAGTTCCCATTCAGGACAGATGGAAAAG
CAAATGAAATGCAGTGAACGACCTCCTCAACGATTCGCCGATGTCTGTGAATGGGATGCGGCTGGAGAAA
TGCATTTATCAAAACATAAAAATAAACACGAATTGCTTCGAAAATAGACAAAGGGCGCAGCGACCAAATG
TTGGCCTCAAAAATAACGAAAAGAAGGAATTTGTATGTGCATATATACATATATATTTAAACAGCTAACC
AAGCAGAGGAAAATGCTTGAGAATGTCCGTGCGTAGGGGTATACATACATATTTATGTACATACATGTGT
ACACTTTCCACTTTGGCTTTTCCTTCGCTTTCCACAGCTGTCAATGAAATACCCTTTCCTCTTGCGTGTA
ATTGCTTGATATTTGGGTAGATTTCGAGGGATAGGAATTTTTGGAATTGGGGGTTCTCCACGCTGACCTT
ATGACTGACAATTTCCTTAGCACATTTATCTTTTTGGATCCCCTGCTATCGCACTTTGTTCCCCACACTC
ACTTTTCAATTATTCCTCTTTTCCTTTGTCTCCGCGGCAGACAAGTTTTCCATATGTGTGTTTGCTTAAT
ACGTTTTAAGCGCTTTTTAAGCGTTCAAGTCACGATTTTCATATTTATAGAGTTTGTCGGGGAAAAGCGG
TGGAAAGCgggggggggggATCGCTGATTGATCAGCTGTATGTATGTATATGTGGATGTGCAAGCTCTTC
TTGATGCCTATCTGCATTCGATTTTTCTTCTCCACTCTGTCGTTGTATTCTTTGCTTTTCTATAAATTTA
ATTATTAGGGTTGCCCGAAGACAACTTTGAACTTTGTCTTATCGTTAATTACATCGCCGACGCTTTTTCG
TTTTTCCCAAGATTTCCATGGTTATTTCTTTCATTCTGCGACCCAATGATGGATCACTCCCATGTGCTGG
GGAATTCCAGCGAAATGGCATTTCATGCTTTGGTGACTCTTCTCAGGACGTTTTAAGTTCACTAAAGTTT
AACAAAACATCTAAAAAATAAAAGTAATTCACAAACACAATAATTTATTAGTGAAATTGTTCAAAGATAA
GATATTAATACTCATTGTTCAAAatatatatattatgatatgatatcattctgatatgatatgatacACA
TGTAATTATAAATATTGCATATTAAACCAAATATTGTTTTAACTCCCACTCTTTTAAATACCATACCATA
TTTTCCACAATACTTAATCTGCATAGATATAATTTTCAATGTGATGTACGGCTTGAGTTTAATGCAAATT
TAGCGATAAAACCACGCAGAGGAAGTAAAGTTTTTCTGTTGACAGGAAGAGGCGAAGACAGCCGACATAG
ATGAACATGGAATGATTTAGCACAAATTACCCACACTAAAATTAATTACTTCGTCTTGACCAGAAAAGAA
CAATACAGAACATCCGAGTCAACCAACCATGTCCTTTTGATGGCTGGGAACGAGCACCGAAAGACCAGCG
AAAACAACTAAAACTATGGCATAGGAAAATTTTCGTTTTCATTGGAAAAATGCATAAATTTTCCACGAAA
CTGGGGAATGAGATGCAAGGCAAGGAAACAACTTGGCTGTCTTTGTTTACATACTTCTTTTAACTGAAAT
TTATCGCCCGTCGCCGCTCCTACGCGTGAAATTGTAATAATTACGGaaaaaaaCCGACTAGGTTGGGGA
AATTGAAAAACCATTACGCCGAGGCATTTTGTATGCCCGGTTTTCTCTTTATTACAGTTATAATTTATCT
GAGGCGATTTTTCAATCGCACTCCACATGCGGTCAGTTTAAGAAGCGCTCCTTCCCAGTTGCAATCAATC
ATGGGAATTGTCAGGGGGTTCCCCCACTCTATAAAAAatatatatatatataCATGTAGTTTCCCTCGAT
CAATCGAAGGGAACATGTGTGTACACAGTACATATCTTGCCCGATTCGTTACGCGGCTCAAAGTCGAAAC

GAAATGGCATTTTAACATGCCACATGAGGCAGTTGGGTTTTCATTAATCAACATTGTAAAGGTTCCACTC
TTCAACCTCGAGGATGCACTTGAGTGGGCGATTTAGATACAGAGTGGGAGGGCATACATTCAACTCTGTC
CAACTGTCCAGTGGAGTGCATCTGGCATTTTAATCGGGGTTATATCATTCTAAATTTAAGAGCACTTCTA
GTCGACATCGTGGGAATATCAATTTTGGGTAGTGTTTCTACCATAGATATAAATTAACTTCATTTGCAAT
AGGTTTTATTTTATAAAACGAGCTTGAATATCATTGCGAGTTTATTAAAATCTGCAGTATAATTCTATTG
GATGTGTATATATATTAGCTTCTTATTTTAATTACATTTTCCACTTATACTCAACTCACCTCATTCTTCA
ATTATCGTTTCAGTTTCCACGCTCTCTCTTTCTGCTAACCCACTCCACTTTGACCCTCGGCAATGGCAAT
TTTCACTTAATCGTTTTTCTTTATGCTGCTGGTGCTCAATTTTCCGGGACTTTGTTGCGTTTTCCTTCGG
CGGCTCCCCTATTTTTCATTATATTTCGTATTTTCTCCTAGTTTCTTTTATGTTTTGttttttttttttGC
TTACCTGAGTTGCATTTTGCTTTGACAGCTCTCTCAATTATGCACAGGTGATTGCCGTGCCATTAGGACG
CTCTTTTCCCAACCCTCCCCGTTTTTCCCTTTAACAATAGAATTAAATAAGGATGCATAGTGGTGCCTTT
ACAGGTGCGTTTCGTGTTTCAACCTGTTGTCCTTTTTGGCATATTGTGTTACATTTGGTGGTCCCTCGAC
GGCATTTTATTCACTTGCTTCCGGAAATTCCTTTGGCATTTTCAGAGTGAAGTAAATTATGCAAGAAAAA
TGCGTTTTTAACAAGCAGAGAATAATTAAATATAATATTCTAATGTATTAAAGAATAAGCCCAAAGGGAG
TGGGTTCCTCACTCTGTTTTTTAAACCAACTTTCGACGACGTAAACCAGGTTCAGACATAAACCATTCCA
TTTCCATATTCATTCTGTGTTTTTATGACGCTATTTTTTTGGCTTGAGGCAATTTCTGAAATGTTCGAGA
GCCAAACTGCATTTTGGTTAATCAATTTGATTAATGTTGCCCTAATTGACTTTCACTTGGCGGAGTTTCA
TTTTGTCCGGGCCTTTCCATTTCGTTAAACGAGGGTATATTATTTGTTGCTGTTGTTTTAACCAGAAGTA
TTTGGCCAACCTGTTTTACAGGGTATTCCGCTTTATTTTTCGTTTTTAGCTAAAGGAAATTTCCATACGG
GTTTGAAAGATCTCAATTTAATTGCCGGCAATTTTTAGCTCGTTTTCACTCGGAATCAGTAGCAGCTGCA
TCTACATTTCGCTTATTGATGCCACATGTCGGAACACATGCTCAACATTTGCGCAACCTTTGGCAACAGC
TCcagatacagacacagatacagatacaTTTGTATCTGCAAGTGCATTGACCTTTTCATCAAAGGGAAGC
ATCTCAAAGTCGGAACTTTGTTCGCTGATATCAAAGCAGAAAGCATAACTAGCAGTTATGTAGTAGTTCT
ACTTTTCATTTCTACAtccatttcggttcagttcagctcaactcagttcagttagttGGCTATTCACGAG
AAAGTGGCACGACATGCTCCTCCTGCTCCTTCTGCAACTCCCCTACTCCTCCGCCTTCACCATTTCCAAC
CAAGCTAACTTGGCGTGTTCAGGAAGCATCGTCTGGCCAACTATACTGAAACTGGGTTGAATTGGGCTGA
CAGACGCCGGATGTCATCTAGCTGTCGGACTATTTTCCTATTTTTTCCCCCCACCCCATCATTTCTGCTT
CTTGCTCCTATTGATGATGGTACCATGCCTCGCCCACTCCATTTACGTAGGATTTTTCACGGAATTCACG
GAAAGTTCTTTTTGGTTTCTAAGGAAACGGGGCATGAACTGCGTTAGCTAATAAGGCATTCTTGGTTGCA
GATTGCAATATTACTTATATAGATAACATTTACAGATTAAAGTAAGAATGAGGTATCTCTAGCGCTCCAG
AAATTAAGTTATATTATTATTTCTGCTATTTAATTCAAGATAAAAGGTAAAAGTGCAAAACATGTTTAGA
GATTTCTCAAATCTATATATTACTACTTCATTGAAACCCAACTTTGGTAACGATACGTTCGTGTACAACC
CCTTAACTACTAACTGCAATATAACCAATTTTTCTAAAAAATTCCCTGCTATCCATATCATTTCCGTACG
CTGACAGGCCCAATAAACCAAGTTAACTGGATGCCGAAAAATGTGCGCCATTTAAGCTCGTTTCGCTCCT
TTTTTTCGGCATCGTCCTGGCATTCCACTTGACGGCACTTGAGCAACGTCGTCAACATCCTTCTTCtttt
ttttttgtttttttGTGCGCAAGGGGTAATTTTTGCACGTGTGCCGCCACTTGACGGCAACTTTGGTGGG
GGAGAGGGAAGGGGCAAAGTCCCAGCCAAGACGGTGCAACAATTGGGGAAGACCTTAAAGCGCTGCCAGC
TCAATTAAGTCAACGTAATTGGCCTAATTGACTCCGACGGCCAACAAAGGCTTGCCTTCTAAGACGTACA
TAACTTTCGGGGCGTCGTAAGGTGGAACACACAGGCAGTTTGGAGAGTGAAATCTTTGAGGACCGGCTGA
TAAGCACGTTGTTGGCTGTCGGGTTAGCTTTTGGCCCCTACTTATCGCAGCAACCCAGAAATAAAAAATC
TATCCAAACACAAAGTGAGGCAAGCAACGGCAGACGCAACATAAAAACCTCATCAACAACAGCGTTGACA
CAAAGACCTCAAATAAACAAGCAGCAATTTCaaaataagaaaaaaataaataaaaacaaaagctgtacag
aaaaaaagcacaaacaaaGGGGATGGGGAGGGGGAGGGTAGTAAAAAACAAAAACTGAAATTCAAGAAAG
AAATTGAACTTCCTTCTTGGCTTTTTGAACTTCAAGTTGTTAAAGAAGTGCAGAGGaaaaaaaaCGAGT

TTAAATTGTTTTTATATTTTGGCCAAGGTTGAGATATTGAAGCATACTTTATAGAAATACAATCCTTTAA
TACAACACTGAAAAAAAGTGATTATAAAAACCAGTTTAAGGTTGATTAGCACTGTGCATTCGGTGGATAT
TTTGATCAATTTTTCGTTGTGTACTGTAAAACTTGTGCATAAAAAGTCAAAACCTATTTGGGTTAAGCCA
AAAGCTAAAAGGTTGGATGGAAAAAGAGGGAGCGAGTGGGGAAGACGGAACCATGAGCCAGCAGAAACTC
AAATTCAACTTCAAATTCGTAATGAAATTGAAAATGCGTATAtgtgtgtgcatgtgtgtgtgGGGAAGGG
GGTGGGGTAGGAAAAGCCAACTTGGCGGCCAATTGAGAATTGAAATTGAAACTCGCACTTCACTCGAAAA
TGAGCAGGAAAATCCACTAGGCGATCCGTTAAATTGGCGCTGCTAATGTGTGAGTTTGTGCGATGGAGTG
GCAAAGATGATAATGCCAATGAACATGGGGCTGCTAATGGGTCCGATGATGATGATTGAAGAAGCCAATT
TATCGAGGGTATGCTGTAATGGAAAGAAAGTCTGTTGCTCGAAATGTTTCTTGTTTGTTGAAAACGAAAA
CGAACTTCCGCATTTGTGGTGGAATTTAATTAGAATGAATTGGAGCAGAACTATATTTTTAATCGATTCG
TATAGTTCACAGCGAACTATTTATTAGGGAATTTGATACGCTTGGAAGTAATTAAAGTTAATTTACTTTT
AATTGGAAGAAAGTGTAATTTGGAGTAGAATCTTAGAAGGGGGGTGGGTTTCTTTTATAGTTCCTAAATA
GGTTGAATTAGATATCACTATTATTTGTTTTACTCATAAATACATGAACATAAACGTGCTAAGTGCTTAT
CTAGTTTATTCGAATAACTACAACTTTGTCATTCATTTGATTCACTTTCAGCGTGAGCCCCCATTGACTC
GATTGATGATGACCCAGTAAACACAATGGCAAGGGAGCCAGCCAAACTGCAGCTGTTTTTGGCCAGATCA
ATCGAAATCGATAAGAAGTAGTCGTGTTCAGTTCAAGAAGCGCCTGTAAATTGGGAAAAAGTAACTTAAA
GCAATCAAGAAGGTTTAAGTGGTTAAAGGCAGATGGATTTATCAGTTAGGGAGACATGGATATAACATCA
TGGAAATAGTGTATTCATCAAAAATTGACAATAAATAATATTATTCATTCCCTAATCTTTATTACTTATA
TTCATTGATTTTCAGTTTTGTGAGGATAGTAATACTCCAAAATCGTATCGCTATCATCCGGGCTTGTGGT
CTGGAAAGAAGTGGAGCTGCTGCAAGGGATTATCTCGAACCACATTTGGTTGCAGAGCAGCCGCCCATTG
GCGTGAGGCCAACAATAATCCAAG
>dme_circ_0001839_junction_seq
ATTGGCGTGAGGCCAACAATAATCCAAGACACTTTTTCTTGTCAAGATGATGGGCA
>dme_circ_0001840
ACACTTTTTCTTGTCAAGATGATGGGCACTAAGCATCGAAACTCGCATGTCAATGGCAGCATTAAGTCAT
CATCATCGCTGCGATCGAGCAGCAAATCCTTTCAAGCAAAAATGGATCTGATGTCCGAAAGGCTGTACGA
TGTCGTGAAAAGTGGATCGATGGTGAAAAGGGCTCAAAACAAGAAGCGTTTCACCCCCGTGAATTACAAA
CATCGATGGTTTGAGTTGACCAAGCGAACTTTCAGCTACTTCGATGTGGAAAATGTGGAGG
>dme_circ_0001840_junction_seq
AGCTACTTCGATGTGGAAAATGTGGAGGACACTTTTTCTTGTCAAGATGATGGGCA
>dme_circ_0001841
GAAGAGTTTGTTGGAGTAAGTCGAGGAGAAGCGGTAGGAGTTGGCCACGATGGCGAACTCGTCGTGGGCG
TGCCTACAGATCTGGCATTGTCTCGATGGAGCCAATGCGGTGAGCATTACGATCATCGAGTAATTCCGTG
GTGCACTCTTCACATACTCCCGAAACTTGGGTCCATTGAAGCGGAGCAGCGGCTTTTTGGCGTTCATGTC
CACCAGGTTTT
>dme_circ_0001841_junction_seq
TTTTTGGCGTTCATGTCCACCAGGTTTTGAAGAGTTTGTTGGAGTAAGTCGAGGAG
>dme_circ_0001842
ATATTTCCTCTTCGATAAAAATCGCAGCTCCTCCTGCCGGAGTCCTCGTATCTTTGTTATGAATATATCC
TCCCAGGCATTGATCTTGATCTGCTTGGCTCCTTGCATTGTTTCCGTCGTGGCAGATAGCCTTGCATCCT
TGGCCGTCATTAAACCACTTGAAAATATGCCAATCCTCTTGGCCAACCATCTATTAATTGGAATCAAAAG
AGCAGCGAATATTACTCCCGCCAGAAATGCTGCTCCTAGTTGAAGATACAGCAAGTACAATGTGG
>dme_circ_0001842_junction_seq
AGTTGAAGATACAGCAAGTACAATGTGGATATTTCCTCTTCGATAAAAATCGCAGC

>dme_circ_0001843
GATTCGTTGTGTGAGAGGCGGTAATGGCAGCCAGAAGACCTGCTCCTCCGACAAGTAGTGTTCCCAGCAA
CTGAAGTCGCAGAGCTAACCATTGTTGCGCAGCCGACTGTGTCAACTGAGCCTTTATGCTCTCCTCGAGT
TTCACTTGGAAGTCCCGCTGAAATCTCGGACTAGCTCGCATACTCCTTATTGTCGTCAAGCCCTGTAGAG
TCTCCGTAAAATGTGTGTAAAGCGGTGACATTGCATTGCTCGATAATCGTTTGATGTCCCTGGAGGCGTG
CCGATATCGCTGCTG
>dme_circ_0001843_junction_seq
CCCTGGAGGCGTGCCGATATCGCTGCTGGATTCGTTGTGTGAGAGGCGGTAATGGC
>dme_circ_0001844
GGTGGATGTGCCACTGATGACGCAGAAGCAGGATCGAACGGCGGACAGCCTAAACAGCGAGCCACTGAAA
GACTTCTGGCTGGTCAAGGACATGATGACATTCGAGAACATCGGGTTCTCCAACACG
>dme_circ_0001844_junction_seq
ATTCGAGAACATCGGGTTCTCCAACACGGGTGGATGTGCCACTGATGACGCAGAAG
>dme_circ_0001845
CGGGAAAGTCTTCGCTGATTAATGCCCTGTTCCGACTGTCCTACAACGATGGATCTGTGCTCATAGACAA
GAGGGATACCAGTGAGATGGGTTTGCATGACCTGCGCAGCAAAATCTCGATCATACCGCAGGAACCCGTT
CTGTTTTCCGGCACTATGCGATACAACTTGGATCCCTTCGACGAGTATAGCGATGATAAGCTGTGGCGCT
CCCTGGAGGAGGTAAAGCTAAAGGAGG
>dme_circ_0001845_junction_seq
TCCCTGGAGGAGGTAAAGCTAAAGGAGGCGGGAAAGTCTTCGCTGATTAATGCCCT
>dme_circ_0001846
GGGAGCGCCACAGCTTATCATCGCTATACTCGTCGAAGGGATCCAAGTTGTATCGCATAGTGCCGGAAAA
CAGAACGGGTTCCTGCGGTATGATCGAGATTTTGCTGCGCAGGTCATGCAAACCCATCTCACTGGTATCC
CTCTTGTCTATGAGCACAGATCCATCGTTGTAGGACAGTCGGAACAGGGCATTAATCAGCGAAGACTTTC
CCGCTCCAGTGCGTCCCACGATGCCTACTTTCTCCTTAGGTTTTATTACGAAACTAAGTGACTTGAGCAC
ATTCTCCGACTTTGGATCCGGCGTATAGCGCAAGCTAAGCTCGTCGAAAACGATTTTTCCCTGCTCTGGC
CATGACTTTG
>dme_circ_0001846_junction_seq
ATTTTTCCCTGCTCTGGCCATGACTTTGGGGAGCGCCACAGCTTATCATCGCTATA
>dme_circ_0001847
GGTGCCCACGGCGCTTACACGACCCTTATCCATGATGACAATCTGATCGGCGTGCTGCAAAAACTGGAGC
TGATGAGTGGCCAATATAACAATTCGCTCGCGTAGATAGCCACGCATGCACTGCTCGAACAGATGGCGGG
CCACATGG
>dme_circ_0001847_junction_seq
CTGCTCGAACAGATGGCGGGCCACATGGGGTGCCCACGGCGCTTACACGACCCTTA
>dme_circ_0001848
TTTCTTTAGAGGCAGTAGAACATGCAGTCGCTGAAAACGGCAGATTTGCCGGAAAATCCCCGGGAACATT
GCAATTTTATTTCGGCAGCATGTTTTTGGTGAGCATAGTAACTACACATTTAATGTAAACCTATTAATGG
CCTTAATTTATTAACAATATACGTAGGTACACCATGCCGACTTTTATCAAGGGTCGGAAACGCACATTGG
ATACCAAGGATCTTTACAGAGCCTTAAAAGAGCACAAATCTGG
>dme_circ_0001848_junction_seq
ACAGAGCCTTAAAAGAGCACAAATCTGGTTTCTTTAGAGGCAGTAGAACATGCAGT
>dme_circ_0001849
GCCGTCGCCATCCGCAAGCACTTGGAGCGCAACCGCAAGGACAAGGACGGCAAGTTCCGTCTGATTCTGG

TCGAGTCCAGGATCCACCGCCTGGCCCGCTACTACAAGACCAAGAGCGTCCTGCCCCCCAACTGGAAATA
CGAG
>dme_circ_0001849_junction_seq
CGTCCTGCCCCCCAACTGGAAATACGAGGCCGTCGCCATCCGCAAGCACTTGGAGC
>dme_circ_0001850
TCCTTCACCTTGGTGGCCACTGCAGCGCGAACCTCGGCCTCGGCATCCTTGAGCAAGTACTGGAAGGCAG
GCACCAAGTCCACCCTAGTAATCTCTGGGCCCACAGCCTTTTGCAGATCAACAAACTTCTCGGCCACCAT
G
>dme_circ_0001850_junction_seq
CAGATCAACAAACTTCTCGGCCACCATGTCCTTCACCTTGGTGGCCACTGCAGCGC
>dme_circ_0001851
GGCCAGCGTCATTGATGCCCAAGTAAAGCCCACACTCGACAAACTGAACACAGACACAGATGTGGATGTC
AAGCATTTTGCTGCACAGGCCATTGCCGGCATAGCTGCAGGTAAGCAATAAACAGTCTAATATAAACAAG
>dme_circ_0001851_junction_seq
AAGCAATAAACAGTCTAATATAAACAAGGGCCAGCGTCATTGATGCCCAAGTAAAG
>dme_circ_0001852
GAAACATGGCGGCAGCGTCGGGCAGCTGGCTGCGCACTTGCTTCAGTGCGGCATCCAGCGCCTTGGTGTT
GTTAAAGGCTTCCAACTGCTCGACCAGCACGGTGGCATTGGGATTGGCTTCGCACAGAGTCTTGGCGCGC
TCCACAACAGTGACCGAGACAGCGGCACGCAGGGCACGTTCCTTATCGTCCAATGTTTTCTTCAAGCCCT
TCAGCAGATTGCGCATCTCGTCCTTTTTCACGTAGGGAATCGTAGCATGCGAGATCTGTTCAGTCAGCTC
AACAATCTCCTTCACATGCGACTTGGAGTCCTTACCGGACTTATCGTTATCAATGGTGGCCTTAAGCCGG
ACGATTTCTTGTTCAAATGCTTCAGATTTCTTCAGTGCCTTCAGAGCTTCGGGTCCTGTAAGAGCCACAA
TTCTTCGGATACCCTTGGCAATGGCCTCTTCGCTGCTGATAACAAAGTCCATTATGTGACCCGAGCGCCT
CAAATGGGTGCCGCCGCAAAACTCGACGGAGGTTTGTTCGCCGGCTTCAGAATCGGGATTCTGCTCCAGT
TCATCTACGGGCACTCCGAAGGAGATTACCCTAACGGGATCCGG
>dme_circ_0001852_junction_seq
CGAAGGAGATTACCCTAACGGGATCCGGGAAACATGGCGGCAGCGTCGGGCAGCTG
>dme_circ_0001853
GCGGGCATCGTTCTGGACAAAACAAATTTCTATGCAGAGAGCGGTGGTCAAATCTACGACCAAGGTGCAC
TGGTTAAAGTCAATGACGAGGCGAACGAGTTCCTTGTGGACCGTGTCTACAACCGCGGAGGCTACATTCT
TCACATTGGCGTTGTAGAGGGCACCCTTAAGG
>dme_circ_0001853_junction_seq
ATTGGCGTTGTAGAGGGCACCCTTAAGGGCGGGCATCGTTCTGGACAAAACAAATT
>dme_circ_0001854
GGTGTCGTTGGTTGGTGGCACACCTTGCTCCTGCAGCTGCGAAATGGCGTGCACATCCAGATTGATCTCC
TCGATTTTGCTAGCACCTTTGCCCTGCGAAAGCACATATGAATTATGCTTGGCTGCCTCATAGCCATCCA
TATCGATTTTCAAGGATTTCTCCTCTGCCATAAGCTGGGTTAGATCGACTGGGAAGCCGTAG
>dme_circ_0001854_junction_seq
CTGGGTTAGATCGACTGGGAAGCCGTAGGGTGTCGTTGGTTGGTGGCACACCTTGC
>dme_circ_0001855
GTGCGCAACGCCTATCTGGACTTCTTCAAGGAGCAGAAGCACATCTATGTTCACTCGTCCAGCACGATTC
CATTGGACGATCCCACGCTGTTGTTCGCCAACGCCGGCATGAATCAGTTCAAGCCCATTTTTCTGGGCAC
CGCTGATCCCAACAGCGAAATGTCCAAGTGGG
>dme_circ_0001855_junction_seq

GATCCCAACAGCGAAATGTCCAAGTGGGGTGCGCAACGCCTATCTGGACTTCTTCA
>dme_circ_0001856
TATGCAGATAACATTAGCTAATTTCGGTTTTTGGGAAATTCTTAGCAGTTGCTTTGGAATGATCTGCCCC
GACCTAGACTACATAGTAAAAGCTAAATAATAATTTAGTTAAGAATACTACGTAAAATCACGTTTGGCAT
TGTGCGGTCATCGAGTTAACACAGAAATAATCTCCAACTGACAAACTGAACAAATGTTGAAAAACTTTCT
ACTTAAAATGCTAGTTATTGTCTGGTACGATTGCAGCTGGCTGATAACAACaaaaaaaaaTGCAACTAAT
TTGCCATAAATATAAATTAATCACACTAAAGCACCGCCACTGGACTGGACCGATACCAGTTGGGAAATGG
TAAACAACTAATGGTAAACATATTCGTAGTCTGAGTGTAACTACATAAAGCCTTCGTCATAAATTCGCAC
ACATTTGTAGGCAGAACTAGCCAGGTTCTGTTTTCGATTCGCTTATTTCGATTAAACTCAGTGGGTTCTG
TCGTTCAGGATTTGAGCTAAGCTGGCCCAGGTGCCAAACACAAAGCCACAGAGTCCAAAGATCAGGATTA
AAACGTCCTTCCATAGCATCCAATTGAAGCG
>dme_circ_0001856_junction_seq
CGTCCTTCCATAGCATCCAATTGAAGCGTATGCAGATAACATTAGCTAATTTCGGT
>dme_circ_0001857
ATAACATTAGCTAATTTCGGTTTTTGGGAAATTCTTAGCAGTTGCTTTGGAATGATCTGCCCCGACCTAG
ACTACATAGTAAAAGCTAAATAATAATTTAGTTAAGAATACTACGTAAAATCACGTTTGGCATTGTGCGG
TCATCGAGTTAACACAGAAATAATCTCCAACTGACAAACTGAACAAATGTTGAAAAACTTTCTACTTAAA
ATGCTAG
>dme_circ_0001857_junction_seq
TTGAAAAACTTTCTACTTAAAATGCTAGATAACATTAGCTAATTTCGGTTTTTGGG
>dme_circ_0001858
GGAAGCCAAGAAGCTATCAAAATTAACGCGGACACAACTGCAGAGCGACGCCGACGCGAACGCCGCTTCT
ACTACAAAGCGATGGCGACAATGTTGACATCGGACGAGGCGGGCAAGCTGCTCGACTTCTCCCAGAAGCT
GGACATTAATCTGCTGGACAAGATCGTCGAGGTTGTGTACACAGCGCAGGGCGAGCAGTTGCGTCTAGCC
CAGAGCATACTGACCACGCTGAAGGAGCATCCAGAGGCATGGACGCGGGTGGACAGCATATTGGAGTACT
CACAGAACCAGCGGACCAAGTTTTATGCTCTGCAAATCCTCGAGGAGGTCATCAAAACGCGCTGGAAGGT
CCTGCCGCGCAACCAGTGCGAGGGAATCAAGAAGTATGTGGTCAGTCTTATCATCAAGACCTCGTCGGAT
CCCATTGTTATGGAGCAGAACAAAGTGTATCTGAATAAGCTAAACATGATTCTGGTCCACATTCTAAAGC
GCGAATGGCCACGCAACTGGGAGACATTCATCAGTGACATTGTCGGTGCGTCGAAAACGAACGAGAGCCT
GTGCATGAACAACATGGTAATCCTGAAGAACCTTAGTGAGGAG
>dme_circ_0001858_junction_seq
GGTAATCCTGAAGAACCTTAGTGAGGAGGGAAGCCAAGAAGCTATCAAAATTAACG
>dme_circ_0001859
GGCGGGCAAGCTGCTCGACTTCTCCCAGAAGCTGGACATTAATCTGCTGGACAAGATCGTCGAGGTTGTG
TACACAGCGCAGGGCGAGCAGTTGCGTCTAGCCCAGAGCATACTGACCACGCTGAAGGAGCATCCAGAGG
CATGGACGCGGGTGGACAGCATATTGGA
>dme_circ_0001859_junction_seq
CATGGACGCGGGTGGACAGCATATTGGAGGCGGGCAAGCTGCTCGACTTCTCCCAG
>dme_circ_0001860
GGTGTTCGACTTTTCGCAGGGTCAGATCACGCAGACTAAGGCCAAGCACCTGAAAGACACCATGTGCTCG
GAGTTCTCTCAGATCTTTACACTATGCTCCTTCGTGCTGGAGAACTCGATGAATGCCGCCCTAATCCAT
>dme_circ_0001860_junction_seq
GAACTCGATGAATGCCGCCCTAATCCATGGTGTTCGACTTTTCGCAGGGTCAGATC
>dme_circ_0001861

GGTTTTAAGTGCGATGGCAATCATTGTGCATAAACTGCGTCAGCATATCACAAATGAAGTACCAAAAATA
TTTGACGCCGTCTTCGAGTGCACGCTGGACATGATCAACAAGAACTTTGAAGATTTCCCACAGCACAGAC
TTAGTTTCTACGAGCTACTGCAGGCGGTCAATGCGCACTGCTTTAAGGCGTTCCTTAATATACCGCCGGC
TCA
>dme_circ_0001861_junction_seq
AGGCGTTCCTTAATATACCGCCGGCTCAGGTTTTAAGTGCGATGGCAATCATTGTG
>dme_circ_0001862
GGTCAAAGTCTTTGTCACTGGCTTATTCAATTTAGACGAGAATGTGCAAGCATTTAAGGAGCATTTGAGA
GATTTTCTCATACAGATTCGCGTGAGTATTTATCAAATGATGGCCATTAAAGCTGGCCAACTGCTAGTAA
ATATCATTTACCTTAATGTATATAAATTGAATTGCAGGAGGCCACCGGCGAGGACGATTCCGATCTGTAC
TTGGAAGAGCGTGAAGCGGCTCTGGCCGAGGAGCAGTCAAATAAACATCAGATGCAGCGTAATATTCCAG
GAATGTTGAATCCGCACGAGTTGCCCGAGGATATGCAGGACGAATAgat
>dme_circ_0001862_junction_seq
TGCCCGAGGATATGCAGGACGAATAgatGGTCAAAGTCTTTGTCACTGGCTTATTC
>dme_circ_0001863
CTGCCTTTTAGCAACAAAGACGACGTTTATCCCGTGACGCCAGTTGGCAACACCTGGCTTATTTCCCAGG
ACATTTTCGAAACCTGGAAGGGCTACTCTAAGGACACGCTCTTTCTACATAACAACAGATTATCAG
>dme_circ_0001863_junction_seq
CTCTTTCTACATAACAACAGATTATCAGCTGCCTTTTAGCAACAAAGACGACGTTT
>dme_circ_0001864
GAGTCTCCACCGAGGCCTATAGTAGTCGCTCACCAAGCCGGACCACTGTTTGCACGCATAGTCCAGGATC
TGACCATCAGGACCCCAGGCGGTAATTTGGTTTCTCGCATTGAATTCGAAGTTTCTTTGTTGCCCAGTGT
TCGGTGCCGCTTGCTTGGCCTGCTGCAGCCAGTTGCCGAGCAGAAAATTCCTGCTGCTCGCCAGGATCAG
CTCCATGTCATCGAACAGTTTCAACAGCTTGACGCTAAGGAATTCGAAACGCGAAACCTGTCGCTTTCTA
TAGGCCGACCTTAGATTGATATACAACTGATCTGCGCTGATCTGCAGGAATTGCCTGGTTAT
>dme_circ_0001864_junction_seq
CGCTGATCTGCAGGAATTGCCTGGTTATGAGTCTCCACCGAGGCCTATAGTAGTCG
>dme_circ_0001865
CATCTGGCTGGACCAGCTTTTCAGGCCTGGCAGCGCATGGGCAACATACGGGGATGGGCGGGACCACTGA
CACCGGCATGGCGACGCTATCAATTACTCCTCCAGCAGGAGATTATCACTGCCCAACGCAATCTGGGCAT
GAGCGTGGCCTTGCCCGCCTTTGCTGGTCATGTGCCACGTGCTCTGAAGCGTCTGAATCCCGAATCGACG
>dme_circ_0001865_junction_seq
TCTGAAGCGTCTGAATCCCGAATCGACGCATCTGGCTGGACCAGCTTTTCAGGCCT
>dme_circ_0001866
TGCCTATTTGGATCAGCCATTGCGGGCTTTGCAGGACTGTTGTCTGGAAGGCACCGCCTCGGATTTGCTT
TCCACCCCGCCCTCCGGCAATAGGTTGACATCACGTAGGCGCACGATGGACTTGCCACCTCTGGAATCGG
CCCCAGCACCAGCTCCAGTTCTGAGTCCCAACGATGCTGTGCACATG
>dme_circ_0001866_junction_seq
TCTGAGTCCCAACGATGCTGTGCACATGTGCCTATTTGGATCAGCCATTGCGGGCT
>dme_circ_0001867
GACGGGCAGACAGCCACCGCCCTAACTGGCGTCGCCTACATGTCTTTGTGTGTGTGGTGTATGTGCTTGC
GTGCCTGGGGTAGTGTGCCTGTGTGCGTGCCTCGCTGCATGTGTGAGTCCTTTACGAATGGAGTGG
>dme_circ_0001867_junction_seq
ATGTGTGAGTCCTTTACGAATGGAGTGGGACGGGCAGACAGCCACCGCCCTAACTG

>dme_circ_0001868
GTTTACACTAGCGACTAAAGGAGGATCAGCAGGAAAAAATGGGTAAAGCTCAGAGCAAGCGTTCAATCGA
CATCACCACCGATCCCAAGAAGGTCGGAGAGGGCGATGAGGTCGCCGGCAAGGTGGAGAAAATCGATGTG
GACCAGAAGACGGACGCGCCGGCTGTGAACGGAGACGCTGCCACGCCCAAAGAGGGTGGTGATGAAGCGG
CGGCGGTAGAGGAAAAAGGAAACTGAGGAGCATTCTGAGAACGACAAAGATCTGACCACCGAAAAGAGTG
CCGCAGTCGCTGAGGGTGGTGATGCGGTCGCTGAAACGGCCAAGGGCGAGGAGGGATCTCCCAAGGAGGC
TGCTGCTGGTGAGGATATAACCCCGCTGGCAGACGAAAGCATCAAGTCCAAGTCCAAGAAGGACAAGGTC
AAGAAGAAGTGGTCCTTCCGAAGCATTTCCTTTGGCAAGAAGGACAAACAGAAACCGGCCAAGTCGGAGG
AGGCTACGTCGCCCACCTCTGGCACAACGTCGCCCACGACGGCCGAGGCAGAGGCTGCTCCAGCTGGGGA
TGCAGCTGTCGCAGAACCATCGGTGGCTACAAATGGCGAGGCTGAAAAGCCAGCTGAGGACTGCCACTGC
CACCAGCGAGCCGGCGTCCAAGGATGAGAAGCCCGCCGAAAATGGATCAGCGACCGAGCAGGAGAAACAG
GCCAACGGAGAAACCGAAAAGGCGGCGCCAGCACCTTCCACAGTTGAGGAAGCGGCGAAGCCCAAGCCCG
CCGAAGAACCAGCCACAGTCACTGCCACCGAATCGAACACCACTGCCACCGAAGAAGTTCCCGTGAAAGA
GAGCCAGCCAGAGCCCGAAGTGGTGACCAATGGACATGGAGCCGGAGAGGCGCTGACCAACGGATCAAGC
AATGGACTGGCCGAGTCCCCAGTGACCGAGACAGCACCTGTCGCTGACAACATTCCGAGCAACGTTGATG
ACGAGCCACCGCACCAGAATGGCACCAATGGCACCACCACGCCCCCGCCCACTCCCGTGGCCACCGAGAT
CGAGAAGGGACAGCAAATTGAG
>dme_circ_0001868_junction_seq
CGAGATCGAGAAGGGACAGCAAATTGAGGTTTACACTAGCGACTAAAGGAGGATCA
>dme_circ_0001869
GGTCGCCGGCAAGGTGGAGAAAATCGATGTGGACCAGAAGACGGACGCGCCGGCTGTGAACGGAGACGCT
GCCACGCCCAAAGAGGGTGGTGATGAAGCGGCGGCGGTAGAGGTAAGCAAATCATATCTTCAAATGTGTA
CGTCTCATGACTGATGTGTTTAATTTACGCCAATAGAAAAAGGAAACTGAGGAGCATTCTGAGAACGACA
AAGATCTGACCACCGAAAAGAGTGCCGCAGTCGCTGAGGGTGGTGATGCG
>dme_circ_0001869_junction_seq
TGCCGCAGTCGCTGAGGGTGGTGATGCGGGTCGCCGGCAAGGTGGAGAAAATCGAT
>dme_circ_0001870
GTGCCGCAGTCGCTGAGGGTGGTGATGCGGTCGCTGAAACGGCCAAGGGCGAGGAGGGATCTCCCAAGGA
GGCTGCTGCTGGTGAGGATATAACCCCGCTGGCAGACGAAAGCATCAAGTCCAAGTCCAAGAAGGACAAG
GTCAAGAAGAA
>dme_circ_0001870_junction_seq
AGTCCAAGAAGGACAAGGTCAAGAAGAAGTGCCGCAGTCGCTGAGGGTGGTGATGC
>dme_circ_0001871
GATACAGGGTTCCATACGCAACTACTTTACGGCAAAGAGTGCTCTGCGAGTTCAACAACCGCACGTCAGC
AAACGTGTCCAATTGGCAATTGACAAGATGTCCGGAAAGATCGATGAGACGCCGGAGAAACCGAAGAAG
>dme_circ_0001871_junction_seq
CGATGAGACGCCGGAGAAACCGAAGAAGGATACAGGGTTCCATACGCAACTACTTT
>dme_circ_0001872
GCGTCCGGAGGATTTTCGAATGTGCGCCCACGCAAATGAAGCGTCCCTCGTATTCGGTCACTGGGGAGTC
TATGCACTTCTCGAAGTTGACCTTCCAGTAGATCTGAAGCTTGAGGACCGGCTGTTCGATACGTTTAATA
ATCAAGCCACTTTGGCAGGCTGAGACAACTACCGCTGTCTCAACATTCTTTCGCTTAATATTGTTGGCCA
CCAACTTGGCTGTGTCCAAGAATCTTAGCACTGTGCGCAACGGAATGTTCTCATCCAGTAGCATTTCGAA
CAAGTGGCGTTGCTCATCATCGATGCACATTTGTAAACCTATCTCTCGGCAAATG
>dme_circ_0001872_junction_seq

CATTTGTAAACCTATCTCTCGGCAAATGGCGTCCGGAGGATTTTCGAATGTGCGCC
>dme_circ_0001873
TTAAACGTCTCCATGGCCGACATCATTTACCTTGGCACACCAATTACCTTCGATCCCTTCGTGGTGGAGT
TCTTCCTAGCTCTTCAAAATGGTGCTACTTTACTGACCAGTCGTCACTCCATGAGGGACTCGCCAAGCAA
GGTGCTAAGTGCCCTCTTCCCCGACAACCTGGCCACACCGGGAATCACTGTTCTGCAGATGACTCCTTCG
CTGTTTCGGCAATTTGGTGCATCTTCCATCAAAGATCGAGTGCTCAGCGGGTCCAG
>dme_circ_0001873_junction_seq
TCAAAGATCGAGTGCTCAGCGGGTCCAGTTAAACGTCTCCATGGCCGACATCATTT
>dme_circ_0001874
GTTCGTGGTCTGGAGAATGTCCGTAAGGACCATGGGGCAGTGGTGATAATGAACCACCAAAGTGCTGTTG
ATCTTTGCGTTCTGGCCTATTTGTGGCCTGTGATCGGAAGAGCAACAGTTGTCTCCAAGAAGGAGGTTCT
CTACATCCCATTCTTTGGCATTGGAGCATGGCTCTGGGGTACCCTATACATCGACCGCTCCCGCAAGACC
GACTCGATCAACTCTCTGCAAAAGGAGGCGAAGGCGATACAGGAGCGCAACTGCAAGCTACTGCTCTTCC
CTGAGGGAACGCGCAACTCCAAGGACTCGTTGCTGCCCTTCAAGAAGGGCTCATTCCACATCGCACTGCA
GGGCAAGAGCCCCGTTCAGCCGGTGGTCATATCGAAGTACTCATTCATGGATGACGAGAAAAAGACATTC
CGTCCTGGCCACGCCCTAATCCACATCCTACCCGAGGTGTCCACCGAGAAGTACAAGAGGGAGGACGTGC
AGCTGCTGATCGACGAATGCCAGTCCATCATGCAGACGGAGTACACAAAGCTGAGCAAAGAAGGCCAAGC
CTTGAGCTCAAAGAAGCAGGCATAGGACTGGATAGGTTACGATCAAACTCAGGTTATAAAAAAGTTACAC
CCAAACGTTTCGGGTTTAAATTTACAATCGATCCACAAGATTTGCCTATCCATGGATGAGAAAAATCAAA
GGCAACAGAATGCCGTTACTTGAGGGACGTAAAACCGTTGGCCTAGTGTTAAG
>dme_circ_0001874_junction_seq
GACGTAAAACCGTTGGCCTAGTGTTAAGGTTCGTGGTCTGGAGAATGTCCGTAAGG
>dme_circ_0001875
GTCGGTGCCCATGTACTCGAAGGCGGACATACAGTTGCCGTCCGACTGGATGATGTAGGCCGAGGGCTTC
AGGACGAAGTTGGTGCCACCGATGTTGAAGGTGACATCGGGCAGGGAGCTGACGCTTGAGCAGTCCAGAT
AGCCATCCTCACCCACGTTCAGGATCTCGGAGAGG
>dme_circ_0001875_junction_seq
CTCACCCACGTTCAGGATCTCGGAGAGGGTCGGTGCCCATGTACTCGAAGGCGGAC
>dme_circ_0001876
GATAGCCATCCTCACCCACGTTCAGGATCTCGGAGAGGGTAATGTAAGCATTGTAGGGAGCCACGATCAG
GGAGGTGCCGGTATCGGCAATAGCCTGGCAATCATCACACAGCGAGTAACCGTCAATGGAGGATCCAGCC
ATGGTGAACTGCCAGTAGCCCTGCTCCGAGATGGGAACGTAGGTCAGAGCGCCAGA
>dme_circ_0001876_junction_seq
AGATGGGAACGTAGGTCAGAGCGCCAGAGATAGCCATCCTCACCCACGTTCAGGAT
>dme_circ_0001877
GCATTGTAGGGAGCCACGATCAGGGAGGTGCCGGTATCGGCAATAGCCTGGCAATCATCACACAGCGAGT
AACCGTCAATGGAGGATCCAGCCATGGTGAACTGCCAGTAGCCCTGCTCCGAGATGGGAAC
>dme_circ_0001877_junction_seq
GCCAGTAGCCCTGCTCCGAGATGGGAACGCATTGTAGGGAGCCACGATCAGGGAGG
>dme_circ_0001878
GGTGCCGGTATCGGCAATAGCCTGGCAATCATCACACAGCGAGTAACCGTCAATGGAGGATCCAGCCATG
GTGAACTGCCAGTAGCCCTGCTCCGAGATGGGAACGTAGGTCAGAGCGCCAGAGTACAGAGAGGCATCGG
AGCCACCGAAGATGAGTTCACCACCCATAGTGGAG
>dme_circ_0001878_junction_seq

GAAGATGAGTTCACCACCCATAGTGGAGGGTGCCGGTATCGGCAATAGCCTGGCAA
>dme_circ_0001879
GAGAGGCATCGGAGCCACCGAAGATGAGTTCACCACCCATAGTGGAGGTGCCATCGCGGGCCAGATAGAA
CGAGAAGACGGAGTTGTCGACCAGACCCTGGGACACCATGTTGTAGAACGGAGGAGCCACACCATCCACG
GCCAGGGACTCATAGGCCATACCGAGAATGCCATCGAAGTTGGCATCGTTGAAGTTGGTGCCCGGCTCGT
TGGTGGATTCAGCAAAGGTCTGGCTCTGGATGCTCAGGCCATTGACGTCGACGGTATCG
>dme_circ_0001879_junction_seq
GCTCAGGCCATTGACGTCGACGGTATCGGAGAGGCATCGGAGCCACCGAAGATGAG
>dme_circ_0001880
GATAGAACGAGAAGACGGAGTTGTCGACCAGACCCTGGGACACCATGTTGTAGAACGGAGGAGCCACACC
ATCCACGGCCAGGGACTCATAGGCCATACCGAGAATGCCATCGAAGTTGGCATCGTTGAAGTTGGTGCCC
GGCTCGTTGGTGGATTCAGCAAAGGTCTGGCTCTGGATGCTCAGGCCATTGACGTCGACGGTATCGGTGG
ACAGGTAGCCAGTTAGGCTGCCAGTGCCATACTGGATGGAGAAGGATTCGCCGTTGGCCACGTAGGTGGA
GCTGGCGCTGGA
>dme_circ_0001880_junction_seq
TGGCCACGTAGGTGGAGCTGGCGCTGGAGATAGAACGAGAAGACGGAGTTGTCGAC
>dme_circ_0001881
GATAGAACGAGAAGACGGAGTTGTCGACCAGACCCTGGGACACCATGTTGTAGAACGGAGGAGCCACACC
ATCCACGGCCAGGGACTCATAGGCCATACCGAGAATGCCATCGAAGTTGGCATCGTTGAAGTTGGTGCCC
GGCTCGTTGGTGGATTCAGCAAAGGTCTGGCTCTGGATGCTCAGGCCATTGACGTCGACGGTATCGGTGG
ACAGGTAGCCAGTTAGGCTGCCAGTGCCATACTGGATGGAGAAGGATTCGCCGTTGGCCACGTAGGTGGA
GCTGGCGCTGGAGTCGTACTGGTTGTGGGTCAGGCAGGCATCGCTCTTGCAGGTGTTCGATGGCACCCAC
AGGTTCGAGGAGCCTGA
>dme_circ_0001881_junction_seq
ATGGCACCCACAGGTTCGAGGAGCCTGAGATAGAACGAGAAGACGGAGTTGTCGAC
>dme_circ_0001882
GAACGAGAAGACGGAGTTGTCGACCAGACCCTGGGACACCATGTTGTAGAACGGAGGAGCCACACCATCC
ACGGCCAGGGACTCATAGGCCATACCGAGAATGCCATCGAAGTTGGCATCGTTGAAGTTGGTGCCCGGCT
CGTTGGTGGATTCAGCAAAGGTCTGGCTCTGGATGCTCAGGCCATTGACGTCGACG
>dme_circ_0001882_junction_seq
CTGGATGCTCAGGCCATTGACGTCGACGGAACGAGAAGACGGAGTTGTCGACCAGA
>dme_circ_0001883
GAACGGAGGAGCCACACCATCCACGGCCAGGGACTCATAGGCCATACCGAGAATGCCATCGAAGTTGGCA
TCGTTGAAGTTGGTGCCCGGCTCGTTGGTGGATTCAGCAAAGGTCTGGCTCTGGATGCTCAGGCCATTGA
CGTCGACGGTATCGGTGGACAGGTAGCCAGTTAGGCTGCCAGTGCCATACTGGATGGAGAAGGATTCGCC
GTTGGCCACGTAGGTGGAGCTGGCGCTGGAGTCGTACTGGTTGTGG
>dme_circ_0001883_junction_seq
GCTGGCGCTGGAGTCGTACTGGTTGTGGGAACGGAGGAGCCACACCATCCACGGCC
>dme_circ_0001884
GCTCCTCGAACCTGTGGGTGCCATCGAACACCTGCAAGAGCGATGCCTGCCTGACCCACAACCAGTACGA
CTCCAGCGCCAGCTCCACCTACGTGGCCAACGGCGAATCCTTCTCCATCCAGTATGGCACTGGCAGCCTA
ACTGGCTACCTGTCCACCGATACCGTCGACGTCAATGGCCTGAGCATCCAGAGCCAGACCTTTGCTGAAT
CCACCAACGAGCCGGGCACCAACTTCAACGATGCCAACTTCGATGGCATTCTCGGTATGGCCTATGAGTC
CCTGGCCGTGGATGGTGTGGCTCCTCCG

>dme_circ_0001884_junction_seq
CCTGGCCGTGGATGGTGTGGCTCCTCCGGCTCCTCGAACCTGTGGGTGCCATCGAA
>dme_circ_0001885
GGAGCCACACCATCCACGGCCAGGGACTCATAGGCCATACCGAGAATGCCATCGAAGTTGGCATCGTTGA
AGTTGGTGCCCGGCTCGTTGGTGGATTCAGCAAAGGTCTGGCTCTGGATGCTCAGGCCATTGACGTCGAC
G
>dme_circ_0001885_junction_seq
CTGGATGCTCAGGCCATTGACGTCGACGGGAGCCACACCATCCACGGCCAGGGACT
>dme_circ_0001886
GGAGCCACACCATCCACGGCCAGGGACTCATAGGCCATACCGAGAATGCCATCGAAGTTGGCATCGTTGA
AGTTGGTGCCCGGCTCGTTGGTGGATTCAGCAAAGGTCTGGCTCTGGATGCTCAGGCCATTGACGTCGAC
GGTATCGGTGGACAGGTAGCCAGTTAGGCTGCCAGTGCCATACTGGATGGAGAAGGATTCGCCGTTGGCC
ACGTAGGTGGAGCTGGCGCTGGAGTC
>dme_circ_0001886_junction_seq
CCACGTAGGTGGAGCTGGCGCTGGAGTCGGAGCCACACCATCCACGGCCAGGGACT
>dme_circ_0001887
ACCATCGCTGTAGTAGTGCTCCTGGCAGCCCTGGCCAGTGCCGAGCTCCATCGCGTGCCGATCCTCAAGG
AGCAGAACTTTGTGAAGACGCGTCAGAATGTTTTGGCCGAGAAATCCTATCTGCGCACCAAGTACCAGCT
GCCCTCGCTTCGCAGCGTGGATGAGGAACAGCTGTCCAACTCGATGAATATGGCTTACTACGGAGCCATC
TCCATCGGAACTCCCGCTCAGAGCTTCAAGGTTCTGTTCGACTCAGGCTCCTCGAACCTGTGGGTGCCAT
CGAACACCTGCAAGAGCGATGCCTGCCTGACCCACAACCAGTACGACTCCAGCGCCAGCTCCACCTACGT
GGCCAACGGCGAATCCTTCTCCATCCAGTATGGCACTGGCAGCCTAACTGGCTACCTGTCCACCGATACC
GTCGACGTCAATGGCCTGAGCATCCAGAGCCAGACCTTTGCTGAATCCACCAACGAGCCGGGCACCAACT
TCAACGATGCCAACTTCGATGGCATTCTCGG
>dme_circ_0001887_junction_seq
ACGATGCCAACTTCGATGGCATTCTCGGACCATCGCTGTAGTAGTGCTCCTGGCAG
>dme_circ_0001888
GGATTCGCCGTTGGCCACGTAGGTGGAGCTGGCGCTGGAGTCGTACTGGTTGTGGGTCAGGCAGGCATCG
CTCTTGCAGGTGTTCGATGGCACCCACAGGTTCGAGGAGCCTGAGTCGAACAGAACCTTGAAGCTCTGAG
CGGGAGTTCCGATGGAGATGGCTCCGTAGTAAGCCATATTCATCGA
>dme_circ_0001888_junction_seq
TGGCTCCGTAGTAAGCCATATTCATCGAGGATTCGCCGTTGGCCACGTAGGTGGAG
>dme_circ_0001889
AAATCCTATCTGCGCACCAAGTACCAGCTGCCCTCGCTTCGCAGCGTGGATGAGGAACAGCTGTCCAACT
CGATGAATATGGCTTACTACGGAGCCATCTCCATCGGAACTCCCGCTCAGAGCTTCAAGGTTCTGTTCGA
CTCAGGCTCCTCGAACCTGTGGGTGCCATCGAACACCTGCAAGAGCGATGCCTGCCTGACCCACAACCAG
>dme_circ_0001889_junction_seq
GAGCGATGCCTGCCTGACCCACAACCAGAAATCCTATCTGCGCACCAAGTACCAGC
>dme_circ_0001890
GGCATCGCTCTTGCAGGTGTTCGATGGCACCCACAGGTTCGAGGAGCCTGAGTCGAACAGAACCTTGAAG
CTCTGAGCGGGAGTTCCGATGGAGATGGCTCCGTAGTAAGCCATATTCATCGAGTTGGACAGCTGTTCCT
CATCCACGCTGCGAAGCGAGGGCAGCTGGTACTTG
>dme_circ_0001890_junction_seq
GCTGCGAAGCGAGGGCAGCTGGTACTTGGGCATCGCTCTTGCAGGTGTTCGATGGC

>dme_circ_0001891
GCCATATTCATCGAGTTGGACAGCTGTTCCTCATCCACGCTGCGAAGCGAGGGCAGCTGGTACTTGGTGC
GCAGATAGGATTTCTCGGCCAAAACATTCTGACGCGTCTTCACAAAGTTCTGCTCCTTGAGGATCGGCAC
GCGATGGAGCTCGGCACTGGCCAGGGCTGCCAGGAGCACTACTACAGCGATGGTCTTGAACATTCTCTTG
GTTTCTA
>dme_circ_0001891_junction_seq
ATGGTCTTGAACATTCTCTTGGTTTCTAGCCATATTCATCGAGTTGGACAGCTGTT
>dme_circ_0001892
ACCATCGCTGTAGTAGTGCTCCTGGCAGCCCTGGCCAGTGCCGAGCTCCATCGCGTGCCGATCCTCAAGG
AGCAGAACTTTGTGAAGACGCGTCAGAATGTTTTGGCCGAGAAATCCTATCTGCGCACCAAGTACCAGCT
GCCCTCGCTTCGCAGCGTGGATGAGGAACAGCTG
>dme_circ_0001892_junction_seq
GCTTCGCAGCGTGGATGAGGAACAGCTGACCATCGCTGTAGTAGTGCTCCTGGCAG
>dme_circ_0001893
GGTGCCCGGCACCCATCATCTGCACCTGGTCACCCCGGATCGAGTGGCGCCCCACATTATTCGATTCCTG
AAGGAGGCGTAAGATTCCATTACTTAAGTAGACGGCTAGATAGAGAGTACTGTGTGTGTATATAGTTAGT
GTATTTCATCCCACTTGTGATGTACAGTGATCAAAGGTAT
>dme_circ_0001893_junction_seq
ACTTGTGATGTACAGTGATCAAAGGTATGGTGCCCGGCACCCATCATCTGCACCTG
>dme_circ_0001894
GAAATGACTGAACTTTCATCCCGCAAACCATTCGTTCGCCCATAAAACCCAGAAACAATTTAAGCCCTGT
TATAAAAAAAGTTGTTGAAcaaaaaattaatcaatatattttgaaataatttaaaatcaatttaaagtaa
aaggtaaatGCAGCTGCAATAACTCTAAAACACGCACAACCAAGCCAAACAAACATCAAATCAAATCCAT
TTAGCTGCAATAAAGTTAGAGGCAAAGGCACACACAGAAAATCAACAATAATAAATGTTGCAAATTTAAT
CAAACCAACTGAAATTTAACTAAATTTTATGCAAAATAGAAATGTTAAATTCACACAACACAAACCACAA
TAACAACAGCGCCAGCAACAGCAACTACAACAAAGGCCACAAAATGCATTTGAAAAGTGCAACGGCAAAA
GCAACAATAATGAAACATAAGTTAAGCAAATTTTACGGATACGGTTGGATGCAAGTCTTCCTACTACTTA
CAGTCCTCGTGATTGGTAATCAAAGTGCCTGGCAGGAGAATATACGACCAAAACTCTACGTTGAATTAG
>dme_circ_0001894_junction_seq
ATACGACCAAAACTCTACGTTGAATTAGGAAATGACTGAACTTTCATCCCGCAAAC
>dme_circ_0001895
GGTCCCGAAGATGTACTGAAGTTTGTGGGAAACGAAAGTGTTGTGGATCACTTCAAGCTCGTCACCAAGG
ATGGCAACAGTCTGCTCATCGGTGCCAGGTAAGAGACCATTTATCCCATAACTTTACAAATATAATATCT
TTGCGATGCGCTTAGAGGAGCACATATTGGGTTAAAGAATTAAATAACCAGCACAATTTAATCTTATTAA
ATGTGTAATCATTTTCCCCACATTTCGACATGTTGGTTCTTTGAAAAACAACTCCTGTCAATTATAAACC
ATTAAAAATAAAATGTGAGCTTAATCCTCATTGACCTAGCTGGCATTAAATTGTAACAAAAAACCATCGA
GTTAAATTATTTATGAGTGGTACCCCCAATTATGTTCGCATTGGCTTTGTTACATTGTCAATTCCCCCAA
GGTTGGATTCCTTGTAAGTCGGCGCCAAAGATTTGGAGTTGGTGGGAAGGGGAGAGTATCTCTGAAATTG
GTAGGAATAGGGGCAAGTGAAAAATACACATAAAGCAAACTTCAATTTATTTTCTGCAGACATTGTATTT
AATTTAAGTAGAAGATACTAGCATGTTCACTTCCCCATATACGTACGTACCAACATGACAAACTTTCTTG
GAGCCGAAAATGGTTAGAACGCATGGGGGTTTTCGGGGGCATGAAGTCTACCCTTAGCTAGTTCTAGTGG
CAACTTTGATTTTGCCGGCAACGCTGGCAAGAGAAACAGAAACAAAAACCAGTGCGAAAGGAAGCGGAAA
CTTTTGCAGCAGGTGCCCCAAAGATATGAGGAACTGTGCTAAAGTTTCCAGTACTTCCTTCTCGAGATAT
AACCCCAGAAGACTTAAAGGGTTGCTAAGGTTTTTCAGGCGAGACCCGGGttttttttggtttttttttttt

tttCGAAAGTGCGGGGTACGGTGGATTTTGTCGTTGTCAGAGACCACAAAATATGTTTGCTGCAAATGTT
GGGTTTATTATATTTTGCCTAGACAATGCCAGCATATATGAGCACATAAACACACCCACATTACCACTCA
CACATGGACAACGCACTGACATACATATTAAAATGTTGCACACTTTTTGGGGCCCAACGAGACTTAATAA
ACTTGCTTCACTCGGGTGTCTTCATGTGTGTGATTGTGAATGTGGCGTCGTGTTAACTTTTTTAGTCGAA
GAATGCTTGCAGCTTGTAATGAATGGACCGAATTCCTTTTTCAAATGGAACAATGGTAAAATGTCCGGAC
AGCGCTTCAACTTTAAATAGTTCGTGATCATTCAATGCACACATTATTAAATGGAAAGAATGCAGTTCTA
TTTTAACTACATAGCAGGCCATGTATCTTAGGAATGAGTTCTCATACATAATCTTATTTACTAAGGCTTA
AATGCTGATGCCTAAATATAGGAGCAGAATTTCTCAATTACGTTCTAAACTAAAATAAATAAAGTCGGAT
ACCGCTTTCTGAGTCGTTTTACTGCCGAGTAATTTACAATGTGAGGATAATGAGGCAATAAAATGCAAAA
CCATAACCAAAGCGGGAAAATTAAAGAAAACCCTCTTACTCATACATATACACACATACACACATATATG
TGCTGGGTTTTTCTCCAGTCTTTAttttttttttGTATTTTCGCACGCATGAACAAATTTGTATGCCGGC
AATGTCGTATCCAGAGTTGCCACTTACGTGATTAATGGATTTTTAACAAGTTGCACACTCGCATTGCGAA
AGGGAATTCACACACGCACATATCCACTTATCAGACCCCAAAAGAGTCAAAGTAACCAAAGGAAAACCTC
TTCTGCGACTGTCGGCGAAAGCCAAAAAGCACCACGTGTGTTTAAGTGTGTGCGTGGGCTTGGGTTTCCT
AGTGTGCATGTATTTGTGGCAGCATGTAGTGTGTATGGCTAGGGTGGACCAACTAAACTAGGATGACAGG
CGGAAATGCGAATGTGACACGCGTTTTGCATGTGAAACGTCATGAACAATGTTTAAAAAAAGGAAGAGTG
TAAAGGGAAAGTTATGCCAGGCAACCATACACATACACCCAAAAACGCACCTTTTCCATGGCATAGCTAT
ACCGTTACATTTCCTGTGGGGCAAGCAGGTACTGCGATGGGTGCTTAAACCCGCACATGATGTCAACCGT
GCCAAAACTCCtttaatttactttctttcatttttattaattttttttttATTCCCAAAAACCGACCAAAC
ATTCTCTCTTGTTCTAATCATATAATATTAAACTAAGCTTTAAACTAAGATTTAGTATATTTAATTACCT
TATCTTAGTCcaaagaaaaacaaacagataaaagaaaaaacaacagatctataaaaTATTTTTACCATTT
CAATCATTTTTCATACAGTAATTTGCAATGTTGCATAAATGGTATAAAAGTAGATTACAATTTCCTTGTT
ATTACGTTTTATTTGTATTACGAATGTAAGCCCCCAATGGAACTGCAAACTTATTAGGCTAACTACCTTA
GCCATTACTGTTCGTATCTCTTACGACACTTATGCAAATAAGCCAGAAGTGCTTGTCTAAACCAGGACAC
CTCCTTCACTATGCGCGTTGCGAAAACTCGGCGGCAAATACTCGTAAAGCTTTTCCCTCGCTTCTAGATT
TTCTCGCTGCGATCTATAAATATTCCACGGCAATGCGCTCGACATGAAAATTGGGTTAACACATTAAGAT
GGAAAAAAATGTGCCAACGTTTTCCCTGCTGTCATTGCCACATTCCGTGCCATGCCATCCCATACCATCC
GTTCCCATTTCCATTCCGTTCCACCCGCGCGCTTCCGTTCCATTCCATGCCGTTCATGTCAGGTTGGCAG
GCAATCGAATGAATCGCGACAAAGGCGGAAATAAAATTGCTAAGCGAACAAAGGAAAATCCATTTCATTG
CCCAACGGGCCTTGCTTCATTCGCCATCTCTCTCTGTCTTACAGTCTCATTGTGTCTGTCCTTGTCCCTC
ACGTTTTGAATTCGAGTGTGTTTTATATGCCAGCCTATCTTTCGGTTTTTAACGTCTTTTTCAACAGCGA
CAGAGCACAGTGGTCTTTTAATGTTTAAATATTAGGTTAAGCCAATAAGACAAACATCCATAAAACAAAA
CCTCTATATCACTATTTCAGCTTCTTGAGTGCTAGAAGTCAAACCAATTTAATTAATAATATTTATTTTT
CACACATAAAGAAAAATTTTTGCTTTAGCCCCACTGTTCGCTTGCAATCGCGTATCCAATTTTTGTATGC
AATTTTCCTTTTGTCGGCAGGTCTGCAAAATTCCTTTGCTCACAAACACGCACACAATCTCACGGCGTTG
TCCATCTTTTATTTCATTTTGTTCTTATCCTGTAGCCTGCATTTGTGTGTGTGCGTCGAGTCCTTTGTGC
AACCGCACAACAATCATTTCGGCACCGCAAATTCATTTCCGTTTGCGCTCTTTTTTGCTCACATCAGCGT
GTGTATGTGTGAATGCGGCATGCATCCGCTAATTTCTGACGATTTAAGTCCATTTCTGCGTGGCCAAACC
CATCAGAAGTTAGGATTGAAATGTAATCGTCCCGATGAATGGATTGAAGGATTTCGAATTGAATTGTCTG
CTTGGCCATTTCACAGGCTTAAAAGCCTTGGGAAAGCGCACGCATTGTTTTCAATGGGCCATTGTTTATA
GCCTAGTCAATGCCTGTAGCACACAAAATAGATTCGTGTAAAAGGTATTTCTTATGGACCAATGGCCATT
GTTCAGAGGACCTTTGTACAGAACAGGATTGATTTCATTAATATATTGGCTTGGCTTCAAGTGAATATGT
GATTTTATAATTAATTATGTGATTGTTAAATATATATTCTAAAATAAAAGGAAATTTATTCCATCATTGA
GAATATGCTATTCTGAATACCACTGTACTTCACTTTTTAAACGAAATATTCTTATTGCGCAGTCAGCACA

TATTTGACAATTTCTAAAATGGACGACTGGGATCGAAGGGTAAGCAGTCTGGCCAAGAGTCATCAAATTA
AAAGTGATTCGAAATGCAGTTAATATTTTATGTGGCTTTGCCAAGAATGTCCAATTTTGGCTACGAAATG
GAGTTTAATCATGCTTATGGGACCACAATGATGAAATGCAATTCGCCTTGCCATGTTTGCCTGTGGCCAG
GAACAATCGCTCAGCACCTGACTTGGCCAAAGGGCAAAAGTCAAATGCCACCACCTGGTTAACGGGACTG
ATTGGTTCGGTGGGTGACTGAGTGCATGGGTGGTGCCGTGGGTTGTACAACTACAACGATATCGAGTTGT
GGGTCAGCTCAGCTCGGGCAATTCACATTTGGGTTTTTGGCATTTGACAGTTTCGACATTTTCTGCCTTC
ATTGAGTGAATGGCACGGTATAACGTGGCTCAGTTCGTAGTTTATTTGGTTGATTTGGTTTGGCTTGTTT
GGGCCGGGCTTGTTGCACACATGCGaaaaaaaaCTTTGGTCAATAACATGGAAATGCAAGCCCATTCAAT
TCATACACTTGAAAAAAGTTGGTGGAAAAAACCTCTAACTTTTGAATTTTTTTCATATATGCAGAGCACA
TTAGTAGCCAATAACATTTTAAGGCGGGAGGCTCATTGAATAAACCATTTGAAATGCTAATTTTGTTGCC
AGTGTAAAATAGCAGCACACAAAGTCCTGAACCCTGAATTGGCTTAGTCAGCATCATCGAATGATGTGGA
ATGAAGTTGAAGTCGAAGCCATATCCCTGGCACAGTCAAAAAGTCAATGAAAAATGACAAAGGCTCAGCT
CCAAATGGTGATGGATGTGAGTGAGAAAGGGGCTACCCCCCCTTTTTCTCTGTTGGACTTCAATATATTC
tttttttttCGAAATGGCTGCCGTTTCAAACATTCGCACACGCACACTAAGACACCAAGCGCATGCAAAT
GGCGGCGACAAACATAAATTCTACCCAAACCAAGGCAAACCAAAAAAAGCCTTGAAAGCGAAGAAACATG
AGATGTTCGCCTGATTTGCTTAGCTATTCAGAAAAGCCATTTACCGTTATGTCAACCCCCATATGCCACG
CCCACGATATACGCCGCCCCCCCTCGCAGGCAGGGCTGCATCGGAATCGAAATCGGAATcaggagcagca
gcagcagcagcagcatcagAATCGgcatcaacgcatcaatgcatcaatgcatTCCGGAAGTCGAGTGTTC
TTTTCTCTGATTGCCTTCGGGCATTCCTTTGGGGCGCTGATGACGTGTGACATTGAACGTTGCTGGCATT
TTTCATGATGAATTCAGTCCTGCAATTTGCCATCTCAATTGGCCATTCAAAGGAAATTTATGGAGATAAG
GAATTGATTTAGCAGAGCTAAGCAGTGTTTATCAAAAGGACTTGGAGACGCACAAAATATTTAAAATGGG
TTTACATTACGTGTGCAAGTGTTATTTGTCTTTTGCGTATTGTTTTTAATATATCACTTGCATTCCAGTA
TACTTTTTAGTCTTAATTTATTCAGATATAATTTTTACTTTCATCTATTAAAGTAAACAAATTGAAGGCA
GAGACAGGTAAAATGTTTTCTTGTTTAATGTAAATTTAAAGCAACATGAAAGTAACAATTTTTAGATGTA
AAATTCAGACTTTGAAATACTATGTTACAAAGTCTTGAAACTCTTTAAAAATTTCCACGAAGGTGTTGAT
TGAAATCGAGTGTCAAGAGAATTTTCATTATTCAAGTGAATTGCCAATTTTGCGCCCGGCAATGATTTTG
TGCTGTACGAACGAATCATTTTTCTTGGAATGCCTGTCTTTTTAATTTACATAATACCCAAAAATGTGCG
TGTCACATTGAAATTTTTTATTTTCGAAAAGATAAAAAATACCAAGAAAACAAGCTCAACAATCCAATCA
ATCATTCAAGCGGTACTAAATGGAGAAGAGAGGGTGTTTTGTTGTGGGCCCAACCAAGGTCGTCCTACAT
TGCGTATGAGTAATTTACGACTTCCTTTCACGTATTTTCCTTTTAAGTCTAGACATATAATATTTGTTTG
GAGATTTTGTTTTATTCTGTTTAGAACCAATACTTTTTGTGGGCCAAACCACAAAAACACCGATTGAAAT
GATGGCTACACCAGTTTTCATCATTCGACGTGCCTTTGAAGAGGTGGCTTCAATCTATTTTATGACTGTT
GTTACCTTCTATGTGTATCCTCTTCTCAGAGGTTTTCGCGATGTCTTCTCGACCACAATGCTGTTATTTA
TGTTCATGTGGCTTTTGTTTCATGAAGAGGGTCCTGCGGGCGTGTTTTATTTTCCTGCTGGTTGTCCTTG
TGGCTTTTGCCTTTCCGATGGCTGGCAACGTTGCGGATGGTTGATTTGCTTGCTGTTTTTCGGTTGGTTA
ACGGCTGGCGAATAATGAGCGAATTTTAAATGAATTCAAACTTATTTTAGGCAAGTAAATTATACATGCT
TTGTGTTTGTCTGGTGTTTTGTAATTTATTTAACATTTCTGGTATTTTAAACGGTAGCGGATTGTTTTTG
ATAAATTTCTTCTTTGCAAAACATATTTTAAATTGAAATTATTTTCCGTATAAATGATTGGTACGTTTAA
AATTTATATTTATATGCATATGTAGCTTCAAATCATTCCGTGTTCAAACGAAATTTGGCACATGTCTGTG
TAAACCACGAATTATTTTGGGGCTTAATGGCATTACCACAAACCGCAGGAATTTCAAACCCATGGTCTGC
CATCAAATGGTTACCACGCAAAGTGTTGTTTTGCCTGATTTGCCTAGTTATCGTTAGCAGACAAGCTCGT
AAATTTGCTTCCTGCATTTATCCTTTTATTATATTTGGTTTCATGCATGTTGTGTTTCGGTTTAGATTGG
GTGTACACATTTAGAAAGTTTATACCGAGTTATTGTTAGATTTTTAGTTAAATAACATACGATAGATTGT
CTAGTTTGTATGAAATTGACTGGAGTGTTAATTACTTATCGACTGATATTTAAAAATTAAATTTTCGAAA

TTTTAAGCATGTTTCCGCCAGGGCCTTTAATATGCTCGTTGGTATTGCAGTTGCCTCATTATCGCCAGCG
TTTCATCCCACTAAAATGTAAACTGTAAACGGTTTTCCAAGAGAGGAACTCCCCACCACATATTCGCCTT
TATATTTGCCAACTGCGCTTAAGCTGCATAATAATAAACTGCAAAGCAGCGGCAACAACGAGCATTGCTA
AATGTGCAACTGCAACAAGGATGGCAAGAAGAACAACAACAGGGCCAGGATAATACGAGCCAAAGCAGTG
GAAATCCTGGCGTAATCGTTGCGTTCACTGCTGGCAGGCAACAACAACAGCAACTGGAACAACACTATGC
CGTGTGGAAAATATTTATGAGCTCGGTTTTCACGCCAAATGCGTTCTTTAAAATGCTCCACTCCACTCCA
CTCTacacacacacacacacacacacagagcacacgcacacaTATATGGCCTTACCATTTCCCTTTGCTT
TCACCTCGCTTTGGCATTTTCCGCGACATTTTCACAAGCGCTTGCATTGCTTGTTGCACTCTGCAATAAT
GTGTGTGTTAGTGTGCTCCTATGTGTGTTAAGCAATGCGCTAAAGTGTTCATGTGTTTGTGAGCGTTGCA
AGCCGACAAGCGACAGCAACAACAGCATTCTGCCACAGGAGCAACACCAGCAATTGCAACACCCTTTGAC
CCAATTTTTTATAGCCTACTCTTACCACATCCAATATACAAATATTTTATTCGAGAATAACATTTAAAAG
TTATTTAATAGATTATCCGACAAAAGCAGAGTTTTTTTGAAGTTGATAAAAAAATTTTAATTAAAATTCT
ATATCAAAGCTTATATAAAATACGCACTGCCATGTATTTTAACTGAAACTAAATGTGTTTTCAAATAATT
TAACATTTAGTCTTGCGATATTTCTTATTTTAAAGCAGCGTATTCGAAGTTTGTCTTCGGTGATACTTCC
TTCCACTACGAATCCCTTAACCTATCACCAACATTCGAGCCATTCCACCTGCCTTTCTAGCCCGGTTCCA
ACCCATTATGTCGACTGTCCCCTTTATATGCTAATTTGGTTCGGCGCATTTTATGTGTACATATGTACCG
GCGCATATATCCATAGCCATATATAGTTCGTCCTGCGAACAGGAGGCGCTGACATTCGTGGGCTGGGGGT
GGGGGAGTTAAGGGAAGTGTGCAGCTGCAGCAACTTTGCTGATTTTGCTGGCATTCTACGATGTTGCTGA
TGGTGCACATTCACGTTCGTTTGAAGTTTCTGTTCGCCGTTGTCATAATTAAAACGCGCTGCATAATTGC
CAAACAAAGGTTGTGTCCTGTTCGGCGTAAAGTCCTGTCCTCTATGCCACCGAAGTTGGTCATTCGTTTG
CCTCAGTTTTAGTGATTTTATCCCAATATTTGAAGAGTACAACTCCAAAACGGACCAATATCAAATACAG
CACGCAAACTAAGCACGGATtatatggaatatatatatatatatatTCATATTGATTTTTGCGTTGTGCT
TTTCTGAGAAAGATATGAATTTATTTCCAAAAAATACCCCTATGGTAAACCTATGCCATGTTTGATACAA
TTCATTTCGTTTCTTAACTAGAAACGAAGCTTTGCGCAATTTGGCCATCTGTATGCAAGCCCACTGCTCT
CACGCAAATAGTATCTGCATATCTATGTATTTATTTAAAATTTTACGCTCCTTTTGCTGCACTCAGACCA
AAGCGTAACaaaaaaaaaaaaaaaaaaaaaaacacaaaaaaaaTGGGGGCAAATTTGAAAAGTTTCGATA
AGGTTATGCAGGTTTTTCGGGTAATATTTGGGTGGTGATGGTGCGATGGCTGATTCCCGTATTCGGTAGT
CATAAAGTGTTCGACCGAAGTGCACCCACAGCAAGGTGGACTAACCAAACccacacagacacactcataa
atacacacacacactcgcactcacACGTAGTTGCGGTTTCAATAATCAATGAGTTGATTTTATGAAATTT
TACCCCCTACACCTCGAATTCCAGGATGTGACACGCCCATCCTTGCACACCACTTCATTTTGTGGGTCGC
ACTTGGACACACGAACATGCACCGCAtgtgtgtgtgtgtgtgCAATTTTATTAACGAGAATTTTGGAATT
TCTGCACAAATATCACATTCAAGCCGACAGTAGGGTGAAAGGTTTAAAAGGAAAACAAAACATAGACCAG
AGGCTTTTTGTGGTCCTGGTCACAGGCTGCGTGTTGATTGCTCGCCGCCCAAAAAGGCCAAATGAATTTT
AATTAATTTTTTAATTAATTCTCCGAAACCGCCAGCTGCCCCTTTTTACTCAACCGATAGGAATGATAAA
ACGAGAACCGCATAAATTCTATCTGCGGAAAAGTTTACCCCTTTAATTGTTGACCACCACAACTTGCGTC
GGATCTTTTCTTATGTCATATTCTTTCTTTGCTTCTTGCAGGGTGAGTGAATAGTCCAATCAATCAAAAT
ATCTAGTTAAGAATTCAGTACATTTATTAATTTTGGTTAGAACATTGACATCCATCAACTTGAATTGGTT
CTGCATTGAAGATTTTTAACTTTACAAAACTTTTAACGGATTTAAAATAGATTTTAGAATTTGCAGTTAA
TTAATCATATACACCTGAAATAATATTTTCGTGCTTTCGCTCCAGTTCATCTGCCGCTTTTTTCGATCTG
TTTGTTAACTTTCCAGCACTTTGGCCTAATGGCCTTGCAATTCCCATTTCCATTCCTATTTCCATTTCGA
TTTCAACTTTGGCTGTTGCTTCCGTTGTCATAGAAATGCAAAAACAAGGCAATAAAGTGTTTCTCTGTCC
TTCTCGCCATCTATCTTAACTTTTTTCGCGCTAAAACATGGAGAAACTATGAAGAAGTAGTACGCCATAC
AAGAAGAAATTGATTTATGCTACTTTTTCTGTTGTTGCTTCGTTCTTGTGTTTTTCTTCTTTGTGGCTAA
GAAATCAAGGAAATTTATTGGAAACTCTTCACCACTGCAGTTGACCCATTAAGAAAATGCCAGTTAAGCA

CTGGCAAACGGGGTAAATTCAGACCGCAGGGAGCGGGCAAATCAAAGCCCGAGAGCAAACTTTTGCCTAA
TGGAAATGGCAAATATTTTGGCTTTCAAATTGTCTTTGTTGCTGGCTTTTTGTTTCGCCTTCCGTTTCCG
GTCAACTAGGCCAAATACTGCGTCTCACTCACTCTAGTGAAACGTTGTTTGTGTAGTTGTCTTGAATTAT
TTATATAGTATAAAAGCTTTTTCATATTAAATGATTGAAATAATATTGTATCAAAACTGCTtataataaa
attgtatatatatataatttttatataacatttttctttatacaatcacaaaatatGTGACCCAGAACTACA
AATCTAATGAATTACTTTTAGGAATATATAGTTTTTAAATATTATGAGAGCACGAAAACCGCACATCGGT
GGTATTTTCTATCCAAAATTTCGAGTGCCCCATCTGAACCCTTGACTGTGTTTTCTTCACCATTTCTGTG
CATGCCCAAGTGCACTTCAAGGTTGGCTTTTTGCTCACGTAATTAGCCAACACTTGTTGGCTGGTCGTGC
CAACAATTGCACGGCGAACAAAAATAGCCGGGGCAACAAAAATACGGCAAGCCAGGGCATTAGCAGGTGT
AATTTGTTGAACTCAATTTGCTTGCCGGCTCCACCTGAGTGGTggggggggggggTCACTTTATATGTCT
GTGCCCGGGTGGGTCGCGTTATTTATGGCATCAAAGGGAGGAAGGGAGAGTGGACTGGAGCAAAGTTTCT
TGGGACCTTTGGCCATGGCGTGCTGCAATGCCATTTGGTACGGAAGGTAAAAACCTCTGAGAATAAATTG
CAATTGTTTGGCTGGGCAGAAGAAATGAAAAGAAAAGTTCAAAATTTACAATTTTAATTAACCGACATTT
TGGCCACTAATTTGGGTGCGAAAATGTCGAGTTAATCAAGCTGATAATCAGCAAAAGGACGCAAAAGCCT
GCAATCTTCTCAATGTGAAATGCATTTAAGTCGATCCGATATATATAATGTTCTTACTCACGCTATATGA
AATGTTAGTCAAGTGGTAAAATAAGTTTTCTTTAAGTCGTGTGATCTGCTAATTTTGATTTGTCCATACA
AGTTTTCTTTTAGTTGTTTGGCAAATGTTTAATTTGTTGATTTTTTAAGTTAAATTCCTTAAAATGAGCA
GTCAATATATGTATCGTAGTTCATTGCCGACATCCACAAAGCTGGTCATGGATGATTTATAAAATATTCA
AAAGTGGTCAACGATGTAAAGCTGACGAACATAAGAGACAAATATTTTTTATTCAACTGGCTGGGTGTGT
TGAACATAAGGAAACCAAATAAATCAAAGAGAAAAATGTTTTTGAAATGGTTCGTAGCCAGCGATGATTT
TCGAAATGAGCAATTAAAGCCAATTAAGTTAAATCAAAGTAGGAATGCAACAAACAATTATGAAGTAATT
TTCAATATTAATACACGAACCACAGAGCAACGAGAAATTATGCTGTAATTTGCATTATTGATTCCCGAAC
TTAAGAGAGTGGTTGTGGAAAACCGTACCGGTTGTTATCCTTATTAAATCGAACTATGACCTTAATCATT
GAACAGGCCACAGAGCATCACCCACGTTCCCATTTGGCCTTCTCTGGTTTTACCATCCAACATTCGATTT
GGTTTGCAAATGGCTTTTCCCAGAATCTGCCCTGCCCAGAAATACAATTCTGGGACCGAAAGTGGAATTG
CAGTCGCCCATTGTTCTATTGGAAAATGCTGTTGAATTCATGCGGAGCTTACCAACACTTTGGCCTTCTT
TGTCTGCCGGAGTTCCGGCAAAAGGGTTCTGAATTACCTTTTACCCTGAATATCCCTAACCGCATACAGC
TCTATTAAGCAACTTGTTCTGGCTCACCATTTAAATTGAGCATTACCAGACTATTTTCAAACTAATTAGG
TATTTAACTCTTACCTTACCATGCTAATTTTTATTAGATGTAATGAAATATATTACGAGTTTCGCCTTAC
ATTTGGCACACAAAATGTAAAAGATAAAACATAGCTTAACAAAGCCAAAATCAAACTTCTGCAGAAGAAA
AAGTTTCATTGTTCATTATGTTTCATTGTCAATGGAATGAAAAGTTGAAAAAGCAAAGGCAGGAAAATAT
TTTGACGGCTAACAAAACGCCCGCGGTTAGACGAAAATTTCGTAAATGATGCCAAAATATGACAAAATGA
ACCAAATTCAGCCGACGAGTAAAAAGCTAATTTCACTTCATAATTTTTAAAAGCTTTATTTGACTGACAG
GAATTAGACAGCCGAAGGCCACTCCTTGATTTTCCAACAACCGCAGATTTATAATTGTAATTAAAATGTT
TGCCAAATCAATATTCCCGCGTGGTATAATATTTAAAACAAAATAAATGTTTTTATTTAAAGAAAAAGAA
ACATATACTCACTCATGTTTTGGGGTGGAGCTACCATTTATGTGAAAAAACAATAATTAAGAATAATTTA
TTAACCATTGTACACAGCCAGTGGCAACCGCAAAATTTTGAAAGCAAATTAATAAATATCACAGTTAATT
GCATTCTAAACATTGTTCGTCTATGTCTGCAATGCGTGTTAATTACAAGCGAAATAAGGGAAACTACGAC
TTCACAATCAGTCCTGGACTTTATGTTATTGTTTTGTAAAATTATTTAAATTTATGCTCTAATAGCTTGC
CACACACAACAAGGATAGAACATTTTAATTATTTCACGAACAATAAACTAAATTAAATGGAAAACGAAAT
TTGTGCAAATACAAGATAGCGAAAGCTTAGGAAAAGTATGGGTATGAGCATAAATTAAAAAGCTAAGCAT
TTCTTAGGCATCTCTAATCAACTTCTTCAAGGACTTTTAAATTAGTGCACTTAAGGGTAATTAGCTATAA
AATTGTATATAAGTGCTATACTTATTAGTGCGAACTTTAGGACCCTTTAATTGGCCTCAAAATGTAGGAA
AATATTTAATAATGACATGAGGCAATACTTTGGTGGAGTAAAATAACTGGCGGTATATGAACGACCCCTC

CTCATAATAAATGCAACTAAAAGACTGGCCTCATTATTAACAGGCAAACCAAATATTTAAATATTTCAGC
GTACACAGGAAATGCAAGAGACACATAAAAGGTGTACAGTGGAATCAGCTGCCTGGGAGTATTTCAAATG
ATTTGTCATGTCAGAGTGCAAACACATCCGGCTGCAATGGTGACACAGGGGGCGCGGGGAGGTTGGTGGG
TTTTGGTATTTGTAGTGGCGCCGGAAATGTCAGGCAACAATTTTCCACCACTATGGGAAAAGTTTGTTCC
TCCTCTCCTGTTAAATGGGAGCAAGTTGCGCATTTGCCTTTTAATATTTATTGAGTTGCAGGTGCACCGC
CCCGGCAACATTCATTGTTTGCTTTCGAAGTGCTTTTGCAACGCATCAAATATTACAATTTGCAAAACAT
TTATGCAAAATAGGTTGGAAGTGGAAAAGCAGAGCAAATATGTTTACACTGTGACCTCTTTAACCATTTT
GGACACACATATTATTGCGCAAAATGACCTTACTTAATAACTAGCGAACTGGTTGAACTTTTCAAAATAT
TTGTTAATAGAGTATAGATGCCTTTTAGAAGTCCTTTTTACTTCTCTTTCTAAAGTACCCTTAGAGTCCA
TTCGCTTAAATTCTTAAACTTTAACATTTTCAGGGAGGCCTGCCAGAACTACATCCGCATCATGGTGGTG
CCATCGCCGGGTCGCCTTTTCGTTTGTGGCACCAACTCGTTCCGGCCCATGTGCAACACGTATATCATTA
GTGACAGCAACTACACGCTGGAGGCCACGAAGAACGGACAGGCGGTGTGCCCCTACGATCCGCGTCACAA
CTCCACCTCTGTGCTGGCCG
>dme_circ_0001895_junction_seq
CGTCACAACTCCACCTCTGTGCTGGCCGGGTCCCGAAGATGTACTGAAGTTTGTGG
>dme_circ_0001896
GTGGACAAGCAGCTGGCCAGCGGAGAGTACTTCCTCAACCAGGAGCAGAAGCAAGCGAAGCGCAACCAGG
AGCGCACCGAAAAGCAGAAGGAGGCGGCCAAGCGCCAGGACGAACGCCGAAACAAGGACTTTGTCCCGCC
CACGGAGGAGTCAGCTGCTTCAAGTCGGAAGAAGGAGGATGGCTCCTCCTCCAGCAAGG
>dme_circ_0001896_junction_seq
AAGGAGGATGGCTCCTCCTCCAGCAAGGGTGGACAAGCAGCTGGCCAGCGGAGAGT
>dme_circ_0001897
GATATTTTTTGCCAGCTAACTGGTACAGTGGGTCACTATGGGTATACATATACATGATGGGGCTTTTATA
TAGATATTATTTTAGATCTGAATGCTCACTATGATtataatatatatatatatTTTAGTTGGTGTTTCAT
TGTACATATATGCACTAAGAATT
>dme_circ_0001897_junction_seq
TTCATTGTACATATATGCACTAAGAATTGATATTTTTTGCCAGCTAACTGGTACAG
>dme_circ_0001898
GGGACCAGCTAAGTGACTCCTCGGCCACGGTCAGCTCCTCTTGCTCCTCCAGCTGCAGCTGGTTGCCCCT
GCACTTTCGCCGAGTGCGCACAAAGGAACGCTGCTGTTGCTGCTGTTCCTGCGGCGGCACTGTCGGCTGC
TGAGACTGATCCTGGTTCT
>dme_circ_0001898_junction_seq
GTCGGCTGCTGAGACTGATCCTGGTTCTGGGACCAGCTAAGTGACTCCTCGGCCAC
>dme_circ_0001899
GGAATAAGAGAGCACGTTTGCATTGTTCCACAGTGAGCAGATCGTTTAGCGGATTAACACGATCAAAGGC
GCCGCGAACGGTACTGGGAAAATTAATAATAAATAAGTCAGTTGATAATGGATTGAATTGGATTCTGTAT
AACTTACTCTAAAATAGAAGATATTTTGGACGGTGGATCCCTGATGCCACCCAGGATATCCTTAGTGCGC
TGCAGACCCAGTGGTATGTTATCCAATACGGGCAGACCGCGTCGGGAGACCAGCTCCTTAA
>dme_circ_0001899_junction_seq
AGACCGCGTCGGGAGACCAGCTCCTTAAGGAATAAGAGAGCACGTTTGCATTGTTC
>dme_circ_0001900
GAACCTGAGAGGCAGTAGTGGCTGCAATATGTGGCGCAGCACCTTGGATCGTCATGCTTGTGTTTTCCGT
CACAATCTTCAGCCACTGCTCTACCAAACGCTTGGCTAGAATGCGTACACCTGGAAAACGGGGTCGAGAA
AAGCCAATTAGTAATCCCTAGGTAATTAAAAGCATAAAGGACAGCCCAAAACATGACCCAAAAACACTAA

TCTACGACTAGAGTAAGACTTATCAAAGAAAACTTACCTTCGTTGCCGCCATCCTTGCACAGCCCCTTGA
CCAGAATAGGTGCTGAGTTTATCTTGAGGCGATTGACATCCACGGGAGAAAGGAGCAGCAGTTCCAGGAT
CTCCTG
>dme_circ_0001900_junction_seq
AAAGGAGCAGCAGTTCCAGGATCTCCTGGAACCTGAGAGGCAGTAGTGGCTGCAAT
>dme_circ_0001901
GCCAAGCGTTTGGTAGAGCAGTGGCTGAAGATTGTGACGGAAAACACAAGCATGACGATCCAAGGTGCTG
CGCCACATATTGCAGCCACTACTGCCTCTCAGGTTCCTGCATCCGTTTCTGGAGCTGGCTCCGCTTCAGT
TCCGGACTCTGCCTCTTCTTCAGACAGCACTGATAGCGCCGGAGCACCTGTGACGTATACCATCACCTCG
GCGGCCAACAGTAGCAATTCCAACAGCATTACCATCAAGTGGAAGCCTTTGTTGGACAACCCAGATGGTG
GCGACGAAACGGATTCTGGCACACAATCGAAAGACAACGAGGTGGAACATCTGGAAAAGGGCTCCTCCGC
AACCCACTTATTAGCAGATGCTAGTCTGGGGAAAAAGTCCGGGTTAGATGATAGTAGCTCCGCGGCGGAG
GTTAAGTCTGGGGAGGACGTGGATAATAAGAAGCATAAGAGCTCAAAGAGCGACAAAAGTAAACGGTCCG
AGAAGGAGCGCGAGAAGGATAGAAAGAAGGAAAGTTCAAGTCATAGGTCCTCCTCATCTAGCCATAAGTC
GTCCTCTTCCTCGTCATCCTCCTCTTCGAAGAGCAGTTCTTCCAAGAGCTCCTCATCTTCTTCCTCTTCC
CATCGCAGTTCTAGCGATAAATACCGCGATAAAGACAAGGCTCGCTCAAGTTCCGGCTCCTCCAGCTCGA
GCAAGGACCGGGAGCGCAGCAGCGGTTCGTCTTCTTCTTCCTCGAATAAACACAAGTCCTCAAAGTCCTC
CtcgtcgtcgtcgtcgtcgtcgtcgAGCAGCTCTGGTTCTAGTTCGTCCAAAGATCGCAAGTCGTCGTCG
TCAACTTCGTCATCATCCTCATCCTCGAGGCAGGACAAGGACAAGGACAACAAGCTAATGCCTCCGCCAG
CTGTGGCAGCATCAAATGCTGCAGGTACATCTCCTACAGCTTCGGCAAAGGAGAGCGACGCACAGAAGCC
CAGATCGATTCCTATCATGTCCCGCAAGGCCTCCATTTCGATAGAAATTCGCCGGGACACCGAAAAGACG
GCCACTGTAAAGACGTACCAGTCCAAATTCAGATCGCATGGCCTGACGGAAGAGGCTCCGCCTCCCCCAT
CTCGCAAGGGCCTAAAGAAGCCCACATCGTCGGCGGTTGCCGCTTCACCCGCTTTAATGGCAGTCCCGTC
TTCACTGAAGCGACCATCGCCGCCACCCAGGGATTTGTCCACCGACAAGAAGGCCAAGATCGACATCAAC
AACGTCGCGGGACATGTGGAGAGGCCCGGAGCCGCCAAGCTGATAGCGCCCAAAAAGAGTAAGCAACAGA
TGCTAGAATCACTATTAATGCTCTTCTCTATTTTATTCCACAGCCCAAGAACTTTGGTGGCTTTTGCTTT
TGTTTTCGATCGGAGTCGAATGTCGAACTCCGCCGTATTATTTGTTGATCTCCATTCCATTCTCAAGGTG
TTTAATTTTGCTTGGGGCGGGTCTTAAATTGTAGAGCAGAAGTGGGGCCTTTTAGTGTGGGTACTTTGCA
TTATAATGTATCAATCGTGGCGGTGTTATCCCCGGATCGAGTTTGAACTGCAAGCTGCCCGAGTGTGAGT
CTATGACCTCAATTCCGGATGACGGCGGATGAAGTTGAATGGAGTTGAATCCCGAAATCTGGGCACTGCG
CATGCGCTGAGACCGAACGAAAACGAAATCTGAGGCACAGACGATGCCGCCGCCGCCGTCGTCGTCGTCG
TTGTCGCTGCCTGATGTTAAGTATTCAAACCTCAAATCTTGAACGTATTTAGCTTAGCTTAGCTATAAGG
TATGTAGATTCACTTGGCGAGCAGCCCACCTTAAAGACTTACAAATAAAATACGGTTTCCTCTAGAAAAA
TACGATTTTTCTATTGAATAAAAAAAATGCATTGAGCGCAGTAAGCAACAGATGCTAGAATCACTATTAAT
GCTCTTCTCTATTTTATTCCACAGCCCAAGAACTTTGGTGGCTTTTGCTTTTGTTTTCGATCGGAGTCGA
ATGTCGAACTCCGCCGTATTATTTGTTGATCTCCATTCCATTCTCAAGGTGTTTAATTTTGCTTGGGGCG
GGTCTTAAATTGTAGAGCAGAAGTGGGGCCTTTTAGTGTGGGTACTTTGCATTATAATGTATCAATCGTG
GCGGTGTTATCCCCGGATCGAGTTTGAACTGCAAGCTGCCCGAGTGTGAGTCTATGACCTCAATTCCGGA
TGACGGCGGATGAAGTTGAATGGAGTTGAATCCCGAAATCTGGGCACTGCGCATGCGCTGAGACCGAACG
AAAACGAAATCTGAGGCACAGACGATGCCGCCGCCGCCGTCGTCGTCGTCGTTGTCGCTGCCTGATGTTA
AGTATTCAAACCTCAAATCTTGAACGTATTTAGCTTAGCTTAGCTATAAGGTATGTAGATTCACTTGGCG
AGCAGCCCACCTTAAAGACTTACAAATAAAATACGGTTTCCTCTAGAAAAATACGATTTTTCTATTGAAT
AAAAAAATGCATTGAGCGCAAAAGAAACGAAAACGAAAATATTGACTTAAGCTGGCGGTTCTATAAGTTG
GTAGGTGGTTCGATCACCACCGTGGGCTTGAGTCCCAGCTGCTTTGCTTTGGCCTCGATTTTGCTGTCGT

GCGTCACCGAGGGTCTCCACAATTGTTCGCCTCGCTAGAGCAGGCAACCCTAGTTTACCCACATCCTCGT
CGTAGTGCGTCATCAGCGGATGCGTCACCTTGGCAGTAAGCTGCACGGTGCGCGTAATCTGATCGCAGTG
TCGGGTTCCTTGGGTAGATTAGTGCTGAGGAAACCCAATGACACTTATCGATGTGCTGACATAAATATAC
TAAAAGCATCAAAGTTTAAAGAGGTTTAACTATTGCGTATAACTAGTAGTCTTAGCAATACACTTTTAAT
AAGCGTACTTCTCAAGTTTCAATCGCAATGAGAGCTTATAACTATTTTAATTATGATGATTAAACACTAT
TTGTGTTGAAAAACTAGTTAAATaaaaaaaaGTCGAATAATTACATTTTGACATTTATATATGAAATTAT
ACCGAATAAAGTCGAGCAAGCCAAAACTTTTAAAGAACTTTTGGGGAAAATTCATGAATTGTTTAGTAGT
TCTAGGTTTTGGTATATATTCGGTGCTTATTCCAGTAGAGAGACTCCCATTTCATTAATTATCGAAATGT
GGGTAAACTTAATCAATCAATCATAAACACAACAAATGTGTGTGTCCAGATCAGATTGTTTCTTTCCATA
ACTTTTTTACTTCTAGCAATTTCCAGCAAAAGCAAAATCCACCAAAGCCAATGGGCCATTCAACGAAGGC
CACTTAAACATTTTCTTAACTAAACCAATTACTGGAGCGATTACTTACTTTACCCTCATTTTTCCAGGTT
CAAACCCTGGTGGAGACCAACCTGTTCTCGGACGCCCTGGCCGCCTCCATCGAACCCAAAAAGGTGGTGA
AGCGCAAGCGTACGTCTGCAACATCGCCCACGGACAAGGACGCGCCACTGGCGCCGCTTAAGTTCTACCA
GGATACTCTGGACGAGTCGAAGAGCGAGGAGAAGTCGGACGACAGCAGCAAGGAAAACGATGATGCGCGC
TCCTCGTCACCTGGCAAGGACACGGATGCCGATGACGACGACATTCCCCTTAAGAGGGTCAAGGAGGACA
TCGAGCAAAAGGTGCAGAAGGCGAAGGCTGCGGCaggcggagaaggcggagaagCGGGTAACACCTCTGA
CAACGCTGAAGATCTTCCCGAGGAGCCCAAGAAGCCTGGACCTGGCTGTGGACCCGATGGACCACCGGGA
GTGCTCACGCTGCACCGCCGCAAGGGCCCGAAAAAGAAGCTAACCTGGCAGTCAGAGGAAAAGCTCACCC
AGATACGATACTTCGAGGTCGATCGCTCAGAGCGCGTAAATGTGATGAAGCAGACCTTCCTGGAAATGAA
GAACATGGAGCGATTCAGCGAGCGGGACGCGCTGACCATTGCTCGGCGTGGATTCGACGATACCATGGAG
CCGCAAATGGAGTGGCGGCCACTACTCGAAGTCGATAATGTGCCCGACCACCCGAACGGAAACCTCTCCA
AGCAGCGCCAGGTGCAGATGGACAGGGAGGCCACCACTCTAAGAGCCCTGTACTTCTCGCCCGACATGAT
TCCCGACAGTCCTGCGGAGGCGGAACTGGAACCGCATTTTGCTCATGACATTCCCGTGATACCCATGGAT
GACCTGACGGGGAATCCGGATGCTGTCAACGACTACAGTGCCTTGGCGTGGCCGGAGCCCAAGAAATACG
CGCCTAGCTCGGGAGTCGGAGCAAATATCAGCGGATCGGACATGGGCTTCAATGACAATATGTTGACCGG
ACTGGGAGCTGGACCGAACCTAATGCAAAATCCATTCGATCCGTTCATGGGAGGCATGCCTGCTCCCACG
GGAATGCCACCCGGACCAAACATGATGCCAAATCAAGTGCCCAACGGAACGCCGCAAATCTGGCAGAACC
AAATGGGTCCGGTAGGTGGGCCGCCCGGTGTACCGCCCATGATGAACGGACCCGGTGGCCCCGGAATGGA
TATGAGCAATATGAACATGAACAACATGCCACCGCAAAACTTTATGGGCAATCAGTTTGGAAGTCCAGTG
TCTCCATTCGGAAGCGGCCCACCACCTGGATTCAATCAGTACCCGCCCATGATGATGAATGGACCAGGTC
CGGGACCGGTAatgggacccaatggtccagtgatgggacccaacggccccatgatgaacggcccacccaa
tggacccatgatgaacgggccgcccaatggaATGGGGCCAGGACTGATGATGGGCGGACCTAGGAATaac
aacaacaacaacaacaggaacaaaaacaacGGCGGCGGCATCTGGCGCAGCGGGGGCAACAACTTCCAGG
AGAACTCCGGCGGCAACTGGCGCTCAGCAGGATCTGGCCCCAATCGAGGAGGTGGAGGAGGCAATGGCAA
CACCGGCGTCTGCAAGCAGTTCATGCGAGGACACTGCAACATGGGCAAGAACTGCAAGTATGTGCATCCG
CAGAAGAAGCGCTTGTAGGACCTACCCGAATCTTCTGCGGAAGATTCAGCTGCATAGATCTCAGACGCGC
AACGGAGGCCAAGATTTCCGAAATCGAGTCCCCACATGAAGACTGTATCCAGGTTAGCCGCCGCAAGTTA
ATCTAAGTGTATATATGTATTCG
>dme_circ_0001901_junction_seq
AGTTAATCTAAGTGTATATATGTATTCGGCCAAGCGTTTGGTAGAGCAGTGGCTGA
>dme_circ_0001902
GTAGCTCCGCGGCGGAGGTTAAGTCTGGGGAGGACGTGGATAATAAGAAGCATAAGAGCTCAAAGAGCGA
CAAAAGTAAACGGTCCGAGAAGGAGCGCGAGAAGGATAGAAAGAAGGAAAGTTCAAGTCATAG
>dme_circ_0001902_junction_seq

ATAGAAAGAAGGAAAGTTCAAGTCATAGGTAGCTCCGCGGCGGAGGTTAAGTCTGG
>dme_circ_0001903
TTGAAAGTGAAAGCGGCGTCGCATCATCAGCGTCGTGTGAAATGTTGTTGCCGGCGAGTCGAGATCACAG
AATGGACAAGTTATGCTAATGGCCACGAAATACCCCATAAAAAACTGACAAACAAATGCAACAAATCGGA
CGAGAATCGGCATATGAAAACTGAAAGCAGCAGTTATCTAGGCAAAATGCGCTTGCAACATCGTTATGCC
AGCAATTCAACTACAAATTCGAGTAATAATACTACCATAagcaccgccagcaacgccagcaacAACAACA
ACATCAAGCGGGCCAAGAAACTGAGTGGCCAGAACATTATCAAATTGCGCTCCGCCAAGCAATCGGCAGC
CAAATCAACCGCCAGCTCCAGCACCACAATGCCACCAGCCACCCCGCAGCCATTGAGTCGTCACCACCCA
ATCATGATGGATAAGGAATTGGAATCGGCCAAGGAAACGGCGGTATTATCATCGAATAATGACCAGAAGT
TGAAGGACGAGGATATCGAGGAGACCACCAGTACGACGACCACATCTGTGGAGCTGCGATCCTCGGCCAG
TTCGCATTATCGGGCATATACCACCATCCGGAGCAATTCGACGTCGGCGATTCTGCACGAGGCGGTACTA
CCGCCCCCGCTCACCCCCGCCGGAATGCTATACGCCCGCCCACGCACCCTCAATGTCCACAATGTCTGCC
AGACGCCCGCACAGATAACGCAAATGTTTTTGGG
>dme_circ_0001903_junction_seq
CGCACAGATAACGCAAATGTTTTTGGGTTGAAAGTGAAAGCGGCGTCGCATCATC
>dme_circ_0001904
TATTCGGCAAATTTTCGATGATCTTGGTGGACTGGAGATTCGTGAAACGACCTTGGATGGCAACCTTCCA
CTAAGCGATATGAAGCGTCTAAAGTTCCACCATGATGGTTCTGGTCCTAGTCACTCAGTTCCGGAGTACT
TTACCAGTCTGCATAAGCCTTTGGCAGCTGATAAAACTCAGGATGCCTCGGAG
>dme_circ_0001904_junction_seq
AGCTGATAAAACTCAGGATGCCTCGGAGTATTCGGCAAATTTTCGATGATCTTGGT
>dme_circ_0001905
TTTGAGTGGCTAGTGGGACCTGTTCCAATTGACGATGAGCTGGGCAAAGAGATCGTCACCATCTTCAAAA
GTGGCATCTCTTCCGGTGGAGTCTTCTACACCGACTCCAATGGTCGTGAAATGATGAGAAGAGAAAAGGA
CAAGCGGGAGGACTTTAGTCCCGATTTAAGTGAGCAGCCTGTGAGCGGAAACTACTATCCAGTCACGTCG
AGGATGGCTCTACAGGACAGTAGCAAGCGAATGGTCCTGCTCAATGACCGTTCTCAGGGAGGCGCCAGCT
TGGAGGACGGACGTTTGGAGATGATGCTGCATCGTCGTCACATTTTCGCCGACGGCTCAGGAGCTGCAGA
AGCCATCAATGAACAACAGTTCGGAAAGGGACTGATTGCCCGTGGAAAACTGTTTCTTTACCTCAACGCT
ATCGAAGATGGAGCCACTGCATCCGAACGGGTGGCCGAGAAAGAGATTCATTTGCCCTTCTGGAAGTTCT
TCAGCAAATCTAATAACATCCAAAGTGATG
>dme_circ_0001905_junction_seq
AGCAAATCTAATAACATCCAAAGTGATGTTTGAGTGGCTAGTGGGACCTGTTCCAA
>dme_circ_0001906
GGTAAAGAAACAGTTTTCCACGGGCAATCAGTCCCTTTCCGAACTGTTGTTCATTGATGGCTTCTGCAGC
TCCTGAGCCGTCGGCGAAAATGTGACGACGATGCAGCATCATCTCCAAACGTCCGTCCTCCAAGCTGGCG
CCTCCCTGAGAACGGTCATTGAGCAGGACCATTCGCTTGCTACTGTCCTGTAGAGCCATCCTCGACGTGA
CTGGATAGTAGTTTCCGCTCACAGGCTGCTCACTTAAATCGGGACTAAAGTCCTCCCGCTT
>dme_circ_0001906_junction_seq
TTAAATCGGGACTAAAGTCCTCCCGCTTGGTAAAGAAACAGTTTTCCACGGGCAAT
>dme_circ_0001907
GTCCTGGCTCTGGAGTTCCGTGATAGCGACACCCAGCACGAATTGGTGTTCAAGGCCTCCGTTGACAAAA
TCGCCAATTACTACATCAAGAAGGTTGATAGCAAGGAGAGTTCCAATGGCGTAAGGGTCATTTCTCAACC
TAAGGCGAATGCTGAGACCTATGATGACGAGGAGACGATTGTGCAGACATCGGCTGATCAAGTTGGTGCT
GGACAACAAAACCGGCCTCTTGAAGAGGGTTGAAATGAACGGAGTATCCGAGAACATTGAGCAGAGAGCTAT

GGTCTTTATAGGACCTATGACTCTGGTGCCTATGTGTTCCGTCAGTACAATCAAGGAGACTTCGTCATCC
AGGAAGATGGTGTCGAGTTCACTGTTTACGATGGAGCTTTAGTCAAGGAAGTTCACCAGCGTTTCAGCGA
ATACATTTCGCAGGTCATCCGTATCAGCGAGGACAAGCCTTACGTGGAATTCGAGTGGCTTGTAGGCCCT
ATTCCGGTTGAAGAGGAATTTGGCACGGAAGTAGTAACTATCTTCAGCAGTGAAATTGCCTCCAATGGCG
TATTCTATACTGATTCCAATGGACGCGAGTTGATCAGACGCGAAAAGGACAAGCGAGAGGACTTCACCCC
CGAGCTTGCTGTCCAGCCTACTTCCGGAAACTATTATCCCATCACATCGCGCATTGCTCTGCAGGACAGC
AACAAGCGCCTTGCGATCCTAAACGATCGTGCTCAAGGAGGCACCAGCATGAAGGATGGCCAAATTGAAC
TTATGCTGCACCGTCGACTCGTCCGTGATGATGGCTATGGAGTGGGAGAGGCCTTGAATGAGGAGAAGTA
CGGCCAGCCCTTGATTGCCCGTGGTAAGGTGTTCCTAATCCTTAACGCCGCCGACGAGTCCACGTCCGCC
GAGCGCGAAGCCGAAAAGGAGTTCCATCTGCCCCTTTGGAAGTTCTTCAGCAAGAACACTGGAAGCACCA
CCGCTGCTGCCAAGTCGGTTCCCAGCTTTGACGACTTCCCCAAGTCGGTGCATCTGCTCACCCTAGAACC
CTTCAACGACGATGAGATTCTGCTGCGTGTGGAGAACTTCAAGGATCACATCGAAGGCAAAGTGGTTAGC
TTTAACATTCGCCCGATCTTTGACTACCTGAACGGAGTCGAAATCCGCGAAACTACCTTGGATGGCAACC
TGCCCTTGAGTGACATGAAGCAGTTCAAGTTCCACGCTGAGGGTTCAGGAATTAGGGGATCAGAACCTGA
GTACTACACCTCGTCCCACAAGCCGCTGTCCGCTAACCAGACGCAAGATGCCGCCGAGTTCGCTGTCACA
TTGTATCCAATGCAGATTCGAACATTCATCATTAAACATGAATAATTGTATTTCAATGAAAATCAAAGAA
ATAAATAAAATTATGTCTGAATAAAAACACTGTCATGTCCCGAAACAAAAGCGAACATGGTTAACATCCA
TTTGGTTCCTCACTCCCACAACGATGTGGGCTGGTTGAAGACAGTTGATCAGTCTTATTATGGTTACAAG
AACAAAATTCATCATGCCGGTGTCCAATATATATTGGATAGTGTGGTGTCCCAGCTCCTCCAGGATCCCA
AGCGTCGTTTCATTCAGGTGGAGACGGCATTCTTTGCCAAGTGGTGGAACGTCCAGTCAGAGTCCATGAA
AAAGGTGGTCACCAAACTGGTCAACGAGGGACGTTTCGAGTTTACTGGCGGAGCTTGGAGTATGAACGAT
GAGGCAAATGTCAATTACCAGGGTGTTATTGATCAATTCACTGTGGGATTAAAGGTTCCTCAACGAGACG
TTTGGCTCCTGTGGTCGTCCTCGAGTTGGCTGGCAGATCGATACTTTTGGACACTCCCGCGAGCAGGCTG
CAATTTTCGCGCACATGGGCTACGATGGACAGTTCTTTTCACGAATGGATCATAACGACAAGGACACACG
TATGAATGACTTGACAATGGAGATGATTTGGGATGCCAGTGAATCGATGAGCGGCGTCAGTCTCTTCAGC
GGCATGTTGTACAACTTTTACTCGGACACTCCAGGCTTTTGCTTCGACATACTTTGCACTGATCAGCCCA
TTATTGATGGTGACAGCGAAGAAAATAATGTGAAACAGAGGGTAGATGACTTTATAAGTTATGCAAAGAA
TGTGTCTGAGGTGTATATCACTAATCACATCATGGTTCCTATGGGCGGTGATTTCCAGTACGAGGACGCA
AAAGTTAACTACAAAAATATGGACAAACTGATCAAGGTACATCAATGAACGTCAAGCGTCTGGTTCAAAA
TACAATATCTTCTACTCCACTCCAAGCTGCTATCTGAACTCCTTGCACCAGAGCCTAGAGTCCTGGCCAA
ACAAGACTCAGGACTTTTTCCCATACGCTCACGAGAAGAATAGCTTTTGGACTGGATTCTTTACTTCCCG
ACCCACTCAGAAACGATTCGAGCGTGATGGAAATCACATGTTGCAGGTGGCCAAGCAGCTTTCTGTACTC
GCGAATCTTACTAGCGAAGAGCATAAAATAGACCTGGATTATCTGCGCCAGGTCATGGGAGTGATGCAAC
ATCATGATGCCATTACGGGCACGGAGAAGCAGGCTGTCTCGGATGACTATGATCGAATGATGTACGAGGC
CATTGTTGGGGCATCTAACAATGCCCGCGATGCCCTGCGTCTTTTGACCAACATGCCTTATGGAGAGTTT
GAGAGTTGCCTGAGGTTGAACATCAGCGAGTGTGCTTTCACCAAGGATGGTGCTGATAACGTTTTGGTGA
CCCTATACAATCCCCTGGCCCACAGCTCCACTCAGTATGTTCGCATTCCTGTTAAGGAGGAGAACTATCA
AGTAACCGATGAGAAGGGCAGAACAGTTCCATCGGAGGTGGTTCCAGTGCCGTGGCAAGTTTTGGCATTG
GAGTACAGATCCAATGATACCCAGCACGAGTTAGTTTTCAAGGCAACGGTAAATAAGGTATCCAGTTACT
CTATCAAAAAAGTCGAGAGCTCAGAGGTCAAACATTCATCGATAAAGAAGATTAATCCCAGAGAAGATAA
CAATTCAGAGACTGTTGTAGAAAACTCGGGTTGTTAAACTAGTGATGGACAACCATACAGGACGATTGAA
GTCGGTCGAAATGAACGGTGTGTCTGAGAAAATAGTCCAGAACTTTGGCGTTTACAAGAGTCACACATCC
TGCGCCTACACATTCCGCCAGGATGGAGATATAGAATTGTTTGAAAATGATTTTGAGTTCACAGTCTATG
AAGGAAGTTTGGTCAAGGAAGTCCATCAGCATGTGAACGACTGGATTTCGCAAGTGATCCGTATCTACGA

GGGCGTAAGCAGAGTGGAGTTGGAGTGGATGGTGGGCCCTATTCCCATCGATGATAAACTAGGTAGTGAA
TTTGTGACAAACTTTAAGAGTGAGATTTCTTCGAACGGAGTTTTCTATACCGATTCAAATGGACGTGAAT
TGATGAAACGTGTGAAGGATAAACGCGAGGACTTCGTTTCCGACTTAAGCCGACAGCCTGTGAGCGGAAA
CTTCTATCCAGTCACATCGAGAATTGCTCTTCAAGACGATACCAAGCGTTTGGTCCTCCTGAATGACCGC
TCCCAGGGTGGAGCCAGTTTGGAGGATGGTGCCCTAGAGATGCTGATCCATCGTCGTCATCTCTTCAACG
ACGGTGGTGGAGTGGGTGAAGCCCTGAATGAGACGCAATATGGCAAAGGTCTGATTGCCCGAGGAAAACT
TTACCTCATTCTCGATTCCGCGACAGATGGAGATACTGTGACGGAACGTAAGACTGAGAAAGAGCTCTTC
CTTCCCTTTTGGAAGTTCTTTAGCAAAACTGGTGGAGTCGAAATAACTCCATCCAAATCCCTTCCCGACT
TTAATGATCTTCCGCAATCCGTTCACCTACTCACCCTGGAATTCTTCTCAGAGCAAGAAATTTTGATCCG
CTTTGAACACTTCTTGGATAAATCTGAAGGAAGGGTGATTAGCTTCAACATTCGGGATATATTCGATAGT
CTTGGCGGTTTGGCGATTAGGGAAACAACCTTGGATGGAAATATGCCACTGAGCGATATGAAGCGATTCA
AGTTCCATGCACAAGAATCCGGAACCAAGCCGTCATCCGTGGAATATTCTACGGCACAGCACAAGCCACT
TGAGGCTGTCAAATCGGATGAGGCCTCACTTTTTGCTGTAACACTATATCCTATGCAAATTCGCACTTTT
ATTATTAAAACAGAGTAAGTCATGTCCCGAAACAAAGTCCAATATGATCAATATTCACTTGGTGCCACAC
TCCCATGATGACTTGGGTTGGCTAAAAACGGTCGATCAGTACTATTATGGCAATAAGCACAACATTCAAC
ATGCTGGAGTTCAATATATATTTGATACCGTTGTTGCCGAGCTGAGCAAGGACTCACGTCGCCGCTTCAT
CCAGGTGGAGACGGGTTTCTTCGCCAAGTGGTGGGAGGATCAGTCGGAAACCAAGAAGAAACTGGTCAAA
ACATTGGTGAACGAGGGTCGTCTGGAATTCACTGGTGGCGCGTGGAGCATGAACGATGAAGCTGCCGTGA
ACTACCAGAGTGTAATTGACCAGTTTGCCGTTGGCTTAAAGGTTCTTGAATGACAACTTTGGGGTCTGTG
GTCGCCCTCGCGTCGGCTGGCAGATCGATGCCTTTGGTCACTCTCGGGAGCAGGCCTCGATGTTTGCCCA
AATGGCTTTCGATGGTCAGTTTTTTGCACGCATGGATCAGAATGACAAGAACAAACGAGTGGATAACCTG
GGCCTTGAAATGGTCTGGGATGCCAGCGAGACCCTGGAGGAAATGGACTTCTTCACGGGCATGCTCTACA
ATCACTACTCTGCTCCACCTGGCTTTTGCTTTGACTCACTCTGCCAAGATGATCCCATTATTGATGGAAA
GAGCTACGACAACAATGTCAAGTCCAGGGTAGATGATTTTCTCAACTATGCTTCCAATTTGGCTGGCTAT
TATCGCTCTAATCACATCATGGTGCCAATGGGTGATGATTTTCAATATGAAAATGCCTACATGAACTACA
AGAACATGGACAAACTGATCAAGGTATGTCAATGAGCGTCAAGCCAGCGGCTCCAAGTATAATATCTTCT
ATTCAACTGCTGGATGCTATCTGAACTCCCTGCACAAGAGCCTCCAAAGCTTCCCGAACAAGACACAGGA
CTTCCTGCCCCATTCCCATGAGGCCAAGAGCTTTTGGACCGGCTTCTTCACCTCTCGTCCCACCCAGAAG
CGATTTGAACGCGATGGCAACCATATCCTCCAAGTGGCCAAGCAACTGAGTGTGCTAGCAGATCTTTCAG
GCGAAGAACAACATAAAGACTTGGACTATCTGCGTCAGATTATGGCTGTTATGCAGCACCACGATGCCAT
CACAGGCACCGAGAAACAGGAAGTGTCCAATGACTACGATCGCCTTTTGTACGATGCCATTTTGGGAGGT
GCCAACACAGCCCGCGATGCTCTTCGAGTGCTTACCAATCAGCCAAATGGAGAGTTCCAGAGCTGTCTAA
AATTGAATATAAGCGAGTGCGCTTTTACCAAGGAAAATGCAGATGACTTCATGGTTACTCTGTACAATCC
ATTGGCTCACACGTCTACGCAGTATGTGCGAGTTCCTGTCAAGGAGGACAGCTACCAGGTTACCGACGAG
AAGGGTGGATTGGTTGCTTCTGAGCTGGTCCCAGTTCCCTGGGAAG
>dme_circ_0001907_junction_seq
TCTGAGCTGGTCCCAGTTCCCTGGGAAGGTCCTGGCTCTGGAGTTCCGTGATAGCG
>dme_circ_0001908
GCAAAGTGGTTAGCTTTAACATTCGCCCGATCTTTGACTACCTGAACGGAGTCGAAATCCGCGAAACTAC
CTTGGATGGCAACCTGCCCTTGAGTGACATGAAGCAGTTCAAGTTCCACGCTGAGGGTTCAGGAATTAGG
GGATCAGAACCTGAGTACTACACCTCGTCCCACAAGCCGCTGTCCGCTAACCAGACGCAAGATGCCGCCG
AGTTCGCTG
>dme_circ_0001908_junction_seq
CAGACGCAAGATGCCGCCGAGTTCGCTGGCAAAGTGGTTAGCTTTAACATTCGCCC

>dme_circ_0001909
CTTTAACATTCGCCCGATCTTTGACTACCTGAACGGAGTCGAAATCCGCGAAACTACCTTGGATGGCAAC
CTGCCCTTGAGTGACATGAAGCAGTTCAAGTTCCACGCTGAGGGTTCAGGAATTAGGGGATCAGAACCTG
AGTACTACACCTCGTCCCACAAGCCGCTG
>dme_circ_0001909_junction_seq
GTACTACACCTCGTCCCACAAGCCGCTGCTTTAACATTCGCCCGATCTTTGACTAC
>dme_circ_0001910
CTTTAGTCAAGGAAGTTCACCAGCGTTTCAGCGAATACATTTCGCAGGTCATCCGTATCAGCGAGGACAA
GCCTTACGTGGAATTCGAGTGGCTTGTAGGCCCTATTCCGGTTGAAGAGGAATTTGGCACGGAAGTAG
>dme_circ_0001910_junction_seq
GTTGAAGAGGAATTTGGCACGGAAGTAGCTTTAGTCAAGGAAGTTCACCAGCGTTT
>dme_circ_0001911
GTGATGGCATCGTGGTGCTGCATCACACCCATGATCTCGCGAAGGTACTCTAGATCCTCCTTCTGTTGTG
TACTCGAGAGCTCCGCAAAGGCGCTCAGCTGCTTGGCCACCTGAAGAATGTGGTTGCCATCGCGCTCGAA
TCTCTTCTGTGTGGGACGGGAGGTGTAATAACCAGTCCAAAAGCTGTTA
>dme_circ_0001911_junction_seq
GGTGTAATAACCAGTCCAAAAGCTGTTAGTGATGGCATCGTGGTGCTGCATCACAC
>dme_circ_0001912
GGTGACGCTGAACTCGGAGGCCTCCTGGGACTGCTCAGCAGCCAGTGGCTTGTGGGCGGAGGTGAAGTAC
TCAACAGCATCTGGCTTCTGTCCCGAGCTGTCGTGGTGGAACTTGAAGCGCTTCATGTCACTCAATGGCA
AGTTGCCGTCCAAG
>dme_circ_0001912_junction_seq
GTCACTCAATGGCAAGTTGCCGTCCAAGGGTGACGCTGAACTCGGAGGCCTCCTGG
>dme_circ_0001913
GGCCTCCTGGGACTGCTCAGCAGCCAGTGGCTTGTGGGCGGAGGTGAAGTACTCAACAGCATCTGGCTTC
TGTCCCGAGCTGTCGTGGTGGAACTTGAAGCGCTTCATGTCACTCAATGGCAAGTTGCCGTCCAAGGTA
>dme_circ_0001913_junction_seq
ACTCAATGGCAAGTTGCCGTCCAAGGTAGGCCTCCTGGGACTGCTCAGCAGCCAGT
>dme_circ_0001914
GTTATCCGCATCTACGAGGGCAAGAACCTTGTGGAGATCGAGTGGCAGGTTGGACCCATTGAGCGGGAAG
AAGAGTTTGGCAGGGAAGTGGTTATCATCTTCAATAGTACCATTGCCTCCGACGGCGTTTCCTACACCGA
CTCCAATGGCCGTGAGATGATCAAGCGTGTGAAGGACCAGCGTGAGACCTTCACTCCCGGTCTGGACAGG
CAGCCAACTGCAGCCAACTACTACCCGG
>dme_circ_0001914_junction_seq
CAGCCAACTGCAGCCAACTACTACCCGGGTTATCCGCATCTACGAGGGCAAGAACC
>dme_circ_0001915
GTAGTTGGCTGCAGTTGGCTGCCTGTCCAGACCGGGAGTGAAGGTCTCACGCTGGTCCTTCACACGCTTG
ATCATCTCACGGCCATTGGAGTCGGTGTAGGAAACGCCGTCGGAGGCAATGGTACTATTGAAGATGATAA
CCACTTCCCTGCCAAACTCTTCTTCCCGCTCAATGGGTCCAACCTGCCACTCGATCTCCACAAGGTTCTT
GCCCTCGTAGATGCGGATAACCTGGGAAAT
>dme_circ_0001915_junction_seq
CCTCGTAGATGCGGATAACCTGGGAAATGTAGTTGGCTGCAGTTGGCTGCCTGTCC
>dme_circ_0001916
GATGATAACCACTTCCCTGCCAAACTCTTCTTCCCGCTCAATGGGTCCAACCTGCCACTCGATCTCCACA

AGGTTCTTGCCCTCGTAGATGCGGATAACCTGGGAAATGTACTCGCTGAACTGCTGGTGGACTTCCTTGA
CCAATGCTCCATCATAAACGGTGAACTCAACACCTTCGTACTGGACGATGAAGTCAGCCTG
>dme_circ_0001916_junction_seq
CTTCGTACTGGACGATGAAGTCAGCCTGGATGATAACCACTTCCCTGCCAAACTCT
>dme_circ_0001917
GATGATAACCACTTCCCTGCCAAACTCTTCTTCCCGCTCAATGGGTCCAACCTGCCACTCGATCTCCACA
AGGTTCTTGCCCTCGTAGATGCGGATAACCTGGGAAATGTACTCGCTGAACTGCTGGTGGACTTCCTTGA
CCAATGCTCCATCATAAACGGTGAACTCAACACCTTCGTACTGGACGATGAAGTCAGCCTGGTGATATTG
ACGGAAGACGTATGCACCGGAGTCATAGGTCCTGTAGACACCGTAGCTCTGGTCGATGTTCTCGGATACT
CCGTTCATTTCGACAGTCTTCAGAAGACCAGTTTTGTTGTCGATCACCAGTTTGACTTGCTAATAAAGGT
AGGAATCAAAATGTTACTTTAGATACAAAATTAACTGAAAATCATTAACATACCGAAGTCTGCACCACAG
TCTCTCCTTCATCGTCGTCAAAGGTCTCAGTGGCATTCTTCAGTGAGTGAACCTTCTTGAAACGCTTAGG
CACCTCCAAGTTAGCTTCTTCAGCCTTGATGGACCTCTTCGATTTAGTGTGTGCAGCGACGTTCTTTGTC
TCCTGGCTGGCGACCTTCTTAATGTAGTA
>dme_circ_0001917_junction_seq
CCTGGCTGGCGACCTTCTTAATGTAGTAGATGATAACCACTTCCCTGCCAAACTCT
>dme_circ_0001918
TATGTGCGCGTGCCTGTAAAGAACGAGAACTACGAGGTGACCGATGAGAAGGGCCGTGTGGTGGCCTCCG
AAGTGGTTCCAGTTGCCTGGCAGGTCCTGGCCCTGGAGTTCCGCAACAATGATACCCAGCATGAGCTGGT
CTTTAAGGCTTCCGTTGATAAGATCGCTAACTACTACATTAAGAAGGTCGCCAGCCAGGAGACAAAGAAC
GTCGCTGCACACACTAAATCGAAGAGGTCCATCAAGGCTGAAGAAGCTAACTTGGAGGTGCCTAAGCGTT
TCAAGAAGGTTCACTCACTGAAGAATGCCACTGAGACCTTTGACGACGATGAAGGAGAGACTGTGGTGCA
GACTTCGGCAAGTCAAACTGGTGATCGACAACAAAACTGGTCTTCTGAAGACTGTCGAAATGAACGGAGT
ATCCGAGAACATCGACCAGAGCTACGGTGTCTACAGGACCTATGACTCCGGTGCATACGTCTTCCGTCAA
TATCACCAGGCTGACTTCATCGTCCAGTACGAAGGTGTTGAGTTCACCGTTTATGATGGAGCATTGGTCA
AGGAAGTCCACCAGCAGTTCAGCGAGTACATTTCCCAGG
>dme_circ_0001918_junction_seq
CAGCAGTTCAGCGAGTACATTTCCCAGGTATGTGCGCGTGCCTGTAAAGAACGAGA
>dme_circ_0001919
GTTAGCTTCTTCAGCCTTGATGGACCTCTTCGATTTAGTGTGTGCAGCGACGTTCTTTGTCTCCTGGCTG
GCGACCTTCTTAATGTAGTAGTTAGCGATCTTATCAACGGAAGCCTTAAAGACCAGCTCATGCTGGGTAT
CATTGTTGCGGAACTCCAGGGCCAGGACCTGCCAGGCAACTGGAACCACTTCGGAGGCCACCACACGGCC
CTTCTCATCGGTCACCTCGTAGTTCTCGTTCTTTACAGGCACGCGCACATACTGGGTAGAAGTGTGGGCC
AATGGGTTGTACAGG
>dme_circ_0001919_junction_seq
AGAAGTGTGGGCCAATGGGTTGTACAGGGTTAGCTTCTTCAGCCTTGATGGACCTC
>dme_circ_0001920
GTTAGCGATCTTATCAACGGAAGCCTTAAAGACCAGCTCATGCTGGGTATCATTGTTGCGGAACTCCAGG
GCCAGGACCTGCCAGGCAACTGGAACCACTTCGGAGGCCACCACACGGCCCTTCTCATCGGTCACCTCGT
AGTTCTCGTTCTTTACAGGCACGCGCACATACTGGGTAGAAGTGTGGGCCAATGGGTT
>dme_circ_0001920_junction_seq
ACTGGGTAGAAGTGTGGGCCAATGGGTTGTTAGCGATCTTATCAACGGAAGCCTTA
>dme_circ_0001921
GCACACATAATACACGAAAGGCACACAGAGAAGTCTCAAATTAGACCGTGAAAGCCATAAAGCAAATAGT

TAGCAAAGCGTGCAAAAAGAAAAGCCTAGAAAATTATTATAGGTGTTGGCGGGGAGGGAGGGGGGCAGAC
ACGTGCACACAGTAGCAGGGCTAGAATAGCTAGAAAATTATTATTATGATAATGAATTAAAGTTGCCATT
AATTTAGCCAAAACGGAACGGAACGCGAACGAGGCCTGGGAAGCGGCCACGAACAGCTAGACGGTAGCAT
AATAACAATAAATAATAATTTGAAATAGGCACACATAATACACGAAAGGCACACAGAGAAGTCTCAAATT
AGACCGTGAAAGCCATAAAGCAAATAGTTAGCAAAGCGTGCAAAAAGAAAAGCCTAGAAAATTATTATAG
GTGTTGGCGGGGAGGGAGGGGGGCAGACACGTGCACACAGTAGCAGGGCTAGAATAGCTAGAAAATTATT
ATTATGATAATGAATTAAAGTTGCCATTAATTTAGCCAAAACGGAACGGAACGCGAACGAGGCCTGGGAA
GCGGCCACGAACAGCTAGACGGTAGCATAATAACAATAAATAATAATTTGAAATAGGCGAGCATCAGAAA
GCAGGAGAAGACGACCACAAAGCACTGGCCAAGTGCAGCCTCCCGTTTCCAGAGGAGCAGCCAGCAAAGC
AGCAAGAGAAAAAACGGAAAAGGCAGCTCGAGCGCATCGAGCGTGCAAATTAAAAGCGCACCACAGTAGC
CCCAGCTCCATCTTCTCAACATGGAAGTGGCCACAACAAACAATCACAGTCAGAGCcatcatcatcatca
tcatcatcTGCAGCCGACGGCGGTGGTGGGGGCAGCGGGGGGCTCCAGATCCCCCTTCCAGAGGGAGGTG
CGTGAATGGCAACGCATCGATCCCAATACCGGAGCCCTCTTCAGCGGACGCTTGGAGGCAGACCGCTGGA
TAAACGGACCGCTGAACAGCTACGGCAAG
>dme_circ_0001921_junction_seq
AAACGGACCGCTGAACAGCTACGGCAAGGCACACATAATACACGAAAGGCACACAG
>dme_circ_0001922
GCACACATAATACACGAAAGGCACACAGAGAAGTCTCAAATTAGACCGTGAAAGCCATAAAGCAAATAGT
TAGCAAAGCGTGCAAAAAGAAAAGCCTAGAAAATTATTATAGGTGTTGGCGGGGAGGGAGGGGGGCAGAC
ACGTGCACACAGTAGCAGGGCTAGAATAGCTAGAAAATTATTATTATGATAATGAATTAAAGTTGCCATT
AATTTAGCCAAAACGGAACGGAACGCGAACGAGGCCTGGGAAGCGGCCACGAACAGCTAGACGGTAGCAT
AATAACAATAAATAATAATTTGAAATAGGCGAGCATCAGAAAGCAGGAGAAGACGACCACAAAGCACTGG
CCAAGTGCAGCCTCCCGTTTCCAGAGGAGCAGCCAGCAAAGCAGCAAGAGAAAAAACGGAAAAGGCAGCT
CGAGCGCATCGAGCGTGCAAATTAAAAGCGCACCACAGTAGCCCCAGCTCCATCTTCTCAACATGGAAGT
GGCCACAACAAACAATCACAGTCAGAGCcatcatcatcatcatcatcatcTGCAGCCGACGGCGGTGGTG
GGGGCAGCGGGGGGCTCCAGATCCCCCTTCCAGAGGGAGGTGCGTGAATGGCAACGCATCGATCCCAATA
CCGGAGCCCTCTTCAGCGGACGCTTGGAGGCAGACCGCTGGATAAACGGACCGCTGAACAGCTACGGCAA
GGGTGAGTGTCTAAAGACTAAAGGTGGTAACGTGATCGGGAGTTGCCCATCATTTGGTAAATATCCGGAA
ATATAGATGATCTGTACCTTGTACACAAGGTCTCAAGCTGCCAAACCTAAATATAAGTACAATATTTTCC
CCGATTTCTATATATGATGGGTAGCATACAAActtttatatatttctttgatctatatttaaattgaaag
atatatagatatattttctatatTTGATCAATCATCAGTTTTCTGAAATGACTACCAGTAAACACCGGTA
GCACTTCTTCCACTCACGAGCTAAGCACTCATCCCTAAAATATGGCCCCAACCACAATGCGATTACTTTT
GGCTCTGATTGATGGAACCAGTGGCTCCAGTCCATGACCAGCCTCATGCTCTAGTCTGCTCAGCTCCCCT
CGGGTCTAGACCTAATTGCTGGGCTCTCTGTTTGTCCATCGTATTTTAAATGAAATTGAAAGGTCCAGAC
TCTCTAAATGGGAAGGCGTTCACTAATTTAGTAGAGTCTGCGGCAATTGCGGCTTGATTGCTAGTTTCGG
TTTCCTTGCTGTTCGATTTTAATCAGTTAATAGTTTATTTAGTTGAATACTTAAAGCTTGGATGCATGAG
TAGGCAACTAGAAACAGAAGTTGATCGATTAAGGTGCATAAGTTCCATCGGAAATCAAAAAGAAAAAAGT
TAAGTGGTTATAACCTAATAGTTCAATTTAACAGTTATAAACAATATTATTTTGTTTAATGTAAGTAGCA
ACTGATGATTAAATAGCTCTAAACTCAATTAAATTCAACACAAATTCTCTAGAAGAAAACTCAAGCATCT
GGCTTTTAACAATTCGGCAAGAACGGAACGCTAACTACGCACAGCTGAGTTAAACAGCTGCTCAATGGTG
GTTACTGAATACCGCAATAACGATAAGCCAGTTGAGATTGTTTTGCGTGTCAGACGGCTTTTCTCCAAGT
TCCTATTAAACCACAGACATCGACTTCGAGATTCGTTGACCGTTTTTACTCAACCATAATTAACAGGTCA
CACGCATGGCTCTGATAAGTTGGATGCGAGTCCCAATTGCTGTTAAAAAAATAGAAATAAAATACATTTA
ATAGAAAGGAAAGGAAAATAAAGACTTTAACGCAGTTGCACACGCTGTGCCACTTACGATTCGATTTCGC

ATCAGAGAGATTTTCAGCAAAAGGCGAAACACTTCTGGCGGTTTCGGTATGGCCGTACTTAATTAATTCG
TGCTATTCTTCTTCAGATTTCAGATTTCACTTGTCTTTGGGCTACGCTTCAGCATTGATTGCTCTAGAAT
TTGTCGGTTAATTATAAATGCCTTTGGCTGGATGGCACAGCAGCACTTCGGGGCCTATCGTTAATTTTAT
GTTATTTCATCCACTGTCAGCCGTCATCAACCTAGCCCATCATCATCATCGTCTGGCCATGTGGCCATCT
AGTCATGTAGTGGTAGCGGTGTGTGGGGCCAAGTTGAAGTTCGCGCCTCGAGTGCTCGGCCCAGAACCAA
ATCGAAATAGAATCTTGGATCTTAGTTTGTGGTTCTGTTCGTGGTCCGGTCAAACTGAAATAGCCCCACA
TACTTAACGAGATCTGGACTTTAAGATACTCTAGCCAACTGCGAACTATTCGCTTTGGACTGCCCGGGAA
CGTCCATCAAATGCAATGACATCATGGCTTTGGTATTGCCGCACTCGAACTGAAGAGATTGATCCAAATA
ATTAAGTGATTTTTTGCTCTAACTGAATACTGATCGGCTTACGAAACAATAGAATATCCTATTGCCACGT
CGATGAGATCATAGCAAAATATCATAAGACTGTTGACCTTAGGATTGCACAGCCATCCGAATAATTTTAT
GACATTTTTAGGCTATTAGAATTTATCTCGACTGAGATTGGGAGCAGCGTGCAATAATTGGGGTTTATTT
CAATGATCCTCCGAATGTTGGAGATTAAAGACTATATATATGCAGCATATTACCTCTTATTTCTGTACTA
ACGAACGATCGAATGCGGTTGAGTTCAAAGTTGACATTTTAAGTGCATTCTTCCTGGAGCTAAATTCGAG
ATCATATAATGGCCAAACAATCACTCAATTTGGCAACTGTTACATTCACCCGATCAGTGTTGATATGCCC
TTTTTGAGCCATACTTTTATGGCTGCAGGGTATTCCGTGGAGAAACACCGAAGCCCGAAAGAAAAATACC
TCGGAAATTTGTTTCATTCCGCTGACTTTGTATCTTATCGCGATTGAACTTTGTTGCCACTGCGGCTGAC
GATGCTGAGAGCCTGGCCAAAAGATGAAAGGGTAATAGAGTGGGGGGATTGGCTGGGGAAACGATCAATA
CAATCAATTGGAAACGATCAGCCGAAAGAAGACACCAAGTCCTTGCCAGTGATGAGAAAAGTTATCCTTC
TTTAGCCTTAATAAGGCTAAGAATAACATGTTTGGCACACTTAAGTTTTAAAAATTATATGTTTTAACTT
GAAAATATATATATTTTTTAATATGTGCTTTGTTAATATATCGAAATACTTCCCCTTAATTCTTTATTAT
CCAAATGATAGTAATAATATAAGACGTTTTTTAATAGCCTCTAAGCCACATTTCTAAGAATAAGAGATTC
AGAGCTAGATGTATGCCATCGCTGGTACTAGCCCGCATTAGTTTCAGTTTAAAGAGCTCCACTCGGCTCT
TTTTGATGGCCAAGTCCGGGATTTATATCACCGATGTCCCGCTGTTCATTCGAAGCGCTTTTGCCGACAA
AGACAACGTCAAAGGCAGCGATTGCCGCCACGGATCGCAGGGAAATCAATTTGGCAATACCCATTATCCA
ATATCGAATTGGCCGGGTCTAGAGCAGAGCAGATCTCTATGCAGACGCACCTAGACACCGTTAGCCAGCT
AGATGGACGGATCGGGGATCTCCTGACAACTTCTTCATTAGCATTATGGTTTGCGGATTGTGAAGAGGGA
AAGGCAAGGCAGAAATTTACATTTTGCAAGATTCTTTTTCGTTTTTGTTTTGCAGCATGCAAGCTCAGTA
GAGTTTGAATTATTTGCTGGCAATTGCAGTTGGTTCGCAGTTGGGGTTTCAGTTGCTGGCCATGTTGCCA
CAATGTTGCCGCCTAACGGTACCGGGCCGCTGGCAATGCCGCCGCCTTTGTGGGTCACTGGGTTGCCACA
AGTCTAGCAAGAAAACATaaaaaaaaaaaaaCAAATACAATCCAGAGATGCTGCCCGCGGAGAAGATACA
ACCGGCCACTCACGGAATTTACCTGTGGTCAACTACCAGCATTTCATTTGTCTTTTTACCCCAGTGCTC
TTCAAGCCATTTGGCCGACACACGTAAATTACTTGCAACTAATTGAGGATGTCAACTGACGTGTCTTGCC
GCAATTAAGTGTTTCCTCTGCCGCTAATGAACCTCACGCCGAGACCGAAGTGAGTTTCTGGTGGCTGGTA
GGGGATTCAATGACGCAAAAATATAGGCACTCTTATGATATACTGACTGCACTCAAAAGCTTGAGCATAT
ATAGAGATATGAAGCCAACGAGATCGTGACAGAAATAATGCATTTACAAATGTATATTATTATGGCTTAA
GCCACAGGTCGTATGGTATGAGGTTCACAAAATGGCACAAACCAGAACAGAAAATACTTCACAGTGCACT
GTGGTCGTGCTCAGAAACATATTTTACAAGAGTCATTCGGCATGACCTTGACAAAATGTTCAATTACATA
TATCTTAATCATAAAAATCCCATGAATTTGTCAAAATAGGATCAGGAATGCCGTTCATAATGCTATAATT
GGCTATAATAAAATAGTGCCACGGAAAATGAAAGTGTAGTTCAAATACACACCAAATAAAACCATATAGT
TATATGAAGAATTGCAATTTATAAATCTTAGACCCACTGTGCATTGCTTTGCCGCAAAGTACCAAAGTCA
GTGCCAGCTAAAAATAAAAAGAGTCAATGGTTTGGCATGTCTATGCCGACGATCTGTGCGAACTGCCAGC
CAGAAAAGCCGGGATAGCCAGAAAAGGTAGAAAAGGCGACATGCATGCCACGAACTGGAGGACATATGGA
CCATGAGCCGGACTAGATGCCACATGGACGACACGGACGTACATGGTAGAGGCAGAGTCACACGGAGTCG
GGAGATAGACAGTCAATCTTATGGCCACAGACAGACGAACACGACCGGGCAGACGACTGCGACTGAGTTT

GCGACTGAGACCGTGATGATGATGGTGCAGCATCATTAAATTCGTTTCAAGCCCAGGCAACAAAATATTT
CGGCATATCCATAAACTGAGCAAAATATTTGGATCTTTAAAAATGAACATTAACATTGTACGAATTTTCT
TGGAAGTTTCCTAATAAGATACGTTTAAAAATAATAATAATTTATGGATTATTTTATGAAACAAGATTGG
TTTGGATATTTTCAAGCGATTTTTATCAGTGTAAAGCCGCACGCCAAGTCTTCCTCTACATCATCACGAC
TTTGGCTGGCATATAATATATGCGAGTGCTGGGCTCCGTGGAAGTCGTGGCAAGTCTCTGCATTTCATTG
AACCTTCAACTTGACTTTGCGCttcggttcggttcggttcggAACGGTTTTGGATTCAGCGCTTTCAATT
ATGTGGTTTCCttttttttgttttGCTGGCCATGTGTACATCATCAGCTGGTCGTCGGAGGCTGTACAAG
ATCATGACACTGATCGCTGGCTGGCTGATTTTGGCCAGGTTGGAATAATTCATTTACAGGCTGGCTTTCT
TTCCGCGCCAGCTGTTGCCATGATTGAGCCACCCCCTGGGCCATGCAGATTGGTAACGAACAGCTTAATT
AGCCTGGCCAAGATCAAGACCAGCCAAACAGCCTACACATGCTGGCCTAAACTAAATCGGCGCAGCGAGC
TGGAAAATCGGCGAGCCAGCGAAAAGAGGTCATCGATAGATAGACAGACAAACACATATTGCAGCGTTCA
CTCCATAGGAAATTGCGAGGCATTACGGAAAAGCGGTCCCAGATCGCCAAGAGTCTCCTTCTGACCATCG
TGAGAAACTCCGGCAGAACGTGCATGGCCGCGGGTGCCAAACTGGGCTTATAATGTTTATGTCCGCCACG
CTATTCTGCACTCttttttttCAGTTCCAAGTCCCAAGGATGAGTGTGACTTTAGGGAGCCGCTGAAATT
ATTATGCATTGTCCGTTTGCCCTCGGGTCAAATGGGAAGCTTGAAGACCTGAGCGCCGAACTAAAATCAC
TAGCCAGCCAGAAGTCgaaccaaactgaattgaactgaactgaactgaataagtgaaACCGCCTGACGGA
GAACTTTGAAAGGGATAACCCTGAACTCGTACAATGTGCATATCATAAAATATAAAGCTGGTGCCGAGTT
CTTCAAAATTCCCAGAATTTGCGTATCCCTTATTTATATTCCATTCTTTGTTCAACGAGCTGaaaaaaaa
TTCCCATCTAATGAAGCTAATTACCAAGTGTATAACCACAGAAATTATTTTCGCCATTCGCCGAGTTATG
AGTTACAGCACAAAAAAGACAAGACTTCAAGTGAGAACCCAGAACACTGAGGTGACTCAACCTAAAACTT
GCATACAATATGCAATTTTCTTTGTTACGAACCGCATGGAGAACATTCAAATTTTTTGTATTCTATATAA
ACAATGCTGCACTTTGACTCATAGTCAAATGTGCGGCTGCATTTTGGTCATTGATACGAATACGCCGCGA
ACTGGACAACCGGCGGGTTAAAGTTTTCATTTGGAGCATATTATGCATTTTAGCACTATAAATCATCAAT
GCAATCGATTGCCACAATCGATCTTTTGGCCAGTTCGATTCGAGTGTGTTTTGTATTTCTCTTTCTGTTT
CTTACCCTCGATGGTGTGTCATGGCACGATCCAATGAACGGATCTGCTAATTGTTCAAATCTATGCACAA
AGAAAAATTAATAACTGAAAGAGTATTTTCTTACATCTAAAGATCTAAATACTTTGAAGGTTTTTAAGAG
CAAATTAATTATTTGTCAAATGCTTGGATCTCACATCCCATTTTTTTGAGTGCATTACCGCAAAAGGGTG
CAGTTTCTGTTTTGTTTGCCGTTGCAATTGCAGTTGCTCTCGAGAGTCTTTTGATTTGTGGCTGGCGTTT
GGTCACGTGTGCCAAGCGGGCGAACTGAGCACGCCCAGCTGTCGAGATAAGAGGCATCTAGGCATTTTTG
AGGGGCAACTTTGAGGGAGGGGCAAAGGGTGGCTGACGGTCAAGTGTTTCGTCTGGCAACTTTCATACGC
CCCGTTGCCAACGGCCGAATGCCATGCGACAAAGCACTTTTACGCCTCGCATGGGACACACCCACACCCG
TAATGAAGTTTAGACAATCACATCGCGATCGCTTGCTTAATGTTTTATTTATCTTAATTTGAACCCATTC
AATTGCTATTATGTGTAAGAAATCGATATAACAAGGCATCTCTTGATGTATTAATTAGATCAGGCAATGT
GTGAAAGTTAAGGTGGCTAAAATAACATTTGAAGATCAGATTAATATATTTTGTAATTTAGGCTTATTGA
TATTTATATATGTTTAATATGTATGGGATGTGTTtaaaataaaaataataaaatggataaaatgaataTA
GAACATACCTAAAGCACACTCCAACTAAATTTAAAAAACTCTTACTAAAATCCTATTACTACATTATGTT
TATATTAATTCCATAAAATATCCCCCTGAAACATCCACTCATTATCCTGCCACCACTGAATCCCCctctc
tctctctctccgctctctcCCCAGATATCCGACTCCCAAAACATCTCACAGCCAAATGGCACACAGCA
CACGCAACGCAAACAGTTGGAGGTGCTGAAGGCCCGCACCGCCAACGGCGCCATGCAGGTGATCCGCACC
CAGACAGTGCAAAAGAGTTCGTCCTCGTGGTCATCCTCCACATCCACAACCACTACATCCACCACCCACC
AACGCACGAGTCAGACCAATGGCCACGATCCACCTCCAAAGTCCACTATCCTATCCCCCATCAGCCAGGG
TGTTGACATCTGCGAGCTTAGCGACGATTGCAGTCTCAGTGGCAGCGATGCTGGCATTGTTCATACACCA
TTCGCCGCCACATCCGCCTCTGCCTCCGCATCCGCAACTGCACTGAGCCAGGAGCTAATCGACGATCATG
TTGATTTTGTTGTGATTAATGGCGAGGCGAGCGATAAAGAGGACGCTGCCGACGAGCCTTTGCACAACGG

CTGCCACAATTTGTTACCCGATACGGCTCCAAGTCTGAAATTAAGCAATCTAATGGTAAGTTTGAGCCCG
GCTCAAATGCAGTAGTCCACAGTCAGGGTCTTGTTTCAATCGCCTTTGTTACACTGGAATACATAAAGAG
TTCTTCTGACAGAATGTATGTAATATTTTCGAAAGTTGCTAATTTTTTCTGCATTCTTATATTTCTATAT
TAGAACGCATAATGATAATAAATTTGAAAGTACAGTCAATTATATGTTGGAAATATTCAACTTGATCCTC
CTGTTTTCCCAGTGTAGTTGGTTTTGTTTAGTTAGCTCATTAGCATGAGCCAATGTTTGCTTTCTTCCAT
TTCGTTTTTTATTCAGGAGTAATCTTTTGACCATGGCCATGGCAATGACCGAAAATGGCCAGTTGTTAAC
AGTCGCCGATTGTCGTGTTTGGGGGAAAACTCATGGCTCTGGGAAATTCAAGGAAATCGTGTAATACACA
GGGATCGAAAAATATCTTGAGAAAGTCCTAAGGTGGTTGTTAACTTCACAACATTAAGATTTTATCGGGG
TGCTTTAAATAAATAATAGAGCTGCAAAATAATTTATAACATTTTCTTGGAAACATAACCGATAATTGTA
ATAACGTGAATTTATGGTCGTTTTTTAGTCGAACATGTCTtataacatctataaaatctataaaaACGTT
TGGGTAATTTTGAATTCAGACCGAATTAGTCTTGGCCAAAAAGATCAGTTATAAATAAATGGAAGTGTCA
TTTCAGAACATTTTACAGCTACCAAAATAAACTCATAAAAGTAGCTTGTTTTCTGCAACTGGCCATAACT
CATATATCAAATTAAACCTTACCAGACTTTCCACAAACTGTCTGGTACAACTGAATGTTTGACTCATCGA
TCGAATCGGAATCGAATGGAAATATTTAATAATAGCGTGGCTCAATTAGGCGTTGCTTAGCACTTCAGCT
GGACATTTGGGTCATGACCACAAAGGAAATTTCTACGCCAACTCTCTCAAAACGCTATTCGCATAGTTAA
AAGTGTCTAATTATTGGGCGGCaaaaaaaaaaaaaaaaTGATGAGAAAGTGTCTTGAGACAAGATCAACG
CCCACAGTCACAGTCAGTCACCTGAGGTTCTGGTCATTAATCTTACTACGAAAACGTATTCGGGGAATTT
CGTTTTGTTTCTCAATTTGTTAAGCCTAGAAAATGGGAAAAAATATATTTAACTCTATGGAGAAAACATT
AAATATATTTATTTTTAGAAAAATATACCCTTTGAGAAGCATAAGTTTTGCATACTTTATAATTGTAATC
GTTACATTAAGACATTAACTTAAACTACTTAATCTGCATTATATTGTACGATTTATTTTGCCTTATAGCA
AGTAGATCTATTTCCATAAGCTACTTATGGCCTGATCTATTCCATTAAAAGTTCTTTATTTATTCGAGGA
CTCAATTCTCTTGAAACCATTTGTCTCCAATGCGGAAATTCATTTGCCACGGCTTATTCAGCGATTAAAT
GCGACTTAAGTGTGCGGCTTCCTTAAGCAGTTCCTGTCAAACACTGCACCCACTCCTATATAGCATAACA
ATTCCAATAACAACGGCTTCAATAACGATAACCATACGAAAGACACCCGCACTCGAGATCGCCATATAGC
CCGGCTATATAATGCGCTCAGTCGGACTGAGATCGTCTTTGTGCTGgtttttgaatatgcttgattttgt
ttaattttgtttttaatttttCGCCTCTCGTTGTTGTGCTTGCCATTTACAGGAAAAGCAGTTCGAGCAT
TTCCAGCCAGAGCAGCAACAATTCGAACCTAACAACTGCAACACAAGCGGCGAGCAACACCCACAGAAGT
GCGGGCAAAATAAGCCTACATGCAATTGTGGGACCAGAATCATCATTATCATCATCATCAACGCCGTCGT
CAGCGGTTTGGGCCAAGCAGGCTGACTTATATGGCCAGCTCCCAAGTGGCGTGggtggttctggtggagc
tggtggtgctgACGTAGCTGTTGCCCCGCCAACGCAGCGTCGTGATTTGGAAAGTTTCCGGCATTATAAC
GATGACGACGACGACGACAACAATCTGCGGACTCACCGCTCGTCACCAGCGTCGCCAGCAGGTGTCCCAG
TCGGTCTACGCCCCTCCATTGCCCTCGATAGGATCCACGCTGCTTCAGCGGTCCCGCTCCATTTCCACGG
ATGATCTCAGCAACGACTGGGAGCGAGAGGATCCCGCCGAACAGCCGGTGGGTGAGTGGCGCAGGGTTAG
CAAACTGCGTCGCTCTTTTCAGTCGCAGGATCACTACAAGAGTCCGGCGACCTCGGCCCGACCTCGACCC
CTGGATTTGCCGAGTAATTCGGTAAGCGTAGCCCGTCTGCGAGCGGAACTGGAGAACGGTCGTCGCCTTC
ACACGGCCATGCGGAATAATCACGTGGATCTGGCCGCACTGGACAACATATTGAACACTCCCTCGGCCAA
GCCCACGGTAGCCAAGAGGAACACCTTCCTAACCGCCGAGTCCCTGCAGGAAATACGCGGCAAGCTAAAG
AAGCTTTCGGACGAGAATCTCTACAAAGAGGACTTTCTGGCCTATCACCAAAAGAAGCCAGTGGTGGTGC
GGGAGATCAGCGTGGAGAAGGTAGATCCTACGCCGCCGGCTCAATCCACTCCCTCGGCCTTTCGACCCGT
CCACAATACGCACAGCTTGGAGTCACGCCAGCGACAGAAGGACACCAGTTCCAGTGAGTGGCATCTGCGG
CGCAAGTCCTACGGCTTCGAGAAGATGTCGCCGCCGGAGGATAAGAGCATCTGCCGGGTAGACGCCTCCA
CGGACAGTGGCCTGGGCAGATCCGGTGAGCAACTTGGCAACTGGTCGCCCACCGAAAGGGAGCGGGAGAG
GAACGAAAAGAATGCAGCTGCGGCACAACAACAGAATGGCGTTGGCACCATTGTTCATTTTGGCAGGTCC
CTTAAACCCATCATCCCGTCATCCAGCCCCACAGAGGGTGAGCTAAGTAAACGTCATTCGATAGCCGTGG

AGGAAACGTGGCGTGATGTCCGCAAAACGTCGCAGGTTCATGTGAATGGAGGCACTGCCGTTACCAGCAG
CTCCCACACCACCACCAGTCACCTGAGCAAAGGTAGCGCCCAGAAGCGAGTGGAGTTCTGCAAGACGGAG
GTGCACTTTGCCGCCGAATCGGGTCGCGTTAATATCGTCGAGACCGACGGCAAGCCGCCGCCAACGCAAA
ACTTTCGCCGACGCCGCCGCTCCAACACCACCACCACCACCAGTGGTCCACTGCAGAGTCTGGTTAAGTC
AGCCAGTACCGTCGGAGGAGGGAGCTCCACCAGCAGCACTGCGAGCAGCGAGGTGACCCACTTTGGTGAC
GATTCACAGCGCCGCAAGACGATAGCCACCACCACAGTGGCCTACCGAGCCACTCTCATGGATCCGCCGG
AGGTGGTTAGCCAGCCGGATTTCCAGTAGCAGTAACAGCACCTCCAGCAGCACTCAAGTGACGGTAACCA
CGGTGGCCAAGCCGGTGCACGAGCCACGTTACAGTCTGATGGAGTCTACGTCGCGGAACAGTTACACCTC
CACCAGTGGCGTGGATACCACGGACAACGAGACGGACGAGCTATCCAACATTCGGGGGATTCTGAAGAAT
AAGCCTGTTAAGCCGAAACCCTATCATCTGGGCGAGAACATAGATAGTGCCGACGTCCTTTGGAGCGTTC
CGGCAATGAAAGCGGATCGCGAGAGTCCTTCGGCAAGGGATAGTGGGTTGGTGCTTTAAATATACCTAAG
TATACTTAGTTGTACATTTGATTAGCTAGTTGGAATGCCTGTAGTTTGTAGTACCTTGTCCACTGAATTA
CGTTGTGTGATCTAGTCTATCTGGGATTTAGCCATGGACTCCTCTACTTGACTCGAACTCTATGTACTCT
GAATTGTATTTTTAATGCATGTTGTGAACTCTGACTGCCGCTTTTCTAAACCACATCTAATGCTACATCT
AACACCACCCGCTAAGCTCAGCTCGATAACCACGCTCTaaaaaaaaaaaCAAACAAAAGAATAAGAAACA
TGAATTTCCAAATCCACCAACAACACTTTCCGCACATTTCCCACACAGGCACTGCAATGCATCACCATCC
CAGCTCGAAATACCGCCAACACCGACGACCCAGTGCCCAGGAGCTGTTGCTGGAGGATCTGCGCCAGCAT
CAGCGCATCCTAGACGAGGGATTAAAGTCCACATCGCTGATTATGAGAACCATGCGATCGGCCAGTGAAT
TCGATGAGGCCATGCGTCGTCTGAGCATAGCTTCGATTGAGTCCGCCTTGGTTCAACCAACAATAGTGGT
GCCCACGCCCATGCTGCGATCGCACAGCTACCAGGAGGAGGGATCCTACTCCTCCTCGCGCCGTCCCTCC
ACCATATCCACCACCTCCATTACGAGCAGCGATCTATTCGGAAGCGCATTGTATGATTCCCTGCCCCGAA
CTCCGTTGGCTCCACCTCGCCTGAAATTATTGGGAAACACCCAAATCCCGGTGAGCCAGCAGTTGACTCA
ACTGCGAAGGCTCTACGATGCCGCCGAGCAGGATCAGGAAGACAGTGCCGATGAGGAGGTGAAGCGTTAC
TTCCGGGACAGCAACAGTGACAGTGGTGGGGGAACCCAGGGTAGTTCCTCGCCAGACCAGCAGCCGACGG
AGACGTTCAAGGGCAGCGAGTACTCCAGCAGCTGGAGTCGCCTCAAAGCCAAAAGAACCATCTGGAAAAT
CGAGGCACAGGATCAAATGCTAAAGCGAGCAGGTAACGCTTGCTAAAACTACAGAGAAATAGAGTTTTGA
GTTAGAGGAGTTCATTTAAAGCAATATAATAAGGAGTAATTGAACCAATGAACCAGTAGATAACTATAGT
CCGATTTGATAGTAGCCATTAAGCTGTTTTAGCTTAATAAATTCATAAATAGTCAAAAATAAGATAGTAC
ATTTTAATGAAAGTATTAGATCAAAAAATCAAAAAGCTCCTTACATATTTTTTTCATCACCTTTTCAGGA
TCTTCCCAAGTCCAATGTGATGAACATAGCGCTCCATGCTCCCCAAATCGAGAAACCTAAGGCAACTCCG
CCCACTCTACGAATTGACCAACTGATACCAGTGGCCAAGCCAAGAACCCTCTTCATTCAACCGCAACTCC
AAGCGCAGTCTACCGTGGATAATGCCGACGAATCTGGCAGCGAGTCCTTGAAGATTGTAAAGGAGGCACG
CGGTGCTCGCAAGTTGCGCGAACACGAACTCTCCTACTTTGGAGTTCAGCATAAGCAGCAGCAGCAAACG
AAGCTATCCACAACGACCACGACAAATCCCAGAAGGACACTGCCCAGTCGCAGTAAAGTGAGCCACAGTG
ATGAGGTGAAGGCAAACACCACGACCACGACCAAGTGGCAATTGCTCAACGATAGACCCGACCTACTGAG
ACACAGCAGTCCCCATCATAACGACAACGGCACGGCCAACGACTACAACCACAATGATGATGATCTGGAT
GATGACGAGGAGCACTGTTACGAGAACATAGCCACCGAACTGACCACCACCTTCAGGGTCAAGTCACCAG
ACCACTCGCCGAACAGATCCCGATCGCCGGGCAGCTATGAGCGGAAGAGCGACCTGCAGAGAGACGCCCA
GATCCTAAGCGAAATGACACGCAACGCTGATCAGACTTTGAAGGGCCCTCTCCGACGAGGCAGCCATCAA
GGATCAGCGGCGCAGATCATGTTCGCTGCAGCGACGCAGCGCCAAACCTTTGGAGACCATTGACGAGAAG
GTTAAGGTATACCCACAATCGATGGGCGTGGCCCGCGGCACCTTCGCCTCTGAGGCGCGCCGCAACTCCA
GACAGTCGACTCCTTCGCCGTCGAGGGCCCGCAGCTCATCGCAGTCTTCCATCGAGTGTTGTCCACGTGA
TCGGTCCCGCTCCAGTTCGCGGGAGTCCATGACCCACGGCGGCGGATCCTCCGATGACCAAGTGGCCACC
AAGCAGCGAGCCGAACGTCTGCGCATGCGTACCCCCAAGCGAGAGAAGTCGCGCCATGAGCCAATCGAAG

TTCGGGTCCATCGGAACAGCAGCAAGCCAAGGAGCAGTTCCAGTGCCACTGGAGCAGGATCATCTTCCCT
TGAGTCGAAGCGAAGCTCTCATCACAGTCGCCACAGCCGGGAGCTGGATCAATCGACTGAGACCAAGACC
TCATCTTCCAGTCGCTTGATAAGATCCTCCCGGGTGGCGGATGAAAGTCGCTCGCGGCAGAGCAGCCATC
GGGATCGGGAAAAGGAACGCGATCGGGAACGCTCTCGAGGCACCGAGAAACACCGCGATGAACTCACGCG
CTCCAGGG
>dme_circ_0001922_junction_seq
CACCGCGATGAACTCACGCGCTCCAGGGGCACACATAATACACGAAAGGCACACAG
>dme_circ_0001923
GAATGCAGCTGCGGCACAACAACAGAATGGCGTTGGCACCATTGTTCATTTTGGCAGGTCCCTTAAACCC
ATCATCCCGTCATCCAGCCCCACAGAGGGTGAGCTAAGTAAACGTCATTCGATAGCCGTGGAGGAAACGT
GGCGTGATGTCCGCAAAACGTCGCAGGTTCAT
>dme_circ_0001923_junction_seq
TGATGTCCGCAAAACGTCGCAGGTTCATGAATGCAGCTGCGGCACAACAACAGAAT
>dme_circ_0001924
GCCGAAACCCTATCATCTGGGCGAGAACATAGATAGTGCCGACGTCCTTTGGAGCGTTCCGGCAATGAAA
GCGGATCGCGAGAGTCCTTCGGCAAGGGATAGTGGTAAGTGTTGTGAATATAATAGTACAGACGGATTAA
AGGTAATAAAATGTATTTTTCACCGAAAGGAATCACCAGTGATTCACCAATTAGTCCCAAGTCTGTGGCC
GAAAGGATTCGCATCGTAGAGCAGCGCCAACAGCAACAGCAATCTTCACCAGCTGGCAATGGGTACTCGA
CCAAGATCAAT
>dme_circ_0001924_junction_seq
TGGCAATGGGTACTCGACCAAGATCAATGCCGAAACCCTATCATCTGGGCGAGAAC
>dme_circ_0001925
GGCACTGCAATGCATCACCATCCCAGCTCGAAATACCGCCAACACCGACGACCCAGTGCCCAGGAGCTGT
TGCTGGAGGATCTGCGCCAGCATCAGCGCATCCTAGACGAGGGATTAAAGTCCACATCGCTGATTATGAG
AACCATGCGATCGGCCAGTGAATTCGATGAGGCCATGCGTCGTCTGAGCATAGCTTCGATTGAGTCCGCC
TTGGTTCAACCAACAATAGTGGTGCCCACGCCCATGCTGCGATCGCACAGCTACCAGGAGGAGGGATCCT
ACTCCTCCTCGCGCCGTCCCTCCACCATATCCACCACCTCCATTACGAGCAGCGATCTATTCGGAAGCGC
ATTGTATGATTCCCTGCCCCGAACTCCGTTGGCTCCACCTCGCCTGAAATTATTGGGAAACACCCAAATC
CCGGTGAGCCAGCAGTTGACTCAACTGCGAAGGCTCTACGATGCCGCCGAGCAGGATCAGGAAGACAGTG
CCGATGAGGAGGTGAAGCGTTACTTCCGGGACAGCAACAGTGACAGTGGTGGGGGAACCCAGGGTAGTTC
CTCGCCAGACCAGCAGCCGACGGAGACGTTCAAGGGCAGCGAGTACTCCAGCAGCTGGAGTCGCCTCAAA
GCCAAAAGAACCATCTGGAAAATCGAGGCACAGGATCAAATGCTAAAGCGAGCAG
>dme_circ_0001925_junction_seq
GCACAGGATCAAATGCTAAAGCGAGCAGGGCACTGCAATGCATCACCATCCCAGCT
>dme_circ_0001926
GTTAAAGTACGTGCACTGCCGGGTCGGTCAAATTAAAGCGAGCGatactgtatactatattatactatac
tatactatatatCGAAATCGGCCTGCTATTAACACTTCCACAAATTGCAGCTGACATCCAAGCGAGACTG
CGAACAGCAAAAT
>dme_circ_0001926_junction_seq
ATCCAAGCGAGACTGCGAACAGCAAAATGTTAAAGTACGTGCACTGCCGGGTCGGT
>dme_circ_0001927
GCATTTCCTCCATGGATCGTCTTTCTATCGTAGACATTCTCCCTCTTTGATCTTTTATAAAGCGTATCGT
AAAGATCCTGTCTCTTCTGATTCCTATGGGCTACCCTATTGTGTCTTTTGAGACGTTCCCGGTGAGGCGG
ACTTTCCGCACTATAATGG

>dme_circ_0001927_junction_seq
GTGAGGCGGACTTTCCGCACTATAATGGGCATTTCCTCCATGGATCGTCTTTCTAT
>dme_circ_0001928
ATATAGCTATTGGCAAGTCGACGACCCAGGCAATATACCATTTTACAACCTGGACAATACAGTTTAATAG
TTTCCTTTTCCAGGGCATCCAAAAGGGGACGAAAGTAGTAGTCTGTGGGCCTACTCAAAAATCCTGGTTT
CATATAATTAAAAATATTGCTAAACACTG
>dme_circ_0001928_junction_seq
ATATAATTAAAAATATTGCTAAACACTGATATAGCTATTGGCAAGTCGACGACCCA
>dme_circ_0001929
GCAATCCTTGCTGGCAGGACGCATTCCGGTGGCCTCCATGACAGGACCCATTAAACGCGGACTGCTCTGG
CAGCAAAGGGATCGGCTCTTCTCTCGCTGGAAGGAGCGGTAAGTACACTGAAAGAAAATGCTATATTTTT
ACATATTTAATTATACCAAATAGATCTATATTATCTGCAAAATGTTAGATACTTCTATAAACAAATATGC
ATAATATATCATGTAACTTCTAAATTTTACTATATTAAATTGATTAGCTGCTGATTACAGACATATAATT
CTTATCCATAAGAACTATGTCCCAATATTGTCCAATAATTTGCTATATTTTCATAAAATACCATTTAAAT
TTGATGCCCAGTTTCTTTTGTGTTCGAGTGGGGATGCATAAATGTTAAGCGCTTTTAAATTTACCTCCAC
CGCAATCTGCATTGGCTGAAGGACCAGACATGAACGCACCCCCACCCCCACTGAAAAATATGCAAAAATA
TTTCACAACCTCCAAGGGTTGTTAATTGCGAAAAAGGGCATAATTGACAGTGGCTGCATGCTGGCCCGTC
CTTTTTTCCAGATAATGCTAATAGGGGGAAAATGGAGCATCGAAATCCAGGGGTTCCACCATTTGACAGC
TCTTTTGTTTTTGTTTCCCCCAAGCTGCATTTAATTGAAATTTAATTATGTTGCAATGCAATTCGGTGTT
TTTGTTTCACTTTCGGCTTTTGCCCTTTCGAACCGATAATTACGTTTATCGCTTTTCATTTAACGGCGCC
ATTTTAATTGACACTTAATGACCAACTAAGCCGGGTATTTCATTGATCAATATTCGATTTGCAGTTTTGC
ATCAAGCTTAATTCGATTTACTAAACAGCTCTGAAAATACAAGCTTAGCACGTTGTTTTAAATAGCTCTA
AATTGTCAGCACACAGTGGGTTTTTATCCATGTTCTTAATCGATTTAATGGAAAATAATATTGTAGACCT
AAatttttatttatctattttttttttttATTTCGAATCATCTTCATTTTGTAGTACTCAGCTTACAAAAA
GACTCCACTTATTGCATTTCTTTGGCTTAGATCTCGGCATTCACCATTAAGTTGCATGCCTCGGACATTT
AACAAGCTAAGCTAATAACAGAACCGCTTATTATCCACGGCAAACAATCTTCTCTTCTCTCTTCTAAAAA
ACCACCCACTGAAGAAAAACGAGAAACCCGCAAGCCATTTAACTCTTTCGCGGAATTGAACATCAATTTT
CTTGGCTCAATTTACTTGACCACGGTAATAAGCCTAACAAAATGATAACTTCCACCTAGGAAGTGAGCCA
TTAGGCGATCCAAAAAATCCAACTAAAAACCATTTATAATTTCTGTTCAGCTTGAGAACTTTTCGCTCGT
GTATCGCTTGCTACGGCTTGTACGCTTCATACATAATAAACAAATGTGAAATTTATGTGCATCAGAATTT
GACTGGCCACATGGCCACATTAATAAAGTCGATTTTCATTGCTGCAAAATAAAAAAATAATACTATTATC
GTTTGAGGAAGTCGTAAAGCTTGAAGTACTTCTCTGCTCAAATCTGAGCAACGGTTTTTAACTTTTTGCC
GCATAATTTATGATTTATTAATAACATATCGAAATGCCATCATCGTTGGGGAAGGGCTCCAAAATTTTAT
TGGCAACACTTAAAGTGCCCGGCTTTTTGATGCCGGTTTTTGATGTGGATTATCTGACCTTTAACTGAAG
ACTGTCGGCAGATATACATACATGAATTTAAAGGTCACTCCTACAATTGTGAACGGATTTGTACTGACTA
AAGCCAATCTCCTATTGCAGATACTTTGTGCTCACTCGTGACTACTTGCACTGCTTCAAAAGAGCCTCGG
GATCGGCCAATGAACGTGCCTCGGATATGGGACAGTTTATCTTCAAG
>dme_circ_0001929_junction_seq
CTCGGATATGGGACAGTTTATCTTCAAGGCAATCCTTGCTGGCAGGACGCATTCCG
>dme_circ_0001930
GTGCTTCATTTACGTTTCGTGCGCGAATTTTATGTGTAGACATCAAATAAACGAATGCaaaaaaaaaaTT
AGCAGCATCATCATCAGCGTCACCAGCAACAACAATAACAACTGCAACAACAACGAAGGGCACATCTAAC
GCTGCGGCTAACAGATGAGCCCCAACAACAGATGTCAATTGCTGCAGCCGAAAATGCAGG
>dme_circ_0001930_junction_seq

TGTCAATTGCTGCAGCCGAAAATGCAGGGTGCTTCATTTACGTTTCGTGCGCGAAT
>dme_circ_0001931
GAGTGTTGTGTGTATATGTGTTTGTTTGCATTTCTGTTTGTTGTTAGGTATTCATTTTCCGCCTCTTTCT
CACTCTTCTCATATTTATATTTACCGTTAGAAGCCGCTAAAGTTTCTCGTTTGACTTTGCCAAGATGGCT
CTTTAGTTGTTGCCTTTTATTCGCTGGCTAGGGCCACCTCATCGACCAGCTTTTCTTTTTTCCATTTGAC
AGACTGTACAAAGGTAGTTTTGGATGTGTGCTTGTGTT
>dme_circ_0001931_junction_seq
AAGGTAGTTTTGGATGTGTGCTTGTGTTGAGTGTTGTGTGTATATGTGTTTGTTTG
>dme_circ_0001932
GTGCCATCAGAACTCCGTCCAAGCAAATAGAAGACCTGCCACAATATGAAAGGCTCTTCAATAACTGGAC
TTATTGCACCTGCCACAGCCATCAAGTCAAACAACTGTCAGAAACTGTGAAACCAATACGAGATTTTGTT
CGTGCCAGCAGTGTGTTTTTTGCGGATAAAACCGGAGCTCAACTGATAATGGGAGTACCCAcataccata
ccataccataccatactgtaccatctcatCCTCCGCCATTCTCGCCTACCATTATCGGTCAGCAAGGCAA
ACAGACACAGCAGGAAGTCGCATGAGCGGAAAATTGGAAAAACTCTGGAAAAATACTGAGCGCAGGATAG
GCGaaaaaaaaaaTACATATGTATATATGTATATGTGGAAAAACGTGGAAAGGAAGAGAAAAATGGAATA
AAATTCTTGCTGACTGGCAACGTAAAGTGAAAAGCATACAAAACGCCGGGCAATTGCGGTGGGCGGAGGA
TATACGAGTATAGGGGGCGTGGCTGGCATATAGAGTGAAATGTTAGACAAAGTGGCAGcacacacacaca
cacacacacacacaACGACCTGCATGCAAAACAGTTTTTGCCGCTGTAAACTTATACTTTTGTGCAAACA
ACAAACGAAGGCAACGTCAGAGTTGCCAGAGTTTGTCACATGAAGACGCACAATTACACGAGGGGGCTTA
AAAGGATATGCTACATGGCTAAAATGTGGGGCCCGTGGAGATGGGTTGGAAAAGCGCGGCGCGGGGCGCT
TGCCTCGTGGAACAATTGCAGCCGTCGCCGTCGTGGAAACTGTGACAATGTTCAGTCGTGTGTGTAAACA
ATTGTAATCCGGTAGAAAAATCACAAGCACCCCATCAGCCAAAGGACAAAGAAACACACGAGCTCAAATC
CACCCATTCCCAAGTCCAGATTTAGGTCCTGAACTAGGACTATGACTAGGCCTATCTATGAGCGGAAATG
TAGTGGAATGCCAGACAGCCAGAGGGGCAAAGTAGCTTTAATGATTGGGCCAAATGACAGAAAATAGAGT
AACACGATATAGAATCAATCTATCAGATACTCTGTAAAGTTGTTGTGCGCGAAAAAACTGGATATATATA
TACAGAATTACTTATGCCTTGGCCTTCCTCAACACATTAATAATATGAATCGAATCCAATAACATAATGC
ACCTTGCAACGAAGTGTTTTAATTCCCAACCCTGAACCCTCTACAGTTTTATATTAATTCAAACCCTAAT
CTGGCTTATCTCGCAGTTTTATCAACCCTTTTCCACATGTGCTAATCGCATCTTTCTCCCTCTGATTACA
GGTGAGTAATGCACGCGAATCAAAGTAGCACGCAACTGAGGAAATACTTAGACaaaaaaaaaaaCTGAGA
AAGTATCCGAAAAACATCACTGTGTCGGTGCAACTTAGTCTACGTAGTTGAAACTCTTGCCTGCGACATG
TAAACAAAATGCAAAGTGCGCAAAGCCAGCAACAAAAGTCGAAAAAACAACAACAGCAACGGCATTGCGA
AG
>dme_circ_0001932_junction_seq
AAACAACAACAGCAACGGCATTGCGAAGGTGCCATCAGAACTCCGTCCAAGCAAAT
>dme_circ_0001933
GGCTTAGCCCAAGTGACTTACCAGATCATTGGGCCTCAGGTACATCAAATCCATCGACCTCGACcagcag
cagcagcagcagcagcattagtgccagcggcagcaacaacaacaaatacaacagcggcaacatatcggtc
tgcaacctgccgcgctcatcgccggccgctgcaacagcagccgttacactatcgtcggccgccggcggta
ttggcagcaacTTGTTGAGCGGCTTGCATCACGGTCTGGCGCCCGGTACGCATCCACTGCAGCGCGCGAC
GGCAGCCAGCGGAGGCGGAGCTGGTGCTGGGGGCGTGGCAGGCGCCtcaacagcagcggcactcacactc
cagcaacatcagcaccagcagcagcaacaacaacagcagcagcaagcgcaacatcagcatcagcaccagc
atcagcagcagacgcaacagcagcaattggcctaccagcaccagcaactgcagcagcagATCATCTCGCA
TCGTTCCATACAAAACAAGGCGGCGGCCACCGCTGCCGCACAAACGGCAAATCTTGCTGCTGCCCTGCAG
CGATCGGCTGCCATTATGAATGCGGTGGCATCGCCGATctcagccaactctgccaactcagcGACGTCCG

GCCGAAAGATACGGCGCAAGACGGATTCAAAGGTCAATCTGGGTAAGTTTCGGCCGGGGTAGAAGTCTCT
CGTAACGAGAAAGGAATTCCGAACACTCAATAAACCTATAAATAAACATGCGAATATTACGTATACGCCA
CATGGACATTCCCATGCTAGCTCCAACGCATATGTTTTGCTGCTTAGCTAAAATATAATTGAATGCTTTA
TCTAGGTACCAAAACCAGAACCTAAGGGATGTAAATTATGTTAGGAAATAAATATTTGTGtttttttttt
ATCATTTTATCACTCCAATGCAAAATGAAATTTAAAGATGTCCATAAATAAAAAGATTATAAATATTTAT
AAAACCTTTTTCTATAGATAATATGCAGATATATACAAAAGTAGCAATAAACTATGCTTAATTGAGGGCT
GAAATTTTAATAATTATTCATACTTCAAGTGGAACACATTTATTAACAACATTCTGCTCTTTGCAGGCCC
CAATCACAAATCAACAAATGCAACAATGAGAAGCGACGTCGCGAGGCGGAGAATGGCTATATCGAGCAGC
TGTCGGAGATATTGACGCTGAACAAGCGTGGAGATATGACCTCAACGAAACCGGACAAGGCGGCTATACT
AAACCAAGTGGTTCGAACG
>dme_circ_0001933_junction_seq
GGCTATACTAAACCAAGTGGTTCGAACGGGCTTAGCCCAAGTGACTTACCAGATCA
>dme_circ_0001934
GGGGTGGTATGTGGATAAATTTTAGCCGCCATGGATCCCAGTGCTCTACAAAACATGGATCGGGACGCAT
TAAAGAAGCAAATCGAGAATATGAAATATCAGGCCTCCATGGAGCGCTGGCCGTTATCTAAATCCATAGC
AGA
>dme_circ_0001934_junction_seq
GCTGGCCGTTATCTAAATCCATAGCAGAGGGGTGGTATGTGGATAAATTTTAGCCG
>dme_circ_0001935
GGCGGCAGAAGGAGAAGGCAAATAAAACACAAACAGAAATAGATATAGATATAGAAGTTGAAGTGCAAAT
AGATTGGAGCAAACAAAAGACACACACATGAGCTGTGTGTCCCAAATAAAATCACATAAAATGCGACGAA
ATCTGCAGCATGCATTAAACAACAGCCTGGCAAACATACAATATTCAAACTAAGTGAGCTCTCGAGTCTG
GCAATTCGCAAGGAAGTGCAACACCGATAAGTCTTGGTCCTCCGAACCGGATCCTGGAGAGATGCCGTCA
CTGGAACCCTGGCTGTGGGGCGACTCCTGTTCCTGGCTGGGGATGCTGGCGATGCTGCGGCTTCGGCTGC
ATAAATCAAA
>dme_circ_0001935_junction_seq
TGCTGCGGCTTCGGCTGCATAAATCAAAGGCGGCAGAAGGAGAAGGCAAATAAAAC
>dme_circ_0001936
CAATCTTGCTGGACATGCTCAGGTCGAGTCCACCAATGGCACTGGTGATTGATGAGACCAAAAGCGGCAC
AATCAAACATTTAAGCATTCTAAAAATATATGTTATTTATTATAGTATTCCAATTCAGTAATCTGATACA
ACTAAGATGAATTTATCCCACTTCTTTTTACTCACCGCAAGAAAATCTCGCCGGGGAAGGATATGTACAT
GATCTCTCTCTTCGACCACTCGCCAGTGCTATTTTTGATGATGAAGCCGATGAGTCCACCCACAAACACA
CCGATAACGGTGGCCATGGTAAGGACATTCTCCTGCATGAAGGCCTTGAACTTGCCGCCATCCTGTTTGG
GTCGCGTCATGGTGAAGATCTATGGATTGGTTCAGGGTCTGGACAACCCTGGGTCCTGCTGGAAACCCCT
TTTTATG
>dme_circ_0001936_junction_seq
TGGGTCCTGCTGGAAACCCCTTTTTATGCAATCTTGCTGGACATGCTCAGGTCGAG
>dme_circ_0001937
GGTACCACCATTGGAAGAATGCGGGAGAAGGGACAACTGCTGCAGGATTTCTTCACCACACTGAGCGAGG
CAATGATGACCATCACCTCATGGGTTATTTGGTAACGCGAGATGTAATTACATATCACCAAGAATTTTAG
GAAACAATTATTGATTTCTTTAGGATTTCCCCGCTGGGTGTTGCCTTTCTGATAGCCGCCAAGATCATTG
AGATGGAATCGATAGCAGCAACGATTCAGTCATTAGGATGGTATTTCATAACGGTCATGATAG
>dme_circ_0001937_junction_seq
GGATGGTATTTCATAACGGTCATGATAGGGTACCACCATTGGAAGAATGCGGGAGA

>dme_circ_0001938
GAATGACAAATCGAGTAACCCGCGGATCGATGCCCATGTTGTCCAAACACTTGATGGTCAGCGGCATGGT
GGCCGAGCTGGAACCTGTTCCAAATGCAGTTGCCAGGACCTGACTAAGCTTGGCAATATAGCGGTACGGG
AGACGTCGGGTGCCCAGGAAGAAGATCACCGCAATCG
>dme_circ_0001938_junction_seq
GTGCCCAGGAAGAAGATCACCGCAATCGGAATGACAAATCGAGTAACCCGCGGATC
>dme_circ_0001939
GTCTTCGCTTGGCGCCTATAACCGCAAACGATGAGTGGACATGTCCCGGCTGTGGGCCTGTTTCCACATC
CTGCCCAGCAACGATAGCGATAGCGATGACTACGACGACGACGAGGAGTTTTAAAGGATCGCCGCAG
GATCGTATTGTATCAGCGCTGGAAAGGAGTCATCACGTTT
>dme_circ_0001939_junction_seq
TCAGCGCTGGAAAGGAGTCATCACGTTTGTCTTCGCTTGGCGCCTATAACCGCAAA
>dme_circ_0001940
GTTTTAAAGGATCGCCGCAGGATCGTATTGTATCAGCGCTGGAAAGGAGTCATCACGTTTGTTTGCGAAT
TATTTTGGGTGACCAAGTCGCATCTAATTTTCAGATTGATCAATTTGAAGCAGCTCCTCCGGCAGAGGAT
AGATATCTGCCAGAGTACAA
>dme_circ_0001940_junction_seq
CAGAGGATAGATATCTGCCAGAGTACAAGTTTTAAAGGATCGCCGCAGGATCGTAT
>dme_circ_0001941
GTTTTAAAGGATCGCCGCAGGATCGTATTGTATCAGCGCTGGAAAGGAGTCATCACGTTTGTTTGCGAAT
TATTTTGGGTGACCAAGTCGCATCTAATTTTCAGATTGATCAATTTGAAGCAGCTCCTCCGGCAGAGGAT
AGATATCTGCCAGAGTACAAGTACTAAGCCAGCAACGAATTTAAGTGACACATTC
>dme_circ_0001941_junction_seq
GCCAGCAACGAATTTAAGTGACACATTCGTTTTAAAGGATCGCCGCAGGATCGTAT
>dme_circ_0001942
GGATCGCCGCAGGATCGTATTGTATCAGCGCTGGAAAGGAGTCATCACGTTTGTTTGCGAATTATTTTGG
GTGACCAAGTCGCATCTAATTTTCAGATTGATCAATTTGAAGCAGCTCCTCCGGCAGAGGATAGATATCT
GCCAGAGTACAAGTACTAAGCCAGCAACGAATTTAAGTGACACATTCGTTAAATA
>dme_circ_0001942_junction_seq
CGAATTTAAGTGACACATTCGTTAAATAGGATCGCCGCAGGATCGTATTGTATCAG
>dme_circ_0001943
GTCATCACGTTTGTTTGCGAATTATTTTGGGTGACCAAGTCGCATCTAATTTTCAGATTGATCAATTTGA
AGCAGCTCCTCCGGCAGAGGATAGATATCTGCCAGAGTACAAGTACTAAGCCAGCAACGAATTTAAGTGA
CACATTCGTTAAATAGTTTGTTTTTTCCAAAATGAATAGATCGAAAAGCAGCGAGAACAGCAATAAGATT
TAAGATGAAAACAAC
>dme_circ_0001943_junction_seq
CAGCAATAAGATTTAAGATGAAAACAACGTCATCACGTTTGTTTGCGAATTATTTT
>dme_circ_0001944
GATCGAAAAGCAGCGAGAACAGCAATAAGATTTAAGATGAAAACAACGTAACATTTATCGCCTAAATTAG
GGATACTTTCAAGGTGTACATAATTTTTAAATATAATTATATTAAATTGAGTTCAAATTTGCATTACATT
CTCCCTATGATTAGTATTACAATTGAGCAGTAAAATTAGTTAAATTCCAAGCAAGCAATCCCAACTTTAA
GCAATTGCATTTGATTAGGTCGCAAATTGTTGAGATTCGTATCTAGTATTTGTTAAAACATATTCTATAA
CTTTTTGACATAGCAAAGCGAAACAAAATAATTAAAAAAGACAAATATTTAACAATAGAACACAGATAAA
TTAACATACTAATAG

>dme_circ_0001944_junction_seq
GAACACAGATAAATTAACATACTAATAGGATCGAAAAGCAGCGAGAACAGCAATAA
>dme_circ_0001945
GCGTGGAAACCGAATTCTTGGATGACAATAATAGAGTGCTGCAATCACACATACAGTCAATTTATACGAG
TAACGAATTATTTATAAAATATTAATAAAACTAATATAAATTAGGTCACATCTTTTCAAAAATCAACCAA
ATATTGACACAAATACACAAAATCAATAAAACAAAGTAAATACTTGAATGTTTGCCATGCTTTCAGCCAA
AAAGGAGAATTTTTTAG
>dme_circ_0001945_junction_seq
CTTTCAGCCAAAAAGGAGAATTTTTTAGGCGTGGAAACCGAATTCTTGGATGACAA
>dme_circ_0001946
CCAGGCGGTAGAGCAGGCGACCCCGCTCCGAGGCATCCATGCGACGCCATGGAGATCCCAACCTAGTGAT
CATAGAGTTGTTATTAATAAGCAAAGTCGATTTATATCCAAATTGCACACTCACTTGAATGCATTGCGTG
CAGCCTGGACGGCAATATCGATATCTTCCTTATCAGCACACTGAATTTCGGCAATGACTTCCGCTGTGGT
CGGGTTGATGGTTTCAAATATTTTGCCCGACTTGCTCTTGTGCCACTCGTTG
>dme_circ_0001946_junction_seq
GCCCGACTTGCTCTTGTGCCACTCGTTGCCAGGCGGTAGAGCAGGCGACCCCGCTC
>dme_circ_0001947
GATCTTTTCCATCTGCTCCTCGTTCACCTGAGGACCTTGCTCAGTGTTCAGATCAAAGGGATTGCCCACG
GTGCGCTTCTTAGCCCGCTCCGCGCTGCGCTCGACAAACTCATCGTAGATCTTGTCCTCCACGAAAGTAC
GAGATCCTGCACAGCAGCACTGGCCCATGTTAAAGAACAGACCAAAGTGAGCCGTCTCGACGGCGTAGTC
CATATCGGTGTCAGACAGAATAATGTTTGGCGACTTGCCTCCCAACTCCAGTGTCACTCTCTTTAGGTTA
GTATTTCCCGATGCTAGTTGGATAAGTTTGCCCACATCGGTGGAACCGGTGAAGGCCACCTTATCCACAT
CGCAGTGGTTCGCCAAGGCAGCGCCAGCAGTTCCAAATCCGGGAACCACATTGACCACACCTTCGGGGAA
GCCAGCCTCCTTCACCAACTGGGCAATGTACAGAGCAGTCAAACTGGTCTGCTCAGCGGGCTTTAGTACA
ATGG
>dme_circ_0001947_junction_seq
GTCTGCTCAGCGGGCTTTAGTACAATGGGATCTTTTCCATCTGCTCCTCGTTCACC
>dme_circ_0001948
TATTTCCCGATGCTAGTTGGATAAGTTTGCCCACATCGGTGGAACCGGTGAAGGCCACCTTATCCACATC
GCAGTGGTTCGCCAAGGCAGCGCCAGCAGTTCCAAATCCGGGAACCACATTGACCACACCTTCGGGGAAG
CCAGCCTCCTTCACCAACTGGGCAATGTACAGAGCAGTCAAACTGGTCTGCTCAGCGGGCTTTAG
>dme_circ_0001948_junction_seq
TCAAACTGGTCTGCTCAGCGGGCTTTAGTATTTCCCGATGCTAGTTGGATAAGTTT
>dme_circ_0001949
CCGGGTAGGCCCTCTGGGCGACTGCCACCGGCAACCAACTTAGCGCCCTGTTTCTTGCCTGTTTTGATCA
TTCCAAGGATCTTTTCCATCTGCTCCTCGTTCACCTGAGGACCTTGCTCAGTGTTCAGATCAAAGGGATT
GCCCACGGTGCGCTTCTTAGCCCGCTCCGCGCTGCGCTCGACAAACTCATCGTAGATCTTGTCCTCCACG
AAAG
>dme_circ_0001949_junction_seq
TCATCGTAGATCTTGTCCTCCACGAAAGCCGGGTAGGCCCTCTGGGCGACTGCCAC
>dme_circ_0001950
GGGAGGAGATCTTTGGACCGGTTCAGCAGCTGATCCGCTTCAAGAAGCTGGATGAGGTCATCGAGCGGGC
TAACAACTCCGAGTACGGATTGGCCGCCGCCGTTTTCACCAAGGATCTGGACAAGGCCAACTACATTGTG
GGTGGCCTGCGTGCCGGAACTGTGTGGGTAAACACCTACAATGTCCTCGCTGCCCAGGCTCCGTTCGGTG

GCTACAAGATGTCCGGCCATGGACGCGAGAACGGAGAGTATGCTCTGTCCAACTACACGGAGGTCAAGAG
CGTCATCGTCAAG
>dme_circ_0001950_junction_seq
CACGGAGGTCAAGAGCGTCATCGTCAAGGGGAGGAGATCTTTGGACCGGTTCAGCA
>dme_circ_0001951
GAGTTCTTCTGGGCAACCTTGACGATGACGCTCTTGACCTCCGTGTAGTTGGACAGAGCATACTCTCCGT
TCTCGCGTCCATGGCCGGACATCTTGTAGCCACCGAACGGAGCCTGGGCAGCGAGGACATTGTAGGTGTT
TACCCACACAGTTCCGGCACGCAGGCCACCCACAATGTAGTTGGCCTTGTCCAGATCCTTGG
>dme_circ_0001951_junction_seq
ATGTAGTTGGCCTTGTCCAGATCCTTGGGAGTTCTTCTGGGCAACCTTGACGATGA
>dme_circ_0001952
AGATGTAGATTATCGCCGGAATTTAGAGCACCTAAGTAGGGCGAACCATATTAAGCGTCCCTCTTGGAGG
AAATATCTGGTCCTTCTTAGGAGTTCTTCTGGGCAACCTTGACGATGACGCTCTTGACCTCCGTGTAGTT
GGACAGAGCATACTCTCCGTTCTCGCGTCCATGGCCGGACATCTTG
>dme_circ_0001952_junction_seq
GTTCTCGCGTCCATGGCCGGACATCTTGAGATGTAGATTATCGCCGGAATTTAGAG
>dme_circ_0001953
TCAAAGGACAACGAGTTGCCGTGGGCCAAAAGTGTATAAGGAATGGGAGAAGTAAGGTAGAGCTAAAACC
ATAAACCCAAACGAACAAGCTCAGCTCTGAGTGCaaaaaaaaCACAAGAGGAAGAGAAAGTACATCCTGC
GCCCAAGTCTAATTAGAAGAATCGCGCTGCGGCAACAGATTGCTCATACGCCGTGTGGATCGCGAGTGTT
TTAGTGTCCGTGTAAACATAAGTACATATGTGCGTGTGACCCTATTTTGTGAAATCTGAAACAGAAAACT
CCTCAAAAACCATTCGAGAGGTGTTAAAGTTTTTGTTCGATTTGCTGGCGGGCCGTCGAAGAATTAGTTT
GGCATGCGGCTGCTGTTGCCACAACTGTTGCATGCAACATGGATGGCGAAAATCGGATCATACTCAATGT
GAGCGGAATAAGG
>dme_circ_0001953_junction_seq
GATCATACTCAATGTGAGCGGAATAAGGTCAAAGGACAACGAGTTGCCGTGGGCCA
>dme_circ_0001954
ATCTCCTCCAGAATCCCGTTGTCTAGCTGCTCGGCGGGCAGGGCTTTCAGTGTGCGCAGCGTCTCCATGT
AGAAGGTCAGCTCTGATGTCACCACCCCGTGCTGCGGACCCCCATTGTTGTTCACATTCAACTTGAGCAG
TTGTTCCCGCAGCTTGTCTTTAAAGTTTCGATGAACAACGCCGTAGATTTCGTGTATCTGTGCCTCCGGC
AGCACACCGGCGATGGCCTCGTGAAGTTTCACCAGGTGACGCGAGATGTGACGGAATGTCTGAGAAGGTA
TAGGTGGTCTGGCTTCCCAGGCATCCAGTTGAGCTGCCAATCTCTCGGATACAATTCCGTGTATTTTATT
CTCGATCTCCTTGATGTGACCCTGGTAGTCCCGCTCGATAGTTTCGTATCCGCTCATCGCTTGGAAATGC
TCTTTCAGCTTGGGCAGTAGCCATAGAACTAGTTGCAGTGCTCGCGAAACCAGAGCCAGG
>dme_circ_0001954_junction_seq
TTGCAGTGCTCGCGAAACCAGAGCCAGGATCTCCTCCAGAATCCCGTTGTCTAGCT
>dme_circ_0001955
GAGATCTTATACCGAATTTCAGTCTTATAAGCGGTTTTGAGTATGCAACTAGGAATTAAAGTTCTACGAC
ATGTAATTTTTAAAAGACTTGAGTCATACTTCGATCATTCTTCCCATACTAAAAGACCGTTTCCATTGAC
TAGACTGATACTGGGGAAGATCCCTTAGTCACTCAACAATTGACCAAGCTACCAAGTTACCCACGAAAGT
CCACCATAAA
>dme_circ_0001955_junction_seq
CAAGTTACCCACGAAAGTCCACCATAAAGAGATCTTATACCGAATTTCAGTCTTAT
>dme_circ_0001956

TATAATACAGAGTTTGCATTTAAGCTTAGAGGGTGCCATTTGTTTTTGTTTTGTTGTTTACTTTATGGTG
GACTTTCGTGGGTAACTTGGTAGCTTGGTCAATTGTTGAGTGACTAAGGGATCTTCCCCAGTATCAGTCT
AGTCAATGGAAACGGTCTTTTAG
>dme_circ_0001956_junction_seq
AGTCTAGTCAATGGAAACGGTCTTTTAGTATAATACAGAGTTTGCATTTAAGCTTA
>dme_circ_0001957
GATAGCGGGTGACGCCCGTGTGGATCAGCAGCTTGACCAGCTGGCCATTGGCCATCAGGAACTTGGGGAT
CAGGTCCACGTTCCAGTCGCGGCCACGCCCAAATCGTTCGCCGGGCGGCTCCAGTCCGTAGCGCTGGAAG
AGCTCCTCCAGCGGCGTTATCGAGGCGGACTCGCCGCCGTA
>dme_circ_0001957_junction_seq
GCGTTATCGAGGCGGACTCGCCGCCGTAGATAGCGGGTGACGCCCGTGTGGATCAG
>dme_circ_0001958
GCCTCGAACACCTTGAACTTTTTTCTGTCAATGCGGCTATCGATCGAGAGCTTGAAATCTTGAAAAGCAG
CTAGGAGGCCAACCAAAGAAAACAACGCAGGCCCACCCACCTGCAGCTTTTGGAGTAGCAAACGCCTCCA
TTTCCACGGCACCCGCGTTACGCTTGACCTTGTGGGTCTCTTTGTGCTCGCCACTGCTCTTTTCGTGATG
CTTTCAGTTTGAATTTTTGGATTATCGGAAGCCCCGAGTCTTGAATCAAGGCCGAGCTTAAAATGCGCGT
TTTAATACCcccgttagtcgtagattaaatgggtatgctagattcgttgaaaagtatgtaaaagttagaa
gcaagcgtttccgGTTATttttatttttgttaatttctattgatatgccaaacatattgttgaactttct
tgtttgcattccaactagctgagtaaagggtatcagatagtcgaggaaatcgactatagcgtacgctctc
gttttttttttattttattatttttgtctttcgttttcgtatttttGGTGCTTGAGTGCGTCGGACGGGT
TCGATAAACTTTGTGGCATTAATTAGGCAAACTCTTTATGTTTTCATAGCTCCGATATAATTTGTATCGA
ATTGAATTTTCTGTGCTGCAATCGTATCTCTCGTTTATCAAGATGATAGATAAATTATTTACCATTTGAA
AACAATATCTTATTTATTTATTTTAATTCATTTCAGGAAGATCATGGCCTCTGCAACTAAGGGGTCACCC
TCAAAAAACGTAACCAGGTGCTAGAATCAACTTTTCGTTAGCTTGGTAGATTGCAATTGGAAACAAAGTT
CACTTTAACTGAGATAAAACGCAGAGAATCCACACATATCCGGCGTAGATCGTGGCCAGGCCGAAGAATT
TAATCTGATGGTTGTGGCTCGCGAAGATGCCTTCAGCGAACACGGAAGACTTGCGGCGAAAGTTTCTAGT
AGTGCAGCAGCAGCGCTCCGCACCGCCCAATCTGACCGGGGACACACTGTCATCGGGAATTCCGAATGCC
AGTGCCAGCTCGGCCACCACCAGCCTGATGATGACAACGTCTACGGGTCTGGGCGGTGTGGCCATGGGTG
CGGGAGAGCCGGAGCGTACGTATGTCTTTCCAGGCGGCAATCCGGTCGTAGGTAGCGCACCTGGTGGAGG
AGGACAAGGCATTCCCGGAGTGGCGATGGCAGCTCCATCGCGTGGTCATGCCCGCTCAATTAGCCATGGC
GGAGGAGCCATTGTTGGAGCCAATGGGCGACCCATTAAGTCGGCCATGAAGGGCCACCAGCGAGCCTTTT
CGCAGGGACAAATCACAGACTCACCACCCGGAAGTGCTGCGCCCGCGGGCAGAGGTCACAGTCGCGTGGG
CTCCAAGACGGATTTCATTCTTCCACCGGGTCACAAGGAGGAACCGGCAAGGGAACCCTCGGCGCCCACA
TCAGCGACCGGTGGGCGTGGCCATTCGCGGCAAGCATCGCGATCCGAATCCATATACACACTCCGGCGGA
CGGAGGCACCGCCCTGGTGGAAACGCCTTACCCTGTGCAATTACAACACCGGCGACAAGTTCGAAGAGCG
AAGCTATCGAACGGTGGTTCCCAATCACACAGTGCCACCGAAGACCCCAAAGAGGGATCATCCCAATGGT
CAGTTTGTTGGCAACAAGATACGGACAACGAAGTATACGCTGCTCTCGTTCATACCGAAGAATCTGCTGG
AGCAATTCCATCGTGTGGCCAACTTGTATTTCATTTTCATTGTCCTGCTGAATTGGGTGCCGGAAATAAG
TGCGTTTGGCAAGGAGGTGGCCATGATACCGGTCCTCTTCGTCCTGGGTGTGACTGCTGTAAAGGATCTC
TTCGAGGATCGGAGAAGAAGGGCGTCCGACAAGAGGATAAACAACACCACATGCCGCGTTTACGATGG
>dme_circ_0001958_junction_seq
ACAACACCACATGCCGCGTTTACGATGGGCCTCGAACACCTTGAACTTTTTTCTGT
>dme_circ_0001959
GAAGATCATGGCCTCTGCAACTAAGGGGTCACCCTCAAAAAACGTAACCAGGTGCTAGAATCAACTTTTC

GTTAGCTTGGTAGATTGCAATTGGAAACAAAGTTCACTTTAACTGAGATAAAACGCAGAGAATCCACACA
TATCCGGCGTAGATCGTGGCCAGGCCGAAGAATTTAATCTGATGGTTGTGGCTCGCGAAGATGCCTTCAG
CGAACACGGAAGACTTGCGGCGAAAGTTTCTAGTAGTGCAGCAGCAGCGCTCCGCACCGCCCAATCTGAC
CGGGGACACACTGTCATCGGGAATTCCGAATGCCAGTGCCAGCTCGGCCACCACCAGCCTGATGATGACA
ACGTCTACGGGTCTGGGCGGTGTGGCCATGGGTGCGGGAGAGCCGGAGCGTACGTATGTCTTTCCAGGCG
GCAATCCGGTCGTAGGTAGCGCACCTGGTGGAGGAGGACAAGGCATTCCCGGAGTGGCGATGGCAGCTCC
ATCGCGTGGTCATGCCCGCTCAATTAGCCATGGCGGAGGAGCCATTGTTGGAGCCAATGGGCGACCCATT
AAGTCGGCCATGAAGGGCCACCAGCGAGCCTTTTCGCAGGGACAAATCACAGACTCACCACCCGGAAGTG
CTGCGCCCGCGGGCAGAGGTCACAGTCGCGTGGGCTCCAAGACGGATTTCATTCTTCCACCGGGTCACAA
GGAGGAACCGGCAAGGGAACCCTCGGCGCCCACATCAGCGACCGGTGGGCGTGGCCATTCGCGGCAAGCA
TCGCGATCCGAATCCATATACACACTCCGGCGGACGGAGGCACCGCCCTGGTGGAAACGCCTTACCCTGT
GCAATTACAACACCGGCGACAAGTTCGAAGAGCGAAGCTATCGAACGGTGGTTCCCAATCACACAGTGCC
ACCGAAGACCCCAAAGAGGGATCATCCCAATGGTCAGTTTGTTGGCAACAAGATACGGACAACGAAGTAT
ACGCTGCTCTCGTTCATACCGAAGAATCTGCTGGAGCAATTCCATCGTGTGGCCAACTTGTATTTCATTT
TCATTGTCCTGCTGAATTGGGTGCCGGAAATAAGTGCGTTTGGCAAGGAGGTGGCCATGATACCGGTCCT
CTTCGTCCTGGGTGTGACTGCTGTAAAGGATCTCTTCGAGGATCGGAGAAGAAGGGCGTCCGACAAGAGG
ATAAACAACACCACATGCCGCGTTTACGATGG
>dme_circ_0001959_junction_seq
ACAACACCACATGCCGCGTTTACGATGGGAAGATCATGGCCTCTGCAACTAAGGGG
>dme_circ_0001960
GATCATGGCCTCTGCAACTAAGGGGTCACCCTCAAAAAACGTAACCAGGTGCTAGAATCAACTTTTCGTT
AGCTTGGTAGATTGCAATTGGAAACAAAGTTCACTTTAACTGAGATAAAACGCAGAGAATCCACACATAT
CCGGCGTAGATCGTGGCCAGGCCGAAGAATTTAATCTGATGGTTGTGGCTCGCGAAGATGCCTTCAGCGA
ACACGGAAGACTTGCGGCGAAAGTTTCTAGTAGTGCAGCAGCAGCGCTCCGCACCGCCCAATCTGACCGG
GGACACACTGTCATCGGGAATTCCGAATGCCAGTGCCAGCTCGGCCACCACCAGCCTGATGATGACAACG
TCTACGGGTCTGGGCGGTGTGGCCATGGGTGCGGGAGAGCCGGAGCGTACGTATGTCTTTCCAGGCGGCA
ATCCGGTCGTAGGTAGCGCACCTGGTGGAGGAGGACAAGGCATTCCCGGAGTGGCGATGGCAGCTCCATC
GCGTGGTCATGCCCGCTCAATTAGCCATGGCGGAGGAGCCATTGTTGGAGCCAATGGGCGACCCATTAAG
TCGGCCATGAAGGGCCACCAGCGAGCCTTTTCGCAGGGACAAATCACAGACTCACCACCCGGAAGTGCTG
CGCCCGCGGGCAGAGGTCACAGTCGCGTGGGCTCCAAGACGGATTTCATTCTTCCACCGGGTCACAAGGA
GGAACCGGCAAGGGAACCCTCGGCGCCCACATCAGCGACCGGTGGGCGTGGCCATTCGCGGCAAGCATCG
CGATCCGAATCCATATACACACTCCGGCGGACGGAGGCACCGCCCTGGTGGAAACGCCTTACCCTGTGCA
ATTACAACACCGGCGACAAGTTCGAAGAGCGAAGCTATCGAACGGTGGTTCCCAATCACACAGTGCCACC
GAAGACCCCAAAGAGGGATCATCCCAATGGTCAGTTTGTTGGCAACAAGATACGGACAACGAAGTATACG
CTGCTCTCGTTCATACCGAAGAATCTGCTGGAGCAATTCCATCGTGTGGCCAACTTGTATTTCATTTTCA
TTGTCCTGCTGAATTGGGTGCCGGAAATAAGTGCGTTTGGCAAGGAGGTGGCCATGATACCGGTCCTCTT
CGTCCTGGGTGTGACTGCTGTAAAGGATCTCTTCGAGGATCGGAGAAGAAGGGCGTCCGACAAGAGGATA
AACAACACCACATGCCGCGTTTACGATGG
>dme_circ_0001960_junction_seq
ACAACACCACATGCCGCGTTTACGATGGGATCATGGCCTCTGCAACTAAGGGGTCA
>dme_circ_0001961
GGCGAGCCATTCTGGCAAATGCGGATGTCTCGGGCGGAACTCCCTGGCAGCGGAGCTTCTCGAACGACTT
CTGGGGCACTCCGCTGACGGACAGCGGATCCCACGGCTCCTGGCGACCGCTGTGCGTGCTCAGCTTCCGG
CTGAACTACCTCATTGGCGGGGGCTTTGCGCCCTGGGGCTTCCACCTGGTCAACAATCTGCTCCACTGCG

TCGCCACGGCTCTGGTGGTGCGGGTGGCGCGCACCCTCCTGGCCTCCGTGTGGGCAGTCCTGGCCGCAGG
CGCGCTCTTCGCCGCCCACCCGATCCACACGGAGGCGGTGGCCGGAGTGGTGGGACGGGCGGACCTCGCC
GCCTGCGTCTGCTACCTGCTCACGTACCTCAGCTACTTGCGGCACATGAGGTGGCGCGAGTCCGGAGATC
CGCGCCAGTGGCTGGCCCTTGGAGCCACTTTGATCCTGGCTGCAGCCGGCCTACTCTGCAAGGAGACGGC
TATTACGGCGCTTCTGGTCTGTGCCCTCTTCGACGTGATGCGCGGTCTCAGCGGACAGGTGGACAAGGCA
ACGTCTACGATCCGTGTGCATAGTTCTTGGAGCTCTGTTCTGCATGGCTTACTGCCGCCTAGTGATTGTC
CCTGGACCGCAGACGGCCTTCTCCAGCGCGGACAACCCGATCGCCAGGACTCCGTCCGCCTGGACGAGAT
TGCTGACCTTCCTCTACCTGCCCGTCTTCAATCTGCGCCTGCTGCTCCAGCCAAACGTGCTGAGCTTCGA
CTGGGGCATGGATGCCCTGCCCAGAGTCACTTCTTTGTGGGATCGCCGCAATGCACAGTCCGCCTGCTTC
TACTCTGTGCTGGTGGGCGTCGCGTGGGGCAGCTGTAGACAACTGCTAAGCGGGTCAAAGGAGGTGACCC
ACTGTGGAGTGAGCTCAACCTTTCCGCAGTATCACATCCAGAAGGTGGCCAGTCGCAAATCGCGCTCCAA
GCGCAAGCGGTTGGCCAACAACACCAAGTACCAAGCCTTTGAGGCAGCCTATCATCAGCAACAGCAGGAA
GCGCTTCCATGTCGCGATTGCAACAACAATAACAGCAGTGGCTACGTCTACGAGGGGAGTTCCCCCGTTG
CACAAGCCCCAGCCCAAGCCCCTCACCTTGTGTCTTCCGCCTTTCGCGGCTCCCgcagcagcagcagttg
cagcaacagcacaaatagctccagttccagctcctccagttcgagttccagcagcagcagcagctccagc
CTGAGTGGCGGCTTCCAGTGCTCCTCCAAGGATTACGCGCTGGAGGGGATGTCGCCCGCCAATCGGCACG
CTTGTGTGCTGATCATGTCGCTTAGCTTCCTGGCATTGCCCTTTCTGCCTGCCAGTAACCTGCTGTTCTA
CGTGGGATTCGTGGTGGCCGAACGACTTCTGTACCTGCCCAGCGTGGGATTCTGCCTGCTGGTGGGCTAC
GGGGTCTCCAAGCTAATGTCCTGCAACCAGAGGACCCGCAATATCCTGCTTCTAAGCTTCAGTCTCCTCC
TGGCCGCCATGAGTCTGCGAACGTTGCGGAGGAACGCCGATTGGCGGGACGAGGAGAGCCTCTACCGCAG
CGCCATTGCCATCAATCCGCCAAAGGGTAAGTGCTCCCCCGCGATCCTTTCCAGCAGAGCGGCCAGCTAA
TTTGATTTGCCTTTTTAGCGTTGGGCAATCTGGGCAGCGTACTGAGCTCACAAGGGCGATACGAGGAGGC
CAAACAGGTCCTGCAGGAGGCCATTCGGTTCAGGCCCAACATGGCGGATGTGCACTTTAACCT
>dme_circ_0001961_junction_seq
CCAACATGGCGGATGTGCACTTTAACCTGGCGAGCCATTCTGGCAAATGCGGATGT
>dme_circ_0001962
TCGTCGTCGTGGTCGTACTTGTGCTACTGGCCAGCAGCTTCTGTCGCTGCTTGCCGTGCTTAAGATTCGC
CGGACTGTTTCCTATTTGCTGCACTTCGATAATCCCACTGGCATTCATCATATCTCGATGAGGGGCGGCA
CCCACGATAACCGTGTTGCAGATGGCCAGTACCACGAGAAATTCTTGAATGCGCTGCGCATGAGGTGTGA
GATATG
>dme_circ_0001962_junction_seq
ATGCGCTGCGCATGAGGTGTGAGATATGTCGTCGTCGTGGTCGTACTTGTGCTACT
>dme_circ_0001963
GGTGGCCAGGTGGAGTACACGCATGTCCATCAGCGCAAATACTTTCCCAAGTACGGAGGCGAATCCTATG
GGGGATTGGTAGGACCAGTGGGCGGACCCAAGGCCTATAAGCCATATCCATTGGTCTACGAACAGGCGGC
ATCAGCTGCTTCACCGGCTTCCATTCCCTCGGTGGCTTCCTCACCAGCG
>dme_circ_0001963_junction_seq
CATTCCCTCGGTGGCTTCCTCACCAGCGGGTGGCCAGGTGGAGTACACGCATGTCC
>dme_circ_0001964
ATCACTCGCCGACttttttttttGGTAAATGACCAAGAATGGCCTACGACATTAGCATAGAGAATCATAA
CCGCAACTGTCAGAGGACATTGACATCGGACGATCGGTTTATGCAACTTTGAAATCGAGTCGGGAAACTC
AAAGGAGAGCAACGACAAATTGCAACTTTCGCGTTTCAGTGAAAAATCCAACAATTTAACcacacaacac
acacacacagacatacacacacaTTAAATAGCCCTAAAGCACCACTAGGTTAAGCAGAGCTAACGAAACC
AAGCGAAACGAACTATTAGACATTAGATCACAACCAGGATCAGATGAGGCAACAGCCGGTGCAGCAATCG

AGCGGAAAATCAATGAGCAGCAGGCGGCGTCACAAAAAGCCCAAGGCAACAACAACAGTATCCACATTTT
TGAACCGCCCCGAACTTTAGAAACCCCCCAAGTCTCGAGCATGCAACAGGCTGGCCAACTGACgccgcag
cagcaacagcaacagcagcaacagctactgcagcagcagcagcagcaaaatggcggcgacCTTGCCGCCT
ACGCCGCCTCCCAGGCGGCCGGCGGTAGGCGTGGCCCCATAAACGGTGGTCGCTTCGATTTCGAGGACGG
CGGCACCTACTGCGGCGGTTGGGACGAAGGCAAGGCCCATGGCCATGGCGTCTGCACGGGTCCCAAGCAC
CAGGGCGCCTACGCCGGAGCCTGGAACTATGGCTTCGAGGTGTCCGGATCGTACATCTGGCCCAGGCGGA
TCACACTACGAGGGTCAGTGGCAGAATGGACGCCGACATGGACTGGGCGTGGAGCAGATCGGCCGGCAGA
TCTACCGCGGTGAATGGTCGAAGGACGGGCACAAGGGACGCTACGGAGTGCGGGAGAGCACTGTGTCGAC
GGCCAAGTACGAGGGCACCTGGAATGAGGGCTACCAGGATGGGTCCGGATGCGAGACCTACGCGGACGGC
GG
>dme_circ_0001964_junction_seq
CCGGATGCGAGACCTACGCGGACGGCGGATCACTCGCCGACttttttttttGGTAA
>dme_circ_0001965
GGAAACAATAAAGGAATAGAAAGTGTATCGGAAAGTCACTAATCCTTAACTAGGGATAGGATAAGTTTAG
TTAGAAGCTTAAGGGGGATCTGAAGCTGGAATGGGTACTAATTCGAATCTGAGCTACTTCAGTCCGCTCA
CTGTTAATGTGTTGT
>dme_circ_0001965_junction_seq
TTCAGTCCGCTCACTGTTAATGTGTTGTGGAAACAATAAAGGAATAGAAAGTGTAT
>dme_circ_0001966
CTTTTAGTGCGAAGATAAGTCGTGTCCTTGTGCATTTCTCTCAGAAAGGAAACCTATTGTGGTCCATCGC
TGCCTAAAAAATGGGGCAAGGCCCAATAAAAAGCCCTCTGTTGAAGTTGTCGACAATTAAATTAAAGTTT
ACCGTG
>dme_circ_0001966_junction_seq
GTCGACAATTAAATTAAAGTTTACCGTGCTTTTAGTGCGAAGATAAGTCGTGTCCT
>dme_circ_0001967
GGACCAGATCATTCATTACTTCCAAGTTTCCACCGCTGAAACGTTCGCCGAATGCCTTTGCCAAGTCGAC
GGCAATGTGGGAACCCACGTCAATGCCAACCTCATCCGCAAGGGTCGCAGCACCCACGGGGAAGCCAAAC
TTCTTCGTGTATTG
>dme_circ_0001967_junction_seq
CGGGGAAGCCAAACTTCTTCGTGTATTGGGACCAGATCATTCATTACTTCCAAGTT
>dme_circ_0001968
ATTCTCGATGTGATCCGTGCTGGCGTGGATAAGGGAACCGATGCCGGTTACGAGGCGGAGCGCAAGGGCT
TCGGCGAGCTATCGGCCACGCCCGAGTCGAAGGGTTTGATTGCCCTGTTCCGTGGACAAACGGAGTGCAA
GAAGAACCGATTTGGCAAGCCAGAGCGTCCGGTTAAGACTGTGGGTGTGCTGGGAGCTGGTCTGATGGGC
GCCGGTATTG
>dme_circ_0001968_junction_seq
GGAGCTGGTCTGATGGGCGCCGGTATTGATTCTCGATGTGATCCGTGCTGGCGTGG
>dme_circ_0001969
TAGAAATTGCCGCATAGTTTTTATAGCGAATCGCGAACACATAACTAACGGTGCATGATCAAAGTTCGT
TAAAAATTAAAACAAATAGTTATCGTAAGCAAAAAGTGACTATCTTATAAGATCGAACTGAGAGCAATGT
CGGCAACGAGG
>dme_circ_0001969_junction_seq
CGAACTGAGAGCAATGTCGGCAACGAGGTAGAAATTGCCGCATAGTTTTTATAGC
>dme_circ_0001970

TCACCAACTCTGGATCACCACCAAAGTGTGATATGTGATCCCTTACCCATTCCAGAGCCATCACCTGATC
GAGGAAACCAAAGTTTCCCTTTGTCTCACTGCTATTTGTGCTTAGAAAGCCCAGAGCTCCAAGGCGATAG
TTAAAAGCCACAAAAACTACG
>dme_circ_0001970_junction_seq
GCGATAGTTAAAAGCCACAAAAACTACGTCACCAACTCTGGATCACCACCAAAGTG
>dme_circ_0001971
AAAACTCATAGGTGGATCAGGGGGACGGCCAGACGTCTATGCGCTGCCCACTTAGATATTGGCCTTGATG
ACATCCTCCAGACGCTTGGCGTTGGCTCCGGCGAACTCTTCGACCTTGACGCCGTTCTTGAGGAACACGA
AGGTGGGCATGCTGGAGATGTTGTATTCCATTGCAATGTCTTCGCATTCGTCCACATCGACCTGTAGGAG
AAGGGCATTTAGTAAGGATTTCCATAAAAAGTGTTAAGCAAATAAAATAAAGCTGAGCACATTTTTAGAT
TTACCTTCAGGACGACGACGTTGTCGGCGAACTGCGTGGAAAGCTCAACCAGTTTGGGCGAGATCATCTT
GCAGGGTCCGCACCAAGTGGCGAAGAAATCCAGCACCACCAGCTTGCCGGATGCCTTGGTCAGCTGTCCA
TCGAGATCGGCCTGCAAATGAAAATGTAAATTAGTACTATCTCTGAGTAATATCATCATACATTAGCGCT
AATAATTTCCCACTCCCTAGAGATGTTTCAGTAATTTTCCCTTTAGCAGGTGGTGGTCATGTTTTCAAAC
TTTATGATTTCCGATTGTGGAGCATTGATCCGGTGCTATTGTAAATATACATATTGCGTTGGGGAGGCCC
CGCTACTATCTCGCCCGATAAGCCTGTTCCCTGCAAACACAACGTTGTCAGACATCCCCAAAGTTGCCAA
GCTGTAATCAGCCCCAATAATCGCCGATTGACGGGTTAAATATGTGTGTGTGCTTCATAAACTTGTGAAA
TCGGGTCAATTTCATTTCAGTTTCTGTATTGCACCCATTGGGCAGCAAAGCAAATCAATATTTAAAGAAT
TTCCAAATTATATGTATGTATGCCGAAAGGTGTGGCGGTCGTGGGCAGTTCCATCCCCTTTCTTCCACCC
ATTTCTCTGCCCGTGACACGTTTTCCCAATGACCTTTAATTAATTGCTAGCGATGCCAATCGCAGAACAG
CACATGCAAGCACAGAGCTCTAATGAAATTGACGATTTCAATTGGAATGGGCAACCGGTTTGGAGGAATT
TGCATAAGTATATGCCCAAGAAATTTAACTTTATACACGCTATTTTTGCTAGCACAAAACCACTTGATCA
TCCCTATTACATATTTTAATCAGGCTTAGATCATTTCTATTGCAGACACATACGTCATGGAATGATTAAT
CTCAAGATGGTGATGGTACTCGATGACTGTTGGCTACAATTCAATTTGTCAACATGTATTGCTGACTCAT
TTTTTAAGACAATATCTGAGTAAACATAAAAAACTAAAATCAGTAGTTAAACTACTTTGTTATATTGTCT
AATTCAGTATGCATAAATGTTTTGATGTTTTCGTGGATTCCTTATGCCTTCGATAAGCATTTTTTGATTT
TTGAGCTTATTGTTGTGTGGTTTGCTGTATAGAACTAGACCGCGAGAGTCAATGTTTGATAGTGTTTAAG
TGTGTATACAAATATAAATAAACTAATATTGAGAAATAAAATAAGAAGGCATGCTTTCACGTATTTACTT
CCCAATATTTCCTGACGTAACTTTGTATTCATGAAATGGAACCGGTTCTTCACCCCGCGCACTCGCGTCT
TTTGTTTTCTTGGCTTTGGATCAACTTGCATGGCGCGCAACTTATGTGTGGAGAGCACACCCTGTGCTAT
AGCAACTATTCGAAATATTTCTAACCAAAAAACCAGGCCTCGACCACGATAATGAATTGCAAATTCATGC
CAGTCGCACACACATCCGTACATATGTGGTTTCTATAGTACGTATATCTTGTTCGCACTATTTCCGATTA
GCTCGGTCCGGAATATTTCAATTTCAAAATTCGGTTTCCGTTGAGACCCGTTTTGCGGTCCACGGGATGA
TATCATTATCATTTCGCTGTGCTGCTATTCGGAACTTTCTCGTTTGCTCTCCGCTTTCTCGCTCGTCTAG
ACCGAACGTGAGTTCCTTCTATTCGCGGGAGAGCGTATGGCAAAAAGCGTAGCGAATCCAATCATTCAAA
GTAAGTGGCTAATTGGAATGCCGCTTCCAATGCCATATTTTCAAAGTCCGGAGCGAAAAAATAAACGTGT
AACTGGAACTGAGGGGTCAAAGGGTAGCGCTATCGAGTTACCCAATAGATGACGGATGTGATGCGTCCAT
AGATCCGGTTGAAGTGACTCACCTTATCTTTCACCTGGTACACCATGTTGGATGGGAGATGTGGAGACGG
AACCGG
>dme_circ_0001971_junction_seq
TGGATGGGAGATGTGGAGACGGAACCGGAAAACTCATAGGTGGATCAGGGGGACGG
>dme_circ_0001972
GTGGATCAGGGGGACGGCCAGACGTCTATGCGCTGCCCACTTAGATATTGGCCTTGATGACATCCTCCAG
ACGCTTGGCGTTGGCTCCGGCGAACTCTTCGACCTTGACGCCGTTCTTGAGGAACACGAAGG

>dme_circ_0001972_junction_seq
TTGACGCCGTTCTTGAGGAACACGAAGGGTGGATCAGGGGGACGGCCAGACGTCTA
>dme_circ_0001973
GGTAACATTGGAGTGGTGTCATCTGCATTCGCCGAACAGCTACAGTGGATTATTCGTGAGGACGAAGCCC
CTGTGGATATTCGCCTACACGGTATTTTGGCTTTCCGAAGGGTTGACTGTGCTCGACACCGAAGCTACTT
CCTGGATAACTACGGCAACTATACATTGAATTCGGAACTGCGTATATACAGCTACTTGCAGGCCATGAGA
TGTCCCGACTACATATCAGTTGGGGTCATTAAATCGATCCTGGAACATGAGGAAATCAATCAGGTGGGCT
CCTTCGTGTGGTCCCATCTGACCAACCTGGCGAAATCAAATTCCCCAGTTCGCATCGAAGCTCAAGGTTT
GCTGCTTAATGATGAGCTCTCTGAACGCTTTAAAATGGACATCCGCAAATTCTCGAGGAACTACGAACAC
AGTTTATTCTTTGACGA
>dme_circ_0001973_junction_seq
ACTACGAACACAGTTTATTCTTTGACGAGGTAACATTGGAGTGGTGTCATCTGCAT
>dme_circ_0001974
GAGAGCTTCTTGCGCAGCAACTGACCGCTCAAAGGTCCTTTGGGACCAAAAGCGTTAGCCGCCAACTCCT
CCAATCCTTCAGCGCGGGCGGTGAATTCAAAGAAGTTAACTGACTGGCCGAAGAGATCGGCAGTAAAGTT
AACACTCGCAATGCGTGGCAAGTAGGAATCGGTTCCAAAGATAACATTCGCATCCGTGGTTGTTCCAAAA
TTGTACTCGTCAAAGAATAAACTGTGTTCGTAGTTCCTCGAGAATTTGCGGATGTCCATTTTAAAGCGTT
CAGAGAGCTCATCATTAAGCAGCAAACCTTGAGCTTCGATGCGAACTGGGGAATTTGATTTCGCCAGGTT
GGTCAGATGGGACCACACGAAGGAGCCCACCTGATTGATTTCCTCATGTTCCAGGATCGATTTAATGACC
CCAACTGATATGTAGTCGGGACATCTCATGGCCTGCAAGTAGCTGTATATACGCAGTTCCGAATTCAATG
>dme_circ_0001974_junction_seq
CTGTATATACGCAGTTCCGAATTCAATGGAGAGCTTCTTGCGCAGCAACTGACCGC
>dme_circ_0001975
AAATACCCGGGACTTTGTATACGTAAACTCCTTGCCGGAAAGTACATTCTTTGCCTGCTGGAAGGGATTG
AATTTGGCAGCCAGGGCCATCACCTCCACCAATGACTCTACGTTGAAGTAGCGCAGATCGTTGCCAAAGA
CCCGTAATCCAAACTGTGCCCGTGGATTATTGTAGTCGTACTTCAGCTTATAACCAAGACTGTCAACGTT
GCGATCGATCTCCTGCTTTCGCGTGGTACTGACATCTCGCTTTTGTCGCTTATTACCTTCTAAGGACTCC
TCAAAGTCGTAATCTTCGTCCACTTCCTCTTCGGACGCACGAGCTTGTTCCTGAGCCTTTCTCTTCAGTC
GCAGATTATCCAGACTCAGCAGATTTTCGAGGG
>dme_circ_0001975_junction_seq
TTATCCAGACTCAGCAGATTTTCGAGGGAAATACCCGGGACTTTGTATACGTAAAC
>dme_circ_0001976
GTTGTTGGTTCAGAAGCGGGAGGAGCAACTGCCCCAGGCGGGCATTGCCAATCGAAGTGCAAACTCCACA
TGCACCTGGCCAGTTCTCGATCAGGCCATTGGATTGCAGATGTGCTCCCACTACAGTGTTCCAGATCTGA
GCAATGCCACCGAAATTTACCCAAGCCTTTTGCTGGCAGGACCACTTAACTTCAGCTTGATTCTGAAGAA
GTCTGATCTAAGTGCTAAGAAGTACGTGTTCGAGTACAAATGGGATCAGCAAGAAGAGGACAACAACTTT
TCGCTGGTGTTTACTACTCCGGGCTCAAAGGTTCCCCGGGTTCTGGTTGCAAATGTGACCAAGGTGCCCG
ATGCCTTCAATGCATCCGTGGCTTTTGTGAATGGACCTAATCGTGTCTCCGCTGGGTGCAGCTATGATGG
AAACCCGGATTTCAGACGCTTGGATATCTACTTAGATACGAATGGAAATCGTTCGTTGGACCTTGGAATG
GAGCTGCGAAGGCACCAGGACTTCACTGCCTGGATCTACAATCCTCGTATGCTGCTGGCCATTAATGGTG
TCAACATTACTGGTCTGGCTGGCACCGTCAAAGTGAACGAGAAGAATGGTATCAAGCAACATGATGTGGA
TCTCTCATTTGAGACCAAGAAGCTGCAGGCTGTGATCAAGGGCAACGTCGTCCAAAGTGAGATAACCACC
TCAACCAACATGACCATTAAATACAGGGTTCCAAGCAAATAAGATTGAGGAGATTAACTTTGCCGGAAAG
CTGGTTAATAATGGAGATAAGAGCAAGACGGAGTATCGCGGTAACATGAAGCTACAAACATCGGCATATC

CCAAGCTAAACTTCGCATCGGAATCCACGTGGCTTAGTCTTCAAGGCCACACCGAAGGAATGATCACTTA
CAATAATGCGCCCGATTATGTCAATCCCAATTACACCAGTTTGGTTCGTTTGATCTTTGCTCGCTCCCAT
TCAGAGGACTCCTTCTTGGATGGAACGCAAACGCGAGCCAGTCTTGAACTGAAGCTTCCAAG
>dme_circ_0001976_junction_seq
GAGCCAGTCTTGAACTGAAGCTTCCAAGGTTGTTGGTTCAGAAGCGGGAGGAGCAA
>dme_circ_0001977
GCGGGAGGAGCAACTGCCCCAGGCGGGCATTGCCAATCGAAGTGCAAACTCCACATGCACCTGGCCAGTT
CTCGATCAGGCCATTGGATTGCAGATGTGCTCCCACTACAGTGTTCCAGATCTGAGCAATGCCACCGAAA
TTTACCCAAGCCTTTTGCTGGCAGGACCACTTAACTTCAGCTTGATTCTGAAGAAGTCTGATCTAAGTGC
TAAGAAGTACGTGTTCGAGTACAAATGGGATCAGCAAGAAGAGGACAACAACTTTTCGCTGGTGTTTACT
ACTCCGGGCTCAAAGGTTCCCCGGGTTCTGGTTGCAAATGTGACCAAGGTGCCCGATGCCTTCAATGCAT
CCGTGGCTTTT
>dme_circ_0001977_junction_seq
CGATGCCTTCAATGCATCCGTGGCTTTTGCGGGAGGAGCAACTGCCCCAGGCGGGC
>dme_circ_0001978
GTCCATTCACAAAAGCCACGGATGCATTGAAGGCATCGGGCACCTTGGTCACATTTGCAACCAGAACCCG
GGGAACCTTTGAGCCCGGAGTAGTAAACACCAGCGAAAAGTTGTTGTCCTCTTCTTGCTGATCCCATTTG
TACTCGAACACGTACTTCTTAGCACTTAGATCAGACTTCTTCAGAATCAAGCTGAAGTTAAGTGG
>dme_circ_0001978_junction_seq
TCTTCAGAATCAAGCTGAAGTTAAGTGGGTCCATTCACAAAAGCCACGGATGCATT
>dme_circ_0001979
GTTATATTGAAGGATGGACTTTCCACAATCATTTAAGATGGTGAAGAGCCTGAACACGCGAGCTGCGAT
CCTTGTAGTTGGCCTTGACTGCGACTGAGTGTCCAGTAAACCATGAGCCGTTAATAAAGATCTAAAGAGA
TTTTAAATTAAATAAAGTTCGTACGCACATTAAACAAAAAAAGTAATTCTTACGATATAATCCTTGGTGG
CTTTCTCGTTCACTTTAAGACTGGCTGTGCACGAATTGAACTCCGGTACGGTCACGTTCATGTAGGCATC
AATGGCAAACAGATTCCGTCTTGGCAAATGGACAGAAAACAGTCCAGTGATTTCCTTCTCAGGAGTGTAC
TTCAGACCAACGATGACATTGTGCTCGGTTCCGTTTTTGATGTGTTCTTTGTGCCTTCAAATAAGGATAA
CACATTTaaaaaactttaaaaaataaaagatttaaacatatacttacttaactaaaatGCGGTAGTCAAT
CTTTGACCTTGGAAGCTTCAGTTCAAGACTGGCTCGCGTTTGCGTTCCATCCAAGAAGGAGTCCTCTGAA
TGGGAGCGAGCAAAGATCAAACGAACCAAACTGGTGTAATTGGGATTGACATAATCGGGCGCATTATTGT
AAGTGATCATTCCTTCGGTGTGGCCTTGAAGACTAAGCCACGTGGATTCCGATGCGAAGTTTAGCTTGGG
ATATGCCGATGTTTGTAGCTTCATGTTACCGCGATACTCCGTCTTGCTCTTATCTCCATTATTAACCAGC
TTTCCGGCAAAGTTAATCTCCTCAATCTTATTTGCTTGGAACTAGATTAGAACAAAAAATTACCTTCatt
aattgattaattaattaattaattaGGCAGATACTAACCCTGTATTTAATGGTCATGTTGGTTGAGGTGG
TTATCTCACTTTGGACGACGTTGCCCTTGATCACAGCCTGCAGCTTCTTGGTCTCAAATGAGAGATCCAC
ATCATG
>dme_circ_0001979_junction_seq
TGGTCTCAAATGAGAGATCCACATCATGGTTATATTGAAGGATGGACTTTCCACAA
>dme_circ_0001980
GTCTGAAAAAATCTGATCCTTGTCAACTCTACGCTCTTCTGGGCAGATGATCAGAAGCTGCAGGTGGGCTA
CAAGAGCGACTACGACGTCAACGATCAACTAATGAGTTTTGATGTTCGCTTCGGCATCAATAGCACCATC
CGGGATATTCCCACGATTAAT
>dme_circ_0001980_junction_seq
CACCATCCGGGATATTCCCACGATTAATGTCTGAAAAAATCTGATCCTTGTCAACTC

>dme_circ_0001981
GTCTGAAAAATCTGATCCTTGTCAACTCTACGCTCTTCTGGGCAGATGATCAGAAGCTGCAGGTGGGCTA
CAAGAGCGACTACGACGTCAACGATCAACTAATGAGTTTTGATGTTCGCTTCGGCATCAATAGCACCATC
CGGGATATTCCCACGATTAATGTAAAGGTAAAGCATTGGATGGATGTTAAAAAG
>dme_circ_0001981_junction_seq
GGTAAAGCATTGGATGGATGTTAAAAAGGTCTGAAAAATCTGATCCTTGTCAACTC
>dme_circ_0001982
AAATGGGCCACCAGACCACCTTTCTCGTAGCCCTTGAATGGACTTACCAGATGCACTAAACCAGAATAAT
TATTGTATCGTTGGTTTTTCTCAATCTCCCAACTGGAGTCCATGCTGTAGGTGTTAAATGTATCATTCTG
TCCACTGTATCCAAGATAGAGCTCTG
>dme_circ_0001982_junction_seq
TGTCCACTGTATCCAAGATAGAGCTCTGAAATGGGCCACCAGACCACCTTTCTCGT
>dme_circ_0001983
TAGTCGATGGTCAAGTGTGTCCTTAGCAGATCACGCAGTCCACGATAGTACGTCGCCTTGCAGTTTATAT
CCTTCTCCAGGAGGGGATGTGCCACCGTCAGCGCGATGGTGCGATGCTCCCGATCTCCACGATGCAGGGG
TTCTGCCTTCCATTGCAGATTGCTGTGTATAATCTTGGGCTGTTGGTCATAGTTGCCGCCCAGCCATTGG
AAAGATAGGTCGGAGTTAAAGTTTGTGTCCGTCATGTAGTACTGTCCTGAGGAGGAGAGATTGCGCTTCG
GATAGAAAATTTCCAGGGCAAAGCTCTGGGACTCATTGCCAACCTAAGGGAAGAATTCCTATTAGGTTAT
CATTCTAATCATGATCGATCGTTTCGCTTACCTTGCTGAAATTACCCAGCTCCAAATTGTATCCAATCCA
TGCTGTTTCCGACAACTCCAACTTTGAGTGATGCCTGACAACTTCGGGGCTGACATCCTCGTACTTAGTG
CTAAGCACTACGGCACGATTCGGATGAATGGCCACCACCTTGAAGTCCTGCGCCTTGAGCGTGGCCCGTG
AGTGCAATTCACCGGTCAGATTGAAGTTCTGCGTGTTCCTTAGCTCATAGATCTCAATGTGCTTCACGTC
CTGGCTTCCTGCGGGGTCGCTGGCATCCCTGGTGCTGACAAAGTGAATTCCCAGCGGTTTCACTTCGTTC
TCCAGCGAGATGTCCGTTGTCTCCTTATATATTGGCGCTTTCGCCTGTTCCAACCGCTTGTACTTGCCAT
CGAGCACTTGCTTCTCATTGTAGACGACCTTGACATTTCCTGCATCCTGATTGCGACGCTTTTGCAAGCC
ATACACAATATCCGCTCGATGCCGTGTTGCCAATGGAACTTCTATTTCCACATTGCCATTGAGATTGTGG
TTAAAGTTGCTGCTGTCGCTATCATAGTTGCTGTTCAACTTGAAGTAGGCAGTATCCTGGGGCCAGAAGC
AACGACAGTAGCGGTAGCTCTTTCCACTGCAAGGTAAATTAATATTTATAAAATGAATTAAGAGTAATTG
TTCGTTAACTCACTTGTTGGTGGTAAAGTTAAAATCTGTCTTTAACCAACTGACATTGAGGAAGGTTGGC
AGTGTGCTGTTGAAGATTCCATGCATATTGCTTAGCGAACTGACGGACACATCCAGATCGGTAATGCGAC
GACTGGCAGGATAATCCAAGGATCCCACCACGTTGTGGTGACCCGACTTTTCCAAGTCATAACTTCCCGT
TAGGGCAAAGGTGTGCTCATTTTTAAATTTGGGCGTGATTATCTTTGCAGAGAACTCTCTTTGGGCTCCC
TGCTTGTGGAGCAACTCAAAGTCACCCTCCACTACAGACAAGTTTTGAATCTTACCCCACTCGACGTGGC
CCAAGCCCTTAAGGTTATTGGTGGCATTTTTATAGGAGATTCGAGATAAAAGCTTATTCTGTGCTTCAAA
ACTCTCGTAGCTGGCCTCGAGGACAAAATCAGGATTGTTGAACTGCTGGCTAATCACGTTGCCCTGATAG
CTGGCACTCAGCTGGTGGCGATCTCCAACAGGCAAAGGCAATGCGGTAATAACTCTGAACTTAAAATCAT
CAACTGACTTATATTCTGCACTACCATTCACGTCGAGATTCCATTTGGAATCGACGCTCAGTCGTCCACC
CGCCTGTAACTGGGTAAATGTTTGTCCCGGGTTCTGGTACTTGGCACTAGCGTGAACAATTCTTGGCTTA
GCATTTGGTCTCTCCAGGTAGTTTTCCAGTACCAGCTGCATGTCCTCGGCTAGAATCCAAGACCACAGGC
AACTACCTCGGCCTATATACTGACCGATCTTATACTCGCCCACTATGTGGCCTCCTTTATTGAGGAAACC
GACGTCGGAGTGCAGGTGCACCGTATCGATTCCGAGCGCCTTCCATGGGGTCTCCAAGCTCAAGTCTCCC
ACCAAGGAACCTTCGGAGTTGGGACTAAGATTGCCGTAGAAATTAATGCGAGCGATTTCTGGTTCCGTGC
TCTCCACTAGAACTGAGAAATCCATCGCTCCGTTGGTCTTGCTGACACTGTAACCTCCTGACATCTGACA
CATTTTGCCATCCTCAATGGCGCTAAAAGAAAAGCGAATCTTCTTCGGTCCCGCCTCATGGATGTTAAGT

TCAATCACACCATCTCGACCGCCAGCTTTGGGCTGTACCCTTAAAACGACATCGATTGGCAGACTATTGG
TCATGCGGACGGCTCCTGTGACTTCATATGTTGCCATATTTCTGTACAAGAACCCTGAGACATCATATCG
GCCGGGATCGCGGGGGCTTCTAATCGCAGTACCGTGCATGGAAATGTTTCGGAAGTTTTTCATTGGAGTC
CACACCTCAATGTTAAAGACATCTTTTTTCTTATTGGCACGCGCTCCGTAGGCTATACTCTGGCCATTCT
GGCTCAATAAGTTAAGATCGAAATTGAGGTGGGGATTCTGACCCTGGATGACCAGAACAGAAGTGGACGC
CATCTGAAGTGGCAACATGGTGGTCTTAATCCTTCCATTAGCGTTAACAATATAAGAAGGATCAGTGTGC
CAATCCGTGGAAATGTGAAGCTAATAAAGAAAATAGGATAAGTAGGCTGTATAATATACTATTACTTTTG
GTATTACCTGGTTGAGTGTTCCATTGTCGGTTACCTCGAATCGAATATCAATGGCTTTATCCACGGCTGA
GAAGTCTTTCTTAAAGTACACCTGGCATCCATAGTGGGGAATAGCGTCCGTCTCCAACTGAATGCCCACG
GAAGTTTCCAAGAAGTTGTCCTCGTTCTGAGTACAAAATTTTGTTGTTTAATATAATTGTTAATAAATTA
GCTTCGTTTTTAGACACTAGTACTAACCTCTATGGAGGCAGCCATCGAAAGATGTGTATGGGCCGTAACC
GCACTGATGTTGCCCTTATAGGCATTGTCATCCAGTTCGATGTGTCCGTGAACGTAGATTTCGTGAAGCA
TTTCGTAGGGTGTGAGGAGCGTAACCTGGACATCATGCGCCTTGGGCTTAACATTATCCTGCAGTGCGGT
GTAGTTAAACACAAAACCCAGCTCTTGAGTGTTGTCCTTGTCATCGTTGACTATAAATTTGACGCGCTCA
TAGAACGCGTTGAAGTTTAAGTCAACATGATACTCCACAATTGACTTTATATTCTTGGCCACCGCAAATG
GTGTAGTCACGGTCAAAAGATTGTGGACATTTATGTAGTCAGGATAGTGGAGGCGATCCTTGAACTCCAC
TTTGCCCTGTGGCAGTTCCACATGGCCTACAACCAGATAGAAATCTATGATCTCCTGAATATCCACATTA
CCGACAATAGTTGGCCACACAAGAGTGTCCACCTGGAAGTTCGTGAAGTAGGAGAAATACTCCTCATCGG
CATCTGGTTTGTAATCCGTCTCAGCATTAAGTGGTTTGAAGTCCGCGTCGTAGAGCATCTCCAATTCCAT
TTTATTGCTGCCCAGTTGATTTACCAAGTCAGACTTTGGTTCTCCATTGATTTTTACACCCAGCTTATAG
TTGGACATCTTGCCGTGCAACTGAAAGACAAACTCCTTCCTCTTCAGTAGCTGAACAATGAAACCGTTCT
CTTCAAAGCCAGCCAGTGGGGTGAATACGTGGTAGGAGTACTCAAAATCGGTTATATTCTGCATGTGCCA
TACGCCAGTGAATCCCAGGGTGACATTGTTCCACAAACCACGCATGTCAACACGCTCCGGATTAAACAGT
GCAAATATTGCCGCCTGTTGGAAGCTTTCGATCGGCGTGGCCACACTCAGCTTGACATCGAAGTTCAGGA
GATTCTTAATATCCGCCAGGACCTCCACTCCAAGAGCTGCCGTGGGAGCCCTGACCTCCGCCACGGCATG
ACGTTTCTTTTCGTTTAGACCAAAGCGAGCGTTAATGGTGCCAAATTTCTCCAATGGAGTGGTGATGTTA
ACGGTTAGCATGTTATCCGTAAACTTTTTGACATATCCGTTCATTGTCAAAGTGAACTCCCGAAGATCGA
AGTTGAGGGAAGCTGCTCCACTGACCACCCGTTGATCGCTCAGTGAGAAATGGGCCACCAGACCACCTTT
CTCGTAGCCCTTGAATGGACTTACCAGATGCACTAAACCAGAATAATTATTGTATCGTTGGTTTTTCTCA
ATCTCCCAACTGGAGTCCATGCTGTAGGTGTTAAATGTATCATTCTGTCCACTG
>dme_circ_0001983_junction_seq
GGTGTTAAATGTATCATTCTGTCCACTGTAGTCGATGGTCAAGTGTGTCCTTAGCA
>dme_circ_0001984
GTCAACGATGACAAGGACAACACTCAAGAGCTGGGTTTTGTGTTTAACTACACCGCACTGCAGGATAATG
TTAAGCCCAAGGCGCATGATGTCCAGGTTACGCTCCTCACACCCTACGAAATGCTTCACGAAATCTACGT
TCACGGACACATCGAACTGGATGACAATGCCTATAAGGGCAACATCAGTGCG
>dme_circ_0001984_junction_seq
CAATGCCTATAAGGGCAACATCAGTGCGGTCAACGATGACAAGGACAACACTCAAG
>dme_circ_0001985
TTTTTCATTGGAGTCCACACCTCAATGTTAAAGACATCTTTTTTCTTATTGGCACGCGCTCCGTAGGCTA
TACTCTGGCCATTCTGGCTCAATAAGTTAAGATCGAAATTGAGGTGGGGATTCTGACCCTGGATGACCAG
AACAGAAGTGGACGCCATCTGAAGTGGCAACATGGTGGTCTTAATCCTTCCATTAGCGTTAACAATATAA
GAAGGATCAGTGTGCCAATCCGTGGAAATGTGAAGCTAATAAAGAAAATAGGATAAGTAGGCTGTATAAT
ATACTATTACTTTTGGTATTACCTGGTTGAGTGTTCCATTGTCGGTTACCTCGAATCGAATATCAATGGC

TTTATCCACGGCTGAGAAGTCTTTCTTAAAGTACACCTGGCATCCATAGTGGGGAATAGCGTCCGTCTCC
AACTGAATGCCCACGGAAG
>dme_circ_0001985_junction_seq
TCCGTCTCCAACTGAATGCCCACGGAAGTTTTCATTGGAGTCCACACCTCAATGT
>dme_circ_0001986
GAAAGACTTCTCAGCCGTGGATAAAGCCATTGATATTCGATTCGAGGTAACCGACAATGGAACACTCAAC
CAGGTAATACCAAAAGTAATAGTATATTATACAGCCTACTTATCCTATTTTCTTTATTAGCTTCACATTT
CCACGGATTGGCACACTGATCCTTCTTATATTGTTAACGCTAATGGAAGGATTAAGACCACCATGTTGCC
ACTTCAGATGGCGTCCACTTCTGTTCTGGTCATCCAGGGTCAGAATCCCCACCTCAATTTCGATCTTAAC
TTATTGAGCCAGAATGGCCAGAGTATAGCCTACGGAGCGCGTGCCAATAAGAAAAAAGATGTCTTTAACA
TTGAGGTGTGGACTCCAATGAAAAACTTCCGAAACATTTCCATGCACGGTACTGCGATTAGAAGCCCCCG
CGATCCCGGCCGATATGATGTCTCAGGGTTCTTGTACAGAAATATGGCAACATATGAAGTCACAGGAGCC
GTCCGCATGACCAATAGTCTGCCAATCGATGTCGTTTTAAGGGTACAGCCCAAAGCTGGCGGTCGAGATG
GTGTGATTGAACTTAACATCCATGAGGCGGGACCGAAGAAGATTCGCTTTTCTTTTAGCGCCATTGAGGA
TGGCAAAATGTGTCAGATGTCAGGAGGTTACA
>dme_circ_0001986_junction_seq
AAAATGTGTCAGATGTCAGGAGGTTACAGAAAGACTTCTCAGCCGTGGATAAAGCC
>dme_circ_0001987
GCTTCACATTTCCACGGATTGGCACACTGATCCTTCTTATATTGTTAACGCTAATGGAAGGATTAAGACC
ACCATGTTGCCACTTCAGATGGCGTCCACTTCTGTTCTGGTCATCCAGGGTCAGAATCCCCACCTCAATT
TCGATCTTAACTTATTGAGCCAGAATGGCCAGAGTATAGCCTACGGAGCGCGTGCCAATAAGAAAAAAGA
TGTCTTTAACATTGAGGTGTGGACTCCAATGAAAAACTTCCGAAACATTTCCATGCACGGTACTGCGATT
AGAAGCCCCCGCGATCCCGGCCGATATGATGTCTCAGGGTTCTTGTACAGAAATATGGCAACATATGAAG
TCACAGGAGCCGTCCGCATGACCAATAGTCTGCCAATCGATGTCGTTTTAAGGGTACAGCCCAAAGCTGG
CGGTCGAGATGGTGTGATTGAACTTAACATCCATGAGGCGGGACCGAAGAAGATTCGCTTTTCTTTTAGC
GCCATTGAGGATGGCAAAATGTGTCAGATGTCAGGAG
>dme_circ_0001987_junction_seq
GATGGCAAAATGTGTCAGATGTCAGGAGGCTTCACATTTCCACGGATTGGCACACT
>dme_circ_0001988
AAAAGCGAATCTTCTTCGGTCCCGCCTCATGGATGTTAAGTTCAATCACACCATCTCGACCGCCAGCTTT
GGGCTGTACCCTTAAAACGACATCGATTGGCAGACTATTGGTCATGCGGACGGCTCCTGTGACTTCATAT
GTTGCCATATTTCTG
>dme_circ_0001988_junction_seq
TGTGACTTCATATGTTGCCATATTTCTGAAAAGCGAATCTTCTTCGGTCCCGCCTC
>dme_circ_0001989
CTGGCCTCGAGGACAAAATCAGGATTGTTGAACTGCTGGCTAATCACGTTGCCCTGATAGCTGGCACTCA
GCTGGTGGCGATCTCCAACAGGCAAAGGCAATGCGGTAATAACTCTGAACTTAAAATCATCAACTGACTT
ATATTCTGCACTACCATTCACGTCGAGATTCCATTTGGAATCGACGCTCAGTCGTCCACCCGCCTGTAAC
TGGGTAAATG
>dme_circ_0001989_junction_seq
CGTCCACCCGCCTGTAACTGGGTAAATGCTGGCCTCGAGGACAAAATCAGGATTGT
>dme_circ_0001990
AGAACTCTCTTTGGGCTCCCTGCTTGTGGAGCAACTCAAAGTCACCCTCCACTACAGACAAGTTTTGAAT
CTTACCCCACTCGACGTGGCCCAAGCCCTTAAGGTTATTGGTGGCATTTTTATAGGAGATTCGAGATAAA

AGCTTATTCTGTGCTTCAAAACTCTCGTAGCTGGCCTCGAGGACAAAATCAGGATTGTTGAACTGCTGGC
TAATCACGTTGCCCTGATAGCTGGCACTCAGCTGGTGGCGATCTCCAACAGGCAAAGGCAATGCGG
>dme_circ_0001990_junction_seq
CGATCTCCAACAGGCAAAGGCAATGCGGAGAACTCTCTTTGGGCTCCCTGCTTGTG
>dme_circ_0001991
TACTGTCCTGAGGAGGAGAGATTGCGCTTCGGATAGAAAATTTCCAGGGCAAAGCTCTGGGACTCATTGC
CAACCTAAGGGAAGAATTCCTATTAGGTTATCATTCTAATCATGATCGATCGTTTCGCTTACCTTGCTGA
AATTACCCAGCTCCAAATTGTATCCAATCCATGCTGTTTCCGACAACTCCAACTTTGAGTGATGCCTGAC
AACTTCGGGGCTGACATCCTCGTACTTAGTGCTAAGCACTACGGCACGATTCGGATGAATGGCCACCACC
TTGAAGTCCTGCGCCTTGAGCGTGGCCCGTGAGTGCAATTCACCGGTCAGATTGAAGTTCTGCGTGTTCC
TTAGCTCATAGATCTCAATG
>dme_circ_0001991_junction_seq
CGTGTTCCTTAGCTCATAGATCTCAATGTACTGTCCTGAGGAGGAGAGATTGCGCT
>dme_circ_0001992
AAATTTACCATATCTCGCCGGGAAGATATCACGCTTGTAGTGGGCTGTAGACTCCGTCTTATAGTAAGAG
TTCATGGCCGCCAAAGTTCCATTTAACTGGACATCCAGTTCGGAAGCAATATGTTTGCCGTACACGTGGA
AGGTGTAGTTGGTGTGGCCCAATTCACTGTATCTATCCGTCAGCTGAGCACCCAGCTCAATAAGTTGGTC
AGGATATTCCGAGTAGTCGATGGTCAAGTGTGTCCTTAGCAGATCACGCAGTCCACGATAGTACGTCGCC
TTGCAGTTTATATCCTTCTCCAGGAGGGGATGTGCCACCGTCAGCGCGATGGTGCGATGCTCCCGATCTC
CACGATGCAGGGGTTCTGCCTTCCATTGCAGATTGCTGTG
>dme_circ_0001992_junction_seq
GTTCTGCCTTCCATTGCAGATTGCTGTGAAATTTACCATATCTCGCCGGGAAGATA
>dme_circ_0001993
AAATTTACCATATCTCGCCGGGAAGATATCACGCTTGTAGTGGGCTGTAGACTCCGTCTTATAGTAAGAG
TTCATGGCCGCCAAAGTTCCATTTAACTGGACATCCAGTTCGGAAGCAATATGTTTGCCGTACACGTGGA
AGGTGTAGTTGGTGTGGCCCAATTCACTG
>dme_circ_0001993_junction_seq
GGTGTAGTTGGTGTGGCCCAATTCACTGAAATTTACCATATCTCGCCGGGAAGATA
>dme_circ_0001994
GAATGCACTGCGTGTATCCGGAATGTAACCGACCATTTGAAGATTCTGACTGGCGTCCTCCGTCAAGTTG
AAGTCCATTCGAGCATAGCGCTCCAAAATGTCCATGTATATGTAGCCCGAGTGGTTTGTATCAGGAGTAT
CCCAAATTGTGGCATTCAATCCGTAAATCGGATACCGTATGGTTGGCAGCAAGTG
>dme_circ_0001994_junction_seq
TCGGATACCGTATGGTTGGCAGCAAGTGGAATGCACTGCGTGTATCCGGAATGTAA
>dme_circ_0001995
TTGAAGTCCATTCGAGCATAGCGCTCCAAAATGTCCATGTATATGTAGCCCGAGTGGTTTGTATCAGGAG
TATCCCAAATTGTGGCATTCAATCCGTAAATCGGATACCGTATGGTTGGCAGCAAG
>dme_circ_0001995_junction_seq
AATCGGATACCGTATGGTTGGCAGCAAGTTGAAGTCCATTCGAGCATAGCGCTCCA
>dme_circ_0001996
TGATTCATCTTTAGATACGAACTGACATCGATCACACGTATCTCCTCGTAGTTGCGCCAAATGTCCAGGA
ATGCACTGCGTGTATCCGGAATGTAACCGACCATTTGAAGATTCTGACTGGCGTCCTCCGTCAAGTTGAA
GTCCATTCGAGCATAGCGCTCCAAAATGTCCATG
>dme_circ_0001996_junction_seq

TCGAGCATAGCGCTCCAAAATGTCCATGTGATTCATCTTTAGATACGAACTGACAT
>dme_circ_0001997
TACTTCTGCACCATAATCTGCACCTGATCAATTTTCTCAAGTTCTTTGATTTCCTGCCAGATTTCGGTAA
GAACCTCGTTCAATTCTGCGCCGAAAGGTACCAGCTTGAAGTACTTCTCCTTGACAAAATTCCAGGCTAT
CGTTGAATACTTCTTAATGTTGGTGATGGCATCGTTAGCCTTGAAGTCCCGGTAAAGTCGCTCCGCATCA
GCAGTAAAACTGGATACCTTGTTGAAGTACGGAGACTCCGCCAGCTCATTGG
>dme_circ_0001997_junction_seq
AAGTACGGAGACTCCGCCAGCTCATTGGTACTTCTGCACCATAATCTGCACCTGAT
>dme_circ_0001998
AACCTCGTTCAATTCTGCGCCGAAAGGTACCAGCTTGAAGTACTTCTCCTTGACAAAATTCCAGGCTATC
GTTGAATACTTCTTAATGTTGGTGATGGCATCGTTAGCCTTGAAGTCCCGGTAAAG
>dme_circ_0001998_junction_seq
CATCGTTAGCCTTGAAGTCCCGGTAAAGAACCTCGTTCAATTCTGCGCCGAAAGGT
>dme_circ_0001999
TTAAGATTCAGACCCACATAACGCTGATCGAAGGTCATGTAGTGACGCGAGTCAATCAGCAAGGCGCGAG
CTATAGAATAAAATAATAATTAAAGTAAATTTAAAAATAAGTATATCTTCAATAAACTCACAGTAGTACG
GTGGCAGCCAGGTCTTGGGATCCAGATGGGTGCGCATGTTATAGAGCTTCATGACGATCGATGAGTTGGT
CTCACTAAAGAAGCTCTGCGCCTTGGCGAGCACCTGGTACTCGGGAATCTCCTCGAACCGGGGCGTCTCG
TTAAATGCATGCCAGGACATGGGTAGCTTCTGTTCAAGGTCGATAATGCCCACTTCCGGATCAAAGACAA
ACTTGGTCTTTGCCTCCCTG
>dme_circ_0001999_junction_seq
AAAGACAAACTTGGTCTTTGCCTCCCTGTTAAGATTCAGACCCACATAACGCTGAT
>dme_circ_0002000
GCTACCCATGTCCTGGCATGCATTTAACGAGACGCCCCGGTTCGAGGAGATTCCCGAGTACCAGGTGCTC
GCCAAGGCGCAGAGCTTCTTTAGTGAGACCAACTCATCGATCGTCATGAAGCTCTATAACATGCGCACCC
ATCTGGATCCCAAGACCTGGCTGCCACC
>dme_circ_0002000_junction_seq
ATCTGGATCCCAAGACCTGGCTGCCACCGCTACCCATGTCCTGGCATGCATTTAAC
>dme_circ_0002001
GAGTTGGATCGAGGACTGCACTACAGGTGGCCAGTATACCGGTGCGGAAGAAGCTGGTACAGGCATCACT
GACCTCCTGGCTGACCTCTTGGGTCTGAGCCAGCTTATTCTGCCTGCACTGCTTCAGGCTCCACGAATCC
GTGAACAGCTGCGTCTCATTGGAGATCACGCCACTGGACATGGTGTACTCGTCGTAGGGCTCGTTGTTCA
AGGTTCCCAATAATCCCGCTGTTCTGCCGAAGTACCAACCACTCACCTCGAACCAACAGAGATCAAACTG
AACATTGCAGTGCAGACTAAACTCGGTATCTGAGG
>dme_circ_0002001_junction_seq
CAGTGCAGACTAAACTCGGTATCTGAGGGAGTTGGATCGAGGACTGCACTACAGGT
>dme_circ_0002002
TAAATATTGCAGGATCGAGGTGTAGTCGAAGCGCATATCCCTGGCCGCACATTTACTGCACGAGAGCAGG
ATGAAGGTTTTCGATACGCCCGGACGGAAGTTCAGCTTGGCAGCCGCGGAAATGGCCATTAGGATATCGT
TGCTGCTGCCATTTCCGGTGTTAATG
>dme_circ_0002002_junction_seq
GTTGCTGCTGCCATTTCCGGTGTTAATGTAAATATTGCAGGATCGAGGTGTAGTCG
>dme_circ_0002003
CTTCTCAAATTAGAGTAAATCGCGATCATAAGCAACCTTAAAGTGTGAAACCAAATGTTAGTCTAAGCCT

AAAGTGTATAACTAGTTTAAGACCTCACAAATTAAAGAAACCAATACGTGCTAAAACCATAACTTAAACG
CGTCTTAACCAAGCGCCTTTAAAACGCAACCTAAACACCAATTTAAAAGGATATTCAGCGAATAAGAGAG
TGAGAGAGCTCCAGTGAGAAGCTCTCTTTGGCCCATAAGGAGCAACTATTTCATCCGCACCGTTGACCCG
TAACTCGGCAAAATAGCTAAGTCCATTTTGCAGTTCCTCAAAAAGAAAAAGTCCCATCATAGTTCCACAT
CCGGCGAGGGCAAGCTACAGGCGCAGTCCAGTTgcgaggaggagggcggagcggcagcgggaggagcggg
agcggcagcgggacctccagcggaCCAGGCTAGCAGCAGCAGCAGCGGCCCCTCGTCAGCAGCCGCCGCC
CACAAGAGCACGCCCCCTGCCGCCACGACCACGCCCGGTTCAGGGGCGGATTcaggaggagccagcggag
gaacaggaggagggggaggaggaCCTAGCACCTCCGGCGACGATCAGGCCCAAGTGTCGGCCAGCGCAAC
CTTCCGTTTGTCATCGCTAACGGGCACCATCTCCAAGATGGGCAACAACGCCACCACGTCGAATAAGAAG
GTCGATGCCGCCGAGACGGTGAAGGAGTTCCTCGAGCAGGCCAAGGAGGAGTTCGAGGACAAGTGGCGAC
GCAATCCGACCAACACCGCCGCCCTCGATGACTTCGAACGGATCAAGACCCTGGGCACCGGCTCCTTTGG
CCGTGTCATGATCGTCCAGCACAAGCCCACGAAAGACTATTATGCCATGAAGATCCTCGACAAGCAGAAG
GTGGTCAAGCTGAAGCAGGTGGAGCACACGCTGAACGAGAAGCGAATCCTGCAGGCCATTCAGTTCCCCT
TCCTCGTCTCGCTGCGCTACCACTTCAAGGACAACTCCAACCTTTACATGGTGCTGGAGTATGTTCCCGG
TGGCGAGATGTTCTCCCACCTGCGCAAGGTGGGCCGCTTCTCGGAGCCGCACTCGCGCTTCTACGCGGCG
CAAATCGTGCTGGCCTTCGAGTACCTGCACTACTTGGACCTCATCTACCGTGATCTGAAGCCGGAGAATC
TGCTGATTGACTCGCAGGGCTACCTCAAGGTGACGGACTTCGGTTTTGCCAAGCGGGTCAAGGGACGCAC
CTGGACACTGTGCGGCACGCCCGAATACCTGGCCCCGGAGATCATTCTGTCCAAGGGCTATAACAAGGCC
GTGGACTGGTGGGCGTTGGGCGTACTCGTCTACGAAATGGCCGCCGGCTATCCGCCGTTCTTTGCGGATC
AGCCGATTCAGATCTACGAGAAGATCGTCTCCGGCAAGGTGCGCTTCCCATCGCACTTTGGCTCCGATCT
GAAGGACCTACTGCGCAACCTGCTGCAGGTGGATCTGACCAAGCGCTACGGCAATCTGAAGGCGGGCGTC
AATGATATTAAGAACCAGAAGTGGTTCGCCTCCACCGACTGGATTGCGATCTTCCAAAAGAAAATCGAGG
CGCCGTTCATTCCGCGGTGTAAGGGTCCCGGCGACACGAGCAATTTCGATGACTACGAGGAGGAGGCGCT
GCGGATCTCCAGCACCGAGAAGTGTGCCAAGGAGTTTGCTGAATTCTAGAGGAGCTCTTCGCCAGTCAGC
CCAATCCCCTCGTTCTGCAGGCGCctgcaactgcaattgtatccgctactgcgattgtatctgcaactgc
TCCTGCTGTTGCCACCGCCGTGGCATCGCGGACCACGTCCTCATCGTCGTTGTCGTCGTCTTGTCTATAT
GTCTACTACTTACAACTACAACAACTGTAACAGCCCCTCCCACAACACAAAAACTACAGCCACATCTTCG
TCTGAATATCTATCCGTATATACTCCACATCCATACTATTGCCAACATTGGATTTCTCACCACACACAAC
AATTTACATCAGCG
>dme_circ_0002003_junction_seq
TCACCACACACAACAATTTACATCAGCGCTTCTCAAATTAGAGTAAATCGCGATCA
>dme_circ_0002004
GTAGACATATAGACAAGACGACGACAACGACGATGAGGACGTGGTCCGCGATGCCACGGCGGTGGCAACA
GCAGGAgcagttgcagatacaatcgcagtagcggatacaattgcagttgcagGCGCCTGCAGAACGAGGG
GATTGGGCTGACTGGCGAAGAGCTCCTCTAGAATTCAGCAAACTCCTTGGCACACTTCTCGGTGCTGGAG
ATCCGCAGCGCCTCCTCCTCGTAGTCATCGAAATTGCTCGT
>dme_circ_0002004_junction_seq
CCTCCTCGTAGTCATCGAAATTGCTCGTGTAGACATATAGACAAGACGACGACAAC
>dme_circ_0002005
GGTTATGCAGAGCATCCTGCGGCACGTCAACTTCGATGTGGTCAAGTGTGTGTTGATTGCGTCGCCGGGA
TTCGTGCGCGATCAGTTCTATGACTACATGTTCCAGCAGGCGGTCAAAATGGATTACAAGCTGCTGCTGG
ACAACAAGAGCAA
>dme_circ_0002005_junction_seq
ACAAGCTGCTGCTGGACAACAAGAGCAAGGTTATGCAGAGCATCCTGCGGCACGTC

>dme_circ_0002006
GACACAGATCCCCCCGAGCAGACTCTTCGCTCACTTGCCCAGCTTGGAAGCCTTGGCAGCTGCAGCCGCC
GCTTCCTTGCGTGCCTCCCGCTTGGCCGTCTTCCGGGCCTCCTTCTCGTTGGCCTCGATGGTTCGTCGCT
TCTTGCGCAACAGCCATTCCTCCTTTTCCTTAGTCTGCTTCTCGCGGATGAGTTTCCGGAACAGATTGCG
ATGACGGGGCTTCACCATACGCGCTTGCAGGCGGTGCTCATCGACCTCGGCCTTGTGCACCTGCCGCTTG
TTGACCTTGTGCACCTTGCCCGACTGCACGGACATCTTCTGTTTCTCCTCCTGCAGACGCTTGGTCTTCT
CGTCGAGCTCTTCCTCCTCTTCATCGTCGTCTTCATTATCATCTTCATCATCCGGATCCTCCTCGTCTTC
GTTGACGCCATCGGGACCGCCGTACTTCTTGTACTCAGCCGTTTCCTGTTGATAGGCCAGCTGCAGTTGC
GCATCGAGCAGCTCTTGGTCCACAGTCTCCTCTTCCTCCTGAGCTTCGTCCTCGGAGTCGTCATCGCTTT
GGGCTGCAGAAAAAGTAATAGTTAAATAGCTGTTCCAAAAAATATTTAAAGATCCTGTAACTTACCATGT
GTTTCGATCAGGGAGGGATCCAGGAGGGCCTTCTCTTCCGGCGGTATGTACGAGTCCCTCTTGGAGTCCA
CGAATGGCGACAGGTGCGGTGGCAGTGTCTCGCCGATGAA
>dme_circ_0002006_junction_seq
GGTGCGGTGGCAGTGTCTCGCCGATGAAGACACAGATCCCCCCGAGCAGACTCTTC
>dme_circ_0002007
GAGGATCCGGATGATGAAGATGATAATGAAGACGACGATGAAGAGGAGGAAGAGCTCGACGAGAAGACCA
AGCGTCTGCAGGAGGAGAAACAGAAGATGTCCGTGCAGTCGGGCAAGGTGCACAAGGTCAACAAGCGGCA
GGTGCACAAGGCCGAGG
>dme_circ_0002007_junction_seq
AACAAGCGGCAGGTGCACAAGGCCGAGGGAGGATCCGGATGATGAAGATGATAATG
>dme_circ_0002008
GGTAATGCGGCCAACTCATCGGAAACATCGATGGCCACCCAGCCGGGCGGTTCCGTAACATCGCGGGCGG
CCAGCGAAACCCGCTCGGGTCCGCCCGGATCGAGCAATGCGGGCGCCACAGGTAATAGCAGCGGCGGGGG
CGGCACCTCGGGTAGTACTCAGACCGGTGCCAAGTTCCGCCACGGCCTGTTACAGCTAATGATACACACG
ATAGATCCACTACACGATGGGTAAGTTCGTTGAGTTGCCACAGTGTGTTTTGCACAAGTCAAGCCAAATG
CCaaaaaaataaaaatgcaaagaaccaaaaaaaaaaaaaaaaTGAATGCTACTAAAAGTCCCCATAACGAA
GTACCCCGATACCCGGCTTACCATTAACCACCCAGAGCTTCCCAACCACAATCCCGATTCCGGAGCCACT
CCAGCACTGATAAGCAAATTGTACAAATCAAAAAGAAATAAAATTTTTGAATTAAATTATATTTATTTTG
ATCCCTTCGCAAATACCAACGCACTTAAGCAATGAATTTTAGAAATGGTATACATGCCGTATGAGTAATA
TTCTTGAACGTTTCCTTTAAAGTCATGAAAGCCTTTAAATACGTGTAACTATAAACAAAATGTTTCACAG
CTTTCCTATGATTAAATTTTCGTCTATTTCCGATAGACTAAATAAAAAAATGAATCTCTATTTACAATTT
GTGACTCAGTGCAATCCAAATCCGCCACGCTTACATCCAAAAATAAGATAAATACAAATGCAATAACATC
CAGAACTCTACTctatatactatatactatactatatatacttactacctgtacttgttatctatatctG
TTCTAAAGTAAAATTGTTATAAATTCAGTTTCCCGTTTTGTGTTCGGGTTATCCTAAAATTCTGTGTAAA
TTCAGTATTCCTTCTAATTGGATGTTTGACTTGCCAGCCAGAAGTTACTTTCGCTCATCCTTCCACTTCT
GCTGCTCTTACCCAATTActgtttctgtttctgtatctatatctatatctgtatctatatctatgtctat
gCTCCGATTTCCACTCGTACTTCCATTTCTCATTTGCGTTGCGTTGTTGCCtttcagttcagttcggttc
ggtttgttttgttttttctttcaattcggttttggctttcgttggCTTTGGTTCACTTTTTTAATGGCGA
CCTTGGAGCCACTTACCTAATTAGCACGCCAGGCCCAGACGTTCCGTCGACTAGAATTAGCACCAAAAGC
aaaaaaaaaaGAGAATAATAAAAATAGGGGAAAATAGGAAAGCGGCGCGATTGACAGCACTAACCCTTTG
ATTTCGGGTGGGGCAGGAAGAGAAAAGGTCATCGCGATATTGTTACTGCCCCCACCATTGAATAGATTCT
GCAATTCTGAAAAGTTTCCAATCGAAACATTGATTTTGGTGCGAATCGAGTGTGGCTACTGAAAATCCCA
TGAATATGTAGGAAAGGCGTCGTCAGTGTCGTTGCCACGGCAATGGCCCACAGAAAACTAAAcgaatcga
atcgaatcgaatcgaaATGAGGCAGCTCAGGGGCGTGTTCGCCAAGGTTCGCAGGTGCCAAGTGTTAGAA

TTCGTGTAGCTCCAAGTGGGCCTACCTCAACCATTAGATATGTGCTAACTTATTACAGGGTCTTTAAAGC
TACAGATACAAAGAATTTGGTTTCAAATCAAGAGATGTTTACTAAATACATTCAGACAGAACTTCGTAGT
AGATGTACAGACATCGCTTGCAGGAGCTAAACTAATTAATACAATTAAATTGTCAGGAATATATTAACTC
AGGCAAAATGCACCTGCATAATTCCCTCATTGTTAACTGACAGCTGAGTTTTCAATTCGAAAATTCCCCA
TTTAAATTGTTTGGATGATTCAGTCAGTTGGAAACTAATGCGGATCTTAGCTTAAGTTTATTAGATCGGG
CCACAAGGGTTTAATCCTTTGCCTTTAGTTGCAATTGCTTCACAAATACGCTACTTACTTACGTTCGGCT
TTGTTTTTCATTTTGTTGcaaacaagaaaaccaaaatataaaacaataaaagcagaaaaacaaacaagaa
aaatgaaaccaaaataacgaaaataTTTTATGTTGTTCAATGTCAGTTTGGAGTTACATTtatatcaata
tatctatatataaatatatatatatacatatgcgttatatatcTAGCATAGAGAACTTATAGTTGCAAAT
TCCTAGAGTTTGTTCGCTCAGTTCAATGGCATACTTACTAGATAAATTCAATGCAGATATAAAGATACAT
TGCAGGTTTAATATATGCCATAGTTCGAGTACCTATGCCATTGAATGCTGGATTGAGTTGAGTTTGACGC
GTGACAAAAACAAAACAATTTATACTGTACATATATTAACTATGCGTTTTTAGTGTTGTATGTGTTAAAT
GTAATCATTATCgtaattgtaattgaaattgtaattgGTTAGCAACTAGTGCAATTGGCTCGCAGCAACG
CTGCTCAAAATCGGCTTCGACTTGGACTAGGGGctcagattcagactcagactcagactcaTACCATATA
GTAAATATTTAATACTATACACCCATCCATCCACACTCCACACCCGAGTAGATAGAAAACTATTTAAACT
ATTAACGAGCTGCGACTGCATGTGCGACTAGTCGAGCTTGTTTCAGGTTGTTTTTGCATAGGTCGGATCG
CTTTTTGTCACTGGAATGTTCGTCATATATTGTTTATATGTGTGTTTCTTTCGGCTCGATATGCTCGTGG
CATGGGAATTGTGTTTTTGGGCCTGCAATCGGTGGAAATCCGGCAGAGACGGGCGGAAATCATCATTTTC
CAACCTCCGATGGCAATGATCTCGTTAGATTTGTCTAATGGCCCTGCCACTTCGGTTCAGTTACCAGTCC
AGTTAAGAGCAACCCAAATCACAAGTCCCATCGCGAACGTTAGTTAGACGTGCTTTTGCTCAGTTATCAG
TATGAGCATGTTCTTCTCTAGGTCTGTATTAAAACCAAAGATAGTCATAGCGATACTATTAAGAGTACCC
TTTAAAATTCATCCATCGCGACACACACATACGATCTGTAGAAATGTAAGCTAAGCTTAGTTGTAAGTCA
GACGGAATCGTGAACATATATACACACATATGTATATGTATATACCTAGAGAAATGCTAGAAAAAGGGGT
CTTTCATTTAGTCAGATGGTCCCCTGAGAAAGTGTAAATTCCAAAACGAGAACTTCACGCCGCATTGACA
ACaaaaaaaaaccgaattgaatcgaatcgaaatgaactgaaatgaattgaattgatatccatccgaaCAC
CCCGTCCGATCCCCAAGCATCATCGAAAAACAGATCAAAATCTTGAATATTTAATCAGCTCCTAGTGCAA
AACACAAGTTCACACTTAATAGATCGAATCGGATTGATTCAGAGACAAAAGTTAGACCAAAAAACCCATG
AGAATCTGACACGGTAAATATTAGTAGTGttttttttAGTATAGATCCTAGTTACATATAGTCCTAGTTG
TAGTCCTAGTCAGCTTACGAACCCCTCCCCTCTGCTGTCCGCTCACCGCTCTCTGCTCTCCCCGCTCTCT
TCCGCTCTAATCTGTCATAACCCGTACTCTACTCTATAGACTAGACTGTACCCCCTAGCTAAATAACTTA
CTCAAGCAACCTCCAAATGCTGTCTCGCAACTGACATCAACATTTTGTATAAAATATTTTCAATATACAT
ATAGACATATATTTGCGTATATGAACGATCCAATCCAATACCCTAAATGAGTGAATTTGATTGTAAATTA
AATGATCTAAGAAAGAAACTAAATCTGAAAATGAAACTCGTAACTGGGTTAATTCGTTTATTTCTGATTT
TAATCGATGCAAttttattctttctttttcttctttttttcttttgatttttcGcaaaaataaaaatacc
aaaccaaaaaaaaatatatcaaataaaaataaacACTTGGATTTTGCGTTCAAAACTCAATTCGTTGTGT
GCCAACAAAAAaccgaatatcgaatatcgaaaaccgaattccgaataccgaataccgTAACCGCACACAC
AACAACCAAATACTACAACGACAACACCGCTACTCGCCACACACCAACACCACCAACACCTCACTCGTGT
TTTGTGTACcaacaaacaataaacaataaacaaaaacaCTGCATTATTTATCGAAACACCCGCaaaaaaa
aaaaaaaaaCCACACACAATTACAATGACAAAGATGATGTTCCAGGATGATGTTCCAGGCAAAGTGGACG
AGAAGGCGACGCAGCTGCACGCCATCGCCTCGCTGAAGGTGTTGCTGGATGCCACCAAACCACAGCGCGG
TGGTGCCACCACCTCGGCGGCCAATCACGTCAAGATCAATCTCAAGGAGACCACGCTCGCCGATCGTCCC
AATGGGAACATCGACACCACCCTCGACTCGG
>dme_circ_0002008_junction_seq
GGGAACATCGACACCACCCTCGACTCGGGGTAATGCGGCCAACTCATCGGAAACAT

>dme_circ_0002009
ACACGCACAACGATAACTCGACCACCACGAAAACGCCACTATTCCCAAAAGATCTATTCACGAAAGAACA
GCTTGAGAACGGCGCTGTCATCCTGCACATCATTGGGGTGATCTATATGTTTGTGGCGCTGGCCATTGTC
TGTGATGAATTCTTCGTGCCTTCCCTTGACGTAATCATTGAAAAGCTCGGCATCACGGACGATGTGGCCG
GCGCGACGTTTATGGCGGCGGGTGGCAGTGCCCCCGAGCTCTTCACCAGTGTGATTGGCGTTTTCGTGTC
GTTCGATGATGTGGGCATTGGTACGATCGTTGGCTCCGCCGTGTTCAACATCCTGTTTGTGATCGGAATG
TGCGCGCTCTTCTCCAAGACGGTCCTGTCGCTCACCTGGTGGCCTTTGTTCCGCGACTGCTCGTTCTACA
GCATCAGTTTGCTGGTGCTGATCTACTTCTTCCGGGATAACCGCATCTTCTGGTGGGAGGCCCTCATCCT
GTTCACCATCTACATCGGCTACGTGGCCTTCATGAAGTGGAACGTCCAGGTGGAGACGTGCGTCAAGAAG
ATGATTACCAAAAACAAGGTGACTCGCGTCAGAAGTACAGATCAACTTATGCCAGCAG
>dme_circ_0002009_junction_seq
AGAAGTACAGATCAACTTATGCCAGCAGACACGCACAACGATAACTCGACCACCAC
>dme_circ_0002010
ACAGGCATCCTTTGAGCCTTAACAGTCATCGAGCGAAGCCTTCGAAAATGTAAACAAAAGCGACGCAGGC
GCAGCAGTACTCTCTCCCTCACTCCTCAAGCAGTTTTGGAACGGAAAGCCGAGCTTCCAGGCTCGCATCC
TGGTCGGTCTTGGTTCGGTCCTCGGCCGTCGTTGGCTGTCGGTTTTCCAGCCCAACTCCTGGCCAGGCTC
AAAAAGAGGCTCAGGCAGCCAGCGCTCACAACGCCAGCAGAGTGCGCAGCGTGATGTAGCCCAGCTGCAG
ACCGACACTGCTACCGATACCGAGATTTAGCTCCAGCTTTCGCAGCGGCCACTCAGGGAAACTTTTCCAC
ACTCGGGCACGCACACAGGGGCAGACCGCGTCGGAAAAGCGGAAAAGCCTCAGACAAATGGCcaggaata
gcagcagcagcagcagcagcagcagcagcagcagtggcacagtagcCGTGAGGGAGCCGTGAAGTGAAAC
GGAAAAGCCTGAAAAAAGGTGGCGTGAAAAATACCACAAGAGGCGGGGGTGTGTGAAAATCTAGGGGAAT
AGCGCGCCCCGTGGAAATGGAAATGCCGAGGCTCCTGTTAGCCTCGCCAGCAGCGGATGTAGCAGCAACA
CCAGCGGAAACAGCAACCTGAATCCGCATCCGCATCCGCAACAGCAACCGCGCCCGCTCCCAATTTCTCC
GCTCTCTCCGTTCCGTGTTAATTGTTATTTATGTATAGTGTATATATAAAGTTGTGTGATACAAATGAAT
CGCTTAGATCTGACAGCAAGCGAGAAACTGTAACCTAAACTCAGTCTAATCAAAAGCGTACAACGACAAC
AAACGGTTTTGCAAGTGAATCGACATaacaccaagccaacaaaccaacaaaccaaacaaaccgatcaaac
aagtaaccaaccaacaacattaatccaacgGGTCCAATGTTGCAGccaacaacatgcagcaaacagcaac
agcagaggcagcaaccagcaatcgcaacagcagcagtgggtggagcagcggtccaggagctgcttcggaa
agccgcagctaaagatcatggagccttagcagcagcaactgcaacagcaacagcagcatcaacaactgca
acaactgcaacagcagcaacagcaagccgcaacagcagcaacacttggtgccacagcgacgacatGTTGT
CCGCAGGCCGCAGCCGCAGCAGCAGCACAACGATAGACTTCAATAGTCGCCGGCGGAGGGGGCGCCTACG
AGGCCACGCCCCCAGTGACCTGGCAATGAACAAGCAACACCAAGCTACGATACTGGACGAGTGTGTAAAT
ATTTTCAAGCGCTTAGTATTATTAACATTAAAAACAATATCAGCAACAACAATAACGAAAGCAAAGACAA
GAAGCAGAACAGCAGCGCAGCTACCAgcaacatctgcagcatctgcaacatcATCTCGTGGTGCCTCCGC
GGAGCAATTGCTCCACCCAAGTAGCCGCAGTCGCTGCAGAAGTCGCCGCTGCCTCCGCCTGCCGATCTAC
AGCATCCTGCTGCTCTGCCTAACCACCCAGGGATTGGGTCTCGGAGATGCCGCCAAGCCCAGGCCGGCCA
AGCAGCATTTCGGTGGCAGCAACAGCAACAGCCCAAATCAGAACCAGAACCACAACGATGTCCTGGGCGG
AGTGGGCGCCCCATCCCAAACGTCCGGCGAGGACGAGGCCGAGATCATGTACCCCTTTCAGTCCGGGGAG
CAGATGTTTGGCCTTGAGGAGGACCAGGAACAGGATCCGGAGCTCAATTCCAATGCAGTGCCCGGCTCCG
ATGAGGATAATGCAGGCAATCAACGTGGTATCAATGG
>dme_circ_0002010_junction_seq
ATGCAGGCAATCAACGTGGTATCAATGGACAGGCATCCTTTGAGCCTTAACAGTCA
>dme_circ_0002011
AGAGCAACCAGTCGGGAAGGCCAAACCAGCCTCCAGACCATTTTCGCCGATCAGGCGCCGAGCAGTGTTC

TCCAGCTCCTCGCAGATTTGGATCATGGTCATGCCCGGCTTAATGTAGCGCTGCATATACTGACGCGTCT
GACGATGGGCCTCAGCCGCCTGGCGCAATTCCTGGTAGATG
>dme_circ_0002011_junction_seq
AGCCGCCTGGCGCAATTCCTGGTAGATGAGAGCAACCAGTCGGGAAGGCCAAACCA
>dme_circ_0002012
CTGGAGCAATCCACTCGCGGGCATGGATACCGGCCTCCAGGAAGATGCCGGGCTTGGCCTTGCCGTTTAT
GGTCTCACCTCCCTTGGTGATCTTAACACCGAGGATGGAGCGTCCCTGGTAGGTCTTGCCGCCCTCAATG
AGGGTGACCACTCCGGGATTCGTCTGGGCCAGGGACTGGAGCCAGGCATAGG
>dme_circ_0002012_junction_seq
TGGGCCAGGGACTGGAGCCAGGCATAGGCTGGAGCAATCCACTCGCGGGCATGGAT
>dme_circ_0002013
TTCTCGGCCAATTCCTTGATGTTCTCCACCTCGGAGGTCAGCAGTTGGTTGATGATAAAGGTGGCGGCAG
CTGGAGCAATCCACTCGCGGGCATGGATACCGGCCTCCAGGAAGATGCCGGGCTTGGCCTTGCCGTTTAT
GGTCTCACCTCCCTTGGTGATCTTAACACCGAGGATGGAGCGTCCCTGGTAGG
>dme_circ_0002013_junction_seq
ACACCGAGGATGGAGCGTCCCTGGTAGGTTCTCGGCCAATTCCTTGATGTTCTCCA
>dme_circ_0002014
AGGTACAGCTGGACCTTGCCCTTCAGTCCCTCAATGAACTTGGACAGTGACAGTGTCTCGATCTCAGAGA
AAGCGGAAGGTCCGGCGTAGGTATCGGAGCAGGCACTGCTGCTGGCACCCACCTCGTTCCAGTGGAAGCC
CCAGTTCCTGTTGGGATCGGCGCCGAAGCAGCTGCCGTAGGGAGTACGGG
>dme_circ_0002014_junction_seq
CCGAAGCAGCTGCCGTAGGGAGTACGGGAGGTACAGCTGGACCTTGCCCTTCAGTC
>dme_circ_0002015
CAGGTATTGGGAGTAGGCATGCAGCGAGAGGTACAGCTGGACCTTGCCCTTCAGTCCCTCAATGAACTTG
GACAGTGACAGTGTCTCGATCTCAGAGAAAGCGGAAGGTCCGGCGTAGGTATCGGAGCAGGCACTGCTGC
TGGCACCCACCTCGTTCCAGTGGAAGCCCCAGTTCCTGTTGGGATCGGCGCCGAAGCAGCTGCCGTAGGG
AGTACGGG
>dme_circ_0002015_junction_seq
CCGAAGCAGCTGCCGTAGGGAGTACGGGCAGGTATTGGGAGTAGGCATGCAGCGAG
>dme_circ_0002016
TAGGCATGCAGCGAGAGGTACAGCTGGACCTTGCCCTTCAGTCCCTCAATGAACTTGGACAGTGACAGTG
TCTCGATCTCAGAGAAAGCGGAAGGTCCGGCGTAGGTATCGGAGCAGGCACTGCTGCTGGCACCCACCTC
GTTCCAGTGGAAGCCCCAGTTCCTGTTGGGATCGGCGCCGAAGCAGCTGCCGTAGGGAG
>dme_circ_0002016_junction_seq
TCGGCGCCGAAGCAGCTGCCGTAGGGAGTAGGCATGCAGCGAGAGGTACAGCTGGA
>dme_circ_0002017
GGATACAGCAGGTATTGGGAGTAGGCATGCAGCGAGAGGTACAGCTGGACCTTGCCCTTCAGTCCCTCAA
TGAACTTGGACAGTGACAGTGTCTCGATCTCAGAGAAAGCGGAAGGTCCGGCGTAGGTATCGGAGCAGGC
ACTGCTGCTGGCACCCACCTCGTTCCAGTGGAAGCCCCAGTTCCTGTTGGGATCGGCGCCGAAGCAGCTG
CCGTAGGGAG
>dme_circ_0002017_junction_seq
TCGGCGCCGAAGCAGCTGCCGTAGGGAGGGATACAGCAGGTATTGGGAGTAGGCAT
>dme_circ_0002018
GAGGTCGACGAGGGACGCAGTTCATAGCAGAAGGCCATGCGCACATCCTGGGTTCCGTAGGCCCAGTCCA
[truncated: 602,785 more chars]
