# Supplementary material for: Age-related ceRNA networks in adult Drosophila ageing
Source: Front Genet. 2023 Feb 28;14:1096902. doi: 10.3389/fgene.2023.1096902 (PMC10012872; doi:10.3389/fgene.2023.1096902)
Supplement: Supplementary file 9 [file Presentation2.pdf]

CAAGTCAACAACAACGAGGTTAACGATAGACCGTTGGGTCTGCTAATCCCAGTGGATGGTGGAGACTTTA
TTGTCCAACAAGATGTTCAGTTTTCCCAACCAATTCAGGTGAATCGGCTGCTCATCAATCAGCGGCTAAA
TCATATCCACGTCGATCGTCAGCGCTTCGATGTCCTGACCAAGCAGGCAAATCACACACAGGTCATCGAG
GGTTCCATGCGATTCGAGAATGTTCGAGTCATGGAACCGATTACCATTGCGGGGTCAAATTATGGGGGCC
GAACTGAGAGCGATGTCACCGGCAAAGGTTACCCACAAGTCGCTTCAACTTAATGGGGATTTCGTAGTTG
ACGGCGATGTGACCATTGGACGGCTGCTGCAAATGAAAGATTTTGTGGACGAATCTACTCAGAAATCTGC
AGCAGAAGCATTGACTCGGGGCCTGCGATTGGATCAACCTTTGGAGGATGTGAATCTCAACTTTGTGGAA
CCACTTATTGCCAACAATACCGAGCTAAGCTTTATCAACACGCAAGATCTACAAAACCTGGTTAAGCTGA
ACGTGGGTGAAGTGCAGTTGGTGCAGGCAAGTAAAATATTTCCTCAAAATGTAGAGATACGCGATGGATT
CGGTGAGATCAAGTGGCTAAATGGGATTGATACGGAAAGACTCCCGGAAATTATGCTCACCAAGAGTGGC
AACCAAACTATTTCGAGTTCAATTCAATTACAAGGCGTAGAACTGAACCATGTCAATTCATCACAGATCT
TATTGAATGATTTGGGAGTTGAGGATTATCTTCAAACAGGTCAGGATCAAAAG
>dme_circ_0003779_junction_seq
TTATCTTCAAACAGGTCAGGATCAAAAGTAAAGGATCTGCAGCTTGATGGGCGACT
>dme_circ_0003780
TTTTCTGGCCCAACTGGATTTCACGCTCATCAAAAGGATGGGAATATTCGCTGAGAAGGCACAGCCCACT
CGCTTGAAAGGAGTCCATCTGATCAATTGCCCCAAGGAGGGAGTGGCGTTACTGAATTTGGCCAAGAGCC
TAATGCCCAGCAAGCTCCAGCAGCGGG
>dme_circ_0003780_junction_seq
CTAATGCCCAGCAAGCTCCAGCAGCGGGTTTTCTGGCCCAACTGGATTTCACGCTC
>dme_circ_0003781
GTGGTGATAAGGCAAACCCAGCGAAAAGTAGCAATGTCGGAGGCCTATTCGAATTCCATACGCAGCCTGA
GTCCAGAGCTGGCCAAAAAGGCGCACGACGAGCTGGGAGAGATACCCGACAGAATCGATGAGGATATCGA
AACGTTGCGCACTTGGATCTCCAAGCAACCTCATCTCAAGGCTCGCCAGGATGCCCAGTTCCTGGTCGCC
TTCCTGCGTGGCTGCAAATACAGCTTGGAGAAAACCAAACTCAAGTTGGACAACTTCTATGCGATGAGGG
GAGCCGTGCCCGAGCTCTACAAGAATCGCATTGTGGGTGAGAAGCAGCTCAGCATTCTGGACACAGGGTT
GCCTCCTCCGATTGCCGCAGCCACTGCAGGCTGATGGTCCTCGCATCCACATCTCCCGCTACGGTCAATA
CGACTCGAAGAAGTACTCCATTGCCGAGGTTGTCCAGGTAAACACGATGCTCGGCGAGATTCAGATAAGG
GAAGACGACAATGCTATGATATCCGGCTTTGTGGAGATCATCGACATGAAGGGCGTTGGGGCAGGTCACC
TATTCCAGTTCGACGCGGTGCTGGTCAAGAAACTGGCCGTTTTGGGCGACAAAGCGTATCCCTACAGGCC
CAAGGGTTTTCACTTTGTAAATGCGCCCTCCAGTGCAGAGAAGTTCATGTCCATTGCCAAGAGCCTGATG
AGCGAAAAGATTCGGAAACGGGTTTCATATACACTCCAAACTGGACAGTCTCTACAAGTATGTGCCCAAG
GAATGCTTGCCAGCTGAGTATGGGGGCAGCAATGGAACCATCCAGGACGTCGTCAGTACATGGAGGACCA
AGCTCCTGGCCTACAAGCCATTCTTTGAGGAGGAGGCTTCTTATGGCACGAATGAAAAGCTACGGCGAGG
ACAGCCCGTTAGCGCCGAATCACTTTTTGGAATCGAAGGATCCTTCAGAAAACTAGACATCGACTAGTCT
AGTGCGTAACATATAAAACGTAGTGATTTTTAATCTTTGTACGAACACAAACAAATCAAAACTAAACAAC
ATAAAACTATACGAATTTGGTTAAATAGTAAGTGG
>dme_circ_0003781_junction_seq
TATACGAATTTGGTTAAATAGTAAGTGGGTGGTGATAAGGCAAACCCAGCGAAAAG
>dme_circ_0003782
GGATCCTTCGATTCCAAAAAGTGATTCGGCGCTAACGGGCTGTCCTCGCCGTAGCTTTTCATTCGTGCCA
TAAGAAGCCTCCTCCTCAAAGAATGGCTTGTAGGCCAGGAGCTTGGTCCTCCATGTACTGACGACGTCCT
GGATG
>dme_circ_0003782_junction_seq

CCTCCATGTACTGACGACGTCCTGGATGGGATCCTTCGATTCCAAAAAGTGATTCG
>dme_circ_0003783
CATGGTCAACAGGTCGATGCCCGATGCCTCCAAAGTCGAGAGAATGGTGCAGACCGCCCTGGACGTTTGG
GCGAACCACTCGAAGCTGACATTCCGCGAGGTCTACAGCGACCAGGCCGACATTCAGATACTCTTTGCGC
GGTAAGTACTCCGAAATCTATGGGGATTGCGAATCGGGCGATTGGGGATGCATGGATACGAGCACTGGTG
GTAGGTGGTGGGGAAGATCATAAAAAAGCGAGTCTTTAGTGCCATAAACAAACGCACATAAAATTGCACT
TAAAAGCAGGCGAACGATAAATAAGCCAAGTATTAAATCCATGGCCATCCAACGACGTCGATGATGAAAT
GCCACCGATGACAAAGTCGATGGCCATGGCTGCCGTGTCTGAATCAAAGAGTTTATGTGGCAGCTGCTTG
TTCTTAGATAAGCTGTGAATATGATTTCCCCCTTGGCCATGCTCGTAATTCCACTGGAACACCGAACTAA
TCCAGGACATCTACGCTGGTCCTTTTCTCTTCACTTTGCAGGGCGCGCGCACGGAGATGGCTATAAATTC
GATGGACCTGGTCAGGTGCTGGCCCACGCCTTCTATCCCGGCGAGGGGCGTGGCGGAGATGCCCATTTCG
ATGCGGACGAGACGTGGAACTTTGACGGCGAATCCGATGATAGCCATGG
>dme_circ_0003783_junction_seq
TTGACGGCGAATCCGATGATAGCCATGGCATGGTCAACAGGTCGATGCCCGATGCC
>dme_circ_0003784
TACAATTACCTCATGCAGTTTGATTATCTGCCCAAGTCGGACCTGGAAACGGGTGCTCTGCGCACCGAGG
ACCAGCTGAAAGAAGCAATCCGGAGTCTCCAGGTAAGAGACACATTCCCTCCCAGCGGCTGCAGCCGTGC
CATGACATCCCATCCCATCCCACTCATCTATTATGCACAATTTCACAACTAACATTACCGTCTGCAGTCT
TTTGGCAACATTACAGTTACGGGCGAAATCGACTCGGCAACGGCCCGGCTAATACAGAAACCTCGGTGCG
GCGTGGGCGACAGAAGGTCCGCGGACAGCTTCTCGCCAGATAACCTGTATCACGAAATCGGCTCCAATGT
GCGGGTGCGGCGCTTCGCCCTGCAGGGACCCAAGTGGTCCAGAACGGATCTTACGTGGAGG
>dme_circ_0003784_junction_seq
GTGGTCCAGAACGGATCTTACGTGGAGGTACAATTACCTCATGCAGTTTGATTATC
>dme_circ_0003785
AGCTGCTAAAGAACGCCGACAAAAGCTCCAAGATCACGCCACTTGAGTTTGAAAAGGACGACGACAGTAA
CTTGCACATGGACTTCATTGTGGCCTGCTCAAATCTACGCGCCGCCAACTACAAGATTCCGCCAGCGGAT
CGCCACAAGTCCAAGTTGATTGCTGGCAAAATCATACCTGCTATTGCCACCACCACGTCGGTGCTCTCTG
GATTGGCTGTGCTCGAGG
>dme_circ_0003785_junction_seq
GTGCTCTCTGGATTGGCTGTGCTCGAGGAGCTGCTAAAGAACGCCGACAAAAGCTC
>dme_circ_0003786
GATTCGACTTCTCAATGAGATCCATGTCTGTGACGAAAATCTGGCCATTGCCAGTTCCCAGGCCCAGCAT
TCCAAAGTTTTTCAATAGCTCACAACCAATGGCTCCGGCGCCGACAATAAACCATTTCGAGTCAGCCAAC
TTCTCCTGGAACTTCTTGCCAAATATGGCAATCTGAGAATCATAGCGCGAGCCCACCGGCTGGGCATCCG
CCTCCTCGACACCCTCTGTGGGCAGACACTCCAGGGCGTCAAAGTACAACCACTGGTAGATGGGTGTAAA
CTTTCCACTGCAGGCCTTAAGCACCTCCTGGGCCACAATTCCACCAACGGCCGCGTCCAGCGGGCACGTA
TTACCCGAGCAGATTTTGGCGAATTGCAGCACCAGTTTTTCGTCGACTTCCGCGTTGCTGCTGGCTCGGA
CTACCTCGAGGAAGCTGTTGGCGTCCTCCTCGTTCCAGGGACGCGGTAAGGCTCCATTATGGGCCTTGCG
GTAGCAGGAGAGGGCATTGAAGGCCACGTGCAGCGTGGCCGGCGAGTCCAGCTTTGCGAAATCGGAGATC
AAAAACTCCGGCTCCTCAGTGGCCTGTGCCAGGGGCTTGAAGCTGATGGTCTTGGGCATCTTCACCTGGG
TGGCCACTCCTCCGCTCTTATATTCTCCAAACTTGCTGGTATCACCGATGCTAAATGTGTAGGGTCCCAG
CACTGTGATCTTAAGGGGCTGACAACCGTTCA
>dme_circ_0003786_junction_seq
GTGATCTTAAGGGGCTGACAACCGTTCAGATTCGACTTCTCAATGAGATCCATGTC

>dme_circ_0003787
GGATTAGAGTGTGGCTGGGAGAGCTCGACCTTAGTTCCACAAGTGACTGCACCACCTACAATTATGAACG
GGTTTGCGCTCCGCCCGTGGAGGAGTTTACCATCGACAAGTGGATTCTTCACGAGGAGTTTAACCTCTTT
TATCCCGGGTATGATATTGCCCTGATAAAACTAAACAAGAAAGTGGTCTTTAAAGGATCACATACGACCC
ATATGCCTGCCCCTGACCGACGAACTCTTGGCGTTTACCTTGCAGCTGGGACAACGCTTCATGGCGGTGG
GCTGGGGAAAAACGGAGAGTCTACGCTATGCCAACAGTACAATGGAGGTGGATATAAGAACTGAGAAGTG
CACCGACGGCAGGGATACTTCGTTCCTCTGCGCCAGTGGAGATTATGTGGACACATGCAATGGGGACTCC
GGTGGACCGCTCTTATGGAAGACCACGTTGTTTGGCAAGGATCGAGCCGTACAATTTGGTGTGGTCAGCA
CTGGAAGCCAAAACTGCGGTGCTGGTCACAAGGCCTACTACATGGATGTACCCACCTATATGCCCTGGAT
ACTTGAAAAGATGGCAGAATTCTCGGATGTTTAAGTAAACTCTGTTAACTAAAGATGCGAATACGCATGC
GCGTTTCACTGGTGTTGATAATTTTCGATTCTTTTTGGTCACCCGAAGGCCTTAACTTCTATAAGCTTAG
TTATAAAGATGTCAGATCTAGTAGCTCACGTTTTTGTGTTGTTCAGATCGGTAGTCGCATCAAGAAATAT
GTATAGCGAAATACGAAATCGGAAAAGTCACATTAGGCACAGTTGTATTGCAAGAATTTTAAAACCAATT
ACACAATATGAGTAATTAGATTGGTGTCCTGTATCTGGGACACTCGGACTCGGTGGTGGAGTAAACTTTT
AAAAGGGACTGCAGTAATGGGTACTTTCgtctctgaaatcttaattgcttaatctcaactttctagcatt
tattgttccaaagattaccatgtacatcggacagacggacggacaacgaacggtcatgaccagagcgtct
cggctattgatccgcatcaataatatatatactttatatagtcggaaacgcatccttctatgagaaacat
gagtttcaacgaatcttatatactcttttactctacgcttaacggttataaaaaaataaaaaaTCTTGAG
TTTTGATGATATTGTACGCTCGGATCATGAACAGTCCTCTCGGGATCACTGCCCTAATTTGGGGCATTCT
CTGCCTTAGCTGTCCACCTTCTTCTCAAGCCGGAAGGGAAGATTGGACACCACACGAGCTTCTGGCTTAC
GAACAGTTGACACAACAGGATTGCGGTGTCCTATCGAATCTGATTCCCGCCCAAAGGCTTCGACGGCGGA
TCACCGGCGGCAGGAAATCTTCGCTGTTGTCCCAGCCTTGGATGGCTTTTCTCCACATTTCCGGTGATAT
AGAGATGTGCCGCTGTGGAGGCTCTCTTCTCTCAGAACGTATTTATGCCAAAATCACTTCCCCTTCTTTA
AACTACTCATTTTGCTTGATTTAGTCTTTGTTTTGACTGCCGCGCACTGCTTCAAAATGTGTCCCCGATC
TAAGGA
>dme_circ_0003787_junction_seq
GCTTCAAAATGTGTCCCCGATCTAAGGAGGATTAGAGTGTGGCTGGGAGAGCTCGA
>dme_circ_0003788
GATCACATACGACCCATATGCCTGCCCCTGACCGACGAACTCTTGGCGTTTACCTTGCAGCTGGGACAAC
GCTTCATGGCGGTGGGCTGGGGAAAAACGGAGAGTCTACGCTATGCCAACAGTACAATGGAGGTGGATAT
AAGAACTGAGAAGTGCACCGACGGCAGGGATACTTCGTTCCTCTGCGCCAGTGGAGATTATGTGGACACA
TGCAATGGGGACTCCGGTGGACCGCTCTTATGGAAGACCACGTTGTTTGGCAAGGATCGAGCCGTACAAT
TTGGTGTGGTCAGCACTGGAAGCCAAAACTGCGGTGCTGGTCACAAGGCCTACTACATGGATGTACCCAC
CTATATGCCCTGGATACTTGAAAAGATGGCAGAATTCTCGGATGTTTAAGTAAACTCTGTTAACTAAAGA
TGCGAATACGCATGCGCGTTTCACTGGTGTTGATAATTTTCGATTCTTTTTGGTCACCCGAAGGCCTTAA
CTTCTATAAGCTTAGTTATAAAGATGTCAGATCTAGTAGCTCACGTTTTTGTGTTGTTCAGATCGGTAGT
CGCATCAAGAAATATGTATAGCGAAATACGAAATCGGAAAAGTCACATTAGGCACAGTTGTATTGCAAGA
ATTTTAAAACCAATTACACAATATGAGTAATTAGATTGGTGTCCTGTATCTGGGACACTCGGACTCGGTG
GTGGAGTAAACTTTTAAAAGGGACTGCAGTAATGGGTACTTTCgtctctgaaatcttaattgcttaatct
caactttctagcatttattgttccaaagattaccatgtacatcggacagacggacggacaacgaacggtc
atgaccagagcgtctcggctattgatccgcatcaataatatatatactttatatagtcggaaacgcatcc
ttctatgagaaacatgagtttcaacgaatcttatatactcttttactctacgcttaacggttataaaaaa
ataaaaaaTCTTGAGTTTTGATGATATTGTACGCTCGGATCATGAACAGTCCTCTCGGGATCACTGCCCT
AATTTGGGGCATTCTCTGCCTTAGCTGTCCACCTTCTTCTCAAGCCGGAAGGGAAGATTGGACACCACAC

GAGCTTCTGGCTTACGAACAGTTGACACAACAGGATTGCGGTGTCCTATCGAATCTGATTCCCGCCCAAA
GGCTTCGACGGCGGATCACCGGCGGCAGGAAATCTTCGCTGTTGTCCCAGCCTTGGATGGCTTTTCTCCA
CATTTCCGGTGATATAGAGATGTGCCGCTGTGGAGGCTCTCTTCTCTCAGAACGTATTTATGCCAAAATC
ACTTCCCCTTCTTTAAACTACTCATTTTGCTTGATTTAGTCTTTGTTTTGACTGCCGCGCACTGCTTCAA
AATGTGTCCCCGATCTAAGGAGTAAGTATGACTTGTATTATTCTTTCTTGACTTTTTATTTTTCTGAGCC
TGGGTCATAAACAGGATAAGAGTGTGGCTGGGAGAGCTCGACATTAGTTCCACAAGTGACTGCGTCACCT
ACAATTATCAACGGGTTTGCGCACTGCCCGTGGAGGAGTTTACCATCGACAAGTGGATTCTTCACGAGGA
GTTTAACCTCTTCTATCCCGGGTATGATATTGCTCTGATAAAACTAAACAAGAAAGTGGTCTTTAAAG
>dme_circ_0003788_junction_seq
AAACTAAACAAGAAAGTGGTCTTTAAAGGATCACATACGACCCATATGCCTGCCCC
>dme_circ_0003789
GGCATATAGGTGGGTACATCCATGTAGTAGGCCTTGTGACCAGCACCGCAGTTTTGGCTTCCAGTGCTGA
CCACACCAAATTGTACGGCTCGATCCTTGCCAAACAACGTGGTCTTCCATAAGAGCGGTCCACCGGAGTC
CCCATTGCATGTGTCCACATAATCTCCACTGGCGCAGAGGAACGAAGTATCCCTGCCGTCGGTGCACTTC
TCAGTTCTTATATCCACCTCCATTG
>dme_circ_0003789_junction_seq
TTCTCAGTTCTTATATCCACCTCCATTGGGCATATAGGTGGGTACATCCATGTAGT
>dme_circ_0003790
GTTCTCTTCGGCACTCCGGAATTTGTGGCGCCAGAAGTTGTGAACTTTGACTGTATATCGTATGGAACAG
ATATGTGGAGTGTTGGCGTTATATGCTATGTGCTGTAAGTTTTATACATTTAATGTACAACATGTATTAC
AAAGCAAAAAAGTTTATACAAGCCTTTGATGTCTAATGTTAGATGTACGCATTGGGTTTTATTAAGTTAT
ATTTAAATTTCTTGAACAGAGAATACTGAAGATCAAAACTGAAAAAATAATTTGAAAAGGAAAGCACAAT
TTTTTTACTTTTAGATCAATTCTTTAAATAAATCCACTAATATTCTCTTAATAAACTTACAAGTCAAATA
GATGTTTATAAATCCTATTAACATCTATAGAAATCATAAAGTGTATTCTTGATCTTACTTACCAAAGTCA
TCATTTAAAAAGAAAAGCCTATCCGAGGATGTTTAGTTTAGTCATCTCTAGAGTAAAGCTAGCTCAATAT
TTTCGAACCATTTATATTTCAGCCCGAGCAATTAGAAATTTAATCAAGACCCGCACTTATATCTTAAGGG
TAGGTGCTTGGGAGAAAACCAATTAAAAGTGTGTACCAGCTAAAAATGTGATAATCAAAAGGTGGTGGTT
GGCGTGGCGTATCTGCTCCCACTCCTTGGCGAATTTAATTTAGTTAATTAAGTGTTGGTTTTGTGTTTTT
ATTTATTCTACTTACGCTGCTTTCACATTACATGTTTATTGACTCTCCCACACCTCGAAACCACACACAA
CCCCATATGATGGTTTGGATACAGCCACCCGGATGACAGCCGCCGAGTGCATGAAGCACAAATGGCTTCA
GCAGCGGCCGGCCACCGCCGCCACGGCCACGCCCATCACAAAGGCCGCCTCCGCGGCGTCGAAGTCGCGC
CTGAAGTCTGTGTCTCCGGTGACAGCTCCCTCGGAATCCTCCGAGGACTCCACGGAAACCATTGAGGACG
AAGACGACGAGGAAGAGGTTGCCGTGCAGCAGGCGAAGCAGAAAGACCAGCAGCAGGACGAGGAGCTGGC
CAATCTCTGCGGCGACGCTGAACTTGAGGAACAAAGAACTGGATGCCACGAAGGACAACCTTAAGAACTT
CATCGTGCGCTGGGAGACGCATCCGAACAGTCCGTACGTGTTCGACGTGGAGGGGAATGTGATTGCTCCG
CTGAGCGAGACGAGCTACCCCCATCCCAGGAGGACCCACGGCGCGGACAGCCTTTCCTCCTCCAGAGG
>dme_circ_0003790_junction_seq
GCGCGGACAGCCTTTCCTCCTCCAGAGGGTTCTCTTCGGCACTCCGGAATTTGTGG
>dme_circ_0003791
GTGCGCGTCTGATCCAGCAGATCCACGGCGAACTTCTGGCACTGCCTTCGCAGGTCCATGTACTCTGACT
TGCACTCCTGCTCGGTGAGGGCCAGGTTCCTCAGTTCCCAGGACAACTGGAAGGCCGTGATGATGGGGTC
GTTGGAGG
>dme_circ_0003791_junction_seq
AAGGCCGTGATGATGGGGTCGTTGGAGGGTGCGCGTCTGATCCAGCAGATCCACGG

>dme_circ_0003792
GGGCGTTGGCCTGCTGTCCGGCAGACTCCTGGGCCTGTCCGGCCGCATCCTGGACGGTCTTCTCGACGGA
CTGGCGCTTCTGGTCGACATACTGGTTGGCCTCCTGCAGCTTCTGGTCCACCGTTTGACTGGTCTGCTTC
AGTGCCCCATCGATGGCGCCCTCCGCCTGCTTCAGCTTCTGGTCCGCCACGTTCTCGGCGGCCTTCACGC
TCTTGTTGATGGC
>dme_circ_0003792_junction_seq
CGGCGGCCTTCACGCTCTTGTTGATGGCGGGCGTTGGCCTGCTGTCCGGCAGACTC
>dme_circ_0003793
GCTTCTGGTCCGCCACGTTCTCGGCGGCCTTCACGCTCTTGTTGATGGCGTCGTTGGCCGCCTTCTTGGT
CTGGTCCACCGCATTGGCGGCCGCTCGCTGGGAGGCCTCCGCCACATTGGCCGCCACCTGCTTGGA
>dme_circ_0003793_junction_seq
CCGCCACATTGGCCGCCACCTGCTTGGAGCTTCTGGTCCGCCACGTTCTCGGCGGC
>dme_circ_0003794
ATTCAAAGGCAGACAGACATGGCCGAGACAATCAACAACAAGCCGAGCATACAGATCCGCAGCGGACTGC
CCTCGCCTCTGCCGGACAAGAGTCCCTCGCCGGCCACCAAGCTGCTGATCAGCCACGGCAAGCCCAACTT
CACCATCGCCAAGTCGCCGAAGATCGACGAGAACGGAAACGGAAACCCACTGTCCGCCCAGCCCAGCGCC
AACAACAACTACAACGCCTACAAGAGCGCCAACAGCAGCGTGGCCAACAGCCAGAAG
>dme_circ_0003794_junction_seq
CAACAGCAGCGTGGCCAACAGCCAGAAGATTCAAAGGCAGACAGACATGGCCGAGA
>dme_circ_0003795
CAATTGAAAATCGATAATAACTTAAGCCGCTTACCACAAAATGTAAATCTGCAGTTGCGCTATCAACTCG
AGGCGGATCTAATTGGATAAACAAAGCAAGTACTTTGATATAACAATTTGCATATTACACATATATACGT
ATGTTACTCATAAACAGAGTTATTGTTCAGATTGATAGGCTCGCAATTGGATAGTAGTAGTTTCTAAATC
GATATGTACATTTCTCAGAAATCCGAGAGCACGTCATCATTGACGGATCGTCGGCTCATTCCCCGATAAG
CAAAACAAAGCAGTAGCCTGAGGAGCTGAACAACCGCGCCCTTAGCAAAGGAGAATCTGTGGCACCATGC
CGATACTATATAGTGTGATATCGCGGGGCACCACAGTGCTGGCGAAGTTTGCGGAGTGCGTCGGCAACTT
TGCCGAAGTGACGGAGCACATAATCGGACGGATCGGGGTGCATAACCACAAGATGACCTACACACACGGA
GACTACCTGATCCACTACACCTGCGAGAACAAACTGGTCTACATGTGCATCACCGACAACGGAGTTCGAA
CGGTCGCGGGCCTTCCTCTTCTTGGCGGATATCAAGCAGAAGTTTATCCAGACTTACGGCCTACAGGTGG
CCACCGCCATCGCCTACTCCATGAACACCGAGTTCTCCAAGATCCTGGCCCAGCAGATGG
>dme_circ_0003795_junction_seq
TTCTCCAAGATCCTGGCCCAGCAGATGGCAATTGAAAATCGATAATAACTTAAGCC
>dme_circ_0003796
GTTGCCCTTGCTATCATGGGCTAAAAAGAAAATAGTTATACTCTAGTCTACGAACATTTCGTTTTCAAGT
AGTTTACAAAACCGTTAGAAGAGTTGTCAACCTACAGTTATAGATGATGAGCTCTACAAGGTCTCGAGTC
TCCGGCCAAGTAGTAATAAGTTCTTTCAGG
>dme_circ_0003796_junction_seq
CGGCCAAGTAGTAATAAGTTCTTTCAGGGTTGCCCTTGCTATCATGGGCTAAAAAG
>dme_circ_0003797
GACATTTGTACATACGAGCATCTATCATGTCGGTTTATGTTGAAGGGTATCTATCCTAAGGTACTCCTAA
CACTTTGGACCAATCGTTTTGGGATTATCGGAATATAGAGCTAATATACATACAATAAACATATGTGATG
TTGTGGTGTTTCAATTTGATTGCATATATAATCATTTTGGGTTTAAGCTAATG
>dme_circ_0003797_junction_seq
ATATAATCATTTTGGGTTTAAGCTAATGGACATTTGTACATACGAGCATCTATCAT

>dme_circ_0003798
ATACATTTAAGTCCTAGGCGGGATCAGCATGTTCATTCCGGCCGCCCAGTCCGCGTACAGGACGCTGCGC
AAGACCCAGCCACGCGTCCGGCTCAACGCCATCTTCAAAAATGGCTACTCCAGCAAGGGTGGAGTACAAG
CCCATCCGGTCAGTGCTGGTGGCTAACCGCGGTGAGATCGCCATCCGAGTGTTCCGTGCCTGCACAGAGT
TGGGCATCAAATCGGTGGCGGTCTACTCGGAGCAGGATAAGATGCACATGCACCGACAAAAGGCCGACGA
GTCGTACATAGTGGGCAAGGGTCTGCCCCCGGTGGAGGCCTACCTCAACATTCCAGAGCTAATTAGGGTG
TGCAAGGAGAACGATGTGGATGCCGTGCATCCCGGCTACGGGTTCCTTTCAGAGCGCAGTGACTTCGCCC
AAGCGGTGATTGATGCTGGACTGCGCTTCATTGGCCCCTCACCCGAAGTCGTCCAGAAGATGGGTGATAA
GGTGGCGGCTCGTGTGGCGGCCATTGAAGCGGGTGTGCCCATTGTGCCCGGCACCGACGGTCCAGTGACC
ACCAAGGAGGAGGCACTGGAGTTCTGCAAGAAGCACGGACTGCCCGTTATCTTCAAGGCCGCTTACGGAG
GCGGAGGTCGCGGCATGCGCGTCGTCCGCAAAATGGAGGATGTGGAGGAGAGCTTCCAGCGAGCCAGTTC
CGAGGCCAAGGCCGCCTTTGGCAATGGTGCGATGTTCATTGAGAAGTTCATCGAGCGACCGCGTCACATT
GAGGTTCAACTGCTGGGAGACAAGGCCGGGAATGTAGTGCATCTGTACGAACGTGACTGCTCCGTGCAGC
GTCGCCACCAGAAGGTGGTGGAGATCGCCCCAGCGCCACGCCTGCCCATCGAAATCCGGGACAAGATGAC
GGAGGCAGCAGTGCGCTTGGCCCGCCATGTGGGCTATGAAAACGCCGGAACCGTGGAGTTCCTTTGCGAC
GAGTCCGGCAACTTCTACTTCATCGAAGTGAACGCCCGTCTACAAGTGGAGCACACGGTCACCGAGGAGA
TAACAGGCATCGATTTGGTGCAGTCGCAAATCCGCGTGGCCGAGGGCATGACCCTACCCGAATTGGGCTA
CACGCAGGACAAGATCGTGCCGCGTGGCTATGCCATTCAGTGCCGTGTGACCACCGAAGATCCGGCCAAC
GATTTTCAGCCCAACACCGGTCGCTTGGAGGTCTTCCGATCTGGCGAGGGTATGGGCATTAGGTTGGACA
GTGCGTCCGCCTACGCCGGAGCCATCATTTCGCCATACTACGACTCCCTGCTGGTCAAGGTGATCTCGCA
CGCCAGCGATCTGCAGAGCTCCGCCTCCAAGATGAACCGTGCCCTAAGGGAATTCCGCATTCGCGGCGTC
AAGACCAATATCCCCTTCCTCCTAAACGTGCTGGAGAACCAGAAGTTCCTCCACGGCGTTCTCGACACCT
ACTTCATCGACGAGCACCCGCAACTGTTCAAGTTCAAGCCCTCGCTCAATCGCGCCCAGAAGCTGCTCAA
CTATATGGGTGAGGTGCTGGTCAACGGACCCCAGACTCCTCTCGCCACCACTCTGAAGCCTGCCCTCGTC
TCGCCCCACGTTCCCGAGGTTCCGCTGGG
>dme_circ_0003798_junction_seq
CGCCCCACGTTCCCGAGGTTCCGCTGGGATACATTTAAGTCCTAGGCGGGATCAGC
>dme_circ_0003799
GCACTGGAGTTCTGCAAGAAGCACGGACTGCCCGTTATCTTCAAGGCCGCTTACGGAGGCGGAGGTCGCG
GCATGCGCGTCGTCCGCAAAATGGAGGATGTGGAGGAGAGCTTCCAGCGAGCCAGTTCCGAGGCCAAGGC
CGCCTTTGGCAATGGTGCGATGTTCATTGAGAAGTTCATCGAGCGACCGCGTCACATTGAGG
>dme_circ_0003799_junction_seq
TTCATCGAGCGACCGCGTCACATTGAGGGCACTGGAGTTCTGCAAGAAGCACGGAC
>dme_circ_0003800
TTCAACCTATAAGTATGAATCGCAGTCCAGCCATAGATCCCTAAAAAACAGTCGAAAATTCAGAGGTGTT
GTATGACGATGATGAAAACTATCTGAACATACGGATTGAAGTAGGGCGAACGAAGTGGATATGTCCGTTT
GGACGTGATCGTAAAACCCGATTCCAACTGCTCGGTTTGCTGACGTGGCGCAACCAGATACTTGTCCGCC
CGCTCGCTGGCCGGTTGATACTTTGCTGCCGCCACAGCGTTGAATATTCGTGTGCTCCATATCTCCTTTT
TCGGTGCCTCGTTCGGCGACGACATTGGGGAGACAGGAACCTTCGCGGATACAGGCGAATCTACAGATTT
TGGATATTCGGGATCAGCATTTACTCTGCCCGGGAAAGCAATCCCGTGTTTGACCTTCATGTGCATCAGG
AGGAAGGTCCGCCGCTTGTAGCGTTTCTTGCATGTGAGGCACTTATGCTGCTTGGTCAAATTCTTCTCTA
ACCGATTCACAGCCAGCTCTTTATTCCCGTGGAAGTGGCTCTCGTGGTATTTCAGCGACTGGCGGGAGCT
CAATCGCTTTCCGCACCAATTGCACTCGAACTGACCTTGATTGAAAGCCAAGGATTTGGCATTTGGGGGC
TTGGGTTCGATCAGAACCGCCGATCGGGACTTCCCTGCACCCGCATCCTGAATCGGATCTTGTGCTTGGG

GTCGGGGATTTTCCAAGACCGGTGGTTCCTCCACCCTTGTGAGAATTCCTTCCGGCACACAAAGGTTTTG
CATTTCAAGCGAAGTATGTTGGTTACTTGGAATCGCGCTTGCCATTGGATGACCAGAAGGCTCCAGAGGA
GATTGCGGTGACATTGGAAGTTTTTGGCGCAGTTCGAGTGGCCGATGACCTAAAAACTGCACTGGTGGCT
CATAAGCCGTATGCTGAGCGGTCATATACATGGGTGGTATCTGAATCGGATGTTGATGAACCGCAGGCAT
TTGAAATGGAATTTCTGGTTCGAGCGGCGGTGAGTAAAAATCCATGGGTGGCGGACAGCCAAACTCCATC
CGATTGCGGTAGTGGGCATTGTGATGGTGATCCATGTCACCATGATCAATGGAATGAAATAAGTGCTGAT
CTGTGGGATGCACGAATGGTTTATTCATGGACTGAACAAAGGG
>dme_circ_0003800_junction_seq
ATGGTTTATTCATGGACTGAACAAAGGGTTCAACCTATAAGTATGAATCGCAGTCC
>dme_circ_0003801
GCATGGATACATATCGCACTCGCAAGCGATTTATTCTGTAGCCTCAGCACTTTGGATCTGGATCTCGACG
TCGAAGTCCCAAAGCCCCAAACTTCTTGTCATAATAAAGAAACTAAAAACGCTGAGCATTGCAATACATT
TTAAGCACTCGGACGCTCAAATTCGTCCGTTTCTGTCATAA
>dme_circ_0003801_junction_seq
CGCTCAAATTCGTCCGTTTCTGTCATAAGCATGGATACATATCGCACTCGCAAGCG
>dme_circ_0003802
CTAACCAGTCAGTAGAGCTCCCGAAGAAGTGGATGGCTCCGTTATTTATTATAGAAACTATGCAATAAAC
CAACGAAACTGTTGTACATTATGCTTTAGCTCGATTTTTGTTCGTTCCAACTGCTTCAAACATTCTGCAA
ACTTCTCAGCCCCACAAATTGTTATCGCTTACCCCAATTGTTTGCTGTTTTCAGTAGTATTTATTTTCCG
GAATCCAGAGAAATCTATATTTATTTATAAGTGTAAGGAAAGGCGTTTTGTTGCACTGTAGTTAGCGAAC
TTATTTCAAACGTATGTACGGATTTGGTATTGTGTATTGCATTCATTGGACTGCTAGGCAGTGAATGTCT
TAAGCTTAGAAGGAATATTATACTGTATCAATAAAGAGACCCCATCCCTGTACCTCAGGTGGCGCAGTTG
TCG
>dme_circ_0003802_junction_seq
CCCTGTACCTCAGGTGGCGCAGTTGTCGCTAACCAGTCAGTAGAGCTCCCGAAGAA
>dme_circ_0003803
GGTAATCGTATTTGATTTATAAGCTATGCGACTGGAAAATCATTGTGCACCGTGTGTTCTATGTCCGTGT
GTCTGTCCTGTGGTCTTGGAAAGAAGCGAGGGTGCAGTATCTGTCGGATACTAGCGTGTCGGTATTGTAT
TTCGTCTATAGCAGAGTCTTTTATTTCAAAAATAGAATCTCTATTATCCTTAAGTAACTATCATTTTGTA
ATCTACAACTTTTGATATATCCCATTATGTATTGGATTTCTTTCTTTGTTGCTAGTTAGTAAATGTTA
>dme_circ_0003803_junction_seq
TTTCTTTGTTGCTAGTTAGTAAATGTTAGGTAATCGTATTTGATTTATAAGCTATG
>dme_circ_0003804
GTCTTTTATTTCAAAAATAGAATCTCTATTATCCTTAAGTAACTATCATTTTGTAATCTACAACTTTTGA
TATATCCCATTATGTATTGGATTTCTTTCTTTGTTGCTAGTTAGTAAATGTTAGTAATGTAAAAGGTATT
GAATAGGTGTCATTGTGTTGCAGTTTACTGCGATTGCCTTTGAAACACACTAGTACTTAGTA
>dme_circ_0003804_junction_seq
TGCCTTTGAAACACACTAGTACTTAGTAGTCTTTTATTTCAAAAATAGAATCTCTA
>dme_circ_0003805
GTCCACCAGTCCCACGCCACCTAAAGGATACAGGCCAGCGGAAGCTCAACACAAAGAAGCGAAATGGAGG
CGTCAGCAAGTGAGCTCCTCTTGGATTTGTCAAAAAACTGTCTGATGGTTTCAATAAAATTGTAGGTATA
AATTTGCAGCCGGTGGTGAACGTTCCTACGATTTATAAGGATCCCTGGTACATGATCACAGTGCTGGTCC
TCTATCTGTACTTCGTCACCAAAGCGG
>dme_circ_0003805_junction_seq

CTCTATCTGTACTTCGTCACCAAAGCGGGTCCACCAGTCCCACGCCACCTAAAGGA
>dme_circ_0003806
TTTATCCATCGGGTTCCATGCTGGAGATGTCGAACGGCTATCCGCATTCACACTCGCCGCTTGTGGGATC
ACCGAGTCCGGGTCCCAGTCCTGGCATAGGTGGGCATAAACATTACATCTTCTCCTTTCCCCATCTAACC
CACATCACCTAACTTACTCCATCGTCGTCTCAATCTGTGGTAATGGTTCCCAACTAACCGAAATCTCCTT
GCAGCCCACCATTTGTCCATTAAGCAGCAGTCGCCGGGCAGCCAGAACGGACGAGCTTCCAATCTAAGGG
TCGTCATACCGCCCACAATTGCCCCCATACCGCCCAATATGTCAGCGCCGGATGATGTGGGATATGCAGA
TGCAACGACAGAGCCAGACATCGCTTAACACGCCAGTGGTCACGCTGCAGACGCCGATTCCCGCCCTCAC
GAGCTATTCCTTTGGGGCGCAGGACTTCTCCTCCTCCGGCGTAATGAACAGCGCGGATATCATGAGCCTC
AACACCTGGCATCAGGGCCTGGTGCCGCACTCTAGGTCTCTCGCACCTGGCTGTCTCGAATAGCACGCCG
CCGCCCGCCACCTCCCCCGTCTCCATAAAGGTCAAGGCTGAGCCGCAGTCGCCGCCGAGAGATCTTTCCG
CCAGCGGTCATCAGCAGAATAGCAATGGTTCCACGGGCAGCGGCGGATCCAGCAGCAGCACCAGTAGCAA
CGCCAGCGGAGGAGCAGGAGGCGGTGGAGCCGTCAGCGCAGCCAATGTCATCACGCACTTGAACAACGTC
AGTGTCCTGGCGGGAGGTCCTTCGGGGCAGGGAGGAGGAGGCGGAGGCGGCGGCAGCAACGGAAATGTCG
AACAGGCCACCAATCTTAGCGTACTGAGCCACGCGCAGCAACATCACCTGGGCATGCCCAACTCGCGTCC
CTCGTCCACGGGCCACATCACACCCACTCCAGG
>dme_circ_0003806_junction_seq
CCACGGGCCACATCACACCCACTCCAGGTTTATCCATCGGGTTCCATGCTGGAGAT
>dme_circ_0003807
TTACCTGGGGACAATTTATACGGAAACACAATTATATAAAATTATATACACAACTATATACAAGTAGAAC
TTGCCGTTTTAACAAGCCGTCCACTTATAAAACAAACAGAAGGGATTATATATAGACAACCTATATAAAT
ATGCCCCACGTCTGCCACGCCAGGAAGAGCCACAGCAATTCGCAGGGCAGCAACTAACGGAGTTTCCCCG
CCAGCTGCCGGAAATCCAGTAGCAAATCCAGCAAACCAGCGACCCAGTCAGCAGGAATCAGCCAGCCGAG
CCGAGCACAAGGACACCACCATCTCCGTTTCCATCGCAGGAGGATAGGAAATCTGTTGCCATGGGCCGCA
AAAAAATTCAAATATCACGCATCACCGATGAACGCAATCGGCAGGGTGAGTGTCACAATGTGTTTGCCAA
TCCGTAGCTATAATAATACAAATTCGCTGAATATCTCTTGTTTGCCATCCTCGAAACAGGCAAATTTGCC
ATGGCGATGAAACTTAATTAAGACGGGTGAATCGATTCATGGCTATTAAATTGAAATCAATTCTGAGTCG
AGATAGCGTTTCATCATTAAAATGTAACAATGGGTATTCAGCGAATTAGTTTTAATATCTTATTAACGCG
TATGATAGTGTAAATGTGATTAATTATTACATTTATAAAGAATTATATTAAGGAACAACTGAGTAGAAAC
TGCAATGCATCCGAATAttgaccaaaatcttcggatgcatagcagttgaccaaaatcttcggatgcatag
cagttgCTACTCAGTTGTTCCTTAATATAATTCTTTATAAATTCCACAAAACTATCAAAATTTAAAGGCA
GACGTTTAAAAGTAGTTTTGCCTCTTTAGCACGGATATAATTTTCCTTTGCCGTACCCATTTTTTAACTG
CCAAGCGTGTGCCGTGTCATCGCAAGGTTTTCCCACCGACTTTTCGTTCCTTTCTGGCTGAGGCTACGTC
TTGGCCAAGCCATAAACGCGTCATCGTCGAGCAGTGaaaaacaaaaacaaaaaaaaaaaaCAGAACAAGG
AACAGTAACAACTTGTTTATGGTGCTGCCCCTTTGGCCTTGACTCGGCCATAATCGATTCATTACCAACT
AGGCAGCTAGGAAACGAGCAAGGGCCTGGGCCACTTTCCCACTTGGCCGGGCCACTTTGACTAAGGCCAT
GCCTCTAATTACATTTTCGAACCGCATAAATATTTGAGCATGAGCAGTAATTGAACGCACTTCCGTTTCC
GGTTCTCTTCCCGCCCGCACAGGGTGACCTTCAACAAGCGCAAGTTCGGCGTGATGAAGAAGGCCTACGA
GCTGTCCGTGCTCTGCGACTGCGAGATCGCCCTGATCATCTTCTCGTCGAGCAACAAGCTGTACCAGTAC
GCCAGCACCGACATGGATCGCGTCCTGCTCAAGTACACCGAGTACAACGAGCCCCACGAGTCCCTCACCA
ACAAGAACATCATCGAGGGTAAGTTGCAGGGTGGAAAATTGGGTGGGAAGTTGGAGGAAAACGGCTCGAA
AGGGAGCGCTATTATGCAAATTCAAGTGAACCGTGAAAAGCACAGCAGAACAGCAGCAGCAGGGAGCTGG
AAATGCAAGGGGAACGAACATACATGAACTGTTGCCAAGACCTACAAGTACCCTCTAATTCTGTTTaaaa
aaaaGTTATTAGGAATTAGGAGCACTGAAACTAGAATGAAACATAAAAGTAATAACGCTGTAAGTAGATA

TAAATTATAAAAAGGATATATATGCAATCCAGTATAGCTTAGAAAACTATAAAAAAAGGAACGAATCTAC
AACGAATCTCCTTAAAATATAAAGTCTCTTGCTAAATTCATACATCCGGGAGTGCTGCAAACCTTTAAAC
AAGGCGACCCGTATCGCCTTTAGCCCGGATTAAGCCAATATCTTAGGGCGACAGAAACATTTGTCGGTGG
GAGAGTTGAGACAAATCCGGCGGAGGAGATGGTGAAATGTCGCCAAAGTACTTTGCATTTCTAATGCGGC
GTTGTCGTCTCGGTTTCGCCGTTTCGTCGATGTTGCTGTCCGCGCGTAAATGGTTTTGTAATGTTAATGC
TGCAGGGGAGCGGAGGTGGGAGCAGGACAAGATTTGCATTTACATAATTTAGCACAACAAAGCGTTAAGT
GGTGGGCCGAGAGAGGAGAGCCTCGGGCAATTATCACGTCACTCGTTGGACTGCGCTTTCAAATTTGCCC
GTTCATTTTCGCATTGCAGAAAGGAGAACAAGAACGGCGTGATGTCGCCGGACTCGCCCGAAGCCGAAAC
GGACTACACACTCACTCCGCGAACGGAGGCCAAGTACAACAAGATCGACGAGGAGTTCCAGAACATGATG
CAGCGCAACCAGATGGCCATCGGCGGTGCGGGTGCCCCTCGCCAGCTTCCAAACAGCAGCTACACGCTGC
CCGTTTCTGTTCCGGTGCCGGGATCTTACGGCGACAACCTGCTGCAGGCCAGTCCACAGATGTCCCACAC
CAACATCAGCCCCCGTCCATCGAGTTCGGAGACGGATTCAGG
>dme_circ_0003807_junction_seq
GTCCATCGAGTTCGGAGACGGATTCAGGTTACCTGGGGACAATTTATACGGAAACA
>dme_circ_0003808
TTATACAACAAATCTCAGGTGAAAGCAGCAGTGTCAACGTCAATTCCAATTAACTGCATACAATAAGCCA
ATGTACAAATCCGTAATATAAGTGATCCACGCGAGTTCTCTAGGACTCTTTTTGGGTATTCACGGGATGC
TCGTTTAACACTGGCCATAGtttttttttCGACGCGAATTACTGCAGGGACAGTTGGCTCCCATATAAAG
TAAGTGTTAACTTTAACAGTTTATAATGCTCTGAACTATTATTACTGTGTTAGATGCCTTATAGAATACT
TTTAAGTAAACACCTTTCTTTCGATCACTATATGACCATATTTTTTTATATTCAGACGCTTTAAAGCTCT
AATATAAAATCTTTTACTTCTTACTTATGTAAGaagtaagtagtactataagtaagtagtattatTTTGA
CTATGAAAGTGTAGATCTTCAAGAACTGGTATCAAATAATAATAATTCTAGTGTCTTTGCTGGTACATAC
TATTTACAGTATTCCTAGCGAAGTTTGATTGTATTACGAATCAATGTTTCATTTGGCATACGTATTGTGT
GGACCGGTAAGAGCTGGTGCTGTACACAAAGTCGTATCCATTTCGGACCAGCTTTCTTGGCTCTCGACCG
GATTCCTGGGCAGTCTGCGACTACGAATACCAGATCGGACCAGACCTACAACAACAGCTGCGTATGCGAG
ATGCGCACAGACACAGATGCACTGGCACTCGTATACACCGACAAGTATCCAATACTTTCAATTAACGTAA
AACATTTTTGCCAAGAATGTTTCCTTCCCTTTGCCATGGAAACAGGGCGGAGAGGGGGGTGTTGGTTGGG
AAATTTGTGGGGGAAACAAAAGCATTGTGTTACAAAGTTCCGTGCAGATATTGATGGTTTTGAATTGCCG
AGCTCCAAGAAGCAACAGCATTTGCAGCTAATTAAGTACATATCTACAGAGCTTCCTCTGTGTGTCAGAA
TTTTTCCTCTGGCATCATATCGAACGTCATTGAATGGCGCCCCGTCCCGCTCTCTTCTTCGTTTAATATA
ACATTATATATTCAGGGGATCATCAAGTATTCAAGCACGCGTCGCCCAAATgtatacacatcggcgtgcg
tacatgtatgtatgtatctatatacttatacatgcatatacatgtacatatatctctatacgacgaACAG
AGGGCCGGAAAAGTTCACCTGAAGATAAAATTTGTTGCAAACGCAGGGTGAGTTTTGTTTCTTTTCCTTT
TTGTTTACGCTCGATAATTGCACTCGACGACCGCAATAAAGGAAAATTAAAGTTTCCCTTTGGCGGGCCA
CTCCGCCGTTTCCCCCATTTTCCGACCCCCTTTGACATTTGTGCAGTTTTGGCTTGACGTCGTCGTCGTC
GACGTCCCGTTCTCTGTCATTTCATTTGCAGCTTTAAGTATTTTGGCCGCGGGCCAAAATGTTTTTCATG
TCTTGTTGTCGCTATGCCACAGGCACAGTCACAGCCACACACTCTCACACACTGACACTCATACTCATCC
ACATCAACATTCACCGGCGGCAGGCGAGAGGGACAGTTAGTGATTAAGCGTGAAAATATCAGATCCATCC
CATCCCCATCCCATGGGGTGGTACAGCAACCGCAACAGCGTCAAGAAAGAAAAATAAAACAAGAAACTGG
CAAAAATTTTCTAGTTTTCTGCAACATTTTCTGCGACCGTTGGTCTCTGGTTTTCTAATTCGTTTTCTTC
GCGTTTGGTTTTTTGGCAAGTGCTGTATGCAAATTAAAATTGTTTCAGCGCAGATAAAATGTAATGTGAA
GAGGGCTGAGCCAAAAGAAATGAATTTAATTCAATTTGAAAGATCCGATTCAATTGCGGCGAAATGGAGC
TTGTTTCGGTGCAGAAAACCCGAATTAATGGAAAAGGGGCAACGAAATGTTAGCTTAAAAGTTCATACTG
ATTCATTGGCAAAACAAAGAACAAATTTGAATAAATATTAGTTTTCACTCAAATGAACAATGTCTTGGAA

AATATAATATAAAATGCttttttttAAAAAGATGTGAGCATTATGGGATTATTTATCTTTTTGATACTCT
TGTGGAAAAGAAGGCAGAACCGCTGATTAAATCTTTTGTTTTTAATACATCATTTTAGGTGAGATATCAT
TTTATTTCAGATCATTCCAGATATTTAGATTAGACTTAGATCTTTAGGGATACTGAAGAAAACAAGCGAA
TTCAGAATGTTTTTCATTCCATTTGTTTTCTTTACCAATATCGCATAGCTCTGGATCATATATCGATCTG
AGGCACACACATTCTGGCATTACGATTTCCATGTGCTTTTCCCCGCCGGGATTATCCAGCTTGCCTGCCA
AGAAATACGAGCAAAGGGGCCAATACAACGATGTGGCCCCAACGTAAAGAAGATATATACACAGATATTT
GTTTATACGGAATGTCAGTATAGCAAACACTGGCATCCTCTCGCTCGCTCCTTGCCCCTTCCCTtgtatt
tgtaaatgtatctgcatctgtgtcggcgtctgtatctgtatctgtatccgtatcCATCCATATGTGTATC
TGCAGCTGCGCAATTGGCGACGACAGACATCAGGCGCTGTTAGCTGACATGGACAGCAACTGGTAGAGCG
GACAATATAGGATACTATATAgaggtggaggaggaggaggaggaatggggagagtggaagggTGACATAC
CGCATGGGTCTTCATTTTGCAGTTACACATCGAGGCGCTTTCGTAACGCATTTTGGCAGCACCTGTCTTC
CCCACCCTCGGTGTCCACCACCCCTTCCGCCCTGTCTCCTCCCCACAGAAGCAGCCATTGCAAAATCTTG
TGTAGAAAGATCGGAAAATTTCGAAAGAGAATTGCCGAGGAAAACTGAAATTTAATCAGCAAACCCACAC
CGATGTAGTTTTGGCAGGGGCCCGAGCATAAGTGTGCGGAAGAAAGGTGTGGGGAAGACTCACACACGAG
CACCCAAAGAGAGGGACCAAAGCAGCGCAACGTTATGTGGCCGcacacacacacacacacCAACGCAGAT
CGTAGAGAGAGGTGTGTAGAATGGGTGGAAAAGGGGGAAGACTGGCGATCGAGGCGAGCACTTTGTCTAT
ATTTAGCCCGAAATGCAAATGGCGTCAGCGATCACGCACACACTCAATTGCACAGACAGGCGAGAGGTGG
GTTCCACTGAGGTGAGTTTACGGTTTTTTGCGCATGCGCTCCTTCCCCACAGACCGCCATTTTGAATATC
GCCGATCGCGATACAGAAAAACGAAAACACAGGAAAACGCAATGAGAGGAGAAAAATTCAAGCTAAAAAC
CTGTATAGATTTTAATAGATTTTTCCTCTTGGGCCATAACTAAATTTAAGATTTTGTTTTGGTTTTTGTT
TAAATTTAACTTGTGAATGAACTGGCCAGCCGATCAGAGACAACAACAGGTCGCCACCCCCCTTTAAGGCC
CCCAAATATTATCAATTTTCTGCAAGCGCCAAGTAAACAATCGATTTGAGCTAAAAATGTTATTGGCCCG
AGTTTTATTTTTACAGAAAGCGCACGGGCACGTGCCGTGTCTAAAATCGTTCAGAAAGCAGATATAGATA
TATAGCCATAGTCGTAGATGCGGTTATAGATATAGTTCTCTGCGTTTGCATCATTTCATCATAATCGCCG
GTCTGTCCGCTCGTCTGTTTTGGTTGTTTCCATGTTTATACGGATTTGTGAGTTTATGCTTGCCTATTCT
TATTGGTTTCTACATCTTCCTAATGCCCGGCTGTTTTGCATTACCGCTCGACTGGGTGGTCCAGAAATGC
AGACAGATGATCTGCCCACCCATCGAAAAACGGAGGCCCGACTTGTCTGTGGATTTGTTTTGGCGGCGTC
TTTTTTGGAAAACTTTTTAAATTTATTTGGAAATGTGGAAAATGAGTTGTTGTAGATGTTGGTATATTAG
GCATTCAAATCTTAACCTTTTTAATTTAACCATAAACTGATTCACTTTGCAAAGAAACTTAAATAGCCTT
TTTCTTTATTTAGTGTTCATAGTTTGGATGTGAGTTTAACGATCTCGTCAAAAAGTCCTATTGCCAATGA
CGATATTAAAAATTCGTATAGGACATTTCGATGGATTTCCTAGGCATCTCCTAATCTATATTTAGATGTC
TCCGCCTTCCGACCAATTGTTTTTCTATACCATTTGGCCATGGTCTCAGCTGTGTAACCCCAAACCGAAA
GTGACAAACAGCATTATTGAAAAATTATGATTTCTTGGGCATGTCCCCAAGGCGTGACGAAGATCGAGGG
ATACACATGTGCATGCAAAGGTGGGCTGGAGGGGGTGTGGCCGTGGCCAAAGGATTGTCTGTGCTCCGCG
GTGCCCAGTTCCCGAGTGCATTCCTCCCTCCCCGCCGCGTTGATTTCAAGCGATCTCCGCTGGAAAAAAG
CCACGTAGAGCACGCGTTGTGTGTGCAGGCAGAGATTTGCCATGGATTTACCCCTCGGCTTCGGTTCTAC
TCGGATATAGCTGCCCCGCTCAGTTTACTAATTGCTTCCGTTTGCCGAtgctacgggtgctacgagtgct
acggctgcCCGAAGTTTTCAGGGGTCTAGAGACTCATTTTAATTGCATTTCTTTTAACGGTAACATCAGT
GGCAGCAGTTTGCCCCCATCCCCCATCGCAGTCCCTCTTGGCTGCCTGTCAGTCTGGGCGACggtctgac
ggtctgatggtctgatggtctgatattctgatTCCCTGCCACGTCAATAACATTTGCGTACGGCAGAGAT
GCGTGTCGGTGAACCTGTCCATACATATGTATATGTGCGCACATGGGTGTGGGTGTTGAGGGGTTCTTCA
TATTTCCCAGCTCTAGACTCCTTTAATAAGTTTTCCTGCACTTAGTTGTTTACAAAAATCATGTTCTTTA
ACAAATGTTGTAAAGCCCCATTGGACTCCGCCTATGTCTATGGCTTTGTTGTAGTTTCGGAGGCTGAATA
AGTTAAAAAAATCTAAGGAAACATTGCGAGTACTTGCACGAAGATATAGACATATGTACATATGTGTATAG

TATGTACTCACATAACCTACACTGAAGTGATACACATTTTGTGTTTTAAAACCAAAATTATTCATACCAA
AAATGTTTGAATACATATTAAACGTTATTTAACATATTGGTATTCTTCTTTCAGTTTTAATATGGTTTTT
TATATATTTTGTGTACTTTTAAGTAGTTTCATAATTTTTTCGCCGAGTGTATGAAAACCCATAAGATGCT
TCTACATCGATGCGCCTTATGGTCGCTGCTGCCGCTTCGGTTGGCTTTAGGTGAGAAAGACAACAATAAA
ATGTGGCTGCAGGCTCCTaaacagatacagatacagaaacagaaacaACCGCACACGGATACGGGGCTCA
AGTGGCTTGGGTGCCTCCATTCCGATGCCCAGACATTCTCGGCCAGAGAATCCGCAGTCGAGTAGAATGT
TATGCTAGATATGCCATTTGACTTCTGGCTCGGCAACTGCAAATGCAATGGCAACCAGGAAACGACCTCG
CCATCCAAATCAGCAGCTAAAGGATGCGCCCACTTGAGACCGGGCTCGCTATCGGTAATCCCGTCCATCC
AGACCGGGGAAATGTGCCTCATTTGGCTTGGGATTTCCTGAGTATCTTTTATGCAACAAACATCTGATGG
AAATTAGTTTGAGAAATAGTCCTCACGTTGCAAATGCTGATTCGATGAACAACATTTTGTTTTCTATAGG
TCAGGGGTGTAAAGATTTTATTTCATCATTATTACTGGGATTATAGAACTCAATCTACTAAAACTTCAAA
GATTATCTATATTTATAATCCAACATGGATCTTAATATAGTTACGTTCTTAGTTCCTTACATTGGCTACT
AAATTAAACAAGAAGAACGCGCGGAATACTTTAGTAGATAGTTAAATAGCAATTCTTAATGGAAGTGCGA
GGTTCTATATATCACTACCCAATCTCCCCCCGAGCAGCATCCCAAGTCGCTGCGTCACTTGTTGAGCCTT
CAGATGGCTCACTGTTCCGCCACCCTACTCCTTGGATTCCCCACGCATCCAGTGGCTTTGTTTGCGGCTT
CGACGAATGTCGCTTTGCATCAGCTTACAAGATTTATGGTATCCTTTCAACTTTAAACCCGCTCTGCTCA
CTTGTCGTCTGGGACTCACGCAGCAGCTTCTCGGTTATCCAACTCGTTATCTGAGTTCTTTTAGTCGATT
TTTGCAGGGGGTATGCCGCACATGTGCGGACCCCGACTTTGCCACCGATTTCTGGGGCTTCTGGAGATTC
TAGTGATTCTGGAGTTTCGGGGCGGGGGGCGCGAACGGCGCGATGCAGGAGGTCAACGTGGTACGCGTGT
GCATTGAACTGAGCATCATAAATCGCTGGCGTCACGGACGCGTTTTCGCAGCTCGGAGTTTTGTCGTAAG
CCGCCCCTTCCGCAGTGCCACCCAATTTTCTAATTTCCATTTTCGTATTTTTCCGCCTCTTTGCAGATCA
ATCTTTTGTACCTGTTGTGTCGACAAAATTGCTGAAAGCGAGCTCCCCGAAGAAGG
>dme_circ_0003808_junction_seq
TTGCTGAAAGCGAGCTCCCCGAAGAAGGTTATACAACAAATCTCAGGTGAAAGCAG
>dme_circ_0003809
attttatCGGGTTCTTGATGTGTCTCGAAGTCAAGTAATACAAGGAAATAACAGCGTAGATAATATATAC
ACTCCCAGCTTTCGGCGAAGTTTGTTTACGATCGGAATACTAATGCGATATTTCGACTTTAAGTCTCCTA
TTGCGTTAGGGTGAAACAAATGATGGACTTCCTGTTTCTATATGTGAGGATGTATTCCATTGCTTGATGT
TCTTCTGCCGTTGTACTAATCAAGAAATTCGAAAGCAAGCACTTATTTCGCTTGGATCATTTTGTGTACT
AAACGATGGCTACCTTACAAGATCCGAACTTAAAAATTTGTATTGCGAAATTTTAAGTTCAATAGCCAAC
GACGCCGGCTTTAAAATAATCTGCATGCGAAATATTTGGATTTATCTAACGGAATCTGAAATGTTTATGC
ACAATAAGGAAAAAGAATGTTAGTATATTCAAttttttttATTTAATTTATCCGCTGACACATGACTTGG
ACTTATTAAACAATAAG
>dme_circ_0003809_junction_seq
ACATGACTTGGACTTATTAAACAATAAGattttatCGGGTTCTTGATGTGTCTCGA
>dme_circ_0003810
attttatCGGGTTCTTGATGTGTCTCGAAGTCAAGTAATACAAGGAAATAACAGCGTAGATAATATATAC
ACTCCCAGCTTTCGGCGAAGTTTGTTTACGATCGGAATACTAATGCGATATTTCGACTTTAAGTCTCCTA
TTGCGTTAGGGTGAAACAAATGATGGACTTCCTGTTTCTATATGTGAGGATGTATTCCATTGCTTGATGT
TCTTCTGCCGTTGTACTAATCAAGAAATTCGAAAGCAAGCACTTATTTCGCTTGGATCATTTTGTGTACT
AAACGATGGCTACCTTACAAGATCCGAACTTAAAAATTTGTATTGCGAAATTTTAAGTTCAATAGCCAAC
GACGCCGGCTTTAAAATAATCTGCATGCGAAATATTTGGATTTATCTAACGGAATCTGAAATGTTTATGC
ACAATAAGGAAAAAGAATG
>dme_circ_0003810_junction_seq

TGTTTATGCACAATAAGGAAAAAGAATGattttatCGGGTTCTTGATGTGTCTCGA
>dme_circ_0003811
TTATACAACAAATCTCAGGTGAAAGCAGCAGTGTCAACGTCAATTCCAATTAACTGCATACAATAAGCCA
ATGTACAAATCCGTAATATAAGTGATCCACGCGAGTTCTCTAGGACTCTTTTTGGGTATTCACGGGATGC
TCGTTTAACACTGGCCATAGtttttttttCGACGCGAATTACTGCAGGGACAGTTGGCTCCCATATAAAG
TAAGTGTTAACTTTAACAGTTTATAATGCTCTGAACTATTATTACTGTGTTAGATGCCTTATAGAATACT
TTTAAGTAAACACCTTTCTTTCGATCACTATATGACCATATTTTTTTATATTCAGACGCTTTAAAGCTCT
AATATAAAATCTTTTACTTCTTACTTATGTAAGaagtaagtagtactataagtaagtagtattatTTTGA
CTATGAAAGTGTAGATCTTCAAGAACTGGTATCAAATAATAATAATTCTAGTGTCTTTGCTGGTACATAC
TATTTACAGTATTCCTAGCGAAGTTTGATTGTATTACGAATCAATGTTTCATTTGGCATACGTATTGTGT
GGACCGGTAAGAGCTGGTGCTGTACACAAAGTCGTATCCATTTCGGACCAGCTTTCTTGGCTCTCGACCG
GATTCCTGGGCAGTCTGCGACTACGAATACCAGATCGGACCAGACCTACAACAACAGCTGCGTATGCGAG
ATGCGCACAGACACAGATGCACTGGCACTCGTATACACCGACAAGTATCCAATACTTTCAATTAACGTAA
AACATTTTTGCCAAGAATGTTTCCTTCCCTTTGCCATGGAAACAGGGCGGAGAGGGGGGTGTTGGTTGGG
AAATTTGTGGGGGAAACAAAAGCATTGTGTTACAAAGTTCCGTGCAGATATTGATGGTTTTGAATTGCCG
AGCTCCAAGAAGCAACAGCATTTGCAGCTAATTAAGTACATATCTACAGAGCTTCCTCTGTGTGTCAGAA
TTTTTCCTCTGGCATCATATCGAACGTCATTGAATGGCGCCCCGTCCCGCTCTCTTCTTCGTTTAATATA
ACATTATATATTCAGGGGATCATCAAGTATTCAAGCACGCGTCGCCCAAATgtatacacatcggcgtgcg
tacatgtatgtatgtatctatatacttatacatgcatatacatgtacatatatctctatacgacgaACAG
AGGGCCGGAAAAGTTCACCTGAAGATAAAATTTGTTGCAAACGCAGGG
>dme_circ_0003811_junction_seq
GAAGATAAAATTTGTTGCAAACGCAGGGTTATACAACAAATCTCAGGTGAAAGCAG
>dme_circ_0003812
GGCAAAACGTTTGGCCAACCTATCGCCTGCAGAAAAGTTGGCTGAGAAGCTGCGATTGCAAAAAATCCAG
GAGGCCTCTGATCTCAAGCATGCCCAGGAAGCGTTCGGTGTGACGAGTACGTGCGGAG
>dme_circ_0003812_junction_seq
GCGTTCGGTGTGACGAGTACGTGCGGAGGGCAAAACGTTTGGCCAACCTATCGCCT
>dme_circ_0003813
ATATATGCGGGCTACATTTATAGATCCTATTCACATGAAGTCGTCATAGTCTTCGGTGAAGTCATTTCCG
TACTTTTGGTAACCGTCAATGTCCTGCAAAAGAGCGGATTAAATAAGTTCATTCGTTGATCTCGAACGGC
GGCGATTGAAACACTCACGTCGTTTTCGGTGCGGAGAGTGACCTTGCCCTTGCCCTTTCCAGCGGGCTTT
TTAGCATTGGCCTTTTCCAGCTTCAGCTTTTCCGAG
>dme_circ_0003813_junction_seq
GGCCTTTTCCAGCTTCAGCTTTTCCGAGATATATGCGGGCTACATTTATAGATCCT
>dme_circ_0003814
GTGATTATTTAGATACGTCGGATTCTGGATCGTGCGATGAAAGTGATACCGATACAAACAAAAAGAGAAT
TCACTGTGTGGACAGCAATGATTTCGAACTAAATATTAACATCTATAAGGCTTTAGAAGCAAGAAAGCAA
TATTTTATCAACAAAATTAAGCCATTTTCAGTTTTTGGCGAGCAAAACCACTCGTCAAATCAACACATCA
AAACATACATAGATTATAATAACGCACAGTTAATAGCACAATATCTGGCCACCAAAAGGCCCTTCAGTCA
ATCTTTTGATGGATGCTTAAAGAAAATTATATTAGTTGTTAAGTGAGCCATCTATAGCAGTAAGGACACG
TGCAATGAAATGTCTTGCCAACATAGTGGAAGTTGATCCATTGGTTCTAAAACGAAAAGATATGCAAATG
GGAGTTAATCAGAAATTCCTAGACACAGCTATTTCGGTACGAGAAGCTGCAGTAGATTTGGTTGGAAAAT
TTGTTCTAAGTAATCAAGATCTAATCGATCAGTATTACGATATGCTGTCTACTCGTATTTTGGGATACTG
GTGTCTCCGTACGGAAAAGGGTCATTAAAATTTTGAGAGATATTTGTTTAGAATATCCAGACTTTTCAAA

AATTCCCGAAATTTGCGTTAAAATGATTCGACGTGTTCATGACGAAGAAGGCATACAAAAACTTGTCACT
GAAGTTTTTATGAAGATGTGGTTTACACCTTGTACTAAAAACGACAAAGGTGAGTACTACTTAAAATTTT
AAGTATTTAAAAAAATATTTTTAACAAAAGagaaagaaaatcgtttcctattttataaagtatatagtct
ggatcaggatcactagcccagtcgattttgaaatgtccgtccgtattaacgtcgaaatcttgcgaactta
aagatagaagcttaagttaaagcatacaggaggaactacccctatcgcaagtttgtttgtttccagatgt
tgccactgtaacgcccaccaatcgaaaatactgtttgttttcttgttttctgttttcttgtttttattag
tcttgtaaatttctatcgatttTGTTGTCACTTTAACACTCACAGGCACTCAactcgtagagtaaaaggg
tatactagattcgttgaaaagtatgtaacgaatcacgaagtacgaacgaagcgaagtatatatattcttg
atcaggatcaatagccgagtctatctagccttctccgtctgtccgtatgaaagtcgagatctcaggaact
ataaaattcagcgtaagtttgctgacccatgttgccacgacccctctagctcccacaaatcgcataaaac
tgtcacggccacacttataaaaaatgtgttgatatttttttcacatttttttttagtcttgtaaatttctat
tgatttgaaaaaaaaaaatttcaacgcccactctaacgccctaaaaccgccaaaaaactgccacgcccacat
ttttgaaccatttttcgatctttttcataattttattagtcttgtaaattttcatcggttttccaaaaaa
aatttttgccacgccctaaaaccgttcaaaccggtcacgcccacactttggaacaattttgaattctttt
ttaatttctcatttcattccccaatatctatcgataccccagaaaaatgatgaaatttcgcgttcgcttt
cacaccagctgagtatctggtatctgatagttttttttttaatttttttttttGTTATTACTATTGCCATT
TTCTACTACAATTTTAGAAATATCGATTTTTGTTTGTTTATTGACTTATAATAATAAATTTCCAATTTAC
CTATGTCTATTGATCTAATAATTCCTCCCTTACACTTTGTGTAAGGGGTATCTGTTAGTACAGATAGTAT
CGTTTACAGCTATACAAAGTAtattacttaatgtttattcttaatggttatttaattaacgtttaAATAA
TTCTTACTCCATAAATTGTTATTTAGAATATTTAAGCCAAGAGATAATATGCTAAGGTCTGAAGGATGCG
TTCAAGAATTTATTAAGAAGAACTCAGAGCCACCAATGGATATTGTGATAGCTTGTCAACAATTAGCCGA
CGGACTAGTTGACAGACTAATTGAATTGGAAGATACAGATAACTCGCGAATGCTGGGTTGTATTACCACA
CTTCATTTACTGGCTAAAGTGCGGCCCCAACTGCTTGTTAAGCATGCTATAACAATTGAACCATATCTCA
ACATAAAATGTCATTCTGCCACAGCCGCCAAGTTCATATGTGCAGTTGCAGATATTCTCGAAAAAGTAGT
TCCTCTGGTTAATAATGCCAGTGAATCCTTTTTAGCATCGTTGGAAGAGCATTTAATGTTATTGGTTGTT
TCACGAAATCAAGCCGAAGTTACTAGCTGTGTTTCTTGCTTGGGAGCACTTGTGAATAAGATTACACATA
ACTTTAAATTGATCCGCGACTGTTTTCAAAAGTAGG
>dme_circ_0003814_junction_seq
TTGATCCGCGACTGTTTTCAAAAGTAGGGTGATTATTTAGATACGTCGGATTCTGG
>dme_circ_0003815
GTGATTATTTAGATACGTCGGATTCTGGATCGTGCGATGAAAGTGATACCGATACAAACAAAAAGAGAAT
TCACTGTGTGGACAGCAATGATTTCGAACTAAATATTAACATCTATAAGGCTTTAGAAGCAAGAAAGCAA
TATTTTATCAACAAAATTAAGCCATTTTCAGTTTTTGGCGAGCAAAACCACTCGTCAAATCAACACATCA
AAACATACATAGATTATAATAACGCACAGTTAATAGCACAATATCTGGCCACCAAAAGGCCCTTCAGTCA
ATCTTTTGATGGATGCTTAAAGAAAATTATATTAGTTGTTAAGTGAGCCATCTATAGCAGTAAGGACACG
TGCAATGAAATGTCTTGCCAACATAGTGGAAGTTGATCCATTGGTTCTAAAACGAAAAGATATGCAAATG
GGAGTTAATCAGAAATTCCTAGACACAGCTATTTCGGTACGAGAAGCTGCAGTAGATTTGGTTGGAAAAT
TTGTTCTAAGTAATCAAGATCTAATCGATCAGTATTACGATATGCTGTCTACTCGTATTTTGGGATACTG
GTGTCTCCGTACGGAAAAGGGTCATTAAAATTTTGAGAGATATTTGTTTAGAATATCCAGACTTTTCAAA
AATTCCCGAAATTTGCGTTAAAATGATTCGACGTGTTCATGACGAAGAAGGCATACAAAAACTTGTCACT
GAAGTTTTTATGAAGATGTGGTTTACACCTTGTACTAAAAACGACAAAGGTGAGTACTACTTAAAATTTT
AAGTATTTAAAAAAATATTTTTAACAAAAGagaaagaaaatcgtttcctattttataaagtatatagtct
ggatcaggatcactagcccagtcgattttgaaatgtccgtccgtattaacgtcgaaatcttgcgaactta
aagatagaagcttaagttaaagcatacaggaggaactacccctatcgcaagtttgtttgtttccagatgt

tgccactgtaacgcccaccaatcgaaaatactgtttgttttcttgttttctgttttcttgtttttattag
tcttgtaaatttctatcgatttTGTTGTCACTTTAACACTCACAGGCACTCAactcgtagagtaaaaggg
tatactagattcgttgaaaagtatgtaacgaatcacgaagtacgaacgaagcgaagtatatatattcttg
atcaggatcaatagccgagtctatctagccttctccgtctgtccgtatgaaagtcgagatctcaggaact
ataaaattcagcgtaagtttgctgacccatgttgccacgacccctctagctcccacaaatcgcataaaac
tgtcacggccacacttataaaaaatgtgttgatatttttcacatttttttagtcttgtaaatttctat
tgatttgaaaaaaaaaatttcaacgcccactctaacgccctaaaaccgccaaaaactgccacgcccacat
ttttgaaccatttttcgatctttttcataattttattagtcttgtaaattttcatcggttttccaaaaaa
aatttttgccacgccctaaaaccgttcaaaccggtcacgcccacactttggaacaattttgaattctttt
ttaatttctcatttcattccccaatatctatcgataccccagaaaaatgatgaaatttcgcgttcgcttt
cacaccagctgagtatctggtatctgatagttttttttttaattttttttttGTTATTACTATTGCCATT
TTCTACTACAATTTTAGAAATATCGATTTTTGTTTGTTTATTGACTTATAATAATAAATTTCCAATTTAC
CTATGTCTATTGATCTAATAATTCCTCCCTTACACTTTGTGTAAGGGGTATCTGTTAGTACAGATAGTAT
CGTTTACAGCTATACAAAGTAtattacttaatgtttattcttaatggttatttaattaacgtttaAATAA
TTCTTACTCCATAAATTGTTATTTAGAATATTTAAGCCAAGAGATAATATGCTAAGGTCTGAAGGATGCG
TTCAAGAATTTATTAAGAAGAACTCAGAGCCACCAATGGATATTGTGATAGCTTGTCAACAATTAGCCGA
CGGACTAGTTGACAGACTAATTGAATTGGAAGATACAGATAACTCGCGAATGCTGGGTTGTATTACCACA
CTTCATTTACTGGCTAAAGTGCGGCCCCAACTGCTTGTTAAGCATGCTATAACAATTGAACCATATCTCA
ACATAAAATGTCATTCTGCCACAGCCGCCAAGTTCATATGTGCAGTTGCAGATATTCTCGAAAAAGTAGT
TCCTCTGGTTAATAATGCCAGTGAATCCTTTTTAGCATCGTTGGAAGAGCATTTAATGTTATTGGTTGTT
TCACGAAATCAAGCCGAAGTTACTAGCTGTGTTTCTTGCTTGGGAGCACTTGTGAATAAGATTACACATA
ACTTTAAATTGATCCGCGACTGTTTTCAAAAG
>dme_circ_0003815_junction_seq
TAAATTGATCCGCGACTGTTTTCAAAAGGTGATTATTTAGATACGTCGGATTCTGG
>dme_circ_0003816
GTGATTATTTAGATACGTCGGATTCTGGATCGTGCGATGAAAGTGATACCGATACAAACAAAAAGAGAAT
TCACTGTGTGGACAGCAATGATTTCGAACTAAATATTAACATCTATAAGGCTTTAGAAGCAAGAAAGCAA
TATTTTATCAACAAAATTAAGCCATTTTCAGTTTTTGGCGAGCAAAACCACTCGTCAAATCAACACATCA
AAACATACATAGATTATAATAACGCACAGTTAATAGCACAATATCTGGCCACCAAAAGGCCCTTCAGTCA
ATCTTTTGATGGATGCTTAAAGAAAATTATATTAGTTGTTAAGTGAGCCATCTATAGCAGTAAGGACACG
TGCAATGAAATGTCTTGCCAACATAGTGGAAGTTGATCCATTGGTTCTAAAACGAAAAGATATGCAAATG
GGAGTTAATCAGAAATTCCTAGACACAGCTATTTCGGTACGAGAAGCTGCAGTAGATTTGGTTGGAAAAT
TTGTTCTAAGTAATCAAGATCTAATCGATCAGTATTACGATATGCTGTCTACTCGTATTTTGGGATACTG
GTGTCTCCGTACGGAAAAGGGTCATTAAAATTTTGAGAGATATTTGTTTAGAATATCCAGACTTTTCAAA
AATTCCCGAAATTTGCGTTAAAATGATTCGACGTGTTCATGACGAAGAAGGCATACAAAAACTTGTCACT
GAAGTTTTTATGAAGATGTGGTTTACACCTTGTACTAAAAACGACAAAGGTGAGTACTACTTAAAATTTT
AAGTATTTAAAAAAATATTTTTAACAAAAGagaaagaaaatcgtttcctattttataaagtatatagtct
ggatcaggatcactagcccagtcgattttgaaatgtccgtccgtattaacgtcgaaatcttgcgaactta
aagatagaagcttaagttaaagcatacaggaggaactaccccctatcgcaagtttgtttgtttccagatgt
tgccactgtaacgcccaccaatcgaaaatactgtttgttttcttgttttctgttttcttgtttttattag
tcttgtaaatttctatcgatttTGTTGTCACTTTAACACTCACAGGCACTCAactcgtagagtaaaaggg
tatactagattcgttgaaaagtatgtaacgaatcacgaagtacgaacgaagcgaagtatatatattcttg
atcaggatcaatagccgagtctatctagccttctccgtctgtccgtatgaaagtcgagatctcaggaact
ataaaattcagcgtaagtttgctgacccatgttgccacgacccctctagctcccacaaatcgcataaaac

tgtcacggccacacttataaaaaatgtgttgatatttttcacatttttttagtcttgtaaatttctat
tgatttgaaaaaaaaaatttcaacgcccactctaacgccctaaaaccgccaaaaactgccacgcccacat
ttttgaaccatttttcgatctttttcataattttattagtcttgtaaattttcatcggttttccaaaaaa
aatttttgccacgccctaaaaccgttcaaaccggtcacgcccacactttggaacaattttgaattctttt
ttaatttctcatttcattccccaatatctatcgataccccagaaaaatgatgaaatttcgcgttcgcttt
cacaccagctgagtatctggtatctgatagttttttttttaattttttttttGTTATTACTATTGCCATT
TTCTACTACAATTTTAGAAATATCGATTTTTGTTTGTTTATTGACTTATAATAATAAATTTCCAATTTAC
CTATGTCTATTGATCTAATAATTCCTCCCTTACACTTTGTGTAAGGGGTATCTGTTAGTACAGATAGTAT
CGTTTACAGCTATACAAAGTAtattacttaatgtttattcttaatggttatttaattaacgtttaAATAA
TTCTTACTCCATAAATTGTTATTTAGATTGGAATACAAAGGAAAATAAATCATATAATTGACGTGGTAAA
TACTGCACATGATACTGGAACTACTTGGTTAGAGGGCCTTCTAATGAGTG
>dme_circ_0003816_junction_seq
ACTTGGTTAGAGGGCCTTCTAATGAGTGGTGATTATTTAGATACGTCGGATTCTGG
>dme_circ_0003817
GTGATTATTTAGATACGTCGGATTCTGGATCGTGCGATGAAAGTGATACCGATACAAACAAAAAGAGAAT
TCACTGTGTGGACAGCAATGATTTCGAACTAAATATTAACATCTATAAGGCTTTAGAAGCAAGAAAGCAA
TATTTTATCAACAAAATTAAGCCATTTTCAGTTTTTGGCGAGCAAAACCACTCGTCAAATCAACACATCA
AAACATACATAGATTATAATAACGCACAGTTAATAGCACAATATCTGGCCACCAAAAGGCCCTTCAGTCA
ATCTTTTGATGGATGCTTAAAGAAAATTATATTAGTTGTTAAGTGAGCCATCTATAGCAGTAAGGACACG
TGCAATGAAATGTCTTGCCAACATAGTGGAAGTTGATCCATTGGTTCTAAAACGAAAAGATATGCAAATG
GGAGTTAATCAGAAATTCCTAGACACAGCTATTTCGGTACGAGAAGCTGCAGTAGATTTGGTTGGAAAAT
TTGTTCTAAGTAATCAAGATCTAATCGATCAGTATTACGATATGCTGTCTACTCGTATTTTGGGATACTG
GTGTCTCCGTACGGAAAAGGGTCATTAAAATTTTGAGAGATATTTGTTTAGAATATCCAGACTTTTCAAA
AATTCCCGAAATTTGCGTTAAAATGATTCGACGTGTTCATGACGAAGAAGGCATACAAAAACTTGTCACT
GAAGTTTTTATGAAGATGTGGTTTACACCTTGTACTAAAAACGACAAAG
>dme_circ_0003817_junction_seq
TTTACACCTTGTACTAAAAACGACAAAGGTGATTATTTAGATACGTCGGATTCTGG
>dme_circ_0003818
ATTTTTCGCAAGGAAAGATATGATAAAATAAGAAACAGTATCTTAGGCGACATTTTGACATCTATTGATC
GGTTACCTTCATCaaaaaaaaaTCTCCGCCCATATAAACTCACAAACAATGGCGGAAATATTCAAATGGT
TACAGCACTTGTATTGCAATTGATTCAATGCGCCACGATTTTACCAGATTCCCTTTGTGATAATGGCAAA
TTTAGTAACAAACCCCAAGAGGGAAATACTTTTGATGAAGAAGGAAAAAAATTGCTTCAGCCTAGTCAGG
ATCTACTCGTTTTACAAAAATATGATGTAGCTGTCAGCATTGGTGGAAATTTTTTAACAACTTTCCTTAA
CAAATGCAAATCTCGGTCTAATGAAACTGATTTTCGACCTCTGTTTGAAAATTTTATTCATGATTTACTG
GCCACCGTGAATAAGCCTGAATGGCCTGCATCTGAATTGTTGCTTTCGCTGTTGGGAACTATGCTGGTTC
GCTACGTTTCTGATAAGGGTATAGAACAAAGCATTCGCTTGGTATCATTGGATTACCTGGGCATAGTAGC
TGCTCGATTGCGAAAAGACACAGTGGAATCTCGGTGTAGAGTTAACATTATTGATTCAATGATTCAATCC
ATCAAATTAGAGCAAGAAAAGGAGGGAGATGTAACATCAAACAATGGATCAATTTGATCTAGAACCCGAG
GAGCAGCGAACGGATTTTTTGCAAAAGATTCTTCTCGACTTTCTAGCTGTAAATGCTCAAGAAGAAAACT
TAATCTGGGACTATGCTAGACATTTTTATTTGGCACAATGGTATCGGGATGTCATATATCAAAGACGTCG
GATAAATGATGGCAAAAAAGGTTTGGCTTTCAGGAAGTCCAAAATTCGTAATAATCGGAGGACTAATGGG
TAAGCTTACGTCAAAAGATGTATTAAAAACAACATGATATTATTAATACAGTTCTATATATTTTTTTATG
CAGTAATCGTTAAATATTGCATTATATGTTTTAACTATGTGAATTTTGGCCCGATTTTCCGGTCAATTCC
ATTTTATTTTTCGTAAGAAATTAATATCCTTGTACATGAACAATGGATTTTGTCCTTTTAAAATCCTGTC

CCTTCCAATCCTGTCATTTCATTATTAACTCTTAAAATAGTCAATGTGGTCTTGAGGTAAAATGTCAATT
ATTTTGGGTGCTTAAAGTTACTCAAATGCTTCCCTGATGGTGCCTTCTTCATCCATCTCCGATATTTAGT
CCGAATGTAAATAGCCATGTATTTTTAATATGGTAGTCCTGAAATGATCATAATCAGAATAAAAGTTTCA
AGCCTCACCATAAAATGAAAGAGAAAACGACAGACAAATCGTAAGTTATAGTTAATTCTAGAATAGCTCT
TTTTTTAGTTGTCCTTGTAAACCACTATGGATAATGGAGACATGTTGTTCATTTGATGTTAAGATATATT
TTCGCCGACCTGATAttatacccgttactagtagagtgaaagggtacataagattcgttgaaagtatata
atagggacaataagcattttcaaccatataaagtatatacattcttcatcaggatcataagccgagtcga
tctgaatctatccgtctgttcgtatgaacgtcgagatctcgggaactaaaaaagctggaaggttggggtt
aggcgtacaaattcttgagacaaacacgcagtgcaagtttgttgacccatgttgtcacgcccagaatgac
cacttttaaaatatgttttggtattttcttatttttattagtcttgtaaatttctatcgatttgccaaaa
aaaattttgaaacgcccactcCTAAAtccagaatgatgcaggccgaccattatatttttttgacaaagtcg
aggcactttaacttgtttcgtggagctattagtggtataactcggctaacatatgttccaaaatcggctt
ccttaaaacggctgcgaaccattctttcactgatatcaatttcttcttcttcactgctaaaaaaaacaaa
tgtttttttgttagattaccaaatcaatatcggaaaaaagtggcatcagtatgtcagagaataagtcattc
aggagggtctgttatgttttggggatgcgtagccttcactggcttgggagatttggttcctgttgatgca
accatgaatcaacgaaaatatttagatgtccttaataatcatgcattcccctctggtgataaattgattg
ataaattcatacttcagcaggataatgctccctgtcataaggccaaactgattatgcagtttctgaaaga
tgtttgcgtgaacacattggattagccaccaacataatagaacataatagaaaacctctggtcatatata
aaagaaaaaaaggcgtgcaaacttatctagaagtcgtgaagaaacgattttgaaaattcaaaccttatgg
aaggatatttcaatagattatatccacagcttggtatagacgctaagagagggtatatttttttttattaa
tttgttataatttttttagttgactttttttcattttgtataaattcgctcaaatcctttttccccatgtt
tacaacagatccatgcgaactaattaaatgtgatgtctaaatacttttgccatcgcccaaattgttggct
atctagctttcttataagcagaaggctaatggaaacggtaaacgacaaaacataccattcgcgtgtaact
gtaattgaactctttgttttaATAGTGTCTAAATACTTGTGACCACTCGTGTATACAAATATCCTGCTCG
AATATTGCAGATATAGAATGTATTACATATATAAGGGTTTGTCTAGTTAATAGCTTAATTGTTCTATTTT
TTCTATAAGAGGATAGTGTAATAATTTTCTGTTTTTTTATTCTATTCGTTGATTCGATGaaagtatgaaa
gttaaaagtatataacagaaagcatttaagaccctgtaacgtatgtccctttgtacccctgtccggccgt
atgaacctcaactagatttgcagacattatgactcctacgcttcgccagtatatatattcttgatcagga
tcaatagccgagtcgatctggacatgtTTTGCCATATTTTTGTATTATTTAGGCATACTCTCCGGTAATC
GTAATTTTTTACGATTTCGATTTGGCAATTTTCACAAAATGTTTGCTATCGGAATGTCTTGTAAAGAGTA
AACAGTTGTGTAAACTGTTAACCAAACAATAGAGCGGCCAACTTTTTCACAGTTTTAATTCCGTTCGCAA
CTACTTTTTTTCTCCACTTTGACCATTTAAGTAATTAAATTAAATAATATTATTTACAAAGCTTTTTGCT
CTCCTTGAAATAAATTAATGAATTATTGTATATTTTATAAGCATTTATACCTGCCTGTTTCTTTTCACCa
acaagatgcgtaacggcatacgatttttttggcccataatttttttggtcgtggttttagaggtcgctccag
gctctctcgaatttttgttcgagagcgagagagcggagagcgctacagcgaacagctcttttcgacgtat
acagtgatagcaggcaatgtatgtgtgcacacgtatgcttatgcattgtaccgcgttaggccttggactc
gagttggccaagggcgcagaggtcgaccggtaacggtgcatgcacaaagaggacgcaccgtgaactaacg
gtacaacttttggaccttgtacccgagcgggccgtgcgcaaaggggaaataacgggatcctcgcggccta
taaagggacggcgcaagtgcagctcgaggcagtcagtACGCGAGGATTGTCGAGGAGAGTACCGGCGCGA
GTCGCGCGGTCAGCGTCTGAAGTCTGAAGGCGGAGGAGTAGgaggcagtcagtgagacagcaagatcgca
cgtggaagaagatagtgcgtcggagcgttaaaggaaggagttcgggagtgagaacgcgcggtcaagtcgg
aaataataaacagtggaagcgtcgggcggcaagaggcccgaaggacatggaaggacgatcaccacaagTA
AAGAGCGATCCTGGGAGTGGAGGAGAAGGATATGGCGTGTCTGAAAGGGTCAGTGAAGTCAGTGGCCAGC
AAAgggtgttttgccaacgtggtgttattagtaagagtaaaaatatcacccggtgcctgctgtgcaataa

ccgtttgggtgagtggacgcacaagcaacgatcgaccacaggtcatttttgtgcgcataaaatttgtat
ggctggaagcgtgacgggtctatgaatgaagggtaaaagggaacacccaaaaaaaatgtcgtgcacatgt
ttttgctttttgatgtactcaatgggtgccacccatttcgaacattaatttttcacacgccgcaaactaa
atactctaatgacttatataaagtcatttttgaaatttattatgtgacattatgtacatggattcggcta
ttactacttctaagctatatttaagtaataataaagttaaggcaatgcaaaacaataatttttTAAatgg
tgccaacggatcaaaaataatataggtttaaagtctaagaacttctaagatgaagagcacattttgtcaa
atttgcaatgcatgagctctctccgctctctcgttcttgaaaaaaaatccgagagagcctggagggacct
ctagagccactccgaaaaaatagtatgcaaaaaaaatgtatTGTCTTTGACTCGGCTGTTTACgagacac
ccaagtattttcggaaacacccatgtgtttaacggtaaaccaataagtgtttttgttactcatcccttgt
taggccttttcctttttctgactgtttgccttctctatcctctgttttgagtggcaagtgtgattggcac
ccgcgacgcagagtaccgtattgattcgggtgcgcgcacaaccgggtgcttgtctgcacgtgcagattta
tacccaaaattgttggctgtcagtcgagtggcaaaaaatgcagaacccttgcagttctcGCGTGCGTACA
CTAAGTTGTTGTACAGTGGGGGAGTGAGTACACCATATTATATTTTGGCTGTGAATGAAGTAAACATGGG
CGGAAAGGAAAACGAAACTCGCTGAACGACCTCGAAATGAACAATGAAATGCAATGAAATTATGGTTCCC
AAAATAATGTTTTTGCGTACTAATATTCATATTTTAAGTACAGAAACATTTTTAGCTtaaaaacaagaaa
taaaatgagaaaatttttttaaattgttcaaaagtgtgggcgtgactggttttgcggctttatggcgtta
gagtgcaaatcgatagaaatttacaagactaataaaatgtgaaagaatatcaacacatttatcaaaagtg
tgggcgcggcagtttttttggcttgtgggcgtggcaacatgggtcaacaaacttgctctgtgtctttgtc
tcaagaatctgtatgcctaatcccaaccctccagttttatagttcctgagatctcgacgttcatacggag
agacggacatggctagatcttccttctgcctgctgttacatacttttcaacgaatctagtataccctacc
ctaccctaccctagtatactctacgaataacgggtataaAAAATATAAATAAAATACTTCTGTTATTACC
TTTTATAAAAGGGACGTATTACAGACAAACCCTGCTGAACCCTGCCTATGTTCTGCCGCAAATCCACTCT
GAAACTAAACCTTCGCCAACATCATTTAGGCCGTACGAGAACACAAAAGTCATGTGGCAGCGAAAAccaa
aacagggtccaggctgtgacctaataactacagaaatgatactgttacttccgtactgtgctattgaggt
tacctgaaaactctacaatggaatcggaggtctctattatcataatgataccaaagcccgggaaagatca
cacgattccgtcaccttacagaccaataattttattatcatacctgtcttaaatattaaaaaaatgtctt
ctatcgcgcataataccatatatggagactcccaacattatcctagcccattaatttggtttcagagaaa
aacatggaacacggacatgttaacagattaacatcagaaatttgcaaagccttcgagcatagagaatatt
tcagcgcgattcctcgatgaatctcaagcattcgaccgcgcctggctcaaaggtctcattcataaaacaa
aaacacacttgcctggggatacacgcataaactccttgagtcgtacctctacaaaaaagccctcgcagtg
aggtgcaacaaaatgatatccgataactacactatcgaagctagagtcccacaaggcagcgcacttggtc
caacctctgcacagcggatatattgcagcacgatattcctcgatgtatctcaagcatttgaccgcgtttg
gcccaaaaggtgtcgtgcataaaatcaaaactAAAACTTACTAAACTCAAAACTTATATTTGAAATTAAA
ACATTCACTGGGATATCGGCATTCACGCAGTAAAGGATAATATCGACAAGCAAAAAGCGTCCCACCACCA
TAATTTGTCTGTTACTGTGACTGTCATTTAGTTAATAGTATATGATATGAACAGAATTctcgtagagtaa
aagggtgtactagattcgttgaaaagtatgtgacagtcagaaggaagcgtttccgaccatataagaaata
tatattcttgatcagaatatatagcagagtcgatctggacatgtccgtgagttcgtataaacgtcgattt
ctcaggaacaaaagcagctagagagttgagattaagcttacagtttccaaaaacataagcgcaacgcaag
ttttttttattcatgttgacatgcccacaaagcgcccaatactgacgcgcccacactttgggacagtttt
aacatttttttatttttttttcaccatgtctaccgatatttcagaaaaattaccaaatttcgcgttcgcata
gccactagctgagtaacgggtatctgatagtagggaaagtcgattataacatCACAACAGAGTCGATCCG
TCCGGATgaacgtcaagatcatacatattctagagacatagacacagcgcaagtttttttgacacatgttg
atacgcccacGAAttattcgtcttgtaaatgtctatcgatttgccaaaaaactttaccacgcacactcta
atgcacacactctattccccaatatctatcgatatcccaaaaaaaaaaaaaaaaatgattcgtattctcact

agatgagtaatgggtatctgatagtcggggaaactcgactatagcaatctctgttgtttTCTGTAGTATT
TGGGATACGTGTATTCTAGTGTAATTTGTTGGTGTTGAAAATTTCACACATTACAAAATAGGTTTTATTT
TCGAGCGTTAACTAGCCCAAAATAATGGCACGTTCTGTGAATTGTGACCCTCCAGTCACTTTCAGTGTTA
CCCAGTGAAGGCAAAACCTTTTCACTCAACAGCCAGTGGAAGTTCCTGAAATGTGCGGACAGATGCCCAG
CGGGAGCGTGGCCTTCAAGTATCACACTATGAAGCCATCGAACAAATCGGACCTTAGCGAGGCGTTAGGG
AAAGCGGTTATGATGATTTTTGAAGATTTTCTGATAAGCAATAAAAGACGCTTACATTCAATTCAAGCTC
TTCAAATTCCAAAATCACAAGAAATTCAAGCATCCCAAAATCAACTATATCTGCCAATCAGTTCTTGATA
CTTTCGGATATGGACACAGACGATGAAGCAAATTTTATTTATCATCTAACCAGCCTCAGCAACCACAACC
ACGTTAGTATGAGCTCCAGCAACATCAATCGCAACACCAACCAAGTGTAGAAAAACTGGCTCTCCAGCAA
CAGTTCTAGCCCAAATCAAGCCTCTGACAGCAATAAAATTTAACCGTTTCACAAACCCCCCCAATTGTGG
TCAAATATTGTGAATCTCTGAACGGTATTTTTAGACTCATAGATAAGATTGTTCACCCAACAAAATTTTC
CATCACATGCCATTTAGATAATTCCATTTCAATCCTCTCTTCAGACCAGATGCGCTCAGAGCTTTTACGA
AATGGACATTAAGGCAAGCCTTACCTTACGTGGCCACATAGTGCGTTTTATCAATAGACCTAGAAGCCGT
TCCGACAAAACAAAGCACCTCCCAAAACTCCTTCCTCAACCTAGTCTTTGTTGAATTAGAGCCCGCTTCC
AGCAATAAGGAAACACATGCGTAATCATACAGCTAGCTACAAGGGCGCTTTGTTACGGGATCGTTGGCGG
TGGTTCTGAGCAAGCAGGTCAGCACCAATGGACAAGGTGCCGGCAGCTACACGAATTTCCCCCAATAGAA
AGTAGGAACCGGAAGCACAACAAAGGAGTCGCAACCAGCAGACGCAGCAAACAATGCAGGATACAAATAT
TAGACCCCATCAACATCAAGGTGCCCAACTTCAGCGGTGCCCAATTTCATCCCATCAACTGCTAAAATAT
CATTACGATCAGTGATGGCAGCAGCGATTAGAACAAATGTTCATGCGATCTGATGAAAAAATGTCGGCCC
TTCTCACACAGCTTGACATAATGTTCCAAATAATGATGTCCATGATGACTCTCATCATTGGCCTAATATC
TTAACACGCGCCAGAAACCGCTCAAGTAATCTTCTCTCCACTCATCAAACTCAACCATTAGTCTAATTGA
ACTTAAAATCGGCATGTGGAACTCCAATGGGCTGACCAACAAGCCTCTGGAGCTGGATCTGTTTGTTCAA
AATCATCACATTGATATTATGTTGGTCAGAGAAACGCACTTCTGCACTCAGACATACTTCAAGCTTCATG
GATATGACATTCTTCAATCAAATCATccagctgatcccgctagtaatcctgatcaagattatatatattt
atcggtttctttcacctgttacattcttgtcaaggaatttagtaaaccTAAACCTTGtatattcttgatc
aagatcaaaagccgatgtggccatgtctcgatgtcaggaactataaaagctagatggttgagattaagca
taaagattctagagaaatagacgGCTTCaactgccctaaactgtcacgccaacacttttcaaaaatgttt
agatttttattcgtttcattgtttgtcttgccaatttttatcggtataccaaaaGCGCTGGAGCCACTCC
CACTTTCAACGCCCAACGCCCAAAACATTTTAAATATTTTCTCATTTTGTTATATGAGTATTACATAATG
TTTTTGCTTGTTTTGAGCAATCTATCACCTTTGTAACATTTCTGATTTttataaccgttactctcatagt
aaaaggggatactttattggttgaaaagtatgtaacaggtaaaagaagcgtttccgaccaCTGGACTTAG
GTTAGATGCATGttttttttttAAATCAAAATCAATTATTCATTTTCATGCTTGTTCTCATTGTCCGAAA
TGCTTTTCTTATCAAAGTTCGAATCATGTTCGAAAAAAAGCTCACAGCTGTTTGATTTGCGAAAAAATAA
CGGTACATTCGAAGATTCTACAAATTTCTTTGGAAATCACACGTGTTTTCGGTTGGTCATTGAATTAGAC
GCAACTTAACTTTCATCATATTGCGTGGACAAGTGTAAACTTTTGTATTGaaaaaaaaaaaaaaaaaaat
aaacaaaaaataaaTTTTTGTTTCCGAGCTACATTTGaaaaaaaaaaTGGGTTAAACTGCTCTTATTGGA
ACGTCAAAAAGTAGACTTCCTACACTCGCAAGGGTTGTCCAATCGGAACATTGCCAAACAGATAAATCGC
AGCTACAGAACTATTGACAGATATTTGAAGGATCCAGAGGCATATGGGAATAACTTCAAAGGAAAAAAAT
CACTATCAAAACAAGCAGAACGCCAAATTGTTAGAATTGCTTCGAATTCTACTAAGTCTGCTGCTAAGAT
CAAGGAATTGGCCGGAGTAAGAGCAAGCTTATCAACTCTTCAGCGTACAATCAGAAATTCAGAACACCTG
AAGCGTCTAAAAATCAAGAAAAAACCCCCCTTGAATGTAATACGCAAAGAAAAACGGCTTTAATTCGCGA
AGGAATACATTACGTGGAATGTCCAATCTGTCACTCGTCTAAATGATTGGCGTTCACTGATGAAAAATTA
TAAAAAACGTTTTAATTTAGATGGCCCGGATGGATTTCAATATTATTAGCACGATTTGCGGAAAGATGAG
CTATATTTAAGCCGCCAGCATAGCCGAGAAAGGGGAGTAATGGAGTGGGGTGCTATCACATTTTATGGAA

CAATTGACTTGATTTTTATCGATCAAAAGATGAACGGCGATCAAAACAATGTTGGAATCCGTATTTACAA
AATTAAAAGATCTTTGTGGACCAATTCCTTGGAGTTTTCAACAAGACAATGCTCCTATTCATAATTCTCG
AGTAGTAAAGTCCACCATATTCTCCAGATCTTAACATAATTGAAAATGCTTGCGGTTGGCTGAGACGGAA
AGTATACGAAGGAGGAAAGCAATACGAAGACAAGGAAGCATTAACTGCAGCTCTTAAACGTGCTTAAAAA
TTAATAAGCCGACGTGGGTCTATTCTAATGACCAAGAAAAAATTCGCCTTATTTTGACTAGCTTTAATAAA
ATGTTAGAAAAACACAATTTGGTTAAATATGTATAATCATTTCAAAATTATATTAAAAGTATCACTAATA
AGAAATGTTTTTGCATATCCACTTCTTTTCTATCGTCCTTTGATAAATAAATATGAAGCAAAGTTGTATG
CAACTCAAGGTGCGTCTAACCTAAGACCAGTGtatatgtattcttgatcaggatcaatagacgagtcgat
tggaccctgtctgtccgtatgaacgtcaagatgtcagaaactataaaagctagaaggcaaacagattcta
gagacatagacgcagcgcaagtttattgacccacgttgactttaacaaacacaaaatgcccagaactgtg
acgctcTATCTATCGACAGAGtctatcgattcgccaaaaaaatgatttgctacggccactctaatgtccta
aCttgaaaaaattgaatattttgtaatattttttattagtcttgtaaatttctatcgttatgccaaaaaa
tttgacacgcccgctctaacgaacaaaatctgtcgaacaaatcacgtacacactgtggaacaatttttag
tatttttttttttgtattttattccccaacatctatcgatatcctagaaaaattatgaaatttcgcgttcg
ttttcacacttgctaagtatcgggtatctgatagtgggaaaactcgatcgggaaagcaatctcttttgtt
CATTGACAATACATTGAGCTTAATATAAATATATTTGTTTATTTTTTTAAGGGTGATTATTTAGATACGT
CGGATTCTGGATCGTGCGATGAAAGTGATACCGATACAAACAAAAAGAGAATTCACTGTGTGGACAGCAA
TGATTTCGAACTAAATATTAACATCTATAAGGCTTTAGAAGCAAGAAAGCAATATTTTATCAACAAAATT
AAGCCATTTTCAGTTTTTGGCGAGCAAAACCACTCGTCAAATCAACACATCAAAACATACATAGATTATA
ATAACGCACAGTTAATAGCACAATATCTGGCCACCAAAAGGCCCTTCAGTCAATCTTTTGATGGATGCTT
AAAGAAAATTATATTAGTTGTTAAGTGAGCCATCTATAGCAGTAAGGACACGTGCAATGAAATGTCTTGC
CAACATAGTGGAAGTTGATCCATTGGTTCTAAAACGAAAAGATATGCAAATGGGAGTTAATCAGAAATTC
CTAGACACAGCTATTTCGGTACGAGAAGCTGCAGTAGATTTGGTTGGAAAATTTGTTCTAAGTAATCAAG
ATCTAATCGATCAGTATTACGATATGCTGTCTACTCGTATTTTGGGATACTGGTGTCTCCGTACGGAAAA
GGGTCATTAAAATTTTGAGAGATATTTGTTTAGAATATCCAGACTTTTCAAAAATTCCCGAAATTTGCGT
TAAAATGATTCGACGTGTTCATGACGAAGAAGGCATACAAAAACTTGTCACTGAAGTTTTTATGAAGATG
TGGTTTACACCTTGTACTAAAAACGACAAAG
>dme_circ_0003818_junction_seq
TTTACACCTTGTACTAAAAACGACAAAGATTTTTCGCAAGGAAAGATATGATAAAA
>dme_circ_0003819
GGTGCTGCGCATTCACACCAAGAACATGAAGCTGCATGATGATGTTGACCTGGAGCAGATTGCTGCCGAA
TCCCACGGACATGTGGGCGCTGATCTGGCTTCACTCTGCTCTGAGGCTGCCCTTCAGCAAATCCGTGAGA
AGATGGACCTCATCGATTTGGAAGACGATAAGATCGATGCCGAG
>dme_circ_0003819_junction_seq
TTTGGAAGACGATAAGATCGATGCCGAGGGTGCTGCGCATTCACACCAAGAACATG
>dme_circ_0003820
TGAGCCATCTATAGCAGTAAGGACACGTGCAATGAAATGTCTTGCCAACATAGTGGAAGTTGATCCATTG
GTTCTAAAACGAAAAGATATGCAAATGGGAGTTAATCAGAAATTCCTAGACACAGCTATTTCGGTACGAG
AAGCTGCAGTAGATTTGGTTGGAAAATTTG
>dme_circ_0003820_junction_seq
GCTGCAGTAGATTTGGTTGGAAAATTTGTGAGCCATCTATAGCAGTAAGGACACGT
>dme_circ_0003821
GTTACGCTGTTTGTTCAAAACAATACGGTTACACTCATATAAAGTAGTTGGAGTCGTTTAGATTTAGATT
TAGGTTTAGGCGTTACCATAGTTTAAGCTCATTCAGTTATATAAATAGAGATATAGGTATGTTTTTAGGT

ATAGGGTAGTAGTACAG
>dme_circ_0003821_junction_seq
TGTTTTTAGGTATAGGGTAGTAGTACAGGTTACGCTGTTTGTTCAAAACAATACGG
>dme_circ_0003822
GTTTAAGCTCATTCAGTTATATAAATAGAGATATAGGTATGTTTTTAGGTATAGGGTAGTAGTACAGGTA
TTGAACAGCAACAAAAACAGTGGGACATACTAAGTACACATTATCCAGGCCATCGATATTGCTTTGATTC
GTTTTCGCCGTGGCCCGAAaaaggatataaaggatataaaTACATATATGGATATATCGAAAAGCGGTCT
AAGCAATTATCCCCTGGTGCGCTGGTGCACTCCACTTTTCCTTTTCCCGTTCTCAACCCGCGCTCTCAGC
TCTAATTTTCGTATTTTTCGCTAGTTTTTCTCAAAGTTTTCTAAGTTGATCCCATGCAACGTATTGATTG
TGCCTTTAGCATTACATATTTGTTTTCATTTCGTTCCGCTTCGAGAACTGAATAACAAAATAACATCCTC
GTGTGCCTATTGTGAGATCGCTTTAGGAAGTGGAAAGCCTTCATCATCTCGGGGACCTGCAAATCTGATA
TCTTTCCCACTCTCCGATGCTTCTTAGTACATAGACTA
>dme_circ_0003822_junction_seq
TCTCCGATGCTTCTTAGTACATAGACTAGTTTAAGCTCATTCAGTTATATAAATAG
>dme_circ_0003823
ATTTGCAGGTCCCCGAGATGATGAAGGCTTTCCACTTCCTAAAGCGATCTCACAATAGGCACACGAGGAT
GTTATTTTGTTATTCAGTTCTCGAAGCGGAACGAAATGAAAACAAATATGTAATGCTAAAGGCACAATCA
ATACGTTGCATGGGATCAACTTAGAAAACTTTGAGAAAAACTAGCGAAAAATACGAAAATTAGAGCTGAG
AGCGCGGGTTGAGAACGGGAAAAGGAAAAGTGGAGTGCACCAGCGCACCAGGGGATAATTGCTTAGACCG
CTTTTCGATATATCCATATATGTAtttatatcctttatatcctttTTCGGGCCACGGCGAAAACGAATCA
AAGCAATATCGATGGCCTGGATAATGTG
>dme_circ_0003823_junction_seq
AAGCAATATCGATGGCCTGGATAATGTGATTTGCAGGTCCCCGAGATGATGAAGGC
>dme_circ_0003824
CCAAAACAACAAAAGCAAGCTAAGGAACAAGGCAAAAAAAGTCAGCGAAGAAAGAGACGGAGGAGCAGGT
GAAACAGGTGGACGCAGGAGACCCACCCACCCGAAAGAAACTATCACCACCAGCAGGTGACAGCAAAGCA
GCAGAAAGTCAGGGTGAGTATCGCGCCGcattcgcattggaatccattccattccattccattcctttcc
attccgctgccattTGTGTTTGCTCCCCGTGCGATTAAATATTTCACGCGCTTATCAATTGCTCAAGGGT
TGGCATTGCTTGATAAACAAACAACTAAACAACTAAAGAGCAAACAGAAATTAAAAAGCCAGCAAAAGTC
GCCTGCTTGGGCAGGTGGGTCGCCAGGTGAGGTTATACTACAGCTAGTGTGGCGGGGATCGAAGTGCTCA
GAAAGCCACTGAGATATTCTGGAATGAGCCCCACCATATATGTACCCATACCTCTACTCGAATATTGTTG
AAATGAGTACGCGGGCCATAAATCAGTGAATACCCCCGGATAACTTTTATAGAATTCGCTTCTATAAGTT
TGAAAATTAAAGAAAAGCATTCAAGTACAACTAGTATGACTAGTTGGCACACTTCACTTCATAATTTAGG
AAAATTATGAGTTTTGCTGCTCAGTCAGGCACCCGTTGTTTCTACAAATGTATATATACTTCACCTTTAT
AAAGCAATAATTACTTTCataaaaacttaataaaagggttggacaagctttataaaaacaaaaataaaca
tataaatGGTACTGTAAAAAGTACTAGGCGTGGACTAAATCGTAAAATCATTTTGCTCAAATAACTTAAA
ATTCTTTGTCTAGATTTAAATGTAACCCAGATTTCTTTGAAAATAACAACAGGAACTATTAAACAACTGG
AAGGTGCCTTTCTATGCAGTGCCATCTATTTTTAAGACTTTTAAACTGCTTGAATTCAAAATTCCCCAGT
TACACCCTTTTTTATGTAATCCGACTACATTTCCCGTTCTTAATATCCTCTTTTCCCCTGTTGCAAAACG
GAATCCAGATAATAAATTCTCGAGCTCCTTCTCGATGAGCTGTATTTGCAATTAATTCCTCCATTTTGTA
AACTTGGCATCTTCGCCAAACGGACAAAGGAGAATAAGAGCCCAGCAACACAGTTGGCCAACAACCAACA
GGCGAGAGACAAACATCCATCAGCagctcaaagaagctgggttcggaaaatcgaatttttaaaatttgaa
agctaaaatcgtttgcccgtttttgcccaccaattagtttttttttgcctacgttttgaaatattatattt
tgaaatataaattttcgaaaatttttggaaatcaaaaaattgactttttaacgtttgcacaccatttaaa

atttgttttttttttttcgtttgcccaataaataatttcgtttgcctatcctttaaaattagttttttttt
ttgcccactcttataaaaCAAAAATGtcgaatttttttggaacttctttgaaatttcatatctcaaaaact
ggacgtggcaaaaaaaccaattggtggggttttccagctttcgaatttcaaaaatgtgatttttccaaac
ccagcttctttgagctTCCATCAGCGGCAAAGCACACAATTTCCAAGCCAAATTCAATTTCGAGTTAAGA
GGGCGAGATTGTGGCCCGAATCTGTGCGCACcacatatacacacacagatacatacacacataAAGAAAG
CCGTGGCAGGACGACTTCAATGCGTATTCGATTCAATGGCCCGGGGCCACCACCCTTGAATGGCCCTTAT
TCCGCCACCATGGCCTCGGGCCCACTAGCACCCACTCCCACTCCCACTCACTCCCTTTACTTTCCTTTGC
CTTCCTTTCTGCTCATTGTCGTAGGACGGCGGGAGATTCCTGTCATGTCCTCACGTTTCCTGGACCCATG
GAGCTTATACTTCAGCTGGGCTTAGTTTCAGCTGGGATTCCTCGTTTATTGATTGATTCGATTCCCCGCA
TTCGTGTCCAGTCATAATGGATACCCCTGCACGGGCTGGAATAGAAAGTCATCAATAATATTTGCTCAAC
ACGGGGAAATGTTCTAAAAATACAACTAAAAACTAAATTCTAGTGGAGCCAGCCCTCCCCCCCAACGAAA
AGGAACTAATGAAAAAGTTGCCAGTCGGATTAAAGGCATTTTTATTGACAGCAGTCCTTTGGCTTCCATT
TATGTTTCATATTTTTTAGGCGGCCTGTAACTGGAATTGACAAGGGTGAATACAGCGGCACGTGAAAAAC
TCATCACAAAAACGAAAGGACAATGAAGGCAAGAAGCTCTCTTTATTGTCCTCGTTTTTTGTTTTTATTC
CCGGCGATATATGTACATCTTTCATTCCATTCTCTTTTATGCCAAACAATTGGCTGGGAAAATTGTTTTT
CAATTCTGATTCGCAATTAATTTCGCAATCCAGAAATAGAACGCATCGCATAAACAAAGGCAACGCGGCG
TATGCGCAATATTTGCTGAATATTCAATTTCCACCTGGTGGTCGAAAAACATGGAAATCAGCTTTGGTGC
GCCGTCAAACTTAAGTTGATATTATTAATTAGTCGCAAAAGTTTTTTGTATTCTGATTTGCGGTGATAAT
GCCTCAAAACTTTGACGGATATTAATAAATTATGTGTAAATATGAAAAGTGAGTCACACACTCTTGGAAA
AAATTATTGCGCATAATTTGCGTCTTCTTCAAAAAGTGGCCAAGAAAATTTCTTGGTGCTGCCTGAATAA
ATAATCATAATTCAAGCaaaaaaaaaacgaaataaaaaGAAATATTCACACAGCGAAAATTTAATTGTTT
GGCCAAAAGGAAAAAAGAAACGAACGGAACGAAACGGTACGGACCGAAAACGGCCAATAAAAGCCACGAC
CTTCGGACAACAAAAAGCGAACAGAAATTGAATCAAACCGCCCAACACTGCCCTCTTTCTTTATGTGCGT
ATATATAGAGTGTGCTAAGGGGATCCAGATTGCTATTCAGATTCGGGAGGCTTTTCAATAATTCCCTTTT
TTATGTCCAACAAGCAATTTTGCTCCACCAATTAGGGCACGAAAATCGATACGAGACAGCCGCAGAAACT
AGGCACATTATCAAGTGGTAATTAGGTTCttttttttATTTCAAAGTAATGGTTTTTTAATGATATTTAA
AATAGATATTAGGTACTTCGCCTTTGAATACGCTGATTCGTGGCAAAGGATGCCGTGTCCATGTCATTCT
TTAATAAATATCTGAATTATGTTCTTATTTCTTGTCCTTTATATTCCAAGTTAGCAAGCTAAAACTCCAA
ACGTATTTGTTTGTCAACTTCTGGCACTTTTGTGTGCCAAATGGGATTTACTTGTCGTGTTTTCGGGACG
CCTCCGACATTACAGCTTCACTTTCTTAGCTCATTTGATTACACAAACTTGGGAAGTTTTCCCTAGTTTT
CTTTTTGCCGCCGTCTAAGACCTTTTCGAACTAATTAGGAGGGCAACCGCATTTTGCTCTGATGGCAGGG
CCAAAGTTTTCACTGGCTTGTCTTGACTGGATGCCAGTCATTTGGCCTCCACGCTCCCGGGGAAACTTAT
CTTGATTAGGTTTTATATTTTTTGCCCAGACTACACACatatatatatatatatGTATGTATTGAATAAA
CGATAAACTTGCTGAGCCGCGAGATAAGCGGACGCGGCACGTGAGCCCAAAACTATTATTAGTTGACAGA
GTTCCAAATCGATAAGTATACGCCACCGCAAGAACATTAACTGGCGATCTATGAGCTCATTGATCCCATC
CGATCCGATGCGAACCGATTGGTTTCACGGATGCGGTTTCAGTTTAGTTTTCCCAGGTTGTCAATATGCC
ACAGATGTCACTTCTTTTTGGCCAGCCGATGACAGGTCCCGTGAAATGTGTCATCGTGGAAAAAGCGAAG
CTAAAAGCTGGCCCCATTTTCATTTCATTTTATTTGTGCCCGCCGCGGTTTATTAGTCGTAAATTTGGAC
TTGCTTCTGTGCGAAACTCATAAATATCATTGTCTCTGTGTGGATTTTAATATGCACGCCGTTTGTCTGC
AGCTCAGCAAAAAATGCCGAATGCTCCAATGAAAAATCCAAATGCCCACGCATAGGATACTCAAGTGTGT
ATGCACATAAAGATATACATACATATAAGACAAGTATATGTCAGCAGTTTTCTGGTGCTCCAGATTGACT
GACCCACTGAGTTTTCGTCTCGAGTCTGTCTCAATCTGAAATCCCAGGCGACTGATAACTTATCTACCGC
CGACTGACGCATTTGCGTGGACTTACTTATATCTAAAGTCTTTGCTTATCGCAAAGATATCTTTTATGTG
CATGCTAAGTAGCTTTGATTTGATGACGCCTTTTGCCGTCAACCGATTTAGTGGCCTCTTCACTTTAATG

CTTGATTTAACAATATTTAAATGTATTTATGCATTTTTCTTACTGCTTGTTCAAGTTTAATGAttttttt
atccaaccttttttttCACTTCATCTACTTGAGTTTCTCTGACTCAGCTGAGTGAAATATTTGGTAAATTA
TTTAAAAACCTCCTTGTTAAGCTATAAATACAATATATTGTATTTAATTTAAAATATCTATTTAGCTTAT
CTACAAGGTCAAAGCTTATCATTTTATCgtgtgtgtgtgtgtgtgCTGGCGAAATGAGTCTGATTTGATG
ACGCCTTTTGACACCTGCGGTTGCGAGTCCTCACTAATTAGAAAAACTTTTCTAGTACAGTGGTAACTTT
TAACTTTTGTTGATTGCAAAACTTTGCTTTTCATAGTGTCCTGGTGAGGGGGTGCTGAATgggggggggg
CGGTATTGAGGGGACAGGTGGTCGGAAATGATTGACTGACTGATGGGCAGCGGCATTATCTGGCTGCTGT
TGTTAGTCTCTTTCACTTTCAGACCGCTCCACTCACGCACTTTATCTCACAAAACTTTGACTTTAATCGA
TTTAACAATTTTTCCCTTAACCCCTGTTGGCCCCCAATAAACCAGCACCTACCCCCCTTACACAAGCTCC
CCTCTGCCCGAAGGCCAGCTGACGAGCTTGTTGTGTTGCCCACAGGCAAACATCAAAAGTCAAGCAGCTG
CGTGACGTCAATTGGAGCCACGAAGCGAAGGCCACCGGAGTGGTGGGTGGGGGGCGGTTAGTTGccccac
cccccctccccccTTCTGACCATGTCTTATGGAGAGTGTGGTCAAAAGAACGACCCACACATACCGCACAC
ACCAAACAGAGCCGGCATTACTTCTATTTGCCCACGTACAGTGTGTTGTAATCTTGGAGCGATTGACGAA
GTACCTAGCTACATAAATATGTATGTATGTGTATATTCGATATGACTACATGTGGCTGTTCGAGAAATTG
GATAATTGTTTTAGATTCGACTGCTCAAGCGAAAGGTAGTCATTTTCTGCTCACGCTTGCCGAGCAAGGT
AAACAATTTTGGCAGAAATGGGTGACATTATTGTTGACCAGTTTGGAGATATATGGGTACACTTGAAGCC
AAGAAAACCTAACTATGAACTATGTTTATATCTAGTAACTAAGAAAATCGTTCGCATAGTTAGCAATTGG
TATTAGAAATATGTTTTGCGCCAGCATGAATGATATAAAATGTCACTGCTACTCTTACATCGAAATAAAA
CGGGTGTACTTTAAAAACTAAAAACTAATGAGTGAGCATTCTTGGGTCCCTGTTAATTAACTTTCCCAGA
TCCCAGCTTGATTAGAGGGTCCGAGGAATGGCTTTAAAGTACGCCCCGTGCTTATGTTCAAAGGAGCGCT
CAATCCAGCTGGGCAAACAGAAAACCCGCCTTGGCATTCGGTGTATTTATGAAATACTTGGGTGAACTCT
TCTCGGTAGCTTCCTTTGAACGACTGCCCACACATGGCCCATGGACTTTGGGGGCATCCTAATTGATAAT
TTAAGGGCTATCTGTCTAGGCCCACACCCCAGAAGCCCGGGGACTTGAGCTTGTTGTAAATTGGGATGAG
AAGTCGTCGGCTTAATTTATTTCTCTTTGAATTCGGCCGCCGGGCCCCAAAGACATTGCAGGCAATTTAA
TTATGGAATATTTATTCAAGTGAGATGTGAAATCTGTTTTATTATTTAATCCTCTGAGCACTTTTGTCCT
GTGGAAAGACAAACTGGGCTGGAATGAAGTTTGCAGCTTGGCAatattttgttttatatcaaatatatat
ataatatttgttttctttattTTGGGGCGTAGGTTCGTTTTCCTCATTGTTTGTTGTCTCAAAGATTATT
TACTTATGCTCTGAACCCATTGTCCTCGTCCATGTTTCTCGACTATCTCTTGTTCGGCGAATTGAGTTTT
TTATTTGCACTTTGTCCCTTTTTTCTTCTTCAGAAAAGGTCCTGGACCTTTGATTTATTGTCAGGCGGCT
TAAAATGTTGAAACATGTTTTTAAAAGCACGCTCTCAAGTTCATCGAATATTTTGACTCGGCAACTTCCT
ACTTTGGCAGCCAACCTTTTTTCGCTGTCTACCTTATGTTTTTCCCTACCCCCAAGTCTTTTGGAACAAC
ATTATTTTTTAGCCAAAAAGTTTGGCCAAATTCTGCCACGTGTAATGGCCATAAAGCAGCGGCCTTGCAG
GTAGAAAATAAAACACAACTGAGTTGAGTAAATTTAATTTTTCATGCCAAGCTTCGGGGGCTGGAAAGTG
AAAATACATATTTGCCAGGATGGGATGGGATATAGCATAGGACTTGGGGGCATGAAAATCCCAGCTACAT
GTTGCGCTTTTGTGGCCCGACTCTTTATGCGATATAAATGTGTGTATATGACTATGATTGTGGTGCACAT
ATGCAAGCTTCACAGTGCCACGCCCACAGAAAAATATATATCCAAACTCACAGAACATATGCAAGCCATA
AATAAACATAAGTTGTCGCTGGTGCTCCCGTTTCGCTGCTCTTTTTCTTTATGGGCGCCAAGTAAGAGTT
TTTCTTTTCCCCATTTATTCATACATATGTACATTTTGtatatatatatatatgtatatatTCATATTCT
TATGTATTTGCATATATAATTTCCATTGTTGCTGCGGTCGAAAAGGGGAGTGCGAAAAAGATAAAGTTTT
GTGTGGGTCGGAAAAATCGTAAGAGGGCAGTCAATTGTGTTTTAttttttttttttttCAGACCCTCGACA
GCTTTTGAATTTAAATATTTTGGCTCTGTTTGGCTTCGTTTGTAATTTTTATCGCATTTTAATGAGCTCG
CCAGCTTATTATTTCGTTGATTTATTTCCTGCCTGTATTGCATTTTATTGATTATTTATTGAAGCGATAT
TAAACAGTTTTAATAGCTGCATTCTATGTAATGCTTCTTTTGAAATAAACGAAATAATTAGTTATTTTGT
TGGTAAATATTGGCAATTCTTCGATATTTATACGGTGTTGCGTATTTTGTTTTACTTTAGTTATAAaatt

gaatgaattgaattgaattgaatttaattAACTTTTAACTTTCGATTAATAttttttttGAATAAATCAC
TTGATTTGTTAGCACATTAACTCCAATCGTTTAGCTGGTGTAATTATTTAAGTACCAACGCTTCGTTTAG
CATTCATTTAGATATCAATTAAAATATTTCCCTGCCAATTTTTCGGACATTTATCACAGCGGTGAAATT
ACCATTTGGTTTTGCCACTCACAttttttttGTTGGACCGATACGCTGCTGCCTAATTATGCAATTAATT
TGCGGCCTTTGTCAATGGCTTGTGTCTCTAATCACTGTCGCATTTGTTTGGGGCGCACATATTTTCGAGT
TAAGTGCTCGAGTACCCCAATCCGTAGATTTCTGGTGCTCGGCGTGGAATGGCTAGAATTTCTGAGCGAT
TTGAATATTTTAAATATGCATTCAGTCACTCGGCAGACGAGTGTCGACTAAATGGCCTTAGTTTTATTTA
TCGCACCACAAATGTCACTCGAGCGTTTCAAATGCCCGTCATATACGGCATACTTACTGTTTTTATTTTT
TCAACTTTCCAACCATTATTTGGACGttttttttttGTGCCGTGACAGGTGTTACACGTCATCGGCTTAT
GAATCGGCACGGCTTTTATTTTTTACGTTTTTGCAACGCCCGTACTGTCCGATTGTTTTGTTTTTTTACA
TTTTCTACATTGGCTTTCTCAGCGAAACGGGCTGGCTAATTCTGAGTGCCAATTTAGTATGGCCCAAAGC
AGGACAGACACTCGGTGTCGCCGCCAGTTGCGATTGCAGTACTACAACAGCCTTGTTCGCCTTGTCAACC
TCACTTCCTGCCACTTGGAAGTATAGGTTGGGTTCCGTAAGTTGGACACTGTTGGCTGGCATAATAAAGC
AAATGTCATTCTTCGAACCTCAGACACAAGGCGTAGAGTTCTAAAAGTTTAACTACTCTAtttttttttA
ATGTAATATAAATTTTCAAATCCGAATTTAAATTTACTTTTACTTTATTTTACTTTGTAACTTTCTGTTT
TATTTCTCTGTGCTTAAAATGTCTGCATTTGAAATTTATTTCGGCTTTCAATTGTTTTAGTTTCGAAAAA
TAGTTTTTGGCACATTATTTCTTGAAAATGTAGTTTACTTAAAAAAATCAGTTTAATCGAGTTGACCTGT
AATTGTATGCACATGACGTCACCGTCTCCTTGTTGGCTAAGTGATTTCCAATGCAGTCGGCTATGCAGGC
CAAGTGACGTTGGTTTGGCCAGGGGGAGGGGATTCGGATCTAGATCTGGATCTGATTTGCATAAATGTCG
GGCGGGGACGGTGGATAATAAAGTGGCGAGTGGCTAGAGTTGCGACCATGGGGGAGCCTCATGCATGCAT
ATCGTCAAAATGGCACAGACCGAAACACAAAATTTGCGTTCGCTTAGCACATTTCGCACATTAACCCACA
TGCaaaaaaaaTGGGGGACGGCCAAGGAAAGCCTTGTTTTGTCTATTGTTTACGTTCTTTTTGCCATTTT
CAAGGCAGAAACCTTTCTCAACCAGCTTTTTGCATAATTATCCAGAAGGTGGAAACGAAAACACCCAGCA
AGGCGACCTGGTTACAGAAAGCACACATTCAAGTGCTTATATTTAGATATTTCAGCTACGAATAATTACA
GTCCGCTTGCAAAGCTTTCAGTTTATTTAACTCGTCTCCTTGTTTGAGGCCTAATTAATTGAGTTGGCTT
AATTGCATTGCGTAGGTGGGCCTACACAAAGTATATACCCATGTGTACATAGGTACATGTATCTATCTGC
GCTGTATGTATATCCTATTTCAGTTTGCCATGCATACTTGCAATCTCTGATGCCACAATGTATGCTGCAG
TAATTGCCGAATTGCAAGCAAATTTGCAAATGCCAAATGCGGCTTTTGTATCTCGAGTTGTAGAACAATG
GTCATGTTGTCTGTAATCGGAAGTTGGCAAAGGATCCATTTGCGTGGCTCCATTAATTTACTGGGAAATG
CAAAGACAGGTCCTACAATCTTACAACTTGTTAAGTAAACTCATATTTGTATTTAATATTAAAGTGCTTA
GGGTTAAGGAAATGTAGAACATTTACGGTGTCAACTCATTTTCTCACACTCATGCAGTGCTTTTCGTCAC
TATGTGGAATGATTTACTGAGCAATACAAACTTGTTAAGGTTATTTTTATTTCGAAAGTGAAAAGATATA
ATAAAAACATGACCTGTATGAGTGATGAGTTCACAAACGTCAAAAAATACGAGGAGTAATGTAACTTAAA
TCAATCTGATTAGTTTAAACAACGGCTAACCACTTTCTATCAGCTATATCCAACTTAAGGACTTAAGAGT
GCTGTAACGGGTAATTAAATTCTATAAATAAGGCGGTAAGCTTTAAAAATAATAATATTAATCCTTGGAC
ACCCCCTAGAAAATAGTCGTTATCAACGTTTTCAACTCTTACTGAGTTGTTAATTGCGTTTTGGAACACT
TTTAATTATCGGCGAACTCGCACTCCGGGGCAAACAGCCGACCAGATAATATCCACAAATTGGCCTCTTT
TGCGCTGTTTGCTCGACTGTGTAGTGTTTGGTAGTAGTAATCCGCGTTTTGGGCCTTTTAGTTTATCGTC
TTGAATTGGGCAATGGCAATCAATGCGGCAAGCGAACTGGAACGAAAAACACTCAGCCACTCGGCTTTTG
TTGCTATTTGCTGTGTGTTTCTGCTTTTATCTATACTGCCATGTTGTTAATTGCCGAAAAACTTGGGGCG
CAAGttttgcttttcttcacgttttgttattcgcttttcagtatttccattttttttttgttttcaccca
atgtttttttttttGCCTTTTGCCGGGCTTGTTTGCTTTGAGCACGCGCCGTTAAAAACTGACACGCGCA
ACAAATTTTTGGTAGATGGTTCGAGGAAAAGGAAGAATCGTGTTTAAAATTAAACTGGGAAAACGACAGG
CGAATGGAATCTAGGAACGTTTTTATGGGGCAAGAGCGTTGGCGGCAACAAATGTAGATAAACGTTTTGT

TACAACACATAATCGCTTTTCCTAGGGTGGCTCTCCCAGATACAGATACAAGCACATCCGTTTACTGTAC
TCACACCGAAGGGTGGATGGCACAGTGAACACTCCATATGGCACAATTATCCACTAGTCTGTGACATTTT
TGGTCAACTTTAGTTTTGATTTGTGTGTAATTGTGAAACAACGTTTTCAGAGAAAAAAGAGTACAACAGC
GCAAGATATTAAATCGCATCCTTAGTGCTCACTGTACTCACTGGAAGGTAATCGCGTTTTATGTATACGC
GGGGGTAGAAGCTGTTGCTTTTGTTGCTGCCACCTTTAAAAGTATCTGAATCTCCATCTATAGACGAGTA
TCTGTATCTGCTGCCCGCCCTAGCGAGCAGGCAACATAATTGCTCACTAATTCAACGCTAATGGACATCA
ATTTAAGCATCCATCCAGCAGTTTATCATTCTGGCCATAATCGCCGGATGGCAGGAAGGATCGGTGGGAT
GCCTCGCTCCGCAGTAGCTGCCTTAAAATTGTGCGTAGATAGCATTGGGAATTTTAACTATTTATAGCCA
ACAGATGGGATGTCCTTAATGGAAAGTGAGACACCTGACGGGTCCTTGACAAATTCATAGAATTTCCTTG
GAAGTCCAAGCAAGGCATCTTGTTAATCAATAGAAGAGAAATTAGAGTCTGGGTTAAACAGGAAAACTTT
AAGATACTCTCTGTTAGATAAATAATTGTAACACAATGAACATAGCATTTAAAATAAGTTATCTTCTTTT
CTATGTTGAGGAAAATTATTTGAGTGTATAACCAAATCCTTAGATATCGCCAGACATATTTTAGAATATT
GTTAAGCGATTTGTGCATCTTGCAGGGCGTCTTCAAATCCAATAGATTAAAAAGTAATTGAATTGACTCC
AGACCGTAGCGAAAAGCTAAGGGCGGAGAACAATTGAGACCGGGTATCTGGCCAGGGAAAAGCCCAGAGA
TGGCAAAGGTTAACTGACAAGTCGTTGTCTCAGTGCCACCCATAGCCCCTCTCATTCCGGTTGAAAATTG
AATGCGAAAAGCGACTTGCACACATACATACATATATGGGCATGCTTCGATATAAAATCGTGTGTATGCC
CTGGTGAATTTTCTTTTTCGCTTCGCGTGGGCTGTAAAATTCCTTTTGACAGCTTCAAGCGTTCCATTCG
CGTTGTATAAAATCCATATGTACATGCACTGGGAAAAATATCAAAACAAATGTGTAGAAAACGACGAATA
ATAGAAAGCAttatttttcttgccaatttccattgataagccataataatttttttcttccaaccagcgga
ataaagggtatctgatagtcatagatatggctaactatagcgttgtctcttgCGCAAATTGCATTTTCTC
TCTGTTAACATGTACACATAATTCCTGTAACAAAGGGCATTTGCAATGTGTGAGTCAGCAAACCGCATCC
GCTTCCCGGCCCCTCCTGCTCTTCCACCAATTTTCCCCGCTTTTCCCATCCGTTTAGTGAGCCCCTCTCA
TTACGCATACGACATGTGTTCTTCTGGGCGTTAATAAATCAATACCCAGGCGTCGTATTTTGACAAACAT
TTTCAAGCTGATGACCTTTTTACTTTAACGTCAGAGAAAGAGACGGCACCTTCTACTTCCAAACCCTTGG
GTGTGTGCCATAGAGGGAGAGAGGTGGAAGAGCAGCGGCAGTTGCAGAAACAAATAAGAAATTCGCCATT
AAAGTTGCGTGACGAAGTGAAATTTGCGTCAAATACTCGCGGGACTCCTTCTTCTTCTTCCTGTCCAGGA
TAAACATACATATATACTCTACAAACATACATACATACTCGTATACGTGGAGTCCCCACACACATGGAGC
AAACGATCCAAAAGTCTGAACCTCTCGTCCTGTGGCGCATTTTGAGCTCAAGTGCCGGCGGCGCCCTGAA
GTATGCCCTAAAATTGCTTTGATGTGAATGCGATACGTTGTGTTGTCAAAATACTAACATATAAATAACG
TATATATTCCAAGAGGGGGTGTGGGGGTGGGTCTGGTCTGGTTGTAGCCAAGCCCACCCGCCTGTGGCCC
CAAATGTCAACGGGAATTAAGTGCTTGCCGCCTTATTTTTCCGCATCTGCCGGGCTGCCACTTTGACGCA
TTGACCGGAAGAGGAAACCTTTTCACCAAGCTTGAAGATGGCGGAAAATTTGAATCGAGATTCGAAAGAT
AAACATCGCTTTCAGGGCCACAGGAAAATCTTCAAGCATCGGAGGTAAACAGGTAACATTGGGGAAATGC
TTTCAGCAGGAAAGTGTTTCTCATTTAATTGGTGCTTCTGGTGTAAAATTAAACGTTGTTGCAAACTTTT
GCTCAAACTTTTCAAAATAATGTTTGTAAAATTTTGAAGTAGCGATGCAGGGAAGGGCCAACTCTAAAAT
AGAATAATATTACAGAGGAAATCTGTTCGGGAATAAAGTTTGTGAAGTGAAACAAAGGTACGCAACATCG
TGCGTGAAATCTAATGAAATGCAAATTGAAAAATTAACTTCAAGCTTAATAAAAACGCCATTGTTCCAAA
ATATTCAATCGGAATGCAATTTGTAACGATTATCAAAAAATTAGTTTCAATTTAGTTTACTATTGTCATA
AAACCAAACCTACATATGAATGATTCCAGCAATAAAATATGCATTTCTAAAAAGAGCATAGCAAAAGTAA
ATGAAAATTGCTACCAAAAATCGATTAAAATTCAATTTCGGACTTTGTCCGAGTATTTCTCCCATTCCTT
TCAAGTTTTTACCATTTTGAATATCAATTTCATTAGTTTCCTAATTGCATGCTATTCAGAGTCATGAAAA
AATAAGTGAGGATATGTCTGGTTGCttttttttGGCAGACTCCCTGCATATGAAACAATAGGCAATGACA
CTGCACTTGAGCTGCAGCCTCGCTGGAAAATCTTTTTCTTGGAGGACCAAATTGTCTCCTGCCGTTGTGC
CATTAAAACACTTAACCCAATTCGAATTCAATAGAGTGGGCGGAGGTCAAGTAGGGTTGGGTTGGGTCTG

GGCTCGAATCTGGGTGAAACACTGTCAATGACACAGACAGACCTCGATATGTGGTCATGGGACATGCATA
ATAATTAGACCGCCTCGAGGGCTTCTTTGTTTGAGGTCTTCCAACTGTTGAATACACGAACTGACCATCA
AATTCGAGATGTGTCCATGTCGTTTGGCTAATTAGGcacacaacacacacacaTTGCTGGTTACTGCCGT
TCCTTAATGAAGTCATTTGGAGCAGACCTTGTGATGGCCGCCGTCGCTCTTGAATCCCCaaaaaaaaaGC
GAAAACCCAAAACCCACGCACAGGTGTCCCATTTCTGAAATGTTGACGTAATTGGATAGGCCAACTGACA
CAGAATTCCGACGAAGGATCCTGAAAACAGGTCGGTTGGGCAACAAAAACAAACGCCATATGGATTTTGT
GTCATCTGGCCTTTCGGGAACAGGAAATCCTCAAGGTTGTTTGCAGGTTTGCAGACCTACCATGTGGTTC
ATCATCGCTGTTTTGAAATTTGATATCGAAGGGCTTTATCATGTCAAAAGAAAGTTAACCAATTACTAAA
AATAATTGATATTTGTATATCAATAGCATACTTGAAATAAACTAGTTTTAAAGCTGTTGTTAGTTTTATC
CACTTAATAAGATGCTTCAAGTTTTTCAGCAAATGTTCATGGCATTCAGCGATGTATCCCTTGAATAATT
CAAACCATTCATTATGAATGGAGAGCATAAAACCGATTCCAAATGTAAATGCAATTTTAAAGTTGGATTC
AATTTCGTTGCCAGCACAAAACTCAATTTCGCAATTTTGTAAAAGCGCAAACATGCGAGGATGATGGGGC
AAAAGGTCGGGCATCCCGAGGATGTCCACCACCCACTGTGGGTGGCCAACGTCGACGGTGGGTGGGTGCG
GTTAGGTGCTTTCACCTTTCACCACCACCTGCATCTATGTACACCTGCGGCTGCAGTAAAAATGACATTA
AAATGAAAGTCATATGCACATGGTTAACTCGCCACGCATAACCAAGCCGTAATCATTTTGATGGCCCTCC
GGATGGAGGATAGCATCGGGTCCTCCGTGGGTGCAACAGTTTACCATGTGGCTGGGTGTGCTGGCAAAAT
TAGTTTGCCTTAATCCCATAAAGCGCTGCCACCGTtgatgatgaccataatgaccataatgacgataatg
atgatgctgGCCAGCGGATAGAGACGCGAACCGAACCGAAACTCGGGATATTCCATTAGGGGAATGGGTA
TGCGAGTGCGAATGCGAATGCAAAACGCGAATATAAATGAACATTTTAATGGAAATGACAACGTAAAACA
GATGCAGCTAAAGTACGATAGAAATATAGATACCCAGCGAAATTCCATCAACTGTTTTTCGTCGaaaaaa
aaGAACTTCCATTCCCATTTGAATAGCTTATAGCCCATAATAAAGTTGCTTTTTATAGAACGTAATCGAT
AAATGAAGAAGAGGTTACACCAGAAGAAGATTTGCATTTGCATTCGAGCTTCAAAGTATTTAGTTATTTT
ACAAAAAATTCCACTATTTGCAATACTTTTTGGCTGGTGTTTTTCATTTTCCAGTGCGAAAAGCGCAATC
TAGTCAAATGTGATTAGTCGATAAAAAGTCATAAAGACCCCTGGCTGTAATCAAGTTATTATCTTTAttt
ttttttATGAAATTTAATCAGCTTAACTCAGTTAAATTCGTAGGTTGCGCGTATTAAAGTCTGAGTCAGC
TGACTTGGCTCGATTTACGAATGTGTAGATATTTCTATACTCGTATATACTCGTATATTCCGTCTATAAC
TGCCGGCGAAATGGTTGTGCAttttttttttttACCTTCCTTTACCGGCAGCTGCGGAAATTGTGAGGAA
ATGAAAATGGTTTAACTTTTCTTTCTTGTGTGCTCTCCGTTAACTTTTCTATTTATAttttttttttttt
ttGCCAACCACCTCGCACCCAAATCCTTGACTGCTGTGGGTGGAAAAGCGAGAAAAGCGAGGGGCCGGCC
GGTGGACTTTTCATTCAGCCTCTTATTACTCGAGCCCAACTATTTATAGCTTCTCAGTTGTCTGTGCAAT
TGCCGTTCGTGTGATTGCAGTGCGTTGAGTGAACCACCTTGACCCACTCTAATTGCAATTGCACCGCCTA
ATGTCCAATGCTCTCAGCCGAGCCaaaagcaaaaagcaaataaaaagtgaaaaactgagcaaaaaaaaaa
acaaaaaGTCAGTCAATAGTCGGCTTTTGGCCAGGCCAATCAGTTGGGGGCACTTAAACGCCCAATTGGT
TATTGAGTCGATAGATATTTCAATTAAATTTTCATCGCAATCGCGACTTGTTTGGTGGGCGGAGCAAACC
TTTATTAGTTTTAAATATTTATTTGTTCCAATGCAGGCCGCAGGGCAAGTTGAATATTAATGAACCTAAT
TGAAAAAGTTCGGATGAAACAACAGCGAACAGAAGAACAATAACCCAAAGTATTTTGCGCAGCCAGGAAT
TAATTTTCAAATTAGGGCGAATAAATGTTGTGGGCAAAAGTTGCTGAAAGCCTCCAGGAAATCATACCAT
TCCCTGGGCCAAGTCTCAATACTCAATTTACATAATTACGCACATTTGACGGAAACTTATAAATATTTAT
GCCGATGCTTAAAATTTAAGTTACTTGTTGGTTGTGCAGACGACAAAATGTAAAAAAAGGGGGCAGATTT
CTAGGATAAAACCAGCGGGGCTGAGAAATAAGGTCTACAATTAATTCACAGGCAGGAAAGTGAGTTCATG
TACAAGTAAACTCTTCAGATATTTAAATATCTTAAAATAATGATATAGGAAGATTTTTCTAACCCATAAT
CAGCAAAATGGGAACTTTATGGTACGCAGTGTTTGCAACTGCTTAGATAAATGTGTTATAATAATTCCTT
GGTTATCTACAGTGAATCACATCACAACACTCATTACTCAAACAATAACCATTCGCTATCTGTGGCGACT
AAAAGTGTTTTATAAGCACGTTTTTAAATGCATCAGCAGTTGCGACCTCATGTAGCCTTTTACTGCGAAT

CGAAAAGACTACGCCATCGAAAGCAATTTCTATAAAAATAAAGAAACCCAAGTGAAAAGTGAATTACTCG
TAAATTATATTTGTCGCAGCTCGTTTTTGGGTAGAACTGAAATTTAACATGAATCTCACGTCAGTCGTTT
CGCCGAACGACCCAGTCAAACCAAAATCTCAAATTGTGCAATGTTGAATCATGAGCCATGAGCAGAGTTC
GGCTTGGATTTGGATTTGGATTGATGGACGGACGGGTGGTGGTGATGAGAAACACGGTGATGATGGATCG
GATCAGGGGATGCGGGCGGGTAATTGCCACCCAAATGAGCGACAGCCAGCAGGTGACCACTGGCCGCAGG
TCGAACTCGGAATACCATCCAATGCCGCATCTGTCCCTGAAACcaaagacactagaataacaagatgcgt
aacggccatacattggtttggcactatgcagccactttttttggtgacggccaaaattactctctttcggc
tcactcccgctgagagcgtaagaaatctaaaaatataatttgcttgcttgtgtgagtaaaaacaagagac
gagaacgcgtataagtgtgcgtgttgtgctagaagacgattttcgggccgaaatcaattctgatcgaaga
aacgaatttacatggtacatattagggtagtttttgccaatttcctagcaatatgataaaataaaaaaat
ttttaaaaattcgcgccctgaatattataattttaaagctttttaaatttgtttgttaaaatcgccgctc
gaattagctaccgtttacacatttatatttatgtttaattctaatttgtctctcatctgacaatttttta
aaagcgaaatattttttttgaaacacttttaatgttaatgttacatcatattaagtcaaatgatttaata
aatatactaaataagtaaatatgataactgtttattgcaaaagtaatatcaaagacactagaattattct
agtgtctttactttgttcatatcttgaggcacgaagtgcggacacaagcactcaacaatcatttccttat
taatttttcacacgccgcaagatgaatactctaatgacaaatattctaatataaagccatttttgaaatg
tatttttttgataatatgtacatagatttggctatttctaacctattttcaaataataataacgttaagg
caatgcaaaacaagaatttttcgcatggtgccaattgatcaaaaataatatagattgaaagtctaagaac
ttctgaggtgaagggcatattttgtcaaatttacaatgcatgagcatacgtgtgcacacatacagttgtc
tgctatcactttgtgcgttgaaaagagctgttcgctgtagcgctcttcgctctctcgctctctaacaaaa
attcgagagagcctggagccacctctagagccacggccaaaaaatcgtgtgccaaaaaatcgtatggcgt
tacgcatcttgttattctagggtctttgCTGAAACCCAAATGGATTCACACTTTTCGGGTGTCCCATTGC
ACTTGCAGTGAATCAAATGAGTGCCGAGCATCGCTTTCAGTACTTTTCCATGTATTTCCACTCGAATCAG
TTGTGCATGAAACTATGTTTCTGATATCTCTTGGCGAAAGCGGATAAGTGTGTGCATATTAATTAAATAA
ATAAGATTCAAGGTGATTACATGGATGGATTTACTCTTTGTAGCAAAACTATAAAGAGTCTATAAAGAAA
GGTGACAGGTTAATCATTACATGTCGCAGTTTTTAGCTTATTTAAACTGTGTTAAATTAACAAATTGACA
AGAACAAGATGTTTATGCATACACCAAATGGCTGAGCTACTGACCATTAAGTCATATTTATTTCGCAGTA
TATTTAGAAAATCGAATACGATCTGCCTACTTTATGCTGTTCTTTCAAAATGTGAATCAATCTAATACTT
ACACATTCATATACGAATATATTCACATTCAATTAAGCACAATCCTCTTGCACTTCCTTTTTGCAGCCAC
AAATATGTCGAGCGATTCGAGTCGAAGGATCCAGGTGCCCGAGGAGCTGAAGGAGGTGCTGCTGCAGTTC
TCGATCTCCTTTCTGGTGGAACAACCGCCGGATGTGATCGATTACGCTGTGGAGTACTTCACCAAACTGC
AGTCGGAACGCCCGAGCGTCTCTCACACGGATCAAAGCACCGATGACCAGCTGAGCGTCAACTCACAGGA
TGCCGATGGGTAAGTGGAGTGTTCTACTTTATTCGAGCAGCTTCTTTAAGCTGCCGGAATCCGTTGTGCT
CCTCTTCtttttttttCTATTTTCGTTTACAAAGCAATTCGATTGCGTGTCCTGTCAACTTTTTCTTGGC
TTAACTGCCAGCCCAAAGTCCTCTACATTTTGCATTTTCTGCTGCTGCGTGTTGGAAAATTCCTGCCGGG
GATAGTTAAAAAGTGCAACTACATACACATACACATACTTTTTTCAGAGCTCTTTTCACCCAGTCCGTGC
TCCATTTCCGGCCAAGCGATTTTCCATGAACTACTGCGTAGTGGATGCAAATCTCTGTCAATTCGGTTCG
ACATATAGACAATCTGCTGGTGTAATTCCCCCGACAATTCTGGCCAATTTTTTTGGCCATGAATAGGTCG
GGGGAAATCAAGATTTATTTGGCTGGCCGAAATTGAAAAGTTTTTTCCGTGCCTCAGATGATTTTACCAT
CCTTTCGAGCTTTTTCCTGCGGCACGTGTTCAGATAATTAGCCTGGGCCACGCATATGGCCATAAATCGT
TGGGCTCCCACCACGAGCATACAAAATAAGAAATTAAATAATGATGCTGATGAAGCTTTGGCCGCGTTCA
ATTtgctcctgatcctgctccagattctgctcccgtttctgcCCCACTGGGTGGGCCTCCTCTGTCTGCG
GGCTTTTGTTCACAGCTTCCTTTTGCGTGTTTATCTTCCGGTTAATCAAACACGCAGCCCTCGAGGGAGG
AGGCGGACGACGCCATGTCAAGTGGGTGGACTGCCGGCCGGGCGGACTTGGGGTGGCAGATAAAATGGGG

CACACTGCTGCTGTCATTAAAAATCTATTAATTGTGCACACAGCAGACCGAAGAGCTGAGCCAACTTGCA
GCTGCCAATCCTTTTGCTACTTGAATACTAGATCCACTCACAGTCAGACTTTCAAAACGCCAACTAGCTT
GTGTCAGTTGAAGGGGCTTTAACTAGGTTGAAGGATTATATTATAAAGATTATACATCTATATCTTGCTT
AAAAAGTAACTGAATGATAAACTTGTACTTACTCTACTCATAAGTTACAACACAAAAGATCAAACTTTAA
AAGGTATTAAAGGAGATGCTTTCTCATATATACAAAATATATTTAGATAGCTTGCTGGAAACATATAGT
TAATTAATGGATACATTTAAATTGTGGAAAACTTTCCATTTGTAATCCATTTCGATAACTAGTGCCTGCT
AGAACCACCAAACTTCCAGCATATTCATTCCGGATTCAAAAGCATTATAGTTTTAGACCTTTGTCTCACT
ATTAACGTCTGGCTGCCATGGCCAACTTCATCCGCTTTGGATGCCCAGTGAAGCCAGCGAAATTGCTTCC
AGCTTCTGCCAGCAACTGAAGCGAAAATGGCACGCAAATAGCTACTGGCCACGCCCAGTCCCAGCCCAAC
CGCCCACTTACCAGGCTTTTCCGCCCTCTTGGATTTCCCATCGATCGGAATCTGTATTTTTGTGCTTTGA
TGTCTATTCGTGGCCGGAAACCTTGCAGAACAGTTTCTGTTTAACTTTGGGCTTTAAACTTGCTGAGACA
TTAATATCGCGCATACGCCGCGTAGGCCTTTGTGGCTCAATAAGgtgtgtgtgtgtgtgtgtgtgtgt
gtTTTGGCAGGTGGTCAAAGGTATGCGCTTGGCATGGTACATTTTATTAACACTCCCGTTGGCCACAGCA
TCAAACCGAGGTAAAAAGCAGGAGCAACGGGCGGATGGATGAGCGTATGGCCAGGCTATGGGCAGGCAGA
GGGTTATGTTTTGCAGGACTTGCTGGACCAGTGAAGCATACTGATGAACAGCTCAGCAGCCGCATACGCT
TCATCGGGCACACAGGGCGCATGACACGCCCCGTGGGAAGGGGGCGGTGGTTTATACTTGAGGGGTGCGG
GGGCTGGGCGGTTGTCGAGTGGAGTGAGTTACATTTCAACATTATTGACATGCCAGCAGCGTTTGTGGCA
GGACTCCAGGCTGGCACTCGGTGGCCCAACCTGAACGGAAATGCCATCTCTTTCCTTTCATCCATCCTAT
TTGCTCTGCAACGGAGCGTCCTCCTGTTGCGCTTAATAATAATGTGTATATGAATGCTTATATGAAAAAG
CTGAGGGAATGCTCATGTTAATCAAGACAAAGCAAAGTGGTGCTGCTATTTATAACGCAGCACGGAAAGA
ATTTAGATGGGTGTCATTGTTTGTACGGAATGATAATAATATGTACGATGGATATGCTTAAAATGTATTA
AACTAAAGCTTTGAAACGTCTTTTTAGCAGCTGTTTTCCTGTAAATAAGTGTTATTTCCAGTACAATAGA
TCGTTTTGTTTGCGAAACCAAGTGTTGTCCAACTGGGGGGTCTTTTCTCTCAGTGCTTAGCCAACAAAGC
GACAGACAGCTGGcacacacccacccacacacacacacacacacacacacacacaGGGAGCTGTATAA
ATAGACGGCCGACTAACACTGGACCGCTTGAGTGGCGTGTTGGGCTGCCTACTTACAGGCACTATTTCCG
CCTTGGTTTGACGTTTGCCTGTCATCAGCGACTCCAGCGGACGTGGATGGCGATGTGGTTGGTGATGCTG
CGATGCGGCGATGCTGTGCCTCTTGTGGCACCATTGGCACTTTCAGTCGCTCGCAGGACGTCACAAGGAT
AGCACGCACATCCAAAACCGTCCTTCAGTCGGTTTGTCACTCAGTTTTTGAGCCCGTTTGCCTGTTGGCC
AGGcattcatttattcactcattcatccattcattcattcCGGCTCACGTCATCAGCGTCGGAAGTTGTC
GTCCtatataaaaatatatatacatatatatCTCGATCTCTCGTTGCCGTCACTCGCAGTCGTTCTCGTT
GCTATTGTTTCACTCCGCTAATGAAATTAATGTAGACAACTTTTTATTTGCCATCCTTAATTGAATTCCC
CAGCTTATTTGCCTGAGTGCATGTGTGTTTGTGAAGCAAGAAACTTACTTAATTGAAATACTTTCGGTGC
ATCAATTACCAAATGATATTCAAAGTTTTGCCAGCGTACTGAGTTGACATCTAAAATCTTTCAAATCTAA
ATATTTCCCGTAAGCAAAACACTTTGCCTCAATTAGTTTTCGTTTTTGACCAAAGCAAGGATTTAGCTTA
GTATACAATCTGATTTCAATTTGAATACCAAAGAAGAAGTTGCACTTTATTTTAGAGTGTTAATTACTTT
TCAGTTGCAAAGAAAGATCTAAATCTAAAGATGATAAAGTTCAATCTTGTGATGGTCTCAAAACAACATC
GAATAAATATTTTGAAAAATATTGTGCGCAGTTTTGACTAGATAGTTCCATGTTGAAAAATCAAATATCC
CATAAATAAGGAAACGTAAGTTTATGTGATTGATAGACGTTTGCAAATCTTCTTCTAATTACCAGACTGT
AAATCAGAAATCTCAACATACCTTGCACATTCGGCTGCAATACAAACCAGAACAGATCACGGAGGCAATT
GCCATCCAAGATTTCGAACGTGGGCGTCTCTACTGCTCCACACACAATGCAAACAGACAGACTAGACGGA
CGAGATGGGTGTATATACATACAGGGCTTGGATAAATACTTATCTGGAGGGGAAATGGACGTGCACTTGT
CCCGTCTAGGTTTACAATTTTTGTGGCCTTTGGCGTTTAGGTAAACTTGAGGGAAATCCAGAATGCGTGC
CTGGGAAACTGTTGCGTAATTGCATCATTAGGTTGACACTCTAACTGGCAACTTGACACGACTGTGAGTG
CAAAACTGCGGGCTGCAGTGTAAACATTTTGTGCCAATCCAAATACAaaaccaaatctgaatccgaatca

gaaaccgaatcagcaacagaaCTATGTGATTTTGATGTGGTGGAACACACAGAACACACATATATACAAA
CATTTTCGACTGACTTGCTGAAGGCTTTGCCAAACATTCGAGTGCATAAATATTTATTACGCATACGCAG
CGTGTGACGGGAAAGCCAAGCGCTCGGTGCTTGCGCTGGATTTGTATGGtttttttcactcttttttttt
GCGTCTACATACATCATTCACCTTGGGTTACATACACACCCAACACATTTTTATTTCTACTTATTTTTAA
ACGTGTTCATGCCTAACTGGTTTTTATTTTCGCGCTCGTTGCAAACGATTTGAAATCGTAACTAAATTCG
GAAACACGCACAGAAAGCAGCACTAGTGCAATTGAAAATGTCCACTCAGGTGTCTGGTAATTTATTGGCT
GGTGAGTAGTGGCTTTGCTTTGCTTTGCTTGGCCAAAGCAAAAACCGAATTTCGTTGACCAAAAATACAT
ACAGTGTGTAGACAAAAACATGAGCACTTAAGGTGTTGTTGTGCTCGGTTTGAATGTATAATTACAGCAA
TCGGTTTTGCGGTCATGTGAACCTGGGCAAACATTTAAATATCTGTAGCAGGCAGGGAAGTGAAACGATC
AGCCTTGATTGCCTCGCATGTTTTAACAAATATTCTGTCAGCTGGACAATGCCTCATATTTGTGCTGTTT
CTGAAAGGCATTCTGCGAACTATAGTACATATCTGGGATTGTCACACGCAAAAAAAGGTCAAAGAGTTCG
CCAACCCATTCTAATTTTAGCAGTGACTCTGATGTTTTAAAGTGTGGAACACAATGGAAAACAGATTCAA
ATTGTTTTTTGTTTGGAAAAACAAACAAATAAATATGTTATAATTCTATAAAGGTACATCAAAGCTGTAT
CGAATTCAATTTCACTTAATAGCCGGGTAATAACTTTCTTTAAAaatatcttaatatctgataatatctt
aatatctGGAAATGAATCTTTTCCGGTGTAAAAGCCTGAAGCTGAGTGAAAAATTTGTGCAGATGCTTTC
TGGGTTTTCCGTACATGGCTCTAAATTCCATTGGATTATGCTGCCTGTCGTTGATATGCGAGCTCCTCTG
CAGTTTCATTTGAATTGCAATTGTGGCGCAGCATGAGATGTGCCAGACCAGCAGATTCCAGGCTGATGCC
AGTGCCCAGATAGCTGGGCATTTTGTCGTTCATTAAATGAAGATGTTGCCCTTCCATGGGAAACACTTTG
AGAGGCACTCAATAAACAATATATACCATATGTCTGATTTATGATTTCGTTTCGTTTCATTGTGATTCAG
CCGGAACCACCAGTGATGGCCAGCTCGCGTCGCAAATCAGTTTTCGCCGAGGCCTACGATCCGGAGGCGG
ATGACGACGATGATGGCGCCACCGCCGTGTTCCCCAAGACAGATGAGCAGCGCGCCCGGCTGGTCGAGTC
GGTGAAGAACGTCCTCCTCTTCCGATCCCTCGAGAAGGAGCAGG
>dme_circ_0003824_junction_seq
CTCTTCCGATCCCTCGAGAAGGAGCAGGCCAAAACAACAAAAGCAAGCTAAGGAAC
>dme_circ_0003825
GATAAACACGCTCAATGTAAAGTCCACACGCCCACACAACAGCAGCCAGCTGCTGGCCCACACAAAACAA
CCGTAGAACAACCAACCGAGCAACCAAACAATGTAAATCTTCGATCACGCCCACAGCAGCAGCAGCCACA
TCTCGAAAAGAAGGCAATGCCGCCCACATGCGCCCGGGACGCGGTTAGTGTTCCTCATGACCAATGCGAG
ATGGATCAGTTGTCCAGCAAGCTTAAGGAGAAGATCGAGGCGGAGACCAAAAACATAGAGGAGTTTATTG
ATAAGACTGTTACCGAAACCGTGAGCGGCATTGTAGAGTTCAAGAACGACCTGATGCGGGACATCGAGTT
CCCCAAG
>dme_circ_0003825_junction_seq
CCTGATGCGGGACATCGAGTTCCCCAAGGATAAACACGCTCAATGTAAAGTCCACA
>dme_circ_0003826
GTAAACTTTTTGATTTGCCAAATATCTGCTCTCTTCCTGGCCTCGCTATTTCGGTCGATGCTGCATCCTT
CGAAGGTTTCCAGCAAATTGCGCCACACATTCGCCCTATCGATTGGCCTGGCCTTCGGCTACTTTTGCTT
TGGTCAGCAGGCAATCCACATTGCCGGATTGCCGGCAATATGTTACATCGTTATCCGCACCCAAGATCCA
CGTATTGTCCAAAGGAGCCGTCCTGCTGGTAGCCATGAGTTATCTGCTGTGCGTACATTTGATGCGCCAG
CTTTACGACTACGGATCCTACGCCCTGGACATAACCGGGCCCCTTATGATCATCACTCAGAAGGTAACCA
GCCTGGCGTTCAGCATTCACGATGGGTTTGTGCGGGGCGATGAGGGAACTCACCAAAGCGCAACAGTACC
ACGCCATCCGCAAAATGCCCTCTGCTTTGGAGTACTTCTCCTACGTGTGGCACTTCCAAAGCATCCTAGC
CGGTCCTCTCGTTTTCTACAAGGATTATATCGAGTTTGTCGAAGGCTACAATTTGCTTAGTACTCCACCG
GGCAATGGTAAGTTTAATTATGTCACCTGACTTTCTATAATCACCGCAGTAATTTAATTAGCGATGTGTA
GGTATGTGAGCCAAGTACTCGACCTCACTCTCGTCTTGTTTATACGAATATAGTCTTGACGCTCTTTATT

CTCAATTCAGAAAATGAAAAAATATTGTACTGCATCAACACAAATTATGTGCACTTTAAAATCAAGTCTT
AAATTACAATTAATTGTGTTGttttttttttttttttCAGGGGAAATCTCGACAGCAGCAAGAGAGAAG
TAGTACTGGAACCTTCGCCTACTAAAGCGGTAATACGTAAAGTAGTTGGTAGTCTTGTGTGCGCTTTCAT
CTTCATGAAGTTCGTGAAAATATATCCCGTGAAAGACATGAAGGAGGATGACTTTATGAACAATACCAGC
ATGGTTTACAAGTACTGGTACGCCATGATGGCTACCACCTGCATACGCTTTAAGTATTATCACGCCTGGC
TCCTAGCGGATGCCATATGCAACAACTCAGGTCTAGGTTTCACCGGCTACGATAAGGATGGCAATTCCAA
GTGGGACCTGATATCTAACATCAATGTGCTGTCGTTCGAGGTTTTCAACGAACATGCGCGATGCCATCAA
CAACTGGAACTGCGGCACCAACCGCTGGCTGCGAACGCTTGTCTATGAACGAGTGCCCCAGCAGTACGGC
ACGCTCCTTACCTTCGCCCTCAGCGCCGTCTGGCACGGCTTTTATCCGGGCTACTACCTGACCTTCGCCA
CCGGCGCTGTCGTTGTAACGGCTGCGCGCACCGGCCGGCGACTCTTCCGTCATCGCTTCCAGAGCACGCA
GGTCACGCGAATGTTCTACGACATCCTCACCTGCCTGATCACACGCGTCGTACTGGGCTACGCCACATTC
CCATTCGTTCTGCTTGAATTTATGG
>dme_circ_0003826_junction_seq
TTCCCATTCGTTCTGCTTGAATTTATGGGTAAACTTTTTGATTTGCCAAATATCTG
>dme_circ_0003827
GTACTATGAATGCCGCGCTGGACGTCGGCTGTTGCGCAAGTGCCGCGCCGGTCTGTGGTACGATTCAGAG
GCAGGAGAATGCCTTCCACGTAGCCAGGTGTTGAACTGCCCGGCTCAACGTAACTGAGACACTTTAGCCG
ACTCGACGCAACTGGAATGGTTTCAGAGATTG
>dme_circ_0003827_junction_seq
GACGCAACTGGAATGGTTTCAGAGATTGGTACTATGAATGCCGCGCTGGACGTCGG
>dme_circ_0003828
CTACCATCCCGATCGGATCCAGTGCCATGTGCGCTGGTCGCGCGGATGGAAGTATAGTCCAGGACCCGAA
TCATTGCAGGAGGTACTATGAATGCCGCGCTGGACGTCGGCTGTTGCGCAAGTGCCGCGCCGGTCTGTGG
TACGATTCAGAGGCAGGAGAATGCCTTCCACGTAGCCAGGTGTTGAACTGCCCGGCTCAACGTAACTGAG
ACACTTTAGCCGACTCGACGCAACTGGAATGG
>dme_circ_0003828_junction_seq
TTTAGCCGACTCGACGCAACTGGAATGGCTACCATCCCGATCGGATCCAGTGCCAT
>dme_circ_0003829
GATTCAGAGCTCGGCGGTGGTTAAGGAATCGGCTCTGAAGACGGCGATGCGTGCCTTTAAAAGCAAACAT
CCAGAAGTGGCTCGCCAGGAGCTGATGCAGCTGACCCACACGACTAAACACCGCTGGTTTCCACGCGCCC
GCGACAGGAAGGCCAAGCAAACGCCGATGGACCGGCCGTACCTATA
>dme_circ_0003829_junction_seq
AAACGCCGATGGACCGGCCGTACCTATAGATTCAGAGCTCGGCGGTGGTTAAGGAA
>dme_circ_0003830
TATATTTTCAAGAACGTGCCATCTGGAAAGTACCTGTTGCAAGCTATCAACCTAGATTCCAAGTTGAAGC
TGCACTTGAGTCCTGAGCTGTTGGAACTAGAAGTGGGCAAGGATACGCTGCAAATCAAGGACGAATTTAA
GATCACAGGCTTCACCGTTTCTGGTCGAGTTCTGACCTCCGCCGGAGGGGAACCGCTAAAGTCAGCTGTT
ATTAAAGTTAATGGAAAGAAGGTGGCCGAGACTGATGCCCAGGGCTCATATACGTTGGAAAACTTAAAGG
CAGGCACAGTCAACATCGAAGTGGAGTCCTCCCAGCTGCAGTTTTCTCCCCTGCAAGTCAAGGCTCAGAT
CAACACAGCTTCCCTGCCCACCATTGTGCCATCTGCTTATGAGGTGTGCGGAAAAGTAGTGTCGCCGAAA
TCACATAATGTGGGACTTACCAAGATCGGCTCCACCTTCCACTCGTCGGCTTCCACAAACGCGGAGACCG
GAAACTGGTGCGCCTTTCTTCCTGTTGGCAAATATACCATTGAAGTGCTGACGACGGATGCTGACAAGGC
CGCTGGAGTGCAGTTCTTCCCTGTTCAGCAGCAGACGGAGGTTCGCGATGCACCCGTCAATGGCATAACC
TTTTCCCAGCTGCGCGCAAAGATTCGCGGCGAACTGCAGTGTCTGCCTGATGCTACCGCCACCTGCACTT

CCGCTGAGGTCACCCTGCAGGCCTTAGATGCCACCGGTCAGCCCACGGAGAACAAGTGGAAGGCCAGGGC
ACATCGGTAAGTAATTATTATTTAGTTTATGCGTACAAGTACAGCAATTTAACCATAGAAAATATAGGTT
TAAATTTAATTATCCTACTTACAAGAAATAACATGCAGTTTTGAGATAACCTATCTTAGAAGGTTTAAAA
ATTATTCAGCAGAAATGGATATGTGCTAATACTTCTAGTTTCAAAACCATTATTATATTTAAATTAAAAA
AGTATCACGAAGTTCACTAGGTAAGAATATTAGTTGACACGGATTTCGTTACCAGTGGCCGCTGATAAGT
AATAAGATTACAACAGTAGATAAAGTATCTTTGGCGTGGTGGTTCTGTTTTAATTAAGATGGTCTTCCGA
CTAACCATGTATCATCCAAAATACCCTATCATCAATCTTATGGTTTTGGATTGTAGGGTGGCAAATACGT
TTTTAAGGATATGCTTCCCGGTCCCTATGAACTTACAATTCCCCAGGGAAATCTCTGCTACGAATCGACC
CGCGTGTTCCTGAACGTTGCTTCTGCTGAAGAAGATGCTCCCCCATTTGTACACAAGGGCTACGAGGTGT
CCATTATCTCTAGCCATCGGGCACTGGATGAAATACACCCACGTCACTGGACCTTCTGAGCCAAAGGCAC
CTACTGAATCGCTGAAGATTGCGTCAGGAGTGAATACCTTTTGCGTGAAGAAGTATGGTAGCTACGATTT
TAAGCTAGAGGGCTGCCACACGTATGATGAATCGTTACCCAGCAAATTCATTACACCAGAACCAGATCAG
TTGCAAACACTAATCATTAATGCAGTGGCCCACAAGACTGGCGTTCGAGTTCTGTCTACCGAACCCACTG
CTGATTCCATTAAGCTTGTGCTCGAATCTGAATCGCTGGGTCAGGAGGTGATCACACCTGTTGCTGAGTC
CCACAAAGTAGATGGCAAGTTCGCCTACCGCTATGACACGTATTTGAAACCGGAACAGGTACTTCGTATT
ACACCAGTGAGCGATGTCCTACTGTTTGCGCCCCAGCAACACGAAATTGTTGGCAGCAGCGATTGTGTAG
ATATTGCCTTCAACTTTGTGGCTACCAGAGGTTTGATTCTACGCGGAAAGGTTGTTCCTGCCATTAAGGA
TGCAAAGATCACACTGTCTTTCCCTGATCAGCCTGAGTTGGAGAGCCTGGAAGTACTCACGTCCGTCACC
GGAGAATTTAAGTTTGGCCCAATTGAAGAGTCTCTTGCTTTTGACCTTAAGGCCGAAAAGGAGTCGTACG
TTTTCAGCGACTACAACCGCCAGACAGCTTCGTTTAGTGCTCACAAACTCTGCGAGATTTCGGTGGTTGT
CAAGGATGAGGCTGGTCAGACATTGGGCGGAGTCCTCCTTTCGCTGAGCGGTGGGGAGAGCTATCGCAAG
AACCTCGTCACCGGCGATAATGGAGCTATTAACTTCCACTCCCTTTCGCCATCGCAGTACTACCTGCGAC
CCATGATGAAGGAGTACAAATTCGAGCCCAACTCAAAAATGATTGACATTAAGGATGGCGAGACTGTTTC
TGTTACCCTTGTGTGGCAAGCGTTTCGCTTACTCGATCTTTGGCACAGTGAGCTCCCTAAACGGCGATCC
CTTCGCCGGCGTTAATGTTCAGGCCACAGCCGATAATTCGTGCCCCCAGCAGCCGGAGGAGGCCACCAGC
GAGGCGAATGGACAGTATCGCATCCGAGGTTTGCAACCTGGCTGTAGTTACTCCGTTCGCGTCGTTCCCG
ACAAGGAAATCGTAGAGCGATCTATTCCTGCCCAGCACACGGTTAAGGTGGCCAACGAGGATGTGCGTGA
CATAAATCTGGTGGCTATTAGCCCCTTGAAGATTGTAGACATCACTGCTAGGGTGACGGCCACACTCAAT
GAACACTACAAGACGCTGCGCATTGTGATGTACCGCAAAGGAAACTCGGATTCTCCTGTGTTCTCGCAAC
GTGTGGGCACCCCGGTGAATCCTAAAGCAAGACTGAACCCTGGTATCACCGTCTTCTTCCCTCGCATTCC
TCTGGACGGCAAGAGCTACGTGGTAGAACTGCAGTCCACCCTCTCCGATAAAACATATACCTACAAGCTG
CCATCGACTACGTTTGTGGCTGACAGGGGTTCCGTGTTCGTTGAGTTGGAGTTCAAGCCGGAGGTGCGCG
CCGCCGAAGCGGATCTCAACCAGAATTCGATTTCGGCTCTGGTGCTCATCGCCTTGGTGGCCATTGCCTT
CTTTAAGCAGGATCTGGCCACCAGTTTCCTCAGCTTCGTGTGGAGCAAGCTTAACGATGTCGCCGCCGAT
CTCGCCCAGCGACAGAAAAGCAAGACACAGGTGCGCAAGAACGAGCCAATCAACCAGCGCGAGATTGAAC
AGATGGCCGACCAGATAAATGCCATCAAGAAGAAAAAGACCAAGAAGATTTAGTTCCGACTGATAGTCGC
ATTAGTTGTTCAATTTAGTCACATTTCGAGG
>dme_circ_0003830_junction_seq
AGTTGTTCAATTTAGTCACATTTCGAGGTATATTTTCAAGAACGTGCCATCTGGAA
>dme_circ_0003831
GTTAATAGCTCCATTATCGCCGGTGACGAGGTTCTTGCGATAGCTCTCCCCACCGCTCAGCGAAAGGAGG
ACTCCGCCCAATGTCTGACCAGCCTCATCCTTGACAACCACCGAAATCTCGCAGAGTTTGTGAGCACTAA
ACGAAGCTGTCTGGCGGTTGTAGTCGCTGAAAACGTACGACTCCTTTTCGGCCTTAAGGTCAAAAGCAAG
AGACTCTTCAATTGGGCCAAACTTAAATTCTCCGGTGACGGAC

>dme_circ_0003831_junction_seq
GCCAAACTTAAATTCTCCGGTGACGGACGTTAATAGCTCCATTATCGCCGGTGACG
>dme_circ_0003832
GTGGAGTTGAACTTCGATGGCAAGACCGACGTTTGCAGCCAGGGTCGCGATGTGAACTTTGTGTTCAAGG
GCTTTGGCATCACCGGTCAGGTGGCTCTCGCCGCCGGCGGCGGTGCCCGAGGCGTAGACG
>dme_circ_0003832_junction_seq
GCCGGCGGCGGTGCCCGAGGCGTAGACGGTGGAGTTGAACTTCGATGGCAAGACCG
>dme_circ_0003833
GCTGAGGATGCACTGTCCGTCTCGCCCTCGCCCTCAGCAGTGGTCTCAGATGTGGGCGTGGTGCCGGTAT
GTGCATTGTTGAGTTTATTTAACATATCGGTGTAGTCCTCGTAGTCTGCGCCAACATCATCCACAGAGCT
ATAGCTAACGTCGTCGTCCTCGTCGTCCGCAATGGAATCCAGACCATCGCCATCGTCGTCG
>dme_circ_0003833_junction_seq
GGAATCCAGACCATCGCCATCGTCGTCGGCTGAGGATGCACTGTCCGTCTCGCCCT
>dme_circ_0003834
GCCGTAAGAGGTGAATTAACTAATTAATAAGTGCTTGCTCGTTTAAAACTAAGTTAAAAGTCGTGTGCTG
AGAGCCCAAGGATTACGCATATATTCGATATTCGCTTGAAAACCCAGGCGCAGTAAGTAGTTTCACCTAG
TGCGTCGTGTGTCGGCTTGCGACCGCCATATTGTCGGCGGATAAAATTTTCCCCGAATAAGTGCGCCCAC
GCACTTTATCGATTCCGTTGGCCTCCTAATTGGATGCCACCTGCCCCTTGGGCTCTCCGCATTTGATTGC
GTGCGGTACTGGCCTTGCGAAAATCCCATTACAATTGGAAATGGACGTAGCAATTTTTCATTACCGCAAC
CAAATAGGACGCCGATGACGTAATTTTTTCCGTCTTGTTCTTTGTGAGCTGTAGTAAAATGTAGAGATGA
GCGCGTGAGCATGTCTTCGCCACGCACGAGTTGAATTGCAATACACTTACACGATTATTTAGAAAATTCC
TCGTACACCTAATTACCGttttttttttttAATGACTTGAACATTATGCTTAATGTAATACAAAAACATC
ATAGAATATTAAATATATTTTCTTATTGTATAATTAACAATAGAAGAAGACTCAAAAGAATATACTCTAA
TTTTGTCGAATTATCCCATATTGACAGGACTAAGTATAATTAGAACTAGAAATTGATTGTTAAATCCTCG
CCTTAACTTCCGGCCCACAATCGTAGGCCCTGCCTCACCTCACGAAAATCATAAGTACGTGGATATCTCG
TTTTTATGTGTGAGTGCACCAATTCAATGAAATCCAAATTAGAATTAAGGCAATTTACCGCCACCCCCTT
ATCAATGGCATACATCTCCCGATTAGTTTCCTCCTTGCGGAGGCCATAAAGTCAAGATAAAAGCGTAATC
TGGCAGCGAGATAAGCGCTCGAAAGTTTCAACAGGTTGGAAACGAACTATAAACTATTACTTCATAAAAC
GATGATAACACAAAGACTTGACAATAAAAGCTGGCTCATTTTAAAGCTCTCTATCTACAAGTTCTGAGAC
GCCTATTAATTAGTATATTTTATCTGAAAGTTTGGTTTCCAAGTTTTACCTGCACACCTGGCAACATTAA
TTTATACAGCAGGTTGGCAACCCTGAAACTCTCTCACTTGTCGCACTTTCTCGCTCTCGCCTGAGCCAGC
CCCATATGCTCCCTCTCATTCTGCGGCGGCGCATGTGCCCCTGCTAGAGTGTGCGTGGGGAGAGAGCGCA
GAAACACAGATACAGCGGCGCGGCGAATGTGTGCAACCTTTGCCGTCCGTCATTCTCATTCGTTCGTTGG
TAGTGTTCGTTGTCGTTTCAGTTTCAGTTAATCGAGAATGTAAGTAACATTTTTGCTGGCCCAGAGTGCC
TTGTACCTATGTGTGTGCGCCTGCCAGTGTGTGTATGGAATTATAAAACGTAAAATTGCTAAAATATCCG
AAGAAAAAACACGTGTAATGCCGCCTATGAGCAATCACTTACATGCATCTATACTTCGTCCCGCAGAAAA
ATCGAAATACATAAATCAGACATCGATCCGAAGCCaaaaaaaaaaaatctacgcaaaagaataaagtgta
aatttgtgcaaaataaggaaaagaaaagaaaatgaaagaaaagaaCATTCCCGAGTTGCCCGAGTATTGG
TGCATGTATGTGATGCGGTGAGATTCGCTTGTAGCTTCGTATTTCTCATCATCATCAGCAGTTCATCGGT
TAAGTTGTAGGCGGCGGACATAGCCGACTTCTATATTTACGCTTATATAGCCAGCTATATGTACACACAG
TACGCCAAGATAGatatatatatatatatatCCAAGTTGATACTTGGATGGGGACACGCGGTTAGGTGGG
TTTCCTCGCCAAACTCAACACACTTGGCGGCCGCCTGTGGTAGATACATTTCGGCCTCTCTCTGCGGCGT
TCTCCGCTCTCTCCCTCCCTCCCACCTCGCTCCTCCTTTCTCCCGATTATTTTTATAATTTTGTAGCGCC
GCACTTGGAAACACGATTGGCGgccccaccaacaccatctcacccatccgcaccaccattaccaccacca

cccaGAACGCACCGACACACGCAGAGTCGCACGCACACAGCCCCAATAAAAGTTAGTAACTTTTTCGCCC
CATGTAGAGCCGCAGTAGCGGTATCTACAGTGTAGAGGAATTTCTACATCATCGGGGATTGGGTTCTTGC
TCGGCTCGGTCTCggggggtttggggggggggggggggTTCTTGAGCTACTGCGCATGCTGTCGGCCGGC
GCAGATAAGGTTTAAAGTACCAAGTATCCATCCGACACGGATTTCGCAGGATTTTCTAGCCCCGAAATTA
GCCTGACCATATGGGTGTTAAACCGTCCTCTAAGGTCTACGTATCTATAACTTCGAGATACCCACCTCCC
ACCTGTTTTTCCCCCTATTTACCAATTTATTCATTCAGGCAGATGAGCAATCTGCGTGGTTGAAACAAGT
TTTTCCTCCTTTGATTTATCCGATAAAAACTACAAACATATTTGTGACCATTTAGGGGAGTGTTACGTTA
ATTTACGTTACGCCTGGCTTAATGTTTTGGCATTTACCGAGCACAGGCAGAAAACATGCCAATTGACCTA
AAAACGCGTCGGGAGACCGCACCGCACCCTCCCCCTTCCATATTTTTGCTACGCCACGCCCCGCCGCCCT
TTTTGTTTTGACAAAGGGTCAATGCTTTTTCAATTTGGCGGTCGGTCGCAACACAAATAATAGCTGTTTT
CATATGGTTTAAACTTCAATTGGTTGGTTTTTAGGCAACTGCGCAGAAATCAGCATTTATAATTGCTTCT
GCCAATGTTACTGGGTCTTTGAAACTAGTTGTTAGCTTCAGTAAAGAACATTTGTATGGGGTTTCAAACA
TTTTGGAGACTATTTTTCACCTAACCATTTCAATGTAGCAACTATTCAGCGGTCGCTGCTAGCCAAGCTG
GACTGTAAATCCAATTGAACGCGGTATTTGTGGGATTGTTGCTGGTGCTTTTATGATGCCGATGTGTTGA
GATTTTCCATTTCAATCATCGGCTGGCTGCATTAGCTGGCATTCTTGCTTGGTTTTCGAGGTTTCCGTTG
CGCGGGCAAACAAAACAAttttttttttttttGTCCAGAGCCGCCAGTTGTCTAAAAATACCAGCAACGC
CCATACATATGCATTTCCACAGACCGATTTTCACCCCTCGTCCTACGCAGTTCTTGTTTCCAAGCTTTCT
CCAGGTCGAGCGGAAATTAATCGATTTTCCTTTCCCACGTCCCTTTGAAAATTCAAGCCGGAAATTGGTC
CTACATTGAAAGTTTGGATGATCCTATCGCAAGGAATTATAATTGGATTACGTGTGAGCGGATTAATGAT
TGTCGCCTGCTTTCCCGTGtttttatttggattttttttttctgtttgttttCTAATAATTACTGCACGT
TTAATCGATAGAAGTTCAGCGAGTGGGCAAGTAATTGAGTGCGTCACTGTGCGGAGAACACAAACAGACG
GTCTCTATGCACGTACATAAATATCTATCTTTGGCACACACACAAAAATATATAAAAACGCATTAAGGCG
GGCCGACTTGTGCTCCATTTCTCCCTCTCTCTTTTTATTCATGGTTTATTTTTATCGCTGTTGTCGAACT
TGTATTTTTTCACATCTATCTGCCAGATACTCTCTTTCCACCTTCGGCCTTCCACAAACCCATAAACTGG
GCTTCGAATAGTTGCATAGTTGCCACACGTGCGGCCATGTCCAGTGCCACATTGTTTTTGTCGCTGGCTT
TTTCCCATTTAAATTAGCCATTCCGTCAGCGATTAGTCAATAAAGACCTGCTGCGAATGCATGAGTCATC
GCAGCTTTGAATCCAACACTTTCGTCCAGCGGAAGCATGACAACAAACTAAATTCGTACGACCTTCCTAG
GAATACTCGCGCAAGTGCATTGCGGGTATATTGGACTTGTCTGAGTGACTCAGATAATATTATTAGTCAC
TCAAGTTCTTTAATTTATAAGGAAAAATCATTTTATAACTTTGTAGTCAAGCAAAACATCTTTTAAATTA
CTTTTAGAGCTAACGAATATAAAATATAGAATCGCGGCCTTCAATTAATTTAAAAGCATAAGCATCTATC
GAACCAAAAACATGGAATATCTTATAATGTCTATTATCCCCTATAAGGGGTGATCGTATATGGCAGTTGA
GTTCACTCCACAGAACATACAGTGCCACACAAAAATTCGCAGTGTTGTGCAACTGCTGGTTGCTGTGGAA
TTTTAGCTAAATATAACAGACAAGTAATCCGCAACAGAACTCTGTTAGTCAGTCAGGTGTTTGTTAAGAG
CTCACAGCCGAGAACCGTTAGGCGAGAACGAGATAACGGGTTAAAGTGGCCTCACCTACATTTCTCCTCC
ACTGAAAAACACACTAATGCTCCTGTTTACGTTCTAATTTATTGCAGAAAGCCATTGTTAGCAACTTTTG
AGGGGCATATCGAACTGTGTGGC
>dme_circ_0003834_junction_seq
TTTTGAGGGGCATATCGAACTGTGTGGCGCCGTAAGAGGTGAATTAACTAATTAAT
>dme_circ_0003835
GAAAGCCATTGTTAGCAACTTTTGAGGGGCATATCGAACTGTGTGGCGTATTTTATTAGTTTTGTGAAGT
AACCGaaaaaaaaaTCAAACAAAATGTCGACAGTCGATAAGGAAGAGCTGGTCCAGAAGGCTAAACTGGC
CGAGCAGTCAGAACGGGTAAGTGGTTCATCTTATAATAAAGTTCATCAACCTCACAGAACTTCAAAAGAA
CCCTACAAACTCATTTGGCAAAAGTTATAGTTGCACATAATATTTATTAATACGTAAAAAAGACATTCTT
GGAAGTTATAAGAACTCTAAGATTTAACATTGTTTTCTGTTTTATTAAATCCATTTAAAATCATCTTCTA

ATGAACAATATATGTATCATTTAAGTTGTTCTTCAAAATTTAAAGAATGCATATATTTCTGTGATCTACA
TGACTAAATATTTTCAATCACTAATGGAACATTTTCTATGCAGGGTCATCGTGGCGTGTCATCTCCTCCA
TTGAGCAGAAAACCGAAGCATCCGCTAGAAAACAGCAGCTCGCCCGTGAGTACAGAGAGCGTGTGGAGAA
GGAGCTGAGGGAAATCTGCTACGAAGTTTTG
>dme_circ_0003835_junction_seq
GCTGAGGGAAATCTGCTACGAAGTTTTGGAAAGCCATTGTTAGCAACTTTTGAGGG
>dme_circ_0003836
GAAAGCCATTGTTAGCAACTTTTGAGGGGCATATCGAACTGTGTGGCGTATTTTATTAGTTTTGTGAAGT
AACCGaaaaaaaaaTCAAACAAAATGTCGACAGTCGATAAGGAAGAGCTGGTCCAGAAGGCTAAACTGGC
CGAGCAGTCAGAACGGGTAAGTGGTTCATCTTATAATAAAGTTCATCAACCTCACAGAACTTCAAAAGAA
CCCTACAAACTCATTTGGCAAAAGTTATAGTTGCACATAATATTTATTAATACGTAAAAAAGACATTCTT
GGAAGTTATAAGAACTCTAAGATTTAACATTGTTTTCTGTTTTATTAAATCCATTTAAAATCATCTTCTA
ATGAACAATATATGTATCATTTAAGTTGTTCTTCAAAATTTAAAGAATGCATATATTTCTGTGATCTACA
TGACTAAATATTTTCAATCACTAATGGAACATTTTCTATGCAGGGTCATCGTGGCGTGTCATCTCCTCCA
TTGAGCAGAAAACCGAAGCATCCGCTAGAAAACAGCAGCTCGCCCGTGAGTACAGAGAGCGTGTGGAGAA
GGAGCTGAGGGAAATCTGCTACGAAGTTTTGGGTAAGTGGTGGTCCGCAAGTCTGCGCAAGTCTCTTTCT
GAAGGATTTATGGGGTTGTAATTGGTCCATCTAATTATTTTCATGCCCACAACTCACTTGGTTGTTCATC
TGCATTGACGGTGATGAAGATGGCAAACCTGTCAACCGCCTAAatatatatatatatatatatatgtata
AAGACCTCCGCCCTTGGCAAGCTCAGCTGTCCGAGGGTGTTGGTTGGATGGTTCAGTGGGTTTTCCAGAG
GGTGGGCCTGTTTTCTGGTCTTCCTTCTTGCTCTAGTGAAATATAAAAATTTGTTACAATCAATAAATCT
ATTTTTAAAATGAACATGTTCCATTTTCCCCCACACACAGAGGTTTTCGCTTGTGTGTATATGAAATTTA
TATTGCCGTTCACGGATCAGAAAATGACCATGGCCAGGGTTGAACTCGGGGGTTTCCCCATTCGCTTTTC
TTTGATTGGGGCTTTTATATTGGATTAAACTTAACTGGTGCTTGATGGATTTTAAAATGGAAAATGATTT
AGAACAAGGTTTACAATAAGCCTGGCGGCTTACTTGGTTAATTGTTGCGTTTGGTTTTCGAAATTGATAT
GAAATTAATTGGAGTACTTGTTTCCTTTGCTTACAGGGACTTCTGGACAAATACCTTATTCCAAAAGCCA
GCAATCCCGAGAGCAAGGTGTTTTACCTGAAGATGAAGGGTGATTACTACAGGTATTTAGCCGAGGTTGC
CACAGGAGATGCACGCAACA
>dme_circ_0003836_junction_seq
GAGGTTGCCACAGGAGATGCACGCAACAGAAAGCCATTGTTAGCAACTTTTGAGGG
>dme_circ_0003837
ATTTTTCGCAAGGAAAGATATGATAAAATAAGAAACAGTATCTTAGGCGACATTTTGACATCTATTGATC
GGTTACCTTCATCaaaaaaaaaTCTCCGCCCATATAAACTCACAAACAATGGCGGAAATATTCAAATGGT
TACAGCACTTGTATTGCAATTGATTCAATGCGCCACGATTTTACCAGATTCCCTTTGTGATAATGGCAAA
TTTAGTAACAAACCCCAAGAGGGAAATACTTTTGATGAAGAAGGAAAAAAATTGCTTCAGCCTAGTCAGG
ATCTACTCGTTTTACAAAAATATGATGTAGCTGTCAGCATTGGTGGAAATTTTTTAACAACTTTCCTTAA
CAAATGCAAATCTCGGTCTAATGAAACTGATTTTCGACCTCTGTTTGAAAATTTTATTCATGATTTACTG
GCCACCGTGAATAAGCCTGAATGGCCTGCATCTGAATTGTTGCTTTCGCTGTTGGGAACTATGCTGGTTC
GCTACGTTTCTGATAAGGGTATAGAACAAAGCATTCGCTTGGTATCATTGGATTACCTGGGCATAGTAGC
TGCTCGATTGCGAAAAGACACAGTGGAATCTCGGTGTAGAGTTAACATTATTGATTCAATGATTCAATCC
ATCAAATTAGAGCAAGAAAAGGAGGGAGATGTAACATCAAACAATGGATCAATTTGATCTAGAACCCGAG
GAGCAGCGAACGGATTTTTTGCAAAAGATTCTTCTCGACTTTCTAGCTGTAAATGCTCAAGAAGAAAACT
TAATCTGGGACTATGCTAGACATTTTTATTTGGCACAATGGTATCGGGATGTCATATATCAAAGACGTCG
GATAAATGATGGCAAAAAAGGTTTGGCTTTCAGGAAGTCCAAAATTCGTAATAATCGGAGGACTAATGG
>dme_circ_0003837_junction_seq

AAATTCGTAATAATCGGAGGACTAATGGATTTTTCGCAAGGAAAGATATGATAAAA
>dme_circ_0003838
GAAACATTTGCTTAGTTGTTTGGTTAGTTGTCGCCGCCCTCCTGTGGCTCAGCTTCGTCGCCTTGGGTGT
CGGACGTCCAGAGAGTCAGGTTGTCCCTCAACAGCTGCATGATGAGTGTCGAGTCCTTGTAGGAGTCCTC
GTTCAGTGTGTCCAGCTCGGCTATCGCATCATCGAACGCCTGG
>dme_circ_0003838_junction_seq
CTCGGCTATCGCATCATCGAACGCCTGGGAAACATTTGCTTAGTTGTTTGGTTAGT
>dme_circ_0003839
CAGTTGCAGATCTTAAGCTACATTTGAGTCGGATTGGCCGGCATCTGTCCAAGCATTTGACCATCTGTAT
ACTGGATTCGCTTGCAACCGGAACCCGGCTAAAAACTACCCGTGACAATCGGGAAAACGGCCTCAAATGC
CGGCCCATCGGCCCCAAATGAACTGTGAAGCATTCTGCCCTCAATTCGTATGTTAATG
>dme_circ_0003839_junction_seq
CATTCTGCCCTCAATTCGTATGTTAATGCAGTTGCAGATCTTAAGCTACATTTGAG
>dme_circ_0003840
GGCTACCAGGGCATGGTGGATGGTGGCGACTGCATCCAGGAAGCCAACTGGGCCTCAGTCTCATCCATTA
TCCATCGTGGTGGCACCATCATTGGCTCCGCCCGTTGCCAGGACTTCCGTGAGCGTCAGGGTCGCTTGAA
AGCCGCTAACAACCTGATTCAGCGAGGAATCACCAATCTGGTG
>dme_circ_0003840_junction_seq
GATTCAGCGAGGAATCACCAATCTGGTGGGCTACCAGGGCATGGTGGATGGTGGCG
>dme_circ_0003841
GCTGTACTGGAGATGGCATCGATTGCCTCGATGATGCGATGTAGTGCCGTGTCCGTGCCGATGGTCATGT
CAGTGCCACAGAAATCGTTGTCGATGGAGCCAACCTACAAAAAGATTCATCCATAAGAAAATATATCCAA
GGAATACTCTGTGTAGCATACCAATCCAACGATGTGCAGGACATTGAACTTCTCCTGCTGCTCGGTGGTA
ATGGTCTTGTTCTTGACCAGCTCGTCCAACAGGCTGGACCACTCCTGACGGAACAGATTGGCGCCCGTGA
GGGAGCCATCGCCTCCAATGACCACCAGATTGGTGATTCCTCGCTGAATCAGGTTGTTAGCGGCTTTCAA
GCGACCCTGACGCTCACGGAAG
>dme_circ_0003841_junction_seq
TTTCAAGCGACCCTGACGCTCACGGAAGGCTGTACTGGAGATGGCATCGATTGCCT
>dme_circ_0003842
GATCCAAAACCGCCACTTCATCAGCGGCTACCACCATCAGCATATTGGATCGGACTATGAGCAAGTGATC
AACTTTGTTGACTCGCCGCCAAACTCAGAGGAATCGTGGACAGGTAAGGCTGCCGCTATCAGTGCACCCA
GATCAGTCAGCTCACCTCAGCTCATCCCCATCACCATCAACTTCCACATCTATCGATCTCGTAGAGAGCA
GCTCGCTAATCGAACAAATTACGATTGTCGTAGACGGACAGTCGAAGGACTCGCCGGGACCGCAGATCAT
CGACGTGCAGACCATTTACCTGAACAGCGGCTCACGCAAAAGACGAATGGATTGGGACTCTTTGGACATT
GGCCAAAGTGAGAACTCACCCACAGCATCGCAGAGCGGCGACTTGCCCTCCAAAGTGGCCCATCAGGAGA
AGGAAAAGCACAAGCGCGAGAAGCACTCGG
>dme_circ_0003842_junction_seq
GAAAAGCACAAGCGCGAGAAGCACTCGGGATCCAAAACCGCCACTTCATCAGCGGC
>dme_circ_0003843
GGTGGAAGCACTTGTGTGGGGGCACCCGACCATATAATTTGGACTGCAATCTAGAGCTCGGCCCTCTCTT
TTTTTGATGGCTTATCTATCATTCGGCATGCGGCACTTGGTGTAGATAAGGCTAAATGCTAGCGTTTGGC
CGTACAACAATCCGCTATTAGGCAAACCACAACGATT
>dme_circ_0003843_junction_seq
ATCCGCTATTAGGCAAACCACAACGATTGGTGGAAGCACTTGTGTGGGGGCACCCG

>dme_circ_0003844
GGTCGCAGCAGCTGGAGCGATGATATCGGCTTCGATCTGAACGCCGAGTTTAACAGCAACTCATATTTGA
ACAAGTAAGTTAATCCCCCCATCCCGTTCACCAAGGGAACTAAATTCAATTTGAGCTAATTTACGTTTTA
CCACCGCGATCAGTGAGAACTTCCTGTCGTTCTCGCCCACGCTGACCACCCTGAAGCAGGAGCCCCAGAC
GGATCAGATCAAGCCGAGCCCCAAGGTATCCCTGGATAATGCCTCCGCATCGCCCTCCATCGCCATTGCC
AAGTTGGATGAGGTCCAGAACTCGCCGTCGCAGGCTATTTCTGGCCAAGACTCGGCTAACGGATCGGGAT
CGGCCGCAAACGGCAAGCATGATGTAAACTCCGGACTCGCCTGCGGGTGTGGCTCTCCTCAGGGTTCACC
ACTAGCCAACGCCGAGTATGAGTTGAACGAAAAGGGAAAGCCCCAGCAACTTAGCGTTTTGGATCCTGCT
AAAATCGAGATCGGTTCGGCCAACGGAGCTACCCATGCCGAGGATCACAAGATTTCAGTACATTTTGGCA
GCGGCCACTTCGATCGCCACCAAGAACAATGAGGAGACTTTGACGTACCTCAACCAAGGCCAGAGCTACG
AAATAAAGCTGAAGAAGATCGGAGACCTGTCCCTCTATAGGGATAAGATACTGAAGGGTGAGTCCAAAGT
CTCTGTTCGCAGCCTCCAATTGATTCGGAGAGTCATGTGATGCGATGAATTATTTGACTGTCGAGTGAGC
GGCTTGGTCATCCCGTCTGTGTGACCGCATGTGTGTATATGGAGGGCTCAATTGATTTTAATAAGCCCCA
ATTAAGCCGAGCGACTTGAGAGATGCGAGGCATTACCATTCCATTGTACATGTGAAATCTGCGTGTGCGA
ATAAACTTTCCAAATGACTTCACTTCTTGCCACTTCTCACAAAACCCCCGATTTTCCCCTCCGATTCCCT
CTTGCAGGAGCGTGATCAAGATCTGTTTCCACGAGCGCCGCCTGCAGTTCATGGAGCGCGAACAGATGCA
GCAATGGCAGCAATCCCGCCCAGGCGAACGCATCATTGAGGTGGACGTACCGCTTTCGTACGGCCTCTGC
CACGTGTCGCAGCCGTTGAGCTCCGGCTCACTGAACACTGTCGAGATCTTCTGGGACCCACTGAAGGAGG
TCGGCGTCTACATCAAGGTCAACTGCATTTCAACCGAATTTACCCCAAAGAAGCACGGCGGCGAGAAGGG
TGTGCCCTTCCGACTGCAAATTGAAACCTATATAGAAAACACAAACAGTGCCACCGCCAGCGGCTCGGGC
GGCAGCAACAACAGCGCCATCGCCAGTGGCAGCGGAAGCAGTGGCTCCGCTGCACCAGCATCTCCGGAAC
GGACACCCAGTGCCGGCAGCAATGGTAAACAGGCGGTCCACGCAGCTGCCTGCCAGATCAAGGGTGAGTG
AGAAGAAGACCTGAACAAGATCACATTGTCCGCTTAACGGAGAGAGTCTCTTATTTTGTCCGTTCAGAGG
AAACAGGATCCTGTTTTTAAAGATAGTAAGCCATTAATGTTCAGTGAGCTATCTATGCTTTTAATAGTAT
TCATTAAATTTATACACATATATGTTCATATTTATGATACATATGTTTAATTGAAGTGTTAAATGTATGT
TGGCCGTTGATATGATCTTAAAAGAGAATGACCTATTTTAATTTTGAGATATGGCCAACTGCGGTCCGAA
AATAGCCAATGGAATTTTACGGAAGAGAACGTAATCAGAATCTACTCTGCATTCACAAACAACAAGTACA
GTAAAAAGTTTATAATTTGTTTGGGCCATTAAAATTATCTACACATGTATTCATGTCTGTGACTTACAGA
TATACCAAACAATGAAAACAAGAATATTATTTAAGGCTAAATCTTTATGTGAAGGTTTTAAGCTGTTAAC
TGTTAAAGTGGCAAAGTACAAATAGCTCTTTTGATAATAAATGTGTGATAATCAAAATTACCAATTATCA
TATTCCGTGCAATCCTTTTAGAACGCTTATATTAAACGCTTATTCTTACAGGGTTTTTAAGCTAAAAGGA
GCTGATCGCAAACATAAACAGGATCGGGAGAAGATCCAGAAGCGACCGCAATCGGAGCAGGAGAAGTTCC
AGCCCAGCTACGAGTGTACCATCATGAACGATATATCCCTGGACCTGGTGATGTCCGCCACCACAACCGG
CTGCTACAGTCCCGAATA
>dme_circ_0003844_junction_seq
CCACAACCGGCTGCTACAGTCCCGAATAGGTCGCAGCAGCTGGAGCGATGATATCG
>dme_circ_0003845
GCATTTACACGCAGTAAGATGGCACATCGAAACCGTTTTCTATATAGGTTTTAATTTGTATGAGATTTCG
CGCAGCAATTTTGAACAAGTAAAAAGCAAGCAGCCTCTAACCCTGTTCCCTAAAATCTTTGTTAAAGAAC
TAGTGCAAAATATTTAAGTTTCGTTTTCAGTTCTCATTTTAA
>dme_circ_0003845_junction_seq
TAAGTTTCGTTTTCAGTTCTCATTTTAAGCATTTACACGCAGTAAGATGGCACATC
>dme_circ_0003846
CCCCTGCACCACGCCCACAACCTGGCGATGATCGACGTGTGGAAACGGACCGCGGCGCTCAGTGTCCATC

CGGAGCAGGTGACCACCACACCCTTGCTGAATCCCTCGGCGCCGGACAGCTCCGAGCTGCGATCCCTGAC
AGCCACGCAAACGCCTACCACCACAACCGATGCGATCGCCACACAAACCCCCATGCCGCCGCATGTGCAG
CATACAGCGACCAATACAAAGGTATTACCGCAGTCTAATTAATTCCATTGGAAGTATGTGATGCAAATTT
AACCACTTTGTACAGTCTTCCGTGCTTAACTCATATCAGTTGGGGTCTGAGAAGCAGCAGCACAATGATG
TTTTTATGACTGAATTAGAGAATAGAACCAAAAAACGTGTTACGTTAAGG
>dme_circ_0003846_junction_seq
TAGAACCAAAAAACGTGTTACGTTAAGGCCCCTGCACCACGCCCACAACCTGGCGA
>dme_circ_0003847
ATAAGATATGACTCATAGAGCGAACTCGAAGCACCCCAACACAGGCACACGTCCACGGATTGGGGATAGT
GGGGAGCTTTCGCGATCCCGTTGTCGATAGCTGACTTCAGCACTGCAATTATCTCGCTCGAGGGGAGCTT
TGAAAAGTTGCCGGAAAAGAGACCTGAGAACTGGCTCTCCCGCTTCCCACATACAATTAGCTGAACAATA
AGTATCTATGATGG
>dme_circ_0003847_junction_seq
TTAGCTGAACAATAAGTATCTATGATGGATAAGATATGACTCATAGAGCGAACTCG
>dme_circ_0003848
GATACGGAAGCATGTAGCCCATACGTCGCGCCTCACTTCTTGGCATCGATGATGATCTCGTCGCCCGACT
GGATGTTGTAGCTGTACAACTGCTTGTTGGGGTACCTCATCTCCTCCGGCCCGAATTCCTTGTAGTCCTG
ATCCAGATAGTAGAGGCGCATCTTGTTGGGCGCCAGACCCACCAGCTTCTCCAGCTTCACCTTCAAATCG
TTGACGGTCAGATAGATGTCCACGAACCGGCTCTCGCTCACATCGTTGTAGGTGAACAGGACCTTGACCC
GCTTGTCTGGTTTCAGGCTCACATTGACCAAGGGATCCAGCTTGCCGTGGATCTGCAGAAGCTCTTGGTA
GCGCGCAGGCCGTTCCTCCTCCGGCTTGTCCATATAGTATCTGACAAAAGCTCGCTCCGAGTCGACGCGC
TCATCGCTACTGATCTTGCCGCCTCCGTTGAGCATCTCCACGTTGGGCAGCCTGGCTATCAGGAGCTGCC
TTCTCTCGTGCTCCGTGCACTCCAGACTTTCCCAGAGGGGCCAGTGTTTCACACGGAGGTTGCGTAGCTC
GCTAAACTTGGCCAGCTCATCGATGGCCGCCCAGCTATCCAGTTGCGCGGAACTTAGGTTCAAAAGCCTC
AGGCTGGGGAAGTATCTATGTGTCTCGCTGCTCTCCTCGGCCTGCAGCGATTTGATGGGACAGTCTGCCA
GAACGAGGGCCTCGAGGTTGGGAAACAGCCGGCCCAGCCGGCAGATTTCCTGCCAGTGCTCGACGGGATT
ACCGGTGAAATGCAGGGTCTTTAGAGCAGGATGTGCTTTG
>dme_circ_0003848_junction_seq
CAGGGTCTTTAGAGCAGGATGTGCTTTGGATACGGAAGCATGTAGCCCATACGTCG
>dme_circ_0003849
TGGGCGTGGGAGTGGCCCCGCCGCTGAGACTGCTGCTCTCCGTCGAGGAGTTATTGCCCAGGGACTGGGA
CTGCGAGGAGTAGCGCTCCAGCAGCAGCTGGCTGAAGGTCTTTGCCGGCCAGTCCGGATCCTCCGGCGAT
TCCCGGGTGGGCGTGGCTATGGGTGTCGGCAGGCGAAAGATGCTGCTGCTGAAGGAGGCACTGGTCTGGG
CGAGATTCTCGAGGGGCTCCAGAAACCGCTTGCCATGGTAGACATTGCAGGTGGTGACCGGGTTCAGGAG
GCTATCGACGGACACGGTGACATTGCGATTGGGCAGGGGCATGGCAAGGGGCATTGAAAG
>dme_circ_0003849_junction_seq
GCAGGGGCATGGCAAGGGGCATTGAAAGTGGGCGTGGGAGTGGCCCCGCCGCTGAG
>dme_circ_0003850
GAAAATGTATCAATAGGGATTTTGACTGTATTATTTGTACTGGAAGCACGACTCTCTAATACACATTCTT
CCGCACTCGTGGGTATAATATTCAAGTTTTCATGTTGCTGCTGGCACGTTTCTTGTAGTAACCCCATGCT
TCTAGCCAATTCTGGTGACTTTT
>dme_circ_0003850_junction_seq
ATGCTTCTAGCCAATTCTGGTGACTTTTGAAAATGTATCAATAGGGATTTTGACTG
>dme_circ_0003851

ATAGCGGGGATTGAGCAACTGCGGCATGAAGTGCGATTCCAAAGCGTTCCGATACTGGGCACGTTTCTGG
CGCACCTGCTCATGCTGCTCTACTCGGCTGCTGGACGGCACCTGCTGATCCGACGGCGATCGCTCCGGTC
CCAAGACTACCGATCCCTCTGATTCCGAG
>dme_circ_0003851_junction_seq
CAAGACTACCGATCCCTCTGATTCCGAGATAGCGGGGATTGAGCAACTGCGGCATG
>dme_circ_0003852
AGACGCTGCTGGAGGCCGAGGAGCTTAGGTAAACTAGCTCCGCATCTTCAGACTGTGGAGACAGGGCGCT
CTGCAGTCGGGTGCAGCTCTCCAGATCGGTTGACTCCTCCAGCAGCTTGGTTTCGAGCTTAAGAGGCGAC
GGTGCCTCCTCCCTACGCTTGTG
>dme_circ_0003852_junction_seq
GCGACGGTGCCTCCTCCCTACGCTTGTGAGACGCTGCTGGAGGCCGAGGAGCTTAG
>dme_circ_0003853
TATCCCAAATCGTTGGCATACCTAGACCAATCGTCCTGCACCACCGGATGGTTATAGTGACCCACGGTGG
TATGCGGACGTGGTGGTGGCTCATTCAGATTGCACAGAGACTCGGCGCGTCGGGAACGTGGCTCCAGCTC
AATGGGAATCTCATGAG
>dme_circ_0003853_junction_seq
GGCTCCAGCTCAATGGGAATCTCATGAGTATCCCAAATCGTTGGCATACCTAGACC
>dme_circ_0003854
GTCTACAAAGTCAAGCACGAGTATATGAGCGAACCGGAGACGGGCAGCGATCGTCCCCGGAAAATGGCAC
AGTTAGGGCGAAGGCAGTACGACGGCATCGGTCCGGTGACCAACGATGGAATGCCCATCATCCTGAGATC
GGTAAAGGAAGCCCCACTCGCTGGGGCACAACGGGGGGTTTTGAACTCGAAGTGAGACGAATTGATACGA
GTATTGTTCCTTATCCTTATCCACATGCAGGAGGTCAAGGAGCCGCATCAGCATGAGTGGTACAAGCGTC
TGTATCAGACGATACACAAGCAGAAGAACGGCG
>dme_circ_0003854_junction_seq
CAGACGATACACAAGCAGAAGAACGGCGGTCTACAAAGTCAAGCACGAGTATATGA
>dme_circ_0003855
GTCTACAAAGTCAAGCACGAGTATATGAGCGAACCGGAGACGGGCAGCGATCGTCCCCGGAAAATGGCAC
AGTTAGGGCGAAGGCAGTACGACGGCATCGGTCCGGTGACCAACGATGGAATGCCCATCATCCTGAGATC
GGTAAAGGAAGCCCCACTCGCTGGGGCACAACGGGGGGTTTTGAACTCGAAGTGAGACGAATTGATACGA
GTATTGTTCCTTATCCTTATCCACATGCAGGAGGTCAAGGAGCCGCATCAGCATGAGTGGTACAAGCGTC
TGTATCAGACGATACACAAGCAGAAGAACGGCGGTAAATGTCACTCACTTTTCCCCTGGTCTGAAGCATT
CAAATTTCCTAGTACACCACACCACACCCCATTCCCATCCCAGCTCATTCCCACTCCCCGAAAATGTCCA
TTCCCCAATCCTTCTAATGCTTCAGCCTGATCTGATCTCCGACTTATCGTTTCGGTGTCTTTaaaaaaaa
aCTCTCGTTTCTCATTACCCCATTCGCTTTCGCCCCAAGATGATTTCGTGATTCGCTACAAGTGTCCTAG
AGGTGATTCTTTGTTCTTTGATCTATAAACTATGCTATCCCCTAGTAGCTCCTCCCCTTTAGTAGCTCGC
TTTCGCCTGTATATATAGACAGGGTCTAGTGACCTCTAACCTCTAACAAATAGAGCGCTTCATTCTATTT
TGGTTTTATTTTGTTTACATGCATGTTGTATTATTTGGTTTACCTATGATTTCTCTCATGTTCCCGTGTG
TAGATCATTGAAAATTTGGAAGCTTCTACTAAATTTCACGACTAACCTAATCTAAGGCCTAACTCACCGA
ACGCAAAACCACAAAACAACGCTAACATCATGTCCGCCAGTACACCACCAAGACGAAGTAATGATTATGT
GAAAGTAGAGCTTGAGGAACCGCTTTGTGCTTGTACAACTGAATACTTACCTCGACTAATCAGTTCTCCG
TCTCCGATCAGGCCCGTCCGTCGTACAAGAGCAACGGATACGTCTCAGAGCCCGAGCCCAACTACGATTC
GGATTACTCGACGGTGAGGTACCGCACCCAGAATCCGCACCGCGTTCAGTCCGTCTCCTCGGCTGTCAAT
GTGCGCAACCTTAATCAGGACGAGAAGGTAAGTATCAGTTCCGATAACATTCACCGTACCGTTTCATCTC
TGTGACCGAAATGCTCACCATTTTGGGAAAATCCACTTCACCACCATCATTTTTAACCCACGCAAAGCAA

TCAGTCGTATTTGCTCAACGGAACCACTTCACTGGCACCGATCAATATTAGCTTATTGCCCCAGCTCATT
TGCAATGCCACGATAATACCCACCAAGCCCCAATTGGGTCCATAATCATTCAGCATTCACATTCACCATC
CATACATAATCTGTCATTCAGATCGCCTCAAGTATTCTAACTCACAGCAATTATTCTATTCACAATTCTA
GTGGTGGGACGAAGTGATGGACATCTTTAACGGGGTATGATCTCTACTATATTATCTAGATTTAGTCTTA
GTTAATTCACCTTCCGATTGAGCTACTCGAGACCGTTTTCCGTTATCTAACGTGAACTAAACCGTTCCCA
ACTATTTGCTTACTATTATATCTTTCCCTGCCTACCATTTACCGCCTACCAAATTGCCTGATCCATATCC
TTCTCAAAGTGGCTTAGGGAGCATACACGCATCCCGCGTATTATCATAGAATTCGTCGACGAGTTTGACG
GCATCGGAGTAAGTTTTCCCGGACAGAATATCCATCGGTCTGTCAGATACATAAAAGCGAGGCAGGAATC
ATTGCAAAAGGCACCAGAACTACTAATAGAAGCCTCTCGATCAGTTTTATCCTTGTGTATGAAATAATGC
TTATTCATATATTTTCTCTTTTCCTTCAATAGGAGCTTTGGCCAAGGAATCCGGCTATACTAGCGATTCC
AATCTGGTCTTCCGTAAGAAGGAGGTACCCGTGAGCAGTCCCCTTAGCCCCGTTGAACAAAAGCAGGCTT
ACAAGAGTCTCCAGGCAGGGGGAGAGCCACCTCTGCTCGGCTTCCGTAAACCAGCGCCCGAGAAACCCCG
TG
>dme_circ_0003855_junction_seq
CGTAAACCAGCGCCCGAGAAACCCCGTGGTCTACAAAGTCAAGCACGAGTATATGA
>dme_circ_0003856
GCCCGTCCGTCGTACAAGAGCAACGGATACGTCTCAGAGCCCGAGCCCAACTACGATTCGGATTACTCGA
CGGTGAGGTACCGCACCCAGAATCCGCACCGCGTTCAGTCCGTCTCCTCGGCTGTCAATGTGCGCAACCT
TAATCAGGACGAGAAGTGAGTCATTATAATTCATACTTGATCGTGATGATCGTGAATGAGCCTTACTAAT
CGAACAACTGCCTTTTACGTTGCAGGTTGTATGGTACTATGCCAAATCCCATAAAATCAGCGCAAAACTC
GTATAAGAATCAGCCAGGTCGCATCGAAAACTATACGACAGGTCATTCGTCTGTTTCCGAAAAGGAAAAG
AAGGAG
>dme_circ_0003856_junction_seq
GTCTGTTTCCGAAAAGGAAAAGAAGGAGGCCCGTCCGTCGTACAAGAGCAACGGAT
>dme_circ_0003857
GCCCGTCCGTCGTACAAGAGCAACGGATACGTCTCAGAGCCCGAGCCCAACTACGATTCGGATTACTCGA
CGGTGAGGTACCGCACCCAGAATCCGCACCGCGTTCAGTCCGTCTCCTCGGCTGTCAATGTGCGCAACCT
TAATCAGGACGAGAAGGTAAGTATCAGTTCCGATAACATTCACCGTACCGTTTCATCTCTGTGACCGAAA
TGCTCACCATTTTGGGAAAATCCACTTCACCACCATCATTTTTAACCCACGCAAAGCAATCAGTCGTATT
TGCTCAACGGAACCACTTCACTGGCACCGATCAATATTAGCTTATTGCCCCAGCTCATTTGCAATGCCAC
GATAATACCCACCAAGCCCCAATTGGGTCCATAATCATTCAGCATTCACATTCACCATCCATACATAATC
TGTCATTCAGATCGCCTCAAGTATTCTAACTCACAGCAATTATTCTATTCACAATTCTAGTGGTGGGACG
AAGTGATGGACATCTTTAACGGGGTATGATCTCTACTATATTATCTAGATTTAGTCTTAGTTAATTCACC
TTCCGATTGAGCTACTCGAGACCGTTTTCCGTTATCTAACGTGAACTAAACCGTTCCCAACTATTTGCTT
ACTATTATATCTTTCCCTGCCTACCATTTACCGCCTACCAAATTGCCTGATCCATATCCTTCTCAAAGTG
GCTTAGGGAGCATACACGCATCCCGCGTATTATCATAGAATTCGTCGACGAGTTTGACGGCATCGGAGTA
AGTTTTCCCGGACAGAATATCCATCGGTCTGTCAGATACATAAAAGCGAGGCAGGAATCATTGCAAAAGG
CACCAGAACTACTAATAGAAGCCTCTCGATCAGTTTTATCCTTGTGTATGAAATAATGCTTATTCATATA
TTTTCTCTTTTCCTTCAATAGGAGCTTTGGCCAAGGAATCCGGCTATACTAGCGATTCCAATCTGGTCTT
CCGTAAGAAGGAGGTACCCGTGAGCAGTCCCCTTAGCCCCGTTGAACAAAAGCAGGCTTACAAGAGTCTC
CAGGCAGGGGGAGAGCCACCTCTGCTCGGCTTCCGTAAACCAGCGCCCGAGAAACCCCGTG
>dme_circ_0003857_junction_seq
CGTAAACCAGCGCCCGAGAAACCCCGTGGCCCGTCCGTCGTACAAGAGCAACGGAT
>dme_circ_0003858

GACGTGAACATCCACTTCAAGACCCCCATCAGGCATGAGCAGCGTCAGAACTTGTCGGAAGAGGAACTAG
CCATTCGCCAAGCGGAGCACATGCAGAAGCTCTACCACGAGGAGCGCCGTCGCAAGTATCTACAGGAGCT
GCAGGACATGAATTCGCGCCGCCACACGGACAACTTCACCCCGTCGCAGAAGTCGCCCATCGCCCTCAAT
CGCTACGATGACTTCCCCACGGACGTGACCCTCAAGTCGCTGGTGGGCCCCAAGACGGTGGCCCGGGCTC
TCTTTAACTTCCAGGGACAGACCTCGAAGTATGTAGCCCGTAAGGAGGAGTCTTCCAAGCAGCACTATAT
CCATATTCATTTCAGGGAGCTATCCTTCCGCAAGGGCGACACAATCTACATCAGGCGGCAGATTGATGCC
AACTGGTATGAGGGCGAGCACAATGCCATGATTGGACTGCTTCCAGCCAGCTATGTTGAG
>dme_circ_0003858_junction_seq
TGGACTGCTTCCAGCCAGCTATGTTGAGGACGTGAACATCCACTTCAAGACCCCCA
>dme_circ_0003859
CACATGGACCACATCTCCCTCGAGCAGTTCCAGTTCGTCGGAGTTCTGGGGCCGGTACTTGTACAGTGCG
CGGTACCTAAAGAAGATCTCATGTTAATAATGGTATTGTTTTGATCTAACCAAAGTAATACACTTAGAAA
GATGTCTAGAAGAATGCATCTGTCTTATCCCCTATTTTCAGTCTGTTATAGTCTCAAAACAGGATGCATT
CTTTTGGACAGTTTCATTGATATTTGATAGGATTACTCACGCCAATGGTTCGGAACTGGTGTCCACATGG
AGGGCGTCCGTCTTGTGAAGTGTCCGCGTTTCCTTGAGGATTCCGTTCGGTGGCTGTGCTCCATTTTGCG
TCAGCGTATTGAACTCATTGTTGATGTTTGTGCGCAGCACGTCGAGATTGGGCCGCAGATTCGTGGTGCT
CTGGCTGGTGATCACGG
>dme_circ_0003859_junction_seq
TTCGTGGTGCTCTGGCTGGTGATCACGGCACATGGACCACATCTCCCTCGAGCAGT
>dme_circ_0003860
GGCGTGAAGTGCACCACAAGCGCCACTTCAGTTAACTTCTCTTCGCCTAACTTTTTAAGCCTTTTGCCGT
GTGTAAGCAGTGCTGGTAGATAGTTCTCACTGGGCACATCAAGGAAAACAAAGGACAGTGCAGTTTCACT
GGCTTCGGTGACGTCGACTGAACGCACCACCTTTCCATCAGGCAGTGTGATATC
>dme_circ_0003860_junction_seq
CCACCTTTCCATCAGGCAGTGTGATATCGGCGTGAAGTGCACCACAAGCGCCACTT
>dme_circ_0003861
GGAGCCCCGCAACAAGAACGCTCAAAAGCAAGAGAGTTGGATGGACATCATTCGGTCGATCCTCGACAGC
AGCGAGTACAATTTAACTCCAGCCGTTCGATCCGCCTTCCAAAAGCTCCAGAGCGTCGACAAC
>dme_circ_0003861_junction_seq
CTTCCAAAAGCTCCAGAGCGTCGACAACGGAGCCCCGCAACAAGAACGCTCAAAAG
>dme_circ_0003862
GGTCGAGGAAGAGGCGCCACCCAAGAAGAAGGCCAAAGTGGAGACCACAGAAGATGCTGCTGCCGAGGAG
ACCTCCAACGGCGTGACCAGCGACTTTGATTGGGCCGCCCAGCTAACTAAGATCGTTACCAAGCAAGCAG
ATGGCATTTTCCTGGAAAAACTTCGTAAGAAGCTGCTCAAGAAGTATGCCAAGCATCTGTCC
>dme_circ_0003862_junction_seq
GCTCAAGAAGTATGCCAAGCATCTGTCCGGTCGAGGAAGAGGCGCCACCCAAGAAG
>dme_circ_0003863
CATCGTCAAGGGGTTTCCACCTGTGTCACCCTTCATTGGCGTTAGTCCCACGCTCTGCTATTTGCTCAAA
GAGAAGAAGCCACTCTGCTGTTTGCAGTTGGCTCAGGTGGGTAGCTATGACAGTCCTTGGGCCGTACAAT
TTCATTTGGAGGAAAACCAATCTCTTTCGCAGGTCTGCGAGCACTGCAGCTATCGAAACGCAAAGGAGTA
CCAGTGGCAGAACAAGACGATTATCCTGGCTGCGGATTACGCCTCCAATGGCATATACAACTTCATCATC
CCGCTGAGAGCTCACTTCCGATCGAAGACCTCGCTGAATCCCATCATCCTGCTTCTGGAGAGAAGGCCAG
ATGTGGCCTTCCTAGATGCGTTGTCTTATTTTCCTTTGGGTCTACTGGATGCTGGGATCGATCGACTGCC
TGGACGATCTGCTGAGGGCTGGCATCACATTGGCAGAAAGTGTGGTGGTGGTCAACAAGGAGCTCTCCAA

TTCGGCCGAGGAGGACTCCCTCTCAGACTGCAACACCATAGTGGCCGTGCAGAATATGTTCAAGCGCGAG
AAGGAGCGCGGGTCGCATATCTCCTATATGTTCCGTCTGCCCTTTGCCGCAGGAGCCGTGTTCAGTGCCT
CCATGTTGGACACCCTGCTGTACCAGGCCTTCGTGAAGGACTATGTGATTACCTTTGTGCGGTTGCTGCT
TGGCATTGACCAGGCGCCGGGCAGTGGGTTCTTGACCTCGGATGCGCATCACCAAGGACGACATGTGGAT
ACGCACCTATGGTCGGCTGTACCAGAAGCTGTGCTCCACCACCTGCGAAATACCCATTGGCATTTACCGC
ACGCAGGACACCTCGAATGCGGACACGTCTCATGTGAGTAACTCGCCGGTCGAGAGATGGGGACCTTTCT
CGGCCTTCAGCAGGCATTGTGTGCGCTTGCGTCCATCGG
>dme_circ_0003863_junction_seq
AGGCATTGTGTGCGCTTGCGTCCATCGGCATCGTCAAGGGGTTTCCACCTGTGTCA
>dme_circ_0003864
CTAGCGAAAATCCGACGGCTGTGATAATCTCGGACTCTAGGCAGAACCTCAAGGACACGACGGTGACCCA
GACGGCGGCAACGATAACCACAACGACTCTGCCGCCACCGCCCCAGACGATGGGATCCCCCAGCATGGCG
GGCGGATCGGGTGGATGTGGTGGAGGCGGCCTCGGCTTGGGCAGTGCCCACAGCATGGGCACCTCGCTCA
GTATCACCCCAGCCACACTGACCACCACGGGCAATCACCTGGATGTGCCCTTTGCCAACAATCCCAACCT
GCTCAGCCCAGATGTGCTCAACCAGCGGAGGGGGTAGTAGACGTCCCTCGATCCTACCCGTGCCCGACAT
GTTCACCTCCTCCTCATTCAGCATCGCCGGCAACGACGATGGTGAAGAGGGGGACGAGAGTGATGATGAG
ATCGACGACGAGATGCCCTGGCGTTCGCCATCCGAGAAGATTGCGGTAAGTATCTCCCCTCTAATCAAAC
CCTCTACACACCCCTTTTTGTCACTATTCCGAGGCACCGTATCCACCTTTCCCAGTGTAGTGTCTCTATC
AATATAGATCTAGACCCCCAAACATATTGTACTACGTATGTGTATCACTAACTATATTTCTTAGCTACTC
TGTTTTCCACGAGAAACTAACCTCTGACATAAATCTGTGTACATAGACCATGCTAGTCAGATCTCAGCAA
TCCGAAACGATCGAAGTATACTTTATTGTACAAAAGAAGTGGATCGTTGCGAGACTGCGAGATCCGACTG
AATGTAAATTGTATATGTTGTATTTAAGCACTAGACACCCGCCCTATTCCTTAATTGTAACCACCGTTCT
TTGAACACATCAAACTAAACGACAATCGAAAACTTCGCCTCAGCTGCTTGGGCGGGCACTTTCCACAATC
GCGCACCTATTCGCTCATCATGAGCTCCTCGGAGGATTCGTATCAGCgcggttgcagcttctgcagtgcc
actgcttccgcttccgctgctgctgttgctgttgctgttgctgctgctcctgctcctgctcctgctcctg
caTATCCAAGTGATTCTGCAGGCGAGTCCGGAACTAATGCCGATTCCGCTGCCATGCCATCCGAGGAGTA
TCTACCGCAGTTGCGCAGGCGCGTCATGAAAAAGAGCTACAGCTGCGATAGTGAGTGCCGCTCGGTGCCC
GGAATGGGAATGGGCATGGCCATGGGTGTGGGTCTGGGTGGAGGAACTCTGGCCAGACTGGCGGCCAGAA
GGCGTCAGTTGCAGCGATGTTCCTCCTGCAGTTGCTCCACTGCCACAACAACAACCACGCCGGCGGTGGC
AGCGACAACAGCAATGGCAGCAGGAAGTGGGGCAACGGCATTCACGTCGAGCAGTTCCGTAGAGACACGT
CGACCGGTGCGTCCGGTTTGGGTCTACGACTACTCCTGGTAAGTCTGACTGATGGCGGGGGGATGGGAGG
AAGGACATTCTGCGGACACACGCCATTTGTTGAAGAGTTCTTGTCTCGTTGTTGCTGTTCGTTCGTTGTT
GTTGCTTTTGTTTGCCTGCCGCAGGACATCTTTTAATGACGACGCCGTTTACCTTGGACCAACTAAGGAG
GAACAACTCGAGATTCTGTGCTGTTTGTCGTGGCTCTCGGTGCCGTGGTGCATCAGCATCTAATCAGCAT
TTGCTTCTGCTTGCTCCTTGGCTTTGTTTTATTTTTCGTCTGCAATTGTGTATTCATTCCATAGGGCTTC
CTGAAAGCTCCAAAGTTTCGCCTCCATCGGCAGTGTTCGTAGGAGGAGCACAACTGCAGGGTCTCGGGAA
TCACAGTTAAAGTTTAAGTTTCACCATTCTGTTAATTCGTAGTGAATGCTTTGGATCTGCTCGTAGTTTG
CATTTCCCTTACCTACCCGACACAATTTCCCAACTTGTAGCTAAATCGTTTGAGTCCCAGCACAACCTGC
TCGTTTTCCCACCCGCACATCTCACCACTCACCTTAAAAACCTGTCGCCCATCGCCTTATCCACGGGGAG
CTCACGTCTAACCCAGAATCGTCTGCCATTCCAGCGTCTGCGAGCACTGCAGCTATCGAAACGCAAAGGA
GTACCAGTGGCAGAACAAGACGATTATCCTGGCTGCGGATTACGCCTCCAATGGCATATACAACTTCATC
ATCCCGCTGAGAGCTCACTTCCGATCGAAGACCTCGCTGAATCCCATCATCCTGCTTCTGGAGAGAAGGC
CAGATGTGGCCTTCCTAGATGCGTTGTCTTATTTTCCTTTGGGTCTACTGGATGCTGGGATCGATCGACT
GCCTGGACGATCTGCTGAGGGCTGGCATCACATTGGCAGAAAGTGTGGTGGTGGTCAACAAGGAGCTCTC

CAATTCGGCCGAGGAGGACTCCCTCTCAGACTGCAACACCATAGTGGCCGTGCAGAATATGTTCAAGCGC
GAGAAGGAGCGCGGGTCGCATATCTCCTATATGTTCCGTCTGCCCTTTGCCGCAGGAGCCGTGTTCAGTG
CCTCCATGTTGGACACCCTGCTGTACCAGGCCTTCGTGAAGGACTATGTGATTACCTTTGTGCGGTTGCT
GCTTGGCATTGACCAGGCGCCGGGCAGTGGGTTCTTGACCTCGGATGCGCATCACCAAGGACGACATGTG
GATACGCACCTATGGTCGGCTGTACCAGAAGCTGTGCTCCACCACCTGCGAAATACCCATTGGCATTTAC
CGCACGCAGGACACCTCGAATGCGGACACGTCTCATGTGAGTAACTCGCCGGTCGAGAGATGGGGACCTT
TCTCGGCCTTCAGCAGGCATTGTGTGCGCTTGCGTCCATCGG
>dme_circ_0003864_junction_seq
AGGCATTGTGTGCGCTTGCGTCCATCGGCTAGCGAAAATCCGACGGCTGTGATAAT
>dme_circ_0003865
CATCGTCAAGGGGTTTCCACCTGTGTCACCCTTCATTGGCGTTAGTCCCACGCTCTGCTATTTGCTCAAA
GAGAAGAAGCCACTCTGCTGTTTGCAGTTGGCTCAGGTGGGTAGCTATGACAGTCCTTGGGCCGTACAAT
TTCATTTGGAGGAAAACCAATCTCTTTCGCAGGTCTGCGAGCACTGCAGCTATCGAAACGCAAAGGAGTA
CCAGTGGCAGAACAAGACGATTATCCTGGCTGCGGATTACGCCTCCAATGGCATATACAACTTCATCATC
CCGCTGAGAGCTCACTTCCGATCGAAGACCTCGCTGAATCCCATCATCCTGCTTCTGGAGAGAAGGCCAG
ATGTGGCCTTCCTAGATGCGTTGTCTTATTTTCCTTTGGGTCTACTGGATGCTGGGATCGATCGACTGCC
TGGACGATCTGCTGAGGGCTGGCATCACATTGGCAGAAAGTGTGGTGGTGGTCAACAAGGAGCTCTCCAA
TTCGGCCGAGGAGGACTCCCTCTCAGACTGCAACACCATAGTGGCCGTGCAGAATATGTTCAAGCGCGAG
AAGGAGCGCGGGTCGCATATCTCCTATATGTTCCGTCTGCCCTTTGCCGCAGGAGCCGTGTTCAGTGCCT
CCATGTTGGACACCCTGCTGTACCAGGCCTTCGTGAAGGACTATGTGATTACCTTTGTGCGGTTGCTGCT
TGGCATTGACCAGGCGCCGGGCAGTGGGTTCTTGACCTCGGATGCGCATCACCAAGGACGACATGTGGAT
ACGCACCTATGGTCGGCTGTACCAGAAGCTGTGCTCCACCACCTGCGAAATACCCATTGGCATTTACCGC
ACGCAGGACACCTCGAATGCGGACACGTCTCATG
>dme_circ_0003865_junction_seq
GACACCTCGAATGCGGACACGTCTCATGCATCGTCAAGGGGTTTCCACCTGTGTCA
>dme_circ_0003866
CTAGCGAAAATCCGACGGCTGTGATAATCTCGGACTCTAGGCAGAACCTCAAGGACACGACGGTGACCCA
GACGGCGGCAACGATAACCACAACGACTCTGCCGCCACCGCCCCAGACGATGGGATCCCCCAGCATGGCG
GGCGGATCGGGTGGATGTGGTGGAGGCGGCCTCGGCTTGGGCAGTGCCCACAGCATGGGCACCTCGCTCA
GTATCACCCCAGCCACACTGACCACCACGGGCAATCACCTGGATGTGCCCTTTGCCAACAATCCCAACCT
GCTCAGCCCAGATGTGCTCAACCAGCGGAGGGGGTAGTAGACGTCCCTCGATCCTACCCGTGCCCGACAT
GTTCACCTCCTCCTCATTCAGCATCGCCGGCAACGACGATGGTGAAGAGGGGGACGAGAGTGATGATGAG
ATCGACGACGAGATGCCCTGGCGTTCGCCATCCGAGAAGATTGCGGTAAGTATCTCCCCTCTAATCAAAC
CCTCTACACACCCCTTTTTGTCACTATTCCGAGGCACCGTATCCACCTTTCCCAGTGTAGTGTCTCTATC
AATATAGATCTAGACCCCCAAACATATTGTACTACGTATGTGTATCACTAACTATATTTCTTAGCTACTC
TGTTTTCCACGAGAAACTAACCTCTGACATAAATCTGTGTACATAGACCATGCTAGTCAGATCTCAGCAA
TCCGAAACGATCGAAGTATACTTTATTGTACAAAAGAAGTGGATCGTTGCGAGACTGCGAGATCCGACTG
AATGTAAATTGTATATGTTGTATTTAAGCACTAGACACCCGCCCTATTCCTTAATTGTAACCACCGTTCT
TTGAACACATCAAACTAAACGACAATCGAAAACTTCGCCTCAGCTGCTTGGGCGGGCACTTTCCACAATC
GCGCACCTATTCGCTCATCATGAGCTCCTCGGAGGATTCGTATCAGCgcggttgcagcttctgcagtgcc
actgcttccgcttccgctgctgctgttgctgttgctgttgctgctgctcctgctcctgctcctgctcctg
caTATCCAAGTGATTCTGCAGGCGAGTCCGGAACTAATGCCGATTCCGCTGCCATGCCATCCGAGGAGTA
TCTACCGCAGTTGCGCAGGCGCGTCATGAAAAAGAGCTACAGCTGCGATAGTGAGTGCCGCTCGGTGCCC
GGAATGGGAATGGGCATGGCCATGGGTGTGGGTCTGGGTGGAGGAACTCTGGCCAGACTGGCGGCCAGAA

GGCGTCAGTTGCAGCGATGTTCCTCCTGCAGTTGCTCCACTGCCACAACAACAACCACGCCGGCGGTGGC
AGCGACAACAGCAATGGCAGCAGGAAGTGGGGCAACGGCATTCACGTCGAGCAGTTCCGTAGAGACACGT
CGACCGGTGCGTCCGGTTTGGGTCTACGACTACTCCTGGTAAGTCTGACTGATGGCGGGGGGATGGGAGG
AAGGACATTCTGCGGACACACGCCATTTGTTGAAGAGTTCTTGTCTCGTTGTTGCTGTTCGTTCGTTGTT
GTTGCTTTTGTTTGCCTGCCGCAGGACATCTTTTAATGACGACGCCGTTTACCTTGGACCAACTAAGGAG
GAACAACTCGAGATTCTGTGCTGTTTGTCGTGGCTCTCGGTGCCGTGGTGCATCAGCATCTAATCAGCAT
TTGCTTCTGCTTGCTCCTTGGCTTTGTTTTATTTTTCGTCTGCAATTGTGTATTCATTCCATAGGGCTTC
CTGAAAGCTCCAAAGTTTCGCCTCCATCGGCAGTGTTCGTAGGAGGAGCACAACTGCAGGGTCTCGGGAA
TCACAGTTAAAGTTTAAGTTTCACCATTCTGTTAATTCGTAGTGAATGCTTTGGATCTGCTCGTAGTTTG
CATTTCCCTTACCTACCCGACACAATTTCCCAACTTGTAGCTAAATCGTTTGAGTCCCAGCACAACCTGC
TCGTTTTCCCACCCGCACATCTCACCACTCACCTTAAAAACCTGTCGCCCATCGCCTTATCCACGGGGAG
CTCACGTCTAACCCAGAATCGTCTGCCATTCCAGCGTCTGCGAGCACTGCAGCTATCGAAACGCAAAGGA
GTACCAGTGGCAGAACAAGACGATTATCCTGGCTGCGGATTACGCCTCCAATGGCATATACAACTTCATC
ATCCCGCTGAGAGCTCACTTCCGATCGAAGACCTCGCTGAATCCCATCATCCTGCTTCTGGAGAGAAGGC
CAGATGTGGCCTTCCTAGATGCGTTGTCTTATTTTCCTTTGGGTCTACTGGATGCTGGGATCGATCGACT
GCCTGGACGATCTGCTGAGGGCTGGCATCACATTGGCAGAAAGTGTGGTGGTGGTCAACAAGGAGCTCTC
CAATTCGGCCGAGGAGGACTCCCTCTCAGACTGCAACACCATAGTGGCCGTGCAGAATATGTTCAAGCGC
GAGAAGGAGCGCGGGTCGCATATCTCCTATATGTTCCGTCTGCCCTTTGCCGCAGGAGCCGTGTTCAGTG
CCTCCATGTTGGACACCCTGCTGTACCAGGCCTTCGTGAAGGACTATGTGATTACCTTTGTGCGGTTGCT
GCTTGGCATTGACCAGGCGCCGGGCAGTGGGTTCTTGACCTCGGATGCGCATCACCAAGGACGACATGTG
GATACGCACCTATGGTCGGCTGTACCAGAAGCTGTGCTCCACCACCTGCGAAATACCCATTGGCATTTAC
CGCACGCAGGACACCTCGAATGCGGACACGTCTCATG
>dme_circ_0003866_junction_seq
GACACCTCGAATGCGGACACGTCTCATGCTAGCGAAAATCCGACGGCTGTGATAAT
>dme_circ_0003867
CTAGCGAAAATCCGACGGCTGTGATAATCTCGGACTCTAGGCAGAACCTCAAGGACACGACGGTGACCCA
GACGGCGGCAACGATAACCACAACGACTCTGCCGCCACCGCCCCAGACGATGGGATCCCCCAGCATGGCG
GGCGGATCGGGTGGATGTGGTGGAGGCGGCCTCGGCTTGGGCAGTGCCCACAGCATGGGCACCTCGCTCA
GTATCACCCCAGCCACACTGACCACCACGGGCAATCACCTGGATGTGCCCTTTGCCAACAATCCCAACCT
GCTCAGCCCAGATGTGCTCAACCAGCGGAGGGGGTAGTAGACGTCCCTCGATCCTACCCGTGCCCGACAT
GTTCACCTCCTCCTCATTCAGCATCGCCGGCAACGACGATGGTGAAGAGGGGGACGAGAGTGATGATGAG
ATCGACGACGAGATGCCCTGGCGTTCGCCATCCGAGAAGATTGCG
>dme_circ_0003867_junction_seq
CTGGCGTTCGCCATCCGAGAAGATTGCGCTAGCGAAAATCCGACGGCTGTGATAAT
>dme_circ_0003868
CTAGCGAAAATCCGACGGCTGTGATAATCTCGGACTCTAGGCAGAACCTCAAGGACACGACGGTGACCCA
GACGGCGGCAACGATAACCACAACGACTCTGCCGCCACCGCCCCAGACGATGGGATCCCCCAGCATGGCG
GGCGGATCGGGTGGATGTGGTGGAGGCGGCCTCGGCTTGGGCAGTGCCCACAGCATGGGCACCTCGCTCA
GTATCACCCCAGCCACACTGACCACCACGGGCAATCACCTGGATGTGCCCTTTGCCAACAATCCCAACCT
GCTCAGCCCAGATGTGCTCAACCAGCGGAGGGG
>dme_circ_0003868_junction_seq
GCCCAGATGTGCTCAACCAGCGGAGGGGCTAGCGAAAATCCGACGGCTGTGATAAT
>dme_circ_0003869
AGTTCGGGTGGAGTACTATGTGAACGAAAACACATTCAAAGAAAGACTGCAACTCTATTTCATTAAGAAT

CAGCGTTCAAGTAAGTAATCACCAGCCAAGTATTGCCTATGAATTAACCAAAACATTCGACTTAATTACA
ACAATCAACAAGGTAGTATTTAATTGAATTTGATTAGTGGCGGGCTGGACGGGAAACGGATGTACGGTGT
ATCCTGCCAGGATACAATCGCCAGcacactgacaaccgcacaaacccacccacagacacacaaacacaca
gccacacaagacagacggacagacggacagacggacggactAACGAATGACATTACAAAAGCGGATTCGA
TAATCTTAAGTAAATATTGATTTGGAGAGAAAATAATTGAACAGGTAAACAGGTCTCGATTGAGGGGGGA
CTTGTTTGTTTGGGCCCGCAGGGAGCAACCCACTCAACGAACTTTCGTAGACGGTATAATTCACTCAACT
GACCCAAATTCTAACGCATTTGCTATTCCACTTTCCTAAAAAATAAGGCTATGGCTGCGAAGTGGGCAAT
AAGACGGAGTTCATCATCTCGGCCAAACTGACGGAGGAGGAGTTCCAGGAGAATCCAATCATTAACTGGG
ACGCAATACTCTGGGTGAATCGGCCAACAGTGCTCTGGGTCCTGCAGCTGCTTCTAGCCATGGTGTCGCT
AACGCAATCCTTGGTTCTCACATATCTAGGCTATAAGGCGTTTGTGGTATCCAGCACTTTCAGCGCGCTG
GCCATCGACATTTGAATCTCTTTCAGAGCACATACTATGTGGTTGTGACCTTCTCCACAGTGGGATACGG
CGACTTTGTGCCGGACATTTGGCCCTCGCAGCTTTATATGGTCATCATGATTTGTGTCGCCCTCATTGTG
TTGCCCACGCAGG
>dme_circ_0003869_junction_seq
GTCGCCCTCATTGTGTTGCCCACGCAGGAGTTCGGGTGGAGTACTATGTGAACGAA
>dme_circ_0003870
GCTGGGTGGTGCTCTTCTCTCACCCCGCCGACTTTACTCCCGTCTGCACCACTGAGCTGGGCAGGATTGC
GGTGCACCAGCCGGAGTTTGCCAAGCGGAACACCAAGTGCCTGGCACATTCCGTTGACGCATTGAACTCG
CACGTGGACTGGGTGAACGACATCAAGAGCTATTGCCTGGACATTCCCGGGGACTTCCCCTACCCGATCA
TCGCCGACCCTACTCGCGACCTGGCCGTCAGCCTGGGCATGCTCGACGAGGAGCAGAAGAAGGATCCCGA
GGTGGGCAAGACCATCCGCGCCCTGTTCATCATCAGTCCGGACCATAAGGTGCGCCTCTCCATGTTCTAC
CCCATGTCCACTGGTCGCAACGTTGAGCCTGGCACTAAGGTCATGATCCTGCCCACTGTCACCGATGAAG
AGGCTCATAAACTCTTCCCCAAGGGATTTGATAAGGTATCCATGCCGTCTGGAGTAAACTACGTCCGCAC
CACTGACAACTACTGACACAAAACCGTTCCCCATTTGTGATCCATTCAATAATGTGCCGTATTTCCTGAC
ATATACTATATACTATGTGCAGTTCATTTATTTAATAAAAAATTATTTTAACTATGGCAAAGTTTATAAA
TGAGATCGGTGAACTTCTTGATTCCTGTAGAGCCCGAGAGGTTTTGATGGAGGTATTATGCCAAAGTGCC
CAATTGGTAATAGGATACCAAATCAATCAGAGTCCAGACCTAAACAATGTGGGTTCAGAAGACTCTGGGG
AGCCACCTGTGCTTCGGTACACTGTGGACAGCGAACTGCACGACTATGAACCAGATCGCATTACGGCTCT
ATTGACGGTGATGTCAAATACGGTGGATCTGCTTTTCTATCCCGTAGACACCATTTGCTGGCTGGCCAGT
CACAAAGTTTTCGATGTCAAAAAACAGAAGACCTGGCGCTTTGTGAACTCGTTACTCAGCATGGTCTCTG
CATACCTAAACGTCGTGAGAATTTCAAGAATTTTCTTACAAGATGGCTATAAACTAAGTCACTTCGATGT
TGTGTCCCTTGCACACTTTTTATTCGATCTGCTTCACAACATCTGCAGTTTACCAAGGAGTTACCTGTGG
GGCATGAAGCATTCATCCCTTTACTTGGGAGTTATTGGCACCGTTTCTGCTGGACTGGGAATTTGCCAGA
TTTTGACCAAAAGAAGGCTGACCAAGTAGAGTATTCAGTAAGCAACAAGTGTGCGAGCTAATAACAGCTA
CCAGCTGAGCAACTAACAAGTTTTTTTCCAAGTGAAACTAAAAATCAAACAGCAAGATGCGTTTGGGACA
GACCGTACCTAACTTCGAGGCCGACACAACCAAGGGGCCCATTAAATTCCACGAGTGGCAGGGCAACTC
>dme_circ_0003870_junction_seq
TTAAATTCCACGAGTGGCAGGGCAACTCGCTGGGTGGTGCTCTTCTCTCACCCCGC
>dme_circ_0003871
GCCTGGCACTAAGGTCATGATCCTGCCCACTGTCACCGATGAAGAGGCTCATAAACTCTTCCCCAAGGGA
TTTGATAAGGTATCCATGCCGTCTGGAGTAAACTACGTCCGCACCACTGACAACTACTGACACAAAACCG
TTCCCCATTTGTGATCCATTCAATAATGTGCCGTATTTCCTGACATATACTATATACTATGTGCAGTTCA
TTTATTTAATAAAAAATTATTTTAACTATGGCAAAGTTTATAAATGAGATCGGTGAACTTCTTGATTCCT
GTAGAGCCCGAGAGGTTTTGATGGAGGTATTATGCCAAAGTGCCCAATTGGTAATAGGATACCAAATCAA

TCAGAGTCCAGACCTAAACAATGTGGGTTCAGAAGACTCTGGGGAGCCACCTGTGCTTCGGTACACTGTG
GACAGCGAACTGCACGACTATGAACCAGATCGCATTACGGCTCTATTGACGGTGATGTCAAATACGGTGG
ATCTGCTTTTCTATCCCGTAGACACCATTTGCTGGCTGGCCAGTCACAAAGTTTTCGATGTCAAAAAACA
GAAGACCTGGCGCTTTGTGAACTCGTTACTCAGCATGGTCTCTGCATACCTAAACGTCGTGAGAATTTCA
AGAATTTTCTTACAAGATGGCTATAAACTAAGTCACTTCGATGTTGTGTCCCTTGCACACTTTTTATTCG
ATCTGCTTCACAACATCTGCAGTTTACCAAGGAGTTACCTGTGGGGCATGAAGCATTCATCCCTTTACTT
GGGAGTTATTGGCACCGTTTCTGCTGGACTGGGAATTTGCCAGATTTTGACCAAAAGAAGGCTGACCAAG
TAGAGTATTCAGTAAGCAACAAGTGTGCGAGCTAATAACAGCTACCAGCTGAGCAACTAACAAGTTTTTT
TCCAAGTGAAACTAAAAATCAAACAGCAAGATGCGTTTGGGACAGACCGTACCTAACTTCGAGGCCGACA
CAACCAAGGGGCCCATTAAATTCCACGAGTGGCAGGGCAACTCGCTGGGTGGTGCTCTTCTCTCACCCCG
CCGACTTCACTCCCGTCTGCACCACTGAGCTGGGCAGGATTGCGGTGCACCAGCCGGAGTTTGCCAAGCG
GAACACCAAGTGCCTGGCGCATTCCGTTGACGCATTGAACTCGCACGTGGACTGGGTGAACGACATCAAG
AGCTATTGCCTGGACATTCCCGGGGACTTCCCCTACCCGATCATCGCCGACCCTACTCGCGACCTGGCCG
TCACCCTGGGCATGCTCGACGAGGAGCAGAAGAAGGATCCCGAGGTGGGTAAGACCATCCGCGCCCTGTT
CATCATCAGTCCGGACCATAAGGTGCGCCTCTCCATGTTTTACCCCATGTCCACTGGTCGCAACGTTGAG
TAAGTACCTCTTTCTGGCTACAACTATTGTTAATCTCAACTGACGATCCTGTAATCTTACAGCGAGATTC
TGAGGACCATTGACTCCCTGCAGCTGACTGATCGCCTCAAGGTGGTGGCCACTCCCGCCAACTGGACC
>dme_circ_0003871_junction_seq
GGTGGTGGCCACTCCCGCCAACTGGACCGCCTGGCACTAAGGTCATGATCCTGCCC
>dme_circ_0003872
GACTGCACCGCCACCCACGACTTCATCGACGGCACCGCAGCGGGACGAGCGGGAGCAGCAGCAGCAGCAA
GAGGCGCCACCATTGCAGCAGGATCAGCAGGAGAATTCGCCCGCGCAGGACCAGGAGCTGCACCCATTGC
TGGATCAACAGAATCAAGAATATCCCGCAGTGCACCAAGATCAACAAGCGGAGCAAAACCAGGAGCTGCA
TCACATAGAGGGGCTTATTCGGCACCGAGAATCACAGAATCCGGAAGAGCATCCACCACAAGCATCGCTG
GAAAACCGGGAGACATTAGGACGAGAAGATACCAACCAAGAACCGGATGAGCCGCAAGTTCGAGATCCCG
AAATCGAACCAGAAGCCGAGCCACCGCCACCGCTGCTTCTCGAAGATCTGGACGAGCAGGACTCCGGATC
GCAGGATCTTAACGAACAGCAGCCGCCACTGATTATAGACGCAAACGCTATAGCCGAGACCATAGACGCC
AACGCCGTGGAGCGCATCGACGACTACGAAGACGACGACGAGGAGGTGGATGAAGAGGAGGAGGTGGTAG
AGGACCGGGTCGACAGAGCCGGCGAAGTCGCTTCCGTTTCCGGTGCCCAGCGCCTGCACTCGGTGGCCGT
TTTGCCGCGCTACTCCTCagcctcacgagccccaaggagcagcaacaataacaacaacaatatcagcaat
cacaacaacaataataacaacagcaacagtaacagcCTCTCGCGACGCACTCGTCACTTCTACAGCAACA
ACGGCAGTCACTTCAGCAACGACATGTTCCCCAGCCACAATCCCCGCAGCAGCACCCAGACATCATCGCC
GCGGGTCGGTGGTCGCCGCCATCACTCGACCCCCGCCGCCAGCTCCAATTCGCCACAGCACCAGGGCGTG
GATCCCCTAAGGCTGCTCTATCCCAATGTCAACGAGAGCGAGACTCCGCTGCCCAGGTGCTGGAGTCCCC
ATGACAAGTGCTTGTCCATCGGTTTGTCGCAGAACAACTTGAGGGTCACCTACAAGG
>dme_circ_0003872_junction_seq
CAGAACAACTTGAGGGTCACCTACAAGGGACTGCACCGCCACCCACGACTTCATCG
>dme_circ_0003873
GAATCAAGAATATCCCGCAGTGCACCAAGATCAACAAGCGGAGCAAAACCAGGAGCTGCATCACATAGAG
GGGCTTATTCGGCACCGAGAATCACAGAATCCGGAAGAGCATCCACCACAAGCATCGCTGGAAAACCGGG
AGACATTAGGACGAGAAGATACCAACCAAGAACCGGATGAGCCGCAA
>dme_circ_0003873_junction_seq
TACCAACCAAGAACCGGATGAGCCGCAAGAATCAAGAATATCCCGCAGTGCACCAA
>dme_circ_0003874

GTGCACCAAGATCAACAAGCGGAGCAAAACCAGGAGCTGCATCACATAGAGGGGCTTATTCGGCACCGAG
AATCACAGAATCCGGAAGAGCATCCACCACAAGCATCGCTGGAAAACCGGGAGACATTAGGACGAGAAGA
TACCAACCAAGAACCGGATGAGCCGCAA
>dme_circ_0003874_junction_seq
TACCAACCAAGAACCGGATGAGCCGCAAGTGCACCAAGATCAACAAGCGGAGCAAA
>dme_circ_0003875
TCTGATTTATGTTGACGTTGCCATTTCCACTGCCGCTCTTGCTGTGCTTGAACGTCTTGGTGGACTGTAT
CACTGACGTCTGGTTTGTGGGCATGGTCTGTGTGGTGGCGGTGACTTTGTTGTTCACG
>dme_circ_0003875_junction_seq
TGTGGTGGCGGTGACTTTGTTGTTCACGTCTGATTTATGTTGACGTTGCCATTTCC
>dme_circ_0003876
GTTGAAATCGTTAAACTGTACATAAGCAAATAAGACATAAAGAAAAAAGTCCAGGAAAATTGGAAAACAA
AAGCCCGAAAACCGAAAAGCCCCGTGTAAATCCGAATCCGAATCCAAATCAGTATCCAAACCCAACCACA
ATGGGCTGCACCACATCCGCCGAAGAACGCGCCGCCATCCAGCGATCCAAACAGATCGAGAAGAATCTAA
AGGAGGATGGAATCCAGGCGGCCAAGGACATCAAGCTCCTGCTGCTGGAGCCCGAAAACCGAAAAGCCCC
GTGTAAATCCGAATCCGAATCCAAATCAGTATCCAAACCCAACCACAATGGGCTGCACCACATCCGCCGA
AGAACGCGCCGCCATCCAGCGATCCAAACAGATCGAGAAGAATCTAAAGGAGGATGGAATCCAGGCGGCC
AAGGACATCAAGCTCCTGCTGCTGGGTAAGTGCACTTTTCGTCGCTTTTTCTCGTTTTTCCACTTGGGTT
TTTCTCACCGGAAATGGAAATCCTGCTCAGCGTTTACCTTTCCACTCGACTCGACTCTATTGTTCGTTCT
ATTTTGGTGGAAATTGCTGGTTTATCTATCGGCCGCCAGATTCCGGGGCACTTGGAAAATTTTATTTTCG
GTTTTATGTGCCGATAGATAGGGGGATATTTATTGCACACACTTTTACTCTGCGGCGGAAATGCTGGATG
TACTGGACGCAATTTTGGATTAGCTGCTCTTCAATTTCCATCAAGGCTTATATCAGAGTTCACATTTTTT
AGAAGATTCAAAGACTGGAATAAAAGTAAATTCAAAAACTATGATTTTTGTATGTACTGACTGGTTATTT
AAATTAAATGTAATGACTTGTTGCCCCACAAAAGGAATCGAACTCAAGGTTATTCCACTCTATTTGAGTG
CTGATGAATCGATTCCATGACAAAACTATTTTTAGCGTGttttttttGGCCAAGGCGAAACCGAAGTAAT
TTGGGTCAACTTTTGGGTGTTGCATGTGGCATGGATATCCGAAAATAAAATGGACTCATGCACCACTGTG
CATGCAACATCTTAGACAATGGCAGCAGCAACAGTTGCATCCGCCCTCGTGGCAGGCGAGTGTGAAAACC
TAAACAGTTTCGAGGAGCCATGTGGCCTGCGGATGACTCGGTCCGATTAAAGTGGTTTCAGTACCAAACA
GTTCGATAGTGATGAAATAATTACAATGTTTTTAAAAAAATATATAAAATAATGACTAGAATTAGCCAAAT
CTAAGAAAACAGTGCAAATTATCTGAACATAGCTTAAGAATTTAAAGATGCTAAAAAATCATCATGTAGA
TATTGTCTTTGCTGTCATCTCTATCCTGCATCAGCCTGAATCTCATCGTCGAGTGCATCTGTGAGCTGCA
GCTGCagccttagccttagccttagATTTTGTCCAAGTGCGTCTGCATCCGCCAAAAGATGAGGGCAATC
GACGCCACTTGACATTGAAACAATTGCACGTGTGTGCCAGTGGGCGGGTGTGCACCATGTAGCATGGTTT
CGAGGTGCAGTGTTTGTTAACCTGGTTGCGCCACTGACACCGAAATGTCAGTTAATCCCATTGACTTTGA
CTTGCCCCCGAAAACGAGATAGGGATGCAGTCAGGCAGAAAAAGGACAAAGCTGCAATATGCACGGGAAT
TTCTCTTTCACTTTAACATTGTACACAAACATGTTGCGAAAAACAAGCAAAATCTCCATATCGTGGATGC
ACTTAAAGCAGCATTCAAACAAACCAAAAGTTAAAGATATAAAGTTTACAAAACATTTAATGCTTAAACC
AAAAATAAACCAGATCAAAGTACTTCTTATATGACTTAGTTAAGATAGATGCCtttttttttttttGGAA
CAAGTTTCTCAAGTTAATACATATTCCCCTGCTCTGTAACCCGTTTTTGTTTGCTGTGTAGGCCCATTAC
TTCCCACACAGTTGCTCGCTGTTGTTCTGTGCAATCAGGCAAAACTTTTAAACGCCCTAACAAGCAACAA
GGAGCAGAGTCGAGTAGCGAGGAGTGCCGAGGAGGGAGTGTATTGCAGCCATGTTGCAACATTGAAAGTG
CGACGGTATGGGCGATTCTCTCTGCAGGGGGTGGTGCCCAGAAGCCGGAGTCGGGGGCAGGTCGAGGGGC
AGACGAATGGCAGGCGGAAGCATCGAATGGCGGCCAGGAAACACTAACGAAACAATGGACACAGCGGCAC
CGGACGGAAGGGAGCGAGAGGAAACGTGTACATGTCCTGGGTGTGGCTGCATACTTCACCACAGCACTGT

GCCTCTCGAGTGGCGCCTCCTGGCGGAAGTTTGAAGAAACCGCCACTTGAGGCATAATTTTCAAGCCGAA
AGGAAAGGCTATTGAGGAAACGTTCGATGCTCGTTTGTTTGCCACTTTGCCGGCCCTTGTGCCGTCGAGT
GATATGGCTACGTGAAACAATCAAGAAACAATTGCTAACTCAAACTGACTAGTTGGCAAATAAACGTCGT
AGAAGTTTCATTTTTGTAAAACCCCAACATTCCTAGATAGATAAGAACAAAAACTATTAGTTCTTTGGTA
GAATTTTAAATAATTATTATGAGTTAAAAAAGATAAGACTCTATGTTTATGAACTCGAAAAGAATTTTGT
TTTATCTTTTCCAATGATAAAAAATAATAACCATATTCCAATCAGAAGAACGTTACAAGTTTAAATAttt
tttaaagttatagtttatatacgagtttttatatagtggaatttttactttAATTCGTCTGAAAAAGTG
CGTAACATCTTAAATATATTTATTGCGATCTGGAGAATATAATTTAAATACGCTCCGGGACCTATTAAGA
CCAATTCTTGGAAAACATTTCGATTTGTAACTTTGGGGAGAATGAAACATTCCGTTTTTCTAAAGCCTTA
AAATGCGTCACCGATTTTTCTTTCAAATTCTTGAATAATTACTTATATTTCTTGATCAAATTATAttttt
ttttATCAATATGGAGCTGTCGACAACCCATCCAATCTGGAACACCAAACATATGTAGCAAAGCAATTAG
GCTGGGACCCGCTTCTCCCAAATTTCGGAGCCAAGCAGTCCACAAAATGACAATAAACAGGCTCGTTAAA
TAAACTATAAAAGATGCCAAATACAGCACAAATCGAACGTAATTGAGCAAAGTCAAATGTCGCCCGAGTG
CCAAAAGGCCTCGCGACCTGCAACCCATGGAACGCCACAGAAAATGGAAAATGGCATTCGATGGATGGGT
GGTTTTGGCTTGGGCTGAGCATGGGGTAGGTGCAGATTGTTTCGGACAAGTGCACAAGTGCTCCACTTTG
CCGCTGTCTGTCACTTATTCGAAAGAGCGCAGAGTTCGCGTGATaaaacaaaaacagaaaaaaaCGTCAA
TTTGCTTGCCGTTTGTCGTTGGCTGCGGTTTGTGGTTCCGGCCCCTCACCAAATCGCatatatatatata
tTGTAGTTCACCCTCTGCTTCTGGATCCCAATTCATTCAATAATTTCATTGGCCCATTCATTGTTTTCAA
ttttttttttttttGCTCTTCTTCAATCATTTTAGTCCATATTCGCACAGTGATAATTTAATTAAATTC
GTCAGGGGCATCGAGGTGACAACAGGGGATCCCTTTTGAATTTCAAATAGTGTGCAAAGAGCAGTATTAA
ATATTGGTGGACTTAGTTCTAAAAGTTGCTaaaaaaaaTGTTGCAAATTCAGAATTTTTAAAAAGTTATT
CGGTTTAAAGATTCATTTATAATAAATTTAAATTTTCATTTTGGGTAACTAAACCGTCTTACATACATTT
CGAAAGTATTCAATTTCAATTTTGATCTAACAACCAGTAAATGTCACTTTATTTTTCCGTGCATGTTCTG
CATTTTCAATAGTCCcttttttttcaacattttctttttGTCACGCCATTTGCTCGTAGTGAATTAAATTG
CCTGCTTTCCTTTAACTCTTAACTCTTTGAAACAACAACAGGTAATTACAACCGACCTTAACTATTTTGG
GGCGGTGGTGCCAGAATTCTTGGGAAAATAAAGCTTTACCGGAAAACTTTCCTCGTCATTGCCTCTTGTT
GTAGGTTCAGAATCAAGATGTCAACTTCTAATTGACTGGTGGATGAGTTCAAGGCAAAATTAAAGCAGTA
GAGCAAAACTGACTATTCTATAACTTTTGGAAATTTGAGATGTACTTGTACTTATTTGCTGAATACTAAG
TAATTAAATAAGTTTATATTGGATTAAGAACAAGTGGGATGCAATCGATTAAATTTTTTTAAATACAACA
AAACAAGCATTTGTTCAATTTGTACATAGGTAGTTATATTAATATTAAAGTTCTTTCCGTCTATTCCAAA
TTATACGAACATTTCTTTATGACAGTGAGGCTGCACTGTACAATTAATCATAAATATTTGTTGGCATTTC
GACCGCCGATGGCCATGTCAATTCCTTAATTGAGGCTCAATGGCATTTTTTATGATAGCCTTAATTGACT
TACCTATTATCTCGATTTGCAGTTGCAGGTATTTGAGGCATTGAGCCATTCAATCACAGTTCCTGTCGAC
AGAATCCCATCAATCGAACGAATGCGATTGTTTTATCTAATGGTGATTTTTCTCGATTTTCCGCTCTACC
GTTATGTAGGCTTGGGAAGAGCGAGAGAGGACACAAAAGATCCTGCTGCTGGCCAACTGTTTTGTGCTAA
TGTGCTTTGGCTTGGCCCATAAACGTGGCAAAGTTCTAAAGCCATCGACTTAATTGTGTGTGGGGAGGGG
GTACCAATGGCATTCTCCATTTAAACTGCAAAGCCACTTTAAGTTGCTaaaaaaaaaaaaaaaaCAAGAA
AACCAGCGAGGAAAAAGGAATATCGCGTGGGCGCACTCGTTGATTTTGGGGTTGTCCTTGTCTCGCGACC
ACTCGCTGTTTTCGTCCTGCGCAGACTCGTTATTGTGGCTCACACACTTTCCAGTGGCGCCGGCGCAAGT
tctctcttatcctctctctctgcgtctctcGGAGCTTTCGTGTATACTATACGTGGGCGGCACCAGAATA
CGAGAGAGAGAGCACTTCCAGCGCATCCAGGCACATAGTTCCGTTAGCTCAGTTGAATTCTGCCAGCTCT
CTAGAAAACTCGCACAAAAGAGAGCTAAACCGTTAAATTAAAGTTTTAAAGTTCAAAAAACGCTGAAAAA
GTGTtaaatatatataacaaaaatatgtaataatatatagtataaattaaaaGCCACCGCAACAATTCAG
TTTGGCGCTTAATATGTGACTTCGCCGAAGTTCTACCCCCTCCTACCTCCTTTTTTTCGAATAAACGCAT

GAATCATTTTGCTGGAATACAAAAACAAATAGCTGCCAAGTGTACAAAAGAAAAGTTTTGTTTGTGCTTT
TCTTATTTGGCTGAAAACCAAGAAAAAAGTAAAACGCCTCAATTTTCCAGTCCTCTCACTCCCTCCCTGT
CTTTTGTTTATGACCACTTGTAAGACAGCGCGTGAATAATAAGTTTAAACAAAAGTTGTCAAATTTGTAC
CATTAATTGTATGTTCTCGTTGCAATGAATAGCAAATATTacaaaaaaataacattttcacataaaaata
taaaataaaagaaaaTGCAGCAGTCGTATATTATTGATTTTTGCCATATTTGTACGCATTTTGAGTTTTG
GTTAATGAAAACCACGCTCCTTAATTACACATTTTTCCATGGCTCGAAAAGCCCTCGCCTAGAGCTTTTC
TATTCGCTGCTTTCACTCTCTCTCTCTTCGTTTCTAGTTTTTCCTAGCCAAAGACAACAAAATTTCCCGA
GTTTCGTAGCGAGTAACGGGTTTCCTCGCTCTTGGACATGCGGGTTTTTCAGTTTACAATTAGACTCTAC
CGTTAGCGGTCAAATCGGGTATCCTTGAGAATCCCTTTtgcgcggatcacaacaaaatgcgcagataaaa
acaaaaCTGAATTGTTTGGTGACATAATTATATATTGCAGTGTTGAATTGAAGAAAACCAAAGTTCAAAA
ACCTGAAAAAACCATAAAGAAGTGATTGAAAAATCAGTTGAAGTGCCGTACTGAAAATTAAAGTCCAGTG
ACACGATCGAATCCCTCGGATAGCGGAGTTAGTTTAGGGTAAGTGCACTTTTCGTCGCTTTTTCTCGTTT
TTCCACTTGGGTTTTTCTCACCGGAAATGGAAATCCTGCTCAGCGTTTACCTTTCCACTCGACTCGACTC
TATTGTTCGTTCTATTTTGGTGGAAATTGCTGGTTTATCTATCGGCCGCCAGATTCCGGGGCACTTGGAA
AATTTTATTTTCGGTTTTATGTGCCGATAGATAGGGGGATATTTATTGCACACACTTTTACTCTGCGGCG
GAAATGCTGGATGTACTGGACGCAATTTTGGATTAGCTGCTCTTCAATTTCCATCAAGGCTTATATCAGA
GTTCACATTTTTTAGAAGATTCAAAGACTGGAATAAAAGTAAATTCAAAAACTATGATTTTTGTATGTAC
TGACTGGTTATTTAAATTAAATGTAATGACTTGTTGCCCCACAAAAGGAATCGAACTCAAGGTTATTCCA
CTCTATTTGAGTGCTGATGAATCGATTCCATGACAAAACTATTTTTAGCGTGttttttttGGCCAAGGCG
AAACCGAAGTAATTTGGGTCAACTTTTGGGTGTTGCATGTGGCATGGATATCCGAAAATAAAATGGACTC
ATGCACCACTGTGCATGCAACATCTTAGACAATGGCAGCAGCAACAGTTGCATCCGCCCTCGTGGCAGGC
GAGTGTGAAAACCTAAACAGTTTCGAGGAGCCATGTGGCCTGCGGATGACTCGGTCCGATTAAAGTGGTT
TCAGTACCAAACAGTTCGATAGTGATGAAATAATTACAATGTTTTTAAAAAATATATAAAATAATGACTA
GAATTAGCCAAATCTAAGAAAACAGTGCAAATTATCTGAACATAGCTTAAGAATTTAAAGATGCTAAAAA
ATCATCATGTAGATATTGTCTTTGCTGTCATCTCTATCCTGCATCAGCCTGAATCTCATCGTCGAGTGCA
TCTGTGAGCTGCAGCTGCagccttagccttagccttagATTTTGTCCAAGTGCGTCTGCATCCGCCAAAA
GATGAGGGCAATCGACGCCACTTGACATTGAAACAATTGCACGTGTGTGCCAGTGGGCGGGTGTGCACCA
TGTAGCATGGTTTCGAGGTGCAGTGTTTGTTAACCTGGTTGCGCCACTGACACCGAAATGTCAGTTAATC
CCATTGACTTTGACTTGCCCCCGAAAACGAGATAGGGATGCAGTCAGGCAGAAAAAGGACAAAGCTGCAA
TATGCACGGGAATTTCTCTTTCACTTTAACATTGTACACAAACATGTTGCGAAAAACAAGCAAAATCTCC
ATATCGTGGATGCACTTAAAGCAGCATTCAAACAAACCAAAAGTTAAAGATATAAAGTTTACAAAACATT
TAATGCTTAAACCAAAAATAAACCAGATCAAAGTACTTCTTATATGACTTAGTTAAGATAGATGCCtttt
tttttttttGGAACAAGTTTCTCAAGTTAATACATATTCCCCTGCTCTGTAACCCGTTTTTGTTTGCTGT
GTAGGCCCATTACTTCCCACACAGTTGCTCGCTGTTGTTCTGTGCAATCAGGCAAAACTTTTAAACGCCC
TAACAAGCAACAAGGAGCAGAGTCGAGTAGCGAGGAGTGCCGAGGAGGGAGTGTATTGCAGCCATGTTGC
AACATTGAAAGTGCGACGGTATGGGCGATTCTCTCTGCAGGGGGTGGTGCCCAGAAGCCGGAGTCGGGGG
CAGGTCGAGGGGCAGACGAATGGCAGGCGGAAGCATCGAATGGCGGCCAGGAAACACTAACGAAACAATG
GACACAGCGGCACCGGACGGAAGGGAGCGAGAGGAAACGTGTACATGTCCTGGGTGTGGCTGCATACTTC
ACCACAGCACTGTGCCTCTCGAGTGGCGCCTCCTGGCGGAAGTTTGAAGAAACCGCCACTTGAGGCATAA
TTTTCAAGCCGAAAGGAAAGGCTATTGAGGAAACGTTCGATGCTCGTTTGTTTGCCACTTTGCCGGCCCT
TGTGCCGTCGAGTGATATGGCTACGTGAAACAATCAAGAAACAATTGCTAACTCAAACTGACTAGTTGGC
AAATAAACGTCGTAGAAGTTTCATTTTTGTAAAACCCCAACATTCCTAGATAGATAAGAACAAAAACTAT
TAGTTCTTTGGTAGAATTTTAAATAATTATTATGAGTTAAAAAAGATAAGACTCTATGTTTATGAACTCG
AAAAGAATTTTGTTTTATCTTTTCCAATGATAAAAAATAATAACCATATTCCAATCAGAAGAACGTTACA

AGTTTAAATAtttttttaaagttatagtttatatacgagtttttttatatagtggaatttttactttAATTC
GTCTGAAAAAGTGCGTAACATCTTAAATATATTTATTGCGATCTGGAGAATATAATTTAAATACGCTCCG
GGACCTATTAAGACCAATTCTTGGAAAACATTTCGATTTGTAACTTTGGGGAGAATGAAACATTCCGTTT
TTCTAAAGCCTTAAAATGCGTCACCGATTTTTCTTTCAAATTCTTGAATAATTACTTATATTTCTTGATC
AAATTATAtttttttttATCAATATGGAGCTGTCGACAACCCATCCAATCTGGAACACCAAACATATGTA
GCAAAGCAATTAGGCTGGGACCCGCTTCTCCCAAATTTCGGAGCCAAGCAGTCCACAAAATGACAATAAA
CAGGCTCGTTAAATAAACTATAAAAGATGCCAAATACAGCACAAATCGAACGTAATTGAGCAAAGTCAAA
TGTCGCCCGAGTGCCAAAAGGCCTCGCGACCTGCAACCCATGGAACGCCACAGAAAATGGAAAATGGCAT
TCGATGGATGGGTGGTTTTGGCTTGGGCTGAGCATGGGGTAGGTGCAGATTGTTTCGGACAAGTGCACAA
GTGCTCCACTTTGCCGCTGTCTGTCACTTATTCGAAAGAGCGCAGAGTTCGCGTGATaaaacaaaaacag
aaaaaaaCGTCAATTTGCTTGCCGTTTGTCGTTGGCTGCGGTTTGTGGTTCCGGCCCCTCACCAAATCGC
atatatatatatatTGTAGTTCACCCTCTGCTTCTGGATCCCAATTCATTCAATAATTTCATTGGCCCAT
TCATTGTTTTCAAttttttttttttttGCTCTTCTTCAATCATTTTAGTCCATATTCGCACAGTGATAA
TTTAATTAAATTCGTCAGGGGCATCGAGGTGACAACAGGGGATCCCTTTTGAATTTCAAATAGTGTGCAA
AGAGCAGTATTAAATATTGGTGGACTTAGTTCTAAAAGTTGCTaaaaaaaaTGTTGCAAATTCAGAATTT
TTAAAAAGTTATTCGGTTTAAAGATTCATTTATAATAAATTTAAATTTTCATTTTGGGTAACTAAACCGT
CTTACATACATTTCGAAAGTATTCAATTTCAATTTTGATCTAACAACCAGTAAATGTCACTTTATTTTTC
CGTGCATGTTCTGCATTTTCAATAGTCCcttttttttcaacattttctttttGTCACGCCATTTGCTCGTA
GTGAATTAAATTGCCTGCTTTCCTTTAACTCTTAACTCTTTGAAACAACAACAGGTAATTACAACCGACC
TTAACTATTTTGGGGCGGTGGTGCCAGAATTCTTGGGAAAATAAAGCTTTACCGGAAAACTTTCCTCGTC
ATTGCCTCTTGTTGTAGGTTCAGAATCAAGATGTCAACTTCTAATTGACTGGTGGATGAGTTCAAGGCAA
AATTAAAGCAGTAGAGCAAAACTGACTATTCTATAACTTTTGGAAATTTGAGATGTACTTGTACTTATTT
GCTGAATACTAAGTAATTAAATAAGTTTATATTGGATTAAGAACAAGTGGGATGCAATCGATTAAATTTT
TTTAAATACAACAAAACAAGCATTTGTTCAATTTGTACATAGGTAGTTATATTAATATTAAAGTTCTTTC
CGTCTATTCCAAATTATACGAACATTTCTTTATGACAGTGAGGCTGCACTGTACAATTAATCATAAATAT
TTGTTGGCATTTCGACCGCCGATGGCCATGTCAATTCCTTAATTGAGGCTCAATGGCATTTTTTATGATA
GCCTTAATTGACTTACCTATTATCTCGATTTGCAGTTGCAGGTATTTGAGGCATTGAGCCATTCAATCAC
AGTTCCTGTCGACAGAATCCCATCAATCGAACGAATGCGATTGTTTTATCTAATGGTGATTTTTCTCGAT
TTTCCGCTCTACCGTTATGTAGGCTTGGGAAGAGCGAGAGAGGACACAAAAGATCCTGCTGCTGGCCAAC
TGTTTTGTGCTAATGTGCTTTGGCTTGGCCCATAAACGTGGCAAAGTTCTAAAGCCATCGACTTAATTGT
GTGTGGGGAGGGGGTACCAATGGCATTCTCCATTTAAACTGCAAAGCCACTTTAAGTTGCTaaaaaaaaa
aaaaaaaCAAGAAAACCAGCGAGGAAAAAGGAATATCGCGTGGGCGCACTCGTTGATTTTGGGGTTGTCC
TTGTCTCGCGACCACTCGCTGTTTTCGTCCTGCGCAGACTCGTTATTGTGGCTCACACACTTTCCAGTGG
CGCCGGCGCAAGTtctctcttatcctctctctctgcgtctctcGGAGCTTTCGTGTATACTATACGTGGG
CGGCACCAGAATACGAGAGAGAGAGCACTTCCAGCGCATCCAGGCACATAGTTCCGTTAGCTCAGTTGAA
TTCTGCCAGCTCTCTAGAAAACTCGCACAAAAGAGAGCTAAACCGTTAAATTAAAGTTTTAAAGTTCAAA
AAACGCTGAAAAAGTGTtaaatatatataacaaaaatatgtaataatatatagtataaattaaaaGCCAC
CGCAACAATTCAGTTTGGCGCTTAATATGTGACTTCGCCGAAGTTCTACCCCCTCCTACCTCCTTTTTTT
CGAATAAACGCATGAATCATTTTGCTGGAATACAAAAACAAATAGCTGCCAAGTGTACAAAAGAAAAGTT
TTGTTTGTGCTTTTCTTATTTGGCTGAAAACCAAGAAAAAAGTAAAACGCCTCAATTTTCCAGTCCTCTC
ACTCCCTCCCTGTCTTTTGTTTATGACCACTTGTAAGACAGCGCGTGAATAATAAGTTTAAACAAAAGTT
GTCAAATTTGTACCATTAATTGTATGTTCTCGTTGCAATGAATAGCAAATATTacaaaaaaataacattt
tcacataaaaatataaaataaaagaaaaTGCAGCAGTCGTATATTATTGATTTTTGCCATATTTGTACGC
ATTTTGAGTTTTGGTTAATGAAAACCACGCTCCTTAATTACACATTTTTCCATGGCTCGAAAAGCCCTCG

CCTAGAGCTTTTCTATTCGCTGCTTTCACTCTCTCTCTCTTCGTTTCTAGTTTTTCCTAGCCAAAGACAA
CAAAATTTCCCGAGTTTCGTAGCGAGTAACGGGTTTCCTCGCTCTTGGACATGCGGGTTTTTCAGTTTAC
AATTAGACTCTACCGTTAGCGGTCAAATCGGGTATCCTTGAGAATCCCTTTtgcgcggatcacaacaaaa
tgcgcagataaaaacaaaaCTGAATTGTTTGGTGACATAATTATATATTGCAGTGTTGAATTGAAGAAAA
CCAAAGTTCAAAAACCTGAAAAAACCATAAAGAAGTGATTGAAAAATCAGTTGAAGTGCCGTACTGAAAA
TTAAAGTCCAGTGACACGATCGAATCCCTCGGATAGCGGAGTTAGTTTAGCCCCCCGAATTCGAGTCCCC
GCACGTTGTACACCTGGTTTTTCTCGCTGGCAACGTAGTCGGCCATTGAGTTGGCCGATACCAAACGACC
TTCAAAACGTTTTGCGTCGAGGCAATACGCACCATGGGCTGCGCACAGTCTGCCGAGGAGCGAGCCGCAG
CCGCCAGGAGTCGCCTCATCGAGCGCAATCTCAAGGAGGACGGCATACAGGCGGCAAAGGACATCAAACT
CCTGCTGCTGGGTGAGTTGTCCCCGGGAACGTAATCGAAACCAAATGAATCGAACGGAAACCAACCCAAC
TCAACTCGCTGAGTGTGCCACACACATGAATATTTGATTCGCGGCACGGCTAAAATTAGTGTAGCATACT
TAGCGGCTGCCCTGGCGTCTCATTAAATCCGCTAAAATGTCCTTAGGGCATGAGCTTCGGCTCTATGGCA
GCCTTGGCCTTTGACCGATGCTATTTGTGGTGCAGAACAGGACAGTCTAAGGTCCTTTGCTTTGACGCGT
TCTTAGCTAAACGTGTTTCTGttttttttttAGTCACGACATTCTTACTCGTTGTTCTTTCAAAAATAAA
ACTTTAACATAATGTAAGTTCCTTGACTCTTTTTAAAAATAAACGAACGGCTTGGAGGAATAAATCATCA
TACAATCAGTCCATCTTGAAAATGAAAGTCATCATGACTGGAACTTTTGCAACCCGATAAATAAATCCCA
CTCAACTTCGCTATTTAATTATTTAATTACTTTATATCGATTGTGCCGACAGAGGAACTGCAAGTGGTGA
TTCCATTTTCTTGGCACAGTTCCCTTGGAGCGAATGTGAAATGCCATCAATTTAATAATCACAATAATAT
TTTTATGCATCAGACAGAAAGTTCAAGGTCACTCGCTTGCGGGTGAATCGAATGGAGAGTTTGTGTGCAA
AAGTCGGGCTATATAGTGTGCGCATATGTACATATGTAAATAATAATATATGGCAGTTGATTGTAATTGC
AATTGGATTCCGATTTGTGATAAGCGAGCGTAATAAACATGTCCGCTCGGAGGCATTAACAAATGAAAAA
TAGACATTGCATCGGCATAAATAAGGTTTGCGAATTGTTTCCGCTTGCGAAAGAGTATGAGAATTGACCA
AATTTGGTCGGAAAATGTAGGAAAATTGCATTTCGCTATTCGTTCAGCTGGGCGCGTGAGTAATTTCCAT
CTATATTTTCATATACTCTGCAGTAGCTCTCATCTCATCTCCGGCTGCATATTTCCAAGCAAGCAACAGC
CCCATTTCGCTGTTTACATTTCGGGCATTTCATTTAATTTCCTCTTTTGCTCGGACTGAGCATTTTCGGG
CGAAAATGTTTGGCAGGGGGAGAAGATTTCAGTTTCTGACAGTTTTGCGTATGTTTCAGAATAAAAATGT
TCATTTTCAGGCAACACCCACTGCGTGTGTGTGATGGCGAGTGTACTGGAAATGTTTATTTaaaaaaaaT
TGTATTTTTTGTACTGCCATGCCGTGCGCATCCAAGAATTAATAATAATAATCAGAGCAACGCGTATGAA
AATAGCTCCATCCTGCTCCGTTGCCGAATCTTTTGTTTTCCTGCAATCCACGAAGGCATTAAAATTAAGC
ACTTTGCCTTCGATTTCCACACAAACAAAACATTAATCTCAGGTGCCTACTCAGTCCCCCAATTGCCTCT
ATAAACAAGCCAGGCAAAATTTCCCTGTTCGCTGCTGCTCTTGAGTTTTCCAAATCTAAACATTTTCTGA
GAGAAGCTCTTTTTTCGTGAACTGTTCTAAGATCTTTTCAAGAAGTGTGGACTTTTTCAAGTGGTGGACA
ATGAACTCTTTATTCTTTTACATGGAAAAACAATTTTGAATTGCAAACTATTTTAGTTTCATGGCGAGTT
GTGCACCTTTTCTTTGATAGAAATCAGAAGGTTAACAAATTTCAGTATCATTATAATATTGAAATAATGT
AAGCTTTGATGTATGTACGTATAATGTTAGTTTATAGGCACTCAAGGAGTAGTTTTATCAAGCATTTTCA
TACTATGGCCAACCAATTGTGTTTTAATTAAAGCTTCTTAGTGAGGATTAAGCTTAGTGGGAAACGAAAA
TGGGCAAATGAAATTAGCATTTATCAAATTATTCCATTTTCCCAAGGACAGCTTGGCTAAGCAATTTTCC
AGCCGAGCTGCCAGTTATCTTGATTATCTCGTTTATCACAAAACCCCCTTCAGTAGACACTCAATTAACA
AATTGCATTCAATTGCATGGAAATTCTGCTGGAATCGCAGCTCGCAAATCCTTCAAAGCCTTCAGCTAGC
aaaaaaaaaaaaaattaaaaaCACCCAATGAAATAGAGATGTAGACGAAAGTAAAGGAGTCTATATACACA
ATCGCCGGATGACCAGGGCCAAACAATTAATCCTTTGTTTGCCATTTATTCTCTGCCATTTATGCCCCAC
CTCCTTGCGTTTTTCTCATTTCTTTGGCCCAACGGCATCTCAAGATGATACATTGACAACGGCGATGATG
ATAATAATAATCACGATGCTGATGTGGGTGGATGGGGCTGGCTGGCTCTGTGTGCTCAAAGGCCATTAAG
TCCAACTTGAGGAATACAAAGCACAGACATTTAGTGCTCGAGAACTAAACGCAGCATAAATATTTGCAAC

CCCCGTCGGGTTCTTTTATCGGCGCCGATAACCGCATAACCAGACGCATAAATAGATCCTATATATGTAC
ATTACATTACATCTGTAATCTTAAATTCCACCACGCATTGCTAACTTTTTGTGCATTTCTTCTAGGGTGC
CGGTGAGTCGGGCAAGAGCACAATAGTCAAACAGATGAAAATCATTCACGAGAGCGGCTTCACTGCGGAG
GACTTTAAACAATATCGACCGGTTGTCTACAGCAACACAATACAATCATTAGTTGCAATATTGCGCGCGA
TGCCAACCCTAAGTATTCAGTACAGCAATAACGAGCGAGAGGAGCGATGCCAAGATGGTGTTCGACGTAT
GCCAACGCATGCACGACACCGAGCCCTTCTCGGAGGAGCTGCTGGCCGCCATGAAACGCCTCTGGCAGGA
CGCCGGTGTCCAGGAGTGCTTCTCGCGCAGCAACGAATACCAACTAAACGATTCCGCAAAATA
>dme_circ_0003876_junction_seq
AATACCAACTAAACGATTCCGCAAAATAGTTGAAATCGTTAAACTGTACATAAGCA
>dme_circ_0003877
GGTGCCGGTGAGTCGGGCAAGAGCACAATAGTCAAACAGATGAAAATCATTCACGAGAGCGGCTTCACTG
CGGAGGACTTTAAACAATATCGACCGGTTGTCTACAGCAACACAATACAATCATTAGTTGCAATATTGCG
CGCGATGCCAACCCTAAGTATTCAGTACAGCAATAACGAGCGAGAGGAGCGATGCCAAGATGGTGTTCGA
CGTATGCCAACGCATGCACGACACCGAGCCCTTCTCGGAGGAGCTGCTGGCCGCCATGAAACGCCTCTGG
CAGGACGCCGGTGTCCAGGAGTGCTTCTCGCGCAGCAACGAATACCAACTAAACGATTCCGCAAAATA
>dme_circ_0003877_junction_seq
AATACCAACTAAACGATTCCGCAAAATAGGTGCCGGTGAGTCGGGCAAGAGCACAA
>dme_circ_0003878
ATCATGGTTCAGGGCATGATTCCTCTGTGCTCCTGGCAGTATGAACGTACCTTCAACACAGCACGCGTGC
CAGGCCTGGAAACCGATCGTATCATCCACTACAGGGACTCCAATCACATTGTGGTTCTCCACAAGGGCTG
CTACTACAAGATGCTCATCTACTACAAGGGTCGCATCCTGCGCCCATGCGAATTGCAAGTGGCAAATTGA
GGAAATCCTGAAGGGCAAAGCAACTCCCGTGGAAGGTGAGGAGCACTTGGCTGCCCTGACCGCCTGGAAT
CGCTCCAAGTGGGCGGAAGCTCGCAACACATTCTTCTCGTGGGGAGTCAATCAAACCTCTCTGCGCACCA
TCGAGTCGGCCGCTTTTGTGCTCTCCCTGGATGATGAGCCCTTCGAGTTCGATTTGGCGCGACCGGAATT
GCTGGACAACTTTGGCAAGAAGCTGCTGCACGGCAATGGCTACAACCGCTGGTTTGACAAGTGTTTCACT
GTCTGTGTGGGCACCAATGGTCGCGTTGGCTTCAATGCCGAACACACCTGGGTAAGTGGATTCCAATCGC
TGCAGCCAATATCTTCCTCTCAGCATCCATAAAATTTCTACGATTTGCCATACTTAACTATCTGAACCGA
TTTGTAAAATTACAATCCAAAAGGTCCGACGCTGCCATCGCGTCACACATGTGGGAGAACCTGATCGTCG
ATGATCTTGTATCGGACGGG
>dme_circ_0003878_junction_seq
GATCGTCGATGATCTTGTATCGGACGGGATCATGGTTCAGGGCATGATTCCTCTGT
>dme_circ_0003879
TGATTACCGTGGATAGGAAGTACAATCTGCGCACCCAGGAAAGTTCCGGTTCTTCGGCAGATCTGCCAAG
TACTTCCAAGCAGAAAGTAGCTGCAGTCCAGCACAAATTGGCTGAATTGGCCAGTTTGGACCAGAAGAGC
GAGAATGAGGAACCCACGGACTTGGACAATGCGGCGAGTCTCAAGAAAGCCATGGCCACCTCGGATGCCA
TGATAGCCCTGCAGCAACTGGCCTCCATTTCCACAGCTAGATCGCTGCAGCATCTCGTCCAGAACATGAG
CAATATAGACAACAGTGCCCTGGTTCCTGGACGGAAGCTTCCGCGGAACGCAGCGAAGAGATCACCCAAA
TATGAGTCCAACCGATGTCCACTCTGCTCCCGTGTCTGTCGATCCCAGGCCTTTCTTAACGAACATATGC
GCAAGGAGCACTCTGTGCTCATTTAGACACACTTTCATTTTAACTTCGTTTGGATTGGAAAATTGGAAAA
TCCAACTCATGTCAGTCTATTCTATACCATTCTATAACTATTTGGTTAGTGTATATAAGAAGTGACTTTA
TTGATGGCTATTTAAATGCATACAATTTAACTTGAAAACACTAAGTCAAATGTGTCCACTTATTGGATAT
TAAAGTTACTTCATTTTATTCAAGTCCAATTTAATTCCAAATAATTGATTTTATCAGAACTGACAGTTAT
GTATTCACAAATGATACATACTTTTATTCCCATATGGATACTAACTTTATTGAAAGAGAAATTCAGCCAA
ATTTAAATGACCAAAAAGCGTAAAATATAATTCTAAAATATGTATAAAATAAGCCATAAACTTGCCCAAT

TTTTAAGAGGTAAAGTCAAGTGCATAAGTATACTCTTCTTTTTAAACAGAAGTTGCGCTGGTAAAATATT
ATGCAGTGTCATAAGGTAGACATTAGGTAGCGGTTATTATCCATACAGTAAACGAGGTCCAATTTAAAAG
TTTTTATACTTATTTCTAATAAAAAAGAAAGTTTGATATTAAAACAAGGTATACAATACACATGGTTTTT
ATTGTGTCATTGTTTAGATAGCTTAATGTTTTATTTATGCATCTATTTTTTATATAATTTCGTTTAAGTT
TTCTGAGGACTTTTATTGCTTGGCCGTTTTTACTAGAAAAGGTTAGTATGTGCTGTACTCAACAACGCtt
tatttttaatttatttttatttttatttttctttttcagtttcatttctcttttatCTCATAATTGGCAA
ATGTGTTTTAGACAAAAGGAAGCAAAGCCAGGCAAGTACCTGATTATATTTCAGAGCCATTCAGTCTTCG
GAATTCTTTGTAATTTTCTTGTAAAAACTGCACTGGGATTCGGACATCCAGACAAGTTGATATGAATCGG
CAAATACCCGAAAGGAATAATCCTTTAACCAAACAGGTTTTTGGTAACAATAGTTGGGTGGTTGGTGCCC
TTGAATCTGCACTGCCGTGCCAAAAGGAAAATCCCTTTCCTCGAGAGTCGGCCAACCACACACTAACTGT
ACTCTCGGGTCTTTTGTCCTCGAAAGAGATATGTATCTCCCAGATACACGCTCTATGCCATCCATGCATC
TCACGCTCTCGCCCACACAAATCCCGAAATGTATTTGAAAAACATTTGCGCACACATAAATCAGGATCTG
TTTTATGTGTTTGTGTGTTTTTCTAAAAATAAGTTTCACATTTTCCAATGCCTGAGCCCAGTTTGCCTGC
CTGCCCGCTTTGTATCTACCTCTATGCACATATGTATTGTACATATGTATCTGTAACTCCGCGCTGGTGC
GGCTTTTGCAGTCGGGCTTCCCACGTGATTTTTACCGATTTTTCCAATTTTGCCTGCTTGCCCTATCGGT
CTCTCCTTTTGGGCTTTGCCTTCGCAAAGCCTGGCCTGATCTATTCGAAGTATCTACACAAATGCACAGG
ACACACAAGTTTTAGGGGCTGGTTTTCAAGCTATTTGTGTGGGACATTCCTGGAAAATAGTCGGAAAACC
TTGATCGACAAGATAATTGTGTGTGCGTGCATCTGAAGATATTTCCAGAAGGAAGCGTCCTCAAGCTGGA
GTTCTGGAAAAACCCAGCATATGTGCGTTTATTTATGACCTAGCTGTTCCTAAGTTTTCTTCATTGCTAA
TGCATTTAAAATGACCAATTTTTTGGAAAAGAGTATAACGACGatcatcatatctatatcatatatatat
cacatatTTTTAATCCCAGCTTTTATTTCGAGAATCATTCAACTATAAAAACAGTCCAGAACTTTTTCAT
TGTACTTCTAATTGAAAATGAGAACTTATATACTTCACCAAAATAATATTTATTTTTTCACATATAAAAA
GAGTTTTCTTAGAGTTATAAATTTAAGGAATCCGAAACATATTATTTTTGTCGAACTATACATTTAAAAT
GGCTAAAAAAGTATCTGATTTATATTCTTGAATCCCAAAGTAACTTATTTAGACATTTCAGAAATGGAAG
TGAGGATCTAAGGAATATAAAATTATCTTAGCCATTAGGGCATGGTTTTTATTTAAACTAAAATACAACT
CCACAATTTTAAAATACAAAAATGTATCTATAACAGTTCTTGTAGAGGATGTCTATACTAATCTAGATTC
TGGAACCCATCCAATCTAACTTTGCCTCCGTTGACCTTGAAAATCCGACATGTAATTTGAACAGATGCAT
TTGGAATAGGAAAATAAACATGGAAAACACCACCGGAAGCTGTGTTTATATGTATTCAACTGCGCCTGCT
GGAAATGCTTTGAAATGCACTCTCTCCGCGCTTAGATAAGCAGAGAGTGGTGTTTCTCTCAGAGAAAGTT
TTCCGAATTGCAGTTTTCCACAAATAGAAAGAGAAGAAGGGACAACAATCAACCCACAACAACAACTGTT
GAGCTGTTTAGGTAGAAAACCCGAAAATAAATCAAGGATTCGAAATGAAGCCGATCCCAAAAAGCAGGCA
GGCTACACTTGCTGTGCAACAGATACACACGGCTCACACACAGATACTCTCTCTcacacacacacacaca
AATGCAGACAGACGAGCATTTTTCAGATGCAGCTGCGTTTCGGAATCGAGGACTTGAGGATTGAAAATCC
GTAAGCTAGATCCAGCCAACCAGTGCAGTGTTGATTGTGTTGCCGTGTGGACGCTTTTTGGTGCGTTGGT
CGTGGGCTGAAATGCAAATTACCAGCGAAAAAAGCTGTAAAAGCTTGCAATAGCCACTGTGATATTAAAT
AAAAAAATTATTTTAAAATTTTAAATGATTATGTTGCATCGGCCCAGGCCAACTTCCAAAGGCAAAAACA
GCAGCCTGCACTAGAAATGTGCTTTTAAACGTGTGTTGTGATTATAAATATATTAAATTAAATATACCTG
GTGACAATAATAAGTGTCTAGATATGTGCCTCATTTTCTAGCACTTTCAGATATTTTTTAATTACCAGGC
TTTATTATCACATAACTACACaaaaaaaaaGCAGTGAAGAAGCACATTCCACAAATTATTAAACATACAA
ATTAATATGCACttttttttATTTATTTATAGGTGCTACACATTTGCGGATTAACACAGTGCTTAATTTA
AATCATTTCATATATAGCTAATCTAtttttttttCTATTTTCTAGATGGCTCAGGTTATGAGTTATTGTA
AAACGGTTTTGGTTaaaaaaaaaaaaaaCTCACccatcacaaaaaaccatcaacacacatcagccacacc
atcaacatccatcatcaaacaaaAATCAATGTGTTCATTTGCCGAGCTTTTCAATTTTATTTGTTTATGC
CAAAGATGTGTTTACCGAATTAATAAAATTTTTTACAATGTGTACTTTTATTTGTTTAATTTTAAAAAAA

TGCGAAATTATGCGTTTTGCTTTACTTATTTATTTACTTGTAGTGTATTTTGCCTATTTATTGCATGCCA
TTCATCTAACAACATTTTTTGCCCTAATCCCAGCAGTCCCAGTGTTAAAACTTGCTTTTTCATTTGACGC
AGCTCAATCCAAACGATTGACATAGACCAAAGCGAAGGAACTTCGAACGGAGTGACTTGAACGAAAAAAC
CCAGAAACTAAATTAACCAATCGCAACCATGTTTCCAATTGCAGAATTTGAGCAGGAAGGAGAACACCGC
ACCCGATGTGGCGTCCACCGCCGAGATTCAGCGCAGTTTCCAGCGCAGCATTCTCAATGGCAAGCAGCGG
GATGAGCAGAAGATCCAGTTGCCCGGATCTCGGCGCAAGCGCTTGTCCGTCACCGAGGTCTCCGACATGC
TGTTCGAGTTCTACAAGACCAAATCGGCCAAGGTGCCCAAGGCGGAGCAGCCGCACCGTCAGGTCTCGCC
GACATCCGGTGAGATCCTAGATCCCTCGACCATTTCGGCCATTGCCGTTTATGGTACGGCCTCCGAGACG
GCCTCAAAAAACTTGAATGCTGATGAAGTGATGCGAGTTCAGAACGCCACAGCTACGCGTGTGGTGGGTG
CTGCCGCTGGCGCCGCCGCCTCATTTCATCCAAGGCCCAAATACACCCTGAAGACAGCGGCATCGTCCAC
GGAGCATACCACGGCCATACCCACTTCGGTGCTGGTGGCCAATAGTGCGGCGGCTCTGACTCCGAAACCG
CAGGCAGCCGTAATTGCCGAGGCCCTGATGCGAAATGGACTGCACAACTTCCAGCAGCAGTTGCGTGCCC
AGGAGATACTGCGCCAACAGACACCCCATCGTCGCATCAAGGAGGAAAACGATGTCGAAATCGCTGGGGG
CGACATAACACCCACTAAAATTCTGGAGAACCTCTTGCGAAAGCAGCAAGAGCGCGACCTCCGCCACTCT
GAGTGTGAGAACGAACCGGGCTATTCGACCGAGGACGACGAAGAGGGTCGCTATCATGCCTTCGATGACA
TACACCTGATGGAGCAAAGCGGTGGCAAGTTCGGCAACAACTCCGGCATGGGCATGTTCAATGCCAATGC
CCATGGAGGATCGGCCAGCTCGATTCTAGATGCCCATCAGGCCTTCCGCAACCTGGAGTTTACACTCAGC
GATTATGGCGGCAGCAGCAGCAATGGCAGCACCACCAGTCCCAATGGCATCGGCCTGGATGGCGAACCCG
TATACGAGTGCCGTCACTGCGGCAAGAAGTATCGCTGGAAGTCCACACTCCGGCGCCACGAGAATGTCGA
GTGTGGCGGCAAGGAGCCATCGCATCAGTGTCCCTACTGCCCCTACAAGTCGAAACAGCGCGGCAATCTC
GGTGTCCATGTGAGGAAGCACCACACCGATCTGCCCCAGCTGCCCAGCAAGAGGAGATCGAAGTACTCGA
TGAATCGCGAAAACGGGATGAGCGGCTCCATGTCCGATGACTCGCAGGGCAAGCTGATCATCGACTTCAA
TGGGAAGGGAGAGCTTGAAACCAAATAAATTTGTACGGGGACCGAGTGATGCTTCCATTTGATATACAAT
ATATTTTACGTCTGGTGTCTTGTTTTGTCCATTGGATTTGTGGAAATTTATTTCTTGTCATTCATGTGTC
ATCCCAAATATTTAATGTTTACTTGTAGTAATTTATGTAATGTAAGCAAATGCAAGCCAATCTAATCAAG
CATATAATCAAATTAGTCTTAAAGCCTTTTTTCTAACGATGCATTTATGCGTTAAAGCATCAGTTAATAT
CTCTTAAATTGTATAAAATGATCATTCACCAAATGTCAATTTTATACTTACGATTAAGTGTGCAAAATAT
TATCAAACAACACTCAAATATTTTCTAAGGTCTCTACTATTTAAGATATCACGATGTTGTCCAGAAAAAA
AGTATTTAGAAAGGGTTCGCCTCGCCGTGTGCGTATTTCCAAAAAGTTCCCAGCATCTTGCCCAGAAACC
CTCTGCCATCCAACGATCTCTTGACTTGGCTTGATTAAGCTACTAAGACCAAAACTTTTGGTAACTACTC
GATAAGCTCAATCGATGCCATTTACAATGATATACATTATGACTGAATTAGTTAAGAGAATTGCATTGAA
CTGACAACTGAACAAACCATTAATTAACCAATTGAAAACCAACTATTCCTCGCCCTGAAGACATTCGTTT
CATATATTTATGGACTTTAAGACTGAGTTTATTGTATCTTAAAAATTATTAACCCATGACCaGCTTGGCT
ATACTCCCATCCCTATCGCCGGTATAAACAACTAACAAAAGCAGCAAAACATTAAGCAAAAATGTCGAAC
TTCGTGAACTTGGACATCTTCTCCAACTACCAAAAGTACATAGACAATGAACAGGAAGTCAGGGAGGTGA
AGATCAGCCAGTCTGAGGGCTTCCATCTGGATGTGGAGGACTATCTACTGGGCATACTGCAGTTGGCGTC
GGAGCTCTCCCGATTCGCTACCAATTCCGTCACCATGGGCGACTACGAGCGTCCCCTGAATATCTCCCAT
TTTATTGGTGACCTGAACACGGGCTTCCGTCTGCTGAACCTGAAGAACGATGGCTTGCGAAAGCGCTTCG
ATGCCTTAAAGTATGATGTCAAGAAGATCGAGGAGGTCGTCTACGATGTCAGCATACGCGGTCTGTCCAG
CAAGGAAAAGGACCAGCAGGAGGAGCCGGCTGTTCCTGCAACCGAATAGTTTACTTCGTACGTAGGATAA
GGCGTTTATTAAAATGAAAAAGTTTCATTTAAACAAGCAAATATATTACATTCATTAAACAGAAGTTTCA
GTCTCTCTTTTGCCACCGCAACGCTTACAACGTTAACATTGCGCGCGCGTCTTTGCATTTTCTTGTTTGC
TGCTTCGTCGTCCGTCGTCCTTCCCGTGTTGTTCTTATTGTTTTTTCCCGCTTTGTGCGCTCTTTATTGT
TTCGCCCTTTCTAGCATTTTGATTTGAAAGTGGAGACTGCAATTGGTTACCATTTCAAGAAAGGTAAGTC

TGGCGGGCAGTTGGCCGTGTGAAAAGTCCGTTGGCCCAGAAACTCACCCGCTCTCTTGACCACGCCCCCT
TTTCCGTCCCCGGCCCGaaaaaaaagctaaaaaCTGAGAGACTCCAACCTGTTTGGCAATAATTGGGTCA
CAAATCTGGTAAAAACTTGTTGCCCGAAATGGTCACTAAACAAATAGCACTGTTTTTATTACAAAACTTT
AGTTGAGTCTGCAGTTGCCttgttgttgtttgtttgttttaattttttttatttCGTTCCGCAGACATAGT
TGTTGTTTGGTGTTTTTCAAAACACGCTTTAATCGAAGCGGTTCAAAGTCCGAAACAAGTTTCCTGAGAG
GCTTAACGGGGCACGCTCTCTCCTTGTTGCTCTTTCCCCTCTTCTTACAACGCACTGAGTGGGAGGAGAG
CGAGAAATACTGAGAGAGCTTTCGACGTAAACATCTGAAAAGCAAAACCACAACAGACACCCACACACTG
ACGGGCAGGTGTTGCTCTCTGCGTCTCTCTTTCTTCCTACCCCCATTCCAACTTTCTTGCATTCTCATTT
CGAAGGGAAAATGCCAAGGAAAAGCCCAGCCGCAAACCAACAACAAAAAGTCAATATAATAAACCCAGAA
TCCGAGTGCTAACTGTTATGGAAAGTAGCGAAACCATTAGCTATGGACCTCCTTCATTGAGTGTTTTGCT
TGCATACCCGGCAATTAAAGACTTGCAGGGTATGTGTATTTTCAATACATCaaaaaacaaaaaaTAATAT
ATAGAATATATTTTAGTATCGaaaaagatttaaaaaaaattgaaaaattttaaaatACGCCCTAATATTA
GTTGTATTCCTCAAAGTCATATTTTTTTGAGTATCGTCTACTGTAAGTATTCAATTAGTAGTGGGTATGT
CTTGAGACAAAATCATTTTAGTAAGTTTGCTTGTTTATTTCGCATGGTAATTACACGTTTATAGAACTAT
TAACCTGCATTGAATATGGTTGTCAAATAAAAATATTAGGTATACCTTGTTATGACCGCTTTTAACGACC
ACACTCAACCAATAGTGTGTTACGTTTTTAAGTGTCGCAGTACTTCAGAAATGTTCACAGCACTTTTAGT
TCAATAACTCATGTGAATCCATGTAATAGTGGATATCTTGTAGTCGAGTATCTCAAAGATACCCCGCTCG
CTTTATCAATTTTCGTTGCGCTGGCCGCGGCAGCTGTGAATGATTTGCAAGGGGTCCCAGCGCAACTGGT
TCCCAGCGCCTTTGTGCTGCCCCTCGGAACCCGACCCTCCAGAGCCACCAATACCTCGATCGTACCGCTC
TCGTTAGACATGCGTGGCTGGCTGCCTCTGTCGTTCGCAGTCGCCGCACATTTCAAGTTTGCTACGGTTC
AAAGTCCAAACCCAAGATACAAATACCAGCACCAGCAGCACCATTTCGTTTTCATCGCAGTGGTTATTTT
AAATCGAAATTCTCCAATCTGGGCACACTTTCGATggggggggggggggggTTACGAAGATCCCGGTCGT
GCGATAAGCAGACGACATTTATGCCAGGTAGACACTTCCAACGCGTCACCGATTGCACTAGGTTATTCTG
ACAGCAATCACCTCTTATCGGAACCGCGTGCTCGAGCAATCCTTATCAGACCACCGAAATTGCGTACCAC
TGGAGTTCTCACAGGCCCCCCGCCACTGCGATTATTGGACTTTCAACAATAAATAGTGAGCGATAGGTTT
TATCGCCTCCCTCAATTGTTGCTTATCCCCGCCACCTCATTGGCCCGTTTTCGGTTATAGAATCAGATCG
TACATATGTACATACATATATTTAAGACTTGTTTTTCAGCTGGTTTATCAAGTAAGCTGGCTAACCATAG
TGATGTAACAGCGATAAGAACAGGTGTGGAGAGCCCATCTTCAGATCTCAAGTTGCTTACCTAAACCGCA
TGCCGATAAAGTGCCATCTGCGCAGAGGTCAGAGTCCACTTGCCGAATTTTAAAGCCTAATCTTTATGAT
ATGAACTCTGATATTACCAAATCGCGCCGTCTGCAATCTCGAATATGAGTTATTTATGTTTTGttttttt
ttttATAGCACAGCGAAAGATATATGGGATAAGCACCAAATACAATCTTATTTTATCATGCCCTTTAGAC
TTGAAAGGAATCTTATCAACTTACAGCTGTGTATGTAGATTTAAAATACTAAGTGAAATCTAAGTGAATT
GTTACCCCTGTTTATTGTCATTATGTATTGAGCGAGGCAAACTAGAATGCTAAGTATATTATAGTATATG
TGTTCATAGGCTCTCCACCTTAGTTTTGACAAGAAATATAAACGACTTTACATAATTGTAATATATATCT
TTCGAATCTTGATAAGCCGACTGATAGGCGACCTAGATTCTTAGAAATTTGTCTAGCCACGTCGAAAAAG
TTGAATTTTAAAAAACGCATTATCGAGGCTAACTGCGCATACCCCTCAAACGATGAGTACACCCATCTTA
TATACACACTTGTAGGTGTTTATTGGACCCAATCACATATCCATTTCGCCACAAGATAAGGCCCAATCGA
TCTGAAGAGAGCGAATTCCCGGAATATCGCCGGTCAATacactccacacgggcacacacccccccaccaca
cactccactTTTCGACGAGCCTCTCGACTGGTGACGCATccataccaataccatactataccatactata
tcatacaataccaaactctaccatacaatgtaccataccaAAAGCACAATCGACTGTGGGCTATACTCGT
AGTATCTCCGTCTCGGCGCTCCGCTCCGAGTGCTTTTAAGACCCTAGCGAACCCGTTGTGCTCACAGTCG
ATTCAGTCAACGCTCGGCGTCCAACCGATCAAGTCGCTTTATAAGAAGGGGGATCCGAAGCATCCCCAGT
GGGAATTTCTCGTGTGTGCATCTGGCCTAGGATTTCGCAATTGTCATCTACCTATTCCCTCTCCCTACAA
CGTTTATAAATACCACGCTCACACATTGCGGTGCAATTAACACAACCCGGAAACAGTTTACACCGAATTG

CTTTAATTCGACTAGAAGG
>dme_circ_0003879_junction_seq
ACCGAATTGCTTTAATTCGACTAGAAGGTGATTACCGTGGATAGGAAGTACAATCT
>dme_circ_0003880
GGTATTTTCTCCCATGGATACGAGAAAATTTGTTATAAAGAGACGTTTAAAATGAACAGCAATACAGATC
TCCATCATTCAGATGATCAGGACTTTTCGGAATTTCTCAATGAGTATTATCTGCAAAGCAATTCCCAAAG
TATCGAACCTGAAGCAAGTTACGAAGGTTTGTATAACAAAAATTATGTGAAAGTCAATAATATTTTCTTT
TGAAATTTAATTTAGATGGAGAAATGGAAGCAGAGTGGCTAGTTTCTGCTGGTTATCCAGAATTGACAAA
ACCGTTTGAACAA
>dme_circ_0003880_junction_seq
TCCAGAATTGACAAAACCGTTTGAACAAGGTATTTTCTCCCATGGATACGAGAAAA
>dme_circ_0003881
TTAGAAATGATTGTTGATTGCTGTTTACGCACGCTTAACGAATAGTAAATGTTGAATGGAATGGAAGCaa
aacgccgaaaaaatgtgtaaaaatgcaactaaaaaaacaaattgcaaacCGATTGAAGTTGGAACATCAT
TTGTTATATAGCTAAGATTGCGCCAACATAAAAATTTACAAAAATTTATACAATACGACAATGAATCACA
TTGCTTTTTGCACTGCAATTTAGATTAACAAATTGAAAATAGTAATCAATATATATTTATGGAAACGAAA
ACGACATTCAAAATGCCAGTTAGACTATGAAAAGTGTGCTTAAACCAATTTAAATGGAACGAAGAAATGT
TAGAACGAATTCAGAGTAAATTTAATTATG
>dme_circ_0003881_junction_seq
GAACGAATTCAGAGTAAATTTAATTATGTTAGAAATGATTGTTGATTGCTGTTTAC
>dme_circ_0003882
TCGCGGTATGTGTGCCGCCATTGCGGAAAGAAATATCGCTGGAAGTCGACGCTGCGTCGCCACGAGAACG
TCGAGTGTGGTGGCAAGGAGCCGTGTCATCCGTGCCCGTACTGCAGCTACAAGGCCAAGCAGCGGGGTAA
TCTCGGAGTGCATGTGCGCAAACATCATCCGGAGAAGCCGCAGCTAGAGAGCAAGCGAGGCCGCAAGGTC
TAGGCGAAAGTTGGGAGGATTG
>dme_circ_0003882_junction_seq
AAGGTCTAGGCGAAAGTTGGGAGGATTGTCGCGGTATGTGTGCCGCCATTGCGGAA
>dme_circ_0003883
AAAACTCCTGGACGATATCCGTGAAATCGGTCACCTCGCTGAACAATGTCTCGCCATCGAACAACTCGCA
CATTTGTCCACGCTGCGAGAAGGCCTACACGTACAAAAAGAATCTCTCCAGGCATCTTCGATACGAGTGT
GGTCAGCTGCCCACGGAGAAGTGCCGACATTGTTCCTACGTGGCGCGCTACAAGCATAGTCTTAATATGC
ACGTCAAGACGCAGCATCCGGAGCAAATCAGTGATACATTCGCGGGTTCTTCGGGATCTAGCGATGGGGT
CAGGGATAGACGGGGCCGATCACTAGTTCGTGGTCTCTTCGATTCGGCTAAGGGGGAAAAGTTCCTTGAT
TACCTTAATAACTAGTATTTAGATCCGATCTCGGGCGAAGGTAAGTTCTGCTGGAAgggggggggggTTA
CTGAAGAGAGTAAGAGACCAGAGACCACTTCTAATGATAATAAAAATGCCTTTTGTAACCAAAATTGAAA
CGTGTCATTATTGAATTCTTCTGTAGTCCTTAAGTTAGTTAAGCCTTTTTAGGGGCGCGGTTTTTATTCC
TTTTTTTGGCTGTGGTATTTTGTTTGTCCTTGGTTATCATTTAATTGTGCTGTACAGCAAACACAACCCA
CCAACACACACCTATACATAAAGCTTTAAATTTAAGAAAATAGCTTAAGAACTAAAACGAAAACGGAACC
CGAAATTTGTTTGCTTGTTTTCATCAACTTTAGTTGTATTCaaaaaaaaaaaaaaaaaaaGTTCAAAAGG
ATACTATTAGTGACAGGAGCTGAGCTTAACTACTTCCACTAAGCGGTAAACTCTTGCAACCCAACCTCTT
ACTAACCACTATTCGTTCAACTCTGCAGCATCCAAGCAGGACAAGGGCGAGCAGACCGAAGGAGCACAGG
ATGAATTCGAACTGGACGACTGTTTGCTGGAGAGCAACGACATAGTCATCACCCAGAACAAGGATGGATT
CGTGTTGCACGTAAAGAAGCTGGGTAACATTACGGCTGCCAAGCTGGAGGAGAACCAGGCGGTGGCACAA
CAACAGGGCCAAGCGGCAGTGACTGTCACCGGGCCGGCTGGACAACCCACGCCCACAATCACGGAACTCT

TGAATGCCGCCGCCGCTTCGCACTCGGAACCCAAACCCACACTGACCACTCTGACCAGTACGCCCATTAA
ATTGCCGTCATCGGAATGCGGTAAGTCCATATTTGTAATAGACAAGCAAAGCTAGAAAAGTGACACAATA
TTTATACAAATGAATGATAGTTTTTATAAATTGAAAAACTAGAGTTTTCTTAAAGTTAAATTGAAAATTT
GGAAATAAATAAATGTAATATATTCTCTCTATAAAATACGTTAATAAACGATTGTATTGTATTGTATTAT
GTATTATGATTTTAACTAATAAGACCTTTTGTACTTTTTTCCAATTCCAGAACTTATCAACATTAAAAAG
ATTATACCGGCAACCACAACAATTGCCACACATCATCCGCACACGAGTTCGACAATCATACATCCCCATC
ACATCATTCAGCATGTGTCCCAGGAACCGCACCACCAGGAGCACCATCAGCAGCATCAGACTATTCACAT
TGAGGAGGTGCCGCAGACTTCGCAACAGCACCACCAGCAGCAGCATCACCACCAGCTTCAGACGGTCCAA
CCGACCCACACCCAAGTACAAAGCATAATCACAGCTCATCCGGGCCAGACTATAAACCTGGTGGGTCTGC
GCAATGTGCAGTTGGCCGATTCGAAGCCCATAGCCTCGAGGATACGATATTCTCGTGGAAAGATCATCGG
GCCGACGGTACAAAACCTGCAGATCGTGGAAACCCATGAGCCCATCCAGCATCAACATCACGAGCTGTCG
GATGGCACCAAGTATGAGATTAGCGAGATTGATCTGAACAATCCCAATGCATCGGCGGCAATCATAAGTG
ACTTAGTGAAGTATGCCGAGATCGATGATATCGAGCTGCCCGACGGCACTAAGATTGGCATCGGCTTTGC
GCCCTCGGAGATTACTGAGCATATGCAGACCTCCGGCGGAGAGACGCACATCACTACAATTGAGCACGAG
CCGCAGGAACTGCAAACGGTCCATCAGCACGAACAGACGCAGCAGACGCATCACATACATGCCGGCCAAC
TGCAGACGCATCATATCCAGACCGTGGTgcagtccagcagtggccagcagcagcacgatcagcagcagca
tcatcagcaccatagcaTTGAGCTGCAGGACGACGATGGTGTGGAGACAATCACACCCGAAGAGCTTGGT
ATGCACGATTCGAGCAAGAGCTACACCATTCTCACCACTCGTCCCATGAAGGAGGAGTCGGAGCACGATC
CTTCGGGCATGACCTACGAACTGTCGCTGAGCGACTCGTCTCTGGGTCCATGCGATGATCCAGAGTCGCG
GTATGTGTGCCGCCATTGCGGAAAGAAATATCGCTGGAAGTCGACGCTGCGTCGCCACGAGAACGTCGAG
TGTGGTGGCAAGGAGCCGTGTCATCCGTGCCCGTACTGCAGCTACAAGGCCAAGCAGCGGGGTAATCTCG
GAGTGCATGTGCGCAAACATCATCCGGAGAAGCCGCAGCTAGAGAGCAAGCGAGGCCGCAAGGTCTAGGC
GAAAGTTGGGAGGATTG
>dme_circ_0003883_junction_seq
AAGGTCTAGGCGAAAGTTGGGAGGATTGAAAACTCCTGGACGATATCCGTGAAATC
>dme_circ_0003884
GTTATTTACGGGGGAGGGGAGGAGGTGAACATGTGCTAATTCCGATAATTGACACTAAATTCACAAATCG
GACCGACCGAATGCTGCAAGGATAGTTGCCAAAATGAATTATTATACTTTTCAATTCAACTAACAATTAA
TCAATTTCTTTAGGCAGATCGGTCAGTTGCGTGTTTTGGATTAGTGTCAAATCGGTCTTAAACTGCAGTT
A
>dme_circ_0003884_junction_seq
GTGTCAAATCGGTCTTAAACTGCAGTTAGTTATTTACGGGGGAGGGGAGGAGGTGA
>dme_circ_0003885
GACTTAAAGTGTATTATTTACTGGCTACATACAGTGGGGCCTCTATATCTGGACACTGGTTTCATTCATC
CAGAAGTTTCACTGTACGTAAGCAGCGTGTGTGTTGGTGTGTCTATGTACCTGGCATAATAACAACTTAA
ACAATAACATTTAGTTATCTTTTGATGATCTTCATGCTCTGCTCTTATTGTTTTTGATGTCATCCACTCG
CATTTCAGGTTTA
>dme_circ_0003885_junction_seq
GATGTCATCCACTCGCATTTCAGGTTTAGACTTAAAGTGTATTATTTACTGGCTAC
>dme_circ_0003886
CATAACTGCTGACACACTAAACAAAACTGAAAAAAGAACCAAGGGCGAAAGCTGTTTATTTTGTTTAGGC
ACCTAAGTTTGGCCAAATTATAATCAAAAAACGAGTGGCGAAAAAAACAGCAAGTCAGAGAGAGCGCTCG
ACGTTCGGCCCCAAGGTCGTGGGCCCAAACCCCCAGGATCCATTTCCAACAACCCCATCCAACCACCCGA
GAAACCAGAATGGATGACGATCAGCAGTTTTGTTTGCGGTGGAACAACCACCAGAGCACACTGATCAGCG

TGTTCGACACGTTGCTGGAGAACGAGACTCTAGTCGATTGCACGCTAGCCGCCGAGGGCAAATTTCTCAA
GGCCCACAAGGTGGTGCTGTCAGCATGCAGTCCCTACTTTGCTGACCTTACTACAAGAACAGTACGACAA
ACATCCCATCTTTATACTCAAGGATGTCAAGTACCAAGAGCTGCGCGCCATGATGGACTACATGTACCGC
GGCGAGGTCAATATCTCGCAGGATCAGCTGGCCGCTCTGCTCAAGGCCGCCGAATCGCTTCAGATCAAGG
GCCTTTCGGACAATCGCACTGGCGGCGGAGTAGCTCCCAAGCCAGAGTCCTCCGGCCATCATCGCGGCGG
TAAGCTGAGCGGTGCCTACACACTGGAGCAAACTAAGCGGGCTCGACTGGCCACCGGCGGAGCGATGGAT
ACGTCTGGCGATGTGTCCGGTTCGCGCGAGGGCTCCTCGAGTCCGTCGCGTCGTCGCCGAAAAGTCCGAC
GTCGCAGCATGGAGAATGGGTGAGTTGTCAAGCGATTAGGATTATCTTACATATTTGCCGAATATaaaaa
aaaGTTCCAATGCGGGAATGTTCTTCAAACGAAATTAGCGTTAATCAAAAATCTTCAATTTGGCACAATT
AATAAATTGAAATTGCTTTGCTAATAATATATTTATGCAACACAACAAAATTTCCTCCTAAGTACAAACT
TATTTATTAAAATTGTTCTTATTTTTTTGCAGAATGCCCACGACAACTCAAACTCGTCCGTGTTACAAGC
CGCCGCCTCGAATCAGTCAATCCTCCAGCAGACAGGGGCCGGTTTGGCCGTCTCCGCTTTGGTCACCACC
CAGTTGTCCAGCGGACCGGCAGCCGGAACCAGCAGCCAAGCGTCGTCGACCCAACAACAGCAGCCATTGA
CCAGCACCAACGTTACCAAAAAGACTGAAAGCGCTAAACTAACATCCTCGACAGCCGCCCCAGCGAGCGG
AGCATCTGCGTCAGCGGCTGTACAACAGGCCCATCTGCATCAGCAGCAGGCGCAGACCACAAGCGATGCC
ATTAACACCGAGAATGTACAAGCCCAGAGCCAAGGTGGCGCCCAAGGCGTCCAAGGCGATGACGAGGACA
TTGATGAGGGTAGTGCCGTTGGCGGACCAAACTCGGCCACCGGACCCAATCCCGCCTCCGCCTCTGCATC
CGCCGTCCATGCCGGAGTTGTGGTAAAGCAGCTGGCCAGCGTTGTGGACAAATCGTCGTCGAATCACAAA
CATAAGATCAAAGACAACAGCGTGTCATCAGTGGGCTCCGAAATGGTTATTGAACCCAAAGCCGAATACG
ATGACGATGCGCACGATGAGAATGTTgaggatttgacactggacgaggaggacatgacaatggaggagCT
GGACCAGACGGCCGGCACCAGCCAGGGTGGCGAAGGATCTAGTCAAAGTGAGTTTGCATTCTAATCTTCT
TGTTTCCTTACAACTAATGGTTGGTTTTTTTCAAATAGCATATGCAACATGGCAGCACGACAGATCTCAG
GATGAACTTGGACTAATGGCACAGGATGCACAGCAACGGGATCCCCAAGG
>dme_circ_0003886_junction_seq
AGGATGCACAGCAACGGGATCCCCAAGGCATAACTGCTGACACACTAAACAAAACT
>dme_circ_0003887
gtgtcaaatcctcAACATTCTCATCGTGCGCATCGTCATCGTATTCGGCTTTGGGTTCAATAACCATTTC
GGAGCCCACTGATGACACGCTGTTGTCTTTGATCTTATGTTTGTGATTCGACGACGATTTGTCCACAACG
CTGGCCAGCTGCTTTACCACAACTCCGGCATGGACGGCGGATGCAGAGGCGGAGGCGGGATTGGGTCCGG
TGGCCGAGTTTGGTCCGCCAACGGCACTACCCTCATCAATGTCCTCGTCATCGCCTTGGACGCCTTGGGC
GCCACCTTGGCTCTGGGCTTGTACATTCTCGGTGTTAATGGCATCGCTTGTGGTCTGCGCCTGCTGCTGA
TGCAGATGGGCCTGTTGTACAGCCGCTGACGCAGATGCTCCGCTCGCTGGGGCGGCTGTCGAGGATGTTA
GTTTAGCGCTTTCAGTCTTTTTG
>dme_circ_0003887_junction_seq
TGTTAGTTTAGCGCTTTCAGTCTTTTTGgtgtcaaatcctcAACATTCTCATCGTG
>dme_circ_0003888
CGTCGTCGACCCAACAACAGCAGCCATTGACCAGCACCAACGTTACCAAAAAGACTGAAAGCGCTAAACT
AACATCCTCGACAGCCGCCCCAGCGAGCGGAGCATCTGCGTCAGCGGCTGTACAACAGGCCCATCTGCAT
CAGCAGCAGGCGCAGACCACAAGCGATGCCATTAACACCGAGAATGTACAAGCCCAGAGCCAAGG
>dme_circ_0003888_junction_seq
CCGAGAATGTACAAGCCCAGAGCCAAGGCGTCGTCGACCCAACAACAGCAGCCATT
>dme_circ_0003889
CCAGAGTCCTCCGGCCATCATCGCGGCGGTAAGCTGAGCGGTGCCTACACACTGGAGCAAACTAAGCGGG
CTCGACTGGCCACCGGCGGAGCGATGGATACGTCTGGCGATGTGTCCGGTTCGCGCGAGGGCTCCTCGAG

TCCGTCGCGTCGTCGCCGAAAAGTCCGACGTCGCAGCATGGAGAATGG
>dme_circ_0003889_junction_seq
AAGTCCGACGTCGCAGCATGGAGAATGGCCAGAGTCCTCCGGCCATCATCGCGGCG
>dme_circ_0003890
GATTTAGGAACAGCAAAATGGTGAAACCCTAACAGAAAAAGTTCATTTCGGTTCGTTACAATGCGACGAT
GATGATGATGATGATCCCCGAAGCCGAGACAATTTCCACCCCAAAAAGAGGTCATTTTCGTggggggggg
ggAGGTGTGTTCAGTGTCGGGGGGTTGGGAGGTAACATCGATTGCGGCAAATAGAGCTTCAGGGCCAGCG
AGCAGAAAACTACCCTGAAAGTAATGTTAAATGAGAGGCGTACGCAATCCCGCTGTTTCACATTCCACGC
AAAAACAAAAAACGAGCCCCAGATCGAAGGGATAAATGCGTGCGCTCTCCCTCCAACGGCGCCTCTCTTG
TTTCGGGGGAGTGACAGAGAGCACGAGATCCACACGAGCCAAAATCATGCCAGAGCTGGAGAGAAAGTAC
GTGGGGTGACAAagagagtgcccgagggagagagtaaacgagtgcgagagagagagagagagagagagag
agagGAGTCCGCGAGAAAGCGCCACGAAGTATGCTCTTGTTTTGTGCTCTCATTTTCACCTTTTGCGTTG
TCTCATCTTAACTTTTCACTTGTGTTTGTGTTGCCAGGCGAGCTTTTTTCTTAAGAAGGAGAAAGGAGAA
ACGAGGATGAAGCGAAGGTAAAGTGATGGAAAAGGAGAAGGGTTTTGTAAGAAGAAGAATTTCGATTACA
AATGGCTAATTTGTGAAGGAAATTAACTTAGTTTTAGAAGTATAAGTAGTTGATCTAATTATAATTATTA
TTCTGTTATTTTATTTTATCAAATTATTTCAATGTAATGTGACATGACACTGTGACATTCTTATACCATA
TACTTTTATATAtttttcattttttttCAAAACTTATATAGATTATGAGAGCTGAACTATTATTTTAAC
CATTGCTGTTGAAAGCACACAAAAAATTGCAATGTAGACTTTCATCTTCATAAACCAACATTTAATCAAA
GCTAATTTGTTGCCGCAATTAAATTGAACTGACTATTAAATTTAAATTGCAAACTGTTGGTAACTCTTTA
ACCAAGTTTAAGCGTTATGTCATAGCCAAGATAAAAAGTAATATGAAATGCTGTCACTTTTATGACAAAT
CCGCATAACATGACCTTGGAATGCTACATTGAAGACTATAGATAAATGTCAAAAATGAAATATTGTATTT
AATTCAattcacacttcctaagtaatgggtatctgatagtcggggaactcgactgtggcattctgtcttc
tttATTATAGAATATTTTGTATCATAATATTCAAACGATCTGCATTAAGTTTCGTGTAGTTTGATTGCCT
TTTAATGCATCGATTGGGGATAACAGCTACTTTAGTTGAGCTGAATATCAAAAAAGGAAGGAATATACAA
AAATGCAATACCTATCGCTAAAACtacatacatgcatacatacattcatatatggacttagacccgcaaa
catgcaaacatacatacatacatacatacatacatacatgtagtatgtacataTGCACGTGGATAATTCT
AGAGGCTAGCTCTCTACATTTGCGTTAAACACACCTGGAAAGTGCGCTTTTGTGTGTGTGTGCATCCATT
GAAGGGCTTCGCTGCAAAAATGAAACACGTCAACACAAACTGCAACTAGAATCCGAGATAGCTAATGGCT
AACAAGCGTGGTTGCAATTATAAGTTCAGCGACAGCACTCAAATGACAAAATGAATGAAATATTGCTTAA
TTACTACTTTTTGGAATCAGTTTTAATTTGTTAGGGCTCACCTTTGTTAACTTATGTAAAAATAGAACAA
TTTTTTAAAGTACTTTAACACGTGGGAAATTGTAGAAATGTACATAAATATAAATTATAAATCTTAGAAT
ATCATTATAATTGAACTAAGTTTGAATCATATGTTGTGATCTTACAGCCCTGATCACTCAACTAGAACCA
CATTACTCAAAAAGTTAGTTTTTAAAATGTTTTAATATAAGCTATTATGTTGAACAATCTGTGCGGATTA
GAATTGAAATCATTTAAAAATGCATGTATCTTATCTTATATCTTTACTAATTCTTGTTAAAATAATGAGC
ATAGCTACTGGTTGTTTTAATCAGCTCAAGCTTAATCATTAGTTCATTTGATCGTGCCTTATAACTATTT
TCAAAATAATTAAATATTCTTCTGAAACATTTCCAAAAGTTTAAGCAAAATATAAAATTCAttttttttA
AACTGCAAGACTTTAATGATTTACAAACAAATACAACTCACTTTCCAACTCAAAACTTAAAGTTCGTAAT
AGCTGTAATCTCAGTTAGTAGTTGCAGCTGCAAGGTGCTATTCCATTGGCCACTACTGTACTTGCCTACG
AACTTTTTATCTACGCGTGTGTGCATAAGTTAGTATGTGaaaaaaaaaGTTCTAGGGCAG
>dme_circ_0003890_junction_seq
GTATGTGaaaaaaaaaGTTCTAGGGCAGGATTTAGGAACAGCAAAATGGTGAAACC
>dme_circ_0003891
GTATACTGGAGTATACACACATTTCCGCAGCGTGTTGAGCACGCCACAAGCATAAACAAAAGTGAGCAAC
AGGAATTCAGCTAGGATTCGGCGTGAAACGAATACGAAAAACCATACGAAATGGCAGCGGTTCGAGGACA

TCAGTATTTCAGTCTGCGCTGGAATAACTACCAGAACACGATGACGTCCGTGTTTCAGCAGCTGCGCGAG
GATCTCTCCTTTGTGGACGTCACTCTGTCCTGCGAGCATGGATCCCTCAAGGCGCACAAGGGTCGTCCTG
TCCGCCTGTTCGACGTATTTCCAAAAACTACTGCTCGAGAATCCGTGCAAACATCCCACAATTATCCTTC
CCGCCGACATTATCTTCACCGACCTGAAAACGATCATCGATTTTGTTTATCGTGGTGAAATCGACGTCAC
CGAATCGGAATTACAG
>dme_circ_0003891_junction_seq
AATCGACGTCACCGAATCGGAATTACAGGTATACTGGAGTATACACACATTTCCGC
>dme_circ_0003892
GGTAAGCCAAGTCGTGACAATGTAATGCTGCCCAGcaaatattcaaaaaataaaaacaaaacacgaaata
aaagtaaaGCCAACAAATGAAATGTAAGTTTCACTCATCGATGGCCAGCAGCAGTGGATACGACTAAAAT
TGAATCCAATATGAGCCGCCAAGCTGCTGAGCCAGAGCAAATATTTTCGATAAATGCGCAAAAATGCAAG
CTGAATCACAATGCAGCCAACGGAGCTGAGATATTGTTTTTCGATCGAGAGCAAGTCGAATTGGGAATAC
ATTTCAATTTTTTGCCGGTTTCAATTCAGTTACTTTGTATTCGCATGAAAGGTTGCACTGCAAATCACAA
GATTTTTTTGTTAATCTGAAACTATTTAAGATACTAAAGTAGGCTTCTTATAGATAGTTTCAAAAGACCA
CAGCCCTTTGGAGATTCATTTTACCAATAGGATAGTGTATGGCCAAAACTCCGTTGAGGAAACAACGAAA
TTCGTGTcaaaaataaaaatcgcagacgtacaaaataaaaaaCGAAAGCAGCTGGACACTCGAATGCCGC
GCTGAGGTCTGCTACGATCCATCCACTCCCAAAAAACCACAGATGTACAAACctatatgggcgatattgt
atatatattttcataaatatatgtatatataCGGTTTACCTGTTGAACGAACGCTGGGCTAAATCCGAGG
CATTCACTTCATGGGCGTGTCGTATCCCCAACCCTTGCCCCAAAAAGTGGTTTGCAGCAGTTtgtgtgtg
tgtgtgtgCCCGTGGTGGCTTGATATTTGTTGTTTGCATGATCAGTTTGGCGTTTTCCAGGAACCGGCGA
TTGCATTTTAGCTCCCGAACATGTGTACTTGGTTCTGTTTTGTTTGTTTGCCAGCCGACTAACTGACTGA
CGGACTGACCAGAGACCAAAGCAAAAAGAGCGAAATCCCCAAACTGTGGCAAGGTCCCAAAGGCAGGCGC
CTCTGGGAGAATTCTGCTTGTTCCCACATCACGTTTACACGATGTGCTAATTGAGTTAGTATGCATGATG
CCaaaaaaaaaaattaaaaaaTAAGAGAAACGAACAGACAATCACACGTAGATACAATTTTCAGGTGCTC
CGGGGGTTTCGAAACGATCCGTGGCTGGTTAAAATTGTGTCTAGTTAGCAAGATTGGGTGGAATATAACA
CCACCGGCAGCACAGAAAATGCAAAAATTAAGGGCCATTACACACGGCTCGATTTCGGTTTCGATACGTA
TTCTGCGTTGTCttttttttttCCTTCGCTTGCCATAGCCATATGGCATCGAAAACCGATAATTACCGCC
ACCATACAAAAGTATCTGTATCTGTGTGCCGGGTTTTTGCTTCAATATGTGAGTGTGTATGTGTGTGCCT
ACCTGCAGCAGAAGCCTCACAAGAGACAAAACTGGACAAATGCCACCATACCACAGCGTACCATATCTTT
TCAAAACACCATCCCATACAACACAACACTCATAGTCAACAAATTACTCACGCGCAAAAATCGCCATTCg
ttattgtttgttgtttttgttagtggttgttgtttttgttgTCGTCGCGTATTTTTCCACATACAATTTC
ACTGTATGACGTATGCTTTCTTGCCGCTTATGTGTCTGCGTTCCATATGCATAAACGCCTGACCGTTTTT
ATTTCCCCACGTTACCTGTATGCGTATGCGGGCACTGGGATTAGGGTGACCCAAGACAAATACTCAAAAT
TCCTCGATATTTGATAAGCCTAATTATAAGCACTTAAGATTAGTCGGTTTAATGCAACTTACAATTTGAA
TATAACAGATTTGGGTGCCTAAAACAGATAATTTAGCAAAGAACTCATAAAATACTCGTTAGTACTGAAA
AATACCAATATTATTTCGCATAAGATCACATTTCCCAGATCATTAAGAAAACGTGGGCGGTTGTGGTTCG
CAGTAGTAGAACCTCTTCAGTTCTGAAAACAATTATCTGATTTCCTAGGCATTTCCCTTTGACTTCATTA
ACGCAGATTGTAAACCCGGCTTTTTCACTGCCTTTTCGCCACACTTTCCTTTGTCTTCTGAGCGGCATTA
TCCCGAAGTCAATTTATTTGCCATAACGTGAGGCACCCTAATGGGCTGCCTTTTCGCTtgtgtgtgtgtg
tgAATCGTATTAGTATTCCGTTCGATGAGTCTGTGCATGTGTGCGCATTTAAGCCGCAAAAAACCAAGTC
GACGCCGCGCCGTCGCTTCTGCCGCCGGCAAAATTAGCAGAGGCGTCAGCAACAACAACTAAATGGGAAA
ATTGTAGCGACTACTTACTATGGAAAAGTGGaaacaacaacagcacaaacaaactaaaacaacaacaatg
acagaagaaacattaacaTTAATGCAACAACATTATCTTAAATAACAGCAGTGTTGTCTACAGCAACATA
ATGACCAGCATAAGCTACAATATCTCAATTAGCAAAGACAACTGCAACATAAGCAGCACTTTTGTAGATG

CATCATCCATAACATAACATAACATACAAATGCAACATAAGCAAGAGTTAACATCATTAGCAGAAGCTAC
AATGTAGCAACCGAAACAGCAGCAGTGGGAGCAACAGTGGTGATTCAACCAATGCGagtaacagttgtat
cagtagcaacatgggcaacaatatcaacagcttgaagcaacagaagcaacatccctaacagaagctgtat
cagaagagacaacaataacagcagctagcaacatcgcaggctacaacagcaacatgagcggccttaacag
cagcagcaacaacaagagcaacaagaacaacaacCGGCATGTGAGCAGCGGTCTACCTGTTCACCGAAGT
TGGGCACATTTTCTTTGCTTACCTAAGGGACTCGCACGCACACAGATACGGTTCACCTCTACCTGTGCAC
AAATcacacacacacacacacacaTGCATACGTGTGCCAAAAAGAGAAGAGGATCGCTATCGGCGGCAAT
TGTGCTCTATCTCGCTCGCACCCGTGCGCCGGTGTCAAGCCAAGCTGAACGGGCCACGCTAAGCTGAGCC
GGCGTTGTTGTTGCCGCTCCGCCGGCGGCGTTAACCACACGACGTCGACGACGCTGGCAGCGCAGAAAAG
AAACCGAACCGAACGAGCAGGGGCGACGTTGAAAGTCTTCGGTTCGGTCGGTCGGTTCTCTGGATATTCA
GCAGAGCGGTCTACAGGTAAAGCGGGCCAAAAGCAACAACAACAACAGCAGCCAGCGGCAGAAGCAGCGG
CGGCGACGTCAGGCAGCAGCGGCAGAGCGGCGTCCAAACTTGGGTTTTGGAATTTTTTTCTTGCTCTTCT
TTGGTTTCAGTTCAGTTCGATTTTTATGCACAGTCGCAACGTTACCTTACCTGTGCGCTTCGCTTTTGTT
TTCTCTTCGTTTTCTCGTCTCGTCTTCGCCGCCGAAACATGTGGGTTCCGTTTTCGATTTGCTTCAATTA
AAAAAAGAAACGAATATTTCCAAAACGTCGCGAAATTACGCGCAAGAAGTGCAGCAAAAGCAAATGTACT
GAAAGCGAGGAATACATTCTTCTTCTTCaaaaaaaaataccatcaaaaagaaagtaagaaaaaGTTTTTT
CTGCAAGAGAGCTTAAGTAAAAAAAGAGCAAGGATGTCGCCAAAATAACAGGAGTTAATccaaaataaat
actaaaataccaaaaccccaaaaaataccaaaCTAGCAGTGACAGAGAAAAGCAAAAGTTACAGCACCAA
AAAAACTGAGCAAAGAAA
>dme_circ_0003892_junction_seq
ACAGCACCAAAAAAACTGAGCAAAGAAAGGTAAGCCAAGTCGTGACAATGTAATGC
>dme_circ_0003893
GGAACACTTTACGGACGCTGCAAACGGGAGGGCATTGAGCTGTCGCGTTCGAATCCAACACCCTGGTCCG
AGGATGCCATGAACGAGGCCCTGAACTCAGTGCGGTGAGTGTGTGAGCCGAAATGGTGGCGTCAATTAAG
TCACTAATCACCATTTCCGACGTGTATCCCCCACAGCGTTGGCCAAATGTCCATCAACCAGGCGGCCATT
CACTATAACCTGCCCTACAGCTCGCTCTACGGCCGCTTCAAGCGCGGCAAATACGACGTGGTGGCCAACA
CGAGTGGCGTGGCGCTATTGAACACCTCGGGCAACACCACCGGCAGCATTGAGATTATCGAGCACAGTCA
GGAGAATTCG
>dme_circ_0003893_junction_seq
GATTATCGAGCACAGTCAGGAGAATTCGGGAACACTTTACGGACGCTGCAAACGGG
>dme_circ_0003894
TGCTTGCAATTAAATCTAAACTTGATGTCCTCGGGCCTGGCCACTCCCGCAATTGTTTCCAACTCCCTGT
GTGGCTGATCATCCTCCTGCTCTCAACGCTTTTGCTTATTGGCTTTCTTGCGCTTGCTGG
>dme_circ_0003894_junction_seq
TGCTTATTGGCTTTCTTGCGCTTGCTGGTGCTTGCAATTAAATCTAAACTTGATGT
>dme_circ_0003895
GGTTTTAGGTTCGCGAAAGTCCTTTGTTCTAGTTAGGTAAGGTAAGGTGGAGAAAGAAGAAAGTTAAGAA
AACATACCTTGAACGTAAGAGAATGATGAATTTTAGATTTGCCTCTAGACTCGAAAGTTAATGGATTC
>dme_circ_0003895_junction_seq
GCCTCTAGACTCGAAAGTTAATGGATTCGGTTTTAGGTTCGCGAAAGTCCTTTGTT
>dme_circ_0003896
GTTAATGGATTCGTTTTGGGCTCGCAGTTTTAAGAGCACTTTGTTATCAATGGATATAGACACGCGTATT
TATTTTGGTCTCGCGACCATTCTGAGAACCTTGAATCGGCAGTTGATTGTGGAATTTCGAACTAGATTGT
TTTCCCTTCAATGTGCTTATGTGCTTTGTTATAATTTTCGCTTAGTGTGAGACTGAAAGTGCGCCTAAAA

CTGTTGAAAAACCCCATGCCCAATATTATAGTGAAGACGCATAAAACACAACCCATACATGAATCAAATG
CTTTATAACCATATTGTTTGAACAGATAGAACGAAGTGGGAGAATACCTTAACGTAGAATTAAACCAAAT
TATCAAGGTTCTAGAACTTAAATCATTAGCTTGTAACGATAAAAGAAAGCAAAACGAGTTATTTGAATTT
TACTGGAGGCGTCCTGAATTTTGGATTGTATATTGATTTGATTTCAACTGAATTAAGAATATAGCCAATA
AATAAATGTAGAAATGAAGTAATTGCAAGGAATCAACAAATGGAAT
>dme_circ_0003896_junction_seq
GTAATTGCAAGGAATCAACAAATGGAATGTTAATGGATTCGTTTTGGGCTCGCAGT
>dme_circ_0003897
GCAGCTGTGTTTGTGCCTTTTCAAATTTTAAAATCCATCATATACCCAGTATTACAGAAGTTGCAATATT
TATTCGGTCAAAATTTTGCAATGGTCTGCAATACCGAAAAATCAAAAACCAAACAGGTGTAACTTTCGTA
ACTAGCCCTGCCAGACTTTTCAATATTGTCGGCTGGCCTCACTATTCCAGCTTCTTCACAACATATAGTT
GTCTCACTTGCACTCCCTTAGAAATCATATGTTTATGAACAGATGAGACAGTTTTAGAAGCACTGCATTA
TTTGAAGCACATGTGCCAAAACAAAACAATTTCGCCGACATTCAATTCGAATTTAGTTGTCATTAAAAAA
TAGAAGTGCAACATTAGCATGCAAGTGGAGAATCTGCATACTTCAGTGGCCTGCAACCGCTGCACGGAAT
GCGCGGATTGGGGTCCAAATGGTTGGATTGCCTATGGGGCATGTAACGCCATAGCCATTATGGATCCAAA
GGTTTCAAGGCAACTCAGCCAAGGTCCTGTTCACGCTGGTGGAGCACACAAAGCGAGTAAACACTGTGAG
GTGGTTGGATTGCGACAAACTTCTTTCCGGCGGAGATGATGCAATTGCTATTCTCTGGGAACTGGATGAA
ACGGGAACGACTAAGAGCTTCACTCTCAAAGGACACACAAGTGGCGTAAATACTGTGGATGGAATTCGCC
AACAGGATGGCTCCTGGCTTTTGGCGACCGCAGCAGCAGATACCACCATCAAGTTGTGGACTTTCCAGGA
TAATAATTATGTGTGCTTTCAAACCATATCTTTATCCGACGGATTCTGCTTCTGTCTTCGACTGCAGCTG
CTGCCCAAGAGCAACCAGGTTCTGCTCGCCTTCAGTGGCGATGACGAGACTGTTTCCCTGTGGTCGGAAC
AGGTAGAAACCGCAGGTGAAGGAGATTCACTAGGTCGGCAGTTCCAACGCAAACACAAGCTCACTGGCCA
CGAAGACTGGGTTCGAGGTCTGGATTTCGTTGTCGATGGCGAGGATTTGCTGCTGGCCAGTGGCTCTCAG
GATAACTTTATTCGTCTCTGGCGGATTGCGCCGCGCAGCAAAGAGCAAATGCAAGAGAATCGGGTTGATC
TGCATCAATTGAGCCACAACGACGATGAGATAAAGGTGGAGGAGAAGATACTACAACTGGGGAAAGAAGC
CTGGTATGCAGTGAGCTTGGAATCGGTTCTATATGGACATGAGGGATGGATTTATGGGGTCCACTGGCAC
AAAACACCCGATCAAGGAGCTCCGCCTTCTATCCGCTTCTATTGACAAGACCGTCATAATTTGGGCACCT
ACGGAAGAAGGCATTTGGTTGGAAGAAGTTCGCGTGGGTGAAGTGGGTGGAAACTCCGTGGGCTTCTATG
GTGGAAAGTTCTCCGGCGATGGACACTCGATCATGGCCCACAGCTACCAAGGTGGCTTTCATATTTGGAG
CCAAGATCCAGATCGGCCACAGCTCTGGACCCCAGGTGTCATCGTTGGTGGTCATTATGGCGAAGTGAGG
GATCTGGCTTGGGAACATAGCGGCGCCTATTTGATGACCGCGTCTGCAGACCAAACAACTCGACTGCACG
CTCCGTGGCTACAGGATGGAGCTAATCCCACGTGGCACGAGCTGGCTCGTCCACAAATTCATGGATATGA
TATGCAGGCGTTGGCTTTGCTTTCACGTTATAAATTTGCCAGCGGAGCTGAGGAAAAAGATTGTGCGCACT
TTTCAAGCGCCAGCCAACTTCATTGAAAACTTTCGTCACATCAGTGGGATAGAAAATGACGATGCGGGAG
ACGTTCTGCTGGATTGCTCTTCCCAAAGGAGCTTCCGTGCCTTCTTTGGGATTATCCAACAAGGCTGTCT
ACAAGGTAGATTCTGAAGTGGAGTCGACCAGCAAGACCAGTAAAGACGAATACCCTGACAATTACTTTGT
GCCTATTGCTCTGGAAACACCACCGCAGGAAGAGACTTTAATGCAGAATACACTTTGGCCCGAGCTGCAG
AAGCTTTATGGCCATGGCTACGAAATCTTTGCACTAGCTGCCACTGCAGATGGTTCCCTCTTGGCCTCCA
CTTGCAAAGCCAGCAATGCCGAGCATGCTCAGATTATATTATGGGAACCCTTCAAACTGGAAGCAAATAC
AGAAGCTGAGTGGACACCAGTTGACAGTGACCCAACTTAGTTTCTCCCCCGATTCGCGATATCTGCTATC
AGTGTCCCGGGATCGTCGCTGGTGTCTCTACGAGAGGCAGGATTCCTCAGTAAGCTATCAACTGGTGGCC
AGCACTGACAAATCCAATGGAGTTCATACACGCATCATTTGGTCGTGTGATTGGTCGCATGATGGTCAGT
TCTTTGTGACGAGCTCAAGGGATGGAAAAGTAGTGGTTTGGAAAAAGGAAGAAGACTGCAAGGAGTCTTC
GCTAAATGGCTGGCAGGCGAATGGAGTTTTAGAACTTAAAAATGAGTCAATCACAGCCGTGGCTTTTTCC

AATTCTTATCTTAGTGGTACAGATGACACATACATTCTTGCTTTGGGCACGGAAACCGGACTGATAAAAA
TATATCAGTTTGTTCGAGGAGCATGGAAGTTACTAAGCGATTTGAATAAGAGGCCAAGCTCATCATCTCA
CCGTTCGACGTCTGCAGTTCCGCCCGGGAAAACAACTCCAGCTTGCGAGTTGTGGTGAGGATCATCTGGT
ACGCATCTACGACATTAAGCTCACATGAGTTTAGCTCCATATGCATTAAAGTAGTCTTATTAACTCCTGA
TTCACAGTTTGTTGTGAACCAATCAAGATGAAGGCAGCAATAATTTTGGCACTAATCGGTACAGTTTTGA
CGGCTCCGCCAGTCAGGGTGATGCCGAAACCAGGCAGTGAGCTTGATCAGTACGCTCACATATTTGCAAA
GGTACGACCACCGGGCCAGAAGCACCAGAACATGGAGATTGAATTCCAGCCACAGCACCACAACTCTCTG
CCGAGCAACCAGGTCAATGCGGAAATCAATCATGTGGAGCCCAAGCAGCCTCAGTACATGGAACAAAGTT
CTTCTCAGGTCGAGTCGAGACCTCCTCACACCGTGGACTTTGAGATCAAGCCGGTGGAGCAGAAGCCACC
CCAGCGATTGGAGTTTGAGGTGAAGCCGCAGCACGAGCAAAACTCTCCTCAGGGACATTTGGTTTCTTTG
GGCGTCAAGCCCGTGGAGCAGAAGCCACCTCAGCACTTGGAGGTGGAGATTAAGCAGCAGCAGCAACAAC
ATTCTTCGCAAAATCATCAGGTGGAACTGGAAATCAAGCAGGTGGAACAGAAGCCTCCACAGCACTTGGA
AATTGAGATCAAGCCTGTTGACCAGAAGCCACCGCACCGTGTGGAGCTTGAAGTCCAACAGCAGCATCAC
CAAAACTCTGCCCAGGGACACTTGGTTTCTTTGGGAGCAGTGGAACAGAAGCAACAACACATCGATCATG
AGCTGAAGAATTCCCATAAAATCGAGCTAGAATTTGTTTAGGAGAATGAGCTTATGGTATTCCAACTGCA
GAAGCAACATTTCTACCTGAATCTGCACCAATAGAGCAGCGGGCCACAAAATGTATCCGTTAAACAAAGT
GCATTAAATATGTATAATTACAAAGCGAGCGTTTTCACTGACCAGTCCTCCCAAGGTGCTTGTAAAATGA
GAAATAAATAAAAATGGTCTAGAGCAGCGAGGACAAGGAGCCGCAGTTTGCAATTTCCGTTTAGCTGGTG
CTGCATAGTTTTCGGCCAAGACAGCAGCCACCGCCACGTGTCCGGGATCGGGAGTTTGAAGCGAGGAGTA
GTGAGGAGTCGGGAGTGGTGCCGGGTGTCGG
>dme_circ_0003897_junction_seq
AGGAGTCGGGAGTGGTGCCGGGTGTCGGGCAGCTGTGTTTGTGCCTTTTCAAATTT
>dme_circ_0003898
GAAATCATATGTTTATGAACAGATGAGACAGTTTTAGAAGCACTGCATTATTTGAAGCACATGTGCCAAA
ACAAAACAATTTCGCCGACATTCAATTCGAATTTAGTTGTCATTAAAAAATAGAAGTGCAACATTAGCAT
GCAAGTGGAGAATCTGCATACTTCAGTGGCCTGCAACCGCTGCACGGAATGCGCGGATTGGGGTCCAAAT
GGTTGGATTGCCTATGGGGCATGTAACGCCATAGCCATTATGGATCCAAAGGTTTCAAGGCAACTCAGCC
AAGGTCCTGTTCACGCTGGTGGAGCACACAAAGCGAGTAAACACTGTGAGGTGGTTGGATTGCGACAAAC
TTCTTTCCGGCGGAGATGATGCAATTGCTATTCTCTGGGAACTGGATGAAACGGGAACGACTAAGAGCTT
CACTCTCAAAGGACACACAAGTGGCGTAAATACTGTGGATGGAATTCGCCAACAGGATGGCTCCTGGCTT
TTGGCGACCGCAGCAGCAGATACCACCATCAAGTTGTGGACTTTCCAGGATAATAATTATGTGTGCTTTC
AAACCATATCTTTATCCGACGGATTCTGCTTCTGTCTTCGACTGCAGCTGCTGCCCAAGAGCAACCAGGT
TCTGCTCGCCTTCAGTGGCGATGACGAGACTGTTTCCCTGTGGTCGGAACAGGTAGAAACCGCAGGTGAA
GGAGATTCACTAGGTCGGCAGTTCCAACGCAAACACAAGCTCACTGGCCACGAAGACTGGGTTCGAGGTC
TGGATTTCGTTGTCGATGGCGAGGATTTGCTGCTGGCCAGTGGCTCTCAGGATAACTTTATTCGTCTCTG
GCGGATTGCGCCGCGCAGCAAAGAGCAAATGCAAGAGAATCGGGTTGATCTGCATCAATTGAGCCACAAC
GACGATGAGATAAAGGTGGAGGAGAAGATACTACAACTGGGGAAAGAAGCCTGGTATGCAGTGAGCTTGG
AATCGGTTCTATATGGACATGAGGGATGGATTTATGGGGTCCACTGGCACAAAACACCCGATCAAGGAGC
TCCGCCTTCTATCCGCTTCTATTGACAAGACCGTCATAATTTGGGCACCTACGGAAGAAGGCATTTGGTT
GGAAGAAGTTCGCGTGGGTGAAGTGGGTGGAAACTCCGTGGGCTTCTATGGTGGAAAGTTCTCCGGCGAT
GGACACTCGATCATGGCCCACAGCTACCAAGGTGGCTTTCATATTTGGAGCCAAGATCCAGATCGGCCAC
AGCTCTGGACCCCAGGTGTCATCGTTGGTGGTCATTATGGCGAAGTGAGGGATCTGGCTTGGGAACATAG
CGGCGCCTATTTGATGACCGCGTCTGCAGACCAAACAACTCGACTGCACGCTCCGTGGCTACAGGATGGA
GCTAATCCCACGTGGCACGAGCTGGCTCGTCCACAAATTCATGGATATGATATGCAGGCGTTGGCTTTGC

TTTCACGTTATAAATTTGCCAGCGGAGCTGAGGAAAAGATTGTGCGCACTTTTCAAGCGCCAGCCAACTT
CATTGAAAACTTTCGTCACATCAGTGGGATAGAAAATGACGATGCGGGAGACGTTCTGCTGGATTGCTCT
TCCCAAAGGAGCTTCCGTGCCTTCTTTGGGATTATCCAACAAGGCTGTCTACAAGGTAGATTCTGAAGTG
GAGTCGACCAGCAAGACCAGTAAAGACGAATACCCTGACAATTACTTTGTGCCTATTGCTCTGGAAACAC
CACCGCAGGAAGAGACTTTAATGCAGAATACACTTTGGCCCGAGCTGCAGAAGCTTTATGGCCATGGCTA
CGAAATCTTTGCACTAGCTGCCACTGCAGATGGTTCCCTCTTGGCCTCCACTTGCAAAGCCAGCAATGCC
GAGCATGCTCAGATTATATTATGGGAACCCTTCAAACTGGAAGCAAATACAGAAGCTGAGTGGACACCAG
TTGACAGTGACCCAACTTAGTTTCTCCCCCGATTCGCGATATCTGCTATCAGTGTCCCGGGATCGTCGCT
GGTGTCTCTACGAGAGGCAGGATTCCTCAGTAAGCTATCAACTGGTGGCCAGCACTGACAAATCCAATGG
AGTTCATACACGCATCATTTGGTCGTGTGATTGGTCGCATGATGGTCAGTTCTTTGTGACGAGCTCAAGG
GATGGAAAAGTAGTGGTTTGGAAAAAGGAAGAAGACTGCAAGGAGTCTTCGCTAAATGGCTGGCAGGCGA
ATGGAGTTTTAGAACTTAAAAATGAGTCAATCACAGCCGTGGCTTTTTCCAATTCTTATCTTAGTGGTAC
AGATGACACATACATTCTTGCTTTGGGCACGGAAACCGGACTGATAAAAATATATCAGTTTGTTCGAGGA
GCATGGAAGTTACTAAGCGATTTGAATAAGAGTGATTCACAGTTTGTTGTGAACCAATCAAGATGAAGGC
AGCAATAATTTTGGCACTAATCGGTACAGTTTTGACGGCTCCGCCAGTCAGGGTGATGCCGAAACCAGGC
AGTGAGCTTGATCAGTACGCTCACATATTTGCAAAGGTACGACCACCGGGCCAGAAGCACCAGAACATGG
AGATTGAATTCCAGCCACAGCACCACAACTCTCTGCCGAGCAACCAGGTCAATGCGGAAATCAATCATGT
GGAGCCCAAGCAGCCTCAGTACATGGAACAAAGTTCTTCTCAGGTCGAGTCGAGACCTCCTCACACCGTG
GACTTTGAGATCAAGCCGGTGGAGCAGAAGCCACCCCAGCGATTGGAGTTTGAGGTGAAGCCGCAGCACG
AGCAAAACTCTCCTCAGGGACATTTGGTTTCTTTGGGCGTCAAGCCCGTGGAGCAGAAGCCACCTCAGCA
CTTGGAGGTGGAGATTAAGCAGCAGCAGCAACAACATTCTTCGCAAAATCATCAGGTGGAACTGGAAATC
AAGCAGGTGGAACAGAAGCCTCCACAGCACTTGGAAATTGAGATCAAGCCTGTTGACCAGAAGCCACCGC
ACCGTGTGGAGCTTGAAGTCCAACAGCAGCATCACCAAAACTCTGCCCAGGGACACTTGGTTTCTTTGGG
AGCAGTGGAACAGAAGCAACAACACATCGATCATGAGCTGAAGAATTCCCATAAAATCGAGCTAGAATTT
GTTTAGGAGAATGAGCTTATGGTATTCCAACTGCAGAAGCAACATTTCTACCTGAATCTGCACCAATAGA
GCAGCGGGCCACAAAATGTATCCGTTAAACAAAGTGCATTAAATATGTATAATTACAAAGCGAGCGTTTT
CACTGACCAGTCCTCCCAAG
>dme_circ_0003898_junction_seq
AGCGTTTTCACTGACCAGTCCTCCCAAGGAAATCATATGTTTATGAACAGATGAGA
>dme_circ_0003899
GCCTCAGTACATGGAACAAAGTTCTTCTCAGGTCGAGTCGAGACCTCCTCACACCGTGGACTTTGAGATC
AAGCCGGTGGAGCAGAAGCCACCCCAGCGATTGGAGTTTGAGGTGAAGCCGCAGCACGAGCAAAACTCTC
CTCAGGGACATTTGGTTTCTTTGGGCGTCAAGCCCGTGGAGCAGAAGCCACCTCAGCACTTGGAGGTGGA
GATTAAGCAGCAGCAGCAACAACATTCTTCGCAAAATCATCAGGTGGAACTGGAAATCAAGCAG
>dme_circ_0003899_junction_seq
TCATCAGGTGGAACTGGAAATCAAGCAGGCCTCAGTACATGGAACAAAGTTCTTCT
>dme_circ_0003900
GGTGGAACAGAAGCCTCCACAGCACTTGGAAATTGAGATCAAGCCTGTTGACCAGAAGCCACCGCACCGT
GTGGAGCTTGAAGTCCAACAGCAGCATCACCAAAACTCTGCCCAGGGACACTTGGTTTCTTTGGGAGCA
>dme_circ_0003900_junction_seq
CCAGGGACACTTGGTTTCTTTGGGAGCAGGTGGAACAGAAGCCTCCACAGCACTTG
>dme_circ_0003901
ATTCCTGGAGAACGGAGGAGAGGTTGTCCGTCAGCATGTGAACAGCTTTTTCGAAGTGCCGCAGAACATT
GACTTGCTCCTTAACTGCACCGGAATGGGAGCCAAGGAGTTGTGTGGGGACCAGCACTTGGTTCCCATTC

GGGGTCAAGTGCTCAAAGTGCGTGCTCCCTGGGTAAAGACGGCTTTCTACGGCGATTACGACACCTACG
>dme_circ_0003901_junction_seq
GCTTTCTACGGCGATTACGACACCTACGATTCCTGGAGAACGGAGGAGAGGTTGTC
>dme_circ_0003902
GCCGTCTTTACCCAGGGAGCACGCACTTTGAGCACTTGACCCCGAATGGGAACCAAGTGCTGGTCCCCAC
ACAACTCCTTGGCTCCCATTCCGGTGCAGTTAAGGAGCAAGTCAATGTTCTGCGGCACTTCGAAAAAGCT
GTTCACATGCTGACGGACAACCTCTCCTCCGTTCTCCAGGAATCTACAATATGTTAATATTTACGATTAT
TTTCAAATTGTTTCTCTTTCATATCATAAAACAGCTACTCACTTTTTAGTGGCATAGGGCAAGAAGAGGC
GGCTTTCCGTTAGGCATGTGGTGAAGAAGGATCCGTACTTCCAGCCGCCGTTGCATAGCCTCAGTTCCTC
CTCGGTGGCTCTTCGGTAGACGGGCAATAGTTTCTCAATAAA
>dme_circ_0003902_junction_seq
GGTAGACGGGCAATAGTTTCTCAATAAAGCCGTCTTTACCCAGGGAGCACGCACTT
>dme_circ_0003903
GCCTTCGTCAACATGACGCTGCCGTCGCCAGCCACGGTTAAGCTGAGCACATCAATCGCCAGAAGCCCCC
TGATCGAGCGCAATCTGGGCCAGAATGCGGCCACCAATGGCGATGCCATCGATGCAAGCGTCGACGGGTT
TGCAGCCGACTGAAGTGCATCCGGTGCAACAGCTGCACTTGCATGCCACGCCCTCAAATCCTTCGGATAT
ACACCAGGATCGGAGTCCACACAACCATAACGCCAAACTGCCACCGCTGGAACTGCTGGTGGAGAACAAG
ATTAGCTACAGCAGCTACCAGCAGCACGCCAAGGACCTCAAGCACCAGAGTCACAATCAAAACCGATCCA
AGAGAAGTTCCCTCAGCAGTCCCTCGTCCAGCGAGGATTGCGTTAGTGCTGCCAGGGCCAATATCTTTGA
TGACGCCGCCGAGAACTTCGTGATCAATTTCCCCTCGGAGTCGGCCACCTCGCAGCATTTGCTGCCACTG
TCGGAGACTGTGCCCCTGCTCACCCACATCGAGGCCACGGTGGTGGATAAGCAGCGCTCCATAAGGCTGA
ACAAGAACAGTGCCTCGCTGATCTTCACGAAGAAGGAACTTAACCAGGGAACCAGGGAACATCATCATCA
GCACAATAATCAGCAGCACCAGCAGCAGCAACGGCGTCCGCACCATAGCAGTTTGTACGGAGTGGGCAGG
AAGCCGACCAAGCACCTACCGCCAACTAGAGGACACCACCAGAGGCGCAGTCTACCTCTTAGCTACAACA
ACAATCTGGTGACCACCGCTCCATCAGCAGGTGGGGCAGGAGGTCCCGGTGGAGCAGCCAAACTTGTGCC
GCTCAAGCAGCAGCATCAGCACCACGGAGCGGGCCACACGGCACCACCCACGGCGAACGTCGTTGTTGCC
GGTGGAGCGGGAGCGGCGGCTCCGTCTGCCCGCAAGCAGCTCTCCTACTCCTGGTACGCGCCCGTCTACA
GTGCGCTCGAGGAGGAGCTGGAGCAGGACTCGCGGGGTGGGTGCACTCTTTGTTATATTCGTTACCTTTA
ACTGGTTTTAGTTTATAAAAACGTCTTTAGAGACAAACAAATCAAAGTTTAGTGTTACAAATCCACCAAC
TTATGCATTAAATTCATTCGCAGGGACTCCTCGCCAATCCACAATCTGGCCAACACAAAGCAGCACCAGC
GGTCCGCCAGCCAAAAGGCATCCACCGCCCTGCATGCCACGCCCCGCCACGATCGCGCCCACGATTCCGC
CGACGCCGAGACGGTTGCGCTGCTGGACACGCAGACGCGCAGCAAGATCATCATTTCCTCGGCGAATGGT
GACGCTGGCGGAGCGGGTGCAGGAGTTGGATTCGGTGGAGGCGGGGGCGGAGGATCTGGAAACGGCGGAG
TACTGGCAGCGAACGGAGGAGGAGATCTGGAGAGCTCGCTGGGACTATCTGGTGGTGGGCAATTGCCACG
AAGGCGTCGCATCGAGAACTTTCTCAAATCGCTGGTGGGACTCAAGGCGAGTAGCGGTCGCGGAGGAGGT
GGTGGTGGCAATGTTGGTGGGGGCGGGGCAGTACCAGATCGGGAACGTCCTGCCTCCCCAGAAATTCGGA
TCACCCGCACTCCATCCGAGCAGGATGTCGTGCTGCGAGATCCTTCTGGCCGACAGCTAGCCAACGGCCA
TGGTCGAGGCAGCCTGAGCATCAGCGGTTCGAGCAGCTCCTTGAATGTGGTCCAGCAGAAGATATGGCAT
ATCATGCGACGCGAGGGTAGCGCTAGCAGCTTGCACCAGGAGAAGTCCCAGTCGATTGTCCAGTACACGG
GACTGCGAAAGTGCGAAACGGTCCTAGCACTCACCCGTCAATCGCAATCGCATGGCCACGTTCACGGCTT
GAGCCAGAGTCGAAGTCAGGGTCACTCCTTGGCGGTCCATGGCAGCGGGATCTTCAGCTCGGGTGGCGTG
GAGCAGATTCGACCACTGAATCGCCTGCGGAACAGCGTGAGCAGCATTAACGGCGTGGGTACCTGTTCGC
GCTGCTCCAGTCTGTTATCCCTGGCTGCCTCCGGATCGCGTTACTCCCTGTCGAATGGATTTGCGCCCCG
GCCAACTTCGGCCCTCAATCACCTAGAAGCTGGATGGGAGGACGATCAACGGCGCGCGCAGGAGCCGCAT

ATCAACGTGAATGCCCAGATCAGACTGAGCGGTGGAGCAACTGCCAACTCGACCAGTCCCAGTCCGCCGT
CCAATGTGACCACGGCCACATTGCACTCCAGTTCCGCCAATAATCTGAATCAGGAACCGCAGCAGTCCAC
GTCGACGCCCGCCACCACGCCCACTGAGACACCTGGGAGCGGCGAATTGGGACCGTTCGGTACCAATCAT
GCATATCCATTGACCGTAAGCTCCCTGCTCTCCATGGCCAGTGCGAATCAGGCGGCGCAGGCATGCTGCG
TTCGTGATGGGATTGCACTGAAGCGACGAGAGATGTTGGCCTCCGCCGATGTCACACCTTCGGTGGGCAC
CCCGGCCACGCCCACCACCGCTTTCAAGCCCTTCACGTGCAAGCTGTGCCTGATCGATGTGGAGGACGTG
GGCGAGGCCATGGCGCTTCAGCAGTGCGGCTGCCAATTTTGTATCGAGG
>dme_circ_0003903_junction_seq
CAGTGCGGCTGCCAATTTTGTATCGAGGGCCTTCGTCAACATGACGCTGCCGTCGC
>dme_circ_0003904
GTTCAACGAGAAATTCGGTCGTTCTAGTGCATTTTCTGGCCATTTGAATTATGGTAAGTTTCGGTATGGT
CTGTTTATTGATTGCCAGCGGTAATTGACTGAAATAATTCAGTGTTTTTGTCGCTCGTTCAGTCGGCTCT
TCATTAATTCCGATTTGGCCAATTTGGACGTATGTCAATTAGTTGGTCTGTTGTGGCGATGATGAAACAA
AACCCCATTTGTTGTGCGGGATTTGCGACTTGATTGCCAGATTTTTGCGTTGGAGATGAAGTTTCATTGC
ATGTCATGACGGGATGACTTGACAGAATGTATCTCGTAGATATAATTAGCACCGGATGCAGGCTGAATGA
GCTGGAAAACAATTGTCGGTTTTGCATTTGGGACTTCCATCAATCTTCGGTTCGGCATTGACTTCTGCCT
GAACCCCCGCCCCCTCGCCCTGTGCCTAAATGGCGTTAAGTGTAAATGTACAAGGGACAAATTTCGGGAA
TTCACAAAGTGCGCCAGAGACGATTAATTTTGCCTGCAGAATCCGGGCAGTCGATGTGAGCCCGGCGGCA
AATGTGTCTTGTAGATGTCCGCCCCACCACCGCCCACCGCCTGCCGCCACACCCCGAACCTCGTCCTTTG
ACTTGTCCTTGTAGCAACGCTTATGTGGGCGTGGGCAAGCAATTTATCTTTCGCTTTTTTCAAGCGCTGC
GGCTTGTGGCTTCTCTCCTTCGCTTCGCGCATCCTGCATGGGAAAGTAAAAATACACAGCAAGAAATATC
TTAGTAGCATGCTGACACTTCTTTTGCATTCCTAAATTTCAATAATATTTATATGTTATACTAATACAAA
ATATGTTATACTTAAATTTTAATTGAAttgaatcaattcctttgaatcaaaatcctttgaTTCTTAAGTG
TACTTTATCTCTTTAACAACTTTTCTCTCGGTGTAATACAGCTTCTCTGTTGCGTCCCACAATTATGCGT
AATTCGCATTTTTTCGTTGCATACTTCGTTGCGTTGCGCTGATGCGGGAGGATGTGGCGACAAAAAGGGG
CGGCAGGGGGAGTGGCTAGGCGCAGGCATGCCAAAAATTAATATAGCCACATACCTCGAATGGCGGAGTG
GCATGTGCAGCTTGAGTCAGCTGCGGCTTCTGTTTCTGCTGCCACCGTAATTAAGTCGAAATTAATGGAA
AATTCCTGCAGGTTGCCACCGAGTTCAGCGAAACAGTGTGGCCAAGCCGGGGGCAAGTGAACAGAAAGAA
ACCAGTGTGACCAAAACGAAAGAAATTATTAAAAGTGTATATTTTTGTCACAATTTCAATTTAAAAATCT
AAATGGCATTCTTTGCCATTCAGAGCGAAAAAGCGCAAGAACATTTATTTCAATTACCGAAAATGCAATT
TTTCGATTTATTATTTATCAATTTGCCGGCCATGGGGTATATTTTTAGGCGCACTGGCAGCGCCACCTGA
TTCGGGCTCAAAGTGGAATTAAACTTAATTGACTGGATGCAAATGGAGAGGCAGATCCTCCAGAGACTTT
CCCTCCGCTGTTGCCCGCTTCGTTTTCGATTTAACTCTGTTTGCCTAGGGAGGAGTTTGCGAATTGAAAT
TGGTTTCCTTGTTTGTTGCATCGCAATGCAAATTTCTGATGTAGAGTTTCTGCCAATTAATTGGCATTAA
ATTGGCTGGATTAGCGGCGCTGGGCACATGGATACCGTGGCAAATAGCAATAGGCCCCAGGAGTTTTTCC
CACCATGATGGATGCGAATGCTCAATCAAATCATTCGCCCGATTGGAGGCGAGCTACATTGAAAGCCACT
TAATGCGGTTATGAGACTCACAATCAAATTATATTCATTCGCATTAGCAGGAGATTCAATTCAGGCGTCA
CGTTAATTGTGATCCATCGATTACCTGGTTTCCCCCTAACTCCTCTATGCCAGCTGCCAAAAAGGAAAAT
TCATATTCAGGCAAATTAAATCCGAAGGGAAAGCCGTGCACAATGCCCAATTCTAATCGGAATACAAAGC
CCCGATTCGATTCCCTTTCGGCCAATTGCTATAAATCCAATAAATCGGAAAATGCAAATGCGGTTGCAGC
TGCTGGCAATTGGATGAAATTCGTAGAGGTAGCTACCGCTTTTTGTGGGGGGGTAGAAGCTCGCTTCCTG
GCAGAAGGGTACATTGGTTTCATATAAATATAACTTTGTGGCGAAAGTTCCTATTTAAGGCTAGGCTTAA
CTTGTCAGATTCGCCTAAAGAGCTAGTAGAACACTTAAATTATTAGGAATTCCTTTTAACTTTTTCTAGT
TTAATATATTATTTTAAGTACCAAAACACCAATACTTACAGACTTAATTAATTTCCAATAGGGCCTTCGT

CAACATGACGCTGCCGTCGCCAGCCACGGTTAAGCTGAGCACATCAATCGCCAGAAGCCCCCTGATCGAG
CGCAATCTGGGCCAGAATGCGGCCACCAATGGCGATGCCATCGATGCAAGCGTCGACGGGTTTGCAGCCG
ACTGAAGTGCATCCGGTGCAACAGCTGCACTTGCATGCCACGCCCTCAAATCCTTCGGATATACACCAGG
ATCGGAGTCCACACAACCATAACGCCAAACTGCCACCGCTGGAACTGCTGGTGGAGAACAAGATTAGCTA
CAGCAGCTACCAGCAGCACGCCAAGGACCTCAAGCACCAGAGTCACAATCAAAACCGATCCAAGAGAAGT
TCCCTCAGCAGTCCCTCGTCCAGCGAGGATTGCGTTAGTGCTGCCAGGGCCAATATCTTTGATGACGCCG
CCGAGAACTTCGTGATCAATTTCCCCTCGGAGTCGGCCACCTCGCAGCATTTGCTGCCACTGTCGGAGAC
TGTGCCCCTGCTCACCCACATCGAGGCCACGGTGGTGGATAAGCAGCGCTCCATAAGGCTGAACAAGAAC
AGTGCCTCGCTGATCTTCACGAAGAAGGAACTTAACCAGGGAACCAGGGAACATCATCATCAGCACAATA
ATCAGCAGCACCAGCAGCAGCAACGGCGTCCGCACCATAGCAGTTTGTACGGAGTGGGCAGGAAGCCGAC
CAAGCACCTACCGCCAACTAGAGGACACCACCAGAGGCGCAGTCTACCTCTTAGCTACAACAACAATCTG
GTGACCACCGCTCCATCAGCAGGTGGGGCAGGAGGTCCCGGTGGAGCAGCCAAACTTGTGCCGCTCAAGC
AGCAGCATCAGCACCACGGAGCGGGCCACACGGCACCACCCACGGCGAACGTCGTTGTTGCCGGTGGAGC
GGGAGCGGCGGCTCCGTCTGCCCGCAAGCAGCTCTCCTACTCCTGGTACGCGCCCGTCTACAGTGCGCTC
GAGGAGGAGCTGGAGCAGGACTCGCGGGGTGGGTGCACTCTTTGTTATATTCGTTACCTTTAACTGGTTT
TAGTTTATAAAAACGTCTTTAGAGACAAACAAATCAAAGTTTAGTGTTACAAATCCACCAACTTATGCAT
TAAATTCATTCGCAGGGACTCCTCGCCAATCCACAATCTGGCCAACACAAAGCAGCACCAGCGGTCCGCC
AGCCAAAAGGCATCCACCGCCCTGCATGCCACGCCCCGCCACGATCGCGCCCACGATTCCGCCGACGCCG
AGACGGTTGCGCTGCTGGACACGCAGACGCGCAGCAAGATCATCATTTCCTCGGCGAATGGTGACGCTGG
CGGAGCGGGTGCAGGAGTTGGATTCGGTGGAGGCGGGGGCGGAGGATCTGGAAACGGCGGAGTACTGGCA
GCGAACGGAGGAGGAGATCTGGAGAGCTCGCTGGGACTATCTGGTGGTGGGCAATTGCCACGAAGGCGTC
GCATCGAGAACTTTCTCAAATCGCTGGTGGGACTCAAGGCGAGTAGCGGTCGCGGAGGAGGTGGTGGTGG
CAATGTTGGTGGGGGCGGGGCAGTACCAGATCGGGAACGTCCTGCCTCCCCAGAAATTCGGATCACCCGC
ACTCCATCCGAGCAGGATGTCGTGCTGCGAGATCCTTCTGGCCGACAGCTAGCCAACGGCCATGGTCGAG
GCAGCCTGAGCATCAGCGGTTCGAGCAGCTCCTTGAATGTGGTCCAGCAGAAGATATGGCATATCATGCG
ACGCGAGGGTAGCGCTAGCAGCTTGCACCAGGAGAAGTCCCAGTCGATTGTCCAGTACACGGGACTGCGA
AAGTGCGAAACGGTCCTAGCACTCACCCGTCAATCGCAATCGCATGGCCACGTTCACGGCTTGAGCCAGA
GTCGAAGTCAGGGTCACTCCTTGGCGGTCCATGGCAGCGGGATCTTCAGCTCGGGTGGCGTGGAGCAGAT
TCGACCACTGAATCGCCTGCGGAACAGCGTGAGCAGCATTAACGGCGTGGGTACCTGTTCGCGCTGCTCC
AGTCTGTTATCCCTGGCTGCCTCCGGATCGCGTTACTCCCTGTCGAATGGATTTGCGCCCCGGCCAACTT
CGGCCCTCAATCACCTAGAAGCTGGATGGGAGGACGATCAACGGCGCGCGCAGGAGCCGCATATCAACGT
GAATGCCCAGATCAGACTGAGCGGTGGAGCAACTGCCAACTCGACCAGTCCCAGTCCGCCGTCCAATGTG
ACCACGGCCACATTGCACTCCAGTTCCGCCAATAATCTGAATCAGGAACCGCAGCAGTCCACGTCGACGC
CCGCCACCACGCCCACTGAGACACCTGGGAGCGGCGAATTGGGACCGTTCGGTACCAATCATGCATATCC
ATTGACCGTAAGCTCCCTGCTCTCCATGGCCAGTGCGAATCAGGCGGCGCAGGCATGCTGCGTTCGTGAT
GGGATTGCACTGAAGCGACGAGAGATGTTGGCCTCCGCCGATGTCACACCTTCGGTGGGCACCCCGGCCA
CGCCCACCACCGCTTTCAAGCCCTTCACGTGCAAGCTGTGCCTGATCGATGTGGAGGACGTGGGCGAGGC
CATGGCGCTTCAGCAGTGCGGCTGCCAATTTTGTATCGAGG
>dme_circ_0003904_junction_seq
CAGTGCGGCTGCCAATTTTGTATCGAGGGTTCAACGAGAAATTCGGTCGTTCTAGT
>dme_circ_0003905
CGGAATCAGAGCCACCGAAAAGAAAGTAAGGCAAACAAACGATATGCGTATAGGCCGGGCCAAAAAGGAC
AAGATAACAAATCCCCCAGCAGCACTTAAGGCATAAATCTCAAACTCAATCAACCATCTGAGGCTGAGAA
AGAGAAAGGAACAGGTCCAAAAGACAGAGACAAACGATCTAAGTGGCTGCCTTTCATCTGGATATTGGGA

CAAAGTCGGCAGCCGAATAGGGAGCAGGATAAAAAGGAGCAGGGGCAGAAAGAGGAGCACCATCGTCATC
CTGCATGTTGATTTCAGTTGAAATTGCGACCGCAACGAAGGACAAACAGTAGAAAGCTTTCAAGGACGTA
ACATGGCCAGGGTAAGTCGATTTTGGCCTTCTACACATATATTTTGCCGGCAATTTATATATGTATATTC
GCTCGCCTGTCAAAATACATTTCCAGTTCAATGCAAACGAAAGCGAAGAAAGAAATCGCATTTATCATTA
GTCGAAATAAATGACAATGTGCCGTAGTTCTCGATATTTTTGGGGTACTTTTTGGTAAATGATAAAAGTG
AATGGCTTTCACTTGCTTTTCGTTGTGGGAGCACATGTAACCGGCGAGAAAAATGTGCACCTGTTCACTA
GAGACATTAAATG
>dme_circ_0003905_junction_seq
TGCACCTGTTCACTAGAGACATTAAATGCGGAATCAGAGCCACCGAAAAGAAAGTA
>dme_circ_0003906
CGGAATCAGAGCCACCGAAAAGAAAGTAAGGCAAACAAACGATATGCGTATAGGCCGGGCCAAAAAGGAC
AAGATAACAAATCCCCCAGCAGCACTTAAGGCATAAATCTCAAACTCAATCAACCATCTGAGGCTGAGAA
AGAGAAAGGAACAGGTCCAAAAGACAGAGACAAACGATCTAAGTGGCTGCCTTTCATCTGGATATTGGGA
CAAAGTCGGCAGCCGAATAGGGAGCAGGATAAAAAGGAGCAGGGGCAGAAAGAGGAGCACCATCGTCATC
CTGCATGTTGATTTCAGTTGAAATTGCGACCGCAACGAAGGACAAACAGTAGAAAGCTTTCAAGGACGTA
ACATGGCCAGG
>dme_circ_0003906_junction_seq
AAGCTTTCAAGGACGTAACATGGCCAGGCGGAATCAGAGCCACCGAAAAGAAAGTA
>dme_circ_0003907
AGCCTGATTACTGCAAGGAAACAAAAGATTCAGAAGAGGCGACTGAAAAAGAAAAGTCGGAGATCAAAAT
CATCAAAGCTTCGAAAGTAGCACAGATACTGGGAGACACCAAGGAAGAGGAAGATCCAAGCCCAACCCAG
GAAGTGGAAGACGTTAAGGAAACAGAAGAGCCAGAAAATAAAAAGGAAATAGAAGACACTGAAGAGCTTG
AGGAACCTGTGGTGATCGAAGGTGAAGAAACAGAAGATCCCCAAAAGAAAG
>dme_circ_0003907_junction_seq
GAAGAAACAGAAGATCCCCAAAAGAAAGAGCCTGATTACTGCAAGGAAACAAAAGA
>dme_circ_0003908
AGTCAATACAAGTGGATGAACCAGAAGAATCCAAGGAATCCGAGGAACTTGAAGAGCACAAGGAAAAAGA
AGAGCCTGATTACTGCAAGGAAACAAAAGATTCAGAAGAGGCGACTGAAAAAGAAAAGTCGGAGATCAAA
ATCATCAAAGCTTCGAAAGTAGCACAGATACTGGGAGACACCAAGGAAGAGGAAGATCCAAGCCCAACCC
AGGAAGTGGAAGACGTTAAGGAAACAGAAGAGCCAGAAAATAAAAAGGAAATAGAAGACACTGAAGAGCT
TGAGGAACCTGTGGTGATCGAAGG
>dme_circ_0003908_junction_seq
AGCTTGAGGAACCTGTGGTGATCGAAGGAGTCAATACAAGTGGATGAACCAGAAGA
>dme_circ_0003909
GGTTCCTCAAGCTCTTCAGTGTCTTCTATTTCCTTTTTATTTTCTGGCTCTTCTGTTTCCTTAACGTCTT
CCACTTCCTGGGTTGGGCTTGGATCTTCCTCTTCCTTGGTGTCTCCCAGTATCTGTGCTACTTTCGAAGC
TTTGATGATTTTGATCTCCGACTTTTCTTTTTCAGTCGCCTCTTCTGAATCTTTTGTTTCCTTGCAGTAA
TCAGGCTCTTCTTTTTCCTTGTGCTCTTCAA
>dme_circ_0003909_junction_seq
GGCTCTTCTTTTTCCTTGTGCTCTTCAAGGTTCCTCAAGCTCTTCAGTGTCTTCTA
>dme_circ_0003910
AGCGAAGAGTCAATACAAGTGGATGAACCAGAAGAATCCAAGGAATCCGAGGAACTTGAAGAGCACAAGG
AAAAAGAAGAGCCTGATTACTGCAAGGAAACAAAAGATTCAGAAGAGGCGACTGAAAAAGAAAAG
>dme_circ_0003910_junction_seq

TTCAGAAGAGGCGACTGAAAAAGAAAAGAGCGAAGAGTCAATACAAGTGGATGAAC
>dme_circ_0003911
GAACTTCCGCACCTATAACCTAAGTGGCCACACAGACGCCATTGTAGCTTGTTTTTCGAGGCAAACAGC
TTGCACTTGAACACCATAAGCCGCAATGGTCAGCTCTGCTTGTGGGAATGCAGCCTAGCTCCCTCGGATT
TAGAGGATGCTGAAAGGCCAAAAATCGCTCCCCCTCAAAGGAAGAAGGAAAAGAAGGCAGTATCTGACAA
TGAGTCAGAAGATGACGTTGAAAAT
>dme_circ_0003911_junction_seq
CAATGAGTCAGAAGATGACGTTGAAAATGAACTTCCGCACCTATAACCTAAGTGGC
>dme_circ_0003912
GTGAATTTGGCAACATGGCTCTTGTGGAAGAACGAGAAGAGCTGGAGGGCGGCAGGGTGGCTATTCGATT
GCCGGGCGTACAACGGGGAGACATGTCATCCAGGAGATTCCAGCAAGAGGTTAGGGTGTTCTCCGTAAAG
TTCTCACCCACAGGCCAGGCATTTGCGGCTGCTGGCACTGAAGGACTATGCATATACGCCTTAGATAAGG
GGTGTTGTCTTCGATCCCTTTGATCTCTCGCTGGAAGTTACGCCGAAAGCGGTCCACGAGTCCCTCAAGC
AGCAGAATTACACCAAGGCGTTGGTCATGTCCCTCAAGCTAAACGAGCCCAATTTGATTGCCCTAGTGCT
GGAGCGAGTGCCGTATAAGGACGGGTGAGTAAAATGCTAAATCGCCTGCTGCATTCAGGGGCCAGTCCTT
GTCAGTTTTGGCAACCTTCATCGCTGCCGCGACTAAAAATAGCGGCGACACACTCCGCCGTAGTGGCGGA
GTGGTTCGCCACTTGCCCTGACTCCGTTCTCCGATCGAATGTGCAAATTTGCACTTTTGCTGTGTTAATA
AACAGTTTCAAGGTCCTCGCCCCAGCTCTTACCTTGGTTAATAACCCCTTCCAATACCGCTTGCAAACGA
ACTATAATGGTAGTACATGCAAATGGCTTGCCGTAAACAGCTTTGGCAGCACGTGTCTGTATTGCATGCA
TATTGAGTCCTTGCTCCTTTCGCTTTTCCAGGTCGAACTGGTTTGTGCGGATCTGTCGCCGGAGTTTGCC
CAGCGACTGTTGCAACAATTGGCCCGTCAGCTCCAGTCCACGCCACACATAGAGTTCTATCTGCAATGGA
GCTGCTGTCTGCTGACCAAACACGGAAACCAAGATGGAGTCTTTCAGCACACGGGCTTGCTGGCACTGCA
CGAGGTTCTTTCGAGGAAATACGAAATGCTTAACAAAATGTAAGTATACTTTTTTATATTGTGTAAAAGG
AGATTTCACTAAGTTCTTTATATATGCAGCTGCGACTACAACAAGTACACGTTGAAAGTACTCCTGGATA
GAGCTGATAAGTTGGAGCAGAAAAACGAGGGCAAACCCAATGCGACGGAGGAAGATAGTGATGAGGAAAT
GCTACTTATCAG
>dme_circ_0003912_junction_seq
ATAGTGATGAGGAAATGCTACTTATCAGGTGAATTTGGCAACATGGCTCTTGTGGA
>dme_circ_0003913
GCACACGGGCTTGCTGGCACTGCACGAGGTTCTTTCGAGGAAATACGAAATGCTTAACAAAATGTAAGTA
TACTTTTTTATATTGTGTAAAAGGAGATTTCACTAAGTTCTTTATATATGCAGCTGCGACTACAACAAGT
ACACGTTGAAAGTACTCCTGGATAGAGCTGATAAGTTGGAGCAGAAAAACGAGGGCAAACCCAATGCGAC
GGAGGAAGATAGTGATGAGGAAATGCTACTTATCAG
>dme_circ_0003913_junction_seq
ATAGTGATGAGGAAATGCTACTTATCAGGCACACGGGCTTGCTGGCACTGCACGAG
>dme_circ_0003914
AAATACGACCGCCCTCGAAGAAGGGCTCACCAGTGATAGTAGACTTTAGCATATTCGTTGTAGATATTAA
CTCAATTAACGTAGAGGATATGGACTTTAGGTAAGTGCCGTCGCTCGTGTTGACACCTCTGAGATCGGGA
GTTCGTAATGGAGCATATAGCGATACTGTGTACATTTTCAGAGTAGACATGTTTATACATCAGCGCTGGC
TGGAGTCGCGTCTGGAGATCCCAGATGACATATTCGAGGAGGGCGACGACTACGTGACGCTGCTTCCAGA
GGTCTTTGAGAATTTTTGGCAGCCGGATCCGTACTTTCTTAATTCCAAAATTGCCGG
>dme_circ_0003914_junction_seq
CGTACTTTCTTAATTCCAAAATTGCCGGAAATACGACCGCCCTCGAAGAAGGGCTC
>dme_circ_0003915

GTAAAATCAATCTCTTCTACCAATTAAAAGCTTGCAAATTATCAACTTAAAGTAAATCAAATGTTTCGAA
TATCAGGCTCCTTGCTACGCGTAATTATACTCACAGTCCTTACAGTTTTCTTGACTCTCAATTAATAGAC
GTAATTTAGTTTATTTCAAAACATTTTTCGACATGCACAGAACAAATTCGAATCGAAGCCTAACTGAGAA
GCTTTCAATAGTAAGCGATACTCTGAACAGACTAAAAATAAATATAATCTAGTCACTTTTTGAAGATCGG
TATATAGTTATTGCCTATCGAGTTTTTCGGGCACCGGAATCGAGCAGGTCGCTATTGTCTTTGGATGACC
TCGCCAGTCCGGCGCCCTCAACTTTGATTTCATTTGCGATTCTTTACAGGTTTTAAGCTAACGATTATAA
TGATAATTTGATTATTTGTTGCCTCCCAGTTGGCTTTATTTGCTTGAAGGAGAGCGCATCACTAGACAAA
AGCACTTGGATTGCAGCTAGGCTAGGTACAGTCTGGTCGGCGGAGTATTTAGGTTATCCCAAATAACTAA
TCCATTACTTTAGCGCCTCCTTGGCCCTCTGCTCCCACTCGGCGCGTCTCTGGTGGATTCCCTTGAACTC
GCCCACGGTGGCTGATCTCTCGATGGGTTTACTGGGCgctactgatgctgttgctgctgcagatgttgca
gttggtgctgcaacaggtgttgctgctgccgGAGACGTTGGAGTAGTTGCTGTCGGGTTGGCCTGCTTCA
GTTGCAGGGAAAACCTTTCAGCAGCGGGCCTTGCACTCCCATTACCATTGCTTATATTGTTATTCAGGCT
ATTGCGGCCCTGATTGCTGGTCTTAATGGGGGGCAGGTAGGTGACCACCTTGGGTGGCACCTTCTGTTGC
TGCTGATGGTTATTGTTATTATTGTTGAGATTGCGGAAGTCTGCAACCGAATTGCGATTGGTGCTGGG
>dme_circ_0003915_junction_seq
CTGCAACCGAATTGCGATTGGTGCTGGGGTAAAATCAATCTCTTCTACCAATTAAA
>dme_circ_0003916
GGTCTCCACTTTGTTCTGCTGTTCCCGCACCAAAGTGGTGGACTGGAAGGTGTCGTTGCCCCGCGAGCTC
TTCGACAGCAAGTTGGTCACATCGTTACCCGAGGTGATCTTCTCCAGCGGAGCAGCTCCCAGTTCCTTGG
GCATGTATCGCTGCCTGGCGGCAGGACCTGATTTCGGCTTTAATGCCGTGGGTGGATGTGGATGCACACC
GTAGTTACTGACCATGGC
>dme_circ_0003916_junction_seq
GATGCACACCGTAGTTACTGACCATGGCGGTCTCCACTTTGTTCTGCTGTTCCCGC
>dme_circ_0003917
AGCATCCAAATcagcagcagcaacagcagcagcagaagcGTCGTCGTTTCCATCCGCTTCGTAACCTGCG
TCGCATCTTCCGGCGACGTACGATTAATAGCTCCACGGACAGTCCAGCATCCTGTCAGTCGCCAGGACCC
GGATCCTGCAACACCTTGCCACCGCCCGCCAGCAGCAGCAGTGAGAAGAATCTGCGCAGCGGGATCGCCT
CGCCAACAGGATCTCCCCTGAATCCCCAGCAGCTGAAGGAGAACTACCAGACGCAATCGCTGCCCAAGTC
CAAATCGTACGGTTCGCATCGCGATGAGCTGCTAAGTGTGCTTCTGGGCAAAAAACGGGCCAGCAAGCAG
GACCACAGCCAGGTTGCAGTCCAACCCGATTCCCAAGCGCAGTCGCAATCGCCGCAGGAAGCCATGTACC
AGGCCCAGCGACAGCATGGCGGCGTGGCCGTCTTCCCGGATTCACTAGTCCTTAACCGGAGCAGCTACTT
TGCCGAGCAGAGAATCCAACAGCGCCATCTACGCAGCGTGGGTGACTCCTCGCAGGAACTGGAGTCCAAC
CTAGGCTTGggatcaggatcaggatcaggatccggaGCTGGTGGCGCCGCTGACATCTCCGACAGCCAGC
GTAGCCTCAGCGAAGGTCGACTCCTGGATGTGGACGGGTGAGTTCATCTCCATTTCGCAGTTACAGCGGT
AGTACAATCTCCGATTTCAGGGATTACTCTCGGGATACCCTGTCCCAGTCACATGACAGCGTCTTCTCTG
AATCGGCCACAGCCAGCAGTCTATCCATTGTACTCAAGG
>dme_circ_0003917_junction_seq
GCCAGCAGTCTATCCATTGTACTCAAGGAGCATCCAAATcagcagcagcaacagca
>dme_circ_0003918
ACCACCAACAAAAGAGATTCGAACAATGTAACGACAACTAAAACGATCTAAACACAATCGAAAGGCGGAA
AATTATTGTATTGCCCAGAAATCAAGTTGAACAGACGCCAAAAAAGCACTTCAGCGAAATCAGGGTGAGT
TGGGCAACGGATTTGGAATTGCTTTTGGGATTGAAAAAAACATTCGTTCATAAATGATGATGTGCAATCA
TAATTCGCTTTGATCATATATTTTTCTGCTTAAATAGAGCAATTTCAAATTGCCATGAGAAATGGTTCTC
TTAGGCAAGAGTGTATTAAACCCCTGAGGATGGAAAACGAACAACCAGTTGTTTATGAGTTTGTTAAAGC

TCTGAAACTTTGGCATTTATTATGTTTCTTTGGCTTTTTCAAAATTTACGTTTATGCGCGCAAGGAACCC
TTCAAAGTACTATATTCACTATACCTAAGTGAAATGTACGGatatatatatgctaatatatatataCTTATT
CGCACCTGGGCGACGTGTCTCTGTTGTTATGGTAATGTGAGCTTCGTTTTGGGGTTTCATTCTCATTACA
ATAACGCTTTTGCCCTCATTGTTTGCAGGCAGCAAAAGACCTTGGTTGCAGATCGACCTTGAATCCGCGC
GTGTAATTCAAACAATATAATCAGACTGATTAACGCGTAAACCATGCAGCTTAACACCTTCTCGCCGGAA
AAAGAGACAATAACTCTGTCAAAGAAAAGTCGAACAACCTTGTGGCGTCCTCAGTTTTTGATGGCTCAAT
TTTTGGCCAAGCGCATAAATTAGACAGTCGGCTTCTCGGATTTCCTTGACTAATTTCAAGGAGGTGGAAA
GCCTGAACATTGCTGATGAATTTCCACCAACTTGGCTGCTCTCCAAAAGCACTTGGTATCCAAGAGCGCA
GGGATTCTGTTGCAGAGAGGAAGGAAGTCGTCGGCTCCTTTTTGCCTTATTTGGTTATTCGGTGACAGAC
TTCTGCCAAATTCGTTGACTAGGTCAGGCTCTCTCTGTTTCttttttttGCATGGAATtgtgtgtgtgtg
tgGGCTTCACTTTTGTTTTCTATTGGCCAAAGTGCGACTGCGCAGGGAGAGAGGGAGCCATTAAGGTCCT
GGCAACATGCCGTTGGCAACATAACTTCTTTAAAGTTTTCGCCTATAAAATACACTGAAAATATAGGCTA
GGCTAAGCTAAAGAGCTATATTAACACTATTAAAATGATGTAAATATGTTTTAAGGTATTAAATAGAAAT
AGTTACATTTAAACCGAGTTGAATAGCGAAATCCTTTTCCTAAAGTTGAGTCATCAGATACTCCAATGTT
CTTTGCAACATATTTCCAGTGCATGCAAAACTGTGTGAGCAATGATGCTGCGATGCCGCCCACGCAAAAA
GAGCAAGCAAGATGGCAGGATGACAGAGGGAAAGGGACGGCGAACTGAGAGCACATCTCGCAGTTGCGCT
CCTTGACCTTGGGCACGAACGCACTCGAAGCCAACAACCCCACCCCTTCCCCAAAAAAATGCTTTACCCT
TCGGTGATGCGAAATCCGTACAGAAATTCCGAGTCAGTCGCTCACTTCCAGCAGCCACAAGTTGTGGCCA
CGCGGATGGAATGAAAAGTGGGAAGAAACAGTGAGGAAAAAAGTGTTGAAAAGTGttttttttttttATG
CTGAATGGTGAACGGAACGATCCCCCGCAATGGCAGCTAGTCCCATCGATTCGCATCTGGATCTGCAGTA
TGTAAAGGGCGGAATCCGGGGTATGGAACTGCAATTCCATCTGTTTGTCAATTAAAACGACGCAGAATAT
AAACAGCAAATAAATCAACAAGAGCATCATCACAGTCAGTTTAGTTCAGAATCGAGCTCAGAATCTCTGA
ATCAGCGATTGCTGCGTCATCATCATCAGTGCTCTTATCGGAACTCTTACTGACCCCCAACCCCATCGAT
TCGCATCGATTTCCATCTATCATACACAGATTGATTGATACGAAGGTGGGGACATTTCCATCAGCTCCTC
GAATGGGCCCTAGGGTTCAAAGTTCGCATTTGTCAAATGGAGCGAAAATAGGGACTGTATAAACACGCCT
GCCGTTTGAAGAGCCCAATCAACTAGAATGAGATGAGATTAAATGGTTCTGTGAAAAACACAGGTTTTCA
CTGGACTCAGTCCCCTTCGAGGGTCGATGGATAATTTATTTGTGTTTGCGAACCTCACTTAATATCTGCT
GCTGTTGAATTTCAATTAATTGGCTAGCGATTGGGCAGCAAAAGTGGATAAGAACTCAAGGGGCTTAGTT
AGAATTGCAAAATTGGGGCTTACATCAGATAAGAAATGTATTCATATAACTTTTAGGTATTTACCATCTA
GTCATAGCATTGTAATGATATTATAGAAACAATATACATACATATGTAGGCAATATTTGCTAATTGATTT
TCTTATCACAACACGCCCACTTGACATTACTTGCTCAACATTGTGCTATTGCCAAGTCGCGTAAAATATG
GCTCATTACATTTGTGTTGGCCAAAAGTGGCAGGAATGTTTATGCTCTTCTTGGCCATTTGTATATATTA
CCTACCAATCTTTGTGTACACAAGATACAGCCATCTCTTGCCTCATAAGTAAGCAAATATTTCATTTTCC
TCGCGTTTTCATTTGAGATTCTTAATAAAGTTGCTTGGTAAATAAAATAAATCATAGAAATAGCAATAGA
TAAATTTGATTTATTAAAGCCAAAATTTGCATTTTCCCCCTACTTCGTCGAGAGTTTTAAAATTGCACTT
TTGCAGCCCTAACTTGGGCATTTTCACACTTAGAGACGACCTTCAAATGGCCCGAATCGCCCTCGATTCG
CATTTTGTCCAAAGTCATTAAAATTGATGACAGATCCATTCAGTTGGCGGCAAATAGGGTGAGGTAATAG
GTACTCCACTCCGGAGGTTGTGCACGTTGTGATTGTGCGATGAGCATGCACTTTTGTTCAGCCCGGCTGT
TTGCTTTTCCTTCTCGGCTTGTTTGAGTTTTCCGGTCAGGGGCACTTTCCTTTTCAATTTGTCCAGCTCT
TTTTTGACTTGGCCATTATGCCATTTGGCCGTTTTGCAATGACGCTCGCTGACCAGTGTTAGCCATTGGG
AATTGTGCTGAAGTGTCGTTAACAAGTGGCTACACTGGGCAAAAAATCAGATTCATTCTAATGTCACttt
ttttttATTGAAATCACAATTACAAGAAAGTAAACTTTGATTTGAAAAATTCGAATAATAAAATAGATCa
tatatatacatatatatTTTTTTAATGGATTAAAATGATATCATAAATAAAACGTAGCTTTTGCAAACAT
ATTTTGAAAGTATCGCACATATTATAAATAATTTTAAACAGCAAATGAATATAATTATGGTATTATCCTA

TAGTTTTTTTCTGCCTGTAGTTAATGGCTTAAACAGAGGCGAGAATCTCTAACTCACGGAGCTGAGTTTG
CTTTAAAGTTAGAGCCATAAGTTGGCTTCCTGGGCCATTGTGTATAGCCATCTTCGTAATTAAGTTGGCA
AATTTGTGTAATGAGCTAGTGCAATTATAGGGGCTGCTCCGTGGTTGGGCCTATGCAAATGCCACACATA
ATTATAGTTTGCGGCCGGCGTAttttttttttttttactttaggctgttttggttttggctttgtttttct
ttgctcgtttcgttttcattttcattttcCCTGCAGTGTTTGCTTGCCCACTTTGCCTGGTGAACCAAGT
GGCGAGTGTGTGCTCATCTGCGAAATGCAAATGAAAGTCAAAGTTAATTTGTAAGATTTTCACGTTGAGA
TACAAGCTCGCGAGACCGCCGGCAAAAACTGCGTGACGGCGATGAGTTACTTTTGCGGCTACGGTTaaaa
aaaaCAGAAATGAAGTGAGATAAAAGTGCCAGGCCACGTCTCAATTTTCCGTCTGCCTGCAGTCTCGTCT
CGGTGGAAAATCGGTGAAAAAAACCCTCAACCGGGGAAATCAACGCCTGCGTTTTGCATTGCGTCTTGCC
TGCCACGAAAATGTGGCATACTTTCGGGCTGTTAATCAATTAGAGCTGCGGAAATTGAATGGATTTTTTA
ATGGCTGGTGCTCAAATAAATGACGAGGGAAACTCCAGCTACTAGCTAATTTATAATTAAGTATTATTAA
GAGAATAAAATTGCCTTTGACAGGACAATCCTTGTTTCATTAAACAATATTCCTTGAATTTGAGTTGAAC
GTAAATTTAACAAGAGGCCAAGCTAATGCTGTTTCAAAGAATACTACAACTACATTTTTAATTTAATTTA
TAATTAACGTATGATGGTTAGCGTTTTATTGAAACAGATTTCTAAGAATTACCTTAGCAAAGAACAATTT
ACCTAGCTGTAAAATAAATCTTTTAAGTGCGGATTCTTTAATTCGAGTGTGTGAATCGTAAACAAACTTG
ACTCATAAACTATTTAGCGTTCTAATTCCCTCCCCATGCCACATTCTCAATTGAAAGTTTTAATGAATTT
CGCCTTGGCATTCCCCCAAGTTCAATGCCAAAATCGCATTCATTTAGTGGACTGATCCCCCCATTTATAT
CATCTATATGCCAACCAGACCCCCAGTTCAGTAATTTCGTTTAATTAACAAACGTTCAAAACGAAATGAA
CATTTTGCAGTTGTGTGGGCTGAGTGCTGCTCATTTGTCTGCCCCAATCAACAtcatcgtcatcgtcatc
gccatcgccatcgCGGTTTCTATCATCATTACGGAGCTCTTCATCATTTCATCAACTGGGGGGTGCAACC
ACGAGTGTCGACACGAATCCCCGGCTGCAGTTTCCATTTCAATGCGCAAAAAACGCTTAGACTGCAATTT
GCGCTTGTTTCAACCGGGAAAAAGGGTTGCCAAggggggggggggCTTCAAAAGGGGCGTGTTCTCCCCA
CTAATTAGCACATCAAATACTCTGTGCACACGGAACACGTACGTATCGCGTAGTGGGTGGGGTTGTTGCC
TGAAAGGGGTCAAATGTCAGCTCAACGCATAGCGCTATTTGGACAGCCCAACTAACTGGGTTAACTTACT
GGCTCTTTGGAGCGTTAGTTTACCACGCTCGCAAATGCACTAATTACCACTTATAAATTAGGCTGCCCAA
AAGTATGCAGTGAAAAAGGTGTCAACTGCATTACTCGCCAGATTGCTACATTTCAGTTAAGGTATAGTAA
TTATTTAAAGTGCTTAAATAATACTACTATTTGAGTAGATTAACAAACGTTTAAGCATGTTGTTTTCGCG
CTTaaaaaaaaaaacataaaaaaGATGGAGCCTGCCAAAAGATCACGATTTTAGAATGATTGCTCATTTG
AATATTTATCGTTACTAAATCAATACATTTTTATGACGTAGACCCGCAAATATTTAACCATCGCATTTAA
ACCACTCGTAAATCATGTTAGTGACCTAAGATGATCTGCCTGATGTAATTTAAATCTCACAACGTCTGCT
GTGAGTGTTCGGCATCCCCGAACTTGGCATGAATCATCCGCTCGAGAAGCCATTACTCACATCCCATTCG
TCCGGCGCCAAAAACAAAGCCAAAATAAAGCCCAAGACAGCAGAGGAGGACCATGAGCCAAAAGGCATAT
GCACATCGACAGAGTCTGCAGGCCACAGAGTTCAGGAAAAGGGGCAAATTCCTAGATGAAAATAGCTTTC
CTTGAAATGCCTTTGGACCAGCCGCGATTTTCGATGCCAGCGATTCGAGGGAATCCGCCGAACAAATGGA
AAATGCATTCTGCTCATGCCGGACTTGTATTTATTGGATGACTCGGGATGATCGAGTGGGTTGACTGAAC
TAAGAATGGGCTGGCATTTTTGGCATTTAAATTATTATGAATCACACTAATTCCTAATGCTTTGAGCATA
TTGTCATCTATGTGAAGTCGAAATGAATTCCTTGCTTTAATACATAAATaaaaaaaaTGATTGATTCATT
TAAAACATTCATTACACATTTTATGGTATTTACAGGCGCAAGTATAGAGATTTTTCCAAATTTTCAAAAA
CAGTATTGTGATTGTTTTTTTAAAGAAAGGGGAGTCACTTTCAAACTGAATTCAGACTCTTACTTGTTGC
CTTCTTCGGTTGACAGGTAATTATTTTAATTGCAAAAAACTGTCATTGTTTATCTTTAATTACAAAGTCA
ACACATCCAATTACCCAGTATTCAATAAGCAATTATGTTTAAGATCCGCACAAATTGAATTAAATGCCAC
AACTTAAGGATTCTCTCAACCAATTTCCCTGTCACTCCGTCATTCTGCAAAAGACTGAGTTTTCCGCCAG
GTTGTGGTGACTTTTATCGCGAATGCAATATGACATTTAATTTCCCCCCAGAACACCGCCATAATGGAGA
TCCATTAGTGCGGTGTCGCAGGATGTCAGTGCAGTTTGAGTGATGGGTATGGGTTAAGTCACGGGAAGTT

CCCGTACGGAGGACTCCGATGCGGAAGATTCCGTCTTCGTAGTCTAGAGAAGATACATATATTTCCATTG
CACAAATATCGCTAATACCGCATACTCGCAGATGGCAATTACAAATAGTTTTCCCtttttttttCCAGCC
ATGTCTTCCGGCCTGATGTCGTGTGTAAGGGAAAACTCGCCGCCGCTGGAATCGGAGTTGGATGCCAGCT
GCGCCGGGGGACCATTGTCATTGCCGGCGGGCCGCGCCCACCCGCACACCCACGGCGCCACCTCAACGCC
CATCTCCAGTCCAGGAAACGATGCCCATCAGTCCCCGACCGCCGG
>dme_circ_0003918_junction_seq
ACGATGCCCATCAGTCCCCGACCGCCGGACCACCAACAAAAGAGATTCGAACAATG
>dme_circ_0003919
GGTGCGAGCCCGCGGTGGCGACGCACTGCAGCTTGTTGCGCGCCATGCCCTCGTATGTGTCGAAATGCAA
GTACTTGGAAACAATATCTGCCACCACTTCCTGCCGGCGATCCTGAATGTGTCGCTTGAACAGAGA
>dme_circ_0003919_junction_seq
GATCCTGAATGTGTCGCTTGAACAGAGAGGTGCGAGCCCGCGGTGGCGACGCACTG
>dme_circ_0003920
GAAAGCATCTACTGAGGAGCGCAACAGCTTATAGGGCTTTCGAAGTCCACTTGGATAGTTCTCAACGATG
GCCATGCTGTCAAGCACTTGACGAGGCAGGAAACTGTGAGATGCAGGCACTTGAACAAGGTTGCTAAAGT
TTTTGGCAACAATCGCTTGCAGCTCGAACTCGCTGCCCTTTGTCTCCAGGTGAATACACTTTAGGTGGAC
GTGGCGTAGGTGAGCGAATATCAGG
>dme_circ_0003920_junction_seq
GACGTGGCGTAGGTGAGCGAATATCAGGGAAAGCATCTACTGAGGAGCGCAACAGC
>dme_circ_0003921
GTTTTCTTTCCGTTCAAGGTCCAGAGCTACTAAACATGTATGTCGGCCAGAGCGAGCAGAATGTTCGCGA
GGTCTTCTCCAGGGCTCGTTCTGCGGCTCCTTGTGTACTCTTCTTGGACGAACTGGATTCCCTGGCTCCC
AATCGAGGTGTGGCTGGGGACTCTGGCGGTGTAATGGATAGGGTGGTGTCGCAGCTATTGGCAGAAATGG
ATGGAATGAGCGATGGCGACACTAGCAAGCCCATATTCATTCTGGCCGCCACCAATCGCCCGGATCTTAT
TGACCCCGCTCTTTTGCGTCCTGGTCGCTTTGACAAGCTTTTCTACGTGGGACCTTGTTCAACTGCCGAA
GACAAGGCAGCCGTTTTGCGTGCCCAAACCCAGCGATTCGCTTTGGACGCCGGTGTGGACATGGAACAGA
TTGCCGAGCGTTTGAAGAGCGAAATGAGCGGCGCAGATCTGTACTCCATCTGCTCGAATGCATGGCTCTC
CGCTGTGAGGCGCACCATAGACGGACACTTGAGTGGAACAATATCAGAGAAGGAACTGGTGCCGGAGAAC
GTAATTGTGCAGGAGGAAGATTTCACGAAGTCTTTCAACAAGTTTGTGCCCTCGATCAGCGCCAAGGATC
TGGA
>dme_circ_0003921_junction_seq
TGCCCTCGATCAGCGCCAAGGATCTGGAGTTTTCTTTCCGTTCAAGGTCCAGAGCT
>dme_circ_0003922
GTGCGCCCCATCCTGTCCGTGGACCGCGAGGAGGCCCGTAAGCGTGCCCTCAATCTGTACAAGGCCTGGT
ACCGCCAGATTCCCTACATCGGTAAGTGCTCCTGGTTATGTCATCAGAGTGTGCATCCGTATCCCGACGC
CATGTAATGCCATCCGTCCGCTTGCAGTCATGGACTACGACATCCCCATGACCGTGGAGCAGTGTCGGGA
CAAGCTGCGCGAGGAGTTCGTCAAGCATCGCAATGTGACCGACATCCGGGTGATCGACATGCTGG
>dme_circ_0003922_junction_seq
ACCGACATCCGGGTGATCGACATGCTGGGTGCGCCCCATCCTGTCCGTGGACCGCG
>dme_circ_0003923
ACCTGCTACGAACTGGAGCGCTATCTGCGGGATGAGCCCAAGCTGCAGAAGAAGATGCACAGCAATCTGG
ACAATCCGTGGGACATATTCTCCACACCCGCCCGCCTGCTCGAGGAGCTGAAGATCAAGGAGCAGCACCT
GGACACGATCTCGACGACCTCCTCGGTCTCGTCCAACTGCTCGGGCACCTCCTGGGACAGCAATGGTTCC
CAGGCACTGAGCTGCTCGGTGCTGGTCAAGCAGGAGCGGATCGACGAGGAGGACGACGATGTGGGCTGTG

ACGACAAGCTGAGCGTGCTGCTCGCAGCTCCGCCCTCGGCCATCTCTCCCAATCCCAGCATCGGCAGTGC
GGGCAACATGACGCCcaccagcatcagcaacagcagcagcatcagcagcagcaatagccacagcaatagc
aacggcaacaccaccggcagcagcaacatcatcgccagcccgctgagcagtctgggcagtggcagcacca
tcaCAGTGAGTTCCCGCAACAGTTCGGCGCCGGGCATCAAGGTGCGCGTCGTCCAGGCCAAGAATCACCC
GGGCAGCAATGCGAGAATGCACCTGGGGCATGTCCAGGACTTTGTGTTGCCCACGCTCACGCCGCCCTCT
TCGCCAGAGTCCAACCTGCGCAGCCACAATTACGCCCAGCAGCTGGAGGCCAACGATTTGGCGGCACTGA
TAcagcagcagcagcaacagcaacagcaacagcagcagcagcagcagcagcagcagcTCAATCCACAGCC
CGGCAACTCCACACCCGCTACAGCAATCCTGCGCTTCACCAGCCAGAGCAATCCCACAATGACgcaactg
cagcagcagcagcagcagctggccgtccagcatttgagcatatcgccacaggcccttggccacagcaaca
tcggcagcaattcgcccaagagcagcagcagcagtagcagcaacagcagcaatagcagcagtagcagcca
cagtagcagcagcagcatcagcggcagcagcgaccagccaacacacagcaacaTTCCGCGCAGCACCATC
GTCCGCCTGACGACGGCCAACGGGAAGCCAGGAGCGGCTGGCATCAGTCTGGCCCGGGTCATCCAAATGC
AGAATAACGGCAATGCCAATGTTGCCGCCGTGCTCAGTgcagcagccaacagcggcagcagcaacagcaa
cagcaacagcagcagcagcaacaccagcagcaacagcatcaacacagcgacgccgcagcaacaggTGCTT
TCGCAACGGTCGGAAAAGTCGGCGAATGGGCTCCTCGAAACCGCACCACCCGCGCCAGCATCACAGCGAT
CACAGCCCGGACGCCAAGCGGCGGATACACAAGTGCCAATTTCTCGGCTGCAAAAAAGTCTACACGAAGA
GTTCGCACCTGAAGGCCCACCAAAGAACGCATACAGG
>dme_circ_0003923_junction_seq
TGAAGGCCCACCAAAGAACGCATACAGGACCTGCTACGAACTGGAGCGCTATCTGC
>dme_circ_0003924
tagcagcagcagcatcagcggcagcagcgaccagccaacacacagcaacaTTCCGCGCAGCACCATCGTC
CGCCTGACGACGGCCAACGGGAAGCCAGGAGCGGCTGGCATCAGTCTGGCCCGGGTCATCCAAATGCAGA
ATAACGGCAATGCCAATGTTGCCGCCGTGCTCAG
>dme_circ_0003924_junction_seq
GCAATGCCAATGTTGCCGCCGTGCTCAGtagcagcagcagcatcagcggcagcagc
>dme_circ_0003925
AAACCAACACGCAAGAGGCCACAAAAATAATTGGAATGTAACAAATCTGAATGAAAGTGCTGAAGTTAAA
TAAATTTTTAAGTGAATAAGAAACCGAAAAATACCACACATAAATGACGAGCGTTTGCAGAGTGTAGATA
ATTACATATTAGTTGTATCTTATTCATATATAGGGCAATATTCAATCGCGAGTGCTAAAATGCTGTCCTC
CGCAGCATCGCTCATCGTCGGGATGCAGAAATCGATCTGAAATCAAAATTAAGAAAAGAAATCAAAATAT
ATATATTTGTCGCTCTAAGTAATCAATCGTCGTAGCCAATCAAATCAAAATTGCATCAAAAGCCAACTCA
AATTGTTGCCCCACCAACAGTTTGAGTGCACATTCATTTCGGCCCCCACTGTTCCTGTTCGTGAAACATT
CAAAAACGGCCTCAAAACGGAGTCCCTAGAGTTTCTGCGTTGCGAGGAAGAGGATATCAAGATGGATATC
CTACCGAGTGGCAACATCTTCAGCGAGCTGGAGCGGATCTGCACCACCGGCTACTACTCGTCGCAGCCGT
CGATTGAGGATCAATGGCAACAGG
>dme_circ_0003925_junction_seq
CCGTCGATTGAGGATCAATGGCAACAGGAAACCAACACGCAAGAGGCCACAAAAAT
>dme_circ_0003926
ATATGTGTAGCTAATTCTCGGGAGGTGCGATTCTCGGACTCAAGTAATTTGAGAACCAGATAGCGGTCGG
AACGTGCCGTGGGAAAACCTACATCCCAGATAAGTTAACACACAGCACTTACTTCCCCCTCTCAACCCCG
CTGGCTGTCACCACTGCAGTTGCGGTCATTGTTATG
>dme_circ_0003926_junction_seq
CACCACTGCAGTTGCGGTCATTGTTATGATATGTGTAGCTAATTCTCGGGAGGTGC
>dme_circ_0003927

ACTCCGCAAACGGAGAAAGCCAATGAAGGTTGCCAGGAATCAAGCAATAAGAAGGATTTGCAAGAATCTG
CAGCTAAAGATGATTTTTCCAAGGAAAAAAGTGGAGAGGATGGTGAAAAAGACACCGACAAGGAGAAGAA
GGCAACTCATTTACATGAAGATAACAACGATGATGCCGAAAAGGATGGCGACGAGGCCGACGCAGAGGAT
GAAGACGATG
>dme_circ_0003927_junction_seq
GAGGCCGACGCAGAGGATGAAGACGATGACTCCGCAAACGGAGAAAGCCAATGAAG
>dme_circ_0003928
GTAAACGATCTTCTATATGCTGTCTGCGGGACTCCCACATATGTAGCACCAGAGATATTGTTAGAAGTCG
GATATGGGCTAAAGGTAATAGTAAATAATAGCTTTCTTAAAAATTAATATACTTATATATTCTTAAAGAT
TGACGTTTGGGCCGCTGGAATCATTTTGTATATACTTCTATGCGGTTTTCCGCCGTTTGTAGCGCCTGAC
AACCAACAGGAGCCGCTATTCGACGCTATTATCTCTGGCATTTATGAGTTTCCAGATCCTTACTGGTCTG
ATATAGGGGACGGAGTGCGCGATCTTATTGCCAATATGCTTCAGGCGGATCCAGACGTTCGTTTCACAAG
CGAAGACATCCTTGATCACAGTTGGACGATTGGAAACAAGGGGAACGAGTG
>dme_circ_0003928_junction_seq
GGACGATTGGAAACAAGGGGAACGAGTGGTAAACGATCTTCTATATGCTGTCTGCG
>dme_circ_0003929
CCGCGGCGTATTATGCGATTATTGCTAAACAAGCGTAACAGCCCGAGCTTCGATCATGTGCTGACCGCCA
TTACGCAAGTGGTTCGCTTGGACACGGGCTATGTGAGAAAAGTATTCACTCTTTCCGGTATCCCAGTGGT
CCGACTATCTGATTTTTTTGGG
>dme_circ_0003929_junction_seq
AGTGGTCCGACTATCTGATTTTTTTGGGCCGCGGCGTATTATGCGATTATTGCTAA
>dme_circ_0003930
GTGGATACTTCTCGTTATTTTTACTACACATACTTTTATTATTTATTTGCCCTTGTAGATTACCTCTCTC
GATGAATTGGAAGATGGACAGAGCTATGTTTGTTCCTGCAATAACGAGAATTTTAAGAAGG
>dme_circ_0003930_junction_seq
TCCTGCAATAACGAGAATTTTAAGAAGGGTGGATACTTCTCGTTATTTTTACTACA
>dme_circ_0003931
AAGTTTCCACTGTGGCAGCAGGAACAGCAACAGCAACAGCGATTTCGGCTGAACGCCCCGTGGTGCAGTC
TTAAGTGGATTTCCACCTTAAGTTGCATTAGTATCGATGTTGTTGCTGCTCACGTTGCACATCGATGTGG
TTGTTGTTGCTGCTGCTGCCGCCGCTGCTGGCGTGTCAATGACGTTGCAAGCCTTTTGTTTGTCTTTCTA
CCTAGCCAGGTATCCTGGCGCCGGCTTAATTTACGAAAAGGTAAAGGTAAACGGCAGGTAAAACGCCTCA
AAG
>dme_circ_0003931_junction_seq
GGTAAACGGCAGGTAAAACGCCTCAAAGAAGTTTCCACTGTGGCAGCAGGAACAGC
>dme_circ_0003932
TTGAACATCCTCGCCCTCGGCCTCGTGCTTGGCCTGCTCTTCCATTTCGGCAGATATTCGCACTTTGTCC
GAAACCGAGATTAGAGTCTTCATGAAACCGCTTTCGTTGCCGGAGGTTCCAAAGATAACATCGTGCATCT
GTTGGGCCTGCGATTTGGGAAGATCATGAGCGGGCACCGAAACAGGCGAGGGGGATATTAATAAGATGGA
GCCGCGTGGCTTAGTGAGATCCATGGGAGCACTCTCCTCGTTGGCCACACTCCGTTTCGGTTTGGACTCC
TCCGGATTGGAGTAATACTG
>dme_circ_0003932_junction_seq
TGGACTCCTCCGGATTGGAGTAATACTGTTGAACATCCTCGCCCTCGGCCTCGTGC
>dme_circ_0003933
GCAATATCCTCCGCGGCCAAAGCCGCCGCCAAAGTGACGAGCGGCCCTAACTCCAGTGCCATCATCGATT

CCCGGCTGTGCTTCAGCATCTTCCAGCGTGATCTTTTGTTCCTCCAGAGGATGGCCGCTGATCATGGCCA
GAATGGCCGCAAAGACGAGGGCAAGCACCAG
>dme_circ_0003933_junction_seq
TGGCCGCAAAGACGAGGGCAAGCACCAGGCAATATCCTCCGCGGCCAAAGCCGCCG
>dme_circ_0003934
GGTGGTTGGACTCCTGGACGTCGCCGATCAGGGAGCGAACCATGCCAACGATGGAGCCCGAGAGGCCCGC
CAGCTCGGAGGATGGGGCggtggatggggtggtcgtggaggatggggcggccgtggaggatggggcggcc
gtggtggatggggtggccgtggaggatggggtggaggatggggcggtcgaggaggttggggaggtcgcgg
aggTGGTTGGTATGGCCGATAGAACGGCTGACGTCGCTGACCACGCCTCCGACCAATCCCACCACTTGCC
AAGTCAGAAGGATACCCTGTCACCAATGTCATGGCGTGAATAAA
>dme_circ_0003934_junction_seq
CTGTCACCAATGTCATGGCGTGAATAAAGGTGGTTGGACTCCTGGACGTCGCCGAT
>dme_circ_0003935
GCTTGCGACACACGTAGATAAGCATTGCAAAGGAGTAACTGCAGACATACCATTAAATCGTATTTGCATC
TGAAGCAACTAGACCTAGAGTATTGCGCATATATGTATGTATGTAATATTCACACTTGTTTCACATTAGG
>dme_circ_0003935_junction_seq
GTAATATTCACACTTGTTTCACATTAGGGCTTGCGACACACGTAGATAAGCATTGC
>dme_circ_0003936
GAACAACAAGCGAGGTGCGTACGCCCTGCTGGGCGCCACCCTATTCTCCATGGGATCCGTTCTGGCCTGG
GCGCTCATCAAGTCCGCCCTGCCACAGGACAATGCACTGCTGGCGACCCTGGCGGGCTTGGGAACTGGCG
CCGCCATCGTGAAG
>dme_circ_0003936_junction_seq
CTTGGGAACTGGCGCCGCCATCGTGAAGGAACAACAAGCGAGGTGCGTACGCCCTG
>dme_circ_0003937
GTCAAGAACGTAATCAACACCTTCACCAAGACCGCCGAGCATCCGCGTCTGCGTTACTTTGGCAACATAT
CGCTGGGTACGGATGTCAGCCTGCGTGAGCTAAGAGATCGATACCATGCCGTGCTCCTCACCTACGGAGC
GGACCAGGATCGGCAACTAGAGCTGGAGAACGAGCAGTTGGATAATGTGATATCAGCCCGGAAATTTGTG
GCCTGGTATAATGGATTGCCGGGTGCGGAAAACTTGGCTCCGGATCTGAGCGGTCGCGATGTCACGATTG
TGGG
>dme_circ_0003937_junction_seq
TGAGCGGTCGCGATGTCACGATTGTGGGGTCAAGAACGTAATCAACACCTTCACCA
>dme_circ_0003938
GGTGGAAAGGATGCATGCCAGGGAGATTCCGGTGGTCCGCTGGTCTACAACAACACACTCCTGGGCATTG
TGTCCTGGGGAACGGGCTGTGCCCGTGAGAAATATCCCGGAGTCTATTGCTCCGTACCGGATGTTCTCGA
TTGGCTG
>dme_circ_0003938_junction_seq
CTCCGTACCGGATGTTCTCGATTGGCTGGGTGGAAAGGATGCATGCCAGGGAGATT
>dme_circ_0003939
GAGGTCCTCCGGAATCTCCCTGGCAGGCATCCTTTCCGCCCTCCGATAGTCCAGCGCAGAGCATGCCCTC
GCTGATCGGTCGCCAGTAGTAGGCTTCCTGGCATTTTTCGGAATCGACAATTGGTACCTTAACCTGCTGC
AGTTGATCGGATGACAAGCCGTTCTCTTTG
>dme_circ_0003939_junction_seq
TTGATCGGATGACAAGCCGTTCTCTTTGGAGGTCCTCCGGAATCTCCCTGGCAGGC
>dme_circ_0003940

CTGCGCAGGCGCAGTTCATCGAGTAGCTCCTACGCCCAAACCTGCGGTGGATGCATCCTGGACGCGGTGA
CCATAGCAACGGCAGCACATTGTGTCTACAACCGCGAGGCGGAGAATTTCCTGGTGGTGGCTGGCGACGA
TAGCCGTGGGGGTATGAATGGCGTAGTGGTGCGAG
>dme_circ_0003940_junction_seq
GGGGGTATGAATGGCGTAGTGGTGCGAGCTGCGCAGGCGCAGTTCATCGAGTAGCT
>dme_circ_0003941
TTTGATCAACGAAGATACTGTTGTCACGGCGGCCCACTGTCTGGTGGGAAGGAAGGTCTCCAAGGTGTTC
GTGCGTCTCGGTTCCACGCTCTACAATGAGGGTGGAATTGTGGTTGCTGTCCGGGAGTTGGCATACAATG
AGGACTATAACTCGAAGACCATGGAGTACGACGTGGGCATACTGAAGCTGGACGAGAAGGTAAAGGAAAC
CGAAAATATTCGATATATCGAACTGGCCACGGAAACGCCGCCCACTGGCACCACCGCTGTGGTCACCGGC
TGGGGTTCCAAGTGCTACTTCTGGTGCATGACACTGCCCAAGACGCTCCAGGAGGTCTATGTCAACATTG
TGGACTGGAAGACGTGCGCTTCGGATGAG
>dme_circ_0003941_junction_seq
GGACTGGAAGACGTGCGCTTCGGATGAGTTTGATCAACGAAGATACTGTTGTCACG
>dme_circ_0003942
ATTAGTGGTTCTTTTAGTTTGCCTGGCTGTGGGTTCCGCCTGTGCTGGCACAGTCGGGGTCTCCAATGGT
GATCCCTTCGAGCGCGAAGGTCGCATTGTCGGCGGCGAGGATACAACAATCGGAGCGCATCCCTACCAGG
TTTCCCTGCAGACCAAGAGTGGCTCCCACTTCTGTGGTGG
>dme_circ_0003942_junction_seq
CCAAGAGTGGCTCCCACTTCTGTGGTGGATTAGTGGTTCTTTTAGTTTGCCTGGCT
>dme_circ_0003943
GGCTTTTGACACATCCAACCTACTGGGTACCAGCTTAGATGCTGTTAGCAGTGCTCACCACCCAAGAGCG
GAGGACAGCAACATCGGCATAGACACCGGGGTAGTTGGAGTAAGCGCATCCGTATCCCCAGGAGACAACA
CCGACGAGGACTCCGCCGGAGACCAGTGGGCCACCGGAGTCACCCTGGCAGGCATCCTTGCCGCTGGCAG
CAGCGCAGATCATGGTGTTGCGGATCTGGCTACCGTATCCGTAGGTGGAGGAAGCACACTGGCTCTGGCT
AACGATGTTCACGTTCACGTACTGCAGCTGGGAGGGGATGGAGCTGGATCCGGACGACTGGGTACCCCAA
CCGGAAACGGCGGCAGAGGCTCCGTTAGCTGGGTTGTAA
>dme_circ_0003943_junction_seq
GGCAGAGGCTCCGTTAGCTGGGTTGTAAGGCTTTTGACACATCCAACCTACTGGGT
>dme_circ_0003944
GATGCCTGCCAGGGTGACTCCGGTGGCCCACTGGTCTCCGGCGGAGTCCTCGTCGGTGTTGTCTCCTGGG
GATACGGATGCGCTTACTCCAACTACCCCGGTGTCTATGCCGATGTTGCTGCCCTCCGCTCCTGGGTTAT
TAGCAATGCCTAAGAAGTATGCGAAATAAATTTCAGTTTGGATATTACAGCATACAAACGATTAAAATGT
TGAAATTCGCGGTACTTCTTTCCGTTTTGGCCTGCGCCTTGGCTGGCACCATTCCCGATGGCCTGCTGCC
CCAGTTGGATGGTCGTATCGTCGGTGGTTATGAGACCAGCATCGACGCCCATCCCTACCAGGTGTCCTTG
CAGCGCTATGGATCACACTTCTGCGGTGGATCCATCTACTCCCACGACATTGTGATCACCGCCGCTCACT
GCCTGCAGTCGATTGAGGCCAAGGACCTGAAGATTCGTGTGGGCAGCACCTACTGGCGCTCCGGCGGCAG
TGTCCACTCCGTTCGCTCCTTCCGCAACCACGAGGGCTACAACTCCCGCACCATGGTCAACGACATTGCT
ATTATTCGCATCGAGTCCGATCTGAGCTTCCGCTCCAGCATTCGCGAGATCCGTATTGCCGACTCCAACC
CTCGTGAGGGCGCCACCGCCGTGGTTTCCGGCTGGGGCACCACCGAGTCCGGTGGCAGCACCATTCCCGA
TCATCTGCTGGCCGTTGATCTGGAGATCATCGATGTGTCGCGTTGCCGCTCGGATGAGTTCGGATACGGA
AAGAAGATCAAGGACACCATGCTCTGCGCCTACGCCCCACACAAGGATGCCTGCCAGGGTGACTCCGGTG
GCCCACTTGTCTCCGGAGACCGCCTTGTCGGTGTTGTGTCCTGGGGCTATGGATGCGGTGATGTGAGGTA
TCCCGGTGTCTACGCCGACGTTGCCCACTTCCACGAGTGGATCGAGAGGACCGCCGAGGAGGTGTAAAGC

CAGACAAACACTTGAAATAAATCGCAATTGAATCAGCAAGCCAGCACTTGTTAGCAACGCCCATCATGTT
GAAGATCGTGATCCTCTTGTCCGCCGTGGTCTGCGCCCTGGGAGGCACCGTCCCTGAGGGACTCCTGCCT
CAGTTGGATGGCCGCATTGTCGGCGGCTCTGCTACCACCATCAGCAGCTTCCCCTGGCAGATCTCCCTGC
AGCGCAGTGGCAGCCACTCCTGCGGTGGATCCATCTACTCTGCCAACATCATTGTGACCGCCGCTCACTG
TCTGCAGTCCGTGTCCGCTTCAGTCCTGCAGGTCCGTGCTGGATCCACCTACTGGAGCTCTGGTGGCGTC
GTCGCCAAGGTTTCCTCTTTCAAGAACCACGAGGGATACAACGCTAACACCATGGTCAACGACATCGCTG
TCATCCGTCTGAGCTCTTCCCTGAGCTTCAGCTCAAGCATCAAGGCTATTAGCCTGGCCACTTACAACCC
AGCTAACGGAGCCTCTGCCGCCGTTTCCGGTTGGGGTACCCAGTCGTCCGGATCCAGCTCCATCCCCTCC
CAGCTGCAGTACGTGAACGTGAACATCGTTAGCCAGAGCCAGTGTGCTTCCTCCACCTACGGATACGGTA
GCCAGATCCGCAACACCATGATCTGCGCTGCTGCCAGCGGCAAGGATGCCTGCCAGGGTGACTCCGGTGG
CCCACTGGTCTCCGGCGGAGTCCTCGTCGGTGTTGTCTCCTGGGGATACGGATGCGCTTACTCCAACTAC
CCCGGTGTCTATGCCGATGTTGCTGTCCTCCGCTCTTGGGTGGTGAGCACTGCTAACAGCATCTAAGCTG
GTACCCAGTAGGTTGGATGTG
>dme_circ_0003944_junction_seq
TAAGCTGGTACCCAGTAGGTTGGATGTGGATGCCTGCCAGGGTGACTCCGGTGGCC
>dme_circ_0003945
GCTTAGATGCTGTTAGCAGTGCTCACCACCCAAGAGCGGAGGACAGCAACATCGGCATAGACACCGGGGT
AGTTGGAGTAAGCGCATCCGTATCCCCAGGAGACAACACCGACGAGGACTCCGCCGGAGACCAGTGGGCC
ACCGGAGTCACCCTGGCAGGCATCCTTGCCGCTGGCAGCAGCGCAGATCATGGTGTTGCGGATCTGGCTA
CCGTATCCGTAGGTGGAGGAAGCACACTGGCTCTGGCTAACGATGTTCACGTTCACGTACTGCAGCTGGG
AGGGGATGGAGCTGGATCCGGACGACTGGGTACCCCAACCGGAAACGGCGGCAGAGGCTCCGTTAGCTGG
GTTGTAAGTGGCCAGGCTAATAGCCTTGATGCTTGAGCTGAAGCTCAGGGAAGAGCTCAGACGGATGACA
GCGAT
>dme_circ_0003945_junction_seq
GGGAAGAGCTCAGACGGATGACAGCGATGCTTAGATGCTGTTAGCAGTGCTCACCA
>dme_circ_0003946
GGAGACAACACCGACGAGGACTCCGCCGGAGACCAGTGGGCCACCGGAGTCACCCTGGCAGGCATCCTTG
CCGCTGGCAGCAGCGCAGATCATGGTGTTGCGGATCTGGCTACCGTATCCGTAGGTGGAGGAAGCACACT
GGCTCTGGCTAACGATGTTCACGTTCACGTACTGCAGCTGGGAGGGGATGGAGCTGGATCCGGACGACTG
GGTACCCCAACCGGAAACGGCGGCAGAGGCTCCGTTAGCTGGGTTGTAAGTGGCCAGGCTAATAGCCTTG
ATGCTTGAGCTGAAGCTCAGGGAAGAGCTCAGACGGATGACAGCGATGTCGTTGACCATGGTGTTAGCGT
TGTATCCCTCGTGGTTCTTGAAAGAGGAAACCTTGGCGACGACGCCACCAGAGCTCCAGTAGGTGGATCC
AGCACGGACCTGCAGGACTGAAGCGGACACGGACTGCAGACAGTGAGCGGCGGTCACAATGATGTTGGCA
GAGTAGATGGATCCACCGCAGGAGTGGCTGCCACTGCGCTGCAGGGAGATCTGCCAGGGGAAGCTGCTGA
TGGTGGTAGCAGAGCCGCCGACAATGCGGCCATCCAACTGAGGCAGGAGTCCCTCAGGGACGGTGCCTCC
CAGGGCGCAGACCACGGCGGACAAGAGGATCACGATCTTCAACATGATGGGCGTTGCTAACAA
>dme_circ_0003946_junction_seq
TCTTCAACATGATGGGCGTTGCTAACAAGGAGACAACACCGACGAGGACTCCGCCG
>dme_circ_0003947
GCTGGGAGGGGATGGAGCTGGATCCGGACGACTGGGTACCCCAACCGGAAACGGCGGCAGAGGCTCCGTT
AGCTGGGTTGTAAGTGGCCAGGCTAATAGCCTTGATGCTTGAGCTGAAGCTCAGGGAAGAGCTCAGACGG
ATGACAGCGATGTC
>dme_circ_0003947_junction_seq
AAGAGCTCAGACGGATGACAGCGATGTCGCTGGGAGGGGATGGAGCTGGATCCGGA

>dme_circ_0003948
GCTGGGTTGTAAGTGGCCAGGCTAATAGCCTTGATGCTTGAGCTGAAGCTCAGGGAAGAGCTCAGACGGA
TGACAGCGATGTCGTTGACCATGGTGTTAGCGTTGTATCCCTCGTGGTTCTTGAAAGAGGAAACCTTGGC
GACGACGCCACCAGAGCTCCAGTAG
>dme_circ_0003948_junction_seq
GGCGACGACGCCACCAGAGCTCCAGTAGGCTGGGTTGTAAGTGGCCAGGCTAATAG
>dme_circ_0003949
GGAAACCTTGGCGACGACGCCACCAGAGCTCCAGTAGGTGGATCCAGCACGGACCTGCAGGACTGAAGCG
GACACGGACTGCAGACAGTGAGCGGCGGTCACAATGATGTTGGCAGAGTAGATGGATCCACCGCAGGAGT
GGCTGCCACTGCGCTGCAGGGAGATCTGCCAGGGGAAGCTGCTGATGGTGGTAGCAGAGCCGCCGACAAT
GCGGCCATCCAACTGAGGCAGGAGTCCCTCAGGGACGGTGCCTCCCAGGGCGCAGACCACGGCGGACAAG
AGGATCACGATCTTCAACATGATGGGC
>dme_circ_0003949_junction_seq
GAGGATCACGATCTTCAACATGATGGGCGGAAACCTTGGCGACGACGCCACCAGAG
>dme_circ_0003950
ATCTCCCTGCAGCGCAGTGGCAGCCACTCCTGCGGTGGATCCATCTACTCTGCCAACATCATTGTGACCG
CCGCTCACTGTCTGCAGTCCGTGTCCGCTTCAGTCCTGCAGGTCCGTGCTGGATCCACCTACTGGAGCTC
TGGTGGCGTCGTCGCCAAGG
>dme_circ_0003950_junction_seq
TGGAGCTCTGGTGGCGTCGTCGCCAAGGATCTCCCTGCAGCGCAGTGGCAGCCACT
>dme_circ_0003951
CACTTGTTAGCAACGCCCATCATGTTGAAGATCGTGATCCTCTTGTCCGCCGTGGTCTGCGCCCTGGGAG
GCACCGTCCCTGAGGGACTCCTGCCTCAGTTGGATGGCCGCATTGTCGGCGGCTCTGCTACCACCATCAG
CAGCTTCCCCTGGCAGATCTCCCTGCAGCGCAGTGGCAGCCACTCCTGCGGTGGATCCATCTACTCTGCC
AACATCATTGTGACCGCCGCTCACTGTCTGCAGTCCGTGTCCGCTTCAGTCCTGCAGGTCCGTGCTGGAT
CCACCTACTGGAGCTCTGGTGGCG
>dme_circ_0003951_junction_seq
GGATCCACCTACTGGAGCTCTGGTGGCGCACTTGTTAGCAACGCCCATCATGTTGA
>dme_circ_0003952
GTTCCTGATCCTGTTGTCGGCTGTAGCCTGCGCCCTGGGAGGCACCATCCCCGAGGGTCTCCTGCCCCAG
TTGGATGGCCGCATTGTCGGCGGTACTGCTACCACCATCAGCAGCTTCCCCTGGCAGATCTCCCTGCAGC
GCAGTGGCAGCCACTCCTGCGGTGGATCCATCTACTCCGCCAGGGTGATCGTGACCGCCGCTCACTGCCT
GCAGTCC
>dme_circ_0003952_junction_seq
CGTGACCGCCGCTCACTGCCTGCAGTCCGTTCCTGATCCTGTTGTCGGCTGTAGCC
>dme_circ_0003953
GGTGATAGCCAGCGCTAACAACGGAGGAGTTGTGCTGCCCACGAGCAACAACAATAATAACAACAATGGC
AGTGGAGCTGGCGGAAATGGAGGACCTTTGAACAGCGGTGTCAATGCCACAGGAGCTGGCGGAGGAGGAT
CTACAGGCGCGGGTGTTGGGGGAGCTG
>dme_circ_0003953_junction_seq
TCTACAGGCGCGGGTGTTGGGGGAGCTGGGTGATAGCCAGCGCTAACAACGGAGGA
>dme_circ_0003954
ACTCCCAACGCATGTTACGCACAATAGTTGGTCCTAAGAACTAGGGGATTTTGAACATGAACAAAAATGC
CGGCGATGGCAGCGATGGCAAAAACTCAAACAAAAACTCGAATGCGGGAGGGGGTGGTGGAGCCGGGGGG

CCACACGACCCCACCGGCCTTTTAGATGCAGCTTCCCTGTTCGGCTTACTGGGGACGTGATCCAACTGGA
GCGGCGGCTGCAGCGGCTTCCAATCCGCTCTTCAACTCGCAGTTCAATGCCGCCGCTGCTGCCGGATTGG
GTTTATTGCCACAAGCTGGAGGCGCCTCTGCCAATGACCGTTATTCCATGGCAGCAGCAGCGGCGGCTGC
GGCGGGAGCCCATCACCACCAGAACACGATGGCAGTGGCCGCTTCCCAGGCCGCCAGTTTGGCCGGTTTG
CATCCAGCAAGGTGAGTAAAATGTCGGCACATTCCGTATTTTAGTAGCACTCCTCTAGAAGACATTTGTG
ACGCCACGAAATTAGCAACTGCTGTGGTGCGGTGTGTTAAGGCGACTTCGTCCCATGAAGAACCGTAAAA
AATCTACGCGAGTTCGAATCCCGCTTGGTGAAAGAAATTTTTATCTGCAAAGTAATTTCCCTAAATTGTG
CGGAGTATAACAGAAAATGTCTTGTCTATCGCGCAAGAACGTTTGAAGAAAATACTCCTAGAGCATTTAG
TAACTTGCTATTCGCTTTACACTGGCCACTACGCTTCACCCAGAGCAGGGCAGTGAAAAATGGTCAAGCA
CAAATATTTTAGTGTTCTTTTGTATGTTATATCTTGTTCTTTAAAATAGTATTTCCTTCGCTCTTATGCG
CCGTAAATTAACTAAAGTTAAAGTATTTATTTGACTCAACTAAGCATTTGCATCCATTTTTGTGAAATCA
CTGTACTAACTCGCTGCTAATCGATTTACAGGGCTGGTGGTCAATGGCCCAGTTAGCTGCCCAGGATTAC
TTCAGTCGCCTGCAAGCCTCGGGTCTTTCCCCCTTTCCACATCCCGATCTGGCGGCTGCCTTTGGACCAG
CTGGGATGGGAATGGGCGGCGGTGCTGGTGGAGCGGGTGCTGGAGGTGGTGGAGCAGGTGTACTCGGCCA
GGGCGGCGGTGGAAATGGCGGAAGTGGCAACGGTGGTGGTGGATCCTCATCGGGTTCTTCCGGCAGCAAG
TCAAACAAGTCGCGCAAGGAGAAGCGAGCCGCCCagcagcaacaacagcaacagcaaaatctggccaaca
gtctaaatgcggcagctgcggcggcagcagcagcagcagccaataatccagcagccgcGCTGGCCAGCAT
GCATGGTTTTGGTGTAGGAGTTCCGGGCACGAGCATTCCGTCGGTGAGCACAGGTAGCGGATCAATATCC
ACAAATAGTGGAGGTCATGGAAGTTACAAGGAGCACGGCCAGCGCTTATGGTAAGCCCTCGACTATGACG
AGCAGCAGCATGGCTAATAATCCGGGCTCTGCCTACGATCCGGTGACCCTGCACAAGGAGCTGCTGGCCA
TGCAGGCCGTGGCAGCCGCTGCTTCCGGCTCAGGATCAAGCGGTTCCTCAGGCAAGAAGTCCGGTGGGGG
TGGCTCCTCATCCTCCTCGATGCACAGCCTTGGAATGGGAATTGGTGGCAGCGGTGGCATGATGTCCAGT
GGCAAAGCTTCGACCAGTGTGAGCGCAAGCAATTCATCATTGGGAGGAATGCACCCCAGTTTGACTAGCA
GCAATCCGCTGATGTCGCCCCATGCGGGCATGGGCTCCTCGTCGTCATCATCGAGCAAGGATCGTGACAA
AAACAATCCCTCGCTCAACGCTCTTAACTCGCTCTCACAGTTTGGAGCCCTGGGAATGACGCCACAGCAG
AGCATGCAGGCGGCAATGAATGCTTTTGCAGCCAGCACAGGAGTTTCGCCATCCGCCACGGTAACGTCCT
CGCCGCACCACTCTTCCCAGCAGCAACAGCAAATGGGAGGGAACAGTTCCACGTCGGGATCGGGCAAGTC
CAGTTCCAAGGATTACATGATGGGGTACTGGAAGTGAGCACCCATCTCTGCTTGGAGTTCGTTTACCACC
GGACACGGAGATAATCAAGTACACGTCCTCGATTGTGGGACCCAAGATTCCTGGTACTACCTCGCGCGGT
CGCAAAAAGACTATTTCCGAAACAGaacagcaaacaacacaacaacaacagaaacaacaacaccaagcag
aacaacacCTACTCCAACAACAGCAACAAGCACAAAAAGAGCTCGACAGCACCAAAAATGCCATTTCCTC
TCTGCTTGCATTTCCAGGTCTCAGTCCCGCGAAGCGGGCTAGACTCGAAATGGAGTACGCGGCGATGGCT
GCGGCCGCTCAAcagcagcatcagcaggcacagcagcaacaccaggcgcagcagcaactccaCGGGATGC
TCGGAGCAGCCGGTATCCCGGGAATGGCTGGACTGGTCGGTCTGCCTGGCATGAGTGGCAATCCCCTCGA
TCAGTTGAGTGTAAGCAAGGCCAGTTCTTCAACGGCGCCCACAACCAGTACCAGTTCCTCCTCAGCAGGG
AGCAACCTGCTCAACCAGAGCAATAGTGATCGCGTGGAGGTAATCAAACTGCCACCAACAATTACTTCAA
ACGGAGCCTATAATCTCTCAAGTAAGGGAAAAGAGGTGCACGACCTCACCACCGACATGGCCACCAACTC
TGGCGGTGTGAATCTCAGCCTAAAGTCCAATGCTGGCTCCAGCGCCTTGACGCCCAGCGGTGCCGTCGGA
TCGGCCAGCAATCCCATTACCATCGATGACTTTGACGCACCACTTAATCTTTCCATGAAGCCCTCGGATA
AGAGCAATAGTAGCTCGTCAAATGCAGCAGCAGGGGGCTCCTCCTCTTCCAGCGCAGCGTTGGCTAACTT
GGCGAGCGATTATCAGGCAGCGTCCAGTGGGCAAAGCGGTAATAGTCTGCAGAGTTTGAGCTCTATTACA
GCTGCTTTGGGTGGAACAGGAGGTATGCCTGGTGGTTCCATTTCCGGCAGCGGAGGAACCTCTCCAGCTC
CAGCTGGTGCCGGATCAGGAGCCACGGGTGGTGGTTCGGGATCTGGTGGATCCGGCGGTGGATCGTCCTC
TTACAAAGAGGGAAGACCTCGTAACCTGGGACGCGGCGTATCCAAGCCCAAGAAGAACACGGTAGCTTCG

CTGTTGGCTCAATCCCGAGCTGTGGGCCTTAAACCTATGCTGGCCACGCAACAACTTCTTCAACAGGGTG
CTGATATTGGAGAAAATCCGCCTGGCACTGAGCGAAGCGAATGCCCATATGGAAACTTCTACAGATTCCG
AAAGCGTGGCAGCCGAAAGTGGTCTTTCGGAGTCTGAGAGTGAGGATGCCAACATTCTGAATGTAGCGGA
GCTAAGGGTGCCACTTGAATTGGGCTGGAAACGGGAGACGGTGATTCGTGGACTGACTAAGCAAGGGCAG
ATCCGTGGAGAGGTCACATATTATGCACCAGGAAGCACGACGCCGCTAAAGAGCAACGGACAGGTTTTTG
CTGGTGAGTTTGTAGGGCGACTTTTTTGAATATATTAAAGCAAATTCTATAACACACAGTAATAACTCTA
AACCACTAAAAATGTATAATTGGAGTTTTGATATGTAATCAGAATAGCTTTAATTTATGGTTTTCTTAAT
TTTAACTTTTCACAAGCATAAAATTATCAAATTGGCTACAATGCACACAAGGATATTCACTTTCTAAGAA
CTTAGCTAATTTATTGAACTCTTCACAGAATCCTGGAGCAGCAGCCATCGAATCTAAGTCGTGAAAACTT
TAGTTTTTCCGCGCGAGCCATTGTTGGTTCATTCCTGCAGCCTGCACCACCGCCGTACGCCAATGATGGC
GAGTACATACGGATGACGGACGAGGACGTGGCCAAGCGGTTAGAGGATCTGAAGGTCTTCACCCGCCAGA
CTCTTAACGTGGAACAGCGAATTGAGATCGCCAAGCAACAGCAGGCGATGCGTGATGCTAAGAAGCTGCA
AAAGGAAGAATTGGCCAGAAATAAGGAGAAGGCGCGACAGGAGAAGAACTCCAAGTTGGAGCAGCAGCGC
AAGGACAAGGAGCTCAAGAATCAGCAGGCTGTTGAGGGTAATTTGAATTTGTAAGACTTGCGAAGATTAT
CTGCTCATGCGAACTCGTATTTTTAGGAGCGCAAAAAGCGTCAGGAGGAGCTAGATCGCCTTAAGCAAGA
GGAATTGCTTAAGAAGCAACAGGTTAGTCTAGATTAATTAAGCTTTGCTGAAATCAGTGCATAATTCTGT
TTTGTTTTGCAATAGGAGAAAGAAAAGCGTCGTCAGGAGGCCATTTTAGCTAAGGAACAGGTACTTAATG
CCTCACCTTATGTCTTGTGCTTGATACCAATCCGACTACTTTCCGTTTAATATTTGGGAGAAAAGAAACT
AGTTCTAGCTTTTCAAAGAGAGATTTATTTATTTTATTTCAAGACTTGTTTGCTTTGCCTTTAATTCCTA
TTTCTTTCTGTTGCCTATACTTTAAACTGTATTTACTGTTGCCAGGCTGGTGATTTGGGCTATTGAATGA
GATATATTCCAAATTGGTTGTTCAACTTTTAATCGTGTTTCGGCGTTATTATTTGAAGTGTCCGATCTAT
CCGCTTGTTGGTTTTTTGTTTAGTAGGGGTCGTAAAAGACCAACTTTGCCATTTCGGTATCAGTTACTAC
TCTCATGTTATTAACCAAAACCAATCTTATTTTATCTAGGGAACGAGAACGTCGCCGCCAACACATGAGC
CTCATTCGAATGTTGGAGCTTCGTCGGAAATTCGAGGATCGTGAAAAGAAGAAACACCAGCTGGTTCTAG
ACCGTTTGCTTCTGCGTGAGCGTCGTATGGCGGAGCGAAAACGAGATGCGGAGATTCTGCAGTTGATAAG
GCGTCCCAATGAGGACTCCGAAATGCCACAAGAATTGGTAATCCCTGAACTGGATAGGATTGCCGGCAAC
CGACTTCCCGGCCAGGCAATGGCCGATTTGCTCATGGTCTTTGAATTCCTACATAACTTTGGGGAGACTC
TAGGCTTTGACATGGAATCACTGCCGTCGCTTCAGAACCTGCACGATGCTTTAATGAGCGATAGCAACGC
GGATGCGGAAGAGGAGTTGTTGTCGGTGATGACGCATCTATTGGTTTGTGCCATTGAGGATCCAGGTGTT
CCCAATCCCGGCAGACACACCACTTTACTGGGACAATCGCTACGCAATGCGGACATCACCAACTCGAATG
TATCCGAAATCTTGAGGATTTATCTGTATGCTACGGCCACAGGTGAAGTGCGTCAAATGCACGGAATCAC
TGTGGATCGTGAGCGAGAGAGAAGGGTGCCGGATCATCATCAACTAGATAGCGATACCACAACCCACTCA
CATTCGGTAAAGAACCAGGAGTACTATAAGCTTCTCCATGAAAACGACACCTGGAAGTTGTCGCAATCGC
TGAAAGATCGCCCCTTCGTTGCGTTAAATCCCACCCGTAAAGCCCAAATGTTGGCCCATCTATGCAACGA
CCTGCTCATGAACAAGGCCGTGCTCCGCCAGATTGATGGAAGTCTAGAAACGTGCGCCCAGATGCGAAAG
GAGAAATACATGACGGACATGAAGGTGCGCAAGTACAAGGCTCTCCACATGCGCAAGGCTCGAATTGAGG
CCTATGAACGAGCACAGGCGGAGCGTGAGGCGGCTATGCAGGCGTTGATGGCACAGCAGAAGCTGGATGC
AGAACGTCTTAAGGCGGAGGAGGAAGCCAAGGCCGCGGCGGCAGAGGAAGCAGCAGCTGCAGCGGGCACA
GATGGAGAGGCAACCAAAGGTGGCTCACCCAATGGCGAGAAGCCGGAAGACGGCGATCAGAATGAAGAGG
GAGCCGCCAAGGAACCCCAGCAGCAGCAACAGCAGCCAATGGAAGTGGATGGCGTGGTCGATGAGGCATC
TCTTGTAAGTCCGGCCAAAACCATCATTCAAACGGACAATAGTCTGACGCCCAGTAAACAGGACATGCCC
ACACCCACCTACCAAATTAATGGATCCAGCACACCTACCACAAGTGGTGTCACTGGAGGCGACATGAATG
TTCTGCTGCAGGCCAAGAAAAGCGGGGCCCGAAACTCCATCAACGATGAACACCACCATGATGTTAGTAT
TATCGACGATGATCTTTCCGACTTGGATTCGGAGATCACGAATGTCGAGGAGGACGAGGACAATCGCTTG

AGTGCCGATGAGTTGCAAAAGAAGCTCGACAAGATTGTCAGAGCTTCCCTAAACTGCAAGGAGGCACTGG
AGAAGAGCACAAATCAGTTGAGAGCTGCGTGCTTTGGGCAGGATCGCTTCTGGCGTCGCTACTGGAAGTT
GCCCAAGGCTGGAGGTATTTTCATTGAAGCACTTGAGTCGGCCCAAAATGATATTTGCGATTATCATGAG
GCATTGGAGGCCATGGATGACAAAAAGGATGCAAACGACGAGAAAGAGAACTCCGAAAATGAGAAGGATG
TTGCAGCGGAGTCCAGTGAGCAGCCAATGGAAGTGGATGAGTCCATTACAAAACTGGAAGATGGTGTACC
AGCTTCGGACGTCGGGATGCCTGAAAGTAATCAACAGAACGCACATCAAGATGAGGAAGATGACGATGAT
GATGTGACAGAAATCAACAAGGTGGAACCGGAAATTGTTGATTTGGGTGATGATGACGATGATGCAGCTC
CCCCGCTGCCCAAGATAGAACCTCCCAGGCCAGAGATTAAAGTTAAATCAGAGATGGAGCTGATGGGTCC
ACCACCCACGACTATGATATCCACTAAGACAGACTTTGAAGCTGAAATTAAAATACCCTCTATGCCTGGC
ATCTTGATGCCGCCTACCCTcaacaacaacaacaccaataataacaataacaacaatgGAAGCGACAACT
GTGATAAGTTGGAAACTGGTTTGGGACTAGGACAGCAGCAGCAGAACTTTAGCCAGTCTGTGATCAAGAC
GGAGGATGTGAAAAAAGAGGATGATTGCATAATAGTTTCTACATCCAGTGTTGACGATACACCAAAGTGG
TTCTCCATTGTCCGGCGGGAGGTTCCATTGATCAGCGAACTGCCAGCGGAGGAGGGAGGCGTGGTGGGCC
AGGAACTTCAGATCAGCTATGCAAATCAGAACTGCTCCGCTCAGCTGCAGCTGCAAGGTCATCCGTGGGA
TCTGATCAACAACATGCAGTACTACTCGATTCCGATGGACGAATGCAAGGTGGACACCAGCAAGCTGGGC
AACGAGTGCATCTTCTCGCTGAGTGGCCTCGATGAAAAGCAAATGCTAGCCAAGGTGGAGGAGTACAAGG
CCCACAAGGTGGAGTCAAAAAACGGTCTGGGCTCTCCTCATCGCCACCACGAGACTAAAGATGATGAGGA
GCAGGCCAAGCTGAAGTTGGACAAGGAGATTGACACCGAAATGGAAACAGATGCAGATGACCTAGCTGGC
AAGGAAAAGTTTTTTCGCTTGCGCTCTGATGTACCCCCGGATACGGGAGGAGGGGTCAGCGAGGGAACCG
ATGTAAAGCCCAAGATTGAGCTTCGTTTGGATGAGGCGTTGTCACAGGCATATTACCACAACATAGCCAA
CATGTCATTGAGCAGTGTTCAAACGTATATACCCATCGATATCCCTCTGCCGCTTTCCATGACCCCCGAT
GAGCATCGTCTGCTGGAGCAGGTCAAGCTGGCAGGTTTTCCGGAACGAGTTCACGGAGTTTACGTGCCCC
GCAGACAGCGCTACGGCTGGTGGCAGTTGGACGATGAACAGAAGCTGCGCCAGCTGCTCAAAACTCTCAA
TCCGTCGGGATTGAGGGAGCGTGAACTGCAGGAGAATCTTCAACGATTCCTCGGACTGGAACAGCCATTG
GGTGTGAATTATAAGCTGAAATCGGACATCGATTTTCCAGAGGAATTCCTAATGCCTGACAAGAAGGGCG
ATTGGAATCCCAAGGTGGCCAAACGTGTGGAGCTTGCCCTTATCGAACAACTAGAATCACTGGAGGACAA
GGTGGCCAGCGCTTCTATGCAATTAAAGAACTGGCAATTGCCCAACCGCGTGGAGAGTGAACTCACCTTG
GACTCGCAGGAGGATGTCACCGAAGAGGACTTCGTCAGCATCATACCCATGATCCGAGAGAGAATCATCG
ACTTGGAGGCAAATATCGAAAGACGTTACTTGAAGCCGCCTTTGGGCTCGCAGACAGGCGATGCCCATTT
GGCGGTTATTGCTCAGAACCAGCATACCACCACCCAGACGCAGAACTCTgcatcggcagcagcatatctc
ctgcagatgcaacagcagcagcagcaacagcagttggcccagcagcagcagcaacagcaacagGGTTCGG
GTGCGGGAAATAGCCTGAATCCCTCATCCTTCAACGAGCGTACTATGGCATTGGCGGCGGCAGCAGCTGC
TTCGGGGCCAGGAAACGCAACCGGAGTAGCCAACTCGGCAGTCGTGGCAGGAGCCACGCCCTGCGAATCG
GGCAGCGGAGAACCGAATTCCGGCAACGCGTCACCGGCCAGCAACTGTGACAGCGATCGGGACGAGAAGG
TGGAGCAGATACCCAAGGGATTGGTGCAGTGGCGGGACGCAGTATCCCGATCGCACACCACCGCCCAGCT
GGCGATGGCCCTGTACGTTCTGGAATCCTGCGTGGCTTGGGACAAGAGCATTATGAAAGCGG
>dme_circ_0003954_junction_seq
GCTTGGGACAAGAGCATTATGAAAGCGGACTCCCAACGCATGTTACGCACAATAGT
>dme_circ_0003955
TATTATCGACGATGATCTTTCCGACTTGGATTCGGAGATCACGAATGTCGAGGAGGACGAGGACAATCGC
TTGAGTGCCGATGAGTTGCAAAAGAAGCTCGACAAGATTGTCAGAGCTTCCCTAAACTGCAAGGAGGCAC
TGGAGAAGAGCACAAATCAGTTGAGAGCTGCGTGCTTTGGGCAGGATCGCTTCTGGCGTCGCTACTGGAA
GTTGCCCAAGGCTGGAGGTATTTTCATTGAAGCACTTGAG
>dme_circ_0003955_junction_seq

TGGAGGTATTTTCATTGAAGCACTTGAGTATTATCGACGATGATCTTTCCGACTTG
>dme_circ_0003956
GCGAAGCTACCGTGTTCTTCTTGGGCTTGGATACGCCGCGTCCCAGGTTACGAGGTCTTCCCTCTTTGTA
AGAGGACGATCCACCGCCGGATCCACCAGATCCCGAACCACCACCCGTGGCTCCTGATCCGGCACCAGCT
GGAGCTGGAGAGGTTCCTCCGCTGCCGGAAATGGAACCACCAGGCATACCTCCTGTTCCACCCAAAGCAG
CTGTAATAGAGCTCAAACTCTGCAGACTATTACCGCTTTGCCCACTGGACGCTGCCTGATAATCGCTCGC
CAA
>dme_circ_0003956_junction_seq
TGGACGCTGCCTGATAATCGCTCGCCAAGCGAAGCTACCGTGTTCTTCTTGGGCTT
>dme_circ_0003957
GTAGTTCTTCAGCGGCAGCAGGGAATTCTCCAGCTGGGCCATCTCATCCCGTGGAATCACCTCCGGATAG
GCCAGCAGCTCCTTGTCCACCACTCCGATGAAGAAGTTTTTGGCCAGCGGCTCTCTGGTTGGTAGTTTTC
GCTGCTGCTCCGGGGACTCCTCTACGGATTCCGCTCTTCCACCCTCCGTGGTCGCCGCATCCTGGTGCTG
TGAGTCCAAGCTGCTGGACGTGGCCTTGGTGCTTCTTCCCCGA
>dme_circ_0003957_junction_seq
GGACGTGGCCTTGGTGCTTCTTCCCCGAGTAGTTCTTCAGCGGCAGCAGGGAATTC
>dme_circ_0003958
GAACAAAAAAAGGAAAACGACAATGTGTAGTTGTCAATTGTATTTAACTAAAAGCTATTAGAAACACCCCC
AACATGCAGCTCATATCGTATCACAGGCACGCTTAATAAACACTCTCAGAACGGAAATACGAAATTAAAA
TCGAAACTAGCGGGTTAATTCATTTCAAACTTGCACTCAACGAAATCGATG
>dme_circ_0003958_junction_seq
TTCAAACTTGCACTCAACGAAATCGATGGAACAAAAAAAGGAAAACGACAATGTGTA
>dme_circ_0003959
GGAGTTTCTTTCGCCACCTGCCCTCCGTATCATCCTGTTTCTCATCCTGCTTCCTCTCCTTCTCCTTCCC
ATTCTACAATTGGTATCTGGGCAACTGCCAGAGCTCCCTTGCCTCCCGCTGCTGCCACTTGTTCTGCAAC
TGCTTGCACAGGCGGCGCAGCAGCTCGAAGGTGCCGCCCAGGGCTTGCA
>dme_circ_0003959_junction_seq
AGCTCGAAGGTGCCGCCCAGGGCTTGCAGGAGTTTCTTTCGCCACCTGCCCTCCGT
>dme_circ_0003960
GTAGCTACTAAATCCTGCCGGCGCTCTCCTGTCGCGCACTGTAGCGCGGCTCGGCGGGCATCATTGTGTC
CGTGACCAGCTCATTGGGTGCCTCCAAAACGCCCTTCGCGTGTGCAGCCGCCGTATCGCAATCGGTGCGG
C
>dme_circ_0003960_junction_seq
GCAGCCGCCGTATCGCAATCGGTGCGGCGTAGCTACTAAATCCTGCCGGCGCTCTC
>dme_circ_0003961
TATTTGGCTTAAAATAAAATATTGATTGTGGGATTATCAAGATAATGATACCAGTGTATACTTCAAATTA
AACTTTTATTTCAAGGTTAAAAAGTAACAAATCGTCCAATTTAAAAAATCCCTCTAATGTAAAATATTGT
TATAGGATATATCCGCAGTCGGTGTCCTTTCAGTTTGCACTTTTATTGATTTGTTTTGGCCACTGCTTGG
TTATAG
>dme_circ_0003961_junction_seq
ATTTGTTTTGGCCACTGCTTGGTTATAGTATTTGGCTTAAAATAAAATATTGATTG
>dme_circ_0003962
GCAAAGACAAAAAAATCAAATCAACGCAGAGTAAAAGACATTGTTTATGTTGTCGATTGAAAGGCAAAAAC
AAAACAAGTGAAAATTTGTGGCAAAAACAAACTTCCCGAGGAAAACCTTCAAGTTCAAGTTCACCTAGCA

AGCTATTGAGCTGATAAGATGAGTGGACGCGACAACAGAGGAGCCGGCGGCGGAGGCGGCGGTCATCAAC
CGCTGAGCAACGCCATGGGCAAGCTCAAGGAAAAACTAACCCGAGTAGGTTGTAAGTATCTCATCGAAAA
CAATATTGGGGTAGTCGACCAGCCTGAAAAGCCCACATCCACTTCAATAGATACATAGATAGTTGGGCAT
TCTTAGATTTTTTTATACTTAATCTACAGATTGAGATTATTTACATCAGAAACAGTTAATCTTATTCTTT
AAGCAAACTTTGAAATCATTAAATATTTAATTACTCAGCAACAAATTGTAGCTAACAGTTCGTAAACTGa
aaattagcctattaaaaacttgaataaaaactatctataaaatttgtaaataaaatattatATGAGCCAC
TTAATTCGCTATGAGTATAGGGCAATTAAAGACACCAGAAGGATCCAAAAACAACATTTCAAAGAGCAGC
ATCAAGTACGCACATGTCCGATAATGCTGGACCTCTTCCAACCTTTGAGATTGGTGTTCAAGAACAAATA
CCGCGACTTAATCGCAAAGATTTCGGGCGGTTTGGAGCGGTTTTATTTTGATGTAGAGATGGGGAGGCTT
CTTAGTTATGCCATGCTTATGATTCGATTTAATTAATTCGCGACTGTCTTTCGATTCGAATCGAATCCAA
TCCACAGGGACGAGCTGGGCTATCATCGTGTGGAGAGCAATTTGTCCACATCGAACACAGCGACCAGTTT
GGACACAATTCTGCCAGAGGATCCGTTCCTATTCCCCCAAGTGTCACCCCAAAGGCATCCACAAAATACA
GTAAGGACACAGCGTTTGCTGGAGGACGAGCCACCGCTATCGTTTCGCCCGCTTTTGGAGGACGACGACA
TCAACGAGCCACCTACACAGCAGCAACAGAGGACTCCCCTGCGCGCCAGCGGTAGTTTGGAGTTGACTCC
CTTGCCACCGCCGCCCACATCATTGGAGATACGTGAGCATCGCGATCGCCAGCAGAGAGGTGCCCAGGGT
GACGAGCTGCAGCGCAGCAAGCAGAGCTTGAAAGGATCACGCGTGTCCTTCGAGCGACGGGATACCGGAA
ACAGCAATACCAACAGTAACAAAGCAGCGGAGAGCAGCGACGAGGACAGTTTCGAGGAGAAGCGAACTGG
TTTCCAGCAACAGAAGGCAACCAGCGTCGATCACAAGGGCATCTTGAAGGACCTGAAGCACATTTTGGCC
AACGACAATCGCCGGCAGTTTCAGGCCAAGAAGCACGTATCCCTGGACGTTAAGGGCACACGCTTCCTGC
AGGATCTACTGAAGGAATCGTCATCGGAAGAGGAGTTCCACAAGACACGGCGGGAGTTCCAGGGTCGCAA
GCACCAGAGTTTGGATCCACGTGTCACATTCAAGCTCGACAAAGTCCTCCAGGGATCGAGTACCGACAGT
GACGAGGAGGGCGAAGATGCCGAGCACAAACGCCTGATCCATCGACCCAAGGACATTACCAAGCCGGTGA
TCATCGATCTTAAGGATCTGGAAAGCGAGAGCGACGAGGACTTTCTCACATCCCGTCAGCACTTCCAACA
GCAGCGTTCCATCAGCACAGATTCCCGCAAGAG
>dme_circ_0003962_junction_seq
GTTCCATCAGCACAGATTCCCGCAAGAGGCAAAGACAAAAAATCAAATCAACGCAG
>dme_circ_0003963
GTTGCTATGGGCGCGCATGGAGCGTTCTGGCTATTCGGCGCCATCTGCTTCGTGGGTCTGTTCTTTGTGA
TAATCTACGTGCCCGAAACGCAGGGCAAGACGCTGGAGGACATCGAGCGGAAGATGATGGGCCGTGTGCG
CCGCATGTCCTCGGTGGCCAATATCAAGCCATTGTCCTTCAACATGTAAATGGCGTGTTGCCAAGGATCT
GAATATGCGATCAAACCAAATACTAGACTAGTGCCTTTAGTGCTTAAATTGTTATTATTATTTATAATCA
TTAAGTGCATAGTTTTATTATGTAAATTATGATTTCTAGCACTAATTTGTTGACATTTTGATGAATGTTG
CGTGAATTTATCGGTGTaaaaaaaaTGACATGAATCGTAGTTAGTTGTAGTGCAAAAGCAAGCGCAAAAA
ACCAAAATCTCCCTGGAAAAATACACCAATTGGCTATATATCGAATATATATTGTACTTTACCTACGCTG
TAAGTGTATCTATATGTAGACTAATTTAAAATACAAATCAAACCAAATCCGGGTTCCTGGGTTGGTGGCA
TCATGCCACTGGCTGCATTAGCAGGTGGCATAACAGGAGGACCCTTGATCGAATATCTGGGCAGGCGCAG
CACCATCCTGGCCACTGCGGTGCCCTTCATCGTATCATCACTGCTGATTGCGTGTGCTGTGAATGTGATC
ATGATTCTGTGCGGACGATTCCTAACTGGATTCTGCGTTGGCATTGCCTCTTTATCCCTGCCCGTCTACC
TGGGTGAGACTTTGCAGCCGGAAGTGCGGGGCACTTTGGGTTTGCTGCCCACCGCTCTTGGTAACATAGG
CATCCTGGTTTGCTATGTGGCGGGATCCTTTATGAATTGGTCGATGCTGGCCTTCTTGGGAGCTGCTCTG
CCCGTTCCCTTCCTGATTTTGATGATCATTATCCCCGAAACTCCGCGCTGGTTTGTGAACCGTGGTCAGG
AAGAGCGCGCCAGGAAGGCTTTGAAATGGCTGCGTGGCAAGGAGGCGGATGTGGAGCCCGAACTAAAGGA
GTTGATGCAATCTCAGGCCGATGCCGATCGTCAGGCCACTCAGAACACCTGTTTGGAGCTGTTTAAGCGA
AACAATCTGAAGCCACTTTCCATTTCCTTGGGTCTGATGTTCTTTCAACAGTTCAGCGGTATCAACGCGG

TTATTTTCTACACGGTCCAGATCTTCAAGGATGCGGGATCCACAATTGATAGCAATCTTTCCACGATCAT
CGTGGGTGTAGTTAACTTTTTTGCTACGTTTATGGGCATCATTCTGATTGATCGGTTGGGCAGAAAGGAT
TCTTCTGTATGTATCCGACATCGCCATGATCGTGACCCTGTCCATTCTGGGCGGCTTCTTCTACTGCAAG
GCGCATGGTCCGGATGTGTCCCATTTGGGTTGGTTGCCACTCACTTGCTTCGTTATCTACATTCTGGGAT
TCTCCCTGGGCTTCGGACCCATTCCCTGGCTGATGATGGGTGAGATTCTGCCAGCCAAGATTCGAGGACC
GGCTGCATCGGTGGTCACGGCTTTCAACTGGTTCTGCACGTTTGTGGTAACCAAGACCTTCCAGGACCTC
ACGG
>dme_circ_0003963_junction_seq
GTAACCAAGACCTTCCAGGACCTCACGGGTTGCTATGGGCGCGCATGGAGCGTTCT
>dme_circ_0003964
TTTCACCCGAAAGTTATATACCGCATCCAGTTGAAATGTCCGCCAAGACGTCGCCATCGTCCACTTCTCG
GGATGCCAACGTGGGATCCTCGAGTGCGATTCCCGTTCAGATCAAAAAAGAACTGCCCAGCGATGAGATA
AAGGGTGAGTGAATGTATGAATGAACACTTTGAGATACTTTCTTGCCGTCTAATTAATTGCCCCTGTCTG
CGCATTATTTCGCCGCGTATTCAATAACAAGGATGCTGACAAAGTTTGCGTTGGATTTAAAAATAAGAAC
GTTCTTCTTTGCTCTTGGCAAAACATTTGGCTTCTATAGCTGCGAGCTGCATATACGCAGAATAATTATT
ATTTGTTTGTTTCCGAGTGTTTGGATGGGGGAAAGGTGAATAACACCCACTCGCCAGTTGCGCgtctata
tcttggagtctatatcttagagtGGCTTTGAAGTGGATCTCGTATCTCGTAGTTGCCATGGAAATATCTA
TAAAAATGTGTGCTCATGATATACGTAACTACCTTTGCCGCAGAACACATATTTCCATTTCATCCTTAGC
GAACGCAAAAATATTTAGTAAACTGCATGTTGCTCATACATAGTTTCCACTTACAAGCTCGAATCCAGCG
ATTCCAGCCAAAGAGCACTTCCAACTAAGCGCTGAAATCAAAGAATCCGACTCCAAATCCCTCGGCCAGA
GAACCAAAAATCGTTTCGGCTttcagttcagcgcagttcagtgcagttcagttcgtctcagttGACTGTT
GGGCTATTTGGAAATGTTTATGCAAGAGCCAATGATAATGCAACGCCCCGCTACCGCCCCCGCTCAAAAG
TGCTACATTAAATTCCATGCAACTTTGTATCTGAATCCGCGTCCGGAATTTGAAAGAGGAGTTGCTAGCG
GTCGCTGCAGCTGAGGAGACtcggattcagattcagattcggattcggattccgattGCCATTGTGATTA
CGCGATTACGGGACGAGCTCGTAAGTCGAGCGGGAGTTGGCGGTAATTTTATATTGATAAGTTTTTATAA
TCATTTTAACGAGTGAGCGGGTCCGATTCCGATTCGGAATCCATCCGCTCCTCTCTGTCCGCAGTCCCTT
ACGCTTCTGCTCCATCCGGCAAATGAGCGAAATTAATTTAATGCCACAGACAGGGACAGAGAGTCGCAGA
GCAACATTTTTGCAGCACGTGTAACCCGATCGGCGAATGGAAATCATACCCCCCGACTTGCAGTTGGGCG
CAATGATACTTATTGTCGGTCATGAGGGGGGAGGGAAACCAACAGCAAACACAAAAACAAAGCCGAGCCA
AAATTATTATAATGAATGGGCTGCTCTGAGGAGGGGCATGGGAACTGGGTATCCGCTGGGAACTGCCAAC
CGAGCTTGGCGACTGTTTTTCTAGGTAAATGTGCGACATTTGCACGGATATCTATATGTGGCTACTCATG
CCTGTATAATGATATTCATAATGGAATTGCAAAGCTTAGGCTTTAGCTGCATGCTGACACAATCAATTAT
AAGATAGTATAAATCTGATGCTAGTAGTTATACCAAACTTACTTACTACAGATATGCAAAGTAATATAAC
AAATATCTCTTAAAATCCGATAGATAGATAGATAGTTTCTTAGATATTTTTGTAATTCATTTCCATAATA
GGAGCTACCATTGCGTACATAACTTAATCATCTCTTTAGGTGTTCGGCTGAATTTCGCCTTCAATCCGGG
CTCTCAATGTCGCCCAATCCCCGCTTTGAGTGTGCAACAACACTTTGCTAACTGAAAATATCAATTAAAA
ATATTGTAATTCATACCTTTTATTTTCGCTTACCTCACCCAGTTTGGGTATCGCTTTCGGTATTTTGGCC
TCTCCGCCAGCTCCTGGCCATAATTATAAGCTGCATTTGCTGCAAAGACAGCAGCCAGGACAAATTTTTA
TATTTAATGGCCCAAAGGCAAAAGCACACAGCGTAATTAAGCAACAACAATATATCGGTTGAGATGATGG
TTGAGGGAATAGAGGATAAGGGCGATGGGGAGGGGATGGGAGGAATATGAGCGCGCTTCATGGGTGCAAC
TTGATAGGACAATGGTAAGGCGATATCCTTCAAGGTTTCCGCTTTGCGACCGATGTCAGGCCAAAGCCGA
AGCTGGGCACTATATATGTACATAGGTATATGTGTATGTCTGTAGCTATGTATCTGCAGAAAAGGATGTC
TTTGACACACACTAGGCATTATCCTGTGAAGTAGGAAGAGTGACAAAGTGGGGAAGGTGGCCATTGATGG
CTGAAATCAATAACTCTTTGTAGGCGGCAAATATGGTAAACAAAGTGGGTATTTTGGGCAAATATTACTT

AAATATACAATTAACTCCTTAAATAGCAAATGATTAAAGAAATGCTGGAGTGACATTTCTACATTTTCTT
TAGGGAACTTTTTCAAACCTCATTAGCTGCTGCATAAAGTGATGAATTCTGTTCTATAATTTGTGATTAA
ATGCATTGTCGCCTCGCCTCTGTTGCACACACAATATTCATCACCTACAAATCGCCTCCGAGTTAAGTGT
TGGCAGACTCCAAGCAACATTCTATGCACATAACCCAGGGTTCCGGCAGCATGGATTGGGTTACACTCAC
ACAACCGGGTGTGTGGGGCTGTGGATCGGATCCAGATGACTATGACGATGACACAATGACAGGCGGCCAC
TCAGGAGCACGGACGGAGTGGAGGCGGAGTGAAGGCGGCTTGGGGCGACGGTGGCAACTGGGCTCAAATG
TTGCCGCAGCAGCAGAACTTTAATAACAGCGACGCAGATTGAGTTACAAACAAAATAGAATATGATAAAT
TCTACACAAAACACCAGGAACCAGAGCTCAAAGGTGCCAGGAGATGGGCGTTTCGGGGGTTCACACTGTA
GTGCACTGGTAACGTGAGGCACCCTGTGCTGCTGCGCTGCTGTGCCGCCTGCCTTCCTGCCACATGCAAC
ACGCCAGCGTCCGCCCATTCACTCCCTTCCACCGCCACGCAGCATCAGCAGCAGCAGCTCGAATTAAAAT
AGCGTTAAATTAATTCGAGCCACTGCTGATGGCCCAGCATTACGAAAGCGAACCCGAGTGGCCAAGAGGC
CATGCGGATGGCTGGATGGTTGGGTGGGTGGATGAGTGGGCGGCTCAAAGCCCTTAACACTGCCAGGTGG
CAGGTGGTGGTTCGAGCTGCAGCAGGTGATGGGATCGTTTCTCGGCAGGAGCAGCTATCAAGTTTTCTAA
CCATTTAATATATTCTTATTCGGCCAAAACGAAATTCAATCGCCATGGATGAAACTTTTACGACACGCCC
CCAAGATAAACTGTAAAAGCTGTCCACTTTCCTGATTTTCCGATGATTGCCATAATTGCCATAGAGCCAT
AAATAGTGTCAGTTGCATTGTTTTTTGTTGATTGATTCATTGGCATTTTGAATGCAAATTGAAACGCAGC
AAGGCAAGTGATATCTAGATATTTATATATTTTTTTAAAAGAGTGCTCTATGTGACCTACCATAAACATT
AATACCAAACCCTTTGGTTTCCGATTTCGTATTCGGTGCCTTGAGTTCGCatatatcatattatatatac
atatattacacatatatgtatatatcTCCGTCTGAGCAGTGAGCCACCCATATCTGGTGTACATTAAAAT
GAAATGGAATATTTCGATGCCGAAGGGAAGGGGTGGAACGGGGCACGGCGATGGGAACTGAAAATTCGCA
GAGCAATAGCAACAAACAACTTGGCAAAGGCCAAGTATGTCATGTAAGAATTCAAAAGAAGGACGCGCGT
CATTCAATCCTGAGCTATCGATATTCGATTACAGCAAACTCAAAACCGACCGACTTTGTGCTCGCCTCGC
AAACacccaatcccaattccaaacccaagcccaaacccaaaacccaagcACATGTACAAACGTGTGTCTG
AGAAAACTAGCGTAATTCTAGAATTTGTTGCCATTGGGTGTTATTTCCACTGGCCGACCATATATACACT
TACGTGCCGGCTGGTATATACTTTTTCGGCGGGGTGGTGGTGTTCGAGGGGCCTGggattggattcggat
tcggattcggatacggattcggTCCGGACAGCAGTTCTACTGCAGCTTGGTTTTAGTTCGCCTTAGTCTC
AGTTCAGTTTACTGGGTTCAGGGTGAGTTATTATAAGCGTATTGGGTTGCCACTACCGCTCCACCCGCTC
CGCCTGGCCCACTAGCCCATCAACCCACCATCCCTCTAGCTCCCCATCCCccagtgagccagtgagccag
tCAGTCTGGGTCTCGTTGCTCTGGCACTGCGAGCATTGCCGCGCCGGGTACTTTACATTATTCTATTAGG
TTTCCTCGCCGGTAACCTAAGACGTGTTTTAATGCGAGCCACTCGCATAACAGGCGGCTCCACTGCAGCG
GGATAAGCCCCTTGGACGACATCGGATGGGCCTGGATGTAGGTCAGGAAACCGAAATCCATTAAGACCGA
TAGTTGGGAGTACACTTAATCAGGTAAAGGAGGAGATATATCGACTTTGGATCACCAATTTAAATACCAG
AAACTTAAACCGAGATCTCTAGCAAGATCTTACTATCTTACTAGTTACTCAGTTTTATATTTCGTATGAA
ATCTAAGATCAACCACTATTTTTTCCCAGTGCACTCAATGCCAGACAAAGAACTTGACTATCGGATTGGA
GTTCCCAGTTCCCGCTTCTCACCCGTAAAGCCGGTTCATTACGATTCATAATAAAAACGAAAATATGATA
TGTTTTTGTAAGTTGAGATTTTGCAAAATGAAGGAGAACACAACAAACTTGAAGAGTCAGAAACAGAATG
AGTGTTGCAAATATATCCACTGGATTCTTGGGCAATAATTACGAAAAAAATTCAAATGAAAAGCGAGCCA
AAAATCCGACAGATCTGATTGCTGGGCGGGAATGGGATTGGTTTTGGAGGCAAAGGAAATTCGATTGGAA
ATATAAAGGTAGACGTGTGTGTTTGTATCTTTCTTAATACAAACGTATTCGTACACAAGTATTTCTGGGC
GGGCAGAGCGAATCAGATCCCAACTCCATCAAGAACAACAGGCGGCATAAAATTTCAGTTTTTAATTAAT
TGATTTTTATTCGCATTTCGGTGTACAGTTTCAAGCATGGGCGAGAGTTTTAATGTCGCTGACGGAAAGG
GCACTGaaaaaaaaTGTGTGTCAATTAGCAGAAATCAAATATAAGCAGAATATGGTTACCAAAAATTATA
TAAGGGGGTTATAAATGTTATGCATAATGCATATAGTTTGTCTACAATGTAAAAAGAGCTTTGCTCAGTG
GATCACAATTTTTGCCAGTGTATGTCCCCACTTTCCCGCCCGCCACACCGGCCCACCCCCTCGAACCCGT

TGAGCCACTGTCAGGCAGCAAAGAGCTGAAAAACTGAACGAACGCACAAATCAGAAATTAAAAACCGAAC
ATAAATTAACAGACAAATGATACTAAAAACCGTGCTGCCGAAAGAGACGGCGCATGCGACAATGAGCGAG
ACGGAGGTAGCGGAGATGGAGTCGAATGTATCTCCGTTCCTCCGGTTACGCTTACTGCAAGATGCTGAAC
TTGAGCCGAGCGTGCGTCGCCATAGcccccccccccccccTCACCGCATCCCACCCGCTCCTATCAGCCA
GCCCATGCCATTCCATCCCACCTGGACGAGGTCGCACCTGAGGCGTGTAATTGATATGCTCGGCTCGGGC
AGCCGGCTCCGTAGATTTTATCGTGCGAATTCTTGATACTCTTTTCGCTTTTCGCAGCGACAGACAGCGT
AAAACCTgaatgcgaatgcgaatgcgaTTGTCACCGATACGCGAATTGCTGGAAAAATAACCAAGCGAGC
GTGTGAGCGCCGTTCATGGAGTGCGAAAGGAAAAGGTGTTGCGCGCCGCGAAAGGGACCGCTTGCTGCGG
TGGGAAAAAGAGATGGCTACAGGGGGCCAGAGGGGATCCGAGATAGACGGCAACTTATTAGACGACTTGC
ATTTGTCAGCAAGTTGGCAGCAACGCTTAATTTGAAGAGTATCGCATGACGCCGGGGATATCCAGTTTGT
CTGGAGCTTGTGATAGATGCAATGGAGGCGATGAATCATCTGGCCAGCATTGGCCAATTGTGGTTGTGGC
TCTTTTGCGCGGACCAATTTTCCCCTGAAACTCGGCATTGGACACCACTTGCGGCCACATGGCGTATGGG
CAATTTGGCATTCCGCAGAAGCAGATAACAGAAAATTAAAACCAAAAAGTTACAGAGCGAAAACGCAAAG
CAGTTAAGGTGGCTAATTTAGATGATTGACTaaaaaataaaaaaaTGATTCTTTTTTGTTAGTATTTTAA
AAAACGGCTAAGATTCAATGAAATATGACTGAAAATTCCTACTCTCTTTTGATTTAAGCTTTCGACCAGA
TATTTGTAGAGCCCCTGGAGCAAAAGGACTTTGCTCGGTGAAAACCACGTCGAATATGAGAATTTCAATG
TAACCCGATCCCCAGTTTGTGCACCTCTCACAGGCTTGTGTGAAATTTTTATGTGCAGCACAGGCAGGTC
CAGAATGATACACACATGGGATTCGAATGGAAATGAGAATGCAGGAGTCAGTCaaaaaaaaaaaaaaaaa
aaaaaaaaaaaaaaaaGAACAGCTTCAAAAGCCGCAAATGCTTTCTGCATCTTCGACTTTAGACTGAGCC
CCTGCATTTTCGGCTTAAATTTAAAGCAATGAGGCAGGAGGCACTGTAAAACGACAAAGAATGTCCGAAG
GTATTGGATATCAATATCGAAGCAGGGATAATCAAAACAGAGGGGGTGGGGGGATGCATGACCATACCTC
CTCCCGAAGCAAGTGTATGCAAATGCCACCCACTCTGGCCAACCAAAAAAGCCAACTAGTCCGGCCCTCT
GGCCGTAATGAAGATCTCTGCCACCCGTTTGTGCACTGATATGTTTGTGGAGAATCCGGAgtgtgtgtgt
gtgtgtgtgtgtgtgAGGTGTCTGATGCGTGGGTGATTCCCCCAGGGTTATCGATTACACTGCAACTTTC
CAGCCTCGGGGAGAGCGCTTTGATACGGTTAGATAAGAACTCGCATAATTGGACATTGGGTTCAGAGGTC
AACGGGCAAAGGTAATCAATAATTAGTGAAAGTTGCCTTCACTCGGAATGGATTGCACCCCTGATCACTC
TACCCCTCAAGCATTTAAGGTCCAGTAGTCGGCCTCAAAGTTTCACCTGCCTGACTCTTTGAATAAAATT
TCAGCTGCTCTTTAATGGCAATGCACTACAACAAAAATTAAATTATTTAAAGTACATATATTATATAAA
TATGGTGAAAAAAACATTTAATTAAATAAGAATATATTCCGAAACTTTTTGGAGTGAACTACTGATGCGA
TCTGCCTGCATATCGGGTTACCCAAAGGCCATGTAAGTGGGATTCAGGGTACCGATGTCAGCAATTCCCT
GTTTGCCACCACTCATCTTTCTCTTTCTGTCCGTCCCTTCCAGGGAATTCGCGCACAACACCATGAACGA
GCTGCTCGGCTGGTATGGATTCGAGGGTGTGGATGTGGATCGTCTCGACCTGACCGCCAAAACGTCGCGC
CTGCTGCAAAGTGCCGCCGCCGCAGCTGTGGCCAATCAGCAGAGCCACCAGCTTCACCAGTTGGAGCCGC
ATCCGAATCTGGAGCGGAAGCGGACCAGTCTGCTTCGCAGTAGCAGTCGCAGTTCCTTGGCAGCGGCCTa
ccacaacaacaacaacaacaataataacaacaacaacggcagcagcagcaagaacaacgGAGGAGTCGGC
GGCGTAGGCGTTGGGAGTGGTCGGAAGAGCGGGTTGATGAACTGCAGCAGCACCACCACCACGCCATCGG
ACCACGACACGCGAAGCTCACGGGAGGAGGATTCCAAGAGTCCGCACTCCATCGGGAAGCCAGCGGGAGC
TGCGCACACGGATGAGAAAATAGG
>dme_circ_0003964_junction_seq
GAGCTGCGCACACGGATGAGAAAATAGGTTTCACCCGAAAGTTATATACCGCATCC
>dme_circ_0003965
GAAAACTCAACCAGGCCACCTTAAGCAGGGCCAGTGCAAAGAGAAGGGAACCCACGCTGGTGATTATCAA
CGAGATGATATCTCTCTTGGCATGCTGATCCACTCTGCCCACCAAGAAGCTGTGTATCACGGTGCTGGTA
ATGTGGGCGCCATCGAAACCAAAGCAGGGAATGGCATTGATCAACGCCAATCCTATGCTGAAGACCAC

>dme_circ_0003965_junction_seq
TCAACGCCAATCCTATGCTGAAGACCACGAAAACTCAACCAGGCCACCTTAAGCAG
>dme_circ_0003966
GGGTTCCCGGGGATCGGTTTACTGCGCGAGTGAACCGGACCCAACTGGTGGAGTCCAGTACGAGTCGGGA
GGGTGCCTTCTCCACACGAAGACGGCTGAGGGCTCCGGCGGAGGGGCGGGCGTGGACGGGTCCGGTGGCG
GAGTCGGCCTCGAATGCGGCTCCCTGTTCTCGGTGGTATCAGCGCCGCCGGCTCCACTGCCCGACATCGT
TGTGCCCACCGAGATGGATGGCAAACACATGGAGTCCGCCGCCTTCCGCTCCGGATGCTGTTCACCCAAC
GCCTCGCCCATGCGACGACCCGCCACCCTCAGCCTGGACACGCAATGCTGCCCGAGGACCCTGCACGCCA
GCCTGCTGGAGCATCAGATCTCCGAGCTGGAGGAGGACGACAATGAGAACCTGCTGACCGTGTCCAGCAT
CACGGCGCGACCGTTGATTGCCAAATCGCATGAGCTTCGCAGCAGTCGGAAGTCGGGTTCAGGTTCCGGA
TCTGGTTTGAATACGGCCCGCAGCAGGTGCGTCCGCCGGGCCAAGGAGCGCGAGCGGGATCGCGTGACCG
TCGCCAGGCGTAGCAAGCAAGTGGTCAAGGATCGGGACTCCTCAACGACCACAACGGAGAGCGGTGGTCC
GAATGTGGAGCCACCGGACGGCGGTTACGGTTGGTTCATCGTCTTTGGGGCCTTCTCCGTGCAATTCTGG
GTGGCCGGACTGGTCAAGTCGTACGGCGTGCTGTATGTGGAGATTATGGAGACATTTCCCAGCTCTACGG
CCACCGTGGCCTCCTGGATTCCAGCGATCCTGTCGGCTCTATGTCTGGTCCTGGCCCCGCTGTCCAGTGC
TCTTTGCCAGCGATTCTCCTGCCGAACGGTGGTCTTCGTCGGCGGCATTTTCTGCGCCATGGGCATGATA
CTGAGCTACTTTGCCACCAGTCTGCTGCACCTCCTCCTAACATTTGGCGTACTCACAGGGTATTGGAGGT
GGCCTCTCGACTACTCCGGGCATAGTGATAGTGTCCCAATACTTTGACAAGCACCGTGCATTGGCCAATG
GAATCTGCGTTTCGGGCACTGCCGCCGGCAGCTTCATCCTACCCGTTCTGATAAAGCACCTCGCGGAGAA
CTGTGGCTTCCACGGCACTATTCTGATCCTGGGCGGTTGCATGCTTCACGTGTGCGTTTCGGCCACACTT
TACCGCCCAATCAGTGCTTACACGGATCAGGACTCGGGCCAGGGACAGGAGGAGACGGCAAAGCCCCTCA
ACGAAGACATCAATCCGAGCACCACGGGCATTCTGACTACATCCACCTACCTAGACACCTGCGATGTGGG
TGCAGGCCACGAGCTGAGCGACAAGTTTATCGAGCATCTGTTCCTGGAGGAGTCCAAGAACCACCTGAAC
TACTATGCCAGCAAGCAGCCAGGCCCAGGACAAGTCAGCGCAGGAGAGTGACGACGAGGTAAAGGATATT
ATTGGCGAGACCACTTTCATCAAGCCCATGAAGAAGGTGCGCAGTTCCGGATTGCTGCACTCCGTCGAAG
ATCTGAGCACGGACTCCACTTGGGTCTATCGCAAGCACTCCGGCACGGACTCCAATCGCGGAAGTCGTCG
TCGGCGCAATGTCTTTGCCAACGACGAGGTGATATCCAAGATACAAGCCCACTTGGAGAAGCCTCTTTCG
CCGCCGAGTGTGGTCAGTCGCGGACTCAGCAAGAGCATGGAGATACCCACTCCGGTTAGCAACCTCAGTG
ATCTCAAGCAGCAGCCGGTGGAGCAGGCGGAGCTCGGCATTCTAGACTCCCAGCTGGTGGAGTCCATTAT
CGATGGCGACGGCCACCTTGCCGCCGATGAGGATGACGATGACGACGAAGAGCGGGGACCACGCACTTGC
TGCGAGCGCATCGAGATGTACCTGGACATCAGTCTGCTGCAGGACCCGAGCTTTATCCTGATGTGCCTAT
CCGTCACCTTGATGTCCGTCGGCTGTCCCTACATGTTGTACTATCTGCCCGCCCACGTTATTTCAATAGG
CATTTTGGGCGCCGGATGTGCCGTGCTCACAATTCCCTTCGCCAAGACTCTGGTTTTGGTGGGTCTATCG
GCCGCTGTCTACGGACTTTGCCTGGGTAGCTGGTATGTACTTATGCCGGTCCTCCTTGCGGACGTCTTTG
GCACGGACAGGATCAGCTCGAGCTACGGGCTGGTGAGGATGTTCCAGAGCATCGGGGCCATATCGGTGCC
TCCGCTGGCAGGACTGATGCGAGACCTTTCCGGAGACTACGAGATATGCTTCTACTGCATGGGATCCTGC
ATGGTGCTCGGGACCACTCCGCTGATTGTGTGGTCCATCCTGGAGGCCCGCAACCATCGGTTGTTTGTTC
AGCGCGAGGAGGAGGAGTGCGAGGAGGCCTAGAAATGATTCGGATTCGTTGGTTGCCCCATATGTTAATA
GTGTTTTAGTAGCGATCAAATACTAGTTGAGTTCCTGAGTTTCAGAGTTCGAATGCCTCACCGATATTGC
TGATTCTACCAGTTACTATTACTACTATCC
>dme_circ_0003966_junction_seq
ATTCTACCAGTTACTATTACTACTATCCGGGTTCCCGGGGATCGGTTTACTGCGCG
>dme_circ_0003967
CTCCTGTTCCATAGCCTGAATCTTCTTATTTTGCTCGTCCAACTCAGCGCTGTGCTTTTGCTTCATGGTT

TCCATTTGCTCCGTCAACTGGGCGCACTGCTCTTCGGCCTCCTCTAACAAACGTTTAATTTCGGAGATGC
CCTGTAGGAGTTCCTCCCGTTGGATCAGATGATCGGATTCGGTGCTCTTAAAGATTTCAAAAAGCTTCTC
TTTGGCATCAAGTTCCTTTTTCAGCTTCCCCACATACTCTTCCGTGGCCTGGTTCTGCTTGAAGGCGGTG
TCGTTTTGCATTTCAATCTTGCTGGTTAGTTCCCCGATGGTCTTGACGGCCTGTTCG
>dme_circ_0003967_junction_seq
TTCCCCGATGGTCTTGACGGCCTGTTCGCTCCTGTTCCATAGCCTGAATCTTCTTA
>dme_circ_0003968
GTTATATTTCGCGGCTCAGAAGAAGCTCGGTCAAAATACTGTGGATCTGGACGACTCCAATCTGGAGCCT
AACGACTCTGCACTGGATACTTCAGAGCAGCCGGCAGCCAATTTTGAGGAGAGCAGGAAACTGGAAAAGA
GGGTGCGACAGCTGGAGCAACAGTTGGAAGGGGAGGTCAAGAAGTATGCCTCACTGAAAGAGAACTACGA
CTATTACACTAGCGAAAAGC
>dme_circ_0003968_junction_seq
AACTACGACTATTACACTAGCGAAAAGCGTTATATTTCGCGGCTCAGAAGAAGCTC
>dme_circ_0003969
GTTCCGTGAATCAATTAACGAGTTTAAGCGACAGGCGGAGACGGCCATCAAGTTGAAAGATGAAGAAAAG
CAGCTGGCAGACAAGTGGCAAGCGGAGCTTACCAGCGTTCGCGAAGAACTTGCTGAGAAG
>dme_circ_0003969_junction_seq
CAGCGTTCGCGAAGAACTTGCTGAGAAGGTTCCGTGAATCAATTAACGAGTTTAAG
>dme_circ_0003970
GTTAACCCGTTACAAGGCAGAATTCTTCAAAGCCAACGACGAACTCAACCAATTAAAGTCAGGTCGGGAA
TCATTGCAAGCCGCATACGATGAACTTCTGCGTTCGAATGCCGAAGCCCAGAAGTTGCTGGACAAGGAGA
GGGAGGA
>dme_circ_0003970_junction_seq
AGAAGTTGCTGGACAAGGAGAGGGAGGAGTTAACCCGTTACAAGGCAGAATTCTTC
>dme_circ_0003971
GGAAGGAACAACGAACAGCTACTAAAGATCATAAAGTTTTTGCGCAAGGAGAAGGATCTGTTCGCTGCCA
AGCTGGACATTCTCAAGGCGGAAAATGCTCGTCTGATTTCAGAACACGCCATTCAACAAAAGAAG
>dme_circ_0003971_junction_seq
TTCAGAACACGCCATTCAACAAAAGAAGGGAAGGAACAACGAACAGCTACTAAAGA
>dme_circ_0003972
GGAACAACGAACAGCTACTAAAGATCATAAAGTTTTTGCGCAAGGAGAAGGATCTGTTCGCTGCCAAGCT
GGACATTCTCAAGGCGGAAAATGCTCGTCTGATTTCAGAACACGCCATTCAACAAAAGAAG
>dme_circ_0003972_junction_seq
TTCAGAACACGCCATTCAACAAAAGAAGGGAACAACGAACAGCTACTAAAGATCAT
>dme_circ_0003973
GAGGACGCCATCGATTCCAGGGCGGCACTTCCAGTGGTGTGAACCTGTTGCTGTGGTGGCACCAAAGCGG
TTTGGGCTACCGCCGAGCTGCCGCCGCCAGCGGCGGATGTTGTGGAAGTGGATGAGGATGAGGATGACGA
GGAGG
>dme_circ_0003973_junction_seq
GTGGATGAGGATGAGGATGACGAGGAGGGAGGACGCCATCGATTCCAGGGCGGCAC
>dme_circ_0003974
ATGACCCTTCCACACGCGGCGACACTAGCGCCACAACTTGCTGTTGGGCTCCGCCTACAAACAACTGCGA
ATCGTTTTGCTGCGGGTGCTGGATACTTTCCGCCTCCTGGGAACTTTCGGCAGCAGATGAGGCGCCCATC
GGCGGAATAGCAGCGACTGCCACAGATGCAGAGCTAGTGGACGGCATG

>dme_circ_0003974_junction_seq
CACAGATGCAGAGCTAGTGGACGGCATGATGACCCTTCCACACGCGGCGACACTAG
>dme_circ_0003975
GGGTGACTCCTCCACCGGCACTGAGGAGGGTGTCGCCGAAAAAATGTCAAAAATTACTAAGAGACTTCGC
GGTCCCATGCACAGCGGAGAGTTATCCGCCGGCCACATTGGTGATTCCGGAATGGATGTCGATCAAATGC
CAACCTCCTCCCAGCGCGATCAGGAGGATGACATTCAAGTG
>dme_circ_0003975_junction_seq
GCGCGATCAGGAGGATGACATTCAAGTGGGGTGACTCCTCCACCGGCACTGAGGAG
>dme_circ_0003976
TCTTCGTAGCCCTCCTGTTCGGCCTCGCCTCCGTCAATGGGACCATCGTCGGCATCGGCCAGAACGTCCT
CCTCGTCGTCTGAGTCAACCACTTGAATGTCATCCTCCTGATCGCGCTGGGAGGAGGTTGGCATTTGATC
GACATCCATTCCGGAATCACCAATGTGGCCGGCGGATAACTCTCCGCTGTGCATGGGACCGCGAAGTCTC
TTAG
>dme_circ_0003976_junction_seq
CTGTGCATGGGACCGCGAAGTCTCTTAGTCTTCGTAGCCCTCCTGTTCGGCCTCGC
>dme_circ_0003977
GGTTGCATGTTTTCTTTCATGGCATCAATGTCAAAGGGATTTTCATCTGTGGGCTGCTCATCCTGGTCAT
CGTGTCCCTCGTCAACATTATTCATATCCCCCAAATCCATTTCCTCTGGTTCCGGTGGCTCCTCCAGCTC
ATTGTGCATTGCATTTGTTTGCTCCTCATCCATTTCCGGATCCTTCATATCGTCAATGTCCTTGGCCTGC
TCCTTGCGTTTGTCCTCTCCGCTGGGTTCATTG
>dme_circ_0003977_junction_seq
GCGTTTGTCCTCTCCGCTGGGTTCATTGGGTTGCATGTTTTCTTTCATGGCATCAA
>dme_circ_0003978
GTTGTTCCAGCACACGCTTCTCTATCCGCTCCATGACATCCAGGCAACTTTGAATTTCACTAATATTTGA
GTCTTTGTAAAAGTCGTAAGGCGAACTGGATACTCCTTCCTCCAGTTCGATATCCCTCAGCTGTTTCTGC
TGCAGAGCCAGTCCAAAAGCCAATCCATTGCATGCCCTTTCCTCTAGACTCGATCCTAGGGCTGTCTTAT
AGTGACCATGTAGGTTTGCAAACACCTGGAAGCGTGCTTGATAAGGCGCGAGCAAATGGAGCTCTAATTC
CTTCGATGACTTGGGTTGCTCTTTATA
>dme_circ_0003978_junction_seq
CCTTCGATGACTTGGGTTGCTCTTTATAGTTGTTCCAGCACACGCTTCTCTATCCG
>dme_circ_0003979
GAACAGAAAGCTAACCAAGGCTTTGAAAACTGTGCTAAAGAACTCGAGAAAATGATTGAAAAATTGGAGA
AAGAAGAAAACAATAATGCCTCTATGACAAAAGATTTTGAAGTTTCGCTAACTAATGCCCTGGTGGGAGC
CATTGAGTTGTGTTTGCTCACGAACACCCCG
>dme_circ_0003979_junction_seq
TGAGTTGTGTTTGCTCACGAACACCCCGGAACAGAAAGCTAACCAAGGCTTTGAAA
>dme_circ_0003980
GGCATTATTGTTTTCTTCTTTCTCCAATTTTTCAATCATTTTCTCGAGTTCTTTAGCACAGTTTTCAAAG
CCTTGGTTAGCTTTCTGTTCCTTCTCACTTAGTTTCCTCACATATTCCAGCTCTTTTAGTAGTTGCCTTG
CATTTTCCTTGGCCTGCAAGAGATTATTGACTTCTCCACTATATCTGGAAGTTAATAGCTGAGAATTCTC
CCAGATCAGGTGTCGTGTTTGCTGCAAAGCGGATCGCCATTCTAGCAACTCGCTTAGGGGAACCGATCGC
AGCTGGCCGTTGGGTTGACATATCAGCGATAAAATGACGCCTGATAAAGCAGGGCCTCGATAACAGAATT
GGCTTGATATATCCGTGCTACCTTCTTCTGAAGGACCTTGAACACAATCCAGACGGGTGGACAGATTTTC
CAATTG

>dme_circ_0003980_junction_seq
CCAGACGGGTGGACAGATTTTCCAATTGGGCATTATTGTTTTCTTCTTTCTCCAAT
>dme_circ_0003981
CCTTTGTTCATGAGCAGGGTGTGCGTGAAATGGAGGCCATAGATTGTATACTGAGAGTGCATAAGGGATT
AAGTCAGCTGACTGAGTTCAATAACTACTCCATATCGCACCTGACACAGTTTGCCTTTTTAAGCGCTGCC
TATAGGAGAATTGGCTATGAGCTTAAACGTGCTATTTATGTGGCCGCCATGGAGGTTTATG
>dme_circ_0003981_junction_seq
ATTTATGTGGCCGCCATGGAGGTTTATGCCTTTGTTCATGAGCAGGGTGTGCGTGA
>dme_circ_0003982
GCACGTTTAAGCTCATAGCCAATTCTCCTATAGGCAGCGCTTAAAAAGGCAAACTGTGTCAGGTGCGATA
TGGAGTAGTTATTGAACTCAGTCAGCTGACTTAATCCCTTATGCACTCTCAGTATACAATCTATGGCCTC
CATTTCACGCACACCCTGCTCATGAACAAAGGCTCGCAAATCATCTACAGAATAG
>dme_circ_0003982_junction_seq
AAAGGCTCGCAAATCATCTACAGAATAGGCACGTTTAAGCTCATAGCCAATTCTCC
>dme_circ_0003983
ATAAATCATACATTCGAAATGTCTTTTCAACCGTTTTCCTATGCGATGCTGAATACCTAAATCAGGTTTC
AAACGATGTCGCTCTCTATTGGACAGCAGATAAAGTATATCTTAACGATGTGGTTTTGAGTCGCAAGCAA
GCGGATGTTCTGGACTTGGTAAATCGACAGGAGCAGTCACCATTGCTGCTGGACAGTCAGAGGCAGCAGC
TTAAGCAAATCATCGAGACTG
>dme_circ_0003983_junction_seq
CAGCAGCTTAAGCAAATCATCGAGACTGATAAATCATACATTCGAAATGTCTTTTC
>dme_circ_0003984
TTTCCATTTAGCTGGCTCGACTCGCATTTTTGCGTGCCAGAATCCTTTAAAGCAAGGCGGCGGCCGTAAG
GGATTACCCCAATCCTTCCTTAATCGTTTTACAAAAGTTTATCTGCGCAAGCTCTCCACTGAGGATCTGC
TACATGTGGTAACTGAGAAGTACGGCAAGTACTTTGATCAGTTGAATGCATATTTTTTGGAACTG
>dme_circ_0003984_junction_seq
TCAGTTGAATGCATATTTTTTGGAACTGTTTCCATTTAGCTGGCTCGACTCGCATT
>dme_circ_0003985
GAAAATCTGCTCCTTCTGTATGCATGTGGCAGTTGAGAATGCGCAGCTGGACATCAGCGATGCTAGCAAG
AAGTTGGCACACGGTAGTCTTTCCACACCCCGTTGGGCCAACCAATAAAACAGGCTCGTCGAACTCCAAG
GCCTTGGCAGTGACCACGGCCATGCGT
>dme_circ_0003985_junction_seq
GGCCTTGGCAGTGACCACGGCCATGCGTGAAAATCTGCTCCTTCTGTATGCATGTG
>dme_circ_0003986
GAACCACTGAACCCTCCGGCTCTAGGATGGTTGAGAGACATTCAAGCGTCTCCGCTGAGGCCAAATTAAT
TTCATCCAACAAAACCCAGTCTCCGTTGCTGATGCAGTTGACCAGAGAGCCGGGTATAAATGCAAAGGAG
ATGTTGATGCTCTTATCCAGTTGCATTTGAAGCTTCTGTAACTTTTCTCTCAGTGTCTGCCAGCGGGGAA
GCAACTTATTCGCCTTCAGTTCCGGATTCTTGGCCACCTGTAGGCAAATATTGATCATGGTACGAATGAT
CACAGCATGACGACCACTGTTGTAGTGTGTGGCGAACAAGCGTAGAAACTGCAAGTTCTTCTCTTGATTG
AAGGTTTCGCACAGAAGATACTCAAATTCATTTCGCAGTGGTGCCAGTACGTAGTTGAGTTCCACGGGCT
TAAAACCGCCTACCAAATCCGACACATCTGATTGGTTGTTCATATTGACCACCACCAATTTATGTTCCGT
CCGCTCGGCAAGGTATTGCACTGAAGATGTCTTGCCCACTCCGGTTTCGCCCACCAAAAGAACTGGCTCA
GAATGG
>dme_circ_0003986_junction_seq

GCCCACCAAAAGAACTGGCTCAGAATGGGAACCACTGAACCCTCCGGCTCTAGGAT
>dme_circ_0003987
CTAAATTCTTTGAACGAGAAAATATCGACTAAAACACGTCTGGAGTTCGATCAGGAAAGGGAGCAGGCAT
GGAACACAGCCAGGAACACACTCTGCAGCTATACACCAGATGCTTCCGACCAACTACTGAACCTGCATTT
GCATGGAATCAATAAATCCGTGACAAACATCGAGGGTG
>dme_circ_0003987_junction_seq
AATAAATCCGTGACAAACATCGAGGGTGCTAAATTCTTTGAACGAGAAAATATCGA
>dme_circ_0003988
GAAGAGGGTGGGGAAGCCGCGCACGTTGAACTCCGGCGGCACATCGTTGGCGGTGGCGTCCATCTTGACG
ATGGCCACATCTTCGTCTTGCAGCTTCTCGGCCAGCTCCTCGTAGATGGGACTCAGCTTCTTGCAGTGGC
CGCACCATGGGGCGTAGAACTCAATCAGCGTGTCTTTGCCATT
>dme_circ_0003988_junction_seq
AGAACTCAATCAGCGTGTCTTTGCCATTGAAGAGGGTGGGGAAGCCGCGCACGTTG
>dme_circ_0003989
GTGGCCGTCGCCAAGAACTTCGACGATCTGGTTATCAACAATGGCAAAGACACGCTGATTGAGTTCTACG
CCCCATGGTGCGGCCACTGCAAGAAGCTGAGTCCCATCTACGAGGAGCTGGCCGAGAAGCTGCAAGACGA
AGATG
>dme_circ_0003989_junction_seq
CTGGCCGAGAAGCTGCAAGACGAAGATGGTGGCCGTCGCCAAGAACTTCGACGATC
>dme_circ_0003990
GCTCCTCGTAGATGGGACTCAGCTTCTTGCAGTGGCCGCACCATGGGGCGTAGAACTCAATCAGCGTGTC
TTTGCCATTGTTGATAACCAGATCGTCGAAGTTCTTGGCGACGGCCACCTTGACGGGAGCGTCGTTCGAC
TCGGGAATAGGCTCACTCTTAATGTACGGCTCCAGCTCATTCGCCAGCAGCTTCTCGACAAA
>dme_circ_0003990_junction_seq
GCTCATTCGCCAGCAGCTTCTCGACAAAGCTCCTCGTAGATGGGACTCAGCTTCTT
>dme_circ_0003991
TTTTTGTTGTAGAAGTAGGCGTTCGACGGCCTAGTTGAACTTGAATTATTCACCGATATCGCTTCAAGTG
AACCCAAATAATGATGTGGCGCCTTGCTGGAGTGCTCCTACTAGGCTTCATCGCCATCTCGTCCGGAGCG
GAGCAGGATGTCCTGGAACTGGGGGACGACGACTTTGCCACCACCCTAAAACAACACGAGACGACGCTGG
TCATGTTCTACGCCCCCTGGGTGAGTTAGTGAGTGGGCGTGGCCGGGCGCCATCCTACAACAAATGCATT
GCGTGTGCGATTCGCGTATAATCCGCTTGTTGCGTCCGCAACTTAATTCGATTCCCAATGCCCAGTGCCG
ACCGATGCCCGATTTTCCGGCCATCGCAAGCGCAAAGTACACGTCAGCGTTTGCCTTGCTTTTGCCGGCA
AATAAAGAAGGAACGAGTGACGGAACGAAACGAGACGCAAAGAGAGGGGCAATGATGATATGCCAGTATT
GGTGCCGTCGGCCATTTTAGAGCGCGGGGTGCCAAAGTGCCTAGTTGGTACAATTTCCGTGCAATTCCTG
CTCCACTTGTCCACTGGCTAATGATAGTGAGCAGGCATAGGGGCCCCAGTGTACATAGCGCATCTTTTAT
TATAGTTTCTTATGCCTATGCATTATACCCATAATCCCAGGAATGTGTAATTCTAAGGGTTAACTTAGAT
TTAGCTTGAAACTGGTTATCATAGTCTGAAACACTCACAGTTCTCATACAAATGTAATTTCCCAACAGTT
TACACCAACCATATTAAAACTTCAATTTTAATTGCCTGCCTACTCACAAAAGTTATGCTGATTGTGCTAT
AAATTTCTCATGTCAGTAGACACATTTTTAACTTCCATCGTAATCCACACTGAACACAATAACCTTTTAA
TACATTTTTGTTTAGTACATAACACATATTTACTGTGCCTATTTACTCTCTTTTGAGCCATTTGTTTGCC
CATTATCAGGGGTAAACTATAAGCTCACACTTGGACAATGAACTAGGTAAATTATTAGCGttttttttGC
CGGCCGGCTTTCGAAATCCCCAAAGAGCTGTTGAACTACTTGGCATGGAGACTTGATTCCCTCATGACGA
ACCTTATCAGTATTCAAGCATAAATACACAGGGATGAAGTTGAAAAAGTTGTACACTGCAATAAAACTGC
TCTTTTCATTATAACATAGTTAACTTAAGGCATCACTTATCTTTATCAAAAATGAATTATTATCTGCACT

GCTAAGAGTGCAGACTATACTCTGCTGTTTCAAATAAGTACCAAGTCGTTTTTCTCTCTGTATGCCTAAT
GGGCACTTGTACTTGTGTGATAAGACAGTTGATAATTCCAGCACCCTTACTTTAATTACAAGCTAGTTTT
TTGGGAACTTGTCTTCCCGTTGCCTTGATAACAAATGATGAATTAGATAGTATTGTTACCAACATAATGT
TTAAGTGACGTGTCCTAACAGAGTGTTTTTTCCTGTTTAAGGGTGCGGCCACTGCAAGCGATTGAAGCCC
GAGTACGCCAAGGCGGCGGAGATCGTGAAAGACGATGATCCGCCAATCAAACTGGCCAAGGTTGACTGTA
CGGAAGCTGGAAAGGAAACGTGTAGCAAGTACTCCGTCAGCGGCTACCCAACGTTGAAGATTTTCCGTCA
GGATGAGGTGTCGCAGGACTACAATGGACCACGCGAAGCCAGCGGCATTGCCAAGTACATGCGCGCCCAA
GTGGGACCCGCCTCCAAGACCGTGCGCACCGTTGCCGAGCTGAAAAAGTTCCTGGACACCAAGGACACCA
CGCTGTTCGGCTACTTCAGCGACAGCGACTCCAAACTGGCCAAGATTTTCCTGAAGTTCGCCGATAAGAA
CCGCGAAAAGTACCGCTTCGGACATTCAAGCGAAAAGGAAGTCCTCGATAAGCAGGGCGAAACGTAAGTG
GGAGGCTCTAATCAAGACTCGTACATCTGTGTATTATGTG
>dme_circ_0003991_junction_seq
CAAGACTCGTACATCTGTGTATTATGTGTTTTTGTTGTAGAAGTAGGCGTTCGACG
>dme_circ_0003992
GAACATGACCAGCGTCGTCTCGTGTTGTTTTAGGGTGGTGGCAAAGTCGTCGTCCCCCAGTTCCAGGACA
TCCTGCTCCGCTCCGGACGAGATGGCGATGAAGCCTAGTAGGAGCACTCCAGCAAGGCGCCACATCATTA
TTTGGGTTCACTTGAAGCGATATCGGTGAATAATTCAAGTTCAACTAGGCCGTCGAACGCCTACTTCTAC
AACAAAAACTGA
>dme_circ_0003992_junction_seq
GAACGCCTACTTCTACAACAAAAACTGAGAACATGACCAGCGTCGTCTCGTGTTGT
>dme_circ_0003993
GCCATAGTGTAATCATCCAACATGGGCAAGGAAAAGATTCACATTAACATTGTCGTGATCGGACACGTCG
ATTCCGGTAAGTCGACCACCACCGGACACTTGATCTACAAGTGCGGTGGTATCGACAAGCGTACCATCGA
GAAGTTCGAGAAGGAGGCCCAGGAGATGGGAAAGGGATCCTTCAAGTACGCCTGG
>dme_circ_0003993_junction_seq
GGGAAAGGGATCCTTCAAGTACGCCTGGGCCATAGTGTAATCATCCAACATGGGCA
>dme_circ_0003994
GCGTACTTGAAGGATCCCTTTCCCATCTCCTGGGCCTCCTTCTCGAACTTCTCGATGGTACGCTTGTCGA
TACCACCGCACTTGTAGATCAAGTGTCCGGTGGTGGTCGACTTACCGGAATCGACGTGTCCGATCACGAC
AATGTTAATGTGAATCTTTTCCTTGCCCATG
>dme_circ_0003994_junction_seq
GTTAATGTGAATCTTTTCCTTGCCCATGGCGTACTTGAAGGATCCCTTTCCCATCT
>dme_circ_0003995
GATTCACATTAACATTGTCGTGATCGGACACGTCGATTCCGGTAAGTCGACCACCACCGGACACTTGATC
TACAAGTGCGGTGGTATCGACAAGCGTACCATCGAGAAGTTCGAGAAGGAGGCCCAGGAGATGGGAAAGG
GATCCTTCAAGTACGCCTGGGTTTTGGATAA
>dme_circ_0003995_junction_seq
CCTTCAAGTACGCCTGGGTTTTGGATAAGATTCACATTAACATTGTCGTGATCGGA
>dme_circ_0003996
GTCGACCACCACCGGACACTTGATCTACAAGTGCGGTGGTATCGACAAGCGTACCATCGAGAAGTTCGAG
AAGGAGGCCCAGGAGATGGGAAAGGGATCCTTCAAGTACGCCTGGGTTTTGGATAAGTTGAAGGCTGAGC
GCGAGCGTGGTATCACCATCGATATCGCCCTGTGGAAGTTCGAAACTGCCAAGTACTAC
>dme_circ_0003996_junction_seq
GTGGAAGTTCGAAACTGCCAAGTACTACGTCGACCACCACCGGACACTTGATCTAC

>dme_circ_0003997
GCGTACCATCGAGAAGTTCGAGAAGGAGGCCCAGGAGATGGGAAAGGGATCCTTCAAGTACGCCTGGGTT
TTGGATAAGTTGAAGGCTGAGCGCGAGCGTGGTATCACCATCGATATCGCCCTGTGGAAGTTCGAAACTG
CCAAGTACTAC
>dme_circ_0003997_junction_seq
GTGGAAGTTCGAAACTGCCAAGTACTACGCGTACCATCGAGAAGTTCGAGAAGGAG
>dme_circ_0003998
GGCCCGTTATGAGGAAATCAAGAAGGAAGTGTCCTCTTACATCAAGAAGATCGGCTACAACCCAGCCGCC
GTTGCCTTCGTGCCCATTTCCGGATGGCACGGCGACAACATGTTGGAACCCTCTACCAACATGCCCTGGT
TCAAGGGATGGAAGGTGGAGCGCAAGGAGG
>dme_circ_0003998_junction_seq
AAGGGATGGAAGGTGGAGCGCAAGGAGGGGCCCGTTATGAGGAAATCAAGAAGGAA
>dme_circ_0003999
GGCCCGTTATGAGGAAATCAAGAAGGAAGTGTCCTCTTACATCAAGAAGATCGGCTACAACCCAGCCGCC
GTTGCCTTCGTGCCCATTTCCGGATGGCACGGCGACAACATGTTGGAACCCTCTACCAACATGCCCTGGT
TCAAGGGATGGAAGGTGGAGCGCAAGGAGGGTAACGCTGACGGCAAGACCCTGATCGATGCCCTCGATGC
CATCCTTCCCCCAGCCCGTCCCACCGACAAGGCCCTGCGTCTGCCCCTGCAGGATGTGTACAAAATTGGC
GGTATTGGAACAGTACCCGTGGGTCGTGTGGAGACTGGTGTGCTGAAGCCCGGTACCGTTGTGGTCTTCG
CCCCTGCTAACATCACCACTGAGGTCAA
>dme_circ_0003999_junction_seq
CCCCTGCTAACATCACCACTGAGGTCAAGGCCCGTTATGAGGAAATCAAGAAGGAA
>dme_circ_0004000
GAAGGAAGTGTCCTCTTACATCAAGAAGATCGGCTACAACCCAGCCGCCGTTGCCTTCGTGCCCATTTCC
GGATGGCACGGCGACAACATGTTGGAACCCTCTACCAACATGCCCTGGTTCAAGGGATGGAAG
>dme_circ_0004000_junction_seq
CAACATGCCCTGGTTCAAGGGATGGAAGGAAGGAAGTGTCCTCTTACATCAAGAAG
>dme_circ_0004001
GTGTCCTCTTACATCAAGAAGATCGGCTACAACCCAGCCGCCGTTGCCTTCGTGCCCATTTCCGGATGGC
ACGGCGACAACATGTTGGAACCCTCTACCAACATGCCCTGGTTCAAGGGATGGAAG
>dme_circ_0004001_junction_seq
CAACATGCCCTGGTTCAAGGGATGGAAGGTGTCCTCTTACATCAAGAAGATCGGCT
>dme_circ_0004002
GTGTCCTCTTACATCAAGAAGATCGGCTACAACCCAGCCGCCGTTGCCTTCGTGCCCATTTCCGGATGGC
ACGGCGACAACATGTTGGAACCCTCTACCAACATGCCCTGGTTCAAGGGATGGAAGGTGGAGCGCAAGGA
GG
>dme_circ_0004002_junction_seq
AAGGGATGGAAGGTGGAGCGCAAGGAGGGTGTCCTCTTACATCAAGAAGATCGGCT
>dme_circ_0004003
GTGTCCTCTTACATCAAGAAGATCGGCTACAACCCAGCCGCCGTTGCCTTCGTGCCCATTTCCGGATGGC
ACGGCGACAACATGTTGGAACCCTCTACCAACATGCCCTGGTTCAAGGGATGGAAGGTGGAGCGCAAGGA
GGGTAACGCTGACGGCAAGACCCTGATCGATGCCCTCGATGCCATCCTTCCCCCAGCCCGTCCCACCGAC
AAGGCCCTGCGTCTGCCCCTGCAGGATGTGTACAAAATTGGCGGTATTGGAACAGTACCCGTGGGTCGTG
TGGAGACTGGTGTGCTGAAGCCCGGTACCGTTGTGGTCTTCGCCCCTGCTAACATCACCACTGAGGTCAA
GTCCGTGGAGATGCACCACGAGGCCCTGCAGGAGGCC

>dme_circ_0004003_junction_seq
GATGCACCACGAGGCCCTGCAGGAGGCCGTGTCCTCTTACATCAAGAAGATCGGCT
>dme_circ_0004004
GGCATCGATCAGGGTCTTGCCGTCAGCGTTACCCTCCTTGCGCTCCACCTTCCATCCCTTGAACCAGGGC
ATGTTGGTAGAGGGTTCCAACATGTTGTCGCCGTGCCATCCGGAAATGGGCACGAAGGCAACGGCGGCTG
GGTTGTAGCCGATCTTCTTGATG
>dme_circ_0004004_junction_seq
GGCTGGGTTGTAGCCGATCTTCTTGATGGGCATCGATCAGGGTCTTGCCGTCAGCG
>dme_circ_0004005
GATCGGCTACAACCCAGCCGCCGTTGCCTTCGTGCCCATTTCCGGATGGCACGGCGACAACATGTTGGAA
CCCTCTACCAACATGCCCTGGTTCAAGGGATGGAAGGTGGAGCGCAAGGAGGGTAACGCTGACGGCAAGA
CCCTGATCGATGCCCTCGATGCCATCCTTCCCCCAGCCCGTCCCACCGACAAGGCCCTGCGTCTGCCCCT
GCAGGATGT
>dme_circ_0004005_junction_seq
AGGCCCTGCGTCTGCCCCTGCAGGATGTGATCGGCTACAACCCAGCCGCCGTTGCC
>dme_circ_0004006
CAGGGGCGAAGACCACAACGGTACCGGGCTTCAGCACACCAGTCTCCACACGACCCACGGGTACTGTTCC
AATACCGCCAATTTTGTACACATCCTGCAGGGGCAGACGCAGGGCCTTGTCGGTGGGACGGGCTGGGGGA
AGGATGGCATCGAGGGCATCGATCAGGGTCTTGCCGTCAGCGTTACCCTCCTTGCGCTCCACCTTCCATC
CCTTGAACCAGGGCATG
>dme_circ_0004006_junction_seq
CACCTTCCATCCCTTGAACCAGGGCATGCAGGGGCGAAGACCACAACGGTACCGGG
>dme_circ_0004007
GCGGCCTCGTCTGCATTTAGCATTCGTATCCGTCGAAACTCCTTTCGATGGAAGAAATTAAACTTATGCT
TCTGCTGAAACATCATCTTATCGGTCATCTTGCGCAGGACAGGTGTTGGTGTTATACTACTATTGTGTTC
GCTGCTGCTGTTGCTGGTATTATTGAGTGGTGTTGTTGGCTGCATTATGATGTTGGTTATATGCTTGTTG
TTGCTGCTACTACTGCGTGTTGTTGTTGTTGTTGAGTGGAAGCAAACCAGCTACTTCTTGCCCTTGGTGG
CCTTCTCGGCGGCCTTGGTGACCTTGCCACCGGAGGCATCCTTGAAGTTGACAGCCTTAATGACACCGAC
AGCCACGGTCTGCCTCATGTCACGCACAGCGAAGCGACCCAGAGGGGGGAACTCCTGGAAGGCCTCCACG
CACAGGGGCTTAGAGGGCACCAGGTTGACGATGGCAGCATCGCCAGACTTGATGAACTTGGGGTTCTCCT
CGGTGGTCTTGCCGGAACGACGGTCGACCTTCTCCTTGATCTCAGCGAACTTGCAAGCAATGTGAGCGGT
GTGGCAATCCAACACGGGGGTGTAGCCGTTGGCAATCTGACCGGGGTGGTTCAGCACGATGACCTGGGCG
GTGAAGTCGGCGGCTCCCTTGGGGGGGTTAGCCTTGGAGTCACCGGCAACGTAGCCACGACGCAGCTCCT
TCACGGACACGTTCTTGACGTTGAAGCCAACGTTGTCTCCGGGAACGGCCTCCTGCAGGGCCTCGTGGTG
CATCTCCACGGACTTGACCTCAGTGGTGATGTTAGCAGGGGCGAAGACCACAACGGTACCGGGCTTCAGC
ACACCAGTCTCCACACGACCCACGGGTACTGTTCCAATACCGCCAATTTTGTACACATCCTGCAGGGGCA
GACGCAGGGCCTTGTCGGTGGGACGGGCTGGGGGAAGGATGGCATCGAGGGCATCGATCAGGGTCTTGCC
GTCAGCGTTACCCTCCTTGCGCTCCACCTTCCATCCCTTGAACCAGGGCATG
>dme_circ_0004007_junction_seq
CACCTTCCATCCCTTGAACCAGGGCATGGCGGCCTCGTCTGCATTTAGCATTCGTA
>dme_circ_0004008
GGGATGGAAGGTGGAGCGCAAGGAGGGTAACGCTGACGGCAAGACCCTGATCGATGCCCTCGATGCCATC
CTTCCCCCAGCCCGTCCCACCGACAAGGCCCTGCGTCTGCCCCTGCAGGATGTGTACAAAATTGGCGGTA
TTGGAACAGTACCCGTGGGTCGTGTGGAGACTGGTGTGCTGAAGCCCG

>dme_circ_0004008_junction_seq
CGTGTGGAGACTGGTGTGCTGAAGCCCGGGGATGGAAGGTGGAGCGCAAGGAGGGT
>dme_circ_0004009
ATCGACGGGGAAGTGGATGAGGAGTGGATGCATAAATGCAAGTGTTTTCCCAGTGTGCACTCTTATCCTT
GGGATCCACCGTTTCCCAATTTAGGCGGCCTCGTCTGCATTTAGCATTCGTATCCGTCGAAACTCCTTTC
GATGGAAGAAATTAAACTTATGCTTCTGCTGAAACATCATCTTATCGGTCATCTTGCGCAGGACAGGTGT
TGGTGTTATACTACTATTGTGTTCGCTGCTGCTGTTGCTGGTATTATTGAGTGGTGTTGTTGGCTGCATT
ATGATGTTGGTTATATGCTTGTTGTTGCTGCTACTACTGCGTGTTGTTGTTGTTGTTGAGTGGAAGCAAA
CCAGCTACTTCTTGCCCTTGGTGGCCTTCTCGGCGGCCTTGGTGACCTTGCCACCGGAGGCATCCTTGAA
GTTGACAGCCTTAATGACACCGACAGCCACGGTCTGCCTCATGTCACGCACAGCGAAGCGACCCAGAGGG
GGGAACTCCTGGAAGGCCTCCACGCACAGGGGCTTAGAGGGCACCAGGTTGACGATGGCAGCATCGCCAG
ACTTGATGAACTTGGGGTTCTCCTCGGTGGTCTTGCCGGAACGACGGTCGACCTTCTCCTTGATCTCAGC
GAACTTGCAAGCAATGTGAGCGGTGTGGCAATCCAACACGGGGGTGTAGCCGTTGGCAATCTGACCGGGG
TGGTTCAGCACGATGACCTGGGCGGTGAAGTCGGCGGCTCCCTTGGGGGGGTTAGCCTTGGAGTCACCGG
CAACGTAGCCACGACGCAGCTCCTTCACGGACACGTTCTTGACGTTGAAGCCAACGTTGTCTCCGGGAAC
GGCCTCCTGCAGGGCCTCGTGGTGCATCTCCACGGACTTGACCTCAGTGGTGATGTTAGCAGGGGCGAAG
ACCACAACGGTACCGGGCTTCAGCACACCAGTCTCCACACGACCCACGGGTACTGTTCCAATACCGCCAA
TTTTGTACACATCCTGCAGGGGCAGACGCAGGGCCTTGTCGGTGGGACGGGCTGGGGGAAGGATGGCATC
GAGGGCATCGATCAGGGTCTTGCCGTCAGCG
>dme_circ_0004009_junction_seq
GGCATCGATCAGGGTCTTGCCGTCAGCGATCGACGGGGAAGTGGATGAGGAGTGGA
>dme_circ_0004010
GGCCCTGCGTCTGCCCCTGCAGGATGTGTACAAAATTGGCGGTATTGGAACAGTACCCGTGGGTCGTGTG
GAGACTGGTGTGCTGAAGCCCGGTACCGTTGTGGTCTTCGCCCCTGCTAACATCACCACTGAGGTCAAGT
CCGTGGAGATGCACCACGAGGCCCTGCAGGAGGCCGTTCCCGGAGACAAC
>dme_circ_0004010_junction_seq
CCTGCAGGAGGCCGTTCCCGGAGACAACGGCCCTGCGTCTGCCCCTGCAGGATGTG
>dme_circ_0004011
GCCTCCACGCACAGGGGCTTAGAGGGCACCAGGTTGACGATGGCAGCATCGCCAGACTTGATGAACTTGG
GGTTCTCCTCGGTGGTCTTGCCGGAACGACGGTCGACCTTCTCCTTGATCTCAGCGAACTTGCAAGCAAT
GTGAGCGGTGTGGCAATCCAACACGGGGGTGTAGCCGTTGGCAATCTGACCGGGGTGGTTCAGCACGATG
ACCTGGGCGGTGAAGTCGGCGGCTCCCTTGGGGGGGTTAGCCTTGGAGTCACCGGCAACGTAGCCACGAC
GCAGCTCCTTCACGGACACGTTCTTGACGTTGAAGCCAACGTTGTCTCCGGGAACGGCCTCCTGCAGGGC
CTCGTGGTGCATCTCCACGGACTTGACCTCAGTGGTGATGTTAGCAGGGGCGAAGACCACAACGGTACCG
GGCTTCAGCACACCAG
>dme_circ_0004011_junction_seq
ACAACGGTACCGGGCTTCAGCACACCAGGCCTCCACGCACAGGGGCTTAGAGGGCA
>dme_circ_0004012
CACGATGACCTGGGCGGTGAAGTCGGCGGCTCCCTTGGGGGGGTTAGCCTTGGAGTCACCGGCAACGTAG
CCACGACGCAGCTCCTTCACGGACACGTTCTTGACGTTGAAGCCAACGTTGTCTCCGGGAACGGCCTCCT
GCAGGGCCTCGTGGTGCATCTCCACGGACTTGACCTCAGTGGTGATG
>dme_circ_0004012_junction_seq
CTCCACGGACTTGACCTCAGTGGTGATGCACGATGACCTGGGCGGTGAAGTCGGCG
>dme_circ_0004013

CGAACTTGCAAGCAATGTGAGCGGTGTGGCAATCCAACACGGGGGTGTAGCCGTTGGCAATCTGACCGGG
GTGGTTCAGCACGATGACCTGGGCGGTGAAGTCGGCGGCTCCCTTGGGGGGGTTAGCCTTGGAGTCACCG
GCAACGTAGCCACGACGCAGCTCCTTCACGGACACGTTCTTGACGTTGAAGCCAACGTTGTCTCCGGGAA
CGGCCTCCTGCAGGGCCTCGTGGTGCATCTCCACGGACTTGACCTCAGTGGTGATG
>dme_circ_0004013_junction_seq
CTCCACGGACTTGACCTCAGTGGTGATGCGAACTTGCAAGCAATGTGAGCGGTGTG
>dme_circ_0004014
GGTCAAGTCCGTGGAGATGCACCACGAGGCCCTGCAGGAGGCCGTTCCCGGAGACAACGTTGGCTTCAAC
GTCAAGAACGTGTCCGTGAAGGAGCTGCGTCGTGGCTACGTTGCCGGTGACTCCAAGGCTAACCCCCCCA
AGGGAGCCGCCGACTTCACCGCCCAGGTCATCGTGCTGAACCACCCCG
>dme_circ_0004014_junction_seq
GCCCAGGTCATCGTGCTGAACCACCCCGGGTCAAGTCCGTGGAGATGCACCACGAG
>dme_circ_0004015
GTCCGTGGAGATGCACCACGAGGCCCTGCAGGAGGCCGTTCCCGGAGACAACGTTGGCTTCAACGTCAAG
AACGTGTCCGTGAAGGAGCTGCGTCGTGGCTACGTTGCCGGTGACTCCAAGGCTAACCCCCCCAAGGGAG
CCGCCGACTTCACCGCCCAG
>dme_circ_0004015_junction_seq
CAAGGGAGCCGCCGACTTCACCGCCCAGGTCCGTGGAGATGCACCACGAGGCCCTG
>dme_circ_0004016
GATGCACCACGAGGCCCTGCAGGAGGCCGTTCCCGGAGACAACGTTGGCTTCAACGTCAAGAACGTGTCC
GTGAAGGAGCTGCGTCGTGGCTACGTTGCCGGTGACTCCAAGGCTAACCCCCCCAAGGGAGCCGCCGACT
TCACCGCCCAGGTCATC
>dme_circ_0004016_junction_seq
AGCCGCCGACTTCACCGCCCAGGTCATCGATGCACCACGAGGCCCTGCAGGAGGCC
>dme_circ_0004017
GATGCACCACGAGGCCCTGCAGGAGGCCGTTCCCGGAGACAACGTTGGCTTCAACGTCAAGAACGTGTCC
GTGAAGGAGCTGCGTCGTGGCTACGTTGCCGGTGACTCCAAGGCTAACCCCCCCAAGGGAGCCGCCGACT
TCACCGCCCAGGTCATCGTGCTGAACCACCCCG
>dme_circ_0004017_junction_seq
GCCCAGGTCATCGTGCTGAACCACCCCGGATGCACCACGAGGCCCTGCAGGAGGCC
>dme_circ_0004018
GATGCACCACGAGGCCCTGCAGGAGGCCGTTCCCGGAGACAACGTTGGCTTCAACGTCAAGAACGTGTCC
GTGAAGGAGCTGCGTCGTGGCTACGTTGCCGGTGACTCCAAGGCTAACCCCCCCAAGGGAGCCGCCGACT
TCACCGCCCAGGTCATCGTGCTGAACCACCCCGGTCAGATTGCCAACGGCTACACCCCCGTGTTGGATTG
CCACACCGCTCACATTGCTTGCAAGTTCGCTGAGATCAAGGAGAAGGTCGACCGTCGTTCCGGCAAGACC
ACCGAGGAGAACCCCAA
>dme_circ_0004018_junction_seq
CCGGCAAGACCACCGAGGAGAACCCCAAGATGCACCACGAGGCCCTGCAGGAGGCC
>dme_circ_0004019
GGAGGCCGTTCCCGGAGACAACGTTGGCTTCAACGTCAAGAACGTGTCCGTGAAGGAGCTGCGTCGTGGC
TACGTTGCCGGTGACTCCAAGGCTAACCCCCCCAAGGGAGCCGCCGACTTCACCGCCCAGGTCATCGTGC
TGAACCACCCCG
>dme_circ_0004019_junction_seq
GCCCAGGTCATCGTGCTGAACCACCCCGGGAGGCCGTTCCCGGAGACAACGTTGGC

>dme_circ_0004020
CGAACTTGCAAGCAATGTGAGCGGTGTGGCAATCCAACACGGGGGTGTAGCCGTTGGCAATCTGACCGGG
GTGGTTCAGCACGATGACCTGGGCGGTGAAGTCGGCGGCTCCCTTGGGGGGGTTAGCCTTGGAGTCACCG
GCAACGTAGCCACGACGCAGCTCCTTCACGGACACGTTCTTGACGTTGAAGCCAACG
>dme_circ_0004020_junction_seq
GGACACGTTCTTGACGTTGAAGCCAACGCGAACTTGCAAGCAATGTGAGCGGTGTG
>dme_circ_0004021
GTTCGCTGAGATCAAGGAGAAGGTCGACCGTCGTTCCGGCAAGACCACCGAGGAGAACCCCAAGTTCATC
AAGTCTGGCGATGCTGCCATCGTCAACCTGGTGCCCTCTAAGCCCCTGTGCGTGGAGGCCTTCCAGGAGT
TCCCCCCTCTGGGTCGCTTCGCTGTGCGTGACATGAGGCAGACCGTGGCTGTCGGTGTCATTAAGGCTGT
CAACTTCAAGGATGCCTCCGGTGGCAAGGTCACCAAGGCCGCCGAGAAGGCCACCAAGGGCAAGAAGTAG
CTGGTTTGCTTCCACTCAACAACAACAACAACACGCAGTAGTAGCAGCAACAACAAGCATATAACCAACA
TCATAATGCAGCCAACAACACCACTCAATAATACCAGCAACAGCAGCAGCGAACACAATAGTA
>dme_circ_0004021_junction_seq
AGCAACAGCAGCAGCGAACACAATAGTAGTTCGCTGAGATCAAGGAGAAGGTCGAC
>dme_circ_0004022
GCGGCCTCGTCTGCATTTAGCATTCGTATCCGTCGAAACTCCTTTCGATGGAAGAAATTAAACTTATGCT
TCTGCTGAAACATCATCTTATCGGTCATCTTGCGCAGGACAGGTGTTGGTGTTATACTACTATTGTGTTC
GCTGCTGCTGTTGCTGGTATTATTGAGTGGTGTTGTTGGCTGCATTATGATGTTGGTTATATGCTTGTTG
TTGCTGCTACTACTGCGTGTTGTTGTTGTTGTTGAGTGGAAGCAAACCAGCTACTTCTTGCCCCTTGGTGG
CCTTCTCGGCGGCCTTGGTGACCTTGCCACCGGAGGCATCCTTGAAGTTGACAGCCTTAATGACACCGAC
AGCCACGGTCTGCCTCATGTCACGCACAGCGAAGCGACCCAGAGGGGGGAACTCCTGGAAGGCCTCCACG
CACAGGGGCTTAGAGGGCACCAGGTTGACGATGGCAGCATCGCCAGACTTGATGAACTTGGGGTTCTCCT
CGG
>dme_circ_0004022_junction_seq
GACTTGATGAACTTGGGGTTCTCCTCGGGCGGCCTCGTCTGCATTTAGCATTCGTA
>dme_circ_0004023
GCATCCTTGAAGTTGACAGCCTTAATGACACCGACAGCCACGGTCTGCCTCATGTCACGCACAGCGAAGC
GACCCAGAGGGGGGAACTCCTGGAAGGCCTCCACGCACAGGGGCTTAGAGGGCACCAGGTTGACGATGGC
AGCATCGCCAGACTTGATGAACTTGGGG
>dme_circ_0004023_junction_seq
AGCATCGCCAGACTTGATGAACTTGGGGGCATCCTTGAAGTTGACAGCCTTAATGA
>dme_circ_0004024
GTTCATCAAGTCTGGCGATGCTGCCATCGTCAACCTGGTGCCCTCTAAGCCCCTGTGCGTGGAGGCCTTC
CAGGAGTTCCCCCCTCTGGGTCGCTTCGCTGTGCGTGACATGAGGCAGACCGTGGCTGTCG
>dme_circ_0004024_junction_seq
CGTGACATGAGGCAGACCGTGGCTGTCGGTTCATCAAGTCTGGCGATGCTGCCATC
>dme_circ_0004025
GTTCATCAAGTCTGGCGATGCTGCCATCGTCAACCTGGTGCCCTCTAAGCCCCTGTGCGTGGAGGCCTTC
CAGGAGTTCCCCCCTCTGGGTCGCTTCGCTGTGCGTGACATGAGGCAGACCGTGGCTGTCGGTGTCATTA
AGGCT
>dme_circ_0004025_junction_seq
GACCGTGGCTGTCGGTGTCATTAAGGCTGTTCATCAAGTCTGGCGATGCTGCCATC
>dme_circ_0004026

GTTCATCAAGTCTGGCGATGCTGCCATCGTCAACCTGGTGCCCTCTAAGCCCCTGTGCGTGGAGGCCTTC
CAGGAGTTCCCCCCTCTGGGTCGCTTCGCTGTGCGTGACATGAGGCAGACCGTGGCTGTCGGTGTCATTA
AGGCTGTCAACTTCAAGGATGCCTCCGGTGGCAAGGTCACCAAGGCCGCCGAGAAGGCCACCAAGGGCAA
GAA
>dme_circ_0004026_junction_seq
CCGCCGAGAAGGCCACCAAGGGCAAGAAGTTCATCAAGTCTGGCGATGCTGCCATC
>dme_circ_0004027
TGTGCACTCTTATCCTTGGGATCCACCGTTTCCCAATTTAGGCGGCCTCGTCTGCATTTAGCATTCGTAT
CCGTCGAAACTCCTTTCGATGGAAGAAATTAAACTTATGCTTCTGCTGAAACATCATCTTATCGGTCATC
TTGCGCAGGACAGGTGTTGGTGTTATACTACTATTGTGTTCGCTGCTGCTGTTGCTGG
>dme_circ_0004027_junction_seq
CTATTGTGTTCGCTGCTGCTGTTGCTGGTGTGCACTCTTATCCTTGGGATCCACCG
>dme_circ_0004028
GCAGCAGCGAACACAATAGTAGTATAACACCAACACCTGTCCTGCGCAAGATGACCGATAAGATGATGTT
TCAGCAGAAGCATAAGTTTAATTTCTTCCATCGAAAGGAGTTTCGACGGATACGAATGCTAAATGCAGAC
GAGGCCGCCTAAATTGGGAAACGGTGGATCCCAAGGATAAGA
>dme_circ_0004028_junction_seq
TGGGAAACGGTGGATCCCAAGGATAAGAGCAGCAGCGAACACAATAGTAGTATAAC
>dme_circ_0004029
TGTGCACTCTTATCCTTGGGATCCACCGTTTCCCAATTTAGGCGGCCTCGTCTGCATTTAGCATTCGTAT
CCGTCGAAACTCCTTTCGATGGAAGAAATTAAACTTATGCTTCTGCTGAAACATCATCTTATCGGTCATC
TTGCGCAGGACAGGTGTTGGTG
>dme_circ_0004029_junction_seq
GTCATCTTGCGCAGGACAGGTGTTGGTGTGTGCACTCTTATCCTTGGGATCCACCG
>dme_circ_0004030
TGGATGAGGAGTGGATGCATAAATGCAAGTGTTTTCCCAGTGTGCACTCTTATCCTTGGGATCCACCGTT
TCCCAATTTAGGCGGCCTCGTCTGCATTTAGCATTCGTATCCGTCGAAACTCCTTTCGATGGAAGAAATT
AAACTTATGCTTCTGCTGAAACATCATCTTATCGGTCATCTTGCGCAGGACAGGTG
>dme_circ_0004030_junction_seq
TTATCGGTCATCTTGCGCAGGACAGGTGTGGATGAGGAGTGGATGCATAAATGCAA
>dme_circ_0004031
TGGATGAGGAGTGGATGCATAAATGCAAGTGTTTTCCCAGTGTGCACTCTTATCCTTGGGATCCACCGTT
TCCCAATTTAGGCGGCCTCGTCTGCATTTAGCATTCGTATCCGTCGAAACTCCTTTCGATGGAAGAAATT
AAACTTATGCTTCTGCTGAAACATCATCTTATCGG
>dme_circ_0004031_junction_seq
TGCTTCTGCTGAAACATCATCTTATCGGTGGATGAGGAGTGGATGCATAAATGCAA
>dme_circ_0004032
TAAACTAAAGATCGACGGGGAAGTGGATGAGGAGTGGATGCATAAATGCAAGTGTTTTCCCAGTGTGCAC
TCTTATCCTTGGGATCCACCGTTTCCCAATTTAGGCGGCCTCGTCTGCATTTAGCATTCGTATCCGTCGA
AACTCCTTTCGATGGAAGAAATTAAACTTATGCTTCTGCTGAAACATCATCTTATCGG
>dme_circ_0004032_junction_seq
TGCTTCTGCTGAAACATCATCTTATCGGTAAACTAAAGATCGACGGGGAAGTGGAT
>dme_circ_0004033
GGTAAGATAAATGATACATATCAAAGTATAAAGGTTAATTTAAACGATATCATAAATGCTACAGAGGATT

CCTATATATGATATGATAAAACGAAGTCAACTCCGTGCATCATACCATATTTAGTAAACTAAAGATCGAC
GGGGAAGTGGATGAGGAGTGGATGCATAAATGCAAGTGTTTTCCCAGTGTGCACTCTTATCCTTGGGATC
CACCGTTTCCCAATTTAGGCGGCCTCGTCTGCATTTAGCATTCGTATCCGTCGAAACTCCTTTCGATGGA
AGAAATTAAACTTATGCTTCTGCTGAAACATCATCTTATCGG
>dme_circ_0004033_junction_seq
TGCTTCTGCTGAAACATCATCTTATCGGGGTAAGATAAATGATACATATCAAAGTA
>dme_circ_0004034
GATGATGTTTCAGCAGAAGCATAAGTTTAATTTCTTCCATCGAAAGGAGTTTCGACGGATACGAATGCTA
AATGCAGACGAGGCCGCCTAAATTGGGAAACGGTGGATCCCAAGGATAAGAGTGCACACTGGGAAAACAC
TTGCATTTATGCATCCACTCCTCATCCACTTCCCCGTCGATCTTTAGTTTACTAAATATG
>dme_circ_0004034_junction_seq
CCCGTCGATCTTTAGTTTACTAAATATGGATGATGTTTCAGCAGAAGCATAAGTTT
>dme_circ_0004035
GATGATGTTTCAGCAGAAGCATAAGTTTAATTTCTTCCATCGAAAGGAGTTTCGACGGATACGAATGCTA
AATGCAGACGAGGCCGCCTAAATTGGGAAACGGTGGATCCCAAGGATAAGAGTGCACACTGGGAAAACAC
TTGCATTTATGCATCCACTCCTCATCCACTTCCCCGTCGATCTTTAGTTTACTAAATATGGTATGATGCA
CGGAGTTGACTTCGTTTTATCATATCATATATAGGAATCCTCTGTAGCATTTATGATATC
>dme_circ_0004035_junction_seq
AGGAATCCTCTGTAGCATTTATGATATCGATGATGTTTCAGCAGAAGCATAAGTTT
>dme_circ_0004036
GTTTAATTTCTTCCATCGAAAGGAGTTTCGACGGATACGAATGCTAAATGCAGACGAGGCCGCCTAAATT
GGGAAACGGTGGATCCCAAGGATAAGAGTGCACACTGGGAAAACACTTGCATTTATGCATCCACTCCTCA
TCCACTTCCCCGTCGATCTTTAGTTTACTAAATATGGTATGATGCACGGAGTTGACTTCGTTTTATCATA
TCATATATAGGAATCCTCT
>dme_circ_0004036_junction_seq
TTTATCATATCATATATAGGAATCCTCTGTTTAATTTCTTCCATCGAAAGGAGTTT
>dme_circ_0004037
GTTTAATTTCTTCCATCGAAAGGAGTTTCGACGGATACGAATGCTAAATGCAGACGAGGCCGCCTAAATT
GGGAAACGGTGGATCCCAAGGATAAGAGTGCACACTGGGAAAACACTTGCATTTATGCATCCACTCCTCA
TCCACTTCCCCGTCGATCTTTAGTTTACTAAATATGGTATGATGCACGGAGTTGACTTCGTTTTATCATA
TCATATATAGGAATCCTCTGTAGCATTTATGATATCGTTTAAATTAACCTTTATACTTTGATAT
>dme_circ_0004037_junction_seq
GTTTAAATTAACCTTTATACTTTGATATGTTTAATTTCTTCCATCGAAAGGAGTTT
>dme_circ_0004038
GATTCCTATATATGATATGATAAAACGAAGTCAACTCCGTGCATCATACCATATTTAGTAAACTAAAGAT
CGACGGGGAAGTGGATGAGGAGTGGATGCATAAATGCAAGTGTTTTCCCAGTGTGCACTCTTATCCTTGG
GATCCACCGTTTCCCAATTTAGGCGGCCTCG
>dme_circ_0004038_junction_seq
CCACCGTTTCCCAATTTAGGCGGCCTCGGATTCCTATATATGATATGATAAAACGA
>dme_circ_0004039
GAGTGCACACTGGGAAAACACTTGCATTTATGCATCCACTCCTCATCCACTTCCCCGTCGATCTTTAGTT
TACTAAATATGGTATGATGCACGGAGTTGACTTCGTTTTATCATATCATATATAGGAATCCTCT
>dme_circ_0004039_junction_seq
TTTATCATATCATATATAGGAATCCTCTGAGTGCACACTGGGAAAACACTTGCATT

>dme_circ_0004040
ACAGCAGAGTTTTGCGTTAAGTACAGTGGAATCTTTGAGTTTAGCACTAGATTATGAAATCTGCGAGGCG
GGCGAAGGCGAGCCGTTCGTTCCTCACGACAGCGCCACCTCCAGACACATCATGATCAGGAAGCCGGATA
CAGTGCCCCACGTGGCGATCGTTCCATTTCCGCTGGCATGAGCCTCCGGCAGAATGTCGTCCGACACAAT
GTATATCATGGCTCCGGCCGCAAAAGACAAGGCGTATGGCAGGATCAGGTTGGCAAAGGTCACCGCCACG
GCGCCCAGAACTCCAAAAATGGGCTCCACCATTCCGGACAGCTGTCCGTACCACAGAGCCCGCTTCACGC
TGAATCCTGCGGCATGCAGTGGCAAGCTGACAGCCAATCCCTCGGGGAAGTTTTGAATGCCAATGCCAAT
GGCCAAATTGCGGGCGCTCTCAAATGTCGATGAGTTCGTGGAGCCAATGGCACCAAAGCTTACGCCCACA
GCCAGCCCTTCTGGAATGTTGTGGACCGTGATGGCCACGACCAAAAGCATAATCCTCTTCCACTGGGACA
GGGCGTCCTCGGCGCGCTGCTGCTCCTCAGGCGTGGTGTAGGTGCACTGCTCCATCTCGTTGATGCTAGC
TCCCTTCTTTCGGCGTCTCGATTCGCCGCTGTGCTGCACGCTAAGGCAGTCGGAGAAGCTGTCCAGACTC
TTTGAGGCCAACCTATCGGGGGCAACTCCATTGCGCTTAGAGTCCTCGATGGCTATGTCCGCCTTGGCTT
TTGATTGCGTCATCTGGATCATCATGTTGGTGCTGTTAAGGCCCAGGTATGACATCAATTTGTCGCATCC
GTAGACGAAGATCGATCCCAAGAGGAAGCCTCCAGCGACGGGCAGAAAGGCGTATACTCCGTACAAATGG
GAACTCTCGGCCATTTCGATGGCCGGCTTGAGGAGTGACCAAAAGGAGGCTGCTATCATTACGCCAGCTG
CGAATCCCAGGGCGGCGTCCAGAGACCTCCGCTGGTTACCCCGGACGAAGATCACTAGGGCGGCGCCAGC
GGCGGTCAGTCCCCAGGTAAGCAGGGTGCCCAGCAGAGCCTGCGTGACGGGTCCATAGCCTGGAATCATC
TTGCACAGTTAGAGCTGCTTTTCCGTGCCCGTCACCGGCTGG
>dme_circ_0004040_junction_seq
CTGCTTTTCCGTGCCCGTCACCGGCTGGACAGCAGAGTTTTGCGTTAAGTACAGTG
>dme_circ_0004041
GTGCACTGCTCCATCTCGTTGATGCTAGCTCCCTTCTTTCGGCGTCTCGATTCGCCGCTGTGCTGCACGC
TAAGGCAGTCGGAGAAGCTGTCCAGACTCTTTGAGGCCAACCTATCGGGGGCAACTCCATTGCGCTTAGA
GTCCTCGATGGCTATGTCCGCCTTGGCTTTTGATTGCG
>dme_circ_0004041_junction_seq
GCTATGTCCGCCTTGGCTTTTGATTGCGGTGCACTGCTCCATCTCGTTGATGCTAG
>dme_circ_0004042
ATTATGAAATCTGCGAGGCGGGCGAAGGCGAGCCGTTCGTTCCTCACGACAGCGCCACCTCCAGACACAT
CATGATCAGGAAGCCGGATACAGTGCCCCACGTGGCGATCGTTCCATTTCCGCTGGCATGAGCCTCCGGC
AGAATGTCGTCCGACACAATG
>dme_circ_0004042_junction_seq
CTCCGGCAGAATGTCGTCCGACACAATGATTATGAAATCTGCGAGGCGGGCGAAGG
>dme_circ_0004043
GACGGTGACAGCGGCAAATATCACCACTCCGAGGATTGTCACCGTCCAACAGAACTACCAACCGGGACAA
AGTTCCAGCAACAGCGGCAGCAACGGAAGCGTAAGTGGTTCTTCATCAGTGCCACCGACACGTAGTAGCC
TGCCACCTCCCTTGGTTCTCAGTGGATCTGGAATCCGCTACCGCCAGAATGCCCCTGGTGGGGTCTCTGG
TGTGGTACGTCGTACTGTGGTTCCCAACTCTGTAAAAACCACTGGGCCAGTCTTTCACACAATCAATGGG
TTCAAAGTAGATTTAAATAGCGCTGCACAACAGGATTCCTATCGCCTGCCCAATGGTAGACTAATTCAGG
TAAAACGTCAG
>dme_circ_0004043_junction_seq
TGGTAGACTAATTCAGGTAAAACGTCAGGACGGTGACAGCGGCAAATATCACCACT
>dme_circ_0004044
ATTTTTTTCGATTTCTCCAATTAAAGTGGGTCTTTCGGAAGATGTTTCATCTTCCATATTTTCAGTATCA
CCTTGTTCCTTTTCGGACCCGCCATATCCTTTTTCCGATAGCATCTCAGTCTCCTCTTCTATTTCTGTAC

ATTCAGAATCCGGGCCTTTTTACAATCGGATTTCTGATCAGTTTTTGATTCATCCTTACTTTCTCCCTT
TACATTGTTTTCTTTTGTTAAAtgttg
>dme_circ_0004044_junction_seq
TTACATTGTTTTCTTTTGTTAAAtgttgATTTTTTTCGATTTCTCCAATTAAAGTG
>dme_circ_0004045
GGTCCAAGTAAATGATTAGGTGAATGTGTTGAAGGTCTGTGATTGTTCTTAGCCTATGTTAAAATTAAAA
CGGACTACATATAATTGCCGTCAGATTAAAATATAAAGGATGAGAAGCCAGCCCTTGAGAGGTTTAAATT
GCAACAATGATGTCCGGGGCCAGCCCTAGAAATCCGCGTCCAGGGTGAACACGTTGTCCAGGGGGTTGCT
GACCACTCCCATGCGCTGGTACTCCCCCACCTTCTTCTCAAAAAAGTTGGTCTTTCCATCCAGCGATATC
ATCTCCATGAAGTTAAAGGGGTTTTTGGTGTTGTAGATCTTTCCAACGCCCAACTCCACGAGCAAACG
>dme_circ_0004045_junction_seq
TTCCAACGCCCAACTCCACGAGCAAACGGGTCCAAGTAAATGATTAGGTGAATGTG
>dme_circ_0004046
CGGACCGTTTGCTCGTGGAGTTGGGCGTTGGAAAGATCTACAACACCAAAAACCCCTTTAACTTCATGGA
GATGATATCGCTGGATGGAAAGACCAACTTTTTTGAGAAGAAGGTGGGGGAGTACCAGCGCATGGGAGTG
G
>dme_circ_0004046_junction_seq
GTGGGGGAGTACCAGCGCATGGGAGTGGCGGACCGTTTGCTCGTGGAGTTGGGCGT
>dme_circ_0004047
GTGGATTTGTCCAAGGACCTTACTGATTGGCACCGCCTGAAGGACGACGAGCGCCATTTCATCTCGCACG
TGCTTGCCTTCTTCGCCGCCTCCGACGGCATCGTAAACGAGAACCTGGTGGAGCGCTTCAGTCAGGAGG
>dme_circ_0004047_junction_seq
AACCTGGTGGAGCGCTTCAGTCAGGAGGGTGGATTTGTCCAAGGACCTTACTGATT
>dme_circ_0004048
ATTCTGACGGATAGCACCAACAACGTTCGCAAGATGTCCATTGGACACGAGGCAAACGGACAGTTGGCCA
AGGAGTCCTCCACCGTGAATGGCATCGGCAAGTCTGCCAATTCCCTCATGGAGAAGTCCGTAACCCCCTT
CGACCCGTCGCTGGAGCCCCTTCTGCGCGAGAATCCCCGACGCTTCGTCATCTTTCCCATCCAGTACCAC
GACATCTGGCAGATGTACAAGAAGG
>dme_circ_0004048_junction_seq
CACGACATCTGGCAGATGTACAAGAAGGATTCTGACGGATAGCACCAACAACGTTC
>dme_circ_0004049
ATCATTTGCCACACAACTTTCAAACTGTACACTTAATACACGTCGTGGTTTAAGTGAATTTTACCAGAGA
ATCAGAGAAGCGCCCCTACCTGCTAATAATAATCCATTCAAAAACATCTCAAATGGCGTCCAAGGAAAAC
ATTGCTGACAACATGGAGAAG
>dme_circ_0004049_junction_seq
GGAAAACATTGCTGACAACATGGAGAAGATCATTTGCCACACAACTTTCAAACTGT
>dme_circ_0004050
CTTCCGTTTTGGCCAGCTCATCGCGTTTCCAGCGAGTGCCCCAAAGTCAGTCCGTGGTGGAAAATGAATC
CGTGAAATTCGAGTGCGAATCGACCGACTCCTACAGTGAACTGCACTACGACTGGTTGCATAATGGTGAG
CAGTGCGATTTACATATCCCATGGTTGTGCATTAAGCATTCGCCTTCCTTGCACTCCATTCCAGGTCATC
GGATTGCGTATGACAAGCGTGTCCATCAAATCGGTTCCAATTTGCATATCGAGGCGGTGCGGCGCACGGA
GGATGTTGGCAACTATGTTTGCATCGCGACAAATTTGGCCAGCGGCGCCAGGGAGGCGTCTCCGCCCGCC
AAACTGAGCGTGATATG
>dme_circ_0004050_junction_seq

CTCCGCCCGCCAAACTGAGCGTGATATGCTTCCGTTTTGGCCAGCTCATCGCGTTT
>dme_circ_0004051
GATGCACTGTTTGTGTAGTTGTGGACGCACTTCCAGCTCCACTTGTTGCTCCATCAGTGGAAGCCGACGA
CTGGCTGTTTACGAAAATACATCGCACCGCGTGCTTTGCTTGCTTGGGGGTTCCAATAAGTGCGAAGTCC
TTGCACACCGGAGCCAGCTCGTCGAGAATAGCCGGTGTAGGGTCGTCAATGAGTGGCTGATAGCGCCCCA
GATGAGTTAGTG
>dme_circ_0004051_junction_seq
GGCTGATAGCGCCCCAGATGAGTTAGTGGATGCACTGTTTGTGTAGTTGTGGACGC
>dme_circ_0004052
AAACGCGATCGTCGCCACTTTAAGCGCATGAGATTCCCGCCATTTGACGATGAAGAGCCGCCTCTAGACT
ATGCCGACAACGTGCTGGACGTGGAGCCCTTGGAGGCCATTCAAATTGAGCTGGACAATGACGAAGACAA
TGCTG
>dme_circ_0004052_junction_seq
GAGCTGGACAATGACGAAGACAATGCTGAAACGCGATCGTCGCCACTTTAAGCGCA
>dme_circ_0004053
TGAAAATCAGCAAATCAATCCTTTTTGAATCTTCGCGTTGTTTATGTAGTCGAAAATCGTTTTGAAAATT
GTCTTCGGAGCGGCTTCATTTTTGGTAGACTTTCAATTATGGCCAGCCACGTTAAGAGCCTGATCGCAAA
TTACTATATTAAACTATATCCAACAACAACaaaaatcgaacgaaaacgtaaaaaaaagtagaaaactaaa
aaatgtcaaaaaaaaaacaaaacaaaacaaagGAATGTATTAATCCTAAGTTTATTCGTGAATCAATATT
TTTTATTTATGTTTTCATGTCTAATCAACATATATATTTTCTTCATGTTTTTTCTATATATTTTATG
>dme_circ_0004053_junction_seq
TCTTCATGTTTTTTCTATATATTTTATGTGAAAATCAGCAAATCAATCCTTTTTGA
>dme_circ_0004054
GGGACGATTTTCGCCACAGAAACGAAACAAAGAAGAAGTAATATCAGTAGCAATCCAATATTCTGTTTCG
TAGTTTCCGCAAATAACTTTTATTAGCCAGCCGTAGATACAATGACATATTGAGCTCAATATGTGTTCAG
>dme_circ_0004054_junction_seq
TGACATATTGAGCTCAATATGTGTTCAGGGGACGATTTTCGCCACAGAAACGAAAC
>dme_circ_0004055
GGCATTACCATAACGATGCCGTCCACCTCCAGCTTCTCCTCGGCGAACTCCAGAAGCGAGATGAAGGTCT
GCTTGGAGCCGGCCGGAGGAAGGTCCTTCGGTAGAGCGACATACAACAAGTTGTTGACTGGGTTCAGAAT
CGTATTCCAATTGGTGTACTGGTCCTCG
>dme_circ_0004055_junction_seq
CGTATTCCAATTGGTGTACTGGTCCTCGGGCATTACCATAACGATGCCGTCCACCT
>dme_circ_0004056
GGCCGAGGGACTGATCGACAGGGGCATTCATCCCATTCGCATCGCCGATGGCTTTGAATTGGCCGCCCAG
TGCGCCATCAAGCAGCTGGACGCGATCGCCCAACCCTTCCCCGTGGACCCGAAGAACAAGGAGCCACTTA
TCCAGATCGCCATGACCACGCTGGGCAGCAAGATCGTGAACAAGTGCCATCGTCAGATGGCCGAAATGGC
TGTGGATGCTGTGCTGAAT
>dme_circ_0004056_junction_seq
CGAAATGGCTGTGGATGCTGTGCTGAATGGCCGAGGGACTGATCGACAGGGGCATT
>dme_circ_0004057
GCTAAGGAACGTCAAGCTGGCCATTCTCACCTGCCCGTTTGAGCCCCCGAAGCCAAAGACCAAGCATAAG
CTCGATGTTACCTCCGCGGAAGACTACAGGGCGCTGCGCGAGTACGAGCAGGAGAAGTTCACCCAGATGG
TCAAGCAG

>dme_circ_0004057_junction_seq
GGAGAAGTTCACCCAGATGGTCAAGCAGGCTAAGGAACGTCAAGCTGGCCATTCTC
>dme_circ_0004058
GGAGGCCGACCAACTGTCCACGCTGGAGCAGTACGCCTTCCGCGCCTTCTCCGTGGCCCTGGAGAGCATT
CCGCTGGCTCTGGCGGAGAACAGCGGCCTGCATCCCATCGAGACGTTGTCGGAGCTGAAGGCCAGACAG
>dme_circ_0004058_junction_seq
GACGTTGTCGGAGCTGAAGGCCAGACAGGGAGGCCGACCAACTGTCCACGCTGGAG
>dme_circ_0004059
GTCGCTCCAAGGCGGGAGGCGCTGTCACGGGGGATTTGATGGCAGACCTGCATAACAAGCTAATGCTGCG
ACGCAAGGGAATTTCTGGGAGCCAGAATCCCGTGGAGGCCACTGCTGGCAATCCCCTGATGCAGCAGCTC
TCCCGCGTCATACCGCCGCCGGTGCAGCCCCGCAAGGGATCTAAGTCGAGTGACGAACACTCCGAGGACG
ACGAGGATGGGTGGAACTA
>dme_circ_0004059_junction_seq
CCGAGGACGACGAGGATGGGTGGAACTAGTCGCTCCAAGGCGGGAGGCGCTGTCAC
>dme_circ_0004060
ATACTCCAGGGACTGGATTTTCTCGTAGCTCAATGACCCACCGCCCTCCACCAAAGCTTCGTTGATCTCG
TCGCGCAGGCGTTTCTGCATCTCTGGATGCTTGGCCATCTCGTACAGGGCAAAAGACATGGTCGAGGAGG
AGGTCTCGAAACCCGCCGTAAAAAACACGCCCGCCTGAGCCACCAGGAAGTCCTGGTTCTTGGCATAGTG
AGGCTTCGAAGGCTCGGCAGCCGCCTCTTTGCGCAGACTCACCAAGACATCTATGAGATCATTGCGGAGC
AGGCCCGATCGCTCTCGCTCCTCCATAACGTGACCAATGGTGCTGCGCATAAAGTGGGAAAAGTCCGCCG
TGAAGAACTGGATGCGCATCAGCGACACCAGCTTGGGCAGGAAGAAGGCCACAAAGAAGTCCTTGGCACG
CGCTACGGTGAAGGTGAACATCTTGCGTCCATAGTTACGGAACTCTGCGTTGGGATTCTCTAGGCTGTTA
GCACGAATGCCAAATGCAATCGTGGCTATGCTGTCCGTGGAGAACTGAGCGCAGATCTCCTTAATCTCCG
TAATGAAACTCCCAGAGTTCTTCTCTCCACGCCTCTGCAGTGCCAGCTCCAGATCCTTTCCAATCTGCAC
AGCATATATGTATGTTAAATCCCTCATAAGGATAAATAAGTTTCAACACAAATCCTTACCTCCTGCATAA
GGGTGTACATCTGCTTGACCTTGCCGCTGGTGAAAACGGGAGTCAGCTTGGTGCGAATTCCCTTCCAATG
GGCATCCCTGACGAAGAACAGGTTATTATAGCCCAATGGATCGCCATGGGGATCGCAGCGGGCGTATCGG
TTGTGGAACCGATTGAAGTCCTTGATCAGAATGGATTTGATCAGCTCTATG
>dme_circ_0004060_junction_seq
GATCAGAATGGATTTGATCAGCTCTATGATACTCCAGGGACTGGATTTTCTCGTAG
>dme_circ_0004061
GATTAAGGAGATCTGCGCTCAGTTCTCCACGGACAGCATAGCCACGATTGCATTTGGCATTCGTGCTAAC
AGCCTAGAGAATCCCAACGCAGAGTTCCGTAACTATGGACGCAAGATGTTCACCTTCACCGTAGCGCGTG
CCAAGGACTTCTTTGTGGCCTTCTTCCTGCCCAAGCTGGTGTCGCTGATGCGCATCCA
>dme_circ_0004061_junction_seq
CCAAGCTGGTGTCGCTGATGCGCATCCAGATTAAGGAGATCTGCGCTCAGTTCTCC
>dme_circ_0004062
GATTAAGGAGATCTGCGCTCAGTTCTCCACGGACAGCATAGCCACGATTGCATTTGGCATTCGTGCTAAC
AGCCTAGAGAATCCCAACGCAGAGTTCCGTAACTATGGACGCAAGATGTTCACCTTCACCGTAGCGCGTG
CCAAGGACTTCTTTGTGGCCTTCTTCCTGCCCAAGCTGGTGTCGCTGATGCGCATCCAGTTCTTCACGGC
GGACTTTTCCCACTTTATGCGCAGCACCATTGGTCAC
>dme_circ_0004062_junction_seq
CCACTTTATGCGCAGCACCATTGGTCACGATTAAGGAGATCTGCGCTCAGTTCTCC
>dme_circ_0004063

GGACTGGATTTTCTCGTAGCTCAATGACCCACCGCCCTCCACCAAAGCTTCGTTGATCTCGTCGCGCAGG
CGTTTCTGCATCTCTGGATGCTTGGCCATCTCGTACAGGGCAAAAGACATGGTCGAGGAGGAGGTCTCGA
AACCCGCCGTAAAAAACACGCCCGCCTGAGCCACCAGGAAGTCCTGGTTCTTGGCATAGTGAGGCTTCGA
AGGCTCGGCAGCCGCCTCTTTGCGCAGACTCACCAAGACATCTATGAGATCATTGCGGAGCAGGCCCGAT
CGCTCTCGCTCCTCCATAACGTGACCAATGGTGCTGCGCATAAAGTGGGAAAAGTCCGCCGTGAAGAACT
GGATGCGCATCAGCGACACCAGCTTGGGCAGGAAGAAGGCCACAAAGAAGTCCTTGGCACGCGCTACGGT
GAAGG
>dme_circ_0004063_junction_seq
AAGTCCTTGGCACGCGCTACGGTGAAGGGGACTGGATTTTCTCGTAGCTCAATGAC
>dme_circ_0004064
ATGGGTATGAACACAGGGGTTCCGTTCTCGAGAGTGTAGTCGTAGAACGGCTTCAGGCTCAAGTCTGGCT
GTCCCTCCACGCTCTCGTACTCGCGGTCCAGGAACGGCAGCACCGGATACATGCGCAGCACCTCGTCCAC
CACCATGGCCAGATACTCCAGGGACTGGATTTTCTCGTAGCTCAATGACCCACCGCCCTCCACCAAAGCT
TCGTTGATCTCGTCGCGCAGGCGTTTCTGCATCTCTGGATGCTTGGCCATCTCGTACAGGGCAAAAGACA
TGGTCGAGGAGGAGGTCTCGAAACCCGCCGTAAAAAACACGCCCGCCTGAGCCACCAGGAAGTCCTGGTT
CTTGGCATAGTGAGGCTTCGAAGGCTCGGCAGCCGCCTCTTTGCGCAGACTCACCAAGACATCTATGAGA
TCATTGCGGAGCAGGCCCGATCGCTCTCGCTCCTCCATAACGTGACCAATGGTGCTGCGCATAAAGTGGG
AAAAGTCCGCCGTGAAGAACTGGATGCGCATCAGCGACACCAGCTTGGGCAGGAAGAAGGCCACAAAGAA
G
>dme_circ_0004064_junction_seq
CTTGGGCAGGAAGAAGGCCACAAAGAAGATGGGTATGAACACAGGGGTTCCGTTCT
>dme_circ_0004065
GTTCTTCACGGCGGACTTTTCCCACTTTATGCGCAGCACCATTGGTCACGTTATGGAGGAGCGAGAGCGA
TCGGGCCTGCTCCGCAATGATCTCATAGATGTCTTGGTGAGTCTGCGCAAAGAGGCGGCTGCCGAGCCTT
CGAAGCCTCACTATGCCAAGAACCAGGACTTCCTGGTGGCTCAGGCGGGCGT
>dme_circ_0004065_junction_seq
AGGACTTCCTGGTGGCTCAGGCGGGCGTGTTCTTCACGGCGGACTTTTCCCACTTT
>dme_circ_0004066
ACATGGTCGAGGAGGAGGTCTCGAAACCCGCCGTAAAAAACACGCCCGCCTGAGCCACCAGGAAGTCCTG
GTTCTTGGCATAGTGAGGCTTCGAAGGCTCGGCAGCCGCCTCTTTGCGCAGACTCACCAAGACATCTATG
AGATCATTGCGGAGCAGGCCCGATCGCTCTCGCTCCTCCATAACGTGACCAATGGTGCTGCGCATAAAGT
GGGAAAAG
>dme_circ_0004066_junction_seq
AATGGTGCTGCGCATAAAGTGGGAAAAGACATGGTCGAGGAGGAGGTCTCGAAACC
>dme_circ_0004067
CTCAATGACCCACCGCCCTCCACCAAAGCTTCGTTGATCTCGTCGCGCAGGCGTTTCTGCATCTCTGGAT
GCTTGGCCATCTCGTACAGGGCAAAAGACATGGTCGAGGAGGAGGTCTCGAAACCCGCCGTAAAAAACAC
GCCCGCCTGAGCCACCAGGAAGTCCTGGTTCTTGGCATAGTGAGGCTTCGAAGGCTCGGCAGCCGCCTCT
TTGCGCAGACTCACCAAGACATCTATGAGATCATTGCGGAGCAGGCCCGATCGCTCTCGCTCCTCCATAA
CG
>dme_circ_0004067_junction_seq
GCCCGATCGCTCTCGCTCCTCCATAACGCTCAATGACCCACCGCCCTCCACCAAAG
>dme_circ_0004068
GGCCGATCAGCTGACAGAGGAACAGATCGCCGAGTTCAAAGAGGCATTCTCGCTATTCGACAAAGACGGC

GATGGCACCATCACAACAAAGGAGTTGGGCACAGTTATGCGCTCCCTGGGCCAGAATCCCACAGAGGCCG
AGCTGCAGGACATGATCAACGAGGTTGATGCCGACG
>dme_circ_0004068_junction_seq
GACATGATCAACGAGGTTGATGCCGACGGGCCGATCAGCTGACAGAGGAACAGATC
>dme_circ_0004069
GGCCGATCAGCTGACAGAGGAACAGATCGCCGAGTTCAAAGAGGCATTCTCGCTATTCGACAAAGACGGC
GATGGCACCATCACAACAAAGGAGTTGGGCACAGTTATGCGCTCCCTGGGCCAGAATCCCACAGAGGCCG
AGCTGCAGGACATGATCAACGAGGTTGATGCCGACGGGTAAGTTGTGGAGGATTGTGGGGGGAAATACTC
TATAAAGTGGGATAGCCTTTTGTGGGGGTAATGTACGTGATTGAAATCCTTCATTGGGGTGATGTAATAA
ATTTCAAGTCAGATTCACATTTCTCATGCCCTCTGAGCATTTAAAACTTACGAATTCGCGAAAGAGATTG
CAAAATGCATACTAATCTGCAAATGTTAGCTAAAGGGTTCAAAAATCGTAAATAATCTGGTTTTGTATGC
CTCGTAACTTCAGTATTTCAAACAAATTGTGAATTTGCCTTAATTTTCTGTGACCCACAACATCTAGACT
ATGAAGTCAGACTTCGTCAGAGCTTACGACCAATGTGCAAATGAGCTGATTTTCTATGCTATGCTGGCTA
TATATGAGAGCTGGCTAGAACCCCTTCTAACCCGATCCCGAGATGTCTAATTTTGTTTACCCCAATCTAT
TTGGTGTTCTGGGCCTAATCCCTCGCACTCGTTCCATTCCCCAACTACCTCTTTCGCAGACAGACATAGA
TCAGACCAACATTTGTACGACAAACAATGGGTGGaaaaaaaaaaaagaaaaggaaaaTCACTTCTTGGCTA
AAAATATGCAACAAAAACACGTCATAGGCATTAAAAATAGATGACTAAAATATTTTGGCTCTTTCCCGTG
TCTTGTCTTTGGCAGCCGCTGCTGTCAGAttttttttcgttggtatttcatttttttttttttAATGTTTA
TTTTTGAGCAAAGGTGGTTGTTCGCTTAGCTCGAAATCTCGTACAAGTTTTAGTTTAACAAACTTTCTTG
GGGAAACGAGGAAGATCCAATTTTATTTTCGATGCAAGAGGAAATCGAGCGACACACGCCTCGCGCCAAA
AATGTGGCAAAATGCGAAAATTTGAATCGTAAAGGGGTTTTTCACCTTTTGCCACAAATAAATAGATTTT
CCAATTAAGTAAGCAGGTTTGTGCTTTGCTGCTTTTGCTAAGGATAATACTTAGCAACTAAATCATAAGA
ACTCAGTGGCTGGCACAAAGTAACTTTTATGCTCATCATCACTTGTTCTTTCATAAGTTTTGCAAGTGTC
CCCGCCCAATTTGTATTTGTTTTCTCGCTGCTGCTCCATTTTCTTTGCCAGCTTTTCTCggctgcctgtt
gttgctgctgctgctgctgttgcgcttgcACGTGGAAATTCTGTGCGCATGTTGTCGAAAATATCCAGGA
CACAATCTGCAAACTCGGAGTCCTGCACACAGAATTTTGAGCGCAGGCGCTGTGAAAAACCCCTGCATGA
TTTTTCTCtttttttttttttttttGCTGGAGCTAAAGTGTTGCAATCATGGACAAGTGGGTGATGTGG
GGTTTGGACAAACGAGGGGGTGGCGATGCGCATACTAACTGTTTATCAGGACTAAAAATAGTAGTTCGGT
ATGTGGGTGGGTGGCAAGGGGTCGCCACTTGGGAGTCCAGCATTTTTCGCCAACTTTATTTTGCTGGAAT
TTGCATATGTGCCAAACTTTTTACATTGAGTGCAAGCAAAATGTTTTTGCCAACTTTATTGTGCAACGTT
TAATATTTAAAATTACTTGCGAAAATTGTTGCAACTCGAAAGTGGCACTTCCCACTGCCCACCACCCACT
GCCGCCTGCTCCCAGCTGACTCCTCTAATTTACGATCCCACACAATGGAATGTATTTTGTGGCCATCCGA
ATCACCCACTCACATGGCCCACTCACGCTGGTCACAACAGCCAGCCAGGCAGGCAGTCAGGAATCTGACG
GAAACATTAAACATTCATTACTTAAAATTTCGCTGGCGTCTCGTCGCAGCCAGTTGACGATGGCCAAAAC
TGCCTGGCTGCCttttatttttttttttttttGGTCTGTGAACTGCATGCAAACGAGTGCAATTAGTTGGC
GGTGGGATTGGTGGGCAGGTAGGAAGGATTGTCCAGCGGAATAAAAATAGCGTTATAGAAGTATAATTAT
ATTATATGGCCATCATAGGTGAACTCCAAAGGTGGTTTTCTTTAAATTGCGCTTGCCAAAAAGTCGCAGT
TACGCTTTTAGCGGAATAATCTTTTTTTATGGTTTGCTTATAATGTCGATCTGAATAAAAGTCGACTATT
TTTGATGAGACCAATGGAAAATGCGCGCAAACTAAATTTAGCCGATGCCTTTGCCTTGCACACACTGTGT
GCGCCAACCaaaaaaaaaaGACAAAACTTATTACAATTTTAAATTGTCTGCAAAATTTTGCGCTCAACGT
TGCGAATCGGCAAGTGGGAGTGTTGATGAGCAGGGAAAGGAGGAGGAGAGAGGGAATGGGCGGAACGGCG
TAATGGAAACCGAAATCCGCGCCAGACACTGGCcatcatcatcatcatctcatcgtcgtcgtcatcatta
tcgccattggcagcgcgatgagcatcataattatGGTAATTGGGCTGCGCGCGGGTTCGTCAAAGGCGTT
TCGGGCCAGTCTAAGAAGCGTCTAACAAAAATGGCAACAAATTTGCCAATGGCTGTGTATGTCTATATAT

GTGTGTGTGTGCGGAGGCTTTCCTTGGTATTTGTTTTCTGATTTTCTATTTTTTTGGCTGGGGAAATCCT
ATCGACAAAGCCAAAAGCAACAGAAAATCGCGTCAAATGTTTTAAATAGAATCATAAACACAAAAACGCA
GGCAAGACAACAAGCAACAGaaaaagaaaaaaaataaaagtgaagaaaaaaCCAATACCGAAATCGAAAT
GAACTAAAGCCACCGGTCGATATTGTATTTGATGTTCCTTTGGCTATTTTCAAAATATATTTTTGCTACT
CAATGCGGCCATATTTGAATAATTATAATATCTAAATTAGCCCAAAGTGTAATGTTTTTTCTTTCGTCAA
ATTTTCGAATTCAATGCACACATTATTTTGAAATGGGAATTGCCTCCAGATTTTTTCAATTCATTTGAAT
TCGTGTGCTCGAGAAGTATTTATTTATTTTAAATCAATTAATTCGGCGAATTACTGTTTACTTTTTTATA
AACCAAATACTTGAAGATTCAAACTATTTATTTTTGTTTCCATTATATGTTTAACGGGCTATTTGTGTAG
CTACATTCAGTTCATTCCATTTTATTTGGCCAAACTCTTTGTCAGTAAATCTAGATTGCCCCAAGGAGGA
GATCATTTGCTATTGAAGTTCAAAAGGGCTTGTGAACCTCTGACTTTGCttttttttttttttttGTTTG
GCAATCATATACGAAAAGTTTCCTCTGGAGAAAACCCATTTCCAGCAAAGTAATAAAAAACACATTCAGT
TCACAAGAAACAAAATGATGGCCCACAATGAACCACGCTCCATTTGGTTTTGAGGGTAGCAAAAACAGAA
AACTGAAAAGCATTTTATACACTTTCATTTTATGGACAAAAGACGCTTCTTCTTCTTTCTGCTGGGAATG
GCTCGAAAAAGTCAGTCAATAACAATTAAGGCCAAAAGTTGTTGCTGCTGCCTTTATGGCGCTGCTGCGG
CCCCaaaaaaaaaaCCAGAACTATTCTCTCAACTTAAAACGAACGAAGGAAACTTTTTCAATTAATATAA
CAAGAATTTCGGTTGGACCACTTCATTTGCGTTTCACTTACGGGGATGATAACCTAAAGAAAATGTATTT
GGCCAAATTCTTTTACAAATTTTCATTCCACTTGGCTGCttttttttGAGCTGCATCTGCGATGAAGCAG
CCGGATATAAAGAGATAATAGATATAGAAAAGAGAGTGTTTCAACCGAAATGGGATTAGGCGGACTTATC
TCTCGCACTCCGCAAGGTGGGTGGTCcacacacacacacacacacacGTGTGGGTGGGGAAAAGTTTGTT
TGGGGCTTAGGCAAATATGAAAATATGCTCGATTATATAGGAGGAACCACAGCACCTCCCAGGGATTTTG
GCGTCGAAAAAGGCAGCGCGTTTGGAAAAGCTTTAAGTAGTGTACACAGCCCCCGGGTGGATGGATATTC
GTATATTTGTATGGCGTTGTCGGGAATGGTGGAGAGCAAACTTGGCCGTAATGAAGAAAAATGCCGGCTG
CCTTTATCCCTGACTTGAATTTATTATAACGCTCCGGGGAAGAATTTAAATTGATTGTATGATGATATGA
GGGTGGTGGTGGAGGAGATGTGATATGGTGCTAGTGGAATCCCCGAAATCCGACCCACAAAGTATGCAAC
GAAATTAATTGTTATTCTTTATGGTTATTTCCCCTCGCACAGGGCAACGGCACAATTGACTTCCCTGAAT
TCCTTACCATGATGGCACGCAAAATGAAGGACACCGATAGCGAAGAGGAGATCCGAGAAGCCTTCAGAGT
GTTCGACAAGGATGGCAACGGCTTCATCTCGGCGGCCGAGTTGCGTCACGTGATGACAAATCTGGGCGAG
AAACTCACAGACGAGGAGGTCGATGAGATGATCCGGGAGGCTGATATCGATGGCGACGGTCAGGTCAATT
ACGAAG
>dme_circ_0004069_junction_seq
GATGGCGACGGTCAGGTCAATTACGAAGGGCCGATCAGCTGACAGAGGAACAGATC
>dme_circ_0004070
ACTTGTTGTTCTTGTTGCTCGAAACGATAACATGCGACTCttgtttgttgctgctggttgttcttgttgt
agttgtagttggtttgtggttttAAACGGTGACTTCATTAAGTATGCTGCTTGGTTGTTG
>dme_circ_0004070_junction_seq
CTTCATTAAGTATGCTGCTTGGTTGTTGACTTGTTGTTCTTGTTGCTCGAAACGAT
>dme_circ_0004071
GCTTTATGCTGGCCCTGGATTTGGAGAGTGGTGACGGCGATGGTGACGCCAATGCCCTGGGCACCTGGGA
GATCGCCGATCTCGCCGAGACCCGTTTCAGTCCGCGCTGCCCAAATCTTGTCGAGAATACCAACACGAAC
GTGAAGACACGCGTGGATGTGTTCTGGGTGGCTCCATCGAGTCCGGGACAGGGCTGCATCCTGCTGAGGG
CTACGGTGATGCAGCATCGAGATGTGTG
>dme_circ_0004071_junction_seq
CTACGGTGATGCAGCATCGAGATGTGTGGCTTTATGCTGGCCCTGGATTTGGAGAG
>dme_circ_0004072

GAACGAAGGTGGTGCTGGGGAACCGGAGGAGGAACAGGCCGGTGGCGACGGAGGTGGATCCGCTGAGAAA
CGCTCCATATTCCGCAACTTCGAGAGTTACAGTCAGCATCGGGAGGATTACATACCACCGGTTTGCGGGG
TCACTCCATGGTCGGATTTCTCGCCGTGCATGGGGCCATGCGGTGGACATGGAAAGCGGCAACGTATTCG
CAAGGTGTGGAATAACAACCAG
>dme_circ_0004072_junction_seq
TATTCGCAAGGTGTGGAATAACAACCAGGAACGAAGGTGGTGCTGGGGAACCGGAG
>dme_circ_0004073
CGTCCTTGTTGTTATAGTCCGAATTGAAGTCATTGTAGCCGGGTCTATTGCCCACAAGCTTGTCATTGCT
GTAGCTATTTGTCGGAGGAAAGCCCTTGTCATAACCACCCATATTGTCAGATGGATTATACGCAGGTCTC
TGAGGATTTTCTATCTGATCGAACGGTAAGTTTGGGGACTCCTGTGCGTTATCTCTAGGGTTGGAAAGAG
ATTTATCTTTCCACGAATTGTAGGGCTTTCTATTCCACGCCTGGTTGCCGGAACCTAGACTGGAATGCTC
CTGTTTACTG
>dme_circ_0004073_junction_seq
ACCTAGACTGGAATGCTCCTGTTTACTGCGTCCTTGTTGTTATAGTCCGAATTGAA
>dme_circ_0004074
GTGGAGGATTCTACAGACATTGTGCCCTACGACAGATCAGCCGCTTCGACGCCAACTGACGAACGCAAGT
CCACCTCACCCACGGAATTGTCCCGCTTGTCGGTGGCCCACAGGGCCAGCATTCGCATGGTGCCTGGCGA
GAGCGATCGTTGGACGCCCACGCCCGAGTGGATCGTCTCCTGGCGCTCCAAGCTGCCGCTGCAGACCATC
ATGCGACTGCTTCAGGTCCTAGTGCCCCAGG
>dme_circ_0004074_junction_seq
CGACTGCTTCAGGTCCTAGTGCCCCAGGGTGGAGGATTCTACAGACATTGTGCCCT
>dme_circ_0004075
ATCTTCAATAACATTATACAGTATCAGTTCGATGGCAACTCGAATCTAGTCTACACCATAATTCGAAAGC
GTCATGTGTTCCATGCCATGGCAAATCTCCCGACCGATATGGCTGGCATAGCCAAGTGCCTGAGTGGCCG
TAAAACAGGTGGAAAGTTTAACTTGCCGCGGGTACCACAGCGAAGAACGCCAGCTG
>dme_circ_0004075_junction_seq
CGGGTACCACAGCGAAGAACGCCAGCTGATCTTCAATAACATTATACAGTATCAGT
>dme_circ_0004076
GGTGAGTAATAGCTTCAGCAACTCCGTGCGCCTGCGCTCCATGTGGGCGTTGTGGGGCGGTGACTGGGCG
AAACCCACGCCCGCTTCCCAAATGTACTCGCAGCTATCGATGTTCGCCAACTCCTCGGCCTTCTCGGGGC
CCGCACGCCGCGTGGCCGTAACCGTGAAGTCGGGACAGAAAAGAAG
>dme_circ_0004076_junction_seq
TAACCGTGAAGTCGGGACAGAAAAGAAGGGTGAGTAATAGCTTCAGCAACTCCGTG
>dme_circ_0004077
GCTTACCAGTTCGATTAGTTAGGATTAGTGAGAGATGCCCAACTAGGCAGCACGCCCAGATTCGTCCAGG
CAGCGTGAGTGTGCGAGTGGGCAAGTGGCGAGTGCCAAAACATATGCGAAGGTCGTCGGGAAGGGAGGAG
GAGCTGCCCTGCCCCACGAAAACTGCAATTTTCAGCGCTAGCCCTGGAACTGGAAGCCAACTAGCTGCAG
TCGTAGAGACACCGCAACGGAGGACACGCGGACACCATGGCGCTTCCAGGCAACGGCATCTACGTGGTGC
GGGGCGAAATGGCCACCCTGATGACGGCGATGCGACGTGGAACGCGTTGGAATGCCACCGCCTACGTGGT
AAATAGTGCCCCTAATGCTCACTGCGAATAGAAAGTATATGGCCCTTTTGCACATCCACAGGACGACGAG
AATGACTCGCTGCTGAAGCTGTTCATTGACCTCAAGCATGAGCTAAATCGGATCGAGGACCTGCGTCAGA
TCGAGCCTCAGGTCTTCCTGGCTCCGTTTCTTGAGGTGATTCGCACGGCGGACGCCACGGGTCCGTTGAC
TAGTCTAGCCTTGGCCTCG
>dme_circ_0004077_junction_seq

TCCGTTGACTAGTCTAGCCTTGGCCTCGGCTTACCAGTTCGATTAGTTAGGATTAG
>dme_circ_0004078
TCGTAGTCGATGTGAGCTCGCATTTATAAAGCTTGCGATGTTCTCCACTTTCTCTTCTGCAGCTACACTA
TTGCCAGAATCAATTACGATTCCCTCCAATCCGCTGTTGTGGCGAGAATGGCGGCTGCCACCTGTTGCTG
GGGCAGCTTCTGGCAAGGACTCTCTGTTGTTGCTGCTATTCTTGCTCGCGGCACAATTTCGCTCGATACT
GTCTATCACACTTAACAGGGTGTCCATTGAGATAATGTGGG
>dme_circ_0004078_junction_seq
AACAGGGTGTCCATTGAGATAATGTGGGTCGTAGTCGATGTGAGCTCGCATTTATA
>dme_circ_0004079
TTTGATCTTCGGCGTACGGCAGGCCAATGGATTTGTGCCCCTGAGGAGCCTTCAGTAGCGCTAGAACGCA
TTTAAGCAACTCCTGCAGCGACTCTAGTTGCACAAACTTTGATTCCTGCAGCATTTGATCCAGCTGGCAC
TCCTTAATGCACTTCCGTCCCAGTTTGATGAAGTCCTGCTCCTCGTACGTTGGTTCTCGCTGACCCTCCG
AGCTGATAAATGAGTACAGGCTGGAAAATAGTCCCGATTCCTGCTTCTCGCGGGGCTTTTCCAGGATTAA
CATGGCCTTTCCGTTCGCCTCACAAAAGTCTTCCACTTCGATCAATGATTTTGGCAGCAACTTTAGACGG
AAAAGCTGCAGATATAGG
>dme_circ_0004079_junction_seq
CTTTAGACGGAAAAGCTGCAGATATAGGTTTGATCTTCGGCGTACGGCAGGCCAAT
>dme_circ_0004080
TAGCTGGCAATTGTACACAAGCGTATTTACTGGAGGAGCACTCAGGCTAGGCGGAGATTGTGGTCTGCAA
AAGCAACTTTGTTGTATTAGATATGAAAGGTTAATGAGTGAAGAATCTCACCTAGAGGCCGTAGTCAATT
CACTGTCCTTGTTATTGACCAGAATCCAGTTCTCAGCACTTGGACTGGAGACTGCAGGTGCTGCAGATGC
TTTCGTGATCTCCGAATCCGAAGTG
>dme_circ_0004080_junction_seq
TGCTTTCGTGATCTCCGAATCCGAAGTGTAGCTGGCAATTGTACACAAGCGTATTT
>dme_circ_0004081
gGCTACAGGAATGGCCAACGCATTTCCCGCCGTTGCCACGCCAGCCAACTACTTTAATGTGAATGTGGAC
CCCAGTAGCATACCGCCCGGATCGGAACTGCTCTACGGCCAGCAGTGAAGAGGAGCATTGAAAGTTCATT
TCAATTTCAATCAAACCACCTGACCGTCTTAATTTGTACCCCGTATAAAATTGTGTCAATTTGTTTTT
>dme_circ_0004081_junction_seq
CCGTATAAAATTGTGTCAATTTGTTTTTgGCTACAGGAATGGCCAACGCATTTCCC
>dme_circ_0004082
GATTACGCAGTGTATGAAGTACATGGGCGACCAGGAGCTGAAGCAGCTTCTCGACCAGAGCAACACCACT
GGCAAGCCCTTTATCTTCTTCGCGCTGAACGACAACGAAACCACCGATGCTGGTGGCAGCCACTGGTCAC
TGTTGGTCTTCTCGCGTCCGGAGAAGACCTTCTATCACTTCGATTCGTATGGGAACAACAACACGGGCAA
CTCCCTGGAGCTGATGAACAAGATCAAGGACCTGCTTGGCGTCCGAATGGCCAAATTCCGGCCCATGCGC
TGCCTACAGCAGGCGAACGGCTACGACTGTGGCATCCATGTTATCTGCATGACCGACCACATTGCCGATT
ACCTAAACAGATACGAGGTGATCGACGGACTGCCGTCGCTGCACATCGACACCGTGAAGGCGAAGCGCAC
CGAGCTACTGAAACTCATCCTGTCGCTGGGCGGCAAGGGCTAGAGGCTAGCGAGGATTGCCCTGCCGAAC
CGCCCACAGCCGAGTA
>dme_circ_0004082_junction_seq
GCCCTGCCGAACCGCCCACAGCCGAGTAGATTACGCAGTGTATGAAGTACATGGGC
>dme_circ_0004083
GATTCATCAGGTGCCTGCAACTGGGCGAGCCGATACAGTTCAAGCCGGTCCTGCTGCTGAAGCAGCTCAT
CCGCTGGTTCGGGTACACACCGCCGCTGGTGCCCGACCGTGTGCTGGCCATGATTATGGAACTCCTTCGA

TCCGAGTACGCCGAGGCGGTGATTCGCAAGATTCCTTACGAACGCTTCAAGGCCGAGCTGCAGAAGGTTC
GCCGTGTGCTACACAAGCAGGAGTCCAAGCGGGTCAGCGAATTGCTGGATGATATGAATTTGCTGCTTCT
CGAGAA
>dme_circ_0004083_junction_seq
ATGATATGAATTTGCTGCTTCTCGAGAAGATTCATCAGGTGCCTGCAACTGGGCGA
>dme_circ_0004084
GCCAAATGCGGATGAGGATTTTGGCGATATTCGCAGCCATACTGGTCTGGATGATCTCAGAATTGGGAGA
ACTATGCCATTGGGATAGCATGTTAATCAGTACCAAGAGATTGCTTATGATACTTTGCGCCTTGTGAGGT
CCCACTCGCTTCACATAGACGGCGGCGTTTCCGATTTTTTCGTCATTGAGAAAATCGTCCAGAATGTGAA
CTACCTTGCCAAGCAGCTTCGATTCTCCGGCTGCTTTCACGGCCCAGGTTGATGCCTTAAGCAGTTCGCC
GAGTGCTTCACAAACCTGCACCTGGCCCACTGCAGGAAGTTCCAAAAAGTTTAAGGCCTTCGATAGATAG
>dme_circ_0004084_junction_seq
CAAAAAGTTTAAGGCCTTCGATAGATAGGCCAAATGCGGATGAGGATTTTGGCGAT
>dme_circ_0004085
ACCGGAAGTGTTGAAGGAAGGAGGTGATCAAACCGTAGCCATCCTATGCTCCTCTGGAACCACTGGTTTG
CCCAAGGCCGTCTGCATTTCCAACAGCATTCTGATCCAAGACAGCATGTAAGTCTATCTTCATAATGATC
CGATTATACCACTTAGGGTCTATATATCTTATCAGGTTGATCACCAGTCAGTCGGTTATCTATGTAGGAA
GCTGTCTGGACTGGATCACGGGCTTGTGGGCCTTTGTGTTCAGCACCGTATTCGGTTGCACCCGCATTAT
CAGTAACAAAGCCTTCACTCCGGAGTACTTTGTGGGACTGGTGAAGAAATACAAGATCAACTATGCTGTG
CTGCCGCCGCGTCATCTGTCCGCTCTGATCACCTGTCCGGATGCGAAGCCGGATGCACTGGCTCCCATCA
CGCACCTCAACTACGGCGGAGGATCGATATCGTTGGCCACCCTGCAGCGGTCGCAGGAACTCTGCAAGAC
AGCCATGTTCAATTCGGGATACGGGATGACAGAGGTGGGCGCCATCACGATCAATATCGGAATCAGCAAC
GTCTCGTCCGCGGGCAGGCCTGTGCCGGGCATTAAGATTCGTATCGTGGATGAGGATGGCAAGAGCTTGG
GCTACAACCAGGTGGGAGAGATCTATGTGCACACGGGTCAGGCCTGGAACGGGTACTACGGCAATCCCGT
GGAGACGCGACGCATGCAGGACTTCGAGGGTTGGTTCCATACCGGCGATCTGGGCTACTTCGATGAGCAG
AACTTCCTG
>dme_circ_0004085_junction_seq
GGGCTACTTCGATGAGCAGAACTTCCTGACCGGAAGTGTTGAAGGAAGGAGGTGAT
>dme_circ_0004086
ATCAACTATGCTGTGCTGCCGCCGCGTCATCTGTCCGCTCTGATCACCTGTCCGGATGCGAAGCCGGATG
CACTGGCTCCCATCACGCACCTCAACTACGGCGGAGGATCGATATCGTTGGCCACCCTGCAGCGGTCGCA
GGAACTCTGCAAGACAGCCATGTTCAATTCGGGATACGGGATGACAGAGG
>dme_circ_0004086_junction_seq
TTCAATTCGGGATACGGGATGACAGAGGATCAACTATGCTGTGCTGCCGCCGCGTC
>dme_circ_0004087
GGTGTCATAGTTGTAGATGGAGTTACGCTTGGCTCCACTCCAGATATTATCCTTTTCGTTGTAGGTGGTT
GGTATCATGGCCATGTTGTGGTTTCCTCGAAAAACTTGCTCGCCGTTAGAAAATGCAACGGA
>dme_circ_0004087_junction_seq
CTTGCTCGCCGTTAGAAAATGCAACGGAGGTGTCATAGTTGTAGATGGAGTTACGC
>dme_circ_0004088
GCTCATTTCCATTCCTATCACGATTGTCGCAGCTCGGGCAGTCCTTTAATTATAACATCCAGCGCCCTCA
GGTCCTTGTTGACGGTATCGCTCAGCACCTCCATGGCGCGCTGCTGCATCGTCAGAAAGGTCTTCATCTC
GTCCTCCGCTTCCTTGTCGAGGGCATACTCGGCACCACCGTTGGCTGCGAATTGATTGCGCTGCATGCGC
ATCTGGGAGAGGAGCTCGCTGAGTCGGCCCTTGAATTGGGTGGGTGCGGAGACGACAGCCAGCATGTTCT

GTAGCTTGGTGCGCAAGGCCTCCTCCTCGGGCGTCAGGGAAGTGCCCACCTTGCGGGTGCACTCTTGTTT
CACTATTATCTGTGGTTGAAAAATGCGGATTTCTGAATGTAAAAATAAAGTTGGGTATTTAACTAGGTGT
CCCTACCCTTAGTATGCGATGACTGAGCTCGGCCAGTTTCCGTTTATGTTCAAGTATCTTCGCTGTGGCA
GTGGCATGGCGCTTCTTGAGTTCTGTCAGCTCACTCTCCACCTTCTTTATGTACAATGCATGCGTGTCCG
TTTCTTGTTCCTGACACTTAACGCGCCACTTTAAGTCGTTAAAGCCCACCATGGGCACTGGTATATATAA
A
>dme_circ_0004088_junction_seq
GCCCACCATGGGCACTGGTATATATAAAGCTCATTTCCATTCCTATCACGATTGTC
>dme_circ_0004089
GGTCTTCATCTCGTCCTCCGCTTCCTTGTCGAGGGCATACTCGGCACCACCGTTGGCTGCGAATTGATTG
CGCTGCATGCGCATCTGGGAGAGGAGCTCGCTGAGTCGGCCCTTGAATTGGGTGGGTGCGGAGACGACAG
CCAGCATGTTCTGTAGCTTGGTGCGCAAGGCCTCCTCCTCGGGCGTCAGGGAA
>dme_circ_0004089_junction_seq
CAAGGCCTCCTCCTCGGGCGTCAGGGAAGGTCTTCATCTCGTCCTCCGCTTCCTTG
>dme_circ_0004090
GACAAGTATATGTTCTAGTGGCCTCCGGTGTGCTCGCTATCCTGGCTGTCGCTTTCTCAGAAGACGAAGC
GGTTGATGTTCATGCCCAGTCCATTGCCTCCACATCCTCCGGGCGGCGGCGGCGCCTTGTCGTCCTCGTC
ACCATCGCCATCAAA
>dme_circ_0004090_junction_seq
TGTCGTCCTCGTCACCATCGCCATCAAAGACAAGTATATGTTCTAGTGGCCTCCGG
>dme_circ_0004091
GCGAAGCCACCATCTACCATTGACAAGACGTTCGCTGGCAAGCTTTCCACCACCTATCGATGTTTAAACT
GCGGCTGGGAGAGCCGGAACGAGGACAGCTTTCGCGAGCTGCAGCTGTCTTTTCCCGATGATAAGGAAGA
CTG
>dme_circ_0004091_junction_seq
TGTCTTTTCCCGATGATAAGGAAGACTGGCGAAGCCACCATCTACCATTGACAAGA
>dme_circ_0004092
GGCGAGGAATGGAACTGGAGCCGCGAGTGGCCATACTGCAGGACCTGCGCCTGCAGACCTTCGACTCCAT
TCGATTTGCCTCCTACCGCACCGCCTCCAAGCTGCGCTATATACAGAAGTCCACAAATCTGCACTTGGTG
GACATATGGAATGTGATCGAGGCCTTTCGCGAGAATGGCCTGAACACCCTGGAGCCGCAGAGCGAGGTGA
GCGTGGCCAGGCTGGAAACCCTGGTCTCCTCCCTCTATCATAACCTCAACAAGCGCCTGCCCACCGCTCA
GCAGGTGCCTGTGGACTCGAAGGCGGGTCTGCTGCTCAACTGGCTGCTGGCCGCGTACACAAGGACATAT
TCTCGCAGATCTCGGAtggcgctggacagctggtggcctggaagctggGCGAGTTCCTGCGCGAAGTGCT
GGCCCTGCCGGCGGCCGTGTACGAGTCGCCCACATTCCACTACAAGGAGGGGCTCGAGGAGGAGATCTTC
CCGGCCGAGAATAAGGTGACGGTGAACGACTTTATGGCCACGCTGATGTCCGAGCCGGGGCCGTCCTGCC
TCGTCTGGCTGCCGCTGGTGCACCGGCTGGCCACCGTGGAGACCATTGTGCATCCGACTGTCTGCTCTGT
TTGCCACAAGGAAAACTTCACCGGGTTTCGTTATCGCTGTCAGCGGTGTCACGCCTACCAGTTGTGTCAG
GAGTGCTTCTGGCACGGCAAGACATCGCTGAATCACCAGAACGATCACGAGGTCAAGGAGTACTCGAGCT
ACAAGTCGCCCAGCAAACAGATCGGTCACTCGCTGCGCAAGAGCTTCCGCTGTGTGCCCGAGAAGACGGT
GCAGGTGCTCCCTCGCTTCCCCGACCAGCCGGAGAAGACGCTCAACCTGTCGCACATCGTGCCGCCCTCG
CCGCTGCCATCGCACAACGGCTTCTCGGACCCCTCGGGCCTCGTACATGGCCACCACGGCCCGCATCCGG
GTCTCCCGGGCCAGCACGGCCTTTTCGATCGCTCCAGCACGCTCGACTCGCGGGCCACAGGCCGCAGTCT
GGACAGCGCCAGCGGCACGGCCGGCACAACCATGTCTCGCGTGGCCGCCGCTTCTGCCAACGACGAGGAG
CACCGCCTGATTGCCCGCTATGCCGCCAGATTGGCCCAGGAAAACCGAGCGGCCATCCAACTTGCCCGAC

AATGCCACACCAATTGGCACAGATAACTCGCGAGCCCAACGCGAGCTAATTGCCCAACTGGAGTCCAAGA
ATAAGGAGATAATGCGGGAGATAGCGCGCCTGAGGCGTCAACAGGAGACGGAGCAGATGGCGCCAGAGAA
TCCGGCGCTAATCAATGAACTGCGTGCTCTAAGGCAGCGGAAAGGAGAACTGGAGGGCCATTTGGGTGCT
CTGCAGGATTCGCGACGACAGCTAATGGAGCAACTGGAGGGACTGATGCGAATGCTGAAGAACCAGCAGA
CAGCATCGCCTCGATCCACGCCCAACTCGAGTCCTCGTTCCGGTAAATCCCCACCAATGCCCGGCGGAGC
TGGAATCCTGGGTACATCCCCCATGAGTGCTCTACACGCCCAGCAGATGCAGCAACATCCGATGGCCGGA
GGAGTCAGAGCCAGCCAGCAGCAACTCCTCCAACAGCAACAGCAGCAGGCTCATGGCCACGGGCCATTTA
GCCAGAGCCAGCTGGAGCAGCTCAACCAGATTAGCAGCGATATGCGCAGCGCCTTCGCCGCCAACGGAAG
TGCAA
>dme_circ_0004092_junction_seq
AGCGCCTTCGCCGCCAACGGAAGTGCAAGGCGAGGAATGGAACTGGAGCCGCGAGT
>dme_circ_0004093
CACCACACCCTGCAAGCGCGCGATTTAATATATGTAGTGTTTCTCAAGAGCTGCGCTCCAAGATCTCAAT
CTTCTCCAAATAGGGTGAGAGCGCGGCGAAGACGTCTCCAAGAAACCGAAGACAACTATTGCCGTTTCTT
TCTTGAGCACAGCAAACGCGGATGCTGAACTGCAGTCTCCACACTTGTCTTCTCGCTCAGAGCTGGTTTC
AGACTTCTTCTCTCGATCAG
>dme_circ_0004093_junction_seq
CTGGTTTCAGACTTCTTCTCTCGATCAGCACCACACCCTGCAAGCGCGCGATTTAA
>dme_circ_0004094
GCTCAAGAAATTCTCCAACACCATACCCATAACTAAGGAGCAGCAGGAGGAGGATGAAAAGCAGCTGGAT
GAGGCCATCGAAGCGGACGCCACTACAGAAGCGGCTGCGGAGTCTGAGGAGGTGGAGGAGGAGGTAGCGT
CAAAGGAGAAGGGTCCCCGAAAACTGCTTGGAAAGCGCGCCGGAAAACCCAGTTTCACCGAAATCGAACA
GGAACTGAAGAAAAAGAAGCGGGAAGAGAGCAAGACCAAGTATGTGGCCAAG
>dme_circ_0004094_junction_seq
AGAGAGCAAGACCAAGTATGTGGCCAAGGCTCAAGAAATTCTCCAACACCATACCC
>dme_circ_0004095
GATCAACCAAACATTCTATTTGTTGTAAGAGATCATTCCTCCGCCAGTAGTTTTGATATAAAAACAAACA
AATTTGTTGGACGCAAAGCCGAAAATCCTTCTCAGATGATACTTGTTGACAGATATTTGGCCTCGGAACA
GCGATTTCAATTTGGAAAATCGCTGTTCGCGGACAAACTTAATAACCTGCAGGGTCGAGAAGTCATCATT
GCTGGATTCGATTATCCACCTTATACAGTGATAAAACATTGTAAGTTTAATAAGTATGTTTTCCAATTTA
AATTATATTAGCCATCCTTCGTATCACTATTTTCATTTTGTAATAGATAACCATAAAAGTTCAAAAGTTT
TTTAAAAGTACTTTATTGAAAAACTCCACTGAAAAATAAACGTCTAAGGTGCCAGTTATTCGACCTAATA
TTCAGATGTGGAAGCCATTAATATATCAAAAAGTTGTCTACCGTAAAAAGAATAAATCGATTGTGCGGGT
TAGATTGGGCGTGTCCAAATGTATTAGCAAAATTGATGATTTGCAAAACAAATAGTAAAACGAAATAAAA
TTAGCAACCGAAAGGGGGCGCGACAGTTTTGGTCATATTAATAGATTTCGGCAGAAAAACATTGAAATAA
AGACTACGGTAATGTTTTACgacgtgttggttttgcgctttatttaggcgttataatgtgcctggaaaag
tcctgaaataaacatgcacaggcgtagttgctactataagctggatgtattttctcaactttctaggtat
taaaatatccataatttcagcattcgtaaggaaagatgaacagacggacggactgacatggtcatGTTGA
CGCTGGCTCGCCACCCAATTTCGTAGACTAAACCCATAGCAGCTATTAATATTTTTAAATTTAACACCAC
AAAATAATccgtgtaatccggctcgaatagcgagaagatacacgcgatgtagaaaagagaacagaatcga
ggaaatgaatataatgtaaaaaacctcaaataaatattaatgttagtaggatctaattatgtattaaaaa
aattaaaattgattgctttaaaagcggcagagagtcaagattacagataatagaataaagtcttttatag
tcagaacataatacactgtaagggtcatgcaattcatagttagatctacaatgatttaagataaggggta
tatagttactagtgaatctagcgggactctcaacatcagcacgaataagcttacaaataaagactgttcc

aagcatagtaaggtcaagcataggaaggtaaattaattaaaagtagtctactggaataaggaggtttgca
ttccaatttaggcgccgcaaggcaaaaagtaatacctttttggaccaattcaatgcggtccgagtggacta
cgtattgtggactccaaacaggtgatccgtacttaagaatcggacggactaacgaaataaataaggtttt
agtcaagtaaggatcatccttagagcacctttttataaaaccaagcacgcccctggccttattgacaata
gtcgaaatatggtcaaaaaattttagtttagggtccagaagaacaccaatatcatcaacccgtgttattc
tatccaaaaggcacccacttagagcgtaagtcgtgcgtatagggttaacacgacaaaaggtcataacttt
gcacttggagccatttaagtttaatagcatatttgaaatctgtctagatcggattgtaagtccaaatggc
gcgaaatgtccttgtgctggaggcgtaatttaacatcgtctgcgtacataagtacacgcgaatgttttaa
ggggaggtcgttaatgaataaggtaaaaataagcggaccaagatagctcccttgtgggacaccggatgtg
actcgaagaatacaagataaagaatttttaaagaggacttgttgcgtcctgccattcagatagcttgaaa
tccaacatagaagatcaacacggaaacctaaaagatcaagtttacttattagaaggtaatgattaacaga
gtcaaatgttttactaaaatcagtgtagatgacagtttgctaccttttttatgaagaggaatcacaaagg
actccttccatatatgagggaattctgaagattccagagataaggtaatcagtttcagtagaggcttgca
caaggcttccgcacagaatcggagcacacagccagggactccatcgggacctggcgaatagaccggttta
acacgctgaagatggttaagcagtgagcttttgtatatagtgaggcaaaataatatattcgacttagata
ccgcataagagtatggctgttcggaatgaggttaatttgaatatgttgtttgaaaaaacttggcgaatag
atcggcttatgcctgatccgatgttaccgtagtgttttcaaaaaataaatcgtttttcgcttagtgctaa
cgaaattataaaactgtttcggatcctgtgcgaactggaacttccaacggtttgaataattcttgtagca
ctgagcattaagcacggtaaagttaaaccgagcgcttaaatattttgaaaggatggactaaggactggta
tttttaaatttttgtataaagtttggagcttacatttcttaggtgtgccaactctttattaaaccagggag
gtttagagattgacggataatacatcagaacagaagggtcaaaaaatgatttaattgcggtataaaacat
attaatcgcatccgccattatgttataggagtaatgatcggaccaactaaagccagctataaaattgttt
agcctccgacaatttgccttgcgaaaacagggaattcgctttgttgacttctctgaatttacttttaata
ccgtacctatgtcgattgccacctcgaaagtaggatgatatggatcttcaggttgagtaagaggtgctac
cctggacagaaacacactttcaggacttgttacaagttatagatccaacagtcgaccaagagagtttgga
atataattaacttgcgacaacgataagtcgagcaagccatgtcatgttgtgctaaaggtaaaaggatatt
caatatttcttttggggaccacattgtgccaggtatattaaaatcacctagaactaccaattggtccctg
tcagacagtttatttaagatggactggatagcggacagatgattctgatattctgagaattcagaagatg
gcggaatatacgagcacgtaatataaactgatctgtcggaaaatgacagctttacgggaatcgtccaaaa
gtaactcctccgacgcgaggatagatctaaccgcaattacctgggaagacttcgaagttaagtatctccg
gcttgatcgctagctctgtgaacactattatatgggatgcaaaggaaagactgtcacaatacaatttggt
tagtttactacgcaagcctctagcatttttataggaaaaagtaagattcgttgttagttattttaagaag
aggaagaggctgacggtgcagtggtctggccacgagggaagtttaattcccacggccccctttttccttt
ttttgatcctagcttcgaactcttttaccactccatggtgggctcactccacagcggattgatcggattg
ctccgaatctttttttagctgccgatttagcaacatttgcggatttgctgcgattgacagctgatcagttg
tggagggaagtttaattcccacgggccccttttttcctttttttgatcctagcttcgaaatcttttaccac
tccatggtgggctcactccacagcggattgatcggattgatccgaatctttttttagcttccgacttagca
acatttgcggatttgctgggattgacagctgatcagttgtggagtccgagcgggacgttcgcctactacc
agcggctcttttgctctttggagattcctctaatagcttaaggccattaaattgtaaatcaagagcggaa
agaagatcatcggctcgacgaaaactatctctagctacgccaaaactgttgatcagttctttgaagccac
tggtcgctgttgaccgctatatttatctttttctttcaggcgtctaggaggttcagctttgtctgcatga
gctcggttgcagcgtcgcccacggtcaggaatgccgagtcatcagcgtatgtggctgccattatggacgg
gctctgcagcacagtcaggtctgccatgtatgcattgtatagcaggggccaaatttcaccaaatttcacc
gcaaacttgcgaccctcgaggaaggtcttcagaaaccgtacttcaaggctagggcagatttcttctgatg

acgccgcctgaagccaaactggtgatcgggaatcaggccagctttgtccataactggcaatgctctactt
aaaaatactctttcggcagcaagcttatgggacgataaaaagcgagattggtctcaggttttccaggctt
aggtatcattactacctcggcacgttttcaatgtgccggaaagtatccaaattcaaggcacttgttaaag
gtacgagtgattaacttaacgctcgagggtgggagtagttttaattcaaccgcatcaataccatcatcgc
ctgatgccttgttgttactcattgcagcaattagtttagcagtttcttcttctgtgaccgggggtgtggg
ctcagagtgtttgaggggttcctccaggagtctaaatgactgcatcagcaggagagcagcggcaataagg
agtaaagaagttttccagatggtcggcgaacgcgttggctttctgcttctcggttctgcaccagctacca
TGGGGGTTCCAGAAATGGACTCAAGTCTCTTCGTGTGTTAGAGAAACGGGGTGCCGCATACGCCGGATTT
GGGTAGTGAGTTCCTCGACTGCCGCATCAATGTCGTCACGAGAGCTTCAAGTTGGGTGGTGTTGTTCAGC
CAGGATTGGTACTTGCCGATGTTGGAGGACTTGTAAATTTGTTTTGTGGCGGGGTCCTTCAGGGGCGCTG
TGGTCAATGACCTTGTCTCTGTGTTATATATAATCCTCCATAAATTCCGATTATTGTTGCCTTAATATTT
TATCATTTACTGTATGATAGGATGAAACTCTTTGTTTTCTAGGAACCGTTTGACTACAAAGCGCACGTCA
ACATCAATCTCTGTCGTTGAGGTTGTATCATATCATATCATCATATCGCATAATATTCTCAAATGACACC
TGTGGTTGTCGGTGCGACGTTATTAAAATTAATTGAAATTATTAGTTTAATCAACCCTTTTAAAAACCCA
ATTAAACTTTCTCCTGGACCATACATTCCATTTCTCTTTTTATTTGGTACTCATAACTTTTGGCCTGCTT
CCTCAGCATCCTCCATCATCTCTTTATGCCGTCTACTATACTTCAAACGCTCTTGTTCTACTTAACAGAC
CATGTAAGGTTCATATCAGCGGCCAAGGCACTAAAACATAGCTCTCTTGACTGACAAAAGTCCTCTTCTA
AGTGATGGCAAGACTAGTAAATTTGCTACACTTTATGAACACTGTTTTTTTCTTGTGCTGTGTCCATCCT
GCTTGGAACATTTTTTCGGATTCCTTGAACATCAAGGGCAAAAAGTGTAAGATAGTTTCTATAAACATCT
AACGTCTCTTCACGTATTTGTAAAAAAGCTAGAAATGTATGATACGGCATGCTGATTCCAGAGTCATAGA
CGCATGtttttttttcacgccaatttttttttttAATATAACAAATGTAAAAACCTATTTTTTGCAGTAT
ACCAGGAAAAATGTCTCCTTTGCACTTTCTCTAGCTGAGTAACTGTCGGGGAGAATGTAACGCATATATG
AAGACATTTTATTAAATGCAAATGTTTCAAGAAGCGCTTGCCTTGCTTTGTAATACTTGTGTGCGCAACG
GCGCTCTGGCAAATCCTGCATCTAACAGGAACGGCCAATTCGACGAGATTAACGTAATTAATGGAGTCCA
AAGAGAATGCTCCAGCAGTACCGGCTTTAAGTTCGTCCTGAATTTAAGCCATTCTGTTTGACACGAATAC
GTTAAACTACCTCGAAGGTTGCCGAATCCACGCCAACACAATAGACGAGTCTGACCAGCAATGAAAGGCG
CAATCGAAATATATAAATTCCTTGACGCTTGCGATTACCTAATTGAACTAAAGCCATGCGGAAAGTTCTA
ATCCTAGAATTGTTAAGGCCTGTAACAATTGGGCACACAGCAGATGTACTTCCGATTCTCCATTACTCTT
CGATCGCTAAAATAAGCAAGCTCCATAAACTGATATGCTAGCATTACATTTCCACGAATCGTGGAAATTT
TAATTTCTTTACGACCAATAACGACCACTCTCTCCATGACGAAAATATTGCCTCTGGCAATGCGTTATCC
CAATCCACTTTAGCACCCCATAAAGATTGCAGAAAGATCTTGAATTGTGTCACAATTGGGGCTATCAGAC
CAAGGGGATCATAATATTTGAATTTTTTGGACACCGTTTTGTGGGTCGATAATTGGACGGTATTTGAGAA
AATTGTAATAAATAATCATCTGAAGCTAGTTCTCAGGCTAATTCCTATGCTTTGGCGATGTCGGATCCAT
CGTAATATTTGATCAAGTGCTCTTTGTCGCACTCAGACTCTCCTTCCAGGGCTGCTGAATCATTCAAAAA
CCATTTGCGGATTGGAAAGTGGCCTCGCGACAGTAGTTCCTCAACTTGGCAATCCTTATAGAAAAGTCTA
CGAACAATTTTAGCTTCAACTGGAAATAATTCCGCAATGGTGCGGCAAAGAGAATTGTGGTATCGATAGT
TCCCTTGAAACATGTGGTCTAGATAGATATACTCCTGTAAAAATTCTTAGTACTGAGCTTTTAGGGTTGG
GTTTTATATGCAGCTCCAAACTCCGGAAACGACGACGAGCCTGGCGAAAAAATCTTTTGGAGATTCGTTC
CCTGAAGAAGATAGAGTATTTACCAAGCCGAGTCCAATTTTCATTGAATAGTTGCTCGCACCGAAAGTCT
TCCGGCAACAGAGATGGCTTTGACGAAGTTAAGGCTTCAATTTCCCAAAATCGAATGATCAGCAGGGTCT
GTGACCCTGTATCCAACCGAGTTTGGTGTTTTGATGAATGAATTTTTCGAAAACGACCACAAACAGTTAA
CGCAAAGGTGCCACTTCTTTGCTTCTCTACATAGAATATTCGGACTCAAATTTTAAAATCAAGAAGACTT
TGGTACATATTGATCTCTCGCATCTTAAAATAAACATAACGGTTAAATGGAGTTAGTAGCAATGCAATAA
ATGTATTTTAGTTATATTTGTGCAATTTTTTCCCACCTTTTTTTCAAAAACAAGTCCATAGTATCCAAGG

TAGATATCTGATTGATTGATAGATCCGATTCTCATATAAATAATAAGTCCATCCAAAATTTGTTGTGTAG
AGGCCATGGATCGAATTGCCCGCAGATGCGAATTACTGCAATCACTAAGCTACCGATGTCCCTTtcgcta
taaaccttatttgcatgggtttcaacaaaacccatttcgttgacccatttccgaacatacgccacttcct
actctgcctctgtgctgcttagggaaaccactgattgtttctctgagtcccacgtaaaggcagctcctgc
agcaatacatgcccatccagtaaaggattttcggtccgtggtgtcaccagcccaatccgcttcaacatag
caatgtatcggcacaccagtacgctcgtagtgtaactactaatcagctgtgcccctcaagtatcgcagaa
ctcgctttgcaccggcttcgttctctttatgtgcgtcagcgtttcgttgtgatagtttaatgaccgaatg
cataatatcaggtcgtgttgtgattgccagatacattaatgagccaatcagcgattgatagctgacttga
ttcaccttctgacaatcggtcttgtcacattttacttcaaagcctgcttccagtgggatgagattcggct
tacaatcctgcattgaataatcattgagaaggctctcaatatactgcttgtggccaatcctgatgccacc
agtttctccgtcacgttcaatttcgatacccaggaaatgtcttagctcgcccccgtctacgacattgaat
tcttttgaaatttatgctttaatatcgcacagttcctccttgattgaactggctatgataaggtcgtcta
cactacaacaaggttcttacttttgccagaattacgggtgtatacacatggctcgctagggcatgaagaa
aacccaatcctttgcagtacttcatttagtagtttatttcattctcggccgctctgcttcagtccatata
tagatttgtgtagcatcagcacctttttttggatatcgttcatcgatgaatccctctggctgcctcatata
taccgtttcgtgaagatcaccattcaaatatgcagacgagacgtccatttggtgcatgtagagaccgttt
tccaccgccaaagcaattactaatgtgattgatgaagtgaagttaacgccgtatcgctgagcgcaccctg
ttgcgacaagtcgagacttaaagcgctcgaccttgcccatcttgttacgcttctctgcaaagacccaaat
gaccagattttatgtgcacgtaggcctccaactccttctgcattgaggttctccattcctcagagttcct
tatggttaaaacctccttgacgcttgttgaaattggaacctcatccgtactcatgccgttgaccatgttg
tattgttttcgcggtcttccaggtttaccagtgtaaatcttcttcggtcgtccaggtcctctgacttcgg
tgacttcgttgtcttcagtggatgacgcttctccctctgaatcgttctgctcgacagacgcaacgtcgtc
atcgtgtttatctgacacctggaaaacatgatcttgtttcttaccctcagcaacacagaccgaaaggtac
tgcatttccacaagttgaacatcgggagaaggtactgcttgccctgccgttataccatcatactggtcgt
tttcaattacaattacgtcacgagcaacacagacattcttcctggtacgattgaacaggcgatatcgctt
agaagcatcagagtatctaaccaaaatattttcttcgcccttcgcagtaaacttctttttagtggatttg
ttcagcacgatagctttcgaaccaaatatcgtcaggtgggacacatatggctttcttccagtccaagact
cgaacggtgtttcatcagataatgctggttttcggcaaatgctttaaactccttgaatttggccagtatt
tcatctctcgtctttataaaatacacaaacaggtatctcgatttgtcatctacgaaggtgacaaaatagc
gtgcacctccaatagacgtcgcttgcattggaccacagatgtcactatgcactagctccagtacttcatt
tgtgcgattaccagaagcttttgggaaactcttcacacatattttgctttttgcgcaactcagttatttt
cttttgcgtcacttaaatatttttttactttagtccgagtaccatttcttgtttgatcattttgttcaagc
ttgcaaCctctgttgggtctaccgttgtaatacaagatcatcttggttttgtcgcagaaaattacgttat
tccagtaatcttctggctgatgttgtaaagtaagccgtttttcgatgttaattgccgacagcatcggttt
ctttcttgctacccgtgagaggtacttataacggagtaaggcttggcgcactgtttcgtgtgacacatcc
agatcgcactcctccttaagcattcactagaaactatatacccccttatctgaaatcattgtagatccaac
tgtgagttgcatgacccttacagagtattatgttttgactataatagactttatcctattatctgtaatc
ttgactctctgccgcttttaaagcaatcaattttaactttttttaatacataattagatccttctagcac
taatatttaatacagttattttacattatattcatttcctcgttcctgttctctttttttatatcgcgtct
atatctttttgcgaatcgagccgtacgatacacggcatttgccctcgtttttttgcgcgattaataaaatt
ataaattgttctccgcgatgcagaaaacatttctgataattccttaacaggttttcccaaatggtgatta
taaaaaatctgttgaactacttcaaaacttaatcgatggccagtttatttttaaatttcacaaagctagc
tggaacaatgctcactttggcaaatgtcacaaccaaatgcattaaaatcggccacccacacaacgcataa
agcgtaaatgactttgattatcgatattcctcagaataggagcaaaatagaaaacaaaagaaaagtgtgc

aaacaatttcttgacagtgtcaaatctgtgccaacttggatttagttaattttctcgttcattgttaata
gttttgaaattatttctttacatattgataaaaaacatataaacaaataaaaatccataaaggtaaacaa
aaggcagagaataaaatttattttcattgtgcaaacaatttcttgtcaaactgTAGCTGTAAATATAaat
taaacaattattaagttatttcaaataataattatttttctttattaagtgcatcagaatacgactgatc
acttttgagccctgttcaaaactgaagtcaatttcatattcaaatatttatagcggcacttctgccgcta
tttctgtcaacgttcctcctcgtgacttctcacaccacaacgtccagaatgctaacgctgcgcctgtggg
aacctgtgggtgtccctttacagtaaccgcaaattccgcCTTTTCCTTGACCTTACTTATGATAACTctt
cccttagttaagtgagggccgttatcgggtttctgcagagctgtctctggttcgacggcgttgtcgttcg
aaccagcgacatgcgatgtaaaaaatgccgaaacaggcgagtgagatggtgaaagtgatgaccctgccta
cgatcggtcttgacaatacttcagcttttaggctaccttcaagcttagctcatggaggtagagcaggctg
aggtgatttttgtggctcgtgacgttgatttgaatcacgacagatgctgctggtttctgttttaaagact
cggtgaacaatgtcccatttagggagacccggtcgtcgtaggttatgaggtaagaccccgtgattgttcg
gttgaggccttcttgatcagtgactctcatggcagcgtcattaatgatgatgatactttcgtcgacttgg
gttattggttctaggtggctggatcgggttccgcaatgtgcgacggttccagtagttagttgttgggcac
aggtgtttttttttttattattatatacgacaggaaaatgaaatcttctaaagataccacctatcgtcct
tggatggcatgcacgattcataaccctcagctgagggcttatgcctacgtactaaacaaaaacctccgct
ctctctcctgtccttgtcaagctcactgccagtcaaggtatttcatcggtgagcaggatgtgatgccaag
ttgcacaccatcttcttttgcggtgcacagcagcgcctttttacgaccaactgagcgtacggtcatagag
ttcttgccccgccaacatacgggagtactccttatctatagttccgattggccatctcgtccttacggac
acgcttcatcatggtgacgatgatcacttgagcctcatcccaaacgattttctccgccatcatagcagca
accaagcctctcgggatgagaggtgacccagcgagcgccacgagttgcgccctctcctcggcgagcctgg
agcagtgcagtaggatgtgctctgcagtttccactgcgtctgtgcaataaaggcactgggctgtatccac
gtggcggaacctaaatagataccctcgaaaacagccatggtccatgaggaactgggtcatgtggtagtcc
accgttttgtgctcgcaatcagcccactccgttagttccgggatgagtttgtaagtccagcgtccccgtt
gtgacacttgccacctggtctgccatactcctcgaatccactctctgatctctaattgagtgtatccctc
actcgcaaggtatttgccctttatgtccaggtcaatgggtgtaatgcctgacagagcgtgcgctgcgtcg
tccgatatggttctaaagcctctgattagcctgagagccattgtccgtgatactgcgcgggcactatcta
gataggttttttttggtggaaacgcagctccagataggcgcagcgtaaagcagcgttgcctttgaaaccgc
cactaacagtcttctagccggcagtctgggtcctcccacgttgggcatgattctcgccaaggctgctgct
gtcattgccgctttttttgccggaataccttgcgtgagccttgaacgagagccttcggtctattagaacac
caaggtatttcaacgtttctgctgaggcgatttcaactcctttgacttccacacgcatgcactcaacgga
ctttctgctgctcagcaggactacttcggtcttatgcgcagttattttaagtccgactttctcgagccat
tctatgaccgccgtgattgccacgttacttttgtcctggagctccggtattgtcttggcgaccgctgtta
ccgcaacgtcgtcggcgaagcagtgcagctccacacctctgggcttgctaatgctcagtattccatcata
cataatgttccacaagatcggtcctagtaccgcacagtacttcttactgccgcctaacaatcggttaccg
tccagtgctttcctggcagcgtcttttctacggctttgagagcgtcgagcgtacttttcccctttcggaa
gccatactgttgaccctgaaggcctgtaggactttcagtgattagctcaatgcgcgcgtacagtatgcgc
tcaaagagcttgccaacaatgtccaacagacacaacggtcggtaactgcgtggagcatttgcgggcccct
tacctttcggcagaaggaccagcttcatctttttccagcgcttggggaagactccgtccagcagacactg
ttggaatgtatccctgaagacctccgatcttgcggtggcggctgctttgacgatcacgcccgggatacca
tcaatgccgggagctttgttcggtctgatcctctttgctgcttcgacgatgcatgacgtgatgcacggga
agtcaggggcgggggggtcaacagcgggttgccataacgtgtgttggcttggaaatagttccgacacgat
gttggccagggcggctgggtcctaaggagatcctgcaccagcggaattgagtttttttccttacgactttg
taggcgagaccccaggggtcgtcgtctacgctatcaatcaggtccttaaatgcattcgcctttgctgctg

atatgccgttcttaagctcagttcgcttcgctttatatacctcaatgcactgggagtggtgcggttgccc
tctagatcgctgcgcttgcctcctcgccttgatgcattccgtccgaagctggtgcaatgagtctctccac
cagtagacagggggctttcgtttcgtgttcctttttctcggcatcaccgcatcacacattgctctgagcc
tatccatgagcgctgtcaccatggtctccgcgtgtccaattatggactccatgccctcgatctggtatgc
cagcatgtcttcgtccagtttcctaatgtcccataccggacctagcgcacatcttcgctgtttgggtgac
ggcctcattgttcgggcaccgaacgtgattaggttatggtcgctcagcgttatgtcctcatggaacatcc
agatcgagctagccactagaactctgctaacaaaggtgacatcaatgaaagatgtacctctgtcattatt
gaacgtcggtttgagctcgacggcacaggtgggtgtgcttgttgttttgcagagagtggtgcacacagtg
gcttcgcattggtttattgcaatggtttttgatccgcaatctgcgactatgttgctgtcttttaattta
atattgtatttttatgttgaacaggataaattttgatctttttacaaactaacttagggcttggaaattt
cattaaaatatatatcatattattgttttggaaatctttaactttggctacttctagaatgtcagctacg
ctggcattcgtgagttgttcattgttaatttctttaatattaatatcttccaaaatgacggggttgatca
aattgatttttgtcaaggtgagtgaagttactatattttctaaactagttatgattattatattttttgc
tagcattgttttaaacacatgttcagtgtctatttcgtcggcttggttgatatcgtttaaggtatttgtc
aattgcttttgtaatttggagtttatttctacctgtctatgttcttgccggaacctaatctgttcccagt
cgtcaaaatctggtatgccggctactacttttaatgcggtctatttctatgttttactcttactgtagcg
atcaacgtgtcaatgtgtccgaggtctgtaataagaatccttttcatgtgatcctttttgtaataagtcaa
tagtccttttggtctcatctgcgtatatctcgtaggatgtcacgtttctcatgtgttctaagtactttta
ctcgtcccaaatcgttgcgtctccgtccacaattggtatatagtccgtatgtgagtagtcatttattttt
gttgtagcgatcgccatcgcaatatttgataatatgatgacagaatgccttatcctagtaaacagcaaaa
tgtaattaagaaatgctcttatttttttatttgagagtgtccttgtggatcaccctccccttaataagaac
cgtcgtacctaaataggcttcgatcgtttcttccgagcataatggtgttaacttggtacctaaccgtttg
tttgattttaacaaactttgtcgcctacttgatactctcggtgagcttttgtcttgatggcttgttcgc
gtgttttgtcttgggtttcccggattctttctttgatttccgattctatctcgattggtgttgaatatat
gacatctactggtttattgtttgtaactgagtggatggttctattataagcggttgttgctaaaaggatt
agatagattgtgtcatcgactaatatacctatttttaagcaacgggcaattagaaagtgttgcttgtctg
tgtagaatatatctacgtgcaaaagctctccggcgtgggatggtattggggtttttcctagtgtttgctt
gacagggtgacgtttgtactttgcctttgaacaagttttacaatttatggacactccagctgctaagcgg
ctcattttcgggaagaagtagtcgcgcaatatctgcttgacgttttcttgggctgcgcgatgtgctctgt
tatgttctgtgtttattatttcagtttgctctgtcctgtcagtaaGAAGTCACGCCAAAGTAGGGCCAGC
AATGCCTAGTATTTTTCCATGATCAGTAAACGGTAACTTGTTTAATTGTTTGTCACAAGCACCGTTAACC
GCTGTTGTTACACCTCTGTCTGCGCACGTCTTCTTGTATTTATGTTCTTTATTGAATCAATTTGTGGAGC
AGGGAAGGTCCGGagtgctacaatgtgattgtatattatgaatacatataggtacagtggttaaccgata
tgtgtgcatactacaacagtgtgccatgggctttatcggtctgtaaattggattatatgtcggagcttgt
ccttgaatattataaaggttttaactgatggtatgtctgtgtgttcaataataacttggattctaaagca
aattgctgctttgtcggtttgttcgccgtgaattttagcaatgttggagtcattgtcggattcgttcgag
acagagtgattgtctagcgcgttaacattttgacgggacagggcatcagctacatgatttccttgcctgg
tttataaataatattggcgttgtgttcgtcgttaaaagctgctcagcggtttatatttgcgtttgggtct
ctgtccgatactgtgaatgtcagcggatgatggtcggtgtagatgttaaggttctcttaatgttctccga
acagatagtttttaggtttttaatgcaagaagttctcgtccgttagtcgcgaagtttatctcattgtcg
cgtagcgtcctggagatcattgtgatcggttttccgtcctgggcctaaaccgcccccagtccatgtgccg
aaggcatgacatgttgtgaggtcaaatggtctcttgatctttgggtatgtaaggagcacatcttctgatg
ccaggacgtttttcaaccaatcgaatgcttgttgttggctttggttatggtttactctaacttttctgga
ttggttgacacttattttcccgttataactttttaatatatccgtaataggtctggtcaggctgcaaat

cctttaatgaaacacttgtagtaactgcgttagactaagacggacctgaggccgaaaagagtatttgggg
gatggaaatttttttatagcctgaactttttcaggagatgtttttaatctactacttgacacaatgaatcc
caggtactccacgctctctttgaagaacttggatttctccatagaaattctcacgccagctgtttataaa
cttttcagagcccaatcggtgtgtttgacatggtcttcttttgtttcggaaacgtttttcctgtttgttt
ccaaagaacgtcatcgattgccctttaaaaaattctaattccgaatgaaagtcaacagaactcgtatttg
ccttcgttaacggagaaagctgtttttttctcgatccctttcagctagttcaatctggtggaaacctagaa
acccagtaaggtccaatgttgtgaaatattgagccttacctatgttggacagtatggtggaaatggcagg
tatggggtatttgtcgtttttatttcgttttgtataatcaataaccagacgtttttttcttgtgaccattt
tcatcatggcctttttttgtcaaccacccagatggggttgttgtatggccttattatgccatccgctagaa
tgggtataattaagaatataccggttctccgtccgtgcgtattgtagctacggtgtttatgttgaaaagt
agggattcgttaggatccgcgaatgcttttgatctttgtttgatcattttgttaaaattttctttgaccg
ccagtggaatgttactgtcgtcaattttgatgaagttgacattttgacacttcataaattgtaatggctc
ggagacgaatttgtgtgcaattaaattgcttttaagattgatggctgcatcaTGGTCGTTGTGGTCGTTG
GTcgttgtttggaactactagccttctgaccctgtgattgattgtttggtccgttgttggACTTCTCGAG
GGCCTGGCCCCTATTAAaatgtgggtttttaccctgatagttgaagccgcctgaattttgttcaccctgt
gcgttgttacgccatccttgggtcttttgagacgaggatttctggcatttttcttcgttatgcttagcat
agacagctttgaatacagagctctcgttgctcttttctgcctcttgtgctaatgctaatgccgaaaaaat
tgtctgttttatggatttttttcaggccatcaatgaaggcatgtagatcatcatttctatgttgttcatta
aggatcattgtgacattattgtcttgttggttagaagtgctagcttcttttctatctcgtcataatactt
tagcaggggcacatcgcactgtctcagtgttgcgagttcttgttgtataacacgcagtggggtttcatct
gcgtatgtgaagtataggcgtgctataatcgcgtcgaaattcatcaccgtactaaatgaggatagtgctg
aattggcagttcctctaactttgttgcgtatacggtatctaagggttcgtcgcacgtaatccctggtatt
actttagctgcctctgatttttatagaaagagtacctatttgtcgttgcatattctgtgtcgtgtcagcta
atctttgttcgaaaaccgccgattggtattcaagcgctgccttgaccgctttttctaccatatttagatc
gagcgcttcgaagtgtccctgcgcttatgctgctgcgggttttcctgttgtgcactgttctgtattcatt
attgtaacttgttcgtttttaagtttctccctgtaactcggtattgtgtcttcttcactactggaattac
tccgattcaagttcctatacattcgtgatcgtttgcaggaaacaatggcttttattgtactgaacaaaat
tttggtattagtttactactactcttactattactctttactctttactctttactctttactattactc
tttttttaatttgtttttgaaaaaaaaggacacgaataatgaatatctcacctgcatttttcgttattattc
tagtgcagctgtatagtgtcgttgtgtgctgatccgctccttggttcttttttggttcgttttgtatttt
tcgttttatatatttcgtttattttgtttttgattcactcagtgcagttgcactggggttttattttttt
ttattAAAGTTCAATATCCAAAAAGACAATTGTCGATTAACACGCTCAGAATACTTGTGCCGaaaaaaaa
aTTAATTGTCTGACTGTATTCTTATCAGTAGAAAGAGAATGCAAACGGTAAACCATATAAAAATTTGTTG
TGCTAAATATTCGTTCGTTGCTTTGCTCATCATCTTCGCACCGTTAGGTAACGGTACAAATTAGCACAAA
TTATGATAACTTGCCGACTAGgtaaagcagcagtggttttgtttttcactcgctgagtgcagttgcact
agggtttttatttttgtttttcgttgttgctgtttttgttttatataaaacgatttcactgttaatttg
ttaggtgttcttgtttaatattttttattaaatacaatattattgcacgactaaaataaatttttcgttt
tgttagtactttttaataaatttgtttgaacttttccgtcgtgagttcttttgatccggtttcagatcac
ttttactgctaagttgggcgccaattaaacaattattaagttatttcaaataataattatttttatttat
taagtgcatcagaataacactgatcacttttgagccctgttcaaaattaaagtacaatttcatattcaaa
tatttatagcggcacttctaccgctatttttgtcaacgtttcgcctcgtgacttctcacatcacaacgtc
cagaattctaacgctgcgcctgtggggacctgtgggtgtccgtttacagtaaccgcaaattacgctgatt
tatcgcaatagattcggaccggatactgacGTTGTGTATAAACTATATTAAGCAATACCAAGGCCGATTT
GAAGACTCCAATGAAAACTCGAGGTAACGAAATGTTTATGGGGAAGACAACCAATATGCCACCCTTAGGC

TGACGGCAGAGTGCGCCagttaccacatcagcagcaactaagtccacttccccgccccgtaagcccccac
gccagaacttttagtttgttgttccctcagctgaccacatcagcagtctacatcttcttccctgcctcct
gcctaaaccatcacgTCAAAGCTGATGGAAAGGTTTGAAAGAACCTTTCTGGGTGTTTTCGATAGTCATA
ATATAAACATTAAATTCGCCTTTTTCATTTCTATTGCAGTTTAACAGAAAGTTAATAGTTGCGCTTGATG
CAGAAAATTCCTCGTCCGAATCCATCACGTTCACTAAAGACTTAAAGAGAAGCGATATAGGTCGTTTCTC
ACAAAGCTCTCACTCTGCCATCTTCTCGCTTTGCTCATAGCTTCTTTCTTCACTTCAGCCTTAGGGTTAT
AATTAATTAGAACGCACAAATAGGTTATATTAATGTTTATAGCTTGCTCTGTTGCTCTGTTTTATTCGGA
GATGCAGACTAGACTGAGCGAAAGCTTGGCTTCAAAATATAAGCTAATCAGGCATAAGCTTACAAAGTAG
TTCAGCTGAATAGATATCGCAACAGCTTTAGCGAGAGAAGAAAGAATGACGAAAAGAGCGACTCGAAGAG
CGCTGCACGCATGACTAAGCTCTGGCGATCTGCTCCTTATACTTACAATCCAGCAATATACTTCTCTTTT
CACTGAATAATAACTAATGTGAGGTGTTAAATCTTCGTACACATAAAGATGTCAACTGCGGAAGGAGGCC
CTAACTGTTGTCAGCTACGCAACAAATCATACATATCATCATATGTTCGTAACAGCAGCGCGAAACGAAG
AGTTTGAGAGAGGGAGCATGGGCGAACAGAAGATGTAGAAAGAGGCTTGGTGAGCAAGAGTAAGACGGCA
CGAATTAGAGCGGGACAACAGTAGAGCCGAGAACGTCGAAGAGCCGATTTATAAGAAGCAACGATACCTA
GCTGGACTTTGGACCAGGCTAACGGTAAAACCATGGACTTTGGATCTAGAGACTAGGAGTAACGGTTAAA
CCAGTCCGAGCGCTGAAAGTTGGATGAGGAGAACGAGAATACGAGTGAAAAACGCGCAGCAATGGAGCGA
CGGCCGTGAAAGTCAACAAGGATCAAAATCGCTACGTCGAGACCTTTGGGATGAAGAAAGTCACCGGATT
GAGACGATGCTGCTCCTTTGTTGTCTTTCCTTAATACCCCTTAAAAACGGATCCTGTTCCGGCTCGAATG
AGACCAAGGATGTTATGGGGCAGGGTGTTTTGTCCTCTAGTTCAGCCCAACGTTCTTTCCGGGACCACCT
TTGCCGACTACTGACTCCACTGTTTCTACAAACCATGCCAGGTATTTGTGTTCTCCTGCTCCTTAGATCT
CAGCGTTCTTCACTTGTTGTATTCGTCCTTGTGTGTCCACCGCGACACTAACTGCttgatatcttctgcg
cattattatttgtcttgtgagtttctatcgatttgccaataaactttttgctgagcccactctcacgccc
taagactggccaaaactgccatgcccacGTGCGTGTCCAAAGTCcgcaccgttaccgttcaacctccgtg
cccttagccggctcgagtccagcgcagtattgttagtttacggtctctccgcCGGGGTCCAAGGTATATT
ATTTCCATTTACATATATACATTTTTTCTCGCATTCCAGCCGTCTTCgctgttgctaggccgcttcactg
cacacgcatttgtgggaagatttaagactcctcTCAAAATATATATTCCAGACTACCCAGATCACATATT
GCTCATTGTCAAGTAAAAGACATTAAATCTTCACAGCAAAAGATACATTTTTCAATTTATCGCGAGAAAC
CATTTGCGTCCCGAAACATATTATATATACTGTAGCGTGGGACTACGATTTCCTTTGACGAGACCAAGTG
GCTGACAGTGTAACTGTCCAGTAGTAAATATAGACTCCAGTAAAAACAGACAGTGGTTCTGCCAGCCAAA
GATGCCCAGTTAGAAAATTTGGAGATTTGGATGGGGTTTACTTCAGGGACGCTTTGTCCTTATTTCAGCT
AATATTCATGTACACTCAAAAAGGTAGATCGTGTAAGTAGAAGTAGCAAGTAATGATCGAAGTTAAGAAT
AAAAAAAGAGTGACCAGCATTTGTGCTACCTGAGCCCGATCTGGCTTGGCATGACACCTGGTTGGATCAC
AGTCGGGGCAGCTCTCGGGCAGGAGTCCGCATCCGAAGCTGATCCTCGACGCAAAACACCACCTCGGCAt
ctgtttccattcatggaccaacacggcattctaagggcatgcagtcgcctcaatgtctgcagaggcccac
aatatgatgaaggccatccaataattcgcctctacgactctagactgtatcgcctcctaatccaatttgc
tctccagattactctgcacggcggtagtcaattaatagtacgcctgatccgatggaagaattggattcct
aaaatcaagaatctggtgaaggctggagtgaatccgtgttcgatgtgcacgatttacaagaagagacttc
aaccccagttgatgggcgattttcagacagatagagtctccttcccgagggccttcacctacagggccct
tttgaaattcaaaaattacacggggatagtgtgtctcataacgaagggatatgtttgtgtgtttttccac
aaagtctatttggaacccacctcagatcttacaactgagaaattgctagcggccattgctcgttgcgaag
cgaggtgcggttgccttccgcaggtccattcgtataatggcaagacctttgtgagggcagcaacattact
ttccaaggacttcctacaagccatcaaagagtcagtgactgatgcatatagccatcaaggactcgtgtgg
cggttcatcataccacatatgggggtctgtgggcagcaggagtgaaaagcttcaaaacccttttctata
catctatgtccgcccgcaagtacacgtttgaggagctggcgaaccttctcgctaagatcgagtcttgcct

caattccagaccactctccccgatgaccgaagaccctcatattaactggccatcacaAGGGCCGAGAAGG
ACAATTTTCAATCAAACAATTAGCGGCTCTGTCGACAAAAGTTCATCGTAGACTTGGAAAACGAGAAGAG
AGAAAACTTGGAAGAAGAAGACCGAGTCCTAACACGAGTATGCGTTAATAAAATTGGCCAAGCCAATAGA
TTTAGATGATGAGAGCATCGAATACTTTGTTGAAAGTATCCAGACTCCAAGGTGAACAAGTCTTTATTAT
ATCAAGCGAAGACTATGAGGCAGTTAAAAATTCAGATGGAAGCATATCAGAAAATAAAATCATCTGTAAA
GCAAGTCAAGCACGACTCTCACTTCATTAAATGTGAGCATGATGTAGCTAGAGTAGAAAAAATGTAAAAAA
TGTTTTAACTGTTGGACGAGTCACACATAAAAGTGAGAAAAAGAGACAACAAATGTTTCACGTGCAACCA
ATCAGTACATAGGGCGACCCAATGCAAGGTCGAAATGGTAGCCAAGCAGAAGAACACAAATGTAGTACAG
GGTGAAGCCAAGACTAAGCCAACAAACGTGTTAACGTATTCAGTCCTGGAGTTAAATTACATTCTTTTCA
GAAAAGTCATCCAATCAGTCAGGGAAAGGAAAGTAGACAGAGCCCGAAAAATGTTTGACTGGGATCGGAG
AAAGTCAAATCGTAACGATTGGAAGCACGACAATCCCAGTTCAGATGGATGACATAGATCTCAATCTAGA
ATTTCACGTCATATTCGATGCGATTTTAGGTAGAACTCTTttactaatggtagacatgaaaagctttcaa
ttcccaataagttaagttcatgctgtagcagatacagatagatgtttaaactgtcccaccccgtaaaagt
actgtcaaaagttcaaagttcacccattcaacagtctcagaaacaatgagatgcctcaaaaaggagaacc
caggaagagatttcaaggaacttttagatagggcagcttatgagtacagctatagttcctattcagctac
gaaaaagtataagtattagaagtattcttcggcagaaaagtgaagacaaacccggactaatacgaagcag
ccagccaagataacattgaAAGCATTTAAAACAAACATTTTCTTCCCctttttgccacgaccattctaag
gcactaaaaccgcacaaaaatgccaACTCAAAGAGGATTGATGTGAGCAACATAGAATTCCACGTCATAG
CCGACGAAGGCATGAAATTCATTGCGATTTTAGGTAGAACTCGTTTTCTAATGATAGACATGAAAACCAG
GAAGGGTCGTACTAAAGTCATACCAAGATCAGGCAGGGAGGTAGTGACGGGGCTCAAAAAGCAGGTCCAG
GATGGAGAGCAGGACCGCAGTTCGTGCATGGAGTTGCTGAACGAATTACAGAGCCTGTGCACGGTATCCG
TAGATCAAACAGGAAGAAAACTTAACGTagagtggtcgcatttgccacactgaaaacaaagtgttgtaga
acggatgatagaagactacaaaccgaaaagagcaataagtggtcctgttgagatacaaattgtgattaca
gacgaaagagtggtcgcatttgccacactgaaaacaaagtgttgtagaacggatgatagaagactacaaa
ccgaaaagagcaataagtggtcctgttgagatacaaattgtgattacagacgaaaCACCAGTGTTTCAGT
ATGCTCGACGCATTCCTTACACTGAGGAGATGGTAGTAGACGAGCGGGCTACTATTTGTGCTCCGACTAT
CGGAAGTTGAATGAGAAAATTGTGAGGGACAACTTTTTAATGATTCACATGGACAGACAGCGTGATTCAG
AAACTACAAGGAGGGACAATTTTTACAACTTAAAACTTAACCAACGGGTATTTCAATGTTCCGGAATTGG
TTAACGCCCAAAAGTATAAAATCGATTATTGATCAGGAACAAAAATGAGACATGTATCCGCTCACAGTCG
AGTGACATGTTTTGATTGGCCAGATAGCGTAATGCATAAGGTAAAGGAAGATTAGAGCCTAGAGAACCCT
GGAAATTGTCTGAAACTATGTTCTAATACATATTGTCGTCTTGCTCAGTTGTTCTTCATCTAAATCGTTA
GTGTCCAGAACTATAGATGTTATCCATTGATTCCAAATGACGATTAATCTCAGTGGTTATTCCAGCACTG
CTTAGGTATAGCTTACAATTAGTACAAACATATGGAGAAATTTCAACAATTGATAAACAACATCGAGCCC
AGGAGCACTAAAGTGTCACCGTTCAAATAATTAACAGGTGTTTACGTGCGAGTTATTGACAATCAATAAT
CTAACGAACAGGTGCAGTAGTCTTAGATATAGGAGTTGGACGAAGAGCGTAAGCAGATGCTTCAGGAAGC
TAAGAATAATATAAAAACCTTTCCGTTGCAGTTAGAACGAATCAGAAGAAAAAGGAAGAAAAGCAGAACA
ATGTTAACGATCTAGTTGCGATCAGACGTACTCAGTACTCATAAATTAAACCGACATGGAGAAGTGAGAT
TAGTATTATATTATTCCAATAGCAGAATATTATTAGATTGTTGGTGAAAAGTCGGttttttttttGACT
ATCAAAAATTAGTAGATGTAGAACTTTAAGGGTAGGATTTGCCTTAAAATTGTTATAGCCGTTACTCGAA
AAATAACAaacgcaagtttgttgacccatgttgccacgcccacaatattttatattttgtatattttaat
atttttttaaactttttgccacgaccactaaaagtgcccaaaactgccacgcccaaacttttaaaaaatg
tttagattttttttttgttttatggtatgtccttcccatttttatcggtataccaaaaacaacgtggcca
cgcgcctcttttagcctacaaaacgcctaaaaggtgtcacgtccacacttttaaacaatttttaatttgt
tctcattttattccccaatatctattgatattccagataaattatgaaatttcgcgttcacattcccaat

agctgatagccgaaaaactcttctatagcattctctcttgtttTTGAATACAGCTTGCGCGAGTAGGATA
TTTGATATAAGAGGCTAATGAAAATTTTACGAACTTGCTCGACTCCAAATGTTTTATAAAATTTAACTGT
TGATATATTTTTTTAGAACATGAGTACTAATGCACAAGACATGGGCGTGAGCGGGGAGTCCGATTTCAAA
AATGTTTACATTGATGGAACTGAAACTCGTATTGTTTTAAATTTCTGTGAACAATTTAATTGTACAATCC
AAATTGATTCAT
>dme_circ_0004095_junction_seq
TTTAATTGTACAATCCAAATTGATTCATGATCAACCAAACATTCTATTTGTTGTAA
>dme_circ_0004096
GGCCTCTATCTGTACACGCTGGTGGTGCAAACATTCTCCAGTGATAACATTAGCTTTATTATCTACGCCC
TCATCGGCTGGGGCTGTCCAGCCGTATGCATTTTGGTGTGGTCCATTGCCAAGGCATTTGCTCCGCATCT
CGAGAACGAGCACTTCAATGGGGGTACTCATCACTAAATTACGTTCTGCTCATACCCTGGAAACGCGGCA
GTATTACAAGGCCTCGAAGGCGCTGCTGGTGCTGATACCTCTCTTTGGCATCACCTATCTGTTGGTGCTA
ACAGGCCCGGAACAGGGTATCAGTCGTAATCTCTTCGAGGCCATAAGAGCCTTTCTCATAAGCACGCAGG
GTGGGCTAACCAATCATTGAGGGGCGACTGCACTCATTTGGCATTCTAGCACATTATTGTTGTTGGATTA
TATTTATATAACCCTGGCTGTCGACATATTGGCTTTTCACTATTTGCACTACCTAGTTGATGTGGCTATG
GCTGATATTGTAGTTGTTCGCTATTGTCGTCGAtgttgttgttgttgttgttgtCATTTAACCAATTAAT
TTCCATTTCAGGCATCGCGCCTCAAAAGACTACTCGCTGAGATCGCGAACCGAAAGTCTACG
>dme_circ_0004096_junction_seq
CGCTGAGATCGCGAACCGAAAGTCTACGGGCCTCTATCTGTACACGCTGGTGGTGC
>dme_circ_0004097
GGTACTCATCACTAAATTACGTTCTGCTCATACCCTGGAAACGCGGCAGTATTACAAGGCCTCGAAGGCG
CTGCTGGTGCTGATACCTCTCTTTGGCATCACCTATCTGTTGGTGCTAACAGGCCCGGAACAGGGTATCA
GTCGTAATCTCTTCGAGGCCATAAGAGCCTTTCTCATAAGCACGCAGGTATATAGATTAGGTGACAGTCT
TTCCATAAACATTGATCTTCGAAAATCCATTCTTTAGGGCTTCTTTGTGGCTCTGTTCTACTGTTTCCTT
AACTCAGAGGTGCGCCAGACGCTGAGGCATGGATTCACCCGGTGGCGGGAGAGCAGGAATATCCATCGGA
ACAGTTCCATCAAGAATCGCAG
>dme_circ_0004097_junction_seq
ATCGGAACAGTTCCATCAAGAATCGCAGGGTACTCATCACTAAATTACGTTCTGCT
>dme_circ_0004098
GGGTATTGCTGGTGCGGTTCAGATAAGCGTGCAACTGGCAGGTGCGCCAACGAATGGCTGCCTATGTGGC
GTGTGTGTCCTATTGCTTTTGCGACTTCTGTGCGGCGACTGGCGTTACATTTTTACATATTTGTATTCGG
CAATCGACTTATTTGTGCTGCAATGTACGA
>dme_circ_0004098_junction_seq
ATCGACTTATTTGTGCTGCAATGTACGAGGGTATTGCTGGTGCGGTTCAGATAAGC
>dme_circ_0004099
GGAGCACTTTCGCCTAAGATTAAATGACTTTTTGTACAATATGAAAAAGAGCTTTCCTTCGTTCATCAAT
CAATTTCCAGAGCCCAATGCCATGCCGGACATTAAACAGTCGATCGCGCGTTGGTATAGCTGCAATCTCG
ATGACCGCGTCGAGGAACTG
>dme_circ_0004099_junction_seq
CAATCTCGATGACCGCGTCGAGGAACTGGGAGCACTTTCGCCTAAGATTAAATGAC
>dme_circ_0004100
ATGACAATACGAAAATGGACGTGGACTCGCCACTGGGTCGCACGGAGTCTTCGACTCGAAATGCCAAGAG
TACGCCCAGTGAAAGTGCCTCTCCGGCGCGTAGCAATGCAAGCACTAGCAGCGTAACACCAGCGGGAATT
AAGAAAGAGACTGATGACTCGAAAGCGACGACAGGATCTTTTGCACCAGCGTCCTCGGCAACGGCATCTT

CAGCGACGCCTGTGGACGATGCTACGCCATCAACATCCTCAGCAGCGGCGGCAGCATCAGCGGCATCGTC
ATCAACTGTTTCAGGAACCGAAGGCAAGCCAAAGGATGACCCCCTATCGTCGCACAAGGAAGAAGGAGCC
GGTAGCACCAGCTCTGGAGTTGCCACGAGTCCCAGGCAAGCCCAAGACACTGCAGGAGCAGGCGTCGATG
TGGAAATCAAACTGGAACATCCTGCGGACTTTTCCAATCCAAAGCTTCTACCGCCGCACGCGCACGGGCA
ACGCGAAGGACGAATCCTACACGCTGTTCTTCGCTAACAACAATTGGTATTTATTCCTGCGGCTACATGC
GATTCTCTGCGACCGTCTGCATGTTATGTATGAAAGAGCGCGTTTGCTGGCCATCGAGGAGGAGCGCTGT
AGGGTGAATCGAAGGGAGAGCACAGCCACGGCGCTGCGGCTAAAGCCCAAACCGGAGATTCAAGTTGAAG
ACTACTACCCCACCTTCCTTGATATGCTCAAGAACGTCCTAGACGGCAACATGGACTCGAATACGTTCGA
GGACACGATGCGTGAAATGTTTGGCATTTATGCCTACATCTCTTTCACGCTGGATAAGGGTTGTCTCCAA
TGCTGTTCGCCAGCTGCAATATTGTGTCACAGAACGAGCCGCACTGGATTGTGTAGAGCTGTTTGCAACG
GAGCAAAGGCGTGGCTGCACCGGAGGATTCTGTCGAGATGCGCACAAGACATTTGACAGGGAGATGTCGT
ATCAGCGGAAAGCTGAGAGCATTCTCAACGAGGAGAATTGCTTCAAAGTGTACATTGTACAAAATCGATT
GCCGTGTAACCATAGAGCTGCTTGATTCGGAGCCCGAGGAAGTCGATAAGCCGGCTGCGTTGAAGGCCCA
GAAGTTCAGCAAGTATGTAGAGCGGTTGGCGAATCCTGCGCTCGGCGGTGGTGGCAATACTGGCCGCTCC
GATTCGGCGCTCGGCAATGACAGCGTTGTAGATGGGTCCGATATCAAGACGGAGGCGGACGAAGATACTG
CCGAGGTAAG
>dme_circ_0004100_junction_seq
AGGCGGACGAAGATACTGCCGAGGTAAGATGACAATACGAAAATGGACGTGGACTC
>dme_circ_0004101
CACAGCCACGGCGCTGCGGCTAAAGCCCAAACCGGAGATTCAAGTTGAAGACTACTACCCCACCTTCCTT
GATATGCTCAAGAACGTCCTAGACGGCAACATGGACTCGAATACGTTCGAGGACACGATGCGTGAAATGT
TTGGCATTTATGCCTACATCTCTTTCACGCTGGATAAGGGTTGTCTCCAATGCTGTTCGCCAGCTGCAAT
ATTGTGTCACAGAACGAGCCGCACTGGATTGTGTAGAGCTGTTTGCAACGGAGCAAAGGCGTGGCTGCAC
CGGAGGATTCTGTCGAGATGCGCACAAGACATTTGACAGGGAGATGTCGTATCAGCGGAAAGCTGAGAGC
ATTCTCAACGAGGAGAATTGCTTCAAAGTGTACATTGTACAAAATCGATTGCCGTGTAACCATAGAGCTG
CTTGATTCGGAGCCCGAGGAAGTCGATAAGCCGGCTGCGTTGAAGGCCCAGAAGTTCAGCAAGTATGTAG
AGCGGTTGGCGAATCCTGCGCTCGGCGGTGGTGGCAATACTGGCCGCTCCGATTCGGCGCTCGGCAATGA
CAGCGTTGTAGATGGGTCCGATATCAAGACGGAGGCGGACGAAGATACTGCCGAGG
>dme_circ_0004101_junction_seq
ACGGAGGCGGACGAAGATACTGCCGAGGCACAGCCACGGCGCTGCGGCTAAAGCCC
>dme_circ_0004102
ATGACAATACGAAAATGGACGTGGACTCGCCACTGGGTCGCACGGAGTCTTCGACTCGAAATGCCAAGAG
TACGCCCAGTGAAAGTGCCTCTCCGGCGCGTAGCAATGCAAGCACTAGCAGCGTAACACCAGCGGGAATT
AAGAAAGAGACTGATGACTCGAAAGCGACGACAGGATCTTTTGCACCAGCGTCCTCGGCAACGGCATCTT
CAGCGACGCCTGTGGACGATGCTACGCCATCAACATCCTCAGCAGCGGCGGCAGCATCAGCGGCATCGTC
ATCAACTGTTTCAGGAACCGAAGGCAAGCCAAAGGATGACCCCCTATCGTCGCACAAGGAAGAAGGAGCC
GGTAGCACCAGCTCTGGAGTTGCCACGAGTCCCAGGCAAGCCCAAGACACTGCAGGAGCAGGCGTCGATG
TGGAAATCAAACTGGAACATCCTGCGGACTTTTCCAATCCAAAGCTTCTACCGCCGCACGCGCACGGGCA
ACGCGAAGGACGAATCCTACACGCTGTTCTTCGCTAACAACAATTGGTATTTATTCCTGCGGCTACATGC
GATTCTCTGCGACCGTCTGCATGTTATGTATGAAAGAGCGCGTTTGCTGGCCATCGAGGAGGAGCGCTGT
AGGGTGAATCGAAGGGAGAGCACAGCCACGGCGCTGCGGCTAAAGCCCAAACCGGAGATTCAAGTTGAAG
ACTACTACCCCACCTTCCTTGATATGCTCAAGAACGTCCTAGACGGCAACATGGACTCGAATACGTTCGA
GGACACGATGCGTGAAATGTTTGGCATTTATGCCTACATCTCTTTCACGCTGGATAAGGGTTGTCTCCAA
TGCTGTTCGCCAGCTGCAATATTGTGTCACAGAACGAGCCGCACTGGATTGTGTAGAGCTGTTTGCAACG

GAGCAAAGGCGTGGCTGCACCGGAGGATTCTGTCGAGATGCGCACAAGACATTTGACAGGGAGATGTCGT
ATCAGCGGAAAGCTGAGAGCATTCTCAACGAGGAGAATTGCTTCAAAGTGTACATTGTACAAAATCGATT
GCCGTGTAACCATAGAGCTGCTTGATTCGGAGCCCGAGGAAGTCGATAAGCCGGCTGCGTTGAAGGCCCA
GAAGTTCAGCAAGTATGTAGAGCGGTTGGCGAATCCTGCGCTCGGCGGTGGTGGCAATACTGGCCGCTCC
GATTCGGCGCTCGGCAATGACAGCGTTGTAGATGGGTCCGATATCAAGACGGAGGCGGACGAAGATACTG
CCGAGG

>dme_circ_0004102_junction_seq

ACGGAGGCGGACGAAGATACTGCCGAGGATGACAATACGAAAATGGACGTGGACTC

>dme_circ_0004103

CCTTTCCATTTTTGGTAGATGACAATACGAAAATGGACGTGGACTCGCCACTGGGTCGCACGGAGTCTTC
GACTCGAAATGCCAAGAGTACGCCCAGTGAAAGTGCCTCTCCGGCGCGTAGCAATGCAAGCACTAGCAGC
GTAACACCAGCGGGAATTAAGAAAGAGACTGATGACTCGAAAGCGACGACAGGATCTTTTGCACCAGCGT
CCTCGGCAACGGCATCTTCAGCGACGCCTGTGGACGATGCTACGCCATCAACATCCTCAGCAGCGGCGGC
AGCATCAGCGGCATCGTCATCAACTGTTTCAGGAACCGAAGGCAAGCCAAAGGATGACCCCCTATCGTCG
CACAAGGAAGAAGGAGCCGGTAGCACCAGCTCTGGAGTTGCCACGAGTCCCAGGCAAGCCCAAGACACTG
CAGGAGCAGGCGTCGATGTGGAAATCAAACTGGAACATCCTGCGGACTTTTCCAATCCAAAGCTTCTACC
GCCGCACGCGCACGGGCAACGCGAAGGACGAATCCTACACGCTGTTCTTCGCTAACAACAATTGGTATTT
ATTCCTGCGGCTACATGCGATTCTCTGCGACCGTCTGCATGTTATGTATGAAAGAGCGCGTTTGCTGGCC
ATCGAGGAGGAGCGCTGTAGGGTGAATCGAAGGGAGAGCACAGCCACGGCGCTGCGGCTAAAGCCCAAAC
CGGAGATTCAAGTTGAAGACTACTACCCCACCTTCCTTGATATGCTCAAGAACGTCCTAGACGGCAACAT
GGACTCGAATACGTTCGAGGACACGATGCGTGAAATGTTTGGCATTTATGCCTACATCTCTTTCACGCTG
GATAAGGGTTGTCTCCAATGCTGTTCGCCAGCTGCAATATTGTGTCACAGAACGAGCCGCACTGGATTGT
GTAGAGCTGTTTGCAACGGAGCAAAGGCGTGGCTGCACCGGAGGATTCTGTCGAGATGCGCACAAGACAT
TTGACAGGGAGATGTCGTATCAGCGGAAAGCTGAGAGCATTCTCAACGAGGAGAATTGCTTCAAAGTGTA
CATTGTACAAAATCGATTGCCGTGTAACCATAGAGCTGCTTGATTCGGAGCCCGAGGAAGTCGATAAGCC
GGCTGCGTTGAAGGCCCAGAAGTTCAGCAAGTATGTAGAGCGGTTGGCGAATCCTGCGCTCGGCGGTGGT
GGCAATACTGGCCGCTCCGATTCGGCGCTCGGCAATGACAGCGTTGTAGATGGGTCCGATATCAAGACGG
AGGCGGACGAAGATACTGCCGAGG

>dme_circ_0004103_junction_seq

ACGGAGGCGGACGAAGATACTGCCGAGGCCTTTCCATTTTTGGTAGATGACAATAC

>dme_circ_0004104

TGATATTCAGAAAGAACTCCAAACGCTACGTGGCAGAGGCGAATCACCGGCGCAGCAACACCAGCAGCAG
ATAACCAGCCGTCAGTCCAATTTCTCTACCTTAGAACTCGAGCGCATTTGCCGCTTGCGGGGCGACCAAA
TGCATAGACAACATATGTCGCATTCACGAGCAGTCCAAGATCAAGAGTTGCGACCCCGACCGTTTGTTGC
ATATTACATACGCTCATAGAGGGCGGCCACTCACCTAGAGCAGCCAGAGTCCTTGCACTTGGGCAGGGAT
CCAGGGAGCAACAAAGTGCTTAGAAAGAAGAAGCAGCAGCACAGGATTATACGCTCCGCCGAGGACAGAG
CAACAAAAAAGATGATGAAACGCACTCGCGTAGACGAGGTGCAATTTGGCACACGACCCGTTCCTCAGAC
CTCCGGAGGTGTGGGCGTTGGTGTCGTCGGCGTAACTGGAGGTGGGCCAACATCCGGCGGCGGTGGCACT
GCTACTGTGGGCGTCAATACGACGGGCGTTACCATTGGTACCGTCGTGCCCAGTGCTCATAACGCCACCA
TCAGCGGGATCGGTTCCATTCACCATCGCATCCTTACTCCACAGCATGGAGGAGCACAAACCATTGCCTA
TTTGCCGTCCACCACGCCGACGGCTACGAATTTGAAGACCACAACCTCGATTGTGGATAGCACTACCGCT
GGAGGACCAGTAGGAGCAGGAGCACAGGTGGCCGTAGGAGTTGGCTCTGCAGCCGGCGGAGGAGGCGTGG
TGGTCTCCACAGGGAGCACGGGAACGCAAACATTACAGTATACAACATGCTTATAGTGTGGCTTCGATAC
AGGCTGGTGGAACTCTGAAAGCCAACACAGCGGATGGCGCGAACACGGTACAGATTCATGTCACAGGAGG

AGGAGCTGCAAACAATCCTGCCAGTGCACAGACTGTTTCCAGCAGCTCACAAACGGGCACAATCCGTCAG
CGTACAATCAGCGGCACCCAGACAGTGGCCACTGCAGTGGGTAATCTAGCCACGATTTCCCAGCAACAAC
CGGTTCAACAATCACCTTTGGGCAAGGCCCAAACGCCTCCATCGTCCGTGGTTGCCAACAGTATCCCAGT
GGGTGGAACCACCCCACCACAAGGACAGTCGGGAAATGCCACGCCACGCTTAAAAGTGGAGGATGCGTTG
AGCTACCTGGACCAAGTAAAGTACCAGTATGCAGACCAGCCGCAAATCTACAACAACTTCCTCGACATAA
TGAAGGAATTCAAGTCGCACTGCATCGACACACCCGGAGTGATTGAGCGAGTCTCCACACTCTTCAAGGG
GCACACAGAGCTGATATACGGCTTCAACATGTTCTTACCGCCGGGTTACAAGATCGAAATTCATTCGGAT
GCTCTTGGCTGTTCGGTGCCGGTGGTTTCGATGCCCTCACCACCGGGAGCGCCAACGAGCACAGGAACGG
TGCATATGCTCACCGGAAATAGTTCGATGTCCGGTGCCGGACATATCGCCATCAAGACGACCAATGCTGC
GACTTTAACACCGGCAGCAGGGGCTGGAGCAGCGGCCGCAGCAGCAGCTGTGGCTCAGATTCAATCCGCT
GGAGCTGTAAATCTAATGACCCACGGCGGAGCCTCGCTTACCCAGACCACTATTCATGCATTGCAACAGG
CGACGCCTCCTCAATCACAATCTCCAGGCGGAGGTCATGTCCACGTGAGTGTAACAAACTCTACGGCAGC
AAACGCCGTAGTGCCCGGACAGCCAGGCATCTCCGTATCGGCGCACAATGTACCGCAGAACTACTCGAGA
GATCGAGAAAGGGCCACCATAACACCTACTGGGCAAGTGGCTGGAGCAGCGGCAAACGTGAATGCGTCTG
CTAGTATAGTTGTCGGTGGACCTCCAACGCCCAATTCTCTAAGTGAGCTTAGCCCTCATGGAGGAGCAGG
AGGTGGGCCAGGTGCGGGAGCGGCGCAGCACAATCTGCATCACATCCAGCAGGCGCATCAATCGATTTTG
CTTGGGGAGACGGGACAGCAGAATCAGCCGGTGGAGTTCAATCACGCCATAACCTATGTAAATAAGATCA
AGGAATCGTTTCCAAAACCAGCCGGCCAAGTACAAGAAATTCCTCGAAATTCTGCACGATTATCAGAGGG
AGCAAAAGGTGATGAAGGAGGGCAGCCTGAACCAGGGTAAAATGCTCACCGAGCAGGAGGTATACACGCA
GGTGGCTAAGCTTTTTGGCCAGGATGAGGATTTGCTTAGAGAGTTTGGCCAGTTTCTTCCTGATGCCACC
AACCACCAGTCCGGGCAGTACATGTCCAAGTCAGCGTCTGTGCACAATGACCATGGCAAGCGTCCCACGG
CTACTTTAAGCGGTGGAGCTCACATAACCATGTCATCTGCTTCGCCAGCGCCAAGTGGATCCCCTCTGCA
TTTGGGTGCAACGACTCTTCCGCAGATCGACAAAAGCGCCCATGCAGCGGCCATAGGAAACCTTTCGGCG
GTGAACACTAGTGTCAGCATTAAGACGTACAATAATAATCAGCAACAGCAGAATCATGTGATCGGTTCAG
GGCTGAATGCGACGCGGAACGATATTCTCTTTGAGAAGGACTATCACGCGGGTCTGCAGCAGCAAGCGCA
TCAGCGAGGAGCTGGAGTTGGAGGCCATCATCATTTGGCCGGGACTGCAGCGGGCGCCAATATCGGACGG
CCTGGAGTGGGAGCCAGCGTGATGGTGTCGTACGATAAAGAGCATCGAAATAATCATCATGTACAAAAGT
ACGTTGGCCACGCGCCAAACCAGAATCTAACGCATGGTCATAACGCAAAGAAATCACCAAGTTATGGCAT
TCCTTCGGTGATTGGTTCCATGCCGCACATTTCGGACAATTCGCTCGACCGCAGTTCACCGGGAATTAGT
TACGCCACGCCACCATTGCCCTCTGGTCCACATGGACAGCATAACTCTGGTTCAGCTACGAGGAGGCCGG
GTGATGACAGTTTAGTCGGACACTATGCTAGTGGAGCGCCGCCTGCTAAGCGACCGAAGCCTTATTGTCG
TGATGTCAGCTTTTCCGAAGCATCGAGCAAGTGCACCATCTCCGACGCCGCGTTCTTTGACAAGGTGCGA
AAGGCGTTGAGAAGTCCAGAGGTCTACGACAACTTCCTGCGATGCCTGACCCTTTTTAACCAGGAAATTG
TCTCCAAAACGGAACTTCTTGGTCTGGTATCACCGTTCTTGATGAAGTTCCCCGACCTGCTGAGATGGTT
CACCGACTTTCTGGGACCGCCCTCTGGTCAACCTGCTGGAGGATTGATTGACGGCATGCCCTTAGCCGCA
ACGCAACGTCAGGGAGGTGGAAGCAGTAATAGTTCCCATGATCGAGGTACCAGTCACCAGAGTGCCGCCG
AGTATGTCCAGGATGTGGATCTGTCTTCTTGCAAGCGGCTGGGTGCATCCTATTGTGCTTTGCCGCAGTC
CACAGTACCTAAGAAGTGCAGCGGACGGACGGCTCTCTGCCGGGAAGTGCTCAACGACAAATGGGTATCG
TTCCCGACGTGGGCCAGCGAGGACTCCACTTTTGTTACATCGCGAAAAACTCAGTTCGAGGAGACAATTT
ACAGGGTAAGAAATATTCCTTTAACCTTTTCACTTAACAGTTCCACTTTAAATAAGTCACGAGTAAAATC
AATACTACTTCTTTATTGGTACTTTTGTTTCCTTTCCGTTTTCATTTGAGTCTAATATGTGCGGTTAACC
TTTTAAGAAATTTGTTTTTaaaaaacaaaaaaaTTGTAATTCTTCATTTGTTACAAAAGAAAAATTTGTT
CCATGtttttttttttttGTAACCGCATATATTTTATACTCTCAACTTACACATCTGTCGTTTGTTTTGG
ATCTTCTGTTTCTTTGGCTTTCGTTTATTTCAATTAACCAATGCGCACGCTCTAAACAAAAGGAATATTC

ACAGAGAATATTCACAGAACGGAGGACGAGCGATTCGAGCTGGACCTGGTCATCGAGGTTAATAGTGCCA
CGATTCGAGTGCTGGAGAACTTACAGAAGAAAATGTCACGAATGTCCACCGAGGAATTGAGCAAATTCCA
TCTGGACGATCATCTGGGCGGCACATCTCAAACGATACACCAGCGGGCCATTCATCGCATCTACGGCGAC
AAGTCGGGAGAGATAATCACGGGAATGAAGAAAAATCCGTTTGTGGCGGTGCCTATTGTACTGAAGCGAC
TGAAAGTCAAGGAGGAGGAGTGGCGAGAAGCGCAAAAGGACCTTCAATAAGCAATGGCGGGAACAGAACG
AGAAGTACTACCTCAAGTCTCTCGATCACCAGGCCATCAACTTTAAGCCCAACGACATGAAAGCCCTGCG
CTCAAAGAGTCTGTTTAACGAAATCGAGACGCTGTACGATGAGCGGCACGACCAAGAGGACGACGCCATG
GAACCGTTTGGGCCGCATCTGGTGCTGCCGTACAAGGATAAAACCATTCTGGACGATGCCGCCAACCTGC
TGATTCATCATGTGAAGCGTCAGACTGGCATCCAGAAGCAGGAGAAGCAAAAGATTAAGCAGATCATCCG
ACAATTTGTGCCTGACCTTTTCTTCGCGCCGCGACAGCCACTAAGCGATGACGAACGCGATGACGGGTAA
GTTCTCGTGGTCAAAATTCCTAAAACCATCATCAGTTCACTGTCAATTAACCTCCAACATATTTAATTTT
TCGGTTGCGCTAACACTATTCTTTAATTTAGTAGCTTCTAAAACTAACTAATATTTTCAAGATCTCTTTA
AGAGTTGACTTAAAACTCATCATCATCAGTACTCATACTAACAATATTTATATTCAtttttttttATTAA
GGAGTAGTGCTTTGTGATTTTGAAACGTTAACTAGTCATGTAAAAAAATTTACTTCTCGATAGAAATTTG
CATGACTATTTTTCTTTCAATACATAAAGTTTCTCCCTAATAACTTTTGCCCGATTGTATTTTTTAACAT
AGTGTATGttttttgtttctttttctccattttttttttGCAATTAAtaacaaaaacaacataaacaaat
aaaaacaaaattttgtaacgtaatcaaaatGCTATTAAATAAAATAAAACCAAAACTGGACTGCAATGCA
CCAACACCCACACACTGACCCCTGAACTGCATCGAATATCCGAACATATGTGTTGTGCACACCGTTTCTC
TATATAGCCCTTTCCATTTTTGGTAGATGACAATACGAAAATGGACGTGGACTCGCCACTGGGTCGCACG
GAGTCTTCGACTCGAAATGCCAAGAGTACGCCCAGTGAAAGTGCCTCTCCGGCGCGTAGCAATGCAAGCA
CTAGCAGCGTAACACCAGCGGGAATTAAGAAAGAGACTGATGACTCGAAAGCGACGACAGGATCTTTTGC
ACCAGCGTCCTCGGCAACGGCATCTTCAGCGACGCCTGTGGACGATGCTACGCCATCAACATCCTCAGCA
GCGGCGGCAGCATCAGCGGCATCGTCATCAACTGTTTCAGGAACCGAAGGCAAGCCAAAGGATGACCCCC
TATCGTCGCACAAGGAAGAAGGAGCCGGTAGCACCAGCTCTGGAGTTGCCACGAGTCCCAGGCAAGCCCA
AGACACTGCAGGAGCAGGCGTCGATGTGGAAATCAAACTGGAACATCCTGCGGACTTTTCCAATCCAAAG
CTTCTACCGCCGCACGCGCACGGGCAACGCGAAGGACGAATCCTACACGCTGTTCTTCGCTAACAACAAT
TGGTATTTATTCCTGCGGCTACATGCGATTCTCTGCGACCGTCTGCATGTTATGTATGAAAGAGCGCGTT
TGCTGGCCATCGAGGAGGAGCGCTGTAGGGTGAATCGAAGGGAGAGCACAGCCACGGCGCTGCGGCTAAA
GCCCAAACCGGAGATTCAAGTTGAAGACTACTACCCCACCTTCCTTGATATGCTCAAGAACGTCCTAGAC
GGCAACATGGACTCGAATACGTTCGAGGACACGATGCGTGAAATGTTTGGCATTTATGCCTACATCTCTT
TCACGCTGGATAAGGGTTGTCTCCAATGCTGTTCGCCAGCTGCAATATTGTGTCACAGAACGAGCCGCAC
TGGATTGTGTAGAGCTGTTTGCAACGGAGCAAAGGCGTGGCTGCACCGGAGGATTCTGTCGAGATGCGCA
CAAGACATTTGACAGGGAGATGTCGTATCAGCGGAAAGCTGAGAGCATTCTCAACGAGGAGAATTGCTTC
AAAGTGTACATTGTACAAAATCGATTGCCGTGTAACCATAGAGCTGCTTGATTCGGAGCCCGAGGAAGTC
GATAAGCCGGCTGCGTTGAAGGCCCAGAAGTTCAGCAAGTATGTAGAGCGGTTGGCGAATCCTGCGCTCG
GCGGTGGTGGCAATACTGGCCGCTCCGATTCGGCGCTCGGCAATGACAGCGTTGTAGATGGGTCCGATAT
CAAGACGGAGGCGGACGAAGATACTGCCGAGG
>dme_circ_0004104_junction_seq
ACGGAGGCGGACGAAGATACTGCCGAGGTGATATTCAGAAAGAACTCCAAACGCTA
>dme_circ_0004105
GCCATCAACTTTAAGCCCAACGACATGAAAGCCCTGCGCTCAAAGAGTCTGTTTAACGAAATCGAGACGC
TGTACGATGAGCGGCACGACCAAGAGGACGACGCCATGGAACCGTTTGGGCCGCATCTGGTGCTGCCGTA
CAAGGATAAAACCATTCTGGACGATGCCGCCAACCTGCTGATTCATCATGTGAAGCGTCAGACTGGCATC
CAGAAGCAGGAGAAGCAAAAGATTAAGCAGATCATCCGACAATTTGTGCCTGACCTTTTCTTCGCGCCGC

GACAGCCACTAAGCGATGACGAACGCGATGACGGGTAAGTTCTCGTGGTCAAAATTCCTAAAACCATCAT
CAGTTCACTGTCAATTAACCTCCAACATATTTAATTTTTCGGTTGCGCTAACACTATTCTTTAATTTAGT
AGCTTCTAAAACTAACTAATATTTTCAAGATCTCTTTAAGAGTTGACTTAAAACTCATCATCATCAGTAC
TCATACTAACAATATTTATATTCAtttttttttATTAAGGAGTAGTGCTTTGTGATTTTGAAACGTTAAC
TAGTCATGTAAAAAAATTTACTTCTCGATAGAAATTTGCATGACTATTTTTCTTTCAATACATAAAGTTT
CTCCCTAATAACTTTTGCCCGATTGTATTTTTTAACATAGTGTATGttttttgtttctttttctccattt
tttttttGCAATTAAtaacaaaaacaacataaacaaataaaaacaaaattttgtaacgtaatcaaaatGC
TATTAAATAAAATAAAACCAAAACTGGACTGCAATGCACCAACACCCACACACTGACCCCTGAACTGCAT
CGAATATCCGAACATATGTGTTGTGCACACCGTTTCTCTATATAGCCCTTTCCATTTTTGGTAGATGACA
ATACGAAAATGGACGTGGACTCGCCACTGGGTCGCACGGAGTCTTCGACTCGAAATGCCAAGAGTACGCC
CAGTGAAAGTGCCTCTCCGGCGCGTAGCAATGCAAGCACTAGCAGCGTAACACCAGCGGGAATTAAGAAA
GAGACTGATGACTCGAAAGCGACGACAGGATCTTTTGCACCAGCGTCCTCGGCAACGGCATCTTCAGCGA
CGCCTGTGGACGATGCTACGCCATCAACATCCTCAGCAGCGGCGGCAGCATCAGCGGCATCGTCATCAAC
TGTTTCAGGAACCGAAGGCAAGCCAAAGGATGACCCCCTATCGTCGCACAAGGAAGAAGGAGCCGGTAGC
ACCAGCTCTGGAGTTGCCACGAGTCCCAGGCAAGCCCAAGACACTGCAGGAGCAGGCGTCGATGTGGAAA
TCAAACTGGAACATCCTGCGGACTTTTCCAATCCAAAGCTTCTACCGCCGCACGCGCACGGGCAACGCGA
AGGACGAATCCTACACGCTGTTCTTCGCTAACAACAATTGGTATTTATTCCTGCGGCTACATGCGATTCT
CTGCGACCGTCTGCATGTTATGTATGAAAGAGCGCGTTTGCTGGCCATCGAGGAGGAGCGCTGTAGGGTG
AATCGAAGGGAGAGCACAGCCACGGCGCTGCGGCTAAAGCCCAAACCGGAGATTCAAGTTGAAGACTACT
ACCCCACCTTCCTTGATATGCTCAAGAACGTCCTAGACGGCAACATGGACTCGAATACGTTCGAGGACAC
GATGCGTGAAATGTTTGGCATTTATGCCTACATCTCTTTCACGCTGGATAAGGTGAGTGTTAAAATTTTA
TTCAAAAAGTTGATATCTATTTTTTCTAAAAACCTTTCCTTTTGAATATAATAAACTAAACTTTTCGCAT
TTGTTCATCTTCAGGTTGTCTCCAATGCTGTTCGCCAGCTGCAATATTGTGTCACAGAACGAGCCGCACT
GGATTGTGTAGAGCTGTTTGCAACGGAGCAAAGGCGTGGCTGCACCGGAGGATTCTG
>dme_circ_0004105_junction_seq
AAAGGCGTGGCTGCACCGGAGGATTCTGGCCATCAACTTTAAGCCCAACGACATGA
>dme_circ_0004106
AACGGAGGACGAGCGATTCGAGCTGGACCTGGTCATCGAGGTTAATAGTGCCACGATTCGAGTGCTGGAG
AACTTACAGAAGAAAATGTCACGAATGTCCACCGAGGAATTGAGCAAATTCCATCTGGACGATCATCTGG
GCGGCACATCTCAAACGATACACCAGCGGGCCATTCATCGCATCTACGGCGACAAGTCGGGAGAGATAAT
CACGGGAATGAAGAAAAATCCGTTTGTGGCGGTGCCTATTGTACTGAAGCGACTGAAAGTCAAGGAGGAG
GAGTGGCGAGAAGCGCAAAAGGACCTTCAATAAGCAATGGCGGGAACAGAACGAGAAGTACTACCTCAAG
TCTCTCGATCACCAGGCCATCAACTTTAAGCCCAACGACATGAAAGCCCTGCGCTCAAAGAGTCTGTTTA
ACGAAATCGAGACGCTGTACGATGAGCGGCACGACCAAGAGGACGACGCCATGGAACCGTTTGGGCCGCA
TCTGGTGCTGCCGTACAAGGATAAAACCATTCTGGACGATGCCGCCAACCTGCTGATTCATCATGTGAAG
CGTCAGACTGGCATCCAGAAGCAGGAGAAGCAAAAGATTAAGCAGATCATCCGACAATTTGTGCCTGACC
TTTTCTTCGCGCCGCGACAGCCACTAAGCGATGACGAACGCGATGACGG
>dme_circ_0004106_junction_seq
CACTAAGCGATGACGAACGCGATGACGGAACGGAGGACGAGCGATTCGAGCTGGAC
>dme_circ_0004107
TGATATTCAGAAAGAACTCCAAACGCTACGTGGCAGAGGCGAATCACCGGCGCAGCAACACCAGCAGCAG
ATAACCAGCCGTCAGTCCAATTTCTCTACCTTAGAACTCGAGCGCATTTGCCGCTTGCGGGGCGACCAAA
TGCATAGACAACATATGTCGCATTCACGAGCAGTCCAAGATCAAGAGTTGCGACCCCGACCGTTTGTTGC
ATATTACATACGCTCATAGAGGGCGGCCACTCACCTAGAGCAGCCAGAGTCCTTGCACTTGGGCAGGGAT

CCAGGGAGCAACAAAGTGCTTAGAAAGAAGAAGCAGCAGCACAGGATTATACGCTCCGCCGAGGACAGAG
CAACAAAAAAGATGATGAAACGCACTCGCGTAGACGAGGTGCAATTTGGCACACGACCCGTTCCTCAGAC
CTCCGGAGGTGTGGGCGTTGGTGTCGTCGGCGTAACTGGAGGTGGGCCAACATCCGGCGGCGGTGGCACT
GCTACTGTGGGCGTCAATACGACGGGCGTTACCATTGGTACCGTCGTGCCCAGTGCTCATAACGCCACCA
TCAGCGGGATCGGTTCCATTCACCATCGCATCCTTACTCCACAGCATGGAGGAGCACAAACCATTGCCTA
TTTGCCGTCCACCACGCCGACGGCTACGAATTTGAAGACCACAACCTCGATTGTGGATAGCACTACCGCT
GGAGGACCAGTAGGAGCAGGAGCACAGGTGGCCGTAGGAGTTGGCTCTGCAGCCGGCGGAGGAGGCGTGG
TGGTCTCCACAGGGAGCACGGGAACGCAAACATTACAGTATACAACATGCTTATAGTGTGGCTTCGATAC
AGGCTGGTGGAACTCTGAAAGCCAACACAGCGGATGGCGCGAACACGGTACAGATTCATGTCACAGGAGG
AGGAGCTGCAAACAATCCTGCCAGTGCACAGACTGTTTCCAGCAGCTCACAAACGGGCACAATCCGTCAG
CGTACAATCAGCGGCACCCAGACAGTGGCCACTGCAGTGGGTAATCTAGCCACGATTTCCCAGCAACAAC
CGGTTCAACAATCACCTTTGGGCAAGGCCCAAACGCCTCCATCGTCCGTGGTTGCCAACAGTATCCCAGT
GGGTGGAACCACCCCACCACAAGGACAGTCGGGAAATGCCACGCCACGCTTAAAAGTGGAGGATGCGTTG
AGCTACCTGGACCAAGTAAAGTACCAGTATGCAGACCAGCCGCAAATCTACAACAACTTCCTCGACATAA
TGAAGGAATTCAAGTCGCACTGCATCGACACACCCGGAGTGATTGAGCGAGTCTCCACACTCTTCAAGGG
GCACACAGAGCTGATATACGGCTTCAACATGTTCTTACCGCCGGGTTACAAGATCGAAATTCATTCGGAT
GCTCTTGGCTGTTCGGTGCCGGTGGTTTCGATGCCCTCACCACCGGGAGCGCCAACGAGCACAGGAACGG
TGCATATGCTCACCGGAAATAGTTCGATGTCCGGTGCCGGACATATCGCCATCAAGACGACCAATGCTGC
GACTTTAACACCGGCAGCAGGGGCTGGAGCAGCGGCCGCAGCAGCAGCTGTGGCTCAGATTCAATCCGCT
GGAGCTGTAAATCTAATGACCCACGGCGGAGCCTCGCTTACCCAGACCACTATTCATGCATTGCAACAGG
CGACGCCTCCTCAATCACAATCTCCAGGCGGAGGTCATGTCCACGTGAGTGTAACAAACTCTACGGCAGC
AAACGCCGTAGTGCCCGGACAGCCAGGCATCTCCGTATCGGCGCACAATGTACCGCAGAACTACTCGAGA
GATCGAGAAAGGGCCACCATAACACCTACTGGGCAAGTGGCTGGAGCAGCGGCAAACGTGAATGCGTCTG
CTAGTATAGTTGTCGGTGGACCTCCAACGCCCAATTCTCTAAGTGAGCTTAGCCCTCATGGAGGAGCAGG
AGGTGGGCCAGGTGCGGGAGCGGCGCAGCACAATCTGCATCACATCCAGCAGGCGCATCAATCGATTTTG
CTTGGGGAGACGGGACAGCAGAATCAGCCGGTGGAGTTCAATCACGCCATAACCTATGTAAATAAGATCA
AGGAATCGTTTCCAAAACCAGCCGGCCAAGTACAAGAAATTCCTCGAAATTCTGCACGATTATCAGAGGG
AGCAAAAGGTGATGAAGGAGGGCAGCCTGAACCAGGGTAAAATGCTCACCGAGCAGGAGGTATACACGCA
GGTGGCTAAGCTTTTTGGCCAGGATGAGGATTTGCTTAGAGAGTTTGGCCAGTTTCTTCCTGATGCCACC
AACCACCAGTCCGGGCAGTACATGTCCAAGTCAGCGTCTGTGCACAATGACCATGGCAAGCGTCCCACGG
CTACTTTAAGCGGTGGAGCTCACATAACCATGTCATCTGCTTCGCCAGCGCCAAGTGGATCCCCTCTGCA
TTTGGGTGCAACGACTCTTCCGCAGATCGACAAAAGCGCCCATGCAGCGGCCATAGGAAACCTTTCGGCG
GTGAACACTAGTGTCAGCATTAAGACGTACAATAATAATCAGCAACAGCAGAATCATGTGATCGGTTCAG
GGCTGAATGCGACGCGGAACGATATTCTCTTTGAGAAGGACTATCACGCGGGTCTGCAGCAGCAAGCGCA
TCAGCGAGGAGCTGGAGTTGGAGGCCATCATCATTTGGCCGGGACTGCAGCGGGCGCCAATATCGGACGG
CCTGGAGTGGGAGCCAGCGTGATGGTGTCGTACGATAAAGAGCATCGAAATAATCATCATGTACAAAAGT
ACGTTGGCCACGCGCCAAACCAGAATCTAACGCATGGTCATAACGCAAAGAAATCACCAAGTTATGGCAT
TCCTTCGGTGATTGGTTCCATGCCGCACATTTCGGACAATTCGCTCGACCGCAGTTCACCGGGAATTAGT
TACGCCACGCCACCATTGCCCTCTGGTCCACATGGACAGCATAACTCTGGTTCAGCTACGAGGAGGCCGG
GTGATGACAGTTTAGTCGGACACTATGCTAGTGGAGCGCCGCCTGCTAAGCGACCGAAGCCTTATTGTCG
TGATGTCAGCTTTTCCGAAGCATCGAGCAAGTGCACCATCTCCGACGCCGCGTTCTTTGACAAGGTGCGA
AAGGCGTTGAGAAGTCCAGAGGTCTACGACAACTTCCTGCGATGCCTGACCCTTTTTAACCAGGAAATTG
TCTCCAAAACGGAACTTCTTGGTCTGGTATCACCGTTCTTGATGAAGTTCCCCGACCTGCTGAGATGGTT
CACCGACTTTCTGGGACCGCCCTCTGGTCAACCTGCTGGAGGATTGATTGACGGCATGCCCTTAGCCGCA

ACGCAACGTCAGGGAGGTGGAAGCAGTAATAGTTCCCATGATCGAGGTACCAGTCACCAGAGTGCCGCCG
AGTATGTCCAGGATGTGGATCTGTCTTCTTGCAAGCGGCTGGGTGCATCCTATTGTGCTTTGCCGCAGTC
CACAGTACCTAAGAAGTGCAGCGGACGGACGGCTCTCTGCCGGGAAGTGCTCAACGACAAATGGGTATCG
TTCCCGACGTGGGCCAGCGAGGACTCCACTTTTGTTACATCGCGAAAAACTCAGTTCGAGGAGACAATTT
ACAGG
>dme_circ_0004107_junction_seq
AACTCAGTTCGAGGAGACAATTTACAGGTGATATTCAGAAAGAACTCCAAACGCTA
>dme_circ_0004108
GTGGGCCAGGTGCGGGAGCGGCGCAGCACAATCTGCATCACATCCAGCAGGCGCATCAATCGATTTTGCT
TGGGGAGACGGGACAGCAGAATCAGCCGGTGGAGTTCAATCACGCCATAACCTATGTAAATAAGATCAAG
GAATCGTTTCCAAAACCAGCCGGCCAAGTACAAGAAATTCCTCGAAATTCTGCACGATTATCAGAGGGAG
CAAAAGGTGATGAAGGAGGGCAGCCTGAACCAGGGTAAAATGCTCACCGAGCAGGAGG
>dme_circ_0004108_junction_seq
CAGGGTAAAATGCTCACCGAGCAGGAGGGTGGGCCAGGTGCGGGAGCGGCGCAGCA
>dme_circ_0004109
GATTCTACAAAACTTGGGCAAAGTCGATCGCACTGCGGATGAAATCTTCGACGATCACCTGAACAACTTC
AATCGCCAGCAGGCGAGTGCCAACAGATTACAAAAGGAGTTCAATAACTACATAAGATGTGTTCGCGGCC
GCACAAGCCGCCTCCAAGACTCTGATGGATTCCGTTTGCGAGATCTACGAGCCACAATGGAGCGGTTACG
ATGCCCTGCAAGCACAAACGGGTGCCTCGGAAAGTCTGTGGGCGGACTTTGCGCACAAATTGGGGGATCA
AGTCCTCATTCCGCTCAACACATACACTGGACAGTTTCCCGAAATGAAGGCTAACCAAAGGACGCGAGCA
GCTGGAGGAAGCTAGACGCACCTACGAAATCCTGAACACGGAACTGCATGACGAGCTGCCCGCCTTGTAT
GACTCTAGGATACTGTTTCTGGTCACCAACTTGCAGACACTCTTCGCCACAGAGCAAGTGTTCCACAACG
AAACGGCTAAG
>dme_circ_0004109_junction_seq
GCAAGTGTTCCACAACGAAACGGCTAAGGATTCTACAAAACTTGGGCAAAGTCGAT
>dme_circ_0004110
GATTCTACAAAACTTGGGCAAAGTCGATCGCACTGCGGATGAAATCTTCGACGATCACCTGAACAACTTC
AATCGCCAGCAGGCGAGTGCCAACAGATTACAAAAGGAGTTCAATAACTACATAAGATGTGTTCGCGGCC
GCACAAGCCGCCTCCAAGACTCTGATGGATTCCGTTTGCGAGATCTACGAGCCACAATGGAGCGGTTACG
ATGCCCTGCAAGCACAAACGGGTGCCTCGGAAAGTCTGTGGGCGGACTTTGCGCACAAATTGGGGGATCA
AGTCCTCATTCCGCTCAACACATACACTGGACAGTTTCCCGAAATGAAGGCTAACCAAAGGACGCGAGCA
GCTGGAGGAAGCTAGACGCACCTACGAAATCCTGAACACGGAACTGCATGACGAGCTGCCCGCCTTGTAT
GACTCTAGGATACTGTTTCTGGTCACCAACTTGCAGACACTCTTCGCCACAGAGCAAGTGTTCCACAACG
AAACGGCTAAGGGTGGGTTTGAAACTAACAACTAAGTAACTACTTAAGATACTATTGCTATAACATAAGA
TATAATCAAAGGATTAAAGAGATCTTGAAACTCCTTATAAATACAAACACACTGTTTCTAAGATGAATGT
CAAAGGAGTGTTGCTTTGTATGTACATAAATACCGACTAATGGCAAATCTTATTTTCATCTTTGCGACAA
CAGATTTACTCGGAACTGGAGGCAATCGTCGACAAATTGGCCACAGAATCGCAGCGCGGCTCCAATACGC
TACGCAAGCAAACAAGTAGGGAGTCCAGCCTGTAATTACTATAACTATAACTATAGTTTCTACTTTTGAA
CAGGCAATCCCATCAAGACATCGAGCCCAGTGCAGTCGCCAGTGAATAAGTTAAACAATGCCAACATCAA
CAGCAACTATCAGAATCAAATAACTACAAACGGTGGCTCCAGTTTGGCAAACAGCC
>dme_circ_0004110_junction_seq
AACGGTGGCTCCAGTTTGGCAAACAGCCGATTCTACAAAACTTGGGCAAAGTCGAT
>dme_circ_0004111
GATTTACTCGGAACTGGAGGCAATCGTCGACAAATTGGCCACAGAATCGCAGCGCGGCTCCAATACGCTA

CGCAAGCAAACAAGTAGGGAGTCCAGCCTGTAATTACTATAACTATAACTATAGTTTCTACTTTTGAACA
GGCAATCCCATCAAGACATCGAGCCCAGTGCAGTCGCCAGTGAATAAGTTAAACAATGCCAACATCAACA
GCAACTATCAGAATCAAATAACTACAAACGGTGGCTCCAGTTTGGCAAACAGCC
>dme_circ_0004111_junction_seq
AACGGTGGCTCCAGTTTGGCAAACAGCCGATTTACTCGGAACTGGAGGCAATCGTC
>dme_circ_0004112
GTCAATTGAATGTATTTTTGGGGCGATGCAAAAAGGCCAGATGTAATTCCTCTGTGGTCCCCGAAGTTTT
GGAAATTGTTTCAAGTAACAACATTAACGTTTAGCGATTACGTGTATCTTTAATTT
>dme_circ_0004112_junction_seq
GTTTAGCGATTACGTGTATCTTTAATTTGTCAATTGAATGTATTTTTGGGGCGATG
>dme_circ_0004113
GGAACTGGCCGATCTGGTGATGAGCATCGATTATGAGACCGTTACCACGTTCGAGGATCCATACTTGAAT
GCCATCAAAACGCTTTGGGACGATGCTGGCATCCAGGAGTGCTATGATCGTCGTAGGGAATATCAGCTGA
CTGATTCAGCCAAATAAGTAAGTTAACCGTTGGCATGCAAAGGGAACGGTTTAAATCCGTCCGCAAAATG
ACCGAAAAACGAACTGAAGAGACTGAAACTACCGAAACGACTATGCAAACTAAATCAATTATCTTGTCTA
TGTATAGTTATCTGAAGGATCTCGATCGTGTGGCTCAACCTGCATATTTACCCACTGAGCAAGACATTTT
AAGAGTTCGTGTGCCCACAACAGGGATAATTGAGTATCCCTTTGATTTAGAAGAAATCAGATTTAGGTAC
ATACCATCTTTCTACCCTTACTTCGTTGCATGTGCATGACCTCTTGTTTGGTTATGGTTAAAATTTATTT
TGTACTTTGTAACTACACACTTAAGAATTTCTTTAGCGCATGTCTCTTCAAGCAAACAATTCAGGGACTT
AGTTAGGGCCTTCAGCGAAATTGATGGACTGAGACGGGTTGCAAAACTATTTTTGCCACTGCTTTTGGTT
CCGTTCATTGTTTTAAAAGTGTATTAATTTATCTAATTTAGGGTACATACCATCTTTCTACCCTTACTTC
GTTGCATGTGCATGACCTCTTGTTTGGTTATGGTTAAAATTTATTTTGTACTTTGTAACTACACACTTAA
GAATTTCTTTAGCGCATGTCTCTTCAAGCAAACAATTCAGGGACTTAGTTAGGGCCTTCAGCGAAATTGA
TGGACTGAGACGGGTTGCAAAACTATTTTTGCCACTGCTTTTGGTTCCGTTCATTGTTTTAAAAGTGTAT
TAATTTATCTAATTTAGCTACTTGAGCGATCTAGCTCGAATTGAACAGGCTGATTACTTGCCAACCGAGC
AGGATATTCTGCGTGCTCGAGTGCCGACAACTGGCATTCTTGAGTATCCATTCGATTTGGATGGCATTGT
GTTTAGGTATCTATTAAAAATATCAATTGGTAATTACCGGCAAGGCTAGTAATGTAAATGGTCATTGTAA
AGCTAGTGAAGACGATTTCTTACAATTTAGTTACTAATAGATTCGTTGCTTGCGCTCTAGAATTAAATGC
TTGTATGTTTGGCCTTATATGTAGTTAATTTGTTGGTTAACTTTTCTTACAATAATATATTTTTCTTAAT
AAACAGGAATGGTAGACGTCGGTGGTCAGCGATCCGAGAGAAGAAAGTGGATTCATTGCTTTGAGAATGT
GACATCAATTATATTTTTGGTAGCGCTATCGGAGTACGATCAAATCTTGTTTGAATCTGATAATGAGGAA
TCGAATGGAGGAATCTAAAGCTTTATTTCGTACTATAATTACATACCCTTGGTTTCAAAATTCGTCAGTT
ATTCTTTTCCTGAATAAGAAGGACTTGTTGGAAGAGAAAATAATGTATTCGCATTTGGTAGACTATTTTC
CTGAATACGATGGGTAAGTGTTCCGCATGATAAAGGTGGCCATAATTATGTACGATTTTATGCGGCAATG
AACCAGGTCCAAAACAGGATCACGCTGCGGCCAAACAGTTCGTTCTGAAAAAGTACTTGGCCTGCAATCC
AGATCCCGAACGTCAATGCTATTCGCATTTCACAACGGCCACAGGTGGATTTTCACCTTATGTACTTAAG
ATCTATGTAATTGCCTTAAATCGCCAATATCATCCGTAGATTATTTGCTAGTTTTCGTAGAACCACGCTA
GACGTAACTTCATATTCGCCTTCTTATCGAAACAGGGTGGATTTTCACCTTATGTACTTAAGATCTATGT
AATTGCCTTAAATCGCCAATATCATCCGTAGATTATTTGCTAGTTTTCGTAGAACCACGCTAGACGTAAC
TTCATATTCGCCTTCTTATCGAAACAGGTCCTCAGCGAGATGCAATAACGGCCCGAGAGTTTATACTGCG
AATGTTTGTAGATTTAAATCCAGATTCCGAAAAAATTATCTATTCTCATTTCACGTGTGCTACAGGTAAG
TTTGCTGCCAGTTCTGTGTTTGGTCAGAAATTGTGTGTGGATGTGATTTTAGTTTTCAGCCTAAAAAGGA
CTGGTCAAAAGGTAAACTTTTGTGTGAAAAACATTTACATTTGCATATTTGCTTTTGCCGCTGATCTAGA
GTAGAGAGTAGTTGTGTATCGCTAGTCAAATTTGCAAACCTAAACGAATTGATTTCTAATAAAACAACGA

TCTCTTGGTAAACAGGGTAAGTTTGCTGCCAGTTCTGTGTTTGGTCAGAAATTGTGTGTGGATGTGATTT
TAGTTTTCAGCCTAAAAAGGACTGGTCAAAAGGTAAACTTTTGTGTGAAAAACATTTACATTTGCATATT
TGCTTTTGCCGCTGATCTAGAGTAGAGAGTAGTTGTGTATCGCTAGTCAAATTTGCAAACCTAAACGAAT
TGATTTCTAATAAAACAACGATCTCTTGGTAAACAGATACCGAAAACATCAAACTTGTGTTCTGCGCTGT
CAAAGATACAATTATGCAAAATGCACTTAAGGAATTCAATTTGGGCTAAGGCGGGCAGTGCGGATATCAG
GAAAAGTGGCAATGCAAATGAATTATTTATTTATAATGTATATGATTTGTTTTTCAAAGTCCCTCAATAA
AAACTAAATATAGTCTGTGAAATATCGTGAATGGAAAATGTGTGCAGTTTACTATCATAGGATTATTTGA
CAAAATCAAACTAACTACGCCTTCTTTTCTTTTGTCTAGGATACGGAAAATATAAGGTTTGTGTTTGCAG
CTGTTAAGGACACAATTCTGCAATCGAACCTTAAGGAATATAATTTGGTCTAAACTGGATTTGGATCGAA
AAACTATTTTTGTGATTTTTATACAAACACACATACACATTCATATAATTCCTTTTTCGCATATTTACTA
AATATAAAAGAAGAATTGAGAAGCGAGCGAATGTGGCCGAAAATTAGGCATAAATTAGACCCGAGAAAGT
TTCTTACGTTTGTTACATCAGGAACCGAAAATTGTAGCATAAATAGAATACTAAACTGAGATGCTAAAAC
CGCATCAGCAGAATGATGAAAAAGATGTATACATTTTAATTTGAGAGAAAAATAACACAAAATCTGATAC
AGTGTGACAACAAATAATCAGGGCAGAGTTTCTCTTACGAATGTGACGATTCGTAAAACATTTATTTATA
TTGCAGTACTTCTATTCTTTCTAGCGGCAAAGTTTTCAGAAAACGTTGTGTGAAttttttttttttGTTT
CGACTAGGAAAACTCTTGAAAGTCAAAAAGACAGTGGCCAGAAATGATTGGTACCCAATCGATAGAGTTT
TTGGTTAGATTTGAATAAATCTGATGGTAGTTTTTCCTTCCTACACAAGAAACATGATTTCCTTGAAAAG
TTTTTATACAAAATATCGTCCGAATTAGTTGTTGTCACTCTGTAATATTTAAATAAATTTATTACTATAG
TAACTTTTGTAATAAAATCCGAAACACAAAATATGAAACGTAAATTTAATCAAAAGCTTGATAAGGAACA
ACAAACTGAAACAACTTTGTCGTACTGAAGCATAACATAATAAAGCAACATTTTACTAAATAACTACAGA
AAAACAAAAACATACTTTTGCGTAAAAATCCCCCGAAATATATATATATTCAAAGAATTATATATGCAAG
AAGTTTCTTATTAGAATTTCAGGGCATATAGGCGTGTAAAAATAATAAACCGaaaatgataagctgataa
aatgataaaatgaATGAAAACGAAATCACAGATAATACTGTATTTTTTATTAAGCTAAACAGTAAACCGT
AAATATTGATACTATTGTTTACCGAAatatttaactatacatgaaagcagatataataattcataaacgc
atatgtaacaattTAAAAATTGATTAATTTGCATTGCGCGAGCTCCATTCATTGTAAATTACGATTCGTA
TTAATACCATGAGAATGAAAAGAAAAGAAATGAAACGCATGCAAACCAATACAAAAACGATATAGGAGCT
AGATTTGTATGAGAAAAGCCAGAAACATTTACATGGAGCATTCATTAAATACGTGTTTGTAAAAAATGTC
GATAACCACCcatgcaacatgcaacatgcaacaGCAGAATCCATACAACTTATTGATATTTAATCAGACA
TCAGGTTAAATCCAATCAAATGCGATATACCACTGGTCGAGAAACACAACTATCAGAAGGGAAAGTTTTG
CTCTCACGTACTTTTGTTGCCATCAAAATTTCAGTCGAAACATTGTTATCAAAAATAGCTTGATTTTCGT
ACTTTTTACATTAACTTTGTTAGTTGTTTGCTTATTTTATAGATCTTAATGTATGCAACAAGGGACTCGC
TTTGGCGGCTAGCCAAAACGGCGATGTCTGGCCAAGTGGGTTCAGtgttttgtttttgtttttataattt
tagtttaatttAATATACAAGGATAACTAAATGAGACGCACATAAATAGAAAACCCCATCAACAAATTAT
TTATGTAAAAATCCAAATAAAAACAATTCTACTCTACGCATATGTGGAAACAAAAAATAAATAAAATGGA
AAACAATTGAAACGTACATGTATGTAAAATAAATATGATGCAAAATGTTTTTCATAATTTATAGTACACT
TATTTTGTTCAACCGAGCGAGATTCCCTCAACCCACATCCCCATCATTTGGCGTTTAGTTCCCTCGGGGC
CACAGAGGAGTAGACACTTTGCCACGACTCCAAGCGGAAGACACTACTCATGGGAGTGGAGAATTGAGAT
ATTCCTATGACCGCGAATAACCAAAAACCAAGACGCATCTTAGTTTAAGTACGATTCAACAGTGTGTTGT
ATAAAGCTTTAATCCTCGTAACCACATATAGTGAAAGTAAAAGTAAAGTAAAAATTAAACCATTTTTATG
ATTAACCAATGGATTAGCTTTTAAAAAGCTTCAATAAGTCAATGTGTATTTTAAAAGATGTAGCCGAGAA
CACAATGAGaaaaaaaaGCCAATGAGTTTTGAGGGCCCGTTCGGTATGGATATGGAATCGGTTTAGGTTC
TTGCGAATATGCGAATACAAAAATTCACAGCTGGCATCCAATTTGGTAGCTCCGATTTTGGCCTGGCCCA
TTTTTTATATCCACGGCATTATAGACATTCACACACTGACATACATATATATAGTTTTCCGTCCAATGAA
TTGCATCAGCATCAGCGAGCAGACAAAGTCTCCCATTTATTTTGATCCTTAACATTTGCGATTTGTTTCT

TCCGAAAATTCCATCCTTTAAAACAAGAGTTTTTCGGCGGTTTCAATGATAAAATGTTCCGACTTTGCAG
CCACACATACGCACACTTTAAATTGATCGATTTCGATATGATTTCCCTAACATTTTGTGCCTTTCCAGCA
TAATGAATCCCCATATTTTATATATAGGACAGACTCCAAATGGGTACTCCAAACTTGATTTAtttttttt
ttttGTACCACTAATGGAGCCTGTTAAAAGCCCCGAGACATTCAGATTATCAGCATGTGTGTATGGCCAG
ATTGTTGTGCTTTGGGTTGATAGATTGGTTCCTTAATTTTATGATACGCACCGATAGTCAGACAGTTGTA
TGTAAATATTTGGCAAATTACTCGCGAGGGCATTCAAAAGGCCTCCCGAGAGATATACCACATAATCGAG
CATGTGTATGTAAGTGTACATTAAACGCAAAATGAACTATAATAAAATGAGAATTTGATATAAACCATTA
ATGTATGTATTTTGTATTTTTATATACAATTTATAATTTTGATTACACTTACAATTGTTACGATTAATAA
CTAAAATTACATGATAAAACCAAAGTAACGCAGATGAATCTACTAGATATGTATTTCCAATATAGAGAAG
CCGAGTACATAATACACAAATCCGACGATTGCTAAAGGAATTTCAGAATATTGTAAGGAATTAAGATTTa
aaaaaaaaGAAATTTTCTAAGCAACAAACTCAAACAGAACTGCAAGCAAAAGGGACAACAATTTAAATGG
GAAATTTATGTAATTGTGTAAATCTAGAGCGGAATGAAAGCGAAGGATGCAATTTGATCCTTCGGGCCGG
ATACTCGATAAGTATGGCCACGATTTTCAGATACTTGAAAACCATTTAGGCATGGCATTCGGTGGCTAGT
TTTTACTTTAACTATGTATGTAAATGAAAGAATGACCACATTCCACATAAGAAAGCTTGAAAAGCaaaaa
aaaatgaaaaaaaaaaaattcttaaaaaaGAATCCCGAATGAAAATCGCCCTTGACGTTGAGGAAAACTAC
ATTATCGATAATAGGAATGGCTAGACGAAGTTTTCGGTTTTGAAATAAAACCCTCTATAAACTCAGCATC
GGGATAAGCAGTAAGAAATGTTAGTTGTGAGTTCGGGCGTCATTAGGGAGAAATAGTAATATTCGACTCT
AGTTTCTCCATTGCACACCTAAGCATATACATACATACGTGTAAATCGGAAGCTAATGAAACATCCACTG
AAACTGCATGCTTAGCCTAGGATTAAGGCAATCCCACACAGACCGATTGCCCCGATTTGGAGACAGAGAC
AGTCCAACATGTCCATGGGCCGACTATTGATAACTGTAGCATttgaattgaattgattgaattgatattg
GACGTACGGGGTTTTCCTGGGTGTCTATACATTTGTCATACAAATCATATTGACGGAGCAAACGGCGATT
GTATGCGTGGCAATTGTAACTAAACAAATTTTTTAGATAATTAAAAGTATCTATGTACACCTTAATATTA
ATGAAAAAACCAATATAATATAAATAAAAGTATATTGAAAAGACAAATTGACAAACAGTTGCGCCTTGAG
AAAAAAAGATCTAAACGGGAACTCAAACTACTGCTATTGGGTACGTGGAGTGGGATGTATTGGGATGTAT
CAAGGATACAATGTGCGTATATCCACAGGCGCATGCGAGTCCGGGAAGTCCACATTCATCAAACAGATGC
GTATTATCCACGGCAGCGGTTACTCGGACGAGGACAAGCGTGGATACATCAAGCTGGTTTTTCAGAACAT
ATTCATGGCCATGCAGTCAATGATCAAGGCCATGGATATGCTGAAGATTTCCTACGGTCAGGGAGAGCAT
AGT
>dme_circ_0004113_junction_seq
GATTTCCTACGGTCAGGGAGAGCATAGTGGAACTGGCCGATCTGGTGATGAGCATC
>dme_circ_0004114
GAATTATATGAATGTGTATGTGTGTTTGTATAAAAATCACAAAAATAGTTTTTCGATCCAAATCCAGTTT
AGACCAAATTATATTCCTTAAGGTTCGATTGCAGAATTGTGTCCTTAACAGCTGCAAACACAAACCTTAT
ATTTTCCGTATCTAGACAAAAGAAAAGAAGGCGTAGTTAGTTTGATTTTGTCAAATAATCCTATGATAGT
AAACTGCACACATTTTCCATTCACGATATTTCACAGACTATATTTAGTTTTTATTGAGGGACTTTGAAAA
ACAAATCATATACATTATAAATAAATAATTCATTTGCATTGCCACTTTTCCTGATATCCGCACTGCCCGC
CTTAGCCCAAATTGAATTCCTTAAGTGCATTTTGCATAATTGTATCTTTGACAGCGCAGAACACAAGTTT
GATGTTTTCGGTATCTGTTTACCAAGAGATCGTTGTTTTATTAGAAATCAATTCGTTTAGGTTTGCAAAT
TTGACTAGCGATACACAACTACTCTCTACTCTAGATCAGCGGCAAAAGCAAATATGCAAATGTAAATGTT
TTTCACACAAAAGTTTACCTTTTGACCAGTCCTTTTTAGGCTGAAAACTAAAATCACATCCACACACAAT
TTCTGACCAAACACAGAACTGGCAGCAAACTTACCTGTAGCACACGTGAAATGAGAATAGATAATTTTTT
CGGAATCTGGATTTAAATCTACAAACATTCGCAGTATAAACTCTCGGGCCGTTATTGCATCTCGCTGAGG
ACCTGTTTCGATAAGAAGGCGAATATGAAGTTACGTCTAGCGTGGTTCTACGAAAACTAGCAAATAATCT
ACGGATGATATTGGCGATTTAAGGCAATTACATAGATCTTAAGTACATAAGGTGAAAATCCACCTGTGGC

CGTTGTGAAATGCGAATAGCATTGACGTTCGGGATCTGGATTGCAGGCCAAGTACTTTTTCAGAACGAAC
TGTTTGGCCGCAGCGTGATCCTGTTTTGGACCTGGTTCATTGCCGCATAAAATCGTACATAATTATGGCC
ACCTTTATCATGCGGAACACTTACCATCGTATTCAGGAAAATAGTCTACCAAATGCGAATACATTATTTT
CTCTTCCAACAAGTCCTTCTTATTCAGGAAAAGAATAACTGACGAATTTTGAAACCAAGGGTATGTAATT
ATAG
>dme_circ_0004114_junction_seq
TTTTGAAACCAAGGGTATGTAATTATAGGAATTATATGAATGTGTATGTGTGTTTG
>dme_circ_0004115
GGTCCAAAACAGGATCACGCTGCGGCCAAACAGTTCGTTCTGAAAAAGTACTTGGCCTGCAATCCAGATC
CCGAACGTCAATGCTATTCGCATTTCACAACGGCCACAGGTGGATTTTCACCTTATGTACTTAAGATCTA
TGTAATTGCCTTAAATCGCCAATATCATCCGTAGATTATTTGCTAGTTTTCGTAGAACCACGCTAGACGT
AACTTCATATTCGCCTTCTTATCGAAACAGGGTGGATTTTCACCTTATGTACTTAAGATCTATGTAATTG
CCTTAAATCGCCAATATCATCCGTAGATTATTTGCTAGTTTTCGTAGAACCACGCTAGACGTAACTTCAT
ATTCGCCTTCTTATCGAAACAGGTCCTCAGCGAGATGCAATAACGGCCCGAGAGTTTATACTGCGAATGT
TTGTAGATTTAAATCCAGATTCCGAAAAAATTATCTATTCTCATTTCACGTGTGCTACAGGTAAGTTTGC
TGCCAGTTCTGTGTTTGGTCAGAAATTGTGTGTGGATGTGATTTTAGTTTTCAGCCTAAAAAGGACTGGT
CAAAAGGTAAACTTTTGTGTGAAAAACATTTACATTTGCATATTTGCTTTTGCCGCTGATCTAGAGTAGA
GAGTAGTTGTGTATCGCTAGTCAAATTTGCAAACCTAAACGAATTGATTTCTAATAAAACAACGATCTCT
TGGTAAACAGGGTAAGTTTGCTGCCAGTTCTGTGTTTGGTCAGAAATTGTGTGTGGATGTGATTTTAGTT
TTCAGCCTAAAAAGGACTGGTCAAAAGGTAAACTTTTGTGTGAAAAACATTTACATTTGCATATTTGCTT
TTGCCGCTGATCTAGAGTAGAGAGTAGTTGTGTATCGCTAGTCAAATTTGCAAACCTAAACGAATTGATT
TCTAATAAAACAACGATCTCTTGGTAAACAGATACCGAAAACATCAAACTTGTGTTCTGCGCTGTCAAAG
ATACAATTATGCAAAATGCACTTAAGGAATTCAATTTGGGCTAAGGCGGGCAGTGCGGATATCAGGAAAA
GTGGCAATGCAAATGAATTATTTATTTATAATGTATATGATTTGTTTTTCAAAGTCCCTCAATAAAAACT
AAATATAGTCTGTGAAATATCGTGAATGGAAAATGTGTGCAGTTTACTATCATAGGATTATTTGACAAAA
TCAAACTAACTACGCCTTCTTTTCTTTTGTCTAGGATACGGAAAATATAAGGTTTGTGTTTGCAGCTGTT
AAGGACACAATTCTGCAATCGAACCTTAAGGAATATAATTTGGTCTAAACTGGATTTGGATCGAAAAACT
ATTTTTGTGATTTTTATACAAACACACATACACATTCATATAATTCCTTTTTCGCATATTTACTAAATAT
AAAAGAAGAATTGAGAAGCGAGCGAATGTGGCCGAAAATTAGGCATAAATTAGACCCGAGAAAGTTTCTT
ACGTTTGTTACATCAGGAACCGAAAATTGTAGCATAAATAGAATACTAAACTGAGATGCTAAAACCGCAT
CAGCAGAATGATGAAAAAGATGTATACATTTTAATTTGAGAGAAAAATAACACAAAATCTGATACAGTGT
GACAACAAATAATCAGGGCAGAGTTTCTCTTACGAATGTGACGATTCGTAAAACATTTATTTATATTGCA
GTACTTCTATTCTTTCTAGCGGCAAAGTTTTCAGAAAACGTTGTGTGAAttttttttttttGTTTCGACT
AGGAAAACTCTTGAAAGTCAAAAAGACAGTGGCCAGAAATGATTGGTACCCAATCGATAGAGTTTTTGGT
TAGATTTGAATAAATCTGATGGTAGTTTTTCCTTCCTACACAAGAAACATGATTTCCTTGAAAAGTTTTT
ATACAAAATATCGTCCGAATTAGTTGTTGTCACTCTGTAATATTTAAATAAATTTATTACTATAGTAACT
TTTGTAATAAAATCCGAAACACAAAATATGAAACGTAAATTTAATCAAAAGCTTGATAAGGAACAACAAA
CTGAAACAACTTTGTCGTACTGAAGCATAACATAATAAAGCAACATTTTACTAAATAACTACAGAAAAAC
AAAAACATACTTTTGCGTAAAAATCCCCCGAAATATATATATATTCAAAGAATTATATATGCAAGAAGTT
TCTTATTAGAATTTCAGGGCATATAGGCGTGTAAAAATAATAAACCGaaaatgataagctgataaaatga
taaaatgaATGAAAACGAAATCACAGATAATACTGTATTTTTTATTAAGCTAAACAGTAAACCGTAAATA
TTGATACTATTGTTTACCGAAatatttaactatacatgaaagcagatataataattcataaacgcatatg
taacaattTAAAAATTGATTAATTTGCATTGCGCGAGCTCCATTCATTGTAAATTACGATTCGTATTAAT
ACCATGAGAATGAAAAGAAAAGAAATGAAACGCATGCAAACCAATACAAAAACGATATAGGAGCTAGATT

TGTATGAGAAAAGCCAGAAACATTTACATGGAGCATTCATTAAATACGTGTTTGTAAAAAATGTCGATAA
CCACCcatgcaacatgcaacatgcaacaGCAGAATCCATACAACTTATTGATATTTAATCAGACATCAGG
TTAAATCCAATCAAATGCGATATACCACTGGTCGAGAAACACAACTATCAGAAGGGAAAGTTTTGCTCTC
ACGTACTTTTGTTGCCATCAAAATTTCAGTCGAAACATTGTTATCAAAAATAGCTTGATTTTCGTACTTT
TTACATTAACTTTGTTAGTTGTTTGCTTATTTTATAGATCTTAATGTATGCAACAAGGGACTCGCTTTGG
CGGCTAGCCAAAACGGCGATGTCTGGCCAAGTGGGTTCAGtgttttgtttttgtttttataattttagtt
taatttAATATACAAGGATAACTAAATGAGACGCACATAAATAGAAAACCCCATCAACAAATTATTTATG
TAAAAATCCAAATAAAAACAATTCTACTCTACGCATATGTGGAAACAAAAAATAAATAAAATGGAAAACA
ATTGAAACGTACATGTATGTAAAATAAATATGATGCAAAATGTTTTTCATAATTTATAGTACACTTATTT
TGTTCAACCGAGCGAGATTCCCTCAACCCACATCCCCATCATTTGGCGTTTAGTTCCCTCGGGGCCACAG
AGGAGTAGACACTTTGCCACGACTCCAAGCGGAAGACACTACTCATGGGAGTGGAGAATTGAGATATTCC
TATGACCGCGAATAACCAAAAACCAAGACGCATCTTAGTTTAAGTACGATTCAACAGTGTGTTGTATAAA
GCTTTAATCCTCGTAACCACATATAGTGAAAGTAAAAGTAAAGTAAAAATTAAACCATTTTTATGATTAA
CCAATGGATTAGCTTTTAAAAAGCTTCAATAAGTCAATGTGTATTTTAAAAGATGTAGCCGAGAACACAA
TGAGaaaaaaaaGCCAATGAGTTTTGAGGGCCCGTTCGGTATGGATATGGAATCGGTTTAGGTTCTTGCG
AATATGCGAATACAAAAATTCACAGCTGGCATCCAATTTGGTAGCTCCGATTTTGGCCTGGCCCATTTTT
TATATCCACGGCATTATAGACATTCACACACTGACATACATATATATAGTTTTCCGTCCAATGAATTGCA
TCAGCATCAGCGAGCAGACAAAGTCTCCCATTTATTTTGATCCTTAACATTTGCGATTTGTTTCTTCCGA
AAATTCCATCCTTTAAAACAAGAGTTTTTCGGCGGTTTCAATGATAAAATGTTCCGACTTTGCAGCCACA
CATACGCACACTTTAAATTGATCGATTTCGATATGATTTCCCTAACATTTTGTGCCTTTCCAGCATAATG
AATCCCCATATTTTATATATAGGACAGACTCCAAATGGGTACTCCAAACTTGATTTAttttttttttttG
TACCACTAATGGAGCCTGTTAAAAGCCCCGAGACATTCAGATTATCAGCATGTGTGTATGGCCAGATTGT
TGTGCTTTGGGTTGATAGATTGGTTCCTTAATTTTATGATACGCACCGATAGTCAGACAGTTGTATGTAA
ATATTTGGCAAATTACTCGCGAGGGCATTCAAAAGGCCTCCCGAGAGATATACCACATAATCGAGCATGT
GTATGTAAGTGTACATTAAACGCAAAATGAACTATAATAAAATGAGAATTTGATATAAACCATTAATGTA
TGTATTTTGTATTTTTATATACAATTTATAATTTTGATTACACTTACAATTGTTACGATTAATAACTAAA
ATTACATGATAAAACCAAAGTAACGCAGATGAATCTACTAGATATGTATTTCCAATATAGAGAAGCCGAG
TACATAATACACAAATCCGACGATTGCTAAAGGAATTTCAGAATATTGTAAGGAATTAAGATTTaaaaaa
aaaGAAATTTTCTAAGCAACAAACTCAAACAGAACTGCAAGCAAAAGGGACAACAATTTAAATGGGAAAT
TTATGTAATTGTGTAAATCTAGAGCGGAATGAAAGCGAAGGATGCAATTTGATCCTTCGGGCCGGATACT
CGATAAGTATGGCCACGATTTTCAGATACTTGAAAACCATTTAGGCATGGCATTCGGTGGCTAGTTTTTA
CTTTAACTATGTATGTAAATGAAAGAATGACCACATTCCACATAAGAAAGCTTGAAAAGCaaaaaaaaat
gaaaaaaaaaaattcttaaaaaaGAATCCCGAATGAAAATCGCCCTTGACGTTGAGGAAAACTACATTAT
CGATAATAGGAATGGCTAGACGAAGTTTTCGGTTTTGAAATAAAACCCTCTATAAACTCAGCATCGGGAT
AAGCAGTAAGAAATGTTAGTTGTGAGTTCGGGCGTCATTAGGGAGAAATAGTAATATTCGACTCTAGTTT
CTCCATTGCACACCTAAGCATATACATACATACGTGTAAATCGGAAGCTAATGAAACATCCACTGAAACT
GCATGCTTAGCCTAGGATTAAGGCAATCCCACACAGACCGATTGCCCCGATTTGGAGACAGAGACAGTCC
AACATGTCCATGGGCCGACTATTGATAACTGTAGCATttgaattgaattgattgaattgatattgGACGT
ACGGGGTTTTCCTGGGTGTCTATACATTTGTCATACAAATCATATTGACGGAGCAAACGGCGATTGTATG
CGTGGCAATTGTAACTAAACAAATTTTTTAGATAATTAAAAGTATCTATGTACACCTTAATATTAATGAA
AAAACCAATATAATATAAATAAAAGTATATTGAAAAGACAAATTGACAAACAGTTGCGCCTTGAGAAAAA
AAGATCTAAACGGGAACTCAAACTACTGCTATTGGGTACGTGGAGTGGGATGTATTGGGATGTATCAAGG
ATACAATGTGCGTATATCCACAGGCGCATGCGAGTCCGGGAAGTCCACATTCATCAAACAGATGCGTATT
ATCCACGGCAGCGGTTACTCGGACGAGGACAAGCGTGGATACATCAAGCTGGTTTTTCAGAACATATTCA

TGGCCATGCAGTCAATGATCAAGGCCATGGATATGCTGAAGATTTCCTACGGTCAGGGAGAGCATAGTGG
AACTGGCCGATCTGGTGATGAGCATCGATTACGAGACCGTTACCACGTTCGAGGATCCATACTTGAATGC
CATCAAAACGCTTTGGCACGATGCTGGCATCCAGGAGTGCTATGATCGTCGTAGGGAATATCAGCTGACT
GATTCCACTGAATAGTGAGTTGGGACACCAGGAGCGATACCCAAAATTGCtttttctcattttcttatt
tcagtttttGGGTGACATAGGTCGAATTGAACAGGCTGATTACTTGCCAACTAATCAGGACATTCTGCG
TGCTCGAGAGCCTACATTTAATATTACCGTTTATCCTTTTGAGTTGGATGGCTATGTACTTAGGTATATA
TTAAATTTTTAGGGGCAGTTTTATTAATTTTATTGTATTAACAAATTTCTCGTTTATAAACAGCATGGTG
GATGTAGCAGGACAGCGAACTGAGAGAAGAAAATGGATTCACTTTTTTTCGAATGTAACATCGATTATAT
TTTTGGCAGCACTGTCGGAATATGATCAGTTCATGATGGAATCCGAAAACGATGTATAACTTGAAGGCAT
TTTATTAATTATTCGCCAACAATAATAAAATCCGATTATTGCAGAATCGACTGGAGGAATCAAAGGCTCT
ATTTCATACTATCATAACCTTTGAATGGTTTAAAAATGCTTCAATCATTCTGTTTCTTAACAAGATGGAC
GTGTTAGAGGAGAAAATAATGTATTCGCATTTGGTAGACTATTTTCCAGAATACGATG
>dme_circ_0004115_junction_seq
TTGGTAGACTATTTTCCAGAATACGATGGGTCCAAAACAGGATCACGCTGCGGCCA
>dme_circ_0004116
GGTCCTCAGCGAGATGCAATAACGGCCCGAGAGTTTATACTGCGAATGTTTGTAGATTTAAATCCAGATT
CCGAAAAAAATTATCTATTCTCATTTCACGTGTGCTACAGGTAAGTTTGCTGCCAGTTCTGTGTTTGGTCA
GAAATTGTGTGTGGATGTGATTTTAGTTTTCAGCCTAAAAAGGACTGGTCAAAAGGTAAACTTTTGTGTG
AAAAACATTTACATTTGCATATTTGCTTTTGCCGCTGATCTAGAGTAGAGAGTAGTTGTGTATCGCTAGT
CAAATTTGCAAACCTAAACGAATTGATTTCTAATAAAACAACGATCTCTTGGTAAACAGGGTAAGTTTGC
TGCCAGTTCTGTGTTTGGTCAGAAATTGTGTGTGGATGTGATTTTAGTTTTCAGCCTAAAAAGGACTGGT
CAAAAGGTAAACTTTTGTGTGAAAAACATTTACATTTGCATATTTGCTTTTGCCGCTGATCTAGAGTAGA
GAGTAGTTGTGTATCGCTAGTCAAATTTGCAAACCTAAACGAATTGATTTCTAATAAAACAACGATCTCT
TGGTAAACAGATACCGAAAACATCAAACTTGTGTTCTGCGCTGTCAAAGATACAATTATGCAAAATGCAC
TTAAGGAATTCAATTTGGGCTAAGGCGGGCAGTGCGGATATCAGGAAAAGTGGCAATGCAAATGAATTAT
TTATTTATAATGTATATGATTTGTTTTTCAAAGTCCCTCAATAAAAACTAAATATAGTCTGTGAAATATC
GTGAATGGAAAATGTGTGCAGTTTACTATCATAGGATTATTTGACAAAATCAAACTAACTACGCCTTCTT
TTCTTTTGTCTAGATACGGAAAATATAAGGTTTGTGTTTGCAGCTGTTAAGGACACAATTCTGCAATCGA
ACCTTAAGGAATATAATTTG
>dme_circ_0004116_junction_seq
GCAATCGAACCTTAAGGAATATAATTTGGGTCCTCAGCGAGATGCAATAACGGCCC
>dme_circ_0004117
GGTCCTCAGCGAGATGCAATAACGGCCCGAGAGTTTATACTGCGAATGTTTGTAGATTTAAATCCAGATT
CCGAAAAAAATTATCTATTCTCATTTCACGTGTGCTACAGGTAAGTTTGCTGCCAGTTCTGTGTTTGGTCA
GAAATTGTGTGTGGATGTGATTTTAGTTTTCAGCCTAAAAAGGACTGGTCAAAAGGTAAACTTTTGTGTG
AAAAACATTTACATTTGCATATTTGCTTTTGCCGCTGATCTAGAGTAGAGAGTAGTTGTGTATCGCTAGT
CAAATTTGCAAACCTAAACGAATTGATTTCTAATAAAACAACGATCTCTTGGTAAACAGGGTAAGTTTGC
TGCCAGTTCTGTGTTTGGTCAGAAATTGTGTGTGGATGTGATTTTAGTTTTCAGCCTAAAAAGGACTGGT
CAAAAGGTAAACTTTTGTGTGAAAAACATTTACATTTGCATATTTGCTTTTGCCGCTGATCTAGAGTAGA
GAGTAGTTGTGTATCGCTAGTCAAATTTGCAAACCTAAACGAATTGATTTCTAATAAAACAACGATCTCT
TGGTAAACAGATACCGAAAACATCAAACTTGTGTTCTGCGCTGTCAAAGATACAATTATGCAAAATGCAC
TTAAGGAATTCAATTTGGGCTAAGGCGGGCAGTGCGGATATCAGGAAAAGTGGCAATGCAAATGAATTAT
TTATTTATAATGTATATGATTTGTTTTTCAAAGTCCCTCAATAAAAACTAAATATAGTCTGTGAAATATC
GTGAATGGAAAATGTGTGCAGTTTACTATCATAGGATTATTTGACAAAATCAAACTAACTACGCCTTCTT

TTCTTTTGTCTAGGATACGGAAAATATAAGGTTTGTGTTTGCAGCTGTTAAGGACACAATTCTGCAATCG
AACCTTAAGGAATATAATTTGGTCTAAACTGGATTTGGATCGAAAAACTATTTTTGTGATTTTTATACAA
ACACACATACACATTCATATAATTCCTTTTTCGCATATTTACTAAATATAAAAGAAGAATTGAGAAGCGA
GCGAATGTGGCCGAAAATTAGGCATAAATTAGACCCGAGAAAGTTTCTTACGTTTGTTACATCAGGAACC
GAAAATTGTAGCATAAATAGAATACTAAACTGAGATGCTAAAACCGCATCAGCAGAATGATGAAAAAGAT
GTATACATTTTAATTTGAGAGAAAAATAACACAAAATCTGATACAGTGTGACAACAAATAATCAGGGCAG
AGTTTCTCTTACGAATGTGACGATTCGTAAAACATTTATTTATATTGCAGTACTTCTATTCTTTCTAGCG
GCAAAGTTTTCAGAAAACGTTGTGTGAAttttttttttttGTTTCGACTAGGAAAACTCTTGAAAGTCAA
AAAGACAGTGGCCAGAAATGATTGGTACCCAATCGATAGAGTTTTTGGTTAGATTTGAATAAATCTGATG
GTAGTTTTTCCTTCCTACACAAGAAACATGATTTCCTTGAAAAGTTTTTATACAAAATATCGTCCGAATT
AGTTGTTGTCACTCTGTAATATTTAAATAAATTTATTACTATAGTAACTTTTGTAATAAAATCCGAAACA
CAAAATATGAAACGTAAATTTAATCAAAAGCTTGATAAGGAACAACAAACTGAAACAACTTTGTCGTACT
GAAGCATAACATAATAAAGCAACATTTTACTAAATAACTACAGAAAAACAAAAACATACTTTTGCGTAAA
AATCCCCCGAAATATATATATATTCAAAGAATTATATATGCAAGAAGTTTCTTATTAGAATTTCAGGGCA
TATAGGCGTGTAAAAATAATAAACCGaaaatgataagctgataaaatgataaaatgaATGAAAACGAAAT
CACAGATAATACTGTATTTTTTATTAAGCTAAACAGTAAACCGTAAATATTGATACTATTGTTTACCGAA
atatttaactatacatgaaagcagatataataattcataaacgcatatgtaacaattTAAAAATTGATTA
ATTTGCATTGCGCGAGCTCCATTCATTGTAAATTACGATTCGTATTAATACCATGAGAATGAAAAGAAAA
GAAATGAAACGCATGCAAACCAATACAAAAACGATATAGGAGCTAGATTTGTATGAGAAAAGCCAGAAAC
ATTTACATGGAGCATTCATTAAATACGTGTTTGTAAAAAATGTCGATAACCACCcatgcaacatgcaaca
tgcaacaGCAGAATCCATACAACTTATTGATATTTAATCAGACATCAGGTTAAATCCAATCAAATGCGAT
ATACCACTGGTCGAGAAACACAACTATCAGAAGGGAAAGTTTTGCTCTCACGTACTTTTGTTGCCATCAA
AATTTCAGTCGAAACATTGTTATCAAAAATAGCTTGATTTTCGTACTTTTTACATTAACTTTGTTAGTTG
TTTGCTTATTTTATAGATCTTAATGTATGCAACAAGGGACTCGCTTTGGCGGCTAGCCAAAACGGCGATG
TCTGGCCAAGTGGGTTCAGtgttttgtttttgtttttataattttagtttaatttAATATACAAGGATAA
CTAAATGAGACGCACATAAATAGAAAACCCCATCAACAAATTATTTATGTAAAAATCCAAATAAAAACAA
TTCTACTCTACGCATATGTGGAAACAAAAAATAAATAAAATGGAAAACAATTGAAACGTACATGTATGTA
AAATAAATATGATGCAAAATGTTTTTCATAATTTATAGTACACTTATTTTGTTCAACCGAGCGAGATTCC
CTCAACCCACATCCCCATCATTTGGCGTTTAGTTCCCTCGGGGCCACAGAGGAGTAGACACTTTGCCACG
ACTCCAAGCGGAAGACACTACTCATGGGAGTGGAGAATTGAGATATTCCTATGACCGCGAATAACCAAAA
ACCAAGACGCATCTTAGTTTAAGTACGATTCAACAGTGTGTTGTATAAAGCTTTAATCCTCGTAACCACA
TATAGTGAAAGTAAAAGTAAAGTAAAAATTAAACCATTTTTATGATTAACCAATGGATTAGCTTTTAAAA
AGCTTCAATAAGTCAATGTGTATTTTAAAAGATGTAGCCGAGAACACAATGAGaaaaaaaaGCCAATGAG
TTTTGAGGGCCCGTTCGGTATGGATATGGAATCGGTTTAGGTTCTTGCGAATATGCGAATACAAAAATTC
ACAGCTGGCATCCAATTTGGTAGCTCCGATTTTGGCCTGGCCCATTTTTTATATCCACGGCATTATAGAC
ATTCACACACTGACATACATATATATAGTTTTCCGTCCAATGAATTGCATCAGCATCAGCGAGCAGACAA
AGTCTCCCATTTATTTTGATCCTTAACATTTGCGATTTGTTTCTTCCGAAAATTCCATCCTTTAAAACAA
GAGTTTTTCGGCGGTTTCAATGATAAAATGTTCCGACTTTGCAGCCACACATACGCACACTTTAAATTGA
TCGATTTCGATATGATTTCCCTAACATTTTGTGCCTTTCCAGCATAATGAATCCCCATATTTTATATATA
GGACAGACTCCAAATGGGTACTCCAAACTTGATTTAttttttttttttGTACCACTAATGGAGCCTGTTA
AAAGCCCCGAGACATTCAGATTATCAGCATGTGTGTATGGCCAGATTGTTGTGCTTTGGGTTGATAGATT
GGTTCCTTAATTTTATGATACGCACCGATAGTCAGACAGTTGTATGTAAATATTTGGCAAATTACTCGCG
AGGGCATTCAAAAGGCCTCCCGAGAGATATACCACATAATCGAGCATGTGTATGTAAGTGTACATTAAAC
GCAAAATGAACTATAATAAAATGAGAATTTGATATAAACCATTAATGTATGTATTTTGTATTTTTATATA

CAATTTATAATTTTGATTACACTTACAATTGTTACGATTAATAACTAAAATTACATGATAAAACCAAAGT
AACGCAGATGAATCTACTAGATATGTATTTCCAATATAGAGAAGCCGAGTACATAATACACAAATCCGAC
GATTGCTAAAGGAATTTCAGAATATTGTAAGGAATTAAGATTTaaaaaaaaaGAAATTTTCTAAGCAACA
AACTCAAACAGAACTGCAAGCAAAAGGGACAACAATTTAAATGGGAAATTTATGTAATTGTGTAAATCTA
GAGCGGAATGAAAGCGAAGGATGCAATTTGATCCTTCGGGCCGGATACTCGATAAGTATGGCCACGATTT
TCAGATACTTGAAAACCATTTAGGCATGGCATTCGGTGGCTAGTTTTTACTTTAACTATGTATGTAAATG
AAAGAATGACCACATTCCACATAAGAAAGCTTGAAAAGCaaaaaaaaatgaaaaaaaaaaaattcttaaaa
aaGAATCCCGAATGAAAATCGCCCTTGACGTTGAGGAAAACTACATTATCGATAATAGGAATGGCTAGAC
GAAGTTTTCGGTTTTGAAATAAAACCCTCTATAAACTCAGCATCGGGATAAGCAGTAAGAAATGTTAGTT
GTGAGTTCGGGCGTCATTAGGGAGAAATAGTAATATTCGACTCTAGTTTCTCCATTGCACACCTAAGCAT
ATACATACATACGTGTAAATCGGAAGCTAATGAAACATCCACTGAAACTGCATGCTTAGCCTAGGATTAA
GGCAATCCCACACAGACCGATTGCCCCGATTTGGAGACAGAGACAGTCCAACATGTCCATGGGCCGACTA
TTGATAACTGTAGCATttgaattgaattgattgaattgatattgGACGTACGGGGTTTTCCTGGGTGTCT
ATACATTTGTCATACAAATCATATTGACGGAGCAAACGGCGATTGTATGCGTGGCAATTGTAACTAAACA
AATTTTTTAGATAATTAAAAGTATCTATGTACACCTTAATATTAATGAAAAAACCAATATAATATAAATA
AAAGTATATTGAAAAGACAAATTGACAAACAGTTGCGCCTTGAGAAAAAAAGATCTAAACGGGAACTCAA
ACTACTGCTATTGGGTACGTGGAGTGGGATGTATTGGGATGTATCAAGGATACAATGTGCGTATATCCAC
AGGCGCATGCGAGTCCGGGAAGTCCACATTCATCAAACAGATGCGTATTATCCACGGCAGCGGTTACTCG
GACGAGGACAAGCGTGGATACATCAAGCTGGTTTTTCAGAACATATTCATGGCCATGCAGTCAATGATCA
AGGCCATGGATATGCTGAAGATTTCCTACGGTCAGGGAGAGCATAGTGGAACTGGCCGATCTGGTGATGA
GCATCGATTACGAGACCGTTACCACGTTCGAGGATCCATACTTGAATGCCATCAAAACGCTTTGGCACGA
TGCTGGCATCCAGGAGTGCTATGATCGTCGTAGGGAATATCAGCTGACTGATTCCACTGAATAGTGAGTT
GGGACACCAGGAGCGATACCCAAAATTGCttttttctcattttcttatttcagtttttttGGGTGACATAG
GTCGAATTGAACAGGCTGATTACTTGCCAACTAATCAGGACATTCTGCGTGCTCGAGAGCCTACATTTAA
TATTACCGTTTATCCTTTTGAGTTGGATGGCTATGTACTTAGGTATATATTAAATTTTTAGGGGCAGTTT
TATTAATTTTATTGTATTAACAAATTTCTCGTTTATAAACAGCATGGTGGATGTAGCAGGACAGCGAACT
GAGAGAAGAAAATGGATTCACTTTTTTTCGAATGTAACATCGATTATATTTTTGGCAGCACTGTCGGAAT
ATGATCAGTTCATGATGGAATCCGAAAACGATGTATAACTTGAAGGCATTTTATTAATTATTCGCCAACA
ATAATAAAATCCGATTATTGCAGAATCGACTGGAGGAATCAAAGGCTCTATTTCATACTATCATAACCTT
TGAATGGTTTAAAAATGCTTCAATCATTCTGTTTCTTAACAAGATGGACGTGTTAGAGGAGAAAATAATG
TATTCGCATTTGGTAGACTATTTTCCAGAATACGATG
>dme_circ_0004117_junction_seq
TTGGTAGACTATTTTCCAGAATACGATGGGTCCTCAGCGAGATGCAATAACGGCCC
>dme_circ_0004118
AATAGAAGTACTGCAATATAAATAAATGTTTTACGAATCGTCACATTCGTAAGAGAAACTCTGCCCTGAT
TATTTGTTGTCACACTGTATCAGATTTTGTGTTATTTTTCTCTCAAATTAAAATGTATACATCTTTTTCA
TCATTCTGCTGATGCGGTTTTAGCATCTCAGTTTAGTATTCTATTTATGCTACAATTTTCGGTTCCTGAT
GTAACAAACGTAAGAAACTTTCTCGGGTCTAATTTATGCCTAATTTTCGGCCACATTCGCTCGCTTCTCA
ATTCTTCTTTTATATTTAGTAAATATGCGAAAAAGGAATTATATGAATGTGTATGTGTGTTTGTATAAAA
ATCACAAAAATAG
>dme_circ_0004118_junction_seq
TGTGTTTGTATAAAAATCACAAAAATAGAATAGAAGTACTGCAATATAAATAAATG
>dme_circ_0004119
GCTAATGAAACATCCACTGAAACTGCATGCTTAGCCTAGGATTAAGGCAATCCCACACAGACCGATTGCC

CCGATTTGGAGACAGAGACAGTCCAACATGTCCATGGGCCGACTATTGATAACTGTAGCATttgaattga
attgattgaattgatattgGACGTACGGGGTTTTCCTGGGTGTCTATACATTTGTCATACAAATCATATT
GACGGAGCAAACGGCGATTGTATGCGTGGCAATT
>dme_circ_0004119_junction_seq
GCAAACGGCGATTGTATGCGTGGCAATTGCTAATGAAACATCCACTGAAACTGCAT
>dme_circ_0004120
GTTTATTGAACAGGAAAGCTCGCTTATTCTTCAGCTCGGGTGTGCAATCCGTCCGCGCCCTTTTGGTGCA
GGAGGAGGAGCTCGGACTAGATGCAGTCTCCGAGATGCCAGAAGAGATCATGCTTTCGTTCGACTCCAGG
GAGAGGTTCTCGGTAGCGGGTCCATCAGCCAGTTCCTCTGTAAAGATACAAGTTGAATTATCCAATACGA
ATTCATTATTGACTAATAAAACCTTTGATCTTTGCCGATACATCGGAATCCGTTTCGAGATCGTCTGCAT
CAGCACACAACGCAGGTGTGTTGCATTCTCT
>dme_circ_0004120_junction_seq
CACACAACGCAGGTGTGTTGCATTCTCTGTTTATTGAACAGGAAAGCTCGCTTATT
>dme_circ_0004121
GTATTGCATTGCCTTTAAGGGATCTTGGCGCGTCATCTCCTCATAGCGCAGTTTACGCACCAAAAACCGA
CAATGCTTCAGGATCTCCTCTCGTTTGGGTCTAAAATATAATAAAATTATCATCGATTACATTCAGCTAG
TAAATTACATACTTTTCCAGACATAAAAGCCAAAAATCATCAAGTCTCAGCTGCTGCTGCTGGCAGATTC
CAGGATTTCCGCCAAACAAATAATGG
>dme_circ_0004121_junction_seq
TCCAGGATTTCCGCCAAACAAATAATGGGTATTGCATTGCCTTTAAGGGATCTTGG
>dme_circ_0004122
CATTACAATTCGAAACAGCTGATAAACCGATAAAGCCATAGCCAAACGGAATACAGAACACACAATACAC
GGAGATCATCAAGAACAATGCTCAAGTTCCAGGTCCATTCTCCGGGCAAGGTCATACTCCATGGCGAACA
CGCCGTGGTCTATCATCGTCCCGCCCTCGCCGCCGTAGTGGGTTTGGGCACTACTCTGAGATTTCGGCAG
CTGGAGGACAGCCAGGTGGCAAGTTTTCAACTGGAGGCCCTTAACTGCACACTGGAGATCCAGTTGCCGG
AGTTCAATGGATTTTTGGCTGAATTTAGGAGTAAATACCCATCAGATAGCACGGCCAATCTGTTGGAAGC
CGTCCGCGCAGAGGTGTCCAAACAACTTGAACGATCCGGAGGCAATGCACCGAAGCAGCACACCCAGCGG
GCATTCATCTCCATATACTATTTGCTGGCCGGCGCTGTACTCTCCGCACCCGGCGACAAGAAACTGACGC
TGCAAACCGGTTTCCAGGTGCAGGTAGACAGCCAGCTAACCGTGGGTGCGGGTTTGGGCAGTTCCGCCTC
TTTCGGAGCAGCTTTGGCCACGACATTTCTCATTTTGGCCGGACACTTTGATAGAGACAGCTACTTGTCG
GAGGCAAACCAGGCGTTGATCTCGTCCTGGGCTTATGAATCGGAGCGTGTTAATCATGGCACGCCCTCGG
GTCTGGACAACACTGTATGCACCTACGGCGGAATGTTGCGGTATGTCAAGGGCCAGGGCTTCCAGTCGCT
GAAGATCCAGAAACCACTAAACATCCTGCTGGTGGACAGTCGTGTGAGTCGCAGCACCGCCGACATTGTG
GCCAAGGTTCGTCATCTGGGAGATGCCTTTCCGCAGCTCATCGAGGCCATTTGGCAGGCTTGCGAGGAGC
TTGTTGCCGCCGCCGTTCCACTCTACGAAAGCTTTGGCAACGCCCAAGACGATAGCTCGAAATTCGAGCA
ACTTGAACGCCTGTTCCAGATCAACAATGATCTGTTGAAGGCTATCGGAGTGTCGCACCCGAAACTGGAG
CAAATCTTCACCATCGCTTTTAAGCGTGGCTTCTTTTCAAAGCTGACGGGCGCCGGCGCAGGTGGCTATG
TCATCGTTCTGCTTCCAGAGAACTACGAGTGCAACGAGGTCTACTGGAAGCTTAAAGAGGAGCTCGAATC
GGCGGGTTTTGGTGTTCATGTTACGACTGCCGGCGGCGAGGGATTGCGCGTTAGTTCGCTGGATGACTGA
TGCGGTTTCG
>dme_circ_0004122_junction_seq
AGTTCGCTGGATGACTGATGCGGTTTCGCATTACAATTCGAAACAGCTGATAAACC
>dme_circ_0004123
GTTACATGGGCTTACGAGTATATCAAAATTCTACTATAGTACCAAAAAACTGGCTAATCTAGCTTGCAAG

TGCCTGGATGCATCAGTAAGTTCGGCTGAACACGTCAAAATTTTGATTAGATTTGCTTGCTTGGTCTTCT
GCTCATCTGCTGAGATTCCTAATCCTACACACCTACTGAGAGATTGAAAGTGGGCAAAGGTTTTGATTAT
AGCCATATGGAGTA
>dme_circ_0004123_junction_seq
AAGGTTTTGATTATAGCCATATGGAGTAGTTACATGGGCTTACGAGTATATCAAAA
>dme_circ_0004124
GAAGGTGCGGTTGGTTTGGTTGCCTACACAAACTTCTGGATCATCGTTGTCCCCGGAATGCTCAACGTTG
GGCAGTGAGTTTAATGGCACCTCACTGAAGCCATGATTTTGCTGATGTTTTGAAAATAATTAGAACAGAA
TGGACACACTTATAATGCCACATATACATACCATATTCAAGGCCTTTACGTATATGTTTACCAAGTTGAG
GACGATTCCAAATGTGAGCGTTAAGTTCTCTTCGTTCAACTTAGGTGCACCAAACTTAACGTCTAGTAAG
ATTGAGTGTTTGGAGGGCTCATATGCACTGCAGCACAGTA
>dme_circ_0004124_junction_seq
GAGGGCTCATATGCACTGCAGCACAGTAGAAGGTGCGGTTGGTTTGGTTGCCTACA
>dme_circ_0004125
GTTGAGGACGATTCCAAATGTGAGCGTTAAGTTCTCTTCGTTCAACTTAGGTGCACCAAACTTAACGTCT
AGTAAGATTGAGTGTTTGGAGGGCTCATATGCACTGCAGCACAGTAGTTCCATTAAATCCGGACTAAATC
TAAGCAAGGCTTTAACGCAAGAAATTATTATATCCGTGAGGCTGTTATAAAATGGAAATATTTAGTCTAG
TATAACAACATTATTCAAGTTAACTTACTCATTGCAGGCGGAGATAAGTTCATCGTCAACGATTAGAGCC
TCTGTGCTGCGCAGAATTTCTAGCTGGGAGCGTCGACCAGCCAGAAGACACTTTAAGTTTTTTTGCTG
>dme_circ_0004125_junction_seq
CCAGAAGACACTTTAAGTTTTTTTGCTGGTTGAGGACGATTCCAAATGTGAGCGTT
>dme_circ_0004126
GGATTGTTTGCTCAATAACAATGCATCCTCCAGGAAAACAGCATGAATGCCCACAAATTGAAAAGCATCT
TCAAGAAAGCACTTCTTGTGCTTCTCGTAAATGATGGTAACAAGCTCAATACCTCTGGCGTATACAGGCC
ACCACTGCTCTACGGTCCAACCTTCTGCATCCGCAGCCGTCGTCTTAGTAAACTCGTGTTTTGACTGCAA
AAGTTCTCTGGGAGGCAACAGTTTCAGCCACAAGTATTCTCCCACATCGCAATGCAGAAACTCAACTGAA
CAGTGGCCCTTGGCAAATACAATGAGCACATCCAGCAGCTGCACACTTAGAACTTGTTTGCTAAACAATG
GAAGGGTTCTAGACACACAGCGCACGAGCCGTTGAAACAAACGCACCGACACAAATGGCAGATCCCAGTT
TCCGGGACCGTCGTT
>dme_circ_0004126_junction_seq
GCAGATCCCAGTTTCCGGGACCGTCGTTGGATTGTTTGCTCAATAACAATGCATCC
>dme_circ_0004127
GATAACTTTGCATCTTTCCAAAGGAGTGTTGCTTGCAGGCGGCAAATATTCTCTTTATCTGCTCCAACCC
CATGGGCAGGTGTTCCGCTAGCACCAGAAGGATGTGCAGCACCTCGTTGATCTCACGAGCATTTCGCTCC
GACCAGGCAACTAAGCGTTCCGCAGAGTGGAAGCATTCTCCCGCCATTAGCTTTCTGGGTGGGACTTCGC
AAATAAGGCTCTCCAGTTGGTCTAG
>dme_circ_0004127_junction_seq
CGCAAATAAGGCTCTCCAGTTGGTCTAGGATAACTTTGCATCTTTCCAAAGGAGTG
>dme_circ_0004128
GCGCCCCACAGCGaaaaaaaaaCAAGACCAAAACGAGAGTAGTTTGTCAACGTGGAGCGCAAATGAAGTG
AATGTGAATGAAAATATTTTTGGGTGCATTGAAAAGTGTCTGTGCGTTGGGAAATGGAAATGCTCGATCG
CGATCGCAGCTGAGGATTATTGATTCCGTCGCATTAGGAATTACGAATTTCAAGTTACGAATTACGGATT
GCACAATCTACAATCCGAGTCCGCGTCTATGGAAACAAATCTCCGGCGAACAGTTGCAGCTGCCTACTTT
GGCTAATTGATTTTCATTTCGGTTTCTGTTTCTGTTTTCCCCATTTTGTTTGCTGCTAGCTGGTTTGTGT

GTGCCTCTTGCAATGAGGCGTCCAAAAGCTGCCAATCGCCACAGCAACAATAGCACCAACAACTCGCCAA
ATGAACAAAAACAATCTGAAAAACAACAAGTACGCGTACATGAAGTGAAGCTGCGCGACAATTGCGTTTG
CGCCTTTTTGCTGCTTTTTTAATTAATTTTAAGTGTCGCCGTACTCCGTTGCTGCCCtttttttttCTTC
GCCGGGAGCGAAAAAGATAGCCCCCGGAAATCGGCAATTTAATTTACAATACAACATTAGTACGGACATA
AAAGCGGATAAAGGCGAGCGGACCAAGGAGAAGCCAAAATGTTCGCGGGAATAAGGAAACGCCTGGCACG
CAA
>dme_circ_0004128_junction_seq
CGGGAATAAGGAAACGCCTGGCACGCAAGCGCCCCACAGCGaaaaaaaaaCAAGAC
>dme_circ_0004129
CACCCAACCGATGGTGCCATCGCCGCCCGCCACCAGTATGCGACACGGGCGAGGACTGGCTTTGGCGGCC
CACTGCAGGGCGTCCTGCGGACCCCTCGAGCCCAGTTCCATCACCTGCAGCGGATGCAGGTAACCGCGGA
GCAGGGACAACACATTGGCACCCGTACTGCTGCCCGACTTGGTGTTGGCGATCACAATAAGCGGCTCCCA
GTTCTCAATGTCTGGCGGCGTAATGCTGGCCAAGTGGAGCCGCATCGAGTCTCTGGTTCGGGCGGCCACA
AAGCTGTATGGCGGGAAGATCATGTCCTTGAACTCGCCCAAGTCGCAGGCTTCCATGGAGTCAGCGCGAG
TGTAGCAATCGTTATGATAGCACCGCTGACACCATGCACATCGCCATCCG
>dme_circ_0004129_junction_seq
CCGCTGACACCATGCACATCGCCATCCGCACCCAACCGATGGTGCCATCGCCGCCC
>dme_circ_0004130
GTGCCACCGGTCTGCCCATCGACCTCGTCTCAAAGGCCAAGCAATCGCGCGGCGAGAAAAAGGCCAGGAA
GATCATGCTGAAGCTGGGACTTAAGCAGATCCAGGGCGTCAACCGGGTGACGATCAGGAAGTCGAAGAAC
ATCCTGTTCGTGATCAACAACCCAGATG
>dme_circ_0004130_junction_seq
ATCCTGTTCGTGATCAACAACCCAGATGGTGCCACCGGTCTGCCCATCGACCTCGT
>dme_circ_0004131
TTCGTCTCAATGGTCAGCTGATAGATACGGAAGTGGTCGTACCGGGCGGCCATGCGACCAGGTGCGGACT
TGGCCGCCACCATGGCCACAAGGGTTATCAGAAGGATTGGTAGTTTCATTTCGGAGGTTTGGTGTCTTGA
ACGTTACAGCGTTCACTG
>dme_circ_0004131_junction_seq
GGTGTCTTGAACGTTACAGCGTTCACTGTTCGTCTCAATGGTCAGCTGATAGATAC
>dme_circ_0004132
ATGTGTTCGTGAATCTTCTTAAGCTCCTCCATCTGCAAGTTCGTCTCAATGGTCAGCTGATAGATACGGA
AGTGGTCGTACCGGGCGGCCATGCGACCAGGTGCGGACTTGGCCGCCACCATGGCCACAAGGGTTATCAG
AAGGATTGGTAGTTTCATTTCGGAGGTTTGGTGTCTTGAACGTTACAGCGTTCACTG
>dme_circ_0004132_junction_seq
GGTGTCTTGAACGTTACAGCGTTCACTGATGTGTTCGTGAATCTTCTTAAGCTCCT
>dme_circ_0004133
GATGAATCCTCTGTTGGCGACGATTCCCAGATGGAATGGGAGACCTACCACACTCTGGACACCATTTACG
ATTGGATCGATCAGGAGTGTGCTGCCCACGATTTCCTGGAGTGCAAGGTGATTGGACAGTCCTACGAGGG
TCGCGACATCAAGAGCATTAG
>dme_circ_0004133_junction_seq
ACGAGGGTCGCGACATCAAGAGCATTAGGATGAATCCTCTGTTGGCGACGATTCCC
>dme_circ_0004134
GAAGGTGACGGTGGCCGACGAGATCCACTCCATGGCATGGATGTTGCCCTCCAGGAAGATGGCCTTGTTG
CCCGAACGCTTCGACAACCTAATGCTCTTGATGTCGCGACCCTCGTAGGACTGTCCAATCACCTTGCACT

CCAGGAAATCGTGGGCAGCACACTCCTGATCGATCCAATCG
>dme_circ_0004134_junction_seq
GGCAGCACACTCCTGATCGATCCAATCGGAAGGTGACGGTGGCCGACGAGATCCAC
>dme_circ_0004135
GGTGATTGGACAGTCCTACGAGGGTCGCGACATCAAGAGCATTAGGTTGTCGAAGCGTTCGGGCAACAAG
GCCATCTTCCTGGAGGGCAACATCCATGCCATGGAGTGGATCTCGTCGGCCACCGTCACCTTCCTGTTGA
ACCAGTTGATCAACTCCGAGGATCCGGAAATGCAGCGACTGAGCGAAGAGTACGATTGGATT
>dme_circ_0004135_junction_seq
GCGACTGAGCGAAGAGTACGATTGGATTGGTGATTGGACAGTCCTACGAGGGTCGC
>dme_circ_0004136
TCAAAGTTGCGGTTCATGTCAATGCCGTAGCAATCTCCACTTTCGTTGCGGTATCCGTTGGGACGACGGT
TCTTGCGCCACAAACGCTCCACCTCGTGGGTGTAAACGAAGCCATCCGGGTTCACCATGGGCACCACAAT
CCAATCGTACTCTTCGCTCAGTCGCTGCATTTCCGGATCCTCGGAGTTGATCAACTGGTTCAACAGGAAG
G
>dme_circ_0004136_junction_seq
GAGTTGATCAACTGGTTCAACAGGAAGGTCAAAGTTGCGGTTCATGTCAATGCCGT
>dme_circ_0004137
GAGCTCACAAAGTTCTGCAGCGAGATGATCTCCACCTCGGTGTTGGGCTCCTCACCACCGAACCAGTGAT
CGCAGGGCTCATCGATGTTCCAGCCGGCACCTTCAAGTAATTGATTATTAATAATCAGCGGATTAATTAG
TAATTATTAGACCATACCTCCCCAGTGGTAGTCAAAGTTGCGGTTCATGTCAATGCCGTAGCAATCTCCA
CTTTCGTTGCGGTATCCGTTGGGACGACGGTTCTTGCGCCACAAACGCTCCACCTCGTGGGTGTAAACGA
AGCCATCCGGG
>dme_circ_0004137_junction_seq
CTCGTGGGTGTAAACGAAGCCATCCGGGGAGCTCACAAAGTTCTGCAGCGAGATGA
>dme_circ_0004138
GGTGGAGCGTTTGTGGCGCAAGAACCGTCGTCCCAACGGATACCGCAACGAAAGTGGAGATTGCTACGGC
ATTGACATGAACCGCAACTTTGACTACCACTGGGGAGGTATGGTCTAATAATTACTAATTAATCCGCTGA
TTATTAATAATCAATTACTTGAAGGTGCCGGCTGGAACATCGATGAGCCCTGCGATCACTGGTTCGGTGG
TGAGGAGCCCAACACCGAG
>dme_circ_0004138_junction_seq
GTTCGGTGGTGAGGAGCCCAACACCGAGGGTGGAGCGTTTGTGGCGCAAGAACCGT
>dme_circ_0004139
TTAAGCAGACCACTAGCTCCATAGGTGAAGGTGGAACCATAGACATCAGCGGCAGCATCGGAGAATGCAG
CGGCAATGCGCTTCATCTGCTCGTAGTTGGGCGGGAACTCGGTGTTGGAGTGTCCGTAGGGCAGGAGGAC
ATACTGTCCGTAGGCATGGTAGGCCATGTACGATCGGATGTAGCCATCCTCGAAGGAGCTCACAAAGTTC
TGCAGCGAGATGATCTCCACCTCGGTGTTGGGCTCCTCACCACCGAACCAGTGATCGCAGGGCTCATCGA
TGTTCCAGCCGGCACCTTCAAGTAATTGATTATTAATAATCAGCGGATTAATTAGTAATTATTAGACCAT
ACCTCCCCAGTGGTAGTCAAAGTTGCGGTTCATGTCAATGCCGTAGCAATCTCCACTTTCGTTGCGG
>dme_circ_0004139_junction_seq
GCCGTAGCAATCTCCACTTTCGTTGCGGTTAAGCAGACCACTAGCTCCATAGGTGA
>dme_circ_0004140
CAGACCACTAGCTCCATAGGTGAAGGTGGAACCATAGACATCAGCGGCAGCATCGGAGAATGCAGCGGCA
ATGCGCTTCATCTGCTCGTAGTTGGGCGGGAACTCGGTGTTGGAGTGTCCGTAGGGCAGGAGGACATACT
GTCCGTAGGCATGG

>dme_circ_0004140_junction_seq
CAGGAGGACATACTGTCCGTAGGCATGGCAGACCACTAGCTCCATAGGTGAAGGTG
>dme_circ_0004141
GCTGCCAAGGATTGGGCCTATGGCGTAAAGAAAATCCCATTCACCTGCACCGTGGAACTGCGTGACAAGG
GCACCTTCGGATTCTTCCTGCCCTCCAACCAGATCACCGAGGTGGGTCTCGAGGTCACCGCTGGTCTTAA
GGCTCTGGTCAACAAGGCTGCCGAGGAGGGCATTTTTGACTAGAGCTTGGTATGTACCAACTTGGGCGCA
GATGCT
>dme_circ_0004141_junction_seq
GGTATGTACCAACTTGGGCGCAGATGCTGCTGCCAAGGATTGGGCCTATGGCGTAA
>dme_circ_0004142
GCTGAAGAAGCTGGCCACGGAGATTGCCAGCAAGAGCGGCGCCGGCGGAGCAGCCGGCTCTTCAGCCGCC
GATGCCGGTGACGATGATGTGCCCGATCTGGTCGAGAACTTCGAGGAGGTGGCCATCGCCGACACAAAGG
AGGAGAA
>dme_circ_0004142_junction_seq
TGGCCATCGCCGACACAAAGGAGGAGAAGCTGAAGAAGCTGGCCACGGAGATTGCC
>dme_circ_0004143
GGTGGCCATCGCCGACACAAAGGAGGAGAAGTCCGGTGAGGTGGCGGCCTCCGCTTAGGCTGGTCCAACC
CCGCCCACCCGGGCAAATCACACCACCACACACCGAACACAAAAACATACATACAAAACATACGGGGCCA
GGGAGCAAGACGAA
>dme_circ_0004143_junction_seq
AACATACGGGGCCAGGGAGCAAGACGAAGGTGGCCATCGCCGACACAAAGGAGGAG
>dme_circ_0004144
CATGACCCCCAAAAAAGTGGCTGGAGGGAGCACGAAGAATGGCCCATTCAGGGCAGAAGCGCTGGTTGCA
GTCCGGAGTGGATCCGTTTCCGATTTGGAACCGGAATGGGTAAGTGGTAGATGTGTTTCCGGCGTGTCTG
GTGTGTGGGTTCTGCCCTGTATGCCAGCCACTTCGTCTTGCTCCCTGGCCCCGTATGTTTTGTATGTATG
TTTTTGTGTTCGGTGTGTGGTGG
>dme_circ_0004144_junction_seq
GTATGTTTTTGTGTTCGGTGTGTGGTGGCATGACCCCCAAAAAAGTGGCTGGAGGG
>dme_circ_0004145
CATGACCCCCAAAAAAGTGGCTGGAGGGAGCACGAAGAATGGCCCATTCAGGGCAGAAGCGCTGGTTGCA
GTCCGGAGTGGATCCGTTTCCGATTTGGAACCGGAATGGGTAAGTGGTAGATGTGTTTCCGGCGTGTCTG
GTGTGTGGGTTCTGCCCTGTATGCCAGCCACTTCGTCTTGCTCCCTGGCCCCGTATGTTTTGTATGTATG
>dme_circ_0004145_junction_seq
CCCTGGCCCCGTATGTTTTGTATGTATGCATGACCCCCAAAAAAGTGGCTGGAGGG
>dme_circ_0004146
CATGACCCCCAAAAAAGTGGCTGGAGGGAGCACGAAGAATGGCCCATTCAGGGCAGAAGCGCTGGTTGCA
GTCCGGAGTGGATCCGTTTCCGATTTGGAACCGGAATGGGTAAGTGGTAGATGTGTTTCCGGCGTGTCTG
GTGTGTGGG
>dme_circ_0004146_junction_seq
TGTGTTTCCGGCGTGTCTGGTGTGTGGGCATGACCCCCAAAAAAGTGGCTGGAGGG
>dme_circ_0004147
GGTAGAGGTTTAGAGCGGCGAAGATGTATTCCTCAGGACTGATGGAGTACTTATGATTACCACCCAGCAT
CAACTGGGTATCGTACACCAAGTAAACGGAGAAGAGCAATGCTCCCAAGGAGGCATAGACCAGTCCGATC
ACCTTGCCCGGTATGAAGATAGCGATTATACCAAAAATGATGAAGACCACCAGACAGGCCACCAACACTC

CTCCGCACATC
>dme_circ_0004147_junction_seq
ACAGGCCACCAACACTCCTCCGCACATCGGTAGAGGTTTAGAGCGGCGAAGATGTA
>dme_circ_0004148
CTTAATCGGGTTATTCAGTAACCTGGAATACACGAATATTGTTGTCGCGTAATCTCATCGGTGTGCTCCC
CCAGTGGAGGTAATCTTCAACTAACGAATTACTCAAATACTTACAAATCTATCTACTTCGTGTTGTGTTT
GCCCATTTTCCCCGTTTCCTTCTAGTTCTAAATGTCAACTAAGGCTATATGAACTACTTACTTTTTGTTA
TGTATG
>dme_circ_0004148_junction_seq
ATGAACTACTTACTTTTTGTTATGTATGCTTAATCGGGTTATTCAGTAACCTGGAA
>dme_circ_0004149
GTATTTGAGTAATTCGTTAGTTGAAGATTACCTCCACTGGGGGAGCACACCGATGAGATTACGCGACAAC
AATATTCGTGTATTCCAGGTTACTGAATAACCCGATTAAGCTGCAAACAATATTTGGAAATAGAGCGGAA
CTTAGTCGCGCGATGCGCCGATTATAGTCAGAAT
>dme_circ_0004149_junction_seq
CGCGCGATGCGCCGATTATAGTCAGAATGTATTTGAGTAATTCGTTAGTTGAAGAT
>dme_circ_0004150
GGTAGAGGTTTAGTGCCGCAAAGATGTACTCCTCGGGACTGATAGAGTATTTGTGCTCGCCGCCCATCAT
TAACTGTGTGTCGTAGATGAGATAAACGGAGAAGAGCAGCGCTCCAATCGAGGCGTACACCAGT
>dme_circ_0004150_junction_seq
CAGCGCTCCAATCGAGGCGTACACCAGTGGTAGAGGTTTAGTGCCGCAAAGATGTA
>dme_circ_0004151
GAGGTTTAGTGCCGCAAAGATGTACTCCTCGGGACTGATAGAGTATTTGTGCTCGCCGCCCATCATTAAC
TGTGTGTCGTAGATGAGATAAACGGAGAAGAGCAGCGCTCCAATCGAGGCGTACACCAGTGTTATGATCT
TTCCCTTTACAAAGATGGCCACGATGCCGAAGATCAGGAACACCACCATGCAGGCGATCAAAATACCGCC
CATCATAGTAAA
>dme_circ_0004151_junction_seq
CGATCAAAATACCGCCCATCATAGTAAAGAGGTTTAGTGCCGCAAAGATGTACTCC
>dme_circ_0004152
GTGCTCAACCCTATCCACAGCCCTACGGACAGGGGCCTCCACCGGGAGGTTATGCTCCCCAGCCGGGATT
TATCCAACCACCACCATCTGCTGGCGGCTACGGAGCCTACGATGATCCGGAGAGCCAGCCCAAGAACTTC
TCGTTTGACGACCAAAGCATCCGTCGCGGATTCATACGTAAGG
>dme_circ_0004152_junction_seq
AGCATCCGTCGCGGATTCATACGTAAGGGTGCTCAACCCTATCCACAGCCCTACGG
>dme_circ_0004153
GATTGAATCCTGCACAGAGCAGTCGCTGGAATTGTCCGTCGAGCAGATATTCGTAATATCCCAGGCCAAG
GCACAGTTGCCATTGCAAATCGAGGATGCATCGCGTCCCGAGAACGCCGATGATGCGGAAGGTCTGAATA
TTCGCGTGAACCAGGACACGCGCTTGGACAACCGAGTGCTCGACCTGAGGACCCCGGCAAATCAGGCCAT
TTTCCGACTGGAAGCCGGAGTATGTCGCCTGTTCCGCGATATTCTCACTGAGCAGGGCTTCACAGAGATT
CACACGCCGAAGATCATTTCAGCGGCCAGTGAGGGAGGCGCCAATGTGTTCACCGTGAGCTACTTCAAGG
ATTCAGCCTATCTGGCGCAGTCGCCGCAGCT
>dme_circ_0004153_junction_seq
CAGCCTATCTGGCGCAGTCGCCGCAGCTGATTGAATCCTGCACAGAGCAGTCGCTG
>dme_circ_0004154

GGTCTATACCGTAGGAGCCGTGTTCCGTGCAGAGGATTCGAACACTCATCGGCATTTGACCGAGTTCGTA
GGCCTTGATCTGGAAATGGCCTTTAAGTACCACTACCACGAGGTGCTGCACACGATTGGCAACACGTTTA
CGTCCATTTTCAAGGGACTGCGTGATAAGTACGCTAAGGAAATCGAATCTGTAGGCCAGCAGTACAAGGT
GGATGCCTTCAAGTTCCTGGAGCCACCACTGATCCTCCAGTTCGCCGATGGCGTGGCCATGTTGCGCGAG
GCTGGTGTGGAGACCGGCGACGAGGAGGATCTGTCTACGCCCAACGAAAAGCTGTTGGGTCGCCTGGTCA
AGGCCAAGTACGACACTGACTTCTACATCCTTGACAAGTTCCCGTTAGCGATACGGCCCTTCTACACGAT
GCCTGATCCAAACAATCCG
>dme_circ_0004154_junction_seq
CTACACGATGCCTGATCCAAACAATCCGGGTCTATACCGTAGGAGCCGTGTTCCGT
>dme_circ_0004155
ATTCGATTTCCTTAGCGTACTTATCACGCAGTCCCTTGAAAATGGACGTAAACGTGTTGCCAATCGTGTG
CAGCACCTCGTGGTAGTGGTACTTAAAGGCCATTTCCAGATCAAGGCCTACGAACTCGGTCAAATGCCGA
TGAGTGTTCGAATCCTCTGCACGGAACACGGCTCCTACGG
>dme_circ_0004155_junction_seq
TCCTCTGCACGGAACACGGCTCCTACGGATTCGATTTCCTTAGCGTACTTATCACG
>dme_circ_0004156
GAACTTGAAGGCATCCACCTTGTACTGCTGGCCTACAGATTCGATTTCCTTAGCGTACTTATCACGCAGT
CCCTTGAAAATGGACGTAAACGTGTTGCCAATCGTGTGCAGCACCTCGTGGTAGTGGTACTTAAAGGCCA
TTTCCAGATCAAGGCCTACGAACTCGGTCAAATGCCGATGAGTGTTCGAATCCTCTGCACGGAACACGGC
TCCTACGG
>dme_circ_0004156_junction_seq
TCCTCTGCACGGAACACGGCTCCTACGGGAACTTGAAGGCATCCACCTTGTACTGC
>dme_circ_0004157
ATACAGCATCACCACACGCTCCATTCCAATGCCACCACCAGCATGTGGAGGACATCCATAGCGGAAGGAC
TCGATGTACGCCGCAATCTTGGAGGTATCTAGAAAACAAATATATACGGCGTTAATATTAAGTATTAATA
GAGTAGATAAGTTTCAACTTCTTATCTGAAATGGGTTACAGTCTTATATAAGCACTTGAGCTATGCATTT
ATATTATTCTCTTATTGCAGTCTTATCATTGTTTACCGAGAGATCTTGTGACTAAGCTGACCTTACGAGT
TATTTACTACGTGTAGGGGGTACTCACCAATGCCATGGTGCTTGGCTCGTTCGATTAAGTACTCGGGATC
GTGGATACGCTGCGCGCCGGACAGGATCTCCTCGCCGCGCATGAACATATCGTAAGAGTTGGAGTAGACC
GGATTGTTTGGATCAGGCATCGTGTAGAAGGGCCGTATCGCTAACGGGAACTTGTCAAGGATGTAGAAGT
CAGTGTCGTACTTGGCCTTGACCAGGCGACCCAACAGCTTTTCGTTGGGCGTAGACAGATCCTCCTCGTC
GCCGGTCTCCACACCAGCCTCGCGCAACATGGCCACGCCATCGGCGAACTGGAGGATCAGTGGTGGCTCC
AGGAACTTGAAGGCATCCACCTTGTACTGCTGGCCTACAGATTCGATTTCCTTAGCGTACTTATCACGCA
GTCCCTTGAAAATGGACGTAAACGTGTTGCCAATCGTGTGCAGCACCTCGTGGTAGTGGTACTTAAAGGC
CATTTCCAGATCAAGGCCTACGAACTCGGTCAAATGCCGATGAGTGTTCGAATCCTCTGCACGGAACACG
GCTCCTACGG
>dme_circ_0004157_junction_seq
TCCTCTGCACGGAACACGGCTCCTACGGATACAGCATCACCACACGCTCCATTCCA
>dme_circ_0004158
GTTCAACCAGGATTTGTACTCCTCCTCGGGCGTCAACTTGGCCGCCTCATCGAGTGCCTCGGGCAGCTCC
CACTCGCCCTGCAGCCCCATCCTGCCGCCATCGGTGAGCGCAAATGCCGCTGCGGCAGTGGCAGCGGCGG
CCCACAATAGTGCAGCGGCGGCAGTGGCGGTCGCAGCGAATCAGGCCAGCTCCTCCGGCGGAATCGGAGG
CGGTGGACTCGGCGGCCTGGGAGGACTGGGCGGCGGACCGGCGAGCGGACTGCTTGGCAGCAATGTGGTG
CCCGGGAGCAGTAGTGTCGGGAGCGTGGGGCTGGGAATGAGTCCGGTGCTGAGCGGAGCGGCGGGACACT

CGCTGCACAGTTCGCACAGgacgcacgcacactccctcgcccacgcacacacgcacccgcactcgcacac
acacacgcatacgcaTCAAACCAAAGAAGAGGACCTCATCGTGCCGCGCAGCGAAGCTGAAGGGTGAGCA
AGGATCACTTGGGTACATCCCTAATGATGGCGATCTAGATCCCAAAAGGAAACTTTCAAATAGTCATTGT
TTGAAATTATCTGAATTGCAAGTTGTTGTTTAGTTTTAGCTTTACTATAACTAAAAACACGACTGTCATT
AATTAGTTACTGAGTAAAGAGAACAATCATTTTAAAATAGATATGATGATTTGTTTAACTTTAGAGATCG
TTTCCATTTAGCCCTTCCACTAATTAATACATTAGTGTCTCAATTAGTACATTATAAAGTTCAGCTCCAA
AGGTGAACATAGTAGCAAAAGTATTGCTACCAAAATTAAAGTATAGTCGCTATAAATGTAATCAATAATT
CATCAGCTAAACACTTTGTTTACACGCGTTCGTTCGAAACGCTTTAAAGCAATGAATTTTATTAGTTTTC
ATGTGCGTGTTCATTGATATTGTCAATGTCAATGTTTGCATAACATTTATTTTTGGCAGCACACGGAAA
ATTCATGCAAGTGAAAAAGCCCATAGTGGGGAAGAGCGCGATAGTCATCGCACACTCGTAGCTAATTAAT
TTGAAAATTCTTGAAATTTCTGACGAAGCACTCGCATTCCAAACCAGTTAGCATTCAATAAATTATATCA
TATTTTCCCGTTGGCGAATTCGCCATTACTTAGCGATTATTTAATAGTTTTTCCGCTTGCCTTTTCTCTC
GCCCTGTCTGATTTCCCAGGCACGCCTGGTGGGCTCCCAACAACATCAGCATCATAATGAATCATCCTGC
TCCTCCGGTCCGGACTCCCCGCGCCACGCCCACTCGCACAGCCATCCGCTGCACGGTGGCGGCGGAGCGA
CGGGCGGTCCATCTTCCGCGGGTGGTACGGGCTCGGGGGGCGGCGACGGAGGCGGCACGGGGGCAATACC
CAAGAATCTGCCAGCACTGGAGACGCCGATGGGCAGCGGAGGCGGCGGATTGGCCGGCAGTGGCCAGGGA
CAGGCTCAATATCTATCGGCCTCCTGCGTTGTGTTCACCAACTACTCGGGCGACACGGCCAGCCAGGTGG
ACGAGCACTTTTCCCGCGCCCTCAACTACAACAACAAGGATTCTAAAG
>dme_circ_0004158_junction_seq
CTCAACTACAACAACAAGGATTCTAAAGGTTCAACCAGGATTTGTACTCCTCCTCG
>dme_circ_0004159
TTATGGGTTATGGGTGCTGGCTTTTTATCCAACTCTGCTACTGTTCCTTCCGATTTCGGTTCTTCCTGGT
TGGTTCCTTTAGGCTAGAAGTAACAAGTCCTGCGTGGTAACACCTGGGATTCGTCTCTCCGTTTCGTTAC
GTCACACTGGG
>dme_circ_0004159_junction_seq
TCTCTCCGTTTCGTTACGTCACACTGGGTTATGGGTTATGGGTGCTGGCTTTTTAT
>dme_circ_0004160
TGGGCTACTCTTGGCGTCTTGGTTGGTTTCCCCGTTTTCTGGGGATCTGGATCCTAGTTAAGGGTCTTAG
ATCCTCCTCGTCTGCTCTGTGGTTCTGATGATCCTTGGGGACACTCGACGAAACGCACACAAATCTAACA
CACACACGCGTACGCACACAGAGACAAGTTATGGGTTATGGG
>dme_circ_0004160_junction_seq
CACACAGAGACAAGTTATGGGTTATGGGTGGGCTACTCTTGGCGTCTTGGTTGGTT
>dme_circ_0004161
CCAGTGGAAAAGTGTCCGGTCGGTCCACCAGATGATCGGCTGCCGTTCCTGCCGCCCTCGCTGTTGCCGC
TTATGGCCGGAGATCCTCCGTTGCCGCCGGCGCTCTTATATCCTGAGCCGTTAGAGCCGCCCTTGCGGTA
ATTGCCGCCCAGCGAATTCGATCCTGACGATGGCGAGGAGCCCTTGTCGATCTTCACCAGATTCTTGAGG
TTGTTAAACG
>dme_circ_0004161_junction_seq
TTCACCAGATTCTTGAGGTTGTTAAACGCCAGTGGAAAAGTGTCCGGTCGGTCCAC
>dme_circ_0004162
GCGAGGGCGGCAGGAACGGCAGCCGATCATCTGGTGGACCGACCGGACACTTTTCCACTGGCTATAGTAA
GAATTCCGTCAAAATTCGCCAACACGACAGTGAGGTGAGCCCAAGACCTCGACGATCTTACCAGG
>dme_circ_0004162_junction_seq
AGCCCAAGACCTCGACGATCTTACCAGGGCGAGGGCGGCAGGAACGGCAGCCGATC

>dme_circ_0004163
GGCGGCAACTCCGACAGCTACAACGGCAGTTCCTCTTCTGGTAGATACCAGCAGCAGGATCAGCAAGGCG
GATCGCATCGTGGTAGTCGCAGCAACTATCACTCAAACTCCGATGGCAGCTACAATCGATCAATAAACTC
AAACTCCGTGGGAGGAGGAGGGTCTGGGGCAGTCGGCTCTGGAAGCGGTGGCAGCAATTATCGCTCCGCC
CGCAACTACGAGAACGGCAATGGGAGCAACGGCACACAGCCACGTTACGGCAGCGGCAACGGGAACGGCA
ACAGCAGCAATTCCAACTCTTCATCCGGTTACAACGGATCTGCAGCAAGCTCAACCAACAGCAAGTCGTC
GTATGGGGCTTCGCGCG
>dme_circ_0004163_junction_seq
AGCAAGTCGTCGTATGGGGCTTCGCGCGGGCGGCAACTCCGACAGCTACAACGGCA
>dme_circ_0004164
AAATGTTCGAAGAATTGGAAGTGGAGCCAGAGATCTGAGCAGACGCACTGCCGTTGCGAAGATTGGGTGT
GCGACTGGACGGGGTTCCGCCGTAACGATCTGCGTCAGACCTGCCGTAGCTGCGGGGTCCTCCGCTCGCT
CCTGCCCCTGATCCGCCTCCTCCGTTTCGCTCTAGGCTGTTGGTGTTGTTGTAGCGCTGGTGCGATGG
>dme_circ_0004164_junction_seq
TGGTGTTGTTGTAGCGCTGGTGCGATGGAAATGTTCGAAGAATTGGAAGTGGAGCC
>dme_circ_0004165
GGCACTCCTCATTCTCAACAGCAGCAGGAACGATCACAGGCTGGCAAGGCAAGCACACCGCCACCGATCA
CCGGACGCTGGATACCACCATCGTTGCGTCCACAGCATGGACTCACCCAAAGCGAAAAGAACGACGCCGT
CTTCCGGCGTGTGAGGGGTATTTTGAACAAGCTAACGCCCGAGAA
>dme_circ_0004165_junction_seq
GTATTTTGAACAAGCTAACGCCCGAGAAGGCACTCCTCATTCTCAACAGCAGCAGG
>dme_circ_0004166
GTTCCAGGAACTGAGCGATGAACTGTTGAAGCTGGATCTTAACTCTATTGCTATTTTGAAAGGCGTGATT
TTGCTGATATTCGACAAGGCCTTAGACGAGCCGAAGTACTCGTCTATGTATGCGCAGCTGTGCAAGCGAC
TTTCCGAGGAAGCCCCGTCATTCGACAAGGAACCAAGCAATTC
>dme_circ_0004166_junction_seq
CGTCATTCGACAAGGAACCAAGCAATTCGTTCCAGGAACTGAGCGATGAACTGTTG
>dme_circ_0004167
GGTAAAGGAAGATGCTGCAATACCTGTGACCACTCCAGATCGCTTCACCAAGCTTGTAGATGGTTTCCTC
GAGCTTAAGCTGCCAGAGAAGGCGCTGAAGGATGTGTGCATCAATCTGCTGATGGAG
>dme_circ_0004167_junction_seq
GGATGTGTGCATCAATCTGCTGATGGAGGGTAAAGGAAGATGCTGCAATACCTGTG
>dme_circ_0004168
GATTTTCAAGCAGGTGGTCAACAAGATGAACGAGCGAGAGGCACTCAATCCCCGAATCGTTAGTCTTGTA
GCCAGCTTGCTGGCAAAGACAATTTGCGAGCCAGCATTGCTGAAGTTAAGCGATATCGCCAACTTCACGG
ACAATGGCCAACACTATCCGCTGTTCCTGCTCGTGCTGCAGCAACTGGTCAAG
>dme_circ_0004168_junction_seq
CCTGCTCGTGCTGCAGCAACTGGTCAAGGATTTTCAAGCAGGTGGTCAACAAGATG
>dme_circ_0004169
TTGTTGGGGTTCGGGTCGCTTTGCAGCTGTTTTAGCATCTCGGCCTGTACCTTCAATAGCGGATAAAGGA
AAGAAAGCTTGCGATCCTCGAGTATCCCGGCCAAGCGCTCCTTGTTCCGATCCGCCTCCGGCAGGCTGTT
CATAAGATCAACTTTGCTCGCCCGGAATTTCTCTTCCAGCGCATCCTTGCCGATGACCTTGACCAGTTGC
TGCAGCACGAGCAGGAACAGCGGATAGTG
>dme_circ_0004169_junction_seq

GCAGCACGAGCAGGAACAGCGGATAGTGTTGTTGGGGTTCGGGTCGCTTTGCAGCT
>dme_circ_0004170
GGCGGATCGGAACAAGGAGCGCTTGGCCGGGATACTCGAGGATCGCAAGCTTTCTTTCCTTTATCCGCTA
TTGAAGGTACAGGCCGAGATGCTAAAACAGCTGCAAAGCGACCCGAACCCCAACAACTTCTACAA
>dme_circ_0004170_junction_seq
GCGACCCGAACCCCAACAACTTCTACAAGGCGGATCGGAACAAGGAGCGCTTGGCC
>dme_circ_0004171
GCGACCCGAACCCCAACAACTTCTACAAGTGGATCAAGGCCAACGTTGACAACAAATACTACAAGGACCC
GGGCTTTATCCAAGCCCTAATGACCGTGGTGGTCAAGTATGTGACCAAGGAGACGACGCTGGCGGACAAC
CTCGACCCCAAGGAGCACCCTGAGAAATCGGTGACCCAGAAGGAGCAACAGCTGCTGGAGAACTACAGCC
AGATGCTGCAGACCTTCCTGGGACAGAACGAGCTTCAGTTGATGGCTCTCTATGCTCTGCAAAC
>dme_circ_0004171_junction_seq
AGTTGATGGCTCTCTATGCTCTGCAAACGCGACCCGAACCCCAACAACTTCTACAA
>dme_circ_0004172
GAGGAGGAGGCATTCGTCCTGTGGAAGGAGGAGATTTCCGACAAATATCCAGGCAAAGGATCCGCTTTGT
TCCAAGTGAACGCCTGGTTGACTTGGCTGCAGGAGGCCGAATCTGAGGACGATGAGGATTAGAAAGCAAC
GCAGTCCCGCATAATAACAGCGCTATAT
>dme_circ_0004172_junction_seq
GCAGTCCCGCATAATAACAGCGCTATATGAGGAGGAGGCATTCGTCCTGTGGAAGG
>dme_circ_0004173
GGTACGCTGGGCAGAGTGAGCGGCTGGCTGGCGATCAGGTGGATGCTGTGCTTGAAGGTCTTGACGTGCT
TTCGCAGCGAGGACGGATCCGTGTAGCGCTTCTGGCAGCCGGCCACTTTGCACATGTACGGCTTCTCCAT
CGAATGGGTGCGCGTGTGCTTGAAACGATCCGAGGAGTTCGAGTACGCCTTCTGGCAGCCCTCGAAGCTG
CACTTGTAGGGTTTCTCGCCCGAGTGTGACCTAATGTGGATCTTCAGG
>dme_circ_0004173_junction_seq
CGAGTGTGACCTAATGTGGATCTTCAGGGGTACGCTGGGCAGAGTGAGCGGCTGGC
>dme_circ_0004174
GGTCCTACTCCTCCCCCGTGCTCCGCCTGCAGTTGCTTGCTTAACGGGAGCACTCGATGGCTTCCAGTTT
CGTTTTGGCTGAGACACTGTGATTTGTCAAGGGGCGTGCAATTGCAATTGAGGTCTGCTG
>dme_circ_0004174_junction_seq
GGCGTGCAATTGCAATTGAGGTCTGCTGGGTCCTACTCCTCCCCCGTGCTCCGCCT
>dme_circ_0004175
ataaatcaaattaaaaatattgtaaaaaATTGTAGTTAAACACGCGCTGGAGCACGGTGAACTGAAAATG
TGAATGAAAACCATCAATAGAGAGAGACGAGCGCGATTTGGTGGCACCAAATGCTGCAGTTGCAACCTGC
ACATGTATGTGTATACGCGTGAGTGTGCGTTTGCGTAGGAGGCACCAAAATAACAACAGCAATCGCAGTG
ACAACAAAGAATCGGAATCGGAATCG
>dme_circ_0004175_junction_seq
TGACAACAAAGAATCGGAATCGGAATCGataaatcaaattaaaaatattgtaaaaa
>dme_circ_0004176
CAGCGCATCGCCATCGCTCGAGCCCTGGTTAGAAACCCGAAGATACTCATCCTGGACGAGGCCACCTCTG
CGCTGGACTTGGAGAGCGAGAAGGTGGTGCAACAGGCGCTGGATGAGGCGAGATCAGGCCGCACATGCCT
GACGATTGCGCATCGTCTGACCACCGTGCGGAATGCGGATCTCATCTGTGTGCTAAAACGCGGCGTGGTA
GTGGAGCACGGCACCCACGATGAGTTGATGGCACTGAACAAAATCTACGCCAATCTCTATCTGATGCAAC
AGGTGTCGGGCTAACACAGCGAACTGGAGTGATAATTG

>dme_circ_0004176_junction_seq
CTAACACAGCGAACTGGAGTGATAATTGCAGCGCATCGCCATCGCTCGAGCCCTGG
>dme_circ_0004177
GCACACAGATGAGATCCGCATTCCGCACGGTGGTCAGACGATGCGCAATCGTCAGGCATGTGCGGCCTGA
TCTCGCCTCATCCAGCGCCTGTTGCACCACCTTCTCGCTCTCCAAGTCCAGCGCAGAGGTGGCCTCGTCC
AGGATGAGTATCTTCGGGTTTCTAACCAGGGCTCGAGCGATGGCGATGCGCTGCTTCTGACCTCCAGAGA
GCTGCGATGTCTTGCCCAGTCGGGTGTCGTAGCCTTGGGGCAAAGCACTTATAAAGTTGTGGATATTCGA
CTTCTTGGCCGCCTCAATAATCTCCTGCATGGACACATCGTCACGGAAATTGTTGCCATAAGCTAT
>dme_circ_0004177_junction_seq
CGTCACGGAAATTGTTGCCATAAGCTATGCACACAGATGAGATCCGCATTCCGCAC
>dme_circ_0004178
GGATCGCTGGCCGAGAAGGAGCTGAAGTCCTACTCCGATGCAGCCAACGTGGTCGAGGAGGTATTCAGTG
GAATTCGCACAGTGTTCGCATTCAGTGGTCAGGAGAAGGAAAAGGAACGCTTTGGCAAGCTGCTCATTCC
CGCAGAGAATACGGGTCGCAAGAAGGGTCTATACTCGGGCATGGGAAACGCACTGTCCTGGCTGATCATC
TACCTGTGCATGGCTCTTGCCATCTGGTATGGTGTGACGCTCATTCTGGACGAGCGTGATCTTCCCGATC
GCGTTTATACTCCGGCCGTTCTGGTCATTGTCCTGTTCGCCGTGATCATGGGTGCCCAGAATCTCGGTTT
TGCCTCTCCACACGTGGAAGCCATTGCTGTGGCCACGGCAGCTGGCCAGACCCTGTTCAACATCATCGAT
CGTCCGTCGCAGG
>dme_circ_0004178_junction_seq
TTCAACATCATCGATCGTCCGTCGCAGGGGATCGCTGGCCGAGAAGGAGCTGAAGT
>dme_circ_0004179
GTATTCAGTGGAATTCGCACAGTGTTCGCATTCAGTGGTCAGGAGAAGGAAAAGGAACGCTTTGGCAAGC
TGCTCATTCCCGCAGAGAATACGGGTCGCAAGAAGGGTCTATACTCGGGCATGGGAAACGCACTGTCCTG
GCTGATCATCTACCTGTGCATGGCTCTTGCCATCTGGTATGGTGTGACGCTCATTCTGGACGAGCGTGAT
CTTCCCGATCGCGTTTATACTCCGGCCGTTCTGGTCATTGTCCTGTTCGCCGTGATCATGGGTGCCCAGA
ATCTCGGTTTTGCCTCTCCACACGTGGAAGCCATTGCTGTGGCCACGGCAGCTGGCCAGACCCTG
>dme_circ_0004179_junction_seq
TGTGGCCACGGCAGCTGGCCAGACCCTGGTATTCAGTGGAATTCGCACAGTGTTCG
>dme_circ_0004180
GGACAATGACCAGAACGGCCGGAGTATAAACGCGATCGGGAAGATCACGCTCGTCCAGAATGAGCGTCAC
ACCATACCAGATGGCAAGAGCCATGCACAGGTAGATGATCAGCCAGGACAGTGCGTTTCCCATGCCCGAG
TATAGACCCTTCTTGCGACCCGTATTCTCTGCGGGAATGAGCAGCTTGCCAAAGCGTTCCTTTTCCTTCT
CCTGACCACTGAATGCGAACACTGTGCGAATTCCACTGAATACCTCCTCGACCACGTTGGCTGCATCGGA
GTAGGACTTCAGCTCCTTCTCGGCCAGCGATCCCTGGAGTCGAGCCACCACACTGGTGGCTGCAATGATG
AAGGGCACACAACTCAGAACGACCAGGGTGAGCTTCCATCCGTAGACGAAAGCAGACACTATTCCAATCA
CAAAGGTCATGATCAGGAATACCACAATCACTATTTTCTCGCCAATGCCTTCCTTCAGTTTGTCCAGGTC
TCTGCAAATTCCAAAGAAAAAAATCGAGGAGTTATAAAAATACTCTAAAGTCTTTAGAATTTAATTCTAG
AATTAAAAGGAATTTGAATAAATACATTGTAAAATTCGCACAAAAAAAGTTCACGTCACTGGCATGAGAT
ATTTAATTTATTTAAAAACTTTCAGGTGGCTTCCTAAAATTGTCTGGGAATCGCGATAGTGATAAATTGT
TTCAATCATATTTAAACCATATTGATGTTAATAAGTCCGATTGCTACTATGATTGCGTAATTTTCTAGCT
CTGCTAATGAATATTCAATTGATATGGTTTTTATAATTTGTTTAATGCAATAAGATTCGGATTATACGCT
TATTAATAATTATATTATTTGAGACGAAACTCACTCTGTCATTTTGCTGGCGAAGTTGGAGCCCGAACTG
GTGTCGTACCAGGCGATGTCCTGTCGCAGCATTGCCTCCAGGAAGAGCTTTCGGATGCGATCGATTTGGT
TGAGGGCTATCCGGTTGGCCAAGTCGATGGCCAGTGTGATGAGCAGGAACATGGCCACAGATCCCACCAG

ACTGCCAATGCCAAAGGCAGTCGCATCATCAATGATCGCCTGGTTGTTCTCCTCTTTGCTGGCATTTGTC
CTGAACGAAAATTTGACGTTATAACATAATCCTGGACTATCTTATGATTAAGAACTTACAATTGCTGACC
GCCACCAAACATGGGCAGAGCAAAGGCTGGCGAGGATGTGCCCACGCCGACTGTTCGGTCCACGAGCAGG
GATGTGAACTCTCCGTAGATAATCATGAAGTAGGGAATAAATGCGGACGCAGCGGTGGCCACCAGCAGAC
TGACTACCAACAGGAAGCGCTCGCATCTGGTTGAGTATCTGAACTATAGAGGCGAGACCAAAAAGGATAT
AGAACCTATAACGATGCTGATGCGGAGTCACCACTCACCAGATCAAAGTAACTGTACTTTCGGGTCGCAT
CCACGACTGATCCTTCCTTAAGCTGAAAGTCACCAGCTTGTGGCAGCCGTGATTCCTCCTTCTTGACCAT
T
>dme_circ_0004180_junction_seq
CAGCCGTGATTCCTCCTTCTTGACCATTGGACAATGACCAGAACGGCCGGAGTATA
>dme_circ_0004181
TGATTGTGGTATTCCTGATCATGACCTTTGTGATTGGAATAGTGTCTGCTTTCGTCTACGGATGGAAGCT
CACCCTGGTCGTTCTGAGTTGTGTGCCCTTCATCATTGCAGCCACCAGTGTGGTGGCTCGACTCCAGGGA
TCGCTGGCCGAGAAGGAGCTGAAGTCCTACTCCGATGCAGCCAACGTGGTCGAGGAGGTATTCAG
>dme_circ_0004181_junction_seq
CAGCCAACGTGGTCGAGGAGGTATTCAGTGATTGTGGTATTCCTGATCATGACCTT
>dme_circ_0004182
GAACGACCAGGGTGAGCTTCCATCCGTAGACGAAAGCAGACACTATTCCAATCACAAAGGTCATGATCAG
GAATACCACAATCACTATTTTCTCGCCAATGCCTTCCTTCAGTTTGTCCAGGTCTCTGCAAATTCCAAAG
AAAAAAATCGAGGAGTTATAAAAATACTCTAAAGTCTTTAGAATTTAATTCTAGAATTAAAAGGAATTTG
AATAAATACATTGTAAAATTCGCACAAAAAAAGTTCACGTCACTGGCATGAGATATTTAATTTATTTAAA
AACTTTCAGGTGGCTTCCTAAAATTGTCTGGGAATCGCGATAGTGATAAATTGTTTCAATCATATTTAAA
CCATATTGATGTTAATAAGTCCGATTGCTACTATGATTGCGTAATTTTCTAGCTCTGCTAATGAATATTC
AATTGATATGGTTTTTATAATTTGTTTAATGCAATAAGATTCGGATTATACGCTTATTAATAATTATATT
ATTTGAGACGAAACTCACTCTGTCATTTTGCTGGCGAAGTTGGAGCCCGAACTGGTGTCGTACCAGGCGA
TGTCCTGTCGCAGCATTGCCTCCAGGAAGAGCTTTCGGATGCGATCGATTTGGTTGAGGGCTATCCGGTT
GGCCAAGTCGATGGCCAGTGTGATGAGCAGGAACATGGCCACAGATCCCACCAGACTGCCAATGCCAAAG
GCAGTCGCATCATCAATGATCGCCTGGTTGTTCTCCTCTTTGCTGGCATTTGTCCTGAACGAAAATTTGA
CGTTATAACATAATCCTGGACTATCTTATGATTAAGAACTTACAATTGCTGACCGCCACCAAACATGGGC
AGAGCAAAGGCTGGCGAGGATGTGCCCACGCCGACTGTTCGGTCCACGAGCAGGGATGTGAACTCTCCGT
AGATAATCATGAA
>dme_circ_0004182_junction_seq
ATGTGAACTCTCCGTAGATAATCATGAAGAACGACCAGGGTGAGCTTCCATCCGTA
>dme_circ_0004183
GCAGGAACATGGCCACAGATCCCACCAGACTGCCAATGCCAAAGGCAGTCGCATCATCAATGATCGCCTG
GTTGTTCTCCTCTTTGCTGGCATTTGTCCTGAACGAAAATTTGACGTTATAACATAATCCTGGACTATCT
TATGATTAAGAACTTACAATTGCTGACCGCCACCAAACATGGGCAGAGCAAAGGCTGGCGAGGATGTGCC
CACGCCGACTGTTCGGTCCACGAGCAGGGATGTGAACTCTCCGTAGATAATCATGAA
>dme_circ_0004183_junction_seq
ATGTGAACTCTCCGTAGATAATCATGAAGCAGGAACATGGCCACAGATCCCACCAG
>dme_circ_0004184
AGAACGGCATTGCCGGGCACATTGCCATGGCTGTCGTGCACCCATCCGTACCCGTATCCGGCCAGCAGCT
CGTAGTCGTGCTTCACAACCTCACCGCCTCCGTAGGGCACATAGGCACAGCCCTTGTTGGGGATCACCTG
TGGTGGATAAGTGGATAGATAAAGTGGGATGCAACAGAACAGATTTAAAGATGGAATGCTCCAAGTGTAC

ATTTTCGGAACACGAGCTGTATATAGTTTAAGGTTCTCACCTTGGCTGGCAGCAAATCGCCCTCGTGGTA
GGCGCGACCCACATAGATCTGATCGCCATCGGCGTCGTGTCCGCCAACCACTGTTCCGGGAACAATGCCG
CGTCCGCTAGAGGCTATCCACGTCTCGGGCTGGACCAGCACTTCATAGGCTTCCAGACGGTGCTGAAATG
ATATAAGATAAAAGTGATTTACTAAGATTTATCACATTGCAAACACTGCTTGTGCGATTCGTCACTACGT
GGTCTTCGAACGTTATTGTTGAGGGGATGAAGGGAATCCATCAACATAACATCAAAGTGAGGGTTAGACC
TACCTCCTGTCCGCCGTAGGGGATGTACAAACACTGGTGCGAGGGATGGACCTTGCCGGGGGTGAGGCTG
CCCTGGAAGTAACCACGGCCCACGTACAGGGGCTCACCCTCGCCAGTCTGGCCCACTTGGATGGCATGGG
GCGGGACAGATCCACCACTGGACGGGATCCAGGAGAAGTGATCACCGACAAGGACCTCGAAGTCATGCTT
GCTGATCTCCTGACCGCCCCATGGCACATAGGCCTGCTGCTTGCCGGGCACCACCTTCGCTGGCAGCATC
TCTCCATTGTGG
>dme_circ_0004184_junction_seq
CTTCGCTGGCAGCATCTCTCCATTGTGGAGAACGGCATTGCCGGGCACATTGCCAT
>dme_circ_0004185
ATTGACGCTTGTGCGAAAAACAAAAGCGAATTTTTAGCGGCGAGAGTGTATTATTACTATGATGACGCCA
GAATCGaaagcaatacagccggcagcagcaacaacaaagcaaacagcagaagcaacagcaacaacaacaa
TGGCTCACACACAACAAAAGTCGCAGTTGTCAACGTTGGCGAAAACAACAACGACAACGGCAACGAATAA
GGCGGCCAAAAGCGTTGTCAGCAATGCAAATAGCAGTGGCAACAATTCGAGCAAAAAGCTGGCTTTGTCT
CAGTCTCAGAAaacaacaacaacaacaacaccaccaacgacgacgacaacaacaacagCAGCAGCAGCAG
CAGAGGCAACAACTAATGCTGATAAAATGCAAAAGCAACAGCAACTGAAGCAGCAACTTTTCGCTGCCTG
CAGCATTAAAGTAAAAAGTGAAAACACATTAGCAACTACTGCGAATGCAGCATTAGCAGCAGCAACAACC
ACAACAACAACAGCAACACCAGCACTTGCCACCGGCAAAGCAGCAAAAACAATATTAGAAAACGGCATAA
AGAAGGAATCCACGCCTCCGGCTGTCGAATCCGTTGAGGcctcctcctcatcgtcatcctcctcctcgtc
ctcctcatcatcatcgtcctcgtGGCCGACGACAAGGAGAGCCACTTCAGAGGACGCCAGCAGCAATGGT
GGCGCCTCCGCCGACGAGGAGAAGTCGGAGGAGGACCCAACGGCAGCTGTGGCTGCGTCTTCAACGGCGA
CAACGACTTCCGATTTGGCCACAACTTCAAGGCCACGCCCCGTCCTTCTAACGGCCGTCAATCCGCACAT
CATCTGTCACCTGTGCCAGGGATATCTGATCAATGCCACCACCATCGTCGAGTGTCTGCACTCCTGGTAA
GTATCCTTCCGCCGATGAACTTCATCAAATTGTATTCTATTTGCGATGCGGACTTGCACAAGTCAATAAC
CTTTGAGTGGCCAGGagatacagatacagatacagatTTTTTGAACTCCTAATGGTTCTAATTCAAACTA
CCCTGTTAATGGAATGAATGAATGCGCTATTCTTGGCTTTACACTTTACTTACACTTGAGCAGTTTCAAA
CGGAAACTTTAAGAGCTTGTACAAAAGAACTTTTGAAAACTTGTCCGATTTTGCTTGATGAAAACCATTA
CAAATTAATCAATGTGGGAGTAAGTTTAAGTTAAAAGAAGAAATTCCTAAATGGTTAAATTCGTTTGGAT
TCACTTTAAATGGTTACCATCCAAAGCGAAATAATTACTTTCCGTTGAGAGAAAATTTTCAAAGTTCTCC
AACAAGTTTACATAAATTTAGATAGAAACTTTTTTCATCAAGATATTAAAAACGAGAGCTACAAGAATAT
GTAATGCGATTAGGTTCGGTATATTTGTATCTCAGAGTAATTAGAATTTTGGGTATTTAGATTTTATCCC
ATAAATCCTTCGGCCAATGTCCAGGGATTGGGAAAAGTTGCAGCTAGTAATCGCTGTGGCTTAATTATTA
TGCCAACTGCCGACTAAGGGCACCATCCCCGCCAAATTCCATTTCCAATACCCATACTCAAATGTCTAGA
TGTTCTGCAAAATTGCCTAGACGCGATGAGTTATGTTTACTTCGGAGGGCGGAGAATTTAGCCAGGGCGA
GGAGCAGTAGCTCTATGTTGTGGCAGCAGAAGGGAGCAGGAGGAGGAGTAGCAGCAGTAGCAGCAAGAGG
GTTGGGCGCTTCGGAGCAGGAACACAGACGACACGCAGATGTTGCCGGGCGATAAGTTTGTGGGCACATT
AATGGTCCCGGCCTCGAAAGTACTTAGCTCGATTGATTGAGAAACTGTTCGAGGTGGCGGGCGAGAGATG
ATCCCTGCTCCTCGCACCACTGGTAAAAATCCCGGATGCATTATCAACGACACAGATATTAACACAACAT
TAGCATTCAGGCGGGGGACTAAACGCCATAAAACTAGCGAATGTGGCCATTCATCAACAGCCGTTCGGTT
CTCCATTCCGTTCGGATATTAAAAGTTGCCCCTTTTGCAGCGTAAGGTTAATTTTCCATTCAGCCGATTC
TTCttttttttttttttGCTTGCCAAACTCCGTTGGCCGTCCGTAAATCTGGCAACCAATGTGTTGATAA

TGGACACTGGATACATTTGGTCAGGCTCATTTAAATCTGTTGGCCGGCAGGGACGCAGAGATATATGTAT
GTATGTACATGCTTACGTATGTAGAGCACCCGGGAGTCCCATCTAACTGTCAAACGAACCGAACCAAAAC
AAACCGATGTGAAGTGGggacgccggacgctggacggtggacagtggacactggacAGAGTTGACAGTCG
GTGGACTGTTTACTGCTGACCGGACCTGGCACTGGCACTGGGACCCAGGCCAAAGCAAAAGCAAACCAAC
GCCGAAACCAAAAGCAAAGGCAACGCAGAGCAAGCAGAGcaaaccaaaccgaaccaaaccaaaccGAAAA
CCGGACTGTAGTAGCAACAGCTGCCTTTGCATGGGGGCACTGCGAGAAATTTGTAAACAAAAAGCAAGCT
AAAATTGTAGATTTTGTTTGGGTATATAATAATGTATCTATTTTGATAGTGGAAGATTGATTACCAACTG
TATAACAAAAGTTTTTAACGAGACAAGGGGTCAGAAAGTTGATTTGGTATGGCTTACGTATTTCTCTCAG
TGCACCGATTCTGCTGCAACTCTTGCAGAAAGCTGGCTGCTGTTGCTCTCCCCATTCGAGGCCATTTGGC
GGGCAGTAAAACCAttttttttttCCTTTTGCTAGCTTGCACACGCCACCGCCCCAAAAGCGCCCAACT
GACGCCCCACTAAACCGCCTGCTTTATATAttttttttCTTCAGCTCAGTGCGAATTTTTCCTTGTATTT
CGCTGGCTGTGTGAGTGTTTTTTTCATTATTATTATTTCGGCATTGGACCAAAACTGACGGACAAGCGTA
ACAGGCGGCGGGCCAGCTTGGACTGACTGACGGTGCTGCGACCCCCAGTGGACTATACTATCCTTCGCCT
CCTGCTTTATGCAGAACCCCACCCACTGGGAATCGCCATGGTCTGCTGCTGGGTGACAGTTGTAGCGGGC
GGGATGTATTCAGGGGAAAACTGGCCGTGGACGTAGCGCGATGGCAgggggtggggagggggagggggaT
GGAGCTACGCAGACACGGAGAAAGAGACAGACACTGCCATTGACATTAAAGTCAAAACAACTTTTTTGCA
GCACGGCACTCACTTTGCCTTGAAGGTTGTATTTCCATGTTTAATGCTCTGCCCCTtgcttggtgcttcg
tccttcgtgcttcgtccttcgtccttagtctttcgGCTGTTTGTGTGtttttttttttttttttttttt
tttGCCCTTCTGTTGGGTTTGATTGCGTTTCCGTTCCATTTTATGTGGGTTGGTTGGATAGCTGGAATGG
TTTTACATCAATCGAATCATCGAAATCTGTTGAGTTTACTTATTTCGAATGCCGTGTGCATCGTTATTTG
CCTATTTCGCATACCAAAGCCCTCTAATTTATACTCGTCTTTTCTGCACTCTTTTCAGTCTGCCACAGTT
GCCTCATTAATCACCTGCGAAAGGAGCGATTCTGCCCGCGCTGCGAGATGGTCATCAACAACGCCAAGCC
GAACATCAAG
>dme_circ_0004185_junction_seq
CATCAACAACGCCAAGCCGAACATCAAGATTGACGCTTGTGCGAAAAACAAAAGCG
>dme_circ_0004186
GGACCCCATTGAAAGAGGCTGCTAGGAGTAAGTATTGAGGCAGATAGAATAGGCAGAAAGATGCACCTGC
AAAACACGCACAACACAATGGAATCAAACGCCGCTATGGATGCCGCATCCCCAGCTTCTCGGGATGTCCG
GCAGTTTCATGATCTGATAACGTGCCGACTTTGTCGCGGTTATATGATCGATCCAACCACTGTGGATTAC
TGTTATCATACCTTGTAAGTAGTCTTAGATTCCAAAGAGTACCTTTAATATACGCATAAAACCAATATCT
TGTATTAATTGCATGTAATTTGATCATTATTTGTTTGATCACTTTTACCATTTTAAAGTTCATTTATAAA
GTTAATTTAAGGTTGTCATGATTTACAATATTGACTGCCCAAGTTAGATCTTAAACTTTAAGATCATTAC
TAAACAATGTAATCACAGCTTCTGGTAGTTCTTAAGCTAAAATACACATTTTCCTTGATCATTAAACATA
TTGTTATTCGGTTTGATCTATCATTTCTCAACAGGATTGCCGGAGTTGCATACTAAAGCACCTGCTGCGA
GCGGTATATTGTCCGGAATGTAAAGCCAGCGGAGGCAAGGAAATCAATGAATTGAATCTGAAATCGGATG
ATACGTTGCGATCGCTAATCTATAAGCTAGTGCCGGGACTCTATCAAAGGGAGTGCAAGGAGCTGGCAGA
TTTCAAGGAGCAGCACGATTTGGTGGATGAACAAACGACTGATGAACCGGAGTTCTTCACAACCACGGAA
CTTATAAGGCTTATCCCTGGAATACCATCCCGCCATGCTGCATCAGTGTGGTCCTGGTGAGGTGCCTCCC
ACTATTTACCTGCAATGTGCCGCCGGATTACCTGTGGAGTTACTGAAAAGGTTCCTCTGCTCCAAGTACA
ACATAGAGACGGACAACGGTCTGGTGGAAGTGGAGGTGACCTACAAGGATGAGGTACTGCCTACCAATTT
CACCCTGATGGACGTTGCCTACTGTTACAACTGGAGCCGGGGTAAGTTTGTTTTACTTAATAAGAAATAT
TAGTGCATTCTTATAATGACAATGGGTTAAAAATGCAAAGGGATTTAACTGCAATGAAGGGACAATGCAA
TTGTAAAATCTTTAAATTAACTTTAAAAAGTACTTATGCTAGTATTTTAGTTCTCAATTAGTGTATTGAA
ATGTATTCTTCATAAGGGTAACTTTTTGATTCCGTAAAACAAAAAAACTACGTAACAATATGGAGAGAAA

ACAGGGTTGAAACTTCTCACTTGATTCTGCGGTCATCCGACTGCCAAATTAGACCTTGAAAACATATCCA
TCTTATCATTATCGGTCAGTTTTAAGTGTTATATACGCCCTTTTTTCTTCTCTTCAGGCGGGCGGTATAT
TGTCCCTTTTTGCGGTTTATCCCAATAACCCGATAGATTTTCGCAAGGGGAAACCCAATCCAATTGTGCC
GGCAGTTTAGCGTTCAATGTCCACTTTCAATTTTCCACCCTTGTCAACCTGCCCTTTTTTCCATCTATTT
CTCCCCTCTCCGAATCCATTTAGGGAATCGCCCATGGCCTTCTGCTACCGGATTCTGCTTTACGACAACG
AGCAAACAAAAAACGATGAGAACAACCTATCCAGGATTAACCAGGACATTGAACCAGAGCATTCGGTACG
TCGCTCCAAGTCGGCCAAATCGGTGACCTTTGCCGAGGACCTGGAATCGGAAATAGACTCCGGTTCTCCG
CGTTCCAAAGTACGGTGTAAGACTCCGCCAAAAGTTTCTCCCTCGTCGAAAAACAAACGATTGACGTCGA
GTAAACGGGAAGCGGAACCTGAAAGTCCGGTGAGCAACTTCAAAAGTCTGCGAAGCAATGACATGCGGTA
CAGTGATTACGCCGTGTCCAAAGTTAAAAGTGAACCGGAACAAGAACAGTTCCTTCTGCCAAGAGAACGG
GAACAACAGCCGCTGGAGGCCAATACTAATATAGTGGTCAGCATTCCACCCTCGCAACTCCGAAAGTCCT
ACGTTGATGCCGAGGATTTTGAGCTGAAGACAGCGAATCGAAAGGGAGTTGGCCATTTGCCCAAACTGAA
GATCGAACTGAACAGCATGAAGAGCAAGCTGAGTATGCCATTATCGGCTGGTCCGCGACTGGAGGATACC
TCCTGCTCCTTATCTTGCTCAGCCCAACAACTCGATCTGGAGACCTACGCCAAGAATATTGGCTTGAAGC
CCATTGAGCAGCCTTTGCAGCAAAGTGCATCCAATCCCGATAGCAAATACAGTCCCAATGCCTCGCCCAT
GTCTTCGTGCTCCAGTTCGACCAATGGATCCAGCAGCAGTCTGGGCACAGCCGATGCCAGTACATCGACC
AGCACGTCCAGTTCCCATCGCAAGCGCAAGAAGAAGCACTCCAAGGAGCCAAAGGATGCGAATGGCAAGC
GGAAGAAGTTGCACGCGGAGATCAGTTCGCAAACCGATGGGAAAATGAAGGTTAAGATAACGGCAAAACC
CAATCATAAGTTGGATTTCAAGAGGTCGCATTCACTGGCCAGTGGAGAGTTGGATCTGCAGAAGTTGAAA
CTGGACAGTACTAGCACTTCCGAGGCACTGAATCGCACATTGGGCGAGGAGGCGCGGAGTATCAACAGCC
TGGTGGTGGGTGGAGCACCCACACCGCCGCCCACGCCCACCGCCGAACCAGaacagcagcagcagcagca
gcagcaacaacaacaaccacaacaacagcagcaacaacagcagcagcagcagcaacagcaacagTTTGTG
GTGCTGCCAAAAATAAAGGACCTAACCTTGCCCACATCGCCACCTCTGCCGCCCAGTTTATTCAAAGCCT
ATACACCCAGCACGACGCCAACTGCTCCACATACGGTGGCCGGCGGAAAACCCAAGCAGCAGCAACAGCA
AATGCCACAGCAACCTCAAGCTGTACTACAGCAAAGTCTGGCTAAAACGAATCCCGCCAAACCGCCGCTG
AGTAGCAACAATAATCGTAAGCCCAACAGTGGACACTTTGCGGTGCCACAGGCTCCAACCCACCGGAATA
TGTATCACATGCAACGCTACCAGTCCACACCCAGTTCGATTGCCAGTGCGGCTAACAAGATGCCAAAGCG
ATCCATGTCCCTGGACGAATCGCATCCGGCCAAGCAGGCGAGATTGAGCCAGGCTCAGGCGATGGCCAGC
AGTTATGCGGCCAAGTTGCATATGCAGACTAGCAACCAGGCCAAAAGTCAAGCGGCCGCCTTTCTGCCCA
ATCCCCAGATGCGATCCTATGGTCTACCCGACCTGGGCAGCAAGCCAACGTTGCCAATGTTGTGCCCGGC
CAGCAGTTCGAGCCAAGTGACGATTACGCCAAGGCCCAGAGCTACGCCGTCGATTTATTCCTTCTCGGAG
CCAAATATCCATGTGCCTGCACTGGAAATAGTGCGATTGCCGGTGAATAAGCAGAGTGCAGGAGGCAAGG
GTCTAACGATGCCACCTCTTAGCCCGCCGGCAACGTCCAGTGCCCGCTTGATGGGACCACCAGCGGCTCT
GCCCAAACATGCAGGACATGGACATGGTGCGGCCAAGCGGAGCTGCCAAATGCCGACAatgccaatgccg
ttgccgctgccgctgcccatgccgatgACCACGATTCCGGCGATTGTCAAGTCGCCGCCACTGTCAGTTG
CGCTCAGTGGCCAGAGGAACAACAAGGGCAACTCCTCGAACTCAAATGCTTATCGCACATCACCGCCTGC
ATTAATAAATCTGCGCAACACAGCCGCTCCACAGCACTCGTTTCCATCAAAGTCGAGCCCAAAGGTGGAA
GCCAATTCAAAGAAATCCCCGCCAGCAGCTGGCTGCCAAGGCAAAACCAATGGCACTGCCGCATTGGACA
AGTCCAAAACGAGTTTGCGCGAGTTTCGGCCAGCAGTGCAGTCGGCTGTAACAGCAACAGCAACCACATC
GGTAACCAcagcagcgggagcaggagcaggagcaggaacaggaacaggaacagCATTAGCCAAGGATGCC
GATATACTTGATTTATCAGCTAACCCGGGTAGGAGCAACAATGACGCCAAATTGGCGCCCAACTCACCGC
CCGCTGGcaacaacaacaacaacaacaacaacaacaacaataacaacaataataataataacaataa
caacagcaccagcaacagcCTGGAGGCAGCCTTAAACAAAATCAAACAGAATATATCGGCAAACAGCAAT
GGTGGACCATCGACCACTTCTGGCAGTAACTCGAATGGTACAACCAACGGCGATGATCTACAGAATCTGC

ATATGCTCTCGGAATCGGCAACGGCCAGGGAGAAGATCTCCATAAAGGCCGCCAGCAGTGGAAACGGCAG
TGGTAGCACCAGTAGTAGTTCCGCCAAACCCAAGAATGCCAATGCTTTGGTAAGACCACAGAATGCCTCA
GTTCGGAGCATACCCAATCCATCGGCTTTGGCATTCCGCAATCAGCCAGCAGCTGCCAGCACAGCAGCCA
GCATTAGCAAACCGTTGACAGTTCGTGCTGAGGAAAAGCCGAAGGTTTCCACCAGCAATCCGGGATCATT
GAGCCCCACCAACACAagcagcagcagcagcagcagcTCAAGCGGCAGCTCTGGATGCAGTGCAGCCACC
TCGCCGCGGGCAATGACCAAGAAACCCACGACCATCGATCAAGTGGCGGCCAATTTGAATATTCGCGCCG
AGGCCAAGGCCGCCGCTCTCGCCGAGGAGGCACCACCCGTTCTCAGTTCCAATGCGGCCAAATCCCCGGA
GTTGGCCAAAACGACCACAGCGGTTGCGTTGAGGCCAGAGCCCAAGGAGACGCCCATTACCGTTTCGGCG
GCCAGTACTCTGCTGACCATCCCATCCGCGGTTTCCAGTGTGTCCGCAGTCCCGGAAACCATGGCCAAGC
CACCCGTACAGATTGCCAATGCTCCGGTGGCGAGTTCCGCTTAGTCACAAG
>dme_circ_0004186_junction_seq
CCGGTGGCGAGTTCCGCTTAGTCACAAGGGACCCCATTGAAAGAGGCTGCTAGGAG
>dme_circ_0004187
GATTGCCGGAGTTGCATACTAAAGCACCTGCTGCGAGCGGTATATTGTCCGGAATGTAAAGCCAGCGGAG
GCAAGGAAATCAATGAATTGAATCTGAAATCGGATGATACGTTGCGATCGCTAATCTATAAGCTAGTGCC
GGGACTCTATCAAAGGGAGTGCAAGGAGCTGGCAGATTTCAAGGAGCAGCACGATTTGGTGGATGAACAA
ACGACTGATGAACCGGAGTTCTTCACAACCACGGAACTTATAAGGCTTATCCCTGGAATACCATCCCGCC
ATGCTGCATCAGTGTGGTCCTGGTGAGGTGCCTCCCACTATTTACCTGCAATGTGCCGCCGGATTACCTG
TGGAGTTACTGAAAAGGTTCCTCTGCTCCAAGTACAACATAGAGACGGACAACGGTCTGGTGGAAGTGGA
GGTGACCTACAAGGATGAGGTACTGCCTACCAATTTCACCCTGATGGACGTTGCCTACTGTTACAACTGG
AGCCGG
>dme_circ_0004187_junction_seq
CGTTGCCTACTGTTACAACTGGAGCCGGGATTGCCGGAGTTGCATACTAAAGCACC
>dme_circ_0004188
GGATTTAGCTTTGAGTTGCGTAAGAAACATGTTTCAGTTGATATCCCAGCGACTTTCAGTCGGTTATAAC
AGATTCGTTTGATCACTTAACGACCCCAGATCTCGACGGTGGAGTTGATTCCGCGGTTCACCTGACCACG
AAGATTCACGGTGGCGAAGCGATAGCCCGGACCGCCGGAATAGAGGCTAGGAGATGCGCCGGAGTTGTTC
TTAAAGTTGTCGTAGACGATCACGGCGGAGATGTTGTAGAATCCGGCGGGATAGTTCACGTTCACGTTCC
AGTAGTT
>dme_circ_0004188_junction_seq
GATAGTTCACGTTCACGTTCCAGTAGTTGGATTTAGCTTTGAGTTGCGTAAGAAAC
>dme_circ_0004189
GATCTCGACGGTGGAGTTGATTCCGCGGTTCACCTGACCACGAAGATTCACGGTGGCGAAGCGATAGCCC
GGACCGCCGGAATAGAGGCTAGGAGATGCGCCGGAGTTGTTCTTAAAGTTGTCGTAGACGATCACGGCGG
AGATGTT
>dme_circ_0004189_junction_seq
TGTCGTAGACGATCACGGCGGAGATGTTGATCTCGACGGTGGAGTTGATTCCGCGG
>dme_circ_0004190
GTGAACCGCGGAATCGACTCCACCGTCGAGATCTGGGGTCGTTAAGTGATCTTTGGGAGCATGTTGTAAA
CGAATTGAAGTCGAATATGAAATGGCAAACATGATTAATTTTATTATACTTGAGAAGAAATTGAGAAAGC
AAAACTATGGAATCAATCAATAAAGAAAGAGTTCTAAGCAATATCGATATAAAAGCTTGGCCATTCGCAG
CAGTTACTATCAAACGATACAAGAACGTCGAAACGAAACATTCAACATGCGTCTTCTGCTCATCCTCTCA
GTTGTCCTGGCCGTGATCCTTGGCTGCCACGCCTACGGCGCCACCTGGGGGCGTCGCAATAACAACGACT
ACCTGCTGTCCCGCACCACGGAAGTCCGCAATCCGATCAAGAACAACTACTGGAACGTGAACGTGAACTA

TCCCGCCGGATTCTACAACATCTCCGCCGTGATCGTCTACGACAACTTTAAGAACAACTCCGGCGCATCT
CCTAGCCTCTATTCCGGCGGTCCGGGCTATCGCTTCGCCACCGTGAATCTTCGTGGTCAGG
>dme_circ_0004190_junction_seq
TTCGCCACCGTGAATCTTCGTGGTCAGGGTGAACCGCGGAATCGACTCCACCGTCG
>dme_circ_0004191
GACTCGAGGCGGACCTGTTCTATGTGCACGAGGGAGCCATCAACACATACGCAATGCACTTCACCGTCCC
AGTGCCCGCGGATGTCCATGAGCTGGAGTTTTCCTGGCAGAGTCTGATTGCCTACCCAGTGAGTAACCAG
GAGCAGTCATCTAGAACCTGCGATAATTAACCATTCCACTCTTGCCCAGCTGCCATACGCCATTAGTATT
GAGTATAACAACGACCAGGAGGCACTGGGCACGCCCACGTTGAGCATTCCGCACAAGGGTCTGGTGCCAC
AGGAGATCGAGTCCTTCCTGGTTTACCTGCCCTGCACCGGAAATGCGAGCCTACAGATGCCCGTCAATGT
CAACATGGTAGTACGGGCTCCTCCTCGATTCAACGACACCCGGCTCCACTTTAAGCGCAATAAGATCTGC
GCCAAAGGGCATCTCACCTGAGCCCAATCAATCGCCGGCGCCAGCCCACGCACCCTCCCAAGGACCCGCC
CTCCTCAGTGCCGCCGCCTGTGCTCTGGGTTTGGTTTTGGCCGTGGGACTAGTGGCCAGCATGATGTATG
TGAGGGCGCGCAAACAGCTTCGCCAGGACTCGCTACAGCACCAGTTTCACCACCGCAGCTTATGGCAGCC
ACCAAAACGTGTTCATCCGACTGGATCCCCTGGGAAGACCGCCGAGTGCCACGGGATCCTATGCGACCAT
TGCCAGCCTCAACAAATACCCTGCGGACAGCAAAAAGTCGTGCAGCATATTCGACCGTAAGTTCATTACC
CACTCGTCCACTCGGGTGACCCCTAACCAACTCCTCCTGGCACTCCCTCGACCAATCCACAGGTTTCCGG
AGCTCACCCACCCCCACGCCGTATGCCACCGCCCTGCTGCCCATGAACCTTGAACAGACCGCAGAGACGA
TTTACTCGAAGCCAGAGTCGATCTGTCCCTCGAGGATTTCCTACTACGCCTCCTCGCAACTGACCCAGG
>dme_circ_0004191_junction_seq
TACTACGCCTCCTCGCAACTGACCCAGGGACTCGAGGCGGACCTGTTCTATGTGCA
>dme_circ_0004192
GGAACTGTGGATAAACGAGGAAGAAATCGGAGGAGGAAGGCCTCTGGAGCAAGGAACATGGAGTGACCAG
CATCAGAAAAGTGGACAAAGTGACAGGCGATAGCCACAAAACAACACAGCCGAAAGAGGAAAGCAGGGCC
AGTTGACAGGAGCTCAGCCAATAAAAGCCAACCAAACTCAGTCTGGGCCAAATTCGGACACACTGATGCA
AAAAGGCGGCGGAGAAAGCCACCAGCAACAGCAACCGCAGCAAAACAAAACTGAAAACGGAAAAGTTGCT
CTGAAACAGACTGCAGGaggagtggaaggaggggaatgaggggaGCAGCCAGCGGAAGCCGAGGCTGAAA
AGTTCCCAAATTTGGCAGACAGACCCCTTTCTGGCCATCTCGTTCCCACTTTCAGCCGGAACAGTTAGCA
GCAGCTGGAATTTGATTTTAACGCTTATTCGAGAGCTTGGGATCTGTTCTGGTTCTGAGTCTCTGTTTGT
GGCACCAGCCACTGACAACCATCACGAAGGCAGGAGCAACTGGCAGGCGAATTAAACTCGGCACCAGGAA
ACTTCACCCCGGAAAGCCCGCACCAACTCCCCCTGAAAGCTACGCATCAATGGCAACACATTGAGATTGG
CTGTCAAAGGGGAGCATCCGGACGACGTGGAGCTGTGGAAATGTTCGCCACTTGCGGCGATACATGCTCT
GACAATTTATTGACTCCTCGCGGCACCCACTAAACAAACAAATCCCGCTCCATCATGGAACTCCTTCCTC
TGAAGTCGCCCCTCCCGATCCTGCTCCTATGCCTAACCATTTCCACTTCGGTCAGCGGCTATCTGAACAT
TTTCATCAGCCACCACGAGGTGATGAAACTGATGGGTAAGTCCAGTCTCAGTTCCGCTTCCTAATCCATT
AATGAAACCCCGAAACCACCCACTCCACCAATTGGACTCCCCGACCCAGGATCTATGATTAATAGTTGAA
GGCCGAAGTGCGACAGGACAAATTCATTGATCCCTGAACACGAAAACACAAACAGTCCCCGAAGTTGGCG
ACCATGTCACAGTCATTAATAGTTCCGAAATTATAGTTGACCATTAAAGAATGGTTGTTGCCGGAGGGAT
GGGGGGAGGTGGAATTATATGGGCATACTTAGTGGGCGAAACCAAAACAAATCGCACACAAATCGATTTC
CGCTGGGCCTAAACCACCCACCTTCACCTTGCTGACAGCTGCAAGTAGCTTGCTCCCCCCGTTCTCCGTT
TCCCTCGAACATTTCCTTTGCCTCCCCACATTTCCATGTTTTCCGTGCTCGCCGTTCCCTGTTTGTTTTC
TTGTTTTTCCGTTTGTTGCTCCACGCGAATTCTCGTTATTGATGGCGCCGGCTCCAGATCGAGCCAAGGC
CAAGTTCCCCCGCCCAGTTCAGCCCACTCCTCCAGATAGGAGTTACACGGATTTCATAGACGCGCGAAGG
AGGAATGGCCTCGTGCTTGTCGTTTCGCTTGCGATTTTCCTGCTCCTCAGGTTGATTAATGGATCCCAGG

AACGGGAATGGAGGTGGAGGTGGGAGTGAAGTGGAAACTCCGATGGGAAGGCATGGAAACTGGCCTAGTT
CGCGGTCGCTACTCCATCATTCGATGTTTCGCTGGTCACAGCATTTTCCGGCACTTAGCTGTAATTGGAG
CATAAATGCTTCCATCTCATTATGCAGGTTCCACTCCACTCCGCTTCCTAGTTGCCAAATCAGGACTCTT
CAATTAGTTGATGGCGTAAAAGTAATCCACAGTCAATTGGATGGAGATATGGATACTAGATTGATGGATG
AATGAGCTGCGCTGCATCCAATTATTGCCACCATCTCCTGACATCCTTCAATTCCGCCTTCAGGCCATTC
AAATGCAACCGGTTCAATGGGGCAAGTGGGCTCTTTCCTGGCAATTGCACTTTATAAACAACTTGTCCCG
CCATTTCAACTATTTATCCCCATGCAAATCGGATTTCATTAGCGACATTTCCAGAGACGCTTTCACTTTG
TTTGGTGCTCCTCGCCATTTCCTTTGCCTGGCCATTCTTTTCAAGCATCCTCCCTTCTTCCTCTTGGAAT
TCCCGCCTCCATTTCTATAAGCTCGTTATGTTTCTAACGACCAGTTAACACCCCTGGAATATTTCCCATC
CAGTCCATTCCATTCAGGCAGGCATCCCGCCTGCCACCCTCGGAGTTGTTTCGATTTGAAAATTAACAAG
CAGCATGAAATCAAACAAAATGACCCCCATCCTCCTCCATTTTCTCCCACCTCACACTCCAGATGAAAAA
TTCCTGTGCATGATTTTCCCTTAGACTTCCACCTCATTTCCCTTTCATCCTTCGTTGCTTTTCGGTCTCC
ATGGGAGTTTCTTCATGTGGAAGGCTCGTCACGGATGCACGAACTAGTCCCCATTCCATGCAAGCCAACT
TTTTCTGGACCCTCTTGCTGCATTTCCCACATGGAAATGAGGGTTTTTCTTCCGACCATTTGTTGTGCAC
TTTTCGGCTTCTCTGGCGACTTTCTTGTACAAGAGTAAACAAAGTTCATTGCTTTCTACCCATTTCGTAT
CAATTGTCACAGCCAAAACCCTGAACTTCCCCTCCCGTGACACCCAATCCCTTCTATGAAGTGTCGAAAG
CATTAAAATTAAATATTTTCTGCGCTTTTGTTGGAGATTTTCCGCCTCTGAGTTCAATTTGGATGTGTTG
TCCCAGCAACGAACCATTTTCCCCGTTGCCACCGACACAGCCACTCCCCCTTCCACCTTCCGCCCTTCCG
CTGAAGACTTTCCTGTTCAAGGGTGCGTAAAAATGCAATTTCAACAAGTTTCGCTTTGATGTGCACACTT
TTTCCTTTACCACCCGATCCCCTCGGACCAGCTTCCAAGTGCCCCACCCTAAGGGTGGACCACAACCTTC
GGCGGGTAAGGTCTGATACTTCCCTGCGAATGAAAAGTTAATGCAAAAGAAAATTATTTTTAATGAATAG
AAAACAGTCCGAAATCCGCACATTAATTTACCCCCCGTTACATGTTTCTTTTCAAGGGTGGCTTGGCATC
TGGAATCCGTCTTTATTGCTCCAAAATCAGGGGGATTCGGGGAGAAGCAATCAGAAACTGTGCCAATTGA
ACGGGCAATCCTCATCTCTGATTCATGGGACAATTAGCAGGAACAGCTTTAATCAGCGACGAGGGGTTCC
TGAACTCCCCTGAGATCGGAAGAGGTGGTTGCAGGTTGGAGGTTGGGGGCTGGAGTGCACTCTCCCAACT
AATGACACTAATTACCCCGGTAATCTCTTATGCAAATAATGTCTCCTTGGCAATGTGAGACTGCAGAACT
AACGTGGAACTGGGATTCACCTTAGCCAACCAGTTTCTGCCACTATTTGGCCTTCACTCCACTTTGTCCC
CATCTCTGGAGCCCCATTTCCCCATCCATGAGAATATGAGTTGCGGTTGCAGCGTCACGTGTGCGCGGCG
GAGTCATTCAGAGACAATCGCAATGGCAGGAATTATAATTATGGCCAGGGAGGCAAGACACATGGAGTGG
TATTACCCAGGGTCTGGAAGAAGAGGTCTTAAAGAAGGGAATATCCAGTAGAGAGCCAAGAAGAAAGGAG
CCTTCATGAAAGCGGCGAAACTTTCGCAGGCAAAGTTGGCGGCACCGTGATATGAAAGACGAGCAAAGTT
TGACACTATAGGGGAAGGGGTCTGATGGAAAGATATCCACCAGGAGCAGGATGAACTTGTGCAAGAGGGT
GAGAACTGCCAATTGATGCTGCTTAAAGTGCAATTCAGGATTGCGCTCCCCAGTGAAGCCAATCGAGGTG
ACAGGCATTACACGGGTTAAGCGAATGGAGTGGCAGAGCAGCTGCGACATCCCAGAAATCAGAACTGAAT
TAATTGAGACAGTCATTTATTCAAATGCCTCCTGCCTAAAACTTCATGAGATAGAAAGGACAGAAAGGAT
AGATAGAAAGGCACAGGATGAGTGGGGGACATTGTCCTTTTCCGTGTTTGTGCATATTTGATGGGAAGCA
CTTTGGGGTTATGTTTGTGTTTTTTCACCAGCTCAAAGAAGTTTCGTGGGGTAGTAGGTAGGACCAAGTG
GTGCACTTGGCCCACTCAGAGAGCCAGGTTAAAAACGCGACGGGGAGCAAAGCAATTTGGGTCTCAAAAA
CAAGCCAGAAATGGTAAAATGGTAAAAGAAAAGTGGAAAGCGCATAGAAAGCTGAGCTGGTGGGGAGAAT
CAGTCTGAAGATGTAGATGAAGCTGAGAGCAGGATGAAAGCTAACGAAAAATGCTTTTTCTTGTTCTGTA
ATTGGATTTAAAATTGTTTTAAGCAGCTAGCGAGCGCTTTGTTCTATCCTAAATGCAGGCGGTTTCGTCC
TGCGCCAATCATCCCCTCGCTCAGTCCGCCGGAAGCGGAATTGCTACATTCAACATCCGGCGGATCGGAG
CACCAGGCAGCAGCAGCTCGAACTTGAAGCTGGTAAACACCCGGCATTCCACTCCTTCCCACTCTGGTGA
ATAGCGAACTTAGCCTGATCCCGTGGAGTAAATAATACGCCCCTCCCCCCAAAGGGGGGCGTCGAATAG

AAATAGGAAGAGCAACAATAAAAGCGAATGAAGAAATTTAATTTCACGTCGTTTTAACTCAAGCAGCTCG
TCTTTCAATGCCAGCGGGTGGGCAATTCGAATGGAGAGTGGAAAAGATCAGGACACGCCAGGACTCCTGA
GCCCGCCAATGCTACTCCGTCCTATGATGAAGCAGTCGCAGTTGACACCTTTTAATATCGCCAACCCGAA
CAGCTTCCCCCAGGACGCCTGTCGCTCCTCATTTCGTCCTGAAGAAGAACTCGAGAAACTCCTTACTTCC
TCGCCTGCTGGTGACGGATCCGATTTCGATTATTATTGTGTCTTGGGCTTTGTTTACCCACCAGCATTGT
CACCTAGTGTTGCCCCCCTTTTTAACCATCCCTTTCCACTGAGAAAATCAATTTCCGCTTAATGGTATAG
AGAAACGCTCGAGGGAAGTGTAAACAGCAAATGCACAAAGTTTGCACAGCAGGAGGAATCTGTAAAGGAC
TGAATTCAGTGTAAACTTTCGCTTTGCATCCGCTTTTACTTTTATATCCTGCTCCCTCTCCATGACCAAC
TCACACCTGATGTTTTCCTTAACCTCCACAGTATGGCTATCACTAACCTCAAATTCCCTAACAATTTAGC
CTTCAGTAGGTATTGACCCAAACAAGGTGAGCATTTCCGAAGGCGAAAAATTCCTGGTCCACCTACAGTG
CACCTGTAGCTTTCCATCAATATGTACATGGACTGCCCTTAAAGGGGTTTTTAACCCATGGCAAGAAGGT
GTTTCAACATCACTCACTTAACTTATCCGCCGAGCATCAATCATCTGAAACCAATCTCATTAACAAGCAT
CTCAGTTGAGTCCAATGACTCACTTCTTATCTCCCTTGATATTCCCAAGATTTGTGATTTGGAAAGGTAT
ATGAAAGCATTGCGGTTCATAACAAATACTTGCGGGAACTAAAAACCAACTAAGCCCGCCAGTCCTTAGA
CCAATCCACAGGTTTCAGGACATAACCAATCCCAACCTTGCACGTTGAACAGACCGCAGAGGCGACTTAC
TCGAAGCCAGAGTCGATCTGCCCCTCGAGGATTTCCTACTACGCCTCCTCGCAACTGACCCAG
>dme_circ_0004192_junction_seq
CTACTACGCCTCCTCGCAACTGACCCAGGGAACTGTGGATAAACGAGGAAGAAATC
>dme_circ_0004193
GGCTCGAACCTTTTTGAATAGAGCATTTCCAGCCAGCTCATTGTCCACACTTGAGAAGCGACTCACTTCC
GACACAGACTCTGTTGACGGCAACGCGTCTGGTTGGCTGTGCTCCGATTCCAAAGCGCTGCAACTACTGC
ATGGAGTGGCTGTGCGCCGACTGCTCTCGATGTCCAGGTTACGTAGTTCGTCATCGTTCACATACAGGCC
GGATATCTGAGTGGCCTCAAACTCGGCGTCTTTGCCCTTGTT
>dme_circ_0004193_junction_seq
CCTCAAACTCGGCGTCTTTGCCCTTGTTGGCTCGAACCTTTTTGAATAGAGCATTT
>dme_circ_0004194
TCACCTGAAGTACAGCTTGCGTTTCCGTGGCGTGGAAGCGCACAATCCCAATGCCTGTGAGTCATTTGTA
AAATGGTAATAGCTTTTGTTGAACTAAAGAAACCATTGTTTTGTAGACGTCATGCATGCGGGCAGGGGAA
TTCATAAGCTGGAGATTGAGGCCCTGGTTGCCGAGGATGTGCAGCCCCAGCTACAGCTAAAGAATGCCGA
AGTGGTGCTAAAACCGACCGAGGCCAAGATCTCGCCGCTAAGCGCCACACGAGACGTCATCCCAGATGGA
CGTCAGGTGTATCAGAACCTGCTGGCCTTTAACTTGAACGTGGCCAAGGCCGCAGATGTG
>dme_circ_0004194_junction_seq
CTTGAACGTGGCCAAGGCCGCAGATGTGTCACCTGAAGTACAGCTTGCGTTTCCGT
>dme_circ_0004195
CGTAACTCCATCGATTACAACGTTTATATAGAGCCCATCTTCTACAATGACAAGGAGGCGGGTGAGTAGA
GACTATAGAGACCCAATTCCAGCTCTTACCGTTCCAATTTTACAGATCCCAAGGACAAGTTTAACTTCAA
TGTACGGCTAAATCTGATTGCCTCGCAGCCGTGGGTGCAGTGTGGAGCTTTCTTGGATCTCAGCTATGGC
ACCCGCTCCATTGCCGTGCGCG
>dme_circ_0004195_junction_seq
TATGGCACCCGCTCCATTGCCGTGCGCGCGTAACTCCATCGATTACAACGTTTATA
>dme_circ_0004196
CTTAAACGGAAAACTTTACTTAATTAAAATTAGTTCGTTTTCTCATCCGGTATGGCATCCAGACCCTGCA
AGAAGATTGGTTCGGTTTTTTGTAAATGATTTAAGCTTATAACTCTTCGAATAAGCGTAAAAAAATGGAT
GGTATGAATTCGTAGGAAATAAAGTAACATTAACTACCGCCACCAATGATCAATAAACAATAAAAACGAA

ATTAACCAAGAAAACTAAGATTTTAAATGCAATTACCTTATAATATAGAAAACTTTAAAACTAATATTTT
ATTAGTGAAGAAAGCTAGTTCATAACACCAAAAACAATCTATTTAACATACTTCAGAGAAAAACATAAAC
AACGAAACCAAAATGAGCGTGCATAATAATGAAGTGCGTGCTGTGAGATAACAAAACTGCTGCCAAAAAT
AGGGTAAAAACGTGATGGTTGGCCTGCCGCACAAAAATCAGTTGTAGGCAGGCCAGACTATAGCACAACA
CTTACGTTCATCTTGTAGGGATTGTCGGTCAGTCGGAAATCGCGCTGGTACATAACTCGGCTGAAACGCT
CGTCCATGTTGCGCCGGAAAGCCTGTTGAGCAAACGATTAGCAAACAATTAGTAACTCCGTAGTGTTCCG
TCGAACTCGTTGCGAAAAGCAGGCGGTTAAATAATGTTTAACAGATTCCGATTGGTGCACAAACAGCTGC
GGTTATATAAGAATTTCGGGCTGCTGGGCCAGAAGGCCAGCGTGGGACTCACATTGCCGATCATCAGCTT
GTCCAGGCCGTACATGGCGTATATGGGCACCGAGAGGAGCGTGGTGATGATCACCGCACCAGGTAGGATT
TCGAACCACATATTGTCAATGGAAATAGATTG
>dme_circ_0004196_junction_seq
ACCACATATTGTCAATGGAAATAGATTGCTTAAACGGAAAACTTTACTTAATTAAA
>dme_circ_0004197
ATATGGATACGAGACGTTGATGATGCGTTCGTTGTGGCAGACTATTCGATCGAGATCGAGAGATCAGAGG
GTATAAGTGTATCAAAATAGTTTCTGTATCGATTAAAGAAAGTTATGTGAGGAAGCATCGAATGAAAGAC
TTGGTAACGGAGCTATCTCTTAAAGATATGGAGTTGTCATTTATACTAACTTAATCACAAACCAGATGCC
CCTCTGATCTCACCTTG
>dme_circ_0004197_junction_seq
AACCAGATGCCCCTCTGATCTCACCTTGATATGGATACGAGACGTTGATGATGCGT
>dme_circ_0004198
ATACCCTCGGCGACGAGGAAGCGACGTGTCTTAGCCGCCTTTTTCGGATTTTTCTGGTCTCGTTTTTCTT
GCTCAATCAGCAGACGTTCTAGATCCTCAACGTCATTGTGCTTAAAGAAAACGATTGTGCTTCGTGAAGC
ATCCAGACCCTTCTGAATGGCAAAG
>dme_circ_0004198_junction_seq
AGCATCCAGACCCTTCTGAATGGCAAAGATACCCTCGGCGACGAGGAAGCGACGTG
>dme_circ_0004199
TGCTCCGTAACTCCGTGACCACCCTGGCCCAATGTGCCAAAGGAGATGCTTTCGTCGATGAACAAACGCA
GCTTGTACTTCTGACGCAAGGCCACCAGGTCGGGAAGTGGGCAAATCTCGCCCGTATTCATATAGATACC
CTCGGCGACGAGGAAGCGACGTGTCTTAGCCGCCTTTTTCGGATTTTTCTGGTCTCGTTTTTCTTGCTCA
ATCAGCAGACGTTCTAGATCCTCAACG
>dme_circ_0004199_junction_seq
AATCAGCAGACGTTCTAGATCCTCAACGTGCTCCGTAACTCCGTGACCACCCTGGC
>dme_circ_0004200
ATACTTTAAGATGAGTTACTTTAACCGATGGAAGACAATGATTTAAAATAGCTTGTAGTACGTACTCTAA
GACAAATAAGCAAAACTGAGTTGCACTTCTGTAAGATATACTGTAACTAATAACGAAGCGAAAAAGCTAC
AAATAAAGCAACTCGAGTAGCCTACATAATG
>dme_circ_0004200_junction_seq
TAAAGCAACTCGAGTAGCCTACATAATGATACTTTAAGATGAGTTACTTTAACCGA
>dme_circ_0004201
CTACGATATATCAGAGGCAGTGCTATTAGCTCTCTGAATAAAACGATCCACACAGAAAGCTGCAGCCAAA
GTACCAAAAATTCTATTTAACGAATACAAGACTCAACACCGAGTTCGCAATGAATACGATAGCTAAACGA
ATCGAGTTTAAAAGTGAATACCACCCAATCATAATCCCCAAACGAAAGCAACGCAAATGGAAGACATTTT
CAATGTTTGCCCAGATCAACGGACAAGCATATAAATAAATAATCCACCAAACTAACTTAGCGAAGCATTT
GTGTACTTACATCCGACACATCACATACTCATAATAGTCGGTTGCTTGAGGAGATCTCGTCTCGTCTCGC

CTTATTCATTGATTCATTCACTATTCCATCGTCACTTGTTGTGTGTGCGTGTGTGCAAGAGCAGCAACGA
GCAAATACCAAAAAAATCGAAAGAAACAAGCAAATAATGATCTCTCCATGaaacataaaaaataaattaa
acatataaaaaaatattGGACAATTAGTGAAAGG
>dme_circ_0004201_junction_seq
aaaaaaatattGGACAATTAGTGAAAGGCTACGATATATCAGAGGCAGTGCTATTA
>dme_circ_0004202
TTCGTCCTCCATCCTGCACACGAACGCCAACACCAACGGAGTGGACACATTGGCCGCCACacagcaacaa
caacaacaacagcagcagcagcagcagcaacaacagcaacagcaCTACCACTATATCACCACCACCGGCG
AGGATGGACAAACCCCGACCACCATAGTGTTCGAGAACTACAACGGCGGAATGCCAGCGGTCAGTTCTGC
CGCACCAACGCCCGTTCCCGTGTCGCAGGCCAACGCACCCGCGACCACGACCACCACTGCCCTGGTCAAC
ACGGACGGCACGATCATCGAGTTCGACGGGAGCACGTACGAGGAGTATCATGTCCTCAAAAGCGAGCCCG
GCAGCAGCGATTGCCTGAGCAACGGCAGCAGTGCCGTGGCCGCCGACATAATCAAGTACGATCCCCAGCA
CGGTGTCGTCGGCGACGAGGAGGACGACGATGAGAACGGCATGCTGCATGTGAAGTACGAGGAGCAGAGC
CTCGAACACGATCCGGAAAACGAGCACGATGCCGAGCAGGAGCATGAGTACGAGGAGTACCAGGTGATCA
AGGCCGAGGTGGAGGCCGAGGCGGCGGAGCTGGCAGCCGGCACCACCAGCTATACGATCAGCCAGGAGCA
GACGGGTTCAGGCAACACGGTGCTGTCCTCAATGGTGCCCAAATCGGGATCAGCGGCGGCCGCCAAGCAG
AAGCGCAAACGCAAGACGCGCGTAATCGCCGACGACGAGCAGGGCGAGTACCTGGAAAAGATGAGTGTAC
GTGGCCTGGACATTGCCCGCTACGAGCACATCATCGACGGCGTGGCTTACTGCCTGGTCTGCGCCAAGAA
CGATATCTTCAAGACGTTTAAGAACAAGTATAGCTTCCAGAGACACGCCTACCTCTTTCACGAGGGCCAA
AACCGCAAGATATTCGCCTGCCCCATTTGCAACAAGGAGTTCTCGCGTCCGGACAAAATGAAGATGCATA
AAAAGGACAAGCACGGCGATGTTCCCATGCCACCATCGGCCACCACGACGCCGCTAAAGGCTGACGGTCC
GCCGGCTAAGCGGGCGCGTACCGCTCGTACTCCGCGCGTCCGCAAGACGAAGGATGGCAGCACGCCGACC
AAGAAGCGGTTAGCGCAGATCGACAGTGTTCTGGACGACGTGCAAAATGGGGAGTCTCTGACCATGGCGC
CAGTCAGCGTGAGTCACTCCCAGCAACAGCAGCAGCAACAGATCCAGCACAGCATCACAACGCTGGCTCC
CTCGACGCCATCGCAGCCGCAGCATCAGCTGCAGACGCTGCCATCGCTGGACTTCAGCGGCATGATCCTG
CCTGGAGGCGCACAGCTGGCACTGGCCAATCCGGGCGCCACCAGCTCCGTGGGACTGCAACGAGGACCAG
CCACGCCGAATGGTCAGCCGGCGCCGCCCACCACCACGTTGATCTACTCGAATCAACTGGAACTGCAGCA
GGCGCTGCTGCAACAGCAGCTGCAAGGTCAGAGCATCATGATCCAGGATCAAGCCGGCAACCTGGTGCCC
CTGCAGCTGGACGGAACCCAGGCGATATACACGACGGCGCCGCAGGCTCAGCGCCAACAGACCACGGCCA
CATATCAGACGACgcagcagcagcaacagcagccagcgcagcagccCGCCGGCCAGGCGCAACAGCAGCC
TCACTACGAGTTGGACAACGTTACCTATTTGAGCTCCACGGCGGCGGCCACACCGACATCGGCCGagcag
cagcagcaacaacagcagcagcagcaCGTGATCGGCACGGTTCAGAGCTATCAGATCCTGACGCCCGACG
GCCTCCAGAACTTCCAACCCAAGCTGGAGACGGCCGAGCTGGGCGACCTGTGTCCCTATTCGCAGTACAC
CTTCACGACGCACGCCGGAGCGCAGCCCACGCTGAATGCGAACGCGCTGGACGCgcagacccagcatcag
cagcaccaccagcagcagcagcaccatcaccagcagcagcaacaccaccagcagacgcaactactgcagc
agcagcCCTTCGCCACGCATCACAATCCGCACCAGCAGTTCATGGAGCTGAAGAACGAGCTGCTGATTAA
GGGCGGCGACGCCTACACCCTGGACACTGCCTCCATGTACCACCTGCCCTTCTCGCCCACCTCGATGATC
AATGCGGTGAACGATGTGAACACCTCGTCGCTGCTGGAAACCAAAGAAATTAGG
>dme_circ_0004202_junction_seq
GTCGCTGCTGGAAACCAAAGAAATTAGGTTCGTCCTCCATCCTGCACACGAACGCC
>dme_circ_0004203
ACAATGTGGTCACAACTATTAGTTCGTCCTCCATCCTGCACACGAACGCCAACACCAACGGAGTGGACAC
ATTGGCCGCCACacagcaacaacaacaacaacagcagcagcagcagcagcaacaacagcaacagcaCTAC
CACTATATCACCACCACCGGCGAGGATGGACAAACCCCGACCACCATAGTGTTCGAGAACTACAACGGCG

GAATGCCAGCGGTCAGTTCTGCCGCACCAACGCCCGTTCCCGTGTCGCAGGCCAACGCACCCGCGACCAC
GACCACCACTGCCCTGGTCAACACGGACGGCACGATCATCGAGTTCGACGGGAGCACGTACGAGGAGTAT
CATGTCCTCAAAAGCGAGCCCGGCAGCAGCGATTGCCTGAGCAACGGCAGCAGTGCCGTGGCCGCCGACA
TAATCAAGTACGATCCCCAGCACGGTGTCGTCGGCGACGAGGAGGACGACGATGAGAACGGCATGCTGCA
TGTGAAGTACGAGGAGCAGAGCCTCGAACACGATCCGGAAAACGAGCACGATGCCGAGCAGGAGCATGAG
TACGAGGAGTACCAGGTGATCAAGGCCGAGGTGGAGGCCGAGGCGGCGGAGCTGGCAGCCGGCACCACCA
GCTATACGATCAGCCAGGAGCAGACGGGTTCAGGCAACACGGTGCTGTCCTCAATGGTGCCCAAATCGGG
ATCAGCGGCGGCCGCCAAGCAGAAGCGCAAACGCAAGACGCGCGTAATCGCCGACGACGAGCAGGGCGAG
TACCTGGAAAAGATGAGTGTACGTGGCCTGGACATTGCCCGCTACGAGCACATCATCGACGGCGTGGCTT
ACTGCCTGGTCTGCGCCAAGAACGATATCTTCAAGACGTTTAAGAACAAGTATAGCTTCCAGAGACACGC
CTACCTCTTTCACGAGGGCCAAAACCGCAAGATATTCGCCTGCCCCATTTGCAACAAGGAGTTCTCGCGT
CCGGACAAAATGAAGATGCATAAAAAGGACAAGCACGGCGATGTTCCCATGCCACCATCGGCCACCACGA
CGCCGCTAAAGGCTGACGGTCCGCCGGCTAAGCGGGCGCGTACCGCTCGTACTCCGCGCGTCCGCAAGAC
GAAGGATGGCAGCACGCCGACCAAGAAGCGGTTAGCGCAGATCGACAGTGTTCTGGACGACGTGCAAAAT
GGGGAGTCTCTGACCATGGCGCCAGTCAGCGTGAGTCACTCCCAGCAACAGCAGCAGCAACAGATCCAGC
ACAGCATCACAACGCTGGCTCCCTCGACGCCATCGCAGCCGCAGCATCAGCTGCAGACGCTGCCATCGCT
GGACTTCAGCGGCATGATCCTGCCTGGAGGCGCACAGCTGGCACTGGCCAATCCGGGCGCCACCAGCTCC
GTGGGACTGCAACGAGGACCAGCCACGCCGAATGGTCAGCCGGCGCCGCCCACCACCACGTTGATCTACT
CGAATCAACTGGAACTGCAGCAGGCGCTGCTGCAACAGCAGCTGCAAGGTCAGAGCATCATGATCCAGGA
TCAAGCCGGCAACCTGGTGCCCCTGCAGCTGGACGGAACCCAGGCGATATACACGACGGCGCCGCAGGCT
CAGCGCCAACAGACCACGGCCACATATCAGACGACgcagcagcagcaacagcagccagcgcagcagccCG
CCGGCCAGGCGCAACAGCAGCCTCACTACGAGTTGGACAACGTTACCTATTTGAGCTCCACGGCGGCGGC
CACACCGACATCGGCCGagcagcagcagcaacaacagcagcagcagcaCGTGATCGGCACGGTTCAGAGC
TATCAGATCCTGACGCCCGACGGCCTCCAGAACTTCCAACCCAAGCTGGAGACGGCCGAGCTGGGCGACC
TGTGTCCCTATTCGCAGTACACCTTCACGACGCACGCCGGAGCGCAGCCCACGCTGAATGCGAACGCGCT
GGACGCgcagacccagcatcagcagcaccaccagcagcagcagcaccatcaccagcagcagcaacaccac
cagcagacgcaactactgcagcagcagcCCTTCGCCACGCATCACAATCCGCACCAGCAGTTCATGGAGC
TGAAGAACGAGCTGCTGATTAAGGGCGGCGACGCCTACACCCTGGACACTGCCTCCATGTACCACCTGCC
CTTCTCGCCCACCTCGATGATCAATGCGGTGAACGATGTGAACACCTCGTCGCTGCTGGAAACCAAAGAA
ATTAGG
>dme_circ_0004203_junction_seq
GTCGCTGCTGGAAACCAAAGAAATTAGGACAATGTGGTCACAACTATTAGTTCGTC
>dme_circ_0004204
GTCTGTGTCCTGTCTCTACTCGAATTGTCGAGAGCATTGGAAAAATCAAATAAATAAGTGCACAATTCAT
TTCAACCGAGGGAGGGAGCTCCGTACAAAACAGTAACGCGTCGAAAAAGGCTCAAAAACAATTGTCGATC
ACGTATTATTTTACTTTATTAGCTAGTTTAAGGTTTTGCAGTTCAAACTTTGGCGCCCAAGCAGCAGTGA
GTTCAATGAGAGCATCAGTGCTGCCCATGCGCGCGCTGACTCTTGGCAGCACTTGCGCGCCAAATTTTGA
AAATAGAGTTTATAATTTATAGATTTGCGTTTATGATTTAGTTACTTTTAGCAACGACACTTTCCCGTCT
TTTCTATCTCTCACTTATTGTTTCCCACTCTTTGGACACAACACAAACACAATCCGTTGCCAGCTCCTCC
TGCTCCGCCCCAAGCGATGGAGGAGGAGGAGGAGTCTACACAAAGTTGGCACCAGTTTCATCCTCTCCTC
TGTCCACCTGTTACCACCACCTCAGTTACCGAAGTCGATTATTTATTTAACTTTATTTAGTACGTATTGA
GTTGCGAGTACTGTACCAGAGTTACGAATTACGATTGCTGTAATGTAGATCTAAGTAAACACAAATTCTA
CCGACAAACAAAAACCAATTCACTCACTTTCTATCTATATGTCCTGTATTGCCTTATCCAATCGATTTGA
TAAAGAGGCCCGCCCGTAAATGCAGCAGATGCACTGCATCTGTGGCATTTGCCTTTTTTGCTGTTGGCCG

CAAGTAGATTTCATTTTGCACAACATTTGTTTTATGCGAACCTGATTTGAGTGAATTGTTGATTGATTTC
GATTTCAATTACGAATTGATTTATTGATTTATGCAAATATTACATTCAAACACATCTTTTATGAACCAAA
CCAAATATGTATTTTCAAAATATATATAAACAAAAGACAATGTGGTCACAACTATTAGTTCGTCCTCCAT
CCTGCACACGAACGCCAACACCAACGGAGTGGACACATTGGCCGCCACacagcaacaacaacaacaacag
cagcagcagcagcagcaacaacagcaacagcaCTACCACTATATCACCACCACCGGCGAGGATGGACAAA
CCCCGACCACCATAGTGTTCGAGAACTACAACGGCGGAATGCCAGCGGTCAGTTCTGCCGCACCAACGCC
CGTTCCCGTGTCGCAGGCCAACGCACCCGCGACCACGACCACCACTGCCCTGGTCAACACGGACGGCACG
ATCATCGAGTTCGACGGGAGCACGTACGAGGAGTATCATGTCCTCAAAAGCGAGCCCGGCAGCAGCGATT
GCCTGAGCAACGGCAGCAGTGCCGTGGCCGCCGACATAATCAAGTACGATCCCCAGCACGGTGTCGTCGG
CGACGAGGAGGACGACGATGAGAACGGCATGCTGCATGTGAAGTACGAGGAGCAGAGCCTCGAACACGAT
CCGGAAAACGAGCACGATGCCGAGCAGGAGCATGAGTACGAGGAGTACCAGGTGATCAAGGCCGAGGTGG
AGGCCGAGGCGGCGGAGCTGGCAGCCGGCACCACCAGCTATACGATCAGCCAGGAGCAGACGGGTTCAGG
CAACACGGTGCTGTCCTCAATGGTGCCCAAATCGGGATCAGCGGCGGCCGCCAAGCAGAAGCGCAAACGC
AAGACGCGCGTAATCGCCGACGACGAGCAGGGCGAGTACCTGGAAAAGATGAGTGTACGTGGCCTGGACA
TTGCCCGCTACGAGCACATCATCGACGGCGTGGCTTACTGCCTGGTCTGCGCCAAGAACGATATCTTCAA
GACGTTTAAGAACAAGTATAGCTTCCAGAGACACGCCTACCTCTTTCACGAGGGCCAAAACCGCAAGATA
TTCGCCTGCCCCATTTGCAACAAGGAGTTCTCGCGTCCGGACAAAATGAAGATGCATAAAAGGACAAGC
ACGGCGATGTTCCCATGCCACCATCGGCCACCACGACGCCGCTAAAGGCTGACGGTCCGCCGGCTAAGCG
GGCGCGTACCGCTCGTACTCCGCGCGTCCGCAAGACGAAGGATGGCAGCACGCCGACCAAGAAGCGGTTA
GCGCAGATCGACAGTGTTCTGGACGACGTGCAAAATGGGGAGTCTCTGACCATGGCGCCAGTCAGCGTGA
GTCACTCCCAGCAACAGCAGCAGCAACAGATCCAGCACAGCATCACAACGCTGGCTCCCTCGACGCCATC
GCAGCCGCAGCATCAGCTGCAGACGCTGCCATCGCTGGACTTCAGCGGCATGATCCTGCCTGGAGGCGCA
CAGCTGGCACTGGCCAATCCGGGCGCCACCAGCTCCGTGGGACTGCAACGAGGACCAGCCACGCCGAATG
GTCAGCCGGCGCCGCCCACCACCACGTTGATCTACTCGAATCAACTGGAACTGCAGCAGGCGCTGCTGCA
ACAGCAGCTGCAAGGTCAGAGCATCATGATCCAGGATCAAGCCGGCAACCTGGTGCCCCTGCAGCTGGAC
GGAACCCAGGCGATATACACGACGGCGCCGCAGGCTCAGCGCCAACAGACCACGGCCACATATCAGACGA
CgcagcagcagcaacagcagccagcgcagcagccCGCCGGCCAGGCGCAACAGCAGCCTCACTACGAGTT
GGACAACGTTACCTATTTGAGCTCCACGGCGGCGGCCACACCGACATCGGCCGagcagcagcagcaacaa
cagcagcagcagcaCGTGATCGGCACGGTTCAGAGCTATCAGATCCTGACGCCCGACGGCCTCCAGAACT
TCCAACCCAAGCTGGAGACGGCCGAGCTGGGCGACCTGTGTCCCTATTCGCAGTACACCTTCACGACGCA
CGCCGGAGCGCAGCCCACGCTGAATGCGAACGCGCTGGACGCgcagacccagcatcagcagcaccaccag
cagcagcagcaccatcaccagcagcagcaacaccaccagcagacgcaactactgcagcagcagcCCTTCG
CCACGCATCACAATCCGCACCAGCAGTTCATGGAGCTGAAGAACGAGCTGCTGATTAAGGGCGGCGACGC
CTACACCCTGGACACTGCCTCCATGTACCACCTGCCCTTCTCGCCCACCTCGATGATCAATGCGGTGAAC
GATGTGAACACCTCGTCGCTGCTGGAAACCAAAGAAATTAGG
>dme_circ_0004204_junction_seq
GTCGCTGCTGGAAACCAAAGAAATTAGGGTCTGTGTCCTGTCTCTACTCGAATTGT
>dme_circ_0004205
ATGCCAAAAAGGAGCGACAGTCGCTGCAGTTTATGCAACAATCGCGTAAATTTGAGGGCAAGATACCAAA
ACTAAGTCAAACGAGTGCAGCAACAGGAGGAGCAACGGCAGCCGCAGAAGCATCCACATCCAAGTCTAGT
CAAGGGCAGCAGCAACAGATTGTCTACGTCAACATGTTGCCCGCGGCCAATACCCTGAATGGCATTCCAC
AGCAGCAGCAGCAGCAACAATATGCGAGCACGGATGGAAATGTCTATCAGCTGCAAAATGAAATACTCTG
TGATGCAAATGGACATGCCGTGACTGCCGCCACGGCCTCATATCAAACTACGGCCAGTAGTCCgcagcag
cagcaacagcagcagcagcagcaacagAATGTTGCAACTAGTGTTGCCGGGTAAGTGTACAACTTGAATT

TGTTCGCTTTTACGTTAATTAATTTGTTTGTTGGTTGTAGTCTTTGTGTATGGTTTAACCTAACCCTCTT
GTGTACTCGTATTTGGTGTTTAAGTTGAAGCTATTACCACTAACCGCTCTCATTGTAAATACAAACGCAC
TCACACAATTATGATTTTGACCGTCAACCGAATTGGCCAAATTTGAATCCAATCAATTGTAAATGTTGTC
GCACATCCATGTCATCCAATCGATATCATCCTGACTTTCCAAATTCAAACTGCATTTTGAGTTTTCAACT
TTTTGTGCCAAAATTCAAATGCGCCACTAAGGGAGAACGTTTCGAATGAGAGCAAACATTGAATTTGACT
TCCATTTGTGTTCCGTGTTCTTGTCACACCTTTGAACCTCCCAAAAAATGAACAAAATACAAAAGAATAT
AAACGAAATAAAAAGGCAATGAAATCTTCAATTAACCATTACCGACCTATCTCAATAAATCGAACTCCAA
TCGCATCGCCCAATTAATAACAACAACCGCTTTCAACTGCTCCACACACTACATGCTTGTCTTTAATTTG
CGACACCATTTCCCAGGTCTGTGTCCTGTCTCTACTCGAATTGTCGAGAGCATTGGAAAAATCAAATAAA
TAAGTGCACAATTCATTTCAACCGAGGGAGGGAGCTCCGTACAAAACAGTAACGCGTCGAAAAAGGCTCA
AAAACAATTGTCGATCACGTATTATTTTACTTTATTAGCTAGTTTAAGGTTTTGCAGTTCAAACTTTGGC
GCCCAAGCAGCAGTGAGTTCAATGAGAGCATCAGTGCTGCCCATGCGCGCGCTGACTCTTGGCAGCACTT
GCGCGCCAAATTTTGAAAATAGAGTTTATAATTTATAGATTTGCGTTTATGATTTAGTTACTTTTAGCAA
CGACACTTTCCCGTCTTTTCTATCTCTCACTTATTGTTTCCCACTCTTTGGACACAACACAAACACAATC
CGTTGCCAGCTCCTCCTGCTCCGCCCCAAGCGATGGAGGAGGAGGAGGAGTCTACACAAAGTTGGCACCA
GTTTCATCCTCTCCTCTGTCCACCTGTTACCACCACCTCAGTTACCGAAGTCGATTATTTATTTAACTTT
ATTTAGTACGTATTGAGTTGCGAGTACTGTACCAGAGTTACGAATTACGATTGCTGTAATGTAGATCTAA
GTAAACACAAATTCTACCGACAAACAAAAACCAATTCACTCACTTTCTATCTATATGTCCTGTATTGCCT
TATCCAATCGATTTGATAAAGAGGCCCGCCCGTAAATGCAGCAGATGCACTGCATCTGTGGCATTTGCCT
TTTTTGCTGTTGGCCGCAAGTAGATTTCATTTTGCACAACATTTGTTTTATGCGAACCTGATTTGAGTGA
ATTGTTGATTGATTTCGATTTCAATTACGAATTGATTTATTGATTTATGCAAATATTACATTCAAACACA
TCTTTTATGAACCAAACCAAATATGTATTTTCAAAATATATATAAACAAAAGACAATGTGGTCACAACTA
TTAGTTCGTCCTCCATCCTGCACACGAACGCCAACACCAACGGAGTGGACACATTGGCCGCCACacagca
acaacaacaacaacagcagcagcagcagcagcaacaacagcaacagcaCTACCACTATATCACCACCACC
GGCGAGGATGGACAAACCCCGACCACCATAGTGTTCGAGAACTACAACGGCGGAATGCCAGCGGTCAGTT
CTGCCGCACCAACGCCCGTTCCCGTGTCGCAGGCCAACGCACCCGCGACCACGACCACCACTGCCCTGGT
CAACACGGACGGCACGATCATCGAGTTCGACGGGAGCACGTACGAGGAGTATCATGTCCTCAAAAGCGAG
CCCGGCAGCAGCGATTGCCTGAGCAACGGCAGCAGTGCCGTGGCCGCCGACATAATCAAGTACGATCCCC
AGCACGGTGTCGTCGGCGACGAGGAGGACGACGATGAGAACGGCATGCTGCATGTGAAGTACGAGGAGCA
GAGCCTCGAACACGATCCGGAAAACGAGCACGATGCCGAGCAGGAGCATGAGTACGAGGAGTACCAGGTG
ATCAAGGCCGAGGTGGAGGCCGAGGCGGCGGAGCTGGCAGCCGGCACCACCAGCTATACGATCAGCCAGG
AGCAGACGGGTTCAGGCAACACGGTGCTGTCCTCAATGGTGCCCAAATCGGGATCAGCGGCGGCCGCCAA
GCAGAAGCGCAAACGCAAGACGCGCGTAATCGCCGACGACGAGCAGGGCGAGTACCTGGAAAAGATGAGT
GTACGTGGCCTGGACATTGCCCGCTACGAGCACATCATCGACGGCGTGGCTTACTGCCTGGTCTGCGCCA
AGAACGATATCTTCAAGACGTTTAAGAACAAGTATAGCTTCCAGAGACACGCCTACCTCTTTCACGAGGG
CCAAAACCGCAAGATATTCGCCTGCCCCATTTGCAACAAGGAGTTCTCGCGTCCGGACAAAATGAAGATG
CATAAAAAGGACAAGCACGGCGATGTTCCCATGCCACCATCGGCCACCACGACGCCGCTAAAGGCTGACG
GTCCGCCGGCTAAGCGGGCGCGTACCGCTCGTACTCCGCGCGTCCGCAAGACGAAGGATGGCAGCACGCC
GACCAAGAAGCGGTTAGCGCAGATCGACAGTGTTCTGGACGACGTGCAAAATGGGGAGTCTCTGACCATG
GCGCCAGTCAGCGTGAGTCACTCCCAGCAACAGCAGCAGCAACAGATCCAGCACAGCATCACAACGCTGG
CTCCCTCGACGCCATCGCAGCCGCAGCATCAGCTGCAGACGCTGCCATCGCTGGACTTCAGCGGCATGAT
CCTGCCTGGAGGCGCACAGCTGGCACTGGCCAATCCGGGCGCCACCAGCTCCGTGGGACTGCAACGAGGA
CCAGCCACGCCGAATGGTCAGCCGGCGCCGCCCACCACCACGTTGATCTACTCGAATCAACTGGAACTGC
AGCAGGCGCTGCTGCAACAGCAGCTGCAAGGTCAGAGCATCATGATCCAGGATCAAGCCGGCAACCTGGT

GCCCCTGCAGCTGGACGGAACCCAGGCGATATACACGACGGCGCCGCAGGCTCAGCGCCAACAGACCACG
GCCACATATCAGACGACgcagcagcagcaacagcagccagcgcagcagccCGCCGGCCAGGCGCAACAGC
AGCCTCACTACGAGTTGGACAACGTTACCTATTTGAGCTCCACGGCGGCGGCCACACCGACATCGGCCGa
gcagcagcagcaacaacagcagcagcagcaCGTGATCGGCACGGTTCAGAGCTATCAGATCCTGACGCCC
GACGGCCTCCAGAACTTCCAACCCAAGCTGGAGACGGCCGAGCTGGGCGACCTGTGTCCCTATTCGCAGT
ACACCTTCACGACGCACGCCGGAGCGCAGCCCACGCTGAATGCGAACGCGCTGGACGCgcagacccagca
tcagcagcaccaccagcagcagcagcaccatcaccagcagcagcaacaccaccagcagacgcaactactg
cagcagcagcCCTTCGCCACGCATCACAATCCGCACCAGCAGTTCATGGAGCTGAAGAACGAGCTGCTGA
TTAAGGGCGGCGACGCCTACACCCTGGACACTGCCTCCATGTACCACCTGCCCTTCTCGCCCACCTCGAT
GATCAATGCGGTGAACGATGTGAACACCTCGTCGCTGCTGGAAACCAAAGAAATTAGG
>dme_circ_0004205_junction_seq
GTCGCTGCTGGAAACCAAAGAAATTAGGATGCCAAAAAGGAGCGACAGTCGCTGCA
>dme_circ_0004206
TTCCAGATCGAAGCACCCTAGCTGTGAAAGAAGTGTCCGACAACAGGTCGGAATTGaaaaaaaagagaaa
aaCTGAGACGAGAATGAAAATGTCAGAGGTGCCACGCATCAAGGTCTTCCGACCCACCTGGGAGGAGTTC
AAGGACTTTCCCAAGTACGTGGCCTACATGGAGTCCCAGGGTGCCCACAAAGCCGGTTTGGCCAAGGTGG
TGCCTCCGCCGGAATGGGTGCCCCGTCGCAGTGGGTACGCCGATCTCGATGCTCTAAATGTCACAATTCC
GGCGCCCATTTGCCAGGTGGTAACCGGCAAACAGGGATACTACCAGCAGATTAACATCCAGAAAAAGCCA
CTCACAGTTAAGCAGTTTAGCGAGTTGGCCAGCACCGAGCGTTACGCCACACCCAAACACTTTGACTTTG
AGGATCTGGAGCGCAAATACTGGAAGAACATAACATATGTGGCGCCCATTTATGGGGCAGATGTCAGCGG
AAGTATCACAGACACAGACCAGGATGTAAGTTCGTTAGGGTTAGGGTTTAGGTTTGGGCATTAGCTGATG
ATTCGTTGCCTTTGCCTTTGCCGTTGCAGAGCTGGAACATCAACCGGCTGGGCACAATTCTTGACTATGT
CAACAAGGATTATAACATCCAGATAGACGGCGTGAACACAGCCTACTTGTACTTTGGCATGTGGAAGACG
ACCTTTGCATGGCACACGGAGGACATGGATCTCTATTCCATCAATTACTTACACTTTGGGGCGCCAAAGA
CGTGGTATGTGGTGCCGCCGGAGTGTGGTCGCAAATTGGAGAAGGTGGCCAACCAGTACTTTCCGGCCAG
TTACAAGAATTGCAACGCATATTTGCGCCACAAGATGACGTTGATTTCACCACAGATTCTCAAGCAACAT
GACGTGCCCGTCAGTAAGGTGCGTTTATCGGTAGTAATCCTGTCCATAGTGGCCGTACTAAATCCGGCTC
TTGCAGATCACGCAGGAGGCGGGCGAGATCATGATCACATTTCCGTTCGGCTATCATGCCGGCTTCAATC
ACGGCTTTAACTGTGCCGAGTCCACCAACTTTGCCATGGAGCGCTGGATCGAGTACGGCAAGCGGGCGGT
GCAGTGCACCTGCAGCAACGATATGGTCAAGATCTCGATGGACACATTTGTCAAGCGCTTCCAAAGCGAT
CGCTACGATCTGTGGATGGAGGGGCGTGACGTGGGGCGGCATCCGGAGGATCCGCCAAACGCGGTGCTCA
GTGCAGCACCCCTGCCACCACATCTCGATGTCTTGCTGTGTGATAAAAAGTAGGTTTCCTTGCGCTACTT
GTGGGGCTACCAGCGAGTCTTATTCCTATCTCCTGCTCCTCCGCAGAATGAAAAAGCAATGCAATCCTAC
CAAGGCCAAGAGTTTCAAAGAGCGTAATCCCGATCTGGACCTGGATGAAATCCAGCAGAATCCCAACGTC
CCCGACGACGTCAAGGCCATGCTGAAGGAGAGCGTGCTGACGCTGGACACGGGCGATCTGGCCACGGATG
AGGCTGATTTTCCCAACGAGGATGCCATGAGTCTACAGAGTCCGGCGAATCTGAAGACCAAGCAGGAGCT
GCTTGAGTACATTGACGACGGCACAGGTGTGTGGGCCGGCAGGTGCCCTTCAAAACGATCATCTTCCCCT
TTCCCCAGCTTAACTATAATATGTTTCCTTTCTCGCATCTGTTCTTTGTCTTCCCCTTTTTGCCTGTTGA
CAGAAGATGATGATGAGGAGGAGGACTTCAAGCGGCGCAAGCAGAAGCGGCGCTATGATGCCGACTATGA
CGACGATTGGCTGGCGTCCAAGCGCAAGACTAACTCGCGAAACAATCGCGGCCGTAGTCCGCGCACCAAG
GACGACCGTTCCATATCGCCGGCCTCCTCCACCTCCTCGACGTCGAGGGGTGCAAGGCGTGGCAAGGCCA
GTGGCACGCCCCGAAAGACTCCTGCGCGGCGGAAAAAGGACTCAATCACCACGTCTCCGGCGGTTAGTTC
TGCAGCAACTGCCGTAAAAACACCGACATCCGCCGTAGTCGCTGGAACAACATCAATAGCCACAACCACA
ACACCACCAGCAGATGGCGGAGGAGGTGAGAGCAGCAGCTTAGGCAGCCACTGCACCACTGCATCCCCAT

AATCACAGCTTCACCTATCCACCTCCAAGCCATCCAAATTCTCTAACCCAATGCCCCAATGTCTCTTTTT
TAGCAAAAATCATTCCAAATTTCCCAGTAGTTTTTTGTTCTAACCAAATTGACAATGTTTCAAGCAGTTT
CACATGTATCGTTCATCGTTTTTGTTTTGCGATTAATGTACAAATAAATCGTTCTATCTAATTGTAAGAG
TGCGCCAGAGAAATATACGATCACGCTTTTAAGTCTATGCTAAAAACTAACCGAAAACCAAAACTGTTTT
ACTAAACCGAATTCAAGGTGTTGGTGCTGTCTGGGCCGAATCCGTTCCTCTTGTTTTGCCGCCCCCAGAA
TCCGCTTCACTTGCTCTTTCGCTTTGACTACACATCCTCATTCCACCCAAATCGCATTGCCTCCTATCCA
GAAAACCCCCCGAGTCCACAtgtgtgtatgtgtgtgtatctgtgtgtTTACACCAACAGTAACCGTATCA
ACCGCATTTATTCACAAACAATTGAAATCCTTGCCTGATCAAGTACTAAACTTTATCGATTGACGTTTTT
CAGATGATCAAGTACTAAACTTTATCGATTGACGTTTTTCAGATGCCAAAAAGGAGCGACAGTCGCTGCA
GTTTATGCAACAATCGCGTAAATTTGAGGGCAAGATACCAAAACTAAGTCAAACGAGTGCAGCAACAGGA
GGAGCAACGGCAGCCGCAGAAGCATCCACATCCAAGTCTAGTCAAGGGCAGCAGCAACAGATTGTCTACG
TCAACATGTTGCCCGCGGCCAATACCCTGAATGGCATTCCACAGCAGCAGCAGCAGCAACAATATGCGAG
CACGGATGGAAATGTCTATCAGCTGCAAAATGAAATACTCTGTGATGCAAATGGACATGCCGTGACTGCC
GCCACGGCCTCATATCAAACTACGGCCAGTAGTCCgcagcagcagcaacagcagcagcagcagcaacagA
ATGTTGCAACTAGTGTTGCCGGGTAAGTGTACAACTTGAATTTGTTCGCTTTTACGTTAATTAATTTGTT
TGTTGGTTGTAGTCTTTGTGTATGGTTTAACCTAACCCTCTTGTGTACTCGTATTTGGTGTTTAAGTTGA
AGCTATTACCACTAACCGCTCTCATTGTAAATACAAACGCACTCACACAATTATGATTTTGACCGTCAAC
CGAATTGGCCAAATTTGAATCCAATCAATTGTAAATGTTGTCGCACATCCATGTCATCCAATCGATATCA
TCCTGACTTTCCAAATTCAAACTGCATTTTGAGTTTTCAACTTTTTGTGCCAAAATTCAAATGCGCCACT
AAGGGAGAACGTTTCGAATGAGAGCAAACATTGAATTTGACTTCCATTTGTGTTCCGTGTTCTTGTCACA
CCTTTGAACCTCCCAAAAAAATGAACAAAATACAAAAGAATATAAACGAAATAAAAAGGCAATGAAATCTT
CAATTAACCATTACCGACCTATCTCAATAAATCGAACTCCAATCGCATCGCCCAATTAATAACAACAACC
GCTTTCAACTGCTCCACACACTACATGCTTGTCTTTAATTTGCGACACCATTTCCCAGGTCTGTGTCCTG
TCTCTACTCGAATTGTCGAGAGCATTGGAAAAATCAAATAAATAAGTGCACAATTCATTTCAACCGAGGG
AGGGAGCTCCGTACAAAACAGTAACGCGTCGAAAAAGGCTCAAAAACAATTGTCGATCACGTATTATTTT
ACTTTATTAGCTAGTTTAAGGTTTTGCAGTTCAAACTTTGGCGCCCAAGCAGCAGTGAGTTCAATGAGAG
CATCAGTGCTGCCCATGCGCGCGCTGACTCTTGGCAGCACTTGCGCGCCAAATTTTGAAAATAGAGTTTA
TAATTTATAGATTTGCGTTTATGATTTAGTTACTTTTAGCAACGACACTTTCCCGTCTTTTCTATCTCTC
ACTTATTGTTTCCCACTCTTTGGACACAACACAAACACAATCCGTTGCCAGCTCCTCCTGCTCCGCCCCA
AGCGATGGAGGAGGAGGAGGAGTCTACACAAAGTTGGCACCAGTTTCATCCTCTCCTCTGTCCACCTGTT
ACCACCACCTCAGTTACCGAAGTCGATTATTTATTTAACTTTATTTAGTACGTATTGAGTTGCGAGTACT
GTACCAGAGTTACGAATTACGATTGCTGTAATGTAGATCTAAGTAAACACAAATTCTACCGACAAACAAA
AACCAATTCACTCACTTTCTATCTATATGTCCTGTATTGCCTTATCCAATCGATTTGATAAAGAGGCCCG
CCCGTAAATGCAGCAGATGCACTGCATCTGTGGCATTTGCCTTTTTTGCTGTTGGCCGCAAGTAGATTTC
ATTTTGCACAACATTTGTTTTATGCGAACCTGATTTGAGTGAATTGTTGATTGATTTCGATTTCAATTAC
GAATTGATTTATTGATTTATGCAAATATTACATTCAAACACATCTTTTATGAACCAAACCAAATATGTAT
TTTCAAAATATATATAAACAAAAGACAATGTGGTCACAACTATTAGTTCGTCCTCCATCCTGCACACGAA
CGCCAACACCAACGGAGTGGACACATTGGCCGCCACacagcaacaacaacaacaacagcagcagcagcag
cagcaacaacagcaacagcaCTACCACTATATCACCACCACCGGCGAGGATGGACAAACCCCGACCACCA
TAGTGTTCGAGAACTACAACGGCGGAATGCCAGCGGTCAGTTCTGCCGCACCAACGCCCGTTCCCGTGTC
GCAGGCCAACGCACCCGCGACCACGACCACCACTGCCCTGGTCAACACGGACGGCACGATCATCGAGTTC
GACGGGAGCACGTACGAGGAGTATCATGTCCTCAAAAGCGAGCCCGGCAGCAGCGATTGCCTGAGCAACG
GCAGCAGTGCCGTGGCCGCCGACATAATCAAGTACGATCCCCAGCACGGTGTCGTCGGCGACGAGGAGGA
CGACGATGAGAACGGCATGCTGCATGTGAAGTACGAGGAGCAGAGCCTCGAACACGATCCGGAAAACGAG

CACGATGCCGAGCAGGAGCATGAGTACGAGGAGTACCAGGTGATCAAGGCCGAGGTGGAGGCCGAGGCGG
CGGAGCTGGCAGCCGGCACCACCAGCTATACGATCAGCCAGGAGCAGACGGGTTCAGGCAACACGGTGCT
GTCCTCAATGGTGCCCAAATCGGGATCAGCGGCGGCCGCCAAGCAGAAGCGCAAACGCAAGACGCGCGTA
ATCGCCGACGACGAGCAGGGCGAGTACCTGGAAAAGATGAGTGTACGTGGCCTGGACATTGCCCGCTACG
AGCACATCATCGACGGCGTGGCTTACTGCCTGGTCTGCGCCAAGAACGATATCTTCAAGACGTTTAAGAA
CAAGTATAGCTTCCAGAGACACGCCTACCTCTTTCACGAGGGCCAAAACCGCAAGATATTCGCCTGCCCC
ATTTGCAACAAGGAGTTCTCGCGTCCGGACAAAATGAAGATGCATAAAAAGGACAAGCACGGCGATGTTC
CCATGCCACCATCGGCCACCACGACGCCGCTAAAGGCTGACGGTCCGCCGGCTAAGCGGGCGCGTACCGC
TCGTACTCCGCGCGTCCGCAAGACGAAGGATGGCAGCACGCCGACCAAGAAGCGGTTAGCGCAGATCGAC
AGTGTTCTGGACGACGTGCAAAATGGGGAGTCTCTGACCATGGCGCCAGTCAGCGTGAGTCACTCCCAGC
AACAGCAGCAGCAACAGATCCAGCACAGCATCACAACGCTGGCTCCCTCGACGCCATCGCAGCCGCAGCA
TCAGCTGCAGACGCTGCCATCGCTGGACTTCAGCGGCATGATCCTGCCTGGAGGCGCACAGCTGGCACTG
GCCAATCCGGGCGCCACCAGCTCCGTGGGACTGCAACGAGGACCAGCCACGCCGAATGGTCAGCCGGCGC
CGCCCACCACCACGTTGATCTACTCGAATCAACTGGAACTGCAGCAGGCGCTGCTGCAACAGCAGCTGCA
AGGTCAGAGCATCATGATCCAGGATCAAGCCGGCAACCTGGTGCCCCTGCAGCTGGACGGAACCCAGGCG
ATATACACGACGGCGCCGCAGGCTCAGCGCCAACAGACCACGGCCACATATCAGACGACgcagcagcagc
aacagcagccagcgcagcagccCGCCGGCCAGGCGCAACAGCAGCCTCACTACGAGTTGGACAACGTTAC
CTATTTGAGCTCCACGGCGGCGGCCACACCGACATCGGCCGagcagcagcagcaacaacagcagcagcag
caCGTGATCGGCACGGTTCAGAGCTATCAGATCCTGACGCCCGACGGCCTCCAGAACTTCCAACCCAAGC
TGGAGACGGCCGAGCTGGGCGACCTGTGTCCCTATTCGCAGTACACCTTCACGACGCACGCCGGAGCGCA
GCCCACGCTGAATGCGAACGCGCTGGACGCgcagacccagcatcagcagcaccaccagcagcagcagcac
catcaccagcagcagcaacaccaccagcagacgcaactactgcagcagcagcCCTTCGCCACGCATCACA
ATCCGCACCAGCAGTTCATGGAGCTGAAGAACGAGCTGCTGATTAAGGGCGGCGACGCCTACACCCTGGA
CACTGCCTCCATGTACCACCTGCCCTTCTCGCCCCACCTCGATGATCAATGCGGTGAACGATGTGAACACC
TCGTCGCTGCTGGAAACCAAAGAAATTAGG
>dme_circ_0004206_junction_seq
GTCGCTGCTGGAAACCAAAGAAATTAGGTTCCAGATCGAAGCACCCTAGCTGTGAA
>dme_circ_0004207
TTCGACGGGAGCACGTACGAGGAGTATCATGTCCTCAAAAGCGAGCCCGGCAGCAGCGATTGCCTGAGCA
ACGGCAGCAGTGCCGTGGCCGCCGACATAATCAAGTACGATCCCCAGCACGGTGTCGTCGGCGACGAGGA
GGACGACGATGAGAACGGCATGCTGCATGTGAAG
>dme_circ_0004207_junction_seq
CGATGAGAACGGCATGCTGCATGTGAAGTTCGACGGGAGCACGTACGAGGAGTATC
>dme_circ_0004208
ATGCCAAAAAGGAGCGACAGTCGCTGCAGTTTATGCAACAATCGCGTAAATTTGAGGGCAAGATACCAAA
ACTAAGTCAAACGAGTGCAGCAACAGGAGGAGCAACGGCAGCCGCAGAAGCATCCACATCCAAGTCTAGT
CAAGGGCAGCAGCAACAGATTGTCTACGTCAACATGTTGCCCGCGGCCAATACCCTGAATGGCATTCCAC
AGCAGCAGCAGCAGCAACAATATGCGAGCACGGATGGAAATGTCTATCAGCTGCAAAATGAAATACTCTG
TGATGCAAATGGACATGCCGTGACTGCCGCCACGGCCTCATATCAAACTACGGCCAGTAGTCCgcagcag
cagcaacagcagcagcagcagcaacagAATGTTGCAACTAGTGTTGCCGG
>dme_circ_0004208_junction_seq
aacagAATGTTGCAACTAGTGTTGCCGGATGCCAAAAAGGAGCGACAGTCGCTGCA
>dme_circ_0004209
TTCCAGATCGAAGCACCCTAGCTGTGAAAGAAGTGTCCGACAACAGGTCGGAATTGaaaaaaaaagagaaa

aaCTGAGACGAGAATGAAAATGTCAGAGGTGCCACGCATCAAGGTCTTCCGACCCACCTGGGAGGAGTTC
AAGGACTTTCCCAAGTACGTGGCCTACATGGAGTCCCAGGGTGCCCACAAAGCCGGTTTGGCCAAGGTGG
TGCCTCCGCCGGAATGGGTGCCCCGTCGCAGTGGGTACGCCGATCTCGATGCTCTAAATGTCACAATTCC
GGCGCCCATTTGCCAGGTGGTAACCGGCAAACAGGGATACTACCAGCAGATTAACATCCAGAAAAAGCCA
CTCACAGTTAAGCAGTTTAGCGAGTTGGCCAGCACCGAGCGTTACGCCACACCCAAACACTTTGACTTTG
AGGATCTGGAGCGCAAATACTGGAAGAACATAACATATGTGGCGCCCATTTATGGGGCAGATGTCAGCGG
AAGTATCACAGACACAGACCAGGATGTAAGTTCGTTAGGGTTAGGGTTTAGGTTTGGGCATTAGCTGATG
ATTCGTTGCCTTTGCCTTTGCCGTTGCAGAGCTGGAACATCAACCGGCTGGGCACAATTCTTGACTATGT
CAACAAGGATTATAACATCCAGATAGACGGCGTGAACACAGCCTACTTGTACTTTGGCATGTGGAAGACG
ACCTTTGCATGGCACACGGAGGACATGGATCTCTATTCCATCAATTACTTACACTTTGGGGCGCCAAAGA
CGTGGTATGTGGTGCCGCCGGAGTGTGGTCGCAAATTGGAGAAGGTGGCCAACCAGTACTTTCCGGCCAG
TTACAAGAATTGCAACGCATATTTGCGCCACAAGATGACGTTGATTTCACCACAGATTCTCAAGCAACAT
GACGTGCCCGTCAGTAAGGTGCGTTTATCGGTAGTAATCCTGTCCATAGTGGCCGTACTAAATCCGGCTC
TTGCAGATCACGCAGGAGGCGGGCGAGATCATGATCACATTTCCGTTCGGCTATCATGCCGGCTTCAATC
ACGGCTTTAACTGTGCCGAGTCCACCAACTTTGCCATGGAGCGCTGGATCGAGTACGGCAAGCGGGCGGT
GCAGTGCACCTGCAGCAACGATATGGTCAAGATCTCGATGGACACATTTGTCAAGCGCTTCCAAAGCGAT
CGCTACGATCTGTGGATGGAGGGGCGTGACGTGGGGCGGCATCCGGAGGATCCGCCAAACGCGGTGCTCA
GTGCAGCACCCCTGCCACCACATCTCGATGTCTTGCTGTGTGATAAAAAGTAGGTTTCCTTGCGCTACTT
GTGGGGCTACCAGCGAGTCTTATTCCTATCTCCTGCTCCTCCGCAGAATGAAAAAGCAATGCAATCCTAC
CAAGGCCAAGAGTTTCAAAGAGCGTAATCCCGATCTGGACCTGGATGAAATCCAGCAGAATCCCAACGTC
CCCGACGACGTCAAGGCCATGCTGAAGGAGAGCGTGCTGACGCTGGACACGGGCGATCTGGCCACGGATG
AGGCTGATTTTCCCAACGAGGATGCCATGAGTCTACAGAGTCCGGCGAATCTGAAGACCAAGCAGGAGCT
GCTTGAGTACATTGACGACGGCACAGGTGTGTGGGCCGGCAGGTGCCCTTCAAAACGATCATCTTCCCCT
TTCCCCAGCTTAACTATAATATGTTTCCTTTCTCGCATCTGTTCTTTGTCTTCCCCTTTTTGCCTGTTGA
CAGAAGATGATGATGAGGAGGAGGACTTCAAGCGGCGCAAGCAGAAGCGGCGCTATGATGCCGACTATGA
CGACGATTGGCTGGCGTCCAAGCGCAAGACTAACTCGCGAAACAATCGCGGCCGTAGTCCGCGCACCAAG
GACGACCGTTCCATATCGCCGGCCTCCTCCACCTCCTCGACGTCGAGGGGTGCAAGGCGTGGCAAGGCCA
GTGGCACGCCCCGAAAGACTCCTGCGCGGCGGAAAAAGGACTCAATCACCACGTCTCCGGCGGTTAGTTC
TGCAGCAACTGCCGTAAAAACACCGACATCCGCCGTAGTCGCTGGAACAACATCAATAGCCACAACCACA
ACACCACCAGCAGATGGCGGAGGAGGTGAGAGCAGCAGCTTAGGCAGCCACTGCACCACTGCATCCCCAT
AATCACAGCTTCACCTATCCACCTCCAAGCCATCCAAATTCTCTAACCCAATGCCCCAATGTCTCTTTTT
TAGCAAAAATCATTCCAAATTTCCCAGTAGTTTTTGTTCTAACCAAATTGACAATGTTTCAAGCAGTTT
CACATGTATCGTTCATCGTTTTTGTTTTGCGATTAATGTACAAATAAATCGTTCTATCTAATTGTAAGAG
TGCGCCAGAGAAATATACGATCACGCTTTTAAGTCTATGCTAAAAACTAACCGAAAACCAAAACTGTTTT
ACTAAACCGAATTCAAGGTGTTGGTGCTGTCTGGGCCGAATCCGTTCCTCTTGTTTTGCCGCCCCCAGAA
TCCGCTTCACTTGCTCTTTCGCTTTGACTACACATCCTCATTCCACCCAAATCGCATTGCCTCCTATCCA
GAAAACCCCCCGAGTCCACAtgtgtgtatgtgtgtgtatctgtgtgtTTACACCAACAGTAACCGTATCA
ACCGCATTTATTCACAAACAATTGAAATCCTTGCCTGATCAAGTACTAAACTTTATCGATTGACGTTTTT
CAGATGATCAAGTACTAAACTTTATCGATTGACGTTTTTCAGATGCCAAAAAGGAGCGACAGTCGCTGCA
GTTTATGCAACAATCGCGTAAATTTGAGGGCAAGATACCAAAACTAAGTCAAACGAGTGCAGCAACAGGA
GGAGCAACGGCAGCCGCAGAAGCATCCACATCCAAGTCTAGTCAAGGGCAGCAGCAACAGATTGTCTACG
TCAACATGTTGCCCGCGGCCAATACCCTGAATGGCATTCCACAGCAGCAGCAGCAGCAACAATATGCGAG
CACGGATGGAAATGTCTATCAGCTGCAAAATGAAATACTCTGTGATGCAAATGGACATGCCGTGACTGCC
GCCACGGCCTCATATCAAACTACGGCCAGTAGTCCgcagcagcagcaacagcagcagcagcagcaacagA

ATGTTGCAACTAGTGTTGCCGG
>dme_circ_0004209_junction_seq
aacagAATGTTGCAACTAGTGTTGCCGGTTCCAGATCGAAGCACCCTAGCTGTGAA
>dme_circ_0004210
TTCCAGATCGAAGCACCCTAGCTGTGAAAGAAGTGTCCGACAACAGGTCGGAATTGaaaaaaaagagaaa
aaCTGAGACGAGAATGAAAATGTCAGAGGTGCCACGCATCAAGGTCTTCCGACCCACCTGGGAGGAGTTC
AAGGACTTTCCCAAGTACGTGGCCTACATGGAGTCCCAGGGTGCCCACAAAGCCGGTTTGGCCAAGGTGG
TGCCTCCGCCGGAATGGGTGCCCCGTCGCAGTGGGTACGCCGATCTCGATGCTCTAAATGTCACAATTCC
GGCGCCCATTTGCCAGGTGGTAACCGGCAAACAGGGATACTACCAGCAGATTAACATCCAGAAAAAGCCA
CTCACAGTTAAGCAGTTTAGCGAGTTGGCCAGCACCGAGCGTTACGCCACACCCAAACACTTTGACTTTG
AGGATCTGGAGCGCAAATACTGGAAGAACATAACATATGTGGCGCCCATTTATGGGGCAGATGTCAGCGG
AAGTATCACAGACACAGACCAGGATGTAAGTTCGTTAGGGTTAGGGTTTAGGTTTGGGCATTAGCTGATG
ATTCGTTGCCTTTGCCTTTGCCGTTGCAGAGCTGGAACATCAACCGGCTGGGCACAATTCTTGACTATGT
CAACAAGGATTATAACATCCAGATAGACGGCGTGAACACAGCCTACTTGTACTTTGGCATGTGGAAGACG
ACCTTTGCATGGCACACGGAGGACATGGATCTCTATTCCATCAATTACTTACACTTTGGGGCGCCAAAGA
CGTGGTATGTGGTGCCGCCGGAGTGTGGTCGCAAATTGGAGAAGGTGGCCAACCAGTACTTTCCGGCCAG
TTACAAGAATTGCAACGCATATTTGCGCCACAAGATGACGTTGATTTCACCACAGATTCTCAAGCAACAT
GACGTGCCCGTCAGTAAGGTGCGTTTATCGGTAGTAATCCTGTCCATAGTGGCCGTACTAAATCCGGCTC
TTGCAGATCACGCAGGAGGCGGGCGAGATCATGATCACATTTCCGTTCGGCTATCATGCCGGCTTCAATC
ACGGCTTTAACTGTGCCGAGTCCACCAACTTTGCCATGGAGCGCTGGATCGAGTACGGCAAGCGGGCGGT
GCAGTGCACCTGCAGCAACGATATGGTCAAGATCTCGATGGACACATTTGTCAAGCGCTTCCAAAGCGAT
CGCTACGATCTGTGGATGGAGGGGCGTGACGTGGGGCGGCATCCGGAGGATCCGCCAAACGCGGTGCTCA
GTGCAGCACCCCTGCCACCACATCTCGATGTCTTGCTGTGTGATAAAAAGTAGGTTTCCTTGCGCTACTT
GTGGGGCTACCAGCGAGTCTTATTCCTATCTCCTGCTCCTCCGCAGAATGAAAAAGCAATGCAATCCTAC
CAAGGCCAAGAGTTTCAAAGAGCGTAATCCCGATCTGGACCTGGATGAAATCCAGCAGAATCCCAACGTC
CCCGACGACGTCAAGGCCATGCTGAAGGAGAGCGTGCTGACGCTGGACACGGGCGATCTGGCCACGGATG
AGGCTGATTTTCCCAACGAGGATGCCATGAGTCTACAGAGTCCGGCGAATCTGAAGACCAAGCAGGAGCT
GCTTGAGTACATTGACGACGGCACAGGTGTGTGGGCCGGCAGGTGCCCTTCAAAACGATCATCTTCCCCT
TTCCCCAGCTTAACTATAATATGTTTCCTTTCTCGCATCTGTTCTTTGTCTTCCCCTTTTTGCCTGTTGA
CAGAAGATGATGATGAGGAGGAGGACTTCAAGCGGCGCAAGCAGAAGCGGCGCTATGATGCCGACTATGA
CGACGATTGGCTGGCGTCCAAGCGCAAGACTAACTCGCGAAACAATCGCGGCCGTAGTCCGCGCACCAAG
GACGACCGTTCCATATCGCCGGCCTCCTCCACCTCCTCGACGTCGAGGGGTGCAAGGCGTGGCAAGGCCA
GTGGCACGCCCCGAAAGACTCCTGCGCGGCGGAAAAAGGACTCAATCACCACGTCTCCGGCGGTTAGTTC
TGCAGCAACTGCCGTAAAAACACCGACATCCGCCGTAGTCGCTGGAACAACATCAATAGCCACAACCACA
ACACCACCAGCAGATGGCGGAGGAGG
>dme_circ_0004210_junction_seq
CAACACCACCAGCAGATGGCGGAGGAGGTTCCAGATCGAAGCACCCTAGCTGTGAA
>dme_circ_0004211
AAGCGCCGCCAGCTGGTCGTCATCCTTCCCGACTTTGAGTGCTTTAACGCAAGCGTGCTGCAGGATCTTA
TTCTAATTTTGAGCGCTCACTGCGGCTCGCTTCCATTCGTCCTGGTCCTGGGAGTGGCCACGGCCATGAC
AGCTGTCCATGGGACACTACCATACCACGTCAGCAGCAAGATTCGTCTAAGGG
>dme_circ_0004211_junction_seq
CACGTCAGCAGCAAGATTCGTCTAAGGGAAGCGCCGCCAGCTGGTCGTCATCCTTC
>dme_circ_0004212

GGACAAATATCCAAAATGCTGAAGCCGGCCCAGAACAAGTCCGAAATGGAGGGAGGTAAGCCCGTGGCGG
TGGTCTACGCCACCGGCTCATCCGCGCGCAACAGCGGAAGTGCTAGCGGCATCGGCAACGTGGGACGCAT
GGGCGCCTTTAGTCAGATGGCAGGTACCACCTACACCACCGTTTCGGCGCAAAAGACTTCGGGTGCTGGC
AGTGGTCATTACGACTTGCCCTTGAAGGGAGATCGCTATGTGAAGTTTACACCAAATCCCATAAAAATGA
AATCAAAACTGCCATGCCATTCAGAGCAACTCGTTGCACTCCATGTCGGCCTCCTCATCGTCCGTTCAAA
GGAAACGCAAGCCGGACAAGGCAGGCAAAGGTTTGCGCCATTTCTCGATGAAGGTATGCGAAAAGGTGGA
GGAGAAGGGCAAGACCACCTACAATGAGGTGGCCGACGACCTGGTCAGCGAGGAGATGAAGAACAATGCG
TACGACAACAACTGTGATCAAAAGAATATCCGACGTCGTGTCTACGATGCGCTCAACGTACTGATGGCAA
TCAATGTTATTTCCAAGGACAAGAAGGAGATTCGCTGGATAGGACTGCCCGCAAATTCGACAGAGGACAT
TTCTGGCCCTGGAAGAGGAAAACTGTCAGCGCCGCGAGCGAATAAAACAGAAAAACGAAATGTTGCGCGA
AATGATCATGCAGCATGTGGCGTTCAAGGGATTGGTCGAGCGGAACAAGCGAAACGAGAGCCAAGGCGTG
GTGCCTTCCCCGAATGCCTCGATCCAGCTACCGTTCATCATCGTGAACACGCACAAGTCCACGAAAATCA
ACTGCAGTGTGACCAACGACAAGCCTACGGCACCGGCAAGACTGGCGAAAATATGTACGAATCGGATGAC
GAGGACAATGAGTTCAACGGGTACCTGGAGTCGGCCAATGAGTCCCAGGGCTTTGCACAACACTCGGCAC
AACACACCACCGATGGCGAGTTCAAGCTGGAGATGGATGATGACGAGCTGGACGACGACATTGATTGAGC
TGATGGTGCCGCCGTCATTGTGCCAGCCATGGCCGATGCGGACACGGATGCGGATGGAGGAGCAGATGCT
GATGCGGAGGACACGTTTAGTTTTACGTTTATCAACGCTTTGTTAGGTTTGCCACGGCCCCCTGGTTAGT
CAGGAGCCGcactccactccaatccactccacacccaatccGACCCACCCACACAATTGGTTTTGTTTAC
TTCTCAATGAGACTCTTAAATTATATATATACAATCTCGCAGCGACGAGAAAGATTCGGTAGTTCAT
>dme_circ_0004212_junction_seq
CAGCGACGAGAAAGATTCGGTAGTTCATGGACAAATATCCAAAATGCTGAAGCCGG
>dme_circ_0004213
CAATTCCCAGCGCCGGAAAGCTGCAACGAATGCTACCGCACGCCCGTCTCCAAGTCGGAGAACTTGGAGA
TCGAATGGAACAAGGACGCCGTGCTGGGCTTCCTCAAGAACATACACAATCCGCAGTTCGTTAGCCGGTA
CGGTGTGCAGCGGGAGGAGCTGCTGCATCCCACGGCGGACAAAATGCGGCAGAAGCGACAGATCTCAAGT
GTCTTCACCGACATGGATATGCGGATGGGCATGCTGCTGTACGCCTTCTGCATTGTGATGATGG
>dme_circ_0004213_junction_seq
CTGTACGCCTTCTGCATTGTGATGATGGCAATTCCCAGCGCCGGAAAGCTGCAACG
>dme_circ_0004214
GTTCTTGATGTACCCGTGCATGGCCTGCAAGACTTCCAGCGGATCCTGGGTCTCCTCATTGTTGGCCGCC
TGCACGGTCATGAAGTGGAAGAGTGTCCACAGGGAGCAACTGAAGCCGCGCAGGCGGGGACTGGATCCAA
CGCAACCCACAAAGTGGTTGGATGAGTAGACGGGCGAGAGATGAGCCTCTAGGCGCTTCAGCTCCTCCTC
GAACTGGCTACCCGTCAGTCGTTGATCGTTGAACT
>dme_circ_0004214_junction_seq
CTACCCGTCAGTCGTTGATCGTTGAACTGTTCTTGATGTACCCGTGCATGGCCTGC
>dme_circ_0004215
TTCAACGATCAACGACTGACGGGTAGCCAGTTCGAGGAGGAGCTGAAGCGCCTAGAGGCTCATCTCTCGC
CCGTCTACTCATCCAACCACTTTGTGGGTTGCGTTGGATCCAGTCCCCGCCTGCGCGGCTTCAG
>dme_circ_0004215_junction_seq
GATCCAGTCCCCGCCTGCGCGGCTTCAGTTCAACGATCAACGACTGACGGGTAGCC
>dme_circ_0004216
GTCTCAGCAGCGCCACGGAGTACGTGGCAGTGGTTCACGAACCGGAGAACACCACTCTAGGCGTCGAGGT
GGCTCTGTTCATGACCCAGTGGCCAGCGGTGAGGGTCCGGCGCGTCATCGATCCAGCCGTGGCCGCCAAA
TTCAAGATTGATCCGACCAATCTGCCCCTCAGCTTGGTGGATCGCAAAGG

>dme_circ_0004216_junction_seq
TGCCCCTCAGCTTGGTGGATCGCAAAGGGTCTCAGCAGCGCCACGGAGTACGTGGC
>dme_circ_0004217
GATAACGTAGCGTTGGATAGCCCATCACCTCGTAGTTGCGGCAGATGCCATTGTTCTCCTCGGCGGCGCA
ATCGATGGCCGCCACGATGAGCACCTCGGACCAGGGCAACAGGTGCTCCGCTACCGACTTGTACGTGGGT
GCGAATCTGCGACAGTGGCCGCA
>dme_circ_0004217_junction_seq
TGGGTGCGAATCTGCGACAGTGGCCGCAGATAACGTAGCGTTGGATAGCCCATCAC
>dme_circ_0004218
CGGACGCAGTGCAGCAGTTCCACGACGACCATGAGCGACCAGATTGGCAACCCCAATGCAACATTcagtg
gcagtggcagtggcagtggcaCCAACGTCGCCTCCATCGCCGAAAGTGTGGCGGAGAGCGGACCGGACTT
CGATGCACTTCGGGCCGCCTGCGAAACGCGGCTAAATGCCAGCGGTCAACTGGCGGGGCTCCGGAGGACC
AGGCGCAGAAGCAGGAACCCACTGTGCCGGCACCTTTGATGGCTGGCTTTGTTGGCCGGATACGGCTGTC
GGCACTTCCGCCTACGAACTCTGCCCGGACTTCATCACGGGATTCGATCCAGCAAG
>dme_circ_0004218_junction_seq
ACTTCATCACGGGATTCGATCCAGCAAGCGGACGCAGTGCAGCAGTTCCACGACGA
>dme_circ_0004219
TTCCGCGGCACACACAGTTGTTCGTCGTCAGCGGTGGTGAACGGAGTGGAGCGGAGCGGTTCGGAGTGAA
ACGGGGTATTTGGGTGAAGGCCAAAGGATGTGCCCACCCCCCTCCCGGAATCAGTACCCTATCACTCAAC
CCAAATGTGAAAGAAAGGAAAATTGGAAGCCCTATGCGGATAGGAGGATAGTATGGCTGCGAGAAAGACC
TCTCAATTAGGCCATATAACTGCTATCAATCGCGTGAAATGTCAAATCAACCAGCTACTTCAGCCCCCCT
CCCACCACACAAAAAAGAGAACTATCCAGCAAATTAAGCGTTCCGCGTCTGTTAATTGAAGTGCCTGGAA
GGCAAACAGATCGAGTGACTGGAACTGGAGATTGGAATTTTATTCGGG
>dme_circ_0004219_junction_seq
GGAACTGGAGATTGGAATTTTATTCGGGTTCCGCGGCACACACAGTTGTTCGTCGT
>dme_circ_0004220
GGCTGAGCCCGAACACTGAACTCCTCTCCATAGGTGGTGCGCCTCATGAAGCCCACTCGGTTGGACAATC
TCCGAAGGACGTCCATATCGAGAGGGGCTTCCTTGACCATGGCCACTCCGTAAACTGCCAGATGCTGGAG
CCATTGCTGCAACGCCGAATCGCAATCGAGCAGTTCTTGGTA
>dme_circ_0004220_junction_seq
CCGAATCGCAATCGAGCAGTTCTTGGTAGGCTGAGCCCGAACACTGAACTCCTCTC
>dme_circ_0004221
TTTTATCCAAAGTAGTGCGAGAGTCCCAGATTGGCACCCAGATCGGTCTGGCCCTTGAAGGGTCCACTTG
TCCACTTGGATGCCGTGCTGCCCAGATCTAGCCTTGTGTTGCGATCCTGCGACTGCCAAAGATTGGCACG
CCCACCCAGCTCCAATTGCTTACCCAATCCGGGAATATTGGCGTGCGTCAGGGTAGCTCCATGACCTTTG
ATGTGGGAGTAGTCCAG
>dme_circ_0004221_junction_seq
CATGACCTTTGATGTGGGAGTAGTCCAGTTTTATCCAAAGTAGTGCGAGAGTCCCA
>dme_circ_0004222
TAGTGCGAGAGTCCCAGATTGGCACCCAGATCGGTCTGGCCCTTGAAGGGTCCACTTGTCCACTTGGATG
CCGTGCTGCCCAGATCTAGCCTTGTGTTGCGATCCTGCGACTGCCAAAGATTGGCACGCCCACCCAGCTC
CAATTGCTTACCCAATCCGGGAATATTGGCGTGCGTCAGGGTAGCTCCATGACCTTTGATGTGGGAG
>dme_circ_0004222_junction_seq
GGTAGCTCCATGACCTTTGATGTGGGAGTAGTGCGAGAGTCCCAGATTGGCACCCA

>dme_circ_0004223
TTTTATCCAAAGTAGTGCGAGAGTCCCAGATTGGCACCCAGATCGGTCTGGCCCTTGAAGGGTCCACTTG
TCCACTTGGATGCCGTGCTGCCCAGATCTAGCCTTGTGTTGCGATCCTGCGACTGCCAAAGATTGGCACG
CCCACCCAGCTCCAATTGCTTACCCAATCCGGGAATATTGGCGTGCGTCAGGGTAGCTCCATGACCTTTG
ATGTGGGAG
>dme_circ_0004223_junction_seq
GGTAGCTCCATGACCTTTGATGTGGGAGTTTTATCCAAAGTAGTGCGAGAGTCCCA
>dme_circ_0004224
TCCCAGATTGGCACCCAGATCGGTCTGGCCCTTGAAGGGTCCACTTGTCCACTTGGATGCCGTGCTGCCC
AGATCTAGCCTTGTGTTGCGATCCTGCGACTGCCAAAGATTGGCACGCCCACCCAGCTCCAATTGCTTAC
CCAATCCGGGAATATTGGCGTGCGTCAGGGTAGCTCCATGACCTTTGATG
>dme_circ_0004224_junction_seq
CGTCAGGGTAGCTCCATGACCTTTGATGTCCCAGATTGGCACCCAGATCGGTCTGG
>dme_circ_0004225
GCTCCTTGCGTTCTCCTATATACATGTGCACCGTGAGATCCAGAGTCCCATCGTTTTCGGTTTAGTTACC
CTCGGGCTTTGGCAGATTCGTATCATAGGCACGTATAATGATAGACTGCGGCACAGCTCCGCCCTCCTCC
TGGGAAAACGCATAGCCATCTGGGAAGGTAAACAATGCCAGTTAGTTAGAATCGTATAGATCACAAATGG
TCAAGTGATTCATGTGTGGTATCTGTTGTAATGTGTGAGTGTGGTCCCAGTGTCTCATACATATGTATGT
ATGTAGTTCTACCTCCAAGGGAGACCCCCTTGCCACAAAATGGAGGCCATACTCCATAGCCCAGCCCCGG
GATGCGGACCTACTGCTCGAGGAGTCCTACCACTAATGGCTGGGTTCCTGCAACTGAGTGGTACTCACGC
GATAGCTCGAGATCCGTTTCGACTCTCGTGTTGGCACGACGGGTAAAGACATTACGTAGCAGCCGGGCCG
TTTCCGTGAATCTGTTGGGTTAATTTTTGAGCGGGTCGTATATGGTTTAGTAAAGGATTTCAATTTCGTA
CGGGGTGAGGTCAGGCGGGTCACTTAATAGATTAGTTTCCATTTTGAATTGTTAGCACTTTACGCAGAGT
TCTTGCAATTTACACTAGAATTCAACGGAATATTGCGATAATCTGGGCTTGGGCAGAGGAGCATACATAT
ATGTATAATCAAATCAGGATGAGCAATAAGGTGTGTACAGGTGTGTACCAAATCGATAGGGTCATGCAGA
ATACGTAATAAATGGAGTAGAGCCTCTACATACTTTAAAGATGGCAAATAAAAGGAATACGATGCGGTTC
TGGATATCAAATTTACAACGAATCTTTGACGCTACGTAAATCATAAGGTTTTGTACCATAAAGTGTTATA
GTTATACAAGTTTGAGGTCCTATCTACCCGTATATATATGTATATATCTCCCTTCTGAGGGAGGGGAACC
CTCTATATCATaaaaaaaaaaCAAATACCCTGTGACTGGGTGAAGTGACAAAGTGGGTGTATGCGTGTTT
TTTGTGGATGGAGTGTGTTGTGACAGTGTAGATATGATTTTTCAGCAATATCTACTTTTTTTACTTACAC
CGAATTATCGATCCCAAGGCATACATGGTACTGCGCACACAGACAGATTAACGCAGAAAACGTTCAGAGA
AATTTCAAATAAACATTTTTCAGAATTTTGCTGTTGACTCTTTGACTTTTATTAAATTATATATTGTTTT
GATTAGTTATTGTTGGGTGTGTTTGGGTTCTGCGAGTAGGTGTTGGGTGTGATTGGTTAAGTCGCTTTGT
TGGGTGTCGAGTAATACAAAATAAAACATAAGTACATACAACATAAGTGCAGAGCATATCGAAACAATTC
GGATCGTATCGTATTCAGATCGGATTGAACAATCAGCAGTCATTGCGTTAGTATAAAGATAGATTATTGG
CCGAATTAAATTGCATGCGAGCTGGGGAGGATTGTGCGGGATACATACATATATTCAAAACTGATACATA
CATATCATCTGGGTACCATCACATTCCAATAACGTTGGGATGATTATATATATATATATCGGTTGGTTAATC
ATAAATTTGTTAATTAGGATCGAACGGAACATTCGTGACTGGGTTTGTTAACGTTATGTAACTTTACAAC
CTGGTAAGTGTCTTAGAAGTATAGTTTAACATCCAGCTAACACATACATATACGATTAAGGTACGATTAG
AGATAAATAAACTCAGCTTGTTGGGAATGATGATAAGAAGACGTCTCACAAGATAGCATTCCCTGATTAT
TGGCCTATCCTACGTGCCTAAACACACCTGTCTACTTCTTAGACACTATCACAGTTGGCAAATAGTTTGT
TAGCACATACAGTATGCGATGTAAAAAATATCCGCTTCTCGGGACTAGCATATAAACATGAAAATATGAT
ATATATGATATGGATGCGGAATCGGGAAATAGTCCCATTCGGAATGCAATTACAATACTATACAATGCTC
ACTTATAAAATACGTTTTATAACTAGAAAAATAATTCGATTTGATTTGCCTTCGATGAGGGAAGAAGTCC

ATCGTCCACATCCTCACATGTGCAGAGTGGCCGTGATGGGTGATGTGGTGCTATTTCTCGCACTCCTTGC
AACTCCAGTGGCCACCTGACCACCTCGCCACTTCTGACGCTGCCTGCTCTGCTGCTGCTGTTCCGTGTCG
GCCTCCATCTCCAGGTTCTCCACCCGCAGATTCGACGTCCTACTGGATTCCGACACGGGATGATGATCGT
GCGAAATCAGAATGTGGCTGGATCGTATCAACTTGCCGAGGGCGTATCCAAAACCGGAGTCACTAGTGCG
TCGCATGGGTGCAGGGATTTCGAATGGGTTTATGGATCGATTGAATGCATGGATTTGGGATTGAGGGGAC
AATCGTCACCGCCATGGGATTGATGGGGGATAAAGGAAGACAGAGAGATTACATTTTAGGGATTTCAATT
CATTAGATACTTGCTCATGGTGATGCAGTCGAAGATTGAGTAAATCTGAATCTGATTGATGCTTTCAGTT
TTTGTCTTAACTCAGTTAAATTGTGTTTGTTTCTTTAAATGCGCCGATTTTATTGAATGACACAGCTAGG
CGAGAATACCTCAACACAGCCAatataattttataaatatatatatataAACCAATTTATGTTTTAATTG
CATTCAAAATTGTTCTATTAAGTCTACAAAAGGCGTATCAAACTTGGGTAAATACATAACATTAGGATCT
AAACATGAGTTTAAACTCGTAAATAACATTATCTAGGTGTGCTATGTTTATTATATGCATTAAATAGGTA
GGTTACATATGAAAGAACACTTGTGGGTGGATATAACAAATGACAAGAAAAAATAGTGTTTAAACTTATC
TTACTCTAGTCTCTAATATTATTTACACATTTATCGGCTAATACAATGCCATTTCTCATTGGTCTCAGTG
GTCTTATTCATAAAGGTCCTTTACTATTTGTGTGTTCGATTCTAGTTATAGGTATATTTGAAGCTAATAT
TCAAGTAGATACTTTGGTTTTGATATAACAGCAACTAACATACATCGAAAGTTGAACAAACACAATTCGA
GGAGGTGAAAGTGCGATAAAGTAATGATTTGGATTTAGTAATGCATTTTTATGACCCAAACAAACTCTAA
ATAATAGCTAAGGTTGGTTTCGATATTGGTGCAATGCAAAAACTACAAGAAGCAGGGTATAAAGTGCAGG
CACATATGTTCAATCAGCCTTGTTTCGGGTTGTGGGATAGGTTTATAGAAATATCAACGTCTTTAGGGGT
CTAGTTAATATTCAGCAAATGCGCAACAATTCAAATTGAAAGTAGGTATAGTTTGATTTCGTTTTTGGAC
AAACAAAAACTAAATACGTATGGAGGAATGTAGGGGAACCTCCGGCCCGGAGGAAAAGCGTGACTACGGG
GGTCTGGTAGGCGATTGCTTCGATACTTACGAAGATCGCGGTTCGTTCATCACTTCGGAGATGTCGCTGC
GTCGGATCTCCGTTTCGCGAACAGCCTCCGTCAGTGTCTTAAACACGGTATTAT
>dme_circ_0004225_junction_seq
TCCGTCAGTGTCTTAAACACGGTATTATGCTCCTTGCGTTCTCCTATATACATGTG
>dme_circ_0004226
AGTTCGTCGGCGAAGGACGCCAATGTGGCACTAGTCATCGATGGTACTACGCTGAAGTACGCCCTTAGCT
GCGACCTGCGCAATGATTTCCAAGACTTGTGCATTCTGTGTCGGGTGGTCATCTGCTGCAGAGTGTCTCC
AATGCAAAAGGCTGAGGTGGTCGAGATGGTCACCCAGAGCACGAATGCAGTGACCCTAGCCATTGGCGAT
GGAGCCAACGACGTGGCAATGATCCAAAAAGCGAACGTGGGAATCGGCATATCTGGAGTTGAGGGTCTCC
AGGCGGCATGCGCATCGGACTACTCGATCGCCCAGTTTCGCTATTTGCAGCGCCTACTCTTGG
>dme_circ_0004226_junction_seq
TTTCGCTATTTGCAGCGCCTACTCTTGGAGTTCGTCGGCGAAGGACGCCAATGTGG
>dme_circ_0004227
ATGCCAGTTTTGACGTTCCACGACCTCCAGGCGGCGGCAACTCGCCCTTGCCGTCACAGGGTCGCTCTGT
ACGCGAATTGGAGGAGCAGATGTCCGCGCTGCGCAAGGAGAACTTCAATCTAAAGCTGCGCATCTACTTC
CTCGAGGAGGGTCAGCCGGGTGCCCGGGCAGACAGCTCCACAGAATCCTTAAGCAAACAGCTCATCGATG
CCAAGATCGAAATCGCGACATTGAGAAAAACTGTCGATGTAAAGATGGAGCTGCTCAAGGATGCCGCTCG
AGCCATTTCTCATCACGAGGAATTGCAGCGCAAAGCAGACATTGACAGCCAGGCAATAATCGACGAGTTG
CAAGAGCAAATACACGCCTATCAGGATGGCGGAGTCTGGTGGTCAACCTGTCGAAAATATTGCCAAAACC
AGGAAAATGTTGCGCCTTGAATCGGAGGTGCAGAGATTGGAGGAGGAACTGGTGAATATCGAAGCTCGTA
ACGTTGCAGCCCGGAACGAGCTGGAATTCATGTTGGCCGAGCGCCTAGAATCCCTAACAGCCTGTGAGGG
CAAGATTCAAGAGCTGGCCATCAAGAATTCCGAACTGGTAGAGCGTCTTGAAAAGGAAACAGCATCCGCC
GAGTCATCCAACGGTAAGTAAATCCGTTTAATAGTAATTCCCCTGTAGAGTAGGTAGAGTGTTTGGTAAT
TGGGTTTGAGAGAACCCTGTATATTTAGGAGGCCATAGACTCTCTTAAGGTTGAATTAGAAGCCTGTAGG

AAAGAGAATCAGGATCTGGTGACCTCCATTCGAACCTTAAAACATGATATGAAGCGACAAGTCCGCTCGA
TGAAGGAGGCCGCCAATACGATGGACGTGCAGCGTCAGTCCATCCTGCTCTTGGAAGTGAGTCGCAACCT
TCCACTTCCTCAGCCATACCCAGCTCAACCTAGCAATCCTTCCCCCTTAAAAAGGCCACAATCAAGCGCA
AGGAAAAGTCGTGCGGAAGTATGCAGAAGAACGTGCTAAACTATGAGGCACTGATAGCCAAGTTAAACGC
CGAACTGGAGACTATGAGGCAACAGAATGTTTACTTCCGTGAGCTCTCCGAAAACCTACAACAGAAGGAG
GTGCGGCAACTGGATCGCGGCGTGGCCATCGTGCAGCCGATGCGCATGACGGCGGATGCCGGGGTTGGTA
TCACATCTTCTCTTGAACGTAGCGGCAGCACATCCTCATTGCATTCAAACTTCTCATCGATCAGCGTTTT
GTGTGGCAGTCAGGGACCATCGTCGCACAAGAGCCTTTGCCATTGGAAAGGCAGTCAGGTGAAGCACTTA
CCATCCAGCGAACAGTGTGGTCAGCTCCTAAAACGTCGTCGTCCACAGCTGCAGCAACAAGCAACGTATC
CGTGTCCGCAGTACGTTTATCATCAGGGCCACCACCAGACCAGACATACCCAGTTAATGGCCATGACCGC
GCCAAGTACGGCCTTCTACAACTATTCGCTGCCAAGCTTGACTGGATTTCGGCAGTTCCTTTGGCGCTGG
GGCACTTAGCCCTCAAGATCATCCTCTTGGCTATGCGGTTGCACTCCTGCCTCCAGAATGTCCATATCCC
TCTTAGGGTGTCCTTTGAGCTGGGGCATTCCATGCCGTTGGCTGGCATTCTGTACTCTATTTGATCATCA
TTTGATCGTTCCGCTTTCGTTGATCGTTCAAAGAAATCCAATCAAATGAATCCGATGTTGTCCATATTCT
ATTAGAACAATGTTGCTCTCTCAGACGTCGACAAATTTTTTGTTAAAGTGTATTAAATTTGCTCACATAT
CCCCCCGTCTGAATTTAATAATTGTTTCATTAAACGTATCAGATAATTCTAAGCTAAGGTCttttttttt
tttATTCCTCCCAATGCTGTAGCCATTTCCGGTTTTCTCTGCTCACTTTCAGGCCATTTCCGGTTTTCTC
TGCTCACTTTCAGGCCAATCGAGATCTGGGCGCCCAACTGGCGGATAAGATTTGCGAGCTGCAGGAAGCC
CAGGAGAAGCTCAAGGAGCGCGAGCGCATCCACGAGCAGGCATGCCGCACCATTCAAAAGCTAATGCAAA
AGCTAAGCAGCCAGGAGAAGGAGATAAAGAAGCTCAACCAGGAGAACGAACAGTCGGCAAACAAGGAGG
>dme_circ_0004227_junction_seq
GAGAACGAACAGTCGGCAAACAAGGAGGATGCCAGTTTTGACGTTCCACGACCTCC
>dme_circ_0004228
ATGCCAGTTTTGACGTTCCACGACCTCCAGGCGGCGGCAACTCGCCCTTGCCGTCACAGGGTCGCTCTGT
ACGCGAATTGGAGGAGCAGATGTCCGCGCTGCGCAAGGAGAACTTCAATCTAAAGCTGCGCATCTACTTC
CTCGAGGAGGGTCAGCCGGGTGCCCGGGCAGACAGCTCCACAGAATCCTTAAGCAAACAGCTCATCGATG
CCAAGATCGAAATCGCGACATTGAGAAAAACTGTCGATGTAAAGATGGAGCTGCTCAAGGATGCCGCTCG
AGCCATTTCTCATCACGAGGAATTGCAGCGCAAAGCAGACATTGACAGCCAGGCAATAATCGACGAGTTG
CAAGAGCAAATACACGCCTATCAGGATGGCGGAGTCTGGTGGTCAACCTGTCGAAAATATTGCCAAAACC
AGGAAAATGTTGCGCCTTGAATCGGAGGTGCAGAGATTGGAGGAGGAACTGGTGAATATCGAAGCTCGTA
ACGTTGCAGCCCGGAACGAGCTGGAATTCATGTTGGCCGAGCGCCTAGAATCCCTAACAGCCTGTGAGGG
CAAGATTCAAGAGCTGGCCATCAAGAATTCCGAACTGGTAGAGCGTCTTGAAAAGGAAACAGCATCCGCC
GAGTCATCCAACG
>dme_circ_0004228_junction_seq
GAAACAGCATCCGCCGAGTCATCCAACGATGCCAGTTTTGACGTTCCACGACCTCC
>dme_circ_0004229
CACCAGTCCGGGAGCCCTGCAAGATGTCACCATGGAGAACTCATGTAAGTTTCACGATGAATATGCCAAA
TATTTCCTGCTTTCGAAATGCTTATATCACTGGTGTTAGAATTGAGAGATCTTTGTGAATTTCGCCTGTT
TTTCCACTGCTTTTCAAGCAGCCTATGAACTTAATGTTATGTTTTATTAGAATGGAAACCATTGCATATT
AGGATTCCGTTTTGGCTACACACACGAGCATTAGCACATTTATTCGGCCGCACTGTTGGCGTTTTATATT
TTATATGTGGTTGCTCCCTAACGGCTAGGAGAAAGCTATAAAATCATGAATAGTAATCGAACGTCGTCTT
CGCACAATTCGAGAAATCTAAAGATTCTTAGTCAGTAAAGATAACCTTATGGATTATCCTAGACACTCTA
AATCAATATCAATCCCTTTGTTTCCTTTGCAGAATGCCAGTTTTGACGTTCCACGACCTCCAGGCGGCGG
CAACTCGCCCTTGCCGTCACAGGGTCGCTCTGTACGCGAATTGGAGGAGCAGATGTCCGCGCTGCGCAAG

GAGAACTTCAATCTAAAGCTGCGCATCTACTTCCTCGAGGAGGGTCAGCCGGGTGCCCGGGCAGACAGCT
CCACAGAATCCTTAAGCAAACAGCTCATCGATGCCAAGATCGAAATCGCGACATTGAGAAAAACTGTCGA
TGTAAAGATGGAGCTGCTCAAGGATGCCGCTCGAGCCATTTCTCATCACGAGGAATTGCAGCGCAAAGCA
GACATTGACAGCCAGGCAATAATCGACGAGTTGCAAGAGCAAATACACGCCTATCAGGATGGCGGAGTCT
GGTGGTCAACCTGTCGAAAATATTGCCAAAACCAGGAAAATGTTGCGCCTTGAATCGGAGGTGCAGAGAT
TGGAGGAGGAACTGGTGAATATCGAAGCTCGTAACGTTGCAGCCCGGAACGAGCTGGAATTCATGTTGGC
CGAGCGCCTAGAATCCCTAACAGCCTGTGAGGGCAAGATTCAAGAGCTGGCCATCAAGAATTCCGAACTG
GTAGAGCGTCTTGAAAAGGAAACAGCATCCGCCGAGTCATCCAACG
>dme_circ_0004229_junction_seq
GAAACAGCATCCGCCGAGTCATCCAACGCACCAGTCCGGGAGCCCTGCAAGATGTC
>dme_circ_0004230
GGAGCAGCTGGACGAAACCATAATGCAACTGGAAATCGAACGAGAGGAGAAAATGACTGCAATACTTCGC
AATGCGGAAATCGCCCAGAGCGAAGACATTTTACGGCAACAGCTGCGCTTGGAGCGCAGCGAAGCCAGCG
ACCTCCAGGAGAGGAATAATCAGCTGGTGAGGGATATATCCGAGGCACGGCAAACGTTGCAGCAGGTCAG
CAGCACAGCACAGGATAATGCCGACAAGTTGACCGAATTCGAGCGA
>dme_circ_0004230_junction_seq
TGCCGACAAGTTGACCGAATTCGAGCGAGGAGCAGCTGGACGAAACCATAATGCAA
>dme_circ_0004231
GTGTTCCAGTCGCTCCAGTACACATAGTCTTGGTACAAGGACACCGCATACGGCTCATCCATATCGCTGC
CAACCAGTATCTGCCGCTTCTTACCATCCCAATCAGCACTTTCGATTTTAGCTGGCCGCGATTGGGTGGC
CCAGTACAGACGTCTTGTCTCCTGGTCGAAGGTTAGTCCGGCGGCATGATTGGCACCCGCTACGATGGTC
TGCAA
>dme_circ_0004231_junction_seq
GATTGGCACCCGCTACGATGGTCTGCAAGTGTTCCAGTCGCTCCAGTACACATAGT
>dme_circ_0004232
GCGAAGTGGGAGCGCAATTAATACAAACCAAACCCAACAAAATTCACCGTCGATGTTTTGGCTAAATAAA
CATAACCAAAACCACTGAGAAACAACCAAACGAAATGAAACTAAACAATAAAGCAGATAG
>dme_circ_0004232_junction_seq
AAATGAAACTAAACAATAAAGCAGATAGGCGAAGTGGGAGCGCAATTAATACAAAC
>dme_circ_0004233
TTATAGCCCAAATGGCGTTGAGGCTCCGCCGCGTTGGACCTGCTTGCTCTGCGCGCTGGGTTTGTTCATC
TACCAGAGTCTGGACTCCATCGACGGGAAGCAGGCGCGACGCACGAATACCTCATCGCCGTTGGGCGAAC
TTTTCGACCATGGCTGTGACTCCATATCGACGGTGTTTGTGGCCCTCTCGGCGTGCATCTCCTGCCAACT
GGGCCACTATCCCAACTGGCTGTTCTTCCAGGTGCTTCTGTGCTATTGCGCTCTTCTACTGCGCCCACTG
GCAGACGTATGTGTCTGGAACGATGCGGTTCGGCAGAATTGATGTGACAGAGGCTCAGTTCTCAATTATA
GCCATTCATTTGGTTTCGGCTGCACTGGGTCCTGAGATCTGGCTCACCAAGG
>dme_circ_0004233_junction_seq
CTGGGTCCTGAGATCTGGCTCACCAAGGTTATAGCCCAAATGGCGTTGAGGCTCCG
>dme_circ_0004234
GTAATAATTTATAGCACAATATGATATAGCAGCAGTTTGAAACTTTTTACACATGTCACAAATGTTTTCT
TCCCGTTTGCAAGATTCATTTTTCGTTCATGGAGTTTTTGTGGTCAGAAGACAGAGTGCAAATTATAATT
ACTGACAATTTTCAACTTTAAAGCGAAGGGACAAAATTTCACATAATCAATTTACAGTCGCCGCAAAA
>dme_circ_0004234_junction_seq
ACATAATCAATTTACAGTCGCCGCAAAAGTAATAATTTATAGCACAATATGATATA

>dme_circ_0004235
GATGGAGATCCGAGAGCTGCTTACCGAGATGGGCTATGATGGTGACAAGATCCCTGTGGTAAAGGGCTCG
GCGCTGTGCGCCCTCGAGGACAAGAGTCCCGAGATCGGCAAGGAGGCGATCCTGAAACTGCTCCAGGAG
>dme_circ_0004235_junction_seq
GGAGGCGATCCTGAAACTGCTCCAGGAGGATGGAGATCCGAGAGCTGCTTACCGAG
>dme_circ_0004236
GCTCATTCTGCGCCTCATCCGGCCAATGGTCCTGGAGCAGGGACAACGCTTCACGCTGCGTGACGGAAAC
CTGACCCTGGGCACTGGTGTCGTCACCAGCACTCTGCCCCCATTGACTGAATCGCAGCGATCCGAGTTGA
CGGAGGGCAAGAAGGCGCGCGAGAAGAAGGTGGCGGCTAAGAAGTAGATCCCCGGAACGCCGTCA
>dme_circ_0004236_junction_seq
TAAGAAGTAGATCCCCGGAACGCCGTCAGCTCATTCTGCGCCTCATCCGGCCAATG
>dme_circ_0004237
GGCAGTGCTTTCTAAGGGCGTGACCATGGCAAGCACGGGTCTGGTCAAGGCGGCTGTTCCTGCCAAAGCA
AGCACCTCGGTACCCGGCTCAGCCATAACCTTAAAGGGCATCCAACCGCTGGCCGGAGGCACTGCGAAGG
CGTCGACTTCCAGCACGACGGCAACCACATCCCCATCTTTGGCACAGCCCAACAAAATTCAG
>dme_circ_0004237_junction_seq
ATCTTTGGCACAGCCCAACAAAATTCAGGGCAGTGCTTTCTAAGGGCGTGACCATG
>dme_circ_0004238
GGGAAAAGTGGGGGATTATATGTGTGATTTTTGGAACAGATCATCTCTAGAATATAGAGCTAAGCTGTTC
CAGAAGACGCTCATATACTCCCCTAACTTTTCTATATTAGAATAGCGCCAAAATTAGTTGTATCATTGCT
GTTTTTTTGACTTTTAAATGTTCTTAACTAAGCTAAATCCGAAAATTATATTCCAAACTATA
>dme_circ_0004238_junction_seq
AAATCCGAAAATTATATTCCAAACTATAGGGAAAAGTGGGGGATTATATGTGTGAT
>dme_circ_0004239
GAGTATGGCAATTCCAAACAACATCCACTCGAAGCCGATGCTCTTCACCAGCGATCCAGATAAGGCGGGA
CCCACGGCGAAGCCAACACAGAAGGCCACATCACCCAGGGCGTAAACGCTGCCGTAGACCGCCGAGTGTC
GGATGTCCACCAGATAGCCCAGTTCCGGCATCATCGACGAGTCCACCATTCCAATGGCAAAGCCAAGTCC
TGCATTCGGGATGATCAGGTGA
>dme_circ_0004239_junction_seq
AAGTCCTGCATTCGGGATGATCAGGTGAGAGTATGGCAATTCCAAACAACATCCAC
>dme_circ_0004240
CAACCAGCGGCTAGCGACGCAATGCAATCATCGACCGATGCGGGCAATGGTGGACCAAGAAAACACACGC
AGAGCACAGCCCCCCAGAAGCAGTCGGAGATTAGCGAGACCACCTCCTTCACCGTAAACGCAAACACCAC
CACCAACAGCCAGCAGCAACAGATTCCCGGCGGACCCGCCAGCAATACGCCCAGCAAAAATCCCTTCAAG
CAGCAGTTGGACAGCAATCAACAGAATGGCAATATGGAGCCGGATTGCGGCATCATGGGCGTCGGCAAGG
AGCCACCGGTACCGCCGCCGCCCACCTACCAGTCGCAGACCTACGCAGGACGACAGGCTCCGCCGCCAGC
GGATCAGTACCGCGAGGAGGATCCACGCGCTGCCGGGTCCTGGGCCGCCGCCAAATCCTGGATGGTCAGC
TGGCGGGGATCCAATCGCCTGGTGCTCGTCATCGTGGCCATAGCCCTGCTCCTCGACAACATGCTGCTGA
CCACAGTGGG
>dme_circ_0004240_junction_seq
TCGACAACATGCTGCTGACCACAGTGGGCAACCAGCGGCTAGCGACGCAATGCAAT
>dme_circ_0004241
CTGGCAGATGAATTAGCCAATTTTAAGCAGCTATTTCAGAAGCAAATCAATGCGCTCGATGTGCAAGCAG
ATTCGATGCAGCTAAAATTGGAACAAGCCTCCGCTCACCTGGATCCCATTATCCTGATCGATTCCTGGAG

CAAACAATGCGTACAGAACTATAGTGTTACTATACCAACGATCACCGTGGCCAGGGACTCCATTATCAAG
TGTAAGGAAAGTATTAGTGGCATTTTAAATGCACCTGAGAGCACCTACAACACCCTGAATAACTACTATA
AAAATAACGTGAAGAATGG
>dme_circ_0004241_junction_seq
ACTACTATAAAAATAACGTGAAGAATGGCTGGCAGATGAATTAGCCAATTTTAAGC
>dme_circ_0004242
CCAAAGCAACGCATCGACGCATAAACGCTAGTTAATAATAAAAGTGCATAAAATTATTCGTATTCCAACC
ATCTTCCGTCATCTAAAGTGCTAAAAATTCGTCACCATGTCGCGTTCAAGTAGTTTGTTTTTTAATTCGG
CTGCCAAGTTAGAGACTAAAAAACTTAATTGATTCGATCAAGAAAAGTGAGCAACCAGCAGTGAAAATAC
ACAACAGCAGCATAATCACTTTCGAAACCAGTTTTTTCGTAAAGAATCGCGAAGAGTGTTCCAGAAAAGT
GCAacatcacattcccacacagtatatgcacacacacacaaacacactatatatacatataggtacatat
atagcaacatcGGAGCATCCAGGGAAACGACCCACAGCACCCACTTCGGTTAATCTCGCTTGGTAATTAA
GGGTTCATCGCCTAATGGCAAGTTCGCTCAACAAGACGAGACACAGGCGCAGACTGGCTGCGATCTCGTT
CCTATCGAATATCTCGTTAGATGGCACCCATCGGGACACCAAATTCGGCGCAACATTCGGCAGCAGCCTG
CTCTGCAACCAGAATAAGAACCCATCCTCGCTGGGACTCGGCTCGGGGGGCTTTCATCCgcactatcagc
agcaccaccatcagcgcagccactcggcgacgcaccacaaccaccagaagcggcatcacacgcagcagca
gcagcaacagcaacaacagcaacacacccaacagcagcagcagcagcagcaacatcagcagcagcatcag
cagcagcagcaacaccagAATGGCGCCCTCTGTTCGCCGCTCCTCTGCAGCGCCGATGTCCACATGGGCA
TCGATGGCGGCATCGGGCTGGGATTGGGACTGGAGGACTGCACCACCGGTGCCAGCGATGGGGACGGGCA
TTTTAGTGAAACTGAAAATATGGGTCAATACCTGATGAACGTCCTGCCGGCCGCCGATCACAATCGCAGC
AGCATTGTGCCGGCCGAGAGACGCAACATAAAGCGCCCGACCTCGTCGGGTCGTCATAACCACCAGGCAC
CGGGCAATGGCAATATGGGCAATAAGCTCcaacagcagcagcagcagcagcacaagttgcagcagcaaca
gcggcaggcaCGAAGTTGCGCCGCCGGCAGCGGAGGATCGGGTGCCGAGAGTGGCAGCGATTCGGACAGC
GTCAAGATACCGCTCAAGGTGTCCAATCTGGGCAGCGGCGGTGGCGTGGCCGGCGGAGCGGGCAGCAAGC
TATTGCCGCTGAGGGAACGG
>dme_circ_0004242_junction_seq
CAGCAAGCTATTGCCGCTGAGGGAACGGCCAAAGCAACGCATCGACGCATAAACGC
>dme_circ_0004243
CACCACCCTGAGTCTCACCGCGCACAAACATAATATTCGAGGCCGCCTTCTCGATGGGCTTCTGTTTGGA
CTCGGCTGCCGCCTGGCGAGCTTTCTCCTCCAGCAATCGCATGGCATCCGGTCCAGCTCCTGCTCCAGCA
TCCGCAGCCGCTCCGTTGTTTGTGCTCAGGAATTGGGCGGCCATCATGTTCACCTGCGTATTGTAAG
>dme_circ_0004243_junction_seq
GCCATCATGTTCACCTGCGTATTGTAAGCACCACCCTGAGTCTCACCGCGCACAAA
>dme_circ_0004244
GTTTTACAAAGCTCGACGAAATGATTTCATCCGAATTTCCCAGCCCTCGTCGACCTCTGCTGACCGTCTA
ACAATTTCCCGGGATCGTTTGCATTGTTGCGGTAAATTGGTAAAATTTACATACCATTGGCTTTTATTGT
TTATGTATCGCTGGCATCATCATGAGCTTGCTTGATGATAGCTTTAAAATTGCGGCGATTAAAATGTCAG
TTGTTGTAGCCACTCCACTCTTTCGGATTTGCTTTTACTTTAATTGGTTTGCTCAGCTCTCAAAATAACA
CTTATTATTGACATTTTCTAATTATTCAACTGTTTAGCAGTGCTTTCGTTTCGCACGATTATTGATTTGT
TCAGTGAACTGAAAGCCAGTCAATGGACTTACATTGCTTGCTGGTCTGGGCCTTAAAGAAAAGTTGAGGA
ATTAAACTTGAAAATTTATAAATCGACCCATAAACAGGATAATGAAGTGCGAAATAGGTGGAAATATCGG
AGCTTTTACTTAAAATTTAGAATAATTATTAATGTCTTAAAATCGCATGTAAAGGTACCTTGAAGTAACC
ATATTTTAATGTGGTGTTTAAGGGTTTTAAGAAATATATATTTCTTGATTTTGTGCATATACTTTCGTTT
TCTTCGCCTTATGCTCGACCACTTTAAAGCCCATAAACCAAGGGGCACTTCACATTTTGACCATAAATAA

GAGAGCGCACCGGCATTTAAGTATTTCGCTCGAAAAAATGTGCGGAAAATGGAAAACTACTGGCTGCGCC
CGCTCGACAGCACCTGGCTTTAATGGGTTTTACCCACCATATGCTTGATTTATGCGGCAAAGAAAAGTCG
AAAGGAAATCTTCGATGCATCTGCTGTTTGTCCGATAACTCGGCTACCAGCCGCCTTTTGGCCAAACTTC
GAGCTTCTGTTAACATAGAATCGTCATGTAATAGTTTGTCACTGGATTTCGTTGTTGGTTCGGCATAATG
ACAGCTGTGCGACATGTCATGGGGTATCGGGCGGGATAGCAGAATACCAGGATAACAGAAATTCGAGGAG
CAGTGGGGGCGTCGGCCATATGCAGAGTCACATATTGATGATGTGCGTCAAATGTCGCTGCTGTTGCTGT
CAATTTTGTGGGCCACAATGGCGGGGGAGTCCCCCTTTATCCGCACCACTTGTTTGCCAGTGTTTTCCCC
CCATGGCAGCGGTTGCTCCATTTATGTTGCCcaggggtcaggggtcaggggtcaACTGCTGTTGCTGCAT
CTATCTATTGTAGCAGTTGATGTTGTTGCTGCAGCTTGTTTGACAAATTGATCGCGTGATATGTTTGCCC
AATGCTGTCATTGTTGTGCCATAGTGGCCGCATAGTTGTTGCACCCATTGTTTGCTGCCGCACATGCTTA
TATTCGAAGTTGCATTAGTTTACACTACACTGCATAGAGTGGTCCACACCCAGTGCATCTCGTATGTACA
TACATGCATGCATTCTGTGCTCGAAATTGCTTTTGATGTAAATCGAATCCTTTGTGTCCGGCAAATAGCA
AAAGCCGAGATGTAACAACTCCAAATTGGTAAGCCCTTGTTGGACAACTTTGTAACGTACTTTTGATTGG
GCTCAGCTCAGGACATTCTACATCTTCATTCCTTTGCCAGATTTCACATATGCATAATCGAGAAGCGTCG
TCCTCCATCATTCGTTGGTTATGAGTGAAGGGGCGAGTCCTTCGCATTCTGCGAGTCCTCGGAGTCCTGG
CAACGTCAATTCGTTAACAGCTCCGTCAGCTTTGCTGCTAATTTTGTTTGCCTTGTGTCGCTACCCAGAA
AACCCGGGCATCCATAGTCACAGTGCTGGTTGAAAGTATGTGCACGCTTTGTGGAGTCCGAGTGGTACGT
TCATCCCCACATACTCACTCCCTcccccccccACCACCCCTCACTAGTCGTTGTCGTCTGTCTTTGTTTG
CCCTGCACTCCAAACACTGCAGACAAAATGCTGTCGAAAATGTTGCCGCCTGATGTCTCTATATACAGCA
GCAGCTCTATACAGCAGCGTTCTACTCCATCCAGTTAATCCGCCTAACGTTATGCAACAAAGTTTTTGTG
GAAAATTAATTAATTTCATGTGCAAACAGTTGTTAGATTTTTCGTTTTCACCTCTCTTACTACGCTCAAC
TGTTTAATTGGCTTAAAAACATTTTTGCTGCTCTCTATTCTAATTAAATCTCCGCTTTGCAGGATTCCTC
TACAAATAGTACACATGTGCCAACCCAAAAGCTGAGCGGTTTCGGACGGAATAAACGTCAAACAGGAAAA
TCCATACCATCGAATCCTTGGATAAGTGATGAAGTCATGAGACCGACATTCGATCGACGCCTCTGGATAT
TATTGTTCATTATTTCAGTACAAT
>dme_circ_0004244_junction_seq
ATATTATTGTTCATTATTTCAGTACAATGTTTTACAAAGCTCGACGAAATGATTTC
>dme_circ_0004245
GGTACCACCTACACACAACCACAAAATATCAATGCCAAGTCAGTGCAGAAGCCAAAGCCCTCCGAGCGTC
GAGAAGTCATCAGCACGAGCAGCTCCAGCAGTCACAGTTGGCCGAGCATATCAGCCGAGCCGGCCGACGA
GGTAGTTGACCATCGCGGCGGCGGTAAGCCGGCAAAATGCGCCAAAAACTGCCAAAAGGCCAAGGCCAAG
TGGGCCAAGTGGCGGACACAACTGAAGCCACATCACCAAGCCCACCGCGCTGTGCAGCACAAGGAGGCCA
GGCAACGGCAGCGCCGCGAGACGGAGGAGGATGAGCTGGACGCGGTTCTGCGAGCACCCAGCTCCACGAG
CACCTCCACCGCAGTGGCCACCACAGTCCAGGCCACTAGCTCCAGCTCCTCGGCCACCAGGAGCTCGGTC
ATCAATGAAACCAAGGCGAAAAGGCCGCGATCCACGCTCCATTTGGCGCCGGAGGATATGCAGCCGAAGC
CCAAGGAGCCGGTGATCATCATCGACGACGTGGAGGAGTTCGATAGTGGCACCAGCACCAGTGACCTAAT
CGCGCGCAAGAGCCGCGAGGAGGCGGAGGAGGAGGAGGACGAAGGTCCGCTGGAGCCCAGAGTACTGCCC
CTGCGACCAGTGCCTCCAAATCCCTACGAGGCGGAGGAAATGTCCGTGGTCTATGCGGAGCAGCACTCCG
AGATCAAGCTGATGTGCGAGGTGGATTTGGATATTGCCACCAGCATGTGGTACAAGAATGGCCAGGGTGA
GTTGGGCATCGGGACAGTTCTTGCCCAAGAGTTTACCCCTCGTTTACAGCTTAAGCTCCTGCGAAGAGCC
TCTCAGCTGTCAGAAGCTGCCAACTTGCGCTGACTTTAATTGCATTCTTAGCCTTCTGCATTCTTTTGCC
CACTTCCCGTCTTTATTCTTTGTTTTTCCCTCCgtctgtctcactctctgtccatctgtctgtctgtctg
tctggggttgggctggttggctgtctggttggctcgctggctCCTCCTGGCTTTATTCCTATTCTGGCCG
AGACGTCTCGAGTGGCAGGGCTTTTGCTCTGTGTCTGACTCTGGTCCTTTGTCGCTTAACTGCCGCATTA

TGCAAATAGGATAATCGTTATTATTTGATAGTTTCTGATGTCAAAAGCCCCGACACAGAGCTCATCTTTC
GAGTGCCGCATTTTTGCTCGATTGCCTCATCATGCAGCTACGAGGGCTTCTAATTACGACTTTTTACCAT
GCCAGAACGCCCACAACTTGGCTACAAATGATGGCTGTAATGGAATTTTCTCTCTGACaaacacaaacaa
aaaaaaaaacaacaacaaaaaaaatcaaaactgaaaacaCGAGGTAAAAGTTAACCCGCCAGCAAAGCCGA
AAGTTATTGAGTTGTAATTGCAAACGCAAACAGTTGCACAAACCTCTCGGCGTAATCCAATTATAACAGT
TTCCCCGAGTGCTAAGAATGTTTTCCATACACTTTTTTTGCACAGCAATATTAGTACGTATATACCTTAA
ATTGTTGTAACACAAGTGGTATCTTCAATAGTGCTGCGGGCAGATATTGTTTAAGATGGTATGGAACAAA
CGATCAGCAAATATAGAATTTAGTTTTCATTCTACCCTGTGTTCATTAATTTATAGCTTACGTAGATATA
AATGTAACACTTGTTTGAAATAAACCCTAAAATCCTAAAGCCAAACGCGAATTCTTAAGAACACTTATTA
TGCCAGCATTCATTCGTTCAACAAGAAGCAGATCTACTTCGAGTTCCAGAAACCGACAGCACTGATCCGC
ATGCCATGAACACTTGCAGGGCAAAGTTGGTAGGTGCAAATATGTGTGGGCCATAACCGAGGTACTCTCC
TGGGCAGGTGTAAATACAACAAGCTGGCAGGCCAAGTTGTTGATGGGCAAATAATAGTTGCAACTAACAC
GGCAGCAACAGCAATGCCACTTTGTGCTCGTGTCGGAGCTGTTTGTTGTTGATGCTGCCGCTGGTGTTGC
TGTTGTCTGTGGCAGTTGACTGGCTGCTATTGCCACTGCACTTGCAACAtgcatcctgcatcctgcatcc
tgcatcctgcagcgtgcatgctgcaACTAGCAACTCGACGCGTGTCTGGTGGCGAAGAGTTGCATACTTG
TGGGCAAAACTGGCTAACATTGTTGGAGCCGCAAGCTGCGATTGTTTGCCTACTTGTTGTTATTTGCATT
TGCATGCCATATTCCGTTGGAAGCCACTAAATTGCCTGTCAGCAGTTTGAACTGGGGATTCGCAGTCGCG
TCAGCGTTGTGATTGCATTTGCATTATAATCTTCCttttgtttttttttttCGGCGAGAAATGTGTCAC
ATCCTTGGCTAGGAGCAAACGTACAGGGTATCCCTAACCGCGTTGTCTTCTCAGGCAATTTGTTGGCTTT
ATAGTGCCATATTTTTCACCCAGCGACAATTGCCAGACATAAACCTTAGCCCAGGCAACAGAGTCAACGG
AAATAGGAGTTTTATGGCAGCAGCCGCCTTGTTGCACACTTTGTGGCGCTGGCAAGCTTATTTATGGCCC
TGGTCTCACGTTTAAGGTCAACAACCCGCAAACCCGCCTGCTGGCTGCCATGAATCCCAGCCAGGCTTGC
ACCGATAAGCCTCCATTAGTCAATTAGGGGCGAACTTAAGACGCAGCATTAATGGGCTAGTTGCTGGCTG
CTCGGTTGCTCGGTTCCCCGGAGTCCTGATGCCTTGAAGTTGCAAGTGCAACTGAGAGCGTAAATCACAT
CAAGCGGGCAAATTAATCAGTCAGCCTCAGAAATCCCAAATGAAAATGAGCGACAATGCGGTGGCACTTA
GCGGGGAAAGTCGGGGGTAGTGGAAAAGTCGGGAAAGCGGGGCTTAAGTTGCTAACACGCCGCCTGCTTT
GTACCTTTGACAAGTTTATTAAGTTAGTTACAATGTCGACGCTGGTCGACGGCTGTTCCTGCCAACGCTG
AGTGCTCCGATGGCAAATAAAATCAACGCCAATGCAGCGGGAGCAGCAGGATCTCGCATCCATCCCATCC
AGTGTATCTCGCATCTTCGTCTGCCATCGCAGCACATTTCCCAGCGGTGCAATTGTATTAGTTGTGTTAA
AACAAAAGCCAGCCATGTGTAAGCGCCGAAGAAGAAGAGAACGAGGACGCGGATATGGATGTGGATGGGG
ATGAGAATGGGGAGGAGGACGAGGACGAGGCTGGGGCATCCTGGAATGGGAGATAAAACAACAGTAACGA
GCAGACTGCGGCAATTATAACAGTGATGCGGCAACAATGTGTGAGAGTGTGTTGCAGGAAGGGAGTCATT
ACAGAATGTTAGCACATCGTCACCTGAATGCCCTTTTCTTCTCGGAAAGTTCCTCTGCAATAAATGTTAA
ACAGTTAAAAATGTTCAAACGTCAGGAGCAAAATTATGGGAATGGCAATAATAACAATATATTATTGTAA
TTCAATAGCAACAACTACGAATTATGAGCAGAATAAGTTTATCTTATATTCAATTATTCTGTAATGATCG
TATAGTAAGATAGTTTCATATTAATACGAACTCGATCTGCAAACTACGAAGATAACTAACAAAAAGCTTA
GAGTTAAATATTATTGATACAAAAGAACTTACTGAAAATAAACTGAAGTCTTACAAAAGAACACTAAATA
GAGAGCAAGAAGAAGCCGATAATAAATGATACCGAATTACCTCAGAGCAGCATACAGAAGAAACCCCGCA
GCAACGCGTTGCAAGTTGAATTGGAGCCAAGGCTTTAAGTTCCCTGGCCTGCAGAAatacatatatatat
atatatatatatatatatatatatCTCGTGATTATCCCCAGTAGCTCCATGTGCCCATTGACTTTCGTCT
TTCGCTCCCGCGCCGCACACTCCTACTCCAACTGCAAAACCCATTGCATTGCAACTGCGGCAATGACATG
CGACAAGTGTTGCACTGACAGCTTGCAACATGAGCGACTTTTGTTGTACTTTCTAGAGCGTTGCACAATG
CATTAGAGTCCCCGAGCGGGAATAACAGCTAAAGGAGTTCATCCCATCCAGGATGCCGGGATACGAGGAT
GCAGGACCTCTGCCTCCCACTTCTGGAGTCTGCTTGTGTGAGTGCCAAGGAATTTATTGTTGAAAATTAT

CAAGAGGCTAATATATTTGCCGAAAGCAATTTCAAGTTAGTAGCTGTGGGTCGGGCAAGGATCAGGACGA
GTATGGGTTGCGAGAACAGCAGGATGGGGGGCCAGATAGCGCGACAGTCAGTGAGCAATGTTGGCTGACA
AGATTTTTGCAATTCCGAGCCAGTGATAGATATAAGTAGCCATTTCTTCTGCTCCTTGGGATGCAGTTG
CTCCCCTCGAAGGAAATAAGTGTCTGTTTGGAATGGTTGCAGCTGAAGGGAAGTCTCTTTCTTCAGCAGC
AGCAGCAGCATGAAGCTAATTGTCGACGAATCTCAATGAAGTGAGTACCCaaaaaaaaaaaGAAAACGTC
ATTTATTATATTTTCCATTAAATGAGAGGATTGTGCATGTTACAGCTAAAATAAAATTGCGAAAAATGCA
CAATATAAATTGTCATGCATTATTCATGAAAAAGGAGTTGGGAGGCAAAAAATTAAATGTAAATCGGCCG
AATCACCGAAAAGTTTGCCAACAATGCAATTAGAGAATTACAGAACCCTCAACTGTCCACCGATAACGTG
GCCACAAATTATGCAAAGTTATGCGTGTGTGCGCCTTCAAAATATTCAGCAATTTATGCGAATCGACGCA
GCTGACAACACACACTACGCCCGCTTTTGCGTCCTGGAAAAACAAAAGCGTCTGAATTCATAATTACCCG
AAAACAGCACTGAAATCCCAGCCAGAGGATCGCCAGCGATGCCATCGATGCCAACAATGGGAGGCACGAA
ACAATCGAGCATGTAATCCGGCCCAAACGCTCGCCGCTGTAAACAAGGATTTGCCACGCTCATTTAACGC
TTTTGTCCGGCGGCCATGCTGCCAAACCAGAAAAATCCAACACGACCAGGTGCCAAAGGCCTGGTCAGCG
GACACTGGACAGTGGACTCGGGACTATGGACGCCAGTTGGAGTGGAAGTGGATGCAGTGGATGCCAAAAA
GCCTCGGATCGCCATCGACCTCATAAATTTCCAACTGAATGCCCCGCTGCACAGAGGCACGCAAAATCCA
GTTTCCCCTTTGCCATTCGCCAGTTGGCCAATGCCAGTTTGCCATTTTGCCAGTTGCCAGTCAAATGCGC
CGTTGGTTAATAAATTTTCTGCAAATGGCCGTTGCGGCGACTGCGACAAATGCGCGCCGGAAGTTGCCTC
ACGCACACATTGATTCGCGGGTAGCGGTCTGCGGGTGATGTTGCCACATTTTTGGGAAGCGCAATTCATA
TACGCATAAATATTTGCACTGCATTTGAAAAATATACACACCAAAAACGGATCGTAAGCTGTAACAAAAA
CGGGAAAAACACACAAACGGAACCGGAAGTGCACCATACAAATATTCTGTTTACAGATTTTTTACGAATT
TTGTATCCTATGACTTTTGGAACTTTTTTCCCGTTCCATTTTGCCTCCTTTTGTAATTTAATTTAATTTT
TGCTTAAAATTAAAAGTTATAGTGGTCAGTTTTTCGCCTTTCTTGCTTTAAAAAAATGAACTAAACTCAA
AGTTCTCAAAAAATATAAACCTTTTAAGGTTCTGAATACAGATAAATACTTATAAATACATAAAATAGAA
AGTTCTATTCACCTTGCACTCAAAACTAAATTTGCAGTATTTATGATGTTGCTTTGGCACCCATGTGGAT
GTAGATACCCTGTAGAGTTGGGAGTGTATAGCTTGGTGATTTAATGCTTCTCGGACAGTACTCATTTTTT
TCGGACCTCCTTAATCCGAGCCCAAAAGTTAATCTCTCCGCTGGCAAATGCAATTAAAATGGTGTTTCAT
TTATGGCCGCCCTCTGCTGACCGCTCTCCTTTTTGGCCCAACATTTAATTGGCACTTAAACGCATTAAGC
GTGCGAAACATTTCCacatatatttattcatatatgcaggatatatatattttctgtttttttgcattct
tcataatttTTACCCGCGCTATGCGGCTAAAGCATTTGAATGCGCACATAAATTTCCGTTTGCATTAAGC
CCGCCggcagacagacggacagatagacagacagacagacagacagacgtacagtcaggcagccagTTGG
CCAACAACTATTGGTAACAGTTTTTAAGCCGCCAGTTCATTGTTGCCACATTACCAGTGGATAACACATG
GCTGGAGTTGGTGCAGTGGGTGCTGCTGGTTCGGACTTTCGTCCTCATTTAATTGTCATTACGAGTATTG
TGTTTGTTTGAAGCGAAATGTAATAAAATTCCAGCGAAGCCCAAGTGTAAATAAATGGATATGAAAACAT
TAAATGCAATTGTTTCGGCCCGCCAAGTGGCGCGTTGAGGGGCGGATTGCATTAAATGACTGAATTTACG
GCCATTTAATGCGCTGCATTAAAAAGTTTCCAAAGGCGCTTTGCATAATTCCGCCGAATGACATTAGTTT
TAACGCCGCACAAACTTATCCCACCATCGTTTTCTGCATTTTCCAGGGTGAGTATATCTACCTACTCCTG
CATCCTGCTGCCCAAACAGCTTAGACAAATATTGTGGGGGTCGTTGTTCCATTTGCCAAATTAGCCCGGG
AAGAGGCCAAGTCAGAAATGGTTTGACGTGGCGCTTGGCAAAATGCTCGCTTTTATGTCCATGGCATCGT
CACCGAAAGCAGAAAGCGGTCATGAAATTAAGATAAGGTCATGTTCTCACAATGGTAAAGTGCGCAATGG
TGTTTAAACTTAAAGGAGTTCAGATCTCAGGGAATAACACTAGCTTAACACGAAGCGCTCCTTTCATTTT
TAATTCGATCATTTTAGGTAACAATAAGCTTAAGCTTAAAAGGCAGAATAACTTCTTTTATGTATTCAAA
ATCCGTTAAGAATAAAGTACATATGGCGAAATGGATTTATATTTAATTGAATGTTTGCTCCTTTCCACCA
CCCCCTCCTTTAGGATCGACCCAAGCCACCGTATTTGCTGATCGACTCCCGCCGCCTGGATGCCAGCAAT
TTGTTCGTGCCGGTGAAGGAGAACTCGGAGCTAAACCTGGCCTGCGTCAGCGAAGGTGGAAATCCACGAC

CCACGCTCACCTGGGAGGTGCTCCTCAGTCCGGGCGTAGACCGGCATGCCCAAAAGGTGTCCGCCGAGGT
GCTGGAGCTGGAGGAGATCAAGGGCGAGAAGGGTGAGACGAGGGCGCATTGAACCCGATGAGTGACAAGT
GGCGGGTTTGTCCGTGTGGGGATTGGTAATGGCTGGCCGGAGTTGGAGTGGAAAAACCACAGCATCGCCG
GACGGCCGGCAAACACATTAGGTGTGGCCAACAACGGCGGCTCCCGGCTCAAACAACGGCTCTTCCGGAC
GACTGACTGAACCCAGTTTGCGGTTGTGCCAAAATTTGTTTGTCTTGCACTCGAGCATAATGAAAAGCAG
ACATTTCGGTCGAGCAATGCAGAAGTTCAGTCGTTtgtgtgtgagtgtgtgtgtTGGGCTCTAACACCCA
GTTTTCCACCCACACTTCCACCTGTCAAacacacacactcacacacaGTGGCCACGTCTTGTGGAGGAGA
GAGAGTGTTTGTCGCTCGGACCCTAAATGCCACAAATTTGCGTGTTCGGCTGGTCACAAACCCAGCCCAC
AACCACTCCGTACACCCCCGATGATTGATTGAAAACTGTGCCAACACAATTTCCATGCTAAAGCCGCAGT
GGGCGACCGACTTGTGGGTGGGTCGATTGTGGGTGGGCGCCCTTGCCCTTGGACCTTAATTTAAATTAAT
GATGTCGCGCTGATAACAGCCCTGACTAATTGGCCGCCCGAACAGCCGTCTGTCATTTGGCCCAATTTGC
CAGTGTTTAGCATACAATTTGAATGGAAATTTTATCAGACTTCCCCCCTCTCCGCGCAGGCTCGACAAAG
ATGGCTACAAAATCAATAGCGGTGCAAAGAGCGAAGCCCGATTACCGGCGGTCTATAGGGCGCATCATAA
CGCCAGGATCCTCTGCGTCATGGAGCATCCCACGCTGAAGATCCGACAGAATGCATCCCTCCTTCTGGAT
GTGCAATTGTAAGTTTTGATAAGATATATATTATACGCTCCTTTCCACGATTGTTTTATGTTTAACATCC
CATTGTAGCATGCATCTTTGGAATACTTTCCTTAAACTAGACTCCCAGCTTGACCTATTAAATTCAAACC
CACCTCATACCTTTTATTTAAAACTAAATTCTATACAGTTATAACTCACACAAATCCATCCTCAGTTTTA
AAATTAAAAAATTTACAAGGCCCAAATAACAAAAGACACGTGATTTTAGTAAGTCATCATGCTAAGGTCA
ACAACTTTTTAATAAAAGCCAAACAGTGCGCTTGGGTATCCCACCATGTTTAATTACCTTAAGGAGGGCA
AAAAACGAGAGTGGAATGATGTAAATAAAAGTGAAAACCAAAAAGCAAATGTTGATTGAAAAGTTAAATT
GCCAAAGTTAACGCACACCACACAAACCTACGGCCAGAATTCCAAGCTGAGGGCCAGTTTGCTTGGGTCG
AGTATGAACAACAGGGAAAATTATTATGCACCTGCAGTGAGAGAAGTCAGTGGAGTAACTGAAAGCAAAG
GTGAAATTATATTAATTgaaaaagcagaaaaaaagggggaaacaaaaagagaaaTACAATCAAATTGCAA
TGAAACTGGGCGAAGGAAAATGTAAAGAAAAGCGCGACAGTTGAGAAAAGGACGCCTACGTACAATTGAA
ATGCAAGGAACAAAAGCTGAAGGGAAAAGGATGGGAATTAATGGAAAGTCGAGGCACGAGAGAATTTCTG
GACTCTCAATTAGCTTAAAAAATAAGAAGGTGTCGCAGCAAAGGACGAAGGACTCAGCAGTTTTCATATT
CACAGTTGTCCGTACTTATTGGCCTTAAAATTTAAATCTTTTATTAAAGCCTGCGGCTTAAAAGATCGCA
AGAATCAATGGATTCAAGCTTATTAAAGTTAACTTAACTTAGTTATATATAACTATGCCACATCAGCCAG
TTAGTTATCAAATTCAATAGTCCATATGTGTGTGGTCATGTTTAAATGTTCTAAACATTGGGCTTAGCTT
ATCACTGTCATCAACCGAATTCCACCTCAGATGAGTCCCTTCACACAACAATAGAATCACTTTAAACGAC
ACACGGCCAACTCCGTTTCGTGATCAATTGAGGTGGACAATATGCATGCCACTGGCAGCCACAACAAAAC
CGGGAAATAAAGATGGGAGTCCTGTTGGACACCGCAAAAGTGACGCAGCCGGACATGGAATTGGGAATCA
ATGCCAATGGTGGATGGTGGATCGGATCGGATGAGATGAGGCTAGATGAGGTGAGGCGACAATTGGAAGA
CTAGCACTAATTGGCAGCAAAAAGCCGCACACACGTTCTGACCACCAGGACATCAGGATCCAGGCATCGG
GACGCCAGGGCATCGTTCTCGTCCTGAGGAGGGTCTCAATCAGCGCCAGAATCCGGGGAGGACCACAGTT
ATTTGCTTTATTAATGTCCGTGAAGCCGCATAATGCCCTGACAAATGCCCAGGTAGTGGAGAGCCCATGA
AAATGATTGGAAAATATAGAAAAGCCGTGCGGGATTTGCATGCCATACACAGTACTTCACGTTTCCCTGC
AGgtgtgtgtgtgtgtgtgtgttggtgggtgtgcgtgtgtttgtgtatgtgtAGGCTTTGGCTTTGGCCAGG
CATGAAAATTGCAAAAACCGCAATAACAACAGCACAAGGCCTCAAACAATAATAACGGCAGCAACAAGAA
CAACCGCAACGTTTTCCGGGCTCAACAAAAGAAGGAACAAGttcgtcctgattcgtcctgattcgtcctg
TTTTTCCTTTTATTATTCCCACTCCCTTTTTTCGTGTGCGCTTGTATAAATTAAAATGCACACACATGAG
AGCATTTGGCCCAGGCCATAGACATTTGTATCCGCATCCCAGTTGGCAtgtatctgtatctgtatccgta
tctgtatatggaccggtagctgtatctgtaATGCTTGCTGCGTTTAATTTGCGCATTTTTATGCAGACAG
TTGACAGGCAAATCAAGGACACGACGGACAGGGATCATGGCTTCACGAAATGCCGAACAATTTAAGCTGG

AAAAACTGAACTGAAGCGCAGGACCCAAAGGTTTCAGGTTTCCCATTTAAATTAGAGCTTTTTAGGACAA
TATTTTACTTTCAATTTGAGCAATTTGCAAAGATGCTAGTGTGCATATTTCAAACAGACAGCATGTTTCA
AACACATTTTTCCCAGTAATTGCTGCCACTTTGACCGAAAACAATACGCCCAAACAGTTAGACCATTTTC
CAGCCTCAATGTTGTGCATTTTGAGTCAACAAATTGATGGACCAGTTGATGGACTGTTCCACAGCGAGCG
GACGTGTAGGGTATACATATCCCAGTCCATTCATAAATATGTATGATGTCTGACGACTGACGGCTGTCTG
CTGGTCACACAAAATATCGACTGAATACGAAGCCGAAGCGAGGCGAGGCGAATTGAATTGAATTGAATAG
TTTGGCAAACAACCGACAAACAAATTTCATTTGTGTCCCTTGGCATCTGCAGACCCATTCCAGGCGCCTA
ATCAAATTGTATTAGTTACAAAAGGCCACAAAGGCGACTGGTGCGCCAAAAAATTGAAAAGATTGAAATG
AATGCTGGGGCGGCCCATTTGGTAATTTGGATATGTCGATAGCATGAAGCATATTTTGGCCAGGCCAGCC
AAATGGCCAGCACTAATTAAAAAACATTTGATTTATGCCTATGAATATGCATTGAATAGAAGTGCACATG
TGTCTGCCCGCACGGGGCGATCCCATTatttacatttacattcagatttacatttTGTCGATGGCTTTTT
GACATGCACTGAATGGATTTGTGATTGGCAGTTTGGGATTGAAATCGAAGTTATATTTTGGATGGCATAT
CGAATTACTTTCGGACTGAATGAATTGTGGCTGCAACACTGAAATTGGTAGCATAAATTCACTGCCGCTG
ACTAAGTAAAATTCACTATTCACAAAATTGAACGCCATATTAGTTGACACGTGGATCTCTGGTAATTGAA
TGCAGTCAGCTTCAACTGGACAATTAATGAATCAGCCAACTTCAATTAGGGAGGTCAATAGAAGGTGCCA
AAACTAAAGGTTTTTATTATTAGATTTAACATGCGACAGCTAACGGTTTTACGTAttttttttttATGTC
GTTGGAGTTTTTTAATTTGAAAGTTAAATACGAAATTCACTTGCTACTCAAGTGACAATTAACTCATCCT
TATTTACCTTCCACCCAATACAGACAGCACTCTGCGAGCCTAGCATTTGCATTTGGCTTAATTATAAAAG
TTTTCCTTTCTTGCCCCCAGTTCGTACCCCCATCCTTTGAGTAATTTTTTAATTAAAAAACTATTTTGTT
GATGTTGCCACAGTCTAGTCGCTGATAGCTCCCAAAATCTGCAGCAAAGTCAAGCCGAAATAACCATCTG
TCCCCCTTGCAGGGTAAGTTGGGTTGCTCCATAAATGGATGCACAGGGAAAAGATTGGGACACTTTTGAA
TCCATAGGCTCTGAAAATATTATGGTTTCTCCTAAAACCATGCACATTTTAGAATAATACATTTAATGCC
TCTTGAAGTATAATATATTTTCATTTTAATTTGTTGTTGGTGCGCTTTGTCACTACGCGTATTGCGTTCA
CTGCGCTTGTACTTACCAAAAAGTTTTAGGTCTTGATTTAGCACCTACATTTTATGTGTTTATCATTTCA
AATTAACTCAATTTTTTTCCCCGTGTAAGGTGCGTGGAAGCCTCGTGCATTCGTAGGTCGCCCAACGGAG
CTCGGCCTTTTGTGTGATTTTTGGCTGTGGAGCAAGTTTTAAATGGATTTTCGTGCAGGCCGAGAAACTT
GTGCGAAACTATTTGCCTTACCTTAATTACCAAAAATTGTTTACGCATAACGAGCCAAACTCATTTCCCC
ACTTGCCACTAAcccccccccccccccccccTAACCCAATGCCACACCGAAAGTGCCAACATTCTGATTT
CCCACTTCTCGATCATTGCAGTGTAAGTATCCCCGTGGTTGAAACCACCGAAAAACCCCAGTGAAACCAA
TAAAACCGTAAATTAGACCGCGTATGCGTGAAATTGCCTTAAAAATGGCGCGTGATAATATTTCGCCCAT
TAGTTTGTGCCTGTAAAATTACACGTAACATCATCGCTCCTACCCCaaaaaaaattcacaaaaaaaagaa
gaaaaaCGATGAACAGAAATCGGCAAACATTTTACGACACCCGCACATGGCGCTCAACGGAGGACAATAT
TCGGTGTGATTTTTCGGGGATGTTCTTGCCACTTTGCGAGGCGCCTTGTGGCGCCACCCTCTGTCATATG
CAAAATGTGGCAAATTTcattggcattggcattggcattgACTGTGTGGGTGGCTGTCTCCCCCTCACTT
TTCCTTTACTATTTATGCTTACGCGCCATTTTGCTGTCAATATTTTCTCGTTTGCGGGTTGGGATTTTGG
TCCTTGTTATTTGCCCAGGGTTTGAGGTAAAGAGCACTTTATGCGCTCAGCGCATGTTAATGCTGGTAAA
TATTTTTATTGAATTTGCTACTCTGACAGGCGGTGGCGGCCTAAAAGCCACCATCAAGCCGACCAATCGA
ATTATATAACTCGAGAAGACTCGAAAACTCATTTACAGCCTCATTAGGTGGGCAATTGCAATCGATGGCA
AACGAAGCTGCTCTCAAGGAATCGAAATACTGCACTGCCAGATCCGAAAATCCATCTTCTCAAAAAACCC
CGCTCATCAATCATGACCGCAAAGGGAAAAACCCAACTAGAGAAATAAAATATTGGTAATAACAACTTGA
CAAAGTGTTGCCAtttttttttttATGCCAACGCAGGGATCAGGCTGGTGTTCCATCCTCATCCTCCCCA
GGGATAGATTTTTTCGCCACTCCTGACAGCCGCCTGTCGCTGCTCATTATGGGAAAACAAATGCTTTATT
AAtaaaacaaaacataaaaaaaaaaaaaaTGTGCTGCCCCAAAAAGGCCAAGAAGTGACGGAGACGGCCG
CCTCGAATCGACATTATCGATGATTGATGGGGAGCGCTTGGGAAAAAGTGGGAAAAGAAACTTTTCCTC

TGCACTTTCCAGCGAAATCTAATTGTGAGAGACGCTGAGCTGGCAACATGTTAAGCACATAAATCAATTT
GTAAAACGTTTTATCGAACTCGAAGGGCCCCAAAATGCCGAACTGCCAAAATGGCAAATCCAACTGGCAT
CCAAACCCCATTTGTGACAACAGGCGTTCGTTCTCTATATGTGTACCTATAAGACATAAGATATGGCAGA
AGCACAGACACGACTTGCCTGTCATAAACTATAAAACAGCTGCTAGACCCATGATAAATGGCGGCCGGGT
AGGATTTTTGAGGCAGCCAGAGAATGGCACAAATTTAAGTCGCTGGaaaacccaaaaatataaaaaaaaa
ccgaaaaaaaaCTGATAAATCGCATTTAAGTGAGGGAAGCTACGAGGCACCAGATTCCCAAATCCCAGAC
AGACGTTTGAATGCGGGCTTAATTGCTGACGATATGTTTCCTCCTCCGAATCCTCTGCCATTGAGCTGGA
AAATGGCGAGGAAAAGCGACGGAGGCGAGAGCACAGGTAGCAGAAAAGCAAAGAAAAACTTCGCAGTCGA
CAACAACGAGGACAACGCTGACAAACTGCAATGAAAAAGCAATCAGCGTTGCCCAACCGAACGCGaaaaa
aaaTGAGGGAAAATGGGGAAGCGCAGGGAGAGGATGCGGAACGTGGAATGCGGAGGGCCCACAAAAGTAG
GCAATTTGACGCAGTTGCTCAAACTTTTCTGCGCCGGCCACTTTTGCGTGATTGATTCGCCCGTCTGGAG
AATGGAGGCTTCGACTTTGGCATCCAACCCATAGGAGGGGCTCACCACTCACATCTCGGAACTGAGGACA
CCAGACACGGCGAGCTTGTTAAAACGTCGCTAATTTGACACGTACGTCAACAGGAGAAGCGACGAGCGTG
ACGCCGATAGGCTGGTAGGACTCCAAGGTCCATCGCGCTCTCGTCGCCCTCGTCCTCGTCCTTCAAGTGG
GCCACGTTCAGGTATCACATAAGCCACGGCCATTGACCAAAGTTCTGTTTGCCAGCCTCATTAATGAGTT
AAAGTTCTCATTGGGGCTAATTAATGTTGAGGGCCTTGCACGCCGTTTGCTCCAACAATGTGCAATTAGT
GAAAGTTTCCCAACTTGAGCAGTTTATATGGCAAACAGGTCCTTCACAAAACTCACATAGCGAGTGTGCG
GCAGCAAATCGAGAGCCGCTTTTAATTGTGGGTTTATTACTTTGACTTTGACTACGCAGCAACATGAGGA
TTTACAGGATTAAATGTGGGACAAAATGAAAACAACGCTTATGTTTAATGGCGTAATAAGATTAGTGTTT
TCTAGGTACTTTAATCCTATTCAGAAACGTCGTAAATTTGAACTTAAGAACCCAAACGAAAGAATAGTTT
GATGCACATATAGCTTGAGAGTAGTCTTGGCGGTCTCCCAGGACAATTGAAATCACCTCCCAAATTACCT
AATTCCCCAGGGTCACTTTTGCGGCCAAGCATTGCCATGCCGAAATCCATGTGCAAATTGCATTCGCCTC
GCAGTGAACTAATTTGCCCAGCCAACTGTTAAGAAAATGCACCACCAAGACCACAAGGACTCTGGCACAT
TGTCAACCACTCAGGAGAAGTGGCAAACAGCAGGCCCACATCCTTTGTGGATGCGGTAGCCAAACGAGTC
CCAGTGGATGCAGGACATTCGAGCCGTGCCGAAACGAAGGCGAATTACAATAAAATCTAATTCTACACGC
TGTCGTAAGCATTTCGATGTCGAGCCATTTCTTACGCCCCAACTGCGCCGATTTCGGGAGTCCTGGGCCA
TGTTATCCATTATCCTTTGTCATGCTGGTTATCGCGGTTTAACTTGATTGTTTATTTCGCCCTCATCCTG
GCGAGAGCTCCCATGTCCTTTGGCCAACTTGAATTTCTGTGTTTATCGGCGCGCACGATTATTAGttttt
ttttttttCTTTCTTTGTTTGCTGCGCCTTTGTTTTTATAGCCGGCGATTATGTTTCGGTATGTGTTGAG
GGTCGCTCGTCCTTCGGCGAGAAGATTTTCAATAAAATTTTATTTGTCCTTGCCCCAGATGAAATCGAAA
CTCATTTCGGCTGGGCGCCTTCGCTTCTGGACAACTTGAGCAAATATTAAAAACTCATTGCGGACAAATG
TCTTGCCGTAAAGTAttttttttATGGCCGCAAAGTTTTCTCCAACATAAACGGAAATTTGTCGAGAGAT
TATCTTTTATGGATATGAGTGCCTTTCGCCCCACCAATTTGCATCGGGCCCCTGCTCGAAATGAACCTTT
TATGTTTCGGTATCTAGAGTTTTTGCATCCACTGCAATCATCTTCAATTTCAACTCATTGCAAATTAATC
GCGTGTTCGCTGAAAGTCCTGGAAATTCCTCGGGCTAgggggggggCGAATTAAAATTTATAACCAAGACA
CAAATGCAATCACAGCAGCCCGAGAGTTTACTCTCAGCCGCTTCCGCTGGCTGTTTATGCAGCTTGTAAG
TTAAGCTGATTGTATTCCGCGATGCTGATGATGATGATGCTGCTGCATCTCGTCGGCCTCCTTCATATTC
ATGACTAACTGCGAAGATGAAGAGCCAGCGGAAAAAAATGCAAATTAATCTTGGGCGTGGACCAGGGGGTG
CTAATGAAAATTGCATGCGAAAAAAAGTGGATAAAGTACCCATCGCTGGGAGTGAGAATTTAATTTTCCA
CCCACCTATGGGCATTCCACCTCGCCATCGAGAGTGAAACTTTGCAGTTCGTTGAAGGATTTATGGATCA
CGCCGGTCCGTCATATTCACACTTTGGACAGGGGACGGGATGTGTCGACACATCGATACACTCTATACGG
CAAAGGACTCGGCACTTCTCATGGGATCCCTGAAAGTGACAGGCCACcccaaccattcaaccacccaacc
atcaagccatccaaccatccaactacccaTTCAGCCAGAGTTCTTCTTGAAGGCGAAACGCCAACATGGC
TGATGATGATGCGTTATGATTAATGATTGGTGGCCAGAATCGGGCAAAACTCCTCAGTCTGGGCCCGGCA

TATTGATTCCCATTGTTTGCATTAGTGTTTACTGTTTGCCCTTCATATTTGCCTTTGTTATGAGTTGGGA
TTTATTCGAGTGTGCCCCCGCCACCCGAAAGATGTGCGGCAAGAATCACTTTCTCCCCATCTTTTCAAGG
ACCTTTCTCTCTCAATCTCCTTCAGTGTCGCCATATGGGCGGTACTCGATGCCCATTTGTTTGCCCTTGT
TTATTTGTTTTCCTTTGCTTCTTTTTCCCCCCaaaaaaaaGTGGTCGGGCGCTTTTATGAATGGCCATTC
AGTAAAATATGCGTGAAACAAGTGCCTAAATTATGAATGCGAGCGTGTGTGTGTGGAAAAACTTGGGAAA
AATATTGAAAGAGTTCCATTTGCTGGCGGGGAAGGCGTGGAAGGGAATCGATTTCTAAGCCGCGTACATC
GAGAGTGGGAGCGTCTAAGTGTTTCATTTAATAATGCTCTTTTGACAGTCTGCACCCATACTTTTGCTTA
CTCTTTTGTCATCTGTATAAAAGGATGTTTCTAGCGTACTTTCACTGCaaaaaaaaaaaaaaGGAATAAGG
TGTAGTGGAACTGTTCCAAAAGCCGCTGCAAATAATAAAGTATATTTCTTAAATGTCTACTAATATGCCA
GGATATGCTTTAAGTTAATGGTTAAATTTTTAGGAATTTTTTCTCTCCCACTCAAACTGTGTTATAAATA
TTTTTCTTATCGCTTCGCCGGAAAACACGCCTGCAGCTTTGGCCAAATGACAAATGTCTGTCAGTTGAGT
CGAGACGAGCTGAGCTCATTAAACATTTTCGCTAGCTTTTCGAGGCGGAGTACGCAGAGAGGGCTCTTAT
TTCAGTCACCAGAATACGAGTACCAGGACTCGTACTGTCCGATCCCCTGCTGGCCATTGAAGTTGTCAAT
TCGAACGACGTCGCCTTTGTCCGCCTTTGTGTTGGGCAATAAAAAATTTTATAACTACGTGATTTTGACT
AAGTAACACGCCCTTCGTCCTGCGAGGGGAGGACTTTTGCCATTGTCAGCAATAAAATCCTCGCTCGTGG
AGGGTTGATTGACATGCGACGAAGGGTCTCTGCAGGCAAAAGGAGCTTACAATTAATTGTCTCTCACTCA
CTCACTCAACAACTCACTCTCGAAAAGGGTCCTTGAAAAATCTCAGTCCTCAGACGCGTAACTTTGGGGT
TTCGCCTTCGATTAGTGCGGCTCGCCCGGTAATTAGTAGTTTCACTTAATGGTGCGGCTCACTTTTGGGT
CTGCTAATTTGCCGTTTGCCCTAAGGAGCATTTTTGAGGCAAAATTCCCTGGGTTTCTCGCCAAGATCCC
CCGCCATTGTGGCCAACATCGCttttttttCATCTCCACCAGGTCGCCGCCACTTGGCAACTTATTGTTA
TGATATCCGGTGGCAGACAAAGGACTCGCTACCTGGCCGCATACTTTTGGGCACATGTCAGGGCAAATTG
TTATTTATACGGTAATATGAATAGCGTCATAAAGTACGCCTCACAAAGGACGCCCTGAAATTTAAAGCCA
TCTTGCTGGCAAGGAAAGTGGAAAATATGCGAGTGTGTGCTCCACCCCGTCTTGGAAATTAGAATGGAGC
ATCGTTATGGCTTCTATGGCCCACTACCCCAACTACCCCAGTCCGCCACAATCCTCCGCCCGCCGGAAAC
GAGCATAAAATATATGTATTTATGAATTTGCGGCAAAGAAGATTGCAGCTCAGGCAATTTTCCAGCTAGC
TGAGCAGCGATTTTCACTCTTAGTTGCCGTCAAAGCGGAAGTGACAACTCATTGCGGAGTGACTCAGGTG
TTCGCATCGCGTCAGCAATTTGTTGCTGGCTCTGTCATTGAAATATTGTCACATTTGACAATTGCCAACA
GCCCTCGGATTTGCACGGCCACAAAATTGCCACAATTTCATGAAAAATTCAAAGAATTTTTTTCACTTTA
AGGGTGTTTTTATCTGTGCAGAAAAGTACCTAATAATGGTAATAATTTTATAAATACCATGGCTCTTGTA
TAATGTATAACTGCTCACTTGACCACTTTTGTTTTTCTGTGTACTATATGCTTAGGCCACATGTGGCCTG
GAGTTTGCAGCCCTAACTGACGGCTTCTGCTTTGCGTACTTTATCTTTGCAGGGTAAGCGTAAGCCGGAT
GGCCAACAACCCAACTCCTAACTTCCGTTGGCCCCAAAAACTTCAAAGGATGTGGGCGTCGCAAAAAGTG
CCGAATTCGTTTGTGCCCATTTAGAATGACAAATGCGTCTGGGCTGTAAttacattttacattttacatt
tCGTTCGATCGAAACGACGAGTCCACGGCCTTTTGTGCGCCTCTTCATTAATTAGCGCAATTGGAAATTA
ATTATGCGTGCAATTTACTTTGCTCACTTTTCGGCCAAATGGCGAGCATAGACAAATGGTTCGGCGTCTG
TGGCAGTGACGCATCAATTAAAGAAATTAAAACTTGCGTGTCGAGTTCCTTGGAAGTCAATTAATTAAAT
CATGCCCTCGGCTTTCATGTTCCTCCCTCGTTCTAGGGCTCCCTGGGCTGCTGGTCGCATTTGGACCCCA
TTTCGGCCCGGTTACGTGGCCTTGGCTACGGCAGCAGTCAGCCCACCGGCCAGTTCTCGCTCAAGGACGT
CATGTACCAGGATGCGGGCATGTACAAGTGTGTGGGCCAGTCGCCCACCAACAAGAAGAAGCTGGAGGTC
CTCCAGAGCGTTACAGTCTCCGTGAAGG

>dme_circ_0004245_junction_seq

CTCCAGAGCGTTACAGTCTCCGTGAAGGGGTACCACCTACACACAACCACAAAATA

>dme_circ_0004246

GATCGACCCAAGCCACCGTATTTGCTGATCGACTCCCGCCGCCTGGATGCCAGCAATTTGTTCGTGCCGG

TGAAGGAGAACTCGGAGCTAAACCTGGCCTGCGTCAGCGAAGGTGGAAATCCACGACCCACGCTCACCTG
GGAGGTGCTCCTCAGTCCGGGCGTAGACCGGCATGCCCAAAAGGTGTCCGCCGAGGTGCTGGAGCTGGAG
GAGATCAAGGGCGAGAAGGGTGAGACGAGGGCGCATTGAACCCGATGAGTGACAAGTGGCGGGTTTGTCC
GTGTGGGGATTGGTAATGGCTGGCCGGAGTTGGAGTGGAAAAACCACAGCATCGCCGGACGGCCGGCAAA
CACATTAGGTGTGGCCAACAACGGCGGCTCCCGGCTCAAACAACGGCTCTTCCGGACGACTGACTGAACC
CAGTTTGCGGTTGTGCCAAAATTTGTTTGTCTTGCACTCGAGCATAATGAAAAGCAGACATTTCGGTCGA
GCAATGCAGAAGTTCAGTCGTTtgtgtgtgagtgtgtgtgtTGGGCTCTAACACCCAGTTTTCCACCCAC
ACTTCCACCTGTCAAacacacacactcacacacaGTGGCCACGTCTTGTGGAGGAGAGAGAGTGTTTGTC
GCTCGGACCCTAAATGCCACAAATTTGCGTGTTCGGCTGGTCACAAACCCAGCCCACAACCACTCCGTAC
ACCCCCGATGATTGATTGAAAACTGTGCCAACACAATTTCCATGCTAAAGCCGCAGTGGGCGACCGACTT
GTGGGTGGGTCGATTGTGGGTGGGCGCCCTTGCCCTTGGACCTTAATTTAAATTAATGATGTCGCGCTGA
TAACAGCCCTGACTAATTGGCCGCCCGAACAGCCGTCTGTCATTTGGCCCAATTTGCCAGTGTTTAGCAT
ACAATTTGAATGGAAATTTTATCAGACTTCCCCCCTCTCCGCGCAGGCTCGACAAAGATGGCTACAAAAT
CAATAGCGGTGCAAAGAGCGAAGCCCGATTACCGGCGGTCTATAGGGCGCATCATAACGCCAGGATCCTC
TGCGTCATGGAGCATCCCACGCTGAAGATCCGACAGAATGCATCCCTCCTTCTGGATGTGCAAT
>dme_circ_0004246_junction_seq
AATGCATCCCTCCTTCTGGATGTGCAATGATCGACCCAAGCCACCGTATTTGCTGA
>dme_circ_0004247
GTTGATTCGGTGGATGTGACCTCCGAGCCGGATGATCAGGATGTTTCCGTGGCAGCTGCCTCGCACAATC
CCAACAAGGGCCAGCTGGAAGTGCAGCTGGGCGGAGCGGTTACCCTGCAGTGCCCTCAAGGTAAGCGTAA
GCCGGATGGCCAACAACCCAACTCCTAACTTCCGTTGGCCCCAAAAACTTCAAAGGATGTGGGCGTCGCA
AAAAGTGCCGAATTCGTTTGTGCCCATTTAGAATGACAAATGCGTCTGGGCTGTAAttacattttacatt
ttacatttCGTTCGATCGAAACGACGAGTCCACGGCCTTTTGTGCGCCTCTTCATTAATTAGCGCAATTG
GAAATTAATTATGCGTGCAATTTACTTTGCTCACTTTTCGGCCAAATGGCGAGCATAGACAAATGGTTCG
GCGTCTGTGGCAGTGACGCATCAATTAAAGAAATTAAAACTTGCGTGTCGAGTTCCTTGGAAGTCAATTA
ATTAAATCATGCCCTCGGCTTTCATGTTCCTCCCTCGTTCTAGGCTCCCTGGGCTGCTGGTCGCATTTGG
ACCCCATTTCGGCCCGGTTACGTGGCCTTGGCTACGGCAGCAGTCAGCCCACCGGCCAGTTCTCGCTCAA
GGACGTCATGTACCAGGATGCGGGCATGTACAAGTGTGTGGGCCAGTCGCCCACCAACAAGAAGAAGCTG
GAGGTCCTCCAGAGCGTTACAGTCTCCGTGAAGG
>dme_circ_0004247_junction_seq
CTCCAGAGCGTTACAGTCTCCGTGAAGGGTTGATTCGGTGGATGTGACCTCCGAGC
>dme_circ_0004248
GACTAGGATTGCGAATATATTGGGCGTGGCCGGCACGGATGTGCCCATCAATGAAATCAAAAAGCTGCTG
TCGCCGTTCACGGTAAGTTGGAATCACTAGAATCCTGGTTATAAACCCTAAAGAATCCATATCTATCCTT
AGCTCGGCGTGAATGGCTATGCGTTTATAGTAACCAACAATGGTTATGTACTATTCCATCCAGACTTTCG
TCCAATTGTAAGTTAAATGCCTTCAAAATCTGTGGATCTTTTGTGTCATACATGGCTTTAAATTCCAGTT
CCAAGGCTACATATTAAAGCCAGCATACAATAGCGTTGACATGATTGAAGTCGAGCTATTGGATGACGAT
CGACCCGCCAGAGACTTTAATCCAGTGCTGATGACGGTAAACCTTTCTTAAAGTCGTCCTCGATGATTTC
AATAACAAATTCTCCGTTCAACAGATAAGAGATTCTATAATCAATCAATCGACAGGCAGCAAATGGATGT
TAGTCAAAAATCATTTCGATGAAATGGTAAGTTCTAAGTGCATATTAGTTCATAGTTATATTCCTAAATT
CGTTCTTAATTAACCTACAGAAACGGGTGGCACGCGTAAAGCGACAATACTACTGGACGGCCATCAAGAA
GACGCCCTTTACTCTGGTCATTTCCTATCCGGAGCAATACGGCGTGAGTCACATGGACATCCGAGCTGAC
CAGGAGATCCATCGCATAAGCATAAAGGGCACAAACTTGCGCAGCGTTTTCAGCGGCAAGCGGTGGAAAA
TTCATCCCGACTGGGCTATTTTGTAAGCACAGCAACAGGACATTCAAAACGCCAGAGATCGAACTGCTGT

ACTTTCTCGAGCGTATGTCTGAGCCTGGCTGGCGATGGCCGGGTAGTCGGAGTGCTATGCCCCCGGAGCA
CGCGGCCGCCATGTTTTTGTAAGTATAGTATAGAACCTccccccccccccACACACCCACTTCCGGTTAC
ATCCATTTCCGATACTCGAATCGATCGCTTCGATAAGCCGCCGCTTTCCCAGACTCTGTTATGCGTTTTT
GTGTTTCGTTGATGTGCGAAAATTACGATTGCGATTTCAATGTTCATGCAAATTCACAGCTAACAACTCA
TCAACCGGCCGTTATCCATCAATCAATGAAAAAGAAAGCTACTATTGTGAGTAACGAATATCAAAAGTCA
TCTTAGTCACAGTTGTGCACCCACAAAAATTAATCACAATCAATCGCACTCAGTAGCACCGTTCTCATTT
CCGACCTACGCACCTGCACATCCGGCGGAAATGCCCGTTGAATTCTGATCCCAGCTCATCTTCAAAACTT
TAGACCAAAAATGAAAGACCCAGTGAATTCTTTAGAATTTCTTTTAAAATTAAAAATCGATCGAAGTTAT
GGATCAAAATGGAGGCTTGCAAACACCAAATTGATTGGACCACTGAAAACCTCCTTTACTTCTATAAGCT
ATCATTTCAATTAGCAAGTAGTTTCTGTAGTTAGCTGCGGAATTTGCACTCAAATCGTTGCATGTCACCG
AGGACTTACTCATTGTTTATGTATCATCTGAAAAGTATGCTCACATTAAGCCAAACATTTTGGAGTAATG
GAGCCAGTAATGCCACTGCAACCATTATTATAACCATGCTGACCACTCATTTTGGATTCTTTTGACGCTG
GAAATTTGATTTTCAGGCGATCGGCAGCTAATGCAGGCTCTGGTCTTCGATGCCCGAGTCACCGGGTGGT
TCTCCAACAATACCAGCTTTAACTCCAAGGACGACAAAGG
>dme_circ_0004248_junction_seq
CCAGCTTTAACTCCAAGGACGACAAAGGGACTAGGATTGCGAATATATTGGGCGTG
>dme_circ_0004249
GCGCCAACTGCCCTCGCATTGCGCACTGCTCTGCTCGATCCTGCGGTCAATCTCCTGTTCGAGCGGCTCA
AGAAGGAACTTAAGGCCACCAAGGCCAAGCTGGAGGAGACCCAGAACGAGCTGTCCGCATGGAAGTTCAC
GCCGGACTCTAATACCGGCAAACGCCTAATGGCCAAG
>dme_circ_0004249_junction_seq
TAATACCGGCAAACGCCTAATGGCCAAGGCGCCAACTGCCCTCGCATTGCGCACTG
>dme_circ_0004250
TTGTGGAACCTTAAGTACTGTGACTAGTTAATGGGGTATTGATTTTAAATTTCCTTCTTTTTCGCCATTT
AATTACTCTCTTATTTTTTCACTATTACGATACCAACGGCTAGTGTTTGTTATTATTTTATAAATTAAAA
TTAGTTAGGTTTATCTACATATGCTCTCGCTTCTCTCTGTATTTATCTTATTATCTTTCCGTATAATTCT
TAATAATATTTAACTAAATAAATATAATGTTATATAATTTATGCAATTTCTCATGTATATATATATCTTT
GTTTTTTGCTTTTGTTTTCTACACAAAATGATGTGTTTGTAACTGGTTTTTATATGTTGGCTAGTGTTTC
TGTTTATTAATATCTTGCAATGTTTTGTGTTGACTGTACCTCGGTTTGTG
>dme_circ_0004250_junction_seq
TTTTGTGTTGACTGTACCTCGGTTTGTGTTGTGGAACCTTAAGTACTGTGACTAGT
>dme_circ_0004251
gtttttagtttttagtttttatttttggtttaGAATGTCTTCCCGTCTTCTTGTGAAGCAAAGCTCAATG
ATATGATTCACGCTGAAAGGTGATTTAACGATTTTTATTATTTCAATAATTTTGCAGTTTATAAAGATTG
CGTGATCTTGTGTTTTATTAGG
>dme_circ_0004251_junction_seq
AGATTGCGTGATCTTGTGTTTTATTAGGgtttttagtttttagtttttatttttgg
>dme_circ_0004252
tcgtttcttcttttgaAAACTCTGGAGCTAAATTAGGTCGTTGTTTGGTTTTCCACTCTTCTACAGTTAG
ATCACTCCACTGATTTATGCCCTTCTTGAAAGATTCCACTCCCAATTCGAACTGCTCATTG
>dme_circ_0004252_junction_seq
TTCCACTCCCAATTCGAACTGCTCATTGtcgtttcttcttttgaAAACTCTGGAGC
>dme_circ_0004253
TTCTAAACTAAACCCCCCAAAAACCACTCCCCAAACTAATCGAGGCTACATTAGCAATGAATGTAGCAGC

AGGAACAGCACACAGTTCTACAAAAGAGACTAACTTTGAAGTGCCCTAATGGATTGGTGGAGCGAAACAA
TTTATAGATGTTTATACTATATATATTTTGTAAAAGACACAGTTAAGATGAAAGTTATGCAGACATATAG
CTAGCACACAAAGTTGATCAGACAAGCAACCTACTTACGAGTACTACTACTACTAATACTACCTTTTGCT
ACTACTAATTTGTAATGCCCACTACCTTTATTCACTAGTTATACACGTTTCCAAAATTATGATTTGTATA
TACTTATAAAGCGAAAAGCAAGCGTAAATTAATGAAAAATACACTCCAAGTGCTCTACGGCAATTaaaca
aacaaaaacaaaaaaaatacaaacaaaaacacataaaaaatatcgaaaaacttaagtgaaaaaaataaaa
CATTCGAATGAAATGCCGCGGTCTCTGCTGCAAACCATCTTGAGTCTCAGTTCTCCACTGGAATCGCCAG
TCCGGTGGAATTCTAGCCAGGAGTGGCACGTGGCAGAGGCGGAACGGCCAAAAAAATAATAACTAAACCC
GTTAAAGAGATAAAATTTGATTTTGTTCTGTTATTAAACTAATAGGAAAGTGAAAATTGTAAGCCGCATA
AAGAAAATATTTAATTATATACTAAAAGTCATTACTATAAGTTTAATTAATATTTAACTGTTAAGCGACA
AacacacacacacacacaGATTTTGTATTGCATAATTATTATGTTTTTATATGCAATATATCAGCGAAT
GAATTACGATGCAGATGCAGAAGGGCGAAAGAAATCGAACCCGTTTCTATAAATACTGTAAAATACTAAT
ACTAAATTATTTTGTTGTACGAAACGAAAGAAATGCATTAAAACTCGTGTTTTG
>dme_circ_0004253_junction_seq
AAAGAAATGCATTAAAACTCGTGTTTTGTTCTAAACTAAACCCCCCAAAAACCACT
>dme_circ_0004254
GTAGTAGTACTCGTAAGTAGGTTGCTTGTCTGATCAACTTTGTGTGCTAGCTATATGTCTGCATAACTTT
CATCTTAACTGTGTCTTTTACAAAATATATATAGTATAAACATCTATAAATTGTTTCGCTCCACCAATCC
ATTAGGGCACTTCAAAGTTAGTCTCTTTTGTAGAACTGTGTGCTGTTCCTGCTGCTACATTCATTGCTAA
TGTAGCCTCGATTAGTTTGGGGAGTGGTTTTTGGGGGGTTTAGTTTAGAACTTCAGTGCGTAGTTAGTAC
GTAGAGTTGAGTGTTTT
>dme_circ_0004254_junction_seq
GTAGTTAGTACGTAGAGTTGAGTGTTTTGTAGTAGTACTCGTAAGTAGGTTGCTTG
>dme_circ_0004255
GGTTGCTTGTCTGATCAACTTTGTGTGCTAGCTATATGTCTGCATAACTTTCATCTTAACTGTGTCTTTT
ACAAAATATATATAGTATAAACATCTATAAATTGTTTCGCTCCACCAATCCATTAGGGCACTTCAAAGTT
AGTCTCTTTTGTAGAACTGTGTGCTGTTCCTGCTGCTACATTCATTGCTAATGTAGCCTCGATTAGTTTG
GGGAGTGGTTTTTGGGGGGTTTAGTTTAGAACTTCAGTGCGTAGTTAGTACGTAGA
>dme_circ_0004255_junction_seq
GAACTTCAGTGCGTAGTTAGTACGTAGAGGTTGCTTGTCTGATCAACTTTGTGTGC
>dme_circ_0004256
CCAAAGGACGCACTAACATAGAGCTACGCGAGCAATTCATATTGGCCGATGGCGTCTCGCAGAGCATGGC
CGCATTCACGCCCAGACGTTCCACGCCAAATGCGGCCGCCACTGCCTCCTCCTCACCCCATGCCCACAAT
GGCGGCAGCTCCAACTTGCCCCCATATATGAGTGGACAGGGTCCCATCATCAAGGTAAATCAATCAGCAA
ATTGAATCTGCAACGGAAAGTAAAGGGGAGCCTAAGTGTCACACCGAATCCATTTCCATCGTTTTTGTCG
CCAATCGGAATTAACTTCTTTTGAAGAACTTAATACCCAATTATCTACTCTTAAATATTCTTTACTTAAA
AACCCTCAAAATTCTAAACTTTGGTCAAATCAATATGATATGTATATTCACTTAAAATCATTTCAACTAA
CCTATTATATATTTCTGTTTGCTTTAAACATTTTAACTTTCTTTAAATTTAACTATAATATCCAAAAAGTAT
TTTAAAAAGAGGTTCTAGCTTTATAGAAGAACAAATATTCTATTATTTCTGAAAATGACAAATTTAAACG
AAATTGAGAAATGAGAAGAGGTGCCAAAGTGTTAAGTGGTTAACGTCAAAGGGTTAAACCCTTGCTCAAA
TCCCGATCCCAATCCATTTACCCATCCAAACATATTTCCATCCAGCGAGGGCACAAGGTAAGTGCTCCTT
TCGTCCttttttttGGGTTCGTTGTGTGTGTTGTTGACAAAGAAAAGCTATTGCTGCTGCGCCGCCGACT
CTTAAAATATAATTTCTATATATCTGCATGAGAACCGAATACTTGGTAACAATGTGTTCACAtttttttt
gtatttgtatttgattaagtaattgcgacatttttttAGTTAATGCTAGAGCATACATATAGTACACCCCA

CACTATTCATTGATGAGGCTATGAGGTTTTTAGGCCAGGTTAGGTTGTTGTCCACGGCTTAAATGATCAT
AAACCGTTTGCCAAATGAAATGCGTTAATTCATTGTTGGTTAAACATATCGAAATTATATCCTTAGCGTA
AATCAATCAGCAAATTGAATCTGCAACGGAAAGTAAAGGGGAGCCTAAGTGTCACACCGAATCCATTTCC
ATCGTTTTTGTCGCCAATCGGAATTAACTTCTTTTGAAGAACTTAATACCCAATTATCTACTCTTAAATA
TTCTTTACTTAAAAACCCTCAAAATTCTAAACTTTGGTCAAATCAATATGATATGTATATTCACTTAAAA
TCATTTCAACTAACCTATTATATTTCTGTTTGCTTTAAACATTTTAACTTTCTTTAAATTTAACTATAAT
ATCCAAAAAGTATTTTAAAAAGAGGTTCTAGCTTTATAGAAGAACAAATATTCTATTATTTCTGAAAATG
ACAAATTTAAACGAAATTGAGAAATGAGAAGAGGTGCCAAAGTGTTAAGTGGTTAACGTCAAAGGGTTAA
ACCCTTGCTCAAATCCCGATCCCAATCCATTTACCCATCCAAACATATTTCCATCCAGCGAGGGCACAAG
GTAAGTGCTCCTTTCGTCCttttttttGGGTTCGTTGTGTGTGTTGTTGACAAAGAAAAGCTATTGCTGC
TGCGCCGCCGACTCTTAAAATATAATTTCTATATATCTGCATGAGAACCGAATACTTGGTAACAATGTGT
TCACAttttttttgtatttgtatttgattaagtaattgcgacatttttttAGTTAATGCTAGAGCATACAT
ATAGTACACCCCACACTATTCATTGATGAGGCTATGAGGTTTTTAGGCCAGGTTAGGTTGTTGTCCACGG
CTTAAATGATCATAAACCGTTTGCCAAATGAAATGCGTTAATTCATTGTTGGTTAAACATATCGAAATTA
TATCCTTAGCCGACGAACACTTGGCCGAACTAATGCCAATATTTGAGAAAATCCGCGCTCAAGACCAGGT
TCCATGCGCCTTTCCCATTCACATGGGTGCCGGCGGCACGGTATTCGTCCGTTGCAATACCAGCCGTTCG
GTGCCGCTGAGTCCACACGTGCTGCATTGTCATCCAACAACACACTGGGTAAGTTCGCAGGCAGCAGGAG
CAGAAGACGCCGAAGCTGCTCCAGCAGGAGTGCCCGATATTGGTTTCATCTTTCACGGGAGAGGGGGTGC
TAAACCTACATGAATGATCCCACATTATAATATTTGTTTATTTATTTTTCTGTAAAGGGTACGCGAGCGC
TCCGTCCGCTCGATTCCCATGTCACGACCGTCGCGCTCCTCACTTTCGGCCAGCACTCCCGACTCCCTGA
GCGACAACGAGGGTAGCCATGGAGGACCTTCCGGCCGGTACACACCACGCAAGGTCACCTACACGAGCAC
GCGCACGGGTCTCACGCCAGGAGGCTCTCGTGCCGGCTCCAAGCCCAACTCGCGCCCGCTCAGCAGACAG
GGATCAAAGCCGCCATCGCGACATGGCTCCACGCTGTCGCTAGATAGCACGGG
>dme_circ_0004256_junction_seq
GCTCCACGCTGTCGCTAGATAGCACGGGCCAAAGGACGCACTAACATAGAGCTACG
>dme_circ_0004257
CCAAAGGACGCACTAACATAGAGCTACGCGAGCAATTCATATTGGCCGATGGCGTCTCGCAGAGCATGGC
CGCATTCACGCCCAGACGTTCCACGCCAAATGCGGCCGCCACTGCCTCCTCCTCACCCCATGCCCACAAT
GGCGGCAGCTCCAACTTGCCCCCATATATGAGTGGACAGGGTCCCATCATCAAGGTAAATCAATCAGCAA
ATTGAATCTGCAACGGAAAGTAAAGGGGAGCCTAAGTGTCACACCGAATCCATTTCCATCGTTTTTGTCG
CCAATCGGAATTAACTTCTTTTGAAGAACTTAATACCCAATTATCTACTCTTAAATATTCTTTACTTAAA
AACCCTCAAAATTCTAAACTTTGGTCAAATCAATATGATATGTATATTCACTTAAAATCATTTCAACTAA
CCTATTATATTTCTGTTTGCTTTAAACATTTTAACTTTCTTTAAATTTAACTATAATATCCAAAAAGTAT
TTTAAAAAGAGGTTCTAGCTTTATAGAAGAACAAATATTCTATTATTTCTGAAAATGACAAATTTAAACG
AAATTGAGAAATGAGAAGAGGTGCCAAAGTGTTAAGTGGTTAACGTCAAAGGGTTAAACCCTTGCTCAAA
TCCCGATCCCAATCCATTTACCCATCCAAACATATTTCCATCCAGCGAGGGCACAAGG
>dme_circ_0004257_junction_seq
CATATTTCCATCCAGCGAGGGCACAAGGCCAAAGGACGCACTAACATAGAGCTACG
>dme_circ_0004258
CTACGCGAGCAATTCATATTGGCCGATGGCGTCTCGCAGAGCATGGCCGCATTCACGCCCAGACGTTCCA
CGCCAAATGCGGCCGCCACTGCCTCCTCCTCACCCCATGCCCACAATGGCGGCAGCTCCAACTTGCCCCC
ATATATGAGTGGACAGGGTCCCATCATCAAGG
>dme_circ_0004258_junction_seq
ATGAGTGGACAGGGTCCCATCATCAAGGCTACGCGAGCAATTCATATTGGCCGATG

>dme_circ_0004259
CCAAAGGACGCACTAACATAGAGCTACGCGAGCAATTCATATTGGCCGATGGCGTCTCGCAGAGCATGGC
CGCATTCACGCCCAGACGTTCCACGCCAAATGCGGCCGCCACTGCCTCCTCCTCACCCCATGCCCACAAT
GGCGGCAGCTCCAACTTGCCCCCATATATGAGTGGACAGGGTCCCATCATCAAGG
>dme_circ_0004259_junction_seq
ATGAGTGGACAGGGTCCCATCATCAAGGCCAAAGGACGCACTAACATAGAGCTACG
>dme_circ_0004260
CACCTCGGGACCGGAACTGGAATTCCGTTCGCCACGCGCGAAGCTTCTGTGGACCAAGTGGCGCGATGTC
TGGATGCTCTCATGGGAGCGCCAGCGGCTGCTTAACGATCATCTTCTGTATCTGAAGGATGTGGAGCGTG
CCCGCAACTTCAGCTGGGACGATTGGCGCAAGCGCGAATTCGATACATCTGGCTTGGAAATGAAGGCTGT
AGCCGATTTGTTCGATCGCAATGGCGAAGGCCTCATCGACTGGCAAGAGTTCATTGCTGCCCTTCGTCCC
GATTGGCAAGAGCGTAAGCCAGCCAATGATTCGGACAAAATACACGATGAGGTCAAACGTCTGGTCATGC
TGTGTACCTGCCGACAGAAGTTCCGTGTGTTCCAAGTTGGCGAGGGCAAGTACAGAGGTAAGTGGTCCAT
CTATCCATCCGAGTTTAGTCAAAACACCCTATTTAGTTGTGTTGCGTACACACAATCTCGAAATTTGTTG
GATTTCATCCATTCCACATCCATACCAGCCACATAAATTCCATACATATATCTAAAATAATAATCTAGCG
TAATCTAGTAGTTCACATTTTTAATATTCCACCCAAATCATGCATTTTGTCTATACCCAGATACTGTGCA
TCGTTTGCTCATATCATTTAAATGCATTCATATCACATCTAGGTACAGTTGTGCTTAACAACTAATTATA
CTATAATGCCAaataataataataataataTTACCAAACGGATATGGTCATTTaaccaaccaacaaacaa
ccaaccaacaaTAAACTACTGTGCCTAAAATAACTAGTCAATGAACCCGGAATATAAAAAGAGAAACCTT
TGTTAAAACCTCATCATTAATTCAGGTCCAAAAGTTGCCTAAACAAGCTAAAATGATATTAACAGTGTGC
CTTAGGGCGAATTTTACCTTTAATTGCCATTCAGCCAGCGCCAAAAGCCACATCATATACACAATTACCC
AGCAACCAAAACCAATTAAATATAAAACTGACCAATCTAGCCAAGGGTTGTACCATCTACTCTACTCATG
CTCATTTTTGTCGCTTCTATCTAACTGCGTACCAACTAGCTGCTATAAGACCATGAAAACACACACAAAC
GCTCACCAAAAATATCCCGCCAATTGTAGGTTCTTCTATTTCCTATTAGTTAGTGTTATTAACCAGGTTT
CTCCACCCAATTCTTAGTCGTAGTTACATTAACCCTTTGTTTTTAACCATTAGTTGAGAATGATATTTTG
TTAATCCTATCCTGCTTTCAAACTAACATCCTGCTTTATTTGTTTAACAGTCTAGCATGCTCTGTTAACT
AACAGGGTTTGTGCACACTGTACTTCATGTCACCCATTTTATGTTTGAATCCTCATCAATTTCGTTGTAC
GTCCTGTATTTAAATTAAAATTGTTGTATTAATTTGTGTTAtttgtttttcgtttttttactaatcaaact
ttatgtttaccccctttttctttttttCCAAAATGGTTTTCTTTATTATACCGAAAATCGATTGAATCTTG
ATAAACCGAACCCGATAAATATACATATATGTATACTCTACGCTTGCGCTGCCCGCTCGCCACGCCCACG
ACCACGCCCGACATGCACCCGCCTGTGCCCACACCAAATACTAAACAACaaaaaaaacaaaaaTGTTGTAA
AACACACCATACTGAAAACAAACTGTGACAAATACTTTGCGGTAACGAACTTTCGTTGTTATTGTTGTTG
TTTATATGTTCGCCCTACAACAAAACATTGCCTATCCGTGTAAATGTGTTATATGTGGGTGCATCTGCCA
TGCCAATGCGTACAAATGCGTATATAAATGCATCTGTGCATCTGCCACTGCAACTGCAATCGCTACCAAA
ACCAATGCCACTGTTATCCGGGAGCAGCCAAAGGACGCACTAACATAGAGCTACGCGAGCAATTCATATT
GGCCGATGGCGTCTCGCAGAGCATGGCCGCATTCACGCCCAGACGTTCCACGCCAAATGCGGCCGCCACT
GCCTCCTCCTCACCCCATGCCCACAATGGCGGCAGCTCCAACTTGCCCCCATATATGAGTGGACAGGGTC
CCATCATCAAGG
>dme_circ_0004260_junction_seq
ATGAGTGGACAGGGTCCCATCATCAAGGCACCTCGGGACCGGAACTGGAATTCCGT
>dme_circ_0004261
CTTTAAGGGATCTCGTGACCAGGGACTCAATGCCCGCAAGGGAAGGTGAGTTCTCGTTGGGCTTGTATTA
ttttttttGTAATTCAAGAATAGATAGATTCCGCATGTACTTCGAAATTCTCAAATCGAATTTGTTTTGT
TTTCAACTTTTCAACTACTTTTGGAAATGCTGCGTCTACAACTATTCGAAATATTTGTATATTTAAATTT

GTAATATCCATGTATTATCTATGTTAATGTGTTTTGCGTATTTTGTTCGTTACTGTACCATTTTGTTTTG
TCTCTTTTTGATTCTACTTTCATCTTGTCTACACACTACGCTACTACGTAAACGTTGCTCATACCGCTCG
AATATCAAATATCAAAACTTTGCATCTTGCCGCTTTATTTGCCTATGTCTATGTCAAAAACCAAAATCTC
TGAGCAGTGTGAGTTCTCGTTGGGCTTGTATTAttttttttGTAATTCAAGAATAGATAGATTCCGCATG
TACTTCGAAATTCTCAAATCGAATTTGTTTTGTTTTCAACTTTTCAACTACTTTTGGAAATGCTGCGTCT
ACAACTATTCGAAATATTTGTATATTTAAATTTGTAATATCCATGTATTATCTATGTTAATGTGTTTTGC
GTATTTTGTTCGTTACTGTACCATTTTGTTTTGTCTCTTTTTGATTCTACTTTCATCTTGTCTACACACT
ACGCTACTACGTAAACGTTGCTCATACCGCTCGAATATCAAATATCAAAACTTTGCATCTTGCCGCTTTA
TTTGCCTATGTCTATGTCAAAAACCAAAATCTCTGAGCAGTCGTATCACGCCCACACGTGACACGCCCGA
CAGAGATCGCCTGCCGCACTATGGCCCCCGATTCTCGCCAAGGTAAGTCCTTGAAATCCTCCAAAATCAT
CCATATCATCTCAAGCTGTGCATGTCCAGTACAATTGTTTGTATTTAGTTTGGTTCATGATGTTCACCGG
GTTTGAAGAACTTCTAATTAACAGAAATATCACATTTTAATATAGCCACCTCGGGACCGGAACTGGAATT
CCGTTCGCCACGCGCGAAGCTTCTGTGGACCAAGTGGCGCGATGTCTGGATGCTCTCATGGGAGCGCCAG
CGGCTGCTTAACGATCATCTTCTGTATCTGAAGGATGTGGAGCGTGCCCGCAACTTCAGCTGGGACGATT
GGCGCAAGCGCGAATTCGATACATCTGGCTTGGAAATGAAGGCTGTAGCCGATTTGTTCGATCGCAATGG
CGAAGGCCTCATCGACTGGCAAGAGTTCATTGCTGCCCTTCGTCCCGATTGGCAAGAGCGTAAGCCAGCC
AATGATTCGGACAAAATACACGATGAGGTCAAACGTCTGGTCATGCTGTGTACCTGCCGACAGAAGTTCC
GTGTGTTCCAAGTTGGCGAGGGCAAGTACAGAGGTAAGTGGTCCATCTATCCATCCGAGTTTAGTCAAAA
CACCCTATTTAGTTGTGTTGCGTACACACAATCTCGAAATTTGTTGGATTTCATCCATTCCACATCCATA
CCAGCCACATAAATTCCATACATATATCTAAAATAATAATCTAGCGTAATCTAGTAGTTCACATTTTTAA
TATTCCACCCAAATCATGCATTTTGTCTATACCCAGATACTGTGCATCGTTTGCTCATATCATTTAAATG
CATTCATATCACATCTAGGTACAGTTGTGCTTAACAACTAATTATACTATAATGCCAaataataataata
ataataTTACCAAACGGATATGGTCATTTaaccaaccaacaaacaaccaaccaacaaTAAACTACTGTGC
CTAAAATAACTAGTCAATGAACCCGGAATATAAAAAGAGAAACCTTTGTTAAAACCTCATCATTAATTCA
GGTCCAAAAGTTGCCTAAACAAGCTAAAATGATATTAACAGTGTGCCTTAGGGCGAATTTTACCTTTAAT
TGCCATTCAGCCAGCGCCAAAAGCCACATCATATACACAATTACCCAGCAACCAAAACCAATTAAATATA
AAACTGACCAATCTAGCCAAGGGTTGTACCATCTACTCTACTCATGCTCATTTTTGTCGCTTCTATCTAA
CTGCGTACCAACTAGCTGCTATAAGACCATGAAAACACACACAAACGCTCACCAAAAATATCCCGCCAAT
TGTAGGTTCTTCTATTTCCTATTAGTTAGTGTTATTAACCAGGTTTCTCCACCCAATTCTTAGTCGTAGT
TACATTAACCCTTTGTTTTTAACCATTAGTTGAGAATGATATTTTGTTAATCCTATCCTGCTTTCAAACT
AACATCCTGCTTTATTTGTTTAACAGTCTAGCATGCTCTGTTAACTAACAGGGTTTGTGCACACTGTACT
TCATGTCACCCATTTTATGTTTGAATCCTCATCAATTTCGTTGTACGTCCTGTATTTAAATTAAAATTGT
TGTATTAATTTGTGTTAtttgtttttcgtttttactaatcaaactttatgtttaccccttttttcttttt
ttCCAAAATGGTTTTCTTTATTATACCGAAAATCGATTGAATCTTGATAAACCGAACCCGATAAATATAC
ATATATGTATACTCTACGCTTGCGCTGCCCGCTCGCCACGCCCACGACCACGCCCGACATGCACCCGCCT
GTGCCCACACCAAATACTAAACAACaaaaaaacaaaaaTGTTGTAAAACACACCATACTGAAAACAAACT
GTGACAAATACTTTGCGGTAACGAACTTTCGTTGTTATTGTTGTTGTTTATATGTTCGCCCTACAACAAA
ACATTGCCTATCCGTGTAAATGTGTTATATGTGGGTGCATCTGCCATGCCAATGCGTACAAATGCGTATA
TAAATGCATCTGTGCATCTGCCACTGCAACTGCAATCGCTACCAAAACCAATGCCACTGTTATCCGGGAG
CAGCCAAAGGACGCACTAACATAGAGCTACGCGAGCAATTCATATTGGCCGATGGCGTCTCGCAGAGCAT
GGCCGCATTCACGCCCAGACGTTCCACGCCAAATGCGGCCGCCACTGCCTCCTCCTCACCCCATGCCCAC
AATGGCGGCAGCTCCAACTTGCCCCCATATATGAGTGGACAGGGTCCCATCATCAAGG
>dme_circ_0004261_junction_seq
ATGAGTGGACAGGGTCCCATCATCAAGGCTTTAAGGGATCTCGTGACCAGGGACTC

>dme_circ_0004262
CACCTCGGGACCGGAACTGGAATTCCGTTCGCCACGCGCGAAGCTTCTGTGGACCAAGTGGCGCGATGTC
TGGATGCTCTCATGGGAGCGCCAGCGGCTGCTTAACGATCATCTTCTGTATCTGAAGGATGTGGAGCGTG
CCCGCAACTTCAGCTGGGACGATTGGCGCAAGCGCGAATTCGATACATCTGGCTTGGAAATGAAGGCTGT
AGCCGATTTGTTCGATCGCAATGGCGAAGGCCTCATCGACTGGCAAGAGTTCATTGCTGCCCTTCGTCCC
GATTGGCAAGAGCGTAAGCCAGCCAATGATTCGGACAAAATACACGATGAGGTCAAACGTCTGGTCATGC
TGTGTACCTGCCGACAGAAGTTCCGTGTGTTCCAAGTTGGCGAGGGCAAGTACAGAGTAAGTAGCAAGAG
TAGTGTGTCACCTGGTCGGCAGTTACTAATGTGTAAAATAACTTATTTCAGTTTGGAGACTCCCAGAAAC
TGCGCCTCGTTCGTATCCTTCGCAGCACTGTTATGGTGCGCGTGGGTGGCGGTTGGGTTGCCCTGGATGA
ATTCCTGCAGAAGAACGATCCTTGTCGCGG
>dme_circ_0004262_junction_seq
TCCTGCAGAAGAACGATCCTTGTCGCGGCACCTCGGGACCGGAACTGGAATTCCGT
>dme_circ_0004263
CAAGCAGTATTCGAGAAGTTCAACATGTGCGAAGAGAATGTCAACGATCTGTTGAAATGGGTTACCACTG
TGGAACAGAAAATCTCAAGTGTCGGCGGACCTCGTGAAAAGATTGATGAGTTACGCAACCAGATTAATGC
CCTTAAGGCAAATCAAGGATGAGATCGAGTCTCAGCAACGGCCAGTTGCCACTTGTTTGGAGCAAATCCG
TCAGATCGTGCTCACCGGTGGTGATGTTTTGTCTGCCCCCGAAGTGACCACTCTGGAGAACTCTGGCCGG
GAGTTGCGTTCGCGTGTGGATCGTGTCAACGATCGCACCGTGCGTTTGCTCCGGCGCCTGGAAGCAGGTC
GAGATGAGCTCACCAAACTGCGCAGCGAATTGGATGTATTCTCCGATTGGTTGCAAGTCGCCCGACGCAC
TTTGGAGGACAAGGAACGTTCCCTGTCCGATTTGACTCGGCTGCCCTCTCAGGCCGATTCGGTTCGTGAG
TTTGTCAGCGATGTGATTGGGCACCAGGCCGATCTGCGGTTCATCACAATGGCCGCACAGAAGTTTGTGG
ACGAGAGCAAGGAGTTCTTGGCCATACTGAACGACTTCCGTACTTCACTGCCCGAACGCTTGCCACATGT
TGAGCCACTGTCCAGTGCCGAGAGTCCCATACGCCAAGAGGTATCGCTGGTCTCGGCACAGTACAAGGAT
CTGTTGAACCGTGTGAATGCTCTGCAAGATCGCGTCTCCGGTTTGGGTGGACGCCAGCGTGAGTACCAGG
ATGCATTGGACAAGGCTAACGAGTGGCTGAGGAGTGTACATCCGCGTGTGTCGCGCATTATCTCTGAACC
GATTGCCGGCGATCCTAAGGGAGTACAGGATCAGATGAACGAGGCCAAGGCTCTACACAATGAGCTGCTT
TCTAGTGGTCGCTTGGTGGACAATGCGCAACAGGCACTTGATAATCTGCTGCGCAGCCTTGGTGGCCAAC
TCAGTCCCATGGAGATCAATCAGTTGGAACTGCCTATTGCCGATCTGAAGAACAACTACCAGCAGCTTCT
GGATAATCTTGGTGAGCACTGCAAAACTCTGGACAAAACTTTGGTGCAATCGCAGGGCGTACAGGATGCC
TTGGACAGTCTTGTGGGATGGGTTAACCAGGCCGAGGATAAGTTCAAGATGAATCTGCGACCCGCCTCGC
TTATCAAGGAGCGTCTGCAGGAGCAGATCCGCGAGCACAAGGTCCTGCTAGCCGATCTTCAATCTCACCA
GGCCAGCATCGACAGCGTTCAAGTTTCGGCCAAACATCTGCTTGCCAGCGCATCGAATGCACGCATTGCC
AAGAAGGTGGAATCGAATCTAAACGATGTGACCGTCAAGTTTGAGAAGCTCTACGAGAAGGCCAACAAAC
GTGGTGAATTCTTAGATGACGTTTACAACCGATTAAGCCGCTATCTGGATGAAATCAGCACCGTGGAGCA
GCGTATGGCCAGCTTACAGGAGGCTCTAGATAGCCGCGAGACAAGCCTGCTTTCAACTGAAGAGTTGGCT
CGCCGCATGAACGAACTGTCGCGTGACAAGGACCAACTGGCTCCGCAGTTTGAGGATTGCGTGCGCAGTG
GCAAGGATCTAATTAGCCTGCGTGATGTCACCGATACTGGAGTTTTGCGCGATCGCATCAAGGCACTGGA
ATCTCAGTGGCGCAACATTAACATCAGCATCGATGAGCGCGCCAAGCTGTCCAAGCAAAAGGCCGAACAA
CAACTGGCCTACGAGGGACTGAAGGATCAGGTCCTCTCCTGGCTGGCCAGCACTGAAGCGCGAGTTAACG
GCCTTCCGCCGGTAGCAATCGATCTGGACAGGATTAAGCAGCAGCATGACGAGCTGAAGCCCATATGCAA
GGATTACCGCGACTATGCACCAACTATTGATAAAATTAACGATATTGGAGCACAGTACGATGCGCTAATC
CGTCCAGAGAGTCCTGCCCGCAAACGCTCCACTTATAGTCCGATTAAGCGGACCAGTCCGTTGCGCCGCA
TGTCCGGAGACGCTAGAAGCCCCAGCCCCACCAAGGGAGGCATCCTGTCGCCACTATCGACTGGTTCCAG
TGGATTCGGTAGCCGCAGATCCTCCCAGGATGGTTTCCAGCTGTCTGAGCTATCTCCGGTGCAGCAGCAG

CTGAGCGAGATCAACAATCGCTACGGGCTGATTGGCGTGCGTCTCAATGACCGCCAACACGAGCTGGACA
ACCTGAACGAGGAACTTCGCAAGCAGTACGAGAATCTGAAGGGACTAGCTCAGTTCCTGGAGCGCATTCA
GCGCCAATTGCCCAAGGAGTCCGTATCAAACAAGGATGAGGCCGAGCGCTGCATTAAGCAGGCGCGCAAG
ATCCTCGAAGACATGTACGAGAAGCAGAGTCTTCTTGACACCACCAAGGCTCAGGTGAAGGATATCCTCC
GCCGCAAGTCAGATGTGCCCGGTGCCGAACAATTGCGCCAGGAGAACGATAGCATCCAAGAGAAGTGGAA
GAATCTCAACGACATCTGCAAGAATCGCATTGCCTTCTCCGAGAAGCTGCGTGACTTTTTGGACACCCAT
GGAAATCTGAAGAGCTGGCTGGACAGTAAGGAACGTATGTTAACCGTGCTGGGACCCATCTCTTCCGATC
CCCGTATGGTCCAGAGCCAGGTGCAGCAGGTGCAAGTGCTTCGCGAGGAATTCCGCACTCAGCAACCACA
ACTGAAGCATTTCCAGGAGCTTGGACATGATGTTGTCGACCATTTGGCCGGCACACCCGATGCTCAGGCG
GTCGAAATCAAACTGAAGGACATTCTGGGCAAGTGGGATGATCTGGTTGGAAAGTTAGACGACCGGGCCA
ATAGTTTGGGCGGTGCTGCTGACAGTTCCAAGGAGTTCGATGCTGCTGTAAATCGCTTGCGTGAGGCTCT
GCAGAACATAAGCGATAATCTGGATACTTTGCCCACGGATGGCGATCACCAGGAGAATCTCCGCAAAATC
GAGAACCTGGAGCGACAGCTTGAGGGTCAACGCCCGCTGTTAGCCGATGTGGAACAATCCGCCGCCACTC
TATGCAACATTCTGGGTGATCCCGCCTCGCGCGCTGATGTCAACTCTCGTGTCGCAGCTCTGGAGAAACA
GTATTTGGCCCTTCAGAAGAAACTGGATACCAAGAAGGCAGAAACAGAGGCTTCCCTTCGGGATGGCCGC
CACTTTGCCGAGAATTGCTCAAAGACTCTTGGTTGGTTGGGCGGAGAGCTCTCCAACCTGACCGACAGGC
TTTTGGTCTCTGCACACAAGCCAACACTGCAGCACCAGATTGACACCCATGAGCCGATCTATCGTGAGGT
AATGGCCCGTGAGCATGAGGTTATTATGCTAATCAACAAGGGCAAGGATCTAACCGATCGCCAGCAGGAT
CGCGGAGTTAAACGTGATTTGGATCGCATCCAGCAGCAGTGGGAGAAACTGCGCCGTGAAGCCGTCGATC
GACACACACGACTGCAGACGTGCATGGAGCACTGCAAGAAGTACTCCCAAACTTCGGAGACATTCCTGGC
CTGGCTGCGTACCGCGGAGGATAAGTTGGCTGATCTTACACCCGGCGTATTGTCGAAGGCAAAGCTGGAG
ACGCGTCTGCGTGATCTGCAGACCTTCCGTAGTGAGGTTTGGAAGCATTCCGGTGAATTCGAAAACACCA
AGGGATTGGGAGAGACATTCCTCAGCAGCTGCGACATCGATAAGGAACCCATTAAGGCGGAACTTCAAGA
CATCCGCGATCGTTGGGAGAGACTCAACAATGATCTGATTGCCCGTGCTCATGAAATTGAAAACTGCTCG
CGTCGTTTGGACGATTTTAATGATGAGTTGCGAAACCTGGATCATTCTTTAGGTCGTTGCGAGGATCGAC
TTGCTGCCCACGATGCTCTCGGTGGAGCTGCCAAGGATCCCAAACTTTTGGAGCGTGTTAAGGCCATACG
CGAAGAGCTCACTAACTTGAGCAAGCCTTTGCAGTCACTTAAGGCTCTGGCCAAGGATATCAGTGCTGAG
GCTAGAGCTGCTGGCGGAGATGCTGATCATTTGACCAGTGAAGTCGATGGCCTGGCCGACCGCATGTCCG
AGCTACAGGGTCGCCTGGATGATCGCTGCGGAGAACTGCAATCTGCAGCCACCGCCGTATCCCAATTCAA
CGAACAAATGAAGAGTTTGGGCATTGATCTGAACGATCTGGAGACCGAGATTGAAAAGCTTTCACCGCCC
GGTCGAGAAATCAAGATTGTCCAAGTGCAAATCGACGATGTTGGCAAGATCCAGACCAAATTGGATCGCC
TTGTGGGTCGTTTGGAGGATGCAGAACGTGCCGCTGATGTTTTGGTAGATGCAGGATTCGCTGCAGACAC
CACCCAGACCCGTGAGCAAATCTCCACGCTGCGTAAGACATTGGGTCGTTTGGACAACAGAGTTCGCGAT
CACGAGGATAATCTTCATTCCACTTTGAAAGCACTTCGCGAATTCTACGACCATCAATCGCAAACTCTGG
ATGACATTCAAGACGTAAGCGATGAGTTCAAGCGCATGAAGCCCGTTGGCTCGGAGTTGGATCAGATTCG
CCGCCAGCAAGAGGACTTCCGTAACTTCAGGGAGCGCAAGGTGGAACCACTGGCCATCAATGTGGACAAG
GTAAACGTTGCTGGCAGAGACCTAGTGCGTTCGGCTGGCAGTGGTGTTTCTACCACGGCCATTGAGAAGG
ATCTGGAAAAGCTAAACGACCGCTGGAACGATTTGAAGGAGCGAATGAACGAGCGCGATCGCCGTTTGGA
TGTGGCTCTGCTGCAATCTGGAAAGTTCCAGGAGGCGTTGGCCGGTTTATCCAAGTGGCTCAGCGACACC
GAGGAGATGGTGGCTAATCAGAAGCCGCCTAGCTCCGATTATAAGGTGGTAAAGGCACAGCTTCAGGAGC
AAAAGTTCCTCAAGAAAATGCTTTTGGATCGTCAAAACTCTATGGGCTCTCTAGCCAATCTCGGCAAGGA
AGTTGCCAACCACTGTGAACCCGGCGAACGTGCCTCCATTGAGAAACAGTTGAACGATTTGATGAAACGC
TTTGATGCCCTGACCGACGGAGCCGAGCAACGTGAGTTGGATCTTGAGGAAGCCATGGAAGTGGCTAAGC
GATTCCATGACAAGATTAGTCCCTTGGAACTTTGGCTGGACAACACGGAGCGATCTGTTAAGGCAATGGA

ACTGATACCTACTGACGAAGAGAAGATCCAGCAGCGAATCCGCGAACACGACCGTCTACACGACGAAATC
TTGGGAAAGAAGCCCGACTTTTCCGACCTTGCCGATGTCACCGCCCAGCTGATGCATTTGGTTAGCGACG
AGGAAGCCGTTAATCTGGGCGAGAAGGTGCGCGGTGTGACAGAGCGGTACACAGGATTGGTGGATGCCAG
CGACAACATTGGGGCTCTGCTAGCCGAATCACGCCAGGGATTGCGTCACTTGGTCTTGAGCTATCAGGAT
CTGGTGGCCTGGATGGAAAGTATGGAAGCCGAGTTGAAGCGCTTTAAGTCCGTTCCCGTTTACGCTGAAA
AGCTGCTGGAACAGATGGATCATCTGTTGGAGCTAAACGAGAACATTGCCGGTCACGCTTCGAATGTGGA
ATCTACTGTGGAATCCGGAGCCGAGCTGATGAAGCACATTTCCAACGATGAAGCTATCCAACTGAAGGAC
AAGTTAGACTCCCTGCAACGTCGCTATGGCGATTTGACCAACCGTGGTGGAGATCTGCTGAAGAGTGCAC
AGAATGCACTACCATTGGTGCAGCAATTCCATGAAGCTCACAACCGTTTAGTGGAATGGATGCAAAGTGC
TGAAGCTGCTCTTGCTCCCAGCGAGCCACGTCAAGCTGATGTGCTTCGGCTGGAGGGTGAATTGGCTGAT
ATGCGTCCTATTCTGGACAGTATCAATCAAGTGGGACCCCAGCTGTGTCAGCTTTCTCCGGGAGAAGGTG
CTGCCACCATTGAGAGCATTGTGACCCGGGACAACCGTCGCTTCGATTCCATTGTGGAGCAAATTCAACG
CAAGGCGGAGCGTCTCCACCTGAGCAACCAGCGCGCCAAGGAAGTGACAGGTGACATTGATGAACTGCTT
GAGTGGTTCCGCGAAATGGACACCACTCTTCGTGAAGCCGATCTGCCTGCCATGGAGCCCAAGTTGGTGC
GTGCCCAATTGCAGGAGCATCGTTCTATCAACGATGACATTTCCAGCCAGAAGGGACGTGTACGAGATGT
TACTGCGGCCTCCAAGAAGGTGCTCCGCGAGTCCCCACAGAGCGAGAACACCGCCACTCTTCGTGAGAAA
TTGGATGATCTGAAGGAGATCGTCGACACTGTTGCTCAATTGTGCAGCGAGCGTCTGGGCATCCTGGAAC
AGGCTCTGCCCCTGTCTGAACATTTCGCCGATTCCCATCAGGGACTCACCGCCTGGTTGGATGACATGGA
GCAGCAGATTTCGCGATTGAGCATGCCGGCTTTGCGTCCTGACCAGATCACTCTGCAGCAGGACAAGAAC
GAACGTCTTCTTCAATCCATAGCGGAACATAAACCTCTGCTAGACAAACTGAACAAGACTGGTGAGGCTT
TGGGTGCATTGGTGGCCGATGATGATGGTGCTAAGATCAACGAGATTTTGGACACGGACAATGCCCGTTA
TGCCGCACTCCGCTTGGAACTCCGCGAACGGCAGCAGGCGCTCGAGAGTGCTCTGCAGGAATCCAGCCAG
TTCTCCGATAAACTGGAGGGCATGCTGCGTGCTCTGGCCAACACCGTGGATCAGGTCAATCAATTAGATC
CCCTGTCCGCTCTTCCGCAGAAGATACGTGAGCAGATCGAGGATAATGATGCTTTGATGGACGATCTGGA
CAAGCGACAGGATGCCTTCAGTGCTGTGCAGCGTGCGGCCAACGATGTTATCGCTAAGGCTGGCAACAAA
GCTGATCCAGCTGTGCGTGACATTAAGGCCAAGCTGGAGAAGCTGAACAATCTCTGGAACGATGTACAGA
ATGCCACCAAGAAGCGCGGCAGCTCGTTGGACGACATCCTAAGCGTAGCCGAACCCTTCTGGAAGCAACT
GAACAGCGTAATGAAGACCCTAAAGGATCTAGAGGAAACACTATCTTGCCAGGAGCCACCAGCGGCCCAG
CCTCAGGACATTAAGAAACAACAGGTGGCGCTGCAGGAAATCCGCCATGAGATCGACCAGACCAAACCAG
AGGTCGAGCAGGTGCGACGTCACGGAAGCAACCTGATGAACATGTGCGGCGAGCCCGACAAGCCAGAGGT
CAAGAAACACATCGAGGACTTGGACAACGCATGGGACAACATCACGGCGCTGTATGCCAAGCGCGAGGAG
AACCTCATCGATGCCATGGAGAAGGCCATGGAGTTCCACGAGACACTGCAGAACCTCCTCAAGTTCTTGA
CCAAGGCCGAGGACAAGTTTGCCCATCTGGGAGCTGTGGGTTCGGACATTGATGCCGTCAAGCGACAGAT
TGAACAACTCAAGTCGTTCAAGGATGAAGTCGATCCGCATATGGTTGAAGTAGAAGCATTAAACAGGGTA
AGGCCTTCtatatatatttatatatatatatTCTTATTCAATCGAATCTATTCAAATTTAAAAATCTAAT
GTTCCATTGCCGTGGTGTCTATCTCAACTTGTGAATTGGCAGAGGTCTCAAAAGGCAAGATAGTTTCAAA
AAAGTCTAGTTCGTTCGTGCGTTGTTGTTGTGAATATTGATTACATTTAAGTGCTTGCAACTGCAATGAT
TTAAGATCTCCGGTATCTTTGAACAAAGTTGAGCCGAGCATGTTTTTTGATTGCCACAGTTTATCCAGTT
GCATGCAATTTCAAAATGAAAACAAAAAACACTTGCCATTTATTGTTTTTGCGCTTGCTTATGTTACACT
GCAGTTTTTGTAAATTCTTCGGCTAATTGTTAATTGAATCTTACAGAACAAGCTGTGGAACTGACGGAAC
GCACTTCACCCGAGCAAGCGGCATCGATTCGCGAACCCCTGTCGGTGGTCAATCGCCGCTGGGAGGCCCT
ACTCCGTGGCATGGTCGAGCGCCAGAAGCAACTGGAGCACGCTCTGCTGCACTTGGGTCAATTCCAGCAT
GCTCTCAACGAGCTGCTGGTATGGATCAACAAGACCGACAGCACACTGGACCAACTGAAGCCGGATTCCT
GGAGATCCGCAACTGCTAGAGGTTGAATTGGCCAAGCTAAAGGTGCTGGCCAACGACATTCAGGCCCACC

AGAACTCAGTGGACACACTGAACGATGCGGGCCGACAGTTGATTGAAACCGAGAAGGGATCTGTGGAGGC
GTCTACTACCCAGGAGAAGCTGCGCAAACTGAACAACGAGTGGAAGCAGCTTCTGCAAAAGGCCAGCGAC
CGTCAGCATGAACTGGAGGAGGCTCTACGTGAAGCTCACGGATATATTGCCGAGGTCCAGGATATTCTGG
GATGGCTGGGCGATGTAGACGCAGTGATTGGAGCTAGCAAGCCTGTGGGCGGACTTCCCGAGACTGCTAC
TGAGCAACTGGAACGTTTCATGGAGGTGTACAACGAGCTGGATGAAAACAGACCCAAGGTGGAGACGATT
CAGGCCCAGGGCCAGGAGTACATCAAGCGTCAGAACCAAATGAAGGTCTCGTCCAGCAACTTACAGCATA
CACTCCGCACGCTGAAGCAACGTTGGGATGCCGTGGTATCCCGTGCATCCGACAAGAAGATTAAGTTGGA
AATTGCACTCAAGGAAGCCACCGAGTTCCATGACACACTGCAAGCCTTTGTTGAATGGCTGACCCAGGCC
GAAAAGCTGTTGTCCAATGCTGAACCCGTCTCACGCGTTCTGGAAACCATTCAGGCCCAGATGGAGGAGC
ACAAGGTGCTGCAGAAGGATGTGAGCACTCATCGCGAAGCCATGCTGCTGCTGGACAAGAAGGGAACTCA
CCTTAAGTACTTCAGCCAGAAGCAGGACGTGATCTTGATTAAGAACCTGCTCGTGTCGGTGCAACATCGT
TGGGAACGAGTGGTTAGCAAGGCTGCGGAACGCACTCGTGCTCTGGACCATGGCTACAAAGAGGCTCGCG
AGTTCAACGACGCCTGGAGTGGCATGATGCAGTATCTGCAGGAAACCGAACAAGTGCTGGATCAAATTAT
CGAGGAAGCCACAGCGTCCAAGGAACCACAAAAGATCAAGAAGTACATTGGCAAGCTGAAAGAGACGCAT
CGTCAACTGGGCGCCAAGCAATCGGTCTACGACGGAACCATGCGCACCGGAAAGAATCTTTTGGAGCGAG
CTCCCAAGGGCGATCGCCCTGTGTTGGACAAAATGCTGATCGAACTGAAGGAACAATGGACTCGAGTGTG
GTCCAAGAGCATCGATCGCCAGCGCAAGCTGGAGGAGGCTTTGCTTCTTTCTGGACAGTTCAGCGATGCT
CTGGGTGAGCTTCTTGACTGGTTGAAGAAGGCTAAGAGCCGCCTGAACGAAAATGGACCAGTCCACGGAG
ATTTGGAGACGGTTCAAGGTCTATGCGAGCACCACAAGCACATTGAGCAGGATCTGCAGAAGCGCGCTGC
GCAAATGCAAGGAGTGCTCAAGACTGGACGCGACCTGGAGCGATCTGGAAACAATCCCGAAGTTGGACGT
CAGCTCGATGAGATGCAGTCCATTTGGGAGGAGGTCAAGAGTGCCGTCGCCAAACGCGGCGAGCGTCTCC
AGGTGGCCCTTGTTGATGCCGAGAAGCTTAATGCCCGTGTCCAGGCTCTCTTTGATTGGCTGGATCATGC
CGAGCACAAGCTACGATATGCCAAGAATGCCCCTGATGACGAGAAGGTGTCCCGTGAAATGATGGACATC
CACATGGACTTCATGAAGGACCTGCGAGTGCGCGAGCGTGAAAAGACCGAGACTTTTGAGTACGCCGAGG
ACATTATCAACAAGGCCTATCCCGATGCCATTCCGATCATCAAGAACTGGCTTTCGATTATCCAGCAGCG
TTGGGAGGAGGTGCGCCAATGGGCCATCAATCGTGAGTCGAAGCTGGAGCAGCATCTGCAGTCGCTCAAG
GATTTGGACGACACCATTGAGGAGCTGTTGGCTTGGCTCAGCGGACTCGAGGGCACCTTGTTGAGACCTG
AAGCACGAACAGCTGCCGGACGAGATTCCACCCGTGGAGAAATTGATCGAGGACCACAAGGAGTTCATGG
AAAACACTGCTCGCCGTCAAAACGAAGTGGACCGCGCATGCAAACCAAGACAGTTGCCACCAGGAGCGCG
CAAGCCGTCGCGCAGCGGCAAGACGCCAGTGGTGAGTAGAATAATAGATTCGCAAGTAGCTGCCGAAACT
GTAGCTCTGCTGTACATATCCTTGGtatattattattattttgtatatttatttttttccaatttcttttt
gtatactttcttTTGTATCCACCACCTTATTGCTACAATATTCCAAACAAAAACCAAAAAACCCCCGCAA
CGTCTTGCACACCTATCTCTCTGCCTATCCAAAAACCACACCAACCAATGAACCAACTACCTACCGACCA
ACAGTCGTGGCTCCAGTCACGATCTGCGTGAGCAGTCACCCGACGGCACCCTGAGGCGTCAAAGGTTTGG
CCGCAAGTTTATACTGTAATGCTTGCTACTCGTTTCATCCACTTGCCAAGTGGCAAATGTGCTTACTCAC
CATCTAATCACGAATGGAGTTAGTGATTGCCTAACATCTGATATACCGAAACCATACCAAGATACTTGTT
TAAATCCCCAAAGTAGCGCTAAAATACGTAGCTGCTAACGCACAATTCATACCAACCGAGCACTCGTCAA
AATaaaaaaaaaaaCACCCTGCTTAATCACTGCCGCTTAACCTATTGCTTGTTTAATCCGATACCTAAGT
ATAATTTTGTAACTAACGATATATATTCTCTTTTTTCACAGCTTTAAGGGATCTCGTGACCAGGGACTCA
ATGCCCGCAAGGGAAGGTGAGTTCTCGTTGGGCTTGTATTAtttttttttGTAATTCAAGAATAGATAGAT
TCCGCATGTACTTCGAAATTCTCAAATCGAATTTGTTTTGTTTTCAACTTTTCAACTACTTTTGGAAATG
CTGCGTCTACAACTATTCGAAATATTTGTATATTTAAATTTGTAATATCCATGTATTATCTATGTTAATG
TGTTTTGCGTATTTTGTTCGTTACTGTACCATTTTGTTTTGTCTCTTTTTGATTCTACTTTCATCTTGTC
TACACACTACGCTACTACGTAAACGTTGCTCATACCGCTCGAATATCAAATATCAAAACTTTGCATCTTG

CCGCTTTATTTGCCTATGTCTATGTCAAAAACCAAAATCTCTGAGCAGTGTGAGTTCTCGTTGGGCTTGT
ATTAttttttttGTAATTCAAGAATAGATAGATTCCGCATGTACTTCGAAATTCTCAAATCGAATTTGTT
TTGTTTTCAACTTTTCAACTACTTTTGGAAATGCTGCGTCTACAACTATTCGAAATATTTGTATATTTAA
ATTTGTAATATCCATGTATTATCTATGTTAATGTGTTTTGCGTATTTTGTTCGTTACTGTACCATTTTGT
TTTGTCTCTTTTTGATTCTACTTTCATCTTGTCTACACACTACGCTACTACGTAAACGTTGCTCATACCG
CTCGAATATCAAATATCAAAACTTTGCATCTTGCCGCTTTATTTGCCTATGTCTATGTCAAAAACCAAAA
TCTCTGAGCAGTCGTATCACGCCCACACGTGACACGCCCGACAGAGATCGCCTGCCGCACTATGGCCCCC
GATTCTCGCCAAGGTAAGTCCTTGAAATCCTCCAAAATCATCCATATCATCTCAAGCTGTGCATGTCCAG
TACAATTGTTTGTATTTAGTTTGGTTCATGATGTTCACCGGGTTTGAAGAACTTCTAATTAACAGAAATA
TCACATTTTAATATAGCCACCTCGGGACCGGAACTGGAATTCCGTTCGCCACGCGCGAAGCTTCTGTGGA
CCAAGTGGCGCGATGTCTGGATGCTCTCATGGGAGCGCCAGCGGCTGCTTAACGATCATCTTCTGTATCT
GAAGGATGTGGAGCGTGCCCGCAACTTCAGCTGGGACGATTGGCGCAAGCGCGAATTCGATACATCTGGC
TTGGAAATGAAGGCTGTAGCCGATTTGTTCGATCGCAATGGCGAAGGCCTCATCGACTGGCAAGAGTTCA
TTGCTGCCCTTCGTCCCGATTGGCAAGAGCGTAAGCCAGCCAATGATTCGGACAAAATACACGATGAGGT
CAAACGTCTGGTCATGCTGTGTACCTGCCGACAGAAGTTCCGTGTGTTCCAAGTTGGCGAGGGCAAGTAC
AGAGTAAGTAGCAAGAGTAGTGTGTCACCTGGTCGGCAGTTACTAATGTGTAAAATAACTTATTTCAGTT
TGGAGACTCCCAGAAACTGCGCCTCGTTCGTATCCTTCGCAGCACTGTTATGGTGCGCGTGGGTGGCGGT
TGGGTTGCCCTGGATGAATTCCTGCAGAAGAACGATCCTTGTCGCGG
>dme_circ_0004263_junction_seq
TCCTGCAGAAGAACGATCCTTGTCGCGGCAAGCAGTATTCGAGAAGTTCAACATGT
>dme_circ_0004264
GGATCGTTCTTCTGCAGGAATTCATCCAGGGCAACCCAACCGCCACCCACGCGCACCATAACAGTGCTGC
GAAGGATACGAACGAGGCGCAGTTTCTGGGAGTCTCCAAACTGAAATAAGTTATTTTACACATTAGTAAC
TGCCGACCAGGTGACACACTACTCTTGCTACTTACTCTGTACTTGCCCTCGCCAACTTGGAACACACGGA
ACTTCTGTCGGCAGGTACACAGCATGACCAGACGTTTGACCTCATCGTGTATTTTGTCCGAATCATTGGC
TGGCTTACGCTCTTGCCAATCGGGACGAAGGGCAGCAATGAACTCTTGCCAGTCGATGAGGCCTTCGCCA
TTGCGATCGAACAAATCGGCTACAGCCTTCATTTCCAAGCCAGAT
>dme_circ_0004264_junction_seq
GGCTACAGCCTTCATTTCCAAGCCAGATGGATCGTTCTTCTGCAGGAATTCATCCA
>dme_circ_0004265
ATTCCTGGAGATCCGCAACTGCTAGAGGTTGAATTGGCCAAGCTAAAGGTGCTGGCCAACGACATTCAGG
CCCACCAGAACTCAGTGGACACACTGAACGATGCGGGCCGACAGTTGATTGAAACCGAGAAGGGATCTGT
GGAGGCGTCTACTACCCAGGAGAAGCTGCGCAAACTGAACAACGAGTGGAAGCAGCTTCTGCAAAAGGCC
AGCGACCGTCAGCATGAACTGGAGGAGGCTCTACGTGAAGCTCACGGATATATTGCCGAGGTCCAGGATA
TTCTGGGATGGCTGGGCGATGTAGACGCAGTGATTGGAGCTAGCAAGCCTGTGGGCGGACTTCCCGAGAC
TGCTACTGAGCAACTGGAACGTTTCATGGAGGTGTACAACGAGCTGGATGAAAACAGACCCAAGGTGGAG
ACGATTCAGGCCCAGGGCCAGGAGTACATCAAGCGTCAGAACCAAATGAAGGTCTCGTCCAGCAACTTAC
AGCATACACTCCGCACGCTGAAGCAACGTTGGGATGCCGTGGTATCCCGTGCATCCGACAAGAAGATTAA
GTTGGAAATTGCACTCAAGGAAGCCACCGAGTTCCATGACACACTGCAAGCCTTTGTTGAATGGCTGACC
CAGGCCGAAAAGCTGTTGTCCAATGCTGAACCCGTCTCACGCGTTCTGGAAACCATTCAGGCCCAGATGG
AGGAGCACAAGGTGCTGCAGAAGGATGTGAGCACTCATCGCGAAGCCATGCTGCTGCTGGACAAGAAGGG
AACTCACCTTAAGTACTTCAGCCAGAAGCAGGACGTGATCTTGATTAAGAACCTGCTCGTGTCGGTGCAA
CATCGTTGGGAACGAGTGGTTAGCAAGGCTGCGGAACGCACTCGTGCTCTGGACCATGGCTACAAAGAGG
CTCGCGAGTTCAACGACGCCTGGAGTGGCATGATGCAGTATCTGCAGGAAACCGAACAAGTGCTGGATCA

AATTATCGAGGAAGCCACAGCGTCCAAGGAACCACAAAAGATCAAGAAGTACATTGGCAAGCTGAAAGAG
ACGCATCGTCAACTGGGCGCCAAGCAATCGGTCTACGACGGAACCATGCGCACCGGAAAGAATCTTTTGG
AGCGAGCTCCCAAGGGCGATCGCCCTGTGTTGGACAAAATGCTGATCGAACTGAAGGAACAATGGACTCG
AGTGTGGTCCAAGAGCATCGATCGCCAGCGCAAGCTGGAGGAGGCTTTGCTTCTTTCTGGACAGTTCAGC
GATGCTCTGGGTGAGCTTCTTGACTGGTTGAAGAAGGCTAAGAGCCGCCTGAACGAAAATGGACCAGTCC
ACGGAGATTTGGAGACGGTTCAAGGTCTATGCGAGCACCACAAGCACATTGAGCAGGATCTGCAGAAGCG
CGCTGCGCAAATGCAAGGAGTGCTCAAGACTGGACGCGACCTGGAGCGATCTGGAAACAATCCCGAAGTT
GGACGTCAGCTCGATGAGATGCAGTCCATTTGGGAGGAGGTCAAGAGTGCCGTCGCCAAACGCGGCGAGC
GTCTCCAGGTGGCCCTTGTTGATGCCGAGAAGCTTAATGCCCGTGTCCAGGCTCTCTTTGATTGGCTGGA
TCATGCCGAGCACAAGCTACGATATGCCAAGAATGCCCCTGATGACGAGAAGGTGTCCCGTGAAATGATG
GACATCCACATGGACTTCATGAAGGACCTGCGAGTGCGCGAGCGTGAAAAGACCGAGACTTTTGAGTACG
CCGAGGACATTATCAACAAGGCCTATCCCGATGCCATTCCGATCATCAAGAACTGGCTTTCGATTATCCA
GCAGCGTTGGGAGGAGGTGCGCCAATGGGCCATCAATCGTGAGTCGAAGCTGGAGCAGCATCTGCAGTCG
CTCAAGGATTTGGACGACACCATTGAGGAGCTGTTGGCTTGGCTCAGCGGACTCGAGGGCACCTTGTTGA
GACCTGAAGCACGAACAGCTGCCGGACGAGATTCCACCCGTGGAGAAATTGATCGAGGACCACAAGGAGT
TCATGGAAAACACTGCTCGCCGTCAAAACGAAGTGGACCGCGCATGCAAACCAAGACAGTTGCCACCAGG
AGCGCGCAAGCCGTCGCGCAGCGGCAAGACGCCAGTGGTGAGTAGAATAATAGATTCGCAAGTAGCTGCC
GAAACTGTAGCTCTGCTGTACATATCCTTGGtatattattattattttgtatatttatttttttccaattt
ctttttgtatactttcttTTGTATCCACCACCTTATTGCTACAATATTCCAAACAAAAACCAAAAAACCC
CCGCAACGTCTTGCACACCTATCTCTCTGCCTATCCAAAAACCACACCAACCAATGAACCAACTACCTAC
CGACCAACAGTCGTGGCTCCAGTCACGATCTGCGTGAGCAGTCACCCGACGGCACCCTGAGGCGTCAAAG
GTTTGGCCGCAAGTTTATACTGTAATGCTTGCTACTCGTTTCATCCACTTGCCAAGTGGCAAATGTGCTT
ACTCACCATCTAATCACGAATGGAGTTAGTGATTGCCTAACATCTGATATACCGAAACCATACCAAGATA
CTTGTTTAAATCCCCAAAGTAGCGCTAAAATACGTAGCTGCTAACGCACAATTCATACCAACCGAGCACT
CGTCAAAATaaaaaaaaaaaCACCCTGCTTAATCACTGCCGCTTAACCTATTGCTTGTTTAATCCGATAC
CTAAGTATAATTTTGTAACTAACGATATATATTCTCTTTTTTCACAGCTTTAAGGGATCTCGTGACCAGG
GACTCAATGCCCGCAAGGGAAGGTGAGTTCTCGTTGGGCTTGTATTAttttttttGTAATTCAAGAATAG
ATAGATTCCGCATGTACTTCGAAATTCTCAAATCGAATTTGTTTTGTTTTCAACTTTTCAACTACTTTTG
GAAATGCTGCGTCTACAACTATTCGAAATATTTGTATATTTAAATTTGTAATATCCATGTATTATCTATG
TTAATGTGTTTTGCGTATTTTGTTCGTTACTGTACCATTTTGTTTTGTCTCTTTTTGATTCTACTTTCAT
CTTGTCTACACACTACGCTACTACGTAAACGTTGCTCATACCGCTCGAATATCAAATATCAAAACTTTGC
ATCTTGCCGCTTTATTTGCCTATGTCTATGTCAAAAACCAAAATCTCTGAGCAGTGTGAGTTCTCGTTGG
GCTTGTATTAttttttttGTAATTCAAGAATAGATAGATTCCGCATGTACTTCGAAATTCTCAAATCGAA
TTTGTTTTGTTTTCAACTTTTCAACTACTTTTGGAAATGCTGCGTCTACAACTATTCGAAATATTTGTAT
ATTTAAATTTGTAATATCCATGTATTATCTATGTTAATGTGTTTTGCGTATTTTGTTCGTTACTGTACCA
TTTTGTTTTGTCTCTTTTTGATTCTACTTTCATCTTGTCTACACACTACGCTACTACGTAAACGTTGCTC
ATACCGCTCGAATATCAAATATCAAAACTTTGCATCTTGCCGCTTTATTTGCCTATGTCTATGTCAAAAA
CCAAAATCTCTGAGCAGTCGTATCACGCCCACACGTGACACGCCCGACAGAGATCGCCTGCCGCACTATG
GCCCCCGATTCTCGCCAAGGTAAGTCCTTGAAATCCTCCAAAATCATCCATATCATCTCAAGCTGTGCAT
GTCCAGTACAATTGTTTGTATTTAGTTTGGTTCATGATGTTCACCGGGTTTGAAGAACTTCTAATTAACA
GAAATATCACATTTTAATATAGCCACCTCGGGACCGGAACTGGAATTCCGTTCGCCACGCGCGAAGCTTC
TGTGGACCAAGTGGCGCGATGTCTGGATGCTCTCATGGGAGCGCCAGCGGCTGCTTAACGATCATCTTCT
GTATCTGAAGGATGTGGAGCGTGCCCGCAACTTCAGCTGGGACGATTGGCGCAAGCGCGAATTCGATACA
TCTGGCTTGGAAATGAAGGCTGTAGCCGATTTGTTCGATCGCAATGGCGAAGGCCTCATCGACTGGCAAG

AGTTCATTGCTGCCCTTCGTCCCGATTGGCAAGAGCGTAAGCCAGCCAATGATTCGGACAAAATACACGA
TGAGGTCAAACGTCTGGTCATGCTGTGTACCTGCCGACAGAAGTTCCGTGTGTTCCAAGTTGGCGAGGGC
AAG
>dme_circ_0004265_junction_seq
CCGTGTGTTCCAAGTTGGCGAGGGCAAGATTCCTGGAGATCCGCAACTGCTAGAGG
>dme_circ_0004266
ACAAGCTGTGGAACTGACGGAACGCACTTCACCCGAGCAAGCGGCATCGATTCGCGAACCCCTGTCGGTG
GTCAATCGCCGCTGGGAGGCCCTACTCCGTGGCATGGTCGAGCGCCAGAAGCAACTGGAGCACGCTCTGC
TGCACTTGGGTCAATTCCAGCATGCTCTCAACGAGCTGCTGGTATGGATCAACAAGACCGACAGCACACT
GGACCAACTGAAGCCGGATTCCTGGAGATCCGCAACTGCTAGAGGTTGAATTGGCCAAGCTAAAGGTGCT
GGCCAACGACATTCAGGCCCACCAGAACTCAGTGGACACACTGAACGATGCGGGCCGACAGTTGATTGAA
ACCGAGAAGGGATCTGTGGAGGCGTCTACTACCCAGGAGAAGCTGCGCAAACTGAACAACGAGTGGAAGC
AGCTTCTGCAAAAGGCCAGCGACCGTCAGCATGAACTGGAGGAGGCTCTACGTGAAGCTCACGGATATAT
TGCCGAGGTCCAGGATATTCTGGGATGGCTGGGCGATGTAGACGCAGTGATTGGAGCTAGCAAGCCTGTG
GGCGGACTTCCCGAGACTGCTACTGAGCAACTGGAACGTTTCATGGAGGTGTACAACGAGCTGGATGAAA
ACAGACCCAAGGTGGAGACGATTCAGGCCCAGGGCCAGGAGTACATCAAGCGTCAGAACCAAATGAAGGT
CTCGTCCAGCAACTTACAGCATACACTCCGCACGCTGAAGCAACGTTGGGATGCCGTGGTATCCCGTGCA
TCCGACAAGAAGATTAAGTTGGAAATTGCACTCAAGGAAGCCACCGAGTTCCATGACACACTGCAAGCCT
TTGTTGAATGGCTGACCCAGGCCGAAAAGCTGTTGTCCAATGCTGAACCCGTCTCACGCGTTCTGGAAAC
CATTCAGGCCCAGATGGAGGAGCACAAGGTGCTGCAGAAGGATGTGAGCACTCATCGCGAAGCCATGCTG
CTGCTGGACAAGAAGGGAACTCACCTTAAGTACTTCAGCCAGAAGCAGGACGTGATCTTGATTAAGAACC
TGCTCGTGTCGGTGCAACATCGTTGGGAACGAGTGGTTAGCAAGGCTGCGGAACGCACTCGTGCTCTGGA
CCATGGCTACAAAGAGGCTCGCGAGTTCAACGACGCCTGGAGTGGCATGATGCAGTATCTGCAGGAAACC
GAACAAGTGCTGGATCAAATTATCGAGGAAGCCACAGCGTCCAAGGAACCACAAAAGATCAAGAAGTACA
TTGGCAAGCTGAAAGAGACGCATCGTCAACTGGGCGCCAAGCAATCGGTCTACGACGGAACCATGCGCAC
CGGAAAGAATCTTTTGGAGCGAGCTCCCAAGGGCGATCGCCCTGTGTTGGACAAAATGCTGATCGAACTG
AAGGAACAATGGACTCGAGTGTGGTCCAAGAGCATCGATCGCCAGCGCAAGCTGGAGGAGGCTTTGCTTC
TTTCTGGACAGTTCAGCGATGCTCTGGGTGAGCTTCTTGACTGGTTGAAGAAGGCTAAGAGCCGCCTGAA
CGAAAATGGACCAGTCCACGGAGATTTGGAGACGGTTCAAGGTCTATGCGAGCACCACAAGCACATTGAG
CAGGATCTGCAGAAGCGCGCTGCGCAAATGCAAGGAGTGCTCAAGACTGGACGCGACCTGGAGCGATCTG
GAAACAATCCCGAAGTTGGACGTCAGCTCGATGAGATGCAGTCCATTTGGGAGGAGGTCAAGAGTGCCGT
CGCCAAACGCGGCGAGCGTCTCCAGGTGGCCCTTGTTGATGCCGAGAAGCTTAATGCCCGTGTCCAGGCT
CTCTTTGATTGGCTGGATCATGCCGAGCACAAGCTACGATATGCCAAGAATGCCCCTGATGACGAGAAGG
TGTCCCGTGAAATGATGGACATCCACATGGACTTCATGAAGGACCTGCGAGTGCGCGAGCGTGAAAAGAC
CGAGACTTTTGAGTACGCCGAGGACATTATCAACAAGGCCTATCCCGATGCCATTCCGATCATCAAGAAC
TGGCTTTCGATTATCCAGCAGCGTTGGGAGGAGGTGCGCCAATGGGCCATCAATCGTGAGTCGAAGCTGG
AGCAGCATCTGCAGTCGCTCAAGGATTTGGACGACACCATTGAGGAGCTGTTGGCTTGGCTCAGCGGACT
CGAGGGCACCTTGTTGAGACCTGAAGCACGAACAGCTGCCGGACGAGATTCCACCCGTGGAGAAATTGAT
CGAGGACCACAAGGAGTTCATGGAAAACACTGCTCGCCGTCAAAACGAAGTGGACCGCGCATGCAAACCA
AGACAGTTGCCACCAGGAGCGCGCAAGCCGTCGCGCAGCGGCAAGACGCCAGTGGTGAGTAGAATAATAG
ATTCGCAAGTAGCTGCCGAAACTGTAGCTCTGCTGTACATATCCTTGGtatattattattattttgtata
tttattttttccaatttctttttgtatactttcttTTGTATCCACCACCTTATTGCTACAATATTCCAAA
CAAAAACCAAAAAACCCCCGCAACGTCTTGCACACCTATCTCTCTGCCTATCCAAAAACCACACCAACCA
ATGAACCAACTACCTACCGACCAACAGTCGTGGCTCCAGTCACGATCTGCGTGAGCAGTCACCCGACGGC

ACCCTGAGGCGTCAAAGGTTTGGCCGCAAGTTTATACTGTAATGCTTGCTACTCGTTTCATCCACTTGCC
AAGTGGCAAATGTGCTTACTCACCATCTAATCACGAATGGAGTTAGTGATTGCCTAACATCTGATATACC
GAAACCATACCAAGATACTTGTTTAAATCCCCAAAGTAGCGCTAAAATACGTAGCTGCTAACGCACAATT
CATACCAACCGAGCACTCGTCAAAATaaaaaaaaaaaCACCCTGCTTAATCACTGCCGCTTAACCTATTG
CTTGTTTAATCCGATACCTAAGTATAATTTTGTAACTAACGATATATATTCTCTTTTTTCACAGCTTTAA
GGGATCTCGTGACCAGGGACTCAATGCCCGCAAGGGAAGG
>dme_circ_0004266_junction_seq
CCAGGGACTCAATGCCCGCAAGGGAAGGACAAGCTGTGGAACTGACGGAACGCACT
>dme_circ_0004267
GTCTATGCGAGCACCACAAGCACATTGAGCAGGATCTGCAGAAGCGCGCTGCGCAAATGCAAGGAGTGCT
CAAGACTGGACGCGACCTGGAGCGATCTGGAAACAATCCCGAAGTTGGACGTCAGCTCGATGAGATGCAG
TCCATTTGGGAGGAGGTCAAGAGTGCCGTCGCCAAACGCGGCGAGCGTCTCCAGGTGGCCCTTG
>dme_circ_0004267_junction_seq
CGCGGCGAGCGTCTCCAGGTGGCCCTTGGTCTATGCGAGCACCACAAGCACATTGA
>dme_circ_0004268
ATTTGGAGACGGTTCAAGGTCTATGCGAGCACCACAAGCACATTGAGCAGGATCTGCAGAAGCGCGCTGC
GCAAATGCAAGGAGTGCTCAAGACTGGACGCGACCTGGAGCGATCTGGAAACAATCCCGAAGTTGGACGT
CAGCTCGATGAGATGCAGTCCATTTGGGAGGAGG
>dme_circ_0004268_junction_seq
GATGAGATGCAGTCCATTTGGGAGGAGGATTTGGAGACGGTTCAAGGTCTATGCGA
>dme_circ_0004269
AATCTTTTGGAGCGAGCTCCCAAGGGCGATCGCCCTGTGTTGGACAAAATGCTGATCGAACTGAAGGAAC
AATGGACTCGAGTGTGGTCCAAGAGCATCGATCGCCAGCGCAAGCTGGAGGAGGCTTTGCTTCTTTCTGG
ACAGTTCAGCGATGCTCTGGGTGAGCTTCTTGACTGGTTGAAGAAGGCTAAGAGCCGCCTGAACGAAAAT
GGACCAGTCCACGGAGATTTGGAGACGG
>dme_circ_0004269_junction_seq
GGACCAGTCCACGGAGATTTGGAGACGGAATCTTTTGGAGCGAGCTCCCAAGGGCG
>dme_circ_0004270
CAGCTTCTGCAAAAGGCCAGCGACCGTCAGCATGAACTGGAGGAGGCTCTACGTGAAGCTCACGGATATA
TTGCCGAGGTCCAGGATATTCTGGGATGGCTGGGCGATGTAGACGCAGTGATTGGAGCTAGCAAGCCTGT
GGGCGGACTTCCCGAGACTGCTACTGAGCAACTGGAACG
>dme_circ_0004270_junction_seq
CCGAGACTGCTACTGAGCAACTGGAACGCAGCTTCTGCAAAAGGCCAGCGACCGTC
>dme_circ_0004271
ATTCCTGGAGATCCGCAACTGCTAGAGGTTGAATTGGCCAAGCTAAAGGTGCTGGCCAACGACATTCAGG
CCCACCAGAACTCAGTGGACACACTGAACGATGCGGGCCGACAGTTGATTGAAACCGAGAAGGGATCTGT
GGAGGCGTCTACTACCCAGGAGAAGCTGCGCAAACTGAACAACGAGTGGAAGCAGCTTCTGCAAAAGGCC
AGCGACCGTCAGCATGAACTGGAGGAGGCTCTACGTGAAGCTCACGGATATATTGCCGAGGTCCAGGATA
TTCTGGGATGGCTGGGCGATGTAGACGCAGTGATTGGAGCTAGCAAGCCTGTGGGCGGACTTCCCGAGAC
TGCTACTGAGCAACTGGAACG
>dme_circ_0004271_junction_seq
CCGAGACTGCTACTGAGCAACTGGAACGATTCCTGGAGATCCGCAACTGCTAGAGG
>dme_circ_0004272
GTTGCTCAGTAGCAGTCTCGGGAAGTCCGCCCACAGGCTTGCTAGCTCCAATCACTGCGTCTACATCGCC

CAGCCATCCCAGAATATCCTGGACCTCGGCAATATATCCGTGAGCTTCACGTAGAGCCTCCTCCAGTTCA
TGCTGACGGTCGCTGGCCTTTTGCAGAAGCTGCTTCCACTCGTTGTTCAGTTTGCGCAGCTTCTCCTGGG
TAGTAGACGCCTCCACAGATCCCTTCTCG
>dme_circ_0004272_junction_seq
AGTAGACGCCTCCACAGATCCCTTCTCGGTTGCTCAGTAGCAGTCTCGGGAAGTCC
>dme_circ_0004273
GTTGGTCCAGTGTGCTGTCGGTCTTGTTGATCCATACCAGCAGCTCGTTGAGAGCATGCTGGAATTGACC
CAAGTGCAGCAGAGCGTGCTCCAGTTGCTTCTGGCGCTCGACCATGCCACGGAGTAGGGCCTCCCAGCGG
CGATTGACCACCGACAGGGGTTCGCGAATCGATGCCGCTTGCTCGGGTGAA
>dme_circ_0004273_junction_seq
GCGAATCGATGCCGCTTGCTCGGGTGAAGTTGGTCCAGTGTGCTGTCGGTCTTGTT
>dme_circ_0004274
ACACTGCAGAACCTCCTCAAGTTCTTGACCAAGGCCGAGGACAAGTTTGCCCATCTGGGAGCTGTGGGTT
CGGACATTGATGCCGTCAAGCGACAGATTGAACAACTCAAGTCGTTCAAGGATGAAGTCGATCCGCATAT
GGTTGAAGTAGAAGCATTAAACAGG
>dme_circ_0004274_junction_seq
TATGGTTGAAGTAGAAGCATTAAACAGGACACTGCAGAACCTCCTCAAGTTCTTGA
>dme_circ_0004275
ATCGACCAGACCAAACCAGAGGTCGAGCAGGTGCGACGTCACGGAAGCAACCTGATGAACATGTGCGGCG
AGCCCGACAAGCCAGAGGTCAAGAAACACATCGAGGACTTGGACAACGCATGGGACAACATCACGGCGCT
GTATGCCAAGCGCGAGGAGAACCTCATCGATGCCATGGAGAAGGCCATGGAGTTCCACGAGACACTGCAG
AACCTCCTCAAGTTCTTGACCAAGGCCGAGGACAAGTTTGCCCATCTGGGAGCTGTGGGTTCGGACATTG
ATGCCGTCAAGCGACAGATTGAACAACTCAAGTCGTTCAAGGATGAAGTCGATCCGCATATGGTTGAAG
>dme_circ_0004275_junction_seq
GATGAAGTCGATCCGCATATGGTTGAAGATCGACCAGACCAAACCAGAGGTCGAGC
>dme_circ_0004276
AGGTCAAGAAACACATCGAGGACTTGGACAACGCATGGGACAACATCACGGCGCTGTATGCCAAGCGCGA
GGAGAACCTCATCGATGCCATGGAGAAGGCCATGGAGTTCCACGAGACACTGCAGAACCTCCTCAAGTTC
TTGACCAAGGCCGAGGACAAG
>dme_circ_0004276_junction_seq
CAAGTTCTTGACCAAGGCCGAGGACAAGAGGTCAAGAAACACATCGAGGACTTGGA
>dme_circ_0004277
ATCAACGAGATTTTGGACACGGACAATGCCCGTTATGCCGCACTCCGCTTGGAACTCCGCGAACGGCAGC
AGGCGCTCGAGAGTGCTCTGCAGGAATCCAGCCAGTTCTCCGATAAACTGGAGGGCATGCTGCGTGCTCT
GGCCAACACCGTGGATCAGG
>dme_circ_0004277_junction_seq
CGTGCTCTGGCCAACACCGTGGATCAGGATCAACGAGATTTTGGACACGGACAATG
>dme_circ_0004278
GGTTTATGTTCCGCTATGGATTGAAGAAGACGTTCGTTCTTGTCCTGCTGCAGAGTGATCTGGTCAGGAC
GCAAAGCCGGCATGCTCAATCGCGAAATCTGCTGCTCCATGTCATCCAACCAGGCGGTGAGTCCCTGATG
GGAATCGGCGAAATGTTCAGACAGGGGCAGAGCCTGTTCCAGGATGCCCAGACGCTCGCTGCACAATTGA
GCAACAGTGTCGACGATCTCCTTCAGATCATCCAATTTCTCACGAAGAGTGGCGGTGTTCTCGCTCTGTG
GGGACTCGCGGAGCACCTTCTTGGAGGCCGCAGTAACATCTCGTACACGTCCCTTCTGGCTGGAAATGTC
ATCGTTGATAGAACGATGCTCCTGCAATTGGGCACGCACCAACTTGGGCTCCATGGCAGGCAGATCGGCT

TCACGAAGAGTGGTGTCCATTTCGCGGAACCACTCAAGCAGTTCATCAATGTCACCTGTCACTTCCTTGG
CGCGCTGGTTGCTCAGGTGGAGACGCTCCGCCTTGCGTTGAATTTGCTCCACAATGGAATCGAAGCGACG
GTT
>dme_circ_0004278_junction_seq
GCTCCACAATGGAATCGAAGCGACGGTTGGTTTATGTTCCGCTATGGATTGAAGAA
>dme_circ_0004279
GAAAGCTGACACAGCTGGGGTCCCACTTGATTGATACTGTCCAGAATAGGACGCATATCAGCCAATTCAC
CCTCCAGCCGAAGCACATCAGCTTGACGTGGCTCGCTGGGAGCAAGAGCAGCTTCAGCACTTTGCATCCA
TTCCACTAAACGGTTGTGAGCTTCATGGAATTGCTGCACCAATGGTAGTGCATTCTGTGCACTCTTCAGC
AGATCTCCACCACGGTTG
>dme_circ_0004279_junction_seq
ACTCTTCAGCAGATCTCCACCACGGTTGGAAAGCTGACACAGCTGGGGTCCCACTT
>dme_circ_0004280
CGACAACATTGGGGCTCTGCTAGCCGAATCACGCCAGGGATTGCGTCACTTGGTCTTGAGCTATCAGGAT
CTGGTGGCCTGGATGGAAAGTATGGAAGCCGAGTTGAAGCGCTTTAAGTCCGTTCCCGTTTACGCTGAAA
AGCTGCTGGAACAGATGGATCATCTGTTGGAGCTAAACGAGAACATTGCCGG
>dme_circ_0004280_junction_seq
TGTTGGAGCTAAACGAGAACATTGCCGGCGACAACATTGGGGCTCTGCTAGCCGAA
>dme_circ_0004281
AGCGGTACACAGGATTGGTGGATGCCAGCGACAACATTGGGGCTCTGCTAGCCGAATCACGCCAGGGATT
GCGTCACTTGGTCTTGAGCTATCAGGATCTGGTGGCCTGGATGGAAAGTATGGAAGCCGAGTTGAAGCGC
TTTAAGTCCGTTCCCGTTTACGCTGAAAAGCTGCTGGAACAGATGGATCATCTGTTGGAGCTAAACGAGA
ACATTGCCGG
>dme_circ_0004281_junction_seq
TGTTGGAGCTAAACGAGAACATTGCCGGAGCGGTACACAGGATTGGTGGATGCCAG
>dme_circ_0004282
GATTAACGGCTTCCTCGTCGCTAACCAAATGCATCAGCTGGGCGGTGACATCGGCAAGGTCGGAAAAGTC
GGGCTTCTTTCCCAAGATTTCGTCGTGTAGACGGTCGTGTTCGCGGATTCGCTGCTGGATCTTCTCTTCG
TCAGTAGGTATCAGTTCCATTGCCTTAACAGATCGCTCCGTGTTGTCCAGCCAAAGTTCCAAGGGACTAA
TCTTGTCATGGAATCGCTTAGCCACTTCCATGGCTTCCTCAAGATCCAACTCACGTTGCTCGGCTCCGTC
GGTCAGGGCATCAAAGCGTTTCATCAAATC
>dme_circ_0004282_junction_seq
TCAGGGCATCAAAGCGTTTCATCAAATCGATTAACGGCTTCCTCGTCGCTAACCAA
>dme_circ_0004283
GGTATCAGTTCCATTGCCTTAACAGATCGCTCCGTGTTGTCCAGCCAAAGTTCCAAGGGACTAATCTTGT
CATGGAATCGCTTAGCCACTTCCATGGCTTCCTCAAGATCCAACTCACGTTGCTCGGCTCCGTCGGTCAG
GGCATCAAAGCGTTTCATCAAATCGTTCAACTGTTTCTCAATGGAGGCACGTTCGCCGG
>dme_circ_0004283_junction_seq
TGTTTCTCAATGGAGGCACGTTCGCCGGGGTATCAGTTCCATTGCCTTAACAGATC
>dme_circ_0004284
GTTCCATTGCCTTAACAGATCGCTCCGTGTTGTCCAGCCAAAGTTCCAAGGGACTAATCTTGTCATGGAA
TCGCTTAGCCACTTCCATGGCTTCCTCAAGATCCAACTCACGTTGCTCGGCTCCGTCGGTCAGGGCATCA
AAGCGTTTCATCAAATCGTTCAACT
>dme_circ_0004284_junction_seq

TCAAAGCGTTTCATCAAATCGTTCAACTGTTCCATTGCCTTAACAGATCGCTCCGT
>dme_circ_0004285
GTTTTGACGATCCAAAAGCATTTTCTTGAGGAACTTTTGCTCCTGAAGCTGTGCCTTTACCACCTTATAA
TCGGAGCTAGGCGGCTTCTGATTAGCCACCATCTCCTCGGTGTCGCTGAGCCACTTGGATAAACCGGCCA
ACGCCTCCTGGAACTTTCCAGATTGCAGCAGAGCCACATCCAAACGGCGATCGCGCTCGTTCATTCGCTC
CTTCAAATCGTTCCAGCGGTC
>dme_circ_0004285_junction_seq
TTCGCTCCTTCAAATCGTTCCAGCGGTCGTTTTGACGATCCAAAAGCATTTTCTTG
>dme_circ_0004286
GATCCTTCTCAATGGCCGTGGTAGAAACACCACTGCCAGCCGAACGCACTAGGTCTCTGCCAGCAACGTT
TACCTTGTCCACATTGATGGCCAGTGGTTCCACCTTGCGCTCCCTGAAGTTACGGAAGTCCTCTTGCTGG
CGGCGAATCTGATCCAACTCCGAGCCAACGGGCTTCATGCGCTTGAACTCATCGCTTACGTCTTGAATGT
CATCCAGAGTTTGCGATTGATGGTCGTAGAATTCGCGAAGTGCTTTCAAAGTGGAATGAAGATTATCCTC
GTGATCGCGAACTCTGTTGTCCAAACGACCCAATGTCTTACGCAGC
>dme_circ_0004286_junction_seq
GTCCAAACGACCCAATGTCTTACGCAGCGATCCTTCTCAATGGCCGTGGTAGAAAC
>dme_circ_0004287
GAGTTTGCGATTGATGGTCGTAGAATTCGCGAAGTGCTTTCAAAGTGGAATGAAGATTATCCTCGTGATC
GCGAACTCTGTTGTCCAAACGACCCAATGTCTTACGCAGCGTGGAGATTTGCTCACGGGTCTGGGTGGTG
TCTGCAGCGAATCCTGCATCTACCAAAACATCAGCGGCACGTTCTGCATCCTCCAAACGACCCACAAGGC
GATCCAATTTGGTCTGGATCTTGCCAACATCGTCGATTTGCACTTGGACAATCTTGATTTCTCGACCGGG
CGGTGAAAGCTTTTCAATCTCGGTCTCCAGATCGTTCAGATCAATGCCCAAACTCTTCATTTGTTCGTTG
AATTGGGATACGGCGGTGGCTGCAGATTGCAGTTCTCCGCAGCGATCATCCAGGCGACCCTGTAGCTCGG
ACATGCGGTCGGCCAGGCCATCGACTTCACTGGTCAAATGATCAGCATCTCCGCCAGCAGCTCTAGCCTC
AGCACTGATATCCTTGGCCAGAGCCTTAAGTGACTGCAAAGGCTTGCTCAAGTTAGTGAGCTCTTCGCGT
ATGGCCTTAACACGCTCCAAAAGTTTGGGATCCTTGGCAGCTCCACCGAGAGCATCGTGGGCAGCAAGTC
GATCCTCGCAACGACCTAAAGAATGATCCAG
>dme_circ_0004287_junction_seq
CCTCGCAACGACCTAAAGAATGATCCAGGAGTTTGCGATTGATGGTCGTAGAATTC
>dme_circ_0004288
TTAAACGTGATTTGGATCGCATCCAGCAGCAGTGGGAGAAACTGCGCCGTGAAGCCGTCGATCGACACAC
ACGACTGCAGACGTGCATGGAGCACTGCAAGAAGTACTCCCAAACTTCGGAGACATTCCTGGCCTGGCTG
CG
>dme_circ_0004288_junction_seq
CTTCGGAGACATTCCTGGCCTGGCTGCGTTAAACGTGATTTGGATCGCATCCAGCA
>dme_circ_0004289
GGAATGTCTCCGAAGTTTGGGAGTACTTCTTGCAGTGCTCCATGCACGTCTGCAGTCGTGTGTGTCGATC
GACGGCTTCACGGCGCAGTTTCTCCCACTGCTGCTGGATGCGATCCAAATCACGTTTAACTCCGCGATCC
TGCTGGCGATCG
>dme_circ_0004289_junction_seq
TTTAACTCCGCGATCCTGCTGGCGATCGGGAATGTCTCCGAAGTTTGGGAGTACTT
>dme_circ_0004290
GTTTGGGAGTACTTCTTGCAGTGCTCCATGCACGTCTGCAGTCGTGTGTGTCGATCGACGGCTTCACGGC
GCAGTTTCTCCCACTGCTGCTGGATGCGATCCAAATCACGTTTAACTCCGCGATCCTGCTGGCGATCGGT

TAGATCCTTGCCCTTGTTGATTAGCATAATAACCTCATGCTCACGGGCCATTACCTCACGATAGATCGGC
TCATGGGTGTCAATCTGGTGCTGCAGTGTTGGCTTGTGTGCAGAGACCAAAAGCCTGTCGGTCAGGTTGG
AGAGCTCTCCGCCCAACCAACCAAGAGTCTTTGAGCAATTCTCGGCAAAGTGGCGGCCATCCCGAAGGGA
AGCCTCTGTTTCTGCCTTCTTGGTATCCAGTTTCTTCTGAAGGGCCAAATACTGTTTCTCCAGAGCTGCG
ACACGAGAGTTGACATCAGCGCGCGAGGCGGGATCACCCAGAAT
>dme_circ_0004290_junction_seq
CAGCGCGCGAGGCGGGATCACCCAGAATGTTTGGGAGTACTTCTTGCAGTGCTCCA
>dme_circ_0004291
GTTTCTTCTGAAGGGCCAAATACTGTTTCTCCAGAGCTGCGACACGAGAGTTGACATCAGCGCGCGAGGC
GGGATCACCCAGAATGTTGCATAGAGTGGCGGCGGATTGTTCCACATCGGCTAACAGCGGGCGTTGACCC
TCAAGCTGTCGCTCCAGGTTCTCGATTTTGCGGAGATTCTCCTGGTGATCGCCATCCGTGGGCAAA
>dme_circ_0004291_junction_seq
CTCCTGGTGATCGCCATCCGTGGGCAAAGTTTCTTCTGAAGGGCCAAATACTGTTT
>dme_circ_0004292
GTATCCAGATTATCGCTTATGTTCTGCAGAGCCTCACGCAAGCGATTTACAGCAGCATCGAACTCCTTGG
AACTGTCAGCAGCACCGCCCAAACTATTGGCCCGGTCGTCTAACTTTCCAACCAGATCATCCCACTTGCC
CAGAAT
>dme_circ_0004292_junction_seq
CAACCAGATCATCCCACTTGCCCAGAATGTATCCAGATTATCGCTTATGTTCTGCA
>dme_circ_0004293
GATTATCGCTTATGTTCTGCAGAGCCTCACGCAAGCGATTTACAGCAGCATCGAACTCCTTGGAACTGTC
AGCAGCACCGCCCAAACTATTGGCCCGGTCGTCTAACTTTCCAACCAGATCATCCCACTTGCCCAGAATG
TCCTTCAGTTTGATTTCGACCGCCTGAGCATCGGGTGTGCCGGCCAAATGGTCGACAACATCATGTCCAA
GCTCCTGGAAATGCTTCAGTTGTGGTTGCTGAGTGCGGAATTCCTCGCGAAGCACTTGCACCTGCTGCAC
CTGGCTCTGGACCATACGGGGATCGGAAGAGATGGGTCCCAGCACG
>dme_circ_0004293_junction_seq
GGGATCGGAAGAGATGGGTCCCAGCACGGATTATCGCTTATGTTCTGCAGAGCCTC
>dme_circ_0004294
GATCATCCCACTTGCCCAGAATGTCCTTCAGTTTGATTTCGACCGCCTGAGCATCGGGTGTGCCGGCCAA
ATGGTCGACAACATCATGTCCAAGCTCCTGGAAATGCTTCAGTTGTGGTTGCTGAGTGCGGAATTCCTCG
CGAAGCACTTGCACCTGCTGCACCTGGCTCTGGACCATACGGGGATCGGAAGAGATGGGTCCCAGCACG
>dme_circ_0004294_junction_seq
GGGATCGGAAGAGATGGGTCCCAGCACGGATCATCCCACTTGCCCAGAATGTCCTT
>dme_circ_0004295
CATCCAAGAGAAGTGGAAGAATCTCAACGACATCTGCAAGAATCGCATTGCCTTCTCCGAGAAGCTGCGT
GACTTTTTGGACACCCATGGAAATCTGAAGAGCTGGCTGGACAGTAAGGAACGTATG
>dme_circ_0004295_junction_seq
GAGCTGGCTGGACAGTAAGGAACGTATGCATCCAAGAGAAGTGGAAGAATCTCAAC
>dme_circ_0004296
GGATCTGCGGCTACCGAATCCACTGGAACCAGTCGATAGTGGCGACAGGATGCCTCCCTTGGTGGGGCTG
GGGCTTCTAGCGTCTCCGGACATGCGGCGCAACGGACTGGTCCGCTTAATCGGACTATAA
>dme_circ_0004296_junction_seq
CGGACTGGTCCGCTTAATCGGACTATAAGGATCTGCGGCTACCGAATCCACTGGAA
>dme_circ_0004297

GGACCTGATCCTTCAGTCCCTCGTAGGCCAGTTGTTGTTCGGCCTTTTGCTTGGACAGCTTGGCGCGCTC
ATCGATGCTGATGTTAATGTTGCGCCACTGAGATTCCAGTGCCTTGATGCGATCGCGCAAAACTCCAGTA
TCGGTGACATCACGCAGGCTAATTAGATCCTTGCCACTGCGCACGCAATCCTCAAACTGCGGAGCCAGTT
GGTCCTTGTCACGCGACAGTTCGTTCATGCGGCGAGCCAACTCTTCAGTTGAAAGCAGGCTTGTCTCGCG
GCTATCTAGAGCCTCCTGTAAGCTGGCCATACGCTGCTCCACGGTGCTGATTTCATCCAGATAGCGGCTT
AATCGGTTGTAAAC
>dme_circ_0004297_junction_seq
CCAGATAGCGGCTTAATCGGTTGTAAACGGACCTGATCCTTCAGTCCCTCGTAGGC
>dme_circ_0004298
GATCCTTGCCACTGCGCACGCAATCCTCAAACTGCGGAGCCAGTTGGTCCTTGTCACGCGACAGTTCGTT
CATGCGGCGAGCCAACTCTTCAGTTGAAAGCAGGCTTGTCTCGCGGCTATCTAGAGCCTCCTGTAAGCTG
GCCATACGCTGCTCCACGGTGCTGATTTCATCCAGATAGCGGCTTAATCGGTT
>dme_circ_0004298_junction_seq
TTTCATCCAGATAGCGGCTTAATCGGTTGATCCTTGCCACTGCGCACGCAATCCTC
>dme_circ_0004299
GATCCTTGCCACTGCGCACGCAATCCTCAAACTGCGGAGCCAGTTGGTCCTTGTCACGCGACAGTTCGTT
CATGCGGCGAGCCAACTCTTCAGTTGAAAGCAGGCTTGTCTCGCGGCTATCTAGAGCCTCCTGTAAGCTG
GCCATACGCTGCTCCACGGTGCTGATTTCATCCAGATAGCGGCTTAATCGGTTGTAAAC
>dme_circ_0004299_junction_seq
CCAGATAGCGGCTTAATCGGTTGTAAACGATCCTTGCCACTGCGCACGCAATCCTC
>dme_circ_0004300
GATAGCGGCTTAATCGGTTGTAAACGTCATCTAAGAATTCACCACGTTTGTTGGCCTTCTCGTAGAGCTT
CTCAAACTTGACGGTCACATCGTTTAGATTCGATTCCACCTTCTTGGCAATGCGTGCATTCGATGCGCTG
GCAAGCAGATGTTTGGCCGAAACTTGAACGCTGTCGATGCTGGCCTGGTGAGATTGAAGATCGGCTAGCA
GGACCTTGTGCTCGCGGATCTGCTCCTGCAGACGCTCCTTGATAAGCGAGGCGGGTCGCAGATTCATCTT
GAACTTATCCTCGGCCTGGTTAACCCATCCCACAAGACTGTCCAAGGCATCCTGTACGCCCTGCGATTGC
ACCAAA
>dme_circ_0004300_junction_seq
ATCCTGTACGCCCTGCGATTGCACCAAAGATAGCGGCTTAATCGGTTGTAAACGTC
>dme_circ_0004301
GATAGCGGCTTAATCGGTTGTAAACGTCATCTAAGAATTCACCACGTTTGTTGGCCTTCTCGTAGAGCTT
CTCAAACTTGACGGTCACATCGTTTAGATTCGATTCCACCTTCTTGGCAATGCGTGCATTCGATGCGCTG
GCAAGCAGATGTTTGGCCGAAACTTGAACGCTGTCGATGCTGGCCTGGTGAGATTGAAGATCGGCTAGCA
GGACCTTGTGCTCGCGGATCTGCTCCTGCAGACGCTCCTTGATAAGCGAGGCGGGTCGCAGATTCATCTT
GAACTTATCCTCGGCCTGGTTAACCCATCCCACAAGACTGTCCAAGGCATCCTGTACGCCCTGCGATTGC
ACCAAAGTTTTGTCCAGAGTTTTGCAGTGCTCACCAAGATTATCCAGAAGCTGCTGGTAGTTGTTCTTCA
GATCGGCAATAGGCAGTTCCAACTGATTGATCTCCATGGGACTGAGTTGGCCACCAAGGCTGCGCAGCAG
ATTATCAAGTGCCTGTTGCGCATTGTCCACCAAGCGACCACTAGAAAGCAGCTCATTGTGTAGAGCCTTG
GCCTCGTTCATCTGATCCTGTACTCCCTTAGGATCGCCGGCAATCGGTTCAGAGATAATGCGCGACACAC
GCGGATGTACACTCCTCAGCCACTCGTTAGCCTTGTCCAATGCATCCTGGTACTCACGCTGGCGTCCACC
CAAACCGGAGACGCGATCTTGCAGAGCATTCACACGGTTCAACAGATCCTTGTACTGTGCCGAGACCAGC
GATACCTCTTGGCGTATGGGACTCTCGGCACTGGACAGTGGCTCAACATGTGGCAAGCGTTCGGGCAGTG
AAGTACGGAAGTCGTTCAGTATGGCCAAGAACTCCTTGCTCTCGTCCACAAACTTCTGTGCGGCCATTGT
GATGAACCGCAGATCGGCCTGGTGCCCAATCACATCGCTGACAAACTCACGAACCGAATCGGCCTGAGAG

GGCAGCCGAGTCAAATCGGACAGGGAACGTTCCTTGTCCTCCAAAGTGCGTCGGGCGACTTGCAACCAAT
CGGAGAATACATCCAATTCGCTGCGCAGTTTGGTGAGCTCATCTCGACCTGCTTCCAGGCGCCGGAGCAA
ACGCACGGTGCGATCGTTGACACGATCCACACGCGAACGCAACTCCCGGCCAGAGTTCTCCAGAGTGGTC
ACTTCGGGGGCAGACAAAACATCACCACCGGTGAGCACGATCTGACGGATTTGCTCCAAACAAGTGGCAA
CTGGCCGTTGCTGAGACTCGATCTCATCCTTGATTTGCTATTTAAACAAAGAATACATTGTTTTCAATTT
TGTAACAAGTATATAAATCAATAGTACCAACCTTAAGGGCATTAATCTGGTTGCGTAACTCATCAATCTT
TTCACGAGGTCCGCCGACACTTGAGATTTTCTGTTCCACAGTGGTAACCCATTTCAACAGATCGTTGACA
TTCTCTTCGCACATGTTGAACTTCTCGAATACTGCTTGCTATATATGTGACATATTATGGGTGGGGGAGG
GCAAAGCAAATAATTTATTAGAAAAGTGGTAAGAAAAGAAATGACTTCTTAGCTCGATACGGGTCGCTAT
AAAGGGATTCATATAGAGATTTCATAGATATCGTATAAGTGTCAATAGATAAATGGAACACAAAATATAT
ATAAAACACAAACATAGCTGGAAGAAACACATTTGTTTGTTGAAAATATTGCAAGGCAAAGACTTAGGGC
AAAGCGGTTTTATAACAAGCATTAAGATACAATGAAGTATATAGGCAGTTAACATAAACCAAAGCAATAA
TATAGACATACAAGCACCAAGGTTCACTACTTTGGGTAACGAGCACTACTGGGGACTAGCAACTACGAGT
TGTTTTACTTTTAGTATGCACTAACTACGGTTTAATGgttttgtttttttgtttttggtttggtttcgtt
ttgttggatttttATATTGGTTAGTGTTAATTGTGGTTTGGGTTTATTTTTTCAAATTAAAACTGCAAAC
GAAATAATATGTAAAAAATATTTATATATGGTTGCTGAAAATAGGTAGAAAATTGGTATTTAAACAAGAT
TTCAAAAATAATAACTAAAGCCTGGGCTATTGCATTGGGGCTGAAAAAGGATATGTAACCTTACTCCAAA
ACTAAGAGCTAAATAGAGACTAGTGAATTGTGGGATATTTCGGGGAAAATCCCACTAACTCAGTCAGATC
AGTTTTTAACCGCATATTAGAGTCAGTTAAAAATTGATCTGATCCTACATAAAATACTGCCTGGGATACA
ATTGGATATAGGGAAAAGAAATAACAAGAAATAGATGAGTGCGAGTTCGAATGAAAAAAGATTCGTATGC
TAGTGTTATAGACAAATCGATAGAATATTGAGTATATAGAGTATTGCATATGGTGTCCTATTGCCGACGT
CATTTATGCGGAGGAGTATGAGACATAACGCATTGCTCTTCGGCTTGATAAAGCGCGATCGATGGAGTAC
GATATTTATGGATGCATGTCTGTTGGATGTGATCTGTGATGTACTTGCTGACTCTGATTGATTGCATTAT
TTTTGGCTGCTGTCGCTTGAGAACTTAAAGCGATCTCAGCGAAAAATACGATATATAGAACATAAAACTG
AATGCCATGCGCTGCaatataagagatttgcaaaataataataaatatgatttgcaaagtaaATTTGCAG
AATGATTCTTAtttttttttCATTGTGGAAAGCTCACATCTTGGTTAGTCTGATGATATAGAATTTGAGA
TTAAGGCAGAGACAGAGATAGACAGAAAGAAATAGAAAGACAGCGCGCTGATCAAAATGTCTCaaaaaaa
aaTCGAAATCAATATCGATAGTACAATACCAAACATGCCACGAACAAAGCTTTTAATTTAGAATTAGAAT
AATTTTGCTGTAATTTCTTGCAATAATTCAGTAATTTCTTGCATTCGCAATTCCGTTTTCGAGATCGGTG
TTGATAGAAATTGAAAGAGTACACCTCGAGTGTTCTGAGACTTACCCTCTGTTCTGCCGGCAGGAGCAGA
CCCTTCTCGGCGGCCTTCACTAGGTCGATGTTGTTCTTCGGCTCGTTGGCATCGGTCAGGCGTCCCTCGG
TGGCATCGATTAGACCACTGCTAATGGCAGCCGTCAGTGGGACAATGACTCCAGTGCTGGGGTCACGCAC
CACTGTCGACGATGGATCAACCAGACCTGAGCCAATGGCCTCGGCCAGCGTGAACTTGCGCCGCCGGATG
ATATCTGGATGCATTTGGAAGGGATCAACTAGGCAGCCATTGGTTGGGTT
>dme_circ_0004301_junction_seq
GATCAACTAGGCAGCCATTGGTTGGGTTGATAGCGGCTTAATCGGTTGTAAACGTC
>dme_circ_0004302
GAGGGCAGCCGAGTCAAATCGGACAGGGAACGTTCCTTGTCCTCCAAAGTGCGTCGGGCGACTTGCAACC
AATCGGAGAATACATCCAATTCGCTGCGCAGTTTGGTGAGCTCATCTCGACCTGCTTCCAGGCGCCGGAG
CAAACGCACGGTGCGATCGTTGACACGATCCACACGCGAACGCAACTCCCGGCCAGA
>dme_circ_0004302_junction_seq
CCACACGCGAACGCAACTCCCGGCCAGAGAGGGCAGCCGAGTCAAATCGGACAGGG
>dme_circ_0004303
AATGCGCTCATGCGAGACGGCTATCGGGCTTATGAAGAATCTCCAGTCGAGCGTGCAAGTGGAGGAATCA

TGGGTGGATGGCACCACGGAGCGACTGTCTGCCATGCCCACCGCCACTTCGGCATACGAACTAGATGGTG
AGTACAAAGACGCTGCCCCTGTAAAAACGAGTTTAGCATTTCGATAAGCGATAGTCTCTAGCTCTAGATT
TACATACATAGATCCTTGTACAGTTTTTCCGAATTTTAGCTATCCCTTAGGGTCTCTGCCAGAGATCTCA
CTTCGCAGGCCAAACACAAAGTATATATACATAGCCTATAAATGGAAACCGTAGTAGCCATTGACCCGCG
TCCAAAGTAGTTTGCTCCCACAGTTGTTGCTCCTATTTTTGTTGTTGGGATAAAACCTGCCGGTGTTGGA
CATGTCTCCGTACTCCtatatatatatctatatatttttatatatgtgtttatctttttttgtattttttgt
ttttgtACTACATTTCCATTTGTTAACTGTCCCCAAAAGCAGAGAGCAAATCTCCAGTCCCTTGTCCGTA
TTCCAGCAGTAGTTCTTGTCTAATCTAACAGCAGTCTTTGACATGTAGTGCTGGCCACCACCCACATAAG
CATACCACATGTACATATATATACACACAGCAGACGATCCTCCAAATGTGGCCAAATCGACCACCTACAC
AACAATAGCCATATTAGTAATCTGTAGCGATATTCCCCCGTCCCACAACACTGTAAATTGATACCAAATT
TGTGTTGTAGTTTTTAAACTTACCTACTTTTATGTTTGTAGTCTTGTCCCCTTAGTATTATAAGCTGCAC
CTATACATAGACCGATATCGAATGATATCGGTTCCTACATTATATATTTCTTCACCCAGTATTATCGTAT
TTGTAGTTGAAAACTAATTGTATATGTATTTTTGTCTAATTTTTGTATTTTCACCAAAATCAAAACAAAA
ATTTTAAACTTTTTCACACAACAATCAAATATTATCACACACTATAAAACGACACAAAACGATCAAATTA
TaaaaaaaaaaaCAAATCGATTTAAAAATCCTGAAAATATTCGAACACCAAAAATACCCCTCGAAAACCC
ATAGAAACTGCTCGGAGCTGCAATCGAACGAAAACCAAAAATCGAAAATGTCAACGTGGCTGGAGGACGA
CTTATACGCGAAGCTAAGGTAAAAATTTGAATGCCTGTTGTGGTATGATTGGTCTGATTCTAATGACTGC
TGATCCATCGTAACCCATCGACTTCAGACATGTGCAATTCGATCGCTTGACATACGATTCGATGGACTGA
CTGGGTCTAGTCAATGTTGGGTTGCGATAGGTCAGTTGGTGGCCGAGTGCCAGCACTTTACGTATGACTG
TGTCGAATGATTGTGGATATTTCGAACGAACACCGAAATCCCTCCTTTTCCGCAAGGAAAACACTTTGTA
GCCCTATAAACACCTCTTAGCTGTTTCTCTACCGAATCTCAAATATATTTAATGCTTTTTAAACCAATTA
CCGGACTGTAAGCTCCAATTCTTAGTTATCTTGCGGGTTAAAGGTGCGTGAATAACACTTGGGTGGAACA
GAAACAGCCTGACAAACAAACTCTTaaacacacaaacaaccaaaacaagaaatcaaccaaacaaacaaac
taattaaaaagcaatTTTACCAAAACGTAATTTCGTATTGATTAATCAATAAAATAAAAAGCACTAAATA
TATCAACAAGCGCCCCCCTTCTTTCCTTGCCTCCTGAAAAATAAACTAAATTTGTTTAACACACAAAATT
ACTAACATTTAACCGAAATTAACCAACCAACCAACCAATCGAACAACAATGTCACCGTGGGACCCTGAAT
TCCGGGAGTTAGATCTACGATAGCAAATGCCTACGCTTCGTGGACTGGCTGGTCGAGGCCCGTCCGTCGT
TCTCGCCACCGCGCCGGGATCTCCGGCCGGCGGATAGCGATCCGGGCGCCACACAGTATTACAGCCAACG
TTTGGACAACCTGAACACGAAGAACGACAGATTGCTGGAACAACTGTCGCAGCGTCTGAAAACAGCAATC
GAAGTCAATGGCAGCGATGGTCTGGTGAGTAATCCAGGTGACCCTTTCCCCTTTTTGGAAGGATCTTTGC
GAGGTGGTGACGTCAGGGTATATTTTTCGCTTCGTTTGTCTACCTCGTTAGCTGGGGTTAGGCTCGAAGT
ATTCAATTGTTTCGTAATCGCAAAGCGGGGTTCAAGTTCAGCAAACGAGTTTTTACGCTTTCGTTTTCTA
CAAGTCAAACCGAGTTTTCAAATGCAAATACGTACGTCTACTTCTCTGTGGTATCATTGTATCTCCCCCT
CCATCATCTGTGCCTGTGTCTATCTGTATCTGTATTATTGTATCTGTTACCGCTCCGAATGCCCGGCAGT
TGGTATATTATTGAATTAACCTTCAGAATCGGTGCACACTGACGGCAGAAGCAGCCACATAATGACATCA
CATCAAATTGACCAAACATTCCGTCAATGGCTTGCCGCTATAAATCACAACTTGATTAATTTATAAAAAT
TTAAACTATTTTTATTTTCTGTCATCGTGACCGTCGTTGATCGCCGCTGTCAACTGATGCAGCAATTTGT
TTAATGTATCAAATTTATTTATAAATATTGCATTATGTCAGGGGAGTTTCAATAGAAAGAGTAACAAAAA
CGATAGACATTGTCATGCGCCTGACCAATTAGGCAAATTGGAGACAGATTCGAAATTAATAATGACGACG
CCGAATTCTATAAATATTAATGAGGCCCAGCATTCGTATGGATTCGTATATATACTCGAAATTCAATGCC
AGGGAACGTCATCAACGTCTTTGTCGCCATCTGTCAACttttcttttatttttttGAATTCCAGTTCCCC
TCTTATTTTGACTATTTTTGCTCAACTGGTTTGGAATTTGTTTATTTCCTAGCAGCTCCATCTTCAGAAA
TTCAATTAAGAATATTATTAAGCAACCAGCTTGTGCCTGCTACGAAAATATTTGTTTGCAAATATTTAGG
AGGCAATTACCATATTGTTTAAATGGAGTTCAGATCGCCGGACATGGTGTAAACATAAGTTCAATAAACT

TTTTAGGCTAATGCTGTAATAAATAGCATCATTAATACTACAGTGAGAAAAACCAATTTTTCTAAATTTA
ATGataaataatataatatatatatatCTGCTTAAAAAACAAACTATAAATGCCATAAAATTGAACATCT
TTAGCCTCAATTTCTTTCTATGTAGTTGATCCATTTCAAAGTAATCCGAATTTGTTGTTTTGGGATCTAT
TGAGGCTTTTCGAAGGTGTTGCCTTCGCCTCGAGCGTTGGAGTGACATCATAACTGCTCCATTACCACTT
CGCCTTCAAGCCAACTTGATATGCATAGCAGGGGCGGGCGCTGAAGCTCCGCTGGCTTAACTAGACTGAA
AAGCGAAATAAGTTATTTAGAAGGTTAGATGGCGGCACAAACTGGTTTGATCTGTTCTTCTGATACTTTG
TTCTCGACTTCCGTGTATTGTTTTCGACTTTAATTTGGAGCCAACTCCAGCAGCGTATACtcccttccct
tccctttccttcccATCGCCAATCTCCACCATTTCATGCCATGCCATTGTAAATGGATTGTTTATAATGT
TTTGGCGCAACAGCTGGGGAATGCTCACCGAAAACTAGCCGGCATTTGTAAGctctccctctctctctct
ctcAGTGGCAATTGCTTTCTCTCAATCTCAACAAAAGCTCGCTCTCGCTCTCCACTTAACCATTTGATTC
TCGGAGTGGCGCTGCTCCATTCAGTACCGAAAGCGACGTGGTTGTGTAACGTCGCGTATCCGCTGTGTGT
GCCGACTTGCTGCCGGTGAACGAAATTTAACAAAGGCCAAAACCTTTAAAACCTCCGGTTTTTGTTGGCA
ACCAAAGTCTATGGAATTTGAATTCGAATCGAGCGTGTAATTATTAGTGAATAGTTGTGATAACTAAACG
GGCAAATAACTTCCCAAAACTCAAATAGAGAACATTGCTCATACGCCGCATGGGCAGACATGATGTCACA
TATACGGCCGGATAGAAAGTGTGTCCGTGTCGTTGTCTTAGTGCCTTAAATTTCAATTAAATGCGAACCG
TGTCGCATTGGTTCGCTTTTATTTTGTTAGAAGAAATTTAAATTCGCTGCCATTTGAATTTGTGCCGCAA
AGAGTTCTATTATAGCCCGAAATGAAGTCACTCGTCGAGTGCTTTGCAACAACTTAAATATAACCGAAAC
GCAAACAGAAGAGTGTGAAAACGAAAATAAAAGAAATACATTATTAATATAGCCTTGAACATGTCGCAAG
AATCGGAATGTTGATATTTTCGGCCGTCTGTTTTGGTGTTAATGAAAGCGCACACACaaaaaaaaaaaaa
aagaaaaaaaggagaaaatgaaaaTCTATGGCTGAAATTTCGTAGTGAGTCAGCGAGTTTTAGGGTCATTA
TAAGTTGCATAAAACCATAAAATGCTGCACTTAAAATGAGAAACCCGAAAAACTGTGGAAATTATTCTCC
CGCTCGATTACTAGCTGACTAGCGTGAATTATGACTTGCTATCAACAGGAGGATCGCGGGAGTGTCAGGG
CTGGCGCTTATCTAATCTATGAGGTGCAATCCATCAGAACTAAATGTGATGCTTGGAATTGAGCGATCGG
TTTACCAGGAAATGCCACCCGGAATGCCGATGGAAGAGTATTTGTTTGTTCGCCTAGCATCCATGCACTT
TCCCTTTGATTTTATCGATTAAACTAGATGCCTTGGTATACCTTCTATATATACTTCGTTGCatatatat
atatatatatTTTTAGCGGCATTCTAACGATAAATCAGGAATGAAAGCGAGCGTTTTGGGGTCTATGCCT
CCACTGAAGCAAAAGGGAGGCGCCTATTTTCATAGTGCCACAAAGGCGAGGTCCAATTGCAGGCTGTGGT
GCATGCTGCACGCTCTCGTTAGAGCTTTTCCTCTCTCGTTTGTTCGCTCTTACCCCCATTTAAAGCCGGT
ACAACGTGGCGTATTAGTCATGTTATCAATGTCAAGCGCATCTCAGCCGATTAGGGCCAAGAATCTGTTT
AATCTGCTTGCGGACTCATAGGCCATTATTTTGTGCTTTAGTTCAGCTTTAGTTACTCGTTGTGCGGGCG
CTCGACAAACTTGTTTCGGTTTCATTTATAATCAAATATTCGCCACTCTGATGACATCATTGTTCTCGTA
CTTGTATATTAACTGTCTAAATGTGTGTCGGTGTGTGTTTCGTTAATTATAATTTATTTGTTGCTTTTAC
CAACTCTAGGGCAACCTTGAAAATCAGCAGCAGCAACAGCAGATTAGTTGTTTATTTTGAAAATGCAAAT
CATAAACGTTGATCACTTTGTAGATTCGCCATTCAATGACTTCATTTGCAGCCCTCTTTTGCAAAGTTGT
ATGTATGACGTCAATGCAATTTTAAGTTGGGGCTACGACCTGCAGCCATATGTCAGTCATTTGCCATTGA
ATCACTGTCTCTGGCAAATGGTAAATAAAGATAAAAATTAAATTACCAGATCCCGTCGATAAGACGCACT
CCGGATCCCCAACTCTTTCCGGGTAATCACCCATTTATTATGGTTGGGACCTCTTGAGAGAGTTTGCACT
ACATATTTGCAGGCCGATTGATTGAGGACTTCTCCGTCATATAGCGTCATTTCTATTTTCATAAAGCCTT
TGGGTTCGCtttttgtttgcgttttttccttatttttttttttttttgttttCACTTAACTATACATATGT
GCTTCGTGTGATTTGTGTGCGTCGGTGCGGGGCTGATAAAAATGTATCCTTGTGTGTGACTGAAAGAACT
GAATCTAGTGAATGAATTAAGTGAATCAACAAACAGAGTAGTATAGGACGGTTTAAGGGCCGATTAATAA
TGAAAGTCCAGCGATGGCTACCGTAAAATTGTAGAGTTCATGTCTTTTGACAATCTTGGAAGTATTTTTA
GTTACTTTATCGCCGGTGACTTTATCACGCGGCCATATCAAATCACGCTCACGCATGCCATAAACATTCC
ACAAAAATGGTTTGAGACTCTAGGGCAACTTCAAAGTGACGCGTCTGCCAAGAATCTCGACTCGTAAAT

TTCTAATTGCTTTTGTCACAATTAAATCTCGCGCAGTGTATTCAAGAAGCCCCTAAGCCAACGAAACAAA
TACCAATTGCTGACTATGACTTTGTCGAACTGAACCAAACGAAAGCCTTAGACATGCCTCAGCATAGATC
GGGTCGCAAGGCGACTAAAACCAAATCCAAAACCCAAACCACGACTGTCCAAGTGGCAGAGGAAAGTGAC
TACGACTTTGCGTTCGAGTTTCAGGTAATACTTCATCTATCCTCCCCATTCCCTGCTGCCCTGttttttt
tCCCCTCGCAAAGATTTCAACGAACCGAGTTTAATTTGCCTGTTGCCCAGTTGTTAACTTTTACTAACCG
AAATTGCTTTCATTTGGCATTTAGCAATACGCTGAGAGTCTACAGAAACCGCTCAAGACCTTTAGGGTTG
ACTTCAGTGCGGGCAGCGTGCCAACTGGCGATGGTTACGCTGCGCGCCAGGAGGATCTCTACACCACTAC
CTACAGCACCACACAGTAAGTACAATTTATACAAAATTTATAATATATATTAAGTGGATATTATTCTCCA
GAAAGAAAGAATTTTACTGCATTCTGAAATCAACAAAATGATTCATTTAAATCTACAATCTACAATATTA
TGCGAAAGAAACGATAACAACTTTTGACAAAGTTGTAGAACTATGAATAATCTTATATGTAGATTTGTAG
TCATACATTCTTTTATTATATACTAATAAAGTTCTAATCGAATCCTTTCAGAATCAGCTCAACGAAGACA
ACGAAAAGCTCTACGAAGAGCGTCTATAGTAGCGATGGCTTGGACGCCGCTAGTCAGGAGGTTAGCACTG
CTGGCCTGCCTCAATCGCAGATCCAATTCAACGAAATCCGTACGCTCAAACGCTCTCAACAGCTGGGTGG
CCATTCCGTGCTGGATATAGCCGGAATACGCGATCCTCGTACGGGTCGCGTTCTAACCATCGGTGAAGCC
ATCCAGCTTCGCATTCTGGACGTGCGCACCGGCGAGATGCTAGTGGGTGATCGCCGTATCACGCTGGAAC
AAGCTGCCGACCAAGGACTGATCGATTTGCAACTGGCAAAGCAATTGCTTGAGCCTGGTGCCGGCCGAGA
TGCCAGCGGACGGGAGCTGTCGCTCCTGGAGGTTATTCAGCGCGAGATTAGCGAAGCGGAATCCGGTTAC
GAGACGGCCGAGAAGCGGATCAAGGTAAACAACACAGTCACCGTAGAGCAAACTTCCGGAGAGCTGGGCT
CTCCCGAGAATCCACGCAACATTGCCGACGCCATTACTGCGGGGAGTGTGGACACCAAAACCGGCCTGTA
TCGCGTCAAATCCGGACAGACAATCAGCCTGGCGGAGGCCTACGAGCGCGGCTACCTCATCCGGCACGAG
TCGGTGACAATCAAATCGAATGCGCTGTGCCTGAGCGATGCGATTGCGCATGGCCTGGTCGATGGCGCCG
GCTGGATAGCGGATCGCAACTCCGGCGACAAGTTCCGACTGGATTCGGCCATTGCCAATCAACTAATAGA
CGCGAGTGTGCGCGAAGTGGTGGACGCGAAACGCGATACGAAGATCACACTGCAGGAGGCACTGCAGTCG
GGCGTACTGAATGCAAAAACGGGTCGTTATGTGAACGAGGTGACCAAGGAGAAGTTAACCTTCGCAGAGG
CGCGAAACCGTCAGTTGATCGTGAAACCGTACACGCTGAAGGACATCTGTGATCTGAATCTGCTCGATAA
ACAGGCTCAGATCACGAGTCCCATGCGCCGCGAAAAGTTAAGCATTATGCAGGCCATCGAGGCCGGTGTA
CTCGACGGTAATTTGCTCAAGTGTATCACCAAACGTAAGGGTGAATTGGTCACCCTTCAGGAGGCCATTG
CCGATGGCATCGTGTTGCCGGCCGAGTGCAAGTACCGCGACTTTATGACCGGTGAGCTAATCAGCATTCC
GGAGGCAGTGGAACGCGGTCTGATTAGCTCGGTGGCGCAGCGTTCGATCTTCGATATTGACGGGTTCAAG
GATCTGAGGAGCAATGACTATGTAAGCTTTAATGTGGCCCTCTCGCGCGATCTTTTGCGTCGCAAGAGCA
CGGGCTTTGCCTTAGAGACGGGTAGGGATAGTCTGGTGCCACTAGAGGTGGCTGTTAGTGAGGGACTGGT
GCGCCAGGAGGTGTACGAGATGTTTAGCCGTGGCATTGGCGTTCAGAATGCCAGTGGTAAGGAGCTTAGC
GTCTTTGATTTGGTCTATCACAATCTGATTGACCCGAAGACTGGTTATCTTTTGGATCCCAAGACCGGGG
AGACAGTGCCCCTGGACACCGCCATCGAGCGGAAGTTTATCACTCCCGAGGGTGCTCTACTACTAAGTAG
TTTGCTCAATATCACTTTGACAACGGAAACGGTGACGCGGACGATCAATCGTTATGTGACAATCAGGGCT
GGTTCCCAGGAGCCAGTCGACAGCGTGTTGCTCACCTTCACGGAGGCAGTGCGTCAAGGTTTCATTGACG
AGGAGAGGCAACTTTTCAAAGATCCCAAAACGGGAAATATTTACTCAGTACAGCAGGCCCTCAACTATGG
CTTGTTGGTTCCCGATAGCAATCAAACAGTTCCGGAGCCAACAAATAGGAAAAAGACAAAATCCACTATT
ACCATTGTGACCAAGCAAATCATACCGGAAGCAGAGCCCATCAAGCTAAACACACAGCACACCAAGTACG
TCGAGAAGTCGGTGGAGATTCCTATTTCTCAGGAACTAGTAAAGCCGCATCGTGTGGTCTCCGAATTTAT
TAACTTGGAAAAGTCTAGCTATATCGAACAAAATGTAACGGAGCGTCAGATCATGGAGTTGCCTCCAGGA
GGATGGCGACTAAAGGACGCCATTGAGCAGCGCCTGTTTAATCCAGATACGGGAGTATTCCATGTCCAGG
GCACAGATCGATTGGTTAATTTTGAGGAGTGCATCAACAAGCAGATAATTAACAATCTATCGCTCTCCGT
AATTGATCCCAACACTGGCGACAAGATCTCCGTGCAATCAGCCTTTGAACGTGATATCTTGGACAGTTAT

GGCAACTATACGAATAGCCGGAAACAGGTGCAGGGTATGCGTAGCGCTATTGACGAGAGTAAGATCATTC
TGGAAACTGTCCCGGCTACGAGAGGAGCCAACCAAAAGACAATTCTTCGCATCACTAAAGTAAACAACAT
TCCAGATGTGTTAGAGGTTTCCACGCCCCTGAAAGATGCACCGCCCAAGTTTGTCGAGGTGCTCACCTGC
CAACGAGAGCTCGCCTCTCCAGAGCCCCTGCAAATTGCACCGGGCGCCATCTATGATCCGTCCACTGCCC
TAGTAATCTTCACGCAAACCGGAGAGACTGAAAATATCTTCGATGCCGCCCGTCAGGGTCTGGTTGATGA
GCAGCTAATTAAAATCGTGGATCCGACAACCAAGCAACCCATTTCCGTTACCGAGGCGATAGCGCGTTCC
ATTTACGATCCCAAGACAGCCACTATTCTTGATTCTGAGGGACAGCCGGTGGATTTGATCACAGCAACTA
AACTTGGACTATTGAGTGTGGTAGGTGCTCCATTGGTTGCCGCCGAGGGAGCCCTGCGAACTGTGCGTTT
CGTCACAGATCCACGAACTGGTGAGCAAATTCCAGTGGAGGTGGCCTACGAGCGAGGCATTGTCTCTCGG
GATCAGTTGCATCGCGGAAGGTCCTTTGACAGTGAACCCGCAACGGTAGAGGACAAAGTTGTAGTCCTTC
AGAGAATGCGTAAGGTCATTCTCAAGCCAAAGGATGCTTTACGAAAGGGAATCATCGACGAAGAGACTTG
TGAAATACTTGAAAACACCAGTAACTTCACTAATCCCAAAGGAGAGGTGGTTAACATAACTGAGGCACTG
AACACCGGTCTAATTGATGGACGACGTGGTCAGGTGATCGATCCCCAAAACAATAGAGTCTTAAATTTGA
GGCAAGCAGTGGAGCAGAAGATTATTGATTCCGAGCAGACTAACCATATTCTTATGCCGTTGGCCAAGAG
CTTATCGGTGCCGCGTCTGGCAGACCAGGGATTAATTGATCCACAAACTCAGACTATTGTCCATCCTGAG
AGTGGTTATCCACTGAGCATCCATGAGGCCATAGTATGTGAGGTTCTGGATCCGCATTCAAAACTGCATA
AACCCGAAAAATGCACACTAGAGGAAGCGATCACCAAGGGCATTATCAATGCAGATGATTCCACATTCAA
CTATAAGAACAAGACACTCAACATTACTGAAGCCATTGAAGCGAAGCTCTTTGATCCCAGTTACGATCAG
CAAAAATCTAAGGTGGAAATTCCGCCGGTGGGTATGATATTCCCTGTTGCTGTTGAAAAGAGCCTTGTGG
AACCGTCCAAGCGTGTTGTTCTGCATCCAAGTACAAAGAAAGCACTTCCAATTAAGCAAGCCATTGAGGA
GAACTTTATCATGTCCATTCCCTATTCTCCCAAGCCAGATGCTATCGAAGTGGTTACTGCCATGGAGCAG
GATCTCATCGATGTAGCTGCCCAAACACTAACTGTACCGTCCACTGGTGAACGTATACCTCTACGACAAG
CTATGGAAAATGGTGTTCTGGTGGTAAAGAATCTCTCAGATTTTGTGATTACCCAGAAACCCATTCCCAA
AACGGAGATCATGGAGACGGTGCGTGCAGTGCACACGGTGACTACTAAAACCATTGAGCTTATGCAAGGC
TACGTCCTCATTTCTAACAATGAAGTGCAGAACGTTAACACAGGCGAGGTATGCAGCTTGGAGGAAGCTA
AGGAGCTGGGAATTCTGCGCGAAGAATCCACAACTCGGGAGACAAAAGCAGCGGCCGGCGAAAGTCCGGA
ATCGGTGGGCGACGTTGGTACCAGTGGCGATCAAACTGTTATTGTAGAAGAGCGCACCCAGACAGTTGTG
GTCAGTTCGGATACCCGCAAGCAAGAGATGCAAGTAGTTAGCACGACGCAATCCACGAAGGATACATCCG
TTCCAAGTGTAAGTGAAATCGAGACAAAGGGCAGCAAGGAGCCATCGAAGACGTTGCAAAATGTGAAAGA
TGCCGCCAAATTGGGTGCATTGGGCGTAATCGCAGCTCCTGTACTGGCCGGAGGTGCCATCGTAAGTGGC
GTTAAAAGCCTTATCAAATCTGTGAAATCTCCCACGGAAGATGCCAAGAGCACTGAAAAACCTAAGGAAC
CTCAAACCAGCTTCATTGAACAGGAGCGCCGTAAAGAGCTACAGCCGCAACAAGTGCCAAGTGAGGTGGA
TAATCTCCTCAGTGAAACGGAGAATTTTATCAGCTCTACGACTGCCAACTTTATTGCAAACGAAAAACAG
CAGGATAAGCCCGATACGTTGCCACAGGAAAGCAATCTGCCCTTGGAAGTTGAATCAACAAGCACTCTGA
CCACAACAACAGTAACAACAATTACTACATCAAGTACAACAACGGAGACCGGAAATCTGGAGCCAGTTGT
GGTAGAAGATGTCTCGACCACAGTTAAGGAGGATAAAGTGGTGGTTAAGCCAAGTACAGTTCCAGAAGAT
AAAACAGAGGTTTCCAAAATAACTGAAACCATAACCACAGTGACTTCTAATGAAACAGATACTGTACCAG
AAAAGATATCACAGAAATCAGCTTCCGAGGAGTCAGCAAAGAAGGATCCAGTGGTTACGAAAGACACAAG
TTCCAGGCCAGCTGAAACGGAGACATCTCCTAAGCCTGTTCAAAAGGAACCAACTCCCGATCCAGCTCAA
AATACAGAATTTCCTGAATCTATTAAAAAAGATCCTACTCCTGAGCCGGATTACAAGGAAACCGCTACCG
AGGCAGCACAAATCACACCAAAGGAAGCTTCTCCTGCTCCAACCAATAAGAACGAACAATCAGCTGAACC
TACCTCAAAGCCAGAGCCAATGCCCCGAACTGCTAAAAAGGAGGAGATTTCTGAAATTGAGCCCCCGTTC
ACAGCACCACTGGCTCCATTTGTTGATCAATTAGCGGAAACCAAGATCACTGAAATTAAAACAGAATTTA
AAGAACCAGTTACAAGTTCCCAAACTATCACAACCATCACCACAACGGTTAAAACAGTCGATGAACACCC

CATAGTCGAGGAAGTAGATGAGGTCAGCAAATCTGAGAAAATTGTGGATGTGGAAGAACCAACAGTTCAC
CAACAAGAACCGATTAAGGTGAATGAGACGAGCGATGATTCCACCAGGACTGAACCTACCAATGAAATAG
TGCATGAAGAAAGAGTTGTCACTGTCGAAGAGCCGACGATCACAGAaactaccgtaacaacaacaactat
cactacaacaacaacATCTTCAAGTGAGGAAACCCTGCCCCAAGAGAATACAGCCACAGTAAAGGAAACG
CCTTTGAATGAGCCTGTGCCTTCATCTCCAGCTGAAACTAAAGAGATTGAGCAGACAACTAATGATGATC
AAAAGAAGCCGGAAGAAATTGTAGATACCGAAAAGAAAACAACAGAGCAGAAGCCGGTGGAAACTATCCC
AACCGAGCCAATAAGCACATCTGAAACAACAACAGTGACTCTTACCACCAAGACAACAATTGTTGATGAA
GGAAAACTACATCCACCAAGTGATATTGAGCAAATTATTCAACCGCATTCTACTTCGGACAAACATCAAT
CTCTTGTTGATTTCATTGAACAGGAGAAGGGCACACAGCCAGCGGAAGGTGGTAGCAATATTTTGCAGAA
TGTGAAGGATGCTGCCAAGCTGGGCGCTCTAGCCATTGTAGGAGCACCAGTTCTTGCGGGCAAGGCTTTG
GTAGATGCTCTAACCACAGAAAAGACACTTAAGCCAGAACACTCCACATCACAAGTTGTAACCGAGACAG
TGGAAGAACTACCAACAGAGATAATTACCACAAGCACTGCTACATTAGTTACCAAAGTCACCACTACAAC
TACCACCACAACGACATCCACCAGTGAGGGCACTCCCGATGATGAGGCTCTGGATGTAGTTAAACTGCCT
GCTGAATTGGTTGAAAGCTCTGTAAAGCGAGAAGTGCCCAGGACACTGGCCATTGGCGATGCCATCTCCC
AAAAGTTGATTAGCCCCGATGAGTGCCAGGTGATCGTAGATGGAGAACAGCAGTCGCAGACCGTTTCCAC
GCTACTCAAGGAGGAGCAGCTTAGCCCATTGGACGAAGTACAGATCATCGACGACAAACTAATTGTTCTG
CAGCAGGTGTCTTACCTGGTAGACGCAGACACAGAACTGACGCCACAGAATTTGTATGAACTTAACATCT
ACGATCCCGAGAACCAATACTTTATTGATCCCAGTACTGGCCAGAAGATTTCCTTCCATACGCTGGTATT
TGACATGAATGTTTTTAACCCAGAAACTATTTTGGTAAAGAACTTTAGTACCGGCAAATACGAGACTCTT
ACAGCAGCCTTGGAGCGTCCACTTCTGGACAGGCACACGGGTCACATGGTGGATCCGAAGACAGGCAAGA
AAATCCCATTCTTTGAGTGCGTTGACCGCCAATGGATTATCCGAGCCAATCCGGAAGAGCAAAAGCCATC
GGGCATCGAGAACGTGACGATAGACACACAGACTGGACAGGTGGTGCTTGACGATGGTCGGGTTTGCTCC
ATTGTTGAGGCCATCAACAGTGGCAAGCTGGACATTCAGTCGATATCGGTGCGCGACCCAGTCAGCGGTG
AGGTGATTCCACTGCGTATGGCAATTGAGTTGGGGGTGGTAGACATGCAGGCGGGCACAGTGATAGACAT
TCAGACGCTCAAAGAGATTCCCATGGAGCTAGCCTTCCAGCTGGGTTTCCTAGTGCCCGGTGCCCGCAAA
CCCATTTCCCTGGAGGCCGCCGTACGCAAAGGTCTCTACGACCCGGAGACGGGAAAACTCTTCGACTCAG
AGAGTCAAAACCAGGTCGATGTTCAGAAGTCGATTGAAATTGGTTTAGTTGACCCGAAGATCTCATTGAT
TGTGGATACTGTCACTAAGAAGGAAGTCAAGCTGGATTGCGCTATCGAAGACGATTTGGTGCTGCCCGCC
ACGGGTCAAATCAAAGACAACAAGAACAACACTTTGGTGCCCTTCGATGTAGGTGTACAACAGTGTTTGC
TGAAAACCGACAACGTCACCTGGAGCCTGCCAGAGTTGCTTCAGCGCGAATACTACACGCCCAGCACTGG
CAAGGTTCTTAATCCCGTCACCGGCGAGGAGATTCTTCTTCAGCAAGCCATTGAAATGGGCTTTGTGGAG
CTAGAAACGACTTTGGTCAAAGATGTCGATCATGATAAAATTTTGCCAGGCAAGGAGGCGGCCAAGGTGG
GTCTTTTGGACACTGTTCGCGGAACACTAAGCTCGCCCAACATAAGTCTCGACGAGGCTTTCGTAAAGGG
CTATCTTATAAGCACGAAAAAGCCGCTTTCCCTGGTCGATTGTCTGCTGCGTGGTCTTTACGATCCTTCG
ACAGCAAAATTCACCATCGACGACAAGCAGTTGGACCTGAAGGCCGCCATTGCCCAAAAGCTCATTAATC
CCGAAGAGCTGGTTTTGCTTGACCCCAAGACAGAGTCTATTATTTCCATTACAGAAGCTATCGCGAAGGG
TTATCTTGATCCGATTGAGGGCTATGTGATTAACCCATATGCCTCCACCAAACTGTCCCTACACGAAGCC
CTGGAGAATCGTATTCTCATCCCGCCCAAACGAAAGCGCAGCTTGCCCGATGCTGTCTACCGCGGTCTTT
ACGACCCCAAGACTGGTCAATTTAGCAATACGGTGACCCGCGAAAAGCTCACAACTGAACGTGCCATTCG
TAGGGGCATCCTCGATCCCGATTCGACGGTCGTCAATGTGGGCTCGGGACAGATCATTCCATTTGGGGTA
GCCACCGAGACTGGAATCGTGGACAGTAAACATGGTACGGTGAGGGATGCCGATGATCACCCGATTGACT
TTAAGGAAGCATTCGATCGTGGTATTTTGGTTGAAGCCAAGAAGCCTCTTCGTCTTATTGAGGCTGTGGT
GAAGAATGTCTACGACGAGGTGGATGGACACTTTGTCGATCCGAAGTCGGGTGAGAAGCTAAACTTTGCC
GAAGCCTTAAACACGAATTTACTGGACGAGCATAGTGTGCAGATCAGAGACTTCAAGACTGGTCTGTACA

AGCAGCTGAACCTAACGCACGCTAAAGACACCGGCATTATTGATGCCCAAAATTCCAAACTACTGTATAA
CAACCAGACAATGACCCTTAAGCACGCCTTTGATATTGGAATCCTAATGGATGTCAATGCGCCCATCAGC
ATTCAGCGAGCAATTCATCAGGGACTATTCGACGACAAGACCGGAAAGCTAAGCGATCCCAGAACTGGTC
GCCAGATTACATTACTTGAAAGCATGAGGAGTTTCGTTGTTAATCCTCACCTGCCCTGTTACTTCGATGA
GCAGACCGAACAGATGTATACTCTGAGCGAGACCTGTAGAAATGGCATCATCAATAGAAGGGAGGGTGTT
TTCCGGGAGCCGGGAAGCAATTCGTTTGTTCCTCTGGGCGAGGCCCTAAGCCTGGGTCTCATTGTGGACA
TTGAGAATGCCGGTTTTGGTTTGTACGAACTATTGTCGATGGGCTTCTATGAAACTTCCACACGTAAGGT
TCTGCATCCAGTCACAAATCGCAAGCTTAATCTCAACGAAGCTTGCGTGGAGGATGTAGTTAGCCTATCA
TCCAGCTTAGTCAAACACCACGAGACCGGCAAGTACCTTCGGCTTGCCAATGCTATTAAGGAGCAGTTAG
TTGATGACTCTAGAGGTTGTTACAATCTGGGCAACGAACAGTTGGATCTCCAGGCGGCTAGGGCCAGGGG
TTTAATTGTTTCCAATCGTCGCCTCCTCAGCCTTGAACTCGTATTGCGCCAACAACTTTATCGCCCCGAA
ACTGGCAAATTCTGCGATCCAACAACGGGAGAATATCTAGACCTAATCGAAGCCATCCATTCGGGTTTCA
TAGATCCCGCGACTACCGTATTTAAAAACCAGCTTACTGGTAAGGAGTTACCGCTAACAGAAGCTATTGA
GAATGGTGATATTGATGTGTCCAAGGGTAGAGTATTTGATCCCAAGTCTAAGTCAGCTTATAACTACGAC
GTGGCACTCTCGCGCGGAATCCTGGTCACCATTACTCGGCCACTAACCGATCGCAATGATGTGGTACGTC
GCCAGGATAGCGTTGAATTACTAAATCAAACACCCGTGAGTGTTGTGATTAGCAAACCGCGTGAAATGTC
TCTCGAAGAGGCCATTCGTTACAACATAATCGATCCCAAGACCGCGCTCGTTCGCGATTTCGATAGCTTT
AAGTTCCTGCCTTACTCGGTAGCCGTGGAACGCGGTCTCGTGGACACCACCAAGCGCACACTCGTGGATC
CAAAGGCCCTGTACTTTGCCTTCGATCCCACTTTAATAGTTTACGTACGTGAACCGGTCACCTTTGACCA
GGCGGCCGAGTCCAAGTGTCTGGACTTGCAGACCGGACTGCTGACCTATGTGCCAGCAAGTGTACCTGGA
TCCGCAACAGCGGCACCAGCCAGCGACGATAGCGCAAACGATTCGCTGACTGTCATCGAGACGCCCAAGG
TATACACGCTCAAGGAGGCCGCCAGTGCCGGTGTGGTGGATCCCGACTCGGCTCTAGTCAAGGACTTGGC
CAAGGCTAAGCTGGTCCGTTTGCCAGAGGCCTTCCGAAAGGGCCTAATGGATGCCAACAAGGCGAACGTG
CTCAACACCCAGACCTCGAAGCTCTGCACCCTGCAAGAGGCCTACGAAAGTGGCCTGATCTGCACGCCTA
AGCGTTCATTCGGTTTGCTAGAAGCCATCACCTTTAACTTGTACAACCCAACCAATGGCTGCCTAGTTGA
TCCCTTCCAAATGCATCCAGATATCATCCGGCGGCGCAAGTTCACGCTGGCCGAGGCCATTGGCTCAGGT
CTGGTTGATCCATCGTCGACAGTGGTGCGTGACCCCAGCACTGGAGTCATTGTCCCACTGACGGCTGCCA
TTAGCAGTGGTCTAATCGATGCCACCGAGGGACGCCTGACCGATGCCAACGAGCCGAAGAACAACATCGA
CCTAGTGAAGGCCGCCGAGAAGGGTCTGCTCCTGCCGGCAGAACAGAGGGTAAGTCTCAGAACACTCGAG
GTGTACTCTTTCAATTTCTATCAACACCGATCTCGAAAACGGAATTGCGAATGCAAGAAATTACTGAATT
ATTGCAAGAAATTACAGCAAAATTATTCTAATTCTAAATTAAAAGCTTTGTTCGTGGCATGTTTGGTATT
GTACTATCGATATTGATTTCGAtttttttttGAGACATTTTGATCAGCGCGCTGTCTTTCTATTTCTTTC
TGTCTATCTCTGTCTCTGCCTTAATCTCAAATTCTATATCATCAGACTAACCAAGATGTGAGCTTTCCAC
AATGaaaaaaaaaTAAGAATCATTCTGCAAATttactttgcaaatcatatttattattattttgcaaatc
tcttatattGCAGCGCATGGCATTCAGTTTTATGTTCTATATATCGTATTTTTCGCTGAGATCGCTTTAA
GTTCTCAAGCGACAGCAGCCAAAAATAATGCAATCAATCAGAGTCAGCAAGTACATCACAGATCACATCC
AACAGACATGCATCCATAAATATCGTACTCCATCGATCGCGCTTTATCAAGCCGAAGAGCAATGCGTTAT
GTCTCATACTCCTCCGCATAAATGACGTCGGCAATAGGACACCATATGCAATACTCTATATACTCAATAT
TCTATCGATTTGTCTATAACACTAGCATACGAATCTTTTTTCATTCGAACTCGCACTCATCTATTTCTTG
TTATTTCTTTTCCCTATATCCAATTGTATCCCAGGCAGTATTTTATGTAGGATCAGATCAATTTTTAACT
GACTCTAATATGCGGTTAAAAACTGATCTGACTGAGTTAGTGGGATTTTCCCCGAAATATCCCACAATTC
ACTAGTCTCTATTTAGCTCTTAGTTTTGGAGTAAGGTTACATATCCTTTTTCAGCCCCAATGCAATAGCC
CAGGCTTTAGTTATTATTTTTGAAATCTTGTTTAAATACCAATTTTCTACCTATTTTCAGCAACCATATA
TAAATATTTTTTACATATTATTTCGTTTGCAGTTTTAATTTGAAAAAATAAACCCAAACCACAATTAACA

CTAACCAATATaaaaatccaacaaaacgaaaccaaaccaaaaacaaaaaaacaaaacCATTAAACCGTAG
TTAGTGCATACTAAAAGTAAAACAACTCGTAGTTGCTAGTCCCCAGTAGTGCTCGTTACCCAAAGTAGTG
AACCTTGGTGCTTGTATGTCTATATTATTGCTTTGGTTTATGTTAACTGCCTATATACTTCATTGTATCT
TAATGCTTGTTATAAAACCGCTTTGCCCTAAGTCTTTGCCTTGCAATATTTTCAACAAACAAATGTGTTT
CTTCCAGCTATGTTTGTGTTTTATATATATTTTGTGTTCCATTTATCTATTGACACTTATACGATATCTA
TGAAATCTCTATATGAATCCCTTTATAGCGACCCGTATCGAGCTAAGAAGTCATTTCTTTTCTTACCACT
TTTCTAATAAATTATTTGCTTTGCCCTCCCCCACCCATAATATGTCACATATATAGCAAGCAGTATTCGA
GAAGTTCAACATGTGCGAAGAGAATGTCAACGATCTGTTGAAATGGG
>dme_circ_0004303_junction_seq
GAGAATGTCAACGATCTGTTGAAATGGGAATGCGCTCATGCGAGACGGCTATCGGG
>dme_circ_0004304
GGTGATGGCTTCTAGCAAACCGAATGAACGCTTAGGCGTGCAGATCAGGCCACTTTCGTAGGCCTCTTGC
AGGGTGCAGAGCTTCGAGGTCTGGGTGTTGAGCACGTTCGCCTTGTTGGCATCCATTAGGCCCTTTCGGA
AGGCCTCTGGCAAACGGACCAGCTTAGCCTTGGCCAAGTCCTTGACTAGAGCCGAGTCGGGATCCACCAC
ACCGGCACTGGCGGCCTCCTTGAGCGTGTATACCTTGGGCGTCTCGATGACAGTCAGCGAATCGTTTGCG
CTATCGTCGCTGGCTGGTGCCGCTGTTGCGGATCCAGGTACACTTGCTGGCACATAGGTCAGCAGTCCGG
TCTGCAAGTCCAGACACTTGGACTCGGCCGCCTGGTCAAAGGTGACCGGTTCAC
>dme_circ_0004304_junction_seq
GCCGCCTGGTCAAAGGTGACCGGTTCACGGTGATGGCTTCTAGCAAACCGAATGAA
>dme_circ_0004305
CTTTAAGTTCCTGCCTTACTCGGTAGCCGTGGAACGCGGTCTCGTGGACACCACCAAGCGCACACTCGTG
GATCCAAAGGCCCTGTACTTTGCCTTCGATCCCACTTTAATAGTTTACGTACGTGAACCGGTCACCTTTG
ACCAGGCGGCCGAGTCCAAGTGTCTGGACTTGCAGACCGGACTGCTGACCTATGTGCCAGCAAGTG
>dme_circ_0004305_junction_seq
GGACTGCTGACCTATGTGCCAGCAAGTGCTTTAAGTTCCTGCCTTACTCGGTAGCC
>dme_circ_0004306
CTTATAACTACGACGTGGCACTCTCGCGCGGAATCCTGGTCACCATTACTCGGCCACTAACCGATCGCAA
TGATGTGGTACGTCGCCAGGATAGCGTTGAATTACTAAATCAAACACCCGTGAGTGTTGTGATTAGCAAA
CCGCGTGAAATGTCTCTCGAAGAGGCCATTCGTTACAACATAATCGATCCCAAGACCGCGCTCGTTCGCG
ATTTCGATAGCTTTAAG
>dme_circ_0004306_junction_seq
GCTCGTTCGCGATTTCGATAGCTTTAAGCTTATAACTACGACGTGGCACTCTCGCG
>dme_circ_0004307
TTGATGACTCTAGAGGTTGTTACAATCTGGGCAACGAACAGTTGGATCTCCAGGCGGCTAGGGCCAGGGG
TTTAATTGTTTCCAATCGTCGCCTCCTCAGCCTTGAACTCGTATTGCGCCAACAACTTTATCGCCCCGAA
ACTGGCAAATTCTGCGATCCAACAACGGGAGAATATCTAGACCTAATCGAAGCCATCCATTCGGGTTTCA
TAGATCCCGCGACTACCGTATTTAAAAACCAGCTTACTGGTAAGGAGTTACCGCTAACAGAAGCTATTGA
GAATGG
>dme_circ_0004307_junction_seq
TACCGCTAACAGAAGCTATTGAGAATGGTTGATGACTCTAGAGGTTGTTACAATCT
>dme_circ_0004308
GATATTCTCCCGTTGTTGGATCGCAGAATTTGCCAGTTTCGGGGCGATAAAGTTGTTGGCGCAATACGAG
TTCAAGGCTGAGGAGGCGACGATTGGAAACAATTAAACCCCTGGCCCTAGCCGCCTGGAGATCCAACTGT
TCGTTGCCCAGATT

>dme_circ_0004308_junction_seq
GGAGATCCAACTGTTCGTTGCCCAGATTGATATTCTCCCGTTGTTGGATCGCAGAA
>dme_circ_0004309
GATATTCTCCCGTTGTTGGATCGCAGAATTTGCCAGTTTCGGGGCGATAAAGTTGTTGGCGCAATACGAG
TTCAAGGCTGAGGAGGCGACGATTGGAAACAATTAAACCCCTGGCCCTAGCCGCCTGGAGATCCAACTGT
TCGTTGCCCAGATTGTAACAACCTCTAGAGTCATCAACTAACTGCTCCTTAATAGCATTGGCAAGCCGAA
GGTACTTGCCGGTCTCGTGGTGTTTGACTAAGCTGGATGATAGGCTAACTACATCCTCCACGCAAGCTTC
GTTGAGATTAAGCTTGCGATTTGTGACTGGATGCAGAACCTTACGTGTGGAAGTTTCATAGAAGCCCATC
GACAATAGTTCGTACAAACCAAAACCGGCATTCTCAATGTCCACAATGAGACCCAGGCTTAGGGCCTCGC
CCAGAGGAACAAACGAATTGCTTCCCGGCTCCCGGAAAACACCCTCCCTTCTATTGATGATGCCATTTCT
ACAGGTCTCGCTCAGAGTATACATCTGTTCGGTCTGCTCATCGAAGTAACAGGGCAGGTGAGGATTAACA
ACGAAACTCCTCATGCTTTCAAGTAATGTAATCTGGCGACCAGTTCTGGGATCGCTTAGCTTTCCGGTCT
TGTCGTCGAATAGTCCCTGATGAATTGCTCGCTGAATGCTGATGGGCGCATTGACATCCATTAGGATTCC
AATATCAAAGGCGTGCTTAAGGGTCATTGTCTGGTTGTTATACAGTAGTTTGGAATTTTGGGCATCAATA
ATGCCGGTGTCTTTAGCGTGCGTTAGGTTCAGCTGCTTGTACAGACCAGTCTTGAAGTCTCTGATCTGCA
CACTATGCTCGTCCA
>dme_circ_0004309_junction_seq
TCTCTGATCTGCACACTATGCTCGTCCAGATATTCTCCCGTTGTTGGATCGCAGAA
>dme_circ_0004310
GAATTTGCCAGTTTCGGGGCGATAAAGTTGTTGGCGCAATACGAGTTCAAGGCTGAGGAGGCGACGATTG
GAAACAATTAAACCCCTGGCCCTAGCCGCCTGGAGATCCAACTGTTCGTTGCCCAGATTGTAACAACCTC
TAGAGTCATCAACTAACTGCTCCTTAATAGCATTGGCAAGCCGAAGGTACTTGCCGGTCTCGTGGTGTTT
GACTAAGCTGGATGATAGGCTAACTACATCCTCCACGCAAGCTTCGTTGAGATTAAGCTTGCGATTTGTG
ACTGGATGCAGAACCTTACGTGTGGAAGTTTCATAGAAGCCCATCGACAATAGTTCGTACAAACCAAAAC
CGGCATTCTCAATGTCCACAATGAGACCCAGGCTTAGGGCCTCGCCCAGAGGAACAAACGAATTGCTTCC
CGGCTCCCGGAAAACACCCTCCCTTCTATTGATGATGCCATTTCTACAGGTCTCGCTCAGAGTATACATC
TGTTCGGTCTGCTCATCGAAGTAACAGGGCAGGTGAGGATTAACAACGAAACTCCTCATGCTTTCAAGTA
ATGTAATCTGGCGACCAGTTCTGGGATCGCTTAGCTTTCCGGTCTTGTCGTCGAATAGTCCCTGATGAAT
TGCTCGCTGAATGCTGATGGGCGCATTGACATCCATTAGGATTCCAATATCAAAGGCGTGCTTAAGGGTC
ATTGTCTGGTTGTTATACAGTAGTTTGGAATTTTGGGCATCAATAATGCCGGTGTCTTTAGCGTGCGTTA
GGTTCAGCTGCTTGTACAGACCAGTCTTGAAGTCTCTGATCTGCACACTATGCTCGTCCAGTAAATTCGT
GTTTAAGGCTTCGGCAAAGTTTAGCTTCTCACCCGACTTCGGATCGACAAAGTGTCCATCCACCTCGTCG
TAGACATTCTTCACCACAGCCTCAATAAGACGAAGAGGCTTCTTGGCTTCAACCAAAATACCACGATCGA
ATGCTTCCTTAAAGTCAATCGGGTGATCATCGGCATCCCTCACCGTACCATGTTTACTGTCCACGATTCC
AGTCTCGGTGGCTACCCCAAATGGAATGATCTGTCCCGAGCCCACATTGACGACCGTCGAATCGGGATCG
AGGATGCCCCTACGAATGGCACGTTCAGTTGTGAGCTTTTCGCGGGTCACCGTATTGCTAAATTGACCAG
TCTTGGGGTCGTAAAGACCGCGGTAGACAGCATCGGGCAAGCTGCGCTTTCGTTTGGGCGGGATGAGAAT
ACGATTCTCCAGGGCTTCGTGTAGGGACAGTTTGGTGGAGGCATATGGGTTAATCACATAGCCCTCAATC
GGATCAAGATAACCCTTCGCGATAGCTTCTGTAATGGAAATAATAGACTCTGTCTTGGGGTCAAGCAAAA
CCAGCTCTTCGGGATTAATGAGCTTTTGGGCAATGGCGGCCTTCAGGTCCAACTGCTTGTCGTCGATGGT
GAATTTTGCTGTCGAAGGATCGTAAAGACCACGCAGCAGACAATCGACCAGGGAAAGCGGCTTTTTCGTG
CTTATAAGATAGCCCTTTACGAAAGCCTCGTCGAGACTTATGTTGGGCGAGCTTAGTGTTCCGCGAACAG
TGTCCAAAAGACCCACCTTGGCCGCCTCCTTGCCTGGCAAAATTTTATCATGATCGACATCTTTGACCAA
AGTCGTTTCTAGCTCCACAAAGCCCATTTCAATGGCTTGCTGAAGAAGAATCTCCTCGCCGGTGACGGGA

TTAAGAACCTTGCCAGTGCTGGGCGTGTAGTATTCGCGCTGAAGCAACTCTGGCAGGCTCCAGGTGACGT
TGTCGGTTTTCAGCAAACACTGTTGTACACCTACATCGAAGGGCACCAAAGTGTTGTTCTTGTTGTCTTT
GATTTGACCCGTGGCGGGCAGCACCAAATCGTCTTCGATAGCGCAATCCAGCTTGACTTCCTTCTTAGTG
ACAGTATCCACAATCAATGAGATCTTCGGGTCAACTAAACCAATTTCAATCGACTTCTGAACATCGACCT
GGTTTTGACTCTCTGAGTCGAAGAGTTTTCCCGTCTCCGGGTCGTAGAGACCTTTGCGTACGGCGGCCTC
CAGGGAAATGGGTTTGCGGGCACCGGGCACTAGGAAACCCAGCTGGAAGGCTAGCTCCATGGGAATCTCT
TTGAGCGTCTGAATGTCTATCACTGTGCCCGCCTGCATGTCTACCACCCCCAACTCAATTGCCATACGCA
GTGGAATCACCTCACCGCTGACTGGGTCGCGCACCGATATCGACTGAATGTCCAGCTTGCCACTGTTGAT
GGCCTCAACAATGGAGCAAACCCGACCATCGTCAAGCACCACCTGTCCAGTCTGTGTGTCTATCGTCACG
TTCTCGATGCCCGATGGCTTTTGCTCTTCCGGATTGGCTCGGATAATCCATTGGCGGTCAACGCACTCAA
AGAATGGGATTTTCTTGCCTGTCTTCGGATCCACCATGTGACCCGTGTGCCTGTCCAGAAGTGGACGCTC
CAAGGCTGCTGTAAGAGTCTCGTATTTGCCGGTACTAAAGTTCTTTACCAAAATAGTTTCTGGGTTAAAA
ACATTCATGTCAAATACCAGCGTATGGAAGGAAATCTTCTGGCCAGTACTGGGATCAATAAAGTATTGGT
TCTCGGGATCGTAGATGTTAAGTTCATACAAATTCTGTGGCGTCAGTTCTGTGTCTGCGTCTACCAGGTA
AGACACCTGCTGCAGAACAATTAGTTTGTCGTCGATGATCTGTACTTCGTCCAATGGGCTAAGCTGCTCC
TCCTTGAGTAGCGTGGAAACGGTCTGCGACTGCTGTTCTCCATCTACGATCACCTGGCACTCATCGGGGC
TAATCAACTTTTGGGAGATGGCATCGCCAATGGCCAGTGTCCTGGGCACTTCTCGCTTTACAGAGCTTTC
AACCAATTCAGCAGGCAGTTTAACTACATCCAGAGCCTCATCATCGGGAGTGCCCTCACTGGTGGATGTC
GTTGTGGTGGTAGTTGTAGTGGTGACTTTGGTAACTAATGTAGCAGTGCTTGTGGTAATTATCTCTGTTG
GTAGTTCTTCCACTGTCTCGGTTACAACTTGTGATGTGGAGTGTTCTGGCTTAAGTGTCTTTTCTGTGGT
TAGAGCATCTACCAAAGCCTTGCCCGCAAGAACTGGTGCTCCTACAATGGCTAGAGCGCCCAGCTTGGCA
GCATCCTTCACATTCTGCAAAATATTGCTACCACCTTCCGCTGGCTGTGTGCCCTTCTCCTGTTCAATGA
AATCAACAAGAGATTGATGTTTGTCCGAAGTAGAATGCGGTTGAATAATTTGCTCAATATCACTTGGTGG
ATGTAGTTTTCCTTCATCAACAATTGTTGTCTTGGTGGTAAGAGTCACTGTTGTTGTTTCAGATGTGCTT
ATTGGCTCGGTTGGGATAGTTTCCACCGGCTTCTGCTCTGTTGTTTTCTTTTCGGTATCTACAATTTCTT
CCGGCTTCTTTTGATCATCATTAGTTGTCTGCTCAATCTCTTTAGTTTCAGCTGGAGATGAAGGCACAGG
CTCATTCAAAGGCGTTTCCTTTACTGTGGCTGTATTCTCTTGGGGCAGGGTTTCCTCACTTGAAGATgtt
gttgttgtagtgatagttgttgttgttacggtagttTCTGTGATCGTCGGCTCTTCGACAGTGACAACTC
TTTCTTCATGCACTATTTCATTGGTAGGTTCAGTCCTGGTGGAATCATCGCTCGTCTCATTCACCTTAAT
CGGTTCTTGTTGGTGAACTGTTGGTTCTTCCACATCCACAATTTTCTCAGATTTGCTGACCTCATCTACT
TCCTCGACTATGGGGTGTTCATCGACTGTTTTAACCGTTGTGGTGATGGTTGTGATAGTTTGGGAACTTG
TAACTGGTTCTTTAAATTCTGTTTTAATTTCAGTGATCTTGGTTTCCGCTAATTGATCAACAAATGGAGC
CAGTGGTGCTGTGAACGGGGGCTCAATTTCAGAAATCTCCTCCTTTTTAGCAGTTCGGGGCATTGGCTCT
GGCTTTGAGGTAGGTTCAGCTGATTGTTCGTTCTTATTGGTTGGAGCAGGAGAAGCTTCCTTTGGTGTGA
TTTGTGCTGCCTCGGTAGCGGTTTCCTTGTAATCCGGCTCAGGAGTAGGATCTTTTTTAATAGATTCAGG
AAATTCTGTATTTTGAGCTGGATCGGGAGTTGGTTCCTTTTGAACAGGCTTAGGAGATGTCTCCGTTTCA
GCTGGCCTGGAACTTGTGTCTTTCGTAACCACTGGATCCTTCTTTGCTGACTCCTCGGAAGCTGATTTCT
GTGATATCTTTTCTGGTACAGTATCTGTTTCATTAGAAGTCACTGTGGTTATGGTTTCAGTTATTTTGGA
AACCTCTGTTTTATCTTCTGGAACTGTACTTGGCTTAACCACCACTTTATCCTCCTTAACTGTGGTCGAG
ACATCTTCTACCACAACTGGCTCCAGATTTCCGGTCTCCGTTGTTGTACTTGATGTAGTAATTGTTGTTA
CTGTTGTTGTGGTCAGAGTGCTTGTTGATTCAACTTCCAAGGGCAGATTGCTTTCCTGTGGCAACGTATC
GGGCTTATCCTGCTGTTTTTCGTTTGCAATAAAGTTGGCAGTCGTAGAGCTGATAAAATTCTCCGTTTCA
CTGAGGAGATTATCCACCTCACTTGGCACTTGTTGCGGCTGTAGCTCTTTACGGCGCTCCTGTTCAATGA
AGCTGGTTTGAGGTTCCTTAGGTTTTTCAGTGCTCTTGGCATCTTCCGTGGGAGATTTCACAGATTTGAT

AAGGCTTTTAACGCCACTTACGATGGCACCTCCGGCCAGTACAGGAGCTGCGATTACGCCCAATGCACCC
AATTTGGCGGCATCTTTCACATTTTGCAACGTCTTCGATGGCTCCTTGCTGCCCTTTGTCTCGATTTCAC
TTACACTTGGAACGGATGTATCCTTCGTGGATTGCGTCGTGCTAACTACTTGCATCTCTTGCTTGCGGGT
ATCCGAACTGACCACAACTGTCTGGGTGCGCTCTTCTACAATAACAGTTTGATCGCCACTGGTACCAACG
TCGCCCACCGATTCCGGACTTTCGCCGGCCGCTGCTTTTGTCTCCCGAGTTGTGGATTCTTCGCGCAGAA
TTCCCAGCTCCTTAGCTTCCTCCAAGCTGCATACCTCGCCTGTGTTAACGTTCTGCACTTCATTGTTAGA
AATGAGGACGTAGCCTTGCATAAGCTCAATGGTTTTAGTAGTCACCGTGTGCACTGCACGCACCGTCTCC
ATGATCTCCGTTTTGGGAATGGGTTTCTGG
>dme_circ_0004310_junction_seq
GATCTCCGTTTTGGGAATGGGTTTCTGGGAATTTGCCAGTTTCGGGGCGATAAAGT
>dme_circ_0004311
GATTGTAACAACCTCTAGAGTCATCAACTAACTGCTCCTTAATAGCATTGGCAAGCCGAAGGTACTTGCC
GGTCTCGTGGTGTTTGACTAAGCTGGATGATAGGCTAACTACATCCTCCACGCAAGCTTCGTTGAGATTA
AGCTTGCGATTTGTGACTGGATGCAGAACCTTACGTGTGGAAGTTTCATAGAAGCCCATCGACAATAGTT
CGTACAAACCAAAACCGGCATTCTCAATGTCCACAATGAGACCCAGGCTTAGGGCCTCGCCCAGAGGAAC
AAACGAATTGCTTCCCGGCTCCCGGAAAACACCCTCCCTTCTATTGATGATGCCATTTCTACAGGTCTCG
CTCAGAGTATACATCTGTTCGGTCTGCTCATCGAAGTAACAGGGCAGGTGAGGATTAACAACGAAACTCC
TCATGCTTTCAAGTAATGTAATCTGGCGACCAGTTCTGGGATCGCTTAGCTTTCCGGTCTTGTCGTCGAA
TAGTCCCTGATGAATTGCTCGCTGAATGCTGATGGGCGCATTGACATCCATTAGGATTCCAATATCAAAG
GCGTGCTTAAGGGTCATTGTCTG
>dme_circ_0004311_junction_seq
CAAAGGCGTGCTTAAGGGTCATTGTCTGGATTGTAACAACCTCTAGAGTCATCAAC
>dme_circ_0004312
GCCCATCGACAATAGTTCGTACAAACCAAAACCGGCATTCTCAATGTCCACAATGAGACCCAGGCTTAGG
GCCTCGCCCAGAGGAACAAACGAATTGCTTCCCGGCTCCCGGAAAACACCCTCCCTTCTATTGATGATGC
CATTTCTACAGGTCTCGCTCAGAGTATACATCTGTTCGGTCTGCTCATCGAAGTAACAGGGCAGGTGAGG
ATTAACAACGAAACTCCTCATGCTTTCAAGTAAT
>dme_circ_0004312_junction_seq
AACGAAACTCCTCATGCTTTCAAGTAATGCCCATCGACAATAGTTCGTACAAACCA
>dme_circ_0004313
GTCCCTGATGAATTGCTCGCTGAATGCTGATGGGCGCATTGACATCCATTAGGATTCCAATATCAAAGGC
GTGCTTAAGGGTCATTGTCTGGTTGTTATACAGTAGTTTGGAATTTTGGGCATCAATAATGCCGGTGTCT
TTAGCGTGCGTTAGGTTCAGCTGCTTGTACAGACCAGTCTTGAAGTCTCTGATCTGCACACTATGCTCGT
CCAGTAAATTC
>dme_circ_0004313_junction_seq
CTGCACACTATGCTCGTCCAGTAAATTCGTCCCTGATGAATTGCTCGCTGAATGCT
>dme_circ_0004314
GACTCTGTCTTGGGGTCAAGCAAAACCAGCTCTTCGGGATTAATGAGCTTTTGGGCAATGGCGGCCTTCA
GGTCCAACTGCTTGTCGTCGATGGTGAATTTTGCTGTCGAAGGATCGTAAAGACCACGCAGCAGACAATC
GACCAGGGAAAGCGGCTTTTTCGTGCTTATAAGATAGCCCTTTACGAAAGCCTCGTCGAGACTTATGTTG
GGCGAGCTTAGTGTTCCGCGAACAGTGTCCAAAAGACCCACCTTGGCCGCCTCCTTGCCTGGCAAAATTT
TATCATGATCGACATCTTTGACCAAAGTCGTTTCTAGCTCCACAAAGCCCATTTCAATGGCTTGCTGAAG
AAGAATCTCCTCGCCGGTGACGGGATTAAGAACCTTGCCAGTGCTGGGCGTGTAGTATTCGCGCTGAAGC
AACTCTGGCAGGCTCCAGGTGACGTTGTCGGTTTTCAGCAAACACTGTTGTACACCTACATCGAAGGGCA

CCAAAGTGTTGTTCTTGTTGTCTTTGATTTGACCCGTGGCGGGCAGCACCAAATCGTCTTCGATAGCGCA
ATCCAGCTTGACTTCCTTCTTA
>dme_circ_0004314_junction_seq
AGCGCAATCCAGCTTGACTTCCTTCTTAGACTCTGTCTTGGGGTCAAGCAAAACCA
>dme_circ_0004315
GCTCCACAAAGCCCATTTCAATGGCTTGCTGAAGAAGAATCTCCTCGCCGGTGACGGGATTAAGAACCTT
GCCAGTGCTGGGCGTGTAGTATTCGCGCTGAAGCAACTCTGGCAGGCTCCAGGTGAC
>dme_circ_0004315_junction_seq
GAAGCAACTCTGGCAGGCTCCAGGTGACGCTCCACAAAGCCCATTTCAATGGCTTG
>dme_circ_0004316
GTGTACAACAGTGTTTGCTGAAAACCGACAACGTCACCTGGAGCCTGCCAGAGTTGCTTCAGCGCGAATA
CTACACGCCCAGCACTGGCAAGGTTCTTAATCCCGTCACCGGCGAGGAGATTCTTCTTCAGCAAGCCATT
GAAATGGGCTTTG
>dme_circ_0004316_junction_seq
CTTCAGCAAGCCATTGAAATGGGCTTTGGTGTACAACAGTGTTTGCTGAAAACCGA
>dme_circ_0004317
GTATTCGCGCTGAAGCAACTCTGGCAGGCTCCAGGTGACGTTGTCGGTTTTCAGCAAACACTGTTGTACA
CCTACATCGAAGGGCACCAAAGTGTTGTTCTTGTTGTCTTTGATTTGACCCGTGGCGGGCAGCACCAAAT
CGTCTTCGATAGCGCAATCCAGCTTGACTTCCTTCTTAGTGACA
>dme_circ_0004317_junction_seq
ATCCAGCTTGACTTCCTTCTTAGTGACAGTATTCGCGCTGAAGCAACTCTGGCAGG
>dme_circ_0004318
CAGCCTTGGAGCGTCCACTTCTGGACAGGCACACGGGTCACATGGTGGATCCGAAGACAGGCAAGAAAAT
CCCATTCTTTGAGTGCGTTGACCGCCAATGGATTATCCGAGCCAATCCGGAAGAGCAAAAGCCATCGGGC
ATCGAGAACGTGACGATAGACACACAGACTGGACAGG
>dme_circ_0004318_junction_seq
GTGACGATAGACACACAGACTGGACAGGCAGCCTTGGAGCGTCCACTTCTGGACAG
>dme_circ_0004319
GAATGGGATTTTCTTGCCTGTCTTCGGATCCACCATGTGACCCGTGTGCCTGTCCAGAAGTGGACGCTCC
AAGGCTGCTGTAAGAGTCTCGTATTTGCCGGTACTAAAGTTCTTTACCAAAATAGTTTCTGGGTTAAAAA
CATTCATGTCAAATACCAGCGTATGGAAGGAAATCTTCTGGCCAGTACTGGGATCAATAAAGTATTGGTT
CTCGGGATCGTAGATGTTAAGTTCATACAAATTCTGTGGCGTCAGTTCTGTGTCTGCGTCTACCAGGTAA
GACACCTGCTGCAGAACAATTAGTTTGTCGTCGATGATCTGTACTTCGTCCAATGGGCTAAGCTGCTCCT
CCTTGAGTAGCGTGGAAACGGTCTGCGACTGCTGTTCTCCATCTACGATCACCTGGCACTCATCGGGGCT
AATCAACTTTTGGGAGATGGCATCGCCAATGGCCAGTGTCCTGGGCACTTCTCGCTTTACAGAGCTTTCA
ACCAATTCAGCAGGCAGTTTAACTACATCCAGAGCCTCATCATCGGGAGTGCCCTCACTGGTGGATGTCG
TTGTGGTGGTAGTTGTAGTGGTGACTTTGGTAACTAATGTAGCAGTGCTTGTGGTAATTATCTCTGTTGG
TAGTTCTTCCACTGTCTCGGTTACAACTTGTGATGTGGAGTGTTCTGGCTTAAGTGTCTTTTCTGTGGTT
AGAGCATCTACCAAAGCCTTGCCCGCAAGAACTGGTGCTCCTACAATGGCTAGAGCGCCCAGCTTGGCAG
CATCCTTCACATTCTGCAAAATATTGCTACCACCTTCCGCTGGCTGTGTGCCCTTCTCCTGTTCAATGAA
ATCAACAAGAGATTGATGTTTGTCCGAAGTAGAATGCGGTTGAATAATTTGCTCAATATCACTTGGTGGA
TGTAGTTTTCCTTCATCAACAATTGTTGTCTTGGTGGTAAGAGTCACTGTTGTTGTTTCAGATGTGCTTA
TTGGCTCGGTTGGGATAGTTTCCACCGGCTTCTGCTCTGTTGTTTTCTTTTCGGTATCTACAATTTCTTC
CGGCTTCTTTTGATCATCATTAGTTGTCTGCTCAATCTCTTTAGTTTCAGCTGGAGATGAAGGCACAGGC

TCATTCAAAGGCGTTTCCTTTACTGTGGCTGTATTCTCTTGGGGCAGGGTTTCCTCACTTGAAGATgttg
ttgttgtagtgatagttgttgttgttacggtagttTCTGTGATCGTCGGCTCTTCGACAGTGACAACTCT
TTCTTCATGCACTATTTCATTGGTAGGTTCAGTCCTGGTGGAATCATCGCTCGTCTCATTCACCTTAATC
GGTTCTTGTTGGTGAACTGTTGGTTCTTCCACATCCACAATTTTCTCAGATTTGCTGACCTCATCTACTT
CCTCGACTATGGGGTGTTCATCGACTGTTTTAACCGTTGTGGTGATGGTTGTGATAGTTTGGGAACTTGT
AACTGGTTCTTTAAATTCTGTTTTAATTTCAGTGATCTTGGTTTCCGCTAATTGATCAACAAATGGAGCC
AGTGGTGCTGTGAACGGGGGCTCAATTTCAGAAATCTCCTCCTTTTTAGCAGTTCGGGGCATTGGCTCTG
GCTTTGAGGTAGGTTCAGCTGATTGTTCGTTCTTATTGGTTGGAGCAGGAGAAGCTTCCTTTGGTGTGAT
TTGTGCTGCCTCGGTAGCGGTTTCCTTGTAATCCGGCTCAGGAGTAGGATCTTTTTTAATAGATTCAGGA
AATTCTGTATTTTGAGCTGGATCGGGAGTTGGTTCCTTTTGAACAGGCTTAGGAGATGTCTCCGTTTCAG
CTGGCCTGGAACTTGTGTCTTTCGTAACCACTGGATCCTTCTTTGCTGACTCCTCGGAAGCTGATTTCTG
TGATATCTTTTCTGGTACAGTATCTGTTTCATTAGAAGTCACTGTGGTTATGGTTTCA
>dme_circ_0004319_junction_seq
ATTAGAAGTCACTGTGGTTATGGTTTCAGAATGGGATTTTCTTGCCTGTCTTCGGA
>dme_circ_0004320
CTACAGCCGCAACAAGTGCCAAGTGAGGTGGATAATCTCCTCAGTGAAACGGAGAATTTTATCAGCTCTA
CGACTGCCAACTTTATTGCAAACGAAAAACAGCAGGATAAGCCCGATACGTTGCCACAGGAAAGCAATCT
GCCCTTGGAAGTTGAATCAACAAGCACTCTGACCACAACAACAG
>dme_circ_0004320_junction_seq
TCAACAAGCACTCTGACCACAACAACAGCTACAGCCGCAACAAGTGCCAAGTGAGG
>dme_circ_0004321
ATGCCAAGAGCACTGAAAAACCTAAGGAACCTCAAACCAGCTTCATTGAACAGGAGCGCCGTAAAGAGCT
ACAGCCGCAACAAGTGCCAAGTGAGGTGGATAATCTCCTCAGTGAAACGGAGAATTTTATCAGCTCTACG
ACTGCCAACTTTATTGCAAACGAAAAACAGCAGGATAAGCCCGATACGTTGCCACAGGAAAGCAATCTGC
CCTTGGAAGTTGAATCAACAAGCACTCTGACCACAACAACAG
>dme_circ_0004321_junction_seq
TCAACAAGCACTCTGACCACAACAACAGATGCCAAGAGCACTGAAAAACCTAAGGA
>dme_circ_0004322
ATGCCGCCAAATTGGGTGCATTGGGCGTAATCGCAGCTCCTGTACTGGCCGGAGGTGCCATCGTAAGTGG
CGTTAAAAGCCTTATCAAATCTGTGAAATCTCCCACGGAAGATGCCAAGAGCACTGAAAAACCTAAGGAA
CCTCAAACCAGCTTCATTGAACAGGAGCGCCGTAAAGAGCTACAGCCGCAACAAG
>dme_circ_0004322_junction_seq
CGCCGTAAAGAGCTACAGCCGCAACAAGATGCCGCCAAATTGGGTGCATTGGGCGT
>dme_circ_0004323
GGCTTTTAACGCCACTTACGATGGCACCTCCGGCCAGTACAGGAGCTGCGATTACGCCCAATGCACCCAA
TTTGGCGGCATCTTTCACATTTTGCAACGTCTTCGATGGCTCCTTGCTGCCCTTTGTCTCGATTTCACTT
ACACTTGGAACGGATGTATCCTTCGTGGATTGCGTCGTGCTAACTACTTGCATCTCTTGCTTGCGGGTAT
CCGAACTGACCACAACTGTCTGGGTGCGCTCTTCTACAATAACAGTTTGATCGCCACTGGTACCAACGTC
GCCCACCGATTCCGGACTTTCGCCGGCCGCTGCTTTTGTCTCCCGAGTTGTGGATTCTTCGCGCAGAATT
CCCAGCTCCTTAGCTTCCTCCAAGCTGCATACCTCGCCTGTGTTAACGTTCTGCACTTCATTGTTAGAAA
TGAGGACGTAGCCTTGCATAAGCTCAATGGTTTTAGTAGTCACCGTGTGCACTGCACGCACCGTCTCCAT
GATCTCCGTTTTGGGAATGGGTTTCTGGGTAATCACAAAATCTGAGAGATTCTTTACCACCAGAACACCA
TTTTCCATAGCTTGTCGTAGAGGTATACGTTCACCAGTGGACGGTACA
>dme_circ_0004323_junction_seq

AGGTATACGTTCACCAGTGGACGGTACAGGCTTTTAACGCCACTTACGATGGCACC
>dme_circ_0004324
GTTTGATCGCCACTGGTACCAACGTCGCCCACCGATTCCGGACTTTCGCCGGCCGCTGCTTTTGTCTCCC
GAGTTGTGGATTCTTCGCGCAGAATTCCCAGCTCCTTAGCTTCCTCCAAGCTGCATACCTCGCCTGT
>dme_circ_0004324_junction_seq
CTTCCTCCAAGCTGCATACCTCGCCTGTGTTTGATCGCCACTGGTACCAACGTCGC
>dme_circ_0004325
GTAGTCACCGTGTGCACTGCACGCACCGTCTCCATGATCTCCGTTTTGGGAATGGGTTTCTGGGTAATCA
CAAAATCTGAGAGATTCTTTACCACCAGAACACCATTTTCCATAGCTTGTCGTAGAGGTATACGTTCACC
AGTGGACGGTACAGTTAGTGTTTGGGCAGCTACATCGATGAGATCCTGCTCCATGGCAGTAACCACTTCG
ATAGCATCTGGCTTGGGAGAATAGGGAATGGACATGATAAAGTTCTCCTCAATGGCTTGCTTAATTGGAA
GTGCTTTCTTTGTACTTGGATGCAGAACAACACGCTTGGACGGTTCCACAAGGCTCTTTTCAACAGCAAC
AGGGAATATCATACCCACCGGCGGAATTTCCACCTTAGATTTTTGCTGATCGTAACTGGGATCAAAGAGC
TTCGCTTCAATGGCTTCAGTAAT
>dme_circ_0004325_junction_seq
AGAGCTTCGCTTCAATGGCTTCAGTAATGTAGTCACCGTGTGCACTGCACGCACCG
>dme_circ_0004326
GAACACCATTTTCCATAGCTTGTCGTAGAGGTATACGTTCACCAGTGGACGGTACAGTTAGTGTTTGGGC
AGCTACATCGATGAGATCCTGCTCCATGGCAGTAACCACTTCGATAGCATCTGGCTTGGGAGAATAGGGA
ATGGACATGATAAAGTTCTCCTCAATGGCTTGCTTAATTGGAAGTGCTTTCTTT
>dme_circ_0004326_junction_seq
GGCTTGCTTAATTGGAAGTGCTTTCTTTGAACACCATTTTCCATAGCTTGTCGTAG
>dme_circ_0004327
GTTCTGGATCCGCATTCAAAACTGCATAAACCCGAAAAATGCACACTAGAGGAAGCGATCACCAAGGGCA
TTATCAATGCAGATGATTCCACATTCAACTATAAGAACAAGACACTCAACATTACTGAAGCCATTGAAGC
GAAGCTCTTTGATCCCAGTTACGATCAGCAAAAATCTAAGGTGGAAATTCCGCCGGTGGGTATGATATTC
CCTGTTGCTGTTGAAAAGAGCCTTGTGGAACCGTCCAAGCGTGTTG
>dme_circ_0004327_junction_seq
AGCCTTGTGGAACCGTCCAAGCGTGTTGGTTCTGGATCCGCATTCAAAACTGCATA
>dme_circ_0004328
GCTGCTCATCAACCAGACCCTGACGGGCGGCATCGAAGATATTTTCAGTCTCTCCGGTTTGCGTGAAGAT
TACTAGGGCAGTGGACGGATCATAGATGGCGCCCGGTGCAATTTGCAGGGGCTCTGGAGAGGCGAGCTCT
CGTTGGCAGGTGAGCACCTCGACAAACTTGGGCGGTGCATCTTTCAGGGGCGTGGAAACCTCTAACACAT
CTGGAAT
>dme_circ_0004328_junction_seq
GCGTGGAAACCTCTAACACATCTGGAATGCTGCTCATCAACCAGACCCTGACGGGC
>dme_circ_0004329
GTTAATAAATTCGGAGACCACACGATGCGGCTTTACTAGTTCCTGAGAAATAGGAATCTCCACCGACTTC
TCGACGTACTTGGTGTGCTGTGTGTTTAGCTTGATGGGCTCTGCTTCCGGTATGATTTGCTTGGTCACAA
TGGTAATAGTGGATTTTGTCTTTTTCCTATTTGTTGGCTCCGGAACTGTTTGATTGCTATCGGGAACCAA
CAAGCCATAGTTGAGGGCCTGCTGTACTGA
>dme_circ_0004329_junction_seq
AGCCATAGTTGAGGGCCTGCTGTACTGAGTTAATAAATTCGGAGACCACACGATGC
>dme_circ_0004330

GGAATCTCCACCGACTTCTCGACGTACTTGGTGTGCTGTGTGTTTAGCTTGATGGGCTCTGCTTCCGGTA
TGATTTGCTTGGTCACAATGGTAATAGTGGATTTTGTCTTTTTCCTATTTGTTGGCTCCGGAACTGTTTG
ATTGCTATCGGGAACCAACAAGCCATAGTTGAGGGCCTGCTGTACTGA
>dme_circ_0004330_junction_seq
AGCCATAGTTGAGGGCCTGCTGTACTGAGGAATCTCCACCGACTTCTCGACGTACT
>dme_circ_0004331
GATTGTGATAGACCAAATCAAAGACGCTAAGCTCCTTACCACTGGCATTCTGAACGCCAATGCCACGGCT
AAACATCTCGTACACCTCCTGGCGCACCAGTCCCTCACTAACAGCCACCTCTAGTGGCACCAGACTATCC
CTACCCGTCTCTAAGGCAAAGCCCGTGCTCTTGCGACGCAAAAGATCGCGCGAGAGGGCCACATTAAAGC
TTACATAGTCATTGCTCCTCAGATCCTTGAACCCGTCAATATCGAAGATCGAACGCTGCGCCACCGAGCT
AATCAGACCGCGTTCCACTGCCTCCGGAATGCTGATTAGCTCACCGGTCATAAAGTCGCGGTACTTGCAC
TCGGCCGGCAACACGATGCCATCGGCAATGGCCTCCTGAAGGGTGACCAATTCACCCTTACGTTTGGTGA
TACACTTGAGCAAATTACCGTCGAGTACACCGGCCTCGATGGCCTGCATAATGCTTAACTTTTCGCGGCG
CATGGGACTCGTGATCTGAGCCTGTTTATCGAGCAGATTCAGATCACAGATGTCCTTCAGCGTGTACGGT
TTCACGATCAACTGACGGTTTCGCGCCTCTGCGAAGGTTAACTTCTCCTTGGTCACCTCGTTCACATAAC
GACCCGTTTTTGCATTCAGTACGCCCGACTGCAGTGCCTCCTGCAGTGTGATCTTCGTATCGCGTTTCGC
GTCCACCACTTCGCGCACACTCGCGTCTATTAGTTGATTGGCAATGGCCGAATCCAGTCGGAACTTGTCG
CCGGAGTTGCGATCCGCTATCCAGCCGGCGCCATCGACCAGGCCATGCGCAATCGCATCGCTCAGGCACA
GCGCATTCGATTTGATTGTCACCGACTCGTGCCGGATGAGGTAGCCGCGCTCGTAGGCCTCCGCCAGGCT
GATTGTCTGTCCGGATTTGACGCGATACAGGCCGGTTTTGGTGTCCACACTCCCCGCAGTAATGGCGTCG
GCAATGTTGCGTGGATTCTCGGGAGAGCCCAGCTCTCCGGAAGTTTGCTCTACGGTGACTGTGTTGTTTA
CCTTGATCCGCTTCTCGGCCGTCTCGTAACCGGATTCCGCTTCGCTAATCTCGCGCTGAATAACCTCCAG
GAGCGACAGCTCCCGTCCGCTGGCATCTCGGCCGGCACCAGGCTCAAGCAATTGCTTTGCCAGTTGCAAA
TCGATCAGTCCTTGGTCGGCAGCTTGTTCCAGCGTGATACGGCGATCACCCACTAGCATCTCGCCGGTGC
GCACGTCCAGAATGCGAAGCTGGATGGCTTCACCGATGGTTAGAACGCGACCCGTACGAGGATCGCGTAT
TCCGGCTATATCCAGCACGGAATGGCCACCCAGCTGTTGAGAGCGTTTGAGCGTACGGATTTCGTTGAAT
TGGATCTGCGATTGAGGCAGGCCAGCAGTGCTAACCTCCTGACTAGCGGCGTCCAAGCCATCGCTACTAT
AGACGCTCTTCGTAGAGCTTTTCGTTGTCTTCGTTGAGCTGATTCTGAAAGGATTCGATTAGAACTTTAT
TAGTATATAATAAAAGAATGTATGACTACAAATCTACATATAAGATTATTCATAGTTCTACAACTTTGTC
AAAAGTTGTTATCGTTTCTTTCGCATAATATTGTAGATTGTAGATTTAAATGAATCATTTTGTTGATTTC
AGAATGCAGTAAAATTCTTTCTTTCTGGAGAATAATATCCACTTAATATATATTATAAATTTTGTATAAA
TTGTACTTACTGTGTGGTGCTGTAGGTAGTGGTGTAGAGATCCTCCTGGCGCGCAGCGTAACCATCGCCA
GTTGGCACGCTGCCCGCACTGAAGTCAACCCTAAAGGTCTTGAGCGGTTTCTGTAGACTCTCAGCGTATT
GCTAAATGCCAAATGAAAGCAATTTCGGTTAGTAAAAGTTAACAACTGGGCAACAGGCAAATTAAACTCG
GTTCGTTGAAATCTTTGCGAGGGGaaaaaaaaCAGGGCAGCAGGGAATGGGGAGGATAGATGAAGTATTA
CCTGAAACTCGAACGCAAAGTCGTAGTCACTTTCCTCTGCCACTTGGACAGTCGTGGTTTGGGTTTTGGA
TTTGGTTTTAGTCGCCTTGCGACCCGATCTATGCTGAGGCATGTCTAAGGCTTTCGTTTGGTTCAGTTCG
ACAAAGTCATAGTCAGCAATTGGTATTTGTTTCGTTGGCTTAGGGGCTTCTTGAATACACTGCGCGAGAT
TTAATTGTGACAAAAGCAATTAGAAATTTACGAGTCGAGATTTCTTGGCAGACGCGTCACTTTGAAGTTG
CCCTAGAGTCTCAAACCATTTTTGTGGAATGTTTATGGCATGCGTGAGCGTGATTTGATATGGCCGCGTG
ATAAAGTCACCGGCGATAAAGTAACTAAAAATACTTCCAAGATTGTCAAAAGACATGAACTCTACAATTT
TACGGTAGCCATCGCTGGACTTTCATTATTAATCGGCCCTTAAACCGTCCTATACTACTCTGTTTGTTGA
TTCACTTAATTCATTCACTAGATTCAGTTCTTTCAGTCACACACAAGGATACATTTTTATCAGCCCCGCA
CCGACGCACACAAATCACACGAAGCACATATGTATAGTTAAGTGaaaacaaaaaaaaaaaaaataaggaa

aaaacgcaaacaaaaaGCGAACCCAAAGGCTTTATGAAAATAGAAATGACGCTATATGACGGAGAAGTCC
TCAATCAATCGGCCTGCAAATATGTAGTGCAAACTCTCTCAAGAGGTCCCAACCATAATAAATGGGTGAT
TACCCGGAAAGAGTTGGGGATCCGGAGTGCGTCTTATCGACGGGATCTGGTAATTTAATTTTTATCTTTA
TTTACCATTTGCCAGAGACAGTGATTCAATGGCAAATGACTGACATATGGCTGCAGGTCGTAGCCCCAAC
TTAAAATTGCATTGACGTCATACATACAACTTTGCAAAAGAGGGCTGCAAATGAAGTCATTGAATGGCGA
ATCTACAAAGTGATCAACGTTTATGATTTGCATTTTCAAAATAAACAACTAATCTGCTGTTGCTGCTGCT
GATTTTCAAGGTTGCCCTAGAGTTGGTAAAAGCAACAAATAAATTATAATTAACGAAACACACACCGACA
CACATTTAGACAGTTAATATACAAGTACGAGAACAATGATGTCATCAGAGTGGCGAATATTTGATTATAA
ATGAAACCGAAACAAGTTTGTCGAGCGCCCGCACAACGAGTAACTAAAGCTGAACTAAAGCACAAAATAA
TGGCCTATGAGTCCGCAAGCAGATTAAACAGATTCTTGGCCCTAATCGGCTGAGATGCGCTTGACATTGA
TAACATGACTAATACGCCACGTTGTACCGGCTTTAAATGGGGGTAAGAGCGAACAAACGAGAGAGGAAAA
GCTCTAACGAGAGCGTGCAGCATGCACCACAGCCTGCAATTGGACCTCGCCTTTGTGGCACTATGAAAAT
AGGCGCCTCCCTTTTGCTTCAGTGGAGGCATAGACCCCAAAACGCTCGCTTTCATTCCTGATTTATCGTT
AGAATGCCGCTAAAAatatatatatatatatatGCAACGAAGTATATATAGAAGGTATACCAAGGCATCT
AGTTTAATCGATAAAATCAAAGGGAAAGTGCATGGATGCTAGGCGAACAAACAAATACTCTTCCATCGGC
ATTCCGGGTGGCATTTCCTGGTAAACCGATCGCTCAATTCCAAGCATCACATTTAGTTCTGATGGATTGC
ACCTCATAGATTAGATAAGCGCCAGCCCTGACACTCCCGCGATCCTCCTGTTGATAGCAAGTCATAATTC
ACGCTAGTCAGCTAGTAATCGAGCGGGAGAATAATTTCCACAGTTTTTCGGGTTTCTCATTTTAAGTGCA
GCATTTTATGGTTTTATGCAACTTATAATGACCCTAAAACTCGCTGACTCACTACGAAATTTCAGCCATA
GAttttcattttctcctttttttcttttttttttttttttGTGTGTGCGCTTTCATTAACACCAAAACAGAC
GGCCGAAAATATCAACATTCCGATTCTTGCGACATGTTCAAGGCTATATTAATAATGTATTTCTTTTATT
TTCGTTTTCACACTCTTCTGTTTGCGTTTCGGTTATATTTAAGTTGTTGCAAAGCACTCGACGAGTGACT
TCATTTCGGGCTATAATAGAACTCTTTGCGGCACAAATTCAAATGGCAGCGAATTTAAATTTCTTCTAAC
AAAATAAAAGCGAACCAATGCGACACGGTTCGCATTTAATTGAAATTTAAGGCACTAAGACAACGACACG
GACACACTTTCTATCCGGCCGTATATGTGACATCATGTCTGCCCATGCGGCGTATGAGCAATGTTCTCTA
TTTGAGTTTTGGGAAGTTATTTGCCCGTTTAGTTATCACAACTATTCACTAATAATTACACGCTCGATTC
GAATTCAAATTCCATAGACTTTGGTTGCCAACAAAAACCGGAGGTTTTAAAGGTTTTGGCCTTTGTTAAA
TTTCGTTCACCGGCAGCAAGTCGGCACACACAGCGGATACGCGACGTTACACAACCACGTCGCTTTCGGT
ACTGAATGGAGCAGCGCCACTCCGAGAATCAAATGGTTAAGTGGAGAGCGAGAGCGAGCTTTTGTTGAGA
TTGAGAGAAAGCAATTGCCACTgagagagagagagagggagagCTTACAAATGCCGGCTAGTTTTCGGTG
AGCATTCCCCAGCTGTTGCGCCAAAACATTATAAACAATCCATTTACAATGGCATGGCATGAAATGGTGG
AGATTGGCGATgggaaggaaagggaagggaagggaGTATACGCTGCTGGAGTTGGCTCCAAATTAAAGTC
GAAAACAATACACGGAAGTCGAGAACAAAGTATCAGAAGAACAGATCAAACCAGTTTGTGCCGCCATCTA
ACCTTCTAAATAACTTATTTCGCTTTTCAGTCTAGTTAAGCCAGCGGAGCTTCAGCGCCCGCCCCTGCTA
TGCATATCAAGTTGGCTTGAAGGCGAAGTGGTAATGGAGCAGTTATGATGTCACTCCAACGCTCGAGGCG
AAGGCAACACCTTCGAAAAGCCTCAATAGATCCCAAAACAACAAATTCGGATTACTTTGAAATGGATCAA
CTACATAGAAAGAAATTGAGGCTAAAGATGTTCAATTTTATGGCATTTATAGTTTGTTTTTTAAGCAGat
atatatatattatattatttatCATTAAATTTAGAAAAATTGGTTTTTCTCACTGTAGTATTAATGATGC
TATTTATTACAGCATTAGCCTAAAAAGTTTATTGAACTTATGTTTACACCATGTCCGGCGATCTGAACTC
CATTTAAACAATATGGTAATTGCCTCCTAAATATTTGCAAACAAATATTTTCGTAGCAGGCACAAGCTGG
TTGCTTAATAATATTCTTAATTGAATTTCTGAAGATGGAGCTGCTAGGAAATAAACAAATTCCAAACCAG
TTGAGCAAAAATAGTCAAAATAAGAGGGGAACTGGAATTCaaaaaaaataaaagaaaaGTTGACAGATGGC
GACAAAGACGTTGATGACGTTCCCTGGCATTGAATTTCGAGTATATATACGAATCCATACGAATGCTGGG
CCTCATTAATATTTATAGAATTCGGCGTCGTCATTATTAATTTCGAATCTGTCTCCAATTTGCCTAATTG

GTCAGGCGCATGACAATGTCTATCGTTTTTGTTACTCTTTCTATTGAAACTCCCCTGACATAATGCAATA
TTTATAAATAAATTTGATACATTAAACAAATTGCTGCATCAGTTGACAGCGGCGATCAACGACGGTCACG
ATGACAGAAAATAAAAATAGTTTAAATTTTTATAAATTAATCAAGTTGTGATTTATAGCGGCAAGCCATT
GACGGAATGTTTGGTCAATTTGATGTGATGTCATTATGTGGCTGCTTCTGCCGTCAGTGTGCACCGATTC
TGAAGGTTAATTCAATAATATACCAACTGCCGGGCATTCGGAGCGGTAACAGATACAATAATACAGATAC
AGATAGACACAGGCACAGATGATGGAGGGGGAGATACAATGATACCACAGAGAAGTAGACGTACGTATTT
GCATTTGAAAACTCGGTTTGACTTGTAGAAAACGAAAGCGTAAAAACTCGTTTGCTGAACTTGAACCCCG
CTTTGCGATTACGAAACAATTGAATACTTCGAGCCTAACCCCAGCTAACGAGGTAGACAAACGAAGCGAA
AAATATACCCTGACGTCACCACCTCGCAAAGATCCTTCCAAAAAGGGGAAAGGGTCACCTGGATTACTCA
CCAGACCATCGCTGCCATTGACTTCGATTGCTGTTTTCAGACGCTGCGACAGTTGTTCCAGCAATCTGTC
GTTCTTCGTGTTCAGGTTGTCCAAACGTTGGCTGTAATACTGTGTGGCGCCCGGATCGCTATCCGCCGGC
CGGAGATCCCGGCGCGGTGGCGAGAACGACGGACGGGCCTCGACCAGCCAGTCCACGAAGCGTAGGCATT
TGCTATCGTAGATCTAACTCCCGGAATTCAGGGTCCCACGGTGACATTGTTGTTCGATTGGTTGGTTGGT
TGGTTAATTTCGGTTAAATGTTAGTAATTTTGTGTGTTAAACAAATTTAGTTTATTTTTCAGGAGGCAAG
GAAAGAAGGGGGGCGCTTGTTGATATATTTAGTGCTTTTTATTTTATTGATTAATCAATACGAAATTACG
TTTTGGTAAAattgctttttaattagtttgtttgtttggttgatttcttgttttggttgtttgtgtgttt
AAGAGTTTGTTTGTCAGGCTGTTTCTGTTCCACCCAAGTGTTATTCACGCACCTTTAACCCGCAAGATAA
CTAAGAATTGGAGCTTACAGTCCGGTAATTGGTTTAAAAAGCATTAAATATATTTGAGATTCGGTAGAGA
AACAGCTAAGAGGTGTTTATAGGGCTACAAAGTGTTTTCCTTGCGGAAAAGGAGGGATTTCGGTGTTCGT
TCGAAATATCCACAATCATTCGACACAGTCATACGTAAAGTGCTGGCACTCGGCCACCAACTGACCTATC
GCAACCCAACATTGACTAGACCCAGTCAGTCCATCGAATCGTATGTCAAGCGATCGAATTGCACATGTCT
GAAGTCGATGGGTTACGATGGATCAGCAGTCATTAGAATCAGACCAATCATACCACAACAGGCATTCAAA
TTTTTACCTTAGCTTCGCGTATAAGTCGTCCTCCAGCCAC
>dme_circ_0004331_junction_seq
CTTCGCGTATAAGTCGTCCTCCAGCCACGATTGTGATAGACCAAATCAAAGACGCT
>dme_circ_0004332
GTCATTGCTCCTCAGATCCTTGAACCCGTCAATATCGAAGATCGAACGCTGCGCCACCGAGCTAATCAGA
CCGCGTTCCACTGCCTCCGGAATGCTGATTAGCTCACCGGTCATAAAGTCGCGGTACTTGCACTCGGCCG
GCAACACGATGCCATCGGCAATGGCCTCCTGAAGGGTGACCAATTCACCCTTACGTTTG
>dme_circ_0004332_junction_seq
AAGGGTGACCAATTCACCCTTACGTTTGGTCATTGCTCCTCAGATCCTTGAACCCG
>dme_circ_0004333
AAACAATTGCTTGCCGCTGACTATGGCAGTGACCTGCCGTCGGTCAAGGAGGAACTGGATCGCCAACAGC
ACGAGCACAAGATCATTGATCAGTTCCACACGAAAATACTCAACGACGAACGTCAGCAGACCAAGTTCTC
TGGAGATGAGCTGGCTTTGTACCAACAGCGTCTCAATCAGCTGCAGAAGGTGTATGCCGAGTTGCTTAGC
ACCTCGACGAAGCGTCTGTCCGATTTGGATTCGCTGCAGCACTTCCTGGGACAGGCCTCGGCGGAACTGC
AATGGCTTAACGAAAAGGAGCAGGTGGAGATTACGCGCGACTGGGCGGACAAACAACTCGATCTTCCCTC
CGTGCACAGATACTACGAGGGTAAGTGGTGGCCTATGCCCCGAATGAATccccccccTTAGAGCTAGTGT
TATATATGTGTTGTTGTGCTTCTATCCGTTTACAAGCGTGCATTTGGTAATGGCGACGAGGTAAGACCCC
AGATTGGTTCATAAATACCATGAGAAATTTTCTATTGCAAAAAAAGAGCAACCAACCAAGTGCACTTATA
AAAAAATTTTTAATATCGTTACTTATAAAACGTTTTTAATAACCACTACAGAAACCTTATGTCCGAACTG
GAGAAGCGTGAGATGCACTTTGCCACCATCCTGGATCGTGGAGAGGCACTGCTGAACCAGCAGCATCCGG
CCTCCAAGTGCATCGAAGCCCATCTGACAGCCCTGCAACAGCAATGGGCGTGGCTGCTGCAGCTAACACT
CTGCTTGGAGGTTCATTTGAAACACGCCACGGAGTACCATCAGTTCTTTGGCGAGATCAAGGATGCCGAG

CAGTGGCTGGCCAAGCGTGACGAAATACTCAACAGCAAGTTCTCACAGTCAGACTTTGGCTTGGACCAGG
GTGAAACTCTACTCAGGGGAATGCAGGATTTGCGCGAGGAACTGAATGCCTTTGGCGAGACAGTGGCCAC
ACTGCAGCGAAGAGCCCAGACAGTGGTGCCTCTAAACAAGCGACGACAGCCGGTAAATCGACAAGGACCC
GTGCAGGCCATTTGTGCCTACAAACAACAGGGTCAACTGCAGATCGAAAAGGGCGAGACTGTTACGCTGC
TGGACAACTCGGGACGTGTGAAGTGGCGCGTGCGCACTGCTAAGGGGCAGGAAGGACCCATACCGGGCGC
TTGCCTGCTATTGCCACCTCCTGACCAGGAGGCTATCGATGCCGCCGAACGTCTGAAGCGTCTGTTCGAT
CGATCAGTAGCTCTTTGGCAGAAGAAGCATCTGCGACTGCGCCAGAACATGATCTTCGCCACCATTCGTG
TGGTTAAGGGCTGGGACTTTGACCAGTTCTTGGCCATGGGTCCTGAGCAGCGCACGGCCATCCGGCGTGC
CCTCAACGACGATGCCGACAAATTGCTGAGCGAGGGTGATCCAAACGATCCGCAGCTGCGACGCCTGCGC
CGTGAAATGGACGAGGTCAATCGCTTGTTCGATGAGTTCGAGAAGCGTGCGCGCGCCGAAGG
>dme_circ_0004333_junction_seq
AGTTCGAGAAGCGTGCGCGCGCCGAAGGAAACAATTGCTTGCCGCTGACTATGGCA
>dme_circ_0004334
GATCATGTTCTGGCGCAGTCGCAGATGCTTCTTCTGCCAAAGAGCTACTGATCGATCGAACAGACGCTTC
AGACGTTCGGCGGCATCGATAGCCTCCTGGTCAGGAGGTGGCAATAGCAGGCAAGCGCCCGGTATGGGTC
CTTCCTGCCCCTTAGCAGTGCGCACGCGCCACTTCACACGTCCCGAGTTGTCCAGCAGCGTAACAGTCTC
GCCCTTTTCGATCTGCAGTTGACCCTGTTGTTTGTAGGCACAAATGGCCTGCACGGGTCCTT
>dme_circ_0004334_junction_seq
TAGGCACAAATGGCCTGCACGGGTCCTTGATCATGTTCTGGCGCAGTCGCAGATGC
>dme_circ_0004335
GTTGTCCAGCAGCGTAACAGTCTCGCCCTTTTCGATCTGCAGTTGACCCTGTTGTTTGTAGGCACAAATG
GCCTGCACGGGTCCTTGTCGATTTACCGGCTGTCGTCGCTTGTTTAGAGGCACCACTGTCTGGGCTCTTC
GCTGCAGTGTGGCCACTGTCTCGCCAAAGGCATTCAGTTCCTCGCGCAAATCCTGCATTCCCCTGAGTAG
AGTTTCACCCTGGTCCAAGCCAAAGTCTGACT
>dme_circ_0004335_junction_seq
TCACCCTGGTCCAAGCCAAAGTCTGACTGTTGTCCAGCAGCGTAACAGTCTCGCCC
>dme_circ_0004336
GTTCCTCGCGCAAATCCTGCATTCCCCTGAGTAGAGTTTCACCCTGGTCCAAGCCAAAGTCTGACTGTGA
GAACTTGCTGTTGAGTATTTCGTCACGCTTGGCCAGCCACTGCTCGGCATCCTTGATCTCGCCAAAGAAC
TGATGGTACTCCGTGGCGTGTTTCAAATGAACCTCCAAGCAGAGTGTTAGCTGCAGCAGCCACGCCCATT
GCTGTTGCAGGGCTGTCAGATGGGCTTCGATGCACTTGGAGGCCGGATGCTGCTGGTTCAGCAGTGCCTC
TCCACGATCCAGGATG
>dme_circ_0004336_junction_seq
CAGCAGTGCCTCTCCACGATCCAGGATGGTTCCTCGCGCAAATCCTGCATTCCCCT
>dme_circ_0004337
GTCAACAAATTGCACCAGCGTTGGGCTCAACTGCGCACCAATTTCCACACGAACCTGGTACAGAAGCTGT
CCGGTTTGAAGTATCCCGTCCACGAGACCACGGTGACGCGTCAAACGCGCATGGTGGTCGAGTCCCGTCA
GATCGACACGAATCCCCATTTCCGCGATCTGCAGGAGCACATTGAGTGGTGCCAAAACAAG
>dme_circ_0004337_junction_seq
GGAGCACATTGAGTGGTGCCAAAACAAGGTCAACAAATTGCACCAGCGTTGGGCTC
>dme_circ_0004338
GTCGCCGCATCGAGCGACTACATCCCGTGGACGCTAAGAGCATTGTCGAGGCGCTTGAAACCGAGATCCG
GCACCTGGAGGAACCCATCCAGGACATGAACCAGGACTGCCACGTGCTCAACGAGGGTCGCTATCCTCAT
GTCAGTGAGCTGCACAAGAAGG

>dme_circ_0004338_junction_seq
CCTCATGTCAGTGAGCTGCACAAGAAGGGTCGCCGCATCGAGCGACTACATCCCGT
>dme_circ_0004339
GATCTGGATGAGCTTGGATTTCTCGCGCTTGCGGGCGCTTACCTCGTCGCTGCGGAAACGCTGCAGATCG
CTCAACAGACGCTTCATCTCAATTAGCGTGGGCGGGAAGGAACGCTCCTGCAGGTAGGCGGTCTTCTCGC
GGCACCAGTAGATGAACTGTTGGGCCAGGTCGCGGTATTCATGAACGCGACGTT
>dme_circ_0004339_junction_seq
AGGTCGCGGTATTCATGAACGCGACGTTGATCTGGATGAGCTTGGATTTCTCGCGC
>dme_circ_0004340
GTTTAATACGTCAGCAAGAGCGTTGGACTCAGCCTCGTCTTGTACATATATCCAGTAGACTAGATAACAT
GGGTACAACAATTGATCGACAGAACCAAATTGCTATTGCACTGCTGATGTGTGATGCAACCAATATACAA
GAAAGTTTTGAATTACACGTGCTATCTGAGGTACTTATTCGTGGGCCTAACTCCCCTTTTTACAAAAACC
TTATAGAACCAAATTTTTCCGGTGGATACAATCAAACCACTGGATATTCATCGGATACTAAGGACACAAC
CTTCGTCGTTGGATTACAAGACCTTCGAGTTGAAGACTTCAAAAAATGCATTGAAATTTTTGACAAAACC
ATTATTAATTCTATGAATGACGGGTTTGATTCCCAGCATGTTGAAAGTGTTCTGCACAATCTTGAGTTGT
CTTTAAAGCACCAGAATCCGAACTTCGGAAATACTTTGCTTTTTAATTCCACAGCATTATGGAATCATGA
TGGCGATGTAGTCAGTAATCTCCGCGTATCAGATATGATATCAGGGCTTCGGGAAAGCATAAGCCAGAAT
AAAAAATATTTCCAAGAGAAAATAGAGAAATATTTCGCCAATAACAATCATAGACTAACATTAACTATGT
CACCTGATGAGGCATATGAGGATAAATTCAAACAGGCCGAGTTGGAGTTGGTAGAACAAAAAGTAAAACT
TTTGGATGAAGTTAAGATTGAAAAAATATATGAGAGAGGATTAATATTGGATTCATATCAGAAGGCTGAG
TCAAATACTGATCTTTTACCTTGTCTGACAATGAATGATGTAAGAGATCCACCAAAATGGCCTAAGCTAT
TCATACAAAATGTTCAAAATGTTCGGACCCAGATTTGCAAGGTTCCGACCAATGAAATTACTTATTTTAA
GTGCATGTTTAATATAACAGGACTAAGTCATGAAGAAACTCAGCTTATGCCGCTTTTTTGCAATGTTATT
AGTGCAATGGGCACCACAAACTATAATTATCGGGAGTTCGATAAGCACATCCTTTTAAAAACCGGTGGAT
TTGATTTTAAGTTACACTTAATTGAAGATGTTAGGGATTCAAAAAGTTACAGTCTAAGCGTAATGATAAA
CACACATGCACTTAATAACAATGTTCCAGAGATGTTTGCCTTGTGCCAAGA
>dme_circ_0004340_junction_seq
TTCCAGAGATGTTTGCCTTGTGCCAAGAGTTTAATACGTCAGCAAGAGCGTTGGAC
>dme_circ_0004341
ACGAACGCGATGCCATTCAGAAGAAGACTTTCACAAAATGGGTTAACAAACATCTGAAAAAGGTGAGTTA
ATGATCGAGCTTTAAATGGTGACCACCACAAAAGCATAAAACTTAACTGAAATTTGTGAAACCTACTGAt
ttttttttttGAAATTTCCATTTAACACCTTTCCCACAAGCAGCATTGGAAGTATGCTAAGGTACTAGC
CAATAATCATAAAATACAATTTATAGTTCTTAttttttttAACATACGATAAAATGAATTTCCTGCGATT
TTCAGACCTATACTATGCTACATGTTTGTGTAACCACAAATGGCATACCCTGTTGTTCTCAGTCTGTAAG
TCGAGCTAAAAAGGATTACCTTCAACTAAATATTTCTTGATGTTGTTTCATAAAGtttttattattttttt
ttCCAATTGTTAATTTTGTTGAGCAAGATTAAAATTTTCTTTTTAACTGCAAGGGTTAATACACCACTTT
TCATTGATGTTGTTTTCTTGAGTTTTTACTAGCCGCTTATTCAAAATGTTTAATTTTACCTTGCGGCTTG
TTTCTTTTGCTGTTCAGTGCCAGCAgtttttttgagttgttttacctgcatgtctgttttttttgttttttt
gtttGGGTTACTCTCTAATTTATTTGAATGGACTTTTACTGGTATTCCACTATAACGCCTCCCCCATCTG
CAGGCCACGCGAGAAGGGTAAAATGCGTTTCCATATGCTGCAGAATGCACAAATGGCTCTGGACTTTTTG
CGCTACAAGAAGATCAAACTGGTCAACATACGCGCCGAGGATATTGTGGATGGCAATCCAAAGTTAACCC
TGGGCCTGATCTGGACCATCATATTGCACTTCCAGG
>dme_circ_0004341_junction_seq
ATCTGGACCATCATATTGCACTTCCAGGACGAACGCGATGCCATTCAGAAGAAGAC

>dme_circ_0004342
AACTAGCCACTCCCACAGATATATATGATATATAATTTTCTATAAGCATTTTAAGTCGCTCCGAGTCATC
AAACCTAACATTTTTAATCAACTCTTGGCACAAGGCAAACATCTCTGGAACATTGTTATTAAGTGCATGT
GTGTTTATCATTACGCTTAGACTGTAACTTTTTGAATCCCTAACATCTTCAATTAAGTGTAACTTAAAAT
CAAATCCACCGG
>dme_circ_0004342_junction_seq
AAGTGTAACTTAAAATCAAATCCACCGGAACTAGCCACTCCCACAGATATATATGA
>dme_circ_0004343
AGAGCATTCGGGAGCGATAGCTCTCAAAATCCTCTTGTGACACTTCATAAAGGAAATAATCGATGCCAAT
ATTTCCAGGTGCTATTGGAGTGTCTAATTGCTGCAACACACCAAGCTTCGACTCAAAAAGAGACTGTTGG
TCAATTACATTTTGATTTGCACGTAACCATTTATAAG
>dme_circ_0004343_junction_seq
TTTTGATTTGCACGTAACCATTTATAAGAGAGCATTCGGGAGCGATAGCTCTCAAA
>dme_circ_0004344
GTTTGTCCCTTGTTGAAATTGCAGTTCTCGTTATTCATTGCCCACTTAACTTATCTGGCCTTGCTTGCAT
TTTtctctctctctctctctctcATTTTGTGTTCGGTTTTTGGTAGAGCCCAACTTACAAACTATTAAAT
GTTCTTGCTTGGTTTTGTCAGCGCCCTAGTTGAAGTATTGAAGTAATTCATTAGTACATGCTAAGTTGTG
TCTACACACTCTTTCAATGCTTGTCGCTTTAATGTCGCTTGTGGTTTTTGTTTATATTTTGGTGGTGGTC
TTCGTTCACAGTACAGAGAATCCGAGTGGTTGGTGTTCATGTGGTGTTTGCTGTGTGAGTCGATCAGCAA
CGTATTCATTTTGTATACTAAAAACCTTTGGTTGTATGCATTCATTCATTATCATTCATCATGTCTTCAT
TTCGTATCGTGTAAAAATATATTGTACAATTTAATCGTAATTTCTATTACAAAACTTGTCTCTCTCTCTC
GTGCTGATGACGCTCGCTTTTCACGTAATTTCACTTCCattttggttatgttttaatttgttttgttttt
ctggtgttggttggtgttgCTGTCGTAGTTTTTGTTCCCTTTA
>dme_circ_0004344_junction_seq
gttgCTGTCGTAGTTTTTGTTCCCTTTAGTTTGTCCCTTGTTGAAATTGCAGTTCT
>dme_circ_0004345
GGAACAAAAACTACGACAGcaacaccaaccaacaccagaaaaacaaaacaaattaaaacataaccaaaat
GGAAGTGAAATTACGTGAAAAGCGAGCGTCATCAGCACGAGAGAGAGAGACAAGTTTTG
>dme_circ_0004345_junction_seq
TCAGCACGAGAGAGAGAGACAAGTTTTGGGAACAAAAACTACGACAGcaacaccaa
>dme_circ_0004346
AATTAAGTTACGTTTTACGTTGAGAGAGCGCGGATGGGTGGAAAAACCAATCAGAATACGAAGATCTTGC
ATGCATTAGTTACGTACACGTACATATATAGTAAGCAGAGTAATGAAAATCccacaaacaccatccacac
actcactcatccacacacacacaaatcagatagccacacagtGAGAAATGATAACTAATCGAATGTCAGG
CACAAGTTTGCGAAATCAAAGAAATGTGAGAATTCTCAAAAACAAGCGTAGCAAACAAATTGAAAGCAAA
TAGGAAATCAACTAAAGGGAACAAAAACTACGACAGcaacaccaaccaacaccagaaaaacaaaacaaat
taaaacataaccaaaatGGAAGTGAAATTACGTGAAAAGCGAGCG
>dme_circ_0004346_junction_seq
GGAAGTGAAATTACGTGAAAAGCGAGCGAATTAAGTTACGTTTTACGTTGAGAGAG
>dme_circ_0004347
ATCCGCATCTGGGCCATCGCCTGTTTTGCCCCACAGCGCACGGTGCGCGAGGATGCGCTGCGTAATTTCA
CCCAGCAGCTGCAGAAGATCTCAAACGATGCAGGCATGCCGATAATTGGACAGCCGTGCTTCTGTAAGTA
CGCCACCGGGCCGGATCAAGTGGAACCCATGTTCCGTTACCTGAAGATCACCTTCCCCGGCCTGCAACTC
GTCGTGG

>dme_circ_0004347_junction_seq
ACCTTCCCCGGCCTGCAACTCGTCGTGGATCCGCATCTGGGCCATCGCCTGTTTTG
>dme_circ_0004348
CTCACCTTGCCCTCGGCGAATCGCTGGTTGTGCTTGGCAATCTTGTGCTTGTTCTCATTGAAGATCTTGA
GGCGGAAACGCTCCTCGGTTTCATCCTGATAGTTCTTGCGGTGCTCCAGCTGCATAATGAAAAGTATTAG
TATTAGTTAGATAATGCACTCTTATCAAAGGTAAGTGCTACTATCGATTGACTTTCGTGTACTTGTTTCA
AGATAAGATAAAGCCAACCAAATGAAAAGTTAACTTTTTAGACGCATTTTTGGGAACTCTATTAAAAATC
AAATAAAATGTTTTACGTAAAATGGATTTACGATCGCTGAGGCAAAAGTTCTTAATCTGCAGATTACACC
TTATCACAGCGCCGTCAACCTGGCACGAGCGAAACGCTCTAGAGGAACGGCCACAGTAGCAAGCTCTATA
AATCTATAAAACATGGTCATAAAATTGTGTTTAACGATTGCCCAACGACAGGAACTTGAAACGGGTGTTA
ATGTTCCAGGTAGTAAACAAACGAGCCAAACTGCAGGTAGGAATAGGAGAAGCGAGGCCAGCACGAGGAA
ATGCGCTCCATCTGCCTGCAGTGGTCCCGTGGATAAGAGACCTCCTCTCCCAGCTCGGGGTTGTTTGTTA
GTCCGACCCGCATATTGACACAGCCACAGCACGAAAATAGCACGAACGCTAAACAAATGATAGCTATAAA
GGGAATTGAAAGGCCAGGGCAAGAAGTCGACTAGGTTGCTATCCATTAGATTCGGCAAATGCCACTATAT
GGGGGTAGTTTTCGATTCAAAGTGAAATTTGTGAATAAAAATTGACTTCCTCTTGTCTGAATACCAAATT
AGCGGCTTTGCATAGAATCGAAAAGTTTATGAAAGTTAAGTTAAGTATATAGAATTATTCAACAAAACTC
ATTATTACTGCTCACGAATATCCAAATAGCAAACATGCTAAACTGAAATGCATTTTCCAAAAGTGTTTAT
TGCTAAACTGGTTATTTTTTTGCGACTCTTCAACGCACTCAAAGTGGCATGTTTTCTCAGTGAAATATGG
GTATAAACCCAAACCTAATCGCTTTTTAAGTGGCTCACCACAAGTCTTAGTAGATTTTGCAATATTCCAG
GGTACTTGGCACATTGAACTGAACAGTCGAGTGGCGCTGTCATGTGAGAAACATGCATGTGTAATTACTT
TTGGACATCAAAGCGGACCAGCATCAGCAATTGTCGCCCCAAATGGACCGGATGGGGTCCACTGGTCCGT
GGTTGACTCCTATAATTGACATAATTGAATCGAATTCGAGTCTTGCAGCAAAATGAAACCCCGCCCGTTG
TGATTTCGAGGGAGGAGGTGGTAACGGCAGAAAAGAGGCGCCTCCCGGCACACTGACCTATTGATTTTCC
TTGCAAACGTCGTTGGTTCTCGTTGCCGCATGATAAAATTCACACATTTATTGTATACTTTTGTAGAAAA
TTTGAATTTGCGATATATGCAGATAGAGTTAACAACTTAAAAACACCGTAATTAAATGCACGTGCGATCC
GATGTGATATCCAGGCACTCGACGTATACGTTTTGTATCTATCAATCTAGATATACTACATATGTGGCTT
AGGAAACTTCGTTGCAGTTGAGAAGCTTGGACGATAGTAGTTGAGTATCAGAGAGCTTAGCTAGTGCGAT
CTCCATCCCTACAGTCGCTCCATCTCGATGGCACAGCTATCGTCCTCCTGATCCGCATCCGCATGCCGTT
CCGTTGTCGAAGTGGCCGCATAGGAGGAATGTGGAACAGGTTGTTGTTGTTGCTGCAGCTCCTCCTCCTG
GCATTTGTATTCCCGCCGCTTGCGCATAATGTAGGCGAAAACGAAGCCATTTATGACCAGCATGGCCAGG
ACAATTATCGTCGGCCAGACCACATCCACCGATACATCCAAATTATTTGCACTGGTATCGGTCATTCTTA
TGCTGTGATTGCTCCTCCGTGGTTTTTATGTAATTTATTTGCTATTACCGCCCGTCTCCCTGCGATTTCG
TAGCACGTTTTTTACGATGACGCGCGTGCTTTTCCGTACTTCTACTACCACCGCTGGGGGCCCCGTCTGA
CTTGGCTCCTTTTATTTCTTTTCTCTCAAATGTTCTATATATTGCGCTCTGCACTGCTGCTGCTACTGCC
TGCCGCTGGCTGCTCGAGCGTTCCGTTGGCGTGGAGCGTCGGTTCGGGAATAACAAAAATACGACAGAGC
TGGCTGCTTCGCTTTtggtggtggtggtggtgatggtggtggtCCGCTCGCAAACGCAAACTCAATCGCA
TGACGGCGGGGCGAAAAAGAGCCGAGTTCACACACGCACACAACACACTCTACACTCTTTCTTCGTATCG
CCGAAATCGAAATGAGAGCCCAATCTACGTAGGCCAACCAATTTGGAGGATGGCAGGCGGGCGTACTCTA
TATATTTCTTGTTTCTTATGCTTTTTCGTATATTTCCTTCTGTTTTGCAGCGCGCGCATTGTGGGTTTGC
TCAAAAAGGCGAACCGGTTTCATTTTGATTTATAAGGATTTCAGGCGAGCTATTCAAATATGTTTGCTCC
AACCGGTTAGCAGAAGTAACAGATTTaaaaaaaaacgaaaaaaaaaaCAGAAACTTATGTATCTTTAATA
AAAAATGTAAGAAAAGAGCCGCATGCTGCTTTTGCAAGTCAGCGAAATTTTTCCCAGCTTGTTTATTTAC
GGCAGCCGATTTGTTTGTGCACTTGTCACTCGGGGAATTAATGCTAGAAATCTCTTTCGGCTCTCGGAAA
TGCCAGCGAAAAATGGCAATTTGATAAGCAACGCCGATTTGTAGCGCGTCATTTCCAAAATGCTCGCGGC

CCCCCGCACGACTTGTTCTTAAATATTGTTGTTGAATATTTGCCGAACTATGCGAATAGCTGCTTAAATT
AAAACGCATTAATCTGGAATTTTAGAGCTTATCAGTGCCACAGTTGCTCAACGCTGACTTTTGCAGCTGT
GCATTTGCAACCGACAACTGTGGCACTTTGGACTGCACACACATTACAGATAATGTCGTTTTTATTAACT
AACTATAAGCAGTGAGAGCTAATGGTTGGCATCGTTATCACATTTAAATTGCGCGCCTTTCATTCCCCAA
GCAAGCATGGGTAGCACTGTTTGCAGCAGTGTGACCGAGCGTTTGCAGTATTCTACATGACACCTAACGG
TCTCGAAATGTAAAATGGCAAATGTAAAGTAATTGTGAGTTTATTTTGaaaaaaaaaaagaaaaCTAAATC
TAAACTAAATTAGATGATCAAAGAAAATTTAGTTAGGTAAATAATATATGAGTGGGGAAATTGCAAATGT
TTAGTTGATTTTCTTTTTATTTTTAATTAAGGATGAGTAACTCTTTCCATAAATATGATTCCTATTCTTT
CAGTTGATCAGACTTTGTTATTTATAAACAATAATTTAATACACCACTCTGTGCTTACTCTAACAAATGA
GTCACTCAATTGAATATGATATTTAACAGCAGATAAAAAAAGGAAGGGAAATAAAGTTATATGCATATAT
TGCATATGCGGGTGCATTTGAATTTGAATTCGAAAAGTGAATATAAAACCATCAAAGGCAAAATATCCAA
AGTTATTAAACCAATATGCATATATAGAGATGTACATATATCCCCCTTATCACATGAGATCTCTGCACAA
ACAGCTAATTATTATTTGTACGCCCCCATTTGGGGCTAATGACGCTTGGCACGGAACTATTCAGTAAAAT
AATGGAAAAAATCACGACCCCTGACTGTTGGCGATAAGAATTAGACGACACTAGGGGCGCATTCAATTAA
TCAATAGACAATCCACCATTGATAAGCAGACGAAGTTGCAAGTGAAGGCTGCGAGACAAAGGATGATTGG
CCCAATCATCAATGGAACCATGGACACACATATATATGCAATAGAAGGGGGCAAAAATCGAAGTCGCATT
AGGAAACATGGCTTGCGAAAAGTGTGATGGCAAATATGCGACCCGCGCCTTATCAGTCGGCCGGCAGTGG
ACCAGTTTGAGTCTTGCACGGCTTATCGGCGGGAAATGAACCGCGAGCGTAGCGAAAGTGGGCGACACAA
CGACTTGGATTATCTGCCCATCCAGCCGATGTTTACGGATGGGCTAAGCAAAGCAGCCCAGCTTGCCGCT
TGCGGTCATGTGAGCCGGCATGTGACTGGACGACAGCCTAGACTTTTGGACTTATCTGAATCCTCCTTCC
GTCTCTTTGAGGCAGCCGCGCAGCAGTGCATTCACACTCGCAATGGCTGCTGACTCAGAAGTCAGAAGTG
CGGAATCGTCACAAACGGAAGAATGCCGCGCGTCCAACTATGCAAACAAAGTCGGACAGAAGACAAAGGC
CCaaaaaaaaaaTTAATGGATTAATCATCATTACACATCAAGCCCTTGTTACCGGAGAACACTTGTTATC
TGCCTCTTCGGCCTGGCGAATTAAATATGATCCCTAGATGATCCGGTTGAAACTCACCTTGAACGTATGC
CATTCCTCCATGACCACGTCGGCGAAGGAAACGGCCTGAGCCACCGCCAGCAGGGCCAAAAGTGGCAGCA
AAACAGCTG
>dme_circ_0004348_junction_seq
AGGGCCAAAAGTGGCAGCAAAACAGCTGCTCACCTTGCCCTCGGCGAATCGCTGGT
>dme_circ_0004349
TGTAGTTGAAGCCGTTCATCAGCTGACGGAATTCGTGGTGCAGCAAATCGGCGTACTTATTGACCGCCAG
TTTGAAGCTCACCTTGCCCTCGGCGAATCGCTGGTTGTGCTTGGCAATCTTGTGCTTGTTCTCATTGAAG
ATCTTGAGGCGGAAACGCTCCTCGGTTTCATCCTGATAG
>dme_circ_0004349_junction_seq
GAAACGCTCCTCGGTTTCATCCTGATAGTGTAGTTGAAGCCGTTCATCAGCTGACG
>dme_circ_0004350
AGTGTAGTTGAAGCCGTTCATCAGCTGACGGAATTCGTGGTGCAGCAAATCGGCGTACTTATTGACCGCC
AGTTTGAAGCTCACCTTGCCCTCGGCGAATCGCTGGTTGTGCTTGGCAATCTTGTGCTTG
>dme_circ_0004350_junction_seq
CTGGTTGTGCTTGGCAATCTTGTGCTTGAGTGTAGTTGAAGCCGTTCATCAGCTGA
>dme_circ_0004351
CGGAAGGCATTGTCCATGAGACCGCCGTTGCATCCATTGTTGCCGTACTTGGTGGAGCAATCGACCAGAT
TCTGCTCGGACAGCGAGACGAGGACACCGGACTTGCGGAAATGCTGACCCTCGAGGGCGCCTGTGCTGGA
GAAGGCCCAGCAGCTGCCGCAGTGTCCCTGATCCTTGACGGCGGTCACAGCTCCCTTGGTGCGCCAGTCC
ACAGATTTGGGCAGCGTCACATGAGCCGGCGAGATGAAGG

>dme_circ_0004351_junction_seq
AGCGTCACATGAGCCGGCGAGATGAAGGCGGAAGGCATTGTCCATGAGACCGCCGT
>dme_circ_0004352
GCATCGATGGCGACGGAAACGGGACCAACGGTGGCCACAGCCTCGGCCATCTTCTTCTCATCACCCTGGG
GAATATCGGTGAATCCACGATCGGTGGCTCCGACTGTGCCCTTGTTAAAGTGGCACGAGTCATCGATGGC
CTCGTAGGGATAGGACTTCTCGG
>dme_circ_0004352_junction_seq
ATGGCCTCGTAGGGATAGGACTTCTCGGGCATCGATGGCGACGGAAACGGGACCAA
>dme_circ_0004353
GAAAAGAAACAGAAAAGATACCAACGCAAGCATCTCAAAAATGAACTTTAATAATTTAGCCCAACCTCAT
ATTTAATGAACTAATTTTGTAATGGGTCCATTGTATTCGCGTTTCTCTGCCATCATGTATGATATAG
>dme_circ_0004353_junction_seq
CGTTTCTCTGCCATCATGTATGATATAGGAAAAGAAACAGAAAAGATACCAACGCA
>dme_circ_0004354
GCCGAAGACCTGGTTGTCTTATTCTCCGAAGGCGGGACGGCTTCGATGCCACAGAGCACTCCGTTAACAC
AGCGCTCCGTGTTAGTGGAGAGCGGATTTGAGGACATCGAAATCGGGTGCTTGGAAGATGAAGGGATTTC
CGGCATTGGAGATC
>dme_circ_0004354_junction_seq
GATGAAGGGATTTCCGGCATTGGAGATCGCCGAAGACCTGGTTGTCTTATTCTCCG
>dme_circ_0004355
GTTCCAAGGTCTGTGGACCACGATTTGGATGAAGTTCGTTCGATAGAGGCCCAACTGAAAGATTTTCAAT
TGGAAACCAAACAGGACGCCGCAGGAACATCGATTTTAGTTACTAATGCTCCAGTTACTAATGCTGCTGC
GCCATCAGTAATACCAAGAGCAGGATTGATGAAGCAGGAGACGCCCGCTTTGCATTCTTCGTCTG
>dme_circ_0004355_junction_seq
GAGACGCCCGCTTTGCATTCTTCGTCTGGTTCCAAGGTCTGTGGACCACGATTTGG
>dme_circ_0004356
GAAGCAAATCGTCGATATTGTCAATGCGCCGATCGCAATTGGCGCTAAAGATACGGGTTAGCGGTCGTGG
CTGGCTCTGTGGTGCTTTGGCGATTGCCCTTAAAAGTCGCAGTGCCACCTCGTCACAAGCCGTATTTGAG
CCAGCCAGCACTAGGATGTGCGTTTCGGGTCGATTTAT
>dme_circ_0004356_junction_seq
CTAGGATGTGCGTTTCGGGTCGATTTATGAAGCAAATCGTCGATATTGTCAATGCG
>dme_circ_0004357
GCTGAAGTACTCCACTGGCCAAAGGTGTATTGGGAATTTCACCGGGAAACAGGATCCGCTGAACATCACT
GTGCCTGGTGAACGACAGCTGCTGAAGTGCTCGGTACTGATAACGCATCACTGCTCGCAATGGACGGAAG
ATCAGGGTGTAGGTGGTGTTGTCCGGTAACTGGCGTTCACAAGTAATGTTAACCCGCTGCGTTGAGACGC
CAAGGATGCGGGCGAAGTAGACTCGTGGCTGCTTGTCCAGAGATCCGACCATTCCGTCAACATTGCGAGC
CCACCGCAAGATGGA
>dme_circ_0004357_junction_seq
CAACATTGCGAGCCCACCGCAAGATGGAGCTGAAGTACTCCACTGGCCAAAGGTGT
>dme_circ_0004358
GTACTCCACTGGCCAAAGGTGTATTGGGAATTTCACCGGGAAACAGGATCCGCTGAACATCACTGTGCCT
GGTGAACGACAGCTGCTGAAGTGCTCGGTACTGATAACGCATCACTGCTCGCAATGGACGGAAGATCAGG
GTGTAGGTGGTGTTGTCCGGTAACTGGCGTTCACAAGTAATGTTAACCCGCTGCGTTGAGACGCCAAGGA
TGCGGGCGAAGTAGACTCGTGGCTGCTTGTCCAGAGATCCGACCATTCCGTCAACATTGCGAGCCCACCG

CAAGATGGA
>dme_circ_0004358_junction_seq
CAACATTGCGAGCCCACCGCAAGATGGAGTACTCCACTGGCCAAAGGTGTATTGGG
>dme_circ_0004359
GATATCCTCCACGCTCATACTGGTGGTACCCATTTGCATTTTCACGGAAAGCTCCCGCCCGAACTGATGC
AGCTCCATTTCTGATTGTTTGAGCGAGAGATAATGCTGCAATCGTTCGATGTCCTCCACCTGGAGCAATG
TGCGCAGTGTCCCGGTCAGATTTTCCTGG
>dme_circ_0004359_junction_seq
GCGCAGTGTCCCGGTCAGATTTTCCTGGGATATCCTCCACGCTCATACTGGTGGTA
>dme_circ_0004360
GGAGAGAAATATGTGGAGCAGCATCACTTCATGCGCACGGAGGCATTGCCCAAGGCTAGCTTCAACATCA
ATCCCTACCGGCTGTCTAGCAAGGAGACCTTCAAGAGCAAGGGTCTTCCGCCAGCCAATCCACCCATTCA
GG
>dme_circ_0004360_junction_seq
CTTCCGCCAGCCAATCCACCCATTCAGGGGAGAGAAATATGTGGAGCAGCATCACT
>dme_circ_0004361
TTACGACAGTTTTACTACGTGTATGGTGCACAAATGCCTCTGCCGGTGAATGGGAGCGGTGAACTTGTGT
TCTTCGTGGACTCTCACGTCTTCATGATCCTTCGGGAGCAGCCCATCGTTATCGGG
>dme_circ_0004361_junction_seq
CCTTCGGGAGCAGCCCATCGTTATCGGGTTACGACAGTTTTACTACGTGTATGGTG
>dme_circ_0004362
TTTCGCGCTATCGCCCAGTCAGCTCAGAAGCAGCCGCTCCCCAACTGAGAGCACAGAGAAACCAGCTCTG
GCCACTTGCTCCTCCGGATCCGGCTCCTGCTCCTGTCCCCGATCCCCTCCCCCACCCGCCAAATCCTGAT
CCTGCTCCAAATGACTGACGGCGAACAGCAGGAGGCGCTGGTCAAGCGGGCCGAGGACGAGCGCGAGGAC
ATCTTCCGGCGCTACGAACTCGGGCTGGATCCCAGCAATGTGGTGGACTCCTGGGAGAATCCCACCTTCG
AGATCTACCACAGGACAGACAAGATATGGCTTCTTGCACGACTCGCGGCTGCCGTCGACGCGTGATGCCC
AGGAGGTGCATCGCAACAAGATTGAAATGGAGCGGGACAAAAAGTGGATGAAGATGCTAAATCAGTGGCC
GCCGCCCCAGGATAAGCTGCACAAGCGCGTCTACAAGGGCATACCGGATCGCGTGCGTATGGTGGCCTGG
AATAAGCTGCTCGACATCCAGCAATCGATAAACAACAATGCTGGCGTCTACCTCCGAATGCTGCAGCTGG
CCAGGAAGTACTCCACGGAGACGCGACAAATCGATGCCGATGTGAATCGTCAGTTTCGCGACAATCTTGC
CTTTCGCGAGCGCTACAGCGTAAAGCAGTGCTCGCTATTCAATGTGCTCAACGCCTATAGCATCTACAAC
TCGGAGCTGGGCTACTGCCAGGGCATGGCCTGTGTGGCTGGCGTGCTGTTGCTTTATTTGCACGAGGAGG
AGGCTTTTTGGGCATTAAATACCCTGATAACAGACCAAAAATACGGCATGCACGGGCTGTTTATCGAGGG
ATTCCCGAAGCTGACGCGATTCATCGATCACCACGACCGCATCATGTCGAAAATAATGCGAAAACTACAC
AAGCACTTTACCAAACACAACGTTGACGCCCTGCTGTACGCTATTAAGTGGTTCTTTGTCGTCTTCGTGG
AACGCGGTTCCTTTTAGTTTGAGTCTGCGCGTGTGGGATATATTCATGTTAGACGGCGATCGCGTCATAC
TGTCCATGGCCATCACCATTCTGTACCTTCACAAAGACGAACTATTGCGCCTGAAGGACATGGACGCCAT
CATTGAGTACTTGCAAGTACGGCTGCACAAGAACTTTGGCTACAGCGACGATGATGCCATTCAGGCTCTG
GAACGCGTGATGAAGAAGCTGAAGGACCTCAAGTTGGATGTGCCGCCGCCAGCCAAGTCAAACGAGTTCC
CCACCCGCAAACTAGGTGACTTCGTGGAGGCGGATATGGAGAAGAAGATCGGGCGCAGACGCAATGATTA
TACAGACGCGGAGAAGCAAGTCATAACAGATGTGATATCAAGGTGGGATCTCTTGGCGTTGTTAACGTTA
ACATATTTATAATAATTTTTTAACTTTATTAGACAAGAACAAAACGCGATCGACGTTCAGTCCACAGTGT
CCTACGAGACTTCGGAGTGCGCAACGGG
>dme_circ_0004362_junction_seq

CCTACGAGACTTCGGAGTGCGCAACGGGTTTCGCGCTATCGCCCAGTCAGCTCAGA
>dme_circ_0004363
GGACTTGCCACAGGATGTGCACTTGGTTTTCTTGTGGCTGCTTTCACCTGCCGCAGCAGCGATATTCCTA
ACACCTCTCCGCGTAGAATAGGCATTTCCGGTCGTGCAGTGCGGAAAcagcagcagcagcagcagcagca
gcagcGCTGCTAGCAACATCGGGCGATATCAGATATAGACAACCTATATACACATATATATTTATTCGCC
GCCGCTGTCGCCGCCTTGTTCTTCGCGATTTCGTCGCCGTCAGCGTTCGCCGCCGACTGAGACGAAAGCT
GCGAAGCAACGAGCGCTAGCGAAAGCCTCACCACGGACGCATTTATGGATGCGGGCGGCCTACCAGTTTT
CCAGAGCGCCAGCCAAGCAGCCGCCGTCGCCcagcagcagcaacagcagcagcagcagcagcaacaacat
ttgaatctccagttgcatcagcaacacttgggcctccatctgcagcagcaacagcagctgcaactgcaac
aacagcagcacaatgcccaggcgcagcagcagcagatccaagttcagcagcagcagcagcaacagcaaca
acagcagcagcagcaacaCTCGCCCTACAATGCAAACCTGGGAGCAACAGGCGGCATTGCTGGAATCACT
GGCGGCAACGGAGCTGGCGGCCCTACAAATCCAGGAGCGGTACCCACCGCTCCGGGCGACACTATGCCCA
CCAAGCGGATGCCGGTCGTCGATCGGCTGCGACGCCGCATGGAGAACTACCGCCGCAGGCAGACGGATTG
CGTGCCACGCTATGAGCAGGCCTTCAATACCGTCTGCGAGCAGCAGAACCAGGAGACCACCGTCCTCCAG
AAGCGCTTCCTCGAGAGCAAGAACAAGCGGGCGGCCAAGAAGACCGACAAGAAGCTGCCCGATCCCTCgc
agcagcatcagcagcagcagcaccaacagcagcagcagcatcagcagcaccagcaacaccagcagGCCCA
GACCATGTTAGCCGGACAGCTGCAGAGCAGTGTTCATGTG
>dme_circ_0004363_junction_seq
CGGACAGCTGCAGAGCAGTGTTCATGTGGGACTTGCCACAGGATGTGCACTTGGTT
>dme_circ_0004364
GCAACAAAAATTCCTGAAACGACCCGCGGAGGATGTTGACAATGGCCCTGACAGCTTTGAACCGCCGCAC
AAGTTGCCTAATAATAACAACAATAGCAATAGCAACAACAACAACGGCAATGCGAATGCCAACAACGGCG
GCAATGGCTCCAATACcggcaacaacacgaacaacaatggcaacagcaccaacaacaacggcggcagcaa
caacaacGGCTCTGAGAATCTAACCAAATTCTCGGTGGAAATTGTTCAGCAACTGGAGTTTACCACATCG
GCGGCCAATTCGCAACCGCAACAGATTAGCACTAATGTCACTGTAAAGGCGCTGACCAACACATCGGTGA
AGAGTGAACCAGGTGTGGGCGGTGGCGGAGGGggcggcggtggaggcggtagcggtaacaacaacaataa
cggaggcgggggaggcggcggaaacggtaataacaacaacaacggaggTGATCATCACCAGCAACAGCAC
CAGCAGCAGCAGCAACAACAAGGCGGCGGACTGGGTGGCCTCGGGAATAATGGAAGGGGCGGGGGACCCG
GCGGCATGGCGACGGGTCCCGGGGGCGTGGCCGGTGGACTTGGCGGCATGGGCATGCCACCCAACATGAT
GTCCGCCCAACAGAAGTCGGCCCTCGGCAATCTGGCCAATTTGGTGGAGTGCAAACGGGAACCGGATCAC
GATTTTCCCGATCTCGGCTCATTGGACAAGGATGGCGGTGGCGGACAGTTTCCCGGCTTTCCCGATCTAC
TCGGGGATGATAACTCGGAGAACAACGACACGTTCAAGGATCTCATCAACAACCTGCAGGACTTCAATCC
CAGTTTCCTCGATGGCTTCGATGAGAAACCGCTGCTGGACATAAAGACGGAGGATGGCATCAAGGTGGAG
CCGCCCAATGCCCAGGATTTGATCAATAGCCTGAATGTGAAGTCCGAGGGCGGTTTGGGTCATGGTTTCG
GTGGCTTTGGCCTGGGTCTGGACAATCCGGGCATGAAGATGCGTGGCGGCAATCCTGGCAACCAAGGTGG
CTTTCCCAACGGTCCGAATGGCGGGACAGGTGGTGCTCCCAATGCTGGTGGTAATGGTGGAAACTCTGGT
AATCTCATGTCCGAACATCCGTTGGCTGCTCAGACACTCAAGCAAATGGCCGAGCAGCATCAGCACAAGA
ATGCCATGGGCGGCATGGGTGGATTCCCACGACCGCCGCACGGCATGAATCcgcagcaacagcagcagca
gcaacaacagcagcaacagcaacaggcccagcagcaacatGGTCAGATGATGGGACAAGGACAGCCGGGT
CGCTATAACGACTACGGCGGCGGCTTTCCCAATGACTTTGGCCTGGGACCCAATGGTccgcagcagcagc
agcaggcgcagcaacagcagccgcagcagcaacacctgccgccgcagTTCCATCAGCAAAAGGGTCCTGG
CCCAGGAGCCGGCATGAATGTGCAGCAGAATTTCCTGGACATCAAGCAGGAGCTCTTCTATTCCTCTCAA
AACGATTTCGATCTCAAGCGTCtgcagcagcagcaggccatgcaacagcagcagcagcagcaacaccatc
aacaacaacagcagcagcagcagcccaaaatgggtggtgttcccaatttcaacaaacaacagcagcagca

acaggtgccgcagcagcaactgcagcagcagcagcagcagcagcagcaacaacagcaacagcagcagcag
cagtaCTCACCCTTCAGCAACCAGAACCCCAATGCGGCGGCCAACTTCCTCAATTGTCCGCCAAGGGGCG
GTCCAAATGGCAACCAGCAGCCGGGCAATCTGGCGCAGCAGCAACAGCAACCCGGGGCTGGACCGCAGCA
GCAACAGCAGCGCGGCAACGCCGCCAATGGGCAGCAGAACAATCCGAATGCCGGACCCGGCGGCAACACC
CCAAATGCGCCGCAGCAGCAGCAGCAACAATCGACCACCACAACGCTGCAGATGAAGCAGACGCAGCAGT
TGCACATTAGCCAGCAGGGTGGCGGTGCCCATGGTATTCAGagcaacaacagcagcaacagcaacaggcc
cagcagcaacatGGTCAGATGATGGGACAAGGACAGCCGGGTCGCTATAACGACTACGGCGGCGGCTTTC
CCAATGACTTTGGCCTGGGACCCAATGGTccgcagcagcagcagcaggcgcagcaacagcagccgcagca
gcaacacctgccgccgcagTTCCATCAGCAAAAGGGTCCTGGCCCAGGAGCCGGCATGAATGTGCAGCAG
AATTTCCTGGACATCAAGCAGGAGCTCTTCTATTCCTCTCAAAACGATTTCGATCTCAAGCGTCtgcagc
agcagcaggccatgcaacagcagcagcagcagcaacaccatcaacaacaacagcagcagcagcagcccaa
aatgggtggtgttcccaatttcaacaaacaacagcagcagcaacaggtgccgcagcagcaactgcagcag
cagcagcagcagcagcagcaacaacagcaacagcagcagcagcagtaCTCACCCTTCAGCAACCAGAACC
CCAATGCGGCGGCCAACTTCCTCAATTGTCCGCCAAGGGGCGGTCCAAATGGCAACCAGCAGCCGGGCAA
TCTGGCGCAGCAGCAACAGCAACCCGGGGCTGGACCGCAGCAGCAACAGCAGCGCGGCAACGCCGCCAAT
GGGCAGCAGAACAATCCGAATGCCGGACCCGGCGGCAACACCCCAAATGCGCCGCAGCAGCAGCAGCAAC
AATCGACCACCACAACGCTGCAGATGAAGCAGACGCAGCAGTTGCACATTAGCCAGCAGGGTGGCGGTGC
CCATGGTATTCAG
>dme_circ_0004364_junction_seq
GCAGGGTGGCGGTGCCCATGGTATTCAGGCAACAAAAATTCCTGAAACGACCCGCG
>dme_circ_0004365
tttccGACAATGATTTTTCTCGCTTTTGAGCCACTGTGACCCACCGTGTGGGGTAAAATTCAAGGCTTAG
TTCTCTCGTCACCCCTCAAGGTGTTTTTCCACATTTCATGTGTGACCATCAGCAAAAGCAATAGCAGCCA
CTACAGCAGGAGTAACATCGAGCGGACTAAGCAACTTTTAGGTCGCGTTTGCCCTTTGGTACCCAG
>dme_circ_0004365_junction_seq
TAGGTCGCGTTTGCCCTTTGGTACCCAGtttccGACAATGATTTTTCTCGCTTTTG
>dme_circ_0004366
GTGTGACTGGTGTTGCTGCTGGGCTGATCGGGAATAGTCTTGGGTTCCTCGACAATGGTCACCGATGGTT
TCTTCTTAATGGTATCGGTGGGCTTACCACCACCTGGCAGTGTAACCCCGGCGGCCAGTGCCAGCTGTTC
CTGCTCGCTGCTAACGCCGGAGCTGCTGTTGTCGCACTCATCTTCGCCTGGCTGCAAAA
>dme_circ_0004366_junction_seq
TCGCACTCATCTTCGCCTGGCTGCAAAAGTGTGACTGGTGTTGCTGCTGGGCTGAT
>dme_circ_0004367
GTTTTTGTTGTAAGGAGATGGTGCTCTCATACTCCGAAACACTGCTGCGTGTTTCCACTTTAACAACCTG
ACCCACTGGTGGTGGCGGAGGTGGGTGATTGGGCGGCGGTAGTACCGCTCCTCCCGCTCCTGTAGCAGAT
CCTGCTCCTAATCCTCCCAGTTTCA
>dme_circ_0004367_junction_seq
GATCCTGCTCCTAATCCTCCCAGTTTCAGTTTTTGTTGTAAGGAGATGGTGCTCTC
>dme_circ_0004368
CTGCCGAATTGGAGGAGCTATTCCAGCGACAGCAAGGCGAGGGCAGTGCAGCAAACGCCAGTCGGTATGC
CACCATGATGACCAGCTCCCGTTTCCAGTCGGGCACGGACAGCGGCGCAGCCACTCCGCCAGCATCCAAT
GGATCTCCCATGAGGAGTGGACCGCTGGTGTACGGCAGTGTGGCTGAGATGAAACG
>dme_circ_0004368_junction_seq
TGTACGGCAGTGTGGCTGAGATGAAACGCTGCCGAATTGGAGGAGCTATTCCAGCG

>dme_circ_0004369
TTCGCATCGTCAGTGCGCCACGTTGCCGAGGAAGATGACGGGTCCGGGAGGAAGTGGTCCTGGCTCCTCC
TCAGGAGGCAGTGTACGAATGGCTCCGATGCCACCGCGCAGGGATCCCAAGACCACG
>dme_circ_0004369_junction_seq
GCCACCGCGCAGGGATCCCAAGACCACGTTCGCATCGTCAGTGCGCCACGTTGCCG
>dme_circ_0004370
GAACTGAAGGAGAGCTTCAACTATGGCCTGTTTGCTCCACCTGCCAACGGCAAGGCGGGAAAATTTCTGG
ATGAAGAACGCCGCCTGGGCGATTATCCTTTCAACGGACCCGTGGGATATTTGGAGGTAAGTGCGGTTAT
AGTCATTCTTTCACGTATTTTTATCATTTATTAAATTTCTGCTTCCATCCAAAAAAAGCTCAAGTACAAA
AGGCGTGTGTACAAAATGTTGACCCTGGACGAACGCCAGCTGAAGGCCCTTCACACGCGGGCCAATCTGC
GTCGCTTTCTGGAGTGCATCAATGGCGGACATGTGGAGAAGATAGCCAAGATGTGTGCCAAGGGCCTGGA
TCCCAATTTCCATTGTTCGGAAAGCGGAGATACTCCGTTGACAGTGGCCACGGGCGCCAAGAAGCCAAAC
AAACTGCTCATCGCTCTGGTCAACGGTGGAGCCTTGTTGGATTACAGGACGAAGGATGGCAGCACAGCTC
TGCACCGGGCTGTCGAACATGACTCCTTGGAGGCGGTCAGGCACACTCCTCGAACTGGGCGCCTCGCCGA
ATTACCGAGATGGGCGTGGCATCACCCCGCTGTACATCTCCATCACCAGGAAGTGTGAGGCGAAGATCAC
AGAGAGTCTGCTGCATGACCATGCCACGTTGGGAATCCAGGACAGCCAGGGCTGGAACGAGGTTCATCAG
GGTGAGTCGGTGGCGGTCCGTTAtgatcctggtgatcctggcgatccCTAACAGCAACTGCTTTGACCCC
GAGCCAATCCCCGGTGAAATGGTGTTGGCCCACAAACGTTGAACGCCAATTAATGATTGAAttctttatc
tctctctctcttctttcttgaaactcttctactctaactctaaaacaaacaaaaccaaaacaaaacaaaa
caaaTTCGCTTGCTAATACTTTGTAACCCGCGGTTTGGCATATGCATCTCTGCTGCATCCTTTGGCTCTC
TCCGCTCACCCGATCGACACGTAGGGCCTGTCGGCATGGCCTGGTCCAGCACCTGGAACACCTGCTGTTC
TACGGTGCTGATATGGACGGCCGCAATGCGTCCGGCAATAGTCCGCTGCATGTGTGCGCTGTTAACAACC
AAGAGGCTTGTGCTAGAATGCTTCTCTTTCGCGGCGCACAGCGCGGCGCACAGAACTTTGCCAATCAAAC
TCCCTACCAGGGTAAAGCCCAGTGTCTGGACTCAAGAACCTACACAGAGaaaaaaaaTCATGCGAATTAC
TTTTACCCGTTTGCCAATGAAAAGATTTAAAACCGCATGCAACGATATTTTCTCTGTGTATATTCTGGGA
CCTAAAACTTTTTGAATTTTGTTAACAACTTAGTAGTTGATAGCCTTTAAGTTGGTACTTTTGTGCTGCT
GTAGTTATGCTTGTAGTTCAGTCTAGTACTTAAGCTAGTTTTTGATTTAATAACTTGGTTTGGTATATGG
AACTATGGTTTTTTAATCCAATTCGATTTCCCATAGGGTAAGTGGTGGCCTACTTTAAAAATCATTCCAT
CATTCACCACCCGAAAAGCAATCGACTCGTACTCGCAACCGAACTAACTTCATACTCATCAAAACTCAAA
TCAAAAATCCCATTTACACGGAATGTGTTTCGAACACCTTGAAATATCATTCTGATTTATACTTTCTTAA
GTTTCTGTTTCAATATTCGTTTGCTCTACTCAGTCTAATTTATTAATATTGGCCACAATCAAAACTAGTT
TCCTTTCCACTTGTTTCGAATCAGTTTACCCCATGAGCGTCTTTATATCTCTATTTGAATACACTTGCAT
atcatcaatcaatcattcgacaatcaatcaatcaatcaatcatccaacttgatcaTCTAGGTCTCATTCC
CCATATCAGTGTATATTACTCGTATTATATAAATATCTGTACTACTCTCAATTTCTATATAAATCGCAAC
CTATATGTACTTTACCCAAAACACTTTTTTCGTATTTTGCATATGCTTGTTTGAGATACGTGCTCCATAA
ATGTAATCAAATCAAAATAGTTTTGTTAATGCCACACTTCCAATATCTTGGCTGTTCTTGATCCATGTTT
TGTTCAATGTTTTTTCGTGTTTTTCCATACATATTATACATATATATTCCATTTCCTAGTGAGTTGATTT
GTTGGCAGGACATTAGTAGAGTGTCCTGCCCTTGGTTTTGCTTTTCATCTCAGTCACTTATCCTTCGATA
AATGAGCAGTAAGTAAGTGTAAGTAAAGTGTGCCAATCATTCCTTGGAATATTCCATAACTTTGCCTTGG
GCATTAGGGTGTTAGATTATTTCTTTAATTTTTTTATATTCAAAGTTTTCACTTTATTAGATTGAGTAGA
GAGCAAACTGAAACAGGGGAATTGGATTAATAATAACTGTATGAGAAAAGAAGGCCTTCTAAAATTAAAT
TTGATCTTCAGCTATAAAAATAAGATCGATTTTTTATATGTATAAATTGATTTTAGAAGAAACTCTATTC
aaaaaaaaataaaaGCTGTGATGACAGCCAGACCTGACGACCAACTAAACACACAACTAAACTGATTACTC
CCAAACCTATTCCTTTCCTAATCCTTTGCCCACCAACTCGAACACATTTTAAGTGAATATCCTTGATCAC

CACACAGTTTCCCTTCCGCGGACCGCCGCGCTACAATCCGAAGCGACGCTCGGGTATCGGGTGGTTGAGC
GCCAATGGAGCCGCCGGCGCAGCTCTGCTGGCAGCGGCTGGAGGTGGTTTCGGTGGTGCGATGGTCGGCT
CGAATGGTGCCGCCAACGGAAACAACGGGGCTGGCGGTGTTGGTCTGATGCTCAGCCATCAGAACCACCA
GCAGCACCACGCCGGCCACCATCAGCAAcagcatatgcaccaccagcaccaccagaatcaccaccagcag
ctgcagcagcagcagTTGCCCCACCTGCACACACTGCAGCTCCACGGCCCGCCATCGCCCTGCCCATCGG
AGCACATGCTGGGAGCCTACAGCTCCGCCAGCTCCAGCCTCTCCGAGGGCTCCTCCGGCCACCGGTCCCA
CGAGGACGACATCAGTATTGTGACAGG
>dme_circ_0004370_junction_seq
ACGAGGACGACATCAGTATTGTGACAGGGAACTGAAGGAGAGCTTCAACTATGGCC
>dme_circ_0004371
TCACAGCCACCACATGTCTGGCCCTACAGTTTGAGCAGCACTGGGTTCTAAGCAACGTGTGGGACGAAGA
GTGAGAAAGCGGTAAGTTAGGCGACTCGTGTTACTTATACAGCTAAACGCAAAACCCATAATTTGTTCCC
CATTGAAACGTATATATCTTATATCTTGGAGTCGTTACTTTTTTTATGGCCGTGTTTTCATGTGTCCATC
TGTAGAGTTGTCGGTTGTCATTTGTTGTTTTGGCTTTGAGATTTGCACTGCATTCGGAAACCGGTCGAAT
CGGTTTCTGTGGTGGTGATCACAGGCATTTGCATAAAGTTTTGTTCTGTTTTTCTCTTTAATCGGTGTGT
CGGTGGGATTTTTCTCCAACCGCTGGATTTTAATTTATTTTCATTGATTGCACATATACAACTTTTGACT
TAATGGCTCGCTGACGTAACTCGAGCTGCTGAAATTAGCAAAATAGTCGTAATATTCACCTCATGGATTT
CTACGACTCTCTGCAGGATTTTTAGCAAATTGTGAAATGGTGCGATTTAATTAATCGATCTGTAAGTTTG
GTTAATTTGCTATTGGATTTACAATTTGTTAAAAAACGCCGATGGGCGTTATATATGTGATTTTAGTTGG
TGGCTAGTGGGAAATGAACACCTGCAAAAGTCGCGTCAATTGAATTACATTATTGCATTAATTTGTTAGG
CTTTCAAATCTTTTTATGGCTTAACGTAAGCtttttttttttAAATGTCTTCTTGGTTTCATTTGAACTT
TCGCCATAAACTAGTTTCAAACTATGAGCAAACAATTCATATGAAATAAAAGCCAAAATGTCAATGGTTT
ATTTGATATTCTTCTCGATTCCCTGCAGATTTATCTGATTCTGTATTAACTTCTGTTTCTGTTTTCTGGT
TCTGAACAGGAAGTCGATGGCTGGCCACCTAGGTACATAAGTGGGTTCTGTGGTTTTTCGGTGCCTCGGT
TTCAGATGATTCGAAGGTAATTTGTTGCCGGTCCGTGGGCCTAGACAAAGGACTGATTGCCGGACTTGAC
TGACAGTTGGGGCAGCAAAGGGTTAAGGTAGTGGCCACACACAGTTACGCCCAAACTCAAGTTAGGGCTA
ATTTTGCCATTGTATTAGCCAATTGTGGTGGCTGTTCCTCGTTTCCATCCTCAGGTTTCCTTTAGCTTGC
GATTTTTATTTCAGCTTCAGTTTGTTTGGCCAGCATAATGATGCCACTTGATTGAAAACGAACGTGACTG
GAAAACAAGTTTCGGGGTTAAAGTTTTCTTCTTTTCCTTTACTCTCTCGGCTTCTTTTGCCACTCTGCGG
TCTTTTCTACCAATTCGAATACTGTTCGAATAAACAGAAAATAGCGAAATAAATTAGCCAGGAGATGCTC
TCAGCCATGAACTTCTGTGACATGTTTTCCTGCCTCAGCTTTATTAGCCGACCGAGGACGAGGAGCCAGG
CGAAATTGCATTTGGTATTATTTAATTAAAGTTTTCTCTCCATCGTCGTCGTCTTCTTCGTTCTGATGTG
GTCCGGGCGCCATTTTGTTGCATGCTTCCTAACAGAATTATGTGCGCATTTCCGTTTCCGGCACATGCAA
CAATGCACCGAGAGCAGTCAGCGCGGAAAACGGAGTCTTCGATTTAGCCCAAGAACAGTGGAAGTaaaaa
aaaTGCAATGAAATCTAAAATATGTCTTATTCTTAAATTACTTTTAATAGTAAATTCATTTCAAATTGCT
CTATTAACATTGGGCAGAGAGGAGAGCTGAAAAACATTCTAATCGTTTGGAAAATTAGCTTTCTCCAAAT
TAAATTCCGTTTTTGATTAAATCCCATTTATTCCACTGTCCACAGCGCTGGATGGTGGAAGAATAATAGT
TTGCCATTATTAAGTTTGGTGCGCGGGTGTATTTTGCATGCGCTTTGTTGATGCCGCCGATGCTGCTTCC
GATTTTGCTGCAGTTTGCTGCTGCCGTTGCCAAGGCAAATTATTTACGGTGCGCACAACCAACAACTGAG
TGTGGCACAATAAGTGGCTACGCAAGGACCTGGTCCTGGCACCCTTCGCTCGTCCGTAAACCCATCACAA
ATCTCCACAAGATTGAGCGGCAAGCCATTGCAATGACAAAAGTAATTTGCTTTTAAAGCACCACCGACTG
CAGCATCGGGCGAAGGCAATTTAAATATGCATGGGCCATTATGCATAAGCGATGCAGTTGCTCCATCCAC
GGATATTTGTGCACAGCGGGAAAATCGAGGTATCAACATAATCCAAAATGGAAGAAATATGGTCTCAGTA
TGGATTATTTGTAGTAGAACAGAATATTACAAACCTGTGTAGTGTGGCGAAAAGCTATCAACTGAAGTGC

GTAATATGAAAGTGAAAGCTATCTTTTCAGTGTACTCATACTTACTTATGGGTGTCGTGTGTGCCACATG
CAACTCCAGTCGCATTTAGTTGCGCATTCGAGCAAACCCATTGGGGCTCTCCACTGACCCACTAAAGTCG
CGCCCGCCCGTTCGGTTCACGGACTCCAGGGACCTGATGCTGTAACTAATTCCAAGCAGAGCCCATTCGA
TGTGGCATATTTAAAACTTAATTTACATGGCTGTCCTTTGGCCGGTTAAACCCTAACCTTTAGCCCCTTC
ATGTTCGTTCGTGCTGACCCCTTCTCTTTCTGGCTTAAAGTCTCGCTGTGGCAGCTGTTTCGAAGCGGAG
CACTTTGTCATTTTCAGTTTGTCACACTTTTGCCCAAATAAAATTTCATCGCCTGTCATAAAGGCTTGAG
AGGCAAGTGTCACAAGTGCTTAAAACTCAAGGAGGTGCATGGCTTGAATATGGGGTGGTGGCGAGAGTGG
GAGCGGGAAAGCAGCACAGTTAGGTAAGAAGTGAAGGAGACAGCACTCAAAACTGCTCAAGTGGGGCAGA
CCCaaaaaaaaaaaaaaaTAAGAGAAATATGCCACATGAAATTCGTCATGAGACAGAAATCAAGAAAACA
TTTATATATTATGAAAGGAAATACATTTTAAAGCCGCTTGCAGAAATGGAATATGTTAGCAAATTTCAAA
ATCTATAAATTATATTTTAAAATTGAAATGGGAATACACTTTATTTCAAACTTTGCGGCTCAATTGATGT
AATACGTATTTTTTAAGACGGCTTCTTCTTTGATGTTTCCACCATAAAACAACCTCATCAACACCTTGTC
TATCTGCTATATTTATCTCTTATCTCTTATATCACTGACTGCAAAACCAATTAGTGTTTACCCCACCCAA
TAAATATGCTTGCCGCTGTCAACGATTGATTCCCTCATAAAAGGTGGGGGAACCATAAAATTGCTCTACC
CTCCCCAATAGATAACATGCACCTTATGCAATCAATGCTTCCTGAGAGAGTCTGGCAAAAAAGTTTGGCA
TTAGCTGGCGTATCCAAGCCAAATAACTGTCAGGGCTAagacaatagaaaggacagacaacagacaggtc
agacaTTCCCGGGTTGATATGAAAACTGGTGTGTGGGGGTAATGGCGATCCAAACAGGGGAGCGCACCAA
AAGTTGCTAGTTTGACAGCCGCAAGACAGCTGTCTGTTTGaaaaaaaaaaaaCCCCCACATATACGAGGG
TTAGCATAAAAAGAAAAACAGCAGCTACATAAATTTTGATTTACTTGCCTGACAGTTGCCCCCCAAATTT
TCACACGCACCCCACCACAGCCACCAAAGGTCGGGCACAATTTTCCTATTAGCTCAAGTGAAAAATCGTT
GCAAAATCGGTGAATTGGGTGGATGGGTCTGAATTTACTGACCATTAAATCAGGCAGGCATTCTTCCATA
TGCCGCAGAACATATTTCATGGCAGACCAGAGAGTAATTCAAATGCCCATTATGGACCAGTAACTTTTCC
AATTAAAAGCAAAATGAACTTTTGCCTCCGGAACTCGCTGGCCGAAGTTTCAATTAACGCCATGACTGGC
TAAGCTGGAATGGCCAATAAAAAAGGCCATCGAATTGCGAATCACCGATTTACCTGTGCGCCATTTTTGT
CTCCAAATTAGGTTGAATGACTTTGAAGCGACCACGTGGCGTATGCTTAATTATCGCAAATCGTTTTCAA
TTAAAGTGCCAAGTTAAAAAGGCACGAAATCTTAGCAAACGCCACTTTTGGCTTATCACTCTTACCACTA
TCACTATTAATTCCAATGCCTTGGCCAGCTTTGCTTATCACAAACAGAAGTCTGGCGACGACACTCGCCC
TCGTGGGtttttttttatttttCGTGGCGTCTTTGTTGATAATTTCACTTCAACAGCTTGAAAATTTTG
TGGGTTTGATTGACAGTCGAGTGGCGGCCAAGGTAAACGGGCCTTTTGTGCCAGCGAGTCACCTGTTTAA
ACGCAGTTTTCATTTCTCGTATTTACTTTTATTGATATGCGTATTTGTGCTCTTAATTTATGTATTTCGG
TGGAGTTTTTTGACCGTGACACATTTGCTTGTTTACTTTCCAGCGAGGCGGTTTAAACGGGGGCAGTGAA
CCAGGGAAAATTGGAGCTGCATATTaaaaaaaaaaaaGAAACCGAAACCACTGATAACaatattgtttaa
aatttaattataaatattaatattaataaatattaagtatagatatgTAATCTTGATTCTTTATAGTTTC
AGTTAAGTTCGAAGGTATAAGCACtgttcacacatgaacacgaatatatttaaagacttacaattttggg
ctccgttcatatcttatgtaaatgaatcgagagcgataaattatatttaggattttgttatctaaggcga
catgggtgcattgctcaaaaacatgtaatttaagtgcacactacatgagtcagtcacttgagatcgttcc
ccgcctcctaaaatagtcccttagtgggagaccacagataaggtcctcgccgctcaagataggcagatgt
gcccgagcgtgggacctcgataaggcggggactatttacttaggcctctgcgtaggccatttactttaag
atgcgattctcatgtcacctatttaaaccgaagatatttccaaataaaatcagtttcttacaaaaactca
acgagtaaagtcttcttatttgggattttacatttggtcaatcgagcctttaatcgactctgcagtttcc
ccctaccaaaggtaaggaactcagagaaaggccagctcctttaagcatcttacagctaaaggtagcaaaa
ataagtgactcttgtttccccctaccaaaggtaaggaacagagtataaatataaaaagcaaaagatacaa
aagaatcttttatgttttaaaacaagcaccttatagtctatagctaaaggttgctttgtgtaccattata
aattgtggtaaggcgtgcttgaggccatacatcagcaattgtgaaattaaaaagtgcataacaaaagtgc

cttataaatgctctaatagcattaaatcagctcataaatagagtgcagtgtataagttattgccctgcaa
ttgattctctaacatcttgtggttccacatagtctccgctgccatcaacgccaacgaacggttaagcgcg
acatcgacacttctgcgctgcgccgcggccgacgcctgctgcgccgctgccgacgacttcacttgattgc
tagggacttagggaaacattttgtacgctagatttagtttcaaatgataaattgcaataaacggtcgctt
gcgatcttcaaaatcaaatcgataactgtaattattaactggcgcccgaacagggaccagcgaataaacg
cgaacgaaagacaaaattctaagtcgcggagcaaaatcaaaattttgctaaaaaatattcgttggttaaa
ttgtgccgaagaaactcccgcgagttattaacaaacaaaattcacggtcggctataaaataaaattgttt
gagaaaaaaacagattttccggaagaggaaaatacttatcggcatagcattgcccattggtggatcacag
tttctgtcaggccagcccgcagaaaaccttctttggagtgctgacgtggattccccagtagcccaggcaa
aaaggacaacattcacccagccaggaggagtgaccgcaacaattctatgttaaataaatgcaaaacgaag
aacagcaatttcatcgaaaagtagaagtagaacgccgagaataacggaactacagaagagcaacacgggt
aacagttaacagtgcaacccaaaacgtaacggcagaggtaaagacgacgaacggcagagaacggctgcgt
gaaccaaagtgcagacaagaaggaaaaaaaaaagaacaacaacagcacagccgtagcagcagcagcagcc
cgccaaaaagaacaggaataagaacacgttgacgacgaagaagctgaagctgaaacaaccgaagaggagg
cagagagactacaagaacctggaaagtacactggaaaaaacatcagcaatttgaaattcaggcatccacc
cataataaaacataagtataccaaaaaaaaaaaaaaaaaaaaaaaaattttaagtatattattattatta
tattattattataatattattattattattatattttattattatattattagtattatattattatta
ttatattattattattgatattattattaatactatattttcaacccagttcctagagatcttctgaaag
gaaaattttcctatttactgttcctttctggtacactgttctcaaagcaaaataaccgcggtgagctaaa
atttattgcatgcaaaaataaaaaataaaaaatataaaaataaaaaataaaaaaacaaaaaacaaaagat
aaataagcaaacacatacacacgcattccatattttctgcccacaacttttgttaagttcaaattggttt
aggcttgttttgtgcaggtgttgtccgaattaaaaatcagtatggcaaatcctaataatataataagacc
atcagcttttagttcaaacgaaagaccggtgccctcagaaagacacgacctgcctgaacagcatcgcgta
gacatgagtgtcgaacagttgacagcattaattggccaaacggtggcccaagttttacctggattgataa
aacaaatgaacaacaatactcttgactttactgacgtgagcgaccaggtcgtcgagcccgaatacagaaa
taatttagcggactttgacagggtgcctgatatcgtaaaatcgatcagggaattctctggaaatccagca
gaattcgggtcttggaaaaagagcgttgatagaatcatggagacttataccccatttgtgggtactccaa
aatattatggtatacttcataccataagaaataaaattgttggaagtgctgatgtggcactcgagtccta
cagcattccgctggactggaaggccatgtcgagatgtctcactctgcattacgcagataaacgggacata
actaccttggaataccaaatgtcaactttggttcaaggccaccagcagtcggtggaagacttccaccaag
acgtctacaaaaatctgtcgcttattctaaataaagtcggctgcatgcaaatgagccgagaatccgagca
ctttgttacaaaaatgtacagagaaaaagctctggatacttttatcagaggccttcgaggagatttacct
cgccttttggcaataaaagagccagctgatctcccctcagctttacattattgccttaaacttgaaaatc
aaactttcaggtcaaaccatgcggcgaataagggtcaacagtcgagtttcagaggaaacgaaaaacccat
tccggcaccccgcaattttcagccgtcgaatgcctttggccatcgacagcctccaccggtccctccacga
catcccatgaggagacctccacaatatttaggccagccattcccggcgccaaggcaacacgtgtctaact
tcggtaatgcaccgttcattccaccccgacaaaactatcaacagcaatggcaacaatacaatgccccacc
ccgtccctttgcgaccaaaccacaaccaagaccagaaccaatggacgtggacggcagtgtacagacgcga
aacattaattatatgaatagaccacactcggacataaacaaacgacaaaagaactacaacatccaaacag
tgaacatgagacagccaagtattgaagtaactgggtccagttcaagcatgacaggctatcaacggtcgat
gcaagattatgaaacccagaacaatataaacggcacactaaatgaatactgtgatggacaaatagacaat
ttggacagcgaacaacgagaccacgtgttgcatttttttagagtagaagactcctcactaccatacttcga
atgtagagtggggagtggaaaggttttaagggtgttgattgacacaggctctaataaaaattacatccag
cccaatttggtgacgaacgcgataccaaataacaagccttacatagctgatactccaggcggtgatgtaa

aaatatcgcattacaaaagagcaagccttttcgatttcgaaataaaattttttttgttaccaacgctgaa
gacttttgacgccatacttggcaaagacacaatgaagggaatgggagcgcaaattgatttgaagaaccta
accatgacactggaaaacgggaaaagagttgttctcaaagaaaaacagttcgaggctgttagcacaatta
gtccgagaataaaacacttagaggtggaacagagaatgatattgaataaaataattgattcgtatccagg
tctcttcgcagacccgaatcaaaaactaacctacacaacaagtgtaagggcagcaatccgaactatatcg
gatacgccaatatactcaaaattctatcagtacccaatgtctcttaaagacgaggtacacaaacaaattt
ccgaacttttacacgatggaatcattcgaccctcaaggtcaccttacaattcaccagtgtggattgtacc
aaaaaaactcgactcctctggcaagaaaaaatacagggtggtaattgactatcgaaaacttaacatggta
acggtagcggacagataccctatccctgacattaatgaagtgttggcccaattgggagacaacaagattt
tctcagtgctcgatctgaaaagtgggtttcatcagattcttttaaaggaatctgatatcgaaaagaccgc
cttctccatcaataatggaaaatatgagtttacacgactcccattcggtctgaaaaatgcaccgtcaatt
ttccagcgcgcactggacgatattcttcacgaacatatcggtaagatatgtttcatttatattgacgaca
tcatcatctttagtaaagatgatgaaacacattaccagaaccttgacactattttcagaactcttcagca
agccaacatgaaatgtcagttggataaatgcgagttcatgaagagaaaagtggagttcctaggcttcgtc
gtgtccgacaagggcattgaaaccagcccaaccaaggtacaggcaatctcagactttccaattccaagga
cactcaaagaactgagatcattcttgggattatccggatattacaggcgatttattcctaactacgctaa
gttagcaaaaccacttagctcgcttttgagaggggaggatggtcgaatttccaggacattatcgtcaaaa
aaatccgtctcccttaatcgcgaagcaatagaagccttcaagaaattgaagagcagcttggtttctccag
acgtaatactccactacccggactttaagaaagaatttcacctaacaacggatgcttccaatttcgcagt
aggtgctgttctttcacaagagaacagacccatctcatttttatcgagaacactctcgaaggcggaagaa
aattatgccacgaatgagaaggaaatgttagccattatctgggctctaaaaaagctaaaaatttaccttt
acggtaaagcaaaggtgaaaatctttactgaccatcagcctttgacccattctctcagtagttggaatgg
aaatgcgaggattaagagatggaaagcataccttgaggaatatgactacgaaattttctacaagccaggc
agagaaaatactgtagccgacgctctgtccagaggaccgacagtcgaacaaattaacacagtagcctcaa
caatgcacagctctgacagttcgagccatgggctgatacctagcgttgaagccccgataaacgcattcaa
gaatcaaattttcttcagggagtccgagtcagagaactactcatttagcattccattcccgacatttcaa
aggcatttagtagatcgcaacttgttcacacccgatagtctcttgtcagatttgaaagaatatcttaacc
catccgtgattaatggaattttcacatccgaggatgtaatggggaaaattcaaattctctaccccatcca
ttttcagggtttcaagattagattctgccaaagcaaagtcaaagaccttgttaacgaagccgaacaagag
gaagaaatacttaggacacataacagagcacacagaaatgctgtggaaaataaagctcagttgtccgaaa
gagtatactttcctaaaatgaggaaaaaagtttcggctatcgtgaatcagtgtttggtgtgtaagactgc
taaatatgacagacatcccacgcatccagaaataagacaaactcccttgccagaataccccggacaaatt
attcatattgacatctactcgacagaacgacatctggtgctcacggcgattgataaattttccaaactgg
ccatgggaagagttatcaaatccaaagctgtagaagacattaggaaagccctaagagatatcgtgtttta
ttatggagtgcctaaattaatagtaatggacaacgaaaagtccctcaactcagcctctatcaaattcatg
ttgacagaccagctgggtattgagctctacaaagcacctccgtataagagtacagtaaatggacagatag
aaagatttcactccacactctctgaaataatgagatgtttgaaaggagatggaacacataggggcttcga
ggaacttctcgatagggccatctatgaatataactacactgtccattctgtcacaaaaaaacgacctcta
gaggtgttcttcggcaggatagctaccgtagctccagaaaaatatgaacaagctagactggacaatatag
accgacttaggcaaaaacaggaaaccgacatagaataccacaaccggacaagaaagcccataaaaaccta
tatcaaagggcaagaaattttcgttagggttaatacaagattaggttctaagttatcaagtagatttagg
aaggaactagttaaggaagaccgaagtaccacaatattaacagaatcagggaaattagtacataaaagta
acataagatcatgattgcttcagaataattactgtggccacatcgatagacaagtagaacatagaaacat
taaacatactgattaacgtaaccacgagaattaaatttgaaacgattcgactttgacagacgagttggcc

ataggtttgcagaattagattgggctcgtgaaagaagagattgcgttgtgaatgcgtttctgcggagcga
gtttaaattgaatgagaccagtagcctacttgaaaacaatataccgatttctgccttgacggtcaaataa
atgttaaaactttcacacgttctcgctttacacaacggaactacaatttgaatgccattgaaatctccat
attgaatcagttaattccttatttcttatattttaaaacatgatcagtaaaggaaactaataataataaa
taaataataatttaatttgaataataataaaatttaccaattaaagacgaaatgtacggaatcaatgcct
tctgcaaaggttccaatggtctaaaaatatgtagatataaattcattagtaatttagtaaaaaccagaat
tttaatagaagcaatcaagttgtcaatgcagaacacatccgagttgcacaaacctcgaacgcacgcaaca
ataaacacctggcattcaaaccattaatgctgtgcccaaactaccacgaatcaccccaccaacgatgcga
ctcaaaaacatcccatccttatgatgaccgctcgaggacaagcgtcaattaagaggggaggagttattgc
cctgcaattgattctctaacatcttgtggttccacatagtctccgctgccatcaacgccaacgaacggtt
aagcgcgacatcgacacttctgcgctgcgccgcggccgacgcctgctgcgccgctgccgacgacttcact
tgattgctagggacttagggaaacattttgtacgctagatttagtttcaaatgataaattgcaataaacg
gtcgcttgcgatcttcaaaatcaaatcgataactgtaattattaacttatatgccataagagcataaatt
aaataaaaagtgcctgaaaacagtgccttataaatgctctaatagcattaaatcagctcataaatagagt
gcagtgtatatgccaaaagagcataaatgccgaaataaatggctaaaaacaaaaaatctgactggacta
caaaaataataaaacgtgccaaaaaaaaaaaaaaaatcatctttaaacatcgacggagccttaaagaaga
gaaggaagtcaaattcaaaggagcctctaccagcagcagaagcagcaacaacagcagcagcagaagcagc
aacagcagtagcaacagcagcaacaacagcagcaacagcagcagcaacaacaacgacatcagctaagtca
aaacaagaattttctgtttatccaaacacacatatatatataaatacatataaaatacatatacacgtac
tatatatattaagaaattacaaaaaattttcaaaatgatgtcagaaaagactattcaattccttaagaag
cagtccgaaattattttggaaattagaaagttggaagtaaaaccaacattaacagatgtagaaattctaa
aattaaatgagcttcaaaaatgtttcattgctaatcatagcaatttgttaaagatcggcgttgtcgatca
tgaatattttaacgcgaagcagtatgatttaataatgatggtgttagaaaaaattaaaaataaaaatgaa
aaaattaagggcgagtcggtagaaaacactttccctaaatcaaacactgtccctaaatcaaaccctcccc
ctacattaaaccttgaaatgcgtggtcaccctgaaaaagagggtatagcacaaaacaacgctttaaaagt
agagcaggcatttcgtaataatgttggccaatttcgagtatatctagaagatacgtctaaactaatagac
agtagtccagatttccttaaaataaggaaaaataaaattgaatttttatggcataaaatagataacctga
ttgaacaggtgaatagtcattttgagagttcgctattcgaagaagaaattagcgaacttgaatttgacaa
acaaaatattcttacagccattaatagtcgactcagtggcacaataaataaagctgaaatgtcgacggtt
gttaaggcggaggagttaccaaccctgcctaaaatacagattcccaccttctttggtgattccaaagaat
gggatctttttaatgaactctttacagagctcatacatgtgagagaggatctcagtccttctctcaaatt
taattatctaaagtcagcattaaaaggagaagccagaaatgtggttactcatttactgctcggctctgga
gaaaattatgaagccacttgggagtttttgaccaagcgatatgagaataaaagaaacatattctcagatc
atatgaataggcttatggatatgccaaatttaaatttagaatccaataagcaaataaagacatttattga
cacgattaacgagtcaatttatattataaaattaaaggcacaattaccagaagatgtggatgcaattttc
gctcacataattcttcggaaattcaataaagaatcactcaatttatatgaaagccatgttaaaaagacaa
aagaaatacaggcactttctgatgtcatggactttttagagcaaaggctcaattctatatcatcattctc
acaggaagtaaaacctgtaaagaaaatgattaataataacaagaataaaaattatagtgacaattgtgca
tattgcaaactaccagggcattatttaattcaatgccataaatttaaaataatgaatccagcagaacggt
ctgactgggtaagaaaaaatgggatttgcctaagatgtctgaggcatccgtttggtaaaaaatgtataag
cgagcagctttgttcgacttgtcgtaaacctcaccacacgttacttcactttgcaggtcataatccagaa
aaagtgaatacgtgtagaacaacaggtcaagccttgttggccacggccttgattcaagtaaagtcgaggt
atggaggctttgaacaattaagagcattgattgatagtggctctcaaagcacaattatttcagaagagtc
tgcacagattctaaaattgaaaaaatttcggtctcatactgaaataagtggagtatcttccacaggaacg

tgcatctccaagcacaaagcggttatttcgataagaaattctccgaaaaatttagaaattgaagcaatta
ttctcccaaaacttatgaaggcacttccagtcaacacgattaatgttgatcagaaaaaatggaagaactt
taaattagccgaccccgattttaataaaccgggtcgcattgatttaatcattggagcagacgtatatact
cacattctgcaaaatggagttataaaaatagacggtctccttgggcaaaaaactgatttcgggtggatag
tttctggatgtaaaaaatccaaaggaaaagaaaccattgtagccacaacaatagaaataaaagagttaga
tcgctactgggaagtggaagaagaagaaaaagatgatatcgagtctgaaatctgtgaaaataaatttatc
aaaacgacaaaaaaagattcagatgggcgatacattgtgtcaattccattcaaggaggatgtcaccttag
gagattcaaagaaacaagcgatagctcgttacatgaatctggagaaaaaactaaaaagaaatgaaaaact
taaggttgactacactaaattcatgaatgaatacatggatttaggacacatgattgaagtgagtgatgaa
ggcaaatatttttttaccgcaccaggcagtgattagagattcaagccttacgaccaaattgagagtagttt
ttgatgcttcagcaaaaactacgaataacaaaagtttgaacgacataatgtgggttgggccacgagttca
aaaagatatttttgacattattattaaatggagaaaatgggaatttgttgtttcggcagacattgaaaag
atgtaccgacaaattaaaatagataataatgatcaaaaatatcaatatattttatggagaaattctccaa
aagaaaaaattaaaacatataaattaaccacagtcacttacggaactgcatctgcaccatatttggctac
cagggttctggtagatattgcagataaatgtaaaaaccaagttattagtgcaataattaggaatgatttc
tatatggatgacctaatgactggagctgattcggtagaagaagctaataaattaataacattaattcccc
atgaattgcagaaagttggattcaacttaaggaaatggatttccaacaattccaaaatattaaccactgt
ggaggacacaggggacaataaggttctcaatattatcgaaaatgaatgtgttaaaactttaggactaaaa
tgggaacctcaaaaggatttatttaagttcagcgtaaattgtaatgatgaatcaaaaaatataaataagc
gcgttgtgttatcaacgctagcaaaaatatttgatccgttaggatggttggcaccagtcacggtttcagg
aaaactttttattcaaaaactttggataaataaaagtgaatgggatcaggaattatccatagaagataaa
aattattgggaaaaatataaagaaaatttattattgttagagaatattcgaatcccaaggtggattaatt
caaacagttcttcagtcattcagattcacggatttgcggacgcctccgaaaaagcatatgctgcagtagt
ctatgctaaagtaggacctcatgttaatataatagctagcaaaagtagagtcaaccctataaaaaatagg
aagacaattcccaaactcgagctgtgtgcagctcacctgcttagtgaattaatccaaagactaaaaggat
caattgacaatataatggagatctatgcttggagtgattccacgattaccttagcatggattaacagtgg
tcaaagtaagatcaaatttataaaaagaagaacggatgacattcggaaattaaaaaatactgaatggaat
catgttaagtcagaggataatccagcagatttagcatccaggggagtggattctaaccagttgatcaact
gtgatttttggtggaaaggtccgaaatggctagcagacccaaaagaactttggcctcggcagcagtctgt
agaagaacctgtcttaataaatacggtattaaatgacaaaatagatgatcctatttacgaattaatagaa
aggtattccagtatagaaaaacttatacgtataatagcatacataaatagattcgtgcagatgaaaacaa
gaaataaagcctattcatcaattatttcagtaaaggagataagaatagcggaaacagttgttattaagaa
acaacaagaataccagtttaggcaagagataaagtgccttaaaatcaaaaaggaaatcaagacaaataat
aaaatattgtcattgaatccatttttggacaaggatggggttctaagagttggaggaagattgcaaaatt
ccaatgcagaatttaatgttaaacatccaatcattttagaaaaatgccacctaacaagcttattaataaa
aaatgctcataaggaaacattgcatggagggataaacctaatgcgaaactatatccaaagaaagtattgg
attttcgggttgaaaaattcgttgaaaaagtatttaagagaatgtgtaacgtgtgcaaggtataaacaaa
atacagctcagcaaataatgggtaacttgccaaaatatagagtgacgatgacattcccgtttcttaatac
tggaatagattacgcaggtccttattatgttaaatgttcaaaaaatcgtggccaaaaaacatttaaagga
tacgttgctgtattcgtttgcatggccaccaaagccatacacttagaaatggtaagcgatctaacttcag
acgcatttttagcagcactcagaagatttattgctagacggggaaaatgttccaatatctattcagacaa
cggaacaaattttgtaggagctgcaagaaaattagatcaagagttatttaatgcaatacaagaaaatata
acgattgcagcgcagcttgaaaaggacaggattgattggcattttattcccccggcaggacctcacttcg
gaggtatttgggaagctggagttaagtcaatgaaataccatttaaagcgtataatcggcgacactatttt

gacttacgaagaaatgtcaactcttttatgtcaaatagaagcatgcttaaattcaaggccattatacact
atagttagtgagaaggaccaacaagaagttttaacaccaggtcattttttaattggaagaccacctttag
aaatagtcgaaccaatggaagatgaaaaaatcggaaatttggataggtggagacttatccaaaaaatgaa
gaaagatttctgggttaagtggaaaagtgaatatttgcatacgctccagcaaaggaataaatggaaaaag
gaaattcctaatatagaagaagggcaaatagttttattaaaggatgagaattgtcatcctgcaagatggc
ctttaggaaaggtggaaaaggtgcataaggggaatgatgataaggtccgagtggctaaagtaaagatgca
ggaaggatatatcactagacccattactaaaatttgtcccttggaaggaataaagtctgttgacaaaaat
gaggctgaccaagagccaaaaagacgaactagagcgacatcgggaatgtccaagatcggaatcattatgg
caatgttgttgtttgtgttaagttgtcaagtttctagcgcattacctaaagatatagcaccaagatattc
tatagacaaaataaataaaacctcagcaatatatctagacccgctaggagatgttgagattgtgagtact
tcttggaatttggttatctatttggatccatattttaaaatgttaacaaagggtaatgcgcttatacaaa
gtatgaggaaagtttgcgaaagacttcatagctttgaagagcaatgtagtctagtcttagataatatgca
aagtcagttatcggaacttgaagaaaacaataaattgtttatgatacagtctagatctagaagcaagcgt
gctcctttcgaatttatgggttccttgtatcatattttatttggtataatggatgaagatgatagagagc
aattagaagaaaatatgaagaatttgttagataaccagaacaaccttgataaactaattcaaaaacaaac
atctgtggttgattcaacttctaatctattaaagagaacaacagaagatgttaactccaattttagaagt
atgcaaataagaattgagaacatgacagaagttcttaaagaaaattattatgtttataaggaatcaataa
aattctttatgattacgaaacagctacactcattgattgaagaaggcgaaaaaattcaagcaggcattat
aagcctgttgattgatattaatcacggtaggctaaatacaaatattctcaggccaaatcagcttaaaaaa
gaaattgccaaaattcagcagagtctttcggagaacctagtaattccaggaaaacggtcaggtacggaac
ttaaggaggtgtatacactgttaacagccaggggtttattcatcgacgataaattgatcattagtgcaaa
agtgcctctgtttagcaggcatccatccaaattgttcaggcttattccggtgccaattcgaaatgaagat
cggataataatggtgcatacaacgtccgaatatttaatttataattttgagatagattcctatcacataa
tgacggaagccacattaaatcaatgtcagaaatggcaactaaataagagaatatgcaaaggaagttggcc
ctggaattcagcgaatgataatgcatgtgagattcagcctctaaagccagataaagcggcgaactgcatc
tataaaacagtagtcgactctaaaagttactgggtagagttagaaaagaaaagtagttggttgtttaagg
ttcctgcgaattcaaaagtccgtctgcaatgtactggctctcaaattgaattgtttgatttgcctcagca
aggagttttaagcattgcgccatattgtacggcaagaaccgacgataaaattctagttgcccaccataac
attcagtccgaaagtgaagaattattatcaacaccttatataggagaagttagtgaagcgccgaagatta
tttgggatccgctgaaactatcaatattaaatcatactgaggaatttgaacgattgaataatgaaattaa
atttatgaaagagaaccatcaaaaattgaaagatttacatttccatcatatttccggacatgctggatta
attattgctttaatactaatgatagtattaataatatatttcatacggaaatgtgctgtgcaacaaagaa
tgcaagcaataacctttgcaggtccgttgccagtactataaatatcaatagtaaataaacaataaaataa
tataacaaataaaaatatacagtccactatatcgttgtttaatagaaaatgtacttctacatagaaaaag
caaaatgtttaaaataagttaattgagtacaaattgttgaattaaaaataatataaaccataattgtaat
ccaataaaattaaaagccagaaaaactaggcccattgaaatcttagttgcaaaataaatgaacatatatc
aaataaatacagtccactactgttataaatgcaactaatatactaatgtacatctcagcttgctggccct
ttggcagaatgttcacacatgaacacgaatatatttaaagacttacaattttgggctccgttcatatctt
atgtaaatgaatcgagagcgataaattatatttaggattttgttatctaaggcgacatgggtgcattgct
caaaaacatgtaatttaagtgcacactacatgagtcagtcacttgagatcgttccccgcctcctaaaata
gtcccttagtgggagaccacagataaggtcctcgccgctcaagataggcagatgtgcccgagcgtgggac
ctcgataaggcggggactatttacttaggcctctgcgtaggccatttactttaagatgcgattctcatgt
cacctatttaaaccgaagatatttccaaataaaatcagtttcttacaaaaactcaacgagtaaagtcttc
ttatttgggattttacaAGCACTTTTAAATGCTTTCCTTTTTTCCACCTTTTTCTAAAGCCTTTCATTTT

GTCACAACCTTTGACTGCAAAACCTTTCATCTTTCAGACAGAGCACCCACAACTTTTATCCTCAATTAGG
ATTTATCATTTCCCGCAAGCGATTTCCATTTTACACTAGTTTTCCCCCATTTCCCGCCATCTCCGTCCCC
CGCCCTTTCGAAATGCCAACTCTAACTAGTTGAATGCAATCAACACGCTCGCAATTAAATGCGACAAAGT
GCAGACTTGCAGATGTGGTTGTTGCTGCAGTTGGTGGCTAACCCGTTCCGCCCACAGTTCCAGTTGCCTC
GACATGTCCCGCTCCGCCCGCTTTTCCGCTCCATTTTCCGccccccccccccTTCCTCCTCCCCAACACA
AAAGCGACAACTGTCAGCGGGTCTTTGGCACACATTTACATGCCTCCAATCCTCCACCCACCGTGTGGCA
CCTCCTCCTTTCCAGCTAGCGTGTGAATTTAATTTGTGCATAATTGCAATTGCAACTGCCATTGCAGTGC
TGCAGCCATAAAATGTCAATATCAAATTGTAATCATCAACGAGAAATGTTTGCGGCAGCCCAAGTGTTTT
CTTTTCACTCTGACAATTTGTTTTTCCTATTAGATAGGCCTGCAGACATGAAAAGGGGCTCGGAAAATCG
GGCCAATTGCCGTCTGACTGATATCATCAAGTGCGTAGGAAAAGTGCAGTGGACAGTTTCGAAAAGTAAA
CCAATTTAAAAATTAAGTGCTGGGGAAACTATTTTATGTTGTTGTCTGGACTTGAGCAGGCTTTTAATGG
ATTGCAATTTGCATATGTGTACTAAATTTATGCGAAAATATTTAGTGCATATGAAAGTGGAGTGAAAATT
AATAGCTTCATTGGGTAATGCATTATGATTCGGAAAGGAAATATTATACATTATAATATATTTTATTCTT
ATACCTCTAATTTGATTCATTTCAGAAACCATCATGCGCCAGACTCAACGAACGCAGCACAGCGAAATGT
CCAAACAAAACGGATTCCAACAGCAGAAGGCCGTAAAAGAAAGCCAAATAGATTGGCAATAAAAAGTTAG
ATAAATTAACTTAGTAGCTCATCACGCCCAAAGGCAAATCACTTTGAAGTGCAACCAAATAAGGAAAGAG
CAAATTAAGCGAATAGAACGGCTAGAAGGAGATCTCCCAGTGGAGCGGGAGTAGCTTCGtttttttttCG
TGGACCGTGCGATAGAAAGCCGTCAAAATTACGACATACCACCCACCCACGCCCAAAGTCAAACAGAACC
CGGCCAATACGGACGCCATGTCCGGGTCGGGTGCGTTTGACGATGAGCCGCCGCCGGAGCCGCGGGACGG
ATGGCTACTGGTGCGCATCCATGTGCCGGAGCTGAATGTCTACAAGTGTCTGCAGTTCCCATCGGAGCGG
CTCGTTTGGGATGTCAAGCAGCAGGTGCTCGCCTCGCTGCCGAAGG
>dme_circ_0004371_junction_seq
CAGCAGGTGCTCGCCTCGCTGCCGAAGGTCACAGCCACCACATGTCTGGCCCTACA
>dme_circ_0004372
GTAATTAAATGGTAATTTCTCATCGATTTCATGGCATATTTAGCGAAGTAAGCCAACTCGCAGCTCGGAT
GATGATGCCTCATAATCGCCTCGCCTCGACTTGACTGGCAATGTGGCTAAGGCAAATAAGGCTCATTAAT
GTAAGCCAAAGACAACAGAAATCTGCTCCTTTGTTTGCAAATTTTTCGTTGGGTTCTGGGTTTTGGTTTC
TGCGAAACATGAGAACGCATATTGTTAGGATAAGGCGAAGGTATGGTATATAAATAGTTATGACTGCCCA
AGTGCCCAAGTGAATGAACTTTGCCGGCTATTGTGTGTGTGTTACTTTTACTTGGCCCGACACTTTCGCA
GGCACATTTCCTGTTCCTAATACTCGGCTGACATGCAACAATTTTCAACCTTTTCATTTGGGCACCCAAA
CAGAATGGAGAGAGAACTATTAAGTCCGATTGCCAACTTTCACTGATAATGCCAGCAATTATGTATTGCT
CCAGTTAATGCCATCACTCGTCTTTTATCCACATCATCATCTAACATTGTAATGACATAATTATGTTGGC
TTTTCATGGGTCATTCTAGGGGGTCAAACACGTGTCTAGTCCACCACGTCATCCTTCAATTAGCAGTCTG
TGTGAAAGGTGACACGAGCGTGTTAAGTTGTCGGGTAAACGCTGGAAAAGAACCCATCTGCAGACCCTTT
GATTTGAGCCAAGAATTAGCATTTTTCCCCCCTCAGCTTGAGAAACAATCTTGGGGCCCAAACAATGAAG
ACGAATTTGTACAGTTTCTGCTTGGCTGATTCCGGCCAACAGATCCCACCGAAAAGCAGCCACTCAACAA
GGGTCTAATGGGTTTTAAAAGTGACCAAAATTTGGCTTGTGCCCCGCGTAAACTGGCTAAATTAAACGCG
GACGCTAAGTTGGCTAAGAGGTGGGCCAACTCGAATTGGGCAAACATTGTGGAATTGAAGTGTATTGAGG
CTTTTGGGGCATGTCGAAGAATGCATTAGCCGAGAATTGAGTACATACCGGTGATGATGGGGAGAAGGTT
TACATAACAGATAGAAGCATCTACAATTTCCttttttttttttGAAAAGCGTGATAATTATTTACCAGAA
TTGATAAGAGAAGCAAAGAAGAGCACTCCGAAATGTAAGTAAATATAAAAATATCTGGAATAAATGAAAA
AGTATTATTTACATTTACCTTGAAAAACGAACACAACAAAATGAATTACTTATTATAAAACATATCATTT
ATATGTTTTTATTGGTTTTTATGTGTATTTTAAGTTTAACTTTAATGCATTTAAATAAATTGGCATTGC
TTGCATAAATCAGGTATATTTATTTATTGCACAAATTGTTATCAACTCCAATCCAATTACGTTTGCTGAA

TGCATTGTTAAAATAACTCCACATTTTATGTCGAATTTAACTTTCGGCTCTTAATTTATTATCCATCATT
TGCCATAACCCGTTTAAATCGAACTCTTATCTAAACTTGTGTGATATATGTTTATAGATTTTATCTTACC
CCGTTTTAGACATAGTTTCAATCGATTTTATTAGTCCACAGCTAAATGATGATGATTTGGAAACAATAGA
ACCGGCTGAGAATTATAATGAACCCGATACGCTTTCAGTTCTATTGGCCATTAAACAAAATGACTTATTT
CCCAGCTTCCATTTGCACTAAAAGCCCAAAATGTGGAAACCACTTTGAACCAGTTCTCAATTGAGTGCAA
ATAAAAGTAAAACTATTGACAAAAGCTGAAAGTGAGCACAGTTTTGCCGATGTCAACCGAGCAGCTACTT
AAGCTACAttttttctttgtttttttttttCTTAACTTGGAGCGTTCAACGATCGCTTAGTTAACTCCC
AGCCCTATTCACGGTGCACTGATATATGAAATTTTGAGTCGAAAACGATGCGATCTCTTTTGTCGCCGGC
AATTTTTGGCACTAATTTGACAAGGTCGTCGTCTCACTGGGCTTTTGTTGGCTTGGCTCGAAGAAATCAC
GACCCAAACACGTTCGAAGTGATTTCGACTGGCCCCtttttatttttttttgggtttgaattcgtattttg
tttattttgCATTTTGAGTGGTCCAAGTCGTCAGCTTATTTCGCAGATATTTGCATAAACATTTGACGGC
GGGGTGGGGTGCGAAAAAGCTGCCGTGAAATGTTGTCATTCATATTAATTTAGTGGGTCACCGGGCGGTG
GTGACCCCGATCGTGCGGATTTCCTGTTAGAAAGGCTCTGACTAACTGACTGCCCGATTGTTTTCCCCCC
CTTAATAAATCGCATCTCTTTTTTAAGTGGGTAATGAATAGTCAAATTTGGCTTCGTGACTACAGATGGC
AGACAATTAACCCTAGAGCACCGAACTATCCCCACTCACCCACCTGCTGTGACCAGCAGTTCGCTTTTGA
ACTTTCATTCCTTTTGGATTATGCAAGTGGATTGGatggtagtgtggtagtatggtaggatgAGGATGAG
GATTTCTGCCCAGATAAGAGCTGGCGAAGGCAGAGCTAATTAAATTATAAGCCAAGTCTGACTCTGACCC
AAGATGAGCTGTGCGAGGGAATGGGGTCACCAGAAGGTGGCCCCACCCCCATTTTCCGCCGAGAGTGGTT
AAATAATATCCAAATGGATGCTTTCGGTGTTTGCCGTTCGTGTTTTGCTGGTGTTGATTTGGCCATAAAT
TTTGTATGCTTTTGACGCCGAAATCGACGACCACAGCCGAAAAACATTCACGGAGAGATGAGGTAATGCA
GCAAATAACTCTTGAGTTACAGTGGGATTATATTGACCAGTCCACTCGAAAAGCCAAATGAACTTCAACT
ATTTTGTCGGCTTTTTATGCTACCGCCAGTGCTGCGGCTTATATATTGATATTTAATATGACCTAAGGGT
CTTTTTCACGGCCTTTACACCAATTGAGTTTCCGTTTTATTACTTCCCGCCTTTAAATACTGAGTAATTT
AATAAGTTATTGTCTGATCATTATGAGTATGATTTTTGTTTTTGACTATTAATAAAACCTTTGAAAGATT
ACGAAATGAGTACAGTTTAACTACCGTCGTGGCAAAGTGATTACTTCTTTGTGTATAAAAAATAGCTGAA
ATTCCAATATGCACGCCCATTTTGGCGCTCGTAACAATATTTCGATTTTTATACTTTTTGGTAGTTTATT
GCGAAAAAATAACTCTGCATGGTTTTAACAAGAACTATTTCAAAATGAGCTTTGACCTTTAAAGCCTTGGT
TTAAAAAATAATATTTAGTGTTGCAACTTTTCATCGTCTTTAAATAGCCCGCACAAATCTATACAAAACA
TTCATAAAACTTTGAAACTCAATTTACCAACCGGCTGTTTTGTGCTCCTCATTAGGACAGGACACCCACT
GTGCCACATGGAGCAGTTGCTGGACGCAAACATGAATTATGCGTAGACAACTCGCGGGTTGGTTAGTTCA
CCACCCACTCCTTTTTCCCCGCTTTTGGGTCTGTCATCGGGCGTTGTCAACGAAATGTTTGTGGCCCGAA
GGCAACTTGAGGCCAAGTGACGGCGGCATCATTTCAATTGCGTTTTATGGCCATAAATAAGCCCAACGGC
GGCTGACAGATTTTCGCAGCGAGAGGGTGCAATGGCTGGCTGCTTAACAAGGAAAATGGGAAAAACTACG
AGTGCATGCAGACGATTTTGCTGGCAAAGTCGTGATCACACTTGACTACCAATTACCTGAGCCCATCAAA
TCGAATGTGAGGTGGATTCTACCGGCAAAGTTAATAATCTCATCAGCTGGAGACGCTGGCTGTGAGATCG
AGAATCTTCACTTCACTGGCCAATTTGCATAGGCAACAATGCTAAATGGACTGTAATGACGTACCAGCCA
GGCAATTCAATTAATTAGTCTGACCAAGAGCGCTTAGCAATTCCCTACATTTTGCTCTCCACCACCGTTG
GTTGGTTTTTCTTGTTGCCACACGCGTGTGATGAACATTTCCGCCGTGGAAAGCGAACTGCTCCACTCCg
tgtgtgtgtgtgtgtgcttatgtgtgtgCTGCAATCAAAATTAATTAAGGCACAAAGAGAGGCGGCCA
GATGCTTTGCATGGCTTTTCCTCCGACCTGCCACTTTTTATTGCCTTTTACCGCAACGCTGCACCAATTT
CCATTGCACCTGCAGCAGGCACAGCTCCGATGACGTTGTATTTGCTTTTCCACTTCATCTCGAGATCTCT
TTTTGTTTCTGTTGTACTACTCCGGGCGGGCACAGTGCCACCAGCGAATGTGCATAATTAGAAGTGGTTA
CATGTGGTCGATGTGATCGCAGAATCAGTTAGTTGCATCGTTTGCTTGTCCAGCAATCAGCTGGAAAACT
GGCAACAGTCAATCAGAGGCGCTTTTCCCGTGGAAAATTGCAGCTGCAGCTCATCCAGTTGAGGGTGCAT

AAATTAATTAGCCACGCTTGCCAGTGATTGTTGGTCCTTTATTGGTACAGAAAATACTGGGTATCATATA
TTGTTCCCCATTTTATTACTAACCACAATAAAAAGTGAAGCATAAACTCCATCAAACATAGAAAATATAG
TACAAGAACTTCTATTTTATTTGCTGCAAATAGTTAAATAATTCTAAAGAATTACGTTTTTATTGACTGG
CTTGAACTGATAAATATGCCTGCATTTTATTTACCAATCATTTGTGGTTCTCACAGTTTTGTCTGACGTT
CCTTTCTACTTTTATTGGCGAAATTTCACAATTTATTTAGCCATGATGGGGAACATAAAATCTTAATTGC
ATTAATAAGCAAATGACTTCACGGGGGGCTTCGCCATTTCCTCAGAGAACTTTCCCAGCACAATTTCCAA
GCGAGAAGGGAAAATGAAACGAGGGACATGGCAGCAAATGCATCCTCGCCCGCAAAGTGAAATGCATAAT
GAGCATAATGTTGGCGCTGCACGACGCAGATTCGGTTGCAAATACATACATACAACTGTCGGTGagatgc
agatagcagatagcagataCGGATGATAATGATAATGCAGTGTTGCAGAGACGACACCTGAGAACCGACG
GGGAGAAATTTGAGAAATGTGAGCAGTAATTAAATGCAAAACTTGAAGCTTGAAGCGCAAGAAGGAGAAC
AGCGACAACGTCAACGACGATCCAAGATAATGCATAATTGCAGAAGACAAAAGGAAATGCCAGCGACTCA
TGAGCCAagttgcagttgcagttgcacttgcagAGTGCACTAGCATGCAAAATATGAGAAGAATATCTAA
TGCCATGCCACCGCATTCTCCATATAGTCGCAAAATGGTAGAATGGTTGTAGTTCGCCAACTGCCACATC
CACGTCCGCATCCATCCTTGGCTGGCTCAAAACGGCGTTCAAATTAATGGCCAAGTTTCACATGGGGCAG
CAAGAAGCTTAGATGCCAGCACAATTGCCAATGGAAATTGCAGTCATCTCACATGTGTGAGCAGTTTCTC
ATTGAgtgtgtgagtgtgtgtgtgtggatctgtgagtgtgcGCCTTCGTTTGTTTGCGAAAGTTGAGTAC
AAGATACAAGTGGTTAGATACAAACTAGAACAGCAACTGTTTGGGTACGATGATGACTCGTGTTTATCAG
TCACGTCAGAGGAATACTCTCCATTTAATAGTTAGCCACAAACTATTATTACGATTATTAAATAATTTAT
AGATACTATTTTTTATAATGTTTATAGCTCGAAAAGAGCAGTTTCTTAACGCATAAATCtttttttttt
tatacttttttttttATAGAGATTGAATATCTGTGCATTTATTTCTGTGCTTTAACTTTTAAGTTCACTTT
AACGTTAATTTACTGATTTTAATTATAAGAAAACAGTTTTAGGAAAGCAAGGCTGTTTAAGCCAAACCAT
TGGTTATGTCACTTGTACCCCTTTGTAGAGCATCAACAAGCATAAGTTCTTGTAACGAAGATGAGTTGGT
AAGAGTGACTTGCGGGCTTCGTTTTTCATTTGTGTTAGAACACGGACTCGCAACACGGTGGCAAAAAACA
GAAGTTGCCACTGGGAAGTTAACTCCATGTCATTCACCAGCCGGCGGAGGAATGTTCAAACAACATTCAA
TCAGAACAATCGGCAAGAACAAGGCGGAGCAGTCAGCGCTGATACAAGTCTAGAGATCGAGATAATAAAT
TTACTGCCAGCCACTATATGCTTCGCCATTTATAAGTTTCGGTTCGCCCCAAAAAGCAGATACTTCCACT
TATTTCACTTTCTAGCCACTGCCACTAGCAAACACCATTCTTTTGGCCAAAACACCTGCTGTTGTGCTCA
GCTTTTTGTTTGGCTAGTTAAAAGCAGAAAGCAAGAAACTTTATTTGGGTTAGCGATTGTTTAAAAATGC
ATAAGGATGTGATACTTGGAGTAGCTGAAAGGGAGCACAAGCCCGCAGGAGTTTGGCCAGAGGAGCTACA
AAGAGCCGATGGCGAAAAGGGGGGGATCAGTGGGGGGATACAACAGCAAATGGAGCTAAAATTGCGGTGC
AGAGAAAGAAATCTTTAATTGTGCATTTTAATCTGCGAACAAAATGTATACAGAATATTACATTGTTTTA
ATACGTGGGTTGTATTGGTTTCAAGTGGGTTTGCTTGGAGAAGAAGTCGTTATACCTAAAATCAAATCAT
AGTTCTATTTTCTTACTGTGCAGTAAATGTGAATCAAAGCTAAGACATCTTAGCCACAAAATGGGAAATC
CAAGTAGCTGGCTTGAAAAGCCCTTTGCAAACCGCAGAACAAGAAGTTGGCACACTTGCAATTTCAGTTA
AATTTGTCTTAGCAGGTGTAGGGATTTTGGCACATAAACTTTGACATCATAAAGCTAAACTGTCTTGAAT
TTAATATGCAAATCAAGCGACGAGCTTTGCGTTATTTAAATCAACTGAACCGCTCACCCACTCACTTGGC
GGCATTCACTGGCAAAAGTTTTTAAATTAAAAAATTATGGTTGGCCAAGTTAATGGCTTTCTCGGTCAGA
AGGaaaaaaaaaTATAGAAAAATGAACGGCAGGTGGGTGCAGTTTTGCAATTTGAAAGAAGAGTGGGCGA
AAGTGGTTCAAGTGGTTTTGTGATTGCGCATGTAAATGCAATTTCATTAAATTGAATATTTATCGGTTGT
CATTGAGCAAATTGCAGGTAAATTGAGTTTTATCCCAATACAACCACACCACCTAATAATCACAGTAATG
TGTGTGCAGTTATTCCGTCAAGATAATGGACGGAAATCATCATTAGCTGGCAGCTCAGGGCTATAAACAT
AGTATTCTAAATCTTAAAATGTGAATATATCTATGTATATTGCTTCTTTCTCTCTGACAAATTGCAACTA
TTTTTGTAACTTCTAAGAATAATGCTCAATGAAAAGAACCGCTTGAACCGGTTACCAGGGAATTCTAGCC
GCACAGAAGCCGTTTGATGAGCATAAATCTTGATATCCGAACATTATTAACGTCAATTAATTCTAACCGT

TCGAAACGAAGGCCATCCATTAGGAGCTGGATGGTGGAATTCGGCAGGGGGAGTCAGGGAAAACTCACCT
GGGATGGATGGATGGTTAGTTGGCATTTCTCCGGAGACCCTCAACCTGTTTTTTCTTAGttttttttttt
ttGTTTATTCGCCCGCTTTTCTGTAATCGATACGAGTGGCTCTTCGGTCTATTTGTATTTGTATTTACAG
CGTTTTCCTGGTGGCCCGCTGTTTTCAGTTTTCCCTCTTTTTCACGTTGTCGTCCCGCCTCACGATTTCC
CAAATCCGATTATTATCAAGTCTCACGTGAGTGCACGCTTTGTTGCTGCTTGCTGCTAAGtttttttttt
tatttttattttttaCCTCTGCTCTCGAGTATTGTGCCTTTTACAGTTGCCTTCTGTGCTTCCTTGGAAAA
TTGCTTTGAACTTGAAAAATTCCCACGTGACGCGGTCAGAATCAATCTCTGCTTCTGGCTTTTCTTTTGC
CCTCTGTTCGTGTCACATGTGCCATGCAACACCACCACCCCTTTGCGAATTTTCCGCCAGTGTTTTCCGT
GCACGGAAGGCAGCTGCACTTTGCATGTCATTCGGCTAACGGTGTCGTCTTTTCCTCGCTTTTCCCCGCC
TTTCTGCGCTTTTGTTTCTTGGCTTAAAGCTTGCATGGTTTTGGTTGCATTTTCTCAGTGTTTTGTGGCA
AGTGTCGATTGAATTTAATTGGATGGTTTTTTCGTGCATTTCATATCTTTCGAGGGGCTAAAAGGCACAT
TTTGAATTCTCAAACTCGTGGGCAATTTCATTTATTGGTTTGTAATTTTTGTGGGAATTTTAGTTGGTTT
CAAATTGGAGCTAAATGAAAGGATTGATTTTATGGTTGAATGCAAAATTCATTTACAAAGGTAATTCTCG
AAAATGCAGTCTTGTTACTTCCATAGTCGTGGTTATTAGTCTTCTGAAATGCATTCCAACTATGGAAAAA
TGAATTGCTAGTTTGTTTTTATTGCAGAATAAAACTTAATAAAATATGCAGCCCCGTTCCGGAGTTTCCA
TTAGCACATCTGCTGCTCTACACTTTCTGTACAACCCATTCGTGGCACTTAGCAGATCTCGGCTAGTCTG
GCAGCCCATCCATTGCACGGTTAAGTGTTAGTCAGTAGAAAAACCCAAACAAAGTGATTTTCGTGGTAAG
CTATccccccccccccTTTTTGGCCAAAAGACAAAAGGCTGCAGATGGCACTCACTTTGCTAGCACGTTC
CATCGAGGGGAAGTGCATAGTTCGCGAGTCGTAAACTGGCTTTTTTACGATTGGTGGCTCAGAACGAGAC
TTGGCCAAGACCGGTGGCAATTAAATATAAGTTAACACTTGGCTTTGGTGGGGGGAAAGGGCGACAGTTC
ATAGATGTCAAGTTCAGGGTTACTTCAAGGGCTCGGAAATTACCTGTCAGAGTCGTCCAAAGACAAAGTG
TGTAATTAATTTGCAAGCGTCCTAATCCATCAAATCTTCTGTTTCCCCTTTTAAAAAATTCATGGGAGTT
GCGCTGGGTTCGGTGGGATATTCCTTTTGATTTAATGAGCTTTTTATTGCCTGCTCTCATAGGATTGAAT
TCTTTGTGCAATCGGTTCATTGAGCTCTGGTCTAATTAATGGTTCATTAAATCCGCACTTTGATTAGGGT
TCCTTGGGTTTGCTTTAATTTAACAGTAGTTAATTACGGTTCTAGATAAACGTTTCAGTTCCGAGTATTT
CCTTTAGCACGCAGCATGTACATACATACTTAACCATATTGAGGGAAATTTGTAAGACTACAAGTTTTTC
TTAAACTACTTTACTGCGATAGTGATTATAGTGATTTATAATTGCTAGTTCATTGAAGTTTTAGTGCCTG
CTATTTAAGTAAACACCGTTTGAATAACCATTTATAAGGCTTTGCATCATCCTCAGTATAAGCCTACTGT
GCTGTCATATGGTTTTTGATGACGATGCTGATGAAATCTAAGCGGCTTAGGTTTTAGAAAACCCCCAAAT
GCAAAAGGCTTCTACTGCCAACTCCCAAGTGGCTGCGTAATCAAATGCATGTAATTTGAGTTTAATGAGT
GTCAAAGTTATTAAAAATCATTTGCTCCGACCAGTTATGGTGGCATTTTCAGCCAATGGCTTTAGCTGTG
GCTTTCGGCCAGTCAGCGGGCCAGATTTTCGGTCCATGAAAGGCAGCAGCTAACGCAAACATTACAAAAT
CGTTTCGAGACATTTTTGGGTTCGTCATCGGGCTTAATTACTACGCGGCCAAGCTCATTCTCCCGCCAAC
GATTCTGGTTAAATGAAATGTGTCAATAAATTCAAATGGCGGCATTCGAGCCGAGTCCAAAATTGAGTTA
TTAGAAGTAGGCCGCTAAATCGATGTGAGATCTTTTAATAAACTTGTTGTGTTGTGTGTCCCAGACAGCA
ACGGCAGCAACAAATAGCAAAATAACAAACAATAATTACAGACGACAGTGTCTGCCATAAGTTGTGCCAC
CAGTTTGGCGATACTCGGCGAAAAAATCACTTTAAAATTCTTGGAAAGCCAATTGTTTATATAACTTAAT
ACACGTTTCGAGTTCGTGGATTTGATTTGTTTATTCAAAGACATGCCAGCGTTTCCAACGAAATTAGGCG
AATTTGTTATGGCTAACAAACTATGAAATTGTAGATTTTGCTGTCCTGATTCATACGTTTCGCAGTGGCT
GCCAAATTCGATAGTACATTTTAAATTCGCAATTGATATACAAGTGCGCACCAAGTGACTTTTGGTTAAT
GGTTAATGGCTGGCGGGCAAGTTATATACTTGGGCAGTCTAAAGCCTCGATAGCTGGCTTGTATTGGCAG
TCTTGGTCTGCGCCTGAAATTGCCAAATGCAATTTTTATGCTCTTTGACGTGGGCGTTGCACACCCACTA
CCCCATCACTTTCCCCAACCCTCCTACTCTGATATTGATTTGATTGTGTGCGTCTGCTGCTCTCGGTTGC
TCACATGGCGTATGATTGATGGCTCTGCGGGCACGAAGGAGCTGCAAACGATGGAGCATTCCTGCCTGAG

CTGCTGCTGCTGGATATATAAATTGCACTTATTCGGGGAATTGACAGAAAAAGTATATGCACGTTAAATA
TAAATTAATCAAGTAGAAGATAAGTGGAGGTTGAATCTGAGAAAATAATCGAGATTAAGATGCCGAGTAA
CAAGAGATCCACTAAAAATTAAACCTTGTTAGAAGTGATTTTTTATGGCGCTCTTGCTATTTTTCAGTGT
AAGAATATAAATAAATATTGCTCACAAATAACCAGTTTGTTTTAATTATTTAATTGCAGTCACAGCCACC
ACATGTCTGGCCCTACAGTTTGAGCAGCACTGGGTTCTAAGCAACGTGTGGGACGAAGAGTGAGAAAGCG
GTAAGTTAGGCGACTCGTGTTACTTATACAGCTAAACGCAAAACCCATAATTTGTTCCCCATTGAAACGT
ATATATCTTATATCTTGGAGTCGTTACTTTTTTTATGGCCGTGTTTTCATGTGTCCATCTGTAGAGTTGT
CGGTTGTCATTTGTTGTTTTGGCTTTGAGATTTGCACTGCATTCGGAAACCGGTCGAATCGGTTTCTGTG
GTGGTGATCACAGGCATTTGCATAAAGTTTTGTTCTGTTTTTCTCTTTAATCGGTGTGTCGGTGGGATTT
TTCTCCAACCGCTGGATTTTAATTTATTTTCATTGATTGCACATATACAACTTTTGACTTAATGGCTCGC
TGACGTAACTCGAGCTGCTGAAATTAGCAAAATAGTCGTAATATTCACCTCATGGATTTCTACGACTCTC
TGCAGGATTTTTAGCAAATTGTGAAATGGTGCGATTTAATTAATCGATCTGTAAGTTTGGTTAATTTGCT
ATTGGATTTACAATTTGTTAAAAAACGCCGATGGGCGTTATATATGTGATTTTAGTTGGTGGCTAGTGGG
AAATGAACACCTGCAAAAGTCGCGTCAATTGAATTACATTATTGCATTAATTTGTTAGGCTTTCAAATCT
TTTTATGGCTTAACGTAAGCttttttttttAAATGTCTTCTTGGTTTCATTTGAACTTTCGCCATAAAC
TAGTTTCAAACTATGAGCAAACAATTCATATGAAATAAAAGCCAAAATGTCAATGGTTTATTTGATATTC
TTCTCGATTCCCTGCAGATTTATCTGATTCTGTATTAACTTCTGTTTCTGTTTTCTGGTTCTGAACAGGA
AGTCGATGGCTGGCCACCTAGGTACATAAGTGGGTTCTGTGGTTTTTCGGTGCCTCGGTTTCAGATGATT
CGAAGGTAATTTGTTGCCGGTCCGTGGGCCTAGACAAAGGACTGATTGCCGGACTTGACTGACAGTTGGG
GCAGCAAAGGGTTAAGGTAGTGGCCACACACAGTTACGCCCAAACTCAAGTTAGGGCTAATTTTGCCATT
GTATTAGCCAATTGTGGTGGCTGTTCCTCGTTTCCATCCTCAGGTTTCCTTTAGCTTGCGATTTTTATTT
CAGCTTCAGTTTGTTTGGCCAGCATAATGATGCCACTTGATTGAAAACGAACGTGACTGGAAAACAAGTT
TCGGGGTTAAAGTTTTCTTCTTTTCCTTTACTCTCTCGGCTTCTTTTGCCACTCTGCGGTCTTTTCTACC
AATTCGAATACTGTTCGAATAAACAGAAAATAGCGAAATAAATTAGCCAGGAGATGCTCTCAGCCATGAA
CTTCTGTGACATGTTTTCCTGCCTCAGCTTTATTAGCCGACCGAGGACGAGGAGCCAGGCGAAATTGCAT
TTGGTATTATTTAATTAAAGTTTTCTCTCCATCGTCGTCGTCTTCTTCGTTCTGATGTGGTCCGGGCGCC
ATTTTGTTGCATGCTTCCTAACAGAATTATGTGCGCATTTCCGTTTCCGGCACATGCAACAATGCACCGA
GAGCAGTCAGCGCGGAAAACGGAGTCTTCGATTTAGCCCAAGAACAGTGGAAGTaaaaaaaaTGCAATGA
AATCTAAAATATGTCTTATTCTTAAATTACTTTTAATAGTAAATTCATTTCAAATTGCTCTATTAACATT
GGGCAGAGAGGAGAGCTGAAAAACATTCTAATCGTTTGGAAAATTAGCTTTCTCCAAATTAAATTCCGTT
TTTGATTAAATCCCATTTATTCCACTGTCCACAGCGCTGGATGGTGGAAGAATAATAGTTTGCCATTATT
AAGTTTGGTGCGCGGGTGTATTTTGCATGCGCTTTGTTGATGCCGCCGATGCTGCTTCCGATTTTGCTGC
AGTTTGCTGCTGCCGTTGCCAAGGCAAATTATTTACGGTGCGCACAACCAACAACTGAGTGTGGCACAAT
AAGTGGCTACGCAAGGACCTGGTCCTGGCACCCTTCGCTCGTCCGTAAACCCATCACAAATCTCCACAAG
ATTGAGCGGCAAGCCATTGCAATGACAAAAGTAATTTGCTTTTAAAGCACCACCGACTGCAGCATCGGGC
GAAGGCAATTTAAATATGCATGGGCCATTATGCATAAGCGATGCAGTTGCTCCATCCACGGATATTTGTG
CACAGCGGGAAAATCGAGGTATCAACATAATCCAAAATGGAAGAAATATGGTCTCAGTATGGATTATTTG
TAGTAGAACAGAATATTACAAACCTGTGTAGTGTGGCGAAAAGCTATCAACTGAAGTGCGTAATATGAAA
GTGAAAGCTATCTTTTCAGTGTACTCATACTTACTTATGGGTGTCGTGTGTGCCACATGCAACTCCAGTC
GCATTTAGTTGCGCATTCGAGCAAACCCATTGGGGCTCTCCACTGACCCACTAAAGTCGCGCCCGCCCGT
TCGGTTCACGGACTCCAGGGACCTGATGCTGTAACTAATTCCAAGCAGAGCCCATTCGATGTGGCATATT
TAAAACTTAATTTACATGGCTGTCCTTTGGCCGGTTAAACCCTAACCTTTAGCCCCTTCATGTTCGTTCG
TGCTGACCCCTTCTCTTTCTGGCTTAAAGTCTCGCTGTGGCAGCTGTTTCGAAGCGGAGCACTTTGTCAT
TTTCAGTTTGTCACACTTTTGCCCAAATAAAATTTCATCGCCTGTCATAAAGGCTTGAGAGGCAAGTGTC

ACAAGTGCTTAAAACTCAAGGAGGTGCATGGCTTGAATATGGGGTGGTGGCGAGAGTGGGAGCGGGAAAG
CAGCACAGTTAGGTAAGAAGTGAAGGAGACAGCACTCAAAACTGCTCAAGTGGGGCAGACCCaaaaaaaa
aaaaaaaTAAGAGAAATATGCCACATGAAATTCGTCATGAGACAGAAATCAAGAAAACATTTATATATTA
TGAAAGGAAATACATTTTAAAGCCGCTTGCAGAAATGGAATATGTTAGCAAATTTCAAAATCTATAAATT
ATATTTTAAAATTGAAATGGGAATACACTTTATTTCAAACTTTGCGGCTCAATTGATGTAATACGTATTT
TTTAAGACGGCTTCTTCTTTGATGTTTCCACCATAAAACAACCTCATCAACACCTTGTCTATCTGCTATA
TTTATCTCTTATCTCTTATATCACTGACTGCAAAACCAATTAGTGTTTACCCCACCCAATAAATATGCTT
GCCGCTGTCAACGATTGATTCCCTCATAAAAGGTGGGGGAACCATAAAATTGCTCTACCCTCCCCAATAG
ATAACATGCACCTTATGCAATCAATGCTTCCTGAGAGAGTCTGGCAAAAAAGTTTGGCATTAGCTGGCGT
ATCCAAGCCAAATAACTGTCAGGGCTAagacaatagaaaggacagacaacagacaggtcagacaTTCCCG
GGTTGATATGAAAACTGGTGTGTGGGGGTAATGGCGATCCAAACAGGGGAGCGCACCAAAAGTTGCTAGT
TTGACAGCCGCAAGACAGCTGTCTGTTTGaaaaaaaaaaaaCCCCCACATATACGAGGGTTAGCATAAAA
AGAAAAACAGCAGCTACATAAATTTTGATTTACTTGCCTGACAGTTGCCCCCCAAATTTTCACACGCACC
CCACCACAGCCACCAAAGGTCGGGCACAATTTTCCTATTAGCTCAAGTGAAAAATCGTTGCAAAATCGGT
GAATTGGGTGGATGGGTCTGAATTTACTGACCATTAAATCAGGCAGGCATTCTTCCATATGCCGCAGAAC
ATATTTCATGGCAGACCAGAGAGTAATTCAAATGCCCATTATGGACCAGTAACTTTTCCAATTAAAAGCA
AAATGAACTTTTGCCTCCGGAACTCGCTGGCCGAAGTTTCAATTAACGCCATGACTGGCTAAGCTGGAAT
GGCCAATAAAAAAGGCCATCGAATTGCGAATCACCGATTTACCTGTGCGCCATTTTTGTCTCCAAATTAG
GTTGAATGACTTTGAAGCGACCACGTGGCGTATGCTTAATTATCGCAAATCGTTTTCAATTAAAGTGCCA
AGTTAAAAAGGCACGAAATCTTAGCAAACGCCACTTTTGGCTTATCACTCTTACCACTATCACTATTAAT
TCCAATGCCTTGGCCAGCTTTGCTTATCACAAACAGAAGTCTGGCGACGACACTCGCCCTCGTGGGtttt
tttttattttttCGTGGCGTCTTTGTTGATAATTTCACTTCAACAGCTTGAAAATTTTGTGGGTTTGATT
GACAGTCGAGTGGCGGCCAAGGTAAACGGGCCTTTTGTGCCAGCGAGTCACCTGTTTAAACGCAGTTTTC
ATTTCTCGTATTTACTTTTATTGATATGCGTATTTGTGCTCTTAATTTATGTATTTCGGTGGAGTTTTTT
GACCGTGACACATTTGCTTGTTTACTTTCCAGCGAGGCGGTTTAAACGGGGGCAGTGAACCAGGGAAAAT
TGGAGCTGCATATTaaaaaaaaaaaaGAAACCGAAACCACTGATAACaatattgtttaaaatttaattat
aaatattaatattaataaatattaagtatagatatgTAATCTTGATTCTTTATAGTTTCAGTTAAGTTCG
AAGGTATAAGCACtgttcacacatgaacacgaatatatttaaagacttacaattttgggctccgttcata
tcttatgtaaatgaatcgagagcgataaattatatttaggattttgttatctaaggcgacatgggtgcat
tgctcaaaaacatgtaatttaagtgcacactacatgagtcagtcacttgagatcgttccccgcctcctaa
aatagtcccttagtgggagaccacagataaggtcctcgccgctcaagataggcagatgtgcccgagcgtg
ggacctcgataaggcggggactatttacttaggcctctgcgtaggccatttactttaagatgcgattctc
atgtcacctatttaaaccgaagatatttccaaataaaatcagtttcttacaaaaactcaacgagtaaagt
cttcttatttgggattttacatttggtcaatcgagcctttaatcgactctgcagtttcccccctaccaaag
gtaaggaactcagagaaaggccagctcctttaagcatcttacagctaaaggtagcaaaaataagtgactc
ttgtttcccccctaccaaaggtaaggaacagagtataaatataaaaagcaaaagatacaaaagaatctttt
atgttttaaaacaagcaccttatagtctatagctaaaggttgctttgtgtaccattataaattgtggtaa
ggcgtgcttgaggccatacatcagcaattgtgaaattaaaaagtgcataacaaaagtgccttataaatgc
tctaatagcattaaatcagctcataaatagagtgcagtgtataagttattgccctgcaattgattctcta
acatcttgtggttccacatagtctccgctgccatcaacgccaacgaacggttaagcgcgacatcgacact
tctgcgctgcgccgcggccgacgcctgctgcgccgctgccgacgacttcacttgattgctagggacttag
ggaaacattttgtacgctagatttagtttcaaatgataaattgcaataaacggtcgcttgcgatcttcaa
aatcaaatcgataactgtaattattaactggcgcccgaacagggaccagcgaataaacgcgaacgaaaga
caaaattctaagtcgcggagcaaaatcaaaattttgctaaaaaatattcgttggttaaattgtgccgaag

aaactcccgcgagttattaacaaacaaaattcacggtcggctataaaataaaattgtttgagaaaaaaac
agattttccggaagaggaaaatacttatcggcatagcattgcccattggtggatcacagtttctgtcagg
ccagcccgcagaaaaccttctttggagtgctgacgtggattccccagtagcccaggcaaaaaggacaaca
ttcacccagccaggaggagtgaccgcaacaattctatgttaaataaatgcaaaacgaagaacagcaattt
catcgaaaagtagaagtagaacgccgagaataacggaactacagaagagcaacacgggtaacagttaaca
gtgcaacccaaaacgtaacggcagaggtaaagacgacgaacggcagagaacggctgcgtgaaccaaagtg
cagacaagaaggaaaaaaaaaagaacaacaacagcacagccgtagcagcagcagcagcccgccaaaaaga
acaggaataagaacacgttgacgacgaagaagctgaagctgaaacaaccgaagaggaggcagagagacta
caagaacctggaaagtacactggaaaaaacatcagcaatttgaaattcaggcatccacccataataaaac
ataagtataccaaaaaaaaaaaaaaaaaaaaaaaaaattttaagtatattattattattatattattatta
taatattattattattattatattttttattattatattattagtattatattattattattatattatta
ttattgatattattattaatactatattttcaacccagttcctagagatcttctgaaaggaaaattttcc
tatttactgttcctttctggtacactgttctcaaagcaaaataaccgcggtgagctaaaatttattgcat
gcaaaaataaaaaataaaaaaatataaaaataaaaaataaaaaaacaaaaacaaaagataaataagcaaa
cacatacacacgcattccatattttctgcccacaacttttgttaagttcaaattggtttaggcttgtttt
gtgcaggtgttgtccgaattaaaaatcagtatggcaaatcctaataatataataagaccatcagcttta
gttcaaacgaaagaccggtgccctcagaaagacacgacctgcctgaacagcatcgcgtagacatgagtgt
cgaacagttgacagcattaattggccaaacggtggcccaagttttacctggattgataaaacaaatgaac
aacaatactcttgactttactgacgtgagcgaccaggtcgtcgagcccgaatacagaaataatttagcgg
actttgacagggtgcctgatatcgtaaaatcgatcagggaattctctggaaatccagcagaattcgggtc
ttggaaaaagagcgttgatagaatcatggagacttataccccatttgtgggtactccaaaatattatggt
atacttcataccataagaaataaaattgttggaagtgctgatgtggcactcgagtcctacagcattccgc
tggactggaaggccatgtcgagatgtctcactctgcattacgcagataaacgggacataactaccttgga
ataccaaatgtcaactttggttcaaggccaccagcagtcggtggaagacttccaccaagacgtctacaaa
aatctgtcgcttattctaaataaagtcggctgcatgcaaatgagccgagaatccgagcactttgttacaa
aaatgtacagagaaaaagctctggatacttttatcagaggccttcgaggagatttacctcgccttttggc
aataaaagagccagctgatctcccctcagctttacattattgccttaaacttgaaaatcaaactttcagg
tcaaaccatgcggcgaataagggtcaacagtcgagtttcagaggaaacgaaaaacccattccggcacccc
gcaattttcagccgtcgaatgcctttggccatcgacagcctccaccggtccctccacgacatcccatgag
gagacctccacaatatttaggccagccattcccggcgccaaggcaacacgtgtctaacttcggtaatgca
ccgttcattccaccccgacaaaactatcaacagcaatggcaacaatacaatgccccaccccgtccctttg
cgaccaaaccacaaccaagaccagaaccaatggacgtggacggcagtgtacagacgcgaaacattaatta
tatgaatagaccacactcggacataaacaaacgacaaaagaactacaacatccaaacagtgaacatgaga
cagccaagtattgaagtaactgggtccagttcaagcatgacaggctatcaacggtcgatgcaagattatg
aaacccagaacaatataaacggcacactaaatgaatactgtgatggacaaatagacaatttggacagcga
acaacgagaccacgtgttgcatttttttagagtagaagactcctcactaccatacttcgaatgtagagtgg
ggagtggaaaggttttaagggtgttgattgacacaggctctaataaaaattacatccagcccaatttggt
gacgaacgcgataccaaataacaagccttacatagctgatactccaggcggtgatgtaaaaatatcgcat
tacaaaagagcaagccttttcgatttcgaaataaaatttttttgttaccaacgctgaagacttttgacg
ccatacttggcaaagacacaatgaagggaatgggagcgcaaattgatttgaagaacctaaccatgacact
ggaaaacgggaaaagagttgttctcaaagaaaaacagttcgaggctgttagcacaattagtccgagaata
aaacacttagaggtggaacagagaatgatattgaataaaataattgattcgtatccaggtctcttcgcag
acccgaatcaaaaactaacctacacaacaagtgtaagggcagcaatccgaactatatcggatacgccaat
atactcaaaattctatcagtacccaatgtctcttaaagacgaggtacacaaacaaatttccgaacttta

cacgatggaatcattcgaccctcaaggtcaccttacaattcaccagtgtggattgtaccaaaaaaactcg
actcctctggcaagaaaaaaatacagggtggtaattgactatcgaaaacttaacatggtaacggtagcgga
cagataccctatccctgacattaatgaagtgttggcccaattgggagacaacaagattttctcagtgctc
gatctgaaaagtgggtttcatcagattcttttaaaggaatctgatatcgaaaagaccgccttctccatca
ataatggaaaatatgagtttacacgactcccattcggtctgaaaaatgcaccgtcaattttccagcgcgc
actggacgatattcttcacgaacatatcggtaagatatgtttcatttatattgacgacatcatcatcttt
agtaaagatgatgaaacacattaccagaaccttgacactattttcagaactcttcagcaagccaacatga
aatgtcagttggataaatgcgagttcatgaagagaaaagtggagttcctaggcttcgtcgtgtccgacaa
gggcattgaaaccagcccaaccaaggtacaggcaatctcagactttccaattccaaggacactcaaagaa
ctgagatcattcttgggattatccggatattacaggcgatttattcctaactacgctaagttagcaaaac
cacttagctcgcttttgagaggggaggatggtcgaatttccaggacattatcgtcaaaaaaaatccgtctc
ccttaatcgcgaagcaatagaagccttcaagaaattgaagagcagcttggtttctccagacgtaatactc
cactacccggactttaagaaagaatttcacctaacaacggatgcttccaatttcgcagtaggtgctgttc
tttcacaagagaacagacccatctcatttttatcgagaacactctcgaaggcggaagaaaattatgccac
gaatgagaaggaaatgttagccattatctgggctctaaaaaagctaaaaatttacctttacggtaaagca
aaggtgaaaatctttactgaccatcagcctttgacccattctctcagtagttggaatggaaatgcgagga
ttaagagatggaaagcataccttgaggaatatgactacgaaattttctacaagccaggcagagaaaatac
tgtagccgacgctctgtccagaggaccgacagtcgaacaaattaacacagtagcctcaacaatgcacagc
tctgacagttcgagccatgggctgatacctagcgttgaagccccgataaacgcattcaagaatcaaattt
tcttcagggagtccgagtcagagaactactcatttagcattccattcccgacatttcaaaggcatttagt
agatcgcaacttgttcacacccgatagtctcttgtcagatttgaaagaatatcttaacccatccgtgatt
aatggaattttcacatccgaggatgtaatggggaaaattcaaattctctaccccatccattttcagggtt
tcaagattagattctgccaaagcaaagtcaaagaccttgttaacgaagccgaacaagaggaagaaatact
taggacacataacagagcacacagaaatgctgtggaaaataaagctcagttgtccgaaagagtatacttt
cctaaaatgaggaaaaaaagtttcggctatcgtgaatcagtgtttggtgtgtaagactgctaaatatgaca
gacatcccacgcatccagaaataagacaaactcccttgccagaataccccggacaaattattcatattga
catctactcgacagaacgacatctggtgctcacggcgattgataaattttccaaactggccatgggaaga
gttatcaaatccaaagctgtagaagacattaggaaagccctaagagatatcgtgttttattatggagtgc
ctaaattaatagtaatggacaacgaaaagtccctcaactcagcctctatcaaattcatgttgacagacca
gctgggtattgagctctacaaagcacctccgtataagagtacagtaaatggacagatagaaagatttcac
tccacactctctgaaataatgagatgtttgaaaggagatggaacacataggggcttcgaggaacttctcg
atagggccatctatgaatataactacactgtccattctgtcacaaaaaaacgacctctagaggtgttctt
cggcaggatagctaccgtagctccagaaaaatatgaacaagctagactggacaatatagaccgacttagg
caaaaacaggaaaccgacatagaataccacaaccggacaagaaagcccataaaaacctatatcaaagggc
aagaaattttcgttagggttaatacaagattaggttctaagttatcaagtagatttaggaaggaactagt
taaggaagaccgaagtaccacaatattaacagaatcagggaaattagtacataaaagtaacataagatca
tgattgcttcagaataattactgtggcccacatcgatagacaagtagaacatagaaacattaaacatactg
attaacgtaaccacgagaattaaatttgaaacgattcgactttgacagacgagttggccataggtttgca
gaattagattgggctcgtgaaagaagagattgcgttgtgaatgcgtttctgcggagcgagtttaaattga
atgagaccagtagcctacttgaaaacaatataccgatttctgccttgacggtcaaataaatgttaaaact
ttcacacgttctcgctttacacaacggaactacaatttgaatgccattgaaatctccatattgaatcagt
taattccttatttcttatattttaaaacatgatcagtaaaggaaactaataataataaataaataataat
ttaatttgaataataataaaatttaccaattaaagacgaaatgtacggaatcaatgccttctgcaaaggt
tccaatggtctaaaaatatgtagatataaattcattagtaatttagtaaaaaccagaattttaatagaag

caatcaagttgtcaatgcagaacacatccgagttgcacaaacctcgaacgcacgcaacaataaacacctg
gcattcaaaccattaatgctgtgcccaaactaccacgaatcaccccaccaacgatgcgactcaaaaacat
cccatccttatgatgaccgctcgaggacaagcgtcaattaagaggggaggagttattgccctgcaattga
ttctctaacatcttgtggttccacatagtctccgctgccatcaacgccaacgaacggttaagcgcgacat
cgacacttctgcgctgcgccgcggccgacgcctgctgcgccgctgccgacgacttcacttgattgctagg
gacttagggaaacattttgtacgctagatttagtttcaaatgataaattgcaataaacggtcgcttgcga
tcttcaaaatcaaatcgataactgtaattattaacttatatgccataagagcataaattaaataaaaagt
gcctgaaaacagtgccttataaatgctctaatagcattaaatcagctcataaatagagtgcagtgtatat
gccaaaagagcataaatgccgaaataaatggctaaaaaacaaaaaatctgactggactacaaaaataata
aaacgtgccaaaaaaaaaaaaaaaatcatctttaaacatcgacggagccttaaagaagagaaggaagtca
aattcaaaggagcctctaccagcagcagaagcagcaacaacagcagcagcagaagcagcaacagcagtag
caacagcagcaacaacagcagcaacagcagcagcaacaacaacgacatcagctaagtcaaaacaagaatt
ttctgtttatccaaacacacatatatatataaatacatataaaatacatatacacgtactatatatatta
agaaattacaaaaaattttcaaaatgatgtcagaaaagactattcaattccttaagaagcagtccgaaat
tattttggaaattagaaagttggaagtaaaaccaacattaacagatgtagaaattctaaaattaaatgag
cttcaaaaatgtttcattgctaatcatagcaatttgttaaagatcggcgttgtcgatcatgaatatttta
acgcgaagcagtatgatttaataatgatggtgttagaaaaaattaaaaataaaaatgaaaaaattaaggg
cgagtcggtagaaaacactttccctaaatcaaacactgtccctaaatcaaaccctcccccctacattaaac
cttgaaatgcgtggtcaccctgaaaaagagggtatagcacaaaacaacgctttaaaagtagagcaggcat
ttcgtaataatgttggccaatttcgagtatatctagaagatacgtctaaactaatagacagtagtccaga
tttccttaaaataaggaaaaataaaattgaatttttatggcataaaatagataacctgattgaacaggtg
aatagtcattttgagagttcgctattcgaagaagaaattagcgaacttgaatttgacaaacaaaatattc
ttacagccattaatagtcgactcagtggcacaataaataaagctgaaatgtcgacggttgttaaggcgga
ggagttaccaaccctgcctaaaatacagattcccaccttctttggtgattccaaagaatgggatcttttt
aatgaactctttacagagctcatacatgtgagagaggatctcagtccttctctcaaatttaattatctaa
agtcagcattaaaaggagaagccagaaatgtggttactcatttactgctcggctctggagaaaattatga
agccacttgggagtttttgaccaagcgatatgagaataaaagaaacatattctcagatcatatgaatagg
cttatggatatgccaaatttaaatttagaatccaataagcaaataaagacatttattgacacgattaacg
agtcaatttatattataaaattaaaggcacaattaccagaagatgtggatgcaattttcgctcacataat
tcttcggaaattcaataaagaatcactcaatttatatgaaagccatgttaaaaagacaaaagaaatacag
gcactttctgatgtcatggactttttagagcaaaggctcaattctatatcatcattctcacaggaagtaa
aacctgtaaagaaaatgattaataataacaagaataaaaattatagtgacaattgtgcatattgcaaact
accagggcattatttaattcaatgccataaatttaaaataatgaatccagcagaacggtctgactgggta
agaaaaaatgggatttgcctaagatgtctgaggcatccgtttggtaaaaaatgtataagcgagcagcttt
gttcgacttgtcgtaaacctcaccacacgttacttcactttgcaggtcataatccagaaaaagtgaatac
gtgtagaacaacaggtcaagccttgttggccacggccttgattcaagtaaagtcgaggtatggaggcttt
gaacaattaagagcattgattgatagtggctctcaaagcacaattatttcagaagagtctgcacagattc
taaaattgaaaaaatttcggtctcatactgaaataagtggagtatcttccacaggaacgtgcatctccaa
gcacaaagcggttatttcgataagaaattctccgaaaatttagaaattgaagcaattattctcccaaaa
cttatgaaggcacttccagtcaacacgattaatgttgatcagaaaaaatggaagaactttaaattagccg
accccgattttaataaaccgggtcgcattgatttaatcattggagcagacgtatatactcacattctgca
aaatggagttataaaaatagacggtctccttgggcaaaaaactgatttcgggtggatagtttctggatgt
aaaaaatccaaaggaaaagaaaccattgtagccacaacaatagaaataaaagagttagatcgctactggg
aagtggaagaagaagaaaaagatgatatcgagtctgaaatctgtgaaaataaatttatcaaaacgacaaa

aaaagattcagatgggcgatacattgtgtcaattccattcaaggaggatgtcaccttaggagattcaaag
aaacaagcgatagctcgttacatgaatctggagaaaaaactaaaaagaaatgaaaaacttaaggttgact
acactaaattcatgaatgaatacatggatttaggacacatgattgaagtgagtgatgaaggcaaatattt
tttaccgcaccaggcagtgattagagattcaagccttacgaccaaattgagagtagtttttgatgcttca
gcaaaaactacgaataacaaaagtttgaacgacataatgtgggttgggccacgagttcaaaaagatattt
ttgacattattattaaatggagaaaatgggaatttgttgtttcggcagacattgaaaagatgtaccgaca
aattaaaatagataataatgatcaaaaatatcaatatattttatggagaaattctccaaaagaaaaaatt
aaaacatataaattaaccacagtcacttacggaactgcatctgcaccatatttggctaccagggttctgg
tagatattgcagataaatgtaaaaaccaagttattagtgcaataattaggaatgatttctatatggatga
cctaatgactggagctgattcggtagaagaagctaataaattaataacattaattccccatgaattgcag
aaagttggattcaacttaaggaaatggatttccaacaattccaaaatattaaccactgtggaggacacag
gggacaataaggttctcaatattatcgaaaatgaatgtgttaaaactttaggactaaaatgggaacctca
aaaggatttatttaagttcagcgtaaattgtaatgatgaatcaaaaaatataaataagcgcgttgtgtta
tcaacgctagcaaaaatatttgatccgttaggatggttggcaccagtcacggtttcaggaaaactttta
ttcaaaaactttggataaataaaagtgaatgggatcaggaattatccatagaagataaaaattattggga
aaaatataaagaaaatttattattgttagagaatattcgaatcccaaggtggattaattcaaacagttct
tcagtcattcagattcacggatttgcggacgcctccgaaaaagcatatgctgcagtagtctatgctaaag
taggacctcatgttaatataatagctagcaaaagtagagtcaaccctataaaaaataggaagacaattcc
caaactcgagctgtgtgcagctcacctgcttagtgaattaatccaaagactaaaaggatcaattgacaat
ataatggagatctatgcttggagtgattccacgattaccttagcatggattaacagtggtcaaagtaaga
tcaaatttataaaaagaagaacggatgacattcggaaattaaaaaatactgaatggaatcatgttaagtc
agaggataatccagcagatttagcatccaggggagtggattctaaccagttgatcaactgtgatttttgg
tggaaaggtccgaaatggctagcagacccaaaagaactttggcctcggcagcagtctgtagaagaacctg
tcttaataaatacggtattaaatgacaaaatagatgatcctatttacgaattaatagaaaggtattccag
tatagaaaaacttatacgtataatagcatacataaatagattcgtgcagatgaaaacaagaaataaagcc
tattcatcaattatttcagtaaaggagataagaatagcggaaacagttgttattaagaaacaacaagaat
accagtttaggcaagagataaagtgccttaaaatcaaaaaggaaatcaagacaaataataaaatattgtc
attgaatccatttttggacaaggatggggttctaagagttggaggaagattgcaaaattccaatgcagaa
tttaatgttaaacatccaatcattttagaaaaatgccacctaacaagcttattaataaaaaatgctcata
aggaaacattgcatggagggataaacctaatgcgaaactatatccaaagaaagtattggattttcgggtt
gaaaaattcgttgaaaaagtatttaagagaatgtgtaacgtgtgcaaggtataaacaaaatacagctcag
caaataatgggtaacttgccaaaatatagagtgacgatgacattcccgtttcttaatactggaatagatt
acgcaggtccttattatgttaaatgttcaaaaaatcgtggccaaaaaacatttaaaggatacgttgctgt
attcgtttgcatggccaccaaagccatacacttagaaatggtaagcgatctaacttcagacgcatttta
gcagcactcagaagatttattgctagacggggaaaatgttccaatatctattcagacaacggaacaaatt
ttgtaggagctgcaagaaaattagatcaagagttatttaatgcaatacaagaaaatataacgattgcagc
gcagcttgaaaaggacaggattgattggcattttattcccccggcaggacctcacttcggaggtatttgg
gaagctggagttaagtcaatgaaataccatttaaagcgtataatcggcgacactattttgacttacgaag
aaatgtcaactcttttatgtcaaatagaagcatgcttaaattcaaggccattatacactatagttagtga
gaaggaccaacaagaagttttaacaccaggtcatttttttaattggaagaccacctttagaaatagtcgaa
ccaatggaagatgaaaaaatcggaaatttggataggtggagacttatccaaaaaatgaagaaagatttct
gggttaagtggaaaagtgaatatttgcatacgctccagcaaaggaataaatggaaaaaggaaattcctaa
tatagaagaagggcaaatagttttattaaaggatgagaattgtcatcctgcaagatggcctttaggaaag
gtggaaaaggtgcataaggggaatgatgataaggtccgagtggctaaagtaaagatgcaggaaggatata

tcactagacccattactaaaatttgtcccttggaaggaataaagtctgttgacaaaaatgaggctgacca
agagccaaaaagacgaactagagcgacatcgggaatgtccaagatcggaatcattatggcaatgttgttg
tttgtgttaagttgtcaagtttctagcgcattacctaaagatatagcaccaagatattctatagacaaaa
taaataaaacctcagcaatatatctagacccgctaggagatgttgagattgtgagtacttcttggaattt
ggttatctatttggatccatattttaaaatgttaacaaagggtaatgcgcttatacaaagtatgaggaaa
gtttgcgaaagacttcatagctttgaagagcaatgtagtctagtcttagataatatgcaaagtcagttat
cggaacttgaagaaaacaataaattgtttatgatacagtctagatctagaagcaagcgtgctcctttcga
atttatgggttccttgtatcatattttatttggtataatggatgaagatgatagagagcaattagaagaa
aatatgaagaatttgttagataaccagaacaaccttgataaactaattcaaaaacaaacatctgtggttg
attcaacttctaatctattaaagagaacaacagaagatgttaactccaattttagaagtatgcaaataag
aattgagaacatgacagaagttcttaaagaaaattattatgtttataaggaatcaataaaattctttatg
attacgaaacagctacactcattgattgaagaaggcgaaaaaattcaagcaggcattataagcctgttga
ttgatattaatcacggtaggctaaatacaaatattctcaggccaaatcagcttaaaaaagaaattgccaa
aattcagcagagtctttcggagaacctagtaattccaggaaaacggtcaggtacggaacttaaggaggtg
tatacactgttaacagccaggggtttattcatcgacgataaattgatcattagtgcaaaagtgcctctgt
ttagcaggcatccatccaaattgttcaggcttattccggtgccaattcgaaatgaagatcggataataat
ggtgcatacaacgtccgaatatttaatttataattttgagatagattcctatcacataatgacggaagcc
acattaaatcaatgtcagaaatggcaactaaataagagaatatgcaaaggaagttggccctggaattcag
cgaatgataatgcatgtgagattcagcctctaaagccagataaagcggcgaactgcatctataaaacagt
agtcgactctaaaagttactgggtagagttagaaaagaaaagtagttggttgtttaaggttcctgcgaat
tcaaaagtccgtctgcaatgtactggctctcaaattgaattgtttgatttgcctcagcaaggagttttaa
gcattgcgccatattgtacggcaagaaccgacgataaaattctagttgcccaccataacattcagtccga
aagtgaagaattattatcaacaccttatataggagaagttagtgaagcgccgaagattatttgggatccg
ctgaaactatcaatattaaatcatactgaggaatttgaacgattgaataatgaaattaaatttatgaaag
agaaccatcaaaaattgaaagatttacatttccatcatatttccggacatgctggattaattattgcttt
aatactaatgatagtattaataatatatttcatacggaaatgtgctgtgcaacaaagaatgcaagcaata
acctttgcaggtccgttgccagtactataaatatcaatagtaaataaacaataaaataatataacaaata
aaaatatacagtccactatatcgttgtttaatagaaaatgtacttctacatagaaaaagcaaaatgttta
aaataagttaattgagtacaaattgttgaattaaaaataatataaaccataattgtaatccaataaaatt
aaaagccagaaaaactaggcccattgaaatcttagttgcaaaataaatgaacatatatcaaataaataca
gtccactactgttataaatgcaactaatatactaatgtacatctcagcttgctggccctttggcagaatg
ttcacacatgaacacgaatatatttaaagacttacaattttgggctccgttcatatcttatgtaaatgaa
tcgagagcgataaattatatttaggattttgttatctaaggcgacatgggtgcattgctcaaaaacatgt
aatttaagtgcacactacatgagtcagtcacttgagatcgttccccgcctcctaaaatagtcccttagtg
ggagaccacagataaggtcctcgccgctcaagataggcagatgtgcccgagcgtgggacctcgataaggc
ggggactatttacttaggcctctgcgtaggccatttactttaagatgcgattctcatgtcacctatttaa
accgaagatatttccaaataaaatcagtttcttacaaaaactcaacgagtaaagtcttcttatttgggat
tttacaAGCACTTTTAAATGCTTTCCTTTTTTCCACCTTTTTCTAAAGCCTTTCATTTTGTCACAACCTT
TGACTGCAAAACCTTTCATCTTTCAGACAGAGCACCCACAACTTTTATCCTCAATTAGGATTTATCATTT
CCCGCAAGCGATTTCCATTTTACACTAGTTTTCCCCCATTTCCCGCCATCTCCGTCCCCCGCCCTTTCGA
AATGCCAACTCTAACTAGTTGAATGCAATCAACACGCTCGCAATTAAATGCGACAAAGTGCAGACTTGCA
GATGTGGTTGTTGCTGCAGTTGGTGGCTAACCCGTTCCGCCCACAGTTCCAGTTGCCTCGACATGTCCCG
CTCCGCCCGCTTTTCCGCTCCATTTTCCGccccccccccccTTCCTCCTCCCCAACACAAAAGCGACAAC
TGTCAGCGGGTCTTTGGCACACATTTACATGCCTCCAATCCTCCACCCACCGTGTGGCACCTCCTCCTTT

CCAGCTAGCGTGTGAATTTAATTTGTGCATAATTGCAATTGCAACTGCCATTGCAGTGCTGCAGCCATAA
AATGTCAATATCAAATTGTAATCATCAACGAGAAATGTTTGCGGCAGCCCAAGTGTTTTCTTTTCACTCT
GACAATTTGTTTTTCCTATTAGATAGGCCTGCAGACATGAAAAGGGGCTCGGAAAATCGGGCCAATTGCC
GTCTGACTGATATCATCAAGTGCGTAGGAAAAGTGCAGTGGACAGTTTCGAAAAGTAAACCAATTTAAAA
ATTAAGTGCTGGGGAAACTATTTTATGTTGTTGTCTGGACTTGAGCAGGCTTTTAATGGATTGCAATTTG
CATATGTGTACTAAATTTATGCGAAAATATTTAGTGCATATGAAAGTGGAGTGAAAATTAATAGCTTCAT
TGGGTAATGCATTATGATTCGGAAAGGAAATATTATACATTATAATATATTTTATTCTTATACCTCTAAT
TTGATTCATTTCAGAAACCATCATGCGCCAGACTCAACGAACGCAGCACAGCGAAATGTCCAAACAAAAC
GGATTCCAACAGCAGAAGGCCGTAAAAGAAAGCCAAATAGATTGGCAATAAAAAGTTAGATAAATTAACT
TAGTAGCTCATCACGCCCAAAGGCAAATCACTTTGAAGTGCAACCAAATAAGGAAAGAGCAAATTAAGCG
AATAGAACGGCTAGAAGGAGATCTCCCAGTGGAGCGGGAGTAGCTTCGtttttttttCGTGGACCGTGCG
ATAGAAAGCCGTCAAAATTACGACATACCACCCACCCACGCCCAAAGTCAAACAGAACCCGGCCAATACG
GACGCCATGTCCGGGTCGGGTGCGTTTGACGATGAGCCGCCGCCGGAGCCGCGGGACGGATGGCTACTGG
TGCGCATCCATGTGCCGGAGCTGAATGTCTACAAGTGTCTGCAGTTCCCATCGGAGCGGCTCGTTTGGGA
TGTCAAGCAGCAGGTGCTCGCCTCGCTGCCGAAGGGTCAGTACACAGCGATTGATTGGCCGGAAAATTAA
CTAAATTAAAGTAAAAACCCTCGAGTGCCAAAGTGGTGTTGAGCAGCAAACACCTTTTAATAGTCCCCCA
TTTGACCTTCACCCATGGACACCCTCGCAAATTGGTAGCACCAAAGTCGGGCTAGCAGTTAACCCTTACC
CTTGATCACCGTTAATTGGACCCCCCTCCTCAGACTATCATGGTCATGGTCAGCAGGTTGTGAAAACGGT
CATTTATTGAGGTGCGTTACCCTTGCATTATATAAGGCACGAAACTCTTCAATGAAAATCTAATTTCAAA
AGGATTTTAACCACTGTAAGAAAAAAAGTTAAAAATGTATTAAAATATCGTATAGAAATGTAAAGGTATT
TGTGCTTTAGATAAATGTAACAATAAAAAGGTGACATCACTATCTGTAACTATTATATTGAATTATTGCA
TTTGTCACTGCAACTGATGATTTCAAGAAAATCGTTAAGACCTACGACAGTTATTATGAAGCAGCTTTTC
TGCAACTTAAATGACTGTTACTTCTGTTCTATGGCAGTTACCGCCTTCTTCGAGTTGCACACACTCATTC
GAAATAAATTCGGACTGGCAGCACGTTTACCATTTGGCTGACTCTTTCTTTAATCGCACCTTTTTATGGG
CCCACCGTTATATTGACCTTCACTCGAGGGGTTCTGCTCTTGAAGAGGCATTCATAAATTTCGTTTTCTG
TGCGAGTGATTTGGAAATTGCACGCTTCCCtttttttttgttttGGAGGGGCTGGCTGCTTCCCCCTTTT
TGTCCCACCAATTGCAGTTATCTTGGTTGTTTTTGTTCAACTTTACTCATTTGTTCATAACGTGACTGGT
TCTTGTTGTGGCCTATTGCTTTTTGGTTGTATTTGTTGCTGCTGCAGCTATTGTTGTTGTTGTTACACAA
GCGTAACAATAAAATTTCAGACTGGCCCGCCATGAAAATAGTTAAAATTTTATATGTGCTGTTATGATCA
TGTTTTATCTGATAGTTGTTCGCTTGTGGCACGTAACACGGTTTGTTTTAAACTTTTTCGTTGATTTGCA
GGCGGCATGAAAGGGGCAAAGatatatgtacatatatgtttatatatatgtttatatgtacCTACATATA
TCACGTAGATCAGCGAGGGGAGAACTATCTGAGATCCAAGAGTTGGGTCAGCGGCTGCTTTTTGTCTCGG
TTACTTAATGTGGATGCGGCTTAATTGTTCCAAAAGATCAGATATCTTGTGCTGCGCCTACAGTTGTGTT
GAGATATAATCGTTACCCAGAAAATAAACATAAATATAAAGGTTTTCAGAAGAGCTTTAAAAATCATATA
CAAATCAGAAGAATATACAGTGCATTTAATATAATTTATTTGTAGAGCATTCCCATATAATTTTTTCTTG
GAAAACGTATAAATTTACATTGAAATATTTATACTACTAGTTAATGCCAAAATAATATAAATATTAATGC
TCCAAAATTTGCTCATGCTCATTTATCAGTCAGAATAATTTCAAAATGTAAATATTCTGAATGTTTTAGT
GTCACGTTGAATTTAAGACGTGATCTCATGCTTCCCTACTCGAACCGAATACTTGCAGCTCTCACTGTTA
ACTGTTTGCGTGATCTTACTTAATTTCCTTACTTTGTGTTATATTCATTAAAAGATTTGACAATTTGCAG
TGATAAATCAAAAGAAATCTGAACTCCTCGTGGCCTGCAAAGAGTGACATTTTTGCTCAACATTTTGACC
AACATTTCGCCCAATTTGCATAAATTTCAAGCATTTGATTCATACGAAACGCTCCAAGTCGAGAAATTCG
ACTTTTATGGGCAAACTCCAAAAGGCCCAGGCAATTTCTGATTTATTTAATGAGCAAAAATGCTTCGTTC
GATTTCTTTGGCTCCCTCGCTGCAAAGTAATCTCTCTACAGACCCGTAGGCGTAGATCTCAGATTGGTTG
GCTGCCGTTGAAGTTTAACAAATTTAAAGCAATTTTCGTAGCTATTTTTGGGGTCAATTAAGTTTCGAGG

GGGAGCCACAGATACGAGGAGGTAGATAGTAAAAGGGGTGCTCGGTGTGGGCGTAGAGATATTTCTTATA
GATTTATTTTGCCAAAGGAAAATTTGGGAGTTAAATTGACGGCTATGACTGCCTCAAttttttccatttt
cttttatttttcccctttttttaATGTATCAAACGATGTGCATTTCTATAAATATAATCTTCCTTGTGAAC
TTGGTCCGAAGGTATTTTAGACTTTAATATTTCTCCGTGTAAAGAGCGTAACAAAAGCTTACAATGGTGT
AAGaaaaaaaaGAACAACAAACCCAGGGAAAATCGATTTAAAACAGATGATTCGGCATCACCCCAGCCCG
ATTTCCTACCCTTTTCGCCCACCTCCTTCTGCTTTATGTATCCCTGTGGCCTGTTTGGGTTCCCCCACAA
AGGATCTCAACAATATCCAGCGCCATTTCCCAGCTCCCGCTCCCGGTTCCCAGTTCCAGGCCAAATGCCT
CACAGGCCGTTAAGATGTGTGCGCAAGGGGAGGGCGGTTGTATTGGGCGGATTTCCCACACCGAATTGCC
TACAGTGGGGGCTGACAAAGTCAGCTGTTCTATTTTTACAATTCCCCAAGCTTCAAAGTAAATAATATCC
GGCAGCTTAGGGAATAAAAGTCAGTTGAAATGTGATTAATAACCAATTAAGGTACAAATAAGATACAATT
GGCGCATGTAAGCTTTAAAATCGATTTTTCTTTGTTCAGAATGATATCAAAATTGACTCAACAATAAAGA
AGTGTATTTTTTTAAATTAATCAACATTTATTAGAACAGCGTTATCTTTTGATGGTTTTCATAAACCATT
TCATATCATGAAGCTCACATTTTTCTAATTTCCCAACATTCGTCCACTGACATTGGTGTTCACTGTATTG
TCCCTCACTTGCCCTTCGCACAACTTCTGTTCTTCTTCAGTTCTGGCCGTACTTTATTTCTGCTCTTGTT
TTCGCTCCTGCTTCTTTGGCTGCTGCTTTCGGTCGCTTTTGTTTTGTTATTCATTTGGCTTGTGCCAAAT
TCTCGTTGAACTTTTGAGGCTCCCAGAACAGCTGTGCGAAATGCCCGGGCCTCGAAGAACAAGTACACAT
CGCTTGCTATCTATCTCGCACTTTTTCCGGATTTTCTCCGGCTGGTGTCCTCTCATTAACACTAGCAGCG
ACGTCGACAGCGGATGATGCGGATTTAATGAGCAAATGATTAGCATTTTTATGTGCCCACCACTTCCACC
CTGCGACCAGCTCCACCGTGCTCCATCGAAAAATCCTTAGAATTCCCTCAAAGTGCCTTCTGCTAAGCTG
GGAAAACTTAATAGTTGGCACTTTCCACCTATTTCCATTGCCGCCCCCTTTTCTCTGTCTGTCGCTGCAC
TGATAAGGGAAGGTGATAAAGGGAATCAACTGGGTGGGGGTAAAGTGGGACACGTGCAACGGATTGTGGC
ACAGAGATGAAGTTCCCGGGCGAAAGCCCTTAAGCGGGTCCAAATTAATGATGCCAACGCAGATTTATGG
CCAACCAGATAAATGGGAAATGGAAAAAGTGTAAAGGAAATCGGAAGAAAACTGCAAAGAAAATTTTAAA
TAAAGTTTACACATACATTAATTTATGAGGAAGTTGCTTTGAAATTATACTTTTCTTACATTAAAAACTT
ACAATTTTTCCAATAAATCAATCTAATTTATTTCGGTTACTTTGACGTATGACTTAAGTCCTTTCTATAA
AGTAACTGCATTTTCATGCACAGAATTAGCCAATTTCCAGAGCTTTTCCCCAGATAGATGCTACAAATCC
TTGATCCGCATTCAGGCAATGCAATGAACGGAAATGTTTGGGAAAAGCCAATCGCATTACCGTGCATTTA
AccccccccGGAAAATCAGTTGAAAGCCAAGTTGGAACTTTTGACGGCCGAACAATGGGATCCAATGGAC
GTAGCCGAGCCCAAGACTCCGATTTCGCTTGCATCCTGTCCACACTCGCAAATGAAATTAACTTTGGCTC
CCCGCCCTCCTTTTGTCGCCCAGTGATTTCACTGCATAATGCAATTaaaaatggaaaaagaaaatatcga
acgcaaaaaatGCGATTTTCCTTTGGCCTTACCGCAAGGGGAACAAAAAACGAGTGAGTAGAAGCaaaaa
aaaaaaaTCAATAAAAGCATGGGGCGTGTaaaaaaaaaGAATCTGCCACCACAATGCGGCACATCGGCAG
CGGATGGGCTGTGGGAAAGTCGGCGAGGAAAATCAATCTGGCCCAAGGATAAAACTACACGTACGAGCAC
TAAATGTGCCCCACATATTATAGATACGATGCTAAGGACACATGTCCTAAAAACGCAATATGCAAAACTT
TTTGGGTCGCATTATGGGCGTGAATCAGGTGTGTGATATTCGAAGTGGAAAGTATGCCTCTTGAATAATT
CGGATGAATGTTTTGTGATTACTATTGATATTTGGTATTCGCATGAAGTATGCAGTAGATGCAACTAGAT
TTTAATCACATATTCATGGATTTTTATCAAAAATACCCTATTTTTGACCGCATTTTCCAAAGCATGGTCA
TATTGAAACAACGAAAAAGAATTGCAAGACTATCACCGTGATTCATGATAATAAAAACTACTTAAGTTTT
GCTGCCAGAATATGAGAGATATATTTTTAGCAAATCAGAATGAAGAATATAAATAAGAACATAAATAGAA
GATAAAGCCTTTTGTAGTTCATGCTGAGTTTGCTTTTAAAGTATTTGCGACAATGATTCACTCATTCACT
TCAAGTAAATTCTGCTGAGCCAAGTTGGCGCTTCCGATTCTGTGGGGGTTATAGATAAGCGACTGCTCCA
AGGAAATCCCTATCAATATTCCCCAGACACATATTATATTTGCAAATAACTTTTTATTTCGTTCGCCAAT
TGTATAATTGTTTGTTCTGCGGGGGAATTTCGCTTGATAATTCAACGCGAGTTTTGCAATAATCTGCCAC
GCGCGCTTCCTCTGCGGCTTTGAGTCAAGGCCAACTGCTGGTGCAAGTCGATATTATCGGTTCATTGCCA

AGAAAAGTGGGGGGAATCTGGGGTCCATATAAGTGGGTATGGAAATGGGATACTTGTGCAAACAAGACTG
TTTATCGAAGTCTTCTCTTCCAGGGATTTTATGCTAATTTTGGCCATGCAGCTGAATTTATGCTGAAATA
ATTCATTTTTTTACTTTGATGTCGTTTTCAGTGGGTTTTGAATTTGAAGTAGCTGAAAAAACAAATTTGT
ATAGTCACGGTAATAATTAAATTGAAAACTTAAATTCTTGGTACTAAGTTGGCATACAATTAGATATAAC
CCAATTACCAAAAATGTCTTTTTAGTGCCACTACACTTGATGGCTCGAAGCCCTTCGGTTGATTTGTGAA
ATTATTAAGGTAATCATAGTCAAATCAAATCGTCATTACCATGGCTAAAACCGAAACAAAAGCCAAAGTT
TATGGCTGCCCATTCGAGGGAGGAAAGCAAAATCGCCTGCAATTGTGGTAAATCAACATTTTCGTTGGCA
GACGATGGCATTTTCTTTTTGTCTTGGCTGAGTCTTGGCACGACATTCATGGGCTTATTGTGGGATGTGA
GATGCGGGGAGGTGTTGGCGCTGCAATAAATAAGTTTTGGCCAACAAAAATAGGAAGAGTAGAAGCCAAG
AACGTGCCAAAGTTGAACAAATACAGGGCTGAATCGTCGACGGGCAggggggggggggggTATGGCATTAT
GAGCACGGGGATTAACAAAGAAGAAAGTTGAATTATGAAGAAAGTTGCGCTAGAACTTTCTCATCTGTGA
ATTCAATTAAAGTTTGGCTCCAAGCAAAATCCAGTTAATTTGTAATAATTTCTGCAATCAAATTAAAATG
GAATTTTCAGCGATTCAAACGGGCCAGAACACACAACGATTTTTGTCAAGTGGCTAAATATGAATTATGA
ATGAAAATTACAATAAAGTTCCCCTCGAATAAATCCAGCTGAAAGGTTGGTGCAAAAAGCAAGCGAAATA
AAAACCATAAACAATAAATTTCTTCAATCGGAACTGCCTTTCTTTTGCaaaaaaaaaaaaaaaCGACGCAG
CGGGAAAAAAACTTTATTATTTCCGCTTCTTAAATGCAACTAAATTACGCTAATTGCTGTTGGCTAAGAGC
ACTGGAATAGGGCATAAAACAAAGAGCGAAATGGCCAAGAAATGTGCAAGGGAAAAGCAACAGCAAAGTG
CGGAATTAGTAGAAAAGTAATGCTTAAACTCTGAGTTATGTAAGTCTGGCCGTGATTTTTCCGGCGCATA
AACTTTTCCTTAGAAAGCTACGCCGTTGAAACAAAATAACATAAAATTAAATGCTACAAAGTGAACCTTC
CCCAAGCTGAAAAGCACTTCAATGGCaaaaacaaaaaaaaaaaaaTGCTGAAAGAAATTAAATGAAATGGA
AATCAAATAGAAATCAATGGACAAAATGAAAGTGAGGAATTTGTTTTTGGGTGTGTCTCTTGACTGCTCT
TCAATTTCTCATTTCCTAAGCAtttttttttCTGTGCCAAACCAAAGTTGGTTTCCCTGTGAGGATAACG
ACGCTACCTACAAAACATGGGAAATCAATAATTAATTTTTAATGTGACGACCTAGaaaaaaaaaaCGTTG
CTAAAAGAGCGATGGCGGAGAAAAATTGGCTTAAATACTTTATAAGCTGAGCGGCATTTGTCAAAAATAT
TTGGCACACCAAACGCTTAAGAAAAGAGTACAACAAAATAAGCTCATCAACACTGGACTTCAAGGTTGAG
GCAATCTGTGAAATTGCAAAAAATTTAGCTCATTATATGTATGCAATATTTTAGGCACTCTTAAGTAATT
TATACTTTAAAGGACATATGTAAGTAGAGTATCTTCATTAATACCTAACTAAAGTAGTAATAAGCAATAA
AATAGCAATCTTGTAGCTTAGTGTTACTTATGCAGGGCTCAAGGCAATTTCCGCACGAGACTTTGGCGAC
AAACAGTCGCAATCCATCCAGCAATCAATCAATCATTGGAACGGATCGATCAAGAACCTCCGCCGATTGA
TAGATGCAAAGATAGTGGCCAAGTTGACTGCCAAAGAGTAGCGTCTCAAAGAAATTTAATAAAAGCTCCG
ACAGGGCTTCCGTGTTCTTTGAAGAGTGGAAAACTGAGGAGGCAGTCCGCTGCTGGTGAAGATTTTTTCC
TGGGCTTCGTGAGCGGTGATTGAAAACCCGTTTGCTGTTGCCAATGGCCGTAGAGCAATCATAAAAAAGA
AATACGTTAATAGAATCTTAACGGAGCGCGTTGGGAGAATCCGGCTTTGTGGAAACCTAGTCGAGGCCCA
GAAGAACGCCCAGAAGGGCGTGGCAGTTGGAGCAACAGTTAGGTAACTGTAATCAATCAGAGCAGCCTAG
GCTAATTAGTTGGTGGACAAGGGGGTGAGAGGAGGGGCAGAGGGTTGGTAAGTTGGCGCCCAAGAACTGC
GAGACTTAATTAAACGCAAAAAAGGCCGCAAGGCAAAGGCATTTACAATTTTCACAAAATTTAAACAACA
GCAAATTGCGTAATTTGCCTCAGCTGCATGGCTAATAAACTGTAATGAGTGGGCGTGCCTAGAGCAGTGA
AGGAAGGGGGCGTGGCTGTGTACACATTTGCCGATTTATGCGAACTTTATTAGAATATAAAAAGTGAAAT
AAAAGCAGCGGATATATTCGAATTATGTACGATTTAATGAGAATCCAGTTTGGAATTTTTATTATAAAAT
TACAGTTTATAATTTCTATTTAATTTTATTATTTTATCTGCACTGCTTTGGTCAGTTTAATTTATGTATC
ATAAAAATTTAACGAATAGTTCGAGCTGTTAATTCAAGCTGAGAACAATATGGCTTTTTCGCTTTCCACT
TGCCGATGTTTTGGCAGCTCATTGAACAATTTGCCCATTCTGAACTTTGACAATATCGAAGTGTAATCGA
TTTCCATAAAATATGAAAATTAATCCTGCGCTCATCTACGcagatacagatacatctacagctacagcta
cagatacaAACGCACATATTTCCACATTTAAATGCAAATCACGTCGATGGTCTGGGCCATGTTTTTGTTG

TAGCTGGAGTTTGTGTGTGCTCATAGCCTTGGCAATTTGAACGAACGTATATGGCCAACAACAAGATGTG
CACATTAAGATACAAGATACATAAACACATTTGCCCTGTATCCATGTGGGTGTCTTTTTAGCGTGTTAGT
TGTTGTTCTGGCATTTCTTTGTTGTTGTCGTGTGACAGGAGAAAAAGAAAAAGTTTATTATGTGACTGAA
AAAGATTTTTTTATGAGGACAAGGCGTGGTTCTGCTGTGTGAACAACATCCTGTTGTGAAAAAAATACCC
TGTGAATTCTATCTTTATGGATCTGAAATAAAAACGAATGGTAAACAATTAAACTAAAAAAGGTGCTAAG
AAAATATGTAAACAATAGCATGATTGATAGCAATTTGTTTTGCATTTATATTTCGATTTCCTTGCAAAAG
GAAATCATGTTTTTATCCTTTAAGATGGTTATGCAATAAAAGCACCACAAACAATTCTATTGTATTTGGA
AAATTATAAAAAATTGTAATAATTATTCATTATTTATTCATAGGTCTTTAAACTTAAACTGTTGCCATAA
AATCATTAATATTCGTATGAGTTACACATTATACGACCCATTTATGGGCACAATTTGGCTCCTTTATCGG
TTGGCGGTTTATCGCAATTATGGGCTTTTACCAAAATTGTGTACATACCCGCTCCAAACAATTTTTTAAG
AACGCAAAAGAGTGCGGTAACATTTTCTAATCGACGCAATAAGGAAGACAAAAACCTAAGATTCTTTGGA
CGACAAGTGGCACATCAAACAAAAGCGCCAAAAAATGAATGAATGGGATATAGAAAATGGAGGGGATAGA
TAAGGGCAAACAAAAACGAAACGAAAAACGTTGATCAAATATTTGCCAAGCGGTTTTCTGTAATAAAAGG
TACACACATTCATGAATACAGGCGAGTGTAGTATTATTCATGTAAATTCTCTACCTACGAATGAAAAATC
ATTGATATTAATTATGCCTCTAAGGTTCAACTGTGTGGCTCTTGTTTCTCTCGTTTTCAAACTAACAAAC
CCATAATTCAAACGAAACTGAAATGTAAACTGAGAAACAAAACAAACAAGGCCCAAAACCAGCTTTTTAC
GTTTAAGGTACTTACAAACGAGTAATAGGAGAAGCTTTGAATGCCTGGAAATTAGTTAGAAGTGTCGGGT
TTTGAGTTGGGAAAAACTCTGTTTACTTATAAATGAAATCAACATAGCCCATAAAATGCCCCTGTCATGT
GGCCAAAACAGCAACGAAACTTCAATTCCAAACTGCGTGTACAATAAATATATTTGGCCAAATTGGACC
CACATGGCCAGCTGGCTAGCTGTCAGTCCAACTTTTGGCCATTTATCGATTTTTGTGGGTGTCCAAGCTG
CGCTTTTTTTGGGTTTGGGTGAGTTGCCATATCAAAATAATGTCGACAATTAATTTTTCATAAATAATTA
TGAAATAGTTTTTGGCCAACATTGATTGACAAACGATGGGACACTTGATGGATGTTTTGGCTGAATGACA
TGAACGAGATGCTTTTGCAGGAGTGCCATATCATTTCGGTTGGGAAATCGAACTTTAATCATAACAATTA
ACACGAAAAATGTGTTAGCCACGAAACGGGCTGGCTGGTCAACTTTCCTAAAGGTCGGTAACCGGTTTTT
GATGGTAGAGGAGCTAGTAGtttttttttttttACCTCCACAACTGGCTAACAAATTTCAAGACTCTTGA
GTCTGGGCCAACATTCTTGGTAAGCCTTTCTCTGCCTCTTTTTTCTGAACATGTTTCTTTGGGAGGGGAA
CGCATTATAAAACCGCTTTTAGCTTAGCGCCAACGTTACGTGTCCGTTAGCACTCAATAATCATCATTTA
CTTGGGCGGCAAGTGCTTCGATTCCGACCAAAAGAGTCAATTCAATTTTCGGCAAAAGAAAGTTGAATGA
ACCGCAAGAAGGTCGTTCTCGTAATGCACTCAGATTTTATTCACCATCGCATCACATCTGTAGGGCCGCA
CAAAGTTTGTGCAAATTAATTGAATCAATGTCAATCAGTGGAGCTTGGAATTGCTTTTTGGTTTCAATCA
AGATTAATGTTTGGGAATTCGCTTTTCGGTTTTAATAATTTATGTGGGAATTTTGCCTGGTCGAGGCTGA
AAGACAACGAAATGTGTTTTAGAAATCAATACAGGCGCATTCCTATCGAAATGGCGATTTATTAAAATAT
TTAGTTAGGCTATTTTCGGGCTTATCACATTCTACTCAACTCTGGCATGTTTCATTTTATTGAAATCAAT
TTAATGGTCAGTTTAGTTTGCGAACCGCAAACCACTTTCTAAATAAAACTATTTCATTTAATTTTAGACC
ATTTTAATTGGTATTTCCCAGCGGAATCCATTCCTTTCATAAATTTTGCATGTCTAGCTTTTTGTTTGAT
AGCTGCGTATTCCACACTTTAAATATTCCAGGACAAATCTCACGCTGCTCAATCGGAGATTTGATGAATT
TTCCCCGCTTTTCTCTTTCAACTTCGCTGAAGTTGAATTCATTCCTAACCCCCCGTCCCCACCtttttca
gcttttttttCTCTTTGCCACCTGTCACTCTCGAATTCTGTTTTTTGTGCATTTCTTGTTTATTTCTTAT
GGCTTATTTGCATACCGTGCACATTTCGTTGGCTCGGCCAAGTTTTTCCTGCTTTTGCATTCGATTTTCA
GAGCCACaaaaaaaaaaaCGGACGTCTTATGTCTATGTGATACCCTGCCCGATGGGAATTATAAGTTTAA
ACGAGAGGAATGACAAGCCGAAAACTTAATATCGCAGTAGAGATATCGAGACTTTAAGCCAAGTTAAGGT
TATTCCCTTTTTTTATGGCTCTTTAGGGCATCTTGGATTCGCTGAGTTCGTTTTCTTTTCGCTTTTTGTT
CGGCTGCTTTTGTTGCGAAGCTTTTGATGGCTCAGCATTTACATTTCATTTTGAATTGGAATTCATTTTA
GTattttatttatttccttgtcgtatttaccccacccccttttttgtgtttgatttattttCAAACTGCG

CTTTTCATAAGCCAACAAACAAGTCGCTGATTGGCGGCCCTGaaaaactgaaaaaaaaaGAAGATGGGAG
AAGAATGCGGCGGCTGAGACCCTTTGAAGTGTTTGTTGTTAGTCATCTTCAGTGCTGCGATAAGGATTAT
TGTATATTTCACTAACAAATAGTTCATTGGAATCCTAATTGCATGCACTGATGCATTATTTATTGCTATT
TAATTTATTCCAACCGAATTCGTGTGTGATGCAAGAGCTCAAACAGCACCAGAAGTGCAAGTTTGCAAAT
TAATTGAAATTGAATTTACTGAAGTTGCTTGTAATTATGTTAATTAGGCAAAATTCACAGCGCACATTAT
AAACTCGCTGgtgtgtgtgtgtgtgtgtgtgCGGCTTTCAGTTAAAATGACAGCTCAGAATACAAGTCGAAA
AGTTCAGTCGTCAACTTTGAAAAGTTGCTCCGAAACGCAGGGGAAAAAGCCCAGTTGAAAATAATTATAC
ACATGGTGGAAATGCCTGGCTCCAGAACGGCGATCCCTTCGAGGTTCTTCACTCCCGATGATGGGCGTTA
AATGAGAAAAAAATCCGAAGAGATGAATGTGGAAAATAATAATAAATGATATTTGGGGCGGGGCCCTTTTT
ATTTAGGGCCCTTAACCTTTTGAAGTGCAAGCGGGGCCAAGTGAACGATTCACTATTGCATCGTTTTCCC
ACAGGTTCTCGAGAAAGTGTTCCTCTTTAATAACTTTAATTGAATTTAGGTGAAACAAATGGCCTAAGGC
ATCAATAACCAAATGAGTTTACGTGCTCAATTAATCATTTTTATTGCTAACAAATATTGAAGAGATTTTT
GAAAGATTCAAGTTATAACTAATCAAGTAATTTCTTGTATTACCTTGTAATTCTTAGAAAACTTAATGCT
TCTTTTTAAAGAGAAGTTTCATATTTAAAACTAAATGAATGCCTTATTTCACAGTGTGCAAAGGTACACA
TACCTTTCTCATTCAGCACATTCAACGCTTTTCTTAGCCCAACTATAATTTTATTGCTGCAAACCGATCA
GCATCATAGAACTCGCTAGATATATCACCTTAATAATTGTCAGAGGCAAAAGGCAACATTTTGCAATTAA
CACGCCGCATGCTCGTTGAAAGCGCTTCACTTTGGTCGGAACGCCTGTGGCGGGATGCAGACGCAGATAC
ACGGCTAGATTCAGATAGCAAGATAGTcagatacagatacagatacagaAAATCTCTGCATGAATCTCCG
CCGAGACATTGACTCCAATTGAGCAATGCAATACGAAGCGGGCGGCAAAGTGGGGGCAACTCGCTCGAGT
TAAGTCATGTGTGGGCTTCATTTACAACTCACTCAACTTTTGTTTTGGATGCTAGATGCTGGATGCTGCG
GGGAATTGGAACTGAGACTGAACATGGGTTGGGGACTTGGGGATTGGAACTGGAGTTTCGCCCACAAAGT
GCAGAGCGTGGCGAATCGCTTCTCCTCTTCTGCTTCGCTCTGAAGTGCTCTGAAGTGTCGACATGGAGTC
TGACAATTGCAAAAGTTGTTTATGACCaaaaaaaaaaGAGAAAATGTATATTAAATGTACGAGTGTTTTT
TAAGCATTTGTGTTGTGGCTCTGACACATAAAGCGAAAGATTGTGCGTCCGAACACTCTTGATTGCACTT
TATATGCATATTGAATGTGGCCAGCCCACTGCATTCGATCCTCTTCTGTGTAAATTCAAGTTCAGCGATA
AGGTTGCCCCAACTATGTGCTCAACTAATTTCCACGCAAACATAAACACCGACATCAAACCGAACTTCTT
GCTGCTCAAAGGGCCCGAGCAGCAGCTAAATATTATTTATATGAATTAGCGCAGCAAGCTAGATGCTGCT
GCGGCTGCTGCTGCTGATGATGATGATGCCGATGTTTATGTTGATGCTACATTTTGCCCCTGGCAAAGCG
GAGTGAAATAAGGCGAAGTAAAGCAGAGCAAACGATGCAATTTTATGCGCAGACGCCTAAATTGAAAGCG
ACGCGTAGCGGACGCAGCAGAACTCAACGAGGCGTTCTATATTTACACTGAGAAAAAATGTAACCAAACT
GAATTGGGGAGCAGAAGATAAAAGATTGGTCGCTTAAAACGTCAGGTTATAGCTTAATAAAGGTGTATCT
AAACATATTATTATTAAATAAGATAAGAAATTATTATTTTTATATAATCTCTTCAACAAATGAAGCAAAG
ATGTTTTTATATTTTATTAAAGCATTATGGTCTAATAGAAAATATATCTGTCTGTGCAAAATCGGCGAAG
GCTCCGCAGTTTGAGAGCAACTGTGCGTGCTCTTTCACTTCACTGCATCCCCTCCACCCCACCCCACCTC
ACTTTCGGGGCAGCCTCTAGGGTCATTAACATGCCCGGCAGGAGAAAAGCAGAAAAACCAAACAGGAGAG
AACAGGCACAGAATGAAAAGTATACAAAGCCAAATCCAGATGTGGGGGAAATGAGGGGGAAAAGTTGAAG
TCGGAGCCGCAAAAGAGAATGCAACCAGATaaaaaaaagtaaaaaaaaaaCAAGCGACGCGACCCTGGAG
CAACAAAGCGCGTCTACATTTGTGGGTGGTGCTGCTGTTGTCAGTGGTGTGCGGTGGGGAGTTGGTGGTG
CGGTGCAGTTGTGCAGCGGTGGATCCCCAATGGTGGTTGTCGGTTCCCTTTGGGGTCCAAGGTGCCATGA
GTTTGCCTATTCTCCAACTGTTTGTTGTACGTGCCGCGTCTCTTATTGCCAGTCGGCGGTTAAAAAAGGG
GGGACAAATGGGTAGATGTAATCCTGGAAGTCTGGGCAAACCATGAGGAGATCCTTTCGAACAGAAAAGG
GTTGGCAATTAAAACTAAAATTAGAATTGCTGGTATTACTAAGCAAATATATATTGATTGCTTGCTAACC
ATTCAATAGAAAGTTTACCATCTAATGATCTATAGAAACATGTTTAGGAAATTTGTACAATACATTTAGA
AACGTGAGCCGGAGAGAAGAAATAATTTTAATGTAAAATCAAATATCTGTGAAATTAACTTTTGTTTGGC

TTTACTGATATAATATAGTAAAGCATCCATAGCAAATTGTAAATTTTAAAGAAAAAACATCTCACAACAC
ACAATTTGTATTATTCAACAGTTTGTAACTTTGTTTACTTAGCCACATGCCTAATTATAGGCCAACTGAA
TTATCCATTTGCAAAACTTGTTCACTAATCGTTTCTGTCTTTCCGTTTCTCTTTGCAGGTAATTAAATGG
TAATTTCTCATCGATTTCATGGCATATTTAGCGAAGTAAGCCAACTCGCAGCTCGGATGATGATGCCTCA
TAATCGCCTCGCCTCGACTTGACTGGCAATGTGGCTAAGGCAAATAAGGCTCATTAATGTAAGCCAAAGA
CAACAGAAATCTGCTCCTTTGTTTGCAAATTTTTCGTTGGGTTCTGGGTTTTGGTTTCTGCGAAACATGA
GAACGCATATTGTTAGGATAAGGCGAAGGTATGGTATATAAATAGTTATGACTGCCCAAGTGCCCAAGTG
AATGAACTTTGCCGGCTATTGTGTGTGTGTTACTTTTACTTGGCCCGACACTTTCGCAGGCACATTTCCT
GTTCCTAATACTCGGCTGACATGCAACAATTTTCAACCTTTTCATTTGGGCACCCAAACAGAATGGAGAG
AGAACTATTAAGTCCGATTGCCAACTTTCACTGATAATGCCAGCAATTATGTATTGCTCCAGTTAATGCC
ATCACTCGTCTTTTATCCACATCATCATCTAACATTGTAATGACATAATTATGTTGGCTTTTCATGGGTC
ATTCTAGGGGGGTCAAACACGTGTCTAGTCCACCACGTCATCCTTCAATTAGCAGTCTGTGTGAAAGGTGA
CACGAGCGTGTTAAGTTGTCGGGTAAACGCTGGAAAAGAACCCATCTGCAGACCCTTTGATTTGAGCCAA
GAATTAGCATTTTTCCCCCCTCAGCTTGAGAAACAATCTTGGGGCCCAAACAATGAAGACGAATTTGTAC
AGTTTCTGCTTGGCTGATTCCGGCCAACAGATCCCACCGAAAAGCAGCCACTCAACAAGGGTCTAATGGG
TTTTAAAAGTGACCAAAATTTGGCTTGTGCCCCGCGTAAACTGGCTAAATTAAACGCGGACGCTAAGTTG
GCTAAGAGGTGGGCCAACTCGAATTGGGCAAACATTGTGGAATTGAAGTGTATTGAGGCTTTTGGGGCAT
GTCGAAGAATGCATTAGCCGAGAATTGAGTACATACCGGTGATGATGGGGAGAAGGTTTACATAACAGAT
AGAAGCATCTACAATTTCCttttttttttttGAAAAGCGTGATAATTATTTACCAGAATTGATAAGAGAA
GCAAAGAAGAGCACTCCGAAATGTAAGTAAATATAAAAATATCTGGAATAAATGAAAAAGTATTATTTAC
ATTTACCTTGAAAAACGAACACAACAAAATGAATTACTTATTATAAAACATATCATTTATATGTTTTTAT
TGGTTTTTTATGTGTATTTTAAGTTTAACTTTAATGCATTTAAATAAATTGGCATTGCTTGCATAAATCA
GGTATATTTATTTATTGCACAAATTGTTATCAACTCCAATCCAATTACGTTTGCTGAATGCATTGTTAAA
ATAACTCCACATTTTATGTCGAATTTAACTTTCGGCTCTTAATTTATTATCCATCATTTGCCATAACCCG
TTTAAATCGAACTCTTATCTAAACTTGTGTGATATATGTTTATAGATTTTATCTTACCCCGTTTTAGACA
TAGTTTCAATCGATTTTATTAGTCCACAGCTAAATGATGATGATTTGGAAACAATAGAACCGGCTGAGAA
TTATAATGAACCCGATACGCTTTCAGTTCTATTGGCCATTAAACAAAATGACTTATTTCCCAGCTTCCAT
TTGCACTAAAAGCCCAAAATGTGGAAACCACTTTGAACCAGTTCTCAATTGAGTGCAAATAAAAGTAAAA
CTATTGACAAAAGCTGAAAGTGAGCACAGTTTTGCCGATGTCAACCGAGCAGCTACTTAAGCTACAtttt
ttctttgttttttttttttCTTAACTTGGAGCGTTCAACGATCGCTTAGTTAACTCCCAGCCCTATTCAC
GGTGCACTGATATATGAAATTTTGAGTCGAAAACGATGCGATCTCTTTTGTCGCCGGCAATTTTTGGCAC
TAATTTGACAAGGTCGTCGTCTCACTGGGCTTTTGTTGGCTTGGCTCGAAGAAATCACGACCCAAACACG
TTCGAAGTGATTTCGACTGGCCCCttttttattttttttgggtttgaattcgtattttgtttattttgCAT
TTTGAGTGGTCCAAGTCGTCAGCTTATTTCGCAGATATTTGCATAAACATTTGACGGCGGGGTGGGGTGC
GAAAAAGCTGCCGTGAAATGTTGTCATTCATATTAATTTAGTGGGTCACCGGGCGGTGGTGACCCCGATC
GTGCGGATTTCCTGTTAGAAAGGCTCTGACTAACTGACTGCCCGATTGTTTTCCCCCCCTTAATAAATCG
CATCTCTTTTTTAAGTGGGTAATGAATAGTCAAATTTGGCTTCGTGACTACAGATGGCAGACAATTAACC
CTAGAGCACCGAACTATCCCCACTCACCCACCTGCTGTGACCAGCAGTTCGCTTTTGAACTTTCATTCCT
TTTGGATTATGCAAGTGGATTGGatggtagtgtggtagtatggtaggatgAGGATGAGGATTTCTGCCCA
GATAAGAGCTGGCGAAGGCAGAGCTAATTAAATTATAAGCCAAGTCTGACTCTGACCCAAGATGAGCTGT
GCGAGGGAATGGGGTCACCAGAAGGTGGCCCCACCCCCATTTTCCGCCGAGAGTGGTTAAATAATATCCA
AATGGATGCTTTCGGTGTTTGCCGTTCGTGTTTTGCTGGTGTTGATTTGGCCATAAATTTTGTATGCTTT
TGACGCCGAAATCGACGACCACAGCCGAAAAACATTCACGGAGAGATGAGGTAATGCAGCAAATAACTCT
TGAGTTACAGTGGGATTATATTGACCAGTCCACTCGAAAAGCCAAATGAACTTCAACTATTTTGTCGGCT

TTTTATGCTACCGCCAGTGCTGCGGCTTATATATTGATATTTAATATGACCTAAGGGTCTTTTTCACGGC
CTTTACACCAATTGAGTTTCCGTTTTATTACTTCCCGCCTTTAAATACTGAGTAATTTAATAAGTTATTG
TCTGATCATTATGAGTATGATTTTTGTTTTTGACTATTAATAAAACCTTTGAAAGATTACGAAATGAGTA
CAGTTTAACTACCGTCGTGGCAAAGTGATTACTTCTTTGTGTATAAAAAATAGCTGAAATTCCAATATGC
ACGCCCATTTTGGCGCTCGTAACAATATTTCGATTTTTATACTTTTTGGTAGTTTATTGCGAAAAATAAC
TCTGCATGGTTTTAACAAGAACTATTTCAAAATGAGCTTTGACCTTTAAAGCCTTGGTTTAAAAAATAAT
ATTTAGTGTTGCAACTTTTCATCGTCTTTAAATAGCCCGCACAAATCTATACAAAACATTCATAAAACTT
TGAAACTCAATTTACCAACCGGCTGTTTTGTGCTCCTCATTAGGACAGGACACCCACTGTGCCACATGGA
GCAGTTGCTGGACGCAAACATGAATTATGCGTAGACAACTCGCGGGTTGGTTAGTTCACCACCCACTCCT
TTTTCCCCGCTTTTGGGTCTGTCATCGGGCGTTGTCAACGAAATGTTTGTGGCCCGAAGGCAACTTGAGG
CCAAGTGACGGCGGCATCATTTCAATTGCGTTTTATGGCCATAAATAAGCCCAACGGCGGCTGACAGATT
TTCGCAGCGAGAGGGTGCAATGGCTGGCTGCTTAACAAGGAAAATGGGAAAAACTACGAGTGCATGCAGA
CGATTTTGCTGGCAAAGTCGTGATCACACTTGACTACCAATTACCTGAGCCCATCAAATCGAATGTGAGG
TGGATTCTACCGGCAAAGTTAATAATCTCATCAGCTGGAGACGCTGGCTGTGAGATCGAGAATCTTCACT
TCACTGGCCAATTTGCATAGGCAACAATGCTAAATGGACTGTAATGACGTACCAGCCAGGCAATTCAATT
AATTAGTCTGACCAAGAGCGCTTAGCAATTCCCTACATTTTGCTCTCCACCACCGTTGGTTGGTTTTTCT
TGTTGCCACACGCGTGTGATGAACATTTCCGCCGTGGAAAGCGAACTGCTCCACTCCgtgtgtgtgtgtg
tgtgtgcttatgtgtgtgCTGCAATCAAAATTAATTAAGGCACAAAGAGAGGCGGCCAGATGCTTTGCAT
GGCTTTTCCTCCGACCTGCCACTTTTTATTGCCTTTTACCGCAACGCTGCACCAATTTCCATTGCACCTG
CAGCAGGCACAGCTCCGATGACGTTGTATTTGCTTTTCCACTTCATCTCGAGATCTCTTTTTGTTTCTGT
TGTACTACTCCGGGCGGGCACAGTGCCACCAGCGAATGTGCATAATTAGAAGTGGTTACATGTGGTCGAT
GTGATCGCAGAATCAGTTAGTTGCATCGTTTGCTTGTCCAGCAATCAGCTGGAAAACTGGCAACAGTCAA
TCAGAGGCGCTTTTCCCGTGGAAAATTGCAGCTGCAGCTCATCCAGTTGAGGGTGCATAAATTAATTAGC
CACGCTTGCCAGTGATTGTTGGTCCTTTATTGGTACAGAAAATACTGGGTATCATATATTGTTCCCCATT
TTATTACTAACCACAATAAAAAGTGAAGCATAAACTCCATCAAACATAGAAAATATAGTACAAGAACTTC
TATTTTATTTGCTGCAAATAGTTAAATAATTCTAAAGAATTACGTTTTTATTGACTGGCTTGAACTGATA
AATATGCCTGCATTTTATTTACCAATCATTTGTGGTTCTCACAGTTTTGTCTGACGTTCCTTTCTACTTT
TATTGGCGAAATTTCACAATTTATTTAGCCATGATGGGGAACATAAAATCTTAATTGCATTAATAAGCAA
ATGACTTCACGGGGGGCTTCGCCATTTCCTCAGAGAACTTTCCCAGCACAATTTCCAAGCGAGAAGGGAA
AATGAAACGAGGGACATGGCAGCAAATGCATCCTCGCCCGCAAAGTGAAATGCATAATGAGCATAATGTT
GGCGCTGCACGACGCAGATTCGGTTGCAAATACATACATACAACTGTCGGTGagatgcagatagcagata
gcagataCGGATGATAATGATAATGCAGTGTTGCAGAGACGACACCTGAGAACCGACGGGGAGAAATTTG
AGAAATGTGAGCAGTAATTAAATGCAAAACTTGAAGCTTGAAGCGCAAGAAGGAGAACAGCGACAACGTC
AACGACGATCCAAGATAATGCATAATTGCAGAAGACAAAAGGAAATGCCAGCGACTCATGAGCCAagttg
cagttgcagttgcacttgcagAGTGCACTAGCATGCAAAATATGAGAAGAATATCTAATGCCATGCCACC
GCATTCTCCATATAGTCGCAAAATGGTAGAATGGTTGTAGTTCGCCAACTGCCACATCCACGTCCGCATC
CATCCTTGGCTGGCTCAAAACGGCGTTCAAATTAATGGCCAAGTTTCACATGGGGCAGCAAGAAGCTTAG
ATGCCAGCACAATTGCCAATGGAAATTGCAGTCATCTCACATGTGTGAGCAGTTTCTCATTGAgtgtgtg
agtgtgtgtgtgtggatctgtgagtgtgcGCCTTCGTTTGTTTGCGAAAGTTGAGTACAAGATACAAGTG
GTTAGATACAAACTAGAACAGCAACTGTTTGGGTACGATGATGACTCGTGTTTATCAGTCACGTCAGAGG
AATACTCTCCATTTAATAGTTAGCCACAAACTATTATTACGATTATTAAATAATTTATAGATACTATTTT
TTATAATGTTTATAGCTCGAAAAGAGCAGTTTCTTAACGCATAAATCttttttttttttatactttttttt
ttATAGAGATTGAATATCTGTGCATTTATTTCTGTGCTTTAACTTTTAAGTTCACTTTAACGTTAATTTA
CTGATTTTAATTATAAGAAAACAGTTTTAGGAAAGCAAGGCTGTTTAAGCCAAACCATTGGTTATGTCAC

TTGTACCCCTTTGTAGAGCATCAACAAGCATAAGTTCTTGTAACGAAGATGAGTTGGTAAGAGTGACTTG
CGGGCTTCGTTTTTCATTTGTGTTAGAACACGGACTCGCAACACGGTGGCAAAAAACAGAAGTTGCCACT
GGGAAGTTAACTCCATGTCATTCACCAGCCGGCGGAGGAATGTTCAAACAACATTCAATCAGAACAATCG
GCAAGAACAAGGCGGAGCAGTCAGCGCTGATACAAGTCTAGAGATCGAGATAATAAATTTACTGCCAGCC
ACTATATGCTTCGCCATTTATAAGTTTCGGTTCGCCCCAAAAAGCAGATACTTCCACTTATTTCACTTTC
TAGCCACTGCCACTAGCAAACACCATTCTTTTGGCCAAAACACCTGCTGTTGTGCTCAGCTTTTTGTTTG
GCTAGTTAAAAGCAGAAAGCAAGAAACTTTATTTGGGTTAGCGATTGTTTAAAAATGCATAAGGATGTGA
TACTTGGAGTAGCTGAAAGGGAGCACAAGCCCGCAGGAGTTTGGCCAGAGGAGCTACAAAGAGCCGATGG
CGAAAAGGGGGGGGATCAGTGGGGGGATACAACAGCAAATGGAGCTAAAATTGCGGTGCAGAGAAAGAAAT
CTTTAATTGTGCATTTTAATCTGCGAACAAAATGTATACAGAATATTACATTGTTTTAATACGTGGGTTG
TATTGGTTTCAAGTGGGTTTGCTTGGAGAAGAAGTCGTTATACCTAAAATCAAATCATAGTTCTATTTTC
TTACTGTGCAGTAAATGTGAATCAAAGCTAAGACATCTTAGCCACAAAATGGGAAATCCAAGTAGCTGGC
TTGAAAAGCCCTTTGCAAACCGCAGAACAAGAAGTTGGCACACTTGCAATTTCAGTTAAATTTGTCTTAG
CAGGTGTAGGGATTTTGGCACATAAACTTTGACATCATAAAGCTAAACTGTCTTGAATTTAATATGCAAA
TCAAGCGACGAGCTTTGCGTTATTTAAATCAACTGAACCGCTCACCCACTCACTTGGCGGCATTCACTGG
CAAAAGTTTTTAAATTAAAAAATTATGGTTGGCCAAGTTAATGGCTTTCTCGGTCAGAAGGaaaaaaaaa
TATAGAAAAATGAACGGCAGGTGGGTGCAGTTTTGCAATTTGAAAGAAGAGTGGGCGAAAGTGGTTCAAG
TGGTTTTGTGATTGCGCATGTAAATGCAATTTCATTAAATTGAATATTTATCGGTTGTCATTGAGCAAAT
TGCAGGTAAATTGAGTTTTATCCCAATACAACCACACCACCTAATAATCACAGTAATGTGTGTGCAGTTA
TTCCGTCAAGATAATGGACGGAAATCATCATTAGCTGGCAGCTCAGGGCTATAAACATAGTATTCTAAAT
CTTAAAATGTGAATATATCTATGTATATTGCTTCTTTCTCTCTGACAAATTGCAACTATTTTTGTAACTT
CTAAGAATAATGCTCAATGAAAAGAACCGCTTGAACCGGTTACCAGGGAATTCTAGCCGCACAGAAGCCG
TTTGATGAGCATAAATCTTGATATCCGAACATTATTAACGTCAATTAATTCTAACCGTTCGAAACGAAGG
CCATCCATTAGGAGCTGGATGGTGGAATTCGGCAGGGGGAGTCAGGGAAAACTCACCTGGGATGGATGGA
TGGTTAGTTGGCATTTCTCCGGAGACCCTCAACCTGTTTTTTCTTAGttttttttttttGTTTATTCGC
CCGCTTTTCTGTAATCGATACGAGTGGCTCTTCGGTCTATTTGTATTTGTATTTACAGCGTTTTCCTGGT
GGCCCGCTGTTTTCAGTTTTCCCTCTTTTTCACGTTGTCGTCCCGCCTCACGATTTCCCAAATCCGATTA
TTATCAAGTCTCACGTGAGTGCACGCTTTGTTGCTGCTTGCTGCTAAGtttttttttttatttttatttt
taCCTCTGCTCTCGAGTATTGTGCCTTTTACAGTTGCCTTCTGTGCTTCCTTGGAAAATTGCTTTGAACT
TGAAAAATTCCCACGTGACGCGGTCAGAATCAATCTCTGCTTCTGGCTTTTCTTTTGCCCTCTGTTCGTG
TCACATGTGCCATGCAACACCACCACCCCTTTGCGAATTTTCCGCCAGTGTTTTCCGTGCACGGAAGGCA
GCTGCACTTTGCATGTCATTCGGCTAACGGTGTCGTCTTTTCCTCGCTTTTCCCCGCCTTTCTGCGCTTT
TGTTTCTTGGCTTAAAGCTTGCATGGTTTTGGTTGCATTTTCTCAGTGTTTTGTGGCAAGTGTCGATTGA
ATTTAATTGGATGGTTTTTTCGTGCATTTCATATCTTTCGAGGGGCTAAAAGGCACATTTTGAATTCTCA
AACTCGTGGGCAATTTCATTTATTGGTTTGTAATTTTTGTGGGAATTTTAGTTGGTTTCAAATTGGAGCT
AAATGAAAGGATTGATTTTATGGTTGAATGCAAAATTCATTTACAAAGGTAATTCTCGAAAATGCAGTCT
TGTTACTTCCATAGTCGTGGTTATTAGTCTTCTGAAATGCATTCCAACTATGGAAAAATGAATTGCTAGT
TTGTTTTTATTGCAGAATAAAACTTAATAAAATATGCAGCCCCGTTCCGGAGTTTCCATTAGCACATCTG
CTGCTCTACACTTTCTGTACAACCCATTCGTGGCACTTAGCAGATCTCGGCTAGTCTGGCAGCCCATCCA
TTGCACGGTTAAGTGTTAGTCAGTAGAAAAACCCAAACAAAGTGATTTTCGTGGTAAGCTATcccccccc
ccccTTTTTGGCCAAAAGACAAAAGGCTGCAGATGGCACTCACTTTGCTAGCACGTTCCATCGAGGGGAA
GTGCATAGTTCGCGAGTCGTAAACTGGCTTTTTTACGATTGGTGGCTCAGAACGAGACTTGGCCAAGACC
GGTGGCAATTAAATATAAGTTAACACTTGGCTTTGGTGGGGGGAAAGGGCGACAGTTCATAGATGTCAAG
TTCAGGGTTACTTCAAGGGCTCGGAAATTACCTGTCAGAGTCGTCCAAAGACAAAGTGTGTAATTAATTT

GCAAGCGTCCTAATCCATCAAATCTTCTGTTTCCCCTTTTAAAAAATTCATGGGAGTTGCGCTGGGTTCG
GTGGGATATTCCTTTTGATTTAATGAGCTTTTTATTGCCTGCTCTCATAGGATTGAATTCTTTGTGCAAT
CGGTTCATTGAGCTCTGGTCTAATTAATGGTTCATTAAATCCGCACTTTGATTAGGGTTCCTTGGGTTTG
CTTTAATTTAACAGTAGTTAATTACGGTTCTAGATAAACGTTTCAGTTCCGAGTATTTCCTTTAGCACGC
AGCATGTACATACATACTTAACCATATTGAGGGAAATTTGTAAGACTACAAGTTTTTCTTAAACTACTTT
ACTGCGATAGTGATTATAGTGATTTATAATTGCTAGTTCATTGAAGTTTTAGTGCCTGCTATTTAAGTAA
ACACCGTTTGAATAACCATTTATAAGGCTTTGCATCATCCTCAGTATAAGCCTACTGTGCTGTCATATGG
TTTTTGATGACGATGCTGATGAAATCTAAGCGGCTTAGGTTTTAGAAAACCCCCAAATGCAAAAGGCTTC
TACTGCCAACTCCCAAGTGGCTGCGTAATCAAATGCATGTAATTTGAGTTTAATGAGTGTCAAAGTTATT
AAAAATCATTTGCTCCGACCAGTTATGGTGGCATTTTCAGCCAATGGCTTTAGCTGTGGCTTTCGGCCAG
TCAGCGGGCCAGATTTTCGGTCCATGAAAGGCAGCAGCTAACGCAAACATTACAAAATCGTTTCGAGACA
TTTTTGGGTTCGTCATCGGGCTTAATTACTACGCGGCCAAGCTCATTCTCCCGCCAACGATTCTGGTTAA
ATGAAATGTGTCAATAAATTCAAATGGCGGCATTCGAGCCGAGTCCAAAATTGAGTTATTAGAAGTAGGC
CGCTAAATCGATGTGAGATCTTTTAATAAACTTGTTGTGTTGTGTGTCCCAGACAGCAACGGCAGCAACA
AATAGCAAAATAACAAACAATAATTACAGACGACAGTGTCTGCCATAAGTTGTGCCACCAGTTTGGCGAT
ACTCGGCGAAAAAATCACTTTAAAATTCTTGGAAAGCCAATTGTTTATATAACTTAATACACGTTTCGAG
TTCGTGGATTTGATTTGTTTATTCAAAGACATGCCAGCGTTTCCAACGAAATTAGGCGAATTTGTTATGG
CTAACAAACTATGAAATTGTAGATTTTGCTGTCCTGATTCATACGTTTCGCAGTGGCTGCCAAATTCGAT
AGTACATTTTAAATTCGCAATTGATATACAAGTGCGCACCAAGTGACTTTTGGTTAATGGTTAATGGCTG
GCGGGCAAGTTATATACTTGGGCAGTCTAAAGCCTCGATAGCTGGCTTGTATTGGCAGTCTTGGTCTGCG
CCTGAAATTGCCAAATGCAATTTTTATGCTCTTTGACGTGGGCGTTGCACACCCACTACCCCATCACTTT
CCCCAACCCTCCTACTCTGATATTGATTTGATTGTGTGCGTCTGCTGCTCTCGGTTGCTCACATGGCGTA
TGATTGATGGCTCTGCGGGCACGAAGGAGCTGCAAACGATGGAGCATTCCTGCCTGAGCTGCTGCTGCTG
GATATATAAATTGCACTTATTCGGGGAATTGACAGAAAAAGTATATGCACGTTAAATATAAATTAATCAA
GTAGAAGATAAGTGGAGGTTGAATCTGAGAAAATAATCGAGATTAAGATGCCGAGTAACAAGAGATCCAC
TAAAAATTAAACCTTGTTAGAAGTGATTTTTTATGGCGCTCTTGCTATTTTTCAGTGTAAGAATATAAAT
AAATATTGCTCACAAATAACCAGTTTGTTTTAATTATTTAATTGCAGTCACAGCCACCACATGTCTGGCC
CTACAGTTTGAGCAGCACTGGGTTCTAAGCAACGTGTGGGACGAAGAGTGAGAAAGCGGTAAGTTAGGCG
ACTCGTGTTACTTATACAGCTAAACGCAAAACCCATAATTTGTTCCCCATTGAAACGTATATATCTTATA
TCTTGGAGTCGTTACTTTTTTTATGGCCGTGTTTTCATGTGTCCATCTGTAGAGTTGTCGGTTGTCATTT
GTTGTTTTGGCTTTGAGATTTGCACTGCATTCGGAAACCGGTCGAATCGGTTTCTGTGGTGGTGATCACA
GGCATTTGCATAAAGTTTTGTTCTGTTTTTCTCTTTAATCGGTGTGTCGGTGGGATTTTTCTCCAACCGC
TGGATTTTAATTTATTTTCATTGATTGCACATATACAACTTTTGACTTAATGGCTCGCTGACGTAACTCG
AGCTGCTGAAATTAGCAAAATAGTCGTAATATTCACCTCATGGATTTCTACGACTCTCTGCAGGATTTTT
AGCAAATTGTGAAATGGTGCGATTTAATTAATCGATCTGTAAGTTTGGTTAATTTGCTATTGGATTTACA
ATTTGTTAAAAAACGCCGATGGGCGTTATATATGTGATTTTAGTTGGTGGCTAGTGGGAAATGAACACCT
GCAAAAGTCGCGTCAATTGAATTACATTATTGCATTAATTTGTTAGGCTTTCAAATCTTTTTATGGCTTA
ACGTAAGCtttttttttttAAATGTCTTCTTGGTTTCATTTGAACTTTCGCCATAAACTAGTTTCAAACT
ATGAGCAAACAATTCATATGAAATAAAAGCCAAAATGTCAATGGTTTATTTGATATTCTTCTCGATTCCC
TGCAGATTTATCTGATTCTGTATTAACTTCTGTTTCTGTTTTCTGGTTCTGAACAGGAAGTCGATGGCTG
GCCACCTAGGTACATAAGTGGGTTCTGTGGTTTTTCGGTGCCTCGGTTTCAGATGATTCGAAGGTAATTT
GTTGCCGGTCCGTGGGCCTAGACAAAGGACTGATTGCCGGACTTGACTGACAGTTGGGGCAGCAAAGGGT
TAAGGTAGTGGCCACACACAGTTACGCCCAAACTCAAGTTAGGGCTAATTTTGCCATTGTATTAGCCAAT
TGTGGTGGCTGTTCCTCGTTTCCATCCTCAGGTTTCCTTTAGCTTGCGATTTTTATTTCAGCTTCAGTTT

GTTTGGCCAGCATAATGATGCCACTTGATTGAAAACGAACGTGACTGGAAAACAAGTTTCGGGGTTAAAG
TTTTCTTCTTTTCCTTTACTCTCTCGGCTTCTTTTGCCACTCTGCGGTCTTTTCTACCAATTCGAATACT
GTTCGAATAAACAGAAAATAGCGAAATAAATTAGCCAGGAGATGCTCTCAGCCATGAACTTCTGTGACAT
GTTTTCCTGCCTCAGCTTTATTAGCCGACCGAGGACGAGGAGCCAGGCGAAATTGCATTTGGTATTATTT
AATTAAAGTTTTCTCTCCATCGTCGTCGTCTTCTTCGTTCTGATGTGGTCCGGGCGCCATTTTGTTGCAT
GCTTCCTAACAGAATTATGTGCGCATTTCCGTTTCCGGCACATGCAACAATGCACCGAGAGCAGTCAGCG
CGGAAAACGGAGTCTTCGATTTAGCCCAAGAACAGTGGAAGTaaaaaaaaTGCAATGAAATCTAAAATAT
GTCTTATTCTTAAATTACTTTTAATAGTAAATTCATTTCAAATTGCTCTATTAACATTGGGCAGAGAGGA
GAGCTGAAAAACATTCTAATCGTTTGGAAAATTAGCTTTCTCCAAATTAAATTCCGTTTTTGATTAAATC
CCATTTATTCCACTGTCCACAGCGCTGGATGGTGGAAGAATAATAGTTTGCCATTATTAAGTTTGGTGCG
CGGGTGTATTTTGCATGCGCTTTGTTGATGCCGCCGATGCTGCTTCCGATTTTGCTGCAGTTTGCTGCTG
CCGTTGCCAAGGCAAATTATTTACGGTGCGCACAACCAACAACTGAGTGTGGCACAATAAGTGGCTACGC
AAGGACCTGGTCCTGGCACCCTTCGCTCGTCCGTAAACCCATCACAAATCTCCACAAGATTGAGCGGCAA
GCCATTGCAATGACAAAAGTAATTTGCTTTTAAAGCACCACCGACTGCAGCATCGGGCGAAGGCAATTTA
AATATGCATGGGCCATTATGCATAAGCGATGCAGTTGCTCCATCCACGGATATTTGTGCACAGCGGGAAA
ATCGAGGTATCAACATAATCCAAAATGGAAGAAATATGGTCTCAGTATGGATTATTTGTAGTAGAACAGA
ATATTACAAACCTGTGTAGTGTGGCGAAAAGCTATCAACTGAAGTGCGTAATATGAAAGTGAAAGCTATC
TTTTCAGTGTACTCATACTTACTTATGGGTGTCGTGTGTGCCACATGCAACTCCAGTCGCATTTAGTTGC
GCATTCGAGCAAACCCATTGGGGCTCTCCACTGACCCACTAAAGTCGCGCCCGCCCGTTCGGTTCACGGA
CTCCAGGGACCTGATGCTGTAACTAATTCCAAGCAGAGCCCATTCGATGTGGCATATTTAAAACTTAATT
TACATGGCTGTCCTTTGGCCGGTTAAACCCTAACCTTTAGCCCCTTCATGTTCGTTCGTGCTGACCCCTT
CTCTTTCTGGCTTAAAGTCTCGCTGTGGCAGCTGTTTCGAAGCGGAGCACTTTGTCATTTTCAGTTTGTC
ACACTTTTGCCCAAATAAAATTTCATCGCCTGTCATAAAGGCTTGAGAGGCAAGTGTCACAAGTGCTTAA
AACTCAAGGAGGTGCATGGCTTGAATATGGGGTGGTGGCGAGAGTGGGAGCGGGAAAGCAGCACAGTTAG
GTAAGAAGTGAAGGAGACAGCACTCAAAACTGCTCAAGTGGGGCAGACCCaaaaaaaaaaaaaaaTAAGA
GAAATATGCCACATGAAATTCGTCATGAGACAGAAATCAAGAAAACATTTATATATTATGAAAGGAAATA
CATTTTAAAGCCGCTTGCAGAAATGGAATATGTTAGCAAATTTCAAAATCTATAAATTATATTTTAAAAT
TGAAATGGGAATACACTTTATTTCAAACTTTGCGGCTCAATTGATGTAATACGTATTTTTTAAGACGGCT
TCTTCTTTGATGTTTCCACCATAAAACAACCTCATCAACACCTTGTCTATCTGCTATATTTATCTCTTAT
CTCTTATATCACTGACTGCAAAACCAATTAGTGTTTACCCCACCCAATAAATATGCTTGCCGCTGTCAAC
GATTGATTCCCTCATAAAAGGTGGGGGAACCATAAAATTGCTCTACCCTCCCCAATAGATAACATGCACC
TTATGCAATCAATGCTTCCTGAGAGAGTCTGGCAAAAAAGTTTGGCATTAGCTGGCGTATCCAAGCCAAA
TAACTGTCAGGGCTAagacaatagaaaggacagacaacagacaggtcagacaTTCCCGGGTTGATATGAA
AACTGGTGTGTGGGGGTAATGGCGATCCAAACAGGGGAGCGCACCAAAAGTTGCTAGTTTGACAGCCGCA
AGACAGCTGTCTGTTTGaaaaaaaaaaaaCCCCCACATATACGAGGGTTAGCATAAAAAGAAAAACAGCA
GCTACATAAATTTTGATTTACTTGCCTGACAGTTGCCCCCCAAATTTTCACACGCACCCCACCACAGCCA
CCAAAGGTCGGGCACAATTTTCCTATTAGCTCAAGTGAAAAATCGTTGCAAAATCGGTGAATTGGGTGGA
TGGGTCTGAATTTACTGACCATTAAATCAGGCAGGCATTCTTCCATATGCCGCAGAACATATTTCATGGC
AGACCAGAGAGTAATTCAAATGCCCATTATGGACCAGTAACTTTTCCAATTAAAAGCAAAATGAACTTTT
GCCTCCGGAACTCGCTGGCCGAAGTTTCAATTAACGCCATGACTGGCTAAGCTGGAATGGCCAATAAAAA
AGGCCATCGAATTGCGAATCACCGATTTACCTGTGCGCCATTTTTGTCTCCAAATTAGGTTGAATGACTT
TGAAGCGACCACGTGGCGTATGCTTAATTATCGCAAATCGTTTTCAATTAAAGTGCCAAGTTAAAAAGGC
ACGAAATCTTAGCAAACGCCACTTTTGGCTTATCACTCTTACCACTATCACTATTAATTCCAATGCCTTG
GCCAGCTTTGCTTATCACAAACAGAAGTCTGGCGACGACACTCGCCCTCGTGGGttttttttattttttt

CGTGGCGTCTTTGTTGATAATTTCACTTCAACAGCTTGAAAATTTTGTGGGTTTGATTGACAGTCGAGTG
GCGGCCAAGGTAAACGGGCCTTTTGTGCCAGCGAGTCACCTGTTTAAACGCAGTTTTCATTTCTCGTATT
TACTTTTATTGATATGCGTATTTGTGCTCTTAATTTATGTATTTCGGTGGAGTTTTTTGACCGTGACACA
TTTGCTTGTTTACTTTCCAGCGAGGCGGTTTAAACGGGGGCAGTGAACCAGGGAAAATTGGAGCTGCATA
TTaaaaaaaaaaaaGAAACCGAAACCACTGATAACaatattgtttaaaatttaattataaatattaatat
taataaatattaagtatagatatgTAATCTTGATTCTTTATAGTTTCAGTTAAGTTCGAAGGTATAAGCA
Ctgttcacacatgaacacgaatatatttaaagacttacaattttgggctccgttcatatcttatgtaaat
gaatcgagagcgataaattatatttaggattttgttatctaaggcgacatgggtgcattgctcaaaaaca
tgtaatttaagtgcacactacatgagtcagtcacttgagatcgttccccgcctcctaaaatagtccctta
gtgggagaccacagataaggtcctcgccgctcaagataggcagatgtgcccgagcgtgggacctcgataa
ggcggggactatttacttaggcctctgcgtaggccatttactttaagatgcgattctcatgtcacctatt
taaaccgaagatatttccaaataaaatcagtttcttacaaaaactcaacgagtaaagtcttcttatttgg
gattttacatttggtcaatcgagcctttaatcgactctgcagtttcccccctaccaaaggtaaggaactca
gagaaaggccagctcctttaagcatcttacagctaaaggtagcaaaaataagtgactcttgtttcccccct
accaaaggtaaggaacagagtataaatataaaaagcaaaagatacaaaagaatcttttatgttttaaaac
aagcaccttatagtctatagctaaaggttgctttgtgtaccattataaattgtggtaaggcgtgcttgag
gccatacatcagcaattgtgaaattaaaaagtgcataacaaaagtgccttataaatgctctaatagcatt
aaatcagctcataaatagagtgcagtgtataagttattgccctgcaattgattctctaacatcttgtggt
tccacatagtctccgctgccatcaacgccaacgaacggttaagcgcgacatcgacacttctgcgctgcgc
cgcggccgacgcctgctgcgccgctgccgacgacttcacttgattgctagggacttagggaaacattttg
tacgctagatttagtttcaaatgataaattgcaataaacggtcgcttgcgatcttcaaaatcaaatcgat
aactgtaattattaactggcgcccgaacagggaccagcgaataaacgcgaacgaaagacaaaattctaag
tcgcggagcaaaatcaaaattttgctaaaaaatattcgttggttaaattgtgccgaagaaactcccgcga
gttattaacaaacaaaattcacggtcggctataaaataaaattgtttgagaaaaaaacagattttccgga
agaggaaaatacttatcggcatagcattgcccattggtggatcacagtttctgtcaggccagcccgcaga
aaaccttctttggagtgctgacgtggattccccagtagcccaggcaaaaaggacaacattcacccagcca
ggaggagtgaccgcaacaattctatgttaaataaatgcaaaacgaagaacagcaatttcatcgaaaagta
gaagtagaacgccgagaataacggaactacagaagagcaacacgggtaacagttaacagtgcaacccaaa
acgtaacggcagaggtaaagacgacgaacggcagagaacggctgcgtgaaccaaagtgcagacaagaagg
aaaaaaaaaagaacaacaacagcacagccgtagcagcagcagcagcccgccaaaaagaacaggaataaga
acacgttgacgacgaagaagctgaagctgaaacaaccgaagaggaggcagagagactacaagaacctgga
aagtacactggaaaaaacatcagcaatttgaaattcaggcatccacccataataaaacataagtatacca
aaaaaaaaaaaaaaaaaaaaaaaaattttaagtatattattattattatattattattataatattattat
tattattatatttttattattatattattagtattatattattattattatattattattattgatatta
ttattaatactatattttcaacccagttcctagagatcttctgaaaggaaaattttcctatttactgttc
ctttctggtacactgttctcaaagcaaaataaccgcggtgagctaaaatttattgcatgcaaaaataaaa
aataaaaaaatataaaaataaaaaataaaaaaacaaaaacaaaagataaataagcaaacacatacacacg
cattccatattttctgcccacaacttttgttaagttcaaattggtttaggcttgttttgtgcaggtgttg
tccgaattaaaaatcagtatggcaaatcctaataatataataagaccatcagcttttagttcaaacgaaa
gaccggtgccctcagaaagacacgacctgcctgaacagcatcgcgtagacatgagtgtcgaacagttgac
agcattaattggccaaacggtggcccaagttttacctggattgataaaacaaatgaacaacaatactctt
gactttactgacgtgagcgaccaggtcgtcgagcccgaatacagaaataatttagcggactttgacaggg
tgcctgatatcgtaaaatcgatcagggaattctctggaaatccagcagaattcgggtcttggaaaaagag
cgttgatagaatcatggagacttataccccatttgtgggtactccaaaatattatggtatacttcatacc

ataagaaataaaattgttggaagtgctgatgtggcactcgagtcctacagcattccgctggactggaagg
ccatgtcgagatgtctcactctgcattacgcagataaacgggacataactaccttggaataccaaatgtc
aactttggttcaaggccaccagcagtcggtggaagacttccaccaagacgtctacaaaaatctgtcgctt
attctaaataaagtcggctgcatgcaaatgagccgagaatccgagcactttgttacaaaaatgtacagag
aaaaagctctggatacttttatcagaggccttcgaggagatttacctcgccttttggcaataaaagagcc
agctgatctcccctcagctttacattattgccttaaacttgaaaatcaaactttcaggtcaaaccatgcg
gcgaataagggtcaacagtcgagtttcagaggaaacgaaaaacccattccggcaccccgcaattttcagc
cgtcgaatgcctttggccatcgacagcctccaccggtccctccacgacatcccatgaggagacctccaca
atatttaggccagccattcccggcgccaaggcaacacgtgtctaacttcggtaatgcaccgttcattcca
ccccgacaaaactatcaacagcaatggcaacaatacaatgccccaccccgtccctttgcgaccaaaccac
aaccaagaccagaaccaatggacgtggacggcagtgtacagacgcgaaacattaattatatgaatagacc
acactcggacataaacaaacgacaaaagaactacaacatccaaacagtgaacatgagacagccaagtatt
gaagtaactgggtccagttcaagcatgacaggctatcaacggtcgatgcaagattatgaaacccagaaca
atataaacggcacactaaatgaatactgtgatggacaaatagacaatttggacagcgaacaacgagacca
cgtgttgcatttttttagagtagaagactcctcactaccatacttcgaatgtagagtggggagtggaaagg
ttttaagggtgttgattgacacaggctctaataaaaattacatccagcccaatttggtgacgaacgcgat
accaaataacaagccttacatagctgatactccaggcggtgatgtaaaaatatcgcattacaaaagagca
agccttttcgatttcgaaataaaattttttttgttaccaacgctgaagacttttgacgccatacttggca
aagacacaatgaagggaatgggagcgcaaattgatttgaagaacctaaccatgacactggaaaacgggaa
aagagttgttctcaaagaaaaacagttcgaggctgttagcacaattagtccgagaataaaacacttagag
gtggaacagagaatgatattgaataaaataattgattcgtatccaggtctcttcgcagacccgaatcaaa
aactaacctacacaacaagtgtaagggcagcaatccgaactatatcggatacgccaatatactcaaaatt
ctatcagtacccaatgtctcttaaagacgaggtacacaaacaaatttccgaacttttacacgatggaatc
attcgaccctcaaggtcaccttacaattcaccagtgtggattgtaccaaaaaaactcgactcctctggca
agaaaaaatacagggtggtaattgactatcgaaaacttaacatggtaacggtagcggacagataccctat
ccctgacattaatgaagtgttggcccaattgggagacaacaagattttctcagtgctcgatctgaaaagt
gggtttcatcagattcttttaaaggaatctgatatcgaaaagaccgccttctccatcaataatggaaaat
atgagtttacacgactcccattcggtctgaaaaatgcaccgtcaattttccagcgcgcactggacgatat
tcttcacgaacatatcggtaagatatgtttcatttatattgacgacatcatcatctttagtaaagatgat
gaaacacattaccagaaccttgacactattttcagaactcttcagcaagccaacatgaaatgtcagttgg
ataaatgcgagttcatgaagagaaaagtggagttcctaggcttcgtcgtgtccgacaagggcattgaaac
cagcccaaccaaggtacaggcaatctcagactttccaattccaaggacactcaaagaactgagatcattc
ttgggattatccggatattacaggcgatttattcctaactacgctaagttagcaaaaccacttagctcgc
ttttgagaggggaggatggtcgaatttccaggacattatcgtcaaaaaaatccgtctcccttaatcgcga
agcaatagaagccttcaagaaattgaagagcagcttggtttctccagacgtaatactccactacccggac
tttaagaaagaatttcacctaacaacggatgcttccaatttcgcagtaggtgctgttctttcacaagaga
acagacccatctcatttttatcgagaacactctcgaaggcggaagaaaattatgccacgaatgagaagga
aatgttagccattatctgggctctaaaaaagctaaaaatttacctttacggtaaagcaaaggtgaaaatc
tttactgaccatcagcctttgacccattctctcagtagttggaatggaaatgcgaggattaagagatgga
aagcataccttgaggaatatgactacgaaattttctacaagccaggcagagaaaatactgtagccgacgc
tctgtccagaggaccgacagtcgaacaaattaacacagtagcctcaacaatgcacagctctgacagttcg
agccatgggctgatacctagcgttgaagccccgataaacgcattcaagaatcaaattttcttcagggagt
ccgagtcagagaactactcatttagcattccattcccgacatttcaaaggcatttagtagatcgcaactt
gttcacacccgatagtctcttgtcagatttgaaagaatatcttaacccatccgtgattaatggaattttc

acatccgaggatgtaatggggaaaattcaaattctctaccccatccattttcagggtttcaagattagat
tctgccaaagcaaagtcaaagaccttgttaacgaagccgaacaagaggaagaaatacttaggacacataa
cagagcacacagaaatgctgtggaaaataaagctcagttgtccgaaagagtatactttcctaaaatgagg
aaaaaagtttcggctatcgtgaatcagtgtttggtgtgtaagactgctaaatatgacagacatcccacgc
atccagaaataagacaaactcccttgccagaataccccggacaaattattcatattgacatctactcgac
agaacgacatctggtgctcacggcgattgataaattttccaaactggccatgggaagagttatcaaatcc
aaagctgtagaagacattaggaaagccctaagagatatcgtgttttattatggagtgcctaaattaatag
taatggacaacgaaaagtccctcaactcagcctctatcaaattcatgttgacagaccagctgggtattga
gctctacaaagcacctccgtataagagtacagtaaatggacagatagaaagatttcactccacactctct
gaaataatgagatgtttgaaaggagatggaacacataggggcttcgaggaacttctcgatagggccatct
atgaatataactacactgtccattctgtcacaaaaaaacgacctctagaggtgttcttcggcaggatagc
taccgtagctccagaaaaatatgaacaagctagactggacaatatagaccgacttaggcaaaaacaggaa
accgacatagaataccacaaccggacaagaaagcccataaaaacctatatcaaagggcaagaaattttcg
ttagggttaatacaagattaggttctaagttatcaagtagatttaggaaggaactagttaaggaagaccg
aagtaccacaatattaacagaatcagggaaattagtacataaaagtaacataagatcatgattgcttcag
aataattactgtggccacatcgatagacaagtagaacatagaaacattaaacatactgattaacgtaacc
acgagaattaaatttgaaacgattcgactttgacagacgagttggccataggtttgcagaattagattgg
gctcgtgaaagaagagattgcgttgtgaatgcgtttctgcggagcgagtttaaattgaatgagaccagta
gcctacttgaaaacaatataccgatttctgccttgacggtcaaataaatgttaaaactttcacacgttct
cgctttacacaacggaactacaatttgaatgccattgaaatctccatattgaatcagttaattccttatt
tcttatattttaaaacatgatcagtaaaggaaactaataataataaataaataataatttaatttgaata
ataataaaatttaccaattaaagacgaaatgtacggaatcaatgccttctgcaaaggttccaatggtcta
aaaatatgtagatataaattcattagtaatttagtaaaaaccagaattttaatagaagcaatcaagttgt
caatgcagaacacatccgagttgcacaaacctcgaacgcacgcaacaataaacacctggcattcaaacca
ttaatgctgtgcccaaactaccacgaatcaccccaccaacgatgcgactcaaaaacatcccatccttatg
atgaccgctcgaggacaagcgtcaattaagaggggaggagttattgccctgcaattgattctctaacatc
ttgtggttccacatagtctccgctgccatcaacgccaacgaacggttaagcgcgacatcgacacttctgc
gctgcgccgcggccgacgcctgctgcgccgctgccgacgacttcacttgattgctagggacttagggaaa
cattttgtacgctagatttagtttcaaatgataaattgcaataaacggtcgcttgcgatcttcaaaatca
aatcgataactgtaattattaacttatatgccataagagcataaattaaataaaaagtgcctgaaaacag
tgccttataaatgctctaatagcattaaatcagctcataaatagagtgcagtgtatatgccaaaagagca
taaatgccgaaataaatggctaaaaacaaaaaatctgactggactacaaaaataataaaacgtgccaaa
aaaaaaaaaaaaatcatctttaaacatcgacggagccttaaagaagagaaggaagtcaaattcaaaggag
cctctaccagcagcagaagcagcaacaacagcagcagcagaagcagcaacagcagtagcaacagcagcaa
caacagcagcaacagcagcagcaacaacaacgacatcagctaagtcaaaacaagaattttctgtttatcc
aaacacacatatatatataaatacatataaaatacatatacacgtactatatatattaagaaattacaaa
aaattttcaaaatgatgtcagaaaagactattcaattccttaagaagcagtccgaaattattttggaaat
tagaaagttggaagtaaaaccaacattaacagatgtagaaattctaaaattaaatgagcttcaaaaatgt
ttcattgctaatcatagcaatttgttaaagatcggcgttgtcgatcatgaatattttaacgcgaagcagt
atgatttaataatgatggtgttagaaaaaattaaaaataaaaatgaaaaaattaagggcgagtcggtaga
aaacactttccctaaatcaaacactgtccctaaatcaaaccctcccccctacattaaaccttgaaatgcgt
ggtcaccctgaaaaagagggtatagcacaaaacaacgctttaaaagtagagcaggcatttcgtaataatg
ttggccaatttcgagtatatctagaagatacgtctaaactaatagacagtagtccagatttccttaaaat
aaggaaaaataaaattgaatttttatggcataaaatagataacctgattgaacaggtgaatagtcatttt

gagagttcgctattcgaagaagaaattagcgaacttgaatttgacaaacaaaatattcttacagccatta
atagtcgactcagtggcacaataaataaagctgaaatgtcgacggttgttaaggcggaggagttaccaac
cctgcctaaaatacagattcccaccttctttggtgattccaaagaatgggatctttttaatgaactcttt
acagagctcatacatgtgagagaggatctcagtccttctctcaaatttaattatctaaagtcagcattaa
aaggagaagccagaaatgtggttactcatttactgctcggctctggagaaaattatgaagccacttggga
gtttttgaccaagcgatatgagaataaaagaaacatattctcagatcatatgaataggcttatggatatg
ccaaatttaaatttagaatccaataagcaaataaagacatttattgacacgattaacgagtcaatttata
ttataaaattaaaggcacaattaccagaagatgtggatgcaattttcgctcacataattcttcggaaatt
caataaagaatcactcaatttatatgaaagccatgttaaaaagacaaaagaaatacaggcactttctgat
gtcatggactttttagagcaaaggctcaattctatatcatcattctcacaggaagtaaaacctgtaaaga
aaatgattaataataacaagaataaaaattatagtgacaattgtgcatattgcaaactaccagggcatta
tttaattcaatgccataaatttaaaataatgaatccagcagaacggtctgactgggtaagaaaaaatggg
atttgcctaagatgtctgaggcatccgtttggtaaaaaatgtataagcgagcagctttgttcgacttgtc
gtaaacctcaccacacgttacttcactttgcaggtcataatccagaaaaagtgaatacgtgtagaacaac
aggtcaagccttgttggccacggccttgattcaagtaaagtcgaggtatggaggctttgaacaattaaga
gcattgattgatagtggctctcaaagcacaattatttcagaagagtctgcacagattctaaaattgaaaa
aatttcggtctcatactgaaataagtggagtatcttccacaggaacgtgcatctccaagcacaaagcggt
tatttcgataagaaattctccgaaaaatttagaaattgaagcaattattctcccaaaacttatgaaggca
cttccagtcaacacgattaatgttgatcagaaaaaatggaagaactttaaattagccgaccccgatttta
ataaaccgggtcgcattgatttaatcattggagcagacgtatatactcacattctgcaaaatggagttat
aaaaatagacggtctccttgggcaaaaaactgatttcgggtggatagtttctggatgtaaaaaatccaaa
ggaaaagaaaccattgtagccacaacaatagaaataaaagagttagatcgctactgggaagtggaagaag
aagaaaaagatgatatcgagtctgaaatctgtgaaaataaatttatcaaaacgacaaaaaaagattcaga
tgggcgatacattgtgtcaattccattcaaggaggatgtcaccttaggagattcaaagaaacaagcgata
gctcgttacatgaatctggagaaaaaactaaaaagaaatgaaaaacttaaggttgactacactaaattca
tgaatgaatacatggatttaggacacatgattgaagtgagtgatgaaggcaaatatttttttaccgcacca
ggcagtgattagagattcaagccttacgaccaaattgagagtagtttttgatgcttcagcaaaaactacg
aataacaaaagtttgaacgacataatgtgggttgggccacgagttcaaaaagatatttttgacattatta
ttaaatggagaaaatgggaatttgttgtttcggcagacattgaaaagatgtaccgacaaattaaaataga
taataatgatcaaaaatatcaatatattttatggagaaattctccaaaagaaaaaattaaaacatataaa
ttaaccacagtcacttacggaactgcatctgcaccatatttggctaccagggttctggtagatattgcag
ataaatgtaaaaaccaagttattagtgcaataattaggaatgatttctatatggatgacctaatgactgg
agctgattcggtagaagaagctaataaattaataacattaattccccatgaattgcagaaagttggattc
aacttaaggaaatggatttccaacaattccaaaatattaaccactgtggaggacacaggggacaataagg
ttctcaatattatcgaaaatgaatgtgttaaaactttaggactaaaatgggaacctcaaaaggatttatt
taagttcagcgtaaattgtaatgatgaatcaaaaaatataaataagcgcgttgtgttatcaacgctagca
aaaatatttgatccgttaggatggttggcaccagtcacggtttcaggaaaactttttattcaaaaacttt
ggataaataaaagtgaatgggatcaggaattatccatagaagataaaaattattgggaaaaatataaaga
aaatttattattgttagagaatattcgaatcccaaggtggattaattcaaacagttcttcagtcattcag
attcacggatttgcggacgcctccgaaaaagcatatgctgcagtagtctatgctaaagtaggacctcatg
ttaatataatagctagcaaaagtagagtcaaccctataaaaaataggaagacaattcccaaactcgagct
gtgtgcagctcacctgcttagtgaattaatccaaagactaaaaggatcaattgacaatataatggagatc
tatgcttggagtgattccacgattaccttagcatggattaacagtggtcaaagtaagatcaaatttataa
aaagaagaacggatgacattcggaaattaaaaaatactgaatggaatcatgttaagtcagaggataatcc

agcagatttagcatccaggggagtggattctaaccagttgatcaactgtgatttttggtggaaaggtccg
aaatggctagcagacccaaaagaactttggcctcggcagcagtctgtagaagaacctgtcttaataaata
cggtattaaatgacaaaatagatgatcctatttacgaattaatagaaaggtattccagtatagaaaaact
tatacgtataatagcatacataaatagattcgtgcagatgaaaacaagaaataaagcctattcatcaatt
atttcagtaaaggagataagaatagcggaaacagttgttattaagaaacaacaagaataccagtttaggc
aagagataaagtgccttaaaatcaaaaaggaaatcaagacaaataataaaatattgtcattgaatccatt
tttggacaaggatggggttctaagagttggaggaagattgcaaaattccaatgcagaatttaatgttaaa
catccaatcattttagaaaaatgccacctaacaagcttattaataaaaaatgctcataaggaaacattgc
atggagggataaacctaatgcgaaactatatccaaagaaagtattggattttcgggttgaaaaattcgtt
gaaaaagtatttaagagaatgtgtaacgtgtgcaaggtataaacaaaatacagctcagcaaataatgggt
aacttgccaaaatatagagtgacgatgacattcccgtttcttaatactggaatagattacgcaggtcctt
attatgttaaatgttcaaaaaatcgtggccaaaaaacatttaaaggatacgttgctgtattcgtttgcat
ggccaccaaagccatacacttagaaatggtaagcgatctaacttcagacgcatttttagcagcactcaga
agatttattgctagacggggaaaatgttccaatatctattcagacaacggaacaaattttgtaggagctg
caagaaaattagatcaagagttatttaatgcaatacaagaaaatataacgattgcagcgcagcttgaaaa
ggacaggattgattggcattttattccccggcaggacctcacttcggaggtatttgggaagctggagtt
aagtcaatgaaataccatttaaagcgtataatcggcgacactattttgacttacgaagaaatgtcaactc
ttttatgtcaaatagaagcatgcttaaattcaaggccattatacactatagttagtgagaaggaccaaca
agaagttttaacaccaggtcatttttttaattggaagaccacctttagaaatagtcgaaccaatggaagat
gaaaaaatcggaaatttggataggtggagacttatccaaaaaatgaagaaagatttctgggttaagtgga
aaagtgaatatttgcatacgctccagcaaaggaataaatggaaaaaggaaattcctaatatagaagaagg
gcaaatagttttattaaaggatgagaattgtcatcctgcaagatggcctttaggaaaggtggaaaaggtg
cataaggggaatgatgataaggtccgagtggctaaagtaaagatgcaggaaggatatatcactagaccca
ttactaaaatttgtcccttggaaggaataaagtctgttgacaaaaatgaggctgaccaagagccaaaaag
acgaactagagcgacatcgggaatgtccaagatcggaatcattatggcaatgttgttgtttgtgttaagt
tgtcaagtttctagcgcattacctaaagatatagcaccaagatattctatagacaaaataaataaaacct
cagcaatatatctagacccgctaggagatgttgagattgtgagtacttcttggaatttggttatctattt
ggatccatattttaaaatgttaacaaagggtaatgcgcttatacaaagtatgaggaaagtttgcgaaaga
cttcatagctttgaagagcaatgtagtctagtcttagataatatgcaaagtcagttatcggaacttgaag
aaaacaataaattgtttatgatacagtctagatctagaagcaagcgtgctcctttcgaatttatgggttc
cttgtatcatattttatttggtataatggatgaagatgatagagagcaattagaagaaaatatgaagaat
ttgttagataaccagaacaaccttgataaactaattcaaaaacaaacatctgtggttgattcaacttcta
atctattaaagagaacaacagaagatgttaactccaattttagaagtatgcaaataagaattgagaacat
gacagaagttcttaaagaaaattattatgtttataaggaatcaataaaattctttatgattacgaaacag
ctacactcattgattgaagaaggcgaaaaaattcaagcaggcattataagcctgttgattgatattaatc
acggtaggctaaatacaaatattctcaggccaaatcagcttaaaaaagaaattgccaaaattcagcagag
tctttcggagaacctagtaattccaggaaaacggtcaggtacggaacttaaggaggtgtatacactgtta
acagccaggggtttattcatcgacgataaattgatcattagtgcaaaagtgcctctgtttagcaggcatc
catccaaattgttcaggcttattccggtgccaattcgaaatgaagatcggataataatggtgcatacaac
gtccgaatatttaatttataattttgagatagattcctatcacataatgacggaagccacattaaatcaa
tgtcagaaatggcaactaaataagagaatatgcaaaggaagttggccctggaattcagcgaatgataatg
catgtgagattcagcctctaaagccagataaagcggcgaactgcatctataaaacagtagtcgactctaa
aagttactgggtagagttagaaaagaaaagtagttggttgtttaaggttcctgcgaattcaaaagtccgt
ctgcaatgtactggctctcaaattgaattgtttgatttgcctcagcaaggagttttaagcattgcgccat

attgtacggcaagaaccgacgataaaattctagttgcccaccataacattcagtccgaaagtgaagaatt
attatcaacaccttatataggagaagttagtgaagcgccgaagattatttgggatccgctgaaactatca
atattaaatcatactgaggaatttgaacgattgaataatgaaattaaatttatgaaagagaaccatcaaa
aattgaaagatttacatttccatcatatttccggacatgctggattaattattgctttaatactaatgat
agtattaataatatatttcatacggaaatgtgctgtgcaacaaagaatgcaagcaataacctttgcaggt
ccgttgccagtactataaatatcaatagtaaataaacaataaaataatataacaaataaaaatatacagt
ccactatatcgttgtttaatagaaaatgtacttctacatagaaaaagcaaaatgtttaaaataagttaat
tgagtacaaattgttgaattaaaaataatataaaccataattgtaatccaataaaattaaaagccagaaa
aactaggcccattgaaatcttagttgcaaaataaatgaacatatatcaaataaatacagtccactactgt
tataaatgcaactaatatactaatgtacatctcagcttgctggccctttggcagaatgttcacacatgaa
cacgaatatatttaaagacttacaattttgggctccgttcatatcttatgtaaatgaatcgagagcgata
aattatatttaggattttgttatctaaggcgacatgggtgcattgctcaaaaacatgtaatttaagtgca
cactacatgagtcagtcacttgagatcgttccccgcctcctaaaatagtcccttagtgggagaccacaga
taaggtcctcgccgctcaagataggcagatgtgcccgagcgtgggacctcgataaggcggggactattta
cttaggcctctgcgtaggccatttactttaagatgcgattctcatgtcacctatttaaaccgaagatatt
tccaaataaaatcagtttcttacaaaaactcaacgagtaaagtcttcttatttgggattttacaAGCACT
TTTAAATGCTTTCCTTTTTTCCACCTTTTTCTAAAGCCTTTCATTTTGTCACAACCTTTGACTGCAAAAC
CTTTCATCTTTCAGACAGAGCACCCACAACTTTTATCCTCAATTAGGATTTATCATTTCCCGCAAGCGAT
TTCCATTTTACACTAGTTTTCCCCCATTTCCCGCCATCTCCGTCCCCCGCCCTTTCGAAATGCCAACTCT
AACTAGTTGAATGCAATCAACACGCTCGCAATTAAATGCGACAAAGTGCAGACTTGCAGATGTGGTTGTT
GCTGCAGTTGGTGGCTAACCCGTTCCGCCCACAGTTCCAGTTGCCTCGACATGTCCCGCTCCGCCCGCTT
TTCCGCTCCATTTTCCGccccccccccccTTCCTCCTCCCCAACACAAAAGCGACAACTGTCAGCGGGTC
TTTGGCACACATTTACATGCCTCCAATCCTCCACCCACCGTGTGGCACCTCCTCCTTTCCAGCTAGCGTG
TGAATTTAATTTGTGCATAATTGCAATTGCAACTGCCATTGCAGTGCTGCAGCCATAAAATGTCAATATC
AAATTGTAATCATCAACGAGAAATGTTTGCGGCAGCCCAAGTGTTTTCTTTTCACTCTGACAATTTGTTT
TTCCTATTAGATAGGCCTGCAGACATGAAAAGGGGCTCGGAAAATCGGGCCAATTGCCGTCTGACTGATA
TCATCAAGTGCGTAGGAAAAGTGCAGTGGACAGTTTCGAAAAGTAAACCAATTTAAAAATTAAGTGCTGG
GGAAACTATTTTATGTTGTTGTCTGGACTTGAGCAGGCTTTTAATGGATTGCAATTTGCATATGTGTACT
AAATTTATGCGAAAATATTTAGTGCATATGAAAGTGGAGTGAAAATTAATAGCTTCATTGGGTAATGCAT
TATGATTCGGAAAGGAAATATTATACATTATAATATATTTTATTCTTATACCTCTAATTTGATTCATTTC
AGAACCATCATGCGCCAGACTCAACGAACGCAGCACAGCGAAATGTCCAAACAAAACGGATTCCAACAGC
AGAAGGCCGTAAAAGAAAGCCAAATAGATTGGCAATAAAAAGTTAGATAAATTAACTTAGTAGCTCATCA
CGCCCAAAGGCAAATCACTTTGAAGTGCAACCAAATAAGGAAAGAGCAAATTAAGCGAATAGAACGGCTA
GAAGGAGATCTCCCAGTGGAGCGGGAGTAGCTTCGtttttttttCGTGGACCGTGCGATAGAAAGCCGTC
AAAATTACGACATACCACCCACCCACGCCCAAAGTCAAACAGAACCCGGCCAATACGGACGCCATGTCCG
GGTCGGGTGCGTTTGACGATGAGCCGCCGCCGGAGCCGCGGGACGGATGGCTACTGGTGCGCATCCATGT
GCCGGAGCTGAATGTCTACAAGTGTCTGCAGTTCCCATCGGAGCGGCTCGTTTGGGATGTCAAGCAGCAG
GTGCTCGCCTCGCTGCCGAAGG
>dme_circ_0004372_junction_seq
CAGCAGGTGCTCGCCTCGCTGCCGAAGGGTAATTAAATGGTAATTTCTCATCGATT
>dme_circ_0004373
AACCATCATGCGCCAGACTCAACGAACGCAGCACAGCGAAATGTCCAAACAAAACGGATTCCAACAGCAG
AAGGCCGTAAAAGAAAGCCAAATAGATTGGCAATAAAAAGTTAGATAAATTAACTTAGTAGCTCATCACG
CCCAAAGGCAAATCACTTTGAAGTGCAACCAAATAAGGAAAGAGCAAATTAAGCGAATAGAACGGCTAGA

AGGAGATCTCCCAGTGGAGCGGGAGTAGCTTCGtttttttttCGTGGACCGTGCGATAGAAAGCCGTCAA
AATTACGACATACCACCCACCCACGCCCAAAGTCAAACAGAACCCGGCCAATACGGACGCCATGTCCGGG
TCGGGTGCGTTTGACGATGAGCCGCCGCCGGAGCCGCGGGACGGATGGCTACTGGTGCGCATCCATGTGC
CGGAGCTGAATGTCTACAAGTGTCTGCAGTTCCCATCGGAGCGGCTCGTTTGGGATGTCAAGCAGCAGGT
GCTCGCCTCGCTGCCGAAGG
>dme_circ_0004373_junction_seq
CAGCAGGTGCTCGCCTCGCTGCCGAAGGAACCATCATGCGCCAGACTCAACGAACG
>dme_circ_0004374
GTAAATGGTCCAGTTCCTGAATGCATGATGAAATTCGTTGTTGAAGGGTACCGCCTATTTCCATGTCAAT
TTTGTCCTCTGCCATATCTGCTACCAAGCCCACTTCACCGATTGCACCCCATTGAATAGCTTTACCTGGA
AAGCCCTCTCTATTACGGTGCTCAATTATACGTTCCATAACTGAATTAGCCATCCCATAATTTGATTGGC
CTGCATTTCCCCGACCACAAGACACACTGGAAAAAACTACGAAGTGTTCCAACTCGGGACAATAAATTCG
AGATAGGTCGTCTAG
>dme_circ_0004374_junction_seq
GACAATAAATTCGAGATAGGTCGTCTAGGTAAATGGTCCAGTTCCTGAATGCATGA
>dme_circ_0004375
GCCCGAAAATTTCTATTCCACCCGCTATGATCCGATCATACATGGGGTCCACATACACATCAAAAACTCG
ATTTTCAGGATCCATTTTGGTCATTAAATCAAAATGATGCACACCGTTTATCTTAAGTTTCCGAATTTTG
GTGG
>dme_circ_0004375_junction_seq
TTTATCTTAAGTTTCCGAATTTTGGTGGGCCCGAAAATTTCTATTCCACCCGCTAT
>dme_circ_0004376
GATCGCGATCTCGCGGAGCTCAGAGGCCGTCGGAACATAGTTCCAAACCATGGAACTTGTCAACTCCTGA
ATAGGCGCAATCCTATTAGCCAGAAAAACGTTGAGTCTCGAAGGTTCATCACGAATCCAGGACAACGCCA
CAGTGGAGTCGCACCAACAGTAAAACCGGACATTAAATATTGTCAAACTAGCTACCTCATGCATAAGTTT
AGCTAAGAGATCTTCATCACACAGTTCCAGATTAGGAACCGTAATTGTGTTTAATGAAGCAACTCGAGAT
TTGGAGCCAAGTAATTGGCTGAAACGATGTTCTCCTTTGGACACAATGTAAGCACGAGCACCGTAAGCCT
CAAGGCTAGCACCTCAAAACCCACGAATTTCAAGATCTGAATCATCCCaaaacgtggaaaacgtggaaaC
GCAGCGATACATCTTGCAGATGTAACGAAACCAGATTAATGTGTGTAACAGCTTAGACTGGATGACTGGA
CCCGGAGAGCAGAAGAagctgattcgccaaatacgactcaaagcttagttgtagagaaagaaagatagct
ttcttcatgccacagctatacggaaaaaagtatcggcaagaaaaggaacaagggacatatgtccaaggcc
cagatactcctttatgaatgcagaatactggtccttcacgacaggattccgattaagcttcctttcaaga
gatagaaacctttgcatggccagctgataggtgtctctcaaaagatctaaatttaatttggtaggaaacc
ggacggaccagtcggcattcgggcgtagtgctttatgaagagcggctcacagtctaactgttcctctgta
gacttatctattgggtccggaccactttctaccttccagaagcgttgcaaaTCTTccagacacaagtttt
ctgcatcatggacaacactggctataaacgtgttctcccaacgaacagaagctcatagaaaagactagct
cctaataacagatccacgcgctgagcttttttaag
>dme_circ_0004376_junction_seq
cagatccacgcgctgagcttttttaagGATCGCGATCTCGCGGAGCTCAGAGGCC
>dme_circ_0004377
GAAAGTCAACCGGATTTTATTAAATCAATTTCCAAAGGATCGTTTAAGGACAGTCTTATAGAAATTGTCA
TTATTTGGCGGCGAGATAAAAAACGTATTAAGTATAATTGGGTCTACGTCGCCAAACAGAATGGCAACAG
TTCACCGGAACACTTGATGAATTCAACTAAAGACCAAATTTCTAATGCAGTGAAAAAGTTATTAAAAAAT
TCGGAAGCGTTATGTTACGAAGCCATTGTAGAGGAACTTATTTAACGAGCATTCACAGGGAATTTTAACT

AAATGGATTTCTCAGTTCTTATATGTGGTCGATTACCTATCTGATAATATTGAAGACGAGCACCTTTTGG
CGGTTTTTTCTCTTTTAGGAACTATTGCATTTATGTTTACTGTCGGTTATGTATTGATCTACTTTGTTAG
AGTTGAAGAGAAATATCTTAAGGCGCATGGCCACCTTATCGATATACAAGG
>dme_circ_0004377_junction_seq
CGCATGGCCACCTTATCGATATACAAGGGAAAGTCAACCGGATTTTATTAAATCAA
>dme_circ_0004378
ATTTGTGAATACGATTAGTAAGGATTCAAGCAATATAATAACTCGGTTTAAGGTCAATACAAGCCTAGAT
ACACTTATTTTATTTAATGAGGATACGACGACCTTTACAGCCAGTGTTTGTATGGAAGAAATCCCAAATC
ACATCTTGGTGAATATGGTATCTACTAATCAATTCTTGGCATTTCCTCGAATATCATCACAGAACATTAT
GGAAAGGTAAGTAATATCTAACATTTTCAGCAATTTCAATCAAAATTTATTCTTGTTCTAATTTAATACA
AATCTTTACCTAAAATCTTGTGTATCAAAATTGGAGAAGTAAAAAACACTGCGCTGGATTTAGGTTACTC
GTAGAATTTTTCACCCAGCGAAAATCTCCCAGTGCACATTGTGCTCATGAAGCGTGTCCTATATACAGTT
CCATGCCTCTTTTCGAGATTTTTATTTTTGATCTCTCTTTTTAATTGGTTTAAATGAGGCAAGCCACATT
GAACGCATTGAAGAATTTGAAAGATATTCTTTTATTCAACGTCGCTAAAATACATCCTTCAGTTTTTAAT
GCATTCAGCCTTCATCTTAATTCCTTAAATTTGGATATGACATTCCAGGGTAGCCGTTAAATTTAACTTG
AGTTCTGTCGAAACGCACCCAACCATGACCAAATGTTTGGACGATCTAAAAATAAATAAAGGCTAACTGC
TTATCTTCTCCCATTCAATGGATAAATTCATATTATTTAGCTTTAGTGTTTACCCTTTAATCTCGATTAT
AATCTTGCTGAGAACCTCCAGTCATTCTTGCCTTTCCAGTCACCCTAGGAGCTCTTCTTAGTTGGAGCTC
GAAAACGTACTCCTGAATTGGCCCCAGGCAAGGACACTCCGATTGTTTACGCTTATCGAGAGCCTGGGGA
TAGAGTTTTATGGGCCTATAGGCCCGTCTATCAgtctgcccgtccgtatgaaagacgaaatctcaggaac
tttaaaagctaaaaggttaagattaagcatatagattctagagacaaagacgcagctcaacttccacgcc
cacacttatgaaaaacgtgttgatatttataaatatttgcattagtctagcaaatttctatctaaaaaaa
acctaccaaaaaaactttttgctacgcccactctaacaccctaatgtcgctaagccaatcacgcccacac
ttttgaaatttttttaaatttttttcttattttattccccaatatctatcgatatcctataaaaatgatca
agtttcgagttcgcattcgagaagctcgactatagcattctgtcttgttatacccgttactggtagagta
aaaaggtatactagattcgtttacaagtatgtaacaggcagaaggaagcgtttccgtctatataaggtat
atatattcttgatcatgatcaatagccgagtcgatcaagccatgtccatctatccgtatgaatgtcgaaa
tctcaggatctatataagctaggaggtttagattcggcgtatcatattggactaatctaccccacgaata
aaataaatgattaggtataacttaacgtcaatacattgtgaccctttgtcttaataaatataaatacaca
aatacccaaaaagactaccaagaactacgtaagcactccagagccccagtaatacgatctaacgcttata
cataagccgatcgcggagcgtgcacgctgagcatgcaagtgttcaatgcctttttgcatacttatgtatat
gtataaatgtaagtaaaatacatagatattagccacaagttgggttagctgaacttcagcacactttgag
cattcaaataaagatcaacgaacagagcagaagctctTCTATTCTTATAAAAATTAAAACATATTACTTC
AAATAAATTAACATATTACTTCCgcatacaaattctagagacaaagacgcagtgcaagttgacccatgct
gcgacgcccactctaacgcacacaaaccgcacaaaactgccacgcccacgctttctaaccatttttcgaa
attttatcataattttattagtcttgtaaatttctattgatttgccaaaaaactttttgcctcgccaact
ctaacgccctaaagcggccgagccggtcacggccacacttttgaacaattctcgatatcccagaataatg
ataaaatttcgcgtttgcatttacaatagctgagtaacgggtatctgtagtcgggagcttgactacttca
ttctttcttgtttTAAATTGAAATTGATTTTTCACCTGAGTTCAATTTAATAAGAACATAAATCGGAAAA
TTAAATGGTTTCGTTTAAGTAAAAATCAATCAAGCAATGTAATTTGCTAATGAATTTTCCATCATACCAT
ATAAGAAACTGTTGTTCTATCATGTGGATGTTGTGTCTGCCTGAACCTCAATTATTATTATTTTCAATga
atgaggtaaagctgaatatgttgtttgaaaaaatttggcgaatagatcggctattgcctgatccgatgtt
accgtagtgtttccaaataatagcgaggaagggtaagaacttgtttttcgcttagtattaacgaaattat
aaaactgttgtggatcctgtgcgaactggaacttacaacgttttaaataattcttgtagcactgagcatt

aagcacagtaaagtttaaccatgcgcttaaatatttagaaaggatggactgagaaccggtatttttaaaa
tttgtatatagtctggactttacatttcttaggtgtgtcaactctttattaaaccaaggaggtttagaga
ttgacggatagtacattgtaacacaagagtcaaaaaatgatttaattgcggtataaaacatattaatggc
atccgtcactatgttgcaggagtaaagatcagaccaattaaagccagctataaaattagttagcctccga
ctgatgattctgatattgtgggaattcagaagatggcggaatatacgagcacgcaatataaactgatctg
tcggaagatgacagctttacgcataagaattcagagttacgggcacgtcaataagtaactatcctgccta
aaatcacccgaacatatggtcgaaaaaagttcattggggacagatatcttTTGTCTTTTATATAAgattt
gctgtgattgacagatgatcagttgtgcggggccttaggcagcctaTggatggactgacggtatttttaa
atttttgtttaaaatctagactttacatatcttatgtttgttaagtctttagtaaaccaatgagaccaag
acttaaaaactgttttaattgcggtatcggacgaattaatgtcaactataaaaatatttagcgtcagaaa
gtaagccttgcaaacacaatgagttcgcttaattgacttctccgatttctcttttataatgattcctatg
tcgagtgccacctcATCAAGaatcatcgagaactatccattggtacctatcggacagtctatttgaggcg
gactcgaaagcggccaaataatttaggtgttctggatatttagaagacggcagaatgtaagaacacgtaa
tataaactgatctgtcggaaaatgacagcattacgcatatgaattttgagttacaggactcgtcaaaaag
ttactcctccaacgcgagggtagatctaaccgcaattaagactccactcccccCCACGAATACTACCCAT
AGCatcattcctttcaagccacctttagtttagttagaacccctagacaagtatcacaacagtattttag
atttcgacacttaggttttataaccaggcggcaattattttgaaagcagacaaaccgaacaactgtacaa
agacacaaaatcaaaagttgcaagcgagcactatttgcatacaaaaacaacaccaaacatcactgctaac
aacgcatgaaagagagagtaaactaaacacaaaaaaagacagaaaggaaaaataattagtaaatttgttt
aaatattctgtattaaaatatatactttgattaactcgcgaagcgaacggggtttcaataaatatgtAGt
ttttttgtttgcatgttttgcttatttattagtcttagtcttagtctttttcattttatgaaatgattcg
ttctgtgtggcctaacaaatttaacgctgggctggttggtcgttccggtgcagtcggctttggctgcata
ctagaataagttttcttgcaagaggatttggatgtgaggctagtttttcattgtattttactttcgtctc
tgctattttcttaatgacaagtttatatgtaagtctttgtgtatattctcgttccgaagataccatggag
ctccagtaatgattctcagaatcctagactgtgctcgctgtactatgtcgatgttgcttctcgatgcgtt
gccccatagctcggagccataaatccatatgggtttcaaaagttatataagaggactttgtactccagtc
ttaggggagagcgaacgtttataagccagtgtagatgccttgcttttaatgtgagttgcgtcgtcttgga
ctctatgtgtttccgacaggtgagccgctcgtccagatgaatagctaagtatgttacgttatcggcttgt
gggatgcgcatgtggttcattaccatgggcgggcagctttgtttgttagggtaaatgtaacctgtttgca
ctttcttcctttactcgtatgcgccaatttgcaagccaatgttttacacaagatagatggcgtgctagtt
gcatcgtagcttttactgggtatctggatcgacttgtattgtagtgtcatcagcaaatgtggatatcgtt
agctggtagtttgttaggatgttcgccgtttatagggtgtatagaattcgtcctagagcacttccttaag
gcactccagcttctatattacaatcgcttgtatgttttgaggcagcagtttgattattttaaacataagt
ccattcagccatactctatcgaatgcttgtgcaacgtctaagaatagagctgcgaatttctgttgtgatg
cggttaacctgttcaagggctccatgttttgcccaaaactcaaaatggtgcgatgggagtgtgttgtgtg
ttttgagatgggggctgatgcgtagcagcagcactttttcaaataatttggatagacaagtaagtaagct
tattggcctgtatgatgatggctgtgttttgtctttcccaggcttagggatcatgattataattgatttc
ttttccagttttgagttgctctttaatgactttcgtaatctcgcttagtcgaaattctacaggatcttgt
ggtgcttagtcagatgcagttaaaggcgggagagtaaatgagttcgtggctggatttggaagatgatcag
cgaagactcttgctccgccgtgtcactgcgagaccagtttccggtagagttacggagaggtacaaccgtt
tctattggtgcctgttgtctttccacaacgagttctttgtacctgtgggcgtaagattctcgatgtatct
tagttgtgcgtccgctgcttctttcttaagcactatggtaagtctgcgagaagctgctttcaatttactc
tttgcgccaggtgatctgtgctcctgccactcccttcgatgtcgtcgtttttcaagcacaagcagtttaa
tatctctacttgttttgttaaactttgtgctgcatacatggttgtcttgtggagttgacgcttttgctgc

tgcgaaaaatatttattctatatcaccaccacactgatcaatgtcttcctcggtttccaggggggctagag
aagccggtatggcaacaatcataGATCATAGAACAtaatccttgccgtaacactttcaaaaataaacttt
atcagcccaataatgtatgttaaacaacgttgatgttaaggagatgacattgaggcatttcactaatatt
aacgtaccagatgtattagaagtagcttctattaaagcatttaaaaatagtgacattttgtatttcctaa
ttaagtatcccatacctaagttagtatgtaaaaaataatatatttcccgtaaaacattctggtaaaattc
tggaatttgaagactcaaacatcgtagcgactgcgttacggatatctttgctgttggcgaatgtaattca
acagtgagcaccactcaaaaacctgaaaacacccagttgtgcgcaccaattaaaagttggaaccctagcg
caatgcagcactcgccctggccatctggataggcacggatgtaatggatgtaatagacgacggcgggatc
atcatcaacgatgatactgTAAACATCTCAGGCTATTCGAGCTCAGCGTCATCTATCTCTGGAAGCTTCC
TGGTGAACTTTGACGATGAAATCACAATTAACGAAACACCGTACACCAACAACTATGGTGTCTTTGCAGT
CACTGCAATCAACGTGACCAAGCCTCGGGAACACTTGCCCTCTTTCATAAGATGAGCCTGACGATATTAC
GGAGTCACATTGACAGCTGTCATGATTTACCTGGTACTGGTGCGAAAAATCCAACAACGCACGAAACTCC
GGGAGCGAAGTGAGATCATTAAGAACCTACAAGCAAATGCTAGAAAAACCGGGGAGGGAGGGAGAAGATA
ACACAAGCAAAGCTGAATCGGGTTTTGCGACACCCAATGGCGGAGTAACTTGTTCCGCACAAATAACAGC
CGGTCAAAAGGGTCTCGAGGCGTTCTAACGCGATGCTGCCGGGTATCCATAGCTTTGGTTAGGCTTCAAA
TGTAAGCTTAAGTCAGTTTTAAGAGAACCATAAACGAGATCCAAACGGATCATAATAAAGCCTAAAAAAC
tttttttttattatttttATTAAGTGAAGAATCCGTACATCATAATATTGTATCTTAACATCACAGAG
TGGAGAAATATTCTTAAGAATTACCAAACACTATATTAAAATAAGTGAATAAAGAACTCAAAtttttttt
tAATATTTGGCGCACAACAGCCTCTAAAATTTTGGAAATAAAAAATTATAGTGGAAAGATATAACAATAA
ACATTTaaaaaaaaCACTCAATGAAAATTAGAATTCTTTGACAGTGACCTTTTTTTCAAATTAAGATTTT
GTCGGTATAGTTGGACTTGACCTGCTTATCCAAGTTGACACACAAATTGATCTGAGCAACGGTATCATTA
GGTTTAGGTTTCCAGTGTTTGAAcaaagacactagaataacaagatacgtaacggccatacattggttta
gcactatgcggccactttttttggtgacggccaaaattgctctctttccgctcgctcacgctgagaacata
agaaatctaaaaatagaatttgcttgcttgtgtgagtaaaaactagaaaacgtgttgtgctagaagacga
ttttcgggccgaaatcaattctgatcgaagaaacgaatttacgtatttggggagttttggccaatttcgt
agcaatatgatgaaattgcgcgcctttttaaaaatatcaaattggaataaacaaaaaaaaaaacaaaaaatg
aaactgagcaattgaatctaatgtgcattttaaataataataatggatcatacatacatcatattaagtc
aaatgacttaataaatatacaaaataattaaagcaaatacaaaggaaattattataactattatatttat
acatatttacataaataattaattatatgtaatagacgtaataattggtacccagggtacagcaggggag
gccattacaattgtaatgcggtcctatatttatatgaccttcgagtTTCTTTttcacaacctatcgcaat
taccatttacaataaaatttatttccatactttactattgcaacaaaacgacAaaatacaaaggaaatta
ttgtgactactgtaaattaaaacgtaaattttgcaaaaagaagtcattaattgagaaaaaaatatttcat
aaaaataatacgttgtagaaaaatttataaaataaataaaaataaattgcaaaagtaatatcttgaggca
cgcagtgcggacagaaggactcaacaatcattgcctcattaatttttcaaacgtcgcaagctgaatactc
taatgacaaatattctaatataaagtcatttttgaaatttattttgttatattaggtacatagattcggc
tatttctaagctatatttaattaataataacgttaaggcaatgaaaaaccagaatttttttacatggtacc
aattgataaaaaataatatagatttaaagtttaaaaacttctaagacaaaggacatattttgtcaaattt
ttatcggctgtcactgtatgcgtagaaaagagctgttcgatatagagctctccgctctctcgctcttgaa
taaagttcgagagagcctggagacacctctaaagccaagccgaaaaaatcgtggaccaaaaaatggaatg
gcgttacgcatcttgttattctagcgtctttgGTTTGaaacagtcaagtcgaaaggtttcacagcactat
tgcggaaattgctcgttgtatggaacttgagaaacagataagcgacactgtttattgttttcaataatcg
aatatactaaaacaattcattcggtaaccaaaatgagaccagttgaactcattcgttcaaACAGTGTGAA
ATTAGGACTGCAAGTAACACATAAACTTGTAGAAGCAGTCTAAAAAATCGAACAGACAAATAAAAAATAA
GAGTTATCAAGAAGGAGAAAGGTCTTGGGTTAGACACTAAACTCGTTTAATCAAGACTATATAGGGAACG

TACTGCTCACTGACCGTGGACACATCGACACACTAAATACGACACTTAAGGTCAATAATTTACTTTGTTA
ATTTAGATAAGTCAGCCTTTCAAGTCATTATAATTATCTAATATTGTTTTTACATTGTTTTATAGGGTGT
GTGCCCAACTGAATGGAACCGACAAAGAAAACATCTGTGTGTTATTCTAATCACAGAGAATAATAGGAAA
AAAGACTTTGAAAGAGTCGCTCTAAGAAATATTGCTTTAAGTGTTGGGTACAATTCAGAAAAAGTTCGAT
TCGCCTATATATTCAAGG
>dme_circ_0004378_junction_seq
AAAGTTCGATTCGCCTATATATTCAAGGATTTGTGAATACGATTAGTAAGGATTCA
>dme_circ_0004379
TTAAAGTTTAAAAGATGATTTCATGCTTAATTAGTAATTATATTATAACCATTCTGGTCCTGTGTACAAC
ATCCTATATACACGTTTGTTCGTCGTTAAATGACCCATACGCTATATTGGGAATAAACAAAAAGGCAACT
ACATACGAAATAAGAGAAGCTTACAAGGAATTAGCTAAGAAATGGGTAAGTCTGCATAACAATTGCGTCA
TAAAAGTTTAGAATGTTTAAAATTAAATGTTCTTCTATCGTTATATACTGGGTATAATGTTGTATACCCA
GGTTGCTTAAGTTAAGTTGCTTTGCAGTAAAAAGGttattaattattattatttatatcccttatcgtag
agtattatattcgtttaaaagtatgtaaacaagagaaggaagttagtttccaaATATTTCAAATGTTGTT
GGTAGCTGCATACCTTATTTAAACATTCTGCGTTATatatttttataattttgtatatcttgtaatattc
tgtcgatttgtcaaaaaactttttaccacgcccactctgacacccaaaaaccgcccaaaactgcccacac
ttttgaaaaatgttttaatattgcttaaaatttttgttagtctttcaaatttccatcgatttgcAATGCC
CAATCTAACGTCCACAAAGCGAAACGACCACAATTCGATCttttttttATTATATTTTCAATTCCAACCG
CTGATGCAAAAAGCGTGACATTTTTGAAGCGCAAGGCGGCGGCCGCAAGTGGAACGATTAGCGGACTTCT
GCAATCATTTCGTTTAATGAGCTTGCTCTTCGAGTGAGGTTGGAGCTGATCGATGAGCTCCAACAAACAA
TTAGAAAAGTTAGCGATGAGCTCGAAGAACTTGATTTTGATGAAACCGAACGTGACTTGGCTTAGAAGTT
TGATGACATTCTTGTAACTCTTAAATCCTCTATAAGGGGGAAGATCTTAAAGGGCGGCTCGCAAATGCAC
TTTTCCACTTAACTTTCGATGACGACATTGGTCCGTTTGATTGAGGCCCCATCAGCTTTCACTCATAAAT
AGGGCACCGTCGCTGCCATCTCTTGAACTTCCAAAATCGCTGGAGGCTATGCTAACTTGGTCAACATGGG
TCCACCCACCAGCACGCTGCCGTAATCCCAATTGCTTCAGGGCGGCCAGGGTCAGGCACAGGGTGCCAGT
GGCGCTCCGGAAAGCGTTGGACCGGACAATATATCGCATGAAAACGAAATAAGCTCCTCGGGACCATTCC
AGAAGattttgtcaaatttacaatgcaagagcaaacgtgtgcgcacatgcagttgtcggctgtcactgta
tgcgtagaaaagatctgttcgatgtagacctctccgctctctcgctcttgaacaaaaattcgagagagcc
tggagccacctctagagccacctccacgttgtacaaatcgtgtgccaaaaaatcgtatggcggcgttacg
catcgttattctagagtctttgCTTGTACTCTCTCTGCAGCTCATTATCATTCGATTTTCGTTATTTATA
CATATCACATTGATGCAAAGTCTTATTCTCTGAACACAATAAAAATAGATCGTTCATAAATCGTTAATAA
AAAGATTATGAGCTTAAAATCCTATTGGAATTTTCCTTCTCCTACTTGTTTCTTTTCTTAAACGCAATCC
AAAAGCCCACAAGATTGACTGTTTTGCGTGCCTTTACTGTGTCTAGGTATTTTGCTAGCTTGATTGTGAA
GTTGGAGTCAGTCAGAAACTGAAATTTAAAGAAGGTAGTTCAAAATCCAGGAAACACAAGGCCACAAGGC
TTGAAACAGGAGGTTGCAATGTTTGAGTCTGGCCATCAGAAGTGtgatcgtctcatgtacacttttaaaa
acgcagcatagcaacctgatcgacgtttagaatgcagtaaaaagtgcacagtcaacgcgaggcgtaaata
aattcgtcatactgccaaaattcaattcctattccgttggcactagtgcagctgcgtggtaagccacggt
ggacctcattctcgcagaaaacccactaatgggcagtgctctgcttgtttccatgagtaaatcacaagtt
tgcttttctggaatcacgtgggtcttatttgaggaattgtttttgcaacaattcgaaggcaacgaaacac
ctgcacctactgttttaatggaaaacggcgagtgatttccctgtatgcaagacacttaataacaacacta
aaagcaaagtggaactcgatgcctacggaacgcggtgtcggtagttctagcgcacacggcctatatcgac
acaagactacaacgaactgtttatacaaccaacatcagaacacgtgacgaattgcagaatgagttgcggg
aatttgcttttgctaagaagaaagaTTAAAGATTAAGAAAGATTAAGAATAATTCTGCGGCCAAAAGATA
ACGTGTTCACGCACCCATTATTTGCCTTTAACGTTGCTAAGCTAcaagaatgatccaacttaaattctgg

acgttcatctgattcaaggaccaaaacaaatacctgctacaagtgtggggcttctggacatattgtttct
gcttgttctgagagaaaggaatcggccactcattataaattcgatgagaggggtgtaaacttgtgccagg
tggccgacccaattggtcctttttttatccggtttttatatttctggagccgaatgttcgcaaagaattt
gtatctgatagactttcgggaacaagaataaataatatcgttgttttaaagggaataggaaataatttag
taataacaactatgcatatatagtctaatgttataatcaattcttattctcttgaagtaatttttcatgt
tgttctcaacagtcacataaattatgacattatagtcgggcgtgaaatttAAGCTCAACTTTTGAATTAT
ACACGACAAAATCTgggtttccgaaaagccgagtcacaactggatagtagcaatacgcttgatcgatcct
aacaagactgtgcgtaggcggcaatcggcaatatcggcttagtatcgcggaaatttgaatcgtaaaagac
aaattaagggaattgttggcttcgaatataatacgccatagctgttagcaatttgcaagcccaattaaaa
agaaaagtgtctcggatagacttagcataggttatcattagttaaatgccaataccgtgtccgaaaatac
ccccttaccccttattaacgaccaagtctctatgttacgtgggtcgagatatggctagaagtttgcacc
agattcctttactaccaaattaattcgaacgaacggcatttgtcactccagagggaatttttgaccatgc
ctttcggcttgaacaatgcaccccccgctgtgttataaaatcgttaggtgaccttgcgcactgctatgat
atcgtttacatggaTTGAAGAAGAATCTTTCTTGAGATTGAGAACAGTATTGGCTGGCGTTTCATTTAAT
GTGACTTCTTTAATGTTAATTCTTAAAATAAGTAGGTGAGTATCTTGGGTTTCTTATTAAGGATGGCGAA
ATTCGGCCCAATCCACGAACCCTCGCTGTCCGATATTAAAGTGAGTCAGCTCATAGGCTTTGCTACAAAG
TAACAAAGTTCTCTGAAATAATGAAATCGCTTTATGGGCTTACATGTTAAAACAAATTATTAAAATGGGA
ACCAAAACATGATCAAATACGTCAATGCGTCATAACAAGCACAGTGTCACCGAGTAAAATCGTCTAAGTC
TCGCTCCCATTCTTATGAGCTCGAACCGTTAGCCGTATTTAATTAAATTAAACATTTCCGACTTATTTGC
ATGGTCGTCATTTTACAGTTTATAACGATTGTAACTCCTTAAGGGCTACAAGAATTATGGTTGACCTTAC
ACCAAGTGTGGGCGTACAAGTTGGTGAGATCCAACTAGAAAAGAGCTTTGATGTTGTTCAATGAGGTATT
CCAGACATTACTAAAGTGTTGAGTTACCAGCGCTAATTGGTAAACCAGTTAATATTTGGACACATTTGTC
GCCTGGTGATCCAATTTTGGTGAGTGTTCTTAATACGTTATAGCATGCGTGTTTGGGAATTCTCTGCCTA
TGGCTATTTAGAGTGTTAAAGAAATATCCTCTGTCACTAGTTTGGACACTTAAAAAAGGGAATTCTTTTA
GATGAACCATGATataaactccatttaattttgaacattattggtacacaagaggacgtgtgatactcag
gaagactgTGTCGCATCGATGCTATCATTGATTTTGTTAGTCGTTAAATACCATTACTACTGAATGATTA
TTGTTATGTCATCATTTATGAAGAATATAAAATAAGGAATAAGTCGTTCTCATTTATCCTCTGCCTAATT
GTGCTTCCTCTTACACACGATCCACAAGCCCAACTAATTGcacagcctcagttcgacaatgcgttagaac
gatacaaatATAGTGTGAATAAGAACTGATGCGCCAATACCAAGTGACACCAATAGACGCGTGCCCTTAC
TGTGTCTAGTATAGTGATAGCATAATTGTGAAGTTTCAGTCAGTGCAAAACTGAACAGTTCAAAAGAAAT
CAATAAACACAAGGCCACGTCCGGGTCTGTTTTTAATCTTGTATTCTATTTTTCTTCGATTTGTTTCGCT
TGTAGTCTGCTGAAGTTTCTCTATGTCCGCTACACCAGGAATCGAAGTTTAACCTCACACACTAGTAGTT
TcttaactttgttcaactcagtgtgtaatcctacttttcgcacgacaatgaatagctaaatttcccACTA
CTTCCCATTTGACCTGTCTGCCCTTGACTTCTCTCGCATTCCAGCCTTCTTCgctatgcagctctcctgc
accctgatttgtggggagtttttagactcctcaCATCTCAACCTTCCATCGGAAGTTTTTGAAAAGTTTG
GTGGTTTTAGACGGTCCTCCGCCTCTGTATCTCCTCGCATAGGCAGCTCCCGCGTTTCTCCTCTCGTCGA
CCCTGCGCCTATGGTTTTTACCAAGACAGTTTCAGCCTAGTATAACCTGTCGTTAAGCGTCTTGACGTGG
AATCTCGATCCTTCATATACAACACTCCGAACTCCTCTCGTCAACCTGGGACTCCATGATCGCAGCGCCC
TATACCGATTTCCTGCATTGCTTTGACGCCAACTGCTGATTTGCTTTGTCTTTCTCCACATCAGCTTTAT
GCTGTCATCCTTGCTCCTGATGCTGGGAACCACCGCTATGCATCCCTGCCTACGCTGTCAGTGCCCACTC
GTCGTCGTCGGTGCGGTTGCGACCTGCTTTTTCTGGCTCGAACTCCTCCTGCGCGTGTGTACCCGTGGTG
CTGTCGCGACCTGCTCCTCCTGGCTTCTGCTTCTCGCTCGAACTCCTTCATGTGTTCAACAGTTGCGAGT
TTCAATAAAATCCTTTGGATTGATGTCGGTCCTTTACATGCTGGCGCTTTGATCAAGACAATCACGTCGG
GCGACACAAATCTTATGAGCTCTATGTTCCCCTGGAGTTACTATATATTGCGTACCTGAGTTACAGAATC

TCCTTAGAAGTATTTTTAATGGTTTTGTtatgagattcatatgaaaggatatcagaatacaattgagcta
gattgctacgcaagcctgataagagatagtaagtcaagatgttagtttttcgatgaagagtggaaagggg
aagaggaggattgtgcagtggttgatctctttcaccaccatactatccgaccataaattaccagagcata
aggttgaaaaaagctcataaaggacaattatcttgaaaaaaaaatgtctctagtgcaagaaaagttaTAT
AGGCTTGATATACAAGCCTTTTTTCTCATCGGGCATTCGTGGTCCTGCTCCCTGAGGGGTTTCTTAATTA
TATCTTGTAGCGAAAATTCTCATTGAATCTGCTTTCCGTACTCTTTGTAGTGCTGCCAGTCTTTGAGTCC
TAATATGAATGAGACTATTGGCTTCTATAACCAGCTTAAAATGCTTGTTGGTAATGGACCAGCTCTCCTT
GTAGCTTTGTATCTGTTTACCATGTGGGTAGAGCTGAACTATCCATATATACGGAGTTGCCCAGATCGTC
TCGGCAAGTAATCCTGATTAAGATTATTTCCACTAGGCTCCGAGTTTTTTATggcaaaaatcctttgtcc
gtgcgtaaaatgtgactttatgcattaaaaatgaacatgtttatggtttgtatgttgtatttcctgcatt
ttaataaacaaatatcttaagtttttacacatgctactcggatgaacatataattattaaatcttcctta
aataggaatgcttcgggggatctagtcataagtgttattctttggacagacttaggtgctatcttagacc
aaCAAAATGTAGTAAAacgtaaagtcgagacatgaaaaatatttagcttccagttgattcaaaaatatca
ttaattctgggtaaaggaaatttatctaaaattaaggtaatactttttatgtcaatgaaacctatttctt
tttctctgaggttaaaactatttttttccgaaataggaaaaggggactgtGGAATTTCATTTCTTCCATT
TCTCTTATTTAGTCGATAATTTACTTTTGCATAATTTTTTTAGTTAGGAACTAAACTATTCCGTATCGAT
CATATGGTTTACAAAACTTTACTAATATCATATCATATAGAAATTTTGCGAATTCGATAAAGCGTTCGCA
GCATACGTAGATACCTGCTTGTATCTCTTGGGATTAATATATTGTTGTCTTTAATGGTAGTGATATGTAG
GTGTATGTTATTGGAACATGGATCGGAAACTTGACTTGATGTTGGGTCACTCAGCTGCTTACAATTTGTT
TGTATTAGGGTTATTCCGTTTGCTCAGCAATAATGTATTTTCTTACTTTCGCTTTCGTAACCCATGCCTA
TGCGCATCGATGCCGAATTTCGCTGTAGCCAACTTaaaaaaaaTAAATTTATTTAAAATCTCTTAAATCG
ATGAACTCGTTTCTTAAAAATAGCTAATAAAAAAACTAATTAGCTCAACAGGTGGGTCTGTTTTAATAAG
GTGAATGGTAATACTCCAGCACATTATATTGGGGAATACTGTTTTACTATATTCAAGTTGTTTGGAATAT
TATATTAATCTGGATGAATATCAATTGATAATAGTCTATTATATTGTGTAACATTTTGTCTGATACCTTC
GATAGTAATTTGGTTGTCAGTTTGTATTATTGTATTGAATTCTTTTAAAACTGAAAAAAATACGCCTGCA
TTGATTAAAAATGGTAATAATTTGGCTACGTTAAGAAATGAGAAAAACGTGTTTAAACTGAGATTGATGG
AGTAAACCTTTGGGTTATCTATGTTGTGTACCTGACAGTTTTGCGAATTGGTTAAAAATACCCTAACGTA
GTTAGGTACAAGTTTCCACATGATCTATTATTATATGTATGTATTGCATATTATAATTCAATTACTGCAA
TAATTACCGGTTCTGTAGTTGTAGATTTTGGACATAGACGTTGcgcccactctaacgtccacaaaccgac
tgaaactgccacgcacacacttttgcaaaatattgtgatatttttTCATTTTGCAAAAAaacgcgctaaa
accgcccaaaaatgctacgctcacatttttgaaaaattgttggataatttttcatagttttattagtcgc
ttaaatttctatcaatttgttaacaaactttttgcaacgcccactctaacgccctaaagccgccgaaccg
gtcacgcccacactcacccaatatcacccaatataaccccgaaaatgaaatgatgaaatatcgcgttcgc
attcacaatagctgagtaacgtgtatgtgatagttagggaactcgactattgcattctctcttacttTGA
AAATAAGAACATTATAGCTAATATAGGATTAATAGAGGATTATTAAATAAATTGTAAGATGAGCATTTTA
CAGCAGACAATTGCCAACTTTAAAGCAATCCTGCTATTATTGTCCAAATTGTAAAATTTAAAATGGTTTC
CAAAGTTGAAGGTATTAACATAGATATATAtttttttttatctttaattaaatgaatctaattttttttgt
gcattgaacctcttttatatgttttgttaatgttttatttagatttttagtTAATATTAGTAAAGTTTTG
TAGTTCCTGTAGTTCCAGACGTGCATGTACAAAAATAGACGTTCGCTGTTTTTCAAAAATTCTTTCTATT
TATACGCGGCCTTGAAAATTGTGATATCTTCTTCGGGTGCAAGATATTGTGATTCCATATAACTCTTAGT
ATTCTTTTTGGTTAGACTTATTTCACTTAGTCATCCGATGGGAAAAAGTTAGTTTTTAGCATAGGAGAAA
TATGCATACCCAATTAAATTATAATTATGGCTTATTCTTTTTAATATATTACAGGGCATCCTGATAAAGT
TAAAAATGATTACGGTGCCGAAAAGTTCATACAAATTAAACTAGCTTATGAAATTCTAGCTGACTTGGAT
CGACGACGAATTTTTGACCGGTATGGAGTATCGGATATTAACTCACAGTATTTTCAGAAGAAACATGATT

ATAGTGAATATAATCGCTTTACATTAAATCAGAATGATGATGATTTTGGACAACGATTCGATATAAAACA
AGACATTGCATTTTATCAGAAGTTATCAATAACAGAAAATTACTTTGAGAAAATGATTTTATCaaaaaat
gcaaaaaaaGTGCACGTCGTCATGTTTTACAATGATTGGTGTTTTAAATGTACTCGAATTGTTGATGCCT
TCAAAAAGATTCTTGAACTACTTCAGCCAATAGGGTATTAACTTTGCAACGGTTAATGCAGTGCATGAAG
AGTCCGTTTTTCGAAAATGTGGCGCCAGAGAAGTGCCACAACTGGTTTTGATTTTAGACAATCAGTATTT
CTTATATCGCGATCATAGCTTTACCCCACAAAAAGTTGTTGGGTAAGTTCTATTTAGCTTTAATCACTTT
ATCACTTAAAATAAAATTTGGTACTTTCGTGTTAGAAAAATAAGGTACAATATACATCTAATATGTAATA
TGATAtatgtaaattattgttgttttaaaataaataataaattatttctaatggacatagaaaaaaagta
ttgcatttgtagttttctacaacatctgcgttgctatgtaaattattgttgttttaaaataaataataaa
ttatttctaatggagatagaaaaaaagtattgcatttgtagttttctacaacatatgcgttgctaCAAAA
ATGTTTACACACATTTTTCAATTTATTTTCAAAGatatttataaaattattactgaaaataaaaatgtca
aatcaaataattattataattattgTTACAAAATATAACTAAAAAATTAAAGATAACGATTAATtcgaga
ctcccgactatcagataccctagtgaaagctagtgaaagtgcgaatgaaaatttcaaaatttttttgtact
gtatggtgaaatttggaaacaaataataataagatgaaaaaaaatgttgaatagtgtgggagtgatagtt
tagagggggttgtgggccttaaagtgggtgtaggaaatctcatttgaaaacctattaaaatatacaagac
aaatgaaaaaaattctttaaaaaaattttaaaatgttcagaagtgtggcgtgacagttttgagcagtttT
TTATACTTATCAGCGGCCGTACACGCAATACAATTCAACTCAATTTAAGAATACAGACAAATATCTaaaa
aaaaaaCTAAATGATCTTCAAGCAAAACTTTACCAAGACATAAAAAACATTTTGACACATCTCAAAAACG
TATATCAGGACTAAACGAATCTCGGGAAAAGCTGATCCGCCTTGAAGAATGCTAACTTCCCGTACCCATA
ATCGCTCAGTGTATTGTGAATTTAGGTGtttttttttttttgacttaaggtaggaaagtgaaatcttcta
aagatacgatctatcgtccgtaggtggcatgcacgatttataaactataatgggattgtggctacgtact
aaaacaaaaacctccgcgtgctcttcagtccttgcttgctcaccgggagtgccagtcaaggtatgacttc
ggggagcaggatgtgaggccaagtcgcacccttcgcctttaacgacaagccgaaaaggctgaggtagtat
tcttcgcccgccaccacgtgggacgactgtttgagcttagccacccactctgttggccatctcgtctgac
cggacccgcttcatcatattaatgataaggctgtgacccagctcccatgcctcgctgttcgccaataaag
ccgcgaacaggcctcaagggctgaacggggtacctgccagcgactttagctgctccctctcctgcgtaaa
cctggagcagtgcacatgtttagctgtttcttcacgaccgacgcagaacaagcatgggctgactctacgt
ggcggacccaagtagataggcccgaaagcaggcatgatccgtgagaaactgggttaggtggtagtccaag
aatttgtgttggcactctgcccaaaccggcaactcagggatgagctggtaagacctcctcccccgtctcg
acgtttgccatctgttctgccactcacccagcagccactcgtgtgcctgagactgagagccttgatgtcc
agatcaattggcggcagtcctgccagtgctagcgccgcgtcttcggatatggtcctgaatcctgtaatga
gcctgactgccatggaccgtagcaccgtgcgcgctcctttccggtatgaacatctgtgcagcgtatagca
gcgaagcctttgctaccgccaccagcagtttcctggccggatatcttgatccaccgacgttgggcatgag
tctggccaatgaagaggctgtgactgctgccttcttgctggcgtaattcacgtgatccctgaacaatagt
ctgtggtctatcattacacctaggtactttagggactcttgggctatcttccttagcttgttggttacga
gcttaaaagcaagaccccatgtttctctgtctacgctatcccccagttccttattccttgaattaaagcg
tttccttctataaggctctatgagcttctctatgcgccgttatcctagccctgaggcattcagaccgtag
gtgacattgggtggcacaggtggttcgggttgtaccttttaattcgttggcttggttgcatggcaacatc
taccaagcatattcatgaggtcagccgccatactctcttcatccccatttgggatttccagggatttcca
gagagagaccgaaggaaatcagagcgtggtcgctcagcgttacgacgacatgggtcatgcagttattgtt
agccactaaccctctgttgataaaggtaacgttatcgtcgcccctatcgttgtcgtcggcttccgtccga
cgttcagcagtatcaggtccatagtttccatagcgtctatacagctcgtcctctggggttggatacactg
ctgccccattccactgcccaggcactaaaaacaccggcaatgatcttcgggcttcgccctctcgcatggc
ccacaagcaccgccagaaactcctcgaacttatcggggccgtcgctgggcggagcataacagctgtacat

atgcacgtctcttacctttggggtgcagaaagttcctgaatgtggagagagctgcaactcttgatagcta
ctttacccgactcgtctaggatcatggaggattgtccccttccagggacgtaaggttcgcttaggagcat
gatgtctacattgcgctccgccgcagttctgagccacgaggctctgagctactgggcaatgattgacatt
gagctggattacgttaatatggacgtccgttatggtggcttttagcttcttttgggctcgctttgtaggt
cggtctcgctaacctacccgtcggatggtttctgtcctcctcgctgctgcagatcagtcattttggtggt
gcccacgctaaccgtatcgaagacatctttctgccctatcagtcgccttgcaggtCTGTAGCATCTCGTC
CTGGGTTATGCGCCCACCCAACGGTATTGGGAACGCATGCAAAAAAGTATGTCCCCTTTATCAGTGCTGG
CGGCGGGTAGATTCTTATTATATTTTAGACTTATATTATATGTGAGTagatcgatctcccggcgcagatg
agctgaaataacagcgtggccttttccgcgaatactttgcaaatgtacgaacacacaaatactttcacct
gaatttcgaatcaaacgactataacagactctttttctcaaatttaacccaaaaatttaaacgcgcactc
tctgaaaatgtacacatcttgagaaaataattagattaggttagaataaataatataaagatataaatat
taaatactaaaaaaaattcaatttaaataaaaattaaaaatagaaaagtaatgtaaaattaatgtgaatt
attgtaaagcattataatatgcaatataaacataaaatcattcactattcaacattcagtgtagaatcgc
agttcggtaatttttcccgccgaagtagggttaggtcagacagagatcattaagaatttttatatatatt
tttgatttttcatgtttacAATTAAAACACTCCTCCCAACCCTGTTATTTTCGCCCGCGTGCTTaattaa
attaaatgcacatcgcagggtctacggttgcttcttaaggcttcggttattcgatgggaagggtgctcca
ctagacggcctgactcggctcttttctctctttctgcccgcctaaaaaattagagccttcgcgtcaacta
atatatcacttttcccgcgatattcactaacctgtgtctgccccactcgaaccttgaggaaaagcatgag
aatttgctggtagcgaaacaccgttgtaattgtgtgcgaggattgactggcctactccaccggtgtttcg
cgagcaacaaattcgtatgctttacctcttcttctataactgtctaccttccgaatatattaatttgctg
ccttcgattttgtatgctagctcctatattcttATATTCGCGCCATGCGGAGATTTTGTCAGATTAAATT
CCCGCCTGGAAAACATACACTAAAACTTAGACCGCGCAccgttttgtagcgaggaaaactcactctaaca
atatatttccgcactaaaaaaatttcacgcccgcaggttataattttacaacgacacgaactagcaacta
catacaagaacaatacaaagccagtctcaaatcccattaaatcacctaacaatgcaactcacagacaaac
aactaactactataatcatacaacaccattccaaccatcatacaacgccatgtatcaaccaattcgccaa
ccaatttcatatataccacctcaattacccagaactaaccctaatccgttttcccaataccaataccgtc
aacttcaccctcaacctaacgtatctgttgtagctcaacctagaccattgaatcgtcaacatacatttga
ccagaaccgaccaggacttagttattcaaacgcactaaatacaagagaaaatataacgaccggtggacca
gcactaaagagacaacgaccgtctgacagtggacaatcacgtatgagttttgatgaggctcattaccaag
aagaattagaccaaactcatcaacagttcaatccttacatgcattacgggaatcatccccaatatccttt
cgatctttacatgccttacgggccgcccccaacaacatcccgatttattacatgccttacgacccccca
caacaacatccggttggcatacaagagatggcagaggcacgagaagagccaactgaacctcctatggaat
taataacagaagctgaaacggctgagaattttcggccccaagcctcggaacaagccaattcataattatt
aaacacaaaggactcaatttaaaatgcttaatagacacaggctcaacagtaaatatggccactcaaaatt
ttttcaaattaccaatccaagagatagcaaagaaaataaactcaagtaatggccagttcattgcacaaaa
gaaagtaacgttacccaaaaataatcttttccctaaaccatacgatttttttgatatatcctttttcaact
aaatacgacttgattttaggacgacaattacttgacgagggaactacatcagtaaattacggaccccgta
cagttacaatatacggacacgtgcacgaaatgatcgatgccttccttccttcagaagaaatacatattca
agatacacagaataattcctttaggctagaccacttaaattccgaagaaaaagcaaaattgataaccctc
ctaaaagaattccaggaccttcaatataagaaaggagaccagctcacattcactaataacgtaaaacaca
ctattagaacatcccataatgacccagtatgcagaaagctttacggatacgcacccggactagataccga
agtagaaaaccaaataaaagaaatgttagatcagggaataatccgagaaagcaattccccttattgtagc
cccattgtagtagtacccaagacaacagacacctccggacagaagaaatacagaatagccatagactacc
gtaacctcaatgaagtaacaatagcagacaaatacccaataccaaatatggacgaaatcttaagcaagtt

aggaggatgcaactattttactacaattgacttaatcaaagggtttcaccaaatagaaatggaccccgag
tctatccccaaaacagccttcacaactaagacagggcattacgagtatacccgtatgccatttggactga
aaaacgccccagctaccttccaacgatgtatgaacgatgtacttcgtccactattaaataaaatctgtat
ggtatacttggacgacattattgtattttcagcttccttagaggaacatcttcaatcccttagagcagtc
tttcaagcattatctaatgctaatctaaaactccaattagataaatgcgagtttttaaaacacgacacat
atttcttaggacatatggtttctccagaaggtataagacctaacccggaaaaactacgagcaatagaaaa
ttaccccttcctactaagccgaaggagataaaatcatttttaggactcacaggtttttatagaaagttc
ataccccatttcgcacagatttcaaaacccctaacaacatgtcttaaaaaggacaaaaaagtcgatagta
aaaacccggaatatattgaggcattcaaaaggttaaaactccttatttctaacgatcccatacttcgatc
accagacttcaagaaaaaatttgtactcacaacagacgctagtaatgtagctctaggagcagtactttct
caagatggtcaccccataagttatattagcagaacacttaacgaccatgaaacaaactacagtacgattg
aaaaagaactactagctattgtttgggcaacgaaaacgtttagacactatttgctaggtcgacattttga
aatagcaagtgatcaccaaccgttgtgctggttgcacaagatgaaggagcccaacgctaaattaacaagg
tggaaattcagacttgcagaatacgactcttcttctataactgtctaccttccgaatatattaatttgct
gccttcgattttgtatgctagctcctatattcttATATTCGCGCCATGCGGAGATTTTGTCAGATTAAAT
TCCCGCCTGGAAAACATACACTAAAACTTAGACCGCGCAccgttttgtagcgaggaaaactcactctaac
aatatatttccgcactaaaaaaatttcacgcccgcaggttataattttacaacgacacgaacttttcacg
acttgactccacgactattttattagaaaagaaatagaacggaaactcaagccggggtctaggcaatcgg
tggaaatcacgatcagctaagtgtaactctatttacgataaccaaattatcccaaaactttccgggtgca
gctgataaagagtctagtatattcccgatgcataatattgcatttaaaatgaccctaaattagaatcttt
tagctgagtgcaagcttcataatgtttgtctattaggaggttcctaacaacgccgcttcaatcgccacta
caATTACAGacatacttttcaacgagtatagtatacccttctaatctacgaataccggatataaATATTA
TACAATGGAAACATCatactatactatatatactatacttatatttaagaatataaaatattatatataa
cattatgtcttatatttagtttaaaatctttatCCAAGATTTATTGATTTTACGTAGCGACCTTTATTAT
ATAATGTATTTAACTTTTCTAAATGAAATTTAAATTTTGTTGCAGAAATTTATACGCAAAAAAATACCAT
TTAATGTTTTCAAACGCATCGAACATGACAATTTCAATGATTTTCTGGGAGGATGGAGTGACAACAGAGC
AAGGGCTTTAATTTTTGAGCCTCGAAGTTTAACTAGGTTACGATACCTTCTAACCGCCTTTGAATTTTAC
GACCGAGTTGCATTTGGGGTGAGTTACataaataaatgttctaactaataaataactaaatgttctaaac
taataaCTACATTTGCATATTATCATGACCTAATAAAAATATTTATTATATGTATTGAGGATTTAGTTTC
TTTAAAAAGCTACTTAGATAATGGGTAAATGTGGGTCACGTCTAAAACCTTAACAAAGCTATAGTTTATA
GTTATAGTTAAAATAAGTGACAGGTAGAAGGATGCGCTTCCGACCATACTTgcagcgcaagtttgttctt
aaatttttccacgcccacgcctaaaaaaagccaattatgccacaccacaacttttgaaaaatgcttttat
atattttcatatttttattaaccttgtaaatttatatcgatttcccaaaaaaaatcgcaacgctactcta
acgcccacaaaccgccggaaacacacacggacgcactttaaattttttttcgattgttatatgtttttttg
tttgtccaaaacggtcacgcccacacttttgaaccatttttaattttttttctcattCATTGTTTGGTTAT
GCAGCCCGCAGCTAGCAGCAGCTCATCATAAATTTGAGTTATTTTATTTTATCACTCTTATACAATGCTC
TGTGCAGCAATATATTTTGCAATCCCCTGTGCTATGTTCCTCTCTCTCAATAGAGAGCGAGCACAACTGC
TTAAATCTTTATTTTGTGCTCATTTGCCTTCTGCTTAGCAAAGTCAGTTAAAAATTATGTGTTGATCACA
CCGGTACTGCTGCGATGCACTTTAACTCTTAATCGGTTCGGTACGGGCACTGCCGCCGTTCATGTTATAC
ACTACAGCCCTGTGTGgcaacaatgtgttacgctttcattctagctcttaaattgaattcttcaatttgt
gtattcTTTTCCTTTCTACCCTCATAAGCAGGAGCAATTGCTCTGCTTAATATAGAGTTCTTAAAAGTTA
CTCTGTCGTGTATTTATTGTGAAGCCACCGGCTTGCGGAAACTGGCGTAAAATACTGTAAACATAACATA
CCAGGAAACTGGTTAATAggcgcccaattaggggtatgtgcattagtcattacccacgagcacaaaaaaa
aaaaCACTTTTATCTACAAGTGAATCAAACTCAGCCACCTtgctgacctacgtcaacgcatgcaacacag

cgagaaaatcaaactcgcctcttgagtacggttggatgaggtgcgctataacgcaaatttTTCTGTGGCC
CCAACAAGCCCAAGGCAAGCGGGTCAAAGAACCCATACTTTAGTAGGCAGCACAACTCGCTCCGGTGCTC
TAGAACCAATGAAAGTGGACCCTTCGCTGTCAAAGTTTGGACAGCCAATCCAGGCCTCCGTTGGTGTCAA
CAGGCGATCCGCCATCTCTGATCGCTCAAACGGTAAGAGCAGACAACGAAGGGTCAGTCGTAtttttttt
tttcgcatgggtcccacgtgcacacctcccgcaaaaccgaccgaggggcgcttccgtgtatcgtacggct
cgattcgcgagaatatatagacgcgatataaaaaagagaacaggaacgaggaaatgaatataatgtaaaa
taactatattaaatattagtgttagtaggatctaattatgtaataaaaaagttaaaatcgaatgctttaa
gagcggcagagagtcaagattacagataataggataaagtctattatagtcagaacataaaactttgtaa
gggtcatgcaactcatagttagatctacaatgatttaagataaggggtatatagtttctagtgaatctac
ttggaatagagaagttaagcagactaatcaagtcgggactctcaacatcaccacgaataagcttacaaat
aaagactgttccaagcattgttctacggttagctagggacggtaaattaattaaaagtagtctactggaa
taaggaggtaatatacggtttgcatcccaatttaggcgccgcaaggcaaaaagaaagaagtttttttgga
ccgattcaatgcggtccgagtggactacgcattgtggactccaaacaggtgacccatactcaagaatcgg
acggactaacgaaataaataaggttttagtcctgtaaggatcatcaaattcctaagaccaccttttttata
aaaacaagcacacccctggccttattgagaatagtcgaaatatggtcagcaaattttagtttagggtcca
gaagaacaccaagatcatcaacccgtgttattctgtccaaagggcacccactaagagtgtatgtcgtgcg
tattggtttgacacgacaaaaggtcaaaactttacacttggagccatttaagtttaataagttatcacgg
caccatatttgaaatctgtccagatcggatggtaagtccaaatggcgcgaaatgtccttgtgctggaggc
ataatttaccaaacacgtgaatgttttattactaaggggaagtcgttaatgaataaggtaaaaagaagcg
gaccaagatggctcccttgtgggacaccggatgtgactcggagaatacaagaaaaagaatttttaaagag
gacttgttgtgtcctgccattcaaatagcttgaaatccaatttagaagatcaacagggaaacctaaaaga
tccagttttctttttaaaaggtaatgattaacagagtcaaatgctttactaaaatcagtgtagatgacat
ctgtttgaagataattttgaaaccctgtattacgaaagaagttagttacaagaggttagtggttgttgat
ctgcgtttcatataaccgtgctgacacggtgatatgattgatctacaaaggtgctgcaaatgaggagtga
taacattttcaaatagttttgggatatccgacaatttagagattcctctgtaattgcttgcatcctgttt
gctaccttttttatgaagaggaatcacaaaggattccagagataaggtaaacagtttcagtagaggattc
aacaaggcttacgcacagaatctgagcacacagccagggatcccatcgggacctggcgaatagaccggtt
taactagctgaagatcgttaagcagtgagctttcgtttatagtggggcaaaatattagatttgactCcgt
ccggttaactcgcaagccaacttgcgttcgatccatggctcaaccaccgtcacacaaaactgctttgttg
cgttgttcaacgtaaaagcaacatttttcattctaccagacttgtcaacctttgaaggcataatcggcct
cgatttgttagctcaggctgggacgtctctctgtttggcctccagtcagctcaaatggggtacggaagtc
gagaaaatcttcttccacaaatgcgctgatgtcaactttaccgatgtggattacgcagaagtgcccggtt
tagtgcgggaggcttttcgaaaattactaaagaccagaaaaaaggctattgcagacccaatcgacgccct
gccatacaatacttcggtgtttgccaccatcagaacagtgagcggagagcccatctatgccaagctttac
ccatttctcatgggcgtagtcgacttcgtcaataaggagatccaaggtcttctaagaaacgacataattt
aaaaatcggcatcccactacggatgcgggcaaccgcaacaagcgcttaatgttagactttcgcaaactca
acgaacgaaccattcccgacaaataccccatgccgaatatatcaatgatattaagcaatttgggtaaagc
tcgctatttttcgacactggatctcaaatctggatatcaggtcgtgctagcggaacgtgatcatgaaaaa
acttctttttcggaaaacgggggaaattatgagttcaagagacagccgttcggcctcaggaacgccgcaa
gcatcttccagcgaacgatcgatgatatcttacgggaacaaataggcaaattttgctatgtttatgttga
tgacgtgattatctcctcgcaagacaagaagactcacattaaacatgtagagtgggtgttaaagagccta
caagaagcgaacatgagagtgtcggacgagaaatcgcgtttttttaagaaaaccgtgagctttcttgggtt
atagtcaccagtaacggtgcaacaacgaacccagaaaaagtcaagggcataaaataattcccagagccca
agacggtattcgaagtcaggtcatttctgggcctcgcgagctactgtagatgtttcatcaaagacttcgc

tgctatagcaatgtccatttcgaacatattaaagggtgaaaatggaacagttagtaggcatagatcgagg
aacattcaggtgcggttctctaagtcccagcgagaggcgttccaatatctttgcaatatataggcgtcag
aagacgtcatgctcagctacccggatctaacgacaaaagcttcggcctacggtatagtagcgttgttttc
ccaaaagtgccataacaatgatttcaaggacacttaaggacagttaagccaactacgagactaacgagcg
ggagttgttggccatcatttgggccctggcttaactgcggcactacttgtatggagtgaaagatataaat
atcttcacttaccatcatcctttaaatttttctgtgtcggaatcaaacctgaacgccaaaattaagaagt
ggaaggcccgcatagacgagttcaatgctcgactattttacaagcccggcgaagataacctggtagcgga
cgccttgtccagacagcagcttaatgtgctggatcaagaatagcccgaatcttgcgcagcaacgattcac
agtgagatgtctctcaccctcacaatcgactcgacagacaagccgttgaactgcttccaaaaccagataa
tactggaggaagcacgtttctcgtctaaagatacctttattctattcgggaataaaaggtgctacacggt
caacttcgtctgcagggagtctttattagacgaactggtagaccaaatttgtcccaaggagcttttcatt
gcgatttgcacacgctcgcaatgatacaggagatagtccggaggttcccagccacacatttctggcattg
tggacgtaatcagatttaaagaaagaaggtaaatcctaactgctgaacacaatagggcacacagagaagg
gcaagaaaatgtaaagcaggtattgtcggaatactatttccccaaaatggcaaggctggccaatgaaatc
gtgcagaactgcaggacttgtgcgagggccaagtatgacaggcacccaaaaaagcaagaactcagtgaaa
caccaataccgtcacacacaggggaaatgttgcacatagacattttctttacttttttttctctcttttt
atttacaatcagtcttaaaaaagtatgacaataagtaaattttataattaatcttaaattaacagaggca
gtagttatccattaatcccttctatataccctactcataacatgttttaatacttaaaacctgtcgggga
tatctagcacgagaaatcttttaagtcttctgacgttgtcattattattttccaaacttaaggctaggtt
attttcatgtgttcttagtctatctatatatttttttaatatgttttgctatttcttctttagcaaaaggg
attcctaagtctttatggatatttgcatttgaaatataaaatggagcattaacaatttgtcttaatactt
ttgattggtagcgttgtagaatctcaatatttgagttgctggcagtaccccaaagctgtatgtcataagt
ccacacgggctttagtatagcttttttttttattttccagagtggttgcagattttcggccaagcaacca
ggtcatcttcaaactttttttgattaaggagctggcgttttgcttttatgtgttttttccacgtcagtctg
cgatcgaggtgcaagccaaggtacttaatgcaattacttccattaaaagacacttctgcgcagtcacctc
ttctaaatgcaaaagttatttgcgcagatttcagggcgttgactttaattttctaaaaaagaaaccagct
ttcaataagtcgtagctctgcttgcaggagttgtgcggcttcctctttagatgagctggtagctaatata
gctgtatcatccgcgtatgttgccacagtgcaggtatttgttaccggcatatctgtcgtgtacagggtgt
ataagacaggtcctaagacgcttccttgtgggactccagcttttataaagtgacagggcgagtattaatt
tttttgttgcatataaaattgtctattggttaagtatgacttaaataataaaaaatatggtgcaggtaa
acactttttgattttatataataagccattacgccagactcgatcaaactcttgttgaacgtcaagaaat
gttgcagtgcagtatttttttgctcccaaatgctgacaaaatttaatttataatcctgtgacattgctcgg
gggttccgtggcctcttctgaatccaaactggtgttcgggtatgatagcgaattcttccaagattggcaa
cattctctttaaaattatttttttcaaaaactttagaaaatagtgacaacaaactaatgcgtacgatgtca
cttcattttctgccttgtttggtttatggatcattataatttctgcacatttccattggcttgggaagtg
gtcaactcttaacatcacattaaatataaacgttagaaatcgtacacactttggtggtagatttttcaag
gttatgccatctattttgtccgaacctggagcctttttactttgcagtcatttaatttctgatgaaactt
cattaatactgactttacgaatgggtaagctcattgggtgactcgagaaagttatgtacttccctttctg
tttgcgggttatcaatatcattttgctgaaatacagagtgcaggtgttgagcaaatgctttggcttgttc
agcatcagatctacacaattcgctattacagtttcggactactgtgtttctttttgcaggtcgcttgaga
tatttggttgctctccataaattatgttcgtggttacaaaaatgcttgataaattgtccagggaattctt
ttttctttgaacttcgGGACAGGCTTGGCGTGGTGTCTTTTTTGGCTTTGATTTTACTCTCTGCCAATTC
AAAGGAGTTAGGTCTTCGTCGCTGTCGCTCTGCCATTTCAAAGCAGTTAGGTCTTCGACGCTCATGATGA
CAGCAAAGATAGTCAACAGTTAATCGGTGCTTATCAGCAACCACAAACTTATTGTTGTATTTTATGATTG

TAAAATACACAAGAAATATCTGATTTTAAGCAAAACAAACGAACTTgacaaaaagttgttcctcacttgc
gttgacaagttttctaaatttgtcatcgtacaacctaccgcctcgagagctattgaagacctgaagccag
cgctgcttgagctcatgaatttttttcccaattttttttttcatcgtcctaacgcaccctgatgagccac
aggtagagatccacaataggGATAATCCCTAATGATcatccacgcatatcatattcttaagcatttttct
tagtattggtgttgggttccccccttcattactgaacagaaaaatcaataccaaaatccagagtcatatca
ataaactaactgccaccgtcaccttaatcctcaagacaccaaaagagttgacatctgtaatttgtatgaa
acattgctcgcgagaaatagaatgcttatgatggagcttcaaaatttaatattagctgtgacacttgaaa
aaatgaacattgttagcccgaatattcttgatcccgtagatttaagatcagtttgcctagaggagcccac
caacacccctataggggacctcatgtcagtatcgtccgtaaaggttctgcaatccaataacactttacac
attacactttatcatcaaatttccggtaattaagttcgcttgtaagaaaattactattgtccgcgtcagt
aacaaaggagccatgctacgtttagacgatggcgttatcgcagagtgcaacaagattacaccgtcaaagc
ttgcaccgaattaagttaacgccgctacggcgagattgtgaaatacggcaacaacggcattgtgcaacgt
aagcattagaccggatgtcattacgtagccggcagacactgcttatttgcgtggccgctacgaatataag
accagaacaatatgtttcgctttcattctagcATTTGTATATTCAGTTCTTTTTTCCCCTCATAAGCAGC
AGCACTTGCTCTGCTCTATAAAGAGTTCTTAAAAGTTACTTTGTCGTGTATTTATTGTGAAGCCACCGGC
TTGCGAAAACCGGAGTAGAAAACTATAAACATAACATACCAGGAAACTGGTTAATTTGTACATAAGTGGC
TGCACTTTTCGCTGCACCTGTTTCGATTGCTCTCGTTTCGTTTTCGGTTTCTTTGCTTACTCACATCAGC
ATTTACGCGTAGCGGCAAATTAACCCGTCTCGCTCGCCCTAATTTTCGGCTTCTTCATTGCCTACCGCca
aattcgtgtcgttttggctgtgcaaaaagaattgactttgtgcataaacacacacTGTTTGGTTGCTGGC
GCAAATACAGTGCTTGTAAGACTGTTCACCATTGTGCGTGCATTTTATACTGAACGGATTAAAATTCAGT
GCTTGAATTATTAAAATAAAATAAAATGTCAAACAACGACACAGAATAACGTTTTTAAAGCGCAAGGAAA
ATTCCATATTTGTCGATGCTAAAGAAATTGCTGCAGGATTAACCACCGAATCGGTCACCTGCCCGAATGA
TGATGGCCTGCATTTAAAGCTGGATTTAATAAACCAGAGGCCTAGTTTAACTTGGCTCATGATTCGCTAG
AGGAATCCCTCAATCAAATGGGAAGCGAATTGCTTGACCAGTTCAAAAACATTGTCTTTGAAATGCGGTA
TGTTGTACAAACGGAACTCAGTAGACGCAAAGTGCGAGAGATTGCTTGTTCGACGACCGAATGTGCCTAA
CCGGAAGCTACCAACCTTTTGGGAAAATTATACTGAATACACAGATCTTATGTCGATGTTTTCCACTGTC
ATCGATAAAAACCCAACACGTGCTGACATGGAGAAGCTTCAACACCTCAAGTCTAGCCTAAGAGGTCCCG
CTTTGGATGCCGTGGTGCACTTCACGACATGGGCACCCCTGTGGAAATTTTCGAGTGTATTCTCGTTTAT
TGTCTTCTCCAAAACGTCGACCGTGAAACCCAGCTATTATGGGAGGATGCTGCTCCCACTGAAAGGCTTC
CCGTCTCAGCGAGTGCAGACGTGTTTCGTTCTGCTTTCCAACTAATGTATAATCAACTAATAAATAAATC
ACAAAATCAGCAAAATTAAAATGcaaaacgaaattcgtgagcaacgtcaaagtgaacaagacgagcgtcg
gctcccttcaatcagaaacactcaaactctcccaagaccagagagcgcactcttgctctccgtcgttgcc
gagcacagttccaaaatcttggagcgaagagtgcacttccccaatgccgcaaccaacaacaaattacaca
caaactccgttaaccattgtaggcaagcctactctcctaagcctggctgtagtaactaccagcacggttg
cctctctcacaactgcaatcgtaacagtctccctcctcaactcaaacgatttagttaacgacctcaccgg
cttttgcattgcaagcaaaacaattaacagctacaataagcgagtgataaagctaataaaacgacagata
gtgcatctaaaaaaccaaaagcctccacaaatatacatccaagagattgctatgaacgcactggttaata
aaaaagtttagcttatcggaaataataattttcacgttatacctctaagaagaggaaaaatgctgaatga
tgaagaggaactatgcttcaggttaaaaccgaagacGGTTTAAACGTACCAACTCAAAAGCAGTAGTTGc
aatgaagttgccaaatgcatttcacgaaaatggctttaacgttaaatcagttattaatattcttaacaaa
gataagaagccccaaccgctcttcaaggtcgagttagacccaagtagccaaacactaaagagaaatgaag
tgcacccaatttacaatctccaaataaattaaaattaaagtaaaattactgtaaaagaaccacataagcg
ttatggtccagtacaatgccccaactgccaagacattttataccсttccttccgtttgcgtcgcctgcgg
ggatctgcacgaatctggcacttgcccggctaataaacttgatcaaaataaaaaaaaatgtggcaattgt

ggtggaaaccacacagctaattaccggggatgacctgtctacaaagatcttaaaagtcgcatgatcaaac
gaattgcttcggctcgtagtacttataatacccacaaatttatagtaagcgtccaatcgtttcctgacat
tttcttctccaacgccaaaatgtcatcccttcctccagtaagcaccataccggatgtacggccataaaat
cgggaatggaaggtcccgcgtccacaacagagttctcaaaagggccgcaacatcaagccgtcccagacga
cctggaaaacccacCaaaaaaagttgacattatgttgctctcagagacccatctaaccagcaagtacact
ttttaaataaatggctacatgttctatgccactaatcactccgatggaaaagcatatggaggaaccggca
tctttatcagtagtcgcattaaacataacttccttgatcgttttgggaaaaactacctacaagctacaag
gcggtaatataattctggccaccgtctactgtccgccccgtttttttaattactgagaatcaatttatggt
attattttgcacccttggggatcaatttttttgctgcgggcgactacaacgctaaacatacacagttggga
tcccgtttagtaaatcctaaactaccagatctgattgattttgcgatcgtaagaaaaattccTTTAGTCT
CATCTGACCACTCACCCGAACTCTTCAATTCTTTTTGGCAGCCAGAAATTAAAGAAAGTCCGCGTCATCT
TACAACATCAGCCACAAACTGGTTAAAGTATAAAAAACATGTCAGCACACACAAAGCCCTAACGAAAACT
ATTAGGAATgagaaggataaagaacatcgccggtatattaagaaactatagccgacaaacacagggcagt
cattctggaaggcaaatttttgcctaagtatacctatggaagcagagatgcctattcgggataacactgg
gaaattggcccgtggtgttgcagaaaaggcaaatgtatttgctgctcatctacaaaaggtctttcagccg
atcccaccatcaaatggctttacgctcccgcatgtctttgacggacaacccactgatccactgaattttc
ctccgaacgaaattgaaaaagttatcaaaaaacaactgaatccaaaaaaaggcaaaaaaaaaaaaaaatg
gccaggcgccatgacttaatacctccaaaaatgattatggagctaccaaactgtgctgtacgatatatca
gcaccttgttcaatgccatagctaaaaacaatcaatttcccaacctatgataaacatccaacataattag
attccaaggccaggaaagccagccatcatcctacagaccaataagtcctctttcatgcatcttaagctct
ttgaaaaattactcatcattcgtatacaagcacacctgcaaactcgtagagtccatcacatcaatttgga
ttccttcgaaatcatggaacaattgaacataacaacagagatcagaacttcttttgagcacgaggaatat
tgctcagccgtattcctagacgtcgcctaagcattcgaccgagtgtggttggaggagggcctcacgtata
aaataaaaccactcctcccaccatatacacacaagctgcttgaatcttatatttaaaaaaaataaaaatt
tgctgaaaagtgcaatgcgaatacctccgaggaccatgtgattgaagcgggaagccctcagggtatacgc
agctgacatcccttcaagtgatcgaacaacactatcaacttttgctgatgacaccgcaattctcagccga
tcgaaatgcctctgcaagcatcagctggattggctggctaactgacgtattgcaatcaacgagcagaata
ggtgtagctgtccccccattaactctaaactccacgaagtgattaggccatttaactcggagttcatgtac
acagaagattatttggcgaaagcacattgaatccaagaaagcgcatttgaagctgaaatccagtaggttt
cactggcttatcaactcgcgctctccattatgtttggactacaaggtgctgttatacaacacagttctaa
aaccaatatggacatatggatgtcaacagcaacattgaaatacaacgagcacaatcaaaaatcctccgaa
caataactggtgcgccttggtacatccgaatctacggattcccgttgtaaaaactgaaattgtccagcag
aacgctaaatacctTTCACTACCTAAAGTGAATAATCACAAGCGCCTCTAACGCAGAGTGGTGGCGTCCA
TAGGCaaaaaatcaaaaaaTCCATCACAACAAATTTTTGCAAAGTATTTGCAAACCGGTTTTAGATATAG
ATATGTAACGGGACTTTTAGAAAAATTTCTCAAAGTCGTATTCATCCAAACGACGCAGCTGCAGTCTGTC
AGCTATTTTCAAAGTAAAATTGTACTTTGAAGTGGCCAAACGTTTGCTATTGTgctccgattaaattgat
ttaaaatggattttaaagttgaaggAATAAACTTGCATTTCATTTTGTCTTGTGAAAATATTTTGATTGA
ATTATTATAATAATAATAATtattttgtgtattttgtgtaCGTCTTTTCTATGTTTAGCTACAGTTTTAG
ATATGTTCCCCCCAAAACCCACCAATGTGAGTATAATCGGTGTTTTCTTTATTGTTTTAAGATAATAAAA
TCGTTGAGTTTTGCTGCCGTTGTATGTATTGTCATGAATAACGAAAAGTTATCTTACTTTTTTTGAACAG
ATCTATGACGCAATCACTAATATTCCCTTATttttgtttgtgctgctgctttgtttttgtcatttgtgtt
ttgtgtataaaaaatatacattatataaattataaattataatttatatatattataaattatattgtgt
atatattatattcattttaaagatttttatctttttcgtgcatttttttccgaatttgttttcgTAGGCG
CTTCTTATAAGTGCGTGATTTCATGCATTCATATTTTTGGGCTTTGGTTCACAGAAGAGGTCTCGTATGC

AGAAAAAGCTCAGGCTGTGCTGAATTATGAATTAGAATTCATAATAAGGGCTTTGCAGCCGAGTTGACTG
AGGCTAAAAAATGTATTGGTTACATTTGCAAAAAAATGGCCACCTATTGAGTAAAAAACAATCCAACGAA
GAATGCAGTTTTGAACAATCAATCCAATTGGCTGACCACCATGGAAATAATTACACTGAAGCCCCCTTTT
TTTCCTCAAAAAATGGATACAAGTAATTATCGTGGCCGCCCTAAAATTGATTTGGCTGAGTCTTCTAAAA
GGACTAAACGCCGCCGAATAGCTCTGCTGGAAGAATATGATGAGTCTGCAGCTTCTATTCTGCGCTCATC
CGACTTCCAGCCGAATTTAAAGTCATCCGAAGCAAATATTGAAAAGGTTGTATAGTTTCTAATGGATACT
GGATTAACAAAACACCAATATTTGCTGATCCGTGAGTTCGTTAATTGCGAAATTGTAAAAATTGTAGCAC
GCTTTTAGGTGTATTTTTTGGTTGTTTCGACAATTTAACCATTCTTAAATTTAAAAGAACTTACAGTATC
AATTTCAAAACGCTATTACCACTAGAAGTATTTTTGGCCGCGACTCCATACAAACAAGCTGTGCAACGTA
ACGGTTCAACTGCCCAGATGCCAAGACCTAGGGCCATTTGGCATAATTTTttaattaagtgagtagatta
agatttgtaaatttattgttaatctctatataccaagagaagattcaataaaATTCACCACCCCATTTAA
ATAAAAAGAAAACTTGGTTTGATGCCTTTCTCCGCTTTTAAGTAGCACAAATTCTCCTACTTTGAGCCTA
AGTACTTTTGCCTAATTTTTATCAAATCGCTCTTTGTCATAAGCTGCAGTGGCTAAAACATTCTTCTCAG
CTTGTGCTCTTACATTTGACAAATCAACATCAGCTATAAATCGGTTTGGTACTCCAAACAACGATACGGC
CGATTGAGCAGCATTGAGAGTTGCACATCACTGTCTAATTTAAGGCTTTGACTAAGATAGACtttttcg
cacgggtcccacggccacacctcccgcctatccgaccgtatgtcgtatatcgtacggctcgattcgcgag
aagatagacgcgatttagaacagaaacgatgaaattaatataatgtaaaataactctaatatatattagt
gttatgtactaaaaaagttaaaattgattgctttaagagcggcagagagtcaagattacagataatagga
taaagtctattatagtcggaacataatactctgtaagtatctaaaatgatttaagataaggggtatatag
tttctagtgaatctacttggaatagagaagttaagttaagactaatcaagtcgggactctcaacatcacc
acgaataagctaactaaccaaataaataaggttttagtcctgtaaggatcatcaaattcctaagaccacc
tttttataaaaacaagcacacccctggccttattgacaatagtcgaaatgtggtcggaaaattttagttt
agggtccagaagaacaccaagatcatcaacccgtgttattctgtccaaaggggttgacacgacaaaaggt
cataactttacactgggagccatttaagtttaatacgttatcacggcaccatatttgaaatctgtctaga
tcggattgtaagttcaaatggcgcgaaatgtccttgtgctggaggcataacttaacatcgtctgcataca
tacagtttgtcaagaaattgtttgcacaatgaaaataaattgtatttttgactttcaatgcaaaaatata
aaggccttttattaaaattcatgaatttttttattaatatctatatattcatttgattttatcaatatgt
aaaaaataataacaaaactattaaaaatgaacgagaaaaacaactatatccaagtttgcacagatttgac
actgtcaggatattgtttgcacaaagtacttaagctatcttagacctttagtttataagaaattataata
acttgaggtaattctaatactttgtatgcatacccttagcattaataaaatattgtatgcgtttttttcat
tgaaaaaataacttttcaagaacatattgttgctggatcttatgccactcttctagtatagcagctctta
gtgctgctccgttgtttggtgaccttttttgccactttttcttcaaatgaacccacaaattttctatgggg
ttcagatctggactctgaggaggcgtatcgatgagcttgccgcagcagccagttccgcacataaagcgtc
ttatgcttggggtcattgtcatggtaaaatttaaaatcaagtctgttgtacttataaaaacagaattttg
gggctgaatgtcagattgcatttcaaaatgtttaaatattgttttgcattcattgtgttctctatgaatg
ccaaattccaactcctcgactagatatgcatccccaaatcataaccgacagtttgccaaatttgactgtc
agtatgatgttgcggttctccagcgtagttagcgccttacgccacactctgttgggtccatcgttgtaat
acaacatcatcttggtttcgtcggagaaaattacgttattccagtaatcttctggctgattttgcatttt
gatagcgaaagtacgccgtttttcgatgttaattgcataattgccggcagcattagtttctttcttgcta
cccgtgagtggtacttatgccgatgtatggtttggcgcactgtttcgtgtgtcacacccagatcgcactc
ctccttaagctccttagcaacagttcttaaggatatttgcggattttcataaatttttcgcataataagt
cggtcggtaaacggttggaaattttatttctacgtcctgctccacattttaaatccaatctgcccacgtt
gtttgcgcgattaataacattataaattgttcttcgcgatgcagaaaacatgtctgacaattctttaaca
aattttcccaaatagtgattataaaaaaacagttgaactacttcaaaactcaatcgtttgccaggcattt

ttaaatttcacaaaacttactggaacaatgctcactttggcaaattttacaaccaaatgcataaaaatct
gccacccacacaacgcataaggcgtaaatgactgattatgtaccgtttgattatcgaaattcctcggaat
aggaacaaaataaaaaacaatttcttgacggtgtcaaatctgtgcaaacttggatttagtttttctcgtt
cattgttaatagttttgtttttattttttttacatattaataaaaaacatataaacaaataaatgtaaat
aaaaaaataaataaaggtaaacaaaaagcccttaattttgcaatgaaaggcagaaaatacaatttatttt
tattatgcaaacaatttctagacaaactgtaagtacacgtgaatgttttattactaaagggaaggtcgtt
aatacgttaatatacgttaatacgtcgttgtggggcactggatgtgactcggagaatacaagatagagca
tttttaaagaggacttgttgcgtcctgccattcagatagcttgaaatccaatctagaagatcaacaggga
aaccataaagatccatttttcttactaaaaggtaatgattaacagagtcaaatgctttacaaaagtcagt
gtagatggcatctgttttaagattatttttgaaaccctgtattacgaaagaagttagctccaagaggtta
gtggttgttgatctgcgtttcataaaagcgtgctgacacggtgatatgattgatctacaaaggtgctgca
aatgaggagtgataacattttcaaatagttatgggatatccgaaaatttagaaaatcctctgtaattgct
tgcatccagtttgctacctttttataaggaggaatcacaaaggactcctttcatatatgagggagctgtg
aagattccagagataaggtaaacagtttcaggagaggcttgcacCgttaccgtagtgttttcaaaaaata
gcgaggaagggttagaacttgtttttcgcttagtgctaacgaaattataagactgtttcggatcctctgc
aaactgaaacttacaacgatttaaataattcttgtagcactgagcattgagcacggtaaggtttaaccga
gcgcttaaatatttacaaaggatgtactgagaaccggtatttttaaattttgtataaagtctggacttta
catttcttaggtgtgtcaaatctttattaaaccaaggaggtttagagattgacggatagtacatcggaac
acaagagtcaaaaaatgatttaattgcggtataaaacacattaatggcaccggtcattatgttgcaggag
taaagagtaaagatcagaccaattaaagccagctataaaattgtttagcttccgaaaatttgccttgcga
aaacagtgaattcgctttgttgacttctctgaatttgcttttaataccgtacctatgtcgattgccacct
agaaagtaggatgatatggatcttcaggttgagtaagaggtgctatcctggacagaaacactgttcaagg
acttgttacaagacatagatttaacagtcgacgaaaagagtttcgaatatagttcatcaaaagtcatgtt
gtgctaaagggagaaggatattcgattgttcttctggggaccacgttgtgccaggtatattaaaatcact
tagaactaccaattggtccctgtcagacagtttatttaagatggacgggatagcggacagatgattacgg
gactcgtcaaaaagtaactcctccgacgcgaaggtacatctaaccgcaattaagactccacctccccacc
tccctcatacctatatattgtgtacatacctgggaagacttcggagtaaagtaacaccgTAAATGGTAAA
TATTAATCTCAAACTTCAGAGTtcgttgccaacatcgcagctttgctatcagcccaacctaaatgaaaat
tctgaagactggaaaataccagctaatctttgcttggccgatccccaattcttcaggccccaaaaaattg
acatgctaataggcgctagtcttttcattgatctgctgtgtgtagcccaaataaaattggccgatggact
cccattctccaaaatactcgttctggatcgattgtcactggtggtggcctctcccctaacaaggttttat
cattgaccgttcagtcaaatgctctcaaatgcctgatgagggcaaattttgggagacagaaaccattgct
gaatccgtctctaagcgcacaaaggaagaactcgactgcgaggcccacttcaagtatacatttctaggc
ttctatctggagaatattcagttcgacttcctgcaaagcgcagcccggaagtgttgggagattcctatca
acaagtctttcgccgtttttttaatctggaaaacaaacttgaacgccatcgccagctcaaagagaagtatt
cagcctttatttacgagtacttatctttaaatcatatgtctctggtaccatttgagtttcagaaactatg
caaattttattggccgcatcactgcgttttgaaggaagacaccacaacaactcagctgcatattgtcttt
gatggctctgccaaaacttcttccgaatactctttaaacgaaatactcatggcaggccccactttgcagc
ctaaactgtttcacaCTTTGTTACAGTTTCGAACTTTCCCTGTTGCTTTAACGATGACATCTGTAAGATG
TACGGGTCAAGGAACCGGACAGCTACTTACAATGCATTTTGTGGCGGGACACACCCCAGGAAAATGTACA
AGTATTTAATCTTGATAGAAGACGAAAAAACGTCGTTTCCAATTGGCGATAAAATTATTCTACAAGACCA
TAAGATTACTGGGATTACTGCTCATTTTAAATAAAGAAAATGGTGCTCAAATGATCCTGGTACTTTGAAT
TCCGAAAAGGATTCCTTTCTTAAATTTGATGATGGCAGCGACATTACCAAAACTCTTGGAATCGCCTGGG
ATTCAATCTCGGATATTCTGCTATTCTCCACATAGCCCATGAAAGTAGTTCTAAAATCCAGCAAGCGCTC

TGTCCTGTCTACAATTCCTCGCTTTTACGACCCTCTTGGTCTTATCTGCCCCGTCATAACGAAGGCAAAA
ATCCTGATGCAGGAAATCTGGGGAGAAAAACTTGATTGGGACGAAAGTTTACCTTCGGCGCTTTATTCTG
CTTGTCTTAATTTGTCTGCTGATATCTGTCTCACCCAGAAGTTGCAGTTTCCTCGTCTCGCACTGGTACC
CAATAGCGTGGTCGATACCATGGATTCAGTGATGCCAGCATTGACGCATATGGAGGATGTGAATTAAAAT
GTCAAACAACGACACGGAAAACTGCATATATGTCGACGCTACAAAAATTGATGCAGGATTAACAACCGAA
TCGGTCAAATTCATGGATTGCATTCCATGATGCCAGAATTAACGCATATGGAGGATGCATCTGCATAGTC
TCCATGTCTCAAAGTCAACGAGTAGCCCACTTTTTCTGCGCTAAATCTTGTGTGGCCCTATTGAAGAGCC
TCCCGGTTCCCAAATTGGAATTATGCGCGGCAGTATTACTGGCTCAACTAATGCAGGAAGTCCTGCAGCA
TGGCTTATTTTCATGCCCTGTTGATTGTTTGTCAGACTCTGCTGTGGTGCTGTCCTGGATTCGAAATGAA
TGTTCACGCTTTCAAACTTTTATTTCTAATCGCATCTCTCTAATCCAGGAGCTGGCTAGAATCTTGTCTC
GCTCCAAAGGTTCTCCCTGAGCTAAGGAAGAAGGCCCATCACTCTCAAGATTCCTGCCTACATCGACATC
CCAACCAGTTGTATATTTGTCAACTCCTGGCAAAAATTAAGCATGTTTTTGGATACATTTTGAAATTTCG
TTATCGTATCAGGCACCCCGGACAGACTGTTAATCATCACCGGAGGCATGAAACTTATCATTCGCTTCCT
TCAGATGGCCACCTTACCGAGGACTATAATGCCGTGCTCAAcattctggaactcgcgcactactcgcttc
cattcgactgtagttctggcccattggcggtcacaagacaaatgcaagtgcagtcagcaaatgcatcata
tgtattcgagtcaagccacatatagcttgtcacattatgagtgatcttcctgaggaacgcGTAACAGGTC
ACTGGTCTGGACTTTTGCGGCCCATTTTATTACAAAAATCCTGTTCGGATCTTAAGCGTTTGTTCCTGAA
CACATCACACCAAACTGCAGTACATGAATTCTGCTTGAGTGATAGCATTGAATGGCGATTCATGCCTCCT
CGATTGCCGCATTTTGTAGACCTCTCGGAGGAGGCTGTCAAAACAGCAAAGCATGATTTTTACCGAGCTG
TTGGCCCTGCTGTTTTGCCCGctgacgacctcgaagtactaacgccaacgcacttcttaggcacagtccc
cctcgtcttgcttgctgagcctgatctaacaaaattaaatctcaaccgatcgagtcgctggcagcggatt
acctcctaccacaaatattctggagccggcggagacgtgaataactaacgctacttagcaacgttccaaa
tggcgagcacagaatacagacgttcttatcgatgatgtcgcttaagtaaaagacgaaaacctgccaccCC
TCAAATGACTACTTGCTCGAGTAATCAATCAATAATTTGAGCTATTTTATTTTATCACTCTTATACAATG
CTCTGTGCTGCAGTAGATATTGCATTCCCCTTTATACTGTGTTCCTCCCTCTCAATAGAGAGCGAGCACA
GCTGTTTTAATCTTTATTTTGTGAAACTTGCTCATTTGCCTTCCGCATAGCAAATTCAGTTACAAATGAA
GTGTTGATCACACCGGTACTGCTGAGATGCACTTTAACTCTTAATCGGTTCGATACGGGCACTGACGCCG
TTCGTTTTATACCCTACAGCCCTGTGCGGCAAAATATATTTGAATAAAATTGAAACATTAACTTTACCGT
GTTTATAAATTGCCACACCATTTCTCAAAATCAATGAAAAAGCTCAAAGCTcattcgaaacaaaaaatat
tctcccttcgtatattattatggaatgcgaatggcgtctcccgcctcaaactctcgctgggctcttgaaa
gataaagcggttgacattatgttgctctcagagacccatctaaacatcaagtacaatttttaaataaatg
gctacaggttctacgccactaatcaccccgatggaaaagcataAAAGGTTGAACTTTCtatcaagtggga
aacccacatactggcctacagacccccgaaaactaccagatctgattgattttgcaatcgtaagaaaaat
tcctttagtctcatctgaccactcacccgtactcttcaattcttttcggcagccagaaattaaaaaaagt
ccgcgtcatcttacaacatcagccacaagctggttatagtataaaaagtatgtcagcacacacttagaac
tcaatcctatgctagacagaaattcagatattgaaagttctgtaaaaacatttgtcgatgataacaaaag
ctgccagtgttgcaacaccttaaataacttctaagcaacacttaagatatgaaactaagaacaggcaaat
cgagaccttagtgctagaaaaacgacgcctccaacgagagtggcaaacccacagatcaccagccacaaag
caacggctaaagacggcaacacgcaccctaaggaaagctattaggaatgagaaggataaagaacattgcc
agtatattgagaaactatcgccgacaaacacaaggcagtcattctggaaggcaaatttttgcctaagtat
gcctatggaagcagagatgcctattcaggataacactgggaaattggcccgtggtgatgcagaaagggca
aatgtatttgctgctcatctacaaaagatctttcagccgatcccaccatcaaatggctttacgctcccgc
atgtttttgatggacaacccactgatccactgaattttcctccgaacgaaattgaaaaagttatcaaaca
ataactgaatccaaaaaaagcacctggccgtgacttaatatctccaaaaagaattatggagctaccaaac

tgtgctgtacgatatatcagcacattgttcaatgccatagctaaaaacaattaatttcccaaccaatgga
acacatccaccataattagattccaaagccaggaaagccagccatcatcctacagaccaataagtcctct
tttatgcttctctaagctctttgaaaaattgctcatcattcgtatacaaacacctgcacactcgtaacaa
agttccatcgcatcaatttggattccgtcaaaatcatggaatcatggaaagtgaacagaataacaacaga
gatagatcagaacttcttttgagcacaaggaatattgcactgccgtattcctcgacgtcgcccaagcagt
cgaccgagtgtggttggagggccttatgtataaaataaaaacacttcttccaccaaatacacacaagctg
cttggatcttatatttaaaaaaaaaaaaaaaattgctgaaaagtgcaatgcgaatacctccgaggaacat
gtcattgaagcgggaagccctcaaggtatacgcagctgacatcccttcaagtgaccgaacaacactatta
acttttgctgatgacaccgcaattctcagcacatcgaaattgtcacgtaactcttgtggagaattagctg
gctaactggcgtattgcaatcaacgaacagaaatgcaaacatgtcacatttacgcttaataggcgtagct
gtcccctattaactctaaataacttaaaattcctcaaagttatttatccatttaccccggagttcatcac
ctgctgaaagacagcaggtttcactggtttatcaactcatgctctccactaagtttggactacaaggtgt
tgttatacaacacagttctaaaaccaatatggacatatggatgtcaactatggggcaatgcatgcaacag
caacatttaaataatacaacgagcataataaaacatcctgcgaacaataactggtgcaccttggtacatc
cggaacgcgaacatccaccaagaccagttgtaaaaactgaaattgcccagcagaaggctatatacctaaa
gcgacttggaacgcatccaactcaattgaccaaatcacttataaaagtgtataatcacacaggcctccaa
cgtaacgattcacctgcccgatgccaagacctagggccatttactataatactataactttttaattaag
tgagtagattaagatttgttaacttattgttaggctctacataccaagagaagattcaattaaatttcac
cacccaattaaaaaaataaaaaaaaGATGCCAGAAATTTGAAAACATCGAACGTACCAATGTTGCACTTC
GAATCATGCCACAGATTGTAAAACCTAGGAAAGCCATCCACAATTcttacaggatgcaaaaaggttggct
ttatgccaaaactgccttaaaggtggacctatgacattgtaatgccgatcgctgtcgtatttgtggaagt
aagcacaacaaattgctacattttggctcaaggccttcgtcatctcgctcccagcctgctcaaccttagc
tagcttctgatgctcattcaaacggattgtaagttgttgcacaggaatcatcacctggttaatcttcttc
cttagtggctcaaaatctcggagcatttttttgtgttcttggccactgccgtcatcaatattcaaaataaa
tcaggatcttggattccttgtcgcgccttgctccggctctcaactacactttgcaattacccaagtacag
ctacagtttctggaattggtgaagatttagATCAGATGAATGTACGGTAAATATTAATCTCAAATCAGAG
TTCGTTGCCAACAACCAGCCCAAACTAACTGTAAGCTCTGAAGACAGaaaaaaaaCCAGCTTATCTTCGC
TTGGCCGATCCCCAATTCTTCAGGCCCCAAAAAATTGACATGCTAATAGGCGCTAGTCTTTTCATTGATC
TGCTGTGTGTAGCCCAAATAAAATTGGCCGATGGACTCCCATTCTCCAAAATACTCGTTCTGGATCGATT
GTCACTGGTGGTGGCCTCTCCCCTAACAAGGTTTTATCATTGACCGTTCAGTCAAATGCTCTCAAATGCC
TGATGAGGGCAAATTTTGGGAGACAGAAACCATTGCTGAATCCGTCTCTaagcgcacaaaggaagaactc
gactgcgaggcccatttcaagtatacattttctaggcttctatctggagaatattcagttcgacttcctg
caaagcgcagcccggaagtgttgggagattcctatcaacaagtctatcgccgtttttttaatctggaaaac
aaacttgaacgccatcgccagctcaaagagaagtattcagcctttatttacgagtacttatctttaaatc
atatgtctctggtaccatttgagtttcagaaactatgcaaattttattggccgcatcactgcgttttgaa
ggaagacaccacaacaactcagctgcatgttgtctttgatggctctgccaaaacttcttccgaatactct
ttaaacgaaatactcatggcaggccccactttgcagcctaaactgtttcacaCTTTGTTACAGTTTCGAA
CTTTCCCTGTTGCTTTAACGATGACATCTGTAAGATGTACGGGTCAAGGAACCGGACAGCTACTTACAAT
GCATTTTGTGGCGGGACACACCCCAGGAAAATGTACAAGTATTTAATCTTGATAGAAGACGAAAAAACGT
CGTTTCCAATTGGCGATAAAATTATTCTACAAGACCATAAGATTACTGGGATTACTGCTCATTTTAAATA
AAGAAAATGGTGCTCAAATGATCCTGGTACTTTGAATTCCGAAAAGGATTCCTTTCTTAAATTTGATGAT
GGCAGCGACATTACCAAAACTCTTGGAATCGCCTAGGATTCAATCTCGGATATTCTGCTATTCTCCACAT
AGCCCATGAAAGTAGTTCTAAAATCCAGCAAGCGCTCTGTCCTGTCTACAATTCCTCGCTTTTACGACCC
TCTTGGTCTTATCTGCCCCGTCATAACGAAGGCAAAAATCCTGATGCAGGAAATCTGGGGAGAAAAACTT

GACTGGTACGAAAGTTTACCTTCGGCGCTTTATTCTGCTTGTCTAAATTTGTCTGCTGATATCTGTCTCA
CCCAGAAGTTGCAGTTTCCTCGTCTCGCACTGGTACCCAATAGCGTGGTCGATACCATGGATTCAGTGAT
GCCAGCATTGACGCATATGGAGGATGTGAATTAAAATATCAAACAACGAAACGGAAAACTGCATATATGT
CGACGCTACAAAAATTGATGCAGGATTAACAACCGAATCGGTCAAATTCATGGATTGCATTCCATGATGC
CAGAATTAACGCATATGGAGGATGCATCTGCATAGTCTCCATGTCTCAAAGTCAACGAGTAGCCCACTTT
TTCTGCGCTAAATCTTGTGTGGCCCTATTGAAGAGCCTCCCGGTTCCCAAATTGGAATTATGCGCGGCAG
TATTACTGGCTCAACTAATGCAGGAAGTCCTGCAGCATGGCTTATTTTCATGCCCTGTTGATTGTTTGTC
AGACTCTGCTGTGGTGCTGTCCTGGATTCGATCTCAGTCTTTGGTCTCATGTTCTTGACAATCTTCAAGA
GAACAAATCGCACTGGCCAGAATCTTGTCTCGCTCCAAAGGTTCTCCCTGAGCTAAGGAAGAAGGCCCCC
ATCACTCTCAAAATTCCTGCCTACATCGACATCCCAACCAGTTGTATATTTGTCAACTCCTGGCAAAAAT
TAAGCATGTTTTTGGATACATTTTGAAATTTCGTTATCGTATCAGGCACCCCGGCTTGACTGTTAATCAT
CACCGGAGGCATGTAACTTATCATTCGCTCCATTCAGATGGCCACCTTACCGAGGACTATAATGCCCTGC
TCAAcattctggacctcgcgcactactcgcttccattcgactgtagttctggcccattggcggtcacaag
acaaatgcaagtgcagtcagcaaatgcatcatatgtattcgagccaagccacatatagcttgtcacatta
tgagtgatcttcctgaggaacgcgtaacaggtcactggtctggacttttgcggcccattttattacaaAA
ATCCTGTTCGGATCTTAAGCGTTTGTTCCTGAACACATCACATCAAACTGCAGTACATGAATTCTGCTTG
AGTGATAGCATTGAATGGCGATTCATGCCTCCTCGATTGCCGCATTTTGGAGACCTCTCGGAGGCGGCTG
TCAAAACAGCAAAGCATGATTTTTACCGAGCTGTTGGCCCTGCTGTTTTGCCCGctgacgacctcgaagt
actaacgccaacgcacttcttaggcacagtccccctcgtcttgcttgctgagcctgatctaaccaaatta
aatctcaaccggtcggatcgctggcagcggattacctcctaccacaaatattctggagccggtggagatg
tgaatatctaactcttcttcagcaacgcaccaaatggcgagcaaagacagacgttcaagtcgatgatgtc
gttttagtaaaagacgaaaacctaccacccctcaaatggagactagctcgagtaatcaatctgattcctg
gacccgatagcgtcgcatgagttgcTGTTCTCAAAACTGGCACTGGAATCTTCAATAGAGCCGTCGCTAA
ACTTTGCGTTCTACCCAAACAGGATGATGTTAGCCCGTGTCCTCCAACGGGAGGAGGATGTTTGCAAAGT
CAGTTACAAATTGAGTGTTGATCACACCGGTTTTGCTGTGATCCACTTTATTCTTAATCGGTTCGGTAGG
GGCACAGCCGCCGATCTTTTTATACATTACAGCCCTGTGTGGCACAATATATTGAAATAAAACTGAAATA
TTAATTGAAACATTAACTTTAGCGTGCTTATAAATTGCCGCGCCACTTCTCCAAGTCAATGGAAAAGCTC
ATTGccccaatatcattattaatgaaattgtgaaatttggcgttcatatttcaacaagctgagtaacgag
gaactctactgtagcattctctcttgttATTTTTATTGTGTTGACCCAGTCGAAACTGCAATTATTTCGA
AACACGCATCTTTACAAAATAAGTGCCAGTTTCATAAATATTTCAATTTTTACTTTATTTAATACTATAA
TGGCAAAGGAAATAAACCACAATTTTATTTTTTCCAGAATTTGTGAATACGATTAGTAAGGATTCAAGCA
ATATAATAACTCGGTTTAAGGTCAATACAAGCCTAGATACACTTATTTTATTTAATGAGGATACGACGAC
CTTTACAGCCAGTGTTTGTATGGAAGAAATCCCAAATCACATCTTGGTGAATATGGTATCTACTAATCAA
TTCTTGGCATTTCCTCGAATATCATCACAGAACATTATGGAAAGGTAAGTAATATCTAACATTTTCAGCA
ATTTCAATCAAAATTTATTCTTGTTCTAATTTAATACAAATCTTTACCTAAAATCTTGTGTATCAAAATT
GGAGAAGTAAAAAACACTGCGCTGGATTTAGGTTACTCGTAGAATTTTTCACCCAGCGAAAATCTCCCAG
TGCACATTGTGCTCATGAAGCGTGTCCTATATACAGTTCCATGCCTCTTTTCGAGATTTTTATTTTTGAT
CTCTCTTTTTAATTGGTTTAAATGAGGCAAGCCACATTGAACGCATTGAAGAATTTGAAAGATATTCTTT
TATTCAACGTCGCTAAAATACATCCTTCAGTTTTTAATGCATTCAGCCTTCATCTTAATTCCTTAAATTT
GGATATGACATTCCAGGGTAGCCGTTAAATTTAACTTGAGTTCTGTCGAAACGCACCCAACCATGACCAA
ATGTTTGGACGATCTAAAAATAAATAAAGGCTAACTGCTTATCTTCTCCCATTCAATGGATAAATTCATA
TTATTTAGCTTTAGTGTTTACCCTTTAATCTCGATTATAATCTTGCTGAGAACCTCCAGTCATTCTTGCC
TTTCCAGTCACCCTAGGAGCTCTTCTTAGTTGGAGCTCGAAAACGTACTCCTGAATTGGCCCCAGGCAAG
GACACTCCGATTGTTTACGCTTATCGAGAGCCTGGGGATAGAGTTTTATGGGCCTATAGGCCCGTCTATC

Agtctgcccgtccgtatgaaagacgaaatctcaggaactttaaaagctaaaaggttaagattaagcatat
agattctagagacaaagacgcagctcaacttccacgcccacacttatgaaaaacgtgttgatatttataa
atatttgcattagtctagcaaatttctatctaaaaaaaacctaccaaaaaaactttttgctacgcccact
ctaacaccctaatgtcgctaagccaatcacgcccacacttttgaaatttttttaaatttttttcttatttt
attccccaatatctatcgatatcctataaaaatgatcaagtttcgagttcgcattcgagaagctcgacta
tagcattctgtcttgttatacccgttactggtagagtaaaaaggtatactagattcgtttacaagtatgt
aacaggcagaaggaagcgtttccgtctatataaggtatatatattcttgatcatgatcaatagccgagtc
gatcaagccatgtccatctatccgtatgaatgtcgaaatctcaggatctatataagctaggaggtttaga
ttcggcgtatcatattggactaatctaccccacgaataaaataaatgattaggtataacttaacgtcaat
acattgtgaccctttgtcttaataaatataaatacacaaatacccaaaaagactaccaagaactacgtaa
gcactccagagccccagtaatacgatctaacgcttatacataagccgatcgcggagcgtgcacgctgagc
atgcaagtgttcaatgccttttgcatacttatgtatatgtataaatgtaagtaaaatacatagatattag
ccacaagttgggttagctgaacttcagcacactttgagcattcaaataaagatcaacgaacagagcagaa
gctctTCTATTCTTATAAAAATTAAAACATATTACTTCAAATAAATTAACATATTACTTCCgcatacaaa
ttctagagacaaagacgcagtgcaagttgacccatgctgcgacgcccactctaacgcacacaaaccgcac
aaaactgccacgcccacgctttctaaccatttttcgaaattttatcataattttattagtcttgtaaatt
tctattgatttgccaaaaaactttttgcctcgccaactctaacgccctaaagcggccgagccggtcacgg
ccacacttttgaacaattctcgatatcccagaataatgataaaatttcgcgtttgcatttacaatagctg
agtaacgggtatctgtagtcgggagcttgactacttcattctttcttgtttTAAATTGAAATTGATTTTT
CACCTGAGTTCAATTTAATAAGAACATAAATCGGAAAATTAAATGGTTTCGTTTAAGTAAAAATCAATCA
AGCAATGTAATTTGCTAATGAATTTTCCATCATACCATATAAGAAACTGTTGTTCTATCATGTGGATGTT
GTGTCTGCCTGAACCTCAATTATTATTATTTTCAATgaatgaggtaaagctgaatatgttgtttgaaaaa
atttggcgaatagatcggctattgcctgatccgatgttaccgtagtgtttccaaataatagcgaggaagg
gtaagaacttgtttttcgcttagtattaacgaaattataaaactgttgtggatcctgtgcgaactggaac
ttacaacgttttaaataattcttgtagcactgagcattaagcacagtaaagtttaaccatgcgcttaaat
atttagaaaggatggactgagaaccggtatttttaaaatttgtatatagtctggactttacatttcttag
gtgtgtcaactctttattaaaccaaggaggtttagagattgacggatagtacattgtaacacaagagtca
aaaaatgatttaattgcggtataaaacatattaatggcatccgtcactatgttgcaggagtaaagatcag
accaattaaagccagctataaaattagttagcctccgactgatgattctgatattgtgggaattcagaag
atggcggaatatacgagcacgcaatataaactgatctgtcggaagatgacagctttacgcataagaattc
agagttacgggcacgtcaataagtaactatcctgcctaaaatcacccgaacatatggtcgaaaaaagttc
attggggacagatatcttTTGTCTTTTATATAAgatttgctgtgattgacagatgatcagttgtgcgggg
ccttaggcagcctaTggatggactgacggtatttttaaatttttgtttaaaatctagactttacatatct
tatgtttgttaagtctttagtaaaccaatgagaccaagacttaaaaactgttttaattgcggtatcggac
gaattaatgtcaactataaaaatatttagcgtcagaaagtaagccttgcaaacacaatgagttcgcttaa
ttgacttctccgatttctcttttataatgattcctatgtcgagtgccacctcATCAAGaatcatcgagaa
ctatccattggtacctatcggacagtctatttgaggcggactcgaaagcggccaaataatttaggtgttc
tggatatttagaagacggcagaatgtaagaacacgtaatataaactgatctgtcggaaaatgacagcatt
acgcatatgaattttgagttacaggactcgtcaaaaagttactcctccaacgcgagggtagatctaaccg
caattaagactccactcccccCCACGAATACTACCCATAGCatcattcctttcaagccacctttagttta
gttagaaccccctagacaagtatcacaacagtattttagatttcgacacttaggttttataaccaggcggc
aattattttgaaagcagacaaaccgaacaactgtacaaagacacaaaatcaaaagttgcaagcgagcact
atttgcatacaaaaacaacaccaaacatcactgctaacaacgcatgaaagagagagtaaactaaacacaa
aaaaagacagaaaggaaaaataattagtaaatttgtttaaatattctgtattaaaatatatactttgatt

aactcgcgaagcgaacggggtttcaataaatatgtAGtttttttgtttgcatgttttgcttatttattag
tcttagtcttagtctttttcattttatgaaatgattcgttctgtgtggcctaacaaatttaacgctgggc
tggttggtcgttccggtgcagtcggctttggctgcatactagaataagttttcttgcaagaggatttgga
tgtgaggctagtttttcattgtattttactttcgtctctgctattttcttaatgacaagtttatatgtaa
gtctttgtgtatattctcgttccgaagataccatggagctccagtaatgattctcagaatcctagactgt
gctcgctgtactatgtcgatgttgcttctcgatgcgttgccccatagctcggagccataaatccatatgg
gtttcaaaagttatataagaggactttgtactccagtcttaggggagagcgaacgtttataagccagtgt
agatgccttgcttttaatgtgagttgcgtcgtcttggactctatgtgtttccgacaggtgagccgctcgt
ccagatgaatagctaagtatgttacgttatcggcttgtgggatgcgcatgtggttcattaccatgggcgg
gcagctttgtttgttagggtaaatgtaacctgtttgcactttcttcctttactcgtatgcgccaatttgc
aagccaatgttttacacaagatagatggcgtgctagttgcatcgtagcttttactgggtatctggatcga
cttgtattgtagtgtcatcagcaaatgtggatatcgttagctggtagtttgttaggatgttcgccgttta
tagggtgtatagaattcgtcctagagcacttccttaaggcactccagcttctatattacaatcgcttgta
tgttttgaggcagcagtttgattattttaaacataagtccattcagccatactctatcgaatgcttgtgc
aacgtctaagaatagagctgcgaatttctgttgtgatgcggttaacctgttcaagggctccatgttttgc
ccaaaactcaaaatggtgcgatgggagtgtgttgtgtgttttgagatgggggctgatgcgtagcagcagc
actttttcaaataatttggatagacaagtaagtaagcttattggcctgtatgatgatggctgtgttttgt
ctttcccaggcttagggatcatgattataattgatttcttttccagttttgagttgctctttaatgactt
tcgtaatctcgcttagtcgaaattctacaggatcttgtggtgcttagtcagatgcagttaaaggcgggag
agtaaatgagttcgtggctggatttggaagatgatcagcgaagactcttgctccgccgtgtcactgcgag
accagtttccggtagagttacggagaggtacaaccgtttctattggtgcctgttgtctttccacaacgag
ttctttgtacctgtgggcgtaagattctcgatgtatcttagttgtgcgtccgctgcttctttcttaagca
ctatggtaagtctgcgagaagctgctttcaatttactctttgcgccaggtgatctgtgctcctgccactc
ccttcgatgtcgtcgtttttcaagcacaagcagtttaatatctctacttgttttgttaaactttgtgctg
catacatggttgtcttgtggagttgacgcttttgctgctgcgaaaaatatttattctatatcaccaccac
actgatcaatgtcttcctcggtttccaggggctagagaagccggtatggcaacaatcataGATCATAGA
ACAtaatccttgccgtaacactttcaaaaataaactttatcagcccaataatgtatgttaaacaacgttg
atgttaaggagatgacattgaggcatttcactaatattaacgtaccagatgtattagaagtagcttctat
taaagcatttaaaaatagtgacattttgtatttcctaattaagtatcccatacctaagttagtatgtaaa
aaataatatatttcccgtaaaacattctggtaaaattctggaatttgaagactcaaacatcgtagcgact
gcgttacggatatctttgctgttggcgaatgtaattcaacagtgagcaccactcaaaaacctgaaaacac
ccagttgtgcgcaccaattaaaagttggaaccctagcgcaatgcagcactcgccctggccatctggatag
gcacggatgtaatggatgtaatagacgacggcgggatcatcatcaacgatgatactgTAAACATCTCAGG
CTATTCGAGCTCAGCGTCATCTATCTCTGGAAGCTTCCTGGTGAACTTTGACGATGAAATCACAATTAAC
GAAACACCGTACACCAACAACTATGGTGTCTTTGCAGTCACTGCAATCAACGTGACCAAGCCTCGGGAAC
ACTTGCCCTCTTTCATAAGATGAGCCTGACGATATTACGGAGTCACATTGACAGCTGTCATGATTTACCT
GGTACTGGTGCGAAAAATCCAACAACGCACGAAACTCCGGGAGCGAAGTGAGATCATTAAGAACCTACAA
GCAAATGCTAGAAAAACCGGGGAGGGAGGGAGAAGATAACACAAGCAAAGCTGAATCGGGTTTTGCGACA
CCCAATGGCGGAGTAACTTGTTCCGCACAAATAACAGCCGGTCAAAAGGGTCTCGAGGCGTTCTAACGCG
ATGCTGCCGGGTATCCATAGCTTTGGTTAGGCTTCAAATGTAAGCTTAAGTCAGTTTTAAGAGAACCATA
AACGAGATCCAAACGGATCATAATAAAGCCTAAAAAACttttttttttattattttttATTAAGTGAAGA
ATCCGTACATCATAATATTGTATCTTAACATCACAGAGTGGAGAAATATTCTTAAGAATTACCAAACACT
ATATTAAAATAAGTGAATAAAGAACTCAAAttttttttttAATATTTGGCGCACAACAGCCTCTAAAATTT
TGGAAATAAAAAATTATAGTGGAAAGATATAACAATAAACATTTaaaaaaaaaCACTCAATGAAAATTAGA

ATTCTTTGACAGTGACCTTTTTTTCAAATTAAGATTTTGTCGGTATAGTTGGACTTGACCTGCTTATCCA
AGTTGACACACAAATTGATCTGAGCAACGGTATCATTAGGTTTAGGTTTCCAGTGTTTGAAcaaagacac
tagaataacaagatacgtaacggccatacattggtttagcactatgcggccactttttttggtgacggcca
aaattgctctctttccgctcgctcacgctgagaacataagaaatctaaaaatagaatttgcttgcttgtg
tgagtaaaaactagaaaacgtgttgtgctagaagacgattttcgggccgaaatcaattctgatcgaagaa
acgaatttacgtatttggggagttttggccaatttcgtagcaatatgatgaaattgcgcgcctttttaaaa
atatcaaattggaataaacaaaaaaaaaacaaaaaatgaaactgagcaattgaatctaatgtgcatttta
aataataataatggatcatacatacatcatattaagtcaaatgacttaataaatatacaaaataattaaa
gcaaatacaaaggaaattattataactattatatttatacatatttacataaataattaattatatgtaa
tagacgtaataattggtacccagggtacagcaggggaggccattacaattgtaatgcggtcctatattta
tatgaccttcgagtTTCTTTttcacaacctatcgcaattaccatttacaataaaatttatttccatactt
tactattgcaacaaaacgacAaaatacaaaggaaattattgtgactactgtaaattaaaacgtaaatttt
gcaaaaagaagtcattaattgagaaaaaaatatttcataaaaataatacgttgtagaaaaatttataaaa
taaataaaaataaattgcaaaagtaatatcttgaggcacgcagtgcggacagaaggactcaacaatcatt
gcctcattaatttttcaaacgtcgcaagctgaatactctaatgacaaatattctaatataaagtcatttt
tgaaatttattttgttatattaggtacatagattcggctatttctaagctatatttaattaataataacg
ttaaggcaatgaaaaaccagaatttttttacatggtaccaattgataaaaaataatatagatttaaagttt
aaaaacttctaagacaaaggacatattttgtcaaatttttatcggctgtcactgtatgcgtagaaaagag
ctgttcgatatagagctctccgctctctcgctcttgaataaagttcgagagagcctggagacacctctaa
agccaagccgaaaaaatcgtggaccaaaaaatggaatggcgttacgcatcttgttattctagcgtctttg
GTTTGaaacagtcaagtcgaaaggtttcacagcactattgcggaaattgctcgttgtatggaacttgaga
aacagataagcgacactgtttattgttttcaataatcgaatatactaaaacaattcattcggtaaccaaa
atgagaccagttgaactcattcgttcaaACAGTGTGAAATTAGGACTGCAAGTAACACATAAACTTGTAG
AAGCAGTCTAAAAAATCGAACAGACAAATAAAAAATAAGAGTTATCAAGAAGGAGAAAGGTCTTGGGTTA
GACACTAAACTCGTTTAATCAAGACTATATAGGGAACGTACTGCTCACTGACCGTGGACACATCGACACA
CTAAATACGACACTTAAGGTCAATAATTTACTTTGTTAATTTAGATAAGTCAGCCTTTCAAGTCATTATA
ATTATCTAATATTGTTTTTACATTGTTTTATAGGGTGTGTGCCCAACTGAATGGAACCGACAAAGAAAAC
ATCTGTGTGTTATTCTAATCACAGAGAATAATAGGAAAAAAGACTTTGAAAGAGTCGCTCTAAGAAATAT
TGCTTTAAGTGTTGGGTACAATTCAGAAAAAGTTCGATTCGCCTATATATTCAAGG
>dme_circ_0004379_junction_seq
AAAGTTCGATTCGCCTATATATTCAAGGTTAAAGTTTAAAAGATGATTTCATGCTT
>dme_circ_0004380
GCATCCTGATAAAGTTAAAAATGATTACGGTGCCGAAAAGTTCATACAAATTAAACTAGCTTATGAAATT
CTAGCTGACTTGGATCGACGACGAATTTTTGACCGGTATGGAGTATCGGATATTAACTCACAGTATTTTC
AGAAGAAACATGATTATAGTGAATATAATCGCTTTACATTAAATCAGAATGATGATGATTTTGGACAACG
ATTCGATATAAAACAAGACATTGCATTTTATCAGAAGTTATCAATAACAGAAAATTACTTTGAGAAAATG
ATTTTATCaaaaaatgcaaaaaaaGTGCACGTCGTCATGTTTTACAATGATTGGTGTTTTAAATGTACTC
GAATTGTTGATGCCTTCAAAAAGATTCTTGAACTACTTCAGCCAATAGGGTATTAACTTTGCAACGGTTA
ATGCAGTGCATGAAGAGTCCGTTTTTCGAAAATGTGGCGCCAGAGAAGTGCCACAACTGGTTTTGATTTT
AGACAATCAGTATTTCTTATATCGCGATCATAGCTTTACCCCACAAAAAGTTGTTGGGTAAGTTCTATTT
AGCTTTAATCACTTTATCACTTAAAATAAAATTTGGTACTTTCGTGTTAGAAAAATAAGGTACAATATAC
ATCTAATATGTAATATGATAtatgtaaattattgttgttttaaaataaataataaattatttctaatgga
catagaaaaaaagtattgcatttgtagttttctacaacatctgcgttgctatgtaaattattgttgtttt
aaaataaataataaattatttctaatggagatagaaaaaaagtattgcatttgtagttttctacaacata

tgcgttgctaCAAAAATGTTTACACACATTTTTCAATTTATTTTCAAAGatatttataaaattattactg
aaaataaaaatgtcaaatcaaataattattataattattgTTACAAAATATAACTAAAAAATTAAAGATA
ACGATTAATtcgagactcccgactatcagataccctagtgaaagctagtgaaagtgcgaatgaaaatttc
aaaatttttttgtactgtatggtgaaatttggaaacaaataataataagatgaaaaaaaatgttgaatagt
gtgggagtgatagtttagagggggttgtgggccttaaagtgggtgtaggaaatctcatttgaaaacctat
taaaatatacaagacaaatgaaaaaaattctttaaaaaaattttaaaatgttcagaagtgtggcgtgaca
gttttgagcagtttTTTATACTTATCAGCGGCCGTACACGCAATACAATTCAACTCAATTTAAGAATACA
GACAAATATCTaaaaaaaaaaCTAAATGATCTTCAAGCAAAACTTTACCAAGACATAAAAAACATTTTGA
CACATCTCAAAAACGTATATCAGGACTAAACGAATCTCGGGAAAAGCTGATCCGCCTTGAAGAATGCTAA
CTTCCCGTACCCATAATCGCTCAGTGTATTGTGAATTTAGGTGttttttttttttgacttaaggtagga
aagtgaaatcttctaaagatacgatctatcgtccgtaggtggcatgcacgatttataaactataatggga
ttgtggctacgtactaaaacaaaaacctccgcgtgctcttcagtccttgcttgctcaccgggagtgccag
tcaaggtatgacttcggggagcaggatgtgaggccaagtcgcacccttcgcctttaacgacaagccgaaa
aggctgaggtagtattcttcgcccgccaccacgtgggacgactgtttgagcttagccacccactctgttg
gccatctcgtctgaccggacccgcttcatcatattaatgataaggctgtgacccagctcccatgcctcgc
tgttcgccaataaagccgcgaacaggcctcaagggctgaacggggtacctgccagcgactttagctgctc
cctctcctgcgtaaacctggagcagtgcacatgtttagctgtttcttcacgaccgacgcagaacaagcat
gggctgactctacgtggcggacccaagtagataggcccgaaagcaggcatgatccgtgagaaactgggtt
aggtggtagtccaagaatttgtgttggcactctgcccaaaccggcaactcagggatgagctggtaagacc
tcctcccccgtctcgacgtttgccatctgttctgccactcacccagcagccactcgtgtgcctgagactg
agagccttgatgtccagatcaattggcggcagtcctgccagtgctagcgccgcgtcttcggatatggtcc
tgaatcctgtaatgagcctgactgccatggaccgtagcaccgtgcgcgctcctttccggtatgaacatct
gtgcagcgtatagcagcgaagcctttgctaccgccaccagcagtttcctggccggatatcttgatccacc
gacgttgggcatgagtctggccaatgaagaggctgtgactgctgccttcttgctggcgtaattcacgtga
tccctgaacaatagtctgtggtctatcattacacctaggtactttagggactcttgggctatcttcctta
gcttgttggttacgagcttaaaagcaagaccccatgtttctctgtctacgctatcccccagttccttatt
ccttgaattaaagcgtttccttctataaggctctatgagcttctctatgcgccgttatcctagccctgag
gcattcagaccgtaggtgacattgggtggcacaggtggttcgggttgtaccttttaattcgttggcttgg
ttgcatggcaacatctaccaagcatattcatgaggtcagccgccatactctcttcatccccatttgggat
ttccagggatttccagagagagaccgaaggaaatcagagcgtggtcgctcagcgttacgacgacatgggt
catgcagttattgttagccactaaccctctgttgataaaggtaacgttatcgtcgcccctatcgttgtcg
tcggcttccgtccgacgttcagcagtatcaggtccatagtttccatagcgtctatacagctcgtcctctg
gggttggatacactgctgccccattccactgcccaggcactaaaaacaccggcaatgatcttcgggcttc
gccctctcgcatggcccacaagcaccgccagaaactcctcgaacttatcggggccgtcgctgggcggagc
ataacagctgtacatatgcacgtctcttacctttggggtgcagaaagttcctgaatgtggagagagctgc
aactcttgatagctactttacccgactcgtctaggatcatggaggattgtccccttccagggacgtaagg
ttcgcttaggagcatgatgtctacattgcgctccgccgcagttctgagccacgaggctctgagctactgg
gcaatgattgacattgagctggattacgttaatatggacgtccgttatggtggcttttagcttcttttgg
gctcgctttgtaggtcggtctcgctaacctacccgtcggatggtttctgtcctcctcgctgctgcagatc
agtcattttggtggtgcccacgctaaccgtatcgaagacatctttctgccctatcagtcgccttgcaggt
CTGTAGCATCTCGTCCTGGGTTATGCGCCCACCCAACGGTATTGGGAACGCATGCAAAAAAGTATGTCCC
CTTTATCAGTGCTGGCGGCGGGTAGATTCTTATTATATTTTAGACTTATATTATATGTGAGTagatcgat
ctcccggcgcagatgagctgaaataacagcgtggccttttccgcgaatactttgcaaatgtacgaacaca
caaatactttcacctgaatttcgaatcaaacgactataacagactctttttctcaaatttaacccaaaaa

tttaaacgcgcactctctgaaaatgtacacatcttgagaaaataattagattaggttagaataaataata
taaagatataaatattaaatactaaaaaaaattcaatttaaataaaaattaaaaatagaaaagtaatgta
aaattaatgtgaattattgtaaagcattataatatgcaatataaacataaaatcattcactattcaacat
tcagtgtagaatcgcagttcggtaatttttcccgccgaagtagggttaggtcagacagagatcattaaga
atttttatatatatttttgatttttcatgtttacAATTAAAACACTCCTCCCAACCCTGTTATTTTCGCC
CGCGTGCTTaattaaattaaatgcacatcgcagggtctacggttgcttcttaaggcttcggttattcgat
gggaagggtgctccactagacggcctgactcggctcttttctctctttctgcccgcctaaaaaattagag
ccttcgcgtcaactaatatatcacttttcccgcgatattcactaacctgtgtctgccccactcgaacctt
gaggaaaagcatgagaatttgctggtagcgaaacaccgttgtaattgtgtgcgaggattgactggcctac
tccaccggtgtttcgcgagcaacaaattcgtatgctttacctcttcttctataactgtctaccttccgaa
tatattaatttgctgccttcgattttgtatgctagctcctatattcttATATTCGCGCCATGCGGAGATT
TTGTCAGATTAAATTCCCGCCTGGAAAACATACACTAAAACTTAGACCGCGCAccgttttgtagcgagga
aaactcactctaacaatatatttccgcactaaaaaaaatttcacgcccgcaggttataatttacaacgac
acgaactagcaactacatacaagaacaatacaaagccagtctcaaatcccattaaatcacctaacaatgc
aactcacagacaaacaactaactactataatcatacaacaccattccaaccatcatacaacgccatgtat
caaccaattcgccaaccaatttcatatataccacctcaattacccagaactaaccctaatccgttttccc
aataccaataccgtcaacttcaccctcaacctaacgtatctgttgtagctcaacctagaccattgaatcg
tcaacatacatttgaccagaaccgaccaggacttagttattcaaacgcactaaatacaagagaaaatata
acgaccggtggaccagcactaaagagacaacgaccgtctgacagtggacaatcacgtatgagttttgatg
aggctcattaccaagaagaattagaccaaactcatcaacagttcaatccttacatgcattacgggaatca
tccccaatatcctttcgatctttacatgccttacgggccgccccccaacaacatcccgatttattacatg
ccttacgacccccccacaacaacatccggttggcatacaagagatggcagaggcacgagaagagccaactg
aacctcctatggaattaataacagaagctgaaacggctgagaattttcggccccaagcctcggaacaagc
caattcataattattaaacacaaaggactcaatttaaaatgcttaatagacacaggctcaacagtaaata
tggccactcaaaatttttttcaaattaccaatccaagagatagcaaagaaaataaactcaagtaatggcca
gttcattgcacaaaagaaagtaacgttacccaaaaataatcttttccctaaaccatacgatttttgata
tatcctttttcaactaaatacgacttgattttaggacgacaattacttgacgagggaactacatcagtaa
attacggaccccgtacagttacaatatacggacacgtgcacgaaatgatcgatgccttccttccttcaga
agaaatacatattcaagatacacagaataattcctttaggctagaccacttaaattccgaagaaaaagca
aaattgataaccctcctaaaagaattccaggaccttcaatataagaaaggagaccagctcacattcacta
ataacgtaaaacacactattagaacatcccataatgacccagtatgcagaaagctttacggatacgcacc
cggactagataccgaagtagaaaaccaaataaaagaaatgttagatcagggaataatccgagaaagcaat
tccccttattgtagccccattgtagtagtacccaagacaacagacacctccggacagaagaaatacagaa
tagccatagactaccgtaacctcaatgaagtaacaatagcagacaaatacccaataccaaatatggacga
aatcttaagcaagttaggaggatgcaactattttactacaattgacttaatcaaagggtttcaccaaata
gaaatggaccccgagtctatccccaaaacagccttcacaactaagacagggcattacgagtatacccgta
tgccatttggactgaaaaacgccccagctaccttccaacgatgtatgaacgatgtacttcgtccactatt
aaataaaatctgtatggtatacttggacgacattattgtattttcagcttccttagaggaacatcttcaa
tcccttagagcagtctttcaagcattatctaatgctaatctaaaactccaattagataaatgcgagtttt
taaaacacgacacatatttcttaggacatatggtttctccagaaggtataagacctaacccggaaaaact
acgagcaatagaaaattaccccccttcctactaagccgaaggagataaaatcatttttaggactcacaggt
ttttatagaaagttcataccccatttcgcacagatttcaaaacccctaacaacatgtcttaaaaaggaca
aaaaagtcgatagtaaaaacccggaatatattgaggcattcaaaaggttaaaactccttatttctaacga
tcccatacttcgatcaccagacttcaagaaaaaaatttgtactcacaacagacgctagtaatgtagctcta

ggagcagtactttctcaagatggtcaccccataagttatattagcagaacacttaacgaccatgaaacaa
actacagtacgattgaaaaagaactactagctattgtttgggcaacgaaaacgtttagacactatttgct
aggtcgacattttgaaatagcaagtgatcaccaaccgttgtgctggttgcacaagatgaaggagcccaac
gctaaattaacaaggtggaaattcagacttgcagaatacgactcttcttctataactgtctaccttccga
atatattaatttgctgccttcgattttgtatgctagctcctatattcttATATTCGCGCCATGCGGAGAT
TTTGTCAGATTAAATTCCCGCCTGGAAAACATACACTAAAACTTAGACCGCGCAccgttttgtagcgagg
aaaactcactctaacaatatatttccgcactaaaaaaatttcacgcccgcaggttataattttacaacga
cacgaacttttcacgacttgactccacgactattttattagaaaagaaatagaacggaaactcaagccgg
ggtctaggcaatcggtggaaatcacgatcagctaagtgtaactctatttacgataaccaaattatcccaa
aactttccgggtgcagctgataaagagtctagtatattcccgatgcataatattgcatttaaaatgaccc
taaattagaatcttttagctgagtgcaagcttcataatgtttgtctattaggaggttcctaacaacgccg
cttcaatcgccactacaATTACAGacatacttttcaacgagtatagtatacccttctaatctacgaatac
cggatataaATATTATACAATGGAAACATCatactatactatatatactatacttatatttaagaatata
aaatattatatataacattatgtcttatatttagtttaaaatctttatCCAAGATTTATTGATTTTACGT
AGCGACCTTTATTATATAATGTATTTAACTTTTCTAAATGAAATTTAAATTTTGTTGCAGAAATTTATAC
GCAAAAAAATACCATTTAATGTTTTCAAACGCATCGAACATGACAATTTCAATGATTTTCTGGGAGGATG
GAGTGACAACAGAGCAAGGGCTTTAATTTTTGAGCCTCGAAGTTTAACTAGGTTACGATACCTTCTAACC
GCCTTTGAATTTTACGACCGAGTTGCATTTGGG
>dme_circ_0004380_junction_seq
TGAATTTTACGACCGAGTTGCATTTGGGGCATCCTGATAAAGTTAAAAATGATTAC
>dme_circ_0004381
TTAAAGTTTAAAAGATGATTTCATGCTTAATTAGTAATTATATTATAACCATTCTGGTCCTGTGTACAAC
ATCCTATATACACGTTTGTTCGTCGTTAAATGACCCATACGCTATATTGGGAATAAACAAAAAGGCAACT
ACATACGAAATAAGAGAAGCTTACAAGGAATTAGCTAAGAAATGGGTAAGTCTGCATAACAATTGCGTCA
TAAAAGTTTAGAATGTTTAAAATTAAATGTTCTTCTATCGTTATATACTGGGTATAATGTTGTATACCCA
GGTTGCTTAAGTTAAGTTGCTTTGCAGTAAAAAGGttattaattattattatttatatcccttatcgtag
agtattatattcgtttaaaagtatgtaaacaagagaaggaagttagtttccaaATATTTCAAATGTTGTT
GGTAGCTGCATACCTTATTTAAACATTCTGCGTTATatatttttataattttgtatatcttgtaatattc
tgtcgatttgtcaaaaaactttttaccacgcccactctgacacccaaaaaccgcccaaaactgcccacac
ttttgaaaaatgttttaatattgcttaaaatttttgttagtctttcaaatttccatcgatttgcAATGCC
CAATCTAACGTCCACAAAGCGAAACGACCACAATTCGATCttttttttATTATATTTTCAATTCCAACCG
CTGATGCAAAAAGCGTGACATTTTTGAAGCGCAAGGCGGCGGCCGCAAGTGGAACGATTAGCGGACTTCT
GCAATCATTTCGTTTAATGAGCTTGCTCTTCGAGTGAGGTTGGAGCTGATCGATGAGCTCCAACAAACAA
TTAGAAAAGTTAGCGATGAGCTCGAAGAACTTGATTTTGATGAAACCGAACGTGACTTGGCTTAGAAGTT
TGATGACATTCTTGTAACTCTTAAATCCTCTATAAGGGGGAAGATCTTAAAGGGCGGCTCGCAAATGCAC
TTTTCCACTTAACTTTCGATGACGACATTGGTCCGTTTGATTGAGGCCCCATCAGCTTTCACTCATAAAT
AGGGCACCGTCGCTGCCATCTCTTGAACTTCCAAAATCGCTGGAGGCTATGCTAACTTGGTCAACATGGG
TCCACCCACCAGCACGCTGCCGTAATCCCAATTGCTTCAGGGCGGCCAGGGTCAGGCACAGGGTGCCAGT
GGCGCTCCGGAAAGCGTTGGACCGGACAATATATCGCATGAAAACGAAATAAGCTCCTCGGGACCATTCC
AGAAGattttgtcaaatttacaatgcaagagcaaacgtgtgcgcacatgcagttgtcggctgtcactgta
tgcgtagaaaagatctgttcgatgtagacctctccgctctctcgctcttgaacaaaaattcgagagagcc
tggagccacctctagagccacctccacgttgtacaaatcgtgtgccaaaaaatcgtatggcggcgttacg
catcgttattctagagtctttgCTTGTACTCTCTCTGCAGCTCATTATCATTCGATTTTCGTTATTTATA
CATATCACATTGATGCAAAGTCTTATTCTCTGAACACAATAAAAATAGATCGTTCATAAATCGTTAATAA

AAAGATTATGAGCTTAAAATCCTATTGGAATTTTCCTTCTCCTACTTGTTTCTTTTCTTAAACGCAATCC
AAAAGCCCACAAGATTGACTGTTTTGCGTGCCTTTACTGTGTCTAGGTATTTTGCTAGCTTGATTGTGAA
GTTGGAGTCAGTCAGAAACTGAAATTTAAAGAAGGTAGTTCAAAATCCAGGAAACACAAGGCCACAAGGC
TTGAAACAGGAGGTTGCAATGTTTGAGTCTGGCCATCAGAAGTGtgatcgtctcatgtacacttttaaaa
acgcagcatagcaacctgatcgacgtttagaatgcagtaaaaagtgcacagtcaacgcgaggcgtaaata
aattcgtcatactgccaaaattcaattcctattccgttggcactagtgcagctgcgtggtaagccacggt
ggacctcattctcgcagaaaacccactaatgggcagtgctctgcttgtttccatgagtaaatcacaagtt
tgcttttctggaatcacgtgggtcttatttgaggaattgtttttgcaacaattcgaaggcaacgaaacac
ctgcacctactgttttaatggaaaacggcgagtgatttccctgtatgcaagacacttaataacaacacta
aaagcaaagtggaactcgatgcctacggaacgcggtgtcggtagttctagcgcacacggcctatatcgac
acaagactacaacgaactgtttatacaaccaacatcagaacacgtgacgaattgcagaatgagttgcggg
aatttgcttttgctaagaagaaagaTTAAAGATTAAGAAAGATTAAGAATAATTCTGCGGCCAAAAGATA
ACGTGTTCACGCACCCATTATTTGCCTTTAACGTTGCTAAGCTAcaagaatgatccaacttaaattctgg
acgttcatctgattcaaggaccaaaacaaatacctgctacaagtgtggggcttctggacatattgtttct
gcttgttctgagagaaaggaatcggccactcattataaattcgatgagaggggtgtaaacttgtgccagg
tggccgacccaattggtcctttttttatccggtttttatatttctggagccgaatgttcgcaaagaattt
gtatctgatagactttcgggaacaagaataaataatatcgttgttttaaagggaataggaaataatttag
taataacaactatgcatatatagtctaatgttataatcaattcttattctcttgaagtaatttttcatgt
tgttctcaacagtcacataaattatgacattatagtcgggcgtgaaatttAAGCTCAACTTTTGAATTAT
ACACGACAAAATCTgggtttccgaaaagccgagtcacaactggatagtagcaatacgcttgatcgatcct
aacaagactgtgcgtaggcggcaatcggcaatatcggcttagtatcgcggaaatttgaatcgtaaaagac
aaattaagggaattgttggcttcgaatataatacgccatagctgttagcaatttgcaagcccaattaaaa
agaaaagtgtctcggatagacttagcataggttatcattagttaaatgccaataccgtgtccgaaaatac
cccctttaccccttattaacgaccaagtctctatgttacgtgggtcgagatatggctagaagtttgcacc
agattcctttactaccaaattaattcgaacgaacggcatttgtcactccagagggaatttttgaccatgc
ctttcggcttgaacaatgcacccccccgctgtgttataaaatcgttaggtgaccttgcgcactgctatgat
atcgtttacatggaTTGAAGAAGAATCTTTCTTGAGATTGAGAACAGTATTGGCTGGCGTTTCATTTAAT
GTGACTTCTTTAATGTTAATTCTTAAAATAAGTAGGTGAGTATCTTGGGTTTCTTATTAAGGATGGCGAA
ATTCGGCCCAATCCACGAACCCTCGCTGTCCGATATTAAAGTGAGTCAGCTCATAGGCTTTGCTACAAAG
TAACAAAGTTCTCTGAAATAATGAAATCGCTTTATGGGCTTACATGTTAAAACAAATTATTAAAATGGGA
ACCAAAACATGATCAAATACGTCAATGCGTCATAACAAGCACAGTGTCACCGAGTAAAATCGTCTAAGTC
TCGCTCCCATTCTTATGAGCTCGAACCGTTAGCCGTATTTAATTAAATTAAACATTTCCGACTTATTTGC
ATGGTCGTCATTTTACAGTTTATAACGATTGTAACTCCTTAAGGGCTACAAGAATTATGGTTGACCTTAC
ACCAAGTGTGGGCGTACAAGTTGGTGAGATCCAACTAGAAAAGAGCTTTGATGTTGTTCAATGAGGTATT
CCAGACATTACTAAAGTGTTGAGTTACCAGCGCTAATTGGTAAACCAGTTAATATTTGGACACATTTGTC
GCCTGGTGATCCAATTTTGGTGAGTGTTCTTAATACGTTATAGCATGCGTGTTTGGGAATTCTCTGCCTA
TGGCTATTTAGAGTGTTAAAGAAATATCCTCTGTCACTAGTTTGGACACTTAAAAAAGGGAATTCTTTTA
GATGAACCATGATataaactccatttaattttgaacattattggtacacaagaggacgtgtgatactcag
gaagactgTGTCGCATCGATGCTATCATTGATTTTGTTAGTCGTTAAATACCATTACTACTGAATGATTA
TTGTTATGTCATCATTTATGAAGAATATAAAATAAGGAATAAGTCGTTCTCATTTATCCTCTGCCTAATT
GTGCTTCCTCTTACACACGATCCACAAGCCCAACTAATTGcacagcctcagttcgacaatgcgttagaac
gatacaaatATAGTGTGAATAAGAACTGATGCGCCAATACCAAGTGACACCAATAGACGCGTGCCCTTAC
TGTGTCTAGTATAGTGATAGCATAATTGTGAAGTTTCAGTCAGTGCAAAACTGAACAGTTCAAAAGAAAT
CAATAAACACAAGGCCACGTCCGGGTCTGTTTTTAATCTTGTATTCTATTTTTCTTCGATTTGTTTCGCT

TGTAGTCTGCTGAAGTTTCTCTATGTCCGCTACACCAGGAATCGAAGTTTAACCTCACACACTAGTAGTT
TcttaactttgttcaactcagtgtgtaatcctacttttcgcacgacaatgaatagctaaatttcccACTA
CTTCCCATTTGACCTGTCTGCCCTTGACTTCTCTCGCATTCCAGCCTTCTTCgctatgcagctctcctgc
accctgatttgtggggagtttttagactcctcaCATCTCAACCTTCCATCGGAAGTTTTTGAAAAGTTTG
GTGGTTTTAGACGGTCCTCCGCCTCTGTATCTCCTCGCATAGGCAGCTCCCGCGTTTCTCCTCTCGTCGA
CCCTGCGCCTATGGTTTTTACCAAGACAGTTTCAGCCTAGTATAACCTGTCGTTAAGCGTCTTGACGTGG
AATCTCGATCCTTCATATACAACACTCCGAACTCCTCTCGTCAACCTGGGACTCCATGATCGCAGCGCCC
TATACCGATTTCCTGCATTGCTTTGACGCCAACTGCTGATTTGCTTTGTCTTTCTCCACATCAGCTTTAT
GCTGTCATCCTTGCTCCTGATGCTGGGAACCACCGCTATGCATCCCTGCCTACGCTGTCAGTGCCCACTC
GTCGTCGTCGGTGCGGTTGCGACCTGCTTTTTCTGGCTCGAACTCCTCCTGCGCGTGTGTACCCGTGGTG
CTGTCGCGACCTGCTCCTCCTGGCTTCTGCTTCTCGCTCGAACTCCTTCATGTGTTCAACAGTTGCGAGT
TTCAATAAAATCCTTTGGATTGATGTCGGTCCTTTACATGCTGGCGCTTTGATCAAGACAATCACGTCGG
GCGACACAAATCTTATGAGCTCTATGTTCCCCTGGAGTTACTATATATTGCGTACCTGAGTTACAGAATC
TCCTTAGAAGTATTTTTAATGGTTTTGTtatgagattcatatgaaaggatatcagaatacaattgagcta
gattgctacgcaagcctgataagagatagtaagtcaagatgttagtttttcgatgaagagtggaaagggg
aagaggaggattgtgcagtggttgatctctttcaccaccatactatccgaccataaattaccagagcata
aggttgaaaaaagctcataaaggacaattatcttgaaaaaaaaatgtctctagtgcaagaaaagttaTAT
AGGCTTGATATACAAGCCTTTTTTCTCATCGGGCATTCGTGGTCCTGCTCCCTGAGGGGTTTCTTAATTA
TATCTTGTAGCGAAAATTCTCATTGAATCTGCTTTCCGTACTCTTTGTAGTGCTGCCAGTCTTTGAGTCC
TAATATGAATGAGACTATTGGCTTCTATAACCAGCTTAAAATGCTTGTTGGTAATGGACCAGCTCTCCTT
GTAGCTTTGTATCTGTTTACCATGTGGGTAGAGCTGAACTATCCATATATACGGAGTTGCCCAGATCGTC
TCGGCAAGTAATCCTGATTAAGATTATTTCCACTAGGCTCCGAGTTTTTTATggcaaaaatcctttgtcc
gtgcgtaaaatgtgactttatgcattaaaaatgaacatgtttatggtttgtatgttgtatttcctgcatt
ttaataaacaaatatcttaagtttttacacatgctactcggatgaacatataattattaaatcttcctta
aataggaatgcttcgggggatctagtcataagtgttattctttggacagacttaggtgctatcttagacc
aaCAAAATGTAGTAAAacgtaaagtcgagacatgaaaaatatttagcttccagttgattcaaaaatatca
ttaattctgggtaaaggaaatttatctaaaattaaggtaatactttttatgtcaatgaaacctatttctt
tttctctgaggttaaaactatttttttccgaaataggaaaaggggactgtGGAATTTCATTTCTTCCATT
TCTCTTATTTAGTCGATAATTTACTTTTGCATAATTTTTTTAGTTAGGAACTAAACTATTCCGTATCGAT
CATATGGTTTACAAAACTTTACTAATATCATATCATATAGAAATTTTGCGAATTCGATAAAGCGTTCGCA
GCATACGTAGATACCTGCTTGTATCTCTTGGGATTAATATATTGTTGTCTTTAATGGTAGTGATATGTAG
GTGTATGTTATTGGAACATGGATCGGAAACTTGACTTGATGTTGGGTCACTCAGCTGCTTACAATTTGTT
TGTATTAGGGTTATTCCGTTTGCTCAGCAATAATGTATTTTCTTACTTTCGCTTTCGTAACCCATGCCTA
TGCGCATCGATGCCGAATTTCGCTGTAGCCAACTTaaaaaaaaTAAATTTATTTAAAATCTCTTAAATCG
ATGAACTCGTTTCTTAAAAATAGCTAATAAAAAAACTAATTAGCTCAACAGGTGGGTCTGTTTTAATAAG
GTGAATGGTAATACTCCAGCACATTATATTGGGGAATACTGTTTTACTATATTCAAGTTGTTTGGAATAT
TATATTAATCTGGATGAATATCAATTGATAATAGTCTATTATATTGTGTAACATTTTGTCTGATACCTTC
GATAGTAATTTGGTTGTCAGTTTGTATTATTGTATTGAATTCTTTTAAAACTGAAAAAAATACGCCTGCA
TTGATTAAAAATGGTAATAATTTGGCTACGTTAAGAAATGAGAAAAACGTGTTTAAACTGAGATTGATGG
AGTAAACCTTTGGGTTATCTATGTTGTGTACCTGACAGTTTTGCGAATTGGTTAAAAATACCCTAACGTA
GTTAGGTACAAGTTTCCACATGATCTATTATTATATGTATGTATTGCATATTATAATTCAATTACTGCAA
TAATTACCGGTTCTGTAGTTGTAGATTTTGGACATAGACGTTGcgcccactctaacgtccacaaaccgac
tgaaactgccacgcacacacttttgcaaaatattgtgatatttttTCATTTTGCAAAAAaacgcgctaaa
accgcccaaaaatgctacgctcacatttttgaaaaattgttggataatttttcatagttttattagtcgc

ttaaatttctatcaatttgttaacaaactttttgcaacgcccactctaacgccctaaagccgccgaaccg
gtcacgcccacactcacccaatatcacccaatataaccccgaaaatgaaatgatgaaatatcgcgttcgc
attcacaatagctgagtaacgtgtatgtgatagttagggaactcgactattgcattctctcttacttTGA
AAATAAGAACATTATAGCTAATATAGGATTAATAGAGGATTATTAAATAAATTGTAAGATGAGCATTTTA
CAGCAGACAATTGCCAACTTTAAAGCAATCCTGCTATTATTGTCCAAATTGTAAAATTTAAAATGGTTTC
CAAAGTTGAAGGTATTAACATAGATATATAttttttttatctttaattaaatgaatctaatttttttttgt
gcattgaacctcttttatatgttttgttaatgttttatttagatttttagtTAATATTAGTAAAGTTTTG
TAGTTCCTGTAGTTCCAGACGTGCATGTACAAAAATAGACGTTCGCTGTTTTTCAAAAATTCTTTCTATT
TATACGCGGCCTTGAAAATTGTGATATCTTCTTCGGGTGCAAGATATTGTGATTCCATATAACTCTTAGT
ATTCTTTTTGGTTAGACTTATTTCACTTAGTCATCCGATGGGAAAAAGTTAGTTTTTAGCATAGGAGAAA
TATGCATACCCAATTAAATTATAATTATGGCTTATTCTTTTTAATATATTACAGGGCATCCTGATAAAGT
TAAAAATGATTACGGTGCCGAAAAGTTCATACAAATTAAACTAGCTTATGAAATTCTAGCTGACTTGGAT
CGACGACGAATTTTTGACCGGTATGGAGTATCGGATATTAACTCACAGTATTTTCAGAAGAAACATGATT
ATAGTGAATATAATCGCTTTACATTAAATCAGAATGATGATGATTTTGGACAACGATTCGATATAAAACA
AGACATTGCATTTTATCAGAAGTTATCAATAACAGAAAATTACTTTGAGAAAATGATTTTATCaaaaaat
gcaaaaaaaGTGCACGTCGTCATGTTTTACAATGATTGGTGTTTTAAATGTACTCGAATTGTTGATGCCT
TCAAAAAGATTCTTGAACTACTTCAGCCAATAGGGTATTAACTTTGCAACGGTTAATGCAGTGCATGAAG
AGTCCGTTTTTCGAAAATGTGGCGCCAGAGAAGTGCCACAACTGGTTTTGATTTTAGACAATCAGTATTT
CTTATATCGCGATCATAGCTTTACCCCACAAAAAGTTGTTGGGTAAGTTCTATTTAGCTTTAATCACTTT
ATCACTTAAAATAAAATTTGGTACTTTCGTGTTAGAAAAATAAGGTACAATATACATCTAATATGTAATA
TGATAtatgtaaattattgttgttttaaaataaataataaattatttctaatggacatagaaaaaaagta
ttgcatttgtagttttctacaacatctgcgttgctatgtaaattattgttgttttaaaataaataataaa
ttatttctaatggagatagaaaaaaagtattgcatttgtagttttctacaacatatgcgttgctaCAAAA
ATGTTTACACACATTTTTCAATTTATTTTCAAAGatatttataaaattattactgaaaataaaaatgtca
aatcaaataattattataattattgTTACAAAATATAACTAAAAAATTAAAGATAACGATTAATtcgaga
ctcccgactatcagataccctagtgaaagctagtgaaagtgcgaatgaaaatttcaaaatttttttgtact
gtatggtgaaatttggaaacaaataataataagatgaaaaaaaatgttgaatagtgtgggagtgatagtt
tagagggggttgtgggccttaaagtgggtgtaggaaatctcatttgaaaacctattaaaatatacaagac
aaatgaaaaaaattctttaaaaaaattttaaaatgttcagaagtgtggcgtgacagttttgagcagtttT
TTATACTTATCAGCGGCCGTACACGCAATACAATTCAACTCAATTTAAGAATACAGACAAATATCTaaaa
aaaaaaCTAAATGATCTTCAAGCAAAACTTTACCAAGACATAAAAAACATTTTGACACATCTCAAAAACG
TATATCAGGACTAAACGAATCTCGGGAAAAGCTGATCCGCCTTGAAGAATGCTAACTTCCCGTACCCATA
ATCGCTCAGTGTATTGTGAATTTAGGTGttttttttttttttgacttaaggtaggaaagtgaaatcttcta
aagatacgatctatcgtccgtaggtggcatgcacgatttataaactataatgggattgtggctacgtact
aaaacaaaaacctccgcgtgctcttcagtccttgcttgctcaccgggagtgccagtcaaggtatgacttc
ggggagcaggatgtgaggccaagtcgcacccttcgcctttaacgacaagccgaaaaggctgaggtagtat
tcttcgcccgccaccacgtgggacgactgtttgagcttagccacccactctgttggccatctcgtctgac
cggacccgcttcatcatattaatgataaggctgtgacccagctcccatgcctcgctgttcgccaataaag
ccgcgaacaggcctcaagggctgaacggggtacctgccagcgactttagctgctccctctcctgcgtaaa
cctggagcagtgcacatgtttagctgtttcttcacgaccgacgcagaacaagcatgggctgactctacgt
ggcggacccaagtagataggcccgaaagcaggcatgatccgtgagaaactgggttaggtggtagtccaag
aatttgtgttggcactctgcccaaaccggcaactcagggatgagctggtaagacctcctcccccgtctcg
acgtttgccatctgttctgccactcacccagcagccactcgtgtgcctgagactgagagccttgatgtcc
agatcaattggcggcagtcctgccagtgctagcgccgcgtcttcggatatggtcctgaatcctgtaatga

gcctgactgccatggaccgtagcaccgtgcgcgctcctttccggtatgaacatctgtgcagcgtatagca
gcgaagcctttgctaccgccaccagcagtttcctggccggatatcttgatccaccgacgttgggcatgag
tctggccaatgaagaggctgtgactgctgccttcttgctggcgtaattcacgtgatccctgaacaatagt
ctgtggtctatcattacacctaggtactttagggactcttgggctatcttccttagcttgttggttacga
gcttaaaagcaagaccccatgtttctctgtctacgctatcccccagttccttattccttgaattaaagcg
tttccttctataaggctctatgagcttctctatgcgccgttatcctagccctgaggcattcagaccgtag
gtgacattgggtggcacaggtggttcgggttgtaccttttaattcgttggcttggttgcatggcaacatc
taccaagcatattcatgaggtcagccgccatactctcttcatccccatttgggatttccagggatttcca
gagagagaccgaaggaaatcagagcgtggtcgctcagcgttacgacgacatgggtcatgcagttattgtt
agccactaaccctctgttgataaaggtaacgttatcgtcgcccctatcgttgtcgtcggcttccgtccga
cgttcagcagtatcaggtccatagtttccatagcgtctatacagctcgtcctctggggttggatacactg
ctgccccattccactgcccaggcactaaaaacaccggcaatgatcttcgggcttcgccctctcgcatggc
ccacaagcaccgccagaaactcctcgaacttatcggggccgtcgctgggcggagcataacagctgtacat
atgcacgtctcttacctttggggtgcagaaagttcctgaatgtggagagagctgcaactcttgatagcta
ctttacccgactcgtctaggatcatggaggattgtccccttccagggacgtaaggttcgcttaggagcat
gatgtctacattgcgctccgccgcagttctgagccacgaggctctgagctactgggcaatgattgacatt
gagctggattacgttaatatggacgtccgttatggtggcttttagcttcttttgggctcgctttgtaggt
cggtctcgctaacctacccgtcggatggtttctgtcctcctcgctgctgcagatcagtcattttggtggt
gcccacgctaaccgtatcgaagacatctttctgccctatcagtcgccttgcaggtCTGTAGCATCTCGTC
CTGGGTTATGCGCCCACCCAACGGTATTGGGAACGCATGCAAAAAAGTATGTCCCCTTTATCAGTGCTGG
CGGCGGGTAGATTCTTATTATATTTTAGACTTATATTATATGTGAGTagatcgatctcccggcgcagatg
agctgaaataacagcgtggccttttccgcgaatactttgcaaatgtacgaacacacaaatactttcacct
gaatttcgaatcaaacgactataacagactctttttctcaaatttaacccaaaaatttaaacgcgcactc
tctgaaaatgtacacatcttgagaaaataattagattaggttagaataaataatataaagatataaatat
taaatactaaaaaaaattcaatttaaataaaaattaaaaatagaaaagtaatgtaaaattaatgtgaatt
attgtaaagcattataatatgcaatataaacataaaatcattcactattcaacattcagtgtagaatcgc
agttcggtaatttttcccgccgaagtagggttaggtcagacagagatcattaagaatttttatatatatt
tttgatttttcatgtttacAATTAAAACACTCCTCCCAACCCTGTTATTTTCGCCCGCGTGCTTaattaa
attaaatgcacatcgcagggtctacggttgcttcttaaggcttcggttattcgatgggaagggtgctcca
ctagacggcctgactcggctcttttctctctttctgcccgcctaaaaaattagagccttcgcgtcaacta
atatatcacttttcccgcgatattcactaacctgtgtctgccccactcgaaccttgaggaaaagcatgag
aatttgctggtagcgaaacaccgttgtaattgtgtgcgaggattgactggcctactccaccggtgtttcg
cgagcaacaaattcgtatgctttacctcttcttctataactgtctaccttccgaatatattaatttgctg
ccttcgattttgtatgctagctcctatattcttATATTCGCGCCATGCGGAGATTTTGTCAGATTAAATT
CCCGCCTGGAAAACATACACTAAAACTTAGACCGCGCAccgttttgtagcgaggaaaactcactctaaca
atatatttccgcactaaaaaaatttcacgcccgcaggttataattttacaacgacacgaactagcaacta
catacaagaacaatacaaagccagtctcaaatcccattaaatcacctaacaatgcaactcacagacaaac
aactaactactataatcatacaacaccattccaaccatcatacaacgccatgtatcaaccaattcgccaa
ccaatttcatatataccacctcaattacccagaactaaccctaatccgttttcccaataccaataccgtc
aacttcaccctcaacctaacgtatctgttgtagctcaacctagaccattgaatcgtcaacatacatttga
ccagaaccgaccaggacttagttattcaaacgcactaaatacaagagaaaatataacgaccggtggacca
gcactaaagagacaacgaccgtctgacagtggacaatcacgtatgagttttgatgaggctcattaccaag
aagaattagaccaaactcatcaacagttcaatccttacatgcattacgggaatcatccccaatatccttt
cgatctttacatgccttacgggccgcccccaacaacatcccgatttattacatgccttacgaccccccca

caacaacatccggttggcatacaagagatggcagaggcacgagaagagccaactgaacctcctatggaat
taataacagaagctgaaacggctgagaattttcggccccaagcctcggaacaagccaattcataattatt
aaacacaaaggactcaatttaaaatgcttaatagacacaggctcaacagtaaatatggccactcaaaatt
ttttcaaattaccaatccaagagatagcaaagaaaataaactcaagtaatggccagttcattgcacaaaa
gaaagtaacgttacccaaaaataatcttttccctaaaccatacgatttttgatatatcctttttcaact
aaatacgacttgattttaggacgacaattacttgacgagggaactacatcagtaaattacggaccccgta
cagttacaatatacggacacgtgcacgaaatgatcgatgccttccttccttcagaagaaatacatattca
agatacacagaataattcctttaggctagaccacttaaattccgaagaaaaagcaaaattgataaccctc
ctaaaagaattccaggaccttcaatataagaaaggagaccagctcacattcactaataacgtaaaacaca
ctattagaacatcccataatgacccagtatgcagaaagctttacggatacgcacccggactagataccga
agtagaaaaccaaataaaagaaatgttagatcagggaataatccgagaaagcaattccccttattgtagc
cccattgtagtagtacccaagacaacagacacctccggacagaagaaatacagaatagccatagactacc
gtaacctcaatgaagtaacaatagcagacaaatacccaataccaaatatggacgaaatcttaagcaagtt
aggaggatgcaactattttactacaattgacttaatcaaagggtttcaccaaatagaaatggaccccgag
tctatccccaaaacagccttcacaactaagacagggcattacgagtatacccgtatgccatttggactga
aaaacgccccagctaccttccaacgatgtatgaacgatgtacttcgtccactattaaataaaatctgtat
ggtatacttggacgacattattgtattttcagcttccttagaggaacatcttcaatcccttagagcagtc
tttcaagcattatctaatgctaatctaaaactccaattagataaatgcgagtttttaaaacacgacacat
atttcttaggacatatggtttctccagaaggtataagacctaacccggaaaaactacgagcaatagaaaa
ttaccccccttcctactaagccgaaggagataaaatcatttttaggactcacaggtttttatagaaagttc
ataccccatttcgcacagatttcaaaacccctaacaacatgtcttaaaaaggacaaaaaagtcgatagta
aaaacccggaatatattgaggcattcaaaaggttaaaactccttatttctaacgatcccatacttcgatc
accagacttcaagaaaaaatttgtactcacaacagacgctagtaatgtagctctaggagcagtactttct
caagatggtcaccccataagttatattagcagaacacttaacgaccatgaaacaaactacagtacgattg
aaaaagaactactagctattgtttgggcaacgaaaacgtttagacactatttgctaggtcgacattttga
aatagcaagtgatcaccaaccgttgtgctggttgcacaagatgaaggagcccaacgctaaattaacaagg
tggaaattcagacttgcagaatacgactcttcttctataactgtctaccttccgaatatattaatttgct
gccttcgattttgtatgctagctcctatattcttATATTCGCGCCATGCGGAGATTTTGTCAGATTAAAT
TCCCGCCTGGAAAACATACACTAAAACTTAGACCGCGCAccgttttgtagcgaggaaaactcactctaac
aatatatttccgcactaaaaaaatttcacgcccgcaggttataattttacaacgacacgaacttttcacg
acttgactccacgactattttattagaaaagaaatagaacggaaactcaagccggggtctaggcaatcgg
tggaaatcacgatcagctaagtgtaactctatttacgataaccaaattatcccaaaactttccgggtgca
gctgataaagagtctagtatattcccgatgcataatattgcatttaaaatgaccctaaattagaatcttt
tagctgagtgcaagcttcataatgtttgtctattaggaggttcctaacaacgccgcttcaatcgccacta
caATTACAGacatacttttcaacgagtatagtatacccttctaatctacgaataccggatataaATATTA
TACAATGGAAACATCatactatactatatatactatacttatatttaagaatataaaatattatatataa
cattatgtcttatatttagtttaaaatctttatCCAAGATTTATTGATTTTACGTAGCGACCTTTATTAT
ATAATGTATTTAACTTTTCTAAATGAAATTTAAATTTTGTTGCAGAAATTTATACGCAAAAAAATACCAT
TTAATGTTTTCAAACGCATCGAACATGACAATTTCAATGATTTTCTGGGAGGATGGAGTGACAACAGAGC
AAGGGCTTTAATTTTTGAGCCTCGAAGTTTAACTAGGTTACGATACCTTCTAACCGCCTTTGAATTTTAC
GACCGAGTTGCATTTGGG
>dme_circ_0004381_junction_seq
TGAATTTTACGACCGAGTTGCATTTGGGTTAAAGTTTAAAAGATGATTTCATGCTT
>dme_circ_0004382

GCATCCTGATAAAGTTAAAAATGATTACGGTGCCGAAAAGTTCATACAAATTAAACTAGCTTATGAAATT
CTAGCTGACTTGGATCGACGACGAATTTTTGACCGGTATGGAGTATCGGATATTAACTCACAGTATTTTC
AGAAGAAACATGATTATAGTGAATATAATCGCTTTACATTAAATCAGAATGATGATGATTTTGGACAACG
ATTCGATATAAAACAAGACATTGCATTTTATCAGAAGTTATCAATAACAGAAAATTACTTTGAGAAAATG
ATTTTATCaaaaaatgcaaaaaaaGTGCACGTCGTCATGTTTTACAATGATTGGTGTTTTAAATGTACTC
GAATTGTTGATGCCTTCAAAAAGATTCTTGAACTACTTCAGCCAATAGGGTATTAACTTTGCAACGGTTA
ATGCAGTGCATGAAGAGTCCGTTTTTCGAAAATGTGGCGCCAGAGAAGTGCCACAACTGGTTTTGATTTT
AGACAATCAGTATTTCTTATATCGCGATCATAGCTTTACCCCACAAAAAGTTGTTGG
>dme_circ_0004382_junction_seq
ATAGCTTTACCCCACAAAAAGTTGTTGGGCATCCTGATAAAGTTAAAAATGATTAC
>dme_circ_0004383
TTAAAGTTTAAAAGATGATTTCATGCTTAATTAGTAATTATATTATAACCATTCTGGTCCTGTGTACAAC
ATCCTATATACACGTTTGTTCGTCGTTAAATGACCCATACGCTATATTGGGAATAAACAAAAAGGCAACT
ACATACGAAATAAGAGAAGCTTACAAGGAATTAGCTAAGAAATGGGTAAGTCTGCATAACAATTGCGTCA
TAAAAGTTTAGAATGTTTAAAATTAAATGTTCTTCTATCGTTATATACTGGGTATAATGTTGTATACCCA
GGTTGCTTAAGTTAAGTTGCTTTGCAGTAAAAAGGttattaattattattatttatatcccttatcgtag
agtattatattcgtttaaaagtatgtaaacaagagaaggaagttagtttccaaATATTTCAAATGTTGTT
GGTAGCTGCATACCTTATTTAAACATTCTGCGTTATatatttttataattttgtatatcttgtaatattc
tgtcgatttgtcaaaaaactttttaccacgcccactctgacacccaaaaaccgcccaaaactgcccacac
ttttgaaaaatgttttaatattgcttaaaatttttgttagtctttcaaatttccatcgatttgcAATGCC
CAATCTAACGTCCACAAAGCGAAACGACCACAATTCGATCttttttttATTATATTTTCAATTCCAACCG
CTGATGCAAAAAGCGTGACATTTTTGAAGCGCAAGGCGGCGGCCGCAAGTGGAACGATTAGCGGACTTCT
GCAATCATTTCGTTTAATGAGCTTGCTCTTCGAGTGAGGTTGGAGCTGATCGATGAGCTCCAACAAACAA
TTAGAAAAGTTAGCGATGAGCTCGAAGAACTTGATTTTGATGAAACCGAACGTGACTTGGCTTAGAAGTT
TGATGACATTCTTGTAACTCTTAAATCCTCTATAAGGGGGAAGATCTTAAAGGGCGGCTCGCAAATGCAC
TTTTCCACTTAACTTTCGATGACGACATTGGTCCGTTTGATTGAGGCCCCATCAGCTTTCACTCATAAAT
AGGGCACCGTCGCTGCCATCTCTTGAACTTCCAAAATCGCTGGAGGCTATGCTAACTTGGTCAACATGGG
TCCACCCACCAGCACGCTGCCGTAATCCCAATTGCTTCAGGGCGGCCAGGGTCAGGCACAGGGTGCCAGT
GGCGCTCCGGAAAGCGTTGGACCGGACAATATATCGCATGAAAACGAAATAAGCTCCTCGGGACCATTCC
AGAAGattttgtcaaatttacaatgcaagagcaaacgtgtgcgcacatgcagttgtcggctgtcactgta
tgcgtagaaaagatctgttcgatgtagacctctccgctctctcgctcttgaacaaaaattcgagagagcc
tggagccacctctagagccacctccacgttgtacaaatcgtgtgccaaaaaatcgtatggcggcgttacg
catcgttattctagagtctttgCTTGTACTCTCTCTGCAGCTCATTATCATTCGATTTTCGTTATTTATA
CATATCACATTGATGCAAAGTCTTATTCTCTGAACACAATAAAAATAGATCGTTCATAAATCGTTAATAA
AAAGATTATGAGCTTAAAATCCTATTGGAATTTTCCTTCTCCTACTTGTTTCTTTTCTTAAACGCAATCC
AAAAGCCCACAAGATTGACTGTTTTGCGTGCCTTTACTGTGTCTAGGTATTTTGCTAGCTTGATTGTGAA
GTTGGAGTCAGTCAGAAACTGAAATTTAAAGAAGGTAGTTCAAAATCCAGGAAACACAAGGCCACAAGGC
TTGAAACAGGAGGTTGCAATGTTTGAGTCTGGCCATCAGAAGTGtgatcgtctcatgtacacttttaaaa
acgcagcatagcaacctgatcgacgtttagaatgcagtaaaaagtgcacagtcaacgcgaggcgtaaata
aattcgtcatactgccaaaattcaattcctattccgttggcactagtgcagctgcgtggtaagccacggt
ggacctcattctcgcagaaaacccactaatgggcagtgctctgcttgtttccatgagtaaatcacaagtt
tgcttttctggaatcacgtgggtcttatttgaggaattgtttttgcaacaattcgaaggcaacgaaacac
ctgcacctactgttttaatggaaaacggcgagtgatttccctgtatgcaagacacttaataacaacacta
aaagcaaagtggaactcgatgcctacggaacgcggtgtcggtagttctagcgcacacggcctatatcgac

acaagactacaacgaactgtttatacaaccaacatcagaacacgtgacgaattgcagaatgagttgcggg
aatttgcttttgctaagaagaaagaTTAAAGATTAAGAAAGATTAAGAATAATTCTGCGGCCAAAAGATA
ACGTGTTCACGCACCCATTATTTGCCTTTAACGTTGCTAAGCTAcaagaatgatccaacttaaattctgg
acgttcatctgattcaaggaccaaaacaaatacctgctacaagtgtggggcttctggacatattgtttct
gcttgttctgagagaaaggaatcggccactcattataaattcgatgagaggggtgtaaacttgtgccagg
tggccgacccaattggtcctttttttatccggtttttatatttctggagccgaatgttcgcaaagaattt
gtatctgatagactttcgggaacaagaataaataatatcgttgttttaaagggaataggaaataatttag
taataacaactatgcatatatagtctaatgttataatcaattcttattctcttgaagtaatttttcatgt
tgttctcaacagtcacataaattatgacattatagtcgggcgtgaaatttAAGCTCAACTTTTGAATTAT
ACACGACAAAATCTgggtttccgaaaagccgagtcacaactggatagtagcaatacgcttgatcgatcct
aacaagactgtgcgtaggcggcaatcggcaatatcggcttagtatcgcggaaatttgaatcgtaaaagac
aaattaagggaattgttggcttcgaatataatacgccatagctgttagcaatttgcaagcccaattaaaa
agaaaagtgtctcggatagacttagcataggttatcattagttaaatgccaataccgtgtccgaaaatac
cccctttaccccttattaacgaccaagtctctatgttacgtgggtcgagatatggctagaagtttgcacc
agattcctttactaccaaattaattcgaacgaacggcatttgtcactccagagggaatttttgaccatgc
ctttcggcttgaacaatgcaccccccgctgtgttataaaatcgttaggtgaccttgcgcactgctatgat
atcgtttacatggaTTGAAGAAGAATCTTTCTTGAGATTGAGAACAGTATTGGCTGGCGTTTCATTTAAT
GTGACTTCTTTAATGTTAATTCTTAAAATAAGTAGGTGAGTATCTTGGGTTTCTTATTAAGGATGGCGAA
ATTCGGCCCAATCCACGAACCCTCGCTGTCCGATATTAAAGTGAGTCAGCTCATAGGCTTTGCTACAAAG
TAACAAAGTTCTCTGAAATAATGAAATCGCTTTATGGGCTTACATGTTAAAACAAATTATTAAAATGGGA
ACCAAAACATGATCAAATACGTCAATGCGTCATAACAAGCACAGTGTCACCGAGTAAAATCGTCTAAGTC
TCGCTCCCATTCTTATGAGCTCGAACCGTTAGCCGTATTTAATTAAATTAAACATTTCCGACTTATTTGC
ATGGTCGTCATTTTACAGTTTATAACGATTGTAACTCCTTAAGGGCTACAAGAATTATGGTTGACCTTAC
ACCAAGTGTGGGCGTACAAGTTGGTGAGATCCAACTAGAAAAGAGCTTTGATGTTGTTCAATGAGGTATT
CCAGACATTACTAAAGTGTTGAGTTACCAGCGCTAATTGGTAAACCAGTTAATATTTGGACACATTTGTC
GCCTGGTGATCCAATTTTGGTGAGTGTTCTTAATACGTTATAGCATGCGTGTTTGGGAATTCTCTGCCTA
TGGCTATTTAGAGTGTTAAAGAAATATCCTCTGTCACTAGTTTGGACACTTAAAAAAGGGAATTCTTTTA
GATGAACCATGATataaactccatttaattttgaacattattggtacacaagaggacgtgtgatactcag
gaagactgTGTCGCATCGATGCTATCATTGATTTGTTAGTCGTTAAATACCATTACTACTGAATGATTA
TTGTTATGTCATCATTTATGAAGAATATAAAATAAGGAATAAGTCGTTCTCATTTATCCTCTGCCTAATT
GTGCTTCCTCTTACACACGATCCACAAGCCCAACTAATTGcacagcctcagttcgacaatgcgttagaac
gatacaaatATAGTGTGAATAAGAACTGATGCGCCAATACCAAGTGACACCAATAGACGCGTGCCCTTAC
TGTGTCTAGTATAGTGATAGCATAATTGTGAAGTTTCAGTCAGTGCAAAACTGAACAGTTCAAAAGAAAT
CAATAAACACAAGGCCACGTCCGGGTCTGTTTTTAATCTTGTATTCTATTTTTCTTCGATTTGTTTCGCT
TGTAGTCTGCTGAAGTTTCTCTATGTCCGCTACACCAGGAATCGAAGTTTAACCTCACACACTAGTAGTT
TcttaactttgttcaactcagtgtgtaatcctacttttcgcacgacaatgaatagctaaatttcccACTA
CTTCCCATTTGACCTGTCTGCCCTTGACTTCTCTCGCATTCCAGCCTTCTTCgctatgcagctctcctgc
accctgatttgtggggagtttttagactcctcaCATCTCAACCTTCCATCGGAAGTTTTTGAAAAGTTTG
GTGGTTTTAGACGGTCCTCCGCCTCTGTATCTCCTCGCATAGGCAGCTCCCGCGTTTCTCCTCTCGTCGA
CCCTGCGCCTATGGTTTTTACCAAGACAGTTTCAGCCTAGTATAACCTGTCGTTAAGCGTCTTGACGTGG
AATCTCGATCCTTCATATACAACACTCCGAACTCCTCTCGTCAACCTGGGACTCCATGATCGCAGCGCCC
TATACCGATTTCCTGCATTGCTTTGACGCCAACTGCTGATTTGCTTTGTCTTTCTCCACATCAGCTTTAT
GCTGTCATCCTTGCTCCTGATGCTGGGAACCACCGCTATGCATCCCTGCCTACGCTGTCAGTGCCCACTC
GTCGTCGTCGGTGCGGTTGCGACCTGCTTTTTCTGGCTCGAACTCCTCCTGCGCGTGTGTACCCGTGGTG

CTGTCGCGACCTGCTCCTCCTGGCTTCTGCTTCTCGCTCGAACTCCTTCATGTGTTCAACAGTTGCGAGT
TTCAATAAAATCCTTTGGATTGATGTCGGTCCTTTACATGCTGGCGCTTTGATCAAGACAATCACGTCGG
GCGACACAAATCTTATGAGCTCTATGTTCCCCTGGAGTTACTATATATTGCGTACCTGAGTTACAGAATC
TCCTTAGAAGTATTTTTAATGGTTTTGTtatgagattcatatgaaaggatatcagaatacaattgagcta
gattgctacgcaagcctgataagagatagtaagtcaagatgttagtttttcgatgaagagtggaaagggg
aagaggaggattgtgcagtggttgatctctttcaccaccatactatccgaccataaattaccagagcata
aggttgaaaaaagctcataaaggacaattatcttgaaaaaaaaatgtctctagtgcaagaaaagttaTAT
AGGCTTGATATACAAGCCTTTTTTCTCATCGGGCATTCGTGGTCCTGCTCCCTGAGGGGTTTCTTAATTA
TATCTTGTAGCGAAAATTCTCATTGAATCTGCTTTCCGTACTCTTTGTAGTGCTGCCAGTCTTTGAGTCC
TAATATGAATGAGACTATTGGCTTCTATAACCAGCTTAAAATGCTTGTTGGTAATGGACCAGCTCTCCTT
GTAGCTTTGTATCTGTTTACCATGTGGGTAGAGCTGAACTATCCATATATACGGAGTTGCCCAGATCGTC
TCGGCAAGTAATCCTGATTAAGATTATTTCCACTAGGCTCCGAGTTTTTTATggcaaaaatcctttgtcc
gtgcgtaaaatgtgactttatgcattaaaaatgaacatgtttatggtttgtatgttgtatttcctgcatt
ttaataaacaaatatcttaagtttttacacatgctactcggatgaacatataattattaaatcttcctta
aataggaatgcttcgggggatctagtcataagtgttattctttggacagacttaggtgctatcttagacc
aaCAAAATGTAGTAAAacgtaaagtcgagacatgaaaaatatttagcttccagttgattcaaaaatatca
ttaattctgggtaaaggaaatttatctaaaattaaggtaatactttttatgtcaatgaaacctatttctt
tttctctgaggttaaaactattttttttccgaaataggaaaaggggactgtGGAATTTCATTTCTTCCATT
TCTCTTATTTAGTCGATAATTTACTTTTGCATAATTTTTTTAGTTAGGAACTAAACTATTCCGTATCGAT
CATATGGTTTACAAAACTTTACTAATATCATATCATATAGAAATTTTGCGAATTCGATAAAGCGTTCGCA
GCATACGTAGATACCTGCTTGTATCTCTTGGGATTAATATATTGTTGTCTTTAATGGTAGTGATATGTAG
GTGTATGTTATTGGAACATGGATCGGAAACTTGACTTGATGTTGGGTCACTCAGCTGCTTACAATTTGTT
TGTATTAGGGTTATTCCGTTTGCTCAGCAATAATGTATTTTCTTACTTTCGCTTTCGTAACCCATGCCTA
TGCGCATCGATGCCGAATTTCGCTGTAGCCAACTTaaaaaaaaTAAATTTATTTAAAATCTCTTAAATCG
ATGAACTCGTTTCTTAAAAATAGCTAATAAAAAAACTAATTAGCTCAACAGGTGGGTCTGTTTTAATAAG
GTGAATGGTAATACTCCAGCACATTATATTGGGGAATACTGTTTTACTATATTCAAGTTGTTTGGAATAT
TATATTAATCTGGATGAATATCAATTGATAATAGTCTATTATATTGTGTAACATTTTGTCTGATACCTTC
GATAGTAATTTGGTTGTCAGTTTGTATTATTGTATTGAATTCTTTTAAAACTGAAAAAAATACGCCTGCA
TTGATTAAAAATGGTAATAATTTGGCTACGTTAAGAAATGAGAAAAACGTGTTTAAACTGAGATTGATGG
AGTAAACCTTTGGGTTATCTATGTTGTGTACCTGACAGTTTTGCGAATTGGTTAAAAATACCCTAACGTA
GTTAGGTACAAGTTTCCACATGATCTATTATTATATGTATGTATTGCATATTATAATTCAATTACTGCAA
TAATTACCGGTTCTGTAGTTGTAGATTTTGGACATAGACGTTGcgcccactctaacgtccacaaaccgac
tgaaactgccacgcacacacttttgcaaaatattgtgatatttttTCATTTTGCAAAAAaacgcgctaaa
accgcccaaaaatgctacgctcacatttttgaaaaattgttggataatttttcatagttttattagtcgc
ttaaatttctatcaatttgttaacaaactttttgcaacgcccactctaacgccctaaagccgccgaaccg
gtcacgcccacactcacccaatatcacccaatataaccccgaaaatgaaatgatgaaatatcgcgttcgc
attcacaatagctgagtaacgtgtatgtgatagttagggaactcgactattgcattctctcttacttTGA
AAATAAGAACATTATAGCTAATATAGGATTAATAGAGGATTATTAAATAAATTGTAAGATGAGCATTTTA
CAGCAGACAATTGCCAACTTTAAAGCAATCCTGCTATTATTGTCCAAATTGTAAAATTTAAAATGGTTTC
CAAAGTTGAAGGTATTAACATAGATATATAtttttttttatctttaattaaatgaatctaattttttttgt
gcattgaacctcttttatatgttttgttaatgttttatttagatttttagtTAATATTAGTAAAGTTTTG
TAGTTCCTGTAGTTCCAGACGTGCATGTACAAAAATAGACGTTCGCTGTTTTTCAAAAATTCTTTCTATT
TATACGCGGCCTTGAAAATTGTGATATCTTCTTCGGGTGCAAGATATTGTGATTCCATATAACTCTTAGT
ATTCTTTTTGGTTAGACTTATTTCACTTAGTCATCCGATGGGAAAAAGTTAGTTTTAGCATAGGAGAAA

TATGCATACCCAATTAAATTATAATTATGGCTTATTCTTTTTAATATATTACAGGGCATCCTGATAAAGT
TAAAAATGATTACGGTGCCGAAAAGTTCATACAAATTAAACTAGCTTATGAAATTCTAGCTGACTTGGAT
CGACGACGAATTTTTGACCGGTATGGAGTATCGGATATTAACTCACAGTATTTTCAGAAGAAACATGATT
ATAGTGAATATAATCGCTTTACATTAAATCAGAATGATGATGATTTTGGACAACGATTCGATATAAAACA
AGACATTGCATTTTATCAGAAGTTATCAATAACAGAAAATTACTTTGAGAAAATGATTTTATCaaaaaat
gcaaaaaaaGTGCACGTCGTCATGTTTTACAATGATTGGTGTTTTAAATGTACTCGAATTGTTGATGCCT
TCAAAAAGATTCTTGAACTACTTCAGCCAATAGGGTATTAACTTTGCAACGGTTAATGCAGTGCATGAAG
AGTCCGTTTTTCGAAAATGTGGCGCCAGAGAAGTGCCACAACTGGTTTTGATTTTAGACAATCAGTATTT
CTTATATCGCGATCATAGCTTTACCCCACAAAAAGTTGTTGG
>dme_circ_0004383_junction_seq
ATAGCTTTACCCCACAAAAAGTTGTTGGTTAAAGTTTAAAAGATGATTTCATGCTT
>dme_circ_0004384
TTAAAGTTTAAAAGATGATTTCATGCTTAATTAGTAATTATATTATAACCATTCTGGTCCTGTGTACAAC
ATCCTATATACACGTTTGTTCGTCGTTAAATGACCCATACGCTATATTGGGAATAAACAAAAAGGCAACT
ACATACGAAATAAGAGAAGCTTACAAGGAATTAGCTAAGAAATGG
>dme_circ_0004384_junction_seq
AGCTTACAAGGAATTAGCTAAGAAATGGTTAAAGTTTAAAAGATGATTTCATGCTT
>dme_circ_0004385
TGCCTTAGCCAATTAAAGAAAAAGTGAAAACCCCTATTTTGGTGAATAGCTGCGTATAGGGGATATTGGA
GGTAAAGCCAGTTTGTGTGTGCTTTCTAAAAACCTAAAACCTTAACCCTTTAATTCTTCTCGGCATTTTT
GGCCTcatcgtgaaagaggaaagcaccacaactaagctttgcgtcgttttcgatggatcagcgtcgactt
cctctggatacttcttaaacgatgattgctggtccagtgattcagccaaaactattccagactcttcttt
gttttcactcgcattcag
>dme_circ_0004385_junction_seq
actcttctttgttttcactcgcattcagTGCCTTAGCCAATTAAAGAAAAAGTGAA
>dme_circ_0004386
gtgcgaggagtccacgaaaggttcccccacttagcccaaggcttatttaggcaaggttaaccttcgctaa
aacctaccgaaactggccatctccaaatggtgtaatatcctttggactgatgggtcaaaaaaagtgctat
ttg
>dme_circ_0004386_junction_seq
actgatgggtcaaaaaaagtgctatttggtgcgaggagtccacgaaaggttccccc
>dme_circ_0004387
tacctttctctgggcccaatttcgcccccacttgagctagaaggtcaaaacctataatgcattcgaaact
tgtcagcgcgtctaaaaaaaggacgcagcggttttcccaaaaatgttaattaaatgttaaaccctgtaag
atccatgaatggaactaactgtgaaaggggaatcgaccgccaccacattttaaagatggactgttgtata
gtctgtgtcatagaggaacgtaactccataggctcagggtactgattgtggtatttg
>dme_circ_0004387_junction_seq
aggctcagggtactgattgtggtatttgtacctttctctgggcccaatttcgcccc
>dme_circ_0004388
GCTCCCGCAACGATAATATCACACCGGTCTTTAACGAAGACGATATCCGATGGACGAACATTAACGAGGA
AGTTGATGTAGTAACCCCAATGATCATATTCCATCTTCAAAAACTTCGAATCGAACATTTCCAATTACCC
GACATAAAAGAATATATTTGCATAGTGAGTTTTAGTTAAGTCATGCAATTCAGCATCTTAATTACTGAGA
CAACATTACTTTCAGAAACCATTCTTTACTACATATAAGGTTGGGTTGCACCTTACCGATGGAATAGTGT
ATAATCTTACAGGTATAGCTCGAAATGGAAATGCCTTTATGACATATAAAAGAAAAGCATTTCTATGCCG

GTTTTATTTAAAAATAAAGAAGCTACAG
>dme_circ_0004388_junction_seq
GTTTTATTTAAAAATAAAGAAGCTACAGGCTCCCGCAACGATAATATCACACCGGT
>dme_circ_0004389
GCTCCCGCAACGATAATATCACACCGGTCTTTAACGAAGACGATATCCGATGGACGAACATTAACGAGGA
AGTTGATGTAGTAACCCCAATGATCATATTCCATCTTCAAAAACTTCGAATCGAACATTTCCAATTACCC
GACATAAAAGAATATATTTGCATAGTGAGTTTTAGTTAAGTCATGCAATTCAGCATCTTAATTACTGAGA
CAACATTACTTTCAGAAACCATTCTTTACTACATATAAGGTTGGGTTGCACCTTACCGATGGAATAGTGT
ATAATCTTACAGGTATAGCTCGAAATGGAAATGCCTTTATGACATATAAAAGAAAAGCATTTCTATGCCG
GTTTTATTTAAAAATAAAGAAGCTACAGGTAATTTTTTTAAAAAACTTGTATTATTTGACTTGTATACAT
CTAACaaaaatataaataataataaaatattaaaggtaaggaaaatGAGAAGTTACAAACGGGTAACAAA
CTTCTGTTAATACGGTTGGACAGTAATTTTAAAGGTTATACGATTTTCTCTTATTTTTAAAGAGTTCCTT
TGGATTGGCTTTGTTTCTTCAATCTCTCTTAATTTATCCAATGTTTTAACaaaaaactaaaaaaaGTGAG
GTCTTGACATTAGGCTCACACAATTTATTCTCTATTACCAAAATAGCACTTGCTTGGATTGCCCCAATGT
GCGAGAGAGCGGCGACCAGCTGATCTCTAGCTCGTTTCTGCGCAGTGCATACGACGGGTATATGGTGAAC
GGAGGAGAAGAAAAAATCAGTCTGAATTTGTGAACGTTTTTGAACGGTCTAATGTGCTATCTGTTTTACA
TACACACGCTTGTACATAGCAATAATTTGAAGTAAGTCAGTGAATAATTATAACGTGTGAAGTTATTCTC
TTTTTTACTCTTACCTGGGAACGCATCTTACAATGACCTTGTGTTAGTGATATACAAGGGGACACAACTG
TTCATGGTGACCCTTGAGGGGAACCTGCGAGTGTTAAACTTTAAAATCGACAGTTTTTTGTAGCGTCTGG
AGCATGTTAAAGGCGTTTCAGGAGACGGATTCCATGATCGTCAATCTAGAAGGATTTCAGAAGAAGAATT
TTCAGTCGGAATTCGAGGAGCGAGTGACTGAACATGCCACAGAAGTcagctcccaagctttccgataaat
gtgtgaactggccaggctactatgacgtctccacaagccacaacaacaatctgggtaatgtgaaatgttc
ggactttctcaaagagtcgatgccaacagggcgcgatgatgacatccgacaaatgcagcgcaaggagaca
aattacgccgtggcttggGGACTGATGATTGAAAGGTACAAAAATCCttggcgcattacttagcagcgaa
gttgcctaaggagacccacaacgcttggtagcaccatcaagaaagctgtaccctcctacttataaggact
tagaatcatttttcaatgatcggctggtaataatggatgaaattaataaccgaaattcgtCGTACAACTC
TTATTTGAAATTTGGCCCACGATTTAACGACCCTAGCAATCGAGTGCATGATGCACAAAAGCGCTCAAGG
CCCCCGGATAGCGCCTGTTACTAGTACGGCGGATATAAGAATATAATGAACAGGGCAAAATTATGTATCA
GCCGTGTcacgctacaccagcaaaaagagctgccaagcttgtgggcagcgtcatcataggtcgttgcatt
ttccagtaTTGGCTCACAAACACAGTTACGGCCGCGCACAGTTACGGCATTCAGCGCCGCCTATTAGTGC
TCTAAGCAAAACCGGCAGACAGCGGAGGACCAGAACCCATGCGCAAATTACATGTGCCATTCGGCAACTT
CTCGAATACCCCTCAAGGAATACGATTCTCATCACTGCACGTATCACCTTACGAAATCCGGTCAATCAGT
GCAGTTGAAGAGGTCTGCCAGCCGAGCAATTATTTTTGGG
>dme_circ_0004389_junction_seq
GTCTGCCAGCCGAGCAATTATTTTTGGGGCTCCCGCAACGATAATATCACACCGGT
>dme_circ_0004390
GTTTGAATACAATTTTAAGCTAGATGTAACGAAATTCGAACGATTGCCACGTGTTACTGGATCATTGGAT
AATACTATTGTGTACGCTGAGCTAGCTATAAATGTGGCAAATTTCAAGCTTCATCTTAACGATTTCCGGA
TAATTCAGTTCAG
>dme_circ_0004390_junction_seq
TTAACGATTTCCGGATAATTCAGTTCAGGTTTGAATACAATTTTAAGCTAGATGTA
>dme_circ_0004391
GCGATAAAAGCCTACTACCAACTTGTAAAACAATTGTTACGCATGTCATCGCACGGCCTTGCTTACACAA
CAAGAATTGAAAGAAAGTCTTATAGAGAACTACAAATCAATAGAGATCAATACTTTGTAACTGCGCCAAA

TGAAGAAGATTTGGTTATGAGTTTATCTCCAAAGGACACACTTATACATACTGCCATTTCCCAGCACCAT
CAAGTGGATACTTCTACTAAATTAAATACAAACGAAACATCGACACAAAATACCGTATCTACAGCAGCCG
CGGCAGCGGTTGCACATCACCACCACAACCTTTCTAGTATTCACCACCTCCAAAACCTGCATAGTCAGCA
TCAAAGTACTTTATTTAATAGTAATCACTCAACACCCTTTAGCGTGACCGATATCTTAAGTCCAATTGAA
GAATCGTATCGCAAACTGGAACTGAACGGAAATCCACCATCTCCGTTTCG
>dme_circ_0004391_junction_seq
TGAACGGAAATCCACCATCTCCGTTTCGGCGATAAAAGCCTACTACCAACTTGTAA
>dme_circ_0004392
GCGATAAAAGCCTACTACCAACTTGTAAAACAATTGTTACGCATGTCATCGCACGGCCTTGCTTACACAA
CAAGAATTGAAAGAAAGTCTTATAGAGAACTACAAATCAATAGAGATCAATACTTTGTAACTGCGCCAAA
TGAAGAAGATTTGGTTATGAGTTTATCTCCAAAGGACACACTTATACATACTGCCATTTCCCAGCACCAT
CAAGTGGATACTTCTACTAAATTAAATACAAACGAAACATCGACACAAAATACCGTATCTACAGCAGCCG
CGGCAGCGGTTGCACATCACCACCACAACCTTTCTAGTATTCACCACCTCCAAAACCTGCATAGTCAGCA
TCAAAGTACTTTATTTAATAGTAATCACTCAACACCCTTTAGCGTGACCGATATCTTAAGTCCAATTGAA
GAATCGTATCGCAAACTGGAACTGAACGGAAATCCACCATCTCCGTTTCGGTAAATATAAGTAGATCGTT
ACACTATATTTACGTTATTCGAAAAACCATTTCATTTTGGGCGTCGTAAATACCaaaaaaaaaaCATTGA
TCAAAAAGTGTCAAAATGACTTTACATATTGGGAAAGACTTTTACACTTAAGTTTAAGGTAAATTGTGAC
TTTTATTGATGTGCAAAAGTCAATTGTGGTCTAAAAACACAGAAACTTCTTATATAAAAATGTTTgttac
gggtggtttatggacgctatgatggacgtagcaatattctgaaataaacttacgctacgtcggcgtgtct
agaatccgaatgtttagtccttacagtctaacatttatagttttcaagatctcgactttcattcggtgag
atgctgttcaacgaatctagtaaacgcttttactctacgagtaTTATGAGGGCAGATTAATAAATACACA
TTACGCAAAACAGATCAATAATTATAAAATTGTTTTCCGTTTGTATAGAATTTTTGATTAAGACTGTGAA
TAAGAATAGTTGTCTTAAGATTTATTCGTACTAAAAGAACAATACGCATCCTTCTGCTAACAAACTTATG
TACACTCACACaataaaaattcaaagtaacgaaaaaaaatgaaaagaaattgattaatttccgtttagttaa
aaattATTTGTGTTAAAGCGCAGTTTGGTGACACGTTCGCAATATGATTACCAATTTACCTGTGGTATTG
CCAATATTGAACATCAGACATATATGTAAATAaaacaatagagaacgctatagtcgagttccccgactat
cagatacgcgttactcggctagtagaaatgcgaacgagaaatatcaaaatttttctggaatatAAAATAA
GTTAATTTTAGTGATTGTGTTTCTAAATGAAATATAAATTTATATTCTGATATCAGTACTATTGTGCGT
TAGTTGCGCGATCGTCCCTAGTTTTAAATTGGGGTTTTGCTGGATTTAGATATTAGGGATGTACGAAATC
AATCTAGTCTTAGGCAATAACTATTTCATTTGAAATTAAAATGTTTAAAAATAATATACAATTACCAAGG
TACAAATTTCCTAGAAAACAAGTTTACCTTAACTGCAAAAAGTTCCTATTTGGACTTAATAGGATAATTA
ATTAGTCTATTCTGGCCTCCTTAGGACCTAAAATCCCTTGTTATTAGTTTATTAATTTTTTAAACTTTAT
TAAATTTTCATACCGAAAGTTGCCTTAAAGCTATGGGCGTTCTTATAATAAAAAAATTTCGTGTATATAT
TTAAACGACCGAATATATTATTTTAAAACGTTTTAAATGACAAAGACTAACTCAACGAAGCGATTAAAGA
AGAATCTGTTTTCTAAttttttttGAGAAactcatatagtaatatgtatactagttttgttgaaatgtat
gtaaaagttagagcgaagcgtctcTAAAAACATTGAAGCTTGAACGCTGAGACTAGATATGTAGATTTTG
ATGACGTCTATAGTGAGAACGTTTGTTTCAGAACGTTGCCATGACACACTTTTGAATAAAGTTTAGATTT
GTTTCGTTTTATTATTTGTTTTGCCTATTTGTGCCCTTGTTACATGTTTCATACTTTTCTTTGATTCAAA
TTTTCCCCTTTACTGCAAGAGTGAAAGTAAACTAATTAATTTATTTTACTTACTTATTTGTACGACTTAC
AATCAAAACATTTTTTAAAAGTTTTCTTTTAGAATGCATGGCTACGTTCTGAAATAAACGTTCGCAGCAT
ATGCGTTACATGACATTTAACGAATGAAATTTAACCTTTCACTCGACGAGTAAAAGTTATATAAAAATAT
TATAAAATATGATTTCTAATCATATAAAAACAAGTTTAAATAGTAACAAAACTAtaatactgtccaaata
gtatttaattcagtcgaaatatgtttaatGCTAATACTTTTTAGTAGACAACTAATTTGAGAGAATATTA
CACATTACTGTAAACTGCTTTCAGGATCAAACTCTAGTAGCAGCAGTATTAATTCTCCTGGAACATTAAC

TACATCAACTATGGCGAATCCGTACGCTATGGGAACCTTATATCACAGCCCAGGTGTACAGACCTATTGC
GGACCTACCGATAACCTATCTCTGGCTGGTCACTACACTGACATGAGAAATTCTGCATCGTGGTACGGAT
CAACAGCTAACGACCCAAGATTTGCAA
>dme_circ_0004392_junction_seq
TCAACAGCTAACGACCCAAGATTTGCAAGCGATAAAAGCCTACTACCAACTTGTAA
>dme_circ_0004393
GATCAAACTCTAGTAGCAGCAGTATTAATTCTCCTGGAACATTAACTACATCAACTATGGCGAATCCGTA
CGCTATGGGAACCTTATATCACAGCCCAGGTGTACAGACCTATTGCGGACCTACCGATAACCTATCTCTG
GCTGGTCACTACACTGACATGAGAAATTCTGCATCGTGGTACGGATCAACAGCTAACGACCCAAGATTTG
CAAAGTAAGTATGGGACAATATACTAAAAGTTCTATAGATTCACATTGCCGATAAATATATTACACAGTA
AATTATTTTAAGACTTAGGCCCGGTTCCTTTAATTTAAAATAAGCGATAGCTAATTACAGATCAGTTAGT
TTTCTAACGAATTAAATTCAATTAAATTGATATTATAaaatttttcaaatttttcaaTAACGCTGAATAG
GCCCTGCGTCTGGCCGctatatatagcggtcataatatatatatattcataaattttaaaaattgtatat
atatatattatatCTTTTAAGTATACTTTTTAGTAAAAAGTTACTTTTTGGCTTCGACATTaaaagtaaa
cttaaagaaaagtaactttaaagaaGTATTCCAACAAGAtttttttttaattttaagctaacattaagcat
ttaaaagtttttttATGAATAGATAAGAATTCCTTTTATAGGAAATTATGAGAAAACCTTATCCCGATTTA
CCAGTTTCTGAACAAATAGGTAAGAGATAGCCATAGAGGTGAGAGAGAGGCGGCTATACTCGTACATACA
TGTATTGCTACCCATCCTGTGATGTCATAGCCTTTGTGTCTTTATACATTAAGAACTTCTATTTCCTCGA
ATATAAGATAGCCAAAACTCAGTTAGTGAAATTCCTGGGATGTATGTTCGCatacctattactcgtatag
tgaaagagtataccagattcgttgaaaagcatTTTAGACCAAATAAAATATGTATTGATCAGAATCATTA
AAAAATACAAAAGATACGATGTTTATATAAAGGACGCAGATTACAGATTTGTTTACTGGTTTTGCCATCT
AATGTACATATCTAACTATgacatgatagtattgggcggtttgcgggcgttattgtggacgtgcccaaaa
tgtgttaCTGCAAAAGATAAAATTTAAAACAACAACAAATGAAATAAAATATAAACAtttttttttGGAAA
CATCTGAAACAAACTTGTCTTGTGAAACTAGAcctgtttacctgtttatcaacgaatcaagaatatccct
tcaatatacgagtaacatggtataaACATTTCTCAAAAGTGATAGCTTTGGGCGGCTTGTAGGCGCGGTT
TGACTGATAGACGTTCTTAGAATCTACAACGTCGTATTATCTCGACgagctctcgaaaattataaagaaa
aacggacattaacagatcgaatgagctgatcagaataatatatacatttaacgatcagtaacgcattctt
ctaccacttacattctgttcTAGTATTAGGTGTATTAATGAAACCAGAGTATGGTGGAACCCGACCTCAA
ACATCCCAGAATCGATTATTGAAAACAATTTTTTAATTAAGAATTCGAACTTTGTTTTTTGTATGGTATT
ATCGCTTTAACGATAGTACGATGGACGAAAGTACTTTTTGAAAGATCTGACTGCAACATGAACTTGTCAC
GCAGAAGAAACACCAAAATTGTTTTTAAAAATATTAGGCCTAATGCTTTACTGCTTTTTAAAGTGTTAGG
AAGGAACATTTGACGTGCTTTTTCTTTGTTGTCATACCAAATTGTTCTtaataaataaataaaaaaaaTG
TATTAAATCCTCGTCTAATGCTTAACAAAACTGTACCGTTTGATCTGATTCTAAGTTATATAAACTAATT
GAGTTCATTGAAAGACTATGAAAATTTTCACCATAAAAAATTAATTGCATAAAATATGTGTTACAAGTAA
TATAATTTGATTTTAATTTCATTTACAGCATACTAGCTTAAAATGAATGTAAACGGGTAAATCAAGGTTT
GCCCGTTGGTCATGAAAATCTTTAGTAGATGAAGTGCATTTTATTTTTCCTTGGAATCGCTTATACACCT
ACATACACAATTATAAATATATTTACAGAGATCTATACAAAAAATATATTCATACATACATCCAAATAAG
GTTATTTGTGTTTATATACCATTCAACAATAATAACCTCTTTGGGAGTAGGGTCATAAAAGCAAAAATCA
ACAATCATCTTTCCATTTCTTAAGTAAACAATGACAACGAAATAATTTTAAATTCAATTGCTTTTTCCGA
TCGCCTTTTTACCTGAAATAGAGTATAACCCTCCTAGTTACACTCTTTAGCAGCTTAAAGGAAAATCATT
TACATTTTTTGAATAACTGCAAAATAACGGATATGCTGTTTGTTACTTGCCAGTTATTTGTTTCAACTTA
ATATCGATAAAAAATTAATAAAATCAAAACAATTTACAAAAGAGTGGACCTTAAAGTTTTTGGCAGTTGT
GGGCGTTGTCAAAATGTGTTTGGTATATTTAAGATTGATACAGGTTTTTATAAAAATAATGAATATAGAA
TGCTAATTAGTGGTATAATAATATGTCaaaaaaaaTAGGTCAATCGGTCCCGTAGGCGATATAACAATTT

TCTTACTATTTACAAAGCTACTAAAAATAGAGAAATCACTAGaaaaaaaaGCGACGAACTCTTTTATTCC
TATACGTACATATATCAGAGACATAGTTGTGGAAATTTCAAAATGTATTAATAATATAAACATTAATAGC
TACATCATTTTTGTTTGAACATTAGCGAGCAAAAATTATTTTAAATTAAGACTGCCGCCCTTTTTTGACA
CGACGTTTTTACGATTCGTTTAACAGTGTTAAAACTTTCACTTTTAAATTAGAATTTGAACAAATAATAG
TTATATAGTTTGAACATAGTTTTAAATAACAGTTATATCTCTGAATAATTTACTCTTGGTTCAGGGCTTT
AAATAAAAACAATTATTTGTTAAGTATCTCATTTCAACAATCTCATTTTTTATTGTTTTTAAATTTCGAA
CCCGCAATTGACGAAAATTACTCTAGCTTACCCAGTTCTTTGGAGGTAGAAAGATATTCATATACATATT
CGTTGTGAATTTTGATTTCATTTAGCATTTTATGGTTTGCGACAAGGAATTACGCAGACAGATGTGTAAA
GGCCCATATAATTGGAAATATATCATGCAAAGGTATTGTATACCCATGCAGAGGATATATGGATTTTAGT
AAAAAGGTTGTAGGGCGGTGTAGAACACTTCACTTTGCATAACTCTTGTTAAAAATACGGATTGATTAAC
ATCTCATCACTGTGGAAGGCAGTTCTCGTAATGATATATATATGAAAACGGTAACATAATCCACAAACGC
TGAAAAATTCCTATTATTTTGTTTGCTTAGCAATAGAATAGGTTTGACAGTTTTGATCAATTTGTGAAGT
GAGCGGTCTATAATATTTTTGGATATCTATAGGAATTTGCAAGACAAATAAAACGGGAAAAGAGAGAGAA
ATTATTCGAAAGTGTGGATTAATATTTAAATGCaaacaagagagagagagctatagtcgagttccccacaat
cagatatccAGCTGATGGCTGAGTATTAGGGGataaaatgagaaaaaattaaaaatttaaaatcgataga
acttaaaagtctaaaaaaactatcaaaaaaatagcaaaacatttttcaaaagtgacttgtgggcgtagAG
TGACTTGGTCCGGATCTCCGGCTTGGATGAACCAAGGACAATATTTCACGACCCCAAACGAAAATGCATT
TCCTTCTTGAGATAGGTTGATCTTAGCTTCTTTCGCTTCTTCTTATTACGCTTCATGTCACAAAACCCGA
AAAATAACCCAGGATGTTAGCGTTACCTTTTTAATCTGGACACCTTAATGCCAGCTTTAAGTAATATTCA
GAACATCTAAGAACAGTTGGTGGATTCATCCGACTCAAAAACTTTATTAATGCCAATTTAGATGTATATG
TTATATGCTGTTTTAATTATCCTTATCTTCAGATTTATTTAAACTTATTTTTTTAGTAATTTGGTCTTGT
CAGAAGAGTATGCCAATACAAAAAATAATCATATTTCATACATTTTATTGTATATTTTTCATTTTCCATG
AAAAAATATTCGTTTGTTAATTTTTAAGTTAATATTACAAACACAAAACAAATAAAAACGAATCAATACT
ACCATATCAAAGCATTTTAAAGTTACACTGATAAAATGTGCAGCGCTTACTTTGTCAAAATACATATTTA
TATGTCTATATGTATATGGCGGCTCTTTTagaaaaaagcgtttctgaccataaaatattcttgataaaga
ttaccagagtagatcttgccatgagtgtctaatgttataaacgctagaaagtaaaattaagcatgtagat
gacgtccacgatatgcaagttggtttcagatcgttgccacgcctactctaacgcccacaTAGACAAATTT
GGTTGTTTACCATTTTGTTACTTGGCCACAAAGTaaaaaaaaaTTCctatcgatatgccagaaaaatgtt
aacatttctttttcacactcccactagcgaagtaacgggtacttgataatcggaaagaaagaaactcgac
tatagcgttttctcttgttataaccgttactcgtgaagtaagaggcataccaggtagaaggaagcgtaaa
aggaagtatatacatatattcttgattaggatcaatagccaaagtcgagttggccatttccctctacccg
aatgtctgtctgtctgtccgtatgaaagtgtataaaagctagaaggttgggattaagcataccgattcta
gagacaaagacgctgcacaagcgtgttgatcgatgttgtcaagcccactcttacgcccacaaaccgcaca
aaactgccacgcccacacttttgcaaaatgtgttgatatttttttcacatttttattagtcttgtaaaaaa
aaactgttagccacgcccactctaacgccctaaaggcgcccaaaactggcacgcaatcattttagaaaat
tttttcgatacattttcataattttattagtcttgtaaatttctatcgttttgccaaaaaacttatTGcc
acacacttaaaactgccatgcccacatttttttttaaatttttggaaatttttttataatattttaatact
ttttctattgatttgcccaaaacaactttttccatggtcactctaacgacctaaaggagcaataaaagcg
atcccgcccacactttgggaccatttttttaatttttttctcattttattccccaatatctatcgatattcc
agaaaaattatgaatgttcgcgttcgcgttcacactagctgagtaactgatagacgggaaactcgactgt
agtattctctcttgtttTTGAAAGAAATTGAAAGGAAATAAATATGAAATGAGTAATGATTTAAATCaaa
actttgtaacgcactaaataaaacgattgcggccatataaagctaatatattagtgattattgtcaacgg
ccgagttgatatggccatAAGCGCCCATTTAGCCTCTCAGCTTTAAAAATATCTTTCTTCATTTAGCTTA
TCTGCCTTCTCTTTATATTTTTGTATAATAAATAGGAATAAATAAATTAGCATTATTATTAAATATTTCA

GGTTTCACGCCTAATGAGTTCATCAGCCAGTGGAACAATGAGTCATATGGGAAACATGAGTGGACTAGCA
GCTTGCAGTGTAAGCGATTCAAAACCGCTACAGTTTCCTTTAGCACAAAGAAGAAAGCGAAGAGTTTTGT
TTACGCAAGCCCAG
>dme_circ_0004393_junction_seq
GCGAAGAGTTTTGTTTACGCAAGCCCAGGATCAAACTCTAGTAGCAGCAGTATTAA
>dme_circ_0004394
GGATAAAATATTTGATAATGACGTTTTGGAAAATATTGAGTCTCTGGATCCACATTCATTGGACGCCCAT
GCTGATATGCCTGGTTCTCGATTGGATGGTGATGGGTTTGGAGATTCTTTTGGACGTAAGTATAATCAAT
ATCATCAATTACAAATGATATTAGCTGGATATATATATGGATAAACCGCGTAAAAGTAGAAATCTAAGAA
ATATATCCTCAAATATATATCAGTCGAAAGTATCCTTAAAAAAATTAATTCAGTTGTAAAATACTCCGTC
ATAAAAGTTGCCTTAGTCTAGTAATACACTTTCTTCAGTCTGTTGTTTTTTTCTTATTTGTCATCTGTCA
TCCATATGGATTTGAATATCTTAAATGCCTCTATAAAATAATTTTTAATCTTATGATCTGATTTATATAC
TAATGATGCGGTAATGAGCATTAGGAGTTGTGATACAAAAATCATTTGGTACAACCATATTTGGTATGGT
GGTATACAGTTACAAAGTATTTTTTAAAATTAATTTGTAACTTAGGCGGACGTAAGTATCTTAAGGTGAA
AAttttctttcttattacattttttttttcttattacattAATGCATAAATATTGTAAAGAGCTTGTTTT
CCTAGTTCTGCCACTACGAGTTGAAATGGCTGGCTCAGAGGGTTTTGTCTGTTTGCCCAAATTTATGATT
TGTGGGTGATTATGATTATGATTTATAATTCTTCAGCTGTATTGTTTGAATACTGGCTCTGGACTTCTAC
TGCCATTGTGTGTCAGCTCAATTGAGACCTACGAAGTTATGGTGGCGAATCTGCAAGGTGTATCGTCCGT
TAGGTCGAACCAAAGCTCTTACCAGAACCACCTTTGCGAACACCCGAAAATGCACGTATGTTAGAACCAC
TCCTGAAACAGGACACCAGATTCCACGATCCCTTCTGACCACGCTATGCCCACGTCGTCTCCCAACGGTC
CGTCCTGAATAAGCCCTAGGATATTATGGTGGCTTGGCCGCTAGGTGTGTCGTTCCTTAGGTCGAACCAG
GGATCTTCCCAGAACCACCTTCGTAGACAGCCAAGAGCACATCTGTTGGGACCGTTCCTCTCACAGGACA
CTAACCAATGCCTTCACAGTCCCTTCTGACCGCGCCAGGTCCTTGTCCTCTTCCGTGTTCTTGCTGAGCG
TTGGTCTGCTCCCTCACTTGCAGTGATCAACCTCGTACTACCGTTCCTAATGCTCACCTTGTGATCTCAC
TCATTGTCCTTTTTTCTCGGTCTTCTACACCAAATCGAAAGGTCTAGGTGTACATTCTGCGcttttcttt
tcttcttctttttctttcttcttggctatgagatgatactgagtgtgtggtcttagtgggcgaacaagta
atgacctaggtaagggtcaaaatctaaaatgttgtgttgcttatctgaaagttccgaatcagatgaaacc
gttaatgggccagaagtgaccaaaagtttaggggtagcttgtaatagttttcgtcgagatgattatctcc
tttccgctccagactcgcagcttTTAGAGGTGACTCCAAAGCCATAACTATTTttatactcgtttctcgt
cgagtgaaaggatataaaaggtacttggaagagtatgtaacagatagacggaggtgtttccgaccagata
aagtatatatattcttgagtaggatcaatagccgagtggacctggccaAAGACATTAGAATAACAAGATT
TTTAATTCGTTTGCTaaggcactactttcatttgtgtacttaatgcatcaacaatcaatttttttatacac
atttgtttttttttttattattttcaaaaatattttcaaaaactttaatgtgtattttgcaaatattaaa
aaatatatcagatggtgcctgtagtttcccgaagtccgagtctattgagtagttattttcatgtataaaa
ggctctgatggagctgttaaatgcacggtacttttaaaattaccgatctgcatttatcacaactggatcc
cttagcaatatatccaacaaaatatctactggaacattattgcacaaaaagttatctatattttgttcaa
agtatgtacctctcattgtcttttacaattttagaaaatgatacagagtattcttgttgttgtattttttt
tttaatgaaaggctgatatattatcgaatcgaactcaattggcatcataacatcatcatctgattcgcaa
ttcgacttttgtgaaaatttaaaaatgtttagagatggcaattattatttaaattcgttaactggaggat
ttctccagttatcaccttttgctaaaatatgttttcaatgggattctggttaactctactagttaaaaaa
tagaaagtggtctgtgtgatccttaaatagatatctttgatgaatccaaatatatctttagCtaaaataa
ataaaaatattaaaactgtttactgcaaaagttatatcttgaggcacgaagtgcggacacaagcactcaa
caatcattgccttataaatttttcacacgtcgcaagctgaatactctaatgacaaatattctaatataaa
gtcgtttttgaaatttattttgtgatattatgattcgggtgttactatttctaagctatatttgaataat

aataacgttaaggcaatgccaagcaagaatttttggcatggtgccaattgatcaaaaataatatataaga
acttctaaggtgaagggcatattttgtcaaatttacaatgcatgagcatacgtgtgcactcatacagctg
tctgctaccactgtatgcgtagaaaagagctgttcgctgaagcgctctctcggtctcgaacaaaaattcg
agacagcctggagccatgtcgaaaatatcgtgtgccaaaaaatcgtatggcgttacgcatcttgttattc
tagtgtctttgATCGaacaaaatgaatttgaatgtttaacaacgtaaatttttttccgatattggacacac
catcgttccgaagcagatatccgataaatttcCAttgacataatatgcacacaggacaatcaattcattt
attcaaggctttacccataccctatggctatagtggatttcgtgaataaagTTGTTATAAAAATAATGAT
GGTTATAGACTTGCGGAAGTTAAACTTAAAGAAAGATTATTTTTTCGAAACGCCGTGAGGACAATTTGCT
GTCGCAATCATCAGAATTGCAATTGCAATTCGTAGTGAGCAGAACCGAAGAAGAGTGGTGTTGATAAAAT
AGCAGCCATTTCGTAAAAGCAAATCACAGTACAAGCCCATAAGTGAGTGATGTATGTCCATAGATTCCAC
ATTACCCAGAATTTAGCGCAAATTAGTATGGGCCCAAACCACAGTTCAAGCCGCTGGCAGCAAAAACCAA
ATTAAATGTATTCATGCATCGTTATAGGAGATAGGTTGTCCCGATCCAACAACTGTTTAAAAACTGAAGT
GAGGGTGCTATTCCAGTACACGAGTTAATCTCTAAGCTGATTAGAGGGTTAGTGTATTGGGTATTGAATG
TTGATGAAACTCCACCGACGACGCGGTTTGAAGCGATAATCTCCAAAACCGCATCTGCGTCCGATCCAGA
GAACGACAAATGAGAAAATGAGAAAAACGCGACAAAAATGAAGAGAAATTTAGATGAGAGTGGCAAAACA
AAAATAGCACTCTGCCGGTGGGCCTACTTGACGTCGACTATGTCAGCTTATCCTTGAAAAATTTAGATAC
AGCTTTCAGCTTTATGGCTACCGTAAATTTATGCCAAGATGCAGAAACCTTTTTCTAAGCAGAACATTGA
ATAGTTCACAAAACCAATTGTATGCCTTTTGTTATTGTTGTGCGGTTTATGAAACAATTTATGACGAATT
TTGGGGTAATGATTCAGCAACTGTTGTTGCAACTAAATTTTCTCATGGTTCAATACAGACGATTTGAAAT
TTAGAATTTAAGATTAGAAAAAGACTTCTTAGTTGTTGTATTTTACCCATCAGCTGATCAGCCTTGATGT
GCGAATCAATGGTCACTACAAAGCTATTAAACTGTCAGAGAAGATATGACACAAGGTTCGAAATTGGATA
GCATTAACTTCGATATTGCACTCAGAACACGAGTAAAGAGACATAGCAGCGCAACAAAGTTTTACCAGAG
TTCGTGTTCTTGGCTGAGCTCCGGaaaaatactaaaaaaaaGTGGATGAACTAGAGAAAATGCGTAAAAG
CTCCGTAAATCAAGGAAACGACTGGTTCTCAAACTTCTCAACACGCGATATCAACGTAGAAACGAAACCA
ACAAAGAGCAACCTCTACTGACCGTCAACAGCAAGAGGACGAGCTATTGTTCTCCCATTCTGCTCACCCC
AACTCCTACATCATCGAGATACCAATACCAAACCCCACCACTGCCGGCTTTGATGGCAGACGCTCCAACA
ACGAACAGGCCTTAAATCCCTGCTGTCGCAACAATAGCACCACCAAATCAACTACCAATATTGCGGCGCC
GACAGCAACAAACCTTGGCCAGCTCCGATAGGCAACAGcgatcctgacgatacagataggtatggatcgc
tacatctagattaagcgtaagctaatcaccctaaacactgttggtaataagccaaaggtcaaccgacaac
agtaccaagaaagcaacgatccaataaggatgataaccgattttctatgttagcagaggcggagaacaat
acagcatggctgcgtcaagagacccaaaagaaaccaaatcccttgtctatttttataagggaaaaaagtt
caaacgaccaggtcaataaaattgctgcgctgggcggggacatgttccgcattGTGTAAAAAAcctggag
gaaacaaaaaagaatttctacaccaaacaactaaaaagcagcaagggattttgctagttgttaaaggaat
tgagattcaagaagcccttaaggacatgggcttctgtgacaaaaatgttagcaccataatgaaacgaaaa
aaatgccgcaaccaGATAACAAAGCCTTAAAGAAAAACGAAGTGcattgaagtatgaccgtggaagaacc
acacaaacgcaacggtcttgtacaatgcaataactgccaggagtatggacacaccaagacacactgcaga
cttaggtcacaactccgtaatgtaccgtaaactccgtaaacaaagacgacgaagacaaaagaatgatgtg
aaaactctggaggaaaccatacgtgaaactacaaaggctCTATCAAAgacactagacactagctactttt
ttggtgacggccaaaatggcccactgtccgctcgcttacgctgagagcgtaagaaatctaaaagtagaat
ttgcttgcttgtgtgagtaaaaacaagagacgagaacgcgtatatgtgtgcgtgttgtgctagaagacga
ttttcgagacgaaatcaattctgatcgaagaaatgaatttacatatttggggagttttggccaatttcgt
agaaatatgatgaaattgcgcgctgtttaaaaatatcgaattggaatataacaaaaaaaaaaaactgaaac
tgaaacaactgaatctaatgggcattttaaataataaaattggatcatttacatacatcatattaagtca
aatgacttaataaatatactaaataattaaggcaaatacaaagaaaattattataactactctaatttaa

agcaaattttccaaaaagaagacattacattgagaaataaatatttcataaaaataatacgttgtagaaa
ataaaaatttataaaataaataaaattatgaaaactgtttattgcgaaagtcatatcttgaggcacgaag
tgcggacccaagactgccaggcccacacttttccaaaatgttacatgttttcatattttattggtgttgt
aaatgtctatcgatttgccaaaaaccttttcgcactaccactcGATTTTTTCTTCGTTCTATTGTTTGTC
TTGCCAATTTCTATGGGTGTATCAAAAACACTTTGTCCACGCCTTTTCTGACATCCGTGTTGGTTATTTT
CCCTTTACTAGTCTACATTACGAGATCACAGAGTTACTGCGGACAGAGCGACAGACAGATAGTGATCATA
ATATTCATATATAAGCCTACTCtagatacgtttcaacgaatctagtaaatccttttactccataagcaat
gggtataaAAACTTTTTATTTCAATATAATTCCGCATATTCTATTATCAGCCTTAAATTTGTATTAACAT
TTTTAGGAACCAGCCCTTTTTGAAGACGATCTTTTTGGTGATCCTTCTCAACCAGTTGAGCAGATTACAA
AAGAATCTACTACTGTTCTCAACGCAGATGATTCTGATGAAGACGCCATTGATAATATTCATAATGTCCC
TTCGCCTGCAACCTCGCTCGTTAATTCGATTGAAGATGAGAAAGAGGGAAAATAATTTAAATGGACATGC
AAGTGTGTCCGAAAATGTGCCAATGAATGAAATAACGCTTGTTCAAAACGAGGATGAGGGATTTGCGTTA
GCTCCTTTAGATGTCTCAATGTATAAAGGCGTTACAAAAGCCAAACGCAAAAGAAAACTTATTATTGACG
AAATAAAAAATATTTCTGGAGAGGAAATGAAGGCTCAGTTAGCCGACACATCTGATATATTAACCACACT
TGATTTAGCCCCACCAACAAAAAGGATTAATGTATTGGAAAGAAACTGGAGGTGTCGAAAAACTTTTTTC
ATTACCATCGCGATCCATACCAGCTAGAGCTTTGTTTGGTAATTACAATAGACAGTTGTTTTCGCATTCA
ACATTCTTTGAGGACTTCTCGAGTGTTGTGCCAATGGAAATTCTTGCACTGGAATTTTATACCAAAGAGA
ACGAGAACGCATTAATTATTTTTAATAAAAAAGGTCGCAAACGTAAGAATGATAATATGTCCAATTTGTT
TTTGGACCATGTCCCCGATTCAGTTGTTCAAAGTTTGGAAGCTCCGGAAGTCCTGAGAGCGAATCATAAA
TCTCTAGGGGTATCTACCGTTTCTGTGGAGATTGTATCAAAAGAGCAAGAAAGCATTTCATGTCAAAATG
AATTAACATTTTTTGATAATATGCGAAGTCCGGACCTCTTGTCCTTAAATGAAATGGAACAGTTCTCATC
AATAAATGAGCTACCTTTAACACCAAGAAACATGAATCATGAAATGGGTGACGATTTTAATCAAGGAGAC
TCAACGCCAGCTGGGTTGGATCATGGTCATGCCACTCCGCAGCATGGAAATATTGGCGAGATGGATCACG
ACTCTGTTATACCGACAAAAAAGACTGCAGTCATTTTAAATGAATCTGTGGGTACATCTGTACTAAGTGA
CAATGGCGTTTCAAAAAGGACAAATAATATTTTAAAAGGATGGGACAACTATGAAATTCCCAGTTTCGTT
GGCCAGGGTACGTTATAAAAATGATGATTAACTATATAAGATTTTTTGCAATGATTATTATTATTTTGTG
CTACACAATAGGGTATAAGACATGCAGGGCACTGTTATCAGCAATATCACTAGGTCTTACGGAACATAAG
TGTATTGGTGTTTGTAAAAGCCAATTCACGTGTATTTTCCCAATCCAACGCTGCTCAAAAGCTGCTACGC
CGGAGAGACGCATAGATGACGATCTTTTCGACGCTATTTttatacacgttactcgtagattaaaagggca
gaaacatgaagcgtttccgatcatataaagtatatatatatttgatcagaatcggtagacgggtcgacct
ggccatgtccatgtgaacgtcccgatcactggaactataaaagctagaagtttaagattaagcatataga
ttctagagacatagacgaagagccagcgcaaatttttttgtatattttctcataattttgttagttattg
aaatttctgtcgatttgccaaacaaccttttgacacgtccactaacgctctgaaaccgcccaaaactgcc
acgcccacacatttgtttgtcttaccaatttttatcggtataccaagaacacggtaaacacgcccctctt
ttcacctacaaaacaccaaaaacggtcaatgtgttcgcattcccctagctaagtaacggctaactcgagt
cggggaactcgactatagcattttcttttgtttTATTTATAACCGGATATTTAGCACTAGTAAAGGCCCT
TGAACTGTTATATTTCTTTTCTGGAAATGTTAATATGTGGCACTGCCGGAAATTTGGAAGAGGTAATCGA
CCTATTTTAGAGAATTATTGCTTACCTATGTCAGACcaaagacacaagaataacaagatgcgtaacggcc
atacattggtttgactgccgaaattgccctctcacagatcgagtagaactgagagcgtaagaaatctaaa
aattgagtttgcttgcttgtgagtgaaaacaataagcgagaacgcgtatatatatgtgtgcctaattgtg
ctagaagacgattttcgggccgaaatcaattctgatccaagaaacgaatttacatatttggaattttggc
caatttcgtatcaatatgatgaaattaaataataattaaaaattcacgccgcgactattataattttaaa
gtttttaatattcgtttgataaaatcgccgctcaaattaccgttcacatatttatgtttgttaataatta
attatttgtaatatatgtaataaGGCTATTACAATTGTAATGCCTCCTATATTGACAGCTGTTTTCATTa

actaattaatacaaaggtatttttttcgagacccgattcgaatcgaatcgatttttttttcacttattatt
gtaaaattgaagatagggaaaaaaggtaaatccggtttgtttttcttgctatcctttttttatttacatt
cttgagcaaaatcacaacttttaggatgttcataagaaaagcagtgcttggttttgaacaacgtaaagtc
ccagaattatcaatattttccaaaaagtgaggttggttaagttaatttgtatatttaaaagggcaataaa
ttaaaaaaaattatattttagtgggtagaggacagaactgctcccaggagaaaagattgttaatgcttaa
gttaagaagggaatgaagaacatataagcacattaaaaaaaaaaccttgaatgctctgccaaaatggtat
ccaatcccattaaatatgaatggaagcccgtaaaccgtggaaacaaacgtggaccgacgcgtagttcgtt
acagcaaagtctatccttttgcatcctctaggtctgagctgaacttggaattcagtgacgttacaattcg
gagacgacgacttattcaaaatttaagtgcgaggaatccacgaaaggtgcccctacttagcccaaggcat
attcaggcacggttaaatttcgctagaacctaccgaaactgaccagtctccaaatggcgtaatatccttt
ggactgatgggtcaataatagtgctatttggtggaactggttcacgacagtatgtccgacgacccccaaa
catggagtaccacccaaaacatacactcaagatgttcaatcatggtggacctaaatcctggtatgggctt
gtatattttacattggtatggtttagggtattatagacatcacttagtgatgtcttactggctgaatgga
atatgcccttaaaatggacatataatgatctgaaacccacaagcaaatcggctaagaatcggttcacccg
aaaaaataaaggtatgccgtggccagcaccatcttccgattcaaacccgattgaaaacctgtgggggat
attaaacaatatgtgtcgaagaactcactaatgtctaaggttcagctttggctgatgtgtaggatgcatg
gtaaaaatctgcccccaaacgttgccaggacctgatggactccatgccgcgtaggtgtaaggctgtgctg
gctaacaaaggatatccaaccaagtattagaccctaattcacacactaaaataaaaaaaatattttagtt
ctaagtcatgttgaattttgttactatttttttcgtagcactgctatttcattgaagaaataaaagtgaac
aatttgttgaattgtttgaaatttaatacctaatgataatataacaaaatttccctttaaaacttcaaat
cataggatttatcctaaactcgcaggtcccaagcactgctatttttatgaacacagctgtttgcgtttat
tacatttccgtactatactattgcaacaaaTTTGggaaattaatattactaatgtaacataaaacataaa
taaataaaaaagtcataaatcataaaagtaatacgttttagaaaacacaaatttataaaataaataaaaa
tatgaaaactgtttattgcaaaagttatatcttgaggcacgaagtgcggacacaagcactcaacaagcat
tgccttattaatttttcacacgtcgcacgctaaatactctaatgacaaatattttaatatagtcattttt
gaaatttattttgtgatattattatatattatattattatagatatagatatagatatagatatagatat
agatatagatatagatatagatatagatatagatatagatatagatatagatatagatatagatatatat
atagatatagatacagatatagatatagatatagatatagatatagatatatatagatattatattatta
catagattcggctattgaaatttctatatttctaagctatatttgaacaataataacgttaaggcaatgc
aaaacaagaatttttcgcatggtgcaaatggatcaaaaataatatagatttaaagtctcagaacttctaa
ggtgaagggcatattttgtcaaatttacaatgcatgagattacgtttgcacacatacagttgtctgctat
cactgtatacagagaaaagagctgttcgctgtagcgctctcagctcgctcttgaaaaaaaattcgagaga
gactggagccacgccgaaaatatcgtgtgccaaaaaatcgtatggcgttacgcatcttgttattctagtg
tctttgATCGaacaaaatgaatttgaatgtttaacaacgtaaatttttttccgatattggagacaccgtcg
ttccgaagcagatatccgataaatttccatatgctaaaggaccgaatgggtgtttttgctgagccagaaa
aaacagtgccatacattacaaatattgttgacacaatatgtacacaggacaaacaaggctatacccatac
cccatggctatagtggatttcgtgaactaagttgttataagacggcattatccgaccctcacggtcacct
gataacaattcagtgtggatgagtaatcgTTATAGGAGATAGGTTGTCCCGATCCAACAACTGTTTAAAA
ACTGAAGTGAGGGTGCTATTCCAGTACACGAGTTAATCTCTAAGCTGATTAGAGGGTTAGTGTATTGGGT
ATTGAATGTTGATGAAACTCCACCGACGACGCGGTTTGAAGCGAAGGGAGCATAATCTCCAAAACCGCAT
CTGCGTCCGATCCAGAGAACGACAAATGAGAAAATGAGAAAAACGCGACAAAAATGAAGAGAAATTTAGA
TGAGAGTGGCAAAACAAAAATAGCACTCTGCCGGTGGGCCTACTTGACGTCGACTATGTCAGCTTATCCT
TGAAAAATTTAGATACAGCTTTCAGCTTTATGGCTACCGTAAATTTATGCCAAGATGCAGAAACCTTTTT
CTAAGCAGAACATTGAATAGTTCACAAAACCAATTGTATGCCTTTTGTTATTGTTGTGCCGTTTATGAAA

CAATTTATGACGAATTTTGGGGTAATGATTCAGCAACTGTTGTTGCAACTAAATTTTCTCATGGTTCAAT
ACAGACGATTTGAAATTTAGAATTTAAGATTAGAAAAAGACTTCTTAGTTGTTGTATTTTACCCATCAGC
TGATCAGCCTTGATGTGCGAATCAATGGTCACTACAAAGCTATTAAACTGTCAGAGAAGATATGACACAA
GGTTCGAAATTGGATAGCATTAACTTCGATATTGCACTCAGAACACGAGTAAAGAGACATAGCAGCGCAA
CAAAGTTTTACCAGAGTTCGTGTTCTTGGCTGAGCTCCGGaaaaatactaaaaaaaaGTGGATGAACTAG
AGAAAATGCGTAAAAGCTCCGTAAATCAAGGAAACGACTGGTTCTCAAACTTCTCAACACGCGATATCAA
CGTAGAAACGAAACCAACAAAGAGCAACCTCTACTGACCGTCAACAGCAAGAGGACGAGCTATTGTTCTC
CCATTCTGCTCACCCCAACTCCTACATCATCGAGATACCAATACCAAACCCCACCACTGCCGGCTTTGAT
GGCAGACGCTCCAACAACGAACAGGCCTTAAATCCCTGCTGTCGCAACAATAGCACCACCAAATCAACTA
CCAATATTGCGGCGCCGACAGCAACAAACCTTGGCCAGCTCCGATAGGCAACAGcgatcctgacgataca
gataggtatggatcgctacatctagattaagcgtaagctaatcaccctaaacactgttggtaataagcca
aaggtcaaccgacaacagtaccaagaaagcaacgatccaataaggatgataaccgattttctatgttagc
agaggcggagaacaatacagcatggctgcgtcaagagacccaaaagaaaccaaatcccttgtctattttt
ataagggaaaaaagttcaaacgaccaggtcaataaaattgctgcgctgggcggggacatgttccgcattg
tgtaaaaaacctggaggaaacaaaaaagaatttctacaccaaacaactaaaaagcagcaagggattttgc
tagttgttaaaggaattgagattcaagaagcccttaaggacatgggcttctgtgacaaaaatgttagcac
cataatgaaacgaaaaaaatgccgcaaccagataacaaagccttaaagaaaaacgaagtgcattgaagta
tgaccgtggaagaaccacacaaacgcaacggtcttgtacaatgcaataactgccaggagtatggacacac
caagacacactgcagacttaggtcacaactccgtaatgtaccgtaaactccgtaaacaaagacgacgaag
acaaaagaatgatgtgaaaactctggaggaaaccatacgtgaaactacaaaggctCTATCAAAgacacta
gacactagctactttttttggtgacggccaaaatggcccactgtccgctcgcttacgctgagagcgtaaga
aatctaaaagtagaatttgcttgcttgtgtgagtaaaaacaagagacgagaacgcgtatatgtgtgcgtg
ttgtgctagaagacgattttcgagccgaaatcaattctgatcgaagaaatgaatttacatatttggggag
ttttggccaatttcgtagaaatatgatgaaattgcgcgctgtttaaaaatatcgaattggaatataacaa
aaaaaaaactgaaactgaaacaactgaatctaatgggcatttaaataataaaattggatcatttccata
catcatattaagtcaaatgacttaataaatatactaaataattaaggcaaatacaaaggaaattattata
actactctaatttaaaacaaattttccaaaaagaagacattatattgagaaataaatatttcataacaat
aatacgttgtagaaaataaaaaatttttaaaataaataaaaatatgaaaactgtttattgccatatcgtc
atatcttgagacacgaagtgcggacacaagcacttaacaatcattgcccttataatttttcacacgtcgc
aagctaaatactctaatgataattattctaatatagtcatttttgaaatttattttgtggtattatgatt
cggctattactatttctaagctatatttaaataataataacgttaaggcaatgcaaaacaagaatttttc
gcatggtgccaattgatcaaaaatatagatttaaagtctaagaacctctaaaatgaagagcatattttgt
caaatgtacaatgcatgagcatacgtgtgcaaattaactttttgcggtataaattatttaaaaaaataat
tttttatttttttaatattttATTGGTGTTAAACTTTTCAACTTATCACTTTATTACAAGACACTTATTGT
TATCTATTTATACATATATAAACCGttttttttATCCTCCCGAATCAGACTGATCTTCAAATTCTGAATC
CTAATCCAATCAACCTCGGCCAGAAGCTTTTCTTTGACACTTTCTGAACTTTTGATAAAAGCTTTATGCT
GAAATTATTCCACTGCATTGTGAAATTCAATTATGCTGATCGATGCAGTTTTTGATTGAAGCGCACTAAG
TTGGCCGTGGTTTGGTCTCCAAGCGGAAGCTGTGTTTACGTTAAGTTTGGGTTGTTTATTTTAGCGGGTA
GCTAAGCGCTGGCAAAGGCAATGACAAAAACAAAGACAAAGGAAATGCCGACGAGATTGAGAATTGAAAT
TTGTTTATAAACTGTGGAAGGGTGCTATGTTTATGGGTTGGATAACTCTCCCCCTTCTAAAATGACGCGT
CCCGCTTCATTATTAATTTCGTTTAaacggccatacattggtttggcactatgcagccactttttttggtg
acggccaaaattactctctttccgctcactcccgctgagagcctaagaaatctaaaaatataatttgctt
gcttgtgtgagtaaaaacaagagacgagaacgcgtataagtgtgcgtgttgtgcacagacgtttttcggg
ccgaaatcaattctgttcaaagaaacgaatttacatggtacatattagggtagtttttgccaatttccta

gcaatattataaaataaacaaatttttaaaaattcgcgccctgactattataattttaaagctttttaaa
tttgtttgttaaaatcgccgctcgaataagctaccgtttacacatttatatttatgtttaattctaattt
gtctttcatctgacaatttttaaaagcgaaatatttttttgaaacacttttaatgttaatgttacatta
tattaggtcaaatgatttaataaatatactaaataattaaatatgataactgtttattggaaaagtcata
tcaaagacactagaattattctagtgtctttgctttggtcatatcttgaggcacgaagttcggacacaag
cactcaacaatcattacttaatttttcacacgccgcaagatgaatactctaatgacaaatattcttatat
aaagtcatttttgaaatttatttttgtgataatacgtacatagatttggctatttctaatctattttcaa
ataataataacgtttaggggcatattttgacacatttacaatgcatgagcatacgtgtgcacacatacag
ttgtctgctatcactgtatgcgcaaaatgcgaaaagagttgttcgctgtcaaacaaaaattcgagagagc
ctggagcgacctctagagccacggcgaaaaaatcgtgtgtcaaaaaatcgtatggcgttacgcatcttgt
tattctaaagtctttgGTCAGACGCAGTATTTAGTAAGACAACACAAAAAGGGTCTCCATTAATAAAGCT
ACTTGTTTTAATTTTCAGAAACATTTTCAATTaaaaaaaaaaTTAAATTATGTATTTTTAGGAATCATGG
TGATGAACAAATGGAAAATGAAACCGATGAGCAATTTGAAGAACGTGTTTTAAACAAGAGAGCCGCCCAC
CTTTTTATTGACGTAAGAGCACATTTTATCGCCAAGGACAGCTTGGAACTTTCTCAGCTTACGTCAGGAA
ATTCCAGAAAACAGGGTAAAATACAGTGGCTTCATTTTGATTTACTTAGTTGTAAGCGTCCAACAGAACT
CAACTCCTGAACTTATATTGTAATAGGTATTGAGCTTAAGAGTTCTTTCTgacgcagcgcaagtttttttg
acccattttgccacgcccccacttttgaactatttcgaatttttttcattttatttcccaatatctatcga
tatccctgaaaaatgatgaaaattcgagttcgcattcacaatagGACACAatcctaatattgaggtgaaa
atcgacacag
>dme_circ_0004394_junction_seq
cctaatattgaggtgaaaatcgacacagGGATAAAATATTTGATAATGACGTTTTG
>dme_circ_0004395
GAACCAGCCCTTTTTGAAGACGATCTTTTTGGTGATCCTTCTCAACCAGTTGAGCAGATTACAAAAGAAT
CTACTACTGTTCTCAACGCAGATGATTCTGATGAAGACGCCATTGATAATATTCATAATGTCCCTTCGCC
TGCAACCTCGCTCGTTAATTCGATTGAAGATGAGAAAGAGGGAAAATAATTTAAATGGACATGCAAGTGT
GTCCGAAAATGTGCCAATGAATGAAATAACGCTTGTTCAAAACGAGGATGAGGGATTTGCGTTAGCTCCT
TTAGATGTCTCAATGTATAAAGGCGTTACAAAAGCCAAACGCAAAAGAAAACTTATTATTGACGAAATAA
AAAATATTTCTGGAGAGGAAATGAAGGCTCAGTTAGCCGACACATCTGATATATTAACCACACTTGATTT
AGCCCCACCAACAAAAAGGATTAATGTATTGGAAAGAAACTGGAGGTGTCGAAAAACTTTTTTCATTACC
ATCGCGATCCATACCAGCTAGAGCTTTGTTTGGTAATTACAATAGACAGTTGTTTTCGCATTCAACATTC
TTTGAGGACTTCTCGAGTGTTGTGCCAATGGAAATTCTTGCACTGGAATTTTATACCAAAGAGAACGAGA
ACGCATTAATTATTTTTAATAAAAAAGGTCGCAAACGTAAGAATGATAATATGTCCAATTTGTTTTTGGA
CCATGTCCCCGATTCAGTTGTTCAAAGTTTGGAAGCTCCGGAAGTCCTGAGAGCGAATCATAAATCTCTA
GGGGTATCTACCGTTTCTGTGGAGATTGTATCAAAAGAGCAAGAAAGCATTTCATGTCAAAATGAATTAA
CATTTTTTGATAATATGCGAAGTCCGGACCTCTTGTCCTTAAATGAAATGGAACAGTTCTCATCAATAAA
TGAGCTACCTTTAACACCAAGAAACATGAATCATGAAATGGGTGACGATTTTAATCAAGGAGACTCAACG
CCAGCTGGGTTGGATCATGGTCATGCCACTCCGCAGCATGGAAATATTGGCGAGATGGATCACGACTCTG
TTATACCGACAAAAAAGACTGCAGTCATTTTAAATGAATCTGTGGGTACATCTGTACTAAGTGACAATGG
CGTTTCAAAAAGGACAAATAATATTTTAAAAGGATGGGACAACTATGAAATTCCCAGTTTCGTTGGCCAG
>dme_circ_0004395_junction_seq
CTATGAAATTCCCAGTTTCGTTGGCCAGGAACCAGCCCTTTTTGAAGACGATCTTT
>dme_circ_0004396
GAACCAGCCCTTTTTGAAGACGATCTTTTTGGTGATCCTTCTCAACCAGTTGAGCAGATTACAAAAGAAT
CTACTACTGTTCTCAACGCAGATGATTCTGATGAAGACGCCATTGATAATATTCATAATGTCCCTTCGCC

TGCAACCTCGCTCGTTAATTCGATTGAAGATGAGAAAGAGGGAAAATAATTTAAATGGACATGCAAGTGT
GTCCGAAAATGTGCCAATGAATGAAATAACGCTTGTTCAAAACGAGGATGAGGGATTTGCGTTAGCTCCT
TTAGATGTCTCAATGTATAAAGGCGTTACAAAAGCCAAACGCAAAAGAAAACTTATTATTGACGAAATAA
AAAATATTTCTGGAGAGGAAATGAAGGCTCAGTTAGCCGACACATCTGATATATTAACCACACTTGATTT
AGCCCCACCAACAAAAAGGATTAATGTATTGGAAAGAAACTGGAGGTGTCGAAAAACTTTTTTCATTACC
ATCGCGATCCATACCAGCTAGAGCTTTGTTTGGTAATTACAATAGACAGTTGTTTTCGCATTCAACATTC
TTTGAGGACTTCTCGAGTGTTGTGCCAATGGAAATTCTTGCACTGGAATTTTATACCAAAGAGAACGAGA
ACGCATTAATTATTTTTAATAAAAAAGGTCGCAAACGTAAGAATGATAATATGTCCAATTTGTTTTTGGA
CCATGTCCCCGATTCAGTTGTTCAAAGTTTGGAAGCTCCGGAAGTCCTGAGAGCGAATCATAAATCTCTA
GGGGTATCTACCGTTTCTGTGGAGATTGTATCAAAAGAGCAAGAAAGCATTTCATGTCAAAATGAATTAA
CATTTTTTGATAATATGCGAAGTCCGGACCTCTTGTCCTTAAATGAAATGGAACAGTTCTCATCAATAAA
TGAGCTACCTTTAACACCAAGAAACATGAATCATGAAATGGGTGACGATTTTAATCAAGGAGACTCAACG
CCAGCTGGGTTGGATCATGGTCATGCCACTCCGCAGCATGGAAATATTGGCGAGATGGATCACGACTCTG
TTATACCGACAAAAAAGACTGCAGTCATTTTAAATGAATCTGTGGGTACATCTGTACTAAGTGACAATGG
CGTTTCAAAAAGGACAAATAATATTTTAAAAGGATGGGACAACTATGAAATTCCCAGTTTCGTTGGCCAG
GGTACGTTATAAAAATGATGATTAACTATATAAGATTTTTTGCAATGATTATTATTATTTTGTGCTACAC
AATAGGGTATAAGACATGCAGGGCACTGTTATCAGCAATATCACTAGGTCTTACGGAACATAAGTGTATT
GGTGTTTGTAAAAGCCAATTCACGTGTATTTTCCCAATCCAACGCTGCTCAAAAGCTGCTACGCCGGAGA
GACGCATAGATGACGATCTTTTCGACGCTATTTttatacacgttactcgtagattaaaagggcagaaaca
tgaagcgtttccgatcatataaagtatatatatatttgatcagaatcggtagacgggtcgacctggccat
gtccatgtgaacgtcccgatcactggaactataaaagctagaagtttaagattaagcatatagattctag
agacatagacgaagagccagcgcaaaatttttttgtatattttctcataattttgttagttattgaaattt
ctgtcgatttgccaaacaaccttttgacacgtccactaacgctctgaaaccgcccaaaactgccacgccc
acacatttgtttgtcttaccaatttttatcggtataccaagaacacggtaaacacgcccctcttttcacc
tacaaaacaccaaaaacggtcaatgtgttcgcattcccctagctaagtaacggctaactcgagtcgggga
actcgactatagcattttcttttgtttTATTTATAACCGGATATTTAGCACTAGTAAAGGCCCTTGAACT
GTTATATTTCTTTTCTGGAAATGTTAATATGTGGCACTGCCGGAAATTTGGAAGAGGTAATCGACCTATT
TTAGAGAATTATTGCTTACCTATGTCAGACcaaagacacaagaataacaagatgcgtaacggccatacat
tggtttgactgccgaaattgccctctcacagatcgagtagaactgagagcgtaagaaatctaaaaattga
gtttgcttgcttgtgagtgaaaacaataagcgagaacgcgtatatatatgtgtgcctaattgtgctagaa
gacgattttcgggccgaaatcaattctgatccaagaaacgaatttacatatttggaattttggccaattt
cgtatcaatatgatgaaattaaataataattaaaaattcacgccgcgactattataattttaaagttttt
aatattcgtttgataaaatcgccgctcaaattaccgttcacatatttatgtttgttaataattaattatt
tgtaatatatgtaataaGGCTATTACAATTGTAATGCCTCCTATATTGACAGCTGTTTTCATTaactaat
taatacaaaggtatttttttcgagacccgattcgaatcgaatcgattttttttttcacttattattgtaaaa
ttgaagatagggaaaaaaggtaaatccggtttgtttttcttgctatcctttttttatttacattcttgag
caaaatcacaacttttaggatgttcataagaaaagcagtgcttggttttgaacaacgtaaagtcccagaa
ttatcaatattttccaaaaagtgaggttggttaagttaatttgtatatttaaaagggcaataaattaaaa
aaaattatattttagtgggtagaggacagaactgctcccaggagaaaagattgttaatgcttaagttaag
aagggaatgaagaacatataagcacattaaaaaaaaaaccttgaatgctctgccaaaatggtatccaatc
ccattaaatatgaatggaagcccgtaaaccgtggaaacaaacgtggaccgacgcgtagttcgttacagca
aagtctatccttttgcatcctctaggtctgagctgaacttggaattcagtgacgttacaattcggagacg
acgacttattcaaaatttaagtgcgaggaatccacgaaaggtgcccctacttagcccaaggcatattcag
gcacggttaaatttcgctagaacctaccgaaactgaccagtctccaaatggcgtaatatcctttggactg

atgggtcaataatagtgctatttggtggaactggttcacgacagtatgtccgacgacccccaaacatgga
gtaccacccaaaacatacactcaagatgttcaatcatggtggacctaaatcctggtatgggcttgtatat
tttacattggtatggtttagggtattatagacatcacttagtgatgtcttactggctgaatggaatatgc
ccttaaaatggacatataatgatctgaaacccacaagcaaatcggctaagaatcggttcacccgaaaaaa
taaaggtatgccgtggccagcaccatcttccgattcaaacccgattgaaaacctgtgggggatattaaa
caatatgtgtcgaagaactcactaatgtctaaggttcagctttggctgatgtgtaggatgcatggtaaaa
atctgcccccaaacgttgccaggacctgatggactccatgccgcgtaggtgtaaggctgtgctggctaac
aaaggatatccaaccaagtattagaccctaattcacacactaaaataaaaaaaatattttagttctaagt
catgttgaattttgttactattttttcgtagcactgctatttcattgaagaaataaaagtgaacaatttg
ttgaattgtttgaaatttaatacctaatgataatataacaaaatttccctttaaaacttcaaatcatagg
atttatcctaaactcgcaggtcccaagcactgctatttttatgaacacagctgtttgcgtttattacatt
tccgtactatactattgcaacaaaTTTGggaaattaatattactaatgtaacataaaacataaataaata
aaaaagtcataaatcataaaagtaatacgttttagaaaacacaaatttataaaataaataaaaatatgaa
aactgtttattgcaaaagttatatcttgaggcacgaagtgcggacacaagcactcaacaagcattgcctt
attaatttttcacacgtcgcacgctaaatactctaatgacaaatattttaatatagtcatttttgaaatt
tattttgtgatattattatatattatattattatagatatagatatagatatagatatagatatagatat
agatatagatatagatatagatatagatatagatatagatatagatatagatatagatatatatatagat
atagatacagatatagatatagatatagatatagatatagatatatatagatattatattattacataga
ttcggctattgaaatttctatatttctaagctatatttgaacaataataacgttaaggcaatgcaaaaca
agaatttttcgcatggtgcaaatggatcaaaaataatatagatttaaagtctcagaacttctaaggtgaa
gggcatattttgtcaaatttacaatgcatgagattacgtttgcacacatacagttgtctgctatcactgt
atacagagaaaagagctgttcgctgtagcgctctcagctcgctcttgaaaaaaaattcgagagagactgg
agccacgccgaaaatatcgtgtgccaaaaaatcgtatggcgttacgcatcttgttattctagtgtctttg
ATCGaacaaaatgaatttgaatgtttaacaacgtaaatttttttccgatattggagacaccgtcgttccga
agcagatatccgataaatttccatatgctaaaggaccgaatgggtgtttttgctgagccagaaaaaacag
tgccatacattacaaatattgttgacacaatatgtacacaggacaaacaaggctatacccataccccatg
gctatagtggatttcgtgaactaagttgttataagacggcattatccgaccctcacggtcacctgataac
aattcagtgtggatgagtaatcgTTATAGGAGATAGGTTGTCCCGATCCAACAACTGTTTAAAAACTGAA
GTGAGGGTGCTATTCCAGTACACGAGTTAATCTCTAAGCTGATTAGAGGGTTAGTGTATTGGGTATTGAA
TGTTGATGAAACTCCACCGACGACGCGGTTTGAAGCGAAGGGAGCATAATCTCCAAAACCGCATCTGCGT
CCGATCCAGAGAACGACAAATGAGAAAATGAGAAAAACGCGACAAAAATGAAGAGAAATTTAGATGAGAG
TGGCAAAACAAAAATAGCACTCTGCCGGTGGGCCTACTTGACGTCGACTATGTCAGCTTATCCTTGAAAA
ATTTAGATACAGCTTTCAGCTTTATGGCTACCGTAAATTTATGCCAAGATGCAGAAACCTTTTTCTAAGC
AGAACATTGAATAGTTCACAAAACCAATTGTATGCCTTTTGTTATTGTTGTGCCGTTTATGAAACAATTT
ATGACGAATTTTGGGGTAATGATTCAGCAACTGTTGTTGCAACTAAATTTTCTCATGGTTCAATACAGAC
GATTTGAAATTTAGAATTTAAGATTAGAAAAAGACTTCTTAGTTGTTGTATTTTACCCATCAGCTGATCA
GCCTTGATGTGCGAATCAATGGTCACTACAAAGCTATTAAACTGTCAGAGAAGATATGACACAAGGTTCG
AAATTGGATAGCATTAACTTCGATATTGCACTCAGAACACGAGTAAAGAGACATAGCAGCGCAACAAAGT
TTTACCAGAGTTCGTGTTCTTGGCTGAGCTCCGGaaaaatactaaaaaaaaGTGGATGAACTAGAGAAAA
TGCGTAAAAGCTCCGTAAATCAAGGAAACGACTGGTTCTCAAACTTCTCAACACGCGATATCAACGTAGA
AACGAAACCAACAAAGAGCAACCTCTACTGACCGTCAACAGCAAGAGGACGAGCTATTGTTCTCCCATTC
TGCTCACCCCAACTCCTACATCATCGAGATACCAATACCAAACCCCACCACTGCCGGCTTTGATGGCAGA
CGCTCCAACAACGAACAGGCCTTAAATCCCTGCTGTCGCAACAATAGCACCACCAAATCAACTACCAATA
TTGCGGCGCCGACAGCAACAAACCTTGGCCAGCTCCGATAGGCAACAGcgatcctgacgatacagatagg

tatggatcgctacatctagattaagcgtaagctaatcaccctaaacactgttggtaataagccaaaggtc
aaccgacaacagtaccaagaaagcaacgatccaataaggatgataaccgattttctatgttagcagaggc
ggagaacaatacagcatggctgcgtcaagagacccaaaagaaaccaaatcccttgtctatttttataagg
gaaaaaagttcaaacgaccaggtcaataaaattgctgcgctgggcggggacatgttccgcattgtgtaaa
aaacctggaggaaacaaaaaagaatttctacaccaaacaactaaaaagcagcaagggattttgctagttg
ttaaaggaattgagattcaagaagcccttaaggacatgggcttctgtgacaaaaatgttagcaccataat
gaaacgaaaaaaatgccgcaaccagataacaaagccttaaagaaaaacgaagtgcattgaagtatgaccg
tggaagaaccacacaaacgcaacggtcttgtacaatgcaataactgccaggagtatggacacaccaagac
acactgcagacttaggtcacaactccgtaatgtaccgtaaactccgtaaacaaagacgacgaagacaaaa
gaatgatgtgaaaactctggaggaaaccatacgtgaaactacaaaggctCTATCAAAgacactagacact
agctactttttggtgacggccaaaatggcccactgtccgctcgcttacgctgagagcgtaagaaatcta
aaagtagaatttgcttgcttgtgtgagtaaaaacaagagacgagaacgcgtatatgtgtgcgtgttgtgc
tagaagacgattttcgagccgaaatcaattctgatcgaagaaatgaatttacatatttggggagttttgg
ccaatttcgtagaaatatgatgaaattgcgcgctgtttaaaaatatcgaattggaatataacaaaaaaaa
aactgaaactgaaacaactgaatctaatgggcattttaaataataaaattggatcatttccatacatcat
attaagtcaaatgacttaataaatatactaaataattaaggcaaatacaaaggaaattattataactact
ctaatttaaaacaaattttccaaaaagaagacattatattgagaaataaatatttcataacaataatacg
ttgtagaaaataaaaaatttttaaaataaataaaaatatgaaaactgtttattgccatatcgtcatatct
tgagacacgaagtgcggacacaagcacttaacaatcattgcccttataatttttcacacgtcgcaagcta
aatactctaatgataattattctaatatagtcatttttgaaatttattttgtggtattatgattcggcta
ttactatttctaagctatatttaaataataataacgttaaggcaatgcaaaacaagaatttttcgcatgg
tgccaattgatcaaaaatatagatttaaagtctaagaacctctaaaatgaagagcatattttgtcaaatg
tacaatgcatgagcatacgtgtgcaaattaactttttgcggtataaattatttaaaaaaataatttttta
ttttttaatattttATTGGTGTTAAACTTTTCAACTTATCACTTTATTACAAGACACTTATTGTTATCTA
TTTATACATATATAAACCGtttttttATCCTCCCGAATCAGACTGATCTTCAAATTCTGAATCCTAATC
CAATCAACCTCGGCCAGAAGCTTTTCTTTGACACTTTCTGAACTTTTGATAAAAGCTTTATGCTGAAATT
ATTCCACTGCATTGTGAAATTCAATTATGCTGATCGATGCAGTTTTTGATTGAAGCGCACTAAGTTGGCC
GTGGTTTGGTCTCCAAGCGGAAGCTGTGTTTACGTTAAGTTTGGGTTGTTTATTTTAGCGGGTAGCTAAG
CGCTGGCAAAGGCAATGACAAAAACAAAGACAAAGGAAATGCCGACGAGATTGAGAATTGAAATTTGTTT
ATAAACTGTGGAAGGGTGCTATGTTTATGGGTTGGATAACTCTCCCCCTTCTAAAATGACGCGTCCCGCT
TCATTATTAATTTCGTTTAaacggccatacattggtttggcactatgcagccactttttttggtgacggcc
aaaattactctctttccgctcactcccgctgagagcctaagaaatctaaaaatataatttgcttgcttgt
gtgagtaaaaacaagagacgagaacgcgtataagtgtgcgtgttgtgcacagacgtttttcgggccgaaa
tcaattctgttcaaagaaacgaatttacatggtacatattagggtagtttttgccaatttcctagcaata
ttataaaataaacaaatttttaaaaattcgcgccctgactattataattttaaagctttttaaatttgtt
tgttaaaatcgccgctcgaataagctaccgtttacacatttatatttatgtttaattctaatttgtcttt
catctgacaatttttttaaaagcgaaatatttttttgaaacacttttaatgttaatgttacattatattag
gtcaaatgatttaataaatatactaaataattaaatatgataactgtttattggaaaagtcatatcaaag
acactagaattattctagtgtctttgctttggtcatatcttgaggcacgaagttcggacacaagcactca
acaatcattacttaatttttcacacgccgcaagatgaatactctaatgacaaatattcttatataaagtc
atttttgaaatttatttttgtgataatacgtacatagatttggctatttctaatctattttcaaataata
ataacgtttaggggcatattttgacacatttacaatgcatgagcatacgtgtgcacacatacagttgtct
gctatcactgtatgcgcaaaatgcgaaaagagttgttcgctgtcaaacaaaaattcgagagagcctggag
cgacctctagagccacggcgaaaaaatcgtgtgtcaaaaaaatcgtatggcgttacgcatcttgttattct

aaagtctttgGTCAGACGCAGTATTTAGTAAGACAACACAAAAAGGGTCTCCATTAATAAAGCTACTTGT
TTTAATTTTCAGAAACATTTTCAATTaaaaaaaaaaTTAAATTATGTATTTTTAGGAATCATGGTGATGA
ACAAATGGAAAATGAAACCGATGAGCAATTTGAAGAACGTGTTTTAAACAAGAGAGCCGCCCACCTTTTT
ATTGACGTAAGAGCACATTTTATCGCCAAGGACAGCTTGGAACTTTCTCAGCTTACGTCAGGAAATTCCA
GAAAACAG
>dme_circ_0004396_junction_seq
GCTTACGTCAGGAAATTCCAGAAAACAGGAACCAGCCCTTTTTGAAGACGATCTTT
>dme_circ_0004397
GAATCATGGTGATGAACAAATGGAAAATGAAACCGATGAGCAATTTGAAGAACGTGTTTTAAACAAGAGA
GCCGCCCACCTTTTTATTGACGTAAGAGCACATTTTATCGCCAAGGACAGCTTGGAACTTTCTCAGCTTA
CGTCAGGAAATTCCAGAAAACAGGGTAAAATACAGTGGCTTCATTTTGATTTACTTAGTTGTAAGCGTCC
AACAGAACTCAACTCCTGAACTTATATTGTAATAGGTATTGAGCTTAAGAGTTCTTTCTgacgcagcgca
agttttttgacccattttgccacgcccccacttttgaactatttcgaatttttttcattttatttcccaat
atctatcgatatccctgaaaaatgatgaaaattcgagttcgcattcacaatagGACACAatcctaatatt
gaggtgaaaatcgacacaggtaccgtaataacaggaaaatgagagaagtaaagcaaacatatacattgct
ttcgcaaggTTATttatacccattactcgtagagtaaaaaggtagaaggaagcgtttccgaccatataaa
gcatatatattcttgactaggatcaacagccgagtggatctgatcatgtccgtctggtcgtccatgtgtt
cgtccgtatgaacgtcgagatctggggaactatagctacagctaaaaagttaagattaagcatacagact
ctagagacatagacgcagcgcgagttttttacctatgttgccacgcccacaaagcacgcaaaaatgtcac
gtccacacttttggaaaatatttgatatattttcatgtttttattagttttgtaaattttcatcgatttg
ccaaaaaaaaatttgccacgcccacaatccaaccgaaactgcacttttgaaacatgttttgaaaacatat
atttttatatttatttattttttatttttccccaattcgatattccagaaagattattcaatatcggt
ttcgctgagaaacaggtatctgatagtcggcgaactcgactatagtattctctcttgttatacccgttat
tcgtagagcaaagaggtatacaaggttcgttgataagtatgtaacaggtacaaggaagcgtttatatata
ttcttgaacaggatcaacagccatatttctgtccgtccatatgaacgtcgagatctcaggaagtgtacat
acagattttggagaaatacacgcagtttggcccatgttgccatgcccactctaacgccaacaaacaacta
acgtctgccacgccaacgcttttaatacatgatttgatatttttggatttttgtattaatcttgaaagtt
tctatcAGCCAcgcccaaaactacgcacacatttttgaaaattatttcataatttataagtcttgtaaat
ttctatcaatttgccaataacaaattttcctacgcccactctaaggaccacaaaccgcccatgttttcaa
tatattgtccaatatttttcgatatgccataaaaattatgaatattgcgttctcactTTAATGATTGATT
ATCCACCCGCGAGCCGAACGATGCACGGCACAGCCCATCGGATGCTTGGGGGAGTTGTGCATTAATACAC
GCGGGACAAATACACAAATTTATGTTCTAGAGCTTTAATATTTCACCATCTGCAGACAACGGGAGCGTGC
TGTTGTGTCGGTTATATTAAATATAGTACTCATTTTAAAGACAATTGAGGTGTTTTACTATTTTGGCTGA
TGTAAATTTGAATACTATTTATTAATTAATATTTGTGCGTACTTTTTTTCAGGGCGGCACAAAAATTTTA
TTCCCTCCTCGTCTTAAAGAAGTTTAAGGTGCTTCACATTGATCAATCTGCTCCCTATGCAGATATTACT
ATTACTCGAGGACCAACGTTCGAAAACCCAAAAATTTAATAAGTTGAAT
>dme_circ_0004397_junction_seq
GAAAACCCAAAAATTTAATAAGTTGAATGAATCATGGTGATGAACAAATGGAAAAT
>dme_circ_0004398
GCCACGCAATTCACGATGCTTGTGGACATGCTTGCAGATCCAGTTAATTTTTGGATCACGACGAATAGCA
CTGTGATGAGTATCAATTAAGATTACTTCAAAATATTTATAAGAAGCATCTTGCGCAATCCAATACGAGT
TCAAAACTCGCAAGCCGCCAAGTCTACGACCAACACGTTCCTACAAGAAAATAAAATTCATGATATATTT
CAGTGTTTTTTGGTGAGGACCTCTTAATTGGACAAGAATATTTCAGACATCTAAGGAAGTTAATGTCAAC
ATATATTGTATGTCAAAAGGGGTTGTCATCAACTTGTTCGAACGTGTGGTTAGCAAATTTCGTTTTTTCT

TACCTCAGCAATGGATTGCAAACCACGATATGGTTTAACTGGTTTACACCATGACTCTTCGGCTTGCCA
TAAGTGCATCCTTTGGGAACTGGACGCTTGCGACCTCCGCGGCGAACACGGATTCTATAAATCACGAACC
CCTGTTTGGCTCTGTATCCTAAACGTCTTGCTTTATCCGGGCGAGTAGGTCTTGGCGAACGATGCAATTT
CGTTAGTTGGCGGTATTGCCAAACGCGAATACGTAGCAAGTAGCGCATCACATCGCTCTGCTTCTTCCTA
TAAAG
>dme_circ_0004398_junction_seq
TCACATCGCTCTGCTTCTTCCTATAAAGGCCACGCAATTCACGATGCTTGTGGACA
>dme_circ_0004399
GAGCCAAACAGGGGTTCGTGATTTATAGAATCCGTGTTCGCCGCGGAGGTCGCAAGCGTCCAGTTCCCAA
AGGATGCACTTATGGCAAGCCGAAGAGTCATGGTGTAAACCAGTTAAAACCATATCGTGGTTTGCAATCC
ATTGCTGAGGGTAAGAAAAAACGAAATTTGCTAACCACACGTTCGAACAAGTTGATGACAACCCCTTTTG
ACATACAATATATGTTGACATTAACTTCCTTAGATGTCTGAAATATTCTTGTCCAATTAAGAGGTCCTCA
CCAAAAAAACACTGAAATATATCATGAATTTTATTTTCTTGTAGGGAACGTGTTGGTCGTAGACTTGGCGG
CTTGCGAGTTTTGAACTCGTATTGGATTGCGCAAGATGCTTCTTATAAATATTTTGAAGTAATCTTAATT
GATACTCATCACAGTGCTATTCGTCGTGATCCAAAAATTAACTGGATCTGCAAGCATGTCCACAAGCATC
GTGAATTGCGTGGCCTTACATCAGCTGGAAAAAGTTCGC
>dme_circ_0004399_junction_seq
GGCCTTACATCAGCTGGAAAAAGTTCGCGAGCCAAACAGGGGTTCGTGATTTATAG
>dme_circ_0004400
GTGCTATTCGTCGTGATCCAAAAATTAACTGGATCTGCAAGCATGTCCACAAGCATCGTGAATTGCGTGG
CCTTACATCAGCTGGAAAAAGTTCGCGTGGCATTGGCAAGGGATATAGATACTCCCAGACAATTG
>dme_circ_0004400_junction_seq
AAGGGATATAGATACTCCCAGACAATTGGTGCTATTCGTCGTGATCCAAAAATTAA
>dme_circ_0004401
GAGATCACAGAGTTCATAAATACTTAAAAAATATTTAATTTCATTGATTTTTTAGTCCAGCTGAAACAAG
TCTCCTCATAAAAATACTAGGAAAGGGGCTGGTGAACACAAAGCTATCTCTCGACATTCAACAAAAAAAT
CCGAATTCACCCTTACATTCCGTGAAAACATTTGAAGCCTTACACCTGTAAGTATATGGATTAGCATATC
TTCGCAAAAACTATTATGAAGCTAAAAAATTTTATTTAGAAAAGCATCTCTGTTAAAGGGAATTTACGCA
ATGGGGTTTAATACTCCATCAAAAATTCAAGAAACAGCGTTGCCAACTCTTCTCGCAGATCCACCGCAAA
ATATGATAGCACAAAGCCAGTCTGGGACAGGCAAAACAGCCGCATTTGTATTGGCCATGCTTAGCCGAGT
CAACGTTTGTTTAAATCATCCTCAG
>dme_circ_0004401_junction_seq
AGTCAACGTTTGTTTAAATCATCCTCAGGAGATCACAGAGTTCATAAATACTTAAA
>dme_circ_0004402
GTCCAGCTGAAACAAGTCTCCTCATAAAAATACTAGGAAAGGGGCTGGTGAACACAAAGCTATCTCTCGA
CATTCAACAAAAAAATCCGAATTCACCCTTACATTCCGTGAAAACATTTGAAGCCTTACACCTGTAAGTA
TATGGATTAGCATATCTTCGCAAAAACTATTATGAAGCTAAAAAATTTTATTTAGAAAAGCATCTCTGTT
AAAGGGAATTTACGCAATGGGGTTTAATACTCCATCAAAAATTCAAGAAACAGCGTTGCCAACTCTTCTC
GCAGATCCACCGCAAAATATGATAGCACAAAGCCAGTCTGGGACAGGCAAAACAGCCGCATTTGTATTGG
CCATGCTTAGCCGAGTCAACGTTTGTTTAAATCATCCTCAG
>dme_circ_0004402_junction_seq
AGTCAACGTTTGTTTAAATCATCCTCAGGTCCAGCTGAAACAAGTCTCCTCATAAA
>dme_circ_0004403
GCTGAAACAAGTCTCCTCATAAAAATACTAGGAAAGGGGCTGGTGAACACAAAGCTATCTCTCGACATTC

AACAAAAAAATCCGAATTCACCCTTACATTCCGTGAAAACATTTGAAGCCTTACACCTGTAAGTATATGG
ATTAGCATATCTTCGCAAAAACTATTATGAAGCTAAAAAATTTTATTTAGAAAAGCATCTCTGTTAAAGG
GAATTTACGCAATGGGGTTTAATACTCCATCAAAAATTCAAGAAACAGCGTTGCCAACTCTTCTCGCAGA
TCCACCGCAAAATATGATAGCACAAAGCCAGTCTGGGACAGGCAAAACAGCCGCATTTGTATTGGCCATG
CTTAGCCGAGTCAACGTTTGTTTAAATCATCCTCAG
>dme_circ_0004403_junction_seq
AGTCAACGTTTGTTTAAATCATCCTCAGGCTGAAACAAGTCTCCTCATAAAAATAC
>dme_circ_0004404
GGTTCTGTGTTTATCCCCTACGTACGAGTTGGCAATTCAAACTGGAGAAGTGGCTGCGCGAATGGGACAA
TTTTGTCGGGAAATAAAGCTAAGATTTGCAGTACGCGGAGAAGAGGGTATGTTCTTTTACCATATTAAAA
AACATCTAAAACAATTTGTTCCGTTCTACATTAAAGTTGATCGCAGTAAAAAAATTGAAGAACACATTTT
GATTGGCACCCCAGGAAAACTTTTGGATTGGGGAATAAAATTTCGTTTATTTGATATGAAGAAAATATCC
GTTTTTGTATTGGATGAAGCAGATGTAATGATAGCCACCCAAGGTCATCATGATCAATGCATTCGAATAC
ACAA
>dme_circ_0004404_junction_seq
ATCATGATCAATGCATTCGAATACACAAGGTTCTGTGTTTATCCCCTACGTACGAG
>dme_circ_0004405
GATTAATGCGTGAAGAGGAATCACTTGAAAACATAAAGCAATATTATGTTAAGTGTAAAAATGAAGAAGG
AAAATATAATGCGATTCAAAATATTTACGGTTGCATAAGCGTTGGTCAAGCTATAATATTTTGCCAT
>dme_circ_0004405_junction_seq
CGTTGGTCAAGCTATAATATTTTGCCATGATTAATGCGTGAAGAGGAATCACTTGA
>dme_circ_0004406
GACAAAAAGAACTGCTGCTTGGCTTGCAGCAAAAATGACGTCGGATGGTCATTCTGTTGCTGTTCTTACC
GGGGATTTAACAGTTGTGCAAAGACTTGATGTTTTAGATCGTTTCCGTTCAGGACTTGAGAAGGTGCTTA
TCACTACAAATATTTTATCAAGAGGTATATGATCAAAACTTGTACGATATGTATTTGTACCACATATATT
ACCGTATTTTTAGGCATTGATATTGAACAAGTTACAATTGTAGTCAATTTCGATTTGCCCGTAGACCTTG
ATGGGATGGCTGACTGTGAAACTTATTTACATCGAATAGGACGGACTGGGAGGTTTG
>dme_circ_0004406_junction_seq
CATCGAATAGGACGGACTGGGAGGTTTGGACAAAAAGAACTGCTGCTTGGCTTGCA
>dme_circ_0004407
GAGACGTTAATAAGCGCATGATTGAGATAACTGCATTTAAACATTTTGAGACGTGGTGGGACGAGCATAC
ATCAAAAGCTCGTTCAAAGCCACTATTCGAAAAAGCAGACTCCACTGTCAATACACCATTAAATT
>dme_circ_0004407_junction_seq
GACTCCACTGTCAATACACCATTAAATTGAGACGTTAATAAGCGCATGATTGAGAT
>dme_circ_0004408
GTGACCAAGAGGAAATGGTGCAACGCTCCGACTCTGATAAGGAGGATTCTAATGTGGAAATCTCCGATAC
TGCACGTTCTAAAATTAAAGGTCCAGTTCCAATTCAAGAGTCAGACAGTAAATCGCATACATCTGGTCTT
AATTCAAAAAGAAAGGGAAGTGCTTCAA
>dme_circ_0004408_junction_seq
AATTCAAAAAGAAAGGGAAGTGCTTCAAGTGACCAAGAGGAAATGGTGCAACGCTC
>dme_circ_0004409
GttttttttCATCTTCGTCTTCTTCCACTTCTAGTGAAGCTGAGTATGAAGCCATTGATTGTGTAGAAAA
GGCTAGGACTAGTGAGGAAGATAGTCCTCGAGGATATGGTCAAAGAAATCTGAATCAACGAACAACAACT
ATTAGAAACAGAAATCTAGTGGGTACAATGGATGTTATAAAT

>dme_circ_0004409_junction_seq
TCTAGTGGGTACAATGGATGTTATAAATGttttttttCATCTTCGTCTTCTTCCAC
>dme_circ_0004410
GTAAAAACTGAAGATGAAAATCCAAACATCAGTATAGCTTATAGAATTGTTGACGAGACCAGTTTTACCG
AGAATTCTGAAATCATTCAAGAAAATGGATGTACACAAAACGGAGATTCATACAATGCCCAAAACACAAT
GGAAGTGGAATCTCTGAGTCACTATTTACTTTCTATTGTTTATATGCCGTTGTCATTAAAATTGATTTGC
GTGACAAATCTTTTTTGTTGGATGGCGCACGTTTGTTATTCGCTATACTTTACGGATTTTGTTGGCGAGG
CTGTTTTTAAAGGAGATCCCAAGGCCACTCTTGGCTCTCTTCCCCAGAAACGTTATGAAGAAGGAGTACG
TTTTGGTTGCTGGGGCATGGCCATGTACTCCTTATCATGCTCTTTTTACTCACTTGTAATTGAATATCTA
ATTCAGCGATTTAGGGGCTAAAACAGTTTACGTTGGAGGACTTCTTGTCTATTGCATTGGAATGGCTTTG
ATGGCTTTAACTAGAGCCAAACTTAGTGTGATAGTTTTTAGTTGGACAGCAGGTATTATGTACTCTACAC
TATTCACAATGCCTTATCTTCTTGTTGCTCACTATCATAATGTTTCTACGG
>dme_circ_0004410_junction_seq
GTTGCTCACTATCATAATGTTTCTACGGGTAAAAACTGAAGATGAAAATCCAAACA
>dme_circ_0004411
ACGCAACTGGCTGCCGCTGATGAATCGCAAGGGAACGGACAAGCCGCCAAGTAGCAAACCGCTGGTGAAG
AAGCCCTCGGAGAAAAACCTGAGAGTGAGTGCTACCGTTTAACGAGCGTTTATTTATATCATTTGACGTT
TCTCCACGCCTTTCCTTTCTATATCTAGACACCCCAGAAGCACGCCGAGGAGCTGGCCGAGCAGCAAAAC
CAGCAGCCAGGTGCCAGTCCCGCCACCGTGCTGCCATCGTCGCAATTCCCCAGCTCAACTCAGCATCCAG
GAGGAGCAGGAGCCGGCGACTATGAGCCGGACGAGGAGGTCGGCCTCGAGCTGCCGCCGCCCATGAAACC
CATCCAAGAGCCGCACCTGATTGCCAACGGACCGCCCGCCTTTGCCAAGGACGCCAAGGAGAGCTCGGGC
AACATGG
>dme_circ_0004411_junction_seq
GACGCCAAGGAGAGCTCGGGCAACATGGACGCAACTGGCTGCCGCTGATGAATCGC
>dme_circ_0004412
TTCGACCACAAAAGGTCCGATGCAGACCGAACGGCCATCTCCAGACGATGGATTCCATGTCGTACCCATC
CATAGTGATGCGGAACAGCACATCCACAGGAACTCCGATCGCCAGCGGGAGCTGATCCTGCAAAACTCAC
AGACAAG
>dme_circ_0004412_junction_seq
AGCTGATCCTGCAAAACTCACAGACAAGTTCGACCACAAAAGGTCCGATGCAGACC
>dme_circ_0004413
GAAACCCATGCTGATGAATTTACTGAACTTATCCTGGGCCTCCCTTAAAGTGGTCCTTCTGGTTTCCGGC
AGGCAAACGAGGCCAAAGAAGAAGCCAAAGAGATAGAGTCCTCCCATGACATAGAAGTAGTTGGCTCCGC
CAGAAGAGCTCCATCTGATCAAAGTCAACACCAAGAATACCAGCATCTCTCCAATGAAA
>dme_circ_0004413_junction_seq
CAAGAATACCAGCATCTCTCCAATGAAAGAAACCCATGCTGATGAATTTACTGAAC
>dme_circ_0004414
CTATTGACGCACTGAGACGACTCCAGAGGCCGTATACCTTGACCAACGAAACCTTCGAGCAGCTGGCGGA
GCACAAGAAGTATTTGGCCCAGAACAAGGAACTTTCCATGGGTCAGAGCATCGGACAGGCGCTGCCCACC
TTCATCCGACTCGTTTTCCTTCGCGGTCTCAATGCCATGAGCATTTCCAAATTTGTAATGATCGGCTTTG
TTTATTCATTTGCCAGTCCGTATGGATATGTATCGCCTTCGGTGGGTTGGTTCATTGGATTCGGCGTTTG
CCGCTGGATGGGAAACTTTATAGCCACG
>dme_circ_0004414_junction_seq
CCGCTGGATGGGAAACTTTATAGCCACGCTATTGACGCACTGAGACGACTCCAGAG

>dme_circ_0004415
GAACTCGAAAATCATTATCAAGACGGTCACTTGGCTCATATACGTATATACATTGTACTGTGAACCCACG
GCAAACGACAAGCCGCAGCTTACGAGCAGGCCAAAGACCGCAGGCTTCTTTCGGCCCAAGGACTCCATGC
AAAAGGATGGGATTAGGGATCCCAACCAACGGCAAAAGGCTAATCCTATGTACCAGGAGCTCGCATTTCT
TAAATCATTGGAGACTAAGATCGCAACGACATAAGTGATGATCACAAAGCTGGAGATGCTCATAGCATTC
AGGGCGCGCAGGTATGCAAGTCGCATGAAGGTGGGCAGCGCTTTGGAAATGCTTTCACTCTTTGACATAT
TCTTGTTGTAGGCCAAGTAGCGTTTGTGCTCCGCCAGAAGCTCGTAAGTTTCCTCGGTTAGCACCGCAGG
CTTTTGTAGCCGACGCAGTGCGTCAATGGCCGCCTGTTCTTCGTTATTGGCCAGCATTAGAACCGGCGAT
TCGATG
>dme_circ_0004415_junction_seq
GGCCAGCATTAGAACCGGCGATTCGATGGAACTCGAAAATCATTATCAAGACGGTC
>dme_circ_0004416
GAGTGCTGAGCACAATCTACGGCGTTTTGGCCCTTATTATGGGCTCATTTTTTACCATCGAATCGCCGGT
TCTAATGCTGGCCAATAACGAAGAACAGGCGGCCATTGACGCACTGCGTCGGCTACAAAAGCCTGCGGTG
CTAACCGAGGAAACTTACGAGCTTCTGGCGGAGCACAAACGCTACTTGGCCTACAACAAGAATATG
>dme_circ_0004416_junction_seq
ACGCTACTTGGCCTACAACAAGAATATGGAGTGCTGAGCACAATCTACGGCGTTTT
>dme_circ_0004417
CTGGGCAACCAGGAGGCCACCTGGGTGGTAGCGGACTACATTGCCACCTCCGGCAGCAATGAGCTGAGCG
TGAGCAAGGGGCAGCAGGTGGAGATTGTCGAGCCGCCCACCGCCGGAGAGCCAGACTTCTGCCTGGTGCG
CCTGAATCCGCAGCACGACGATGCCGCCGTGCAGGAAGGACTCGTTCCTGTCTCAGTGCTCAAACCGCCG
CCGGGCTCACACAAGCACGGTTCCGGAACCGCAGCGAATGCAGCCTCATCAGCGGGCTCCCAGAAATCCG
ACATGCAGGATCAGAGTAAGTGCATCGATTTTATATACATTTATGGCCCTGTCTGAAATTATATCATTCT
GATTGTACAGGCAATCGCAGCAAGGCGGATGCACTCTCATCGTCCACTAAACGTCGCGGTTTCAGCGGG
>dme_circ_0004417_junction_seq
GTCCACTAAACGTCGCGGTTTCAGCGGGCTGGGCAACCAGGAGGCCACCTGGGTGG
>dme_circ_0004418
GGTATGTTTTGCTCAAAGAACCATTGGGAAATCATTTAAATGCTATCGGTGAATCTTATTCCACACTTTT
ACTAAACTGCTTTAAGCTAGTTTTAATAACAGCAATATACTACTTTACTACGCTAATATTATATTTGTAG
CTTTAACTTTTTTATGCGGAAATTTCTGGTAGAAAGGTGGACAAGTTGGGAAGGGGGGAAGTTGAGCCAT
TCTCATTTACTGCGCCTTAATTCCACAAATTAAAATGCTGTACATGTGGTACGATTGCCATTGGTCGCTT
AATGAGCAAACTGCTGCCATATCGACAACGTCTGGAGTCTAATTTTAGATGCCTTTGGCTTCCGACTTCG
GATGGCCAAGGGATCTGGTATAGGCTGTTGGTTCTGCTTCTGTTTAACCATAAGGTTGTGGGGTTGATAC
ACTGGGAGCACTTTTCAGGCTAGTATCAAATCACTTTAAAAATAATAGATGTTTTTAAAGGGTTGAAGAA
GTGACGAAATTTCTACTTTACTGAACTGAATTTCAAGGATATTACGAAGTCAAGTATATTATGTATTATT
AAAAATGTCCAGCAACGAAAGGCGTTTTTTTACAGTGTAGCACAGACAGTCAAAAACGCAGCATGGGGTT
GTCTGTTTCAACAGCAGCAGAAAGGCaaaaaaaaaaTGAGAAATAAACAGACTCAAAACCGAGTATAGGC
GCTGCCAAAGAGACTGAGGAAACTTTATTAAAGAGGTAGACGCTGCTGCGATGGACGAGAACACTCCACT
CCACAATTACGACAAATCCACAGCTCTCTCGGTACCAAGGCACTCGGAATATCCCAACTCTTTGGTTGAT
AATGCGAACGAACCCCAGAGAACTCAGCGAAACTCGGTGCTGGGGGATAAAAATAGACAGCAGAAAGGAT
ATTGTAGCAAAGTGGAGGAACTCTGAGTGCCaaaagaaacgtaaaagaagaaaaaaaaacgtagcagtgga
aaatctagaggaaaagcagcggcaaaagggaaaaTCTCCGCAGTCGCCATGTGTTGCATTTCAAAGTGTG
TGAGTTATGGGCAGGGGGTAAATGGAATGAAAAACTCCCCGAGCAGAAGAAACACTGCCAGATGAGACGA
CAAATTACAAGATGTCGAGGGGAGACTGTACTATGATGCACTGCACCTGCGAATCAATCAAATTCCAGTG

CAGCGAAACAAGTTAGCTTTCACTAATTGGTGTCTGTATGCGAG
>dme_circ_0004418_junction_seq
CTTTCACTAATTGGTGTCTGTATGCGAGGGTATGTTTTGCTCAAAGAACCATTGGG
>dme_circ_0004419
GCACATGGTGCAGGCGGGCGTAGCGCCTTTTCCGGGCGCTCCGGCCGGCTATGCCGCCGCACCGAATCCT
GGagcagcggttgttgccgcagcggccgccgcccaacaacagcagcagcagcagcagcaacaacaacagc
agcagcagcaggcccagcagcaacagcaacaacaggtggccggaggaccacccagcgccgcggacagttt
gtccatggcggtggcagcggcagcggcCAAACAGTCTGCTGATCCAGTGACCCAAATGAAATCGGGCAGC
GAGGCCGCAGGATCTGGCAACAGCAATAACAACAACACAGCTGGTGCGGGAACAGGAGCGCCAGGCGCAG
CCGGAGGATTGACCACAGAATATTCAAGTGGCGGCTGTGGGGGCGGTGGTGCATCAACGGCCAATTCGGT
GGTGGTGGCCACCAGTGTTTCCGACGTTGTCAATGCCTCGCTGTACATGCAACAGAAG
>dme_circ_0004419_junction_seq
CAATGCCTCGCTGTACATGCAACAGAAGGCACATGGTGCAGGCGGGCGTAGCGCCT
>dme_circ_0004420
GCACATGGTGCAGGCGGGCGTAGCGCCTTTTCCGGGCGCTCCGGCCGGCTATGCCGCCGCACCGAATCCT
GGagcagcggttgttgccgcagcggccgccgcccaacaacagcagcagcagcagcagcaacaacaacagc
agcagcagcaggcccagcagcaacagcaacaacaggtggccggaggaccacccagcgccgcggacagttt
gtccatggcggtggcagcggcagcggcCAAACAGTCTGCTGATCCAGTGACCCAAATGAAATCGGGCAGC
GAGGCCGCAGGATCTGGCAACAGCAATAACAACAACACAGCTGGTGCGGGAACAGGAGCGCCAGGCGCAG
CCGGAGGATTGACCACAGAATATTCAAGTGGCGGCTGTGGGGGCGGTGGTGCATCAACGGCCAATTCGGT
GGTGGTGGCCACCAGTGTTTCCGACGTTGTCAATGCCTCGCTGTACATGCAACAGAAGGTGAGTTTGCGT
GGACAATTGTTTGACCTATTCCAAcccccccccccccccTCACCCATCCTCCGTAAACCTACCGACCGCC
AAACATCCCACTTTCAAACCACCCACCAATACACCGATTCAACCCACTCGCTAGCTATCAGTCTATTTGT
GTTTGTGTGTGTTTGTTATGCATTTGTAGCTGTGTTTGGTTTTTGGCTTTTGGGTTCGCTACGGTTTGGT
TCGGTTTGGTCTGTTTTGGTTTGTAGCTCTGCTTTGAATTGGGTacacacacacacacacacacacacac
acacactacacagatggcgacacgcacacacaGATGCATAGACACATAAACCGCTACACTGAACAAAaac
tgacatacacagaactgacatacacagaaATAAGTGAATTTTTGTTTTTACTATGATTTTAAAATATGGT
GAAAATCTATGACATACCTTTAAATTTATTTAAGTTTCGTAGTGGTTTGCTTTGTAGTTTTAGTTATCAA
TACAAATAACTTTAACATTTATTTAAATAAACTCTTTTTAATGTTATATTTGGTAAATATTTTAGAATTC
CGTTTAAAAGTAATAGAAAGCAATATTTTTGTATGTATTGTACACTGTTAAATGGCTCTAACGGCTTGAA
ATCAAACGTAAAAATATTCCATAATATCGTTGACAATATTTTTGCCAGTGTAAGCCAGGGCATACAAATA
AAAGCGGTGCTTGTTATCGCTATGGCACCTGCTCTTGTTTAGTCGGTATTTGTTGTTGGTGTGAGCGCTT
TGCTTTTACATTTTTAATTGCAGCGAGCTGCACAGAGAAAAATGCCAACAGCAAACAATACTCACGCACT
TTGGCATTcacaaacacacccactaacacacacatgcacacacacacacaTACCGTATTCACCTGTCTCA
CTGCGTTTGTTTGTGTTGGCCGTTTTTGTAGCATACTTTTAGTGGCTGGTCGCTCTCGTTTCTTATTTGA
CCCTGGAGCTGGGTTTCAGTTTGCTGTGTAGTTTATTGTTTCTTCGCTTCTAATTAAAAATTGATTATAG
CACACACGTTGCCATAAAGATGCAAGTGCTTTTCAGAGAATCTCTCAAAAAATAAAAGCATATATTTATA
TTTTTAAGCAACTTTCAAAAGAGCAAAACAGAAATGTTTGTGGCAGATGATGGTCGAGATAGAAAATAGA
AAATCAGCTGAATAAATACAAACCCTTGTTTGGTCATTGACCAACAGATATTTACTTATGAAAATGGATT
CGTGTATTCGAATAATTTAACAGCTAAGAGCATTACTGTATTTTTCACATGTAGGCCAACTTTAAACACA
CGAAAATTGAATTTAGTTGTGCCTTGCAGGTGTAATAAATATTCAAATATTTAATTAGGCGACATAAATG
TGTTTTGTGTTTTTGTTCTTTTGCAACCATTGACTGGCTTTACCCTCAACGTAGTTCACAATTAATTAAA
TTGTGCGCTCTTCAATAAGAACAATACGCATACGCCATGTTAAACAATATTAAACCTCACACTTTCATAA
CTCTATTGTTTTTGTTGTTGTTTGATCGGCCTGCGGCTATATGCACCGGAAATGGCAAATTATTTTATAC

TAAAACATGGGTTGGGAACTTTAATATGCATATTCTAATACGACAGAAGACCGTGGTCAGTTAAAGCAAA
TCATAACTATTAAATCCAGTGGTTAAGTCTCTTGAATGTGCAAGCACTTTGGTCAACACATTTCTATTTA
TTTCCTCTCGTCCAAACATTCAATTACTCAGAAAATTTCATTTAATCAAATGTTTATCAATATTAATTGG
CTAAGCAACAACTTAAAAGGATGTCAGAACTGGAAATATAGATGAAATGTAAATGAAAAGTGGAAATGGA
GACCGAAAAATGAAGAGATGATCTTGGGAAatgtatatatatgttttttggttattattttatgtttttg
tttCAAAGTATTATTTAGATTCCAGCAATGGTTTGTTCAATGCCTTTGATAATCCCGATAATTGTATTT
TCTAATAATGTTTAGAAATAATTGATGACATAATTTGGGAATGGAAATTCATAAGCAAGCATATAGGTTA
TTTATTAATCTGCATTTTGTCACAACTCCAACTTTAAATTATATAAATCTCTATATGGTAACTGTTCAat
atatatatatatTCAAGGCATGTTCCACGCAATTAAGCAGCTGTAAAGCGGTTGAGAGTAAAAAATTAAG
CTACCCATTTAAGCAAATTGTTGGTGTTGGTCGCCTGCCTGTCTGCCTGTCTGGTTTTTAGCCCATTTCT
ACACTCTGTCTCTTAATCCAAGTCCTCCACACAAACACAATCACGCAGATAAACAGAGAGGctgcaactg
caactgcaactAAAACGCAAATCCTTGTTGTTATCTGAAAATTTATCACAGACAAATCTGTCAACACAAA
CACAGACACagtacgagtacgagtacgagtacAATGGAACCAGCCTCGCAGAACCAAGAGTCCCAGGTCC
CATTCCGCATCCACTGACAAATGGACATGCCGTGGACCAAGTGCAGCAGCCACCAGTACAACAGCAGCAT
CTGCAATCATCGCTGCCCCCACAAAATGTGCTCAGCATA
>dme_circ_0004420_junction_seq
GCTGCCCCCACAAAATGTGCTCAGCATAGCACATGGTGCAGGCGGGCGTAGCGCCT
>dme_circ_0004421
GCACATGGTGCAGGCGGGCGTAGCGCCTTTTCCGGGCGCTCCGGCCGGCTATGCCGCCGCACCGAATCCT
GGagcagcggttgttgccgcagcggccgccgcccaacaacagcagcagcagcagcagcaacaacaacagc
agcagcagcaggcccagcagcaacagcaacaacaggtggccggaggaccacccagcgccgcggacagttt
gtccatggcggtggcagcggcagcggcCAAACAGTCTGCTGATCCAGTGACCCAAATGAAATCGGGCAGC
GAGGCCGCAGGATCTGGCAACAGCAATAACAACAACACAGCTGGTGCGGGAACAGGAGCGCCAGGCGCAG
CCGGAGGATTGACCACAGAATATTCAAGTGGCGGCTGTGGGGGCGGTGGTGCATCAACGGCCAATTCGGT
GGTGGTGGCCACCAGTGTTTCCGACGTTGTCAATGCCTCGCTGTACATGCAACAGAAGGTGAGTTTGCGT
GGACAATTGTTTGACCTATTCCAAcccccccccccccccTCACCCATCCTCCGTAAACCTACCGACCGCC
AAACATCCCACTTTCAAACCACCCACCAATACACCGATTCAACCCACTCGCTAGCTATCAGTCTATTTGT
GTTTGTGTGTGTTTGTTATGCATTTGTAGCTGTGTTTGGTTTTTGGCTTTTGGGTTCGCTACGGTTTGGT
TCGGTTTGGTCTGTTTTGGTTTGTAGCTCTGCTTTGAATTGGGTacacacacacacacacacacacacac
acacactacacagatggcgacacgcacacacaGATGCATAGACACATAAACCGCTACACTGAACAAAaac
tgacatacacagaactgacatacacagaaATAAGTGAATTTTTGTTTTTACTATGATTTTAAAATATGGT
GAAAATCTATGACATACCTTTAAATTTATTTAAGTTTCGTAGTGGTTTGCTTTGTAGTTTTAGTTATCAA
TACAAATAACTTTAACATTTATTTAAATAAACTCTTTTTAATGTTATATTTGGTAAATATTTTAGAATTC
CGTTTAAAAGTAATAGAAAGCAATATTTTTGTATGTATTGTACACTGTTAAATGGCTCTAACGGCTTGAA
ATCAAACGTAAAAATATTCCATAATATCGTTGACAATATTTTTGCCAGTGTAAGCCAGGGCATACAAATA
AAAGCGGTGCTTGTTATCGCTATGGCACCTGCTCTTGTTTAGTCGGTATTTGTTGTTGGTGTGAGCGCTT
TGCTTTTACATTTTTAATTGCAGCGAGCTGCACAGAGAAAAATGCCAACAGCAAACAATACTCACGCACT
TTGGCATTcacaaacacacccactaacacacacatgcacacacacacacaTACCGTATTCACCTGTCTCA
CTGCGTTTGTTTGTGTTGGCCGTTTTTGTAGCATACTTTTAGTGGCTGGTCGCTCTCGTTTCTTATTTGA
CCCTGGAGCTGGGTTTCAGTTTGCTGTGTAGTTTATTGTTTCTTCGCTTCTAATTAAAAATTGATTATAG
CACACACGTTGCCATAAAGATGCAAGTGCTTTTCAGAGAATCTCTCAAAAAATAAAAGCATATATTTATA
TTTTTAAGCAACTTTCAAAAGAGCAAAACAGAAATGTTTGTGGCAGATGATGGTCGAGATAGAAAATAGA
AAATCAGCTGAATAAATACAAACCCTTGTTTGGTCATTGACCAACAGATATTTACTTATGAAAATGGATT
CGTGTATTCGAATAATTTAACAGCTAAGAGCATTACTGTATTTTTCACATGTAGGCCAACTTTAAACACA

CGAAAATTGAATTTAGTTGTGCCTTGCAGGTGTAATAAATATTCAAATATTTAATTAGGCGACATAAATG
TGTTTTGTGTTTTTGTTCTTTTGCAACCATTGACTGGCTTTACCCTCAACGTAGTTCACAATTAATTAAA
TTGTGCGCTCTTCAATAAGAACAATACGCATACGCCATGTTAAACAATATTAAACCTCACACTTTCATAA
CTCTATTGTTTTTGTTGTTGTTTGATCGGCCTGCGGCTATATGCACCGGAAATGGCAAATTATTTTATAC
TAAAACATGGGTTGGGAACTTTAATATGCATATTCTAATACGACAGAAGACCGTGGTCAGTTAAAGCAAA
TCATAACTATTAAATCCAGTGGTTAAGTCTCTTGAATGTGCAAGCACTTTGGTCAACACATTTCTATTTA
TTTCCTCTCGTCCAAACATTCAATTACTCAGAAAATTTCATTTAATCAAATGTTTATCAATATTAATTGG
CTAAGCAACAACTTAAAAGGATGTCAGAACTGGAAATATAGATGAAATGTAAATGAAAAGTGGAAATGGA
GACCGAAAAATGAAGAGATGATCTTGGGAAatgtatatatatgtttttggttattattttatgtttttg
tttCAAAGTATTATTTAGATTCCAGCAATGGTTTTGTTCAATGCCTTTGATAATCCCGATAATTGTATTT
TCTAATAATGTTTAGAAATAATTGATGACATAATTTGGGAATGGAAATTCATAAGCAAGCATATAGGTTA
TTTATTAATCTGCATTTTGTCACAACTCCAACTTTAAATTATATAAATCTCTATATGGTAACTGTTCAat
atatatatatatTCAAGGCATGTTCCACGCAATTAAGCAGCTGTAAAGCGGTTGAGAGTAAAAAATTAAG
CTACCCATTTAAGCAAATTGTTGGTGTTGGTCGCCTGCCTGTCTGCCTGTCTGGTTTTTAGCCCATTTCT
ACACTCTGTCTCTTAATCCAAGTCCTCCACACAAACACAATCACGCAGATAAACAGAGAGGctgcaactg
caactgcaactAAAACGCAAATCCTTGTTGTTATCTGAAAATTTATCACAGACAAATCTGTCAACACAAA
CACAGACACagtacgagtacgagtacgagtacAATGGAACCAGCCTCGCAGAACCAAGAGTCCCAGGTCC
CATTCCGCATCCACTGACAAATGGACATGCCGTGGACCAAGTGCAGCAGCCACCAGTACAACAGCAGCAT
CTGCAATCATCGCTGCCCCCACAAAATGTGCTCAGCATAGTGAGTCAGCAACCAAACaaaaaaaaaaaa
ttataataaaataaacagacgaggaaagcatacaaaaaatgaaaaacaaaccatacaaaATAGAGAAGAG
ACGGGTGGACAATTGAAATTTACGTGCCAGTTTCTCGGTCTTACCACGTTTTTCTTTCTCACATTTTATT
CTCCTATTTGTTTACCAAGCTCATCTTCATTAGGCGACCAACCGACGACTGGCCATGGGTAAAGCTGCTT
TTTTGCCTCAACTTGAACTCTTTTGTAACTGCAAttttttttATACGCATAGTCATCATCAGGGAAACAT
TGTTCGTTATGCGTGGCAAAAGCAGATGCTGGCCAAGTTTCTTATAATTAATTGAGTCGGTGAAATTAAA
TTCCAACTGTGGGTACTACAGCTCAACCAAAATGCGATTATAGGGATTTGTGTACACGTTATGTAATCTT
CATCAAATATGATGTCCTGGTGCGCTTCGTCACTATGCCAAAGGGAATAATTTATTTGATATTACTTGGG
TTTTTATCATTTTCCTATCAATAATCCAATCAAATCTCGCCATCTTGCCGTGCTAGCTTAAATTAAGTTT
TAGCAAATAAATAGCTCCAATCTGATTGGATTGTAGTGCAAAAGTAAGGCCACACAATGCATTCAAAGTT
CAAGAATAATACCCAAGTCAAATATTCAAATCAAAACAATTTAGTTCAAATTTCTGGCCCtttttttttt
GCCGGGCAGAAAGTTTTCAAATTTATGCAGAAATTCTTTTAATACCAATTCAAGCCGAGCCGCAGAGACC
TTTTTTCAGCCTTGTCGCGTGCCGCACAAAAAGCATTTCGATAAACTAAAAAGCGTTAAATGTCATTTAC
GTGTAACCCGATACCCGAAACAATAAACCCTCCGCCACCTCCGCCAGTTGGCCATTTATGGGGCCAAAGT
TTTTCACGCCATTTCACTTTGGGCCCGAAACGTTCGGTAAACATGCAAACCGAGTACAAAAATGAATAAA
AAAAGCATAGGAGGAGGATGCAAAGCGTGGAAAAAATATATTTTGTTACAGGGCGGTGGAGAAATTTGTT
TTAGATTTATGTGTCAACAATGCGCAGCAAATTAAGTTAAGCGTTATTTCTATTGCCTACATTATGGGGC
GAGCGCAAAATCTGTTTATATTCCTTGTTTTTTTCTCTGTCTACTGTTCATTCTGGTTTTTATTGCATTG
TCATCGAATTTGGTCGCCGGCGTAAATTGAGAATATTTCATATTATTCCGGATCGGGGAAACTGCCGTAG
GTGGTGGGGGAGTTGGGGCATTGGAACAACAATAGCATACCATGCCATGCCATCGATGGCAAGTACATTT
TCAGTTTTAATAATGCAGAAATACAAAATGCAAACATTTTCGCGGGCACGTTGGTGGATTTTCTGCTGCG
CTGTGGTGGATTGGGTATATTGCAACAGTTTGCGCGACAGTTTTATTGCATAAGTCAACACAAGTTTGTG
CTACAAAATACGTACGTACATTCATAAgtgtgtgtgtgtgtgtATGTATGGGACTTCCAGCTACTATTTT
CTCTGTTCTGTGTCTGTATGTATTTATATCCCCGTGTGTGCATATATATGACttttttttATTGGGTCAA
ACCcacatacacacacacacacacacttacacaTGTATGTATATACAGATGTGTATGCCGAGTTTTTCCG
CACCTCTCGCAGGATTTTCATCTCTCCCGGGATGTAAACCACTTGAAAATTGTATTTTCATTTCAATTGT

TTATTTATTGCGGCACTTGTTCGCATTCGATTTGCCAGGCAGCACTGAAAATTCAACTGCAGAAAAAATA
CACGCAAATCAACTGCAAACAACAACGGCATGTAGGTGGAGTGCTTTTGAAAGTTTCACAAAAATCGCTT
TTCCACCAAATGATATTGGAAATATTTAATTTTCACTTTTGCCGGTTTTCGCGAACGCAAATTTTTAAGG
CAAGCAAGATAACATCGTCAAATAGAAATAGAAATCGGAAAGATTTATTATTTTAATAGAATTGCAGTAT
ATTGTTAAAATATGGAACATTGGAAAATTTAAGCATTTTTCTTTTAAGTTATTATATTTTAAAGTAAATC
GTATTTTAAATTATGTTAATGTTAATACAAACCCCCACTAATTCATTTATTATTTTATGTTCTTCCTTGC
AGGTCCACAGTATTGATCGCCAATGAGGCAGCAGAATCACAACAGAGCTCCGCCATGCAAAATGCcggcg
gcggtggaaatactggcggcggcggcggcggaggaggcggcggcACTCCCAGTTCTCCGCTCAGCAACTC
CCCGTCCAGCGCAACAGCCTCGCAGGCGGGCGGATGCGGACTGACCCTCAATGGCAGCGCCACCGAGGGC
AGCATGTCCGGTGATACATCGCCGGTGGCCAGTGGTGAACCGCTCCTGCAGACGCCGCCCGCCCACcaac
agcagcaacagcaacaacagcCGCTCCTGTGCAGCAGTCCCACGTCGATGCAATCGTCGGGCACCTCCGT
GACGGGCAGTTCGATAGCTTCCGGCACATTGGCGGCCACCAGCAGCAGTGGCGTTGGACTTTTGCCCACC
ACCGGATTGGACAGCATTGCCAATGGTGGTGCACCAACAGGCTGTGCCGTTGTGCCGGCCAGCACATCGC
AGGTGATCGCCCACCTGAATGCAGCTGCCGCTGCGGCCAGCGGCATCGTGTCGCCCAGCGCCAATGTGGC
CACTAGTCTCAGCAGCGCCCTAGTCCCGGCCCAATCGGTTGCCGCCGTTGCGGCCGCCTCCCTCGATGCC
AAAAGTCAGCCGAAACGGCTGCACGTCTCCAACATACCGTTCCGCTTCCGGGATCCCGATCTGAGAGCCA
TGTTTGGGGGTGAGTACGGATGTGGTGGATTGAATATATTTTATTTTTAGTACCCTTTATGAAGAATCAG
ACCTAGAAAATCAGACCTTCAGGAAAGAAATGAAATATTGAAAAAGTAAATAGAAACTGACTGTGTCTCT
TTAAATAAAAATGTTTACCTCTTTCTATCGCGCTCATAAAATTCAAATTATACACGGTACGAAGATCTGC
GTTAGACACTTCTTACAAACTTGACTATCCCTTTGAAAGCATTTCGAAAATTAATATTTATTATTTTTCT
ATTCCCCAGGGTAAGTTGCGCCAAACTTTCTATACTTTCTAGAAAGAAGCACGCCATCTCTTTCTCTGCA
ACTGTATCGCCTGCCTGCTCGCTCTCTGTGTACCTCTCTGTCTCGCTGGTGTCGCCATCTCTCTCTGCCG
CCAATTGTGCGCGGCATTTTGCATCTAATTGTCGCACAATTCATTTGAATTATGCACTTTAAGAATTCCC
AGATGAGGAGAGATGTCTTTGTCCGTCGCCATGGCAACCTTGTCGACTTGTCTTTTTTGCAGCATTATTA
TTATGCCCGGACTGCAGCTGGAGCCAGGTGAAAATAATTGCATAGTTGCCATAGCACCAGGAATTGTGCA
TAATTCAGGAACGAAGCCCCCAACACCTTGAGATGCTCAAGGAGTTTTTGGCGAAAGAATGAGTGGAGAG
CGTGGCAGGCAGGCGAGGGAAGTGGCGGAATGAGTAGTAGGTTGCAAAAGCAGGGCACGTGCATGTCGCA
GTCGCCAATCCaaaaatataaaaaaaaaaCACCCAGCCCCAAAAATCCCAATCCAACAATCCCGACCCAC
TTCAAACCACCCCCGGAGTACAAAGATGTGCCAATGGCCCCTGTCCGAAAAGCATGTCCTTGGCCGCCTT
GTTGATGTATTTGTAGCTTTAGCTTTATCTATTTAAAatatacataatatatatatatatataCATTTAA
ATATACTACGACTTCAACAAAACCCGAAGAAGAGAGCTGACTTTGTTGTACCAAATCGTTTAAttttttt
agttttttGAAAATCGTAATCTATATACTCTATATGTTTCTTTAATTGCATGTTTGATTGCGAATGGCAG
GGTGGGTGCATGCCATATCATATATACAACTTACCAACCAacacacacacaccacacacaACTAATATAT
ATTATGTAGAATGTATCACAGGAGTGGCGGAAAGAAATTGATTTTCAACTTTTAATCCTTGGGTGCCGAA
GAGATATGGCTTTAAAAATCCACCATCCAACTACCCTTTTTTATTTATCTGTTTTCATTTAATCCAAGGA
ATAGCGCACAGTGATTGATTCTTATTTGCCTTTCATTAAAAATTAATTACGCATTTCTCATTTTCGCCTT
CCCTTTTGATTAATGCTCACTGGGTGAGCCGCAAATAATTGGCATTTAAAATATGCTGAAAATGCGCAAA
GTACGAAAAAGTACGTCTGTCCGTCGGTTGGTTAAACAAATTAACTGAGCCGCATAAAAGGCTGAAATGG
AATGTTATTTCCCCAGCAAATGGCAACGGAAATCGCGCGAGTCTAATTAAGACCCAGTGTCCACAAATGG
ATGCCATACATGTATGCATTTTCATGTGTGTGCGGAGCAACCGGAAACGGAAATGATCGTCCGTCCGCTC
ACTGAAAGCGTTCAAAAATTAATTTTCTCAGTCGAAACAAATGGAATTCAAAAAGTATTCAGTATTTATG
TAAATACTCTTCTACATAAATGTTTTCTTTGTTACTTTTTATATGAGCTTAAACGGATAATAATCATTCG
GTTTTTTCATAAAATTACAATAAATGAACTTTTAAAGGCCTTTCCGACACACACGCACACTTCCACAAAC
ACTTTCAACACTATTTGCAAAGATTTTCGTAATTGTTAACTAACACATAATTTATTTCTACATTTTCTAT

ACATGCGAAATTTaaaaaaaaaaaTTATATATATATATACTCTGTTCATATGTGTGTATATGATATAATa
aaaaaaaattttaaaaaTTCATCACCAAACCAACAACAACAACTACAACCACCACGCTCGCTGATCTATCA
AAAAATTAAAACAAATAACCAATTGTGAAGGTGAATAACGCCACTGCACGTGTACAAACCAAAAAAGTGA
CAGCAGTACCCAACG
>dme_circ_0004421_junction_seq
ACCAAAAAAGTGACAGCAGTACCCAACGGCACATGGTGCAGGCGGGCGTAGCGCCT
>dme_circ_0004422
GGGATTCGGTTTTGTAACATTCGCTAACAGCAACGATGCAGAACGAGCACGCGAACGTCTACACGGCACC
GTGGTTGAGGGACGCAAAATCGAGGTGGGTGCATGCCATATCATATATACAACTTACCAACCAacacaca
cacaccacacacaACTAATATATATTATGTAGAATGTATCACAGGAGTGGCGGAAAGAAATTGATTTTCA
ACTTTTAATCCTTGGGTGCCGAAGAGATATGGCTTTAAAAATCCACCATCCAACTACCCTTTTTTATTTA
TCTGTTTTCATTTAATCCAAGGAATAGCGCACAGTGATTGATTCTTATTTGCCTTTCATTAAAAATTAAT
TACGCATTTCTCATTTTCGCCTTCCCTTTTGATTAATGCTCACTGGGTGAGCCGCAAATAATTGGCATTT
AAAATATGCTGAAAATGCGCAAAGTACGAAAAAGTACGTCTGTCCGTCGGTTGGTTAAACAAATTAACTG
AGCCGCATAAAAGGCTGAAATGGAATGTTATTTCCCCAGCAAATGGCAACGGAAATCGCGCGAGTCTAAT
TAAGACCCAGTGTCCACAAATGGATGCCATACATGTATGCATTTTCATGTGTGTGCGGAGCAACCGGAAA
CGGAAATGATCGTCCGTCCGCTCACTGAAAGCGTTCAAAAATTAATTTTCTCAGTCGAAACAAATGGAAT
TCAAAAAGTATTCAGTATTTATGTAAATACTCTTCTACATAAATGTTTTCTTTGTTACTTTTTATATGAG
CTTAAACGGATAATAATCATTCGGTTTTTTCATAAAATTACAATAAATGAACTTTTAAAGGCCTTTCCGA
CACACACGCACACTTCCACAAACACTTTCAACACTATTTGCAAAGATTTTCGTAATTGTTAACTAACACA
TAATTTATTTCTACATTTTCTATACATGCGAAATTTaaaaaaaaaaaTTATATATATATATACTCTGTTC
ATATGTGTGTATATGATATAATaaaaaaaattttaaaaaTTCATCACCAAACCAACAACAACAACTACAA
CCACCACGCTCGCTGATCTATCAAAAAATTAAAACAAATAACCAATTGTGAAGGTGAATAACGCCACTGC
ACGTGTACAAACCAAAAAAGTGACAGCAGTACCCAACG
>dme_circ_0004422_junction_seq
ACCAAAAAAGTGACAGCAGTACCCAACGGGGATTCGGTTTTGTAACATTCGCTAAC
>dme_circ_0004423
GCCGCCGTTGCCGCCGCAATGCGTGGAGTGGCCATCCAGCGCGGACACGTGGGCGTGGTCGGCGCCACCC
CCTAccatcatccccatcatccgcatcatcATCCGGCGCTGCTGGCGGCCTCCGCCGCCGCTGCCCAACA
ACAACAGCAACGCCAACTGGCCGCCGCCGCCGTGGCCACGGCAGCAGTCGCCcaacagcagcagcagcag
cagcaggctgttgtacagcagcaacagcagcaagtggctgccgccgcccagcagcaacaccagcagcagc
agcagcagcaacaacaggccgtgcagcaacaacaggcggtccagcagcagcagcaacatcaacaacaaca
gcagcagcagcaacaacagcaacatgccgccgtagcagccgccgcagcTGCCGCTTCGCATCCGCACATG
CATGCCGCTcatgcccatgcccatgcccacgcccTTGGTCCACAATTGGCGCAACTGCAAGCGGTCGCCG
TGCCCACGGCCGCTAGCAATGCTGCTGCACTGCAGCAATCCCTCGCCGCTGCCATCCAGAATCCGAGCGG
TAATCCGAACGCTGCTGCCGCCGCCGCCGCCTATGCCGCCCGTCTATCGGCGGCCACGGGTGCCACACAA
TCGCCGCAAACAGCCGCTGCTGCTGCGGCTGCTGCTTCCATGGCTGCGTCGGCCAATGCGGCCAACAATG
CGGCCGCTTTGCATGGATTCGCGCC
>dme_circ_0004423_junction_seq
ATGCGGCCGCTTTGCATGGATTCGCGCCGCCGCCGTTGCCGCCGCAATGCGTGGAG
>dme_circ_0004424
GCCGCCGTTGCCGCCGCAATGCGTGGAGTGGCCATCCAGCGCGGACACGTGGGCGTGGTCGGCGCCACCC
CCTAccatcatccccatcatccgcatcatcATCCGGCGCTGCTGGCGGCCTCCGCCGCCGCTGCCCAACA
ACAACAGCAACGCCAACTGGCCGCCGCCGCCGTGGCCACGGCAGCAGTCGCCcaacagcagcagcagcag

cagcaggctgttgtacagcagcaacagcagcaagtggctgccgccgcccagcagcaacaccagcagcagc
agcagcagcaacaacaggccgtgcagcaacaacaggcggtccagcagcagcagcaacatcaacaacaaca
gcagcagcagcaacaacagcaacatgccgccgtagcagccgccgcagcTGCCGCTTCGCATCCGCACATG
CATGCCGCTcatgcccatgcccatgcccacgcccTTGGTCCACAATTGGCGCAACTGCAAGCGGTCGCCG
TGCCCACGGCCGCTAGCAATGCTGCTGCACTGCAGCAATCCCTCGCCGCTGCCATCCAGAATCCGAGCGG
TAATCCGAACGCTGCTGCCGCCGCCGCCGCCTATGCCGCCCGTCTATCGGCGGCCACGGGTGCCACACAA
TCGCCGCAAACAGCCGCTGCTGCTGCGGCTGCTGCTTCCATGGCTGCGTCGGCCAATGCGGCCAACAATG
CGGCCGCTTTGCATGGATTCGCGCCGTAAGTAGAGTCCTCCGTCGTGGACTTCACTCCGTCACACCCGCG
ATCCCGTCGTAGTTAGTCCGTTGTAGCTGTTATTTTGTACTTTAGTTGGTTCCGCTGCGAATGTAGTTTT
AGAGCACCCAAGTTTACAATTATCATCACTCCGTTCATAAATAATACTACGTTTATACGCACGCCTTACA
ATTACGAATGCTTTTATTTAATCTGTTTCATTTCAATTTGCGAAAGAAAATTTTTAGCTTTCTTTAAACT
TTTTATTGGGCCAATTTTTTAGATGTTACCGTTCTTACTCTGTTCAGTTAAATATTGATATTAATAAAGC
AATCAAttatgaagttaaaatgtataaattatttataaaaaaaaattacaaatatttatttattaaCCATG
AATTTTGGCGTATAAGCGTAGTAATATCCATAAATTGTATAATTATTTCCCAACCCCTTAATTTTTTCTG
TGGTGTCTGATTCGTAACCGCAGAGGAACATCCCGCTCATCTGTCATGATTTTAACCTAAAGCTTAAATT
GTTTATAGCTTAAAGATCGTTTTTCCTACCGTCTCTATTTTTATGTGTTACTATATACGTCTCTATCTTG
TTGGTGACAAATGTTTTTTAGCTATCTTGTGTGTGTAAAGTTTTAATATCGCATATAATGAAACCTAGAC
TTTGTCTTATTTTGTATACATAAATCGAGCAATAGAAGAGTATAACGATCCGAACGCAAACTGAATACAA
AATTTGCAAAATCAGCAACCGATGAACGCACGCTAACACACAAACATCATTCACGTAAACTATCTAAGAC
TCTATATATAAATACACACTCGCATATATTCTTTGATCATACTTTGATTGACAGAAGTGTGTTTTTGGCA
TCGTTAATTAATCGTTACTTTGGTTAAGGGACCTACTCTACACTCGGTTGTATATTGTGTACCACTAGGC
GTATGATGAATTTCGATTTGAATTTGACTTGAAAGTGTTTAAATGCGACTGCGTCGCTCTCTACCAGCGC
CTTGCGTGCATGTTCTGCCCCGACATtatatatatatatatatatttatataGACCTTTGATTAACTTAT
TCTTTTGTGGCTGCTCTCTacacacacacacacacacacatacatacacaaacacacactcacCCGCCCA
GCCATATATCAAACCTTACACCATAGCGTACGAATGAATGCTCCATTAAGAACTGTATACTTATCAACAC
ACATCAAGACATGCCAAACCAAAGAACCAATTAAAAATATTaaaaaaaaaaaaaCTACAAAACTAATTAG
CTAGGTGCATGAGTGTCTGTTCTGTCTAAAGATTAAATGCAAATTTAAAGCAAGCAAGCAAGACAAATTT
TCGTAGTGTTTGGGAAATGGAATGACCCTAATGTATGTGGTTGGTTGGTTGGCTATCTTTGCTAGTGCAA
GTGCTCTTTATAAACGACAATATATCTAAACCTTTTAATTTCCCTTTTGTTTCGTTTCCTCTGTCTCAAA
CATTTCCTCCAAAATCAGTTCGTCATCAAGTGGGGAATCAGGTCAGATCAGGTCAGTTTAGCACAGGCTA
TTTAGCTAGGTAGGTGGGGTGTCTATAACAATTAGGTGCCAACTTTTGTTTTTCAGTTGCCCCTTAAGTT
CCAGCCATTTACCCACAACAGCATCCGGTACCAAGaaaaacaacaacaacatttaataaaaaaaaaataa
acaaaaacaactaaaaCCGGATGTGGATGAAGTTTTATTGGCCAGCGAGAAATATTTGAATCAAACtttt
ttgtatttctttttttttCGAAAACAAATAAACTTTATCCCGTCTGGTTTTCCATTGTGCCCAAATCAAT
GCCCACACAAGTGCGTTAAAAAAATGAACCAAAAAGAAAATTTTTGAATTTTGCTTTTAAGTGCCAGTAC
AGTGAAGCAGAGGTAAGCCAATTAAAATGGATATAGCGTATATATTCCTTTTGATTGGATGCCTCTGGCT
TGACTCACCTACCCAGAAATGATCCTATCACAAACTCTTTATTAATATATGATCTTCTCTTATCATCTTG
TCCCAAATGCAGTGTATACTATGATCCCTTCTTAGCAGCCGCTGCTTCCGCTGATCCAAATCTACGCTTC
CAG
>dme_circ_0004424_junction_seq
TTCCGCTGATCCAAATCTACGCTTCCAGGCCGCCGTTGCCGCCGCAATGCGTGGAG
>dme_circ_0004425
TGCTCCAAATAAATAAAACAACAAATTCCAAAAAAGTGCTAATTAATAACGCAACGAAATAGAAGCAAAC
ACCAATACTACGAAATCCCCCAAAACTACAAGTATTAGTTAATCAAAGAAAATGGGAAAGCTGCTGAGCC

TGCTGTCACGCGACGACTCCAATTGTTGCGCCGCCAAATCCTACGATGTCTTCCTGGATTTCGAGAACGC
CGCGCCCACGGAAACGGAGCGTGCCGTCTACGACGAGGTGGAGCGGGTACTCCGGGAATCGGAGGTGGTG
CTCGACGAGATACAGATCTATCGGGGTGAGTCTCAACTGGTCATATGCGTCATTAGCCACGGGCTAAACC
GCAACCCCTGCCAACCGGTTATTGGGCATTTGTCCGATGATTCCTATAGTTCGAGTCACTAAATGGTGAT
CAAAACGATTTTTTAAAGAAAATATCAGTACTTTATATATAACATAAATGTAAAAAGTACTTTTAAAAGG
ATTATAAGCATTATAAAATTTTAAATGATATATTTTCAAGGCATTTATGCATTATCAAGCTAGTTTAAAA
ACTATTTTAGGTAGTTTGATTATGCCTGGGCTTCCCCGAAAAGGGAGGTACTGTATAAGACTGATAATTT
CAACAACTTGCTAAAAATAACAACAATCGCCGGGAAATTGAAAGCAACAAATAAGAGCAACCATAAACGA
GCAACAAATTGAGTATTTAACCTTTTGCCAGGGTGAGAAAAGAGCGGCACTGAGCAGAGCGCTATCTTTT
GCTCTCTTTAAATGCTGTTACTCCAACGCCCTCTGAAACTACTAAAAATAAATGTAATACTATTATTAAA
ATAATAAACAAATGATTGTCATTATGTACACGCTATTTTGGTAATAAAACCTAGCATTTTATGGCCGCAT
TAAAATTAGCATGTCAAGACTGTAAGTAAGCGTAAATATTTTGTTAACTATAGATCACTAGGCTTTAGGC
GCCTATACTTAGAATCCATTTATTAAATATCCCTCAGAGTAATAGATTTGAGTAGACTAATTAGTTAAAC
TGGCCCTAAGAAGAAGAGCATTTTACGATGTATCCAATGTGGCTCCTCCAATGTGAAATGCCCCCCCAAA
CATGGATCTCTTTGATTGCCTGACTCACGCACGTGCCCACATGATCCGATCCGGTGAGCGGACTTCTTTC
ATTATCGTCGCTCCACTGACGTCATTAACCAACTTTTGCCGCCGCTCTTCGCACTCCCATAATTCTCCTT
CTCCGTCTCGCGGCGGGAAGAATTAGTCACCCCATTCTTCGTTACTCACACAGaaaaaaaaaTGTCTAT
GATGGGTTTTGTAACTACTCTTTTATCAACAATAACTTACGCCTTTTAAAAACTGATTAGCATAGTTTTC
TTTTCTGTCAAAAAAAGCTGACCTAATAATGCTGTCATTAAATAGTTTGGACATCTTTGATTTGATAATG
ATAAGATATGATAAACTTATGTGAATCTGATAAAGATATTTGCGATGTGTAAAGCTAAAAGTCCTTAACT
GAGAACCCTAAAAATAACTACTACAATATATGCAATTTATAACGATTTAAATTGAATACAAAATTATTGG
AGCATATAttttttttCTGTGTGCATTTGACCTTTTGGCGACAAATTCTAACTCACATTGGTGTGTGCAA
ATTATTAATTGGGTTTCAGACGCAAAAATAAAAAGAAGACGGTCAAAGCGGAAAAAAGGGTAGTTAAAgg
gggggggggTAAAGGGTGGGGGTAGGTGGAAATGAGGGGCGGCAGACAAACAAATGTCAGGCCGAAAAGC
TTTAATCAACGCAAATGAACTGGAAAATGTATACCAATGAGCTCATTTCCTTTGTGTTGACCCACTTAAA
TGGGACCGAAAGCAACAAAGACGACCTCGTGGGGCATTTAACCCTTTGGTGCAGCGATTCCCGTTCGCAT
TTGTTTCTTTTTGGTTCTACACCAGAGGTATTTAAAATGCCCGCTTAATTTGCGGGCTGCATTTTATTTT
CATTGCAATTTTTATTGGACTGCTCTGTGCGCGTTCATTTTGTTAGCCCCAAAAAAGAATGAGTGGGTGG
CTTTTGGGTTGGCACAACTGGTTGTCAAGGCAAACAAACTGGTTGTGGAGTAGGTGATTCTCCACTCACC
CCCTCATCCCCCTCACCATCCAAACAAAAATAGTTGGCCAGGCGCCAAAAGCTGGCAAAATGGCAAAAAC
TTCATGTTCTGTTTGGTTGTTTTTGTTGCACTTATTCCTGCAAGTCGGTTGCTATAAATAGAAAGAAAAG
TGCAAAGAGGAAAAATCAAAGCttttctttttatttttttGCACCCCGGCATTTCTTGTTTCTTTAAGTTG
GTAATTGACATGGCTTTAAAAGAAATCGTTTATTGCGATTTCTGCTTATAAATATTTAACATTTCACGCC
TTTATAAAATATTAGTGATTTGTTAGGATTAGGGTACAAAAATTAGATCAATCCAAAAAATCATCTACAA
TGGCATTCTTTTCATTCGTCGCAAACATTGTGCGACTTTTTCCCAGCGGTAATAGGCTTTTCTATCTGAT
TTATCAGCACTTGTTAGTGACGCTTTTGTTAAATATTGGCCTAGACTAGATATGGCATACAAGGCGCCCA
TTGTGTGTGCGTAATAAACAGTAAACAACAGGGTCAACAAGGGGCCGCAAATAATAAGAGTAACAGAAAA
AGTCCCATTTCGGTGCAACAGATGATAATCATGATGATAATGCTTACGCACTCACCTGGAGGAAAATGAG
AAAAATAACAATAAATGTGAAATGACTATATTATATTAACAAGCAACAACGTGTGGGTGCTGCACATGTA
ATAAAAGTTTGTCGGTTTTGTGGGCCAAAGTCATTGAAATCCAATAAAATGCTTTGATATATATGTACAT
ATATAGACTATGTGAATTAAGCCATTAGTTTTGTGGTTTTCCATACCGCTGACAGTCTGGGCTAGATAAC
GATTACATATGGCCAGACAAACATATGTCAAACACATGGAGTAATCCAATACACAACCTTGGATGGCCAA
ACCCACATGAGAAACAAGTGCAAAGTGCTCCATATCAGCTAGGTTTATTGTGGTTTGGATGGCCATATTA
CGCACGATATGGCTTTAGATTTGATGTATAATACATATGTATATACTTACTCTCAGTCGAAATGAGTTTG

ACCACAATTGAGTCATTCTGCGAATGCTTTTTTATAATTTAAAGAATATGTATCCAACAGTTTTTGAGTC
ATTAAAATGATATCCGCCCTTTTTGCGTTACGCAAACTTAAGATTACATATTTATCAATTTTAAGAAAAA
AATGTATATTTTATTCTGTACTAGGAAACTATTCCAAAACATAACTCATATAGCTTCATTTCAATTTCTA
GCCTAAAGCAACTGAAACTTGCCTATCGAATAGCATAATGATTTGTCATATACTTCCAAATTTTCCCACA
AACTCAAAACTCAGTCACAGCTGCTTTCACTCGAAAAAAAGGGGAAAAGCCCCATTTCAAATTGGCGACC
CATTTCGAAAGATTGCACCACATACAAATaaaaaaaaGAACGATTTGGCATCGTATGAATAAAAATATAT
GCACATTTAAGTATTTAATGAGAGTTATACACTCGAGGGCGAAAGGCAGACAGACAGACAGACAGCCAGC
GGCAATCAGAATAATCACAGAATCGGAATGAGCAGTCATTAGATAGAccccccccccccTCCCAATACCA
CCCTACCATATAGAATACCTCTCATCTCGGCACTCCCACCTCTTCCAGTTTCCAGTTCCCATTCCACGAA
TAACTTGAATGATGTGCGCGAATGCAAAAGCACAAGCGGGAAATAAAAGCCAACTTCCGGTTGGTCATTC
AGTTTGGTCAGTCGATATAATCGCTATAATGTGCGTGCCAGAGATCGGCATTTGAATATTATAAAATTAT
GATACGGCAAGAACTCGAAAACGGTTGTGTTCAAAGGGAATGTTATCAAATTTGCAGTTTAAAAGTATTT
ATTAATTATCTCAGACCATATGCAGATATTTTATGTTACCTTATTATAGGTATTATTGATTAAAAAACTA
AGTTTCAAACTTTAATGCTCAATATATTCATTAAAAAAGGTGTAATAATAGATAATCACAATTGCATTTA
GCTTAACTAAACAATTTTTAATGACGTTATTCGTAAAATTTGTATAGCTTTTCACTGCATAAATGTTTTC
TCTTTTCTTCCACGTCATAAACCACAAATTAATTTCGACGAAAATAGAAAAAACCGCCGACATAAACAAA
TCGAAGTGTAAACAAATACATAAACCGCCTTTTTAAGGTCAGCAGTTTAATCAATCTATTAGCTTCAACA
AATTTCCTTACAAATCAACTTCCGCATTTGAAACATTTACAAAACTTCCCaaaaaaaaGTGGGTTAATCC
GCAAATGGAAATCCCCGCAAGGTTTAATTTCTACAACCAACATCATAAATGCTTTCCAAAACAGTTGAAT
TTGATTTTGTAATGCCTAACCTTTATTGTCCAAATTAGCCGCAATTTAAATTGAAATTCATCGTTCATTG
CATTTCACTTTTTCAAGTCCGTTTTTGATGGTTGGCCACTGCCAAATTGTTTATAGCACACGTTAACATA
TTTATTTTCGAATTTCCGACGTCATCAGCCGCAAATCAATTTGCGCCAAAAATAGACAAGTTTAAAAACG
TAAACAAATGGAAGTAAATTAAAGTGTATACACTTTTAGAGTGTAAAGCTCAACGAGGAATTTGTTTTAA
CAGCTATGCATCAAGTATCATTTTCTCGAGTTTTTGAGTGCACTTAAAATAATTTCGTGTAGCCTGAAAA
TTAAATCACAAATGTAGCATTTCAGTGCGCCcacacacacacacacTTGAAGTGCGTTTAAAAATTATAG
GCTAATTAAATAAATATTGCATGCATAATGAAAAATAAATGAAATTGCAAGGTGCACGTGTGAAATGAAA
TAAATTTATTTTGATTTTCATCGATGCATTTTACAGTACTGAAACGCACTGTGCGTTTGTGTGACACATT
TTTGTGATTTATTAAAATGGCACTGAGCTGATTTCATTTAACCGAATTTAATTTAATTGAATAACGCCCC
GGACAATCGTGGTTTATTGTATTTTACTTTGTTTTGATGGCTTTGAACAAGGGTTATGCTATACTAGCTT
AAACGTCAGACTAGAACATGGCCTCGAAATTGAGTTTCCACGGCCGTCTGGACAGGATGGTCAATTATTT
TAAATACTCGCCTCGTTGAAATGGCCTTAAAGTTGCCAAGTGAGCCATTTACGACTTCTGGGCAGGAACG
TATGTATGTACGTCCAGGTCTGGGTGAATCCCTCTAAGCAGCTTAGAAACCCAGTGCATTGCATGCACAA
AGTGCAATGGCGCAAAGTTTTCAGCAGCttttttttttctttttACTGCGCGGCGTCTGTGCTGCAGAAAT
GTATTTACATTATTTTATATCTTTTTTACGAGCAACTGTTTGGCATCTCCGTTTATGCGTGTAATTTGCA
CAAAATAAATACGCCTTTCGGACCGGAGAAATGTGCTGttttttttcaggaatttctttgatttatttatc
ttgtttttttttttttcttttcaaccattcagcaacacagaaggtttttcatttttttttACGCCGCTGGT
AACCCAATTTTCAACAGCTGTTTATGAGTTTTCAGCCATTGGGTATAGTTGTGAGAATCGATAACATAAA
AACCTTGTGAAAAAGAAATGTGATTTACACGAGTTAATTGAGAACGAGGCAGTGACACATGTTGTATGGG
TATGTGacagatattttcccactccttctgccaatttccacatggtttttgcccactccttcggctcatt
tccacagcatttttcccaTGGGGGGACATTGACATTTGTTTGGCTGCACCGATGAAGTGCGTCAAACTCG
GGTTACCGATAAGCGAACTTTTGTCGATAATTAATTGCTATTATTTTGCATATCGTTGCGTTTGTTTTAC
GTACTCCAGGCTAGAGATTAATTAATGTCAACTTTATTGAAGGATCACAATCGAAGAGATTGTTAAAAGG
ACATATGGTATTATATTCCTTAAAAATCCTCTTAAAGCTTAAGATCGTTAAAGTTTTTCTTTATTGAAAG
CAATGAAAGTAATCCCTTGGATTGGTATTTCATATTCCCATGAATTTTCCGTGTTTTCCAGCGAAAAACC

GCACATTATCCCCTCGCGTTCACACCTGACATTGATAATGCCCGGGTTTATCTTATCTTTATTGCAATCG
CAGCGTACTGTTGTAATTGCTGCTGTTTTTCGACTCttttttttCTGCTTTCGCTTCGATTATCTTGATA
AATTATGTCCGTGCTCGTGACAAACAAAATCGCCATTGGCCGTCGAGGAAATAAAGCTATTATGTTTTAT
TTAAATGTAAATGGTAATTGAATTTACGACTCACGCACCAGAAGAATTTGAATGAGTTTAGTTTTATTGT
TTTCGGTTCATTATATGATTCTCAAAGACCCTAATGATTCACACAGAGCTGATTTCCTCCCGTTTCTGTG
TAATGCCCGAATATGTCGAGAAGATTGTTTTCATTGGAAATCATAATCACATTTGGAATTTACGCAATAT
TGGTTAGACTAGCAAATCTCTATTTATAAAGCGATGCATCAACTTAAAGTGATCGTTTTAATTGTCAAAT
GCTATTGTTTAGCAAATGACTTTTAATGATGCACAGCATCGTTGCATTCTTGTGAAAATGAAAAGTTAAA
CTTGCACCTCTGGTTTTCTATCTCTATAGCACCCTCTCTCTCTCTCACTTCCCCGTCTCGCTTCCGCAAC
AAAAGCAGCAGTCAACGATCGTTCAGATTCAAAAAATGACTCACAAAAGCCATAGTTGCGAGTTCTTGTC
CGAGTCAGCAGTTTTACAAGTGTAACGCAACATCAACAATAACAAAGAATTTTGCAAGGCCTACACAGGG
AAAAAAATTGTGCAATAATCCTGATAAATTATCCTTAAATTAAGCAAATATTTGCTTAATATATCTGCTT
GAACGAAAATTAATAATTTAAAATATTTTTTAGAATACTTTGATCAAAAATCATTCATGTAAACTTTGTT
ATTTCACAATTTGATGCTTGTTTATTCATAAATAAATATGTATAAAAATTGTTATGACTTGATTTTTAAT
AACTTAATGACTTAATAATATTTTGAATCAAATGACTGAAAACAGCTCATATGAAAATAATTTGTTGCGC
GTATTTCAAAATTTAAAACTTATCTTTTTGACAGCATTAACTATAAACAAAGCCAAATGTATTCAACATA
AGCTACAAAGTTTATGAATAAATATGCATTAGGTAACTTATTTATAGTTTTAAACTACACATTTTTTCCC
CACTTATTTATATGTTCGTGCTGAGATTCCAGTTTAAAACTGGAGCAAAATATAGAAGAACTTTTATTTT
CCATGTTCAACATTGAATTATTAAAAGATTTTCGTCACCGGAAGGCGCAGATTCAAGCAGCAGAATTTGT
TGAAAATTATGAAACATTTACGATTAGGTTAGTCATCTGAGTGGCTCTATAAGAAAATTGTATATTCAAT
ATGTCGTCGTCGTTGTCGCCATAATCTTCATCGTCATCATTGCACTTGCACTTGttttttttttttATTA
TTTTATTGTGTATTTTATTTATACCCTTTCGTAATTCATGGTTGTCATGAACGGCGACCGAATGTCTGTA
GAATCAGCTTCATTTGTTTCGTTTTCTGCTTTTCATTTGAGTTTTTCTTATTaaaaaaaaaaTTATGAGA
AATTATCGGTCATCAAAGAGATTCGCCCCTCTGGGAAATGGTTTTTGTTTTTCGAAATTCCCGGAATTCA
TTTGCATTGATTTGTGGCTCGCCAATTTTATCACAAGCAAGAATGAATATTTTGTCCTTTTTAGTTTGTC
TGCGGTTCATTCATTGATTCATTCACTTTAACGTGCTGGGAATTCGAATGCGGTGTTTTGATGATGCAAG
CAAAGCATGGCGAAAATTAGAAAAGCGCAGTCATATGCGTTATTTTTTATTTATTTTATGAAAATGAAAA
TGTCAGCAGAAGCCAGCGAAAAATGTGCGGCATGTTTGGCATGTCTGTCTGTTTGTCTTCCCAAGAGCAC
TGTATTTTGTTGTTTAACACACTGCAAAAGGGGTGTAAACAACTTCTGTGGCATTCACGAAAACATACGC
AATTCGCCTGCTGCTGCAACACCACCCACACTTTTCCGGCGAACAccccccccccccccTTTCCGTGTAG
CCCAAACGCATGACATCAACTGTTTTATAGCGTCACGCGTTTCCATTGTGGGCGAAAGTCCAATGGCAAG
TCGGAGTTGCACTTTAGCGCCACTTGGGATAAACACGTGCACAGTGCGAAAATTCTACTAAAAAACTTTA
TAATTATACTAAAATAAACATCTTACTCATAACAAGGATTTGCATTAGGCCTTTAATTGTGGGTTTAACT
ATAGCAGGGTGTATAACTTAAAGTTTATAACGGAAATGAACGAGTTTTTGAAAAACATAATAAAAAGTGG
AGTGGTTTTTTGAACAATTCATTTATTCAAGACATTATAGTATTAAACATCCTATAAATTGTTAAAAACA
GAACATTAACATTATTTTATAGATAAAATTTAAAAGTAGGTGTACTCTTTGTAAGTTAACATGCATTTGC
ATGATTACATTTGTGTTCTGATCTAATAATTTCTTTCAAAATTACATCTCCTCTAAAAATAAGTCATACT
CTTACTCAGAATTGTAGCTCTGCAAGATCTCCCCCtttttttttttAATGCTGTAGCTGCAAGATAAAGC
CATCGCTGTGTAGCAGGTTCGAAGTTTAGTTAGTGAGTGAGTGAGTTGGGGGGCTGCGATTAAAACACGA
TCATGTTCGCTAATGCTAAACAACACTTGCCAAGTGTCAATGGCAATTATCTGGGGCACAACGACCATTC
TCTGCACAATTCACAATTTCTGCATTTCCGCATTGCTGCCTCCGATTTCGATTTCAATTaaaaaaaaaaC
ACTGCCATTCTGCGGCAGATGCAACACTCGCCTAAAGTTAATTAACTATTCATCTAAgtgtgtgtgtgtg
tgtgtagcatgtggtgcttatgtgtgGTTGGCATTGTGAGTGCTTTGGGGTTTGGTTTTTTCATCTTTCA
TCCGTTTGCTGCATGATGGAGCAAATGAAACGCTGACTGATTGCGCTGCTATGCTGCTGCTCGCAGCTGA

TGAGCAAGATGGGCGCAAGTGTTTTAATTAAGCCCCAAACTAGAAACAGAAACAGTTACAAAACGAGGCA
AACGGTTCTTGGCTTATTATGATTACTTTTCAACCGAACAAGCGCTCGACTATAAAAGTCTTACAAGTTC
AAGTTTGTTGTGGTCTTGTGGGTGATTTCCCCCAACGATTTCCCTCCCCACATTTTCTTGGCAAACAGAC
AGCACGTGCTTATCCAAATGGAGCGCATCGGTGTTTTGCTGTGAACTCAAGATGTTTTGCAGTGATGAAT
CAACACAATTGGTCACTGGACAGAAGATGCAATGCGGCATCTCGATCCCGTTTCCTTGAGTTGCAGTTCT
GCCCCACAACAAACACCTACGCCTTTTGTTTTGAAACTGAAATGCACATGACGCAGGATAAAATGCAGGC
GACATGCCAAAGTAGTTGGGGAAAGTAAACTTACATTTTTACAAGAATTTTAAACAATTTTTAGTaaaaa
aaaaaTTGTTTAAATTGCAAATGTGCTCATTTTTTCAAGCATCTACCGTATTTTTATGATTTTTCCGAGC
AAGTGAGTTAGTTGCGTCTGCCCGATTCGAATTACAATTACAATTTTTCTCCAGCAGTGCTCAATATTTT
CTACATACACCAACAATTATTATTCCCCCAAGAAACGAATGAACTTGCTACCACCCCTAATCATAATATG
CCCCCCGCAAGGGTAATAAACAAAAGTGAGCTGCCTAATGACACTTGGCCATATGTACATGTAGGCAACC
CCAAGAAGTCCTCCTTTGCTCCGACAATCTCAGCCAGCAATCTCTGGAATGGAATGCACAGCCGAGAACA
GTCCGCCCACACAGGCCACGTTTATTTCAAAACCAGACACAATTTTTGCCACCAACTCAAAGGAGAAGCG
AGCAGAAAAGACCTGGccagaaccagaaccaaaaccaaaaccaTTTCCGTTGCATGACCGTGCGCTTGGT
TGGTGGCATCAAGAGTCCTTAGAGAGTCACTGGCGTAAATGAGACAATGCCGGTGAATAAAGTGGGATGA
ACACTGGGAGAAATACTTGTGAGCAAGATGAGGGTACTTTGAATATACCCAAGAATATAATATATCAAGA
ATTATATGAAGCTATTTCCATTCTCTTAAAGGACTATATAGGAATATATTAAAAAAATGTTCAAGCATTA
TTCTATTCTTATTCCTAGCTGTGTATAATGGTCCATTGAGTCCAAGTCAACGACAGAAGTCCTCATCCTT
CTAACTTGTATATATATACATCTACttttttttGTAACTCCCCCGCACATTTTGTTTACTTGTTTGCGCG
CGTTAAACATTCTGGGCCGCTTGAAGAGCAGCGGCCAAGGACGTGGGATCTTGACTTACAGACAGGTGTA
AATTATGTAGAGTGAAAAGAAATGCCAACACATAGAGTCCGAGAAGGAAAAACAAGAGACCAAACATCAC
AGATGTTGTGTGTTTTTGCTCGGCGACGAGCTGCAGACAGTTGCATTCGTATTATTATGATTATGCCAAA
GAACTCGGCAGCTGTATTACACTGAAAGAAAAATGTATGTCTTAAACTATAATATATTTTAGAAAATACA
GAACTCCTGTTAAGATGACAAGTCTTTTGATACTTTAAGTAATATACTCTAAGTACTGGACTGTTTCAGT
TCTTGGCAAGTTCTTAATCTTTCAGTGCAGCGCTGCATTTTGCCATCCACTCAGACTGTTTGTTGATATG
CGCAGCGATTTAATTATAAACACACAACACGTGCTAGTCCATTACAGTACAAAGAGGTGTGCAAACCAAA
AATAACATGCGTTTACTTTTTAATAGAGTTAACCCACCACTCCGACATATCCATGAGCTTGAACTTTGCA
GGCCACTGAGCCGTGTGTAATTCGCATGTTATCTAAGCCATCCAAACTGGAGTTTTGGGTCGAATCGAAG
GCGGGGTTTGGCTATATTTAATTCTACATAATGTATGCCATAATATGCTATAATCGCTTAAACACGCGAA
CCCATCAGATGGGGGCATATATATATAATTATCCAATTATGTGCGTTGTTTGACCAAAAGAAACTGAATC
GAAGGCCTATAATTAAGTTAACTAACTTTGTTTAGGAGCATCATATACATTAGTAGTAGAAATATACTGT
ATTCCAACAGCGAATATTTACGATTAAATTTATTAATCGCACAGGGCAGTCAATTCTTGAAAAATTATTC
ACTCAACGGAAATATGACTTTTGAAACTATTTCAAATTTTAAAAGCGGAAAAAGTATATCAGACTAACAA
TCATGAAAATCTACTATTAACAGAGTCGAAAACGGTTGCACTCATTATTTTCATTCCATAAATTATATCT
TTAACACTAATTTCGCTGACAATTTAGCTGGCCGTCTAAAGAAAATAAAATATTTACAGTATTATTAGCC
ACTTAAAATGCATTTGAAAAATGCCAAAAGACCTCAATTGAAATAATCAACGCCATTTGCTGATTGTACG
ACTGTACGAAGTGAACTCTTCACAACATGCAATGACCGTTCGTTAGCTCCAGTTTAGTTGTTCCGCTGCC
GTGTGGGTTTTCATTCGACTATTTTATGTTATTTCATTATTATTTTTTTGTAGATCATAAAGCATCGATA
AAAATCGCTGCGCTGCCGGTCAATGGACGCGGCTTGCCACATCGTTATTGTTGCATGTGCCACAGAAATA
AAGACGCAGAGTTTTTCTCGATGAAAACTCGCCGGCCACAATGGCTTGGCACGCAGTTTGCTGATTGTTA
TAAAACAAATGCATGAAATGTGGTCAGCAGGCGTAGTTTTTCTCCAGGGTACCGTAAAAACTAGAACCCC
TAAAAGAGTGCACTGTAAGAAAAAATTATGGATATTTTAGATTAGTGTATTACATACGTAAGACCTAGTT
TATCTATCAGAGGTTAAACTATAACTATATATAGGAAAGTCTATCTGTATCTGTAGTCTGTATCTATAGA
TACAATTAACAttttttttttAAATTGCAAAAGGTTTCGAGTGTAAAAATGTTCCAAGTCTAAGGTTCGC

ATGCCAAGCTAGCATTTAATCCTTCTTAGAGCCAAATGTATTGACTTACGCTTTTCCGCCTTCGTTCCTG
CGAATATTTACAGGGGTGCCGGCAAGGAAATCCGAGAAGCCATCGCCGATCCATCGCCGGAGGTGACCGC
CAAAGCCTGGGCGGCGGTGCTGCCGTTGGTGAACAAGCTGAAGCGCTGCTACGAGCATTCCATGGAGCTG
GACAAGATTGTGCCCAAGCTGCTGGGCCAGCTGGTGGGCGGAAAACTGAATCCGACACAGCATCTGGAGA
CGCAGCAGGCGCTGGTCAAGCAGCTGGCCGAGATCTTGGAGTTCGTGCTGAAGTTCGATGAGTACAAGG
>dme_circ_0004425_junction_seq
TTCGTGCTGAAGTTCGATGAGTACAAGGTGCTCCAAATAAATAAAACAACAAATTC
>dme_circ_0004426
CTTCCTGCCGTTTCCAATGATAAGTTTCTATAATTTACTTTTCCTGTCGTCGATCAGTTCACAAACAAT
TTCTACCCCCACCCCATTGAGGTTAATATCATTCTACATTCGTCCGCAAAATGCTTCGCCTTAATTCAAG
TTAACTGATACTCCCTGCTTCTGCTGCTCCTTGAG
>dme_circ_0004426_junction_seq
ATACTCCCTGCTTCTGCTGCTCCTTGAGCTTCCTGCCGTTTCCAATGATAAGTTTT
>dme_circ_0004427
GTTTCAACCTGTTCGTTACTTTTTATGCGATTTATTATTGAAACGCTGAAGGCTTAATGCAAATGCTTTA
CAAACTGAAAGAGTAAATTATTCAGCGCAAAGTTTCCCCTTTGGGCCTTGATCCTTTACCCCACTACCTT
TGATATTTCACTCCACAAAAACTCACTTAGCGACCGATGGAAATAAATTTCCGACAAATGTGCGCGGCAA
ACAATAGCGAAAAGTTGGCAGAATAATCAATTACACCAAAAATGTTAGCAAAAACAAAGTTACTAAACTA
AAGACACACGTCTGGCCGAAGCCAAAACAAATATTTATCACAAAATGAGAACCCATGCGAGTTGAAGATA
AAGCCGCAACAAAGTAATTAAGAAAACTTAAGCGTAAATTTTGTGACTATCAGCAAACGCCGCTTGTGAA
GAAATGCAAATTAGAAGCGGATGTGGAAATTTTTAGGGCGAAAAAAGAAAAGACTGAAAAACTGGCGACA
ATTTCGAGTCAAGATGTCGATTGGTGAAATGTAAACAGCTCGCGGTCAGTGAATTTGCATAAGTGTAAAT
GTACACATTCGTCAGAGTATCATTTAAATGCATATGAGTAATATATATATACATCTGTTCGTATCTACGG
GCTCATATGCGGATTTATGTGTTTGTTTATTCCAATTGCATTTTAAATATATGCTTCCTCGAAATAAAAT
GGTAAGATAGTGAGGAAAACGACTAGTTAGTAACCTTTTAAATCCTTGAATAAATGAGTAAGTATGTATA
AATACTAAAACCATTTAAGAGGTCAATTTGCTAAACAAACTTGAAAAGATTTTCCAGTTATTTAACTTGA
TTTCAACACTTATCCATATCTAGTCCAATCTACCCCGTTCATTTAATAGGTATCACCCTCACACCGAAAA
TGCCACTCCATGGAAATGAGTTGTGGTCAAAAGGTCAGACAGTTTATCGGGCATTAGTTTCGGCTACTTG
GGCTACTGCCATGCCCAACTGCATAAAATATTCAAAATTTTAATTAAGCAGCGTGAAATTTATGAACAGA
TGGCCGGCGCTCCAAACTTGCTGACCAGataaatatatatatatatatacatatatTCAGATTTTGTACC
AGGCATTTGGGCATGGATATATGTTAATGCGGTCAGTACTAAATCATAATTTGTCAACAATTTCGTTTAT
GCAAAATTATTTGGCTTTGTTTGAAATTTGTATTGTTGTTTGTTGTGGGCTGTTCAAATGTAtttttttt
ttCTGGTTTAGAGCTTTGGAGCTCGTACCTCTGGTTCTCTACCCTTTGGATTGCATTATTGTTATTTTTC
CACATGGCTGGTCGCTGATTTTGTGGGTATTCTTTTTTCGTGCCTTTGCACTTGTCAAATTTATAAATTG
TATCTGTCAGATACGCGCTCATTAGCATCGAGAACACATCCATTCAAGACAAGAAGCTGCGGATTCGGAT
TATTCTGCATCTGATGGAAACTTTCCTTTGCCCTCGGGGCGAATACAAAATTTCTGTTGTTGGCCTGACA
CAttttttttttCCAATCTTGTCAACAACACACACAAACACACCGATAGAGAATCGAGTTAATAATAAATG
GCAACAACAACTCGGTGAAAATTATTTGGAACACGTCAAAATATTTGAATTCAAAAAGAGAACTTCTTGT
CgtttgtttgtttgtttgtttgACAATAAAGAACTAAAAATGATTTTTGGTTTTTCCATTTTCCCATATG
TGTGTGTGTGCTAGGCATTATTAATAAAGTGGCAGTTTAACAATTTCACAGTTCCTAAACAAATCATATA
AAGATATAGACAAAGAGATATAGATAAAATGCGGGGAGACACCACCCTAGTGGTCTAAAAATATTTGAAA
TTTTGTTTGATTTCGTTGACAAATGCGTTCTATTTAGAGTTATGCCATGGATCATTTGCTATGCACGTGC
GGGGGCTGAGTTATAAAATGAATGAAGAGTCGTTTATGTGATGGATAATTTTGGTCAAGAAATGGAATTT
GTGGTGGCTCAGCACTTGGCTCTGATTGATGGACGTAATATGGCCTAAAATATGGACTTACTGAAGCTGA

AAATTAAAGGAATGATTGTTAAATG
>dme_circ_0004427_junction_seq
TGAAAATTAAAGGAATGATTGTTAAATGGTTTCAACCTGTTCGTTACTTTTTATGC
>dme_circ_0004428
GAGAAATAAACCAAATAGCAGCATCAGTTTATTGGGCTCGTCGTTCAATTCCAGCGACTCTTCTTGGCTC
TTTTGGCATCCTGGTTGCTGCTGTTGTTGGCCGATGAGGCGGAAGCACCCTGTGTTGATGATGAGGGCGG
TGCCATTGGCGCAGCGAAAACTCCCGACTCCGGCAGAGTTTTCTCCCAAGTGCGATCGCTGGACGCACGG
AACTGATTGTTATACTGGGATCGGAATGCCTCCCTCATTGCTGTATAGCGATCTGTGGCTGGTCCCGAGC
TCGAAAAGGCTTTGCTAGAGGGTCCGTCGGAAGGACTGTGGGCAGACACACTCGCAGTGGACTCCGAGCC
ACCAGCCGAACCATAGCCACCACCTCCATTTCCTTTCTCCCGATAGCCCAATCCTGTGTGGGTATTTGTT
GGTCTCTTGCCTTTGCCCTGCTTGAAGCGTGAGCTGCGGAACCAGGAACTCTTCATGGCCAGCTCCATCA
GATCGTCGGGCACCAGCTGATCGGCTCCCTCCAAGTTACGCACCAGATGTCCGGCGAACTCCTTGTCCTT
ATCGGTGACTAGAGTGTATGCATTACCCTTTTCACCGGCTCTGCCCGTTCTTCCAATTCTGTGCGTGTGG
GTTTCAATGTCCCTGGCTGTGTCGTAGTTTACCACATTCCTAATATGGGGTATATCCAATCCTCTGGCTG
CCACGTCGGTGGCCACTAGAATATCGCACTCTTTTCTCTTAAACTGAGTGATGACCTTGTTTCTGTCCGC
TTGGTCCATATCGCCGTGGA
>dme_circ_0004428_junction_seq
CTGTCCGCTTGGTCCATATCGCCGTGGAGAGAAATAAACCAAATAGCAGCATCAGT
>dme_circ_0004429
TTTAAGAGAAAAGAGTGCGATATTCTAGTGGCCACCGACGTGGCAGCCAGAGGATTGGATATACCCCATA
TTAGGAATGTGGTAAACTACGACACAGCCAGGGACATTGAAACCCACACGCACAGAATTGGAAGAACGGG
CAGAGCCGGTGAAAAGGGTAATGCATACACTCTAGTCACCGATAAGGACAAGGAG
>dme_circ_0004429_junction_seq
CACTCTAGTCACCGATAAGGACAAGGAGTTTAAGAGAAAAGAGTGCGATATTCTAG
>dme_circ_0004430
GCGACTCACGGGCCTTCACGCTGTCGGGTTTCTCCACAGTCTCGCGCTTTTCCTGCTTCTTGGGCAGCTC
GGCGGTGTGGGCCACAGCGGGCAGGGCATGGGGCAGCGAAAGATCCTCCACCTGAGGACGATCACCTGCC
TCATCCTTgtggccctcctcgtg
>dme_circ_0004430_junction_seq
CTGCCTCATCCTTgtggccctcctcgtgGCGACTCACGGGCCTTCACGCTGTCGGG
>dme_circ_0004431
GCGACTCACGGGCCTTCACGCTGTCGGGTTTCTCCACAGTCTCGCGCTTTTCCTGCTTCTTGGGCAGCTC
GGCGGTGTGGGCCACAGCGGGCAGGGCATGGGGCAGCGAAAGATCCTCCACCTGAGGACGATCACCTGCC
TCATCCTTgtggccctcctcgtggtgagcttccctcttttccttcttgtcctcat
>dme_circ_0004431_junction_seq
gcttccctcttttccttcttgtcctcatGCGACTCACGGGCCTTCACGCTGTCGGG
>dme_circ_0004432
ctcaccacgaggagggccacAAGGATGAGGCAGGTGATCGTCCTCAGGTGGAGGATCTTTCGCTGCCCCA
TGCCCTGCCCGCTGTGGCCCACACCGCCGAGCTGCCCAAGAAGCAGGAAAAGCGCGAGACTGTGGAGAAA
CCCGACAGCGTGAAGGCCCGTGAG
>dme_circ_0004432_junction_seq
GAAACCCGACAGCGTGAAGGCCCGTGAGctcaccacgaggagggccacAAGGATGA
>dme_circ_0004433
gacaagaaggaaaagagggaagctcaccacgaggagggccacAAGGATGAGGCAGGTGATCGTCCTCAGG

TGGAGGATCTTTCGCTGCCCCATGCCCTGCCCGCTGTGGCCCACACCGCCGAGCTGCCCAAGAAGCAGGA
AAAGCGCGAGACTGTGGAGAAACCCGACAGCGTGAAGGCCCGTGAG
>dme_circ_0004433_junction_seq
GAAACCCGACAGCGTGAAGGCCCGTGAGgacaagaaggaaaagagggaagctcacc
>dme_circ_0004434
gaaaagagggaagctcaccacgaggagggccacAAGGATGAGGCAGGTGATCGTCCTCAGGTGGAGGATC
TTTCGCTGCCCCATGCCCTGCCCGCTGTGGCCCACACCGCCGAGCTGCCCAAGAAGCAGGAAAAGCGCGA
GACTG
>dme_circ_0004434_junction_seq
CCCAAGAAGCAGGAAAAGCGCGAGACTGgaaaagagggaagctcaccacgaggagg
>dme_circ_0004435
GGACGATCACCTGCCTCATCCTTgtggccctcctcgtggtgagcttccctcttttccttcttgtcctcat
gtgtttcgggagcctgcttcacttcctccttatgtggagctgcatcatcaccatggtggccttcctcgtg
gtgagcctctcgcttttccctttttggcatcatgagcttcgggggcctgcttcacttcctccttatgagga
gcctcatggtcttggtgaccctcctcatggtgagcctctctcttttccttcttggcgtcatggtcctcgg
gagcttgcttcacttcctccttctggggcactgcatcgtcaccatggtgaccctcctcgtggtgagcttc
cctcttGCGCTTGTGGTCCTGTGGCCCAGCCGGAGAAGGCTTCTGGTCATGAATAGGAACGGCCGGCTGC
GAGTGATGCAGCTGTTC
>dme_circ_0004435_junction_seq
CGGCCGGCTGCGAGTGATGCAGCTGTTCGGACGATCACCTGCCTCATCCTTgtggc
>dme_circ_0004436
GGCTTCTGGTCATGAATAGGAACGGCCGGCTGCGAGTGATGCAGCTGTTCGTGGGCCTCCACGCTGTCGG
GCTTCTTGGGTTCATTACGCCTCTGGCGATTCAGGACGGGAGCATCCTTCAGCAAGACTACCTTGGCG
>dme_circ_0004436_junction_seq
AGCATCCTTCAGCAAGACTACCTTGGCGGGCTTCTGGTCATGAATAGGAACGGCCG
>dme_circ_0004437
GGAACGGCCGGCTGCGAGTGATGCAGCTGTTCGTGGGCCTCCACGCTGTCGGGCTTCTTGGGTTCATTAC
GCCTCTGGCGATTCAGGACGGGAGCATCCTTCAGCAAGACTACCTTGGCGGTGGGCTTGGGATCAACGAG
CACATCGGGGCTGATGGGACTGGGCCTAGAGAGCTCCTCAGAG
>dme_circ_0004437_junction_seq
GGGACTGGGCCTAGAGAGCTCCTCAGAGGGAACGGCCGGCTGCGAGTGATGCAGCT
>dme_circ_0004438
ATTATTCTCTTAGCCCTGGTGGCTTTCTGCCACGCAGCACCTCTGGATGTCAAGGAGTCTACCTCTGAGG
AGCTCTCTAGGCCCAGTCCCATCAGCCCCGATGTGCTCGTTGATCCCAAGCCCACCGCCAAGGTAG
>dme_circ_0004438_junction_seq
GTTGATCCCAAGCCCACCGCCAAGGTAGATTATTCTCTTAGCCCTGGTGGCTTTCT
>dme_circ_0004439
GAAAAGAGCCATTGTGTGGCAGCGTCTGTAAATCGAGATCGTTGATCTGTGATTTCTAAGGCGCAGATAA
GGAGAGGCCTTTACTCATAAGTTGGCCTGGGTTATAGAGCACCTGGGTCGTAAAACCGTA
>dme_circ_0004439_junction_seq
TATAGAGCACCTGGGTCGTAAAACCGTAGAAAAGAGCCATTGTGTGGCAGCGTCTG
>dme_circ_0004440
GTTTTAGATTTTGTTTGTTTAGTTCCATTTCGTGTTTGCAAAAAGTGCTATTCGCTCAAAAATTATTACA
TGTTGTTTCCGCCTACTTTTGCGTCGTGTTATAGATCTCGAATAATAATAAGTTATCGCTTAGCATTGAT

CGAGTAGCGTTTTTGTTTTCTTCACCTCCTCTCCACTCGCTCTGGAGTAAACATATAAATTTAGTTCCGA
TACACTTACTAAAGACATAAATGAACGCCTTGACTTAAACTTAAACGAAAATGGGGCTATGTGGGGGATG
CGGGGTAACATAAGTATCGCTTAACCAACATAATCTAGAAGACTGCCTGTCTATTT
>dme_circ_0004440_junction_seq
CATAATCTAGAAGACTGCCTGTCTATTTGTTTTAGATTTTGTTTGTTTAGTTCCAT
>dme_circ_0004441
GTTATCGCTTAGCATTGATCGAGTAGCGTTTTTGTTTTCTTCACCTCCTCTCCACTCGCTCTGGAGTAAA
CATATAAATTTAGTTCCGATACACTTACTAAAGACATAAATGAACGCCTTGACTTAAACTTAAACGAAAA
TGGGGCTATGTGGGGGATGCGGGGTAACATAA
>dme_circ_0004441_junction_seq
GCTATGTGGGGGATGCGGGGTAACATAAGTTATCGCTTAGCATTGATCGAGTAGCG
>dme_circ_0004442
GCAGTCTTCTAGATTATGTTGGTTAAGCGATACTTATGTTACCCCGCATCCCCCACATAGCCCCATTTTC
GTTTAAGTTTAAGTCAAGGCGTTCATTTATGTCTTTAGTAAGTGTATCGGAACTAAATTTATATGTTTAC
TCCAGAGCGAGTGGAGAGGAGG
>dme_circ_0004442_junction_seq
GTTTACTCCAGAGCGAGTGGAGAGGAGGGCAGTCTTCTAGATTATGTTGGTTAAGC
>dme_circ_0004443
TCTTTAATGAAACTCTTCTAAGCACAGGGATGTCTAAGTTAAGTTTTTAACTAATTTTAGGGCTAACCAA
AGTTTTCTAGGACTCCGAAACCTCCCGCCTCGCCCCCTGGTTATCCTATCCCGCCCCATCCTTTCCAACT
CGATCCGCCACGATCCCTGatatatatatatatatatatctatatatatgtatCCACATCCATAACCCCC
ATAGGGGGGTATCAGCAATCAAACAACAAAAGTATTTCGATTTAGTCGGTGCCACGCCCTCAAGGAGTTG
CGGCGGTGCGAAGGGGGCGTGGCATTAAGGTTACTTAGAGAATTCTTGTAGAAGTGATTTTGTATCGTAG
TGATTTTGACAACAACTTAGTCGTAATTAGTTCCTACTCGAACTAAGCTAAAACAGACGGACGCAGCTGA
AGTTTGTTTTCGTTCTTCAATTATTTTG
>dme_circ_0004443_junction_seq
AGTTTGTTTTCGTTCTTCAATTATTTTGTCTTTAATGAAACTCTTCTAAGCACAGG
>dme_circ_0004444
GTTCATACGTTCGGACATGGCCGACGTACCCGTGCAGCAGGCGGCAGGATCGACGCTTCCGTTGGTAATC
TCTCGGAAAAAAGGCGACGACTCCGAGGATGGCAACTACAATCCGTTCGAGCACCGCAAGGTGGAGCATC
CGACATCGGTAAGTACTCTGTGTAAAAGTCGATTTAAATTAAAAGGGGATTTGTAATTTATAAAGATATA
TAGGGGAAAATTCAAGACTTTCAAACCAATCTGTCCGTTAAATATTCTTGCAATTGTTGAAAATGTTACT
TAAATTTTTAACAGAGTTTAAGAGGTTTAATTTAATATGTGAGCAAGAAACATACTTTGTTGCTAAACGA
TTTTGTTGATATTTTATAGAAAATAGTGTTCTATTTCTTTAGTTCTGCTGGCACCTAAAATAAATGCTGT
AATATCATGCCTTACTTAGTATTTAAAACCTTAACGTACACAACTTTTGTTGGCACCCCCGTTAAGCAGC
TGATCCAAGTCGTTAAATGCTCAATGACTAATTCCCTTAATTATAAACCACCATTCGCGTCATTGACCTT
TAACCGATCTAATGACTTTGACTTAGCATCGATCTAAAGCGAAAAGAACGGAAATGCAATTGTATTGACA
TTGGCCTTGGACATTTTTGGCAAACTACTTTTTCCAAACTAAAAATAAGTAAAATCGCAGATGAAAATCT
CGAGCGAACGAGCTAATAAAACGAAAGCGCCGACAAATGTTTTGCTCGACTCGTTTAGCAAGTGGAATGA
GGAATCCACCCAGCCTTCCACCCTCCGATTTTTCATCTGATAGAGAAAAACGCATCCGCTAGGCCTTGCA
TTTTGCAATTGCGTGAAATGAAAATGCCGAGAACTGTGAACTGCAATAGCTCACTAGAATAATTATTAAC
CGAAATATACCAAAATATTCGGTGCTATATACTTTAAAGTATACAGATGAGTGATTAATAGGATAACTAC
GTCATTGACGTGCTTTGCGGGAAAGTCCCTCATTTCCAACACAATGAGATCTGCAGCAGGGCATCACGCC
ATCATGCATCCAATAATTTAATTGAAATAGTTTTTTTGGCAACGAAAAAGTGATCTATATTTATTTCAGT

ATGATGATTTTAAGAGCATTTACTTTTTGTATCTACCTATTTCACTAACTGTCTAGCTAGTAAATATATT
GAAACTATTATTAAATTAAATTATAAGAATGATACCTATATGTATACATATATTTAGTATGAATGGAAAG
TTGTTTTTCTATATTTGGCTTAGTACGGAAGTTTTGAAAGAACATAAACTAATTAAACACAATTTTTTCA
GGGGATCTCGAGACATTTGTACACTTGCTAAAGGGATCGCTTGGCTCCGGAATATTGGCCATGCCAATGG
CCTTCTCCCACGCTGGATTGTGGTTCGGTTTGGTGGCCACCTTCGCGGTGGGCACCTTGTGCACCTACTG
CGTCCACATTCTGGTCAAGTGCGCCCACATCCTGTGCCGACGTCGTAAAATCCCGATGATGGGATTCGCC
GATGTGGCGGAACAGGCTTTCCTCGACGGACCACCTGCCCTGAATCGCTGGTCACGCTTCATCCGGTTCA
TGGTCAATACG
>dme_circ_0004444_junction_seq
ACGCTTCATCCGGTTCATGGTCAATACGGTTCATACGTTCGGACATGGCCGACGTA
>dme_circ_0004445
GTATTCCTCTCCAGCCTCGCCATCATCTCCCTCTTCTGCGGCGTCCTTCTTGATCTCCTTTTGCTTTTTG
TTCTGCGCCACAATTTCCGCCTGTCTACGTTTATGCTCCAAGAAGTTGAAGATGAACATCTCGTCCTCGG
TACAGTTGGCCTTGGTCAGCGATTTCACTGG
>dme_circ_0004445_junction_seq
AGTTGGCCTTGGTCAGCGATTTCACTGGGTATTCCTCTCCAGCCTCGCCATCATCT
>dme_circ_0004446
GTAGATGACGATGAGTTCGAGGCCTATTTGGATGGATATTTCGGCAAGAAGTTCAAGGAAGGTGTCGACG
AAGAGCAGGATGAAGAGGAGCTGAACTTCCTGCAGGAGCTGGGTGGCGAGATAAAGAAGGATAAGTCCAA
GGATAAGAAAAAGAAGAAGCAGTCCGACAAGGCGGAAGATGAAATGGACGATATTGATGACGACTGGG
>dme_circ_0004446_junction_seq
GAAATGGACGATATTGATGACGACTGGGGTAGATGACGATGAGTTCGAGGCCTATT
>dme_circ_0004447
GGAAGGTGTCGACGAAGAGCAGGATGAAGAGGAGCTGAACTTCCTGCAGGAGCTGGGTGGCGAGATAAAG
AAGGATAAGTCCAAGGATAAGAAAAAGAAGAAGCAGTCCGACAAGGCGGAAGATGAAATGGACGATATTG
ATGACGACTGGG
>dme_circ_0004447_junction_seq
GAAATGGACGATATTGATGACGACTGGGGGAAGGTGTCGACGAAGAGCAGGATGAA
>dme_circ_0004448
CAACGAGTTTACCGACCAAGTGCCCGAGGATCCCGAGTGCGATGCTCCGCCCAGCCGGATACGACTATTT
CGCGGTTACACATACGTGGCGCCAGAGCATTTGGAGCAGATGCGAAGGGATAACCACTGTGAGATTCAAT
ACTTTAATACAGGACTTCAGG
>dme_circ_0004448_junction_seq
ATTCAATACTTTAATACAGGACTTCAGGCAACGAGTTTACCGACCAAGTGCCCGAG
>dme_circ_0004449
TAAAATTGTACAGTGATGAGGCCGTTAGTCTGAATGACTTCAAAATAATACGCGTTCTCGGAACAGGTGG
TAAGTAAAGTATTATTGAAATATAAATACAAAATGTATGGAATATTGACTTGTTATTGTTTACATATTTT
TCATAGCCTATGGGCGGGTTTTCCTCGTTCGCAAGCTAACTAGACACGATGCCGGAAAATTGTATGCAAT
GAAGGTTCTCAATAAGATAACGGTGGTCCAGAAGCGAAAAACCGCGGAACACACCAAAACGGAGAGAGTG
G
>dme_circ_0004449_junction_seq
GCGGAACACACCAAAACGGAGAGAGTGGTAAAATTGTACAGTGATGAGGCCGTTAG
>dme_circ_0004450
ctcaacgatgcgccacgatcagtccctggaccttttttatttttatttgttaattcctggaatatccatcg

attacgtgaggagcacgtagagcattattaaagGCATAGGATCTTTTACGGAAGAATCTAGCCATGAGTC
GCTTGCAAAAGCAAAACTACGAGATCTTAAGCGGTACAAGTACGTCGCGCCTCAAGAATCATCaacatcc
ccgtgaatcggagtcactagcctacgaggagccagatcaaatggtgcggaatcatctaaatggccagctg
gtagccaatggcaatggcaaaacgcgaaagaatagcaattccgaaacgatgaccaatgggaaaaagtcca
aactgaatacggagggatccggctctggttctggaaagaccttgaactacaataataataacaataacaa
caacagcatcagtgccaccaacggtcagtacaccaatagcagcagcaagaccacttcagcatctgcaagg
gattacacttaccgcgagaccatatcaccgcccacacctccatccccgcccaccacaaatgtggcggata
ttgtttgcatatcggatgcggagagcgaggacggacgcgatcccgagcgggagtactacgaccaggacat
ggaggaggatgagccgaacgggatagagatcgatgaatccagctccagcttgtccaaggccaagtccaac
aatgcagctgcagcggcggcggcggcggctgcagcagcggcagcagcagcctcgaaggcctcctcgtcga
ccacgccctcctacgccatgcccaccagcaatagtacaccccttgatctggacaatgaggcccatcaacg
ggacctggaggcggtcaccgatctgaaatACTACGG
>dme_circ_0004450_junction_seq
aggcggtcaccgatctgaaatACTACGGctcaacgatgcgccacgatcagtccctg
>dme_circ_0004451
GCATCATCTTCGAATATGGCTTTCAGGCTTTTGGGAACCTGTCCCGCTCCAAAGGCTGTATTGACCGCCT
TGATGGcctcttcatagttttcctcatccgccttcatctcctctctaatggtctccttcaactgattctt
ctctttgtaatttcgaggcgtttgagtcccatccgcttgttg
>dme_circ_0004451_junction_seq
gaggcgtttgagtcccatccgcttgttgGCATCATCTTCGAATATGGCTTTCAGGC
>dme_circ_0004452
gaatcagttgaaggagaccattagagaggagatgaaggcggatgaggaaaactatgaagaggCCATCAAG
GCGGTCAATACAGCCTTTGGAGCGGGACAGGTTCCCAAAAGCCTGAAAGCCATATTCGAAGATGATGCCT
GCGAACAGttaaacaagaag
>dme_circ_0004452_junction_seq
TGATGCCTGCGAACAGttaaacaagaaggaatcagttgaaggagaccattagagag
>dme_circ_0004453
gattatcgcttagaccttggcaatgccaataacgccgcacccgatgcgggcaccagcgttgcccgttgac
ttgctcagctcgtgtccaccctggccaagatcatcggcatcggcgtgcacgacaacggtgcgtccgatga
tgctgtcggcgccgaagagc
>dme_circ_0004453_junction_seq
tccgatgatgctgtcggcgccgaagagcgattatcgcttagaccttggcaatgcca
>dme_circ_0004454
gtcaacatcaccgactccaagattacgctcttcggcgccgacagcatcatcggacgcaccgttgtcgtgc
acgccgatgccgatgatcttggccagggtggacacgagctgagcaagtcaacgggcaacgctggtgcccg
catcgggtgcggcgttattggcattgccaagg
>dme_circ_0004454_junction_seq
gggtgcggcgttattggcattgccaagggtcaacatcaccgactccaagattacgc
>dme_circ_0004455
aaattaaaggaaaaacctcacaggggcagcgtcaacttgatggcctcctccttgggcggagccggtggcg
atatggtggtcaTGGGCGGCAATCGGCGTATCTtgcgtccgtgtttgccttccttgcgcttccacagacc
ggaacgtggtcgattgcccatctcccgcatccgctgggcactgcccacatgctccttg
>dme_circ_0004455_junction_seq
ccgctgggcactgcccacatgctccttgaaattaaaggaaaaacctcacaggggca

>dme_circ_0004456
GATCCACGAACATCTTTATTCACGTGCCGAAGCCACAAGTTGTTTGTTGATAACTCACTGGGCAAACAAC
AAACACGACCCGAAGACCCAAGAAACCCGTCGAGAAACCGAAAACTGAAAACAAGAAACAAACAAATCCC
AAAACTCTTGGCTGGCATTTCATTTGCTTGGATCGCGTTACGTGGTGCGGTTGATGTTGGTCGTCTGCCC
GCCAAAAAGAAAGTCACGACAGAGAAACGAGATTTCCAAGATTCTGCAACGAAAGTCAAAGTTACAAGTT
TACTCCAAATTCGAGTCTTTTGGCCCGGTTCAACGTCTTTGCAACACATTTTCGGTGTCTCCCCgtgtgt
gtgtgtgtgtgtgtgCCACTTTAGAAAGTGAAAATCGAGTGCAGTCCGCTACTTTGTCTGGAATTGAATC
TGAATCTGAATCCCAACCCATCGGCGGCTGCAGTTGAAAGTGAATTTTTTTCGACCTGCAAGTGATCACC
ATTTTCGTGATTCGATTTTTGGTGTGGTGTTTTTGGCTTTCGGGCGCTTAATACATCACTATCGATCAGT
AGCCCGAAGTTTTCGGCTACTTGAATACACTTCACATCCATCGATAGGAGATTTGACCGGCAGCAAATCG
GAGAGGCAACTATGGAGGCCGAGGAAGTTACAAAACTCGTCGATGGCGTGTATCGG
>dme_circ_0004456_junction_seq
TACAAAACTCGTCGATGGCGTGTATCGGGATCCACGAACATCTTTATTCACGTGCC
>dme_circ_0004457
GAACATACTGGATAGATTCAATCCGGGTGCCAGGCAGCTGATCGCAGCGGGCAAAAGTTACCTCAAGGCG
TTGCACGGTAAGTTTCAGTGCTGCTTTATGCTGGTCTATAGATACACCACTGTAAAGTAACACCATAATC
GACTGCCGCAAGTGCAGTACAGTTAACTAATTTGCAATGCATTCCGTGCCATTTCATGCAACAGGTGCCG
CAACCGCCTCGCGTCTATTCAATGAAGCATTGGCCAAGATTGCGATGAACGCCCAGCAAAGCGGAACGGG
CGATATTGGTAAGTCAAGCCTAAGATATTGCGTGGGATGAGTCCAAAACCACAAAAATTGATCATTAATA
TCAACTATTCTGTACAGGTTCAGCTTTGATGAGCGTTGTGAATGTGAACAAGGAGATACAGGATCAGCAG
ATGAATATTGTATGTGAACTTAAGTTAATTCTAACCTAAGAAGGCCTATTCAAAACTTGACTTCTCTTTA
GCTGAAAGCCTTCTACGTGGATCTATTGGTGCCATTGGAGACAAATCTGGAGAAGGACACAAAGGTGGTG
CAGCATGAGCAAAAGAAGTTCCTGCAGCAGCACAAAGTGCGAATGGAGAGCTATCAGAAGGCCGTGTCCA
CGATGAAGAAGCAGCGCAAGAAAAAGGCCACGCCCGAGAACACCGAAAAGGAGCTTAGGTGTGAGTTCAT
ATGGCTGCCGGCCGAATCTTGATCCACCCATTCGCGGAAAAGAATTCTGTACTTGAAAAAGTGACGGATC
GATTACACTGATAAAAACAAAAAATGTCTTCAAAGTAGTGGAAGTGATCTGAATACTAGCTAGCAGGCTT
TAAATGTATTATAATTCCAAATTTGTTCATGATAGCTACCTATCTTAAAGTTCTGATTTTACTAAGCACC
GCAAAAGATTGAAATAATTTTAGAAATGATCTATGATTTCTTACCGTGTATAACTCTACCACTCTTTCCC
ACTTCCGTTCCGGGCGAAAAGGGCCATGACACAGGAGCGACGACGGTACGGATTCGTCTTGGAGCGCCAG
TGCTCGATCGCCAAACACTGGATGGTCTACCACACCACCGGCAAGACGGTAATTGATAATAATTTCGAAA
ATTGGCAAGAGATTGCCGCTTCTCGTGAGATCATTCCACCGGCGGCCTATGAAagtggatacagtaccag
caacggacacaacaacaacaacatcagcagcagcagcagcagcggcaacaacaacaccagcGGCAACATG
CGACGCCTGGGAACGAATTAAGGACGGCGACGATGACCACCTCCAGGGTCACGGAGCTCAGCTGAAGAAA
TCGCGCAGCATCGATGCCCCCTATGGTGATATGCGCACCCTGCACGAACGGGAGAACCTTTCCGGATTGG
GCGGTACTGGGCCAGCGCACTATGCCCAGAACTCGCTGCCACGCGCCAAATCTGATTTCAATCTGGCTTT
GGTGGGCACTGGACAGAGCTCAGGCCAACAAACACAAGATCATTGAGGAGCAGCTAGCCACTCTGGGCGA
CGATCAGCGCGGTGATCAGCGTCCTCTGGTGAAGGCCCTCTACGCCTACATGCCCTCCGGCGAGAACCAG
TTGTCCTTCGAGGAGGGCGACCGCATTGCTTTGGTGGGCGGAAAGGCCAAGGGCTGGCAATTCGGTGAGA
ATCTGCGGACACAGCACTTCGGCTGGTTTCCGGTGGCCTACACCAATGCGGAAGTGGCAGAGGTGACCAC
TTCCGGTGGTCGGCGATACGAAAACATGCTGATGAGCTATGAGCGAAATGGTGGCGGATCAGGATCCGGT
TCTGGCGGAGGTGGTCTGCGTAGCTATCAGCAACAGCAGGATTACGAGGCGCAGCTGGAGCTGATGGAGA
GCCATGAGGCAACGTACCGCAGGCGAAGGAATCACAGTGCCGAGGATTCATCACCCACGCGGATGTTCGG
CGACACAATCAAGCAGCAGAAGAAGTACCGCCCCGGATCCGGGGCCAATCCGCGACCGGGACCGCCACCC
ACACTGCCCGCTCCGGTGCCCAATGGACCGCAGAGCCAGTCTTCCAGAATGCTGAACAGCTCGCAAAGCT

TCTGCGCCTCCAACGGAGGAGGAGTCAATGCGGCGGTGGAAAGACGCAAACAAAAGCTACTTAATGGCGT
TCAGGAGCTCGAAGAACCATCCATCGGGCAAATCCGGAGTGGCGGCCAAGACCTCGCTGCATAGCAGCAA
TGACAGTGGATTCGCCAACGAGCCTCCACCACAGCCGGAAGTTGACTATTCGGATGAGGAGCCAGCCACC
CAAAGAGTGGCCATTCG
>dme_circ_0004457_junction_seq
AGCCAGCCACCCAAAGAGTGGCCATTCGGAACATACTGGATAGATTCAATCCGGGT
>dme_circ_0004458
GAATGCCGTTTAAAGGAACTACCATATGATCCTTTGAGGAGTGCGGTAGTTTTTCCAGTAGAACTCCTTT
GTAAAGTACACGTAAAACGTGGCCACATTGAGCAGCAGATGAGCTCCGAGCTCCAGCCACTCGGCGTAAC
CCTTGCGAGTGTGCCGTGTGTTGAGTCGAAACAGAATGTACGGCACCAGGAAG
>dme_circ_0004458_junction_seq
TCGAAACAGAATGTACGGCACCAGGAAGGAATGCCGTTTAAAGGAACTACCATATG
>dme_circ_0004459
GGTAGCGATGCTCGACGCCGTGGGCGTACAACTTCCAGCCGTTGTCCGGATCCCCCTTATTTCGGTACGC
CAGACGCATGTCGATTGTCAACTGAAGTTCCCTGGGCGGTTCCCTTAGCTCCGACGAGGAATCATAGCCG
AACTCCACCT
>dme_circ_0004459_junction_seq
GACGAGGAATCATAGCCGAACTCCACCTGGTAGCGATGCTCGACGCCGTGGGCGTA
>dme_circ_0004460
aaagaaaaaCCCAGCCAGACAGTCTGGAAAATCCATAGAAATACAAACACAATTTTAAGTCGGCACTAAA
AACCGAAAGTGGCTGTATCCCCGATTTTGATGAGCAGCCAACGGAGATTTCCGGTATCCAGCCAAATGGC
GAGGTGGAGTTCCACGTTCCAGGGTGAGTGCGATATAGTGCCACATGCCCACCCCACCCCTTTATAATTT
CACCGCACACGGTGAGAAATACCATTTTCTCCACTTTGCAAACATAATCGTATCGAACGCAATTACCAAA
AATCATTTGACTTGAAACAGGAAACACTAGCTGGAAAAAATTTTATTTATTATCAGCATTTCAAGCATAT
TCACtttcatgattgattgcacttaaaccgttgccattgaacgaggtcaaaaaatagtatcttaaaggaa
aggtaaaatttaaagatacgacaataacattttcagttccaattaaactgttttcaatgatgaaaaagta
gtttgaaagcatactaatttttaaaaacaaaaagtgcttgcaactttaagaaaacgtttcatgattgatt
gcacttaaatttttgccattgaacatggtcaacaaatagtatcctaatggataggtaaaattgaaagata
cgacaaaaacattttcagttcaatcgaactgtttttaatcatgaaaaagttattttaagcgtactacttt
ttaaaattaaaaagtgtttgctactttaggaaaacATTCATTGTTATTTATATTTATTAATATTTAATAT
TAAACAAATATTTTCATAATTTATAACTACTTATAGCAAAGGCAATAAGAAATATGAATATTGGATAATA
ATATTAAATTACGGTACTATAATATTAACTTAACTTAACTAAAATATTAAGTTACGGTAGTTAATATAAT
TCTTTACTGCTCTTCAGTAACTTCGAAGCTGGCTTCACTAAATGTGTCTTCTCACACAATATATTAATTA
TATATTTGCATATTACCTGAAAACCAAGCCGAGTCTTAGCCTTGAGTTGTCAAAACTGCTCACTATCAAT
ATATTATATGCGGTATACATACGTatttatttatttattttttttCCGTGGAGTTAAGTTCGCATGTGTG
TGCCGATTGCTTGATATTTTCTATTAGCGGGTGCTGGATTTTCCTTGCTCGTCGCCGTGAGCAGCTGCTA
ATGGGTTGTGAAAAAAGTGGGCGAACGAAGCGGGCTGGAAGGCTGTGGAAAAGTTAGGGAGGTGTGTGTG
TGTTTgggggaggggggggggggggTGTGGCCAAGCATGTGTGAAACTTCCTCACAGTGGAAAAACATATG
GTGGCAAGTTCAGTGCTTAAGGTTCGAGAACGCAAGTTTCCACACTTGATTATATGGCCCATAAATTTGT
AGTACATTTAAATAAATGCCATATCTTTGCAGCTTACGTGTTTTGGTATATTATTTTATTACTATTAGGT
GAACACAATTAAATTTTAGTTACAAAGTTCTGTTGTTAAGATGTATAATGCTTCTTAAAGATAAAAACCA
GTTACATATATACCAGATTAAGCTAGAAGTTTTAGGTTAGGCAAATAAAAATCTAAAGTGTAACACAATT
TAAAACTATTATTCCAGTGGATAGTTTTGCATATGTTCCTGATTTTTTGTGTGAAGTTCTTTTTTATTTG
TTTGTAGATTTTCGAGATGTTTCCTGCAGGCGAATGCCTCGCAAAAATTTACCAACTGGACCACAAGAAA

CATGTGGTAATGATAAAGAATTCTTAAGCAGCTTTCTATTTTTGGAATAAACATGTTTAGCGTGTTTGTT
AGTTGAACTAGTTAAAGTTTAAACATCAGATTTCCATCAGCTTGGTGACCCAGAACAAGAGTCCGTCGCT
TGAAATGTCATTGGCTTCCTATGAAGAGGACTTCTTCAAGGGCTTGGAAGCCCTATATCAAGTCTACATC
CCGGCAATCATCATAAAAGAAACCAGCTGAGCTGGCCCGGGGCAGGTGGATGGATAATGGTGCACCTCTC
TCTGGAAGGCACATATGCAAATGTTGGCCCAGTGCCCCCCACTTGGAGCTTCACCTCCGTTAAACCCCTC
CTCCGCTCCACGACCATGTGCGTGCGTGCGAAATCTGCGAGCAGAGGCAAAATGATTAACAAAGGCTCCC
CGAGGTGCGGCAATATGTTGGGGCGGCACCAGCAGTGGGTGGCTATTTGTACTAGACGTGCACTGGCAGA
AATTACGTGTGAGAAGATCTTGATCTTTTACGGACATAACTTATTGGGCAAAACTAATTCCGGGTTTCAA
ATTCCCTTTGCTTCTTAAAAACTATGAAAAGGAATGAAATAATTTATATGTACGTAATAAGTAGCTAACT
TAGCCTACATTGATAGTTGTTAGTTAAGTTAACTAGCTACCTTTTGGTAGGTTTGTCCGTTAAGTTCGAA
ATCTCGTGCAATTTCTTTCCGTGACCATGGGCTTTTGCCGGCGTACGCGCATGAGGAATTACATCCATCG
TCACTGACACGGACACCGACCGAGACGCGGACATTGGGACAGATCCTGGACAGAGGAGCGCCCCTCAGGA
CAGGCGCAGTGCGGTTGGAACAACCAGGGCCCAGCCCAGTGCTCAGCCTGCCCGACGTTCCTAGTTTCCC
TCCCCTCCACTCATCATCTTTCTCGGACGCCTATGTCCTGGTTAATGACATTTGAGTGAGCGAGAAGAAG
GTGGGCGGTAGGAGGGGGCAAAGGACACCCGGCGGCGACGCAGGCCGAGCGAGTAATTTGTGTCACGTTT
TAATTTTTGACGCCGCAAAGGACGTTCAAGTGTCTCCCCTCGTGATTATGACAATTAGGATTGCGTTGAA
ATCCTTCTTGCTTATTTTGCTACGCGTATTTAAAAAGGTTTATTGTGTAAGCCTCGCCTGGGGAGCTTTT
GTCCAACTTATGCTTGTGGCCCATTAAAGTAAATTTCGCGGCGAGTTTGCGCTGCTCGGAATTAAAATTA
ATCACTTTTAAAGCTAAATTTGTAAATACAAATGTGTTGTGAGATTACAAAGCTTAAACTCTAATGTGCT
AAAATATAATTAAATGGCAGTAATTTAATTAAATATTCTATGCAGCAACAATTAATAACAATTAACCGAA
TTTAATGATGACTAGATGGCGTAGATTATGTTATAAAACCCGTGAACGATGGAATGTCTTTAATACGGAT
TTTTATTGTTTACGTTTTGCCGGTATAACAATTTTAAATTTCATTTATATGAAACAAAATGCTTTGATAT
AAAATTAATAGTGCACCGTATGCGCGGCTTTTTATCGCTCGCCATTCGAGCGAATAACTTTTTATGCTGT
GAACCATTTTTGATAAACATTTTAAAAATATGCCATCGCAGTAATTGGATAGATTTGCATATCAATCAAT
TTGTGGCTTAATTGCCATTTTATTATTCATTTTCCGCCGCGAACGTCGAGTGGGTTGGCTCAAAGAGCTG
CAAATTCGGCTGAAATGCGTCAGTGGAAAGTCCGCACTCATTAGCCGTGATTTTCTTGCTACCCTGCTCT
TGACCGAAACGCATTTCACAAGGCAAATCCTTCTCTCAGCGAACTGTCAAATGTCCTGCACACTTCGTTG
GCTCCTGACAGGCAGCTCAAAAAGTGGCCAAAGGATGTTCCAACTTTGCTCGCAAAAGTTACCTTTGAAA
AGTGGTCTAAGAAGCTTTGTTTCCGTGTTTCAAGCCATTTTCAACATTAAATTTATTAAATATTAAATAT
GCGAAAAGCTTTTATAGTTTTATATATTTCAATCTAGAAAGAGTCGGGCCAACGCAATTTCAATTAACCT
TAAGTGGGATTTCCTAACCAGTTTCCGCATTATTCATCGAGAAACCCTCGTAAATGTCATAGGCATGGCT
TGCACTTTCCTATCCTCTTCGCTATTCCTCTGATTTCCTTTCCATTGCCTCAGCTTTTCTTTTCTGCCAG
TGGCCTCAAGGAAACCAACAACTGCGTAaacagaacagaacggaacagaacagGAGCGACAAGGAGCGGA
CAGGACATTAACAAGATGAGCAGGGGAATGGTGGGCTTGTAGGCGAAAGGACTTCACTTCACTTCAGTCA
ATTTGACAAACATGACACAGAAACGAAAACGAGAACGCCTCATGGCCCACCGTCGTTTGCACGGGGAAAA
GTGTATTAAAACTTTAAAAGAAAATATGTTTTAGTAACTTATAGCAAATACTTTCCTTATAAATGAAATA
TTCAATTTAAATTAATAATGCTTCTCATTCATATTGCTTGCCTTTGTGCTTTCCAATAAGTAAATTCTTC
TAGAAATAGTTTCATATTAGTAGTTAGAAATTTGCCATTTTTATTGAAAGTTTTTCTTTTTGTGTAGCCG
GTGACTGGCAATTTGGCGAAGCAGTTGCTCTAATGGACAGGACTCTCAGACGCTGCTGGAAGGCATCTTC
CATCCTTGGGTTGTTGGCGGGCCAGAGCTGGCAATTAAATTTTTAAAATGCCATTTCAAAAGCAATTTCG
AAAGGCAATGGAAATTAAAAATGCCATCTGTGCATGCGTGTGTGTATGAGTGTGTTGCAAAATAGAAAAC
AGAGCGTGAAAAGGAGCTTCGCAGGAGAATGGGGTCGGTGGAAAGAAGGGGGGCGGGGTGCGCCAGGACA
CCAAGGACAGCGGCAATATTTTCGCAAATAAACGTAAATTGAATTTGCGGCCAAGAATGGCAAGTTTTGT
GCGGCCGAGCAGCACAAAATCGCCCAAAAGTAGGAAAACAAACTGAAAAGAAAGTTTTTACAAGTTTGCG

TGTGCTTTCTTTAGTTCttttttttttttttGTATTTGTGTGTGTCATCATCGAGGAAATCCGGCGCCAA
GTGCCGGCGCCTCTGCGGCTACTTTTTCGTTTTCAGCCAACTCATGTGATTTCCCCCATTTTCCATCCAT
TTTTCCCCTGTTCCGCATCCTGAAATGCGCTTTTTCCTTTTCCTCTGTTTTGCTTGCGACTTTCGCAACA
GCTCAGCTGTTGTCACCACACCACACCACCCCACTCCACCGCCCGGGAAAATGCAATTTTAACTGGGCCC
ACGTTGTAGCCGTTTTTGGTTTTTCGACTTTTATTGGACCCGTATCCGCATTGCCATTTCTCACATTGTT
TTCTGGCCATGCCTTGTAATTTAATTGGCAATTTGGACAGTTTTTCACTTTCCAGCTTCTTTTGGCCAAG
AGCCATGAACTTCGACCAAGTCTGGCGCAATCTCAATTTAAGCAAGACCAAATGAAAACAGTGTTTTTGT
TCCATTCATTTTGCCTTTAGCAATTTTGGCCAGAATGAAATCTTTTGTAAACTCAATTTGATTATATGCA
CTGGACACATCCCAGATTGCCAGCCATGTCTCCTCCTCACGAAGAATATCAAACGATACCATTTAAAAGA
AGACTGCCACTTTTTGCCAGGTAATGGAAGACAAACATGAGATGGTTTGATTTATTGCAGCTTTTCTCTG
TGCGAGAAATTACAGGGAAAATGAAATATTGCACTAACTAAGGCAGTAGAGACTTTTAAGGGTATTTAGA
AAACGGAGAATCTGAATAGAGTGACGTAGTGTATAGATTTCGAACTCGATATAAGAGGTCGATTTTTCTT
TGTTATCGCATTCTTTTCATGGACTGCTTCCGGCCATTTCTAAACCTACAAAAAACTAATGTGTTTTATC
TTGAAGTTTCCCTGAGATTTGTCAACCATATCAGTAGTTTAAATTCGTGTGAAATCAGTGCAAAAGAACA
TTAATAATaaaatgttttgtttaatgattacccataaaaatatatttttttaatgagtaagcgtaaaatta
tatAAAAGTCATCAATTATATTTGAATAATaaaaaaaacaaaaCACGTATGTATGATGTAGTCACCGCTT
TTGATTTCCATATATATATTTATTGGGTCGTTTATTTTCATTAAGTTTCGAGAATATTTCTCGTGATTTT
CTTCCATAAAATCAATCTGAAAAACTGATTACTAATTGATGTGGGAATCGAACCGTCGGCTATCAACGAA
TCAAATTGGCTTGGTCGCTAATTAATTGTCTTAAGTGTTTTTAGCCGCTGGTGATTAAGAAAGGTGAATG
TGATTTAACATGCAAATCACGAAGAAAGTTTTAGTCACACCATTGATTATAACTTTTAAATAGTTGAGCC
AACAAAAGAATTCTTGTGGGACGAACTTCAACTTACTATATCAGCTGTTGCGCCCACAAAAACAAAAGAA
CCAAAGGCGCACCCAAATTCACGCAACAGTTTTCCCAACTTATAGCTCCCCATTCACCACCCACAAAGTT
CGATTGATGATAGGCCTAGAATTTGGTAGTACCTGGTGGCAACAGTGGATTGGTAACCAACTTGGGCAAT
AGCGAAAATTGCGCAAAGTTCTAGGAAAATATACGAAAGTTTGAACAGCTGTGGATATAACAATTATATT
TGACTTTCTATATGTTTACTTGCTTAACCTTGCTGTCATTGTTAATTTATGTAAGCAGCTGCTGTGCTGC
TTTTTCGAAATACAACTAAAAACTAGTTGATGCTAATGCTAAGGAAATTAAATTAAAATTAACATCAAAT
TGAATACCAGTTTCTTATTTTTCTGCTGTTTTTAATACGAATTTCCTGCGGAAATGTTTATAAGTATTTC
CGATTGTCCTTAATTCTCTCCACTGTGTCCTTTATTGGCATTAGCCTAATAGCTCATCGAATCGAGCCCA
ACCCAGACATGCCTAGATGAGCCATTAGTCGCAGCTCACTAATTTGTTGTCATAAATTTTATGGATGTGC
TGTAAAGTTGATTTTATGCGAGGCACACTTTCTCCCTTTATTCGCACGATATTCGGGAAAATTGGATCTG
AATAGGTGGTATGGTTGTGTGGCAGATATGATTTGATAAATGCCCCACTGTTTGACTCCATTATGGTTAA
AAATCTCTGCCGCCACACCGATATGCGATATGTAGCATGGTTATTAATCAAATTGATGGGTAATTTGCAA
TGAAGCTTATCTATAGAGGTTTCCCTTTTTGGTTCAGTATATTGAAACCTCTGTACGAAGTAGCACACAC
CTTGTGTGAAAAATATGTTTACTGATAATTTTGCCATTTGCGCTGAGGCAAAGCTAAATATAGCAGATAC
TAAATAGAAGTGTTAAAATTGTGTTAATTTCCATGGTATATTCAGCGTGAACAATGAATTTGACACATGT
TAGCTAAAACCAATATGATCGTATCATGTTCTGGTGGGTTATTTCAGTATAAACAGAATCATGTTTAGTA
AACACCCATTTAGTCGTTTATTAAACGCTGTTGTTAGTTGTATTTGTTCGGTGATTAAAATAAAATATAA
CTACGTGTATTTGATGCTAAACTCTTTAAAGAAATTAGTGTGTTGAAATTTGTTTAATATTTTACAAAAT
ATTGTGTGCCTGGTACTTTAAAAGCGCAAACTCTATATTCGTATGCACATGCAATACTTCAATTTTGATG
GATTGCATACATGCACTTACTTTCCCACTTCAACAAGCACGAAGTGCAGGGAACTCCAAAAAAgtatata
tgtatatatgtacatatAGAAGCACCCGAGCGCGGCTCTTAAGAATTTGTGGCACGTTTCCATTGATTGC
GTTTGAGAGCTGCCACCAAACCGGATATAAATGACTCTATGCACTATAACTCACATGACCATAAAGGCAT
TCAGATACGGCCACCTCCGTTCTTCGTCCGCTCGGGAAATCCATTCGTTAATCCATTAAATTTGAAATAT
GTTCTGGCGGGTGGTCGTTGTTGGCTCGAAGAATCCTGCTCGATCCTGGAAATCCCTTTCGCACTTAGAT

AAATGCCATGCGCTTGGGTAATGCCGATGTGTCAAGTTCGGGTTGAAGCTCCTCTTAAGCTTTGCAACAC
AAATCAAAATGCTAGCAACTTGCTGTTTCACCTCTGCCCTTTTCAAGCGAAGAACTTCCCAGAATTTTCT
CTCTGCTTTGCCAGAAGAAATAGGGGAGCGTTGAAGTGTCTAAGAACAGCTATATTTCATTTAAATAAAT
AAGAAAATATTTCTCAAAAGGAAGTGCTGAATGTTACGATCTAAATTAAAAGAAATATGCATTTTTTGCA
TTCCTCTAAAGTTTTTCTTTGTTAAAGCTATAAAAGAAACCTAAAATACTTTCTCATGTTTATACAATTT
GTGGAAACCTTAAACCACTACAATTAAAATAAAATAAAATGCACTGGAAAGTTGTTATCTCAACAGGTAA
TTTTAGTAAGAACAACTTAAAAAAACGATTAAGTTCGCACTCCAACCGAAATGGTTGAAATAGCTGGTCC
AGTTACCACGGCTCCCAAATGCACATGCCCCTAGTATTATGCAATTGTTTTCGCCAGACACAGACAGACA
CACGCATACCAACAAACACTCTGAGCACCAATACAATGATGAAAAAAGAAGAAATTTCGTTGCCGGCGTC
ATTTTGATGGCTGTCCACAGTGGGATATTTCATTTGGCGCTTATGGCCGAAAATGGTTGAAATTGAATGG
ACCGAGATGGAAAATAGAAATAAAGGCGAGGCACTTTCATATCCGAAATAACTCCTAAATGCGTGTCCTT
GCCGCAAAACGAGCTAGTGGTCGGACATTCAAAAAGCACTTTGAATTCCGCCATGCCACAAAAATTGCCA
ATTCAGTCGTTCATAGCGTGTTTTCACTTTAATTATTGAAAGTGTGATGGGGGTATAAATATGCAATGTT
CTTTTACTTAGTATATCCTTAAAGAGTCCTTTGGGCATATCTCTTTAAAATGGACTTTGAAAGTTATTGC
TTTTTAAGATTTTGAATTTGTATAATTTATTTGTAAGTAAAGTAAAGTGACTGCTTTTGTTACATTGTTT
ATTGGGAAATGAAACATATCATTAACTATTTGCTTTAAAATTAATTTATATGCTGGTCTGtttttttttt
ttttATTAAATCTCAGGTTCAGCTCTTTGTACTAAAGAATTTCAAAAAGGCAATTGTTTATAAAGACCGA
GAACATGTTTATCAACATTTTGTGCCACAAGTCAGGCTCGATGAGGATGAGCTGTCAACGATACGTGACA
AATGCCAACATCTTTAATTCTGGTATTACaaaaaaaaaaaaaaaaTAATAATAAAATATTGTGATATTGC
ACTCTGACATTTGTGCCCCATTGTGTTTGAGCACTCGCCACCCACTGTGCCGCCCATTGTGGAGCTGTGT
GGGTGTGTATGTGTGGGTGGATGGTTGCGTCCATTCAAGGACACGGCGTGGCACCGCTCTCGAATGGCAT
CCGGGTGCTCGAGAGCCTGTGCCTGGTGCTCGCTGCAGCTCCTCGACGCCGCTCTCCATAGCCGTGGCAA
TGGCAGAGTGCTCCATTTCGTAACTTCCACACCCATACACCCACACACAGCCAACATTTTCCGGCTTTCC
CTTCGTCAGCACTTGGCACCCACTTGTGCGGTATTTGGTTTTCGCACTCCACCGCCCACTACGCAGACTA
CACAGGAAAATTTAGCAATAAAAACAAGCAATTTCAAAAGTTTTTTAATATTCAATCACATTGTTGGATA
TGATATAATTCCTAATGAAGGTAATAAACTTAATGAAGGTGAACTGAATAGTTTGAAGTTCTGCTAAGAA
AAGTGATAAAAGTACATGTTTGTCTGTTTTCTGAACATTTCTAACTAATTTCTTTCTAACTTCAGTCCAT
TTAATTTCCAATAAAACTAAATGAATATACATGATGTGCCTGATCATTTTGCACAGTGCACATAACCATG
CCCACTAAATGTGCCCCGTCTGGCTGGGATGCATTCATATATGTATACTCACATCCAGTTGTTTCCATTT
CCATTGCGGCTTTATTTGTTGGCCTCGGTGTGCTGAAATTGTCATGTAAATGGGTTAAGTTCTGACATTA
AAATGTGCCCGTCCAGATTTTTCATTTGTGCTCTTGCTTTGACATTTGGCCAAAATGATTTCCCATTTCC
GGGCGACCATGGGCGCCATGGTTCGTGATCCTGTTTCCGCATCCTTCTATccccccccccccTTCACCAC
TCGGTTAACCCTACGTCCGTTATCCTGTTTATTCATTAAAAACCAAAGCCGAGAAAATGTCTGAAATTTC
TGCCAACGGGCACAAAGAGATTTGCAGTATTTGTTGATAATTGCATTTTCTACTCTCTTTTGGTCTGACA
AGGCATTCAAATTTAATAAAATGGCTTCTTCTGTTGAAATTTGTAAGGATTTTCTAGCATGTTACCAATT
CAACATTCATTAAAAATGTCATGACATTGAAATGAAATACATATCTTGTaaaaaaaaaaaaTAAAGACGC
ATCTATCGAGGATGTTGACAGCCAAGCAACAGAATATTTTATGTTTATTTTCAGAAGGGAAATATAGCCT
ATATATAGGTCCAAATTCGAAATATATATTAACCCGATAAGAAGTCATATAATTGTACCCTTGGTTAATA
GTCTTAGTAATCAAATTTATTGCAATCATAAATATGAAATTGTGGGCAATTCCCTAAACTCTGTCAAAAA
AGTGGTATATCTATAAGCAATTAGTAGTACTATGTTGTAACATCATACATAAGTCTGTATAGATAAAAAC
AACATATGTTCCGATAAATTGATTTAATGCATTCCATCTGCTTCGTTTTTTGAGCTGTATAGGCTTTAGT
TGAAATTCCGTACTAACACCTACTGAGTGTATTATTAAAAGTATTTATGAAATTTCCATTAAatatataa
atataaatatatatTTAGCTTATAGACACACTAATGGTGGCTTGCACTTCAACATTAACAACAGCTTAAC
AACTGATTTGCAAAATAGGTTTTTTTGTAACCGCGGGCGCACACTTTAATTGCATACAGATTGAAAATAA

TGCAAAACAGAAAAGAGTCGAGAACAAAACCAAATGCAACTAAAACGATGAACAAAACGGCGCCCAACTT
TGTTGGAATCATGCAAAGCACAGCACATTTCCAATTGGCCATTGAACTTTCATATCAAAAACAGCGACAC
CACCACCACCGGCGAAAATGGTTTTTAAAATGCTTAAACACGCGAGACCCAACAGCCGCGCCCCATCATA
ATTGGAAAAGTCTGTAAGAATCGCCAATGTTCGTACCCCAGGCGTACtatacgtactatgtgttgtctac
gtgcatactcgtatgaatgtgtaaatgtatgtgtgtgtatatgtgtatatgtataagtgcatgtgcttgt
aGGTCGGCGGGAAAACAACTTCCGCTACCAGCTGATAGCCAACCCTTCCCTTGCTCTCATTTCCCCAGGC
TGTTGTTAACAACTTTGCGCAAATTACAATGGGAGCGTGGAAAGTGGAGTCGAAAGGGTTAGGAGGTCTT
GGCTTTTGGCCACAGGTGAGATTAGCACAACAGCGGGAAAAACGCAATCTATTAAAGCGATGCACTTATT
AGGTATGTTACATGGATAGCTTTAATCAGGACATACATACATATGTAAGGCATAACTCATCTCACACTTA
CTCATTTTTAAACAATATAAAACTTTTGGTTTAAACTTCAGTATTTAACAAGCTATTGCACCCAAAACGA
ACTAAGAATCGGTTTTGAGATATGCTATCATAATAATACAATTAGCAATAGCTGATAACAAATTTGGTAT
TGTCATTTGCGTTAGCATTAAGATTTTCCGAACTTATTTTTCAGCGCTTTAGATGCGTCGCAATAAGCTA
ATTAAATTAAAACCTTAACGACAAAACCGAAATCAATCAAAATTTAGTTCGAAACCGTATCGAACTATTT
TGTTTTTCAACCGCACAGCAATGAAATGATAGCACAACATCAGTGACTCAGATAAATGCTTTTCGCCTTA
TCTCTTCTGAAATTAAttttttttGTACGAATTTTTCTCTTGCGAAAAACGAAGAGCTGATAATGATGGA
TATGTTGGACATGTTTATTaaaaaaaaaaaaCAATCAAAGTTGAGTGGCTTTTTCATGTTTCGGGTTATT
GATAAACTTTAATAGGGGTATTATGTGTGTTCGTAAATATATAAAACGAAATTATTTAGCCTTTTAAATA
TATTTTTTATGTCATCTTGGTTTTAAGAAAACTTTTGCTGATAGTTTCGTATTTACATTTTTAATTACCT
TTGTCATAAGTTCTTATTTAACAACACTAACTTCCCTTTAATTTTCTACATTTTTTTCAGCGTAAGTTTT
AGCTTCGCCATTCAGCTAAGTTGGAGTACGATAGTAAAACCATTAACAAAAACCGATATCGCTGCCTAGT
ACTTATATACATATATATGTACATATATAGATGCCCATATGGATACATGGAGAGGCAATTGCGATAAAGC
CAGTATAAACATAATAACCATACAATTTCAGAAATTCATTGCGTACCGACCAGCACGCCCCATATCGTCG
ACCGTCCCGCCCCCTTACGACGCTCCCTCCCCCCCTCGGGCCACGCCTCCGCCACCGTCACGCCCCCTTC
CGCTCGACACAGCGTTGAAGGCAGATAGTCAGTAAATGGAATGGAATAAAATAAATCAAAACCGCAAAGG
CAGCGAAACGGCGGAGTTGACAAACAAAAATATTATACAAAATTCGAATGCGAACGAATGCTATCGAAAA
ACCAACGCAGCGTAACGCGGCCAACACGAAAGAAATCAAAACACCACTtgcaacacaggctgcaactgca
acacaggctgcaagcgcaacaGGACGAGAATTCCTCGAACAGATCCAGCGACTAGGATGAAGGGCGGCAA
CTACACCAGCCTCGGCACGTGCAGTGGCATCAATGTCAGTGGCAACGTGGCCGGCACCCGCAAGATGTCA
CTGGGCAAATCCATTAAAATGTACCTGACCATTTTCATCCTGACCACCTGCATTTATATGGCGCTCTATC
AGTACCACATATCCCGTGAACCCTTCGCCGCCAGCGAGGTTGTCAAGGGTGAGTTCATATCTGCACTGCG
AGAAAATTCAAAATTAAATGAGAAATTTGAACGGGAGCATGGCATCAATATGTTGACATGATCCACATAT
TTAGTTGATTGTAAAATGTACATCATATTATTTATATTTTGGAACAAAAAATCTAGCACTTTAAAAAAGA
GTTTGTAGAAGAAATCCAAATAAGATTTAAGATATGTTTCCCCTTTTTCTCTGCGTGACTTGGATAATGT
TTGTTTACTTAAAAGTTGCCAGTTTTCTATTGCACCATATTCCTCTACAAATTTCTTCATCACATATATT
TCGTAATACCAGTTTCACCTAACATGATTTTGGCCTTTGAACGATATCGTTAGCAGATATCATTCTCTGG
TTGCGAAAGTTTGATTAAACTAATCTAAAATATGGAACACTCATTGCTTGCCTTGGCCCAATAACAAATG
AAATCAAAAGTAAGTTTATACCTTTTAATTGGCATCTATTTAAGATAATGCCTCCTAATTGGTTAAAACG
CTGGGCAGCAGTGAAATAAATCGTGGGAACATGTACTGTTTACTTTGTTTAAAAATAACAATAGAACGTG
AACGATATAAGCAACAAAACCATAAAGATAACTAAAAATCTCATGAAGAGAAATAAGGCAATAATAAAAG
AAGAACATGTGTAGCTACAGATTGTGTATTTATACTTATAATTTACTCGTTTGAAGTGCAATAAAAAATG
GATAGCAACATAAACAGGTAGAAGCACGAAATAAAAAGTCAGGTGCTAAATAGAGTAAAGCAGAATTAAG
ATTAGAGTAAAACGTGCTTTTAAAATGAACTTTATATGGGTTAAACTTTGTGTTGAGAAAGGTGGCTCTT
TTGTTTTTTACGATCCCAAACTCACCGAAACAATAACTAGAGTAAATACTATTGAAATATTTTCTCTGTG
TTCTTTTCTCCATTTCCCTCGATCTTCACTTACCCAATTATTGTGAACCAAACTGTAGCATCAGGAGAAA

TCATCTTCATACATCGCTTCATATTTGTGGTCACCCATTTCCCTGCTAATGGCCAATAGTTCCTCCAATA
CCAATAACAATTccacaacaacaagcaccacaacaacaacagctccaacgacaccaacaaccacaacaac
cacgacaGTGGGCAGTGTGGGCCAAAAACTGGGCGCATCCTCCATCTCCTCCATTCGCATGGTATCATTA
GCGGCCACAATACCGTCATTTAAATCGACCTTATCGGAATCGCGATCGGTATCGTTGGGTGGCCATCAAA
AGACTGCAACAGTGAAAACAAGCACCACAATCACAACAAGAACAACAGCCAGTGGCTTGGCCACAACCAA
ATTGTCAGCAACAACAAGAACAACAGCCAAAACAAGCGCCAAACTGTCCGCGGCAACCACGCCCACTGCC
TCCCACATGGAAAACGGCTATAAGACTCGACCAACATTTGTTGCCGCATCCCTGCCCGTAAATATATTGA
CACAATCAAAGTCCTAGTCGAGCACGTAAAAAATGCACAGAGAAAAAATTCGAGCCAATAGCAAATATTA
AATATCAAAATTTTGAAAAATATGAATATATATTGAAATCATTCTGTAATATGAATTTATTGAAATTTAT
AAATGATATTTAAATTGATTTCTTTACCCACATAATTGGAGAAACAAATAGATAAATTTAGATAAATTTC
AAAAAGTATAAAAATTGTACAGTGCTGAACaaaaaaaaaGTTTAAATAATGTAACTAAGTAATCCACAGT
AGTGGCAGTATTTCAACTTAAAGATACATTTATCTCACAACACCCACAAATAGTCAAATTTAGCATATTT
ATCGCTTTTTTCTCCCGCATTTCACTTTTTCCGTTTTAATCTTTGAAAGTGCATTTCGATCTTATCTGCA
TATTATTTTCACTTGACGCGTGCATGTCTGGCTGCCTTTCATTATTCGATAACTGAACGTTGCACTTGTT
GCCCCATTCAATAGCAACTGCAATGTTTTACCCTTAGCTCACTCTCTTATCGCTTTTCCTCAGCCCGTTT
TTTCCAATTTCCTAATTGCCCCATTGGTTGCTGGTCATAAATAAATTGAAATGGGCTAGTTATAATCATT
TATATATAATATAAAGAGAAGAATTAGATTTCATAGACTaaaaaaaaGCGAATTAATGCTTGAGCATCTT
AACTAAGATCGTATATTTAGTATCTATGCAAATATAGCGACAAAATGTGCTGCTCCCCACATTTTGCGAA
CCAAGTTTGCTGCTCGTACGTTAATTATGGTCTCATTAAAGTCCAAAATGGATCTATATTACCAACCGAC
TTGGCCATGCTAATAAACACAAAGGGACGCTGTGGCGTACAAATTACGCTGTTTATCTAACCGACTTTTG
TTGGCCAGCATGTGCAAATTGGTTGCCAGCTATACGTATCACGATTTCTGGGTTAATCACATGCATTATA
TTAACCAACAAGCGGTGCCAGCTGCGGGTGTGCCACTGCAACTGGTGGCAAACAAAAAGATACTCATTTA
CCATTTGGCATGAGCCTAATTTGAAAATTGCTCTACAGCATTACATAGAACCCAAAAAAACCAAAACCGC
ATACAGCTGCAGTTGAATGCAATGGAAAAATATAGAAAAGTGAGTAGCATTGAATGCGGCTGTTGGGTTG
GCTGTTGATCTAGATTCCAGATTTATGCAAAATTCAAGCCAATTGAAAATATTCACGCGCCAGTTGCATC
CACTAACTGCGATTCCCCAGTGCCCTGCACTCAATGTCAACGGTCAAGCCCATCAGCAAAGAGATAAAGT
TGTTCAGGTGGCAATATAATATACCTTGTATATAATACAATTGCAGTTTAAACTTTCTGTTTGTCCACGT
TTTTATTAATTTCAAAAGGTTCTCAAGCGAAGTTTTGAAATTGATTGATATAATCTCGCAACTATTTTTA
ATTCACAAGTGGATTCAAAAGTCTAAAGATATAATGATATGTCTGCTTAATTTGATATGACCTTCCATAT
AAGCAATCAAATGGGTATCTATTACTTGAAATAAGCTAATATTGTGATAGACCAGCTACTTTGCATGCCA
TCCTACCCCTTGATCCTCACTATCTTTTTCCATTTCATTTGAATATCCGAGTTTATTTTGCAAAATGAAT
GCATTTATATTTGCCACCGCCTTGCATGTGAGTGGGTGGGTGTTATGGGGTGGTATTGTTCTGTGGGTGC
ATGTGTGTCAACAATGGACAATTTGCGAAGTGAGTGCGCCAGTTTATTTATTTTCAACGTCAGTCGTTCA
ATGGGCCACTGGTTCACAGGCACCACATCATATCTGGCACGATGGGAATTACACTCGAAACAAAAGGGGG
GGTGTAACCAATTAATATACAACCCAAAACGTCATGCAACATGCTTAACTTTTTCAGTTTAATAAGGTTC
GAGAGCTGTCTTTAAGAACTTTTCGTTAAATAAGGTCGTATTTACTTGCTTAGTTTTTAATTTGTTAACT
TTCTTATATGTTATACTTAACCTTTATTTTTTCGAGTGCCTTTGACTAGCGAAAGGCGACCTATATTGCT
TAACCAGAGCCTGGGCAATCCGAGTTGGGGCAATGATGGGCGGATGTGGCTCGCTAGCTTTCGCCTAGTC
AAGTCATTGGGCTGTAAATTAATTTGCATAATTGCTCATAAAAAGTAGCTGCTCACGCCGCATTACGCAC
CACCCAATGGCACCTAACAAGGCACTCAGCCAAGCTGGCACCCCAATCACCCAGCCACCTAGTCATCTAA
CTACCATCTAGACCTAACCAGTCCAAGTGCAGTGCACACACACATACGCCCCGTAGTCAGTTCGCAGCGT
TAGATAACCTAATGACACTCAACTCAAAATGAACTCGACCCACAGCCCGCCCCTGTACATAATCACGCCC
ACCTACCGACGACCCGAGCAGCTGGCGGAGCTCACCCGGCTGGGATATACGCTGAAACATGTGGTGAATC
TACTCTGGCTGGTCATCGAGGATGCCAACAAGACGAATCCGCTGGTGGGCCACACTTTGGACCGCATCGG

AGTGCCCTACGAGTACATGGTGGGCGCCCATGCCGGAGAAGTACAAGCAGACGAAGAAGGCCAAGCCGCG
TGGCGTCTCCAATCGGAATCGGGGCCTCGAGTATCTGCGCGAGCATGCCACCGAGGGAGTGCTCTACTTT
GCCGACGACGACAACACCTACGACATTAGCATATTCGAGCAGGATGCGCTACATCAGCAAGGTGGCCATG
TGGCCAGTGGGCCTGGTCACAAAGACCGGAGTGAGCAGTCCCATTATTCAGGCGGGCAAGCTGGTTGGCT
ACTACGATGGCTGGATCGGAGGACGCAAGTATCCAGTGGACATGGCTGGATTTGCAGTGAGCGTCAAGTT
CTTGAAGGAGCGTCCCAATGCCCAGATGCCCTTCAAGCCGGGATACGAGGAGGATGGGTTTCTCCGCAGT
TTGGCACCACTTGACGATGCGGAAATCGAGTTGCTGGCGGACGAGTGCAGAGATGATTCTCACGTGGCAC
ACGCAGACGAAGAAGAACGCACCCGCTCAGGCGCTGAACAGGACGCGCTACAAGAACACGAACCTGGAGC
ACATAGATAGGCTACTGGTGCGGCCATAGGGATATTTACTGTAGTTAGCTAGTTAGGATTAGCAAGGAGC
TCGCCGTATCCAGGTTGGCTCGCTAGCATATTGACCGTGGCTCAGACGGATTGTACAGGTACAATCCGGC
GCACGGAGATACCAATCAGAATTGATCAGATCATTAGTCGGTGTACATTATTATCATAATCATATGATTA
GTTAGGCTCTAAGCGCACACATACCTATGATAATTCTTTGCACCGTAGTCTTTGTTTGTTCGCACACCTA
ATCGAAACTTGAGTAAGCTATTGTATCATATCTCAGCGTCCATGACTACTTTGTAAATAATTTAAGATTA
AAGTTGTTTTACGCTCTAAGTTGAAACGGAATTCTCAGCCATAAAAGCAAAAATCAGCAACGAATTGTAT
AAGATGTTCCTAGACCAAAGAAAAAGCCCCTCAAGTTTTGCATTCAGCAATGCCCCTGGGAAAATGCAAT
CACCTGCGACAGCCAGGACCTCCAACTGCCGTCTGCAGGATTCGTGCGAGGTCAGGGTCTGAACCACATC
AACAATCAATTAGGGCAAGGTGTCAACAAAAAATGGTTGTTCTTCGAATTGTTCTGAAACCGGGGCAAGC
GTTCAAATAATTTATGCCGACATATGGATGAATGTTCGAGGCGGCACAGATAAGTCGTGCACGGATGAGC
CTCCTGCCCCGGCGAAACCAAACGGAATCGCGTGCTTCGTTTAAATTGCACGAAATAAAAATTGGATACG
AACAGGACGAAGGAGACATACCTAGTTTAAACAATGTAAGGGGTTTTGGGCCCTGCTCTAATGGGACTGA
ATGGCTTGAAAGTTTGGCATAATCAATTTCATCCTAAACGCTGCACGATTTTTTGTCGAATGAGAACTAC
TTTTTGTATACCTAAGATATGCGTTTTGTTCTTCGTCCGAAACGGGTTTCCAAAGCAAAACTTTTTCACT
ACTCATAAGTATGCTAAGTTAAGTTTATATAGAAATTTATATTCAAGTTATGAACTGCACTGAAATCGCC
TACATGTAAATACTAATCACCAAATATTGTAACCAAATTTTTACAAGTCCCGCTAAAGCAGGGAGATGCG
CAGGGATTGGAAAAAACTCTGACAAATGG
>dme_circ_0004460_junction_seq
AGGGATTGGAAAAAACTCTGACAAATGGaaagaaaaaCCCAGCCAGACAGTCTGGA
>dme_circ_0004461
CCGCTCCTGTTGGCCAAGtttttttttGTTGTTGCTTTGGCAACCGTTGGaacaacaacagcagcaacaa
caacataaacaacaggacacataAGGGTACTTCCATTTCTGAAAACATTTGCTCTTTTGAAACaaaaaa
taaaaagaaagaaaaaCCCAGCCAGACAGTCTGGAAAATCCATAGAAATACAAACACAATTTTAAGTCGG
CACTAAAAACCGAAAGTGGCTGTATCCCCGATTTTGATGAGCAGCCAACGGAGATTTCCGGTATCCAGCC
AAATGGCGAGGTGGAGTTCCACGTTCCAGGGTGAGTGCGATATAGTGCCACATGCCCACCCCACCCCTTT
ATAATTTCACCGCACACGGTGAGAAATACCATTTTCTCCACTTTGCAAACATAATCGTATCGAACGCAAT
TACCAAAAATCATTTGACTTGAAACAGGAAACACTAGCTGGAAAAAATTTTATTTATTATCAGCATTTCA
AGCATATTCACtttcatgattgattgcacttaaaccgttgccattgaacgaggtcaaaaaatagtatctt
aaaggaaaggtaaaatttaaagatacgacaataacattttcagttccaattaaactgttttcaatgatga
aaaagtagtttgaaagcatactaatttttaaaaacaaaaagtgcttgcaactttaagaaaacgtttcatg
attgattgcacttaaatttttgccattgaacatggtcaacaaatagtatcctaatggataggtaaaattg
aaagatacgacaaaaacattttcagttcaatcgaactgtttttaatcatgaaaaagttattttaagcgta
ctactttttaaaattaaaaagtgtttgctactttaggaaaacATTCATTGTTATTTATATTTATTAATAT
TTAATATTAAACAAATATTTTCATAATTTATAACTACTTATAGCAAAGGCAATAAGAAATATGAATATTG
GATAATAATATTAAATTACGGTACTATAATATTAACTTAACTTAACTAAAATATTAAGTTACGGTAGTTA
ATATAATTCTTTACTGCTCTTCAGTAACTTCGAAGCTGGCTTCACTAAATGTGTCTTCTCACACAATATA

TTAATTATATATTTGCATATTACCTGAAAACCAAGCCGAGTCTTAGCCTTGAGTTGTCAAAACTGCTCAC
TATCAATATATTATATGCGGTATACATACGTatttatttatttattttttttCCGTGGAGTTAAGTTCGC
ATGTGTGTGCCGATTGCTTGATATTTTCTATTAGCGGGTGCTGGATTTTCCTTGCTCGTCGCCGTGAGCA
GCTGCTAATGGGTTGTGAAAAAAGTGGGCGAACGAAGCGGGCTGGAAGGCTGTGGAAAAGTTAGGGAGGT
GTGTGTGTGTTTgggggaggggggggggggggTGTGGCCAAGCATGTGTGAAACTTCCTCACAGTGGAAAA
ACATATGGTGGCAAGTTCAGTGCTTAAGGTTCGAGAACGCAAGTTTCCACACTTGATTATATGGCCCATA
AATTTGTAGTACATTTAAATAAATGCCATATCTTTGCAGCTTACGTGTTTTGGTATATTATTTTATTACT
ATTAGGTGAACACAATTAAATTTTAGTTACAAAGTTCTGTTGTTAAGATGTATAATGCTTCTTAAAGATA
AAAACCAGTTACATATATACCAGATTAAGCTAGAAGTTTTAGGTTAGGCAAATAAAAATCTAAAGTGTAA
CACAATTTAAAACTATTATTCCAGTGGATAGTTTTGCATATGTTCCTGATTTTTTGTGTGAAGTTCTTTT
TTATTTGTTTGTAGATTTTCGAGATGTTTCCTGCAGGCGAATGCCTCGCAAAAATTTACCAACTGGACCA
CAAGAAACATGTGGTAATGATAAAGAATTCTTAAGCAGCTTTCTATTTTTGGAATAAACATGTTTAGCGT
GTTTGTTAGTTGAACTAGTTAAAGTTTAAACATCAGATTTCCATCAGCTTGGTGACCCAGAACAAGAGTC
CGTCGCTTGAAATGTCATTGGCTTCCTATGAAGAGGACTTCTTCAAGGGCTTGGAAGCCCTATATCAAGT
CTACATCCCGGCAATCATCATAAAAGAAACCAGCTGAGCTGGCCCGGGGCAGGTGGATGGATAATGGTGC
ACCTCTCTCTGGAAGGCACATATGCAAATGTTGGCCCAGTGCCCCCCACTTGGAGCTTCACCTCCGTTAA
ACCCCTCCTCCGCTCCACGACCATGTGCGTGCGTGCGAAATCTGCGAGCAGAGGCAAAATGATTAACAAA
GGCTCCCCGAGGTGCGGCAATATGTTGGGGCGGCACCAGCAGTGGGTGGCTATTTGTACTAGACGTGCAC
TGGCAGAAATTACGTGTGAGAAGATCTTGATCTTTTACGGACATAACTTATTGGGCAAAACTAATTCCGG
GTTTCAAATTCCCTTTGCTTCTTAAAAACTATGAAAAGGAATGAAATAATTTATATGTACGTAATAAGTA
GCTAACTTAGCCTACATTGATAGTTGTTAGTTAAGTTAACTAGCTACCTTTTGGTAGGTTTGTCCGTTAA
GTTCGAAATCTCGTGCAATTTCTTTCCGTGACCATGGGCTTTTGCCGGCGTACGCGCATGAGGAATTACA
TCCATCGTCACTGACACGGACACCGACCGAGACGCGGACATTGGGACAGATCCTGGACAGAGGAGCGCCC
CTCAGGACAGGCGCAGTGCGGTTGGAACAACCAGGGCCCAGCCCAGTGCTCAGCCTGCCCGACGTTCCTA
GTTTCCCTCCCCTCCACTCATCATCTTTCTCGGACGCCTATGTCCTGGTTAATGACATTTGAGTGAGCGA
GAAGAAGGTGGGCGGTAGGAGGGGGCAAAGGACACCCGGCGGCGACGCAGGCCGAGCGAGTAATTTGTGT
CACGTTTTAATTTTTGACGCCGCAAAGGACGTTCAAGTGTCTCCCCTCGTGATTATGACAATTAGGATTG
CGTTGAAATCCTTCTTGCTTATTTTGCTACGCGTATTTAAAAAGGTTTATTGTGTAAGCCTCGCCTGGGG
AGCTTTTGTCCAACTTATGCTTGTGGCCCATTAAAGTAAATTTCGCGGCGAGTTTGCGCTGCTCGGAATT
AAAATTAATCACTTTTAAAGCTAAATTTGTAAATACAAATGTGTTGTGAGATTACAAAGCTTAAACTCTA
ATGTGCTAAAATATAATTAAATGGCAGTAATTTAATTAAATATTCTATGCAGCAACAATTAATAACAATT
AACCGAATTTAATGATGACTAGATGGCGTAGATTATGTTATAAAACCCGTGAACGATGGAATGTCTTTAA
TACGGATTTTTATTGTTTACGTTTTGCCGGTATAACAATTTTAAATTTCATTTATATGAAACAAAATGCT
TTGATATAAAATTAATAGTGCACCGTATGCGCGGCTTTTTATCGCTCGCCATTCGAGCGAATAACTTTTT
ATGCTGTGAACCATTTTTGATAAACATTTTAAAAATATGCCATCGCAGTAATTGGATAGATTTGCATATC
AATCAATTTGTGGCTTAATTGCCATTTTATTATTCATTTTCCGCCGCGAACGTCGAGTGGGTTGGCTCAA
AGAGCTGCAAATTCGGCTGAAATGCGTCAGTGGAAAGTCCGCACTCATTAGCCGTGATTTTCTTGCTACC
CTGCTCTTGACCGAAACGCATTTCACAAGGCAAATCCTTCTCTCAGCGAACTGTCAAATGTCCTGCACAC
TTCGTTGGCTCCTGACAGGCAGCTCAAAAAGTGGCCAAAGGATGTTCCAACTTTGCTCGCAAAAGTTACC
TTTGAAAAGTGGTCTAAGAAGCTTTGTTTCCGTGTTTCAAGCCATTTTCAACATTAAATTTATTAAATAT
TAAATATGCGAAAAGCTTTTATAGTTTTATATATTTCAATCTAGAAAGAGTCGGGCCAACGCAATTTCAA
TTAACCTTAAGTGGGATTTCCTAACCAGTTTCCGCATTATTCATCGAGAAACCCTCGTAAATGTCATAGG
CATGGCTTGCACTTTCCTATCCTCTTCGCTATTCCTCTGATTTCCTTTCCATTGCCTCAGCTTTTCTTTT
CTGCCAGTGGCCTCAAGGAAACCAACAACTGCGTAaacagaacagaacggaacagaacagGAGCGACAAG

GAGCGGACAGGACATTAACAAGATGAGCAGGGGAATGGTGGGCTTGTAGGCGAAAGGACTTCACTTCACT
TCAGTCAATTTGACAAACATGACACAGAAACGAAAACGAGAACGCCTCATGGCCCACCGTCGTTTGCACG
GGGAAAAGTGTATTAAAACTTTAAAAGAAAATATGTTTTAGTAACTTATAGCAAATACTTTCCTTATAAA
TGAAATATTCAATTTAAATTAATAATGCTTCTCATTCATATTGCTTGCCTTTGTGCTTTCCAATAAGTAA
ATTCTTCTAGAAATAGTTTCATATTAGTAGTTAGAAATTTGCCATTTTTATTGAAAGTTTTTCTTTTTGT
GTAGCCGGTGACTGGCAATTTGGCGAAGCAGTTGCTCTAATGGACAGGACTCTCAGACGCTGCTGGAAGG
CATCTTCCATCCTTGGGTTGTTGGCGGGCCAGAGCTGGCAATTAAATTTTTAAAATGCCATTTCAAAAGC
AATTTCGAAAGGCAATGGAAATTAAAAATGCCATCTGTGCATGCGTGTGTGTATGAGTGTGTTGCAAAAT
AGAAAACAGAGCGTGAAAAGGAGCTTCGCAGGAGAATGGGGTCGGTGGAAAGAAGGGGGGCGGGGTGCGC
CAGGACACCAAGGACAGCGGCAATATTTTCGCAAATAAACGTAAATTGAATTTGCGGCCAAGAATGGCAA
GTTTTGTGCGGCCGAGCAGCACAAAATCGCCCAAAAGTAGGAAAACAAACTGAAAAGAAAGTTTTTACAA
GTTTGCGTGTGCTTTCTTTAGTTCttttttttttttttGTATTTGTGTGTGTCATCATCGAGGAAATCCG
GCGCCAAGTGCCGGCGCCTCTGCGGCTACTTTTTCGTTTTCAGCCAACTCATGTGATTTCCCCCATTTTC
CATCCATTTTTCCCCTGTTCCGCATCCTGAAATGCGCTTTTTCCTTTTCCTCTGTTTTGCTTGCGACTTT
CGCAACAGCTCAGCTGTTGTCACCACACCACACCACCCCACTCCACCGCCCGGGAAAATGCAATTTTAAC
TGGGCCCACGTTGTAGCCGTTTTTGGTTTTTCGACTTTTATTGGACCCGTATCCGCATTGCCATTTCTCA
CATTGTTTTCTGGCCATGCCTTGTAATTTAATTGGCAATTTGGACAGTTTTTCACTTTCCAGCTTCTTTT
GGCCAAGAGCCATGAACTTCGACCAAGTCTGGCGCAATCTCAATTTAAGCAAGACCAAATGAAAACAGTG
TTTTTGTTCCATTCATTTTGCCTTTAGCAATTTTGGCCAGAATGAAATCTTTTGTAAACTCAATTTGATT
ATATGCACTGGACACATCCCAGATTGCCAGCCATGTCTCCTCCTCACGAAGAATATCAAACGATACCATT
TAAAAGAAGACTGCCACTTTTTGCCAGGTAATGGAAGACAAACATGAGATGGTTTGATTTATTGCAGCTT
TTCTCTGTGCGAGAAATTACAGGGAAAATGAAATATTGCACTAACTAAGGCAGTAGAGACTTTTAAGGGT
ATTTAGAAAACGGAGAATCTGAATAGAGTGACGTAGTGTATAGATTTCGAACTCGATATAAGAGGTCGAT
TTTTCTTTGTTATCGCATTCTTTTCATGGACTGCTTCCGGCCATTTCTAAACCTACAAAAAACTAATGTG
TTTTATCTTGAAGTTTCCCTGAGATTTGTCAACCATATCAGTAGTTTAAATTCGTGTGAAATCAGTGCAA
AAGAACATTAATAATaaaatgttttgtttaatgattacccataaaaatatatttttttaatgagtaagcgt
aaaattatatAAAAGTCATCAATTATATTTGAATAATaaaaaaaacaaaaCACGTATGTATGATGTAGTC
ACCGCTTTTGATTTCCATATATATATTTATTGGGTCGTTTATTTTCATTAAGTTTCGAGAATATTTCTCG
TGATTTTCTTCCATAAAATCAATCTGAAAAACTGATTACTAATTGATGTGGGAATCGAACCGTCGGCTAT
CAACGAATCAAATTGGCTTGGTCGCTAATTAATTGTCTTAAGTGTTTTTAGCCGCTGGTGATTAAGAAAG
GTGAATGTGATTTAACATGCAAATCACGAAGAAAGTTTTAGTCACACCATTGATTATAACTTTTAAATAG
TTGAGCCAACAAAAGAATTCTTGTGGGACGAACTTCAACTTACTATATCAGCTGTTGCGCCCACAAAAAC
AAAAGAACCAAAGGCGCACCCAAATTCACGCAACAGTTTTCCCAACTTATAGCTCCCCATTCACCACCCA
CAAAGTTCGATTGATGATAGGCCTAGAATTTGGTAGTACCTGGTGGCAACAGTGGATTGGTAACCAACTT
GGGCAATAGCGAAAATTGCGCAAAGTTCTAGGAAAATATACGAAAGTTTGAACAGCTGTGGATATAACAA
TTATATTTGACTTTCTATATGTTTACTTGCTTAACCTTGCTGTCATTGTTAATTTATGTAAGCAGCTGCT
GTGCTGCTTTTTCGAAATACAACTAAAAACTAGTTGATGCTAATGCTAAGGAAATTAAATTAAAATTAAC
ATCAAATTGAATACCAGTTTCTTATTTTTCTGCTGTTTTTAATACGAATTTCCTGCGGAAATGTTTATAA
GTATTTCCGATTGTCCTTAATTCTCTCCACTGTGTCCTTTATTGGCATTAGCCTAATAGCTCATCGAATC
GAGCCCAACCCAGACATGCCTAGATGAGCCATTAGTCGCAGCTCACTAATTTGTTGTCATAAATTTTATG
GATGTGCTGTAAAGTTGATTTTATGCGAGGCACACTTTCTCCCTTTATTCGCACGATATTCGGGAAAATT
GGATCTGAATAGGTGGTATGGTTGTGTGGCAGATATGATTTGATAAATGCCCCACTGTTTGACTCCATTA
TGGTTAAAAATCTCTGCCGCCACACCGATATGCGATATGTAGCATGGTTATTAATCAAATTGATGGGTAA
TTTGCAATGAAGCTTATCTATAGAGGTTTCCCTTTTTGGTTCAGTATATTGAAACCTCTGTACGAAGTAG

CACACACCTTGTGTGAAAAATATGTTTACTGATAATTTTGCCATTTGCGCTGAGGCAAAGCTAAATATAG
CAGATACTAAATAGAAGTGTTAAAATTGTGTTAATTTCCATGGTATATTCAGCGTGAACAATGAATTTGA
CACATGTTAGCTAAAACCAATATGATCGTATCATGTTCTGGTGGGTTATTTCAGTATAAACAGAATCATG
TTTAGTAAACACCCATTTAGTCGTTTATTAAACGCTGTTGTTAGTTGTATTTGTTCGGTGATTAAAATAA
AATATAACTACGTGTATTTGATGCTAAACTCTTTAAAGAAATTAGTGTGTTGAAATTTGTTTAATATTTT
ACAAAATATTGTGTGCCTGGTACTTTAAAAGCGCAAACTCTATATTCGTATGCACATGCAATACTTCAAT
TTTGATGGATTGCATACATGCACTTACTTTCCCACTTCAACAAGCACGAAGTGCAGGGAACTCCAAAAAA
gtatatatgtatatatgtacatatAGAAGCACCCGAGCGCGGCTCTTAAGAATTTGTGGCACGTTTCCAT
TGATTGCGTTTGAGAGCTGCCACCAAACCGGATATAAATGACTCTATGCACTATAACTCACATGACCATA
AAGGCATTCAGATACGGCCACCTCCGTTCTTCGTCCGCTCGGGAAATCCATTCGTTAATCCATTAAATTT
GAAATATGTTCTGGCGGGTGGTCGTTGTTGGCTCGAAGAATCCTGCTCGATCCTGGAAATCCCTTTCGCA
CTTAGATAAATGCCATGCGCTTGGGTAATGCCGATGTGTCAAGTTCGGGTTGAAGCTCCTCTTAAGCTTT
GCAACACAAATCAAAATGCTAGCAACTTGCTGTTTCACCTCTGCCCTTTTCAAGCGAAGAACTTCCCAGA
ATTTTCTCTCTGCTTTGCCAGAAGAAATAGGGGAGCGTTGAAGTGTCTAAGAACAGCTATATTTCATTTA
AATAAATAAGAAAATATTTCTCAAAAGGAAGTGCTGAATGTTACGATCTAAATTAAAAGAAATATGCATT
TTTTGCATTCCTCTAAAGTTTTTCTTTGTTAAAGCTATAAAAGAAACCTAAAATACTTTCTCATGTTTAT
ACAATTTGTGGAAACCTTAAACCACTACAATTAAAATAAAATAAAATGCACTGGAAAGTTGTTATCTCAA
CAGGTAATTTTAGTAAGAACAACTTAAAAAAACGATTAAGTTCGCACTCCAACCGAAATGGTTGAAATAG
CTGGTCCAGTTACCACGGCTCCCAAATGCACATGCCCCTAGTATTATGCAATTGTTTTCGCCAGACACAG
ACAGACACACGCATACCAACAAACACTCTGAGCACCAATACAATGATGAAAAAAGAAGAAATTTCGTTGC
CGGCGTCATTTTGATGGCTGTCCACAGTGGGATATTTCATTTGGCGCTTATGGCCGAAAATGGTTGAAAT
TGAATGGACCGAGATGGAAAATAGAAATAAAGGCGAGGCACTTTCATATCCGAAATAACTCCTAAATGCG
TGTCCTTGCCGCAAAACGAGCTAGTGGTCGGACATTCAAAAAGCACTTTGAATTCCGCCATGCCACAAAA
ATTGCCAATTCAGTCGTTCATAGCGTGTTTTCACTTTAATTATTGAAAGTGTGATGGGGGTATAAATATG
CAATGTTCTTTTACTTAGTATATCCTTAAAGAGTCCTTTGGGCATATCTCTTTAAAATGGACTTTGAAAG
TTATTGCTTTTTAAGATTTTGAATTTGTATAATTTATTTGTAAGTAAAGTAAAGTGACTGCTTTTGTTAC
ATTGTTTATTGGGAAATGAAACATATCATTAACTATTTGCTTTAAAATTAATTTATATGCTGGTCTGttt
ttttttttttATTAAATCTCAGGTTCAGCTCTTTGTACTAAAGAATTTCAAAAAGGCAATTGTTTATAA
AGACCGAGAACATGTTTATCAACATTTTGTGCCACAAGTCAGGCTCGATGAGGATGAGCTGTCAACGATA
CGTGACAAATGCCAACATCTTTAATTCTGGTATTACaaaaaaaaaaaaaaaaTAATAATAAAATATTGTG
ATATTGCACTCTGACATTTGTGCCCCATTGTGTTTGAGCACTCGCCACCCACTGTGCCGCCCATTGTGGA
GCTGTGTGGGTGTGTATGTGTGGGTGGATGGTTGCGTCCATTCAAGGACACGGCGTGGCACCGCTCTCGA
ATGGCATCCGGGTGCTCGAGAGCCTGTGCCTGGTGCTCGCTGCAGCTCCTCGACGCCGCTCTCCATAGCC
GTGGCAATGGCAGAGTGCTCCATTTCGTAACTTCCACACCCATACACCCACACACAGCCAACATTTTCCG
GCTTTCCCTTCGTCAGCACTTGGCACCCACTTGTGCGGTATTTGGTTTTCGCACTCCACCGCCCACTACG
CAGACTACACAGGAAAATTTAGCAATAAAAACAAGCAATTTCAAAAGTTTTTTAATATTCAATCACATTG
TTGGATATGATATAATTCCTAATGAAGGTAATAAACTTAATGAAGGTGAACTGAATAGTTTGAAGTTCTG
CTAAGAAAAGTGATAAAAGTACATGTTTGTCTGTTTTCTGAACATTTCTAACTAATTTCTTTCTAACTTC
AGTCCATTTAATTTCCAATAAAACTAAATGAATATACATGATGTGCCTGATCATTTTGCACAGTGCACAT
AACCATGCCCACTAAATGTGCCCCGTCTGGCTGGGATGCATTCATATATGTATACTCACATCCAGTTGTT
TCCATTTCCATTGCGGCTTTATTTGTTGGCCTCGGTGTGCTGAAATTGTCATGTAAATGGGTTAAGTTCT
GACATTAAAATGTGCCCGTCCAGATTTTTCATTTGTGCTCTTGCTTTGACATTTGGCCAAAATGATTTCC
CATTTCCGGGCGACCATGGGCGCCATGGTTCGTGATCCTGTTTCCGCATCCTTCTATccccccccccccT
TCACCACTCGGTTAACCCTACGTCCGTTATCCTGTTTATTCATTAAAAACCAAAGCCGAGAAAATGTCTG

AAATTTCTGCCAACGGGCACAAAGAGATTTGCAGTATTTGTTGATAATTGCATTTTCTACTCTCTTTTGG
TCTGACAAGGCATTCAAATTTAATAAAATGGCTTCTTCTGTTGAAATTTGTAAGGATTTTCTAGCATGTT
ACCAATTCAACATTCATTAAAAATGTCATGACATTGAAATGAAATACATATCTTGTaaaaaaaaaaaaTA
AAGACGCATCTATCGAGGATGTTGACAGCCAAGCAACAGAATATTTTATGTTTATTTTCAGAAGGGAAAT
ATAGCCTATATATAGGTCCAAATTCGAAATATATATTAACCCGATAAGAAGTCATATAATTGTACCCTTG
GTTAATAGTCTTAGTAATCAAATTTATTGCAATCATAAATATGAAATTGTGGGCAATTCCCTAAACTCTG
TCAAAAAAGTGGTATATCTATAAGCAATTAGTAGTACTATGTTGTAACATCATACATAAGTCTGTATAGA
TAAAAACAACATATGTTCCGATAAATTGATTTAATGCATTCCATCTGCTTCGTTTTTTGAGCTGTATAGG
CTTTAGTTGAAATTCCGTACTAACACCTACTGAGTGTATTATTAAAAGTATTTATGAAATTTCCATTAAa
tatataaatataaatatatatTTAGCTTATAGACACACTAATGGTGGCTTGCACTTCAACATTAACAACA
GCTTAACAACTGATTTGCAAAATAGGTTTTTTTGTAACCGCGGGCGCACACTTTAATTGCATACAGATTG
AAAATAATGCAAAACAGAAAAGAGTCGAGAACAAAACCAAATGCAACTAAAACGATGAACAAAACGGCGC
CCAACTTTGTTGGAATCATGCAAAGCACAGCACATTTCCAATTGGCCATTGAACTTTCATATCAAAAACA
GCGACACCACCACCACCGGCGAAAATGGTTTTTAAAATGCTTAAACACGCGAGACCCAACAGCCGCGCCC
CATCATAATTGGAAAAGTCTGTAAGAATCGCCAATGTTCGTACCCCAGGCGTACtatacgtactatgtgt
tgtctacgtgcatactcgtatgaatgtgtaaatgtatgtgtgtgtatatgtgtatatgtataagtgcatg
tgcttgtaGGTCGGCGGGAAAACAACTTCCGCTACCAGCTGATAGCCAACCCTTCCCTTGCTCTCATTTC
CCCAGGCTGTTGTTAACAACTTTGCGCAAATTACAATGGGAGCGTGGAAAGTGGAGTCGAAAGGGTTAGG
AGGTCTTGGCTTTTGGCCACAGGTGAGATTAGCACAACAGCGGGAAAAACGCAATCTATTAAAGCGATGC
ACTTATTAGGTATGTTACATGGATAGCTTTAATCAGGACATACATACATATGTAAGGCATAACTCATCTC
ACACTTACTCATTTTTAAACAATATAAAACTTTTGGTTTAAACTTCAGTATTTAACAAGCTATTGCACCC
AAAACGAACTAAGAATCGGTTTTGAGATATGCTATCATAATAATACAATTAGCAATAGCTGATAACAAAT
TTGGTATTGTCATTTGCGTTAGCATTAAGATTTTCCGAACTTATTTTTCAGCGCTTTAGATGCGTCGCAA
TAAGCTAATTAAATTAAAACCTTAACGACAAAACCGAAATCAATCAAAATTTAGTTCGAAACCGTATCGA
ACTATTTTGTTTTTCAACCGCACAGCAATGAAATGATAGCACAACATCAGTGACTCAGATAAATGCTTTT
CGCCTTATCTCTTCTGAAATTAAttttttttGTACGAATTTTTCTCTTGCGAAAAACGAAGAGCTGATAA
TGATGGATATGTTGGACATGTTTATTaaaaaaaaaaaaCAATCAAAGTTGAGTGGCTTTTTCATGTTTCG
GGTTATTGATAAACTTTAATAGGGGTATTATGTGTGTTCGTAAATATATAAAACGAAATTATTTAGCCTT
TTAAATATATTTTTTATGTCATCTTGGTTTTAAGAAAACTTTTGCTGATAGTTTCGTATTTACATTTTTA
ATTACCTTTGTCATAAGTTCTTATTTAACAACACTAACTTCCCTTTAATTTTCTACATTTTTTTCAGCGT
AAGTTTTAGCTTCGCCATTCAGCTAAGTTGGAGTACGATAGTAAAACCATTAACAAAAACCGATATCGCT
GCCTAGTACTTATATACATATATATGTACATATATAGATGCCCATATGGATACATGGAGAGGCAATTGCG
ATAAAGCCAGTATAAACATAATAACCATACAATTTCAGAAATTCATTGCGTACCGACCAGCACGCCCCAT
ATCGTCGACCGTCCCGCCCCCTTACGACGCTCCCTCCCCCCCTCGGGCCACGCCTCCGCCACCGTCACGC
CCCCTTCCGCTCGACACAGCGTTGAAGGCAGATAGTCAGTAAATGGAATGGAATAAAATAAATCAAAACC
GCAAAGGCAGCGAAACGGCGGAGTTGACAAACAAAAATATTATACAAAATTCGAATGCGAACGAATGCTA
TCGAAAAACCAACGCAGCGTAACGCGGCCAACACGAAAGAAATCAAAACACCACTtgcaacacaggctgc
aactgcaacacaggctgcaagcgcaacaGGACGAGAATTCCTCGAACAGATCCAGCGACTAGGATGAAGG
GCGGCAACTACACCAGCCTCGGCACGTGCAGTGGCATCAATGTCAGTGGCAACGTGGCCGGCACCCGCAA
GATGTCACTGGGCAAATCCATTAAAATGTACCTGACCATTTTCATCCTGACCACCTGCATTTATATGGCG
CTCTATCAGTACCACATATCCCGTGAACCCTTCGCCGCCAGCGAGGTTGTCAAGGGTGAGTTCATATCTG
CACTGCGAGAAAATTCAAAATTAAATGAGAAATTTGAACGGGAGCATGGCATCAATATGTTGACATGATC
CACATATTTAGTTGATTGTAAAATGTACATCATATTATTTATATTTTGGAACAAAAAATCTAGCACTTTA
AAAAAGAGTTTGTAGAAGAAATCCAAATAAGATTTAAGATATGTTTCCCCTTTTTCTCTGCGTGACTTGG

ATAATGTTTGTTTACTTAAAAGTTGCCAGTTTTCTATTGCACCATATTCCTCTACAAATTTCTTCATCAC
ATATATTTCGTAATACCAGTTTCACCTAACATGATTTTGGCCTTTGAACGATATCGTTAGCAGATATCAT
TCTCTGGTTGCGAAAGTTTGATTAAACTAATCTAAAATATGGAACACTCATTGCTTGCCTTGGCCCAATA
ACAAATGAAATCAAAAGTAAGTTTATACCTTTTAATTGGCATCTATTTAAGATAATGCCTCCTAATTGGT
TAAAACGCTGGGCAGCAGTGAAATAAATCGTGGGAACATGTACTGTTTACTTTGTTTAAAAATAACAATA
GAACGTGAACGATATAAGCAACAAAACCATAAAGATAACTAAAAATCTCATGAAGAGAAATAAGGCAATA
ATAAAAGAAGAACATGTGTAGCTACAGATTGTGTATTTATACTTATAATTTACTCGTTTGAAGTGCAATA
AAAAATGGATAGCAACATAAACAGGTAGAAGCACGAAATAAAAAGTCAGGTGCTAAATAGAGTAAAGCAG
AATTAAGATTAGAGTAAAACGTGCTTTTAAAATGAACTTTATATGGGTTAAACTTTGTGTTGAGAAAGGT
GGCTCTTTTGTTTTTTACGATCCCAAACTCACCGAAACAATAACTAGAGTAAATACTATTGAAATATTTT
CTCTGTGTTCTTTTCTCCATTTCCCTCGATCTTCACTTACCCAATTATTGTGAACCAAACTGTAGCATCA
GGAGAAATCATCTTCATACATCGCTTCATATTTGTGGTCACCCATTTCCCTGCTAATGGCCAATAGTTCC
TCCAATACCAATAACAATTccacaacaacaagcaccacaacaacaacagctccaacgacaccaacaacca
caacaaccacgacaGTGGGCAGTGTGGGCCAAAAACTGGGCGCATCCTCCATCTCCTCCATTCGCATGGT
ATCATTAGCGGCCACAATACCGTCATTTAAATCGACCTTATCGGAATCGCGATCGGTATCGTTGGGTGGC
CATCAAAAGACTGCAACAGTGAAAACAAGCACCACAATCACAACAAGAACAACAGCCAGTGGCTTGGCCA
CAACCAAATTGTCAGCAACAACAAGAACAACAGCCAAAACAAGCGCCAAACTGTCCGCGGCAACCACGCC
CACTGCCTCCCACATGGAAAACGGCTATAAGACTCGACCAACATTTGTTGCCGCATCCCTGCCCGTAAAT
ATATTGACACAATCAAAGTCCTAGTCGAGCACGTAAAAAATGCACAGAGAAAAAATTCGAGCCAATAGCA
AATATTAAATATCAAAATTTTGAAAAATATGAATATATATTGAAATCATTCTGTAATATGAATTTATTGA
AATTTATAAATGATATTTAAATTGATTTCTTTACCCACATAATTGGAGAAACAAATAGATAAATTTAGAT
AAATTTCAAAAAGTATAAAAATTGTACAGTGCTGAACaaaaaaaaaGTTTAAATAATGTAACTAAGTAAT
CCACAGTAGTGGCAGTATTTCAACTTAAAGATACATTTATCTCACAACACCCACAAATAGTCAAATTTAG
CATATTTATCGCTTTTTTCTCCCGCATTTCACTTTTTCCGTTTTAATCTTTGAAAGTGCATTTCGATCTT
ATCTGCATATTATTTTCACTTGACGCGTGCATGTCTGGCTGCCTTTCATTATTCGATAACTGAACGTTGC
ACTTGTTGCCCCATTCAATAGCAACTGCAATGTTTTACCCTTAGCTCACTCTCTTATCGCTTTTCCTCAG
CCCGTTTTTTCCAATTTCCTAATTGCCCCATTGGTTGCTGGTCATAAATAAATTGAAATGGGCTAGTTAT
AATCATTTATATATAATATAAAGAGAAGAATTAGATTTCATAGACTaaaaaaaaGCGAATTAATGCTTGA
GCATCTTAACTAAGATCGTATATTTAGTATCTATGCAAATATAGCGACAAAATGTGCTGCTCCCCACATT
TTGCGAACCAAGTTTGCTGCTCGTACGTTAATTATGGTCTCATTAAAGTCCAAAATGGATCTATATTACC
AACCGACTTGGCCATGCTAATAAACACAAAGGGACGCTGTGGCGTACAAATTACGCTGTTTATCTAACCG
ACTTTTGTTGGCCAGCATGTGCAAATTGGTTGCCAGCTATACGTATCACGATTTCTGGGTTAATCACATG
CATTATATTAACCAACAAGCGGTGCCAGCTGCGGGTGTGCCACTGCAACTGGTGGCAAACAAAAAGATAC
TCATTTACCATTTGGCATGAGCCTAATTTGAAAATTGCTCTACAGCATTACATAGAACCCAAAAAAACCA
AAACCGCATACAGCTGCAGTTGAATGCAATGGAAAAATATAGAAAAGTGAGTAGCATTGAATGCGGCTGT
TGGGTTGGCTGTTGATCTAGATTCCAGATTTATGCAAAATTCAAGCCAATTGAAAATATTCACGCGCCAG
TTGCATCCACTAACTGCGATTCCCCAGTGCCCTGCACTCAATGTCAACGGTCAAGCCCATCAGCAAAGAG
ATAAAGTTGTTCAGGTGGCAATATAATATACCTTGTATATAATACAATTGCAGTTTAAACTTTCTGTTTG
TCCACGTTTTTATTAATTTCAAAAGGTTCTCAAGCGAAGTTTTGAAATTGATTGATATAATCTCGCAACT
ATTTTTAATTCACAAGTGGATTCAAAAGTCTAAAGATATAATGATATGTCTGCTTAATTTGATATGACCT
TCCATATAAGCAATCAAATGGGTATCTATTACTTGAAATAAGCTAATATTGTGATAGACCAGCTACTTTG
CATGCCATCCTACCCCTTGATCCTCACTATCTTTTTCCATTTCATTTGAATATCCGAGTTTATTTTGCAA
AATGAATGCATTTATATTTGCCACCGCCTTGCATGTGAGTGGGTGGGTGTTATGGGGTGGTATTGTTCTG
TGGGTGCATGTGTGTCAACAATGGACAATTTGCGAAGTGAGTGCGCCAGTTTATTTATTTTCAACGTCAG

TCGTTCAATGGGCCACTGGTTCACAGGCACCACATCATATCTGGCACGATGGGAATTACACTCGAAACAA
AAGGGGGGGTGTAACCAATTAATATACAACCCAAAACGTCATGCAACATGCTTAACTTTTTCAGTTTAAT
AAGGTTCGAGAGCTGTCTTTAAGAACTTTTCGTTAAATAAGGTCGTATTTACTTGCTTAGTTTTTAATTT
GTTAACTTTCTTATATGTTATACTTAACCTTTATTTTTTCGAGTGCCTTTGACTAGCGAAAGGCGACCTA
TATTGCTTAACCAGAGCCTGGGCAATCCGAGTTGGGGCAATGATGGGCGGATGTGGCTCGCTAGCTTTCG
CCTAGTCAAGTCATTGGGCTGTAAATTAATTTGCATAATTGCTCATAAAAAGTAGCTGCTCACGCCGCAT
TACGCACCACCCAATGGCACCTAACAAGGCACTCAGCCAAGCTGGCACCCCAATCACCCAGCCACCTAGT
CATCTAACTACCATCTAGACCTAACCAGTCCAAGTGCAGTGCACACACACATACGCCCCGTAGTCAGTTC
GCAGCGTTAGATAACCTAATGACACTCAACTCAAAATGAACTCGACCCACAGCCCGCCCCTGTACATAAT
CACGCCCACCTACCGACGACCCGAGCAGCTGGCGGAGCTCACCCGGCTGGGATATACGCTGAAACATGTG
GTGAATCTACTCTGGCTGGTCATCGAGGATGCCAACAAGACGAATCCGCTGGTGGGCCACACTTTGGACC
GCATCGGAGTGCCCTACGAGTACATGGTGGG
>dme_circ_0004461_junction_seq
TCGGAGTGCCCTACGAGTACATGGTGGGCCGCTCCTGTTGGCCAAGtttttttttG
>dme_circ_0004462
GAGCGCCATATAAATGCAGGTGGTCAGGATGAAAATGGTCAGGTACATTTTAATGGATTTGCCCAGTGAC
ATCTTGCGGGTGCCGGCCACGTTGCCACTGACATTGATGCCACTGCACGTGCCGAGGCTGGTGTAGTTGC
CGCCCTTCATCCTAGTCGCTGGATCTGTTCGAGGAATTCTCGTCCtgttgcgcttgcagcctgtgttgca
gttgcagcctgtgttgcaAGTGGTGTTTTGATTTCTTTCGTGTTGGCCGC
>dme_circ_0004462_junction_seq
GTGTTTTGATTTCTTTCGTGTTGGCCGCGAGCGCCATATAAATGCAGGTGGTCAGG
>dme_circ_0004463
GACAGCTGAATAACGCTGCTGGTAAGCTTCTCCGTTATAGCTCGTATTTGACAGCCAAAAAGGAGACGCT
GCACTCGTCGCAAACCGGGCAGATCCACCAGTGTGTCGCTGCTTTGGGATGAACACACATCATCGTCCTC
TTCTTCGGGCTCCGCCTCTGATTCGACTGATTCTGCAGTAGCAGTAGCATTAGCCGCCGATTCTGATTGT
TC
>dme_circ_0004463_junction_seq
TAGCATTAGCCGCCGATTCTGATTGTTCGACAGCTGAATAACGCTGCTGGTAAGCT
>dme_circ_0004464
CTTGTCACCTCGTTCGTTGGCACATGGTTGGGTTTGCTGGGCGCCGCAATTGAAGTTCGTTTTTGACGCT
GCGCCGGCTTCAGTCGCACCTGCAGTTCGTAGTCCTCGCAACCGAGATCCGGCACTCCAGTGTTCAGGAA
TGTTATG
>dme_circ_0004464_junction_seq
CGGCACTCCAGTGTTCAGGAATGTTATGCTTGTCACCTCGTTCGTTGGCACATGGT
>dme_circ_0004465
TTGAAAGTTGTTTACCTTTTAGCACATGCCTTGTCTGGAAATTTGCATTGAAAGCCATAATATTTAAAAA
CTCACTTTTGTCTGCTTAAACCACTGTGTCGACCAAGttttttttttACATTTTGTATTAAATTTTGTGT
TGTGCTGAGTTTTGGAATCAAGCCAATTTAATAGGAAATTAGTTGCAACACTTGAGAACCATTTAATGCC
GAACAGATAAATCAATCAATGTTTTTGAACTTGTAAACATGCGATTAAAGAAAAAATAAACAGGCCTAAA
TTAACATTCTATATTGCTTCTATTTATAATTGTTGTTAAATGTTGTGCGCAGCATTGTTGCACAAAACAT
CACATG
>dme_circ_0004465_junction_seq
GCAGCATTGTTGCACAAAACATCACATGTTGAAAGTTGTTTACCTTTTAGCACATG
>dme_circ_0004466

TTTTAAAATCTTTCTGGAACTTTCCCGATTCTTCCCTATAGACACAAATTTCTCTCCGAATTTTCGGTAC
ACTTACCTTTATTTTTGTTTGATCCCTGCAGGCTACCGTAGATGATTTGTTCCCGAGCAATAACATCCAT
TTTCATCGGTTTTTCTGTGTTTTGTTGGCTTGGAAAGTCGAATCGAATCGAAAGTCGTTCGTTCGCTCGC
TCGCTCGCAATTTTCCGACGGCTTTTGTCGCCTTCTTTGTATATCACTTTTACAATTTAATTAAGCTTAT
TTAATTTATCACAATTTGCTGTTTGTTTTCTTGCTGATTGCTTAAATTCTTCCTCTTTACGTTCTGGTTG
CAGTTTAAATTAACATttgttgttgttgttgttggttggttggttggttggttggttgttgttttgtAGT
GGAAATCACAGCACTTATGTTG
>dme_circ_0004466_junction_seq
tgtAGTGGAAATCACAGCACTTATGTTGTTTTAAAATCTTTCTGGAACTTTCCCGA
>dme_circ_0004467
GTGCTATAACAAATAACTAGAGTTTTAAGTTGTTTCTCCTTTTtcaaaaacgcaaaagcaagaaaaacaa
aacaaggcgacaagagcggaagaaaagtaaagaaaaaAACCAACATGAAATTCAAAGACGACAGCCCCAC
GAATCAATCCCCAGAATCATATTTCTGGGGCATTCGCTTTGGGGCTCGCCGGTCGACAACTCATGAATAT
>dme_circ_0004467_junction_seq
GGCTCGCCGGTCGACAACTCATGAATATGTGCTATAACAAATAACTAGAGTTTTAA
>dme_circ_0004468
GAGTTTTAAGTTGTTTCTCCTTTTtcaaaaacgcaaaagcaagaaaaacaaaacaaggcgacaagagcgg
aagaaaagtaaagaaaaaAACCAACATGAAATTCAAAGACGACAGCCCCACGAATCAATCCCCAGAATCA
TATTTCTGGGGCATTCGCTTTGGGGCTCGCCGGTCGACAACTCATGAATAT
>dme_circ_0004468_junction_seq
GGCTCGCCGGTCGACAACTCATGAATATGAGTTTTAAGTTGTTTCTCCTTTTtcaa
>dme_circ_0004469
GCCAAGATGATCGAGAGTGGAGGCCACGGTGGATGGATGGGAGCGGCAGCTGCGGCAACTGGAGCAGCTT
CTGTGGCGGGTAAGTTTCAGGTCTAAGCCGATGCCATTTCCAGCCGTTTCATTATGTCCGCGTGACGTGC
TTCTTCCAGCAGATAGCTACCAGTACCAGCTACAGTCCATGTGGCAGAAGTGCTGGAACACCAATCAGCA
GAACCTGGTGCAGCAGCTCAGATTCCGCGAGCGCGGCCCATTGAAGTCCTGGCGACCCGAGGCCATGGCC
GAGGCCATTTTCAGTGTCCTGAAGGAGGGGCTCTCCCTGTCACAGGCTGCCCGCAAGTTCGACATACCCT
ATCCCACCTTCGTCCTGTACGCCAATCGGGTGCACAACATGCTGGGACCCTCGCTGGATGGCGGAGCTGA
TCCGCGGCCAAAGGCACGCGGTCGTCCCCAGAGGATCCTGCTGGGCATGTGGCCGGAGGAGCTCATCCGT
AGCGTCATTAAGGCCGTGGTGTTCCGGGACTATCGCGAGATTAAGGAGGACATGAGCGCCCATCAGTACG
CCAATGGACAGGGTCATGGTG
>dme_circ_0004469_junction_seq
CAGTACGCCAATGGACAGGGTCATGGTGGCCAAGATGATCGAGAGTGGAGGCCACG
>dme_circ_0004470
GACTGGCAAGCTTCTACGGCGCCGCTGGAGGAGGAGGAGGAGCTGACCGCAGGCGTGAACGGGAACACAG
CCGCCGGCGAGGGCATTCTGGACGTCGACGTGGTAGATGGCCATCCAGCCTCTGTGCTCCACATGCGACA
GCACCAGGCGCTCAACACCCGCCCATCGGCCACGCCTCCATCGGCAGGGGGCGGTGGTCCGTTGGCGGGC
GGAGGATCCGTCGGTATGACCACTCCCAAACAGGCCACGTCGCCGGTGGCCGCGGCCAGTTTTGAGGCGC
CCCTTAGCGGTGGCAGTGCAGCCGCCTATCATCACGCCTACATGACCAACGTGCTGTCCAGCACCGCCCA
GCAGCACCACCCACTGCCCGCATCGCCGCTGCAGTCGACCGCCTGCGCTCGGTTTGGGGCCGCCGACAAC
TTGGACGATGTGAGCGCCAGCGCAGTGCGGGAACTGTCGCAGCAACTGCAGGCTCAGCTACGGGATGCCA
AGCGTCGCCACCTGGCCTGTACCGAGGTCACGCTGCCCAACGACCTCACGCAGCGCATCGCCGCCGAGAT
CATCCGGATGTCGGAGCGGGAACCATGCGGCGAGCGGGCCTGCACGCTCTTCATTGAGTTCGAGAGCGAG
CCCAACAAAGTCAAGAAACCTTACTCGTAGCAATACCATCAACATCAGTCCCGACTTCACGCTGACCAAG

AAAAAGCTCTACTCATCCGAGTGAGTCGCTCGACGGAGGAGCGGCATGGAGTGCGAACCAGAATCGCTGG
GCTGCTCAGAACTCAATCCCTCCGAACAACAACAGTAACAGCAGCAATCGCAATACGAACATCTATTGGT
TGTGCCTCCTTCGTCGCAATAGCCAAGATTCATGATCTTCATTATGGAATATAGAGAGATGATGTTTAAC
CCTAGAGGCAAACTTTTGGACCACAAACTATCAACTTACTCCTGCAAATAACAAATCAACTCAACGCTAA
AGCAAAAACAATTATTTTGGAACCTAAGCTCTaaaacaaaaccaaaaaaaccaaaacgaaaacaaaaaca
aaTCAACTGCAGTTGCAGGCAACTCAAAACTCTTATTGGACTGACTTTTTGGCTTaaacaaaaaag
>dme_circ_0004470_junction_seq
GACTGACTTTTTGGCTTaaacaaaaaagGACTGGCAAGCTTCTACGGCGCCGCTGG
>dme_circ_0004471
GAGAGATGATGTTTAACCCTAGAGGCAAACTTTTGGACCACAAACTATCAACTTACTCCTGCAAATAACA
AATCAACTCAACGCTAAAGCAAAAACAATTATTTTGGAACCTAAGCTCTaaaacaaaaccaaaaaaacca
aaacgaaaacaaaaacaaaTCAACTGCAGTTGCAGGCAACTCAAAACTCTTATTGGACTGACTTTTTGGC
TTaaacaaaaaaggtactttttgaaaaacgaaaaaaaatcgcaaaaaaaGAAGTATTGAAAACGAGGATA
GAGTGAGGAGAACACAAAAAACCATAAGGAATATTCAAAATATTGTAAAACGTTAAGGCAGACAGACAAG
CAGAAGAAAACTTCACTCAGTGAACGGTTGTCGGTGATCCGTTTTAAATTGAATACAAAAGTCAAAACCC
CCGGATGGAAGTATAGAGTATTATCCCAATTT
>dme_circ_0004471_junction_seq
ATGGAAGTATAGAGTATTATCCCAATTTGAGAGATGATGTTTAACCCTAGAGGCAA
>dme_circ_0004472
AAAACCACACAGAAAGCGTTGCGAATTCTACAATCTTACAAAGCTGGCAACATTGTGAAGAAAAACTGTA
AACAATTATTTTGGAAAAATGTTCGGCAAATGATCTTGAAAATTCTACAAATCAAATTCGTCTACAACTA
CTTGATTAATAATTTCATAAATTGAACAAAAG
>dme_circ_0004472_junction_seq
ATTAATAATTTCATAAATTGAACAAAAGAAAACCACACAGAAAGCGTTGCGAATTC
>dme_circ_0004473
CTGATCTCAAAGCCCTAGTGGATCTTCCAGCTGGTTTGGACTACAATGAATGGCTAGCGTCACACAGTAA
GTTTCTAAAACTTGAATATATACATACATTACCACCCACAATTGGCTAATTGAACTTCACGTGATTCTCT
GTGTTTTTCGGATTCCTTTCGAAATTGCCTTTATTATTATTTACTCACATCTGTCATTTGCCTCCATTGT
TGGCAGCAGCTTTATCGTGTGATTTACAAATGAGTTTAACGAGCGTCGTCAGCTGCTGTTTGGCAAAAGT
TTGATAGCCTAATCGCCAGTGGCTACGCTCGAGTACCCCGATTTACGAAACGCTGCCCACGGTGCACTAT
ATGTATGTACATTATCGTCCAGCCAATGAACTTGAACAGAAGAATTCAGACTGAGCGATAGCATTATCTG
TATGCAGTTTAAGAAGTGCAAGGGCACCCTGCAAAGTTTGAGATCAGGCAGTTTTCTTTGTATCCAAAAG
TTTGCGATACTAGAAATCCAATTAAAAGGATATTTTCCTTTGTATTCATAGTGAAATTATTAAGTGTATA
CATACTCTTTTGCCTTGAGTTTCTTGTTTCGTGCTGAATAAAGCCACTCACTGCTTTAATTACATATCAA
TAAAACGTCTAATATCATCTTCGAAAGCCAATATACGGTGAGTCACATCTTAATTGGCTGGTTGCCCCAC
CGCCTGGAGGGATCTTTTTTGGTTACATCATGGGTGGAGAACAGAATCGATTTTGAGAAATTACATCATC
TTGTAAATAATCTGTGTGCGCTGGGTGGCAGAATGCAAATTACACAGTTCTTTCTTAACTCAAATGAGGC
AAATAATTGGCAATGTATCAGCAAATTGTAGCCACCAAAAGCAGCCGGAGGGCATCAGAAGCAATCCCTC
AGACCTCCATTTGCTTCTCCCATGTCCACCAACGTTTTCATTATCATCCTCCTCTCGTTTTGGAAAAACA
ATCAAAGCCAAACTGTTTGTCGTGGGCGATGATCAGGGTCATTTCACGTCGCCCGATGGCGTCAGCTGCA
AGTTCATATGCAAATTTAATATTTGAAATTGCTGACTCGAGAGGCACAGGCCCTCGACTCCTGCTCTGGT
TATCTGGGACTGGGACTTGTTTGGGCTATAAGGAAAGCTAAAAGTTAAGCCATCGGTTTATTTTGCTCCA
ATTCTGCTGGCATTTCAAAATAATGCAGCTCTTCTTCCGCTGACGTAATGGGAACACTTGTGCAAATATT
GGGTCGGCATTGACCTTACCCGGGTGGTTCCGCTCAATAAATATCGTCGTATGTCCACTGCTGTGCCTTA

TCACTGCAAACCAGACACTGAAAGAAACCAATAAAACCCGCAGCCGTCTCATTTCGATGATAAGATTGCT
TCTGTTTTGAAGGTAATTTGTGCACCAGCTATTATTAACCGAAGAGTTGGCCATTATTTAGCAGGTTGTT
GAACCATTATTTTTAAGACGAACCAACTTTCGCTATTGTCTTAGAAGCACATAAAGAAGTGCCTACTTCA
ATTCTAATCAATATTCGCTTCttttttttAGCAACGTACTTATGGTTCGACGAGAAGGGCAAGAAGACGC
GCGTCGCAGCACCACAGTACATCGACTATGTGATGACGTTCACCCAGAAGACGGTCAGCGATGAGTCGAT
ATTCCCAACCAAATACGCCAACGAGTTCCCCGGCTCGTTCGAGTCGATTGCGCGGAAGATACTACGCTTG
CAATTTCATGTGATAGCGCATCTGTATGCGGCGCATTTCAGAGAAATCGCGCTGCTCGGCTTGCACACCC
ATCTAAATCTGACTTTCGCGCACCTCACCGCACTGCACCGGCGCTTCAATTTGATCGACGAGAAGGAGAC
GGACGTGCTGCGCGACCTTGAGGTGGCCCTGCGCCTCACCGACGATACCGGTGGATGCCAGGATGCTACC
TCCACGACGTCGTCGGTCCATGACCACTCCCACTCCGGCGATCTCCAGCATCAGTCGCTgcaacagcagc
agcaacaccacaacagcagcagcaacagcacCTCCTCGGCGGAGGCGTTTCACGTCAATTCACAAAGCAA
CAATGGCAGCACATCCGCCTCGGCATCGGTATCCCTAATCGATGGCGATGCGGTGGCGCCGCCCATTTGC
ACGCAACCAGAGGCGGGTGCGGGCTGCAAGCCAGCGGGCAGCAGCGGATTGCTGGGCGGTATACTGGGCG
ACCTGACCAGCGGCGAATTTGG
>dme_circ_0004473_junction_seq
TGGGCGACCTGACCAGCGGCGAATTTGGCTGATCTCAAAGCCCTAGTGGATCTTCC
>dme_circ_0004474
GTTAACGAGCTGGGTGGCTACGATAGCGATGCAACAGACATGAGTAGCACCAGCTCGGTGGGACACAGCG
GTGGCAGTTCATCGCCGAGCACAGCTGCAAGCCAACGTTCGGGCAAGGGATCCAAGTCTCAACGTCGCGC
TACGGCCCTCAGAAAGAAGCGACGACACGAAACTCGCGAGACGACTTTGGCTAGCAAGAGG
>dme_circ_0004474_junction_seq
TCGCGAGACGACTTTGGCTAGCAAGAGGGTTAACGAGCTGGGTGGCTACGATAGCG
>dme_circ_0004475
CACGAGAATCGGGAATATTTCGGTCAGTTTGTCACCGAGGACATCAACAGCTATATACAGCGTAAACGGG
CTCGTGATGCTCATGGCAACCACATTGAGATCCAAGCTATTTCCGAGATCTACAGCCGTACCGTCGAGGT
ATACTGCTACCAGTCAAGTAAG
>dme_circ_0004475_junction_seq
CGAGGTATACTGCTACCAGTCAAGTAAGCACGAGAATCGGGAATATTTCGGTCAGT
>dme_circ_0004476
GTCACCGAGGCTGTCAAAGTTGCCATTGATGCCGGATACAGGCACATTGACTGTGCCTATGTATACCAAA
ACGAGGATGAGGTCGGAGATGGTGTTGAGGCCAAGATCAAGGAGGGCGTTGTCAAGCGGTGAGGATCTGT
TCATCACCAGCAAACTGTGGAACACTTTCCATCGCCCGGATCTTGTCAAGTCGGCATTGGAGAACACATT
GAGCTCCCTGAAGCTGAAGTACCTCGATCTGTACCTTATCCACTGGCCCATGGGCTACAAGGAGGGATGC
GATCTGTTCCCCACCGACAAGGATGGCAAGACGCTGTACTCGCCGGTTGATTACGTCGACACGTGGAAGG
CCATGGAGAAGTTGGTGGAAGAGGGTCTGG
>dme_circ_0004476_junction_seq
ATGGAGAAGTTGGTGGAAGAGGGTCTGGGTCACCGAGGCTGTCAAAGTTGCCATTG
>dme_circ_0004477
GCGCGTAAGACGCCAGTGCGATCAAAACAAAACGGGTAATAAATCTGCAATCACACGGCCATGCGTGCat
aaagaggaacaataacaaaatcaaaagtcagaagaaaaaacaaaataaaaatataaaaaaaTCACAACAA
TAATAATCGCGAAGCTGGCAAAAGTACAACAAAAAAGACAAGAGCAAGTGTAAAATAAATAAATAAATAA
CCGCGCGGCAGCCAGCACAGATCCAGCACAcattcccattcccattcccattccaattccGACTTTGGTG
CGGTAGGTAATGACACATTCGGCAGCCAGCACACGTATCTAGATGCGAGCGAGCATAGTACGTATCTGAA
TCCGTCAGTGAAAAGCCGGGTAGACACCATCGCCATCAGAACCATCGGAGCCAACCCTTGCCACCCACAT

CGTTCATTAATCTATGAGCTTTTGAAAGCGGCATTAAAAAACGATAAACAAATTCATTAGAAGTCGCCAT
GGAACAGTCGCAGAACTTACTCGCCAAACAGTCCAAAAATGTGGAGTTCCAAGATGTGTACTACACCGTC
AAGGAGCGCAAGAATTTCT
>dme_circ_0004477_junction_seq
TACACCGTCAAGGAGCGCAAGAATTTCTGCGCGTAAGACGCCAGTGCGATCAAAAC
>dme_circ_0004478
AGTTTGTTTGAACTGGAGCTGGCTGGCTGCAACGAGGTGACCGAGGCGGGACTGTGGGCGTGTCTAACGC
CCCGAATCGTATCCCTATCCCTGGCGGACTGCATCAACATCGCGGACGAGGCGGTGGGCGCGGTGGCCCA
GCTCCTGCCCTCCCTCTACGAGTTCTCGCTGCAGGCAAGTCCAGATTGATGAGGATCTGCCACAGCTCCT
AGAGCTGCTGCTCCTACACCCTTTTCCCCTCTTATGCCGCATCTCGTATCGAATTAATTGAATCTCCTTC
CGCCCCTCAACAGGCTTACCACGTCACGGATGCGGCTCTCGGCTACTTCTCGCCCAAACAGAGCCACAGC
CTCAGCATCCTGCGACTGCAGTCGTGCTGGGAACTAACCAACCATGGCATCGTTAATATCGG
>dme_circ_0004478_junction_seq
TAACCAACCATGGCATCGTTAATATCGGAGTTTGTTTGAACTGGAGCTGGCTGGCT
>dme_circ_0004479
GTGAAACTCGCTAATTGTCGGGTGGAAAAAGGTTAGCAATACACAGAGCAAACGGGATGTAAAGGATAAG
CAAAGCCGACACCATAAGCGATTGAAAAACGAAGCAGAGGAGAGGACGAAGGACTCCACTTTTCCAGAGC
GCTTCGTTTCCCAGGAGGCAAGGTGAATCTGTTCCAGCACACGATGTCATCGATCTCGGCGCAGGGCGTG
GTCGAGCGAGCATCCGCGGAGTTGTCGAAGCGGATCAATGGCCTGGGCCTGCGCTCGAAGCACCATCATA
GCAGCACATCCAGTGGTGCTGGTGGCGCCGGCGATGCTGCATCCCCGGCAGGAGCCACGCCCACTCCGGC
AGCGCCCAGCGGCAAGACGTCGGTGATGGAGCGCGTAACGAACGCCCTGTGCGGCGGTGGCAACTCAAAT
TCTAACTCAGGATCGAATAGCTCCAATAGCAACACCTCTTCAGCGTCTGCCACCGCTGCCACATCGCCCG
CCAGCAACGCCAATCCTCCACAGACGCCGGACAAACCGTCGCGTGGCAGTAGCCCCAGTCCCGGCGGTAT
CACAATGCCAGGTGGCCAGTCGCAGGTCCAGAACTCCACACACCACCTCctgcagcagcaacaacagcaa
cagcagcatatgcagctgcaacaatcgcagcagcagcatctccagctgcaaGCCTCCACGCTGATCAACT
CCAACCACCATGTGATGGTGGGTCCTGCTCCGCCCACTGGCATGCCTCTGGGTGCCCCGCCCACGCCGAC
AGTGAAGTCCATTGCCAAGCAGATGAACATAACCATACCGGGTGGCGGAGTAAATCCCGGTTCGCCCACC
TTCAGCACCATGGGCATGGTGGCCGCCCAGAAGGCGGCGGCCAGTGCTGGAGGTACACCGTTGCAGTTGC
GGAAACAACTGCCCAATCCTCACCTGCACCATCCGTATGGCAGTATGGGCATCAACGCAGCATCGCCGCT
GATCATGCAACACCAGCTCCACCCGCCGCCTATCCACAGCATCGAGCAGCTGATGCTCGACGATCGCTTT
CTGAGCCGCTTCTTCCAGTACTTCTCCCCCTACGAACGGCGCATCCTTGCCCAGGTGTGCATCAAGTGGC
GGGACACACTGTACCGCAGTCCGCGCTACTGGAGCGGCCTGTTGCCCACGCTGCAGTGCCGCGAGCTGCG
CCAAATGCCAGGCTGCGATCGCGGCAAACTCTACAACTCGCTCATCCGGCGCGGATTCCACGCTCTCGGA
CTGGTCGGCGCCTCCGACGAAGACGCCCTGGATGTCGTCCACTCCTTCCCCCTCGCATCCAAGCATGTCC
ACTCGCTCAGTCTGCGCTGCTCCTCGATTAGCGACCGGGGACTGGAGACCCTGCTTGACCACCTGCAGGG
TGAGCTTGATTCTTTATACTTATGGCtttagtttttagcttttagattttttttaTGTAATTATATTTCAA
TAGTTCGCTATATATTTTGTCTCTTTTCGCTTTAATTTACCTGTGACCAATTTAATTTCAATTATTTTTA
CAATTTTACAAAATGAATGCGTTCATTTGCTTGAAATGTCCAATTAATAAAAGTGCATTGGCAATCTGGC
TTTTATTAAAGCATAATACAATTTAAAATTTCCATCCACTATGTGAATTTATGCAAAGAGATTAACTCTA
AAGTTGTAATTATATCTCTTTAAAATGGTTATTCATCTCAACCCAGACGGCTCATTAAGATATGTCGCTG
ATAAAAGTGAGACAGAGCCGACTAAAAATGTTTGGCAAACGGCTTTCGTATCTTTTCGCCCTTGAGATAC
AAGTGCAAACTACTTATGTGTGTGTAAACAAAATTGCCATCTGCACATAAGCTGGAATAATGGGCTGTCA
TTTGTTTAAAATGCAAGACAACTTGGCCCATGGGCAGACGGCAATGTCCGGAAATCTGGCCCCAAAGGGC
ATCCCATTCCAGGGCAACTGCTCCCAGCTCTGCAGCATCTCTCCCTTTTCGGGGCCCAGCTGGTTTTCAT

TTGGCCCCACATCCGACTTGATTTGGTTTTCTTGTTTTCCTGGGACCCCCGACGGAGATGGGGTCGGAAA
ATGTCTGAGAAAGAGAGCATTTTTCGCCTGTGGGCGTTTCGAGTTTTGGCCAGCGGCAGCGGCAAGTTGA
CAGTGGTCAGTCCCGCTTTTGGGCCACATGCACAAGCAAAGTAGAAATAAAATGATACAGATTCGCACAC
GAATCCGTATCTGTGCGCTGAAATGAAAACTATTTTCATTTTATTTCGGGACTTGTGCCGCGCGCCAGAG
AAATGAAACGTGTCAAAGCTGAAAAGTGTGTCATCGGCTGGCGATATTTTGGCGAAATAAACCCTCCACT
TTCGTCACTCGACTCGACTCGCCGGCACATAATGAATTGATGCGATCAATTTCAGATGCAAATCACATCA
AAGCTCCGTCATAAATTTCATTTAATTTAGCCTTTTATTTAGTTGCGCTAATTTGTGCGAAATGTGCGAG
TAGCTCAATCGAATACACTCACTGTGCATTCATAATTTCTACGCGCCGCCAGGTGAATCATTTATGAGTT
GCGCTCCGATGGCAGGAGAAAATTCCATATTTTATTAGAATAAAAGCAGGAAGGCGTGAACTGAAATCTT
TTGCGGAATTTATTATAAACAATTTACAATTTAAGAACTTTATTTTTGGCTGAACACATTTATGTGAGAA
CCATTTAAATTAACCAATATGTATATATACCTTGATTGGTATTCCTTTCACAACAATCTCATTCACACTT
CTCATTCATAACAAGCTATCATTTTATCCTCGGCCATTCCACTCGCTTTGTCTTTTTTACTACGCTTCCA
ACGAATTCATCTCTTTCGGTTTGCCGGACTGCCTCAATAATCCCGAGAAACCCAATCGAATTTGGACGAA
CAAGTGGGCAGCTTAATTGATGGCCAAACTGTTTTGGGGATAGTCCTGGGCAAACTAATCCATTCATTTC
ATGGACCAATCCCGAGCAATCTGGCAACCTAATCCACCAGGGGCCCAGCATGGGTCACCGGAAATGGACC
ACACAGACACTGGGATTTGAGACAACACCAATACAAATACATACACACGAGCGGAGCCAGTAAAGTGGAT
TACGTTGGTGAACTGGCTGTGGCCAAGTCCTTTATGAAGGAGCGTTCTACATGAATTTGCGGTCCTGTCA
GCTCAGTCAGTCAGACACCCATTGGGTTGACTGCATCACTTTTCCCCACAATTCAGTCCCTTTGATTTTC
AGCTTGTTTGATTGCTAGGGTGATCCCCTCTCTATCATATGCTGATATATTTACTTAGTAGTAATATTCG
CAATTTACAATTTTAATTTATCTAACCACAACATATGATTGATTTATATCAGACTGCACAATAATCTTGA
ATGCTTTAGTTTTAAGGAATTCAACTTTTGTTGTATATGAATAGCTAACACTGCGTATGAGTAATATGGT
CTAGCTTCAACATTCACATCTTTCTAAGCTCCTAAGATATCTTTCTCCTAGAAATGAGTACCCTTTAAGT
AGAAGAGTTTCAGTGACTTAGGTTTAAGTTGCGACTAATAAGGTCCACCCTGCTGTGCATACGTCATACG
TCGGAGTGCTGATGTCAGAATTAATCCACTGCGAGTGCATGTCATGTTTATGAGCCTAGTTACATTGCCA
GGGTCTAAAATTTTCACAtgtgtgcgtgtgtgtttatgtgtgtgcgtgtgtgtgggtgCCCAGGAAGCGC
CCACAAATGGTTTGATTGCAAATTGTTGTCGCTGGCAATGTCAACAACAATGACACACTGGGGACAAGTT
GGATTTGGCCATGATGCGAAAGGATCACCCGCAATGGGCTCAGGAATTTGACCTTGGGGACTCTGCGGAA
AGTGTGTATGGGAAGTGCCCATATGGAATTTGCCCAACCCTTTTGCACACTGACTTTCCAGGAAAACCGC
CCCAAAACATGCAACGCGCTAAATTAATGCTAGTTACTGCTGCTGAATGAGCGGCGGAAATGCGGCGACT
TTCCTGCATTCCCATACATATTTAATGGCAAATGCACTGCAGTCGCCTCGGGCGAGTGCAAATATAGTGT
ATATATGCGATTTCCGCATTGCCGCTCCAAAGTTGCCACTCCAGACTTTTCAAAGGGGGGTATTTTACAA
GTGAGTCCGGAACGGAGCTCGTTGAACCCAACCCCATTGACCCAGAAACTTAGGCTACGCTCTGGCACCT
CTGGCTATTTAAATGCGAATTCCTTTTCACCAGCAGATGCAGTAATAAATTTATTAATTTATTTAAAGTG
CATCTCTCGTTTGAGGGATTATATCACCAAGCACCCAGCTATTTATTCAAAACGAAATTGAACATAATCA
AGTTTTAACTTGAGAAAAAGTAGCTTAATAAAGCAGGAGATACTTCACTTAATATAATTTAATACATAAT
TCATTATATTTTATGTAAGCAGTAAAATTTCTAATTTGAAAGTCTGGCAAACGAATATATTTTCCCTCAG
TTTGACCACTCGCTCATAAATTGCCATCGTTTTTAGCGAAAAAACCTCAGAAACCGAGGCGAAAACACGG
GAAAACTTTTTCCGGCTAAATAGTCACACATCCTGGCAtgtgtgtgtgtgtgtgtgtgtgtgtgCTTCCA
TTAGCATTTCTATGTGGAACTCTCCTTTGCGTGGTTGACTTTTCCTATCGATTTGGATTTTCTCCTGCCC
GACGAGAACAACACATTGTACCGAAAAAAGTTTCCCATGGCTACGGCTACGTGTTTTGTGTTGTTCGGTT
TGAACAAGCCATGGGGCAAAAGTTCCACAATTGTCCATAAAAACGAAATGCGGAAAATGGTTGGCGGTGA
AATCTAAACAACTAGCGAGCATTTACCATCCATAATGAAGTCATGCCAAAAGGCTTGGCTTCCTTGTTTG
TCGGGCTTCCTTTTGCTGATTGTTGGCCAAGTTAACCAAGATGTACAGAATCCAATTACTGGCCATGTAT
TCAACACATCCGTAATTAGACCATTTAATTTGCTAATGAGTTGGATGACTGTCCTTTACTTGTGCCATGT

TTCGTTGGTCACTAATTGGAACATCAGATGCGATTAGGATTAGAATACTGAGAGGATAAAAGGATATCTT
AATGCATTTGCCACAACATTCCTATACAAAGAAATATTATAAATCAGAAATCTATAACTAAGCCTTTGAT
CTTGTTTAGTAACCTTTTGCTCTTCTCGCTGCGAAAGTAAATGAATCCATGGCGAATGTCGAATGGATTC
AATTTTGATTTATTACTCGAAGTCACGGGGATTCGCATTAAGGATTCGGATTAAATTGGTATCTTGCTGT
TTTAGCTGTTTACGTTTTGTTATTTCTCCAGTTTTGCGTCAACAATACAAAAGTAATTGTTGTTCAGCCG
CGGCAGTGGGAAAATTTCTCGCTTAATGCGTTTTGTGTGTGGCCATCCTTGGGATTTTCATTCTTCtttt
ttttATATATATTGTGCTTCCGGCTGCATTTGCTGTTTATCGCCTTTGTTTCCGTGTTTCCGGTTGTTCG
AAGGATGGGGTGGGGGTTGGTTCAGGCAAATGGGTTGAATTCGAGGGATCCAATGGCAATGGCGCCTGTA
TCCCGACACAAAGTCTGCCACTCGAATCGGGTCGCCTCCATCGAGATGTTATGTGTTTTTTCCCCGCTTT
TACTTCGTCGACAATGTTTTGCTCTTGAAAAGGTCAAAGTTAATCCTGTTATGGGGACTTAAATTGCTCG
TAGAACGCATGGaaaaataaaaaaaaGACAAATTCAGCTAGGAGAGAAGTTTTCCCGAGTTTGTTGACTC
GTAAATTTTGGCATCAAAAGTTGGCAGTGCTGAAATTTAATTTCATTTTTAATGGATACGCGTCGCACCC
ACCAGGTGGCAGCTGTAATTTGTTTGAATCACTCTGGTCTATTTTTCCAATAGAAAATTCTTTCAATTTT
CCCGCATTCCACGCACACAATCCAACACATCGCGAGATTTTCAAAGTGCAATTTTCCTTGTTTTGTCTAC
GGTTCACCGAATGATTTATGAGGTAAATGCAAAAATGCAATTAGGTTTTCCTAGCTTTGGGACATTCCAT
TTGCACTTTTGCGTTTAATTAAGCACATTGATTAGTTTAAGCTTCAAAAACCTCGAAATGATTGCTTTTC
TATTTTGTTTACAAAGAAGTACATTTTTATTGGCCTCGATTTTCATGGACGTCAAGGAGAATTGAAAGAA
ACGTTTAATTATGTGGCAATATTAAATGTTCTTTAGCAGATACTTAAAAATCCAAACCACCTGATGCACA
AGTTTTCCATATTGATATATGTATATAGGTGTTTTATGTAAAAATATATGTGCCGAGGTATTTTCGTTTT
GCTATTTTTCACTCGCCTCCGATTGGCCtttttttttCCTGCTCGCCAAGTGTCAACGTTTATGAACGCT
GTGAAATGTCCAAAATTATTTCAGAATTAAATATGGCAGATCATAAAAACATTTGACGGTTTCATGACAG
AAAAGTCACCCTTATAGACGAATATACCGAATTCGAGACCAAGAGAATGAGCAGCGAACTCATGAATTCC
TTAAGTAATTTTCTTCAATATTTGCGGTTTCAAGTTCATTGCCAAGAATAACAACACACCTCTTGTTGAA
AATATATTTGAAGAATTATTACATTTCTGTTCAAGAATTTTGAAAAACTTTAGTCGAAGCACGtttttttg
cattttttttCCATGGAAAACATTTGTTTACAAGTGGTTTTTTTGTTTTCGCTTGTGCTACATGTAAATTA
ATATCGATTCGTGTTGATTAGTCTGACTTAATTTAAACAATTTTATTTATTTAGAGTGGCATTCCTTGAT
TCCTAACTAATGTTTAGAAGTACTATAAATTTAATACACATACTAGAGTAGATGTCTGATATTTTTAATA
ACATCCTTTGTGCAAAGTGTCCTTGCTGCAGTTGATAATACTCAATCCTTTTAAATTGCAGAAGTTTGTT
TGAACTGGAGCTGGCTGGCTGCAACGAGGTGACCGAGGCGGGACTGTGGGCGTGTCTAACGCCCCGAATC
GTATCCCTATCCCTGGCGGACTGCATCAACATCGCGGACGAGGCGGTGGGCGCGGTGGCCCAGCTCCTGC
CCTCCCTCTACGAGTTCTCGCTGCAGGCAAGTCCAGATTGATGAGGATCTGCCACAGCTCCTAGAGCTGC
TGCTCCTACACCCTTTTCCCCTCTTATGCCGCATCTCGTATCGAATTAATTGAATCTCCTTCCGCCCCTC
AACAGGCTTACCACGTCACGGATGCGGCTCTCGGCTACTTCTCGCCCAAACAGAGCCACAGCCTCAGCAT
CCTGCGACTGCAGTCGTGCTGGGAACTAACCAACCATGGCATCGTTAATATCGG
>dme_circ_0004479_junction_seq
TAACCAACCATGGCATCGTTAATATCGGGTGAAACTCGCTAATTGTCGGGTGGAAA
>dme_circ_0004480
GTGAAACTCGCTAATTGTCGGGTGGAAAAAGGTTAGCAATACACAGAGCAAACGGGATGTAAAGGATAAG
CAAAGCCGACACCATAAGCGATTGAAAAACGAAGCAGAGGAGAGGACGAAGGACTCCACTTTTCCAGAGC
GCTTCGTTTCCCAGGAGGCAAGGTGAATCTGTTCCAGCACACGATGTCATCGATCTCGGCGCAGGGCGTG
GTCGAGCGAGCATCCGCGGAGTTGTCGAAGCGGATCAATGGCCTGGGCCTGCGCTCGAAGCACCATCATA
GCAGCACATCCAGTGGTGCTGGTGGCGCCGGCGATGCTGCATCCCCGGCAGGAGCCACGCCCACTCCGGC
AGCGCCCAGCGGCAAGACGTCGGTGATGGAGCGCGTAACGAACGCCCTGTGCGGCGGTGGCAACTCAAAT
TCTAACTCAGGATCGAATAGCTCCAATAGCAACACCTCTTCAGCGTCTGCCACCGCTGCCACATCGCCCG

CCAGCAACGCCAATCCTCCACAGACGCCGGACAAACCGTCGCGTGGCAGTAGCCCCAGTCCCGGCGGTAT
CACAATGCCAGGTGGCCAGTCGCAGGTCCAGAACTCCACACACCACCTCctgcagcagcaacaacagcaa
cagcagcatatgcagctgcaacaatcgcagcagcagcatctccagctgcaaGCCTCCACGCTGATCAACT
CCAACCACCATGTGATGGTGGGTCCTGCTCCGCCCACTGGCATGCCTCTGGGTGCCCCGCCCACGCCGAC
AGTGAAGTCCATTGCCAAGCAGATGAACATAACCATACCGGGTGGCGGAGTAAATCCCGGTTCGCCCACC
TTCAGCACCATGGGCATGGTGGCCGCCCAGAAGGCGGCGGCCAGTGCTGGAGGTACACCGTTGCAGTTGC
GGAAACAACTGCCCAATCCTCACCTGCACCATCCGTATGGCAGTATGGGCATCAACGCAGCATCGCCGCT
GATCATGCAACACCAGCTCCACCCGCCGCCTATCCACAGCATCGAGCAGCTGATGCTCGACGATCGCTTT
CTGAGCCGCTTCTTCCAGTACTTCTCCCCCTACGAACGGCGCATCCTTGCCCAGGTGTGCATCAAGTGGC
GGGACACACTGTACCGCAGTCCGCGCTACTGGAGCGGCCTGTTGCCCACGCTGCAGTGCCGCGAGCTGCG
CCAAATGCCAGGCTGCGATCGCGGCAAACTCTACAACTCGCTCATCCGGCGCGGATTCCACGCTCTCGGA
CTGGTCGGCGCCTCCGACGAAGACGCCCTGGATGTCGTCCACTCCTTCCCCCTCGCATCCAAGCATGTCC
ACTCGCTCAGTCTGCGCTGCTCCTCGATTAGCGACCGGGGACTGGAGACCCTGCTTGACCACCTGCAGG
>dme_circ_0004480_junction_seq
CTGGAGACCCTGCTTGACCACCTGCAGGGTGAAACTCGCTAATTGTCGGGTGGAAA
>dme_circ_0004481
GGAGGACAGCGCCGCCATGCCCAAGAAAGACTCCCGCCTAATGCTAGCGCACTTCAACGAGCAGCTGCAG
CCACTGTACGACCTGCTCCGTGAGGTGGAGGGCATCAAGCTGGCGCCAGAGGTGCTCGAACCGGAGCCC
>dme_circ_0004481_junction_seq
GGCGCCAGAGGTGCTCGAACCGGAGCCCGGAGGACAGCGCCGCCATGCCCAAGAAA
>dme_circ_0004482
aaaaaaaaaGATAAATGCGTCCAATGAGTAAAGGAAGCAAATTACTACTATACATTTAGCTGCAAATATT
ATTTATAAATATACAAATATTTATGTTTAACATTAAGATGGTGTGGAGAAACAATAGTTGAACAATCACA
GCGCCAAGGCAGCGGCTTATTTAAACTAAAATTCGCGG
>dme_circ_0004482_junction_seq
AGCGGCTTATTTAAACTAAAATTCGCGGaaaaaaaaaGATAAATGCGTCCAATGAG
>dme_circ_0004483
GTGTAGTCGTCCACGCAGCAAGCTCCGTAAGTCACATCGCCCATTATCACAGTATCTGCGCTGGTGAATC
GCTCCACAATGTCGCTGATAACCATGGCGTACATCAGCAGGCCCTCCGGCAGTTGCAGGGCCACACGCTT
GGCCTTCATCTCCCGTATGCGCCAAATCGTCTTGTGCATCTCGAAGTTGTAGTTCGAGGGCAGACGGTCG
ATGGCCTGCTGCAGCAGTGGATCATCGAGCAGCTCCTGGGGAATCCGATTGATGCGCGCCTTTGGCTTGA
ACACCTTCTTTGGTCTGGCTCGGACCACCACAATATCGGTGCTTTCGCTGGTCGGGAG
>dme_circ_0004483_junction_seq
CAATATCGGTGCTTTCGCTGGTCGGGAGGTGTAGTCGTCCACGCAGCAAGCTCCGT
>dme_circ_0004484
GTATTTAGGACACGCAAAACGAACTGCCACAAAACGAGAATAAGCGAATATTTTTTGTCAAACTGATTAT
TGCAAAACGTGTATCGAGAAACTATTTTTAATGTACACCAGTATGTATAATTACCGATTTGTTCATTTTT
TGAGACAAAACGAGAAATACACTTTTATGAATGACTTTTGAGCGATCTATTGTAGCGGTTAAGTCTAGTT
TCTTTAACGTTTATCAGCTGCTTTTGGTGCTAAATCG
>dme_circ_0004484_junction_seq
TTTATCAGCTGCTTTTGGTGCTAAATCGGTATTTAGGACACGCAAAACGAACTGCC
>dme_circ_0004485
TTTATTGAGAAAATATTCATAAATGTACATTCTGTATTCAGTAGCATATGCTACTAAATGCATATATTTA
CAGCGAAGATGCACATGTAAAATTATCGAAAATGTCCGAGCTTCGAGCAAGGGAAATGCGCTAATAATAC

AAAATTTACATTATAAATATTTCATGTTCACTACATATG
>dme_circ_0004485_junction_seq
TATAAATATTTCATGTTCACTACATATGTTTATTGAGAAAATATTCATAAATGTAC
>dme_circ_0004486
CTTTGAACTGCTCCCAGGAAAAGTTCTCGTTTCGCCCCCGAAAATGTGTTAGAAATCCCGCAGAAAACCT
GCAAGACAACCGATAAGAAGGAAAACTCTATAACAGCACCATGGACATGACCAAACAGATTGTGGACTTT
GAAATAAAGTCGGAACTGATCGGCGAAATCGATCAGTTCGAGGCGAGTGACTACACAATGGCTCCACCGG
AAGAGCCTAAGATGGTGGAAGAGTCCCCCCAGTTGGGTCATCTAGAGGACCAGAACAGAAAGTACTCACC
CGAAAGGGAGGTTGAACCCACTCTGCAGGATCCAAGTGAGGTGGTTGATCAAATGCAAAAAGATACGGAG
AGCGTTGGAGAAGTCAAGTCACCCGAGAAGGATGTGGAAACGGAGCTGGTGAAGTCCAAGGCGAGTCCGA
TGAACGACCAAGCTTTGACTCCCCCACCACGACCTCTGACCTCCAGTGAAGTGGTGGGTCTCCGGGATCC
CGAACATACCGAGCTGCGCATGTGCCTGGAGGCCAAGAAGTCGCGCTCCCTACCAGTTTCCCCACAGCCT
CAACCAAATCTTAAGCTAGCCGGATCGGCGCTCTTTGAATTCGGTCAGAGATCCTCTCCCGTGGAGACCA
AGATCAAAACCAATCCAGAGACAAAACCGCCGAGGCGCAAAATAGTTCCTCCCAGCGGCGAGGGGCAGCA
GTTCTGCCTGAGGTGGAACAACTATCAGTCTAACCTGACCAATGTCTTTGACGAACTCCTTCAGAGCGAG
TCCTTCGTGGACGTGACCTTGTCCTGCGAAGGCCACTCGATCAAGGCACACAAGATGGTGCTATCCGCCT
GCTCACCCTACTTCCAGGCCCTGTTCTACGACAATCCCTGCCAGCACCCCATCATCATCATGCGGGACGT
CAGCTGGTCCGACCTGAAGGCCCTGGTGGAGTTCATGTACAAGGGGGAGATCAACGTCTGCCAGGATCAG
ATAAACCCCCTGCTCAAAGTGGCCGAAACCCTGAAGATCAGGGGTCTGGCGGAGGTCAGTGCGGGCAGGG
GCGAGGGAGGCGCCTCCGCACTTCCCATGTCCGCCTTCGACGATGAGGACGAGGAGGAGGAACTGGCCTC
GGCCACTGCAATTCTGCAGCAGGACGGTGATGCCGATCCCGATGAGGAGATGAAGGCCAAGAGGCCCAGA
CTGCTGCCCGAGGGAGTCTTGGACTTGAATCAGCGACAAAGGAAGCGGTCCAGGGATGGCAGCTACGCCA
CTCCAAGTCCATCCCTTCAGGGCGGAGAGTCCGAGATCTCGGAGAGGGGCTCATCCGGCACTCCGGGACA
GAGCCAGAGCCAACCCCTGGCCATGACCACCTCCACCATTGTGCGCAATCCCTTCGCCTCCCCCAATCCT
CAGACCTTGGAGGGCAGGAACAGCGCCATGAATGCAGTAGCAAACCAGAGGAAATCACCAGCACCAACAG
CGACAGGTCACAGCAATGGGAACAGCGGCGCCGCCATGCACTCCCCACCCGGGGGCGTGGCCGTCCAGTC
CGCCCTTCCGCCCCACATGGCCGCCATCGTGCCGCCACCCCCCTCCGCCATGCACCATCATGCCCAGCAA
CTGGCCGCCCAGCACCAGCTGGCCCACTCGCACGCCATGGCCAGCGCCTTGGCAGCCGCAGCCGCCggag
ctggcgcagcgggagcgggcggagcaggatctggcAGTGGATCGGGCGCCAGTGCTCCGACTGGAGGAAC
AGGAGTGGCGGGAAGTGGAGCCGGCGCGGCGGTGGGATCCCATCACGATGACATGGAGATCAAGCCAGAA
ATCGCCGAGATGATACGCGAAGAAGAGAGGG
>dme_circ_0004486_junction_seq
GCCGAGATGATACGCGAAGAAGAGAGGGCTTTGAACTGCTCCCAGGAAAAGTTCTC
>dme_circ_0004487
GAGGAGGAACTGGCCTCGGCCACTGCAATTCTGCAGCAGGACGGTGATGCCGATCCCGATGAGGAGATGA
AGGCCAAGAGGCCCAGACTGCTGCCCGAGGGAGTCTTGGACTTGAATCAGCGACAAAGGAAGCGGTCCAG
GGATGGCAGCTACGCCACTCCAAG
>dme_circ_0004487_junction_seq
CCAGGGATGGCAGCTACGCCACTCCAAGGAGGAGGAACTGGCCTCGGCCACTGCAA
>dme_circ_0004488
GGTCGTATTAGAGTCTGCTTGTAACTTTTGTGTCAGTAACCACTCTAAAATAGTATAATGCTAGTAATTC
TACCCATCAACCCATTGTATACATACTTATATTCAAAACCCTTTCACCACATTTCTAAGCCTAGATTATG
GATAATGCCTCTAATATGTAACGAGTGCTTAG
>dme_circ_0004488_junction_seq

ATGCCTCTAATATGTAACGAGTGCTTAGGGTCGTATTAGAGTCTGCTTGTAACTTT
>dme_circ_0004489
TATTATGTTTGCTTGAAGGGAAAAGCGGTTTTGCATTACTGCAGTCCGGGAAATTGGTTCGACCTTCGCA
GCCAGAAATGCATTGACCAGCGGCTGGCTAAGGTGACCTAAGCACTCGTTACATATTAGAGGCATTATCC
ATAATCTAGGCTTAGAAATGTGGTGAAAGGG
>dme_circ_0004489_junction_seq
ATCTAGGCTTAGAAATGTGGTGAAAGGGTATTATGTTTGCTTGAAGGGAAAAGCGG
>dme_circ_0004490
GATTATGGATAATGCCTCTAATATGTAACGAGTGCTTAGGTCACCTTAGCCAGCCGCTGGTCAATGCATT
TCTGGCTGCGAAGGTCGAACCAATTTCCCGGACTGCAGTAATGCAAAACCGCTTTTCCCTTCAAGCAAAC
ATAATACTTGTTATGCTGCTTGACGTCTCCAAATCGT
>dme_circ_0004490_junction_seq
GTTATGCTGCTTGACGTCTCCAAATCGTGATTATGGATAATGCCTCTAATATGTAA
>dme_circ_0004491
GATTATGGATAATGCCTCTAATATGTAACGAGTGCTTAGGTCACCTTAGCCAGCCGCTGGTCAATGCATT
TCTGGCTGCGAAGGTCGAACCAATTTCCCGGACTGCAGTAATGCAAAACCGCTTTTCCCTTCAAGCAAAC
ATAATACTTGTTATGCTGCTTGACGTCTCCAAATCGTGTATCCTCTTTCACTTTGGTGCAATCGGGTACT
CCCTCCGCCA
>dme_circ_0004491_junction_seq
TTGGTGCAATCGGGTACTCCCTCCGCCAGATTATGGATAATGCCTCTAATATGTAA
>dme_circ_0004492
GTAATGCTCCGTGTGCATCAGCCTGAGATTCCTCCAAGGAGCACTGTACCAAGATTGGGTAGTTACAAAA
GTTGGATTTTATGTCAAAATGTAGGCCCTGGGGACACTGTCGAGGTATAGCCCGTCCATTGGCACAAACA
TAAAACCTGCTGCACGACTTTGGATCTCTAACATAGGCACCATTTCGCAAGGACTTACAGTCAGGAGTCG
ATTCGGTGTCCGAGGGTTTTTTCGTTACATTTTCTGGGGCCTCATCGAGTGGGCAATCCACTAGACTGGG
AAAATTACAGACCTTCCTCTTAATGTCGAAGTGCAGATTTCTGGGACAGTGGCCGGCAATTGCTTTGCCA
TTTAGACACACATAATATTTATTGCACGACTGGAAGTCCCGAAGAAAGATTCCATTCGGCATGTTGCTGC
AGTCAGTTCGATCTTCTTGATGACCAGGTGTCGTTCCAGTTGGTGATTTTAAAGTTGAGGTTTCAGCTTT
ATTTCCCTCGGTTGTATATTTTGGAGTCGGATGGGCG
>dme_circ_0004492_junction_seq
GGTTGTATATTTTGGAGTCGGATGGGCGGTAATGCTCCGTGTGCATCAGCCTGAGA
>dme_circ_0004493
GGTATAGCCCGTCCATTGGCACAAACATAAAACCTGCTGCACGACTTTGGATCTCTAACATAGGCACCAT
TTCGCAAGGACTTACAGTCAGGAGTCGATTCGGTGTCCGAGGGTTTTTTCGTTACATTTTCTGGGGCCTC
ATCGAGTGGGCAATCCACTAGACTGGGAAAATTACAGACCTTCCTCTTAATGTCGAAGTGCAGATTTCTG
GGACAGTGGCCGGCAATTGCTTTGCCATTTAGACACACATAATATTTATTGCACGACTGGAAGTCCCGAA
GAAAGATTCCATTCGGCATGTTGCTGCAGTCAGTTCGATCTTCTTGATGACCAG
>dme_circ_0004493_junction_seq
CAGTCAGTTCGATCTTCTTGATGACCAGGGTATAGCCCGTCCATTGGCACAAACAT
>dme_circ_0004494
GATTTCTGGGACAGTGGCCGGCAATTGCTTTGCCATTTAGACACACATAATATTTATTGCACGACTGGAA
GTCCCGAAGAAAGATTCCATTCGGCATGTTGCTGCAGTCAGTTCGATCTTCTTGATGACCAGGTGTCGTT
CCAGTTGGTGATTTTAAAGTTGAGGTTTCAGCTTTATTTCCCTCGGTT
>dme_circ_0004494_junction_seq

TGAGGTTTCAGCTTTATTTCCCTCGGTTGATTTCTGGGACAGTGGCCGGCAATTGC
>dme_circ_0004495
GGTGTCGTTCCAGTTGGTGATTTTAAAGTTGAGGTTTCAGCTTTATTTCCCTCGGTTGTATATTTTGGAG
TCGGATGGGCGGTATAAGAACTTGTGGTACTCTTTTTTGGTGCATTGGGCGTACATG
>dme_circ_0004495_junction_seq
CTCTTTTTTGGTGCATTGGGCGTACATGGGTGTCGTTCCAGTTGGTGATTTTAAAG
>dme_circ_0004496
GCTTTATTTCCCTCGGTTGTATATTTTGGAGTCGGATGGGCGGTATAAGAACTTGTGGTACTCTTTTTTG
GTGCATTGGGCGTACATGGTGTCGCGCCTTCAGGAGGAGATTCTGATGAactgtttccggaatcgccagg
attttccggggcacttggcgagggctcttggcttgcgggattatcggtc
>dme_circ_0004496_junction_seq
gggctcttggcttgcgggattatcggtcGCTTTATTTCCCTCGGTTGTATATTTTG
>dme_circ_0004497
tttagaaccttcgcctagtaccccaggaaatgatgacgactccggaaacagtgggtctgagaatggaaac
tcctccacaagtggatctccatgtaccaccgataatccctctgacccagaatcttcgtcaagtaccccag
gaaatgatgacgactccggaaacagtgggtctgagaatggaaactcctccacaagtggatctccatgtac
caccgataatccctctgacccagaatcttcgtcaagtaccccaggaaatgatgacgactccggaaacagt
gggtctgaaagcggaatcacttccactactggagctccgtatacgaccgataatcccgcaagccaagagc
cctcgccaagtgccccggaaaatcctggcgattccggaaacagtTCATCAGAATCTCCTCCTGAAGGCGC
GACACCATGTACGCCCAATGCACCAAAAAAGAGTACCACAAG
>dme_circ_0004497_junction_seq
CCAATGCACCAAAAAAGAGTACCACAAGtttagaaccttcgcctagtaccccagga
>dme_circ_0004498
GGAGATTCTGATGAactgtttccggaatcgccaggattttccggggcacttggcgagggctcttggcttg
cgggattatcggtcgtatacggagctccagtagtggaagtgattccgctttcagacccactgtttccgga
gtcgtcatcatttcctggg
>dme_circ_0004498_junction_seq
gtttccggagtcgtcatcatttcctgggGGAGATTCTGATGAactgtttccggaat
>dme_circ_0004499
GGAGATTCTGATGAactgtttccggaatcgccaggattttccggggcacttggcgagggctcttggcttg
cgggattatcggtcgtatacggagctccagtagtggaagtgattccgctttcagacccactgtttccgga
gtcgtcatcatttcctggggtacttgacgaagattctgggtcagagggattatcggtggtacatggagat
ccacttgtggaggagtttccattctcagacccactgtttccggagtcgtcatcatttcctggg
>dme_circ_0004499_junction_seq
gtttccggagtcgtcatcatttcctgggGGAGATTCTGATGAactgtttccggaat
>dme_circ_0004500
GGAGATTCTGATGAactgtttccggaatcgccaggattttccggggcacttggcgagggctcttggcttg
cgggattatcggtcgtatacggagctccagtagtggaagtgattccgctttcagacccactgtttccgga
gtcgtcatcatttcctggggtacttgacgaagattctgggtcagagggattatcggtggtacatggagat
ccacttgtggaggagtttccattctcagacccactgtttccggagtcgtcatcatttcctggggtacttg
acgaagattctgggtcagagggattatcggtggtacatggagatccacttgtggaggagtttccattctc
agacccactgtttccggagtcgtcatcatttcctggggtactaggcgaaggttctaaacttgacggatta
tcggtggtacatggagttccTTGCGTTGAACTATTTG
>dme_circ_0004500_junction_seq

catggagttccTTGCGTTGAACTATTTGGGAGATTCTGATGAactgtttccggaat
>dme_circ_0004501
taccccaggaaatgatgacgactccggaaacagtgggtctgagaatggaaactcctccacaagtggatct
ccatgtaccaccgataatccctctgacccagaatcttcgtcaagtaccccaggaaatgatgacgactccg
gaaacagtgggtctgaaagcggaatcacttccactactggagctccg
>dme_circ_0004501_junction_seq
cggaatcacttccactactggagctccgtaccccaggaaatgatgacgactccgga
>dme_circ_0004502
gggattatcggtggtacatggagatccacttgtggaggagtttccattctcagacccactgtttccggag
tcgtcatcatttcctggggtactaggcgaaggttctaaacttgacggattatcggtggtacatggagttc
cTTGCGTTGAACTATTTG
>dme_circ_0004502_junction_seq
catggagttccTTGCGTTGAACTATTTGgggattatcggtggtacatggagatcca
>dme_circ_0004503
atcctttgtctactgaatcgtctacagaagctacttatgagtcttcttcgacggaatcttcacaagatag
tacgacccaagagagtagctctagcactgaaggtcctttgtctactgaatcgtctacagaaggttctaat
gagtcttcttctacagaatcttctcaagatagtacgacccaagaaagtagctcaagcacggaaagccctt
tgtctactgagccatctaccgaagctaatgagtcttcttctacagaatcttcacaagatagtacgaccca
agaaagtagctctagcactgaaggtcccttgtctactgaatcatccacagaagctaatgagtcttcttct
acagaatcttcacaagatagtacgacccaagagagtagctctagcactgaagg
>dme_circ_0004503_junction_seq
cccaagagagtagctctagcactgaaggatcctttgtctactgaatcgtctacaga
>dme_circ_0004504
gtcctttgtctactgagccatctaccgaagctaatgagtcttcttctacagaatcttcacaagatagtac
gacccaagaaagtagctctagctctgaaggtcctttgtctactgaatcgtctaccgaagctaatgagtct
tcttctacagaatcttcacaagatagtacgacccaagaaagtagctctagcactgaagatcctttgtcta
ctgaatcgtctacagaagctacttatgagtcttcttcgacggaatcttcacaagatagtacgacccaaga
gagtagctctagcactgaaggtcctttgtctactgaatcgtctacagaaggttctaatgagtcttcttct
acagaatcttctcaagatagtacgacccaagaaagtagctcaagcacggaaagccctttgtctactgagc
catctaccgaagctaatgagtcttcttctacagaatcttcacaagatagtacgacccaagaaagtagctc
tagcactgaaggtcccttgtctactgaatcatccacagaagctaatgagtcttcttctacagaatcttca
caagatagtacgacccaagagagtagctctagcactgaagg
>dme_circ_0004504_junction_seq
cccaagagagtagctctagcactgaagggtcctttgtctactgagccatctaccga
>dme_circ_0004505
ctactaatgaatcttcttcgacggaatcttcacaagatagtacgacccaagaaagtagctcaagcacgga
aagccctttgtctactgagccatctaccgaagctaatgagtcttcttctacagaatcttcacaagatagt
acgacccaagaaagtagctctagcactgaagatcctttgtctactgaatcgtctacagaagctactaatg
agtcttcttcgacggaatcttcacaagatagtacgacccaagagagtagctctagcactgaaggtccttt
gtctactgaatcgtctacagaaggttctaatgagtcttcttctacagaatcttctcaagatagtacgacc
caaaaaagtagctcaagcacggaaagccctttgtctactgagccttctaccgaagctaatgagtcttctt
ctacagaatcttcacaagatagtacgacccaagaaagtagctctagcactgaaggtcctttgtctactga
gccatctaccgaagctaatgagtcttcttctacagaatcttcacaagatagtacgacccaagaaagtagc
tctagctctgaaggtcctttgtctactgaatcgtctaccgaagctaatgagtcttcttctacagaatctt

cacaagatagtacgacccaagaaagtagctcaagcacggaaagccctttgtctactgagccatctaccga
agctaatgagtcttcttctacagaatcttcacaagatagtacgacccaagaaagtagctctagcactgaa
ggtcctttgtctactgagccatctaccgaagctaatgagtcttcttctacagaatcttcacaagatagta
cgacccaagaaagtagctctagctctgaaggtcctttgtctactgaatcgtctaccgaagctaatgagtc
ttcttctacagaatcttcacaagatagtacgacccaagaaagtagctctagcactgaagatcctttgtct
actgaatcgtctacagaagctacttatgagtcttcttcgacggaatcttcacaagatagtacgacccaag
agagtagctctagcactgaaggtcctttgtctactgaatcgtctacagaagg
>dme_circ_0004505_junction_seq
ctttgtctactgaatcgtctacagaaggctactaatgaatcttcttcgacggaatc
>dme_circ_0004506
gtcctttgtctactgaatcgtctacagaagctactaatgaatcttcttcgacggaatcttcacaagatag
tacgacccaagaaagtagctcaagcacggaaagccctttgtctactgagccatctaccgaagctaatgag
tcttcttctacagaatcttcacaagatagtacgacccaagaaagtagctctagcactgaagatcctttgt
ctactgaatcgtctacagaagctactaatgagtcttcttcgacggaatcttcacaagatagtacgaccca
agagagtagctctagcactgaaggtcctttgtctactgaatcgtctacagaaggttctaatgagtcttct
tctacagaatcttctcaagatagtacgacccaaaaaagtagctcaagcacggaaagccctttgtctactg
agccttctaccgaagctaatgagtcttcttctacagaatcttcacaagatagtacgacccaagaaagtag
ctctagcactgaaggtcctttgtctactgagccatctaccgaagctaatgagtcttcttctacagaatct
tcacaagatagtacgacccaagaaagtagctctagctctgaaggtcctttgtctactgaatcgtctaccg
aagctaatgagtcttcttctacagaatcttcacaagatagtacgacccaagaaagtagctcaagcacgga
aagccctttgtctactgagccatctaccgaagctaatgagtcttcttctacagaatcttcacaagatagt
acgacccaagaaagtagctctagcactgaaggtcctttgtctactgagccatctaccgaagctaatgagt
cttcttctacagaatcttcacaagatagtacgacccaagaaagtagctctagctctgaaggtcctttgtc
tactgaatcgtctaccgaagctaatgagtcttcttctacagaatcttcacaagatagtacgacccaagaa
agtagctctagcactgaagatcctttgtctactgaatcgtctacagaagctacttatgagtcttcttcga
cggaatcttcacaagatagtacgacccaagagagtagctctagcactgaagg
>dme_circ_0004506_junction_seq
cccaagagagtagctctagcactgaagggtcctttgtctactgaatcgtctacaga
>dme_circ_0004507
gattcagtagacaaaggaccttcagtgctagagctactttcttgggtcgtactatcttgagacgattctg
tcgacgaagactgatctgttactgctgttgatgattcagtagacaagggaccttcagtgcttgagctact
ttcttgggtcgtactatctggttgagattctgttgaagaagattcatctgtcgcatctgtagttgattct
gtttggaaagaagtttcagtgcttgagctactatcctgagtcgaactatttggtaaagattctgaggatg
atgactcatttgtcgcttctgtagataattcaaaagaaacagggctttctgagcttgagctactttcttg
ggtactatctggtagggattctgttgacgaagaatcatctgtcgctcctgtagatg
>dme_circ_0004507_junction_seq
gaagaatcatctgtcgctcctgtagatggattcagtagacaaaggaccttcagtgc
>dme_circ_0004508
gagctactttcttgggtcgtactatcttgagacgattctgtcgacgaagactgatctgttactgctgttg
atgattcagtagacaagggaccttcagtgcttgagctactttcttgggtcgtactatctggttgagattc
tgttgaagaagattcatctgtcgcatctgtagttgattctgtttggaaagaagtttcagtgcttgagcta
ctatcctgagtcgaactatttggtaaagattctgaggatgatgactcatttgtcgcttctgtagataatt
caaaagaaacagggctttctgagcttgagctactttcttgggtactatctggtagggattctgttgacga
agaatcatctgtcgctcctgtagatggttcgttggacacaggactttccgagcttgagctactttcttgA

ATCAAACTATATAAAGCTTCTATTAACAAGGACTCAACTTTTGATCCTATCGAAGAATCTGTGGAAATGT
GACTTTCCCAACTACCTTCCGTCGAACTTGAAATAATG
>dme_circ_0004508_junction_seq
ACTACCTTCCGTCGAACTTGAAATAATGgagctactttcttgggtcgtactatctt
>dme_circ_0004509
gattctgttgaagaagattcatctgtcgcatctgtagttgattctgtttggaaagaagtttcagtgcttg
agctactatcctgagtcgaactatttggtaaagattctgaggatgatgactcatttgtcgcttctgtaga
taattcaaaagaaacagggctttctgagcttgagctactttcttgggtactatctggtagggattctgtt
gacgaagaatcatctgtcgctcctgtagatg
>dme_circ_0004509_junction_seq
gaagaatcatctgtcgctcctgtagatggattctgttgaagaagattcatctgtcg
>dme_circ_0004510
gattcatctgtcgcatctgtagttgattctgtttggaaagaagtttcagtgcttgagctactatcctgag
tcgaactatttggtaaagattctgaggatgatgactcatttgtcgcttctgtagataattcaaaagaaac
agggctttctgagcttgagctactttcttgg
>dme_circ_0004510_junction_seq
gctttctgagcttgagctactttcttgggattcatctgtcgcatctgtagttgatt
>dme_circ_0004511
GATAATCTGGCTGATAAGGATAAGAAGGATATTATATCCAGCTCTTCGGATTCATCCCCAACTGAAGATG
CAACATATAGTTCGACACAGGTATCCTCCACACAAGTGCCGGAAGATGCTTCATCGGCTGAATCTATACA
AGAAAGTACAACACAAGGAAGTCGTTCAAGCACAGATATTTCTCTGTCTACAGAAGCATCCTTAGATGAT
ATAATATTAAGCTCTGAAAGTATCGTGCCTACGGAATCATCAACAACCATTATTTCAAGTTCGACGGAAG
GTAGTTGGGAAAGTCACATTTCCACAGATTCTTCGATAGGATCAAAAGTTGAGTCCTTG
>dme_circ_0004511_junction_seq
TTCGATAGGATCAAAAGTTGAGTCCTTGGATAATCTGGCTGATAAGGATAAGAAGG
>dme_circ_0004512
GGACTCAACTTTTGATCCTATCGAAGAATCTGTGGAAATGTGACTTTCCCAACTACCTTCCGTCGAACTT
GAAATAATGGTTGTTGATGATTCCGTAGGCACGATACTTTCAGAGCTTAATATTATATCATCTAAGGATG
CTTCTGTAGACAGAGAAATATCTGTGCTTGAACGACTTCCTTGTGTT
>dme_circ_0004512_junction_seq
ATCTGTGCTTGAACGACTTCCTTGTGTTGGACTCAACTTTTGATCCTATCGAAGAA
>dme_circ_0004513
GGACTCAACTTTTGATCCTATCGAAGAATCTGTGGAAATGTGACTTTCCCAACTACCTTCCGTCGAACTT
GAAATAATGGTTGTTGATGATTCCGTAGGCACGATACTTTCAGAGCTTAATATTATATCATCTAAGGATG
CTTCTGTAGACAGAGAAATATCTGTGCTTGAACGACTTCCTTGTGTTGTACTTTCTT
>dme_circ_0004513_junction_seq
GAACGACTTCCTTGTGTTGTACTTTCTTGGACTCAACTTTTGATCCTATCGAAGAA
>dme_circ_0004514
GGACTCAACTTTTGATCCTATCGAAGAATCTGTGGAAATGTGACTTTCCCAACTACCTTCCGTCGAACTT
GAAATAATGGTTGTTGATGATTCCGTAGGCACGATACTTTCAGAGCTTAATATTATATCATCTAAGGATG
CTTCTGTAGACAGAGAAATATCTGTGCTTGAACGACTTCCTTGTGTTGTACTTTCTTGTATAGATTCAGC
CGATGAAGCATCTTCCGGCACTTGTGTGGAGGATACCTGTGTCGAACTATATGTTGCATCTTCAGTTGGG
GATGAATCCGAAGAGCTGGATATAATATCCTTCTTATCCTTATCAGCCAGATTATCCTGCGACAAAGGTT
CAATTCCAACGGCACTGTTTATAAATTGGAAATGGGCAATTGGGTTGGGTTTCAAGGGCGAAGGTGTCGT

CTCATGAGAGTTGCCCCACAAACTGGCGAAGAAATTGAAGTTTCCGAAAATGTTGAATATAAGGGGTCTG
TCCACGCTGATGTTTTGCTTGATG
>dme_circ_0004514_junction_seq
TCTGTCCACGCTGATGTTTTGCTTGATGGGACTCAACTTTTGATCCTATCGAAGAA
>dme_circ_0004515
GATACTAGAATCCATCCCTGCTCCACCTCCCAGGACCATGAGAGGAGCTGCTGCATCCAAGCCTTCAGGA
CCTGCAGCTTCTGTCGACACAGCTCCCGAGCCTCCTAGCGAACTGGGAATGCCCAACTTCGATCACTTTG
CTGAAAAGTACGAGTTGTTTAGT
>dme_circ_0004515_junction_seq
CTTTGCTGAAAAGTACGAGTTGTTTAGTGATACTAGAATCCATCCCTGCTCCACCT
>dme_circ_0004516
GGTGATGAGTCTGAAGAGGATGAGCCGATGCAACAGCCAACTCCCACGCAGCGTTCGATCATTTTTGAAC
CGAGTAATACTGGAGTGCCCAGCCTATTCGGATCCCAGGTCTCCCAGGAAGACAGTGATGACGATAATGG
CTTTGGACGAGCTATCAGCCAACATCGCTCCATGACTGCGACACTTAGGACAAACGCTGTTAGAAACAAT
ACCGATGACTAGTCCGAAAACAGGAAACTGCCGCAAGTCTTGACTTCCTTAGTAAATACA
>dme_circ_0004516_junction_seq
GCAAGTCTTGACTTCCTTAGTAAATACAGGTGATGAGTCTGAAGAGGATGAGCCGA
>dme_circ_0004517
GTAACGGACCTGGGTAACCTGAGACTCGTCATACTCCTGATCCACCATCGCTGCTCCAATAGAGTTGTGG
GGATCGTCAGGCAGACTGTGTACAAGCAGCCAATACATGTGATGGGCAAAGCGCGGTGACTCCAGGCATT
TGGACAGCAAGAATCGAGCCATTGCCGAGCCTTC
>dme_circ_0004517_junction_seq
GCAAGAATCGAGCCATTGCCGAGCCTTCGTAACGGACCTGGGTAACCTGAGACTCG
>dme_circ_0004518
TTGAAATAATTCTTTATTCACAGCCCATTGGTCTTCTGGAGTGGCTGGCACCCACCTCGAGACTGAGTCA
GCTGGAGTGCGTGCACAATAGCTTCCAATTGGAGAAGGATGTACATTTGGGCCTGTGTCTTAGTACGGCG
GCAAACATGCAGGCTATTGCACGAACTGAGCGGGATGATGAGCACGATGCGGATTTGCTGCCGGAACATC
TTCTTCCCAACGAGGTGGTGCCAATTGTGACCTACGACAATATGATGATACTCATCGAAACGCTGGAGAT
GGAGATTGACAAGCTGGAATCGGCGGCCGACGGAGTACCCGGACGGAGTGTCGTGAGCTGCTCCGGAGTT
GTCCAAGCAGTGAAGGCCATATGCGCACTGCTCGGTTCAATCGACACAATGGAAATTGCACGATGTGTTG
CCGATCTGAAGCGCATTTGCGAGGTGGAGCAAAAGAAGTACTCGACGGGCGCTAGCAACCCAGAGATTGT
GAGTGACTATGGTGATTACGCTCAAGTTGTACTCCGCCCGCGCTCCATGCTGGAGCAGATCAAGGTCAAG
TGCAACGAGCTGCGAGATGCAGTGCAAGAGCTGGTTGAATTGTATGCGAATGTTTTCCGGGTGGCATTCT
CCGTGAAGACGCCCGATTACTCAACAAGTAAGAG
>dme_circ_0004518_junction_seq
AGACGCCCGATTACTCAACAAGTAAGAGTTGAAATAATTCTTTATTCACAGCCCAT
>dme_circ_0004519
AGTAGTCTCCTCCTGCAGTTCCAGCTGCTGTTGGATCTCAATTTCACGCTTCTTGCGCTCAGCGATCTCC
ACCAGCTTCTTCTCCAGCTCTATCTGGCGCTCACTGTACGTGTCCAGCAGGTTTTTCCGCCGCGAACAA
GTTGACCCTCCAATTCTGCGAGTTTGGCTGCCAGTTTTGAGTTCTCGACGCGCTCCCG
>dme_circ_0004519_junction_seq
CCAGTTTTGAGTTCTCGACGCGCTCCCGAGTAGTCTCCTCCTGCAGTTCCAGCTGC
>dme_circ_0004520
GGTGCCATTGCCGAGATATTGTAGCCTACCCGAGGGACCACGGAACGAGATCTTGTCCCCCAACTCGAGC

TGCTCCAGGTGTTGTGTCATTTTGCCGCCGGCCGGGAACTTGGGATGCGAATCCTTGAAGTACACCTTGA
CGACTAGGTCCACGTAGCCCACATCCTCATCAGACGATATCGGAGTGTATGGCCGAATTATTAGCTCATT
GTCGATGGTGGCGATCAGATGAATGTGCTGGCCAACGGGCAGGCCAAGGACATGCTGCTTGGAGGGCAAT
CCAAACCGGAATCTCCTA
>dme_circ_0004520_junction_seq
GGAGGGCAATCCAAACCGGAATCTCCTAGGTGCCATTGCCGAGATATTGTAGCCTA
>dme_circ_0004521
GGTCGAAGGTTTCGCGTGATACGCTGTATGAGGGCGTCAATGGACTCCTGGAGGCTTCGGCGAAGAAGAA
GCGCGGCTTCCTCGAGACGGTGGAACTGCAGATCGGCCTGAAGAACTACGATCCCCAGAAGGACAAGC
>dme_circ_0004521_junction_seq
AAGAACTACGATCCCCAGAAGGACAAGCGGTCGAAGGTTTCGCGTGATACGCTGTA
>dme_circ_0004522
CCTCGGCATCCATGAAGTCGACGTTGTTGGCCTTGGCTTCGTCGCAATGCTGCTGATCGCCAAGGATGCA
CACCTTCATCTTGGGACGAGGAATGTGCTTCAACCTGTGGGATGACAAGAAAGTAACCATAAATTAGCCA
GTGCTTTGTGGATTCAAATAGATGTAGATATAAGTGGGGCCTCATGGAAAACTTACAAGGCTCTCAGAGG
AGCACGTTTTCATAGTTATTAAGAAAATGGGGGTTCGGTGTCCATTGCTGGAGACCTACTCAAATTTTAA
AGACCCTGAGTGCAGGTCCGTCGAGATGTGACAGCCACATGTCCTCGACTACCTGATGACGTGATGAACT
CAACTCCCATGGGAGCTTCTATCCAAGCACGTGCGCAGGTGACTTAGCCAACGTACTTGACGGTGCCGGA
GAAACGCTTGTCCTTCTGGGGATCGTAGTTCTTCAGGCCGATCTGCAG
>dme_circ_0004522_junction_seq
GATCGTAGTTCTTCAGGCCGATCTGCAGCCTCGGCATCCATGAAGTCGACGTTGTT
>dme_circ_0004523
CTTCTTCAGAGCCTCGGCATCCATGAAGTCGACGTTGTTGGCCTTGGCTTCGTCGCAATGCTGCTGATCG
CCAAGGATGCACACCTTCATCTTGGGACGAGGAATGTGCTTCAACCTGTGGGATGACAAGAAAGTAACCA
TAAATTAGCCAGTGCTTTGTGGATTCAAATAGATGTAGATATAAGTGGGGCCTCATGGAAAACTTACAAG
GCTCTCAGAGGAGCACGTTTTCATAGTTATTAAGAAAATGGGGGTTCGGTGTCCATTGCTGGAGACCTAC
TCAAATTTTAAAGACCCTGAGTGCAGGTCCGTCGAGATGTGACAGCCACATGTCCTCGACTACCTGATGA
CGTGATGAACTCAACTCCCATGGGAGCTTCTATCCAAGCACGTGCGCAGGTGACTTAGCCAACGTACTTG
ACGGTGCCGGAGAAACGCTTGTCCTTCTGGGGATCGTAG
>dme_circ_0004523_junction_seq
GAAACGCTTGTCCTTCTGGGGATCGTAGCTTCTTCAGAGCCTCGGCATCCATGAAG
>dme_circ_0004524
GCTGAACAAGAACAAGAAGCTGGTGAAGAAGCTGGCCAAGTCCTATGATGCCTTCCTGGCCTCTGAGTCG
CTGATCAAGCAGATTCCTCGTTTGCTTGGCCCTGGCTTGAACAAGGCCGGCAAGTTCCCTGCCCTGCTGT
CGCATCAGGAGTCTATGATTGGCAAGATCGAGGAGGTTAAGTCGACCATCAAGTTCCAGATGAAGAAG
>dme_circ_0004524_junction_seq
GTCGACCATCAAGTTCCAGATGAAGAAGGCTGAACAAGAACAAGAAGCTGGTGAAG
>dme_circ_0004525
GCTGGCCAAGTCCTATGATGCCTTCCTGGCCTCTGAGTCGCTGATCAAGCAGATTCCTCGTTTGCTTGGC
CCTGGCTTGAACAAGGCCGGCAAGTTCCCTGCCCTGCTGTCGCATCAGGAGTCTATGATTGGCAAGATCG
AGGAGGTTAAGTCGACCATCAAGTTCCAGATGAAGAAG
>dme_circ_0004525_junction_seq
GTCGACCATCAAGTTCCAGATGAAGAAGGCTGGCCAAGTCCTATGATGCCTTCCTG
>dme_circ_0004526

TTCTTCTTCAACAGCGACACCAAGAAGTTGATCGACAAGTTGACGTTTTGGGCCAGCTCGTCGGACTTCA
TGCCAACGTGGCCGACGGCGACGGACAGGCAGAGCACCTTCTTCATCTGGAACTTGATGGTCGACTTAAC
CTCCTCGATCTTGCCAATCATAGACTCCTGATGCGACAGCAGGGCAGGGAACTTGCCGGCCTTG
>dme_circ_0004526_junction_seq
CAGCAGGGCAGGGAACTTGCCGGCCTTGTTCTTCTTCAACAGCGACACCAAGAAGT
>dme_circ_0004527
GGTTAAGTCGACCATCAAGTTCCAGATGAAGAAGGTGCTCTGCCTGTCCGTCGCCGTCGGCCACGTTGGC
ATGAAGTCCGACGAGCTGGCCCAAAACGTCAACTTGTCGATCAACTTCTTGGTGTCGCTGTTGAAGAAGA
ACTGGCAGAATGTGCGCTCCCTGCAC
>dme_circ_0004527_junction_seq
GAACTGGCAGAATGTGCGCTCCCTGCACGGTTAAGTCGACCATCAAGTTCCAGATG
>dme_circ_0004528
CAGCCGGATATTAATACTAAATCGAACGTGAATTTGTTTTATAAGTTTTAAGTGTTTTGAGCCGCTGTCA
TGGCCAAATTGTATATTAGTTTAGTGTTTCCATGTAACATTATGTAGTTTTAGTTATAAGTGTACACAAT
GGATCAATTGCAGTTCGGTCGCAGTCAGCCAGTCGTATAAAAGCTTCGCATTGGTTGGCCGATTGACAAG
CTCCCGTTGCAAG
>dme_circ_0004528_junction_seq
TGGCCGATTGACAAGCTCCCGTTGCAAGCAGCCGGATATTAATACTAAATCGAACG
>dme_circ_0004529
TACAAACTGGACACGGAGACGCTCTATCTCTCGGTCTTTTATCTAGATCGATTTTTGAGCCAAATGGCGG
TGGTGCGCTCCAAGTTACAGCTGGTGGGCACGGCAGCTATGTATATTGCCGCGTAAGTATTCTTGATAGT
CCGGTAATAATGGTGTGTGTATTCATGGGGtttattttttattttttCAGAAAATACGAGGAAATCTATC
CCCCAGAGGTCGGTGAGTTTGTCTTCCTCACCGACGACAGTTACACCAAGGCGCAGGTGCTACGCATGGA
GCAGGTTATCTTGAAGATTCTCTCCTTCGATCTGTGCACACCGACTGCTTACGTCTTCATCAACACCTAT
GCTGTACTTTGCGACATGCCTGAGAAGCTGAAGTACATGACGCTGGTATATTTCCGAGTTGTCGCTCATG
GAGGGAGAAACGTACTTGCAATATTTGCCATCACTTATGTCATCCGCTTCAGTCGCACTGGCGCGTCACA
TTTTGGGCATGGAGATGTGGACGCCAAGGCTGGAGGAGATCACGACTTACAAGCTGGAGGACCTAAAAAC
GGTTGTGTTGCATCTGTGTCACACCCACAAAACGGCAAAGGAGCTCAACACGCAGGCCATGCGGGAAAAG
>dme_circ_0004529_junction_seq
GCTCAACACGCAGGCCATGCGGGAAAAGTACAAACTGGACACGGAGACGCTCTATC
>dme_circ_0004530
GGTCCTCCAGCTTGTAAGTCGTGATCTCCTCCAGCCTTGGCGTCCACATCTCCATGCCCAAAATGTGACG
CGCCAGTGCGACTGAAGCGGATGACATAAGTGATGGCAAATATTGCAAGTACGTTTCTCCCTCCATGAGC
GACAACTCGGAAATATACTGTGTCAGGTAAACATTAAGTTAAGTTTCGGCTTTGTTTCTTTTTTCTTTTG
TATTGGCAATAGCTTACCAGCGTCATGTACTTCAGCTTCTCAGGCATGTCGCAAAGTACAGCATAGGTGT
TGATGAAGACGTAAGCAGTCGGTGTGCACAGATCGAAGGAGAGAATCTTCAAGATAACCTGCTCCATGC
>dme_circ_0004530_junction_seq
AGAATCTTCAAGATAACCTGCTCCATGCGGTCCTCCAGCTTGTAAGTCGTGATCTC
>dme_circ_0004531
TTTCCAGATCCACCAAGACATGAGCAACAAGGAGAATCCGGGCATTAAGATTCCGGCCGGAGTGAAGAAC
ACCAAGCAGCCGCTGGCCGTAATTGGGGGAAAAGCGGAGAAAAATGCGCTTGCGCCGCGGGCCAATTTCG
CCGTGCTCAATGGCAACAACAATGTGCCGCGTCCGGCCGGGAAAGTTGGTAAGTACCGTTGCCGATTTCC
CGTACATCCAGTGCCTAATTCCAATGCAGCTGCCTTTTTATATTTTTATGGTGGTGGGATCGTCACACCA
CTGCGACCGTGCGCATGTGTGAGATCTCCTAGTGGTGGTGGTGGTTTTCTCTCCCTTACCTCTCCCCTAC

CATCCCATCCTTTTCACTTCTCCATCTTACGTTACTCTCTTCTTAGAACGACCAATGCACTTACTTGCGC
CTAACCTAGAACCTTAACTAACCTAGAAAAAGACACAGGCCTTGCAATTACGCGCCTTTTTGATGATTTG
CAACAACAAAGCGAAGCAAGGAGGGCAAAAGCAATTGCGACATGCAACGCACACATGCGTATAGGTGcac
acatacacacacacacGAGCAATACGGGAAGCCAGGGAAAGGCGCGCAACTTAAAGTTGGATCGATTTAT
ggggggggATAATCATTTTATTTCTTTTTTGCTTACTATATTAAGGATGCATATGTAAAAGGAATTATCT
TTGTGTTTTTACTTTTATAATCCCACTGCAATATTACCTAATATAAAATTATTACCTAATATAGTAAGTT
GACCATATTGTTGAATTTGTCTTCTTCGTCTTCTGTTCCTTCCAGCAGGCAGGTCTTCCGTGACGTCAGA
AATCTTAATGTTGATGAGAATGTGGAGTACGGCGCCAAGAAATCGAATGTGGTGCCCGTGGTGGAGCAAT
TCAAGACCTTCTCCGTGTATGAGGACAACAACGATACCCAGG
>dme_circ_0004531_junction_seq
GTGTATGAGGACAACAACGATACCCAGGTTTCCAGATCCACCAAGACATGAGCAAC
>dme_circ_0004532
CGCACCATGGCCAGTTTCCAGATCCACCAAGACATGAGCAACAAGGAGAATCCGGGCATTAAGATTCCGG
CCGGAGTGAAGAACACCAAGCAGCCGCTGGCCGTAATTGGGGGAAAAGCGGAGAAAAATGCGCTTGCGCC
GCGGGCCAATTTCGCCGTGCTCAATGGCAACAACAATGTGCCGCGTCCGGCCGGGAAAGTTG
>dme_circ_0004532_junction_seq
AATGTGCCGCGTCCGGCCGGGAAAGTTGCGCACCATGGCCAGTTTCCAGATCCACC
>dme_circ_0004533
CAAGAAGGCAACGACGCTAAGAGAAAGAGAGAAGGAAGGGAAGAGCAGAGCATAATCGGACTCCATTTTA
CAAGGCGAAAAAAGGAGTAGGAAAGAGCGCACCATGGCCAGTTTCCAGATCCACCAAGACATGAGCAACA
AGGAGAATCCGGGCATTAAGATTCCGGCCGGAGTGAAGAACACCAAGCAGCCGCTGGCCGTAATTGGGGG
AAAAGCGGAGAAAAATGCGCTTGCGCCGCGGGCCAATTTCGCCGTGCTCAATGGCAACAACAATGTGCCG
CGTCCGGCCGGGAAAGTTG
>dme_circ_0004533_junction_seq
AATGTGCCGCGTCCGGCCGGGAAAGTTGCAAGAAGGCAACGACGCTAAGAGAAAGA
>dme_circ_0004534
GAGAATCCGGGCATTAAGATTCCGGCCGGAGTGAAGAACACCAAGCAGCCGCTGGCCGTAATTGGGGGAA
AAGCGGAGAAAAATGCGCTTGCGCCGCGGGCCAATTTCGCCGTGCTCAATGGCAACAACAATG
>dme_circ_0004534_junction_seq
TTCGCCGTGCTCAATGGCAACAACAATGGAGAATCCGGGCATTAAGATTCCGGCCG
>dme_circ_0004535
CAGCACAAAACAACAACAACGGCAGCAGCAGTAACAACAACAGCAAGAAGGCAACGACGCTAAGAGAAAG
AGAGAAGGAAGGGAAGAGCAGAGCATAATCGGACTCCATTTTACAAGGCGAAAAAAGGAGTAGGAAAGAG
CGCACCATGGCCAGTTTCCAGATCCACCAAGACATGAGCAACAAGGAGAATCCGGGCATTAAGATTCCGG
CCGGAGTGAAGAACACCAAGCAGCCGCTGGCCGTAATTGGGGGAAAAGCGGAGAAAAATGCGCTTGCGCC
GCGGGCCAATTTCGCCG
>dme_circ_0004535_junction_seq
GCGCTTGCGCCGCGGGCCAATTTCGCCGCAGCACAAAACAACAACAACGGCAGCAG
>dme_circ_0004536
GTATGTTAATGAAACCCCAGTCTCAGCAAGTCAATATCCTTGCCAGACAAACACCAAACAATGACCAGAA
GCCAAGAGAAAACTAACCTAATGAATTTATGTACGGGGTGAAAGGTGAAAACGCACAAGGCCAGCCAAAG
GCTCCAACTGAAAGTACTTAAGCCCATAAAATATTCAGCCTGTGGACATGTGGGGAAAATTACAAAGAAC
GCTCGGAAAAGGCGCACGAAAATCCAGAATAGGGGAAAAATGGGAGGTGGGAAATAGATGGACTGATGGA
AGGACCCTCGACCAAGTAACAAAATCTTAAACGGATACATAAGGAAAATAAAGAAAATATTAtttttttt

tttaattttttGCACCTTTTAAAACGAGTTTTCATTTGATTGATATGTTAAAAGCTGAGTTTTTCTACGTT
TCAATATTGAGCTTGTGCCATTTTATACATATAAACATTTTCCACTACCTATATTTTGCGTGTACTTCTT
TAAACTTATCAAGTGCTCGGTTTCAGTCGCACAATTTGCCACTGTAGTGTAGATAAATGGAGGGAACATG
GAGATATGGGGATATGGGGCCTTGCTCCTGCCCTCGGGAATAGGCAGCCAGCCACTGATTGAAAAGAAAA
TTACGGCAAGTAGTCGGTTGGGTGGCCGCCAGATGGTCTCCGCATCGACAGCGCTGCTGATGAGCAGCAT
AAATGTCCTGCGGAGAGACGGGCGGAAACGGAAGCGAATGGCGCCGCCATTGGGACCAGCGGCCTTGAGT
TCCGGAAGTGGTAAGAGCCAAAGGACCGGGAGCACACGGCTGCCAGCCATTTGCATTTAGGAGTACACAT
AGATACGTACGTATGATGGCAACTGTTTCCCACAGCTCCACAGAGCAGGTTTCTTTTTCACATTTACTGT
GGAGTTTGTAAGCCACTTTGAAGTGGAAGTATAGCTCGTATACAGATTCTGGGAAATAAAAGCAAAGCAA
GCGAAACAAATTAAATAGCAGATATATATGTAAAAGTTTAATCTCaaaaaaaaaaaaaaaCACGAGTCTG
CTGCTTGCCAAGTATTCAGGTAGTTGAATATTGTGCGATCTTAAAGTTTTAAGAATGGGTATTACTCGAC
TTCATTACATTCAATACGTTTTCGTTACACATAAATTGTGTAACAATTTCCAACtttttttttCGTAATA
AACCCATTCTTGTTATAAGAAGCTAAAACCATTTGATTTTTCCACTGTGCCGACACTGGAGCAGCCCGAG
AACTTTAAAGGCGCAGAGGAGAAGCAGTTCGGGTGGCACGTTGTGGATATTAAGCATGTCCTTAAGCATA
AAATGTCAATAAGTGCAGCCAATCGATGCTAAGCAGTTTAAGTTGCGACAAGCCACTGAGGCACCGTCTC
AATCCGAGTCCTTTACGTCCTTCGATATTTCCACTTTCCAGCAGGAACTGCGCTAACAATGCAAAGGACA
CGGAAAAGCACAATCACCCTCACACACACATTCAAACTTATCTTTATGCATGCGTCTCATATTTTTCGAT
AATCGTATTTGCGAATTTTTGTTACAAGTTACACTaaaaaaaaaTACATTACATTTGACATCTAAATAAA
ACAAGGCAAAGAAAAATATTACATTAAAAACCGAAACATTTTCATAATAGATTTGTACTCTTAACCGAGC
TTCGACGAAAGTTCGATAAACATTAATAAATTTCTACTTTATCGCAGTATACAtatcttttatcttatat
ctttcttttcggcttatcttttttgtctgcagaattttcCCATCTTAGTGAAAAGTAGTACAGAATATG
CTAGTGTTTTTTCTGTACTTAAGTTTACTCCTACCTGGTTACATTTTGGTAGCAAGCAACTTGCGTAAAA
GTACATATTTAGTTTAAATAAAATTGAATTCAACTGCTGACTTCGATATCATTTTAAAGTGACATACTTT
ACCACCGTGTAAAATTCAAGTTCAATTCAACTTTATCAATTGAGCAATTGAAAGGGTTTCCCAAAAACGA
TTTTGGCAAAAACATTTTGAGTAATCGACACCTTTCTTATCCGCTTGTCACTCATCCGAAACATTTCTAA
TTTCATTGTGAAAATATGTTTGCCCTTATCAGCTTGTTTCGATATCTAACAACAAGACACGACAAAAATC
CAAGATAAGTGGCTGTACTTAAATGTAGCAATAGATTGGGAAACGAGTTCAGTACGCGTTTAGTTCCGGA
ACAGTTGGCGGTCTTACCAAAATGAGAGGTACTAAAAGCGGAGTGATGAATGACCTGAGACTAAAGATAG
CTCATCGTAGTTCCGCCATGTGGATTCCATTTGTGTGTCATTTTGGCCATAGTCGCGGAGATTAGAGGAT
TTTCGATGGAGGACAAGTGCAAATTGTGGGCTGGAACTGGCTATATTGGTGATCCCAGTGATTGCCAGGC
CTGGGGATATTGCCAGGACAACAAACTCATCGATAGACGTAGCTGCACTGAAGGATTACTATACAGTTTC
CGAGATGGCACCTGCAAGAGGGCATCCGATACGATTTGTCATTCCCAGCTCTCGGAGATTTGCGCCAGCC
TCGAGCCTTGGAATTACGTGGCCAACCCAGCTGATTGTCGGAGATTCGTCAAGTGCGCGGATTTGGATGA
TCCCACTTGGGGCGACTGTGGTGTGGGTCAGGTGTTTAGCAACAAGAAGCAGACCTGCCTCGAGGAGGTT
GCCGGCTGTCCACAGGATAATATCTGCTCCCATATGAAAGATGGCTCCTTTGTGGGCGATCCCAAGAGCT
GTCAAATCTACTATAAATGCCATAATGGATTTGGCACCATGTTGAATTGCTCCGTTGGGCGATACTTTAA
TCGCAAAACGGGTAATTGCCAATCCTGGATGCCGCATTACTGTTCCAAGGATGATGAGGATAACATATTA
ACGCCACCATCCACGGACCATAATATCTGCTCCAAGTATTATCAACGGGATAGAGATGGAGTGCAACTCC
TGCCGGATCTGATGACCTGTTACGGATATTACTCCTGCACATCCCAGTTCGACGTGGGCAAGTGGAGCAG
CTGTCCTTGGGGACTGCACTTTGAGTGGTGGAGCCAGCGGTGTGGCTCGCCGAAAGACAATAGCTGCTCC
TACGATCGCTGCGCCAATAGGAACCAACTAATGGTGGCCACCATTAACACGGGCTGCAGGGAGTTCACGA
TCTGTCAAGATAATCGTTCGAAGAGCTCGCAAAAGTGTCCGGAGGACTATCCATATTTCAATGAGCTATT
GCGACAGTGCACCGATGAATATCCCAATCATAGGGTGTGCTACATGGATGGATAAATTAAATAAATTAAT
AATATTCCACTAATAATTAACAAGAAAGTAAG

>dme_circ_0004536_junction_seq
TTCCACTAATAATTAACAAGAAAGTAAGGTATGTTAATGAAACCCCAGTCTCAGCA
>dme_circ_0004537
GTATGTTAATGAAACCCCAGTCTCAGCAAGTCAATATCCTTGCCAGACAAACACCAAACAATGACCAGAA
GCCAAGAGAAAACTAACCTAATGAATTTATGTACGGGGTGAAAGGTGAAAACGCACAAGGCCAGCCAAAG
GCTCCAACTGAAAGTACTTAAGCCCATAAAATATTCAGCCTGTGGACATGTGGGGAAAATTACAAAGAAC
GCTCGGAAAAGGCGCACGAAAATCCAGAATAGGGGAAAAATGGGAGGTGGGAAATAGATGGACTGATGGA
AGGACCCTCGACCAAGTAACAAAATCTTAAACGGATACATAAGGAAAATAAAGAAAATATTAttttttttt
tttaattttttGCACCTTTTAAAACGAGTTTTCATTTGATTGATATGTTAAAAGCTGAGTTTTCTACGTT
TCAATATTGAGCTTGTGCCATTTTATACATATAAACATTTTCCACTACCTATATTTTGCGTGTACTTCTT
TAAACTTATCAAGTGCTCGGTTTCAGTCGCACAATTTGCCACTGTAGTGTAGATAAATGGAGGGAACATG
GAGATATGGGGATATGGGGCCTTGCTCCTGCCCTCGGGAATAGGCAGCCAGCCACTGATTGAAAAGAAAA
TTACGGCAAGTAGTCGGTTGGGTGGCCGCCAGATGGTCTCCGCATCGACAGCGCTGCTGATGAGCAGCAT
AAATGTCCTGCGGAGAGACGGGCGGAAACGGAAGCGAATGGCGCCGCCATTGGGACCAGCGGCCTTGAGT
TCCGGAAGTGGTAAGAGCCAAAGGACCGGGAGCACACGGCTGCCAGCCATTTGCATTTAGGAGTACACAT
AGATACGTACGTATGATGGCAACTGTTTCCCACAGCTCCACAGAGCAGGTTTCTTTTTCACATTTACTGT
GGAGTTTGTAAGCCACTTTGAAGTGGAAGTATAGCTCGTATACAGATTCTGGGAAATAAAAGCAAAGCAA
GCGAAACAAATTAAATAGCAGATATATATGTAAAAGTTTAATCTCaaaaaaaaaaaaaaaCACGAGTCTG
CTGCTTGCCAAGTATTCAGGTAGTTGAATATTGTGCGATCTTAAAGTTTTAAGAATGGGTATTACTCGAC
TTCATTACATTCAATACGTTTTCGTTACACATAAATTGTGTAACAATTTCCAACtttttttttCGTAATA
AACCCATTCTTGTTATAAGAAGCTAAAACCATTTGATTTTTCCACTGTGCCGACACTGGAGCAGCCCGAG
AACTTTAAAGGCGCAGAGGAGAAGCAGTTCGGGTGGCACGTTGTGGATATTAAGCATGTCCTTAAGCATA
AAATGTCAATAAGTGCAGCCAATCGATGCTAAGCAGTTTAAGTTGCGACAAGCCACTGAGGCACCGTCTC
AATCCGAGTCCTTTACGTCCTTCGATATTTCCACTTTCCAGCAGGAACTGCGCTAACAATGCAAAGGACA
CGGAAAAGCACAATCACCCTCACACACACATTCAAACTTATCTTTATGCATGCGTCTCATATTTTTCGAT
AATCGTATTTGCGAATTTTTGTTACAAGTTACACTaaaaaaaaaTACATTACATTTGACATCTAAATAAA
ACAAGGCAAAGAAAAATATTACATTAAAAACCGAAACATTTTCATAATAGATTTGTACTCTTAACCGAGC
TTCGACGAAAGTTCGATAAACATTAATAAATTTCTACTTTATCGCAGTATACAtatcttttatcttatat
ctttcttttcggcttatctttttttgtctgcagaattttcCCATCTTAGTGAAAAGTAGTACAGAATATG
CTAGTGTTTTTTCTGTACTTAAGTTTACTCCTACCTGGTTACATTTTGGTAGCAAGCAACTTGCGTAAAA
GTACATATTTAGTTTAAATAAAATTGAATTCAACTGCTGACTTCGATATCATTTTAAAGTGACATACTTT
ACCACCGTGTAAAATTCAAGTTCAATTCAACTTTATCAATTGAGCAATTGAAAGGGTTTCCCAAAAACGA
TTTTGGCAAAAACATTTTGAGTAATCGACACCTTTCTTATCCGCTTGTCACTCATCCGAAACATTTCTAA
TTTCATTGTGAAAATATGTTTGCCCTTATCAGCTTGTTTCGATATCTAACAACAAGACACGACAAAAATC
CAAGATAAGTGGCTGTACTTAAATGTAGCAATAGATTGGGAAACGAGTTCAGTACGCGTTTAGTTCCGGA
ACAGTTGGCGGTCTTACCAAAATGAGAGGTACTAAAAGCGGAGTGATGAATGACCTGAGACTAAAGATAG
CTCATCGTAGTTCCGCCATGTGGATTCCATTTGTGTGTCATTTTGGCCATAGTCGCGGAGATTAGAGGAT
TTTCGATGGAGGACAAGTGCAAATTGTGGGCTGGAACTGGCTATATTGGTGATCCCAGTGATTGCCAGGC
CTGGGGATATTGCCAGGACAACAAACTCATCGATAGACGTAGCTGCACTGAAGGATTACTATACAGTTTC
CGAGATGGCACCTGCAAGAGGGCATCCGATACGATTTGTCATTCCCAGCTCTCGGAGATTTGCGCCAGCC
TCGAGCCTTGGAATTACGTGGCCAACCCAGCTGATTGTCGGAGATTCGTCAAGTGCGCGGATTTGGATGA
TCCCACTTGGGGCGACTGTGGTGTGGGTCAGGTGTTTAGCAACAAGAAGCAGACCTGCCTCGAGGAGGTT
GCCGGCTGTCCACAGGATAATATCTGCTCCCATATGAAAGATGGCTCCTTTGTGGGCGATCCCAAGAGCT
GTCAAATCTACTATAAATGCCATAATGGATTTGGCACCATGTTGAATTGCTCCGTTGGGCGATACTTTAA

TCGCAAAACGGGTAATTGCCAATCCTGGATGCCGCATTACTGTTCCAAGGATGATGAGGATAACATATTA
ACGCCACCATCCACGGACCATAATATCTGCTCCAAGTATTATCAACGGGATAGAGATGGAGTGCAACTCC
TGCCGGATCTGATGACCTGTTACGGATATTACTCCTGCACATCCCAGTTCGACGTGGGCAAGTGGAGCAG
CTGTCCTTGGGGACTGCACTTTGAGTGGTGGAGCCAGCGGTGTGGCTCGCCGAAAGACAATAGCTGCTCC
TACGATCGCTGCGCCAATAGGAACCAACTAATGGTGGCCACCATTAACACGGGCTGCAGGGAGTTCACGA
TCTGTCAAGATAATCGTTCGAAGAGCTCGCAAAAGTGTCCGGAGGACTATCCATATTTCAATGAGCTATT
GCGACAGTGCACCGATGAATATCCCAATCATAGGGTGTGCTACATGGATGGATAAATTAAATAAATTAAT
AATATTCCACTAATAATTAACAAGAAAG
>dme_circ_0004537_junction_seq
AATATTCCACTAATAATTAACAAGAAAGGTATGTTAATGAAACCCCAGTCTCAGCA
>dme_circ_0004538
GCCATGCGAGAGACGATTATGGACTATCTAAGCACCACTCGGCAGTTTTTGCTCTCTTTGCAAGCCGATA
GCCTTTTCCAGCTGCTAAACAACACCTCCACAGCGATTCAGTTACTCTGCCAACTAGATAGGATGTTCGA
AAATGAACCAAGATG
>dme_circ_0004538_junction_seq
ATAGGATGTTCGAAAATGAACCAAGATGGCCATGCGAGAGACGATTATGGACTATC
>dme_circ_0004539
ACAATGCCCTCGGCCATCAGTGGCGCGCCACATGCCCACATACCCAGCAACGAGGAGTGCGGCATCCGCG
ATGTGTGGAAGCAAAACCTGGAGGAGGAGTTCCGCACCATACGGAAAGTGGTGCAGAAGTACCACTATGT
GGCCATGGACACCGAGTTTCCAGGCGTGGTAGCACGTCCAGTCGGCGAGTTCCGCTCCACGGCGGACTAT
CATTACCAGCTGCTGCGCTGTAATGTCGATCTGCTGCGGATTATCCAATTGGGCCTTACCTTCATGGACG
ACGATGGAAAGACACCACCCGGCTACTCCACCTGGCAGTTCAACTTCAAGTTTAACCTGAGGCGAGGACA
TGTATGCGCAGGACTCGATCGATCTGCTGCAGAACTCCGGAATTCAGTTCAAGAAGCACGAGGAGGATGG
CATCGATCCCATCGACTTCGCCGAACTGCTGATGAGCTCTGGCATCGTGCTGGTGGAAAACATCAAGTGG
CTGTGCTTCCACTCCGGCTACGATTTTGGCTACCTTCTAAAGCTGCTCACCGATCAGAACCTGCCCCCGG
ACGAATCTGAATTTTTCGATCTGCTGCACATCTACTTTCCCAACATCTTCGACATCAAGTATCTGATGAA
GTCGTGCAAGAACCTGAAAGGCGGCCTGCAGGAGGTTGCCGATCAGCTGGAACTGCGGCGCGTGGGCCCC
CAGCATCAGGCCGGCTCGGATGCTCTGCTGACGGGCATGGCCTTCTTCAAAATGCGTGAGG
>dme_circ_0004539_junction_seq
GGCATGGCCTTCTTCAAAATGCGTGAGGACAATGCCCTCGGCCATCAGTGGCGCGC
>dme_circ_0004540
GATCCAGGGCTGTGCGTCCATTGTGATCCTCGTGGTGGCGATTACAGCCATGTTCTATGAGCGTCTTGGC
CGCCGCCAAGTGGCCACGTAAACAGGCCCAGCTGAGTGCAGTGAATCCCTCGTGATCCTGAGCTTCTCGT
TGCGCTCCCCGCGCCAGCAGCAGATCGACCAGCTCCAGGT
>dme_circ_0004540_junction_seq
GCCAGCAGCAGATCGACCAGCTCCAGGTGATCCAGGGCTGTGCGTCCATTGTGATC
>dme_circ_0004541
GGATCGGAGCTCCACCCATGTAATCCGTGCGGTGATTCGGTGAAGCTCCTGCCAAGAGAACTAGTCGGGA
AACCTTTAGGTTCGGACTGTAGACATTTCGCAGGGCGCCCAGCGAGGATGAAATGTTATCCGCCGCTCCC
GCCAACCAATAAGATTGCAAATCCCGCGGAGAAAGCAGCGTCAGACTAGTTCCTCCGTATAAAT
>dme_circ_0004541_junction_seq
AGCGTCAGACTAGTTCCTCCGTATAAATGGATCGGAGCTCCACCCATGTAATCCGT
>dme_circ_0004542
CATGTTAATACTGAAACTTCCCAAAATGTTTAGCCAACTGTGGCTTCTTCTGATTCTATCGCTCTTAACC

CTAGAAGGCCCACAGCCTTCGACGGGCAGTGGTTATACGACAAAATCAAGTGTGGATTTGCTGGAAAACG
ATTCGCGATACATCTCATATCAAGGGTGAGTTCTCGAGGTGGGCGGATTATCAAAAATTACTCGAAACTG
TGTCAATTGCACGGCAATCAATCAAGTTGGCCACTTGAAGCGGTGGAACTGTAAATTGGGTTGATGTCTG
TCAAAGTTCTAAGTGATTCCGAGAGTCTGCTGCAATCGATTGCACAAATGTTCGGTTCGATCGAACAGGT
GCCATTTCGAATACAACGAATACACCCATCTGCCCAACTGTTTGTATTTGCACGGAATGTCAACAATGAT
CTCGATTTAACCGAGTTCCCCAACAAACCGCTTTTATGACACCCCAATACATTCGTGTATCTGCTGCTTT
TAATCAGAAAAAGTTTCGCATTTTGTTAAAATATTTGTTCTTTTTTTGTGATTTTTGCACTGAAATGTAT
CTCGAAGTCCGTCTTAAAATGCGAACTCAGCCCAACGTTGTGTTGACAATGattttaatttttgtttagc
gctttttttattttatttCGCACTTAAATATTGAAAAGTATATGTTTTACCAATGAATCAGTGAATCTTTT
AAAAACCTTGAAAAATATGGGTTAAATAAAATTGAGCTTAATTGTGCCAAGATGCCAAAATGTTTtaata
aatttgcaatataattgctgagataataattgcttatataataattgctaatataattgctaataAAACT
TCAATTAAATTGCTTAGTATTTGTTTTTACGATTTTGTGCAATCTTTTTCTTACAAAAATGTTTTTATTT
TCTTAACAGATTTAATGTCGACGGCCAAACGATTTTACGATCCCGAAACGACCTGGTATTCGGAAATGCT
ATTCGACGTGGCCAGGAACCAAGTGATTGTCGGCGCCAGG
>dme_circ_0004542_junction_seq
CAGGAACCAAGTGATTGTCGGCGCCAGGCATGTTAATACTGAAACTTCCCAAAATG
>dme_circ_0004543
TAGTAGTTGTTGATCTGGAAGATTCCGTAGTCGTTGGAGCCGTTGTAGTTCTCAGGACCCACCACTCCGG
TGCGGTAGGAGGACTCGTGCTCGGCAATACAGGCCCAACGAGCCAATTGGTCACGAGGAACGCCCAGGTT
GGACATCTCGCGGGCCAGGGAGCAACGGTCCAAGGTGCGACCAAGAGCAGGAGCGGCCATAGCCAGGGCA
ACCAGAACGATGAAGGCCTTCATTTTGATTTGAATACCACAGTAAACTGATGGAGAGGTCCTCGCAATGG
GCCCTTATATACCCTGAACCATCACCTTATTAAGAAGAACGTATAAGTACTCAACCGTGATAAGGTGATC
ACCATATTGCGGCTGTCAATCGCCCCTTCATTAGCCATGAAAATTCAGCGATAAGGACTTAGGTGTAGCG
AATGGTTCCAAATCAGATACGTTACGCGACCTTATCAGCGAAGTTTGTTTACAGATTTCTTTAACGTTGG
CCATTTTTAGTTCGACTGACTGTGATTACTTACTAAATGGGATGGAAAGCCGTTTTAAGAGGGCAAGAAC
AGCTATGGCGGCTTCTTGTACCATCTTTAAAGATTCGTGGCCATTATCCTAACAGTTCCCAAAAGTTCTC
AAAGTTCGGCTGACAATTAGCGACAAAATTTATGGAAATGAGATTTAATTGGCCTGGGTCGATTGTCAGT
TCCACAGGCACAAAGTTCCAAAGAGAACTCATGCATATGCAATGAGGTCTGGTCTGGTCGTCAGAAAATT
CTTCTTGGTCAGAACTGTTAGCTGACACACTTCGGGGATAGAAAAGCTATTAGTTATACAGCGGCGATTC
AATAAATCAGCAAAAAGTAAAAGTGATAAGCAGCATTCTCCGCCGACAAAAGAATCTACAACTCATCTCG
CCGATGAGCGCCCCACTCGCTCACCAACCAGCATTCATTTATGAATTCATCTATAATTCATGGGACTCTA
AGAAGGTCACGCTGGCAAGCTGGAGAGCTCCAACCACCTGAAACGACTTTGTCTTGGGGCCTCCCTTTCC
TCCACAAAAATCTCGCAACTGGCGTCTTTCGAATGCCTCCACAGAAGCCTTTTGTCATTTGTCTTTGCTG
TTAGCCACACGCGAAAGGTTAACAACTTTCTTGGCATTGCACAAATCAGTGGAAATCTGGGCTCCCAACC
TGCCCCCACCAGACGCGCCTGGTTTTAGGCCCACTGGTAACCCCCAGGAGTACCGCTAAGAGCCCCTCCC
CGCGGAGACGAATGGGGTTAATGTAACAGCCCGCAGTTTGCATACTTGTGGTCGGGTCGTTAGTCAGAGT
CGGTACTTGAGTCTCAACTCCAactccatccactccatccgcttcatccacCGAGAAAGCAATGTGCAGC
CAGGTTGTTGTGACTGTTGATGATGTGTGGTAGCCTTGGGGTCTTTGAAGAGCCAGTGGCTTGGCGATTG
AACGCACGCCCGTTACAAGAGATGAACCAGCCCGCACCAAATAGAGTTCTTTTTAGCTCGCACTCTCTGT
CCAGTTACAGCTCACTCAGGTGCACCAAAGTGGAGCTTCTGCCCACCAACGACTCGTAATTGCCCACTCC
AAAAGCTAGCGGAGATGCTAACAAGGTTAATGGCTCGGATAGCCAACGTTTAAGTCAGAAAATTAATACC
ACTTGCTCTAAAAACCCAAGGGTCTTGCTGGCTGGGATGGGGCTAATCTACTTTTTACCTACGTCTATGC
TTGCGAATCTTCAGGGAGCGGCTTGAGACCATTTGCTAGGTGCTGATAAGCGATGTAGATTGATGAGCTT
GCGTCTGGCCATTTGCAATAGCCAGTTATTTACTGTCTCGTGTGCACTTGCGATAATATATACATAAGTAGC

TAAAGACTTCGCGGCGCACTTGAGATAATAACATTGTGAAGATCTACCAGTTAAGATAATACCGCCCTAT
AAAAGCACGGCCTACGCTCCCTCCCGTCATTAGTTTCAATTCGAATCATGAAGGCTTTCATCGTTCTGGT
TGCCCTGGCCTGTGCCGCCCCAGCTTTCGGTCGCACCATGGACCGTTGCTCCCTGGCCCGCGAGATGTCC
AACCTGGGCGTTCCTCGTGACCAATTGGCTCGTTGGGCCTGTATTGCCGAGCACGAGTCCTCCTACCGCA
CCGGAGTGGTTGGTCCCGAGAACTACAACGGCTCCAACGACTACGGAATCTTCCAGATCAACGACTACTA
CTGGTGCGCTCCTCCCAGCGGTCGCTTCTCCTACAACGAATGCGGATTGAGCTGCAACGCTCTCCTGACC
GACGACATCACCCACTCCGTCCGTTGTGCCCAGAAGGTCCTCAGCCAGCAGGGATGGTCCGCCTGGTCCA
CCTGGCACTACTGCAGCGGATGGTTGCCGTCCATCGATGACTGCTTCTAAACTGATTTTGACCCCGATTA
AAACGATTGCGCACCAGTAGTAGTCGTTGATCTGGAAGATTCCGTAGTCGTTGGAGCCGTTGTAGTTCTC
AGGACCAACCACTCCGGTGCGGTAGGAGGACTCGTGCTCGGCAATGCAGGCTCACTTGTTCAGCTGGTCG
CGGGGAACGCCCAGGTTGGACATCTCGCGGGCCAGGGAGCAACGGTCCATGG
>dme_circ_0004543_junction_seq
TCGCGGGCCAGGGAGCAACGGTCCATGGTAGTAGTTGTTGATCTGGAAGATTCCGT
>dme_circ_0004544
gttttttttgtatgtttgtttgCTGTGATTTACATGATTTCGTTCTTCTGTTTGTTTGCTCTGCTTTGC
TTTCATTTCTTTTCGCCCGATGGCTATGGTTCTCGATTTCGGATGATGCATAAATGGGACAAAGTGGTGG
TGTGGTTATTGGTATAG
>dme_circ_0004544_junction_seq
CAAAGTGGTGGTGTGGTTATTGGTATAGgttttttttgtatgtttgtttgCTGTG
>dme_circ_0004545
GGCAACGAGGCGGCCAACGTGACCGGTCCCTCCGGTGAGCCGGTGCGGGGCAGTCAGTTTGCAGCGGACA
AGCGCCGTAACTTCCGTCCCTGGATGAAGAAGAATCGCCGCAAGGATGGCGAAGTGGAAGGCGAAGACGC
CGAATCGTCGGCCCAGCAGCAACAGCAGCAGGCGGCACCAATCGTTGACGGGCAGCCGCAGCAGCAGGTG
CAGTCCGGCCCGCGTCAACCACGACAGAACTTCCGccgaggaccacccggtggaccgcccggaggacctc
gaggtggcccccgaggaccgcctggtggagctcccggtggcccccgGCGCTACAACAACTATTATCTGCG
TCAGCCGAGACGCGGCCTGGGCGGCGGTGACGGCAGCGCCGAGCCCGGTGTCCACGATCAGAATCCCGAG
GGTCTGCAGCGCGGCGAGGGTCAGGGACCACGTCGCGGTGGAGGCCCACCGGGCGGACCCCAGAGGCGCT
TCTTCCGACGCAACTTCAACAATGGTCCACCGCCACCACGCCGCGACGGCGGAGAATACAGTGAGTGAAC
TTTGTGCTATTAGATAATTGGTATATATTATTATTAATATTATTCTGTTGCAGTTCAAGGCCAGGGACCG
CCACGCCCACAACAGCCACGTCCACGTCGCCAGAGGAAACCAAATGGCCCTGGCGGTGGTTCGGAGCAGC
AGCCAGAGAAGG
>dme_circ_0004545_junction_seq
GGTGGTTCGGAGCAGCAGCCAGAGAAGGGGCAACGAGGCGGCCAACGTGACCGGTC
>dme_circ_0004546
CCACCAAAGTCACCGGCACCGTCAAGTGGTTCAACGTGAAGAGCGGCTACGGCTTCATCAACCGCAACGA
CACCAGAGAGGATGTCTTTGTGCACCAGAGCGCCATTGCGCGGAACAACCCCAAAAAGGCGGTCCGCTCG
GTGGGCGACGGTGAGGTCGTTGAGTTCGACGTGGTCATTGGTGAGAAGGGCAACGAGGCGGCCAACGTGA
CCGGTCCCTCCGGTGAGCCGGTGCGGGGCAGTCAGTTTGCAGCGGACAAGCGCCGTAACTTCCGTCCCTG
GATGAAGAAGAATCGCCGCAAGGATGGCGAAGTGGAAGGCGAAGACGCCGAATCGTCGGCCCAGCAGCAA
CAGCAGCAGGCGGCACCAATCGTTGACGGGCAGCCGCAGCAGCAGGTGCAGTCCGGCCCGCGTCAACCAC
GACAGAACTTCCGccgaggaccacccggtggaccgcccggaggacctcgaggtggcccccgaggaccgcc
tggtggagctcccggtggcccccgGCGCTACAACAACTATTATCTGCGTCAGCCGAGACGCGGCCTGGGC
GGCGGTGACGGCAGCGCCGAGCCCGGTGTCCACGATCAGAATCCCGAGGGTCTGCAGCGCGGCGAGGGTC
AGGGACCACGTCGCGGTGGAGGCCCACCGGGCGGACCCCAGAGGCGCTTCTTCCGACGCAACTTCAACAA

TGGTCCACCGCCACCACGCCGCGACGGCGGAGAATACAGTGAGTGAACTTTGTGCTATTAGATAATTGGT
ATATATTATTATTAATATTATTCTGTTGCAGTTCAAGGCCAGGGACCGCCACGCCCACAACAGCCACGTC
CACGTCGCCAGAGGAAACCAAATGGCCCTGGCGGTGGTTCGGAGCAGCAGCCAGAGAAGG
>dme_circ_0004546_junction_seq
GGTGGTTCGGAGCAGCAGCCAGAGAAGGCCACCAAAGTCACCGGCACCGTCAAGTG
>dme_circ_0004547
GTAGTAGGCCAAATCGTGATCGTAGTTCAGGGAACCGTGCACGTTCGAATAGTTCGTACGGATCACCACG
TTGTAGATGTTGTCCATCTGCTTGACATGATGGAAGCCGTGCATTTTGTAGTGCTCAGCCCATTCCAGTG
CCTGGCTATCGAAGTAGCTCCATGGGTGGATCTCGTAGTGGGCCGGCAGGACGACTCCCTGCATATCATC
ACGGAACAAACTGGCAATGGTCACGGCGTACACGAACAACTGCTTGTTTACGTGCTGGCGGGCCCAGTAG
ACGACGTGCTGGAAGGTCTCCCAGTTCTTGGCGAAGTAGAAGAGACGCACCAGACCCACCACATACTCCT
CGTGCTTGTCGTACATCACCGACCACACCATGTCATCGGTGAAGGGTTGGTGTTGCCACAGCTCGAAGAA
GTGCTCCAACATCTCCGGGTGCACATAGTCGGCCACGTGCTCCCACGGCTTGTACTCCACGTAGCTGCCC
CCGAAGGGCGTGGTGAACACATCGTCCTGGTAGATGTGCTGCAGGATCTGGTACATGAACTTCTGTTTCA
TCAGAAAGTCCTTGTCCGCCACCTTGGAGTCCAGGTGCTTGGCCTGGCCGAGAGTAGCCAGCAATGCCAC
TGCAGCTAACGCAATCACCGTGAACGACTTCATCTTCTCGAG
>dme_circ_0004547_junction_seq
TCACCGTGAACGACTTCATCTTCTCGAGGTAGTAGGCCAAATCGTGATCGTAGTTC
>dme_circ_0004548
GTAGTAGGCCAAATCGTGATCGTAGTTCAGGGAACCGTGCACGTTCGAATAGTTCGTACGGATCACCACG
TTGTAGATGTTGTCCATCTGCTTGACATGATGGAAGCCGTGCATTTTGTAGTGCTCAGCCCATTCCAGTG
CCTGGCTATCGAAGTAGCTCCATGGGTGGATCTCG
>dme_circ_0004548_junction_seq
ATCGAAGTAGCTCCATGGGTGGATCTCGGTAGTAGGCCAAATCGTGATCGTAGTTC
>dme_circ_0004549
TTAAATTACGATCTCTATGGGGTAAGTGGATGCAGACAAAGATATAACAAGGAAAACATATGTAATCTAT
CCAATATCGTTGGTTCGTTTTGGGGTTCGTATTCGTTGGGTCCAGGATCTAGACCACATTGGTGTGCTCG
TCGCGATGGAAGATCTTCACGTCGACGAACTTGAAGTTGTGGACATCGAAGTAG
>dme_circ_0004549_junction_seq
GAACTTGAAGTTGTGGACATCGAAGTAGTTAAATTACGATCTCTATGGGGTAAGTG
>dme_circ_0004550
ATGTCGCCGGACACCCAGTTGCTGATCCACCAGCTTCAACTCGCTGGCAATCTTGCAGGCAGCCAGATAG
TGGCGCATATGCACCAGATTTTGGCGAAGTTTCTTTACCGCACACAATTTTTCTACGAACACAAACAGCT
TGGGATTGTCCTCCTCCAAGCGAATGAGCGGTTTATTTAGGAACAACCTAATCTCTTGGAGCGCCGTGCG
CGACACTCTGCGTGGCGAAAAGTCCCAATTGTGGATAATGCGGGCTGG
>dme_circ_0004550_junction_seq
AGTCCCAATTGTGGATAATGCGGGCTGGATGTCGCCGGACACCCAGTTGCTGATCC
>dme_circ_0004551
GTAATTTTGGAGCATAATGGCAAGTTCGAGAGTTCAGATGAGGAGCAGCAGGAAAAGGAGCACGAGGAAC
TCCAACGTGCGCAGTCACCGAGCGCCGTGGAGGAGGAGCGCCGTCTAAAGGCCGAGTTCCGGAAGG
>dme_circ_0004551_junction_seq
CGCCGTCTAAAGGCCGAGTTCCGGAAGGGTAATTTTGGAGCATAATGGCAAGTTCG
>dme_circ_0004552
ATTTACGCAAAACTACTAGAAAATGGTCGGTTGGAGCCGTTTGCTGTCGCCGGCGGCGAAGTTCGCCAGC

TACCGGGCTGTCCTGCAAGAGCCCGCGTGCCGGAATGGTGAGCATTTCTTGAAAATATCCGCCGTAAACA
CACTTCACATTACGCAACCATATATTCTGGATTTCTAGCCCTGCACCAACAGCTGCGCAGAATGGGAGGC
GATCATGGACACCACCAAATGATCATCAAGCCATCCCGCTTCCAGTGGGACAAGTTCAAGGATCTGTTGC
ACTTTTACGTAATGCTCGGCGTCATCCCAGTCACCGCATTGGTTCTCTACGCGAACATCTTTGTGGGACC
CGCGCAGCTCGCCGAGATTCCCGAGGGCTATGAGCCCAAGCACTGGGAGTACGAGAAGGACTCCTTGAGG
ACGAAGTACGTCGCAAGATGTCGGAGCGCAATGATTACCAGGCCTACTACTACCGACCATCGGTGGCCAA
GTACCACAGGATTTCGAAAGAGGCTGCTGACGAGCTGGAGGCTCTGCGCGGAGACTAGAACCCTTG
>dme_circ_0004552_junction_seq
AGGCTCTGCGCGGAGACTAGAACCCTTGATTTACGCAAAACTACTAGAAAATGGTC
>dme_circ_0004553
GAACTACTACGCCGCCCGGATGCCGGACAACGTCAAGTACTTCCTCATAAACTTCTGCCAGGCGATCAAA
GAGGGCAACTTGTACGACATCCAAAACATGTACGAGAACACGTTCCCGCAGATCAGCGACCACCACTTCG
ACAAGACTGCTTGGCCCGAGGAGCAGGAGGTGGCTGCTATTGTAGACAACGACAAGGTCTTCCTGATCTT
ATACAAGGAGTTG
>dme_circ_0004553_junction_seq
GGTCTTCCTGATCTTATACAAGGAGTTGGAACTACTACGCCGCCCGGATGCCGGAC
>dme_circ_0004554
TACTTCCTCATAAACTTCTGCCAGGCGATCAAAGAGGGCAACTTGTACGACATCCAAAACATGTACGAGA
ACACGTTCCCGCAGATCAGCGACCACCACTTCGACAAGACTGCTTGGCCCGAGGAGCAGGAGGTGGCTGC
TATTGTAGACAACGACAAGG
>dme_circ_0004554_junction_seq
GTGGCTGCTATTGTAGACAACGACAAGGTACTTCCTCATAAACTTCTGCCAGGCGA
>dme_circ_0004555
GTTATATTGGAAGACCGCATCATGATGAACGGGAGCAGGTGAAGGCCTTTATGCGTCACTGGTGTCTCAA
GGAGGCGTACGTCAAGGAGCTGGGTGTTGGCATCACTGTAGATCTGCAAAAGATCAGCTTCTCAGTGGAC
ACTACTCGCAGTTTGGAGACAGATGTTTCCCCTCTGATCGGTACTAGTCTGCGTTGCCACGACCAACCCA
TGGACAATTGGCATTTCGAGGAGCATTTACTGCAGGAGGACTACTGTGCGGCCATTGCATTCCGGAATTG
CTTGCCACAGGAGCGTGGAAAGTTCAAGTTTCTGCAAGTTGAGGAACTTCTCGTGAAGAGTGAAGATTCG
GAACTTGAGGAAGTGATCAGCTACTGCCAAAAGGCTCTTCTTAAGCCCTACAAGCGATCATGAT
>dme_circ_0004555_junction_seq
CTTCTTAAGCCCTACAAGCGATCATGATGTTATATTGGAAGACCGCATCATGATGA
>dme_circ_0004556
GGTGGCACGATCGTATTTCCATGCGTGTGGAGCTCTACGGCTGCGATTACAGTAAATTTTGAATGGATTC
ATTGATATTGTGTTGTTTCTAATCCCAAACCTTTTCTTTCAGTCTCTGAAAACCTATACTTTAATGGCAC
TGGCTTGGTGCGCTATGATCTGAGGCGGGAACCCATTACCTCCACCAAAGAGTCCATCCGCTTCCGCTTC
AAGACGGCCTTTGCCAACGGAGTGATGATGTATTCGCGTGGCACACAAGGGGATTACTATGCCCTGCAGC
TTAAGGACAACAAAATGGTGCTGAATTTGGATTTGGGATCGCGGGTGATGACCTCCTTATCGGTGGGCAG
CCTGCTGGATGATAATGT
>dme_circ_0004556_junction_seq
CGGTGGGCAGCCTGCTGGATGATAATGTGGTGGCACGATCGTATTTCCATGCGTGT
>dme_circ_0004557
CACCATTTTGTTGTCCTTAAGCTGCAGGGCATAGTAATCCCCTTGTGTGCCACGCGAATACATCATCACT
CCGTTGGCAAAGGCCGTCTTGAAGCGGAAGCGGATGGACTCTTTGGTGGAGGTAATGGGTTCCCGCCTCA
GATCATAGCGCACCAAGCCAG

>dme_circ_0004557_junction_seq
CGCCTCAGATCATAGCGCACCAAGCCAGCACCATTTTGTTGTCCTTAAGCTGCAGG
>dme_circ_0004558
GGTGGTGTGCCCAACGTACAAGAGGGCTTGATTGTCCAACAAAATTTCTCCGGCTGCTTAGAAAACATCT
ACTTTAATTCGACCAACTTTATTCGAGTGATGAAAGACAGCACTGAACTGGGAGAGGGATATCTCTTCAC
TAGGGTAAACACGATCTACGCGTGTCCTTCGCCTCCGATTTATCCGGTGACCTTCACTACCCGTTCGTCG
TTCGTCCGTTTGAAGGGTTATGAAAATTCGCAGCGTCTTAACGTCTCCTTCTATTTCCGTACTTACGAGG
AAACTGGAGTAATGCTGCATCACGACTTCTATTCGGGTGGTTATCTAAAGGTCTTTTTGGAGTTTGGCAA
A
>dme_circ_0004558_junction_seq
TCTAAAGGTCTTTTTGGAGTTTGGCAAAGGTGGTGTGCCCAACGTACAAGAGGGCT
>dme_circ_0004559
GTGATGAAAGACAGCACTGAACTGGGAGAGGGATATCTCTTCACTAGGGTAAACACGATCTACGCGTGTC
CTTCGCCTCCGATTTATCCGGTGACCTTCACTACCCGTTCGTCGTTCGTCCGTTTGAAGGGTTATGAAAA
TTCGCAGCGTCTTAACGTCTCCTTCTATTTCCGTACTTACGAGGAAACTGGAGTAATGCTGCATCACGAC
TTCTATTCGGGTGGTTATCTAAAG
>dme_circ_0004559_junction_seq
CGACTTCTATTCGGGTGGTTATCTAAAGGTGATGAAAGACAGCACTGAACTGGGAG
>dme_circ_0004560
GCTACAACGAGGACGAGATCCCAAGTGTGCGTGGTGGTCCTTTGGCGGAGAAAACTCCTCTGGGCTATCA
GTTCGAACAGTTCCACTTTCACTGGGGCGAGAACGATACGATTGGCAGTGAGGATTTGATCAACAATCGT
GCCTATCCCGCCGAGTTGCACGTGGTATTGCGGAATCTAGAGTATCCGGACTTTGCCAGCGCCCTGGACA
AGGATCACGGCATCGCCGTGATGGCCTTCTTCTTTCAGGGTGGGCGACAAATCAACTGGTGGCTATGAGG
GCTTCACCAACTTGCTCTCCCAGATTGACCGCAAAGGCAAGAGCGTTAATATGACCAATCCACTTCCACT
GGGCGAGTACATCTCGAAGTCCGTGGAGAGTTACTTTAGCTACACTGGTTCTCTGACCACACCGCCTTGC
AGTGAGGAGGTCACCTGGATTGATTTCACAACGCCCATTGACATCACGGAGAAGCAGGTAAATAAATATT
TAAGTGAACCTTGTTTTTATGTTTTACACTATACTCTTCCCTTGCAGTTGAATGCCTTCCGTCTGCTGA
CAGCCAACGATGATCATCTGAAGAACAATTTCCGACCCATCCAGCCGCTGAACGATCGCACCTT
>dme_circ_0004560_junction_seq
CCATCCAGCCGCTGAACGATCGCACCTTGCTACAACGAGGACGAGATCCCAAGTGT
>dme_circ_0004561
GCAATCCTCCTCTCCTACCAATTTGTTGGCCCCAGGCGTGAACTGATCCCACTGCAGGCACGCCACCGGG
GTTTTGGTGGCATCGAATATATCCGACCACTTTTGTTTGTACGGCTCTGGAGCCTCGAATCGTAGATCGC
CAGTGGGCGGTTCGGCGTAGGGAATCGATTCGTAGCTGTAGTAGCTTCCATTATCGCGACCACGTAGCTT
GCCCTGGGGCAGCTGCACCAACAGAGGGTCATCTG
>dme_circ_0004561_junction_seq
GGCAGCTGCACCAACAGAGGGTCATCTGGCAATCCTCCTCTCCTACCAATTTGTTG
>dme_circ_0004562
GCGTGAACTGATCCCACTGCAGGCACGCCACCGGGGTTTTGGTGGCATCGAATATATCCGACCACTTTTG
TTTGTACGGCTCTGGAGCCTCGAATCGTAGATCGCCAGTGGGCGGTTCGGCGTAGGGAATCGATTCGTAG
CTGTAG
>dme_circ_0004562_junction_seq
GGCGTAGGGAATCGATTCGTAGCTGTAGGCGTGAACTGATCCCACTGCAGGCACGC
>dme_circ_0004563

GATAAGCTATCGCCTGGGGCCATTGGGTTTCGTGAGCACCGGCGATAGGGATCTTCCCGGAAACTATGGA
CTGAAAGATCAACGGCTGGCTCTCAAATGGATTAAGCAGAATATAGCCAGTTTTGGTGGAGAACCGCAGA
ACGTACTGTTGGTTGGTCACTCCGCTGGAGGAGCTTCGGTCCATCTGCAGATGCTTC
>dme_circ_0004563_junction_seq
GGAGCTTCGGTCCATCTGCAGATGCTTCGATAAGCTATCGCCTGGGGCCATTGGGT
>dme_circ_0004564
GAATATAGCCAGTTTTGGTGGAGAACCGCAGAACGTACTGTTGGTTGGTCACTCCGCTGGAGGAGCTTCG
GTCCATCTGCAGATGCTTCGTGAAGATTTCGGCCAGCTGGCCAGGGCGGCATTCTC
>dme_circ_0004564_junction_seq
TCGGCCAGCTGGCCAGGGCGGCATTCTCGAATATAGCCAGTTTTGGTGGAGAACCG
>dme_circ_0004565
GATCCATGGGTTATACAGAAGGGAGCAAGAGGACGAGCCTTTGAACTGGGACGCAACGTGGGATGTGAAT
CGGCTGAAGACTCGACCAGCCTGAAGAAATGCCTAAAGTCAAAGCCAGCCAGTGAATTAGTCACCGCCGT
CCGTAAATTCCTTATATTTTCCTATGTGCCCTTTGCTCCATTTAGTCCTGTATTGGAGCCATCGGATGCT
CCAGACGCCATTATCACCCAGGATCCCAGGGATGTCATTAAGAGCGGAAAGTTCGGACAGGTTCCGTGGG
CT
>dme_circ_0004565_junction_seq
CGGAAAGTTCGGACAGGTTCCGTGGGCTGATCCATGGGTTATACAGAAGGGAGCAA
>dme_circ_0004566
GAATTTACGGACGGCGGTGACTAATTCACTGGCTGGCTTTGACTTTAGGCATTTCTTCAGGCTGGTCGAG
TCTTCAGCCGATTCACATCCCACGTTGCGTCCCAGTTCAAAGGCTCGTCCTCTTGCTCCCTTCTG
>dme_circ_0004566_junction_seq
CAAAGGCTCGTCCTCTTGCTCCCTTCTGGAATTTACGGACGGCGGTGACTAATTCA
>dme_circ_0004567
GAAAATATAAGGAATTTACGGACGGCGGTGACTAATTCACTGGCTGGCTTTGACTTTAGGCATTTCTTCA
GGCTGGTCGAGTCTTCAGCCGATTCACATCCCACGTTGCGTCCCAGTTCAAAGGCTCGTCCTCTTGCTCC
CTTCTG
>dme_circ_0004567_junction_seq
CAAAGGCTCGTCCTCTTGCTCCCTTCTGGAAAATATAAGGAATTTACGGACGGCGG
>dme_circ_0004568
AGAGCCATTATCACTCGAAGCAAAATCTGCCAGCATATTGATAAAATTTCTCGAAATTATCTGCTCATCC
GGACGCATTTCCACATCTCGTACGAAATTTTCGAATATCAAAAAGTAGTCGTCACCGTGTACAGTTCCTA
TAAAAGTAATGATTTAACTAAGTCCATTTAAAAGTACGATTTCCTTACCAAAATCATAATCGGTTCGATT
GGCCAGGACCTGTGCGATTCCTTTTTCGGCTGGATTGTCATAGACATAAGCGTAGGCAGGACTCTTTCCA
TATTTGCGATGAAGATCCAATGACTCCTGCGTGCTATTCTTGAAGAGAATATCCGTGAATAGCCGCTGCA
ATTCTGAATAGCTTTCGATGTCAAATCTCTGATTGCCTATATACTCCTGCTTAATTTTCCGCGAGTAG
>dme_circ_0004568_junction_seq
ATACTCCTGCTTAATTTTCCGCGAGTAGAGAGCCATTATCACTCGAAGCAAAATCT
>dme_circ_0004569
GATTTTGCTTCGAGTGATAATGGCTCTCTAAAATATGGTGAATGCGATTTCAAAGATAATGTAGGTAGTG
AGAAATTCCAATTATTAGCTATTTATATTGATGGCTGCCAGAATAGGCAGCATGTGGAATTTCCGTAAGT
TACATGAATAAAATCAAAAATTTTTCGTTCTGTGTAATTTTTAATTATTTAATTATTCTCAACTGGCTTT
AAATATCATTTGTACAAAACGTGTTTGTCGCTTTATATTTTGGTTTTTGTT
>dme_circ_0004569_junction_seq

TTTGTCGCTTTATATTTTGGTTTTTGTTGATTTTGCTTCGAGTGATAATGGCTCTC
>dme_circ_0004570
GGTGGAAAATTGAAGCTTTGGGTCCACCACCGCAGAGCAGCCAATCCTCGTTGACAGCCACTGCTCCTAT
CCATTTGCCCCAATCCGGACGTAACAGATTTGGGTTCTTGTAAGGCTCCAGCATTGAAGTGTGCTGCTGC
TGCTTGGTGCTCCACACTCGCACAGTGCCATCCTCGGCGCCAGAGAAGATCTGTCCGTTGGCATTGCCCA
CCACACTGTGCACGTAGTCTGTGTGTCCGCGGTACTCGCGCTGAATTCGTCCGTCCTCCAAACTCACTTG
GTAGATCACTCCGTCGCCACAGCCAGCAAACAGGATGCTATTTTCCGAGTCCAGCCACATGGAGTTCACA
TCCGGCACTTCGACGGCATCCACTTGCATGGGTATCTTTACCTCCCAGGACCGTTTGGTGGCCAGAGATT
CCTCCTCCTCGTTCCATTCCAATCCGTAAATAAGACCCACTGCACCAACTATCAAGAAATCGCGATGGAA
GGCCAGGTAATTGATGTCCACATCGCTGCCCTGCGGGAAGATCTTCAGTTTGCCAG
>dme_circ_0004570_junction_seq
CCCTGCGGGAAGATCTTCAGTTTGCCAGGGTGGAAAATTGAAGCTTTGGGTCCACC
>dme_circ_0004571
TGTATAATTTTTGTATTTAAGTTTTTACAAACAGCAGAAACAGAAAGGGGTTCGTTTCGCAGTTCAGAGT
TTAAAAAACATTTGCGGTGTAGCCAGAAACCGAAGAAACAAAGAAAAGAAAGCCAACTAGAAATATCCTG
CAATATACGTGAAGTTGAAATTGTCGAGTCGATGAGAAAATGTTAACAGCATAAGAGTTAAAAGCAACAA
TGTGGTGTATTAAATG
>dme_circ_0004571_junction_seq
TAAAAGCAACAATGTGGTGTATTAAATGTGTATAATTTTTGTATTTAAGTTTTTAC
>dme_circ_0004572
GTGGGAAACTGCGTGGGCAAGCGGAATTATCGGTTCTTTTATTTGTTTCTAGTCTCATTGGCATTTTTAG
CTGTATTTATATTCTCGTGCTCTGTGACGCATTTAGTTTTATGTGAGTACAACGGAATAGCAATCCATAT
GCGATATGAATGATATGATTTGTTTATACCACCGGCAGTGATGAAGAAGGAGCACGAGGTCTTTAATGTA
ATCAAGGCGGCGCCCTTCACTGTGATTGTTGTGTTCATTTGCTTCTTCTCGATATGGTCGGTGATCGGCC
TGGCCGGCTTCCATACCTATCTGACGACTAGCGATCAGACAACCAACGAGGATGGTAAGTATACAGAAAC
AAATATATTATATATTGGTAATTAAATCAGCCACAAGCTTCCTCTTTGAAAAGAAATGATTATGAATTAT
GAAAGTATACACATTCTAAAGCATTATTATTCGAATTCTTTCCTAGGAAATTTTTAAAATTATCCAGTGT
TTACACACATGAATTGTGTATTACTTTCGTACTTTCGGTATACTTGAAAATGACCTTTACACAATATAAA
ACTGCTCTTTTCAACAGCACGCGGCATCGCCACCGACGAGTTCATCCAGCAGATGCAGCACCAGTCGAGT
CCGCGGCACGCACTGAGCGATGTGCTGAGTGCCTCGCACATGGTCACCACCTCGCAGCCCATGATGGGTG
GTTTAGGTGGTGGTGGCATCGGTGGTGCCGGCGGCGGGATCAGCATTGGTGGAGCGGAGCTAAAGCCCCG
ATTCTACGACGAGGTAAGCGGCCAATCCAGCGGCCACATTGAGTCAATCCAAGAGCACCCTTTCACCACC
GAAATCTAAAAATATCTAAATGTCTGAGCACACTGCTTGCTTGATATCCTTtatatatttatatatatat
atCAAATCCAATCAAATCAGTAAGCGGCCAATCCAGCGGCCACATTGAGTCAATCCAAGAGCACCCTTTC
ACCACCGAAATCTAAAAATATCTAAATGTCTGAGCACACTGCTTGCTTGATATCCTTtatatatttatat
atatatatCAAATCCAATCAAATCAAAACTCTCGTACGATGAACTCTTTTGTGCACATCATTTTGACGAT
CTGTAATTCAAAtttttttctttttGTGTTCTATGCAATGTTCTTTTTGGTAATctactactacctacta
ctactactCGTATCATACACCACAATCGACACAATCACACCACGAATACGACCACGATAACCAGTCCAAT
CCCTCCTCCTCGACACTGGAGGGCAACGGAGGTGCCATCAATGGCCATGGGAATGGCCATGGCAACGGCT
TCGATCATCCGCCGCCCAGTTACGATCTTGTCCAAAACGG
>dme_circ_0004572_junction_seq
CGCCCAGTTACGATCTTGTCCAAAACGGGTGGGAAACTGCGTGGGCAAGCGGAATT
>dme_circ_0004573
CTGTCCCTTTCTGGCGGACAGCATAAATCCGGCCATTCCGATTGTGGGAGCGGTACTATACTTCTTCACG

ATGAGCTCCTTGCTGCGCACAACCTTCACGGATCCGGGCGTCATACCGCGAGCCTCCAACGATGAGGCCG
CCTATATTGAAAAGCAAATAGGGTACGTTACTCATCCGCCCCGTAGTCTCGCGATAGCAATAGCAATAGC
AGTGGCAATCGCAGTCGAAGTCCTCGCGTCCATCCAAAAGGAAATGTGGCCAAGGTCGTTGCCTGCTGCT
GCCTGTTGCCTGAATATCCTTCTTCTCCATCTGCCACGCCCCTACTCACCCGTACTTGCCAAACACTCGC
CCACTCAAACAAATacacacacacacacacaGACAGCACTTGCGTATATATTTGTTTTCACTTCATTAAG
TTtgtgtgtgtgtgtgAGAGAGCAAATGGGAGGAACGTAAAAAGTAATGAGCGTGGCATGACATAGAATG
aaaaaagcgctaaaaaaatgggaaaatatatatgttccgtataaaaaTCATCAAAGGCGAACGCTGCGTT
GCCTTTGCGCTAATGAGTTGCTGTTGTTGCCTTTGTATAAAATATTTAAAATGAGAAAACTTTGGCCCTT
TCCTCCAAtttttttttttcatttttCTTCCACTTGCATGTCAACATCGTCGATGTTGCTGATGCTGCAT
TTCCTCTGCGTCGCTCTGGATGAATACGGGAATATGTGCGTATGTACGTGTGTGGATAACCCAAATCGAC
GTGAGTAGAAGGTGCCAAACTCGCTGAACAGTCCCACATATCGACCGCCGCCGAGGACCAAGGAGGTCCT
GGTCAAAGGACAAACGGTCAAGCTGAAATACTGTTTCACCTGCAAGATCTTCCGGCCGCCGAGGGCCTCG
CACTGCAGCCTGTGCGACAACTGCGTGGACCGCTTCGATCACCACTGTCCCTGGG
>dme_circ_0004573_junction_seq
GACCGCTTCGATCACCACTGTCCCTGGGCTGTCCCTTTCTGGCGGACAGCATAAAT
>dme_circ_0004574
GACTGGACTGACCCGCTCAAACAGGACTAAAACCAGCCAAACCAATTCGACGCCAATTTTAACTACTGTT
GGTGGCGCCAGCAGCGAATAGCGAATAATTGAATCATAAAGGACACGGACGGGCCACCCAAAAGGATCCA
GAATTCAGGATCAACAAAGCCGAGCAAATAATTGCTATATAGTCtgtgtgtgtgtgtgCGCGCACTGAAC
GTGGTCTCCCCACCCTCGATTCAGTACTCACCCCCATTTCCGGTGGGTGGAAATAGGGGAGTGGCAGCAG
CACTGGCAAACGAAGACGGCTGCAGGGAAATGACAGTCGTACTGCTTTAATTAAAGCCGGGACGTGGGCG
TGTGGGCGTGAGGCCCGAAAGTGTGCGCTGCAATAAAAGGAGGAAACTGAAACGCAATGTGGCTGATGCC
GCGGACCGTGGACAACGCAACAAAGTGGACGAAATTGCTTTTGGAACATTACAGGATACCACTCGAGTTG
GACAGCAGTAGCGGCAGCAGCGAGCCAGGCATTAATTTTGATTAAATTCACCTGAGAAAATTCGCCTTCA
AATAGCAACTAACAAACGAACGACAAAAACTACAAACTACAAAATAAAGCTAAATCAAACTTTAAGTGGT
GAATATTTCAAGCAGCGGCGCGGTGTGCATGAAATTCACAGGAGATAGCAGCAAACAGGTTCGGGAGCAG
CAGGAGACATCTTGTGGAAGTCGGTTTTGCGTGCCGCTGGCATTGCAACAGCTACACATGAACACTTCCC
TGTGACACGAGGTGGTGCGGAGCGAAGGGAGGCGTCGTCGGTCGCCAGGACGCCAGGAAAACAGCATAAT
TTCGTCAACGGCAGCCATTTGAACTGTCGTCGCCAGAGCATAACCCACCATTCCCCAGGATCCCACAACC
CCAAAACCCGAATCCCCATACCGGCCCACATCATCATCAGCAGCAGGAGCAGAAGATTCAGGAAAGGCGT
CACTGTCACAGTCAGCAATTGTTGTCAGCAAAGGAAAAGTAACAGAATCAGTGGAACCACCAGCAGGACC
AAGACCTGGCATAACAACACCATCCTATCCAAAACTTAATACCCCATTCGCCACGACCGCCCCGATGAAT
CTGCTGTGCTGCTGTTGTTGCAGTAACATGGCACCAAACCAACGCGTCACACGCAAATGGGAACTGTTTG
CCGGACGCAATAAATTCTACTGCGATGGATTGCTAATGTCCGCACCGCACACAGGCGTCTTCTACTTGAC
GTGCATCCTGATTACCGGCACCAGTGCGCTCTTCTTTGCCTTCGAG
>dme_circ_0004574_junction_seq
CACCAGTGCGCTCTTCTTTGCCTTCGAGGACTGGACTGACCCGCTCAAACAGGACT
>dme_circ_0004575
GACAGGCTAAAATAATAACTTATGTATTCGTGTTTATTTGAATTACGCCTAAGCATAGCATGTTGGCCAA
CTATTTGTAATATTTCGTATAACGTGTACATCTATATAATGTAGACCATATGTTTAGTTATCCATTAACA
AGCAAAGTGGAGCGGGGAAGGCCCAAGGATCGAGGATGTGTGTATGTGGGTG
>dme_circ_0004575_junction_seq
AAGGATCGAGGATGTGTGTATGTGGGTGGACAGGCTAAAATAATAACTTATGTATT
>dme_circ_0004576

TATATCTGCTGATTGCTGCGTGACTTCTCCCTTCCGATGGCTGCTTCGTTGAGGGCTTCCGGATGCGCTG
AGGCGAAGGCCGCCTCCAGGCGCTCCACGTTCTTGGTGACTCTCCTGGCCTGCAGGGCAGCCGAGATGTC
TGGCTTGGGATACTTCTGAGGTGTGTTCATGCCGGTCATGAAGAAGACCCCGTTAAACTGCTGGGCTGGC
GGAGTCGGAGGCGGTGGTGCCGGTGCTTTGACCTTCACCGGGTGATGGGGAATTGTCACGGGCGCCGCAT
CCTGGACACTTTTCCGCAGGGCCTGCATTGTGGGATCGTGATCGTCATGCCGCGCATTCATAATCACGTC
CAGCAACTCTCGATTTGTGCTCGCATTGTACGCCGTGGCCGTGGCATTCGCCTCTGGCATGTCCGGCGTT
GGTGATTCCATTTGGTAGGCAGCAGGCTGATCTGTCTCTGGAGCATCGTAGTCCACTTCGTCGAAGAAGC
TTTGCATGGCCGCTAGTCGCTCAGCGGCGTCGGGATCTGCTGGTTGGTGTAGAACCATTCCATAGTCCGA
TCCCTCCTGATCGGAGCAATCGGTGCGGGGCACCAGATGTGCCAAGGACCGAGATTTTCTGGGAGGCTGT
GGTACGATG
>dme_circ_0004576_junction_seq
AGATTTTCTGGGAGGCTGTGGTACGATGTATATCTGCTGATTGCTGCGTGACTTCT
>dme_circ_0004577
TTCTTCATCTTCCATGTAGCGCGTGGCCCATTCGTGGAGGAGTTTTACCAGTGCGTCACCCACGGATTCT
ACACGGCGGATTGGCAGGAGCAGATGTACGCCACCTTCACGCTGGTCTTCACCTTCCTGCTGCCGCTGTG
CATCCTGTTTGGCACCTACATGTCCACCTTCCGCACCATTTCCAGGCAGCGAAAAGATGTTCCAGGGATC
AAAGTTGGCCAACTACTCAACGGCCAAATTGCCCACACAGACGAATCGCCAGAGGCTGATACACAAGGCC
AAGATGAAGTCGCTTCGCATATCCGTGGTGATCATCATAGCGTTTCTCATCTGCTGGACGCCCTACTACG
TCATGATGATTATGTTCATGTTCCTCAATCCGGACAAAAGGG
>dme_circ_0004577_junction_seq
TTCATGTTCCTCAATCCGGACAAAAGGGTTCTTCATCTTCCATGTAGCGCGTGGCC
>dme_circ_0004578
CTTATTTATCCAACCTAAGCCAACTAAATGATGCAATGCTGGTGACATATAAAACGAAAGTGGAAACGGA
GTTCATTCCTTAAGTAACCAATTTAAGATGTTGTGCTCCCAAGATCCCTAAACCACCGTCCAAACCCTTC
GAATAACTTATTTAAGCCAAGATATAGACAAAATGGAGGACGAGTGGGGCTCCTTTGATCGTCTGCCCAG
TGTTCCGAGTGCCTCGATGGATTTGGAGACGGAAAACGAGGTGGTCAGCAATTGGTCCACACTGGCCAAC
TTCACGCGACTTGTGGCTGGTGCCGCTCCTGAAATCGTCAACTATACGCTCAACATGATCGACGTGGGTG
TGGGCATGGCCACGGATATATCCAATTTGAGCGTGAGCACCACGCCCCTGCCCGCCTACGCAATCTCCAA
TAGCTCCTCACTGGCGCACACCAATAGTCGCCATGAAGCACCACCGATGGCGGAACAGGTTCCGGAGCAC
GTGATGGATCACGCACCTCAACTATCCCGATCCGGGTTGCTGAAAGTGTATGTCCTGGCGGTAATGGCAC
TGTTCTCTCTGCTGGGCAACCTGCTGACCATCTGGAATATCTACAAAACCCGCATCTCAAGAAGAAACTC
ACGGCACACGTGGAGTGCTATCTACTCACTGATGTTCCATCTGTCCATCGCCGATGTCCTGGTCACCTGG
TTCTGCATTATCGGGGAGGCCGCGTGGTGCTACACCGTCCAGTGGCTGGCCAATGAGCTCACCTGCAAGC
TGGTGAAGCTCTTCCAGATGTTTAGCCTCTACCTGAGCACCTATGTCCTGGTCCTCATCGGAGTGGACCG
CTGGATAGCGGTCAAGTATCCGATGAAGTCGCTCAACATGGCCAAGAGGTGTCATAGGCTACTTGGCGGT
ACTTACATCCTGTCGCTGGTGCTCAGCTTGCCACAGG
>dme_circ_0004578_junction_seq
CTGTCGCTGGTGCTCAGCTTGCCACAGGCTTATTTATCCAACCTAAGCCAACTAAA
>dme_circ_0004579
GGCTTGACACTTCAGTCCACATTCAATTGCCGGAAGGAGCCCTGCAGACCAAAAAGACTCTCGAAGTCAA
CAGGCTGACCCACTCGGAGGCTTTCATCGGTTCCGTAGTTCTTCTCCTCCTCCCAG
>dme_circ_0004579_junction_seq
GGTTCCGTAGTTCTTCTCCTCCTCCCAGGGCTTGACACTTCAGTCCACATTCAATT
>dme_circ_0004580

GTTAACACATAGCCACGAACTGGCTCATTATTCTTGCCATAACCTTTGGCAAACCACACAATCTTCGGAT
ACAGCTCATCCTTGCACAGAGCCTGTGGATATGGATATACTTATAGACATTTGGCCTCGATACGGCAACT
GGATTATCCCACCTGGAAGACCTTCGGGGCAGACACCAAACTGGCCAATGCCGAGGATAACGTGGCAGCA
TAGCAACCGGCGTAAATCAGAGGGCCAAACGCGGATACCAACTCAATTACTTGAAATGAGTTTTGCAAGC
CGTAATTGCATTCACCTGGTTGGTTACGAAAGTGTTGCAGTTTAAATTATGTTTCATAAAACAATCTCT
GTAAATCGATTCGTTACCAGGCTGACAGTCGAGGAATGCAAAGGTGCCGTTTACGACATCTGAAAGATTT
CCCGTGGCATCACGAGCCACTGTGGCACCACACTGGAGTACCATAATCAAATAGGTTCCGGTGGTGATAA
CGATGGCCAGGATCGTGCCTTTTGGAATGGATTTTTGTGGGTCCTAGTATTTAATATTTTCAATTATTTT
TCAAACGTCTTATTTAGGTTCAAAAATTACCTTCAGATCGCCCGAAATATTGGCTCCAGCTAAAATGCCC
GTTGCGGCAGGGAAGAATATAGCAAACACTGAGAAAAAATCGTGTTGAATGCCTCCCTTTTCCGGCCGAT
AGTCCGCGAAAAGGTTGTTCTTAAACAAAGTGGCTATGGAAAATTATAGTATATTATTAGTAAATACAAT
TGTATACATCTATCTCTACCGACTCACCATTATATCCCAAAAATCCCTTGGCCATTTCCGCATCGCTCTT
CGGTCCGATAAAGCTGCCAATAACAAAGTCTCCAATGGCCACCAACAGGATGATGAGCAGTCCGATTTGC
GCCTGGTTAATCGGAATAGAATTAGGTCCATAACTAGATTGCATATTTCGCATACGCACCTTGGCCTCCC
ACTCCATGCCGACGACCACAATGATGAGGAGCAGCAGTATGGTAATGCAACCAATGATGCGCACATCCTG
GACGCCACCATCTATGATCTCCCAGCCAAAGGTCGTCATCATGGCCAACATAGACTCGCAGAAGCCGACC
ACATACATGGCACACGCCACCGCATTTGCCAGGGAGAATATCAAACCGATGGATCCACCAAACTCCGGCC
CCAGAGACCGGGATATCATATA
>dme_circ_0004580_junction_seq
CCGGCCCCAGAGACCGGGATATCATATAGTTAACACATAGCCACGAACTGGCTCAT
>dme_circ_0004581
GAATTAATACGAATCCCTCGATGACGCCCGCCTGACCCACCACCCAGCTGAGCCGGAGGAACAGCATCAC
GCCCCAGATGTTCAGCAGGCAGCGGACGAGCACACCTTTGATCCAACCGAATTTCAGGACCCCATTCAGG
ATGCCCGACTCCGGGTCCTGATTACGTGTCAAGCTGTGCGTGTTCTATGTTTGAATACAGAGAGCGAAAA
ATGTGTTGATATCTGATTTGATATATTTTGCATGCTAAAATCATGAACCTAAGCTGCCACCTCTCCATGG
CTTGTACATATTTTATTTCAACATAATATATTGTTGTACAGATTACTGAAAATAAAATTGTTTTCATAAG
TTTTATTCTCTCTGTACGTGGGTGTAAAGTGCAAAGGGTAGAGTGTTCTTGAGAAAATTTCAATAAAGCT
GTCTAGTGTTGATAAATGATTCGGCAAACAGCGTGAAGTTGGAGGTTGGGGTAAATATCTTTCAATTCTT
TGATAATTTGCAGGCCATGGAGGCACATGGAAATTTTTCGGGAACTTAAAAGAAATCCCGCTGAAAAAAG
ATGACTTCAGCCTTCGGGAATTCTGGGCAGCCGTCATTGCCGCATTCAACCATTATTAATAGATTAATCA
TACGCCCCCGAAACCGCAGCACCCGTTGTGCCAATTGATCTTGTCTTGAACCTTTGCACTTTTTAACGAG
ACCCCATCTTAATTGCTTATTATTACAAGATGCCCCAGTGACATTGAGAAAGCTCTTTTTTATAGTTATA
CTTAATTACTACGACTTGCTCGTCAACCCATTTATGTGGACTCCAGCTAATAAGAAAAGAGCTTGCCACA
TGGCATGATATTGTGCAACGCATCGCATCAATTTCTCAATGGACCTTCGGATGCAGCTCGTAGAAATTTA
ACTTTTCATTTCTTATTGACAATCATTTGCCCAGTCAATTGTGAGGCAAAAACAAAAGCAATTACTCTAA
AATGTGTGGAAATCAATTAGTGCCAATTGATGGCTTTGGATCTTTGGGATCTATTGAAAACTTTTTAAAA
TGATTCACAGCAGAGCAAGGGCTTAGAAGTCAAATGAAAAGTCAATGGACTTGTCAGATTTAATAGCCGC
ACAGTCAAATTTAATCGGTTAAGTCTGCCAAAGTTATGCTATTTTTGGAATAAATTTCGGGACCGCACGA
ATTGCATTCGACAAGCAATTTCCCAGGGATTTCCCAGCCTGCAGTGCCAACGACCGAGCTATTTGACTCC
TGGCGGCACTCGCAGTCACGCTTATGTGCCTGCGCAAAAATGAaaatgggaaatgggaaatgccaagtgc
caaatggcaaatggcTTTCTGGCTTTTTGGATCAGACGGGCTGACACTTTCCGCCGTCGATTTGACACTG
CCCCGTCCGGGTCAATTCTCCCGTAAAGGGTATTGAATTTGCAtttttttccgaatttttttCGCGGGTGC
GTATGAGCGTAAATTTCAATTTGTCTTTCCCTGCGACTCGTAAAGCATTTATGTGTCCTGTGAATTTGTA
ATTTGTGGTTTTCGATTATCCTGCGGTATCTTTACGACTTGCGCGTTATTTCTCATTAGTTAACCCCAT

CCGAAAGGCAAGTATGTGTGGAACTCACCTTGCCCACCAGCGTGGCGTTATGCAGCTCGTCGAGCGTTGG
ACGGTAGGCCGCCTGGATGGACATCATGTTGCGGTAGTTGTCCAGGCGGGGTAACGCCTCTCTGGTGAAA
TGCCT
>dme_circ_0004581_junction_seq
GGGGTAACGCCTCTCTGGTGAAATGCCTGAATTAATACGAATCCCTCGATGACGCC
>dme_circ_0004582
GCAGCTTTAAGCGACCGACTGGTCGACTGCCACTTTAATCGTCAGCGACGGATGTCCTGATTTTACGACG
AAGGGATACCCATACCCATACATATACATACACAATTAGCCACAGTGGATGGGTCTGAAAGTTGCGACCC
GCACACGGAAACCGCATAAGCACTCGGCGAAATCTTCCCGGGGCGAGGACCCCAAAAAGCACCGGCCAAA
CAAACAAAAGCAGTCGCCCCAATGTCGGACACAATCTCTTTCGAGTTGGGCTCAGCTGCCGACCGGCCCC
CCAACAGGTTTCAAGTGAACCCGGTCAACGGCAACAGTCGTAAGTCGCAGGGAGCGGATGGCCcaggatc
cggatcaggagcgggagctggagcaggagcaggagctggGGAGGATGGGCCGCACGAGGTCTACCGCCGA
CTAACAAACGCCGAGGGCGAGCTGCTCGAGGACGACACATTCGATGCGACACAAATGCTCAACCAACGAC
AGCCCAGGCAGCAGAGG
>dme_circ_0004582_junction_seq
CAACCAACGACAGCCCAGGCAGCAGAGGGCAGCTTTAAGCGACCGACTGGTCGACT
>dme_circ_0004583
GACCCCAAAAAGCACCGGCCAAACAAACAAAAGCAGTCGCCCCAATGTCGGACACAATCTCTTTCGAGTT
GGGCTCAGCTGCCGACCGGCCCCCCAACAGGTTTCAAGTGAACCCGGTCAACGGCAACAGTCGTAAGTCG
CAGGGAGCGGATGGCCcaggatccggatcaggagcgggagctggagcaggagcaggagctggGGAGGATG
GGCCGCACGAGG
>dme_circ_0004583_junction_seq
ggagctggGGAGGATGGGCCGCACGAGGGACCCCAAAAAGCACCGGCCAAACAAAC
>dme_circ_0004584
ACGACTGTTTTTCCATTTGACTTGCCAAGTAGTGCAAAATGAGCCGGCCATAAGAGCAGAGCAGCTGATA
GCCCAACACGTAATCCCCCAGAGAATCGGAAACCGAGCAAACCCAGAGAAATCCTTCAGCTGCCAGTTAA
AGAACAGAAAACTCAAGGTAAACTGTAAACACGAGTGTGGAGAATGGTCAGAAAGCAAGTTGGAATCGAA
AACAGTGCATCCTTAGCAGTAAGCAAGCAGCAGGATCAAAAGGAGCAGGACTATAAGCCAGAGGATCAAA
AGCAACAGCACATCGACATCGACTTGACGCCCGATCCGGCCAGCAACTTCAACTTCGCCCAGCAGGCGCA
GGCCAACTACTCCGCCCGCACGCTAGCCGCCAGCTATGAGCACGGCCAAAATCTCGGCCTGCAACATGCC
CATCTTGCCACCAGCAGCACTACCAACACGCCCTTGGCCTATGATCTGCACGAGGTCGCCAGCTCCGGCT
CCATCCTGCCCACCGAGATCCCCATCGAGGACGAGGAGCGGCTGGAGCGCATCTTCAACAAGCTGGACCG
CGATGGCGACGGCAGGATCGATATCCACGACCTGTCAGCGGCACTGCACGAGTTCGGGTTGTCGAGCGTT
TACGCGGAGGGTGAGCAAAGCCTATTTATAGGATGATATATGGAAGCCATCACATCTCATCTAGCTATTT
CAAATGTATCATGGATAGGACATATCTGAGGCTCCCATGATTTTAACAGATGTATCTTAGGGTAGAAAAC
AAGCAATCTTGATTAGCATAGTTGAACTACACGTGCCCTGGGCTCGCCATTTTCACAGGGAACTAACAAT
GCCTTTTTCTATTTGCTATTCTTCGCCAAACAGAAGGCAGACGGTTGAGATCAATAATATGCGGCCTTGA
CGGGCAAAAGTATCTGAGAATCTTAATTTATACATTTTGCATTGTTTGCTTGCTCTGATCAAGGCGAAAC
ATTTGGCACTGCTGCTGACCCTGGGCAGCCTGACCCTTTCATTGTGTCATTTTTGCAGTTGCCAAAAAAG
GGCTTATTAAATCAAAAGAAATGTGGGGTGGCAAACCAGGCGAAGATGAATATTCTATTTTTAGATGAGC
ACAATATTATTTCGCGTACAAATCTCTTTGTTTGACTAACAGATATTGTTTGTGGTATTATTAGCAGCTT
TGTTTATATTTTAGCCAAAACAAACTAGATATAAAGAAATTTAAACTATTACTTTTATCCTGTGACCTAA
ATTCTGCTCCATTATACTTCTAGTGAAATCCTATATTAGTTTATTTATGTTGCACAAATCGCCAGATTGT
GAAATTACATGCAACAAGTATTGTGACAACTTCCGAAAGCAATCAGAATTGGATTAGATGGTATATAGAG

GGTTAATGTGCAGACAACTGAGTAGATTTATATGGTTTCCGCCGGCGAAGCCCGCATGATACGAGAAATT
GATTTGCTTTCACGATTCAGGTCGATACTCTAGTCAGCGGCTCACTATGCGAAAGGCCTGCGAGTGTTCG
ACACAAATTTTCCAGTAATATTCCGGCAGTGAATCTAGTAAATTTTCCAGGAGCAATGCCAGTAATATTC
CGCACTTGCTTTCCATTACAGAAAATTCCTTCAGCAGTCGGACAAGGATCAGAGCGGCAACGTGGGCTTC
GCCGAGTTCCTGCACTACGTGCGGGAGCATGAGAAGAACCTGGTCCTGCAGTTCTCGCACCTAGATAAAA
ATCGCGATGGTGGGTAATTGATTAAACCTTAATTGGATTAGTTCTTATAAGTGAATTGTATTACAGGCAA
AGTGGACTTGGAGGAGCTGATATCGGCGTTCAAGGACCTGGGCCTGGACATTGACATGGACGAGGCCAGA
AATCTGCTCACCAGGTGGGTTAATTATCCTCAACTTTCTTTTGTGCATGTGTTAATACGCGTGACTTTTC
TTGCAGAATGGACAAGGATGGCAGCCTGAACATTAGCTTTAACGAGTGGCGGGACTTCATGCTCTTGGCG
CCCTCAACGGACATTCATGACTTGATTAAATTTTGGCGGCACTCTACTG
>dme_circ_0004584_junction_seq
TTGATTAAATTTTGGCGGCACTCTACTGACGACTGTTTTTCCATTTGACTTGCCAA
>dme_circ_0004585
GCGGGCGGCAATCGCAATGCGCGCGAGTTTCTGGAGGATCAGGAGGACTGGAACGAACGGGCGCCTATCA
CGCAGCGTTACAACTCGAAGGCGGCTGCTTTGTACCGCGATAAGATAGCCACCTTGGCCCAGGGCAAGAG
CTGGGATCTCAAGGAGGCGCAAGGCCGCG
>dme_circ_0004585_junction_seq
TGGGATCTCAAGGAGGCGCAAGGCCGCGGCGGGCGGCAATCGCAATGCGCGCGAGT
>dme_circ_0004586
GCGGGAATGCTTTGTCTGGACCAAGGCGTTGCCAACTGTTCGATGCTTCAGGTGCACATGAAGAATCTCA
ACCAGAAATACGAAAAGGAAACAAATGAGAATGTTGGTTCCGTTCGAGTGCACCACAATCAGG
>dme_circ_0004586_junction_seq
GGTTCCGTTCGAGTGCACCACAATCAGGGCGGGAATGCTTTGTCTGGACCAAGGCG
>dme_circ_0004587
CTGAAAAAGACCGAGGAGAACCTACTGAACACGAAGGGTACTTTGCTCCTGACCAAGAAAGTGCTGACCA
AGACCAAGCGACGATACAAGGAAAAAAAGGAGTTGGTGGCCTCGCATATGAAAACGGAGCAGG
>dme_circ_0004587_junction_seq
GTGGCCTCGCATATGAAAACGGAGCAGGCTGAAAAAGACCGAGGAGAACCTACTGA
>dme_circ_0004588
GGTGCAGATCAACGGGCAGACATTCTTCTGGAGCATCATCACCAGGCGTTCCGTGCAGCGGGCGGGCACA
CGGCTCTTCTGCCGAGGCAGCGACGAGCAGGGCCATGTTGCGAACTTCGTAGAGACCGAGCAAATA
>dme_circ_0004588_junction_seq
TGCGAACTTCGTAGAGACCGAGCAAATAGGTGCAGATCAACGGGCAGACATTCTTC
>dme_circ_0004589
GGCTACAATGAGCCACTTGGGCCTTGCGGATGTGCACCAGTCCGAGGATGCCACTCCTGACCTGGAATCC
TTCACTGGCTTCGGCAACTCCGCCGCCGAGGCCTTCATTCAAAGTCTCGCAGGAATGGTGAATCAGGGCG
ATGTGGAGACCATGATACGGGCTCAAAAGCAAATGGCTGCAACGCTTTGAGAAAACCAACGAGATGCTGC
TCAACTGCAATGCCTTATCGCAGAGTCGTTTGAAAAGTGCCAGCGAGGATTTCAAAAGACACGTGAAATG
CCTGAGCGAAATGAAGAAGGATCTGGATTACATATTCCGCAAGATACGGATCATCAAGCAGAAGCTACAG
TCGCAGTTTCCCGCCATTTACGCCGAAGTTCAGCCGCAGCGTAGCAGTTTGGCTGAGGAAGCCGAGGATG
ATACGGAAGCCCAGGCTAAAAAGACTGCAGAAACACCTGCTCCTGCTGCCGCAAAACCTGTCCTATCCAC
CAAGAAAAGTGCCGCCACCATAGAGTACGTGCAGATGGAGGAGGCAGTGGACAATGGCACTGTGGAGATC
GAAAACGAGCTGATCAAGCGAGTTTGTTCCGTGGAAACTGCCAATCCCAATGATTCCTCCGACTGCACAT
CCGAGGATACAGGTTAAGCCTAATTAGCTTTAGTCCCTCGATATATTTATCTTCATATAATAACTTATTG

GTTTAATATTTGTATAACCTGCTACTACATACAAGGACACAATTCGTTTAGTTAGATTTAAATGAAATAT
GCGTAATAATTTAAGAAACTAATGGCTTAAAATATAATCTGGTACTTGTCCTGTGCCCAAACACAGTTTC
TTCTCGTTCTCCTTCCATTTTGTTTTCACACAAAAGTGaaaaaaaaaaTATCTGAAAAAGCAACAGACGC
GTATTCCGACCGATCAAACTAAATTTCCACTATACAGTACATATAATCACAATTGAGAATCGAAAACCCG
ACCGCCACGAAGCGCGCTAAATTACACGCACATACTGAAAGCCAAACAGCGGATAGCACTAGCATCCTAC
ATATATAGACGTAGATATATAGTCATGGCGCAGAATATCAGCCCCGAGCAAAGTGGTGGAGCAGGCGGCG
GCGGCAGCAAGCACAGCGATGACTCCATGCCCGTGAAAGACAATCACGCCGTGAGCAAAAGGACTGCACA
AGGAACTGATGAACCTGATGATGGCCAACGAGAGGGGCATCTCAGCGTTTCCGGACGGCGAGAACATCTT
CAAGTGGGTGGGCACCATAGCGGGTCCACGGAACACGGTGTATTCGGGGCAAACGTATCGTTTGTCACTG
GATTTTCCCAATTCCTATCCGTATGCAGCACCCGTGGTGAAGTTCCTGACGTCCTGCTTCCATCCCAATG
TTGATCTGCAGGGCGCCATCTGTTTGGACATACTGAAGGACAAATGGTCGGCCCTGTACGATGTGCGCAC
CATTCTGCTGTCCATACAATCCCTGCTGGGCGAACCGAACAACGAGAGTCCACTGAATGCGCAGGCCGCG
ATGATGTGGAATGACCAAAAGGAGTACAAAAAATATCTAGACGCCTTCTACGAGAAGCACAAGGACACCT
AGAGTTTGGCAATTCGTTGTCATATATCCTATGCTCATATTGCGGCGTATTTATTTACATATAGTGCAAG
CAACTCCACTCATCCGAAGACCTACTGCCAACCAACAAACCAATCATCAATCACCACCGTAACACAGTAC
CATCAGTTAAGTGGATCACCAGATCAATTTCAACAACGTTCAATGCTGATAGTTCATTTGGCAAAAGACC
CCGTGCGTTTCTTATGTAATTGTCGATCGATTGACGGATAATCTGGTTTTTATTCTCATTTCTTTCCTCC
TCCCTACAAATTAATGATAAATGAATGTATTGGTAATGTAATTTTAAAACTGTCATCCGTTCTTTAAGCT
TAAGGATAAATTTTAGTTAATTGTTTTATATACGCATGATTACTTTTTTGAGAACTACTGCGAGTGCGCG
CCATCGCCATCGCCATTTTTTTGTACTGATGATTTTTAAGCGACTATGCTGCTGCGCCGGCCATTATGTG
AATGTTAGCTAATTTTAGGGCAGCCTCATCGATTCGTCAACCATTCGCGTCGTCTTAGTATCTCCAATTA
TGCGGCATTCTCCCACAATACTGGCATAACAAAATTCCAAGTAATTCGAATCATTGTAACAGTTGTGTTA
GCTTGATGTTATTGAAACCACAAATAAAACGAATTATTGAACTACGTTCATTTTCGACGGGTAGGTTTTC
GCAGACGGACGGAGCAAAGATTTGTGCATCGAAAATTGCAATTTTCACATTTGGAGCCGTGTGTGCAGCA
TCACAGACATTGGAAAAGCGCTTGTCGCACTTGTGCAAAAACACAGCTTGATAAAGCAAACGCGGCTAGA
AGGCGATTGGCCGCAAATTCGCCCTGGAACTGGAACGCAATTGCGTGTgaaaaaaagacggaaaaaaagt
aaaaGAGCAGAGACTGAAACCGTCGAGAAATCGTTGTCGTCGCGCTCGCTATAATTGTTTGCTTTTGACA
GAGAAATTCGTGTGCCTGTTGGCAGAAACATTTAATAATAGTTTGTATTTTCGGCGTGAGTGCGCAGTGA
AGCATCGAAGTAGTTCAAGATGAGCGGGGGATTACCGGAGTTGGGCTCCAAAATCAGCCTCATTTCCAAG
GCGGACATTCGATACGAGGGCCGCCTGTACACGGTGGATCCGCAGGAGTGCACCATTGCGCTCTCGAGTG
GGTAAGTATTTAATACGAAAATCGCCCACGTCGCCCATATATGTACATATACGGGATATACATATGTGGG
TGCCGTAATCTGCGCCaaaaaaaaagaaaaaaaaaatcgtgtaaaaaaaCGGTTCATCGCAAAAGAAAAT
GGATTCTGTCCATTTCTCTTGTGTTGGTGGCTCTACTCTTTCTTTCCTTTTGGTTCGTTTCTTTTTGTTA
GTTTTTACAGTAATTTCATACTCGTGGAGACGGTGAGTGCAATTGCCGCCACCGCTCTCTCCATTACCCA
TTTCGACAGCCGTCTCTCGCTCTCCCACTTATGCTTTTTGTTTGTCAtgttggctttggcctgtttttgt
ctttggttgttgttgtACAGTGTTGCTTTTGCAATTTACTTGGCACACATACACCCGCGCACAAAGTAAC
GAGCACCAGCAAGGCCCCAATGCAATTTGTTTATTTGCAAATACAATTTCGATTTGGCGCCAGATAACGG
GATCCAAGGGCTCCCGTTACGGATGTCAGTGCCCTGGAAGTCGAAAAGGAGGAACGGAGAGCGGAGAAAT
ACCGGCAAAACATGCGTTCCCCAGTTGAGGTTAAATTGGAAGCGCGGCCTAACCTTAATGGGTTTACAAG
TTTGCCGCAATCCAAGTAATTCGTCTTTTGAACAATTGCATTATATAATGCGGTGCATTCATTTTAAAAC
AATTGGAATATAAACTTGAAAGATGTGGGCTCGGAAAGTTCCACTTATTAGATGGAGCACTTTTAAAAAC
CGACTTAGAATACCGGTTAAGCAAAAAAGGTAGCTTATTTGCAATGGaaaaatttaaaaatttaaaagtt
taGTTTTGTGAAAGATCTTTCTTAAAGCAATGGAATAAAAATGGCGGCTGGGATAAGAAAACTTTACGGA
AAAAATGTTCACTATTGTACTCTTTATACGATTTAATATTATAACTCAATTCATTTTAACCCTCTTTTCT

TTTTCAGGTGCGATCCTTCGGTACAGAAGATCGGGACACCCAGTTCCAGATAGCGCCGCAATCGCAGATC
TACGACTACATCCTATTCCGTGGCTCGGACATCAAGGACATTCGCGTTGTCAACAACCACACGCTGCCGC
ACCACAATGACCCGGCGATCATGCAGGCGCAGCTCCAGAACGGACCGCCCCAGATGCCGCAGCACTTCCC
CATGCCCAGCGGGATGAGCGGTCCGCCGCAGCAGCAACAGGTGCCGTCGCAGCAACCGCCCCAAATGCCA
AGCGGTGGAGGCAGTGGTGGTGCTGGTGGAGCAGGAGCTCCTGGCGGCGGCGGACCCGGCTACGGCAATG
GCAATCCGTTCGGCAACCTCGGCGGTCCCAATCTGGCCAACATGGTGGGCAATGCGGGCGGATCGCTTGC
ACCCGGTTCCGGTGCTCCGGGCAGCGGACCCTTCATGCACATCGGTGGCAATCAGCAGCAACCGAAGCCC
CAGCAGCAGAAACAGCCAAAGTGAGTTGGTGTAGACCACACATTACTAAAATTACATTGCACACAAGTAG
AACTTCTCATTTGTGATCTTAAATCAAAATTCGTTTCCTGCTAAAAAGCGATTGCTATTTTCAAAAACGC
AAATTCCGGGAATAGTTTAAGATATGGTTCATTTTGTGGCTTTATCCGATAAGCGAGCATGTCGATTTAA
ATGCTTAAAAATTACGCTCTTTTACTCAGCCCTTTAAAATAGTTAATGTCACCTTTTGATACAAAGTATT
TGACACAAGTATTTGTTTGGACTAATGTTTTTGGTGCCCACACGCATTACTCTCTCCTTTACTTTCGTCC
TCGTTGCGTCCTCTATATGTGTACAGTTTCCGTTCTAGGTTTCCGTTCTAGACATGCTGGCCGGTGCCTC
GCGATCGACGACGCCCATTAGCCTGATTGTGAGTCCCACGGCGGAGCTGACTCAGCAGCAGCAGCTGCAC
CAGCAACAGAACGCTGGCCAGGGTGGCAACGGACGCGATGCAGGACATAAGCGCCAGAACCACCAGCAGC
AACAGAATCAGCAGCATCAGCAACGTGGCGGACCCAGCCACAACAATATGCAGCAGCAGCAGCAACGAGG
CGGCGGCAGTGGAACGGACTTCTATAACCAGCAGCGGGATCGTCGGGACTCCGGACGTCAAATGGACAAC
AACTATAGCAACAACTACAACAACAATAATAATAATCAGCGCAATCGCGGCGGCGGCAACGGAATGCAAC
AGCAGCAGCGAGGAGGAAACGGCGGCAGCGGCGGCGGCGGTGGAAACGGAGGTGGAAACAACCCGGCCTG
GAACATGCATCGCGGCAACCAGAACTCGAACAACATGATGAACATGCGCAACCGCGGCATGGGATCCCGC
GGCCCCATGCGACCCAATCAGGGGCTATCGTCCGCAGTCGGCCAATAATCAGAACAAGCCGCGGAACAAG
ATCAAGTTCGAGGGCGACTTCGATTTCGAGCAGGCAAACAACAAGTTCGAGGAACTGCGCTCCCAACTGG
CCAAGCTCAAGGTGGCCGAGGATGGTGCACCCAAGCCAgccaccaatgcaacggccgccactgcaactgc
aaccaatgAGCAGGTGGGTGAGAAGGTTGAAGGCGTTCACACAGGTGGGTGAGAAGGTTGAAGGCGTTCA
CACAGTAAGTCCACATACACTTTTTATTTTTATACACTACAAATTAGTTCTAAATGAATAAGAGCAGTTT
ATTAACAAATAAGGTAAATCTTCTTCTTTATTTATTGCTTGTTTAAGTTCAGTTTAAGTAACATTTTCTA
TACGATCGTTTTAAGAGTTACTCACTATTTGCATCATCTGCTTGCAGGCTGAATGGCGAGACCGACAAGA
AGGATGATTCTGGCAACGAGACCGGCGCTGGAGAGCACGAGCCTGAGGAGGATGATGTTGCTGTGTGCTA
CGACAAGACCAAATCGTTCTTCGACAACATCTCGTGCGAGGCTGCCCAGGATCGCAGCAAGAACAAGAAG
AACGATTGGCGCCAGGAGCGCAAGTTGAACACGGAGACCTTCGGAGTGTCCTCCACACGACGTGGCAGGC
AGTTACCGCGGACGCAACCAATACTACAACAATAATGGCAACGGCGGCATTAACTCGGGATATGGAGGAG
CGCCAGGCTACAACAGGAACAATTATCGCATGGGTGGCGGCGGCGGAAACTTCCGAAACAGGAGCAACAA
TCGCAACAACGGCGGCGGTCGTGGCGGAAACGGAATGCCAAACATCACCAATGGCAACACGGCTGCTGCG
CTGAAGGCGGCCAACAATGCTGCTGGCCACGGATCCAATGCCACGGACTCCAGTGCACCAAATGCCACAA
CCGCGACGACAAAGTCGACGTCCCTCTTGCCAGAGCAGACGCAACAGGTGGCGGCAGTTT
>dme_circ_0004589_junction_seq
GAGCAGACGCAACAGGTGGCGGCAGTTTGGCTACAATGAGCCACTTGGGCCTTGCG
>dme_circ_0004590
GTGGAGACCATGCTAAAATCGGAGCAGGAACGAAATAAATCCCAGAAATCTCAACTTGATACCCTTCATG
AGAAACTCAGAGAGCGCAACGATCAATTAACTGATCTACGAAAGCAACTAACTACGG
>dme_circ_0004590_junction_seq
ACTGATCTACGAAAGCAACTAACTACGGGTGGAGACCATGCTAAAATCGGAGCAGG
>dme_circ_0004591
CTTGATGTCACGCCACGGCCCTTTCGATGAGGACTTGGCTCGTTTCTATCTGGCCGAGCTCACAGTGGCC

CTGCACACATTGCACGAGATGGGCTATGTGCATCGGGATATAAAGCCTGAAAATATACTGATTGATCGTT
TTGGCCATATTAAACTGGCTGATTTCGGTAATGCCGCTGCCTTGGATCGCGATGGCCATGTGCTGAGTTT
GTCGCCGGTGGGAACACCGGATTACATAGCTCCAGAGCTGCTGCAAACCATATCCACCTACAAGCTGTCC
AAG
>dme_circ_0004591_junction_seq
AACCATATCCACCTACAAGCTGTCCAAGCTTGATGTCACGCCACGGCCCTTTCGAT
>dme_circ_0004592
GTTTGTATTTTCGGCGTGAGTGCGCAGTGAAGCATCGAAGTAGTTCAAGATGAGCGGGGGATTACCGGAG
TTGGGCTCCAAAATCAGCCTCATTTCCAAGGCGGACATTCGATACGAGGGCCGCCTGTACACGGTGGATC
CGCAGGAGTGCACCATTGCGCTCTCGAGTGGGTAAGTATTTAATACGAAAATCGCCCACGTCGCCCATAT
ATGTACATATACGGGATATACATATGTGGGTGCCGTAATCTGCGCCaaaaaaaaagaaaaaaaaaatcgt
gtaaaaaaaCGGTTCATCGCAAAAGAAAATGGATTCTGTCCATTTCTCTTGTGTTGGTGGCTCTACTCTT
TCTTTCCTTTTGGTTCGTTTCTTTTTGTTAGTTTTTACAGTAATTTCATACTCGTGGAGACGGTGAGTGC
AATTGCCGCCACCGCTCTCTCCATTACCCATTTCGACAGCCGTCTCTCGCTCTCCCACTTATGCTTTTTG
TTTGTCAtgttggctttggcctgtttttgtctttggttgttgttgtACAGTGTTGCTTTTGCAATTTACT
TGGCACACATACACCCGCGCACAAAGTAACGAGCACCAGCAAGGCCCCAATGCAATTTGTTTATTTGCAA
ATACAATTTCGATTTGGCGCCAGATAACGGGATCCAAGGGCTCCCGTTACGGATGTCAGTGCCCTGGAAG
TCGAAAAGGAGGAACGGAGAGCGGAGAAATACCGGCAAAACATGCGTTCCCCAGTTGAGGTTAAATTGGA
AGCGCGGCCTAACCTTAATGGGTTTACAAGTTTGCCGCAATCCAAGTAATTCGTCTTTTGAACAATTGCA
TTATATAATGCGGTGCATTCATTTTAAAACAATTGGAATATAAACTTGAAAGATGTGGGCTCGGAAAGTT
CCACTTATTAGATGGAGCACTTTTAAAAACCGACTTAGAATACCGGTTAAGCAAAAAAGGTAGCTTATTT
GCAATGGaaaaatttaaaaatttaaaagtttaGTTTTGTGAAAGATCTTTCTTAAAGCAATGGAATAAAA
ATGGCGGCTGGGATAAGAAAACTTTACGGAAAAAATGTTCACTATTGTACTCTTTATACGATTTAATATT
ATAACTCAATTCATTTTAACCCTCTTTTCTTTTTCAGGTGCGATCCTTCGGTACAGAAGATCGGGACACC
CAGTTCCAGATAGCGCCGCAATCGCAGATCTACGACTACATCCTATTCCGTGGCTCGGACATCAAGGACA
TTCGCGTTGTCAACAACCACACGCTGCCGCACCACAATGACCCGGCGATCATGCAGGCGCAGCTCCAGAA
CGGACCGCCCCAGATGCCGCAGCACTTCCCCATGCCCAGCGGGATGAGCGGTCCGCCGCAGCAGCAACAG
GTGCCGTCGCAGCAACCGCCCCAAATGCCAAGCGGTGGAGGCAGTGGTGGTGCTGGTGGAGCAGGAGCTC
CTGGCGGCGGCGGACCCGGCTACGGCAATGGCAATCCGTTCGGCAACCTCGGCGGTCCCAATCTGGCCAA
CATGGTGGGCAATGCGGGCGGATCGCTTGCACCCGGTTCCGGTGCTCCGGGCAGCGGACCCTTCATGCAC
ATCGGTGGCAATCAGCAGCAACCGAAGCCCCAGCAGCAGAAACAGCCAAAGTGAGTTGGTGTAGACCACA
CATTACTAAAATTACATTGCACACAAGTAGAACTTCTCATTTGTGATCTTAAATCAAAATTCGTTTCCTG
CTAAAAAGCGATTGCTATTTTCAAAAACGCAAATTCCGGGAATAGTTTAAGATATGGTTCATTTTGTGGC
TTTATCCGATAAGCGAGCATGTCGATTTAAATGCTTAAAAATTACGCTCTTTTACTCAGCCCTTTAAAAT
AGTTAATGTCACCTTTTGATACAAAGTATTTGACACAAGTATTTGTTTGGACTAATGTTTTTGGTGCCCA
CACGCATTACTCTCTCCTTTACTTTCGTCCTCGTTGCGTCCTCTATATGTGTACAGTTTCCGTTCTAGGT
TTCCGTTCTAGACATGCTGGCCGGTGCCTCGCGATCGACGACGCCCATTAGCCTGATTGTGAGTCCCACG
GCGGAGCTGACTCAGCAGCAGCAGCTGCACCAGCAACAGAACGCTGGCCAGGGTGGCAACGGACGCGATG
CAGGACATAAGCGCCAGAACCACCAGCAGCAACAGAATCAGCAGCATCAGCAACGTGGCGGACCCAGCCA
CAACAATATGCAGCAGCAGCAGCAACGAGGCGGCGGCAGTGGAACGGACTTCTATAACCAGCAGCGGGAT
CGTCGGGACTCCGGACGTCAAATGGACAACAACTATAGCAACAACTACAACAACAATAATAATAATCAGC
GCAATCGCGGCGGCGGCAACGGAATGCAACAGCAGCAGCGAGGAGGAAACGGCGGCAGCGGCGGCGGCGG
TGGAAACGGAGGTGGAAACAACCCGGCCTGGAACATGCATCGCGGCAACCAGAACTCGAACAACATGATG
AACATGCGCAACCGCGGCATGGGATCCCGCGGCCCCATGCGACCCAATCAGGGGCTATCGTCCGCAGTCG

GCCAATAATCAGAACAAGCCGCGGAACAAGATCAAGTTCGAGGGCGACTTCGATTTCGAGCAGGCAAACA
ACAAGTTCGAGGAACTGCGCTCCCAACTGGCCAAGCTCAAGGTGGCCGAGGATGGTGCACCCAAGCCAgc
caccaatgcaacggccgccactgcaactgcaaccaatgAGCAGGTGGGTGAGAAGGTTGAAGGCGTTCAC
ACAGGTGGGTGAGAAGGTTGAAGGCGTTCACACAGTAAGTCCACATACACTTTTTATTTTTATACACTAC
AAATTAGTTCTAAATGAATAAGAGCAGTTTATTAACAAATAAGGTAAATCTTCTTCTTTATTTATTGCTT
GTTTAAGTTCAGTTTAAGTAACATTTTCTATACGATCGTTTTAAGAGTTACTCACTATTTGCATCATCTG
CTTGCAGGCTGAATGGCGAGACCGACAAGAAGGATGATTCTGGCAACGAGACCGGCGCTGGAGAGCACGA
GCCTGAGGAGGATGATGTTGCTGTGTGCTACGACAAGACCAAATCGTTCTTCGACAACATCTCGTGCGAG
GCTGCCCAGGATCGCAGCAAGAACAAGAAGAACGATTGGCGCCAGGAGCGCAAGTTGAACACGGAGACCT
TCGGAGTGTCCTCCACACGACGTGGCAGGCAGTTACCGCGGACGCAACCAATACTACAACAATAATGGCA
ACGGCGGCATTAACTCGGGATATGGAGGAGCGCCAGGCTACAACAGGAACAATTATCGCATGGGTGGCGG
CGGCGGAAACTTCCGAAACAGGAGCAACAATCGCAACAACGGCGGCGGTCGTGGCGGAAACGGAATGCCA
AACATCACCAATGGCAACACGGCTGCTGCGCTGAAGGCGGCCAACAATGCTGCTGGCCACGGATCCAATG
CCACGGACTCCAGTGCACCAAATGCCACAACCGCGACGACAAAGTCGACGTCCCTCTTGCCAGAGCAGAC
GCAACAGGTGGCGGCAGTTTGTGAGTAGCAATTACATCTAAGCACTTCATTTGCAACATGTTTGTTTACA
TGTGTGTCTGTTTACTTTTGCAGCACAATAGTGCCGCCCGGCCAGTGGAAAGCCACTTTAAATATACACC
AACCACAACCAACAAACCACAAATTGATAGATGCGAATTGAGAAGAGCGTAAA
>dme_circ_0004592_junction_seq
GATAGATGCGAATTGAGAAGAGCGTAAAGTTTGTATTTTCGGCGTGAGTGCGCAGT
>dme_circ_0004593
GACATGCTGGCCGGTGCCTCGCGATCGACGACGCCCATTAGCCTGATTGTGAGTCCCACGGCGGAGCTGA
CTCAGCAGCAGCAGCTGCACCAGCAACAGAACGCTGGCCAGGGTGGCAACGGACGCGATGCAGGACATAA
GCGCCAGAACCACCAGCAGCAACAGAATCAGCAGCATCAGCAACGTGGCGGACCCAGCCACAACAATATG
CAGCAGCAGCAGCAACGAGGCGGCGGCAGTGGAACGGACTTCTATAACCAGCAGCGGGATCGTCGGGACT
CCGGACGTCAAATGGACAACAACTATAGCAACAACTACAACAACAATAATAATAATCAGCGCAATCGCGG
CGGCGGCAACGGAATGCAACAGCAGCAGCGAGGAGGAAACGGCGGCAGCGGCGGCGGCGGTGGAAACGGA
GGTGGAAACAACCCGGCCTGGAACATGCATCGCGGCAACCAGAACTCGAACAACATGATGAACATGCGCA
ACCGCGGCATGGGATCCCGCGGCCCCATGCGACCCAATCAGGGGCTATCGTCCGCAGTCGGCCAATAATC
AGAACAAGCCGCGGAACAAGATCAAGTTCGAGGGCGACTTCGATTTCGAGCAGGCAAACAACAAGTTCGA
GGAACTGCGCTCCCAACTGGCCAAGCTCAAGGTGGCCGAGGATGGTGCACCCAAGCCAgccaccaatgca
acggccgccactgcaactgcaaccaatgAGCAGGTGGGTGAGAAGGTTGAAGGCGTTCACACAGGTGGGT
GAGAAGGTTGAAGGCGTTCACACAGTAAGTCCACATACACTTTTTATTTTTATACACTACAAATTAGTTC
TAAATGAATAAGAGCAGTTTATTAACAAATAAGGTAAATCTTCTTCTTTATTTATTGCTTGTTTAAGTTC
AGTTTAAGTAACATTTTCTATACGATCGTTTTAAGAGTTACTCACTATTTGCATCATCTGCTTGCAGGCT
GAATGGCGAGACCGACAAGAAGGATGATTCTGGCAACGAGACCGGCGCTGGAGAGCACGAGCCTGAGGAG
GATGATGTTGCTGTGTGCTACGACAAGACCAAATCGTTCTTCGACAACATCTCGTGCGAGGCTGCCCAGG
ATCGCAGCAAGAACAAGAAGAACGATTGGCGCCAGGAGCGCAAGTTGAACACGGAGACCTTCGGAGTGTC
CTCCACACGACGTGGCAG
>dme_circ_0004593_junction_seq
TCGGAGTGTCCTCCACACGACGTGGCAGGACATGCTGGCCGGTGCCTCGCGATCGA
>dme_circ_0004594
gctacaaaaaacaaaacaaaacgaaaGCAACCATTTAGCAAGGAGAGGAGCAAGGAGATTTGGCGGAGGA
GCCGATAACAACAATAATGAAATTAATGGAGTAAACCAATTAAATCCATGGAATTCCCAAATCTAAAATA
AAAGCTAACTTAATACGTTTTTATTTGAATGAAAGA

>dme_circ_0004594_junction_seq
CTTAATACGTTTTTATTTGAATGAAAGAgctacaaaaaacaaaacaaaacgaaaGC
>dme_circ_0004595
TTAGCTTTTATTTTAGATTTGGGAATTCCATGGATTTAATTGGTTTACTCCATTAATTTCATTATTGTTG
TTATCGGCTCCTCCGCCAAATCTCCTTGCTCCTCTCCTTGCTAAATGGTTGCtttcgttttgttttgttt
tttg
>dme_circ_0004595_junction_seq
GGTTGCtttcgttttgttttgtttttttgTTAGCTTTTATTTTAGATTTGGGAATTC
>dme_circ_0004596
GTTGGCAAAGGAGGTCTTCTTGGTTCCCACGCGCAGCACCTGCGGCGGTCGCATCACGAACTTTGGCTTT
CGGCCGGCGGCCATGTCCGGATTCTTGTCGAGAATGATTTCGAAGACCCGCTTCAGCAACTCGTCGTATG
TGTAATCGCGGTCGGAGCCAAACCAGGTTGAGCTGTTGTCCTCGTCT
>dme_circ_0004596_junction_seq
AAACCAGGTTGAGCTGTTGTCCTCGTCTGTTGGCAAAGGAGGTCTTCTTGGTTCCC
>dme_circ_0004597
GTTCCTTCTTCTTGCTCTTCTTCTTCTTTTTGGCCATCGAGAAGTCCATGTCCAGATTGATCTCCTCCTC
CTCGGGCTCCTCGCTGGCGGCCACATCATCGCCGCCAAAGCTGGGTATGGCCGCTTCGATTTCGTCCAT
>dme_circ_0004597_junction_seq
TGGGTATGGCCGCTTCGATTTCGTCCATGTTCCTTCTTCTTGCTCTTCTTCTTCTT
>dme_circ_0004598
GGGcttcttcttctttttcttcttcttGCCGAAACTCTCCAGATCTAGATTATCCTCCTCGGCGGCGGCA
CCTCCTTCCGCCGATGCCTCATCCTGGGGATCCTCCTTCTTGGTGTCATCCTCCAGACCCAATGCCGCGT
CCAGATCGAACGTG
>dme_circ_0004598_junction_seq
ACCCAATGCCGCGTCCAGATCGAACGTGGGGcttcttcttctttttcttcttcttG
>dme_circ_0004599
TTTCCAGTCAACTAGTATTCAAACAGCCCAGACACAATGGACGCGGAAGACGTAAGTTCCCGGAACACCG
GCTAGTGCGTTTCATACGCGGAAAACTTGCGTGGAAAATTCGCATATTATCGTACATGTGCGCCCTTGTT
TGCGTATGTGTATGTGTGTATTTACACATTTGTGTATGAGAACTGGGCGAACATTGAGGTTATGTTTGCT
TGATCCGGATTTAAACCATATTGAAATTCCAATGATTTTCGCAGGGGTTTTGATCCCACTTTGCTGAAAa
agaagaagaagaagaagaCCACGTTCGATCTGGACGCGGCATTGGGTCTGGAGGATGACACCAAGAAGGA
GGATCCCCAGGATGAGGCATCGGCGGAAGGAGGTGCCGCCGCCGAGGAGGATAATCTAGATCTGGAGAG
>dme_circ_0004599_junction_seq
CCGAGGAGGATAATCTAGATCTGGAGAGTTTCCAGTCAACTAGTATTCAAACAGCC
>dme_circ_0004600
GTTCCATGAGATAGCCGGCGATTAGGGTGGCTATGCGATCGCCATCGAGCAGATGGAACTCACCCTTGTC
GTCGGTAAAGAAGTACACCACCCGATCGGCATCGCCATCCACGCTGACGCATCTCGTGTACGGCTTCACA
TCCTCGGGCATGGATTTCGGCGGACTTTGCTGCACCTTCACGTAGTCGGCGCCGCAGTCCTCGTTGATCT
TGCCGACACCGATGCCCTGATTAATGACCGTCACGTTGAGGGTGCCCTTCATGCGCTTGATGAACTGCAG
CATCTTGCGCGCACCCACACCGTTGGCACCATCGTAGATGATGCTATTGCGATAGTTGCCGTTCTCCAGG
CGCCCGTTGCGCAGCAGTTCGAATGCCTTGATCAGCTTGTCGTAGTAACCCTCCTCGGTGGGCTTGCCGT
ATGCCTCCTTGGTATTCGCCGCCACCACGAAGTAGTGCAGCATGGGTGTGGTCACAATGCCGTACTCCTT
GACGTTACCCTTTAGCGCTATCACGCCATCGGCCACCGCTTTCAGCAGCCGCGGACTGTGGTATCTGTT
>dme_circ_0004600_junction_seq

TCAGCAGCCGCGGACTGTGGTATCTGTTGTTCCATGAGATAGCCGGCGATTAGGGT
>dme_circ_0004601
TTCCAGCGATGACGCTTTGCGTGACTTCGGAGAATGAGCTTGATAAATGCATCAAGATGAGGGTGAGTTG
AAGAGTGAATATATGGATAGCAAGATGAGTTGATGATCCTTTACAAAAACACTTTTAGACTGCCTTGAAA
GCTCACCTCCTGAAACCAGAACTCATCTGCAAGAAAATGCACTCCCACATCAACTGCATGCAGTTCATCG
AAGCGGGCAAAGCAGACATCTCCGTTTTCGATGCCGGCGATGTCTACACTGGTGGCCTCAACTACGACCT
GGTGCCCTTCATGTCGGAGGTGTACAATCTGGGCGAGCCCGAG
>dme_circ_0004601_junction_seq
GGAGGTGTACAATCTGGGCGAGCCCGAGTTCCAGCGATGACGCTTTGCGTGACTTC
>dme_circ_0004602
GTGTTCGTGCCGTCCTGCTGGTTTCTTTCCTCTGCAAAACTGCTGTCCAATCGTTCGCTGCGATACTGAT
CGTATTGATTCTGCGTTCTGTACGGATCATTATTATCGAACTGGCCATAGGCATTCTGATCGTTGATGTT
GTTGCGGCTGTTGAAGCTTTGTCCTCCCTGCCTGCTCTGATCATCACGTGTGCCATCGGAGTACAGCTCG
GCAATGCGCTTCAAAAACTGCTGATACTGCTTGCGATCGCTGAAGCTGCGAGCCGAAGATGTGACAATGG
CATCGGCGGGCACTTGGCCCCAATTACACTGGCGATAATCGTTGATGGAGGCACGACGACCATCACGACA
CAGCAGCTCAAAAGTGTCGGGTGCTATGTTCTCTAAAAAACGCAGCTTAGATCAGTAATGGGTTTTCAGA
AGTTTCCCTTTGGTAACTCACTGAATTCGGTGGTTTGCAGCATTTCATTCACAGTCGAATGGCGCAAGAA
GGCAACATCTCCTTTTTCAAGCAGACATTTGAAGGCGCCTTC
>dme_circ_0004602_junction_seq
TTTCAAGCAGACATTTGAAGGCGCCTTCGTGTTCGTGCCGTCCTGCTGGTTTCTTT
>dme_circ_0004603
GGTTCACAGCGATAACGTGACTAGAAGTAGTGCATATATAGCGGAAATGATAAGTAGCAAGTACTTTAGG
CCGGAAATATTCTTGGGCGATGAAAAGAGATTTAGTGAGAACAACGATATAATCCGCGACAACGACGAAA
TTTGTCGGAAATGCTTGGAGGGTACTCCATTTCTGAGGAAACCCGAGATCATTCACATACCCCCACAAG
>dme_circ_0004603_junction_seq
CCCGAGATCATTCACATACCCCCACAAGGGTTCACAGCGATAACGTGACTAGAAGT
>dme_circ_0004604
TCCTCCTCTATGTCTGAGTAGCAGCGCTTGGCAGCCGAAGTTCCCTGATGATTCGTATCGCTGGATTTAC
CATCGTTGCTGCTCTCCTCCGCTGAAGATTTGTCTTTCGTGTCCAGGACCTCTTGCTTCTCATTCAGGCT
GCCCTCGTCAG
>dme_circ_0004604_junction_seq
TGCTTCTCATTCAGGCTGCCCTCGTCAGTCCTCCTCTATGTCTGAGTAGCAGCGCT
>dme_circ_0004605
GATCTGATCGAAGCGCACACCGTGGAGCGGGAGAAACTATGCATGAACGTTGAGCAGGAGATAATACGCG
TTCACTCGAAGGCGGCCAGGAGCATATCTGGACAGCCGGCGCCCTATTCCGTTTGCACGTATTTGAAGGA
CGACGAG
>dme_circ_0004605_junction_seq
CGTTTGCACGTATTTGAAGGACGACGAGGATCTGATCGAAGCGCACACCGTGGAGC
>dme_circ_0004606
GCCAGAAAAGCAAACTTAGACACGTGCACTCTCACCAATTGAATCTACACCAGATTTTCGTACAAATCCA
TGGCTGGACATTGATTGGACCCATATCGGTGGACAAAGTGGGCATGTTTTTCCGCACCACAAAATTGGAC
TCGCAGTTTTTGACGAAAAGTCGGATAGTCTTCGATATATCATTGATTGGATCTGCCCAGAAGCTCATCA
AAGTCAA
>dme_circ_0004606_junction_seq

GATCTGCCCAGAAGCTCATCAAAGTCAAGCCAGAAAAGCAAACTTAGACACGTGCA
>dme_circ_0004607
GATACATAACATTTTGCAGTCCTTCGGTTGAAGCAACATAGTTTATAGCCGATGCGTTATTGAACTTATT
TAAAATGGTCTCCATTTCGCGTATTATGTTTGGCACCGCATCCAAAGGCTGTTGTTCCGATCCGCTCAAC
TTCGAATTGCATATTTCCTTTACAGTCTTGATCACCAATTTTCTGAGTTCACTCAAATCGGTACTACAGA
TTTGGTACGAACTTGTCACGGCATCAATTTGCTGTGTTGCTTGGGTTAGTTTTACCTCA
>dme_circ_0004607_junction_seq
CTGTGTTGCTTGGGTTAGTTTTACCTCAGATACATAACATTTTGCAGTCCTTCGGT
>dme_circ_0004608
GTGTCCTGTGCGACAGTGAGAACAACAGCGGTTCTTGCAGCTCCCACGAGGACTCCGATGACTTTGTGCT
GGTGCCAAAGAACCTTCCGGAGGACCAGCGACAGGGTCTGGCCCAAGTCCAAGCCCAGCCCGCTTCCGGT
GGCCAACG
>dme_circ_0004608_junction_seq
AAGCCCAGCCCGCTTCCGGTGGCCAACGGTGTCCTGTGCGACAGTGAGAACAACAG
>dme_circ_0004609
GATTATAAATAGGTATATGTATATCACGTATGTATATGGCTAGTTAGTTAACTACTTTGACGCGGTATCT
AGCCTAACTATATAGTCTCTCCTACAATGTTTCCCGCACAACAACTGAACCGAATCCGTTGTTAGATTCT
A
>dme_circ_0004609_junction_seq
ACTGAACCGAATCCGTTGTTAGATTCTAGATTATAAATAGGTATATGTATATCACG
>dme_circ_0004610
GGTCAAGACAGCGTCAGATCCAGAGCCCTTAAAGGAGGTCAATCCTCCTGTGAAAAGGGCACCTATCAGA
TCCAAAAAAGCTAAAATTAATCTAGTGCAACGCACCAAAAGACCAAGCATCCAAAAGCCAATTGAA
>dme_circ_0004610_junction_seq
AAGACCAAGCATCCAAAAGCCAATTGAAGGTCAAGACAGCGTCAGATCCAGAGCCC
>dme_circ_0004611
GATTAAGAATTATCGAAGAAATGCTTGCAAACACCTTAAAAATCCGAACAAATGCAAGTATGGCTTAAAA
TTTTGTAAAACAGATTTCGTTGGTTGAATGAATAACACTAAATAAGCATACATACATtatatggatatat
atataggtatatatGTTTTTCTTAAATTGCTGTTGTCTTTTCTATGGCTGCAAAAAAATATTATCGCGG
CTTTTCTGCAGTTGACCTTCA
>dme_circ_0004611_junction_seq
ATCGCGGCTTTTCTGCAGTTGACCTTCAGATTAAGAATTATCGAAGAAATGCTTGC
>dme_circ_0004612
GTCGACCGGTTGCCAGCCAGCTCCTGCTGAATCTCGTTGAGACTCTTGCCCTTGGTTTCCGGCACGGCGA
AATAAACGAATATCACACCCACAACAGTCAGACCAGCGAATAGCCAGAAGGTTCCGCCAATTCCCAGGCC
ATCGTTCAAGTTCACAAACGTCTTGGTCACCACGAATGCCAAAAGCCAGTTGGATGTTCCGGCCAGCGAT
CCAGCAAATCCCTTGAT
>dme_circ_0004612_junction_seq
CGGCCAGCGATCCAGCAAATCCCTTGATGTCGACCGGTTGCCAGCCAGCTCCTGCT
>dme_circ_0004613
GCGACGAGGAGCCAATGAACTTGCTGACAACTTGAGACGACCACAAGGAGGGGAATCCCCTTTGGGCGGT
CAAGGAATCGGGGCACCATGGGTGACCTACAGGCCACCATTGAATTCGTCGTGGAGCTGCACAAGTTCTT
CAATGTGGATTTGTTTCAGCGCGGGTGAGTCTGTCCTCAGTTCGAGTTTAAAAGCACTCAAACTCGGTGA
TGTATTAATCCAAAATGCCAGAAAAGTTTCCAAAGAACGCCGTGAAAATTGATGGATTAGCTGGAAAACG

CGTTAGTCAAACTTAGAGAAGCTCCAACTTTTCCATTTAAAGTTTATCCCAATCTACATTTGTCACCGTC
GAATAAAAGTTACATTTGCCATAAGATCATTAAACCCTTCCGAATTGGCTTCCTGCAATTAGTGCCTTGG
TGTGTGTGTGCAGATTGCCAACGATGATTTGCACTTCGTGATTACTAGTTCGCAAACAGTTAAGAAATGC
GGGCCAATAGTGGCATTTCACAAACGCCACCCACTGTTGGCACAAAATATTTCAAATAAAATTGTTAATT
TTTCTCCAATTTGCCGAGCACGTAACACGGACCTTTGAAGTGTCGGACAAACAAAATGAAATGCAGTGCA
ATAAGCAAACAAAGAGTGCCCCACCAACCAACCAATCAAGAGGCAGTaaaaaaaaGGGGCATAGTGGGCT
GCGCAGACATGTGTACATACACATAGTGACCGTAGCACTCACTAATTGTCAGCCAAGTGTTTATGGGATT
TCAATTTGTTGCTGCTGCTCTTGAAGAAACAAACGAATGaaaaaaaaaGTATAGAACACGAAATGAAAAG
AAAAACAAAGCATTTTGTTTGGGCCGATTTCTGTGTCTGTTTTCCTCGGATTTCCCTTTGTTCGCTTGTC
ACTGCAAATGAAGCTGCTGACGACCAGAGTGTCAGTGGCCCCCAAATCTGCTGGGAATCGAGGACTCCTG
ATCTTGAAATTTTTCGACAGCAAGTGCATTGAGGGAGAAATTCGATATCTTGGGGGGGAATATATACCTT
TAAACAAGAGTAATATAAATTACAACTGTTCCCTATTGATAAAGCGAATTTAATTAATCCGCCCACCTTA
CCCCTTCCGTCATCCACAGGGTGAGTGCACTCGGAAAAAATCATGTGTGATAAATCAATGTTTTGGTAAAA
GGTTCTTAACCCCTGTTTTTTAAGAGTGTGGCCATGGGCCAAGTAGGCTGCCGCATAAATTGCACTTTCG
AGTGCCTCTTGCCAAGAATGCGAAAAGGCATCGCGTTCTTCCGCAATGTTGGCATTGCTAAGTTTGTTGC
AAGAGTGACTAATTCTACTCTATTAATTATATGCTCTCAAAAAAAGAGAAGAATATAATTTACAGAATCT
ATTGAATTGCTTTAAACATTCCAAATTAAATTAAGTTAGTGTCACGAACATTTGCCAAACGGACTGGGCT
AGAAATGTGCACTTTGGCGAGTGGCAAGAATGCCGGGAATTCCCGGTTGCCGCATTGGTGGCCTTTTGAT
GGGAATCCGGGGAAAAGCCCCAGCGCGGGTAACCAGACACCAAATCACTCGACCAGCCAGCGGACAGCGA
AACTTTCGGAGTCGAAATGAGTTTATTGTTCGATTGGAAATATAAATCAAATTGTCAGAGAGCGGTCCAA
AGTTGCGAAAGTTTGGAGCCCTGGGGATCGGCAATGGAGCTGCTGACCACACGGGACAGATGCCAAAGAC
GTATTGGCAGGACACGGAAAAACTTCGGTTCGGAAACTTCCTTTCCACCTAATTGCATTTATTGCTGACT
GCAGCTAAAAGGATTTTTGCTGCCCTGGCTGCAATGCAATTACGGCAAGTTGGGAAACTCCTTTTTGGCA
AATTCTCGGCCAGCAAAGGAAAAACTTGGCCCACACCATGACCCAGTTTACATTATTAAAAGAAAAACAA
GACGCCGAACGAAAGACAGACGGCCAAGTTTAAGAAATCTAAAGTAAACAATTTAGTTATGCATTACAAG
AAGTGGATGATGGACTAGAGAGTGACCTATAAAAACGAATTAGGTTTCAGGCACAAAAGGCATGGTATAC
ATATGTGTATATTATATACATTATGTTCACATTGTGTGTGCACTTAAGTGTGCGTTTATAAAATAATCCA
ATTTTAATAATATTTTGTGTACATGGTAACCGCACCATAAAACCCAATAAAAGCGGCATAAAAACTTAAC
GGCGCAAAAACATAAGAGAAAAATGGGCAAATTGAAAGTAAATTTCCACATGATGCGGAGAGTGCGATGC
TGGGAAGGCGGAAAATGCGGAAAAGACCATGGAAATATGTACACACGCGTCGGCGGCAAATGGAAATGTT
GGATAAATATATAAAGAGATATTTTTATTCGCATATTTATGGGTATTGTGTGAGGAAATAAAAGAGCGCC
AAAGATTTCAAGGTGTTGAAGTGCCAGAATGGCGATGAGCACTTGACGGACGCCTAAGCCCAGGAACCTT
ATCCGCATCCTTGTAGCTGTTGCCTTTTATTTTTCCCGCCATCGCATCCTTCGACAACAATAGAAACATC
ATCCACTTGGCTGCCGAGCAAAGTGATTAATTCATTTGGACAAGTTTCCCAAAGCAAAGTGCGGGGAATC
TAGAAAGAGCCACCAACAAAAGTAACTTTTCAGTTGACAGCTCGCAGTCTGTTGTGGATGCCTCCTCGCA
TGTGTGTGAGTGATTGTTGTTGGgctttccccccttccggttttccgctttcccgctttcccgcttttccg
ttcttccgctcttccTAACTGTGCAATTTATCTTTCGTATAGTTCGTACAGTGGGTTACTGCACCGCAGA
CAATAAAATAAACTGTGTCGAGCGGCGGAGAGGAGACCCTTGTGAAGCCCTCAGCATTTAAATTATTCCA
GCCAAAAGCAATTGTCACCACGGACCACATGGCGGATGAGTGATGCAGCGGAGAAGCCAGCAAAAATCTC
AACACGCCAAAGAGATCTGGCTACTCTCCGCTCGTTTTATTGCCTTTCGCCATAAGTAAATCCTTATTTT
ATGAAAACAAATGAATTTTATGGTCCCACGGAGGAGCAGGCTGCTGGCATCCTAAGTGGGAGCCCCATAA
ACCACCATCGACATGTGGAGCAAATTGAATTTGATTAACATTTTCGGCTCGGAAACGACGTGGGTGGGGC
TTACGGACCTAAAGGTAGCCATGGGTTAGATGGACCCTTCAAAGGGAGGAGCGCAACTCTGATGGTTATT
ATTATGGTGTGGCAGGTAGAATAATAGCCTGTATGGATGAATATACAAAGAAAGGTGAGAGTCTGTGATT

CGATTCGACTCACTTGTTGAACATCCAAAAAGGGAAACCCCATCCAGATGATGTCCCAATGGGTCTAGAA
TTTAAAATCCACACCTTAAGTTAAGTTGCCCTAACTTTCTCACAATCAAACATTTAAATCCATCCATATT
TAACACTGGAACATCTGCTTGAATCGGCAATGGAAACTAATTGATTGAGTTCCCAAACACAACTAGGACA
ACATTTTCCTGAGTCATCGATTCACTCGGTTTCCGGCCAACGATTTGCGGTCTTCGCAGAATTTTGATTT
GTCTTTGTCGCAGCACAACTACATGTACTTTAACCCATTTTGCCTGTGGGTGATGGGTGGTGGCAGACCG
ACCTATCCCGAACCACCACCCACTTGCCACCCGCTTGCCACTATTTCGAACTAAACGAAAATCAATAGAA
AAACAAATAAGTCAAGACGGAATTGAAATAGAAGGGATTTCGGAGGGGCATGGGGTATGCAGGATGTTTG
CATGCTGGATTTTCTGCCGCATTTATCCCGGGGATTTGCAGTTCACTCAACCTCAAGACGGATACTCACT
TTTTTCCCGCTCTTCTCATCATTCCTCTCCATTTTTTGCAGGTTGACAGTCGTCACCTGAAGGAGTCCAT
CGAACGAGCCGAGTTTTCGCTGCAGTTGGAGCTCTGGTTCGGGGAGCAGAACGGGTCGAGCAGCCTGAAC
GGCAATGGAGGCGGCAGTGGGATGGGCAGCTTCTCCGGACCCGCCAGCTCTGCCCTGACACTGGCCTCCA
CCAGGAACCTGCAGCTGAACTTTCATCCGGGACGTGGCCTTCACTACCATCTGCCTGTGCTGTTCGACTA
CTTCCATCTGGCGGCCATCAGTGTGGGCATCCACGCCAGCCTCGTGGCCCTTCATCAGCCGTATATCAA
>dme_circ_0004613_junction_seq
TCGTGGCCCTTCATCAGCCGTATATCAAGCGACGAGGAGCCAATGAACTTGCTGAC
>dme_circ_0004614
GTGATCAAGACCAAGAACTGCATCCTGTTTCCACCTAATCCGAACGCACCTCCTCCCACAATAAGGGAGC
GACCACCAGGATGCCGGACGGTGTTTGTGGGTGGACTGCCGGAAAACATAACCGAAGAAGTGATCCGGGA
GATCTTCGAATCATGCGGCGAGATCACCACATTGCGCATGTCCAAGAAGAACTTTTGCCATATTCGCTAC
CGCCAAGAGGG
>dme_circ_0004614_junction_seq
TTTGCCATATTCGCTACCGCCAAGAGGGGTGATCAAGACCAAGAACTGCATCCTGT
>dme_circ_0004615
GCACCACCTGCTCACCAACCAGCACCGCAACAGCCGCAGGCACCGTCGTCGAATGAACCCAATTGGTATG
GTTTTTCAGCGCCGCCGCCGCAGCAGCAACAACAGCAGCCGGGCTATGGGCCACAAGGATGGGCCGGTGG
CTATGTCCAACCTCCGCCGTACGCTTCATGTGCCCCAAATTGTCCACCACAGCCGCCGTACGCCGCTCCG
GCTCAGACGTACAATGGTCCACAGCCATATGGTGGATACGGCAATGTTCCTGGCGGATTTAATACCCCCG
GATTTCAGCAACCGAATGGAAATCCAAATGGCTATCCAGGTGGACCACCGCCACCCGGACAACAAGCAGC
AGGAGCAGCTGGAGGAGCCGCCGCATCTGGAGCCAGCTTTATGGGCTTCAGCTTGCCCCCCTGGAAGTGAG
TAGAACTCATCATCCCATTCATCACTTTTTTAACATTTCCCTCCCACAATGCACATTTCTAAATTGGTTT
TATTCTagcaaaagataaaaaaatatgagcaaaaggacaagaaaattatgaACATCTTTAAAATAGTCGA
AACATCTAAAGATCTAAAATGTTATCAAGAATTTAATTGAAGTATCATGAACCACCTTTTGCTTTCGGAC
GCTCAAAGTATTCAAGAGGCATCCAAAAACCATTCAGAAGCATTCGTTCTGTTTATTTTTCTCACTTACA
AAATTATTCAATTTGTCCAAATAATAAATGATTTGTTAGCATAATTCATTTGACGGTGTAATATTCCttt
tttggtagtgtttatatttgtttttttcccatttttGAGTTCCCTTATCAGGTTTCTCACCTAATATCCA
AATAACGATTAACCTATTACTAATCAAAACAAAATTATTTCCCGAATCTaaaaaaaaGCATTTATCTTTT
GACCTATTTTACAGTGTAATTAAAAGCAAACTACTGTACCTCAAATAGCCATCCCGACAA
>dme_circ_0004615_junction_seq
ACTGTACCTCAAATAGCCATCCCGACAAGCACCACCTGCTCACCAACCAGCACCGC
>dme_circ_0004616
GCGCCGCCGCCGCAGCAGCAACAACAGCAGCCGGGCTATGGGCCACAAGGATGGGCCGGTGGCTATGTCC
AACCTCCGCCGTACGCTTCATGTGCCCCAAATTGTCCACCACAGCCGCCGTACGCCGCTCCGGCTCAGAC
GTACAATGGTCCACAGCCATATGGTGGATACGGCAATGTTCCTGGCGGATTTAATACCCCCGGATTTCAG
CAACCGAATGGAAATCCAAATGGCTATCCAGGTGGACCACCGCCACCCGGACAACAAGCAGCAGGAGCAG

CTGGAGGAGCCGCCGCATCTGGAGCCAGCTTTATGGGCTTCAGCTTGCCCCCTGGAAGTGAGTAGAACTC
ATCATCCCATTCATCACTTTTTTAACATTTCCCTCCCACAATGCACATTTCTAAATTGGTTTTATTCTag
caaaagataaaaaaaatatgagcaaaaggacaagaaaattatgaACATCTTTAAAATAGTCGAAACATCTA
AAGATCTAAAATGTTATCAAGAATTTAATTGAAGTATCATGAACCACCTTTTGCTTTCGGACGCTCAAAG
TATTCAAGAGGCATCCAAAAACCATTCAGAAGCATTCGTTCTGTTTATTTTTCTCACTTACAAAATTATT
CAATTTGTCCAAATAATAAATGATTTGTTAGCATAATTCATTTGACGGTGTAATATTCCtttttggtag
tgtttatatttgtttttttcccatttttGAGTTCCCTTATCAGGTTTCTCACCTAATATCCAAATAACGA
TTAACCTATTACTAATCAAAACAAAATTATTTCCCGAATCTaaaaaaaaGCATTTATCTTTTGACCTATT
TTACAGTGTAATTAAAAGCAAACTACTGTACCTCAAATAGCCATCCCGACAA
>dme_circ_0004616_junction_seq
ACTGTACCTCAAATAGCCATCCCGACAAGCGCCGCCGCCGCAGCAGCAACAACAGC
>dme_circ_0004617
GCATAAAGATGAACTGTTTCTTGGCCAAACCGAGTCGTAATCGTTTTAGCCAACTAAGTTTTGTTACTTC
AACAAGCTCTAAAAATTCCAGATTCGAATTATCCAGAATTAAGGATTGAAGATTACCTAAAACTTTCAGT
TCCTTAAGAAGCTGCCACAGGGATATACCAAGAGGATTATCGATTTGCAA
>dme_circ_0004617_junction_seq
ATATACCAAGAGGATTATCGATTTGCAAGCATAAAGATGAACTGTTTCTTGGCCAA
>dme_circ_0004618
TCTCCACTTTCTCCCAGTTGCCCCCGTTGAAGGTCTGCCATTGAGGCGCCACATTGATGAACAGGAATGT
CGTGTGCTGAGCCGCCCCGAAGATCTGATCCGTTTTGGCGATCATATGTCCCCGAGACAGGATGCGGTCA
TCGCTAAAGTTGAAGTAGGGTGACGCATCCAGACCGAGTATG
>dme_circ_0004618_junction_seq
GTAGGGTGACGCATCCAGACCGAGTATGTCTCCACTTTCTCCCAGTTGCCCCCGTT
>dme_circ_0004619
ATATAGCTCCCTCTCAATGCCATCCACATCGGGAAGGGTTGAAACACCCCAAGTGCCTGTATAGCAATCA
GTGGTTAGATTACGATCTGCCACGAATTTTCGAACACTAGTCTCCACTTTCTCCCAGTTGCCCCCGTTGA
AGGTCTGCCATTGAGGCGCCACATTGATGAACAGGAATGTCGTGTGCTGAGCCGCCCCGAAGATCTGATC
CGTTTTGGCGATCATATGTCCCCGAGACAGGATGCGGTCATCGCTAAAGTTGAAGTAGGGTGACGCATCC
AGACCGAG
>dme_circ_0004619_junction_seq
TGAAGTAGGGTGACGCATCCAGACCGAGATATAGCTCCCTCTCAATGCCATCCACA
>dme_circ_0004620
AAAACCGGCAGCCACATCGAAGCCGATGTGCACCACATCCGTACCACCAGCGCAAGATTGTCCCGATCGG
ACTGCCGCATATGTGGGCCAACTCTGGCAGACGAATCCTCGAATATTGACCAGCTGTCCTTCGGCCTCGT
ACAATTGATCACCAACACAACGAGCGCTCAGAGCTGTATACCTATTAAATGGTGATACCAGAGCTCCAGT
GCAGTGCAGATCAATGGAGGATCCTTCTTCCACGGTGTAAAGCCCACTTTCATCGGGCAGCAGCCAGCTG
AGAGCTCCTTGCGGTGTTAGTAGTGGAGCAGGATCCTTGAGATCACCATTTGTACTAAACCTACAGGGAT
TGGCAATGGGATTGGCAGTAGTCACCGGAGGACTTGAAGTAGAGGTAGACGTTGGAGATGTTGAAGTTGA
TTCTTCGGTTGATAAAGTTGGTTGTGAAGTGGTAGAGGAAGATGTAGTTGAAGTAGGTTCCAGAGTGGTA
GACGAGCTtggttctgttgtggatgtaactgtggaggtaggtggttcagtggtgggtgtaactgtggagg
tgggtggttcagtggtggatgtaactgtggaagtgggttgctcagtggtggatgcaactgtggaggttgg
tggtttagtggtggatgtaactgtggaggttggtggttcagtggtggatataactgtggaggttggtagt
tcagtggtggGTGAAGCGGTAGATGAGCTTGGCTCTTCGGTGGTAGAAGAAACGCTAGTGGAACTAGGTG
TAGTATCAGTGGTTGATGGAATTTCGGTGCTTGTAGTTGAGCTCGGCTCTTCAGTAGTTGTGGAACTTTC

GGTTGTCGAAGGCTCAACAGTGGTCACGTCGTCCAATCCAAGGATACCATTTGTGTGCAGGTCCTCCAGC
GGCAAGTCCTTAACCACTTCAATGAAATCCTCGACAGAGCAAGCATAGGAATAACCCTCGTGCAGATCTT
CTTTGTACCAAGTTAGCCAGCTTAGCTGATGGCCAATATCCTGGCACAGTATGTAGTCCTTTTGGATTTG
CTCCAGTG
>dme_circ_0004620_junction_seq
ATGTAGTCCTTTTGGATTTGCTCCAGTGAAAACCGGCAGCCACATCGAAGCCGATG
>dme_circ_0004621
GATCCTTCTTCCACGGTGTAAAGCCCACTTTCATCGGGCAGCAGCCAGCTGAGAGCTCCTTGCGGTGTTA
GTAGTGGAGCAGGATCCTTGAGATCACCATTTGTACTAAACCTACAGGGATTGGCAATGGGATTGGCAGT
AGTCACCGGAGGACTTGAAGTAGAGG
>dme_circ_0004621_junction_seq
GTAGTCACCGGAGGACTTGAAGTAGAGGGATCCTTCTTCCACGGTGTAAAGCCCAC
>dme_circ_0004622
GTCCTCCGGTGACTACTGCCAATCCCATTGCCAATCCCTGTAGGTTTAGTACAAATGGTGATCTCAAGGA
TCCTGCTCCACTACTAACACCGCAAGGAGCTCTCAGCTGGCTGCTGCCCGATGAAAGTGGGCTTTACACC
GTGGAAGAAGGATCCTCCATTGATCTGCACTGCACTGGAGCTCTGGTATCACCATTTAATAGGTATACAG
CTCTGAGCGCTCGTTGTGTTGGTGATCAATT
>dme_circ_0004622_junction_seq
TGAGCGCTCGTTGTGTTGGTGATCAATTGTCCTCCGGTGACTACTGCCAATCCCAT
>dme_circ_0004623
CGTATCTGGAGATCTGCTCCTGCTGATCGTAGCAGAGCTCAACATGGCTAAGAAAACCGGCAGCCACATC
GAAGCCGATGTGCACCACATCCGTACCACCAGCGCAAGATTGTCCCGATCGGACTGCCGCATATGTGGGC
CAACTCTGGCAGACGAATCCTCGAATATTGACCAGCTGTCCTTCGGCCTCGTACAATTGATCACCAACAC
AACGAGCGCTCAGAGCTGTATACCTATTAAATGGTGATACCAGAGCTCCAGTGCAGTGCAGATCAATGGA
GGATCCTTCTTCCACGGTG
>dme_circ_0004623_junction_seq
ATCAATGGAGGATCCTTCTTCCACGGTGCGTATCTGGAGATCTGCTCCTGCTGATC
>dme_circ_0004624
GGCCGAAGGACAGCTGGTCAATATTCGAGGATTCGTCTGCCAGAGTTGGCCCACATATGCGGCAGTCCGA
TCGGGACAATCTTGCGCTGGTGGTACGGATGTGGTGCACATCGGCTTCGATGTGGCTGCCGGTTTTCTTA
GCCATGTTGAGCTCTGCTACGATCAGCAGGAGCAGATCTCCAGATACGCTCGCTATGAACTGACTCCGGC
CAATGTGGCCTACCAAAAGGGTGTTGCTCAACCGGGCTATCTTCGAGGTGACTTCTATAGCGGCGAAGAT
GTCAATGTTCTATATGACCAATCACACCAGCTGGATGCATTTAGTACGGCCCTGGGATTAGATGCCAGTC
AGTACTTTGACAGCACTAAGGATCTCTATTTGACCAGAGGACAGCTGGCAGCTCGTCTTGATTTCGTCCA
TAGTTCCGCCCAGAGAGCCACCCACTTTTATGTGAACGCAGTTCCTCAATGGAGAAGCATCAATATTGGA
AATTGGCTTGCAGTGGAATCCAGTTTGAAGCAATTTGTGGCGGATGAGGCACTTAATGTGAGTGTACATG
CCGGCAGCTATGATATCTCAACTCTACCCAATTCACAAGGTGTCCAGACACCTCTGTATCTGAATGCCGA
GACCAAACAACTGCCAGTAGCTAAGATCCTCTATCGCATTGTTATCGATCAGGAAAGTCGCAAGGGAGTA
GTTCTAATTGTGGTCAACAATCCACATATAACATTGACGGAGACCCTCGAGGATTATGTGATCTGTGAGG
ATGTGGGAGCGCAAATCGATTGGTTGGACTGGGACAAAGCAAACCTGCAGAAGGGCTACTCCTATGCGTG
CTCTGTAGAAGATTTCATCGAAGTG
>dme_circ_0004624_junction_seq
GTGCTCTGTAGAAGATTTCATCGAAGTGGGCCGAAGGACAGCTGGTCAATATTCGA
>dme_circ_0004625

TTGAGATATCATAGCTGCCGGCATGTACACTCACATTAAGTGCCTCATCCGCCACAAATTGCTTCAAACT
GGATTCCACTGCAAGCCAATTTCCAATATTGATGCTTCTCCATTGAGGAACTGCGTTCACATAAAAGTGG
GTGGCTCTCTGGGCGGAACTATGGACGAAATCAAGACGAGCTGCCAGCTGTCCTCTGG
>dme_circ_0004625_junction_seq
TCAAGACGAGCTGCCAGCTGTCCTCTGGTTGAGATATCATAGCTGCCGGCATGTAC
>dme_circ_0004626
GCTATGATATCTCAACTCTACCCAATTCACAAGGTGTCCAGACACCTCTGTATCTGAATGCCGAGACCAA
ACAACTGCCAGTAGCTAAGATCCTCTATCGCATTGTTATCGATCAGGAAAGTCGCAAGGGAGTA
>dme_circ_0004626_junction_seq
TATCGATCAGGAAAGTCGCAAGGGAGTAGCTATGATATCTCAACTCTACCCAATTC
>dme_circ_0004627
GCTAAGATCCTCTATCGCATTGTTATCGATCAGGAAAGTCGCAAGGGAGTAGTTCTAATTGTGGTCAACA
ATCCACATATAACATTGACGGAGACCCTCGAGGATTATGTGATCTGTGAGGATGTGGGAGCGCAAATCGA
TTGGTTGGACTGGGACAAAGCAAACCTGCAGAAGGGCTACTCCTATGCGTGCTCTGTAGAAGATTTCATC
GAAGTGGTTAAGGATTTGCCCACAGATCAACTGCAAACAACAGGAATTCTTGGTTTGACTGATCAAATCT
TGGACAATGAAGTGTGCAGCTTTAAAGTGAACGGTGATCTTAAGGATCCGGCTCCTTTATTTGTAGTCAG
GAGTGAATTGGGTCATGCGAGATATTTGGAACCGAATCAAGAAGGAGTAGTTCAGCTAAAACATGGAGAA
GCCCTTGAGTTCCACTGCATCAATTCTTTCAGTGGACCCTTCTCTGGTTATACCTTCGTCAATTCAACAT
GCTGGCAACAGCAGAGCATCCTGGCAATGGGCAGTTTGTATCAGCTGCAGGACTTTGTCTGCACTTCCTG
GCCAACATACACTGCTCGTCGCTCTGGTCGTCCCTGCAATGGTG
>dme_circ_0004627_junction_seq
CGTCGCTCTGGTCGTCCCTGCAATGGTGGCTAAGATCCTCTATCGCATTGTTATCG
>dme_circ_0004628
GATCCTCTATCGCATTGTTATCGATCAGGAAAGTCGCAAGGGAGTAGTTCTAATTGTGGTCAACAATCCA
CATATAACATTGACGGAGACCCTCGAGGATTATGTGATCTGTGAGGATGTGGGAGCGCAAATCGATTGGT
TGGACTGGGACAAAGCAAACCTGCAGAAGGGCTACTCCTATGCGTGCTCTGTAGAAGATTTCATCGAAGT
GGTTAAGGATTTGCCCACAGATCAACTGCAAACAACAGGAATTCTTGGTTTGACTGATCAAATCTTGGAC
AATGAA
>dme_circ_0004628_junction_seq
TTTGACTGATCAAATCTTGGACAATGAAGATCCTCTATCGCATTGTTATCGATCAG
>dme_circ_0004629
GATTTCATCGAAGTGGTTAAGGATTTGCCCACAGATCAACTGCAAACAACAGGAATTCTTGGTTTGACTG
ATCAAATCTTGGACAATGAAGTGTGCAGCTTTAAAGTGAACGGTGATCTTAAGGATCCGGCTCCTTTATT
TGTA
>dme_circ_0004629_junction_seq
TCTTAAGGATCCGGCTCCTTTATTTGTAGATTTCATCGAAGTGGTTAAGGATTTGC
>dme_circ_0004630
GATTTCATCGAAGTGGTTAAGGATTTGCCCACAGATCAACTGCAAACAACAGGAATTCTTGGTTTGACTG
ATCAAATCTTGGACAATGAAGTGTGCAGCTTTAAAGTGAACGGTGATCTTAAGGATCCGGCTCCTTTATT
TGTAGTCAGGAGTGAATTGGGTCATGCGAGATATTTGGAACCGAATCAAGAAGGAGTAGTTCAGCTAAAA
CATGGAGAAGCCCTTGAGTTCCACTGCATCAATTCTTTCAGTGGACCCTTCTCTG
>dme_circ_0004630_junction_seq
ATCAATTCTTTCAGTGGACCCTTCTCTGGATTTCATCGAAGTGGTTAAGGATTTGC
>dme_circ_0004631

GATATTTGGAACCGAATCAAGAAGGAGTAGTTCAGCTAAAACATGGAGAAGCCCTTGAGTTCCACTGCAT
CAATTCTTTCAGTGGACCCTTCTCTGGTTATACCTTCGTCAATTCAACATGCTGGCAACAGCAGAGCATC
CTGGCAATGGGCAGTTT
>dme_circ_0004631_junction_seq
AGCAGAGCATCCTGGCAATGGGCAGTTTGATATTTGGAACCGAATCAAGAAGGAGT
>dme_circ_0004632
ATAGACATTACCAGTGGTGTTGAAGTATGGTGAGGCATCCATGCCCAAAATCTCGGAAACCGTGATATTC
TGTTGAACTTGGGTGTACAAAGTGTTCACATCCTTGCCTCCGTAAAAGTCGCCGGGTATAAAGGATGGCC
TGGATACAGATCGCTGATATTGGTCACTTCCGGGGTACAAAACATGATGCACATAGCGGGTGACTTCGCT
CAGTTCATCGTGACACACGTCATAGGTCTGCAGGAAGTCGTCGCTCGAGGAGGATGACAGCTGAAAGCCC
ACTTCAAGCAGATCAG
>dme_circ_0004632_junction_seq
AGCTGAAAGCCCACTTCAAGCAGATCAGATAGACATTACCAGTGGTGTTGAAGTAT
>dme_circ_0004633
ACATTACCAGTGGTGTTGAAGTATGGTGAGGCATCCATGCCCAAAATCTCGGAAACCGTGATATTCTGTT
GAACTTGGGTGTACAAAGTGTTCACATCCTTGCCTCCGTAAAAGTCGCCGGGTATAAAGGATGGCCTGGA
TACAGATCGCTGATATTGGTCACTTCCGGGGTACAAAACATGATGCACATAGCGGGTGACTTCGCTCAGT
TCATCGTGACACACGTCATAGG
>dme_circ_0004633_junction_seq
CTCAGTTCATCGTGACACACGTCATAGGACATTACCAGTGGTGTTGAAGTATGGTG
>dme_circ_0004634
TTTCCGCCATTAAAGGTCTGCCACTGAGGTGCCGCATTCACAAAGAAGAAGCTCGCCTTTTGAGCGGCCC
CAAAGACAAAGTCGGTCTTTGCAGAAAGGTGACCCCGAGCCAGATAGACATTACCAGTGGTGTTGAAGTA
TGGTGAGGCATCCATGCCCAAAATCTCGGAAACCGTGATATTCTGTTGAACTTGGGTGTACAAAGTGTTC
ACATCCTTGCCTCCGTAAAAGTCGCCGGGTATAAAGGATGGCCTGGATACAGATCGCTGATATTGGTCAC
TTCCGGGG
>dme_circ_0004634_junction_seq
AGATCGCTGATATTGGTCACTTCCGGGGTTTCCGCCATTAAAGGTCTGCCACTGAG
>dme_circ_0004635
CAATCCACTGTGATGTTTTCATCCGCCACAAACTTTCTCACACTGTCCTCGATTCGTTCCCAGTTTCCGC
CATTAAAGGTCTGCCACTGAGGTGCCGCATTCACAAAGAAGAAGCTCGCCTTTTGAGCGGCCCCAAAGAC
AAAGTCGGTCTTTGCAGAAAGGTGACCCCGAGCCAGATAGACATTACCAGTGGTGTTGAAGTATGGTGAG
GCATCCATGCCCAAAATCTCGGAAACCGTGATATTCTGTTGAACTTGGGTGTACAAAGTGTTCACATCCT
TGCCTCCGTAAAAG
>dme_circ_0004635_junction_seq
AGTGTTCACATCCTTGCCTCCGTAAAAGCAATCCACTGTGATGTTTTCATCCGCCA
>dme_circ_0004636
TTTCCGCCATTAAAGGTCTGCCACTGAGGTGCCGCATTCACAAAGAAGAAGCTCGCCTTTTGAGCGGCCC
CAAAGACAAAGTCGGTCTTTGCAGAAAGGTGACCCCGAGCCAGATAGACATTACCAGTGGTGTTGAAGTA
TGGTGAGGCATCCATGCCCAAAATCTCGGAAACCGTGATATTCTGTTGAACTTGGGTG
>dme_circ_0004636_junction_seq
AACCGTGATATTCTGTTGAACTTGGGTGTTTCCGCCATTAAAGGTCTGCCACTGAG
>dme_circ_0004637
GTGACGAAAGCAATCCGTTTACCGTTGTCTTTGCCTCTTCATCCTCCCCGAGAACGGCGGAGAACGCGAA

AAATATCATGAAATGTAAAAGCTGGTCCAACAAAATTAACAACTACGTTGTGCGCAAAATAAAGTCTATC
AAG
>dme_circ_0004637_junction_seq
CGTTGTGCGCAAAATAAAGTCTATCAAGGTGACGAAAGCAATCCGTTTACCGTTGT
>dme_circ_0004638
GTCGCGATGTGTGGAGAACGCCTCCCTTTCGTTAGCCACCCACTCGGGCTCCTCGTCTTCCGCGCCCGTC
GATCGGTACATGTCGCCAATGTACTCGTCCACCGAGATCTTGCCATCGTGGTCCTTGTCCAGATCGGTAA
TGGTCTCGCGCAGAACCACACCCTTCATGCTGGGATGGTCCTCGGGGTGCAGGAAGGCGG
>dme_circ_0004638_junction_seq
GGATGGTCCTCGGGGTGCAGGAAGGCGGGTCGCGATGTGTGGAGAACGCCTCCCTT
>dme_circ_0004639
GATGGATGAAAGGCCAATTTTAGGTGGCGGTGGACGGGGCGGTACAACTTTTCCTTTGCTTTGTTGGGCT
GGAATGGCTTTAAGAAGCTCCACTTCATCATCAGAGCTGTCGAATGGGTTGGCACCTTTAGATGAGTCAT
CATCTTTAAAGGGATTTAGATCCTCTGGATA
>dme_circ_0004639_junction_seq
CTTTAAAGGGATTTAGATCCTCTGGATAGATGGATGAAAGGCCAATTTTAGGTGGC
>dme_circ_0004640
TTTCGGGTTTGTGGGTGTGTTACATACTAATAAAAAGCATAGAATTCATAGAATTGTGAGACAGTGTCCG
GAGGCGAGATCTACTCCAATTTGGCCAGGATGTCGGACTCGCGGAAGAGGAACAGCTCCTTCTGGTCACC
CTCTAGGTTCACCTTGGTGCCACCGAATTCGGGCAGCAGAACACGATCGCCCTCCTTCACGCCAATGGGA
ATGTGGTTGCCAGTGGACTGCGGATGAAGTGGGATGTTCAGTGATTAGATGACCATCTCTGCATTGATAA
TGGCGAAATACTCACGGCATTACGGGTGCCAGGGCCTACGGCCAGAACGGTGCCCTCAAGTACTTTGCCC
ACCGCTTTCTCCGGCAAAACAATGCCTCCTTTCGTCTTGGTCAGCGCCTCGGCACGCTGGATTAGGATGC
GG
>dme_circ_0004640_junction_seq
CGCCTCGGCACGCTGGATTAGGATGCGGTTTCGGGTTTGTGGGTGTGTTACATACT
>dme_circ_0004641
GCTGGTTACCGCCCTGTGCAACGAGCACCAGATCCCCCTTATCCGCGTGGACTCGCACAAGAAGCTGGGC
GAATGGTCCGGTCTGTGCAAGATCGACAAGGAGGGCAAGCCCCGCAAGGTGTGCGGCTGCTCCGTGGTCG
TGATCAAGGATTTCGGTGAGGAGACACCCGCTTTGGAC
>dme_circ_0004641_junction_seq
TTTCGGTGAGGAGACACCCGCTTTGGACGCTGGTTACCGCCCTGTGCAACGAGCAC
>dme_circ_0004642
GGCTCAAGAAGAAGCTGCAGCAAAGGAAGAGCAAGGAGGCGGCGGCTTTAAAGGGAAAAGAGAAGGAGCA
TGAGAAGGAGAAGGACAAGCGGCGCTCGGACAAGAAGTCCAAGCGGGGCGCAATCTCCAGCGGCGATGAG
GA
>dme_circ_0004642_junction_seq
GGGGCGCAATCTCCAGCGGCGATGAGGAGGCTCAAGAAGAAGCTGCAGCAAAGGAA
>dme_circ_0004643
GCATTTGAAGCGTTTAGCCGCCCCCAAGGCATGGATGTTGGACAAGCTGGGAGGCGTCTTCGCCCCGCGT
CCCTCGACCGGTCCACACAAGCTCCGTGAGTCGCTGCCCCTGCTGATCTTCTTGAGAAACCGCTTGAAGT
ACGCCCTCAATGGCGCCGAGGTCACCAAGATC
>dme_circ_0004643_junction_seq
CCTCAATGGCGCCGAGGTCACCAAGATCGCATTTGAAGCGTTTAGCCGCCCCCAAG

>dme_circ_0004644
GAACTCCCTTGGCTCCCAGCTGGGTCTTCTTGACCTTGCACAACTTGTACTGCAAAAGAAGCAAAGAAAA
TGTGGGTTAATTTTAAGACGTTTTATTTCTACACAATTGCACTACCACTTTCATTTAATTGCATTGTAAT
TGCACGTAAAAGTTACTCAGACGACTGTGGAATGCCGAATCAGAGACCGTCGTATCAAAAATTGCTTTGG
GCTTCGATTTGAACCAATAGACACATGGCAACAAGCGGAAATCGGTCTTCGACGTAGCCCACAGAATTAC
AATATAATGCTTTATACTCCTTGGTTTCTTTTGTAATTGCACGTAAAAGTTACTGAATCAACTGGGATTT
GTCGAATCAGAGACCGTCGTATCAAAAATTGCTTTGGGCTTCGTTTTGAACCAATCGGCGCACTATTCTA
CACGCAAAACGGTCTTCGACAAAGCCCTTTCGATATTTAAGTTAATTACTTTCGGTAATTAGCATAAAGA
TTGATTTTGTATTCCAAAATAGGTGAACTATTTATGTTTTAAAGATGAAGTTAGATGAATCTAATTCAAG
TGCTCTTGCATGAAAAGAATTCCTAGTTGAGGATGAAACCTCAGTTAATAATTTTAGATTTTAACTCCAT
GGTTGGAAAGCGGGAACCTCTGGAAGCACAGACTAGATAACTCACCTTGGCCTCCTCGGCGGAGATGCGG
TGGATGACGAAGCGTCCCTTCACGTCGTAGACCAGACGGAAGAACTCACCGGTCTTCTCGAGGGTGATGA
CATCTAAAAAACGGGCAATGGAGCCCATTAGTATGGAGCACATTTGAAGCACACTCTTGCCGAGCACACA
ACTCACCCATGTAGCCAGCGGGATAGGTGGGGTCGGTGCGGACCTTTCCATCGACCTTAACCAGGCGCTG
CATGACGATCTTGGTGACCTCGGCGCCATTGAGGGCGTACTTCAAGCGG
>dme_circ_0004644_junction_seq
GGCGCCATTGAGGGCGTACTTCAAGCGGGAACTCCCTTGGCTCCCAGCTGGGTCTT
>dme_circ_0004645
GATGTCATCACCCTCGAGAAGACCGGTGAGTTCTTCCGTCTGGTCTACGACGTGAAGGGACGCTTCGTCA
TCCACCGCATCTCCGCCGAGGAGGCCAAGGTGAGTTATCTAGTCTGTGCTTCCAGAGGTTCCCGCTTTCC
AACCATGGAGTTAAAATCTAAAATTATTAACTGAGGTTTCATCCTCAACTAGGAATTCTTTTCATGCAAG
AGCACTTGAATTAGATTCATCTAACTTCATCTTTAAAACATAAATAGTTCACCTATTTTGGAATACAAAA
TCAATCTTTATGCTAATTACCGAAAGTAATTAACTTAAATATCGAAAGGGCTTTGTCGAAGACCGTTTTG
CGTGTAGAATAGTGCGCCGATTGGTTCAAAACGAAGCCCAAAGCAATTTTTGATACGACGGTCTCTGATT
CGACAAATCCCAGTTGATTCAGTAACTTTTACGTGCAATTACAAAAGAAACCAAGGAGTATAAAGCATTA
TATTGTAATTCTGTGGGCTACGTCGAAGACCGATTTCCGCTTGTTGCCATGTGTCTATTGGTTCAAATCG
AAGCCCAAAGCAATTTTTGATACGACGGTCTCTGATTCGGCATTCCACAGTCGTCTGAGTAACTTTTACG
TGCAATTACAATGCAATTAAATGAAAGTGGTAGTGCAATTGTGTAGAAATAAAACGTCTTAAAATTAACC
CACATTTTCTTTGCTTCTTTTGCAGTACAAGTTGTGCAAGGTCAAGAAGACCCAGCTGGGAGCCAAGGGA
GTTCCTTTCCTG
>dme_circ_0004645_junction_seq
GCTGGGAGCCAAGGGAGTTCCTTTCCTGGATGTCATCACCCTCGAGAAGACCGGTG
>dme_circ_0004646
GATCACCGACTACATTAAGTTCGATTCTGGTAAGCATCCGCTATCGTAGATCATCGTGTTCATTGGCTGG
TTCGAAGACCACAAAACGCTCCTCGAAAGTTGCGTGGTTCAAAAACAATAAGCCAAAAGCAATTTTTGTC
ACGACGGTCTCTGATGCGGCATTCCAAAGTCGCTTCAGTAACTTTTACGTGCAATGACAGTCGCCCAATA
TTATTAACCAAGTCGTATGATTCCAACTAACTGGTATATTTCTCCGTCTCCACAGGCAACCTCTGCATGA
TCACCGGAGGCAGGAATTTGGGACGTGTCGGCACCGTTGTCAACCGTGAGCGTCATCCCGGTTCCTTCGA
CATTGTGCACATTAAGGACTCGCAAG
>dme_circ_0004646_junction_seq
GACATTGTGCACATTAAGGACTCGCAAGGATCACCGACTACATTAAGTTCGATTCT
>dme_circ_0004647
TCCTTAAACTCCTGCGCCTTAGTGGGTCTTGGCGGCCAGACGCTTGTCGCGCTCCTCCGCAATGCTCAGC
TTGACACCCTTGCCCTTAGGCAGGGAGATGTAGGGCTTGTTGCCCTTGCCAATGATGAACACGTTGGTCA

AACGGGTGGCGAACACATGACCTTGCGAGTCCTTAATG
>dme_circ_0004647_junction_seq
GAACACATGACCTTGCGAGTCCTTAATGTCCTTAAACTCCTGCGCCTTAGTGGGTC
>dme_circ_0004648
GCTTTGCGGCAGCCGGCGGTTCGTCCGGCTTCAGTCCGACGGAATTCATGTCTCTTAGTGAGGACATTGG
CCACAACATCACTGCCATCCACAGCAGCACCAAGCAGTTGGAGAAGCAGCTGAAACTGATTGGCACATCC
AAGGAGCAGCCAAATCTGCGGGAGAAGGTGCACACCATCAACACCAAGTGCAATGCCCGGGTGCAGACCA
CCAGTCAGGACTTGCAGAGGCTACAGGCCGTTGTGCGGCACGGAGATCGCCAGCAGAAGCTGCAGCTGGA
GAAGCTCACCCGGGA
>dme_circ_0004648_junction_seq
AGCTGCAGCTGGAGAAGCTCACCCGGGAGCTTTGCGGCAGCCGGCGGTTCGTCCGG
>dme_circ_0004649
GAAGGCCAAGCTACCAGCAGCGATACAGAAAAAGAAGAACCAAAAGCAAGCTGCTTTCACCCGTCGATCA
AGTGATTACCCACATGCTTAACAATCTTCTCCAAATTCTATGTTTTTGTTTTATTAATTTACAGATGCGC
CAATTCAGGCGAAAAAGGCCAAGTTCAATGAGACGCAGAAGATCAAGTCCGTTATTTCGAAGTCGGTCAA
CAAGAGCGTGGAGAGCGAACTGCGCTCCCGCGCCCACGAGGGCTACATCCAGTTGAGCAAGGCCCAGGAG
GCG
>dme_circ_0004649_junction_seq
CATCCAGTTGAGCAAGGCCCAGGAGGCGGAAGGCCAAGCTACCAGCAGCGATACAG
>dme_circ_0004650
GATTGGCGCAAATACTTCGATTTCGTGGTAACATACGCCAAGAAACCAGGGTTCTTTACCATGCAGCGTC
CGTTCATGCAGGTGGATGTGGAAAAACTGGCAGAACTGCCGCAAGAGGCAGACAACATAAAGGCGGGCGA
GG
>dme_circ_0004650_junction_seq
GAGGCAGACAACATAAAGGCGGGCGAGGGATTGGCGCAAATACTTCGATTTCGTGG
>dme_circ_0004651
ATGAAAAATGATTTTGTGAGGACTACCTAAGTGATAGTGAATTTCGGCTTAATTTTAAATTTAGATCACG
CTTCGGACAAATGAGATGTGCTAGTTGATAAGATAAGATGCTGGCGCTGGGCAGAGCAGCCACCGTGGGC
GCCTTTTCCGCCCCTGCCCTTTCACTTCGACTGGCCacgaaaacgaaaacgaaaacaaaaacaaacg
>dme_circ_0004651_junction_seq
aaaacgaaaacgaaaacaaaaacaaacgATGAAAAATGATTTTGTGAGGACTACCT
>dme_circ_0004652
ATATTGCATTACCTCGACGTTGTTGTGCTTGTAGAACTTCTTCATGCAGTTCTCATACATCTGAATAGCG
CTGATGTACTGCTTCTGTTCGACATAAACGTGCGCAATGTTCAGCCACACGTCGCAAAAGTCGGCGGTGG
CTTCGCGCACCTGGGCGAAGATATCGCGCGCCTCGATCACGCATCCCTTATGTGCCAGCACGGCACCGAT
TCCATTGGTGGCCCAAATATTTCGGGGATCATTGCGAAGAACCTGGAAAGCGGATGCATTTCGTTACAAA
TTGAGCTATAATTGGTCTATTTTAATCTGTCCCACTTGTTTGAATATGGCCAAAGCCTTCTCCTGGTGCT
TCCTCTCCTTCTCCTTATCTCTACTCGGCTGATGCAGTGTTTGCAACGAAAAGTTTCCCAGAGCAATCAG
TGAGTAGGCGTCGGTGGAAGTAGACGGATTCTTGAGGATCGTTTCGAAATTCTTCTGTCCCAAAGCGAAT
TGCATCTTGGCCAAATGGAGATTTCCCAGCAGTGATCTCGCATCTGGATTGTCGTTGTTGATGTTCAGCG
CGTCCTTGAAGAAATCGGAAGCCACGAAAATGAGACCCTTGTCCCTTGCCATGCAACCCAGCCGTAAGTA
GCAGTCAATATAGTTGGGATGCTCCTTGAGGATCTCCTTGTACAGCTTGTCGGCCACATCGTAGCTACTC
ATTGCCTCGTTCAGACGGGCCAGATTGTACTTCATGGTGACCTGAATGGACTCGTAGTATTTTACATCCT
TGTCCATCTCAGAGGTTGCATGCTTCAGGGCCGACTCCAAAGTGAGCTTTGCCATCTTCAAGTTGCCCAG

GCGGTAG
>dme_circ_0004652_junction_seq
TGCCATCTTCAAGTTGCCCAGGCGGTAGATATTGCATTACCTCGACGTTGTTGTGC
>dme_circ_0004653
CAGGAATCACCAACTGTAGCCGTGGCCGAGAGGAACTGCGGCTTGGTATAGTGGTTCAGCTGGGGATTCT
GCTGATGCACATCCCGACCCATGGTGGCCGACTTCATGTGCAGCACAAATGAGGCACTGTCGTCGATGGC
TCCCTGTGAAACCAGGGCCTTGTTGTTATTCAGCTTCTTCTCACTGATCATTGTGGCGGTGGGCACTACC
AACTGTTGGAACTTCTGCTGGGGATCGATGGCGCCCAGGTCATTGACAGGCATCGGGCACATTCCATAGT
GATGGG
>dme_circ_0004653_junction_seq
GGCATCGGGCACATTCCATAGTGATGGGCAGGAATCACCAACTGTAGCCGTGGCCG
>dme_circ_0004654
ATCCAGATCCACTTCTGCTCCTGCTGCGCTTGCGGCTAGCCTCGCGATCACTGCCGCTTCTAGAGGCACT
GCCACTTCGGCTACCACTGCGATTTCGACTCCTGCTTCTACTTCTGCTCTTGCTGCGCTTGCCGTCAGAC
TCGGATTCGCTGGTGGAGATCGTCTCCTTGGAAAGCACCTTGAGGCGCTGCTTGGCAGAAAGCTTCTCCT
TGGACTCTTTAACCGGCTTCGGCTTCTTGCCCTTCTTCTTTCCCGACTTGCCACTGGCACCTGGCTCTTC
CTCGCTGTCGCTCTCGTACTTCTCCGTTTTGCGCTTGCGACCGCCACCCTTTTTGCGTTCCCCCTTGCCC
TTCTTCTTCGAGGGACGTTCTCCCTCACGACGAGCTCCATCG
>dme_circ_0004654_junction_seq
ACGTTCTCCCTCACGACGAGCTCCATCGATCCAGATCCACTTCTGCTCCTGCTGCG
>dme_circ_0004655
GATTTCAATATTTGCAACTATTCATTCATTTTCGACCAGTCTGCTAAAACAGCGTTACTCCAAGCGGATC
AGGCGCTCCAGATGCACTCCGCCATGGCCAATGCGGCTACCATGGTGGGTTAATGACGCCGAACAGAAAT
GATTTATGAGTTAATAACTACTTTTTTATCCAGCAGGCATTCTCCTTTTTCAACTACGGGATGCCCATCT
CGCAGTTCATTGTGCTCAACGTGACGCGTGAGAATCTGGTGCAGGATTCCCTTCGAGAGTTGCAGCATTA
TTCGCAGAGTGATCTTAAGAAGCCGCTGAAAATCAAGTTCCATGGCGAGGAGGCGGAGGACGCGGGTGGC
GTGCGCAAGGA
>dme_circ_0004655_junction_seq
CGGAGGACGCGGGTGGCGTGCGCAAGGAGATTTCAATATTTGCAACTATTCATTCA
>dme_circ_0004656
ATCGAAGACTTCCTTAAAATCGTCGCCCTGGTAGTCGAGCAGCGATTGCATGGAGTTGGCCTCCGGAGGG
GAAAGCTGACGGAGATCACTGAGATCCACTGGCTTGCCCAGCAGCTTTTTGAACAGAGCCAGTGGGAATG
GCAAGTTGATGATGGTGAAG
>dme_circ_0004656_junction_seq
TGGGAATGGCAAGTTGATGATGGTGAAGATCGAAGACTTCCTTAAAATCGTCGCCC
>dme_circ_0004657
GATTATACCGGCAGAACGTCAGTGGGGCTGGGAGTGATTGGATACACATGTGTTAGCGGGTAAGTGGTCG
AATGAATGGGCTTTGGCTATATCGATACGATACTATATGTCGGATTAAAAGAGCTTCGAAACGGGGGCTT
AGGTTAATTCTCCATGATCTCGCACATGTGTCAGACTGTTTTGGAGCATTTTAGTCTATTTTCGAGGGAT
TTAAAAGCTATTGGGCAGGCAAAGATCTGGTAATGTTGGGAAATATTTGTTAAGTGCGAGATCATAACTA
TGAGATGCGTaaaaaaaaGTCTTAAGTACACATTTTAAAATGCACTTAACTACTCCCTATAATTAAGACA
TAATCGCCTCTTAGTGGACTTTGTGTTCGAGTGCTTCAAAACGGGCTACCAAATGCCACTAAACGTGCCC
TGGACTCGAACATAAACCCAACGTTAACCCCATTCAATCCGTACTTAAATCAAGTGAAACTCCACCCACC
TGTCAACCGGGCCGTTGGCACCTTCAGCCCAGCAGATAACTCAACTTGGCTCAGTTCGGCTCAACTTGGT

GCAATCGCTGAATGCCAGCCAGCCCAAAACTGCAGCCGACTTGATGTGAAAATTCACACTATCCTCGCCG
GACACAAAAAAATGTAGAGTCGCGCATACGTCCCCAAAAATCGTTGGCGCTCTGGCACTTCATTAAATAG
ACGCCGTGGCGGCGCTTTTCCCCACAAAAACACAGCGCaaaaaaaaaaataacagaatgaagaaaaatcc
aaatgaaaaaaaTTCGTTGACGACCACACCCCCCACATTACTTGGGGGCTGAGCTCGGGAAACTTGCTTA
TTTTTCACACATGCGCGTCAACaaaaaaaaaaaaaaaaCAAAACCGACGAGAGGCAGAAATTTAAGCAAC
AAAATCGTGGCttttttttttttttGCTTTACTCACATTGTTTATGATGACAATTTGGCGTTGTTGTTGT
CGTCGACATGTGGCCATAAAGTTGTCGGTAAATAACAAACAGGCCTTTCCTCTCATCCAAGTTTTTCCTT
TAAAAATACCGAAACTGTCGACGACAACTTTTGGAGTATCCTTTGTAGTCTGTTGAGAGGATTCCGTCGC
TGGAAGTCCTGGGGCTAAAAGCCACTCAGTCTAAAAGTATCTCATGGGTTGCAACCACAAATTAAGCGCA
TAGGCCTCACAATTGTGACAAAGTTCGAATGGATAAAATACAAGTGAATATTTCGTTGCTTACTTTAAGA
GCTTCACTCGATGTATTTTAAAGAAATATTTTCTGATTATTAAAAAAACAATTGAACAGTACACGTTTGC
GTTGCTTAATATTCATGCGTAAATAAACATCTaaaaaaaaaGTATTGCCAAATTAAAGCTTCTGGGTAAT
GACATCGAAATTACTACCTGACATCATCATAAAAATAATCAAATATTTAACTTTGCTAAAATCTGTGTTT
CTCGTTTGCTTTTTACAAAATTTGTTTTCGGCTGAGCTCACCTTAGACGCAATCCAAACCTGCACTTGAC
TAAGTGGAATGAATTATAGCGGCGACCTTGAGGCGCTCGATTTCAGGTGGAAATAATTCCATAATTTGCC
ATGCTGATTACTTCCAGCCAAAAGCTGACAACGCAATTTGTTTTTTTGAAAATTCTTAACGTGTAATTGT
CATGTTGTCGGGCAAACAATGGACTCACGAAAAGGCAGCCCAAAAAGCGCGCTCACAGaaaaaaaaaaaa
caaacaaatcggccaaaaaccaacttgcggccccaccctaaaaagctaaaaGACGTCTATTGTTTGACTT
TTTCCAACGGCTTCGGTCGCATAAATAACCAGAAATTAGCAGGCAAATAAGTCAAGTCGCATGTAAACTC
GTTTTGGCATTGAAACACCTGCAAAGCAAGCGATTCTTTGCATATTCCATGTGCTAAATCACCTGCCCGC
CGCCAGACTGGAGGTAAAAGTTTAACTTGGCTAATTTAAATTTATATACTAGAGATCTAAGCCGAGATGC
GGCCCCCTGATTTATGCCCAACAGATTGTCACTGGCGATAGGGTAAATGAtatactatactatactatag
tataTAGGAAATGCGCATCGATGGGAATTGTATGCAAATGCCAGCCGCACATAATGATGATGATGAGCCG
CTGATAAAGATTTGGATGCTGGCATTGATGTAAGCAGAAGCAACTCGACAGGTGTAACAGCTCTTAAGAG
ATCTAACCAAGTGCCACTAGTTGAACGGATTTGTCATCCGGCTCTCCATCGGTATCAGTTCGGTTTGGTT
TACTTTTTGTAAGCTGGCTACGATTGAAGGTAAACCGATGATGACGTACTGTTGGGTCTTGTGACAAAAG
GTGCTCGATTTCGGATTAGCTTGTTTGTCGGCTTAGTGAAGCTGAACTTACCTTGATATTGAACTCGGCT
CGTTGCGGCGGAATCAATCAACCGAGTTGCGAGGTAATTGGAGCTACGCAACAGCAGGGGGACTCTGAAC
GGGGCAGGCACGTCTAAATCACTTataaaaaagaaattcatcttcataaaaacataaaacacagcgaaaa
agggaaaacaaaaaCGGGGTTACATATGAAAACCGGCAGCTCGTTTCCCCCCAACAAATGAAACGAAACA
GCGAAAACATCCAGCTGCGAGAGATAGTTTACTTGCGGTAATCACACCGAAACCAATGCCCTCTACTCTA
CCCATCGTAAACACTTGAAAAACTGTTGGAAATTTCTGTTGTACCACCGACAAGTGGAACTCGGCCTGGA
AAGGTTTTCTTTCGGCCTCAGTTGTCTTCCCTGTCCATATTTCGTTCGAAATTACATTTAATGTGTGCCT
GAGATTTCGATAGATGCGAGGCGTGAACCTGTTGAGATACTTTCAAAACTTCAATCGTTGGCTCATCCTC
AGCAGACAAAAAAACAACAGAATCGGAGAGAGAAGCATCATTTGTTAGAACCTGTCGATTTGGATTCAGT
TGGCAGCTCATCGACCCTAATCGCGTTTGAAAGTTATAGCATCTACGAAAGCTATGCCACTGTTAATTGT
CAAAGAGTTTCGACTTTGCTGGTCTAACTTTTTTCCCTCATTCGGCTTTCTTTTGGCCAAGTTGGCTAAT
TGCCGTATTAACCTTTTGGCCCCCTGCGATGATGATGACGATGATAACCAAAGTCCGGCTGCGTGCTCGA
GTTGTAACTGCGCCTCTGACTTTGACTTGGCTTATTTGCAAGTTGGATTTGATGACGGGCCCTGGTCCAG
TGAAAGTAATTAGTGGCTCGCCGGCCACTGGAAACAAGCCTTAGGGTGAAAGCCGAGCTTTTAATTGCGC
AGCTGTCGCTGCTGTTGCCGCATAATCAATCAGTTGGTTGTGTAATTGGGGTCTTATGTGTACACATGTA
GGCACCCAAAACGAAAGTGATCTGTTGGAAAATTCTGGTCGAGTCAGAGGTCAGAGAAAGGGTTGACCTT
TAACACTGGAATTATAATGGTGTGCCAGCCACTTAAAGTTAGCTACGTCTCAGGGAGTAATATATTTGAT
AATTGCATGGGCTGACGAAAAATAAGGGATATAACCCTGGAATAAATATCCCTTGGATATAATTATATAT

TCCGCGATCAGCCCCATGCGGTCATAATTCCCAACCTATCCAAGTCATCCGGAATCCCAGAACAAAAAGC
TCTCACGCTCCCTATACCTTCAAAAGAACAATCTCCCCCACCTGGTTCCCATCCTTGGCGGCGAGCAACC
CAAATCAACCCCCTGTGCACGGCATTCCGAAGTGTTTTCGAAAACTGTGCCAAAGATCTTTTACTGCGAT
CTTTCGCCTTTCCTCTCCCAAGTCGATTATATCGAGAAGTGACCCCATACCAACACACACTCACTTGCGG
ATACAACCGATCTGGATCGGCGATCAGCAGCCGCGAAAAAGAAAAGCAAGTCAAGAAATGAACATCAAGC
TGAGATGATTGTCATAATTAAGGATGCTGGATGTTGGATGTTGGCATCTGCCAGACGCATGGAGAAGAAT
GGTGCCGGGGGAGAATCTTCTGCAGCACTCGAGGCACTTGTAAAACCAACAGATAACCGTAAGAACGAGT
TATTCTTTCttttttttCTTGCCCGCTCTGCTCTGCTCTGCTTTTGATTCACAAAAGACCCGCGCACAGA
AGACGAAGCTTCCGGTGCAGTTCAGGTTTCTCCTCATGTGAGCGAGCACCCCTCCTCGACCAATTCTTCT
GGCTCGTTTGGTTCAAGATCAGCCCCGCACATCTCATAATTGCAGCTAAGCAAAATTATATAGCCCAAGT
CAAGTCGAGATCCAGATCAGCACACAGAGAGAAAAGAGCAGCATGTGTGTGGAGTGGTCTCAGCTTGCAG
CTTGTCTGTGCAAATATTTCCAGGCTATCGGTCTTTGGACTGGACGGACTCACTGTCCTCCCTTCCAGCT
GCAGTCCACAACAATTACCGGCCGACTTGCGGCAATCTCTGTGGCATACCAAGGCCTGCGAGAGGGTTAA
CGCAAGAGGGTACTACGATCAGGGGCAGCCGCGGGGCGGTTTGCCTCGCACTCGAGACAAATGTATATCA
TTCATCAAATTAAAAGTTCAACTGCATTGCAGCGTTTTGCCAGCCGGCCGGGCCTGACTTCTCAGCTCTG
ACTGCCTGTCTGCCAGCCTTGAGTTAAATTGATAAACGGGTGGCTTCGCAAGTTCTGGCAAAAGCGAATC
GGTGATTGATATCGATATATCACAGTTAACCTCATTCTTTCCATTGCACATCCTCTTTTAGGTGCCTATA
AATTTAAGACAAAGTCCTTACTTAACCTTACGATAAACAAGAATACGCTTTAATATAAATTGCTAGTTAA
AAAGCTGTACAAAAAATCCTTCTGCAAAACTAAAGTGCCTAACTGAAAAAGCAAATTTCTTTAATATCCA
CAATgattttagtttgatatttttaaagatttctcataaagaaagacttttaacttttaaagtttttaAA
TTATTTTATGCTGTTAAATAAACTTTTAAGCCAGCCCTCGTTTACTGGCAGCCAGGCTGTCATCTTGAAC
ATGCAACATGACCGCAGCTCTGAGGCAACAACTGTTGCCGCCGCCGACTGTTGCTTTATTTTACAGCCCG
TTCTGCGGCGTTTTCCCCTCTCTGTCACACCGCTATAGCCGACTCTTTCTGCTGCCAATCATTGGCCAAG
GCTTGCGCTGCTCATCTTTCGACTTCATTCACTCAAACATCAGCTGAAGTGGTTTCTCCTTAACCCTGCC
ACACTCCATCTGGGACCCTTCGTTTTCCACTTGTTGCAGCTCGCTCGAATGCAAGtttttatttttattt
ttattttGCTCGAGTCTGGCAAAAATGCTTATTAAAAGGGCTTTTCAACTCGAATGGGTTTCTTTACGGA
GCCAACTTACACTTACACTCATTCACACGTAGTTGGATTATTTTGTGGCCCTAGAACCAACAACTGGCTG
CCCTTTGATTCCCGACCACCGCTGTGTATTTGAAAATAACATTTATGAGCTCAGGAAGGCTTAAGACTTT
TTATTGGTTATTAGTTGTTGCCATTTCTGCTGCCAAGCTGTGTGTTTTGAGTCCGGGATTTATGTCTTGA
ATTTTCGGTTGCAAAGAGTTTGGAGCGGAAGGTCTTACGAAGCTTCATGTTGCAAAAGAAATCAGGGTTC
CTAATAAGCTTAACACGTGCTGGTCATCCATTTCCAGCGCCTTTGGTTTTATTTTCCCCCCTGAAAGACT
TTAATCTTTAATTGCTTTTCTGAGGGCTTTTAAGGATGTGTGTTTGTTTTGGCTTAGTTTATGTTTACCC
AAGCGATTTGGAAAGATCAAGGTAAAAAGAACAAAAAAGGCAAAGATTCGTGGATAATTTAGAGTAAGGA
GGCAGAAACGATGCACGGTAACAGATACTTTAAATCCCAAATGAGGTTCGGGAAAAAATTAAGAAAATCAT
ATAAATTTAAAAAGTCTTTAGTTTTTATGTTCAGGCAGACTAAGAGGAAAAAGCTTTTAACTTTTGTTAT
GTCTTCACTGAGCGTTGAACACAAGAATTATGCCAAATTTTGGAACCTCCCCTTTGATTACGCCCCATTA
ATCCGCAACCAGTTTTCTGCATCTACATCTTTTTGATTTCGAGGCAATCGGGCAATCCGTGGGATGATTC
ATCGTCGACAATTATCCCCAACATGGCAATTATGCAATTTTCCACCGATCCCGAGCACTTGTCGCACTTT
AATGGCCAGTTGCCAGTGGCCATACGACCGTATCCGATGATTCACACGCCTCCCAGCCATTTAGATATGT
AGCGAATTCCACATGCCTCGATGCCGATGCAGATCTACGACCAGAGCAGAAATCTTTGCACTTCAGACAA
TGGAAATCAAAAAGTCTCGACTGGTATCCAATAGCATTGGCGACCGCACATGACTGACTCAAAGATGGAG
ATGTGAGATGCGAgaagatgaagatgaagatgaagatACATACCTGGAGGCGGTCGAGCGGACTTCAAAC
GAGTGCCGCCGTTCGCATAGCTACTTCAGCTAACTGTAATTGTATCTAACAGCTACGTTTGACAAGCCGG
AGAAGCAGAACACCACAAAAAGCCATCCCAAGTTGCAATGGCAACGCGTCGCCCTGGAACTTGTCTGACT

TTTTATGGGCAACACAACGACAACAACGGCATCAACGCGTGCTGGCAGCTTCCAGGCTATAGATTCTATA
GACACCAACTCTGTACCCCCCAACTCTGAAACTCCGACAACGTTGGCGGGGCCACCCAAAAATGGTCAAC
GTAGCGCCACTCGAAAGAGCGAACAAGCCCGACAGATACAGATAGTTCGAGCAACCAGCAGATACAACTT
CCCTCCGGCCTTGGACAGCACTTTGCTGGTTTAGATACATTTGGCCATCCAGATGCATTTGGCCACGCCA
AACTTCGATTTCTGCCGTTTGCCTTTGAATTATGGGTTGGAATCAAGATGAAACCATTATTTAAGCCTTA
AAAAAGTGGGAGTGTCCTTAGAAAAAATAAACAAAGCCAAATTCTAGAGAACTTGAATGCAAAAAGTTGG
GGGAATTGCAAACTAAAGAATGATTGAAGCTATAGCTTCGCACAGAGAAAATATAAATGTTATTGGAAAT
ATTTATTATTATATATTAAGGAGTGGATTTTAGTGCAAAAATAGTTAGTTGGTTAGCATTGAATATTTAA
AATTTTTATCTCTTTGGCCAAGCTATCTTGAAAACGAGGACATTTTTCAATATCGATTCTTCCTCTGTAC
GTAAAAATTATACATGAAAATAACTAGGAACAACCCTGTGGACTTGAGGGAACTATAAAGTGGAAAACTG
AAATGGTAAAGGAGAAGGAACTCGAAAAAGGAACGGAATCGGGAGTGGAGGGGAGTCAGAGTCAGAGTGT
GAGGGATGAAGTCAGTTTGGGGAGCCAAGTCAACACGACAACACTTGGCAGGCCGACAACAGCCCGGGGG
AAATTCTGGAAGGATGGGCGACGGACTCCATCGAGCGTCAATTGAGTGGCAAACTGGAGCTGGAGGAGCT
CTACATCCTGTCCCCCGAACACTCCACTATTGTCAGAGTAAATAATAACTTGTGTGTATGGTGTCGGGGG
CCAAAATGGATCAGGATAGCCACGGAAATCCTAGCTATGTTCGCTCTTCCTGCCCCATCGATGGCAGATG
GCAAATGTAAATGCAAAGATAGTCGAACTTTAACTTCGATTCACGCTCCGGCTGATAGACTTTCATCAGT
CGGATATTTGTAACCATCGAATGCAGTCAACTCGAATCACAGAGTCAAGCACATAATGTTAAGCCCTCaa
aaaaaaaaCACAAACTTTCTGCCAGAATTTGTCAGCGGAACAGGCAGGAAAAAGAGGAAAGGACCGGGGA
AAAATGTTTTATGAAAGGGAATTTTGTATGAAAGTAACTGAAGCCAGGAACGAGGACCCAGCCAGAAACT
CTGGCAGTTAAGTGCAAATTATTGGTTCGGAATTAGTAGAGCGAGAGCAGGCGAGATGCAGATATAGAAC
AGGGGAGATACAGCCAAGCTTTTGTGGTTGCATTTGCTAATGGAAAACTGTGCTGGCCCTTTTCCATTCC
CGCGCTCGTTCGTTGCttttttttttttgtttttggtttggcccccttttttGCAGTGAACCTAAGCGTG
CCAAATAAAAGGGAAAAATTTGCATGGCCCTGCAGATAAATTTTCAACACTTCGCAACCAGTTATTTCCA
GAAAACCCCGGAGTAAACATTTTGCACCCATCCGACATTTGATTACTCAATGCGAACTGCGAACTGCGTT
TGCGTTTGCCAATGCGAATGCAGCAGGATTCGTATGTAAACACTCTTATTAAAGCGAAAGTAGCAGCTTT
TAATTCCCGGACTCGATGGAACAATATTTATGTGGAATCGACTTTATCATAGAGTGGTTCAATTTCCGAA
AAGCCCGAATGGCTAAGCGCCTTAAGAGTTGTAAATAAAATCAATTGATTTCTTATTTATAAAGCGAATT
CGAGAACTGCCACCTACTAAGCCCTTGGCAAACATGACAAAAGAAGCCGACTCAGATCCCGAGCCGGAAC
AAGTAAACAAGCCCATAAATAAATTATTTGCTTAGGAGCACTCGGCAGTTGGACGTCAGAAGTCAGCAGT
GGGAAAGAATCTGGAGAGTCGGAAGTCAGAACTAGGCCTTATTTTAGCAACTGGGGGAGGTGTGCAAata
tatatgtacatacatatataaatacccacagccccatatatatatatatTTATTTTTTCGTGTTTCCGCT
TAGTAAACACAACTGAAATGACAAAAAGGCAGCGATGTCTTGACCAACTTGAGTCTCACTCTGCCCGCCT
GGAAAAATTATCAAGGCAAACAGCGGCATAATGGAGGACCAGCCTAAGGACTAGaaaaaaaaGTGAGACG
AGCTCGGGAACTATTACGCTGTGTGTGGTCTCCTCGATGGGAATGGGCTAGGGGAATAGGGCTGGGTCAT
TGTGATAAATAAGTAAATAGCCGGAGACAGGCTCAGACCTCAGATCGATTTATGCAAAGCTACCCCCCGC
CCAGCGCTTTTAGTGACATTAAGGGGCGTGGTCCTAAATGTTTAATGGCCTGCAAATAAAAGTTATGCCA
TCCCATCCATGGCCAGGATGGCAGGCTGCGGACATTAACGAAGCGGAAGATTATATCGGGCAAAAAACAC
GCGGAGGAAATGGAAATCCGCTGATAAGCCCAACTCATTTGAGGCTTAGCGGTAATAGTAAGGGTATAAT
TAGCCCAAATCACATATGACTGGCAAAAGTAACGAACGCTGTTCTACAGTCGCCGAAGATATATACTAGA
TATGGTATTGAATTATAATTGGAGGAATACTTCCGAGCTAAGCTGATTTACTAACTTTTGCAAATTTTAA
GCATATGCAAAAGACCTAAGATACATTCAACCGCAAAAGATGCAAATTAGCAATGCTGTTTGGCACCTGA
AAAGTTTCAAAGTTAAAATTAATCACTCTTTAACGACAAGCGCACCTTTGGATTTTTAAGATTATGCATG
ATTTCTGTGGAAACAGCGATAGGTGAAACCAGAAATTTATAAAGATTAAACTTTGTGTCTTTATATATAA
ATAAAGATCATTATGTCAACGCTTCGCACAAACATCAGCAGCCAATTAACCTTAGCAATAAATTTTTATa

aaaaaaaaaTTACTTTGCTTATCAAGGGAAACGTTCCTGCGTGTTTAAATATATAGTTGCATATTTACAC
AGATTCGTGTGGAGGGTTTGCATTGGCTGCCGATCCGTAATTACGCTAAACGTTATGCGCATTTATTACC
CATTGATTTTGCATTTTAAATTTCGTTCGTGATTTTATTTGTGAGCTCCCAATGAAATAGATTGTTGTGT
CGGAAATGTGGAGCGTAAATTTATATAGTTCAAAGTTGATTACTCGACATGATTTTGTTTAAATATGCAA
ATCTGGTGCTTGATAAACTTTAATATGCAGCCGGTCAAAGAGCCAAAAACTAGTTTATGGTCAACGGttt
tttttttttCGGATGCATAATTCGCTCAAAATAGCTGACCAAATGACTTTTAGTTCGGCTCACTCATTT
TGAGCTCATAACAAATGAGAGCAAAGTGGGGGTGGCTGCGATTTTGGTTCACCCTAATAGGAGGCCCAAA
ATAGGAGGATTTGGTCAGTCTCGAATGttttttggttttttCGCAGATACTTTCGTTCGATTTTAGTATT
TGTGTGCGAGCATTTCTCTCCCTGTTTGGTTTGCCGGTTGCATTGACCCAATTTGTTTATTTATTGAAAT
TGCTTTTATGGGTGAGCCGAGACAGAGTGTCTACATCGCTTTCGTCCCCCAATAGTATCTCTTCAATCTA
CAGTCATTTTCTGGCCTCTGCACACTCGACTTCAATTATTATTTATCGATTCCAACTCAATTTGTCAAAT
ATATATACATATTTTACTCTGCTCTGCTCGGCTCTCCAGTGGGTCAGTGCTATAATTTGGCATTCGATCC
AATGCATGATTCATGGAGGCTGAGATTCCATCGGATTCTATCTAAAATGTGCTCCACATCAAATGAGGTT
AGTAATGTCCGAGCTTGCAAGACTTCGAGGACACTCGCGTGGAAATTAAATTATAAATTTACACAGATTT
ATTTTGCCAAAGTCCTCTGGGCATGATTCTGGCCGCTTGGCCAAATCTCTGACCACCCACCCACAAATCG
GTATAAATGACTTTAGTGCCTGAGGGCGTTTAATGTTGGATTTTGGATAACGTGTATGGAGTGCTATTTT
GTGGATCCAAAAACGTGCATCTCCCTTCGCCCAGCCCCTTGACATTGTCACCTCAGACTATTGTCTGTTG
TTTGTAATTGCTTAGTCTAATTTATGCCAGCGGACGGAGCAAAGTCAATGTCGTTGACGTACTGGGCAAA
ATGAAAACGAAATGGAAAAGCGACCAAAACAAAAATAAACACACAAACGAACACCGGGCACCTAGACAGC
GACATGGAAAAGCGTTTATCTAAGCAAAATGTTACCGAAGTCCGTAATTACAATTTATTGCAGGTAATTA
TGTAATTTTCGTGACGGATTTTGTTGTTTAGAGTTTCAACGAGGGGAGACAAGCTATGTGCGTGCGTACA
CAGCGAGAAATAATTGAGAATTTACATCTCAAAATGTATTGACTATTTGTTGAGCGAATATCCTAAAAAA
TAGTACTATTACATTTTAGTTAGTTGTTTAGATATACttttttttGGTTCAAAGATAAAAATTTATTGTA
AACAATACATTCATTGATTACATTTTTCGAAGATGATTATTTTCTGTGTGCGAAAGTACATTAAATTCCC
TCACCTCGCTCGATGCGTACCTACTTTAAATGAGTTTTCGGATCGGAAAACTTGGACGTCTGATTTTTAG
GAGCCCCTTGCATGTGGCATGCAACGTTTGATTGCCGTTGACCATGGCTGGAAATCTTGGCTGGAGTTGC
TCCTTGTTTTTGCTGGCTCGCAAGTGGTTTGATTTCTTGTATGCTCGGTCAGCTCGTTGTCATTTCCTGT
CCGAGGTTGGAAAATGGACATACGCCATGAAGAGATGACTGATTTTCCAGCCGTTTATTCTTACCACTTT
AAGATGTTTGGAGCTGAGACACGCACATCCTACTGGGATTAACAAGGATTGCGACAAGTCAATCGATTAA
GCAGTGTGGGAAAGAATCTTCGATAGGAGTTGGAATAAAAGACACTGCAAAAATACTTCTTGATAGCTAA
ATTTAAGTAAATTGCAATATTGTAACAAGATTTCACTTTCATAAATTTCCCATAACCAATGACGCAAAGC
TCTTCTTCACTCATCCTACCCCGTTTTTTATGTTTCTTCGCGCCAATTAAAAACCCAAAGCTATCTAAAT
CACATTAGACCCGAAGAATGAAAGAGAGAAATCCCTGTTCGAACCGCTTCCGCTGTAAATTGAAATTGAA
ATTTAATTTGCGAAGCGAGCTGGCaaaaaaaaaaaaGCGCCAGCTCATCAACGGCGGCATTAGTTTGCAC
ATATTCAAATCAGTTTTTTATTTGCCGAACAAAGGCTGCTAAAGAAACGGGGAAAATCGTTAAAATAAAA
ACAGTTGAAGAGGTGTTGGGCAAGCGGCGGAAAGACAGGAAATACGTTTGACCAACTAAACAGGGAAAAT
GCCGTGGAGAAAAACTGAAAATCATTGTGTGCCCCGTCACCACCCCCTTCTGCTGCATCATTAACATATA
TTTGAAAACGCAAAGGAATTCCGCTCTGGCCCTCAGTCGGCGGGCTTAGTGGGTCCTCATGAAAATTTGT
AAGTATGTTGGACTTGTCACTTGCACACTTCATGACCATATTTGTCACATTTGCGCCATGTTCGCGTGGC
CAAGGAGCCGGCGGGGTTACCAGCCAGGGGTCCAGCACTGGGTCCGCATCCTGTAGCTCCACTGCTCGTA
GCCCCCAATTCCTCAGCTCCCGTTCCATGGCTCTTCCCTCGACGGGCAAATCAGATGCGCAACTAACGAG
CAACAACCACGACAACGACAACGACGACGACATGGAAATTCGTTCGCTCTGCGTTTTGCCATTCCGAGTG
ACCGCTGGGTAGCATGCGGCATGATGAAGCCAAGGAAATTTAGAGATCTACACaaaaaagcaaaaaaaaa
GTAACTTAAGTATTAAAGGATAAAGGTTATGGCAAGTTTAGCCAATTGAAATACGTTTTAACAGCTCTAA

CTCTTTATTCAAAAACCCTTGTTTAAAAATTGGCTATCCTTTCATTCGACTCATTTAATATTAACAAATG
AAATGTGTGCATAATAATAGGATGTTCCACATTCGACAGCCAGTTAAGTTGAAGCATTCACAACTTGCTT
TTATTGTTCTGCCGGCTGCGACTCCAACTGCTCTCGACTGCTGATGCTGATGCTTTCTGCGGTTGCCGCA
GACGCCTTCTGGCCATATTTGTATGAATAAATGCGTATTTAAGCACATACTTACAACACTTGCCATGTAT
GGAGCCCAAAAGACAAGGGACACAGGCTGGTCCAGGAGAAAAGGAtcgggagtcgggagttgggagtcgg
gGGAATTGGGCGGTTGGGCTCCCAGACAAGGGACAGTTTCCCAGTGGCAATAGGCGAGCCCCGGCATTTT
GCATTTTCCCTTGTATGCTCCTTGCCTTTGCCCACATTTTCAGATATATACATACATATGTATATATTTT
TTATGCTTCGGCCAAAGCTCACATTTGCTTTATGGTGCCTCACCTCTGTGGCTCCTTTTGCAGCATATTA
AATTGCGGAAATATTTCACTTGCGAAAACAGTTGCACAGCCAAATTGCAGAGCAAACAATTTATTAATTA
AGACCGAACAATTTGTTAAACGCTTTTTTTACCACCGATTGGGAACCTATTCAAATGCAAAATTGCTGTT
TGAGTAATTGAATATTAAATAATTATCTAGAGTTTATCTAGTCGAATTCGGATGGGAATTAACGTAGGAA
CGCATGAAGGTAAAAAATCTAATTAATGCATAATTTTAAATGTAATCAAGTGTTTCGGGAAATGTATGGA
AAATCAATATCATCGAACCATTAAGGCATATAAGTATAAATAAATAATTAAAATTGAAATTATAAAAGAA
AATTGCTGGCAATGGGAGCaaaacaaaaaaaaagatagcgcaaaaaaTATGGATATTATGTAAAACGTGA
GTGTCCCCAGAGCGATTTTACTCGGAAAGCATTTAAATGAATATTTTAAGCAAATCATAAATTGCCGACA
TACAAACCTCTTTGCCATATTATTAACAATTTACGATGCCGCTGCAGCTGGATTAAATAATTTATGGttt
tttttttGCACATTTCTCTCGTTGCAGGCGGCCCTTTATCAACAATATGTTTGACAACAAGTTCCGGCAT
TGGAATTGAATTTAATAAAATAAATTAAGCCACCAGGCAGTCGGAGTGGGTCAAAAGCACACAGTTTGAA
TTGTTAATGAGCGGCGGCAAAAAGTGTTAAATGCTGATGACGCTGCGCCTCTGCCGACGTCACAGTCGCT
GATTCAACGGCGCTGAAAAGTCAATTAAAG
>dme_circ_0004657_junction_seq
TTCAACGGCGCTGAAAAGTCAATTAAAGGATTATACCGGCAGAACGTCAGTGGGGC
>dme_circ_0004658
GATTCGTGTGGAGGGTTTGCATTGGCTGCCGATCCGTAATTACGCTAAACGTTATGCGCATTTATTACCC
ATTGATTTTGCATTTTAAATTTCGTTCGTGATTTTATTTGTGAGCTCCCAATGAAATAGATTGTTGTGTC
GGAAATGTGGAGCGTAAATTTATATA
>dme_circ_0004658_junction_seq
TCGGAAATGTGGAGCGTAAATTTATATAGATTCGTGTGGAGGGTTTGCATTGGCTG
>dme_circ_0004659
GGCGGCCCTTTATCAACAATATGTTTGACAACAAGTTCCGGCATTGGAATTGAATTTAATAAAATAAATT
AAGCCACCAGGCAGTCGGAGTGGGTCAAAAGCACACAGTTTGAATTGTTAATGAGCGGCGGCAAAAAGTG
TTAAATGCTGATGACGCTGCGCCTCTGCCGACGTCACAGTCGCTGATTCAACGGCGCTGAAAAGTCAATT
AAAG
>dme_circ_0004659_junction_seq
TTCAACGGCGCTGAAAAGTCAATTAAAGGGCGGCCCTTTATCAACAATATGTTTGA
>dme_circ_0004660
GTTAACGTAGAAAAGGAGAGCGGCACAAACGAAGCGACGACGAGTGCAAAGAGCCAAAAGACAGTGAGCG
TGTAATGAGCAGACGACGGGCAATTACCATGTCGTTGGCTCCCCATCTTGGCCAGGCTTTTAgcctctgc
ctgtgcctgtgcct
>dme_circ_0004660_junction_seq
CTTTTAgcctctgcctgtgcctgtgcctGTTAACGTAGAAAAGGAGAGCGGCACAA
>dme_circ_0004661
GGCCAAAGTGGTTATTAGAGCGGCACAGGCCACCACCAGGATCCCGATGATCGCCTGCTTACTGGCGGCT
CCATGGGCACAGCCTAAAAGTGGTTCTGCCAAATGGGCCAGTGGCAAGTCCCTCAGAGCCGTTGGATAGG

CACAGATGACGGGCGCATACTGTCCACTGGCATTCCTGCTGACCAGCAAATGGCGCAGCCATAGTAGTCT
GCAGTCACATTCAAATGGATTCTCCGAGAGATCAAGGGTTTG
>dme_circ_0004661_junction_seq
ATGGATTCTCCGAGAGATCAAGGGTTTGGGCCAAAGTGGTTATTAGAGCGGCACAG
>dme_circ_0004662
GCCTGGTCCACTGCACTGGGCACCTGGAGCTGCTGTACGGCGCCGCTGGCGGTGCTGGCGCCACCACCAC
CACCCCGACCCCGCAGCTGAGGCGGGGGTGGTGCTTCTAGCTAAAGCTCGGTGACCGGTATCATCCGGCT
GTTGCCAGCGATGTAGGGATAGCCCCCAGCGGCAGGATGAACTCCACCGCCACCAGGAGGTGGTCGCGAC
ATGTACTCTTCATCGGAGAAGGTCTTTTGG
>dme_circ_0004662_junction_seq
GTACTCTTCATCGGAGAAGGTCTTTTGGGCCTGGTCCACTGCACTGGGCACCTGGA
>dme_circ_0004663
GAATCCATTGACACTGTAGGTGACCTCGCCGGCATAGTGCACCAAGCGGAACTCATCGCGCAGCATGATT
TTCTTGATGTGGGCGGGCGCCTTTTCGTGGCAAACGTAGTGGTGGTGCTGGGCCAATTTCTGGGTGAGCT
TCTCAAGGAAGGTCTTATCCGTGGGCTCTCCTGGTCGCAAGCACTCCTCGTCCAGTATGGAGATGATGCC
CTTGTGCTTCTCCTCGATCAGATTGCAAATCACCTTGTTGTCGAAGTACTCCACAGG
>dme_circ_0004663_junction_seq
TCACCTTGTTGTCGAAGTACTCCACAGGGAATCCATTGACACTGTAGGTGACCTCG
>dme_circ_0004664
GATAGTAATTAGTAAGCAAAAGTCACTGTGATCACTGGAATGCAAAATTGTTCCCTCCTCCGCCTTCTAG
CGGCATCCTCGTCGGGTGGCGCACATGTCAGCGAGTATTGAATGTCGTCGCTGGCTGGTCTAGCTATATC
AGCTGCCAAGGTTGTCGCTGGGAAGGAAACACCATCCGTGGCAGAAGCACTCCCAGTTTCCATCTCAGAG
CTCCCATCTGTCTCCGAGTATCTTTCGCACGCACACGCATTGCATCGCAACTCGGCATCCAGCAGCTCCG
CATCCCAGCTTCTCTGAATCCAACGCCCCAGCATCCAACATCCGTGGCAGAGTCAAGGAAATGTGCGAAA
CTTGGGCACTGGCAAATTTATAGCGACAAATGTTGACTTGAGATACGACATCGGGTGTGTCGGCAGGGAG
GGACTGACTTTAGTCggggggggggggAGCTGCCTGTTCAAATTGAATGTCAATCGATTTTAGTTTTTAAT
TTAAAAAACCATGCCTCGTCTTCGATCCCTTGTAATTTGGTCTGGCGTGAGTGCACTTGCTTAGTGGTGG
CACAAACTAGTTACGGATTATAAGAGGGCAAATGTATATTTAGCATAAGATGTAAGTTGTAAGTTTAAAA
TGGAAGTGGACTTTAATAATATCTACCATTTACAAAACTTAAGAGTACTTAACATTATTATTATTATAGT
TCATGCCCAGGCACGACTAAGGGAAACTATGCCATCACCATTGCAAAAAAGAGGTGCATCTTTGAGATAC
TTGAAGGCAAGCGCTTGATTGCAAGTAGTGGGCGTGGCAAACAGGGTCTCCAGAGGTTTTCCGCCCGAGA
GCCAACGGTAAATGACAGTGGGGGAAACACAATCGCGATGTGTGAGAAATGTTTATTCGACAAGCGAACG
AGTGGGAGCTGTGATTCTCTGGTACAGGGCATTCAGGCGTTCTCCATACGAGCCTATTCAAATAATGGTC
CGCTAATTGTTTAATTGATAACCAGCCAAATCCCACCACAAGCTTGTCTCGCATCCTCAATGCGGTCTGT
CTGCAATTTGTGCAAATTGCACGCACATAGAAACCATTCATGGCCCATTCACCACCCATCCCCCCCACCG
ACAACGCCGCCCATTAGGCACTCTGTAAACATATCAACACCTGTTATGGCGCATGGGCCTGTGCCCAGGT
AAGCCCGCTGCAAATATTGAAAATCACTGGTCTGTTCGCTCCTGCAACCTAAATAAGTTTTATTTGCTCG
ATTCGCCCAGCTCAGTTTTATGCAAAAATGTCGGCCGCTGATGTGTGCAGGTGTCCGTCCCAGTCAATCA
ATGTGGCACGTCGACAGGGGACGGCATACAGGAAGCCGGACACAGGACATTTTCATGAG
>dme_circ_0004664_junction_seq
GAAGCCGGACACAGGACATTTTCATGAGGATAGTAATTAGTAAGCAAAAGTCACTG
>dme_circ_0004665
GCTGAAGGTCCTGGCGGAGAAGGTCTTTAAGGGCAAGAAGAACAACTACGCGAGCAGTGTGTCCACTTGG
TTCCAGGAGGACCGCATCCCCAAGGAGCACATCCAGCGAGTCAACGACTTCGTGGCCAGCACCTTCGGCA

GCGAGCAGCTTAAGTACCAGTCCTTCTGCACCAA
>dme_circ_0004665_junction_seq
AGCTTAAGTACCAGTCCTTCTGCACCAAGCTGAAGGTCCTGGCGGAGAAGGTCTTT
>dme_circ_0004666
CGAGGCTATCGTGTGGCCCTTGAAGTTCTCCCGCAGAACGAGGGCGTCCCGCGCATCTTCTCGCTCCAGC
ACGGCTCCGATGGCCATTCCTGTTGGCAGGGCTGTAGGCAGATCCTTGAGGGAGTGCACCACAAAGTCAA
CGCCACCGTTGCGCAGCGCATCCTCGAGGTCGCGGGTGAACAGGCTCTTCTCGCCAATCTTAGGAAGGGA
AACG
>dme_circ_0004666_junction_seq
CTTCTCGCCAATCTTAGGAAGGGAAACGCGAGGCTATCGTGTGGCCCTTGAAGTTC
>dme_circ_0004667
AGAGCTGCGCAAGCAGCCGCATCCTCACAGATCACTGGAGGGCTGCCTTGCTGGCGCGGACTGCTGGAAT
TCGAGTCCGAACCCGAACTGTCGTTGCCATTTCTGGCACGCTTCGTGGCCGGGGGAGAATCGCAGCTGCT
GCTGGTCTCCTCCTGCAACTGGCTGGTGGCCTCCTGGGCCCCTCGTTTCCGTTGG
>dme_circ_0004667_junction_seq
GGCCTCCTGGGCCCCTCGTTTCCGTTGGAGAGCTGCGCAAGCAGCCGCATCCTCAC
>dme_circ_0004668
GAGTATATGCCCTCCAATCGTCGCACACGCTCATCGATCGAGTCCAATAGTTTGCTGATCTCGTCCAGCC
GCGCGGAACAGGAGCACAAGTCCGACCCAGATTCGATGGACACATCCAGTACCTGCGGAATGTTCGGCTG
GTTATGTGCGGCAAAGGCTACTGAACGTGTTGGTTAAAAGAAGCGCGGACTGGAGCTATGCTGTTTGAAG
TCCATGCCGTGACCGAGCACCTTCTCCGGCTGCGTCGAATGTTTACT
>dme_circ_0004668_junction_seq
CCTTCTCCGGCTGCGTCGAATGTTTACTGAGTATATGCCCTCCAATCGTCGCACAC
>dme_circ_0004669
CACCGAGTGCTGCGGCGTCGGCTGCTCCTCGTCGGCGTAGTCGAACATTTGGACCGTTTTCTGCAGGAAC
GGGCACTTGGCTGTGGCTGCGTTTCCGTTGTCCTTAGCCGGCGCCTCAAGACCGCTGCCCTGATGCATGG
CCGCAAAGGGACACTTGGAGGAAG
>dme_circ_0004669_junction_seq
ATGGCCGCAAAGGGACACTTGGAGGAAGCACCGAGTGCTGCGGCGTCGGCTGCTCC
>dme_circ_0004670
GATCTACGTGACTGGTCTTCTTTGTCTTTTTAGCTGGCGACCGAGGTGCAATCATGCCTCCATCGACCTG
GTGCTGAATGTTGTTCCTGGATCGTATAATCTTTTCTTCTGCGGGTCCTTCATCATCCTCCGCGGTGTTG
TCAGTCTTAAAAGCGTCCACAATTTCCACAACCTCATCCTCTTCTTGCAGAATCTTTCGGTTGGTGAACT
TTGCGGCTTTAGTAGGGCTTTGTTGTGATTTCTTAGGTCCTTCAACCTTCTCGGCCGTGTTTTCAATCTG
AGTCGTACCCAGAATGTCTACGTCCTCATCCTCGTCCTGCAGAATCTTTCGCTTGCTGGAGTTCGGCGCT
TCAGCACCGTCGGCATGTAGAGTCATGTGCGTTTTCAGCGACTGTTTGGTAGCCAAACTCTTCTCGCAGA
CGTGGCACTTGAAAACCTTTGGCTTGTCGATGCTGTGGACGGTCTTCATGTGCTCCTGCATCTGCTTGGC
GGTCATCAGCTCACAGTCGCAGATCTCGCACTGAAAATTATTCGAATCCGCCCTGGGAGTGGAGGCGCTG
GATCCATTGATGGCAAATGTATATTCCGAGTCGATCACGTTCTCTAACATCCTTTTCTTCATTAATGACT
CATTTAGCGCTTTAAAGGAGGCGATTTTGGTAGCCTGGGTCTG
>dme_circ_0004670_junction_seq
AGGAGGCGATTTTGGTAGCCTGGGTCTGGATCTACGTGACTGGTCTTCTTTGTCTT
>dme_circ_0004671
GACGTAGACATTCTGGGTACGACTCAGATTGAAAACACGGCCGAGAAGGTTGAAGGACCTAAGAAATCAC

AACAAAGCCCTACTAAAGCCGCAAAGTTCACCAACCGAAAGATTCTGCAAGAAGAGGATGAGG
>dme_circ_0004671_junction_seq
CGAAAGATTCTGCAAGAAGAGGATGAGGGACGTAGACATTCTGGGTACGACTCAGA
>dme_circ_0004672
GTGAGGAGCAGACGGTCAACTCACTACTAGCCTGCGCCGTGGGTGCAGAAACTGAGGCCGCTCGCATGGA
GCATGATGTCCAACCCTATATGGAATAAAAGAACTGAAACTAATCGAAAATAGCCTGTGATATTGTGCAT
TGTATTTCGAAAATAAGTTATACAAGTCGAGAG
>dme_circ_0004672_junction_seq
TTCGAAAATAAGTTATACAAGTCGAGAGGTGAGGAGCAGACGGTCAACTCACTACT
>dme_circ_0004673
GGATCCGCCACATTGAGGTAGGTGATCACGCCCGAAAGCGAGACGGTGATTAGGTTGTCTCCTTGCCACA
AGCAGGACACCTGCTGGTCGTCCACAGTGGTGCCCATCACAAACTCGCTAACCAGCTCGCGCGACTCCAC
TGTCCAAAGGCGGCACGTCTTGTCGCCGGAGCAGG
>dme_circ_0004673_junction_seq
AGGCGGCACGTCTTGTCGCCGGAGCAGGGGATCCGCCACATTGAGGTAGGTGATCA
>dme_circ_0004674
GAAATTTGTGAGACCTGCGACCGGGAGGCGAAGGAGAAGCAGTTCAGCACCTCGGAATCGGAACGCCACA
CGAACCGCGGCCTGAATCGCTATCGGGATGTGAATCCGTACGATCATTCCCGCATTGTTTTGAAGCGCGG
CAGCGTAGACTACATCAATGCGAACCTGGTTCAGGCTGGAGCGCGCCGAGCGCCAGTACATCCTGACCCA
AGGACCGCTGGTGGACACAGTGGGCCACTTCTGGCTGATGGTATGGGAGCAGAAGTCCAGAGCAGTTCTC
ATGCTCAACAAGCTGATGGAGAAGAAACAGATCAAGTGCCACCTCTACTGGCCCAACGAGATGGGAGCCG
ACAAGGCCCTGAAACTGCCCCACGTCAAACTCACCGTGGAGCTCGTCCGTCTCGAGACCTACCAGAACTT
TGTGCGGCGATGGTTCAAGATTAACCGATCTCGAAACGCAGCAGAGTCGTGAAGTGATGCAGTTCCACTA
CACCACATGGCCGGACTTTGGCATTCCCAGCTCGCCGAACGCATTCCTTAAGTTCTTGCAGCAGGTTCGC
GACTCCGGCTGCCTCAGCCGCGACGTGGGTCCAGCCGTAGTGCACTGCAGTGCCGGAATCGGGCGGTCGG
GCACCTTCTGCCTGGTGGACTGTTGCCTGGTACTTATCGACAAGTACGGAGAGTGCAATGTGTCCAAGGT
GCTCTGCGAGCTGCGCAGCTACCGCATGGGCCTCATACAGACCGCCGACCAGCTGGACTTCTCCTACCAG
GCGATCATCGAGGGCATTAAAAAGCTGCACGATCCCGGTATGTATGCGCGACCAGCGGTCGGCGTTTGGG
AGTCAAACTTGGGGGCTGCCAAGCACCGCCGTATATATTGCACATCGCTATTTATTCTGGGGGGTCACAG
AAATCTCTGGGCTTGGTTATGGTTACTAGAATTCATATTGTTTAATGGAATTATGATTACTTTTGAAAAC
TGTATTATGTTTAGTTTTGTTTTTTATTATGTGATCTAGTATCAGCTTAATGATTATTGTTTAATTGATG
CGAAAAAGAAACCATTGTTTTTGGTTTGCAGAGATTTCTGTGAAGGCCCTGATTATTTTCCGCGACTAAT
TAGAACATTTGATTTCTCTTCAGAACCTTTCTTGACGCTGAAGAACCCCTCATTAGCAACGACACAGAGA
CTCACACACTGGATGAGCTGCCGCCGCCATTGCCCCCACGCGTCCAATCGCTTAATCTGCCACTGGCCCC
CAATTCCGGCGGTATACTTTCGCTCAATATGCGCGCGGCCCAGGCTAATGGAGCTGAAAGTATCGGCAAA
GAGCTGAGCAAGGACGCCCTGAACAACTTCATTAATCAGCACGACATGATCCACGATGCCGAGGTGGCCG
ACAGTCGTCCCTTGCCGCCATTACCCGTAAGGGCTTTCAACGATTCGGACAGCGATGAGGATTACTTGCT
GgatgacgatgacgaggacgacaccgatgaggatgaggagtacgagaccATTAACGAGCACGATGCCGAT
CCAGTTAATGGACACGTGCCCGCGACAACACAGCCACATGCCGACGATGTTAATGCCAACAACGAGAAGC
CAGCGGTGCCAGTCGACGAGCAGCACAAGGCGAACGGCATCGATCCAATTCCAGGCCAGCTCCCAGCCAG
GTAAGAATCTAGTCTTAGATCTGCTCAGTATCTATTTTTCAACCGCCTAGTCAAACAAAAATCCCTCTAA
TCCCCTTCCAAATTCGTACTTTTCAGTCCCGAGAACGAGCTGAAGCGGCGAAAACGCAACGAATACCAGG
CCAGCCTGGAGCAGAAGGTCAACGACATGAAGCGGAAGCAGCGGGAGAACGAGGACAAACAGCTGGCGGC
AAAGAAACGAAGG

>dme_circ_0004674_junction_seq
CAAACAGCTGGCGGCAAAGAAACGAAGGGAAATTTGTGAGACCTGCGACCGGGAGG
>dme_circ_0004675
GAACAGGAAACGACTGACGGCAAAGGGAGTTGGCCAAGACGAGGACGCAATTAACCAGACGGGAGTGGGC
ACACCCACAGCAAAACAAGTAGGAAGTGGATTCGAAGGAGAAGGGAGCAATGCAAACTCAACAAAAATGT
TGTTGCATCTCCTCGAAGGATCAACATCTGTTGCCAAGGACACTGTCACACAAAGGAGAACCCTCATCAG
TTGCATGAAATAATTCCGTTTAAATTAAGCGCCCCCCTGACAAAAAGGAGCAGCATTTCACGCGATTCCC
GTCCCCGCGTAATGTTTGCTCACCCCTGTCCTGCTCCAGCTGGAAACTACCACTCAGGACTGCTGCATCC
ACTGCTCCTGCTCCTCTTTCTGCTCGCTTTTAGCAACTTCCGGCCGACTCATGGAGAGGTCTTCACGCTG
GGTTACCTCACGGCCTCGCAACGGCGGCCGGGAAACCTGGACTACAACCGACCCGGTCTCACCATATCCG
GCGCCATCTCGCTGGCGGTGGAGGAGGTGAATGCCGGACGACTCAGGGATCGGGGTCATTCCCTTCAGTT
CGTCGTGGCCGAAACTTACGGTGAGGAGGTGGTCAGCATCCGTCAAACGGCGGCACTGTGGACGCAGCAG
GTGGCCGCCTATATTGGCCCCCAGGAAACTTGCGTACACGAAGGTCGCATGGCGGCCGCCTTTAACCTGC
CCATGATATCTTATGTACTGCACCCATCGGGATCCCTCGAACAAGGCTGATTTTCCCACCTTTGCCAGGA
CACGTCCACCGGACACGCAGATCTCCAAGTCCGTAGTGGCCCTTTTGCTGGCGTTTAACTGGACGCAAGT
CAGCTTCCTTTACTTGGACGACGCCAGTGGTCAGTATCAGCCCGTGGCGGAGACCATCCTAAGCACACTG
ACTGATGCCGGAGTCAGTATCCGGGACATTCGCACTTGGAATACCATCTACCATCACGGATTTATGGACA
ATCCATTTGAGGCGCTGGTGGAGCAGACCTATGCCAACACGAGGA
>dme_circ_0004675_junction_seq
GTGGAGCAGACCTATGCCAACACGAGGAGAACAGGAAACGACTGACGGCAAAGGGA
>dme_circ_0004676
GCAGCCGACCTTTGGATTCGAACCGAACATGGACATGGACAACATGTTCCCGCGCTCGCACCTGGGCAGG
ATGCACGATCCCTTCGCCGGCTTCGGCGACTCACGTAAGCGGAGCAGTTTGCACGGTCCCAGTGGTCAAG
TCCATGCTGATGATGACTTTCCATCCTATTTCGACGATCCAGACTTTGGTTTCCCCCGCTTCTCAACGAT
GGGCCGCCGAGGTCGAATGGCCGGTGGGGCCAACACTCACATGGACCACGATGATGACTTCTTTAATCGG
CTGCCCTCGGAGTTCCGCCAGTATATCCCAGATGGTTTCGGAGGCAGACGTGGTACCGGAGCTGCTCCTG
CACCCGGACAAGTTCCACAGCAGCAACAGCCGCAGCCACAGCCGCAGTACACCCAACAGCAACCCCAGAC
GCACTTCCAGTTCGGTGTGCCGCCCCAGCAACAGCCAGTCTATGCCGGACCAACCCAGCAGCAGGTGCCG
CAGACGCCTTCAAAGCGCCTTTGCGATGCTGCCATCCAGACGGAGGATCCTGCCGGTCGCTCGGAGGTTG
ATTGTGCCCCGCCGACTAACTTGAACCAGCACGGACTGCGAAACACTGTCGACATGGGCGTGAAGAGTGT
CGCCGAGCAGGATCAGGGACTGCGATCCCACTCCGCCCCTCCGCcagagcagcagcagcagaatctgcag
tatcaccagcagcagcaacagcatcagcaaccgggaGTACATAGCCAACCGTTTGGCACCCAGACAAGTC
CGCCCGTTCAGGGTCAGCAGTTCAAGACCTACTATGCGCCGCACCAGCAGACGCATCCCCAGCAAAAgca
gcagaccccgccgccagctccacagaccccgggtggcacctatgtgcgcaccattcccatctttgttgag
ggtcgctccgagcccatcatcaatgccccacaaggagataccgaaccagaacgctccgcccagtgctcagg
cccaagctcaggcacaggcatatgcgcaggcgcagccacaggcacacgctgctccacagcagcacaacag
accgacgccactaaacacccaggcgcaacctgagcagcaggtgccagttgagggagccgctggattgccg
ccacagacaccacatacccctcaactctattaacaaaatccaggacattcagcgcgacgtactggagctca
tgggcaaggtggagCAGTTCAAGGGAACGCGCGAGGAGAAGGAGTACGCCTACCTGGACGAGATGCTGAC
CCGCAACCTGCTTAAGCTAGACACCATCGACACAAACGGCAAGGAcagcattcgtctggcccgaaaggag
gctatcaagatgcattcaggcttcgataaatgtgctggaggccaaggccgaggagaatgccagagcggca
tcaggagcagcacctgcacctagcgcaacagctccagcggttgacacaggtgcagttgctgcctcggaag
cggccggacaaaatgcggagccagtgactccacagccacaggatgctacaaagatgcaggaacccatccc
tctaccagcaccaccaaatgctgctgccgtcgagggcgcagctgcttcggaagccaccgccgcggaggcg

acacagtccgccgttcagtcggagacaaccacaacgacgtcggaatGATGAACACAGCGCCTCTACAAGG
CGACCGTTTCGAAGCAGCTACGCAAATTAACCAAATTTAAGTGACAACAGGCGGAGGATAGAGAAGAGTT
CCGATCCCGACTCATCAACGGACAGCGACAGACTTGATTTTACTGAATTCAAA
>dme_circ_0004676_junction_seq
CGACAGACTTGATTTTACTGAATTCAAAGCAGCCGACCTTTGGATTCGAACCGAAC
>dme_circ_0004677
gatatttagcaaaagggcgtcccagctgcttggcacttgggtttccctagttgatgatctcgacatagtt
tgccggaaacagtccataggttccatcagggcccagtccctgccaccatccctcgtcgatttgatcgata
tgcgtaatgacatcgcctggatcgaaagtgatctctgtctcgtcggctgcctg
>dme_circ_0004677_junction_seq
aagtgatctctgtctcgtcggctgcctggatatttagcaaaagggcgtcccagctg
>dme_circ_0004678
GGCGCATCCTCCAGCCTGGTCGTCAAATACGCCGACACGGAGAAGGAGCGACAAATAAGGCGCATGCAGC
AAATGGCCGGTCACATGAACCTGCTGAATCCGTTCGTCTTTAATCAGTTCAGTCCGTACGGCGCCTACGC
CCAGGTAAGTCCGCTTCCCCATTACCGCGGCAATCATCCACCTAAATATAAAGCAGCTTCCCTCTCCTCC
GGAATTGCAGCAACAGCAACAGGCCGCTCTGATGGCCGCCGCTGCCACGGCACCCGGATCCGCAGCCGCC
TACATGAACCCGATGGCGGCTCTGGCAACGCAGATACCTCATGGCCTAAACGGCACCGGCCAGCCCCCCT
CGCTGCCGTCGCCGACCATGCCCAACTTCAACATGGGCGCCAACACGCCCAACGGACAGCCCGGCGGAGC
GGCCGCCGCTGCTGCTGCGGCGGCTGCCGCGGACGGCGTCTTcacgaacggcattccacaaacggcattc
cCAGGACGTAAGTAACACAGTGAAGGAACACTATCTTTTACACTTTAGATGCAAATACCATACTAAGATT
ACCATATTCAAAGAACGTATCTGATGCGAGTTTCTAAATGTTTTCTATAGCCACTGAACTGTTTGTTGCA
GATCCGCTGCACTTGACTATACCGCCACAAGGTTTGCCAAACGGCGATGCTGCTACCCTGCAACATGCCT
TCCCTGGTCTGCCACCGTTTCCAGGAGTTGG
>dme_circ_0004678_junction_seq
CTGGTCTGCCACCGTTTCCAGGAGTTGGGGCGCATCCTCCAGCCTGGTCGTCAAAT
>dme_circ_0004679
GATCAACGGTACACAGCAACAGCTCCAATTGCCAGGCAGCGGCGCTAGCGGAATCGCAGCGGCAGCCGTA
ATCACGGTAGCATCGGATTGCGGCGAAAACTGCAGCAGTAACGGCACCGAGCATCAGCAGCACTTCAACA
TTGCTACCACCACAGCAACTTCGGCGACAGAGGCAACAATGCCGGCTATGGCCAAGGAGAAAGCATCAGC
AACAGTGTCCTTGGGCGAGTCGAATTTCAGAGATATCAACCTAAAAGACTTGGCCGTGGTCGTCGAAGCG
GCATCGCGGCTGCACCACCAGCAAAACGTATGCGGCTGCGGAGCGGTATCGTCTACTGAGAACAACAACA
ACAGTCGCTACGGCAGCAGCAAGTATCTGACAAACGGTAAGCTTAATTCTGAGTTACTCAAATAGAGTAT
GTTTCAAGTTGTTCTTAAAACACTGAACATTGTCCGTTTGCAACATGTTTTTGCTTCGACGCTAACACAA
TCTCTGCTTTTCTCTGGCAGGCCACACGTCGCCTTTGGCGGCAGCGGTTGCTAGCAACAGTTCGTCGGTG
GCCACGACACCGCATTGCAGAATGTTGCACAACTGCAGTCTGCAGCAGTACCAGAATGACATAAGGCAGC
AGACGGAGATATTGGACATGTTgcggcagcagcatcagcagggctaccagtcgcagcaacagcaacaaca
gccgcagcagcagcaggaacaacagcagcagcaggagcaatcgcagcagcagcaacagctacagAATCCT
GCGCCCAGGAGGTCTCCGAATGATTTCATTTTCGGTCGTTACATAGGCGAGGGCAGCTATAGCATAGTTT
ATCTGGCCGTGGATATACACTCTCGCCGCGAGTATGCAA
>dme_circ_0004679_junction_seq
GATATACACTCTCGCCGCGAGTATGCAAGATCAACGGTACACAGCAACAGCTCCAA
>dme_circ_0004680
CTATCGGCCTGAGAAGCATCGAAACGAAGGCGTTCAACTAAAGACGACGTTCACAATGGAAAACACTTGG
AAAATCCATCCGAGCCCAATTACCAAATAACTCGACGATTGCGTAAATGCAAGAGGAGAGCCCACAGCAA

ACGTCCATAAAAAGTAGTTCTAAAATCAATGCTTTTAAGGCATCAAAGTTTGTTAATGTAAACAGTTTCA
TTAGGCCGTGATAAGTAATCGTAATCGTAATAAGCCGTGCCTCAATTAGACAGAGCTTTAAGCCCTCTAG
TTCAAACCGAAACCAAGTGCTGAACTATAATCCACCACGACTACAGGGATATCCACTGAAATTAAAATAA
ATGTAGGAAAATTAAAGTAATTAAAAATACTCTGAAATACCAGCAATAAATGAAAGCACGGTACATGAGT
TACTAATGCAAACATCTAAAGTCTAAATCACAAAGTAAGCATTAAATATTTACATAAAGCCTACTCATAC
ATATAATTTCCATAAGAATAAATTCGAGTAAGAAAAAACATATACGAGCTTAGTCAAATTTACGGCCGTG
TCCCTTGGCCATTTCAAAAATCTCCAACGAAGTTATAAAATGGGACAGACCATATAATTATACCAGCGTA
TGTCTAGCGACTATAAACTTTAAATTCCGCTTTATCGTGAGTTGCCCCTATTATACACACATATCTTGTC
TATACATATATACGAAATAAAAATCAAATCTGTACAGACATAAACGACAGCCACAAAGAGGACATTTTCC
ATTTTCCCTAGCCGGGCTCCTTTTTTTCGCTGCTCTCCTTTTTGCTTCTTTAAGTATTTTCACGTGAACG
GCGGACACTTTTATGCGTTTGAAATGAAAATGCCATGTGCCATTAACATTATCCCTGACGCGTCACTACT
TAAAATAAAAATTTCTATATTTTTTACACATTTATTTATATAAAAGGAGCGAAGACGAGAGCACCTTTTG
GTCGTCGCTCGCCTTTTATAGCCAATTTCTGGTTACCCACAGTGTGCAGCCAATTTCATGTCAAGCTGCA
GCCGCAGCAAAACCAGCGCAAAATGATAATCCCGGGGTCATAAAAAAATTTTTACTTTATATTGACTTTTA
GTGCGATAGTCAAGCCAGCGGAAAAAAGCGGAGACAAGGTGCACATGAAATGTTAATGTCTTGTCAAGAT
TGCCATTCCAAGTGGCGGGCTGGTCTCTGGATGCGGATGCTCAAGGTGAAGAAGCTCAAATTTATCACCG
CCTGCAAAATGGAATGCAAATGTTTTCGGTACCGAAAAGAGAAAGGCACGTTTCAAAATCACGGCTAAAA
TACGGCTAATACTGGAGCTGTTGATAGTTCAAAAGAACATTTAATTACCATTAAAATTATTCCATTCAGC
GTAAGAAGCAACCCCTTTAAAATTTTAAAGTGTGATTTTTGTTGGTGAAAGCACAAATGGCATTCTTGCA
GTCCTTTCTTCGCTTCACTCAGAAAGTCAGACAAAAGTTTATCCCTTTCAATATTAATTAACACTTCAAT
ACCACAGCCACGAGGATAAAGTTTCCGTCTTCTTCCTACTTCCGCTCCGTCTGCCTTTGACTTTTGGTCC
TGGCCAAACAAAAACTTTTGTGCCTAAAAATGTTACTATGAAAATGTCTGGCACTGCAGCCGCACACAAA
TTCACGCTGACACTAATTAACAATTAGGCGCATTTTGGCGGCTCTTAGGCATTTCGAATTCCAAGAAACT
GAAACAACGCCAAGTTTAGCTGTTAGCTGACTTAACTTTTGAACTACTTACAAGGCTAATAACTAATTTA
CTTCTAACTTTGGCTAAGGTCTAGAGCCTGCTTTCGGCAGGATTTTGTACAATTTCACCAGTTTGTCCCC
AGCCTATAGTCGAGTTTTACGCTTATTACGAAAGAATTTTCAGCTGACAAATAAACTCGAAAAGCTCAAA
GTCGGCGGAAGGAGGCCCAAAGGAATCGCGACACAATGACAACAAAGGCGAAAAGAGCAGAGACCATAGA
GCATAGAGCCTTTCGCCAGCTGAGTTATCTCTAGTCATATTTAAACGTCAAGAAACGAGGGGAGACTTTT
GACAATTGCCACACAATTGTCGGGGCGACGACACCAACTGTTTGCCGGCGACGAAGCCGCAGCTGAGGGA
CTCATAAAGGGAGCATTTAGCTTATTTGCTGTATAAATACTGACACCTCTGCAGCAGCAGGGATGACTTT
CAGAGTCTGGTGCCATAAAACTGGCATGTCGACGGCATCCTGCCGAGCGGCCTGCAGGCACACTTGCCCA
GGACGAGACCGGCACAATTGGCGGCGATATTGATGGGGCAGCATAACATTTGCCACATCTGATCCCAGTG
ATTGGGCATAAACATATGGCGCTCGAATTGATTGCACGCGTAGTCATTTACATTTTAATCAAAAGCTTTT
GAAGAGGACTCGTTTCTCGTTCCTTCCGCATATGTATGCCCATGCCCCTATTTTAATCTGAAGGATAGTC
AGGAACATTTTTACATTAGCCCCGTTACATGTCAAATGAGTGTCACTAATTACATATTAATTAGTTTCCT
TTTACCAGTCAGTCACTCACACTCCGaaaaaaaaaGGCACATCATCAATCAAACCGAGTGAAGGGCTCTAA
TAAAATCTGGAAAAATGCAAATGAAAAACTATTCTAGGCTTGTGCACTAAGTGCTGTTTGCTTTAATCAA
AGTAAACATATAGTATATTTCTAATTAAATTTTCATTACAAGCTGCTAGGAAAATCGGGCATATCAACGT
GAAGAGCCAGTTGGGCAGCATTAAGTATACGATTTGGTGCTCATTTACCCATTTCCTGCCTAAGTAATTA
CAATTTCAATAATTTATAGCAAACTAAACGTGGTCAGACGAGATACAAACACATAAATCTCGTATAAAGG
CAGTGAATGGGGGTTGATTTCGTTTTCAATCAGTTTTTGGCAAGCAAATATTATTCCAAGGAGGCACTCG
CCTTTCACAAAGTTTTGTAATTATAACGAGATGCTTGAAAATCCCCACAAAATAATTAATAACTTAAATA
TATTTACATACCGTTTTAAGCTATTTTGAGTAATCAGATTAAAGCATTTTGAGATATGAATTGGTCGCAT
TACGGCAGGATCCTCATTTGTAGAGCTAAGTTGCTGCTAAGAATGTTGGCTAAGCTGTGAAAATTAATGG

AGTATTTATTTGTTTGTTTAATGATTTATTAGCTGCACTGGGATAGTAAAGGGTTCACAAGATTGAGCTG
AAAGTAATCCCCCATCAAAGTCAGCTATTTCGCAGAGAAATTTCATCGTAATTCGCACACCTGATTCAAC
ACAGACCTATGCACTTCCGTCTCCACAGGCAAATGTCTTCAGGTATTCAAATTGCCCCACCTGTCCACTC
ATCTTCGCTGGGGAGAAGTAGAACTAATCCTCAGACGCTCATTAGCAGCCATTAAACGTCAATTTACATG
TCAATTGGCAACAGTGCCCAGCCAGCCAGCCATTCAAAGGAAGCCGGTCGGTACAGTaaaaaaaaCCGGA
GAAGGGAGTTCAGGGACACATGAGCTTTGAGCTTTGACTTTCGCTTTGTCAGCGACTTTGTGTTTAGTTT
TTTCCTTTATTTATCCTCTGGCGCCGCTGTCTCCACAATTTTTTGTATTATTCATAGACGCGACTGGCTT
TTTCACCTTTGGCCAGGATTTAAAATACAATTACTTTGCCCTGGAGGACGGGGAAAACCCGGAAAGATGC
AGCTGGGAACTGGAAAGTGGATGGTGGATTAAGGATGCAGGATCTGGGTTGGGAAGATAAGCAAAAACAT
AACGTGCAACAATTAGTGGCCTGTCCTTGTCGTCTTCTCCCGTTGGAGCACATGTACAAAAGTTGCCATG
TGGTTAGCTAATTTAATTTGGCTTTCTTTTTGCCGCCGCCGGTGCCGCACACCCCCTTTCACTTTGGTTG
CGGCATCAAAGACAACGACACGTTAATGACTTCTTTTCGCCGGGACAGTCATAAAAATAAAATCTAAAAA
CTGCCACACATGGCTAGCAAAGGCATCAGGAGAAACGAGCACACAGGACCGAGGATCCGGACCAGGAGCC
ACTTAACTTGTCTATATGAGCCTAATGGCCTTTAATATTATTTTCACTGCTCTTTCCTTTAGCTCAGAGA
CCGaaaaaagaataaaaaaggaaagtaaaagcaaaagcaaaagcataagAGGAGAACAGCCAAAAGGGAA
TCACTGAAAGCCCTGTGTGCATTTTATCCCCTGCCGGTCTGTGTCGTTGTGCCCCAGGTAATTGTAGAAA
ATTGCTGCAACGCCAGGCTAAACATTGTGCAAATCCTTTTGTCCTGCCTGCCTGCCGCTCACCACCCACC
ACTTCTACTTAGCTCCACCGccccccccccccTATTTTTTTGCCCTTTTTTGGCCGCAACTGTTAACAGC
CAGTTGAACTATGCCATTTCATATGTACAACAGCAGGCGCCGCAAAGTATGCTGCACTATTTTTACCTTG
TCCTGCCCTCGGTTTCAACGTGCTTTACATTTTTATTAAGCCAACCCTTCTACTGCTTCCAACATCCTTT
TGCCCTTCTCACTGCTGTTGATGTCGTTGTTGTTGTTTAGCATGCCAGGGGCGTTGTTTGGCTTATTGCA
CGTGCTTGGGCCGGCAAGGATGGTAAGGAGCAGGAAGGCCAGCGCAGGACGAAACGGAACCCAAATGCCA
TTAGGCTTAACCCGGACACAAGACAATTGCCTGCAGGCCATGTGCATGGGTCCAACGTGTCGTATTAGTT
ATTTTCTACTTCGATTGCTTTTGCCACTTTAACGCGACAGCAGCTGGGGCAGCTCGGCTGTCTGTGGCAA
GTGTTTTGCCAATTATGTGACGCTCAATTTTCGATATGATACCAAGTGCGAGTGCGAGAccttggtgtcc
ttggtgtccttggCTGGCCTTTCAGATTATACGAAAAATGTGAAAATACGAAATTGAGAGGGTGGACAGT
CGTGCGACAGCTGCCGTTTGTGCGACCTTATCCATTTACCAGTAGATATTGATTAGATTTTAGAGAATCT
CTCGCTGATCACCACCATCCAAGGCTCACCACCACCACTACTAGTCGTATATACCTAGACTCTAAGTACT
TTTAAGCCATACATTTCCTCATGCTCGCCCGTAATTTTACCAACTATTTTTTCAAGTAGTGGCTAGCGTG
TTCAATTACCTTTCAATGTCTATCCTTAACTCAGTAGCTCTGTGCGTGGCCAGCTTGTAAGTGTGTGCGA
GATGGTTTGCTTGTGTGTGCGTGTGACTGTtaaatataaatatacatataaatatacatacttatatatt
acgtatatgaataGATTATTTTAATTCTCGTAAGAGTTGTAAGCTAAACCAAAAACAAATTGTACGACAA
TTTTAACTAAATATCACCCATTTTTATCTTCTTTTCTTTCCTTTTTATGTAACACTTGTTCGTGCTCTAT
ATCAAATAAATATGATTGAAACGaaaaaaaaaaaTACATCAAAATTGGCAAAATTGGAAAACTGACAATT
CTTCTCTTATGATTTTCAAAAACTGATTTCACttttttgtgattttattcgtatgattttgatttgattc
gattttttcCATATCCAAATTTCTTCCTTCTTGATGCAACAATTTCAACAAAAAATTCGTTCCGCTTATA
AATCGAAATTTGTACTCCCTCTTCCCACCCAACaaaaaaccaaaaaaaaaaaaaaaaTACCTAACCACAT
TAATAAATGTTGTAATTGTATTTGCTCTATTGGTGCTCAAACTATCCACCCAAACACCAACGATTTCTCC
TTAACTAAATGTTGTTGCTTTTACTTTCCTTTGGGTATTTTGCGCCTGTTGAAATTTTaaaaaaattacc
aaacaaaaaacAATTACGTAAATGTGCCTGCTGCGTTCGTTACTCGTTACaaaaaaaaaTGTTGCGTAAT
GTGTAACCCTGTTGCATTTACATCTCTTTTTAAACGACACACCAACACATTATGATCGAGGATATATATT
TTTTCCGTATTCATTTTTCGGATTTCAATATGATTTGTTGCGAATTTTCTCTGGCTATGGCTTCTTGATT
GAAAACTTATCATTTTCACACACCGAATACCAAACACGATTGAACACTGCCGCTCACAATACAAAATACA
AACACATTGCCACTTGTCCATTTTGCCGTCTCTTTTGGTGCAACAATCAAATTCGTAAATCAATCGTAAT

CAATCAAAATCAATCGGATCGATCGAACCACATGACATATGAAACATGAACAAACCCGAAACCAAAACAG
AGCATTTAGATCGCAAGCTGTTCGTTGGCATGCTCAGCAAACAACAGACCGAAGACGATGTTAGACAAAT
CTTCCATCCCTTCGGCACTATAGAGGAGTGCACCATACTTCGTGGGCCGGACGGTGCTAGCAAAGG
>dme_circ_0004680_junction_seq
TTCGTGGGCCGGACGGTGCTAGCAAAGGCTATCGGCCTGAGAAGCATCGAAACGAA
>dme_circ_0004681
GTTTCGCACAGGACGTTTCAGTCCTGGAGATGTTCTGCAGCAATGATGGATGGATCATAGGCAACTCCGT
GTTTGCGGAGGTGCCACCCTGTCAGGCACAATGTACACCTCCATGCCAAAACAATGGCATCTGCATTTCC
GCCGGAGTGTGCCA
>dme_circ_0004681_junction_seq
GCATCTGCATTTCCGCCGGAGTGTGCCAGTTTCGCACAGGACGTTTCAGTCCTGGA
>dme_circ_0004682
TTAAAGGTTCCGCAAAGGCCCTGAGTTTGGCCTCGCAAACTGGGCGGTGCATCGATATACACCCGTGAAA
CTCCGTCCCACCAGACGCGAATGCCATCTGCGAACTCCACAGTCAGGAAGGTGGAACTAGCCCGCCGGAT
GAGGACCTCGCCCAGGCCCAGCATCTTGGGTAACTTGGCGATGGGCTTG
>dme_circ_0004682_junction_seq
CATCTTGGGTAACTTGGCGATGGGCTTGTTAAAGGTTCCGCAAAGGCCCTGAGTTT
>dme_circ_0004683
GTGGCACATGAATCCGTAAAGGTAATGTCACCTTCGTAGAATGTCTTTCCATCTCTCACCATACACACTG
GCTTACACTCGGCCTTGGGCACACAATCGCGGTAGGTGTCGTTTAGGTAAATGAATCCATCCTCGCACGG
CTTGATGTGGCATCCCTCGAGGCAGTTCTCACGG
>dme_circ_0004683_junction_seq
GTGGCATCCCTCGAGGCAGTTCTCACGGGTGGCACATGAATCCGTAAAGGTAATGT
>dme_circ_0004684
GTTTATTCGCACAGTCGATGGATTGTGTGGCTACTACAATGGAAACCAAAAGGATGACAAGAGATCACCA
GATGGTCAGATTATTCCAAATACCGAAAAGTTCGGCGACAGTTGGTATGATAAGCGGATACCCAAGGACC
AATGTGGAGATCTTAAGTGCCCCAGGGAGATGCAGGCAAAGGCCTTGCAGCTGT
>dme_circ_0004684_junction_seq
GAGATGCAGGCAAAGGCCTTGCAGCTGTGTTTATTCGCACAGTCGATGGATTGTGT
>dme_circ_0004685
GTTCTACAATGCTGAATTATTTGGCAAGTGTCCTTTGGCTGTGGATCCCATTGCCTATGTCTCCGCCTGT
CAGCAGGACATCTGCAAGCCCGGAAACACCCAGCAAGGAGTCTGCGTGGCTCTGGCCGCTTATGCCAAGG
A
>dme_circ_0004685_junction_seq
GCGTGGCTCTGGCCGCTTATGCCAAGGAGTTCTACAATGCTGAATTATTTGGCAAG
>dme_circ_0004686
GGAATGTACCCAAATAGGGTATCGTTCCATGCGATGTTTGTGTTCCCAAATTCAGAATAAGCTTCTGCAG
ATGCCTATCGCTGTGATCACTGCCTGGATCTGGATTTTTGGCCGTTCCTTCTCGTTTCAACACTTCTCGC
AGAGTCCAAGCATTGTTATCCTCCGAGCAAATATTCGCCAGCTCCGTAAAGACTTCCATCTATGAGCAAA
AGCATAATTAGTCTACAAATTAAGAATGTGTATGATACAACACAACTTACCCTTTCTTTAGGCAGCACTT
CCCAGATCTTGGAGAGGCGGTATATTGAATTGGAGTTTAGAGCCGAAATGATGGCCTTGAGCGAACTAAA
GTTTTTGAGCATGCGCAACTCTTGGGCAATATCAATCCATCGTGAAATGTTCAGAGCGCGCTCCTGGTAA
TTAAAATCCAATCGTTAATTTACACTGTAGTCGGCAGAGAATCATGTATAAAAAGAGACACCCACCTGAG
GTTTCAGGCGATCGATCAGGATACTGGACACCACCCGGAAGAGCACTGCATTAAACTGGTTGATGGTGGC

CACCACAGTCTCGGATCCTCCACTATCACGTCGTGCCCACGTGTGTCCGAGGCACTGGTGGGGAATCAGT
CGCTTAAAGAGTTCCGTATCCATACGGGTCAACTGCTCAGCAAAATGCCGGACGGGCACATGCGGAAAAC
GGAATGCCTGCAGAAAGTGGGTAGGTCCACGATAGATGGGCCCGTGGCCCATTGGCGAGAG
>dme_circ_0004686_junction_seq
AGATGGGCCCGTGGCCCATTGGCGAGAGGGAATGTACCCAAATAGGGTATCGTTCC
>dme_circ_0004687
GCAGCTGGCTGAAAAGAAAATGGAATTGGAAGACAAAGTAAAACTGCTCGAGGAGGAAATTGCCCAATTG
GAGGCCTTGGAGGAAGTGCACGAACAGCTGGTGGAGAGTAACCACGAACTGGAGCTTGATCTGCGCGAGG
AATTGGATCTCGCCAATGGGGCCAAAAAGGAG
>dme_circ_0004687_junction_seq
GGATCTCGCCAATGGGGCCAAAAAGGAGGCAGCTGGCTGAAAAGAAAATGGAATTG
>dme_circ_0004688
TAGCACTCGCAGTGGGTCTTTTGAATGTCGCATTTCCATCGACTGGCAGAGACTCAGACTCTGATTGCAG
CGCAGAAGTTGTAAGGCTATCCACGAGGTTAAAAGTTGTGTCGCCGATCATCTCCTCCCCTAAAATCATG
GTACGCTGTAGGGCAGGACAAACTGCCGATACCACAACGGGCATCGTTCGATCTGCGGCCAGGGGAATGG
ACATACTGTCTGTGCTGTTGTCCATTGTGCTGCAGCCGGAAGCAGTTTTGCTCAGTGTCAACGATATTTC
GTCAATTTGCCCGATGTGCGAGTCTTTGATAAGAGTCCCATCGTCCTCACACATGGTCAGCGACTCCAGC
GCCTTGCGATCCCGGATCATGTCACTGAGCGTATATCCGCCTATGCTGCCGCCGATTCCCATGTTATCAG
TTGTCTGGTTGGTAAGCGTAGTGGTAAGGTTTGGATCCTGCTCAAGACCAATTGTGGATTGAAGGAAGTT
CAAGTTCGTAAAGTCTGCTGAGGTGATTAGCGAGTTGTTCATCCGTTCAGTGTTCATGGAGGAGCAGATG
GAAGACGGCGGTGAGGTGTCTTGCAGGAAGGATTTGTGCATATTCGTCGTGACAAGACCGCCAATTTCCA
GCTGAAGCATTGCTGGGGCACCCGCGACCGACCCGGTTCCAACACCGCTGTCATTTCCCGCTCCATTTTG
GCTCTGAAAAAATAAGATACTTTAATATACAAATTAGTTGCAATTGCTTATTTGTACTTACGAAATCCAG
ATTCCGACGCTTTGGGCGAGTAAAGGTTTTCGAATTGTTTTTGAAGGCTTTCGTAAAATATAAAAGTTTA
AAAATAAATTAGATCCTTGAGTTCTGCACTCGTTAAAGTAATGAATAGTAATGTATGGTGTCTTAACCGA
TAGGTGCAAGGTTTCTAAAAGCTATTTGTTTGAATTGTTTAAttttttttGTTCATATTTAAAGG
>dme_circ_0004688_junction_seq
TTTAAttttttttGTTCATATTTAAAGGTAGCACTCGCAGTGGGTCTTTTGAATGT
>dme_circ_0004689
GGCGCTCGAATTCTTCCGAGTCCTGACCTTTGTGCTACTCTTCCTGATCATCTCCCTGTATCTATATCGA
TTCGCATTCTAAAGGACTAGCGCTGACTGCCTGGGATTTTTGGGTGAGGATCGAGTCCGTTGTGTAACCA
GACCAAAAGGGGAGACAGTGATTCTAATTTCATTTTCGATTTCTATTCCGTTTCGGCTCGCCTTATTCTC
GTTCTCGATCTCTCGGCGGGACCTTGGACGTGGTGGTGGGGTCTAATTAAAGACAGTGATTCTAATTTCA
TTTTCGATTTCTATTCCGTTTCGGCTCGCCTTATTCTCGTTCTCGATCTCTCGGCGGGACCTTGGACGTG
GTGGTGGGGTCTAATTAAAGCCACAGCCAAGATGCCCAAATTACCAGCGGTTGGCATTGATCTTGGTACC
ACGTACTCGTGTGTCGGTGTCTTTCAGCATGGCAAG
>dme_circ_0004689_junction_seq
GTGTGTCGGTGTCTTTCAGCATGGCAAGGGCGCTCGAATTCTTCCGAGTCCTGACC
>dme_circ_0004690
CTCGGGATAACGAGCCGCGGCAGACCGAAAGAGGACCAACCCAGATTCGCAAAGTTCGTGCCATAAATCT
GCAGTTTGCATGGAATTAATACCCGACTAGAAGGCGGCGGCAGACGTGCAGCGGTGCTGCACATGCAACA
CACATTAGTGGGTCACTCGAAGATGATGACGCTGCTGACCGCAACGGCAACGGTAGAAGTAAAACCAACA
AAAACAAGCGCCACTCGAGCCGATTTGTGGCAGCGGGGGCAAATTATATAAAATTATCATGCAGGGCAGG
CAGCTAGCTAGTTGGCCACTTGACAACGGCAGGCGGGACAAGGCTAaacatcatcatcaggagcagcagc

agcagcagcagcagaagcaccactaccatcaccaccagcaccaaaagcaacaacagcggcCGGCGACGTA
TACGTAATGTGCAGCATGACGTATACGTAATGTGCACCGGGGTTTGGCCAATTTGTCCAAAGCGAGCTAT
AAACCACTGAGGCAGCGGAAACGCAAACCAAAAACCGAAACTCGAGCTGCAAACTATAACATAAACTGAA
ACTGCTAACGACGCAAACGGAAACTTCTTGGCCAAATGGCGAAAGTTCGCAAACAATTGCGAATGTGTTG
AGAAGCGGCTGGCGGTGCGAAAATGACAAAATGGCGAAGTTAACGTTGCCTTGTCTACTGCGGCGACTGC
AGCTGATGGCGCAACAACATCAAAAACAGCAGCAGCTGCATCCACACGGCTGCCACTTGATGGCCATGCA
ACTGGGCCAGGCCGTGCTCCTCTACGGTTGCCTTCTCCTGCTCCACGCCTCCGTGTCCGCCTCTGTCCTG
GCCACGTCCTCCGACTCGGCCGCCATTCCGATCCTGCTGAACATCTCCAGCACGATGGATCTGGATGGCG
TACCGCAGCAGCAACAGCATGAGCATACCGAGAAACGCACGCGGAAGCGTGATGCCGCCAATGTGCCTGA
TGATGACGACTATGGCAGTTATCCGGACGACGTCGACGAATTGGAGGCCTTGCTTAATAATAAGTCAGG
>dme_circ_0004690_junction_seq
TGGAGGCCTTGCTTAATAATAAGTCAGGCTCGGGATAACGAGCCGCGGCAGACCGA
>dme_circ_0004691
CGCACGGGAATGGGCATTCGGCTGGAGGGATAATGCGGCGACTTCTTGTACCGCACCCACAGCGTATAGA
TGTTCTTCTTTTGGCCAATTGTCGACTCCAGGCGGAACTTGTCGCACGTGTAGCCCAAAAAGGATTCGGT
GCCCACCAGGCTGAAAGGTTTCGCATCGGGCAGGATGCTCTGAATATCGACAGCCTGGTCGGCGGTGCCA
TTCACCTGCAGACAGGTTAGCTTGTTGTTCTCCGTCTTGGTGGTAATCGGTGCCAGCTTCAGCAGGGTTC
CGTACTGACCCTCGCCAGCCAGTTGGTATG
>dme_circ_0004691_junction_seq
TACTGACCCTCGCCAGCCAGTTGGTATGCGCACGGGAATGGGCATTCGGCTGGAGG
>dme_circ_0004692
TAATGATCGTAGTGCGATCCCAGCAGGGTGTTATAGCCCCTCATCTCGTAGCGCACGGGAATGGGCATTC
GGCTGGAGGGATAATGCGGCGACTTCTTGTACCGCACCCACAGCGTATAGATGTTCTTCTTTTGGCCAAT
TGTCGACTCCAGGCGGAACTTGTCGCACGTGTAGCCCAAAAAGGATTCGGTGCCCACCAGGCTGAAAGGT
TTCGCATCGGGCAGGATGCTCTGAATATCGACAGCCTGGTCGGCGGTGCCATTCACCTGCAGACAGGTTA
GCTTGTTGTTCTCCGTCTTGGTGGTAATCGGTGCCAGCTTCAGCAGGGTTCCGTACTGACCCTCGCCAGC
CAGTTGGTATG
>dme_circ_0004692_junction_seq
TACTGACCCTCGCCAGCCAGTTGGTATGTAATGATCGTAGTGCGATCCCAGCAGGG
>dme_circ_0004693
GCCTTGTCCACATGCTCATCGGTTCCGGATATAAACTCCTGCATGGGATTGAAGGTGGCATAGTGACCGG
TGCCGGGTCCGGGGAATCCGACGCACTGCAGGCTGTCATCGATCTCGAACACCTCGTTGGGAATATCATC
GTGCTCGTAGCTGTCATAGTCCAAGTAG
>dme_circ_0004693_junction_seq
GTGCTCGTAGCTGTCATAGTCCAAGTAGGCCTTGTCCACATGCTCATCGGTTCCGG
>dme_circ_0004694
TTTGGCCCTGTTCTTTGAGTGGATGTAGCGCAGGTTCTGACGGAAGATGTTCTTGCGATGCTCGTGTTCC
GTGTCGCTGTGATAAGCAACTCCGTGCTTGCGCTTGAAATGGTGGAAGGCCTTGTCCACATGCTCATCGG
TTCCGGATATAAACTCCTGCATGGGATTGAAGGTGGCATAGTGACCGGTGCCGGGTCCGGGGAATCCGAC
GCACTGCAGGCTGTCATCGATCTCGAACACCTCGTTGGGAATATCATCGTGCTCGTAGCTGTCATAGTCC
AAGTAG
>dme_circ_0004694_junction_seq
GTGCTCGTAGCTGTCATAGTCCAAGTAGTTTGGCCCTGTTCTTTGAGTGGATGTAG
>dme_circ_0004695

GCTACGAGCACGATGATATTCCCAACGAGGTGTTCGAGATCGATGACAGCCTGCAGTGCGTCGGATTCCC
CGGACCCGGCACCGGTCACTATGCCACCTTCAATCCCATGCAGGAGTTTATATCCGGAACCGATGAGCAT
GTGGACAAGGCCTTCCACCATTTCAAGCGCAAGCACGGAGTTGCTTATCACAGCGACACGGAACACGAGC
ATCGCAAGAACATCTTCCGTCAGAACCTGCGCTACATCCACTCAAAGAACAGGGCCAAACTCACCTACAC
GCTGGCCGTTAATCACTTGGCCGACAAGACCGAAGAGGAGTTGAAGGCACGACGAGGATACAAATCATCG
GGCATTTACAACACCGGCAAACCGTTCCCCTATGATGTGCCCAAGTACAAGGACGAGATTCCCGACCAGT
ACGACTGGCGGCTATACGGCGCTGTCACTCCGGTGAAAGGATCAATCTGTGTGCGGATCGTGCTGGTCAT
TTGGCACCATTGGTCACCTGGAGGGCGCCTTCTTCCTGAAGAATGGCGGCAATCTGGTCCGGCTTTCCCA
GCAGGCGTTGATTGACTGCTCGTGGGCCTATGGCAACAATGGCTGCGATGGTGGCGAGGATTTCCGCGTG
TACCA
>dme_circ_0004695_junction_seq
ATGGTGGCGAGGATTTCCGCGTGTACCAGCTACGAGCACGATGATATTCCCAACGA
>dme_circ_0004696
GTTTATACATCAGGCAGAGTCCACAACTGCTGTTCCTGCTCGGGAACAATTGCACCTACCACTACAAAAC
AATTCGTCAGTGAAGAAGCGCTGGTTGCGACAAGCTATCAGCGAAGAGACCACACCAGTCGATGAACTTC
AGCAGTCACAAAATCAGTCGGTAACTGCCACTCCCTCTCCACAGCCGGTACCAACT
>dme_circ_0004696_junction_seq
CACTCCCTCTCCACAGCCGGTACCAACTGTTTATACATCAGGCAGAGTCCACAACT
>dme_circ_0004697
TTGTCTTTTTCTTGGTGTCAACAATTTGCGAAGTTGAATTAACATTAACTGCTGCCttttgcttctcctt
atccttcgatttttttcttctcctttttcgatttttttGAGCTGCTTCTCCTTGTCGCGCTTTTCATCCTTA
TCCTTGGATGATTTTTTCGACTCAGTGCCACTACCGCCACTGCTGCTATTGCTCTTCTGGCTGGGGGAGG
CGGAGG
>dme_circ_0004697_junction_seq
TTGCTCTTCTGGCTGGGGGAGGCGGAGGTTGTCTTTTTCTTGGTGTCAACAATTTG
>dme_circ_0004698
GTGTTTACCAATCTCTTTTGAGGGGCAATAGTATATATAATCTTGTACATATGCAATTTTAATTCTAACA
AGCAAATCATGCAGGTGGTCTTTGGCGAAGCGCTTTTTAGTTTTAATTTAACATTTATTTTTGTAAATAT
GCATATGCAACGTTTATAATGTAATCATCGCGTAAGTGTAAAATATTTTACTAAGCCGATTTTATGAAAA
>dme_circ_0004698_junction_seq
ATATTTTACTAAGCCGATTTTATGAAAAGTGTTTACCAATCTCTTTTGAGGGGCAA
>dme_circ_0004699
GTTTTTGCTCGTTGCCGTAAGGATCGGATAAGTGTCGCTTGACCCGTTCGTGTGCCTCCTGAGTTAGATT
GATGGGCGCCTTCCAAGCAGCTGTTAAGAAACAAATCGTCAAATGATTTTACAATATAATACGAGTTTAG
TTTGTACTTACTTAGCAGATGAGCCACCAGATTGCATTCGATGCGGAAGGGAGCGACCAAGTTGTACTGC
TGATACTTGGGATTCTCATACTGGGAGAGGCCACTAAGGTTACCGATGCACACGTCGCTTAACCAAAGCG
CATTGACCATCGGTAT
>dme_circ_0004699_junction_seq
TTAACCAAAGCGCATTGACCATCGGTATGTTTTTGCTCGTTGCCGTAAGGATCGGA
>dme_circ_0004700
GATTGATTGGCCAGTGCGGATGGCGATTGAATTTAACGGGGGAAACACGGAACATGGTGCTAGAATTTCA
GTTTAAACTGTCGCGAAATGCCCGCACAATCCGGCAGCAGCCATCTTAGCATTAGGTTTTTCATTAAAAT
TAGGTATAGAGCATTTGAAGTGGGGAACGGAGAACTGGCTGCACTTGAG
>dme_circ_0004700_junction_seq

GGGGAACGGAGAACTGGCTGCACTTGAGGATTGATTGGCCAGTGCGGATGGCGATT
>dme_circ_0004701
TGAAATACTATGCAGTCTTTGCCTGTCCCATTGTGATAGCCGATCCTACAACTGTGCCGCGAGTCCGGAG
GCAGAGATGTTGTCGCCAGCCCCAGCCGTTTTTCTGGCTTCGCGACACACCAGCACCGGGGCCACACAGA
TTTCCACCTCTAGCCGTTGCCGGTGACGACCGTACTGGATATACTCCCGCCAACAGGGCACGGGACTGGC
TGCACCAATGCGCATTCGCGGCGCATCCGCCTGAGCCGAGGTGGCGAAACTGTCATCTAGAACCTGCAGC
ACTGCCTCCGGGTTAATCTGCGGATGAAAGAATTATCAGTTCAAAACTAAAACATACTACACATAAATGC
GTACTCACAAATTGGGATTTGCAGACGTATCGGTGAGCAGTGAGCGCTGCCTTTGCAGCGGCAGCACGTG
>dme_circ_0004701_junction_seq
AGCGCTGCCTTTGCAGCGGCAGCACGTGTGAAATACTATGCAGTCTTTGCCTGTCC
>dme_circ_0004702
GCTCCTTTGTGGCCTTCGATGGCAAGAAGCGTATCATCGGCGTGGCCGCTAAGAACCAGCAGGTGACAAA
CATGAAAAACACAGTTGGCGGATTCAAGCGCCTGCTTGGCCGCAAATTCAACGATCCCCATGTACAACAC
GAACTCACGAGCATTCCAGCGCGCGTGGAGGCGCGCGGTGATGGCAGCATTGGCATCAAGGTGAACTACC
TCGGGGAGGATCAGCATTTCGGACCTGAGCAGCTGACGGCGATGCTCTTCACCAAGCTGAAGGAGACATC
GGCGGCTGCCATGCAGACGCAGGTTAACGACTGCGTCATCGCCTGTCCCGTGTTCTTTACCAATGCAGAG
CGTAAGGCTCTCCTGGATGCCGCCCAGATCGCCGGACTGAACGTCCTGCGATTGATGAACGAAACGACGG
CCACCGCGCTGGCCTACGGCTTCTACAAGAACGATCTGTTCGAGGACAAGCCACGTAACGTGATCTTTGT
TGACTTTGGGCACTCGTCACTGCAGGCCAGTGCCTGTGCCTTCACCAAGGGCAAACTGAAGATGCTGGCC
AGCACCTGGGACCAGATCGGTGGACGCGACATTGACTTGGCTCTGGGCGATTACTTCGCCAAGGAGTTCC
AGGAGCGTTACAAGATCAACGCCAAGACCAATGCCCGTGCCAATCTGCGCCTGTTAACCGAAATCGAAAA
GCTGAAGAAGCAGATGTCCGCCAACAGCACCAAGCTGCCGTTGAACATTGAGTGCTTCTTGGATGATATC
GACGTCTCGTCGTCCATGCAGCGGTCGCAGATGGAGGAGCTGTGCGCCCCCGTCTTGCAGCGCGTCGAGC
AGACTTTCAAGCGTTTGCTAGCCGAGTCTAAGCTGCAGCTGGACGACATTCACTCCGTGGAGATCGTTGG
CGGAAGCTCACGCATTCCGTCCGTAAAGCAGCTTATTGAGCAGGGTCTTTAACAAGCCGGCTAGCACCAC
TCTTAACCAGGACGAAGCTGTGTCTCGCGGAGCAGCCCTTCAGTGCGCCATCATGTCGCCAGCTGTACGC
GTCCGAGAGTTTGGCGTCACTGATATACAGAACTACGCCGTTAAGGTGCTGTGGGACAGTGAGGGCTCCG
CTGCTCCGGGAGAGATCGAGATCTTCCCGCAATACCATGCTTCTCCGTTCAGTCGCCTGCTGACAATCAA
CCGCAAGGGACCGTTCAACGTTTCGATTGTGTATGGCCAACAGGTGCCCTATCCAGATCAAACGATTGGC
GTGTGGAAGGTCAAGGATGTAAAGCCGACGGAACGCGGCGAGGGTCAGGATGTAAAGCTGAAAGTGCGCA
TCAACAACAATGGCATTGTGCTAATCTCGTCCGCCACTTTGGTGGAAAAGAAGGAGGCCGAAGAGGCTGC
CGCAGCTGCTGAGCAGGCTGCCAGTGAAGAAAAACCAGGCGATCAGACCAACAATACTGGAGAACCTGCG
GATGGTCAGCAAGAG
>dme_circ_0004702_junction_seq
TGGAGAACCTGCGGATGGTCAGCAAGAGGCTCCTTTGTGGCCTTCGATGGCAAGAA
>dme_circ_0004703
CACGCGAGCCATAGCCAGGAACGATGACCGGCTAACCTTCCACCACATTTGTGGTACTCACGCCACCGTC
ACCCAGAGCGGGCGGACAGCGCTGCGGCCCAAGTGAGCATTGTTGTTTACGGCAGAAGATTTTTATGAAT
TTCAATTCCCCAATTTGTATGCAGTGCTGCCGATGATTTTAACAATGGTGTAGTGCTCACAAGGAGACCT
CTGCGCCCCAACGAACTCTTCCAAG
>dme_circ_0004703_junction_seq
CCTCTGCGCCCCAACGAACTCTTCCAAGCACGCGAGCCATAGCCAGGAACGATGAC
>dme_circ_0004704
GAAACGGAAAATGGTCGGCGAGTCCACGTCCACCCAGGTGCTCCGCGATCTGGAGATCTCGCTGCGCACG

AACCACATCGAGTGGGTGAAGGAGTTCCTGGATGACACGAACCAGGGTCTGGACGCCCTGGTCGACTATC
TCAGCTTCCGACTGCAGATGATGCGACACGAGCAGCGCCTTCAGGGTGTCTTGTGTGCCTCGGAGGAGCG
TCTGAATCTCACAAACGGCGGCGATGGCGGTGAGATAGTGATGGGAAACAGTAGTTCTGTTAGTCCTGGT
GGAGGTGGTGGTTTACTATCACATGGAAACAGTACGGGACATGGTCTGGCCAATGGCACACTTGACTCGA
GGCAGCAGCACACAATGTCCTATGGATTCCTACGACCTACCATTGCCGATGCTCTGGATAGTCCTAGTTT
GAAGCGAAGGTCACGACATATTGCCAAATTAAACATGGGTGCCGCCACGGACGACATCCATGTGTCCATT
ATGTGCCTGCGAGCTATCATGAACAATAAGTATGGGTTCAACATGGTTATCCAGCATCGCGAGGCCATCA
ACTGCATTGCCTTGAGTCTTATCCACAAATCGCTGAGGACGAAAGCCCTGGTCCTGGAGCTGCTGGCAGC
CATCTGTCTGGTAAAGGGAGGACACGAAATCATTTTGGGTTCGTTCGATAATTTTAAGGATGTGTGCCAG
GAGAAGCGACGCTTCCAAACGCTCATGGAGTACTTTATGAACTTCGAGGCCTTTAACATAGATTTTATGG
TTGCCTGCATGCAGTTCATGAACATCGTTGTCCACTCGGTGGAGGACATGAACTACAGGGTGCACTTACA
GTACGAGTTTACAGCCCTGGGCTTGGATAAGTATCTGGAGCGAATTCGATTGACAGAATCGGAGGAACTG
AAGGTGCAGATATCAGCCTATTTGGACAACGTCTTTGATGTTGCTGCCTTGATGGAGGATTCCGAGACAA
AAACTTCAGCCCTGGAACGAGTCCAAGAGCTTGAGGATCAACTTGAGCGAGAAATAGATCGTAACTCAGA
GTTCCTCTATAAGTATGCGGAATTAGAGTCCGAGAGTCTAACGCTGAAAACGGAACGCGAGCAGCTGGCT
ATGATTCGGCAGAAGCTGGAGGAGGAACTTACAGTGATGCAGCGAATGTTGCAGCACAACGAGCAGGAGC
TGAAGAAACGGGACACACTGCTGCACACAAAGAACATGGAGCTGCAGACGCTTTCGCGTTCCCTGCCACG
ATCCGCCTCCAGCGGCGATGGTTCTCTGGCGAATGGTGGCCTCATGGCTGGTTCCACATCGGGGGCAGCC
TCTCTAACATTGCCACCACCTCCGCCGCCAATGCCCGCCTCGCCTACTGCAAGTTCAGCTGCTcctccac
cacctccgccgccagcaccaccggctccaccaccaccgccggGCTTCAGTCCGCTGGGCAGTCCGAGCGG
CAGCCTAGCCTCGACAGCGCCATCGCCGCCACATGCCCCGCCCATGCTAAGCTCCTTCCAACCGCCACCG
CCTCCAGTGGCCGGCTTTATGCCCGCTCCCGATGGCGCCATGACCATCAAACGCAAGGTGCCCACTAAAT
ACAAGTTGCCCACCTTGAACTGGATAGCACTAAAGCCTAATCAGGGTACGTGGTACAATATTCAACGAGC
TGGATGACGAAAAGATCTTCAAGCAAATCGACTTCAATGAGTTTGAGGAGCGCTTCAAGATCGGGATTGG
CGGTGCTTTGCGCAATGGTAGCAATGGAACCGAGGTCGATGGGTCGCTGCAGTCCAGCAAACGCTTCAAG
AGGCCCGACAATGTCTCGCTGCTGGAGCACACGAGGTTAAGAAACATTGGCAATCTCCCGTCGCAAGCTG
GGTATGCCCATTGATGATGTCATCGCCGCCATTCATAGTCTGGACCTGAAGAAACTTTCCCTGGAGAACG
TCGAGCTGCTGCAAAAAATGGTGCCCACGGATGCCGAGGTCAAATCCTACAAGGAATATATCATCGAGCG
CAAGGACCAACAGCTACTCACCGAAGAAGACAAGTTTATGCTGCAGTTGTCGCGTGTGGAGCGTATCTCG
TCCAAGCTAGCCATTATGAACTATATGGGCAATTTTGTCGACAGCGTTCATCTCATTAGTCCGGGTAAGA
AAATATATATATATAACTTGGAAAGAATTCAATAGTTAGTTATCTAACTGTCGTGTAAATATAATATCCG
TTTCTATGACTAATAGTTAAGTCATGTGGCAGCTGGTTTTCAATTCGTTTTGTTTATTTTGTTGGACAAT
AAAAAGAAAAACTATCTCTGTTTATAAAAATCCGTATGCCGTCAACATAATTGCACTTTGATCAGCAAGA
AATTTGAGAACTTTATTATTGACATATTTTTGATGAATCAAATAAGAATATATATTTTTTCTTAATTCTA
GGCAAGTGCAATCGATAGCAGGAGCGTCGACTTCCTTAAAACAATCTCGAAAATTCAAGGCGGTTTTGGA
AATTGTCCTGGCTTTCGGCAACTATCTCAACAGCAACAAACGGGGACCAGCCTATGGCTTTAAGCTGCAA
TCGCTGGACACGCTGATCGATACAAAATCCACAGACAAGCGATCGTCACTGCTTCACTATATTGTGGCCA
CCATACGGGCCAAATTTCCGGAGCTGCTGAACTTCGAGAGCGAGCTGTATGGAACAGACAAGGCTGCATC
GGTGGCACTAGAGAATGTGGTGGCCGATGTTCAGGAGCTTGAAAAGGGCATGGATCTGGTGCGCAAGGAG
GCCGAGCTGCGAGTGAAGGGTGCCCAGACGCATATCCTGCGTGACTTCCTGAACAACAGCGAGGACAAGC
TGAAGAAGATCAAGAGCGATCTGCGGCATGCACAGGAAGCGTTCAAGGAGTGCGTTGAGTACTTTGGCGA
CTCCTCGCGGAATGCAGATGCGGCTGCTTTCTTTGCGTTGATCGTACGCTTCACGAGAGCGTTTAAGGTA
GGACTATTTCAAATGATTCCCTAATACTATGCTAATATGATATCTTTGGTAATTCTAGCAACACGATCAG
GAGAACGAGCAGCGTCTTCGCCTGGAAAAGGCCGCTGCGCTGGCCGCTTCCAAGAAAGAGAACGATCAGG

TGCTTATGCGCAACAAGGTTAACCAGAAGAAGCAACAG
>dme_circ_0004704_junction_seq
CAACAAGGTTAACCAGAAGAAGCAACAGGAAACGGAAAATGGTCGGCGAGTCCACG
>dme_circ_0004705
CAGCTGAACTTGCAGTAGGCGAGGCGGGCATTGGCGGCGGAGGTGGTGGCAATGTTAGAGAGGCTGCCCC
CGATGTGGAACCAGCCATGAGGCCACCATTCGCCAGAGAACCATCGCCGCTGGAGGCGGATCGTGGCAGG
GAACGCGAAAGCGTCTGCAGCTCCATGTTCTTTGTGTGCAGCAGTGTGTCCCGTTTCTTCAGCTCCTGCT
CGTTGTGCTGCAACATTCGCTGCATCACTGTAAG
>dme_circ_0004705_junction_seq
GCTGCAACATTCGCTGCATCACTGTAAGCAGCTGAACTTGCAGTAGGCGAGGCGGG
>dme_circ_0004706
GCTGGTCCCCGTTTGTTGCTGTTGAGATAGTTGCCGAAAGCCAGGACAATTTCCAAAACCGCCTTGAATT
TTCGAGATTGTTTTAAGGAAGTCGACGCTCCTGCTATCGATTGCACTTGCTAGAATTAAGAAAAAATATA
TATTCTTATTTGATTCATCAAAAATATGTCAATAATAAAGTTCTCAAATTTCTTGCTGATCAAAGTGCAA
TTATGTTGACGGCATACGGATTTTTATAAACAGAGATAGTTTTTCTTTTTATTGTCCAACAAAATAAACA
AAACGAATTGAAAACCAGCTGCCACATGACTTAACTATTAGTCATAGAAACGGATATTATATTTACACGA
CAGTTAGATAACTAACTATTGAATTCTTTCCAAGTTATATATATATATTTTCTTACCGGACTAATGAGAT
GAACGCTGTCGACAAAATTGCCCATATAGTTCATAATGGCTAGCTTGGACGAGATACGCTCCACACGCGA
CAACTGCAGCATAAACTTGTCTTCTTCGGTGAGTAGCTG
>dme_circ_0004706_junction_seq
TAAACTTGTCTTCTTCGGTGAGTAGCTGGCTGGTCCCCGTTTGTTGCTGTTGAGAT
>dme_circ_0004707
GCTGAAGAAGATCAAGAGCGATCTGCGGCATGCACAGGAAGCGTTCAAGGAGTGCGTTGAGTACTTTGGC
GACTCCTCGCGGAATGCAGATGCGGCTGCTTTCTTTGCGTTGATCGTACGCTTCACGAGAGCGTTTAAGG
TAGGACTATTTCAAATGATTCCCTAATACTATGCTAATATGATATCTTTGGTAATTCTAGCAACACGATC
AGGAGAACGAGCAGCGTCTTCGCCTGGAAAAGGCCGCTGCGCTGGCCGCTTCCAAGAAAGAGAACGATCA
GGTGCTTATGCGCAACAAG
>dme_circ_0004707_junction_seq
GAACGATCAGGTGCTTATGCGCAACAAGGCTGAAGAAGATCAAGAGCGATCTGCGG
>dme_circ_0004708
GAAGACGCACTACAAAATCATGGAGAACGCATTTAAGCGATTTGTGATAGAGAGAACTCAGCACAAGCCG
ATGCTCTTCAACCAGGAGGAGGTGGTACGGCTGGAGGACGATTTACTGGTTACTGTTGGAATTTTACCAG
AACACTTTCGTTATTGCGTGGTGGACGCGCAGTTTCTGAAGGAGTCCAAGATCTACGCAGCAGATCTGGT
GCGTCCGGTTGGCGAGATTATTAAGGAGGAGACGCCTCCGACATCGCCACTAAGTGTTCAGGATCTCATC
CAGTTACCGGAGTACGATAAGATTGTGGATCAG
>dme_circ_0004708_junction_seq
ACCGGAGTACGATAAGATTGTGGATCAGGAAGACGCACTACAAAATCATGGAGAAC
>dme_circ_0004709
CTACGACCGATGTCAAACCTTGACCGCGTGATCCACCCCACTTCAGTTGGCTTTTTGTCTCAAAAACAGC
GTGAAAGATGGAAATTATTAATTCACAGCAGCAACCAAGGCAAGTCGCTACCCTTTCCCCTACCCCAAAC
CGAACCCGCTGGCCATACGAACGCATCTTACAGATACAAATCACTTTCGCGCTGCATCTTTCAGCACGTT
CGTACGCATCAATCACAACAACTTAGCCTGCATATGaaaatatgaccaaaaaaacaatacaaaagccaga
aacaaaaaaccaacaaaatATTTACGAGTTTTTGTTGTGCTTCACGCCTTTAAGCTGACAGATACAGga
aagaatgaaaatgaaagaaaaatgtgaaataaaagaaaagccctacaaataatgaaaataTCCCTTGTTA

CATTGATTCAGTAAGTTTCGGCTTTCTTTGACTGACAAAAACGCAAGGTAATTGTAAATTATTTATGTGA
ATTCTTGCAACGTCAGTCGCATTAGAATCAGAACAACATTGCCATACCAAATATGCATTAGTTTTTAAAA
GTTTCAAAGAAACTATAGGTGTATACATAATAAACATTTACTATATTATTTAACATCATTTTAGTCTAAT
CTATCTTATGAAAGTCATGTTTAACCTTTCATTTGGTAATAATAACTTTTAAGTAGTTGAATAAAATTTA
TTCAGGTTTGTTCACGTTTTGTACAACTCCTAGTTTAAACTTCATTTTCCTGTGGCACCATAAACATATA
AAATTCGCTATGCTACAAGACGGACTGCTTTTCCAGCTCCTTTCCCTTTCATCTGAGGCCTCTATAAAAT
TGCTACAAATTTCCTTTACAGTTTATATCCttttttttAGCAAAGATGTCATCGGGCCGTTTGGGTCAGT
CAGCCAGTTTGTCTATTGTCTGGTTTGAAACTAAAACTGGGCTGGGCAGCAACCACCGAGGGAAAACACA
TTTTCTCAGCTTGTAAGTTGAAAATTTTATGGGCCTTGACTTGGGGTAGCATCGTCATTAACATTAAATT
AGGGTAAAAGAAAGGAAATTTATGAAGGCACCGCAATGGGATGCGCTAAATTTTCGTTTGACTCCTTGCA
CCTCTCTCCATCCTGtttttgattttttttCTGACTTCTTCTTAACATTGGCGAACTTGTAATTTATGAGG
CTGAGAACGGAGAAGGGACAGAAAACTGTCTTCTTTTTCAAGGTGTGGCCAATTAGTGGCAGCAAATGGT
AGGAATGGATAAAAGGGTCCCAAGAAACCAAGGACAAGGAGGTAATTTTCCAGCCCAACCACCCACAGAA
TCGGTTTCCCAGTCTGCAACCTAATTGAATTGGGACAAGTGCCAAAATCGCTTTTGGGCTAATCCGATTT
GCGGTGAGGAGTATCTGAAGAACCCTCCAAGGAAACCCCACCAGACTGGACACCCGCAAAATCTTGGCTT
TCTTTCTTTGATAGTCCAGTGaaaaaaaaattggtcatcgcaaaaaaaaaaaTTGGACAAATACTCATGTG
CACTTGAGTTTTCTTTTTTATCCAGCCCGTCCCTTTGTGACAGATTTTCTTTCATATTTTTCCATTTGTT
GTCCAAAAGAAGAGAAGGTTGAGCGTAGGTGGAGCTCCTTTGGCTGGTCAGTCAGGCGCAAATTATAAAG
GAATTAATGGTAGATCCAGTCAGAGAATAGAAGCACAATGTGCATAAGGAAAACCACACAAAAAAGAGAG
AGATTCAGGACAAAAGGGAGTTTGAGGTTTGGGAGGGATTACGGCCTGACAATCTTGACGCAAAAATTGC
CATTTAAATATGCCCATAATCGTAATGAAGCCCAGGCTGTCCCGGAGCTAAGCTGATTGATGGCTCTTGC
CAAAGATGATGTAAGAACTCCAGCTATCCATCAAGGGCTGCCAGCCACTGCATCGATTTCAAATGCAAAT
TTTGTCCGACTCCGGGATCGAGTTTGCCTGGAAGACTAACCCACTTAAGACTGTCATTATGATTATTATT
GATCATCAGCCGCGAAGAGGAGTGAATGCAGAACTGGATCTCGGATTGTCTTCATTTGCATGACGAACAA
GTTCTGCAGATTAACTAGCCAATGTTTTGAGAAAACTTTTCAGCAAACAATCCACCCAGGGGCAATAAAC
AACAAATGAAAATGAAATTGAAATGGAAATGGATAGGACATGGCACTCGGAGCTTACCGTATGACGGGAT
TAGCGTTCTTGCTTAATTTTTTCTTCGAATACAGTTTTGCTTCTCttttttttttACCAGGTATCTTGAG
AGGACTGCCAGCTGTCAGCTTAGATGGCTAACTTGATCTTAAGCTGTTTTGGTCATGTGAAAATTATGGA
AAGCAGGCAAACAAACTTCGGACTGGGCCGGCGGACAATAAGTAACAAAAATTGCACAAGATTGCAGCGA
TCGCGTTATCGCAGAACGATCGCAGAACTCGCCAGGTAGGAAGGAAGGTGAGTGCGTATAAGGATTCCCA
CACGGGACATCGGGAAGACTTGAAGTGGTTCAACTAATCAAAAGTGACAAGGCAGCAGAGCGGTTCGAGA
TCAGGTCCCGACATCCCTCCGATGATCCAGATGAGTCGATGCGAGGAGAGGCGAGGGATACGGAGGCTGG
CCGGAAGTGCGAAAGGAGTTCATCAAATGGTTAGGTGACAAAAGGGTTGCTCCGGACTTACAAGGACCAC
ATCCCGACCGAGG
>dme_circ_0004709_junction_seq
ACTTACAAGGACCACATCCCGACCGAGGCTACGACCGATGTCAAACCTTGACCGCG
>dme_circ_0004710
GCTGCAGTAAGCTTTCCACCAAACATGGCACCAATGCCCCGAATTCAGTTACCCAATGGAAATTCTTCCA
TAAAGGCTAGCACAAATGATTTTCTCTTTGCGGAAACAATGTCCGGCGACAGTAGTCCCACCCCCAGCAG
TCCGCCATCCAGTACAGGCAGGCGTTGCCAAATCTCAGTGTTCTTCCCTTTCGGATGGCGAATCCTTTGA
GGGATATGGCGAAAATGAGTATCCCACCCAACTTCGCGAAGCCCGCAGCTCCAATTCCAATGGGAGCCAT
AATATGAGCAATCATATTAACAATAATTCTAATATCAGCGTGGGcaacaacagtggacacaaccacagtg
gacacTCGAACGATGGAAATAACAACCTAAATGGCAGCACTGGCGTCGAACTCGATTTGGCTCCACACGT
CGGCTCATCAACACCTCAAGA

>dme_circ_0004710_junction_seq
CACACGTCGGCTCATCAACACCTCAAGAGCTGCAGTAAGCTTTCCACCAAACATGG
>dme_circ_0004711
GCTGCAGTAAGCTTTCCACCAAACATGGCACCAATGCCCCGAATTCAGTTACCCAATGGAAATTCTTCCA
TAAAGGCTAGCACAAATGATTTTCTCTTTGCGGAAACAATGTCCGGCGACAGTAGTCCCACCCCCAGCAG
TCCGCCATCCAGTACAGGCAGGCGTTGCCAAATCTCAGTGTTCTTCCCTTTCGGATGGCGAATCCTTTGA
GGGATATGGCGAAAATGAGTATCCCACCCAACTTCGCGAAGCCCGCAGCTCCAATTCCAATGGGAGCCAT
AATATGAGCAATCATATTAACAATAATTCTAATATCAGCGTGGGcaacaacagtggacacaaccacagtg
gacacTCGAACGATGGAAATAACAACCTAAATGGCAGCACTGGCGTCGAACTCGATTTGGCTCCACACGT
CGGCTCATCAACACCTCAAGAGTAAGTTTTTGAAACATTTATATAGTGCGTTTCAAAAGTGTCATATCCA
TTTAACATTGCGATAAATTCAAAATATATACTTATGTATATGAACACAGTTGTTCTATAAACTGTTAATG
AATTTATTGCATATAAATATGTAACTCCTGTAAAGCAAAGAACATTAGTCTTGCGGAATTACCTCTTGAT
TACTGTCAATTAATCTTTTAAACTAACTGACACTGAAACGCCTTGTGTATTTCCATTGTCACAACTGATG
AGCATTTACTAACTCGATTTCGATTGTTGCAGTGATGACGAACTAAACATAATGCCCCGGGATAATTGGG
CCAGGCGCAGTCTGCGACGAACTCCCACCAG
>dme_circ_0004711_junction_seq
GGCGCAGTCTGCGACGAACTCCCACCAGGCTGCAGTAAGCTTTCCACCAAACATGG
>dme_circ_0004712
GCTGCAGTAAGCTTTCCACCAAACATGGCACCAATGCCCCGAATTCAGTTACCCAATGGAAATTCTTCCA
TAAAGGCTAGCACAAATGATTTTCTCTTTGCGGAAACAATGTCCGGCGACAGTAGTCCCACCCCCAGCAG
TCCGCCATCCAGTACAGGCAGGCGTTGCCAAATCTCAGTGTTCTTCCCTTTCGGATGGCGAATCCTTTGA
GGGATATGGCGAAAATGAGTATCCCACCCAACTTCGCGAAGCCCGCAGCTCCAATTCCAATGGGAGCCAT
AATATGAGCAATCATATTAACAATAATTCTAATATCAGCGTGGGcaacaacagtggacacaaccacagtg
gacacTCGAACGATGGAAATAACAACCTAAATGGCAGCACTGGCGTCGAACTCGATTTGGCTCCACACGT
CGGCTCATCAACACCTCAAGAGGTAATCTCGCCCATTTTGATCGGATCACATCTCTTAAAATGGCAATCG
AAGCGAATGCGAATTTGCTTAGTTGTCGGGCATTTATAAGGTTGCGCACATAATCCAGCTGAATACGTAA
TAGCCGTGAATATAAAATAAAGCTGACCAGGCCCAGAGACTTCATCAATTCAAAACTCTCAATTTGTCTG
TCCGAGGCAGCATAAAAAAAGCGTATTTGAATAGTTAACCTTTTCTTTGTGTAAACCTGTGTCTGTTGGT
TTCAACGCATGTATGTGTGGCATGAAAAATGCATACACAGACTGAAACCAGTTTTTTCTATGCTCTGACT
TGGGCTCATGAATCGCTTTGGGCCGTGATTCATGCGTGTCTCTGTCTTCGGGTCTCGGAATCTCTCACAA
CTCAGCGATCGTGCCTCTCTTTCTGGTCGTGTTCCGCCGTCTCTTTTACTCTATTTCCGCCTTTGTTCAA
CAAGTTGCCATAGCCGTGGTGAATTCACAGTTCAACTGATCGTAAGAGAGTGCAACAATTCTCTTAAATT
ATACAGTGATGAGTGTTACCTAGTTATAGCTAGAATAAATCATTTATTTAAATAACAATTTTATATAATT
ATATTGGCAATTTTAAATAATTCTAAATAACTGCCTATATAAATATTTGGAAAATTAGGCGTAAATTTCA
TTCTATATATGAACTTATATAAAAGAAAATAAGATGATTAGAAAGATTGCTATATTGCTATGTTTCTATT
GCGGTTAAAACTGAACCGAATAAGACTATTATTAGATATCCCAATTGATAAAAAATTTTTCTTACAGCAAT
CCTGATAGTTTATCCCAGCGTCGTTGGGGATCCATGAGACA
>dme_circ_0004712_junction_seq
CCCAGCGTCGTTGGGGATCCATGAGACAGCTGCAGTAAGCTTTCCACCAAACATGG
>dme_circ_0004713
GCTGCAGTAAGCTTTCCACCAAACATGGCACCAATGCCCCGAATTCAGTTACCCAATGGAAATTCTTCCA
TAAAGGCTAGCACAAATGATTTTCTCTTTGCGGAAACAATGTCCGGCGACAGTAGTCCCACCCCCAGCAG
TCCGCCATCCAGTACAGGCAGGCGTTGCCAAATCTCAGTGTTCTTCCCTTTCGGATGGCGAATCCTTTGA
GGGATATGGCGAAAATGAGTATCCCACCCAACTTCGCGAAGCCCGCAGCTCCAATTCCAATGGGAGCCAT

AATATGAGCAATCATATTAACAATAATTCTAATATCAGCGTGGGcaacaacagtggacacaaccacagtg
gacacTCGAACGATGGAAATAACAACCTAAATGGCAGCACTGGCGTCGAACTCGATTTGGCTCCACACGT
CGGCTCATCAACACCTCAAGAGGTAATCTCGCCCATTTTGATCGGATCACATCTCTTAAAATGGCAATCG
AAGCGAATGCGAATTTGCTTAGTTGTCGGGCATTTATAAGGTTGCGCACATAATCCAGCTGAATACGTAA
TAGCCGTGAATATAAAATAAAGCTGACCAGGCCCAGAGACTTCATCAATTCAAAACTCTCAATTTGTCTG
TCCGAGGCAGCATAAAAAAAAGCGTATTTGAATAGTTAACCTTTTCTTTGTGTAAACCTGTGTCTGTTGGT
TTCAACGCATGTATGTGTGGCATGAAAAATGCATACACAGACTGAAACCAGTTTTTTCTATGCTCTGACT
TGGGCTCATGAATCGCTTTGGGCCGTGATTCATGCGTGTCTCTGTCTTCGGGTCTCGGAATCTCTCACAA
CTCAGCGATCGTGCCTCTCTTTCTGGTCGTGTTCCGCCGTCTCTTTTACTCTATTTCCGCCTTTGTTCAA
CAAGTTGCCATAGCCGTGGTGAATTCACAGTTCAACTGATCGTAAGAGAGTGCAACAATTCTCTTAAATT
ATACAGTGATGAGTGTTACCTAGTTATAGCTAGAATAAATCATTTATTTAAATAACAATTTTATATAATT
ATATTGGCAATTTTAAATAATTCTAAATAACTGCCTATATAAATATTTGGAAAATTAGGCGTAAATTTCA
TTCTATATATGAACTTATATAAAAGAAAATAAGATGATTAGAAAGATTGCTATATTGCTATGTTTCTATT
GCGGTTAAAACTGAACCGAATAAGACTATTATTAGATATCCCAATTGATAAAAATTTTTCTTACAGCAAT
CCTGATAGTTTATCCCAGCGTCGTTGGGGATCCATGAGACAGTAAGCACCCTTGAATATAATTTCTTTCG
GGGTTTCATGTAGAGCTTCATATAAATCATAACCCCCATTCTTGCTCCAttttggttttggtttttttgat
ttgtaaataaaaaatatttataatttatcttaattaaaaaaatTATTGGTGCACTGCTCCTTAAAAAATAT
ATATATATAGTTTTTAATTCAGTATTTGTCCTCATCACATTTGATGTTTTCACAAGCGTTTCCATGACTT
CGCTTATCTAAGATTGTTAATTTCAGCTATAAGTTGCCTTTGGTTTTTTTCCCAATTTTCCATTAGCTGG
TTTGCTCAATTGCTTGTTTTTCTTCTGCCTAAATTAGTTATCAGTTTTTCCCATCTTATTCGCCTGTTTC
AATTGTTGTACAACTACATAGATGTTCAATTGGGCAATTTTCAAGAGCTTTCCTAACGCTGCCACCCCCA
AAAACTAAAATTTATGATTATTGCCGGCGGGGGAGGCTGTCATAAAAGTTACAACCGaaaaaaaaaTTGC
TCTCTTTTCTGGGACTTAATTGCAACTGAAACCAAAACGAAAGTAAAATAAATATATCAGATTAACAGCA
TGAGATTTATGTTAGTACAATGAGAGAGCTCCAAGATGGAATCTGCTGCAATTTATATAACTTCCTTTGG
CAGTTCTGCTTGTCAACTGGCGACCGTGCTTCCTGCTGCCATCTTCTTGAGCCAAAAGTAGTTTGGCCGC
TTTTGTGAACTTTTCTCGGGGACCGAGCGAAAAGGTTGAAGGTAGAAGATGCGGGTCTTTTCTTGTCTCC
CCTCAATTTAGCCAACTCCTTCCCTCTTGATGTGGCTTACAAGTTGGCTGACTCGATGGGGTTGTCATGC
TGTTGGAATTTATTTTTTCGCTCATCCGATCGGAGGAAGTGAAGTGAGAGGGTTCCAGAAACAGGTTTAA
CTTGCCGCAATTGTTAACTCTTCAGAATTTAGGCCGGCACTTTTGTCAAGTCGCGAACAAAATTTGACCC
AAAATAAAACTCAGTCTGCTGATGTTATCGATTCTGACACATTTATCTCGTATCGTTATGTACTGTTATA
TACttttgatcattttgatcattAAACTAAGGCTGTAATAGTTAGATCATTACTAGAGCATCGATCAATA
TCGATTAGCATTTCAATTATTAAACAAATTTTTTTCATAAACATTTCTTCGAAAATTATGCACGAAATCG
GATAAAGTAATTAAAATCGTCTGCATTTAAGGCGTGTGCAAATCAGTTAACCCCACGCAGGCTATAAATT
GTGGTTGCTCTGTCAACTTTCCGGACCTTGATGTTCTTCCCATGGCTTCTACTCTTTTCGAAGCAACTAC
AACTTGGGCCGACTTGAATTTGTCATGCAAATGCCTTTGTTTTTGCTCCGCTGCTGGGAACATGTTTTTC
CCACTCTCTGCACTTGGCATTCAAAAAAGCTGCACTGGGAAATGGTGGGGGAAAATGCATTAAAGAACCG
AGAACAATTAGGGAAAATTGTGGGAAGAGGTTAAAGATTTTTATCTGAAAAAGGAAAGGGAATGAGTGGA
ATTCTACAATTATTTTCTTTAAAAGAACGAATATTTTTCCACATAGGAACATTTTAAATGTTGTCATTTG
AATTCAGTCTAGCAAGCAAAGAAAATACTTTTCAAATCACATTATAAAAGTGATAACATCACCGGTTGGT
TATCCGTTTTAATACAACCTAAATTACAGATAGAAACTAACAAAAAGTCAGGCCCAAAATGTGGAACTAT
CGTCAGAAAGTTGATTTGGAAACATCGGTAAAGATATCGCCGTATTGCAATTCTTGGCCATCGGCTTGTT
GGTTTACTCTTAAAGGCATTGATTTGTTGAAACTTCTGGTATTGGAAAGATCACCAACTTACCTGGTGAT
TCTTACGATTGTCCTTATCGTACTCCTCTTACTTGTATTTAAAAGATTCAAACACAAATGGAACGATGTA
AGTTAAAggggggggAAAACGTACAGTAGAAGGGGCTTAATAGGGTTTTGTTCTAGGTGCCTATGTCGGA

GATTGAGTCACTGAGAAAACGTGTTAAGTTTGTAACCAAGGATATGGCCATGCTGCAAGAAGCCCTAAAT
TCGACCGTAATACCCAAGCCGCCGCTAGCGATTTCTGATGATATTAGGAGTAATATTAATTACTTGGATG
AACCAGAGTCCAACGCAAATGATGACAAACTTTCAGAATTTAAAGAAATCTTTGATCCTTTCCCCCCAGA
ATTCGACAAAATTAGTGAAACTAAAGCGCTAGTACGAGAAGAAAATAAACCGGAGCCTGTGATTCCAGAC
GCCGAACCTCGAGAAGATTCCGTAGTAAAAAATAGGGTGCGATTTGCTCTAACAGATCAGGGAAAACCAG
CCAAAGATGAGACCAAGCACATTAATATAGCGAAACCCACGAAGATTATAGCTGAAAATAATGTATCCGT
GGAAAATAGAAATATGCGTAAACGGTCATCTCCTTCAAACACGAAAACGGGATGGAAGCTCTAGATCTAT
TTTCAGGAATTTTCATATTGAAATTTCATTAAAATTGTAGTGCTCGAATTTGTAATAAACCATTCATAGT
GATTTGTATAAAACTTGTTGTGATCGTAATTCATTATAGCCCTATGCCAACCTTGACATCTTGCCTTCGC
AAGCGTTTTGGCTTTAAGTGATTTCCAATTGTCTTTCGTGGTAATTTTCACCTATTTCATTGTGTATCTC
TGTTTTCCCGTGCATAGTTCCCAAGTCTACGTGGTTGGCCCTCAATTAGCCTTAAAGGTGGCCGGCTGCC
TAATCGAGGCAGTTTCCAAGCCACTTTGCGGGGGCCCAACATTGTTTGGCAGTTGGCTTGCATTTCTTGG
CTATTTGGCAGACCTGGCGACAATTTCCCGTTTTTTTCCCCCTGCATGTATTTGAATTATTTGGCAGCTG
AAAACGGCTTGAAAGTACGTCTGTCGCCAATTTGGCTTCGGTTTTAGGTAATTGCCATCTATCTAATAGG
TACATGAGAATGTTTATTGTTGGCCAGATTTGTAGATGTTGCATAGTTTTAGTTTTTAGTATCGCCGAAC
AACATGTTGGCAGCAAACAAGTTGCATGGGAAAGTGTATGGGATTGTAAAAGAGTTGCCAGATAAAAAAG
AAAAACAGTACCATCATCAAAGACCTTTCAACATTACTAATGAGACTATAAATGTTGACTTAGGTGGCAA
CAAAGAATAGTAAATTAAATATTTAACAGTATTGAAATGTTATCTTAAaatattatttttagtatatta
ttttaagaaaagaatatatttattgatatatGTGAAATCGTATTATTGAATGCTGAAAGAAGAGGAATTT
GAATGAAGATGATTAGAATTAGAAATTATGTTATTAAATATATAAATTATAAATATTCTACCATTTTCTC
TTAGGAATAAGCAAATATATTTCTCCATTTTCATTATAATTATATTTTTTTATGTCACAAATGATTGACA
TTTGTAAGTTCACACTCGCTGTGTAATTATTTTAGTCTGTCTGGCAAGTTCGAACTGCAACAATGGTTTT
AGCCCTCTCCCACACACACTTGTTCGCTCTTTTGGCCTGCTTTTCCGTTTTGTTTATTATTTGGCTAGTT
GGCTCTCTCGCATCGAACCTGTTTGCTGGTCGCCTTTAGATTGGAGCAGGCGGCTCAAAAGTCGGAATGT
GGTATTCGGTTTTAGTTCGAACATCGCTGTAACCGCGTGCGGATGTCAGGAAATTTGTGTCGTCCTTTTT
TGGCCTGATTTGATTATTTATTAATCGCCCGAGAGTCTGTGACTTGTGTTCCGGTGTAAAACCGAATAAA
AATCATACAAAATTCCGCCGCATCCATTGAGAAAACCAAAGTATGTGAAAgtgtgtgtgtgtgtgtgtgG
CATTTGTTTATATACAAATGTGTATATAGCATACATATGATTTTTGGGCGCACCAGACAACGCGGCGTAT
GCGAAACGTTTGTGCAAATAGATTTAATTGTGttttttttttgtcggttttgtttttGGCTATAAATTTCG
CTTTCCACGGAATAGAAAACGTTTTTAATTCGAGCTGCGCGCTTTTATGAGATTTCGTACATAGTTTTGT
GCTGTAGTTTACACTTTGGTAATTTACGGCGTCACGCCTTAATGACGTCCAACTACCATCATAATAGCCA
CAATAACAAGCGCAGTCTTTGGCTATATACTGCACATTCCCATGCAAAATCGGAGGGTCTTCGCCGTCTT
CTCGTGCGTTGTCCTCATCTCCATCTTCTTCGACATGTGTGTCTCGTCTTTTGCACGTGACTTTTCCCGG
GGTTTCCACGGGCCCACGCCCTTTTGTGCTGCACTTTTCCCATGCATTCATCCAGATTAAGGCCCATTTG
TACACAGACGGGTAAAAAAAGAGGTTGTTCAGAATGTATTAAAGTGTAGGGTTTGATACACAGGTGGTAT
TCCAATTCGAATAGACTTTCATTCATTTCTGAAAGTGCTTTATAAATAAACTATTACATGAATTAAAATA
ACGATTTTTATCTTATTTTGAGTGTACTACCAAAACAGTTCTTATTGAAAATCCAATTATTTGTTAACCA
CAAAGTTTTCCACGAAAAAAGAATAAAAAACCTCCAAGAGCGTGTTTGATAATTCCTAAAGCATACTAAA
ATATTGATAAACAACTCAGAACTGGTGTTAGTTATGTTGGATCATTTGAATTTTAAATTGTGAGAAAGTT
ATTAAAATATTGCATACACAAGTTAATGTTTAAAGCTGCGTATTCTTTTACAAAATAAATAACATTTTAA
AGTAATTTGAACTTTAGCTTTCTAATAAAGTATATGTTTTAATCATTTCCTTTAACATTACACACTTATT
TCAACACCTTTTATAAGCAGTAACGACATGGCCAATCAACCGAATCTCTAGATTGCGTTTAGATCTAAAA
GAGTAATCGACTTTTTAAAGTGCTTTCGTTTCCATCTATTGCCACACTCATCGCATCATCTTCAACTGGG
CTCTATAGCGTTTCATCCCTTTTCTACACTCGCATTGTCGAGAGTCATCAATCAAAATCGAATCAGTTTT

ATATCGCCTTAATTATCCGAAGAGCAGCCATATAAATGCTGCCCGTTCGCGGCTGTACGATTTCATTGTT
AGTTGGTCAGTGCAGTCGAGAGTGCAGTTTGGCTGCAATTTTCTTCTTTTCGATTTTTGAGTTTGTGTT
CGTTTTCGATTTCGCTGGCAGAGAGCGTTTAACGACGTTATCAGAGCGATTTCCAGCAGCGTGTTGGCTA
AATATTAACCATCTCATCCGCTCATCGAGTGCAAAAATTGCGGCTTGGCTTGGATGTTCGGCTTGAGAAG
ACTCGTAATGAAAAGTGAAGTGTGGCTGGCGGATCCGTCAATTGCGAAAGTGTGTCTAATTTGCCGATGA
TGAGTGCGAGTGCGTTTTCTCGAATGAAAATTTCCTTTTTGTGGCAAATAACGAGCTAACAATTTGCTGA
ACGGCTCATAAACCAAATCAAAAGTGAAGTTAATGGAGGTGCAAACATCTGGTGCACCAGTGTAAATAGT
CATACATCGTGGAGGGTCTTGGAAAAATGTTAAATTCACCAGATTTTTGCACGTTTTACATACAAGTTCC
TAATTGATTTACCAACTTTGTATTGCCTTTAATCATAAAGAGCTGTTAATATTGTTGTAACACACAAATA
AAATAAGCAAAGGAATTCAAGCTTTTAATCTTATCAAAAAGATAATAAAGTTTACGTTATCGGTTTAAAT
CAACATATTAATGTCAATGACGTTCACAATGCGGCCAATGGCATTTAAAACTGATAACCAAGTGAGCTAA
CCCAACGATCAAAGATAAACATCTAAATAAACAATTCAGTTAACGAAAACAGATCTCACCTAAATGGATG
ATGAATATTTTGAGTATTTTCCCTATGACCGCCGTCGCAGTCATTTGAGAGACATGTGCGTATTAAACCC
CTTACTAGAATTCGAAATCCTTCACCAACTTGTTGTTACTGTTGCACTTAGCTTAATTCATATTGTTTGC
GATATCCATATTTAATTCTCAAGAAAGCCACCGAGCCACGCCCACCACAAGTTAGCCGGAGAAGCCGACG
CCCACATGTCCATAAATACTTTCACTTGTCCGGTAACAAAAGACGCACATTTCTACTTTATATTTTCTGT
GACTCAACGGCATTATTTCTTTGGAAATCGCAACAATCGCGACATTTCTTGACCCCCAGTTGTTTCTTTA
ACGCTTCTGAAGAAAATAACTGAAGCACAACGAAATAGTTTGGGTAAAAGTTTTTTGTAAGTCGGAAAAA
CTATTTGGGACAATTTTTGGGTTTCAACAATACTACCAATGAGTGTATTAATTTATGAATTCTGATTTTC
TTAGTGTTTTTATATTTATTACTTAAATAATTAGAAAATAGACATAACCTACGTATTATTATAATTCCtt
tttttttttAACTATCTCCTTCCGAAATCTTTTTATATTGCCGCCCTTTCTCTGGCTCTGTGGAACTTTA
ATGAATACCTTACCTCCTCTTTCCATAATTTAATTATCCACAAAAATCTGAAAAGAAACCAGATACTCTC
GTAAGAAAAAACCGGTCTAGGGTGCCGAAAAAAATAGTTGTTTTTAATAGTTTCAATTAGCTGAACTTCCCA
GGCGCTTAATGAGCGGTTTTCAGGCTGAAGTGGCGGAGGGGGCGTGGCCAGGTGAGTGGGCAAGGTGTGCT
TCACCCTTTGGCAAGACTGCTGAAATAGTAACAGTAACAGTAAGTCCGAATCACAAAACCACTGTGCATT
GAGGCGTTTCGCCGTGAACAATAATTTCTGCAATAGAAAACTTGATTTGGGCAGGCCAAAATAAAGCTTC
CGCCAGATTATGTGGGTCAATCTCACCTGACGGGGACAGGTGTTTGTCTAACTTAtttttaccctgtttt
tgtttgccttttccttttttgtttttttattttttGCATAATCCGATCGTGATTGATTGACGTGCTTTGAGG
ATTGGCGCGAAAATATGAGACATAAATTGCACTAAAGAAAGACAGGGAAAACAAAGAGAAATACTGAAAA
TGGAAGAAACTCGAGGGGATAATGGCAGTAAACACACAAACTTGACGGGTATTCATTGTCTGTTTTTCTG
TTATTGTTTTGAATTTTAAGTAGTAAAGTTCAAGAAACTTTTGCCACCTTAACTTAGTTGATTATTTAAT
TTCGGAAGGCCCTCAGACTTGAGATCATTCAAGTTGGACTTTTACTTTAATTTGCGTCGAGCCTCGTAAC
AAGTATATACATATGTATGTACATACGTTAAACTGGTTGCCACTTCTGTGTAAAGAGTTAACTCGTTATG
AGACGAGTCCCTTTGTCAGGTGACTTACGATGAGGAGAGGGGGGGCTTTCCCTGCATTCAATTAACAATTT
CTTGCTAAAAGCTGCAAGTGACGTACCTCAAGGTAGTTAAGTGAATTTAACTGAACCGCTTTTTTGCCAT
CTTCGTGTGAGAGACGCTCTGTCATCAACTCGAGATATTTAAAAAATGAATATTACTAGTTATTAGTTAC
TTTTAAAGTGCTTACACTCATGTTTCGCTTATTTTTACGTCTTACAAAAACTTACTGAAGTTTACTTTAT
GAAAATTTAATTACATCTTAAACGTTAATCTACCTTGCACTCAATGTGCTATATATGCATTTAATAGTTA
TAAATTCTAAATAGGAGTTTTAATTCTATAGTACAGCCTTTTTACCATTACATTCCACTTGTTGCTGATT
ATACAATCTACATAGGCCAACGAATACCTGTGGAAAATTACATAAACAAATATATATATATTTACATTTG
GTTATGACTGCAAATCATTCTAAATAACAATTGAAATACCTTTGCAAAGCTCCCACTCCGATTTTCGTGC
CTACCCCACTCCCATCCTATTCAGGTGACCCCTCGCAAAGGTCAACGAAGCGAAACGCATTGCGAGAGGG
AATAGAATAAATGAGGGGTGGTGGGGGGTGGTCAGGGAAATGAGCATGCAATCAGTAGTAATTGGTAAAA
ACGTAAAAATAAATGCTTAGAAGCGGTAGACTTTAGTATTCAACAGGTGTTTGCCAAAATGCATTGACCA

TTTTTACCTAACCATAATTTATTTGAACATTCATATTGTCTCTTTTGATTTATTATCTTTGGAAACATGC
AAAGAATCGTAAAAAGAACCCAAAACGATGAAAGAGTTCAAATGAGTACAAAGGAAAACCGCTGCATGAT
TTCCGGTCAAAGTCTCGAATTTCATTGAAATTTTCCAGCACTCCCTCTCCGAAATTCAAGGTTACCCAAA
TGCCGAAAACTGATTCAGTAATTCAGACTCCACTCCCCTTTGATCAACACTCCTCAGAATACTGCCAACG
ACCTTAATCATATTTAAACAtttttttttattttttGGTTGCTATAGAGATTACATGCCATTACCTTGAT
CGTTTTTTGTCTACACTTAACTGTCATTTTTTGATAGCTTATTTTCTTTTTCTGGCTTGTTTTATGTGGA
CTTTTCGTTGGCTTTAATGTGAGGAAACAACAAATTAACaaaaaaaaaaaGTTGTTTGCGTTATACAAGT
TTATGTTTTATCGCTGAATTTTTCATTTTTAATGCTGTAGTTTAATTGCTTTTGCATCACATAATAAAAC
AGTCGACTGTAATTTTGCCAATTTCCAAGGTTATAATCAATGAAATCTGTTATGCTAATACATATATTTA
ATCCACCAGTTTTTCTTGATTTTCTCAGATTAAGTACGTATCTTAATTCTTTAGTTCTCATTTATTCATT
ATTTTGTGAATAGCACCAGTAAAATACAACTTTTGCTCGACGAGTATAAAGTTGCAACTTTTTGGCTGCC
ACTTTATGTCAACTTCACTTTGCATCAATAAATTGCCAGCCGGACCAATGACTCATAAAAAGCGGCTATT
CAACTAACCGCAAATTCCtttttttttttGCATTTTCTGCTTCTGCTTTTTGGCTGCAAAAATTGCGCAA
CATTTTGCAGCAGGTCCCCTCAACACTTTTTGGCTCTGGTCAATTTGCAATAATTTGCTGGCTTGttttt
cgatttttttttttttttGCATTTTCTACGAGCACGCAACTTCAATTTGTATGTTTGCTTTTTACATAAA
GTAAATTAAAACATAAAATAAAGTTGTGTTCGTAGAGTGCCATAAACCCAGATGAGATAGATTTTTATGA
ATGTCTTATTGtttttttttttttttttttttttttttGAGCTCAACTACACTGAAAAGTGGGCTAATGA
GTTCAGTTGGGGCCGCAGTGAGACAGAAAGAGTCGAATAAAAAAGTGGAATAACTAACGTTTTGTTTGCA
AACTTTTCTGGCTTTGCAAATTTTGCTTAATATCTTGTTTTCAATTTTCCAAAACGGAAACTATTTGCGG
TTTTTAAATCCACGGTTTTAAGGGCACGGCCTCACTAAAATACAAATATATTCCATGAATTTGCCATGTG
AGATCACCTCAGATAGAAACCGCAAAAttttttttttttAGCCCATAAAGAGAACAAAATACTAACAATT
GCAAGTGATGTTATAGTTTGATGGGTATTTTATGTGTTGGTAAAAATAAAACTGTGCATAATTTTGTTTT
TAATTTTAAGCGAGTCTTTTTCTATACGCGTATAATATTTTGTTAATATGCAACAACTCAAGTATCCCTA
CTGAATATTTATTTAAAAAGCCTTTCAGCAAAAGCATACAAATAAATGAAGACACTTTTCCGGCATTTCT
TTCTCCTTCATTTGGCTTTCATCTGCTCATCTGTAGTTGCAAAATGTCTCCCACCTTTGACCCATTTTCC
CGATATGCACTTTAGACAAACACACACATTTTCCCCCACTTTTCCACTTTTACTCGAAATTTAGTCGTCT
GCTTCTTTGTGTCTTTTGAGTCTCTTTATCTGTCTACATCAACATCTTTCGCAATTATTTTGTCTTTGAC
GTTTCTTTGCTGCTACAATTTCTTGGCTGCATTTCGACTTTTCCCATTTTCTTTTTACCTGTTCGCGTGG
GTGGCGCAGTTTTTGTTGTATTTTGGCTGCTACCATTGGTTGTGTTGTTTCCTTCATTATCACGTGTTTG
GTTTCTTTTTCCTTTTGTTTGGCTGCCTGCGTCAAGTTGTTATTCAAGTTGCCTTTTGTGGATAATTTCT
TTTTTGTTGGCCATTCAATGCCAGCCAATTTATTTGCATTTCGAATTGCTGGAATTTCATCCATTTTGAA
GGGCTTCTATGGGTTGCGGCTCGCTTAGGCTTAGGGTTAAATTTGCATTTATCTATTCTTAGTGGTTTGG
TGGTTTGTTGGTAGAGTGCGGAAAGTATGTTTTTATTTACTAATCAATGGTTTATTTTCCGTTTTCAGTT
CCGGCCGCAGGCAAATTAGTTCAAATGCCTTAGCCAGGTGAGTTCGCTACTTATTTATAACATTACCAAG
AAATATTATAAACACGATTTTATGATTAGTTTTTTTGTGGAGTTTTTTCCAATACAGCAAGAAAATGATA
TTTTGCTGTACGAAAAACATTAAAATCTTAAAACTTTTAAGATGTTTTATACAGACTCTTTTTAGTTTAT
GTAAATTTTACATTTTACATGCTGGGCTCACAATGTCACATGGGCAGCACTGGTTGCGTTAAATTTTGAC
AGCACTGAAACAGAAGTCGCTTACAGTGCCCATTCGCAGCCGGCAAGTATGCCGCACATATTGAATTCGC
ATTGTGTGCTAACATTGTGCTACAGTTTGAGATCAATTGTTTAGTTGGTTAATGTCTTGGCTAAATCTTT
ATATCTCAGTTTGCGGAAAGAAATGCAAGATTCTATATTATAACTTAAAAAGATAGCTAAAATTAAATTT
AAATTAATTTAAATTTCTTTTTAAGTTGTAAATGTTTCGTTTTTGTCTTTTGGCATTGTTTAAGTTTTTT
GCTGTATGTGATGGAAATATTAGAGACTTGCACCAATTTATATACATTTTCATTTACATTGTCAAGTAAA
AATGTATTACACCGAGGCGAATTCGAAACGGTATTAATCGAATTCTAAGGCGAATTCGTCCAATGGATTG
TTTTAACttttttttttttattttttttGTTATGTTTTAAAAATAACACTAACTAGAACAAAATTGAAAG

TTTAGAGATATACAGACACAGATATATATATCAATATAATGTGACTTtaaaaatttaataaacagttaaa
actaaatataaaaattatttgatttaattacttacaaattCAACTCCTTTTAGCCAACTCTATAGATCAT
CGAGCTTTAACTCATCGGGCCGTAGCTCCAACTGTGATACAACAGAAGACATGTACAGCGACATAAGCTT
GGAGAATCGTCATGACTACGATTATAGG
>dme_circ_0004713_junction_seq
GGAGAATCGTCATGACTACGATTATAGGGCTGCAGTAAGCTTTCCACCAAACATGG
>dme_circ_0004714
GGTAAGCAAAACAATAGCTAAACACAAACTACCTCGAGCACTCTTATCTTACCAATTCCACTTGGTAAGT
GCTAAAACGAACCCACAAAAGTGTTTCTTGTCTACCTTTTATCTAAGCTGAAAAATCGCCCGAAGAATTG
GCCATAATGTGAATTTATGTTCCCACACACACTCATACACTCGCAAGTCCATAAAAGAGGCGCCTATTTC
TGGGGGCGTGGCAGTGGGAGCAGTTGGCAAGTAGAAATCCTACGCTTTCCATTACGTCATGTTGGCTTTC
CGTATATTACTTTTTGCACAAGGTTGTCCAAGGCGAACGGGGCGTTACATTAATGGGTAACTAGTCAAAA
CGAATCGAATCGGTTTCGAGTTCGAGTTCAGTTGGGGCTACACTTGTCTTGGCCAACTATGGATTGTATC
TTTCTTGTCTTCGCTATTCGGTCGCACTACAATTTATTATAAGTAATGAGACAGTGGAGAGTCGAGTTAA
CAATTACGACCTACGTGCGAATAACCTTCTTTCGTTAAATCATCTTGAACTTTTGGCATTTTGAGGGGAG
ATTATCTAAGGGTCTTGAAGTAGCTTGtttttaatttttatttttatttaTGAGCGAAAAATACATAAAA
AATGATTATAAAGCTTATAAAGGGAAAGTCCGTGCAACTTATTAAATATGCCAAACCTACTTTATCGAAC
ATTGTGTTTACAACGTTCTCGGTTACTAGCCAATCTTATCGTACTTCAACCTTCCTTTATTTTCCCCTTA
TAACAATTCTTATGTATATGTATACCCAAATAGCAATTGTTGTCTTGGAAACTCACTAGGCCCTGGACTT
TAATATATACACAAAATGTGCTAAACCCAAAGAAGTCGGGGATAGAAGAGTTTTAAAGGAGTGCTATGCG
TTCATATAGACAACAAACAAATGAATAAATATAAATGCAATAGCAGCTTCTTTAAAACGTGGATAATTCT
CACAGTTTGTATTTGCTTTGGCAAATAAATATTGACTAttttttttGCCTAATTATACAAACATACACTC
ATCGCGTGGATGGATATATAAAGTTTGTACACAACTGCAAATGAAAGGTAAATATAGCCGGCTTTAAAAG
TATCTGTGAGTTTTGCGTTGATTAAGGCGGCGGTTAAACAAATTAGAAGAGTAAATAAGGTCTGTAAGAT
TAGACACGGATAAAGGTATTTTAAGGACAATATTACCTTAAAATATCCAAATATACCGATCATTTGCAAT
GTATTAAAAATTGTGGAAGCCAAAGCAGGCAtttttttttGCTACTGGATTTTATAAAGAAGGCTGTGAA
ATGAATTTCAACGAGCCCTTACTTCAGATCCCTGACTAGCCCAGAATATTCGTCAGTTCAGTTCCAGCTG
TTCTACTGTACGGTTCTCTTGAAAGTCGTTACttttttttATCATAAAGCTGACACCACCAAGTTATCCG
GTAAATATATTTCTTGTTTTTGAACAAGGCATCGTTCGATTTTAAACCTAATAGATCTGGCTTTTCTCCG
AAAATTTTCGGCATCTGCGAAGATGTCGTTGCCACGCGTTTACTTCGATATAGCCGCAGGAGGTGAGAAG
TTAGGTAGAATTGTGATGGAACTACGTTCGGATGTGGTGCCCAAGACGGCGGAGAATTTCCGGGCCCTCT
GCACCGGAGAGAGAAGGGGTACGGCTACAAGGGATCACCATTCCACCGCGTCATCCCGAATTTCATGTGTCA
GGGCGGTGATTTCACTAATCAAAACGGAACTGGAGGACGCTCCATTTATGGAAACAAGTTTCCCGATGAG
AATTTCGAGCTAAAGCATACCGGAGCTGGTGTACTTTCGATGGCCAATGCTGGGGCCAATACCAATGGAT
CTCAGTTTTTCATTTGCACCGGGAAAACCACATGGCTGGATAATAAGCATGTCGTGTTCGGCAAGGTCGT
TGAGGGAATGGATATCGTCCAGAAGGTAGAATCTTACGGTTCACAAGATGGGAAGACCAGCAAGAAA
>dme_circ_0004714_junction_seq
TTCACAAGATGGGAAGACCAGCAAGAAAGGTAAGCAAAACAATAGCTAAACACAAA
>dme_circ_0004715
GCTGGAACTGCTGCAGCGCAAGGTGGACGATCTGTCAGATACACAGAATATAGCTGAGGATCGGACAACC
CGCACCAAGACCGAATATGCAGTCCTTCAGGCGCGTTATCACATGCTCGAAGAGCAGTACCGAGAGGGTT
TGTATTTAATAATTCATTCGAGTTTTATTTATTTAAGTATTAAATATATACTTGTTTAATTATAAAAAAA
TAGTTAATGGTAGATAGGAAGTACGCTATATGAAAGAGGTCAAGAAAAACAAAACAAACTTCTTCGACAA
ATAATAATTTTGTTtttgtgtattcagtttgtagagtttgtgagtgagtttttagaAAGATAGTAATTAA

ATTTATCAAATTTAAAGCAACTAAATTACTTTGCTCAGAAAAATATATTTATGTAATGTCATCAGATTGA
TTAATCCCAATAACAATCAATTTATGAGATTATGAGATTTAATCATAACTCTTTTCTTGCAGGTCGGAAC
TGCGTGCCGAGGAGCGTTTGGCAGAGGAGCAGAAGCGGCACCGCGAGATTTTGGCCCGCGTGGAGCGTGA
AGCCTCGCTGCAGAACGAGAACTGCCAGATGAAGATCCGTGCCACCGAGATCGAGGCAACGGCACTGCGG
GAAGAAGCCGCCCGACTGCGTGTCCTGTGTGACAAGCAGGCCAATGACCTCCATCGCACCGAGGAACAGT
TGGAGCTGGCGCGCGACCAAATCGGCGTCCTGCAACAGGAGCACGAGGAGCAGGCACAGGCACTGCGCCG
CCACGAACAGGAGAAGAAGTCCACCGAGGAGCTGATGCTGGAGCTGGGCCGGGAACTGCAGCGTGCTCGC
GAGGAGAGTGGAGCCCGGGCAATGCCGACCACATCGCCGGAGAGCATAAGACTGGAGGAGCTGCATCAGG
AGCTCGAGGAGATGCGCCAGAAGAACCGAAGCCCTCGAAGAACAGAACGAGGAGCTGCAGGCCACGATGC
TGACCAACCAGGCCACCATGCTGACAAATGGCGTGGAGCAGGGTCGCCATCTGCTGAACGGAACACTCAA
CAGTTTGGCTCAGGAGCTCGAGGAGATGTCACAAGCCCAG
>dme_circ_0004715_junction_seq
GGAGCTCGAGGAGATGTCACAAGCCCAGGCTGGAACTGCTGCAGCGCAAGGTGGAC
>dme_circ_0004716
CTCCTCCAGTCTTATGCTCTCCGGCGATGTGGTCGGCATTGCCCGGGCTCCACTCTCCTCGCGAGCACGC
TGCAGTTCCCGGCCCAGCTCCAGCATCAGCTCCTCGGTGGACTTCTTCTCCTGTTCGTGGCGGCGCAGTG
CCTGTGCCTGCTCCTCGTGCTCCTGTTGCAGGACGCCGATTTGGTCGCGCGCCAGCTCCAACTGTTCCTC
GGTGCGATGGAGG
>dme_circ_0004716_junction_seq
CTCCAACTGTTCCTCGGTGCGATGGAGGCTCCTCCAGTCTTATGCTCTCCGGCGAT
>dme_circ_0004717
GTCCTCGGCGTCCTTATCAAAGCCTTGCGGGAAGTCCACGGCGCAGTCAAGAATCTCTTTAAAAATGACA
TAATCGTTGCTGCCACGGAATGGCGGTAGGCCGGCGATCATCTGATAAACTATACATCCCAGTGCCCACA
GG
>dme_circ_0004717_junction_seq
ATAAACTATACATCCCAGTGCCCACAGGGTCCTCGGCGTCCTTATCAAAGCCTTGC
>dme_circ_0004718
GGGAAAATAAATCAAATAAGTATTCAATGGTGTATAAGTGAGTGCAAGTAGATGAGCAACATGTGTGTGT
TTTAAAGTGTTTAAAGAGATCGAGAAAAAGCCCCAAAAGTTATTGACAGCGATATCGAAAAATTCCAAA
ATGTGGC
>dme_circ_0004718_junction_seq
GCGATATCGAAAAATTCCAAAATGTGGCGGGAAAATAAATCAAATAAGTATTCAAT
>dme_circ_0004719
GGTGCTCACCTTCTTGATTGACTCGTCGCGTTTTCGCTACCCGGAGAGGGCCATTGTCTTCTTGGCCGTT
TGCTACTTGGTAGTTGGATGTGCCTACGTGGCGGGACTGGGAGCGGGCGACTCTGTGTCGTGCCGCGAAC
CATTTCCGCCGCCCGTCAAACTCGGCCGCCTGCAGATGATGTCCACCATCACCCAGGGTGAGATGAGCCA
AGAGAAGCATCTCttttttttttttttttttttttttATTAATTTAtttattaaggcatacggggaagt
cttgaatcccctttgtgtccttcttatctattaattcagcccatttgggctcgccggctaactatagtta
gctttttcaaatttacagtaatttaactacaattacataacaatcacatataacaaataggatttaagat
aagcgtcagcttctgcggacccttgtcaaaggagatcgggtaatgagatccctcggttgccttctattga
gcctccttggtgggcgagatctgtttaaagcgctggccagccgatttctgtggcgctccagcctgtcatg
gtatctgctcgagtgcttgtttatttcgtcaaataccgttgcgagtttcaggtctctgtgcagggtggtg
tttcgaacaaaccactcgcagccggtgatgagtcttgctaccttattttgaatagcctggatccttttga
tgtggctgtcgcaggctaagccccagatttggcacccatacttccagtttggtgctaggatctgtttgta

aattgtcagcttgttggatagcgacaatttactgcgcgaagaaagtagccagtagtgcttcgccacctta
gcacgtaggcgcgttctgatgtcggtcacatgcttggaaaatgtgagtctgcgatccagaagcactccaa
ggtactttgctgcgttcggctgtggtacggggacttcctcgatgtagacgggcggtggcgttctccgctt
taaagtgaagcagacgttgttggatttactgctattgatgccaatattccatcgtcttgcccattccgag
aaccggtatgcaaagtcctggataccatcggctgcgtcgtgctcgcatcgggacctgtaggtgacgcaca
cgtcatcggcaaatgtggccaacatagatttcccgtaaaggcttacatctggctgcggcatgtcgtggct
atacaggcagtagagcagggggccgaggacactaccttgtggaacaccagctgccacgttgtgctcggtg
gaaattgctgaatgaaagcgcacagcgaacctccttccttccaggtacgatttcagcaacccaaaatatg
gagcaggcagcgtctgcttgattttcagttggagtccaatgtgccacactcgatcaaacgcttctcgaat
gtccaagaagaggctgttacaatattccttactgtcgtaggcagtcagaatttgctcgacgactcgatgg
agctgctcgacagtacagtggccagcacggaaaccgaactggtgctcaggggtaatcccctgggcttcca
taatccttacaatccggacagcaatcagtctctcaaacaccttcgaaattgaagggaggagactgatcgg
ccggtaggaggcgggctccctttccggcttgccaggtttgtggatcatggagattatcccgagcttccac
tgataagggaagtattgcaacctcacaatagcgttgaaaaccaacgttatgtagaggatcgcttgtctgg
gcagggccttcaatgtggcgttgcagataacgtcatgtccaggtgctttgttgttactctggcgcacaat
gacttcggctacctcgcagggttcaaacggcgtgattggcatatccatttgaagcgcttggttcagctgg
tcctgagtttcttcaacctgttgcaggctggcaagcttgaatggttgaaatcgctcggcgaggtgcgcag
cgaatacctccgcttgtcccaagtcggtccgacaccatgtcccgttgctatcgactagaggcgccttcct
cgtgcaacgccttttgattgcgcgcgtggccttccacagcgaatgcgtcgcttcgctttcgactccggta
ttggcaagcctttcgtcgaaccatgcggctttgtgcagcaccaacgcgcatcggagcctactcagagctc
ggttccattccgttttgtccgacgggtgccgagacctcatccacctagtgcgtaagcgcctcttctcagc
tataagcagcaggatctccctgggtattgggattcgatctgccgcgggctgccgagatatgctgcgagtt
gccaatctagctgctgacttgatattgttggtgagcagttcgatagcatcatcaacgtcctgtccagagt
ttatggcagtgttgaggggcagtgtggactccagttgcgattggaatacctccaggttagtacgcctagt
gataagccgctccatcttagggtaggatattgcccccgcatccagcgtaattaccagaggcaggtggtcg
gagctaagctcaacaaccgcacttatgttagcgtggatgcccagcacaccctttgtcagtgcaaaatcga
tgtaccctggtgagcctctacagccgtacgggtatcttgttggtcctcctgttgctagcgagtccacctc
cgaatttaggacgagattcgctaatgcaatgcctctttggttgctccttcccgcaccccagagccagtgg
gacgcgttccaatcgcctgcaacaatgaacttcgtctggaactcttccaaaatgtccttaaactcgtccg
tagtccatgcaaatctcggtgggcagtagaccgctccaaagcttataggaccaagtgccgtttcaataac
cgccggcgcaagttgcaccttggcagtggctattggtgttagcggaaagtgggcaaggctatttttgatt
aagatagctgctccgcctttggcgttgccaccactcggatcattggcagtgtaggctacaaatccgaata
gcttaggcgtctctgccccccttgcagtgtgtctcgctgagcagtaatacatcgatttcgtgacgtcgcac
gaagcactctacttcaggcaacttcgtggatacgccgtcggcgttccacactaggatgcgcagcggcatc
attatggcgcttgacgcgcttggagagcagtgagcagattgataatctgcccctgctgatgtgactgttg
ttgctgttgcttcatcatcatcatcattaattccatttgcttctcttgaatcgagcgcaaagaattaatg
ttctcattaagcgtctgcaggatggcttcaatgctgctgctagcatccgtcgagcgctggagtgtacccc
tagacgcccttgtccggctgcggctcctttgacgactggggcttccatcatagggctgttgctgttgctt
ctgttgctggtgcgtcaggctcattggttgcttgtggtgaatggaccctctagccacatctgcataagat
attccgcctcgagaagaggggatctccttacctccagctaagccccgtggagttggtcgctcgcttactt
ctgttcttccagccactccacttctgggtctggatcgctgcaggaacgcttgtagcttgctgcaaccttt
atagtttgcaggatgctggccgccacagttgtagcaagtgcacagctcgatgcgtggcctggtacagtcc
ttggctgggtgttcgccggcacatttcacacaaatgtgggccttacggcagtatttggctgtgtggccaa
actgctgacaacgatggcactggacaatagcgttacgcttgcggagaggctcaataactactctcatatg

gcatagtgcctttaccgccaggatctccttattgtttgccgcagcagcaatatttaggaagaacatattg
actggcgacgcttttgaggtacccggttcgtttcgcctaagcggtgtgtgaatatcgagaaccttgtgcc
cgatcttttccagctcatccttgatttggtgatgtagcgtactgtggtgcaggcctttcacgcataccct
gtaaggcctgtcctctttaagctggtagcagtggaactcatactcttcgatctcgagccagttcactatg
gtgcgaaatgtattagcgtcagccgcgtaaagccttaggacaccgctcattgacgccctagcctcaatct
tttctaggtccactatattttcgatgctctgcatcatcaggtatacgtcgtccactccctccattactat
aggaggtggtttggactgcgcctttttggctgagctagtggtaccagcggaggctgcatcccgcttagcg
gcagcaattgcagcttgatgactggtactgggctggtcatacagatcgctaatttcattgcctacttgtt
tgtcagctgtgagatgcgcaagtaaaccaaatcggtttgaattgccgccatatctaggttctgtagccga
agggttttccccttgtttgccgatggttgatttgggttttttcctttcagtctccggagaagcacgagac
tttcgctttatttttttgaaagcgctgcggggcagcacattgggggctcgagagaaacgcagtatccaacg
attcatcagccgcagcggtttcatttaaagtgttcatattgaagtgtgcaccaaatcaccagggttctag
ccaaaacttgaaccctgggatagaaagctttaagctcgcaaacagctgtttgtgcgatcgcgtggcagct
gggcagcggagccccagttcgagaaacttaactttgtatttttgaatgaaaatgttttcgtttcactatt
atttcactgttagtgacttggggggtcactatttgctatgtgtgccagtattctggtcttgtccataagt
ttattaccgcgaaaactaatttcacttaatccactttgtcaggtgcgtattgttagacacgaacctttTT
GTATGAAGCATCTCTGTAGCGACTGGGAAACCGGTCGTCAATTATTTTTAATGCCACCGAAAGTGCAAAT
TTTATTTGCTGAAGTTAAACCGAAATTCAGAACTACAGATATAGAGaataataaaaataaaaatataaaa
atgttaataaaaataTTTATCCTCTTAGGATTATATAAATTTAATATATATTCTCAGCATGAGTACCTTA
AAACTGTGGCAGGTCAAAGCTTTAACTTAATTGTTATTTTATTGACCGAGTTTCTTTTTCTTTTCTCCTA
CAGGGGCCACCGACAAACCACGTCCTGCACGGTTTTATTCATGGCACTCTACTTCTGCTGCATGGCGGCC
TTCGCGTGGTGGTCGTGTCTGGCATTCGCCTGGTTTTTGGCCGCTGGCCTCAAATGGGGCCACGAGGCGA
TTGAGAACAAGTCGCACTTATTCCACCTGGTTGCCTGGGCGGTGCCCGCCCTTCAGACCATCTCCGTTCT
GGCCCTGGCTAAAGTTGAAG
>dme_circ_0004719_junction_seq
TCCGTTCTGGCCCTGGCTAAAGTTGAAGGGTGCTCACCTTCTTGATTGACTCGTCG
>dme_circ_0004720
gtctctggaggcaagccagtggagatcatgacccatgccgatgacggtcttattcccgacatgtccgttg
cttcagacgccgacgtgacccccaagcagactgttgccggtctcaaggccctgccccagttgaccaagg
>dme_circ_0004720_junction_seq
ctcaaggccctgccccagttgaccaagggtctctggaggcaagccagtggagatca
>dme_circ_0004721
gttgcagctagcctcaagaccctgcccaaaatgaccaaggtctctggaggcaagccagtggagatcatga
cccatgccgatgacggtcttattcccgacatgtccgttgcttcagacgccgacgtgacccccaagcagac
tg
>dme_circ_0004721_junction_seq
gacgccgacgtgacccccaagcagactggttgcagctagcctcaagaccctgccca
>dme_circ_0004722
gtctctggtggcgagccagtggagattaagacccatgccgacgccgatcaaattcccgacatgtccgttg
cctccgatgctgatgataccccaaggaaggttgcagctagcctcaagaccctgcccaaaatgaccaagg
>dme_circ_0004722_junction_seq
ctcaagaccctgcccaaaatgaccaagggtctctggtggcgagccagtggagatta
>dme_circ_0004723
GAAAATGTGAAATAAAGTTAAGATTCGATTTCTCATTAATCATTTTTCTTGTGCAAATTTTGAACAAAAC

TGTCGGTTTTCTATCGTCTAAAAAATTATGAAcaaaatagaaaaagttaaaaactttttccaaaaaaaaa
aaaTTCGAAATTCTTAAAATTATAGGGAACTATAAAAAATGTTTAACGCAACATTATAAGTGCATTAGAT
AGCTTAACAATTTTTCAACGT
>dme_circ_0004723_junction_seq
ATTAGATAGCTTAACAATTTTTCAACGTGAAAATGTGAAATAAAGTTAAGATTCGA
>dme_circ_0004724
AGAAATAAAATTGAATCCTCGGAGACAGCAGCCACTATTGCTGCGGATGATTTTTCGCTGGACAACCTTA
AGCGAGAGCCAGTGGATCCCGCGGAGGATCACAATGACATACTCGGTGTTTAAGACCAGGCCAAAGAAAT
TTCCGAGTATTGATTAAAGAGTAAAGAATTAAGACAGATTCATTAAATCG
>dme_circ_0004724_junction_seq
AAAGAATTAAGACAGATTCATTAAATCGAGAAATAAAATTGAATCCTCGGAGACAG
>dme_circ_0004725
ATAAGAGGTTGTTCAGGTTAGCGGCTATGGGTGGTAAACCGAATGGCAACTTGAGTGCCACTCAGGAAAT
TTTGCGCAAGGATAGTGTTGACAGTAACCATAGTGTGGTCAAGGTGGCCAACCACGCCCCAACAGGCAGT
CCCAAAGGAGCTGCTGTAGTGGCTGCTATAAGGCCGCAGGGTTCATTGATCAACAG
>dme_circ_0004725_junction_seq
TAAGGCCGCAGGGTTCATTGATCAACAGATAAGAGGTTGTTCAGGTTAGCGGCTAT
>dme_circ_0004726
CAGCAAGGCCACCACTGGTGCCTCTGGTGCGGTGATAACTAATATACCCACAACCACCACAGCTGGAACA
TCCGTTCCGGTCGGAAAGAGTATAACCCAATTGAAAATGACTGAGCGAGGCACTCAAACCGGAGTCCAGA
ATCCTTTCTCCGATAATTACTTTCTCGAAATGATCAAGCCCCAGATGCAGGAAATGAACCCCCG
>dme_circ_0004726_junction_seq
AGCCCCAGATGCAGGAAATGAACCCCCGCAGCAAGGCCACCACTGGTGCCTCTGGT
>dme_circ_0004727
GATTGCCTCTACGGGAGTTTCGGGATCCCCTTCGTCGTGCTCCTCCTGTCCCTTGGGTCTTGACCTACGA
CCTCTGAGCGGAACGGGCACTCCGGGAGTGATGTGCTTTTGCAGGAACTTCAGCTCGCTGTTAAGATAG
>dme_circ_0004727_junction_seq
CAGGAACTTCAGCTCGCTGTTAAGATAGGATTGCCTCTACGGGAGTTTCGGGATCC
>dme_circ_0004728
GTCAAGAGGAGGATAACGACAGCACCGATGGTGAAGCCTGTTCCCTGGCCAATCGTAGAATGAAGCCGAT
TTCCAGCTATAAGCTCCAGCAGCAACAGATCCAACTGGGCAAACAACTGGTAGTGGATAAGCTGATCAAT
GTGGCCGCTTATGTGGAACTAACCTCGGATACGGACGACAGCAGCCGTAGATCTGATACGCCTGCCAAGA
TCAG
>dme_circ_0004728_junction_seq
GTAGATCTGATACGCCTGCCAAGATCAGGTCAAGAGGAGGATAACGACAGCACCGA
>dme_circ_0004729
GCTCTAACTTAATACTGCTAAAATGGCCTCCACACCAAAAGCGAATGGCACGACAGCGAAGACGGCAACC
ATAAATGATGACTACATAACGGTCGTGGAAGTGGATGATCCGACGTCGAACCCGAAGGATCGCCTTAAGA
AGCTAGTCAAGCGTCAAAGCTCTGTGGCGGAGGACTCCTCGTTCATCACAGTGCTGACGATTAACGAGAT
GGAGTTGCTTCAACAGCGAAAAAAGGAGGAGGATGTTGGTAGTGGCTCAAGCCCTACGATGGAGGAGCAA
ATAGAGAATGTCACAGTGTTCAGATTACCGGGAGAACGTCTCGGTTTTGGCCTAAAGTTTCAAGGAGGCA
CGAGGAGCACAGAGCTCGTCCAAAAACTGTACATACAATCCTGTGCTGCAGACAGTCCTGCCTCCAAAGT
GTCCACCAGCTGGGGTAATCTACGAGAGGGTGATGAGATCGTCAGTATCGATGGTCGTGATGTCCGGGAA
TTGACTCGAATCGATTGCGTTCGCGGCCTCAAAGACAATGTGGCCATTAAGCTGGTGGTGCGAAATGGTC

ACGGACAGAAGCCTCCCAGCGAAGAGGACCCACAGCAGCAGGAACACCTTTCGATCACACTGAATGCCCA
ACCACCGCCTCCGCCACCAGTGCCGCCAAGAAAGTTGGTGAGACGGCAAAACTCCAAAGAGAACCAGGCT
CAGTTGCTCATTGAGAAGCCATTGACGCCACCACCGGATGCGGAGTACTACCTGAATCTTTTAACCGAAT
CCATTAAGGCGGGCTCAGAGTCGGATGATACCGCCAGCACAATTTCCACTGTTATCGACAAGTTTTCCGT
GGGCTCCAATTACTCCTCGGACAGTAGTCTCAATGGCCACGAGCTGGCCAAGGTTTTGCAACCTTTTACC
CTTCTCGAGCAGGAGTTTCTGCCTTTGGAGCAGCCGCTAGGACATCCCAAGTTGCTTATTCCTGGCAATA
ACTATGAAAATGTGGAGTTCAAAACGGAGAAGGTCAATGTATACGAAAATGTGGAGCTCAGGAGTCCGGA
AACGACGCCGACACCAAAACCCAGAGTCCAATTGGCCACCGTGGAGCCAAAGAAGCGTTCGATCATACCC
ATGCCACGAAAAATTCCTACTCCAACCAAGCTGCCCATTGAGGTGACCCCACCCCGGGTTCCCATATTGG
AGGAACCCAAAACGCCCACAAATAACAAAGTTGATTCTCCCAAAACACCAACAAACGAACTCAAGGAGTC
TCCCATCAGTGCTACAAAAATACCTAAGGCCAAGTTCTCGAGGGCCAAAACTGAAGGAGAGATCAAGTTG
CATTTGCCAGCCCCAAAACAGCAATCACCACAAAGTAAATCTCGTATACCCATAGTCAGTACCCCTACAG
CACAAAAAACTGTTCCCAAACCAGTATCACCCACAATGAATGGTACACCACTCAGTTCAAATATACCACG
GCTGTTGCAGAAGCAGAAATCCGAAACGGATCTGAAATTGAACTTGTATAGGAGTAAATCTAAGGAGTCG
AGTCCCAGACCTCCCTTGCAAAGAGCGAACTCGGCAGAGGCTCCAGAAAGGACCTTTATCCCTGTACTTC
TTAATGGGAACTCCAAGAGCTCAAACTCCTTGGAGAGCGTCAGTTCGATAGGAAGTAGTAGCAGTGGAAA
TAGTGTAAGATCACCAAAAGGACCAAAGCCCAAGCCACCAGAGCGAGTTCAATCGCTTCAGAAAACCCAA
ATACCCAAACTGCAGGCACTCCCAACTACTCCACCTCAACAGATACCCAAGTTGAGTATGCAGACGTTCA
AGCAGAGCCCTCCCACACCCCGATCAACTCCAGTGGGAACTCCATCTCCAACAAGTAATCGAGAGATACG
CTTCAAGATTCAGACGTACGAAAGCAAAACCCAGGACGAAGATAAACTACCCAGCTTATTTGACCTAGTG
CACAGTCAGGAGAATGATAAGGATAAAGATAGGGATAGTGATTCGCTCAAGACCCACAATAGCATCACTG
CTCTTTTGGCAGCCGCCGCTGCCGAGGCCGCTGATCTCTCTCGGGAGTCGCCACCTCCGTTGGTCGTTGG
CAAATGCATGAAGATCGTCGATGACACCAATACCACCACCTGCTATACCAGTTCCAGCAGCGGGGAGGAG
GACGAAGACGATCTGGATGCCTCCAACCGGGAGTACATATGCGAGGACGGCGAGAAGTTGGGACCTCCGG
AGCTGATAAATGGCCCCGGTCCCTCGGAGGCCTACTTCAACATGTTCTGGCACTCGAACATGCTGCCCAC
AATCGGCGAGGTTGAAGAGGAGTTCTCCTCCCTGGAACCCCAATCCCTAACAAACGGGGTGAGTAATTGG
TTTTGAAAGTGTCAGTCGGGGGATTAGATTAGGTTTAAGAGTGTCTATAAGCCATTTTTCGAACCTCTTC
AGCTGTGCAGTTTTCTATAGTAAAATCAAATTCAAACAGGTCGATGGTAGAAATATTGAAATTGAATGCA
TTTAGATAAAGTAAATTCTTtaataaaacaattaatcaattaacaataaattaattaatgcattaataat
aataaatatttattaaagtaaatgcctaataaaaagtaGTCCATTACAGTCAGGTTAGGAAGGAAATGTG
TCTAGTCGTTCTAATCAGGAATTTTTATTATATGACTTAGACATTTGCCAATACTATTAGCCAGTAAACC
AAAAATATAGGATTACGCATTGCGTAGTGCCTAGTAGGTACTGCGTACATTAGAATAAACTTAATTTCTT
TAAAGTAATATGTATGAATATGAATAGTTTACGAATGGAAACATTGACTCTTTCCAAGGCATTGTTTCGT
GCAGTGGCCAACTCCATTGATTAGGTCTAGCTGCAAGTTCTTGTATAAGTGCTAGGGCTACTTTTCTATA
GTTTGACTAATACACGATTTTTGCAAATAATATACTACAAAACGTGTAGGGAGAACAGCCAGCAGGGGTA
AACGGGCAAAGAGGTGGCTCCTATCCCTCCCCCACACaaaaatgaaatagaaaacaaagcaaataaaaaa
cAAAGTCTGATAAGACAAAATGCCGGTGTGTTTAATAACACACTGATAATGGGGCTTCAATCAGCCGATA
AGGTGAAATGGCAATAGGAAAAAAGGTATCAGTGTGGTTTTGGTAACGTTGGGCGAAGGTTCGGAACTGA
TCGAACCTGTCTCTATTGACGTTTTGCTTAGCAGGTGTGATAGCAACAAGTCAATTAGCATATCTATTCC
ACCGATAAGAAGCGTAATTGTCATTGCTGAGGTTCTCTTACCATGTAtttttttttATCCAGAAAACCTT
GTCAAATCGGAGGAGTCGAAGAAGTTACCCAAAATGGAAGTAAACGCCGGTGGACTGGCCCAGCTGACTG
ATGATAAGATTGCTGACCATGACGCAAGTGCAACCAGAGCGGATACTCGCGACGAAGTCACAACAAACCA
AACCCAGGAAATCAGCTCCAGCAGTTCGAGTTCTGTTAAAAGTCAAAAGACCACCACAACCACTCGAGTG
ACCACCACCAAGACTAGTTCCAGTTCCAGTTCCTCCAGTTGCTCATCCACTGTTATCCCAGATGTTGCCT

TCAAGCTGCAACCCTATGAGGAAAGGGACGATATACTCTCGGAACCCATTACCACCATCCACGAAGAACG
ACGTGTGCTAAGTGAGCAGAAGACCCTCAGTGAATCGAGGACTCGCGATGCATTAACCGGCGAAGAGCAG
CTGGTAACTAGTGCCAATTCGAAGTCGAGCAGTGCCCGCTTCAAGAAGATCAGCAGCAACGACAACCTAC
TCGACCTTGGCGACGAGCAGGACATGCAGGAGCAGCCGTTGACCGCCACGATCACCCAGGAGTCGAGTCT
GAGGGAGCGGTTGGAGTGTCCTCAGGATAACCAGAGTATCGACAGGGGTACTCTGACCCTATCCGAGCGG
GGAAAGCAGCTGAGCGAGAAAGAAGGCCTCACCACCTACACGGAATGTGAGACGAGTGAAAAGGAGTCCT
ACCTAGAGGGCCAACAAGTCCTCAACGGCAAAGGCGGTTTCGTTAGCCAGGATGAGAATAGTGACCAACC
AACTTACGAACGAGAGGTCAGCGAGACACTAACGATCGTCAACGATGGCGAAAAGCAGGTCACCAAGAAA
TTGGAACAGAACAAGGAAATTGCTGGCAAAAAGGGTGCCAAGCTCCTTAAGAAGACCGATGAGGAGCGAC
GTCTTGAGCAGGAGGCCCAAAAGCTGATCGAAAGCTATCAGAAAGTCAAGAAAGAAGCGGAGAAGCTGTA
CAAGCTGGAATTGGCCGACGATGATCAGGGCTTTGATCTGAGTGCCTTCGAGCAGGCGGAGGAAGAGGAT
AAGCCAGAAGTTTCTGAGGAAATCAAAGAAGTGAAGGATTCTCAGCCAGTAAAGGTCGATATTCAAATTG
AAGAAGAGGTTAAAACACCTAATCAAGTCATAGGGGAAGTCAAAGTGGAAGATGTGAATAAGGAAATTAA
AGAGACGGTACAAGTCTCTCAACAGGAAATTAAGAAAGAGGTCAAGGTCTCACACCAAGAAACAAAAGAA
ACCTTTAAGAGTGAAGAAAAAGATGAGAGTAATGAACTCAAAATAGTTAAGGAGGAGGTCAAAGTAACAC
ATCAGGATCTTAGGGAACAGATCCAGGAGATCGAGGCTGTCAAAGTGGTGCACAAAGAAGTAAAAGTTAT
TAAGGAAAGTGCTAAAGTCACTACGACCACGAGTCACAAGCAAGCAACTAAAGAAGTAATCATCTCGAAA
GAAGATCACAAAAATGATAGCGCACTCCCTGCCGTGTCAACCACAAAAGTAGAGATCATCTCACCTCCCC
TGGAAGACGACTTGGGTTATGTCCTCCACAAGCACATTATTTTTCCCCAGGAAAAGGTCAAGACGGCTCC
TACGCCACTGCCAAAACCGAAACCCAAACCTCCAGTGCCCACGAACAAACCCAAAATGCCGGTCAATTTG
ACCAAACCGAAATCAGCACCACCACCGGTGCCCAGTAAGAGGAGTGAGCTATCCGGATCCGGTGGCAAGC
CCACACCCACCCAAAGACGCGGCAGCCTGGAGACCGCCCAGCCTCCAAAACCACTAGAACGTATAATTGT
CGGTGTGGAGCAGCGGGATGAGCCAGAAAAGCCCCAGCCAATTATCAACTTGGCCAGCGTGGATCTGCCA
AATGTGGCGCCCAACAGCAAGCAAGCGACAGTGGAAACGACTCCCGTGGTCGAAATGCCACCGGATGAGC
TCCAATTCGAGAGTCACCAACTGCCGGATGACGGTGAAAAGGAAAAGGACTTGCACCAGGAGTCAAATGA
CCTGCTCAGCGGGTCGTCATCGTCGTCGGTAACCACGCCCATAACAACGCCAATTTTGGTATCTGCCACC
TCGTCGATGGACTCGGTGCAAAGTGTCATCGAGGTCGTCAATGGCATGCCCATCATCAGCAATCAGCAAA
CTGACGACGACGACATTGATGAAGTTGCAGCGTATGACGATGGGATGCATTTGGAGGCCGAAGGTACGAA
GGATGTTCGTCCCGTCCAGGAGGTGACAACGTTGACGCTCGACAGGGAACACGAATCAGG
>dme_circ_0004729_junction_seq
TGACGCTCGACAGGGAACACGAATCAGGGCTCTAACTTAATACTGCTAAAATGGCC
>dme_circ_0004730
GAGGTGCGGTACAGGTAGGCGTTCTCCTCGCCAATGATTTCGGCGTACTTCACCTTGAACAACTCGTACA
GACCGAACTTGCACAGACCCTGAAAGATACAGAAACGCGGATTAGCCTGCTGTTTCTCAGAAGTTATTTT
TCAGTTTGCAGTAGTATCCAAGATTTTATCTTCGGTATCTGTTTCTCCCGTAACCATAAAGTTGTCAAAG
GTGTGTTGTTATTCTGAGGTATACTAAGATATCAGCCTAGGAATCAAACTTCCTAATGTTCGATTCGAAT
CACTTTTATCTGCTTCAAGCCCGGAACCGATGTAATTAAGGTCAGCCGACCTGGTCACGAGCATATAATT
CGCCTCCAGACGATTATGCAACCCATAAAGTGAAGCCCAATCTCACCTGTGCCGAGTAGCCGAGCAGAGT
GGGGAACCAGCCCTTAGCCAGTCCGCGGGCGCCCTCCTCCGCCACGGTGACCTTGAATCCGTGCACCAGA
TTCTTG
>dme_circ_0004730_junction_seq
GACCTTGAATCCGTGCACCAGATTCTTGGAGGTGCGGTACAGGTAGGCGTTCTCCT
>dme_circ_0004731
GGTGAAGTACGCCGAAATCATTGGCGAGGAGAACGCCTACCTGTACCGCACCTCCTTGTATCTGGCTGCT

TCCGCTTCGGCCGAGTTCTTCGCCGATATCGCTCTGGCGCCGTTCGAGGCCGCCAAG
>dme_circ_0004731_junction_seq
CGCTCTGGCGCCGTTCGAGGCCGCCAAGGGTGAAGTACGCCGAAATCATTGGCGAG
>dme_circ_0004732
GGTGAAGATCCAGACTATTCCCGGATACGCCAACAACTTCCGCGAGGCAGTGCCCAAGATGCTAAAGGAG
GAGGGCGTCAATGCCTTCTACAAGGGTCTGGTTCCCCTGTGGATGCGACAGATCCCATACACCATGATGA
AGTTCGCTTGCTTCGAGCGCACCGTGGAGCTGCTCTACAAAGTAAGTAACATTTCCTCTTTAGGTATCCT
CTAGTTTCTACAGCGGAAAAACTTACCAGTGCTGTGCACAATATCTGTATTCGTATTAGCGTTTTGCTAT
TACAATAGTTTAAAGGTAGACTGTTAATCTGTGAAATTTCAAAAGTGATATGCTAATTGATCAAAACTGA
AAGTATTCATAATAAAATAAACTTTTAGCGCGTCAGCAGATAAGAATGCCATTCGTATCTAATAAATGGC
ACGCCAGAATAAAAACCAACGACGAATGCTTATCTTGTTCAAATATAGGAAAATCATATTATTTCAGAAC
TAGTTTGTTACTATGGATAAAATAAAAAAAGCAAAGCATTCCTTTTGAAGTGCAAATTAGCAGGTAGCCA
CTGAAAGTTCAAATGTAAAAACTAGAGCAATACCGTTCACAGTTCGGGTTGTCAAGCCCCAACGAACCCC
GTTAAATCAATACTAATGATGCAACTTTTCCCAGGGTATGTGGTGCCCAAGCCTCGTGCCGACTGCACCA
AGGGCGAGCAGCTGATCGTGACCTTCGCCGCTGGCTACATCGCCGGTGTGTTCTGTGCCGTGGTGTCGCA
TCCCGCTGATGTGGTGGTGTCCAAGCTGAACCAGGCCAAGGGAGCTAGCGCCATCAGCGTGGCCAAGTCG
CTGGGCTTCAGTGGCATGTGGAACGGATTGACTCCTCGTATTATCATGATCGGTACCCTGACCGCTCTGC
AGTGGTTCATCTACGATGGT
>dme_circ_0004732_junction_seq
CGCTCTGCAGTGGTTCATCTACGATGGTGGTGAAGATCCAGACTATTCCCGGATAC
>dme_circ_0004733
GATCCAGACTATTCCCGGATACGCCAACAACTTCCGCGAGGCAGTGCCCAAGATGCTAAAGGAGGAGGGC
GTCAATGCCTTCTACAAGGGTCTGGTTCCCCTGTGGATGCGACAGATCCCATACACCATGATGAAGTTCG
CTTGCTTCGAGCGCACCGTGGAGCTGCTCTACAAAGTAAGTAACATTTCCTCTTTAGGTATCCTCTAGTT
TCTACAGCGGAAAAACTTACCAGTGCTGTGCACAATATCTGTATTCGTATTAGCGTTTTGCTATTACAAT
AGTTTAAAGGTAGACTGTTAATCTGTGAAATTTCAAAAGTGATATGCTAATTGATCAAAACTGAAAGTAT
TCATAATAAAATAAACTTTTAGCGCGTCAGCAGATAAGAATGCCATTCGTATCTAATAAATGGCACGCCA
GAATAAAAACCAACGACGAATGCTTATCTTGTTCAAATATAGGAAAATCATATTATTTCAGAACTAGTTT
GTTACTATGGATAAAATAAAAAAAGCAAAGCATTCCTTTTGAAGTGCAAATTAGCAGGTAGCCACTGAAA
GTTCAAATGTAAAAACTAGAGCAATACCGTTCACAGTTCGGGTTGTCAAGCCCCAACGAACCCCGTTAAA
TCAATACTAATGATGCAACTTTTCCCAGGGTATGTGGTGCCCAAGCCTCGTGCCGACTGCACCAAGGGCG
AGCAGCTGATCGTGACCTTCGCCGCTGGCTACATCGCCGGTGTGTTCTGTGCCGTGGTGTCGCATCCCGC
TGATGTGGTGGTGTCCAAGCTGAACCAGGCCAAGGGAGCTAGCGCCATCAGCGTGGCCAAGTCGCTGGGC
TTCAGTGGCATGTGGAACGGATTGACTCCTCGTATTATCATGATCGGTACCCTGACCGCTCTGCAGTGGT
TCATCTACGATGGTGTGAAG
>dme_circ_0004733_junction_seq
GCAGTGGTTCATCTACGATGGTGTGAAGGATCCAGACTATTCCCGGATACGCCAAC
>dme_circ_0004734
GGCAGTGCCCAAGATGCTAAAGGAGGAGGGCGTCAATGCCTTCTACAAGGGTCTGGTTCCCCTGTGGATG
CGACAGATCCCATACACCATGATGAAGTTCGCTTGCTTCGAGCGCACCGTGGAGCTGCTCTACAAGTAA
>dme_circ_0004734_junction_seq
GCGCACCGTGGAGCTGCTCTACAAGTAAGGCAGTGCCCAAGATGCTAAAGGAGGAG
>dme_circ_0004735
GATGCTAAAGGAGGAGGGCGTCAATGCCTTCTACAAGGGTCTGGTTCCCCTGTGGATGCGACAGATCCCA

TACACCATGATGAAGTTCGCTTGCTTCGAGCGCACCGTGGAGCTGCTCTACAAGTAAGTAACATTTCCTC
TTTAGGTATCCTCTAGTTTCTACAGCGGAAAAACTTACCAGTGCTGTGCACAATATCTGTATTCGTATTA
GCGTTTTGCTATTACAATAGTTTAAAGGTAGACTGTTAATCTGTGAAATTTCAAAAGTGATATGCTAATT
GATCAAAACTGAAAGTATTCATAATAAAATAAACTTTTAGCGCGTCAGCAGATAAGAATGCCATTCGTAT
CTAATAAATGGCACGCCAGAATAAAAACCAACGACGAATGCTTATCTTGTTCAAATATAGGAAAATCATA
TTATTTCAGAACTAGTTTGTTACTATGGATAAAATAAAAAAAGCAAAGCATTCCTTTTGAAGTGCAAATT
AGCAGGTAGCCACTGAAAGTTCAAATGTAAAAACTAGAGCAATACCGTTCACAGTTCGGGTTGTCAAGCC
CCAACGAACCCCGTTAAATCAATACTAATGATGCAACTTTTCCCAGGTATGTGGTGCCCAAGCCTCGTGC
CGACTGCACCAAGGGCGAGCAGCTGATCGTGACCTTCGCCGCTGGCTACATCGCCGGTGT
>dme_circ_0004735_junction_seq
CCTTCGCCGCTGGCTACATCGCCGGTGTGATGCTAAAGGAGGAGGGCGTCAATGCC
>dme_circ_0004736
GCAGCTGATCGTGACCTTCGCCGCTGGCTACATCGCCGGTGTGTTCTGTGCCGTGGTGTCGCATCCCGCT
GATGTGGTGGTGTCCAAGCTGAACCAGGCCAAGGGAGCTAGCGCCATCAGCGTGGCCAAGTCGCTGGGCT
TCA
>dme_circ_0004736_junction_seq
ATCAGCGTGGCCAAGTCGCTGGGCTTCAGCAGCTGATCGTGACCTTCGCCGCTGGC
>dme_circ_0004737
GTGGGCTAGTCCAATTGGTTACCTTAAGGACAGAGTTCACTAGTTGGAGCGATTGTGGGCAGACACAGGA
GCAACATACTTTTACTTTCTAACGAATTTCGGTTTCAGTATAGCGAAAATGCTTTATACTTGTTTCTCAG
CAAGAATACATTAAT
>dme_circ_0004737_junction_seq
ACTTGTTTCTCAGCAAGAATACATTAATGTGGGCTAGTCCAATTGGTTACCTTAAG
>dme_circ_0004738
TATAACAGAATATATATGGAATTGAATACTTTGAACTTGAGTGTAGCTTAGTTTCGTACGATAGAATGTT
TGGCTTTTAAAGTTAGAAAGATGTGTTTATAAAAAAGTGTGTATAAGTGCTGTGTTAGAGAAATGCGTAA
AGTGTTGTTTTGGAATGTACATGTGCAATGGCACACACGCAACATTAATGTATTCTTGCTGAGAAACAAG
TATAAAGCATTTTCGCTATACTGAAACCGAAATTCGTTAGAAAGTAAAAGTATGTTGCTCCTGTGTCTGC
CCACAATCGCTCCAACTAG
>dme_circ_0004738_junction_seq
TGTGTCTGCCCACAATCGCTCCAACTAGTATAACAGAATATATATGGAATTGAATA
>dme_circ_0004739
GCGAAAATGCTTTATACTTGTTTCTCAGCAAGAATACATTAATGTTGCGTGTGTGCCATTGCACATGTAC
ATTCCAAAACAACACTTTACGCATTTCTCTAACACAGCACTTATACACACTTTTTTATAAACACATCTTT
CTAACTTTAAAAGCCAAACATTCTATCGTACGAAACTAAGCTACACTCAAGTTCAAAGTATTCAATTCCA
TATATATTCTGTTATACTAT
>dme_circ_0004739_junction_seq
CAATTCCATATATATTCTGTTATACTATGCGAAAATGCTTTATACTTGTTTCTCAG
>dme_circ_0004740
AATATATATGGAATTGAATACTTTGAACTTGAGTGTAGCTTAGTTTCGTACGATAGAATGTTTGGCTTTT
AAAGTTAGAAAGATGTGTTTATAAAAAAGTGTGTATAAGTGCTGTGTTAGAGAAATGCGTAAAGTGTTGT
TTTGGAATGTACATGTGCAATGGCACACACGCAACATTAATGTATTCTTGCTGAGAAACAAG
>dme_circ_0004740_junction_seq
CATTAATGTATTCTTGCTGAGAAACAAGAATATATATGGAATTGAATACTTTGAAC

>dme_circ_0004741
GTATCAGTTATATAGATGTAGACGATCACACAGGCATACAGGTAAACATATAAATCTATCTTTTCCAAAA
ATTCTTTACAGTCAAACATAAAACATTTTAAAAAGTTATACATAGATTTTGAAATTCCATCAAGTTGACT
AAAGACAAGACATACAAATTCGGGCTTCGCTCAGTAGAAGTAGTTATAAGCCCTCCTATCTCATTTTATA
TTGAGTAAACCTCAAGTCAATGGCACATTAAAATTGGGATATTTGTACCGG
>dme_circ_0004741_junction_seq
CACATTAAAATTGGGATATTTGTACCGGGTATCAGTTATATAGATGTAGACGATCA
>dme_circ_0004742
GTAGTTATAAGCCCTCCTATCTCATTTTATATTGAGTAAACCTCAAGTCAATGGCACATTAAAATTGGGA
TATTTGTACCGGGTATTAGCTTAGGTCAAAAAGTTTCGTTAGTGGTAAAATCTTTTGGACTCTCCCTCTG
TCAATGAAACTGATTATAACACTTTAAATCTTTTCCAATTCTGTTTCTTTCTGTTCATGTGGCCTAAATG
AGAAACTTTATTCAATCGCTTGGGTCAGGAAAATCCAATCCCGTTCCCAGGCGATTTCACCAATATGGCG
TATGTGGCTCCTGGTGCCAGCCATTTTGGTCTTGCACCTTGGCCAAGGCATCGGTCAGGGTGGACCTTAT
TTGCTGGCTCGAGTTGCGATTCTGACCCTTATCATAGGGCGACAAGGCGTTGTAATAAAAGGGCGATCGC
TTGCTAATGGTGTAGTTTTTACCCTGTAAGCAAATACAAATTATGTAAAATATTTTTTAAGTTATTTTGA
TGCCAAACTCACATCATTATTATCCCAATATTCTGTTTCGTTGGTGCGATAGCAGATGCAGAATTCCAGG
CGCTTGGAGGATGGCGGCAAGGTGATTTTGAAGGAGAATGTATCGAAGACTACATGAGCACAAGTAGCCG
GTCCATAGGCCCTGGCGTATGTACAGAAGATATCCTGCTGGCTCTTCCAATCATCCCAGGTCACACGGAC
AATGATCTCCTTCTGGAAGTCGATATTCTTCACCTTGATGGTGCCCACGACAATCGATTCCTCATCCTTG
ACGATCACATTCTCCAAGGAAACAAAGTCGCGTTCTATCTTTTGTCTGTGTAAATACAAATATTTAGAAT
ACTCGTATATATTTTACAGTTGTGTAAATAGGGTACCAACCTAAATGATAGATAGTCTGAGGCTGGCTGC
TTGAAGTCCACTGTCCACTGATCCGGAGGATGTGGGCTGACCAGGCCCTGTGTAATTTGCTCCAGAAACT
TCATGCTCCAATATGGCGGCACATTCGAGGGTTCGGACATCACACGAACCTCGGTTAGCGATAGACCCTC
ATCATCAGCGAAGATCACATGCTTTTTGGGGCCACGGCTGCaaaaagtaaaaaaaGAAATACCATCCATT
GAGTACATCGCAATCTATTATAAGCAAATTATTAATTATTAAAATATACATTATCAAATATAGATTTGAC
CCTGGCTTTATCAGTTTGACTTGACCCCCCATTCAGAATTATTTATTTTATTTCTTTGTGGTCATTGACT
CAGCATCTTTCTTTGCCCGGATTTCTATCCTTAACAGTTTCATTTTATTGGTTTGTTTTTACTTTAGTTA
AGCAAACTGCGTCTTTGCCAAATGCAGTTTTTAAAACCATTTTTGAAAGCTCCTACTATATGTGCCAAAG
TTGTCGATTGTCTGACAATTGGTAATATAAAATCCCGGTCGAGTGAAACTTTTCCTTTAGTGtttttttt
tttCTATTCTGCTGATCATTTTTATTCCTTTGCGTTTCCCGAAAAATTCCCTGCGTCATAAAAAGAGTAA
TGTGTATTGTATAtttttttgttggttttatttttAGAAGTCACGTACGGCTTTGCCAAAGATTTGCCTT
AATCTATATGTAAATCACGCCTACTTTTGCTTTTCCTTTCTTTTTGCACCCATGTTATTGGGCGACTCTT
CCCACGGTTCTTTTGCTTAAATGTAAATAAAAACACACCCACTAAGATATATAAAATATAGAGGATAACT
CGTGATTCAAAATATATAAGTTATTCAACGAAATTTAGTTGGAATCTGAAATTCATTGGACTTTTAAAAT
TTTACCTAATTTAAATGCAGATACGAATAATACAATATAATAATATATTGTGTTCAATACAAATGTTTGC
TGCAGTTAATTACTTTCCAAACTCTTCGAACAAATGTTCCGCCCCTTTTGACTTTCATATTACTGAAAAAA
ATACGCATACAAGTTCTATTTAGAAAGTTGGCCATAAATTCGATAAGATATAGCGACTAATTTTGGCTAA
TCAGCTTCGGTAGGGGCTCGATTTTTGTATGGTTCTTTGCTGTTGCCAAACCCAAAGCGAAAAGTTTCCG
TCAGTTGGAGCAGACCTACGACTAATGGCCCCACACAGAGCAGACTCTATTTGGTCTGCAAATTGGAGGA
GAGTCGGCGGGAACGCATCGAGTGCTAAATATATATGGGAAGTACAAATAGCCAACAATTGAAATGGCTA
TCGACAGACTGGCTCTCTGTTGATTGTACCCCAGCCGTATTGCCAGAGATCGGTGATGGTGATGGTGTAT
CTGAGATGGCAGATCGTACCACCCGACTCGGCCAGAATCGGATCGTAACGACTACCGACTACCGACATCG
ACGACATCACGCATCGAGGGCATGATCGGTGTGCGCCTGCTGTTGCTGCATTTTCTTCAACAGGTTTCTT
AGCGATCGCTTATCTGGTGTGTGTGGGGTGGGTCTCAACGCCCAGCAGGCCGCCAACCAGAAACCGGAGA

CCGCGGACGGGTCTCATTTTTTACCGACCGCTTTCCTAAAACTTGTCAACTTGAAAGAGATCACCCGAAA
AATCAAATTaaaaaaaaaaaaaTCATTTACATTTTCATTGATTTTCCCCGACTTGGCTTACATTTTCTCCA
AGGGAAACGAGATACGACTATCAACTGTTTTTCTTAAAACCCAAAGGCAGTCACAATTCCGTATGCGTT
ACGGAACGCATTTATTTTCGTGGGTCCGGTTTAAAGTCCATAAATTTGTAAACTCCTCAATATGCAAGCC
GCATTAAGATCGTTGATGGGTGCAGGAGAAACAAGTTTTGGCTGCACCAAAAGGGCTTTTTAAAATCGAA
ACGCTTGTAATGTAAATGAAAATTGTGTTTAAAAATACTTTTTAATTTCCTCAGTTTTCAATTTGCAGAC
CTTGTTCGAGTTCCCTCATTTTCTAAAGTCAATAACCTGTCCGATAAAAATATTCTGGCAAGACCTGTTG
AAATTTTTACAACTGACTGATTTGATGGTCATTCTGGTTGACCTTACAATCGAACTACTTTTTTGTAAAC
GAATCAAGGTAGTTCCGTATTGTATGTCGCCAAATTTTCTGTATTTTCTATTTTATTTCCCCCATAAGAA
TTTCAGTTTCGCCAAATATACCTATCGTTTATATTTTTCTGAAATTTTAAGCAAAGTCCAATAGTAAGAG
ACTGAAAATAGTTTTAACTCGGAAAATTCTGTCGTGGATTTGGTTTTTCTTAATTATCTCGATCGCTACC
AATAAATATAAATTTAATTACGAATGAAATAAAATTGTTGTAAATGTACGAAATTCTTTCACATTTTTCA
GCTAAAGGTGCGCCAACATTTTTACAGCAGACCCATGTGCCCCAGACTCACTTTTCCGATCGATTCACGT
GATCTTTGCATGAACATCAGACATATCTCCCCATCTCTATATATAACCTTACGTTTGTGTGCATTCGTAT
TCCGAGGCTTGTAATGTTGAAATATTTTTAGTTACATTACACCCGAAAAAATTGTGTCTTTTATGAGATG
ACGCCTTTCGCTTGATCACGCTTCTATGCTAATAATTCTTCGGCCTTGACGTGAACCGTCTGCCTCGTTT
TGTGGTTGATCTTGATGATTTTTGGGGAGGCTAATTAAGACAAAAGAAAGATCTTTCATTGGCTGTCAAA
ACTCATTGTAAAATGTGGGGCCAAATGGGTGACGAGCAGATGCGATATGCTTTGGAAGGTGATGAATAAG
GTCTTCAATTCTTAAATCAAGCCTCCAATAAATATAATTCTTAATAATTTTTCTATTGCATTGCTTTACG
TTCTTGTGTTAACTTAAATGAATTATAATTTACTCTGAAGATTTGAATTGGTAAATTAATTTTTTCCCAG
TGGATTTCGTAACTGATAAAACGAGGCATACAAAATACGCAACCGCAAAGTGTACACAAAATCTCCAACA
GCAACAAGCACACGTCTGGTTTACATTGATAATATTATGGTTAATTGCATTCGTTTGAGTAGCTGCGTAG
CTTTAAGCCTGATCTCTTGTCTAGAGACGCAGCCCAAATAGAAATAGTGTTCGCATTTGACGCAAATGAA
CGTATAAATTATTTTCTCTATCTCGTGGCATATGTATTTTTAGGCATTTCTCAAAATGTCAGATTCAAGT
GCAAAAAGGAAATTATTTCTAGGGCTCGTTGAAAGCGAAATATACATATGTATACAATTTTACAAAATAT
CTGCGAGACATCGATAACCCAAATATTTTCATGGCCTGCACATGAATCGATAATGTATCTTCCCTATATA
TAGCGAATAAATGGAAATGGAAAACGAAAATAATGGCGCCTCGCTGTTGGGGTCTATACTAAATACCAGT
CTATTCCCATTTGAGTCTTAAAATGTGAGATTTTTTATCTCAGATATACGAGATACAGATTCTCGACTCG
CGATGCAGTGGCGTGTTTTTATTTTGGCTTATTTCTACAGTCATTTAGATAATTTATATTTTGTTTTTGT
GAAATTGCATGCTCTTAAAAATATATAAAATATATTTTCAAGTATATTTCATGATCTTTTTGCATTTGTT
TAGCCTGGCGGCCGTCTGCAATTGGCTTTCTCAGCTTATTTAATAAATGACATGTGCTCGGCTATGACGT
CTGTGGGAAAGTTGTTCGACTGCCAACAGATTTTCTTATTCCTTTTACTAGAACTATTGCAAATGTCTTA
TAAACGATTAATCTTTGTTTATAAAATATTATGGCGGAAGTGGTATTTTCGCAGTTTTATAAGAAATGAT
CTTTTCACGTTCCAATTTTTAGAACCAAAATCCTATCCTCCCCATATTAAAATTCAAAGATTTAAGTTTG
AATCATAAGCTTTCGATTTCTTTTTGACCACAGTCTACATAGTTAAGATGCGCTATGAAAACCATCTACA
GCTTCTTGTGTTACTTTCTCGGACTTTCCTTAAGATTTCCCCTCAATTGACATCTTACTATTTAATGAAT
CTGTTATAGGTTGATACACCCAATTCTGACCACAATTACCCAGGCTCTCACACCACTAAGTTCCCATCTA
GTTCAGTTGGTGGTTTAATAAGTGCGGCGCCATAATCTTTGAAGGCCTGACGTCAAAGTGACTGAGTCCG
CCGGCATTTACAGCCTTCTTGATCGTTTCGGCGCCGCTGTATCGAGTTACCTACATTACGTAATCTTATC
GCCCGCACACTCACCTCACTTCGCCGTTGCTGTCACTATCCTTGCTGCAGATACAGCTATGACTGGCACT
GTCCGCGACATCGGTGTCCGTCGGCGAGAGATCTGCGGAACTGGCTGTAGTGTTCTGGGATTTGTGCAGG
CAGGATTTGCGGCTGAGACAGGATTTGGGCTGATTG

>dme_circ_0004742_junction_seq

GCGGCTGAGACAGGATTTGGGCTGATTGGTAGTTATAAGCCCTCCTATCTCATTTT

>dme_circ_0004743
GGTCAAAAAGTTTCGTTAGTGGTAAAATCTTTTGGACTCTCCCTCTGTCAATGAAACTGATTATAACACT
TTAAATCTTTTCCAATTCTGTTTCTTTCTGTTCATGTGGCCTAAATGAGAAACTTTATTCAATCGCTTGG
>dme_circ_0004743_junction_seq
AAATGAGAAACTTTATTCAATCGCTTGGGGTCAAAAAGTTTCGTTAGTGGTAAAAT
>dme_circ_0004744
CCGTGGCCCCAAAAAGCATGTGATCTTCGCTGATGATGAGGGTCTATCGCTAACCGAGGTTCGTGTGATG
TCCGAACCCTCGAATGTGCCGCCATATTGGAGCATGAAGTTTCTGGAGCAAATTACACAGGGCCTGGTCA
GCCCACATCCTCCGGATCAGTGGACAGTGGACTTCAAGCAGCCAGCCTCAGACTATCTATCATTTAGGAC
AAAAGATAGAACGCGACTTTGTTTCCTTGGAGAATGTGATCGTCAAGGATGAGGAATCGATTGTCGTGGG
CACCATCAAGGTGAAGAATATCGACTTCCAGAAGGAGATCATTGTCCGTGTGACCTGGGATGATTGGAAG
AGCCAGCAGGATATCTTCTGTACATACGCCAGGGCCTATGGACCGGCTACTTGTGCTCATGTAGTCTTCG
ATACATTCTCCTTCAAAATCACCTTGCCGCCATCCTCCAAGCGCCTGGAATTCTGCATCTGCTATCGCAC
CAACGAAACAGAATATTGGGATAATAATGATG
>dme_circ_0004744_junction_seq
GAAACAGAATATTGGGATAATAATGATGCCGTGGCCCCAAAAAGCATGTGATCTTC
>dme_circ_0004745
CTACAATGACGAGCCCAACAGAGCGTCGCGCTACTGCCGGCCCCAAGTGATAACGCTGGCCTCCAAAGCC
ATGGTCAACAACGCCTCGGCGGCGGTCCAGGGTCAGGGTCCCAGTCAGCAGAAGGCGGGTGCCACGCCCC
TGGGCGTGGGCAGTCGTTCCGGACGGAGTTACCTGGAGCGAATGGCCTCGGCGCCGCTACTCACCAATCA
GCCCAAATCCTGTCTCAGCCGCAAATCCTGCCTGCACAAATCCCAGAACACTACAGCCAGTTCCGCAGAT
CTCTCGCCGACGGACACCGATGTCGCGGACAGTGCCAGTCATAGCTGTATCTGCAGCAAGGATAGTGACA
GCAACGGCGAAGTGAGG
>dme_circ_0004745_junction_seq
GGATAGTGACAGCAACGGCGAAGTGAGGCTACAATGACGAGCCCAACAGAGCGTCG
>dme_circ_0004746
GCAAGCAAAAATTAAAAACATTAGATTAGAAAATCCCCAACAAATAAAGACAGCACTAAAATGGTAAGCT
TTTTTCGAATTTAACCAAATAAAACACTCGTTTTGTCGCTTCTCGGTTTGCAATCATCGGGTTTATTTAG
ACACTGATATTAATTAGAGGACTTCAACTTTGGCTGTTCATACACTTGGATGGGAAAAACGATGGGAGTG
GAATCTCCGGAATTAGTAGTCCGGCAGGATCGGTGGTTCCGGTGACCTTTTGCCGCCCCACGAGTGGAAT
CGCTGCTTCTTGCCCAGCACGACGGAGTTGCGCTTCTCGTCGCGCTGCAGCTGGGATTTGATGCGGCACC
TTTCAATCACCGCATCCCGGATGATGGGCGAGAACTCCTTGTCACCGAACTGGGCCATTAGCAGGCGGAA
GAAGGCCCAGATCTCGCCACGCTGAGATGTGGGTGGTGGGGCGTAGTGCCGGAAGGGCCATCCCTCCGAG
TCCGAATCCTGGCCACCGGGCCTGGAGCTGTACGCGTCGCAGACATCCTCAAAGCTCAGTATGGCCTGCA
GGATGAACCTGCGGTACTTGGAGAGCTGCAGCTCACAGGTTGCCAACTCGGGATCCTGTTCAGAAATCGG
TGCCGGCACCAGGCTGGCTCCATAGACTTGACGGCCAAAGGCCAGGAGCACCATACACAGGACTATCTTT
GCCATCTTCTCCAGCGGTTTTAGCCGTCTTAATCCTCTCGGCCAACTGCGTTGTTTGGCGCCCTGCAGCC
CTGCTTATATAGCCACTCGATCCGTAAGCCCACCACCGAAACCACCACCACTAGCACCCACCAGCCAGCA
ACTTTGAAGTCCATAAAATAATTATAAAACGTTTGTCGTTCGCATTTTCTAAGCCGTTACACACGTCGCT
AAATAAATACGAGTGCGTATTAATAAGCGTCTCTACGTTTTCCACACACTTTTCCTTTTTCCCTCCAGAC
AATGGCAACAAGTGTCATAAATTTTCACTCGCTTAACTCGCTCTTCCCTTCCGAACTCGAGTTTATTAAT
CCTGCCCAGAGATAAACGTGACGTGCACTTGAAACTGAGACGGGCTCCCCAAACGCTCCTTCTTTATATG
TGTggggggggAAAACTCGCAGCCGGAAAATGCCCGAACCACGAAACATTGCAACCCCTTTTCTCCGCTC
GCCACTTGTACAAATTGCTCGCTGATTAGCAGGGAAGCGTATGAAATTAATCATATGCAAATATCGTCCA

CTTTTCTTTTCCTTGACGCGAATTTGTTGCTGTCGTGGGAGTTCTTGGGACCTATGAAATGCTATCCCTA
TAGGGTATGATTAGGAAATTCATGTTTGACCCGAATGTGCTTAGATAGCTTGTACTTTTCTAAGAAATTC
TAGAGGCAATGCTTTCTTTAAATGAATTCCCCAAAATTACATATCTTATTTCTTATTAGCTTTGTCCCTT
CAATTTACATGGGTACATTTAGTTTTTGATGTATATATAATTTTGCtttttttttCTAAGACACAAACAG
GGCAGTTAAACAATTTTTTATCTTATTTAGTATATAGATGACAAAACTTATTTTGTAAAAGCTTCTTAGC
ATTTAGTCGATTTCCCAGGGTATTTTCAGTTGCGACGCCCCTTGCTTTCTGTTGTTCTTAAGAGCTCCTC
ATTTGGTTGAAGCCCACTTGGTCTGGTCTTTAATTTACTCAAAAACGTGATGGGTTAACTTGGCTGTGGG
CGTGCGCCCAAGTACGAAATGTGAAATGAACTTAGATTATTTTCTGTTTTTTAAATTACCGACATTTGGT
TTGCCCTTTACAGGAAATTCCGCTTCTGTGGCGAAGGCGATTGCCCCGATTGGGTCCTAGCTGAGATCAT
ATCAACACTCTCGAACTTGAGCATTGAAAACTTGGAACAACTTAGCGATTTAGTGGCACAACGAATTTGT
GGAGAGACATTTGAG
>dme_circ_0004746_junction_seq
ACAACGAATTTGTGGAGAGACATTTGAGGCAAGCAAAAATTAAAAACATTAGATTA
>dme_circ_0004747
GATTTGAACAGACAATATGGCGACATAGTGCTGGAGGTGATGCCCTCCAATGTGCCAATAGTGCACCTGT
ACAATCGCGATGATCTGGAGAAGGTGCTGAAGTACCCCAGCAAATACCCATTCCGACCTCCCACTGAGAT
CATCGTGATGTACCGTCAATCCCGACCGGATCGCTATGCAAGTGTTGGAATTGTGAACGAGCAAGGACCA
ATGTGGCAGCGCCTACGATCTTCCCTGACCTCCAGCATTACTTCTCCCAGGGTCCTGCAGAATTTCCTGC
CAGCCTTGAATGCGGTTTGTGATGATTTTATCGAACTACTGCGAGCCAGGCGGGATCCGGATACACTGGT
GGTTCCCAATTTCGAAGAGCTGGCCAATCTGATGGGTCTGGAAGCTGTGTGCACTTTAATGTTGGGCAGA
AGGATGGGTTTCCTGGCTATCGATACCAAGCAGCCGCAAAAGATCAGCCAACTGGCAGCTGCTGTTAAAC
AGCTTTTCATCTCCCAAAGGGACTCGTACTACGGTCTAGGTCTGTGGAAATACTTTCCCACCAAAACGTA
CAGGGACTTTGCCCGCGCCGAGGACTTGATCTATGAGTGTGATCTCCGAGATCATCGATCATGAGCTGGA
GGAACTCAAAAAGTCGGCTGCCTGCGAGGATGACGAGGCTGCTGGATTACGAAGTATCTTTCTGAATATT
CTGGAGCTCAAGGATCTGGATATCAGGGACAAAAAGTCAGCGATCATAGACTTTATTGCCGCTGGCATAG
AAACGGTTAGCCAACACTTTGTTGTTTGTACTGAGTTCTGTTACTGGAGATCCCGGTGCTATGCCACGAA
TCCTAAGTGAATTCTGCGAGTATCGGGACACGAATATCCTGCAGGATGCACTAACGAATGCCACATACAC
AAAGGCCTGTATACAGGAGTCCTACAGACTGAGGCCCACAGCCTTTTGCCTGGCCAGAATCCTGGAGGAG
GACATGGAGCTCTCGGGCTACTCGCTTAATGCAGGG
>dme_circ_0004747_junction_seq
GCTCTCGGGCTACTCGCTTAATGCAGGGGATTTGAACAGACAATATGGCGACATAG
>dme_circ_0004748
CAAGAAGACCAGCGAGGTAACCAAGGCAATCGAGGCGGAAGCCATCACGAGATCCAAACGATGCATATTC
GGGTGCCAGTAGTTGCGATCCTTGGTGGTGGACCAATCCCGGAAAAGCAGGGCGGTTACGGCCACGAAGA
GCACAAAGGCCACTAGATTCCACAAAGTAGCCGTACGCCATGGAG
>dme_circ_0004748_junction_seq
TTCCACAAAGTAGCCGTACGCCATGGAGCAAGAAGACCAGCGAGGTAACCAAGGCA
>dme_circ_0004749
AACTTTAAACCTCCTCGGACGTCGATTTTGTACATTTTCGATTCGATGATGGCAATTTGTTTTCTTGTGG
GATTACAATTAGCTGGGAGACGGGTATGTGAACGGGGCTCTTACTTCTAAGCTGGAGCTATAAATATTAC
AG
>dme_circ_0004749_junction_seq
TTCTAAGCTGGAGCTATAAATATTACAGAACTTTAAACCTCCTCGGACGTCGATTT
>dme_circ_0004750

GAACGAGTCCTACGATATTGCGAGTCCCCTTCGGTGGGCACTTGTTGCACCTACAACATGGAGACCCGCA
TGGCGATGCAATCCCGCCAGCAGCTCGAAGGGCACACCAAGGACCAGATCTCTCGTATGTCTGGAATCCT
GGGCAGCAAGGCCACAAAATTCAAAGGGTAGGTCACCGATTCAGCCTAAAACTCGTTGCGTCAAGGAGCC
ACTTGAAAATGCCACAGAAGCCACCGATTAGAGATCTTTCGGGCGTCAAGTGGTTCTGCCCCCACACAGA
ATAGAACCCTGATCTATATATATAGATATATGATCTCCATTGAGAGACGATGATGCGGGCATCGAGTTAC
TGACAATGTCAAGTGGCAGGCGACAATGCGGATGTGCCGGCATGCTATATTTTCCAGCGGATGCTGGCTA
GAAATAAAATCGAACATCTAAACATTTAGTCGGGTAGAGCGGATGTGTGCGCTGATTGATCCCCTCCCAC
CGCACAATCGATTCTCGTTACATTTTTCACTAATTTGATTTTCCGCTTGCAGGATATTTTCACGGCCCTG
CTCAAAGAGTCGCGAACGCAGTTCAATAGTATGTTCATCCGAACATACGGCGTCATTTACGAGAGAAATT
CGTACGTCTTCTCGGATCTGTTCAAGGAGTTGGAAACCTACTTCGCCAACGGTCGCGTGGACTTGCTGGA
GGTCATGGACAAGTTCTTCAATACGCTATACCAGAAGATGTTCACCGTGCTGAACACCCAGTACACATTT
GATGAAAA
>dme_circ_0004750_junction_seq
TGAACACCCAGTACACATTTGATGAAAAGAACGAGTCCTACGATATTGCGAGTCCC
>dme_circ_0004751
GAACGAGTCCTACGATATTGCGAGTCCCCTTCGGTGGGCACTTGTTGCACCTACAACATGGAGACCCGCA
TGGCGATGCAATCCCGCCAGCAGCTCGAAGGGCACACCAAGGACCAGATCTCTCGTATGTCTGGAATCCT
GGGCAGCAAGGCCACAAAATTCAAAGGGTAGGTCACCGATTCAGCCTAAAACTCGTTGCGTCAAGGAGCC
ACTTGAAAATGCCACAGAAGCCACCGATTAGAGATCTTTCGGGCGTCAAGTGGTTCTGCCCCCACACAGA
ATAGAACCCTGATCTATATATATAGATATATGATCTCCATTGAGAGACGATGATGCGGGCATCGAGTTAC
TGACAATGTCAAGTGGCAGGCGACAATGCGGATGTGCCGGCATGCTATATTTTCCAGCGGATGCTGGCTA
GAAATAAAATCGAACATCTAAACATTTAGTCGGGTAGAGCGGATGTGTGCGCTGATTGATCCCCTCCCAC
CGCACAATCGATTCTCGTTACATTTTTCACTAATTTGATTTTCCGCTTGCAGGATATTTTCACGGCCCTG
CTCAAAGAGTCGCGAACGCAGTTCAATAGTATGTTCATCCGAACATACGGCGTCATTTACGAGAGAAATT
CGTACGTCTTCTCGGATCTGTTCAAGGAGTTGGAAACCTACTTCGCCAACGGTCGCGTGGACTTGCTGGA
GGTCATGGACAAGTTCTTCAATACGCTATACCAGAAGATGTTCACCGTGCTGAACACCCAGTACACATTT
GATGAAAAAGTGAGTAGTAGTCTGCGAAATCCAAAACCCGATGCTCCAAAGCGAGACCGATTTTTAGAGG
CCTACTACTACGCCTTTAGTTGGCAAAAACCAAACGTGTGAAACCGCAAAGTGTCTCGGGCTGTCGACCG
ACGACAACTAACTTACTTGACGGCCCGTCGAAACTTTGACACGGCCGGTGTGCGATTTCAGTTAGCTGAT
ATTCCTGATATGAGTATCCTATGGATTTTTTCTTTCTCCGCTCTTTAGAGTTTTAAGTTGCTCTGGTTTT
TGCCAGATTTTTCGATTGCGGTTGAAACGCTTACAGCAAAGAGAAAATGAGGAAATTGTCGGGTAATTTA
GAAATCCTACAATATGATGAGCAAAAGCACAAATTTTAGATTTTCCAATGGTTGAGATTTAGCATTCAGA
TACAGACTATCTGAACCGACCTCCTCTTAGTCTACAGACAGATTGTCTCCCACATGGGTGATATTACATC
ACGTCCCACTGCCAATCTACCTACTGCGATTTATTCATCCCGGCCAACTCATTCCGGCCCACCACGTATG
TATAGTATGCACCATTTCCCATCCTATCCCATAACACAGCCAAAACCAAAAACCGCACTTTATGCCAGTT
CCTGTGGCAGCTGCTCGAATTTCCGCCAAAATGGTGAGGAGGAGGaaaaaaaaaGCGAAAATTGCGCCCA
CTTGGCTCATTGCCCCGGAACGGTCTCCACTGGTCTCCGGTAACCGGCGACTTGGCCAAATGACTTCTGC
TCCCTGCACGGCCACTCCTCGTTGGCGAAGGGCTCGTGTTACCCACGCGCTTTCCAATTGATGCCGCTGA
CTTAAACTCCGTCTCCAACTCTTAGCTCAACTCCAGCGATTCGCTTTTATTGTCATCGTTTTAGTTTGTC
GTACGTGCTTTGGCACGTTGTCGTTTATTGCCATTGGACAGCAGCAACGACAACAACGATGTCGCGTAAT
TGCCGGCACTTGCCAGCCGCCTGCCCCTTCTACACTGCCCAAAAATAGTGCCCATTTAGTCATTAGGATT
TTTGTCATCGCGCAAATGGTTATGATACGAAATGGACGTTGAGTATTCCAACATTAAGGCTAAAGTGGTT
TAAGTTCTCAGTTTATATTCTTCTTTAAAATATTAATGATCTACACATTTTCTTCCTGTGTATTGTCCCC
CTGTTATTTGTTTGTGGTTGTAGTTTGTTCTGTGCGGTTGGTCGGTCGCTTGAACTGTGTTGGTTGTGCC

CTTAGAATGGATCAGATGCCCCGCACAACACAGAGCTGGACCTGGGAACTAAGGTTCTCCTCCTGGCTAT
TTAATGAGGGGGTAGCTTAAGCAGTTAGTTTGTCTGGCAGACTTTGGGGGGTTTGGCCAAAACAGTGAAG
TAAAGTATGGGAAGTCAACGGGTGTTTAAATAGAACCCTGATATGCAATATGTCCTAGGGATGAGAAGTC
AAATGGCGTGACAAATATAGAAACAAATAGGAGTGAAACCATAGATAAATGATGGTAAAGGGCTGTTTAA
ACTCTAATAAGCACGCTCTATGTTTTTAATTAATTTAGTTCTTACCAACTTTTGCTACCTAACAGTAACA
TACATAGAACAAACTATTACATTGATGGAGTTCTGAATGATTAGACATCTAGGGCATCCGAACTCCACAA
TCTCCCCTCCCATCAGCGATTTGCAGCTGCTGGTGCTGCCGCTTGACTGATCTGTCACCTTTGTGAATGC
GACTTGCAGTAATTCCTAAATTGTTTAGCAAAAGTGAAGAAGGAAAACGGGGAACCCAGCGACTTCTTGA
CCATAATGGCGAAGGCAGTGACAGTGAGGGCCTGAATGCAGCCGTACAAGTGCAATGACTGTCATGCTCG
ATGGCCAAACTGTAGGACTAGGCACATACTTACTGCATAGAGGTGCTGCAGGGGGAAACAAAAACATCAA
CAAAGCCAGCACATGGCGAGCAACTATTTTTATGATTTTTCCGCATAAAAATTTTTAGTTGTGCGGCGGC
GGCAAAGAATGCGCCGATGATGATGTTGGTGAAGTCGGGAGCGGAGGCGATGGCGTTGAGCCGTGCCATT
TACAAGTGCAGATTTCTGCCGCCATCAGTACGCCCAATGAAAATATTTTTCCCAACATTCCGCACTCATT
TTCCACCGATTTAATCTTATTTTCCAGGCTACATGCGATGCGTCAGCGAGCACATGAAGGAGCTGAAGCC
TTTCGGAGATGTTCCGGACAAGCTGTCCGTGCAAATCAAGCGATCCTTTGTGGCCACGCGCACCTATGGC
CAGGCTCTGACCACCGCTTCGGAGGTGGCGAAGAAGGTCCTCAATGGTGCGACTGAATGCCGATTGTACT
GGGGCTCTTACCAAGATGCAGCACTGTGGTGCTTGCAAGGGATATACGGAAAAGCCCTGTACCAACTACT
GTGTGAACGTCATCAAGGGATGCCTGCACTATCAGCACGAGTTCGACAGCGAATGGGAGAACTTTGCGAT
GGCCATGGACAAGGTGGCGGAACGACTGCTGGGATCCTTCAACATTGTGATGGTGGTGGAGCCACTCAAC
ATCAAGATCTCCGAGGCCATCATGAACTTCCAG
>dme_circ_0004751_junction_seq
GATCTCCGAGGCCATCATGAACTTCCAGGAACGAGTCCTACGATATTGCGAGTCCC
>dme_circ_0004752
GCTACATGCGATGCGTCAGCGAGCACATGAAGGAGCTGAAGCCTTTCGGAGATGTTCCGGACAAGCTGTC
CGTGCAAATCAAGCGATCCTTTGTGGCCACGCGCACCTATGGCCAGGCTCTGACCACCGCTTCGGAGGTG
GCGAAGAAGGTCCTCAATGGTGCGACTGAATGCCGATTGTACTGGGGCTCTTACCAAGATGCAGCACTGT
GGTGCTTGCAAGGGATATACGGAAAAGCCCTGTACCAACTACTGTGTGAACGTCATCAAGGGATGCCTGC
ACTATCAGCACGAGTTCGACAGCGAATGGGAGAACTTTGCGATGGCCATGGACAAGGTGGCGGAACGACT
GCTGGGATCCTTCAACATTGTGATGGTGGTGGAGCCACTCAACATCAAGATCTCCGAGGCCATCATGAAC
TTCCAG
>dme_circ_0004752_junction_seq
GATCTCCGAGGCCATCATGAACTTCCAGGCTACATGCGATGCGTCAGCGAGCACAT
>dme_circ_0004753
GCAGATACTCGTACTTGTTAATTGGAGGGAAGCCAACTGAGACACCACTAGAGCAGCGAAAGGTTAGAAA
ACCCAAGGGACCAGAAAGCGATTCCCTGACATGCAAGCCAGAGTTTGAAATCCGTGACGACAATCATTGC
ATCATCGCGATTAGAAGGCATTATTGGGAGTAGCCAAGCAAGATGAGTGCTGCCGGCTGGCTTGATATGA
ATGCAGCTCTTCAGAACCTCGACTCTCCGCCGGAGACAGTGAAAAAGGCCAGAACCAAACGGAGGAGCAG
CTATGCCGGACGAACTACTGCAACGGCCGGAGTACATGGACGTGGCAGCATTGGTACCATAAGTTCTGGA
ATAATCAAATCATTTCGGGAACGTTTCCTCAGCAACTCATCTGGGGACTCCGGCAAGTATAGAAAACTTA
GGCAAAGGGATGCCGAGTCTGGCAGAATGGAAGCCAGTGGATCAAGTTCATCGGGAGAAAATGAGGACCC
CAGCAGCAGTAGTAGTGTCGAGGATCTCTTTGCCAATGAGGCAGAACGTCGCAATTGGCACAGGAAAACC
TCGGAGAAGCGACGAAGGAATGCCAGAAAAAAG
>dme_circ_0004753_junction_seq
GAAGCGACGAAGGAATGCCAGAAAAAAGGCAGATACTCGTACTTGTTAATTGGAGG

>dme_circ_0004754
GAGAGCCGTTGCTGGTGGAGATTTCGTGACCGTGATGCCGGTTCCGTCGGCCCTCAACACGGAAACGGAC
TACATTGTCACCGTGGTCAGTTGGGTGGACGATGACAACGTTCTGTCCATCTGGATGAACCGCATTCAAA
ATGCCGCCTATGTCGTCACCTTCGACGGACTCAACAGGAAA
>dme_circ_0004754_junction_seq
CGTCACCTTCGACGGACTCAACAGGAAAGAGAGCCGTTGCTGGTGGAGATTTCGTG
>dme_circ_0004755
AAGTAGGTCTGTTGCACATCCCCGGACTTGATAAGGTTGCACGTGAGGCACTTGGGACTCTGCTTGGCCA
GTGCCCGAATGGCGTACAGATGCAGCTGCTCGGGATGATCCTCTGTGTTGGCCGTG
>dme_circ_0004755_junction_seq
CTCGGGATGATCCTCTGTGTTGGCCGTGAAGTAGGTCTGTTGCACATCCCCGGACT
>dme_circ_0004756
GTCCCAGAGAGGTGATCACAATGTGGTTATTGTCGTTGAAAGTGGCCGAGAAGTAGGTCTGTTGCACATC
CCCGGACTTGATAAGGTTGCACGTGAGGCACTTGGGACTCTGCTTGGCCAGTGCCCGAATGGCGTACAGA
TGCAGCTGCTCGGGATGATCCTCTGTGTTGGCCGTG
>dme_circ_0004756_junction_seq
CTCGGGATGATCCTCTGTGTTGGCCGTGGTCCCAGAGAGGTGATCACAATGTGGTT
>dme_circ_0004757
AGAATATCTTGACGCTCCAGATTCTTGGCCAATATCATGGCCTGCTGGTAGTGCACATTATCATCCAGTG
TGCCATGTACCAGAAGGTACTTCTTACCCCTCAGCTTCACGGCCATTGTGCTCAGTCTAGAGTTGGCATA
GCCCAGCTCATTCGTGTTGGGAAGACCCATATAGCGCTCTGTGTAAATGGAG
>dme_circ_0004757_junction_seq
ACCCATATAGCGCTCTGTGTAAATGGAGAGAATATCTTGACGCTCCAGATTCTTGG
>dme_circ_0004758
GCTGAGGGGTAAGAAGTACCTTCTGGTACATGGCACACTGGATGATAATGTGCACTACCAGCAGGCCATG
ATATTGGCCAAGAATCTGGAGCGTCAAGATATTCTCTTCAAGCAGATTGTGAGTATAAGACTAAAGAGCT
AGTCTCAAAAGGATTTTTCGTTACAATTTTGTAATCTTTTAGAGCTACGCTGACGAGGATCATGGACTGT
CCAATGTGCGACCCCATTT
>dme_circ_0004758_junction_seq
ATGGACTGTCCAATGTGCGACCCCATTTGCTGAGGGGTAAGAAGTACCTTCTGGTA
>dme_circ_0004759
CAACCAGTGGCAAGAAGTCTAGTAGCGGATCCAGCGGTTCAGGCAGTGGGGCACTGAGCTCCAGTGGCAG
TGTGCCGCAGGTCCAGACGGTTCAATCCCTGCACACTTTACAGGGTGTGCAAGTCAAGAAGGATCCGGAT
GCCcagcaacagcagcagcagcagcagcagcaacaacaacaacaacagcagcaagcaatgacagtgagcg
gtgcgacgggcggacaagtgcaacaacaggtgcagcaagtgcagcagcaggtccagcagcaacagcagca
gcagcaacaacaacaacaacagctgcaacatcaccaAATCATAGACTCGTCGGGCAACATTACAACAGCA
ACAACGAGTGCTCAAGCGGCAGCTGCCGCACAGCAACAGGCGGCTGGCCAGCAGCAGCAACTGGTGGCAC
AATCCGATGGCAGCGAGAGTGGAGCTCCGCTTTCAATTGCCCAGGTGCAGACGCTGCAGGGCCATCAGAT
CATAGGCAATTTAAATCAGG
>dme_circ_0004759_junction_seq
CATCAGATCATAGGCAATTTAAATCAGGCAACCAGTGGCAAGAAGTCTAGTAGCGG
>dme_circ_0004760
GACCCACCGATCGAACTTTTGGCGGCGAGGAGGAAGAACATAGCTTTCCGGGCACTTGGCCATGTAAGTT
AAGTCTAGGTCTATTGCCTGCCAACGTCTTGGGCGCACATTACATGTACATAACAATTATTATTATTCTA

ACTCACGGCCAGGTCATGTGGCGAGAATTGCGACAGCTGCCACCACCGACGACGTCGTTAATCCAACCCG
GGTAAGGCAAAAGACGGATACGGCTACAAAGAAGCGTTAAACGGTGCGACCTTCGGACGCAGCCCCCCAT
GGGCCACCCCACGGCACTCTTCAAAAAAGCCAATAAAATTAGCAACTACACTAGGGATTTATAGAAGAAA
GCACACATGTGAAAACACAAGTCCCACCTAAATGGTAAGGCAAAAGACGGATACGGCTACAAAGAAGCGT
TAAACGGTGCGACCTTCGGACGCAGCCCCCCATGGGCCACCCCACGGCACTCTTCAAAAAAGCCAATAAA
ATTAGCAACTACACTAGGGATTTATAGAAGAAAGCACACATGTGAAAACACAAGTCCCACCTAAATGTAG
GAAAAGTTTAGAAAAGTTTTAATAGGGCAAGCAGGAAGGGCTAAATGCAAATAAATTTAACATATTACAA
TGTGAAAAAGATCTTGAAAGTTGAAATTAAACTCTGAACAAGCTGGGAAATTTTAAATAATTACTTAGCA
AGTTTTAAATGAGTTTAATATAACTGATTACTTATCATTCTTTTAATTGAATTATTAAATATTAGAGTTT
AATTTTTGTTAGAACTAGCATATACAAATCATTCTTGATTTGAATATGTATTTACTTCCCAATTTTTGAC
AGTGTACAAAACTAAATGCCTTGTTTTTGTTGCAGGCGCCTGCATTAGTCATAGGAGGAATGAGGATGAC
AGCCGGCCCAAGGGAACGCCACCAGCACAACGAACCAGTGACCGAGCCAGAACGAAAAGCCAACCCAGAA
GAGGAAGCAAAGCAGCCCCAAGGCAAACAGTTAGCTAATTAATACGACACAGCCATCGGAGGAATCCTGG
GCAGAGCGAACGATGTTCAAGATTAGGCAGCGGAACTGCATGCTGATAGTGACGGCCCTGGTGCTGGCAC
CATGCATAGTGATCCTGATGGTGATGGGACCCGAGTTATCCACCGAACAGTCGCTGACTCAGCGCCTAGC
CGAATGCCATATGCGGCTGCAGTATTTGGAGTCCATGTACCGCGCTCGCCAGGAGGACGTGGCGCTGCTT
TCCCAGTACCTGGGCCAATTGCAGATCGTCAATGTGACGGGAGCCAGTGCACCACCGCCGCCGCCACCTT
TGCCCGTGGTTAATCTGCTCGACGGGCTGAGTCCCGAGGCTAGGCAACTGCTGAGAAATGCGTCGCATAT
CAGTAAGGGTGGCAGTGCAAATGGCTCGATGGCCAGGAGCCAGAATATACGGCTACCGAATGCTTATCAT
TTCCTGCCGCATCTTCTGGACGATGCCGGCTCCCTGCGACCAGCATACTTGCAAAGCAAGGGCCGTTCGG
ATGTGAGCATTGTCCTGGGTGTGCCCACCGTGCGGAGGGAGAAGCAATCGTACCTCCTTGGAACCCTCCA
CAATCTCATCGAGAACATGAACGACGAGGAGCAGAACGAAACACTGATCATAGTCTATATAGGAGAAACG
GATATGGAGTCCGTGCAACTTATTGCCCGTCAAATCGAGTCCAGTTTCGATCAGCATGTGGAGAGTGGAC
TGATTGATATAATAGCACCTGCACCTAGTTACTATCCGAATTTCGAACGACTTCGCATCACACTGAATGA
TCCTTTGGAGCGCGTTAAATGGCGCAGTAAGCAGAATTTGGACTTTGCCTACCTGATGGCCTACGCCCAT
TCAAAGGGAACCTTTTACGTTCAACTGGAGGACGATATACTGACCAAGCGGCAGTTCATAACCACCATGA
AGAAATTCGCCCTTATCAAGAGCGCTTTGACGAAACCCGATCAACCAGAATGGTTTGTGCTAGACTTTTG
CCAGCTTGGCTTTATTGGCAAGATGTTCAAGAGTGCGGAACTACCCTACCTGATCACCTATTTCCAAATG
TTCTATAATGACAAGCCAGTGGACTGGTTACTTACGTATTTTATCGAGTCCAAGGTTTGTCGCACGGACA
AGGATCAAAAGCACTGCAATGCGGAGAAGGCAAAGTACTGGCAGCATTTTCGAAAATCCCTTTTCCAACA
CATTGGTACAAGTTCGTCGCTGAAAGGAAAAGTTCAAAAGCTGAAGGACAAGCAGTTCGGCAGTAAGGTG
CCGCAGTATTATGCCCATACACACAATCCACCGGCTCTGGTGAAGTCCAACATTGCGCCGTATAAAAACT
ATCTGTTGCAGCGCGCCTACAGAGGAGAATCCTACTTCTGGGGCTTGCTACCGCAGCCTGGAGATCTGGT
GCAGATCATATTCGAGCGACCCACGCAGCTGAGGCACTACCTCTTCCGAAGCGGCAACTCGGAGCACCCG
TCCGACAGGTTCTACAACACAACGGTGGAGCTCCTGCCGGCGGATACGCTTAGCGAAAATTCGTCCGTTT
GGAGCAGCTACAACACCACTACGGACGGCTATTTGGTGGTGGGTTCATTTGATAGCATGGGCGTGGCGGA
GGGCCTTCTGGATGCCAAGATAGGTGCTATCAAGGAGCTGCGCCTTCACGTCCACAGTGACAGCGAGAAT
TGGGCCCTGCTCAGCGAGATCGAACTACAGGAATGGGGCAGTGATTCCAAATCCGAGGACAATGCCGCAA
AGCCACG
>dme_circ_0004760_junction_seq
AATCCGAGGACAATGCCGCAAAGCCACGGACCCACCGATCGAACTTTTGGCGGCGA
>dme_circ_0004761
GTAGAAAGTGTGGCAGAGCACGCTGCTCCCGCAACAGCGGAAATTCCTGTAGCAGCCACCGAGGGAACTG
CTCAAACTCCAGTTACCATTCCTTCGGGTCCGAAGTGTGCCAGTTTAGATAACCTATCCAACTGTTCCGA

CCTAAGTTCCCTGGAGAACTTTGACTGGGACTCGGTATCCG
>dme_circ_0004761_junction_seq
GAGAACTTTGACTGGGACTCGGTATCCGGTAGAAAGTGTGGCAGAGCACGCTGCTC
>dme_circ_0004762
ATGAATTTGCAGGGCCAGTCGCAGGATAACAACCAAGCAGGCATGGCTTATAATTACGGGTATCCAAGTG
GGTCTGTTCCTCCTCTAGCTTACGCTCCACCCGGAACAGTGCCCGCAAGCTCAGCTTCCCAGGGATTGAA
TATCCAGCAGCCTCTGTATGTGGCTACATCGGGCAATCCAG
>dme_circ_0004762_junction_seq
CTGTATGTGGCTACATCGGGCAATCCAGATGAATTTGCAGGGCCAGTCGCAGGATA
>dme_circ_0004763
GCGTAAGCTAGAGGAGGAACAGACCCACTTGGATACCCGTAATTATAAGCCATGCCTGCTTGGTTGTTAT
CCTGCGACTGGCCCTGCAAATTCATCTGACCGAGCGCTCCGTGGAGGGTCTGAGAGTTTGGCGATGCTTG
GGCCTTGTAGGCCGGTGGAGCAATGTGCTGCTGTTGCTGCTGATACATGGGATTCGTGTAGCCGGCGGCA
CTGGGATCAAAATACTGCTGCTGTGTGAGACTATAGGGTGGAG
>dme_circ_0004763_junction_seq
TGCTGCTGTGTGAGACTATAGGGTGGAGGCGTAAGCTAGAGGAGGAACAGACCCAC
>dme_circ_0004764
CTCTATGACCAGCCTCACTTCGATGCCACAGGACTTCTTCATTCCAGCCGCTTATATGGACCCCGTGTTT
CAATCCTTGTGGATGAATGGTAAGTTAAGCAGTAGGATTAATCCTCCAAGGAAGCGTTTAACTAATCGTG
CCGTTCTTTTGGTTTTCAGTGGAGCCACACCGCTAGAGGACGATGTCGACAACTTTGAGCATGTCCTCAA
GATGACCACGCACAACGACGCTCTATATGCCTATTACTATACTGACAGCAACACGAACAAGGTGAAAGTA
ACCGAGATCTTTGACTTTGACGAAATAACGGAGCACGACAAGCTGGCCACCGATCTGCAGATCTACGAAG
GCTCTGTCGAAGATCACCCCAATCCCCACCAGCACAGCCACAATACGGTGGTCAGCGAATGGAATGGCGT
CGAATCAGG
>dme_circ_0004764_junction_seq
TCAGCGAATGGAATGGCGTCGAATCAGGCTCTATGACCAGCCTCACTTCGATGCCA
>dme_circ_0004765
GTTTACGAAGCGTTGATCGAGGACAAAGGCAAGAATGTGATTTACTTGAAACTATCCGCAAAATGTGTGG
AGGCAATGGCTCTGCAGGCGGATACCGAACTGGAGGTGGACATACAATTCCAGCTGAACCGAATGCCATA
CTGTGAGTGGCACAATGCGGTGGATAAGATCACAGATTTCCGGTTGATCTTTCCGGCTACGGAACTGGAA
CCGAGTATACCGTGGACACCGAAGAAGCAGTGGGCCGATTCCTGTGAACCGAAGTTGAATGCCAAACAAA
GGGAGGCCGTGAATGCCATTACCACAGCTCTGAGCATTAAGTTGCCGCCAATCCTGCTGATAGGACCCTT
TGGCACTGGAAAAACCTACACGCTGGCCCAAGCTATCAAACAACTGCTCACGCAGCCGGAGGCCAAAATT
CTGATCTGCACGCACTCGAATTCAGCCGCTGACTTGTACATCAAGGAATACCTGCATCCGTGGATCGAGG
AGGGTTTGAAGGAGGCCACACCGTTGCGAGTTTACTACCATAAGAGATGGTCAGCCACGGTAAACGGTGT
GGTGCAAAAATACTGCATCACCGATGGAGTTGGCAACTTCCTAAGGCCCACTGTGGAGGATATAATGCGT
CATAGAATTGTAGTTGTTACGCTGAGTATCAGCATGGAGTTGGCCACTTTGGGTCTGCCCAAGGGATTGT
TCACTCACATCTTTCTGGACGAAGCTGCTCAGGCAATGGAATGCGAGGCCATTATGCCGCTTGCTTTGGC
CAATGATTCTACAAGGATCGTTTTGGCTGGAGATCACATGCAAATGAGTCCGGAACTGTTCTCAGCCTTT
GCCAAGGAACGCAAGCTGCACATTTCGCTGTTGGAGCGGCTGTACGACCACTATCCCTCCAACTTCCCCT
GCAAGATCCTGCTGTGCGAGAACTACCGTGCCCACGAGGCCATCATCCGTTTCACTTCGGAGCTTTTCTA
CGAACAAAAGCTGGTGGCCTCCGGAAAACAGCCACGGCACGATCGCTTTTATCCCTTGACCTTCTTCACT
ACTCGGGGAGAGGATGTTCAGGATAAAAACTCAACTGCTTTCTATAATAATGCTGAAGTTTATGAGGTGG
TGGAAAGGGTTTCCGAGCTGCGGAAACGTTGGCCCAGTGCTTGGGGTAAACTGAATGATACCAGCATTGG

CATTATGACTCCCTACTCGGATCAGGTGTTTCGCATTCGTTCCGAGCTGAGAAAACGGCGCATGGGTGGA
ATATCCGTAGAACGTGTGCTCAATGTCCAGG
>dme_circ_0004765_junction_seq
TCCGTAGAACGTGTGCTCAATGTCCAGGGTTTACGAAGCGTTGATCGAGGACAAAG
>dme_circ_0004766
GTTTATGAGGTGGTGGAAAGGGTTTCCGAGCTGCGGAAACGTTGGCCCAGTGCTTGGGGTAAACTGAATG
ATACCAGCATTGGCATTATGACTCCCTACTCGGATCAGGTGTTTCGCATTCGTTCCGAGCTGAGAAAACG
GCGCATGGGTGGAATATCCGTAGAACGTGTGCTCAATGTCCAGGGTAAACAGTTTCGGGCGGTATTCCTC
TCTACA
>dme_circ_0004766_junction_seq
ACAGTTTCGGGCGGTATTCCTCTCTACAGTTTATGAGGTGGTGGAAAGGGTTTCCG
>dme_circ_0004767
CCACTACCATTGTGCCTCAGATAGGGAGGTGGCGGCGGTCCGTTCTCCATGGGCGACATGGGTCCGTTCG
GCACACTGCTGCCCCTCTGCATACCCATCGGCGGTGCCTGCGGCTGTTGCTGCGATGGAGGCGGCGGTGG
TCCTGGTCCGGG
>dme_circ_0004767_junction_seq
ATGGAGGCGGCGGTGGTCCTGGTCCGGGCCACTACCATTGTGCCTCAGATAGGGAG
>dme_circ_0004768
ATTGCTGTGGGGGCTGCTGCTGCTGTTGCTGTTGACCCTGTGGATACATCATGGGATACTGTGGTCGCTG
CACGTGCGGATTATTGAGCATCGGATTGTTAGCCATCGGTGGCTGCGGTGGCTTATGTTGCTGCTGCTGC
TGTTGCATTGTGGCCATCACCTGGCGCAGCATTTCCGCAAACTTGTTGGCACCATCCTGACCCACGGTTT
CAAGCAGTTTAACGTACCACTTGTATTGCTCGTCTTTCGGATTAAAAGCCATGTCATAGATGCTTACATT
GGGTGGGAG
>dme_circ_0004768_junction_seq
TGTCATAGATGCTTACATTGGGTGGGAGATTGCTGTGGGGGCTGCTGCTGCTGTTG
>dme_circ_0004769
GCTAATCATCACCAGCAGTCCTCTGGCTCATCGGCAACCACCTATGCGGCTGTGCTGAGCCAGGGACCAC
AGGCGGTGGTTGGCGGAGGCGTTGGAACAGGACCATCGCCGGGACAGTCGCAGGCGCAGGCGGGCGTGGC
TGGTTCAGGAACGGGCGGAAAAGTCGCGCCGGCGGTTG
>dme_circ_0004769_junction_seq
ACGGGCGGAAAAGTCGCGCCGGCGGTTGGCTAATCATCACCAGCAGTCCTCTGGCT
>dme_circ_0004770
GGTGACATCTGCCCAAGCGCGTGCCATGCCAAGGTGCTTCGAGGAAAGTGCAAACAGCAGATGTAATGGA
TATGGAAAGGCAAATCGAAAACTGATTTGGAGCGGAAGTTCGTTTGCACCTTTCTGCAGTTTCCTGCCAG
AACAATAAACATATCCACAATCCGCAGCTACAGCCACTTCCATTCCATACCCACAATCGCAATCAGAATC
GCCGAGTGTTCCATATAGTGCCACGACTTGTCTTGGCCTTTGACGCCGTCTTTTGTACTTAGTTGAGTTG
AGTTCAGAAGGCATGGTGATGCAGATGGAGCTGGAGGTGAAGTCACCGCCCGCTGGCTTTTCAATGAGCT
GAAAATCAAAAGCTATACGCGATTTCACACACAGTACGACGGATTAAGGATATGCGTGTGCACGCAGAAA
AATATTGGATGAAAGTTTCAGTTTCGATGGGTTATAAGTTTAAATACGAAATATAACTGGCTGGATTGAT
ATATGACCATATTGATATATTACCCATGCTAACTGTATGTATATGATCCAATACATTTATACTTGAAATC
TTCATTATTTTCTCCATGTATGCAAAGCCAAGGACCAAGGATCCCTGGAGCAGCTGCTCCTTCGGAACGC
TGTATTTGATTTGCGATTCGAGGCTGGCGGAAATGGAATGACACGCGAGGCACTCGTACTTTGTTTGACG
ATTGGCAAAAATGCTGCCTCCATTGCCCGACaaaaaaaaaaaaaaaaaaaaaCAGAAGGAACAGAGCGAG
CGACCAAGGACCCGCCAGCCGTAAGAGACTTCCACTTGAGCCGCACGCATTGTTTGCCCGGCGAAGCGAA

GGGGCGTGCTTGGAAATCGGATATAGGATCGTTCGTTCGGGACAATCAAATAGCGCAGCGGAGTTTTCCA
ACCCACTGAGCAGCCGCAATCGGTGGCGAATCAGCTGCCTATATAGCCTACCTTTAAGAAGCGTCTACGG
CGATGAGGTGCCAACCCGAGCCACGcgtgtcccgtgtcccgtgtcccgtgtccTATGGCCATTGACCAAT
GGTCAAAATTGGACACTGACCGTCTCATTAGCGCACCAAAGGTTGGAGGGTTAATGATGCGCACCCCGTG
TTTGGGCTTCCGTCCACCACTCCACACCACTGCTCCGCCTCCAGGCAATTATGCGACGCACTTGAGCTTG
AAGGGCCTCGAAGATGAGGAGTACACTTGGATAAATCGAACAACAACTGGGATGCACTGCACAAAAGTGT
CATATAACTAATGTTGCTTACTTAATCGTATGACATACGAAATACTTCGATTAAGTAAATAAGCGGAACT
TATGTATCAGCAGTTGGGAATTATAGGCACATGCTTGTACAAGAACTCCTGTTAACATAACCGTTTTTCT
TCCAGTGTACGCGTAGGAGGACTGCAGTGGCCATTCATTGACCCCGTCTGTGTAGTAGCTCCGGTTTTAT
GGTGTCGTCGTAAATGTCAGGTGCGCACCCCATTTAAATTTATTCAACGTAATTAGTTGTCTCCCCATAA
AGTGCCTGTAATGCCAGTGCACGAATCTAATTTGATTGCCAACTGTAAACTACAAGTGTTAGGGGATTAC
GATGCAGTGCTTCCGCCCGTTCACGAAAAATTCGATTTCCCCTTTGGAAGATAAATTTTTTGTGGCGCGG
ATTAAACGGCACATAAAACATATAAATGGGACGCAACACACACCTGCAGGTGGAAAACATGCAAAAACGA
TCATTGCGAGATATGCGAACGGGACATGACGCCGCAGGATGCCAAACGGGCTGCTCCATCCCGCTGCCGC
TCCACTTGGGTCACCATTCTCGACTCGGTTATCGGTAAATTCCGTGACGATGGCAAAAATATTGCAACGC
AACCAAAAGGGGCAGAAAATGGCTGGCGGGGAAACAGGgggtctgggtcctgggtcctggatcctggTCC
ATTTCGTGTGCACGTCGTTATCCTTGCCCCGGATGCACAACAATCAAAATAAACGGCTGCCAAATTGACC
ATTGACCAAACATCGTCAGCAGGGAACGGCGAGGAGGTGCCGGCGACCACTGCAGCAGGATGTGGGAACG
GCCAAGGACCTCCAGGGGCAAGGGTGCAAAGTGAAGCACCGGATAGCGACATCATCTTTGGCCCCGTCAC
GTCCACTTTCTGCTCCACTCAGGAGTCAAGCAGCCATCTCGAAATAAACATGAAATGCTCTTGTTTTTGT
GCAGCGGGAAAAACGATTAAAGGTAAAAGTGTAGAGAACTTCTAGGACATTTCGATTGGGAAACGAATTC
AAGGTGTTCCATTGCAGGCTGTTAAAGCAAGGACATAAAAAACTAGACACAGATCAAACTATAATCAAAG
AAATTCAGGAATTAAAATTAAAACTATCTGTGATATCGTTTAAGCCAGAAACATGTGTAATGGGCCATAA
TTGATACCCTATAAGCCAGATGCCATCCCCAGAGTTAGAGAACTGCCACTACATAATTAACCAGAAATGT
TCGCTCATCCCGCGTCCCCACTCGTTTTACGATATTATTGCAGAATAATGAAATCGCCAAGAGATTCCAA
TTCAATCTACGCGCCTCATAATGGCGTAGGCCATTTATGGATTGGCATTGATTTAATACCGCGTCGCGAG
AGATCGCCTCCAGGTTCGGTTCCCCCGAGTCCGAGGCGCAAGGCATTTTGCACCTACTTCAGGCCCAAGA
CGTCCCCAAGTCGCGCTCGCGGCTCCAAAGTCCGCATCTGATCTGATTCCGCCGCCTATCGCTCCCCAAG
ATCAGCGGCGCAGACGCCACCTCGTCGGTTGCTGCATCGGCGGGCAGGAGGGCATCGCAGCACTCAGGGA
AAATGCACCTCCGAATG
>dme_circ_0004770_junction_seq
GCACTCAGGGAAAATGCACCTCCGAATGGGTGACATCTGCCCAAGCGCGTGCCATG
>dme_circ_0004771
GACTTTCTACGATTGTAACGAATGATTTGTATGAATTTGAAGGCAAACTTGGAGCTGAAAGGATTAAGCA
GTCGCATACTTTACGAGCCCAGTAAGTACGGCAAACTCATTAACATCGAATCCCCATCGAGATATGGAAC
AGTCGATATATCATTTTACGGGACCGCCATCAAAATATACAACTTGACCAAAATTATACCCTTAAGCGGG
CTGCAATAGCTGGCAATTTACCTCCCATAttttttttttATTTGGGCGGGGACCGTAAATTCTGGACA
GACTGGGAGGAATCGCAAAAAATTTATAGACAAACTTGTGCTATTGCAGTGGTTCGGGTGCTTTTGGATG
GCAGAACTTTTGCTTGAATATAATATTCATAAATTTAATCCGAGCAAACACTTTTCGCGTATTTCGTGCG
CTATATTTTTGGTTTTTCCCATTCTGGAGCCGTTTTTGTTTTTATTTGGCCGCTCACGTTTATCACTGAG
ACGACGTCAAGTATTCCACTTTTGCAACATTTTCCGGGTTCGGATTTTCCCCACCATCTCGCCCCCCCTT
TGCCTAACTCTCTGTATCTGTGTTTTCGTGGCAAAGAAGGAATAAACAAAATAAATAATTCATAATGCAA
ATAGGCCAAAACACACAGAGACAAGCCGAATGCCAAGGCAACGGCGAAATAAGAGCCCGGCGGCGGAATT
GCAACCCGGCCAGGACTCGATCCTGACTCCTCGGACTCCTTCATCATGGTCCTCGATCCTTGGACGATTC

GTCGGCAATCTGGCCTGTGTACATAAGCACTTTTCGGGCGAAAGGATTATCAAACGAATCAGGTATTCTG
TCCATTGTTGGCATTGCCCAAATGAGGGATCCTGTCGGGCACATAAAACAGTAAAACAATCGCAAAAAAA
TGTCAATTGTTGATTTTACTAGGCACTGCTATAAATTTTATGTATCTTTTAATCTTGCAACCTGTTGTGA
TATAGGCTTGAGGCGGTATTTAAGCGTTGAAACTTAAAAAAATGTTTTTCGATTAAATTTAAAGAATGTA
TAAACTTTTAATAGCCCGATAAACGGATCTATCCAAAGAAGATACTTTTTCGCTGTAATAGAACACGTTT
TCTAGATTAAATAAATATGTTATTAATCTTTCCCGCAAAGTAACCCACCTCTCTATCCAACGCTACTAGT
TACCCCTACTTGTTAGTCTATCCACCTAGTCTATAGTCTAGTCTCTATCTAGTCACCTCCACCGAAAACT
ATTTCAAGTTCGATGAACCGTTTCTCGCTGTGCACTCTCATTCCGGCTGAACCTATGGCCCGTGCAGCTA
AGCCACGGTTAAGGGTTTTACGGCTTTAAATAGTCAACCAAGGCTAAGCAGGGGTCGCAACATCTACGCC
AGAGAGGTGAGAGCGGTGGGGAAAAGGGCAACACCGCATATATTAAATAAAAAATGCAAAATCGTTAGGG
CCGTCTAAGGGTGATGAGGGTGACTCTCTCGGCCGAAGCGTGTGCAGCTGAAATAATCAAAAAAACTCGG
AAAAGTAGGTGGAAAAGCCAGAAGAAGAAGCAGCAGCAGCGGTACCAGTAGCACAAAATAAGAAGAAGCG
GCAAGCGTAATCGCCAGCAACACTCCcacacacacacacacacaTACTCAGCGTGGTAAAAAATGCAACA
AAATCGGATTAAAAAGTGCTCACGGTTTGAGCAACCGAGCAAAAGACCAAAGCCAAAGCCAAAACCGGCT
GCCAGAACCTCAGAAACAGTAGTAAAAACAACAAAAGTGACAAGAAAGTGCAGAAAAGTCAAGTCTTTCA
ACTATCAGATACTTACTATCCTGTTGTGCTATGCAGAATTGCACAAAATATGAATATATAATATTTGAAT
AGAAAACATTTTGATTGTAGGTTTTCATTGAATTGATTATGGTTGATTATTTTAAAGCATGGTCAAGAAA
TGTTATTTTTTAGAGCCTAAAGAGGATGCATCCCAAAATGGCAAACTTACTTATACGACATCTCAGGATC
TTTTGGTTTGACTCCATTAATTATTCACAGATTGTGTCAGTTACGCAGAATGCAAAGCAAAACGCTATTT
AATCCTTTTAACATAAATTTTCACATTCGCGGTATGCCCGCGTAATCTACTAGAATCGGGTATTATAAAC
CCCCCATCCAAAACCTTTTTGCCACCCAACCAACCGGCAAAAGAGATACAAATTGCAGTAAGACGGTTTT
TTATAGTTCAACGATGGATTTACGATGTTGCACGACTTTGAACTACATTTGTGGGGGTTTGGCCTGCAAT
GCCACCAAATCCCCGCGGACTGGCTAAGTTTGTTGTTGGGGGTAGAATGGTAGGTATGGTGGGTACGGTG
TTATGGTGTTCATCTAGAACGGGTGCTGAATAAAACTATCTTTCCATCTATCCAGCTAGTTTGTTAACAG
CTAACCAGTCGGGTGTGTTGCTCCACCGGGTGTAACTGTACTTGCCTGCCCGTTCGACCGTTACGGCTCG
TAAAAGTTGAAACTTTTTCCTTAGCGGAGGGGTGGGCTTTGGCCCCATCTATTTTTTATTTCCAGCCAAA
CACTCGATGAACTACAAGTAGATGCAAAAAGGCTGAGGCCGGGCTGCAAGGAGCACATAATTCCCGCAAA
CTGGGAATGCTACGTCCGCTGCAAGTTGCAACAAAATTGCAGCAGCAGCCATGTTCTAAGCCGGTTAACG
GTCGCACCCACTGGGTAAATATTGTGAAAGGGGCTGCAGGGCGGGGCGGGGCGCAGGGTTGAGGGGGCGT
GGCGCATGACGCCTCCCAAGGAAATTACGGATTCAAATTAGCGGCACTCAATCTCAATCGGGGTAGAGTT
CCGACCACCCACTCAACCGCCAGCTAATGCGTTTTATGATACCCACTTTATAGACCGAATGCTTTCACTA
AGAATAATTATGCGTCTGTAATATTGTAAAATTAATATGGGCGTTTTTAAAATATTTTAAAAATTTGTAT
CTTTGCCAGTAAAAAGCTACCATAAAAATTAGAGTCGTACGATTGAATCAACTTCAAAATCACGATTTCG
AACCGCTTGATATCACATTTTTTTACAGTGCAGCCTTCTTATATGCCACACAGCCTTCGATTCGTTCGGC
CCACTTTTATGCAATTTAATCAACTTATCGCGTAAAATGCACAAAACGTTTTTCTTTCAGTTCGCCAAGT
GCAGCGCTCAACTTGCAACCCAAAAAAAGAACGCCCAGCAAAGTAATCAATGATTTTTCAGTTTGTGCAT
TAAAATGCAAAGCCCTGAAAACACCAGAGCCACCCACCGAAAAACCCAACCCATTTCAGCCACATTTTAC
TCATTGGCTCTGTTGCTTATTTAGGGCTAATTAAGTTTCATTTAATGGCTGCCATTGGGAGGCGACCGAT
GGAGAGGTTCCGAGGTCAGTGGGTGGCTGTTGGCGCTTTGCAGTTTAGCAGTCAGTTGTTGTTGATGGCT
CGCTATATAGCAAAAATACTAAACTGAAAACAAAAAGAAAATAGAAAATTTCCGCGTGCCGCTTTTTAAC
TTTTTCCAGAGTTGGCAATTGAAAGTTTCTTGCGGCTGTGGAAAGGGGATTTTGGATTGTGGATTGGATA
GCATTGGAATCTATCCCCCTTATGCAATTTAGGGATTTTAACCAAACCAGGGTAAACTTTTGTTTTGAAT
GATCCCACTTTTATTTTGACAGAGTTTTGTATTCCATCTCTGACAGAGTTTTTTATCGTTTAAGCTGGGT
ATCGCTTTGAAATGGTAATTATCCATATAGTTTTTTGGACAACATGCAAACTATATTCTGTGCGGACTAA

AATAATAGGTTAAAACATAATAAAAGAAATCGGTGCAATGTACTAATATTTTAGACATTGTGGTCATATA
GGTTGTGCCTGAACTTCCATGTGAATCTAACCTCAGAAATCAAAGCAAAAGAAAGAACAAAGTGAAACAT
TTGTTAATTAAATAAATTAGCTAGCCGCAAGCCAGCCTTGTTGTTTCCTCTTTGGATGTCATTAGTTGGC
GGATTTGATTCTATAGAACGATATTAACAGAAATAGCTGGCAATTTTCGATGGGCATTTAATGTCTGTCT
TCAGTACAACATTAATGATGACCTTGTAGGGTCAGATCGGACCTGTCCGAATCAGCAATTATGGGTCTCT
CTCATGATGGATATCTGCCTGTGAAGCGAAAAGCGCTATGGAGATGATGCTGGGTACCGGTTCTCGGTTT
CCATTGATTTTCCTTCATGATTTGAATCATTCATGGTTGCATACAATCAGCGATGATCTGCTGTATAACT
GGGCGAGCTTAATGATTTATAATTGACAGAAAACACTTGGTTTATTCGTCTCCGGTTGAATTGAATCCAA
ATCCATCCACTTGTAACGCCAACAAGCTGCCTCAATccagccagccagttagccagtcaggcagtcagTC
GTTTGTCCATTTTTCATAGCCAACTTGGCTAATATATAGATTTGTGGTTGAGGCAGGCAGGCAAACCGGT
TCTTTTCCGTAAGGCATTTCTTTCAAGTGCACATACTGTAGGTTCTCACTCACCCTTTACTGCGCGAAAC
CAAACCAAATCCAAATCCACACACTTGTGCGAAACTAGTTTTCAAACAAACTGGCTCGGTCGGCCATTTG
AATATTTATGAATTCTAGTGCAAAAGTTGTaaaaaaaaaTTAGAAAATGAAATATTAATCTTAGCGTACC
GAATATAGTAATACCCTAGGGAATAGGCTTCCTAGAAATATAACTATTCAAAACCTTCAATCGCGTACTC
ATATTCACAGTAGTCTAATGAAAAGATTGATTTGAATGAATGCAATTTTACTAAACAATACTAACTGAAT
TAAATAATAGAATTTTTTAATTATTGCAAAAGATAAAACAAAGTGTAGCCGAGCATCAACCGTATTAAAC
TCCTAACTGAAACAAAATATATGCGGATATATTACCTATGCGTTGTAATAAATATATTAACTTTATGGGT
TCATGGAATAATTAGACTACCCACTGCAAGTGTGTCTAAAACCACCTTGCTTAAGGCAGGCTAAATCAAT
ATCGACAATTGTACGTCGTGAACATCTTTATTGGTTTTTAAACGCTATTTGTTGGACTTCTTCATTCACA
CCTTCAGTGGAGAACAAAACCATAGGTAGGTAATGGAGGTACTTAAAAATGCTCACAGTAAATGGCTGGT
AAATACCGCGGGACAATTAACCGAGACAAAGTACTTTGAAAACAGTTACAAGGCCAAGGCGACCCAAAGC
CAGTCACCCACCAATCCACTCGGCGAACTCAACATCAATTCAACAATCCAACAATTTGAATTCTCAAAAG
CTGAAACAGCTGTTTCATCTATTTGTCGCG
>dme_circ_0004771_junction_seq
GAAACAGCTGTTTCATCTATTTGTCGCGGACTTTCTACGATTGTAACGAATGATTT
>dme_circ_0004772
GACTTTCTACGATTGTAACGAATGATTTGTATGAATTTGAAGGCAAACTTGGAGCTGAAAGGATTAAGCA
GTCGCATACTTTACGAGCCCAGTAAGTACGGCAAACTCATTAACATCGAATCCCCATCGAGATATGGAAC
AGTCGATATATCATTTTACGGGACCGCCATCAAAATATACAACTTGACCAAAATTATACCCTTAAGCGGG
CTGCAATAGCTGGCAATTTACCTCCCATAtttttttttttATTTGGGCGGGGACCGTAAATTCTGGACA
GACTGGGAGGAATCGCAAAAAATTTATAGACAAACTTGTGCTATTGCAGTGGTTCGGGTGCTTTTGGATG
GCAGAACTTTTGCTTGAATATAATATTCATAAATTTAATCCGAGCAAACACTTTTCGCGTATTTCGTGCG
CTATATTTTTGGTTTTTCCCATTCTGGAGCCGTTTTTGTTTTTATTTGGCCGCTCACGTTTATCACTGAG
ACGACGTCAAGTATTCCACTTTTGCAACATTTTCCGGGTTCGGATTTTCCCCACCATCTCGCCCCCCCTT
TGCCTAACTCTCTGTATCTGTGTTTTCGTGGCAAAGAAGGAATAAACAAAATAAATAATTCATAATGCAA
ATAGGCCAAAACACACAGAGACAAGCCGAATGCCAAGGCAACGGCGAAATAAGAGCCCGGCGGCGGAATT
GCAACCCGGCCAGGACTCGATCCTGACTCCTCGGACTCCTTCATCATGGTCCTCGATCCTTGGACGATTC
GTCGGCAATCTGGCCTGTGTACATAAGCACTTTTCGGGCGAAAGGATTATCAAACGAATCAGGTATTCTG
TCCATTGTTGGCATTGCCCAAATGAGGGATCCTGTCGGGCACATAAAACAGTAAAACAATCGCAAAAAAA
TGTCAATTGTTGATTTTACTAGGCACTGCTATAAATTTTATGTATCTTTTAATCTTGCAACCTGTTGTGA
TATAGGCTTGAGGCGGTATTTAAGCGTTGAAACTTAAAAAAATGTTTTTCGATTAAATTTAAAGAATGTA
TAAACTTTTAATAGCCCGATAAACGGATCTATCCAAAGAAGATACTTTTTCGCTGTAATAGAACACGTTT
TCTAGATTAAATAAATATGTTATTAATCTTTCCCGCAAAGTAACCCACCTCTCTATCCAACGCTACTAGT
TACCCCTACTTGTTAGTCTATCCACCTAGTCTATAGTCTAGTCTCTATCTAGTCACCTCCACCGAAAACT

ATTTCAAGTTCGATGAACCGTTTCTCGCTGTGCACTCTCATTCCGGCTGAACCTATGGCCCGTGCAGCTA
AGCCACGGTTAAGGGTTTTACGGCTTTAAATAGTCAACCAAGGCTAAGCAGGGGTCGCAACATCTACGCC
AGAGAGGTGAGAGCGGTGGGGAAAAGGGCAACACCGCATATATTAAATAAAAAATGCAAAATCGTTAGGG
CCGTCTAAGGGTGATGAGGGTGACTCTCTCGGCCGAAGCGTGTGCAGCTGAAATAATCAAAAAAACTCGG
AAAAGTAGGTGGAAAAGCCAGAAGAAGAAGCAGCAGCAGCGGTACCAGTAGCACAAAATAAGAAGAAGCG
GCAAGCGTAATCGCCAGCAACACTCCcacacacacacacacacaTACTCAGCGTGGTAAAAAATGCAACA
AAATCGGATTAAAAAGTGCTCACGGTTTGAGCAACCGAGCAAAAGACCAAAGCCAAAGCCAAAACCGGCT
GCCAGAACCTCAGAAACAGTAGTAAAAACAACAAAAGTGACAAGAAAGTGCAGAAAAGTCAAGTCTTTCA
ACTATCAGATACTTACTATCCTGTTGTGCTATGCAGAATTGCACAAAATATGAATATATAATATTTGAAT
AGAAAACATTTTGATTGTAGGTTTTCATTGAATTGATTATGGTTGATTATTTTAAAGCATGGTCAAGAAA
TGTTATTTTTTAGAGCCTAAAGAGGATGCATCCCAAAATGGCAAACTTACTTATACGACATCTCAGGATC
TTTTGGTTTGACTCCATTAATTATTCACAGATTGTGTCAGTTACGCAGAATGCAAAGCAAAACGCTATTT
AATCCTTTTAACATAAATTTTCACATTCGCGGTATGCCCGCGTAATCTACTAGAATCGGGTATTATAAAC
CCCCCATCCAAAACCTTTTTGCCACCCAACCAACCGGCAAAAGAGATACAAATTGCAGTAAGACGGTTTT
TTATAGTTCAACGATGGATTTACGATGTTGCACGACTTTGAACTACATTTGTGGGGGTTTGGCCTGCAAT
GCCACCAAATCCCCGCGGACTGGCTAAGTTTGTTGTTGGGGGTAGAATGGTAGGTATGGTGGGTACGGTG
TTATGGTGTTCATCTAGAACGGGTGCTGAATAAAACTATCTTTCCATCTATCCAGCTAGTTTGTTAACAG
CTAACCAGTCGGGTGTGTTGCTCCACCGGGTGTAACTGTACTTGCCTGCCCGTTCGACCGTTACGGCTCG
TAAAAGTTGAAACTTTTTCCTTAGCGGAGGGGTGGGCTTTGGCCCCATCTATTTTTTATTTCCAGCCAAA
CACTCGATGAACTACAAGTAGATGCAAAAAGGCTGAGGCCGGGCTGCAAGGAGCACATAATTCCCGCAAA
CTGGGAATGCTACGTCCGCTGCAAGTTGCAACAAAATTGCAGCAGCAGCCATGTTCTAAGCCGGTTAACG
GTCGCACCCACTGGGTAAATATTGTGAAAGGGGCTGCAGGGCGGGGCGGGGCGCAGGGTTGAGGGGGCGT
GGCGCATGACGCCTCCCAAGGAAATTACGGATTCAAATTAGCGGCACTCAATCTCAATCGGGGTAGAGTT
CCGACCACCCACTCAACCGCCAGCTAATGCGTTTTATGATACCCACTTTATAGACCGAATGCTTTCACTA
AGAATAATTATGCGTCTGTAATATTGTAAAATTAATATGGGCGTTTTTAAAATATTTTAAAAATTTGTAT
CTTTGCCAGTAAAAAGCTACCATAAAAATTAGAGTCGTACGATTGAATCAACTTCAAAATCACGATTTCG
AACCGCTTGATATCACATTTTTTTACAGTGCAGCCTTCTTATATGCCACACAGCCTTCGATTCGTTCGGC
CCACTTTTATGCAATTTAATCAACTTATCGCGTAAAATGCACAAAACGTTTTTCTTTCAGTTCGCCAAGT
GCAGCGCTCAACTTGCAACCCAAAAAAAGAACGCCCAGCAAAGTAATCAATGATTTTTCAGTTTGTGCAT
TAAAATGCAAAGCCCTGAAAACACCAGAGCCACCCACCGAAAAACCCAACCCATTTCAGCCACATTTTAC
TCATTGGCTCTGTTGCTTATTTAGGGCTAATTAAGTTTCATTTAATGGCTGCCATTGGGAGGCGACCGAT
GGAGAGGTTCCGAGGTCAGTGGGTGGCTGTTGGCGCTTTGCAGTTTAGCAGTCAGTTGTTGTTGATGGCT
CGCTATATAGCAAAAATACTAAACTGAAAACAAAAAGAAAATAGAAAATTTCCGCGTGCCGCTTTTTAAC
TTTTTCCAGAGTTGGCAATTGAAAGTTTCTTGCGGCTGTGGAAAGGGGATTTTGGATTGTGGATTGGATA
GCATTGGAATCTATCCCCCTTATGCAATTTAGGGATTTTAACCAAACCAGGGTAAACTTTTGTTTTGAAT
GATCCCACTTTTATTTTGACAGAGTTTTGTATTCCATCTCTGACAGAGTTTTTTATCGTTTAAGCTGGGT
ATCGCTTTGAAATGGTAATTATCCATATAGTTTTTTGGACAACATGCAAACTATATTCTGTGCGGACTAA
AATAATAGGTTAAAACATAATAAAAGAAATCGGTGCAATGTACTAATATTTTAGACATTGTGGTCATATA
GGTTGTGCCTGAACTTCCATGTGAATCTAACCTCAGAAATCAAAGCAAAAGAAAGAACAAAGTGAAACAT
TTGTTAATTAAATAAATTAGCTAGCCGCAAGCCAGCCTTGTTGTTTCCTCTTTGGATGTCATTAGTTGGC
GGATTTGATTCTATAGAACGATATTAACAGAAATAGCTGGCAATTTTCGATGGGCATTTAATGTCTGTCT
TCAGTACAACATTAATGATGACCTTGTAGGGTCAGATCGGACCTGTCCGAATCAGCAATTATGGGTCTCT
CTCATGATGGATATCTGCCTGTGAAGCGAAAAGCGCTATGGAGATGATGCTGGGTACCGGTTCTCGGTTT
CCATTGATTTTCCTTCATGATTTGAATCATTCATGGTTGCATACAATCAGCGATGATCTGCTGTATAACT

GGGCGAGCTTAATGATTTATAATTGACAGAAAACACTTGGTTTATTCGTCTCCGGTTGAATTGAATCCAA
ATCCATCCACTTGTAACGCCAACAAGCTGCCTCAATccagccagccagttagccagtcaggcagtcagTC
GTTTGTCCATTTTTCATAGCCAACTTGGCTAATATATAGATTTGTGGTTGAGGCAGGCAGGCAAACCGGT
TCTTTTCCGTAAGGCATTTCTTTCAAGTGCACATACTGTAGGTTCTCACTCACCCTTTACTGCGCGAAAC
CAAACCAAATCCAAATCCACACACTTGTGCGAAACTAGTTTTCAAACAAACTGGCTCGGTCGGCCATTTG
AATATTTATGAATTCTAGTGCAAAAGTTGTaaaaaaaaaTTAGAAAATGAAATATTAATCTTAGCGTACC
GAATATAGTAATACCCTAGGGAATAGGCTTCCTAGAAATATAACTATTCAAAACCTTCAATCGCGTACTC
ATATTCACAGTAGTCTAATGAAAAGATTGATTTGAATGAATGCAATTTTACTAAACAATACTAACTGAAT
TAAATAATAGAATTTTTTAATTATTGCAAAAGATAAAACAAAGTGTAGCCGAGCATCAACCGTATTAAAC
TCCTAACTGAAACAAAATATATGCGGATATATTACCTATGCGTTGTAATAAATATATTAACTTTATGGGT
TCATGGAATAATTAGACTACCCACTGCAAGTGTGTCTAAAACCACCTTGCTTAAGGCAGGCTAAATCAAT
ATCGACAATTGTACGTCGTGAACATCTTTATTGGTTTTTAAACGCTATTTGTTGGACTTCTTCATTCACA
CCTTCAGTGGAGAACAAAACCATAGGTAGGTAATGGAGGTACTTAAAAATGCTCACAGTAAATGGCTGGT
AAATACCGCGGGACAATTAACCGAGACAAAGTACTTTGAAAACAGTTACAAGGCCAAGGCGACCCAAAGC
CAGTCACCCACCAATCCACTCGGCGAACTCAACATCAATTCAACAATCCAACAATTTGAATTCTCAAAAG
CTGAAACAGCTGTTTCATCTATTTGTCGCGGTACGCTTGCGCACAAAATATGCAAATTAAGTGaaaaaaa
aaCTGTGCGAATCGCAGAAACTTGAACAAAAGATAAACTGCAACAGCTGACTAACTAAGAATTTATGTTT
GAGACAAACTTCCATAATTCTAATGCAAATTTCTATGATTATGGGAGAAATAATTAAGGTACTTTTGAAG
CGAGGCGGTTTAAGTTCTTAGGTATTACAAAATAACATGAAGTAAAAAATCATTTATTGATTATCAAAAT
AAGAAATTAAGCGAGTGCCTAAATAATAAATCGCAATTAACATTCTAAGAAATTTTCCATCAAATTCCAG
TAGAAAACCTAATTACAAAATCCCTCAACAAAATCATAGAATTCACGATAAATATAATTCAATTAGGAAA
TGTCTACAATCTGTGCTAAATCAAACTCTGACTGACATATCAAAATGTATTCAATGACAAACTAATTGAA
TCTTTGTATCCCAGACAAAGTGAAACATTTGGCAACATTGTGGGCAAGGTGAAAACGTTGTTATTTTACA
CTTGAATTACAAATGCTAGTAGCAACATGTGTATCTGTCCAATTGCAGGCAAAGACAACAGTGCGTATTA
GTAATTTATTTGCTTTACTTCACCGAAAGTCTCGGCTTGTTCAAATATATACATATATTTGGAATATATA
TGTATAAACCGAATTATCGGTGAACTGAGTGAACATCTGGGTTTGCAAGAGGGTCGTCTGCCGAAACATT
TGAACTCAGGCGGGAATTATTTAAATGGTTCATCTTTAATGCTGTTAATAGTCTGCAATCAGGCGGTTAT
TTGTTGTTCTACTTGCTGATGTCATGGCATAATTTACCCTAGCCCCAAAACTCCCCTTGCATATTAATGC
AATTTATGAgggggggggggCTTACAAAAGCGTTGCTTCATTTCCCTAAGCTAAGGATTCCCCGGCAACCT
GGCAACGTCTTCGTTGTCCTGGGAACTGATGTTCCTCGGTCCTGTCCCTGGTGTACCTTGATAATTCTGC
ATTTATAATGCTGTCAAATGCATTCAGTGTTGTTTTTGGCTTTCACATAATTTAGTTAGCAGACGAGAAA
GCGGTCAGGTGAATGGCCGATAAGTGACAGGATTTGTGGGCCAACGACAGCTGTGTGAATTGGGTTTGAA
CTTTGGCTCAGCAATTGAAAAGTGGTGAATTCTTTCTCTTAAAACAAATTCTGTTGAATTTAAAGTTGTC
CAAGGATGGTCGAATTTAGGCGTTAAATTTATTGATATTTGCATTTTGAATAGTCTCTGTAGAGCATTAC
TGCAATCATTAAGAGGTCGTGATTCGTGGTAAATTGCCTCCCCCAAGTTGCCAAAATAATTAAACAATTT
GCTATATTATGTTTCCTTATAAATTAACTTTCTCAAATTAGTGCTAAAATTACTAAGCAATTTGCTTGTA
TGCTGTAATTTTGTCCACAGTCGTTTGTTAGGCTCTCGTACACATTTAATTGCATTTTCGTTACTTGAGA
TCTGTAATTCTTACCTTTGAATTCACACGACTATTGATGTCTATACGATAGAAGTTATAAAGGTAAACAA
AACCGGTTTTAAATACGTTTTCGACCAAacaatatacacaatatacacTTATCGAGCTATCTATTTGGAC
AGAAAACACATATAAGCGAAATGTTATACGTACTTATATATTTGTCTGTTAATTGGGAACAAAATTAGTA
TCACATTGTTTGTGCGGCACATCACAAAGCTTATTTTTATTAGTCATCAATTGTGGGGGCTATTGTCAAT
TCGATTTATTTTGATGTTATTGAATCTAACGTCTTCTTCAAACCCTTTAGGCAATACCATAAATTCCTAA
GGATATCTGCAGTAACCATGTCTTAGGCTTAAAAGGGGTTAACAAAATGTCTGTGTGTAATAAACATTTA
TGGCTGCTAATATTGAGTACGTTGAACTGGCGGAATGAGATAGCTGGTTCTAAAGGGGCAAGTTCAAGAC

ACATAAATTACGTTTGATTATCAAAAACAGTTACCAAAACTACTTACAGCCATTCTCATACTTTCTGCCA
TAACTTTTAGCCCGGCCCACAACACAGGGCaaaaaaaaaagaaaaaaCTCAACTAGATGAGAATACAAAA
GACGCCCACAAAACTATGTCAGTCAGTATCTTGGCTTTTCCCGCCTCCCCCGTCGACGGTTCGATGTCAA
GTTGCCTTGTCAACGGCAAAGTTTTCCCCACATGTCGGGGTGGCTTGGCGATTTTCTCGGGGTTTTCCGA
CGGGTTAGTGAAGTGTGGCGTCTGCATAATACGTATTTAGGTTTCAGGCGGACGGGTATAAATTTCACTT
TGTATTTCACTGGGCCCATATTTATTTCGCCCCATCCCCGTGCCCCCTCCCTACGTATTGTGCGGATTTG
TGTGCGAGTATTTTCAATTATATACACACATTTGTACACATAACCACGCATGCATAAACAAAGTAACAAC
AAAGTCGACAGGTTGAAACATAGCGCAAAAAGCCAAATCGGAAGGAGAACGGCTGCCTGGCAGACAAAAG
TTAACAAAACACAAAACCAATTTAACTTCTTGATTTTTGGTTCATTTTCATAAATAAGTGAAAAAGCGGC
AACAGCAGTAGACGGAGCTGGAGACCCAATCCCTGTCCCATCCACACGGATATATCCTTCGAACAGGACC
CATTCCAGCTAACCTTAATGCTTTTGCACGCCCTTACGCCTTGACACAGGAAAATCCCCGATATCTCCCC
GCCCACGAAATCCTTACCCAAAGTTCCCCAACCCAATTGTCCTTCGGTTGTACACTGCAAAAAAATTAAA
TCTTTTATCCTTGAGAATATAATCAAAAAGAAATATTTTATAGGCCGCCTATTTAAAAGTTGTGGATTAT
TTAAAGATAGTTGGGCTTGAAAATAAAATTTCTTTATGTCAATTTATTTGCAGTTGTCATTTTATGGCAA
AAAAATTTTTGCTTATAACCAATAATAAAATGCAAAATATGTTGACCTAACTTATATATAAATTTATATT
TCCTATAATTTGTATAACAATTCTAGAACCTCATTGTAAAATTCCGCTTAGCTTAGCACAGAGTAGTATA
GTAACTTTAATAACGAAAAAATTTTTCTCGGTGTAACTTTTCCAAGCATCATCAGCTGCTTTTTGTGTGC
TCATTACGGCAACTTGACATTAATAGGCAGCAAACAGCAACACAAACACACAACCGTGCAGATAGAcata
tatgtatatgtatgaatatatatatTACATGTGTATTTTCGCACACACCCACAAATACAGTGAACAAATA
AGCCCTGCTGGGCTATCCTTTCCATTGAAAATAATTAAATTTTCAATTATAATCAGGAGTAAATGCGGGA
GTTTCTTTATTTTCGTGGCCTTTTTCTGGCTTTGTGTTCTACAGCGTTTTTCGTCCTGGGATTGGAATGA
TTGGTGTTACATATACATACATATGTACTTTGTTCATATGGCATATTAACGATGTTGATGTGCTGGAACA
CCATTACGTTTTATTATGGTTTTAACATTGAGAGTCATTAAGTTTAAGTGGAATTAAGTTTAAACAGtaa
aaaagtaaaataaaaaacgtccaaaaaagtaaaaaTGTATACAATAAATCAAATGATTCCTTTAAAATCG
CTTTTTATAATATTAAAAATCTAACTATTTACAATCGTCAAAAAATCAACCCAAAATGAATATTaaaaaa
aaaaTTTTTGAAACTATGTAAACTGTACAGCACAACTACAAAATTACCATAATTTTGTGACATATTTGCT
TTGTATATTTTGACTATCATAGCGTTTTGCTAATTTTTTATGTCCACATGTGCCGCTCACAAACTGAGGA
ATGTATAAAAAAAGCCCGGCACAATGTTAAAAGTTTTTGCTAAACCGATTTCCATTTGCAAACAAAAGCC
TTTGCTCATGTCTATGGTCTGAAATATTATTTGCTTTGCTGCGTTCCGTGCCGATTCTGCATATTTTCCC
CCACAGAGTGGAAAATTTAATTTTCTATTAATTATTATTCGCACTTTTATGTCTGTGTGTGTGGTTGTGG
GTGTGTAGTGGGCAGTCTGGATTGCGTCCTTTGCTCCGGTCTACTAAAGTGGTCAAAGTTTTATTGGCTT
CTCTTCGGTTTTTGTTTAATTGCAAAATACTAGGATGCTCTGGACTGTGGACTTGTGGACCAACTTTAAC
TGCAGACCAGTGACCAACGTCATCTGCTTGGTTTGGCTTTGCTGCTGGTCCAAGACAAGGAAAGTTGTTT
ATGGCACAGGTTGAGTGAGGTAAAAGTTTTCTTATTTACCTGTTGGAGCAGACAATACTTTATGGCCTGA
TGATGATTATAAACCTGCTCCCCGACCTCGGATTTATGAGTTCTCTTTGAGAGTTGCCGAGTGGGAGTTG
ATCCAGATTGGAAGCGTGGCTACTTGATTGTGGCAGGTGCTCCTGCTAAATATCCTTCTTTATGAGGATA
AAGGAAATTGGCTTAGTTGTCTGTTAGTTTAGCGAGTCTCAAGAATGCGTCTGTGGTGGCCTATTTATAA
GTAGGTGGGTATTAAAAAAATAGAGAAAGTACGGGATATAGTAGAAGTTTCGTATTGCATTCGTGGGAGC
AACTTATAGCTTATATTATCAAGAAATAATTACTATTTTATAGTTAAAGCATTTTGTATGAATTCCTACT
TCATTTTGCTGAATTCTTATCTTGTGTTTCAGTTAGTTTTGTAAGACTTTGCCATATTTTTGATTTGAAG
TATGTTTTGTTTAAATAGAATCGTAAAGTATTATTCCCCACATAAATTCTCTTCGATCGAACGCAGTGAA
TTATTATTTTCCCTCGACGAGCGATTAATCGTTGGACGAAAAGCTCAATGCGCCCCAAGTAAATACTGTC
TATCTGTACAAGAATGCTTTCCCATCGATTATGTCTAAGCCCCAAGATGTCCGAATGGCTTATCTGTAAA
TATTTCGCAGATACAACAGAATGGGAAGATGAACAAGGACACAAAACAATCGAGAACCATTTCGTCAACG

TTCATTTGCGATAAGTACTTGTTCTAGGGATAAGTTTTGGGGCCATGTCGAAAACTATCAATATTGCAAT
ATCATTTAGCGCTAATCCCCGATACAATTTGGCCATGTTAATGCCCCGACAGCTGACCCGAAAGACTATT
TCAACACTTTCAATCAAGCGGGGCCAAAAAGGTTTCCCCATTCATTTTCATTTTCAAGATGCCAGACATC
AGCTGCTGGTCCCTTCGGAACTTTTCCCTGGCTTCATCTCTTGCCAAATTGTTCGTTGTTCGCTGTCTGT
TGGTTTTGTTGGCTTAAAGCTGCTTTTCAAGTGGCTTTCTGGGTAGGAAGCTGGCATTCACCGCGCCCAC
TTCTGGCCTTCGGTTGGGTCGGTTCGGCTGGCCGTTTTGAAGATATTCGCTTATAAGTTGGAAAATTACA
AACATTTTTGCGTATTTGCCTCGGCGGTTGCTGTTCTGGCGGTTGCCTTGACTCAGTGTCTGTCGTACAC
ATGCTCTTGTCTCTGGATCTTTGGTTGTTGATGCAACAAAAGCCAGCGGCAAAATCAAATAAGCAACAGC
CAAAACATCTTTTCACCAGCATCCAGACGCCGATAGAATAAAACTTGCTGCAGAAGAAGGCTAAAGAAGC
ATGCAGCCCAGCCATCAAAAGCCGGAAACTAAAATTTTGTGGCAGAAGTTAAGGCAACGCGCACAACTTG
TCGATTGTGCGTTGCGTTGGTTACTCCCCGATTTGCTGCACTGTAAAAATGTCATTATCCTTATTCTAAT
TTAGCTTAAAAAACCATTTTAGAACAACTAATATATAGTGTGACTATAAATTCTTATTGGTATTAATCAA
ATCGCTCAATTAAAATAAAAATTACATATTTAGAAATGATTCAATAATATCGTATAGAAACCACACGCTA
AAAAATCTATTTTAAGGAAGTAAACTATATTCCATTACAATATGTACACAATAAATTATTTTTCCAGTGT
GCCTTTCTGCTACTCTCTTTTTTTGGCGGTCGCAGTGCTGAAActttgtttttccttttttatagagtttt
tcttcgggcctttttcgatttatttttGCTTGCAAAAATCGTGCCAACAACTGATTTGACTGAGGACTTT
GCCGCACTCGCTGAGGCGGTGAGTGGAAATTGGGGATCGGAAAGTGGGGTTGGTGGGTGTCTGCTTTGAT
TTGGTGGGTGTCGTGGGCGGCGATTTGGAAGTGGGAAATAGAAGTTGGGAGTTCAGCACTGGGAGGCGGA
TAAGGCAGGTTCTTATTTAGTTTTTTGCAGCTACTTTGGCACTGGGCAAAACAAATGTGTAGGAACTTAA
GTGATGGATTAGTGCCAATTCGGCAGTGTGGTATTTAATCTTGCTATAGTTGCAATTCTCATAAGCCAGA
TGTGCTATTGTAATAAAGATAGATGCAGGCCAACTTCTTTTTTAATATCGTATTCAAATGCAAATAAAAA
TATAAAGAACAATATGAAATTGAAATTTTGCTAACTGAATTGAAATTTATTGCGTAATAAGTCGATAATA
TACTATTATGGAATATTAAGAGCAGAAAGGAAAGCTATGCCAATTATAGAAGTTCAAACTTATATATTCT
TAATATTTTATTCCGAAAAACACAATTCTGATTTTTTGCAGTGAACTTTTAACTCCATTTTCTGTGCATA
TTGAAGCCATCTATCCAGACACCAACTCGATGGCGGCGGGAATGGATGAGGGAAATGCTTGGCACTCAAC
TCAACTACTTTATTTGTGCTTTTGTATCTGGTCGCGTTATTATTTAACTTGCGAATGCCCTCAATGATGT
GTTTTTCTCTGCGTATTTTATGCTGATTTTGCCTCAGCTCTTGGGGCCATCTATTGTGTGTTTGGATTTG
CACATTTCCAGATTTTCTGGGGGCAAACACACAGGAACAGCTCCAAATCGAGGGTAGCACGCGCCAAAAG
ACGGCATCTGGTTGAATGGCTTGGCGTACTTTTAACTTTCCTTGGCCATTTGGCGAGCATGTTTTTCTTT
TCCATTTGTTGACGGTCCGAACACAACTTTGTGCCGCAAAAGTTTGTTTATAAATCAACTTTTTTGTTGA
GGGAACTTTTCTTTGACTCTGGATCCTTGGCGCTTAGGAGCCATTCCTGCCTCCATCCATCCATCTCCCA
GTTGGATAGTTTGCATACAACGCTGCGTATGCGCAACGTCAGCGAGTCCAGCAGAATGCGCTCGGCATTT
GGGGAATTTCTATGCAAAATGTACATTCATTTTGGTGCAAATTATTGAGTAAACAGATTGCACCTCAGCA
CCTAAGTGGGTGAGAACTCGTTTTGGAAATTTTCGCGCATTTGCTGGCACTAAGCAGCAACATCCTTCGT
CCTTTACCCATTTTTCCctctccctctctctctctctctctctctacctctctGTAAGCAACAGTTTT
GTGTAGTCTACTGCATtgtttttcgtttttagttttcagttttctgtttgcagttttGTACAAAAGTTGC
TGTTCAAGGCGATTATGATCTTTGCTTAATGTAGTGAAGTCTCCAAAGTGGTTTATCCTCTTAAAATGTC
CTTGCCGCTATGAATGAATGGCTCGACCGCATTCCGCATTTCGCAACATTTTCTTTCCGAATTAAAATCT
TATTTAATCCCTCAATCCTGTCTGTATAAATAAACCATTAGTTTGCCACTTTGACTGATGGCCCGTCCCT
CCAGTTTCGTGATTAAAACCCACCAGCAAAAAAGTATAAAAGTGAAACGGATAAAACCCTTTTCTCATTA
AAAATCGACGAGCAACGAAATGTTTTGGCAAACAGAAACAGTAAAACGTGTTAAAACCAAATGCTATTGG
CTTTGCTCGTCCGACGAGTCTGACAACGCTGACAACTGACTGAAGATTGCCCGCAGCGGAATGGGTTTCC
AGTTCAGTTCGGGCCGGGTCAAAATCGGAATGGTATATCTACTTCCTACAGTTTCAGCCATTGGCCAACT
GACGTACCATGATTAATTATTTGCGGAGTTGAAGTGTTATTGAATCAACAATTTCGATAGGCTGACATCT

GGATGTACTTCGCAGAGTTAAAAATTGGATATCAATGTATTTCGATACGGTTGTCGAGACCAAAGGTTTT
TATTTATGGATGGGTTCGAATAAATCAATTATTAATGACATGAATAATATAAAAAAATTATTTCTGGAAA
TTTTATAATATTCAAAAAAAGTCGCTATTTCAGTATTCTTATTCAAGAATTTGTAGTTGCACTTTAAAAT
GATTTTGACTAGAAGTGTATAAGATTTTTTTATGAATACTATTTGAATATAAAGGTAGCAAAAGCCTGGA
AGTAACTATAATAGTATTTCAGTAAATATAATTAATCAAAATACGCACTGTCATTGTAAGAAATAGACTT
TGCAAAGTTTTTCTGTCTTATTAATACCAAGAACTTATTTCGAAAACTTTATGAAGTCTGTCAACTTTAT
GGCACTACAGACGCCACCGCCTCCCTTTCGCTTCGTTTTAATTAAAAATATTTTTGTATAGCCCAAACAA
ACTTAATGCCCAACAACTTGTATTTACATTTTTCATTCTTTGTCCATCGTTTGTATTTTCACTGTCTCTG
GCTCACTCATGTATTTATTTGTTAATTTTCTGAAATGGCTTTTGCAACTGATTTCAGGAAAGATGCCTCA
TTTTGTTTTATATCCACTTTTTGCTCTCGCCTGTTAAATTGTAGGCACAATTGTTGGTGACAGAAAATTA
ATTGCATTTAATCAGTACGGGCCAAGTTCAGGGTGTACCAGCAACTCGAAAATCGTTAATGTGTTTTTGA
TTAATTGTCCAGACGGTCGGACAATGGACTTTGATTTGCATTTTACAAATTTAATGAATGACCCAAGAAT
AGGCCTACGATGTCAGGGTTGAATATAACAAATACAAATATTTATTATTTTTTCAGCCAAGTACTTTAGT
CGAAAATCCCTTGTAATCTAGTGAAATTTCACTTAATTTGCTTGTACAAGGGCATTACATAGTCGAATTG
ATGGGACttttttttAATGACTGAATTTTATCATAAGCTATTAAAATGATTTTAATTAATTTGATTAAGG
ATTGGATGTTGCGAGTGTAAACAAAAATAAAATAATTTTTTCAGCACTACCTGGTATTTGTTTATTAAAC
AATTGTTTCGTGTCAGCAAGGGGACATTGCAAACAGCTGCAGGGATTACATTATTAAATCGCATATATTT
ATTTAATTAAAAAATAACAAGACCGCCGGCGAAGTGAACAAATGGATGGCCGCCATTGGATCGACAATAA
ATTAAATGTTTTCTCAGACCTTTTAATTGCCTGTGCAAATGGAATCTATCAAATATGTATAAGGTTTGCA
TACACATAAAATATGCACGAATTATAATATTAAATTGAAATAGTTAAAGGGCAAATTGGTTCCCAAAGAG
ATGTCAACTTGGTTTTTACTCGTGATTTATAATAGCATTCACGACGGGATAAAGGGATAATCCCCTGACT
GTCTGAGACATCAGCAGCTTCAAAGCGCATAAAAGTCGATCAACGAATTTCCCGCTGCTATCGGCGAAAT
CATGGCCAAAACGTGGAAAACTTTTCCATTTTCCGTATTTTAATTGACGCGCATCATGTCAGCGGAACAA
AGCCCTGAGAACACAACACGGAAACGAGTGGGTGGCTTTTCGATACGAATGTGGGTGAGTGGGCGAGTGG
GTGGGTGAGGTGCCAATCCGATGACAGTGTGCCCTGCGCTATAGTTTCACAGTTAGCAAAATCATCGATA
GATGACGCGACGGCGGGTGCTTGGACTTGATAGATTGGGAGCAGaaaaaactagaaaaaaaTCTGTACAA
AATGGGTGGGCAGGGGTCCAAGTAGATGGAGCGATGAATTTTGCCAGCGCAAAATGCAGTTGACAGCCAT
CCAACGTAAAAACGGCGACACTTCATCGAAAAAAGAAAatatatacatatatatatatataAAATCGAGG
CAAAAGTTTAGACTACAAAACACCTACCAACTTAAATTAATTGCAACGTGATACTGGCTACAGATTACCA
ACTGATTTTAATGAGAAGCTTAAGCAATTAGAACTATTATGCAATTGAGTTTACTCATGTTTCAATGCTA
GAGGCGCGGAAATACCTTTTGAGTCTTCCGTGAGAAGTTGTCTATCATACCTGTGAAATAAAGGGTGATG
AATGGCGATGGTAGATATATGTGAATATGTAATGATCTGTGCACACTGGGCCATTTAATATTTTAATATT
AGATTTGTAAAAGTATTATAATTTGTTATATGTAATATATACCACGACTTAGTGCAAATTTGATATCTTA
AATATAATAATATTGACTTGTATTAGGACATTTTTTAATGCATATATTTAAGTATCGTTGTATATTGCCA
CGCTGCAAGTGTGCACAGATGTAAATTATCGAATTTCCGCACCGCAATTATGTGGTCCAATATGAAGAAA
GCCATTGAGCGGATGCTCGTTGGCTGAGGAAATGAGAAACTCATTCATTTGGCAGCGGCGGCAGTGGCAA
TATACCTACGTCCACAGTTACCTTCGCAAAATCGTTGACATGTGAAATTTTAATTaaaacaaaaatgaaa
ataatacaagcaaaggaaaggaaaaacaaaaaaGCATCAGCAGCAACATCTCTTGTACAAAGTGCAGTAA
CAATAACGAGAACGCCAAACGAAAATGTCAAAAATATCAACAGCATAAACACACGTCACCAAACAGAGGG
CGGAAAATTGGGGAGGGGAAAACGGGGTACACAAATAAAAAATATGTAGTAATTTTGCAAAAATGACTTA
TTTTTTATGACTACATATTTTTAGAGTAGGTATTCTTTAGTTTTAATAAAATGTAAGTCATCAAAGTAAG
TCTAAATTGTTGGGCTTGAAGATCATAACACTTAACTTTGATTATTCGACTGGAGGCCTCGCTTGTTTCG
AGTGTATGTGTGTGTGGCAGAAAGCTTTTAAGTGCTGTCAGCACGGAAAAGAGCCAAAGTTGGTAACAGC
AAAATGGCTGCCTGCTAACATCTTCAGTGACTGCAAACTGCAGCTCATTAGCACCTCGTGGTGCTCACAC

AGATACTCACGGATACTCACCGTGCACACCAACACTGCGGCACGCGAACTTGGCACTTTCGGTCGCGGTT
GACTTTCCTCGATGGCGGCGACGCCTTCAGGCCTCACTTTTATCCTCATGCCAGGACTCAGTTTCGGATG
CACCGCCTCCACTTTGGCATTACTGCCGCAAAACTCTTTGCACATTGGTGCGGAAATTTTGTTGCAATGA
AGTTTAGCGCTGAGTGCATGTTGCTCTTGAGCAAAATTAATGTAATTGCTCTTTGAAATCAGGTTCGTAC
TTTTTTCTTTCGCCGCTGTCGTCCTCAGGCACGTGCTGAAATTACCGCGATGGCTCATAATTAGATTTTC
CATCTGCTGCTATCTTGCCTGATTATAACTAATTTATTTATGAGACTTTTTACAAAGGAATTGAGAAGAT
ACGGAAACTATAGGTGTTTAGTTTCTTCATACCTGGAAATGATTTATTCGAATATTTTACACACAGAATA
ATCCAAAAAGAAGAGTACATTAAAATATAGCGATGTTCTTACTATTTACTTCGGTTGTTATTTTTTCATA
TTTATTTAATTTTATTGCAAACTGACATTTTGCCAGTTCTTATAATCCCAATAAACTTTCCCCACATTGA
CCAGAAAATAGTTTATCTCCTTAGCTGTCTAAATCCTTAGTATCGCAGTTGATTTTCAATGGCGAAATTA
GGCCAAAAAAGTTTTGAGCAAAACTAACACCGCATTGCGACCAAAAGGATGACCAGTCCAATTGAACCAC
TTAGAAGTTGCTGTTCTGCCACAAATGTCCGTAATAAAAGTTTTCGTTTTGTTCACATTTCTTTCTTGCT
ACACTCCTTCCTGTTCTGCTGATGACCATAAACTTGTAACACTTTGGTAAAAGCCACACTCAGCTGGTCT
CTGATATTTCCAAGTGATTAAGTCGTTTACAAACACAGATATGATGGGGTAAGAGGAGCAATATTTTTTA
TTGATATCCACAGTTGCTGTTGCTTGTGCAACCAATTTGGTGCGGAGAACAACAAAAGTGTTGAAACTGC
CCCTAAAAAGCCAAAAAGTCAACATTCCCATGAGTAAATTTAATAGTACAACGGGGTATACGAGCTCCAA
AGTGCCCCAAGCATTTGTTTTACTTGGTATCttttttatgattttttttcttttttttttggttttttGGGA
AAAAGTTGACTTGGCTTAGATGAAGTTTTTGGTACTTTCCAGCAGGGTAGCTGGGTTTCAGGCTTTTGGG
GTTTGTCGGGAAACTAGTAAATCTAATTTCTAACCACGCTCACACACACTCTAGCACCAATACACAGGCA
GGCAGCCATGTAACTGCATCCGAGGTCCTTCGAGGATGTCTGAGCCAGTTCGTCAGCTTTCGTCAACTGC
AGAAACTCCTCGGAGCGTCGGTTGTTGGCATTTTCTGAGCTTACTGACTTAGCCAACTTGTAGCTGTTGG
CCTGGTAAGCACTTCAGTTAACCCAAACACCCGTCACAACCACACATACATATGTGTGTTCGTGGCTCGT
TTGCTGCGGAGTTTTACTATGTTTTATTTGTTGCCAACTACTAGAAACAACGGGCAAAGCCAAAAGCAAA
GGCAAAAGGCAAGTGCAACTGCGTTTGCCACTGCAGGTTGCATATTGCTCTGCTATGCTCTTTACAACTT
GTATCAAGTTGTTTTGGTGCTTTCTAGTTCTGTGGCAGCCACATGTTGAAAGTCTGAAGTGGCCACATCT
TTCCCATCAGGAATGAACGTTGATACGCATATGGAAAATGCATTTGACCTGCACGCAGTAAAAGAAAAAT
GTTTGAAAATTTAAATTAATTTAATCACTTTGTAAAACCTGGTGTGAATTTATAAAATTTGACCTAAACA
ACTTTCTCAGTGCAAATGAAAATGAAAGAAGCTTTATGGTAATCCATTACAATATGTCAACGGGTGTTGA
ATCGTTAAATGTTTTAAGCATTTTTAAGACGTGCAAAATGACAATCATCAATCTACTTTTTACAAAAGAT
ATTTTCAACAAAAATGCTATCCTGGCATGCCATGGTTTTCATAGAACCTTAGTAGCTGCAAGTCCAACAA
GCTTTTGCTTCCATGTGATTGAATATATTCTTTGCAATAATTTGTACATTAAATGTTATCATCTGAAATA
ACGAGTTAAAGATGTAGAAATTAATAAGAAATATATTATGTAACAGCTTGACTAAAAATTCAGATTAGGA
AACAATAAACATTTAATTCTTATAGATTTACATAATAACACAATGAAATATACATAATTTAGTAAGAATT
TTAAAAACATATGAAACTTAAATCATGTTTTCTTTAGATTTCGTTGCTAAAAAATGAACCAGTTGCTTTT
CAGTTTATAATTATGTATTTATTCGCAAGGAAGCATCCTATTGTACGCAACCAGCTTGCATAAATTCCCA
GCTCCCCAGTGAAAGATGAGGACGTCTTTGGTTGCACATCCCTGGCTGCCATTGAAGTGCATCTGTCGCA
CATTTGTCCCGTTGCCTGCAATCACTGAGCCCCAAAACGAATGCACCCACACCCATAACAGCTCACGTTT
GTTGTGCGTCCTTGTTGTTGGCCtgtgtgtgggtggatgtgtgagcatgtgtgtgCGGGGCAAAACAAAA
ATGCTGTGATGCTGGGTGTGTAATGTGTGCATGTAGTGGTGTTGTGGAAATGACGGCAGCGGAAGTTAGC
AGGCAAAGCGGCGGAAATGTAAAATCAATGTTGTTGCTGTCGCCGTAGAACTGTAGCTGGCAGTTCTTTG
GTTCGGCTGAATATGGCTGAACATATAGTTGCATATAGTTGGGGGTTTTGGAGAAAATGCGGCATTGTTG
TCGCTGCTCAGGCTTGAGCTCGAGTCAGTGTGCTGTGCATTTCGATGCTCCTGATTGTTATGTCTGTTTG
TGCTGCtattgttattattattattcttattattattattGTGCATTTGCGCTTGAAGCGAATTATGTGT
ATGAAAAATGCATATAAAGTTTTTCCTCTTTATCCATAGCGAAATGTGCAAACAAAATAAACAATCCGAA

AAACAAACTTATGCACACCAACACACACCAGCACACACATGTGCATGCAATTATGTCAGATTTACAAATA
CTCACATGCTTGCTTAGACACAAAAACCCCGCCCTTCCCTCTGCACCGACGACCATGTCTTTCCTCTTGG
CATTTGCATATGTAAATATATATTTTAAAGTAAATCTTTCATCTACCCCTTCTTAACTGCTTGTGCAGAA
TTATTTTTTATCATTGCTTGGGTTTTGTTTTGCGCCGGAATATTTGTTTAATGTGCCGCTTCTGTTATTG
TTATGCTTGAGCATTTATTTCTGCTCtttttttttGATATTTACCAATCTTATCGAGGGGTATTTAAAGA
ACGTTTGGGTATTTTTTATTTGTGTGAACCAAGACTTAGGTATAGAATAAAACATTCTGTTAAATTAAAT
AACTAATATAACATCTTTATAAAAAGATATTCAATACCTTTCTCAGTTAAACTGTCTTGGACACACAGAT
TAGCTTTGGTATATAATAGTATTACGTTTGTACTAAGCTCTTTAGCTACAATTATAATGAGTTGCCCAGC
AACAAATGAAAGCTGCAACCTTACACCTAAGTAAACTACCATAGGGCATTTCCCAGTCGAGCAACAAATG
GAAACCACACTTTGCCATTTTGCGGCTCTCATTGTTATTGTTCATTATGTTTTGGTCCTTTGTTGTAACA
TGTTTTGCTTATTCGCTGCTTACAAACCTGCGATTTTCCCAACATCTGTGCATCCCCATCGTTTTTATGC
CTCTTTGGGGAGATTTTTACATTGAGGTGTGTTGATTTTGTTTTAATGCTAAAAACAAATTTTGCTTTTT
TCCCTTTTGTTCCGGCTTAATGCCACAAGCCGATTTCGTTGGTCGGCTCTGGCATTTGCATTTGTTGGGG
TTTTTCTTGCTGTTCTCCGTATTAATTTTAATTACAAAATTCGACTGAGGCTTCCTGAATAATTTAACAC
TTTCTTGTACATTTTTGCGCATGATGTACATAATAGCCAGAGTCTAATCAAATTCGTGGAATTAACTCTT
TAATCAAGGTGTGTTCTCGATGTAAACCAACATTTCCATTTGGGCTCTAGCTGAACTGGACCCTAACAAA
AACAAACAAAAGTTGTGCCTCTCTGTTTTTAGTTGGGCTTTTGTCGCCTTATCGCCCGGCCTAAAAGTTT
TGCACAACTTGTGCTCTCACGGCGTATGAGTAATTTTTTTGTTTGATTATCATATTTCTCACTCAGAGTA
GAAGCCGGAAAAGCCAAACAAAAGGCCTAAACACACTTTTACCTTCCCTTCGCACGGCAAATGAATGGAA
AAAGGTTGACAATGTTCTTCTTCTCTGCTGTTTATTTTTTACACTCAAGTTCAAGTTTTCCGGGCAAGAG
CAAGAACAGAAATATGCACAGCTATGAACATTAGTTTCAATTTGAAAAAGAAAATGTGTGTGTGTGTTTG
GGTACGTTCTGTAGGTCTTTTTGGCTTACCTTGCAGCCTCGACTTTTCACCTTGCCCGACCCTCCTCGAT
TTCTGTGAGTGTGTGCGTTTTCAAGAGGTTGTCGCAACTGGAAAGTATGCAACACTTTTACTTTTCGCCC
CGCACTCCGTTGCGTGGGAAAACAAGAAACGAACAAAAGCTGCTTAAAAACACCAAACCATAACAATGTA
AAGTGTTGTAGGAGCTGTCGAAAATATTTTACACCTCTTGCACTGTGACTCACAATCTATGGGTTGTTGT
TTTCGAAGGATGCGGACCTTATCTGGGACTACCTGAAAAATATACCACCAAAGCCGAAGCACAAGCAACA
AGGTAAACATTTTGCGGTAATTAAGAAAAATTACCTTGCCTGGCAGTGGAGaaaaaatcggaaaaaaaac
aggaaaaccaaaaaagaaaaacaagaaaagcacaacaacaaaGCCCATAAAGTAGTCCTGGCCAAAAGCC
GTACGGACACACACAGAGACAGACAAAGTTGGCCGGAACGAAAAGACAGTTGGAGCGTCAGCTGTGGGAA
ACCCTCGTTCAGTTTTCCCCATTCATCCCCCTCACCCTCCATTGCTCACCTCACCTAAGCCACCCTCGCA
TTTTCACCCCTGTTCTTCGGGTCTTTCACCATTTCTAGTGGCTGCGACTTTTTGGTGTTAACTTTAAAT
TAGCAGCAAAGTGGAAAGTGTTGTCGGTTGGCTTTTAAAATGTTTTGCGGTGTATTTTATTTATCATGAA
GTAGATCTGGTGTGGCAATTGCCAGCCCTGCTCCTTTTTGCCGCTGCCCACCTTGTCTCGGCTGTCATTT
GTCTGTCTTGGCCATGTTATAAAATAATGTGTAAATGTCCCTGGCCttttttttCCATTTATCCCGCCAT
TCTTCGTTTTTGTTCTTTTTTTGAGTTTAATTTACTTAATGTCCTGCCTGGGCCCTTTTATGATGCCTTC
TTGGGGACTATTTTCCGCTTTTTTTGTCTTTGGCTGTTCATCTCAATATTTTGTTGTTGTTTGGGGCCCA
TTTACCTTGAGAGTTGGCCCCGGTTTTATCATTAATGTGTGTGCCCAGTTCCTTAATTAGCGGCGGGCAC
CGTCaaaaaatatcgagtgataaaaaatggaaaaaaagaaaTGTGGTGTGGTATGCTAATTTATGCACTT
GATAAAAATTTCCAAGGGGAAGGGACCCACAAGGAAAATGGGCGGAGAGCGAAAATTAATACCAAGATCA
GAATATAATTGTTCGCCATTTGAAAAAATTCGTAAAAAGAACACAATTTTAATAGTTTTTAATGATTTAC
TATTAATTGTTTGACTTCCCAAAATCTCATTAACAGTGATTTCGGTAAATTGCCAAGAATGGCGGAGTAA
GATCTTTTGCTCTTATTAAATCGCTATTACCCTTAATATCGTTGAAATAAAATTAACTGACAGCTTACAC
ATACCACTTGAGAATTAAATTCAGAACTTGAATTTCGGTTGTGCTCTTGTCACATTGTCTCCCAGGGAAA
TTTAATTAAGGGGAGCGCAACTAAGCGCTCCACTTTACCGCTCGAGTCAAAAACGTATACGTAATTTCAT

TAACAATTATGACCCGAGCATTAGGGTTGTGCATCTTACATCATAACCCATGGTTTCAGATGCGAGGAGG
TTGAGAGATTGGATTGATGTGTGCCACTTGACATGCCAAGAAGTTGAGGGAGCAACACATATAACATGGA
TGGAAGACATCGTAAAAAAAGATTCGAGGCGCTTACCGGTCATTTTTGGGGTGGCACGCCAGCTAACTGT
CTGTGGCAGCTCCTTCGCTGCTGTGCTAATAAAATCAAAACTGATTACCACATCATCTTCAACGAGCTGA
GCGGAACAGAGCACCCAGCACGGAGCACAACACAGCGTAAAGCTTCGAATAGAAGCATGCATATCATATT
ACTCATACGCCACGTTATACAATTTACTTACAACGTCAACGAGCAGAACGTTGACAGCGCACCAAGACGG
GACAAGACGAGATGAGATGAAATGGGAAGCCAGGAGGTTTGGTAGTTGTGGCCCAGATGAAGACAAATAG
ATGGCAACATCATGGAGGACATATAAACACACGCTTACACTAAAGGCAAAGCTAACCTACAATGGTGTTA
AAAATGGCAACTTTGAATAAATTTTAAAAGCCGGAAGAGCCGTTTGCATGCTATGCAATAAATTAACATT
AGGCCGGAAGTATAAATCATATTTAATAAGGCAAATGATTGTACTAAAGAGAACTACAATTATttttcaa
tttttttaattgtttatattaatacatagacatattattattattatataattatatttaaaatatCAAA
GCAAACTCCAAAATATTTGTTTCCTTGTTTGGTTGCAGAAAAACTTCACTTAGTATTAAAATGTAGTGTC
AGAATCCGATAAAATATTTTTAAATGTAATTTATTGTTAACATATTTTATTTGGGATCCAGAATGTAATA
TTTTCCGTGTATCCTGGCATACACGTACGTGTGTGTCTGTGCGCAGGATGTGCGTGCCTGGATGAGTGCG
CAAAAATGGCAGCGGAACGGCAAAGAGCAAAAGCCATGGGGCCAAGAGCAAAAACAATTAATTGAGTGGG
TGGCAAGTGACACCAGAGTGCAGCGAAAGATAAATCGTTTTGCAATAGCGCTATAATTTAATCAAATTTG
TCACTTTGCAATTGAGCAACTCGCGAGTTGCACGCCAGACGACTGCAGTTGCTCCAGTCTGTTGGAATTT
ATGAATGGCATTTGAGGGGAGCATCTAGACAAAGGTACTTCCACCTGCACTGGTAACTCAATCTTCGCAT
CGTGGAAGGAGGCAAGTGCGTGCTCGGGCATCGCGTTCGCACTTTGTAAATTCAATTTGAAATGCGCTTG
ATGCGCACAAAGGCAGGTACAAGCCCACCTTGTAAACCTTATGCTCGCGTAGGTACAAATACCCACCCAC
CACACATTCATCTACCCCCATCACCCACTTCGCACCACTCTGATAACATACAAGTGCCGCCCACTCGGAA
AAGGGTGGCCTTTTTCAGTGTCGCTGATGGCCAAACAAATGCCAGCCAGCTAAGCGGTTTGTCAGCGCAA
TGGGGGTCAGAAAATGTGTGGGGTCGGGTGATAAACGTCATTGATGATGACCCAGGCAGCAGGTGACCCC
ATCCAACCCTTCGGCGATTAGGTGAATCGATTAACCGTTTTCGTAATTGAATACAAGCGCAGCAGCCACA
CGCACTTCTTTTGCTGCAGTGTACGCAATTTGTTGTTGCCATTACCCGTGGAATGGTCGCTAAAACTGCC
AACAAATTCTCAGTTACGCTCTCACGTACTGGGCACTTACACAGCTGCAAGAGTAAacacacacacacac
TCACAGGCACAATCAGCTCGAGCACCTTTCACAGAATGCACGAACCAATTTCAACAAATCTAGACCCCCT
ACACACTGAGAAAAATAATCTAAGCGCTGATATTTTCTAAAGAGCTAAGTGCATTTGGAACTTAACCAGA
AAATATATAACTAGCTTTTATTAGACCAGATTTAATTTTAACGTTGTATCTATAAGCCAATATACATTTT
TCTGAAACCAAAAAGCCAACACCAACAAATTGAATTGTTGGGTATACCTGTACCTTCTATTGTACAAATT
TACGATTAAAGTATACCAAAATCCTAGTTCAGTTGATATATTTTTAAAATTTGTAAATTAATTTTGTAGT
TTTAACAGGAAAATACATTTTCCTTTTCATTGTGTAGCCTAGCACTGCAATGTTAAACATTTTATCAACG
CAGaaaaaaaggtaaagcaaaaaaCTCGTTCGAATTTTTAAACAAAAATCCAAAAACGTTGGCCATAAGC
GAACAGGTAACCAAACCAGGCATCCAGCCTGCGGGAAAACTGCGCGGGAAAAGTGCCGTGGGGCGTTTCG
AGTTTCGAGAGGGGTTGTGAATGTTATTTTTCAAACATCAAATGTTCTGGCAACGTTGACGTCAACACGG
GATCCAAGGCAAAAGTGACGCTGCTTTCAGCTGTTTGTGCTCGCTTCCTTCCTCTTGTCCAACGTTTCCC
AATTGGCTGCATTTCCCAGGTCAGCCATCTGCCACGCCCTCCTCTGCCAACGCCCTCGCTTTTACCGTCC
CCATCATAGTTGCCACTGGCATTTCGCCTCACCCGGCCATTTATTGCAATATCAAAATGTTTCCTTTCCG
GCGATGAAAATGATTGAAAAATATTCTCTGTTCCATGGCTTCGCTCTTTAAAGTTTTGGAGAAAAAATGTT
GTTTGATTTGCGCATTACAGGTGATTCGCAAAATTTAGTAAACGTTTTTCCTTTGAATTCTGTTTTGCAG
GGAACCGGCGGACCTTCAATTATTTTGGAGAGCAAAGATGCTTTATTAACCTGCGTAGTCTCAGATAACT
CCTCGAACAACACAGTAATATGGAAGAAGGGTGACGAAATCCTGACCGCCGGCACAGTGCGGGTAACCAA
GGATCATCGTGTGCGAATCCTGCACGATGAAAGATCCTAAGGGGAAGAACCTGGAAACGGGGGGAGAGGT
TTGGGTGCTTTTGATTAAGACTCTTAAGCCCAACGATTCGGGGGCGTATATATGCGAACTTAACTCGGAT

CCCGTTCTCCGCAGCATCCACATCCTGACAGGGTGAGTACCGACAAAAAATCTATACACTTGAAGAAACA
ACATGCGTTTTAGCAAATTTGTATTTAAGCCCTTATATACAAAAAGCAAACGATTTATAAATTGTTTTGG
TTAGTGAGATATCAAACAAATATTACATTTTAAAAGGTATTACTTAATTTTCACCGCTTATGTTTTAATC
CCTTAAAGATCGGTCAATCTTTTGTTATATCCATTTAAATACCTTAAGAAATTGGTATATATCCATTGGT
AAACTATAAAATGGCAAAACATTTTATTTATTTTATGGATTAAATTTTTCCCTGTACTGAAATTAATTTC
ACGCCTTAACCCCTGACAGGTTAAGGAGCTTCATTCGTCGACGGGTGGTCCGGGAAATTCCACGGACACC
TCAACCGCGGAGTCGAGTGATGTGTTCCTGCTGCCGCCACCGTTGGACGCGCAGGATAATCGCAGCTTTT
GGAGGCCCAGTCAAGTGCCACCGCTGGTGACCCACGACTTTACGGAGTGCTGCGAACGGGCTAACGTGTC
CACCCAATGCATGGGCTTCTGCAACGTGCACAACATCCTCGACGGCACCACTGGCATCGATCCGGAGGCC
TGCGAACGGGACTTCCCCGGCATTGTTCGCTGCATGGCGGATGGCCGCAACCATGTGCCCTGCTGCGTGG
ACAAGCAGATTCCGGACTTGTGCCAGGACATGTGCCGCGGCGAGTACACTCCATTCACGGACAAGTTGAA
GACCCGTGTATCCTGCGTTCAGCACACACTGAGTGGGCTGCAGTGCATCCTGAAGGGTGTGCAGTTGATA
CCAAGTTCGCCCACCTTCGTGACGATCGAAAATGTTAGCGAAACGAGTGTGACCATCGGCTGGACTGCAC
CGGATAAACTGGCTGATCGCGTTTCAGGCTATACCATTAATCTGACCACTTTGCACAGTTTCGATGAGGA
TGAGTTGAGTGGCGAGATTGTTCACCGAACGGCGTCTGATTCTCCAGAATCGATTAGTCTCTATTCGGTG
CCCACGAATCAAACTCAGTTGACGCTCAGCAATTTAAGTCCGCTGACCATGTACGCCGTAATTGTCACGG
CTACCAACGAATTTGGATCGAGTTTACCCACCGAGCGGCTGAGATTTTTCACCCACACCAGCGCCTCGGC
GGCGGAATCGAAAGCCGGAACCACGGACAAACAGGCAATGCCCCAACTACCGGATACCCGACAATGTTGC
GAGGACAATGGGATGACCCATCGCATGTGTCTGGACAAAATGTGCGATCCGCAGAAGACCGATCAGGCCA
ACTTGCCCGATTTCATGGTGTGCGCTCCCTGGTCAAATATCACCTTCAATTGTTTGGCCAACAATATTGA
TCATACGCCGTGTTGTCGCGCCAGGGGAATTCCATCAGCTTGCTTTCCACTATGTGCTGGCAAGCTGACG
TCACTTGACTTTAGTTTATTCAAAGTAAGTAGATCATTTTTTATAACTACATTTTTGAATAATTAAATAA
AACTAAGATGATTGGTGCTTAAAATGTAATGTATGGATTCAACCAGCAAATAATGTATTTGCCTCATCAG
TAATGAGCAACAATTGTGTTCATTGAAACCATTGCGCCTATTTCTCCCAGAAATTCATTTTTTTCAATTT
GCCTTTATTTCATATATCTTCCTTTGTATTGGTACTTCTCATAAGGACTCTCAAATTTGAATCGATAATC
ATCAATGAGCTTCTCATTTCGCAGTTAATCGCTTAGCAAGCCCATCCTTCCCCATCATCCATGTTTAGGA
AACCCATAGGCCGTTAAGCCCTAATAACAATcctattcctatgcctatgcccatgcccatgcctatgcct
acacccatTCCAATTTTTTTCTTTTCCACAACTCCGCACCCTTATCCTTATCCAGCGCCCAAACACAATG
CTACTTGCTAATGACTCCGTAGGATGAGTTTTTATGCCGAATGCCAATTACGGAGCTTTGCTTCTCTCCG
CTCTCCGGTTATTTGCAGGATGCCTGCGCTTCATGTCCGAGTACAACAGCTGTCTGTTCCAGGGCTACGG
CGTACTGGCCGATCCGCCGTCAAAACTCCGGACCGTGGCCAAGACGGACAGCTTCGTGATTCTGGACTGG
AACAAGCCGAAGCGCTTGGCCGACACCGTCAGCACATATCACGTCAAGTTCCGACGTCTAGGAGTGGGTG
ATGACTATCTGACCGTGGAGAAGGTATTATTACACTGGACATTTTGGaaaaaaaaTTCTTTATAATATCA
AGATGTTTAAAAGAGCATATATACATTATACCTAAATAAATAAATTGCGTTGGTATTATTACACTGGACA
TTTTGGaaaaaaaaTTCTTTATAATATCAAGATGTTTAAAAGAGCATATATACATTATACCTAAATAAAT
AAATTGCGTTAAATAAGTGTTGGATTAGGAACAGTGGTATAACTACTGGTCATGCTAAGTGGCAATAGAA
AATATTTCATTTGTATCTACAGGATACGATTTTGTGCTTTTTATAGTTCCAAAAAGTTCTTGATGTTAGA
AATTGATTTTTCCGCAAGTGCAGGCAATCTGCCGGCGCGGGCAACCATTCCCTAAATGGTTTGGCTAATT
CATTAAGCTTTGCAGGCGCCAACCGCCGCTGATTCTCGAGGGCCTGGAGTCGGACATCTACTACGAGTTC
TACGTGGTCTCGATAAACGCGTACGGCAAGAGCGAGGCCTCCCCCCGCCTGATCACCCGGACTCTGCCGG
CGGAGCCTGTCCAGCAGACAGCCGCCAGATACAACATGACGACCTGCTGCCAGGCATCCGGACTACTGCC
CCAGTGTGCCCCCCTCTGCTCCTACAACATTCGACTGTCCGATATCGAGCGACTGGGTCCGGCTTGTCGA
GCCCAAATGCCGTAAGTAACAGATGGATTTTCGGGGGTTTTCCCCGAACCTCATGATATCGCTCCCTTGG
AAAACACTAATTAGCGAGTGGATAGACCAGTCATTGTCCCGTGGACTGTTGTTAATTTAACGAGCTCATT

TTGCATTTTTAGTTTCGTATGGCGATGCAATTTGGAATATTAAGTGACGCGCTTGGCCATTTGGGCATCA
TGAATTTTTGCACTTATGCAAATCAATTTTAAGTTAATTAGACGCATAGAAGCACCGAAAATGGCCAACA
GGGATGTTTGATGTTACTAAATACACGTTAATTTTGCGAAATGGTTATATCATTGAAAACTGTTTGGCAA
GAGATTGCCAGTGTTTGCCTGGGCTTATGTAATTTTAGAAGAGATGTGCCTCTGTGCCTCATTGTTTGGC
CTCGTTAGAGTAAACAATGCTTTCCAGAACAGATGCAAGAGCCCCCTTGGTTTCCCATAACATTTAATGG
TTGCATTTCATAACTAGATTCTCTTGCCTTCTCGAATAGCTAAGACAATACCCAACAACACCTAATGTTT
TTATAAAAGTAGATAGGTGGCTTGCAGTGTTAATTTATTTGTTTAAAATGCCTTAATACATAAGTAGTAG
GGAGTATTGATTTATACAGCAATATTGAAAATATTGTTATTAATCTTTTGGCTTAGGTCTTGTTTAATTA
AAGTAAGTTTAGGTCAACATTTTCTATAAATTGTTATATTATAATACGCCTTTTCAACGAAAGCGCTTTA
AATAAGATACAAATATTGATTTACGGGCAATCAGAGCTCAGCCAAAATTTCAAAATTCCTTGAATTTGTA
CATTATCttttttttttttttGTGTTTGCTCAGAAACACTCAGTGCCAAAAATATCCCCGTCATTCTCAA
TCAAATAAATTGAATCTGCTCCAATGCCACCGATGAGTGGGCAGAATTGTGCCCCAATTTACCCGTCACC
TAGTCAATGATAGGAAATCTTTCGGTTAATTTCCTGTAAATTGCTGCCAAGTTGCCGACTCATGGCTCAG
ATCCTTTAAATTATCATCTCAACCGAAGTGGCAAACGGGGCAGAATTGATGAGTTCATGTCACAAATTGT
TCCATAACATAGCACAATGAGCCGTATCGCTGGCCGGGGAAATTTATGCAAATGATGCCACAATATTAAC
CCATCTGGCACTCTAATCCTTAGGCAATCCTGGCCCGCTGTGCAGCCGGAGGACGTGATCATAGTCCGTG
CTGCAGTCGCCGTGGAGTCATTAACGCCTGCATGCCCCTCTGTCGTGGCGTCATGCCGCTCGCATTGACC
ACCAAATCGtcatcagcatcggcggctgcagctgcaacatcatcatcatctggagcaacagcagcaacat
cTGCATCTGCCACCAGTAATAGCAATGCGGCAGGAGCACCATCATCATCATCATCGTCTGCCTCATCGGG
AACATCAAGCAATGTGCCCGATTGTTTATCATATGCCGGCAACATTCTGCAGTGTTTCGAGGAAGGGTGA
GTGGTAACCATGGCAGTGCTCCCTGCCTTCCGTCCACATCCTGGTCTTTGTCTGTTGGAGCTGAGTGCCT
TGTGTTGAGATTGGATAGCGAACTGTGACTGTAGTGAATGCAATCAGCTCATTTGAAATTATTCCGTGCA
ACGTAGAGTTAATTAGGCAAGATGCATGCGAATCACCAAAAGTCACAGATGCCAGCTGTCTTCCAATGTT
TTCAAACAAGAAAGAAATGAACTTTATTCTCTAAGTTAAAAGAATAAAAGAAAACGTTATTTTATTTAAA
ATCCTAAACTTGGATTTTGAAATTTTAAATTTATAAACACATAAATCACTTTTAACGATAATATCAAATT
GATTTGTATTTGTTTTGAATGGTATTTGAATAACCATCAAGTGTTTTATGTAATGGAAGAACTATATGAC
TTTTAAGGATGATTTAGCATGTGCAAAACGATATTAAACTTCATTTTTCTCATTAAATGCGTGACTGAGG
GTCACTGGAGGTCACAGCTAACCAGCCCGGCAGGCAGATAAACTGCACCACTGGCCACACCCTGTGGTGA
ATCCATCGGTGGGTCTTCTCTGTGGATGGGATGTCGCTGGCCTTGCTGTGTTTGCAGCTAATTCTGTTTG
TTTAATGGCTTGTCTGTGCACGTCGAGTATACGCACTGTTGCACGCCATTGTACGCTAAATTATTTGGTC
AATGCTTTGGGGCAACCAACCACAGCCCGTCAGACAAAACCAATATTGGCCAATTAAAGTTGCATTATAA
AACAAAACAATGCCGTTTGCTCACTTCACTATCCCCCTATTGGTCTATTCAGGGCACACAGAATATCCCA
GGACCACCGGAAGATGTGCATGCGACATTCGTGAATCAGAGCAGCGTCAGTTTGGCCTGGACGCAGCCTG
ATGTGGATAATAGCAATAGTAGCAGCAGTGGCAACGCATCGCCAGGTGACATGATAACCGGCGATGAACT
GGCAGCTGGTGCCGTGGATCCTGCTAACGCTGGAAAGGATGTGCCGAATGCTGGCGATGCCACAAGTGGG
ACAACTGGAACGGAGGATATTGAATTCATCGTACAATATGGCAAAGTCAATAACATGACCATGTACGAGA
CAGTTGCCAAATTGGAAAAT
>dme_circ_0004772_junction_seq
GTACGAGACAGTTGCCAAATTGGAAAATGACTTTCTACGATTGTAACGAATGATTT
>dme_circ_0004773
GGTTATCCTACAAGGAATAATGCTCAGGCTTTTGCTGGTTCACATAATTACGATATGTGTGCTAATAACT
ATTGTTAAGTGTGGTGTGCCAATTGGTAAGTAGTATTATTAAGTGAATGCTTTTAACTTATATTTAAGAT
ATATATATTTCTTTCTGTGAATTCAAGGAACCGGCGGACCTTCAATTATTTTGGAGAGCAAAGATGCTTT
ATTAACCTGCGTAGTCTCAGATAACTCCTCGAACAACACAGTAATATGGAAGAAGGGTGACGAAATCCTG

ACCGCCGGCACAGTGCGGGTAACCAAGGATCATCGTGTGCGAATCCTGCACGATGAAAGATCCTAAGGGG
AAGAACCTGGAAACGGGGGGAGAGGTTTGGGTGCTTTTGATTAAGACTCTTAAGCCCAACGATTCGGGGG
CGTATATATGCGAACTTAACTCGGATCCCGTTCTCCGCAGCATCCACATCCTGACAGGGTGAGTACCGAC
AAAAAATCTATACACTTGAAGAAACAACATGCGTTTTAGCAAATTTGTATTTAAGCCCTTATATACAAAA
AGCAAACGATTTATAAATTGTTTTGGTTAGTGAGATATCAAACAAATATTACATTTTAAAAGGTATTACT
TAATTTTCACCGCTTATGTTTTAATCCCTTAAAGATCGGTCAATCTTTTGTTATATCCATTTAAATACCT
TAAGAAATTGGTATATATCCATTGGTAAACTATAAAATGGCAAAACATTTTATTTATTTTATGGATTAAA
TTTTTCCCTGTACTGAAATTAATTTCACGCCTTAACCCCTGACAGGTTAAGGAGCTTCATTCGTCGACGG
GTGGTCCGGGAAATTCCACGGACACCTCAACCGCGGAGTCGAGTGATGTGTTCCTGCTGCCGCCACCGTT
GGACGCGCAGGATAATCGCAGCTTTTGGAGGCCCAGTCAAGTGCCACCGCTGGTGACCCACGACTTTACG
GAGTGCTGCGAACGGGCTAACGTGTCCACCCAATGCATGGGCTTCTGCAACGTGCACAACATCCTCGACG
GCACCACTGGCATCGATCCGGAGGCCTGCGAACGGGACTTCCCCGGCATTGTTCGCTGCATGGCGGATGG
CCGCAACCATGTGCCCTGCTGCGTGGACAAGCAGATTCCGGACTTGTGCCAGGACATGTGCCGCGGCGAG
TACACTCCATTCACGGACAAGTTGAAGACCCGTGTATCCTGCGTTCAGCACACACTGAGTGGGCTGCAGT
GCATCCTGAAGGGTGTGCAGTTGATACCAAGTTCGCCCACCTTCGTGACGATCGAAAATGTTAGCGAAAC
GAGTGTGACCATCGGCTGGACTGCACCGGATAAACTGGCTGATCGCGTTTCAGGCTATACCATTAATCTG
ACCACTTTGCACAGTTTCGATGAGGATGAGTTGAGTGGCGAGATTGTTCACCGAACGGCGTCTGATTCTC
CAGAATCGATTAGTCTCTATTCGGTGCCCACGAATCAAACTCAGTTGACGCTCAGCAATTTAAGTCCGCT
GACCATGTACGCCGTAATTGTCACGGCTACCAACGAATTTGGATCGAGTTTACCCACCGAGCGGCTGAGA
TTTTTCACCCACACCAGCGCCTCGGCGGCGGAATCGAAAGCCGGAACCACGGACAAACAGGCAATGCCCC
AACTACCGGATACCCGACAATGTTGCGAGGACAATGGGATGACCCATCGCATGTGTCTGGACAAAATGTG
CGATCCGCAGAAGACCGATCAGGCCAACTTGCCCGATTTCATGGTGTGCGCTCCCTGGTCAAATATCACC
TTCAATTGTTTGGCCAACAATATTGATCATACGCCGTGTTGTCGCGCCAGGGGAATTCCATCAGCTTGCT
TTCCACTATGTGCTGGCAAGCTGACGTCACTTGACTTTAGTTTATTCAA
>dme_circ_0004773_junction_seq
TGACGTCACTTGACTTTAGTTTATTCAAGGTTATCCTACAAGGAATAATGCTCAGG
>dme_circ_0004774
GGTTATCCTACAAGGAATAATGCTCAGGCTTTTGCTGGTTCACATAATTACGATATGTGTGCTAATAACT
ATTGTTAAGTGTGGTGTGCCAATTGGTAAGTAGTATTATTAAGTGAATGCTTTTAACTTATATTTAAGAT
ATATATATTTCTTTCTGTGAATTCAAGGAACCGGCGGACCTTCAATTATTTTGGAGAGCAAAGATGCTTT
ATTAACCTGCGTAGTCTCAGATAACTCCTCGAACAACACAGTAATATGGAAGAAGGGTGACGAAATCCTG
ACCGCCGGCACAGTGCGGGTAACCAAGGATCATCGTGTGCGAATCCTGCACGATGAAAGATCCTAAGGGG
AAGAACCTGGAAACGGGGGGAGAGGTTTGGGTGCTTTTGATTAAGACTCTTAAGCCCAACGATTCGGGGG
CGTATATATGCGAACTTAACTCGGATCCCGTTCTCCGCAGCATCCACATCCTGACAGGGTGAGTACCGAC
AAAAAATCTATACACTTGAAGAAACAACATGCGTTTTAGCAAATTTGTATTTAAGCCCTTATATACAAAA
AGCAAACGATTTATAAATTGTTTTGGTTAGTGAGATATCAAACAAATATTACATTTTAAAAGGTATTACT
TAATTTTCACCGCTTATGTTTTAATCCCTTAAAGATCGGTCAATCTTTTGTTATATCCATTTAAATACCT
TAAGAAATTGGTATATATCCATTGGTAAACTATAAAATGGCAAAACATTTTATTTATTTTATGGATTAAA
TTTTTCCCTGTACTGAAATTAATTTCACGCCTTAACCCCTGACAGGTTAAGGAGCTTCATTCGTCGACGG
GTGGTCCGGGAAATTCCACGGACACCTCAACCGCGGAGTCGAGTGATGTGTTCCTGCTGCCGCCACCGTT
GGACGCGCAGGATAATCGCAGCTTTTGGAGGCCCAGTCAAGTGCCACCGCTGGTGACCCACGACTTTACG
GAGTGCTGCGAACGGGCTAACGTGTCCACCCAATGCATGGGCTTCTGCAACGTGCACAACATCCTCGACG
GCACCACTGGCATCGATCCGGAGGCCTGCGAACGGGACTTCCCCGGCATTGTTCGCTGCATGGCGGATGG
CCGCAACCATGTGCCCTGCTGCGTGGACAAGCAGATTCCGGACTTGTGCCAGGACATGTGCCGCGGCGAG

TACACTCCATTCACGGACAAGTTGAAGACCCGTGTATCCTGCGTTCAGCACACACTGAGTGGGCTGCAGT
GCATCCTGAAGGGTGTGCAGTTGATACCAAGTTCGCCCACCTTCGTGACGATCGAAAATGTTAGCGAAAC
GAGTGTGACCATCGGCTGGACTGCACCGGATAAACTGGCTGATCGCGTTTCAGGCTATACCATTAATCTG
ACCACTTTGCACAGTTTCGATGAGGATGAGTTGAGTGGCGAGATTGTTCACCGAACGGCGTCTGATTCTC
CAGAATCGATTAGTCTCTATTCGGTGCCCACGAATCAAACTCAGTTGACGCTCAGCAATTTAAGTCCGCT
GACCATGTACGCCGTAATTGTCACGGCTACCAACGAATTTGGATCGAGTTTACCCACCGAGCGGCTGAGA
TTTTTCACCCACACCAGCGCCTCGGCGGCGGAATCGAAAGCCGGAACCACGGACAAACAGGCAATGCCCC
AACTACCGGATACCCGACAATGTTGCGAGGACAATGGGATGACCCATCGCATGTGTCTGGACAAAATGTG
CGATCCGCAGAAGACCGATCAGGCCAACTTGCCCGATTTCATGGTGTGCGCTCCCTGGTCAAATATCACC
TTCAATTGTTTGGCCAACAATATTGATCATACGCCGTGTTGTCGCGCCAGGGGAATTCCATCAGCTTGCT
TTCCACTATGTGCTGGCAAGCTGACGTCACTTGACTTTAGTTTATTCAAAGTAAGTAGATCATTTTTTAT
AACTACATTTTTGAATAATTAAATAAAACTAAGATGATTGGTGCTTAAAATGTAATGTATGGATTCAACC
AGCAAATAATGTATTTGCCTCATCAGTAATGAGCAACAATTGTGTTCATTGAAACCATTGCGCCTATTTC
TCCCAGAAATTCATTTTTTTCAATTTGCCTTTATTTCATATATCTTCCTTTGTATTGGTACTTCTCATAA
GGACTCTCAAATTTGAATCGATAATCATCAATGAGCTTCTCATTTCGCAGTTAATCGCTTAGCAAGCCCA
TCCTTCCCCATCATCCATGTTTAGGAAACCCATAGGCCGTTAAGCCCTAATAACAATcctattcctatgc
ctatgcccatgcccatgcctatgcctacacccatTCCAATTTTTTTCTTTTCCACAACTCCGCACCCTTA
TCCTTATCCAGCGCCCAAACACAATGCTACTTGCTAATGACTCCGTAGGATGAGTTTTATGCCGAATGC
CAATTACGGAGCTTTGCTTCTCTCCGCTCTCCGGTTATTTGCAGGATGCCTGCGCTTCATGTCCGAGTAC
AACAGCTGTCTGTTCCAGGGCTACGGCGTACTGGCCGATCCGCCGTCAAAACTCCGGACCGTGGCCAAGA
CGGACAGCTTCGTGATTCTGGACTGGAACAAGCCGAAGCGCTTGGCCGACACCGTCAGCACATATCACGT
CAAGTTCCGACGTCTAGGAGTGGGTGATGACTATCTGACCGTGGAGAAGGTATTATTACACTGGACATTT
TGGaaaaaaaaTTCTTTATAATATCAAGATGTTTAAAAGAGCATATATACATTATACCTAAATAAATAAA
TTGCGTTGGTATTATTACACTGGACATTTTGGaaaaaaaaTTCTTTATAATATCAAGATGTTTAAAAGAG
CATATATACATTATACCTAAATAAATAAATTGCGTTAAATAAGTGTTGGATTAGGAACAGTGGTATAACT
ACTGGTCATGCTAAGTGGCAATAGAAAATATTTCATTTGTATCTACAGGATACGATTTTGTGCTTTTTAT
AGTTCCAAAAAGTTCTTGATGTTAGAAATTGATTTTTCCGCAAGTGCAGGCAATCTGCCGGCGCGGGCAA
CCATTCCCTAAATGGTTTGGCTAATTCATTAAGCTTTGCAGGCGCCAACCGCCGCTGATTCTCGAGGGCC
TGGAGTCGGACATCTACTACGAGTTCTACGTGGTCTCGATAAACGCGTACGGCAAGAGCGAGGCCTCCCC
CCGCCTGATCACCCGGACTCTGCCGGCGGAGCCTGTCCAGCAGACAGCCGCCAGATACAACATGACGACC
TGCTGCCAGGCATCCGGACTACTGCCCCAGTGTGCCCCCCTCTGCTCCTACAACATTCGACTGTCCGATA
TCGAGCGACTGGGTCCGGCTTGTCGAGCCCAAATGCCGTAAGTAACAGATGGATTTTCGGGGGTTTTCCC
CGAACCTCATGATATCGCTCCCTTGGAAAACACTAATTAGCGAGTGGATAGACCAGTCATTGTCCCGTGG
ACTGTTGTTAATTTAACGAGCTCATTTTGCATTTTTAGTTTCGTATGGCGATGCAATTTGGAATATTAAG
TGACGCGCTTGGCCATTTGGGCATCATGAATTTTTGCACTTATGCAAATCAATTTTAAGTTAATTAGACG
CATAGAAGCACCGAAAATGGCCAACAGGGATGTTTGATGTTACTAAATACACGTTAATTTTGCGAAATGG
TTATATCATTGAAAACTGTTTGGCAAGAGATTGCCAGTGTTTGCCTGGGCTTATGTAATTTTAGAAGAGA
TGTGCCTCTGTGCCTCATTGTTTGGCCTCGTTAGAGTAAACAATGCTTTCCAGAACAGATGCAAGAGCCC
CCTTGGTTTCCCATAACATTTAATGGTTGCATTTCATAACTAGATTCTCTTGCCTTCTCGAATAGCTAAG
ACAATACCCAACAACACCTAATGTTTTTATAAAAGTAGATAGGTGGCTTGCAGTGTTAATTTATTTGTTT
AAAATGCCTTAATACATAAGTAGTAGGGAGTATTGATTTATACAGCAATATTGAAAATATTGTTATTAAT
CTTTTGGCTTAGGTCTTGTTTAATTAAAGTAAGTTTAGGTCAACATTTTCTATAAATTGTTATATTATAA
TACGCCTTTTCAACGAAAGCGCTTTAAATAAGATACAAATATTGATTTACGGGCAATCAGAGCTCAGCCA
AAATTTCAAAATTCCTTGAATTTGTACATTATCttttttttttttttGTGTTTGCTCAGAAACACTCAGT

GCCAAAAATATCCCCGTCATTCTCAATCAAATAAATTGAATCTGCTCCAATGCCACCGATGAGTGGGCAG
AATTGTGCCCCAATTTACCCGTCACCTAGTCAATGATAGGAAATCTTTCGGTTAATTTCCTGTAAATTGC
TGCCAAGTTGCCGACTCATGGCTCAGATCCTTTAAATTATCATCTCAACCGAAGTGGCAAACGGGGCAGA
ATTGATGAGTTCATGTCACAAATTGTTCCATAACATAGCACAATGAGCCGTATCGCTGGCCGGGGAAATT
TATGCAAATGATGCCACAATATTAACCCATCTGGCACTCTAATCCTTAGGCAATCCTGGCCCGCTGTGCA
GCCGGAGGACGTGATCATAGTCCGTGCTGCAGTCGCCGTGGAGTCATTAACGCCTGCATGCCCCTCTGTC
GTGGCGTCATGCCGCTCGCATTGACCACCAAATCGtcatcagcatcggcggctgcagctgcaacatcatc
atcatctggagcaacagcagcaacatcTGCATCTGCCACCAGTAATAGCAATGCGGCAGGAGCACCATCA
TCATCATCATCGTCTGCCTCATCGGGAACATCAAGCAATGTGCCCGATTGTTTATCATATGCCGGCAACA
TTCTGCAGTGTTTCGAGGAAG
>dme_circ_0004774_junction_seq
GGCAACATTCTGCAGTGTTTCGAGGAAGGGTTATCCTACAAGGAATAATGCTCAGG
>dme_circ_0004775
GGTTATCCTACAAGGAATAATGCTCAGGCTTTTGCTGGTTCACATAATTACGATATGTGTGCTAATAACT
ATTGTTAAGTGTGGTGTGCCAATTGGTAAGTAGTATTATTAAGTGAATGCTTTTAACTTATATTTAAGAT
ATATATATTTCTTTCTGTGAATTCAAGGAACCGGCGGACCTTCAATTATTTTGGAGAGCAAAGATGCTTT
ATTAACCTGCGTAGTCTCAGATAACTCCTCGAACAACACAGTAATATGGAAGAAGGGTGACGAAATCCTG
ACCGCCGGCACAGTGCGGGTAACCAAGGATCATCGTGTGCGAATCCTGCACGATGAAAGATCCTAAGGGG
AAGAACCTGGAAACGGGGGGAGAGGTTTGGGTGCTTTTGATTAAGACTCTTAAGCCCAACGATTCGGGGG
CGTATATATGCGAACTTAACTCGGATCCCGTTCTCCGCAGCATCCACATCCTGACAGGTTAAGGAGCTTC
ATTCGTCGACGGGTGGTCCGGGAAATTCCACGGACACCTCAACCGCGGAGTCGAGTGATGTGTTCCTGCT
GCCGCCACCGTTGGACGCGCAGGATAATCGCAGCTTTTGGAGGCCCAGTCAAGTGCCACCGCTGGTGACC
CACGACTTTACGGAGTGCTGCGAACGGGCTAACGTGTCCACCCAATGCATGGGCTTCTGCAACGTGCACA
ACATCCTCGACGGCACCACTGGCATCGATCCGGAGGCCTGCGAACGGGACTTCCCCGGCATTGTTCGCTG
CATGGCGGATGGCCGCAACCATGTGCCCTGCTGCGTGGACAAGCAGATTCCGGACTTGTGCCAGGACATG
TGCCGCGGCGAGTACACTCCATTCACGGACAAGTTGAAGACCCGTGTATCCTGCGTTCAGCACACACTGA
GTGGGCTGCAGTGCATCCTGAAGGGTGTGCAGTTGATACCAAGTTCGCCCACCTTCGTGACGATCGAAAA
TGTTAGCGAAACGAGTGTGACCATCGGCTGGACTGCACCGGATAAACTGGCTGATCGCGTTTCAGGCTAT
ACCATTAATCTGACCACTTTGCACAGTTTCGATGAGGATGAGTTGAGTGGCGAGATTGTTCACCGAACGG
CGTCTGATTCTCCAGAATCGATTAGTCTCTATTCGGTGCCCACGAATCAAACTCAGTTGACGCTCAGCAA
TTTAAGTCCGCTGACCATGTACGCCGTAATTGTCACGGCTACCAACGAATTTGGATCGAGTTTACCCACC
GAGCGGCTGAGATTTTTCACCCACACCAGCGCCTCGGCGGCGGAATCGAAAGCCGGAACCACGGACAAAC
AGGCAATGCCCCAACTACCGGATACCCGACAATGTTGCGAGGACAATGGGATGACCCATCGCATGTGTCT
GGACAAAATGTGCGATCCGCAGAAGACCGATCAGGCCAACTTGCCCGATTTCATGGTGTGCGCTCCCTGG
TCAAATATCACCTTCAATTGTTTGGCCAACAATATTGATCATACGCCGTGTTGTCGCGCCAGGGGAATTC
CATCAGCTTGCTTTCCACTATGTGCTGGCAAGCTGACGTCACTTGACTTTAGTTTATTCAAAGTAAGTAG
ATCATTTTTTATAACTACATTTTTGAATAATTAAATAAAACTAAGATGATTGGTGCTTAAAATGTAATGT
ATGGATTCAACCAGCAAATAATGTATTTGCCTCATCAGTAATGAGCAACAATTGTGTTCATTGAAACCAT
TGCGCCTATTTCTCCCAGAAATTCATTTTTTTCAATTTGCCTTTATTTCATATATCTTCCTTTGTATTGG
TACTTCTCATAAGGACTCTCAAATTTGAATCGATAATCATCAATGAGCTTCTCATTTCGCAGTTAATCGC
TTAGCAAGCCCATCCTTCCCCATCATCCATGTTTAGGAAACCCATAGGCCGTTAAGCCCTAATAACAATc
ctattcctatgcctatgcccatgcccatgcctatgcctacacccatTCCAATTTTTTTCTTTTCCACAAC
TCCGCACCCTTATCCTTATCCAGCGCCCAAACACAATGCTACTTGCTAATGACTCCGTAGGATGAGTTTT
TATGCCGAATGCCAATTACGGAGCTTTGCTTCTCTCCGCTCTCCGGTTATTTGCAGGATGCCTGCGCTTC

ATGTCCGAGTACAACAGCTGTCTGTTCCAGGGCTACGGCGTACTGGCCGATCCGCCGTCAAAACTCCGGA
CCGTGGCCAAGACGGACAGCTTCGTGATTCTGGACTGGAACAAGCCGAAGCGCTTGGCCGACACCGTCAG
CACATATCACGTCAAGTTCCGACGTCTAGGAGTGGGTGATGACTATCTGACCGTGGAGAAGGTATTATTA
CACTGGACATTTTGGaaaaaaaaTTCTTTATAATATCAAGATGTTTAAAAGAGCATATATACATTATACC
TAAATAAATAAATTGCGTTGCGCCAACCGCCGCTGATTCTCGAGGGCCTGGAGTCGGACATCTACTACGA
GTTCTACGTGGTCTCGATAAACGCGTACGGCAAGAGCGAGGCCTCCCCCCGCCTGATCACCCGGACTCTG
CCGGCGGAGCCTGTCCAGCAGACAGCCGCCAGATACAACATGACGACCTGCTGCCAGGCATCCGGACTAC
TGCCCCAGTGTGCCCCCCTCTGCTCCTACAACATTCGACTGTCCGATATCGAGCGACTGGGTCCGGCTTG
TCGAGCCCAAATGCCGTAAGTAACAGATGGATTTTCGGGGGTTTTCCCCGAACCTCATGATATCGCTCCC
TTGGAAAACACTAATTAGCGAGTGGATAGACCAGTCATTGTCCCGTGGACTGTTGTTAATTTAACGAGCT
CATTTTGCATTTTTAGTTTCGTATGGCGATGCAATTTGGAATATTAAGTGACGCGCTTGGCCATTTGGGC
ATCATGAATTTTTGCACTTATGCAAATCAATTTTAAGTTAATTAGACGCATAGAAGCACCGAAAATGGCC
AACAGGGATGTTTGATGTTACTAAATACACGTTAATTTTGCGAAATGGTTATATCATTGAAAACTGTTTG
GCAAGAGATTGCCAGTGTTTGCCTGGGCTTATGTAATTTTAGAAGAGATGTGCCTCTGTGCCTCATTGTT
TGGCCTCGTTAGAGTAAACAATGCTTTCCAGAACAGATGCAAGAGCCCCCTTGGTTTCCCATAACATTTA
ATGGTTGCATTTCATAACTAGATTCTCTTGCCTTCTCGAATAGCTAAGACAATACCCAACAACACCTAAT
GTTTTTATAAAAGTAGATAGGTGGCTTGCAGTGTTAATTTATTTGTTTAAAATGCCTTAATACATAAGTA
GTAGGGAGTATTGATTTATACAGCAATATTGAAAATATTGTTATTAATCTTTTGGCTTAGGTCTTGTTTA
ATTAAAGTAAGTTTAGGTCAACATTTTCTATAAATTGTTATATTATAATACGCCTTTTCAACGAAAGCGC
TTTAAATAAGATACAAATATTGATTTACGGGCAATCAGAGCTCAGCCAAAATTTCAAAATTCCTTGAATT
TGTACATTATCttttttttttttttGTGTTTGCTCAGAAACACTCAGTGCCAAAAATATCCCCGTCATTC
TCAATCAAATAAATTGAATCTGCTCCAATGCCACCGATGAGTGGGCAGAATTGTGCCCCAATTTACCCGT
CACCTAGTCAATGATAGGAAATCTTTCGGTTAATTTCCTGTAAATTGCTGCCAAGTTGCCGACTCATGGC
TCAGATCCTTTAAATTATCATCTCAACCGAAGTGGCAAACGGGGCAGAATTGATGAGTTCATGTCACAAA
TTGTTCCATAACATAGCACAATGAGCCGTATCGCTGGCCGGGGAAATTTATGCAAATGATGCCACAATAT
TAACCCATCTGGCACTCTAATCCTTAGGCAATCCTGGCCCGCTGTGCAGCCGGAGGACGTGATCATAGTC
CGTGCTGCAGTCGCCGTGGAGTCATTAACGCCTGCATGCCCCTCTGTCGTGGCGTCATGCCGCTCGCATT
GACCACCAAATCGtcatcagcatcggcggctgcagctgcaacatcatcatcatctggagcaacagcagca
acatcTGCATCTGCCACCAGTAATAGCAATGCGGCAGGAGCACCATCATCATCATCATCGTCTGCCTCAT
CGGGAACATCAAGCAATGTGCCCGATTGTTTATCATATGCCGGCAACATTCTGCAGTGTTTCGAGGAAGG
GTGAGTGGTAACCATGGCAGTGCTCCCTGCCTTCCGTCCACATCCTGGTCTTTGTCTGTTGGAGCTGAGT
GCCTTGTGTTGAGATTGGATAGCGAACTGTGACTGTAGTGAATGCAATCAGCTCATTTGAAATTATTCCG
TGCAACGTAGAGTTAATTAGGCAAGATGCATGCGAATCACCAAAAGTCACAGATGCCAGCTGTCTTCCAA
TGTTTTCAAACAAGAAAGAAATGAACTTTATTCTCTAAGTTAAAAGAATAAAAGAAAACGTTATTTTATT
TAAAATCCTAAACTTGGATTTTGAAATTTTAAATTTATAAACACATAAATCACTTTTAACGATAATATCA
AATTGATTTGTATTTGTTTTGAATGGTATTTGAATAACCATCAAGTGTTTTATGTAATGGAAGAACTATA
TGACTTTTAAGGATGATTTAGCATGTGCAAAACGATATTAAACTTCATTTTTCTCATTAAATGCGTGACT
GAGGGTCACTGGAGGTCACAGCTAACCAGCCCGGCAGGCAGATAAACTGCACCACTGGCCACACCCTGTG
GTGAATCCATCGGTGGGTCTTCTCTGTGGATGGGATGTCGCTGGCCTTGCTGTGTTTGCAGCTAATTCTG
TTTGTTTAATGGCTTGTCTGTGCACGTCGAGTATACGCACTGTTGCACGCCATTGTACGCTAAATTATTT
GGTCAATGCTTTGGGGCAACCAACCACAGCCCGTCAGACAAAACCAATATTGGCCAATTAAAGTTGCATT
ATAAAACAAAACAATGCCGTTTGCTCACTTCACTATCCCCCTATTGGTCTATTCAGGGCACACAGAATAT
CCCAGGACCACCGGAAGATGTGCATGCGACATTCGTGAATCAGAGCAGCGTCAGTTTGGCCTGGACGCAG
CCTGATGTGGATAATAGCAATAGTAGCAGCAGTGGCAACGCATCGCCAGGTGACATGATAACCGGCGATG

AACTGGCAGCTGGTGCCGTGGATCCTGCTAACGCTGGAAAGGATGTGCCGAATGCTGGCGATGCCACAAG
TGGGACAACTGGAACGGAGGATATTGAATTCATCGTACAATATGGCAAAGTCAATAACATGACCATGTAC
GAGACAGTTGCCAAATTGGAAAAT
>dme_circ_0004775_junction_seq
GTACGAGACAGTTGCCAAATTGGAAAATGGTTATCCTACAAGGAATAATGCTCAGG
>dme_circ_0004776
GATGTGCATGCGACATTCGTGAATCAGAGCAGCGTCAGTTTGGCCTGGACGCAGCCTGATGTGGATAATA
GCAATAGTAGCAGCAGTGGCAACGCATCGCCAGGTGACATGATAACCGGCGATGAACTGGCAGCTGGTGC
CGTGGATCCTGCTAACGCTGGAAAGGATGTGCCGAATGCTGGCGATGCCACAAGTGGGACAACTGGAACG
GAGGATATTGAATTCATCGTACAATATGGCAAAGTCAATAACATGACCATGTACGAGACAGTTGCCAAAT
TGGAAAATTGTGAGTATATATCGGTGTTGGACAATGGAACGTCTCGTTCTGCACAAACTTTTCCAGTGCA
GAACTATTCCCCCACTTTGCCTTCCCTGTTCGCCCCACCTGTGAGCTGGCTCGGAAAAGGttttttcctc
tttttttGTGTTGGACAGGCTCAGAGATTCACACACACAATGGGGCACTTCATTTGCATTGGGGCCTGCG
GGCGGTCGCCTTATTACGATTTGCCTTTTGGGCCAAAATGCTGACATTATATATATTCGAGTGCCAATGG
AATTGAAAAGAGCTTACAAATTGCCTTGTCTGTGCGCCGGAGGCCGGGGAGTCAGGACAATAACCGTTTC
AACAACTTTCCGACCGGGGAGAACTTAAATTGCCAGCAGAGTTAATGAAGAAGTTCGGGGACAGGAAATT
GCTTCCATAACAAGCAGTCACATAAGCATATATTGTGGCCGTTTGAGCATACATGGCGTATGAGCAACGT
GAGAGGCTATACTAATTGTTCAATTAAGACGACAGCTGAAAATTGTCTATCTGGATTCTATTGTTCAATT
GAATGTTGGAGCACCTGGAGCACCTCTATTCAAATATCTTTTAAATTATTGCATTTATAAAGGTTTTGTA
ATAGGTATTATTTAAATCACGTTAAGAGGAGACAAATCTATAAAATAGGAAACGAAATGTTACTCCAAGG
CTGTGATAAAAATTACCAAACACATGGCGGATGAGTTATATTTGCAGTTAAGCCAATTATTCAATTATTT
ATTTAAATCCTTGGCCATCGGCTAAATCTCACTAAATGCATATATCGATGGCCATTGTTTATCCATTGAA
TCGACTGAAATCAATTGGATTTTTCCTGAATTTCAAGTGCATTCCTCTCTAACCAAAATATTCAGCTTAA
ATTTAGTTttatatgtatataatattatatatataAATTGGCAAATAGTTTTGGTTGGCCGCCGCCGCCG
CTAATCCAATTCAGTATAATAATCTTTGAACTCTGTCCAAAAAGCATCGATTGAAGGGGGAATGGCCAGG
TTTAGATGTACGTAGAGCAATGGCAAAAGTTTTTGACGCCTTTTCAACAgggggggcggggctgggggACT
TATGGCCGCCATATGCACTGGCAACGTGCAAAACGTGACTATAAACTGGTAAAAAGTGCAGCCGATCCAC
taaaaaatacaaaaatagatgaagaaaaatccccaaaaaaaGAGTAACCAAAACCAGACAACGGACCAAA
CTTGGCTCTGGCTTTGCCATTGGCACTGTTGTAATTTTCTatgttgttgccacttgttgttgttttagtc
attttgttgttgcaattgttgttgctcttgctatttggttgttgcatttgttgttgtagttattgGCCGT
TGTTGTCACACATCAACGCGCTTTGCTGGCAGCTGGGCCTGGTtgatggtgctgctgcgatgttgttgct
gctgcactgttgttgctgctgcTGCAGCATCAGCCATGGCCATCAACAGAGTTATTGGCCTCGAAAAACT
CGCAAATGCTAAAAAAATACAGCAATACGGCAACAACAAAAGAAGCCTGGCCAGAGTTTGTCGCGTGTTGA
TGCGCACAGAAATGTATTTAAAGtgaggtcgatgttgccgttcttgatgttgttgctgttggcattgctg
ctactgctgctgttgctgctgctgGGGGCATTGCAACCTGTCGTCGACGACCTGCCAATGATGCTCCAGC
CTCGGCTGCTCCATGGGAGGTGTAGTCGTGAGATACGCTTAGGGAGTCCAATAGGACTATTTTCGGTATC
CAGAAAATGTTTAACGATAATAGCTAGCTAGCAGTAGAGCAAGAATTAACTGAttttttttATAAACTCT
CAAAGCAACATACATTCGTTATCATCATCACCTAAACAACCCTTCAAAGTTTATCTTAAAATAAATAAAA
TTTGTGTCAACTTACTTTCTATTAtttttttgcatttttttcatttatgtattttGCGATGGCAGACATTG
GTTATTTTTCTCTACAAGATTCATTGCCATTCGATTGATTAAATATGAATGATTGATCATGAGGTGTGGG
AATCACAGTTTCTATTGACATCTaaaaaaaatgaaaaaaaaTCCCCTTAGCAAGCGTGACAGAATCTTTCG
TCACGTGAGCAACTCTACACCAGAAAACCAATCGATATGGAAGATACTGTGCGGTGTCAGCTTTGGTTCG
ATTTTTTTCTTATACTCTGAGCTGTTTTTTGTTGATTGTGTTGTGGAAAATTCATTCGCCCTGTTGGCTG
CTTACTTTTTCGTTTTCCCTCCACttttttttttttagtttttttgttttattttttGCTTGCTTTGGTCAGC

AGTGCACGCAGCTGTTGCGGTGATGCTGTTGCTGTTGGCTTAACTTGGGGCTGGCTTCGGCTTGGCTTAA
ATGCTTTTCAGTTTTGCCGTTTTTGCGGTGCCCCCCCTCCATCAGCTCACCATCGCATCGCATCGCCATC
GCCCGTTGGTGTCAAATGGTTTAAACGCGCCTCGCGACCCCTTTTGAATGGGTTATTGTATCGCATGTAT
ATCTATAAGTTCGTGGCTATCGGGGGAAACTGTACCCCAGCAGCAGGCTGCAATGCGTTGACTCAGTGTG
ACACTTTTATGGACAAACTTAATTGCTTTTGGACGTGTCAGAAATATGTAGAGAATGGGTTCAAAGTAAA
ACCTCAGTGGGCGACAAACCGCTGGGCCAAGACGACTAATGGTTGGCGGAAAAGTTGTGCTCAAACTAGC
AATGGCAATTCCACCATTTCGATATTTAAAGGTAATTTCGCCGGCAGCCCGCTGTGTTTTTGCAACCCTT
CCTGATTGAATCAGTGCCAATAATTTGGCAATTTCTATTCGTCGCCAATGCACGGCCGGCATTGTGTCAC
CAGCAGCATCAGCAACAAACAGTCCCACAAAAACATTATTCGAATCTACAGGATATTACATACCATCAGG
ACGAGGAATGGTGGCGGTGTGCGGAATGGGATAGGGGGGATTTCGGGAAAACGGAAATAAAAGGTTTTCC
TCCACAGTCGCTGCTTTTCAAATGATTCCTCTGTTGACGCTGGCCGAAAACGAACGTAACTTTTGTGATG
ATTTTTAGTGCCGCCGCTCTGCGTTAGTTGGAGTCGGACTTTTATTCCTATTTGTGTTTGTGTTTGCCAA
ACACACGACCATAAAAAACTTTTTAATATAGCCAGCAACACTGAAAAGTAAGGaaaataaaaaaaaaaaC
GTTGGGAAGGGACTGACCACGATGCCAGAAATTAGACACATTAAATATTCTGATACAGTTGTGAAGGAGC
CAAAGGGGTTTGGGCCACGGCGATTATGTGCTTTGTCACTGCAGTCGACTTCGGGCCAAGACAACTGGTA
AATGAGCGACGAGGGACCTAGTTTTATGGCCGGAATGTGACTCCCGTTAGGGGGAAGGACTTTAGATACA
GTAGATACACACTCGAAACAAAAGTATGTCCTTTGCATTGATAGTAATCCAATAAAATAATATACCCTTT
CGCAAATCAGACGGGTATAGGCTAAATATTTGTTGTATAATGATCTTACAACGAATCATACAATTTTTAA
TAGAATGATTCAATAAAAATATTTTGCTTTGGCTTTAAAATACCACCAGTAGTATATTCCAGTGGAAGTA
AAGGTTAAAGGGAACCCTAATTTAGCATTTAATTTCTGTAAGTGCACTAGCTTAGCACCGAAGGACATCG
CGCTATGCATTTTGAGCAGCTATTCGCTCAAGTAGTTGCTGGGGACGACTCACCTCCATTTCTGGAAGGT
AACCCCATTAATTATTCAGTTAGGACCAGCTCGTCATGCCAATTGTGGCACATTTAAGTTGCGGTTAATG
TAATTACGAAAGGGGACTGAAAAAGCAAAGCAAAATCGCGACACCCACAGGACAACCGCCCAACTAAAAG
TCCTTTGGCTGCTATTAGTGTTGCCCttttttttGCTGTAAGGGCTGTCGAGAGCATAATAAATCGTTTT
TTCCTTCTTTGCCTTGACAATCTTTTTCGGCTTTGAATTTAATGCGTTTTAGATAAGTTTCTAATGGAAG
GAAATTAGTATTGAGCCAGGCAATTTATTCTCTCGGGCAGTGGCCACTAAATTCTAGTTAGTTTATCGCA
AACTACCTGTGTCAAATGGATGTCTCTATCTCTGCCTTTAACCCCTACTCAGCTTTCCTGTTTCGGACGG
GTTCTGAGGTTAAAATGGGTCCATTGCTCGGCATTACATATTTGACACAAGTTGTCACAAGCAGGGCAAG
CAAGCCCCGCTGAAATTGAGATTTACGACTTGACCCCCCCAGGCATGACCaaaaaaaaaaaaaacataga
aggaaaaaaaaaaaaatacagaaaataaaaCCTCTAACCATAAGCGAGATGAACCTCCATACATATTTAG
TGAGGTTGGCCAAACTGGCTCCACGAACAGATGTCCAACATCGCTCTGACATTGACTTACATACTCAAAA
TGACTCGTTACGCCGGATAAGCTCATCGTGTGAAGCCGCTGCTATTAGGGGCGGCTGCTCGGTAGGGGGC
GTGGCACTCAAATCAAATAATTTTAGCGCATTGCCAGCACTAAGTAGCTGAAATAACAAACTCTGTTCTG
GTCGCCGATGACGTTGATGGTGATGATGATAATGCCGCCACGGCAGCTTCTGCTGCCAAGCTGCTGATAA
AAAGAAGTTAATGCTGCCCCCCAGAACTGACTTTGCTTTAAAAACAAACAGCGCTAGGCCATAAAACGAA
ATAAATTACTTAACGCGCCGCATTTTTTCGCACTGGCCACGCAGCCAAAAATGCAATTAAAGGCCAAAAA
ACCGCACCGCGACTGCGACATCTGCAGGAGATTaaaaaaaaGAAACAGAATGAAACAACgcaacagcagc
agcaacagcagcatcggcagcacaagcagcagcagcagcagcaacatcaacgcagcagcaacattaattc
agcagcaacatcaggcagcCAAGCGATTCGGTGTGTAAATGATTGAGATGGTTTTGAGATTGTAGGGTAT
GATGCAAAGATTAGGACGTTTGGATTTTTATTATAGCTTTGTGAAAAGAAGATCATAACTAGATCAACTT
GATTTATTCATCGGGTAAACTAATTTTGTATACCAAAAATCTGTATACTTTAAATAATTTAATACTTGAA
TATAGTAATATTTGTAAAAAATAATACTGGTGGGTTTTATTTTTAAAGAATGTAATGAACTTGCTGACAA
TGTTTAATAAATATTTCATTATTATAATGTGCATTCATACAATTGAACCATGTTAAATACTTATTTCAAT
GACTTATTCATAAGTCCATTTCTCTTTTTTCACACTGTCTCCATGGTAAATTATTAAACAGATTCGTTAA

AATCATCACATTTATTTTTAAGGAGACAGTGAGATTTCAATATAATTATGAGGATTACCCTGAACAGAAA
TTTAATACTTTCAATCCCAACCCATATGCCGCCCAATTCCCACAGTTCAGCGCATCTATCTGCCCCCAAA
GCTGTAACGCCAACAGATTACGCTAACGATTTTTAATGTGGATTTGCGCGAAGTTTTTTTGGGAGTTGGG
GTCACGTCGAGTGTTGTGGGGGGAATACTCCATGCATAGACCAGAGTCCGAGATATAGCTGTCAATTTCG
AGGATGGCTTTTGAAACAAAATAGCGCTAGTGCGGACAGTGGGGACATGGTGCTGGAACTGGAACTGGAC
CTGAATCACAGATGGCGAGATAGGGGCAGGCAGAAAGCCCGATGAGTTGAATGACTGCGACAATGGCACA
CTGCGAGTGACTCTGATGATGATGCAGCTGGTTGGGTTGGACGCTGTCGGAAACGGGGTGTGGTGTCATT
TTGTGGGCTAAATACCACAAATTGCGCTGCGTGGCAGCAATTACAAAAAGTACTAATCATGCCGACATGT
CTTTCAGCGAAATCAAGCTAAACCACAGCCACGAGCAGACGACAGCAGTGCAGACTGGACAAATTTTTTA
TTCGCTTAAAGGGAAATGGAGGACCCTCAATAGGAGGAGTAGTGGAAGTTTGGGGTTAATTGGAAAATTA
AAGCACAGTATGTGGGGTATATGGCTGTGTAAAGATTTACCACCGAGTTTTTAGGTGATTTATTATTGAC
TAAGAATGGATACTTGGGGGAGTAATATAAAACATTGCAATTATAAGGCGTATGTACATAGTCGTGTTTA
GCTTAATACTTTTTTATTCAATTTCGATTGCTCTTTATATTTAAGGGTATTTCCGAGTCGACTCTGTTTT
GCTTATGATTCTTGAGTTCACTTTATTCATTGCAATTTTCACACATTTTTTCTCGTTCTCGTTAACGTCT
CGTCGTCGGCTACTCACATGCAATTATACACTTAGGGAATTGACCACCAATGAGACGAGTATCGATTTGA
CCAATTTGGATGCGAATGCCTTGTACCGAATTACGGTTGTGACGCGCGGAAAAATTTGGCACATCGCTGCC
GGCATCCATGCTCTTGCTGAATACAACCGACCAACAACGGACCGGCGAAGGGTGAGCAAGGAAACGTGCC
TTAAGCAGCCACTGCTAAAAAATTGGCGGACATTTTAAAAATGTTTTTAATAGTTCAGTTGTTACATACG
TATTTGTGTTTTAAATCTTCAAAGTAACAATTTTTGTATTTATTTTAGAATAAAATGTGGTTGCCACTTT
GAAGGCAAAGACTCAAAGAAAATTGTTAATAACTTAAGTTGGATAGACTTGGAACTGTATATAATGATTT
TAGAATATTTGATTTTATTTTTGAGTCATATTTCAAAAAAGTGGCAAACATCATAAATTATTTAAAGACC
TATAAAGTGTTTTGGTGCAGGTTTTAAAAACCAATTTGCAGTCCTTTCTCCAGTTTGGTCGGGATCTAAG
ACTCCCTCTTCCCCTCTTTTTGTTCCTCCTGCAGGAGTTTACCACAAGGCCATGAGAGCCGAGCAATTCA
TAAAGATTGAAACGAAGCTGGCCTGGCTGAGGATGAACAACCTGAAGCCGAGCTCCCAGCATGTGCTCTA
CGTGGAGGCTGTGGGTGAACGGGGTGATTCCCTGCCCTCGGAGACGCTTGTGGCCTGGACGGATCCCGCC
CTGCCCGCCTTCGTGGATGCCCCCCACTGTGCATCCGGCGGACAACATCCCGGAGGGCGGTTCCATGACC
ATTCTCTGCCTGGCCTTCGGCAACCCGGCTCCCACCATTTCGCTCTACGTGGGTGGACATCTGGTGCGGC
AGGACGCCTCGCGGCACATGGTCACGGTCATTCACAACGTCTCGGCCGACATGGAGCACGTCTCTTGCTA
TGCGGACAACGGCTACGGAGTGCCCATGCAGGCCACCAGGAGGGTTAACATATGCTTGTAAGTAATGCAA
TGAGGGTTAATTCGCTTAGCTGCCTTATAGTGTCAAGTGTCCTTCTGGTGCAAAATGTAAAATACTGCAA
GTAAATACTCACATTTAAATTGCTGTTGAAAAGATTTATGTAAAACTAATTACGGAGAGGTTCTAAAATT
GCATTTATTTTATTATTTAATTTCATTGGTTGATTTGAAGTACTTTATTAGCGCAATCGCAAAAAATATC
CCCTTTTAAATGCAGCATGATATAATTTAAAGTTTTAAAAAAGATTTGCAATATAATTATTATACATTTC
CTCTTGCAGGATCCTCCGAAAATCCAGGCTGCTGGCATCACCGTGGCCAATATTGGTGATGAGATCGAAC
TGCGGTGTACCGTGGACTCCAAACCGGCGCCTAAGACAATTTTCTGGAGAGAAAAGGATGGAAGGGTGCC
CGTTATAAATGGCGGAAACTATTTCATATCCGTAAAAGCCAATGAATCCCGACCCTCGATATATACAATG
TCGTTAAGGATCAGCAAGCTGCAGGCCAACGATGTAGGCGATTATTTCTGTCATGCGGATAATCCCTTCG
GAGCCACCACCACGCCGGTATCGGTGCGTATACGCAATACGCCGGCACTCAATCACAACGTTTCCGAATG
CTGTGCGGCGCAAAAT
>dme_circ_0004776_junction_seq
CGTTTCCGAATGCTGTGCGGCGCAAAATGATGTGCATGCGACATTCGTGAATCAGA
>dme_circ_0004777
AAAATTGTCTTAGGCGCCGGTTTGGAGTCCACGGTACACCGCAGTTCGATCTCATCACCAATATTGGCCA
CGGTGATGCCAGCAGCCTGGATTTTCGGAGGATCTGCAAGAGGAAATGTATAATAATTATATTGCAAATC

TTTTTTAAAACTTTAAATTATATCATGCTGCATTTAAAAGGGGATATTTTTTGCGATTGCGCTAATAAAG
TACTTCAAATCAACCAATGAAATTAAATAATAAAATAAATGCAATTTTAGAACCTCTCCGTAATTAGTTT
TACATAAATCTTTTCAACAGCAATTTAAATGTGAGTATTTACTTGCAGTATTTTACATTTTGCACCAGAA
GGACACTTGACACTATAAGGCAGCTAAGCGAATTAACCCTCATTGCATTACTTACAGCATATGTTAACCC
TCCTGGTGGCCTGCATGGGCACTCCGTAGCCGTTGTCCGCATAGCAAGAGACGTGCTCCATGTCGGCCGA
GACGTTGTGAATGACCGTGACCATGTGCCGCGAGGCGTCCTGCCGCACCAGATGTCCACCCACG
>dme_circ_0004777_junction_seq
GTCCTGCCGCACCAGATGTCCACCCACGAAAATTGTCTTAGGCGCCGGTTTGGAGT
>dme_circ_0004778
GCCAGGCCAGCAACGTAGTTCTTTGCTGGGCAACCAGCGACTGCGTGCTCGAAGAAAACCTCCAAGAAGT
TGCCCCCCTTTTGGGAGCTCCAAGTTTTCCTTTGCGTCGACGCGACGCTGCCCTTAGCGATTCCGTTTGA
CCACAACAAACCGAGCACGCAAGTTGAGCCAATTCCTTAAGAGAAAGCTGACGTGACTGCTTTAGGAGCA
GGACAAGCAACAGTACCAACACCAGGACAATGTCCAACGAAGAGAACGACGACTATATGGCCAGCTTGAG
TCAGGCCCAGACCGGCGAGGATGAGGTCGATGGCGGTGGAGAGGCAGCTGGCAGTAACGACTCCGTTCTG
GGCGGCCAAACAATGCCACAAATTAGTCCGAACGCACCGCTCGAGGGAAACGTTTCGCCGCCACCGACGC
AGCCCCATTTGGATGATAATCTGCACaacgggaacgggaacgggaacggCATCCACAGCAGCAGCAGCAA
CATTAACAAGCACGGCAGCAACATCAACGGCAACAACGATAGCAACCGCAGCAGTCCTGCGAAGAGTCCC
AAGCGCCCAGTGTCCTCCAGCTTCCGGATTCAGAACACCAGCCGGTACGATAATGATGAGGAGGACTTAG
CAGCCAGCGAGCAGGTGGCTGGTGGAGATCGCCAGGTGCCGGATTATTGGCAGCAACGGAACGGTGCGAC
CACTGGTGCAACCAGTGGGCGGCAGAGTCGCGCCATTAGTCCCGGGTATCTGGACAACATGAGCGAGAAC
TCGGAGCAGCCGCCAGTGGTGCCGCTGGTGCGATCTAAATCCCGTCCAGAAATTTCAAGTGCAGCGGCAT
CGCGATATAATAATCTCTCCTATTGGAAGGCACGCCGTGTGGTGTTCTACCGGAATGGGGATCCATTCTT
TCCGGGTGTGGAGCTGCGCTATCGTCCCGGACGGGATGTAACCTCACTGGACAATCTTCTCGATAAGATC
TCGCCCAAGATGGACTTGCCACGGGGTGCTCGATATGTGTTCTCCATGGACGGAGATCGAAAATATCATC
TCGATGAACTGGAGGATGGAGCCTTCTATGTCGTATCCTCGTTCAAGGCCTTCAAG
>dme_circ_0004778_junction_seq
TGTCGTATCCTCGTTCAAGGCCTTCAAGGCCAGGCCAGCAACGTAGTTCTTTGCTG
>dme_circ_0004779
GTAGGGAAGGACCCTCCTAGTCATTGCACCACCATCGGGTATAAAAGGCCGGGCAGCTAGACCTACAGCA
TCATTCGATCAACAGTTCTCCATCCGAGCAGATCATAAGTAACCAACCTGCAAAATGTTCACCGAAACCG
CTCTTGTTTCCAACTTCAATGGAGTGACAGAGAAGAAATCTCTTACCGGCGCCTCCACCAACCTGAAGAA
GCTGCTGAAGACCATCAAGAAGGTCTTCAAGAACTCCAAGCCTTCGAAGGAGATTCCGATCCCCAACATC
ATCTACTCTTGCAATACTGAGGAGGAGCACCAGAATTGGCTCAACGAACAACTGGAGGCCATGGCAATCC
ATCTTCACTGAGTTCTTCTGGGACATCCCCCTCCATCGAGTATCTGTGATGTGACCCGATCAAAAGGTCT
ATAAATCGGCACTCCGGCTTTAATATCCAACTGTGATGACGAGAACACAAGACTGACTGACTTGTGTGCC
TTGGAGGTGACAAAGTTCGTCGCCTCTGCCAACTGTACATATCAAACTAGCTGCTAAAATGTCTTCAATT
ATGCTTTAATGTAGTCTAAGTTAGTATTATCATTGTCTTCCATTAGTTTAAGAAAATCATTGTCTTCCAT
GTTTGTTTGTTAGGGTaaaaaaaaCTAGCTTAAGAATAAAAATCCCTCGCGGAAAGAAAACAATATTTCA
TCGTATTATTTTTTCATCCTGTTTATTATTAGGTTCACTTATTTGTTTTGTGTTTTTAATAGCCTTGAAG
ATACATTTGATGAACACAATGTCTTAATTATATTGCGATGAAATAAATTGGTTCGGCAGTTTTCTGTACG
AAGGACATGTACTATACACTGTTCGTCAGAAAATGTTCTGATCGAATTGGTCTCGCTCAAAATAAGTGTA
TTTTTGCTATTTATATGCCTTTGATAAAAATGATTTGAAAAATACAACCGAATATCGTATCATTTGCGTC
AAAACTAGAAAGTTTGCTTGATTCTAAATATTGTATTTTTATTGTTTTTATACAAATGCAATTTTCTCAC
ACTATGTAATTCATTAAAGGGGAACCCTAGTAATCGGGCGCAATGCAGATGTACCGAACAGTCGTATTAG

AATATATATTTGCAAAGTTAAAAAATTTTTATTGAATCAATTTTTTATTTAATTACAATATTTTACACGG
GTCACACAGTTAAAGTCGAAAACTATTCAGAAATTTAACATTTCAAAAACTCAAGTTTTTTACTTTGTCC
ATAGTTAGCAGTCACAAATCTAATTGATTGCTCGCTGTGTTGCAGACATTTTAAATAGTTCTGCCCGTCC
CTTGTCGGCGAGGGCACACTGTACAACAGCCGAGGGCAAACAGTACACTTAGTGATAAGGGGCTCTTGAA
CACATACAACttttaagtttttattgttgtttgtttgttgttgtttttATAAGATCGTTATTGTGTGC
TCCCTGTTATTTAACGGTAAGTTTCAGACATAGGGGTAAAGATTTTTATTTTCTATTGCCAACATTACCA
AAATATGAATAAACTAGAAATCTAAAAACTAAAATTATTCAAATTAGGCAGGTGTAATATAACTCACATG
CTAGCCCTTCAAGTAGGCAACACATTGTGCAAAAGTACAGCAAGCAGCTAAATTTCTAGCTTGTAACTTT
TTTTATGCTGTAATTGTATTCGTTTTAGCCAACTCACCATCTAAAGAACTGCTAATTCTACCTGCAAAAC
GGTTTTCATCCCCTTAGCCACCGAAATTTTACAAAGCAGG
>dme_circ_0004779_junction_seq
CTTAGCCACCGAAATTTTACAAAGCAGGGTAGGGAAGGACCCTCCTAGTCATTGCA
>dme_circ_0004780
AGTTTATTGTCGGAGAATACCGTGGACGATCAGCATTGGCCCAAGGAGCTGATCTTGCTGCGCGAGAAGT
TTACGGCCAAGAGTCAACTGGAGATCACTCAGCTGAACATTAAACATGCCGAAGAGGATGTCGCGCCTGA
AATTAGAATTTGAAAAGCAGCTGAATCGAAAGAACAAGCGCCATCTGACCTTCGATGCAGCCCGTGACCT
GGAGCAGGTGATCTGCGAACGAGATGGATTGAGGGAGCTGTCCAAGAGTTTCCGCAGCGTACTTTGTCGG
CTGGCCAAGTGTGTGGCCCACTGTGAGGAGGATTTGAATGCCACACTATCCGAGGAGGTGCAACGCCTGC
TGTTCCACAGTCGCAGTCAAGATGGAGGTGATGATCTGGAGGCTACCATTAGTAGCTCCCTAAATAATAC
GAAGCACATGCTCCGTGTGCCGGATGTCCACAGCCTGCTGGAAGTTGTGGAGGATCCGAGTTTGGTGCAG
TTCATCGACAGCAAGAGCAACGAAGAGCCAAGTGAGGACTTCGATTTGAATGATTGCCTAGAGCGTTTGA
AGTCGGAAGCCTCCTACCTGTTGCATTTGTCAGAGGATTTGCATAAGCAGCGGACACACGATGAGTCGTC
GGAGCACTTGGACGAACCGGAAAAACAGGAGCATGAGCTCTGCTGCGAGGCGGAAGATGGTCTGAAGACC
ACAGGTGCAGTGAATCAACAAGTACTGTCGAAATTCCTGCGCACAAATTCCTTGAACGATCAGCAAATGG
GCGTGGCCAATCAGAGGAAGAACAGCAATCCCGAGGCGGGAAAAACACATTCCTCCCTACCTCCAGATCT
TCAAGAGCATGCGGGAAATGCATCAGAGCTGTCGTTCCAGCTTGTGCAGCTAAAGAATCGCTTGATTAAA
TCGGAGGCAGATCGTCAGAACTTGCAACAGCAACTCAGTCGCACTATTGATCGCAATGCGGAGCTGGGAC
AGGAGCTACAAGCACTTAGGGATCAGCTCTCCCAGTTGAACTCTCTGAACCACACGGATTACAATGAGGG
ATACGGCTTGGGCACAATGAAGAGCTTGCAGGAGCAGGGATTGGATCAATCATCGGCCAGTTTTCTTGCC
CTCCAAGAGAGAGCGCGTCATTTGCTTTCATCCTCTCCGGTCAAGGAGCAACCATCAAGAGACCACGGCA
ATTCGACCGTTATCTTGCTACAAATGATCGAGGACTTTTGCCGCGAGGGCGACAAAGTGGTGGAGTTCAG
CAAAAAGGATCGCGAGGATCTGCAGTCCCAGGGTAAGTTAAGAAACGGTCTGCACACGGATCATCTTCCC
CTTTTTAAGTTTTCCCTTTCCCACCTTTTAACATTGAGTGCTAAACGCATGATCCAAAAATAAATTCGAG
TAGTCCGTAAGCCAGGTTAATTATGAATTAAACACCACTTCGATGCCCTCTCCTTTGACCTATAACCACA
AACACAAACACAGATCGATACCGCTGACAAGCAGCTGAAGGATACCAGGCGATTCCTGGAGGATCAGGCC
GCCGAACGCGAACAGGAACGCGATGAGTTCCAGCGCGAAATCGAGCGGCTGAAGGCTCAGTTGCGCGACA
AAGAGAAGGAGCACAGCTCCTATGCCAATGCCTCCGAGGAGGTGAGTCCAGTCCATGGTTCTTATCTATG
ATCCCTCGGATCTTGATCTTGATTTTGGGCTTGGTTTGACTTTCGTTTGCGTTTTTGCCTGGGTTCACAA
AGTCCTTTCCTCTGTCCTTTCAtatctctttatttgttttctatctatctttttattattgttattatta
ttattattattTCAAGGCTTAAGTTTTTGGTTGTAAGTTTGGCTATAGGCATATAAATATTTAAGTTGTC
CCAGCATGTGGCCTTCCTTTTTTATTAACTGACACAATTCCGCACATTCTCCAAATATTAACACAGAACT
ACTTCAAAGGCACTGAATTCTGGAAGCCATCGAAGTCATTTCCTCGAAGAGTTTTCCAAATCGCATGGGG
CACCTGTTGGGCATAATTCAATTGTAAATCGCCATGGGGGCATTTCAACGGAGTCAAGGGCAGCCTCCGA
TTCTTGTGTATATATGCCGGAGCTTTTGTAAGCATCTGCCGACATATTTATAGACCCCAAGAAATGAGCA

CTTAAGTGCGAGCGAGTCAGAGCCTTTCAATCAGTTTCCATTTAGCCGAACTACCTCAAGTTTAAGATCT
CTGGGGGGTTGAGATACTTTCAATGTCGCACTGATATATGAACAAAATACTTATGTTTGAATTTAATTTG
CTATGTTCTTGTGAATGTTTCAATGGTTTTCCAATTAAATTGCTCTGCTAATTAAAATTTTAATTAAGCG
GGGATGTATTCATCTGTACTTTCAAAGAGTTTTTATAAATTTAAATGGGTTTTTTGTTATATTTCGACCC
ATATTATATAAATTATAATTACCATAAGTGTTCTCTGTGCTAATCAATATCATATATTGAAAATAATATT
AAACTGATTAACGTATTTGTAATTCTTTGCATGCACCCAATAAACAAAACCGCAACTAAAATTACAAACT
AATGCACCTGTACCCTTTTGCCACCGTGTGCACCCaaaaaaaaaaaaaaaaaTAGTTGAAGGTAGCGAAG
CGAAAGCATGTAAGTTTGTCACTAACATCGAAACCCGCACCCGCACACTTTTCATCTCATTGGTCCATTA
ATTCATATTGTATTTTAAAACATTACTTACTTAGCTTTTATTTTTATACAGTTATGCACAACTGGAAAGC
CAATTTAGGGAGGTAAATCAACAGTTGTGCGAGTCGAATGCGAAGCGGGATAAATTCGAGGTGGAACTGA
AGGCTTCAATTGACAAAATCTTCGTGCTGCGGGAGATCATCTCAGAGCTGGAAACCCAAGTCCAGACAAA
GGCACTCAATGAGGAAGTTTTGGCCGAGAAGGCCCAACAACTGGAGGAATATGTGAGTTTGCAGATGCGG
GACAACGATATACTGCAGCAGGAGGTGCACAGCCTGAAGACAGACATTGGAGAGGGCTATCAATCCCGTA
TCAGAGTGCTGGAGGAGAAACTGAAACAGAGTGGACCAACGGCAGAACAAGGTGTGGTCTTGAGTCAGGT
TGCGGAAAAGTTAAGAGACATTGAAACCACACTGGATCAAAAAACCAAAGCTCTGGAATCTCTGCATAAC
TCCAACGCGACCTCCAATTCGGCCAGCTTGAGTGTCACCGAAGATGTGTCTATTCATGGCAGCAAGGAAC
CCACGGCAGTGGGTTCACCCTCGCATCCATCGCTCACCGTGGAAGGTGTTCAACGGGTTACCGAAAAGCT
GGACCGGCACACTCGCGTCGAGGAGGCGGCCATTAAGAGGATTCGCGACCTGGAGATGCAGGTTCATCAA
ATGCGCGCCGGTTGCGTGG
>dme_circ_0004780_junction_seq
GTTCATCAAATGCGCGCCGGTTGCGTGGAGTTTATTGTCGGAGAATACCGTGGACG
>dme_circ_0004781
AGTTTATTGTCGGAGAATACCGTGGACGATCAGCATTGGCCCAAGGAGCTGATCTTGCTGCGCGAGAAGT
TTACGGCCAAGAGTCAACTGGAGATCACTCAGCTGAACATTAAACATGCCGAAGAGGATGTCGCGCCTGA
AATTAGAATTTGAAAAGCAGCTGAATCGAAAGAACAAGCGCCATCTGACCTTCGATGCAGCCCGTGACCT
GGAGCAGGTGATCTGCGAACGAGATGGATTGAGGGAGCTGTCCAAGAGTTTCCGCAGCGTACTTTGTCGG
CTGGCCAAGTGTGTGGCCCACTGTGAGGAGGATTTGAATGCCACACTATCCGAGGAGGTGCAACGCCTGC
TGTTCCACAGTCGCAGTCAAGATGGAGGTGATGATCTGGAGGCTACCATTAGTAGCTCCCTAAATAATAC
GAAGCACATGCTCCGTGTGCCGGATGTCCACAGCCTGCTGGAAGTTGTGGAGGATCCGAGTTTGGTGCAG
TTCATCGACAGCAAGAGCAACGAAGAGCCAAGTGAGGACTTCGATTTGAATGATTGCCTAGAGCGTTTGA
AGTCGGAAGCCTCCTACCTGTTGCATTTGTCAGAGGATTTGCATAAGCAGCGGACACACGATGAGTCGTC
GGAGCACTTGGACGAACCGGAAAAACAGGAGCATGAGCTCTGCTGCGAGGCGGAAGATGGTCTGAAGACC
ACAGGTGCAGTGAATCAACAAGTACTGTCGAAATTCCTGCGCACAAATTCCTTGAACGATCAGCAAATGG
GCGTGGCCAATCAGAGGAAGAACAGCAATCCCGAGGCGGGAAAAACACATTCCTCCCTACCTCCAGATCT
TCAAGAGCATGCGGGAAATGCATCAGAGCTGTCGTTCCAGCTTGTGCAGCTAAAGAATCGCTTGATTAAA
TCGGAGGCAGATCGTCAGAACTTGCAACAGCAACTCAGTCGCACTATTGATCGCAATGCGGAGCTGGGAC
AGGAGCTACAAGCACTTAGGGATCAGCTCTCCCAGTTGAACTCTCTGAACCACACGGATTACAATGAGGG
ATACGGCTTGGGCACAATGAAGAGCTTGCAGGAGCAGGGATTGGATCAATCATCGGCCAGTTTTCTTGCC
CTCCAAGAGAGAGCGCGTCATTTGCTTTCATCCTCTCCGGTCAAGGAGCAACCATCAAGAGACCACGGCA
ATTCGACCGTTATCTTGCTACAAATGATCGAGGACTTTTGCCGCGAGGGCGACAAAGTGGTGGAGTTCAG
CAAAAAGGATCGCGAGGATCTGCAGTCCCAGG
>dme_circ_0004781_junction_seq
AAGGATCGCGAGGATCTGCAGTCCCAGGAGTTTATTGTCGGAGAATACCGTGGACG
>dme_circ_0004782

ATCACATCATTGCTCCGGCCGCACGGTGCCAGTGTAAGCTATAAGGCGATCGAAGCGGCGACTCTCGAAG
AATTGCCAGCAACATCCGTGGCAACAGCAACATCCACGGCTGCAACATCACGCGCCACTGGAGGAGCAGC
AGCAACTTGTGTGGACGTTGAGTTCATACCCACGCTAAATATCATATTTGAGAAGCCGACTTCGTCGAGT
GAGCAACTACAGTGCGCCGAGAAATCGGAACATGTGGCCGGTGGTGTGTCCGATAAGGAACCAGCAGATG
ATTGTGACTCCGACAATGATTCTACGCGCACATATATAATCTCGCGAAGCAGTTGGACGGGGGTAACATC
GAGTAGCAGCACCCCATATGCGGTTACATCCACGGATACCGCCGAATTACTCAGGCGCCAGAACAATCTC
AGCCTTACGGAGGAAGATCAATCTGTGTCGTCGTTTAGCATCGAGCAGCCCACCAGTTCCATAACCATTG
AAATGGCCGATCAACGGACAACCACGACCACCACAACAACGGCGACGACGGCAACAACGCCGAGCAGTGC
TAACATTCTGGCCACAACAGCAACGACAACGACCACAACTAGCAGTAGTATTGAGGAGGAGATCGAAGAG
GCCATCGAGATCAGTGAGGTGGAGGAGCAGCTAAATACTCCACTGGAATCTCTATCGGAGGCGGCTGCCT
ATGCCCGGCCAAAGGATGAGCAATCAAGTGCCAAAAGTTTGAGTAGCAATCCGGCAGAGGATGAGGATGC
TGATGAGTTTCGGATCGATGATGCCCAGTTATCTGGTGGCCAGTTGGCTCAGCACTTCCTGGTCGATGAG
GATGAAAGCGAGGAGGGTTCGGATCTTCCAGCTGCCTCCAAAGTGGAGGTTAGTCTCTCCTCTAATGATG
ATGACTTTGATATCAGTTTGCCATTGGAGCAGAGGAAACCTGTGCATTCCCTGCACGAAGATGATGAGTC
TGTAGATCAAAGTTTAAGTAATCAAAGCACTACGGATGATGTTTCCGAATTGGTCGAGGAGCCAGTCCAT
GAGATGGACGATAAAACCGATGGCAGTGCGGCCATTAGTGCCAGTGCACCTCAAGAAACTCAGGTGGATG
AAAGCCAAATAACCGACGAGCAGGATCACTCTATGGAAGAAATAGTTGCCACAAATGAATCAATTGAGGT
GACGTACGAAGCTGAGGAAACCGTCAATACTTCACAGAAACAAGATTTAATCACCGATTTAGATGAAGAG
TCAGAGGATCAGGTGCACGTGGACAACAGGCCCACTTCGACTTTGCAAGCATTGAAGCTTCCTGCGTTGC
AACAACCTCAGATTGTAGAAAGGAAAGCTGCTACAGCATTGACCACATTACGTCTGCAAGCAGATGTTTT
GGAGCCCTTAGAGTTAACCATTCTGGAAATGGATGATGATGAGAATGAAGAGGATGACGACGATGAGGAG
AGTTCCTTGCAACTGATGAAGCTGCGATTAATGGCCATGAATCAGCAAATGATTGTGGACAATGCACCCA
AACTTTCGCCCACGGAAGCGGAACAAACACAAGTAACCAGCAGCAGTAACAATTTAGAAATCAAACAAAT
GGAGCGAGTGCCACTCACAGAGTTCTCCAAGGATGTTCTGGAGGATATTACCGAAGAAAGCGAGAGACTT
CTCTCGATGAGCACAACTATGGAAGAGGAGCAGGATCCGCCTTCCCTCTCCTTGGATGAGTCTAAAACTC
TGCTGCAGCCAGGAGCACATAAAACTGGGGGCAGCAGTTCCAGTTTGGTGAGTTTAAATATGCTTAAGCA
GCTGGAGGCCAAGGTACAGGAACTACACACCCAACTGGAGACCAAGGACAACTGCTTGGCATCGCTTAAT
CTGCAGCTTGAGACAGCAAGAAGAGAATCCAGTGCGGGTCCAGCTTCAGCAAGGGATTCCAGTTCGCTAA
TGACCAATTCGACAGAGTATCGCACTCTTCAGGAAGAACTAGGCGGCCCAGACATTGGACATTTATGTGG
AGATGTCACGGAGGGATGAGTTGATTGCCAAACTAACCGATTCGCTGCAGCAATCGCTGAATGTGCGTGA
CAAGCTGCAGGTGGATGCGGATCGTCTGGGTGGCGAAGTACAGAATCTGCGTAGGCAACTCCAAGAGACG
CTCGATGCAGTGAAACGCTCGAGTGCCGTATGGCCCGATCAGGAATGCAATCCCGGTCAGCGTATCTCAG
AGATCTCCATGGATTTGATCAGCGAAAGCGATGATGATCTGGATCGTCATTTTCTCACGGACAACGAAGA
GCGCGGATCACGCAGCTCCAAGGAAAGGCAGCTAACTCAGTTACAAACCAACGAAGAATTGGGACTGCAG
GCGTCAAACCCGGAGTGGACACCGACATTTAGCAAGCAGATCGAACAGTTCCACACATATCTGTTGCCCA
CCGAACAGCGCATCTTCCAGATGGTCCAGCGCAAGTTCGACGACTATTTGAACCAACAGATCACCTTGTG
TCGTGATCTTGGCGCCCAGGAGCTGAAGATTGCCCGCGATCAGTGGGAGAGCGAGAAGCTAACCAGCGAA
CAGAACCAGCAGGCAGCTCATGCCAAGCAAATGGAGGATCTGCGCAAGTACTTTGAGCATAAGTGCGCCG
ATCTGGAAAAGCAATTCTCGGACGATGTCTTCTCGCACAAGTCACAGCATCAGACAGGGGACAGTTCATC
GGAGTGCTCGGAGGTAGATCAGTTGCCCGAGGAAGGGGCCGCCGCGGTGTCCTCCAAGGAACCATCTCCC
CGCAAACGGAAGCGAGCTGAGCTGCTTCTGAGTCCCAGTCACAGGCAGATGACTCCTTGTGGCTTGGATT
CGCTGGGAGAAAACACTGGAAAAACTGAAAAAGACGAATACTGCGGATATAGCCGACCTAAAGATCTTTT
ACCAAACCCACATCAAGGAGCTAAAGCGCGCGCATGAGGATCAGGTGCGCAAGCTGAGCGATCGGATAAA
GTTCTATGAGCGACAGCAGGGTGATGATGACTATAAGG

>dme_circ_0004782_junction_seq
CGACAGCAGGGTGATGATGACTATAAGGATCACATCATTGCTCCGGCCGCACGGTG
>dme_circ_0004783
GTATTACATTTAAAATTTTATCGTTTTCTTTAATTCTTCATTAAAACTGAATTTTAAGTTCATGCAAACA
GCGGACGTGTAATCATCGTTATCTGCGTCGCAAATGTACTACATACAACGGAAGTATGCGAATAAAAACC
GTGGGCGGGCAAAAAGCTTCGATGGCTACACTGGCGACCAGCTGACCCCATCCTCCGACTGATAAAAACT
CAGCCGGCGAATGGGGAAACTCCGGTGGCCCACATGCGCCGAAACTTAAGAAGTAAACTACAGAATGTAC
GTTGCTAGTTGTGGCTTTTTGTTTCTGTTCATAGTTGTTGGATTTTTGTGT
>dme_circ_0004783_junction_seq
TCTGTTCATAGTTGTTGGATTTTTGTGTGTATTACATTTAAAATTTTATCGTTTTC
>dme_circ_0004784
TTTACTTCTTAAGTTTCGGCGCATGTGGGCCACCGGAGTTTCCCCATTCGCCGGCTGAGTTTTTATCAGT
CGGAGGATGGGGTCAGCTGGTCGCCAGTGTAGCCATCGAAGCTTTTTGCCCGCCCACGG
>dme_circ_0004784_junction_seq
GCCATCGAAGCTTTTTGCCCGCCCACGGTTTACTTCTTAAGTTTCGGCGCATGTGG
>dme_circ_0004785
GGTTGAAGTCGATGACCTTGTTGTCCTCCTTGCGGAAGTATTTGATTGTGGGGAAGGACGAGATCTTAAT
GCTCTCCAGCTCGTTGGCAGTGGAGTCCATCTTGGCAATGACAATATCCTCATTGTCCTT
>dme_circ_0004785_junction_seq
TGGCAATGACAATATCCTCATTGTCCTTGGTTGAAGTCGATGACCTTGTTGTCCTC
>dme_circ_0004786
TTCCTGGACGGCAAGCTGAAGCAACACCTCCTTTCCCAGGAGCTGCCCGAGGATTGGGACAAGAACCCCG
TCAAGGTGCTGGTATCCAGCAACTTCGAGAGCGTTGCCCTGGACAAGAGCAAGAGCGTGCTGGTTGAGTT
CTACGCCCCATGGTGCGGACACTGCAAGCAGCTGGCCCCCATCTACGACCAGCTGGCTGAGAAG
>dme_circ_0004786_junction_seq
CCCCATCTACGACCAGCTGGCTGAGAAGTTCCTGGACGGCAAGCTGAAGCAACACC
>dme_circ_0004787
CTGGAAGAGGACATGGCCAAGTACAAGCCCGAATCGGACGATCTGTCCGCCGAGACCATCGAGGCGTTCC
TTAAGAAGTTCCTGGACGGCAAGCTGAAGCAACACCTCCTTTCCCAGGAGCTGCCCGAGGATTGGGACAA
GAACCCCGTCAAGGTGCTGGTATCCAGCAACTTCGAGAGCGTTGCCCTGGACAAGAGCAAGAGCGTGCTG
GTTGAGTTCTACGCCCCATGG
>dme_circ_0004787_junction_seq
CGTGCTGGTTGAGTTCTACGCCCCATGGCTGGAAGAGGACATGGCCAAGTACAAGC
>dme_circ_0004788
GAACTCAACCAGCACGCTCTTGCTCTTGTCCAGGGCAACGCTCTCGAAGTTGCTGGATACCAGCACCTTG
ACGGGGTTCTTGTCCCAATCCTCGGGCAGCTCCTGGGAAAGGAGGTGTTGCTTCAGCTTGCCGTCCAGGA
ACTTCTTAAGGAACGCCTCGATGGTCTCGGCGGACAGATCGTCCGATTCGGGCTT
>dme_circ_0004788_junction_seq
CGGCGGACAGATCGTCCGATTCGGGCTTGAACTCAACCAGCACGCTCTTGCTCTTG
>dme_circ_0004789
GAACTCAACCAGCACGCTCTTGCTCTTGTCCAGGGCAACGCTCTCGAAGTTGCTGGATACCAGCACCTTG
ACGGGGTTCTTGTCCCAATCCTCGGGCAGCTCCTGGGAAAGGAGGTGTTGCTTCAGCTTGCCGTCCAGGA
ACTTCTTAAGGAACGCCTCGATGGTCTCGGCGGACAGATCGTCCGATTCGGGCTTGTACTTGGCCATGTC
CTCTTCCAGCTTGATCAGACGGATGGTGGGCACCTCCTCCTTGTTCATGCCGAAGAACTCGAAGATGCGG

>dme_circ_0004789_junction_seq
GTTCATGCCGAAGAACTCGAAGATGCGGGAACTCAACCAGCACGCTCTTGCTCTTG
>dme_circ_0004790
TTCCTGGACGGCAAGCTGAAGCAACACCTCCTTTCCCAGGAGCTGCCCGAGGATTGGGACAAGAACCCCG
TCAAGGTGCTGGTATCCAGCAACTTCGAGAGCGTTGCCCTGGACAAGAGCAAGAGCGTGCTGGTTGAG
>dme_circ_0004790_junction_seq
GGACAAGAGCAAGAGCGTGCTGGTTGAGTTCCTGGACGGCAAGCTGAAGCAACACC
>dme_circ_0004791
GCACGCTCTTGCTCTTGTCCAGGGCAACGCTCTCGAAGTTGCTGGATACCAGCACCTTGACGGGGTTCTT
GTCCCAATCCTCGGGCAGCTCCTGGGAAAGGAGGTGTTGCTTCAGCTTGCCGTCCAGGAACTTCTTAAGG
AACGCCTCGATGGTCTCGGCGGACAGATCGTCCGATTCGGGCTTGTACTTGGCCATGTCCTCTTCCAGCT
TGATCAGACGGATGGTGGGCACCTCCTCCTTGTTCATGCCGAAGAACTCGAAGATGCGG
>dme_circ_0004791_junction_seq
GTTCATGCCGAAGAACTCGAAGATGCGGGCACGCTCTTGCTCTTGTCCAGGGCAAC
>dme_circ_0004792
GGGCAACGCTCTCGAAGTTGCTGGATACCAGCACCTTGACGGGGTTCTTGTCCCAATCCTCGGGCAGCTC
CTGGGAAAGGAGGTGTTGCTTCAGCTTGCCGTCCAGGAACTTCTTAAGGAACGCCTCGATGGTCTCGGCG
GACAGATCGTCCGATTCGGGCTT
>dme_circ_0004792_junction_seq
CGGCGGACAGATCGTCCGATTCGGGCTTGGGCAACGCTCTCGAAGTTGCTGGATAC
>dme_circ_0004793
GTGCCCACCATCCGTCTGATCAAGCTGGAAGAGGACATGGCCAAGTACAAGCCCGAATCGGACGATCTGT
CCGCCGAGACCATCGAGGCGTTCCTTAAGAAGTTCCTGGACGGCAAGCTGAAGCAACACCTCCTTTCCCA
GGAGCTGCCCGAGGATTGGGACAAGAACCCCGTCAAGG
>dme_circ_0004793_junction_seq
GAGGATTGGGACAAGAACCCCGTCAAGGGTGCCCACCATCCGTCTGATCAAGCTGG
>dme_circ_0004794
GGAACTTCTTAAGGAACGCCTCGATGGTCTCGGCGGACAGATCGTCCGATTCGGGCTTGTACTTGGCCAT
GTCCTCTTCCAGCTTGATCAGACGGATGGTGGGCACCTCCTCCTTGTTCATGCCGAAGAACTCGAAGATG
CGGGTATGGTCCTCCTCGTCGGACGAGATGGTCACGAACAGGATGTCGTCGCG
>dme_circ_0004794_junction_seq
AGATGGTCACGAACAGGATGTCGTCGCGGGAACTTCTTAAGGAACGCCTCGATGGT
>dme_circ_0004795
GGAACTTCTTAAGGAACGCCTCGATGGTCTCGGCGGACAGATCGTCCGATTCGGGCTTGTACTTGGCCAT
GTCCTCTTCCAGCTTGATCAGACGGATGGTGGGCACCTCCTCCTTGTTCATGCCGAAGAACTCGAAGATG
CGGGTATGGTCCTCCTCGTCGGACGAGATGGTCACGAACAGGATGTCGTCGCGGTACTTCTTGGCGATCT
CCTTCAGGGGATCGACGTACTTCTCGATGTGGCCGCCCTCCCTGGACACGAAGAACAGCAGATGCGACTT
GATCGAGCCACCGAAGATCTTGGAAGCAGATTCGTGGTTGAAGTCAACGATCAGGGGCAGAGACTGCACC
TGGGCGAACTTCTTCAGGTTTTCCTCGTTCAGCTCACCCTCGAACACGGACTTCTTGTCGTCGAAGGGCT
TGAACAGAACCACTCCATTGTCCTTAGCCTC
>dme_circ_0004795_junction_seq
ACAGAACCACTCCATTGTCCTTAGCCTCGGAACTTCTTAAGGAACGCCTCGATGGT
>dme_circ_0004796
ATCTTCGGTGGCTCGATCAAGTCGCATCTGCTGTTCTTCGTGTCCAGGGAGGGCGGCCACATCGAGAAGT

ACGTCGATCCCCTGAAGGAGATCGCCAAGAAGTACCGCGACGACATCCTGTTCGTGACCATCTCGTCCGA
CGAGGAGGACCATACCCGCATCTTCGAGTTCTTCGGCATGAACAAGGAGGAGGTGCCCACCATCCGTCTG
ATCAAGCTGGAAGAGGACATGGCCAAG
>dme_circ_0004796_junction_seq
GATCAAGCTGGAAGAGGACATGGCCAAGATCTTCGGTGGCTCGATCAAGTCGCATC
>dme_circ_0004797
GGTGAGCTGAACGAGGAAAACCTGAAGAAGTTCGCCCAGGTGCAGTCTCTGCCCCTGATCGTTGACTTCA
ACCACGAATCTGCTTCCAAGATCTTCGGTGGCTCGATCAAGTCGCATCTGCTGTTCTTCGTGTCCAGGGA
GGGCGGCCACATCGAGAAGTACGTCGATCCCCTGAAGGAGATCGCCAAGAAGTACCGCGACGACATCCTG
TTCGTGACCATCTCGTCCGACGAGGAGGACCATACCCGCATCTTCGAGTTCTTCGGCATGAACAAGGAGG
AGGTGCCCACCATCCGTCTGATCAAGCTGGAAGAGGACATGGCCAAG
>dme_circ_0004797_junction_seq
GATCAAGCTGGAAGAGGACATGGCCAAGGGTGAGCTGAACGAGGAAAACCTGAAGA
>dme_circ_0004798
GGGGATCGACGTACTTCTCGATGTGGCCGCCCTCCCTGGACACGAAGAACAGCAGATGCGACTTGATCGA
GCCACCGAAGATCTTGGAAGCAGATTCGTGGTTGAAGTCAACGATCAGGGGCAGAGACTGCACCTGGGCG
AACTTCTTCAG
>dme_circ_0004798_junction_seq
GAGACTGCACCTGGGCGAACTTCTTCAGGGGGATCGACGTACTTCTCGATGTGGCC
>dme_circ_0004799
GACAATGGAGTGGTTCTGTTCAAGCCCTTCGACGACAAGAAGTCCGTGTTCGAGGGTGAGCTGAACGAGG
AAAACCTGAAGAAGTTCGCCCAGGTGCAGTCTCTGCCCCTGATCGTTGACTTCAACCACGAATCTGCTTC
CAAGATCTTCGGTGGCTCGATCAAGTCGCATCTGCTGTTCTTCGTG
>dme_circ_0004799_junction_seq
GATCAAGTCGCATCTGCTGTTCTTCGTGGACAATGGAGTGGTTCTGTTCAAGCCCT
>dme_circ_0004800
TGGAGTACAGCGGTGGCCGCCAGGCCGCTGACATCATTGCCTGGGTGACCAAGAAGACCGGCCCACCAGC
CAAGGATCTGACCAGCGTCGCTGATGCCGAGCAATTCCTTAAGGACAACGAGATCGCCATCATTGGATTC
TTCAAGGACCTGGAATCGGAGGAGGCCAAGACCTTCACCAAAGTCGCCAACGCCCTGGACTCGTTCGTCT
TTGGTGTGAGCAGCAATGCCGATGTGATTGCCAAGTACGAGGCTAAGGACAATGGAGTGGTTCTGTTCAA
GCCCTTCGACGACAAGAAGTCCGTGTTCGAGGGTGAGCTGAACGAGGAAAACCTGAAGAAGTTCGCCCAG
GTGCAGTCTCTGCCCCTGATCGTTGACTTCAACCACGAATCTGCTTCCAAGATCTTCGGTGGCTCGATCA
AG
>dme_circ_0004800_junction_seq
TTCCAAGATCTTCGGTGGCTCGATCAAGTGGAGTACAGCGGTGGCCGCCAGGCCGC
>dme_circ_0004801
GATCTTGGAAGCAGATTCGTGGTTGAAGTCAACGATCAGGGGCAGAGACTGCACCTGGGCGAACTTCTTC
AGGTTTTCCTCGTTCAGCTCACCCTCGAACACGGACTTCTTGTCGTCGAAGGGCTTGAACAGAACCACTC
CATTGTCCTTAGCCTCGTACTTGGCAATCACATCGGCATTGCTGCTCACACCAAAGACGAACGAGTCCAG
GGCGTTGGCGACTTTGGTGAAG
>dme_circ_0004801_junction_seq
GTCCAGGGCGTTGGCGACTTTGGTGAAGGATCTTGGAAGCAGATTCGTGGTTGAAG
>dme_circ_0004802
CAATTCCTTAAGGACAACGAGATCGCCATCATTGGATTCTTCAAGGACCTGGAATCGGAGGAGGCCAAGA

CCTTCACCAAAGTCGCCAACGCCCTGGACTCGTTCGTCTTTGGTGTGAGCAGCAATGCCGATG
>dme_circ_0004802_junction_seq
GTCTTTGGTGTGAGCAGCAATGCCGATGCAATTCCTTAAGGACAACGAGATCGCCA
>dme_circ_0004803
GAACTTCAGAGTGGGGTAGCCACGGACGGCGTACTGCTCGGCCAGCTCACCCTCGACGGTGGCATCGACC
TTGGCCAGCTTGATGGGCGACTCCTTCTCGGCCAGTTGCTGGGCAGCCTTGGCGTACTCGGGAGCCAGAG
CTTTGCAGTGTCCGCACCATGGGGCGTCT
>dme_circ_0004803_junction_seq
TTTGCAGTGTCCGCACCATGGGGCGTCTGAACTTCAGAGTGGGGTAGCCACGGACG
>dme_circ_0004804
GAGTGGGGTAGCCACGGACGGCGTACTGCTCGGCCAGCTCACCCTCGACGGTGGCATCGACCTTGGCCAG
CTTGATGGGCGACTCCTTCTCGGCCAGTTGCTGGGCAGCCTTGGCGTACTCGGGAGCCAGAGCTTTGCAG
TGTCCGCACCATGGGGCGTCT
>dme_circ_0004804_junction_seq
TTTGCAGTGTCCGCACCATGGGGCGTCTGAGTGGGGTAGCCACGGACGGCGTACTG
>dme_circ_0004805
TTGGGGTGTTTGTTATGGCGTCGTCAGATCGAAAGCTCATTGTAGTCTCCGGATTTGGTCCATTTCTCGG
TCACGAAGCGGTCAACGCCAGCTGGGAAGCTGTGAAACTGCTGCCGGAAATCCTAACTCACGATGGCATA
GAATATGATCTGGAAAAGCGGTTGGTTTCGGTGGAATACGGGGCTGTGGACGAGGCGGTGGCGGAGATTT
GGAAACGCCAGCCATATG
>dme_circ_0004805_junction_seq
GCGGAGATTTGGAAACGCCAGCCATATGTTGGGGTGTTTGTTATGGCGTCGTCAGA
>dme_circ_0004806
GATTCATAAGTGCGTCGGAGCAGCGGAGCTTGCTACTCAGCGGTATCAACTCATATAGAGCCGGTGAACC
AGGAAAACCACGGACTTAGCGGCATGCCTCTGCCTACGAGCTCATTTTCCCAGCAGGGACCAACGTGCTT
CGATAAGATGAAGACGGGCTTCATCATCGGATTCTGCGTGGGCATGGCCAGCGGTGCGGTCTTTGGCGGG
TTCTCGGCGCTCAG
>dme_circ_0004806_junction_seq
CGGTCTTTGGCGGGTTCTCGGCGCTCAGGATTCATAAGTGCGTCGGAGCAGCGGAG
>dme_circ_0004807
GTGAACTCCTTGAGATTGGGCTGCAGGAGGAGCTCGTAGTGCCTTGGCACCACATTGGTGGGCAGCCTCT
CGAACTTTGCCTTCTGGGCCATCTTGCGCTCCTGCTCCAGCTGCTGCTCCTTGCTCAGTAACTGTGTG
>dme_circ_0004807_junction_seq
CTGCTGCTCCTTGCTCAGTAACTGTGTGGTGAACTCCTTGAGATTGGGCTGCAGGA
>dme_circ_0004808
CAAACATGTCATCGATCAATCCCAGACGATCTAGAGGCGGCAGCTCCATCTTCTCTACTGCGGGCATCAG
CTGTTCTAGCATTTCTTGCGAATAGCGGGTGCGATAGTAACCCACTGTTCCCGGATTGATTTTGATCCAA
TCGTCCTCATCGACGTTGTCGAGTGTCACCTCCATTGAGGACTTGTCCAGCAGGAAGGTCTTGGCGATCC
CTGTGGGATTCTTTGAGGTAGACACCGAAATGGGCACCACCCATAGGCAGTTCTCGTCCGCCTGCGAACC
ATCAGCCGTAAACTTGCACTGCTTAAGGCGCAGCAGGCGTTGATTCTTGCTTGTTTGTTCTGACTCGACG
CTAACCACTGGGAAGCCCTTGTGCTGTGTCCACGAGGTCATCACGTCGCTCACGTTCTTGGAGCTGGCTT
CTTGCAGAGCAGCCCACAGGTCCTCGGTGCAGGTGTTTCCGTACTGATGACGAGTTAGATACAGATTCAT
GCCCTTTCTGAAGGTATCCTCGCCGATG
>dme_circ_0004808_junction_seq

GCCCTTTCTGAAGGTATCCTCGCCGATGCAAACATGTCATCGATCAATCCCAGACG
>dme_circ_0004809
TTCTCGTCCGCCTGCGAACCATCAGCCGTAAACTTGCACTGCTTAAGGCGCAGCAGGCGTTGATTCTTGC
TTGTTTGTTCTGACTCGACGCTAACCACTGGGAAGCCCTTGTGCTGTGTCCACGAGGTCATCACGTCGCT
CACGTTCTTGGAGCTGGCTTCTTGCAGAGCAGCCCACAGG
>dme_circ_0004809_junction_seq
GCTGGCTTCTTGCAGAGCAGCCCACAGGTTCTCGTCCGCCTGCGAACCATCAGCCG
>dme_circ_0004810
AAAACTCATACTGTTCTTGGCCATATTCAGATGGGTCAGTCGGCCCAGAGCCGATAACATTGCCGTGGGC
AAGCTGACCATCTTGTTGGCACTCAGATCCAAACTTTGCAGTGTGGAGCCGCAAACTCGTGCCTTCCGCG
AACTGAGCGAGGCACTGAAGTAAAAGTTGCTGATGTCCTGAATTTTGTTGTACGAAGCATTCAGGTGCTG
CAGGGATTTCAGGGGGCACACCATGCCATCCGG
>dme_circ_0004810_junction_seq
ATTTCAGGGGGCACACCATGCCATCCGGAAAACTCATACTGTTCTTGGCCATATTC
>dme_circ_0004811
ATATTCAGCCACACCAAGTTGGGCAGTTCCGTGAAGAGGCCAGCAATGGATTTTAGCTGATTTCCATCCA
GTCTGATGGCCTGAAGCTGACTATTCCTCTGCAGTGAACCAGCCTCGATGGACTTTAGCTTGTTCTGGGA
CAGGTTAAGGATCTGCAGGGAGCTCATGCGATCGAAAACTCCTCGCCGGATGTGGGTGAGAGAGTTCTCC
GTCATCCGGAGGCCATACAGACTCTCCAGCTGGGTGATGCTAG
>dme_circ_0004811_junction_seq
TACAGACTCTCCAGCTGGGTGATGCTAGATATTCAGCCACACCAAGTTGGGCAGTT
>dme_circ_0004812
GATTCACTGCCGGTTGGCTTACGCTCGAGGATCTTCACATGTCTCCCTGATAGAGGCCAAGTCCGATGAT
TTCCTTTGCAAATACGCCTCACATTGCTTTGCCCTGTGCCATTGCTGCGATTTCCAGGCGTGTGACTGCA
AAATGGAGTGCCCGGATAGGTGTTCCTGCTACCACGATCA
>dme_circ_0004812_junction_seq
CGGATAGGTGTTCCTGCTACCACGATCAGATTCACTGCCGGTTGGCTTACGCTCGA
>dme_circ_0004813
GCGTCGATTGGAAGTCGCTTACGATCCCCGCCTGTCTGCCCATCACCACGGACTACTTCCCGGACAAGCG
TTCGCTGAATAACGACTATGTGATATCGGATTACACTCTGCTGCCCGACGATGTGAATCACGATTACGCC
CAGAGCAGAGCGGTTTACCGGAAACCCCTCTCCACGGAGGAGGTGTGCAAGGAGATCGTGTCGCAGCGCT
TGGCGCAGGGCTTCCAGCTAATCGTGGTCGACGAGAAGCCTCCGACTGCGAGCGGTTGTTCCTCGGGATC
TGCAGTGCAGCCGGTGAAGCTTTCGCGCGAGACCAACAAGGAGTATCTGCTCTCCATTGGCCGCATCTTT
CACAAGATCTCGTTGAGCGGCTCGGTGATCACGGTCACTGGCTATAGGCCCAGGACACCCGTATCCTCCG
ATAAATGTGGACTACCGCTACCGTTTCCATGCCCCACAACACGAGACCTACGAAATATCCGGCGTGAACT
TCACCACCGAGAAGTTGGAGAACTTCAACTGGAACCACATGGACCTGTACATCTGCACGCGTGGCGATGT
GGATTACCCACTCATGGAGGAGCCTGAAGTACTGGCGCTATCGCATGTACCTGCTGCCCAGGGAGAACAT
TGTGAGCAAGATCGCCAGTTGCCAGCGATGCGACATATTCCCCGACGTGACTGCGGATAACACGCGGGAG
CAGGTCGAGGACTTCGTCCGACTGATCGAGGCGGTCAGCAAGCTGAAGCGGCAATATGCGCGCAAGGCGA
GAGGTAAGATAGAATCGATTCCTTGGCTTGGCTATTGTGCATTCGTGATCCGTTGCGTTAATTCTGTAAT
TGCGTTCTGACTCCTAATTGACTAATTGTCCCGACAGCTGTCTTGAAATCGGATTACAAGTGGATGGATT
AGGCAACTAACAATTTCAGATATTATTGAGGGGGATGACTCTTGAATGACTATTAAGTAATAAGAAATGT
TTCATGATCAAGATATGTTAGTTAAATTGGACAGTTTGTGAAAGTTTCTGTACATACAATAGAATATTTG
TACACGTGCAGTAAAAGGAATTGGATGACCTTTAGTCTTGCTTATAGATTAGGCAAGCAATTAGGAAGAA

GACGCATAACCACTATTGTTGAACTTTGAACTAACCTTGCATCATGAAGCCCGCTTTTAGTGCCCGTTGC
CTTTTCCCTGATGTATCGAGTATCTCGCCTCACTCTTGCAGGACAGCCCCATCGCCCACATAACCAAGCG
GAGACACAGCACCAGCATTATATCCAGGCCTCAGCCTAACCAGGTTATCTACTTAACTTATCTCAAGaaa
aaaaaTTAACTTTTTGTTTCATCCAAACTGCGCACATTTGCCTAATTTCAAAACTTTATTTCTTTATCAA
ATGCGCACGTTCGAGTTGATTAGTTTTGATTATTGCGAGAGAACTGCCGATGGATGGTTTTATCTAATGC
TTTTGTCTCTCTTTCAAACGTCTCTGTCTGCCCGCCACCCACTTTCGACCTGCCCCAATCCGCTCCGAAT
CCGAAAACGAACCCATCTCCTCAGGGACTCACGAACTCTCCGTTCCGGGAGCGAGTCGGCAGCAATCGAC
TGCCGGAGAAGCGACCCAGG
>dme_circ_0004813_junction_seq
CAATCGACTGCCGGAGAAGCGACCCAGGGCGTCGATTGGAAGTCGCTTACGATCCC
>dme_circ_0004814
GCGTCGATTGGAAGTCGCTTACGATCCCCGCCTGTCTGCCCATCACCACGGACTACTTCCCGGACAAGCG
TTCGCTGAATAACGACTATGTGATATCGGATTACACTCTGCTGCCCGACGATGTGAATCACGATTACGCC
CAGAGCAGAGCGGTTTACCGGAAACCCCTCTCCACGGAGGAGGTGTGCAAGGAGATCGTGTCGCAGCGCT
TGGCGCAGGGCTTCCAGCTAATCGTGGTCGACGAGAAGCCTCCGACTGCGAGCGGTTGTTCCTCGGGATC
TGCAGTGCAGCCGGTGAAGCTTTCGCGCGAGACCAACAAGGAGTATCTGCTCTCCATTGGCCGCATCTTT
CACAAGATCTCGTTGAGCGGCTCGGTGATCACGGTCACTGGCTATAGGCCCAGGACACCCGTATCCTCCG
ATAAATGTGGACTACCGCTACCGTTTCCATGCCCCACAACACGAGACCTACGAAATATCCGGCGTGAACT
TCACCACCGAGAAGTTGGAGAACTTCAACTGGAACCACATGGACCTGTACATCTGCACGCGTGGCGATGT
GGATTACCCACTCATGGAGGAGCCTGAAGTACTGGCGCTATCGCATGTACCTGCTGCCCAGGGAGAACAT
TGTGAGCAAGATCGCCAGTTGCCAGCGATGCGACATATTCCCCGACGTGACTGCGGATAACACGCGGGAG
CAGGTCGAGGACTTCGTCCGACTGATCGAGGCGGTCAGCAAGCTGAAGCGGCAATATGCGCGCAAGGCGA
GAG
>dme_circ_0004814_junction_seq
AAGCGGCAATATGCGCGCAAGGCGAGAGGCGTCGATTGGAAGTCGCTTACGATCCC
>dme_circ_0004815
GTGGTTCACAAGTCCGAGGAGCAGCAGCACTATTTTAACACACCCTTCCAACTGTCATTGCCGCCGCCGG
GACATGGGCCCAATGTCCTCAGCGACAGCCCTGAGTCGGCGGACACGATGAGCTTTCCGGTGCGGGATGG
CGACGTGATTCTCATTGCAACCGACGGAGTGTTCGACAACGTGCCCGAGGATCTAATGCTGCAGG
>dme_circ_0004815_junction_seq
AACGTGCCCGAGGATCTAATGCTGCAGGGTGGTTCACAAGTCCGAGGAGCAGCAGC
>dme_circ_0004816
GAGTACTGATCTGAGGACATGAATTTATACTATAGGCCATATTAATAATAACTCCGTCGAAATCGAAATT
GAAACGAACTAAGAACCAAAGCCGTTCAGTGCATGACTTAATCTAATCGCGTCGGATGCTCGTTATGGGA
TTTTTTCTTATCTATAGATAGTTTGGGACTCAATTGAATGGGCAATTTGTGCGTTCTTTAGAAAGG
>dme_circ_0004816_junction_seq
TGGGCAATTTGTGCGTTCTTTAGAAAGGGAGTACTGATCTGAGGACATGAATTTAT
>dme_circ_0004817
GTCCAGATAGTTGATGCCTACTTGCCCGGGTTTGCCGATTGTTCCGCGCTTAATGCTCCCGGCTGGCTGA
GGCGGTAATGGTAATGGGGCAGCCTGCTGTTGCTGAGGTGGTCCACGAGATTGGTATTGGCCTTGGCTCT
GGGTTTGGTTTGgttgttg
>dme_circ_0004817_junction_seq
CTTGGCTCTGGGTTTGGTTTGgttgttgGTCCAGATAGTTGATGCCTACTTGCCCG
>dme_circ_0004818

GAATAATCACAATTGCCAGATCGTGCTGGCTGCGCTTCAGATCCGCGAAAATAGTGTCTAGGCTGCGTTC
GTCATCCGTGAACGGTCTGTATGTCACATCAGAATCCAAGCTTATGTTGACTGCCTTGCCTTGGGATATT
ATTAGGTTCCCGAAGTCATTCAGCTGGGTATAGTTCATTTTGCGACCACTCCTCGGATCGCAATACAAGA
CCGCACACTTGTGCGCCTTGGGCTTGGGCTCCAGAAACTTCATGCCATCCATGCGCCACGACCCGTTCTT
CACCATAGTAAACCTCTTACTATGATATTCAACCTGAGGTGGGCTTAGGACGCGGGTGCTTACCACAATA
AAATCGTTGGCAATGCGGATGCCAAAGCGACTGATGGTCGGATCCAGGTTGTGCTGGAAG
>dme_circ_0004818_junction_seq
GATGGTCGGATCCAGGTTGTGCTGGAAGGAATAATCACAATTGCCAGATCGTGCTG
>dme_circ_0004819
GAGCAGCAAAATCCAATGGAGCAGCTCCAGCGGAATCGAAGAAGCAAAAGAAGGCCAAGGGCAAGAAGAT
GGTTCCCCTGTTCGCCACGGGCATGAACAGAGCTCCATGAAGAGCTAGATGATGATCGCTTATGAAG
>dme_circ_0004819_junction_seq
AAGAGCTAGATGATGATCGCTTATGAAGGAGCAGCAAAATCCAATGGAGCAGCTCC
>dme_circ_0004820
GAAACGTCCAACTTGCTGCGTATGCGTCACTCCTCCTTGGGGAAGTCAGCTCCATGCCTCACGGGCAACT
ATTTTCGCCACGAACTGACCGCTCCGTTACCAGTACAGCCACCTGGCTTTGGGGCTTCTCCCTTGGGCGG
TCACAACATTTCGCGGTCGGGCAGCTGTGCAGGGATTGGCTTGGCCAAACACCACCATCACCATCACTTG
CATGGCGTTGTGATGCGGGGTTCAGCTGGAGGAGGTGCTGGCTCCGGTGGTGGCTCCTCCAGTGGAGTTG
CCCATCATCGTCTTAGTTTGGTCACCAACTCGGCTGCCGTGGCAGCGGCTGGCGGATCCCGAACCCATTC
CCCATACTCGGCCAGCCCCGTGGATAGTCCGCGCCTCAATTCACCCATGCCATTTGCATTTGCTCCGATT
AAAAGAATCGCCTCGTGCCGGGGCGTTGTCGCCGATGGACGTCGTTGGTCCGTCGCCTCGCTGCCCTCTT
CAGGTTACGGAACCACGCCTGGTAGCTCCAATTTATCGGTCTCAGTGCTCGAGTCAGGAGGGTCTCAACC
AGTTGGCGCACAACATTCCGCCCGGAGTGCAGGAGGCGGATGCCGCTGCCGTGGCCGCCGGTGCCTGCTG
TGCCGAGCACCTCAAACAGCATTGCCCCAAGCACTGCGCTCTGTTGCTAGCCGTTTCCCAGAAGCAACTG
ACACCCCAGCCACCGAAGCCAACGCAGCTGCAGCCCCAGAAATCGATGACAGCGGCCTGCGTTAATTGCT
GTGCCGAGCTGAGCGTAACCTGTGGTGGTCAGCAAGCAGGTGGTGGTGCTGCTAATGGTGGTTCATCCGC
GAATAGCTCGGGCTCGAGTGCAATGCAAGTTGGTGGTCGGATGTCGCCCTATTTTCGTCCGCGCTCCCGA
TCGCTGTCCAGCCCATCGCGTTCACCAATCGTGGACAATGAGATTGCCGTGATGAACACTTTGTACAAGG
AGCGTTTTCCCAAGGCCACACAGCAAATGGAAGAGCGGCTGAAGCTCTTCATCAACGAAAACAAGAGTGC
TGCATGCAATAGCTTTAGGGACTCGCAGCCGATAGTGCG
>dme_circ_0004820_junction_seq
GCTTTAGGGACTCGCAGCCGATAGTGCGGAAACGTCCAACTTGCTGCGTATGCGTC
>dme_circ_0004821
TTGGTGACCAAACTAAGACGATGATGGGCAACTCCACTGGAGGAGCCACCACCGGAGCCAGCACCTCCTC
CAGCTGAACCCCGCATCACAACGCCATGCAAGTGATGGTGATGGTGGTGTTTGGCCAAGCCAATCCCTGC
ACAGCTGCCCGACCGCGAAATG
>dme_circ_0004821_junction_seq
CCCTGCACAGCTGCCCGACCGCGAAATGTTGGTGACCAAACTAAGACGATGATGGG
>dme_circ_0004822
GCGGATGCAGATTTAACAATTGACCTGGACCGATGATGCTTCACACTGCTTCCGCTAGTGCTGCCGTCCA
TGGATTTTGGTTGATGCTGCAAAGCCAGCGGAGATTGTTCTCTTGGCAAGTTCATGTTCTCCG
>dme_circ_0004822_junction_seq
TGTTCTCTTGGCAAGTTCATGTTCTCCGGCGGATGCAGATTTAACAATTGACCTGG
>dme_circ_0004823

GTCTTGACATCAATAGTGTACAGATTGCGCCAGGCATTTTTCAGGGCCAACAAGGCATAGGGTATGAAGT
TGGGATGTCCATTGATGACGACATCCTGCAGTGGCACCTGGTTTTGATTTATTGGAAATATTATTAAATA
AAGGTGATGGAACTCTCTTTTAAGCTAAGACTTTTCAAAGCTATGTTACACATAATTAAATCCTATTGCA
TAAGCTTCTAAATATTGTTTACTTTGATTATTATTTAATTATTTCTTATTTAACCTTTGAACTCCTAGTC
TTGACCTTGACATATTGATAGTCGTACCTCCTTTAATCCACCATTCTGGAAAGCAGTCGTGTGCTGGAAA
G
>dme_circ_0004823_junction_seq
TTCTGGAAAGCAGTCGTGTGCTGGAAAGGTCTTGACATCAATAGTGTACAGATTGC
>dme_circ_0004824
GGGACCTGGCATCTATGCCATGACTTCGTCGCCGTTGCCGGCGAAGCGGAATGGCAATGGCAACGGAAAT
GCCACGGACTACGACTATGATGACTATGCCGACGATGATGAGGACAACGATGAGCTGGACGGCGTTCACG
ACTTTGGTGCCGAATTGGAGATCAGTAGCATAGCTCCGCCGCAGGGAA
>dme_circ_0004824_junction_seq
ATCAGTAGCATAGCTCCGCCGCAGGGAAGGGACCTGGCATCTATGCCATGACTTCG
>dme_circ_0004825
GAGTGGAAATCAGTTCTGGTCCTTCGACTCCAAGACCCATCAGGTGATCTCCGGCCAACAGCAAAACTTC
AGACATTGCCTGGAGGCCCAGCCGGAATTAAATGCGGTTACTTCGAGTGTCTGTGATCCGAAGAACCATA
AGCAACAGTGGAAATTTGGATATCTGAATAGCCAAAGACTGCAACATTTTTGGGATAAT
>dme_circ_0004825_junction_seq
CCAAAGACTGCAACATTTTTGGGATAATGAGTGGAAATCAGTTCTGGTCCTTCGAC
>dme_circ_0004826
GGAAATCACTGCTGGAGATGGCATCCAATGTGGTGATGGTCGGGGTGGAGGCTTCAGGAGAACTTCTCCC
GCTGTTCAGAATCTTCTGAGCTGCGGCACCGGCGTAATGATCGAAATGCAAATCGTGCGAGAGTTCGTAG
TTTAATGCGCGCGGCAATTCCTCCATGGTTTACTTTTATATCGGTCGACTGGCGGGT
>dme_circ_0004826_junction_seq
TTACTTTTATATCGGTCGACTGGCGGGTGGAAATCACTGCTGGAGATGGCATCCAA
>dme_circ_0004827
GCGAAATTAAACGGAATTTGCAACTGTGAAAACGCTCTAAAGCGTGGAGAGTTGCATTTGAAAAAAGGTA
ATTAGCGTACCAATTAAAAAAGGAATTGTTCAGTTTGTGCGTGCAGGTTTTCTGCATGTGTGTGTGTTTG
TGTAAAAGTTGTTTTCCCTGAAATGGCAGGTTGTGGATTACCCGATTCGATGACGAAATTATTGAGCTCG
TTTTCTGTGTTTTCGTTGTTGTCAATGTACTTTTGCATTGATAAGCAAGgtgttagtgtacttgtgtgtg
tttgtgtgtgCGTGAGTGGAAGAGCAATGAAGATAAGCTCGAGTCTTCTTCTTTCCCCACTCTCCAAAAG
GTCGTTTCGCATATGGTTTATTGACGCTGCATATGTTTTTGTTTTTGCCTGAAATTCAATTTACCCACTG
AAGCCCCACAGCAAGGCTTCCGACCTCCAACACACCCTGCTCCATAATTACCAACTTACCGCGCCTCCTT
TTCCCTCACAATCATCGCAATATGGCTTTTACTCGTCTCTTGCCACCTTTGGAGACTCTTCTTCGCTTGG
AGACTTTTGCTTGGCCAATTTCTGTGTATCTCCGCCAGCAGCAACTTGTTGTTGCTTCTTCTTTGTGCAG
CTGATTGTGTTTCGCTTAGCCTTCTCTCACTCTTCTTCTGCCTCTTCTTTTGGCCATTTACTTCTGGCTT
TCGGTGTCGCCGAGATTCGTAAGCAAATTTCAAGCGTTTAACATTTAACGCTTTGCGTTTTGCTCACAAA
TTCGAGTTCAACAATGCGAGTATTATTAACTTTTGCACGAGTTCGTGACTCATTCGTTATCATTAACGTG
ATTTTTATTCCCCTAATGGGAAACTCTTGTCAGCACAACTGGAATACTCACAATATACCATAAGTATTCA
AAACATCCAAAGAGTTTTTATATTTGAATACCATTTCTGGAGATAAAATGACTCTATAATCACAAAATAT
CCCATTTGTTAAAACTAAATCATAAAAAAAGGTGTTTATTACACCttttttttGTTAAGTTTATTAAGTT
TTTAGTGTTTCCCAACCCTTTAGTGGTGAGTCAGTGTTTCGAGCTATCTTGTACTTGATGTTATTAAAAA
TGATTGGTAAAGGCACTCCTCAAAAAAAGAGttttttttttAAATGGTGTTGAATAAATAATATCGGAAT

TTACCATGGGCGATACCCTTTTTTCCCACTGTTCAGCAAGGAACTTTGACATTTCATTTTACTTCCTGTT
CGTCATTTCCGATATTTTCAATTCTATTTCACTTCGATTTGTAGGTTTTGCCTTTTTGGAATATTAGAGT
AAGTGGATTCCACGCAAGATTTTAACATTATTCTGTATTTCCCCCCTGTTTCCTCGAATGCTTATCGCAT
TTCTTGGCCCCCATAATCCTCTTGAACTGCCATATGTTTCCGTTCCACCAGAACTTTCAGTTTATTTGTA
TCCATTGCACTTTCGGTTATTTTTGTACGCACAATAGCATCGCTGCTCAAAAAGCTCCCCATTCGAGATT
ATTTaaaaaaaaaaaCTTCACTCAAATGGACTCAAGATCTATCAAATATGTAATTCATTCTGCTTGCTTT
GCACTCGCCTATTGATTGGTTGGCATTCCCAGGCGGGTGAAATCGATATGGAGCAGTTTGAAGTGACCCT
GAGACAAGGTGGAAGGTGGGTGTGGAGGTGGCCATAACTTTCCTTGGCTGCGCCAGAGTCCATTATTAAT
ATTGGATTGCGTGATGATCTAATGCGCGCGCCTCTGTCCCTCTGCTCCCAAAATCCCAACCCAATCCCGT
TCACCACCCGCCGCCCGCCGCCAACTCttttgtttgtttttgtttttgTTCCTGCTGCCGCTCCACATTC
CCCACTTTGCTTCTTGCTGACCCAAATCCGTCCAAACATTGTTGCAGTGCTGTCACAAATTTTGGGGCAA
CATGGAAAAGATTCATAGAACACATATGAACTTATCAAGATAAGAACATTTTCATAATAAACCTTAAAAA
TCCATATTCCAGGGCAATAAAAACACACAGATATTAATGCACATACATAATGGATGTTTATTTAAAATCA
TAATTATTCAATGACTATTATTCAACATCTTGCACATGACTGGATTCACAATTTCTGACCAAGCGTGTTG
TTTAAACCTTTATATAAGTGCGAATAATTCGAAAGCATATGAAAATACAAAGAAATAATTCACTAATGAA
TTTAGATTATGAGTTAGGAGCTTGAATAAATCAGGTGTAACTATAATATAGCTAAGAGCGCAGCACAGCA
GTGCTGAAATGGGGTTTCGTGTCTCTGAGTAAGAATGTCATTTTCGGAGCAATGTTCGTGTGAAAGTCGA
GACGAGAACGTAAACATTTTGCCTGCCTAGCTGTCTGTGCTGAGCACTTCAACTGGCCGCACAATAGCTC
CTGAACCTAGCCCAGTTACACCCACGCTAATCGGTCTTCCTCGTTCTGGATTCCATTGCAGCATGAGCAC
AACCGCCGCCAGTCTCATCGAGGCGGCTCTAAATGCCAGTAGTGGAAACGCCACGGCTACAACTGCGGGA
TCGCTTGAGGCTACAGGGAAGCCCAACTACGTGGTGCAG
>dme_circ_0004827_junction_seq
TACAGGGAAGCCCAACTACGTGGTGCAGGCGAAATTAAACGGAATTTGCAACTGTG
>dme_circ_0004828
GCACAACCGCCGCCAGTCTCATCGAGGCGGCTCTAAATGCCAGTAGTGGAAACGCCACGGCTACAACTGC
GGGATCGCTTGAGGCTACAGGGAAGCCCAACTACGTGGTGCAGGTAAAGAACACTCCGATGAGCACGCCG
CTGGACCCGCTGCTCCACGTGGGCGATGGCATCTTCCTGCAGACAAAGACCGCCCAGGTTCTAGCTGGGG
TGTGCGTCTGGGCCGCCCTCTTCATCACATGTCAACAG
>dme_circ_0004828_junction_seq
GGCCGCCCTCTTCATCACATGTCAACAGGCACAACCGCCGCCAGTCTCATCGAGGC
>dme_circ_0004829
GTGCTGATGGCGGTTACATTTACATCACGATTATCTACAACATCTCCGTGTCGCTGGCGTTGTATGGCCT
CTATCTGTTCTACTTCGCCACGCGTGATCTGCTGACTCCGTTCGAGCCCGTGCTAAAGTTCTGTACCATT
AAGTCCGTCATCTTCTTGTCCTTCTGGCAGGGCGTGGGTCTGGCCATCCTGGAGAAGGCCAATGTTATCT
CGCCCATTGTAGACAGTGCAGGCACTGTTACCGTGGAACCCGGTACCGTCTCCGCCGGATACCAGAACTT
CTTCATCTGCATCGAAATGCTGTTCGCCGCCATCGCGCTGCGCTATGCATTCCCCTATCAGGGTGAGTTT
TGGAATGTCGGAGTGGGCTAGATTTTACGCAGCTATTTACGATTTTTGGTGATTAGATAGTGCTGACTTG
AGTGCTTTTTTTCGGTTATTTCGGTGTTTATTAAGCGGCTTTCCTTACACTTGTTAGATATGTGAAAGGT
ATTCGTGTAATGCTGACTTGGCTAGAAGAAATCAGCTAAAACGAAGTGCTTGCAGTAAAAATAACTTAAA
TGTTGTATGCTCTTTGTAGGTCTACGCACGCAGCTGCATCAGCGATGGCCACGGACGGTCTGTCACCATG
CAGTCCATTTCCAGCAGTCTTAAGGTAAGCTGGAATATTTCCCTGGGAATTCCCGTATCTAAAACTCCCG
CAATCCTTCGCAGGAAACAATGAATCCCAAGGATATTATGACAGATGCTATACACAACTTCCATCCGCAG
TACCAGCAGTACACGCAGTACAGCTCAG
>dme_circ_0004829_junction_seq

TACCAGCAGTACACGCAGTACAGCTCAGGTGCTGATGGCGGTTACATTTACATCAC

>dme_circ_0004830

TTGCCATTCTTTTCGGCCTGTGGAATGTGTTCTCCATCATCACGCTCAGCGTATCCTGCCTGGTGGCTGG
CATCCTTCAAATGGTGGCAGGATTTGTGGTGATGCTCTTGGAGGCTCCCTGCTGTTTCGTCTGCTTTGGG
CAGGTGAACGAGATCGCGGAAAAGGTGGAATCTAAGCCCTTGTACTTCCGTGCCGGGCTCTACATTGCGT
GAGTAACCAAAGAAATTTGTCACAAATTGTAATATTTCCTAAAGATTATGTTTTCCATTTCAGCATGGCC
ATTCCGCCCATTATTCTGTGCTTTGGACTGGCCAGCTTATTCGGCAGTGGGCTGATCTTTGGCACTGGCG
TGGTTTACGGCATGATGGCGTTAGGCAAGAAG

>dme_circ_0004830_junction_seq

TTACGGCATGATGGCGTTAGGCAAGAAGTTGCCATTCTTTTCGGCCTGTGGAATGT

>dme_circ_0004831

ATAACGGGTCTGCTGGCACGGCCGAATCAACAAGATCCAATTGGACCGGAGCAGCCGTGGTATCTCAAAT
ATGGAAGTCGATTGCTGGGCATTGTGGCCGCCTTCTGTAAGTTGTAGGATATATAATAAATTATCTACTA
GCTTAACACATTAAAAAGTTGGGAAAAACATCCGTGACATTGGGCGGCCCAAAAATATGTTGCATCGATT
TTTGTGTACCACCCTTTGTTGTTGTTGCTGTTATCTATTCGTAGGTGTAGGTGTGCAGTTGTGTTCGTGT
GTGTACGCACGCTCTCTGACATTGATAAAGGAAAAACAAGAGAGGGTGAGTGAGAGGTGAGAGGCAGTGA
GAGCGGAGAAAGGAAACATGGAAAAGACAACACAAAAAGGATACACAAATTCACACACTGAAAAAAATAC
TAGTTATGTATGTAGCTAAAGTTTATTTTTACATCAAAATCAAAGAAATGTAGGGTAAAGTGTGAGATTT
CCAAATGAATTAAGAATGAAACGTATAATTTTAAGGTAAAACAAAGTCCAAAAAAAGGtttttttttgttt
tGTTATTGAAATATAAAATAATACATTCACCCACTTAACTAAGTGGGCTTCCTGCACTTAATATCTCTTA
TCTAACCATTTCGTTTGATAACTTTTTATTTCGTGTGTAGGAAATTGCAAGTATGTATAGGAGCTTTTCT
GTGTCAAGTTTGGCGCTCTGCTTTTTCTTTCCGCATCGAATGTAATTCTCTGTGCCCATTCAATCGAAAT
TCGCACTGCCTTTTGTTGTATCAGCAGCTTCCCTCTTTCCATCTCTATTTTTAACTTTGTTTTACCCGTT
TGCGTATTTGCTTTTTTATCTGGACTCAGTTAACCTTAACCCTTAGGCTGCCACACGTGTTATAAGTGCA
ATATCGTAATAAGTCTATGCGTATCTTAAATTCAATTGGGGCATCTTTTTTGTAATTTCATCATCACCTT
CTGAATGCATAAAAAAATGCAGTTCAATAAACTTTTAAGTAGTCGTGATCTACTTTGATTAAGTCCAAGT
CAATCTTTTTAATCGCAGTTGGGGTTTTGATTATGCTATTGGGTTTCGTGTCGTTAGAATACTTACAAAA
ATTATTTAAAAATCAATCTAAACCATACATATGTATATAAATGAAAATTCCACAGAAACAATATGCCTC
ATTGACATATTATTCCTGGAATTTTTAGAGATCAACCAAAGGAATATTTATTTATTCAAAACGTTTTTAA
CATCttttttttCTCTGTACAAAAGCATCTTTTGTTGTTGGATATTCTGGCAATGATTCTCACTTAGTGA
AATGCAGAAATTGCCCAACAAATGAACACCATCCACTTAAAATCAATATAAATAGCTGAATACCTCAATT
AAGGGATCACAACTCACTCGTCCGGCTTCCACAATGAACCGGTTTTGTTTGCGTAAAATAAAACCAAAGT
CTATGATTAATAAATAATTTTATAAGCCGTTCCGTGGAGCACAACGACTTCTTCCCCAGGTTGGGTCAGT
TTGCCTGGCTTTAAGAACCGCAATAACCTCAAAACCCAGATCCGATTTCGCAAAAGGAAAAACCTAACTT
GCGAAGCTAAATATTAAAAGTGGCCTAAATCGTGAAATCTTTTTTATTTTATTATTCATGTATTTAGAAT
TTTAGAATTCAACCTTTCCTCGTGATCTTTTGCTGCATTTATACCAGCCACTTTTATCCAATTATCACGC
CCCAGGCATTTGTTTATCTGCGAATTATAGTCGTATGTGCTGTTTACTCTTTGTTTATGCTTTTATCATA
GACCAAAAGCAGCTGATAAAATATTTTAATGTAATTCACTTGTACGCCCCTCTAGCGATATCGAATGGAA
TTTAGTGAATTCTACCTGTGAACTGAACTACTTACTATCAACTAACAATTTGCCCAACTGTTTTTCATGC
TGTTTGTCACGCTTCTGTGGCTCGTTTTTGTTTAAAAAATTTTTAAATAAAAGAGAAGGCCATAAAAAGT
TAGAAATTGGCACAATATGAGTCAGCGTTGTAGATTAGATTGCGTTTCCTCGTTTTTACCATACGGGGCG
GTTGGCCCGACATAATGGGTCTAGTGGGAGTATGATATTGGTATTTTTGGTCAGTGTTTAGCGATAAAGA
TATACGCCTTTTATTGGAAATTACAAAAATAGCTTGACCTCTGCATACATTAAATACAACATATGATAGG
AATGTAATTTTTTTAAATAATAAAAAATCAATAGAAGTTAGCAGTAAGCAGTAACTTGAGCTACATGGAA

ACAGTAGATTGAGGAGCAAATTCATAATAGCCAAGGAAAATATTCATTATTAGTAGATTATAACATTGCA
TAACTAACATCTTTTATCCCATGTTTTCCTTCCAGTTTGCCATTCTTTTCGGCCTGTGGAATGTGTTCTC
CATCATCACGCTCAGCGTATCCTGCCTGGTGGCTGGCATCCTTCAAATGGTGGCAGGATTTGTGGTGATG
CTCTTGGAGGCTCCCTGCTGTTTCGTCTGCTTTGGGCAGGTGAACGAGATCGCGGAAAAGG
>dme_circ_0004831_junction_seq
GGGCAGGTGAACGAGATCGCGGAAAAGGATAACGGGTCTGCTGGCACGGCCGAATC
>dme_circ_0004832
CGTTTATCTTTAGACTAAGCAATGACCAGCAATGTCTCCAAGGTGGTAACATCAGAGCCAATAGGTTTAC
ATCACCGAAAATCAAAAGAAGTTCCCTGTAACTCGGCCTTTTGTGTTCAATTCGAAAGTAGTTCTCTAAG
CGTATCGGCCGAAAACAGTAAGAAATTTGCATTTGCTTTAAATCTTTTACCATTGACTTTTAAAGCTTTT
TGAAAAGCCATATTAACTTTTATTTTATATGTTTACTTAATGAGCTGCAAATCTGTTTTAAACACTTCTC
TCCTTATTTGGATTTGGATTATACTTCATTTTTATTAAAAGCCTATAGTTTAAAGTTTTATTTTTTTGGA
AACACTCAAGATCCGTTTCCAGCCTCAGACAAATGCCCAATGAAATTGATAATCTTGTCTTTCTATCCTA
ATTTAATGCCTTCTTTCTTCTCAATACCAAAACGAACTTAACTTCTTTGGTCATTTGTCTGGGCCAAGTG
ACAAACAACATCAAACTTATCATCCATCAAGCAATGAATATCAAACTACAAGAACAGCGTTGTTTACACA
GCTTGAAAATTTCAACAAACCCAAGAATAAAATATTGTAACATTTTGTTGTAAACGAAGAAAGAAACTCA
AAAGTGCAACAATTTAAATATGAACCCAAAACAGaaaaaaaaaCGACTCTTCATTTGATGAACTGCAATC
CCATTACAATTTTACCAATCCAGAGACACACATTATATTTACTTTCATTTTTTGAAGAACCATAGACAT
AGCATTAATTAACTTTAGTAAAGGGCTCCTGGAACAACAACCGACCACATTAAGTCTAAGTGAATGTTGG
ATGCGTGAGGACTTGCGCTATATTGAAATCTTGCCAACACCTGAAAGTCAGAACCGCCCGACGATTGGAG
TGATCCGAGTACTGGCTCCAAGCTAAATTTGCTATGCATTTTAACCATTGTATCGAACATAGACAAACCa
aaaaaaaaaaaaaaCAAATCTGTAAACACGTCCAAAACAAAAGCGAGGACGTGGATGCACAAGAATAGAT
TTCCTGAAAATAAAGTGGAAAGTAATCCAGATTCTTTGG
>dme_circ_0004832_junction_seq
AAAGTGGAAAGTAATCCAGATTCTTTGGCGTTTATCTTTAGACTAAGCAATGACCA
>dme_circ_0004833
GACTACGTTTGAAGGAGGAATTAGTTAACATGCTAACGTGACATGCTTACGAATTATAGGAAACTTGGCC
TGGCTGAATAACCATCACGAATTCTACGCGTAGTTATCAAAAGACTTAACATTTCTTAAAAGGCTTCTCT
CCCCCGTAACTTACGCACTA
>dme_circ_0004833_junction_seq
GCTTCTCTCCCCCGTAACTTACGCACTAGACTACGTTTGAAGGAGGAATTAGTTAA
>dme_circ_0004834
TATTTCTTAGCACAATTGTGATCTTAGCAAACGGCTGGCAACTACAAAAACTTTTATAAGCGCAACCACA
ACACCATTTTAATCAATCAAAAGAATGCCCCCGCACACACACCGTTCGAAATGAAGAGCCACTTTCCCAC
AATCAGGGGCAATATCAGGCATATCTCGGCAGCAAAGTGTTTCGTCAATTTTTGATTGACCACTTGGGAA
TGGGATTGTGAAGCTAAACATGAGAAAACAAAACATTTAACGAATTTAAATTAG
>dme_circ_0004834_junction_seq
AACAAAACATTTAACGAATTTAAATTAGTATTTCTTAGCACAATTGTGATCTTAGC
>dme_circ_0004835
GCTTATACAGAAGGGATTCTAACATAATTATACTCAATTTGTTCGCTCTCACTTGGATCTTAAAGTCGAA
CAGCGATTGTAGTGTTATTACAAAATATTACTTTACGTGTGCTGAGCGGAAGACTCTCGTTTATGtttta
cttttcttttttttctttttGGTATGAAAATCTTGTTCTTTCTCGTATTAAAAAT
>dme_circ_0004835_junction_seq
AAATCTTGTTCTTTCTCGTATTAAAAATGCTTATACAGAAGGGATTCTAACATAAT

>dme_circ_0004836
TCAACAATCTAGCGTTGCTCGCGTCGCTGACTTTACAAAAAGTGCTAAATATCAACAATAACAATAACCA
GCACAGCAAGAACAACAACCACAGCGACAATAGCAATAGCAAACAACACGAGCAACAAAAACAGCAGCAG
CAACCACAACGACGAAACCTCGACATTAAAAGCGAGTATGATGTACTAAACCAATTGCTAAAAACTGGAG
CTGTTTTCACCGCCACGCCCACAGCGCCAATTGCCATCGAAAGGAGGAAGCAGACGCAGCAGCAGCAACG
TCCATATGCACAACAGGCACATCAACAGCAGCAGCAGAAGCAACGCCTAAACTCATCCATATCATCGACC
CACTCATCGACATCGACAAACTCCTCCTACGGCAGCTCATCATCGGAGGATGCTGCAGGCACATCTGCGC
CAACGGGAGCAGGTGAAGTTGTCACATCAGCAGCATTAACACCAAGAGCAAGCACCACGAAGGCGTCCAG
CAATAAATTGCCAGAGAAATCGAAAATTCCGTCCGATATACTTGATGATCTGGCCAGTCGATTTATAATC
AATGTTCCGGACATGGAGCTAAACAATCTAATACGTATGTGTTTCCAAATCGAACTGGCCCATTGGTTCT
ATTTGGATTTCTTTTGTGCCCCCGAATCTGGCGAGGATGGCGAGACCCCGAAGTGCGTGCAACGAAAGCT
GCCATCGGTGGGCATCAAGCAGTTTGCCATGCAGTTGTTCCAAGCACATTCCCTTCCTGAACAAACATTT
TGGCACCGTGGATCAAATCCTGGACGAGTGGAAGAACTACAAGCTATCGGTGCCCACTTATGGCGCTATT
CTGGTGTCCGAGGATCACAATCACTGCTTGCTAGTGCAGTCCTACTTCGCGCGCAACTCCTGGGGATTTC
CCAAGGGCAAGATCAATGAGAACGAAGACCCAGCCCATTGTGCAACGAGAGAGGGCAAGTCGGGAATATA
TTGGAATTCTAAAAAATATTATTGTGAAAAATGAATGCATTCAATGTGGTGTTaaaaaaaaaaaGAAGTG
AAATGGAATTACTTTACATGGTTTATGGACTTTGAAAACAAATTTATTAATCTCCGGTGATTTTTTAGGG
TTTATGAGGAGACCGGGTTCGATATCACGGACCTAATCGATGCCAACGACTACATCGAGGCGTTCATCAA
CTACCAGTACACGCGGCTGTACGTGGTGCGAAACATACCGATGGACACGCAGTTCGCGCCCCGCACTCGC
AACGAGATCAAGTGCTGCGACTGGTTCCGTATCGATGCGCTGCCGGTGAACAAGAACGATGCCATATCAA
AGGCCAAGTTGGGCAAGACCTCCAACTCCTTCTTCATGATTATGCCGTTTGTGAAGCGCCTGAAGAAGTG
GGTGAACGATCGGAAGGCTGGAATCGAGCCGCGTCGCCGCAAATTCTCTGGTCAGCAATCGCCAAAAGCG
GCCTCGCCCACCAATATGAACGGAAATTGCAAGAAGGACGTCACAGGAGTGCGAAATGAATCGCAACGAC
AGAGGCACAAGTCCATGGGCGACTTGGATGGCGTCAAATTGAATAATCTCAATGGCAAGGGGACCGTTGG
AACGGGAGCGGGATCGGCAGCTTCCGCCTCCGCCACCGCTGCCATCAACTCGATGAACATATGCAATAGC
AATGGGAAACGCAATAAGCAAAATGCGGCTGCGGGCAGCAATCAGGCAACGCCAACTACAATAGCAACGC
CAATGACGTCAAATGGCGCCGTCGTAGGAGGTACTGTATCCTCGAAGCGTCAACTGTTCCATAGCCAAAG
TCAGAACGATGCGCAGCAATCGGCAAGCAATGAGAAGGCAGATTGTCAGCTCCTTCGATTTAATACGCAA
GGAGAAGCAGCAACAACTTAAGGCAGCTAAGGCGCAAAAGCAGCAGCAACAACAGCAGCAACGATCGCGC
ACCAAGTCGCAGAGTGATAAGCAGTACAATCCACAGCAGCCAGTCAAAATCCTGGGACAACAGCAGCCCA
CAACGGGCATGGATTTGATAGCCATGCTATCGGCAGCGGCGGCAGCTCAAAAGACACCAAACAACGAGCA
GCAAACACAAGAGAAGCAGCAGCAGCAACAACGACCACGACCTGCTTCAGTGTCGCTTAACCTTGGAGCA
GGCAGTGCCACAGGAGCAACAACATCAGCTCCGGATGCCCAGTACATGCACAGGCTGCAGCGCATGGCAT
CGGGCACAAATTTGAGGATTCTGAAGCGGGCAcagtcgcagcagccgcagcagcaccatcagcaacagtt
gcaactagaacagcaacaacagcagcagcagcagcTGGGAATCGACTTTACGCCAAATTTGAATTCGTGG
ACGAATTTCTCCTTTACCAAGAACTTCATAGCAAATGTGTTTTGCTAAACTTTTCTTTGAGTCAATCACA
CACACTTCCTTTTAAAACTCTCAATTAATGCATTTCCGCGCAAAAGGCCCGGTTACCGGGAACACCGAAT
AGTATAGGAGGCTCTGTGGAGTTTAAGGACTCGCGCTTTGGG
>dme_circ_0004836_junction_seq
TGTGGAGTTTAAGGACTCGCGCTTTGGGTCAACAATCTAGCGTTGCTCGCGTCGCT
>dme_circ_0004837
GGAAAGAGAGCGAAAATCGAGAGAACAATAGCAAAAGGATGTAATGCAACCGATGGAATAACTAGAGTAC
AAGTACAAAAATTGCATTGGAAATTTAAATTCGTGCGACAAAAGCGGACGATTGCAGAAATTTTCGAAAC
TTGGCAGCCATATTTACTACAAAAGCAACGCCGTTGTAAAAATACGTGACAAAACGCTTacacacacata

ctcacattcgtacacccacacacagttgcgctctctcacacacatacacacagttgcgctctctcacaca
taaacacacaAGCGTATCGAAAGACTGGTGCAGACAGAGAGAGACGCGAATGTGAATTAacaaacaaaca
aaaatattttgcgaaaaagacaaacacaaaaaGTAAAGCCAATAAAGTG
>dme_circ_0004837_junction_seq
aaacacaaaaaGTAAAGCCAATAAAGTGGGAAAGAGAGCGAAAATCGAGAGAACAA
>dme_circ_0004838
GTTATTCCATCGGTTGCATTACATCCTTTTGCTATTGTTCTCTCGATTTTCGCTCTCTTTCCCTCAACAT
GTTTTGCATTTGCATTTAATTCTGTTGTTGCTATTGCTGTTTTTGTTGCTGTGTCGTCGTTTTTGTTATT
GCTATAGTCGTTGTCGTTTTTGTTGTTT
>dme_circ_0004838_junction_seq
GCTATAGTCGTTGTCGTTTTTGTTGTTTGTTATTCCATCGGTTGCATTACATCCTT
>dme_circ_0004839
GCGAGTTTTGAGCAGTTGAAAAGCCCGTTAAATAGTGCAAAATAGTAGCTAATTTTAAGTGCAACGTGTA
AATAAAGGCAATTTAGATCGCGAAAAAGTTCGGTGAGCAAATAATAATTTGAGATTGGAAAGTGGAAAG
>dme_circ_0004839_junction_seq
TAATAATTTGAGATTGGAAAGTGGAAAGGCGAGTTTTGAGCAGTTGAAAAGCCCGT
>dme_circ_0004840
GCGAGTTTTGAGCAGTTGAAAAGCCCGTTAAATAGTGCAAAATAGTAGCTAATTTTAAGTGCAACGTGTA
AATAAAGGCAATTTAGATCGCGAAAAAGTTCGGTGAGCAAATAATAATTTGAGATTGGAAAG
>dme_circ_0004840_junction_seq
GAGCAAATAATAATTTGAGATTGGAAAGGCGAGTTTTGAGCAGTTGAAAAGCCCGT
>dme_circ_0004841
GACATGACCGCGTTGAAGACCTGCTCCTCGGAGCGCACGTTTAGCTCGTCGCTGCAAATGATGTCCACCA
GCTGGCCGACGGGGAGCAGCAGAAACTCCTCGCTCTCCATCACCTCCTGGAAGTTGTGCTGCGTGAATTT
GTCGGCGATCCGTAGAAGTTCCCGGCAGGAGTGTGTGTCGGCAAAGGCGCGTATGCCCAGGCAGTTGGTG
GGATCCAATTGCCGTTTGAGGAACTCGCAGCAGATGTCCTGAATCTCAACCAGTTGAAGCAGGCAGGCGG
CGGGAAGGAGGGTCTGGACATTCGACTCCTCGACGATTATGTGGGCGGTG
>dme_circ_0004841_junction_seq
CGACTCCTCGACGATTATGTGGGCGGTGGACATGACCGCGTTGAAGACCTGCTCCT
>dme_circ_0004842
CAATGGCACGTGGACACCAGAAGATCCAGTCGCAGGCGAAGGCCTCCGAGAAGCAGGCCAAACTAAAGAA
GCAACAAGGACACAGTGCCAACGACCAGAAGAAGGCGGCCCAAAAGGCACTTGTCTATGTGTGCGCCGTT
TGCAAGGGTGAGCCAGCTACAGTGAAGCCTCTGTAAAGCGAACAAACTAGAGTTTGAAAGTGCCTATTGC
ATTTCATCCAGAAGGTTAACTTCTGTATGATGGAGTTATTAGTCAACTCAAATAATAACAAAATGTAGTG
CTCCTTAAAACTATCATAATCAGAAACACTTTTACTTGTGATATTGTTTTAACACTAGTTTCCGCTCTTA
CCCATGTTTTTGAGTGTATaaaaaaaaaGTATTTCAATTTATATGCACTTCCTACTTTATTAACATTAAC
TGCTTTTACTTGTTGTCGTAAGTAATTCAAATTAATTTAATTGAACATGAATATTTACCAATATCAAATG
AATATGTTTGTCCAACGTTATCTTAAATTAAGTGATGACCAAGCACTGCTGATATTTGATTACTTTAAAT
GATAACATATTGATATACGATCACTGTTTTCAAGTTGCTATATTTGCACCCCTAGTTCCTTTATGTATGT
GGATAACAATTTGGGTGAAAACCTAATCGAATCTCGCCTCCATTTCAGTTCGCAAATGCCCGATCCCAAG
ACGTATAAACAGCACTTCGAGAACAAGCATCCCAAGAACGACATACCCGAGGAGCTGAAGGAGGTCTGAT
GATCGTTTCACTTTAAGCTGAACGAGATGGACGAGTTGCCTACCAGTACGAAACATTTGCAACAATAAAT
TTGCATCAGCCCTAAGAAGAAAGCCCACAATCAAGCCGAAGCCAAGAGGATGGATAATAGTCGTAATAAT
AATATACAAATGCCAGTTTTTTCGTATACACACTGCTGAATTTTGGTTTTGTTTTATACATTGAAATTAA

CACAGTATGCGATTGTTCGATGTGTGGCTAAACCAATACAAAGACACCTACCTACCTTAAAAAATGAATA
GAAATTGTTTAATTTTGATATTTATTGCCTAAGCCACAGGAAGATATCTAAAACACCATAAATACTCTGA
GCTCCGTTTTGGATTTTTGTTTACACGTGAAAGTTTTATACGAATTATGCCACAATATAGTTCAGCTCTG
GTCCCATTTAATCCTGGCAGCGAAACAAAACCCGACAAATTGATACAAATTTACGGTTAATCGTTAATAA
TTTCGGGTAAAAAATCAAAACAGTTTGTAACAACGAATTTTACGTAACTGCAAAGGCTGCATCACACCTG
CATTTAGTACAGCCAACCAAATTTAATCGGGTTAATTAGG
>dme_circ_0004842_junction_seq
CCAACCAAATTTAATCGGGTTAATTAGGCAATGGCACGTGGACACCAGAAGATCCA
>dme_circ_0004843
CAAGCAATGGCACGTGGACACCAGAAGATCCAGTCGCAGGCGAAGGCCTCCGAGAAGCAGGCCAAACTAA
AGAAGCAACAAGGACACAGTGCCAACGACCAGAAGAAGGCGGCCCAAAAGGCACTTGTCTATGTGTGCGC
CGTTTGCAAGGGTGAGCCAGCTACAGTGAAGCCTCTGTAAAGCGAACAAACTAGAGTTTGAAAGTGCCTA
TTGCATTTCATCCAGAAGGTTAACTTCTGTATGATGGAGTTATTAGTCAACTCAAATAATAACAAAATGT
AGTGCTCCTTAAAACTATCATAATCAGAAACACTTTTACTTGTGATATTGTTTTAACACTAGTTTCCGCT
CTTACCCATGTTTTTGAGTGTATaaaaaaaaaGTATTTCAATTTATATGCACTTCCTACTTTATTAACAT
TAACTGCTTTTACTTGTTGTCGTAAGTAATTCAAATTAATTTAATTGAACATGAATATTTACCAATATCA
AATGAATATGTTTGTCCAACGTTATCTTAAATTAAGTGATGACCAAGCACTGCTGATATTTGATTACTTT
AAATGATAACATATTGATATACGATCACTGTTTTCAAGTTGCTATATTTGCACCCCTAGTTCCTTTATGT
ATGTGGATAACAATTTGGGTGAAAACCTAATCGAATCTCGCCTCCATTTCAGTTCGCAAATGCCCGATCC
CAAGACGTATAAACAGCACTTCGAGAACAAGCATCCCAAGAACGACATACCCGAGGAGCTGAAGGAGGTC
TGATGATCGTTTCACTTTAAGCTGAACGAGATGGACGAGTTGCCTACCAGTACGAAACATTTGCAACAAT
AAATTTGCATCAGCCCTAAGAAGAAAGCCCACAATCAAGCCGAAGCCAAGAGGATGGATAATAGTCGTAA
TAATAATATACAAATGCCAGTTTTTTCGTATACACACTGCTGAATTTTGGTTTTGTTTTATACATTGAAA
TTAACACAGTATGCGATTGTTCGATGTGTGGCTAAACCAATACAAAGACACCTACCTACCTTAAAAAATG
AATAGAAATTGTTTAATTTTGATATTTATTGCCTAAGCCACAGGAAGATATCTAAAACACCATAAATACT
CTGAGCTCCGTTTTGGATTTTTGTTTACACGTGAAAGTTTTATACGAATTATGCCACAATATAGTTCAGC
TCTGGTCCCATTTAATCCTGGCAGCGAAACAAAACCCGACAAATTGATACAAATTTACGGTTAATCGTTA
ATAATTTCGGGTAAAAAATCAAAACAGTTTGTAACAACGAATTTTACGTAACTGCAAAGGCTGCATCACA
CCTGCATTTAGTACAGCCAACCAAATTTAATCGGGTTAATTAGG
>dme_circ_0004843_junction_seq
CCAACCAAATTTAATCGGGTTAATTAGGCAAGCAATGGCACGTGGACACCAGAAGA
>dme_circ_0004844
GTTACAAATAATTGCAATTACACCAGCATACTGGCATTATTATCGCAAAACTCGTAAATATTATCAACAA
ACTGCATTTCCTGCGTATTTTTAGCAACTTGAGGGGTATTTTCATTTTCCTGTAAGGGATGTGTTATTTT
TGCTGCGCCTTTTTTACAGGCGGCATCTGCCTCCGATTCCTCCAACGTCAGACGCTTTAAGTTATTAATC
AGCGATATATCATGTTTAATTTCATTTTTCAATTCGAGAAGTTCGTGTTCAGTTTTAATAAT
>dme_circ_0004844_junction_seq
CGAGAAGTTCGTGTTCAGTTTTAATAATGTTACAAATAATTGCAATTACACCAGCA
>dme_circ_0004845
GATCTGTTTGTAAATATATAACATCTATGTATATATGTGTGTATAAACAGGTTTGCATCATTTCGACTAC
TCAAATAGGTAAAGCCTTAAAGCTGTTTGGTTTGCGTTTCATATTTCATAGTTATAGGCTAG
>dme_circ_0004845_junction_seq
CGTTTCATATTTCATAGTTATAGGCTAGGATCTGTTTGTAAATATATAACATCTAT
>dme_circ_0004846

GCGTGACAACAATGCTGAATTTCTTCACTACCTCGAACGGTTTCCGGAGCACTCTGCCGGTTGTTTCTAA
TCTGACGGCGATGAACGTGTGGGACGGCGTGTGTATGTGCTTCATCTACGCCTCCTTGCTGGAATTCGTC
TGCGTCAATTATGTGGGGCGAAAGCGACCCCTGCACAACGTCGTCTATCGGCCCGGCGAGAATCCCGTAA
CACAGGGTAAATTAAATAATCATTTAATCCGATTCACACCAGGCAGCCCCGACCCAAGAAAAAAGCTAAT
TTCGTATGCATTTTGAAAGCAATTAAATATGAATTTTACATGAGCGTGCAAAGCCAACaaaaaaaaTACG
TATCTACATATATAGTATTATATGTATAAATAAAACCAAGCAAATGATATAAAAAAGAAAACACACAGCG
AACACAACTATCAACATTCGTACTATATAAGTTATTCAAGTTATTTGTTCTTCCTTTGTTTAAGAAGCCA
GCAACACCTGAGCCCAAGTCATGAAAACAAACAGaaaaaaaaaaaatatatatatatatatataAAAAAC
TCAAACGAAAACAATTCGATATCCTCCATTGTGTTGCGTCTCCctctttctctctgtctcgctctatatc
cgtctatatctcttctttcctctatatctgcGTACACCCAATTTTGATCACATAACCGTGTACTGTCCAG
TCTTATTTAATGGTCCTTAGTTAATAGAACCATTGCCATATCTACATTATCAACTACCAGATGCTCGATA
TAAAAACTCACTTTGTGTTAATCCCGAAACTTAGCATTTTGTATAACAACCTTTTGAAACTATAGTAAAC
GTAACTTTGTGTGCCCTAAGAATAATGAAATAACTCAAATGAACATTTGATTTTTGTCCACACACTTCTG
ATTTGAATTTTTGTTTTTTGTCCTATATGAAGATACTGTCGATGTATGTGTATTTATTGTGCCGATGCTC
AGTTGTATTATTTGCATTTGCATTTATTAATCCAATGAAGTGACATTTACCCCGAGAAATGCCCACAGAC
AATTCTTCCTCTTTGATAGAAATCTTAACATTCATCTTCTGTTATGTTTCGATTATTTTCTAAATGTTGT
ATTATACAATCGTTCGTAAGGAAAACAATGCGTTTTATTCATCGCCAATTAGTTTTAAAATAACTTAACA
AAACCCAACCATAAAATGAATAATAATAAAATAATTGAAGTATTTTACATGCCGTAGGTAGGTATGTTAT
TTATGTTTTCATTGTTTTCTAACGcaaatgaaacaacaaaaactaaaattgattatacaaaataaaacca
ataaaatATGCATTTTGTTTTTACGTAGGCACTTTTCGAATTAAGATCAAATCGTTTAACAATGCACGTT
AAGCATTTTTGTTTGATGTGATTTACGAATATGTAATCCTATTTATGGGAATTCATTTAAGTGGCATGTT
ACGTAACGAGATTAAAAACTAAAATCATTTTAAAGAGCTCATGAATCAACAAAGCTGTTGATGCCACTTA
AATAAATGCCGCATTTTAGTCCCATGCATTATACAATTACCCCATTTTTATCCAAGAGAGTCGCAAGACA
AAAAGCGTGAACGACAAAAAAAGAATCGAACATTGTTCCCAATTTCAGTGATACGCAACTATTCGAAACT
ATAACCTTCAGGCAAATTAAACTTACTCTCAATAAATAAATTAGCAGCTAAGTGAGAACTGAGCAAGCGA
TAGCaaacagaaaaaaccagaaaccagaaaaccaaaaaaccaaaacttagcccaaccaaaaTGACAACCA
AATCAAACTTAAAGTGTGTTTTGAGTATGAACATTGTTTTGTGTACATTTTTGCAGCGTCTTCCAGCAGT
ACTAAGCAGGATCGGAGTAATTCTTGCAAGTCCTTTGGTAAGCTATCAACCACAACTCATGCACCACCCC
CGCCCCGCCCCCTGAGAAAACAGAAAACTTTAACAAAACCATTTAGCGGCAAACGGCAGCAAACCAAAAA
ATAAAACCAACTTAacacacacacacacacacacaATCCAAGTAAAATACACACCCACACTGGTACACCC
ACCGAAAAGTCCTTTATACCCCCTTCTCCTGGCCaaaaacagcgaaaagtaaaaataagaaaaaaTGGCA
TGCGAATTTCGCTTTGATGAAATGCATTGAAAAAGTTTTGTTTCGATTTTGGTTTTTGTGTTTCCCAAAA
ACAAAAACAGTGTTTAATTTGATTTTCGAGCCAGAACGGAAAACCAATGAAAATAACAATAACTATAATT
CAAATTTAAATAATTACCTAGTAGATTCGCACTCGCTTTCAATGTATATAACTTCATATTTATTACGACG
ATTATTTTCATAATTTAATTATTTTAATTCGTTTTCAATCATAAACAAACTCGTTATGTACTTATACACC
TAACAGCCACTCGTGCGTATACTTAATATGCGTGGCTTAATTGTTTTTGAAGAATCTATTTCCACACGAC
CAACAGCAGAAACGACAACTACAACCACAACAAATAAATACTCATGATGaaaatgaaaaaaaaaagaaaa
TTATATCTATATCAATAAAACACACTTTAAGGTCACTTACAATTGGGAAAAATGTTACGAATGAAAATTG
TAACaaaaaagcgaaaaaaaaattattaccaattatggaaaaaaattCTTTCTCCTTTTGCATATATGACA
AAATTATATCGAAAGAGTTATAAATCAATTCAGTTATTTGCCCCTGATTTACATTTTAATTTTTGTATAT
GAAATTCGCCTTGAAAAAATATACATTTAAATTAAGTTCTTTATTGATTTTCTGACACGCCCTCTACCAC
TAAAAAAATAAACAAATATTTCGTTGGCTTACTTAAATAAAAATATGGCGATATATCGTGCCGGAAATGT
TGGCCACTATTATTTATGGTACTAAAAAGTAGTAGCTGAAGCATTTTTGTTCTTTGGCTATTTTCTGTTG
TCAGTGTTTGCTTGAAAGCTTTTTGTTTAGGCGCTTAGTTTACTGCATTTTGTGCATTTGAATGTTTTTC

AATAAATATaaaaaaaaaaCACTAATGCAATCTACATACGAGACACCAAAAACATCGAATGTGTATAGCT
TGGAACGAAGAAGCTAAAAACAATATAAATATGGATTATATCTGTTTTTCTCATAGGCGGGTAATATTTA
TACATTTAGGTATAATAAAAATTTAATGACTGGAAATCATATTGAAAATTAACGAGGTCGCTTTAAAAAA
CTTTAAGGACCTTCTGCTTGAATCTAACAGCAACATGAAGTGAAACAAGAGTCCATTATCTACTGGAACG
ATTTAACCAACTTAACTTACCATTTCACTACAACTGCTTAATTCCAAGTAACCTCTCCTGTTAATGCCCA
TCAATTAACACAATTACTTTTGTGGCCTTCGATTATCCTGACTAAAACCACCTATGTGTCTCTCTCCGAA
AATTGTCTTTCTCTTTCCCGCGTTCAAAAACCAAACACTATAATGAAACCAATGCTAGAACCAATCCAGA
ACAAATAAAAAACAAACCAAGACAAAAACTATCCCCATTCAACCAACCTGCTCTGTAGGTAATAATCGAA
CGCGAATTTCATGCCGCTTTTGTCCTCCGAAAAGGCAAGCGCCGCTGCCACGCCGGCCATGTCGATGATG
GGCGGGGCCACGCCCATGTCCAGTGGTACCCCGGCCGCCTCATCCTCGGTGGCCACGCCCGGTACCCCAA
GGATCACCGATCCGGAGGAGTTCTTCGGCGGCGGCATGCGTTGGCAGGAGGCCCATGGCGGAATCATTGG
AGCTGCTGTCCAGAATTTACGGGCACGTTCGATAAAACGTAAGGCGGCCAGAAGTCCATCCACTTCCATA
TCGCCCCATCCCAGTGGGCGACCCACTGAGCATATCTACGAAGAGCCCGGTGGATCCACATCCGGTGTCA
CAAGTACGAAAGTAAAGTTCAAGGACGAGCTGAAGGAGGAGCAACCGGAGGAGGCGGAGTCCAGCACCAA
ACTACGACGATCGGTGGCCACCAGTACACCGGCTTTGGTTGTAAGGATCGAGGCTGAGATCGATCCGGAG
GCGGAAGACAAGCCCATCCAGGATATGGAAATGACAGAACGTCAGTTGTCCTTGCCGTTGAAACCGCCGC
GTCGCAAGGCATCGCGCTCCAATTCACCAGCCTCGGTTTACAGCGATGCATTCGAGAATGTGGAAGCTAC
AG
>dme_circ_0004846_junction_seq
GATGCATTCGAGAATGTGGAAGCTACAGGCGTGACAACAATGCTGAATTTCTTCAC
>dme_circ_0004847
GTAATTACAGTTGCCTACAGGTCGAGTTGACATTTACCCGTGATCGTGCGTATTATTTTACAACCGTTTT
CATACCTGGCATTATATTGGTCACCTCGTCGTTTATCACATTTTGGCTGGAATGGAATGCAGTCCCAGCC
CGGGTTATGATCGGTACGTaaaaaaaatgactaaaatcaaattgaaaaCTTTCGAAAGAAGTTCCAAGTT
CTCAATAAAGCAAACAGAAAACCCAACAAAAATCGAAGTCTTCCAAACCAATGAAAAAACAGTCCAAATC
ACATTTTAGTCCTTAACGAAACGTAGCAGTCCAGTAAGTTTTAGTCCAAATTTAAACGCAACCACGAGAC
AATTGGCCAAATCTCAATGATAATTTAGAAGTTTGTTAGCTTAGAACAGCTTAGGAACATTTAACTCATT
AAAAGTTCAAGTTTAGCTAGCCGCTCGATTTAAATGGATTCCTTTTTTTACATTTCGATCCATTACTTGG
ACTGATTTTACCAATGTTAATTTTCTCATTTTGTATTTTCTACTTCCTGCACTCGTATTTGGTAAACGCC
CAACTCCTTTGAGTCCTTGTGCATTGCATGACTTCTTAACTTAAACTAGGCAATTTTTGGCTTTTTTACT
TTGAGAAGCTTTGGTAAATCCATATACGAAAATCAACTGAAGCATACATAAATCATGCTTAAACTTAAAA
TGACTACTCCAATTCATGTTCAATTAGATTGCAACACACATACATTTGAATAGAAAATGTTCCACTACTC
CGaataacaaacaaaataatggtattaataaccaaaaataaaataaattaaaaatataaaaaattatgat
attataaacagtaaaaCTTATTTGTAATAACAAAACGGAAGATAAGGTAATAAAAAAATGTATTATCGTG
GTTTTAAGAGCAGGAAATAATATATTCCATATGTAGTATATTGTTCTCAACCGTTCACAAAGTGAGGCAA
GGATAATGCGATTACTATGTCCTAGTCCTGGCGAAATGTGGGCAAGGGAAACCGAAATGAAAATAATGAG
AGCGGCAAAGGGAATGTCAGCCGAAACATCAAGATCCTACGTCGTTGGCAGCTGCATCGGGACAGGAGAC
ACAGAAACACGGACACGGCCGTATCCTGGCATTTTACACGGCTTGTGGCACACTCCACATCCCTGGGATC
ATGATGATGATGACGTGGAGCGGTGGTCACATCATACACGATGATTTATGCATAAGCCATATGGTCAGCA
TGGACTAGGCAAATAGAACAGAAAGCGCTCTGTGCGTGTAATGCAAAAACTACAAAGTTACGGCAAGGAT
GGAAAACTTGCAGGCGGCGTTAGAAAAGCTTAGTTCTTGACTTGACTTTAAAATATAGTAAAATATATAT
ACTAAAGCCATGAAAATTTGTTCAACATCATTGAATACTATCAGTTTTAAAGTACAAACCTATATTGAAA
ATGACATTCGCGCTTTTAAAGATAAAACCATTTGAATTTAATATCTTAACTTCTTTGATTCTAAAAATAT
TCATGTGTGTGAATAGTCCAAAGTTTATAACCAGAAAATTGCATTCAATTTCAATTTAAGTCGTCTTTAA

ATTGCTGCAATGAGTTTTGAACAATTTTCCAGTAATAATGCTCTTGAATCGTTCGATGACAAAACGACGA
CATGGCACTCAAATAGCATTGTGGAAATCGAAGACGGGGAAGGATAATTCCATTTCTGGCCGTCATTGTC
TGCGCAAGCCAAACACAATCAAACGACATTTCAATGACAAAAGGAAACTAAATAAATAAATAGTCAAAAA
ACGGCGAAATGGCTCCAAGGCAAGCGATGCCGTTTTGTTAGTCAAAGTGCTGACATAGCATGCATTTTAC
AGCAGCTCCTTCTTAGCATGGCAGCATCCTTTTTGCCTCATTTGTGACCCGCTCGCAGCTCGAGCTTTTC
CTCGAAGCATTTGCAGTTGAGTGCAGCGTTGAGATTTTCCTCCGATAtttttctttttttgatttttttgGT
AAGAGTAGGGCGGATAGCAGAACGCTGGCAAATTGAAAATCAATGTGGTTCGTGGATGCAGCGATGCGAA
TGCAGCAAGAGCTCCTTGCCACATGCCAAACTAGTTGGAGAATTGTTGACGCAACTAATTAGGCAACCAA
GATGGATAATGTTGCATATCGAGCTGTTTTTTTATGTCGCGCGTTCATGAGCTGAATAATTAATATTAAA
TGCTCCAGCATTACCTCTGATTAATTTTATTTGCGCTAAAATGGAGAAAATTCCAATGGAGTAAGTTGAT
TAATGAAGCAAGGAACTTAGTGATTTGCAGCACAATGCTGTGTTAATGGATTTACATAATTTAACACATG
ACTTTGATTTATAACATTTAATGCGTAATCAAATATTATTTATTGCTCATTAGCCTTTAGGTTTATCACA
ACGAAATAATTATTATGAATTAACTTATTAACTTTATTAATCAGAACCATCAATTATAAAGTGCAAAAAT
GTGAATTAATTGATTACCATTACGCAAGTTATTGCATACAATTATTGTAACTATTTGATTAGTTCTGTTC
GTatattttaaaaatataaatatattttgaaacaaaatataaaacataataatataaataattaaacata
acaaatacataaatGCTTTCTAGATAAAAGCGTTTATTGAACCAATAGACGAACTTAAAATAATAAATCA
CATGGAACAATACTCTGAAAAATTAACATTGATAAAACTACAGAAACCTATTACTGCATTACATTTTCGC
ATATATTAGAACTACTGCTCCTTATCAAATAAGTAGAATTTAATAATTTTCTTTAGCTTCTGCACAGTTT
ATAAATCACAAATTAAAAGCAAACTGATAAACCGAACACCATGTGATATGATAACTACCTTTTTACTCTC
CGTAAACCGGacttaaaactaaaaactaaaaactTTTGTACATATTCCAAAACCAATGAAACAACTCAAT
TAATTGCCACATAAAATATATTCTACATACGTACATCCCTTCGTCCTTGACACCCGTTTGTCCCCAGCCC
CAAAGTATGCAATGCTTTTCTAATTACGTGTCAAACGGCTAATACAACTTTCTCTACAATTACCATCCAC
AATCGGCAACTTTAAACGACTCGAAACAACTTGGAACAACTTTTGACAACCGGAATGAAGGGCGTGACAA
CAATGCTGAATTTCTTCACTACCTCGAACGGTTTCCGGAGCACTCTGCCGGTTGTTTCTAATCTGACGGC
GATGAACGTGTGGGACGGCGTGTGTATGTGCTTCATCTACGCCTCCTTGCTGGAATTCGTCTGCGTCAAT
TATGTGGGGCGAAAGCGACCCCTGCACAACGTCGTCTATCGGCCCGGCGAGAATCCCGTAACACAGGGTA
AATTAAATAATCATTTAATCCGATTCACACCAGGCAGCCCCGACCCAAGAAAAAAGCTAATTTCGTATGC
ATTTTGAAAGCAATTAAATATGAATTTTACATGAGCGTGCAAAGCCAACaaaaaaaaaTACGTATCTACAT
ATATAGTATTATATGTATAAATAAAACCAAGCAAATGATATAAAAAAGAAAACACACAGCGAACACAACT
ATCAACATTCGTACTATATAAGTTATTCAAGTTATTTGTTCTTCCTTTGTTTAAGAAGCCAGCAACACCT
GAGCCCAAGTCATGAAAACAAACAGaaaaaaaaaaaatatatatatatatatataAAAAACTCAAACGAA
AACAATTCGATATCCTCCATTGTGTTGCGTCTCCctctttctctctgtctcgctctatatccgtctatat
ctcttctttcctctatatctgcGTACACCCAATTTTGATCACATAACCGTGTACTGTCCAGTCTTATTTA
ATGGTCCTTAGTTAATAGAACCATTGCCATATCTACATTATCAACTACCAGATGCTCGATATAAAAACTC
ACTTTGTGTTAATCCCGAAACTTAGCATTTTGTATAACAACCTTTTGAAACTATAGTAAACGTAACTTTG
TGTGCCCTAAGAATAATGAAATAACTCAAATGAACATTTGATTTTTGTCCACACACTTCTGATTTGAATT
TTTGTTTTTTGTCCTATATGAAGATACTGTCGATGTATGTGTATTTATTGTGCCGATGCTCAGTTGTATT
ATTTGCATTTGCATTTATTAATCCAATGAAGTGACATTTACCCCGAGAAATGCCCACAGACAATTCTTCC
TCTTTGATAGAAATCTTAACATTCATCTTCTGTTATGTTTCGATTATTTTCTAAATGTTGTATTATACAA
TCGTTCGTAAGGAAAACAATGCGTTTTATTCATCGCCAATTAGTTTTAAAATAACTTAACAAAACCCAAC
CATAAAATGAATAATAATAAAATAATTGAAGTATTTTACATGCCGTAGGTAGGTATGTTATTTATGTTTT
CATTGTTTTCTAACGcaaatgaaacaacaaaaactaaaattgattatacaaaataaaaccaataaaatAT
GCATTTTGTTTTTACGTAGGCACTTTTCGAATTAAGATCAAATCGTTTAACAATGCACGTTAAGCATTTT
TGTTTGATGTGATTTACGAATATGTAATCCTATTTATGGGAATTCATTTAAGTGGCATGTTACGTAACGA

GATTAAAAACTAAAATCATTTTAAAGAGCTCATGAATCAACAAAGCTGTTGATGCCACTTAAATAAATGC
CGCATTTTAGTCCCATGCATTATACAATTACCCCATTTTTATCCAAGAGAGTCGCAAGACAAAAAGCGTG
AACGACAAAAAAAGAATCGAACATTGTTCCCAATTTCAGTGATACGCAACTATTCGAAACTATAACCTTC
AGGCAAATTAAACTTACTCTCAATAAATAAATTAGCAGCTAAGTGAGAACTGAGCAAGCGATAGCaaaca
gaaaaaaccagaaaccagaaaaccaaaaaaccaaaacttagcccaaccaaaaTGACAACCAAATCAAACT
TAAAGTGTGTTTTGAGTATGAACATTGTTTTGTGTACATTTTTGCAGCGTCTTCCAGCAGTACTAAGCAG
GATCGGAGTAATTCTTGCAAGTCCTTTGG
>dme_circ_0004847_junction_seq
ATCGGAGTAATTCTTGCAAGTCCTTTGGGTAATTACAGTTGCCTACAGGTCGAGTT
>dme_circ_0004848
GCAGTATTTTGTGATGAAGTCATGTGGAAGTAGCAGTACAATTATAGGCAAAAGTTAATTAAACGCTGCA
TCATTTGCAAACTGCAAAAAGGAATTCTTAACAATCGCAATAAAACTTTTGTCGAACAAAAGTAATTAAA
GGACCTCACacccatactcaagcccatacccaatacccatactcaCTTGCAGCTATGCGACAACAAAGTT
GGTAGGCGTGGCAAGGGGGCAAGGCGGCGAGGAGGCGAGTGGGGCCAGGTGGAGCACAGGAGTACATGGC
CAGGAGTCAGTTGACGCTGCCAGCCGCAAACAGCTAACAGCTAACACCCAGGAGCCCCAAGCAGCGGGCG
CCAaggacgcagggcgcaggacgacaggacgtaggacgaAAACGAAGCGACGGCCAGCAAAATGCAGAAG
GAGAACAATGTCACGGCGGAGAATTGCCAATTTGTGGGGCCCTATCGCCTGGAGAAAACCCTTGGCAAGG
GTCAAACGGGTAAGTGCAGGAAAACTCTTTCCTTTTACGAGAAACTAAGTGGGTGGTGGTGGCTGCAACG
GGTGAAACTATCATTGCATTTTCTAGGCGGAGTGGCTATTCACTCGAGACTTATTTACTGTAATTGCACT
TGAGTCAGCTATAACTATGTGTTTGAAACCCTAAAGCAAGTTATTCAATAGTCATCATATTGGACATAAA
AATAATAATTCAATAATTGAAATGAAATCAAATTGGCTGCAATGTTTGAAATTCAACAGCGCACGGTAAT
AAAACTGTTCAATAATCATTAAAGGCATTTAATTATATTAAATTTATGCAATCCGACAGTAGATAATTTA
ACAATTATATTAGTAAATTTTATATTAAATCTTTTCAGCCCAGTTAGACTATAATTGCAATTAAATTGTA
GTTTAGTCACTCATAAAGTTCACTTAATTAGCCATATTAATGGATTTACCTTCTTCTTACTAAAACTGAG
AAACCAATTAAGATATCTGCAATTCTCGGAAAAGTAATTGCAATTACGATTTCTAACTTATTCTTATTGT
TTCGCAGGTCTCGTCAAGTTGGGCGTGCATTGTGTGATTGGCAAGAAGGTTGCGATTAAAATAATCAATC
GCGAGAAACTCAGCGAATCGGTGCTAATGAAGG
>dme_circ_0004848_junction_seq
AAACTCAGCGAATCGGTGCTAATGAAGGGCAGTATTTTGTGATGAAGTCATGTGGA
>dme_circ_0004849
GCACGGGCACCTTTGGACGAGTTTGCTTATGTCGCGATCGCATTTCGGAGAAGTACTGTGCCATGAAGAT
TCTGGCCATGACCGAAGTCATTCGTCTCAAACAGATTGAGCACGTCAAGAACGAGCGGAATATATTGCGC
GAAATACGACATCCGTTTGTCATCAGCCTGCCAACTTCTATGCGGCTGAGATCGTCAGTGCCCTAGAATA
CCTGCACTCACTGCAAATTGTTTATCGCGATCTGAAGCCAGAGAATCTGCTGATCAATCGGGATGGTCAT
TTGAAGATAACCGATTTTGGATTTGCCAAAAAGCTGAGGGATCGCACTTGGACATTGTGTGGAACGCCCG
AGTATATAGCACCTGAGATAATTCAGTCCAAGGGTCATAATAAAGCCGTAGATTGGTGGGCATTGGGGAG
TTCTCATCTACGAAATGCTCGTGGGATACCCACCATTCTACGATGAACAGCCCTTTGGGATCTACGAGAA
AATATTGAGCGGTAAAATTGAGTGGGAGCGACATATGGATCCCATTGCCAAAGACTTGATCAAAAAGCTG
CTGGTGAATGATAGAACCAAACGACTGGGCAACATGAAGGGTGAGAATCATAGATTCAGAATTTTTAGAT
CTATAAGCTTAAAAAAGTCTCAAAGATCTAACATGGGAATGGAATAGATAAACAAATAATTTGGAGCATA
AAGTTTGTTCAGCTGCCGTACTATCCATCAAGTCATTCGGtttttttttAATGAAAATTAAAATTAGCAC
TGTCAAAGTTTATTTATTTGTTTTCACCCCTTCGATCTCCTTTACTCTTGCCTTAGATAGTCTCATAAAA
ATACTTGGCAAGGCTTTCGTTAACATTTTCCGGCTTATTAGGCAGCATATTTATAGAGAATTGTTTACAC
TTGTCGCGGTCAGATTTTGTTGCTGGCATTAAAAATTGCATCGCATATATCAAAAATTAATTGTTGCCTA

TCACAAATAGATCTGCACATGCTCGTTGTCAGTTAGCCTAATACATTTTCTTCGAAATCATTTTTCACCT
TCCCCCGAATTTTCGTTTTTGTCGCCATAACTTTGCTTTTATTTTATTAGATTTGTCGCGCGtttttttt
tttttttttGCAACACGAGCTAGCCAAATGCAAAACGTTATTTATGTCCAATATTTTGTATTTCACTCCA
CAGAATGGCGCCGATGACGTGAAGAGGCATCGCTGGTTCAAGCACTTGAATTGGAATGACGTCTACAGCA
AGAAACTTAAGG
>dme_circ_0004849_junction_seq
AATGACGTCTACAGCAAGAAACTTAAGGGCACGGGCACCTTTGGACGAGTTTGCTT
>dme_circ_0004850
GCACGGGCACCTTTGGACGAGTTTGCTTATGTCGCGATCGCATTTCGGAGAAGTACTGTGCCATGAAGAT
TCTGGCCATGACCGAAGTCATTCGTCTCAAACAGATTGAGCACGTCAAGAACGAGCGGAATATATTGCGC
GAAATACGACATCCGTTTGTCATCAGCCTGCCAACTTCTATGCGGCTGAGATCGTCAGTGCCCTAGAATA
CCTGCACTCACTGCAAATTGTTTATCGCGATCTGAAGCCAGAGAATCTGCTGATCAATCGGGATGGTCAT
TTGAAGATAACCGATTTTGGATTTGCCAAAAAGCTGAGGGATCGCACTTGGACATTGTGTGGAACGCCCG
AGTATATAGCACCTGAGATAATTCAGTCCAAGGGTCATAATAAAGCCGTAGATTGGTGGGCATTGGGGAG
TTCTCATCTACGAAATGCTCGTGGGATACCCACCATTCTACGATGAACAGCCCTTTGGGATCTACGAGAA
AATATTGAGCGGTAAAATTGAGTGGGAGCGACATATGGATCCCATTGCCAAAGACTTGATCAAAAAGCTG
CTGGTGAATGATAGAACCAAACGACTGGGCAACATGAAGG
>dme_circ_0004850_junction_seq
AGAACCAAACGACTGGGCAACATGAAGGGCACGGGCACCTTTGGACGAGTTTGCTT
>dme_circ_0004851
GGTAACTGGAAGAGATAAGGATTACACCGACTTCCTAAGAGAACGCAAGCAGTGCACATAGTCGTATTCT
GAATCTCGCCGACCCACTCTCGAGTATCGATTTAGCCTTCGCTATTTTCCCACTTGCTATTTAAGTTGGC
AACCGTTGACAGTTGTTAGCTGCAGCGGGAGACGCGGGGGCGGGCCTTCAGCATGTGGCCCTAGACAATA
GCAACAACCATAAAACGCAACAAAAAAA
>dme_circ_0004851_junction_seq
GCAACAACCATAAAACGCAACAAAAAAAGGTAACTGGAAGAGATAAGGATTACACC
>dme_circ_0004852
GAAAAGCGACAGGAGACAACGGGACGACAGGAACTCCGGCAAGGACAATCGGAAAACCGCAGGCGAGGAT
TGCGACAGCGATGTCCACGGCGACTTGTGCCCGCTTCTGCACGCCTTTGTCATCCGGCACGGCAGGATCC
ACATCAAAATTGACAACCGGAAACGGAAGCGGCAACACGATGACGTCGGCGTACAAAATTCCATCAAACA
ACAGCACAACAGCAAATGACAGCAGCAACACAGAGACAACATTCACATTCAAATTGGGCCGCTCGAACGG
CCGAAGTAGCAGCAATGTAGCCTCAAGCGAGTCATCCGATCCACTGGAGTCCGATTACAGCGAGGAGGAT
CCGGAGCAGGAGCAGCAGCGACCCGATCCGGCGACCAACAGCCGGAGCAGCAGCACCGCCACCACCACCA
CCACCAGTTCCGCTGACCATGACAATGACGTCGACGAGGAGGATGAGGAGGACGACGAGAACGAGGGGGA
GGGCAACGGCCGGGATGCGGATGACGCCACTCACGATTCCAGCGAGAGCATCGAGGAGGACGATGGCAAT
GAAACCGATGACGAGGAGGACGACGACGAGTCGGAGGAGAGCAGCAGCGTCCAGACCGCGAAGGGAGTGC
GCAAATACCACTTGGACGACTACCAGATAATCAAAACAGTGGG
>dme_circ_0004852_junction_seq
ACGACTACCAGATAATCAAAACAGTGGGGAAAAGCGACAGGAGACAACGGGACGAC
>dme_circ_0004853
GTCGTCCAAGTGGTATTTGCGCACTCCCTTCGCGGTCTGGACGCTGCTGCTCTCCTCCGACTCGTCGTCG
TCCTCCTCGTCATCGGTTTCATTGCCATCGTCCTCCTCGATGCTCTCGCTGGAATCGTGAGTGGCGTCAT
CCGCATCCCGGCCGTTGCCCTCCCCCTCGTTCTCGTCGTCCTCCTCATCCTCCTCGTCGACGTCATTGTC
ATGGTCAGCGGAACTGGTGGTGGTGGTGGTGGCGGTGCTGCTGCTCCGGCTGTTGGTCGCCGGATCGGGT

CGCTGCTGCTCCTGCTCCGGATCCTCCTCGCTGTAATCGGACTCCAGTGGATCGGATGACTCGCTTGAGG
CTACATTGCTGCTACTTCGGCCGTTCGAGCGGCCCAATTTGAAT
>dme_circ_0004853_junction_seq
TCGGCCGTTCGAGCGGCCCAATTTGAATGTCGTCCAAGTGGTATTTGCGCACTCCC
>dme_circ_0004854
GCAGCTGACTATCAACGAACTGAAGCGCGAGCTGCAGCTATTCAAGGACTTTATGCGTGCCATGCGCGTC
TCCAATCCTGCGATGCGGGCCATAGCCGACCAGATGGAGCGCGACGAGGTGATCCGCTGGAGCAGCACAC
TGCCCCGGGCCAGGGTTACGCGCTGGGGGGGAATGATCTCTACGCCCGACGATGCACTGCAG
>dme_circ_0004854_junction_seq
GATCTCTACGCCCGACGATGCACTGCAGGCAGCTGACTATCAACGAACTGAAGCGC
>dme_circ_0004855
GTTTCAGTAGATCGGGAATGGATGAATCCTCGCTAAGCTGCAGGCAGCCCAGGCGTTTGGGGGAGGATTT
GCTCAAGATGTTCCTGCAGGAAATTGCCACGGATATGCTGGTGGAGGTGCACGGTCGGCGTATCCGTGCC
CACAAGTGCATCCTACGCTCCCGTTGTCA
>dme_circ_0004855_junction_seq
ACAAGTGCATCCTACGCTCCCGTTGTCAGTTTCAGTAGATCGGGAATGGATGAATC
>dme_circ_0004856
GCTACGCAAGAAGATTCAGACGCTGGCCCGTCTCACAGAACCGCTGGACATGAAGCGTCAAAACGGCAAG
CCAGTGGGTAAGGCCTTTTCCTTTGGCGGAAGCACACGCAGCCAGGACCTTGTGCAGATCAAGTTGGCCA
TTCAGGCGCAGACGAAGCGAGCCAATGCTCAAGAGACACTAAT
>dme_circ_0004856_junction_seq
AGCGAGCCAATGCTCAAGAGACACTAATGCTACGCAAGAAGATTCAGACGCTGGCC
>dme_circ_0004857
TAGTTCATCCAGTCCACTCGCTTGGGCTGCACCAATCCGAAGCAGCTGCACTCGAGGAAGTCCACGAGGT
TCTGGATGGGACCACGTGTGAACGGGCTGTGGCCGCCCTTGGCCTGGAAGTGGCGATACCGTCCCCGGTT
CATGCGCTCATTCGTTGTCATGCCCAAGCAAATCACCTGATATGTCTGGCAGATCGTCAACAGGATGACC
CAGGACATGTGCAGCAGGGCGTTACCCATTACCCAGCCCACCCAGGCATCGCAGTTGCCAATGGCCCGCA
TGGCACCGAGAAAG
>dme_circ_0004857_junction_seq
GCCAATGGCCCGCATGGCACCGAGAAAGTAGTTCATCCAGTCCACTCGCTTGGGCT
>dme_circ_0004858
TTCATCCAGTCCACTCGCTTGGGCTGCACCAATCCGAAGCAGCTGCACTCGAGGAAGTCCACGAGGTTCT
GGATGGGACCACGTGTGAACGGGCTGTGGCCGCCCTTGGCCTGGAAGTGGCGATACCGTCCCCGGTTCAT
GCGCTCATTCGTTGTCATGCCCAAGCAAATCACCTGATATG
>dme_circ_0004858_junction_seq
GTCATGCCCAAGCAAATCACCTGATATGTTCATCCAGTCCACTCGCTTGGGCTGCA
>dme_circ_0004859
GCGCACACAACAAAAGGAAACACCAAAAGTAAACTGTCAAAGTTTTAAGAGATTTGCACGGAGGTGTGTG
GGTTCTGAGCGAAGTTCGTCCTAGGCGGGCTGCTCATTGGAGCCTTATCTTATGGAGAAGTGTATAGTTC
CGAAAATTCAGGACTACGCAACCTTCACAGCCGACAGCTCTCTAGGATCGACTACTTCGCTTCGGGAACG
GCACGCAAAGACTTTTCAAATATCACTAATTAACGTGGCAAAAGCAGCGAAGACAACAATTTAATCGATT
GATCCCAAGCACTAGCATACTTAAATCAATAATGAATTAATTCTAAATGCGATCCAATTCTAATTATGCA
CTAGTCTAGTCAGAAAATTATTTAAGTAAAATGTTAAAGTGCAACTACAGCCACAAATTCGATAAATAAA
TTGAATATTTATATAAACCCATATAGCTATGAAATGATCTAATTATAATGTAAAAGGAATTTCAGCAACC

TGAGAACAAACCGCATAGCAATGAATAAAATATGTTGTAATTGTAACACGTTACTTAAATGCATTTAAAT
ATTAATTCAAACGAAGTCTACAAAACCATTTTAATACAAACAAACGCTAAGCAAACCTTTTCAACTATGT
ACTGTACGAAATATTGATAAAACAGAAAAAACACCACTTTTTCGACGTATTTTGTATTTGAGATTATACA
AACGGATGTGCGGATGAAGGATTTGCTATGAAGTTATATGTTTCAAATCCATTTTGTATATTCCTTTCGC
TCACACTGCTAGAAGATGAAAGCTTTCAAAAGACTATATAAACCGAACAAGATCTAACATCAAGCAAGCC
CCTACGAAGCATTGCGCATATTAACTTATGAACTAAGTGTTTTTGGCCTATTTAATAAAGCAAAAAAAGC
AAATCAGCCCCATTGCCTACATCTTTAGTTTAGCGACTAAGTTCAAGACAAATATATTTAACTAAAGATA
TATAAATGAATGTATTAAATAGTGTGTAGAACGATGGAAATTGTGAGCTCGACGAGGTGTATGTGTAT
>dme_circ_0004859_junction_seq
TTGTGAGCTCGACGAGGTGTATGTGTATGCGCACACAACAAAAGGAAACACCAAAA
>dme_circ_0004860
ATATTATCGTTCAGCCTTTGCTGCGGAGGTAGTGGACGACCGGAGAAGGTGTCCGTGGCAGCTGGAGCGA
TGGCAACAGTGGGCTTGCCACTGCCCGACGATCGTTTCATGGAAGCTGCAGCAGCGGCAGCTTGTGCCAA
TGCCTCCGGCCATAGCGTGGCCATGAACAACACCGTGGTTACTCCAGTGGCGGGCAGCAGCAATCAGTTG
AATAGCCAGAATAATCTCCTGGCCGCTGCAGCAGCCTCTTCGAATG
>dme_circ_0004860_junction_seq
CTGGCCGCTGCAGCAGCCTCTTCGAATGATATTATCGTTCAGCCTTTGCTGCGGAG
>dme_circ_0004861
GGTCAAATCAGCTCCATTGGCAGCCAAGAAGCATGCAATCGAAGCAGAGGCCTTCTTGTTATTAGCATTT
CTCAGGCCCATCAAGAGCTTACCAAATCCTTCCACATCTTGCAGTTGCTTCAGCTGCGACAGAGTGTGAT
GTCGCAACGCTTCATGCAGCGGAGTATCCCCGTCTTTGTCGGGTATGTTAAGATCTGCCCCATCCTGCAC
CAGCAGCTTGACGATTTGCACATGCTGACGCTCCACCGCAAGATGCAAAGCCGTCTGGAGATTCACATTT
TGCCTATCCATATTGGCCTTACCCATATGCACTAGTAGCTCAGCAATTTCAACGTGGTTGTTAAGTGCTG
CCAAGTGCAGAGCTGTGTAGCCATCATCCTTCTTCTCTTCCACAATCCAAGGACGGTTCGTCTTG
>dme_circ_0004861_junction_seq
TTCCACAATCCAAGGACGGTTCGTCTTGGGTCAAATCAGCTCCATTGGCAGCCAAG
>dme_circ_0004862
ACATCACTCTCATCGGAGGAGGCCGTTTCGGGGGATGTGGCTCCGTCCGTAGCTCCCACGGCAGCCACAC
GCATCTTCAACAAGATCGTCGAGGCGACAGCGGTGGCTACTCCCTCGACAAACAGCAGCGGCTCCACCTC
CATACCCGAGGAAAAGTTGTGCAAGATCTGCTACGGCGCCGAG
>dme_circ_0004862_junction_seq
GTTGTGCAAGATCTGCTACGGCGCCGAGACATCACTCTCATCGGAGGAGGCCGTTT
>dme_circ_0004863
ATAACAGAAAACGTGTTATTGTGTTTTTCCGTTCGTGTTCACGGAAAATCAATAGTGATTGTGAAAGCGC
ATTATTTACACGATTAACAAATCGTCAAATCTCAACGCAACGGAAATTTTCCAGTGCAAACTTGAGTTTT
TCAAGAAAAAGAGAAAAGCCGTCAAGGTGAGCCCCAGTTTTATAATCTAGTATGGTATTTTGTGTACCCC
AAGCGAAAAATAACCCCAAATTAACGAAAACGTGTCATCAGAAGGCAAAGGCAACAGCTAAAATATATTT
TGTTTGCTTTGCTTGTATCTTTACTCGCACACTCACACACACACTATATATCAGTaaaaaaaaaaCCACA
CAGGCGCACACGATCATACGAGTGCCCACAAGACAAAGTTGTCACATGTGGTTTAACTGCATTTACGTTT
CCATTTGTTATTAATAAAACGAGTATACATACTTTTGCATCAGTATACATACTTCTGCATACATAAATGT
AAATTACCAGTAAGGGGTTTAAATTTAAATTCCTTTTAAATCGAAAACGttttattatttttcgtcgggt
gtgcttcgttttcttttgtgtttttttgtgCTTGCATAAAAAGATGGCGACCGTAGAGTTGCTGAATACT
CGCTCTCTTATTTCATTTTAAATTATTTACGGAATTACAAACTTGTGTTTACCCAGTGAATTCGGCGAAT
TTTTCGGTAATGGTTAACAACAATTCAAAATGACACACGTTTGTTTGCACGTCTTTTCTGGCCATATGGT

AACCCTGTTTTCACATCGAGTGGTTTCATCCAAAATTCCGCACCCCGCACTTGCCCCTTGATCATCAATG
CTGCTTTAAAAACAACATACTCAATTGGATTGGATGATGTGGATGGAGAAAAATAAGGGGCGGTCATTGT
ACCATACAATGCTATAATTTTATATATTCGCCCAAAGTTGGGACTACGCAATACACAGTATTCGTCTACG
CTTAAATTAAGCGATGACTACATACATATAGCATCCAAAATATACCAGTTTAGCGGATTCGAAGGTCATT
TTATGTGTCATCCTACCATCAATAGAGAGCTTTATAATGTTCTAAATAAATTTAATTGTTTTCGAGAGGA
AAAATGCTATTATATTATTGTGAATCCTATAAACGAGTAGTTTGCTAAAACAAGTAAAACAACAGTTTAA
AATATAATTTCTTAACAGTATTTTTTACCGTGCATTCGCATAAACAGATGACGCGTCAGTTTTCTGGGTA
TATACACATACACATACAGTACTCCATTTGCTATATACGATTACAATGTGTGTAACGTACTTCCTCTTTT
ACGCACACACTCATAATCCGGCCCAACCCACCACCACCACCATCGTCATCGGAGGCACCACCAATTCCGG
ACTATGGAGCATTAGCTTCTTTGTGGAACATCCGCTGCCAACGGAAGTGGTGCACTCATGCGTGTTTTTC
ACTCTGCAGAAAAGGACAGAAAATAAGTCGAAAGACAACACATTACCATTGCCATAGAAACGGACTCTGA
AATGCGTTTGAGGAGCATTCATTTTTGCACACTGGTGACTCttttttttGCTTCATTACTTTCAACTTAT
GTGTTCGTTTTTCGCCGAACAAAGAGAAAGTCTCTCAGTAGCTCAATTCGAGCTGAGTCTGAGCTTCGGC
TTACAGCTCAATAAAGTCCGACTTTTGTAAATAGAGCTGGACTTCTCTTATCTGAACTTATGGTAACCAT
AGCAATCGCAATATCATGTTCTTGAAACCGTTGTATCAGTACTAAACTGGTGTTATATGCTCGTAGTTTT
TAATACTGCTCATAGGATTTTATTATTATTATTCCATTTTGATGTTAAGAAAATGTATTAATACTTTAAG
GTATAAATGTGTTTTAACCGAACGAAGAAACTTATTAAAAAAGCCTGAACAAGCTGTTGTTTCCTTGTAG
GGATTTCAAGTTTTGACCTGTTTTTCGATGGCGACGTCGCAGGGCAAACTTCTTCAACTTCCATTATCCC
CGCACCCACACATCATCGATGGTGGTCCCTGTGCCCACCTTTTCCCACTCCCACGAAACGGATTTCGAGA
GGAGTCGAGACAATTCGTTAATAAAACGATAGATAAGGTGCTTAAGACACAACAAACAGTTCTCACGTAG
ACAAATGCTGGGAGTAGTATTTTTTTCGAAAAGCAGAATAGGATCTATTGTTTGTTCGTCTGGGTTCCAT
TTGTTATCGCCAGCAGACGCAACGGCAATCAGCACATGAATGGAAATGGACCAGTCCACCGCGGCCCTTG
AAAGAGAATCCCGGAAAAGACCAGGCATAAGATGAGAATAATTACAATTCGGAATTCCCGGAATTTTCGA
TGCTCGCCCAGCTGAATCATCGCGTCGCCTCCACGACCTTTGAGCGTGGACCGCGTGAAAAACGACCTTT
CAACCATTATATCCACTAACGAGGGGACATCCCCCACCCCTCCCTCCCCTCTCCACTAACCACGCCTGAT
CTTCATCTCACTTCTTTTCCATGAGATCGTACCCACATTCCAAGTCCCATAGAGATGCTGCTGTGATGAT
AAATGCTTCGCCATCTCATTCGCACACGACTTGACTCTTTGGGTTTGAGAACAATTTTCCCTTGGTTTTC
CGAGGAAAAACGCTGGCGGCGTCGGCGGTTCTGGTACTTTGAACCAAGCCGCCGTTAGTTATTTCGACAA
TCGATCAATGTTCAATGGGAATgagagagagagagagagagGGATATATGGCTACGAGACGATCTTAATG
CATTCTTTAGCAGCTCGGATCGGGGCGCATTTATTATGATTACAATATTGCCCTTTGTTGGCCAGCATCC
CATCTCTGTTTTCTTCTCGTATTATCGACGTCGGCGGTCGTGGTCGTTGTCGTTGTGTTGTTGCTGCATT
TCTTGGAAGGCCACAGCACTCACGcacacacagacacacacacacacaGTTTGCTGGAGATCATGCAA
GAATGTTCCTTGCCTGTGCTGCATTTATTTATTTTTCTGCATCCTCCTTCTGTGTGCTCCGCTCTTCCCT
CTTCATCGTCCTCCTCATCGGATTCTTTCACTTCGGATTCTTTAGCATCATCATCTTCCTCTTCTTCACG
TTGTGAGCACGATGCGCTCCGCTTCTCCCCTTTTAACCCTAGCTTGCTGTTGTTGTTGTTGCCCCTACAG
TATCTGGTATCACCGCATTCCAATCTCGttttttttattttGTGGAAGGCAACAACAACAGTAAGAACTA
AAAGAATGGTAAAGTGTTTATAATAGTTTCCCACAGTCGAGCTCAAACAGCTGGGGTAAAAACTTTAGAA
CCAATCAATTTTCAGATAAAAAGGTATTTTTCAAGTATGCAGCAAACGAATCCTTACTTCGCTTACTCTG
ATGATAAGTTGTTTAATTAAAAATAGTAGTATTAAGAACTATTTCatatatatatatatactacagatat
ataaatttatacataATTCCGCAAACTTTGTAGGCTGTATACACTACGTGCAAGCCTATTAATTTGCCCT
TGTCTGTAGTTGCAGAACGCAGACCTTCTCCACGGCTTCGGACCTCTGCGGTTACCTTCACTTACTGCAA
CGATAGCAGCAGCATAACTCCAATCACTTGGGCCCCAGCTTTACTTACCGACTGGGAATGGAATGAAATG
GCCTGGCCTGGCTGACTTGGCTTGCGTTGGTTTGGCTGGGCTTATTCCAATCAGAAAACTCGAAAGGCAG
CTCCAACCTCGATGGCACTCCAAGCCAGTTTGATTTTCTGTGGCTGAGTTTTGTAGAAAACTTGCCCGAC

TGCATTCCATTGGAGGTTCgttggtttggttttggttttggtttggtcttcgtgttcgtttgtttttttt
ttttttttGAGTTTTCTGTTTGTTGCCTTGCCTTGCCTCCTGGGCCTAGCCTTTTGCTTTGGCCTTTGAT
TATGATTTTCTTTGCTGCGGAGTGGTATTTTCTTCTGGTCTCTGGGATCGAGCTCCCATGCCATTCATGC
GCCGCCCAGGAGGCCTTGTCTCTTCAGTCGGTCTTCGGTTGGTGTTGGGTGAAGGCGCTGCTCACGGTTT
ATCCATTGCTCGATATATATATAAATAGTGTAGTTCCTTGTCTGCCGCACAATAATTTTGTGGAATATAT
CAATTTTAAAGTTCATCGAGTTTTTTGTATTTTGATCGACACTGGTAAGTGTATGAAGTAGTAGTAACCT
GTATAATTGAGTATATGCAATTGTCTGGCTCTTGAACTTTTTACGGAATTTCCTGATAGCCCCGGTGAAA
TTCCAATAGTCGGTTTGCATTACAATTGTTAATGGGCTTGGCATTCCACAATAACAAATAGCAGACGACG
GCAGAGGCAGCTGTAATTGCACTTTGTAATGGCTGAAGAAGCAAAACAAACAATAATGATAATAAGCTGC
ACTAAAGACCGAAGACTCGAATACTCTCGCAAAAACAATTTTCACTTCGGTTTAATATGAAACTTTGTAA
AAGCGTAAATTAAAATATTATCATAATATCTTTCAAGTATTATGAAATTATCTGTACTAATGTGCGAGAT
ATTTGATTTTAAAGCTACAAGGTATTGCAGAATTGCAATGCCAACAAATTGCAATTACAATCTCAAGCTC
TTCGTGTGCGTTTCTGTCTGCTGCTGTCGGCTGCTCCACTTCAAAACAAAGCGCAATCTCTGCAGAGAAA
CAGACCCCCATTTATATAGCATCCCCCGATCTGGAAGCATCCTCAATAAGTATGTATGTATACACCGAGG
TCCAGAAGTCAGCATTTGTAAATTACAAttgcttttgttttttttttgtttttttttttttttgCTGCACTG
TATGTGTGTGTACTGTTTTTCCATCTTCTTGTTTTTGTGTTGCGCTTGCTGCTGCACTCTGTTTTCTCTG
TGGTTAGTTGTTTTTCCCATTGATGGTTTCGTCTTTGCACCATATGGCCGTCTCGCTTGCACCGCCAGGC
TACCAATATGCATTATATGCTGCGCTTTATGCTGCAAGTCGGGCTCCATGGTGGTGGAGCTGTGGAGCTG
TGCAACTACAGCCAGAGTCGACGAGTGCGCCGTCGCCAGAGTTTGACTGCATTCCATTAAAACTTTCTCA
CAGTCGGCTAGCTCGCAGTGGTGCTCCACAGTGCATGGCACTGCCAGCTAACCCACCGATGGCCCCCTGT
CCTCCTAAACCTCCACCGCCCGATCCGTTGCAATTGTTTGGCGTGTTTGGCTGGTTTGTGGTTGAAATGG
ACTTTGAACAGTCGGGAGTCCAACTCAGCGGTCGTGCTGAAtttttcttttttttttGGCCCATGCACAAG
CTCAGGCGAGTAAATGTACAATCTCCTTTAAAGAGACCACCGCGAATACAGTTTAGGTTCCCAGTCAGCA
GTCGATATGTTTCTTACGGTTTCAGAAGCTTTATAAAGTAATTTATTGTTTTTCGAATTTTGCGCTAATT
AAAAATATATCGATTGGCAATATCTGATCTAAAATTACGTCGTAAATATACATACATACTTGCACTGAAC
AGTAATCGATATATTGAACTATTTCTTAAACGCATACATGTACATATATGATTTTCCTGTCATTACTATA
ATTGCCAACACAGCAACAATCTACTTCAAGTTCTTGCTATTATTATTGAAAATATTTACCAGGTGAGTCA
GGTCGAGTATCGTTATCGAGCAACGTCTGGCTGGCGCCACGAACTTTTCTAAGAAAAAAAGGAAACACCA
CCACCCACTTAGCTTTGCCTTTGGTGGTGCTGTTTTGGATAGGATTAGGATTTGATACTAATACTCACTG
CGATTCTGCGCACTGGAAAAAGTAGCGGTTGTAACATTGCGTATTCGATGGCACTACGAATATTTGtttt
tttttttttttGTAGTCGCAACTCGGGAGTTTTTCCCGAAGGGAAAAACCACCCTTAGCTCGAGTGCGA
GCAGGACAACCGCTATGTTTTCCATCATATAAGGGCTCTTAAAGTTCGTTGATGCTGCATCCCCAGTTTC
TTGAACTGCGTTTCAGTTTTTCCCTGCATTGTACATACACACTCTGAAAGACGTAGAAGTCGTACTACAA
AGTGTCTGGAAGCTTTGTTTGTGAAAATTTTGGTCCTTTTTGCAGAAAAAAAGGTAAAAATAAGACCAAA
ATGTGGCTGATCGATTTAAATATAAAATCAACGATAACGAAAACTTGTTGAATTATTTTTAATGGAAATT
TTATTTAAAAAAGATAGGTATATTGTGCTGCCCTCTAATTTACTTGTTGTAATAAGGAAATAGGCGTGTT
TTAGGAAATTATGTAATTCACACAAAAATGTATAGGTTACTTAGGAATTTATTTTACTGTATTGATATCT
CCACTAACGACTGAAATCTTTGTTAAACACCCCCCCTTATCTGTGATATAAAAGTACGCTTGAATTTGTA
GTCCGTGACAGTTGACCATTATAAGCAGCATGAACCAGTATACAAATATTAACGGATTCTCTCTTTTTTA
TTCAGTTTCGACTTCAGAGGAAAGGAGCCAGAGAAGAAATTAAGAGTCAGGCAAACAAAGGAGCTAACAA
CCAGAACACAAAATGGCATCTGTTGTAGCTGATCTTCCG
>dme_circ_0004863_junction_seq
AATGGCATCTGTTGTAGCTGATCTTCCGATAACAGAAAACGTGTTATTGTGTTTTT
>dme_circ_0004864

GGCGTGCATAGACGACAATCTGGACATGGTGGAGTTTCTAGTGGAGCGAGGTGCCGACATAAACCGCCAG
GATAACGAGGGCTGGACGCCACTGCACGCCACTGCCTCCTGCGGGTGAGTTTTAAGTCCAAATTGCAATC
ACTTTCATTACTCATCTTGTGCTTAATTAATTTTCTAGATTTGTGAGCATAGCTCGGTATTTGGTGGAAA
ATGGCGCTGATGTGGCCGCTGTGAACAGCGATGGTGATTTGGCCTTGGATTTGGCCATCGATGTACAGCA
CATGGCCATGATTGACTACATGGAGAAGATGGTGCAGGAGCTGAACATCAACGTGGATGAGGCTCGAAAG
GCCGAGGAGCTGGCGATGCTGAACGATGCCAAAAAGTGGCTGAGAAGCGATGCCGCCGAGGTGGATAGAC
CGCACCCGAAAACGGGAGCCACAGCCCTCCACGTAGCCGCCGCAAAGGGTTACACGAAAGTGCTGGGTCT
GCTCCTGGCCGGACGTGGCAATGTGGATCGTCAGGATAACGATGGCTGGACGCCACTGCACGCGGCATCG
CATTGGGGTCAGCGGGAGACGGCCGAGATGCTCGTGGAGTCACTGGCCGACATGGATATACGAAACTATG
CCGGGCAGTCATGCATCGATGTGGCCGATCGCAAAATAGTCAAATTCCTGGAAGAACTGCGGGCCAACAA
ACGCAACAAGCGGCGACCATCTAGTCAAATAAG
>dme_circ_0004864_junction_seq
ACAAGCGGCGACCATCTAGTCAAATAAGGGCGTGCATAGACGACAATCTGGACATG
>dme_circ_0004865
GCTGAGAACGTGAAGCCCAACCAGCAGATCCACGCCGTCGAGCATCCAGTCGAGGAGGAGGCGCCATGGC
GACGCAAACTGCCCCGCACACCCAGCGATAGTCCCACTAATAATCGTAAGTCCTAACGGAGCTATGAAGT
GCGTTTTAGGTGTGGCTGTTTAGATGATATTTGAAAATGAAGTTAAATTGTTTCAAAAAGTAAAAACTAT
ATACGAATCTATTTCGAGATCATGTCGTGTCCTACAATGGTCATAGCTATCAGCAATCTCTTGAAGCGCA
CTGACTGGCAACGTTCGTAATCGAATAAGATAGTAATATTCAATATAATTACCATTTTGCAGAAGTTCCT
GATAGAGAGCTCGGCACCAATAGCTCGGAGACCGCCAACGACGTCATTTTGCGGCGCACGCAAAGCTTTG
AGAACGATCAAAA
>dme_circ_0004865_junction_seq
GCACGCAAAGCTTTGAGAACGATCAAAAGCTGAGAACGTGAAGCCCAACCAGCAGA
>dme_circ_0004866
GCTGAGAACGTGAAGCCCAACCAGCAGATCCACGCCGTCGAGCATCCAGTCGAGGAGGAGGCGCCATGGC
GACGCAAACTGCCCCGCACACCCAGCGATAGTCCCACTAATAATCGTAAGTCCTAACGGAGCTATGAAGT
GCGTTTTAGGTGTGGCTGTTTAGATGATATTTGAAAATGAAGTTAAATTGTTTCAAAAAGTAAAAACTAT
ATACGAATCTATTTCGAGATCATGTCGTGTCCTACAATGGTCATAGCTATCAGCAATCTCTTGAAGCGCA
CTGACTGGCAACGTTCGTAATCGAATAAGATAGTAATATTCAATATAATTACCATTTTGCAGAAGTTCCT
GATAGAGAGCTCGGCACCAATAGCTCGGAGACCGCCAACGACGTCATTTTGCGGCGCACGCAAAGCTTTG
AGAACGATCAAAAGTGAGTTGAATCCCAAGAAAATAAAACATTGCGTGCCGCTCACACACAAACACCCAT
TCGCATCTCAGATTAACCCACATATACAATGATTATGTGCTACAACGATCGGCCAAATTGCACCGAAAGG
TCTTGATTTGATTTATTGGTTGATTTTGCAGTTCGCTTTTCACTTTTGCCGATTGCATTTGCCTATCATT
ATCATCACACACACGTTCACGCTAAAATTGTTAACCACTCATTCAGCTAATCTCGAAATTCATGTACTGA
TAAATTGTTTAAATTTACCTATGATTGTATCATGGATCTGGGAAAGCTTTGAGAATCTTTATGAATCACA
AGCTTTGCGTTTATGAGCTTTTTCCCATAACACCACTCGCATTTTGGCAAAGTGAATCGTTGACTGCAAA
AGACAACAAATAAGGGAAAACAAAGTATTGTATTGattttaaaatttttgttttgctttattttttttC
CACCCAATTTTTTCCAAACAACAATCAAAAATCGATAAAAAAATAATTGCATTGCCAACGCACCACAACC
AACCAAAAACAACAAACTCATCAAAACGCTTATCCCGAAATCTAACCTGCCCGCTCGCCCACCTGCTCGC
TACGACCACCCGCCCGCCCACCTGTACGCCCGCCCGCCCGAACGTTTTGAAAAACGTTCGCAAACGCACG
CTCCTCGCTCGAAAACGGCCAAAATCGAAAATCCGAATGACAGGTTCTATCAAAAGTACAATGAGCTGCG
GGCACGCATAAAGGCCAATTCCTGTCCCATTTTGCCGGCGAATGCGAATGCTAATGCCGCTGCTGCCAAC
AAATCCAATAACAATAATAACCTAAGTAGCAATAACTATCATaataacaacaataacaacaaCAAAAGCG
ATCTGCTATTGGGATATGCTGCTGGCACcacaacgacaacaagcacccccacaacaacaacaacaacaac

atccagtacattcaacaacacccactcgaacaccaacaacacaagcacaacacaacaacaaccagcagca
gcagcagcagcatcggcagcaAATCAATTATATAGCGTACAAAGATCGGCCTCACTCAAAGATAACTCAA
TGTATTACAGGTTTGTTTTGCATTCAACACATCCCAAAAAAAGAACAGACTAGATCAGATTTGTTAGTTA
CGTTAAGTAAAAGTTGTTAGTGTAAAAAGGGAACTAGTATAGTATATATATTTCTATGCTTTATTTGAAT
TTTTAATGAGTTGTAAATGGGTATTTCAAGTAGAGTTGatataaataaaaatagaaatataaataaaaGG
TATTAAAGGCCACCTTTATACTCTAGTATAATTCAAATTGTTTAATGTATTTCTAAGGTACTTGTTAGAT
ACAATTATGTAGTATTTAATACTCAATAGGAGCTAATAAGTATTCGAATTTGTCCGTCCTTAAATCTGAT
CTAGTTTAAAAACCATGCCTACTCTCATAGCTGTTAACCCAAGCCTAATTTGTTGTAACTATTAACCTGA
CTGTGCTTTTGCTTAGCTTCTTCTCGTCTACACCTTCAGTATAATAACTCATTGCACCAAACTATTTAAC
ATATTTTCCTAGGCCACGCACCAATGTCGTCTACCTACTCTTGGCTGTATTCCCAGTTCAACTTTGAACC
CTAAAGTCTAAAGTTTTTCCTTTTTTGATTACCCCCAGGGTAAGTTTACTTGTGTAAAATATGATGTATA
TAACTCttttttttaaaaaaaaaaCTCCTATGTCGATCAATTAGCCAAGTTACAGCGAAAATAGCAGAAA
AGAGCAATAATGGCAGCTCGTCCACGGCCAGTGTCAGCGATGCGGAGAGCTCCAAGCCACCGCCCAAACA
ATCCGCGAGCAATATGATCAAGAACTTCTTCAAGTAATGAGCAAGATTTAATGGTTGTTTTCGTTGACGT
GTTGAGCTAACCCCGCTAAAAAGCTGCTAACTCGATTTTTGCTCCCAAGAAAGCGAAGTTTTTTGTAGCA
TGATTTTATGAGATTGACTTTGAACTTTCCGATCGATTGATTAACCGTGTGTGTTCAAAAAGATTTTAAG
GCTCGAAGTGTTGCTACTGCCTTCCAAACGCTCTAGCCGCTATTTAATTTGATGCAATGTTTAATAGTTA
GTGTTGAAACAAATTCTAAAACTACATATTTTCATTCAATCCAAACGTGTAGGATCCTTTGTGCCACCGG
TGCGTGATGAAGAGAGCGAAACGCAGCGGAAGGCACATGCTAAGCGTGTGAGGGAGACTCGACGGTCTAC
CCAAGGTGTCACCCTGGACGAGATCAAGAGTGCCGAGGAGCTGGTTAAGAAGAAGAACATGGGCATGGCC
AACAACAACAATAACAACAATATTAGCACCACCACCACGAACACGATCAGCAACAGCGGC
>dme_circ_0004866_junction_seq
CACCACGAACACGATCAGCAACAGCGGCGCTGAGAACGTGAAGCCCAACCAGCAGA
>dme_circ_0004867
GCTGAGAACGTGAAGCCCAACCAGCAGATCCACGCCGTCGAGCATCCAGTCGAGGAGGAGGCGCCATGGC
GACGCAAACTGCCCCGCACACCCAGCGATAGTCCCACTAATAATCGTAAGTCCTAACGGAGCTATGAAGT
GCGTTTTAGGTGTGGCTGTTTAGATGATATTTGAAAATGAAGTTAAATTGTTTCAAAAAGTAAAAACTAT
ATACGAATCTATTTCGAGATCATGTCGTGTCCTACAATGGTCATAGCTATCAGCAATCTCTTGAAGCGCA
CTGACTGGCAACGTTCGTAATCGAATAAGATAGTAATATTCAATATAATTACCATTTTGCAGAAGTTCCT
GATAGAGAGCTCGGCACCAATAGCTCGGAGACCGCCAACGACGTCATTTTGCGGCGCACGCAAAGCTTTG
AGAACGATCAAAAGTGAGTTGAATCCCAAGAAAATAAAACATTGCGTGCCGCTCACACACAAACACCCAT
TCGCATCTCAGATTAACCCACATATACAATGATTATGTGCTACAACGATCGGCCAAATTGCACCGAAAGG
TCTTGATTTGATTTATTGGTTGATTTTGCAGTTCGCTTTTCACTTTTGCCGATTGCATTTGCCTATCATT
ATCATCACACACACGTTCACGCTAAAATTGTTAACCACTCATTCAGCTAATCTCGAAATTCATGTACTGA
TAAATTGTTTAAATTTACCTATGATTGTATCATGGATCTGGGAAAGCTTTGAGAATCTTTATGAATCACA
AGCTTTGCGTTTATGAGCTTTTTCCCATAACACCACTCGCATTTTGGCAAAGTGAATCGTTGACTGCAAA
AGACAACAAATAAGGGAAAACAAAGTATTGTATTGattttaaaatttttgttttgctttattttttttC
CACCCAATTTTTTCCAAACAACAATCAAAAATCGATAAAAAAATAATTGCATTGCCAACGCACCACAACC
AACCAAAAACAACAAACTCATCAAAACGCTTATCCCGAAATCTAACCTGCCCGCTCGCCCACCTGCTCGC
TACGACCACCCGCCCGCCCACCTGTACGCCCGCCCGCCCGAACGTTTTGAAAAACGTTCGCAAACGCACG
CTCCTCGCTCGAAAACGGCCAAAATCGAAAATCCGAATGACAGGTTCTATCAAAAGTACAATGAGCTGCG
GGCACGCATAAAGGCCAATTCCTGTCCCATTTTGCCGGCGAATGCGAATGCTAATGCCGCTGCTGCCAAC
AAATCCAATAACAATAATAACCTAAGTAGCAATAACTATCATaataacaacaataacaacaaCAAAAGCG
ATCTGCTATTGGGATATGCTGCTGGCACcacaacgacaacaagcacccccacaacaacaacaacaacaac

atccagtacattcaacaacacccactcgaacaccaacaacacaagcacaacacaacaacaaccagcagca
gcagcagcagcatcggcagcaAATCAATTATATAGCGTACAAAGATCGGCCTCACTCAAAGATAACTCAA
TGTATTACAGGTTTGTTTTGCATTCAACACATCCCAAAAAAAGAACAGACTAGATCAGATTTGTTAGTTA
CGTTAAGTAAAAGTTGTTAGTGTAAAAAGGGAACTAGTATAGTATATATATTTCTATGCTTTATTTGAAT
TTTTAATGAGTTGTAAATGGGTATTTCAAGTAGAGTTGatataaataaaaatagaaatataaataaaaGG
TATTAAAGGCCACCTTTATACTCTAGTATAATTCAAATTGTTTAATGTATTTCTAAGGTACTTGTTAGAT
ACAATTATGTAGTATTTAATACTCAATAGGAGCTAATAAGTATTCGAATTTGTCCGTCCTTAAATCTGAT
CTAGTTTAAAAACCATGCCTACTCTCATAGCTGTTAACCCAAGCCTAATTTGTTGTAACTATTAACCTGA
CTGTGCTTTTGCTTAGCTTCTTCTCGTCTACACCTTCAGTATAATAACTCATTGCACCAAACTATTTAAC
ATATTTTCCTAGGCCACGCACCAATGTCGTCTACCTACTCTTGGCTGTATTCCCAGTTCAACTTTGAACC
CTAAAGTCTAAAGTTTTTCCTTTTTTGATTACCCCCAGGGTAAGTTTACTTGTGTAAAATATGATGTATA
TAACTCttttttttaaaaaaaaaaCTCCTATGTCGATCAATTAGCCAAGTTACAGCGAAAATAGCAGAAA
AGAGCAATAATGGCAGCTCGTCCACGGCCAGTGTCAGCGATGCGGAGAGCTCCAAGCCACCGCCCAAACA
ATCCGCGAGCAATATGATCAAGAACTTCTTCAAGTAATGAGCAAGATTTAATGGTTGTTTTCGTTGACGT
GTTGAGCTAACCCCGCTAAAAAGCTGCTAACTCGATTTTTGCTCCCAAGAAAGCGAAGTTTTTTGTAGCA
TGATTTTATGAGATTGACTTTGAACTTTCCGATCGATTGATTAACCGTGTGTGTTCAAAAAGATTTTAAG
GCTCGAAGTGTTGCTACTGCCTTCCAAACGCTCTAGCCGCTATTTAATTTGATGCAATGTTTAATAGTTA
GTGTTGAAACAAATTCTAAAACTACATATTTTCATTCAATCCAAACGTGTAGGATCCTTTGTGCCACCGG
TGCGTGATGAAGAGAGCGAAACGCAGCGGAAGGCACATGCTAAGCGTGTGAGGGAGACTCGACGGTCTAC
CCAAGGTGTCACCCTGGACGAGATCAAGAGTGCCGAGGAGCTGGTTAAGAAGAAGAACATGGGCATGGCC
AACAACAACAATAACAACAATATTAGCACCACCACCACGAACACGATCAGCAACAGCGGCCGTAAGTAGC
CCAGTGTCCAAGTGGACACTCTAACCAGATCACTCACCCCAGGCTATCACAACAAACAACCAAACCGAAA
CATTCACACTCGAGGAAGCTCTCTTCATAGAATCATTGTCAGATCTCATACCTGTAGTCCTAGCCGGGAT
TACAGACCATCCACACACGATGTCCACATGCAGTTCACACCCACTCAAACATTGAGCAATACACACCAGT
TCGAAAACCGAAAAAAATATATGCTTATGGTTATGGTGTCCCCATTGTTTGGATATCCCTTTTTGTTTAT
GTGTTCCATTCTGTTCACTCACTCAAACGCAATCATTATAAATTTCAAGAATACCATTAGTTGTTCGTGT
GTGAGTGAGTGAAACATGTACTAAAATAGTGATTGTAGTTTAGAAAATATCATTTAGTAAGCAGAATGCA
TCAGGTTCCAAAATGCCCAAGGAAGTATGCTATGAATATACTTAAAAAGTATTTAAATTCAATGAACTCA
TTGATATGCTCTTAGTAAATCGAGTACTTCAACGGATTGCTAGCACCTTGAGTATCGTTCTTAATACAAA
ATTTGAAAGTAGCTTTAGCACCTTGGTTGTTATAATTGAAACCTGCTGCATTCAGTCTTCTCATTTCACA
AAGAAAGCAGCCCCAAGCTGTCATCATTTCAATCAGATCCAGTCTCATCGATCAATCAATGCTCATTTAG
CGTACGTTGGCTCCCGTTTGATTATTATTATTATTATTCGTTCGTGATCGATCGCAGTATCGCATTTAAC
CAATCAAGTTGAGTTCAGTTTCTGCGCAACGCGTAATGcgcctccgcccagctgcgcctccgccctgcca
cgcccCTTTGACCCGCCTCGCATCCGCCTCATCGATTTTGAGTACTCTATATAGCGTAGCTTTATAATTAT
GTTTTCCGATCATCAGCAGCTCCAGATCAGCGAACCATAGCGTAATTACCATTCCAACTTTTATTTATGT
TACAGCTCATAGGTTATATCTATGTTCAGTTCTATACTACTCACTAGGTTCTATATTTATATAGCAATAA
ACTGTATGTCTATGGAATATGTTGATGATTATCATTTTGCATGTCCCATTTGCATGCGTAATCAAATAGG
GTCACCCTAAGAATCATTATAGTATGCAATTGATATTTACACATATATGTAATGTAAATAGTGAGAAATT
ATTTGATTTTTGTTTGAAACACATTCGCGTCCATCGCCTTAGGACAACTGTTTTTTATTTGTTCCGAACT
AATATTATTAACAGAACGAAACAAGCTCTGCATCATCGACATCAGCTCCAACACCATCGATCCTCGGCAG
CAATGAGCAGCAGGACGAGCTGCAACCACCGCCACCGCCCACCACACCACCGCCAGCTGTAATACCCACC
ACAACAACGGCCACCGATGAGACAGAGATCGAGGAGGTGGTGTCCAGTCCTCCAGCTGGCCAAAGTCAAA
ACGATACCACTGCTAGTTTTACGCTATCGGCTCCCGTGCGAAGATCGCTGTCGACCGAGGGCGAAGCCGA
TGCCAATAGTGATCCGGAGGGTGATCAGGATCAGACGGTGGTCAGTGCCAGCTATACGATAATGCCCAGG

CGGGAGAGGCTTCCCGAAAGCTCCGAGAATGTAGAGAGCATAAGATCGCCACAAAAGACTGAGCCAGTTG
CCAAAAGTTCCAGCGAGGATAAGTCCGAGGAGCCACCACATGCCAGGCCCACGGATCTGCCACTAATTCC
GGCTCCAGCTGCTCCCAGCACAGAAGCTATCAAATCATCCTCGGCGGCCACTACGCCCGCTGCCCTCGAG
AGTCCAGTGCGCTTGCGGGATAAGCGGGGTCTGTCCGGCAGCCAAGAATCGGAAACCAAGTCCGATACCG
CCTCGCCCGTGTCCTCACATCCGGACTTCAATGCCCGGGACTCGCTACTCAGTCTATACGCCCGTCGCAC
AACGGACAGCAGCGCGGGAGGCGGAGGAGTTGGTggtggtgcaggtgtaggtgcaggtggtgcaagtgga
gctgcaACCTCGTCTTCAACTTCGGCAGCGGAACGGCGTCCGTCTTGGCGCCTGAAATTCGATGCCGGCT
CCAAG
>dme_circ_0004867_junction_seq
GCGCCTGAAATTCGATGCCGGCTCCAAGGCTGAGAACGTGAAGCCCAACCAGCAGA
>dme_circ_0004868
GAACGAAACAAGCTCTGCATCATCGACATCAGCTCCAACACCATCGATCCTCGGCAGCAATGAGCAGCAG
GACGAGCTGCAACCACCGCCACCGCCCACCACACCACCGCCAGCTGTAATACCCACCACAACAACGGCCA
CCGATGAGACAGAGATCGAGGAGGTGGTGTCCAGTCCTCCAGCTGGCCAAAGTCAAAACGATACCACTGC
TAGTTTTACGCTATCGGCTCCCGTGCGAAGATCGCTGTCGACCGAGGGCGAAGCCGATGCCAATAGTGAT
CCGGAGGGTGATCAGGATCAGACGGTGGTCAGTGCCAGCTATACGATAATGCCCAGGCGGGAGAGGCTTC
CCGAAAGCTCCGAGAATGTAGAGAGCATAAGATCGCCACAAAAGACTGAGCCAGTTGCCAAAAGTTCCAG
CGAGGATAAGTCCGAGGAGCCACCACATGCCAGGCCCACGGATCTGCCACTAATTCCGGCTCCAGCTGCT
CCCAGCACAGAAGCTATCAAATCATCCTCGGCGGCCACTACGCCCGCTGCCCTCGAGAGTCCAGTGCGCT
TGCGGGATAAGCGGGGTCTGTCCGGCAGCCAAGAATCGGAAACCAAGTCCGATACCGCCTCGCCCGTGTC
CTCACATCCGGACTTCAATGCCCGGGACTCGCTACTCAGTCTATACGCCCGTCGCACAACGGACAGCAGC
GCGGGAGGCGGAGGAGTTGGTggtggtgcaggtgtaggtgcaggtggtgcaagtggagctgcaACCTCGT
CTTCAACTTCGGCAGCGGAACGGCGTCCGTCTTGGCGCCTGAAATTCGATGCCGGCTCCAAGGTATGCCC
ACACTACCCAACTACGCCCTCTAAGCGAATCCGATAGCCTAATCCTCAATATACTTCAAGACTATTTTTT
CGATCGGTTGCCAAATTCGTGTGCGCCGTAAATACTTATGTTAATCTCTAAGTGTGTAGCATGGATTTAT
TTTGATTAGTGTATGTACTTAGTGTATGGAGAACTTCTTCTTaaaaaaaaaTGTGGAATTACCAATCGCT
TTGGTCGAATTCATAAAATAACCCCATCGTTTCCATAATGATTTTTGAATTTTCGCAATTTAATATAAGA
TCATTATTTTATATCATTTCGATTTCGCCGCTCTTTTAACACCTACCTTATTCATATTTTATGATTTTAG
AAACTTTGCTTATCGACGGAAATATAATTAATAATGTGATTCGCTTACTTAAatatatattttatgatat
atattttttttACAACGCCTGAATGATTAATTTTAAGATCTTTTGCATTTAGTTGTATCTACGCATCTGT
ACTTATTCATGATTTTATTTGCGCGTAACTTGTGCTTGATGTACTCaaaaaaaaTAAGTAATTTTATCAT
TAAGTAAATTCGTATGTATTATTCACTTTTTATTTCTAATAGTTTTCGTATGTATGTACTTAGATACCAA
TACTATACATTTGGCTGATAAACATTTTGTATAAATATTACATGCTTAAAATAATCTTACCAAATTGGCC
AATGCATTTGCATTTATGTCTCTTAACGTATTAAAATTACTTTTTTTATTACATCTGGCTTACCAAAAGT
TACTAATTATATGTTATAAAATATAAAAAACTACTTGCATGTTACTTTATAAACACATTTACACTTTTGT
TCAACTAATTTAACTACACTATACTGGTTCAGGGAAGTGCAGAAGTGCTTAGCTTAGGTAATGGGTTGCG
GGCTCTAACTGATGGATCCAAATTGGTAAAGGGTGTCTGATTTGTGTTGATTACTACTAAATGTTTAGTA
TGAACTAACTCCCGCTGCATTCGAATGATTCAAGACACGGCATAGCTTCTTGTAAGAACAAATACATCGC
ACACAACCCAAACAAATACGACAATTATAGAGAAACATTTCAAAACATATATTGATATTTAACATTTTAT
CTTCATAACTGCATTGAGCAATTAGTAAACGGTTCTTTATCCATGCAGGTTCAAGTTGGAGGACATAACC
AGCGGTGGCACATATCCGCCCAACAACAGCACCATCATACCCAGTGCTCCGGCGGTAATGGCAGCCGCCA
ACTTATCAACCACAACTGGTGTCCAGCGGCGCATCAGCAGCGGTCCCATCGCACGTAAATGCTTCCAATC
AGTCGCTCAACTCTGTGGGTCGTCCAGTTTCCGCGCCAAGCGAGGGCACCAACAACAGTGCCTATGTCAC
GCCCTCAGCTCGCAAATTCGAGACGAACGCCACGTCCACGGGAGCGACCACTGCAGCGACCACGACCAGC

AACTCAACCTCCAATTCAGTGTCTGCAACCAGTGCCAATCATACAACGGCTACGGCACCAAATGCCACGT
CAAACCATGACGATAAGGGGTAAGGAAAAGTCGAGGAAAGCTAGGGAACCAGTCCCAAAAACTGTTGAGA
TGCACATACCTCTTATAGGAGTATCATAATCAAAAGTGAAAAGTAGAAGTACTAATTCCGTTGTAATTAT
GTCTAAAGTAGTTGTAAAGCTAGAGCCTAAGAATTTGTCTTATATCTTGAGTATTGAATTAGTTATTCTC
TGAACAAAAGAATATTGTAGTATCAGATTTTTCGCTTACTAACTTGCttttttttttaaatttttttAACA
GTGTAAGTATGGTTGGGAGATACATCATTTGTGTGCCTAATTGCCAGGCAGTTTGTCAGAATAAATGAGC
CTTGGTTGAAGAGCCAACTATGCCAACTATTCTCAGTCAAATTGCTTATTAGTCGGAAGAATGGGAAAAT
GCTAAATGCTAAATTGAAAATATCTTTTGTTCTGCTGCAGGGTGAGTCTGTCCACCGATAATTAGTGCTT
ACCATATGTGAACTACCAACAACAACAACTAGAGCAACAGCATCAATATTATTGAATATTTCACACCTTG
CCAAGCAATTAGAAAATGTATTTGTGTCAGCCTTTTATTTCCGAGCTGCACCTGACCACCACATCTGTTC
GTTGTGCCTTTGCCTTTGCCTTCGTCACACCTTGTCACAATTTTAGAGCACTCCGCGTCCTTCAAAGTAG
TAGCTAGTGTCGTGCCCCTCCTCCATGCATTTGGCCCACAATTTTCTCGTCTCCATCGTATGTCTGGTCC
AATTAGCGGACGACCCAAAAAACGAAATTATGATTTGCCACTTTGAATGTGCGCACGTGGCATTGTGGGT
TCTCGGTATGATTTCGTGTGGGAATCCGAGTGTAAGTGCAAGGCAACTGCAAATCCTTTCATGGCCGAGG
TCAGAACTATTAGTGCGCAACCAATTTGCGTCCATAGAAATCACGAGTCTGTCCCGGCCCGAGAACCTGT
TAACAATTTATCTGAGCAAGTGTCAGCGCCGGTGTCGCCTATTATTAAAATCTGACACAATTTCTGAGTG
CATCTTGTCATTAACGTGCGCTCGCTCTGCTTGTTTCTATAAAAAGTTATTAATTGTTTCGAACATTGGT
CAATTAAAGACGTACCGTAAATCTTACATTCGGTTTAGCATTGATAAATGCTAAATATTTGCATTTTATA
TATTGTAGTTTCGTTTGGCAGTTTATAATGATTGAATTTAAAAATGGTATGGATATGGGGAAAAATGTGG
GTTCTTTAAGTAGAAAGAAAAAAACATAGATATTCTTTATAATTAAACTAAACTTTGTTTAACAAATTTC
AAATAGCAATGCATATATACCCATTGCAAATTATTGCTACAAATACACACAATCATTAAAAGTGAATTTA
ATTGGTAATTTACTTGTCTGAAATTTTGCTGAGGTCTACATCCACATAAAGTGAATGATGCAACACAATG
TCTAAATTTGTTTGATACCTTCAATTTTGAAATGGAAATTTCATTTTAAGAACCATATTTTCCTGGAATT
TGTGGCTTTAACTATTCGTTTTTCTGTGGCGTGAAGAAATAAGTAATAGTATGCAATTTTCAACATGTGT
TCCCACAAACGATCCATCCAAAGTGACGGCAGACCATCTGACGCTCTTTGATTGACGATGTCAAGTGTGC
CATTGTGCATTTATTCTACTTTTTAGTTTTCATCTCGCAAATGCTTGGGAATCTGGGGAAATCACTTTCA
CAATCGTCTTCGCAGACAGTTTTGCATTTTATAATAAATTGGGCAATTACAGAGGCACGACCCAAGCCGA
AGGAAATATTTGATTCTATATTGAGAAGCGCATAATAATAAGAAAAGCTGGCACACGTCCGTTGAACTTT
GCAGAAAAAGCGAGAAAATGTGCGGCTAAATTTATAACAGCTTGCTGCAGGCATTTGCTTCCTCATTCTC
TCTCCCTGCGGCAAAAGGATCGTCATGCGGTATTTATAGCCGGAAAATGCATCCAACGAAACCATAAACT
CTGCACGGAGGGGTCTGATTTTGGCCCAGTTATGAGAGGGGAGGGAGCAACTCCCCAAATTGGATTCTTC
TGTCTCCTGTCGCGCACTTAATGCTAAATTTAAACGCAAACAAAAATAGTTTTTCCTATGCTCGCGCTAT
TCTATGTCTGCTTTTTCACGAATTTTCCTAGTTTCGCTGCAGTCATGTGTCAATGACTGTGCTTCGCCTT
TTGGCCCAGTTTGCAGTGCTCTCGTTTGCGTTTGTATTTCCCTGATTTCTTCTGACACCAGCATAGTTAA
CAATTTTTCGTCTCGGACATTGGGCCAAATACGTGTGCGATTTACTAAATTTAGATGTGATGTCTCAACC
AGGTGAAAAACTCGCACAACGAATTTTTCGTGTGCGCAATTTCAAGTGAAAATTTGGCTCCTTTCCCTGC
TCTTATTATTTCTTGTGTTCATATTTGAGTTGAAAATATGCCGCCACCTTTTATTTCTCTATGttttttt
tttttttGTTTAATTTTTCCGGCTTTTCCGCTCTTCATTTAACTCTTAGCTCGATGTGGGCACTCTTTCG
CTGCTCTTTCGTGTTTATTTCCGATCGTTCGCTTTCAAATAAAACATTTCAAATCGTCAGCGAGCAACAC
AGTTGAGTTTTGTGCGCCCAGCACAGCGGATCATCTCAGTTCGATTCAGTTCAGATCACCCAAACCCAGC
TAATCCAATCAAACAAGTCTAAATCTCAATGTAAATCCCAATTAAGCTCAAGAATTCAATACGTCAAGTG
CAACGAGAGACCAAAAACCACAAGACCTGTCGAATGTGTCAAAGAAGAATTGTTGGGGGGGTCAAAATCA
ACTGCTATTGCAGCTTAGATCAGTTTACCCCTGTGATCAAAGGGGCAGCTGGAGGGGATTTGCCAAACCA
AAGTTGATGATTTGACTGCCGGCCGCAAAAAGTTGAATCGTTTTCAGTTGATGGAATCGCTTCGCGTTGC

CTTTTTTGGCCAACTGCGCATAATTTTTGGACATTTCAAGTTGATGCTCTTACCAGGTGGTTTTTGCAAA
TATGGTCAACTATTTTGATAAGTTTTTAATTTGATTTCGTGAACGTGCCTAATTTATTTGTATTTTTTTC
ATGACCACAAATGTTAAAGTTTAACACGAGAACGGGGAAAAAAATTCATGCAACATCTATGTAGTTTAAA
GTGTGTATTTTAAAAGTCAAGTTTTAATTCAAAATATTAATTTGTTATACCCCACTTACATTGAATTTTT
CATTTAAAGTATGCAAGTCTTAATCATGTTTTTATAAATACTTGTCTTGAAAATTGTCGAATCATTTCGC
TAGTATTTATCCTTTGACTTGATTTACGTCTATTTCCTAATCTTTTTTATGATGCCTCTGCGACCCGGCT
AGTTCATTTATCTTCGCCATGACACAACAGAAAATTGTATTTTTAAAAGCTTCCTCTATATACATTTTAC
GCGATATTAGCCAAGCAAAATGTTTGAATTACCACAATACAGAAAGGCATAAAAGGGGGGAAATGATTGC
AAAAGACCAAACATTAAAAAGGCGCTTCATTCATGGGCGATTGTCCTATAGATAGAGCCATTTAAACGGG
CAGATACACAGAATGAAATGAGTCTGGTAGGAAAACGAAACGGTTCGTCATGAATTATATGCATTGGAAA
ATCGCTTGAAAAGAAAAACGTTTTCTGGTCTGACGGTCGGTTGGTTTCATGTTTGTTGTTTTGTATCGCG
TGGGCGGAGGATGAGAGGCATCAACTTAGAGAAACAAATAAATACTAGGCCGGCAGCGGGATATTCGCAT
CCATATATCACGTTTTCTATGTGTTTTCAATCCCAACAAGAGCATCCCGTCGAGATCCAACAGACCCAGC
TAGTGCCAGTGGAGGTATATACGTCGAAAACTGAAATGAAAATTGTCAAAATGTATGCTTTGACGTCATT
TTCGGTTTTCCCGGTGCACGTCCAAAACCGATTCCCGAACCACATGTCAATATTGCGTTTTCCAAATCGA
AAATGTATCCCTCTAGTATACAGCCGAGATATATGGACACATGTAAACACACCGAGAAGCTGTTTGTTTT
CCTTTATTTTTGTTTATGCCGAATTCAAGAACATTTTGTATGTTCGCTTTGATTATAATTGTAATTGCCT
TTTGTGTGCGCGTCTGTAATGGGAACACAAATCGATCAGAATCGAAGTTGAAATGCCCTCTAATTGCAAA
TTATTCCCAGGGGTATCGTTCGTTCAAATGCGTTCTTTATATAGCATCTTGAATCCAAAGTCGAACTAAA
GTTCAAAGAGTCTTTTTTGAATGACAGCTGTCCATATTCGTGAAAGATTTTTCGTGTTAATTAAAGATAT
TTTAGCGATTTCTTTCTTTGAGTAATTAAATGGATTTGATTTTAGTTTATAGAGTACATTTTATCGAGTC
TATTAATTATTCAAAAACCTACTTAAAGAAAGATGTGTCTAAGAAATTATTGGTTTATTTATGCGAAACT
ATCAATAGCTAAAATGGATTGGGAACGATTTACCAAAAATATAAAGGCTTTGCAAACGTGTGGCCAGATG
GCAATTACCCTCCAATAGGAAAGATCATTCAGGTCATGTGGTTTTTGGTCGAGCTGTTTAAGCGTAATTG
CACTTTGGCACTGACGATTGATATACGAAGTGCCTGGCGAGATGGCAAACTAATGGAGCCCACGTTCTTA
TAGTTTCCATCAGCGAGATGTCCTTTCGCTCGAGATCAAGAACCCAGGCGCCCCTGTCCAGTCGGCGGCG
ATCGTTGTCCTCCAGTTCGCGAATGGCTCCATCAAGTGGTGGTTCCGGGGCCTATAACTACAACGGAAGT
AGCTATAGCAGCTCCGGCAGCACTGGTCGTCCCCTGAGCACGGGCTACTTTCCCAGCAGCAGTAGCGCCT
CCAGTTACCAGAGTCCCTATGCCAGTGTGTACAGCTCGAGGGAGAGCTTGTACGGCGGAGGAGGTGCTGG
TCGGAGTTCTTATGGTAATGCGGGCAGTCGCTACGATTACGGGGCGTCCAAGTatcagatccatcagcag
cagcatcatcatcatcttcatcagcatcagcaGTTCACCAGCACCACCAACCACTATTCGAATCAGCATT
CCCATCAGCCAACCGCAAGCAGTGGCCCATCGACAGGTGTAACCTCATCCGCCTCCACATGCCATGCCGC
ATCCACAGCTTCTGCGTCCAATGCCGCCATGCCAGCAACTAAAACGCAAACGCACCAGGGCAATCGCTAC
TATCTGCAGCTCCAGCCCACCGCCACTTCATCTGCTTCGGGTTCGGCGGCCACAAGCGGTGGTCATCACT
ATGGCCACCTGGTGGTGGGCAAGCATATAAGCCAGAGCCATAGTTACGGCCATTCTAAAGCTTCTGCTCT
CTCCTCCTCTCTGTCCACCGTCTCGTCGTCGTCCTCCTCCTCGGCTTCGAATGGAACAGGCAGCGGTGGT
GGTGGATACGACAGTTCCAGGTACAATCCCAGTTCCTATTCCTCGGCGGGATCTTCGTACTCCACAAGCG
ATCGATATGTCAGCCCCTATTCGAGTAGTTATGATAAGGGTGTGACCACCGCTTCGCTGACCTTCAAGTC
GCCCGGTTTGAGTTCGTCCAATTCGTTCAAGAGTTCTCGTTTACTGAAGACCAAATCACTGTCAGCATCA
AATAGCAGCCTGAATGGTGCCTATGGTGGTCCATCCTCGAATGGGGTAACCAGTGCCGGAGCCACTGTGG
CCGCCATCGCAGCCACAAGGAGCAACTCTTTGCGGGAACAGGAGCGCAAGTCTCGCAATCGCACGCGGTC
GAAGAGTGCGGCACAGAGATCCATTAGTGCTTCCTCAGAGAAGAGTGAAGGATATGAAGTAAGCAGGTGT
ACTGGTTAAGTCGGTTTCTATTTAATTACTTTATGCTGTTGTTGTAGTTGTTTTTATTGGATTCACATAT
CTACACTATTTACTTAAAATCATATTTGAAATTTATTTAGAATAGAAAAGATATTTGAAAATTATAGAAT

ACTTAAATATTATTTAGTATATGCGCAAGGATCTTAAGAGCAGTTGCGCATGAAACTTTGAAAATTTTCA
AGAAATATGAAGTAGCAGAACAAATAATTTAAACTATCTAAAAGTTGATCTCCCAAATTTGACTTTCCCC
AGTTGCTAAAATTAGTTAAATTTTCTTTTTCTCCTTTACAGGAGTGGCAGTGAGCGGACATCCCGTTCCC
GGCTGGGCAGCACCGCGAGTACGGCCACCACCAGCGAGTCAAAGAGCTCCAGCAGCAATGACAAGACGGA
GAATGGCGATGGCATTGACTACAAGGCGCTCTGGGAAGCGGAAAACGTAAGTACATGCCACGCCCCTAAC
ATGCCACGCCCACATCAGCCCACTATAAAAACTAAAAGCCGATAAACCAATACCGAAACTGCCAGCAGTA
ACCAACAACAAAACAACACGCAACACGATAAACTTAGTTCAAGGATGCCACCAAAATATATTTACTTTAA
TTCTTTAACACACAAAAACCACCCACCTCGCTGCCTCTTTTTCCCCCTCATGTGAAGCTTACTGCTCGCG
CTCTCTTTCGCTCTTTACACCCCGCTCTGTCGTAcatatatacatatatttatatatatatGTTTTTTCG
ATCCGTTGAACAAAAATATGAATTCCCAACAAACAACAACTACACCACAAAACTGAACAAGAAAAACCAA
GACCCGAATcaaaatcgaaaaacaaaaaatcgcaaaaCCACATCTTGTTTCATTTCACATTCGCAGACAA
CGAAAAATTCACTATCTGAACTTGAGAAACGCGAAAGAAGAGCTATGGAACGCAAGCTTTCCGAGTTGGA
AGAAGAGCTCAAG
>dme_circ_0004868_junction_seq
GCTTTCCGAGTTGGAAGAAGAGCTCAAGGAACGAAACAAGCTCTGCATCATCGACA
>dme_circ_0004869
GGCAGCGGTGGTGGTGGATACGACAGTTCCAGGTACAATCCCAGTTCCTATTCCTCGGCGGGATCTTCGT
ACTCCACAAGCGATCGATATGTCAGCCCCTATTCGAGTAGTTATGATAAGGGTGTGACCACCGCTTCGCT
GACCTTCAAGTCGCCCGGTTTGAGTTCGTCCAATTCGTTCAAGAGTTCTCGTTTACTGAAGACCAAATCA
CTGTCAGCATCAAATAGCAGCCTGAATGGTGCCTATGGTGGTCCATCCTCGAATGGGGTAACCAGTGCCG
GAGCCACTGTGGCCGCCATCGCAGCCACAAGGAGCAACTCTTTGCGGGAACAGGAGCGCAAGTCTCGCAA
TCGCACGCGGTCGAAGAGTGCGGCACAGAGATCCATTAGTGCTTCCTCAGAGAAGAGTGAAGGATATGAA
GTAAGCAGGTGTACTGGTTAAGTCGGTTTCTATTTAATTACTTTATGCTGTTGTTGTAGTTGTTTTTATT
GGATTCACATATCTACACTATTTACTTAAAATCATATTTGAAATTTATTTAGAATAGAAAAGATATTTGA
AAATTATAGAATACTTAAATATTATTTAGTATATGCGCAAGGATCTTAAGAGCAGTTGCGCATGAAACTT
TGAAAATTTTCAAGAAATATGAAGTAGCAGAACAAATAATTTAAACTATCTAAAAGTTGATCTCCCAAAT
TTGACTTTCCCCAGTTGCTAAAATTAGTTAAATTTTCTTTTTCTCCTTTACAGGAGTGGCAGTGAGCGGA
CATCCCGTTCCCGGCTGGGCAGCACCGCGAGTACGGCCACCACCAGCGAGTCAAAGAGCTCCAGCAGCAA
TGACAAGACGGAGAATGGCGATGGCATTGACTACAAGGCGCTCTGGGAAGCGGAAAAGTGAGTGAAGCTC
CCCTCCAAGAACTTTAAAGTATATAATCCGAATCTAAACGATTATCCTTTGTAGATTGGAGAACGATAAG
CTGAGGCAGATGCTCAAGCAGAAGGACGATGAAGCCGTCCAGACACGTGCAACACTCGAGAGATTCGCCA
ATGCC
>dme_circ_0004869_junction_seq
TGCAACACTCGAGAGATTCGCCAATGCCGGCAGCGGTGGTGGTGGATACGACAGTT
>dme_circ_0004870
GGCAGCGGTGGTGGTGGATACGACAGTTCCAGGTACAATCCCAGTTCCTATTCCTCGGCGGGATCTTCGT
ACTCCACAAGCGATCGATATGTCAGCCCCTATTCGAGTAGTTATGATAAGGGTGTGACCACCGCTTCGCT
GACCTTCAAGTCGCCCGGTTTGAGTTCGTCCAATTCGTTCAAGAGTTCTCGTTTACTGAAGACCAAATCA
CTGTCAGCATCAAATAGCAGCCTGAATGGTGCCTATGGTGGTCCATCCTCGAATGGGGTAACCAGTGCCG
GAGCCACTGTGGCCGCCATCGCAGCCACAAGGAGCAACTCTTTGCGGGAACAGGAGCGCAAGTCTCGCAA
TCGCACGCGGTCGAAGAGTGCGGCACAGAGATCCATTAGTGCTTCCTCAGAGAAGAGTGAAGGATATGAA
GTAAGCAGGTGTACTGGTTAAGTCGGTTTCTATTTAATTACTTTATGCTGTTGTTGTAGTTGTTTTTATT
GGATTCACATATCTACACTATTTACTTAAAATCATATTTGAAATTTATTTAGAATAGAAAAGATATTTGA
AAATTATAGAATACTTAAATATTATTTAGTATATGCGCAAGGATCTTAAGAGCAGTTGCGCATGAAACTT

TGAAAATTTTCAAGAAATATGAAGTAGCAGAACAAATAATTTAAACTATCTAAAAGTTGATCTCCCAAAT
TTGACTTTCCCCAGTTGCTAAAATTAGTTAAATTTTCTTTTTCTCCTTTACAGGAGTGGCAGTGAGCGGA
CATCCCGTTCCCGGCTGGGCAGCACCGCGAGTACGGCCACCACCAGCGAGTCAAAGAGCTCCAGCAGCAA
TGACAAGACGGAGAATGGCGATGGCATTGACTACAAGGCGCTCTGGGAAGCGGAAAAGTGAGTGAAGCTC
CCCTCCAAGAACTTTAAAGTATATAATCCGAATCTAAACGATTATCCTTTGTAGATTGGAGAACGATAAG
CTGAGGCAGATGCTCAAGCAGAAGGACGATGAAGCCGTCCAGACACGTGCAACACTCGAGAGATTCGCCA
ATGCCGTAAGTACATGCCACGCCCCTAACATGCCACGCCCACATCAGCCCACTATAAAAACTAAAAGCCG
ATAAACCAATACCGAAACTGCCAGCAGTAACCAACAACAAAACAACACGCAACACGATAAACTTAGTTCA
AGGATGCCACCAAAATATATTTACTTTAATTCTTTAACACACAAAAACCACCCACCTCGCTGCCTCTTTT
TCCCCCTCATGTGAAGCTTACTGCTCGCGCTCTCTTTCGCTCTTTACACCCCGCTCTGTCGTAcatatat
acatatatttatatatatatGTTTTTCGATCCGTTGAACAAAAATATGAATTCCCAACAAACAACAACT
ACACCACAAAACTGAACAAGAAAAACCAAGACCCGAATcaaaatcgaaaaacaaaaaatcgcaaaaCCAC
ATCTTGTTTCATTTCACATTCGCAGACAACGAAAAATTCACTATCTGAACTTGAGAAACGCGAAAGAAGA
GCTATGGAACGCAAGCTTTCCGAGTTGGAAGAAGAGCTCAAG
>dme_circ_0004870_junction_seq
GCTTTCCGAGTTGGAAGAAGAGCTCAAGGGCAGCGGTGGTGGTGGATACGACAGTT
>dme_circ_0004871
GACAGCAAAGAAGAGTTTGCGCATACAACACTGGCCGCAGATGTGATGCAACCAAAGTATGTATAAAGAA
AACCTTTTTTTCCTCAAGATCATCTAACAAAAACTTCATTTTAGGCGGAATGAAACTTTGAATGTCATTA
CCCAAGACTCCATGACGGTGGACGATGTTGAGAACCTTCTTAAAGAAACGGAACACAATGGCTATCCGGT
GGTGGTGTCGCGGGAGAATCAATATTTAGTGGGCTTT
>dme_circ_0004871_junction_seq
GCGGGAGAATCAATATTTAGTGGGCTTTGACAGCAAAGAAGAGTTTGCGCATACAA
>dme_circ_0004872
GGCCGGTCAACCGCTGCTCAATTCAATGCTGCCGGCTGGCGTGGTGGTGGGCATGCGCCAACAGGCGCCG
TCACAGCAGCAGCAGAAGAATGTGCCCACCAACCCGCTCAGTCGCGTGGTGATCAACTCCCACATGGCGG
GCGTGAGACCGCAGAGTCCATCGGGTGAGTATGAAGGATAATATTGCTATTACTATGGTTGTTGTGGTGG
GTTCTTCCCTACTAACTCACAGAGCTCCCTAACCCAGCCACGCTCCTCACAAACACGCATGTGCACAGCC
CACCATTCACTCACACCATACACCATACACACCATGCTCCAGCAGATGAAAACCGAGTCTCTCTGTCTTC
TCTCTCTACTTTTAGATAACTTTAAGCACACTTAATACGGGTCAGACCCCGGCATTGCTGGTCAAGACGG
ATAACGGATTCCAGCTGTTGCGCGTGGGCACGACGACGGGTCCGCCGACGGTGACACAGACTATAACCAA
CACCAGCAATAACAGCAACACGACAAGCACCACAAACCATCCCACAACCACACAGATCCGTCTGCAAACT
GTGCCGGCTgcagct
>dme_circ_0004872_junction_seq
CCGTCTGCAAACTGTGCCGGCTgcagctGGCCGGTCAACCGCTGCTCAATTCAATG
>dme_circ_0004873
GATAACTTTAAGCACACTTAATACGGGTCAGACCCCGGCATTGCTGGTCAAGACGGATAACGGATTCCAG
CTGTTGCGCGTGGGCACGACGACGGGTCCGCCGACGGTGACACAGACTATAACCAACACCAGCAATAACA
GCAACACGACAAGCACCACAAACCATCCCACAACCACACAGATCCGTCTGCAAACTGTGCCGGCTgcagc
t
>dme_circ_0004873_junction_seq
CCGTCTGCAAACTGTGCCGGCTgcagctGATAACTTTAAGCACACTTAATACGGGT
>dme_circ_0004874
CGGCGGAAAAACCTACAAGGTCAAGGAGGCCGACAACTTCAGCTACACAGATCCTGTCGACAAGTCGGTG

GCCACGAAACAGGGTCTGCGCATTGTGTTCGAGGATGGCAGCCGCATTGTGGTGCGCCTCAGTGGAACTG
GAAGCTCGGGAGCAACCGTTCGGCTTGTACATTGATTCCTATGAGAAGGAGAATGTTTTGGGCCAGGCCA
GCGTGATGCTGAAACCCTTGATCGACATCGCCCTGGAGATCTCTCAGCTGCCCAAGTTCACTGGACGCAA
CGCTCCAACGGTTATCACG
>dme_circ_0004874_junction_seq
TGGACGCAACGCTCCAACGGTTATCACGCGGCGGAAAAACCTACAAGGTCAAGGAG
>dme_circ_0004875
GCTGAAGTTGTCGGCCTCCTTGACCTTGTAGGTTTTTCCGCCGCTGGAATAGCTCTTGCCGACGAACTCC
GGAGCAGTTATGGTCTTCTCCATGGTGGCCACCATCTCGTTGCAAGGATCGGAAGCGCACTCCTCATAAT
CGTAGCGG
>dme_circ_0004875_junction_seq
GGAAGCGCACTCCTCATAATCGTAGCGGGCTGAAGTTGTCGGCCTCCTTGACCTTG
>dme_circ_0004876
GTTGTCGGCCTCCTTGACCTTGTAGGTTTTTCCGCCGCTGGAATAGCTCTTGCCGACGAACTCCGGAGCA
GTTATGGTCTTCTCCATGGTGGCCACCATCTCGTTGCAAGGATCGGAAGCGCACTCCTCATAATCGTAGC
GGGTGAAATAGTTGC
>dme_circ_0004876_junction_seq
TCATAATCGTAGCGGGTGAAATAGTTGCGTTGTCGGCCTCCTTGACCTTGTAGGTT
>dme_circ_0004877
GATTTGGGTCAGGATGCAGACCACCAAAGTCGGGCAGTGGAGTGGTGTGGACCACCGATGATTCGGTGGC
ACCCAACCGGTTCAGGAAGATCTCGCGCACATATGAGCCAGTTACTCCGTTCATTGCATCGATGCGCATC
TTCAGAGGCTTTCCAGTGGCTTTGCCACTTACGAAGTCCTTCAGCTTGGCGAAGTCGAAGATTTCCTCCA
TGTGGCGCACATAGTTGGCCACCGAATCGATCACCTCCACGGTAAAGGGCTTTCCGGCGATGTCGAACGA
GGTTACACCAACCTTTGAGAT
>dme_circ_0004877_junction_seq
CGAACGAGGTTACACCAACCTTTGAGATGATTTGGGTCAGGATGCAGACCACCAAA
>dme_circ_0004878
TGACATGTCGCTAACGGTGGAAATTGTTGCCACAAAGCCCTATGAGGGTCAGAAGCCAGGAACCAGTGGA
TTGCGCAAAAAGGTAAGGTCTTCAGGTGGCGGAAAAGCTCGCACTTGCATTTTATAACTTCTGAAATCGT
TTCCGAATCTCAGGTTAAGGTTTTCACCCAGCCCAACTACACGGAGAACTTTGTCCAGGCCATCTTGGAG
GCAAATGGAGCTGCTCTGGCTGGTTCCACCCTTGTAGTTGGTGGAGATGGACGTTTCTACTGCAAGGAGG
CTGCCGAGCTAATTGTGCGGCTATCCGCTGCCAATGGCGTCTCCAAGTTGCTGGTGGGTCAAAACGGCAT
CCTGTCCACCCCTGCCGTATCCAGCCTGATCCGTCACAACAAGGCTTTGGGGTAAGTTCAACGCAGGCAG
CCTTCACTGTGCTCGTAAAGATTTTAATTGATAAGAAGCATCTTTACACAATCAACGGGTGGCGGCTGTT
CGTTGACAGAGGTCCCTGGGGTCGAATAATAACTATGGATTCAATCGGTTAACAGAGAGTTCGTTACTCG
GGCAACCAAATTTGAATCGGGTAAAATTGATTTAGGGACTCGTGCAATCGCTAATCAGACTAACCTTTAA
TAAAATTGTATGGTGTAATTACTTAAGCGAATTTGATATACAAATTTGCTTTTAATATCTATCTTATACT
AACAAAAGACCATCTGTCAAGCTAACATTATTATTTCGCATTATAGAAGCAATATTGCAAAATCTCTATT
ATCTTAATAATATGAAATATTTAAATCTAAGCTTTTGTTGTACTTACTCAAACAGTTTATTCACATTAAA
GGTGGCCTCTTTGGAATATAAAATTGTTTTAGATGCCTAGGCACACCCAACTAAAAAATATACACGATTT
AAAAATGCTTTTTTAAACCTTATAGGGCGGCATTGTTTTGACTGCATCCCACAATCCCGGTGGTCCAGAG
AATGATTTCGGCATCAAGTTCAACTGCGAGAACGGAGGACCTGCCCCGGATGCGTTCACCAACCACATCT
ACAAGATCACCACCGAGATCAAGGAGTACAAGCTGGTGCGCAACCTGCAGATCGACATCTCAAAGGTTGG
TGTAACCTCG

>dme_circ_0004878_junction_seq
CGACATCTCAAAGGTTGGTGTAACCTCGTGACATGTCGCTAACGGTGGAAATTGTT
>dme_circ_0004879
GTTTTCACCCAGCCCAACTACACGGAGAACTTTGTCCAGGCCATCTTGGAGGCAAATGGAGCTGCTCTGG
CTGGTTCCACCCTTGTAGTTGGTGGAGATGGACGTTTCTACTGCAAGGAGGCTGCCGAGCTAATTGTGCG
GCTATCCGCTGCCAATGGCGTCTCCAAGTTGCTGGTGGGTCAAAACGGCATCCTGTCCACCCCTGCCGTA
TCCAGCCTGATCCG
>dme_circ_0004879_junction_seq
CCACCCCTGCCGTATCCAGCCTGATCCGGTTTTCACCCAGCCCAACTACACGGAGA
>dme_circ_0004880
TTTAACAGATTTATTTAATGTAGGTCTGTCGCCAACTTTTGCGCTGGTACGAAAAGACTGTTTATTAGTC
AGAATTTAATTTGTAAATTTGAGTAACGAGAGGAAAGAAAAGTTAGAGAATGATACGGAGAAGACATATG
TATAGTTGCGCTGCCGCCTTTTAAGCACTCACCACAGACCGTAGAAGGTTCATTTTTAAG
>dme_circ_0004880_junction_seq
CACAGACCGTAGAAGGTTCATTTTTAAGTTTAACAGATTTATTTAATGTAGGTCTG
>dme_circ_0004881
GTCGTTTGTTGCTAATCGCCATTAGTTTATCCGTGTGCCTGGCCTGTGGCAGTGTGTTGGTGGCCGCGAT
TGCCCTGCGCCGTTCGGCCAACgcaacagcagcattagcagcaacactagcggcagcatcagcagcagcg
acatcagcatcagctggagctggagcagcagcagcagcaacatctgcagcaacattagaagcggcaacaa
cgcgcaggatacgccacaagcgtcgctcgtcgcaacgacggcgcagcaccaacaacaacGCGGTGCTGTc
atctgggatcgggaccggcatcgggatcgggatcgggCCTGGACTGGAAACCAACTCGGCTCTGGCCCTG
CCACTGgcaaccgcaccaccaacatcggtctccacagcagcggatcagacagctatagccgccgctaccg
ccaccgccgcctccgctgcagcagaagcagcagtagcgacaacaacagcagcagcagccgcaacagcatc
gacagccgcagcaacagttacaacaacaCTTTTGCCAACCGAGCCGGAACATTCTGTACCCG
>dme_circ_0004881_junction_seq
CCAACCGAGCCGGAACATTCTGTACCCGGTCGTTTGTTGCTAATCGCCATTAGTTT
>dme_circ_0004882
TTATAATCACGGCACATTGATCGGGTATTTGCACCGCAATTGACCATGCGATTTGGGCCATTTCCGGACG
AAAGCACGGCGTAAACAATCCGATCGCCGGCGGCGGACGACGCTGTGGCCCCCACGCATGCAGCCAGTGG
CGCCGCGGGGGCCCGCTGCACATTGTGTTGGTGGTGCACAGTGGTTCTGGTTGCGATCAGCCTGACGACG
CTCCTCCGGCCGGGCTCAGTTTCAGCCTCAGCCCTCGCGGTGCCAATGCCAATGCCGGAATCGGGGATTC
CCCGTTCCAGTCACGCCCTGCAGCAGGAACACCAGGTATCCGCCACACCGACGTGGTGGTCGCTAATCGA
GATACTAAGTCGAACTGAGCGCACTGAACGGCCAAGTAGAAGCGGTGGCAAGTGTTTGAGATGCCAGAGC
AGCGATTCGATCAGCAGTTCAATATCGAATGCAACTGGAAGCGGCGGCGAAAGTGTGAGTTCGAGTTCTG
TGCCAAGTGCGCCGGCGCAGAGTGCTGTATATAGTGGTAGTGCAATTTTGTGCAGTGTAAATAGTGCAAA
TAATACGCGTAAGTTAAATTTAATTCTGAGCAAGTCACAGCAAAAGCGAGGGGAGCGCTTCGCTGTTGGC
ACAAATTTGCAAAATAAATTACCAGCATCGAGTGCGGATTGCCACTTAAGTCCAAGTGCCGAGACAAATA
TTCGTACCACAACCGCAGCCGATCCACCGCGATCCCCGTTCTGTCCGCCGTGTTCATATAGTCATCTCCA
TCATCGTCGTCACTGGCATCGGCATCGGCATCGCCATCGTCCTCGTCATAGCCAGCATCATCAACGTCAT
TATCGTCATCGTGGCAAGTGTTGTCGTTGTCGCGACGCGTCGCAGTCTGCATCCGCATCGCCCGCGTCTA
TGCTCTTGCCATATCAAATTAATCAACGTGCTACATTGTTGCAGCAATCTGCAACAGCAGCAACAACATC
AACTACATTGCCACATGTGCCCGGGGCAGCAGCAGCAAGAGCAGCAACAACAGCAAACGTATCTCTGCGT
GCCGTGCCTCGTATTTTTAAACGCAAACGTGATACGGTCCAAACAGCGACCACTGCATCACCTTTGACCC
CGCAGCAACATCCAACAGTCGCAGCAGCAACATCAGCCACATCCACAACAACCACGCGCAGATCGCGACT

CGAATTCATACTGTACACGGAAGTCTACCAAGTCGACGAGGATTCGGTTTCCTCCAGCGATTTCCATTCG
AAGTCGAACGTGGGCAGCGACGAGCCCTTCTGGCAGAATTACGG
>dme_circ_0004882_junction_seq
GCGACGAGCCCTTCTGGCAGAATTACGGTTATAATCACGGCACATTGATCGGGTAT
>dme_circ_0004883
GGCTGATTCCATTGATGCAATGGCGCCTGGTCTCGGGTATCAGGTACTCATTAGTTTCACAGGTGAATAT
CCGATTGACCAAATCGTACTCCACCAGCTGGCCATTGGCGTAGGCGACCACCACGCGTGGGTAATCCTCG
TGCATGGATAGGGCGGTGGTACCTGCTCGGTGTCTTGGTAGGTTCAGCAGGTGCTTGTATTGTTTTCCAT
>dme_circ_0004883_junction_seq
TTCAGCAGGTGCTTGTATTGTTTTCCATGGCTGATTCCATTGATGCAATGGCGCCT
>dme_circ_0004884
GGGATTTCACATAGTGTTTGTGGGCATCGCGCTGTAGGAATTTAATCCAACGCTCGCTGGTGGACTCTTC
CCGCTTTATCTTGGTTTTGCTGAAAATCCGGATGAGCGGCGGCTGCATTCCACTGCAAACTAATCGATCC
TCCTTATTATTCACAGCCAGGGCAAATACGTTTTTATCCAGAACTCGTGTGGAATCTATCTGTGTCGCGT
TATCCGCATCCCAAACGGTAACAAATCCCGCTGAATCACCGGCAATAATCGTATTATCGGATAGCACCTG
CAGGGACCAAACTATTACGTTCTTTTCGGATAGGGTCATCGTGTGCAGAGTGGTTCCCTTGAGGACGTTC
CAAATGCGAACAAATCCCTCAGTGCCGGTCACCA
>dme_circ_0004884_junction_seq
CGAACAAATCCCTCAGTGCCGGTCACCAGGGATTTCACATAGTGTTTGTGGGCATC
>dme_circ_0004885
AAGACGTAGAGGAGAAAGCTTTTTGTGGGTTATTAGGTCCAGAAAGGGAGTTAAGAATAGAAGTATCATA
GCTTTGGCGAGTTCATCGTGTTAATCTGTTGAATGGATCCTTAGTTGCGGATGAGAACCACTCGGGGATC
GGAGGCCACGCTCCACAGAAGTGGTGGCTGCTGGCGAATCACCTGGGTGGTGCGAACAGCGGGTGCCACA
ATGGGTGTGACCAATCGGCGGTGATCGTGCACCACCGTCTGGGTCTGATGCGAAACGGCAGTGGGCACGT
GCTGCACCACCTCGCCCACCTTGGCCACGGCTGGCTCCTGGATGGTCAATACGG
>dme_circ_0004885_junction_seq
ACGGCTGGCTCCTGGATGGTCAATACGGAAGACGTAGAGGAGAAAGCTTTTTGTGG
>dme_circ_0004886
GCTAAACAAGCAGAGTGCCGCTGATGTCTCTCGATGCATTCTGAATGTCCTGGAGAAGGACAAGAACGGA
GCTGTCTATGTCATCGAGGGTAAGCGCGTTTACCCTTTGGAAATTAAGCCCCAGTGGACGGGCAAGGAAC
AGGCCCTTTAAGTGGGTTAATTCGTTCACATCCTAAACACATGCATTTTACAAATATGAAG
>dme_circ_0004886_junction_seq
TAAACACATGCATTTTACAAATATGAAGGCTAAACAAGCAGAGTGCCGCTGATGTC
>dme_circ_0004887
TACTATCAACGCTCGGGCATCAAGTTTGTGACTGTTTGTCCTGGAGCTACAATGACGGACATGTTCACCA
ACTTTACGGAGAAGATCATCTTCCCGGAAACGAGTGACGAGACCTATAGGATCCTGGATAGGCTAAACAA
GCAGAGTGCCGCTGATGTCTCTCGATGCATTCTGAATGTCCTGGAGAAGGACAAGAACGGAGCTG
>dme_circ_0004887_junction_seq
GTCCTGGAGAAGGACAAGAACGGAGCTGTACTATCAACGCTCGGGCATCAAGTTTG
>dme_circ_0004888
AATTCAGCTCAGACTCGTAGATCCGTAGGAAACCACAAAAGAGGCGAATGATCAAGAGTGATGAGGATGC
GGATGAGATATGCAAATGCTTGTTGAGATCGGGCAACATCCAGTTTTGTATAGCAGCGAAGATTTTAAAG
CAATTTTCTGAGATGAAATAGAGATACTG
>dme_circ_0004888_junction_seq

AATTTTCTGAGATGAAATAGAGATACTGAATTCAGCTCAGACTCGTAGATCCGTAG
>dme_circ_0004889
CCGATGTGGGGCAGGAAACCGCAACTGATGTCGGAAGACAGTTTCGAGGGCGGCATCGATAGTGGAATGA
TGCTGCAGCTGGAGAAGAATCTGGTGGACATCGTCGATCCCGATGACAGTGGGATCAAGGTGGACTAGGA
GCTGCAGACGGGCCCACTCAGCCAGAGGATATTTCTATATAGG
>dme_circ_0004889_junction_seq
ACTCAGCCAGAGGATATTTCTATATAGGCCGATGTGGGGCAGGAAACCGCAACTGA
>dme_circ_0004890
GTTTGAGCACCTCCTCCTGCATGGCGGGTCCAAGGAACTGGCATCCTCTTGTCCGCACTGCAGCCCACAG
ATTTGAGCTCAACTGCTGGGGATTTTGGTGCTGCAGGATCAGCTCGGTGACTGTGCGTTCGCCTAGGGAT
CTGGCTGCTCGAAGCGCTCTCACCCGTCCCTCCTCCTCCATCAGCTGGCAGTTGACCAGAGTAACCA
>dme_circ_0004890_junction_seq
ATCAGCTGGCAGTTGACCAGAGTAACCAGTTTGAGCACCTCCTCCTGCATGGCGGG
>dme_circ_0004891
GGAACTGGCATCCTCTTGTCCGCACTGCAGCCCACAGATTTGAGCTCAACTGCTGGGGATTTTGGTGCTG
CAGGATCAGCTCGGTGACTGTGCGTTCGCCTAGGGATCTGGCTGCTCGAAGCGCTCTCACCCGTCCCTCC
TCCTCCATCAGCTGGCAGTTGACCAGA
>dme_circ_0004891_junction_seq
CTCCTCCATCAGCTGGCAGTTGACCAGAGGAACTGGCATCCTCTTGTCCGCACTGC
>dme_circ_0004892
CCTAGGATGTCGCATCTCCGGGGTTTGGGGCGACTGCTGATCGCCGTGACCTTCTTCACATGCGGATTCT
TCGTCAACATTGGCCAGCTCCTGCTGATTCTCCTCGTGAGGCCCTTCGATAAGAAGCTATCCCGCAGTCT
GGCCTACTACCTGCACTACTCCTTCTATTGCAGGTAAGTAACCATCATATTAATATAGATTATCATATCA
TTATGAGTATAAACGTTTCACATTTCATTCACATTAAGTATAGTGTAGTTTTCAGCACACTTCCTGTTTT
CCTGATTAGATTAGGTATATGCAAGGTCTTCTTTTGTATAAATCACTTAGTTTTTACATTTGATGATTAA
ATGAAATAACGTAAAAGTGGTACTTTTTGATAATTTAAAAACTATCCTCACACTTTAGTTCCTTGTCTGT
GTGGCGGAATGGTATGCGGGCAGCAAGCTGAGGGTGTACATCGATCCCCAGGACGAGCAGAAGTTCTTCG
GCAAGGAGCATGGACTGCTCCTGATGAACCACACGTACGAGATCGACTGGCTGACCGCCTGGATGATCAC
CGACAAGCTGGGCAATCTGGGAGGCACCAAGGCGTATGCCAAGAAGATGCTGCGCTATGTTCCGGTCCTG
GGATGGGTCTGGTGGATGGCCGAGTTCATCTTCCTTGACCGCAACTTCGAGAAGGACAAGGTGGTGATCA
AGACGCAGCTGAAGGAGGTCTTCTCCTATCCGGATCCCGTGTGGCTACTGCTGAACGCGGAGGGCACCCG
TTTCACACCCGCCAAACACGAGCTGTCAGTCAAGTTCGCCGAGGAGCGAGGTCTTCCCCTCCTCAAGCAC
CATCTGATTCCGCGCACTAAGGGATTCACGACCAGTCTGCCCACCATGCGTGGCATCTGTCCTGCCATCT
ACGACATCAATCTGGCCTTCAAGAAGAATGCCGAGGCCAAAGCCCACCATGCTGTCCCAGTTGAACGGAG
AGCCCGTGGAACCGTACATGTACATTCGCCGAGTGCCACTGGACGTGGTTCCCGATGACGAGAAGGAGGC
CGCCGCCTGGATGCAGGACTTCTTTGCGGAAAAGGACAAGATCATCGACAGCTTCCACGAGACCGGCAGC
TTCTTTAAGAATTCCGGAGTCAAGGAGGTGCCCGAGAAAATCTACAAGCCGCGCCTGAGCACGCTCCTCA
ACTTCTTGGGCTGGGCCACCTTTGCCGTCCTGTGCATTCTGCACTACTTGGTCACCTCGCTGGTTGCCGG
CAACTGGTTTGGCTTCATCACGGTTCTTTCCATTTTGGGAGGATGTCTATAGCCTCATGGAATACGCTGT
GAATGCTTCGAAGATCAGCAAAGCCTCTGcgtatggagcagctgctgccaagaaatagattttcatacct
gagttttataaactctgcaacagttttGGAATATACGACCTATGGACTAATAACTCAACTGCACTGACCA
TAGAAATATTATGTACATTGAATATGTACGATAAACCATTGTAGTTAACTGAATAAAATAACATTTTATG
CGATAACTGCTTATCGCAATCAGCTGAGAGCGCCTAGTGCGACCCTTCGTGTAAGCAGTGCAGCAAGACA
TTCAACGTTTTCAGCCAGCTGGATTCAGTATTCCGCCGCTCGTCACCTGCGGTCATACTGATTGCAACTT

CCCAAATTTCTTACAATAATCCAATGAAAATTAAAAAACATATTTTCGTACCTTTAATGCAATAATATGT
GCATTTTGAAGG
>dme_circ_0004892_junction_seq
TAATGCAATAATATGTGCATTTTGAAGGCCTAGGATGTCGCATCTCCGGGGTTTGG
>dme_circ_0004893
GTTGCCGGCAACCAGCGAGGTGACCAAGTAGTGCAGAATGCACAGGACGGCAAAGGTGGCCCAGCCCAAG
AAGTTGAGGAGCGTGCTCAGGCGCGGCTTGTAGATTTTCTCGGGCACCTCCTTGACTCCGGAATTCTTAA
AGAAGCTGCCGGTCTCGTGGAAGCTGTCGATGATCTT
>dme_circ_0004893_junction_seq
CGGTCTCGTGGAAGCTGTCGATGATCTTGTTGCCGGCAACCAGCGAGGTGACCAAG
>dme_circ_0004894
GGACGGCAAAGGTGGCCCAGCCCAAGAAGTTGAGGAGCGTGCTCAGGCGCGGCTTGTAGATTTTCTCGGG
CACCTCCTTGACTCCGGAATTCTTAAAGAAGCTGCCGGTCTCGTGGAAGCTGTCGATGATCTTGTCCTTT
TCCGCAAAGAAGTCCTGCATCCAGGCGGCGGCCTCCTTCTCGTCATCGGGAACCACGTCCAGTGGCACTC
GGCGAATGTACAT
>dme_circ_0004894_junction_seq
CGTCCAGTGGCACTCGGCGAATGTACATGGACGGCAAAGGTGGCCCAGCCCAAGAA
>dme_circ_0004895
CAAGGCATCTAATTATGGATCCTCCAAGTCGGTCGCAAAGTAACTTTCGAATTCGCATTTAGTTTCGATT
GTCGTCGAACTCAATTATCTACAAATGACTTTGTAAAAGGCAGTCCTCTCACAAATCTCTCTGCTAAATG
GATCAATTCGTGATTCCTATCCGGACGATTTAAAG
>dme_circ_0004895_junction_seq
TCGTGATTCCTATCCGGACGATTTAAAGCAAGGCATCTAATTATGGATCCTCCAAG
>dme_circ_0004896
ACCATCATCACCGAACAGCTCAAGGTGGTCTTCTCCTATCCCGATCCCACCTGGCTTTTGCTCAACGCCG
AGGGCACTCGCTTCACTCCCGCCAAGCACGAGGCATCCGTAAAATTCGCCCAGGAGAGGGGCATGACGGT
GCTGAAGCACCATTTGATCCCGCGCACCAAGGGATTCACCGCCAGCTTGGCACCCATCCGCGGTCTCTG
>dme_circ_0004896_junction_seq
CCAGCTTGGCACCCATCCGCGGTCTCTGACCATCATCACCGAACAGCTCAAGGTGG
>dme_circ_0004897
ATCGATTGGCTCAACGGCTGGATGATCTGCGAAAAGTTGGGCGTGCTGGGCAACTGCAAGGCCTATGCGA
AGAAGGCAATCCGCTACGTGCCCATCATCGGCTGGGGCTGGTGGCTGGCCGAGTTCGTCTTCCTCAATCG
AAACTTCGACCAGGACAAGACCATCATCACCGAACAGCTCAAGGTGGTCTTCTCCTATCCCGATCCCACC
TGGCTTTTGCTCAACGCCGAGGGCACTCGCTTCACTCCCGCCAAGCACGAGGCATCCGTAAAATTCGCCC
AGGAGAGGGGCATGACGGTGCTGAAGCACCATTTGATCCCGCGCACCAAGGGATTCACCGCCAGCTTGGC
ACCCATCCGCGG
>dme_circ_0004897_junction_seq
TCACCGCCAGCTTGGCACCCATCCGCGGATCGATTGGCTCAACGGCTGGATGATCT
>dme_circ_0004898
GTTTCGATTGAGGAAGACGAACTCGGCCAGCCACCAGCCCCAGCCGATGATGGGCACGTAGCGGATTGCC
TTCTTCGCATAGGCCTTGCAGTTGCCCAGCACGCCCAACTTTTCGCAGATCATCCAGCCGTTGAGCCAAT
CGATCTCGTATTTATGGTTCATGATCAGAAGGACGT
>dme_circ_0004898_junction_seq
TATTTATGGTTCATGATCAGAAGGACGTGTTTCGATTGAGGAAGACGAACTCGGCC

>dme_circ_0004899
GTAACCAGGACATGGCAAGCTCCTCGACACATTTGTCAGCCAATCTTTCAGAGACGCTTCGCAAGATTAG
GGCTTGGAAAAATTATTCGCATTTTAGTAACGTCTCAATGGAGGACAATAATTCGGATAATGAATCACAG
ATCGAACCGCTGTTCAACAGCAGTCCGATGGCCTCCCGTCGCCAACTGGCCAATAGACAG
>dme_circ_0004899_junction_seq
CTCCCGTCGCCAACTGGCCAATAGACAGGTAACCAGGACATGGCAAGCTCCTCGAC
>dme_circ_0004900
GAatgtacatacatatgtacatacatatAAAAGTAGTTACAATTGCGTACAAAATAAATGGTAAATTTTG
TTCGTTTGGATGGCCGAGTATTCTTTTTGGTTCGAATTGATTTGATTTTGTGTATGGGGGTGAAATATCG
ATGAGCATCTCATACTAAGGGATTTTGGTTAGCAAAATCATAATACACAGTACAATTACAATAATATTTA
TAGAAATAAA
>dme_circ_0004900_junction_seq
CAATTACAATAATATTTATAGAAATAAAGAatgtacatacatatgtacatacatat
>dme_circ_0004901
TTTTCATTCAAAAGCGATCTATATAATacactcatcgacactcacactcactctcacacacactcaacaa
aaaacacaacacaaaacaacacactctacacacaTATAGCATATGCAGCAACAATTGCAACTCGATGGTA
TTGTTTACTCTTCCAAAATTGATTAAGCTTaaagtaacgaaagtaacgaaaggaacCGCTACCAGTACGA
TAGGAAGATACGATAGTGATGGTTAGTAGGGATGGCATGCAATGGGGATGAGAATGCAAATGCAAATGCG
AATGCGAATGGTATAGGAATGATATAACGGAATGACGGCAATTAAGTTTATTTTTTATATATATATGATT
GCAAATAG
>dme_circ_0004901_junction_seq
TTTTTTATATATATATGATTGCAAATAGTTTTCATTCAAAAGCGATCTATATAATa
>dme_circ_0004902
TGCTTCCCCGACTGGGACGAGATCTGCACGAAGCTGGACCTCAATGCGCACATTCCCAAGCCAGAGTGAG
TAATCTTGGATGAAAACTTTCCAATGGATGATCGGTGTACTAATTCATTTTTCCCGCTTTTCCGACAGGC
CCGAGACCAAGGAGGGCAAGGAGGGTGGCGAGCAGGAGAAATCAAACGAACAGGAGGGCACTGAGGGCGA
CAAGATCGAGAAGGAGTTGGAGAAGGACAAGG
>dme_circ_0004902_junction_seq
ATCGAGAAGGAGTTGGAGAAGGACAAGGTGCTTCCCCGACTGGGACGAGATCTGCA
>dme_circ_0004903
CAACGATGGCGATGGTGGTGCGGGTGGAGAGGAGGGTGCCGGCTGGGATGTGGGCGATGATGACCTGGTT
GTGCCCGAGGAACTGGCCTCCAAGATCAAGGCCTCAGCCCTCGACAGCAACTACTATGCGGCTCCCAACA
AAGGCTTGTCTCCTGCCCAACAATGGGCCAACAACTCGCCTCTGG
>dme_circ_0004903_junction_seq
CAACAATGGGCCAACAACTCGCCTCTGGCAACGATGGCGATGGTGGTGCGGGTGGA
>dme_circ_0004904
GATAAGCTAACGAAAGTTGTCCGCAGTTTTTGAGGATGTTTACCCGCTCCTTGACGTCACCGAGAAGGAG
GGCACCCTGGTACTGGGCGGAAACATCCTTGCGAATCTCGGCAATCTTGTTCATCTTCTTGAGCTTCTCC
AAGTTGCCGGTGATCAGGTAGAGGAAGCTAAGCTT
>dme_circ_0004904_junction_seq
CGGTGATCAGGTAGAGGAAGCTAAGCTTGATAAGCTAACGAAAGTTGTCCGCAGTT
>dme_circ_0004905
GTCGTCGCCGCCAGAGACGAAGAGCGGCATCTGCTGGTGGAAGGCCACGCCTCGCACCGGTCCGTCGTGC
TCGTCGAACTTCTCGAGGAGCGTGTGCATCCGGTAGTCCCACAGCTGGATGACGCCGCTGTGCAAGCTGA

CCAGGATCCAGGGGCGCTTAGGGTGGAAGGAGAGGCCCTTCACCCTCGCCGACTTCGACTCGAAGTTGGT
CAGCATGGTGGCACTTTTTCTGTTCTGCCTGTAG
>dme_circ_0004905_junction_seq
GGTGGCACTTTTTCTGTTCTGCCTGTAGGTCGTCGCCGCCAGAGACGAAGAGCGGC
>dme_circ_0004906
GGATCTTGTTCATCAGCGGCACAGCCAATAGCATGGCAATCACGTAGGCCGATGACTTGAATGTCTTGAA
GTTGCTGTACGCACTCACATCCCAGTCAAATTTGCCCAGGGTGTACATGTAGAGATACTGCCCCTCGTCA
CGCTGGAAGGTGTACAGTGCCATGCTGACCAGAAGGATAATGAGAAAGCTGCGACGATGACCCTTTCGCG
GCTTCACCAACACCGCCAACGA
>dme_circ_0004906_junction_seq
TTCGCGGCTTCACCAACACCGCCAACGAGGATCTTGTTCATCAGCGGCACAGCCAA
>dme_circ_0004907
CATCCTTTTTGCGGACATCTGCGGTTTCACCAGCCTCTCGGATCAGTGCACCGCCGAGGAATTGGTGCGT
CTGCTGAACGAGCTCTTTGCCCGGTGAGTATTCGGATTCAGATCCGGACTCGGATGCGAACTGGATGTTG
CTGGGGCGGCCAGAAGTTGTGGTCATAATTAATGGCAAACAACTCATGCACTTGCCAAAAAAAGTGGGAT
GCTAATGGTCATTGAAAACGTTTATGGTAGATACATAATAACTGAAAATATTACATTCAAAACTAACTGA
CATTCACATTGACAAGTACTGAAACATGAAAAAAACTATATAACGGGTAGCATACATTTTTTGCAAGTGT
AAGTAAGAAAAGTAAGCTCGTTAAATTCTTAAAttttttttttCACAGTGCACGTCTATCTGCCTGAGTC
CTTTTCTTATTCATGAAATCGAAACCCAGACAGAGCCTGTGGCTGTATTAATTGCTCCAAGTGAACGAAG
AGAAAACTGACCTTGGGGCAAACAAATAAACCTGCTGACCTCTTTGTAACCGAGCTTAGGCACCAGTAAC
AAGTTGGTTTAATGGTCGAGGAAAAGATATAATTTAAGAAGTTAGGAAAGGGGTATGAAGATGGGATATA
TATATTATTCGGTTATTCTCGTTGACCTCTTAATAATATTAGTTTACAGGAGACTCTTTAATGACCATGG
TGGCAAAAGTAAATTTGTAGTGAGCCTTCGGAGGAAATTTAATCAAATTATTTAGCCTTTAATTAATTTA
AAGTTTATGATACCCTGAAACAAAAGCTTGGCTATTCAGTGGATGAGGATATTCTTTTTTCATTTCTTTT
GTGTCAGAAAAAGCTGAGATGAGGTGATGGCCATTGCTTATTGTGTATAGACTTACAGCTCTTGTGGCGA
TTTTCCTGTCCATTTTCCAACTTCTCCGTTTCCATTTTCCACTACCACTACCATTGCAGCGTGAGTATTC
GTGTCAGCAAACAGAAACACAAAGGAAACTGCTGTTTTGTGGCTTTGTTGTGGCTGCACCGCGGGAAAAT
CGAGAAAGGAGTTCCTTTCCTCGCATTCCTTTGCTTCTTTTAGACATTCTCACTTTGCCATACTTTGTAG
ACCTATGTAAAATCGGTGCTTATATACAAAATAAAACAATGCAAATTGGTTTTTTTGCAGAACTGGTTCG
CGAAGTGATGGCCGTGAACGTGAACATGCGAGTGGGCATCCATACGGGCCGTGTGCACTGCGGCGTTTTG
GGCCTGGTCAAATGGCAGTTCGACGTTTGGTCCAATGACGTCACGCTGGCCAATCACATGGAGAGTGGCG
GAATTCCCGGG
>dme_circ_0004907_junction_seq
TCACATGGAGAGTGGCGGAATTCCCGGGCATCCTTTTTGCGGACATCTGCGGTTTC
>dme_circ_0004908
CGCAAGTGGATCGAAGCACCCAGATGAGCGGTGGCCCAGGAGCCGGATCTGGTGGCGCCGTAGCTGGTGG
AGTGGGCGGTGGTGCCACCCACATCTTTCGCTCGCTGTCGTCGGAGAGCCAGAAGCGGGAGTGGTCCAAG
CGGCTCTCCCTCCGGGGAATGCGGCATGGAGGAGCTGCCGGTGGTCCTGGCGGATCCGGCACTTCGAGCC
TGGCCAGCAGCAAGGAGGGAGAGCAAACACAGAAAGTTCTTCAGCCGCTCGGCATCGCTGCGTCAGGCGG
GAAGCGGTGGAGGAGGAGTAGGAGTTGGAGGCGTAGTAGGAGGAAGTGGCAGTCACCAGGCGTCCATGGA
GCTGCATGTGCCTGGCCAGGAGGGTAGGGGCACCTCCTCCGGAGCGGGCTCGCCACAGACGCAGACCCAA
CCGTCGACGCCAAAGAAGTCCAACTGGGAGGTGATCGAACATTTCAATACGAGTGCCAAGGGAGGCAAGG
CCGTGGTTAGCAGCAGTTTGATTGCGG
>dme_circ_0004908_junction_seq

GCCGTGGTTAGCAGCAGTTTGATTGCGGCGCAAGTGGATCGAAGCACCCAGATGAG
>dme_circ_0004909
CTATACACCTCGACGGGCACCGTCGACTTGGCCTGAAAGATCTTGTTGTAGCGTCCCTCCATGATGTACT
GCTCGAGCGCCAGGATGGGCCGAATGAACTGATTGTGCTGGATGACGTCCACGGAGAGCAGCTCCAGTTC
CGTGTGAAAGTCGGATACCCGGTTGCCAGACAAGAGGTACAGCAGATTCAGACCGAGCAGCTTATACTTG
CTCTCCGATTCGCCAATGATCTTAGCGTAGTCATAGTAATAGCACTTCAGCTGCGCCATGTAGCGCTCGA
AGGCGAGCAGGTCCTTGCTGAGCACGCTATGCTCCACGGCCACCTCAAGCACGCTCCTAGCCAGGATCAA
TTGCTTCTTGGAGCTCTGGGCATCGTTTCCGTCCGTCGGCAGGAAGGCCATCTTCACGAGCGCTACTTTC
AGTTGATCCAGCAGCTGGCCGCATTTGACGGTGTTTGGTGGCCGCTTTGACCACTCGGCGGTCAGTTCTT
TGTACAGCGATTCCACGTTGGCCATTCTTTAGATTGGTATATTGAAGCTTAAATG
>dme_circ_0004909_junction_seq
TTTAGATTGGTATATTGAAGCTTAAATGCTATACACCTCGACGGGCACCGTCGACT
>dme_circ_0004910
TAATAGCACTTCAGCTGCGCCATGTAGCGCTCGAAGGCGAGCAGGTCCTTGCTGAGCACGCTATGCTCCA
CGGCCACCTCAAGCACGCTCCTAGCCAGGATCAATTGCTTCTTGGAGCTCTGGGCATCGTTTCCGTCCGT
CGGCAGGAAGGCCATCTTCACGAGCGCTACTTTCAGTTGATCCAGCAGCTGGCCGCATTTGACGGTGTTT
GGTGGCCGCTTTGACCACTCGGCGGTCAGTTCTTTGTACAGCGATTCCACGTTGGCCATTCTTTAGATTG
G
>dme_circ_0004910_junction_seq
ATTCCACGTTGGCCATTCTTTAGATTGGTAATAGCACTTCAGCTGCGCCATGTAGC
>dme_circ_0004911
ggaaagacaacgatctagggagaggcagcggtctaaggaaagacagcggtcaaaagaaagacagcgctct
attgaaagacagcgatccaaagaaagacaaagatctaaggaaagacagcggtctaGG
>dme_circ_0004911_junction_seq
aagatctaaggaaagacagcggtctaGGggaaagacaacgatctagggagaggcag
>dme_circ_0004912
TCTTCGGCGACTTGTCCAGCTTCAGTAAAAGTGGAAGGAGGAGCGGCACTGGTCGGGACTCTCCTGCCGT
CCACGAGCGGCTCCGAGAAGAACGACTGCGTGCGCTGGAGACACTGGAAAATCCCCGACCGTCGCGTTCC
AGCCAAGCAAAGAGCACTGCCCTCCAGATGCGCTCGTCCACAAACATCTCGGAGCTGGTAACAGACGACA
CCATCGATCTGAACAGCAGCAGCTCCTTTGCTTTGGTCATGCCCGCCACCAAGGTCACAAACATCTCTTT
TGAGCCTGCGGAGATCACCGGCAGGTCCACACTCTGCCAGGCGGGAAAACAGCGCCGCAGGGAGAAGCCA
TCCCTGTCGATGGCCGAAATTCTAAAGTCATCCTTTGTTGAGAAGGCTCGCCTGTTGGAAAGGAAGCTGC
AGGATGCCAGCCAGGCGGAGGCCAGCTATCACTTATCACGTGGCAGCTCCAGCAGCCTCTCAGATTCATT
CAACTGCTCGCGCAGCTTACCGCGCACTGCGGACATGAGTGAACTGAGTCTTAACGGAGGTGGGGGATTT
TCCTTTGGATCTGCCTCTTTGGCGGCGGAAGGCCAGGATGAGAGCTTTGCTCCCGCCGAGCTAATGCAGT
CCAAATTGGTGCTCGGTGAAATCTCCTGGGCGCAGGAGTTCACGGCCATGCCGACGGCCACGTTGAGCAC
ACAGGCGTTGAAGAAGATTGCCGCACCAACATCCGAGATAGAAACCTCGGTGTCCAAGTTCTGTCTTGGC
CGGAGATCCAGACTTTTCGCTGGGCAACTACTTCCAGACGCGTTCGGAAAATATATGGAACATTGTGAGC
AATGGCAGCCCGAATCAGAAAGTAACGTGACACTGGATTCGGTTGGCGAGAAGACCCCACAGCCAGACAA
TAAAACATACACTAAAACGGATGCCATAACTGGCTCGAATCTCGGACGCAATCTTATGCGTAAGATGCAA
CAGGATCGGATTGAAACTGCGCTAAAGAGCAGGAATGGTCTGGCAGCCAAAGAGACCAAGCGTCCGCCGT
CGAGCTCCGAGATCCTAAGTCTCTCCGCGATCGACATGGCGCTAAGGGACATAGATCTCAATTCGGATAC
ATCCACTGTAGAGGTTGTCAACCACCTGTGGGAGCATGGTCGTGGAAACAATTATGACGATGGGGAAAAC
AAGGAGAACCAGAGCAGCAACTCCCATGCGGAGCGCACTTTGTGCGGCTCCAACAAGCTTACCGACACCA

TGAGTTTCACGGACTCTGTGCTAAACTCAACGGATTTTCGTCATTTGCAGCAGAGCATTTCGCGCAAGCC
GCTCAGTCCAAATCCTGCCGCAGATCCAATCGTCACGCGCTGGAAGCGTGACGACCAAGGAGCAATTGTG
GCCAACCCGACAACCGGGAATATCATACAGATGGTTGCCATACAGCGGTCCGACAACAAGTTGTGGGCCA
TTCCCGGTGGAATGGTCGATCCCGGCGAGAATGTCAGTGTCACCCTTAAGCGGGAGTTCACCGAGGAGGC
ATTGAATTTCACGGACAAAGCCAACATGGTTGAGCGATTTTTTCAAGCGGGAGGCGTTCAGGTATACCAA
GGCTACGTAGACGATTTTCGAAACACGGACAACGCTTGGATGGAAACCACTGCGCTGAACTTCCACGATG
AAGACGGGAGCCAGGTGGGACAATTGGAGCTAATGGCCGGTGACGATGCCTCCAATGTGCGCTGGACGGA
CGTCGACTCGAATCTCAAGCTGCACGCCAATCATGCCGACATAGTCCGTGAGGTTGTCATCCGACGAAAT
GCACACTGGTAGAAACTAGACTGAAACTATAAGAGTTAAGATCAACTGCCTAATGGTTGAAGAAAAACCA
ATTTTAAATAATAACATAGCCTTCAATGAAACAAGTgttttCGCGTTCGCACTGCAAACTGTAACTGTTT
AAGGACAAAGCGGAATGGACAGTAGCAGTGGAAGCCAAGGAAATGGCAGTTTCATGGACCAGAACTCGCT
GGGCATCCTGAACATGGACAATCTAAAGGGTAAGCAGG
>dme_circ_0004912_junction_seq
AACATGGACAATCTAAAGGGTAAGCAGGTCTTCGGCGACTTGTCCAGCTTCAGTAA
>dme_circ_0004913
TCTTCGGCGACTTGTCCAGCTTCAGTAAAAGTGGAAGGAGGAGCGGCACTGGTCGGGACTCTCCTGCCGT
CCACGAGCGGCTCCGAGAAGAACGACTGCGTGCGCTGGAGACACTGGAAAATCCCCGACCGTCGCGTTCC
AGCCAAGCAAAGAGCACTGCCCTCCAGATGCGCTCGTCCACAAACATCTCGGAGCTGGTAACAGACGACA
CCATCGATCTGAACAGCAGCAGCTCCTTTGCTTTGGTCATGCCCGCCACCAAGGTCACAAACATCTCTTT
TGAGCCTGCGGAGATCACCGGCAGGTCCACACTCTGCCAGGCGGGAAAACAGCGCCGCAGGGAGAAGCCA
TCCCTGTCGATGGCCGAAATTCTAAAGTCATCCTTTGTTGAGAAGGCTCGCCTGTTGGAAAGGAAGCTGC
AGGATGCCAGCCAGGCGGAGGCCAGCTATCACTTATCACGTGGCAGCTCCAGCAGCCTCTCAGATTCATT
CAACTGCTCGCGCAGCTTACCGCGCACTGCGGACATGAGTGAACTGAGTCTTAACGGAGGTGGGGGATTT
TCCTTTGGATCTGCCTCTTTGGCGGCGGAAGGCCAGGATGAGAGCTTTGCTCCCGCCGAGCTAATGCAGT
CCAAATTGGTGCTCGGTGAAATCTCCTGGGCGCAGGAGTTCACGGCCATGCCGACGGCCACGTTGAGCAC
ACAGGCGTTGAAGAAGATTGCCGCACCAACATCCGAGATAGAAACCTCGGTGTCCAAGTTCTGTCTTGGC
CGGAGATCCAGACTTTTCGCTGGGCAACTACTTCCAGACGCGTTCGGAAAATATATGGAACATTGTGAGC
AATGGCAGCCCGAATCAGAAAGTAACGTGACACTGGATTCGGTTGGCGAGAAGACCCCACAGCCAGACAA
TAAAACATACACTAAAACGGATGCCATAACTGGCTCGAATCTCGGACGCAATCTTATGCGTAAGATGCAA
CAGGATCGGATTGAAACTGCGCTAAAGAGCAGGAATGGTCTGGCAGCCAAAGAGACCAAGCGTCCGCCGT
CGAGCTCCGAGATCCTAAGTCTCTCCGCGATCGACATGGCGCTAAGGGACATAGATCTCAATTCGGATAC
ATCCACTGTAGAGGTTGTCAACCACCTGTGGGAGCATGGTCGTGGAAACAATTATGACGATGGGGAAAAC
AAGGAGAACCAGAGCAGCAACTCCCATGCGGAGCGCACTTTGTGCGGCTCCAACAAGCTTACCGACACCA
TGAGTTTCACGGACTCTGTGCTAAACTCAACGGATTTTCGTCATTTGCAGCAGAGCATTTCGCGCAAGCC
GCTCAGTCCAAATCCTGCCGCAGATCCAATCGTCACGCGCTGGAAGCGTGACGACCAAGGAGCAATTGTG
GCCAACCCGACAACCGGGAATATCATACAGATGGTTGCCATACAGCGGTCCGACAACAAGTTGTGGGCCA
TTCCCGGTGGAATGGTCGATCCCGGCGAGAATGTCAGTGTCACCCTTAAGCGGGAGTTCACCGAGGAGGC
ATTGAATTTCACGGACAAAGCCAACATGGTTGAGCGATTTTTTCAAGCGGGAGGCGTTCAGGTATACCAA
GGCTACGTAGACGATTTTCGAAACACGGACAACGCTTGGATGGAAACCACTGCGCTGAACTTCCACGATG
AAGACGGGAGCCAGGTGGGACAATTGGAGCTAATGGCCGGTGACGATGCCTCCAATGTGCGCTGGACGGA
CGTCGACTCGAATCTCAAGCTGCACGCCAATCATGCCGACATAGTCCGTGAGGTTGTCATCCGACGAAAT
GCACACTGGTAGAAACTAGACTGAAACTATAAGAGTTAAGATCAACTGCCTAATGGTTGAAGAAAAACCA
ATTTTAAATAATAACATAGCCTTCAATGAAACAAGTgttttCGCGTTCGCACTGCAAACTGTAACTGTTT
AAGGACAAAGCGGAATGGACAGTAGCAGTGGAAGCCAAGGAAATGGCAGTTTCATGGACCAGAACTCGCT

GGGCATCCTGAACATGGACAATCTAAAGGG
>dme_circ_0004913_junction_seq
GCATCCTGAACATGGACAATCTAAAGGGTCTTCGGCGACTTGTCCAGCTTCAGTAA
>dme_circ_0004914
GAATCAAATCGAACATGCTACAGGTCCTCATCGCGGTGAAGGAAAAGACCGTGAAGGGCACGGTGGCCTT
TATAATTGGATCGCTGGTGCGCCACGAGGAGGGCGAGCAAAACTCCTGGAGGGAGGAAATACTGAAGTTT
ATTTACGAACGTTGCAGCAGCCCCGATCCAATAGAAAGCGAGCGGGGCAGCTCAATCTTTTCCTCGCTCA
TGGACGCTGCTCCCGATCAGTTTTCGGACCACACGGATACTATGTTCCCATTGCTTGCCGGCATTCTAGT
TACCGCCGAGGCGAATGGGAACATGGCCACACCCACCGTGCATAACATGCTGACCGGTTCATGCTTCTTG
TTGCCTTTCGTAAGTGGACATAGTAATGCTGAGCAGATTGTGGTTAAGGCTGTGCCGCTCATACTCAAGG
CCCTGGCCGCCTTTGTCGAAAAGGGGTACAGTATTGAGTTCATGGGTGCCTTTGATATCATCGACAGCAT
GGCCGAGCATGTGCCCCACTTGCTGACCGGTAATGTGAAGCTGCTTCTGGAGTTTTGTCTGATGATTGCG
AGAAACAAGCAGTTCGACGCTTCGATTCGGGTTCAGGTGCTTACCTTTGTAGGCAGCCTAGTTCGCCTCA
AGAAGAAAATTATTATGAAGCAGAAACTACTACAGCCCACACTATCTGTTCTTTTCGAAGTGATTTGCCA
GGACGACCTCGAGGAAGGTGGACGATGATTACTTTTCCTCGGAGAGCCTGAGTAGTCCATCCAATGCAGC
TGCGCAGACACTGGACCTGATGGCCCTTCACATGGTGCCGGACAAGTTTATTCCGCCACTACTGGATTTG
CTGGAGCCTGCGTTGCAGAGCCCGGAACCTGTGCTTCGCCGTTCCTCTTTTATTTGCATGGGCGTTATTG
CCGAGGGCTGTTCCGAGGCCATTGGGAAAAAGTATCTCGAGGTCATGCTAAATATCATCAAAGCTGGAGT
GTTGGACTCTGTTATGTTCGTGCGGACCGCCGCATTCTTCGCCCTCGGTCAGTTTTCCGAGTTTCTTCAG
CCGACGATCTGCAAGTTTGCTCCCCAAATTCTACCCGTGTTGTTCGACTATCTAAACCAGCTGGTACTGG
AACTCAAGGGTAGGTAATCATTGTGGCCATCTTCGACCAACTTGGAATTTGTATATTTTACATTCTCGTT
TATTTGTTTGTGCTGCTTCTAAAGGCTGGCCATAACAGCAATGGGCCAAATCTCGATTCCGTACCTGCCA
AAGATTCCATAATGTACTTAGACAAGATACAGATCAAGCGAAGATCGGTCCTCGCCAAATATAGGGTGCT
TTTTCGATGTCTTACTGTTTACTATGTACTTGATTGCACATCAAACACCTAAAATTTGTTGAATAGTTTG
CCAGATTGATTACATTAAGAGCTCTGTTGTAGATATATCATTTCGCACTGGTTTATTTAACTTTTTGGCG
TATGTTAAATGTTCATTGACTCGTTTCTTAATTGTTATTTAAATGAGTAATAAGTTCGTCGGTTACATAA
AGAAGGTGTCTTCAAATGGGGTTCTTTTCACGTTTTTGAGGACTGCATTCCGTTTTGCACCAACAAAGC
CAACAAATTGTTTGGCAGAAACATACTAAATGTATTTTCTTTCAGGTCTGTTTGGGGTTATGGAACCGCA
GAACTCAAATCAAATGCGTGAAATGGCTTTGTCGGCCATAGCAGCGGTGTCAGCAGCAGCTAAGGAAAAC
CTGATGCCATATTTCCCCAGGATCATGACCGTACTACAGGGCTGTCTGGTGAAGGATTGCCCAAAAGAAA
TGTACAGTCAGCGCATCCAAGCTATCGACACTCTGGCCGCGCTGTGTCGCGAATTGGGCAAGGATAATAT
TATTCCTCTTGCCGACGATACAATGAACTTCTGCCTGATGATGCTGGAAGACGGTCCGGATGATCCCGAG
TACCGCAGGAGTATATACAACCTGATGTCCTCCCTGTCATCAGTTGTCAACGAGAGCATGGCCAGTGTGT
TCCCCAAGTTCATTGATCGCATAATGGAGTCTGTGATTTTTTCGGAGGACATGGTGCCAAATGTGTCGGA
CAATGCGGACGATGATTTGGCCCTAGTAGATGCCCCCGACATAGAGATTGATTTAGAGCACACAGACGAC
GAAGATGACCAAGACGCTTACCCGGTGGAGAATGATTATATCGTCGAAAAGGAGGAGGCCATTCTGTCAC
TCAAGGAGTTTGCTACCCATACCGGCGCCGCCTTCGCGCCCTATTTGCAATCTGCATTCGAGAACGTCTA
CAAGATGATTGATCATCCGCAAGGCGACGTTCGTATGGCATGTATTGACTCCATTTGCTCGTTCATTACG
GCTCTCCACAAGTTGGATGATGCCGCCGGCCTGAAGCGCGCCTGCGAGATTGCCATTCCAAAGTTTGCAC
ATATAATGCGCACCGACGACCAGGTTGCGGTCGTACTCCGCATGCTTGACGTCCTCTACGATGTCTTCAA
GTACGTGCCGGCGATAAACAGCCAGGAGCACGCCGAACTTATATTCGGTTGCATCAGGGACATCTTCACA
AATAAAATGGCCTGTCAGTTTAACGAGGAGAGCGGCGGCGGAGATGATGAGTGTTCGGAGGAAAGCGAGA
ACGACGAGATGCTGTTCGAGAACGCTGCCAATCTGTTCCCCATGTTTGGCTTAACCCTTCAGCCGGAGCT
GTTCTCGCTTTATTTTGGACGCCTTTACCATTTCTATATTCAAAGGCTGGGCGAAGGTAAAGGAGCGCGA

TTTACCAGAACAGCGGGCATACATCTATGGCGCACTTGCTGACTGCTGCAAGGCGTTAAAAGGTTGCTGT
GCTACTTATTTCGATGCCCTACGTCCCATTTTTATCGCCGGTTCCAGGGATTCTGATGCCAAAGCGCGAC
AGAACTCCTATTTTGCACTAGGCGAAATTGTCTTCCATTCCGAGGAAAAATCATTCGAGGTCTTATCCGA
CAATTTTGCAAGCCCTTTCCGAAGCAATTGTCAGGGAATCCGTTCCTGCCGCCATGGACAACATTTGCGG
AGCAGTTGCTCGTCTCATAGTTACCAACCCAGATTCAGTGCCGCTTGGCCAGGTGCTGCCCGTGTGGCTC
AACCATTTGCCACTGAAGGATGACACCGTGGAGAACGATGTGATTCAGAAGGCGTTCCGTGTGCTATACT
TGAAGGCCCGCCCCAGCATTGAGGCACATCTTGAGCAAATCCTGGCCATCACCATAGAGGCGAGTTACAA
GAGACAGATGCCTGACGTCGAGACAACCGAGAGCGCGGTGGCCCTCATCAAAGAGATTCGCGCCAACTAC
CCGGAACTGTTCAGCAAAGTATCGAACATGAATCCAGAAGTGTTCAATTATGTGCAAGCCCTGTAAGCCC
ACATATCCGTGTGAAAACCACCAAACAGCTCAGCACACCCAACGTCCTCCGCCCGCAGATTCATTTATTG
GAAGTGATGCAAGCTTTTTATACGAATACTTTCGAAATAGTCTTAAATTTCAGTTGTGCTTCTGTGGAGT
TAAATCATTCAAGATGAGCGATCTTTAAAATGGCAAGATGAAGGTTTCTACGCGCCAAAAAACGGAGGCA
AATGTTTTCTAACTTATAACCACATCCAGTGAGACATTAATATGCATTGAAATAATATAGGGATTACTTG
TTTCATTGACAACGTGCAACGCCAACGCGCCTCCAGCCCTGGTCTCTTTTTCGCGAATTCCTCCCCGGTA
TCCCACTTTTCAATTCTCAATTCCGCGTAACGTCGGCTGTAATTTGTACCGGATTGCATTGGATTATCCG
CCTGGGCGCGAACAGTAACTCCCCAAAAATGGAGGCAGCTATTCTGGATATAATCAACGGGATCCTGGCC
ATAGACACGGAACGAATCCGTGAGGGTGAGCAAAGCGGGTCAAATTGACCATCCGGCAATGAGAAAGGAG
GTTATCCTGGGCGCCAATGGATGTCTTTCATGTATGCTTTCCTTTCTGACTGCTCCATGTCGGTCTGACC
GCGTGGCTGGATCGCATTCCCTATATACATGCGTACATATCTATCTATATATGTACGCCACATGAACACA
GTGCCATATAGTTCGGCATGGGCACATTCCTCTATACATGAAATGTATAGAAATGTATACATGATCAGAA
CAAACTTCCGAAAACTGTTGAaattttttagaaataattatttatatatttataattaatattaattaat
taatattatatttttatttgcattaaattCGTGGATATACCATCATTCTTTTCCAAGAATCTTTGGTCTC
TCATGTTCTTGAAATGCTTCCTAATTGAAAAATTACTTATGTTGATAGCATGATAGCATAGGGCCATAAA
TAATAAAGTTCCATTTCTTTCGCTTCCTTAGGTCCACAGCCAAGATGCTAAAGGCCTACGAGAATCCCGA
TTCTCTGCTGGTTCTCACCCAGATCGTGATGTCGGACAGGCCGGTCCAAGAGCGGCAGGTCGCCGCCGTG
CTCCTCAAGAGGCGGGTCAAAAAGTTACGCCACTGGCAGTTGGTTCCCGCCGAGCACCAGGCAGC
>dme_circ_0004914_junction_seq
AGTTGGTTCCCGCCGAGCACCAGGCAGCGAATCAAATCGAACATGCTACAGGTCCT
>dme_circ_0004915
GACGATGATTACTTTTCCTCGGAGAGCCTGAGTAGTCCATCCAATGCAGCTGCGCAGACACTGGACCTGA
TGGCCCTTCACATGGTGCCGGACAAGTTTATTCCGCCACTACTGGATTTGCTGGAGCCTGCGTTGCAGAG
CCCGGAACCTGTGCTTCGCCGTTCCTCTTTTATTTGCATGGGCGTTATTGCCGAGGGCTGTTCCGAGGCC
ATTGGGAAAAAGTATCTCGAGGTCATGCTAAATATCATCAAAGCTGGAGTGTTGGACTCTGTTATGTTCG
TGCGGACCGCCGCATTCTTCGCCCTCGGTCAGTTTTCCGAGTTTCTTCAGCCGACGATCTGCAAGTTTGC
TCCCCAAATTCTACCCGTGTTGTTCGACTATCTAAACCAGCTGGTACTGGAACTCAAGGGTAGGTAATCA
TTGTGGCCATCTTCGACCAACTTGGAATTTGTATATTTTACATTCTCGTTTATTTGTTTGTGCTGCTTCT
AAAGGCTGGCCATAACAGCAATGGGCCAAATCTCGATTCCGTACCTGCCAAAGATTCCATAATGTACTTA
GACAAGATACAGATCAAGCGAAGATCGGTCCTCGCCAAATATAGGGTGCTTTTTCGATGTCTTACTGTTT
ACTATGTACTTGATTGCACATCAAACACCTAAAATTTGTTGAATAGTTTGCCAGATTGATTACATTAAGA
GCTCTGTTGTAGATATATCATTTCGCACTGGTTTATTTAACTTTTTGGCGTATGTTAAATGTTCATTGAC
TCGTTTCTTAATTGTTATTTAAATGAGTAATAAGTTCGTCGGTTACATAAAGAAGGTGTCTTCAAATGGG
GTTCTTTTCACGTTTTTTGAGGACTGCATTCCGTTTTGCACCAACAAAGCCAACAAATTGTTTGGCAGAA
ACATACTAAATGTATTTTCTTTCAGGTCTGTTTGGGGTTATGGAACCGCAGAACTCAAATCAAATGCGTG
AAATGGCTTTGTCGGCCATAGCAGCGGTGTCAGCAGCAGCTAAGGAAAACCTGATGCCATATTTCCCCAG

GATCATGACCGTACTACAGGGCTGTCTGGTGAAGGATTGCCCAAAAGAAATGTACAGTCAGCGCATCCAA
GCTATCGACACTCTGGCCGCGCTGTGTCGCGAATTGGGCAAGGATAATATTATTCCTCTTGCCGACGATA
CAATGAACTTCTGCCTGATGATGCTGGAAGACGGTCCGGATGATCCCGAGTACCGCAGGAGTATATACAA
CCTGATGTCCTCCCTGTCATCAGTTGTCAACGAGAGCATGGCCAGTGTGTTCCCCAAGTTCATTGATCGC
ATAATGGAGTCTGTGATTTTTTCGGAGGACATGGTGCCAAATGTGTCGGACAATGCGGACGATGATTTGG
CCCTAGTAGATGCCCCCGACATAGAGATTGATTTAGAGCACACAGACGACGAAGATGACCAAGACGCTTA
CCCGGTGGAGAATGATTATATCGTCGAAAAGGAGGAGGCCATTCTGTCACTCAAGGAGTTTGCTACCCAT
ACCGGCGCCGCCTTCGCGCCCTATTTGCAATCTGCATTCGAGAACGTCTACAAGATGATTGATCATCCGC
AAGGCGACGTTCGTATGGCATGTATTGACTCCATTTGCTCGTTCATTACGGCTCTCCACAAGTTGGATGA
TGCCGCCGGCCTGAAGCGCGCCTGCGAGATTGCCATTCCAAAGTTTGCACATATAATGCGCACCGACGAC
CAGGTTGCGGTCGTACTCCGCATGCTTGACGTCCTCTACGATGTCTTCAAGTACGTGCCGGCGATAAACA
GCCAGGAGCACGCCGAACTTATATTCGGTTGCATCAGGGACATCTTCACAAATAAAATGGCCTGTCAGTT
TAACGAGGAGAGCGGCGGCGGAGATGATGAGTGTTCGGAGGAAAGCGAGAACGACGAGATGCTGTTCGAG
AACGCTGCCAATCTGTTCCCCATGTTTGGCTTAACCCTTCAGCCGGAGCTGTTCTCGCTTTATTTTGGAC
GCCTTTACCATTTCTATATTCAAAGGCTGGGCGAAGGTAAAGGAGCGCGATTTACCAGAACAGCGGGCAT
ACATCTATGGCGCACTTGCTGACTGCTGCAAGGCGTTAAAAGGTTGCTGTGCTACTTATTTCGATGCCCT
ACGTCCCATTTTTATCGCCGGTTCCAGGGATTCTGATGCCAAAGCGCGACAGAACTCCTATTTTGCACTA
GGCGAAATTGTCTTCCATTCCGAGGAAAAATCATTCGAGGTCTTATCCGACAATTTTGCAAGCCCTTTCC
GAAGCAATTGTCAGGGAATCCGTTCCTGCCGCCATGGACAACATTTGCGGAGCAGTTGCTCGTCTCATAG
TTACCAACCCAGATTCAGTGCCGCTTGGCCAGGTGCTGCCCGTGTGGCTCAACCATTTGCCACTGAAGGA
TGACACCGTGGAGAACGATGTGATTCAGAAGGCGTTCCGTGTGCTATACTTGAAGGCCCGCCCCAGCATT
GAGGCACATCTTGAGCAAATCCTGGCCATCACCATAGAGGCGAGTTACAAGAGACAGATGCCTGACGTCG
AGACAACCGAGAGCGCGGTGGCCCTCATCAAAGAGATTCGCGCCAACTACCCGGAACTGTTCAGCAAAGT
ATCGAACATGAATCCAGAAGTGTTCAATTATGTGCAAGCCCTGTAAGCCCACATATCCGTGTGAAAACCA
CCAAACAGCTCAGCACACCCAACGTCCTCCGCCCGCAGATTCATTTATTGGAAGTGATGCAAGCTTTTTA
TACGAATACTTTCGAAATAGTCTTAAATTTCAGTTGTGCTTCTGTGGAGTTAAATCATTCAAGATGAGCG
ATCTTTAAAATGGCAAGATGAAGGTTTCTACGCGCCAAAAAACGGAGGCAAATGTTTTCTAACTTATAAC
CACATCCAGTGAGACATTAATATGCATTGAAATAATATAGGGATTACTTGTTTCATTGACAACGTGCAAC
GCCAACGCGCCTCCAGCCCTGGTCTCTTTTTCGCGAATTCCTCCCCGGTATCCCACTTTTCAATTCTCAA
TTCCGCGTAACGTCGGCTGTAATTTGTACCGGATTGCATTGGATTATCCGCCTGGGCGCGAACAGTAACT
CCCCAAAAATGGAGGCAGCTATTCTGGATATAATCAACGGGATCCTGGCCATAGACACGGAACGAATCCG
TGAGGGTGAGCAAAGCGGGTCAAATTGACCATCCGGCAATGAGAAAGGAGGTTATCCTGGGCGCCAATGG
ATGTCTTTCATGTATGCTTTCCTTTCTGACTGCTCCATGTCGGTCTGACCGCGTGGCTGGATCGCATTCC
CTATATACATGCGTACATATCTATCTATATATGTACGCCACATGAACACAGTGCCATATAGTTCGGCATG
GGCACATTCCTCTATACATGAAATGTATAGAAATGTATACATGATCAGAACAAACTTCCGAAAACTGTTG
Aaattttttagaaataattatttatatatttataattaatattaattaattaatattatatttttatttg
cattaaattCGTGGATATACCATCATTCTTTTCCAAGAATCTTTGGTCTCTCATGTTCTTGAAATGCTTC
CTAATTGAAAAATTACTTATGTTGATAGCATGATAGCATAGGGCCATAAATAATAAAGTTCCATTTCTTT
CGCTTCCTTAGGTCCACAGCCAAGATGCTAAAGGCCTACGAGAATCCCGATTCTCTGCTGGTTCTCACCC
AGATCGTGATGTCGGACAGGCCGGTCCAAGAGCGGCAGGTCGCCGCCGTGCTCCTCAAGAGGCGGGTCAA
AAAGTTACGCCACTGGCAGTTGGTTCCCGCCGAGCACCAGGCAGCGAATCAAATCGAACATGCTACAGGT
CCTCATCGCGGTGAAGGAAAAGACCGTGAAGGGCACGGTGGCCTTTATAATTGGATCGCTGGTGCGCCAC
GAGGAGGACAAGCAAAACTCCTGGAGGGAGGAAATACTGAAGTTTATTTACGAACGTTGCAGCAGCCCCG
ATCCAATAGAAAGCGAGCGGGGCAGCTCAATCTTTTCCTCGCTCATGGACGCTGCTCCCGATCAGTTTTC

GAACCACACGGATACTATCTTCCCGTTGCTTGCCGGCATTCTAGTTACCGCCGAGGCGAATGGGAACATG
GCCACACCCACCGTGCATAACATGTTGGCGGGTACGTGCGTCCTGTTGCCTTTCATAAGTGGACATAGTG
ATGCTGAGCAGATTATGGTTAAGGCTGTGCCGCTCATACTCAAGGCCCTGGCCGCCTTTGCCGAAAAGGG
GAACAGTAATGAGTTCATGGGTGCCTTTGATATCATCGACAGCATGGCCGAGTATGTGCCCCACTTGCTG
ACCGGCAATGTGAAGCTGCTTCTGGAGTTTTGTCTGATGATTGCGAGAAACAAGCAGTTCGACGCTTCGA
TTCGGGTTCAGGTGCTTACCTTTGTGGGCAGCCTAGTTCGCCTCAAGAAGAAAATTATTATGAAGCAGAA
ACTACTACAGCCCACACTATCTGTTCTTTTCGAAGTGATTTGCCAGGACGACCTCAAGGAAG
>dme_circ_0004915_junction_seq
GTGATTTGCCAGGACGACCTCAAGGAAGGACGATGATTACTTTTCCTCGGAGAGCC
>dme_circ_0004916
GACGATGATTACTTTTCCTCGGAGAGCCTGAGTAGTCCATCCAATGCAGCTGCGCAGACACTGGACCTGA
TGGCCCTTCACATGGTGCCGGACAAGTTTATTCCGCCACTACTGGATTTGCTGGAGCCTGCGTTGCAGAG
CCCGGAACCTGTGCTTCGCCGTTCCTCTTTTATTTGCATGGGCGTTATTGCCGAGGGCTGTTCCGAGGCC
ATTGGGAAAAAGTATCTCGAGGTCATGCTAAATATCATCAAAGCTGGAGTGTTGGACTCTGTTATGTTCG
TGCGGACCGCCGCATTCTTCGCCCTCGGTCAGTTTTCCGAGTTTCTTCAGCCGACGATCTGCAAGTTTGC
TCCCCAAATTCTACCCGTGTTGTTCGACTATCTAAACCAGCTGGTACTGGAACTCAAGGGTAGGTAATCA
TTGTGGCCATCTTCGACCAACTTGGAATTTGTATATTTTACATTCTCGTTTATTTGTTTGTGCTGCTTCT
AAAGGCTGGCCATAACAGCAATGGGCCAAATCTCGATTCCGTACCTGCCAAAGATTCCATAATGTACTTA
GACAAGATACAGATCAAGCGAAGATCGGTCCTCGCCAAATATAGGGTGCTTTTTCGATGTCTTACTGTTT
ACTATGTACTTGATTGCACATCAAACACCTAAAATTTGTTGAATAGTTTGCCAGATTGATTACATTAAGA
GCTCTGTTGTAGATATATCATTTCGCACTGGTTTATTTAACTTTTTGGCGTATGTTAAATGTTCATTGAC
TCGTTTCTTAATTGTTATTTAAATGAGTAATAAGTTCGTCGGTTACATAAAGAAGGTGTCTTCAAATGGG
GTTCTTTTCACGTTTTTTGAGGACTGCATTCCGTTTTGCACCAACAAAGCCAACAAATTGTTTGGCAGAA
ACATACTAAATGTATTTTCTTTCAGGTCTGTTTGGGGTTATGGAACCGCAGAACTCAAATCAAATGCGTG
AAATGGCTTTGTCGGCCATAGCAGCGGTGTCAGCAGCAGCTAAGGAAAACCTGATGCCATATTTCCCCAG
GATCATGACCGTACTACAGGGCTGTCTGGTGAAGGATTGCCCAAAAGAAATGTACAGTCAGCGCATCCAA
GCTATCGACACTCTGGCCGCGCTGTGTCGCGAATTGGGCAAGGATAATATTATTCCTCTTGCCGACGATA
CAATGAACTTCTGCCTGATGATGCTGGAAGACGGTCCGGATGATCCCGAGTACCGCAGGAGTATATACAA
CCTGATGTCCTCCCTGTCATCAGTTGTCAACGAGAGCATGGCCAGTGTGTTCCCCAAGTTCATTGATCGC
ATAATGGAGTCTGTGATTTTTTCGGAGGACATGGTGCCAAATGTGTCGGACAATGCGGACGATGATTTGG
CCCTAGTAGATGCCCCCGACATAGAGATTGATTTAGAGCACACAGACGACGAAGATGACCAAGACGCTTA
CCCGGTGGAGAATGATTATATCGTCGAAAAGGAGGAGGCCATTCTGTCACTCAAGGAGTTTGCTACCCAT
ACCGGCGCCGCCTTCGCGCCCTATTTGCAATCTGCATTCGAGAACGTCTACAAGATGATTGATCATCCGC
AAGGCGACGTTCGTATGGCATGTATTGACTCCATTTGCTCGTTCATTACGGCTCTCCACAAGTTGGATGA
TGCCGCCGGCCTGAAGCGCGCCTGCGAGATTGCCATTCCAAAGTTTGCACATATAATGCGCACCGACGAC
CAGGTTGCGGTCGTACTCCGCATGCTTGACGTCCTCTACGATGTCTTCAAGTACGTGCCGGCGATAAACA
GCCAGGAGCACGCCGAACTTATATTCGGTTGCATCAGGGACATCTTCACAAATAAAATGGCCTGTCAGTT
TAACGAGGAGAGCGGCGGCGGAGATGATGAGTGTTCGGAGGAAAGCGAGAACGACGAGATGCTGTTCGAG
AACGCTGCCAATCTGTTCCCCATGTTTGGCTTAACCCTTCAGCCGGAGCTGTTCTCGCTTTATTTTGGAC
GCCTTTACCATTTCTATATTCAAAGGCTGGGCGAAGGTAAAGGAGCGCGATTTACCAGAACAGCGGGCAT
ACATCTATGGCGCACTTGCTGACTGCTGCAAGGCGTTAAAAGGTTGCTGTGCTACTTATTTCGATGCCCT
ACGTCCCATTTTTATCGCCGGTTCCAGGGATTCTGATGCCAAAGCGCGACAGAACTCCTATTTTGCACTA
GGCGAAATTGTCTTCCATTCCGAGGAAAAATCATTCGAGGTCTTATCCGACAATTTTGCAAGCCCTTTCC
GAAGCAATTGTCAGGGAATCCGTTCCTGCCGCCATGGACAACATTTGCGGAGCAGTTGCTCGTCTCATAG

TTACCAACCCAGATTCAGTGCCGCTTGGCCAGGTGCTGCCCGTGTGGCTCAACCATTTGCCACTGAAGGA
TGACACCGTGGAGAACGATGTGATTCAGAAGGCGTTCCGTGTGCTATACTTGAAGGCCCGCCCCAGCATT
GAGGCACATCTTGAGCAAATCCTGGCCATCACCATAGAGGCGAGTTACAAGAGACAGATGCCTGACGTCG
AGACAACCGAGAGCGCGGTGGCCCTCATCAAAGAGATTCGCGCCAACTACCCGGAACTGTTCAGCAAAGT
ATCGAACATGAATCCAGAAGTGTTCAATTATGTGCAAGCCCTGTAAGCCCACATATCCGTGTGAAAACCA
CCAAACAGCTCAGCACACCCAACGTCCTCCGCCCGCAGATTCATTTATTGGAAGTGATGCAAGCTTTTTA
TACGAATACTTTCGAAATAGTCTTAAATTTCAGTTGTGCTTCTGTGGAGTTAAATCATTCAAGATGAGCG
ATCTTTAAAATGGCAAGATGAAGGTTTCTACGCGCCAAAAAACGGAGGCAAATGTTTTCTAACTTATAAC
CACATCCAGTGAGACATTAATATGCATTGAAATAATATAGGGATTACTTGTTTCATTGACAACGTGCAAC
GCCAACGCGCCTCCAGCCCTGGTCTCTTTTTCGCGAATTCCTCCCCGGTATCCCACTTTTCAATTCTCAA
TTCCGCGTAACGTCGGCTGTAATTTGTACCGGATTGCATTGGATTATCCGCCTGGGCGCGAACAGTAACT
CCCCAAAAATGGAGGCAGCTATTCTGGATATAATCAACGGGATCCTGGCCATAGACACGGAACGAATCCG
TGAGGGTGAGCAAAGCGGGTCAAATTGACCATCCGGCAATGAGAAAGGAGGTTATCCTGGGCGCCAATGG
ATGTCTTTCATGTATGCTTTCCTTTCTGACTGCTCCATGTCGGTCTGACCGCGTGGCTGGATCGCATTCC
CTATATACATGCGTACATATCTATCTATATATGTACGCCACATGAACACAGTGCCATATAGTTCGGCATG
GGCACATTCCTCTATACATGAAATGTATAGAAATGTATACATGATCAGAACAAACTTCCGAAAACTGTTG
Aaattttttagaaataattatttatatatttataattaatattaattaattaatattatatttttatttg
cattaaattCGTGGATATACCATCATTCTTTTCCAAGAATCTTTGGTCTCTCATGTTCTTGAAATGCTTC
CTAATTGAAAAATTACTTATGTTGATAGCATGATAGCATAGGGCCATAAATAATAAAGTTCCATTTCTTT
CGCTTCCTTAGGTCCACAGCCAAGATGCTAAAGGCCTACGAGAATCCCGATTCTCTGCTGGTTCTCACCC
AGATCGTGATGTCGGACAGGCCGGTCCAAGAGCGGCAGGTCGCCGCCGTGCTCCTCAAGAGGCGGGTCAA
AAAGTTACGCCACTGGCAGTTGGTTCCCGCCGAGCACCAGGCAGCGAATCAAATCGAACATGCTACAGGT
CCTCATCGCGGTGAAGGAAAAGACCGTGAAGGGCACGGTGGCCTTTATAATTGGATCGCTGGTGCGCCAC
GAGGAGGACAAGCAAAACTCCTGGAGGGAGGAAATACTGAAGTTTATTTACGAACGTTGCAGCAGCCCCG
ATCCAATAGAAAGCGAGCGGGGCAGCTCAATCTTTTCCTCGCTCATGGACGCTGCTCCCGATCAGTTTTC
GAACCACACGGATACTATCTTCCCGTTGCTTGCCGGCATTCTAGTTACCGCCGAGGCGAATGGGAACATG
GCCACACCCACCGTGCATAACATGTTGGCGGGTACGTGCGTCCTGTTGCCTTTCATAAGTGGACATAGTG
ATGCTGAGCAGATTATGGTTAAGGCTGTGCCGCTCATACTCAAGGCCCTGGCCGCCTTTGCCGAAAAGGG
GAACAGTAATGAGTTCATGGGTGCCTTTGATATCATCGACAGCATGGCCGAGTATGTGCCCCACTTGCTG
ACCGGCAATGTGAAGCTGCTTCTGGAGTTTTGTCTGATGATTGCGAGAAACAAGCAGTTCGACGCTTCGA
TTCGGGTTCAGGTGCTTACCTTTGTGGGCAGCCTAGTTCGCCTCAAGAAGAAAATTATTATGAAGCAGAA
ACTACTACAGCCCACACTATCTGTTCTTTTCGAAGTGATTTGCCAGGACGACCTCAAGGAAGGTG
>dme_circ_0004916_junction_seq
ATTTGCCAGGACGACCTCAAGGAAGGTGGACGATGATTACTTTTCCTCGGAGAGCC
>dme_circ_0004917
GAATATCATACAGATGGTTGCCATACAGCGGTCCGACAACAAGTTGTGGGCCATTCCCGGTGGAATGGTC
GATCCCGGCGAGAATGTCAGTGTCACCCTTAAGCGGGAGTTCACCGAGGAGGCATTGAATTTCACGGACA
AAGCCAACATGGTTGAGCGATTTTTTCAAGCGGGAGGCGTTCAGGTATACCAAGGCTACGTAGACGATTT
TCGAAACACGGACAACGCTTGGATGGAAACCACTGCGCTGAACTTCCACGATGAAGACGGGAGCCAGGTG
GGACAATTGGAGCTAATGGCCGGTGACGATGCCTCCAATGTGCGCTGGACGGACGTCGACTCGAATCTCA
AGCTGCACGCCAATCATGCCGACATAGTCCGTGAGGTTGTCATCCGACGAAATGCACACTGGTAGAAACT
AGACTGAAACTATAAGAGTTAAGATCAACTGCCTAATGGTTGAAGAAAAACCAATTTTAAATAATAACAT
AGCCTTCAATGAAACAACGTGCCttttttttgttttCGCGTTCGCACTGCAAACTGTAACTGTTAAAGGA
CAGAGCGGAATGGACAGAAGCAGTGGAAGCCAGCGAAATGGTAGTTACATGGACCAGAACTCGCTGGGCA

TTCTGAACGTGGACAATCTAAAGGAGTCTTCGGCGACTTGTCCAGCTTCAGTAAAAGTGGAAGGAGGAGC
GGCACTGGTCGGGACTCTCCTGCCGTCCACGAGCGGCTCCGAGAAGAACGACTGCGTGCGCTGGAGACAC
TGGAAAATCCCCGACCGTCGCGTTCCAGCCAAGCAAAGAGCACTGCCCTCCAGATGCGCTCGTCCACAAA
CATCTCGGAGCTGGTAACAGACGACACCATCGATCTGAACAGCAGCAGCTCCTTTGCTTTGGTCATGCCC
GCCACCAAGGTCACAAACATCTCTTTTGAGCCTGCGGAGATCACCGGCAGGTCCACACTCTGCCAGGCGG
GAAAACAGCGCCGCAGGGAGAAGCCATCCCTGTCGATGGCCGAAATTCTAAAGTCATCCTTTGTTGAGAA
GGCTCGCCTGTTGGAAAGGAAGCTGCAGGATGCCAGCCAGGCGGAGGCCAGCTATCACTTATCACGTGGC
AGCTCCAGCAGCCTCTCAGATTCATTCAACTGCTCGCGCAGCTTACCGCGCACTGCGGACATGAGTGAAC
TGAGTCTTAACGGAGGTGGGGGATTTTCCTTTGGATCTGCCTCTTTGGCGGCGGAAGGCCAGGATGAGAG
CTTTGCTCCCGCCGAGCTAATGCAGTCCAAATTGGTGCTCGGTGAAATCTCCTGGGCGCAGGAGTTCACG
GCCATGCCGACGGCCACGTTGAGCACACAGGCGTTGAAGAAGATTGCCGCACCAACATCCGAGATAGAAA
CCTCGGTGTCCAAGTTCTGTCTTGGCCGGAGATCCAGACTTTTCGCTGGGCAACTACTTCCAGACGCGTT
CGGAAAATATATGGAACATTGTGAGCAATGGCAGCCCGAATCAGAAAGTAACGTGACACTGGATTCGGTT
GGCGAGAAGACCCCACAGCCAGACAATAAAACATACACTAAAACGGATGCCATAACTGGCTCGAATCTCG
GACGCAATCTTATGCGTAAGATGCAACAGGATCGGATTGAAACTGCGCTAAAGAGCAGGAATGGTCTGGC
AGCCAAAGAGACCAAGCGTCCGCCGTCGAGCTCCGAGATCCTAAGTCTCTCCGCGATCGACATGGCGCTA
AGGGACATAGATCTCAATTCGGATACATCCACTGTAGAGGTTGTCAACCACCTGTGGGAGCATGGTCGTG
GAAACAATTATGACGATGGGGAAAACAAGGAGAACCAGAGCAGCAACTCCCATGCGGAGCGCACTTTGTG
CGGCTCCAACAAGCTTACCGACACCATGAGTTTCACGGACTCTGTGCTAAACTCAACGGATTTTCGTCAT
TTGCAGCAGAGCATTTCGCGCAAGCCGCTCAGTCCAAATCCTGCCGCAGATCCAATCGTCACGCGCTGGA
AGCGTGACGACCAAGGAGCAATTGTGGCCAACCCGACAACCGGTAAG
>dme_circ_0004917_junction_seq
AATTGTGGCCAACCCGACAACCGGTAAGGAATATCATACAGATGGTTGCCATACAG
>dme_circ_0004918
GAATATCATACAGATGGTTGCCATACAGCGGTCCGACAACAAGTTGTGGGCCATTCCCGGTGGAATGGTC
GATCCCGGCGAGAATGTCAGTGTCACCCTTAAGCGGGAGTTCACCGAGGAGGCATTGAATTTCACGGACA
AAGCCAACATGGTTGAGCGATTTTTTCAAGCGGGAGGCGTTCAGGTATACCAAGGCTACGTAGACGATTT
TCGAAACACGGACAACGCTTGGATGGAAACCACTGCGCTGAACTTCCACGATGAAGACGGGAGCCAGGTG
GGACAATTGGAGCTAATGGCCGGTGACGATGCCTCCAATGTGCGCTGGACGGACGTCGACTCGAATCTCA
AGCTGCACGCCAATCATGCCGACATAGTCCGTGAGGTTGTCATCCGACGAAATGCACACTGGTAGAAACT
AGACTGAAACTATAAGAGTTAAGATCAACTGCCTAATGGTTGAAGAAAAACCAATTTTAAATAATAACAT
AGCCTTCAATGAAACAACGTGCCttttttttgttttCGCGTTCGCACTGCAAACTGTAACTGTTAAAGGA
CAGAGCGGAATGGACAGAAGCAGTGGAAGCCAGCGAAATGGTAGTTACATGGACCAGAACTCGCTGGGCA
TTCTGAACGTGGACAATCTAAAGGAGTCTTCGGCGACTTGTCCAGCTTCAGTAAAAGTGGAAGGAGGAGC
GGCACTGGTCGGGACTCTCCTGCCGTCCACGAGCGGCTCCGAGAAGAACGACTGCGTGCGCTGGAGACAC
TGGAAAATCCCCGACCGTCGCGTTCCAGCCAAGCAAAGAGCACTGCCCTCCAGATGCGCTCGTCCACAAA
CATCTCGGAGCTGGTAACAGACGACACCATCGATCTGAACAGCAGCAGCTCCTTTGCTTTGGTCATGCCC
GCCACCAAGGTCACAAACATCTCTTTTGAGCCTGCGGAGATCACCGGCAGGTCCACACTCTGCCAGGCGG
GAAAACAGCGCCGCAGGGAGAAGCCATCCCTGTCGATGGCCGAAATTCTAAAGTCATCCTTTGTTGAGAA
GGCTCGCCTGTTGGAAAGGAAGCTGCAGGATGCCAGCCAGGCGGAGGCCAGCTATCACTTATCACGTGGC
AGCTCCAGCAGCCTCTCAGATTCATTCAACTGCTCGCGCAGCTTACCGCGCACTGCGGACATGAGTGAAC
TGAGTCTTAACGGAGGTGGGGGATTTTCCTTTGGATCTGCCTCTTTGGCGGCGGAAGGCCAGGATGAGAG
CTTTGCTCCCGCCGAGCTAATGCAGTCCAAATTGGTGCTCGGTGAAATCTCCTGGGCGCAGGAGTTCACG
GCCATGCCGACGGCCACGTTGAGCACACAGGCGTTGAAGAAGATTGCCGCACCAACATCCGAGATAGAAA

CCTCGGTGTCCAAGTTCTGTCTTGGCCGGAGATCCAGACTTTTCGCTGGGCAACTACTTCCAGACGCGTT
CGGAAAATATATGGAACATTGTGAGCAATGGCAGCCCGAATCAGAAAGTAACGTGACACTGGATTCGGTT
GGCGAGAAGACCCCACAGCCAGACAATAAAACATACACTAAAACGGATGCCATAACTGGCTCGAATCTCG
GACGCAATCTTATGCGTAAGATGCAACAGGATCGGATTGAAACTGCGCTAAAGAGCAGGAATGGTCTGGC
AGCCAAAGAGACCAAGCGTCCGCCGTCGAGCTCCGAGATCCTAAGTCTCTCCGCGATCGACATGGCGCTA
AGGGACATAGATCTCAATTCGGATACATCCACTGTAGAGGTTGTCAACCACCTGTGGGAGCATGGTCGTG
GAAACAATTATGACGATGGGGAAAACAAGGAGAACCAGAGCAGCAACTCCCATGCGGAGCGCACTTTGTG
CGGCTCCAACAAGCTTACCGACACCATGAGTTTCACGGACTCTGTGCTAAACTCAACGGATTTTCGTCAT
TTGCAGCAGAGCATTTCGCGCAAGCCGCTCAGTCCAAATCCTGCCGCAGATCCAATCGTCACGCGCTGGA
AGCGTGACGACCAAGGAGCAATTGTGGCCAACCCGACAACCGG
>dme_circ_0004918_junction_seq
GAGCAATTGTGGCCAACCCGACAACCGGGAATATCATACAGATGGTTGCCATACAG
>dme_circ_0004919
GAGTCGCCGGTGTAGATGTCGCGCTCAGCGGCAGAGATGAAGGTGTCGGAGGCAACGCTCACGGCCCGCT
CCTTGGTTAACTTGATCTTGTCGGCGTCTTCCAAATTCATGTTCTTGTGACCAATCTGGTTGTCCAGCAC
CG
>dme_circ_0004919_junction_seq
TTGTGACCAATCTGGTTGTCCAGCACCGGAGTCGCCGGTGTAGATGTCGCGCTCAG
>dme_circ_0004920
TTCTTCCGCTACGTGGAGGTTTCCACCTTTGACATTGCCAGCGATGCCTTTTCTACGTTCAAGGAGCTGC
TCACGCGCCATAAGCTGCTGTGCGCGGAGTTTCTGGACGCCAACTACGACAAGTTCTTCTCGCAGCACTA
CCAGCGCCTGCTCAACTCGGAGAACTATGTGACGCGGCGTCAGAGCTTGAAGCTGCTTGGGGAACTGCTG
CTGGACCGGCACAATTTCACCGTGATGACGCGCTACATATCCGAGCCGGAGAACCTCAAGCTAATGATGA
ACATGCTCAAGGAAAAGTCGCGCAACATTCAGTTCGAGGCGTTTCACGTCTTCAAGGTGTTCGTGGCCAA
TCCCAACAAGCCGAAGCCCATTCTGGACATCCTGCTGCGCAACCAGACGAAGCTGGTCGACTTTCTGACC
AACTTCCATACGGATCGCTCCGAGGACGAGCAGTTCAACGACGAGAAGGCCTATCTGATCAAGCAGATAA
AGGAGCTGAAGCCGCTGCCCGAGGCTTAGTTGGACCTCCACCTCGgctccaacttcagctccaactccag
ctccaGCGTCCCGGGGGCAAAAGGACCGTAGCCAGAAGCCAGAAGCGATCGATACGGAAGCAGAGAGAGC
GATGGTGGGTTAGCAGGATGTGAACATGTTTTG
>dme_circ_0004920_junction_seq
TGGGTTAGCAGGATGTGAACATGTTTTGTTCTTCCGCTACGTGGAGGTTTCCACCT
>dme_circ_0004921
TTCTTCAAGTTCTTCCGCTACGTGGAGGTTTCCACCTTTGACATTGCCAGCGATGCCTTTTCTACGTTCA
AGGAGCTGCTCACGCGCCATAAGCTGCTGTGCGCGGAGTTTCTGGACGCCAACTACGACAAGTTCTTCTC
GCAGCACTACCAGCGCCTGCTCAACTCGGAGAACTATGTGACGCGGCGTCAGAGCTTGAAGCTGCTTGGG
GAACTGCTGCTGGACCGGCACAATTTCACCGTGATGACGCGCTACATATCCGAGCCGGAGAACCTCAAGC
TAATGATGAACATGCTCAAGGAAAAGTCGCGCAACATTCAGTTCGAGGCGTTTCACGTCTTCAAGG
>dme_circ_0004921_junction_seq
CAGTTCGAGGCGTTTCACGTCTTCAAGGTTCTTCAAGTTCTTCCGCTACGTGGAGG
>dme_circ_0004922
GCCAACTAAACCACAACGACAACCACATCCGCAGTCAAATCCCAATCCAAATCCACGTCCAAATCCCATT
TCGAGTGAAACAGCGAGTGAGACAGCGAAATGCCACTGTTCGGGAAGTCACAGAAGTCGCCAGTGGAGCT
GG
>dme_circ_0004922_junction_seq

AAGTCACAGAAGTCGCCAGTGGAGCTGGGCCAACTAAACCACAACGACAACCACAT
>dme_circ_0004923
GGTTAACTAAATAAATGCCTTCCCCTAACAGAATCGAACTTTTTGTATTGCAGCATGACTGACACACTGG
TTAAAACTATACTGACTGACTGGAATATATGTTAAAATTTCAAAAATATTTATTGGTGACTATGACAAT
GCTTCGACTTAATCCAGTCAGTCAGCAACATTTTATCAGACGTCTTTTCGACT
>dme_circ_0004923_junction_seq
CAACATTTTATCAGACGTCTTTTCGACTGGTTAACTAAATAAATGCCTTCCCCTAA
>dme_circ_0004924
AAGAAAAAAACAGACGAACCTAAGCTAACCAAAACTCAGAGTCGAGTCAGAATAAACTCTATGTAATTGA
GGGGCAAAGAAGCGAGCGCATGAAAAGCTTGAATTGAAATCTCTAGATCTCTCTAGGGATATGTGAAAGG
AACGCAAGGAATTTAGCTGCTCACTAGTTTGCATGCGTTTAATTAATAG
>dme_circ_0004924_junction_seq
CACTAGTTTGCATGCGTTTAATTAATAGAAGAAAAAAACAGACGAACCTAAGCTAA
>dme_circ_0004925
GCTTAGGTTCGTCTGTTTTTTTCTTCTTTTATATACATGTTCAGATATATGGTTCGTACGCCTGTCCACA
CGGTACAACTTTTGGCTTACAAAATTAAACTCTCATCCGACCGGTCGTGTGCCACCCAGGTTTAAAATTA
AAAAACAACTTGTACACCGCTACATAAATAAAGGTCCTTAGCCGTAAACAGGGGGAGTGGTGATGATGGG
AGTTAG
>dme_circ_0004925_junction_seq
CAGGGGGAGTGGTGATGATGGGAGTTAGGCTTAGGTTCGTCTGTTTTTTTCTTCTT
>dme_circ_0004926
ATTCCAATTGCGCGGCCCGCTGATCATATGGAACACCTTGCTGGCCATGTTCAGCATCATGGGTGCTGCC
CGAACGGCGCCGGAGCTGATTCACGTGCTGCGTCACTACGGACTCTTCCACTCCGTCTGCGTGCCCAGGT
TGGTATTGAACTCCCCATCGGCCTTGCATCGACAGGTTCCAGTGCCTGTCTGTCCGCATGACCTGCTTGT
GCGGCTGGAAACTTGATTGCCATATGCAAATCAACGATCACTGACCGATATTCCCAACCTTCTCCCACAG
CTACATCGAGCAAGATCGCGTGTGCGGCTTCTGGACCTGGCTCTTCGTGCTCAGCAAACTGCCGGAGCTG
GGCGACACGATATTCATTGTGCTCCGCAAGCAGCCACTCATCTTTTTGCACTGG
>dme_circ_0004926_junction_seq
CAAGCAGCCACTCATCTTTTTGCACTGGATTCCAATTGCGCGGCCCGCTGATCATA
>dme_circ_0004927
GTTCAGTGTGGCAGACTCACCTAGGCCGATTTTGCATAAAGTGCTGACCACCGAAGATGACCAGCATGTA
GATGCCGCAGTAGTAGAACACCCATGTCCAGTTCTCTAGCATCCATTTGCGCGTCCGCTGGTGTATGAAA
TCGTTCTCGAAGTCGAAGATGTACGA
>dme_circ_0004927_junction_seq
AATCGTTCTCGAAGTCGAAGATGTACGAGTTCAGTGTGGCAGACTCACCTAGGCCG
>dme_circ_0004928
GGGTGTGAGAGGGAAGAAAGATTGAGTAAGAGAGAGCCATGGACCAACGGTGtttttttttGTTGCCTGGC
AAAATCAATGAAATGGATGAATGAGAGGAGGAGTGGTGTGGGACGAAGGGGAAAGGAAGGTCGCCGAGAA
ATTTATGCGACAAAACGCGGGCTTTTTATGTGCCTGATtgtgtg
>dme_circ_0004928_junction_seq
GCGGGCTTTTTATGTGCCTGATtgtgtgGGGTGTGAGAGGGAAGAAAGATTGAGTA
>dme_circ_0004929
GAGTACTGCGACCACGTCACTACTTTGTGGAACGATGTGGGTATTCGCGCCTGCTATGATCGCTCAAATG
AGTTTCCCCTGCTGGATAGTGCGAAATAGTAAGTATCTAATTGGATTGATTCAAAATCAGATTTCTTTCC

CATTTATTCAAAAAAAAACCTTACTAAAGTTTGAAtttttttttACCCCAGCTTCCTGGACAACTTTGTG
CGGATCAGCGATGCTGAATACATACCCAGCACCGAGGACATTCTGCACAGCCGTAAGATCACCACCGGCA
TCTCGCAAATCACCTTCCGCGTACCCATTCCCAAGAGCATGGGTGGTGGCGAACAGCAGTTCCAAATGTA
CGATGTGGGTGGCCAAAGGGATCAGCGGAATAAGTGGATTCAGGTATTCGAAGGCATACAGGCCGTATTG
TTCCTGATCTCGTGCAGCGAGTTCGATCAGAATCTGCGCGAGGATCCCAGCCAGAATCGTTTGCAGGAGG
CTCTGAAGTTGTTCAGAGCCGTGTGGCAGAATCGTTTCCTCGCCTCCGCCGGATTGATCGTGTTCCTCAA
CAAGTACGACATCATGGAGCGAAAGATTCGGGCCGGCAAGCACATTGTGGACTACTTTCCCGAATACGAG
GACTTCTGCAAGAGGCCGCAGCAGGACAATTGCTTTGGCGAGAGTGACTGGACGAAGATGTTCATCAAAC
AGAAGCTGGTGGATATCACACAGGAGCCGTTCAAGCGACACTCACGGAATCAGGTGGATCTGGGTACCTC
GGAAAGGGAGTGCTACTACCACTTCACCGTCGCCACGGATACGCGATGCATCCGTGATGTCTTCTGCGAT
GTCCAGAAGATGATCCTCTCGGAGAACGTGTCCAGCATGGGCCTATTCTAGTCCAGATCCCGTTGCGGAT
CGGAGAGCTTCTCAATAAAGTTAAGGGAGGGCTGTCCTCAGGCCCCCACCAACTGTGATCTAGTAGGATA
TGACTTACATAAGTAGCCATGTAAGACCGTAGATCGTAGTGGCAACCGTATGTTGCACAATGGCTGGTAC
AACGCTTTAATATATTTAACTGCATTGGGTGTAGAGGAAGAATCCTCATACCGAATGCTCTCACTACTCC
TTCAACCAATGTCTAAACTCTAATAGAATATAGCATCTACAATAAACCAAAACACACCCAGACATAAACG
TTCAGTTCGCTTTGCGCCGGCAAATTCGATAGTTTTTTGAGTTTGCTGAACCGAGTCGCTGGGGCAGAAG
TGTGTGACAGCCAGCCGTGTGTGTTGTGATCCAGCCAATTGATACTGCGATAAGAGGCGGCGAGCAAATG
>dme_circ_0004929_junction_seq
TACTGCGATAAGAGGCGGCGAGCAAATGGAGTACTGCGACCACGTCACTACTTTGT
>dme_circ_0004930
ACTTTTGCGGTGTTTGCGCCAACAGCCGGCGAAACCAGCGGCGGTTATGACCCACAAGGAGGATCAGTAC
CCGGTATCACTTGACCACCACGTGCTCAAGGACATGGCCAAGGGCGTGAGGGACACCACCGTTAAGATCC
TGCTACTAGGCACCGCCGAGTCGGGCAAGACGACCATCATCAAGCAAATGCGGATACTGCACATCAACGG
ATTTACAGACGAGTGAACGGCGAGAGAAGATACCCGAGATATATCAGAACATCCACGAGTCCATCCTGCA
ATTGGTGGGTCAGATGGGAGTGCTGGGCATTGATTTCGGCAGCTGTACCAGCGAAAGATCGGCGGACTAT
ATCCTCTCTCTGCCCGGATCGGCACCCGAATATATGAACGAGG
>dme_circ_0004930_junction_seq
GGATCGGCACCCGAATATATGAACGAGGACTTTTGCGGTGTTTGCGCCAACAGCCG
>dme_circ_0004931
TTCAGTGATACAGACAAATGGATATTATTATAGCAGTGACAAGAACTCAAGTGCTCGATAAACACATTCC
CGGCCAAAGAAAACAGAAACTCAGTGGAATCCAAAGTATTTAATAACAAATtgtgtgtgtgtgtg
>dme_circ_0004931_junction_seq
ATTTAATAACAAATtgtgtgtgtgtgtgTTCAGTGATACAGACAAATGGATATTAT
>dme_circ_0004932
tactaccagcaggtacagatgatgcagcagcaggagcacccgccgcagcaacagcagatgcgccagcagc
CATCCTACTCCTCCCTGCAGGAAAAACAATCGCGCCAGAGCACAGCAATGGTAAGTTTTGTTGGCTGTCT
ATTGTTTATTACAAAGTTATTTTTAACTATATGTCCCATATTTTAGCGCGTCTATCGGGCCATCTATGAC
TATGAGGCGCAGGACGTCGACGAGGTCAGCTTCCGCGAGGGCGACGTCATCTTTGAGGTGGAG
>dme_circ_0004932_junction_seq
CGAGGGCGACGTCATCTTTGAGGTGGAGtactaccagcaggtacagatgatgcagc
>dme_circ_0004933
caccagcagtactaccagcaggtacagatgatgcagcagcaggagcacccgccgcagcaacagcagatgc
gccagcagcCATCCTACTCCTCCCTGCAGGAAAAACAATCGCGCCAGAGCACAGCAATGGTAAGTTTTGT
TGGCTGTCTATTGTTTATTACAAAGTTATTTTTAACTATATGTCCCATATTTTAGCGCGTCTATCGGGCC

ATCTATGACTATGAGGCGCAGGACGTCGACGAGGTCAGCTTCCGCGAGGGCGACGTCATCTTTGAGGTGG
AG
>dme_circ_0004933_junction_seq
CGAGGGCGACGTCATCTTTGAGGTGGAGcaccagcagtactaccagcaggtacaga
>dme_circ_0004934
ACACATCCCGAAGGCGAAGGCAACGGCCATTGCCGATACGCCCGAATTGAAGCGCATCGCGGAGAACACC
AAAATCCAGTCCAACGTCAAGTACCACGCGGACTTTGAGAAGGCCAAGGGCAAATTTACACAGGGTCAGT
GCGGAAAAAAGTTAAGATAAGCTTAAAGAACTTATACTTCTGATGGAAGTTTATTGCCTAATTAGGCTTAA
TTTACTTGCGCGATGGATAGTTCTTTGAAATATTCAATATTCTGTTTGACATGTTTAACTAAATATAACT
AAAGCGCGGCCCTTGTGAAGTTCACTTCAGTACAAGAACATTGATATATCCAACCCAAGTTTGAGATAAT
GCTTTTGATATATGTAAAGTTCCATCCGTCCATTTGGAGTGTGCCCTTACCTAAATTGCTTTTGCATTCG
ACAGGGTGGCCGATGACCCGGAAACGCTGCGTATCAAACAGAACACGAAGCACATATCGAATGTGGCATA
TCACGGGGATCTGGAGAAGAAGGCGGCCATGGAGAAGCAGCGAGGATCCGCCGAGGTCTCCGACAGCAGC
AG
>dme_circ_0004934_junction_seq
GATCCGCCGAGGTCTCCGACAGCAGCAGACACATCCCGAAGGCGAAGGCAACGGCC
>dme_circ_0004935
GGCCTTCTTCTGGCCATCGGGCCGGATGAGCACGTTCACCTTGGCCACCTTGATGTCGTAGAGCTTGCGC
ACTGCGGCACGCACGTGGTTCTTGTTGGCGCGCAGGTGTGTGAGGAAGACCAGGGTGTTGTTGTCCTCGA
TCTTCTTCATGGCCGCCTCGGTGGTCAGTGGGTACTTGATGATGTTGTACGCGTCCATGCGGTTCCTGGT
GGGCACCGATTTCCTGGGGTACTTGGGACTCCTGGGCAGCTTCAGGGTG
>dme_circ_0004935_junction_seq
CTTGGGACTCCTGGGCAGCTTCAGGGTGGGCCTTCTTCTGGCCATCGGGCCGGATG
>dme_circ_0004936
GCACGTTCACCTTGGCCACCTTGATGTCGTAGAGCTTGCGCACTGCGGCACGCACGTGGTTCTTGTTGGC
GCGCAGGTGTGTGAGGAAGACCAGGGTGTTGTTGTCCTCGATCTTCTTCATGGCCGCCTCGGTGGTCAGT
GGGTACTTGATGATGTTGTACGCGTCCATGCGGTTCCTGGTGGGCACCGATTTCCTGGG
>dme_circ_0004936_junction_seq
GGTTCCTGGTGGGCACCGATTTCCTGGGGCACGTTCACCTTGGCCACCTTGATGTC
>dme_circ_0004937
GTCCTCGCCGGCAAGAAGCCCCAGTCCGTGCTGGCCAAGCTCTCTGCCAAGGCTCGCGCGGCCGCCAAGG
CCAAGAAGGGCGTGAAGCCCGTGACCAAGCCCGCCAAGGGAACCGCCAAGGCCAAGGCTGTGGCTCTGCT
GAACGCCAAGAAGGTCCAGAAGAAGG
>dme_circ_0004937_junction_seq
CTGAACGCCAAGAAGGTCCAGAAGAAGGGTCCTCGCCGGCAAGAAGCCCCAGTCCG
>dme_circ_0004938
CCCCAGTCCGTGCTGGCCAAGCTCTCTGCCAAGGCTCGCGCGGCCGCCAAGGCCAAGAAGGGCGTGAAGC
CCGTGACCAAGCCCGCCAAGGGAACCGCCAAGGCCAAGGCTGTGGCTCTGCTGAACGCCAAGAAGG
>dme_circ_0004938_junction_seq
GCTGTGGCTCTGCTGAACGCCAAGAAGGCCCCAGTCCGTGCTGGCCAAGCTCTCTG
>dme_circ_0004939
AAGGTCCTCGCCGGCAAGAAGCCCCAGTCCGTGCTGGCCAAGCTCTCTGCCAAGGCTCGCGCGGCCGCCA
AGGCCAAGAAGGGCGTGAAGCCCGTGACCAAGCCCGCCAAGGGAACCGCCAAGGCCAAGGCTGTGGCTCT
GCTGAACGCCAAGAAGG

>dme_circ_0004939_junction_seq
GCTGTGGCTCTGCTGAACGCCAAGAAGGAAGGTCCTCGCCGGCAAGAAGCCCCAGT
>dme_circ_0004940
GCTGCTCCCTCGGCTGCCAAGCCAgctgccgctgcgcccaagaaggctgctgccccagcggctaagaagg
ctgctccggccgccaagaagCCAGCGACCGCCGGTGCTGCTGCCAAGAAGCCCGCGGCCGTAAAGACGAC
CGCGGCCGCCAAGGCCAAGAGCAAGGATGCCAAGAAGAAGG
>dme_circ_0004940_junction_seq
GCCAAGAGCAAGGATGCCAAGAAGAAGGGCTGCTCCCTCGGCTGCCAAGCCAgctg
>dme_circ_0004941
GATCGCAATGATCCGGGACGCTTTTTCGGCGATGGCGTTCAGTTCAAGGCAAAGCTCATCGGCATTCTGG
AGGTAAGAGTTTACTTTTTGGATAAATTTCAGTATCTAACATTGTGATCTTTCGAAATTTAAGGTGGGCG
AGGCCCGAGGTGATCGGATGTGCCAGGAGGCGCTGCAAGATCTCAAAATGGCCATCCGGGCGGCTGGAGA
GCACAAGCAGCGGATAACCATCCATGTGACCATCGATGGTCTGCGGCTGAGGGATGAGAAAACGGGTGAC
TCCCTGTACCATCATCCGGTGCATAAGATCTCCTTCATCGCGCAGGATATGACTGACTCGAGGGCCTTTG
GCTATATCTTCGGATCGCCGGACAGTGGTCACCGGTTCTTCGGCATCAAGACGGACAAGGCGGCCAGCCA
GGTGGTGCTGGCCATGCGCGATCTCTTCCAGGTAGTTTTCGAGCTAAAGAAAAAGGAGATTGAGATGGCG
CGCCAGCAGATCCAGGGTAAGTCCCTGCACGACCATTCCAGCCAGCTGGCCTCCCTGTCGTCGCTGAAGT
CCTCCGGCCTGGGTGGCATGGGTCTGGGCCACTCGGATTTGGCCAGCGGAGGAATCTCATCCGGCCATGC
CCTCACCCTGCTGGGCAGTAGTCTTAGCACCACGAATGGAACGAGCAGGCTGGGCGTGAGTCTTGACGTG
GCCAAGGCATCCGGATCGGCGGCCAAAGAGGGTCTCTCCGGAATCCGTGGCCGATCTTGTGGACTTGGAG
CAGGAGCTTACCTCGCTTCAGCGGGGAATCAGTCAGATGGAACGGATAACGCCAAACGAGCCGACCACCA
GTAGCACTGGTGGTGCTGGCCATCCATCTCTAGCGAAATCAGCCAGCGAGGACGATCCTTTCGGAGACTC
GTTTATCTATGTGCCCTCGTATAGCATACTGCCACCGCCACCTGATTCCGGACGCAACAGGCACAAGCCG
CCCAACAAAACACCTGATGCGGTGACCAGTTTGGATGCAATGCTTTCGCCACCTCCTGGTACAAGTAGCT
CCCACGGCTCAGCCTCCGCTGGACTACAGGCTGCAGATAACGACGATGACAACTGGCTGCAGGAACTGGA
CCAACAAAATGATGTCTTCGACACGTCCAAAGTGGTGAGCAGCAGTGGATTGGGTTCGGTTTTGGCCATG
GCTCCGCTGGCCTCCAGCGAATCCACGGCCACTCCCACCCAACAACTGACGGAGGTTGCTGCAGGATCTG
GACCTCTGGCTGATCTGGATATTGGACTAAGTACGGCCTTGGGAAATGAGGAACAGACCTCCACCATTTT
GTCATTGG
>dme_circ_0004941_junction_seq
GAACAGACCTCCACCATTTTGTCATTGGGATCGCAATGATCCGGGACGCTTTTTCG
>dme_circ_0004942
GTCCAGATCCTGCAGCAACCTCCGTCAGTTGTTGGGTGGGAGTGGCCGTGGATTCGCTGGAGGCCAGCGG
AGCCATGGCCAAAACCGAACCCAATCCACTGCTGCTCACCACTTTGGACGTGTCGAAGACATCATTTTGT
TGG
>dme_circ_0004942_junction_seq
GGACGTGTCGAAGACATCATTTTGTTGGGTCCAGATCCTGCAGCAACCTCCGTCAG
>dme_circ_0004943
GATCACAGGGATAGGGAACGAGAGCGTGATCGCGACCGAGAGTATCGGGATTATGCTCGTCCAAGCTACG
ATTTCGACTACGAAAACGTCTACGAGGAGCGTGGAGGTCGTTCACCGTTGGCCTACAAGCCAGGAAGAGG
TGGTGGTGATTATCTGTATGACAGAGAAAGGGACAGGGATCGGGAGCGCGATCGAAAATCCTTCGATCGG
GAAAGTCTCGAGTCCTATGAGAGCGCCACCCGTCGTCGTCGCAGTTTTGGTAGTGGAAACGAT
>dme_circ_0004943_junction_seq
TCGTCGCAGTTTTGGTAGTGGAAACGATGATCACAGGGATAGGGAACGAGAGCGTG

>dme_circ_0004944
ATAATCACCACCACCTCTTCCTGGCTTGTAGGCCAACGGTGAACGACCTCCACGCTCCTCGTAGACGTTT
TCGTAGTCGAAATCGTAGCTTGGACGAGCATAATCCCGATACTCTCGGTCGCGATCACGCTCTCGTTCCC
TATCCCTG
>dme_circ_0004944_junction_seq
GCGATCACGCTCTCGTTCCCTATCCCTGATAATCACCACCACCTCTTCCTGGCTTG
>dme_circ_0004945
AAATGAAAAGTCCGTGTCGTTGACCACATCGATCTCCGCATAGTTGTGGTTATCGATGTCCGGATCCGGT
CGCTGCCCCGATGCCTTGGCAAACAGTTGGTCAAATCACATGTTTTCATCGAACTTTGCAAAGTTCGCGT
CCTCGCTCCACTGAAATTTGCCGCCCTCAGTGTCATCATTGTGCTGATCCATGGCCTG
>dme_circ_0004945_junction_seq
TGTCATCATTGTGCTGATCCATGGCCTGAAATGAAAAGTCCGTGTCGTTGACCACA
>dme_circ_0004946
GTGGAAGAACCAGAAAAGCCCCTAGAAGTTGTCCAAGAGGAAGATGTTCCGTCTAATGAGTTTCAAGTTC
TTGGAGGCGATGATTCGGCGGCCAAGAAGAAGAAAGTGCAGATGCAGCTGCCCAATTGGCTGGCACATCC
AACCATCATTGAAGG
>dme_circ_0004946_junction_seq
GGCTGGCACATCCAACCATCATTGAAGGGTGGAAGAACCAGAAAAGCCCCTAGAAG
>dme_circ_0004947
AACTGCATGGCTCGCTCGGAGGCCTCACGATCCTCGGCGGAGTTGGGATCCCTGGGCTCTGGCCACGACG
TATCCAGTGTGATGCCCATGCGTCCACCTTGACGCGGCTGGAAGAGCTCCCGGTACATGTGCACCACCTC
GGCGTGAGCCTTCAGCAGATTGTGACCACAGAGGTAGGCGGGAATGCCTGGATAGTTGTACGAAGG
>dme_circ_0004947_junction_seq
CGGGAATGCCTGGATAGTTGTACGAAGGAACTGCATGGCTCGCTCGGAGGCCTCAC
>dme_circ_0004948
GAAACTTTACGCCAAGGCCATTTACGATAATTATGCGGACACGTCGGACGAGCTGCCCTTCAAGAAGGGT
GATATTCTCACAGTCCTTGAGCAGGACACGGAGGGCTTCCAGGGTTGGTGGCTCTGCTCTCTGCGCAACC
GACAGGGTGAGTTGGGTCCTATGATTTCATGATTTGTTATTAGTTTGGGTCTACACGAGCCCAGGTAATG
GTACTGCATGCAAAATAGTCGCTAATAGAACAATTATGAACCATTCGATCCTAAGTCATTACCCAGTCTA
TCAAACGTTTTCGGATATACAAGACTTTGTTTTGATTTGAAAACAAAGTTGTGGAAAATTTTAAATTTTC
TGCACGTCGATCGACGGCAATTTTTAATTAACAACTGAATTGGCGCCACTTAAAAAATCTCGTTTGTGTA
GGGGCCGGCCGGCGATGGTGTCAAAACATATATCAAAGTGGAAATTAGAAGTGCTGGGTGAGATATGGAA
ACATGAACGCGATAGCGGTACGTTATACATATGTCGGGCAATTGTCGCTAATTAAAAAGTAGCCGAGCCT
TTTAATGGAAACAATTAACCCAAAAACGGTCAATTGAGTGCCGACAAGAGCAACAACGTGTGGCATAAAT
ATTAACAGCAACATAAATAACTGTGTCAAAAAGCGAAATCAACTTTCGGCACACACTTTTAGCTAAAACA
GATGTTGACAAGATTTTCTGTTGACTTCGAGTTTTAAGGAGAATCGCACGAATTTCTGTCAAAACTTGCA
TTGAGATGGTGAATTGGGCGAGTATAAAAAATGAGCCGTCCAATTAAAAGCACGCACGACATTCTTTTTA
CAACAttttttttttAACATATTGTGTCCATTTATTTGTATACGCAGAAATAAATAATTCATACGTCGTC
GGTTGATAAAAAAACAATCAAAATCAATTGGCATCATACAAAAGCGTATTAATGTTTTGTTATGAGAAAA
CTAAAAAATGGGGTTGCTATCTTGTGGTTATAGGAAACTTTTAGTTTAATATTTTAATTTGCTTTCCCTA
CTTAATGTCGATTTATATTCAACAAAACATATTCTCATTGCTCTATATATATTTCAGGGGTCTTTGCCCG
GGCAACCGGTTGCGCGTCTTGAACTCGTACGACTCGGGATGCTTCTCTCCTTCGCCGGCCAGTTCTCCAG
TTCCGTCCCTGGCGGCCAGCACGGCCACGCTGGATAGCTCCATCTGCTCGGCGGAGATATACGAGAACGG
TTCCGTCATCAGTGCGGTATCGTCCTCAAACTCAAATTCGGGAGGGGCTAGTAGTGGACTCGGATCGGAG

TCGGTGTCTCGTGTCTCGAGGAGATCGTGGCACGCCTCGCCGACAAAG
>dme_circ_0004948_junction_seq
GAGATCGTGGCACGCCTCGCCGACAAAGGAAACTTTACGCCAAGGCCATTTACGAT
>dme_circ_0004949
GTTTTGTTCTGTTTTGGAAGCCGCACGCGTTTTCCCTCGCTCCGGTTGTGCTCGCTTTCGCATCGcacac
acacacacacTCTCGCCGCACAGACGCGACCAAAAGAAAACAAAATCAAATTCGGCGCAAACAAGGAATA
ACTTTTTATATTCCTATTGTGCATCGTATGTTGTATTAAGTGCAAGAAATATATTTACACAAATCTAACA
AAAACACCGCAACTACTTTTGTGCATTTTGTTTTGCTCTGTGCCGAAGGAGCGGAGaaaagaaaaaaccg
caacaaatcaaaaaaTCTGTGC
>dme_circ_0004949_junction_seq
aaaccgcaacaaatcaaaaaaTCTGTGCGTTTTGTTCTGTTTTGGAAGCCGCACGC
>dme_circ_0004950
GTGGATCAGCCAGGTGCGTTGCTGCAGGGCCTGGATGGTGCTGAAGTTGGCGTTGTCAATGTAGTCGCGC
AAACGCGTCAGATCCTCCAGAGCAGTGTTCCAGTTGAGGGTCAGAATCTCGGCGGCCAGCTTGCCCCACA
GCACATTAAGGTAGTTCTGCAAAATTTGGGAGACAATATCAATTAATATTCTTGTTTTAGTGGTTTTTGC
CGCTTTTCATGTTAATTTAGCTCCATACGCACCTTGTCGTTGGGCGACATGACGATGAGGCAGAAGTACA
GGTAAGAGGTGGACTCCTGATAGTTGCCGCACTCGTAGAGGTACTTGGCCAGCTTGTAGGCGCTCTCCAG
>dme_circ_0004950_junction_seq
ACTTGGCCAGCTTGTAGGCGCTCTCCAGGTGGATCAGCCAGGTGCGTTGCTGCAGG
>dme_circ_0004951
GGCAGAAGTACAGGTAAGAGGTGGACTCCTGATAGTTGCCGCACTCGTAGAGGTACTTGGCCAGCTTGTA
GGCGCTCTCCAGGTGCTCCACCTTAAAATTGTAGTCCTTCTGAAGGGCGTTGACGAAG
>dme_circ_0004951_junction_seq
GTAGTCCTTCTGAAGGGCGTTGACGAAGGGCAGAAGTACAGGTAAGAGGTGGACTC
>dme_circ_0004952
GGTGGACTCCTGATAGTTGCCGCACTCGTAGAGGTACTTGGCCAGCTTGTAGGCGCTCTCCAGGTGCTCC
ACCTTAAAATTGTAGTCCTTCTGAAGGGCGTTGACGAAGGTCTTCGAGTCCTTCATGCTCTCACCGTTCT
TAAGAATGTCG
>dme_circ_0004952_junction_seq
CATGCTCTCACCGTTCTTAAGAATGTCGGGTGGACTCCTGATAGTTGCCGCACTCG
>dme_circ_0004953
CAGGAGCTGCTGGAGTACATTTTGGAGACGGTGAACAAGACGAACATGATCGATTACACGATGGACACCC
GCAAGCGTCTCAATCTCAGCCAGGAGATGCCCGAGGAGCTCGTGCAGCGCAAGGCGGAGGTCCTGGCCAC
GCTTAAGCAGCTGCAGAACGAGGTGGCACCCATCATGAAGGCCACCGACATTCTTAAGAACGGTGAGAGC
ATGAAGGACTCGAAGACCTTCGTCAACGCCCTTCAGAAGGACTACAATTTTAAGG
>dme_circ_0004953_junction_seq
GCCCTTCAGAAGGACTACAATTTTAAGGCAGGAGCTGCTGGAGTACATTTTGGAGA
>dme_circ_0004954
AAGTACTTCCCCGCAGACCTTATTCTCCTGGCCATGGGCTTCCTGGGTCCGGAGAAGACGGTGCCGAGCG
AACTGGGCCTGGAGCTGGACCCGCGCGGCAACATCAAGGCATCCAACGGACAGTATGGCACCTCGAACTC
CAAGG
>dme_circ_0004954_junction_seq
GGACAGTATGGCACCTCGAACTCCAAGGAAGTACTTCCCCGCAGACCTTATTCTCC
>dme_circ_0004955

GGAGAATAAGGTCTGCGGGGAAGTACTTCTCCGAACCGGCCACCTCCTGCATGCGCCACTGGCCCGTTTC
CGTCTTGGTCCACTCAACCTCGACGGTGTTCACGCCCTTGATGGCTCCATTTTCGCCCACGAACTCCTTG
GTGGTGGTGCAGTACTGGCGCGGATCTTTGCCCCACTTCAGCTTCACCTCCTCGTGACC
>dme_circ_0004955_junction_seq
CCCACTTCAGCTTCACCTCCTCGTGACCGGAGAATAAGGTCTGCGGGGAAGTACTT
>dme_circ_0004956
GAATAAGGTCTGCGGGGAAGTACTTCTCCGAACCGGCCACCTCCTGCATGCGCCACTGGCCCGTTTCCGT
CTTGGTCCACTCAACCTCGACGGTGTTCACGCCCTTGATGGCTCCATTTTCGCCCACGAACTCCTTGGTG
GTGGTGCAGTACTGGCGCGGATCTTTGCCCCACTTCAGCTTCACCTCCTCGTGACCGTA
>dme_circ_0004956_junction_seq
ACTTCAGCTTCACCTCCTCGTGACCGTAGAATAAGGTCTGCGGGGAAGTACTTCTC
>dme_circ_0004957
ATGCTGGAGAGCGACAGAAGGATTGGGATGAAGTCTACAACTTCCCACATGTGCGCAAGAACCTCAAGGT
TCAAGCAGCTCGATGCATGGAATGCGGTGTGCCCTTCTGTCAGTCCAACTCCACGGGCTGTCCGCTCGGT
AACATCATTCCCAAG
>dme_circ_0004957_junction_seq
CTGTCCGCTCGGTAACATCATTCCCAAGATGCTGGAGAGCGACAGAAGGATTGGGA
>dme_circ_0004958
GAAGGGCACACCGCATTCCATGCATCGAGCTGCTTGAACCTTGAGGTTCTTGCGCACATGTGGGAAGTTG
TAGACTTCATCCCAATCCTTCTGTCGCTCTCCAGCATCTCGGTACGGAGCCGATTCCCTCTTGTACTTTA
CAAAGCCACGGGTCTTGTCCAGGACGCGATCGGCACGCTTCTGCTCGAGCACCACGTCCTGAATCGCTTC
CTCAATGTCCTTGATGTGCGGTTCATGCTTTCCGTTTCCATTCTCGATGGCGGACTTCAGGGGCTGCTCC
ACTGCTTGTTGTTCGGCCATGTCCTTTAGAGCCTTTTG
>dme_circ_0004958_junction_seq
GTTCGGCCATGTCCTTTAGAGCCTTTTGGAAGGGCACACCGCATTCCATGCATCGA
>dme_circ_0004959
ATGGTTCCTTTAAGCCCAAGGTAAACCCGGAAAGTGTGGAACTCCTGCCGTTGGAAATTGAGAAGGATGT
GCTGCTCGTCAAGGAACTGTTGGCTGATTTCATTGAGAAGACAGGCTCGAAGGTGGCCAAGGAGTTGCTT
GACAACTGGGCCGAAGCCCAGGGCAAGTTTGTCAAGGTCTTCCCGTATGAATACCAAAAGGCTCTAAAGG
ACATGGCCGAACAACAAGCAGTGGAGCAGCCCCTGAAGTCCGCCATCGAGAATGGAAACGGAAAGCATGA
ACCGCACATCAAGGACATTGAGGAAGCGATTCAGGACGTGGTGCTCGAGCAGAAGCGTGCCGATCGCGTC
CTGGACAAGACCCGTGGCTTTGTAAAG
>dme_circ_0004959_junction_seq
CCTGGACAAGACCCGTGGCTTTGTAAAGATGGTTCCTTTAAGCCCAAGGTAAACCC
>dme_circ_0004960
GGTTCCGCTGGTCAGAGCTTCTGCGCGTTCCTGGCGCGGGGTGTTAATGTGACATTGAAGGGCGATGCCA
ACGATTATGTGGGCAAGGGTCTTTGCGGTGGTAATGTTGTCATCATGCCACAAGATACGGTTCCCTTCGA
ATCACATCTCAATG
>dme_circ_0004960_junction_seq
ACGGTTCCCTTCGAATCACATCTCAATGGGTTCCGCTGGTCAGAGCTTCTGCGCGT
>dme_circ_0004961
GCTGCACTATGATGCGCAAGTGTCACTTGAACACCTGTCCCGTGGGCATTGCCACCCAGGATCCGGAGCT
GCGCAAGAAGTTCACGGGTAAACCAGAGCATGTTATCAACTTCTTCTTCATGCTGGCCGAGGATG
>dme_circ_0004961_junction_seq

AACTTCTTCTTCATGCTGGCCGAGGATGGCTGCACTATGATGCGCAAGTGTCACTT
>dme_circ_0004962
GATTCTTATTTACGGCCGCTTCCTGCAGGCTGCCAATGGAGGATGGCTCGTTAATGTGAGCTTCGCCACC
GTGACGCCAGTGGTACTGGCCAGGATTCCGCAGGATGCGTGTGTCCGGCGTTGCTTTGCCATAGGTTAGT
TGATAGCGTTGAAGGCCCTCCTTAGCCAGTATCTCCAGG
>dme_circ_0004962_junction_seq
AAGGCCCTCCTTAGCCAGTATCTCCAGGGATTCTTATTTACGGCCGCTTCCTGCAG
>dme_circ_0004963
TTACCCTGGAGATACTGGCTAAGGAGGGCCTTCAACGCTATCAACTAACCTATGGCAAAGCAACGCCGGA
CACACGCATCCTGCGGAATCCTGGCCAGTACCACTGGCGTCACGGTGGCGAAGCTCACATTAACGAGCCA
TCCTCCATTGGCAGCCTGCAGGAAGCGGCCG
>dme_circ_0004963_junction_seq
TCCATTGGCAGCCTGCAGGAAGCGGCCGTTACCCTGGAGATACTGGCTAAGGAGGG
>dme_circ_0004964
GGGTAACTCCACCGATGCGACTTTGTGTGCCGCGGAAGCACTTGGCCACCAGGTCGGAACCCAATCCCAC
AGCCTCGAAGATCTGGGCGCTCTTGTACGATTGCAAAGTGCTGATGCCCATTTTGGCCATTACTTTTGCG
ATTCCC
>dme_circ_0004964_junction_seq
CATTTTGGCCATTACTTTTGCGATTCCCGGGTAACTCCACCGATGCGACTTTGTGT
>dme_circ_0004965
GGGTAACTCCACCGATGCGACTTTGTGTGCCGCGGAAGCACTTGGCCACCAGGTCGGAACCCAATCCCAC
AGCCTCGAAGATCTGGGCGCTCTTGTACGATTGCAAAGTGCTGATGCCCATTTTGGCCATTACTTTTGCG
ATTCCCGTATCAATAGCTTGGGCATAAGCGGCATAGATCTGCTT
>dme_circ_0004965_junction_seq
CTTGGGCATAAGCGGCATAGATCTGCTTGGGTAACTCCACCGATGCGACTTTGTGT
>dme_circ_0004966
GGGTAACTCCACCGATGCGACTTTGTGTGCCGCGGAAGCACTTGGCCACCAGGTCGGAACCCAATCCCAC
AGCCTCGAAGATCTGGGCGCTCTTGTACGATTGCAAAGTGCTGATGCCCATTTTGGCCATTACTTTTGCG
ATTCCCGTATCAATAGCTTGGGCATAAGCGGCATAGATCTGCTTGTCGTTAACCTCAGGCGCAATTACTC
CATCATCACGCAGAGCCTGGGCCAGTTCAAAAGCCAGATATGGACAAATGGCATCCGCTCCGTAGCCAAG
GAGTACGCAAATATGGTGAACTTCGCGGGCCTCAGCAGTTTCCACCACAATGCCCACCTTCATGCGCTGC
AGTGTCTCAATCAGATGATGATGCAAGGCGCCGAGGGCCAAAAGGGCTGAGACTGCCACCTTACCATCGA
TTCCCGCTCCACGATCGGAGATCACCAACAACTGATAGCCAGCTTGAGCTGCAGCATATCCTTCGCGGCA
GATACGATCGATGGCATCGATATAACCCTGAACGCCTTCGTTATACTGGAAT
>dme_circ_0004966_junction_seq
ACCCTGAACGCCTTCGTTATACTGGAATGGGTAACTCCACCGATGCGACTTTGTGT
>dme_circ_0004967
GTTAACGACAAGCAGATCTATGCCGCTTATGCCCAAGCTATTGATACGGGAATCGCAAAAGTAATGGCCA
AAATGGGCATCAGCACTTTGCAATCGTACAAGAGCGCCCAGATCTTCGAGGCTGTGGGATTGGGTTCCGA
CCTGGTGGCCAAGTGCTTCCGCGGCACACAAAGTCGCATCGGTGGAG
>dme_circ_0004967_junction_seq
CGCGGCACACAAAGTCGCATCGGTGGAGGTTAACGACAAGCAGATCTATGCCGCTT
>dme_circ_0004968
GTTCAAAAGCCAGATATGGACAAATGGCATCCGCTCCGTAGCCAAGGAGTACGCAAATATGGTGAACTTC

GCGGGCCTCAGCAGTTTCCACCACAATGCCCACCTTCATGCGCTGCAGTGTCTCAATCAGATGATGATGC
AAGGCGCCGAGGGCCAAAAGGGCTGAGACTGCCACCTTACCATCGATTCCCGCTCCACGATCGGAGATCA
CCAACAACTGATAGCCAGCTTGAGCTGCAGCATATCCTTCGCGGCAGATACGATCGATGGCATCGATATA
ACCCTGAACGCCTTCGTTATACTGGAAT
>dme_circ_0004968_junction_seq
ACCCTGAACGCCTTCGTTATACTGGAATGTTCAAAAGCCAGATATGGACAAATGGC
>dme_circ_0004969
GGTTATATCGATGCCATCGATCGTATCTGCCGCGAAGGATATGCTGCAGCTCAAGCTGGCTATCAGTTGT
TGGTGATCTCCGATCGTGGAGCGGGAATCGATGGTAAGGTGGCAGTCTCAGCCCTTTTGGCCCTCGGCGC
CTTGCATCATCATCTGATTGAGACACTGCAGCGCATGAAGG
>dme_circ_0004969_junction_seq
CTGATTGAGACACTGCAGCGCATGAAGGGGTTATATCGATGCCATCGATCGTATCT
>dme_circ_0004970
GTAACCAATCCGCCTATCGATCCATTCCGCGAGAAGGTGGTCATGTCGATGCAGTGTCCTCTCGGTCCGG
AGGCGAATCTTCTGCAGCCATCGGCTCAGCAGGTGCATCGCATTTGGCTCACCAATCCCATACTGAGCAT
TCCAGATACCCAGCTGCTCAAGCGGAATACGCACCGCGGCTGGCGGACCAAAG
>dme_circ_0004970_junction_seq
AATACGCACCGCGGCTGGCGGACCAAAGGTAACCAATCCGCCTATCGATCCATTCC
>dme_circ_0004971
GAAACGCGATGGACGCAGTCCATTACGATCCAAAACGGCGCCAATGTAGCGACCATCGGTGAAGCTAATA
AGAGCCGGACCGTCCCAGGGTTCCATGACACAGGCGGCCCACTGGTAGAAGTCACGCTTCTCCTGCGGCA
TAGTCTTGTCATTCTGCCATGCCTCCGGCACCATGGTCATCACCGATTCCGGCAACGACCTGTCGCTGGC
CATGGTGAGGAACTCCAGCACGCAATCGAAGCTGCCGGAATCGGAGAGATTCGGCTCCACCACTGG
>dme_circ_0004971_junction_seq
AATCGGAGAGATTCGGCTCCACCACTGGGAAACGCGATGGACGCAGTCCATTACGA
>dme_circ_0004972
GAGCCGGACCGTCCCAGGGTTCCATGACACAGGCGGCCCACTGGTAGAAGTCACGCTTCTCCTGCGGCAT
AGTCTTGTCATTCTGCCATGCCTCCGGCACCATGGTCATCACCGATTCCGGCAACGACCTGTCGCTGGCC
ATGGTGAGGAACTCCAGCACGCAATCGAAGCTGCCGGAATCGGAGAGATTCGGCTCCACCACTGGGTACA
>dme_circ_0004972_junction_seq
GAGAGATTCGGCTCCACCACTGGGTACAGAGCCGGACCGTCCCAGGGTTCCATGAC
>dme_circ_0004973
GAGCCGGACCGTCCCAGGGTTCCATGACACAGGCGGCCCACTGGTAGAAGTCACGCTTCTCCTGCGGCAT
AGTCTTGTCATTCTGCCATGCCTCCGGCACCATGGTCATCACCGATTCCGGCAACGACCTGTCGCTGGCC
ATGGTGAGGAACTCCAGCACGCAATCGAAGCTGCCGGAATCGGAGAGATTCGGCTCCACCACTGGGTACA
GTTTCTTCAGCTGATCCCCAAACAGATCCGACTGCATGACACCCTCGCGGGCCTTCATGAGGTTCACATT
TCCCCGAAGCGTGTTGATCTCACCATTGTGGGCCAGGACTCGCAGAGGATGAGCCCTTTCCCAACTGGGA
AAGGTGTTGGTGGAGAAACGA
>dme_circ_0004973_junction_seq
ACTGGGAAAGGTGTTGGTGGAGAAACGAGAGCCGGACCGTCCCAGGGTTCCATGAC
>dme_circ_0004974
GGAACTCCAGCACGCAATCGAAGCTGCCGGAATCGGAGAGATTCGGCTCCACCACTGGGTACAGTTTCTT
CAGCTGATCCCCAAACAGATCCGACTGCATGACACCCTCGCGGGCCTTCATGAGGTTCACATTTCCCCGA
AGCGTGTTGATCTCACCATTGTGGGCCAGGACTCGCAGAGGATGAGCCCTTTCCCAACTGGGAAAGGTGT

TGGTGGAGAAACGA

>dme_circ_0004974_junction_seq

ACTGGGAAAGGTGTTGGTGGAGAAACGAGGAACTCCAGCACGCAATCGAAGCTGCC

>dme_circ_0004975

GGTAGAAAATGCCCGTGGCATAGTCACCCAACTCCGGCAAGGTTACACCCTGCTTGGCACTGCAAACGGA
GAATATGTAGGGTTAGTTGAGTTTGAGGGATATGCAGAGTTGAATTTGTCATAGCTTATGAACAGTTTTT
TAAGATTTTAAAGGAATTTCACGGAATCAGACTACCGATTTGTCAACGTTTAAGCATTTTTCCATTATAT
TCATTCCGAGCTCCAGTATTAATGCGCCATTAAGTCGCTCTCCGCAACTGGCGCAAGTCAATCCAAGTCA
CCCAGTTCAGTTACTCAGTCATTCATTCATCGCGCCATTCCATTGAGTGCTGGTGACGAAACGAGTCCGC
CACAAGTCGCACCTTATCAGCATCTCTCTATGGAGAAGCTGTCAGCGAACCAGCCGTCACAACCTGATGG
AACCAATCCCATACCTCCCCTATTCCGTCTACCGATGCGCATTATTCGCCTGTCCACCTGTTTTCGTTAT
TCAAGCATGCATTCGTACACTGCGAGAAAGCCGTATGAACACACATAATTTGCCATACTCATAttttttt
tttAAACTATACTATAATAGACATAAAAATTTGGATTTCAACACAAATGGAAAACTGTAAATGCAAAGTT
TTTTCCCAGGTGCAATTGAGATTGAACTGAGCTTATATGTCTGTAGCTAAACTGCGTTCTTATTACGTCT
CAGAATTCCGGTCGAACCGATGGGCGGCGTACTGTTTAAGTGTTTTGCCATAATTTCGCAATTAGTCCAA
GACTTGAAGCCGGTCCTTGAACTTGGCAATCACCGACTTGTTGCGTTGCTTTCAGATCGCAATCTTCTTT
AAGGCTCTTGACCGTTTGCACGTGCGTGTCTAGAGTGAATTTCTTTCTTCTCGATTGGGTCACAATTTGT
TGACCAAAGCGAATCTCCGAATCGTCCAGAATACCCCGAAATTGCTCGGACATATCAATAAAGCCGTAGG
CTAGACGCTAATTTAACTAGATTTATGGGACTGTCGTGCGACATTACGCGTTTTAATGACCCAACCAGTT
GTGAAAATTCATCTGTCACCTCCTCCTATTTGTGTATATTATATATAGTATATACTCGGGCATCAGACGG
CTGCTCTACTAATTAAATAGCAAATAGTTGCAAATTCGCGCCATTGATTAGCGCATATTGAGTTGTAAAT
CGCTTGGACAGTGTCGTTTTTATTAGCTGGGGTTCGGGAAATGGCTTGGAACGCATTAAAATCAATCGGT
TCCATTGGAAACTATCAACCATTTTCAAAGATTCTAAAAGCAGCACCTAGCAACCGAAAGAGCATAACAA
ATTTAACTGATATATTTAATAAAGGTCAGCGTAATTGAGCTCTGATTAAACTGCTTAAAATCATAGAGGT
CTTTTGCCTTATTTGACTTTGATTTGAGCGATTTAGGTCAGTTAACCTTAATAAACTTTTCAAATATTAT
TTTTATGAttttttttttttCAACCGTTTCAAATGCCTAATTGAACCTCTCGGTCTCGGTTTAAAGTTCT
AGGAAATACAATAAAAACTTTATACCTATTATTTGCAATTAATTGTTTAAAGTTTTCAAATCTTTCTTCA
TTTAGAATATCTGAATCTTACCCAACCAAACCTCATTACTATGCACATCTGGTACACTGGATTAAGTGAA
GAACCACCCGAGGGGTACGAATAGGGGACCTTGCCGTGTTTTTCCATTTGGCGTATTTGAGCTAAATAAT
GTAGAATAATTTGCACAAAGTAATTGGCTGCAAATGGCTTCTATTCTGCTATCCACTTAGACTGGTCCCT
AAAAACATCGTTTAGTCCCATATATAGCCAATTAAAATAAATAAATATATATATTACGGCTACGTATGTT
TACCGATTCTGGGCAAAGCGTGACGAAAACAAGAACCGTGCCAAAAGCTGACGAAATAAACAGAAAACAA
AATACATAGGTCGGTCGGGAGCAGAGAGCAGATGTTGGATTGTCGGATCTCTATAGGTAGGCAAATACTC
CTCGACATATGTACATACATACATTCTATATGTACTTGTATGTGAGCGCCACTGGTCGTGGATATGATAT
ATCTTTGTGTTCCTGTGGATGTGCCTCATTTTAACAAACTATTTTTGACATGCTCGATCATTTCCCCACG
GTACTTTGAAATTCAGCAAATAAAGGGACATCAATGTTGAGATCCTCCCTTAAACCAGAAAGTACATTAA
GTGTTTATCATTAATAAATTGTATATCGCAATTGAATTGGCTAAGCTAAGTGATCTTTCTTCTATTACTG
CATATAACTGCAAATAAAAGACAATTTACTGATACTTTAAAAGGAAAAATCAATCTTACGTGTATTATCG
ATTATACTTCTCGGAGGAATAAATTAACTTTGTTATATAATCATTATACTCGTTTTAATAATGATTGAGT
AAGTTATTATTATGAATTATAATTATTATTGAAGAAAGAGTAAATATGAGGTTATTGCCTCTATTGAACC
AACTTAATCTCAAATGAATTATACTTTTATTAAGAGAATAGATCTCAACAGCTTTGAGCTATTTTCGTAT
TTCGCGCACGAGATCGCGGCGATATTGCTCCGTCATTTCACCCTGCCCATCGACAAGTCCCGTGTTCGTT
CCGTGTAAGCAGTTCGTTCATCAATGGTGCCAGAGGAACCGGCACAAATAAACATATGTATATTCGATGC
tatatctatatatataaatatatTAGTGATACTTACAGAGCCTTCGAGTAGAGTCCGTGAGGAATGGAAG

CCAAGACGCCAGCGCCATCGCCGGTGTCATTGTCGCAGGCGCAGGCGCCGCGGTGATTCATCCGTTCTGA
GAGGGTCTGGGCATCAC
>dme_circ_0004975_junction_seq
ATCCGTTCTGAGAGGGTCTGGGCATCACGGTAGAAAATGCCCGTGGCATAGTCACC
>dme_circ_0004976
GCTTGAGGAGATGGACAGCGAACGGCTGAACGAGCTAGAATCAATTCTATATGCCTCGATCCACTACAGC
GATGGATTCGAAGAGCACACTGCCATGGATCAGCCTGCGAAGGAGTCAACCAGCGACCAGGATGCCAGGA
GCATGCCACCGCCTCCTCAGCAGAAGCATCCCATTCCCGGACAGCGTTTCGTCAGCGGAACACGAGTGAT
CAACAACAGCATGCAGAAACCACGCTACTGGATCGATGGTGCGGCAGCTGTGGGGGAGGGGCAGGTGGTG
CAGGCGGAGGAACAGCAGGCCACCACAAACAAAACAATGCCAG
>dme_circ_0004976_junction_seq
CAGGCCACCACAAACAAAACAATGCCAGGCTTGAGGAGATGGACAGCGAACGGCTG
>dme_circ_0004977
GTTTGCACATGACTGTGTGTAAATCTCACTCGTTTGCAGAAAAATGCCAAATATTTTTGGTGAGATTTCG
CAGATTTGTATAACACACTTTCCTGTCGAATGCGAAAATCGTGGCTGACCTGTATGTTTGTATGTTTAAT
CGGTTCGATTGGTACTCTAGTTCTAGGACATGATTTTTCA
>dme_circ_0004977_junction_seq
TACTCTAGTTCTAGGACATGATTTTTCAGTTTGCACATGACTGTGTGTAAATCTCA
>dme_circ_0004978
ATAAACGGTGGGTCCACCAGTGGATGCTGATCGCGGGCCGGGCTTAACAGGTGCATTGTAGTGGCCGCCA
AAGTAGGTCGTTGCCTGTGGTCTACTCTGTGGTTTATTGTCATACAGCTGGATTCTTGTTGGCGCCCTGC
CTCGGGTTGGTGGCGTCATGGTCTTACTCTGGTCGGATACAATGTGCACCAGGGTTCGTCCTCCCTTTtg
ctgctggctgaggtggctgggctggtgctggtgctgctgctgctggtgaCCCACACTCAACCGGGTGGGT
GAGTTCTGCTGGCGGTACCATCGAGAGCCCTGGTTTTGACTTGGACTGGGGCTCATGCTGGTTCTATTCG
CGAGCAGTCTTTGCGCATAGTACTCCGTGGTGGGCATGGGCTTGGCCACGCCCACGGGCACAAATCTGCC
CGGAGAATGCGGTGATATGGAGTTCTTGATCTCGATGCGAATGGAGGGAGCAGGTGTGGGTGTCGGCGAT
GGCGACATATGACGCACTGAGGGTTGCGTGACCCGGAAAGTTTCGTTGCGTTTCACCGGAGAACGGGTGG
CGGAACGAGAGGGACGATCCCGGAGTGGAGAACGCGACTGAGGGCGGTAGTTATCATTGTCGAAGACCAG
GAAATCGGGACGGTTCTCCTTGGACTCGCGACGTTCCAGAGGTTTCGTGGTTGTTGGCTGGCCGGGAACT
TCCACAAACCAGGCGGGATTGTGGGGCGTTTCCTTCTGCTGCTGCAGCAAGCGGTCCCTGAAGGACTTCC
TAAAGCTATTTAGGGTGACTGTCTTGTACTTCTCACGCTCATCCTCCTCCCGCTGCTGATCCTCCAGCAG
ATCGTCGTTGGAGTGGGTTTTAAGCTGCTCCAGCTGCTCTCGCAAATGCGTGGGAGTTTTGCTCTTACGG
TAGGTGGATGAATCAAAGCTGCCACTGGAAGTGGAACCAATGCTGGCCTGCCGGTGGATACCACTGGAAT
CACCCGTTTGCAGGGCCCTCAGATTGGAGTGCTGCTCG
>dme_circ_0004978_junction_seq
CAGGGCCCTCAGATTGGAGTGCTGCTCGATAAACGGTGGGTCCACCAGTGGATGCT
>dme_circ_0004979
GGGTGCCATTATAAGAGAGGTGGAAGATGGAGTACGGTGTGAGCAATGTAAATCAGATTGCCCAGGCTTT
GCAGCGCACGATTGGAGGTAAGAAATCATCACCTTTGAATATAATCTACAATATTTTCAAGCAAGAAGTT
CGAATGCATTTTTGAAAATATCAGTTAATCCTTTACAATAGTTCCCGGTTTAAATTTTTTTAAAATTTGT
AATGCGTTTTCTTGGCTACTCAGCAAAAATGTCTCTTAGATTCAATTCAAGATTGCTTTATTTTAGCAAA
AAGAACACATCGCCCCTCAAGGTCTGCCAACTTTTTGTGCCTTCACTGTAAAGCTCACTGAAAAGCTTTA
CTGCTTGATAAGATTAGTTGCAGGAATGGCCGCTAGATGGATCGGTCTGTAGTTTCGTAAGCAACTTAAC
AAACAAAGTAAAAATCAACAAATCTATAAGATACAACAAAGTGAGCGCATTGTTTAAAAAGAAGACTCCA

ATATATTAATTTGAAATGAATTAAAGAGAACATTTAAAGATCATTTTCCTGTGCTTGGCTCTGCATTCGT
GTGTCTCGATAAATAAATTATGCGTCAGCGGTGCCAAAAACAGAGAATCCTCCTCGCAAGCAGCAGGAAA
TCGAGAAAAAGGCAAGAACAAATCACCTACAATCTTGTGGTTCATAAAAGCCAAGATAGAAAATGAACCA
AAAACCGCAAGACGGTGGCAAAGGCGATTTGGCAGCGCCTCCATATATTCCCTCAATGAAatatatatat
ggatatatacatatGTATTAGAATCGATGACATCGGTGCAATGTGCAATGTGGATAATGACTGGGGCCGC
AGCGTTAATTTATTGCTCGCCTTGTGGCCGCGAACCAAATAAATATTAGCTCCTCTGATTATTTTCACTA
TTTATCATTGATATTTGCGAAAGCCAAATAGTAAGCCATGCGGAACTAGTTTAATTCCACTAGCATGAAT
TATGCAATTATATGCCGCATTTTATAATGCTCCATATATGTTTGTTATGTATGTATATATATACGCAAAT
CGGGAATCGACAATTGGCCAAACACTTAGCCATTTCGTTTGCTCCTGCGCCCAACCACAAAAACCATTTC
AAATTACGCCATTAATTTGTGAACTGAACCGAATGTCCGACTGTCTGTCCGGCGTCTCTTTTTGTCTAAC
TGCGGTTGTGTTTTCTATTTGTGTTTTCATTTTGATGCTGGCCGCACGCCAATTCCATTTGATGATATAC
GGGGCGTATACGTGATACACTTTAAGGGTCATACGCTTATCGCAATAGTTGGTTTAAAAGCCAAAAATAA
AAGGTAACATAAATGAAAATATGATTTCATTTTGTTGTAGATTGCTTGAGCAACAATTAAATACTTTCTT
TGATATAAAATCTTTaaaaaaaaaaGAAAGAGTATTCCACCATAGTTATGTTTACCTCTAATTCGTGTTT
TCTATTGTACAATTACTTAAATACGGCATGATTGGATTGGTTTGCTTTTGGCATTTAAAAAGTATTATTT
GCTAAAAGCAGCTAAAATATCTGTTTTTGGCTTTGGGATTTATGTCTTTATGTGAATTTTTTTGTGGGGT
AGTAAATTAAAATTAAAATTTCTTCAACAGAGCATTTTATACACACATACTCAAATCACAAGAACTATAA
AAACGATATTCGGAAATTTTCTATTTATAATTAAAACGTAGGCAATTTTTAATGACCATGTTATCAAATT
CATTTGATGAAAACATAGATCAAACCGTATCGTGGGAGAACAATTTATTATGTGGGAACACGCACTTCAG
GATATTATGGCTCAATGAGAATTATTAAGGGCAGTGATTCGGTTTTATAAATATAAAAAGAAAATCGTGA
AAGATAATGCTAGAATGCACTACTATGCTTTATCGCTTCAATAAATAGCAGTGTAATTTGCACTGGAAAA
AGTTGAGATTGTATTTGATTGGACAATTAAACCATTATTTTAATTGTGCAATAATGATGGCAAAAAGGTG
TTAATTATTAATCTCAGTACACGGAGATAATTGACTAAGTAAATAATTGTTTTCATGCCCAAGTGCGTAC
ATTAACCCCTTGTTTCCCCTTCGCCTCGTTGTAACTCGATTTCATTTCGTTGCAGCGGCAATTAGTTGGC
AATTAAAGAAAGACCTTGACTTAAATTTCTATAAATTCACCCAAAGGGTTCACCGCACATTTGTTTGTCC
GCCCTGGAGATTCAGATTGAATTGATTGCCAAACAAGATTGAAgattgacgattgcgattgcgattgcga
ttgcCATGTGGTTGCTACATCAATTTGCTGGGTTCAGTTCAAGTTCAAGCTGTCGAGCGACGCGTGCAGT
TTTTTATGTCTCTTATTTTTTAGGGCTTATGCTTGAGCAACGAAGACCGACATTGACTAACTGACTGAAT
GACCGACTGACAATCAGTTTGcatatgcacatatatacatatgtacatatatatgtatatgaatataGCT
TAAGCTCGTGTTTGTATTTGCGTATATTATGCATTGACGATCTCTCTAACAGCTGCGTGCTCGTTTTGAG
TTTGTCGATTTATTTGTGTCttttttttttgtattttatttttCGTGCTCAGAGACGAGCTCAAATTGCA
TTGTTTAACTGCTAAAACAGTTGTTACTCAGTTGCCCGCACGATCGGTCTGTCCCACAGAGAGCGAATGA
ATATTCAAGTTGAATTTCaaaaaagaaaatattgtaaaaaaaTACGAGGCTGTGGGGAAAAGAGCACGCG
ATGTGTCAGCACCGTCACCTTATCGCGTTTAAAAGTGCGCTCATTTCCACGGCGATTGTTTGCCTAAACG
TCAATTGTGCGTATCAAAATCGAAATCTCTTGCGGGCAACAGTCTGTTCAAAAATATAGAGTGCAACAGT
GGTCTCAAGCCATATTATATACCCAAATACGCAAGAAGTGAGATAATCGTTAAAAACCCACTGAGGTGTA
TTCTAAGAAAGGCCGAAAAGCTTCCACAAACTTAAAGTATAAGAAATCTTAAATATATCTAGAAATAAAG
TACATACGGCTATGTTTTAAAAGCAAAAAAGTTGAAACCAAATTAAAATTATTGCGTACCCAATTATTTG
TGCAATGTGCTCATCTGAAAACGACCTTAAAGTTGTGAAGCCCGCTGCTCAAAGCCACCAAACTGAAAAC
GATTTTGACAGCCAGAGTTGCTGAATGTTTTTAAGTTGCGTGCCGAAGTCGTTAGCTGAAACTGAAGTTG
AACTCTGTGGACAATATGCTGTTGAAGGTGTGTCCATGCGACGACTTCCAAGTGCAGCCGAAAGTGGTGA
AGAAGCAGCCTAAGTTTCCCACCCGCGAACGCCACATGGCCATTCGGCGGAAAAAGTCCACGGCCATGGT
CACGACCACGTCGGAAAACACCGCCAAGTGTCCGGAACAACGGTGAGCATGTGAAAAACGGGAAACGGGT
AAAGTGGGGGCAGAAGAATGGGGGAACTGGGGTTCTGCCGGGTCAGCTGTTTATGAGTTATCGAGGCGGT

AATTATCGGCTAATGGATTGTGCCCCGCGGCCCACAAAGTATTTTAATTGGGCATCAATCTTTCGGCACT
CGCCTCGGGCTGAAAAGGCCAAGAACAACAAAAACTTAAATAAATTCCAATTTGACTTTGGCATCTTCTC
TTTTCATTCCTCGACTGCTGTTTTGCATAAGTTCACACATTGCGGTATCAGATTTTAATAGTATCCCATT
TAACCGGCGCAAAGTGTGTGCTGAGGGAGCCAAATCAAATTAAAAAACCGTCTTAAATAATTATAATTAC
GTATCTACATTTTTTCTAACATTTTTCCAAGCGATTTGAAGATAAATTTATTCCTGATAACTCCTAGATA
CAAATTGGTTGATTAAAAACATTTCTTTATTTACATGTTACACTTAAGTTCTAAATAAAGCTGTCGAAAA
AATGTAGATTTCTATGTTCTTCAGAACTTTCCACTGACCCAACTTTCTACTCGAAAAAACTACAAATGGG
TTATGCAAAATCCACGAGATCTTACCCATAAATAACCCGTGCCCCACGTGAGATGCCTTTTCAGCCGAAA
GGCGATTAGAGAAACACATTTTTGACAACAAAGCAATTTAAAACATTTGCCGGCCAGAGGCTAACGCAAA
AGTCGAAATAGTGACGAATGATCATTGTACTTGGAGCAATAATAAACATTGGGAAATTTGTGTGTTTACT
TAATGTGACGGTAGCATTAACGTGAATTTCCACGAATCAAAGCAAACGTCaaagcctaaaaaatatatat
gtgtacagaaataaataaatatacataccaataaaaaGTAAGACAGCATTTGTAGCCACCTTTTTTAGAG
GGGTTACGCGCCCCGAACACGCTGCAAAAATGCCTCAGATGTAGAAAGATGGCCTTAATGCTTGGCCGCT
AGAGCCATTTACATCTGCGGCCGGCGACAACAGCCAAATGTAGCCGGCAGATACATGTATCTACAGCATC
TGTATCTCTTTTTTCCCCCCCACAACGGGGGAAATGTAATGTAATTTCCTTTTGGCTGTTTTTGCCTGTT
CGTGTAATTAAAAGCAACAAACGACAGGCTCGTGCATCAAAGGTGCCTCTGATGGTTGGGTTAATaaaca
aacaaacaaacaaacCGGTTTCCCCACAGCTGTTCACCTGGCCCCGGGTGCCAATGATAAATGGGCTGAA
TTATGCCCAATTGCATCTGCATGTGCACTTCATATTGGCTTTTGAATGGGAAATCAGAATGGACCTCATG
CGGGTGTATATTTATTCAAAAGAATTGATATGAACCAATTAAAAGGGTTTGCTCTCAATTTATTTTTAAT
GACTGCTGTTTATATATCCTCTTATTAAATAAGTAGTTTTTATTGAGAAAGCTAATCTTAAGCTGTTTTA
CCCAACTTTATAACACTCTATGCGACGAAAGCGTAAATTGTGCATTTAAGAATTGTCTGGAATGTTAAAA
AATAATAATAAATCACAACACATTAATTGCCTATCTGTCACCATAGATCTCTATACATGCAGTCTTACAA
CAAAAGCTCAATATCTTGCGATAATAGCACGTGGACGTGCATTTAAAAGATACGGATATTTAGCCGTAAT
CGATGTGCTGCCAAAAGAAGATACATTATCCAGATACATTAAAAACATTGGCTATGCACTGCCGGGTTGT
TCCGGCAGTTGGTCGATTTTAGCTGGGCTAGTCTGCGTGTCCATTAATCAGCTGCCTCGCCAAAGTCACG
TTTTGCATTTCGCCAGTGTATCTTTGTATCTGCGAGACAGGTCGTGTGTGGTTGTGGTTGTGGATGTGGA
TACACTTAATGCGGCTGCTTATCAAGTTGACAGACATTGCGGCGACACGGCACAAACTACCTGGATGTGT
CTGGAGCCTGGACTTTTGTGAGCCTCTAATTAAGTGGTTTCAAACTGGAACTTGAACTTTGCCAGGCGAT
GGTCATCAATCTTGCCCAGATAGAGATACAAACATGCACGCAGATACAGACGTCAGTGAGCGCACATTAG
ATGCCTGATTTTATTGTACAATTTACGAAATTAATCATTAAAATTTACGATTAAGCGAGGCAAAAAATCT
TCATAAATTACAGCCAGCACAGTGACAGAGATGGCAATGGGTGTGTTGACTCTCTTCGTGCTCGTGCGCC
ATGAATGAAAACACTTGGCCACACCAATCAAACGGATCCCTCTACTCCGCCTCACACCCGCCTCTGGGCA
CACCAACCACCCACTCACTCAAAGGCTTTGTGTATTTTTAGTGCAAATATACAAATTTGACCAAGTGCTG
CTAGCAGTGGCGAGCAAAGCGAATCCAATGGCATAATAATATTATTAAAACAGTTGCGTAGCCCAAAGTA
GCTAGGCGAGAAGGGGTGTTGTTTTTGAAAACATCGGGTAAATGATTTAATAACATTTTGCGGTCTAATC
AAGATATGTTCTAAGAACAAATAAATAAAGAGTTAGTACAACTTTGGTTCTTTTCGAAAATGTGTGTGTT
ACCAAGTAAACTATGAGGTGAAACGAAACTTTTGGTTATTCTTTTAAATTCCGATATGAAATAAATGATT
TCTTTACAGAAACTTCAGTCAGCTTAAGAAACCTTGAGTTGTTGGATAATTAGATATATTCCTTAACTTA
CATGCTTGTTATAGTATTGTTTTAAATACAGTTTCTTCCTTCCCTAAGAGATCCCCTCTTGGCAACGTCC
TTTGATTTTTTATGGCCCATTTGCGCTGTCTGTATGGATTTTGTCCAGCTTATCCCGCCTTCCCTGCCCA
GCGACAGTTGGCCTTTTAGCTAGTTGGTTCTAGTTCTTTGGTTATAAATAATTGCAGTTCGGAATTTGTT
TCATGTCTGCGCACTGCTACATGTGGCAATCAAAGTCAAAGTGTGGAAAAAATGTCGCAGACGCTTTTGAT
AAATGGCTGGGGCAACTGCTGCGGAGGTCGGTCATCCAATACGACCTTGACCGCCTGCCGAGGGGCGGAA
GGCAGAAGGTGCATATGCTTGGTTTACATTTCGAGTAATAAGTGCAGGGAGCGATGGCCATTAATTTGCC

GGAATTACCTTACCTTACCCTCCAGTCATTTACTTCCAACTTACTGCTATTCCCCCGAAAAACCCACTAT
CTAAGCGCTTCGCCAGCCAAGCGCGCTACCTGTTGGACTCGTTAGCTTAGGTTAGTGGCACACTGTGAAA
ATTATGTATATTTGTTTTATAGTGTGATTAGATAAAAATGGCTTAAGTAACTCATGAAATGGCAGAAAGA
AATAAAGTTCTTAGAATCAAACACTAATCTTCTTTTGAATTAACCTTCAAATTTGAACTATTATGGCGTT
TCTCTCAGTGCTGGAGTTGCTACTCCCACCACATTGTCACTACCCTTGCACGTGTGTGTGTGCTAATGAA
GAGGGCCATAAAAAAAGCAACGCAATGCATTGCTAATAAGCGGTTCTTGCGGCTCTCTTGCGCCCCCCTTC
TCGCACTCCCACTCCCCCTCCCTAGCCCTTTGGATGCAGCAGAGCACACACTTGTGCGGTGCCATTATCG
CACACCTTTTGGAGCACTCCCCTGACCAACGGCAACCCAACCACCCGCTCTCTCTGCAGCGCAGCGCGCT
TTCTTCGTTCGGCAGCTTCGGGCTTTTATGCTGGGCTTCGGGCCGCTACTTCGGCTGCCGAACGGTGCGC
GCAGACAGCGACGCCGACGCAGCAGAGTTCAGTTAGAATTGGTCTGCTTAACGGTGTGGTTGTCGGCGGC
GGCGTTTATTCCCCGCGATTTGCGCGCGATTCGCATGCGTTTTCCCAGTCGGTTGGCAGTGTTGTGGAAT
AAATTTAAAATAAATTGAATTAAATTTAATTGCACATTTTTCCGTATTCTCACCTCGGGACTGGGCCATT
GACTTGGCGGGGGAGTGACGTCGGTGCGCTTATATTTGCTCGATCTGAAGTGTTTAACATTTATTTGAAT
CGTAAACAAAAACAAATCGCCACGATTTGTTTACATTTTGTTGTGCGGTCTCTGGTTGCTttattattat
tattattattaCGGAAAAATGCAAATGTCAATTGCCGGAATCAAAATATATCCCAGTGCAGAGAGGAATT
TTTGAAAGAAATATTCCTGTGGATTATTCATTTTTCACTTGTCCCGAGAATGAGTCAGCTTTTGGATTAC
ACTCCCACCTATTTTAAAATCTTATCCCACACACTGACAtttgttttttcctttttccctttttCACGGCC
CATTGCGCTTTTCTCAATTTTGTAACACATTGTGACCAGATTTCGCGGAACTGTAACCTCCAGACAATTT
TTGCATCAGTGCTGAGTGATAAAAATTAATGCGATGGGATTGGGGATCTTAAGCCCCATAATAGTTCCCG
GAAATTCGACGACATTTCTACAATTCCACTGATCCAGATTTCTGGGTCAGCCTATTCGCGCTAGATTGAT
GAGACAATAAAGTGAAGATGATTGTGAGCTATCATCTAACTCACATACTATAAATCATAATAAATTACTG
AGTATTAACGGTGTTAAGTCGAGCAAACAGAATAACTGGGTAATGAGGTGTTTGCCCTCCGATCGGTCAG
ATTGATACTGGGTTCTCTTCGAAGCAATGCAGGCGGATTGGAAAAACAGAGTTATCAGTTTTCTATGACG
TTATGTTATCTACGTAAGTGCTATCTGGATGCCAGGTTAGCGACCCATTAAAACTACGGATTTCGAATTT
TCAAATTACTTTATGGTATTTCGATTTCACAAGTAAATTATAAAGAAAGTATCTCTTCCAGTAAAGTCAA
TTATAATTATGGGGAATTGCTGAGATAGTTACTCAGCTAACTTTTCTTTTATGCATTTTATTGGAATTTT
CTTTTTACTATTCCTATGTGGCCCTTTAGAAATTGGTTTAGTCGTGATTTATGCAGTGACATTTGCGGTT
CATCTCTGTGGGAACCCCGCAAGGTTGAAATAATTCACTATGCAATTTGTTGCTAAAGAATATTCTATCT
GCTTTCCGTCGGGCAGCCCCAACAACTTTCCGTACAACCCCGTGCTCGTAAATATCAACTAAGCTGCCCC
TCGGTTATGGGAAATATAGGAAGTGGGGTTGTCAGTggggggggggggggTAGAGTGCAAAAGGGGCTT
GTGCATCCTGTAGTGCATATGTTAATTATCCCACCTGCCCACCGATAGTGGGGTAAACCTTTTAGATGAG
GCGAGTGGAGGGGGTAATACTACTCACTGATTCAATTAGCGGTAGCCATACGCACATAACCCATATTTAC
GGGTGCACAGGGAAAAAGTACACTTATGCACAAAAAAGAACAATAAAAAAGAAATCATTTTATAAGGTGG
TTTGAATATTCGCATAGGATTAAGAATAAATGTCACGTTAAATAACTTAATAAGGTGATTTTAATGCAAA
ATTACATCTTTAGTTTGTTTTAGTGTGTCTCATTGTTCCAATTTGTTCTCTGTGCATCTAGAAGTGTGGG
GCAATGCAGCTCTACTATAACTATATAAATATATAGGGCAATCTGAGCGTCCAACAGTAAAGTGCATTGT
TCTGTTGTTGTTGGGGTAATCGCATTAAAAGTCGTTTAATCGAAAGCAAAAAGAGAACTTTCCCCACCCC
GCCCTGTCCCACATTGTGGGTTAAGTGGGTGGGAGTGGGCGGTATAAAGCAGGTGGGGGGTGATAAAAGA
CGGACCACAGCTCATAATTGAGATTTGTTGCACTCCAGCGTTGACTTTTATCCGAGCGCTTTTATTGTTT
ATTGTTTACAGCGGCAACATGTAAGTGCAATTACTGCGGAAAATTGGATTTCCTCGCTGCGGAAAAGCTC
TCTGGCTGGCTGGGATGGTCTTAGCGCTTGAGCTTCGCTTTTCGGTAGGTGGCAAACAAATTGTTGGTCA
AACAATGCTGATTGCTCTCTAGCCGAAACAGGGCGATACTATATACTGAATAGGATATAGCGATTGGGTT
TGGGTGGGCGCATAGCCCCAAAAGTTTTCCAATTGCGGTTGCCGCACGCCGCTTGCCTCTATAAACGAAA
GTGACTTTGGGGCTTATCAATTGTAATTGTGGGTCCGGGTTACCTACACCTACATGCTGCTTCCACCCCC

AGCTCCACGCCCATCGCGAAATGCTGCGGGGCCTAACAAGAAAAGTTGTCGCCGAGTTTGACTGTCTATC
TGTGCGAGAGTGTGAGAGTTTGGTTTCAGGATACAGTGCTTGACGAAACCATAAGGCCATGTACCAGACA
CAGCCTAAACAAATCCCAGATCTCGTTAAAAGTGCTGAAGTGACAACTTTCCGACTAAAATGGAGTGTAT
CAAAAATTAAAAGATTTTTGTTGTTTAGTTTGTCATTAGTTTACTATAATAATAAACACTATTCGATTAT
AGATTTGAATTCCCTGCAAACTTAATTTTCTAAATCTTTTATAGAGTTTCCTTAATTGGCTCATTAAAAG
TTTCAAAGTACCAATCCGTTATGCCTCGCACTGTAGCAAACTTTTGGCTTTTAATTACATTTATCTGGCA
AAAGTTTCAGCCCCGAAACTAGTTTTATTTGTTTGTCATACGCATTTCTGTTGATTGTACACCATACGAT
ATCAGTTATTATTATATTATGAAGCGTTTTTTATGCCATTTCCATTGCCATTGCTCCATGTTGCCCATTT
CAATTAAATGAAAATCTTTGCCAGATGTTAAAAACGCATTGCGTTCGCTTTTTCGCGTCTGTTCTGTTCG
ATGTGACAAAAAAGCCTTGTCACAGAGAAGGTTCTAATCTGAAATGAACACGTTTTAATGACATTTTAAT
TGTTTATTCTGCGATAACACTGCGCATCTTCTCTCCGCTGAAGACTGATAGCAAACAGGGAGATACAAAG
TCTGTTTTAAAGGCGAATAAAGAAAAGTCGCGAGAACAGGAGTCATTCAATATAGACCATATTAGCACAT
ATGGCCCGGCACGCATTGGTGCATTATAAGCTGTCGCTGGGCAAACAAATGTGTTCCTCAAGGTCCCAAA
AAGCATTGAAATCCAGAGATGTGAGCTCCCCATACAGCTGCGGGCATTCTAAATTCACTTGAATATTTAA
ATATAGCCTCTGCCCGTGTGATCTATCTTGTACTTGGAAATCTGTCAAATTGAATTTGTTTCTACATCAA
TTACGAAAATGACATTCATTCGGGGAGGGATTTTTTGGTTAAGTCTTGTCTCCCTTTTATTTTCGTTTGC
TTTAAATTAAAATCGATTTGTATCAATTTTCTGAGGGGCTTGTAATCACGTCGGATGTTTCAATCTACAT
ATGCACATATTTGTTTGATATAAAAGTATCTATTAAGCAATACTTACGATTTAACTTATACATCTTATGA
TTTTTATGCTTTGGGGGAAAGCAAATATATTAAAAAATTAATGAAACCGTAATAGCATACCGCAAATCAA
CAATTATTTTCATTTAATGCTTCCTCAATCTAGAATTTACTGATAAATATTATAAAGATAACTGCGTAAG
CCAAAATGATTCCACCCTCCGTGTAAAATGTGTCAGCCGAATCTTATCAACATGTTATTCTGAGTGGCCC
CCTTAATTATAGAGTATGCTTCATTTCTATGGACTTCAAATGTAAACATAACACGATATATATCTAGATC
TTGCGAGAACCTTGACTTTGTGTGTCACAGAAACATGACTCAGCTGCAATTGCATTAAATTATTCAATTT
ATTTGCATTACCTTCTCCCAGGTCTCCTCGTGCCAGCAAAAGCATAGGCAAAACCTGTTGCACTTTTCCG
CATCAAAGTCGTGAGCAATTAGCAAACAGTTCCTCATTTTCCAGACAAGTCCATAAAAGGACAGCTGGCA
TTTCGATACCATATACCCTCCGTTTTCCGTTCCACATAGCCATTTGGACAATCACCAGTCCCTGTTTCCA
TTTGATAGCATTTAGGTGGAAGAACTCACATCGGCTTATCTTATTTTGAACGCTAACGCAGTTTGGCCTC
CGTCGATGATTGAGCAAATGGAATTTGGCAATTTCGGTTTTGGGCTTGGAGTTTTTCGTTTTCAGCTCCC
CCCGATGACATcacacacacacacacacTTATGGCCAATCCCAACCCAGGATTGCTGGGTGCCtgtgtgt
gtgtgtgtgtgtgtgtATAGGGGGAGAGACCTTTATTAATCTTATCAAAAGATGCTTTTCTACCTTTT
ATTTTATTTCATTGCTTGGCCGTTTTTATTTTCGCTGAATGTCAGACCAACGGGCATGCCATAATCGTGA
GCTCATTCTATCAGACCTTCTTTTTTATTTTTGTAATACTCTTCTGAACCTTTGACTGATAAATAATATT
TTAAAATTAGTTAGTTTATCCGATTTACTATACAATCTGCAATTTATCTTTCTGTATACTGGTAATGGGC
CTACGCACAATTTCACTGGTGTGGGCGTATTTATGATTGTGATTATTTTGCTTGTATCTCTAATTGAAAA
ATAAGTAAACAAGCAATTAGAGAAATTCCCAGACCGTAGAATACCGCGCAGAGGTAAAATTAAATTCTTG
GATAGTAAACAAATGCTATCAGAACAAAGGTCAAATTAGCACTTTAATCAAAATGTCAATGAGCAAATAA
CGCGCACCGACGTGCCGAATGAAAGGTGATTTAACCTAAGTTGCGAGTTTCTCTTATTTTCGTGGAGGTG
GCAGCGCGAATCACCAAAGAAGAACCGACAAATTGTGCCGAGTCAGGCAATAAGAAAAGATATTTAAGCA
GAAACCCACGAGAGCCAAGTCAAGTTCATTAAGACAAACGGTAACAGTAATAGGAATaaaaaaaaaGAGA
AGAAGCGAATATAAAGCCAAAAACCCTTAGTTCAAAGTGCTGTTGAAAGTGGCGTAAAATGGTGAAAATG
CTCGATCTGGACCGTCACGCAGAGTGCCCACTGTGGGCGGCACTGGCCTCCTCGGATGTCCCATGGACGG
GAATGGACTACGCCTGCCTGAACGTGCTCAGTATCTACTGCAAAGTATTCGATAGGAAGGAAATATACGA
TCTCATCGTGAGGTAAGGCAATAGCCGGGTTCGCAAAGCTGCCGCTGGATGGCGCCACGCGAACTTACGT
TGAAGCATAACGAAGCTGCGCACAGTGGAATGAATTTGGAATTTATTGATAAAATATTTTCGAATTAATA

TTATCTTCAGCAATGTTGTTACATTGTATATAAAGTATAATGTATTAAACGACCTTATTTCCCTCTTAAT
TTCCAGGGAAAACCTGCCAGTCCTGCAAATGTCCTCGCGAGGCGCATGCCATCTATCAGCAACAGACTAC
CAACGTGCATGAGCGACTCGGCTTCAAATTGGTCTCTCCGGCGGATTCGGGAGTGGAGGCGCGGGATCTG
GGCTTCACGTGGGTTCCGCCCGGAGTGCGTGCCTCCTCGCGCATCAACCGCTACTTCGAGCAGCTGCCCG
ACGAAATGGTACCCCGGCTGGGCAGCGAAGGCGCCTGCAGCCGGGAGCGACAGATCTCTTACCAGCTGCC
CAAACAGGACCTCTCGCTGGAGCACTGCAAGCACCTGGAGGTGCAGCACGAGGCTTCCTTCGAGGACTTT
GTGACGGCGCGAAACGAAATCGCACTGGACATAGGCCTACATCAAGGATGCCCCCTACGATGAGCATTGT
GCGCACTGTGATAACGAGATAGCCGCCGGCGAGCTAGTTGTGGCGGCGCCCAAGTTTGTGGAGAGCGTGA
TGTGGCACCCAAAGTGCTTCACCTGCAGCACCTGCAACTCGCTACTCGTGGACCTCACCTACTGCGTCCA
CGACGACAAGGTCTACTGCGAGCGCCACTATGCGGAAATGCTGAAGCCCCGCTGCGCTGGCTGTGATGAG
>dme_circ_0004979_junction_seq
GAAGCCCCGCTGCGCTGGCTGTGATGAGGGGTGCCATTATAAGAGAGGTGGAAGAT
>dme_circ_0004980
GGAAAACCTGCCAGTCCTGCAAATGTCCTCGCGAGGCGCATGCCATCTATCAGCAACAGACTACCAACGT
GCATGAGCGACTCGGCTTCAAATTGGTCTCTCCGGCGGATTCGGGAGTGGAGGCGCGGGATCTGGGCTTC
ACGTGGGTTCCGCCCGGAGTGCGTGCCTCCTCGCGCATCAACCGCTACTTCGAGCAGCTGCCCGACGAAA
TGGTACCCCGGCTGGGCAGCGAAGGCGCCTGCAGCCGGGAGCGACAGATCTCTTACCAGCTGCCCAAACA
GGACCTCTCGCTGGAGCACTGCAAGCACCTGGAGGTGCAGCACGAGGCTTCCTTCGAGGACTTTGTGACG
GCGCGAAACGAAATCGCACTGGACATAGGGTAAGACTAGATCTTTAATTAAGTTGGAACTATTGTGTTGA
TGTTTTTAGTAAATTCTAAAAACGAATATATACACTCTTTCACATAATTAACTTAGGCATTACACATTAT
GACAAGCCTGCGAATGGCTTTAGTTTAAATTGCCTGACCTCACCCAGAGTGACCTAACTAATTGCGTTTT
GCATTATTTTCCTGTTGAATAGGCCTACATCAAGGATGCCCCCTACGATGAGCATTGTGCGCACTGTGAT
AACGAGATAGCCGCCGGCGAGCTAGTTGTGGCGGCGCCCAAGTTTGTGGAGAGCGTGATGTGGCACCCAA
AGTGCTTCACCTGCAGCACCTGCAACTCGCTACTCGTGGACCTCACCTACTGCGTCCACGACGACAAGGT
CTACTGCGAGCGCCACTATGCGGAAATGCTGAAGCCCCGCTGCGCTGGCTGTGATGAG
>dme_circ_0004980_junction_seq
GAAGCCCCGCTGCGCTGGCTGTGATGAGGGAAAACCTGCCAGTCCTGCAAATGTCC
>dme_circ_0004981
GGAAAACCTGCCAGTCCTGCAAATGTCCTCGCGAGGCGCATGCCATCTATCAGCAACAGACTACCAACGT
GCATGAGCGACTCGGCTTCAAATTGGTCTCTCCGGCGGATTCGGGAGTGGAGGCGCGGGATCTGGGCTTC
ACGTGGGTTCCGCCCGGAGTGCGTGCCTCCTCGCGCATCAACCGCTACTTCGAGCAGCTGCCCGACGAAA
TGGTACCCCGGCTGGGCAGCGAAGGCGCCTGCAGCCGGGAGCGACAGATCTCTTACCAGCTGCCCAAACA
GGACCTCTCGCTGGAGCACTGCAAGCACCTGGAGGTGCAGCACGAGGCTTCCTTCGAGGACTTTGTGACG
GCGCGAAACGAAATCGCACTGGACATAGGCCTACATCAAGGATGCCCCCTACGATGAGCATTGTGCGCAC
TGTGATAACGAGATAGCCGCCGGCGAGCTAGTTGTGGCGGCGCCCAAGTTTGTGGAGAGCGTGATGTGGC
ACCCAAAGTGCTTCACCTGCAGCACCTGCAACTCGCTACTCGTGGACCTCACCTACTGCGTCCACGACGA
CAAGGTCTACTGCGAGCGCCACTATGCGGAAATGCTGAAGCCCCGCTGCGCTGGCTGTGATGAGGGTGAG
TCGTCTTTGGCATAACCCCACCTATCCGATCCCTAATTGACAAATATTGTGTGGCACACTTTTGGGGCAA
CGTCTGCAGATGGTATCTGTGTGTCGCCTGGCAAATCAAGAGTGTCCGTGTGAGTGAGTGCTCGCTGTCA
CGGGCTCTGGTTTTTATCGCCCGCCGCACGCAGTTCACGTTTATTGCATAGGTGCGGCAATCGCCCTTTC
CAAAGGTTATGGCATTTTCATTGAACTTCACTTGGCTGCCTGTGGGAGTCGGGGATCCCGGGGATATCTA
TCCTCCAGCTCCGCAAATGCAGCGACTCGCGAGTGGCGATGGCAACAAAGAAATGAAGGCAGTTCATTGG
CAGCACTGGCTGTTGACGCGTTGTCATCCGTCGAGCGGAAGTTCATTGCGAGTGAGCCGGAAATTTGTGG
CATTCGGTTAAGGTTAGAGATTGCTAAAAATTGCTGCAGGTATTCGCAATTTGTTATTGATGTGCACAAA

CTGCTTTAGCTCGATTGAAAATATCCTTTATCCATTTATTTTGATTTTAAATAGATTGGGGGTGTAATAT
GCAAGTAAATAAAttttttttATATGCGTGTTGATCGATTTTTAATTAGAAAATGTTGGATAAAGAAATT
GCATTGTCTGTCGCCTTTCGGTGTTATTGTACAAAAAACCATGTTTAAATTTATTCAAAATAAATTTAAA
TTACCATCAGAAACACTTTCGCTTTCGTTGCGAAAAGCAAAATAAACATATGAATATATCAGAGCCCAGA
TGACCCATTTATTCATATAAATCTATCTATTTTTGCCACAATTTCTAATGACAATCAGATGATGAGAATA
AACAAAAGAATACAAAAATAGTTTGGCTTTGTGGGGCAAAGAACACAATTATGCCGCGTTTGACAATAAT
TATACTGATTCTGAAAACGCAGAAGAGAACCACAGCGAAATCGGCGATAAAGAGCCTCTGAAAGCTGCTG
GCAATGTTAGTGTCAGTGCCAAGACGAAATTTATGGCAAACATTTGTGTGCAATCCGCTCGGAGGCGGCG
AAATGGGGCGTGGCGACCCTGGCCCCAGGCGCTGGCAATGCCAAAGAAAACTGATTTGCAAACACCAGGA
ACAGTAAAAGTCATGCCCATAAGAATTACGAATGCTAAAATATATTTTTCCCACTTTCGAGAATACCAAA
TTTTATTGGCAAAATAAACAAAAGAATTGGGCATTTTATAAACAAAATTGTATCTCAACATTGTACTCTT
AAATGTTATTTTTTCTAAAATCGGAGATACGATGTGACCATTAATGCCAAATTAAGCATTTTTATGGAAA
TTGGCACCTTAGCTCTGTATATTATTGCAGCCAACTGTGCGTCTGAATAATATGTGTGGTATGCAGTATA
GAAAGATCATGTTTTTCAGAAAATACTTGATTTTTTATTATTTTAAAATATCAAAAGAAACATTTTCTGA
TTAATTTTGATAAATTGAATTTTTAAACCTGAATAAAAGGATAGGAAAGAAAAGATCCCTAAATCTTGAT
ACTTGCTTGAGATTAAAAAACTCTTCATTATGTTATCGAAATGACAGTTGCATTTGATTTTCCCTATCCG
ATTTATGGTCATGTCGCTAACTGTTCTCCCGCACGAAAACGGCGAGCAAAGCAGAAAGTGGCGAAAAGTG
TTTTGTGGAAAACAGCAATCGAAGCATAGACCAAAAATAGTTCCCCCCAAACGAAAAGGGGGCGCGGCCG
GCGGGGTGTCGACTAAACTGATTAGAAAATCGGCTTTGGCCAATTCCGATTGCTTTGGCATTTGACACGG
AGGCAAGGCAGTGCAACAACGGCAGCGGACAGAAGCAGATACATTTGTGTTGCCAAATTGCCAGCGACAA
AAGTCGGCGATGTCTTGTCAGCTCTACAGGCCTTTTGCTCTCATCGCTGGCCGTCAATTAGCGCCGTTTG
CCATTTGAACGGATTCGGTTCTTGGCGGTTCTCTCGCCGCTGATTCACTTGTTCTCTAGAAATCGGGGGA
CCCATGGGCATTGGCTGGGGCATTAATCAGTTGCAAGACGATCGATTAGCGGAAAACAAGAGCTCGCCGC
CCGAGCAACTGCATCCGCCTGGCGAGTCCGCCCATCTCCGATTCTTCTTGCTTCGCGCTCTCATAAATCG
TCTCAAAACGTAGATAAATGAATATATATCGATGATTGGTTCAATGATTTGTGAGCGAAAGTGAAATAAA
TATTTTTCGAATTACCTAATCGGCTGGCCATTAACGTGAGTGGTGTGAATTTTCGAGTGCGTGGTGAATA
TATTTTATATAATACGTGTTAGCCGATTCGGTTCTCGTCGACACATACTCTCATTGCATCGGCGAACGTT
GGAGGATTCGGATTCCGTTTCGATTCTGGTCATTACGCAATGGCCGCTGAGGAGACGGCGCTAACCGGCG
TCGGCGAGCTACTATTCTCGCTGCTAATTGGATACCTAGGTGCCCTGGAATTCGCCTTTGTGGCGCGCCA
TCTATACCACTGGACTTTCCAACATCCCGGCGATTCTTCTGTAGATGCAGCAGCTGACGCAGAAGAATTG
CCTCGCATGCGAGAGCGATTGGATAAGCAGATCAAGGAGGCGGCGGAAAGGGAAGCCCTGGCAGGAACCA
ATGTCATGCAGGATGGTGTCCTATATGTCAATGGAATACGAGTAGATCCTGCTGGAGATAAAAGACAAGC
TTTGGCAGCCGAACTGGCACGTCAACAGCAAATTGAAGCCGATACGAGGCGTCAGCTGGCCGAAGCCGAG
GCCAAGCTGGTAGAAGAAAGACTACGAGTCCAGAGGGAGAAAGAGGAATCGGAGGAGCAGCAGAGAAAGC
TCGTAGAGGCGGAAAGACAGCGCGAAAGAGAGCAGGCGGAGAAAGAACTGCAGGAACAAAGAGAAGCCGA
ACGCAGACAGCTAGAAGCAGAAGAAAATCAGCGTAAACAGCGCGAAAACGAGGAGAAAGAACGCCTAGAA
AACGAAAGAAGGCTGATAGATGCCGAACGGGAAAGGGAGGAGAATGAGCGAAGACTGCAAGAAGCTGAAG
AGCAGCGCGAGCGAGAAGAAAGCGAAAGAAGAATCGTCGTGGCAGAGCGTCAGAGAGAGCAGGCCGAAGC
CGAAAAGGAGCGAGCCGAACAACAAAGGATCCTGGCTGAAGCCGAGGCAGCGCAAGCCGAGCGTCGTCTC
TTTGATGCCGAGATTCAGCGAGAGCGCGATCAAGCTGACGAAGAAGGGCAAGCACTGAGGGATGCGGAGA
TCGTAGAGCGTCTTCTGGCAGCAGAAAGAGAGCTCAGTCCAAGCGCTACAGAGAGCGAACTGGAGGAGGA
TGCTGCCATCGCAGAGCAGTCGCGTCGCCTGATCTCCAGCAGAACTGATCTGGAGCAAAAACAGCGAATG
ATTGAGGAAAACGCCAGACGCTTTCTTGAGGCAGAAGAGGAGATGGTAATGTTACAACAGCGCCAACTTC
AAGCCTCACACAGCAAAGAGGAGGCAGACGAGTATGCTGGCATGGAACCCGTGGTGGAGGAGCTGTGTGT

GAAGAAAGTGGCTCCACCCAGGTTTCAGGAGCCTCCAGAAGAACCCATGGTGCATAAAGTGAAAACTCAA
TTTGGTCAAGGAAGTGGTCCTGAAGACGCCTACTCAGCAAAGTCAAAAACACTCACCTTCCCTGGCGTAT
CGGATGAAGGATCCTCTCTTGAACCCAAAACTCCGTTCTCCATCCAGCAGTCGTCCTTCAGCACGCAGCC
CTCTGATGAGGGCAACAGAATCGAAAGTGAACGCCCAGAACCTGAAGGAGAAGATCAGATACAGGAAGTC
CAGTACACCGAGGACTACCTTCGTTCTCTAGATGGCATTAAGAATCGTCCTTTGATGCGAGAGGATGGTT
CAGGGAGAAGAAGGGCTTTTAAGAAGCGACGATCCAGTGGAAGCTCGAACTCCTCGCGGGAATCGCGAGC
CTCCCGCGACGAAGAACTAAAGATGTTCACCTCGCTAGAGGAGGAGGAGTTGCGCCACGGCGACCGAGAG
GACTATAACCCCATCAAGTATACGAGCGAACCTACCCTGAGGGTTAAATCCCACCGACGTCACAAAAGGA
GTCCAGCAAAGGATGTTAGACCAACGGCAGAGTCGACCACATCCTTAGAGATGCTCGGTGAGGAGAGCAC
AAATCCCTGGGGTGAGGTTGTGCCGGAGCATTATAAGGATACCGAGTTTTGGAAGCGGGAAAAAGCGCTC
TCCATAGACGAGGAGGAAATCGAACTAGAAAGACCATCCAGGGGAGAGGATGTGGAGGAAGAAGCTACAA
ATGAGCCAAAATCGTCCTCCTTTGAAGAGGCCACTGAGGCACAAAACGAACAGGCAGTGGCTGCCCTAAA
ACAACAACTTTCCAAGGAGCAGTTGGTGAGTAAATGAATCTACAAGCCGAGGATCTATATCAATGATTTA
TTATGTGTGTGTTAGGACAAAGAAGCGGAGAAGGACAACCCGCAAGCAGTGGATACCAGCGACAGCAACA
AATCCAATGTAAGTGCGTAGCACAGACACCTGGCATCATTTCTCACCCAATAATTTCGACCACAACCTAA
TCCACCAATCGCAGACACCACTGCCGCATAACTAACTGTCCTAACTGCTTTACCTCTTTGCTTTTCATAT
TGCTACTGCTCCCACATTCCGCTTGCTTTTCCGCTTCACCTTTCCCCCAAAAAGCTGCAAACCTCTATCG
CAGCAGAGGAGCAATCAAGGGGTGGTTCACCTGGCTTGCGACGCAGTCCACGTATGGATGAAATGGAGCA
TTATCCGGAGCGCTGGTCCGCCAGCCAGGAGCAGCAGTCAGTGCAACAGCAGCCACCGCGAACAGCACCA
GCCATCAATGTCCAGCAGCCGGATACGGCTCCTTGGCGGGATCAGTACGGATTGCTAATGGAAGGACTTA
GCGATTTCTACGACTTTACCGCATCCGTAAACCCATCGAGATCCCGTTCGGCATCGCGAAATCCCTCACC
AGCTCCCAAGAATGTGGCAAATTTGATGGATGGGGACACGGAGCGGTCGCTGGAGGTTTACGACGACACT
CAGGAAGGTGTAATGGCAGGCGAACTAAACTGCGAGTCTGTACTTCAGCTTCTGGAGTCGAACTTGAATG
AAACCGAATCTACAAATGACCAACTTCAAGTGCAAACTGTAGCCGATGATGCCAGGGATGGTAAATCGAT
CTTACCCATGGTTCACGAACCACTTGAAAGCTCTGCACTTGGTGCTGTTTTGGAAGAAAGGGGACGGGAG
CCTTCTGTCGGAAGGATTCAATCCAGATCCCGCTCCCGCTCCCGTTCACCACTTCCCACCTCAGAGAACC
TAGAAAAAAGCATTCATAAGCGCAGGGAGCGCTTGATCAAACAGAACGATGAGCTCGCCGAACGTCTATC
CCACTCAAGTCCAGAAGATTCTATGGAAATCGAAACTAGTGACAATGGAAGATTAAGTCCAACACTAGAG
GTCACCCTGCCACAGCCTGTAATCGAGATTAATGAAATGGCTGACGAGCCCATGGAGGATGCGGAAACTA
CACCTGAAGAAATGCGACTTTTTGTTGAGACCCTGAGAGCTGAGAGGAATGCCCGCTCAAGATCCCGATC
TCGTTCCCGTTCTCCTCTGCCCACTGCGGGTAATATAGAAAAGAGCTTGGAAAGTCGTAGGCTGAAACGC
ATTCAACACAATGATGAAATCTTTGAAAAACTACACGATCAAATGTCTTCGCCTCAGATCATAATAGAGT
CCGCTTCACCGCCATCTTTACCGCTTCCTCTACCCACAATATCCATTGAAATTGCCGAACCACCGGAGGA
AAATGCTAGCCACACTGAAGCAGAACCAGAGGATACACCGGAGAGCATGGCTAGACTCTTCGAGCAACTT
CGTCTGAATCGAGTGCGATCCCATTCTCGATCCCGGTCGCGTTCGCCACTTCCGACACCCGCCAATTTGG
AGCAGAGTGTGCAGAAGCGCCGGGAGAGGCTGATTGCTCAGAATGATCAATTCTTCGAGGTACTCCAGGA
AAGAGCAAGAACTCCGGAGCCAGAAACTCGTTTGACCGTTGAGTACGTGTATGAATATGTTCAAGAGAAA
GATGAACAGGTTGGTGAAGGTCGCGACAAGAGATCCTTGTCCCCATCTCGATCCCGGTCACAGTCACAAG
AAAAAGCGCTAGATGATTTTCACTTGATGCTTAACCTGGAGGGTAGCGAGTTGCGCACACTACGCAAAAA
TAGTGAGGAGTTGGAAAGTGTCCTGGCCGTGGGTGCAAAGCAACGAGAGGTCGACATGGATGCCCTGGAG
AAGCTACACAATGCCAATGAGGAAATAGAGCTTCGCGTGCTCAGTCCCACTAATTCGGAATTAGAGAGAG
TGGTTGAGATGACACGTGAGAATCCGGACATGGACCGCACGGTTTCTGAGGTGGCACGGGATCTACAGGA
CGAGCTCATGGCCAGTGGATTTAAACGTTCCACCTATGTTGGGGAGTCCTTAGACCTAGGCAGCGATCAA
TTTAAGGAGCTACGACGAGAGGCAGCTCAATTTCAGAGATCCCGAAGCCCATCTCCAGGATCGAGCATGG

ATTACGCTAGAGAGCAGGCTGCAGCTCAAAGGGAGCTGCAAACAGCCATTCTAGACTCTCTGACCGAAAG
TCGCCTAGGAGCCAAGCCCAAGACCTACTCCGAGGAGAGAACTGGCGAATTCGAGGATATATCTGCAGCC
CAACTGGACGATTTGCGTCATCGTACGGCCGAGTTCCAGCGATCACGTAGCCAGAGTCGGTCGCCATTGA
GGGAAGTGGAGCTAACCCAGTTGAGGGACATCGAAGCGGAGTCGGCTAAGAGGGAGCTGCAGGATCAGTT
ACTAGATAGGTTAGCCGCCTCGAGCATTAATTTCGAGAACTTCTCGCGGCACTTGAACGCAGACTTCTTG
GACAGCGAGCGTCGGGCCTCGGACTCGGCTTTAATGGGTGACATGGAACCGGATGAATACCATCACTTCC
GTCGGTATTCACTTGCCCAGGAACAGCCCGTCGAGGAGTATCCCAGTGATGATTACGTTTATATCTCACA
GATCGAAGTGCGAACTCTAACTGGAACAAGGACTTGGTTAAAAGAGGATTTTTACACCCATGAACCAAAC
GATTTTTCTGACTCAAAAAGTCCGGTTAGGGATGAAGACTCCTGGGCGAGAGCTGTTTTGGCTGCAGAGG
CAGAAGCTGCCGAATTCGATTCCACCAATGCCATCTATAGCTATGATATTGTGGGAGACCACGAGAATTT
GGATCAGGAGAGGAGCAGCTCGAATGACAGCGAAGACACACGACAGACGGTGGTGGAAATTGATGACGAA
GATGAGTTTGAGTTTGAGTTGGACGAGGGAATCTCGGATAACAACGAACGCACCCAACCCACCGATGAAT
CCCACCATGACACTTCCGAATGTGAAGAGGCAGAAAGGAATGCAGATCGCTATGAAAATCGGGGAGCTCA
GGATTGGGAAGAAGTAGATGATGTGGAGGATTTCCTCGACTACGGTTTCGACGATGGTGCTGTGGGCGGA
GCAACACCGTACTCCGATCCCGACTCCTTTATCGAACAGATCTACAATGAGGCCATAAAGAACAGAAAGC
AGTCTGGAATTCTAAGGGAGTACGAGACCATTGTTCAGGAGCAATTGCCTTCGGATCTCAGTGAATCTGA
GAAGGAAAAGTCCGGTTCTGATAGTGAGAAATATGCCTTGTGGGCCAAGACCAGGGAACTGGAAAGATCA
CATTCCGGTACCCGACACTCAGATGTTGATATTGCGTTGGGGTTGATACCAGAAAGTGATCATCGCGATT
CTGAGATGCGTTACCTAGGTGATGCGCATGTGGTGGCACGACAATCCACAAGGTTACGTACCCGATATGG
CTTTAATCCTAAACTTGAAGTTGGCGACCTAGAAGATGACATCGAGGTGGACCTTAATTACAAGCCCAAG
AGGGAGTACAACTGGCGCAAGAACTTCCGACTGGACGACAATGAAGAAGGTGAAAGAAGTGTCCAAGTTA
AGACAGAAGAGGATGAAGAACAGGAAGCCAGGGATCAATTTGAAGAGGAATTCGGGTGGCAAGATCGAGA
GCAAGAACTAGAAGAAGAACTTTATCAGGATCATGAGCAGCCACTGTATGCTAATGAAGAAAACGGCGAA
ATCGAAAACGTAGAGCTGTTGGAAAATCGCAGGATCAGCCTAGGCGGAGAAAGTATCACCCTCTTTAGCC
GTAGTCCTGTTCGAGAAATTTTACCAGATGTCCCTGAAGAACTTCCGCAGGAGGAAGAAGCACTCGAGGA
ACAGCCAGAAGCAGTGGCTCCATTGGAAGATTTGCCAGCCGAAAAGCCCAAGAAGAAGAAGAGCAAGAAG
AAGCTGCGTAAAGGATCTAAAACCGAAGAGGAACTTCGAGATGATAATGATTCGGACACCCAAATGGGAT
CCTCTCGGCGCAGTGATGACCAGGAGCCAACCGATCCGCCAAAGAGAAAAACACGCTCAAGGCGAAGCTC
ACGAAGTAGCTTGACCAATGAAGAGCCCAATGGAGGATCGGGACCCTTTTCACCTAGGAGCAGTCTTGCC
TTTGTGGGAGAATCCTTGGAGGGCATAGCTGAATTTGAACCACAGGAAAGAGAGGAAGAGCAGTCCACCA
AGAAAAGGACTAAAAAACGCAAGAAGACCTCAACAAAGGAATGCAAGGAGGAAGCCAAAGAAGAGACCCA
GACACAGGCCATTGACGATTCTCAATTGGATCGGGCACTAGCTGCTGCTCTAGCGGAGATTGCCCCCGTT
TCGGTGTCCACAAATTTAACCCCAGAATCCACGCACCAGAACCTACTTTCCACCATTAGCACCGGTCAGA
GTGTCTGCATAACTCCAGCGTCATCCATCGAAGAGATTGGTGGATCACCATCCTCGACTTCAACTACGGT
AGCTGCAGTGGTACCAGCGCGATCCGTTGTGGACACCTTCGATATACTCAAAGATGCCAATTTCTATCCG
CTCTTCTAGATCGCTTGGTTTGCAAACTAAAACTTCAACCTTCACACTCTATCACCCATTTGATTTACTC
TCTATCTGTTTTCTTATAGACATGATCATAAGCCAAGTCAAGCCAAGCACAGCCCAGCACAATCCAGTCC
AAGCCACATCCAAAACCGAGCACGCTGTACACGAATTTAGTCCCAGTTAAAGATCCAGTCTCCAGAATCC
AGCATCCGCTCCTCTGACTGAGGGTAAACACCCGTCCACGGGATCCCAGAGATTCCGATCTGTATAAACT
ATGTAATTATACTAACGACCCAGAAAAAAGCCTTGAACCCAAGAAAAAACGAACGATTGAAGGATTGTTT
ACCATAGCTGTTAGTTTACTTAACACTTTTGTCTTTATAAAAATGACGAGAATAAAAGCCAACTGAATAA
ATGAAGAAAACATTTGGTTGTACTCTGGTTGCTATTAATAGTCATACACCCCACCGAAAATCGCATCTGC
GGTTTGTGCCAATTGTGCACCGGTCCTCGAAATACTCGGGCATTGAGATCATTCATGCCGCGGCAGCGGT
GTCACCCATCAATGGGCCACCAAGTGCAACCGCAGATGAAGAGGCTTCATAAATCTTCCCCCACAATTGC

CCATATTATTTTGTACAAATTATATATCCCAATTTGCATTTTCGCATGATGTCATCGCTTGACACACGGC
TAATACCAATGAGCCAACACTTAAGTCCCAAAGGAAAATATCCGCCATATAGATTTACTCGATTTTGAAT
GCTGTTGTCATTACGACTATAAACGAAACaaaaaaaaaaCAATCTGTCATGAACATGAGTTATATGTGTG
TATAGTATTTGGGCTCACACACTTGGTTTTCTTGGGTCTTATGTCTGAATGATGGCACTAAGGTCAGGGC
TAAGTGGCATTATGTTGGTCTAGAATTTGTCAATGAAAATATGGGAACATCTGTTGTTCTTGATAGAAAG
CTAAATTGTGTCACCATTCTAATTCTAAAGTTTGATCAAAATAAACTTTGAACATAAATTTAAACCATTC
TAACTTTTTGTTTTGTAAGATTGTATTTTGTTTTATCCCAAAAGTATGCTAATCTATGTGGCCAACTTTG
CATTTCATTCATGAAGAGATCGTTTAGTTTATGTCAGTTTAGGGAAAAACATTATGTTTGCACTTTTCTC
ACTCGTGtttttttttttttttttttGGCGAGATGCCGCTGTCAAGTTTATATGTCATATCCTTAGTCGA
ATTAAGCAAAAGTCAGCCGCAGAATGGCGACAAAGGTTCGCGGAGATAACAAATTATCTCAGCGACTGTC
AGGCCGCAAAAGGGGGAAATTGAAAATGAAAAATTCCCAAAATATTTATGACAGGCCAACAGGAAAGTGT
GTCACTCGAGTGATTTGTTTACGCAGCTCACTTGGCTTTGTTCTCCATTTTTGCACTGGGAACACGGGCC
ATTTGGAATTTATTTCTGTAAAACCCATTGCGTAATGCATTTTGTACGGCAAATAAATGTATCTCTCCGA
ACTTGCGTTAGAGCCACATCCAAGCAAACACGGCCAAAACGTTGCCAAAAAGTCTTATTCAATAGTTTTG
TCTTTGACATGAATTCATTTTTCTTGTGGGGCGCACTTAAAAGCCTGCGGTTATATTTTCTATCATATCT
TCCCCTCAAAGTACAAGTGTGTATATGTATATAGTATTTGCACTGCGCGCGGAAAATTGCCTTAAATAAT
TTTCTAAAAAATTCACTTGAATTTCGTGCGTTCAGCTGGTAAATGAATTTTTCATATTCAGTGTCAGTTA
AATGAAACACCttttttttGCACACCGCCATACATTTGTTTTTGCGTTTCGAAGATACATACATATTAAA
GGCGTCATCTTAGGTGCATCAATAATTTTCCAAATAACTTGATTTCATTTGATTTGCATCGACCAAATGA
AAGAAATATTCAAAATTTATCAAATTAAAATTATATCTTTTCGGGGCGCAGCTGTTCTTAAACTTGAACT
TATAAAATTTACACAGAATTGGCTTCTTGTCTCGGGAAAGTTTCCCCGTTTGCTTTTGATTTTCGCTCAA
TACTCCTGAAATCAGAAATATAAAAATACGCTGGAATATtctatactgtctatactgtctataTGTGCTT
TTGCCTGAATTTATGACCCAATTAACGCTTTAACGCCTGCGTGAAAAATGCCAAATAATCGTAATGAGGA
TCCAAGGGAGGGGGGACAGCGGTCACATCACGCAAAAACTTTTTATCAAAGATTCGCGAAATGCGTCTTA
TTTTTAGACCAACTTGCAGATTGCATTTCACTCGGCTCCATTGCAgttgttgttgttgttgttgttgttT
CTCGTGTCTGCGGCGAAAATAGCAACAACTAATATACACATGCGATGCATTATTTTATAGGTATGTGCAT
ATAGCCAAGCACGAACATATATCATACTGTATATGATAGATAGAAGGAGCATGGCCAGCGAAACTTAATT
CGCACGAAGAAGTTGAACCGGAAGCGTACTTATGTATTTTCATTTGAAATCAAGTTAACTCAAGCCAAAT
GATAGTAAAGTGTGAAATTATTCTGCCAATGACCTATAgattattattatcttattattattattggatt
atacaatggattattattaCTAGATATGTTTATGCTAAGCATTATTGAATTTTTATGATTTTTGCTTAGA
TATCTTTCTTTTTTATTCATTTGAAGAAATTAATCCACAAAAACTGATTAATATTCAGAGATTTTATAAG
AATTTTTCCCTGTGGTTTAAAATCATCCCACGTCACTGGCCACATCATCATTAGAACCATAAATTTCCCA
TCTTAGCAGTGAATCAGTTTACATCAACATTTGGATTCTTAAACAATGTGCATGTATTTATTGTGTTGGC
TGCCCTCTAGCCAGAACATTTCACGAGCTGTTGATTTTCTATATTAATCATTTTAATCGGACTGGCTTTT
CGTATTCCCCGCTTGGTTTAGACGGCCTTGTTAATAGGTTTCTACCACTGAACTGTCACTAGAACATTCT
TTCGGCCCATTTGGCCATTTGTACTTGTTAGTTTTCGCCAACTTACCTTGGATGGCTGCCCTTTGGTGTC
GTTTCAATTTGTCTAATGGAAATTTGTTCTGCTGTTTAATAAACATCGGTTATTAATTGGGGGGAAATAT
ATTAATTTGCCAGAAAATTTAAGAACTGAATTAGGACCTGATGAGGTCACGTTGTATAAGTGTTCTATTA
AAAGATACTGAATATTGATGGATTTTTTTCTTTGAATGCATAAGATCATTGAATTTTTGCCATAATTATT
AACTTGCCCTTGATGAACACTACTTTCAAATCATAAAACAAACAAATTTTTGAAATGTACAATGTACAAA
CATAAATTCACAAAATTAAAATGAACTGATATAAACGTACCTATTTATGGTATTTCACAATTTCGGAATT
CTTTCTTACATTAAATTGAGATTATTTTTCCAATTTGCCGGCACACCTGTTGGTTCATTTCAATTCAATA
AACATTTTCGCCCATTGGCAACTCAATGTCTGTCTGTATGCCAAATGCTGTAATAATGCAAGAAAATCAA
GAATGCGAAAATAAATTCCATATATTAATACAATTTCGATAATGTATGGCCTGGAGATCAATTAGGGAAT

CTATTGGAAAATTCAAAAATACCGCAGGGTGCGATAAGCCAAAATGCCAGACGCACGCAGATCATCGTTT
TAGAATCGTTTTAGAACCGATCCAAGGGAAAAGCGATCTATGCTCAACTAGATTGCCGATCGTGTCGTTT
GTAGTGCAGTGTGGGAAGCGAGAGAATTTTCCTCGATTTTCCATTTGCGCTAGTGTTTGTTTGGGTGTGC
TGCTTGTGTGTCGTGTGCACAAGCGCCACAGCAACAACAAGAAAAACACACTACATTTCATTTGGTGAAA
AATTAAACAAATTTTCAAATCTCTGACATGCGATTTAAggggggggggggggTCGATTTTTGAATGTGGA
GGGGACACTAACGATTTTCGAGGGTGGCCAAGCATGCCACAACCCTTTGATCGCCGGCAGGCAGGTGGGG
CAAACGCCCACAACTTTTGGCACCTGAAATGCGTTCGATTTTTCATTTAGTATTTTTAGCGGTAAGCGCG
AACGATATATTGCATGCAAATTGGCCGAAATCGCTGAGAAAATTACCCCAATTGCATTGCAAATTCAATA
ATGCCATTAATTATTGGCGATATTTACATTGTCTACCGCAATAATTCAGAACGCCCGTCTCTGGTGCTCC
GTAAAATAAATCTCTATTGAGATCCAAAGATCTCGACAGATCGTACATGGTTCCCTCTCTCCATTCTGCG
CCCATTTGGCTCTGGCTCTCCGAGATTTTATTTTTACACTTGGCGGTGGCGAAACTAGATACAGAGCGGA
CTGAAAGCGGTGCAGCAGGCGTTTTGAAAAACATTTTCGGAAACCGCTGCGCCAGTTGAGATCCCGACTC
GGCAAAGTTCATACCGTTCTGGACCTCCGATCGAAATCGAAAATCGAAAATACACAAAAACTAACACACA
CATCGATATACAGAAGACAGACGGCCAGACAAATCCACAAGATACAATAATTATAGAAAGTTCAGTCGGT
CATTAGAGCGAGCGAGATAGCGGGCGCGATGGCGGACGTGGAAATTATGCAAACGGTGCAAACGGAAGTG
AAGAAGACCACGAAAAAGGTTAAGAAGTCGTCGAAGCGACGCGAATCCGAGGTCCAGATCAGCGAGGTCG
AGGTGGATGCCACCATCGAGGAAAGAGGGTAGGTGACCTGTGTCATGTGTGTCCCAGCACAGTTTTGTGG
CAGTTCGTAACGAAAGTCTTCTTCTGTAATCATTTATCAATTCTTTTGTGCTCTTTGTCCGATCATCCCA
AATACTTTGTTGTACCAAACAAAATAATaaaaaaaaaaCATTTGCACATTCCAATCGAAAAGATTCCAAA
ATCAATATATATGTATACAAACCAATGAAAACTCTTGCATATCGAACTTTTACAAAACGACACACGTTAC
TACTCTACTTTCCTCGGCCAAAATGTTTACTAAAAGGTATCCAACAAGTCTATCTAAGCACACCCACCAC
CAAAACCAGTGCCACAAAGTAAAACAAACCAAGTAACACCCCAAAACTACACTGCACAAAACGAAAACGA
AAACTATACAGAAAACCCATGATGAAACAGATCCCCGAAACACACAATCCATGTGATATATGTATACCCC
CTTATATTTTGTTTATGCCCATGTGCGATGTGAATCGATATCTATATCCTCCCCGATCTCTGTGTGTGCT
TGCAGGGTAGGTGACCTGTGTCATGTGTGTCCCAGCACAGTTTTGTGGCAGTTCGTAACGAAAGTCTTCT
TCTGTAATCATTTATCAATTCTTTTGTGCTCTTTGTCCGATCATCCCAAATACTTTGTTGTACCAAACAA
AATAATaaaaaaaaaaaCATTTGCACATTCCAATCGAAAAGATTCCAAAATCAATATATATGTATACAAAC
CAATGAAAACTCTTGCATATCGAACTTTTACAAAACGACACACGTTACTACTCTACTTTCCTCGGCCAAA
ATGTTTACTAAAAGGTATCCAACAAGTCTATCTAAGCACACCCACCACCAAAACCAGTGCCACAAAGTAA
AACAAACCAAGTAACACCCCAAAACTACACTGCACAAAACGAAAACGAAAACTATACAGAAAACCCATGA
TGAAACAGATCCCCGAAACACACAATCCATGTGATATATGTATACCCCCTTATATTTTGTTTATGCCCAT
GTGCGATGTGAATCGATATCTATATCCTCCCCGATCTCTGTGTGTGCTTGCAGTTAATTTTCAGGTTAAT
TTTCAGTGGCGAGTACACAAAAGCCATGGACAAGGACTGGCACTCCGGACACTTCTGCTGCTGGCAATGC
GACGAGAGCCTCACCGGACAGCGTTACGTCATCCGGGACGATCATCCGTACTGCATCAAGTGCTACGAGA
ATGTGTTCGCCAATACGTGCGAGGAGTGCAACAAGATCATTGGCATCGACTCCAAGGGTACGTGAGTGTA
TTTGGGACAATTTCGACTGGGGATCGTCGATTGCGGACAATAAACTGTTCGTAAACCGAATTTATGGCCA
TATGTGGGTAGTCAACAAATTTAATAACCATCAAGTAGGACAGAAATGCACGCAGTGGGCAAATATGGAG
CACACTTCCCTTGAGAAGTTTCGAAAACCAGTTCGAGAGTGCAGCTCGTAAAACCGAAAAGAAATGGAAT
CGATAAATATTTTTGAAATCGCTCCAAAGACTTCAGATTACAACAGTTTAACCGaaaaaaaaaaTTGAATT
TTAATGCGATTTTTATGTTTGCGCTGTGAACTTTATGTCCATTTTATTATCATCGCGCTTTTATTGAGTT
GGTTTTATGCGTCTAGCCATAATTTGTTTTAATTATAGCGTTAAAATTAGAATGACAACTGCAACACGAG
AAGAAATGCAATAGCTTATGTTTAATTTGTTTTCGGAATGTGTGTAGAATATGCGAGCAAGGTTTTTTTC
AAGTTCTGAATTTATTTAAGGGTTTCATTGCGTAAATAATTTCTCACGGTCTATGCAGTCTCTTTGTGTC
AAGTTTGAATAATGTATTTTATATAAAGGCAATTAATATGTCTGTCGACCAAAAATTTAATTCTAGGTAA

TTTATTTTGCTTGGCTAAAAGCCCAGCACTCAAAAGCCCAACGGAAGTCCACATTCTATTTCAAATGATT
ATAGTTAGTGAGAAAAGCAAAATTTGAAGAAGAATATTATGGCCTAAAGTTATCTAGTTATAAAAGTAAT
TTAAACAAGTTTTCAAAATAATTCCTATTATAAATTCACTATTTAAGGAAACACCTGAGTGTAGGAATCT
AGACTCCGTTCTGATCCCATTTGTTATCCCTCCCTTCACAGGGATCTGTCCTACAAAGACAAGCACTGGC
ACGAGGCCTGTTTCCTGTGCTTCAAGTGCCATCTGTCGCTGGTGGACAAGCAGTTTGGAGCCAAGGCGGA
TAAGATCTACTGTGGAAACTGCTACGATGCCCAGTTTGCGTCCCGCTGCGATGGCTGCGGCGAAGTTTTC
CGCGCAG
>dme_circ_0004981_junction_seq
GATGGCTGCGGCGAAGTTTTCCGCGCAGGGAAAACCTGCCAGTCCTGCAAATGTCC
>dme_circ_0004982
GTTAATTTTCAGTGGCGAGTACACAAAAGCCATGGACAAGGACTGGCACTCCGGACACTTCTGCTGCTGG
CAATGCGACGAGAGCCTCACCGGACAGCGTTACGTCATCCGGGACGATCATCCGTACTGCATCAAGTGCT
ACGAGAATGTGTTCGCCAATACGTGCGAGGAGTGCAACAAGATCATTGGCATCGACTCCAAGGGTACGTG
AGTGTATTTGGGACAATTTCGACTGGGGATCGTCGATTGCGGACAATAAACTGTTCGTAAACCGAATTTA
TGGCCATATGTGGGTAGTCAACAAATTTAATAACCATCAAGTAGGACAGAAATGCACGCAGTGGGCAAAT
ATGGAGCACACTTCCCTTGAGAAGTTTCGAAAACCAGTTCGAGAGTGCAGCTCGTAAAACCGAAAAGAAA
TGGAATCGATAAATATTTTTGAAATCGCTCCAAAGACTTCAGATTACAACAGTTTAACCGaaaaaaaaaT
TGAATTTTAATGCGATTTTTATGTTTGCGCTGTGAACTTTATGTCCATTTTATTATCATCGCGCTTTTAT
TGAGTTGGTTTTATGCGTCTAGCCATAATTTGTTTTAATTATAGCGTTAAAATTAGAATGACAACTGCAA
CACGAGAAGAAATGCAATAGCTTATGTTTAATTTGTTTTCGGAATGTGTGTAGAATATGCGAGCAAGGTT
TTTTTCAAGTTCTGAATTTATTTAAGGGTTTCATTGCGTAAATAATTTCTCACGGTCTATGCAGTCTCTT
TGTGTCAAGTTTGAATAATGTATTTTATATAAAGGCAATTAATATGTCTGTCGACCAAAAATTTAATTCT
AGGTAATTTATTTTGCTTGGCTAAAAGCCCAGCACTCAAAAGCCCAACGGAAGTCCACATTCTATTTCAA
ATGATTATAGTTAGTGAGAAAAGCAAAATTTGAAGAAGAATATTATGGCCTAAAGTTATCTAGTTATAAA
AGTAATTTAAACAAGTTTTCAAAATAATTCCTATTATAAATTCACTATTTAAGGAAACACCTGAGTGTAG
GAATCTAGACTCCGTTCTGATCCCATTTGTTATCCCTCCCTTCACAGGGATCTGTCCTACAAAGACAAGC
ACTGGCACGAGGCCTGTTTCCTGTGCTTCAAGTGCCATCTGTCGCTGGTGGACAAGCAGTTTGGAGCCAA
GGCGGATAAGATCTACTGTGGAAACTGCTACGATGCCCAGTTTGCGTCCCGCTGCGATGGCTGCGGCGAA
GTTTTCCGCGCAG
>dme_circ_0004982_junction_seq
GATGGCTGCGGCGAAGTTTTCCGCGCAGGTTAATTTTCAGTGGCGAGTACACAAAA
>dme_circ_0004983
GTTTCCAAGGGTCGTGGAGCTCTTTGGATGCTATTGGTCACATCGGCCAATTAGTTTATTATGCCCATTT
AACAGATAATCACTTCCTGTACTGAGATTTTAATGGACAACACCGAACACCTCAAGTCTGTAGTCGTGCT
TCTTCTATGCTAAATTGTTTG
>dme_circ_0004983_junction_seq
TCGTGCTTCTTCTATGCTAAATTGTTTGGTTTCCAAGGGTCGTGGAGCTCTTTGGA
>dme_circ_0004984
GTTCTATTTTGTAAAGCTACAAAATGCATAGAGCCCACACAGCCTTCAGCCTGGCCTTGTCGCCGATGGC
GCACAAGAACTTCGCCACATGGCTGCTCAAGAGCAGCAGCAGTCAGCAGGTGAGTTTGGTCGATATAACC
ATAACTCCAAAGAAAACCCCAACTAATGAGCCCCCAATCTTTGGCAGATGGCCAAGGTAACTGCAGCGGC
CGCTGTGCGCACCTACAACTCCGCAGCTGCCGAACCTTTTGCCAACGGCAGCACCGCCTCCTACGTGGAG
GAGATGTACAACGCCTGGCTTCGGGATCCCACTTCCGTGCACACCCGTAAGTATCCCTATTAAACAGGGT
TTTATTTCTGCGCCTTTTTAAATTCAACTCTTGATTTTGAGTCATCGATAAACCAGTTTCTAGTGTTTAT

TTCTGAAGCGGAAGTGCTTTATTACCTTTTAAAAACCACTTAAAAATGTTAAGAGCTTATACTAAAGGGA
ACCAAACTTCTCTGCAATAAAAAATATAATAAAAGCCTAATAAAACGGTGATCCTGGAATAATGCATTCA
CAATAAATGACATGACAATAAATCCTTATTGATTCATTCTTCTTTTAGGTCCTGGGACGCGTACTTCCGC
AGCAACAGCTACGTTAGCCCGCCCAACTTGGCGCCCGTGCAGGCCAACACTCTGCCGCTGACCGCCTTCA
ACTTTGGAGGAGCCGTCGCCGGCGCTGCGCCCGACTCCAAGACCATTGACGACCATCTGGCCGTCCAGGC
CATCATCAGGAGCTACCAG
>dme_circ_0004984_junction_seq
CGTCCAGGCCATCATCAGGAGCTACCAGGTTCTATTTGTAAAGCTACAAAATGCA
>dme_circ_0004985
GCCTGGCCTTGTCGCCGATGGCGCACAAGAACTTCGCCACATGGCTGCTCAAGAGCAGCAGCAGTCAGCA
GGTGAGTTTGGTCGATATAACCATAACTCCAAAGAAAACCCCAACTAATGAGCCCCCAATCTTTGGCAGA
TGGCCAAGGTAACTGCAGCGGCCGCTGTGCGCACCTACAACTCCGCAGCTGCCGAACCTTTTGCCAACGG
CAGCACCGCCTCCTACGTGGAGGAGATGTACAACGCCTGGCTTCGGGATCCCACTTCCGTGCACACC
>dme_circ_0004985_junction_seq
GCTTCGGGATCCCACTTCCGTGCACACCGCCTGGCCTTGTCGCCGATGGCGCACAA
>dme_circ_0004986
GATTCGAGGACATAATATTGCTCACCTCGATCCACTTGAAATTAATACACCAGAATTGCCTGGCAACTCC
TCGACGAAATCCATTTACGCTAATTTCAGTTTTGGTAAGTAAATCCGGCGTTGGTCGTAACACCTGGCTA
TTAGCCCAAAGCATAACCTGTTTTAGTTCTTAACCCTTGATTTGTAAATACTTTTAAAACCAAATATTGT
GTTAGTGACGTTCTCTGTCTTTCACCCGCACACACGATCCCCGGCATCAAAAATTCGATTATCGAACCCA
ACCACACAAATGTATCCAATGATATATAGAACCAAGTAAATATGCGCTATATATTGCCAGTAAATTTGTG
TTACATTGTTTGCCCATTTCTGCCTTTAATTGTTATAATTTGGATGTCGTGCCAAAATATATAAATATAT
TTAAGTATACCTCACCGATTGATTATGTCATAAATAGATTGCATGATTGTTTTGCATTTCTCAAGCGAAA
TAAAGGGAAATCGATTGATAAGCCCCCTCAGTCTGATTGCAAGTGAAAGATTATGAGCCCATTAAAAACA
AATTTAAGTTATCACCGATAGTGTCTTATGCATGTCGAACAAATGTCCGAACGAGGAACCCGTGAACAAA
CCAGCTGAGCATGAGCATCCGATCCCAATTTAAACTCTGTACTGCGTACAAACACCTCCGAATAGAAGCA
ACAAAAATCATTACACTCCGAATTTCAAACCAACATCTGCCGCCTACAATTTACTAATCCCACACCATGA
CACAATCACTCAAAACCCCTTACTAAAGTCTTAACCCATTGTTCCAACCCACTGCGCAAAAAACAGGGGA
AATCCTGAACCGCCTGGAGAATGTCTACTGTAATAAGATTGGCGTGGAGTTCATGTTCATCAACTCTCTG
GAGCAATGCAACTGGATCCGCAAGCGTTTTGAGACCCCCGGCGTCCTTAACTTCTCGCCCGAGGAGAAGC
GTCTGATCCTGGCCCGTTTGACCCGTGCCACCGGCTTTGAGGCTTTCCTGGCCAAGAAGTACTCTTCTGA
GAAACGTTTCGGTCTGGAGGGTTGCGAGATCATGATCCCAGCCCTGAAGGAGATCATCGACGTATCAACG
GAGTTGGGAGTGGAGTCGGTCATCATGGGCATGCCCCATCGTGGACGTCTTAACACCTTGGCCAATGTAT
GCCGCAAGCCCTTGAACCAGATCTTCACCCAGTTCGCTGGACTGGAGGCTGCTGATGATGGGCTCTGGTG
ATGTCAAGTACCATCTCGGTACATACATCGAGCGATTGAACCGCGTTACAAACAAGAACATCCGCCTGGC
TGTCGTGGCCAACCCGTCCCATCTGGAGGCTGTGGATCCCGTGGTGCAGGGCAAGACCCGTGCCGAGCAG
TTCTACCGTGGCGACCAGGAGGGCAAGAAGGTTATGTCCATTTTGATCCACGGTGATGCCGCCTTCTGCG
GACAGGGCGTCGTCTATGAAACTATGCATCTTTCGGACCTGCCCGACTACACCACCCACGGAACTATCCA
TGTGGTGGCCAACAACCAGATCGGTTTCACCACCGATCCCCGTTTCTCGAGGTCCTCTCCCTACTGTACG
GATGTGGCTCGTGTCGTCAATGCTCCTATTTTCCACGTCAACGCTGACGATCCAGAGGCCGTGATGCATG
TGTGCAAGGTGGCTGCTGAGTGGCGTGCTACTTTCCACAAGGATTGTGTTATTGATTTGGTCGGATACCG
CCGCAATGGACACAACGAGATCGACGAGCCCATGTTCACGCAACCCTTGATGTACCAGAAGATCCGCAAG
CACAAGAACTGTCTCGACCTGTATGCCGACAAACTGATCGCCGAGGGCACTGTCACCGCCGAGGAGGTTA
AGTCGGTTGCCGCTAAATACGAGAACATTTGCGAGGAGGCATTCGCTCTGGCCAAGACCGAGACCCACGT

CAAGTACAAGGACTGGCTCGACTCGCCCTGGTCCGGCTTTTTCGAGGGCAAAGACCCATTGAAGGTAGCG
CCCACTGGAGTGAAGGAGGAGACCCTCATCCACATCGGCAACCGCTTCTCGTCGCCACCACCGAACGCTG
CTGAATTCGTGATTCACAAGGGGTCTATTGCGAGTGTTGGCCGCTCGCAAGGCAATGGTGGACGAAAAGG
TCGCTGATTGGGCTTTGGGAGAGGCCATGGCCTTCGGCTCTCTGCTGAAGGAGGGAATCCATGTGCGCCT
TTCGGGACAGGATGTCGAGCGTGGTACCTTCTCGCATCGCCACCATGTGCTGCACCACCAGCTGGTCGAC
AAGGCCACTTACAACTCGCTGCAGCACATGTACCCAGATCAGGCGCCCTACTCCGTCTCCAACAGCTCGC
TGTCGGAGTACGCAGTGCTTGGTTTCGAGCACGGTTACTCGATGACCAATCCCAATGCCCTGGTGCTGTG
GGAGGCTCAGTTCGGAGACTTCAGCAACACGGCCCAGTCGATCATCGATCAGTTCATCTCCAGCGGTCAG
TCAAAGTGGGTGCGCCAATCGGGTCTGGTAATGCTCCTGCCCCACGGCATGGAAGGCATGGGCCCCGAGC
ACTCCTCGTGCCGCGTGGAGCGCTTCCTGCAAATGAGCAGTGACGATCCCGACTACTTCCCACCAGAGTC
CGATGAGTTCGGAGTGCGACAGCTGCACGACATTAACTGGATCGTGGCAAACTGCTCGACGCCCGCCAAC
TACTACCACATCCTGCGCCGACAGATCGCATTGCCTTTCCGCAAGCCCCTGATTCTGTGCACGCCCAAAT
CGCTGCTGCGTCACCCCGAGGCTAAGAGTCCTTTCAGCGAGATGAGCGAGGGCAGCGAGTTCCAGCGCAT
CATCCCCGACAACGGTCCCGCCGGCCAGAATCCCAGCAATGTGAAGAAGGTCGTCTTCTGCTCGGGCCGC
GTCTACTACGATTTGACCAAGACACGTAGGGAAAAGCAGCTGGAGGGCGAAATCGCCATTGTGCGCGTGG
AGCAGGATCTCTCCCTTCCCCTTCGATCTGGTCAAGGAGCAGGCCAACCTGTACAAGAATGCCGAACTCG
TGTGGGCCCAAGAGGAGCACAAGAACCAGGGCAGCTGGACCTATGTGCAACCTCGTTTCCTCACCGCCTT
GAACCACAGCCGCGACGTCAG
>dme_circ_0004986_junction_seq
CCGCCTTGAACCACAGCCGCGACGTCAGGATTCGAGGACATAATATTGCTCACCTC
>dme_circ_0004987
GGCGAGCAGGACATGGATCGCCAGTTCAAGCTGCCCAGCACTACCTTCATCGGAGGCGATGAGGCTTCGT
TGCCCCTAAAGTGAGTAACCCCACTAAAGGATATTGAATGACCCAACTAATATGTGACATTCCCTTTATT
AGGGAAATCCTGAACCGCCTGGAGAATGTCTACTGTAATAAGATTGGCGTGGAGTTCATGTTCATCAACT
CTCTGGAGCAATGCAACTGGATCCGCAAGCGTTTTGAGACCCCCGGCGTCCTTAACTTCTCGCCCGAGGA
GAAGCGTCTGATCCTGGCCCGTTTGACCCGTGCCACCGGCTTTGAGGCTTTCCTGGCCAAGAAGTACTCT
TCTGAGAAACGTTTCGGTCTGGAGGGTTGCGAGATCATGATCCCAGCCCTGAAGGAGATCATCGACGTAT
CAACGGAGTTGGGAGTGGAGTCGGTCATCATGGGCATGCCCCATCGTGGACGTCTTAACACCTTGGCCAA
TGTATGCCGCAAGCCCTTGAACCAGATCTTCACCCAGTTCGCTGGACTGGAGGCTGCTGATGATGGGCTC
TGGTGATGTCAAGTACCATCTCGGTACATACATCGAGCGATTGAACCGCGTTACAAACAAGAACATCCGC
CTGGCTGTCGTGGCCAACCCGTCCCATCTGGAGGCTGTGGATCCCGTGGTGCAGGGCAAGACCCGTGCCG
AGCAGTTCTACCGTGGCGACCAGGAGGGCAAGAAGGTTATGTCCATTTTGATCCACGGTGATGCCGCCTT
CTGCGGACAGGGCGTCGTCTATGAAACTATGCATCTTTCGGACCTGCCCGACTACACCACCCACGGAACT
ATCCATGTGGTGGCCAACAACCAGATCGGTTTCACCACCGATCCCCGTTTCTCGAGGTCCTCTCCCTACT
GTACGGATGTGGCTCGTGTCGTCAATGCTCCTATTTTCCACGTCAACGCTGACGATCCAGAGGCCGTGAT
GCATGTGTGCAAGGTGGCTGCTGAGTGGCGTGCTACTTTCCACAAGGATTGTGTTATTGATTTGGTCGGA
TACCGCCGCAATGGACACAACGAGATCGACGAGCCCATGTTCACGCAACCCTTGATGTACCAGAAGATCC
GCAAGCACAAGAACTGTCTCGACCTGTATGCCGACAAACTGATCGCCGAGGGCACTGTCACCGCCGAGGA
GGTTAAGTCGGTTGCCGCTAAATACGAGAACATTTGCGAGGAGGCATTCGCTCTGGCCAAGACCGAGACC
CACGTCAAGTACAAGGACTGGCTCGACTCGCCCTGGTCCGGCTTTTTCGAGGGCAAAGACCCATTGAAGG
TAGCGCCCACTGGAGTGAAGGAGGAGACCCTCATCCACATCGGCAACCGCTTCTCGTCGCCACCACCGAA
CGCTGCTGAATTCGTGATTCACAAGGGGTCTATTGCGAGTGTTGGCCGCTCGCAAGGCAATGGTGGACGA
AAAGGTCGCTGATTGGGCTTTGGGAGAGGCCATGGCCTTCGGCTCTCTGCTGAAGGAGGGAATCCATGTG
CGCCTTTCGGGACAGGATGTCGAGCGTGGTACCTTCTCGCATCGCCACCATGTGCTGCACCACCAGCTGG

TCGACAAGGCCACTTACAACTCGCTGCAGCACATGTACCCAGATCAGGCGCCCTACTCCGTCTCCAACAG
CTCGCTGTCGGAGTACGCAGTGCTTGGTTTCGAGCACGGTTACTCGATGACCAATCCCAATGCCCTGGTG
CTGTGGGAGGCTCAGTTCGGAGACTTCAGCAACACGGCCCAGTCGATCATCGATCAGTTCATCTCCAGCG
GTCAGTCAAAGTGGGTGCGCCAATCGGGTCTGGTAATGCTCCTGCCCCACGGCATGGAAGGCATGGGCCC
CGAGCACTCCTCGTGCCGCGTGGAGCGCTTCCTGCAAATGAGCAGTGACGATCCCGACTACTTCCCACCA
GAGTCCGATGAGTTCGGAGTGCGACAGCTGCACGACATTAACTGGATCGTGGCAAACTGCTCGACGCCCG
CCAACTACTACCACATCCTGCGCCGACAGATCGCATTGCCTTTCCGCAAGCCCCTGATTCTGTGCACGCC
CAAATCGCTGCTGCGTCACCCCGAGGCTAAGAGTCCTTTCAGCGAGATGAGCGAGGGCAGCGAGTTCCAG
CGCATCATCCCCGACAACGGTCCCGCCGGCCAGAATCCCAGCAATGTGAAGAAGGTCGTCTTCTGCTCGG
GCCGCGTCTACTACGATTTGACCAAGACACGTAGGGAAAAGCAGCTGGAGGGCGAAATCGCCATTGTGCG
CGTGGAGCAGGATCTCTCCCTTCCCCTTCGATCTGGTCAAGGAGCAGGCCAACCTGTACAAGAATGCCGA
ACTCGTGTGGGCCCAAGAGGAGCACAAGAACCAGGGCAGCTGGACCTATGTGCAACCTCGTTTCCTCACC
GCCTTGAACCACAGCCGCGACGTCAGGGTAGGTGCCAGGGCTATATTTAAGCGGTCAGCAACTATCCACC
CACAAGAGCAGGAGCACTCCGACCACGTCCTCACTCCCTGATTTTTCTCTCAGGCTCAGATTCTTCTATA
GTCTCAGTTTATCTTTGTCTATTTCATAAAGTGCACTATTTTCATCGTGTACTGTACTTTTTATCTTTGA
TTCTCTAAACATTTTACTCTCTTTTGCTATTGTTTCACTACTTTTGCATTAAATTAGGCTTTAATTTTAC
CATTTCATTTTTCATCGCAGTCGCTATCATTTTAAATGCCCCAGCCGTATGATATTAACTTAATATCAAA
TAAAACTTAATTTAATATAAATAATAGTTCATAAAGCTTACGAAATGTGTTCAACCTTAATTCCGACAAC
AAAAGGTACCTTCATTTACTATCTTTATTGATAAGAAAGTATCATTCGGCCAAGGTGAATTTAAAATTTT
TATTAGAGCTTCCTCTCACTCCTCACATGCCTCCTAGCTGCTGTATTTATCACGCTCCCACTTTATATAT
CTCTCTACCTATCTTATTAGCTAGTTATTATTAGTCCCAACAAATGATAAAACACCCTATACACAAAACT
CAAACACTCATTGAACGGATATCCGCTTCTGATTTCTATTTTCGTTTGAATGAGCATGTCCAGCACCGCA
AAAGAAAACCAAAAATCATTTTCACTACCAAGTTTAAGAATCCATTACTGAAAATTTATCGCAGCAATTC
TCCAAGCGAGTCCATCAGCAAATTGGCTTCATCCATACAATATTTCATCCTATTTATTTTTACTGTGCAT
GGCTTTTTATTTATAATTTAGTTTAAACTCTTGAGTTACGTTTATTTTCTTATCGAAACTTGTAAAAGAC
AAGCAAACCAGTTGTGTGTTATTTTGAATCTCCCAAATTCCCACCCCAAAATCCACTCGTTGTGTAAATA
TGTACGTGCAATTGGCaaaatctaaagaagaaaaaatttgaaaaaaaaattcgaaactacccaagaaacg
aaaaaagcaaccaaaaacaaaTCCTttctctctttctctctctctGTGTATGTGTTATTGTTACAGCCAA
AGCGATGAACAATCCTCCTCCACCAATACTACCACTACTACTGATCATACTAATCATGAATCCGACACCG
ATTCCGATTCAAAATCTAAACCGTGGCTGAGTCGCATGTTCGCCGCCCCGAACTCCTCGACCGGCGGCGA
TCCTAAGGATCCGGCGCAAACCCTTGGCGGCGACTTCAATGCCGCCAAGCACTTTGACTTAAAGAATGTA
CGACACGATTTCAATAGGCCCGCGGGCATCGCGGCCGCTCCGGGCGCCCGCATAGCGAAAACCGGACGCA
AAATTAG
>dme_circ_0004987_junction_seq
GCATAGCGAAAACCGGACGCAAAATTAGGGCGAGCAGGACATGGATCGCCAGTTCA
>dme_circ_0004988
GGTTGCGTGAACATGGGCTCGTCGATCTCGTTGTGTCCATTGCGGCGGTATCCGACCAAATCAATAACAC
AATCCTTGTGGAAAGTAGCACGCCACTCAGCAGCCACCTTGCACACATGCATCACGGCCTCTGGATCGTC
AGCGTTGACGTGGAAAATAGGAGCATTGACGACACGAGCCACATCCGTACAGTAGGGAGAGGACCTCGAG
AAACGGGGATCGGTGGTGAAACCGATCTGGTTGTTGGCCACCACATGGATAGTTCCGTGGGTGGTGTAGT
CGGGCAGGTCCGAAAGATGCATAGTTTCATAGACGACGCCCTGTCCGCAGAAGGCGGCATCACCGTGGAT
CAAAATGGACATAACCTTCTTGCCCTCCTGGTCGCCACGGTAGAACTGCTCGGCACGGGTCTTGCCCTGC
ACCACGGGATCCACAGCCTCCAGATGGGACGGGTTGGCCACGACAGCCAGGCGGATGTTCTTGTTTGTAA
CGCGG

>dme_circ_0004988_junction_seq
CAGGCGGATGTTCTTGTTTGTAACGCGGGGTTGCGTGAACATGGGCTCGTCGATCT
>dme_circ_0004989
GTCGAAATCGCGTCGTTGAGCAGCGCATTCAGCTCACGGATGTGCTGGGCCTTGGATCCGGTGGCGGTGG
AGGCTCCGCAGGCGCGTCCGACGTAGCTGTGGATTAGGAAAATAGTTGGGATTAGTGGGGATACTTGAGG
TATCGAGCGGTGAGTTGGCACAACATTCGGTATTCGATTTGCTGATTCGGTTAGACACGTGCACTGAACT
TTAGTCTAGTTTCTCTTATTGCTTTTAGCACAGTTATGGCAAATGTTCAATGAAATGAGGGTTAGGCAAG
CGCAGGATGTTAAATATTAAGATTAAAATGTATAGAGATCTTAAGTCGAGAGTAATATTAGGGGAAACCA
CACCGAACCCAAACATTGAAATGGACCCCAATTAATTCTGCGATGCAGAATCACTTAGATTTTTATCATC
AATTCTCAAGCTTCGTGAAACAGCAAATTGCCTAGATCAGGATGGCATATGTATGAGATGTCTGCCTATA
AGCGCTAAAAACTCTGTTTTTAAGGTAGTTGTGGAAGTTTTTAACTTGTTTGTGATCTATATAGACAGCA
CACACACATACAAACAAACAAATTCAAGTACAGATTACAGCACAACTTTGGTTAAATTGGAGCAGTACAA
ATGTCGGGGTTAACTGTGGttttttttgttttACGATGGAAACTGGACTGGCAAACAGCGAAATAGGGCA
AACTTATCAAAAACAAATGCAAAACTTTCGAAACGCGCGAGGTGCAAACAAAAACTCAGAACTGAAAAGA
AGAACAGTGCTTTAGGATTAGAGGATTAGATTGTTGAGCTGGAGGTTAAGAGTAGTAAGTAGGGTTTGGT
GTGTTGATTTCTTTTTAACCGGGGGTACCATGAGGCGCTGGATCATGGAACAATCATCATGCCAAACAAC
AGAAATCAGAAACGCAATCGAGGAAACTGATAAAAGGGAAACGAGGAGACAAATGAAGAGTTAACGGAAC
TGAGAGTTAATTTCCGTTTGGTAGGATAGTTAAGTTGCAGATAGTAGTTGGTAGTTGATAGTTGATTAGA
TTAATTGTAGATATACAGTTCATTGACCGCGCGCATCGTTGCGGAGCTGAGCTGAGCTTGCCCTCTCACC
TAATTTTGCGTCCGGTTTTCGCTATGCGGGCGCCCGGAGCGGCCGCGATGCCCGCGGGCCTATTGAAATC
GTGTCGTACATTCTTTAAGTCAAAGTGCTTGGCGGCATTGAAGTCGCCGCCAAGGGTTTGCGCCGGATCC
TTAGGATCGCCGCCGGTCGAGGAGTTCGGGGCGGCGAACATGCGACTCAGCCACGGTTTAGATTTTGAAT
CGGAATCGGTGTCGGATTCATGATTAGTATGATCAGTAGTAGTGGTAGTATTGGTGGAGGAGGATTGTTC
ATCGCTTTGGCTGTAACAATAACACATACACagagagagagaaagagagaaAGGAtttgtttttggttgc
ttttttcgtttcttgggtagtttcgaattttttttttcaaatttttttcttctttagattttGCCAATTGCA
CGTACATATTTACACAACGAGTGGATTTTGGGGTGGGAATTTGGGAGATTCAAAATAACACACAACTGGT
TTGCTTGTCTTTTACAAGTTTCGATAAGAAAATAAACGTAACTCAAGAGTTTAAACTAAATTATAAATAA
AAAGCCATGCACAGTAAAAATAAATAGGATGAAATATTGTATGGATGAAGCCAATTTGCTGATGGACTCG
CTTGGAGAATTGCTGCGATAAATTTTCAGTAATGGATTCTTAAACTTGGTAGTGAAAATGATTTTTGGTT
TTCTTTTGCGGTGCTGGACATGCTCATTCAAACGAAAATAGAAATCAGAAGCGGATATCCGTTCAATGAG
TGTTTGAGTTTTGTGTATAGGGTGTTTTATCATTTGTTGGGACTAATAATAACTAGCTAATAAGATAGGT
AGAGAGATATATAAAGTGGGAGCGTGATAAATACAGCAGCTAGGAGGCATGTGAGGAGTGAGAGGAAGCT
CTAATAAAAATTTTAAATTCACCTTGGCCGAATGATACTTTCTTATCAATAAAGATAGTAAATGAAGGTA
CCTTTTGTTGTCGGAATTAAGGTTGAACACATTTCGTAAGCTTTATGAACTATTATTTATATTAAATTAA
GTTTTATTTGATATTAAGTTAATATCATACGGCTGGGGCATTTAAAATGATAGCGACTGCGATGAAAAAT
GAAATGGTAAAATTAAAGCCTAATTTAATGCAAAAGTAGTGAAACAATAGCAAAAGAGAGTAAAATGTTT
AGAGAATCAAAGATAAAAAGTACAGTACACGATGAAAATAGTGCACTTTATGAAATAGACAAAGATAAAC
TGAGACTATAGAAGAATCTGAGCCTGAGAGAAAAATCAGGGAGTGAGGACGTGGTCGGAGTGCTCCTGCT
CTTGTGGGTGGATAGTTGCTGACCGCTTAAATATAGCCCTGGCACCTACCTGACGTCGCGGCTGTGGTTC
AAGGCGGTGAGGAAACGAGGTTGCACATAGGTCCAGCTGCCCTGGTTCTTGTGCTCCTCTTGGGCCCACA
CGAGTTCGGCATTCTTGTACAGG
>dme_circ_0004989_junction_seq
CCACACGAGTTCGGCATTCTTGTACAGGGTCGAAATCGCGTCGTTGAGCAGCGCAT
>dme_circ_0004990

GGCCCGGAGGCAACAGCAACATAATCATCATCGGCAGCAGCAGCAGCAACATCTTGGCCGGCTGCATCTT
GGATAGCCGAGAACGTGATCGCTTTCTGGCCAAGATCGGAGCAAGCCAACGCCATGGCGATGTCACCTGT
CTTGGATGCGGCTGCCAAAGCCGCTGGCCGTGATCTCAATGGTAATAGTAATgcagcaagtgcaacagca
gcatcaacagcaacaccaactgcagcagcagccgcagcagcaacCACACCGCGCAAAACGAAAAAGACAA
TTATCAATTGGCTGGTGAATAGTGACAGTGAGAACAACAATCGGGCGACGTGCGATAAAGCCGGCAAAGA
TaatccaaatccaaatcaaaatccgaatAGCCAAGCAAATCCGAATCAAAACTGCAGCTGTGAGTCGCGG
GCATTTCGCGATTTCCGTGGCGTGCAAACTTTGCGATTATTGTGGAGCAGGCGCATCAAAAGTGCCAGCG
AGGATCAATGTCACTCCCCAGTCCACTGCCACACCTCCTCCTCTTCATTGGGCACATTAAAAAAGTTAAA
TAAAAGCGTTAGCTGTCTGGTTAACGCCTTTAACAACTCGCGTGATTGCGCCACGCCGGAAAAAACGCTG
GCAAAACGCGCTGCCAGTTTGCACAATTTGCAGGATTCGCAAGCGAAGCATAAGCAATTGCATAGGGAAA
AAGATAATTTCTACAAGTACAAAAACGCCGAGCAGGCCAAAATGCAGGCCAAGTTAAGAAGCAGCGCCGA
CGAGGCGATTTTAAACGCCAGCGAAACGCGCGCGAAACGTGACGACAGCGGTTGTTTACCTGGCGTCAAA
TCGTATAATTCGAAAGCCTTATCAGCCGCAGATAAGGTTGCAAAAGTGGCAAAGGGGGAAGCTAAGACGC
AGCGGAGGGCGGAGGAGCCGGTGGGAGAGGCAGAGGTGGAGGTGGAGGTAGAGTTCCGAGCCAAACGGGA
GCACAATCGGCTTGAGGGTCTCAGTCTCAGCGCGGCGAGCGCCCAAAGTGGGGGCAGCAGCGAATCGCTT
CAAACCACCACCACCTCCTCCTCCACGCCCACCACCCACAGCAGCGTGACGTCATCGACGGGTCGGTCGG
GCGGACGTCGCAAGTATAGCTTTAAAACCCATGCCGGCAAGTCATATCATCAGCACCATCATCCGTCGCG
CAGGACTAGCAACATGGATTCCTCAAATGGTGGCATTAGTATGGTGGCCAAACTTACCCATCAGTTCAAC
GAGATTATTCAAAAGGATGCGAAACTCCTGGAACAGGTCAAGCGGAACAATGGCGTCTGGATATCCCGGG
GCACTCATGTCTACAAGATCGTGGAGAAACCAACGTCAGAGGGAAAGGGATTACCTAACGCCGACGATAA
TCGCATCTCGACCGTTCAGCGGAATATTAAAAAGTTCGAGAGGCTGGAGAAGCCGAGTGTGCCCTTGAAG
AGTGAACAGTTGATGCGGAAACATCTCGAACTGATGAGGGGCAGTAGTCTTTCGCGGGGTCCGCGAATCA
AAAGGAATCTCAAGCTACCACCGGGCAGTGTGGGCATTCCAGAACTGGCAATAGTCAGCGAGGAACTGAA
GACTCCTACTTCCGAAGAGCAAGCTAAGAAACCAAAGTCAGTGCAAAATGAATCACAGCCCAATCATCCC
GAAGAATTGAATCAGTCGAATGACAAACCCAGTGATGAGCTGGATTTAATTATGGGGGAGGAAGGCGACG
AGGAGGAGCTGCGCCAGAAGCAGCGGAAGCACAAGTATGCCTCCATATACGAGAAACTCCGATTCACCTT
CGCGAAAGGTCGGAAATCAGCGAGTCCCTCACCCACGAAGGCCCAAATCCGGGAGGAAGTATCAATAGAT
GAGGAGTCCATCAAGGATAGCACATCCGAAGATATGGTTACTCGGAAAGAAGATTTCACATTGGATCTTC
CCAAAGAGGTAATACCCAGAGCAGAAGACCTAGAAGATTCCTCACACGCCCTGCTTGTATCTCCGTGCTT
TGAAGCCGATGCCAAAATCCTCGACGCCCTTGAGGCAGTGGATCTAAAACTCAAGGTGCTCAACCCCCTC
AACGATGTGACCACCGCAGACGGTGAGCGGGAGCTCCTCCTGGCCGCCAACGATGACTTCGAGCAGCGCA
TCCTGCCCAATAGCTCCTTTGTTTACCAGGCGGCCAACAAGCAGTACTCGAATAATCAAATTCTGCTGAA
CCAGGCGGTTAATGTTACCCTAGTTAATGCCATCGAGGGTGAGCAGATGATCATGGAGCAAAAGCAACTG
ATGAAAGTCAGAGATTTGGAGCTCACGGAGAGGGACATAGATCAACCTCTCAAACGGGCGGAAAAATCGC
CAGAGAAGGACGATACCCTGGAACCCGAGTCCCTCTACGAACCCATCACACCTGTTATGGATGAAACCAA
GACTGAAGCCCAGACATCCAGCCTCTTTAAAATATACACCAAGAAATCAGAGCTGGTGGAGGACATATAC
CAAACTGTTGAGGAGGCATCCACTGTCGAGGTGAAGCCGAATAACTGGCTGGAGGGCTACGAATCAATCG
CTGGATCCCAGGAGGCATGTCCCTTGACGTACGATGGCTACGAGTCCTTCGCCCCAGAAGAGGTGGTAAC
AACACCCACGACTCCATCTGCCACTGGCACATTGGGCCGGAATACCCGCGATGAGCTGCCGGAACTGCCG
AAGCCCAAGAGAGTACTCAACAAATCACCCATTCCCTTGCGACCAGCACCTCCAAAGCCACAAGCCAAAG
AGTCGGAAGAGGATGAGAATATATACGACACCATCAAGGGCTGCTACGAGTCCATGCACTGCAAGGCAAC
ATCCTCCTCGTCCAGTGGAGAAGCCACACTCACCACGGACACCATTTCCCTGAGTTCCAACTGCTACGAG
AGTATCTCCCACATCCGGAGAAGCCGAGTGGCAACTACTCAAAATCAGGGCCACGGTTCGGCGGGCAGTT
GCATCCAACTCTCGAGCAGCGGCTCCACATTGACAATCTCCTCGGATCACAAGACCAACAGCCTGTATGA

ATCCTCTCTGGCGGCCGCCGGATGCGTGGTCTACGGAAGTGCCAGTGTGGGATGCAGGTCGTCCTTGGGC
AGTAGTGCCGGAAGCAGAGACAGCGGAAAGCCTGGCGATAAGAGATCCTCAATAGCCGGCTCCAGTGACA
ACAGCGATGCGTGGGTGGACATTTCGGATGGGGAGATTGGCCAGGCGACGACCGATGCGACCGCCAACGA
GGCCCAGTTCATTGT
>dme_circ_0004990_junction_seq
CGACCGCCAACGAGGCCCAGTTCATTGTGGCCCGGAGGCAACAGCAACATAATCAT
>dme_circ_0004991
GGCCCGGAGGCAACAGCAACATAATCATCATCGGCAGCAGCAGCAGCAACATCTTGGCCGGCTGCATCTT
GGATAGCCGAGAACGTGATCGCTTTCTGGCCAAGATCGGAGCAAGCCAACGCCATGGCGATGTCACCTGT
CTTGGATGCGGCTGCCAAAGCCGCTGGCCGTGATCTCAATGGTAATAGTAATgcagcaagtgcaacagca
gcatcaacagcaacaccaactgcagcagcagccgcagcagcaacCACACCGCGCAAAACGAAAAAGACAA
TTATCAATTGGCTGGTGAATAGTGACAGTGAGAACAACAATCGGGCGACGTGCGATAAAGCCGGCAAAGA
TaatccaaatccaaatcaaaatccgaatAGCCAAGCAAATCCGAATCAAAACTGCAGCTGTGAGTCGCGG
GCATTTCGCGATTTCCGTGGCGTGCAAACTTTGCGATTATTGTGGAGCAGGCGCATCAAAAGTGCCAGCG
AGGATCAATGTCACTCCCCAGTCCACTGCCACACCTCCTCCTCTTCATTGGGCACATTAAAAAAGTTAAA
TAAAAGCGTTAGCTGTCTGGTTAACGCCTTTAACAACTCGCGTGATTGCGCCACGCCGGAAAAAACGCTG
GCAAAACGCGCTGCCAGTTTGCACAATTTGCAGGATTCGCAAGCGAAGCATAAGCAATTGCATAGGGAAA
AAGATAATTTCTACAAGTACAAAAACGCCGAGCAGGCCAAAATGCAGGCCAAGTTAAGAAGCAGCGCCGA
CGAGGCGATTTTAAACGCCAGCGAAACGCGCGCGAAACGTGACGACAGCGGTTGTTTACCTGGCGTCAAA
TCGTATAATTCGAAAGCCTTATCAGCCGCAGATAAGGTTGCAAAAGTGGCAAAGGGGGAAGCTAAGACGC
AGCGGAGGGCGGAGGAGCCGGTGGGAGAGGCAGAGGTGGAGGTGGAGGTAGAGTTCCGAGCCAAACGGGA
GCACAATCGGCTTGAGGGTCTCAGTCTCAGCGCGGCGAGCGCCCAAAGTGGGGGCAGCAGCGAATCGCTT
CAAACCACCACCACCTCCTCCTCCACGCCCACCACCCACAGCAGCGTGACGTCATCGACGGGTCGGTCGG
GCGGACGTCGCAAGTATAGCTTTAAAACCCATGCCGGCAAGTCATATCATCAGCACCATCATCCGTCGCG
CAGGACTAGCAACATGGATTCCTCAAATGGTGGCATTAGTATGGTGGCCAAACTTACCCATCAGTTCAAC
GAGATTATTCAAAAGGATGCGAAACTCCTGGAACAGGTCAAGCGGAACAATGGCGTCTGGATATCCCGGG
GCACTCATGTCTACAAGATCGTGGAGAAACCAACGTCAGAGGGAAAGGGATTACCTAACGCCGACGATAA
TCGCATCTCGACCGTTCAGCGGAATATTAAAAAGTTCGAGAGGCTGGAGAAGCCGAGTGTGCCCTTGAAG
AGTGAACAGTTGATGCGGAAACATCTCGAACTGATGAGGGGCAGTAGTCTTTCGCGGGGTCCGCGAATCA
AAAGGAATCTCAAGCTACCACCGGGCAGTGTGGGCATTCCAGAACTGGCAATAGTCAGCGAGGAACTGAA
GACTCCTACTTCCGAAGAGCAAGCTAAGAAACCAAAGTCAGTGCAAAATGAATCACAGCCCAATCATCCC
GAAGAATTGAATCAGTCGAATGACAAACCCAGTGATGAGCTGGATTTAATTATGGGGGAGGAAGGCGACG
AGGAGGAGCTGCGCCAGAAGCAGCGGAAGCACAAGTATGCCTCCATATACGAGAAACTCCGATTCACCTT
CGCGAAAGGTCGGAAATCAGCGAGTCCCTCACCCACGAAGGCCCAAATCCGGGAGGAAGTATCAATAGAT
GAGGAGTCCATCAAGGATAGCACATCCGAAGATATGGTTACTCGGAAAGAAGATTTCACATTGGATCTTC
CCAAAGAGGTAATACCCAGAGCAGAAGACCTAGAAGATTCCTCACACGCCCTGCTTGTATCTCCGTGCTT
TGAAGCCGATGCCAAAATCCTCGACGCCCTTGAGGCAGTGGATCTAAAACTCAAGGTGCTCAACCCCCTC
AACGATGTGACCACCGCAGACGGTGAGCGGGAGCTCCTCCTGGCCGCCAACGATGACTTCGAGCAGCGCA
TCCTGCCCAATAGCTCCTTTGTTTACCAGGCGGCCAACAAGCAGTACTCGAATAATCAAATTCTGCTGAA
CCAGGCGGTTAATGTTACCCTAGTTAATGCCATCGAGGGTGAGCAGATGATCATGGAGCAAAAGCAACTG
ATGAAAGTCAGAGATTTGGAGCTCACGGAGAGGGACATAGATCAACCTCTCAAACGGGCGGAAAAAATCGC
CAGAGAAGGACGATACCCTGGAACCCGAGTCCCTCTACGAACCCATCACACCTGTTATGGATGAAACCAA
GACTGAAGCCCAGACATCCAGCCTCTTTAAAATATACACCAAGAAATCAGAGCTGGTGGAGGACATATAC
CAAACTGTTGAGGAGGCATCCACTGTCGAGGTGAAGCCGAATAACTGGCTGGAGGGCTACGAATCAATCG

CTGGATCCCAGGAGGCATGTCCCTTGACGTACGATGGCTACGAGTCCTTCGCCCCAGAAGAGGTGGTAAC
AACACCCACGACTCCATCTGCCACTGGCACATTGGGCCGGAATACCCGCGATGAGCTGCCGGAACTGCCG
AAGCCCAAGAGAGTACTCAACAAATCACCCATTCCCTTGCGACCAGCACCTCCAAAGCCACAAGCCAAAG
AGTCGGAAGAGGATGAGAATATATACGACACCATCAAGGGCTGCTACGAGTCCATGCACTGCAAGGCAAC
ATCCTCCTCGTCCAGTGGAGAAGCCACACTCACCACGGACACCATTTCCCTGAGTTCCAACTGCTACGAG
AGTATCTCCCACATCCGGAGAAGCCGAGTGGCAACTACTCAAAATCAGGGCCACGGTTCGGCGGGCAGTT
GCATCCAACTCTCGAGCAGCGGCTCCACATTGACAATCTCCTCGGATCACAAGACCAACAGCCTGTATGA
ATCCTCTCTGGCGGCCGCCGGATGCGTGGTCTACGGAAGTGCCAGTGTGGGATGCAGGTCGTCCTTGGGC
AGTAGTGCCGGAAGCAGAGACAGCGGAAAGCCTGGCGATAAGAGATCCTCAATAGCCGGCTCCAGTGACA
ACAGCGATGCGTGGGTGGACATTTCGGATGGGGAGATTGGCCAGGCGACGACCGATGCGACCGCCAACGA
GGCCCAGTTCATTGTTGTAAGTACTATATCTCACAATGAATGTGCTTATCTTTTAATACTTTTAAAAAGA
GCTAATCAAAAAATAAGTAAGGAAAGTGTAGATAAATGATAAATGAGATTGGAATTTTGTTTATAAAAAG
AAAATATTTAAGTAATGCGTTatttttttcagtgcatttttttttttATTTTACTTAAAGTATATAAATAT
TGTTCAACGTTTCATGAGTCTGTAGGTCAGGTTGATATGAATAATGTTTTAAGTATACACACATTGCAAA
CCGGGAGAAAATATCGCAATGTTATTATTATTTATTCACTGCCCGTTACGCAAAACCAATTGCGAGTGAA
AAACGATTGGAATTGCAATCTTCCTACAGCTGGGCTATATAAATTCTGCGTAGATTGCTTATCAGTTTGT
TTGGGTTTGCTTATCTAATCAGTTGATAAAGGACAGGATACCGCAGGGGTTTTTAGTGTCTGAAATTTTA
TTTGCCGCCAATGAATGGTGCATAATGGGTTGCAACATATACATGTATATAATAGTCATTCATTTCGGTG
TAGAGGTTGACCCCAAAACTATACTAATATGCAGGTCAAGGTAAATTTGGCTTTGATATCAAGATTATAT
AGCGTAGCAGGATTAAAGTCGAAAGATTCGTCGAGCACCAATGTGAAGTGTATTACGCAAACGAAATTAA
TATATTATTAAGAAAAATGTTCATATTTGGTATTAGTATTAGTATTATTTTTGCACATACTTAATCTCCT
TTAGGGGTAAACAACTGGCGCAGATTTTTTAACTACGTAATGATAGCAAAATAAATGACGTTTATGTTTT
CCTACATACGCTTTTTATAGCTTATCTAAGTTGTTCAGCAACAATAATACACACAATGAAACAATTTAAA
ACAGTTGCTGACTGCAACAGGCATTTAACGATCTTTTAATAGTTCTTCTGATCAACTGATAATTATCACC
ACCAGAACTTATTGTTAGCACTTAATTAGTACAAAGTCATGCTGCGTTCCATTTGGTTGTTTCGACATTT
GCCGGCTGATTGAAAGCCCAATTCACAGCCCAATTCTCCCCCACTTATTAGCGGCCCCTATCAATTAGAG
CGCTGACCTACAATTTCTCTGTTATCAGCTGATAAGATTGGCGCGCCTCTTGAGTGAATCTCTGGAAATG
CTGAGAGCCCAAAATCAATACAACTATTCAAGACAGTCAATTGTTACAACTACCCAAAGGAGATCAGTTG
TCTGGAATTGGTTTGATCGATCGGCTGGTTAATTATAAAGTACTTAGCAAAACCCATTTTCAGTCAATTA
AATGTGTATTATTTTTGATTTTGTTTTTAATACCTCTTGTTGCACTTGCTTGTTTTCAGTGTAAGGGAAA
GGTTTAAACCACATCGCACCCGGAGTCCGGATTGGTCGAAACGGATCAGAGACAAAAGACTGCAACAAAA
GAAGGCGATTAGCTGCATAGAGG
>dme_circ_0004991_junction_seq
CAAAAGAAGGCGATTAGCTGCATAGAGGGGCCCGGAGGCAACAGCAACATAATCAT
>dme_circ_0004992
GGACATGCCTCCTGGGATCCAGCGATTGATTCGTAGCCCTCCAGCCAGTTATTCGGCTTCACCTCGACAG
TGGATGCCTCCTCAACAGTTTGGTATATGTCCTCCACCAGCTCTGATTTCTTGGTGTATATTTTAAAGAG
GCTGGATGTCTGGGCTTCAGTCTTGGTTTCATCCATAACAGGTGTGATGGGTTCGTAGAGGGACTCGGGT
TCCAGGG
>dme_circ_0004992_junction_seq
GGTTCGTAGAGGGACTCGGGTTCCAGGGGGACATGCCTCCTGGGATCCAGCGATTG
>dme_circ_0004993
GATATGTTCTGGCGATCGATCGATCCGATAGCAGCCCGTTTCGAGTGCTGCTAAGCCGGAGTCGTCATAA
TCATCGTCACACTATCGCCAACACAATCTGGCCCGGCTAATTCTAACCCAGATGATTTACAATACTTCTT

CATGTGACCGCGATGAGTGGATGTGGTAAATTTCGATGAAGGACCCATTTACACTGGGCGATTACAATAG
TCAGAAACCAATCGTTGAAGTTCAGTTTGGATTGGTTTCGTTCGAGGGCTACGGCTAAGCTACGGCTCAT
AAACGTATCCTGCATTTAGGCGCTAGATTCCTACACATATATAAATATATCACAAAATCAGTGAAATTAA
AATTCGCTGGAGTATTTGCTTGTTTCGCTTTGGATGATGCATGAGAAGAGCTCTTTGAAACGTTGAGTGA
TAAAATGTTTGGCAATGTCCTAGCTCAGCAGACGGCAACAACCTGTCACCTGAAGCTGATGCCGCACTTG
CGGCTGATCTTGGGCGTGTGAATATATGCTATTGACAGCAGCAATG
>dme_circ_0004993_junction_seq
TGAATATATGCTATTGACAGCAGCAATGGATATGTTCTGGCGATCGATCGATCCGA
>dme_circ_0004994
GACTCAGTCCCTCCTTTACACCTGCTACCGGCACCATGTTCCAAAAGAAATTCTTTTTCGGAGGTGCCTC
CACGTTGTCTTCATAGTCGATGGCCTCGATGGCACCGTCATCCACGGAACCTGTACTTACGGAGCCAGTG
CCGCTCGACTGCTCCTCCTCCATGCACAGCTCCACGTTATCCGGCGTGGTTACTGTCAAACGGATCCGGC
CGTCTTTGCCAGTACGAAAGTACTGCTGTGCCTGCCGCGCCAGCAGCATCTTCTTGTCAATACCTGT
>dme_circ_0004994_junction_seq
CCAGCAGCATCTTCTTGTCAATACCTGTGACTCAGTCCCTCCTTTACACCTGCTAC
>dme_circ_0004995
GTTTCCGATACGCAGCTCCTGCTGCACAAAGTGAGAGTCTCGGAAGGCCAACACAGTGGGAATCTCATTC
TTTTTCAGAGTCTGCATGCCATCGCTGGCGTCCAGGGACCCAGAGTCCAAACCATCTGGGTCCCCATCCT
CGTTGTCCCTATCGTACATGTTCAGTTCGCCATTCTCATCGTCCGCATCATCAGCAATTGCATTTAGATT
CTGTTTGCAGGTTGCCGCCCTGCTCAATCCGCGCTTCTGCGTCGGTGGACTCTTGTGCCTCTCCTGCGTA
CCCGTTTGATTTTTCGTCTGGCTCTGGGCGGCAAGGTCGTTAATAAGCTGCTTGTTAAAGTCCTCCCGCT
TGGCCGCTCGCCGCACTTCGCTGCACCGAATAAAGTAT
>dme_circ_0004995_junction_seq
CCGCACTTCGCTGCACCGAATAAAGTATGTTTCCGATACGCAGCTCCTGCTGCACA
>dme_circ_0004996
AATCTCGCGTAATTTGCGCACCTCCTTCGAGAACGAGCATTCCAAGAACGATTTTATCGGCTTTCTTAGC
GACAATTACGCATTGAATGGCCCTGTGGTAGCACCAGCAGGAGGCGGAGCTGGAGGGGGCAGCGAAGG
>dme_circ_0004996_junction_seq
GAGGCGGAGCTGGAGGGGGCAGCGAAGGAATCTCGCGTAATTTGCGCACCTCCTTC
>dme_circ_0004997
GGACATTAGCTTTAAGCTGTGCAAGGCACCGAGCCAAACGCAACGTACCTCCATTACACATGACTCTCCC
TGGAAGTTGCAGCAGGTCCAGGATGCGGCCAATCATCTGCAGACCGCCATCAACCACATAGACGATGTCG
ACGACTCTTATCACTTCAAGAACCTCCGACGAAGTGCTGCATGTGATAGGCAACATTCTGGATGCCCTGC
AGCGGGGCAGAAACAGTTTGCTGGTGCCCAAAAAGAAGCCCATCGATGAGCTGATCAAAGGGCGCAATAT
GAAGTCGCTGGTGCCCAATCTGCCCGAGGATTTGGCCGTTAGCTTCTACCTGCAGAGCCACAAGCTGATC
ATTGCCGTCTACCAGCTGCTGAACAACCAGGGCACAATGCGCTTCGATTCCCGCCAAGCGGAGGCCTCTG
TCCAGTGGCTTAACGACGTGCTACTGCTGCTGATGAATGGCCAGAAACTGTGCCAGCAATTAAAAGACAA
G
>dme_circ_0004997_junction_seq
GAAACTGTGCCAGCAATTAAAAGACAAGGGACATTAGCTTTAAGCTGTGCAAGGCA
>dme_circ_0004998
ACATCATTGATGCCCGTATCATCGGTGACAAAGACGGGCAGTCCATTCACCGCCTTATCGGACACCTTGA
GGGCCTTATAGGCGCAGATGTGACCCCAGGCCACATTCCCGGCGTAGACCAACTGCTGCTTTCCGCCCAC
GCCCGCGATCCTTGGGAACACCCATCCCCTAGTCGAAAGGAAG

>dme_circ_0004998_junction_seq
GAACACCCATCCCCTAGTCGAAAGGAAGACATCATTGATGCCCGTATCATCGGTGA
>dme_circ_0004999
GATCTAACCACCGGGTGAAGTGACTGTTCCGGCTGCCTACTTTAGAAATTTCGGACTGGAACTAGGCGAC
GCCGTCGACACGTTGTCCAGGTTGGTGTTCGTGTTGTCGAAGCACTCGTGTTGTGGCTCCGGTTCCGTGG
GCGGCAGCGGTTTGTTCAGCAGCACAGTCATGTTCTTCACGTTGCTGGGCTTCTCCTCGTCCTG
>dme_circ_0004999_junction_seq
TCACGTTGCTGGGCTTCTCCTCGTCCTGGATCTAACCACCGGGTGAAGTGACTGTT
>dme_circ_0005000
GCGTTCATTGCGAGACCAACTGTCGGAGCGGTTACTATGGTGAAAATTGTGATAAAGTTTGCAGATGCCT
GAATAACTCCTCCTGCGACCCCGATTCCGGTAACTGCATCTGCTCAGCCGGTTGGACTGGCGCCGACTGT
GCGGAACCCTGCCCACCTGGATTCTACGGCATGGAATGCAAGGAACGGTGCCCGGAGATCCTCCATGGGC
AACAAGAGCTGCGATCACATCACAGGTGAAATTCTCTGCCGCACGGGTTACATAGGTCTCACCTGCGAGC
ACCCATGTCCTGCTGGACTCTACGGACCCGGCTGCAAGCTCAAGTGCAATTGCGAGCACGGCGGCGAGTG
CAACCACGTTACAGGCCAGTGCCAGTGCCTGCCAGGGTGGACAGGTTCCAACTGCAACGAGTCCTGTCCC
ACAGACACCTACGGACAAGGATGCGCCCAGCGGTGCCGCTGCGTGCACCACAAGGTTTGCCGCAAGGCCG
ACGGTATGTGCATCTGCGAAACCGGCTGGTCGGGCACCCGGTGCGATGAGGTCTGTCCGGAGGGATTCTA
CGGCGAGCACTGCATGAATACCTGTGCCTGTCCGTCGGCCAATTTCCAGTGCCATGCGGCCCACGGTTGT
GTGTGCAGGAGTGGCTACACCGGCGATAACTGCGACGAGCTAATCGCCTCTCAACGTATTGCAGACCAAA
GCGAAAATTGCGAGCCGGGCGAGTGTAGCTCTGACCTTAGTCCTGATGACCCTGTTTGCGTGCATAATCT
TCGCAGTGTTCATTTACTACCGCCGACGTGTGTCCAATCTAAAAACCGAAATCGCCCATGTACACTACAC
CCATGATACGAATCCCCCGAGCTGGCCGCCCAACCACAACTTCGACAATCCCGTGTACGGCATGCAGGCG
GAAACGCGTCTCCTGCCCAACAATATGCGTTCCAAGATGAACAACTTTGACCAGAGGAGCACGATGAGCA
CGGACTACGGCGATGACTGCAATGCCAGTGGCAGGGGTGGGTAGCTATTCGATCAACTACAACCACGACC
TGCTAACGAAGAACTTGAACGCGGACCGGACCAATCCGATTGTGTACAACGAATCCCTCAAAGAGGAGCA
TGTCTACGACGAGATCAAGCACAAGGAGGGCTACAAGGATCCCGG
>dme_circ_0005000_junction_seq
AGCACAAGGAGGGCTACAAGGATCCCGGGCGTTCATTGCGAGACCAACTGTCGGAG
>dme_circ_0005001
ATTGCCCACCCGGCTGGTATGGCCGGAACTGCTCGATGCAGTGCGACTGCCTGAACAACGCGGTATGTGA
GCCCTTTTCAGGCGACTGCGAGTGCGCCAAGGGCTACACCGGAGCACGCTGCGCGGACATCTGTCCGGAG
GGCTTTTTCGGGGCCAACTGCTCGGAGAAGTGCCGATGCGAGAACGGCGGCAAGTGTCACCACGTGTCCG
GCGAATGCCAGTGTGCTCCCGGTTTCACGGGTCCCCTGTAAGTGGACGAAGGGGCCTCTTTGGCGGCACA
AACTTCTGGCCGAGGATCAGGCAAAATGAAAGGCAGAATGCAATCAATTATTTTTCGTGGCTGGGATTTT
AGGCCGTTGCATTCTACGTTTCATTTTGCTCCTGCTCCAACGCAAAACCCATACTAATACTCCGCTCCAA
CTAGATGTGATATGCGCTGTCCGGATGGAAAGCATGGCGCCCAGTGCCAGCAGGACTGTCCCTGCCAGAA
CGACGGAAAGTGTCAGCCAGAGACCGGGGCCTGCATGTGCAATCCAGGCTGGACGGGCGACGTGTGTGCA
AACAAGTGTCCGGTGGGCAGCTACGGCCCTGGATGCCAAGAGAGCTGCGAGTGCTACAAGGGAGCGCCCT
GCCACCACATCACCGGCCAGTGCGAGTGTCCGCCCGGATATCGCGGAGAACGCTGCTTCGACGAGTGTCA
GCTGAACACGTACGGATTCAACTGCAGCATGACCTGCGACTGCGCGAACGATGCCATGTGCGATCGGGCG
AACGGAACGTGCATCTGCAATCCCGGCTGGACGGGTGCCAAGTGCGCCGAGCGGATCTGCGAGGCGAACA
AGTACGGGCTGGACTGTAACCGCACCTGCGAGTGCGACATGGAGCACACGGATCTGTGTCACCCGGAGAC
GGGGAATTGCCAGTGCAGCATCGGATGGAGCAGTGCTCAGTGCACCAGACCCTGCACCTTCCTGCGCTAC
GGCCCGAATTGCGAGCTGACCTGCAACTGCAAGAACGGAGCCAAGTGCTCGCCGGTCAATGGTACGTGCC

TATGTGCGCCGGGCTGGCGCGGACCCACCTGCGAGGAGAGTTGCGAGCCGGGCACCTTTGGTCAGGACTG
CGCCCTGCGCTGCGACTGCCAGAACGGAGCCAAGTGCGAGCCGGAGACGGGCCAGTGCTTGTGCACGGCC
GGATGGAAAAACATCAAGTGCGACCGGCCGTGTGATCTGAACCACTTCGGACAGGACTGCGCCAAGGTGT
GCGACTGCCACAATAACGCCGCCTGCAATCCGCAGAACGGAAGCTGCACCTGTGCCGCCGGCTGGACGGG
CGAGCGGTGCGAAAGGAAGTGCGATACCGGCAAATTCGGACACGACTGCGCCCAGAAGTGCCAGTGCGAC
TTCAACAACAGCCTGGCCTGCGATGCCACCAATGGAAGATGCGTGTGCAAGCAGGATTGGGGAGG
>dme_circ_0005001_junction_seq
GATGCGTGTGCAAGCAGGATTGGGGAGGATTGCCCACCCGGCTGGTATGGCCGGAA
>dme_circ_0005002
TTGAATCCGGTTTAATCTCGGCTTGAGTCATCCTTTAGTTTATACTGACTGTGTTTATCAGTTCAATGTG
ATCCGCCAGTGAAGCTGCATCAGAACGAGTAACTCATTCCAGGACCTGGAATCCACTGCCACCATGTTGC
CGGTAATCCTCATAGCCTGCCTGGCCCAGCTGGTACTGGCTCAGGCGGATCTTAAAGATCTGGATGGACC
CAATATCTGCAAAAGAAGAGAACTGATATAACGTAGACGTTGTCTACACAGAACTGCAATCGTTCCAGGA
ACGGGGCTCCACCTGGTGCGTCACATTCCCGCCGAGGTGCTCCACCTATCGCATTAAACACCGAGTGGTC
AACAAAACCAAGACGATTGCAAAGAACCGTATTGTGAGGGACTGTTGTGGATGGCTACATCGCGAGCGCA
GGAGAATGCGTGCCGCATTGTTCGGAACCCTGCCAGCACGGTAGATGCATCTCCCCGGAGAAGTGCAAGT
GTGATCATGGTTACGGAGGACCCGCCTGCGATATAAG
>dme_circ_0005002_junction_seq
GTTACGGAGGACCCGCCTGCGATATAAGTTGAATCCGGTTTAATCTCGGCTTGAGT
>dme_circ_0005003
GGTTGGCCACATATTGCGACAGTTTGTCCGTGGATATGCGCTTGATGACCTCCGAATTGCTGTCCCTAAA
CTGAATACCGCCAAACTCCTCAATCGAGGTGGGAGCATTTTCCGTAGAAACAGCCGGAGGTGGCGATAGT
TGATAGATGGTGGTCTTGAGCATATAGGCCGCATTGTTGCAGTAGTTCCAGAAGGA
>dme_circ_0005003_junction_seq
CCGCATTGTTGCAGTAGTTCCAGAAGGAGGTTGGCCACATATTGCGACAGTTTGTC
>dme_circ_0005004
GGGCGATACGACGAAGGCATTCCTTTGCAGCCACCAAGCATTCACGTGTTTTTGTTGTTGTTTTTATTAT
TACTGAGGTCTTTTCGAAGGTGTTGTGTCGTAATTTGCAGTTGTCGTTTTTGCTTTTTGCCATTATTAGT
TGTTGCTCATATTGCTCATTTTTGAATTCGTTTGTTTTCTCGTTACAATT
>dme_circ_0005004_junction_seq
TGAATTCGTTTGTTTTCTCGTTACAATTGGGCGATACGACGAAGGCATTCCTTTGC
>dme_circ_0005005
ATCCAGGGCGGCTACCATGACATGGACCACGATGCGGTGTCCAAGAACTTCTCTATATCGGCTTCCGCAC
ACAACATCACCACGGCCAGCAATGCCAAGCTCCTGCCCGCCGTTGAGGATGAGTCCAACAAGCAGGGTGA
GTGGAGCAGACAGGTGGGGTAAAGCAGCGTGCTTAAATGATTGTGATTTATTAAAAATGTAATTCGGCTC
TACGATTGAGTAAGTAACATTTAAGTAAGTAAAAGACATAACTTTAATTTAGCCAAGTGAAAGTTTATAT
TCCTATTGATAGTAGAGAATATCTTTTTCTTGTATTAAGCATCTTTATAATGTTTTAAATAATTGATATA
GCGAATGCTTACTAGACCTTTTGCAAAACTATAAAATGTTAATGATTTTATTTCTTTCAAGTGGAGGAGT
TTGTAAAGGAATCTTCATAGAATATATTCCACATGCCTTTTATTTCGTTGCCAAGCGTAAAAAATGTATG
TTAATTAATTAGCGTTTTTGAGATAAATTTAGCATTCCGATTGATCGAACTTTCAAAAAGTAGTTCATCT
TAGAAGCCGGAGCTCGTGTATGCAATTTATTTTGGCTTCCAGTCAAGTGGTGGTCTTACCTGTTTTGGCA
ATTTAGAAGTTATCAAGATCTCCCCAAGTTGAACCTCTTTTGTTCAATGCTACGGATTAGCCAGAAGCGA
TTGGAATCATCTTCCTCTTCAGATCTTTTGAGAATAAATAAGAATGAATCTGAATAAGAATTATGTCGAG
GTAATTCTCAGCTAACTGCTGCTTATCATCAGGTGAGACGAGTCTTTTGAATGAAGCTCCGGTGATTCCG

TTTATCGATTACTCACGAGTCGCTATAAATTTTATTTCTCTGCTTAATAATGCTGAAAATATTCGAAATG
AATATGTGTACGTGTCTCAGGCCCCAAGATAATATTTATATTATTGTTTGCTGAGCTCCTATCGATTGAT
TTGTCTAAACCAATGCTATTGGTTCTTAACCACATTTAAGATATGTTTAAATACCTCCGCCATAAAATAA
ATGCATCTTATTAACAAAAATTGTAGAGGACCTAATCTGCAATTTAATGATTTTGCGTACAGTTCTACTT
GTATTATATTTTTCTGCGCGTTTGAAGACTGTTTCTACTCACTGTTATTCCTTCTAGCGTTGAAACGCGT
TAATTTTCGCGCGATTTGTTTGCTCTCCTCTTTTGTTTGCTAGTTTACGATTTGAGGTGTTTAAATAGTT
TTCAAGATGAAGATTACCCAAAGATTACACAAACTCGGTTGCAGCAGAGGGCGTGGCGACGGCAACCTTC
AAACACCTGAAGCTCTTTTGTCGATCGAACCAGGCGGGGAATGCAGGTATTCAACACAGCCCATCCTTGT
GTCTTGGTCTAATAGTCATTGTTTATAGGGTTGTTCGGTGCGGCGGCATCATAAACACGATCATAAAACT
AGTTTCGGCATCTGTGAGCTCGTATTTGTCTCCTATTTGGGTTAGTAAGGCAGCGGGCACCGGACTGGAT
CGGATCGGAATGGACCGGATAGGGCCCAGCTGGTCGCTTGGTCATTGTCTTTCGCATCCACACACCTCTC
CTATTGTTTCCCGCAATTTTCACCCATTCAAGCAACGTTCGCCTGCCGATtgcaagtggaagtgcaagtg
gcaagtgcaaTATGAATTGCGTCGAACAGATGCTAATTGGCCTTAACTAGCTGCACACATACACACACAA
ACATGTCGTCCCAAGTGCGTTCAGCTAATTTAATAACACAAATTAATCAACAGTTTTGAAAGTATTAAAT
TATGTGGGATTAGTCATTGAGTTAGACATTTACAATGTTCATCTTATTGAATTAAAACGGATCAGCAGCA
ATTAAGTTATATTAAGCCATCCTTATTGTGTAAAATATTTACTTAATTACAGAttttttttATGCATATT
GTGACATACTCAATATGTTTTACATATTTATGACAGACCTTCTCAAACAGAATATTTTACATTAATTGCA
CATTACCGTTTCTACACAACACATTTTTATATTTGACACAAGTCAAATAAATAATTTTCAGCCTTAAGTT
ATGCTTTAATTTTGTATTGAAAGCCAATGGTAACTTAAGTTTGGTAAATGAGTTTGTTGCCTAAGTCTTT
TGTTTTGTTGTGATTAAAAGAATTATTAAAATTAATTTTACAATAACAAATGGCTTTAAAGGAACATTAA
AAAATTCTATTAAATTGCAAATCATGATTATAGTAGAATTTCAAAACTTTTTCTTAAATTTTGATTAGTT
TAAATTTACATAAAGATTAAGTAGTAAAACAATTTCCTTTATTGCTTTCCTGCTTATCAAAGTGATTTAA
AACAATTACAAGGAACTAATTTATCTGTTTCCTTATTGTTTAGGCATAATAATTAAATTAAAGAAATCAT
TACTCACTTAAAAGCTTACTATTGAGATAATTAAACAATTTTCTTCCGAAATCCTGCAATCAAATGAACA
AGTTATTGACCAATTTCATGGTCTCGCAGTTTAAACTGTATCTGCAACTGGGCTCGGCTAATTCCATGTC
TGCCGGCAGTTCGCATATTTCTTTGCTTATTTTTACTTTTAACTTGGGACTTTTTGAATTTGCAATTTGC
TTACCACAATCGATGGCCCCCCGTCTCTGGACACTTCCCTTCCATTACTTTTGCGGCTCATGCGAGTGGC
CAAAACCCAAGACCCAAAAGCGAAAACCTCTGCCCATATGCATGTTTCTCGGGCTGTTCAGTTACTTCAT
CTCTTATGTTATTTCGTTTCCAATTCGCTGATTAACAAGTTCAACAAAACACAGTGGCAGCCAGTACAAT
CTTGCAGATACTTTCGGTGCAAATTCGGCGGTGGCAAGACTCACGTTTCGGCCATAAAGAGAAATGAAAA
TAAGAAATGACGTCAGTGGCAAACTGGTTCGATGCAATACACTAATTGCGTGGGGCTATTAGAATTGCAT
CGATAAATCGGGAAAAGTGTATCCAGTGAAAGTGGACATCGGTGGATAATCGGACAGAACTCCCTTGGGA
TTTACGATATACAATCCCAATCCACGGTACACTGCTCTACCGGGTTTGGGTGTAACCGAGACCATTTGAC
GAGTTAACGATATCACACACAGCTGCATGGCTTCATGACTTCTCCTTTCACTGGCCACTGATTAGAGGCC
CTGAGGCGCAATCCAAGTCGTAAAACAATAGCCCAGATGCGAGTACCATAACTAAAACTCCCCGGGTACA
CTTGTCCATGCTTCAGTGCCCAGATCCGTCCAGTCAAAGAGATTGTCGAGGCATCGCTTATTCAACATCA
AATACTGCTCACAATAGCCTTATTTCGGACACCTTTTGTGACCAGCCCCATTGTGGGGACATTTTCACAC
TCCAGTTTCATGTGACACTAGACTGAAGGTAATTATTGTGCAGCCTACAAGACCTTTCCATTCTGATACA
ATCGCGGAATCAATTCTCGATAGAAGAAACCCGGAGAAACCTTGGCATGTATTTATGGGTTAAGTTTCTC
AATTAATTGGCTCCAGCCCACGCTTTTACTTCTGGCTTTTTGCTTTTGGCTTTTCATATTTCATATATTT
ATAATACCTCTTTAAAGCTCATTCGGTATGCCAGGCACAGGCCAACCGTCAAAAGGCCaaaaaaaaGAGC
CATTATTTATGCTAAAAGTGTTTAAGTTCTTGATGTCATCATCGTTGAGAAAGTCAACGCAAATCGATTC
GATTTTATTAGCATTTTAACGGACTTCCGCTTGATATGGCAGAACTTTGATGTCAAATATAAATTCTAGC
TAGTAATAATATTTGTATTTCCCATTAGCGCCCATTCAATCCACCTATTTCGCTCGGCGCTACAGCGATA

ATTTTTTCTCATTCCCTCCttttttattttttGATCACACAGTGAGTGCTGTTAATTACGCGACCAGTGC
CAGTTTCGCTCGCATGCTTACATAATAATTAGAAATTGCCACGCTCTTTTCTCTTCAGCGCCAAAGAACG
TGTTAAAATAAAATTTAATATAAGTATGAGAAGCGGCTCAAAGAGCCTAGACTAATAGACGGTTTCTTCT
CTTTGCGGTGGCCATTTCCTGCTGTGCAGAAGACAGGTTAATTATGCAGATGGATTTCGAGACATGGGAA
CGCGACCTGAGGGCATCATTAagcaatagcaatagcaatagAATTTCATTCAGCCAGCATCTTTGTGTTC
CCTGATCTTTTTTTCAATTAACCCACGTGGGCGAGCTTAGAATGGAGAAGAGCTTTAATAGGCCTGTGAC
TATTAGGTACTCTGAGAAAAGTTAGGTGCACTTGGAAAAAATATTTGCATTTATAAATTTGAAATAACGA
ATCGTTTAAAGCTTGGAATCGGAAATAAATATATCATTTTCCTTGTAAAGTTTAAAGTGTTATATATATA
ATTTCGTGTGGACTAAAAATCTATATTTGGTTGCAAGCTATTTTTTCTAGTGCTGAAAGAAGCATGCATA
AATTTCCAAAGAAACCCAGTGAGCCGTGCGGCTGGCTCGgcgttttcagcgttttcagcAGATGCGCGGA
GGCGTGCGCTAACTTTGCGCAGCCACTGTGCATAGAAAGCCACGCTTTGGTGCCGTCGCTGCTTGGAGCC
GCTGCTGCTGGTGGTGCTGTTTTATTTTGCTGCCGCCGGCACTACGTCAGTCGTCAGTTCTGCATTAATG
AGAGAGCAGTTAGGTACGCTCGCTCGCACGCTCGGTGTGGTCCAAAAACAACATACGAGCTACAGAGTTT
CATCCAGATGAAAACCCAGCCGGAGGAAAATCGGGCAGAGAGAGATCCCAAATAAAAGTTTGCCTATTGG
GGGGAAAACTTGTTTGGCCATCTCTCGGTCGCCTCTTTCTCCTCGGGCACTCCAATTCGATTTCCCCCCC
AATCAAAATCGTCGTCTGCGCAGTCGCAACTCGACGCTGCAGCGGTGATGAGTGGTGCATACGCTCAGCC
aaaaggcttcgaaaagcgaaaaaacatatagtaaaaggaaaaataaaagcagcttagcgaattgaaaaaa
ggaaacgCAGTGCAATCGGGGAAAAATGCAAAAGTGTGAAGTGAATAATCGCTAATGGCCAAAGCGTTCT
GTGCAATGAAATGTAACTAAATTCCAAGCAATCCCGCGACCACAAGCCACTTACATATTGTTTATAGAAA
AGGAGCTCGCGCACTGTAATTCCATGAAAAGTGTCACTGATATGCGTTGGCGATTTGTGTAAAATGCGaa
aaaaaataaagaatacaaataccttatgtgatataaaaacaaaacGCGACGGCGAGACATTCAATAAGCG
GTTCAGGGCCAGGCTCTGAAGGTGTTGCCAATTTATTTTAAAAGTCTGCGCTTTTTGTTGGCTCGCTGTC
GTCACGTGATAACTTGTTCCACTTGCATAAAAATATTGTTATTTTCTTGACACAAATGCCCCGGACCGAG
GCTTTAATTAGCATGATGTTTAAAAAATAAATACGAAATGCTTAAAATACACACGAGTGCCCGCGCTAAT
TGTAATTTATTTATTTTCGAGTGCAGTGCAATTAAAATTTCAGCTCGAAGGGAAACACAGCTCACAGAAC
CTCGCAATAAAATCCAAAGCATAACAATACAAAACCTGATTTGTATGCACAAACAAACAAATGCAAGAAA
ATGACAAGTTCTTCGACACCTACAAGCAATACAAAGTCTGTCCCAAGATGGAGATACCAACGACAAAGTA
TTTAGCCGACTATCTGTACGTTGTGCACGAGGACGTACCCTTCCGATACTATCGCAAAGTGAGTGCTAGG
GCGGGGGTTTGCGGGGGCCATCATCATGCatgatatggcatacgatatatgatatgttatgatgcgatac
aatgatgatatgctatgCCCATGGAGCGCTCGTCATCGGCACGATCATAAACTCTAAACGCCGCTTGGCT
GGTCGAttttttttttGTTTACATTTTCACCGTGCGGTTTTCGCTTTATTAGCCGCCTTCACCTGGGCGT
CACGTTGTTACCGCTCTGCCCCTTCGGATGCTGAGTTTGCCCTGTTGGCAGTTCGTTTGCCTAACTAAAC
GAATTTCACTTAGATGCGCTGGGCATACCCAGTGGCATTAAATTAGGGTCTGGAAGTCGGATGAGCCTTA
AGAAGCCTTTCAATTTTACaaaaaaaaaaaaaaaTATGTTTGTGAGCTTACTTCTGCGTGGAACATAAAT
TTACTATCTTACAGCTTTGCGGTTTTCACAATAATACTCGTTTTAGGTGCTTGGATCTACGTTAATAAAT
TGTCATTTGTTATCTTATTAATTAGTCATTCAGTTATATTGATTTAATTAAATTAAAATCTTTTTAAAAA
TGCGAATGCTTAATATGTTGTTTACTTATGTTCTATTATGAATTCAAATGTATTATCACTATTTTGATGA
TTTATAAAAAATATTTCAATACTAGAAATTAAAATTATTGCCAAATCCAAGGTATTCTTTGGTTGCTACG
CTGTATTTTCCGCCTCTTGTGCTTGGAAAGTGAGCAAAGTGAGGAGCGTTGTTAGTTCGCTGATGTTTTT
GCACGGGCACATCTAATGGGCTTATGCTGTACATTTCGCAGATGACTTATTAGAGCCATGAGCGAATTTT
GGCCGAAGCTTAATAAGATCCCCCGTGACACCCTGCTGCGGTGGGCACTTTCTTTTGCCGCTCACTGTGC
GAACACAAAGAACTAAGCCAGTTACGATGGCTGTGGATGGGTGGGGAAAATTCGAGGAATATGAGTCGTA
AGACAAATAAACAGAAAGCAAAATTGTGTAATTAATGGAAATAAAGCGAAGCACGGCGAAATTACACAAG
TCGAAAGCGGAAGTACCGGAAATGACAGAGACGCTCCGTAGGAGTATCTTTTTGTTTTTGCTTGATGTCG

GCGGATGCGGCAACTGTGACTGTGACTACCGCTATTTGTCCGGGTTACCGGCAAAAGAAAATCCAAATGG
GTTTGCATATGACAGACATTTGTAGATTTGGGCTTTCTCATCAGCTGCGAAAATTTTTCGGACATGGTTC
ATTGCTAACTTTTACTTTCTTCGAAGGGAGTTCAGGGTTAATTGCATTCACGAAAATTAGTGGAGTCTTT
GAACCTTAGAAAAGCTTTTTACAATAAGTTGTGCTAACTCATAATTCAAATTTTAGTTATTACGTGTTCA
GCTGTGCTCAATTTCATTTAAAACTAAAATTTAGCTATGGTATATGGTAAGCATATTTTGTACAGGGAAA
CAGAGCATGTCGCAATACATAACATCAGATTTATATTCAAATATAACTTAGTGGGTGACGTTCCACTTTA
GGTGGGTTTAGTTAATGGGTCCACTTTAGGCCAGGTTTATGCCTATTCCATGTCGTCTAAACTAATTTCC
ACACTCGTCCCATTTCTTTTCATTGCAGAACGAAATGCTCGGTGTTCCGCGGGCTCGTGCTCTGTCTGTG
CCTGAACCTGAGCTATGCGAACGTGGTCCGCTTCCCACGCGAGTTGGACCGCTACGGGTCGGCGTACCTG
GTTCCGTACGTGGTGTTGCTG
>dme_circ_0005005_junction_seq
GTACCTGGTTCCGTACGTGGTGTTGCTGATCCAGGGCGGCTACCATGACATGGACC
>dme_circ_0005006
GTACTGAGTCACATACGCTTTTAGCGCATCGTTATAACACTTCTTGGCCTCCTTTTTCAGGGCGTCCAGA
CCAGGAACCTTGAGCTGGGCTAACAAAGCGTACATGTGGTGGTAGTTCTCCATGCGCACTACCTGAACTG
GT
>dme_circ_0005006_junction_seq
GTTCTCCATGCGCACTACCTGAACTGGTGTACTGAGTCACATACGCTTTTAGCGCA
>dme_circ_0005007
CTTCGTAATTCTCGGGAGGATCTGGACATGTCTACGTCTTCAAGAAAGTCCGCAGTTTCCACAATACCTT
ATGGCACACTGGGAATCAACAGGGATCAGTGGGGTCCCGGTGTAGAGACAGCGGATCGAATGCGTTTTGA
TGCATTGTTGGAAAAGG
>dme_circ_0005007_junction_seq
ATGCGTTTTGATGCATTGTTGGAAAAGGCTTCGTAATTCTCGGGAGGATCTGGACA
>dme_circ_0005008
GGCATCTCGCTGAAGGACTTCGAACAGCATCGCCGCATGATCGAGGAGCAGAACAAGCAGAAGAAGCAAA
TGCTGTACCAGGCCATCGAGCAGCACACCCAGAAGACGGCGGCGGAGTCGCGAAAGATCGAGGAGATCCG
CCACGAGCTGTCCAAGCTGGAAAGTGATCTGGCCGTGGACGTAGCTCTGCTGAGGAAGCAGATCGACAAT
GCCTGCATACATTTTGCCAATG
>dme_circ_0005008_junction_seq
GACAATGCCTGCATACATTTTGCCAATGGGCATCTCGCTGAAGGACTTCGAACAGC
>dme_circ_0005009
GAAATATCAGTGGCAGCGGAAAGATCTTGGCGAAGGTATTCCTCTGCAGAGGGGGAAAGTTCACCAATTT
CAGGCGCTTGCCCATGCCCAGGACCACAATGCTAGCAGTAAGTTGCCCGAGGCTGAGGAACAGGAACGAG
GGGAAGTGGTAGGAGGTAAGCACCGTCTTGTTTACCACCGTAATCATGAAGGAGGACAAGCCATAGAACA
AGGCGCTCCCGATCTTCTTGACGAACAACGCCGAGTCCTCGCGTTCCCGGTGACTCAGCTCCTTCGAACC
GGATCCATCGAGCAGCGATGACCGATCCGCCGATCCGCCCATCTTCTCCTCCAGCTCCCTGTTTCCGACA
TCGCTCTCCGCCAGGAGCGGCTGCAAGTCCAGAGTTGTGTTGCCGCCGCGCGACATACTCATCGTATTTG
CGGGAGTGCTGGTAGTTGAATTAAATCACTTTCGTCACTATCGTTTGTTCGGCGGCGACTATCTTGCGGC
GCGCACATTTGCTCACTTTCTTCGGGGCCACTTTGGGGCCGGGGAGGAAGCGTTCCCACAGCACATTAAC
GA
>dme_circ_0005009_junction_seq
AGGAAGCGTTCCCACAGCACATTAACGAGAAATATCAGTGGCAGCGGAAAGATCTT
>dme_circ_0005010

GATGTAGGCCAGCAGGAAGACCACCATGAAGACGTGCGTGGACGCGAATCTTCTGGCCCACCTGGGCCGA
CAGCCGAAAAGGCCGCAACTGGAGCTGGATTCCGCGGAGGAGCCGCCATTGAGGTCGGAATCGAAATCGG
ATTCCTCGGACTCACTGGAGCGAGGTGGCAACTTGTAACCTCCGGTCAGGGGAGCCTGTTCATGGGCATA
CTTTTCGCTCAGACTGTGCCCATTGCCGGTGCCATTATTATTACTGCTCAGTATGTTGCCCAGGCCATTC
TTCAGGTGTCCCAGATCCTCCGGGGCCTTCCACTCG
>dme_circ_0005010_junction_seq
TCCCAGATCCTCCGGGGCCTTCCACTCGGATGTAGGCCAGCAGGAAGACCACCATG
>dme_circ_0005011
GTCCCGCCTTGATCGAGCCCACGTGCAGTGCCGCTCTCAACTGTACCTGTGATAAGGAGAACTTCGCGCC
CATCTGCGCCGACGGCAAAATGTACATCTCGGCCTGCCACGCCGGATGCAGCAGCTCCTCACTGCGGCCC
AGCGACAATCGCACACTCTACTCCGATTGTGCTTGCATTCCAGATGGCTCCGGAGGCGGTCAACGGTTAC
TGTGATAATAACTGCAAGAACTTCATCTACTTTATACTGATCTTTGCCATTTGCGTATTTATGCATTCCA
CCTCCGAGGTGGGCAGCATGCTGCTCGTCATGCGCTGCACACACCCCAAAGGATAAGGCCATGGCCATGG
GTGTGATACAGTCGGCCATCGGCCTGTTCGGCAACGTTCCCTGTCCCATCATCTACGGCGCAGTGGTGGA
CTCCGCCTGCCTCATCTGGAAGTCGGTGTGCGGCAAGCACGGCGCCTGTTCACTCTACGATGCGGACACT
TTCCGGCAATATTTCCTAG
>dme_circ_0005011_junction_seq
GCGGACACTTTCCGGCAATATTTCCTAGGTCCCGCCTTGATCGAGCCCACGTGCAG
>dme_circ_0005012
AGAACGCTTTGGGGTTAcaaatcaaatcaaatcatatcatatcaaatgaaatcaaatcagagcaGTGATG
GAGTGTGTCAGAGTTAAATATGGTGGAGGCTTACCCCAAAGCGGGACGACAGCGATGCACAACACACGTT
GACTTCGACTGGGTGAACGTGATCGTTAGCTGAGAAGAGTACATGG
>dme_circ_0005012_junction_seq
GTGATCGTTAGCTGAGAAGAGTACATGGAGAACGCTTTGGGGTTAcaaatcaaatc
>dme_circ_0005013
AGAGGTGCGTGGTGCGTAGTTAGTTAGTGGCGAAATTGCGTATTAGAAACGAAAACAACAAGCAGCTCTG
CGCGGGCCAGCAAACATTTCGAACAACCATTAGCCAGGGCAGAACAAGCCGATCCAACGTGTTGTCGCAT
TCGCAAAGGGAG
>dme_circ_0005013_junction_seq
CAACGTGTTGTCGCATTCGCAAAGGGAGAGAGGTGCGTGGTGCGTAGTTAGTTAGT
>dme_circ_0005014
GCAGCGCCAAATCCCGCATCAAGGGCACACTGCGCATCTACCACGCCTTTATCCGGGAGACGCGGGAGCA
GAGCGAGCCATCTAGTGGCAATAGCGATGGCGAGTGGGAGCACGTTGAGGCCACCAATGCCGGTGAGACG
TCTGCCCAGCCGTGTAAGTTGGGCGTGCAGATATGAGTTTAAATCAGAAAGACTGTTCCATAAATTTCAC
TGATGAATACATATTTGAGTAAGCCATTTTCTGATTTCGGTAAACATTAACAGCAACTTGATTTTTGTAC
GCTTAATATTAAATTTTATCTAGTTTCGTTAAATTTAAAGAATAAGATAAACATGCGAAAATGCTTCTCA
GAAGAAATGAGGAACAGCTTTTTACAATGTAAATAATAATTTATTTATAACCCTGAAAATGTATAATCCT
TACTCAGCTTAAACAGGGTAAGTGGAAAAACTTGGATCTGTATTCCCTAGGGTGCACGTTTAAGTTTAAG
TTGGTTAACCACAGGCAAATTTGTAAGTTTCGatatatatatatatatTTAATCAAAGACGTATAACTAT
TTATTATGCATGTGTGATGAGCATTTGATTTGATTGATTTTTGTGCATGATCTCTTAAACTTCCGCATCG
AACAAAAACCCATTACACAATGCCCACACAAAACGCACTCGAACCGACTAACCCAATAACAAAACACCAC
AAACCTCAGAGGCCAAGGAACGCTGCTCACATGGAGACTGTGGCCAGCGATGAGAGCAGCGAGGATGAGG
ATGTGGCTGAGAATGGCGAGGAGATGGTGGATCTTCAGAATAATCAGGCCAATTGTCTGCACTGCGGCGA
GCTGCATGACAATGACGAGGCAGAGGAGTATGTTGATGAGGAGGAGTATAATGCAGAGCTGCAGCCTCCA

ACACCTGACCCAGATCAGCCGTCAGCCACATCCCAAAGCGCGCAAACATGTGATATACTCGATAGGAACT
TGTACTTGAAACGCATATTCGTAATGATATATAttttttttAACTACAGATTAAGTGTATCGCTAGTTTC
TAGATAAGATCTTAGTAGTATGACATCAATATTGGGAGCCAGTTATAGGTTTCTAATCCTAAGATGCCCA
CTGCCGTTGATCGTTGTGTCTTTGCCTTCACCGCCTTGTTTGAAACCCCACTTAGAATTTTAGATGTGTA
GACGTCTAGAGCCCTTCAATAATAATCCCCTGTATATACGTAGGACTCCATCAGTCACAATAGTATAGAG
GACAATAACAATGCTGCTGGCTTAGCATATACTCCAAAAACAGCAGCCACCTCATCGGCACCACCCAACA
CCCCAACAAACAACAATGGAATTCTCGCACAAATTGCCATGCAATATCGCGCAGAGGAAGATCAAGATCC
AACAGTGGACCATACCAGGTTAATATCATCCGCTATGCGCTAGCAGCACCCATCCAAAAGATCCCTCAGC
CTTTTGTCGGTATTTTGTCCGTACCCGAACAATCCTCCCAACCCCCAAGGCTCCTTCACTTCGCAGCCAG
CTTCCTAACCGCCATACGTTGTGCTCTATCTTTCATGACCTCAGTTTTGTTTACAATTCCCTGCGACATC
CTGTCGCCCATAGGCAGCCCGAAATCTCAGCGACCAGTTTGCAGAACGACCTGCGGCCGGTACGGGAGGC
TCCCGGTGTGCCGGATATAGCCATAACCAACCCGTTCACGCGGCGGGCCGCGGGCAATATGGCCGGCGGT
GCGGGATGGCAGCAAGAACGGCGGCGACAGCAAATGCAACTGCATATCCAGCAGCATCAGCAGCGGCAAC
AGCAACAACAGCAGAATAGAATATTG
>dme_circ_0005014_junction_seq
ACAGCAACAACAGCAGAATAGAATATTGGCAGCGCCAAATCCCGCATCAAGGGCAC
>dme_circ_0005015
GATTTTCGACGAGCACGAACTGGAGCTACTGATGTGTGGCATACAGAACATCGATGTGAAGGATTGGCGC
GAGAATACTCTGTACAAGGGCGACTACCACATGAATCATATTATTATTCAGTGGTTCTGGAGGGCTGTAC
TTTCCTTCTCCAACGAGATGCGTTCTCGTCTGCTGCAATTT
>dme_circ_0005015_junction_seq
CGAGATGCGTTCTCGTCTGCTGCAATTTGATTTTCGACGAGCACGAACTGGAGCTA
>dme_circ_0005016
GTATGTGCCCTTGGAAAGTTATTAGGAGTGCCCCACTTTTCGATAGTAAACATCTGGGGGCCATTTGAGC
CGTACAGCTCCTTGAAACCGTTCATGGGCACGCGTGAAGTGCCCGTAACAAATTGCAGCAGACGAGAACG
CATCTCGTTGGAGAAGGAAAGTACAGCCCTCCAGAACCACTGAATAATAATATGATTCATGTGGTAGTCG
CCCTTGTACAGAGTATTCTCGCGCCAATCCTTCACATCGATGTTCTGTATGCCACACATCAG
>dme_circ_0005016_junction_seq
CATCGATGTTCTGTATGCCACACATCAGGTATGTGCCCTTGGAAAGTTATTAGGAG
>dme_circ_0005017
GAGGCGGATGGATGTGGGACCGGCTGCTGCTGCGGCTGCTGGGGCTTGGAGCCCGAAGTCATCTTGTGGC
TGCCGTGAGGTGGCATGCCCACGCCTACCGGCGGCATGCCGCGAGAGGATTTGTGATAGTCCACGGGCAT
AGACG
>dme_circ_0005017_junction_seq
GATTTGTGATAGTCCACGGGCATAGACGGAGGCGGATGGATGTGGGACCGGCTGCT
>dme_circ_0005018
ACACCGAAATTCCTATTGTCAAGCAATCGACAAGTCCGgcaccgcagcagcaactgcagcagcagcacca
cctgcaacagcagcaacagcagcagccgcaCAACGGCTCCACCTTCGCCGGCGCCACCGCTCTGCTCCAC
ATAAAGACGGAGCAAAATACGCTGCTCACTCCGCTCCAGCTGCAGCAGCAGCAACAACAGCAGCAGGGTC
TACACGGTGCGGCAGGCAACGGAGGCAGCAGCAACGGCAACAATGCCCACCAGCAGCAACAGCCGCTGGC
CATCCCACAACGCCCGCTGCTCCACAATCTGCTTAGCGGCGGTGCCATCCACAATCCGCACCACAGAAAC
TACACGACGGCCACAACAGGGTGATTATACACAAGGCAAATTAATGTCACATTCTAAAAGCAACTTGATC
TTAAAGGATATGTGCAACAATTTGTTAAAATTGATTCAAAAACTTTTTGATTTTGATTTGAGAACTTTTT
AAAATTTTAGAAACTACAAATGTAATGTTTACTTACATATGTACAAAGTTGTAACCCTTAATATGCACCA

ATCTTTGAACTAACTTTGCTACTTTAAATTAATCTCGAAAGTCTTTATTAAAGTAACGCTGCATTTAAAA
TGACATTTAATAGATAACTTAACGTCCGTATAGTTTCGAATGCATTTGTTATAACAATGCCAGTTTCAAG
TTGAAAGAGATCTTGTTTTTTCGAGTGCATTCAGTAGTGAGGCGAGCGTGTCCAGCAGCAAGGTGAACCA
CTGCACACGCCCTCGACTCGATTCGAGCCATATCGATTGCTAATTGCCCCTTTTCTTGGCTCGTCCTCGC
TCCGCGCGGCTCTATCCCGTTCGCAGGGTTCATTTCCGCCCAGTCCCGCGGACAGCGGCGTCTCCGACGT
GGACAGCTCCAGTTCCGGCGGCCAGCCCTGCGCCGACGAGCTGAAGGCGCGTCTCGGGATGCCACCAGCC
ACCTcggcgtcggcagcggccgcggcagcagcggcggcggcggcagcggcacacctgcacacgggcaccT
TTCTGCATCCGAATTTATACCAGAACAATGCGGCAAATTCGCTGCGGAACATATGGAATCGCAGTGTTGG
GGG
>dme_circ_0005018_junction_seq
GGAACATATGGAATCGCAGTGTTGGGGGACACCGAAATTCCTATTGTCAAGCAATC
>dme_circ_0005019
AGGCCGCACAGCAAGCCGACATAGGGCGACATTGGCTGATTGACAATTCGGCTGATTGGGGCAATCGGAA
ATTGGGAGCCATTTCCACGGAATCCACTTGTCTTCGGGGCACCGCCCACCGACGCCCACCGCCGCCCGCC
CCCAAATGCCCCAGCGCACCTTCAAACACCATAAATGCGTGCCAAAAGCAGCAAACTGGGAACTAATGCA
AATTTGGTCAATGCCATCGATATCGATGCTAATGGCAATAAAACTTTATACATCTATCGCATTCGCTGTA
AGATAAAATCGGCCAAGTCGGCAAACTGTGAGACTGCTTCCCCGAACTCCATCATTTACCTGTGACAAGT
GCGGCGGCCACAAAAGACCAAAATAATTAACTAAACTATATAAAATGCGCCCGAGTGCGCGGCCAAGCAA
ATACAACAACATCGAGTGGCACGTCAATGTGCAACAAGTGAAGCAACCCAGAAATGCAGAAAAACACTTT
TAGATAACTTTACACTGCCTTACGGTTAACATAAATCGTTAATATTAACTTACAAATTACGCGTAGTTca
atatacaaactatacaacaagcaaaaaacaatcaaaatttatatatttacaacaacaacGAAACCTTTTA
CAAATAAGTGaaaaaacaagaaaaaaaCATTCAGCAACGTCGAGGATCGTCAGCGAACAACGACAACAAA
AAGCGAAAGG
>dme_circ_0005019_junction_seq
GCGAACAACGACAACAAAAAGCGAAAGGAGGCCGCACAGCAAGCCGACATAGGGCG
>dme_circ_0005020
GCCGTTGCCGAAAAGCTCTGGCCAAGAACACGCAACGTACTGGACACCATTTCGGGCAGACCGTTGGGAA
ACAGGAATCCTCCTGCAACACCCAGCAAGGTCATCAGCAGCAACGGGTTAAAGAACAGTGCAATTAGGGT
GTTGAAAACCAGTATAGACCGTTCACCCAGGCAGCGATTCCGTTTGGACATCTGTTCCGCGGGACAAG
>dme_circ_0005020_junction_seq
CGTTTGGACATCTGTTCCGCGGGACAAGGCCGTTGCCGAAAAGCTCTGGCCAAGAA
>dme_circ_0005021
GTTTATTTTGAATGTAATTTTGACTGCTGTGATTGTACGCCAAACAAGAACGCACACACAATGCAGTCAG
TTGTGTGTGGGTAACAGTAATGGCGAGTGGGAGTGGGAGGATGAGCTAGTGAATTT
>dme_circ_0005021_junction_seq
GGGAGTGGGAGGATGAGCTAGTGAATTTGTTTATTTTGAATGTAATTTTGACTGCT
>dme_circ_0005022
GCTCCGGCTGCCGAGGGCGCAGCCGAGGAAACCACCGAAGGCGCTGAGGGTGAGCTGTACGATCCATCGG
AAGCCACTGGCGATGACGAGGACGTGGAGATGGTGGATGATAATGCTGAGGGCGAGGGCGAAGGCGAAGG
TGACGAGGAAGCCGAGGCTGAGG
>dme_circ_0005022_junction_seq
GAAGGTGACGAGGAAGCCGAGGCTGAGGGCTCCGGCTGCCGAGGGCGCAGCCGAGG
>dme_circ_0005023
GGCGATGAGCTAAACGAGAGCCAAGAGGAAGAGGAAGTGGCACTGCCCGTGGACCCCGAGGACTGCATCC

TTGACTTCAACGACGGCGATGAGATCCCCAGCGAGGTGGATACCCGCCTACCTAAGTACAACTGGCAGCG
TGCTGTCGGTCCCGGTCTGATCTCCAAGCTGGAGTGCTATGAGTGCTCGGTGTGCAGCAAGTTCTTCGAC
ACCGAGGTGACCGCCGAGATTCACTCCCG
>dme_circ_0005023_junction_seq
CCGAGGTGACCGCCGAGATTCACTCCCGGGCGATGAGCTAAACGAGAGCCAAGAGG
>dme_circ_0005024
ACCCGCGATTTGCGCCTGCCCCAGAAGCGCAAGAAGAAGCCGGTCAAGAAGGAGGGCGAAGCAGCCGATG
GCGAGGCTAAGAAGGAGGGTGCCGGCGATGGCGAGGGTGCCGAAGGTGATGAAGCCGAGGGAGAAGAGGC
CAAGGAGGGTGAGGAAGCTGCTGACGAGACAAAGGAGGGCGATGAGCTAAACGAGAGCCAAGAGGAAGAG
GAAG
>dme_circ_0005024_junction_seq
CTAAACGAGAGCCAAGAGGAAGAGGAAGACCCGCGATTTGCGCCTGCCCCAGAAGC
>dme_circ_0005025
CGTAAAGGTCAACGGAAATCCGCAGAATCGTTTAGTGAATAACGCAAAGGTCAACGGCAACATGGCTTTC
CGTGGTAACCAGAACCGCAACCGCAATTTTGGAGGTGGCAACAACAACTATGGTGGACCCATGGGCGCCA
ACCGCATGGGCGGTATGAACATGTCGCCTTGGGAATCGCAGAATCCCGGCGGCGGACAGTTTGGTAACAA
CATGCGACAGGGTGGCGGTCAGATGAACGCCCAGGCCATCAACTTGGCCAACAATCTGCTGAACAATCTG
TTCAGGAACCAGAATCCACCATCGCTTCTCGACTTGCCCCGCGGCGGCGGTGGCATGGGAAATCGCAACC
AACGTGGTGGCCCGGGTATGTACAAAAGTTTACTTATTCTCGAATTAGTTGACAATGCCAACCGAATGAA
TTGTTATACTTACCTATGTTGTTATTATTTATGGGTTCGTGAACTGCGAATCGTATTTCTGTTGTGGAGT
GTCATATGTTGAAATAATTTAAAAAATTCTACTAAACATGTAGAATTCAATTCAAAGCACATAGTAATCT
TAATATTTTGTCTGTTTACTTTTGTTGTACTCAATATAAACTCAACTTAGGATGCCTAAATCGTATAAAC
ATTTTGTAATTACGTGTATACCTTTTGGTAATATACTTGCTCTCCACAGCAGAACCTTTTATTTTGTGTT
TTACCCTTAGTTAGATCGTAATATTGCTAAATGTAGCTAATGCTTTTCCTTTAAAAGATAAATCAAAATG
ATCTTAAACCTAATCTTAGCTTGTAGTTTACTAAACTAAACCGACGACTGAAAAATAACGAAAACCAGCG
AAAAGAATATTGAAAAAAGTAATAAGAACTTTGTGCAGTGTAGGATGTAAGGTTGTCAAACATTAGGATT
AATGTGGTATTAGGAGGATTTCGATAAAAACGCATTTATTCCATTTAAAATGCAATGCTTTTTTGTTTCG
TAGAAACTGCACAAACGAATAAGAACCTGATTATTACACCGAAAATAAGAAATGACAATTTTATAAAATA
ATACACACGTATTGATAGCGAAATGTACCAATTAAAACATGTACCGCCAACACTTGTTCCACTTAACGAT
GAAAAGAGTATGATGATGATCTCCATATGTCAAGATTGAATCATAAGTAATTTATATGCAATATTTTCTT
ACTAGTTGTACTTAAGCATTTAATTACATTTTTACCATATTTATAATTCGATTAATTAATTTCCGAAGTT
CCATTGCCAAGTAAAAGAAACTTTCTTGGCCGAACTCAATTTGCCACAACGCTTTTCTAATTTATCAATT
ATCTATTTTTATTCTCTCTTCCAAAACCAAACAATGCCAAACTCAAAAATTCGATGCTTTTCAATACACC
TCCAAATATCGAATATCGAAACCGCATCGTCTTGCATATTTCGAATAATGCATTCCATATTGTTGTTGCT
GCTCCGGCATATATACTTGCTGTTGTtgttgttgctgccgccgctgctgtcgttgtggttgttgttgtcg
ttgtcgctgttgcgcgttgtTGTTGCCGTCGATGATGACTCTTCCCATTTGCAATAATCCATTGCAATCA
ACAAAAATCTAATCCCCATTGACGGCCAACAATATTTTCGAATATCCAAATGAAATTGAATGCAAAACGC
AacgcacacgcactcaacacacacgcacacgcgatgcacacacacacgTTGTGGCCAATATGCGAATCTT
CACCAACGATGCGATACTGTAAACTGTACGATGCTTCCCGTGCGACGCCactgattgattgattgattga
ttgattgattgaccgattgccaaatcgattgacattgatcgCCATGCAATCTTCTAATCTGCTCTGCTCT
GAAACCGAAACTGATCCGATCTGGTACTGGTACTGCTTGATGGTGCAACATAATTACATCCAAAACCACA
ATATGAAATACCTATTAATATTTTATGTCTAATGCCAATTCATCGCCAAATTCTTCTTCGACGTTCCTGC
CTCTGAAACTCAAAATCCGATTTCCATTTTTAAATGCCACTCGCCGCCACACGCCAACGAATTAatatat
atatatgtttatgcaatatatatCAACCTATCCCCGTCGACTGATTGGCCGCCGCAACTGCCCCGTCGAT

CTGACCAAAATCAATATATATATACCATGTGCTCCATGTACTTATGTATACTTTACTATATATCAACGAT
TTATTCCCACAATTATCTATAATGTGTTCAATCTGATCGCTCCGCCCCTCACACAATATTGTAAATGGCC
AACCGCGCCATATTGTCTCCGATCTTTCGAAAATGCTTTGTATCATTAATGCCTCTGTTTGTAAATCAAA
ACGATATCAATTCACATATGCCCCATGTGCGCATTCCGAATTGTGCAAATATTTTAATGTCAATCCCAAA
TGATAAACGAAACAATGTATAGATGGTCAGTCGCGGCGGCGGTGCCGGCAATCGTCTCAATAACCGTCGT
GGCCAGGGAGGTGGCTTCCAAAATCGTGGCGCCACTGGTAGTGGACCCAAGCCCCCGCCAAAGCAGGGCG
GCGGCGGTATTCGCAAGCAAAATGCTTTTGATCGTGCCAAGAAACTTTTGGCTAAAAATGCCAACCAAAA
TAAAAAGAAGGAACCCACTCCTGGCGAAAAGAAAATCGAGAGG
>dme_circ_0005025_junction_seq
CACTCCTGGCGAAAAGAAAATCGAGAGGCGTAAAGGTCAACGGAAATCCGCAGAAT
>dme_circ_0005026
TCATGCCGCACAACAACCAGCCGTCTCCCGGGGATGATCAGTTGGGACCGCCAAAGGCGGACTTCAGTGC
TCCGCCACCGTACGAGGCGAACGTTGGCCATGGCCAGCAGGTAGCACTGCCGGCTCAGATGCCACCGCTG
GCCTTGGCCACACCCGATCCCAGTCAGATCCCGATACAGATGCAAGTGCAGCTGCCGAGCCAGGCGGACC
TTATGAATGCACAACTGCCGCCAGAAATACACGGGAAGTTGCCCACGTACGAGGAGGTTCAAATGGAAAA
GTCGCTGAACGGAGAGCTGCCGCCCGCCTTTTTGACTTTACCCTCCTCGCAGCAGCTACCGCCGCCGCCG
AATCCGCTACTGCCGCCAAATCCGCTGCGCGATGGAGCCGCTGCTGTGCGATCTGCCCAGCCGGCACTCA
CGTTCATCGCCATCGATGCCAGCGATCCGGAAAATAGTCTGTCCACCACCGACAACTTGCTCGGCACGGA
CATCATGTTCATCACGGCCTTCATTGTGGCCTTTTTGTTTAACTGGATCGGCTTCCTGATGCTCACCTGT
TTCTGTCACACAATCGCCGCCCGATATGGAGCATTGTCGGGATTCGGATTGTCGCTGGCTAAATGGACGC
TGATCGTGAAGCACTCGACGGACTTGGCCTCGCACGAAAACTCCTGGCTCTGGTGGCTGATCTGTGCCTT
TGGCTTCCTGATCAGCATACGCGCTTTAATACAGTATGTCAGCATCAAGCGATCGTGGCGCCTCCTTTCC
GCCTCTGCCCAGGAACGGCTGCTATTCTTTTACTAGGTGCACCTGGAGGCTTTCCAGTGGTCGTCTTGTA
AATACGTTGTGGGTGGCTAGGAGGCTCGAATGCTACCAACCAACCGATCCCAACACCCTCTATTCCCCAG
ACCCAGCACACGAAAAATCAACGCGAACTTATTTTGCAGAGTTCTCGAAATCACTATGGACTTGAAAGTA
TTCCCTCAAATGTATTAGTCAATCAATTTCTTTAAAATAGAAGTAATGTTATTCAAAAATATACTTTGTA
TATCCGTTTACTACACTTTCAAGTCACAAACTTATTGGGATATCTCGCAAGTCCAAAATTGCATTAGCTT
TCAAGAAAGTTTCAATTTTCTTTTAAATACTAAATGTATTGAACAGTTTTACATTAAGCCAATTGTAAAT
TGTAAACGGTGCAGCGGCTTAGATGTTGCGAAGAAAATTCGATTTCGAGAGCTATCCGAGTTGTGGGTTA
TCATTTTTTGTAGTTTCTGTGTTAACTAGAGACGTTAAAAATGTGTGTTAAAAAGTGAAAGGCGAATGCA
TCTTCGCATCTGCTCTCCCCCAATCGATTATAATTGTAAATGTATGTGAAATGTGTATGGATATATAAAT
ATCTCACCCTAATTGATCCCTCTACACCCCCACAAAATCTCCCTAGGCCTTTTAGTGGCGCCCATGCACT
TGaaacaaagacacaaaacaaaccaaaaaataaatgggatacaaaattataaagGGTCGAGATAGAAAAG
TATGCCACCCATATCTTTTGTTGAGCCCATTTTACGCTGTGGTACTTAATTAATTAACTACACAAGAGCC
AGCAGTAATACGAATGCTAAACACACACAAACAACTATTGCTAGAACATATGATTATTGCCTATTATAAC
GTTTAATTACTATGTTTAATCCCTTTGTATGTAAATCAAACGATGCTTATGCATTGCAAAGGGTGCAGAG
TCGCATTATCTGCGCCAGTTTTATGCAAATTCAATAAAATCGAATGATGTCATTCAACATCAGCAAAAGA
AGCGGAGGTTCACTTAACAAATCCTCCAACAGAATGCAGACTGGTGTGTAATCCAGGGTATCATATCGTG
GTTAAAAAGGGTACCTTGTATCATAATGGTAGGAAGCTTGACGATAATGCACGGTATGCGCCGAAGATGG
CGTGTGATTTGGAGAACAGTTGCAACTTTAAATTTACGCCACAAACATTAAGTTACATGAATACAGCAAT
TAATGTAGGCACACAACTAACTATTAATTACGACTGCAAGCGGGATAACTTCCAAGGAGATCCGAGAAGG
ATAACAGATTATACGAAAACGCAAGGATGTCCGCAAGACTCTTTTGTTCAGAAACACGTCGCATATTTTA
ACGATGAAAATATTGCTCCCAAGAGGGATACACCTGTCGCGCAAAGGGAAAGGGAAATGATTGCCATGGG
CGTATGTGCTCAGGTCAAACGTTTTAGGCAAAACAACCCAAGAACGCCCATCGATCCACATCTTGGCACA

GTGTACTTGATATTTGACAATAGAACAAGTAAAAATGATTTCATGCCGAGTCCTGATGTTAGAAAGTGCC
AACTTAAGCCCAATTCTCTGACCAAGACATACAAGTATGATTGCGAGAGGAAAGCGGATAAATGGACATG
TACTTTTGCCAGCAAGGGAAGGGACTTGGCCATAAATGACAAGATTACAAGTCACTTAGGATGATTATCA
TTTTATTGTATAGGACTCAAGGCTCAAATGGGAATGCAAGACACAGCGGACTAATTTTATTCCAAACGAA
ATTTTAAATATACATATATGCAATTGATCAAATTCGTAATAAAAATATGAATAGACGTGATTCGATTTTC
TTTCGTCGTTCGGATCGGAGCGTAATATTGGATAGGCGGAGTGTTTAGACAAGGGCGGCCATCTTTCTGA
AGTGAGGAAAACTGTAGTCCAAGTAGTCCAGTCATTTTGACAAATCGCCGTTGCAACACG
>dme_circ_0005026_junction_seq
CATTTTGACAAATCGCCGTTGCAACACGTCATGCCGCACAACAACCAGCCGTCTCC
>dme_circ_0005027
GGGCGACCCGGGACCTGCTCCCCCTCCTCCACCCGCCGGACTAAACGGACTGGGCGACTGATCCCGTTCG
CGGCGGTGATTGTGCGGAGACATGACTGGCGACAGGGTttgctgcaactgctgttgttgctgcgacggct
gttgctgggattgctgctgctgctgctgGGGTCCATAGTTTTGAAGGGCCGTGAGATCGGGCAGGGAGCC
GGTGTTGGCAAA
>dme_circ_0005027_junction_seq
GATCGGGCAGGGAGCCGGTGTTGGCAAAGGGCGACCCGGGACCTGCTCCCCCTCCT
>dme_circ_0005028
GAAAATGCTGATACTGCTGCCGCTGCTTGGAATTTTTGGTAATGGTTTCGTTGCTGAAGTCGAGGCAAAT
CTAAGTCTGGGCTTTAAGGCCATCAAAGTGAGTTTTTGAACAAAAGCGAAGCAGCCATGAAAATCTTTAT
GAATACCATTAATGCTCTTTCCTTAATTATACAATCCTATCCGCCTTCCATGGATCAGTTGCCCAGGCAT
CCGAATCCGG
>dme_circ_0005028_junction_seq
GATCAGTTGCCCAGGCATCCGAATCCGGGAAAATGCTGATACTGCTGCCGCTGCTT
>dme_circ_0005029
GAAAATGCTGATACTGCTGCCGCTGCTTGGAATTTTTGGTAATGGTTTCGTTGCTGAAGTCGAGGCAAAT
CTAAGTCTGGGCTTTAAGGCCATCAAAGTGAGTTTTTGAACAAAAGCGAAGCAGCCATGAAAATCTTTAT
GAATACCATTAATGCTCTTTCCTTAATTATACAATCCTATCCGCCTTCCATGGATCAGTTGCCCAGGCAT
CCGAATCCGGGTAAGTTCCTTGGGATTTTGGCAGTCCCTACGAATGAAATTGAATTGCACGAAGTTGATG
CGTATTTGCAATTGATTTGGGAAAAATAGATTTACGAATCACATTTTCTGTGTGCTTCATTTATCAAAGA
CCTGTCCTCTCATCATCATCATCATCAGCAGAATTAAATGGTCCACACCAGAATGATTTCATTAAAGTGC
AATTTCTAGCTACGACTTTAAAAGCTACAGGAAAATTTGTTTATTTCCGATATATTGTTTTTTGTACCTT
AATATTAGAATTTTCCCTTTTGGTGCAATTTACCATTTGTTATATGTCACTAATTGTTCATTGCTTGTAC
TATTTTGCCACTTTTGAAACTCTTTGGCAGGAAAACGATATAGAGGAACTATTTTGTCCAATAAAGGCAA
ATTCAAAAGGAAATAAGGGAATGCCAAATAGCCGTATGCAATTGAAGAACGAACCATTTCATTAAAACTT
GCAGACAGGAAATGTAATTTTTTTGTTCACATTTGCGAAAGTTGTAGACCCGTTTCCGAACACTTATTGT
TCACCTCTACCCATTATATCCCGGCTTAGCAAATCCCATCTCCTGGGCAAGAGTCCCTCGCACACCGGCT
CAAATGCCTAACTAACGAGTAAAGTGATTTTAATGGTTTTATGTTCGATTCTCTTTCTTCATTTTCCATT
TGGCCCAACATTTTTGTCGACGGGATGTGTGTGTTCAATTCGGTTCGACCTGTGCCATTTTCCATGCTCT
TTTTCTGTTGCCAATGCTTTCCATCCAGTACCGCCCACCTTCCACCGCGATGTCGTAGAAAGCTGTTCAA
AATTAAACAGCAGTAGTCCGAACTGTGCCAGAAACTTTTCGATTCGAGGGCGTTACAACAAATTCAAGCC
ATTTCGTGCAGCCGAATAGCTGCATATACACTTGTATATGTATTATACTGGCCCTTTAGACCCACCGCCC
CCCCGCTGGAGTCAAAAGTTTTGAGTCAAATGCCAATACAGTTTGTGTATGGCCGGCTCCAAAATAAAAG
TTCTCCGACCAAGCTGAGCTGAGAGCTCCGGGCTTCCTGGCACGTAGACCACCGACCAGTTGTGCCCCGG
GTCCATATCAATGGCCCACACGATTGGCTGACTTGAACACATTGAAAGAAATCACAGGAATCGAATATGC

TGTAATCCTTTAAAAAATGTTTGTAGCATATTTCATATATTTATAAAATCATCATATCGTTTAGTAGTAA
AATATAATTTGTGCATAATAAAAACCAGTTCTCTTTTGGATTGTACAGATTTGCTTAGATTTTAAGCATG
tttttattttttAATCTGTATGTCCAAAAGCTGTGGATTTTGCAGTGCTAAACTACAATTTTTTCGACT
TTTTCGTCTGTACATATTACAATTGCTCAGGGCCTTGATGCATTGTCCACTCAATGCGGAACAAGTTTAT
TTAAACTTAAGAACTTGCCCTTTACTGGCATGTTTTTCCTGGCCAAAATCGGAAAGTTATCGGAACACGA
AAAATTCAGAGACGCTGTGAACTTTGCCGAACATATTTGCGAATACGTGAAGCAATGAAGAGTTCTTACA
ACCCAGTCAGGAACTAAAAAGTTCACTTCGAATGAGCTGCAAGGATATCGTTTGCGAACGATTCCACAGT
AAGGCCAATTCATATGGAGCTAATGTTGAGCAGAGTTCACTCCCAGGGGAAAAACACTTCCTaaaaaaaa
aTACAAATAATAATACAAATAATTCATGGGCGTTAGGCTCTTCTGAGTGTAATACTCTttaagtattgga
tattaagttttagatattaaAACATTTCTCTGTAGAACTTTAATTAAATTAGCCAATAGTGTTTTGGCAA
GCTCTTTTGGGGTCACTGAACCTTACAGTACTGGCCTATACACTTTATTCCCTTCGGAAATCACTGCCAA
CTGTGTTCAAACATACCCTCGAGCCATTGGCAATCTTTAGTGCAGATTTTAGTTTGTAAAGTAGAATTGG
GCACAGTTTTCAGCTCTCAATCACAACATGTCCCAAGCAAACACTCGAGCAGCAAAAATTGCGCATACGC
AGCATGGGCTGCAGATTGAAGCGTTCTTTTCGAACTTTTGTTCGGACTGCCTTTGCCGTAGCCCTTCCAT
GATCCGCTCCGGGGCCAATGTCGGATGGCACTCAAAACTTGTCGCACACGCTCAAGTGTTGTAAATCTTT
GCACAAAATGAAACTTTATTTGGTCATGGTCATCAACGACTTAAGTAAATCTAAAACTCTAAGTAAAACA
AACACTGCACACAGCCAAAGAAGAAACGAGTCCGAAGGCAGAGTCTCGCAGAGGCCGCATAAAACAAATA
AACTGCGCACACGTGACCCCAAAGAGGGCGCAAGGCGGTGGGCGGGGCCGGTGCTGATGACCCCAACAAA
GGAAGACGGGGTCGCAAAAAAATGTGTCAAGCCATCAGAAAGGGTAGTCAACCAGAGGCTAAGAGTTCATT
TCGCACGGCAAGACATCGAGCTTAAGCAGGCAATAGTCGGTGTATGTACTATGTTTATTGATGGGGGAGC
TCTTCGGTGCACTTTGCCAACTAAATGAAAGCCCTAAATTTGTGTCAGAAATCGGAAGGGAAAAGCACTG
ATAATGAAAGCTCATGCCTCATGCGATAAGGGATATCTGTAAGTGTATATATCTCCTGAGCGCTATTATA
CTGTCATTGTAAGTAATTTCTACAGAATATAACATATGCTTAATAGAGTAAATACAAGTAAAAATATGCA
AGCTTAAGATTTAAGCTTACAATTAAATCAATAACTTATTTTCTGTCTTTTTTGGAATATTTATATATCC
AAGTATAGGTTCGCTTGtttcattttgttaactttttattatttgttatttttttattttCTATACAAAAA
TAGTGAAGAATGGTTATTAACTCCTATTAGTAGAAACCAATCAATTGTGGTAAATAAAGGGAATTGGTAT
AAATAAGTTATTCGATTTTAAAAAAATCCCCTTTTACCTCAAAGCAAATTCCATTTGGTCGCGTAACATT
GTTCTCATCAATTGCTACCAAATTAATTACCATTCGATTCCTTCCAGAAGGAAGCGTCAAACCCAATCCA
AAATGCATTTGTAAGGTATATGGTTCCACGAAGTAAACAGTCTGTAAATAAAAACTTGTAAGAATAGCAT
TGAATAGCATTGTACAGCGCATACTCCTTTATAGTTTAGACATTTTTGTCGAGCCTCGTCCTTGTTTATA
ATATGTTTTGGAAAAACATTGTACCAATATTGATTCGGATCCAGAAACGTTTTGCAAAAATCTTTGCACA
CCATATTTAGTACCGTCGGTTGCCAAGTGCCGCGGTCCATATGAACGACATTTAATCGACCCTCAATGCG
ATCCGTTGGTTGTATATCCCACGTGGAGGTTACGTGTCCGTCGATGTGAACACCTTCTGGCCCCATTTCG
AAGTTGACGGTTGAAACATCAAATAGCCCGCTTACATTCGCATATCCAGGCGGCGTATTAAAACATTCCG
AAAACACTGATTCGTCATCCAAAACATACTCGAAGTAACCTCCAATTGAAGGCTGTGCAGCGGTTAGAAA
AATTAATATTTGACAAAGCACCGAAAATTGAAAATCCATTTCACACTTTTTTCCAAATGATAAAACGTCT
ATTTGATGGAGCTTTTATACTCAAATTCGTTACTTGTAAAACTGAAGGGTATGTTGTGCTGTGAAAAAGA
ATCTAACATGCAGATGGGGAATAAACATGAAGATATAAGTTCTTATAAATGTTTAATATTTAGGAATCTA
GAATAAATATTTAAATCAATTTGCTATTTTGTGTGTTTTTAAATTACCAACAGCCCTCGATATAGTTAAA
ATAAATTTGAGCATTTTTCCATTTCGGGTGCAAAAGTACTGGGCACTTTATTGTCGTGACGACTATAACA
CTAATATTATTATCAAGAATCTCCTGTATAGATTGTTTTACATGATTGTGTAAAGTTGATCTTGATAAGA
GTTGATAAAAACTGTAGTTGTTAATTGGAGGAAAAGATTTATGATAAAACCTGCGTTTAACCTAATATTA
AAAAGCAGATAATCAAACAATGTGGAAGGTTGTTGCGAACTTTTGTTTGATATATAGATTTTGATTAATA
GTAAAGAAAAAATAACCTAAGCACAATGAGCaaaaaaaaaaaaaaaCAATAAAATCTATACCAATATCTT

GTCAAAAATCGTTGTGCAGCTGTGTCTCTGCCTGGCCTGATTTTCCAAGGATAAGGGATCCTTGCAGACA
AAGCACCCACATAGATGCTTACTTAGAAATATCCTTGCTAAGGGATGCATGAGTATACCGTGTACACATT
AAGATGATTGCACTCCGGAAAATGACGCGATATTGCAGGAGTGAAGGGCGCACCACACGTACATAGATAC
TTCCGGCATTGTTCGACCTTCGCTGGGAACTTTTCAATAATAAACGTAGATTGCTAATGTCATAAAGATG
CGACAACGTGGCCATATTCATTACAATCAGCTAGCAATGCCAATTTTCGCATTAACCATTGATGCCAAAT
GAATGATGATGCCGAAGGAGGCGGGCGGCCACGTCTGCCTCGTCCTTTCGCAGTAGTCACGTCGCCAGTG
AAATCAATTCAAAGTGGAAAAGTACAGGGCACGTACCCGATCCCCTTGCACACAAAAACACAATTGGGTG
GCTCCTCCTTCGCTCGCTGCTCCCACAATCCTTGTCTATGGACCAAATTCAGCACCACTGTGCGATTGCA
AATGGTCCGCCACAAGCCCGAGTAATTGTGATTGGCGGCAACACATACGAGTTTTTGGCCAGGCCAAAAC
AAGAACCTGCTCAAACGCAGCCACGGGAACAGTGTCTGTGAAAGGGCACTGCAAAAAATGTGCCCAAAGT
TGAAATTAAATGTATTAATATTTTTACACTTAATAAAAGACTCaaaaaaaaGGGTGTAAATTAATAAATA
TTCCAGATTTTATGGGATTTTCTAACGATTATTATTATCAAATATAAAATAGCACTGCACATTTTGTTCC
GTGTAGAACAAAGTATGAGGCAAAAACAAACTTTAGCGGGCTAATGGGATGCCATAAAGCAGCGGCATTT
GTCAATATCAATTTTGATGAGACACGAAGGCAAACGGTGGAATGGTAGCAGGTCTATTGTTTGCGCTGCA
AATATGCGATTCTGGCGAAATTTATAACAAACAGCACTGACATTAATTGGGTTCCCATTCATTGGAATTA
CACATACGACATGTATTACGCATTTGATGACATAAATTAGCAGTGAAATGAAGGCAAACTGAAATGAAAT
GAAATGAATTGTGTAATAAACAAGTCAGAGTCACTGATTGCCTAAGAAATGTTTGAACCGCGTACTCTTT
ATTTAAAGATATAAGCTGTTTCAATTCATTTTGCCTTGTAGAATTTTCCCGTTTTCATGCTCATTTGAAT
CTTCTATTTTCTATTTTTTCCATCTACATTTGCTGGCGACAACGATGGAATCCAACACAACATCTGGATG
CGCACACGGTCTTCGCTTCGGATTCGCCTGCAGCCAATTGCAGTGTGGGCAACACAACATTCGCACATGG
AGTCACATTCAAATTGGATTGTAAAACCCAATGCGTTTGCGAG
>dme_circ_0005029_junction_seq
GGATTGTAAAACCCAATGCGTTTGCGAGGAAAATGCTGATACTGCTGCCGCTGCTT
>dme_circ_0005030
GCCAATTGCAGTGTGGGCAACACAACATTCGCACATGGAGTCACATTCAAATTGGATTGTAAAACCCAAT
GCGTTTGCGAGGTGAGTCGCGGGTACCAAAACGCAGGTCGCAGGTCGCAGCCGTTGGCCAAATGCTTGGG
GCCATCCGCACAAAAGTTCGCCACAAACCACAGACATCCAGAGATACGTACTACATATACATAGATACAG
ATACAGATTTTGGGACACAGAAGGCATTCCGGCCAAAAGCCAAATGCATTTGCGTTAAACGCTAGTCGTT
GCCATCGCTGTTGTTTAACCTGTGCTTAGTCTGGCTCCCAGCGCTTTTTAGTTCCAGCCTTCATTTTAAC
CCTTTTTTCTCATTTTTGGTGGGATTAGAGGCAGCACTTGCAGCATCTAAAACCAAATGGCTTTGCTGGG
TGGCGATTTTAATTAAAATTTAAATATAGTGTGAAATGCCCGGAACTACAATTGCGAAATAGCACAGTAG
AATTTAATAGAGACTATAATGGGGGAAAACTATATTTCGCTAATGAGATTAAAACAACGCTTACCAGGCA
TTATCAAACTTAGAATAGGGATGGGAATTAAGGCTATTTAGTTGGATATAAAAACATAATACCTTTTAGC
TAATATCATAAATTAAATGAAATTTATTTTTAATGTTTTCAAACTTTCAAAATGGTAAAACAATTTGCTG
TAAAATATTTTTGGATTTGGCTTATAAACTTATATAACCACCATATAACAAAGATGGAGCTATAGGTTTG
GCTACAGTGAATCCTTGCATATAAAACTACGATTAAAGTTCACTTTTAAGTTGCTGTAGCCCCAAATCCT
TGACATTGCTTTATGGCCACAGAGAAGTTTTTTAACTGCTCCTACGTTCCTGGGTTTCCATGTCTGGTTT
GGTTTTTGCACCTTATTTCCAGCTGCGCTGGTAGTTTATCATGTGGCATAATACAAACACGAGCACTTGC
AATTTATGCAATGCAATTTCGTGACGATGCGAAAGACTCGGAATGGGTATAAAAAAGTATCATCGGGCTG
TGTGACCGCTTAGAGACGAAAAGTGGATTACAAACAAAGCTGTGAGGGTCTGCACGAAATGTTAATTTTA
CCATATACCAAAGTACACATAAGGAAACAAACACCAAGCAAAAGTTCGTGCCCAAGTCGAGGACCCTGCC
AACTTTCGGTCGTCCTTGTGCGATTGGGCCAATATTTTCTTTACATTATGACTTTATTTTCGGCTGCCCG
GCCAAAGCTAAATAAACAACCCCACAGCAGCAACAATGGGCCACATTTAATGTATTTTAATGTGGCATTG
CAGGAATGGCCGCCACGCCTGCTCCACGCTCTGCCCCAATGAGCAGCTCCCGGCGCCGGAGGACACGATT

TCATGCCGATCGCCGCGCCTCGTCGAAGTTCCCGGCCACTGTTGCAAGATGTGGCTATGTGAGAATCCAA
CAGCTGATG
>dme_circ_0005030_junction_seq
TGGCTATGTGAGAATCCAACAGCTGATGGCCAATTGCAGTGTGGGCAACACAACAT
>dme_circ_0005031
GCCAATTGCAGTGTGGGCAACACAACATTCGCACATGGAGTCACATTCAAATTGGATTGTAAAACCCAAT
GCGTTTGCGAGGTGAGTCGCGGGTACCAAAACGCAGGTCGCAGGTCGCAGCCGTTGGCCAAATGCTTGGG
GCCATCCGCACAAAAGTTCGCCACAAACCACAGACATCCAGAGATACGTACTACATATACATAGATACAG
ATACAGATTTTGGGACACAGAAGGCATTCCGGCCAAAAGCCAAATGCATTTGCGTTAAACGCTAGTCGTT
GCCATCGCTGTTGTTTAACCTGTGCTTAGTCTGGCTCCCAGCGCTTTTTAGTTCCAGCCTTCATTTTAAC
CCTTTTTTCTCATTTTTGGTGGGATTAGAGGCAGCACTTGCAGCATCTAAAACCAAATGGCTTTGCTGGG
TGGCGATTTTAATTAAAATTTAAATATAGTGTGAAATGCCCGGAACTACAATTGCGAAATAGCACAGTAG
AATTTAATAGAGACTATAATGGGGGAAAACTATATTTCGCTAATGAGATTAAAACAACGCTTACCAGGCA
TTATCAAACTTAGAATAGGGATGGGAATTAAGGCTATTTAGTTGGATATAAAAACATAATACCTTTTAGC
TAATATCATAAATTAAATGAAATTTATTTTTAATGTTTTCAAACTTTCAAAATGGTAAAACAATTTGCTG
TAAAATATTTTTGGATTTGGCTTATAAACTTATATAACCACCATATAACAAAGATGGAGCTATAGGTTTG
GCTACAGTGAATCCTTGCATATAAAACTACGATTAAAGTTCACTTTTAAGTTGCTGTAGCCCCAAATCCT
TGACATTGCTTTATGGCCACAGAGAAGTTTTTTAACTGCTCCTACGTTCCTGGGTTTCCATGTCTGGTTT
GGTTTTTGCACCTTATTTCCAGCTGCGCTGGTAGTTTATCATGTGGCATAATACAAACACGAGCACTTGC
AATTTATGCAATGCAATTTCGTGACGATGCGAAAGACTCGGAATGGGTATAAAAAAGTATCATCGGGCTG
TGTGACCGCTTAGAGACGAAAAGTGGATTACAAACAAAGCTGTGAGGGTCTGCACGAAATGTTAATTTTA
CCATATACCAAAGTACACATAAGGAAACAAACACCAAGCAAAAGTTCGTGCCCAAGTCGAGGACCCTGCC
AACTTTCGGTCGTCCTTGTGCGATTGGGCCAATATTTTCTTTACATTATGACTTTATTTTCGGCTGCCCG
GCCAAAGCTAAATAAACAACCCCACAGCAGCAACAATGGGCCACATTTAATGTATTTTAATGTGGCATTG
CAGGAATGGCCGCCACGCCTGCTCCACGCTCTGCCCCAATGAGCAGCTCCCGGCGCCGGAGGACACGATT
TCATGCCGATCGCCGCGCCTCGTCGAAGTTCCCGGCCACTGTTGCAAGATGTGGCTATGTGAGAATCCAA
CAGCTGATGGGTAAGTTGTCCTGTACACTTGGAGAAAACAGAATAAAGCATATCTTCATCgtttttattt
ggtaatttttgtttttacatacattacattacatttttgtttaTGAACATAAGATAGTTTAAATGTTCAT
GGTGATTAGGTGAATAACTTTACAGCTTAGAAAAAATGTGCAAGATTTTTGTTTTAAGTGCATCCTTATT
TCCACTGCCAATGCCTTGTTATTGTTTTATAAGTGTGTTTATGGCTGTGGCCATCCCATTGTGTCCACTT
TCTGACCCACCCACCGCCCACTGGCCTGAGTTAATGGCGCTGAGGGGCGTGTCGTGCTGTGTCCGATGCG
CAACTTAATGCTTTTTAAGCGCCCGGGAAACCACATCCTACCCAAAAACCGAGTGGAGAATGCAGCGCAT
ATTTATGAAGATGAATTATTCCATCACGAGACTGGAGGCCACGAGCAGGAGTAAATGCTGGAGCAAAAGC
GTAATTACTTACACAGGGATTAAGTGTTAATTAAACGCGCAGAATGCAATAAATAAACAAGATATTAAGC
GCTCCTGCCAGCAGCTGCAAGGAATAGCCTCTGGCGAATTAACTGGGAAATCGAGCATTTGAGACATCTA
CGGAGCGGTTGGCAGGACCTGAGGAGTCAGTCCTGGGCGGCAATTAGTACACATGGCATTGAAGAATGTT
TTCACAGACAGGCAAATGTAGAAGGTAGCTTAGGGGCTTCTAATAATCAAAACACGTCCAAATatgaaaa
atgtataaaaaaatgtatttgaatttacaaaaaccaactaacaacgcaaaaaaTTATTTAGCAACACCAA
CGTTTCCTGAAATAAGATATATTTAAAACCTGACTGAAATATGTATGTATAAGCACCCACCGTTTAGTAA
AGATATCTGAACTATCTCGTTTTGAAGCAAAATCCAAAAAGATTTCCTCCAGCCGTGTTTGCATAATTAG
GTAATCTTCGATATTCAGTTTATGCGAGTTAGTTTCAAGGAGTTGAAATATTTTCGACAATGTTTTTGAG
TGTGGTATATAGTATGTAATCAGTCCATTATATTCCTCTTTGAGCTCTGCGTCAGGTATCTCTTTTGATA
TAAATCGATTCACCTTTAAAATTCGATCTGCTTGAGAACTCTCCATAACAGAAGCCATTTGAAGAAACTT
TAAATCAGTTTCTGAAATGGATTTTTTGTCATTTGAGGAAGAAGAATCTTCTACAACACGCTGAAAAGTT

TTCTTTTTGTGCTTGACTTTAACTTTAAGAATCAGGCCCTTTGAGAACTGATTCTTTAAGCTTTGCACCG
AACCCAAGCACTTGAATTCGCCATCGACCATTATGGCCAATCGGCTGCACAGAGCCTCGCACTCATCCAT
ACTGTGCGAGGTTAGGACAATGGATTTTCCAGCAGTTCGAATCAGGCCTATTATTTGCCATAGTTGTCGT
CTTGCCGCAGGATCCATGCCACTGGTGGGCTCATCCAGATATAAAACCGATGGATTTCCAAGTACCGATA
TGGCGGTGCTCAACTTTCGCCTATTTCCGCCACTGTAATGCTTCGTTTGCTTATCCATGTGCTTGGCGAA
TCCGAAGGATATGGCCAGTCCCCAACATATGGGCGTTACATGTCGTCGTTGCACACCTCGCAACAGACAA
TAGATATTTAAGGTTTCGCGACCAGTCAAGTCCTCAAACAGTGCATCAAACTGCGGACAATAACCGATTT
TCCGATAAACCTTATGAGTGGATATGTTGGTGCCATCTATATAGGCTTCTCCAGAGGTTATTTTCTCATC
CCCAGCTAGCATCTTAAAGGTTGACGTCTTACCAGCTCCATTGATACCCAGAAGTCCGAAACATTCGGCA
TGGGGTACATGAAACGAAATGGCCTTAACTGCAAGTTTGCTTCTATAGCGTTTGCTTATTTTGTAGACCA
AAAGAGGTACATCTTTTCGTCCTGAGTTTATCATTTCTCGGACCACACGTTGTTCGGCGACTACACTATC
GTTTTCGATAGAAGTTCCTCCTTCAACACGTTTTCGCTTCGAAAAACAATTTCCCAATTTTTCGGCAATA
AGATTGCATACCTTGGCGTCGTGCATGACTATTAGTAAAAATAATGTAGAACCAAGCATAATCATATATG
TGATTTCTACCAAAACACCAGGCTTTCGCCAGCCATAATAGCCTGGAATATCACAGCATATGGGAACATT
TCGGCAGCGCAGTTGTTTTGGTAATTTTCTGATGCCACCTATACTGCAACCGCGTCGAATATTATATCCT
ATATGAATGCTGTGAGTGGAACTGGCCAAGCTGTAGTGTGGAGATAACCGAAAGTACCAGGCTAGTTTTT
CCGCCACATCCTTCAGCTGAAAGGCCTCAAAATTCAGGGTCATGAACATCAAAAAGAGACCAGTTCCCAT
CAAGGTATTAATAATGGATATGCGAGTAAACCCTGTGGCAGCATCGGAGAAGCATCCTGATGCAGCGTAA
CTTAGCATAATGCCGGGAAATCCAAATATCAGCAGCATATAGAAGTAACGACTTAGGTCTAGTGGTGTTA
TGTAGCCAATCTCCTTGAATGCGGCAATGGTGAGTATGGAGGATAAAGCTGAGAGAACAAATACTAAGTA
ATCCCAAACAATGTGACTTGTCCAGTAGGCACACACATCAACTCCACTTAAAAATTGCAAAAGTTTGGCT
CGAGTTTCTCGTTCCCTTATGATGGGAATCGCATAGAAGGCCGTGATGAAGCACATGGTTAGGGATAAAT
TTATAGATATCTCAGCGCCCAACACTTGTCCTTTATTCAGTCTCAAAGTCCTTGTATCATCGCTATATGG
CAACGGCTCATTTGTTACGTAAGTAGAGGCATCCTGGCCGAGCAATTTAATAGCCAAGGCGTTGTGCAAT
AAATTGAGCGTCAAAGGAGCCGTATGCAGGGGCTTATTATTTAGCCACACGATAAAATTATTTTCCGATA
CAGTGAGTCCTGCTAGGAAATGGAAATCAATTCGCCGGCTATGGTTAACGTCATGGCTCAGTATGTAGTC
CTCAAACTTCAGATCGCCTGTTCTTAGCAACGTGCACCTTCTGGCGTAGTGCTCCAAGTGTTTCGAGTAA
GCATCGGCCAAGGACGAAGTTTTATCAGTTGTATTATCCTCCAATACGACATAGGCGGAACTATACTGAT
TGATTGATATGGGCATAGCAGGTAGTTCGTAGTAAATTCTACCACCTCGTGAATTGAGTATTGTCAAAGC
CATGACTGCTATGGGCAGAACTAGCTGAATACCCAGCAGCCAGTAATTCCTAGTCGTGGTATAAAACTTC
TTAAGAAAAAGAGCCTGCCAAAACATAAAACAACGGCGGATACGTCTTTTCTCTCTCGTTTTGCTATCAA
AAACTAGTTCTTTAAAATCAATACTTCCGTCTACCGGAGGCCCTTCTGCTCCACCTTGAACCCTTTTATC
GGTGTTGACTGCCATAAAAACATCTTCCAAAGTGGCTCCGCTCAAGCCGTAACCCACCAACTGCAGTTGG
GCACACTTTTCATCCAAGTCCTGCAGTAATTCTGCAAACTTCTTTGAGTACTCGGTGGGCAGGCGATAGG
TTAGCTCAGTTCCCAACACTCGCTCCGGTTTTATGGGAGGAACGTACTTTCGTATCAACTGGGTTACGGC
TCCTACATCACAGCCACTCTGCATTATACAGACCAATAGGTAGCCGGTGCCAAATCTCTTTTTCAAATAA
AAGGAGGTGCCCTGGCACTGCAGTTTTCCTTCAGATAGGATAGCTATGCGATCGCCCAGGACATCGGCTT
CATCCATATAGTGAGTGGTTAAGAGTATGGTGCGTCCATCCTTTTCGCTTTGCAATAGATCCCAAAGACT
CCTGCGACCGGCTGCATCGATTCCTGAACTGGGTTCATCACATAGGACCACCTTGGCATTTCCACATAGA
GCACAACAAAGGGACAACTTACGCTTCATGCCACCAGATAGATTTTTGGCAGCCACATATGATTTATCCT
GCAATTTCAGCATGGTCATATACTTACCCACCTCATTTTCCACGGCCTTGGTACCTCGAATTCCTTTCAG
CTTACTAAAGAATATAATATGATCCCGTACGCTCATTTCTTTAAAGAGTATATTATTCTGAGGACAAATT
CCTAAAGATTCACGAGCTTTTGCCAAATGGGTTCGTATATCATACCCGTTTACTATGGCCGTTCCACTTG
TGGGCTTAACTATACCAGTTATCATATTGATCGTTGTGGTCTTACCAGCACCATTGTGGCCGAGTAACAC

GGTTATTTCGTTACGATGCATCTTTAAACTTATACCCTTGACCGCTTTTAGTTTGCCATAGTTTTTTTGC
AGGTTAAACAATTGTATTCCCACCTCCCTTGCACGTCGACAGCTCTTTGCTTTTGCGGATCCATGTAGGA
TGGTTTGGAAACTGGGAACAATATTCAAACTTGGCACGTATTGTCGCCAGTAATTTTTGTGGCACATGAA
GTTCCACCTACGGGGTACACCGTATTCTCCAGGAAACACCTGCTCCACATACAGGCAAATAGATATGAAC
ATTATTGATCCGAAACTCATTGTCATGATTATATAAAACAGCGTAAGACTATCATCTGTTGAGACGGGGT
GAAACATATTCCTCCAAGTGATTCCCTCTCCGGTTCCTTCCCACATTACTATGACGTGGATGCCAAATCC
CAAGGCCGAGTTCGAAAATATCAGCGAAATGAGCAGCTTACTCATGAGGCTAAGACGTTCGTAATAATAG
TAGCCAAAAGAATAGGGTATATAGGTGAGAAACCAGAAAATGGCAGCCACTGCCGAAGCTGTGCTGGCTT
TTGAAAATAAAACGGCTAGCATAAAGCATAGACATACGCTCGTAACAACATAGGTGTGTAGAAATAATAG
AACCGGAACCCAGCTGGAAAATGTAAGTACGGCTACGGAAGCGTAAAACTTCACCATTATCAGGCTCATG
ATAAGAAATACCACCAGCATGAGCATTATGTAACTCTTAACGAACCACGCCACCCAGTGAAGCCAGTTGT
GTACGCCGATCAATTTCATTATCTCCTTGAGCTGAAGTTCCTTTTCGCTGGTCACATACTTGGTCACAGT
CGAGGCGGGATAGATGAAGCTCAGCAGGATTACGAAGGGCAGTAGTTGACGCAGGCCACTCAGCAATTGG
TCATAGGTATAGGCCCTATAGGGAAAGCGCTGAAGGTGTATGGCTGGAATACCTGTATCCGTAACTCCCG
ATGCCAATGCCAGCCAGGACATGGTTAGGGCATGCTGAATTGGCAGAAAGCCCTCCCTAATATATCCGAC
CGGAACTCCTCCATCGTTATCCAATCTATTACGGGATCCACTTGTGTCGTAGGACAGAAATAAACGAGAA
GTACGCCAGGTGTCTATGATTGGGCCTTGCATGGTTCGCAGCTCACTGGGAAATCGGAGCGAGTAATTAA
GATTAAGTGGATACCCTGCATCATTGGTGTTCACGTTCTCGTTGTCTTCAAACTGAACTCCTGCCAAGAA
GTTGTGCAATACCATATCCTGTTCCATGTGCAAAGCGGTGTCATAGGCTCTAGTGCCCAGAAGTCTTAGT
CTTATCGCAGTGGCACCCACAATGGCACTATTGATAGGGTTATTTGGAGTATAGCAAAGGAAGTTTCGAG
GTATATTAAGGGATCCATTCTCGTGCAATATCCTATTTCCAAGTGGTACCATCCTCATATAAAGGTTTAG
GTCCGATATGGATACGGTTGGGTAGCGGATCTCATCCATCTTTTCGGGCAAAACTATTATGCGAAGTACT
AGAATAAGACAAAGAAACAAAACTGGCAGGACCAGGGCAAATACAATTTGCATTTTTTGGTTCCACTGAA
GAATCCAATTCTTCCACATAAGCAGCTTTAGCTTATCCCAATCTCCAACAGCGTATATTTCACTGGTTGA
AAATGATTCTCTCTCGGACTTCATTATGCAGAAGAATTCATATAATTCGCTCAAGGTTTGATTAAttttt
ttgatttttttgatataattttctaagcactatatatatatttgtttAGCATTTTTAAATATTCAAAATAT
GTTCAGGACAAACATTGTGACCAATAAGGTTTGGTTCTTATTGCACTAAGAATCATGTTTCCTAACTGCG
TTTCTGGCGGTGATTAATGAGCACCCCGATCATGAGCTGGCTCATTAGTAATCCTCTCCAACTTCCCACT
CGTTGCAGGTTTACGCCACCTGCCACAACAGCACCACCAGCGGCAATTGGACGGCCTGCAGCAGGAGCTG
CGGCCTTGGCACCGCCACGCGGCACACCACCACCCACGCGGGCTGCCATCAACTGAGCAATTTGCGGCTC
TGCGAGAATCGCAGGTGCGACAAGGACGACCACGAGGACAATAAGTGGAGCAGGAGCCGCCCGTTGACGC
ACAAGCGGAAGCAGCATCGCGCCCACAGCCACTCCCATCAGCGTTATCATCACCACAAGGAGCACGAGGA
GCCGGCCCACAGGATACGAGGTGAGTGGGATGGGGATTCCCCGGATACATATCCAACACCTGCCAAGTGG
CAACTGGGCTAAGACGGGCTGAGCTTAAAGTCCGACCACCTGACGCCTCAGGAGGAGAGATGAAAATCCT
CGCGGATTGAGTTTGCTGCATTTCCTTTCCTTTCCATTGCTCCTCTGTTGCAGCTCGAAGAAATTTACAT
TTTCCGCATCCTTGCTTGTTTTTCGCCATTTGTCCTTTTCAATTAGATTACCGTTAATTTGAAAGCAATC
CCATCGACAGCCAATCGACAATGTTTTGTAAAGCATGAACTGTATTTACTAAAATTGTGATTAAAAAATT
AATAAAGAGGAAAATATTCTTTATGATATGCATATTATTATTTTGTTAGCATAAAAAGTTAGTAGCACAG
ATTACAACATATTATTTCATTAGTCAATCAAATCAAAATATCTTCACATCCGAAGCCAATCAGGCAAATA
ATATTTTCCATCAATTTTATTTGCCTGCATAAGCAAGTCAATCGGAAATATATTATTCAGACTTTATTAT
TTATATATTCTGCTGTTTATATAGtgtagttaattagaattcccattacttctgatgtagttaataaagt
ctcaaaacgcagttggtcattttatctttatttggtttttattaagcgagtgtacattccagatctattc
ctgatttataactgatcttagtgtggtctacaggcagatctcttgtgacagccaagtgctgacactagca
aattctgcaatatgtatgtatgaagttcatactcgataaccaatgggagtcgagtgcgacccctaaaggc

gtacatgctgaattcgcatatttagtattagggtgtatctaaagatctactagggtgaccctaaggaatt
agggtggtcctaagtttacttattagtcgacttattattatgattcatattataattattattaatatta
ttattattattgttattattattgttattatgttcgtatatactacatctccctccttttgaaaaataac
gtaataacatgaagttattttcaagtatataataatttttttttttttttttaaatgtacattaatatt
tgaaatgaatgattaaagagatattaaaaaaatatatatattttcattatattttgcaaagtaaaagttt
aggaaaatgtaactatattgaacaaaaaaaaaaaaaaaaaaaatcaaaacaacatatttttaaagtaaaa
taaaccaaaacccaaaaataaaacaaaaaaaaaaaaaaaataaaaatatacaaaaatgggttggtttgga
tctgacgatagtcagacaaaagataatacggccaatgtggtcaataacttaaaaatagtcgaccatacag
atgacattcagtcactgtggttactccttttgatcatgacgatcgtaacagtcgctcagtttatattaac
gctatatgttaagcataacaagataataaagaggcgttatataggcaaagcagagaatagtttggataaa
atttaaaaaaaaaaaaaaaaaaaacatccaaaatgtattttgaattaagatgatctaaaatttttatttt
taatatcagaaaactagaatgaaaaaaaaaaaaaaaagattattgaagaaacctatttgagagaggccaa
aatttataaagttcgattgcatagataaatccatagtattctttaaatggactggcaagaaatacaaaac
gagcttaaagaaattaaaacaacttttgacaagtcttataaatgcatgacaccaaatagagaagtgcaac
aagacactctcaacaagcatgcgcagatattggtaagatgctttaatggagcacgccaattaatttacag
agaaaggaaaagattaacaaaaaatcatttatcacaagcagtaaaatttctaaacaggttccgtgagaac
ttgttaaacgtcaagtacagacacaacttaaatattacaatcccaacgattttaagcacacctatagtgg
ctgagatcggtgaggatatcgaaagtgtaggagaatcagaaatagaaataaaagaagaggatctccacga
tcttgcaattccagcggtaataacattacccgaattacttgaagaagaactttcagattcaaatacagga
ataagaatacaggaaacggacaaaatgacagactctgccgcaacagcaagggaatatgtgcgacaaattt
cgtccacaatacctgagtttgacggcaaaaagttaaacttgaatagattcctcacggctctccggctaat
agatctgacaaaaggagatcaggagatgctagcggttgaggtaatcaagacaaagatacttggtccatta
tcacacaaagttgaaaatgaaaagaccattatcggtataataaatctattaaaagcatcagttaaaggcg
aatcgcccgatgtcatcaaagcaaaaatgcttagtacacaacagcgcggcaaaactgcagcgcaatatac
cacggagatagaaaacctacgtgggttgctcgaagcagcctatatagatgatggtttagattccaacaat
gcagacaaattcgctacaaaggaagccatatctgcaatgaccaagaactgtgggcacgataagctcaaaa
ccatattggaagctggaaatttcaacacgatgaatagcgtgattgaaaaatacatacactgcagtacaga
aatgaccggcaattcaaatagtgtattattctataataatagaggacactatcgaggtaataattaccga
ggaaattaccaaaacagaggtaatggccgaggaaattataactcctacaataacaactatagaggcagag
gtggttaccatggtggaaacagaggacgaggtggtaaccaaaattataatagaggtggaggttactcaag
aggtaaccaaaaccataactataaaacaagtcatgcccacaatgtccgaaacatacaatcggaaaacgaa
catacccccttgagcgacaatctacaataaaattatacaaaattaatctcaatttaagcatttttatacg
attgaagaatatgagtaccaattcatgggtaactcttttaatagatacaggtgcagaaatttccctgctt
aaatgcagaaacaataatcttaacgatttaaatccaaaaaatacaacaaatatatcaggaatagggcaag
ggacaattcagtctctaggtacactacatttagaaatgtgtattgctaatgcagcaataccatatgaatt
ccatatcgtacctaacaattttcctataccaggggatggtataattggcttggatttcattaagaaatac
aattgtattttggaattccacgaccaagaagattggttcactttgaggcccaaaaatttcaggaacataa
acattcctattatacattcactagataatgaaataattttgccagctagatcagaagtgattcgaaagat
tcaactaacatctactgacacacatgtcctcatccccaaccaggaattacaacctggcataataatcgca
agtgcactcgtaaacactcagaacgtcttgattcgaattattaatacaactgaaaaagacgctatagtta
gtagcgcaaatataaaaagcgaatcattggatgattatgatgtatacaacgcaaatatagaaaatagtgc
acaaagaactttagaagtattaaaacttcttaaatttccatcgttattcaaaagcgatttaacaaaatta
tgcaccgaatatagcgatatttttggtcttgaaacagaaaccatatcagctaataatttttacaagcaaa
aattgagattaaatgacaaaactccagtctatatcaaaaactatagaatgccagaaagtcaaaaaccaga

aattcaaaggcaagttgacaaattaataaaagatggcattgtcgaaccatctatttcagaatataatagc
cctcttctcttggtacccaagaaatcactgcctaactcggaggaaaagagatggcgattagtagtcgatt
atcgccaaataaacaagaaactgctagcagataaattcccacttccaagaatagaagatattcttgatca
attaggccgagcaaaatatttctcgtgcctagacctgatgtcaggattccatcaaatagaattagacgaa
aggtcaagaaatataacatcattctcaacttcaacgggagcataccgctacacgcgattaccatttggtt
taaaaatagccccaaattctttccaaagaatgatgacccttgcattttcaggtttaacgccttcgcaagc
atttctgtatatggatgatttagtagtcataggctgttctgaaaagcacatgcttaaaaatctaaccgac
gttttcaaattatgtaggcaacataatttaaaattacatccagaaaaatgcactttctttatgaaagagg
ttacttatttaggtcacaagtgtactgacaaaggtatattgccagatgactctaaatatgaggtaataaa
gaactaccccaaaccagtaaacgcagacgaagctagacgcttcgtggcattttgcaattattacagaaga
tttattaagaacttttctgagaaatcacgccacttaacgaggctttgtaaaaagaatgttccatttgaat
ggacaagcgaatgcaatgatgcattcgaatatctcaaaagggaattaatgaaaccaacactccttcagta
cccagatttcagcaaacaattttgcataaccacagatgctagtaaacaagcatgtggagcggtactatct
caagaccataacggtcaacagctaccagtggcatacgcttcaagaagctttacaaaaggcgaaagtaata
agtccactacagagcaggagctagcagctattcactgggcaataaatcacttcagaccatacgtatatgg
tagacatttcttagtacaaagtgaccataggccactatcatatctttttttcaatgagaaaccccagttca
aaattaaccagaatgagacttgacttggaggagtttgaattcacagtagaatatctcaaggggaaagata
atcatgtcgcagacgcattgtcccgaataacaatcggagaacttaaagcaataaatagacagatactaaa
ggtaacaacaagatcaacaacaagacagaaaaatacctgcgcaggtgaaaaattgcatgaaccaaatgag
aaagaaaatataaaaatgcccaatatctatcaggtaatcaataacattgatgccaaaaaatatgttatac
tcaaaatagacaagcataagtgtttgttgaaaagaggaaaacaaattataacacgttttgatatgactaa
tttttattctaatgaaataatcgatttagatcaattctttcaaaggcttaatgaagaagcaagaataaat
agcatcattcaaacacaattgtcaccaagtgaacaaatcttcgaatttgtcactataaagaattttaaag
aaaagggcaataaaatactaaaaaatttaaaaatagcgctattaaacaaggtgactaagatagataaaaa
tgataaggttcaaataaaagcaatactgtctaaatatcatgatgatccatcagaaggaggccattcagga
atttctagaaccctgaggaaaataaaaaactattattattggccacaaatgacaaaggcaataagtaaat
atgttaaaacatgtttaaaatgtcaacaagccaagactacaaaacatactaaaacaccgttgacaataac
agaaacgccagcaacagcatttgataaagttttgatagataccattggtccactgccaagatcagaaaac
ggaaatgagtatgctgttactatcatttgcgatttaacaaaatatttggtaacggtaccaattccaaata
aaagtgcaaaatcagttgctaaggctatattcgaaaattttattctaaagtacggtccaatgaaaacaat
cacaacggacatgggaacggaatataaaaaccaaattatagacgacctatgcaaatatatgaagataaaa
aacattacttcaacagcacaccatcaccagacattaggaacaatagaacgaagtcacagaactttcaacg
agtatgttcgctcatatatatctgttgacaaaaccgattgggatatatggatacaatattttacttattg
tttcaacacaacaccatcggtagttcatgaatattgtccatatgaattagtatttggaagattaccaaga
cagttcatagattttaacaggatagacagaatagatcctatttacaacatggatgactattcaaaagaag
ttaagctacgattagaaatagcatatagaagagctaaaaatatgttagacaaggcaaaagccgatagaaa
gataaaatatgatagaaatattagtaactttgaattaaagataggagataagatattacttaaaaacgaa
acgggtcataaacttgacaataattatttaggaccatatttagtttcagaaataggagataatgacaaca
ttacaattataggaaataaaaataaaaaacagatagtccataaagataggttaaaaatttttaattcata
atacattttgtttggttggccaaccacaaataaaaaaccacaaataaaataaaaaccaataaaaacatta
taaacataaaaacatggctttacgggccattcttctcatcgggcagaaaaagttccgctcaatgttacaa
tattttgtgtaatattaatatgcagcgcagtgcatcgagggtggtgtgccacgcagggcaacaaaaaaaa
atattttatttttcagctcctatttgcttcactctgaccctccttgcgtcagctggacgtcgccggggtt
ggcacgctgacactccctcgatgacgtaatgagcgggctgctcaccacgttgatgatgatttcctcattt

aggggttatgtggtggagctgcaatgtctgcacgtatgttcacaatgcggtgtgaacagtggtccctcgc
agtcgttcgggcatcttttcttgaaaacaggcaggcttttcggattgggagttggagttttgttaattgt
taacgattttatttgattttctgagtgagcgcgtgtggtggggatttttttcttgagatattcgacacag
tggtctagctcagcttgtagtttttgagctttcgaccattggggtggtggagtgttcgtatataatagcc
actttatgtgcgctattctctttttttatattatgtcgaactccgcccctgtttttttcccatcacactgac
actctactcactcagatcgctttttttcttcataattgccgccgtcttggtggatgagcatcgaagttgaa
aattatcacaagcggtttcctttagcttcATTTATCATTCAGGTCATTCATTTAggtcattttatcttta
tttggtttttattaagcgagtgtacattccagatctattcctgatttataactgatcttagtgtggtcta
caggcagatctcttgtgacagccaagtgctgacactagcaaattctgcaatatgtatgtatgaagttcat
actcgataaccaatgggagtcgagtgcgacccctaaaggcgtacatgctgaattcgcatatttagtatta
gggtgtatctaaagatctactagggtgaccctaaggaattagggtggtcctaagtttacttattagtcga
cttattattatgattcatattataattattattaatattattattattattgttattattattgttatta
tgttcgtatatactacaATAGCCACTCACCGCAATTTTTGTACCTTTTACAATATTTGTTGATGCACTTG
CCATAATTTGAGGCCGTATTTATTTGATAGCCGGAAATTTAATGATAGTTTTTCCTGCCGGATATACCCA
AAAGACCATTGTACAATGCCATTGCTGTTTTGTTTATTGACTCTCCTTTCTCGCCTTTCTGCATCCTTTT
ACTTTTAATTAAAAGGTAAATGGCATTAAAACGTCAATTCGCAGAGCTGCAACAACAAGAGCCAATGCCG
AATGGCAGCAAAATTAAAAGTCAAATGCTACCCAGATGTCAAAATCAATTAAACAACTTTTCACCAGCCC
GCATTCTACGAAATGCATTTTTTCCCAAAAAAAGGAAATTCTCTCTGCTGAGATTTCGACATCTTCAATC
TTTTTGCGAAAATTCTctttttttctttttcccttatgcactttttttttGTGCTTCAGGGTTGAGTTGCTG
TGTCCTTTAAATGCTGTCGATCCAATTAACTACGTACAGCGACGTCAAGCGAACAAAAAAAGGCAGATGA
AGGAGCCCCAGgatgatgaagatgatgatgatgatgatgatggggatgataatgTGGAGCCTGAGCCGGT
GCTGCAAAGTGCACAGTTATGGGACACGGTCGCCTTGGAGCCAATCGATCAGGAATTCCTGCAATCACAT
CAGATACAAATCGAGAATAAATTCATTGCCGTCGAATGGATACTGAAATGTGAATGCAGCACGAAG
>dme_circ_0005031_junction_seq
GATACTGAAATGTGAATGCAGCACGAAGGCCAATTGCAGTGTGGGCAACACAACAT
>dme_circ_0005032
GTTTACGCCACCTGCCACAACAGCACCACCAGCGGCAATTGGACGGCCTGCAGCAGGAGCTGCGGCCTTG
GCACCGCCACGCGGCACACCACCACCCACGCGGGCTGCCATCAACTGAGCAATTTGCGGCTCTGCGAGAA
TCGCAGGTGCGACAAGGACGACCACGAGGACAATAAGTGGAGCAGGAGCCGCCCGTTGACGCACAAGCGG
AAGCAGCATCGCGCCCACAGCCACTCCCATCAGCGTTATCATCACCACAAGGAGCACGAGGAGCCGGCCC
ACAGGATACGAGGTGAGTGGGATGGGGATTCCCCGGATACATATCCAACACCTGCCAAGTGGCAACTGGG
CTAAGACGGGCTGAGCTTAAAGTCCGACCACCTGACGCCTCAGGAGGAGAGATGAAAATCCTCGCGGATT
GAGTTTGCTGCATTTCCTTTCCTTTCCATTGCTCCTCTGTTGCAGCTCGAAGAAATTTACATTTTCCGCA
TCCTTGCTTGTTTTTCGCCATTTGTCCTTTTCAATTAGATTACCGTTAATTTGAAAGCAATCCCATCGAC
AGCCAATCGACAATGTTTTGTAAAGCATGAACTGTATTTACTAAAATTGTGATTAAAAAATTAATAAAGA
GGAAAATATTCTTTATGATATGCATATTATTATTTTGTTAGCATAAAAAGTTAGTAGCACAGATTACAAC
ATATTATTTCATTAGTCAATCAAATCAAAATATCTTCACATCCGAAGCCAATCAGGCAAATAATATTTTC
CATCAATTTTATTTGCCTGCATAAGCAAGTCAATCGGAAATATATTATTCAGACTTTATTATTTATATAT
TCTGCTGTTTATATAGtgtagttaattagaattcccattacttctgatgtagttaataaagtctcaaaac
gcagttggtcattttatctttatttggtttttattaagcgagtgtacattccagatctattcctgattta
taactgatcttagtgtggtctacaggcagatctcttgtgacagccaagtgctgacactagcaaattctgc
aatatgtatgtatgaagttcatactcgataaccaatgggagtcgagtgcgacccctaaaggcgtacatgc
tgaattcgcatatttagtattagggtgtatctaaagatctactagggtgaccctaaggaattagggtggt
cctaagtttacttattagtcgacttattattatgattcatattataattattattaatattattattatt

attgttattattattgttattatgttcgtatatactacatctccctccttttgaaaaataacgtaataac
atgaagttattttcaagtatataataattttttttttttttttttaaatgtacattaatatttgaaatga
atgattaaagagatattaaaaaaatatatatattttcattatattttgcaaagtaaaagtttaggaaaat
gtaactatattgaacaaaaaaaaaaaaaaaaaaaatcaaaacaacatatttttaaagtaaaataaaccaa
aacccaaaaataaaacaaaaaaaaaaaaaaaataaaaatatacaaaaatgggttggtttggatctgacga
tagtcagacaaaagataatacggccaatgtggtcaataacttaaaaatagtcgaccatacagatgacatt
cagtcactgtggttactccttttgatcatgacgatcgtaacagtcgctcagtttatattaacgctatatg
ttaagcataacaagataataaagaggcgttatataggcaaagcagagaatagtttggataaaatttaaaa
aaaaaaaaaaaaaaaacatccaaaatgtattttgaattaagatgatctaaaattttatttttaatatca
gaaaactagaatgaaaaaaaaaaaaaaaagattattgaagaaacctatttgagagaggccaaaatttata
aagttcgattgcatagataaatccatagtattctttaaatggactggcaagaaatacaaaacgagcttaa
agaaattaaaacaacttttgacaagtcttataaatgcatgacaccaaatagagaagtgcaacaagacact
ctcaacaagcatgcgcagatattggtaagatgctttaatggagcacgccaattaatttacagagaaagga
aaagattaacaaaaaatcatttatcacaagcagtaaaatttctaaacaggttccgtgagaacttgttaaa
cgtcaagtacagacacaacttaaatattacaatcccaacgattttaagcacacctatagtggctgagatc
ggtgaggatatcgaaagtgtaggagaatcagaaatagaaataaaagaagaggatctccacgatcttgcaa
ttccagcggtaataacattacccgaattacttgaagaagaactttcagattcaaatacaggaataagaat
acaggaaacggacaaaatgacagactctgccgcaacagcaagggaatatgtgcgacaaatttcgtccaca
atacctgagtttgacggcaaaaagttaaacttgaatagattcctcacggctctccggctaatagatctga
caaaaggagatcaggagatgctagcggttgaggtaatcaagacaaagatacttggtccattatcacacaa
agttgaaaatgaaaagaccattatcggtataataaatctattaaaagcatcagttaaaggcgaatcgccc
gatgtcatcaaagcaaaaatgcttagtacacaacagcgcggcaaaactgcagcgcaatataccacggaga
tagaaaacctacgtgggttgctcgaagcagcctatatagatgatggtttagattccaacaatgcagacaa
attcgctacaaaggaagccatatctgcaatgaccaagaactgtgggcacgataagctcaaaaccatattg
gaagctggaaatttcaacacgatgaatagcgtgattgaaaaatacatacactgcagtacagaaatgaccg
gcaattcaaatagtgtattattctataataatagaggacactatcgaggtaataattaccgaggaaatta
ccaaaacagaggtaatggccgaggaaattataactcctacaataacaactatagaggcagaggtggttac
catggtggaaacagaggacgaggtggtaaccaaaattataatagaggtggaggttactcaagaggtaacc
aaaaccataactataaaacaagtcatgcccacaatgtccgaaacatacaatcggaaaacgaacatacccc
cttgagcgacaatctacaataaaattatacaaaattaatctcaatttaagcatttttatacgattgaaga
atatgagtaccaattcatgggtaactcttttaatagatacaggtgcagaaatttccctgcttaaatgcag
aaacaataatcttaacgatttaaatccaaaaaatacaacaaatatatcaggaatagggcaaggacaatt
cagtctctaggtacactacatttagaaatgtgtattgctaatgcagcaataccatatgaattccatatcg
tacctaacaattttcctataccaggggatggtataattggcttggatttcattaagaaatacaattgtat
tttggaattccacgaccaagaagattggttcactttgaggcccaaaaatttcaggaacataaacattcct
attatacattcactagataatgaaataattttgccagctagatcagaagtgattcgaaagattcaactaa
catctactgacacacatgtcctcatccccaaccaggaattacaacctggcataataatcgcaagtgcact
cgtaaacactcagaacgtcttgattcgaattattaatacaactgaaaaagacgctatagttagtagcgca
aatataaaaagcgaatcattggatgattatgatgtatacaacgcaaatatagaaaatagtgcacaaagaa
ctttagaagtattaaaacttcttaaatttccatcgttattcaaaagcgatttaacaaaattatgcaccga
atatagcgatatttttggtcttgaaacagaaaccatatcagctaataatttttacaagcaaaaattgaga
ttaaatgacaaaactccagtctatatcaaaaactatagaatgccagaaagtcaaaaaccagaaattcaaa
ggcaagttgacaaattaataaaagatggcattgtcgaaccatctatttcagaatataatagccctcttct
cttggtacccaagaaatcactgcctaactcggaggaaaagagatggcgattagtagtcgattatcgccaa

ataaacaagaaactgctagcagataaattcccacttccaagaatagaagatattcttgatcaattaggcc
gagcaaaatatttctcgtgcctagacctgatgtcaggattccatcaaatagaattagacgaaaggtcaag
aaatataacatcattctcaacttcaacgggagcataccgctacacgcgattaccatttggtttaaaaata
gccccaaattctttccaaagaatgatgacccttgcattttcaggtttaacgccttcgcaagcatttctgt
atatggatgatttagtagtcataggctgttctgaaaagcacatgcttaaaaatctaaccgacgttttcaa
attatgtaggcaacataatttaaaattacatccagaaaaatgcactttctttatgaaagaggttacttat
ttaggtcacaagtgtactgacaaaggtatattgccagatgactctaaatatgaggtaataaagaactacc
ccaaaccagtaaacgcagacgaagctagacgcttcgtggcattttgcaattattacagaagatttattaa
gaacttttctgagaaatcacgccacttaacgaggctttgtaaaaagaatgttccatttgaatggacaagc
gaatgcaatgatgcattcgaatatctcaaaagggaattaatgaaaccaacactccttcagtacccagatt
tcagcaaacaattttgcataaccacagatgctagtaaacaagcatgtggagcggtactatctcaagacca
taacggtcaacagctaccagtggcatacgcttcaagaagctttacaaaaggcgaaagtaataagtccact
acagagcaggagctagcagctattcactgggcaataaatcacttcagaccatacgtatatggtagacatt
tcttagtacaaagtgaccataggccactatcatatctttttttcaatgagaaaccccagttcaaaattaac
cagaatgagacttgacttggaggagtttgaattcacagtagaatatctcaaggggaaagataatcatgtc
gcagacgcattgtcccgaataacaatcggagaacttaaagcaataaatagacagatactaaaggtaacaa
caagatcaacaacaagacagaaaaatacctgcgcaggtgaaaaattgcatgaaccaaatgagaaagaaaa
tataaaaatgcccaatatctatcaggtaatcaataacattgatgccaaaaaatatgttatactcaaaata
gacaagcataagtgtttgttgaaaagaggaaaacaaattataacacgttttgatatgactaatttttatt
ctaatgaaataatcgatttagatcaattctttcaaaggcttaatgaagaagcaagaataaatagcatcat
tcaaacacaattgtcaccaagtgaacaaatcttcgaatttgtcactataaagaattttaaagaaaagggc
aataaaatactaaaaaatttaaaaatagcgctattaaacaaggtgactaagatagataaaaatgataagg
ttcaaataaaagcaatactgtctaaatatcatgatgatccatcagaaggaggccattcaggaatttctag
aaccctgaggaaaataaaaaactattattattggccacaaatgacaaaggcaataagtaaatatgttaaa
acatgtttaaaatgtcaacaagccaagactacaaaacatactaaaacaccgttgacaataacagaaacgc
cagcaacagcatttgataaagttttgatagataccattggtccactgccaagatcagaaaacggaaatga
gtatgctgttactatcatttgcgatttaacaaaatatttggtaacggtaccaattccaaataaaagtgca
aaatcagttgctaaggctatattcgaaaattttattctaaagtacggtccaatgaaaacaatcacaacgg
acatgggaacggaatataaaaaccaaattatagacgacctatgcaaatatatgaagataaaaaacattac
ttcaacagcacaccatcaccagacattaggaacaatagaacgaagtcacagaactttcaacgagtatgtt
cgctcatatatatctgttgacaaaaccgattgggatatatggatacaatattttacttattgtttcaaca
caacaccatcggtagttcatgaatattgtccatatgaattagtatttggaagattaccaagacagttcat
agattttaacaggatagacagaatagatcctatttacaacatggatgactattcaaaagaagttaagcta
cgattagaaatagcatatagaagagctaaaaatatgttagacaaggcaaaagccgatagaaagataaaat
atgatagaaatattagtaactttgaattaaagataggagataagatattacttaaaaacgaaacgggtca
taaacttgacaataattatttaggaccatatttagtttcagaaataggagataatgacaacattacaatt
ataggaaataaaaataaaaaacagatagtccataaagataggttaaaaatttttaattcataatacattt
tgtttggttggccaaccacaaataaaaaaccacaaataaaataaaaaccaataaaaacattataaacata
aaaacatggctttacgggccattcttctcatcgggcagaaaaagttccgctcaatgttacaatattttgt
gtaatattaatatgcagcgcagtgcatcgagggtggtgtgccacgcagggcaacaaaaaaaaatatttta
tttttcagctcctatttgcttcactctgaccctccttgcgtcagctggacgtcgccggggttggcacgct
gacactccctcgatgacgtaatgagcgggctgctcaccacgttgatgatgatttcctcatttaggggtta
tgtggtggagctgcaatgtctgcacgtatgttcacaatgcggtgtgaacagtggtccctcgcagtcgttc
gggcatcttttcttgaaaacaggcaggcttttcggattgggagttggagttttgttaattgttaacgatt

ttatttgattttctgagtgagcgcgtgtggtggggattttttttcttgagatattcgacacagtggtctag
ctcagcttgtagtttttgagctttcgaccattggggtggtggagtgttcgtatataatagccactttatg
tgcgctattctcttttttatattatgtcgaactccgcccctgtttttttcccatcacactgacactctact
cactcagatcgctttttttcttcataattgccgccgtcttggtggatgagcatcgaagttgaaaattatca
caagcggtttcctttagcttcATTTATCATTCAGGTCATTCATTTAggtcattttatctttatttggttt
ttattaagcgagtgtacattccagatctattcctgatttataactgatcttagtgtggtctacaggcaga
tctcttgtgacagccaagtgctgacactagcaaattctgcaatatgtatgtatgaagttcatactcgata
accaatgggagtcgagtgcgacccctaaaggcgtacatgctgaattcgcatatttagtattagggtgtat
ctaaagatctactagggtgaccctaaggaattagggtggtcctaagtttacttattagtcgacttattat
tatgattcatattataattattattaatattattattattattgttattattattgttattatgttcgta
tatactacaATAGCCACTCACCGCAATTTTTGTACCTTTTACAATATTTGTTGATGCACTTGCCATAATT
TGAGGCCGTATTTATTTGATAGCCGGAAATTTAATGATAGTTTTTCCTGCCGGATATACCCAAAAGACCA
TTGTACAATGCCATTGCTGTTTTGTTTATTGACTCTCCTTTCTCGCCTTTCTGCATCCTTTTACTTTTAA
TTAAAAGGTAAATGGCATTAAAACGTCAATTCGCAGAGCTGCAACAACAAGAGCCAATGCCGAATGGCAG
CAAAATTAAAAGTCAAATGCTACCCAGATGTCAAAATCAATTAAACAACTTTTCACCAGCCCGCATTCTA
CGAAATGCATTTTTTCCCAAAAAAAGGAAATTCTCTCTGCTGAGATTTCGACATCTTCAATCTTTTTGCG
AAAATTCTctttttttctttttcccttatgcacttttttttGTGCTTCAGGGTTGAGTTGCTGTGTCCTTT
AAATGCTGTCGATCCAATTAACTACGTACAGCGACGTCAAGCGAACAAAAAAAAGGCAGATGAAGGAGCCC
CAGgatgatgaagatgatgatgatgatgatgatggggatgataatgTGGAGCCTGAGCCGGTGCTGCAAA
GTGCACAGTTATGGGACACGGTCGCCTTGGAGCCAATCGATCAGGAATTCCTGCAATCACATCAGATACA
AATCGAGAATAAATTCATTGCCGTCGAATGGATACTGAAATGTGAATGCAGCACGAAG
>dme_circ_0005032_junction_seq
GATACTGAAATGTGAATGCAGCACGAAGGTTTACGCCACCTGCCACAACAGCACCA
>dme_circ_0005033
GATTAACTGGAAAGGAGCTGATGAGCTTACCGTGCGTTGCTGGAATGGCGAGGTAATCACAGCGGATCAT
GTCATATGCACTGTTTCCTTGGGAGTTCTGAAGGAGCAGCATCCGAAACTTTTTGTTCCCGCCCTGCCAG
CTGCCAAAGTGAGAGCGATCGAGGGTCTTAAGCTGGGCACCGTAGACAA
>dme_circ_0005033_junction_seq
AGGGTCTTAAGCTGGGCACCGTAGACAAGATTAACTGGAAAGGAGCTGATGAGCTT
>dme_circ_0005034
GACCCTCTCCTCGGTGAGCGTTTCCATGTGCCGGGCGTGTGGGCCAATGATCCAGCCCTGCAGGATGCGT
GGCTGCCTGGAGACTGGATAAAAGCCGAACACACTCTCCAGCCAAAAGAGTTCCGAAGCCCGCAGCTCCT
CCAGG
>dme_circ_0005034_junction_seq
GAGTTCCGAAGCCCGCAGCTCCTCCAGGGACCCTCTCCTCGGTGAGCGTTTCCATG
>dme_circ_0005035
GCCGGTCTCTTGGTTTTAAAAAGAGAACCGGAGCGAGAACTTTATCTTTATATTGTGTGATATTGCTGCC
TTCTCATTAATTCTAACCCACTGATAAAACATGAAGAAAGAAAACCAAGGGCTGATTGTGCAGCGAGCGA
TTGATTGAAGTCCGCCGTCGACGTCGGCGCTCCGACGAGGGATCATAGAGGGAGGTTCTCCGGTTCGAGT
TCTGGATCGTTGGAATCAGTCGTTGGGAGGCACTTTTGGCGTGAAGATCGCGGCGAGGAAGTGCTCGGAA
TCTGAAATTTTAAGTCATATTGTGTGGTCTACGCTTGTGGTAAAGTTTCGTTTGATTATGTACATGGTGT
TGAAATCAAATACAAATATTGTATAATTAAAGATAGCCAAGAAATTTAGATTTTCAAAAAACACTAGATA
AATACAGCTGAAACTCAATTGATGATTATGCGCCATAAATATTTTTGAATGCAAACAATAAGCAAGACAA
AGGGCATAAAAAACAAGGGAGTAAAAGTCACATAATTGATTCATTTATGCAAACTCTGCGAAATTAATCT

ATGAATATTCACCACGTTAAGCCATTTAAAAACAACAAAAACTCCAACCGATTGGCCATTTAACATTTTG
TTTATTTGATTTAATCACTTTGTTATGCTTGCTTATTTGTTGTAAAATCAGCCTGCTGAATGAAAATCAC
TAGATATTaaaaaaaaaGAGCGTGCAGCGATGTCTTTGTGTTTTTGAGAACCACCAGCTGATTACTTATT
TTTAACTCAGCTTTCTTTTATGTCCAAATTTTAGTTCTCTTTAGTTGTGTCTCTTAAAAAATTGAATGTG
TCGGAGAGTGTGTTATCACACGTGATATATGTACG
>dme_circ_0005035_junction_seq
GTGTGTTATCACACGTGATATATGTACGGCCGGTCTCTTGGTTTTAAAAAGAGAAC
>dme_circ_0005036
GCCGGTCTCTTGGTTTTAAAAAGAGAACCGGAGCGAGAACTTTATCTTTATATTGTGTGATATTGCTGCC
TTCTCATTAATTCTAACCCACTGATAAAACATGAAGAAAGAAAACCAAGGGCTGATTGTGCAGCGAGCGA
TTGATTGAAGTCCGCCGTCGACGTCGGCGCTCCGACGAGGGATCATAGAGGGAGGTTCTCCGGTTCGAGT
TCTGGATCGTTGGAATCAGTCGTTGGGAGGCACTTTTGGCGTGAAGATCGCGGCGAGGAAGTGCTCGGAA
TCTGAAATTTTAAGTCATATTGTGTGGTCTACGCTTGTGG
>dme_circ_0005036_junction_seq
AGTCATATTGTGTGGTCTACGCTTGTGGGCCGGTCTCTTGGTTTTAAAAAGAGAAC
>dme_circ_0005037
GTATCCCAGAAACCAGCGGACGGAGACTGTCGGAGCCAAGGTCTTCTTCTACAGAGCATCCCTGCGGTCC
GGCCAGGTGCCCGAAAATCTGACCAAGTTCGAGTGGCTTCCCAAGGAGGCGCTCAATGAAAAGCTGACCA
ATACAGCCTATGCCCAAAGCGTTAAAAA
>dme_circ_0005037_junction_seq
ATACAGCCTATGCCCAAAGCGTTAAAAAGTATCCCAGAAACCAGCGGACGGAGACT
>dme_circ_0005038
GCGCAGAAAAAGGAAATGGATGAGGAGTCGCGGAAGAAGTTTGAGACCGAGCTCAAGGAGAAGGAGGAAA
AGCATAAGGACCACGAGAAGCTGCACCACCCTGGCAACAAGGCCCAACTAGAGGATGTGTGGGAGAAGCA
GGACCACATGGACAAGAACGACTTTGATCCGAAGACATTCTTCTCCATCCACGACGTCGACAGCAACGGC
TACTGGGACGAGGCTGAGGTCAAAGCTCTGTTTGTCAAGGAACTGGACAAGGTCTATCAGAGTGATCTTC
CCGAGGACGATATGAGGGAGCGAGCAGAGGAAATGGAACGTATGCGCGAGCACTACTTTCAGGAGACGGA
CATGAACCACGACGGCTTAATCAGCATCGACGAGTTCATGG
>dme_circ_0005038_junction_seq
GGCTTAATCAGCATCGACGAGTTCATGGGCGCAGAAAAAGGAAATGGATGAGGAGT
>dme_circ_0005039
GCTCGGTCTCAAACTTCTTCCGCGACTCCTCATCCATTTCCTTTTTCTGCGCCTCACGCTCAAACTCTTT
CTGCATTTCGTACTCCTTGAACTCGCCACGTCGCTTGCGGTCCGCCTCGGCCAGGTCGTCGGAGGTCTTC
TGAATGAGCTTTCGCAGATCTTCGATCTCGAAGGTATGCTCGTTGTCGTGGTCCAGATGCTGAGACACCT
TCAG
>dme_circ_0005039_junction_seq
CGTGGTCCAGATGCTGAGACACCTTCAGGCTCGGTCTCAAACTTCTTCCGCGACTC
>dme_circ_0005040
GATCACAAGGAGGCGGCGGAGTCCTCCACTCCGGCCACCGCCGACGTGGAAACGGCCCTGGAGTACGAGC
GCTACCTGCGGGAGGTTGTCGAGGCCCTTGAGGCGGACCCTGAGTTCCGTAAGAAGCTGGACAAGGCGCC
CGAGGCCGACATTCGGG
>dme_circ_0005040_junction_seq
GACAAGGCGCCCGAGGCCGACATTCGGGGATCACAAGGAGGCGGCGGAGTCCTCCA
>dme_circ_0005041

TCACGCCCAGTCGCCGGCAATCCTAAAGCGTCACGGAGACGTGATTGCCAGGTGGCAGAAGTTGCGCGAC
GCCTCCAACACGCGCAAGGACCGACTGCTGGCCATGCAGGAGCAGTTCCGCCAGATCGAGGAACTCTACT
TGACATTTGCCAAGAAAGCTTCGGCCTTCAATTCTTGG
>dme_circ_0005041_junction_seq
CAAGAAAGCTTCGGCCTTCAATTCTTGGTCACGCCCAGTCGCCGGCAATCCTAAAG
>dme_circ_0005042
CTGCGCCTGAAACTGGACAACCTGTCCGCCCTGGCTGCCCGCCGCAAGGGTGCCCTGCTGGACAACTCGG
CCTATCTGCAGTTCATGTGGAAGGCCGATGTGGTCGAGAGCTGGATCGACGACAAGGAGAACTATGTGCG
CTCCGATGAGTTCGGACGCGACCTGTCCACCG
>dme_circ_0005042_junction_seq
GATGAGTTCGGACGCGACCTGTCCACCGCTGCGCCTGAAACTGGACAACCTGTCCG
>dme_circ_0005043
GGTACTCCCACTGATCGGCGATCTGCGTCAGCCGCTTCTGCACCGCGTCCTCCGACCCGCTGCACTGCTT
CTTATCGATGAGGTTTCCGCCCATGGCTAGCACGCTCTGGATGCGATCGGCGTTAGCCGCCAGCTCCGCC
TCGAAGGCCTGGTGCTTCTGGTGCTTCGACTGAATGTTAGCCGGGTCCTTGTAGCTCTCTTCG
>dme_circ_0005043_junction_seq
GTTAGCCGGGTCCTTGTAGCTCTCTTCGGGTACTCCCACTGATCGGCGATCTGCGT
>dme_circ_0005044
TTGCCTCCTTCTCCACGCTGACCGTCTTCATGCCCATCTGGGCGGCCTTGATGGCGGCGACATAGCCACC
GGGTCCGGAGCCAATCACCACGATGTCCGCCTCGTGGGTGCTGGAGTAGCAGCGGGCGTTGAGGGCACCC
AGAATGGCGGCATTGGTGCGCAGAGGAGTCTG
>dme_circ_0005044_junction_seq
TGGCGGCATTGGTGCGCAGAGGAGTCTGTTGCCTCCTTCTCCACGCTGACCGTCTT
>dme_circ_0005045
GATGTTCTTGGTCTTGACAGTCTCCGTGGAGCCGTCGGATTTCTTAACCTCCACCTCGTTGGGATTGACG
ATGGTGCCGAAGCCCGTCAGCTGTGTGACCTTGTTCTTCTTGAAAAGCATGGCAATGCCACCGGTCAAAG
CCTTCACGGCGTTGGACTTTTGACCCATAAGCTTCTCCAGATCCAGGCTAACGCTGCCGCAGCTAATACC
ACGTTTCTCCAGATCACCGGAGTGGGCCATGTGGTAGTAGTGGGAG
>dme_circ_0005045_junction_seq
GGAGTGGGCCATGTGGTAGTAGTGGGAGGATGTTCTTGGTCTTGACAGTCTCCGTG
>dme_circ_0005046
GGTTAAGAAATCCGACGGCTCCACGGAGACTGTCAAGACCAAGAACATCCTCATCGCCACCGGCTCGGAG
GTTACCCCCTTCCCTGGCATTGAAGTAAGTGCAAAACCTCTACAGCGCGCTTGATTACTAATGCATGCCA
TTCAAAAACCTTCACAGATCGACGAGGAAGTTATTGTGAGCAGCACTGGCGCCCTGAAGCTGGCCAAGGT
GCCCAAGCATCTGGTTGTCATCGGCGCCGGTGTCATTGGCCTGGAGCTGGGTTCG
>dme_circ_0005046_junction_seq
CGGTGTCATTGGCCTGGAGCTGGGTTCGGGTTAAGAAATCCGACGGCTCCACGGAG
>dme_circ_0005047
GGTGCCCAAGCATCTGGTTGTCATCGGCGCCGGTGTCATTGGCCTGGAGCTGGGTTCGGTATGGTCGCGT
CTGGGCGCTGAGGTCACTGCCATTGAGTTCATGGATACGATCGGCGGCGTGGGCATCGATAACGAAGTCT
CCAAGACGTTCCAAAAGGTGCTCACCAAGCAGGGTCTTAAGTTCAAGCTGGGCACCAAG
>dme_circ_0005047_junction_seq
GGGTCTTAAGTTCAAGCTGGGCACCAAGGGTGCCCAAGCATCTGGTTGTCATCGGC
>dme_circ_0005048

GTTCATGGATACGATCGGCGGCGTGGGCATCGATAACGAAGTCTCCAAGACGTTCCAAAAGGTGCTCACC
AAGCAGGGTCTTAAGTTCAAGCTGGGCACCAAGGTGACGGCTGCCTCTCGCAGCGGCGACAACGTCACCG
TGTCCGTGGAGAACGCCAAGTCTGGCGAGAAAGAGGAGATCCAGTGCGACGCCCTGCTGGTCAGTGTTGG
ACGTCGTCCCTACACCGAAGGTTTGGGTCTGGAGGCCGTGGGCATT
>dme_circ_0005048_junction_seq
AGGTTTGGGTCTGGAGGCCGTGGGCATTGTTCATGGATACGATCGGCGGCGTGGGC
>dme_circ_0005049
GTGAAGAATTTTGCACCGCGTTTGAAATCTTTGAATAACCCCTATTCGAAAAATTATCCTGACGGCCGAA
ACGACAGTCTCCGCATATTTGTAACTCTCAATGTAGGAACAGAAAAGTGGAAAATATAAATAATGGTAAT
GGCGCCAGGCGTTAATTGCAATGTAATAT
>dme_circ_0005049_junction_seq
GCGCCAGGCGTTAATTGCAATGTAATATGTGAAGAATTTTGCACCGCGTTTGAAAT
>dme_circ_0005050
GGTGAGTATGTGGATGATGAGAAGCGTGTCTAATCGCGCATTTCGCAGGCAAAAAAAGTAAATGTATAGT
GTGGTTAATATCAATATATATTTAGGATGTACTGTGCATCAATATCAGGGGCACCTCGTTGCTGTACTTT
GAGTTCTCCTGGCCGCCCAACAACGCCGAATTGCTGCAGTCCCGCATCATGGCAATGGAATTGTTGCGCT
GTTCCCGCTGCCTCAGCCGGCTGTGACGGATGCCATAGGCCAGGAAGATTGCCAGGCCTATGGCGATCCA
TATACTGAAGCGAATCCATGTGAGAATGTCCAACTTGATCATCAGGTAAATGTTGATCAAAATGGAAATG
CCGG
>dme_circ_0005050_junction_seq
TAAATGTTGATCAAAATGGAAATGCCGGGGTGAGTATGTGGATGATGAGAAGCGTG
>dme_circ_0005051
GTTCTCCTGGCCGCCCAACAACGCCGAATTGCTGCAGTCCCGCATCATGGCAATGGAATTGTTGCGCTGT
TCCCGCTGCCTCAGCCGGCTGTGACGGATGCCATAGGCCAGGAAGATTGCCAGGCCTATGGCGATCCATA
TACTGAAGCGAATCCATGTGAGAAT
>dme_circ_0005051_junction_seq
ATATACTGAAGCGAATCCATGTGAGAATGTTCTCCTGGCCGCCCAACAACGCCGAA
>dme_circ_0005052
GTCTTATCCGCCTTCGATCTTACCGCCCTGGGAATCGGATCGACGTTGGGTGTAGGCGTTTACGTCCTAG
CTGGCGAAGTGTCCAAGCAATATGCCGGTCCAGCCGTGGTCGTAAGCTTCCTCATCGCTGCCATCGCCTC
GATTTTCGCCGGGCTGTGCTATGCGGAATTCGGAGCTCGGGTTCCAAAGGCGGGATCGGCGTACATCTAC
AGCTATG
>dme_circ_0005052_junction_seq
GCGGGATCGGCGTACATCTACAGCTATGGTCTTATCCGCCTTCGATCTTACCGCCC
>dme_circ_0005053
ATCAACGAGATGTGGGACCAGATCATCACCAAGTCCACCGCCCGCAAGGAGAAGCTGCTGGACTCGTACG
ACCTGCAGCGTTTCCTCAGCGACTACCGCGATCTGCTGGCCTGGATCAACTCGATGATGAGCCTGGTCAC
GTCCGACGAGCTGGCCAACGATGTAACAGGAGCCGAGGCTCTGATCGAGCG
>dme_circ_0005053_junction_seq
TAACAGGAGCCGAGGCTCTGATCGAGCGATCAACGAGATGTGGGACCAGATCATCA
>dme_circ_0005054
GTCAACGACCGCCAGGGCTTCGTACCGGCCGCATACATCAAGAAGATTGACGCCGGCTTGAGCGCCTCCC
AGCAGAACCTAGTGGACAATCATTCGATTGCCAAGCGCCAGAACCAGATCAACTCGCAGTACGACAACTT
GCTGGCCCTCGCCCGCGAGCGCCAGAACAAGCTAAACGAGACTGTGAAGGCGTACGTTCTTGTTCGTGAG

GCTGCCGACTTGGCCCAGTGGATCCGGGACAAGGAGAACCACGCTCAGATCGCTGATGTTGTGGGAGAGG
ATCTGGAAGAGGTGGAGG
>dme_circ_0005054_junction_seq
GTGGGAGAGGATCTGGAAGAGGTGGAGGGTCAACGACCGCCAGGGCTTCGTACCGG
>dme_circ_0005055
GTCTCGATGAAGAAGGGCGATGTTCTGACCCTGCTGAACTCGAACAACAAAGACTGGTGGAAGGTGGAGG
TCAACGACCGCCAGGGCTTCGTACCGGCCGCATACATCAAGAAGATTGACGCCGGCTTGAGCGCCTCCCA
GCAGAACCTAGTGGACAATCATTCGATTGCCAAGCGCCAGAACCAGATCAACTCGCAGTACGACAACTTG
CTGGCCCTCGCCCGCGAGCGCCAGAACAAGCTAAACGAGACTGTGAAGGCGTACGTTCTTGTTCGTGAGG
CTGCCGACTTGGCCCAGTGGATCCGGGACAAGGAGAACCACGCTCAGATCGCTGATGTTGTGGGAGAGGA
TCTGGAAGAGGTGGAGG
>dme_circ_0005055_junction_seq
GTGGGAGAGGATCTGGAAGAGGTGGAGGGTCTCGATGAAGAAGGGCGATGTTCTGA
>dme_circ_0005056
ATCAACTCGCAGTACGACAACTTGCTGGCCCTCGCCCGCGAGCGCCAGAACAAGCTAAACGAGACTGTGA
AGGCGTACGTTCTTGTTCGTGAGGCTGCCGACTTGGCCCAGTGGATCCGGGACAAGGAGAACCACGCTCA
GATCGCTGATGTTGTGGGAGAGGATCTGGAAGAGG
>dme_circ_0005056_junction_seq
GATGTTGTGGGAGAGGATCTGGAAGAGGATCAACTCGCAGTACGACAACTTGCTGG
>dme_circ_0005057
GTGGAGGTCAACGACCGCCAGGGCTTCGTACCGGCCGCATACATCAAGAAGATTGACGCCGGCTTGAGCG
CCTCCCAGCAGAACCTAGTGGACAATCATTCGATTGCCAAGCGCCAGAACCAGATCAACTCGCAGTACGA
CAACTTGCTGGCCCTCGCCCGCGAGCGCCAGAACAAGCTAAACGAGACTGTGAAGGCGTACGTTCTTGTT
CGTGAGGCTGCCGACTTGGCCCAGTGGATCCGGGACAAGGAGAACCACGCTCAGATCGCTGATGTTGTGG
GAGAGGATCTGGAAGAGG
>dme_circ_0005057_junction_seq
GATGTTGTGGGAGAGGATCTGGAAGAGGGTGGAGGTCAACGACCGCCAGGGCTTCG
>dme_circ_0005058
TTCGGGCGAGAACATGCTGAAGGACCAGCCCTTCGCCAGCGACGATATCCGTCAGCGTTTGGAGGCGCTC
CAAGAGCAGTGGAACACGCTGAAGGAGAAGTCCAGCCAGCGAAAGCAGGATCTGGATGACTCTCTCCAGG
CACACCAG
>dme_circ_0005058_junction_seq
TCTGGATGACTCTCTCCAGGCACACCAGTTCGGGCGAGAACATGCTGAAGGACCAG
>dme_circ_0005059
GATCCTGCTTTCGCTGGCTGGACTTCTCCTTCAGCGTGTTCCACTGCTCTTGGAGCGCCTCCAAACGCTG
ACGGATATCGTCGCTGGCGAAGGGCTGGTCCTTCAGCATGTTCTCGCCCGAACTGATCACATTCAGCAGC
CTGGCCTCATGGTTGTTGATCTCGGCCAGCACAGCCTGGT
>dme_circ_0005059_junction_seq
TTGTTGATCTCGGCCAGCACAGCCTGGTGATCCTGCTTTCGCTGGCTGGACTTCTC
>dme_circ_0005060
GGAAGGAGATTGGGCATTTCCTAAGGATGATCGTTGGCCCTAGTAACCGATTGGGCTGTGCCATAGCCAG
TATTGAAAAAGACGGATGGACCCATCAATGGCTAGCCTGTCTGTACAGCTGTTCGCCCCAGAAGAACTCG
CTTCTTTATGAGTATTCCGGAAAACCGGGAGTATATTGCACCACTGGGATTAATGGAAAATTCCAAAATC
TATGCAATGACACTGAACCTGTGAAAGATTGCATGCACTCTGAACTATTCCAAACAATAACTGCCAACGA

CACTACATCACTAATCAGGAGCATGTTGAATAGACAAACGCAATCCAGATCTATTCATTCTTGGTTTTCG
AATCGGGAGACAACAGTTTGGGGCTTGATGAAGAATGCTTGGAATGCAGTTGTGGGTTTGCTGTAATGGT
GGTGGTGGTGATGATGGTGAATGAATTCGACTTATAATCAATAAATTTATAACATGAAAATTAGGGCTAA
AATAAAGGTAAGAATAATAGTTTGGAAACAACAACGTAATTTCATTCTTCTCATAAAGGGCGTTGCTAAT
TCTGTCGGGGTTTGGATTTTTTCAAATCTTTCTAGCAATATGGCTGTTGTTATCTAAAGATTTGAAAATT
TGTTTTAGCTAATACCAATATGAATTGGATTCTGTACGTATTACTATGCTATTATGTAAAAAATTTTTTA
GTAAGCTAGCGTTAAGTTACACATCCCAATTCCTTACCCGTTACTCGTATGGTAAAAGCAAGCATTTCGT
aagcatttccgaccgtataaagtatataaattcttgatcaggatcaatatcgagtcgatctgaccatgtc
cgtctgtccgtatgaacgctatactatatggaaccataaaatctagaaagttaaattagacatgcagatt
ctagtgacgcccacgctgtgcaatttttcaaacccattatatagcccacaaacccatgttgccgcatcta
ctccaacgcccacaaatccacacttttcaaatctgtttagttttttcttcgttttattgtttgtcttgtc
attttctatcggtataccaaaaacacttcgaccaggcccctccctacacctacaaaacgcccaaaacgtt
cacttttaaacaatttttaagttttccctcattttgttctccaatatctatcgatatcccagaaaaatta
tgaattttcgccttcgcattcacactagctgagtaaaggatatttgatagtcggggaattcggctatagt
attctctcttgttATTGTATTGCAGGAGCTGGTCGCCAAAGTGCGGAAAAGGACCAAAAATCATTGACGT
AACCAAGCATATAAAGAAGTTAATTCTAAACTATCACAACACATATCGCGATAATGTGGCCGGGGGTCAA
ATGCACAGGCTACCTATTGCAGCTCGTATGCTCAAGTTGAAGTGGGACCAAGATTTGGCCCTTTTGGCTA
CAATCTTGGTAAAGCGATGCGATCTCCAACCCCACAGACCACTGCATATCCACGGAAGAGTTCTCGTCTCC
AAGCTACCATGCGGTATATAACAAGTTCAAGGCGAAGGAAGATACATTCCGGATAGTTCGATCTCAGCTG
AACGCATGGTATGATCAGTACAAGCACGTTTCTTCATCCAGTCTAATTGATGGATTGTCCACAGCTAAGT
AAGCTTTAATTGTAATTTTAGTTTTCATTTAAATATAAATATTTAAATAAATAACATTTATCGGTTTCAT
GAAGGAGATTGGGCATTTCCTAAGGATGATCGTTGGCCCTAGTAACCGATTGGGCTGTGCCATAGCCAGT
ATCGAAAAAGGCGGATGGACCCATCAATGGCTAGCCTGTCTGTACAGCTGTTCACCCCAGAAGAACTCGC
TTCTTTATGAGTATTCCGGAAAACCGGGAGTATATTGCACCACTGGGATTAATGGAAAATTCCAAAATCT
ATGCAATGACACTGAACCTGTGAAAGATTGCATGCACTCTGAACTATTCCAAACAATAACTGCCAACGAC
ACTACATCACTGATCAGGGGCATGTTGCATAGACAAACGCAACCCAGAACTAATGAGGCGTATATGAAAG
CGGGGAAAAAAGTTGGGCAGTTTTTCTTCATCCTATTTCAATGCGTATGTCGAGCCATAAAAAAATATTT
AGACTCGACaaaaaaaaaaaaaaaaaaaaataaaaaaaaTTAGTTTTCTTGAAGATGTAACCAGTAACATT
TTGTAGTCATATTTGGGCCATGAACTGAACTAATGAACTGAGCTtaatttaatataaatgaatataatat
aaactaatataaataatgtaatataatataataataataacaataataataataatataatataata
aAAACTAATTTCCACACCGAGAAATGGTCAACACAATTAGTTTCAAAGAAAAGTGTGAAGATGTGGAGTT
GTCCTTTCGTTGCAGCTGGGTAGCTGCTGTTATTCGGTCTGAATTTGGTATTTTTGCTGCCAGGAGCCAA
CTATTGCTATTTAAAAAACTGTCCATCGGATAAAAAACTGCGATACATTGGCATCTGATACTCTAGAAAC
TCTTATACATATAGCGTAAGAAATATACTACATAAGAAATTTCATTCTAAACTATCACAACACATATCGC
GATAATGTGGCCGGGGGACAAATGCACAGGCTACCTATTGCAGCTCGTATGCTCAAGTTGAAGTGGGACC
AAGATTTGGCCCTTTTGGCTACAATCTTGGTAAAGCGATGCGATCTCCAACCCTCAGACCACTGCATATC
CACGGAAGAGTTCTCGTCTCCAAGCTACCATGCGGTATATAACAAGTTCAAGGCGAAGGAAGATACATTC
CGGATAGTTCGATCTCAGCTAAACGCATGGTATGATCAGTACAAGCACGTTTCTGCATCCAGTCTAATTG
ATGGATTGTCCACGGCTAA
>dme_circ_0005060_junction_seq
GTCTAATTGATGGATTGTCCACGGCTAAGGAAGGAGATTGGGCATTTCCTAAGGAT
>dme_circ_0005061
GTCACGACCGCGGTTCGTGGATGCGGCAATGGGTTCCTTCTCACGGATCCAGGCAGCCTCGTCCTCCAAG
TCGCGGAAGAGCTGCTGCACCTGAAGGGAGTCAAGAAGGTGTTGTTTCCTCTCGCCCATGGGTGCAGCTA

GGGCGGCATAGCGAGCCGACAGATTGCCCTCCTTGTTGCGAATGTTGTCCGCGTCAAA
>dme_circ_0005061_junction_seq
CCTTGTTGCGAATGTTGTCCGCGTCAAAGTCACGACCGCGGTTCGTGGATGCGGCA
>dme_circ_0005062
GCTCGGTGCCCACGTTGGTGATGTCCTCGATGCGCGACTTGTTGGCATTCAGCTCGTGCTCAAAGTTCTG
GTGCTTCTGCATCTTTCCGTTAAGGTTGGTAGGATCCAAATAGCTGTCATCGGTGGCAAACTTCAGCTTC
TCGCTAATCCAGCCCTTGGTCTCGTCGCAATCGCGCTCGAACTGCTGGTAGCGGTTCGAATCCTCCAGCA
ATTGGCGACGCTTGGACGATTTTTCTTGGAGAGCAGCCCGCCGGGCTAGGAGCATCTGACGGCGCTGGGC
CACATCATCGGCGGCATAATGCTGACCATCGATTAATTTGGTGGCAAAGATGTCTAAAGCCTTGATCTTC
TCCTCCTGGGCAGCCAGACTCTTCTCGAAGTCCTCGTGCTTCTTAATTAGCGCCTCCACAGAGTCCAGGG
AGTCGCCAAGGTCCTCGTTGGCCAGGAAGGCCTCCTGTTTGGCCATCCAAGTGTCAGCCTGCTCCGTATC
GCGGTAAAACAGTTGCAGATCCATGCACTGCTCGTACAGGATGCGACGATCTTCCCACAGCGAGAGCAGT
GAACTCTTGTCGTTTTCCAGAGCGGCAAGCTTTTCCTGGATTTCGGCGGCGGCGTAATGCTCACGCTCCA
GCAGCTTCTGTCCGGATTCAGTGGTCAGCTTGAAGCTATCCTCACGAGCGTCGATCTCGCCCTTGTGCTC
CTGGTGACGTTCCAGAAGAGCCTCTGCTCCAGCCACATCCTTAGCCAGCTCATCGGCCGAGATGATGGCC
TTCATGCCATTGATCCAGGACACGAGGTCGCGGAAATCGGCGAGGAAACGGTGCAGGTAGTAGGACTCAT
CTAGCTTCTGCTTGCGCTCGCGAGCCTTGGTGGTGAGACTCTGCCAGTAGTTGGCAATCTCGGCCTGTTT
GTCACGGATCTGGTCACT
>dme_circ_0005062_junction_seq
CGGCCTGTTTGTCACGGATCTGGTCACTGCTCGGTGCCCACGTTGGTGATGTCCTC
>dme_circ_0005063
GAACTTCATATTTGTCGAACTTTCGCAGGGCCAAGCTCTCGTGCTTGATGATGCACACCTTCTGGCTGCT
AAGGAGCAGGGTGCCGATTAGGATGCCTCCATCGGTCTTAGCACGGCAGCAGAACACCCGCTCATTTTTG
ACCGTCGTGCCCACGGCAACGGCTCCCTTTTCATAGAATCCATCGAAGCTCCTGACCCACACGTAATTTA
CATCCCGCTCAGCCACTAGGATGTCGTAGACGAGGAGCTTCGAGGATGTGGTTTCC
>dme_circ_0005063_junction_seq
GACGAGGAGCTTCGAGGATGTGGTTTCCGAACTTCATATTTGTCGAACTTTCGCAG
>dme_circ_0005064
GGTAGTAGGACTCATCTAGCTTCTGCTTGCGCTCGCGAGCCTTGGTGGTGAGACTCTGCCAGTAGTTGGC
AATCTCGGCCTGTTTGTCACGGATCTGGTCACTGTGATCGGCGTGAATGGAGCACAGCCGCTGGGCCTCG
GCTCCCAGTGTGGAAACCTTATCCTCCAAGGCGGCCAAATCCCGCTCCACTCCTTCGTGTTTGCGTTGCA
GCGCCTGAACACTGGCCAAATCGCGGCCATAATCATCGGACGACAGGACCACATCCTTCTCGGCAATCCA
GGCGACGGTTTCGTCGGCATCGCGATTGAAACGCTGGATCTCGTGGGCACCGAATAGCTTCTCTTGGCGC
ACAATGGCTAACTGCTTAAGTCGCTGCCAAGCCTCGTTTAGCTCCTCCTTGCGCTTGGTAATAGTGTCGC
GCTCGGGATGACCATCTTGCACCAACTTATCGGCCAACTG
>dme_circ_0005064_junction_seq
CATCTTGCACCAACTTATCGGCCAACTGGGTAGTAGGACTCATCTAGCTTCTGCTT
>dme_circ_0005065
TCGATTCAGGTCCGCCTCGACGAGCTGCACAAGCTGTGGGAGCTGCTTCTTAGCCGTCTGGCCGAGAAGG
GTCTAAAGCTGCAGCAGGCTCTCGTCCTGGTGCAGTTCCTGCGCCAGTGCGAGGAGGTGATGTTCTGGAT
TAAGGACAAGGAGACCTTTGTCACCGCCGACGAGTTCGGCCAAGACCTGGAACATGTAGAGG
>dme_circ_0005065_junction_seq
TTCGGCCAAGACCTGGAACATGTAGAGGTCGATTCAGGTCCGCCTCGACGAGCTGC
>dme_circ_0005066

GGCATCCCTGGTTGTACAGATTCGTAATGGTCGGGAAGATTGTGCACTCCTTACGCTGTCCACCGAAAAG
CTCCTGGCAACAGGACTCAGGCACTGGTCCGTACCTATTCCAGTCGTGCCACGTATCCACTCCGCAGCAC
TGTAGTCGCTCCTGGGTCAAATCCCAGGCCTGGGTGATCTCCCTCCTGCTTCCGTATA
>dme_circ_0005066_junction_seq
TGGGTGATCTCCCTCCTGCTTCCGTATAGGCATCCCTGGTTGTACAGATTCGTAAT
>dme_circ_0005067
TTTAGCATATTTGCGATGCATGCGCCAGAAAAGTGTTAATCAATTTTTTATTGGCAACAAGTGATACAAC
AATAACAAAGGCGCTATCAAAAGTTGAAGGTGCCAACAAATCGACGGCAGTATAGACGACTACAAGAACA
ACAACTACAGCAACAGCTGTACTCGTTGGCAACCGAATTGAGTGCCAACTAAAAATTAATGTAGTTTCTC
AAAGG
>dme_circ_0005067_junction_seq
ACTAAAAATTAATGTAGTTTCTCAAAGGTTTAGCATATTTGCGATGCATGCGCCAG
>dme_circ_0005068
GTATCCCACATTGTCCGCCGACGTCACAGTGACAGCCTCCAATGGCGGTCCTCCGTCGGCGGCGGCGAGT
CCGGCGCCCAGCAGCAGTCCGCCGGCGAGTGTGGGCTCCCCCAATCCGGGCCTGAGCGCCGCCGTGCACA
AGGTAATGCTGGAGGCGTAAGAGCGGGAGGAGGTAGGTGG
>dme_circ_0005068_junction_seq
AGGCGTAAGAGCGGGAGGAGGTAGGTGGGTATCCCACATTGTCCGCCGACGTCACA
>dme_circ_0005069
CTGTGCGTTTTGGACGCGTGCCGAAGCGCGAAAAGGCGCGTATCCTGGCGGCCATGCAACAGAGCACCCA
GAATCGCGGCCAGCAGCGAGCCCTCGCCACCGAGCTGGATGACCAGCCACGCCTCCTCGCCGCCGTGCTG
CGCGCCCACCTCGAGACCTGTGAGTTCACCAAGGAGAAGGTCTCGGCGATGCGGCAGCGGGCGCGGGATT
GCCCCTCCTACTCCATGCCCACACTTCTGG
>dme_circ_0005069_junction_seq
CCCTCCTACTCCATGCCCACACTTCTGGCTGTGCGTTTTGGACGCGTGCCGAAGCG
>dme_circ_0005070
GTTTCAAATTCCCCGACGAACCACAGACCAATTTCGGGTCTTATGCGTACTCTCTTCCAGGCCCCAGTTT
CTTTTTATATATTCTTTTTGTCTCACTAATTATTATGCTGTGGCCAAAAAGACAGCCGAAAGGGTTGCGA
ATTTTCGAAAGAAACGAGAAGTGAGAAGACGCGGCGGCGAAGAACGCACAATTCTACAGAAGACAAGACT
TCGGTTAATGGAGGTCATTCACTTTCGCACGAATTG
>dme_circ_0005070_junction_seq
TGGAGGTCATTCACTTTCGCACGAATTGGTTTCAAATTCCCCGACGAACCACAGAC
>dme_circ_0005071
GTCTACGATGTCGGCCTGGACAACTATGTGCTGAATCGCCAGTGCCGCTGTACAGACCAGAAAATGCTCA
ACGATTCGCCAACGCCCGAGGAACCAACCAATTTGTGCCAGCCCAGCAATGTGCTAGAGGCCGCGCCGAC
TCCGCTCATGTCGCTGCCACCCTGCGAGGACGAGGATCACGAGCCTGAGCATGAGCACGAGCACGAGGAG
AGCATGAAGTATCGGATCGATAAGGAGAGCAGTGGAGCTGCCTCCGCAGAGCTGGGCTTGCCCGCCTACG
AGGCCTGCTCGCCCAAGATGGACTACGACACGCAGGACGAGCTGCCGGACTCGCCGCACTCGCAGCCGCT
GCCACCACCGCCTTTCCCATTGTCCACGGCCTTGCCGCTGCCGCCTCCGCCCACCTCGCATCACCAACAT
CTgcaacagcagcagcagcagcagcaTGAGggagcgcatccagcaagagtacatcatccagcagcagcag
caacatcagcagattcagcagcaacaacaccacctggctctcctgcagcagcagcagcagcaggagcaac
agcagcagattcagcagcaccagGGAGTCAACATAGCCATAGGCACCACTGCGACAAACAACATTCGAC
>dme_circ_0005071_junction_seq
GGCACCACTGCGACAAACAACATTCGACGTCTACGATGTCGGCCTGGACAACTATG

>dme_circ_0005072
GATGACGGTCACCACAGCAGCAGCTTCGACCATCAGTGGAGCCAGTGGGTCCACCACGAATAATGGTCAG
TCTGGTGGCGGTGCACGCCCACTGGAGACGCTAAACGGAATGACCAGGCTGACCCCCGCCCGCGACTTGA
CCCTTGGTGGGCGCGGCGCTGGGACAGCCAGCAAAAAGG
>dme_circ_0005072_junction_seq
CGCGGCGCTGGGACAGCCAGCAAAAAGGGATGACGGTCACCACAGCAGCAGCTTCG
>dme_circ_0005073
GTGGGCGTGCACCGCCACCAGACTGACCATTATTCGTGGTGGACCCACTGGCTCCACTGATGGTCGAAGC
TGCTGCTGTGGTGACCGTCATCCTCTTCGCTGGCATGGCGTTCCATTCGCGCCGCCCGTCACCTCGAAAA
TTCGACCGAAGTCGCTGGGTTCTGGTTTCCCC
>dme_circ_0005073_junction_seq
ACCGAAGTCGCTGGGTTCTGGTTTCCCCGTGGGCGTGCACCGCCACCAGACTGACC
>dme_circ_0005074
GGTCTCATCTGAAAAGTCGTATAACTATGGAGCGGGAAATTTACAGTGCGGAATACTTCGAGAGAGCTTT
GGCGAGAGCTTATGGTTGTGAAATGCTTCGCGTTGAGAACTTTCACATCAAGGCGGTGAGCCAGAAGGGC
GAGAACTTTTGCAGCGTCATCTACCGGGTGGCGTTG
>dme_circ_0005074_junction_seq
TTGCAGCGTCATCTACCGGGTGGCGTTGGGTCTCATCTGAAAAGTCGTATAACTAT
>dme_circ_0005075
GGTATCCGTGGAGCTCTATTCAAGCCCCAATGGCGATGGGGAATTCAAGAGGATGGTCATGGATGTGCCG
CAAACTAGCATTTGCGAGTGCTTCAAAAAGTTCTATGTCCAGTTTGTGCAACCCTCTCTGAAGACTGGAG
AAACCACAAATTTCCCCGTT
>dme_circ_0005075_junction_seq
GACTGGAGAAACCACAAATTTCCCCGTTGGTATCCGTGGAGCTCTATTCAAGCCCC
>dme_circ_0005076
CTATAAATATAAAACCCAAATACAAAATGGCAAACTTCGTGTGCTTCGTTATCCTTTCGTTGGCTCTCTT
CGCCAGTGTTGCTGTGGCGCGACCGGGATATGCCCTGGATTACTATGTGAGTTTTGAAGTGTGATATATA
TATTCGTGTTGAAAACCATAAAAGAGTGCTAAAAACGTTTTATGAAATATTAAAAAAAGTAATAGATTTC
AAAGATACCACTGCTCTCGTATAATTGAAATACAGAGATTCATAATCCGAATGTTTAATCTAAATCCTTT
TGCTTTATATTCCTTAAACAGGATCATCCAAAATATGCCTTTAACTACGGTGTGGCTGACCACAGTACAG
GAGATGTGAAGTCACAGCACGAGACGCGAGATGGAGATGTGGTCAAGGGTAAG
>dme_circ_0005076_junction_seq
GCGAGATGGAGATGTGGTCAAGGGTAAGCTATAAATATAAAACCCAAATACAAAAT
>dme_circ_0005077
TTGAGGATGTCCGCCCTGTCCGCGCCCACCACATGTGGCTGCTCGCCTGTCCACTGGGCCATTGTCATTG
TGCTCCTGTCAGTGGCGATTGGACCGGGAGACGCCATGGCCCGTCCAGCGCGCAACACGCAGTTGTTGTT
CAGCGAGCTCTTGGGGGGCGGAAACGATGATAACAACTACTACGGCGACCAGTTGGTAAGAGCAGGCCAC
CCTACATGGTAGTGTGTCAATGTGCGAGGAAAACTTAGCCATGTGTCCACACTTCCAATCCGCAGAAGTA
CcagcaacagcaacagcaacagcaggaacagaagcagcagCGGGTGCCGGCTTTCGCCAGGAAGTGGCCC
TCGCTGAGGGATCTGTTGCTCACCGTGGACTACGATGACTTCGGGGTTACCCAGGAGAGCGAGGAGCAGG
TGGCGCCCAGTTCCCGCCTGCTGGCTCGACTGCACAGGCTCGGCGATAATGGAGGCGGCGAGGAGCTGCG
GTACAACGTCGTTAACGAACTGACCAATATGCCCAGCAAGAAGGTGATGCCAGGACATCCCCTCAAGGAT
CACAACACCAAGAAGAACGTTCAGTG
>dme_circ_0005077_junction_seq

ATCACAACACCAAGAAGAACGTTCAGTGTTGAGGATGTCCGCCCTGTCCGCGCCCA
>dme_circ_0005078
ATATACGAAGGATATACAAACAGCACAGCAGCCAGCATGCGTGAGTACAAAATCGTGGTCCTTGGAAGCG
GCGGCGTGGGCAAATCCGCGCTGACAGTCCAGTTTGTCCAGTGCATCTTCGTGGAGAAGTACGATCCCAC
CATCGAGGACAGTTACAGGAAGCAGGTGGAGGTGGACGGACAGCAGTGCATGCTGGAGATCCTGGACACG
GCGGGTACGGAGCAGTTCACGGCCATGCGGGATTTGTACATGAAGAACGGCCAGGGATTCGTGCTGGTCT
ACTCGATCACGGCGCAATCGACGTTTAACGATCTGCAGGACCTGCGCGAGCAGATACTCCGGGTGAAGGA
CACGGACGATGTGCCCATGGTGCTCGTGGGCAACAAGTGCGACCTCGAAGAGGAGCGCGTCGTCGGCAAG
GAGCTGGGCAAGAACCTGGCCACCCAGTTCAACTGCGCCTTCATGGAGACCTCAGCCAAAGCCAAAGTGA
ATGTGAACGATATTTTCTACGACCTGGTCCGGCAGATCAACAAGAAG
>dme_circ_0005078_junction_seq
CGACCTGGTCCGGCAGATCAACAAGAAGATATACGAAGGATATACAAACAGCACAG
>dme_circ_0005079
GTGGAGGTGGACGGACAGCAGTGCATGCTGGAGATCCTGGACACGGCGGGTACGGAGCAGTTCACGGCCA
TGCGGGATTTGTACATGAAGAACGGCCAGGGATTCGTGCTGGTCTACTCGATCACGGCGCAATCGACGTT
TAACGATCTGCAGGACCTGCGCGAGCAGATACTCCGGG
>dme_circ_0005079_junction_seq
CAGGACCTGCGCGAGCAGATACTCCGGGGTGGAGGTGGACGGACAGCAGTGCATGC
>dme_circ_0005080
GTCGGTGCCTTTTGTGCGCTTCAATTCGACTCGATTCCGATTCGTTTCAACTCGATTGAAAGCAACTTTG
CAATCGAAAAATAAAGCCAACTAACAAAAACGGAAAGTCTCGCCTTGCAATTGGACTTGAATGGCTCTAA
AGGCCTTTTTTTCGCACCCGGACTCGAGGGTAAGCCTGTTTAATTTTCATGCCGACTTCGCATTTTAAGG
CAGGCGACAAATTATTTGGGTATCGTTTGCAACGACACGTTTCGTTAACTTGTATGCATGCGATTCGTTT
TAGTGGTCACAAAAACATTACCAATATTTCAAGTAGAAACTTGAAAAAGTAAAAAATTGTTTAATTGAtt
ttttttttCGATTATAATGGTGCTATTTTTGCTTGAATTTAGCTGATCGATGCCGTAAAACCTCTTCTCA
GTTAAATTTTACACACTTGTAATTGTTAATTGGTTTAAAGAGTGCCAAATTGAAAATATACACGATTCTA
AGAGTCTAAATGGAGTATTTTCTATTTAATATTGGGAATTTTATAATTGGGACTGACTGGAAATATTATT
TGCAAGTAAGATATTTAATGATCTCAAACTTTCTGGATAGATCATAAACCGTACAGTGTTGCAAACTGCA
AGTAACACTGGTGTATGAGTGCTTTCTGCACTAGTTAGAACGGGATAGGGCAATCAAGAGTGTAAACAAC
AGTTAAATGTTGTTTAACTCGAATCCGGCGACTGCTTTCGTCACATGTTTTGCGCCCGCAGTTCCACTCT
TTTTCGCCCTTTGTCGACGAGTTTTGTGCCTTATCGCAACCCTTGTTCGttttttttttttttttGTATT
CTTCTTGCCGTATTAAATGGCCGGTTCTGCTGCGGCTTTTGTATAACTTCCGCTATTGTTATTATTTTTT
AGTTGTTCGTTGTTGGAAGAAGTGTAATTAAACAGTGGAGTGGTCACGGTGGCTGGGGAAGGGGCGCGGC
GGAGGTAACCCTGCAGCAGCAAATGCAGCAGCCGCCGTCTCACGGGAGTTGCCACCTCGAAAACCGGTTA
CAAATCATTAAACTAAAATTCTGACTCGCCATCCCGCTCGCACCGTGCTGCCTCTCGCTCCCACCTGCAC
GCCTCACTGCACAGTGGCACATGCAAGTGCAACTTGTTGCAGCAACTGTTGCCGCCGCCGTCCGTGTTGC
ATGTTGCATGCTGCTGCGAGTGCACTTTCATTAGACAGACGAATTGAAAGATTTATGAATAGAAAGAAAG
TGTGTGGTGGGGAACAGGTCTAAAACAAATCCATATTTTATCAATAAAATTAAGACCAACCGATTTCCGA
GGCACTTATAAAGTGGGCTAATTAACAAAAATTTACTTGAGCAGGTTTTTCGGAAATCGTAAACAGTGCG
GACTATATTATATTATACAGAGCAACAAAATGCACCTGTTGTGAAAAATATTTAAGTTATTTATAAATTT
TATTAAGATATAGTAAATTCCTTATAAACATTTTAATTATTATATTACAGGGTATTACAGACTTTTGCAG
AGCATCTCAACTTCTTTATCAGCATTTAGCTGAGCTTTCCCGTTTTTCCTGTTTCGGTTGCaaaaaaaaa
aaaaaaaaCTAATTCAATTTGTCTAATATTCAAAATCGAAAGAGGGAAAAACTCGTTTATCGGCATCCTT
GTGACACACTGACATCTTTAGCTAATCTCGGGCCCTCCCCCTTTTCCTCCTCCGCCTCACCGCTCCCATC

ACACTTTGCTTAATTAATTCTGTTTTCGGTTTTGTGTGCCGCCACCTATCAAAGCACCCCATcccccccc
cccccTCGGTTCCCAATCGCCCCTCGCACAAACGATCGTTGCTGGCCCCGTCATTTGCATTGATTTGCAA
ATTTTTTATTGGCCGTTTCGACTCGAGCCTGACGTAATTTGTTAAACTTTGTTATTTACCTGGCTGACAT
CGATGGCCAACTAATCAGCGCCAGCTTTTGTTGGCTTTAATTTATGGTGTGTGGGCATTTCAAGCGCTTT
AATCGTCAAGGATTCAGTCCCACACTCCCGTTCCTAATCTTTCGGTACTATTTTCCAGGTTTAATCATCT
CATTCGGGGTCAGAGATTAAGTATATTTCTCGAAAAAGTTGTCCATAAAAGTGGTGAATGAATTAAACAA
TATCACTTCAAATCAAAGGAGAGATAAAAGTGACGATGCTTCAACTGGATTATATTTAAATTATACAAAC
TAGATTTAATGGGCTAAGACCATATTTATTTCAGCTTGATTAGCTTCTGTTGCTGTGCCATTTTATTAAG
TCCTTATATTTATTCTACTTATTTCTTATTCAGCGTTTAAATTCCTTAATGTGGAGATCCTTTAACTGAG
TATCCCTTACACTTGTTAACAATGTCGATACAGAATTAACTTAATATAAAACGTCGTCATAGAAGGCACT
TTATATTTATATTTATATAGTCATTTGTTAATGATTAATTCCTTCCTTAATGTTTCTAACTTGCGCAGTG
CAtttttaagttttttttttACAAACATGAAATACAAGGGATATATATGACGGTCGAACTAAAGATTTAA
ATACCAACTACTCGAAATTTGTCTGCGTGTACTTCAAATTGGGCTCGAAATTCGATGCCTTAAATTGAAA
TTTGACAATGGGGAAGGAAGTCTCGAAACCCAACCCCCTCCCCCTTTCCACTTGTTTGCTAACATATGTT
CATCGGTGTGCAAGGCAGCAGCTGTTGAGCTGAGACAGAAGTTCAAGAACAAGAACCTCGAACATGACAG
ACTTCGCAACAGGCCCTTCAGTTAGTTGGTTAGGGTTTACTTATTAAAGCGGCCACTCGTTAATTGCGCA
CTTTGCATTCTATTGCAAGTTCATCATGCCTAACGGTTTCGTTTTCAAGTACAGGGATAAAGACTCGACT
GACTGAAAAGTCAGTTAACTAATTCAATTAGTGCCACGTGAATTACCCGTCCGAAAGTCATGGAAAATGG
GCCAAGAAAGACGAGCGAGGGAAACGCAGGCAACAAACTGGACAGAGACGAGGCGAAACTTTTGTTATCC
CATTGTGTCTGCTGTCGAGATGATTGCTCGTACTCGAGGGAAGCGGAGGGCACCGGGAATGGACCCGTCG
AGAAATGCTAAAGAAAAGGCAACCCAGAGGTGGACAATTCTCGGAACCCTCCACCGAACAATATGATTAG
ATAAGGTTCTGAATATAGGCGTCTTGGATTGAGAACGACACGAGTGCCGATGAATGTTCTTTTCGGAATG
CAAAGAAAACTAGTAACCTGCGAGGTGATAGAATGTTTTTCATACCTACCTAAAAAGTAATTCAAAATGG
GAATGATCAAGCACATAAAATATGGAGTGTTTATTATATATAAAGACATAAAGCTAGAACTGCAGGCATT
GGATGTTTAAGTTCGCAAAGTAACTAATGAACAAAGTTCATCCAAGAAATATATCTCTATTTGAATTTAT
AGACATAAAGAATCTCGATTAGGATGTATATTTATAGATACATACATATATTGAAAGTTGCAAGTATCTA
AACATCTTAATAGGGACTACACTTAAATTGCAAGTTAGAAGTTAAATAAGAAGAGCTGAGTATTGAGTTT
TGCAGAAAGTTTAGTTACACAGTCCCTCGTTTGAGTTGTAAAACTTTGTTTAAGTTTCGACTCAAGTCGT
AGCGTATCTCTGGCCCTCAAATTATTGCTATCGAATTCGCAAATCGCCGTTGACCTTGTTACCACTTGCT
GACCCATCTGTGGCCCTTGGTTCAACCAGCTAGAGTTTCAGTGCAAATTAGCAAGCCAGCCAGTCAGCCA
ACAGTGTTAAATAGGCTTGCTTTGGCCCGGCTTGTTCCGCTGGCCATTCTGGCTCATTATTATCACATGG
AAGTGGAAACTTTGTACCAGTTTCGTTTGGCATTGTTAGCCGGGCTTCTGTGAATTCTTTTTTGTTTATC
CCAGAGTTTACGAAGCCCGAAAATTGCACAACAGCAAGAACAACAACATTGGCACACAACTTTTGGTGGC
GGATGGTGGGTGTGGGTGTCAACGTTGTTTTGGTTTTTGTTACTGCGAAAGTCCCACGCCCACAGCCACG
CCCCGCGCCGCGCCCCCATCGCCCTTAAACATGGCAAACAATTGTGCTTTTGTTTACTAATTTGCGGCTG
TATCAGTATTTTTTGTTGCTGCGACTGCTAGCAGAAGTAGAAGTTTGGGGGCTTTGACTTTATTGCCGGT
TTGTTTACTTTCTTTGGCTTGGTTTAGTTTTTGGCTATTTGCTGCTGTCTGCCGAGTTGAAAGTTCATTT
GCCGTTTTAATTGCCAGCTAATAGCTCGAAAGCTACTCGTATTTTCATTTTATAACGCTTTTTGTTTTGT
TTATGGTGTTTTCCCCTATTTTTGACCCCTCATTTCTGGGGGCGAATGGCAAAAGTTATGCGGGCAGCAA
GGGAGATaaaaaaaaaCGGAAAGAGAGAGTGAGATCAAAGCGAAGTTTTTGACTTTTGGGGCTAGCGAAA
TTTGAATTATTTTGACATTTTTTTGGACTTCCGGCCATTTCATAGTTTCTCTGACAGTTTGTGTGTTCAC
TTAATGCATTTATCTGGAAACAATAGATAGTTTTGGGATAGAATTTGTATATGCTAGTCAACTTTTTGTA
CTTCTATATATTTACTTCATTTTAGTCTAATTGTAGAAAATATGTGCAATAAGTAACAAATTGCACTTTA
TGGAAATTCCCATTAACGTAATGCCAGCAAATTCCACAATGCGCATTGCATTTTACCCAAATCGGAATGG

ACATTTCGAGCAGACATTTCTTTCCAACTCAAGAATTCGACCTCTGACCTTTACCTGTTGCTTGTGCCCC
CTGGAAATTTAATTTCACCTCTTATCTGCCTGAGCTCTTCTCTCGTCAGCCATTTTATCATCCAAGTCCG
GCAATTAGCCTGTTGGAAGCTGGCCTTATACCTCTGCAGAGCAATTTCCTTGTTTTGAGGAAActttttc
atttctttcataattttttttCTTTGCCCAAGTTAGGCGATCTCCCCCCTTTTCACCCAATGTATCCATT
AATCAAACTTCTGGTCTTTGTTCAATCTCTTCTACTTTGTGTGGCTCTAGCATTTTTGCTGCTTCTTTTT
CCTGtttttttttttttGTCTAGGGGCTTACATTTGTTGTTATTCATCATTGTTGCGGCATAAAGTTTAT
TCTTTAAAGGGGGGCATATTGCCCTTGGGGTCTCAGCCAGTTGATGACCCCGCACGCGATTTTTACCTCT
CTATTGCTAACCGCAATTCCCAGTCTGTCTCGTGAATATTTCACGCTTTTTCTAATTGCCAACCGACTTT
GTGATACGTTTTTATGTGTGGCTCTTTTTTCAGCTGATTTGCGATAGAGAAACAAATACTGTCAACGCAG
TCGGAAGATAAACAAAATGAACAGAAttttttttGCCATTGTTGTTGTGATTATTTAACTGttttttcag
ttttttttttcagttttCATTGCTGTTCTGGCATGCTAAAACAAAGGCTTGTCATTCAACTTCGGTCATA
TGTTCCAGATATGATTAGAGCCATCTAGCAAAAAGAGCAAACAACAGCCAAATTGAAAATATTTTAGCTG
CAATCATACACATTTATATTAATATATACATATGGTTTTTGGTCATGGGGTTTTTCTTGTCGCCTGAGCT
AACTGATATTGATAACCGAGTGTCAAGTTATCGCTACATTTTTTTATTGGTCAATTAATAAAGTTGAAAA
ATCGCAAAACAATAAGGAAATATTTAAAAAAATGCGTGATAACATTATTATCAAGCTTTGGAATTTAATG
GGAAAGAAATATACAAAAGTGTGAGCAGCTAGCAATTAGAATAAAAATTGTATAAATTATTTTAATAGCT
TAGAGATATACGTTTTTATATAGCAACAAGCTACAATTATGTCTTATTTGCTTATGGTACATATGTGATA
AAATTAGTTAAATAGAAAACTATTCCATATAAACATGAACACATGTGCACATGTCTAATAAGTATTTATA
GTTAATTAATTCTGTATACCTTAGTAATTCAGCAACAGCTCTTAAGTACTAAATATAGCCGAGCAATTCG
ATGTCAACCGAAATAAAAATATTATAAAAATTTATTGTCCATGGGCGACTGGCAGATAGAGGGAAAATGC
AATATCTAGAAGCGCACACGATCCGATTTGGCTGTACATTAGTCTAAATATAGGCGAGTGTCATAACACT
GTGCCCCGAAAGTACCACACCCCCCCACGTCTCCCCCTTCCCTCGACCTACCTTTGGGCCTTATAAAGCG
GTTCTGTTCCAGTGACAAGCCAACAAAAACAATAACATTACCGCCAACAACAATTCAAATTCAACTTGTT
TTTGATTAGATCATTCGAGGCGAAATGTCAGTCAATAAAGGGTGGCGTCGATGGGAAGGTGCGGGGTGGT
TATACTTGGACCCCACCCCATCCCCTTTGTCTCAccccccccccccccccTCCTCATCAGTTAACCTGCC
CGTGGCTTGCGATAATTAACTGAAATAGACCCGTGACTGTCAGTCAGCCACAAGACATTCTACACAGATA
TAAATATGTACATAAATCCATACTCTACAGTGGGTTTCAAAGGTGCTGAATGGATGATCTTGCATTTCAA
ACATCACTTTTGGTTTCTCTGAAAGTTAAGTTAGTTAGTTAGTTGAATATTTTACCTTTTTGTGATTAGT
GAAACCGGTATTTTTGAACAACTTAAAAGTTCAACATCTGTCTATGCTTTGAAAATGTGTTTAATCCTAA
TATTAGGTTATTTTGCTTAAATATTTACTAAAATTATAAATTAAAATGTATAAATTTGTAGTAGTAAGCA
TAAGCACCTCGATAAAATTGCAACTTTGGTATGATTAATAATATCAATACTGCAACGGACTGTAAGTGTG
CAGATATGAGAACATGTGTATGCATTTATTTCTCTTCATATGGGGGTGTCGTTAAGCACTTGAGTTGCAA
TTACATTTCTCGACGAGACTGCGACTGCAGAAGTGGGCTAGTTAAAAATGTCAAGAAGAAGTACCGCACT
GAAAGACATAAAAGCCATAAAAAACAGCCAGCAAAGAAGAAGACCGATTGAGTCAAGAAGAGAGAGGGCC
GAAAAGCCGGGCCAAAAGACAGGCTTTGAAACTTTTTGGCATGCACTCAAAGAAATGACTAGCATATTAC
ACAATTTTTACAATTTTTTACAATAAAATACTTTTAGTTTGAATATAAGATTATATATTCAAATAAACAC
ATTATTATTTAAAGATATATATTACCAATTAAAATAGCTACTAGTATTATAGCTTATATAATGTATTATA
GTTATAATATCCGTTTAAATCAGATTGGAACTCATTCAGTTTTCTTTCTGCAGCAATCGTGGCAAAAGTT
TTTGCCTCCCTTATCCCATTGCCAGCTACCTTCGTACCAGGTGCTTAAGACCATTCACTGATTTTTTAAT
AACTCACGACTTTGGCCATGGCTTTGACAAGCTGTTAATGGTAATAAACGAGGGGCGAGGGGAGAACTGG
AAATCTCGACTGCAAAGGAGGAAGCAAATTCTTTGACGAGATGGAGaaaaaaaaTTGTTGGTCATTTAAC
AAAAAGCAATTTCGCTCAGCTCACAACTTGCTTTGAGAATTATTTTTCTATTTTTCAACTTTTCTCCGGC
CGTAATTGGTTTCAATTTGAAATCGAAACGCATTTCAAAGAATTTCTTTGGTATGCTCTTTGAAATCTAG
TTTGCACTTCTCTCGCTGAATTTTAGCCAAATATTTTTCTGTGGTTTTTGTGATAGAGATCGCTATCTAT

AGGCTGTCCTAGGCCGCATTTCAGTCGACTTTTCGGCCAGGAGAAACAGTTTATGAGATACCACTAATTG
GCCATAAAATTGTATGAAATTCATGTGCCGGTTTTTCCCACTTTCGTGGCCAATTAGTCACCTAAAATGT
GGTTTTTGGTCAATTTTTGTGATACCCTATAATCCAGCAGAGAAAAACCATATGCTGATAGCTGATTAAA
TCAAGAGATTTCGTAAATAAATTGTGATGAACTGAACTGAATAAGTGTACAACAAAGTTTGATTTCGTTT
GATCATACATTTTGTATGTTATATATATTATATAGTGTATGCCAATAATTTTTTAATACTAAATAAAGCA
TTTAAAACTTTTAGGGTATTATCTAGTCATTACCCATCAACAGCTATATATTTTTGATTATTGTTTTCTT
TCTTTCGAACTCTTTTGATAAGCTATAAGCTGATATAAAAGCTGACCATGCTGATAATTTAATTTCCCCC
GAATGTTATTGCTCGCTATAAATCATAAATGCTTGCGTGAAATACACAAAAAGTACACATATTTATTAAT
GCTGATCAATTGCATTTTATTTGAATTACTTTGGGAAATGGTTAACCGAATGCAGTTAAATATGCCGTAA
ATTAAAACCAACAATTGCATAAATCGTTGGAGCAATGTTTTAAAGAAGGCGTAAAATATCTGTGCttttt
ttttCAATTATTTCTAGCAAATCTCTTTTCGAGGCAAACCAATCAAATATTTATGTTCCATTAACAATTC
GCTGAGCGACACTTGACAGGACGATaaaaacaaatattcaaaaaaaaTCGTTTGAAATATTAATGCCATT
AAAAATGTAATTTCAATAAAGACGGCGAGAAAATTCAATTAAAGCAAAATTTGTGTGCCACCGCAGAaaa
accaaaacgcaagccaaaacaaataaaaacgcggagagaaaaaacagtgccaaaaTGCTAAAGCTACTGG
ATAACTTTTCGTTCTATTTTCCACCCGGTTTTATTTACttttttttttGCCATTTTAATGAACCCGCCGG
CCAATAAATAAACCCAATTCCCCCAATAGCCGCTAAAAATTCCATACTAATTAGTGGTCAAGGGTTGGTA
GTAGCCTTTTGATGGCCATATGCCATTAAAAATAAAATGGGCGCATCTATTGGACCCGCTTAGCCAAGTG
AGCCAACTTTTAACCCAGTTAGCTACCCTACCTTGACGCACCTGCAAAAGAGAAATAGAAGAACCCGAAC
AGAGGCAAAACTTGAAACTAAAACTAAAGCCATTTCCCTCACAGAAACACGTGTCGGCAAAACAATTTTC
CAACTATCCCCCGCAATTGCAACTCCCCATCAACCCACGAAGACAAAACTTTGGCGAATTCTTTACAACG
AAAACACCCATCAGCGAATCCATCTCCATGCGCCCCTTGCTGCAATATTTGCGCATTTCCTGTCGTACAA
ATATTTCCCATGTAACAGCTTCCCTCGCGTTTTTCCAACGTGGATTCCACTCTGCTCCACCGGTAACTGT
AATAGTTACAATGTCAACAACAACACGGCCGACGTAACCGGCGCAACACTGTTGCCAACTAACTTCTGGC
CAAAAGCTGTACACAGATAGTCAAAATTGCGGAGTGGAGTTTTTGTTTTTTAAACATGACGGATATTTTA
TACCCTCTCCAACGAGCTTAGACATTACTTTCTTTTAGTTTTAAAAAATATTTTCAAGTGCTGTTATCAT
TGGGTATTTTTTATATTTTTATAAAAACTTCCATAAAAAGCCTTATAAAtatttttgtattatttattatt
attattttaGAGTCCCGATTGTATTATTATTGCAGACATTTGAGTGTAATGCTATAGGCTTACAGAATAA
ATATTTATTGAATTTATTGTTTATCGCAATAGAATGGATTATTTATTATCAATATCCCAGTCAAGGGCGA
CCGGTTTGTTTGGGATTTTTGTTTAACTCGTAGTGTCCTATTCCCCGGAGCGTAATTTGGTCAAAAGTTT
CATCTGCAGCTGCATTCGTCGGAAAAGAGAAAGAGTCGCAGACAAAGACGGGCGGAAAAGAGGTGGCCGA
GTGGTGGTCTATACCGCTAAACAAAGGCAAACAATGTAATGCCAGCAGCAGCGACCACGCACAACGAAAA
CAACAAAGTTGGGGCAAACCGCGGGCGTCGGGAAAATGAggggggggggggTTAAGGTCAAGGGGGCTGAG
GGGTAAAACCGAGTTGGGAGTCACAAACTCAGCTGCAGTACTGCGACAAAAAAGGAACAGATGTTCCATA
CCCGATGATAATCTTTTAGATTTCTGATAGCAAAATCTTACTTTTTTGAAGCAATATCTTAAGGATGTTT
TTCAAATCTTCTAAAATCGCTTGATATATTCAGATTTTTTGATTTAAAATATTTCCTAGCATACTTTAAA
GCACTATTTATTTCTGTAATATTTTGTAACAATAAAGAACAATTTAGATTAGTAAatattatatattata
ttgattatatattatatatatatcgattatataATGCCCTGTTAACTACTCTTCAATAGGATATTATTAA
ACTACCCTTAGGGAATTTAATAAGGATTTACTATAAGTATTAATACGACTATTATTATTTGCCGTGTGGT
AGAAAAAGAAGAGCAAGGCGAATGTTGCCACAGCTagaaaaaagaaaaagcacatacatttgcggcaaac
aaaaaaatagaagcaaagTGGAAACAAAGCGACATGTTGCGCCTGAAAGGTTCTTTGGGGTGGGAACCTC
CCCCATCCCAACCACTCGTACTTCCACACCGGCAGAGACGCAGCGGTTGACCTTCACTGGCAGCACGTGG
TGTCGGAAATGTTACCAATCCGAGACAGCCGAATGTCCTGGTATCGAAGGGAGGTAACGCCTGGATGCGG
TTGTCTGAGCAGAAATCCGGATTTAGCTTTAAGATGGATGTATGTTCTTGCTTGTTTGCGAATTTCTAGT
GCGAAGGAATGAGTATTGGGTTTGATTTAATTTAGATATAGGAGTTTCTTTGAAAGGGATTCGCTTGAAA

GTTTGCTAAACATAGGGAGCTTAATAAGATAACATTTCACTGATACAGTATGCAAATAAATATACTATAT
GTGTGAATAGCTCTTCTGAAATGAGCAAATACGCATTTGGGAAAATCCGCCTAGATAAGAATTTGTGATT
CAAAAAATGTTTCCACTGGAATACTTCGTTCTTGACGTTTTAAAGTGGTTTAGTTTCGTGACTAATCCTC
TCCAGCTGTTTGTTTTAGAAAAACCTATTCAAACATATATTAAATGGTTGATTAGATTAGCGAGCAAACA
CATTCCCATTTTGGCACATCCCATTTAGGAAAAGCGGGACCCTCAAAATGCTTTATAAAGACCAGGCCCA
TTAACGGTCAACGTCATCCATTTATCGCTATTTATATTACGCAATCCCAGCCCTCTTATCGCCAATCAAA
TAATTAGAAAAGGTGAATCAGGTGAAACAGGGCGAAACACCACAATGCCAATACAAATTATGGAACTTGC
CCTTTTGAGGTCTTTAAGGCTTCTTTATAACACCAGAAAATAGAGAGACAAAGAAAGCGGCGTGCCATGG
GAAATAGTGATAAAAGAAGGCAAAACAAGTTGGCTACACAGAGAGAAATCGCTGATTTACTAGATATAAT
TAAAGAAATTAAAAACAAAGTTGTTTTCGAGAATTTATGTCGTCCTACAACATAATTATTGttttttttt
ACGCCCAAAGTTTTCATATGTATCATATATATAACATTTTAGTTTAGCTTTAATTAGTTTCGATTTCGTT
GTTTCAAAGGTAATTTTAAATCGATTATTGCCTTTTGGTTACCACAAAATTGGCATTCGACATTAATTTA
AAACGCATGGTCTGTTTTGTGCAGAAAAAGAACAGCTACAAGTTTGTTTAACAAAGTGGTTTTCAACACC
CCATAAAAAACATTTGCAAAAAGCAACAGAAAAATACTGGGAAGAGGAAGAAGGCGCAGGCAAAGGCGCA
GGCCGTAACAAAAGAGCaaaataaaaaaaaaaataaaaacaaaaacacagaaaaaTCCAAACCCAGGCGAA
TTTCCCTCTGTTTCCGCACGTAAAAGTAAAAGGCAAATAAATAACCGTTGAGAGAAATGTAAACAATGCA
GGCCAAAGATGCACAGCAGATGAACAAGAAGAAATAGCAGCAGATGAAAAGATAAAAAGAGGGAAATGCC
AAAAGCACAAATGGCAACTGATGCCGGTTCAAGTTGAATTTTCCCCGCAAGGtttttcttttcctgcgat
tttttattttttttattttGAGCTTGCGCATGCATACTTTGCAAAGATTTACACTCGAGAAGGGCAGACCA
AGTGGCAGACAGTTGGAAAATGAAGTGGAAAAGAGTGGAAAACTAACTACACCATTCCTAATAAACGAGC
TAAGCAGAGAGAAGGAGCCAACCGAGCAAAATGGAGATGGGATAAACAAAATACAGAGAAAATTGCTTAA
TTTATGGGGCTTGCTCGGGTGGAAAATCCTAGACATTTTTAAAGCACGTTGCTGAAGAAAAATTCATGAT
TCGACACTAAATAATGCAAAAAACTTTCTTTTAATATTGGCAATCTGCTAAATCACTAATGTCTTAATTA
ATTGTCATAGAtttttttttCAAATGTGGATTTAAAATGTTATAGATATAAATAGTATATTAGTAAATTA
AACAAATTCATCATTATTGATTGCCATTTTATACTTTAATAACCTTCCTTATTTGCAATAACTAATACTT
AAACATATTTATTTTAGCACGTTCTAATTTGGTTCAATTTGTTTAATTTATTATCTAGAGTTGTGCAAAT
TGCTATTTTTAAAGGTTTATTGTAAGAGCATCATGAATATTTTTGATGACATTTAGTGTCAACGAGAGCA
CAAATTTGAATAAAAAATATATTGTTGATCACTTGTGACAAGTAAGAGTTaaaaaaagagtaaaaaaaTAT
ATAACTTAAAATTACGTATACGCAGAGTGACTATTAAAGTAATGCTTTGCGCGTGCCTAAGTAGGATTTA
TTTTGAAAACCAGCTAATAGACCTACACTTCGACCTACTTTAAAAATGTTGAATCGAAATATAATATAAA
ATATTACGTATATGCCAGCTGTACCAATCGAGTATTTTGTGCACAAGCATGGAAATTTTTAAACCAATTG
AACAAACCaaaaaaaaaaaaaaaTGAAACAAAATCAGTGATACAAAAAACGGGAAACGGCAAACAAGTGA
AATGGAGCAAATCTCTAAGCAAATGTCGAGTGGGCGGGAATAGATAAACTGCACCAGATAGAGTAGCAAT
AACAGAGGTCGGGGGAAAACACGGGAAAAGCTTGAAAAGTGGGAGGGAGGGAGAAACACACAGTGGAACA
CTTTTCCCCTGCCCCTTTTCCCGCCAGGACAGGCATTGCCAATAGCGCAACGTTTCGGAAACAATTTGAA
GGCCATAAAACTTGATGTGGCGGCTGTCATAAAGGAGGCGACACCAGGCACAGATTCTACCCCCATTTTC
CGACCTCCAGAAGCTAGGTGAAAACTGCGACGGCACTTAAGATGTGGCAAGTACGGCAGGCGGAAAATTC
AGAAACGAGTACGGCAAAGCAAACTCCGGCGGAGGAAAAGGCGGCAAACGTCGACAAAAACAAACAAATA
AACAACAAGCAGCTGTTTTTGGCGCCGCCACCgagagaaaaatagagagcgaattgagagaaaaagaaaa
atggtgagaaaGGATGATTTTATAACATCTCTATGTCGGTTACAGTAATCTCTCGGAAAAAGGAAAGAGA
TTTGTTCATTATCATAAATAGACATTCAATATTGCAGCTTATTTATTGTTAGCTAGATTATTTAAAATAA
TATCAAGGCTAAAAAAATCACCAGGTAACTCAATATTCCATCCCACCACTGTAAGCACTATCTTCTCTCA
CCTGCCTGCTTTCTCTCACACTTTGCCAGGCTCTCTCCCGCTCTGTAGCAAtctctctcgctgtccctct
ctctctcttcctctGTTGGGCACTTCCTGTGCAATTGCTCACCGCCCACCTATCGTACGGCACTCACTCA

TTCATTGCCAGACTGTAGGACTCTCCTCCCCGCCTTTCTTCCTAAAAGTCAGCTGCGTCTATTGGCAAAA
CCAATACTACACTTCAGGTCAAAATAGTAGCAGAGTTGGTTAAATACTATTTATTTATTGTTTTAATGTT
TGCATTGAAGAATAATAATTTGTTTACTATATGAACGTTGAGTTTTTAATGTTCGTATAAAGTTTATAAC
TTATACTGCTTTCATAAAATCCGTAAATAGATTGATCAACGTAATTTATTAAGATGTACTGTTAACATTA
AttttttattttaatgttttttttttACGAAAATATTATAATTTTGCCCTTGCCTGTGCGGGCAAGCACAC
GAATGGGAGAACGAAGACGGCGGCGCCGACTGCAAAACGACGCTGCGGCATTTTGGCGCTTGGCATGTTG
GCCATGTTTCGAATGGTTTGGAACGGCATTTGCTCCGCAGTCGCCGTTCTGCTGCTGCGAAGCCAGCAAC
AAAGTTCCTCAGCGGACTCGCAACTAGTTGAGTTTAGTGCCGTAAATACGAGTACGTACGGATACGGAAT
ACGTCGTTTGCAGCACGGGCCTCAAGCCGTTTATTACGTATACGAACCGCGTGCCGCCGCCTGGCAAACT
TTAATATTTTCCATGAGTGTCACAAGGCAAATATGAAGCCGAAATGTAAATGAAAATGAGCGGGCTAGTC
TAGAAGCGCaaaaaaaaaaGATAACAGCTGGGCACACAACAATAGTTGAGCGAAATCAATGACGATAAGG
CGTCAAATACTTGaaaaaaaaGAATGGCAAAATAGAAATAGCAAGTGCTTGAAGTGaaattaaatgtaaa
aaatgcatcaaatcaaataaaggaaacccaaaggcatacaacaaaaatAAACACAAACTATTATTCGAGG
taaaataaataaaaagcaaaataaaacacaaaaaaagaccaatgtcaataaacgagaaacacaaaaaaag
taaaagcagcaaaaatcagagaaaaaatatgtgaaaaGGCAGCTAGCAAATCAAGCCGAATATAAAGGGA
AATTGTAATTTTTTGCCACTTCAAttttttttttGCCCATTCACGTGATAAGAAAATGGTAAAAGACCTC
AGATAGACATATAGATTGAAGGCACAATTTTCAAGAGGTGGGGACACATTTCCCGATTTCGATTTCGATT
TAATGTGAGTGGCGGGCGGGCAAATACAAATAAACAAACAAATCAAGCACAATAGAGTTCTCTTACATTC
CGTTCCGATTCACACTCCTCCATTCCGGCGCGTTTGATTCTACGGAAAATGCAACACAAATATTAGCAAT
GCCATAAAACAATAAACCCGAACGTATTAATTGCAATCTTGTGATAGTGAAAATATGGAAACGAAAAGCC
AAGCGAAACGGGAATAACAAACAAACAGGAGGTAATAATACACGCAAACTATTTATTGTAGTTCCATCAC
AACAGGAAACTTATATACACAATAATAGAATAACAAAAGACACAATTTGCACAAAGTTTGTTTATCTAAA
GCCGGATATAATAGAAAATCTCTTTATTAAATAAGTACAGTGACATTAGAGAATAATTGGATTCTGGCAA
TTAATAATAGCAAATAAAATAATTGAATAACAAAATAACTCTTCATATACCAAAAATTTATAAGTCTACA
ATTTAAACCAACCTTATCGTCACTAGTTAAAAATTTGTCCATCGTACATTTTTAATAAATGTATAGTCTG
TTGCTTTATTAAGTAGAATGTTATTGCGGACTAAATTAACAAAATATGCCTTTTAACACGAAAAGTTTCA
TTTTGCACTCGAATTTCACATTTTCCACATGCTTATCATTCTTTCATATTTGTCAACAGAAAATTCGGAG
AATAATTTACTTTTACCCGTTCGGTTGCCACTTTCTAGCCAATAATTGGACTATATGTTGACCAATTGAC
GATTGCTGTCGATGACAGCTTTAAACTTTTGGCAGAATTAATATGAATTTCGGCCGTATGCTGAACAAAT
AGAGATGCCACGAAAATGAGTTAAAATTCCAATGAAATAAACTTATAATTTTGCCGAAAAAAATAAAATA
TACCCATTTATTATAAAATGACAAATGAAAGTTCTCATTCGTATTAAGTTATTATGGAGTTCTTTTTTCC
AAAATGAAATTAATCCAAAAATTGTCAATTGTATAATCTGTGTTCAAACAAATATTTAAATCAATTCAAG
CACAATTATTTGTATGTCTAAAAAGCACTCAATATAATACATTAAATTTGAATAGCTAACAGAAATAGGA
AATTAAGTTAACTAATTAATTAGGACATTTGTGTTATTGCTGCTTGAAATTCATGGAGCAGCCAAGGACG
ATTGCTACCAATTTCGGTTTAAAACTTTTAATTAAAATATTTATTTATTGCCCAAACCAGAAATTTCACC
AAGTCCTATTCCAGTTCCGAAGCTTTGCCGCAACGAAAACAAAACTTTAAATACTTTAATTTCATGGGAG
AAAACTTTCGGAATAAAAGCCAAACCATACAAAAATCGAGTGCCAATAACAACTTGTCCTTAGACACCAA
ATTGGCCTCCATGAAAGTTCAGTTGCCAAAAATGTGGGAGGAAACAGATGGAGAATAGTGCAATGCGGTC
ATATGGGGAATGTCAAAATACCCTGTAAAGTGCAGAATATAGCGCATACATATGTGaaaatggtaaaaat
atgaaaataaatacttgtttaatatagttaatgtaaaaatatatTGAGATAAAATAATATCCTTATATAA
TTCATTAAACTCAACGTTTTAACATGAGAATGTAAATATTGTACTTTCTGTCCTTATTATGATTAAATAT
TTAAACTCGATTGGATTTTTATGATACGTGTTATAAGCGTTAGATTTCGAATTTTATCATACCCCGCAAA
GTTGAAGCCCaaaaaagaaaaataagaaaaaCAATAACACGCAGCAGGAATAACTTTTTCAATTGGCTTC
CGCTCTTACAAGATTTATCCAAATATAGATATATATTGTATATGTCGTGTGTGAGTGTGTGGGATTAGAT

GGAATCATTCATTCCCTTGGATCATTGAGTAAGTGAGTTTCTGTTGGCTTTCCTTGGGGTTTTCCTTTTG
CTTTTTATTTACGCCCGCAATTTGTGCTGACAGCTCCCATAATTTATGGCAGCCCACTTATTGATTACGG
CAGAATCGCTCAAAGGTAAAATTCATTTTCGGCTTTAAAAGATTGTTATCAAACTGGGCAATAGATTTGC
TGCTCTTTCCACTTCGCTTTTCCACTCTTTTGCCGTTTAACTGCTCTTCCGCAATTGGGGTCGTAATTTG
TTGGTTCGCCGTGTTTTCGGAAAACTCAACAGTGGAATCAAAAAGTTTGAACTTTATGCACAAAATATGA
AGTTCAATAAAGTTATTGATGATACGTTTTTATGTTTCTGTTCAAACGTACTTTATTTGTAATATTTTTA
AATCCAGAAAATACACTTATTACCTGATAATTATATTAACTGATTTTAGTTTTCCTATTAAAATATGTGA
ACTATTCCAAAATTTCGCATTATAATTTGAACGCAAAGATTTTTGAGTTTTGAACAGGTAAATTCATCTC
AGAAATTTATTATAAATTATTCTAAGATTGACTTTTCACAATATAGGCTGAACTTCATCACCAAGTTAGA
GCTATAGAAGATGGACTGTATTTCAGTTTGGACCACTGGCCAAGCGGAAACGAGAGCTGCCTCGGTTTGG
CAGTAGCTTCCAGCCGTTTCCGTTTTCCGCTAGCTGGACAAATTCTGAGCGACAttttttttttttttGGT
TTATGCGAAACGAAAAGCGGGGGCAGGTTAATGTTTAGTTGCAATTGTTTGCAATTTTGTTGCCACCACA
AAATGGGCAACTGTTTTCGGGGCTAATCTGTTGCCAATTTGTTGCCCTATTTGGCCGTCAATTTTGGTGT
GGTCGTTATGCAAAGGCTTTAAGTGCTTTTCAGTAGTTTTAAAGGTTTTTCCAGCTGTTAGGTTGAGGGC
TGGAAAAATGAGATTTACATATTTTCCTTTGCCTGCATTTCACTTTGCATTCGCATAAAATGGCATTATGC
TAATTGTAAGCGCTATAGCCTGCTTTTAGAATTTATTCAAAGCAGGTTTTATTTTGTTGAATATTTTTGT
TTTCATACAGATTTTCGTTCATCACTCATTTGTTGTTAGCTTTGGCTAATTAGAAGCGTGCTCCAATTGC
CTTGCCGAAATTTATGCAGATAAAGTCAGATCTCAAGGGAAAGAAAAAAATAAAAGCACGCTTCAAGGAAA
TTCCCTGTGTGTGAAGAAATGGGAAATGGTAAGAGTTTTTGTGTTAAAGTTTCAAGAGGCAAAATTTGTG
GGGTTTAATTCTTAGCCAAGTGCTCCATCttttttttGACATTATTTCGGGAAATCTGCTCACTGTTTAT
GCTTGTGAATTTATATGCGTGTGAATAAGTTGTTTCTTATAAGCTGAGATTTTCATGACATAAGCATCTT
AATAAAAATGGTGAAGTAGACAAGAGTGACatttttatattttatattttttatGATAATCAGAGATTTA
GATATGCAAAGCTTCTTAAGTAAATATATGTAAGATGTTAAAGTTACAAAGATTGAGTCACTCGTATCAT
TTTAAACCTAACATAAGTTACGAATTTCTTTAAAAAATACCTGTATATGGATATACGTTATTTATAATAC
CATATTTATTGAGTTAATCATGCTAGTTGCAAGGACGTGACTCTTAAATTTCAGCTATATTCCATTCATA
AAGTCATGCATGCTTAAAGATTCATTTCATATACAGCAACGATTGGAATAATAACAGGCATTCTTATAAA
AATGCGCTATCTGATTGCCTTTGGCTGTAAGCCAAGCCACTTAAGTGGTAATGTTTCTACTGTCTGGGTT
ATTAAAGTTAAGCCAGCCCATTGTTGTGGCCATCTTTGCTTTCAAGGCGACTTAGGGGCATGACATTAAA
TTGACAGCCCATTCCCGTGATATCTCTCAGGAGATGATCAATAAAGCCAATCCCTAGCAGCCAATTGGCA
GGTCTCTCACCGAACTTAAAGGCGGAATATTTACGACTCGGGGTTATGCAACCAACAGGCGGGCAAAAAA
ATGAAAATGATTTCAAAATTCATTCGTGTGTAAAAATGGCAAGCGAGTAGAGACATTGGCTTAATGGATG
CCGTACCCACAAAAGCGGGCTATACAAAATTGCGACCAAGAGGCCTTGGGTGTCGGGAAAAATGTGGGTG
AAGTTCGCCGAATGGAAAATCCATTTATCAAAACGACGTCCAAAGAAACCGCAAACGCATCGCACAATTT
GACGCCAAACAAGCGGCAACAACACGAAACAAATGATTTAAATTAATCCACTTAAGGCCACTTAAACCCC
GATGCTAGGAGTCGAAAAATGTATGCTGAAAATTCCCACGCTCCGGCAAACATATAAATTCATATATTTG
TATCCCAGTTCATGTTCGAATTATCTGTGGATGAGTGTAAAATGTCAAACTCAGGCAATGGTGTGTACTT
CAGTGCGGCAGGGAATGGCACGAGATGAATGCAAAGGAAACCAATATGGGAAAAATGTGAGAGCATTCCC
AGCATTGACCCAAGTGGACTGTGCACTTTGAAGGTGAATCTGGTTGAATATTATATGAACTTTGTGAGTG
CCAAGTGGGCGAAGTGCAAATAAAGACGGACCAATCCTTTTGTCGGGCTTAAAAGTGTTTTAAATTGGTT
ATTTACACGTAGCTTTCGAGGTTGAGGTTATTTTAAAGGACTCCCTTGTTACTATCTTCCTCAGCTGAAA
TGTTATTCTTCGTTTTTAGAAATCTATATGTGATATTATTAACAATTATCATTATACATAAAATTTATAA
AAATCCTATCTTATTATTTAAAATTCAATTTTTTAGCAAATTTAATGATCCATTTAAATGAGCTTCTGTT
TTTTCTGCTCAAATTGTTTATTAAATCTCAAATAAATTATTATAAGTCATTCTGAACCAATGTTTCTGCA
ACATTCGCGTTAGTATAAAACATATAAATAAGAGGAAAAATCTGTACCATTCCACTTTGTAAATCAGAAA

CTGTTCATTCCCCCAAAGGGTATAGCCCACAAATAAGTGGGTTTGACGGCGTGACATATTGAAAAAGGAA
GCCGTAGGAAAAAATATTTCGCAATTTCAATATAAAGCCTGGCGGCATGCTAAGGCGGCTAAGGACAATC
GTGGGCGGCGGCACGCCCCCCAATGTGGGTCAAACTACACTCGCACACTCTCACGCAATTGTTGTTTGCC
TGCTGCAGCGGAGGCGGAAGAGAGGGGCCCGCTAggaaggttggaaggttggaaggttgTCAACGTAACG
ACCCCAAGCCGACCCGCTTTTGGCCAAGGCGCCGGCACCAGAAGCGGCAATAAAAACAATCGGGTCCTGC
ATATGCTGGACTTCCCCCGCTGAGGGTGGCAACCCTGTCAGTGGTCGCCCTCAGCGCTTTTAAAGCGACG
TTATCGTCTCTGCGGGGTATTTTGTGGTCCCTGATTTCTATGCTCCTTGATCAGCCAGCCAAAGGGTATG
ATGTTCCAGAAGAGCACTTAGTTTCATATCTTTGTACAATATATAATATGATTATAGTAAAGTGGAACAA
ATTAAAAAAATATATTTGTGGAGAATGTGGAAATGCCGAAATCAAAATATATTCCATTATAAAAAAATAC
AATAAATATCACAGCTGTATTTGACCAAAAGAGCAAAACTAAAAGCTTACTTTTCCAGTTTACGCCATTT
TTATTGACCTTGATTTCGACTAATAACTTCAGCGTgcaaaaataataaataattacaataataattaaca
ataattacaattTTAGGATTAAAATTGTGCAATTTAAGTTTATTAGTTAAAAACCTCTTTCGAATGATGT
TCGCCTCtaaatactgtagataagctattaaataatgttgattagctattaaataatgttcgttggctat
taTTATATTATATAAATATTTTTTGATAGCATATTTAAATTGTTGGACTGGTGTACTGAGTGTTTTCTGG
TTTGAAGGTCTCTAGTGTGGCATGTGTTTCCTGATTTGGTTCTCCTGGTTTTTGTTGCTGCTTAACCGTT
ATAGTGCCGACATTTTATTGCTGCATCCTTGCAGTTTTTATAGACCCATCCACACATGGCTTCGTCCCAC
AATTCTTGTATCCCCTCCCTTTCCCTGCCCCTCTCTGTCTGGCACGCTTACACAATGAACCCACATGCAT
ATGTACATATATGTGTGCCCGTATGCGAGTCCATATTTATCCGTGTGGGTGTTGGTTATACGTGTGTGCT
TGCGGTCTTTCACACATCCTGTTGCAAATTCGTTACTTACTGTTATTTTAGTGCATTGTGCGGCCGACGG
AAGTTGAAATGAAGGAACTGCCAGGATTCGGATCCCAAACATATTTTAAAGCTCGCAGGCACAGAAAATA
ACATAACACACATATGACAAATTCTTCTTTAAAAAATTGTAGATTGTAGATTAATTTAGTAAATGATATT
TAATTAGTAAATGAAAAACCGTGAGTAATAGATAGCATTTAGTTTGAAATTTGTTTCTCAAAATAGTTTC
TTTCATTACAAATGTATTGTGTGTAAGCATCGTAAGCACACCTCGCATTTCGCACATCAGGTTTTTGCAT
ATCGGCAAATGCCATGTGCAGCCCACATTTCTTTCAAGGTAAATTTCCCAGTTCAAAGGAATGAAATTTT
CGGTACAAAAACTGCAACAACAGGGAACTCTAGATGCAGACGGAAATGTCAAACAAAAATAAGTAGGAAA
AGTAGTAGAACCGGAAATGGGAGAAGACTTGGGGGCATGGGCAACCATATTGGATGGCAAAATGTGCCAG
AAAGGAGCTTCGGCAGAAGCACACACACGGTTCCCTCTTCCGATTTCGCTCTTTATCACTTTTTCTGCCA
TCTTCCGCAGATTGGAGTCCATATGCTCATCCTTACAGCAATTTGGCTCGTCAACGGGtttttttcacag
cttttttttGTGTTCGAACGGGCAAAAAATGTGAATCCGAAGGAAGATAAAAAAGCGTGCTAAATCCGA
AGTTAAATGTACACTAAACCAATAAAGAGAATAATAAATAATAAACCATATTGAAAATGAAAATGTAACT
TCACTTAATTTTTACTTTATTTTATCCACTTATTTTAAATGCTATATAATACAATAGTTCGATATTATTT
TATTTACTGAATAAATTTCTATTTATGGTTCGGCTTAATTATATTAGTAAGAGAATAACAGTTTCAATGT
TAAACTTATGGCTGCTTAGTCTCTATGCTCGATTCTTCACAATGCAAAGATACCTTTTTTAAGGGGACAA
CATAAAGCTCGTTAGGCTAAATAGAAAGTAAGAAGATAATCGAATGGAATTTTTACAGTACTCGTTAAGC
TTTCGTACTCCATTAGATAAGTTACGTATTGAAAGTTGAGATAAACGAACTGCCAGGTGATATCAAAGTT
AGATAGTGTGCTACAGATAAGGGTTTGCCAATAGATAGCAACGTAAATGCACAGTAAATTAAGATAAAGT
GATTAATAGACCTTTTAGTCCGCCCGCTATAAAGTAAATTCCCAACCCATAAAATGCCACATACCACATT
CAATCCCCCCCAAATATTGGCAGTTAAGTCTGAAAATGTGCGAATCGCTCGCTGAACCCAATAATTCGTT
TCGATTGCTGCCAATTTTCAACGTCCTTCATCTGCTTCCAATTTATAACTGGGCGAAATATCTCCATAGC
TCTGCAATTTTAATATTTTAATCTCAGAAACAATCGTGGCGTGTGCAATAAGAATGTATATATGTAAACA
AATTAAATAGCAAATTGACGATTATATTAATGAACTTTCAAATTAAAACGCCGATAACCGCAAAATGGGC
CATAAACTGCAGGTGAAGCGTATTATTTTTATTTATTTCCTTCAGTTCGCAAAGGACCGCGTACGACCTT
GAAAGTATTTTCGAAATATTTTCCGTGCCTTCATTTTTTGCTGCTGATTAAGTGGGCGGAGGAAATTTTC
ACTACAATTGACAGTTTCACAAAAAGCATTGAAATTTGAATAATTTATGCAAAAATAAGACTCGAATTGA

TAAAATTACGACTTGCTGAATGGGAAAAATATGCATGGCTGATTTACAAAATATGAATGGGTACCAAAAT
GTATTAAACATAATTTACACACAAATTGTATCAACTGTAAAGAAGAACTAAATTAAATATAAACGTATCA
TAAACAATTTAGGGAATTATCTGCTAGTTTTAGATGTTTAAGAGCTAAACTTAATACGGTTAAGTACAGA
GATTTAATTTTGTTTAGTCCAACAAGCAGCTTTTCTTGAAGCAGGAAGAAGAAGGGATTGATGGGAAAGT
TCAAATTCTAAACCTTGATGTTGATTTTTAATTTAATCCTCGGGGGGCTTACCCTGGCAAACTTTATATT
TTCTCTTATTCTCACTCTGTGATCCTGTAAACTCCGGTTTAAGCCTTCAATTGTTGCCATTGTCATGGGC
tcatcatcatcatcatcatcattatcatGGGCCAAACAACAGTTTGAAATCAAAACAGGAAGCACGGCAG
ACGACCAGGTGGCCAAACTACTGTCACACCACCCAACACACCCATTTCCAGAAAACCGACCCACCCAATT
AAGTCACCCACCCACTGATTGCTACGTGCCACCGTCTGGTGCGGCTCCTCTGAGGTCCGTCTGTCCCTCT
GTCCATAATAAGCCCATATAAGCTCCTCTTAAACCCTTTCAGTGGACGAAGTCGCTTTTAATACAAATTT
GCCATGGTGTGTGTACTCTCAAAAGCCAATTACGTGCCCCAAATGAAGGCCGAGCAGTCCGGGAAATGGT
AGCCAAACCGAACATAACATTTATAAGATCACGTGCTCAATTATTAAGCCACTAAATAAATAAATAGCGG
ACCAGCGACACGTGCCCCATCTAACAAAATCACTCCTATACCCTGACAGTTATGCGACCTGCCCATCCAA
CCGTCCGTATCATAAACTCTTCCACACACACAGGCGGGCATGAAATACaaaaaaaaaGAGGAATTTCATT
CATGGGCTCATTACTCATACGCAGTGGTGGCCCGCTGCCTCGAATGACGGCTACGTTTTGCGGCTTCCGC
AGTTTAATTAATCACAAGGATAGCTAATAACGTGATGTAATGGACAAAAGAATGCGATTATTAAGCGAGC
CTTTAATAGTTCATTTAAGCTTATGCGTTAATTAAGAATTCAGCGTAATAATGTGGTCAATGATGTGGTT
TCAGTTTTAAAAAATATATCAAGCTTAACTGAATGCAAAATTTGTGGTTAGACCTTCAGTTGCACGTTAT
TAAATAGGTCCTATTGTATTTTCATTTTCTTTTCAAGCAAATGCGGCTGTTTGAGTAGTCCTCTTGTAAA
TTTTAAATGGGCACACTTTTATGAAAGAAAAACCAATCGCATTGCAATCCCAGGTGGCATTGTGTGCAAt
tttttttGCCATAATTTATGCCCTCCCCAAGGTGTTGCGACCCCGTTCATATTGGGATGATAAAAATCGC
GCAATTAGCCGGCGTTGATTTTCGCACACAGGACGACGACCAGCGCAGAAACACACTATATAGAAGTATA
TATAGAACTAGAACGATGCCGAAGCGGTCCTATGTAGGTCTAATTTACGAGCAGGGTGCAAAAATAATTT
ACGTTCCGAACGCAAAAGGGGGGTGACAATGGTTTTCCCTAGATTTGGGAGGTACGGGGCCTGGAAGAGT
TTAAACGAAGGTGCAAATTAAATCAACATGCATATCCGTTTGACACCGTTTCGCCAAAGCCTCCAACTCA
CCCCTGCGCACCTGCCTCCCTTTAAAGGGAATACTATATCACTGGGAGGGTGGGGGTGTTGTTGTGTATT
CTCCGGGGTTGGGGTTTGACTAGCGGGGCTCTACAAAATGGCGTACACATTGCAAATGACCGAAAAAGAG
AAATTGGATGGCAGTGTGGGTGTGGGGTGGGCAGTGAAACGGTAACGGCAAATGGGGGAAATGGAAAATG
GAAAAACTTTTGGCCATGTGTTGTGACTATGTGCACGTCTATGCTAACCTTGTTTAAAATCTGGGACGCA
TAGATATATGGGAGGTGGCATGCGAAGGCATCGAAAGTGGCAGCAACAATAAGGGGCAAAGGACGATAAC
AACTTTTCGGCTCTCCTACTGTGGGAAAATATTACGAATCTCGATGTTTATAGATTATTATTTAACTAAA
GATCAAATTGAATATGTTACTTTCTTATAACTGCTACCAATTTTGAAATCAAGTGTAAACCTGGTTAGAA
ATAGTCAAAGTATTCAAGATGATCGTAAGAATACAAATAATTCATAATTTTTAATGATGCATTTTCTGCA
GTGCATTTGCCATAAGGGATATGTACACTCCCTCAAAGGGATTTCGCAACTTTTGCTTGTGGTTTTTAAG
AACAACAAGCAAACTGCATTAAGGCTTATACGTTAAGAAAAAAATACGGATAATATGCACGCAGATATGG
AAAATAAAAGATGATGGGCTGTAAAGGGAATGCAATTAAGTTCGCTTACCATATTGAACAAAACAAGCTA
TGATAAAATGGCAAACATTGGATGATGGATTGTCGGAACTTTGAATAATAGTTTTTTAACCAAGCATGGG
AAAGAATTTATTTGCGATACTTAAGATACTGAATAAAAGGGTCTGTTTTGAAATAGCTGAGCGTTTGCtt
attcaaaatatattaaaaacaacacaaattataaaaatatatataataaCTAATTATATTTTTCTCTTTA
TTTCAGGTATGTTAACAGTCAATTAATTAAGAGACGAAACGGAATAGGTAAAGTTAATATGTGAAAATAA
CAAATTTAACCTATTAAAATCATAATATTTTACTATGCAACCTAATGTTCGACTTATAATTATTTATTCT
TTCAAAATAAAAAATATTTTTTCTTTTTGCTATGTTACTATCATATTAGTTTTCATGTTCATCCCATCCA
CTTGTGCATTTAAAATTCCTTCAACTTTTTCCCCATTTACCGTGTTTTTCGACTATTCAATTTGGCCACG
AATATTAAATAAGTGACTAATGTTGTTAATAGCCAACACGCGGAGTGTAAAAGAGGCAAAAGAGTTGGCT

AACAAGAAATTTGTTTGCGCATATGCCAAAAACACTAAGTGAAAAAGGGAAAAAGTTGTGACGAGGGGAC
GAGGTGTTTATGACGACGCGAGCGCTTGACACTCGCTTAAATAAAAGATTTTTTAATTAAAAATATTTCA
CCTGCAAAGTAGAAAAAAGGCAAGCCGGGaaaaaaaaaCGAGAAAAGCGCCGACACAATTTTCCTCTTTG
CGCTCGCATAAATAAATGCAACCACGCACAAGAACAACGATACCAACAACAATATGAACAAAGAACGCCA
ACAATATCTTAAAGAACACTaaaaaaaaCTACGTACCAAACTGTTTAAAAATGTTATCTGTTCCAAACAA
TTAAGATGGCGTTGTAGGCGTTTTTAATAGAAATGTTGGTCATATTGGAATGTTAACACTTTCTAAGCAA
TTATCTCTAATAAATAGTAAAAACACATCAAAATTATTTAGTGTTTGTTCTATCATGGGCTAGTTCATCA
TGTTGATATTTATGAAAATATTAATAATGTTTTTCCCGTGTACAACTACAATAACAACAACGAGAACCGC
GGCAAAAACAAACACCCAGGACGCAAAGAGCAATTTAACAACAAAGACACGTGCCAAATGGGCAAAGGGC
GGTGGAATAAGCGAAAGTGGGAGGTGGAAAAGTGGGCGGGGCATCGGCAGTGGGTCAGCGAAATGAAAAT
AAGCCGCGGGCGTAAATATAAATAAATGAACGGGCCAGGCGACAAGCGGTAAAAggggggggggggTGTG
tgctgccagctgcctgctgcctgcGGGTGCAAGCGAGACAGCATCTGGGCGGTAAATGATAGGAAGGGGC
AAAAGGTAATAATTAATTCGATACGCAAACAATTGAACTGTAGGTGGGCGCTTTAAGGTCATCAAGACCG
CAATGACAGTTGCGATAAACAAAAAAGGTCAAATGGGCGAGAAGAAAATGCGAGGCAGGTGGACAAAATG
GGTTTAAAACAGCAGATTGTAACTTAATTCGCGAAACAGTCAACTTCCTCGACTTTTGAACTGTCTCTGT
aaaataaaaaaaaaGAACTATATTTCCTTTAATAATAATTTGTACTTTATTGCTACCGAATCATGTAATC
GTTGAAAAATCTGCTATTAGAACTGCAATCTGTCATTAGAAAAGTATCATTTTGGTAATACCTCTGCTAT
TTGTGCTCAATCGATTTTCCGAACTCCAAAATGGCGTGACAATAATAAGTTTAATTCGTAATCTTTCACG
AACTGTGTTTATAGATTTAGCTATCAACGGGTTGTTTGTTTATTAGTTTTAAGCAATACCTATACCACAT
ATCATTCAACTCCCACAATACTCTGTATTTATAGAAATATAAAAAAGGACCAACCAACACATCGTTAAGC
TGGTCAAgaaaaattacaaaaataatggcttaaacaaaaatgaaatgaaatGAGAGTGGGACGCTTGGAG
TTCGGGGTCCAAAAAGAGGAAGTGGGATCACAGCAAGGACATGCCACGTCATGCTGACAGTGGGCGGAAT
TCGTTGGGATCCTTCGCTATATATATACATGTGTCATCAGTAAACTTGATTGAGTAGAGAACGGGCGGAG
TGCGGAGGGTGGGATTGGTGGTGCCGTGAAGGGTCTTAACACGACGACGTGCTAAATTTTGAGAGATGAC
CAAGGCTTCTCTTAAATTGTGTACGCCAGTTGGCCAGTTGACTGCCGCACAACGAATTGCACTGGGGGAA
ATTCGCAACTTTACACGGAAAATTCAAGACTTTTCAGTTATGCGAATGCCACGGGGAAATCGCAGAGATG
GTTGAAAATGCTTTCATACTCTCTGAGTGAAATCCTTCAGGTGTTTCAACATTTTGCACTTGCAGTGAGA
CAAGTGACTATAAATGCATTTTTTTCATCACACCAATAAGTATTCAGACATTAGACAAAGTAAAAATCAT
TCCCTTAACATTAATATACTTATTAAGTTCTTTTTAAGAACAATAAATCAATGAACACTGTGGTGCGTTG
TTTAAAACACGCATGGAGATGGAGATTTAGTCTATTAACATTCGGGAATTTTTAGTTTACGTTGGATCAA
GATCTTGAACATTTCTGTAATTTTTGATAGTTTTCTCTCAGTTTATAAGGACGATGTGGCAAGCGAAATG
AACACATGACTGCAGGACCAAATGGAATATACGACACTCCATGATAATCAGTTGTGGACACTTTTGCTCT
GAAGGAGTGTGTGTTACTGGGAAATTGGAAGGCCATGGGATGTAGCCGGAAAATTTAAGGATTGGGGGAA
GTTCGTCCGGCAGTTTTAATATATTTACTCACTGCTTATGCATATTCTGCTGGAAATTACTAACAGGAAG
TTGCATTCGAAAAGCCATTTTCAGCACACACATACACACTTTTTGCGTTGTGGATAACAGTTTAGAATAC
GATTAGGAACTTCTTTAACTCAATGCACTCCATTAGATGGTACATACATTCATAACTATTTTAACTATCt
ttttttttAAGGATATGCGGTTTAAATATTGATTCAATAATAACTTAGCGTTAGATAATCTGAGCAAATT
AATTTAATATCAAGCTTAGTTTCTGTTTTGTCAGCTGCCAGGTGCAATGAAAAGTAACACGCACTGCTAA
ATGTTGAATACGCTCTATGTAAATAAACAAAGTTTAAATGAATAGACCAGAAGTTTAAATGATGACAAGG
CTGTGGAATTCCAGACATGGAGAATAACATTTATTGTACAATTCCTTCCAGCTGCTGGCACATTGCCTGA
ATGAGGTTAAAACTAAGTTGAAATTCCTGGAAAGCACCACAAAACCTACGAGAGTGCTTTCAGTGACAAT
AGTACGTAGCTAAATTCAGGCGGCAGCAACTCGGCAACTCCGGCAAACATTCGGTTGCCAAGGAATCCCA
TGCCCTAAGAGCAATTCATTTCAACTCGTTGAAATCTCATGTGAAGCACACTCATTATTTTGAGTTTTTG
CTCGTAAGCATTATTTTTGTGTCGCTTATGGTAAGCTGCTCAGCTTCGGGTCGTTTAGACCCCAAGGTGA

CATGCCCTGTCCATTTTGGCCCAGTCACTGCAAggttccttggttccttggttccttggGACCCGAGCCA
GAGAGTCAAAGTCCTAAAAACCCAGGGCGACACaaaaaaaaaaaaaaaaaaaaaaaaaCCGAACAAAACG
AAGCGATGCGAAGGGAACTTAACTGGCACAACATACACCTGTGACCCCGTTCGCCAGACGCAGCGGGCCA
GGCACTGCAGACAAATCAGCACTGAAATTTAATTTGCATGAGCCACGTCTCTTCCACTCTTGGCCCAAAA
TGGGCGTCGAACCGGCCGGGCGGAAACAACAAAGTTGGAAACCCAATTCGCATTTTCCCAGTCTCGGCGC
CGGCTCAGCCCCATCCCAAACCGTATTCCGAACCCATCTCGGCCAAAACTCGACCTGACTCGAGACTTGG
CTCACGTGCAATCGCTTTATTTGATTTTCATTTTTGGGCCAAACACAACAGCAACAGAAGCTACATGCCA
GTGCCACTGCCAATAGCAAAAAAGCAACACGATTGTTACAAAGCACAGCACGGAATCAGCCTATACCCTT
TGCATTCATGGTAAGAGTGCTGGAAAAATTCCCAGAATTTTTGATTATTAGCAATAAGTGAGTTTAGTAC
TTTTGAAATGCTGATGCTCTTTTCAAGAATTTAAAAACTTAAAGATATATTTGTGAAAGTGCAAATTTCC
GAATTGTTCACATATGTTTGCCATATGCTATTTCGATTTGCACATAAACACGCATTTGTCAAATGGATTT
GGAGCAACCGAAATTTAAAAAGGATCTGATGCTTGGCTGATTTTGCCAAAGAAATGTGATTGCCATCATC
CAAAGTGCCACGTGGCCATCTATTTTCAAGGTAAACTGGGAAGAAAGATTCTTAATGCTACATAAAACTT
GATGTCCTGCAGGGTCCATTGAAATTTTGTGAAATCGCCAAAATTCTTATGAATTTAAGTGTTTGTTTTT
GTTTCATAAATTAATAATAAGCTCCACGGGAAGTAACCGAATTTGAATTTGTACAATTGTGCATGTATGC
CTTTATGGAAGTAGTTACTTTTCTTTAAGAACTTTAGTTCAAAGTTTTGCTGAGAAGTGTACTTTCCTAG
GAGTTTTATTAGCACAAGTTTCCGGTAGAACCTTAGCTAATGATATTTGGCAAGTGCTGCTGATATATTG
CAGTTCGCAATAAAACCGCACATAGATTGCCCAAAATTATAATATCCCCAACGAAGGGCACAGCAACAGA
CGCAGCAACTAGAATTTTCATTGCCCTGCCGTATTTGGCCAGTGAGTCTGTGTGTTTTTCGTGGCACACT
TTTCGGCCAGGCCAAAACTAGTCGTACTGGGCCCATTCAGTGGCAGTGGCAGACATTGCAATGGCCTCGC
TAGGAAGCAGAAGCACTTGGGCACCAGTTAAGGCAAACATCCTGTTCCACCGTCTAATCGGCAGTTCCAT
CTCCCCGGCACCACTGTAAACCACGTACGTACGGCCAAAACCCCCAAATTCCCCACATCCCCTCTTGGCG
GGTGCTAATGCACTTGCACTCAGTGCAAGCACATAATTCGCTGCCCTCGAGCACACATGCAAGAGCAGCC
GGCACTCGAGAGCCACAATACAGTACAGTGCGACCCGGCACCCACTCCAGTAGCCCCCCCACCCAATCTC
TATACCCTTGGGGAGCACCGGTGCCCGCTTACAGTGGCTGCTAATTAAGCGGAACTCGAACGCTTCGATG
GCCATAAACCATACGAGAAAATAATGAGCAACGCGTGGCTCTGAATAAATTTTTAATTGCACATTTTTAT
TGATGAGAAATAAAAAACGGATAATAATTATGTGAAAGTGAGAGGAAGGTAACACAGGTCCTGAATGGAC
GGGTTTAATTAATTTTTGTAAATGAGTTTCCAATTCAAAGGGGTGATATAGCTGCCAAGTGGAATTGCGG
CTCTTAAAATCGACATATTGGTTAAAAAAAGTGCTTTTTTTAAATTATTCCAATATAAATTCATGCTAGA
ACCACAACGGTATTTTCCATATCGAAATAAAATCAGAAATGTTACAAGATAGTAGATAGAACAGGTCTAC
AAATCTTAAAAATATGTTTTAAAATACATGTCATACAATTATCATTGGGAATAGGACTAAGAGTCATGGA
CAATCTAGGACATAACGATTCCCTCTTAGTGGATTACTACTGTAGCCATCGAGCAAAGGCATGTGCAGCT
GGTGCTGTTTCGGATTCTGCTGGTGGTGCTGGCGCTCATTAGAAAGGACGAGGGAATTCCTTGGCCGCTG
GCCGTCGGCAGATTTCGTCGCCAGCTAGATCCCCTGACCTATCCGAAGGATAATGCCTGCAAGGTGATTT
TTCTACTCTTTTTTGTTCTATCCATCCCTTGTTGTTGTTGCTATCTCGTGTTGCACATTGCGCCTGTCGA
GTGCCATTCATTTGCCAGGCCCACAGAACCCCCTCCACTCGCCCGCCCTTTCATCCGGCCCTTGAGGTCA
ACGTGGCTTTCGTCACTTGCCTTGATGTTTTGGGAGCACTTGGGCACCGAGAGTGGGAAAAAGACAAATT
AAACTGTTCTGAATGTATTTGAGTTTCGTTCAGTTAAGCTTTCTTTTATCTTATCGGTTAATGTCTGCCT
TTAAGTCGTAAATTATATGCACTTTTGCAGGGTAAACTAATATTGCAAAGTGTATGTTCTTGCAAAATCA
ATATATATATTATTTGAAAGGTAAAGGCAATAACTAACTTTTATACGAGGAATTATACATTACTGGCTTA
TTTTTGAATTTGTAATATTCTTAACATTAGGCTGGATATAATTAATTACATTTAGCCAGTGGCTATAATG
AAGTTAGGTAGAATATAAAATAATTCAATTGGTATCAGCACTTAACATCTTTTTCTCAACTGCTCTAATT
TCAGTGATTTGAAAGCTGTTTTGGTTTTTGTGCTGCGGCATGGGCTTCCTTATCTTAAAGTCATAAATTA
TTTGAGCTCTTGCCATTATGGAGTTGCATAAATATTGAGCTTAAAACGAGGCGTGCAAAATTAATAACAC

CAAGTTGCAGGCACGCACAGTCAAGTGCGAAAGAGCAAAAACTGCTGTCTGACCGCACAGATAACACGCA
TACGCCATGGTGTGCGTTGTAGATGGGATGGTCCCCATTGTTATTTCAGTGATGCGTAGAGCTGTTATGC
TATCGACAATTTTTGGCACACACACTCACACATTTACGTAGTCACGTCGGATGGCAATGCCTTTTGTATT
GCAGCTGACAAAACGCATAAATGAACATTTCCTTAATGCCAGCAATTTCCCATCCATCCACTTCAAGGAT
ATTCATGTGCCGTAACAGGATGCCGAAAAGTGTGTTACCAGCAGCCATAATCGATATTCGATTTAAATTG
TTCATTTAATGACTATTACTGCAGCCATATAGCAATATGGAAGAGGAAAGTGCACACGCACACAATCTCC
TGTCGCGCATGCAGTATTTCAAGGCAAACAAGAATTACAATTTTCAATTTATGATAATGAACTTTATTCT
CGCATGGAATAGGAATTGATACAGTTGGCCGAGTTACTCAAATGATGAAAGCGCCGGATTAAGTGTTTTC
AGGCGAAAATGTTTTCGAATGttttttttttttttGGGAGGATGATTGATTGTTGGTTTAACAAATGTTT
CGTTTTACTCAGCTGTGAAATTGGGCTTGAGAATGCTCATACGGAAGGATGGAAAATTAATTTTATTGGT
TTTGGTCAATGACTTGTATTGTGCAAGAAAACAGGCTAATAATTAATGAATCGCATTAATTAATTGTCTT
GATTTGTAATGGTGATGCTTGATGCTTGTTTATCTGTAAAGTAACTTAGTAAACCAAGTGCGGTTTCAAT
TATTGGATTATTAATTAATTGGTTAAAGGAATGTTTTTGATGGACTTTAGCTGGAAGATTTTGTTCGCAA
AGGACTTGAGATAACTCAATATGTTTTCTATGCACTACTGCCGTGTAATCATCCTTTAAAATAATCTTGT
AGTCTTTTTTCTATTTTCCGCTTTTACCGTTTTTGGCGCTCTTGGCGCATAGTAAATTAGCCACCTAATG
TCTTGGTGCTTTTTACCGAGCCAACTCAGCGAGGTCTTTAAAAAACTTGCTACTTTTTCTGAATTTTCGT
TACAAGTCCACTTAGTAATTCCCTTTGTTGACTGTACCAGGATAAGACACATTCACAGGATCAAGCTGGC
TGCACATGAGATTGGGCTTGTGGCTTCAGTCGAGTAAAGTAAACTTAACTCGATGATGGCTCCTTGGCAT
GCAATTTCCACGAAGCAGAGGCTAATACTTATTCAAGGCATTGGCCAGGCTATCGATGACAGGTGGGGAG
TGGGGGAGGGGGCCTCAGAGGGTTAAAGCAGGGATCCAAGGACACATGCGTCATCAGCATTATTCTTGCG
CTTCAACGCACCCAAAAAGAGACCATTACTGGCGGAAATTTCCTCGGGCACATCAAAGTTCAAATACTCT
AGGGAAACTACAATCCTTTGATTAAATTGTAACTAACAAATGAAAAATGAGCGAAATTGTTTTAATTTAG
AGCGCTCGAAGGATATCTGAATCAAATTAAATAAGTACTAACTAGCAAAATTAAGAATTACAACATAAAT
ATATAGCACTTCTTTTTGTATTGAATGGCATAATAACCGTGTTTTATACCTATATATTTCTTTTATATTT
TAACACTTTCCTCAACCTAAGCTAAGAAAATCATCTTTGAGCTATAAAATAAAGTGCACCAAAAGGAATA
ATGGTTGTTGCTTCATGAACTTTGAATCCATTAACTCGATTGCCCAGGCCCCACCCGTTTAGGATTCATT
TATCATGCTTAATTTTATCGGACAGCTAGATTTTAGATGGGAGTAACTAAGAAACTTTACAGTTTTATGA
AATATTATATGTattttattttatttttattttttttCGATTTTCCCCTTACAGGAGTTTACAACGAAAA
GTTGCAGAGAAACGAGCTACGGCAAACATCAACAGCTGGGAAAAGCAAATCATAAGTAAACACTGAGGAC
AGGACCAGACAACCGCACAGGACGAGGGATAAACGTGGTAGTTGCCATGAGAGCAGCCTCGGGTGTTGAC
CAGGAGTGAAAGGATCTGGGAAAAGTGAACCgcagccacagcagcagcagcagcagcaatagcaacagcg
aaacagctgctgcagcaATCCAAACAGCTGCTACATCCAAGAGCAACGAGCATCGAGCAACTGGAAACAA
CGAGGGCAACGATAAGAGCCGCCAGAGACAACACCCGCAATCAAATCAAATTCAAAGATGCAAATTGCAA
TTTGCATACGAATGCATGCGCACCAGACGAGCAACATCACCACAAACTGCAGCACAAATAACAAAAGCCT
CGCAAATTGTTGTCAAATGCTATAAAATTGAACGGAATAACTCTGGAGCACACTTGCAAAACATATCATT
GAGCGGGCAGGATAGGGTTCAAGTCAACAAATCAACATGATCAAGGGCATTCTGATACAGCGCAAGAGCC
AGGAGGATGCCAGCTACACCAAGCAGCTCAAGATGGAGCACGCCGATCTGGTGGCCGAGATGCAGACCGT
GGAGAGCCTGCCCACCCACGAGCGACTCCAGCTGGCCAGACTTGTGAGTTCCACCATGCACTTGATTAGT
ATATTAGTATATAGCATAAACTTTataggatacacatggatacataggatacaCATGAACTTGAAATTTT
TAAATATTATTACCTGATTTTCGTAGAGATTCGAATATATTTTATTAACAGCATTATGTTGGCTATTTTG
ATTAAGTAGATATTTCTCAAGCCAACAATTCGATCACTTATGTAcattattttgtcaaatttttatttc
atttttatttttcgcttgcttttcttttatgtttaattcactttattatGAGCAGATATATTTAACAAA
TGATTTAATTGCGAAGTTTGATAATCGTCGGTTATTTTTTCCGGCAACTTAATAAAGGCGAACCTTTCCA
ATTCAAATCTTAGTTAATTTGGATTAGACAATAAAAATTCGCCACCTAtttttttttttttCAATAAAAC

TCCGAATAGGAAAAGAAATTGATGGCTTTTGTCTAAGTAAATCCCCTAACTTCCGCCTCTTCTTCATTTT
GAAAGCCATTAAACTTCCACAACTAATCCCAAATCTCATCCTCAATCCCTTATTGTTAATTAGGACGTCG
TGCGCAGCAGCTGAAGCTGTCCCGCCAGAAGGACAAGGAGTGGGCCAAGTTGCAGCGGGCGAAGGGCAAC
ACGGCGAGCGGCGGCAGCGGAACCGGATCGCTGGGCAACGGGCTGATGAACGGCAACGGAGATACCACGC
ACCACTTCCGGCACGCCAATGGTTCGGGCACGTTGAGCGGCAGGCGGCACATATCCTTCGAGAACAGCGT
GGTGCTCCTGGAGGCGGCATCCCGGAACGACATGCCCGAGGTGGCCGCCCTGCTGCAGCACGGAATCACG
CCGGATGCCGCAAACGAGGATGGACTGACGGCGATGCATCAGGCGTGCATCGATAACAACGTCGAGATGC
TGCAGCTGCTGCTGGAGTACGGTGCGAATGTGGATGCGCAGGACAGTGATAAGTGGACGCCGCTTCATGC
GGCTGCCACCTGCGGCCACCTCGAGCTCGTCCGCATCCTCATCCGGCACGGCGCCAATCTGCTGGCCGTC
AACACCGACGGCAACATGCCCTACGACCTGTGCGACGATGAGAAAACCTTGGACTTCATCGAGGGCGAGA
TGGCTCAACGGGGCGTGACCCAGGAGCTCATCGACGAGACGCGCTCCTCCACGGAGCGGATTATGCTGCG
CGACCTAATGGAGTTGGCGCGCACAGGAGGAGATTTGGAGGAGCCGGACTACCAGTGTGCAACGCCGGGT
GAGAATATGCGAAAAGGTTGCGGCACTGAATCAACGGTTATCCGGCTAGAGCAAAATAACCATGAATATG
AAATTGAGCTATAAATTGTTGTTAATGCGACACGGAAAATGTGTAAATCTAGACTATTTGTAGTAGGAGT
AGGAGGAAAGCGCTCTCAGGTGTGAAGAAAGATTGCATTAATTTTTTAGCTTGGCAGGATCAACGATTTA
TAATGGCTAACTCGAAAAAGTGTTACCCATTTCCGTAAAGTTCCTCCTAGCTCAGTATTTCAAATAAACA
CATATAGTACATACGTTCATTTAGATGTTAGTTGCCATAAACATAAGAATACACTTAAACGTTTTAACTG
GATATTAACAAATCAATCCATCCGCCTTTGTAGGAGCCCAATCAGTGCGACGCACTAGTTTGCGAGACAA
AACGCTGACAACCAAAAAGGATGCCGTCGAGGAGACCCGGCTGCGTCTGCAGGCGCAGGAGGCGACGGAC
TCGGAGGCTCCCTCATCGGCAGGGGATCCGCTGACCAATGGCTATGGTTACGGAAACAACAACGGAGAGA
AGCGACCCTCGACGGGCAGTTCGAATGGCCAACCACTGCCGCACTCCAAGTCCGCCGATGCGCTGGATAA
TGCCACCGCCGCTTTGTCGGCCAACGCCACACATTCCTATCCGTCGCGCCGTCCACCGGATGGCCGGGAA
AACGATGAACTGCTACGCCTGAAAGCTGCAGGTGCTGCGGGAGAGATGCAGCGAGCCACCTCGGCGGCGG
CCGTCATTCTCACCGAGAAGGGTACGGCGGCCAGTGCCGAGGCCGTTCAAATCCAGTCGTCGAAGGAGGC
TGCCAACGGCAAGATCAATGTCCAGGTCACCGTCCTCGTGG
>dme_circ_0005080_junction_seq
ATCAATGTCCAGGTCACCGTCCTCGTGGGTCGGTGCCTTTTGTGCGCTTCAATTCG
>dme_circ_0005081
GAGTTTACAACGAAAAGTTGCAGAGAAACGAGCTACGGCAAACATCAACAGCTGGGAAAAGCAAATCATA
AGTAAACACTGAGGACAGGACCAGACAACCGCACAGGACGAGGGATAAACGTGGTAGTTGCCATGAGAGC
AGCCTCGGGTGTTGACCAGGAGTGAAAGGATCTGGGAAAAGTGAACCgcagccacagcagcagcagcagc
agcaatagcaacagcgaaacagctgctgcagcaATCCAAACAGCTGCTACATCCAAGAGCAACGAGCATC
GAGCAACTGGAAACAACGAGGGCAACGATAAGAGCCGCCAGAGACAACACCCGCAATCAAATCAAATTCA
AAGATGCAAATTGCAATTTGCATACGAATGCATGCGCACCAGACGAGCAACATCACCACAAACTGCAGCA
CAAATAACAAAAGCCTCGCAAATTGTTGTCAAATGCTATAAAATTGAACGGAATAACTCTGGAGCACACT
TGCAAAACATATCATTGAGCGGGCAGGATAGGGTTCAAGTCAACAAATCAACATGATCAAGGGCATTCTG
ATACAGCGCAAGAGCCAGGAGGATGCCAGCTACACCAAGCAGCTCAAGATGGAGCACGCCGATCTGGTGG
CCGAGATGCAGACCGTGGAGAGCCTGCCCACCCACGAGCGACTCCAGCTGGCCAGACTTGTGAGTTCCAC
CATGCACTTGATTAGTATATTAGTATATAGCATAAACTTTataggatacacatggatacataggatacaC
ATGAACTTGAAATTTTTAAATATTATTACCTGATTTTCGTAGAGATTCGAATATATTTTATTAACAGCAT
TATGTTGGCTATTTTGATTAAGTAGATATTTCTCAAGCCAACAATTCGATCACTTATGTAcattattttg
tcaaatttttttatttcatttttttattttttcgcttgcttttcttttatgtttaattcactttattatGAGC
AGATATATTTAACAAATGATTTAATTGCGAAGTTTGATAATCGTCGGTTATTTTTTCCGGCAACTTAATA
AAGGCGAACCTTTCCAATTCAAATCTTAGTTAATTTGGATTAGACAATAAAAATTCGCCACCTAtttttt

tttttttCAATAAAACTCCGAATAGGAAAAGAAATTGATGGCTTTTGTCTAAGTAAATCCCCTAACTTCC
GCCTCTTCTTCATTTTGAAAGCCATTAAACTTCCACAACTAATCCCAAATCTCATCCTCAATCCCTTATT
GTTAATTAGGACGTCGTGCGCAGCAGCTGAAGCTGTCCCGCCAGAAGGACAAGGAGTGGGCCAAGTTGCA
GCGGGCGAAGGGCAACACGGCGAGCGGCGGCAGCGGAACCGGATCGCTGGGCAACGGGCTGATGAACGGC
AACGGAGATACCACGCACCACTTCCGGCACGCCAATGGTTCGGGCACGTTGAGCGGCAGGCGGCACATAT
CCTTCGAGAACAGCGTGGTGCTCCTGGAGGCGGCATCCCGGAACGACATGCCCGAGGTGGCCGCCCTGCT
GCAGCACGGAATCACGCCGGATGCCGCAAACGAGGATGGACTGACGGCGATGCATCAGGCGTGCATCGAT
AACAACGTCGAGATGCTGCAGCTGCTGCTGGAGTACGGTGCGAATGTGGATGCGCAGGACAGTGATAAGT
GGACGCCGCTTCATGCGGCTGCCACCTGCGGCCACCTCGAGCTCGTCCGCATCCTCATCCGGCACGGCGC
CAATCTGCTGGCCGTCAACACCGACGGCAACATGCCCTACGACCTGTGCGACGATGAGAAAACCTTGGAC
TTCATCGAGGGCGAGATGGCTCAACGGGGCGTGACCCAGGAGCTCATCGACGAGACGCGCTCCTCCACGG
AGCGGATTATGCTGCGCGACCTAATGGAGTTGGCGCGCACAGGAGGAGATTTGGAGGAGCCGGACTACCA
GTGTGCAACGCCGGGTAAGTGGCGTCTTCTTTGAGGAACAAAACGTAGGAATTTTGATCCCAGAAAGAAG
CAAATAAAACTTAATTCAAAGAATAATTCCTACACAAAAAAGAATCCACAATTCATTAAATCCTCACTTA
TTCCCTGTGTGCAACATTTAACTTATCTCAATTGTTCAGCTTCACATAGCTGCTGCGAATGGATACGTCC
GCGTTGTGGAGTTTTTGTTGGAGCAGCATGTCAACGTGGATGCCATGGACAAGGATCTGTGGACACCGGT
GCATGCCGCCGCTTGTTGGGGTCATGTAAGTGAAGCCTCGAAGCCGAAAAATGGGAATAATAATTTTAAA
TATTTCCTTCTTTCAGCTTGAGGTGCTGGAGATGCTGGCCCAGTGTGGTGCTGATCTGAATGTTCGCAAC
AAGGACGACGAGACTCCTTCAG
>dme_circ_0005081_junction_seq
CGCAACAAGGACGACGAGACTCCTTCAGGAGTTTACAACGAAAAGTTGCAGAGAAA
>dme_circ_0005082
GAGTTTACAACGAAAAGTTGCAGAGAAACGAGCTACGGCAAACATCAACAGCTGGGAAAAGCAAATCATA
AGTAAACACTGAGGACAGGACCAGACAACCGCACAGGACGAGGGATAAACGTGGTAGTTGCCATGAGAGC
AGCCTCGGGTGTTGACCAGGAGTGAAAGGATCTGGGAAAAGTGAACCgcagccacagcagcagcagcagc
agcaatagcaacagcgaaacagctgctgcagcaATCCAAACAGCTGCTACATCCAAGAGCAACGAGCATC
GAGCAACTGGAAACAACGAGGGCAACGATAAGAGCCGCCAGAGACAACACCCGCAATCAAATCAAATTCA
AAGATGCAAATTGCAATTTGCATACGAATGCATGCGCACCAGACGAGCAACATCACCACAAACTGCAGCA
CAAATAACAAAAGCCTCGCAAATTGTTGTCAAATGCTATAAAATTGAACGGAATAACTCTGGAGCACACT
TGCAAAACATATCATTGAGCGGGCAGGATAGGGTTCAAGTCAACAAATCAACATGATCAAGGGCATTCTG
ATACAGCGCAAGAGCCAGGAGGATGCCAGCTACACCAAGCAGCTCAAGATGGAGCACGCCGATCTGGTGG
CCGAGATGCAGACCGTGGAGAGCCTGCCCACCCACGAGCGACTCCAGCTGGCCAGACTGACGTCGTGCGC
AGCAGCTGAAGCTGTCCCGCCAGAAGGACAAGGAGTGGGCCAAGTTGCAGCGGGCGAAGGGCAACACGGC
GAGCGGCGGCAGCGGAACCGGATCGCTGGGCAACGGGCTGATGAACGGCAACGGAGATACCACGCACCAC
TTCCGGCACGCCAATGGTTCGGGCACGTTGAGCGGCAGGCGGCACATATCCTTCGAGAACAGCGTGGTGC
TCCTGGAGGCGGCATCCCGGAACGACATGCCCGAGGTGGCCGCCCTGCTGCAGCACGGAATCACGCCGGA
TGCCGCAAACGAGGATGGACTGACGGCGATGCATCAGGCGTGCATCGATAACAACGTCGAGATGCTGCAG
CTGCTGCTGGAGTACGGTGCGAATGTGGATGCGCAGGACAGTGATAAGTGGACGCCGCTTCATGCGGCTG
CCACCTGCGGCCACCTCGAGCTCGTCCGCATCCTCATCCGGCACGGCGCCAATCTGCTGGCCGTCAACAC
CGACGGCAACATGCCCTACGACCTGTGCGACGATGAGAAAACCTTGGACTTCATCGAGGGCGAGATGGCT
CAACGGGGCGTGACCCAGGAGCTCATCGACGAGACGCGCTCCTCCACGGAGCGGATTATGCTGCGCGACC
TAATGGAGTTGGCGCGCACAGGAGGAGATTTGGAGGAGCCGGACTACCAGTGTGCAACGCCGGGTGAGAA
TATGCGAAAAGGTTGCGGCACTGAATCAACGGTTATCCGGCTAGAGCAAAATAACCATGAATATGAAATT
GAGCTATAAATTGTTGTTAATGCGACACGGAAAATGTGTAAATCTAGACTATTTGTAGTAGGAGTAGGAG

GAAAGCGCTCTCAGGTGTGAAGAAAGATTGCATTAATTTTTTAGCTTGGCAGGATCAACGATTTATAATG
GCTAACTCGAAAAAGTGTTACCCATTTCCGTAAAGTTCCTCCTAGCTCAGTATTTCAAATAAACACATAT
AGTACATACGTTCATTTAGATGTTAGTTGCCATAAACATAAGAATACACTTAAACGTTTTAACTGGATAT
TAACAAATCAATCCATCCGCCTTTGTAGGAGCCCAATCAGTGCGACGCACTAGTTTGCGAGACAAAACGC
TGACAACCAAAAAGGATGCCGTCGAGGAGACCCGGCTGCGTCTGCAGGCGCAGGAGGCGACGGACTCGGA
GGCTCCCTCATCGGCAGGGGATCCGCTGACCAATGGCTATGGTTACGGAAACAACAACGGAGAGAAGCGA
CCCTCGACGGGCAGTTCGAATGGCCAACCACTGCCGCACTCCAAGTCCGCCGATGCGCTGGATAATGCCA
CCGCCGCTTTGTCGGCCAACGCCACACATTCCTATCCGTCGCGCCGTCCACCGGATGGCCGGGAAAACGA
TGAACTGCTACGCCTGAAAGCTGCAGGTGCTGCGGGAGAGATGCAGCGAGCCACCTCGGCGGCGGCCGTC
ATTCTCACCGAGAAGGGTACGGCGGCCAGTGCCGAGGCCGTTCAAATCCAGTCGTCGAAGGAGGCTGCCA
ACGGCAAGATCAATGTCCAGGTCACCGTCCTCGTGG
>dme_circ_0005082_junction_seq
ATCAATGTCCAGGTCACCGTCCTCGTGGGAGTTTACAACGAAAAGTTGCAGAGAAA
>dme_circ_0005083
GACGTCGTGCGCAGCAGCTGAAGCTGTCCCGCCAGAAGGACAAGGAGTGGGCCAAGTTGCAGCGGGCGAA
GGGCAACACGGCGAGCGGCGGCAGCGGAACCGGATCGCTGGGCAACGGGCTGATGAACGGCAACGGAGAT
ACCACGCACCACTTCCGGCACGCCAATGGTTCGGGCACGTTGAGCGGCAGGCGGCACATATCCTTCGAGA
ACAGCGTGGTGCTCCTGGAGGCGGCATCCCGGAACGACATGCCCGAGGTGGCCGCCCTGCTGCAGCACGG
AATCACGCCGGATGCCGCAAACGAGGATGGACTGACGGCGATGCATCAGGCGTGCATCGATAACAACGTC
GAGATGCTGCAGCTGCTGCTGGAGTACGGTGCGAATGTGGATGCGCAGGACAGTGATAAGTGGACGCCGC
TTCATGCGGCTGCCACCTGCGGCCACCTCGAGCTCGTCCGCATCCTCATCCGGCACGGCGCCAATCTGCT
GGCCGTCAACACCGACGGCAACATGCCCTACGACCTGTGCGACGATGAGAAAACCTTGGACTTCATCGAG
GGCGAGATGGCTCAACGGGGCGTGACCCAGGAGCTCATCGACGAGACGCGCTCCTCCACGGAGCGGATTA
TGCTGCGCGACCTAATGGAGTTGGCGCGCACAGGAGGAGATTTGGAGGAGCCGGACTACCAGTGTGCAAC
GCCGGGTAAGTGGCGTCTTCTTTGAGGAACAAAACGTAGGAATTTTGATCCCAGAAAGAAGCAAATAAAA
CTTAATTCAAAGAATAATTCCTACACAAAAAAGAATCCACAATTCATTAAATCCTCACTTATTCCCTGTG
TGCAACATTTAACTTATCTCAATTGTTCAGCTTCACATAGCTGCTGCGAATGGATACGTCCGCGTTGTGG
AGTTTTTGTTGGAGCAGCATGTCAACGTGGATGCCATGGACAAGGATCTGTGGACACCGGTGCATGCCGC
CGCTTGTTGGGGTCATGTAAGTGAAGCCTCGAAGCCGAAAAATGGGAATAATAATTTTAAATATTTCCTT
CTTTCAGCTTGAGGTGCTGGAGATGCTGGCCCAGTGTGGTGCTGATCTGAATGTTCGCAACAAGGACGAC
GAGACTCCTTCAG
>dme_circ_0005083_junction_seq
CGCAACAAGGACGACGAGACTCCTTCAGGACGTCGTGCGCAGCAGCTGAAGCTGTC
>dme_circ_0005084
GCCACCACCACCACCTCCCTGGTTGCCCGCTCCGCCACGACCACGATCGCCCATGGGCCTGCCGCCGCCA
TACGAGCCATTTACATTACGCTGCTGGTTGCCACCGCGCTGGCCTCGATAGCTTCCACCGCCGCCGCCGG
AGGATTCGTAGCGCGACTCCTTGTTGTAG
>dme_circ_0005084_junction_seq
GGATTCGTAGCGCGACTCCTTGTTGTAGGCCACCACCACCACCTCCCTGGTTGCCC
>dme_circ_0005085
GCCACCACCACCACCTCCCTGGTTGCCCGCTCCGCCACGACCACGATCGCCCATGGGCCTGCCGCCGCCA
TACGAGCCATTTACATTACGCTGCTGGTTGCCACCGCGCTGGCCTCGATAGCTTCCACCGCCGCCGCCGG
AGGATTCGTAGCGCGACTCCTTGTTGTAGGTGCCACCGCCACCTCCTCCGTTCTGGTA
>dme_circ_0005085_junction_seq

TGCCACCGCCACCTCCTCCGTTCTGGTAGCCACCACCACCACCTCCCTGGTTGCCC
>dme_circ_0005086
GCAACAACTACTACCAGAACGGAGGAGGTGGCGGTGGCACCTACAACAAGGAGTCGCGCTACGAATCCTC
CGGCGGCGGCGGTGGAAGCTATCGAGGCCAGCGCGGTGGCAACCAGCAGCGTAATGTAAATGGCTCGTAT
GGCGGCGGCAGGCCCATGGGCGATCGTGGTCGTGGCGGAGCGGGCAACCAGGGAGGTGGTGGTGG
>dme_circ_0005086_junction_seq
GAGCGGGCAACCAGGGAGGTGGTGGTGGGCAACAACTACTACCAGAACGGAGGAGG
>dme_circ_0005087
GTGGCGGTGGCACCTACAACAAGGAGTCGCGCTACGAATCCTCCGGCGGCGGCGGTGGAAGCTATCGAGG
CCAGCGCGGTGGCAACCAGCAGCGTAATGTAAATGGCTCGTATGGCGGCGGCAGGCCCATGGGCGATCGT
GGTCGTGGCGGAGCGGGCAACCAGGGAGGTGG
>dme_circ_0005087_junction_seq
GTGGCGGAGCGGGCAACCAGGGAGGTGGGTGGCGGTGGCACCTACAACAAGGAGTC
>dme_circ_0005088
GTGACGAGCGGCGCAACGGCGGCGACAGGGGCAACTACCGGTCGCGTCAATACGGCAACTCATCTAACAC
GAACGGACGCAATTCGGGCAATTCGAGCGGTGTGTACTTCCGCAACAATGAGTCGGGCAATGGTGGAGGC
AACAACTACTACCAGAACGGAGGAGG
>dme_circ_0005088_junction_seq
GCAACAACTACTACCAGAACGGAGGAGGGTGACGAGCGGCGCAACGGCGGCGACAG
>dme_circ_0005089
GAGGGCAGCCCGGGGTTCCCTGTTTTTATAAGGCTTGACATCCTTGGCCAATAGACTATCGTCGAAGTTC
AGGAAGCCGCCCACCGTAACGCCAAAGATAATAAAGCACTTCTCGTTGAAGAATGGCAGTCCGTCGTACA
GCTCCCCATAAACCTCATC
>dme_circ_0005089_junction_seq
CGTCGTACAGCTCCCCATAAACCTCATCGAGGGCAGCCCGGGGTTCCCTGTTTTTA
>dme_circ_0005090
GGGGTAACTCCGTGCTGAGGATGCCGCGCGGAAAGCTTGTAGATGGTAGATCCCTATACGGAGGACCCGT
ACTCTCCACGGATGCCCACCAAAGGGAGGACTTATGGCTGAGCAAGCGAAAGATATCCCATTTCGGCGAT
GACTTTCACACCTACAGCTTGGATTGGAGCAGCAATAGGCTGCTCTTCTCCGTAGATGGTCAG
>dme_circ_0005090_junction_seq
TAGGCTGCTCTTCTCCGTAGATGGTCAGGGGGTAACTCCGTGCTGAGGATGCCGCG
>dme_circ_0005091
GGAGGAAGTTCTAAGCAAACTAACTTCGCTTCATAGCGAATTTACCGTGGAGGATATGAAATCCCTTCCG
TATCTCAGGGCGGTCATCAAGGAATCGCTTCGATTGTATCCAGTCACCTTTGGTAACGCCCGATCCGCCG
GAGCTGATGTTGTCCTCGATGGCTACCGAATCCCCAAGGGCACCAAGCTTCTGATGACCAACAGCTTTTT
GCTTAAGGACGACCGACTGTATCCCCGGGCAAAGGAATTCATCCCGGAACGCTGGCTGCGCCGAAAGGAC
GATGACAAG
>dme_circ_0005091_junction_seq
CTGGCTGCGCCGAAAGGACGATGACAAGGGAGGAAGTTCTAAGCAAACTAACTTCG
>dme_circ_0005092
TGTTCTCGAGCAGTTGCTGCAAATCGATAGAAAGCTCGCCGTTGTTATGGCCATGGACATGCTGATGGGA
GGAGTTGATACCACCAGTACGGCCATCTCTGGAATATTGCTCAATCTGGCCAAGAATCCGGAGAAGCAGC
AAAGACTCAGGGAGGAAG
>dme_circ_0005092_junction_seq

GAGAAGCAGCAAAGACTCAGGGAGGAAGTGTTCTCGAGCAGTTGCTGCAAATCGAT
>dme_circ_0005093
GTTATAACGTATACTATATATAAATTGTAATTTATTGAAATTTAATTGCACCAAAAACGCCGCGAGGCGT
GCGAAAATGTTTTACGATTTTCAAAACCAACACTCAAGAAACTAAAAAGATTAACAACCGTACACCCACG
AATCTATGTATGTATATGATTAGTGAGCGTCTGTCCAGGGATGCGCCTTAAAGAACTAGATCTAGACCTT
AGTCAACGTCGAATTATGATTATGATTAT
>dme_circ_0005093_junction_seq
GTCAACGTCGAATTATGATTATGATTATGTTATAACGTATACTATATATAAATTGT
>dme_circ_0005094
GATTACGAACGGAGTGCCACACAGGCTTGGCGACAGGAACAGCTCCCTAATGTCGCGACTTCTTCGACTT
GTGCCCATTGTCGCGACTGTCATCCCGCGAGCGCTTGCGATCCTTGTCCTTGCTGCTGCTCTTGTTTCCG
GACGATGAGGAGGAATGATGCTTGTCCCGGCTGTT
>dme_circ_0005094_junction_seq
AGGAGGAATGATGCTTGTCCCGGCTGTTGATTACGAACGGAGTGCCACACAGGCTT
>dme_circ_0005095
GTGTAAAAAATCAGAGGTAGCAGAAAGATTCACGAAAATAATATAAAATTCTGGCAGACGCAAGTTTAGT
AGGGGACAAAAAATACAAAAAACGAAAGCACGACAACATCTATATAGGTTTGCCTAGGTATATAATATAT
ATTTAGGTCTCTAGCTGGTATGTCTAACTTCTATATGTATTATAAAG
>dme_circ_0005095_junction_seq
ATGTCTAACTTCTATATGTATTATAAAGGTGTAAAAAATCAGAGGTAGCAGAAAGA
>dme_circ_0005096
CAATAGTAACGTAGGCAAAAGAACACTGGTTTTCTATAAGCAAAGGTGCCTGCAGCCTCGAGCGAAATCA
CACAAAACAGGCCGAAGCAAGATGCCATCTAAAACAGTACTGTTTGGTAGGTGTGATTGCATTCCAGATT
CCGATCGCGGAACGATACACAACGCCCCGGTCCACCTAGACTTTCTGTATTCTTTTGTCGCCAATAG
>dme_circ_0005096_junction_seq
ACTTTCTGTATTCTTTTGTCGCCAATAGCAATAGTAACGTAGGCAAAAGAACACTG
>dme_circ_0005097
GTAGCGCCTGGCATTTGGGTCCGATGACAATGGAGTTCTCCCGGAACTCTAGGCCCATGGCGAAACCAAA
GCTTTTGGGCAGATTGTCCACCATACTCGGCTTCTCCTTCTTCACGAAGTCCAGGGTCTTTTCGTAGACG
TCGCACAGCTTTGTGCCCGGAACCAGCAGCTTGAGAATCTCCTCCTGCACACTTACCAGAAATGTGTAGT
TCTCCTGCATGGCCTCGGTGGGATTCACCAGAAAGGTGCGCGATATATTTGAGCAATACGACTTGTAGCG
GGCGCCCAAGGAGCACACAATCACACCGAAGTGCAAGGGATTTTTGTCCGCCACCGCGGAGAACTTCAGG
CTGTAGGCTCCTCCCGATTGGATGATGGGTGGGTAGGCCATATCGAGGAGTCTGGGATCCAGACCACTGG
TGTACTTCTTCTCGCCAATGGCAGCCTCGCAGCCATCTGAGAGCTTGTTGTGTTTGACTTTCTAAAAAGG
AGGTTAAAAAGGTTAATATATTAGGAAAACACATAATATTATAGCCGCCAGGTGCTCACCCTATCCGAAT
CAATAATGTCCATGATCTCATCTTTCAAATACTTGTTGAAAATGTCCATTGACACTAGCGATGCCTTGCG
TATGTTGTTGATCTCCGACTCATCCTTGGGACACATGAGGTAGGCGATAATAGTGCTAATGTCCACATGC
TCGAACTTGGACGCCGTCAGGGATTTCTTCCATGCTTCGCTGAACTCCCCGGGATAGGCGTCCTTGGCGA
AGACGCCCAAACGCTTGCCCTTCTTCGAGTTTTGCAGGGCTTTGATGAGCTTCTCGAAGTTGCCCTGGTC
CTTGTCCGTCTTCTCGAAGTCAAGATCAGAAAGGATTAGAGAACTTTTGGCCTGGAGCAGAAATCGAAAC
TCACCCGATCCCTAACAAGCAGATTGATTTCCGGAAAACCTTCTTCGGTGATGTTCTGCGTCTGCTTCAG
GAATTCTATTTTCTTTTTGCTAGTGAGAAAATACACGGCATCCGAGCAAAACACTGAGATGGTGTCCGTC
AGCTCGTAGCCCAGCAGCCAGAGCTGCAGGGCCATGGACTTGGAGTACATGACATCCTCTTCGACGCCCA
CGATGCTCATGATGCAGTCCAGATTCCTGAGCGCGTCATCGTGTCCGATGCTGGGAGCCTGCGAAAGACA

AAGAGTCCGATATTATTATGCAGTAATCCTCTTTTCCCCAAACAACCTGGTCTATGGGTAATTAATTTCA
CCACATCTCCCAATCTTTCATTATCGTGACCCGTCTGCAGAGTCTCCACGTGCTCCCCGGTGCTCCTGCG
GCCCCAAAAACACGCAGATCGCCGACCGGCGGACAGACTCACCCTCCATTCCGTGTAGAGGCGTTTCACG
CGGCGCACGAACGCCTCCTTGTCCAGCACAAAGCTCGACATATTGCCGCCGGATTTGTTACTGTGTATGC
>dme_circ_0005097_junction_seq
TTGCCGCCGGATTTGTTACTGTGTATGCGTAGCGCCTGGCATTTGGGTCCGATGAC
>dme_circ_0005098
CTGTTCGAGCGGATAGAATGCGTGGTGGACGCAACAAATTCGGACCCATGTACAAACGGGATCGCGCGCG
GAAGTTGCAAGTGATGCGGCAGCGGCAGTTGGCGCTGCAAGCGCTGCGCAACTCGATGGGTCCGGACATC
AAGCCAACGCCGATCTCGCCGGGCTACCAGCAAGCATATCCAAATATGAACATTAAGCAGGAAATTCAAA
TACCTCAGGGTATCCTCACTCACCCAATCTCCGGACTCGTCGCCCAGCCCCATAGCAATTGCGTTGGGAC
AGGTGAACGCGAGCACGGGCGGTGTTATAGCCACGCCCATGAACGCCGGCACTGGCGGCAGTGGGGGCGG
TGGTCTGAACGGACCAAGTTCCGTGggcaacggcaatagcagcaacggcagcagcaacggcaacaacaac
agcagcacgggcaacggaacgtCCGGAGGAGGAGGTGGCAATAATGCGGGCGGCGGAGGAGGAGGAACCA
ATTCCAACGATGGCCTGCATCGCAACGGCGGCAATGGCAACAGCAGTTGCCACGAGGCTGGAATAGGATC
TCTGCAGAACACGGCCGACTCGAAATTGTGGGTATTTAGCCCAAAAGCCAGAACAAAACTCAATGAATAT
TCGAACTCAAACCAAATTAAACGACAAACGAACTTCAACAAACATTGAAAAGAAACTTTCGTTACATAAG
CATATTTGTGGAAAAGGATTCAACCTGATCATGCAGCAAGTGTGGAAAAGGGCAGTCatatcaatataat
aatcaatataatataataattAGACTTGaaaaaaaaaGATGTATTGTAAAAAATGTACAAACCTTGAACG
ATTTCCTTGTACAGAAGAAGAGTATATACATTTAATTGGAAATATTTGTATTCATTATCAGATATTATAT
GAGCTTCGGTTTCTATATTTAAATATATATCTGATAATGATATTATAGATCTCCAATTAAAGTCTTCATC
AAACTGCCCTTTTACGAATTGCTTAGTTATCACTTGATGAGCTCTCCAATTAGAAGTGATCTGAACTTTA
ATTTATAACTTTTCCTATATCTTTTGTTTTCTACCAAAAAGCAGTCTCACCGATGATTCGTGAATTTGTG
CAATCTATTGACGATCGGGAATGGCAGACGCAACTGTTTGCCCTGCTGCAGAAGCAAACCTACAACCAGG
TGGAAGTGGATCTCTTCGAGCTGATGTGCAAAGTGCTCGACCAGAATTTGTTCTCGCAAGTAGACTGGGC
ACGGAACACCGTCTTCTTCAAGGATCTGAAGG
>dme_circ_0005098_junction_seq
AACACCGTCTTCTTCAAGGATCTGAAGGCTGTTCGAGCGGATAGAATGCGTGGTGG
>dme_circ_0005099
GGAAGTTGCTGGTGGAAACGGAAGTCGACGAATTGACTTCAAGTGGTTTCAATTGATGATATTCGACGGG
CGGCACAATTATGCTACTGTTGTTGCCATTTGGATTTTGGGGATTCTTTTGCAACAGGGCGGGATCAATG
CACTCGTTGGCGCTGCGATGGGTGGGAAATAGAATATTGGCAAACGATGGCATCGTCACAGATTCGATGA
TCACCGAATTGCCGTTCGATGGCCTCACTGTCTTAGCCTCCAcgttgttgttattgttattgttagtgtt
gttattattgttgttgttggtagtg
>dme_circ_0005099_junction_seq
gttgttattattgttgttgttggtagtgGGAAGTTGCTGGTGGAAACGGAAGTCGA
>dme_circ_0005100
AAACGACAGCAGCAGGAACAGCGAATTGCCGCCCGCTTGGCGCCCAAACAGGGCCAAAGCAGTTCATCGG
AGAGCGATACCACAGAGCTTCACAAGCGGAATACGGCGCGGCAAAAGAAACCACTACTGCGAAAGGGACG
AACGAGGCAAAGGTCTCCGGAGGCAGCTAGCATTCAGCAGAGCAGCGATGAGGAGCAGGAGAAGG
>dme_circ_0005100_junction_seq
CAGAGCAGCGATGAGGAGCAGGAGAAGGAAACGACAGCAGCAGGAACAGCGAATTG
>dme_circ_0005101
GCACCGAATTCGGAACTTTGGCCAGAGACATGCAAAGAGGGCGCACTTTGGTGCGACGATGACTGACTGC

CGCTGACGTCCGTAATGCTCGCGGCAGTCTCCCTGGCCATCTTGTGCCGTTTGGCATTGTCGTTCTTCTT
CCGGTAGAGTCGCACCAGGTCCGTGTAGCACTTGCAGCAGATGAGG
>dme_circ_0005101_junction_seq
GTCCGTGTAGCACTTGCAGCAGATGAGGGCACCGAATTCGGAACTTTGGCCAGAGA
>dme_circ_0005102
GCAAAATGGCTGGACGCGATGCGGCTTCCAATCAGTTGATTGACTACAAAAACTCCCAAACggtgagtgt
gttggtgtgcgagcgcgagagagcgcaactgcatgtgtgtgcgtgtgtgtgCCGGCaggaaaataagaaa
acaagaaaggcaaaaaagacaaaaagagaaggGAGAAGCATTGTACGAAATGAAATAAACAAAAAACAAG
ACAATTTCAGTAACGATTGCTATGAGCCTGTACTttccattaattccattaattATTAAAATGCGAAAAA
CAACAAAGGCTTGTCCAACAAATGCCAGTAATGATGAAGTCGTTTCCCCAGAGTTCGAAGTTCAAAAGCC
GCCTCTCTGGGTCTTCTATTTATTTGTAGAAATCTTATCAGAAGTGAACTGATAAACCTTATCAGTGTGG
CAATTCTTATTGGATTTGTGTTTTCTGTACCTAATCCAAAGTTAattttattttcccttttatttttatt
ttCCTTCTTAAGGAAATCCAAAGTTTAGTCTAGCTAACCTATTTTCTAACCTTGGGCGACCTCTTACCTT
AGTCTATTTTCATTTTTCAGCGCTGTTTTCACTTAGCACTTCCAATTTCATTTGGCACTTGCATTGTGCA
TTGGATTTGAGTATTCGAATTTGTTCGTTTGCACTTTTGTGTTAATTATTCGCTTTCGCTTTTGGTTTAG
ATTAAATGCATGATTTCAATTGTGGAATCGTGCCCTCTTAAAATTTTGAACAAATATTGTTTGTTCACAC
TCGGAAAATTGTACTTTCCTAAATTCGTGGCATTCCACAATTGCAACATTTTATACACTGCAATCATTTT
TGAAATCAAATATGCTGTTACATTTTATAAAAAGGAATGAAATATAACAAAATCAATAGTAAGCCTCACC
CATAGATACTGTATCTATATATAATTTACTCGGCAATTTCCCTGTGGTTTTCCCAATGCCCTCTAAAAAT
CGATAAATGAAATAATAATTCTGCATAGCTATTTGTCAGCCAGTTGATAGGCATCCAAAACAGCTAAGTA
ATTGGCCCAAATAGATTAGATGTATCAGCCGTATAGATATTGTTGAGTGCCCACATTTGGTTAGTAACTA
AAGTAAATTTCCATTGGATTTCGATACTTTGTAAGTATGAATTGTCAGTTCTCAAAGAATACAATCAttt
tttgtttttaatttagtatacctttttttACCCTATTCAATTAAGGCTAAATCTAGAACTTAGTTCTC
GCATCAGCGATTTAAGTGCATTTGTGCATTGAGTCACGAAGCCAATTACTCCACTAACTAAAAGCAATCA
GCGGTCATTATTTTATATAAACAAATATAGATATGAACTTTTTGAGAAACGCGATCGCTGGATTGCACCG
CTTGTAAGTTGGAAAAATTCGTTGGGAGTCAATGGACTCTGCGGCGACCGGCTCAATGTTGCACAAACTGG
TTGCAATGCGCATCGACTGCGAGTGATTCGAGTGACAACGAACCCGGCTTTAAACCCGTTTTGGGTTGAG
TGCGGAAGGCTAGACCGCTTCGGAAAATGCGTGTGCCAGTGCCATAAGTCAACGCAAATTTTCTTTCTCC
AATTTGGTTATGCAACCTGGCTGGAACGCCCGGCTAAAAGCGACTGTATGTGAGAATATGTTTCGAACTG
ACCCAGAAAAGAGAGCTAGCTCCACAGTTGGTTGGTTGGTTCGTGTTGTTTTAGTCGATTTTGACCTTCT
ATGACGAAACTGTGAAAACTGCATTCTGTCAGGCAATTAAATGTTTGCCTTTGTTTCGGTGAATAATCAT
GGCAACTGGGTTCTTTGTTCTTTTGGGAATCTCGCAATCGAGTTAATTTGTTATCCAAGAAATTCGAAAA
TGCAAGGAAAATACTTGGAATATTTTGCAACGCAATGCATATTGCGTAAGGTCATTGTGAGTCGGTTAAG
AACCCTATAAAAATCCTTATTTAAAATCCTTTAGCTTATTTTCTATGCATTCTCATTCTCGAATGAGTAA
CCTTCTGCTGATAAATTATGTCTAGAAGGCAAATTTATCTCATACTATCAGGTGCACTGATCTTATAAAG
CCGCCTCATTAAGTTGCTGTTTGCCATCTTTGTGTCATTGGCATCGCCAACAAGTTGCAATTAAAAACTT
TCCTGTAGACTGTGTTCTTTGCATTTCTATTCGAGATCGGTTGCGGTTCTCTTATCAGTTTGCTCTTCGC
TGGAGATATCTACAACCAAATACATACATACATATATAAGAACGAAGGCTCTGATAGGAACCAGATTGAC
TTGTTGGACGTGGATCGCAGTTCCAGCGGTTCCACTCGCATTGTCATTCCCGGTTTGCTTCACTTATCGT
ATCTGCCCCCTGAAGTGGCCCCATATTAATGTCTCAAAGTGCAAAACATTCCGGCTGAATCGGCCGAATC
TAAAGGGTGACTCCAGTATAAATTCCAGAATGATAATTTCGAAATTATATTCATTTCTTCGTTTTTATGT
AGCGCTTTTTGTTTGTCAATAAGTGGGTATACTACTGGCCTGTCTAAGCTGATTTTATTATTAAATAGAT
ACATATATACTTCTAGGAACTAAGATTTGATAAAGTCAACTACTAATTCCTTAAACAATTGTAAAAGTCT
TAGTGCCCAACCGGGTAATCCCAACCCAAGTGCTATATGTATCTAAATGACTTTGTTGTGAAATATAAAC

AGTAGCTTAATTTATATGTTTGAcacaccaccaccaccaccaccGGTGGCTTCACTTTGATAAACAACCT
TCTGTATAAAATTCTATATGCATTGTTCTAGAAATGCTTTCGAATTAGAATTTCGCATGGCGTTGTTCAG
GTTTCAAATACATTAGCTAAACAGTGCGCATTGGCCGGTGACAGTTTTGCAAATTGTTGGCTGAGTTAAT
TTTATAATGAGTGGCTGGCCTGCATGCGAAATAAACGATAAGGTGATTAAGGAGCGGTCTCGGATTACTC
TAGGGAAATGAATTGAAAACAAGGAGTATGATTAGATCGACTGATAAATGACAGGACAGCTGGTGGGCTA
ATTGATATTTCAGATGGACAATACACTTTTATTAGCTTAATGGAATGAAATAATTAATTGATCGCCAGCT
AATAAACTGTGTAACATCATTTTGATGTTTGAATACTTTCATTTAATTGGTTTTTTCCTTGCACACGGAT
TTGATATCTTGACATGTTAATGCAAAAAACCCATACATCATCACTATATTAAGGCCTCAAGCTCTTAAGA
ACACCTGGGATCTCTTATTTTAAATTTATATGCACTGCAAAATGACCCCCCGTGTAGAGATAACGCTCTG
CAATCTTTATGTTAATGTTTTTTTCTTTTGGTGAAAATCGAATGATTAAATGAAAGTCCGCGGGCCAAAA
ATGAATTATCGCAATAGGCCCGATAAGATTGTAAGACACTTGAACCAATATATTGTTGCTGAATGTTGCA
TAGCATTTTAAAATTAAAATACATTTGATTAAACTTGACTTTCATTTGCTTTTGCAGGGTTTCTCCTGGT
GCCATTACCACCGGCAATGGAGCACCCATTGGAATCAAGGATGCCTCCCAGACGGTTGGTCCGCGAGGAC
CTATCCTGCTGCAGGATGTGAACTTCCTGGATGAGATGTCGCACTTCGACAGGGAGCGGATTCCAGAGCG
TGTCGTGCACGCCAAGGGAGCTGGTGCTTTTGGTTACTTTGAGGTGACCCATGACATCACCCAGTATTGT
GCCGCCAAGATTTTCGACAAG
>dme_circ_0005102_junction_seq
GTATTGTGCCGCCAAGATTTTCGACAAGGCAAAATGGCTGGACGCGATGCGGCTTC
>dme_circ_0005103
AAGACCGGCGTGTTGTTGCCAACCAAATCCCAGACGCCATCCTCAGTGTAGAACTTGACGGCAAATCCTC
GAGGATCGCGGGCGGTGTCCGCAGATCCGCTCTCACCACCCACGGTGGAGAATCGCACGGCCAGTGGAGT
GCGCTTCTTGACCTTGTCGAAAATCTTGGCGGCACAATACTGGGTGATGTCATGGGTCACCTCAAAG
>dme_circ_0005103_junction_seq
CTGGGTGATGTCATGGGTCACCTCAAAGAAGACCGGCGTGTTGTTGCCAACCAAAT
>dme_circ_0005104
GTTGGCTAGCACTGATCCGGATTACAGCATTCGCGATCTGTACAACAGGATCAAGACCTGCAAGTTCCCC
AGTTGGACGATGTACATTCAGGTCATGACCTACGAGCAGGCCAAGAAGTTCAAGTACAACCCCTTCGATG
TCACCAAGGTCTGGTCGCAGAAGGAGTACCCTCTGATTCCTGTGGGCAAAATGGTGCTGGATCGCAATCC
CAAGAACTACTTTGCTGAGGGTGGAGCAGATCGCCTTCAGTCCCGCTCACCTGGTGCCCGGCGTTGAGCC
CTCTCCGGACAAGATGCTGCATGGTCGTCTGTTCTCCTACTCGGACACCCATCGCCATCGCCTGGGACCG
AACTACTTGCAGATCCCGGTGAACTGCCC
>dme_circ_0005104_junction_seq
ACTACTTGCAGATCCCGGTGAACTGCCCGTTGGCTAGCACTGATCCGGATTACAGC
>dme_circ_0005105
ATCCGAAAAGCGTTCGCCTTTCTTAGTCAATTTCGGAAtgggtggtgttggtggtggtggtgttggtgtt
ggaATAATTAGTTCCATTGGATTAATCGTAATTAAGGCCAAAAACCAAGTCTGATCCGTCGAATCCGCTC
GCTCAGCTTAGAACTTCGAGGACTTGGCCAGGTTGAGTTCCTCGGTCAGCATGCGACCGAAATCGGCGTG
CACCTGGGTGAAGTTCTTGACGGCCCGCTCCTGCAAGAACTGGCTGGCGTTGCTCAAATGGCCGGCAATG
TTCTGCACCAGACGCTTCTTGGCGCACTTGTCGAGCACATGCACCCAGAAGTCGGTGACCTGGCCGAAGT
TGTCCTCGGTG
>dme_circ_0005105_junction_seq
GGTGACCTGGCCGAAGTTGTCCTCGGTGATCCGAAAAGCGTTCGCCTTTCTTAGTC
>dme_circ_0005106
GGTCACCGACTTCTGGGTGCATGTGCTCGACAAGTGCGCCAAGAAGCGTCTGGTGCAGAACATTGCCGGC

CATTTGAGCAACGCCAGCCAGTTCTTGCAGGAGCGGGCCGTCAAGAACTTCACCCAG

>dme_circ_0005106_junction_seq

GGAGCGGGCCGTCAAGAACTTCACCCAGGGTCACCGACTTCTGGGTGCATGTGCTC

>dme_circ_0005107

GTATTGAGTGAGTTGTTCATTTAAAAATTTTTACAAGTTGAAAAACATGTATGAATATGTAAGTTCTCTC
AGTCTAGTTATTTAAAATATTAGCAGGCCAGGGCTGCCATTGAACAAACTGTTCTAACTTTTCCAAATCT
TTTCCATTTTTATGCTAAATGCAACAATTAAGTAACATTAATTTGTATTTAAATATTTAGCTGAGTGCGT
AGACCAGGTGTTCAATTCCTGATTAACAGTATATTGCGATACGttttttttttAACACATTTTTCTAAAC
AGTAGGTAATGCTTTTGTATCAAAACTTTTCGCAACTCTGAAATGCAAGTATACATAGTTAAATATTTTT
CACATTTTTCGCTGTCAGCAAAGGCACTTTTCGTTTAAAATTCCCTAAATTTTCCTCAACTTTATCGCTG
TGACGGGTATGTTAGTTATTTACACTAATTACTAACTATCTAGTGCGTCTTGTTTCCCAGTGCTGCCGCG
GCATAAATCTGAGCCCATTCGGGGAACGAGTTAAGGTTCGGATAGACGACCAGGCCCCAGGTTTGGCAGA
AAACAAACAGGGTGATGATGGTAATGATG

>dme_circ_0005107_junction_seq

AACAAACAGGGTGATGATGGTAATGATGGTATTGAGTGAGTTGTTCATTTAAAAAT

>dme_circ_0005108

AACTCCACCGAACAGATGGACTTCCCCACCTTCATGTTCTACTCGGTGCCATCCATGTTGGTCTACACCT
TGCTGACATTCGTGTTCCTGCAATGGCACTTCATGGGTCTGTGGCGTCCCAAGAGCAAGGAGGCACAGGA
AGTCCAGAGGGGACGAGAGGGCGCCGATGTCGCCAAAAAGGTTATCGATCAGCGCTACAAGGATCTGGGT
CCCATGTCCATTCACGAGATCCAAGTGATGATTCTGTTCATTTTTATGG

>dme_circ_0005108_junction_seq

CAAGTGATGATTCTGTTCATTTTTATGGAACTCCACCGAACAGATGGACTTCCCCA

>dme_circ_0005109

CTGCAGGCTCAGGGTGTCTGCAAAATCAACCATGAGCCTCAATACCAAATCGTTGGAGGCAACAAGAAAA
ACAACGAGGATGAGTAAGTAACAATTGGTATTGGTGGCATCAAGCTTTACACAATACTTGTATAAACTAT
ATACACCTTTACTTTGATTATTTGTTTAATAAATTCATATTTTTGTAGGCCACCATACCCCACCAAGATC
ACTCTGTGCTACTATCTGGGCATTGCCTACGCCTCCTCGCTGGGTGGCTGTGGAACCATCATCGGAACTG
CCACCAATCTTACCTTCAAGGGCATCTACGAGGCTCGTTTCAAGAACTCCACCGAACAGATGGACTTCCC
CACCTTCATGTTCTACTCGGTGCCATCCATGTTGGTCTACACCTTGCTGACATTCGTGTTCCTGCAATGG
CACTTCATGGGTCTGTGGCGTCCCAAGAGCAAGGAGGCACAGGAAGTCCAGAGGGGACGAGAGGGCGCCG
ATGTCGCCAAAAAGG

>dme_circ_0005109_junction_seq

CGAGAGGGCGCCGATGTCGCCAAAAAGGCTGCAGGCTCAGGGTGTCTGCAAAATCA

>dme_circ_0005110

GGTGGGGAAGTCCATCTGTTCGGTGGAGTTCTTGAAACGAGCCTCGTAGATGCCCTTGAAGGTAAGATTG
GTGGCAGTTCCGATGATGGTTCCACAGCCACCCAGCGAGGAGGCGTAGGCAATGCCCAGATAGTAGCACA
GAGTGATCTTG

>dme_circ_0005110_junction_seq

GCCCAGATAGTAGCACAGAGTGATCTTGGGTGGGGAAGTCCATCTGTTCGGTGGAG

>dme_circ_0005111

ATATAGGGGCGTCTGCGTCCATTTCGCGTTGATTAGCGTGGTGATGGGATAGCTCTGACTGGATTCCCCA
GAAGTTGGCGTATATAGTGCTATGAGCACCACAGACACACACAAAGCCACGGCACGTAACATGG

>dme_circ_0005111_junction_seq

ACACACAAAGCCACGGCACGTAACATGGATATAGGGGCGTCTGCGTCCATTTCGCG

>dme_circ_0005112
AAGCCTCTGACGCAGTTGATCCAGCGCTCTCTTAAGTGTAGGATGCTTCTGCTTCAGCACCTTGAAATCA
AAGCCCTGGACGTCCGATTCATTAGCCAAATCCTCATCAGAAGTGGAACCAGCTTCGGGCTTTGGAGCAT
CATCCTGACTCTTGTATTCCGTTGACTTCAGATGGAGTTCCACTCCATAGCCCGAAAGTCGTACCGGTCG
CTTGTCCTTCTTGGCCAATTGATGACGCAAGATGTAACGAATCCGGCCAGCATTGGCTTCCTTTTCCAAT
AGTTTGTGATAGG
>dme_circ_0005112_junction_seq
GCTTCCTTTTCCAATAGTTTGTGATAGGAAGCCTCTGACGCAGTTGATCCAGCGCT
>dme_circ_0005113
TATTTAAGATTATCCATTAGCGTAGCGGTCATATCCCGGTTAGACAGACGATTTAAAACTCCAACATTGC
CAAGATTTTTGTAGGCCACGCCGTTAATATCAAGATATTTCACATCCTCCTGGCTTAGGATTCGCTGATT
CAATCTGGGCATCACGTGAGGTTGATTCATCAGATAATCAATGGCTACATCGTTGTCTGTCAG
>dme_circ_0005113_junction_seq
AATCAATGGCTACATCGTTGTCTGTCAGTATTTAAGATTATCCATTAGCGTAGCGG
>dme_circ_0005114
GTTTTAAATCGTCTGTCTAACCGGGATATGACCGCTACGCTAATGGATAATCTTAAATACTTTGGTGGCA
AGAAGTCTACGGAGCTTATTGGCCGAGCATCCCTACAGTTCCTAACGATTTGGGTGTTTGCTGATTTGGA
AACTGACCAGGGTCGAGATCTGCTCACCCATGCCCTGGACTATGTCCAAAGTGGAGAGAGTGTGCGAGTC
GCATTCATTCCAAACACTGAAAGCTCTTCCGCCTCAAGCCGGAGGAATCTTAATCGATTGGTTTGGGCTG
CCATGCAGAGTCTTCCACCAACTCAAGCCACGGAGCAGGTTCTCAAGTGGCTAAAGAAACCAAAGGAGAA
AATTGAGATACCCACTCAGCTCGAGGATATCCTGGGATCTACAGAGCTGCACCTGAAGAT
>dme_circ_0005114_junction_seq
TGGGATCTACAGAGCTGCACCTGAAGATGTTTTAAATCGTCTGTCTAACCGGGATA
>dme_circ_0005115
AACTGAACCTGGCTAGCAAAGCGAAATCGGCGCTATCAAAGCTTTCATCCGACGAAAGGGGCCCATAAAG
CCGCCCATTACCGATGACCAAACGCTGGGATTTATTTAGACCCAACACTCGCTGGGAATAAACTCTCAAC
ATCTTCAGGTGCAGCTCTGTAGATCCCAGGATATCCTCGAGCTGAGTGGGTATCTCAATTTTCTCCTTTG
GTTTCTTTAGCCACTTGAGAACCTGCTCCGTGGCTTGAGTTGGTGGAAGACTCTGCATGGCAGCCCAAAC
CAATCGATTAAGATTCCTCCGGCTTGAGGCGGAAGAGCTTTCAGTGTTTGGAATGAATGCGACTCGCACA
CTCTCTCCACTTTGGACATAGTCCAGGGCATGGGTGAGCAGATCTCGACCCTGGTCAGTTTCCAAATCAG
CAAACACCCAAATCGTTAGGAACTGTAGGGATGCTCGGCCAATAAGCTCCGTAGACTTCTTGCCACCAAA
G
>dme_circ_0005115_junction_seq
AAGCTCCGTAGACTTCTTGCCACCAAAGAACTGAACCTGGCTAGCAAAGCGAAATC
>dme_circ_0005116
TATCTACCAAGGTAGGTTGACTCTGGGTACCCAACACCAACTGAAGTCCTCTGGGCGGAGCGCCGCTAGC
AGCATCAAAGCAGTGACCCTCCAACAGCAGATACTCCAGATCGAATTCGCTGTGCACAGGTCCACCAATA
TCGGTCAACTTAATGTTGTCCAGATCGTAAACTGCTCTCACAGCTTCGACCAACCAGTTCTCGGGAACCT
GCAGCTGCTGGGTCAGCAGAGGATTGGCTGGCAATCCACTGAATTTGGCCAAAGGACCATCAGATCGGCC
TCCATTCGCCTCGAATTGGACCTCCGGTTCCACAACGTATCTGTAGAAGTTCTTCACGGGCATATCGCTG
TGCTGGGGGACGGGAATCAGGTATAAG
>dme_circ_0005116_junction_seq
GTGCTGGGGGACGGGAATCAGGTATAAGTATCTACCAAGGTAGGTTGACTCTGGGT
>dme_circ_0005117

GGTTCTTATAACCTCCTTGCGATCCCATGTTGTCAAATTAAGGGTGTCTAAGAAGCCAGGCATGCAGCAG
GCGGAACTCCTGTCAGATGACAACGAACAGGCAGCGCAATCAGGCATGTGGAACAGCATCGCCAGCAGTT
TTGGCGGCGGCAGTGCCAACCAAGCAGCCACTGATGAGGATACGGAAACCATCAACATTTTCTCTGTGGC
ATCGGGACACTT
>dme_circ_0005117_junction_seq
ACATTTTCTCTGTGGCATCGGGACACTTGGTTCTTATAACCTCCTTGCGATCCCAT
>dme_circ_0005118
CTGTTCGGATCCTGGCTAAGTGCCTGGTATTGGCCTCTTAGCCTATCTCCTGCCGCAATCTTGCGGAATC
TCTTCAAGTCCACCACGTACAAGGCGGAAATGTGGTAACGCCTGCCCATCAGATGGCTTCGCCAGTATCC
CTGCTTCCAGAATCGGAAGCCCTCCATCTCTTTGCGGGAATCGCAGAATGGCGTGTAGGCATAGGG
>dme_circ_0005118_junction_seq
AATCGCAGAATGGCGTGTAGGCATAGGGCTGTTCGGATCCTGGCTAAGTGCCTGGT
>dme_circ_0005119
CAGTCTTGAAGTTGCTGTCGCTGCACCACGTTTGGCACCATAGCCAGTCGTCGGGCAGGGATTTGATGGC
GACCTGGTGGATCATGTTGTTGGGCAAGTCCTGATCCAAATTGGATAAGCTGTTCGGATCCTGGCTAAGT
GCCTGGTATTGGCCTCTTAGCCTATCTCCTGCCGCAATCTTGCGGAATCTCTTCAAGTCCACCACGTACA
AGGCGGAAATG
>dme_circ_0005119_junction_seq
CAAGTCCACCACGTACAAGGCGGAAATGCAGTCTTGAAGTTGCTGTCGCTGCACCA
>dme_circ_0005120
GCTTATCCAATTTGGATCAGGACTTGCCCAACAACATGATCCACCAGGTCGCCATCAAATCCCTGCCCGA
CGACTGGCTATGGTGCCAAACGTGGTGCAGCGACAGCAACTTCAAGACTGCTAAAGTGATTGATTTGTGC
AACAACCCGCAGACCAAGGAGGCCAAACTCACGGCCGCCCAGAGGATTGTGCCCGAATGGAAGGACTACG
ATGCCGAGCTGAAGACCCTGATGTCTCGCATCGAGGATCATGAGAATTCGCATAGCAGGGACTCGGCAGT
TGATGATTCGGTTGACGATTCGGTGGAGGTCACCACTGTGACGCCTTCTCATGAGCCCAAGCACGGCGAG
CTGTGATGAACGCGTA
>dme_circ_0005120_junction_seq
AAGCACGGCGAGCTGTGATGAACGCGTAGCTTATCCAATTTGGATCAGGACTTGCC
>dme_circ_0005121
GGCATATTTGAATGAATGCTTCCCAGTTTCTTGAGTTCGATTCGTTGTTGATGTCTATGTATGCATATAG
GCATTGCTTTAATCCAGGTGTTTATACATATCCTCCAGCTGGGGCTTAAAATAGCTCTTAAGCTGCGGTC
TCTTGGCTTTGAATGTGGGCGTCAGCAGTCCGTTTTGCACCGAGAAGGGATCCGGATGCAGATAGATATC
TTTAACCTAAAATTAAATATCCCGGATATTATCTCGAAACCTATAGGTATGCTGATTCAAAAGCTCACCT
GTTCGAATGACTTGAGTCCACTCTGCTTGCCCCAGTTGAGCATGTCGTTCATGATCAGTTCCTTGACATT
CTTATTATTGCACAGGACGGAGAGTGTGCCGCGTACATTGTTCTCCGTGGCCCATTGCTTCAGTACATCG
GTATCAG
>dme_circ_0005121_junction_seq
GCCCATTGCTTCAGTACATCGGTATCAGGGCATATTTGAATGAATGCTTCCCAGTT
>dme_circ_0005122
GACATAAACTTGATTTACGTATTGACTGAGCGTGTAGATATTCTCGATCTTCTCGGGCACTATGTACTCG
CCCTGGCTAAGCTTGAAGATGTGCTTGCGGCGATCGATGATCCGTAGTGTTCCATTTGGCAGCCACATGC
CCACATCGCCCGTATGATGCCAGCCCTCCGAATCGATGGCCTCCGCCGTCTTTTCGGGATCCTTATA
>dme_circ_0005122_junction_seq
CCTCCGCCGTCTTTTCGGGATCCTTATAGACATAAACTTGATTTACGTATTGACTG

>dme_circ_0005123
GCCCACACAGCCGCCCACATAGTACATTCCATTCTCGCAGCAACGCTCGAACATGTGGGCCAGGGGCAGG
AATGACACCATCACATCGCCCGCCCGGATGCGATGGTCGCCCATCTGCAGGATCACAGAACAGACGCCGG
CCACCACGTTGCCGTGGGTGAGCATCACGCCCTTCGGATTGCCCGTCGTCCCGGACGTGTAGCACACCGT
GCAAAGATCCTCCGCCGTCGGCGGCACCTCCGGATGGTTGCCTTTGGCACCTAGTTTCTCCACATCGATA
AATGAGAATATCTGTATGCCTCGGCTGCGTGCCCGCTCCAGGGTCGTCTGCCGGATGGGCTTGATAGCCA
CTATGATCTTCAGACTGCGCGGCGCCTTCTCCAACAGCATGGCCGCCTTGCCATCGTCCTCCACAATCAC
CACCTGCATATCCGTCTGGCGTATGATGAAGGCGCAGGCATCGGGGCCCAGGGTGTCGTACAGCGGCACC
ACCACCAAGGAGAAGGAGTAGCAGCCCTGCTCGTACAGGATCCATTCCGGGCGGTTCTGCGAGTAGATCC
CAATCAACTGCTTTGGCCTGGCTCCTAGCGCCAGCATTCCGGCTCCAAAGTTCTTGGCACGCAGCAGGGC
CTCGTCGTAGTTGATCCACTAAGTTGACAAGGATTGGAGTAAATTAAATATGTAAATGCATATTACATAT
AACTAGAACTATATCAATTTCCAATTATGACTTTTGGGACTTTTACCTGGTAGGGGGAGGTCAGGGTCTC
GCGCCAGCCAAGACATGGTCCATTGTTGGAGGCGTAGGCGCCCTCGCGGAACGTCTGGTACAGGGTGCGG
ACATTCTCTGTGATGTAGGACACGAACTTGCCGTTCTTCGACTCCTTGTAGAACTTGGAGACGTGTATTT
GTTCTGGACCCTGCAATGCAAGTGAAGTGACCCCAAAAGAAACAGAGAAGTCGGAGGACATGCAATGAGT
GAGATACGTTCAGATATTGAGGTAGAAAAGAATATATACAATCAAGAGTTCGGTGGGGTACAGATAGTAT
TTTGTTGGGTTAGACAGATGCAAATACATATGTACATATGTTCAACGTTAGTGAGTTGCTTGTCTTGAG
CTaaaaaaaaaaaaTAAATAAATATTTCTTCGTCATTTAATTGCCATTAACGGAAATGGGAAGCCATATA
AAAGATTTAGGTACAATGGGATATTGTCGTAAGTTCAAGTTTTAATTGATCTCATACGATCGGAAATCTG
TTTCCCCTAAATCTAGAGCTATTATAGTCCGTCCATCCATTGAGTGTGTTTATTTTCGGTTACTTTAGCC
AATTGTTAGAATAAAATCAATAACCATTCTAGTTTATGTACGCAGCCAAAAGGCTATGATTTATCATTTA
TTTTGTGGCCAGAGACATAAACCCATCACTCGGCGGGATTTCATTAGTCCGCTCGAAAGTAAAGCACATC
GAGCCACTTCCTTTATGGTAAACAGCTGCGAAAGAAACAACAACCGCAATGGCCAAAAGTGTTGATGTAC
GAGAACGAATATCAAAATACTCTGCGATGGGGGAATATTTTTGTGGGGGGTACTGGTCCGCGGATCCAAA
GAGTTAGTTTGTTTATTTTTGCTGCCCGCTTTTAATGAGCAAAATGCGAATGTATTTTCAAAACTAATTG
TTTTCCACAGATTAAAGTGCTTTGCAAATTGATTTTGTTTATGGATTTCATTTTCATTATTGACGCAAGT
CTAGGGGAAATTGTTAGTTAGCTAGTGCATACTACGAGGGGCTTAGTTTCATGGGGCATACCCTATGGTG
GATATCTTGACGATGATCGTATATGATCTAGCCCACGAAATGTAAGAGACGGTAAACAAAAATAAAAGAA
GCAAAATAAAGTATATATTAGTGATTGCTTAGTTAATCCTGGTCAGGCAGTTTTGTCATTTCATTCTATT
TGTTTAGGTCCTCATAAAGGGGTTTACCTTGAGCACATCGCTCTGATTGCTTAGCTCGATGGGCACTCGT
ATACTGGTGATTTGGCCGCAACAGGACATGGttttcttttttttttCTGTATTATCACTTCTTAGGTTTAC
TCAATGTTTGCTGACTTTTGGACACTTTCTTCACTTATGGCTCTATTTATTGATCGTTCAGATTGCATCT
GACATTTTACACGCACATTTTCCAAGCGACTGTAAAACACAAACGCGAATTTAAGCACATTTCCAATTCA
TTGACCAATTTGCATTCAATATCTGGCGTTTAATTGCCAAGTACGCCTCGCGTTTAAACGGTCCAATGTA
CATTTCAATTATAGATTTTCCCATAAGGCACATTTCATAACCGCATCGAGATCGTTTAAACGCTAATTAG
CGCGCGCTTAAAATAGATTTGTAATTGAAGGGTGTGCGGAAAATGTTGTTCAGATATTAAACAGATATTA
TTGTTTATTTTATATCGATAGTTGCTCCATTCTATGTAttttttttACTATTCAAAATTTTTGGTTCACT
TCTTCTACTAATAATTTTTGGTATTTAATCTTATATTGATCGTATAGTATCATTCCTTATTTTATTAATC
CACTTAGCATCTACTTAAATTCATTTTAAAACCTCACATAATATCTGAATTACACCAATTagaacaaaaa
ttaaaagcagaaccaaaagtcaaaagcCATAGCATAATTATGACGCCTGGCTGTCCGTTTGCATATTTTT
GGCAAGTGCGGCCCAGTCATTTTGTTTATAATTTTTCAATATTGGCAGCTGCAGGGCGGCCCCaaaaaaa
ggggatagcaaaaaaaaaaGGCAAGAGAACGGTGTAAAATGGCCAAGACAAAAAGAGAGAGTGGCCAACA
CATTATCGACAAGGCGTCGTCTGCGGATTTTGCTTGGCCTGCCCCAAATTATTTTTATAAATAGTTCGTT
TGTGGGTAAAAACGTTCCACTCTGTTGTTATCTATGGGACCCGCCAAATGTGTGTGGCGGCATTAAATAA

AAGAAAAAAAACCAAACTATGCTTTCACACACACACTCGAATACAATTAACACTTTATGCAAAATGCTAAG
AAATAGAGGAAAATTTCAATTTTCTAAAGACGATCGAGTACTGAGTCATGGGGAACGCGTTTCGGGCCTC
ATTGGGATGTTTTGAGCCAATTGGCAGAGAGCAAACATTTTCCTCAATGAGCAATTCATCTTTGAGTTAT
GGTAATTGTGGTGGTCATAAAATCGGACATAAACTATTGGCCCAACAAAAATCGAGAGCCAAAGAAAAGA
AATGAAAATAAATGAATGAAACAGCCGGGACCAAAAAGAATTTCAATTGAATCTGCAAGGTATCAATAGT
TTGGCAGCATATTTTTaaaaataaaaaaaaataaaCGAAAGCCAGGAAAAATATGCGAGAATTTCAAGGTC
GTTTGGGAAAAGCttttttttCGGTTTCCAGGTGGCTGGCATATTTTTTAAAGCGTTCTGAATTTATGTA
TTTTCTAGTACCAACTTCAAGTGCTCACAAAATTCATTGTTCTAACTTATTCCTTACTAACCATTATATT
TTAGAAATTATCTTAAATAACAAACCACAGGGAATCTTTTGAATGCATTGGTTGTAATACAAAAGAATAT
ATTTAAAGATATATGTGCGGTGGTTTTACTTCCTGGCATTTTGATTGATTAAGATAGATTGCAGTCGTGT
TATATAGAGCCAAAGTTAAAATGTAAGGGGAAAAAAATATAAAATTGTAAAATTTGATTTATTTTTCAAT
TAGTGGCTATTTCTGTAAATCTCTTTGAATTTGTTGATACTTTCGACATATTTGTACAAATGCATTCACG
TTCAAAAGTGCACATGGCTCAAAAATTCTTGAAAAATATAAAATGTATCTCTTAAATATAAACAGCTATA
AAATGATATGGGCAATCTGTGTGTATTGTTAAGATCAACCGTTTACAACTGAAAAACCCCACCAATTAAG
CCAACAACATTCATCAATCTAAATATGAATCAAAATCGTGTTAAAATTAGCTATATAACCAATCTGGGGG
CTGAGTTTTTCTTAGATGTTTCAAGTATAGAAACTGCGTGAACTTGAACTTGAGTGGCTGAGTTCACGTG
GCAACACGACGGCACTTTGACActttggcaaatcgatttcctttggaaaatcatttcatttTTCTTCGTT
AATTTCCGTCCACtttttttgaacattttttttttGGGGGTTCTTGCGCTTTACGTAATGAGATTAAACGCT
TGTATTTTTGTCAATTTGTTGCAAATTGTCGTGCGTTTTTTCGCTCTTTATTTCGTTTTGTTTAGTATAC
ACGAGCTATTATTTATAAAAAAATACGTTTGTTTCATAGATATACATATATCTGCGACATGTACATTATG
TTGTTGTTGCTTGTTAtttcgttttttttctttttcaaatttttttttgggatttttCTAACTTGAAATATA
TTCTTGCGACTTGAGCCCGCCACGATCTGCGCGACACGATGTTAAATTTCGAACTGAGTGGCAGAAGTAA
AATATTGGCCGTGGCTAAAAATGATTTTGGCCGGCCCGAGGAATCTGCTTTTCATTTGGGAGAGAGATTG
AGAAAAATCGAGCGAAAGATTTTGTGGGTTTTTGGGGGTAATTTCGGGGGGATCAAGTAGAATGCAGCGT
TCGGCCAGAAATTTTATTCGTTTATATGTTCTTGTTTTGATATTAAATGGTTTTTCTTTTTACTCTTTGC
CAAATAGATTTGTTCTAATTTTTTGTTGGCTCGATCAAGTTGTCCACCTGTCTCGATTTTCCCTTTACCA
AACGAGCAGTATTCACATATTTATGTGAATGTGTGTAGTAAGTAAGCAATAGAGGTGTTTCCTTATGGTA
GTGACAGCAATTTTCAACTCTTTAATAACCAAAATGATTTATGGTCTATACGAGAGGACCACGCCCACTT
TTGCGACTGGTAAAAATAGAAAGCATTTGTTTACTTTTGGGCATCCCATCATGGAATGCTTTATACTATG
CAAAGTTTCATTGGTCATTAAATAAAAACAAAATATTCTATTTTATTTTTAAAAATAAATAATATTTCAG
ATATGTTTGCCACACACTTCCCATACCAATTAGACTCGTTGCTAATTGTTTTAAATTTATAACCGATTGC
TTACATGCTCTCAATTACTCAATATTCAATGAATGTTGTTTAATTTATGCACCTTTCAGTCTAAATCCAC
TCAACATTAGCTCATAGCCACATATTTATCGTTTCATTTGCACCTCAATTTGCGCACATTAGCAATTTCA
GTGCTCATACACACTTATGTATATTGGAAAACACTCTGTGAAGAAGAAAGAGTGTGCCAAATGTTGGTGC
ATTCTAATCTGTACGCCATAGCAGACTAAACAAGCACACTAAGTGAGTGAAATTCCGAAATGCATAATTA
TACAAAATACTTGTTTTACAACCGCACAAGCAAATGTATCTGCTCACATGTAGACAGGTTGGCCCTGTCT
TGTCAAAAGTCTGACATTTCCTTATTATTCCGTTGTCTTTCATTGTTTGTTTGTAATTGGGCGGGGACTT
TCGATGGCATTTAACATGGAATaaaaaaaaaaCTTCAATATTTTTCAAATTTTTCTTTCGATTTTAAAAC
TATTTCAAAGCTAATTCCTGTAATACAACTAACTTGCTGTTTGAAAATTTATATCTTTCGACTGCTTTTA
AATAGATTTATTCGCCGGATATGAATGTGAAAACAGGTTATGAGAAAAATAATCAACACTTAAGTGGAAG
TGCTGTATGGTTTCGAAATGTATCACTTACTTAAAAAAAGACAAGTTAGAAATACCTTAGTATTAATAAT
GGAAGAAGATGAAATTTCTACAATTTCATTCAACTTTTCCGTTTTTACAACGCTAGGAACAAAAATGTAT
TCACTTAGCATTCAATAAATCATTTGTTTAATCAACGATTATCGCGTTTCACAGTGCCTTCAGCCTTCAA
CCCACGAAAACTGGGGCTGCGATTTTGGCAATGTCGAATATTGCGCGAAATATGCTATTGTGTCAATAAT

TGTTTATTTACATGACATCAACACAAAAGGAAAGGTCCGTTACAACAATTCGAGCGGGGAAAATGTCGGC
TAGTTCAGTTGAAGGAAATTCCTCTGACAATCGTGTGTGTGGGGTACAATCGGCGCGGAAAATATCTGCA
TAGATCGAAATTGAGAGAACCAGGATACTAATTAGGGGAAAATGCCAATTGAGCCATGACAAGATTTATT
ATTGTCGTGCCGCTGTCATTCGATAATTTATCAGCGAGAATGTTGCAGTCTTGGCAAAAGAAATTCATAC
ATTAGAGCATGAGGTGGTGGCAGCTATTTTTAATCATTTTCGCACCACTGTGGCACAGACGTCACGGCTA
AAAGTCGAAGAACAAAGGCGAAATGAAAATCGAAATGGTGAAAATGTGTGAAAATCATGTGACGACAACA
ACGAAGCAGAGTCCGTTTTTGGGAAACCGACTCGTGATCGTGGACGTGTGCACATCAAAGCAGCCAAGCT
CTTTTTGGCCCACATGCCTCGGGGGATTCTCCTGTTCTACCTCAAACACAACCGAGCCTCAGAGAAAACT
CAAGACAAACAGTTCGCAATTGAATATTGAATGATGTAATTGATGAGATCAAAGTCGATCAATTGCGAAA
TCAGCATACCTCTAGCAATGGCGATTGGTTCTCCAGAGGCACTAACGGTCGTTCCGGAACGGGCCTCGTT
GCAAAGTAGTATGCCACACTAGCTGCCAGGCTTCCTAGGGTTAAT
>dme_circ_0005123_junction_seq
ACTAGCTGCCAGGCTTCCTAGGGTTAATGCCCACACAGCCGCCCACATAGTACATT
>dme_circ_0005124
GTGCCAAAGGCAACCATCCGGAGGTGCCGCCGACGGCGGAGGATCTTTGCACGGTGTGCTACACGTCCGG
GACGACGGGCAATCCGAAGGGCGTGATGCTCACCCACGGCAACGTGGTGGCCGGCGTCTGTTCTGTGATC
CTGCAGATGGGCGACCATCGCATCCGGGCGGGCGATGTGATGGTGTCATTCCTGCCCCTGGCCCACATGT
TCGAGCGTTGCTGCGAGAATGGAATGTACTATG
>dme_circ_0005124_junction_seq
CGTTGCTGCGAGAATGGAATGTACTATGGTGCCAAAGGCAACCATCCGGAGGTGCC
>dme_circ_0005125
GGGTCGTCTGCCGGATGGGCTTGATAGCCACTATGATCTTCAGACTGCGCGGCGCCTTCTCCAACAGCAT
GGCCGCCTTGCCATCGTCCTCCACAATCACCACCTGCATATCCGTCTGGCGTATGATGAAGGCGCAGGCA
TCGGGGCCCAGGGTGTCGTACAGCGGCACCACCACCAAGGAGAAGGAGTAGCAGCCCTGCTCGTACAGGA
TCCATTCCGGGCGGTTCTGCGAGTAGATCCCAATCAACTGCTTTGGCCTGGCTCCTAGCGCCAGCATTCC
GGCTCCAAAGTTCTTGGCACGCAGCAGGGCCTCGTCGTA
>dme_circ_0005125_junction_seq
TCTTGGCACGCAGCAGGGCCTCGTCGTAGGGTCGTCTGCCGGATGGGCTTGATAGC
>dme_circ_0005126
GCTATCTTCTGTAGGCAATCATTAAGCTAAACACGTAACATTCACTCAGGTTGAAAAGAAAAACAACTAT
TAACTACTACTGTAATACCACaaaaaaaaaaaaCAATAATATATTAAGGAAAAAACGATGACGCCAGAGA
AATCGGTTATGGCCTACGTCAATCATTTGATTGTCAGCAATTTTTCATATTTCTGGGGACGAGATCGATG
GGTATCTGTACTACCTGGGAGGCACTTTGGGAACATTAACCCTAGGAAGCCTGGCAGCTAGTGTGGCATA
CTACTTTGCAACGAGGCCCGTTCCGGAACGACCGTTAGTGCCTCTGGAGAACCAATCGCCATTGCTAGAG
G
>dme_circ_0005126_junction_seq
CTGGAGAACCAATCGCCATTGCTAGAGGGCTATCTTCTGTAGGCAATCATTAAGCT
>dme_circ_0005127
GGCATCGGATCCTGCACCGGGTTCGGTCAGGGCGAAGCTACCGGCGTACTCCTGGGCCAGCTTAGGCAGA
TACTTCGCCTTCTGCTCGGCATTGCCGAACTTGATCATCAGCGAGTTGACCAGTGT
>dme_circ_0005127_junction_seq
ACTTGATCATCAGCGAGTTGACCAGTGTGGCATCGGATCCTGCACCGGGTTCGGTC
>dme_circ_0005128
ATCCAAAATTCAAAAAAGTCCAGTTTGGCCTCTTGATTAAATCAATTCTTCCACTTCATTCAAGATGATG

AACAGCCTAAAGAAATTGCCCGTGCGCATGGTGAGTTGGCCACGCCCCCCTAACCGACACCTTTATACAT
ACATATATGCCTACAACTAATTCCTTTTTCTTTATTAATTAACTTTTTAGCTGGCTAACGCTGCACGTCA
GCAAAGTGCTGCGATGTCCTCGGCAACTGGTCTCCCCCCACCCCTCACCTTTCTCACCGACGATGAGAAA
ATGATGAAGGAGACTGGTGAGTATACCCTTTGGATCTAAATAGATCATAGTGTTAATGGTACTGTACACG
GAGTTGTAGTCCCCATTTGATAGGCGTGTTTTTTCTCCATGTCGAATTTTTAGATATTTTTATGGTGTTT
AGAACTTGCTTGGTGTTACGTAACACCAAGCATCGCATTTTAATGATGCTCTTGTTATTTATTAATTTTC
CATTGTGCTAACCAGCTGAAGTCTTGCTGCACTACTATGTGTGTATTCGTAtttttcgagttttgttctt
ttttttttcctttttttGCTGATAAAGTTTATTCAGTTGAGGGAAACGCGGAGGAGGCCGCTTGCCAGATA
AGCACATTTTTATTTTGATAGTAGAAGTAAAATGTCACTAAGTATTAGTGGGTAGGTTTGTCCATTTTCT
TTATGACGTTTGTATGTTTAAATATTACAAGATATACAATTTCCGATTTGATCCTTATTGTAAAATGACG
ATCAACGATTATTACCTTATAATTAATTCCTTATTAAATTAATTTTTCTGGCGATTATTATATAATTTGT
AATGATTAATCCCTTAAATTTTTTGTAGTGTAATAAACCTAATATTTTTAAGGTAAATGAGGTCCTTGAA
TGTGTTTCTATGTTTATAATTTATTCCCTTTGGTGaaaaatatttttatctatataaaataatttttaat
aaaactatttcagtatttaataatagtatGCTTAAAAGTTACAAAATATTAATGATTCATCCCACTGAGG
TCCTTAGGTGACAATTTTGTTATGTACTGCCATAATAAGGTTAGCTCTTAAAAATCATACGATTTCTTAA
AGCTGGTTACATGCATGTTTAATGAATATAATTAAATTTAGAAAGTTACAaataatataataatataata
tatataataatatTTCGAATATCGTGTTGTGTCAAATGTGTTTAAAAGTGTTCACTTAAACTAAATGAAT
ATATGTATTCTTAAAAAGTGCACAATTAATGGCCGAAGAGCTAAAGCTGCCAGCTTGGCAAGTTCAAGGT
CTAACCTTATAACAAGCTTTTTTCCCGATGGAAAAATTTTCCTCCCACCTGCTTAATTCTTTAAAAAATT
ATAATTAATTGATAATAATAATAATTTACCAGTGAGAAAACTTACCCAGGTGGGCTGAAGGAAAAAGGGG
TGGGCCCCGcacacacgcccaccctgctcacacacacacacacGCGTGTTCTAAAAATAGCTTTTGCCCA
CCGCCAACGCTGAACATCGAGTTTCGTCGCTGATATTTCTCGGTTGGCCGTGGTGTTCCTCGTTGAGCGG
CCGGCACATAAAGAAAGCAATTTACCAAAGCGCGAGTTCGAGCCCTCAAATTATAAGCCTCTCTCATCCA
AGATCCTCTCATTTCCCCTCCCACAGAGTACTGTTATTGCGTGCGCGGAATGTTTCTTATTAAACTTATA
TAGTGTCCTTAAGGATATCCTTTAACCACGAGTTTTCACAGGTCAGTCAGCTGTACGGTTCTGGTTTTAA
ACGTCAACCGAATAGCGTTACACCAGTGATAGAGCAATAAATGCGGCTGTGCCATTCCCCCTGTCCACCT
GTGGCCACTGTTCTAGTGGCCACGCCGGGCAATCCGGGGCGTGGCACTTGGCCGGGCGCCCTTCCCTAGA
GAGTTGAATTACCTGAGTGGGGGGAGGACGACGACTTGTTGTTGTTGTCCCCCTGCAAACCATCTAATTC
CTAGTTGGGAAGGTTACATTTCCTTTTTATCACCGATCACCTTGTTTTTCCTCCATAATAATTAATAATC
CTCGTCTCGCTTATCAGTCCCTCAACTGGACTCAATCTATATGGCTTCCCATATATATACATATGTACAT
ATGTATGTGTGTTACATACAAATAATTCTAAGTGGGAAAGTATGTGTCTATATGGCCTAACTGTTGCGTA
ATCTGAGCTGAATGCAAAGTGGCTAAGTGCATGAAGTCAAACTGGttttttttttatatttttCAATCATA
TATCATAGGCTTTGGCACAGTTAACACATTTTAAATTAAATTATTACATTAAATTTTCTGTTTTAATATG
TATTTCAAAGTGCTTGATTTTTTAAGTAAGCTTTAAGTACGTAAAAGAATAGCAAACACACATTTACTCG
AACGATTATTACCTTCAAGAACCAAGTACAGTTGCCCaaaaaaaaaGCAAAATTTCAATTTCAGTCTATA
TCAGACTAAAGAAATGACCTGATTTTATTGAATTTCATATTTCCACTATAAGCCAAACTCCAAAAAGGAC
ATGTAAAATTTACCATCAAGCCCATTGGGACACGACACATCTGTTCGAAATTTTCCGACTGACCCCTGTC
CATATGTTGGAAAACTAAATGAATATATAAAGTTAGTTAATTATTTAAGCAATTCCCATGAAAAGTGAGC
GAAAGAAAGTATTTCCAAATGTGTGCAAAACGGCAGGAAAGTGATTGAAGAAGGGGCGACGGAGGTGTGG
GAGCTTCTGTGGGTGTGTACCAGTTGGATTTCCAAGCAGaaaaacagaaacacaaagtcaagaaaaaaga
aaaaaatgcttaaaaaaGATCAATAATAAATACGCTGAGAGAATAATAGAACTGCCGAAAGAAGACAGCT
GCGAGAGCCCCACGCGATCGTAGAATCTAGTGATAAAAAACGAACACACGAAGACTTTTTATCTGAGAGA
CAAACGCGTGTAGAAAAATACATACCTCAACCTACACAAGCAAGAGATCAACAGCAAACATCGCGCCCCT
TAGAAGAAAAAGAACTCCaaaaaaaaataaaaatcaaaaaaGCTTAAGTTTTTACCTTTTTTCAAAAACGT

GCAGTTAAAAGCTTATCGAAGCTTTACAAAAACTTTGACATTAAGCTCTTTCACCGATCGTTACATTCTA
TATCTACACATATACATATACACATATGAGTTATACTTAAGGTCTAGGAATTCTTATGTGTTATAATATA
TTTaaaaaataaaaaaCCTATTTTAAATGTATCTAAATGTATGCAAGCATATTTGACGTATATGAGTTAT
TTCACATGTTTCATAAACCATGGAAACTTTCCTTTAAACTTTCAGTTTGCCAAACTGGCTCAGGAGCAGA
TCCAGCCCTTGGTGAAGAAAATGGACTTTGAGCACAAATTCGATCCCTCCGTGGTGAAGGCGGTTTTCGA
GAACGGCCTGATGGGCATTGAGATCGACACCGAGCTGGGAGGCAGTGGCTGCAACTTCATGACCAACATC
GTCGTGGTGGAGGAGCTG
>dme_circ_0005128_junction_seq
GACCAACATCGTCGTGGTGGAGGAGCTGATCCAAAATTCAAAAAAGTCCAGTTTGG
>dme_circ_0005129
GTACAATGTGCGTTCCATGCCCATCCGCCGCGACGATGAGGTCCAGGTGATCCGCGGCCACTTCAAGGGC
AACCAGGTGGGCAAGGTGGTCCAGGCCTACCGCAAAAAGTTCGTCGTCTACGTCGAGAAGATCCAGCGCG
AGAACGCCAACGGCACCAAC
>dme_circ_0005129_junction_seq
CCAGCGCGAGAACGCCAACGGCACCAACGTACAATGTGCGTTCCATGCCCATCCGC
>dme_circ_0005130
TGAGCGTTTCAGGTCACTGAACTGCTGCTGGATCTTCGGTCGCTCCTGGCGGTACCGCTCCAAATCCTCA
CGCAGCCGGCGATCACGATACTCTTTGCGCTTTTCGTCCATTCGCTTGTCTATCGAATCGTAGATAGCGT
CCGCCTCCTCGTCATCCTTGTCGTACGGATCCTTGGAGAACAGGGAGCCGCTG
>dme_circ_0005130_junction_seq
CGGATCCTTGGAGAACAGGGAGCCGCTGTGAGCGTTTCAGGTCACTGAACTGCTGC
>dme_circ_0005131
GAAGGTTGCCCTCTGCTCGGGAGCGTAGTCGAAATCAGCCTTGGCACCCAGGTGACCACGTGCCAGGTAG
ACATTGCTGCTACTATCGAAGTACTTTTCCGCATCTCCGCCCAGTTCATTGTTGATGGTCTCCAGCTGGG
TGGCTTGGGTGTACAGTTTGTCCACGTTCTTTCCATCAAAGAAGCCAGCCGTTTGGAAGGTAATTCTAGC
CACTCCGGTCTCGTAGTAGTTGCTTCCGGGCTCCAACTTGTGGCGAGTGTATCGGGTCACTTCCTCCTCT
TCGTTGAAGCAAATCTGCATCTGCTCGGCAAACCTCGATGAGGTGATCTCGAAACCTACGCGGATCACAA
TGCCACCGTTGCAGGTGGCTCCGGACTTAACAGCCTTGAATCCCGGCCAGGATTTACAG
>dme_circ_0005131_junction_seq
AGCCTTGAATCCCGGCCAGGATTTACAGGAAGGTTGCCCTCTGCTCGGGAGCGTAG
>dme_circ_0005132
ATAGACTAGCTGTGATAATTATTGGGATAATTACTTTAATCTTAGGCATTATTTTGTCCTCCATGCCGTG
GCTGGATTATTTTATACTAAAGGTAAGTTCTACAGAAATCCAAGCTGCAAATGAAGAAATTAACTAAGTA
AAAACTTTAGAACTTAAGGCTGTGGAATGATACACTGAGCTATCATTATTGGCAAAGGCCTGGCGTCATC
CGACTGACCAAGCTATATATCTACAATGTCACCAATCCGGATGGATTCCTTCGAGGGGAAAAGCCGCACC
TGCAAGAAGTTGGTCCTTTTGTGTACAGG
>dme_circ_0005132_junction_seq
GCAAGAAGTTGGTCCTTTTGTGTACAGGATAGACTAGCTGTGATAATTATTGGGAT
>dme_circ_0005133
TGTGCCGCCTTCGTGGACGGAGCCTCCATCACCTATGGAACCGGAAACGAGCTAGAGTCGGGATCTGGAT
CCTCCGCCGCCTGTAAGCCCACTCTGCGGGAGTTCAATGTGCGGCTGGAGGTTCCCCTGGCCGCCGAGGA
GCGACTGGGGCTTACCGGCGATGTGAAAGCCCTCGGCGAGTGGCAGCTGTCCAGAAGTGTGGCTCTAGAA
TCGCTGGACGAGCTCAACTGGCAGGCCACGGTGGCCCTTCAGTCCTGCCGCCAGCTGGAGTACCGCTACT
TTGTCTACGTGGAGGATCTCTCTGGCTACAAGCAGATCCGCCGTTGGGAAACCCATTTCAAGCCCAGGTC

CCTGGGACCCTGTACGGAGCTACAGTGCAGCCAGTTGGACGTCTTCGGTATTACGTCGGACAACTCCGAT
CTAAAGCCGCAGGTGCACCGCGGCTGGCTGAACCATGAGGCCATTCTGCAGCTCAAGTTCAATGGCGAGA
AGATGTTCCAAGTCCACGACATCGAGACCTTTGACCCCCAGCACGTCCAGCTGAAGATTGTGCCCGTGGA
GAAGACCGCTGGCCTGCATGTGGAGTACTCCAAACAGGAGTACGGCAAGAGCCAGCTGGAGCTGCAGCCT
ACTTTCGGAGTGCCCTACACCAAGGGGGACATTGTCATCTTTCACATCACGCTGCCGCTGGAGAGGATGA
TGGAGCAGCACTTCCGCTTGGAGTGCTACAGCATGTCCAACGAACTCCTGGGCAGTGCCACTCTGGTCAC
CTCGGACCTGACTGGCAGCGAGGGAGTGCTCCACCTGCCGATTAAGTCGGCCAAGAATGCAGATGAGACC
CTGGCCAGGCTGAGGCTTCCCTATGTCGCGGTGCAGCCTTACCGCTACTCGCCGCTAGACTTCAAGAACA
CCTATGCTCACTACTGGCCCAAGAGCTGGCCCAACCTGGATGTGGGTCATCGTGGAAATGGCAAGAGTTA
CATTGCAGACGCTCCTGCGGAAAGGGAGAACACTATCGCGTCTTTCCTGAGCGCCCATGAACATCACGCA
GACATGATCGAGTTGGATGTCCATTTGACTGCTGATGGTGTGCCTGTGATTTATCACGATTTCGGACTGC
GAACTGCTCCGCCTGGCAAGCAGATCAGCCGCCCAGACCAGCTGGAGTACGTGCTGATCAAAGACATAAA
CTATGAGCTGCTCAAAAGGCTGCGCATCTTCTCTGTGATCGCAGGCCAAGTGAGGGAGTATCCCTCGCAC
AACGCCGAGCCCAGGATGGAACACCGCATATTCCCAACGCTGGTGGAGGTACTGGAGAAGCTGCCCAAGT
CACTGGGCATTGATGTGGAGATTAAGTGGCCACAGCGTCGCCAGGGCGGAGGATCAGAGGCTGAGCAAAC
AATCGACAAGAACTTCTTCGCCGACAAGGTGATCCATCAGGTGATCCAGAAGGGCTGTGGCAGACCGATA
ATCTTCTCCAGCTTCGATGCTGACATGTGCACGATGCTGAGGTTCAAGCAGAACGTCTTCCCAGTGATGT
TCCTCACGCAGGGAGAGACGAAGAAGTGGCAGCCGTTCCTGGATCTGCGAACACGGACCTTTATTGCAGC
CGTAAACAATGCTCAAGCTTTCGAGCTGGCTGGCACAGCTCCACACGCCGAGGACTTCCTGGGCGAAAAT
GCATCAGAAATGCTGCGAAAAGCCAAGGATCTGGGCCAAATAGCTGTGATCTGGGGCGACGATTGCAACT
CCAAGGAGCGGGTGCAGTACTTCACCCGGATCGGGGCTACCGCCACGTGCTACGATCGCAGTGATCTCTT
CATGCCGGAGGGCAAGCGCGAAGCCTTCTTCAAATCACCCGCACTGATGGCCGAGTTCGCGGCCCAGTGC
CGGATCTAAGAAATGGGGTTACTAGAATGAAACGGGCGGGTACAATTAAGCTATGGCTAATCATTAGGCT
TTTTAAGTTAGCTTGTATATTTATTTAAACACTAACCGGGAAAAGAGAAATGTGGGAAGCTTTAAAGTGG
TTGGGTAATTTGTAGTTTCTCGTTGCCCAACGGAAACAACGAAACGGAGACCCATAAATAAATCATCTCT
ATGCACGTTAAGCCGTCATTTGGCAAAATTACACATGCAAAAAAGGAATTCCCCCAAAGTTAAGTAGCAC
CACAAAAGACAATGGGAAGAAAGCGAAGCGACGAATCGATAATAACAAAAATATATACACCTACATTACG
TGCTTTAAATCAATCTATCTATACCTCAATGCAAAACTTGCGAAAGAGATGTTGCTAATAAGCTTTTGCT
ATTTAAATCGAAACAACAAATTACTATTAAAATGTGGACGGAACAGATATCCACACCGATGCTTGTGTAA
CTTGTGCAGAAACACTCTATTGTAAATTAGAAGACATCCTATTTAACTGTTGTAAATCTAATTTGCTGCG
TCTTTGTACAATTTGAAAATTTACGTTTGTTTTCAAAAATTGTTTATAATGTATACATTCGTGTAAACCA
TTTTTAAGAATCAATTGTATCGACTTAAGGGTAAACACAAATATTTAAAGAAGTCGCTTACACCTATCCA
ACTCTAAGAAAAAGCAATAATATATTTTGATCTATATTACAATCAGTTTTATGACAAAAACCAAAAGTGA
TTGATTCGTATGTAAATAAAATCACACTAAATATTTTCATAGTAAAAAGGTTTGGCCCTCGTGCTGGAAG
TCAGGCCTAAAGTCCGTTCGGTTTATGACCAATTAGAGAGCGCCTCATTTGCATCGGTCTTGGCATGGCC
AAATGCGAATGTCTTCTCGCTGCGGGCCTGGTTCCATTGATGCTGACCAGCTCCATCGTGGCCACTTGGA
CTTGGAGATGTTTGTACAAAGAATTTCCAGTGGACGTTGAAAAGGTGGGAATGATCTTGAAATTCGATAA
GCCAAAGACCGCTAATAGAGGTAACTTGATTTGACTTTTCTAGATCAAGGGCGGCTGGTATGTTTATGGG
ACTAGTCCCGGGAGAATTAAGGTCTGCTACTTTGAAGAGCG
>dme_circ_0005133_junction_seq
GAATTAAGGTCTGCTACTTTGAAGAGCGTGTGCCGCCTTCGTGGACGGAGCCTCCA
>dme_circ_0005134
ATTAAGTGGCCACAGCGTCGCCAGGGCGGAGGATCAGAGGCTGAGCAAACAATCGACAAGAACTTCTTCG
CCGACAAGGTGATCCATCAGGTGATCCAGAAGGGCTGTGGCAGACCGATAATCTTCTCCAGCTTCGATGC

TGACATGTGCACGATGCTGAGGTTCAAGCAGAACGTCTTCCCAGTGATGTTCCTCACGCAGGGAGAGACG
AAGAAGTGGCAGCCGTTCCTGGATCTGCGAACACGGACCTTTATTGCAGCCGTAAACAATGCTCAAGCTT
TCGAGCTGGCTGGCACAGCTCCACACGCCGAGGACTTCCTGGGCGAAAATGCATCAGAAATGCTGCGAAA
AGCCAAGGATCTGGGCCAAATAGCTGTGATCTGGGGCGACGATTGCAACTCCAAGGAGCGGGTGCAGTAC
TTCACCCGGATCGGGGCTACCGCCACGTGCTACGATCGCAGTGATCTCTTCATGCCGGAGGGCAAGCGCG
AAGCCTTCTTCAAATCACCCGCACTGATGGCCGAGTTCGCGGCCCAGTGCCGGATCTAAGAAATGGGGTT
ACTAGAATGAAACGGGCGGG
>dme_circ_0005134_junction_seq
ATGGGGTTACTAGAATGAAACGGGCGGGATTAAGTGGCCACAGCGTCGCCAGGGCG
>dme_circ_0005135
ATTAAGTGGCCACAGCGTCGCCAGGGCGGAGGATCAGAGGCTGAGCAAACAATCGACAAGAACTTCTTCG
CCGACAAGGTGATCCATCAGGTGATCCAGAAGGGCTGTGGCAGACCGATAATCTTCTCCAGCTTCGATGC
TGACATGTGCACGATGCTGAGGTTCAAGCAGAACGTCTTCCCAGTGATGTTCCTCACGCAGGGAGAGACG
AAGAAGTGGCAGCCGTTCCTGGATCTGCGAACACGGACCTTTATTGCAGCCGTAAACAATGCTCAAGCTT
TCGAGCTGGCTGGCACAGCTCCACACGCCGAGGACTTCCTGGGCGAAAATGCATCAGAAATGCTGCGAAA
AGCCAAGGATCTGGGCCAAATAGCTGTGATCTGGGGCGACGATTGCAACTCCAAGGAGCGGGTGCAGTAC
TTCACCCGGATCGGGGCTACCGCCACGTGCTACGATCGCAGTGATCTCTTCATGCCGGAGGGCAAGCGCG
AAGCCTTCTTCAAATCACCCGCACTGATGGCCGAGTTCGCGGCCCAGTGCCGGATCTAAGAAATGGGG
>dme_circ_0005135_junction_seq
GGCCCAGTGCCGGATCTAAGAAATGGGGATTAAGTGGCCACAGCGTCGCCAGGGCG
>dme_circ_0005136
GATCACTGCGATCGTAGCACGTGGCGGTAGCCCCGATCCGGGTGAAGTACTGCACCCGCTCCTTGGAGTT
GCAATCGTCGCCCCAGATCACAGCTATTTGGCCCAGATCCTTGGCTTTTCGCAGCATTTCTGATGCATTT
TCGCCCAGGAAGTCCTCGGCGTGTGGAGCT
>dme_circ_0005136_junction_seq
GCCCAGGAAGTCCTCGGCGTGTGGAGCTGATCACTGCGATCGTAGCACGTGGCGGT
>dme_circ_0005137
ATTAAGTGGCCACAGCGTCGCCAGGGCGGAGGATCAGAGGCTGAGCAAACAATCGACAAGAACTTCTTCG
CCGACAAGGTGATCCATCAGGTGATCCAGAAGGGCTGTGGCAGACCGATAATCTTCTCCAGCTTCGATGC
TGACATGTGCACGATGCTGAGGTTCAAGCAGAACGTCTTCCCAGTGATGTTCCTCACGCAGGGAGAGACG
AAGAAGTGGCAGCCG
>dme_circ_0005137_junction_seq
GCAGGGAGAGACGAAGAAGTGGCAGCCGATTAAGTGGCCACAGCGTCGCCAGGGCG
>dme_circ_0005138
CTGCTCAAAAGGCTGCGCATCTTCTCTGTGATCGCAGGCCAAGTGAGGGAGTATCCCTCGCACAACGCCG
AGCCCAGGATGGAACACCGCATATTCCCAACGCTGGTGGAGGTACTGGAGAAGCTGCCCAAGTCACTGGG
CATTGATGTGGAGATTAAG
>dme_circ_0005138_junction_seq
GTCACTGGGCATTGATGTGGAGATTAAGCTGCTCAAAAGGCTGCGCATCTTCTCTG
>dme_circ_0005139
GTTTATGTCTTTGATCAGCACGTACTCCAGCTGGTCTGGGCGGCTGATCTGCTTGCCAGGCGGAGCAGTT
CGCAGTCCGAAATCGTGATAAATCACAGGCACACCATCAGCAGTCAAATGGACATCCAACTCGATCATGT
CTGCGTGATGTTCATGGGCGCTCAGGAAAGACGCGATAGTGTTCTCCCTTTCCGCAGGAGCGTCTGCAAT
>dme_circ_0005139_junction_seq

TCTCCCTTTCCGCAGGAGCGTCTGCAATGTTTATGTCTTTGATCAGCACGTACTCC

>dme_circ_0005140

GATTTTCCTTGAGAGAAATTGGAGATGCGAGTTTGAAATTGGTTAACCTCTTTACCTATTCAGGAACCTA
GCTTAACTTCACTGTGTTAGGTTTAATATTTATCCTAATTTACATATTGGTTTTACAAAATCAAAGAATA
ATAATCAGTTATTTTATTTAGTATCTAGCATTTCTATCAATTGATTTTTGTCAGCAATCGTTCTCAATTT
GTCTTTGGTTAACCAAACTCCAAACTCACTTAACCAACCCCACT

>dme_circ_0005140_junction_seq

ACTCCAAACTCACTTAACCAACCCCACTGATTTTCCTTGAGAGAAATTGGAGATGC

>dme_circ_0005141

TCCAGGAATGCACCACAGACTCCGAGGAGCTGATGTACCACCAGGTGCGGGCCTCCTCCTCCTGCAGCGC
CCCGCCCGATTTGCTGCTGGTCAGCGAACGTGACAATAATATCCAACTGCGATCGCCGGTAGTGAACATA
ATCACCACGCCGCCGGGCAATGCGTCTGGTGCGGGAAGTAAGCAGCAGTCTCATCACCAGACGAACCACC
ACTCCTCGGGCAGGAGTCATCCCGGGCACACGGCACATCCGCAGGATGTGAGCAGCGGCGGCAGCCATAG
CAAGCATCTGCGCATCAGCAGCACTTCCAATGGCAAGCACGGCAAATACTCAAATATGCAGCAGCAACTG
CCGCAGGATGAGGATGTGGTGGATGCGGCTGCCACGATGCAGCAGCAGCAGCACACTGGCCACGCCCACT
CGCGCCACCTGCACCACCACAAGGAGGAGCGCATCCGACTGGAGGGTGAGTTAGCAATGCCATTACCCAA
ATTCCCTGCCAGCAGGCACTCAATGCATTATCCCATGGACACAATCAGATTCCATCTTCCGCACCCATAA
ACACTGCGCCTAATAGTCCATTCTGatttcaatttcaatttccatttccattCATTAATTAGTGAATTTA
TGGAATGGATTTGTCTTGTTTTCAACGTTTCATAACACAAATATTTATGGAGCCATTAATTGGCGCTCAC
TTTAAAAGGGTTGTGCGCAATTAGGTAGAATTTTGCATTTACTCAGAGTTCATCATACTTCAAGGCGACC
CAGATGAAATTTGGAATGTATCTTGAGCAGATAGTCAATCTCGATTGTTAATTTTGTCTGAGATTAAGTA
TTTTCCCTTTAAGTACTTTTCATTTAGTGTAGTGCTAACAAATAAAATAAGGTTTGATTAATATTATTTG
ATAGCTTTAGTAGGGTATTCTCGTTGCTTGTATGCCCTGTAATTTTCAATTAGCCAAACAATCGATTTGC
TTATAAATACGAGATGATAAATTAAATGATGTTTGCTTTTCCATTTGCGGGATTTTTCTGACCCCCAGCC
AGGTGTTTGGTGTTGGGCCAAGTTTAATCAACTCGCCCGGTGTTATTACCACGATTCCCGGCCATAATTC
ATGGCCCACAGGGCACATCGCACATCGGCCATAAACTCAACATTTCAATGTGCTTTTAATTAAAATTTCA
ATGGCTGTTCGCTCACCGCGATGGCCTCAGATTCGGCTTCAGCTTCATTTGTTGTTCCAGCGCACCCACC
CACGaaaatgtagtaaaatcgaaaaaaaatagggaaaaaTATTTCATTAACACAAGGGCGCCGGCAATTT
TCCATTACCACCCACTGCGGATGGTCGTTTTCTGTTTCGGACGCCGCTGCCACTTGTGCCAGTTTCGCCA
AAAAATATcaaaacattaaaattcaataaaacccattaaaagcaaaacaacaaacacgaaaagCACTCAG
GGGCAATTAAAAATCTACAAGCCGagcagcagcagcagcagcaAAGGAAATTATATTTATTTATTTGCGA
CTCTGAAGGGGCAGCCATGTGGGGGAGTGGAAGTTCTGGCAGCGAGCTGTTGGCTGCTGGGATCTGCCAC
TTTATCAGTGGTAATGGGAATTGTTTTGTGCCCCCTGTGAGGATGACGTTGCTGCTCCTTGTGCAGCGGC
AAATTAAATATTTAGTCAAATTATGGCCACAATTTTCGACTGGTTTTAGTGGTCCTGAACATTGTTCGCT
ATTGTTGTTTCCAGTGGCTTCGGTTTCATTTTCGCCTCCCCTTAGCCATGTGGCATCAGCATCCGCAACA
TGTTGCATTTGCAAAAAGTGTTGCCTACTTTCGGGTCGTGTGGTAAATTTGCAGTGCGAtttttattttt
ttttttttttttttattacgatgatgtttttattatttgatcaacgcactgttacccatgcggcaactta
atttgatctgcttaaattattttattttacaatgtgtcttggttacttaagactaacagatttttaatcc
taaaaatgatagggagaattataatagttgatataacatagtaattattatttatttgattattctaatg
aattttaaaactaagactttctaggtcaaaaccaggttagggaggtcatgagggtggtgtGCGATTTTTA
TGCCCGAAAGTTTATTAGCAGCGTTATTTTTTTGTTGTCTATTTTTAGTTAGTTTTCCATTGCATCAGGC
TTATGAAGCTGCTAAAAATTTGTGGGCAAAAAGTTTTAACACATTTGCGGATAGAGGAATTTAAGTCTTT
GAGTTTGTAGAACATTTTTAATGGAATGGTATTTATTTGTCTGAGTTGTTGGTGAGGAGATTTAAATGGC
TGGCATGGTAATTTAAAAGCTATAATTATAAGTTTTTTGAATAGCATTCAGTATAGCTACAATTTATTAT

AATGCCTCTTTGAGTTGTTCAGGTAATTAAACAGATATTCAAAATGTTTATCTTTGAATGCCAATGAACA
TCCATGATTCCTCTAAACTTTCAACTACATTGTGATAACTAAACAAATAAGATAATTGAGAATCATTTGA
ATGGTGCTTAAGTTAGCTTGCAGGTTCTTCGTTTTGATAGAAAAATATGTTTAAACAAGAATCTTTAATT
AAGGCAGTGTTAGAGAATCTCTTGCAAACCCTTGGGAAAATGCAATTACCATAGAATCTTGATTTGCACG
CTCTTGATTCAAATCAAATTCAGTCCGAGACAATCAATCACTCATCCCGCAAAATGTATTTGTATTCCAA
AACAGGGAATTCACCTGCGACGTGTCCGTGGAGGGCGGCAAGTCATCGCAGCCGCTGCAGTTCTCGTTCA
CGTTCTACGACCTGGACGGGCATCACGGCAAGATAACAAAGGACGACATCGTGGGCATTGTGTACACCAT
ATACGAGTCCATTGGCAAGTCGGTGGTGGTGCCCCACTGCGGCAGCAAGACAATCAACGTGCGCCTCACC
GTCAGTCCCGAGGGCAAATCGAAATCGCAGCCGGTGGTGCCCGTTCCGGTGGCAGCCGGATTCAGCAGCA
GCCACGCCAGCAAACTGAAGAAGTTGCCCACGGGTCTGGCGGCCATGTCGAAACCCCTGGCCGGCGGAGG
AGTGGGATCCGGCGGAGCGTCGGCGCTAACGACATCCGCCGGCAACCGCCGCCAGCATCGCTATCGACCA
CGCAAACTGATTAAGTCCGATGACGAGGACGATGACAGCAACAGCGAAAAGGAGAAGGACGCCGCCCACG
CCCCTGCCGCCGACCAGCCCAGCGGAAGTGGAACAAAGGCGACTGGGAAGAGCCATCACCACCAGTCGCA
GTCCGCCAGGTATCACCAGAAGAACAATTCCCGGGCGGAGCAGTGCTGCACGGAACAGAATACGCCCGAC
AATGGCCACAATACCTACGAGAATATGCTGAATCTCAAGTGCTGCAAGCCGGAGGTGGACCAGGTGGACT
GTCCCTCGCACCGACAGCACCACCAGAGCCACCCGAACCATCAAATGCGCCAGCAGGACATCTACATGAA
GCAGGCCACCCAGCGGGTCAAGATGTTGCGAAGGGCGCGCAAACAAAAGGTAGGCACAAGGGTAGGCACA
AGGTAACAAACGAAATTGTGGCAAAAACAAATCAATTGCTAAACTTAACTGAAACTGCTGCCAGTCTAAC
TAACACAAGCGAAGTTATGCATGAATATTTTGACTAAAAAATATTAATATGTACTCTGCATCCATGATTA
CTCAAATTTAAATTTATGTTTAAATAAAGTTATGCATATAAGTACAGTTTAAACCAAAAAGATACATTTC
CTGTTACAATTTATGTAGCAAATTGCAAACTTTTCATACGGCTGAATTGAATTTTCAGTACAGATTTGTA
TATAATAATTGAGAAAATGATGGATATCTTGTGTAATCCCAGCTTGTCTGGCAGCAGATGCAAAACTGAA
TCCCCTCCCTTTGAAATGTCGTCTTTTGTTTTTGTTACACCTTTGTTGCTTACCAAATGCGTTGAACATA
CGTTATTTTTTGCATTATTATAATTTAATTAAATTTAATTTCCTTTTATTTTTGGTTAACTCATCGCCAT
CAATCAAACACCAACCACAGTACCAGGACCACTGCCTCGAAACGCGACAGCGCAGCCTGTCAGTGGGCAA
CGATTCCGCCTGCCCGAATCGCCATCTGCAGCTGCAGCAGCCGCCGGTGGGTCACCCCCAGCCCCAGTCG
CTGAACCACAAGAGCGCGTCGGGGTCACCACCGCTGGGCGTGGGGGGTGGTGGCGACATGATGCTCGATG
GGGTGCAGCTGCGTCAGCCGCGACCCCATTCCCTCACCCCgcatcagcatcaacagcaaaatcagcagca
gcagcagcagcagcGAAAATCGGCCGAGTGCTGGAAATCGGCGCTGAATCGCAACGATTTAATTAGCATC
ATCAGGGAGAGCATGGAGAAGAACCGCCTGTGTTTTCAGCTGAATGGG
>dme_circ_0005141_junction_seq
GAACCGCCTGTGTTTTCAGCTGAATGGGTCCAGGAATGCACCACAGACTCCGAGGA
>dme_circ_0005142
TCCAGGAATGCACCACAGACTCCGAGGAGCTGATGTACCACCAGGTGCGGGCCTCCTCCTCCTGCAGCGC
CCCGCCCGATTTGCTGCTGGTCAGCGAACGTGACAATAATATCCAACTGCGATCGCCGGTAGTGAACATA
ATCACCACGCCGCCGGGCAATGCGTCTGGTGCGGGAAGTAAGCAGCAGTCTCATCACCAGACGAACCACC
ACTCCTCGGGCAGGAGTCATCCCGGGCACACGGCACATCCGCAGGATGTGAGCAGCGGCGGCAGCCATAG
CAAGCATCTGCGCATCAGCAGCACTTCCAATGGCAAGCACGGCAAATACTCAAATATGCAGCAGCAACTG
CCGCAGGATGAGGATGTGGTGGATGCGGCTGCCACGATGCAGCAGCAGCAGCACACTGGCCACGCCCACT
CGCGCCACCTGCACCACCACAAGGAGGAGCGCATCCGACTGGAGG
>dme_circ_0005142_junction_seq
CACAAGGAGGAGCGCATCCGACTGGAGGTCCAGGAATGCACCACAGACTCCGAGGA
>dme_circ_0005143
ATGGCTTCAACAATTTTCGCCAGGCATTTCCGGAATGGTGCGAGGACGATAATCAGACGCACAGCAAAGA

GATCGAATCTAGTCGCAATGTTCAAACCGATCAGTTAATGGGTCTTAGGTAAGTGATGCATCCTGATTTT
TAAAGGAAGATAATATATAGATTAAAGAAACGAATATATGGAATAAATTTAACCCTGCCAAATTTTTCAG
GTCCCTGCGCATTTCCACAACGCAATCCGATTCCGCGTGCAGCAGTTCGGCGGAATCGTCGGATTGCGAG
AGCTCCAGCCACCATCACCACCACCACAGTCACCACAACTACAACGAGGCGCCCGTGGAGATAATCCCTG
GACTACTCTTCCTGGGAAATGCCACACACAGCTGCGACTCGGAAGCGTTGAAAAAGTACAATATAAAGGT
ATGTTTTGAATGTGACACCAGATTTGCCAAATAAGTTCAAGGAGTCGGGCGACATCAAGTATCTGCAGAT
TCCGATCACGGATCACTACTCACAAGATTTGGCCATACATTTCCCGGATGCCATACAGTTTATAGG
>dme_circ_0005143_junction_seq
ATTTCCCGGATGCCATACAGTTTATAGGATGGCTTCAACAATTTTCGCCAGGCATT
>dme_circ_0005144
AATTTATGCCTGTGCAGTTTTCCATCGATTTTCGTCTTGCGCCACAACGGACTGGGGATGAAAAACCAAA
CACATAAAACGGATTTGATATTAAAAAAATATGCAGGTAAAACATTTGGATGGGTAGGGTGCACCTATAA
TTACTCACACTTTGACAAATTATTATTTAAATATTTAAGAGCGGAAACATGTATTGAAAACTTTTTGCAT
ATCATTGCTGAAAATTTGTCATTATTAGTAAAGTATTTATTATCTTACCAGAACGCATTGCAACCAGAAT
AATTTATGCGGAACTTTTAGCGAAACACAATTTAATTTAAATTGCAAATAGTACTTGTGCAAAAGTTTTC
GGTTTTTGTTTGATTAGCAAAACCACGATGTTTTTTAGCTTTAAATACAAAGTGTTACCATTTATGGCC
ACAGTGCGTATGAGTGATATACCAGAACAAGCTTATCGTTATTGACTTTCTTTCATTTTATACCGAAATC
AGAGTGAGTGAATCGCTTAAATATGGGCTTAAAGGTTCTGTTAGAACGTAAACTATTTAAATTACCAGAA
GTATAAGAAAAGAAATCGTAGTTTTCTTTGTTTTAAGTATTCCATCGAAAGCAGTAGAATTTGGATTTCG
AAATTGGTTTTGCTCATAAGTTGCTCTATGGGAGATTTGAACTAGAAGCAACCTAGCCACATGTTGGCTG
TCCCATGGAACTCGAGCACATGGAGGAGCGAACTGAACGCCAAACGGCGAATAGCGAGCACCGAGTGGCA
AACATCGGCAACAAACAAAGGCAAACAATGGCTTCCAGCGACTGCGGCTTATCGCCAAATGCAACATGGC
GGCGGCCAAGTCACGTCTCAGTTGGCTCCTGACGGTTGTTGCTGCTGTTGTGGCACGGTGGTTGCCATTT
GTTAGCGGAAAACAGCACACTAGCCTTTTTAATACAGACACACATTCGGCGGTCGACATGCATACGAATT
GGGGAATGAGGAGCTCGAACAAGGGGCAAGTGCCACAGTTTAAATTGAAAAAATAATGCTCGAATTTGGC
CAACAGCAAGCAAGGTCGATGATGATGGTCATGATGATGGCAACGTTGATGGCGCACAAAGAGTCGCTGC
ATTTCGCCAACGCAAATATTTATCGCCACCACACGAAAATGTCAAAGGCCATGGGCCTTTCCACGCGAGG
GTTAAAATTTAATTACTCCGTGACAAGAGCACCTGTTGCGGTCAATTAATTTTAAATTAGCAGCAGAGTT
TTTAAATTATTGGGACTTTGAACCGGAAACCGGAATCGCATAAACAGGGAGAGCTGCAAACGGTTGGGTA
ATTTAATATTGCAAATTTTCTGTTCCAGCAAAATGATTATTTTGAAATGAATTTTAATATTTTAGAATGG
GTGTAAAATTCAAATTTAATTTGCAAGAGAATTAATGGTTACAACAGCCACGTGTTTCACTATATTTATC
ATAAACAGCAATAATACTTCTTAACCTATTTGAATGTCGTTCAAATATTCAGCGATTAATCAAAGCTACC
ATGAAATTTTTTGTCATGACGAACTTGATTTGCCAGGAAATTAAATGACACTTTTGAATCAAACGAGCTT
CATGAGCCATGCCAATTAATGTCTTTCATTTATTCAACTAATTGGAATGTGAattatttatttaattata
attattatattattattattattcatttaCTCGCTTTAAAGAATATGAGGAGCAATGTAGTTCTTTTCAA
AACAAAACACAGAATAATGACGAAAATTAATTATTAACTAAAGAGAAACTAAAATATTCAACACATATAT
AAAATAAAAACAAGAATAACACAGAAGAGGCATAATTTTTCTTTTTCGGTTATTAATTCGGTTAAATAAA
TTGTCAATTCTTTTAAATGCAATGTATTTAtttaaatggattttaaatggattttTATTGGAGAAAATTT
AACATTTCAAAAATACCGTAGGTAAAATGCACACGTGCAAACAATTTCTGTGTGCATCCAAAAGAATTTC
CCGATGTTTCCAGCCGTGCAAACAACACTAATGAACACCGAAGCACAGGAAAATTCTGTTGAAAAGCCAA
TTAATCAATCCGCGTTTCCACTTGCAGAAAACAAGAAAAAAGGCGCGAGCTGCGCCTGAAGCGATTCTGG
CGCTCCCCCAAGACCACGTCCTCGGAGGACTCCAATGGCTATGAGAGTCCCACGAGAACGAAAGCGTCCA
GTGCCGGCGGCAGCAGCGATGCCCAGAGATTCAACCACCTGCACTACACCAGCCTGTCGCAGGGATCGGG
CACCTCGTCCTCGGCGGGCGGAGCCACTGCATCCGGAGCGGGGCCGACGACTCTGGGGCAGTCCGGCAAC

AGCAAGCCCTCCTGCTCGCTGCCACTTTTGCAGGTGTGGCCCAGCCAGG
>dme_circ_0005144_junction_seq
CCACTTTTGCAGGTGTGGCCCAGCCAGGAATTTATGCCTGTGCAGTTTTCCATCGA
>dme_circ_0005145
GTAAACGGATTATTGGGTGTAGTTGCAGTTTGAACGGGAACTGTTGAAAGCGCAGGCTGTTGTACAGAAA
TCATACCCAAAGTAGTCGCGGAATAAGGTGTTCCGGGGTACTGGTTGACCTGAGCCGGTGGAGGTAGTGT
AGTGGACTGCACAGCGCCTCCAACTCCCCCATTGCCGTATAAGCTTGAAGGCGGTTCTGTAGGAGTTGTC
TGTGGATGCATGATACCCGCTAGTTGGGGGTAGATAGTTCCGTT
>dme_circ_0005145_junction_seq
CCGCTAGTTGGGGGTAGATAGTTCCGTTGTAAACGGATTATTGGGTGTAGTTGCAG
>dme_circ_0005146
GTGTTTACGTTAGGTTAAGGTTAATAGTGTAGTTAGTTTGATTAAAAGTTAGTTGTAATTTCGAAATATG
GCTCGTGGCTCTGACTTGGCAGTGTGGGCAGATTCCGCTCGCTACTTTACCCCGAGTGCTCTACGTGGCG
GTTTATTAATTGGTTTTGCGTTTCTCAGTCTATTTCGTTGAGGAAAGTGTTTGTGGAAGGGATTAACAAC
AGAAAAGGTGGCAATATGAATATTCAGATAGGAAATCAGTTAATGGCAGGTAGAACAAACGCGCTTCAAC
TGGATTCGATCTTGAATTTTTACT
>dme_circ_0005146_junction_seq
CAACTGGATTCGATCTTGAATTTTTACTGTGTTTACGTTAGGTTAAGGTTAATAGT
>dme_circ_0005147
GGAACCAGGTGAAACTGAGGATCACCCACCAGATGGACGAGGCCATGCCAAAGAAGTAGGTAAGCAGGAA
GACCAGGGTGCACGAGTGCGGACCCGTGGAGCTTTCCCGGAGCAGCAGGCCGTCGCAGGCGATCTCCTCG
TTCTGCAGGAAGTTGCGCGACAGGTAGCCCACTGCCACCATGAA
>dme_circ_0005147_junction_seq
GCGACAGGTAGCCCACTGCCACCATGAAGGAACCAGGTGAAACTGAGGATCACCCA
>dme_circ_0005148
GTGCAACCGCAGAGAAGGTCAGCAAAGAAAGAGCCCAAGGGGTTCCAAGTCACACAACCGAACTAAGCTA
AGACGCACAAAATGAGACACAATCGACTGAAGGTCCTGATCCTGGGACTCGTCCTCCTGCTGACATCTTG
TCGAGCGGATGGACCGCTGCACAGTGCGGATCACGGCATGGGCGGAATGGGCATGGGTGGTCACGGCCTG
GACGCGAGTCCCGCACCCGGTTACGGAGTGCCAGTCATACCCAAGGATCCCAATCTGCGATGCGAGGAGA
TCACCATACCAATGTGTCGGGGCATTGGCTACAACATGACATCCTTCCCCAACGAAATGAACCATGAGAC
CCAGGACGAAGCGGGCCTGGAGG
>dme_circ_0005148_junction_seq
GAGACCCAGGACGAAGCGGGCCTGGAGGGTGCAACCGCAGAGAAGGTCAGCAAAGA
>dme_circ_0005149
CAAGCCAACCGATGACGCTAACAAGGCAAGAACAACAACGACAACGGCTCGGAAGAATGATCATGATCGA
AAGGGGTTTTCAGAAGAGTCGCTGAGCGAGGGAGAGGGCTAGAGAGCGCACGAGAGAGACGGAGCTCGTG
GGCTGACGGCGTTATCTGGTAACAACGTGTTACGAATTACTTTTAACAAGACCCTTAGCAGATAAAAACC
AGAAAACCGGTCGTGAGAGATTGAAGCCGAGTCGGGAGAACAAGGTGAGAAAGAGAAAGAGCGGTGCGCC
GGAAGAGACTCTTCGGAAGAGAGAGCGTGTGTTTGGAACGTGCGCTCATTAGCAAGGTGAGCACACCAAC
AGGAAGGAATTGCAATTAAAGCCAGGCTGTCGGAGTTGCTGACTTTTTCTCGTGGGTGATGGGGAGGGGG
AGTAGGGCACCCTGTATTCCCACCACCGCCGAAGAAAGTCAAAGAACTTGCTGAGTTTTCAGCAGGGTGC
ACTGGAAAAATCAGGGAGAGGAAGACGCAGAAAGAGAGAGTGAGTGAAAAACCTGAAAATATGCCAGACA
ACAACAGATGCGTCTGAAAAACTGGGTTGGAAAAGGGGCCGACGGGCGGGGGGCTAGAAGTGGAAGGTTG
GCATGCGAAACGCAGCACATGTGTAACGGATAGAGGAAGAGCAGTGTTGGTAGAGGAGGGTCGTCGAAAG

GGGGTAAAAGAGAGAAAAGTAAGGGAGAAGGACGAACGACAGCGGAAGGAAACGTGGCTGTCAATCCATC
CAGTCAGAGATTTGTCACCCGGAGAGTGGAAAATATTCCACGGGGGTTGGGAAAACGCAAGTGGGTTAAA
AATAGGAAAACAATAGGTGAAGCCCACGAAACTCGGAAGGACAAACTCTAGTAAGAGTAAATAATATGAT
TTACAGAAAGTAAAGTGTATAAAGTATAAATTTCTATTTGAAAGATGCATCTTTACGCAGTGATTACGCA
GTATGTAAGTAGAATTGAAGGCCTACAAATATTTCCAACCATAATAATAGTCCTCTTTCTTGATAATGGT
ATTAAATACTCAACAGGAAATCACCTTCATAAAACCCGATTTACATCGGCTATAAAAATCACCTAAGGAA
AGCTAATGTAACAGTAAATGCTCTATTAAAACATGAGTTCCTTCCCGTATTTATTTGGTTTGTGCAAAAA
GTAACGAAATGCAGGGCAAGACAAACAAAAAGAAAGCAAACCGTAAACAAAACATTTATTTTGCTGCATT
TATCGCATGCTAGAGAACTCTCTCCAGCTTACGCTCTCTCTCACTCTCGCACTCACAATCAAAACACCCG
TTGCCCTCCCCCACAATTTGTACCAGCGAGCGAGCGAGCTCTGGAACTCAAACTCAGTTGGCATCAGTTC
TCTTTCGACGATCGGAGTGCTCGGTTCTGCGGACGGAAGACTGCCGCTCGAATCATATCGAAATAAGACT
CGCGAGTCACAATTGCACCGTTGAAACGGCAATTAATCAATGAAGAAAACGTAATAATTTATTTGTATAC
AAACACCTCTCTTTTGTGCGCAAATCGAAAGCAGTGACAATCAATCGTAAGCATTCGTGAATAAAAGAAG
TTTCTTCAAGCTAACACCGTAGAGTGGCGTCCTCGAAAAAGCGACAGAAAATACAGAAAACGAGTTATGA
AAATACAAATTGTGGCCGTGCCATGTGAGAATGTGTGTTGGAAATTGTTATAAAGAAACAAGATTTGCTG
TTCGTTGTTATTGTTGCTATTCCGATCTGTGATCGCTGATCGACAAGCGTCAACTGAAATTGGATTTTCG
GGGCTCGGGGCTCACCTTTAAATATATCTGTAGGAAAACAAGCATCCCACACCGTAGCACCTGAAAGATA
GCGACAACATTTGGCGCTACATGAAATATTAATTAAAAAATCTGTCCGAAAATAAAGTAGAAATTACTTG
CAGCGGCAAAAAGTTTCTATAAGAACCGGCGCACGTATTTATATGTGTGTCTGTGTATCCGTCAGATACT
TGCTGTATCCGTGTGTGCCTGCATCTCCAGGTGTTCGTGCGCCAGTGTGAGTGTTTGATGTAAGCAGACG
TGAAATTGGATTAACTTTGACAGACGCACGGCGTTTTTTAATTACCACCAAAGAGTTTTCGGGATTAACG
GCGGAAAGTTGGACCAGCTCCCCAGG
>dme_circ_0005149_junction_seq
CGGCGGAAAGTTGGACCAGCTCCCCAGGCAAGCCAACCGATGACGCTAACAAGGCA
>dme_circ_0005150
CAAGCCAACCGATGACGCTAACAAGGCAAGAACAACAACGACAACGGCTCGGAAGAATGATCATGATCGA
AAGGGGTTTTCAGAAGAGTCGCTGAGCGAGGGAGAGGGCTAGAGAGCGCACGAGAGAGACGGAGCTCGTG
GGCTGACGGCGTTATCTGGTAACAACGTGTTACGAATTACTTTTAACAAGACCCTTAGCAGATAAAAACC
AGAAAACCGGTCGTGAGAGATTGAAGCCGAGTCGGGAGAACAAGGTGAGAAAGAGAAAGAGCGGTGCGCC
GGAAGAGACTCTTCGGAAGAGAGAGCGTGTGTTTGGAACGTGCGCTCATTAGCAAGG
>dme_circ_0005150_junction_seq
TGTTTGGAACGTGCGCTCATTAGCAAGGCAAGCCAACCGATGACGCTAACAAGGCA
>dme_circ_0005151
GGAGGATCATTGGATTCGGACACTGAACTAGGCGAGCTGCTTCTTCTCTCAGCGATAAGATCGCTTTCGT
TCCACGGCATTCCCCCGAACGGCGACTGCATTCGTGTGCTCTCATCGTAGTCGTTGATCTTCGCATTGAC
TCCCGTTCCGGATTGCGTGGGAACGGTTGCTGCTCCGCGAAAGCTTCCGAGAGTGTTCTTTACCGCTGCT
GCTGCCAAGTCTACAAAGTCAGGAGTGGGTGCAGGTTGCCCTACATTAGCTCCTCCGTTGCTGGATAGGG
GACAAGCCCCTTTTTGGTGGGGTGATTTTTCCAGGAAGAGACCTAACGTTGGAGGTGGCTGAGAGTTCGG
AAAATTTACTTGATACCCAAGATTGTTCATTGTTGCGTTGGTGGGT
>dme_circ_0005151_junction_seq
AAGATTGTTCATTGTTGCGTTGGTGGGTGGAGGATCATTGGATTCGGACACTGAAC
>dme_circ_0005152
CAGCGGTAAAGAACACTCTCGGAAGCTTTCGCGGAGCAGCAACCGTTCCCACGCAATCCGGAACGGGAGT
CAATGCGAAGATCAACGACTACGATGAGAGCACACGAATGCAGTCGCCGTTCGGGGGAATGCCGTGGAAC

GAAAGCGATCTTATCGCTGAGAGAAGAAGCAGCTCGCCTAGTTCAGTG

>dme_circ_0005152_junction_seq

GAGAAGAAGCAGCTCGCCTAGTTCAGTGCAGCGGTAAAGAACACTCTCGGAAGCTT

>dme_circ_0005153

GTCTACAAAGTCAGGAGTGGGTGCAGGTTGCCCTACATTAGCTCCTCCGTTGCTGGATAGGGGACAAGCC
CCTTTTTGGTGGGGTGATTTTTCCAGGAAGAGACCTAACGTTGGAGGTGGCTGAGAGTTCGGAAAATTTA
CTTGATACCCAAGATTGTTCATTGTTGCGTTGGTGGGTGTAGCATTTGGAGTGTTGGTGAGACTGTTTGC
ATAGCTTCCACTGTTGGCTGTCATAGGAGAATCCTTGATAGTCAATGATGCAACAGAGTTAGGTCCTGAT
GAGTTTCCATAAATACATGTTTGAGATGATTGCAgctgttgttgctgttggtgctgctgttCTTTTTGCG
CATGAATGATATCCTCTGGGTCCGCCGCATTGTGAAAAGGAAAAACCGATGGGATGGCTGGAGTTGGAGG
TAATAGATCGACCGCCTCCTTGCTACTGCAGTCATCCTGCGCCTTGCGCTTTGTTTTGCGCAGTTTCTCT
AAGAGATTTCGGGTTGCTTGGATGCTATCCTCCTCACTTATTTGCTTACTCCTGTCTATACTAGACCCGA
TTAGACTCATCTGATTGGGAGGATTTAACACACTGGCTAAGTTTGACGCGAAGTTGTTAGCAAACTTACT
GTCCTCCATCTCAAGTATTGTCTCAACGATGGGTAAGTGCTCGACAGGCAGTTGCTCGATTAGAGGCCGT
TGTGAGGGCAGCAGCTGTTGCTGCATAACTGTTGGTACAACATCTGCAGCAGGGGATTGTACAACAAGCG
GAGGCGGCAGGGGTTGGTCATGTAGATGCTGCTGTTGCTGCTCTGCAGAATTCGAGTCCAGTTCAATTAT
TTCCGGCGGATCGGGCGGAATCACAGTGACTGCTGGAGAGGATGTAGACGCCGAGTACTTACTCGCATTT
GCAAGCAGCTCGTTTTTACGTTCCTCTTCCAGCTCCCTAGCCATTTCCATCTCGTTGAGGAGATGTTGGG
TGTGATGAGTGTGTTCCTCGTCTGGCTCGTGACTACGTCTCCTTCTTCTCCGTCGGGATCGCCAGCGCAT
ACGCCGCCGTTCGCTGAGATCACTTCCAGCGTCAGACATCTCGTCATTAGAGGTAGGGGTAGGAGTGGGC
GAGGCGGGAAGCGGAGCCGCCGGTGGACGCTCATCATCGATTATGTCTGCCAGTTTACTCGCGCTCGCAG
ATAGTTGGGTTCGCGTTGGAGCAGTGGTAAGCTCCTCATCAATATCCATTGGTAGTTCACCAGCTTCTGC
CGCAGACTCTGGGTCCAGATCGGGTTCGGGTTGCGCATCTGCTCCCAAGTTAATCTGTTGGATGGACACC
TCATCGGGTTTAAGATCACATTGATCTTTTACGATACGTGTTTCAGAAGCAGGTGGAGTTGGTGAAATTG
GTGGGGGTGTAGCTTGAGCAGAATGGTTCATCTGCTGCTGTTTCTTATTTAACAGCTTGTCCAACGAACT
AGTCCCTTTTCCGCTAGCTACTTTTCTCTGGCTTATTTCTTGTTCCAAATTTGCAACCTTCTTAGGAGAT
TCTTCCGCTCGAGATTTACATCTGGATACTGTCTTTTCTGGAGACGATTTAGCAGTAGGCGAGGGCGGTC
GCGACTTGGAACCGCGAGTCGCTCTTTTGGTAGCCTGTTCCAACTGGACCTGCTTTCCACATTCGTCCAT
AGAGCCCTGCAGACGATTTGTTTTCGGCGTACATTTGACGTCATCCTCAACTGATTGCACTGCCTTCTGC
AGTGCTACCTTTGTCGATTTTGTTGTAGAGTTCTGACGAGACCGCGTAGACGGTATGTTCAATACATTGA
CCGATGCTGGCGCAATAGTTAATGCTGAAGGCTTATCCATAGACTTGCGGGGTGAACGTTTTGGCACAAG
AGAGGAATCAGATGGCACTTTCGCAGGGGCTACAGGAGGCTCCCTACTAGATGGGTTTTCCGAATTGCTG
GCCTGAGACTCGCTGTCCGAGTCGCTGCCGCCACTGCTGGAACTACTTCCTGAGCTGCTAGAGGAGCTGG
AACTTTCACTGTCGCCAGAACTAGAACTTGAAAGATTTGGGACAACTACGCGGCGCTTAACCAGGGGAGT
TGTTTTTATAGGTGTCGCCAGCAAATCTTCTTTTGATTTTCTTCTGCCACTTAAGGGTTCCTTTGGAGTA
ATTGGTGCTTCTTTAATTGAGGACCGTCTGGTAGGCTTAACTGGCGTGCTAGCCGCTGGAACAGATGATC
CTGATCCGGATATCGTTGCCGACGTCACTGTTTCTTTGTCCGAAATGTCGTTCCCCGTTGAGAAAGATTC
TAGAACGGGTTTGCTACTCTTTAATTGCTCCGCTGCCTTCTTAGCCGCTTGCCGTTGAGGAACCAATGAG
ACTGCAAAGTTCGACTTGGCTGAGACTGGGGTAGCGACGACAGATGGGGGCTTGCTTTGAGTATGCGTTG
GGAGGGCAGGCTTGTCATTTTCCGTAATAGATGGCGGACGTGCATCCTTTGGGACCTTTGGTGGCCTTCC
GCGCTTTCCACGTTCAGCAGGTGCGCTTTCGGACGTTTTTCCAACAGTCTTCTGTAAGTGTTCTTTTGGT
CGTACTTTTTCAAGACTTGATTTATCCGCTTCATTCGACACACGAGAGCTTGAATCCTTCACCTTGCTCT
TTGCAGTTCCTGAAATGGAGTTTATGTCTGGCTCTCTGACTCTGTCGGACACATCTGGCTGTAGCGTCGT
GGCGAGATTTGTGGATCGCATGTTCTCAGAAGCCTTTTTGGCTGCCTGGCGCTGTGGCACCACCAAAAGA

TCTGCCGGATACAACTTGGCATTCGGGTTCGGTTTGGTTTTTGTACTGTTGCCGATACTGGCAGCCCCTT
CCTTTCCTTCCCGCGTGTTCTTCGCTTTACTAGTTGAGGACGCTGCTTGGGAAGTAGAGGATGTTGTGGC
TGCTGTTCCTGGCACAAACTCTTTCATAGCTGCCTTTGTTCTAATCGTCTGTTGGTTTTGTGAGTCACTA
ACATTACTTTCCACGGTGGCCTGCTCCTCCTGGTCTTTTTCTTGTTCAGAGGAACTGCTCTCAGAGTCGG
AGTAGATATGCTGTCCCAA
>dme_circ_0005153_junction_seq
CAGAGTCGGAGTAGATATGCTGTCCCAAGTCTACAAAGTCAGGAGTGGGTGCAGGT
>dme_circ_0005154
GTTCGTCAAATCAAGCGGATCTCTCCAGCCAAAATAATCAGCAATCTGATGTAGCAAACGCCTATATATA
GAATATACGAAAATGCAACCATTCCGTTCCGCGCCGTGCTAATGCAAACGTATACGATCCGAAACCGATA
AATATAGAAATACAATAAACCCAAGTGAAATAGCAATTTTAAACGCCAATAAAAACAAAATATACCAATT
ACACTGCCCAAGAGAACATCAAAGGAACTCATTTTGGAGG
>dme_circ_0005154_junction_seq
AGAACATCAAAGGAACTCATTTTGGAGGGTTCGTCAAATCAAGCGGATCTCTCCAG
>dme_circ_0005155
GATCATCGAGTGCTTCATGAAAGAGAAGATCCAGGCTGGCGATGTGATCACCATCGACAAGGCGTCCGGA
AAGGTCAACAAGCTGGGTCGCAGCTTCACCAGAGCCAGGGACTACGACGCCACTGGCGCTCAGACCAGAT
TCGTCCAATGCCCCGAGGGTGAGCTTCAAAAACGCAAGGAGGTGGTGCACACTGTGACCCTACACGAGAT
CGATGTTATCAATAGTCGCACCCACGGGTTCTTGGCCCTGTTCTCCGGCGATACTGGAGAGATCAAGCAG
GAGGTTCGCGATCAGATCAACAACAAG
>dme_circ_0005155_junction_seq
GGAGGTTCGCGATCAGATCAACAACAAGGATCATCGAGTGCTTCATGAAAGAGAAG
>dme_circ_0005156
GTCACTTCCTCCTATTGCTTCAATCCTACTCAGACTTCTTATCTTCTGGTTTCTTTTCCTCTACGAGTTT
GTCCGTGTTGCTGTCCTGCAACTTCTGATCCTCTCCAGCTAGTTTCCGGGAACTGCTCCGGAAAAAGTGA
TTGAAGGTATGATAGAAGGCAGCCCAGAGTACTAGGAAAGTGAATCCATAGTAGCTGACCGCTCCGTACA
CCACGATCCAGCGCTCGTATTTCAGAAACACAAATGCGGTCAGGCACCAGCCCAT
>dme_circ_0005156_junction_seq
ACACAAATGCGGTCAGGCACCAGCCCATGTCACTTCCTCCTATTGCTTCAATCCTA
>dme_circ_0005157
GGAAACTGGAGGAGTACCTGCAGGGCGTTGACGGTTTCGAGCAGCCCAAGATCCTGTTGGAACAGTACCC
CACTCCGCCGCACATAGCCGCGTGCATGGCTCATCACATGCAGTCGCAGCACGAGGACATCGAGGGAAAG
CTGGTGGGAGATTTGGGCTGCGGCTGCGGAATGCTCAGCATTGCTTCCACTCTGCTGGGCGCCCAGCTCA
CGGTGGGCTTCGAACTGGACGGCGATGCCGTGGACACCTTTAGGGGCAATGTGGTGGAGATGGAGCTACC
CAATGTTGACTGCGTGCGGGCGGATGTGCTGCAGCTGATCGGCAGCAAGTGGGAGAAGTCCTTTGACACG
GTGCTGATGAATCCCCCATTCGGCACGAAACACAATGCCGGCATGGACATGCG
>dme_circ_0005157_junction_seq
CGAAACACAATGCCGGCATGGACATGCGGGAAACTGGAGGAGTACCTGCAGGGCGT
>dme_circ_0005158
GGTGGCCCTACGGTTGGCCAACAGAGCAGTTTACTCCCTACACAAGACGTCAACACGGTAAGTCTGTATA
AAATTGAttataatcgatattcgtagaggaaaacttaagttttcttgcaaagaatgtaacaggtagaggg
aagcgatttccgctttacaaagtatatttttaATTAGGATTACCAGCCGTGTATAACCAACGttttgaaa
aaatcttttgatataacttatcttatttcctttacaatttctatccatatctaaacaaaatttggaattc
ctctctagcaatccctctagatgagcggcgtatctgatattcgaaatcttggttataccattctctcttg

tttAATAGTGTAGATCTCTTGGTTAAACAGATACTCAAGTAGTATGCTTACTGGTCTATATATAGGTATA
TATTTTAAAATATATTGATACTCAATATTCGGCATATGAATGTACATATGCAGATTTATACTTTAAGAAT
ATCTGCTGGTATAAACAGATGCGCAATCTTTTGTTATTGGCACAGTAAACAAGAGATACAATTGTACCCG
CTTTTCGGTAAATGTGCTTCCTTACCGCTGTTTCTGCTTTTGTTGTTGTTTTGTTAAGAGCGCTGCTCAC
AACTATAAAACGCCGCAGAAACACCAACAAGTTACAGATAGCAACTAGAAGTCAGCAGCAAGATGATCCT
CTCACGTAGCCTTCTAATCATCACTGTGGTGCTGGCGATTCTTCTGGCCCCCATGCCCACTGATGCGCAA
ATCCTGGGCAGCGGCCAATGCCGTCTCACCCCCATTCGAGTGTCCGCTGGCGGGACAGTGTGCGAGAGAC
TGTGCTTCCGAAGAGACTACCGCGGTCCGCCCAGCCTTTGCCGCCGCATTCCCAGAGATGCAGTGGTTCC
TGTCTGCGGGGGTGGTTGCTGCCGCAGCGGTCCCAACTGCCTGCAGCTGCTTTACACATAAAGTATGTGG
ATAAGATGCAGCACGTTCCATCAATAGTTTAGATTTAGTGAGAAATTATGGAAATTAAATATTTTAAAA
TATATATGCTACTCATATTAAAACGATGAGTTCTTTAATTAAAAGGGTATCTTATTATTCGACCCCTTCC
TGTTTTTTATGGAAGAATAGCCAAGCCTAAACAACTGTGGAATAGTACATCAAAGCCAATTTAAATATCG
ATTTTAATTGTTCGCAGGTCGTACATTCAGAAAAAGGCACTGGAATGGGGCGCCCGCGGCTCAGTGGTCG
CCGAACTCCGGTACAACATCGACGCCAGCTACAAGTTTCACAAGCAGAAGTCGAAGGATATTGAG
>dme_circ_0005158_junction_seq
TCACAAGCAGAAGTCGAAGGATATTGAGGGTGGCCCTACGGTTGGCCAACAGAGCA
>dme_circ_0005159
TTAACATCAACACCACCACCAACGGCGCCGAGATTGGTGGAGCTTTCGGAGGCGAAAAAGCCACTGGAGG
AGGTCGCGAGTCCGGATCCGATGCCTGGAAGCAGTATTGCAAGCGGGCCACCATTACAGTCAATCACTCC
GGAGAATTGGCCTGCGCCCAGGGTGTTG
>dme_circ_0005159_junction_seq
GGAGAATTGGCCTGCGCCCAGGGTGTTGTTAACATCAACACCACCACCAACGGCGC
>dme_circ_0005160
GGTATTAATCACGTAGTAGGGCAACAACTGATCCGTGGCTGAAATGCGTCCAGCAGTCAAGGGATCGCAG
TCCGCATAGTGGGTGAAGACCATCAGACCCGTGTAGAAGTTCAGCAGGAAGACCACAATTAAGCCGATAA
TGGCAAAGCCCAGAGCAATCTTGGCTAGCCTCAAGGACTTCAAGGACATGCATTTCTGAACGGACGCCTG
GTTGGTGCAGAACAGCGAGGTCCA
>dme_circ_0005160_junction_seq
CCTGGTTGGTGCAGAACAGCGAGGTCCAGGTATTAATCACGTAGTAGGGCAACAAC
>dme_circ_0005161
GTATTTCATCCGCCACGCAATATCCGAGACGAGAAGCCCTTTGAGTTCAGCATAAGATATAAATTTATAA
GCCAGTCGGATGCAGTTGTGAGGTAAGTTGCAGCACCGAATTGCACTGGCAAACTGACAAATATCTGTGC
AGCTCGAAAAGTATTCAATTGATATTGGTTCGGTGCATTTCCCTCTTACGACTTCACCCCTCTTCTCGAC
CAAACAAGGGTAATGTCATCAGTTGCCTCAGCTTTTCCCGCTCGAGTTTTCATCTTTCCATAGATGTTTC
TATTCCTGGCACTGGCTCTGGATTTGGCTCGATTCAAAGGCTCTTGCTTAGAACTTAAATCTTTCTGCCC
GAAAGAACAATGCCACTTGGTTGCCCTGGGGATGGCTTTTCGGGAAATTCCGAACCGAAGAAGGTTGCCT
GCATAAAACATGAGCCGTGTTCTGTCTAGTTTCTCAATACAGGTTTATCCTCACCTCATGTATTTGCACT
GCAATCCATTTGTGCCTGTTGCCTAGGCATGTGTCAAATTTGTCTCCGTATCGCTTTTGCTCCCACTTCC
ACCCACTTCCTCTTCCGAGTGCCGCGCTTTTTGTTTGCAGGTGGAAAATTAGAGCGAGAGGAAAATAGAA
ATTGAAATGTATTTTTACACCTGAATAAGTTGCACAGAATTTACAAGTCATTGCTACACTGCGAAAACAA
CGTTGTTAGGGGTTTGAAGACATCAAAAAACGTGTTAAGTAGAGCTTAAGAATCTGCAATAATGTATCTT
GACTGTCTTGCATCCATTTAAAGTGACTATTTCTAAGTTACTGTTACTGTAATCAATGTTACGAAACATA
TTTCCCTTGTTTTTCAAGAGGTAAATTGGAATATTTTTCAAGTGCAAAGTCCGTTCGTTGTCCGTGAATC
GTGTGCACACCATTAGCAAACTAAACAATGATCAGAGCCCAGGAATGAGCCCGTCTGTACATGCATATGT

AGAATGGTAGGCGTGTGTGCGTTTGCGTCCCGTAGCACATTTTATGCAAATTGCAGGTCTTTGTGTTGTT
GGTGGTGCTGGCCCTGCCTTAAATACCATTCAAAATGTAGTCGCACGGACCAAAAACGAAAAGGACGCAG
AACCTAAAACTCGCACAACACTTAGTGAACTGAGAACTGAACCTTTTGGCCTCTATTCCAGAATACGGCC
TGCCCGATAAGCCGCTCGAACTGGGCCGTGTGACACCCGGCACCTATTGTACCAGGCAATTCGACGAGTG
CTATCGGAACAAGTGTCGCCTGCAAAGTCCAAATTATCCTGGCATGTATCCGAGGAACGTTACCTGCTAC
TGGACGATCCGGCAGAAGGAAGTCCCGACCAGCAAGCACGCCATGATTGCCGTTAGCCAGGAAAATCAGC
ACAAGGCCTTGGTAAAACGGGTAAGTTGGAGCTGGGAATTGTGGGAGGGGGGGATTGGCCCGAAGAATCT
CGAATGAATACCTGCATTGGAGAGCATTAAATAAGCATTACCATTCTGTCCGACGAATTCGAGTCCCGTC
CCGAATTGAATGGGATATGGGCATATACATACATATCCCGGCCAGACAGAGTGCATCTGCATCTGCACTG
CATTTGTATGACAAATTTCAATTTGGCAAATTGCTAGGCGAAGTACCAGTTTGCAGTTTGTTAACTGACG
GCACAAATGAAACTGTTTCGTGTCCGAGAATGTTAAGCATTCGTCGCCCAAAAAGTTTTGTATATTCCAA
TTAAATGCTGCAAACTGGCAGCCGATGCAATTTGTATGCAAAACCGTATTAGAGGACAGTATGTGAAGTT
TGTAATTGGATTGATGGGATTAGGTAAATGTATTTTTGATATTAGCTTACATTTATTTTCAACTTTCGAA
GAGAACATCTGGGGTTTCTGCTCAGGTGGAAAATCAGCTCTTATATATGCACCAACGAAAATAGTCATTG
CAGTACTCCAAACTTTTTTAAAAATTTACAAAGTTTGATTCTAAAAAGTTTGTCGTTGAAATAATAAGGA
ATAGAAAAACAACACAGTTATTGGAAACAAACGTATATACTATAAAATTGATTGAAAAACATTTGTTACC
ATGTAACCATTGTTTGTCTACAATTTTTCGCATACTTGTAAATATTATTTGTATTACTTTAGCTTTTACT
ATTATTTTGACCCCAGCTGTAACAATTTCCTAGCCTGGCTTTGGCAAATGAAAATGTTTTCATTGCATTT
CGATTTGATTGCTTTTCTCCGTTTTTCAATTGTACATATTGTTCGTGCTCTTTGGCTAACCTCTCCAATA
TGATTTCTATTTTTTCCGCCCACACTCACAGCTCAATAGCCAGCTTTAATAAAACATCCCGGTCCATTCG
AGCCTGGTCGGACTGTACAGGTGAACGGGACCATTTGATTTTCTACGACGGCAGTTCCACCAACGATCCT
GTTTTGGCCAAATACTGCGGAGGAGATTGGTTGCCACGCGTCGTTTCACGG
>dme_circ_0005161_junction_seq
AGATTGGTTGCCACGCGTCGTTTCACGGGTATTTCATCCGCCACGCAATATCCGAG
>dme_circ_0005162
CTCAGCGAATTGGGACGCCCATTCACCGGCGGCTCCTGGTGCGGCAAGGCCACCGGACCGCAATTGTACT
TTAGCGAAACATCCACGGTGACAGCTTCGGTGAAGGTGagtgcaagtgcaagtgcaagtgAGAGGGGGAA
CATGGGGGAAAGAGACGGGGCGCCTGGAACTGGAGCTCTGGGAGCCTCGGCTCACCTGTGTGTGCTCAAA
TTACATACCCTGCCAGGACTTAAGGCCATTAAGTTTGGAGCATCTGTTTAGATTTGATTTAAATaaaaaa
caaaaaaaaatatataaaatatatataaatttataTTTTACTCTTAATAAGTTTGGTTAATTGTATTTCA
TTTTACATTCAATGTTAGCGAGTTGAGCGAGTTTCACCATTGTCCACGATGCATTGTAGGCTTTATATGT
CCATAATGGGCGGATCTGCATCCTCTTTAATTACCATGCAGAGAATGAAATCTTCAGCTTATCCCGAAGC
TTACGTGTCCAGCCTTTCATTTTAAATTGAACAACTTTCCCGCGTACACCACACGGCGTATGCGTAATAT
TTTCTTAAGGCTGTCAGGCGACGAAGCAGTTCCACTGCCACTTTTTGATTAATTTTTAAATAAACGCGCC
GCCATAAAAACGGGCAAATGGACAAGGCATCGAAGAGGACACGTCCATGGGAGGATCTTGGCCGGAAATA
TGGTTTCAGTTACTCCCATCGAGTTGCCGCGCATAAATTATGAAAATTAATAAAAGTTCACTCGCCATGT
GAGAGGAAACTCCTTCGGACACTTCTTGTCTGCAAAAGTGTTGACGAGCAGTTGACATGGCAATGGGGGA
AAATCAAAGGAGAAATCGCAGATGGCATAATTGAAGAGAAATTGCGTGTCGAGATGGAAATTTGCACTCG
TTAAGCATCCAATTGTATTGTTTTCTATCGAGAGGCAGGTCTTCTTTTCAGCAACTTAAGTAGTAATGGG
AGAGTCATGTAAATGTCAATCGAACTCCGTTTTAAAAAGACCTACCTCAGTTTACATATATTTCGTTCCT
TTCATTAACTTGTATTTTAATCAGTTTTATGTAAAGGGAATTGCTTATGTGTACGTTAAAGTTTGGTTAG
ATGTCGTTCCTGTTGTTTCACATCACAGGAAACTAGCTTGAGTCACGTTTCCCGAGAAACATTTTCACAT
TGATTGCTCTTTTTGGCGTTGTAACATTTCAAATTGCATTCTACGACCGCTTTCCATTTTCTTGCAGGGT
AAGTTGCAGCACCGAATTGCACTGGCAAACTGACAAATATCTGTGCAGCTCGAAAAGTATTCAATTGATA

TTGGTTCGGTGCATTTCCCTCTTACGACTTCACCCCTCTTCTCGACCAAACAAGGGTAATGTCATCAGTT
GCCTCAGCTTTTCCCGCTCGAGTTTTCATCTTTCCATAGATGTTTCTATTCCTGGCACTGGCTCTGGATT
TGGCTCGATTCAAAGGCTCTTGCTTAGAACTTAAATCTTTCTGCCCGAAAGAACAATGCCACTTGGTTGC
CCTGGGGATGGCTTTTCGGGAAATTCCGAACCGAAGAAGGTTGCCTGCATAAAACATGAGCCGTGTTCTG
TCTAGTTTCTCAATACAGGTTTATCCTCACCTCATGTATTTGCACTGCAATCCATTTGTGCCTGTTGCCT
AGGCATGTGTCAAATTTGTCTCCGTATCGCTTTTGCTCCCACTTCCACCCACTTCCTCTTCCGAGTGCCG
CGCTTTTTGTTTGCAGGTGGAAAATTAGAGCGAGAGGAAAATAGAAATTGAAATGTATTTTTACACCTGA
ATAAGTTGCACAGAATTTACAAGTCATTGCTACACTGCGAAAACAACGTTGTTAGGGGTTTGAAGACATC
AAAAAACGTGTTAAGTAGAGCTTAAGAATCTGCAATAATGTATCTTGACTGTCTTGCATCCATTTAAAGT
GACTATTTCTAAGTTACTGTTACTGTAATCAATGTTACGAAACATATTTCCCTTGTTTTTCAAGAGGTAA
ATTGGAATATTTTTCAAGTGCAAAGTCCGTTCGTTGTCCGTGAATCGTGTGCACACCATTAGCAAACTAA
ACAATGATCAGAGCCCAGGAATGAGCCCGTCTGTACATGCATATGTAGAATGGTAGGCGTGTGTGCGTTT
GCGTCCCGTAGCACATTTTATGCAAATTGCAGGTCTTTGTGTTGTTGGTGGTGCTGGCCCTGCCTTAAAT
ACCATTCAAAATGTAGTCGCACGGACCAAAAACGAAAAGGACGCAGAACCTAAAACTCGCACAACACTTA
GTGAACTGAGAACTGAACCTTTTGGCCTCTATTCCAGAATACGGCCTGCCCGATAAGCCGCTCGAACTGG
GCCGTGTGACACCCGGCACCTATTGTACCAGGCAATTCGACGAGTGCTATCGGAACAAGTGTCGCCTGCA
AAGTCCAAATTATCCTGGCATGTATCCGAGGAACGTTACCTGCTACTGGACGATCCGGCAGAAGGAAGTC
CCGACCAGCAAGCACGCCATGATTGCCGTTAGCCAGGAAAATCAGCACAAGGCCTTGGTAAAACGGCTCA
ATAGCCAGCTTTAATAAAACATCCCGGTCCATTCGAGCCTGGTCGGACTGTACAGGTGAACGGGACCATT
TGATTTTCTACGACGGCAGTTCCACCAACGATCCTGTTTTGGCCAAATACTGCGGAGGAGATTGGTTGCC
ACGCGTCGTTTCACGG
>dme_circ_0005162_junction_seq
AGATTGGTTGCCACGCGTCGTTTCACGGCTCAGCGAATTGGGACGCCCATTCACCG
>dme_circ_0005163
ATGCCCAGGATGGGGAGACGTCACAAACACTGCCATCATCGGTTGTGCGCTTCATCTTCATCTCCGCTGC
AGCGGCTGGTGGAGCTGCAGCTCCTTCTGCCGGCGCTCTTGGTCGTCCTGCTGGCCACCACTCTGGGACT
TGCCTGCCGTGTCTCCGAGTTCTCCTGCAAGGGCAGCGGGAACAGCGGCACCATCTGCGTGCCCCTGGAC
AAATACTGCGACGGACGCAGCGATTGCGCCGATGGCAGCGATGAACCCAAACATTGCTCCGGGTGAGTGA
ATGCCGCCAGCTGTCGTCCGCTTCTCCGACGAGTCGAGTGGACAACAGTACAGTAAACAATCGAAAGAGT
GAACTTATGAAAGAATGTTTGCACTAGCATTTTTTGCACTAGTAGCTTAACAGGGAGTGCAACACCATGT
TATGAGCACAAAAAATAAACTATTTGAAGAGACAACACTGCCTATGAGTACTGAGTACCATGCCAGTTAA
CTATCGGTTAACGTATATATAAATATTGCTACACATTCGATTGTTTACTTTGCTTAACCCACTGTACCCC
CAATTCACATTCTTTTTTCCGACCCCCCGCAGTGTGAGTAGGAGGTGGTGGGGAGTTCGGGATGTTGGAC
AGTGATGGTCGATGGTTGCACTTACTATACTTACAATCCTATACTCAAAACATTtatatatattataaaa
gataatagaataatagaatatatataGAAAACAACTTTATTTGCTTGTTTATTTTCCTAAATTAATGTCT
TAATATTGTATTTCTATATTTAAGTTTCTGGACAATATATACGCGCTTACCGCGGAAATCTTTTTCATAA
CCAGAAATGGTGAAAAAGGTACAAAACGTGAATGGAACAATTTTGTTTCAATGGCACTATTAATCATTTT
TGTCATTTAAAGTAAGCTTTAAAATAGTTTTGATTCGCAGTAATGGCTTACGGAGGTAGTCGAAGTTGGG
CACTTAAGTTCAGAAGGAAGAAGTGGTACCTTCGCATCACTGGTGCTGTGAAATCAGAGCGGAGTGTTTG
TCCAGCTCGTGTTGTAGTCCACTGTCCGTTGTCCTTTGTCCGTTTGTTGGCTTTAGTGCGCCGTAGGCCA
AAGATTAGCCGTGCGTCAGCGAAATAAAAGTGCAATGcacacacacccacacacacaTGCTCTTGAGGGC
ATAAGGAGGAGTGTCCTGCGGGAGGAGTGCACTCTAACGGGAACCAACCCACACACATGCACACACATAC
ACGCCGCCAGCAGACAGCGACACGCACAAATGCACCGCCGTGAAGTATTCCGAGACACATCCCCCCTCCC
AACAATGGCAATCCGATTCGCACATATCACGCGAGGGGCTTTATGTGTCCGCCATCCGGACAAGGTATAT

GGGTTCTGTTTGGCGCGGACACTGGCCACAGTCGGGACTGCAGGGATAAAGAAGGTACATTCTCAGTTTG
GATGGTATATAGAAATTACATAAGTCTTAGCTATATACATATATTTACCAAAATGTATGAACAGATTGGG
CAAAAAATACATTTCTAAGTTTTGTGAAAATATTTAAATACCGTAACGAAATCGTAGACGCAAAATTTAA
AAGGACTTAGTCTGTTGTTTAGGTGGGTATCGTGCATCCAGTGATATTTGCCAAGCTACCAGGGTATTCT
GAAAACACGTACATATGCATATCCCATGGTCGCATTCGCCGCACTCTGTTCGTGCTTGTTAGGCAGAATG
TCTCCATTTCTCGCCTTGGCGGCATATCTCCTGACATTTTTGGCGGCGCTGGCATTTTGGTGCGTGCTTA
ATTAGGACTTCCAGCGATTGCAGTTGAATCCCAGCCATGCCGCCTGCTTTTTAAATTAACCGGACCGGAT
CTTTAAAATGCCTTGGGCACACCCAGAATGGGAGACCGAAAAGCAGTCGCCGAAATAAGCAAACAAATGG
AGCGGCTCGAGTGGCTAGGGAGGAGTGGAAACACCCACGTTGACAATGAAATTAGACACGCTTCAAAACA
TTTTTGGCGAGCTGTCAGAGAAACGTCAATTTCAATTTCGAAAATAATTGATTTCCCCTGCAATTTGCGT
AAACATCGCAATGAGCAGTTGAGTCCCAGGAAGTGGTAGTCAGCAAATCTGTTCCCCAGCTCCCACGACG
CCCCATTTGCTCCTTAAATTTCCCCAATCCGCTTGGGAGTCTCGCACATCCATGtatatgtatatactat
ataagtatataagtatatatgtatatatgAGTAAATGTGGAATGATGCATGCAAAATGTTCCTTGGTGGG
CTTTGCACCTTGCACAAATCTGTCATGCATAAGTTATAACTTTTTATTTCGCGTTGCCGAATTTGTAGAA
CACTTTTCGATAGAACGTGGATACACCTAAAACAGTTTCCTATCTGACTGCAAGTAAATTCACCTTGATT
GTCATTAGATGAGTTTATTGTTTGGTATTTACCTTAATATTACCCCTTTTTATTATGTAGGTTATTTCTT
ATTTTGTATCTATACTGCATATAGCTTATACCCTTTTTATAGCAGTGTAGCTCAGTGTAAATTTTTTCTT
CACCTCTTCATCGTGGCATATAAATCATGCGGTTGGCTTTCGAGGCTTCCCGTTTTTTCCGCTTTCCCT
TCGATTTTCATATATAttttttttttGGATCTCACCCGTGTGCGTCTGTGTGGGAAATTGCTTGAAATA
ATAATGTTAGACATCGGCGGGGCGAGGATTCGATGGAAGGATGCGATGTTGTCGGGGCTGTCTGTTTGTA
CATATTGATTGATGATTGTCAGCCGAAGTCCCAGAACTTCGCGGCAGGGAGAAGTTTAAATTGCCTGGGA
CTCAGCCATGCACCCATGGGTTAATTTTTAATGGAAATACCTTAACCCAAACTCTGAAATATGTTAGCTG
TAATCAAATAGCATATAGCGTCGGATTTTTAATAATTAAAGCTTGTTTTAATCACCCTCTAATGGACGGG
AAAATTTATGCAACAACGCAACCAAAGGGAAACTTTCCACTGGTAAAAGGCAGACAAAACATTTCATAAA
TCAAGTAGTTTTTTATTTACGAAATATATATTTCTGTCATTTATTTAATTTTGCATTGTAAATTGCTTGT
ATGTGCAAGAAGTTGTTGCCCATTGTTTTCGAGGAAATTTTAAAATGTGCGCTTTTCATTTTCCCAATAA
TTTGTTGCATTTTCATGCCCAATTAGTGTGACAATTAGAATAAAACTTTGCTCAGTTAAGCAGAGCTGAT
GGAATTTCGGAAAATATTGGTATTTTGTGGTCGAGCTAATAATGGATTTATTTTGTGATAATTTTAAATG
AAGACCTAATTTACTGCTGCAAACAAATCGAAATCAACACCACTGACCACTCTATTTAAAGTGTCTGTTA
ACGGAGTCAGTAAGTTTTCCTTAAAGTCCTTAAAGTTTCCCAGTTTGTCACGTCGAAGACCAAAAAAGTT
TGAGAGGAAAAAAACCATAAAACATGAGAGAACTCCTTAAAAAGCTACACTACACATAAATTATGCTTCC
TCATTTGCCCAGGAGAATTTCCGGGATGAGAAGTCTACGTTGAAGTCCAGAGCAAGCTTGCAGATGCAAA
AGTTTTTCACAGAATTTCCCTCTGTCGTTCAGTTTGCCGCTTCTAATGAAGCCACAAATGCCATGTGCGA
TGTGGATTTCCCTTCCTCATTTTCATTTTTGCTCGCTGTCACTTTTGCCTTTGTTTAATATTTCAAATTG
GAAGTGAAAATCTCTTCGTGTGCGTAGGTGTTGGGAAATTCGGCCAAATGAAAAGCAAGTGGCAGGCAAC
TGAAACCCGAGGCCGAACCCTTTTATTGGAGTTTTCTTTCCAATTTTCGACACACTCTCTGGCAGGCATT
TATTTGCATATTTTGTTAGGAAAACTCATGTGCCGCACGAGTACATAATGTACATATTGCATACGGGATT
AGAACACTTGACTCTCTGACTTTTTGACTCACACAACTTCCTTACTTTGACCTTGACTTGACTCCCTCTC
GAGGCAGTGTGAATGTGGTATTATCtttttttttATGGCCAATCAAATATGCAAACAGTGGGCGTTGTGC
TGTTCCCCACATTTTTCGCAAGTGGAATTTCATTTGCATAGCTTTAAGCAAATATAAAATATAGTGTTAG
TACCCTGAAATGCCGAGCTAAACTGTACTCTATAGATTTGGAAAAGGATTTTAATTAATTTAGGATTATA
AATAGTAAGTCGTATAAAAACGGAGGTTGTATTGAACCTTTTAACAAGATTTTAATATTAATTTTCAGCT
ATTCATAAACAACTTACTCTAAAATGACTTTCAATAAGTTCAGTTTTAATGTTATTCTTATAATCTATTA
TCTTATAATATAAAAGTATAGCTAACTATTATTTTACGGAAATTTTTTCCAAATGATTACAATTTTAAAT

TCGACTCCTACAGAATTATCTTCGATGGCTTCACGGTGGGAAGGTTCGACGAGGGCATGGTCGACACGGA
TGTGGATGGTTACGAAACGAACCTCAGTCCCACGGGCGAATTGCCAGGATGTCCCGAGGGCTTCATGCAG
G
>dme_circ_0005163_junction_seq
CCAGGATGTCCCGAGGGCTTCATGCAGGATGCCCAGGATGGGGAGACGTCACAAAC
>dme_circ_0005164
GACGTCCTTGTCTTGGCCTGAACTGCTTGGTGCGCCTTGGCCTACATTGCTAACGTGGCTTACTCTCCTC
CTCTGGTCTGACTGTCGTAGTAGCTGGTCTCTCTCCAGTTGTGACCGCTGCCAGTTGTCAGGTTAATGAA
TGAAATCGCTCTTGCTCCTGTGATTGCTCCCGTGGCTGCTCCTCTGGCTGTGTCTCTCTTGGCCCTGTAC
GCAGCCGCTCCTCTGGCTGCGCCATGACCTACTCCTCGCCTTTGGCTTACTCCGCACCACTGAGCTACGC
CGTTCCATCTGCACCGCTCCTTATTTAAATTACTAAGCTGTGATCACCTCGGAGACAACGGAAATAAATG
GATATTCAATTGAGAGGCTCAACTAAAGCTTGATAACAGCAATGGCGAAGAAGTTTAGTAACATAATATT
CTATCTGACCCTGATAATCATAGCCATCTGTGTGGCCTGTATCTGGATGTTCTCGGGCTTCCGTCACGAA
TATGGCAAATTCAACATGATCCTGGTTGCCTTCTTGGCCGTTACTTTACTTCAGATCCTAATCTTTACGC
CCATTAAGTTCACAATTATGTCGCTGGATGCCGCCTTCTGGCCGGCCCACCAAGCACCGGAGACTCCCGA
CGAGAATGCCAATGTGGACACGTTCATGGACAATCTCAGACTGCGCCTCCGGACGCTGCGGAGTGAGCTG
ATGATCACGGAGAGGCATCGGAACGAGAGGGTCAACCTGAAGTATCGCCTGATTACCGAGGAACTTTGGC
TCACGGGCAAGCTGTTCCTGGTTTACTTCTTCATGGCCTTGGCTTTCTTCGATGAGTTGCTGTACTTTAA
CACAGAGGCCACGGAGTTACTGTTCCAATGTAATCATGGGGATGCGTTCGGACTATTGAGTGTGGGGGAT
GTGCCGGATATATATTTTTTTGTGGTGTCCTCGTTAGTGCTGGCCTTTACGGATGGCAAGAACACCAGTG
GCGGAGCTCCTTGGATCCATGCGGAGGGCACTAGACTACTGGGCGTGGTGAGACTGAGGCAGCTGAGGAC
AGAGAGCAACCGATTGGGTCTATCGCTTCCTGTGTTCACTGAGCGGGACTTTTCGGAGAGCTGGACACTG
CCCTACGAAAGGGTTCCCTATACGGACAAGTACTGGCCCATCTACACACCCTGGCTGCCCAGTGTTTCTG
TTGCTCGGGACAACCTACTTATGGGCATTAACCACGTGGGGCACATGTTCAACTATCCGGAATCGAAGGG
CTACAAGGTACTGCTCTCGGACACCCGCCACAAAAGCCTGAAAATTATTGATTACTTGATGAAGAAAAAC
TGGCTGGATGCGAATACGACCGCTTTGTTCATGGATTTCAGCTTGTACAACGCGGATGCCAACACCTTTA
CCGTGTGCACCTTGTGGGTGGAAAAGTTCCCTTACCAGTATCCTGATGGCCACACGAGGATCGAGAGTCA
CACCTTTGTGGAGCAACTGCGAGAGTTTACGAAGTTCGGAATGCTAATGGTGTTTGTCTTTGTCGTCACG
TGGCTGCAGTTCACCAAGGCGTTCTTCCTGAAGGTCTGGTACGATCCTCGACAGTTGAAGACCCTGTGGG
TCCAGGTGGATGCAATGATCGTGGCACTCAGCCTCGTTGTGGGGATGATCATGTCGGTGCGGGATAACTT
GGTTCAAAAGATGATCAAGAGTGTTGAAATCGCCGTTGTGGTGGACTTTCTGGACTTTCGGGAGCCAGCC
CAGCTCGCCTACCTTGAGGATGTGGTCACGGGATTGGCCGTGGCCTTGGTCACTATGCGTTTGTGGAAAG
TGTTGCAGTTTTCCGCCACCTTCCAACTGTTCACCAAGACAATCGCTATGGCGTGGGACGCGTTGCTCTG
CACCTTCGTAATAACCGTGATTTTCATCATTGCAATTGGCATTGCGACGGTAACCATCAATGGCAACTAT
ACCAGCAACTTCAGGGACTTCCCCAAGGGCATTGTTACGGTCACCTGCTTTGCCTTTGGCTATACGAATT
TGGTATACCCACCGGATATCTTTTACGGCGGAGAGTGGCTGGGCATCTTATTGTATACCATAATGGGATT
CGTGGTCAAGTACATGCTGATCAACTTGATCGTTTCCATGATGAGGGATCAGATGGCCAGTGTCAAGGCG
GATAGGGATAAGAAAGTGAGGCAGCGCATAACATTCTGGCAATTCCTGCGCGTTGAATATGCCCACTTTA
TTAACTACATCGTAAAAGTGTTCCACTGCCAAAAGGGATATCAGCGAAAGAATCGCACAGTTGCACAGAA
CATCCAACGAAAACTGAATAGTAATGAGCTTAAGCGACGAAAGACGAAGATTAGTTCAATTTATTCCGAG
ACAATATATGTCATGCCGATAGACAAGGATCTGGAGCAGATGAAATATAGGGAGCGGATCGAGCGAACCT
TCACTCTGGCCGCCATCCTCCACACCCAAATGGAGCTCATGGAGAGGCTTATGTTCGGTGACGAAGAGGG
GAACCTGCCAAGTTTGGACCAGGAAGACGAGCAGGTTCCAGAGACTGAGGAGGACTGAAGAACGACAGCT
TAACTGCAGTTTATACAGTGCAAGGGATCATAGGGATTCTAAGAGATTCATGTGAAATAGAACTTATGGT

AGTACCTTCATCAAGGTAAAATGATGATGTGTACATACGTGATGTTTGGTAACATTTTCTGCACAGTTGA
AACCAAATCTCGGCAAAAGTCCCTAATAGTTAAACACTACTTAGGATAGTTTTGCAGGATCCTTCCCCAT
CCGTGCAGTTTTGTGATTTTTTGTTTATGTCACGGCTAAATAATTATGCGCAAACAGGCAAAACTTTTGA
CTTGAAAAGGCTTTTAGGGCAATTTGGCGCTTGCTTTGTTCGGCCTGTTTGCCATCCGAGGATTCTGGCT
GAGCTCCAAGTGCCCGAACCAAGTGACTTATAAAAGGGGGAGTGGCACAGCCCTCACTATTGTTATGAAG
TAAGCAAATAAAAATTTAATACTGCATCACGCTGCCACCACCATCATTTTTGACATACATTTGCAAATTT
ATTTCGTCAAGTGATCACATGTTGATAATTTAGATGAGGACCTGCGCAGCAGCAGAGGCGCAGCTGATTG
GAGCGGAGTTGGCCAAACGCGAAGGGTAGGCAAAAGGAAGCAATCTCAGAAGCAGCATCGATACCATTGC
TGTTCCTGGCGATAACCTGGCTACTGGTGGCGGTCACAACTGGAGCGGGAGCAGCTACCACGGCAGCagg
agcagagtaggccagaggagcagagtaagccagaggaccagtgtacgccagGGCAGCACCCAGGACTCCA
GGCTTGGCAGCAGCGCAGGCGACGGTTATCAGGACAAGGACGGCCTGTTAACAAATCAGCTTGATTAGTT
TAATCATCTGGGTCCTTTCTGATGAACGGTACGTATTGGAACATGCTGGAATATTTCTTAGCTTTGCTTC
TTCGGAAGGCTAGAAACTCTTGATATCCTACTGCTCTCCGTGGAGCAAAGTTATACCCGAATTTGTGGTC
AGCTCTGACCCATGATCGCACGCATTCAGTTCATTTAAACAGTGACAGTCTCCCGTTACCATTTATCATT
TACCATTTTCACTTTGCCGTAGTTTATGTGATTTCCCACTGCTGACTGCAGAATGTGGAATAACTCTTGG
TGGCCACGGAATGCGATGGCCTGAGGTCACAGGCGCAGTCCCGATGCGGCGCATAAATAGTGGAATGGAA
TGTGTTGGGCGACATCAGGTTCAAGTTTTCGAACCGAAAGCACCAGATCCATAATCCAAATGTTTAAATC
CGTAAGTTTGAGATCTTAGCTTGCTGCCCTATATACTAATTGTATTTTATTCACAGGACGTCCTTGTCTT
GGCCATCGTCTCCTGCTCTGCTGCCAAGCCTGGACTGATTGGTGCGCCTTTGGCCTACACTGCTTACGTG
GCTTACTCTCCTCTGGCTGTACTAATGGCCTACCCCTCGCCTTTGGCCTACTCCGCACCACTGAGCTACG
CCGTTCCATCTGCACCGCTTCTTATCTAAATTACTAAGCTGTGATCACCTCGGAGGCGACGATAATAAAT
GGAGATATAATAAGACGTTATTCAATTGTATATTCTTATTTAAGCACGCTTCAAATTAATATTCATTTAT
TTCAATGAAAAATTAGCTCTGAACGTACACGAAAATGATTGCTAGAAAATTTCCCAGCATTGATTAACAA
TTATTCCAATTATTCTTATTCTTATATTATAATTTTAACGACTGATGCATTTCTCAATCACAGCCATTTC
TAAATCAATCGTCAAATCTAAACTAACTGCAAATTTTAAAAGTATTGTAGTGATTTAGAAAATATAGGTC
TTTCGCTAAATGATTACCGAAATactcgtgttgcgtctacgtttctggaatctccctgctgcattttaac
tttttagcttttatagctttcgagatcacagcattcataggaacagtcggatacatagtcgtgaaaacag
tgattcttattaaaagtatatatacaatacgggctcgaaaactttagcttccttttacccattacgtact
tttcaacgaatttagtatacatttttactctactagtTTGATATGCAAACTACAACATTTTACTAAAGTT
TGGGCATTTAAGAAGTTACTAGAATATTACTTGTATATATCAGAAATTTCGGGCTTTCAAAATCCCCAGA
TCTTAGACGGTCAAAGATCCCAATGAATTTCTTTTTTGTAAGAAAGCACTTCCAATACAGCCTTTTATTG
TAAAACTGTTTTTTTAATTTGATATACAGATaaaaatgtgggcgtggcagttttgggctgttttagggcg
ttagagtgggcgtggtaaaatgctcttttgcaaatcgatagcaattaacttgactaatggaaatataaaa
atatataaaaacatttttaataagtgggagtggcagttttgggcgtcttgtgggcgttgcaacatgggtc
aaaaaacttgcactgcgtctaagtctctagaaactgtattaatcttaatcttctagcatacggacatggt
cagatccattcggctactgatcctgatcaggaatttatatattttatatggtcataaacgctttcttcag
ggaactcaactatagcattctctcttgctttcatagttcccgagaattcgacgttcatccggccgaacat
gtgttacgtacttccaaacgaatctagtataccctgttattctacgagtatcgttttttttttGCAAGT
CCAAAAATCTAATACTTCAGCAATTTATGGGGTCATATAATCGATATAAGCGTTCATTTTTATTATATTT
ATCGTCCTAGATAATCACAGCATAAAAATTTAGATGAGAAATGGTGCAGGAGCAGCAGCGTAGCTCAGTG
GTGCGGAGTAAGCCAAAGGCGAGGAGTAGGCCAGTCGTgcagccagaggagtagccgcgtacttcgccac
cacaggagcagccagaggagtagccgcgtacttcgccaccacaggagcagccagaggagcagcaacggga
gcaatcacaggagcagAGGCGATTCCATTGTAATTCCTGGCGATAACCTGGCTACTGGTTGCGGTCACAA
CTGGAGCGGGAGCAGCTACCACGGCAGCaggagcagagtaagccagaggagcagtgtaagcaaggggagc

aCCCAGGAGTCCAGGCTTGGCAGCAGCGCAGGCAACGAGAGCGAGAACGACGACAGCCTGTGAACAAATA
AACTTGATTAGTTTAATCCTTTGGGTGTCCTTTCTGAAGAACGTACGTATTTGAACATGGTGAAATATTT
GTTTGGTTTGCTTCTTCGGAAGGCTAGAAACTCTTTGATATCCCACTGCTCTTCGTGGAGCATATTTATA
CCCGAATGTTTGCTCAGATCTGACTCATGATCGCACTCATTCAGTTCATTTAAATGCGGTGACAGTCTCC
CGTTACCATTTACCATTTGCACTGTGCCGTAGTGCCGTTGTTATTCTGGCTATCGTTGCCTGCGCTGCTG
CCAAGCCTGGACTTCTGGGtgctccccttgcttacactgctcctctggcttactctgctcctctggctta
ctcagctcctGCTGCCGTGGTAGCTGCTCCCGCTCCAGTTGTGACCGCCACCAGTAGCCAGGTTATCGCC
AGGAACTACAATGGAATCGCCGCTGCTCCTGTGATTGCTCCCGTTgccgctcctgtggtggccaagtacg
ctgctgctccattggccgctcctgtggtggccaagtacgctgctgctccattggctgctcctgttgtggc
caagtacgctgctgctccattggccCACATTTCCTCCTTGGCTTACACCTCGCCTTTGGCATACAGTGCC
TTGCCATCAGCTCCTGTTCTTCTTTAGAAAACTTTAAAAGTAATaaaaaaaaTCAGCTGTCGTCGTTCTC
GCTCTCGTTGCCTGCGCTGCTGCCAAGCCTGGACTCCTGGGtgctccccttgcttacactgctcctctgg
cttactctgctcctGCTGCCGTGGTAGCTGCTCCCGCTCCAGTTGTGACCGCAACCAGTAGCCAGGTCAT
CGCCAGGAATTACAATGGAATCGCCgctgctcctgtgattgctcccgttgctgctcctctggctgctcct
gtggtggcgaagtacgcggctactcctctggctgctcctgtggtggcgaagtacgcggctactcctctgg
ctgcACGACTGGCCTACTCCTCGCCTTTGGCTTACTCCGCACCACTGAGCTACGCTGCTGCTCCTGCACC
ATTTCTCATCTAAATTTTTATGCTGTGATTATCTAGGACGATAAATATAATAAAAAGGAACGCTTATATC
GATTTTATGACCTCACAGCCGTTGTCATTTGCGCCCTGATCGCCTGTGTGGCTGCCAAGCCAGGATTGCT
GCACACTCCTCTGGCAGCTCTGCCCGCTCCGGTGATTGCTGCTCCCGCTCCCGTGGTCACTGCCGCCAGT
AGCCAAGTCGTGGCCAGGACCTTCAACGGCATCGCCGCTGCTCCAGTGATTGCCCAAGTTCCGGTGGCTC
CTGCTCCAGTTTTGCGGACCGtagcagctcccctagcagctcccctagcagctcccctagcagctcccct
GCGCGCTCCAGCTCCCGTTGCCTTCGCCGCTCCAGTCGCTGCTCCCCTGGCCGCTCCAATCCTGAACAGA
GTGGCTCCATTTGCTGCTCCCATTGCCACCCCCTTCGCTGCTCCGTACGCCCATCCCTATGCTGCGCCCG
TTTTGGCCAAATACGCTGCTCCACTGGCTTATGCCCCAGCACCTTTGAACTATGCCGCGCCCGCTTTGTG
GTAAAGGAAAATCGCATACCCCGGGATTAAGCAACCGTTTGTGTGCTCAGTGACTGATAGCCCTTAATAA
AACTCTTTAAAATTGGTaTATGCACAAttttttttttGTTAAAAGTGCTATTAATTTTATGCTTTAACTG
GATGTAAATGGACCGTTAAAGCCCAGCTCTGCTTGCTGATGCCTGCGTGTATCGAAAACCAAAAACCAGC
CGCAGAGCAACGAACCCGCGACCGCCGAAACATTTCAAAGACAATCCACCAACAGCGGCAACACCGTCAA
CAGTCGGCCAcaccaccatccattcaccgtcaccatcaccatcaccgtcgccaACAGGACGAGGGCGAGG
ACAATGGATCAACTCAAACTCAGACCTTGTAACTGCCTGACCGCACATCCACTGGCAGAGTGGGTGTGGG
TGGAATGTGAGCGGCTGGGTAAGTGGGTGGCCAAAGAAGTGAGGGCCAGTTGATGGCCACATGTCACAGA
CATTATGGAATCATGAACTTATGCGCACCTGTGCGTGCTTGCGGGGGCGCGGGCGGTGGGCCCAGAGCTT
CATTCTGGTTATATCCGTGTGGACTTGCGGTCGAGGACAGCGATTTGAGGTCCGCCTGGTGATGGTTTCG
TGACTCGGCAAGGATTTCATCGGTTTAATGTGGAGCACTTAGAAGGAGAAACATGGCAACGTATCAGAAG
TATTATTAACTTGAAATTATGTGATATATTATTAGTTCTATATTTTTCTATAACTTCCTTCAAGCCATAA
CATTCGAGCTTCTCACGCAACCATCGCCAATTAGGAGCCGGGCCATTTCAATCATCAATCAAAGTGTTTT
GTTACGACTTTCATACGTCATGTACATGGCTCGGAGCCACCGCTTTTTGCTTTTGCAACCCATAGGGGGC
TCCTCGCTGTAGCTCCTCCATATCCTGCCTCAGTCTGGGGAGCCAGTGATGCGGAGGTGTGACTGCGACA
CAACGAACGACATTTATGGTGTCGTCAAGAAGAATTCCCATTACAGCCACAGACAATCAATCACTGTGCA
CCGAAATCAATTGTCGCAGCTCCTATTTTCGTTTTCAATCTCATTTCGCTAAATATTTATGAGGCCAACA
CTTGGGCCGTTCGATTTGTAATCCTAATGAGGCGTAATTAGAAAACATTCAATTACATTTCATTGGCATC
AATCAATGGCGCCCCATCCATGGCTATTTCAAGGATGACAACTTTTAGTAAACCGAGTTTCAATTTCCCA
ACGCATTGTTAAGATGCCTCTGGTGCTTTAATCCACGTTCCCAGCTACTGTTTCTGTTTGTTGCTCCTGA
TTGCTGCTTAAACTTTTCCCTCGCTCGGACGACTTGTTTGTGATTAAGCTTTATTGTTAAGAGGCTTAGT

ACGCCTTTTGATTTATAGATATTGGATCATACCACTCACTCTGCTCCGCCATCAAAGGAGACTGTGGTCC
TTTGAGAAACCGGCAATGTGAAGTTGGCCGGCGCGCGTAGCTTCATAGAAAAACTTCCAGAAGGAGTGAG
GTATTCGTAGGAAAAGTCTGTACACAGAAAGGATAGGATATATGCCTAGTACTTAAGTTGTAAGCCGCTA
TTGTTGTCCTATAACTTAAAAAATTTGTTGTCCATTAAGATACATTTTTGGTTTGATTTCCTGGTTTAGA
ATAACAAAAGAATATTTCTTATAGATACACTGATAAGACCTTAAGATTTAAATTTTCTTTCATTTTTTTC
AAGCTTCAGCAAACTGGTTTGCATTACCCTTTATCTGTTATTTAACGTATTGTTCATGCACCTTTAGGTT
GATAAACCTTTTTTATCTGTACTTTCCGCAGTGCATTGGGAATTCGCTGATCCTTTCTATGTATGTCCAT
TTAGCGCTTCTCACTTGTGCCTTTGTTGCATACTCTTAGACGCGCGGCTGAACTTGTTTACAGGCACACT
AAGGACGAAGGACCCAGGATCCTGTACATTGGCCCAAAAGTGGACGCTGGCTATTCGGCATCCTTGACAG
CCGCACAATTTGCTGCCGCGTTTTTGTATCTCTCAGTGCTTGTTTGTCTGCACCACAGCGCCTGGGCTCT
GTACACAGAAAGAAACATTCGAGTCGCAAGCAGGATATTGATATTAGAGCTGAATGCCCTATTAATTCTG
TATGCAACCCATGTATAATATTTATTTAGCTTTGTTCCCGGGAAATTTGTATTCCCTGATACCCTAGATT
TGCCCGAAAATAGGTATTTTTCTCTCTGTGCATGTTTGTGTTTGTGTGCAGATGGCCAGCAAAAAGTTTG
CGCACTGATTGCAAACTTGTTTCAATTGTTTCGTAAACCCAACCGAAGGGAAGAACCCAGAAGACTCCCG
GTGGGTGGATGAGCCTGGTGGGCTGGGTTTTTGTGGACCGCTTTTTCCGCCATGAAAGTCAAGGCGAACG
CTACGAGTTCCCAGCTACGAGCTCCGAGACCAAGGCAATTCGCCTGCATATTTGTGTGGCTTAGTTTCCG
TTTCACAAACGAAACGAAATGAAACGGGCGGCATAAAAACCCATTGAAAATTTGCATACGAAAATGAAAT
TCAAATAGGTTTCCCATGTCCTTCGCAATCAGTTCCGGCTAGTCCAATTGCACAAAATAGTGAACGAAAA
CTTTCGCTTTTCCTCCGCCGTCTAAGCCCTTTATTTATGATCCACAATAATGCAAAAAGATACATATCTG
AATATCTCATCTTTGGGGTTTATCTCCTGCGCCAGTTGCCAGTTTTAATCTAAATCGTTTGGCTGACTTT
GTTTATCGATGCTTTCTTTGGACGCTTCTACCTCGGATTTGCGATTCGAACACTAAGTGATTTTTCATTG
ACTTTGTAATGCAAATTGCCTGTCTGTCCTGCCCCTTCCTTACCATGCTTCTCTTTGCGATTGGCTGGAT
CTTTGGGCCTTTTATCACTGGAGGCCTGTGCTACGGACCCTTCAACAGCTCGAAAGCCAATAAAATGCAA
ATTTTTTCATCGCGTAAACAAATGCTAGACAAAGATGCCTAGAGATGGGGACTCTACGTGTCTGAAAGCT
GTGAAATAAACCTGTGATTTATGCGGATATCGTTGTGGGGACCTAAAGCCCCTTTAAATCTTTCGCGAAA
TATACGTAAAATAACTTTAATGTGGTTTCATTCTTTGCTGTTCAAATTCAATAATCGTACTGAAACAAAA
CCCATATAATGAAATTCATATAGTCCTAACCGTATTAACTTATACCAGTGTCCTATTTGATTGCTTGGTC
ATCCTGACTGCTCACGATTCCATCTCCAGTGGCCGATGAAGTCCAAAACTGAAAACCCAAACTCATTTCC
ATCGGCATCAGACTTGACTTGAGCCGGGGCCCTCGACTCGCAGTCATTGATTTAATTGTAAGCTGTTGAA
TTTTGATGGCTTTTAATTGTCTTAAGACCGGTTCAGCAAAAACCAATCAGGGATCCTTTGATAAGACGtg
gtggttggatggttgcatggatggatggatggatggacaatgggttgggttCTGTTGTCAGCAGAATAGT
TGGAAATTCCACTTAGTGCTGACTACTGACCCACTTATTGGTCACTTCTGGGTCTGTGTTTCCTTCCGAA
CCCCCATCCATCTATCCCCACTCCTTGCAAAGTCAACCACTTGACTAAATAGCACTCAAATTTGATTTTG
ATTTATGCTGCCCAGCAGTCCGCACCGAATTTCCTCCTCTTTCCTCAAGCTCCAACTTTCCCTCCCTCCG
ACTTTTTCCCCCTCCCCcacaccacacacacacaTAAGTACAATTGCTGGCTCATAACTTTGGCATCTAT
CGCAGTATGCCTTTCATATTTACATGCTCCTTACACGCGCATCGGCGTGTAACGGAAACGGAGTGAAACT
AACACTCAAAGAAATATCTATATCTGCTATATTTGAATATCTTGATTAATACCATTGATATCCCAAGCTC
AAATCAGTAAGTCGAAAACCATACAATACGACGAATATAATTGGCTTATTGAAAGAAATACTCTATATAT
AATGAATCTATTTTTTCAGTTGCACCCTTCGTCTTGCCAGTTTTCGAAAAGGGTTCTCCGAAAAGGGTTG
CCACCGCACCCCGATGCCCATTGAGGACTCATGGAGCAAACGGTTTCGGCATTTCCCTTTTGTTTCCGCC
TGCATCCTTTGGCCAAGACACGGACACCTTCCCCTTCCCACCACGCTGCCACAATGGAAAATACAATTTT
CGCAATCTTTGCTTATCTTTTCATCTCTCTGTGGCTGCCTCGTTTGCTGCACGATTCGTTGTTGCTGCTG
TTGGCCATCCAGAAAATCGGTAAAAAGCTAAGATCCAGGGGGCGTGGCTGATACGAAGGCGGAGGAGCAG
AAGAATGTGTTGGCCGTGCATCCTTTGTTTGTCGGCAGTGCCGGTAATGCCGAGTTGGAGCAGTAAGCTC

TAAGCATCGGTTATGGGCGAGGTTCGTTTGAAAAATAACAACCGTTTCCCATCTGGATTCTTTTTTATGG
TGGCTGAGTCCTTAATATTTATTGAAAAAGGCGACATTCCATTTTATTTCCAAGTAAGAAAGATAATCAA
GTGCACTTTTCATGACTTACTATCTTTATTATACTATCATAAAACTTATAGCATACTATTACTAATACAC
CATCATTTTTGACATACATTTGTAAATTTATTTCGTCAAGTGATCACATGTTGATAATTTAGATGAGGAC
CTGCGCAGCAGCAGAGGCGCAGCTGATTGGAGCGGAGTTGGCCAAACGCGAAGGGTAGGCAAAAGGAAGC
AATCTCAGAAGCAGCATCGATACCATTGCTGTTCCTGGCGATAACCTGGCTACTGGTGGCGGTCACAACT
GGAGCGGGAGCAGCTACCACGGCAGCaggagcagagtaggccagaggagcagagtaagccagaggaccag
tgtacgccagGGCAGCACACAGGACTCCAGGCTTGGCAGCAGCGCAGGCGACGGTTATCAGGACAAGGAC
GGCCTGTAAACAAATCAGCTTGATTAGTTTAATCCTCTATGGATCCTTTCTGAAGAACGGTACGTATTTG
AACATGGTGAAATATTTGTTTGGTTTGCTTCTTCGGAAGCCTAGAAACTCTTGATATCCCACTGCTCTTC
GTGGAGCATATTTATACCCGAATGTTTGCTCAGATCTGACTCATGATCGCACTCATTCAGTTCATTTAAA
TGCGGTGACAGTCTCCCGTTACCATTTACCATCTACCATTTTCACTTTGCCGTAGTTTATGTGATTTCCC
ACTGCTGACTGCAGAATGTGGAATAACTCTTGGTGGCCACGGAATGCGATGGCCTGAGATCACAGGCGCA
GTCCCGATGCGGCGCATAAATAGTGGTATGGAATGTGTTGGGCGACATCAGGTTCAAGTTTTCGAACCGA
AAGCACCAGATCCATAATCCAAATGTTTAAATCCG
>dme_circ_0005164_junction_seq
AGATCCATAATCCAAATGTTTAAATCCGGACGTCCTTGTCTTGGCCTGAACTGCTT
>dme_circ_0005165
TTTATGTGATTTCCCACTGCTGACTGCAGAATGTGGAATAACTCTTGGTGGCCACGGAATGCGATGGCCT
GAGGTCACAGACGCAGTCCCGATGCGGCGTATAAATAGTGGTATGGAATGTGTTGGACGACATCAGACAA
GTTCAAGTTTTCGAACCGTAAGCACCAGATCCATAATCATAATGTTCAAATCCGTAAGTTTGGAATCTTA
GCTTCCTGCCATATTTACTCATCGCATTTTATTGACAGGCCGTTGTTATTCTGGCTATCGTTGCCTGCGC
TGCTGCCAAGCCTGGACTTCTGGGtgctccccttgcttacactgctcctctggcttactctgctcctctg
gcttactcagctcctGCTGCCGTGGTAGCTGCTCCCGCTCCAGTTGTGACCGCCACCAGTAGCCAGGTTA
TCGCCAGGAACTACAATGGAATCGCCGCTGCTCCTGTGATTGCTCCCGTTgccgctcctgtggtggccaa
gtacgctgctgctccattggccgctcctgtggtggccaagtacgctgctgctccattggctgctcctgtt
gtggccaagtacgctgctgctccattggccCACATTTCCTCCTTGGCTTACACCTCGCCTTTGGCATACA
GTGCCTTGCCATCAGCTCCTGTTCTTCTTTAGAAAACTTTAAAAGTAATaaaaaaaaTCAAACGAACTCC
AATTTCTCCCATTTTTTGGGGCTTCGCTAATTGGATTTACCGCTGGCATACTAAAGCGTCGAAATCGACA
ACCTACTGATGGGGGGCATTTGAAAATTTCGAACGCAGGTCACGTAATGGTCATAAAAATTATTGTCAGC
TCAGCGGCAGTTGCTGCTGTCCAATGTTTGTTACATTAAATTGTGCGAAATTGATTTGTATTATAGGTTT
ATTTTATTTTTCGGCTTTGAGTACAGACCTTGGCAATTATGTCAAGGTCAAAGCTGACCTGTGACCGATA
TGAGCCACAGATTTGGCACAATCAACCTGTCTTAGAACCGATGGCCCAATTTGCGTTTGGTTGCCTATTT
ACAGAATATCCGTGTCTTTAAAAAGGTTTTTATTGCGATTGCGGaatatatgttttaaatatatgttaaa
tatatgttaTTGCCCCagagtaaaatcggtatatgtactatattcaaggaaaagtgggtgaaaggtagaa
ggaatagtttccgaccCCAGATTTGTAGCGCTTGAGGCAGCTAAAGTTGGTGACAGTGCCTTCTAGGGGG
ATAGAAGGTTTTGGGCCGCCATCTGTGTCTGGTTTCTAAAACTATAATCGGAACACAAGAAGAATTCTTC
GGGTCAGGCATTTTGGACTGAAAGAGGCAACAGAGCTGGTTGTCAATTTCAGGAGAATAGCGTGGGCACC
TGTAGAGAGGTGGTTCGACCAGGATCCGAGTGCCAAGATGGGTCTTTTCCATTTGCGGCGGTCCAAGAAA
ACAGTTGTGGCAACAATGTATGTGGCTAGGAAAAATATTGGACGGCGCGTATGGAATTCGGAAGAACGGA
GAAAGTTCAACTTCTTGTCTGGCGCAGAATGAGAAGAGGGTATACGAGTGTAAAGAAAGTGAAGGGATTT
AAACATTATGCAGAATCGGCGCTTGATATTGTATAACTAATTGGAAGCGGATGCAGTTAAAGTTTCATTG
GATTTGGAAGTCGTAGTATTTCTCCACCCTAAATAAGGGGACACTGAGGCCAGAGCCTCATACGTGGGGT
TCGAAATTCCAAGTCGATCGAATTCAGCAGCTAGCAAGAAATAATTAAAAGCGTCAAAGTAAGGTTCGCG

ACCAAATTGGTCTCTAACTTATTTTCTATCAATCAATAATCTATCATTTATGTTAAGTAAAAACTCGAGA
TAGCTGTGTGAAATTTGATTAGTATTTAAATATATTTTTATTTTTTGAAGTCTCTAATTGTTTGCCTCTG
ACTAGGAATGAAACTAGGCTGAGGATCCGGGACATGACCACCGCACATAACCGATTTCGAACATTTTCAC
CCGAGGTTTACGAAATCGTTACACAATTGAAAAatatttctgatcaggatcaccagtcgagttgatttgt
ccatgttcgccACTTGAAAGTCACCAGAATGTATATTGGTCTTCGGACAGTTGGGatggacggcgggatc
ttgggaaccatttaagctacacagtttggattgggcattcagattttagaggaggaggcgcagcgcaagg
taatttgaagattaaacgcccactctcacATTGTTTACACGCTGTGTGTCCTTTAAATTCGGTGATATCG
AAAGCGATTTGAGCTTAAAAGTTAAAATTCAGCAGGGAGATTCTAGAAACGTAGAAGCAACTCGAGTATT
TCGGTAATCATTTAGAAAAACACCAATATTTTCTGAATCACTGCAATAATTTAAAATTTTGCAGTTAATT
TAGACACAACAGATAATAGGGCTGTGATTAAGAAATGTATCAGGTGGCGCTAGAGTGCATTCTCGGAATA
ATTGTTCATAAATGCTGGGAAATTATCGAGAAATCATCTTCGTGTACGTTCAGACTCTGAACTTCAGAGC
CAATTTTGTATTGAAATATATATTAATTTGAAGCGTGCTTAAAAAAGAATAAACAATAGAATAACGTCTT
ATTATATCTCCATTTATTATCGTTGTCTCCGATGTGATCACAGATTAGTAATTTAGATAAGGAGCGGTGC
AGATGGAACGGCGTAGCTCAGTGGTGCGGAGTAAGCCAAAGGCGAGGAGTAGGCCAGTGGTgcagctaga
ggagtagccgcgtacttcgccaccacaggagcagccaatggagcagcagcgtacttggccaccacaggag
cGGCAACGGGAGCAATCACAGGAGCAGCGGCGATTCCATTGTAGTTCCTGGCGATAACCTGGCTACTGGT
GGCGGTCACAACTGGAGCGGGAGCAGCTACCACGGCAGCaggagctgagtaagccagaggagcagagtaa
gccagaggagcagtgtaagcaaggggagcaCCCAGAAGTCCAGGCTTGGCAGCAGCGCAGGCAACGATAG
CCAGAATAACAACGGCCTGTCAATAAAATGCGATGAGTAAATATGGCAGGAAGCTAAGATTCCAAACTTA
CGGATTTGAACATTATGATTATGGATCTGGTGCTTACGGTTCGAAAACTTGAACTTGTCTGATGTCGTCC
AACACATTCCATACCACTATTTATACGCCGCATCGGGACTGCGTCTGTGACCTCAGGCCATCGCATTCCG
TGGCCACCAAGAGTTATTCCACATTCTGCAGTCAGCAGTGGGAAATCACATAAACTACGGCACAGTGCAA
ATGGTAAATGGTAACGGGAGACTGTCACCGCATTTAAATGAACTGAATGAGTGCGATCATGAGTCAGATC
TGAGCAAACATTCGGGTATAAATATGCTCCACGAAGAGCAGTGGGATATCAAAGAGTTTCTAGCCTTCCG
AAGAAGCAAACCAAACAAATATTTCACCATGTTCAAATACGTACGTTCTTCAGAAAGGACACCCAAAGGA
TTAAACTAATCAAGTTAATTTGTTCACAGGCTGTCGTCGTTCTCGCTCTCGTTGCCTGCGCTGCTGCCAA
GCCTGGACTCCTGGGtgctccccttgcttacactgctcctctggcttactctgctcctGCTGCCGTGGTA
GCTGCTCCCGCTCCAGTTGTGACCGCAACCAGTAGCCAGGTCATCGCCAGGAATTACAATGGAATCGCCg
ctgctcctgtgattgctcccgttgctgctcctctggctgctcctgtggtggcgaagtacgcggctactcc
tctggctgctcctgtggtggcgaagtacgcggctactcctctggctgcACGACTGGCCTACTCCTCGCCT
TTGGCTTACTCCGCACCACTGAGCTACGCTGCTGCTCCTGCACCATTTCTCATCTAAATTTTTATGCTGT
GATTATCTAGGACGATAAATATAATAAAAAGGAACGCTTATATCGATTTTATGACCTCACAAAATTGCTG
AAGTACTAGATCTTTGGCTTTGCAAAAAACCCGATACTTGTAGAATAAGTCTgttttgagcggtttgtgg
gcgtggccaatacatggaccaacaaacttgcttttatagttcgtgagatctcgacgttaatacaaacaga
tggacatggctagatcgactcatctattgatcctgaATGCCTGTTACATACTTTTCAACGAGTAACGGGT
ATAATTATATTATCACTTGTCTTACAAATATATATCGAAACGTTAAAAAACGTCCAGAGTCCAAGCAAAT
CTTTTTTAGAAAATGGCTTAGATTCGTTTTAATTTATTAGCTTGAGCTGTGCTGGACTATAGAATTTTCT
TTTGGTATATCCAGGAGTggtagaagaaagcgattttgaatttataaggtacaaatattcttgatcagga
tccctagccgatacggtccagccataatttctgtccgGTAGGTCTGTCCTTTCGTATAAACATCTCGAGt
ttttcgcacgggtcccacggccacaccgatataaaaaagagaacaggaacgaggaaataaataaaatgta
aaataactatattaaatattagtgttagtaggatctaattatgtaatataaaagttaaaatcgattgctt
taagagcggcagagagtcaagattacagataataggataaagtctattatagtcagaacataaaactctg
taagggtcatgcaactcatagttagatctacaatgatttaagataaggggtatatagtttctagtgaatc
tacttggaaccgagcagttaagccgactaatcaagtcgggactctcaacatccccacgaataagcttaca

aataaagactgttccaagcatagttctacggttattaattaaaagtagtctactagaataaggagttcat
atacggtttgcatcccaatttaggcgccgcaaggcaaaaagtaagaattttttttggaccgattcaatgc
gatccgagtggactgcgtattgtggactccaaacaggagtgatccccactcgagaatcggacggattaac
gaaataaataaggttttagtcaagtaaggatcatcaaattccttagaccaccttttttataaaaccaagca
cacccctggtcttattgacaatagtcgaaatatggtcagaaaattttattttgggtccagaagaacacca
agatcatcaactcgtgttattctgtccaaggagcacccacttagagtgtatgtcatgcgtattgggttgg
cacgacaaaaggtcataactttacacttggagccatttacgtctaacacgtcatcacgacaccatatttg
aaatcgatctagatcggattttaagtccaaatggcgcgaattgtccttgtgatggaggcattatttaaaa
tcgtctgtgtacataagtacacgcgaatgttttattattaaggggaggtcgttaatgaataaggtacaaa
gaagcggaccaagatggctccctttgggacaccggatgtgactcggagattacaagataaagaattttta
aagaggacttgtagcgaccggccattcagatagcttgaaatccagtttagaagatcaacagggaaaccta
aaagatcaagttttcttattagaaggtgatgattaacagagtcaaaagctttactaaagtcagggtagat
gatatcggtttgaagattatttttgaaaccctgtattacgaaagaagttagctccaagaggttagtggtt
gctgatctgcgtttcataaaaccgtgctgacgcgatggtatgattgatctacaatacaaaagtgctgcaa
atgaggagtgataacattttcaaacagtcttgggatagccgacaatttagagattcctctgtaattgctt
gcattcagtttgctacctttttttatgaagaggaatcacaaaggactccttccatatatgggggaattgtg
aagattccagagataaggtaaacagttttagtagaggcttgcacaaggcttccgcacagaatctgagcgg
aacacagccagggattccgtcgggatctggcgaatagacaggttcaacacgctgaagatcgttaagcagt
gagctttcgtttatagtggggcaaaatattagattcgacttagataccgcataagagtatggctgttcgg
aatgaggtgaagttgaatatgttgtttgaaaaaacttggcgaatagatcggctattgcctgattcgatgt
taccgtagtgttttcaaaaaacagcgaggaggatttagattttgtgtgggagagcgaaagagagagagag
agagagagtgagagcgaaaagtgcagtttatgtgtatgcaaaaaacaacaccaaatagcactgcgaacaa
cgcacaaaagagagagtaaacaaaaaacaaagacaaaaaatactttgtatattatttgtttaaactactg
cacaaatcacaaaagaatcactcgcgaagcgaacggatctttaataattatgtttaaaattaatgataat
tacagatgaccacggctggtttggcaaacacaaatataataatAAATTTGAAAGCCAAATGAAACTTGAA
ACTTAATTAATATAGAaagattaagattaagcataattattccaatgtctttgttggacgcaacacaaag
tgccaagaccactctaacttccacaaatcgctcaaaactgctacggtcacactgtggaaaaatgatttga
ttttttttatatccgtactcgtagagtaaaaggctatactatattcgtttaaaagtatgtagcaagcaga
aggaatcaattcctgatcaGGATCAATAGATTAGgagattaagcatacagtttctatagacgcagtgcaa
gttttttttacccatgttgcaacgcccacaagccgcccaaaactgccactcccacttattaaaaatgtttt
tatatattttcacatttgcattagtcaagttaattgctatcgatttgcaaaaaagcattttaccacgccc
actctaacgccctaaaacagcccaaaactgccacgcccacatttttatctgtatatcaaattctttcgcc
tgcaaaacgacaaaacggtcacgcccacacttttaacaattttaaatttttttctcattttattcccaaat
atctatagatattccagaaaaatgaataatttttttcgttcgcattcacactagctgagtaacgggtatct
gatagtcggggaactcgtttatagcattctctctttttATTTTTTCTACCCAGAACACGCCCCTTGTAAA
GCCCACAATCCGTCAATAAACTGATTCTCTCACACATTTGATAAATGTGTATGATAGATGTtgaaatttc
tcgttcacagttcgactagctaagtaacgggtatctgatagtaggggaactgtactatgacgttctctct
tttttttAGTTTATATGCGTTCGGTTTTAATAAAACATTAGTTTTACAATAAAAGGCCTTATTGGATTCT
ATAAAAATTATGTAAAACGTTGAATAAAGTGCTTTCGCACAAAAAGAAATTAAGTGGAATCTTTGACCGT
CTAAGATCTGGGGATCTTAAAAGCTCGGAAGTTCGGATTTTTTGTAGTTAGATTCAACATATTATAGGGC
TGTGATTAAGAAATGTTTCAGTCGTTGGGTGGCGCTAGTGTGCATTCTCGGAATAATTGTTCATAAATGC
TGGGAAATTATCGAGGAATCATCTTCGTGCGTTTTTAAAGCGTATTTAAAAAATATACAATTGAATAACG
TCTTATTATATCTCCATTTATTATCGTTGTCTCCGATGTGATCACAGCTTAGTAATTTAGATAAGGAGCG
GTCCTGAAGCAGCGGCGTAGCTCAGTGGTGCGGAGTAAGCCAAAGGAGAGGAGTAGGCCAGTGGTGCAGC

CAGAGGAGTAGCCGCGTACTTCGCCAccacaggagcagccagaggagcagcaacgggagcaatcacagga
gcagcGGCGATTCCATTGTAATTCCTGGCGATGACCTGGCTACTGGTTGCGGTCACAACTGGAGCGGGAG
CAGCTACCACGGCAGCaggagcagagtaagccagaggagcagtgtaagcaaggggagcaCCCAGGAGTCC
AGGCTTGGCAGCAGCGCAGGCAACGAGAGCGAGAACGACGACAGCCTGTGAACAAATTAACTTGATTAGT
TTAATCCTTTGGGTGTCCTTTCTGAAGAACGTACGTATTTGAACATGGTGAAATATTTGTTTGGTTTGCT
TCTTCGGAAGGCTAGAAACTCTTTGATATCCCACTGCTCTTCGTGGAGCATATTTATACCCGAATGTTTG
CTCAGATCTGACTCATGATCGCACGCATTTAAATGCGGTGACAGTCTCCCGttaccatttaccatttacc
atttaccatttGCACTTTGCCGTAGTTTATGTGATTTCCCACTGCTGACTGCAGAATGTGGAATAACTCT
TAGTGGCCACGGAATGCGATGGCCTGAGGTCACAGACGCAGTCCCGATGCGGCGTATAAATAGTGGTATG
GAATGTGTTGGACGACATCAGACAAGTTCAAGTTTTCGAACCGTAAGCACCAGATCCATAATCATAATGT
TCAAATCCGTAAGTTTGGAATCTTAGCTTCCTGCCATATTTACTCATCGCATTTTATTGACAGGCCGTTG
TTATTCTGGCTATCGTTGCCTGCGCTGCTGCCAAGCCTGGACTTCTGGGtgctccccttgcttacactgc
tcctctggcttactctgctcctctggcttactcagctcctGCTGCCGTGGTAGCTGCTCCCGCTCCAGTT
GTGACCGCCACCAGTAGCCAGGTTATCGCCAGGAACTACAATGGAATCGCCGTTGCTCCTGTGATTGCTC
CCGTTGCCGCTCCTGTGGTGGCCAAGTACACTGCTGctccttttgcctacgcttctcctttggcctactc
cgctcctctggcttacacttctccattggcttaTAAAACTCTTCCGGCTGCTGCTCCAGTTCTTCTGTAA
GAACGTCGACTGATCAATAAAAATACAAAAAAATGAGATAAATCAGTAGAAATTACTTCAATGGGCCAGT
GGTACTGAGGACTACCGCACAATTTAAATTGAATTAGCCACTGACAGCTAGCCGTGCTCATAAACAACGG
TAATGACTGGCCTCTGGAACTCCAACGCCTCGTTTGCGGATGCATATCACGTAGCCAGTGGACAAGTTAT
TGGCCACCGGCAGCCGTCGCTGGATGCGTGATATTTAAAAGTGGCCAAAATGGTTTGTTTTGAAGTTGTT
GGGCGACTGAAATATGTAATTAAGTCAAGGTCGCGGAGCAGTCGAGGTCGGGCTAATGAACTTTCCAGTG
CTAATTGAAAGAAATCAAGGGCTTAAAAAGTAATGACATTTTAATTTAACGGTACTAGGCAGTTAAGTAT
TAACACTCAGATGGTAGTTACGAACAAATTTTAAGCCATGGAAAAATAAGCCATTAGGCTGAAAGAAACA
ATCTTTTCTTGGTTGCTTATCAAATTCCAGGTAGGTGTTAAGATTTACTCTATAATGTAGATATGTACT
CAGTTTTTAAGTTTTAATGGAGTCCGAATATTGCCATGATCAACATTTTATTTTTAATAATTGATAAGTG
TGCTGAAAACAGAAAAGGTTAGGAACGAGAAATGTATATATGTATGTAAATACCATTAATAAAATGTCTA
CCTACTTATGGCACACTTATATTTGACTTAATAatacccattactcgggcatactagattgtttaaacgt
atgtaacagaggaaaggagcccttctgatgtcaattacttatgaattattgatcaggatcaagtctgttc
attcgtctgtctgtccCAGCGTCCCTGTCCTTGTTTGTTCCCTTAAATCGATAATAGGTACCTAATAGTC
GCAACACTTGCCTTTAGCATTGTCTCTTGTTTAAATTGTGGATTCCGCAGAGAACGTGACGGTCCTCTAA
AGAATTATCATTCATAGAGCACACCTCCTATAGTGTAGTAAATAAAATTGAAAGTGAATTGAGATCAAGG
CTTTACCAAATATTTTCTTAAATTATACAACAGTTAGTAGAAATGGAGCTTGCTAAAGGTCAATATATTA
TTAGTAtttttttttAAATCAAACCCGGATGTCCAAAACAATAAATGTTGCAATGTCGCCGGCCAGCAGA
GGAGTTCCCCAAATCATTCGAGTGTGTTCATGTGTGCTTTCTACTGCTGACCGAAGCCGGCACGTGTTCA
AAATGCTTAATTGGCCGGGAAGCCTGGACTAGTCTCTATATACATACATATATATTTTTGTGGCTGCCAA
CAGGTTTTCGATGAAAATTCCTGGGGCCAAAGAGCAAGGGTTAGGTTAAAGTGTGAGAAATGTTGACTTA
AATTATTGTTATCACTATTTTTGCCTAATTTCTGACCCGGAAAACCAATAAATCTCTACCTGTGCCTTTG
TGGTCAAAGGGTCTACATTGTGTCTATACCGATGGCTTTGGTTGTGGAGAACAGTTGTAGAAAATGTACT
AATATGTTTGCCAGCAGCAGCTATTAACCCACTTGTTGGCATCGACCACATCCACCCAATAATAGAAAAT
TCCCCATGAAATCCGAATTGCATTTAAAAACCATTTCTGTATAATTAATCGGCGAGTGCAATTGACTGCC
CTTCGGGTTTTATGCCGTCCATCGAAGACCTAAAACTTGCGAACTACCAACTAATATTTTTCAGACACTA
ATAATGGCCCAATGGGTTGCGGCGCACTTGGAATATTAAGTAACCGTCGCGTGGGACACTGAGTGGTATA
AATACGCCGCGGTCTGCCTTCGATTGTCATCAGTCAGCTCTTTGCTTTCAAGTCAATCCAAGCTTAGCTT
TCAAATCCGAAAAACAAAAACCATCATGTTCAAATACGTTAGTTTGGGATTGCCAAGTAGCTGAAGTATA

TGTATTGTAGTTTACTATCTTATTCTTCTACTTTTAGGCCGTTGTCATTTGCGCCCTGATCGCCTGTGTG
GCTGCCAAGCCAGGATTGCTGCACACTCCTCTGGCAGCTCTGCCCGCTCCGGTGATTGCTGCTCCCGCTC
CCGTGGTCACTGCCGCCAGTAGCCAAGTCGTGGCCAGGACCTTCAACGGCATCGCCGCTGCTCCAGTGAT
TGCCCAAGTTCCGGTGGCTCCTGCTCCAGTTTTGCGGACCGtagcagctcccctagcagctcccctagca
gctcccctagcagctcccctGCGCGCTCCAGCTCCCGTTGCCTTCGCCGCTCCAGTCGCTGCTCCCCTGG
CCGCTCCAATCCTGAACAGAGTGGCTCCATTTGCTGCTCCCATTGCCACCCCCTTCGCTGCTCCGTACGC
CCATCCCTATGCTGCGCCCGTTTTGGCCAAATACGCTGCTCCACTGGCTTATGCCCCAGCACCTTTGAAC
TATGCCGCGCCCGCTTTGTGGTAAAGGAAAATCGCATACCCCGGGATTAAGCAACCGTTTGTGTGCTCAG
TGACTGATAGCCCTTAATAAAACTCTTTAAAATTGGTaaaaaaaaaaaaaTTGTTCGCTCTTGTTGGTTT
GGCTGTCTGGTTTTTCAGGTATTTGGTTAAAGCCCTTTGACAAAAAGATTTATGGTGCAGAAAAAGATCT
GGCTATTAGCTTTTTTCACAAATGTATTTTTTTCAAATTACATCACACAATCGCACATCGGTAGAAAAAA
TTTAACAGGCATTCAAAAACTCTTAATAATAGTTCTTAAATAAAAAGCTGGAACATTGTTTAACACTTTT
AAATATTAAAAGAACTTTAATGCACATGATCTGATACATTTAATATTTAAATTGTTAATTTTTTATATAT
ATAAGAAGTAATGGCATTTTAAAACAACTGGTTTCGTTACATTTACATTCGCTCAGTTTTCATAGCAATA
TTAGATACATATATTTTATATTTTTAAAACTTGAAGGGTATTTGGATGCCTGGCTATATTCTCCGCATCC
CATACATACTGAATCCCCTaaaaaaaaTTCTGGCCACTCATAGAGAGTCAATCGCTTGTGTCGCTTCGTC
GCTTTTATCCACGTCATTTGTGTTTCATTGTCCCACATTGGCTTCGTCTATGGTTCTAAGGACGAGTTAT
GGCCCATTGGTGATGTGTGTGTATGTGGTTTTCTGGGGATAAGTGGGTGCCCGAGTGGGTTTATAGGTGG
CTCAGGTTGTGGAGAAGGCCACTCTGGGATCGGAATCGTTTTTCATTTCCCTTTCATCGATATTTGCCAT
TCATTTGGCATTTGACAAACAATGCAAGAAGCGAAGCGCAATGAATGCCCAATAAAAGTTCAAATTATGG
TCGTGTTTCCACTTTTCAAGTTAAAGGCAAAATGGTCATGGAGCTTTAAGGTAACCTAAAGCTAAAATTT
CGGCAGAAGCAACGCCACATAGGGAAAAAAGGAAATTAAGAAAGTAATGTCGGGTAGAAAGCGAAAACTT
AAACGGCAAAAGAGATAAGAGGCAAACATACATGCAATTCCTACACTACAAAAGCCTCTTTCCGCCAACC
TCACCTccccccccccgccccccAACAAAGTTTGCCACGATTTTCCCGGGTTGGGGAAATTTTCCATGTC
GACGTTGGTTGATTTTCAACAGCAGCGCAAACGCAGCAAAGTTTATCCTCAATTAAACTTTTTGTTGTTG
CGTAAAATTGCGCTTGAAGTTCGCCCCGCCATCTGTCTCTTTGCCTCTGAGCTTTGTACGCGCGCGTGGG
GCTGGGCACTCACATGTACACTGAGAGGAAATTCGGCATTAAATAAAATCTAACCTTAATAAGAGTGGTA
TGGGAAATTATTGCAATATTAAAACATTTCCATGTGTATCTACACATTTTTTTCCTATGTATCTACATAT
ACATATGTATGTGCGATAGGCCGTACATCAGGAGCAGGGTCCCTGCACTTCCCATTCATCCGAATCTTCC
TTTCCACTTCCAGTACCATCCACCATCTTCATTTGTTGGCTTAACTTTTAACTCCACTTGCCTTAATTTT
TTGCGCCAAGTTTATTGTTGTTGTTCATCTACTCGGCACAATTTTTCGGTAATTTCCGCCCTTGTCCATA
GAAGAAGTTGGGGTATGGCCCCAAACTCCCCCATTTCTTTGAGGAAGGTGGCTTTCCCAACCATTTGAGC
CTTAGAATTTCATTAGCTCGCTTCGTATGCGTTTTAATTTGTTGCATCATTGCCTTGCCGAGTTTTCCTT
TTAATTGATTCAATATCAAGTTCTGTGCCTGGCTTCCATCCACACCCCACCCTGGTTTATTATCATCTTC
TGCCGCTGTGGGCGAAAAGTTTTTAATGCATATTTTCGAGTAATTAGCAAGACTTTTTCATTAGGGTTCG
TGTTTATTGGTTTCATTATGACTCAATTACATATTTTCCCAAGCCCACCTTGGtacaattatacaataaa
attatacaatacaatacattatacaatTCTTAATTCGAAAACGGCCTAGATTTTGAGAAATAACTATATG
CCAAAACTGCTTGTTACATCAACATATTTGCTATTGAACAGTGTGTGATATCTCGGTTTTATAAAAACCA
TTTATTTATGTACCGGTTTTATTTTTTATTGAAATTGTTTAACATTTCAATATCATTCGCTGCTCATTTC
AATATTTACTTTAGCTCCAAATGACTCCGCTTGCACATGCCAATGGTGCAGTAAATTCTTTAAGGACTCG
CACATCGAATCCGCACAGGAAATGTGTGTATGCGCACGTGGGGAGCTACATCGCAGGACTTCGAGCAGAC
AAAGCGGGCTAGACGTTTGGTCGCACCCGTTTCCGGTTGTGGTTGCTTAGGCCCTGGATCCTGATTCCAG
GATAACGGCAACCGTGGGGAAACGCCGGGCGTGCGAGGGGGACTTGGCCAAATAACGGCACTCAGTCACT
CAGTGCGGTTGCTTAGCCTTTCAGCTACAGACCGCAAAAATCCAGCAGCCTTTTCTGGCTTTTGATTCGA

CGACAACGGAACGGGAACGAGAACGGGAGCGAAAcgcatcgcatcgcatcggatcgcatGGCGTATACGT
AATGCACAGAGACGAATTTTGGGATTTCTTGGGTCTCGGTTATAAAATGACTGTTAGTTTCATAAAAACT
AACCGTGCATGGCAGCTATTGTGTATTAAGTTCCTCAGTTAATGCCATTTTCACAAGAGCCAAGTCTACA
TCAATACCAGATCTTTTCGGCCAGTGTGCGTATACGTGATTTGTTTGCCAGAGCAATTTACATGGCCCGG
CGGTGGATGAGATGCGAGATGTGCGACTGAGATGGCGCTGCCGATGCCAATGGAGATTGTGATTCGGATG
GAGATGTCGAACGGCGGATTCTTTTGCGTGTGATTGTGGGCAATGTGGGCGGCTGTGTGGGCGGGGGTCG
TGTGGGTGTGGCTGCTGGCACTTTGACATTTGGCACCCGCGCGAAATTTTCAATTTCCGAGCGCCAGGTG
GCGTCCGGGGCGCAACTTTAATCGCCCAAATGCAAATGCAAAACGACGCGTGGTTAAGCGTCTAAGTAAT
GAGCAGCTGTCGGATCGCAATTTGCATCCACGCGGATGCCAAATCCGACGCCAAATGCAATTGACAATGG
AACGAATCGACAAAAGCTTAATCAGAACTAATAGAGAAAAAGGCGTCGGGTGGCTGGCGAAACaaaaaaa
aaaaaaGCCATGCCATGTCTGTTTCTGGATGCCGGAAACGCTTCACTTCCTGTCAATTGCCATGAGCTGC
GGGATTTAGCTGAGCAAAGCCTTTTTCATTGAATGCGGATTATGCATTGATTTTTCGCCCATATTACGCA
TACGCAGGGTGGGCGGCAGGTGAGCCAGCGAACAGGAACATAGAAGGTAATAAAAATCCCCTAAGATAAC
ATCGTTATTCCAAAACTTAGTTCCGAGCTTAAAAGTGTGTAAATTGGTCGCGCCAATTAAACGAATGGTA
ATACTCAAAAAAACCGGTATTAAATCAGTTTATAGTTCATTTGTAAAAGTTCAtttttatgttttccgtt
ttttttttCCAGCTCATGTTAATTATGTGTAAAACATTACGTTTTTAATTGTTTTTGGTGGCAGTGTAAA
AATCCTAGGAGAGGATCAATGTTTCTCAGTTGAAAGGAAATAGGGTCAAAGGTTGAATAGGAATTTTCAC
TTAAATGCATTCAGCAAAGGACATTGAGCTAACAGCATATCCAGCAAATTTCCTTGCTCGCAGCGCCTAA
ACAAAGCAAAGAATTTCCCGGGTTCCGTCTTCCGGTTAATAACCCTTTTCTGTACCAGCTCCATGAAGGG
GAAAGTTTTTGCCGAAGTTTGTTTTCCTGCGCGTGTGTGCTGGTGGGCAAAGGGATTCCCCTTTCCGGAC
CACCCACACACATACATACATATGTGCGTGCTGACGCAGCTCCTTGAAATCCGCTTTGGGTGCCGAAAAG
GAAAAATAATTAATTAAGAAGTGCAAATCATAGCAAAATTATGCGGGCTACCAAATTAAAACGAAAGTAA
ACGCCACCAACAGAAGCATCTCAAAGGGAGCAGCTGGTGGCAAATTGCGGGAAGTAAGCAAAATTAAATG
CTTGGCGTCACGAGATGAAAGATACGAGATACTCACATGCAGGAGCTAATGAAGGATTGATGGGCCTAAC
TGATTGGCAGTGTGGTGTGTTCTAATCAGTCCAAACAGGGCAGCTTCAAGGTGTTTCAGTTTCAGTTCGC
TAACACCATAATTCTACCAATTTTAAGTGGCAAATAATGGGCATTTCACTTTTCCCACTTTCACAGGATT
TCATACTAAATGTATATGTGCTATTTCCAACCCAAAGAAGACCAACAAATAACTTCCCCGGTTTAAGTTT
ATAAATTTGCAATCTGCGAAACATACTGTCTGGCTCTAATAAACTGCTGGCCAAACGATTTGCGGTCTGA
TGACATGCGTTTTCCTCCGCTTTCCCACTTGCATTTCCCAACAACTCTCGAAGTGAAAAAAGGGAAACTG
CTTGGGGAAAACTGTGGAAACAACTGCAACTGCCAAGGCTATTTAAAAGCTTCAACTCGAACTCAATTCA
ACTCCTTGAGTGTCAGTTTGGATTCCGTCTAGTTGGCCACCATTTCACCCATCTCACTCGGAAACCTGTG
GGTCGACAAGCAAAACTCAGTCACAACACCTGAGGGCAGCCAGCGAGAACTGCGGCAATATAATCACAAG
AGGTTCCCAGGGTCGACTCTATCTGTCTATGTATTTAAACTTTTGTAATAATCTACGGCAAATGCGCAAC
GTCAGCGTCTTTAGCTGGGTGAAATTGCTGTACCAACCCCAACCCAATGGTTTTCCCGGCAAAGTTGTGG
GACGCCATTTCGCTTTTGCTATTTGTGTTGCGACTTTCGTTGACGCTGTCTCCAGTCCCAGGTCTCAGAA
ATCCAATGCCAACTGCATCTGCCGCTCCCGGCGTACAAACTTTACCTTCCCCAAATTTCATTTGCAAACA
TTCTGGCACAGACTGCAAATTGAGTGTTTAATTGCAACGAAGTGGCATTCCAGGTGGCAGCAACATATGC
ACTGGGCAGTCGAAGCCTTTGGGCCTCTTTATTGGCCTGCTTTGCACTCTGCATTCCCCAGTTTGGTTGT
GAAAAATTGTTGGCTATTCAAGTGTGGCAGGGCTGTAAGTGTTGCAAGCACAATTTGCTGCAGTGGTCTT
TGTCTTTGTCTTACATAATGTATACTCTTTAAAGCATACTAGCACTATCTTTTATATAAAGATGGAATAA
ATATTTATTTTGACAAGCTAGAACAAACTGCACAAACCATGCCATTTAAGGGAGATTAGCATATCTTATT
TAATTGTATTTAATATAATTTTTTCGCTTTGAAGCTGCCAATCATTCGGGCAAATGGTGCTATCTTTAAC
ATTCCCCTTATTTCGAGAATCCAACACCCACTGCCTGCGACGGTGAGCAGCCAATCATTTCACTTCCGTT
TTCATTTTATCAAGCGTCAAAAGTAATTTGCATTTAGCAAATATTTGCCGGCACCCAAGCCTCTTTTTCC

CGCCCAGCCACAGCGTCCTTCCCGCAGGACGAACATCAAAGTGGCATCCCGCCATGCGTGTCAAAGGCGT
TGGCGTAGCTTTAAGTGTAACTCGCTTACCGTTCCGACTGTCAGTCATTCGAGAGCGGAAGAGAGTGGCC
GTGGAGCCCCTTTTCCttttttttCTTCCACCTCCGGCGATTTTCGCGATGCCAAGGCGCCAAAAGTGAA
GCCGTCAAATGCGGCGGCAACGTGGCCGAAATGAGTTTTCAACTCAAGTGCTGGCAGGACATGGCCTTCG
GCTAAGCAGAAGGGGTAATTTTTTCTATGGATTTCTGGCTTGCCTTCTTTTGGGCTGGAAAGTGAGAGCT
GCGCATTCTTAGCCAACTCTAAAATCAAGTATACCGTTAACTAGATCTAGGCTTAAATCTTACATTTTTA
ACAAATCTATGGTATTTAAATGTTGTGTTAAACTAGTAAAAAGCTCTTCATGTATCATTTTACATACATA
CTCCTACTCCTACACTCCATATTTTAGCATAAGACTAAAAATTCGTTTATGGGATTGCAAGGATGAAATG
TTTTCGCCCCCCCGTGTAAAATCGGCTTACCCCTGTTTTCACCTGCAAACTGAAACCGGATGGAGTTATTG
TTTTGTTGCGTGTATGGTGGCGATAACTTTGTGTGATTTTTAAGAGGGATCGTGAAAAGAGGAAAAAACT
CAGCGAGCGGCAAGTGCAAAAGCTTCGCCGATGGTCGTTGTTGTTTGCTTTGAGTTTTTCAAACCGTCCA
AGCCAACAACCATTTGCCAAAGCTGTTTCCCAACCACCGATGCAGCAATGCTCCTGTGCATTTTATTGCA
CTTCGGCAAAGGATGCCCCGTGGGTGGATCCATAAATTGCGCATCCATAAATCCATAAATGTGTTAAACT
GGTGTTTAACACATAAGGCGGCAGTACTTAAAGTAACCATTAAAATTCACAGAATAGGAAGGTTCAGCAA
GATTTCCCCGTATGCCTtaaaaaataaatttaattataaaataaaaaaaaagttatattttaaataagtt
taaagtGACATATCGTAAACTAAAAATTTAGTAAGCATCAAGAGGAGTTCGCCAACTCAAAGTTCTCTTA
AGTAGTTAAATAGTTTTATCATTGCGACGCACAAAGTATTTGATCCACCCTCTTGCTCCTCCTATTTGCT
ATACACGGTTTTTGTAGCAAACTTTTTAGCATTTTTAACCATCGTATTTGAGCTGGGGAAAACCTCTTTG
TTTTCCTTTTGACGATGGCTCTGTCTCCGCTCCACCCGCAACCCTCTCCCCCTCCCACCTTCTCGGCCAC
CCTAAACACGCCCCTTTTCCACCTGACACGGATGCACAATGCGCACGTGTTCAATAACAATTGTGTTGCA
TACTTTAGTGCGCCGGCACAAAAGGATGGCATTAAATTTTAAAGTTGAGCTGGCTGGCATCGGCGAGCAA
AGCGTCAAACGGTTTACAGCAAATCAATTAGAGCTAAGAACTTTTCCAGCGCTAATTGCAAGGGATTTTC
TTCCTCTTCTGCTGCTGGTTTTTCTTCGATTCCCCCTCCGCCACCGACACTGCCAACACTCTTCTTGTTG
ACTTTGTAGTTGGCCAAAAAACTGAGCAGGAAATAAAATAAGTTCCAGATGGACAAAGGCACTGCCCCAT
TCCTCCGACGCTCGGCCACTCGGTTGAACAGAATTTTCGTGGATTTATCATGGAGCGCTTTGTAAGGCTT
TTCGGGTCTTTGCCGGCCATGTCTGCATTGCGTATTTCCTATTCGTGCAGTTGAAATTAATGTGCAAGCC
TTTTAATTACACGACAAATAAGCAAACAGCCCAAAAAGTTTTGCTCACTTTGTGAGCAACTTCAAGATGG
AATGGAATGGAATGACGCCGAGTGAGCTATGGCCAAAATCACTTTGAATTCGAGGGCTGCGAGTCAAGAA
ATTGAAACTTATTCGGAAACTCCAGGGATGACAGGGATGTTTTATAATTTAAGATTTAATAGTACGCAAA
ATAAGTTTTAACAGGTTTAACTTGGAAAAAGACTACTTAAAAGTTAGAACATACATGTTCAAATCTCTGT
TTTAAAATACTATTGAAATAAATGCGAGAAAGTTTTAATTTGAGAAATGGAAATGAGATCATGTTCCATC
CCTGATTAACTAGAGCCTGCAACATTAGTTGAGTTTACATGTCTCtgtgtgtgtgtgtgtgtgtTAGCCA
CTAATTAATTTGGCTTTGACTGAGAGAGCTGAGAAATCGCCGGCAGCTTTTGGCGACGAATTTTTCTCGA
TGCCAGCGGCAATGCGGTTTATTCACGACTGCTCTGCAGCAAGTTTTCTTATGCCAATGCCCAATTTAAG
TGTAAAATCGAGGCAAAGTTTATCAAAATGCTGCTTAATCAGCTTTATTTACAATGATAGCATTTGCATT
GCATTTTCGCAGAGAGCAAAAACAAAAAGGACTCGAGGGCAGCAGGATGGAGGACGCAGCACAGCGTAAA
ATGCATTAAAAGCGAGGCAAACAAGTCTGGGGTAACTCTCGCAGGACTTTTTCCACCGCACCTCATTCAG
AAGTTATGCATTATCAAGTGCCTCCGTGAAGGTCCTGTCGCACGGAGAATAATCAATGGAATTAAAAGAA
AATCCTGTAGATTTTAGTTTACATAGTAGTTGAAAATAGAATTTCAATTTAAATAAAGGATAATAAAACA
ACGCCCATTAGTCGTGATCTATTCAAAAGAACTAGGTGTGTGTAATTAAAGACTTATGAATGAAAAATAT
AGCAAGTAGGTATGAAATGTTCCTGGAAGGACTTTTCTTCTTTTTATTCAATTTATTACATAACTGTGTC
TCTGAGTAACTAAAATTTTTCCGTTAACATTCAGAAAGGAACTGttttttttttCTTGTGCAGGACAACC
ACATCCCAGGTACTGGGAGTCTCGATGGGGTCCTGTTCCCGACTCCCAGGCAGCTCCTGTGGCCGTCCCT
GCTGTTTACAAAGCTACTTAATCGAAGTGCTGCCGATGCCGACGCCGATGTTGGAATTTCCCGAAAATCC

TTGCCCTTCTCCACTTTCTGTGTGCATTAAATGCAATTTTTTGCGTTTGGCTTGCAGCTTTCCAttttttttttGTTTGCCTGCATCCATTCGGTTTCTGTTTGCTTCCGGTTTTCATTAATGGTCTTAACATAAAAATACAATTTGCTGACGTTTAACTGGCTTGGCGGACGTTAATAAATTTTTAAGCGATATTTGATTTAATTTCTTCACCACCACTTTCGAGAGCCGAACCCCCCCGAAATGTGCCGGGCACAGAATGATTGGGTTGGACTTTCCAGCCGGAGGCTCTCGAGCTGGCCAAGGTGTTGGAGCTGGTTAAGTGTGTGGTCCGGAGGGTTTTCCGCCTGATCACCCGTCCAATCAATCTGTTTACGCTTCTGGATTCGCCAGACGGATTATTTACACAGGGGCAGGTCCGGATAAGCAAGGTTCCTCATAAGTGGATATTTGGTATGATATGGAGTTTGTTAGCAAAACATCCACAATGATAGTCTAGGTAGCAATTCTAGAACCATACATCTCAAAACAAATGAGTAAGTACTAGTTACTAGTACCATTACGACATTGGAAGAACTTGCAAGAAAATATTGCTTATATGCTTTtatatatatatatatatCGTTACAAAATTTATTTTTAGAAGTAAATTTTTTGATTTCAAAAGAATAGTATGCTTATTTCATTTTAAAACACACAACATTTAATCTGATATACGAATGAGCTTAAATTGAGTATGAGACCAAGCGGGCCAACTACTATATTTGAAATTTAATTTATTCCATCTGCGAAATCCATCATCTATCTGCGTCAAAGCTCTCGAAATTTTTAATTAGCGCAATAAACCGTCAAATGTTGCTGTTAATTCGATTAAATAGTGGAAACAACGCCCGGATTCCGGGTCATCTGACATTGCTGACAACTGCAGTGGCTGGGCAGAGCggaaagctggaaagctggaaaATGGGAAATTCGAACGAACAATGCTGCACCTGTTACTGCAGCAGCAATAAAGCAAAGCAGCAGCTCAAATTTCTCTGCCCCACACACACACAGTTGTGGGTGTTGGTAGTTCGAACCACCCCCCACACCCCCTGTTTCCATTCAGCTTTTCATTTAATTGCGGTCGACTTTTGGGGTTTTGACTTACACAAGGCGAGCAAATCGGGCAGGAGAGTGGCACTCGCCAGCAAAATGCCAATCATCAATCAAGGTGATTTGTTTCAATGCCCCCCCAGCCACACATTTCACGCTGTTGTGCATTTCGAAATGGACAGACACTGATAGCGGTCGAGGGGGGCTGCTTTTCGAGGGGGGTTCCGGGGGTCGGAGGTCGGCTAGGGTATGCAGAGGTTCATTCGGCTTGGCACATGTGGCCAATCACTTGCATACACTTCAATTGCTGTGGTTAACTCACTCCCAGTCGCATTCGTCACGCGCATACGCAACGAAGTTCGAGAATAGGTACGTTAAAACATGAACATGTTATTCTTAATTAAACATAGACAGTATAATAATGTGTTCTTAGTTTGTATCTAATACTTCCCGTGATGTCTAATAGTATAAATAATCAAACGTTAAGTTAGCCAAAAAACGTATGTACTTAAGCAACTAAAACATTTATGGTCACATATTCGTAAGCACTTTATATTAATTTAACACAAACTGCAAATGTGTGCGCATAAACGTGTCGAAAttcgactgttcgactgttcgactgttGTTCATCAATGAATTCAATCACGAAAATGCTGCCCCATTTGGCAGAGTCCCTTTCTCGATGTCTactcacactcgcactcactcaaacagtcacaacactcacagtcacagcactcacagcACTCGAACCCCCCCGCAGACAACACTCCTTAGCGCACTCATTCAGTCGTTGCATATTCATGAGCCAAGCACTGGCCTCCTTGTTTGAGTTACCGACGTGGCTTTGATTTAATTGCCTCGATTTTGTCTATAATTTATTTTAAGCCTTATCCTCTAGCCTTTTTCACCGCCAGCAACTTCCGCTTCGTATTTCGGGCTTGGGTAAATATTTCGTTTAATTCGCACAGTTAAAGTTTTCCGTTGCGTTCAATTCCTTTCGTTCTACAGAAAATAAATATATTAAATTATAACTTCTTTGCACTCAGTCTTGAAACAATGAATTGATGGCAACATAGAAAGTTACTTCAAAAGTGTGAACCTGTAACTTTTGGGTGGAAATTCAATAGTTAACATTTTCCCGCCAGCCTTGGTCGTCTTAAGTTGTAGGAATATTGCACTTTATCCTGGTTCCGCCAAGGGATCCTGATCCAGCTCCTGTTCCTGGAAACCGCAGCAGAACATTTTTGACGCTTTAAAAAGTTGTGTGGCTACCGTTATTTTTATGCGCCATTCACTTTTGTCGCCATTCAGTGCGCGCTAAGGAAAATGGGAAGAAAAGCCCACTGTCTCGCAGGATGCCCTCTCTCTCTTTCTGGCCAAGTTAGCGCTCTCTTTCACGTGCTTGCAGCCATGTTTCATACTTTTTCGCAGACATTTTTAGCACTTTTTTAAAATTCCGAAAGAGGTGTGCGCATAAATTTTTTGGTGCCCTGCAGGCGGAATTCTTTATGCAGAATATTCCGCGAAAATAAAACGCTGCAAATTGTTGAAGAAGGAACTAAATTCAGTTTAAGGCCAGACTGATTGTAATTTGATTTGGCCCTGCCCGCTCTTTCCACCGCCCATCCACTAGGTGATTTTGGTGATTTCGTTTTTCCAGCGACAACAAATGTCATTTCCTGTCGCCGTGCGACTTGTGACCCGAGAAATCGTAATCGCACTTTATCTATTAGTGACATTTTCTTGCCACGCCCCTCGTCTCCCAGACGGCCTTTAATGAAATTTACTCGGGTCGCCCCGTTTTCTATTTACTTTTGTGGCACGAAATTAATAAAGGTCCTCGTTGGCTGACCCACCATCTTCGCCATCGAACCGAACAAGGCCAAGTGGAAACTTAATCCCCAAGGAAATTATGTGAAAAATGTGCGTTAGGTTTGGCTTTTGTCCGGCGTTTTCCTTTTACTCTCAGGTTGGCTGGGGA

TTTTGTATTATTTTTTTGATGCTCTAAGTTGTTAAGCGGCGAATAGCTGTAAATATTTTTGTTGGTTGGC
TTAAAACATTCAGCGTATGGCCGGAAATAAAAACTAAGGGTTTGGGGGTGGGGTCAAGCAAAGGGTATAT
CAACAAAGGCAGCAACGTGACCCCTTTTACATTTCGAGTTTGGGCTGTCCTTTTCCGCTTCTTCTCCGGA
ATTGGAGGTTCATATTTAAGCTGCGCAATTATCATTAAGTGGTGGCCGTTCTGGAGATCAGAGGCGTTTC
CCTGAGGGGGGATTTAAGGGTTCCGAAGGAATTTTCCAAGGCCTATTGAGCAGAGGAATGCCAGAGAAAA
ATTCATAAAGGTATCAGAAAAAAATCTAAATAATTCTTGGATTCCCCAATGGATAACTAAACTGTTTTTA
AAGAAGTGTTTGTCATTAACAGAACAACCCTTACTTTTCAATTTTAAAATATCATAAATTTATAATTCTC
TTATTGTAATATCCTTTCCAATTGGTATTCCTGAACACTTACAACGCGCCCATGACCTCTATCAGCATCC
TTTTTTCGGAACCCTCCGGTGGTTTTTACCGTCCTCCAACATTTCTCTTTGAATGCTTAAGGtttttttt
GTAATTGCATAAAAGTTGCCGGTAATTTTGTTCAATTTGTTTGAGTCCTTATTTCCGGCCTCTGTGCGGA
TGTTTTTACGCAACGGATAACTTGCGGACCACGGTGCGTATAAGTTATGCGATGACTGCATAATACGTCT
TACACATAAGCTCACCGAAAATGGAGGCATGGAGTAAATGGATAGATTTCCTCCATTGGCAGGACATCCA
TTTAATCAGGACGATTGTCTTTCGTCCTTTGTGAAGGTCCTCAGACGCTCCAAGGAGTCAATTACACTCG
TCGTTCACGTGTTCGAAATCGTGAAGGGAAGGCCGTAGTTTACCTAATGCTTATTTAATTTGTCAGGTGA
TTGTTTCGGCGCAGACATAACGCTCTAATTAGGCCACAATACAAGAATTAATTAGGTAAATTTAAAGCCG
AACGCGATAACGAAGTATGGAATTTATATAATCTTGTGATGTGCGTCCAAAAGCTTTACATTTATCCCTA
AAATCATCAAATTTCCCATTAAATTTCTAAGTTGCAATTTTAACTGCAATTATCGATAGGAGGAAAGTTT
TAATACATTTTAATTTGGCAACTTGATCTGCTTGTAATTTAAAATCTTCTCTACATGATTAAAATAAACA
TTCAAGCCACACTCACACTTTCAATTAGGAAATATTTACTCGTATCTACCCTAAACTAACAAAGAACTGC
AACAATTTCAGAGACAAGTCAAGTGCAAAAAATATGATTAAGAAAACGTAATTAAAGTGAAGTCATCTCG
AAAACGACTTGGCCATTTTCCCATTGTATGTGATAATTAAATATGCCGGATTTAATCCATGAGCTGGTCA
CCGGACGCATCTAAGTAATCGCCAGGCCAGACACTTTGGGGATCCCCAGGACCTTTCACCTGCACTCTGG
CACTCTGTTTTCTGTCTTTATTTCATTTTAATTACATTGCAGTTACATTTCACAATTCTCGGGGCCATTT
GATGGCCGGTTTCAGGGCTCTGGCAGTCAAATCAGGCGTATGATAAATTGATTGCGAACGCATACTTATG
TGCATACGAGGATGTCCTTGCTCATGCATGCATTTATGGGTACACCCAGGATAAGTGCAATCAAGATGCA
ACAGCAGCTCATGTGCAATTTAATTATCTTCATTTCAAAAATATTATTTTGCAGAAAGACCTAATGACAA
AACGAATCCTTTTAGAAGACTGTATTTACGATTTTTCAGCAttttttttCTCCCAGTGTATATTACTATA
TGTGTGTTGTGTTTGTCTGGCATTGAGACTTTTCTGCTTCATCCTGCGAAAGCTGGGCTTTAATAAAATT
TTACGCATAAACTGCCTTTGATTGAATTACATTTAATTATTTTACCAGACATTTTGCGTGGAGTTCCTGG
CCGAAATCGTTTTGACACTGCTCTTCGTTTTGTCATTTGTCTATTTTCTTTCCTTTTCCATCGAACTCAT
CTTTATTTGTTTCTCTGTTTTAAATTAAATAATTTGACAAACATAATTAAACACTTAAATTACTAAGAAT
GTTAATGATAGAATATTTACTGGGCGAAAAATTGTTACAGTCGAACAAAAAAGTAATAGTACTGCTATTA
CTTTTCTTATATTTTTAGTGAAGTCTTGGAGCAAAATAACTATATATTAAAGATAGCTGCGCTTAGGCAA
GCTTGAAAAGTAAACTTATAAATTAACAATTTGGTTTCTGTTTCAAATTCATTTGTTGATTTGTACCCAA
TGGCGGTTATTCCTTATCTCACATTAATACTATACTGCTTGGGGTGGCTCGATTTGGAGAGGGATATTGA
TTGGAGTAGTCGATTTCCTTTCGGATTCGCTTGCCCTTTCCCATGATGCTCTCGACGTCCACCTGGGACT
GCTGCGACTGCTTCTTTTGCTTCTTTGGTAGCTTCGGCTGACTTTTTCCCATCAGGTTTTCCCAGTAGAA
TGGGTCAGTGTTTTCAGCATCGTTATTGTAGTCATCATGCTCCTCGTGATCTTCCTTAGTGGCATAAGAA
GCGACCTTAAAGGACGAAAGATACTCGTTGGCCCAAGACTCCTTCTCTTCAATGCCGCGATTGGTGCGGT
CAAGCAGATCGGCCACCGCTTTGTCGTCGTAGTGAATAGCCTCACTCTTGCCGTCCTTAAAAAGATCCTC
GGTGCCAAAGCGAAGGATGTCTTCGAGCTCGTCCTTTGAGAAATTTGTTGTCATGCCACCCATTCCCGGG
CGCACCACAAGATGGGTGAGCATCATCTTGTGCTTGGCAACCTGCATAATGCGCTCCTCCACCGAGTTGT
GGGTCACAAATCGGTAGATCATTACCTTCTTTTTCTGCCCCATGCGATGGGCTCGGGAAAAGGCCTGAAC
GTCGTTGTGCGGATTCCAGTCCGAGTCGAAAATAATGACGGTGTCGGCTGTTGCCAAATTGATACCCAGT

CCGCCTGCACGAGTGCTCAGCAGGAAGACAAAGTGCTCCGATACGGGATCATTGAATCTGTCAATGGCCT
TCTGACGTAAATCGCCTTTGATCGACCCGTCGATGCGATCGTACTGGTATCCCTCCCCTTCGAGGAAGTG
TTCGAGAACATTCAGCATCTTGGTCATCTGGGAGAATAAAAGGACCCTGTGATTGTCCGCCTTTAGCTGT
TTTAGCATCTTTGACAGTAAATCTAGCTTGCCGGAAGCTTTGGTCAGCGAACTCATCTCGTAAAGACCAC
TTGGCGAGATAGTGGCCTCCTCGGCGGCGGAGGGGAACAGGTACGGATGATTGCAGCACTTTCTCAGGTC
CATCATGATGTTGAGCAGCGAACACACGCGACCGCCGCCTTTCTGATTTAAGGCCTTGAAGTTTTTGGTC
AAAATATGCTTGTAGAAttttttttGCATAGACGACAGCTCCACGCGCACAATGAACTCCGACTTTGGGG
GCATGCTCTTCAATACATCCGCCTTAAGGCGACGGAGCATATGCGGTTCCAAGATCTCGTGCAGTCGCTT
CACTTGCTCTTCCTTCGAAACGTCAGTGAACTCGGCCTGGAAGGTCTGCAGGTCGTTAAACTTTCCAGAG
CTCAGGAAATTGAGCAGATGGAACAGCTCCTCGAGATTGTTCTGAAGCGGAGTGCCGGTCAGCAGCAGCT
TGTAGCCGATGCGGTACTTGCTCAGGATCCTAAAAAACTTGCTCTGGTTGCTCCTTAGCCGATGGGCCTC
GTCCACGACCAGCGCCGCCCAATCGATGCACCCGAGAAAGGCTGCGTCCACAGAGATAAACTCGTAGCTG
GTCAGCATTACGTTAAACTTATACTGGGTTTGGTTTTCGCGCATGGTTTTGGTGGTTACCTCCTCGAAGC
TTATCTCATGTTTCCGGATAACCGCTCTAGCAGTCTTGCCGCCCACATAGGTGACACAGTATAGTTCTGG
AGCCCAGAGCTCCAGCTCTCGCTCCCAATTGGTCAGTGTGGAAAGCGGCACGGATATGAGGAAGGGTCCG
CGGCAATGTCCCTCCTTAAATAGTGAGTAAAGAAAAACCACGGTCTGAATGGTCTTGCCCAGACCCATCT
CGTCAGCCAGTATGGTGGGGATGCCTTGGCCCCAACTATAGCGAAGCCAACTGACGCCCTCTATCTGAAA
GGGATGCAATTTAAGGCCAGCCTCCTTCAAGAATACTGGCTGATCCTCGTACTTCTTATTCAGGTCTATT
GTGGGTGCAGGTCTATCTCTTTGACGTCCCTTATTACTGGACCGTAGTTTCTTGTAGAGAGCGATAGCCT
GGTTTAGACCCGGGATACTGTCGCTTTCTCTTTCCCAGCTGCTATCGTTATAAGACAACTCACGCCATTT
CACCAAGTACATAGTCCCTCCATTGGGCTCCTCACTATGATTGATAACCCGCTGGACAAGGAGCCACTCC
GGCTTAATGCCGTATCTGTAGAATCGCTCGTGCAGATTGCCGTCCTGATCATCGAGCTCCTCGAGACTTG
GCTCTTCCATGTCGCTCCTTCTCTGGAAGCTGGCAACCATTGAGGCATGGTGCAAGAGCATTTGACCTTC
GGGAATCCATTCGCAGTGCCAGTAGGACATGCCATGCCACTTGATAAAGTACTCGCGCCGCTTTTCACCT
TTAGATGTACGAAGCTCCACGCTTCGATCGAGGGCCCAGCGCCATGACAATATCTTCTCGGCTTTTCCGG
GCAAGGGAATGCAGCGCGGGCAGATCCAATCGCCTTTGGGAATCGATTTCAAGGGAGGACTCAGACAGGT
TCTGTGGTAAACGGAAGGGCAGGAATCGCAGCACAGCAGATCTCCTCCATCACTACAAACTTTGCAGTAT
TCTTCGTCGCGAAACTTCTGCTTCTTGGCATTGGTTTTCGGCCGCTTGTCCTTGGAAGCCTTGCCAGGTG
TTTTCCAGTCTGGATCAGCCCCTCTCTTAGACGACATGCTATTTGAGTTTAGTTTTTGCAATCACTGATT
CTGAAATGGAAATTGTCAAATGCGCGCCAGAAGAAAACGGTACTACCAATTATAATTCCAAGCAAAGAAG
CATTCACACTGATCTTCCGAAATAAATTGGTCTGATGCGCCGTTTACACTTGCATCTGGTTAGGATGCGC
TAACACTATTGCGAGGTAAAAAGGTGATTCtatatttatttatttatttatttattatttatttattatt
atttatttattttttatttatttattatttatttatttatttattatttattttCAGCCACTTGTGTCAC
CATAAAAAACCCTAGCATAATAGAACAATACGAACAAATAAATGTTTAAGTCAGGGGTCCGTCTATATAT
TCATTTTTTATTGGAAAATATCAACGTTTTTTCTAAACGATTCGGATGGTAAATATCGAACAAAAGTCGG
CTTGCATTTATTTCGATATTATTCTAGGTATTGAACGTCGCtaataccaaatacactataataattccag
tgtctttgctAATACCCTGTTCGCACGGGCGGGCTTGCTTTGATCGGCATTCGATAGGTAAACAATTATT
AAGATCGTAAAAACTTGGGCTTGCTCTTCATGATATTATCATAAAGATAATAGAATTTACTCATGTAAAA
TTTACTTAATTTAGCGGGAAGTTCTCTGAAACTAAAAAATCAAGATTTTTTAAATAAATGCTAGTTTAGA
CTTCACATTTTGTAGAAGTGTTTAAACATTAGCGTTAATACACAGACAACAATCGAAAACCTTTCGAAAT
ATCGATATACATATATTTAGGTAATTAATTTAATTCAAATTAAGGGAAACAGCGTAAGTTTATATGCACA
AttttttttttGTTAAAAGTGCTATTAATTTTATGCTTTAACTGGATGTAAATGGACCGTTAAAGCCCAG
CTCTGCTTGCTGATGCCTGCGTGTATCGAAAACCAAAAACCAGCCGCAGAGCAACGAACCCGCGACCGCC
GAAACATTTCAAAGACAATCCACCAACAGCGGCAACACCGTCAACAGTCGGCCAcaccaccatccattca

ccgtcaccatcaccatcaccgtcgccaACAGGACGAGGGCGAGGACAATGGATCAACTCAAACTCAGACC
TTGTAACTGCCTGACCGCACATCCACTGGCAGAGTGGGTGTGGGTGGAATGTGAGCGGCTGGGTAAGTGG
GTGGCCAAAGAAGTGAGGGCCAGTTGATGGCCACATGTCACAGACATTATGGAATCATGAACTTATGCGC
ACCTGTGCGTGCTTGCGGGGGCGCGGGCGGTGGGCCCAGAGCTTCATTCTGGTTATATCCGTGTGGACTT
GCGGTCGAGGACAGCGATTTGAGGTCCGCCTGGTGATGGTTTCGTGACTCGGCAAGGATTTCATCGGTTT
AATGTGGAGCACTTAGAAGGAGAAACATGGCAACGTATCAGAAGTATTATTAACTTGAAATTATGTGATA
TATTATTAGTTCTATATTTTTCTATAACTTCCTTCAAGCCATAACATTCGAGCTTCTCACGCAACCATCG
CCAATTAGGAGCCGGGCCATTTCAATCATCAATCAAAGTGTTTTGTTACGACTTTCATACGTCATGTACA
TGGCTCGGAGCCACCGCTTTTTGCTTTTGCAACCCATAGGGGGCTCCTCGCTGTAGCTCCTCCATATCCT
GCCTCAGTCTGGGGAGCCAGTGATGCGGAGGTGTGACTGCGACACAACGAACGACATTTATGGTGTCGTC
AAGAAGAATTCCCATTACAGCCACAGACAATCAATCACTGTGCACCGAAATCAATTGTCGCAGCTCCTAT
TTTCGTTTTCAATCTCATTTCGCTAAATATTTATGAGGCCAACACTTGGGCCGTTCGATTTGTAATCCTA
ATGAGGCGTAATTAGAAAACATTCAATTACATTTCATTGGCATCAATCAATGGCGCCCCATCCATGGCTA
TTTCAAGGATGACAACTTTTAGTAAACCGAGTTTCAATTTCCCAACGCATTGTTAAGATGCCTCTGGTGC
TTTAATCCACGTTCCCAGCTACTGTTTCTGTTTGTTGCTCCTGATTGCTGCTTAAACTTTTCCCTCGCTC
GGACGACTTGTTTGTGATTAAGCTTTATTGTTAAGAGGCTTAGTACGCCTTTTGATTTATAGATATTGGA
TCATACCACTCACTCTGCTCCGCCATCAAAGGAGACTGTGGTCCTTTGAGAAACCGGCAATGTGAAGTTG
GCCGGCGCGCGTAGCTTCATAGAAAAACTTCCAGAAGGAGTGAGGTATTCGTAGGAAAAGTCTGTACACA
GAAAGGATAGGATATATGCCTAGTACTTAAGTTGTAAGCCGCTATTGTTGTCCTATAACTTAAAAAATTT
GTTGTCCATTAAGATACATTTTTGGTTTGATTTCCTGGTTTAGAATAACAAAAGAATATTTCTTATAGAT
ACACTGATAAGACCTTAAGATTTAAATTTTCTTTCATTTTTTTCAAGCTTCAGCAAACTGGTTTGCATTA
CCCTTTATCTGTTATTTAACGTATTGTTCATGCACCTTTAGGTTGATAAACCTTTTTTATCTGTACTTTC
CGCAGTGCATTGGGAATTCGCTGATCCTTTCTATGTATGTCCATTTAGCGCTTCTCACTTGTGCCTTTGT
TGCATACTCTTAGACGCGCGGCTGAACTTGTTTACAGGCACACTAAGGACGAAGGACCCAGGATCCTGTA
CATTGGCCCAAAAGTGGACGCTGGCTATTCGGCATCCTTGACAGCCGCACAATTTGCTGCCGCGTTTTTG
TATCTCTCAGTGCTTGTTTGTCTGCACCACAGCGCCTGGGCTCTGTACACAGAAAGAAACATTCGAGTCG
CAAGCAGGATATTGATATTAGAGCTGAATGCCCTATTAATTCTGTATGCAACCCATGTATAATATTTATT
TAGCTTTGTTCCCGGGAAATTTGTATTCCCTGATACCCTAGATTTGCCCGAAAATAGGTATTTTTCTCTC
TGTGCATGTTTGTGTTTGTGTGCAGATGGCCAGCAAAAAGTTTGCGCACTGATTGCAAACTTGTTTCAAT
TGTTTCGTAAACCCAACCGAAGGGAAGAACCCAGAAGACTCCCGGTGGGTGGATGAGCCTGGTGGGCTGG
GTTTTTGTGGACCGCTTTTTCCGCCATGAAAGTCAAGGCGAACGCTACGAGTTCCCAGCTACGAGCTCCG
AGACCAAGGCAATTCGCCTGCATATTTGTGTGGCTTAGTTTCCGTTTCACAAACGAAACGAAATGAAACG
GGCGGCATAAAAACCCATTGAAAATTTGCATACGAAAATGAAATTCAAATAGGTTTCCCATGTCCTTCGC
AATCAGTTCCGGCTAGTCCAATTGCACAAAATAGTGAACGAAAACTTTCGCTTTTCCTCCGCCGTCTAAG
CCCTTTATTTATGATCCACAATAATGCAAAAAGATACATATCTGAATATCTCATCTTTGGGGTTTATCTC
CTGCGCCAGTTGCCAGTTTTAATCTAAATCGTTTGGCTGACTTTGTTTATCGATGCTTTCTTTGGACGCT
TCTACCTCGGATTTGCGATTCGAACACTAAGTGATTTTTCATTGACTTTGTAATGCAAATTGCCTGTCTG
TCCTGCCCCTTCCTTACCATGCTTCTCTTTGCGATTGGCTGGATCTTTGGGCCTTTTATCACTGGAGGCC
TGTGCTACGGACCCTTCAACAGCTCGAAAGCCAATAAAATGCAAATTTTTTCATCGCGTAAACAAATGCT
AGACAAAGATGCCTAGAGATGGGGACTCTACGTGTCTGAAAGCTGTGAAATAAACCTGTGATTTATGCGG
ATATCGTTGTGGGGACCTAAAGCCCCTTTAAATCTTTCGCGAAATATACGTAAAATAACTTTAATGTGGT
TTCATTCTTTGCTGTTCAAATTCAATAATCGTACTGAAACAAAACCCATATAATGAAATTCATATAGTCC
TAACCGTATTAACTTATACCAGTGTCCTATTTGATTGCTTGGTCATCCTGACTGCTCACGATTCCATCTC
CAGTGGCCGATGAAGTCCAAAACTGAAAACCCAAACTCATTTCCATCGGCATCAGACTTGACTTGAGCCG

GGGCCCTCGACTCGCAGTCATTGATTTAATTGTAAGCTGTTGAATTTTGATGGCTTTTAATTGTCTTAAG
ACCGGTTCAGCAAAAACCAATCAGGGATCCTTTGATAAGACGtggtggttggatggttgcatggatggat
ggatggatggacaatgggttgggttCTGTTGTCAGCAGAATAGTTGGAAATTCCACTTAGTGCTGACTAC
TGACCCACTTATTGGTCACTTCTGGGTCTGTGTTTCCTTCCGAACCCCCATCCATCTATCCCCACTCCTT
GCAAAGTCAACCACTTGACTAAATAGCACTCAAATTTGATTTTGATTTATGCTGCCCAGCAGTCCGCACC
GAATTTCCTCCTCTTTCCTCAAGCTCCAACTTTCCCTCCCTCCGACTTTTTCCCCCTCCCCcacaccaca
cacacacaTAAGTACAATTGCTGGCTCATAACTTTGGCATCTATCGCAGTATGCCTTTCATATTTACATG
CTCCTTACACGCGCATCGGCGTGTAACGGAAACGGAGTGAAACTAACACTCAAAGAAATATCTATATCTG
CTATATTTGAATATCTTGATTAATACCATTGATATCCCAAGCTCAAATCAGTAAGTCGAAAACCATACAA
TACGACGAATATAATTGGCTTATTGAAAGAAATACTCTATATATAATGAATCTATTTTTTCAGTTGCACC
CTTCGTCTTGCCAGTTTTCGAAAAGGGTTCTCCGAAAAGGGTTGCCACCGCACCCCGATGCCCATTGAGG
ACTCATGGAGCAAACGGTTTCGGCATTTCCCTTTTGTTTCCGCCTGCATCCTTTGGCCAAGACACGGACA
CCTTCCCCTTCCCACCACGCTGCCACAATGGAAAATACAATTTTCGCAATCTTTGCTTATCTTTTCATCT
CTCTGTGGCTGCCTCGTTTGCTGCACGATTCGTTGTTGCTGCTGTTGGCCATCCAGAAAATCGGTAAAAA
GCTAAGATCCAGGGGGCGTGGCTGATACGAAGGCGGAGGAGCAGAAGAATGTGTTGGCCGTGCATCCTTT
GTTTGTCGGCAGTGCCGGTAATGCCGAGTTGGAGCAGTAAGCTCTAAGCATCGGTTATGGGCGAGGTTCG
TTTGAAAAATAACAACCGTTTCCCATCTGGATTCTTTTTTATGGTGGCTGAGTCCTTAATATTTATTGAA
AAAGGCGACATTCCATTTTATTTCCAAGTAAGAAAGATAATCAAGTGCACTTTTCATGACTTACTATCTT
TATTATACTATCATAAAACTTATAGCATACTATTACTAATACACCATCATTTTTGACATACATTTGTAAA
TTTATTTCGTCAAGTGATCACATGTTGATAATTTAGATGAGGACCTGCGCAGCAGCAGAGGCGCAGCTGA
TTGGAGCGGAGTTGGCCAAACGCGAAGGGTAGGCAAAAGGAAGCAATCTCAGAAGCAGCATCGATACCAT
TGCTGTTCCTGGCGATAACCTGGCTACTGGTGGCGGTCACAACTGGAGCGGGAGCAGCTACCACGGCAGC
aggagcagagtaggccagaggagcagagtaagccagaggaccagtgtacgccagGGCAGCACACAGGACT
CCAGGCTTGGCAGCAGCGCAGGCGACGGTTATCAGGACAAGGACGGCCTGTAAACAAATCAGCTTGATTA
GTTTAATCCTCTATGGATCCTTTCTGAAGAACG
>dme_circ_0005165_junction_seq
ATCCTCTATGGATCCTTTCTGAAGAACGTTTATGTGATTTCCCACTGCTGACTGCA
>dme_circ_0005166
TTTATGTGATTTCCCACTGCTGACTGCAGAATGTGGAATAACTCTTGGTGGCCACGGAATGCGATGGCCT
GAGGTCACAGGCGCAGTCCCGATGCGGCGCATAAATAGTGGAATGGAATGTGTTGGGCGACATCAGGTTC
AAGTTTTCGAACCGAAAGCACCAGATCCATAATCCAAATGTTTAAATCCGTAAGTTTGAGATCTTAGCTT
GCTGCCCTATATACTAATTGTATTTTATTCACAGGACGTCCTTGTCTTGGCCATCGTCTCCTGCTCTGCT
GCCAAGCCTGGACTGATTGGTGCGCCTTTGGCCTACACTGCTTACGTGGCTTACTCTCCTCTGGCTGTAC
TAATGGCCTACCCCTCGCCTTTGGCCTACTCCGCACCACTGAGCTACGCCGTTCCATCTGCACCGCTTCT
TATCTAAATTACTAAGCTGTGATCACCTCGGAGGCGACGATAATAAATGGAGATATAATAAGACGTTATT
CAATTGTATATTCTTATTTAAGCACGCTTCAAATTAATATTCATTTATTTCAATGAAAAATTAGCTCTGA
ACGTACACGAAAATGATTGCTAGAAAATTTCCCAGCATTGATTAACAATTATTCCAATTATTCTTATTCT
TATATTATAATTTTAACGACTGATGCATTTCTCAATCACAGCCATTTCTAAATCAATCGTCAAATCTAAA
CTAACTGCAAATTTTAAAAGTATTGTAGTGATTTAGAAAATATAGGTCTTTCGCTAAATGATTACCGAAA
Tactcgtgttgcgtctacgtttctggaatctccctgctgcattttaactttttagcttttatagctttcg
agatcacagcattcataggaacagtcggatacatagtcgtgaaaacagtgattcttattaaaagtatata
tacaatacgggctcgaaaactttagcttccttttacccattacgtacttttcaacgaatttagtatacat
ttttactctactagtTTGATATGCAAACTACAACATTTTACTAAAGTTTGGGCATTTAAGAAGTTACTAG
AATATTACTTGTATATATCAGAAATTTCGGGCTTTCAAAATCCCCAGATCTTAGACGGTCAAAGATCCCA

ATGAATTTCTTTTTTGTAAGAAAGCACTTCCAATACAGCCTTTTATTGTAAAACTGTTTTTTTAATTTGA
TATACAGATaaaaatgtgggcgtggcagttttgggctgttttagggcgttagagtgggcgtggtaaaatg
ctcttttgcaaatcgatagcaattaacttgactaatggaaatataaaaatatataaaaacatttttaata
agtgggagtggcagttttgggcgtcttgtgggcgttgcaacatgggtcaaaaaacttgcactgcgtctaa
gtctctagaaactgtattaatcttaatcttctagcatacggacatggtcagatccattcggctactgatc
ctgatcaggaatttatatatttatatggtcataaacgctttcttcagggaactcaactatagcattctc
tcttgctttcatagttcccgagaattcgacgttcatccggccgaacatgtgttacgtacttccaaacgaa
tctagtataccctgttattctacgagtatcgttttttttttGCAAGTCCAAAAATCTAATACTTCAGCA
ATTTATGGGGTCATATAATCGATATAAGCGTTCATTTTTATTATATTTATCGTCCTAGATAATCACAGCA
TAAAAATTTAGATGAGAAATGGTGCAGGAGCAGCAGCGTAGCTCAGTGGTGCGGAGTAAGCCAAAGGCGA
GGAGTAGGCCAGTCGTgcagccagaggagtagccgcgtacttcgccaccacaggagcagccagaggagta
gccgcgtacttcgccaccacaggagcagccagaggagcagcaacgggagcaatcacaggagcagAGGCGA
TTCCATTGTAATTCCTGGCGATAACCTGGCTACTGGTTGCGGTCACAACTGGAGCGGGAGCAGCTACCAC
GGCAGCaggagcagagtaagccagaggagcagtgtaagcaaggggagcaCCCAGGAGTCCAGGCTTGGCA
GCAGCGCAGGCAACGAGAGCGAGAACGACGACAGCCTGTGAACAAATAAACTTGATTAGTTTAATCCTTT
GGGTGTCCTTTCTGAAGAACGTACGTATTTGAACATGGTGAAATATTTGTTTGGTTTGCTTCTTCGGAAG
GCTAGAAACTCTTTGATATCCCACTGCTCTTCGTGGAGCATATTTATACCCGAATGTTTGCTCAGATCTG
ACTCATGATCGCACTCATTCAGTTCATTTAAATGCGGTGACAGTCTCCCGTTACCATTTACCATTTGCAC
TGTGCCGTAGTTATGCACAAttttttttttGTTAAAAGTGCTATTAATTTTATGCTTTAACTGGATGTAA
ATGGACCGTTAAAGCCCAGCTCTGCTTGCTGATGCCTGCGTGTATCGAAAACCAAAAACCAGCCGCAGAG
CAACGAACCCGCGACCGCCGAAACATTTCAAAGACAATCCACCAACAGCGGCAACACCGTCAACAGTCGG
CCAcaccaccatccattcaccgtcaccatcaccatcaccgtcgccaACAGGACGAGGGCGAGGACAATGG
ATCAACTCAAACTCAGACCTTGTAACTGCCTGACCGCACATCCACTGGCAGAGTGGGTGTGGGTGGAATG
TGAGCGGCTGGGTAAGTGGGTGGCCAAAGAAGTGAGGGCCAGTTGATGGCCACATGTCACAGACATTATG
GAATCATGAACTTATGCGCACCTGTGCGTGCTTGCGGGGGCGCGGGCGGTGGGCCCAGAGCTTCATTCTG
GTTATATCCGTGTGGACTTGCGGTCGAGGACAGCGATTTGAGGTCCGCCTGGTGATGGTTTCGTGACTCG
GCAAGGATTTCATCGGTTTAATGTGGAGCACTTAGAAGGAGAAACATGGCAACGTATCAGAAGTATTATT
AACTTGAAATTATGTGATATATTATTAGTTCTATATTTTTCTATAACTTCCTTCAAGCCATAACATTCGA
GCTTCTCACGCAACCATCGCCAATTAGGAGCCGGGCCATTTCAATCATCAATCAAAGTGTTTTGTTACGA
CTTTCATACGTCATGTACATGGCTCGGAGCCACCGCTTTTTGCTTTTGCAACCCATAGGGGGCTCCTCGC
TGTAGCTCCTCCATATCCTGCCTCAGTCTGGGGAGCCAGTGATGCGGAGGTGTGACTGCGACACAACGAA
CGACATTTATGGTGTCGTCAAGAAGAATTCCCATTACAGCCACAGACAATCAATCACTGTGCACCGAAAT
CAATTGTCGCAGCTCCTATTTTCGTTTTCAATCTCATTTCGCTAAATATTTATGAGGCCAACACTTGGGC
CGTTCGATTTGTAATCCTAATGAGGCGTAATTAGAAAACATTCAATTACATTTCATTGGCATCAATCAAT
GGCGCCCCATCCATGGCTATTTCAAGGATGACAACTTTTAGTAAACCGAGTTTCAATTTCCCAACGCATT
GTTAAGATGCCTCTGGTGCTTTAATCCACGTTCCCAGCTACTGTTTCTGTTTGTTGCTCCTGATTGCTGC
TTAAACTTTTCCCTCGCTCGGACGACTTGTTTGTGATTAAGCTTTATTGTTAAGAGGCTTAGTACGCCTT
TTGATTTATAGATATTGGATCATACCACTCACTCTGCTCCGCCATCAAAGGAGACTGTGGTCCTTTGAGA
AACCGGCAATGTGAAGTTGGCCGGCGCGCGTAGCTTCATAGAAAAACTTCCAGAAGGAGTGAGGTATTCG
TAGGAAAAGTCTGTACACAGAAAGGATAGGATATATGCCTAGTACTTAAGTTGTAAGCCGCTATTGTTGT
CCTATAACTTAAAAAATTTGTTGTCCATTAAGATACATTTTTGGTTTGATTTCCTGGTTTAGAATAACAA
AAGAATATTTCTTATAGATACACTGATAAGACCTTAAGATTTAAATTTTCTTTCATTTTTTTCAAGCTTC
AGCAAACTGGTTTGCATTACCCTTTATCTGTTATTTAACGTATTGTTCATGCACCTTTAGGTTGATAAAC
CTTTTTTATCTGTACTTTCCGCAGTGCATTGGGAATTCGCTGATCCTTTCTATGTATGTCCATTTAGCGC

TTCTCACTTGTGCCTTTGTTGCATACTCTTAGACGCGCGGCTGAACTTGTTTACAGGCACACTAAGGACG
AAGGACCCAGGATCCTGTACATTGGCCCAAAAGTGGACGCTGGCTATTCGGCATCCTTGACAGCCGCACA
ATTTGCTGCCGCGTTTTTGTATCTCTCAGTGCTTGTTTGTCTGCACCACAGCGCCTGGGCTCTGTACACA
GAAAGAAACATTCGAGTCGCAAGCAGGATATTGATATTAGAGCTGAATGCCCTATTAATTCTGTATGCAA
CCCATGTATAATATTTATTTAGCTTTGTTCCCGGGAAATTTGTATTCCCTGATACCCTAGATTTGCCCGA
AAATAGGTATTTTTCTCTCTGTGCATGTTTGTGTTTGTGTGCAGATGGCCAGCAAAAAGTTTGCGCACTG
ATTGCAAACTTGTTTCAATTGTTTCGTAAACCCAACCGAAGGGAAGAACCCAGAAGACTCCCGGTGGGTG
GATGAGCCTGGTGGGCTGGGTTTTTGTGGACCGCTTTTTCCGCCATGAAAGTCAAGGCGAACGCTACGAG
TTCCCAGCTACGAGCTCCGAGACCAAGGCAATTCGCCTGCATATTTGTGTGGCTTAGTTTCCGTTTCACA
AACGAAACGAAATGAAACGGGCGGCATAAAAACCCATTGAAAATTTGCATACGAAAATGAAATTCAAATA
GGTTTCCCATGTCCTTCGCAATCAGTTCCGGCTAGTCCAATTGCACAAAATAGTGAACGAAAACTTTCGC
TTTTCCTCCGCCGTCTAAGCCCTTTATTTATGATCCACAATAATGCAAAAAGATACATATCTGAATATCT
CATCTTTGGGGTTTATCTCCTGCGCCAGTTGCCAGTTTTAATCTAAATCGTTTGGCTGACTTTGTTTATC
GATGCTTTCTTTGGACGCTTCTACCTCGGATTTGCGATTCGAACACTAAGTGATTTTTCATTGACTTTGT
AATGCAAATTGCCTGTCTGTCCTGCCCCTTCCTTACCATGCTTCTCTTTGCGATTGGCTGGATCTTTGGG
CCTTTTATCACTGGAGGCCTGTGCTACGGACCCTTCAACAGCTCGAAAGCCAATAAAATGCAAATTTTTT
CATCGCGTAAACAAATGCTAGACAAAGATGCCTAGAGATGGGGACTCTACGTGTCTGAAAGCTGTGAAAT
AAACCTGTGATTTATGCGGATATCGTTGTGGGGACCTAAAGCCCCTTTAAATCTTTCGCGAAATATACGT
AAAATAACTTTAATGTGGTTTCATTCTTTGCTGTTCAAATTCAATAATCGTACTGAAACAAAACCCATAT
AATGAAATTCATATAGTCCTAACCGTATTAACTTATACCAGTGTCCTATTTGATTGCTTGGTCATCCTGA
CTGCTCACGATTCCATCTCCAGTGGCCGATGAAGTCCAAAACTGAAAACCCAAACTCATTTCCATCGGCA
TCAGACTTGACTTGAGCCGGGGCCCTCGACTCGCAGTCATTGATTTAATTGTAAGCTGTTGAATTTTGAT
GGCTTTTAATTGTCTTAAGACCGGTTCAGCAAAAACCAATCAGGGATCCTTTGATAAGACGtggtggttg
gatggttgcatggatggatggatggatggacaatgggttgggttCTGTTGTCAGCAGAATAGTTGGAAAT
TCCACTTAGTGCTGACTACTGACCCACTTATTGGTCACTTCTGGGTCTGTGTTTCCTTCCGAACCCCCAT
CCATCTATCCCCACTCCTTGCAAAGTCAACCACTTGACTAAATAGCACTCAAATTTGATTTTGATTTATG
CTGCCCAGCAGTCCGCACCGAATTTCCTCCTCTTTCCTCAAGCTCCAACTTTCCCTCCCTCCGACTTTTT
CCCCCTCCCCcacaccacacacacacaTAAGTACAATTGCTGGCTCATAACTTTGGCATCTATCGCAGTA
TGCCTTTCATATTTACATGCTCCTTACACGCGCATCGGCGTGTAACGGAAACGGAGTGAAACTAACACTC
AAAGAAATATCTATATCTGCTATATTTGAATATCTTGATTAATACCATTGATATCCCAAGCTCAAATCAG
TAAGTCGAAAACCATACAATACGACGAATATAATTGGCTTATTGAAAGAAATACTCTATATATAATGAAT
CTATTTTTTCAGTTGCACCCTTCGTCTTGCCAGTTTTCGAAAAGGGTTCTCCGAAAAGGGTTGCCACCGC
ACCCCGATGCCCATTGAGGACTCATGGAGCAAACGGTTTCGGCATTTCCCTTTTGTTTCCGCCTGCATCC
TTTGGCCAAGACACGGACACCTTCCCCTTCCCACCACGCTGCCACAATGGAAAATACAATTTTCGCAATC
TTTGCTTATCTTTTCATCTCTCTGTGGCTGCCTCGTTTGCTGCACGATTCGTTGTTGCTGCTGTTGGCCA
TCCAGAAAATCGGTAAAAAGCTAAGATCCAGGGGGCGTGGCTGATACGAAGGCGGAGGAGCAGAAGAATG
TGTTGGCCGTGCATCCTTTGTTTGTCGGCAGTGCCGGTAATGCCGAGTTGGAGCAGTAAGCTCTAAGCAT
CGGTTATGGGCGAGGTTCGTTTGAAAAATAACAACCGTTTCCCATCTGGATTCTTTTTTATGGTGGCTGA
GTCCTTAATATTTATTGAAAAAGGCGACATTCCATTTTATTTCCAAGTAAGAAAGATAATCAAGTGCACT
TTTCATGACTTACTATCTTTATTATACTATCATAAAACTTATAGCATACTATTACTAATACACCATCATT
TTTGACATACATTTGTAAATTTATTTCGTCAAGTGATCACATGTTGATAATTTAGATGAGGACCTGCGCA
GCAGCAGAGGCGCAGCTGATTGGAGCGGAGTTGGCCAAACGCGAAGGGTAGGCAAAAGGAAGCAATCTCA
GAAGCAGCATCGATACCATTGCTGTTCCTGGCGATAACCTGGCTACTGGTGGCGGTCACAACTGGAGCGG
GAGCAGCTACCACGGCAGCaggagcagagtaggccagaggagcagagtaagccagaggaccagtgtacgc

cagGGCAGCACACAGGACTCCAGGCTTGGCAGCAGCGCAGGCGACGGTTATCAGGACAAGGACGGCCTGT
AAACAAATCAGCTTGATTAGTTTAATCCTCTATGGATCCTTTCTGAAGAACG
>dme_circ_0005166_junction_seq
ATCCTCTATGGATCCTTTCTGAAGAACGTTTATGTGATTTCCCACTGCTGACTGCA
>dme_circ_0005167
GTACGAGGAGGAGACCGAAAGCGAGGAGGACAACGAACCCCGCCTGAAGCCGCTCTTCGTCCGAAAGAGA
GATCGTGCCACCATCCAGGAGAAGGAGCGAGAGGCTCAGAAGCAGAAGCAGCTGGAGGCGGAGGCCAAGC
GGGCAGCCAAGGAGCGCAGAAGAGCCACCCTGCGAATG
>dme_circ_0005167_junction_seq
GGAGCGCAGAAGAGCCACCCTGCGAATGGTACGAGGAGGAGACCGAAAGCGAGGAG
>dme_circ_0005168
GGCGGAGGCCAAGCGGGCAGCCAAGGAGCGCAGAAGAGCCACCCTGCGAATGGTCGAGGAGAGCGTCAAG
AAAGATCTGGAGAAGACCAAGCCGGAGACCAATGAGGCCTGCATCGAGGACGTGTGCACGGACGACGAAA
ACGACGAG
>dme_circ_0005168_junction_seq
CGTGTGCACGGACGACGAAAACGACGAGGGCGGAGGCCAAGCGGGCAGCCAAGGAG
>dme_circ_0005169
TTATTGGAACTTACCAAGGACATAATTCCCGATGCGCCTTATGCAGAGTTGTGCAAAGCGAAGGGAACTC
AAGGCGGCGCTAAGATAAGCTATCACTAGAATGCTGATTAATCATGATTAGTGATCTAGTATACAG
>dme_circ_0005169_junction_seq
TAATCATGATTAGTGATCTAGTATACAGTTATTGGAACTTACCAAGGACATAATTC
>dme_circ_0005170
TGCACCGGAGGACTGGATTCCCAGCAGCGAGCCTCGGCGAAGAGCAGGCAGAGCTATGCCCGCGGATTCA
GCTTGTCCTACAACTCAGCCTTCCGGAAGATGACTAACAAGGGAATCGGCGGAGGTGGATCGGAGCGGGT
AGATGAAGCAGGCAACCTGCATCCAACCAGTTCTGCCTCCTCAGTTAACTCGTCCAGCATTTCCTCCGGC
GGGGATGCTTTCCAGCGGGACGGTGCCGATCTTAATTGGCTGGGACCCACG
>dme_circ_0005170_junction_seq
TGCCGATCTTAATTGGCTGGGACCCACGTGCACCGGAGGACTGGATTCCCAGCAGC
>dme_circ_0005171
CCGGATACATACAACGTGATCTCCTGGGGCTATGATGATCGCATTGTGAGAATCCAGCCACTCAACAAGC
CACAGGCCAAGCCAAAGAATCTTCTACACAATGGGACCTTTGACGACATTACCGCCTG
>dme_circ_0005171_junction_seq
ATGGGACCTTTGACGACATTACCGCCTGCCGGATACATACAACGTGATCTCCTGGG
>dme_circ_0005172
GGAAGAGCATAGACCACATTGGTGTTGCAGAAGCTAACCAGGTGCTCGGTGCCTGGTATCTTGTGAATGT
TTGCCCATGAAGGAGCTTTCAGCTGTGGCGATCCCACATAGACGCCCCAGCGCAGACCGCGTACGGTAGA
CACTATGGGTTTGCCCACCAGTGAGTAGTCTAGCGGCTTGGACGCCGGATGTGGCGACTTAAAAAGCTGC
CTGGGCATTTGACCATAT
>dme_circ_0005172_junction_seq
AAAAAGCTGCCTGGGCATTTGACCATATGGAAGAGCATAGACCACATTGGTGTTGC
>dme_circ_0005173
GAGCATAGACCACATTGGTGTTGCAGAAGCTAACCAGGTGCTCGGTGCCTGGTATCTTGTGAATGTTTGC
CCATGAAGGAGCTTTCAGCTGTGGCGATCCCACATAGACGCCCCAGCGCAGACCGCGTACGGTAGACACT
ATGGGTTTGCCCACCAGTGAGTAGTCTAGCGGCTTGGACGCCGGATGTGGCGACTTAAAAAGCTGCCTGG

GCATTTGACCATATGTCTTCACCATTGTCTTGACTGCTTCCCTTTCGATGGGATCGCTGATCTCGGAGTC
CAGAAAAACGGCGTAAGTCTGTGGATAAATTCAAAAGAAGATAAGCATCTATTGCATACATTTCACAGGA
CATCATCACTCACTGCTGGGTGAAACACATTGATGGCCTCCACGGCACTCTCCCCGCTCTGTTTGTAGCC
GAAGATCAGGTCTATCCAGTTATGAATCTGATTCCGTACCAATTCCGACTCCAAAGCCTGGCGGTGGATA
AGGACAAATAGCCGGGAGTCTCGCTGACTCCATGGTGGTAGTGAGACATCCTCCACACGCTCCCCATTTT
GTCGGCAACCGAATTTAAAGCGTTCGAAGTTTTCAAACATCTCCGGCAGGCAGAAGAATTCCGGAATGAG
TTCCTTCACATCCGTGGGACTATCCCGGCTAGCCAACAACCAAGTGGTGTTCAGGGCATGGAACGTGCGA
TCCGGTAAATCAAAGTCGTTGTCCTGGTAACGCAGAAAGTAGCTGGTAAACGGAGGAACCCGTACCAGGA
AGTGCAGCACAGTGCCGGAATTCGAGTAATGGGAGCTGTAATGATAGGGCTTAAGGATCAGCGAGCCCAT
GGTGGTGTTGGTACTGTCAATGTACTGGAAGGGGAGAAGAGAGATTATTTTCCAATGGATTGCTGAATAA
GATCGTTACATACTGTATAGTTGCTGATGTAGTGCTTCTCGTTCTCCTCCACCTGCACGGCAATGGGTCG
TCCAAGTCGCCTGAAGTTGTGTCCCTCCCGCAGATCCAAAACCTCCGAGTTGTAGTTGGCCAGCACCCAG
GGAAACACTGGGTACTGCATGAGATCGTTGTAA
>dme_circ_0005173_junction_seq
CACTGGGTACTGCATGAGATCGTTGTAAGAGCATAGACCACATTGGTGTTGCAGAA
>dme_circ_0005174
GCTCCAGTGGTGACCAAAGTCGCCACTGGATATGGAGGAAGTGGCTCGGGATACAGTTCGGGCGCTGTTT
CCCATCAATATGTCTCTAAGCCAGCGGTTGCTAAAGTGGCAACCTATGCTGCTCCTGCCATTTCAACATA
CTCAGCAGCTCCGGCAGTGACCAAGATCGCGACAAGCTATGGAGGATCGGGTCATGGAGCCGTCTCCCAT
CAGTATGTCTCCAAGCCAGCAGTAGCCATCTCAGCAGCCCCCGCAATCGCTAAGGTAGCGACATACGCTT
CTCCGGCAATATCAACGTATGCCACTGCTCCTGTGGTCTCAAAAGTGGCTACCTACGCAGCTCCCTCCAT
AGCCACTTACTCCTCCGCTCCGGCTCTAGCCAAGGTTTCCTATAGTCAGGCAGCGGATGTATCCCACCA
>dme_circ_0005174_junction_seq
ATAGTCAGGCAGCGGATGTATCCCACCAGCTCCAGTGGTGACCAAAGTCGCCACTG
>dme_circ_0005175
TTCTGCAAGTCAAATAGTAACATCACCTTGGACCACCTCTGCAATTTCCTGTTCAAGCAGCTTTATAGTG
TCCAGCACTCAGTTCGATTAAGTGCTGTACACTCGCTTCGCCAATTAACGCCACACTTTGTGGCGGACGA
CATAGAGCTGAATGAGAAACAAAGTGAGAGTCTAGACGCCAGCACAACGATTTGCAAGTGGCACTTCCTC
AATCGCTTTGAGGACTATCTAACACGCTACGATGCACTGATAACTAAATACCTGGAAGAG
>dme_circ_0005175_junction_seq
TGCACTGATAACTAAATACCTGGAAGAGTTCTGCAAGTCAAATAGTAACATCACCT
>dme_circ_0005176
GGTATTTAGTTATCAGTGCATCGTAGCGTGTTAGATAGTCCTCAAAGCGATTGAGGAAGTGCCACTTGCA
AATCGTTGTGCTGGCGTCTAGACTCTCACTTTGTTTCTCATTCAGCTCTATGTCGTCCGCCACAAAGTGT
GGCGTTAATTGGCGAAGCGAGT
>dme_circ_0005176_junction_seq
AAGTGTGGCGTTAATTGGCGAAGCGAGTGGTATTTAGTTATCAGTGCATCGTAGCG
>dme_circ_0005177
CTCGTTTGGGACATCCTCAACTTGCTATCCTTTTACCAGGACAAGCAACCACAGTTGATTATGCGCAGTT
TTCGAACAGCCCAGGCACAACTTCAACTGGAGTGGCTTACCAAGTTCTTTGAGCCAAATAGTTTCCGCGT
TGCAGTGACCAACGATTCCTCCCTGAGTCACTCCGAGCTGCGAACCCGAGTGGAGCTGCTCATAGATCGC
TGCACTCAGTTCTTCACCGTTGGCGTGGGACAGACGACCGGACCCAACTACGTGGCCAGTTCCCAGGAGC
TGGCCCTCTTCCAGCTGCTCGTTTCGTACGGGATCTCGAGCAATCAGCGATGCAACAACTTCATTGCCTG
GGGACTTCAACCTTCGCGTCCACGCGATCTGCGCTCCTACATTGTGGATGCCCTGTGGCGATCCCAACAG

GATGAGTTCCAGCGGGCTATTATCTG
>dme_circ_0005177_junction_seq
AGGATGAGTTCCAGCGGGCTATTATCTGCTCGTTTGGGACATCCTCAACTTGCTAT
>dme_circ_0005178
TGCTGCACATCAACATAACTGGAGAACACGAGAAACTTTTCATATCCCCGCAGTGAACGATAAAAGCAAA
AGTAGCAGCAGCCACAGCAAAATGACAGCAATAAGTTCAAGCAATAAAACACAGCTAGCCAGAAGTTGCA
AACATGCACTCGCAGCACAATAAGGACGGGTGGCGGTCGAAAGCTTTAAAATAGGAGTTAATCACCGGAC
TCTCCATCCCGCAGAGCGCCCGGTACACCCGGGACACCCCAATAAACTGCCAAATAATATAGGATATTAG
CCAGCCGGTGGACGGAACCCAGCGAGGAGCGCAAGAAAGAAGCCCTACTGTCAGCCGGGACACTGAAACA
TTCCACCCCATAGCATGGCCTCGGTCGCCGCTTGGCTGCCCTTCGCCCGGGCGGCGGCCATCGGGTGGGT
GCCGATAGCCACCCACCCACTGCCACCGCCCCCGATGCCCAAGGATCGCCGCAAAACGGACGACGAGAAG
CTCCTGATCAACGTCTCCGGGCGGCGTTTCGAGACGTGGCGGAATACTTTGGAGAAGTATCCGGACACCC
TTTTAGGGTTCCAATGAAAGGGAGTTCTTCTACGACGAGGACTGCAAAGAATACTTCTTCGATCGGGACC
CGGACATCTTCCGGCACATACTGAACTACTACCGGACGGGCAAGCTGCACTACCCGAAGCACGAATGCCT
CACCAGCTACGACGAGGAGCTGGCCTTCTTTGGAATAATGCCGGATGTCATTGGCGATTGCTGCTACGAG
GACTACCGGGACCGGAAGCGGGAGAACGCGGAGCGGCTGATGGACGACAAGCTGTCGGAGAACGGGGATC
AGAATCTGCAGCAGCTGACCAACATGCGCCAGAAGATGTGGCGGGCCTTCGAGAATCCGCACACGTCGAC
GAGCGCCCTGGTGTTCTACTATGTTACGGGTTTCTTCATCGCCGTCTCCGTGATGGCCAACGTGGTGGAG
ACGGTGCCGTGTGGCCACCGGCCGGGCAGAGCGGGAACTCTGCCCTGCGGCGAGCGCTACAAGATCGTCT
TCTTCTGCCTGGATACCGCCTGCGTGATGATCTTTACGGCGGAGTACCTACTTCGACTCTTCGCCGCCCC
CGATCGCTGCAAGTTCGTGCGCTCGGTGATGAGCATTATTGATGTGGTGGCCATTATGCCGTACTACATT
GGCCTCGGGATCACCGACAACGACGACGTGAGCGGTGCTTTCGTCACGCTGCGCGTGTTCCGTGTCTTCC
GCATATTCAAGTTCTCGCGCCACTCGCAAGGACTTCGGATCCTCGGCTACACGCTCAAGTCCTGCGCCAG
CGAACTGGGCTTCCTTGTCTTCTCGCTGGCCATGGCCATTATCATCTTTGCCACCGTCATGTTCTACGCC
GAGAAGAACGTCAATGGCACCAACTTCACATCGATTCCGGCGGCCTTCTGGTATACCATCGTCACAATGA
CGACGCTGGGGGTAAGAAAAGGCTATCTGGGGAATCCCGAGGAATCCGTTAGTGTCACAAGAACTTTAGA
CTGGAATTACCAGTAATTATAGTTACAGATTTGGCCAGCAGGTTCTGTGACACTGATATTAAATCGGTGA
AAATTTTATCTGGGAGCCAGTGAAAGTCATCCATTTTTCCGTGTGCATCCCAACTAAATGCCACCCACCA
CCCGCAAATACTCATTATGTGTTATGACCTTGCAGAAAAGCTCGCCTGGCGCGCATCCGCATTGCCAAGG
CCTCGTCCGGAGCCGCCTTTGTTAGCAAGAAGAAGGCCGCCGAGGCCCGGTGGGCTGCCCAGGAGTCGGG
CATCGAGCTGGATGACAACTATCGGGACGAGGACATCTTCGAGCTGCAGCACCATCATTTGCTGCGATGT
CTGGAGAAGACAACGG
>dme_circ_0005178_junction_seq
TTGCTGCGATGTCTGGAGAAGACAACGGTGCTGCACATCAACATAACTGGAGAACA
>dme_circ_0005179
ACTAGCGAATGCTCGTCGAGAATCCAAACCAGTGAGCCAAACGCCAAAGAAAAACTTGCAAAAGTCTGTG
GATAAACAACGAACAATAACAAAAGGCAGCAAGCGGAAGGTGGCAGTGGAAGTGGACTAACTCGCCCGTG
GAGAAGTGCGGAGCACACAGGACTGCAAAGTGGCGTCGCTACTGGATATTAGGGCTGGGGCAGCAGCGGC
GTCCATGTCCACGGGGCACAAAGGGTGGACCACCAAGAATCACGACCGCACCACATCCAGGAGAGCGCAG
CAGCAGCAGCCGGTGTGGTGTCGCCAACTGTGGCCTTAAGCATGTAGGTCTTGCAGCAAAGAACCCGTTC
TCTAACAAGCACAAGACACAAGGGACAAGGTACCAATCGAAGCAAAAACACACAAATATCCAATCAACAC
CAATGAAAAATGTCACAAAGAGGTAAGCGCGGTAATCAGCAGCATCACCAGTCGCACCATCCGCCGCCGC
AACAGCATCAGCGCAAGGATGTTGAGCCGCAGCCGCCGCCCACCAAGCGGCGCAAAGGTAGGCCACCTAA
TGGAGCCACTACGGCAGCAGTAGCTGAAGTGACTGGATCGGGTCCAGCTACAGGATCGGAGCGTGTGCCA

GTTCTGCCTTTGTGCAAAAGCAAACATGAAGAGCCAGGGGCGGAAGCGGGGGGAGGAGGACAAGGAAGAG
CCGCGGCAGGCGCTACCAGCACTAGCAAATCGAAGTCAACGAAACTGGCAAAGTCGGCAAGCAAGTGCAA
GTCGCAGGGGGCGAGTTCTAGTAGCTCCTGGCAGGCGCGGTCTGTAGCGGACATCAAGATGTCGAGCATC
TACAATCGAAGCTCAACGGAAGCTCCAGCTGAACTTTACCGGCAAGGATCTCATTAGCGCAATGAAATTG
CCAGACTCTGAGCCGTTGGCCAACTACGAGTATTTAATAGTAACAGACCCATGGAAGCAGGAATGGGAAA
AGGGTGTGCAAGTGCCCGTTAACCCAGACTCCCTTCCTGAGCCATGCGTCTATGTTCTGCCCGAGCCTGT
TGTGTCTCCAGCGCACGACTTTAAGCTGCCCGAAAAATCGCTATCTGCGCATTACCAAAGACGAGCACTA
CTCGCCGGATCTGCATTACCTGACGAATGTTGTTGCCTTGGCGGAGAACACATGTGCCTATGATATAGAT
CCTATTGACGAGGCCTGGTTGCGTCTCTACAACAGCGATCGTGCCCAATGTGGTGCTTTTCCCATAAACG
CGACGCAGTTTGAGCGCGTCATTGAGGAGCTAGAGGGTTCGTTGCTGGGAACAGATTCAAGTCATACTTA
AACTGGAAGAAGGCTTGGGCATCGAATTCGATGAGAATGTCATCTGCGATGTTTGCCGATCGCCCGACTC
CGAGGAGGCGAACGAAATGGTATTCTGTGATAATTGCAATATCTGTGTGCACCAGGCGTGTTATGGCATT
ACAGCAATTCCATCAGGGACAATGGCTATGCCGCACTTGTTCGATGGGAATTAAGCCGGACTGTGTCCTC
TGTCCAAATAAGGGCGGCGCTATGAAATCCAACAAGTCGGGTAAGCACTGGGCACACGTCTCTTGCGCTC
TGTGGATACCCGAGGTCAGCATTGGGTGTGTGGATCGCATGGAACCCATCACGAAAATCTCTAGCATTCC
TCAGTCGCGTTGGTCCCTGATCTGTGTACTCTGCCGAAAGCGAGTTGGCAGCTGCATCCAGTGCTCAGTA
AAGCCCTGCAAAACAGCATACCATGTGACTTGCGCGTTTCAGCATGGCCTGGAAATGCGTGCAATCATAG
AGGAAGGTAATGCTGAGGACGGCGTGAAGCTGCGCTCTTATTGTCAGAAACACAGTATGAGCAAGGGTAA
GAAGGAAAATGCTGGTAGTCATGGGGGAGGCAGTGCCTCAGTCGCAAGCGCCATGCAGAAAGCAAACAGA
TACGGCAGTGGGGCTGGTGGAGGAGCCGACGACGGCAACAACGCGTGCGGGACAACTGGAGAGGATCCAC
GCAGGCGGAAAAATCACCGTAAAACCGAACTGACCTCCGAGGAACGTAACCAAGCCAGAGCTCAGCGCCT
CCAGGGAGGTGGAAGCTGAGTTCGATAAGCATGTTAACTTTAATGACATCAGTTGCCATCTATTTGATGT
CGACGACGATGCTATTGTTGCCATATACAATTATTGGAAGCTTAAGAGAAAGTCTCGACACAATCGCGAG
CTTATCCCGCCCAAGTCCGAAGATGTGGAGATGATAGCCCGCAAGCAGGAGCAACAGGACATGGAAAATC
ATAAGTTGGTGGTGCATTTGCGACAGGATTTGGAGCGAGTTCGTAATCTCTGCTATATGGTTAGTCGAAG
AGAAAAGCTTTCGCGCTCTCTCTTTAAGCTACGTGAGCAGGTATTCTACAAGCAGCTGGGAGTTCTGGAT
GAGATGCGCTTGGAGAAGCAGCAGACCAAACAAGAGCAGCAACAACCTGTGATGGATCTGAACGCTGTAA
TCTACGCCAACGACGGACCTACTTTGTATGACCGTTTCTACAGCTCTGTGGGAGGACAAACTGTGCCGGC
GCAGTACCAGGATTTGAAATACATCCTTGAACAGCTTATGGGTAAGCTGCAGAGTGGTAAACAGGGCCGT
GGCCGTGCCTCGCAGTCTCCTAACAAACGCAAGCAGCCTGCTAAAGCTTCGCCAAACAAGAAGCTTAATA
ATGGCATTCTTAGTTCACGCACGTCATCGCCTGAGAAGACTGTGGCAGGGAGTAAGGTGGGAACGACTAC
ATCCAAGGTACGGTCTCCGCCAGGGAAGAATCCTACAGGGAGGCGTGCCTCGAAGAGCAGCGCGGCGGCG
GCGACGTCGACCCACAACAAAAGTCAATTCCACTCAAATATCCGCAGTAGCACGACCTCCCATTCGTCAA
GCGGCACTATATCATCAGG
>dme_circ_0005179_junction_seq
ATTCGTCAAGCGGCACTATATCATCAGGACTAGCGAATGCTCGTCGAGAATCCAAA
>dme_circ_0005180
GGTCAAGATGAGCGAGTACACAAGCATTCTGCAGCGCGAGTTTCCCAAAATCGACCACGAGCTGCTCACC
TATGTCGTTGGCGTGCTCACCGGAAGTAAGTGCCTTGCACTAGCCTGTGATCTTTTATTCTTTACCCCGC
ACACAAATTCCAATTGCCAGGCGAGGAGGACTTCGAAAGCGGCGATGACATCTTCGAAGCGGTGGGCGAC
ATCCTGCAGAGCGTCGACTGTGATAGGTCGGAGGAGAGTGTACGTACGCTTTGCGAGCAGTTCCTCAACA
TCATGAAGAACAACGAG
>dme_circ_0005180_junction_seq
GTTCCTCAACATCATGAAGAACAACGAGGGTCAAGATGAGCGAGTACACAAGCATT

>dme_circ_0005181
GGTTCTCAACGCGCCGGTCAACATCGAGGAGATGGCCAAGAACATGGAGCGGCTGGACAAGGATATGCAA
AGCATCTGGGTGGCCAACAAAGATGGCGCCAATGTAAGTGTTCCCCTTGACGAACCACTCCAATAAACTG
AATGAACGGCGCCATCAATACGACTTTCATTAAATAACAACTAAACTATTCTTATTTTAACAGAAAGTGG
ACTCCAAGAAACTAGGAAAGGCGGAGGCCAAGCTGCAGCAGAAGCAGGAGAAACGGCAGGAGGTGAACAA
ACATGGTATGATTCCTGTTGCGGTGAAGCTACAAACGGCCACAGCCTCACAGGTGACCAACAAGAAGAAC
ACCAAGCTGGATCAGAAAG
>dme_circ_0005181_junction_seq
AAGAAGAACACCAAGCTGGATCAGAAAGGGTTCTCAACGCGCCGGTCAACATCGAG
>dme_circ_0005182
GATTCTGGCTGCCCTTAACAATGGAGTTCAGGATGCGACGCTGTCCAATGAGCTGAGCGAGACCTACGCC
TCTCTGCAGAACATCGAGGCGGACAAGGCTGTGGCCCGCGCATCTGTGATACTTAAGGGCTTGGGATTCG
ATGCAGATATGCAGCTGCGTCCAACTAAATCCTTCTCAGGCGGCTGGCGCATGCGTCTTGCTTTGGCCAG
GGCACT
>dme_circ_0005182_junction_seq
GCATGCGTCTTGCTTTGGCCAGGGCACTGATTCTGGCTGCCCTTAACAATGGAGTT
>dme_circ_0005183
GGTTACTTTCCGTTACAATCCAGAAGATCCACTGCCCATCTTCAAGGGCGTCAATCTCTCAGCCACATCG
GACTCTCGAATTTGCATTGTCGGAGAAAACGGAGCGGGAAAGTCGACGCTGCTGAAGATTATTGTCGGTC
AACTGAGCACTATTCACGGAAACATTGTCCTGCATCGCGGTCTTCGAATTGGATATTTTGCGCAGCACCA
TGTGGACCATCTGAATATGAATGTAACGTGTGTGGGAGTTTTGGCAGAACT
>dme_circ_0005183_junction_seq
TAACGTGTGTGGGAGTTTTGGCAGAACTGGTTACTTTCCGTTACAATCCAGAAGAT
>dme_circ_0005184
GAGGAGCAGCTAAACAGAAGGTGTCGGCAGCGAAGGACCTCGTTCAGCTTCAATCCCCAGCTGGTAGCTA
TCCTCCCCCCGGTGTGTTCGAGCCATCTGTGGAGCTGGAAATCCAAATTCCTCTTAGTAAACTGAACGAA
TCCGTCATAACCAAAGCAGAGGTCGAATCTCCACTGCTCTCAGCATTGGACATTAAGGAGGATACGAAAA
AGGAGGTTGGCCAGCGCGTTGTCGAAACCTTGCTACACAAAACGGGAGGCAATCTTCTTCTGAAACGCAA
GCGGAAAAAGATAAATCGAACTGGATTTCCCACTGTTCGCAGGAAGAAACGTAAAGTTAGCG
>dme_circ_0005184_junction_seq
GTTCGCAGGAAGAAACGTAAAGTTAGCGGAGGAGCAGCTAAACAGAAGGTGTCGGC
>dme_circ_0005185
TAAATTGAATGTACCATCGAACTGAAGACAGTACTGCGTATATCCTAGACATCTGCATTAGCCAAAATCC
AGTCCAGGTAGCGATCCACGCGAGTGTAGATACCCGGGCGACCCCGCTGTCCGCAGCCGACGCCCCAGGA
CACGATGCCGATCGTGACCCAGCGTTGGTTGGGAAGTTGTACCAACAGAGGACCTCCGCTATCGCCTTGA
CAGGAGTCCTGACCTCCTTCCGGAAATCCAGCGCACATGGCAGTGTCCGGAACGTGCTG
>dme_circ_0005185_junction_seq
CGCACATGGCAGTGTCCGGAACGTGCTGTAAATTGAATGTACCATCGAACTGAAGA
>dme_circ_0005186
ATGAGCCCATTTGGAACAAGATTGAAGAGATGCTTGTGATCACTGCTGCCAATAATAAGCCCTTTGTCTT
TAAACCCTCGAGATATTTGTACACTAAACAGCAGCCTGCATTGGAAACTCCAGTTGCCGTGCAAGG
>dme_circ_0005186_junction_seq
CATTGGAAACTCCAGTTGCCGTGCAAGGATGAGCCCATTTGGAACAAGATTGAAGA
>dme_circ_0005187

TGTGGTCATTGTTCGGACTGCCGGCAGCTCTCAACATTCCATGGCCAGTGAGTTGGAGTTCTCGTTCAAG
GCCTTGATTCCGTTCTCCATTGGCATGGCCAAACTATTATCATCTTCGGGATGGATAACTGCTGAAGCCG
CCTCCGATGTGGACGGTCCATCGGTGGCCATCTCATCTTGATGGCCGTTCTCCACGCCATTTCCATTCCC
GTTGATG
>dme_circ_0005187_junction_seq
CTCCACGCCATTTCCATTCCCGTTGATGTGTGGTCATTGTTCGGACTGCCGGCAGC
>dme_circ_0005188
GTTGGTGGCAGTACGATGACCGCACTAGTCAGGACATCGAAGATGCCTTTAAGAAGGGCGACAAATCCTG
TACAATTTTGGTAGCCGGCTACGTCTACATTGTGGACTTGGAGCAGCTAGTCCAGCAGCGCCAGAATGAG
CCCACTCGCTGCCGGAGGGTTAAGCGAGACCTAGCCACCATACCCAAAAAGGGGGTGGCCGGTCTGCGGA
TCGAAGGCAACCAGGTGACCAGCGACACCGTCTTCAGCAGGCCAACCAACACAGCCAATCCAACCACCGT
GGCAGCAGCTGCCTCCAGTTTCATATCCACCATAGCAGCCACAGATGCGGCCATTAGAATCGCTAGCGAC
ATAATAGGCTCAACGCTGGCTCATGCAGATGAACTAACTCGGGGACTGACGGCTAGCAATCTCAACGATG
ATCTCAGCAGCAGCGGTGAGCAATCCAACTCCACAAATTCAAACGATGCAGGGCGCTCGCCTGCCTCCTC
GCCTGCCTCAAGTTCCATGCAGGATCTGATCACGCGTGACCTGAGGAACACCCAACAGGTGATTGCCCAC
AACCAGCACACCATTGATCTCTTCGAGCAGGCCTTGAACGACTTCCAGGCGCTGGCCATGCGCAACTATG
TTGACTCCAGCGATGAGGAGAACAACGACCAGGATCAGGAGCATGAGCTGCGGGAACGGGAGCAGCTGGA
GGCGGGCGAGCAGCCACAGTCCAACGATAATCCCCAGAGATTTTAGAAATAATCTACTTACCAGAAAGCT
TATATTGTTGCTCGCCTAATCGATACATTTAAAGAAAATATCAAATGTTCCTTTAAGCAGATTTCGATAT
ACGGAAGGGGAATGTTAACTAGAGAAATGCAGCGCGTACCTGAGAATATATCAACTAACATATATAACCA
TAGGCTCAGAGCGTTGGGCGTCGAACAGGaaaaatagaaaaaaatagaaaaGAATGTCTACAGGGTAAACA
AACATATTCCTTAAAACATAAAATCAGATATTGAAACATAGACGTTACAGCAGAAACATAAATAATCTAT
TTAATACGATATTCAATTTTCCCCAAACAAACTTAACCCAATTAAAGCAAAAAAATGGTATTCTTAGTAG
GAAATGGTAAGGACAGCAGTGGCATTGGTTGAATCCATTTGTGTGGCCTGTAATGCTCTAAGAAATCTCT
GTAAGACCTCCGAATAGGATTTCTCTGAATTAACCTGTACATAATAATTACAAAATCAAGAGTTCTACAA
GAATTCGGAAAGTTAACCCTCCCTATGGTCTCATTTGCAAGCTGGCAAAAGTAAATATAAGCTCCTCCAC
CAAGAAAGAGTAGTGTGAAAAAGACAATATTTATGAATTGGGATTAACTAGACATGTGAGTTCAATTAGC
AGGAAGTTCCCACTCACATAATTGATATTCAGTGCGTTTCTTAAATCGGAATCGTTTAATTTGACTTACT
TTCCACAGTATCTATGTTCTGACACAGTTTTTAGATTTAAATTAATGCAAGCTGTTTTCGAAGCGCACTG
TCATCAGGGGAATCAGATGCAGTATTAATTATTAAGTCGCCGTGAAGTGCGTTAAACAAAAACATTTCTG
TGACAGTTGAATATATTGATATTTTGTATACTCTAAGTTGCTTTAAATAATAGATTTAAAGGAAAATTGT
TAGACAGACCTAATGTTGTATACTACAACTATGTGCTACGGATACGATATACGCCCGATTTAATAATCTC
AATTTTGTGACTACTATTTATAGATTCGTTTTCGATATGTACTCCGCCATTACTTCTAACAGCGAAGTGC
GTATTCGTTGCTTCCCTAATATCTACGGTATACCTAAAGCTCTACTGAACCCGACTCCCGACTTCGAAAA
GCCCCTCCTGG
>dme_circ_0005188_junction_seq
ACTCCCGACTTCGAAAAGCCCCTCCTGGGTTGGTGGCAGTACGATGACCGCACTAG
>dme_circ_0005189
CGCTGGACGACGACGAGGATACGGATGTGCAATTCGTTGCAGCTCTGCGTCCCACCGCACCAGTCATCGA
TCTTTGCGTCTCGCCCTCCACTTCTGCTGCTGCTGCTGCGGCGGCGGCAGCTGCAGCCCACTCAGGAGGC
GGAAGTGGCGAGGATCCGGCGGTTGGATCCGCATCTGGAGCTGCTGCTTCGGATACCGATTCACTTGCTT
CTTCGCCTGACGAAGGAGCGTTAGCCACCGGTGTGGATGCAGG
>dme_circ_0005189_junction_seq
GAGCGTTAGCCACCGGTGTGGATGCAGGCGCTGGACGACGACGAGGATACGGATGT

>dme_circ_0005190
ATACATACATACATACTATCGCCAACATGTTCGCCAAGCTATCAGCCCGCGGGATCGCCACCCGCATGAG
CTTCTTGGCACAGAAGACAGCTTCCCAGGAGACGACGGCCGCCGCCGGATCCCGTTCCGAACTCGTCTAT
GCCAGGGAGAACAAGTACGGCGCCCACAACTACCACCCACTGCCCGTGGCCCTGACCAAGGGCGAGGGCG
TGTTTGTCTGGGATGTGGAGGGCAAGCGCTACTTCGACTATCTGAGCGCCTACTCGGCGGTCAACCAGGG
TCACTGCCATCCCAAGATCGTAGCGGCACTCACAGCACAGGCCTCCAAGTTGGCCTTGACATCGCGGGTA
AGTAAATAGGAAAATGGGAATACAATCAGAGAACAAAGAGACGTGCCCCCTGAAAAATTATCGGATCTGT
GCTTTATCAGTTATATGTATTTGTTTAGTGTTGTGTGCACTTTAGGGTGGACTTTGTTCCACAAATTTAT
GCTCATAAATCGTTTTACAATACTGTGCATTTAAATTGATCATGTCACACGAGGCGTATGCGCAATTAAA
ATATTTAGAAGCGATCACTCGCTGCTAAATTGCTGTTTCCAACTGAAGAATTGAACTCATCATTTGATTA
ATAACttttttttttACTGAATAATAAAACTTGAAAATAGAGATTCGAATGTCGCCGATGCTTATTTTAG
TACGTCTGGACAAGATTACATAGGCTATAAATACAATTTCCGGATTATAAAAAGTACATTTCGAAAGCAA
CTGCTTATTTTTAAAGATGTCATGCGATGGATTAAGACTTTTTCGCCTAGACCAATAAAAAGCTTACAAT
CGGAAACACACATCATTTGTTTATGTTTTTACCATATATACATTTCTGACAGTGATAACCCCCCATGAAG
TGGACGGAATCTTATCAGCAGTCAGTACATGTATAAATTCTCACTTTACCTAGTGATTCTACATTTGTCA
CAGGGAATTTATTTAATAATTGTACTTTACTTGTTTTTCAATTTTTGATGCCGGTTTTATTTGGTTTCTC
TTGGGTTGCCAGGGCCAGATTGTCAGGTTGAGTGGGTCTATAAAGTCAAAAGAGGTTCGTTGATAACAAA
TCGAGGGTTCGATGACTTGTTATTGTTTGTTGCAATTTCTAAATTTGGAAATTGGAGGGGAATGATTACG
GATAAGCCAATGGAAAAATATTAGCGGTTTATTTTTGGTTGATCCAAATGGTTATTGTCCTACTTGGGCA
GTTTAAAAACAAAACGTGACGTAGCAATTAAATACAATAATACAATATATTAATCATTTAAATAATTTTT
CTAAATTGTCTTTTCTATTTTCCGCTGTGCAGTTGCCTTCTATTCGGATGTCCTTGGAGAGTACGAGGAG
TACGTGACCAAGCTCTTTGGCTTCGACAAGGTGCTGCCCATGAACACGGGCGTTGAGGGCGGAGAAACCG
CCTGCAAGTTGGCCCGCAAGTGGGGCTACCTGGAGAAGAAGATACCTGCCAACCAGGCAAAGATCATCTT
CGCGCGCAACAACTTCTGGGGACGCACCCTGTCGGCGGTGTCCGCCTCCAATGATCCCAGTAGCTACGAG
GGATTCGGACCCTTCATGCCCGGATTCGAGCTGATCGAGTACGACAATGTGTCAGCGCTGGAGGAGTCCC
TCAAGGATCCGAATGTGTGCGCCTTCATGGTGGAGCCCATTCAGGGAGAGGCTGGCGTGGTGGTGCCCAG
TGATGGCTATCTGAAGAAGGTGCGAGAGCTCTGCACCAAG
>dme_circ_0005190_junction_seq
GAAGAAGGTGCGAGAGCTCTGCACCAAGATACATACATACATACTATCGCCAACAT
>dme_circ_0005191
AAGATACCTGCCAACCAGGCAAAGATCATCTTCGCGCGCAACAACTTCTGGGGACGCACCCTGTCGGCGG
TGTCCGCCTCCAATGATCCCAGTAGCTACGAGGGATTCGGACCCTTCATGCCCGGATTCGAGCTGATCGA
GTACGACAATGTGTCAGCGCTGGAGGAGTCCCTCAAGGATCCGAATGTGTGCGCCTTCATGGTGGAGCCC
ATTCAGGGAGAGGCTGGCGTGGTGGTGCCCAGTGATGGCTATCTGAAGAAGG
>dme_circ_0005191_junction_seq
GTGCCCAGTGATGGCTATCTGAAGAAGGAAGATACCTGCCAACCAGGCAAAGATCA
>dme_circ_0005192
GATAGCCATCACTGGGCACCACCACGCCAGCCTCTCCCTGAATGGGCTCCACCATGAAGGCGCACACATT
CGGATCCTTGAGGGACTCCTCCAGCGCTGACACATTGTCGTACTCGATCAGCTCGAATCCGGGCATGAAG
GGTCCGAATCCCTCGTAGCTACTGGGATCATTGGAGGCGGACACCGCCGACAGGGTGCGTCCCCAGAAGT
TGTTGCGCGCGAAGATGATCTTTGCCTGGTTGGCAGGTATCTTCTTCTCCAG
>dme_circ_0005192_junction_seq
CCTGGTTGGCAGGTATCTTCTTCTCCAGGATAGCCATCACTGGGCACCACCACGCC
>dme_circ_0005193

GCCAAAGGTCACCAGCAGCGCTAGTGATTCCTCAGTGCCTCGTTGATGACACGCAGAATATCAATTTGCA
GTTCGGTAAAAGCCACCGGCTCCAGCTGAGCATTGCGTATCTTCATGGACACAGCCTTGAAGAACGCACC
TATCTCATCGTCCTCTCCGGAGTTGTGGCCATTGCTGGAGGGATACTCCATATGGGTGGGTTTGCTGGGC
GTAATGGGCGCCGAGTGATAGCGAGAC
>dme_circ_0005193_junction_seq
CGTAATGGGCGCCGAGTGATAGCGAGACGCCAAAGGTCACCAGCAGCGCTAGTGAT
>dme_circ_0005194
GCTCCTCAATGACGCGCTCAAACTGCGTCGCGTTTATGGGAAAAGCACCACATTGGGCACGATCGCTGTT
GTAGAGACGCAACCAGGCCTCGTCAATAGGATCTATATCATAGGCACATGTGTTCTCCGCCAAGGCAACA
ACATTC
>dme_circ_0005194_junction_seq
TGTGTTCTCCGCCAAGGCAACAACATTCGCTCCTCAATGACGCGCTCAAACTGCGT
>dme_circ_0005195
CTCCGGTTCAATATAAACATTGTTAATATACCTTCAACGAAAAGCGTTGAAATCGATTCCAAGGACTATA
TGTTTCAGGGTGAAACACGACCCACATATTTCCTTCACCAAAATTGTCCTTCACAACAAAAGAAGTTATA
TTTATTTGACGGAAATCAAAATTAATATACCTGGATCTGTGGCTGCGACTACACCAACAATTGCAGCACA
TACTTACACACTATACAACAAACATATCGCG
>dme_circ_0005195_junction_seq
TTACACACTATACAACAAACATATCGCGCTCCGGTTCAATATAAACATTGTTAATA
>dme_circ_0005196
GTCCATGTCGTTGAGGATGATGTCCAGCATGTCCGAGTGCAGGTCGGCGTCGAACGGTTCGAAGACATCC
TCCGCTTTGAACAAATCCATGGACTCGTCCAGCGGAGCGTCTATTAAGTTCATCAGTGTCGTG
>dme_circ_0005196_junction_seq
AGCGTCTATTAAGTTCATCAGTGTCGTGGTCCATGTCGTTGAGGATGATGTCCAGC
>dme_circ_0005197
GTACCTTGCCCTGGACTTCCATAGCGGCAGGTGACGGAGCCAGTGGTGCCACTCGCACCTTGCTGGCCCG
ACTACCTGTGCTTGTCGGTGGTGGAGCCACAGGTGGAGATGGGGTTGGACTGGCTACCGTGGGCTGCAAG
AGGAAGTACATGGCTTCATCGGAAGTCATTCCGGCCTTAGAGTTGTACG
>dme_circ_0005197_junction_seq
GAAGTCATTCCGGCCTTAGAGTTGTACGGTACCTTGCCCTGGACTTCCATAGCGGC
>dme_circ_0005198
GAAGCGCTGCACCTCCATGATGAAGGCATCCATGTAGTCAAAGGCTTCCCGACGGCTCAAGTGAGGAAAC
GCATCTGTGCTGGCAGCAGCCACGCTGGCGGTCACTTGGCTGAACAGCTTCTCCTGCACATCCGGGCGCA
TAGCAA
>dme_circ_0005198_junction_seq
TTCTCCTGCACATCCGGGCGCATAGCAAGAAGCGCTGCACCTCCATGATGAAGGCA
>dme_circ_0005199
GGGAAACTGGGCCAGCAGACCACCGCAGATGTCAAAGAGTTTGGAACGCCGATTCATCAGGTCGAGCAGA
CGCCGCAACGTCCCATCCTCCTGTCTGGCGATCCTCTTTCCAGCTATCAGACACCACAGTACATTGA
>dme_circ_0005199_junction_seq
CCAGCTATCAGACACCACAGTACATTGAGGGAAACTGGGCCAGCAGACCACCGCAG
>dme_circ_0005200
GTTGGACACCAAGGTTCGCATGGTCACATCCTCCTCCAACGACAACAGTGGTAGCGGCGGCGCCAGTGGC
GGTACAAGCGGAGCGGGCGGAGGAAACGGCGGTGGCGGTGTGGTTTCGGTGCCCGTATCCCTGCCCATCG

GTTCGATGATAACCGGCACTACCTTCAATGTGATCACGCCCGACCAGCTGCCCCCTCACTTCAAGCCGAT
GCTGTGCGTCGACAACAATGGCTACCTGTCCGGCAGCACGGTCAGCATGGGCAACGACCTCAAGACGATC
GTCATCCAGCAACAGCAGACGCAACCAGGCGGCGGCGGTGGCGGCGCCAATAATGCGGGCACCAATACGA
CCGCCACAAATACGATTGGACTGAATCACGATGGCTCCGGGAGCAACAATAGCCACGACAGCCTGGCCAC
CTTGGAGCACGCCGCGGGCGGAGCGAGTGGCGTCGGCGGTGGCGGCGGCGGAACGGGTGGTGGCTCCTCG
GGATGGTCGGAGAACCCGTCAACCCAGCACAATGAGGTCTTCCAGATCCGCTGCAAAACCACCTGCGCCG
AGTTATACCGCAGCAAGCTGGGCTCCGGCGGCCGTGGACGCTGTGTGAAGTACAAGGACAAGTGGCATAC
GCCCAGTGAGTTTGAGCATGTGTGTGGCCGGGGCTCCAGCAAGGATTGGAAGCGTTCGATCAAGTACGGC
GGCAAATCGCTGCAGTCCCTCATCGATGAGGGTACGCTCACGCCGCACGCGACCAACTGCAGCTGCACCG
TCTGCTGCGACGACGAAGCAGGTGAGTCAGGGTGAGTCAGGTGAGACGAACAACAATTACTACACACTCC
CCCCTTCTGCATGGCGCACCGAGTTGACCTGGTCATCGGTCCGTCATGGTGTCCCAGGTGCAGGACTCGC
GCACAAACCGAATTCATAACACCACAGCTTTAAAAACCCACCTGACTTCTGTTTATCAACAATTTTTTGT
TTGCAAACTATAGGGTTTAAACAATTCCAAGTAATTGGAAATTTTACTCAGCAAGGGCTTCTTGGGCAAA
AATGTTGCAAGAAATAATATTAAAACTTTCTTATTCTCAAGTGTGTATGTCATATTGTAACCCTATAGAA
CATCTCATTAAGGAATGAGAtgtaatgatgaactccaattaagaacacaagtccgttttaagttgaaggc
agccgtcgggctgacatatatttattcaatttcttcttttgattttagagtaggcagaaatggttctgtc
agctcaacgtctacagagtctctccgattttacagtttttttatatatttggacttaggctaatccgcgta
gctgcgctgccgggtacgccagcagcggagcggcgttcttatgtatatcttatatatctaggcttatcgg
gagagcgagatatgtatgtacgtatgtaatatgtatttggtcggcgtgtgaatgctgaccatgcacttat
actttttgccttcgagtgggtgatcatgttacatccgccctggcctaactgcgtgcataaacataaacat
aaacatagcaacaaagtgctgccataagttaatatgctattttatttaattgttcttaatattgcatagg
cacatactacatctccctccttttgaaaaataacgtaacctagtgaagttattttgattattaaatgcaa
attattttatttttatttttattttgtttgtgtttttgtttgaacaacaatagttgattttataatgcaa
agataaaaatatacgtagtgtatggaaaatttgtataaatgattaaggaaaatataagtttaaattcaat
catgaatgaaatttctttaatctatctttatgaactatttgtttttttgtttttattagtaagtagcgtaa
tattgttattatctcctatgctttctatcttatagggccccgtatatttaaagtctaatttatgacctac
ctcatttcttagtaaaactttatctcctacttctaattctatgtcttttatttttaagtcataattttct
ttatttttttctttgtgtgcttcaagaagttttcttgctcgagcatatgctacctctaacctatatttac
tctccttagcgtaatcatctatgttatatattggttctatgctatgtagtttattaaaatgttttggtaa
attacttgttctaccgaaaactaattcatatggacaataattatgtaccatagattgggtcgtgttgaag
cagtatacgaaatattgaagccatacgtcccaatcggttttgtccgtcgatatgtaggatcgtatatact
cgtttaaagttctatgacttctttctactactccaactgtctggtggtgatgagctgttgatgttatatt
ttttattttcaaatatttacacaggtcagtaattattgaattcttatactctgttcccatgtccgttatg
aacgtcttcattggaccgtactttagaataaaagattcaaatatagcttttgcgactgtttttgcgcttt
tatttgctattggtatggcaactaagtacttggttaaatcacatatgagagtgactgcgtactcgttacc
attttctgacttgggtagtggaccaattgtgtccacaacaactctatcgaaagcatgttctggtgtttca
gttatcgtcattggagtctttgtgtgctttgttgtttttgctttttggcatttttgacattttcttacgt
actcttttatgtatttactcatatttttccagtaataatgtcttttgaccttggccaaggtttttgtaat
gcctgtatgccctccttgtattggatcatcatgtaatgtagacaatatagcttctttttctttttcatta
tttatttgggtcaccgggttaagtagcgctacttttaaattctttaatattttattgctcatatttttaa
atttatctattgaaacgtgttcaaagatttttttccacggtgccattttgatttggctgatatcatatat
accggcctgcaattcaagcctttggagaaattgatctaaatcaagaattccattagtataaagatcacca
acatcatatcttgcaataattttctttccatgtttaaataaacatatcgagtcattcaattgcaatgtca
ctacttttcgtacctcgtcatttgttatgacttcgtatacgttgggctctgaagctatttctttggtttg

cttttgcaaatccaattgttcttttcctgcgcaggatttttgtctactttgaaatcttgtagtgactttt
aatatatttccagttatatcttttagctctttgatggttattcttgataacgcatctgctacatgattgt
ccttgccctttagatactctactgtaaaattatattcctctagttcaagccttattctagttaatttaga
gctggggttcaccatcgagaataaatatgtcaatggtctatggtctgttttcacagtgaaatgttttccg
taaatgtatggtctgaaatgtattattgcccaatgaattgctgctaactcttgttctgttgtactcttat
tgctttcacctttcgtaaaagctctggatgcataagcaactgggagttggtggccattatggttttgagt
taaaactgcgccacacgcttgcttgcttgcatctgttgttatgcaaaattctttgctgaagtctgggtac
tgcaagagtgttgggttaattagctgagattttaaatgtatgaatgctttttgacattcatctgtccact
cgaatggaacattctttttacataatcttgttatgtgccgcgaatagtcggcgaaatttttgataaaacg
tctgtagtaattgcaaaatgctacaaaacgtctagcgctgtccgcatcatgtggaactgggtagttctga
atgacatcatatttttttgtcatccggcaaaattcctttgtctgtgcatttgtgtcccaaaaaatgtgactt
catgcatgaaaaatgaacatttttcaggatgtaactttaggttgtattccctgcatttaccaaaaacttc
agtgaggtttttaagcatatgtttttcggaacaacctatgactattaagtcatccatataaaggaatgct
tgagacggttctattccggagaatgctatagtcatcattctttggaatgaattaggcgctatttttaagc
caaatggcaatcgcgtgaaacgatatgagccattgctggttgagaaagatgttatatctctcgagccttc
atccagttcgatttgatgaaaacctgacattaaatcaaggcaggagaaatattttgctcgaccaagttgg
tccaaaatatcatctattctcggtagtggaaatttgtcagctaaaagtttcttattaatttggcgatagt
ctattactaatctccatttctttttatcagaattcgggcttgactttttgggtactaatagcaaagggct
attgtactgtgaaactgatggttcaactattttatcttttattaatttctgaacttgggcttgtatttct
tccacttgactatgaggacttctataatttttcgtgtatactggctcatcatcttttaatctcaactgtt
gtttataaaaattattaactgttataggttctgattctaatgcaaatatatctatatattcgctgcatat
attttctaattgtgatttaaacaattcggggaaatttttctttaattgagataagacagttttatttctg
tgttcactatttgcctgaactacattgtagttcgaaagtggctcatattttagagtgtccatattgacta
attggtcggaatcggttgtatttaaaattcggacaaatgtattacttgatgttgcgattgtatttgcaac
ataaataccagtttgaatttcctggtttggaattaaaatgttatcatcttttgatgatactattaatctt
cggacaacttgggatcttgctggtaataacgttgtgttaataccagagctgtatgctatgggaatatata
ttggaaatttcaaattgtttggtctaattataaaccaatcttcttcttggtttaaatcgatttggcaatt
atattttttttatgaaatctattccgattattccatcacacggtattggaaagtttttatctactaaatga
aaatcgtgtgggataacgtatttacctgtctgtatctcaataaaagtctgtcctcgagactgaattttct
gttggcctatgccttgaatgtttattttattggttatttgaatattagaaaatttgtcagagttctcttt
gagaatagagatatctgcacctgtatcaagtagaaaaactagttttacgcctgttttggcatgaatgaat
gtagaaaatatgttgagattataattgatggtgtatactctacgatcttcttctactgagtacctaaagg
ctgttgcgagtttccctgttcttggattactcgtacattggcattgtttctgttgtagttgttctgactg
ttgttacggcctctgtttccattataacggcctctgttatagccattataacggttattatttccattat
agcggttttgataaccgttattctggtaaccgttattttggtaaccgttattttggtaaccgttatggta
gttgcctctaatactattgttattaaagcctctaccgcggttactacctctattggcatttccgtatcct
ctattgggtctgttacctctaaagtacatcactttattgatgtcaccggtaactcttgtactcgttctta
tgtatgcgcttatcgctgcatccatagtggtgcaagtccctgcttccagtactgttttgatactttcgtg
ctcagcacgatgtatcatggtttcaattgcctctttggtgcttagaccagtggcatgttctagaggtata
ccctcatcgatataggaagcttctaaaagttttctcaggctatcaacttcagcggtaaattgcgttgcag
ttttacctctctgctgaactgttgctagctttgctttgacattactggatgtctcaccgacaactacttt
ctgcaatttgtttataattgctggaattgtcgtttcattgtcaactgagttcaaaattgtgccaactatt
tttgacttaatgacctcaactgcaatgtcttcaaatggtcccttagctaggtctaccaatttgattgcct
taatgaatctttgtaaatggatcttttgcccatcaaattctggcactgtgcaggcgacttccctaatgta

agacctaatggcagctgcttcttctgccatggttaagttattttcggagtcggctgcgttatttgtttta
tcgtcgtttttcagcattaattttgctggtatagttagatcgtgaagatcttcctctttaatgtcttcct
tgactatagtcctatcttcctcgtcagagttagtgctgtcttcattcaaatttattcttagtggggtgtt
tactatggttggaatttctatctggagactgaatttatttttataaatattaatttttctcttaatgtc
accagtaggttcaaagccttagcacactgtttgcgttccaaattctttctattttgttggatcaactctc
tgatttcattataactatttaccaagacttgggcattcctattgattgttacagcttgtgttgatctgtt
ttgcgttaagcacttataggattttctgaaattttcattttgatcttttatctgatcaacaagcttttcc
cagtccatgctgtggtatactcataacttcaaatttattcatttgtatatattttttttttttaatgta
gataatttcaatattacgttttatattttttttttatttattattttttttttcatataatttttctca
tattttcattcataattgactttattaaagatcttccttaaatattatctataccgttagcagtgctacg
gtatcgtttcttaagacacttgttatgcagtttgtatatttttattaatgcattggataccataattaat
gcaattacaattagtattatggttatccagaacaagtctgcattttggacttctactttatttattacat
tggctgtagagtccacagttttagtgtctattagacccattttgggtatactaatttttatattttggaga
atagaatagaaaatttcctttgtatatacttttattttcagttttacttatttcatttgtctctatagac
atttatgtttggaattatctatttataaaatctttcaatgttcgcaagagtaaaaaaaaaattgtatttt
acattgttgcctgcctgattctagctatggcttcacgggccatattgggttaacagggcataatagtata
tgctccgttttacaatatttcttacaatttttcttatctatatatatttttatatttactttatatttata
tatatttattattatattctgctgttgatgatgctcatcaggtgtcagctgggcgtcgccgggggtggca
cgctgacactccctcgctgctgtctatttgttgggtgtcagctgggcgtcgccgggggtggcacgctgac
actccctcgctgctgtctatttcttcgccgctgaagacgtcgatggtcgctcacaagtccccaggcagtg
gggactccagattccttcgcagccagggggggcaggacttctttggttttgttaatgctggtgctggtgct
ggacatgcctggtattttggaacttcattaattgtgggtatatttttaatattatctgagtgagttttct
cgagcttatgggtctttaattcttgctgatattgcaacaagtggtctagctctgcatgtagctttagagc
tttcgaccaaaccggtggcgtttgctgaccgtatattagccaccttacgtgggctatacgtttattcagc
ttagttttcaaaccgccacggtgtttgcccatactcaatagaactctactcactcagatttttccttggt
ttttgcagttccatttaattagtccagtcttcttccagtccagtgttcctttgttcctttgttttcacca
gctttggtcaattcgtccagcgttggacgactgtcacggtcgccatgtaatgatgaactccaattaagaa
cacaagtccgttttaagttgaaggcagccgtcgggctgacatatatttattcaatttcttcttttgattt
tagagtaggcagaaatggttctgtcagctcaacgtctacagagtctctccgatttacagtttttttatat
atttggacttaggctaatccgcgtagctgcgctgccgggtacgccagcagcggagcggcgttcttatgta
tatcttatatatctaggcttatcgggagagcgagatatgtatgtacgtatgtaatatgtatttggtcggc
gtgtgaatgctgaccatgcacttatactttttgccttcgagtgggtgatcatgttacatccgccctggcc
taactgcgtgcataaacataaacataaacatagcaacaaagtgctgccataagttaatatgctattttat
ttaattgttcttaatattgcataggcacatactacaGTCAGCTCTCTAAAAGAGGTTTTTGAAATGCAAT
ATTGCATAATAAATATAGATTAATAAGAAAGAGCGTAGTAACTAGCAATAGAAAAAATAGTTAAAAATTT
GTTGCGCTTGCTGATATGCAAACATATTGACAGATGTACTCTCGCCGGTTAATTAAAAGCAATTTAAAAT
TGATTGCTCTTGACAACTAATAATGCTACTTTTTAAGTCATTCGGACAGGACATTCGGTTCTGATGTCAT
TTTGATTTGATTTTTTATTAGCAACTCATTACTTTGATCGGCCCTAATTCGTAATAGTTTGATAACTAAT
TAATAACTAAGGAAAGCAAATAGTCCTTTCTACAATAGTTAACTTCTATGTGAAGCCCTTGTTATGTCTT
CTCCAGTTGTGCCGAGCCCTATGTGCACCCAACTGCACCCAACTACAACCATTTGTTTAACCCCCCTTACA
CGCTCATCTTGTTTAGGCTTCCGGCCCTGTCCGTCTCTTTACACCGTACAAGCGGCGGAAGCGGAATCAA
ACTGATCTCGATATGGAGTCTGGACCCAAACGCAAGCGTAACACCCATCATagtaacaacaacaacagta
acaccaataacaacaacaCGAGCGGGAGCGGCGCCAACAACTGTGTGGATGTAACTGCAGCTGTGGCTGC
TGCAACAGCCAGCGTGGTGGACGAGAACAACATGTTCCTGTCGGAAGAGAACATCACGTCCAAGGATGAG

CCGTGGGCAGCGCTCAACGACAGCCTGGACACCTCCACCGAGCTGGTCGATCAGTCGCAGATGGGCAACA
CGTACGAACGGGAAACGTTTGTGGTGAACATCAACGATGGATCCTCCATCGCCGTCCTGGACACCAGCCA
GTCGATGAAGAACATCGAGCACGTGTACTGCACCATGGTCAAGGCCACCAATGACTTCAAACGCATGCTT
AACGACATGAAGCAGTCCTTCGAGCGTCGCATCGAGGTTCTGCAAAAGGAGCGCGATGCAGCGGTCAGCG
CGATGCGTGTCCAAGTGCACGCGGACATCGATGATCCAAATATTTCAGGCTCCCTGCACGGCAACGAGAT
CATTTCGGCTAAGAAGGTGCGCCAACTGTAACCGCGAAGCATTGGCCGAGTGCTCACTGTGCCGAAAGAC
GCCGTACTGCTCGGAGTTCTGCCAGCGGAAGGATTGGAATGCTCACCAGGTGGAGTGCACAAGGAATCCA
CAAACGACAACGCAGCAGGTCATGCTGTTGATCGACGATCAGTCCTAAGCAACGTAACGAAAT
>dme_circ_0005200_junction_seq
CGATCAGTCCTAAGCAACGTAACGAAATGTTGGACACCAAGGTTCGCATGGTCACA
>dme_circ_0005201
TATTTCCAATACTTAAGCGCATTGCGACTATCTTCGAACTGGCTTCCAATTCCAGAAATTGTAGAGCCTG
GTGGAACTGCAGCTCCTGCACCCGCTGCGCCTCCTACCACAGAAGACCCCGGCCCACCGACTACTCCGCC
AAGGCTTCCCGTCGTCGGCATGACACTGCCGGCATTCACTCCTCCGCCAGGAGCTGCCTGTTGGCCATTA
GCTGGGCTATGAATCGGTGGCAGTGGACTGGCCATGCTGGG
>dme_circ_0005201_junction_seq
TCGGTGGCAGTGGACTGGCCATGCTGGGTATTTCCAATACTTAAGCGCATTGCGAC
>dme_circ_0005202
AAGATGACGATCGCAGCTCAGCCGAATGCTAAAGTCGTTTCCCACAGCGTGCAGTGGATTCGGGGAAGTG
CTTACCGAGTACATGCCAGGTTGAGGAATCTCCAAGGTCTTAAACAGCTTCAGCACCAAGTGACATGTAA
GACGAGCACCTGCCTCTGCATCTACACTCAGTTCGGCTCCACCAGCGCTTACCGATCGGGCCAATGTAGG
CAGGGCGAACTTACTTACAAAGTCGGCTAGTGAGAAGCAATGTCTGGCCACCAGAATGCATACGAACACG
GCCAGGGCGTTGTGAGTCTTAAGTTGTCCAATGTCGACCTGTCCGCCCAGGTGCGGATAACGCGGACTCT
TACTGCCGCTGCACAGACTCTGCAGTGCG
>dme_circ_0005202_junction_seq
ACTGCCGCTGCACAGACTCTGCAGTGCGAAGATGACGATCGCAGCTCAGCCGAATG
>dme_circ_0005203
GTACAAGGAAAACTTACTGGTTTCGCTCTATGCCCAGTTATCACAATGTCTGCAATCCTTTGCTGAGGTG
AGTGTTTACTTTCTATCCTTAAAGTTAAACTTACTAGTAACATGACTCCTTTTAGCTGGACACAATCGGT
GGCATTGATGAGCCGCAGGCGCGCGAGGAGATCCTCGATGCGCTGCAGTTGCGCTTTTCGCTGGTGGGCG
GCATGTTCGAGGCTATCCAAAAGAACAGTACCCCCACCACAGACTGGGCTATTCTTCTGGCCCAATTGGT
TTGCCAGGGCGTGGTAGATCTCAGCTGCAATCGTGAGCTGTTCACCACCGTTGTGGACATGTTGGCCACA
TTGGTGCACTCAACGCTGGTCTCTGATGACGAGAGGCACTACATGAACCTGATGAAGAAGCTAAAGAAGG
AAATCGGAGAGAAGAACAACGCCTCCATTCGGGTTATTCGGCAGCTTCTTCCACTTTACAAGCAGCCTAC
TGAGGGTGATTGCGTGCGAACACTCTGGAATGGACACCAAGGGCAACAAGATCTGCGATATAGACAAAAA
ACAACTACGCATTTCGGACAAACAGCGTATCAGCGTGTGGGACATCCTTGAGGGTCACAAGAACCCGGCT
CCTTTGTCCTGGGTGTGGTTTGGTGCAGTCAAGCTGGAGCGCAAGCCCCTTACTTACGAGGAGGCTCACC
GCAACCTGAAGTATCACACGCACAGTCTGGTCAAGCCCAGTAGTTATTACTATGAACCGTTGCCACTGCC
ACCTGAAGACATTGAGCCTGTTCCCGAGAAGATTTGCATAGAAGGATGAAATGAAGGCGGACACGCCCTC
GTCGGTGGATCAGTCGCCAAGCGCTGTGGTTGGCGGCACGGGAAGAGGTCGCGGCAAGGGAACGACCACG
CGGAAACGCAAGCCGAAGAACCCGAAAACGCCACCGGTGGTCAATACTcagcaacagcagcctcagctgg
cacagcagcctcaacagccgcagaatgtgcagcaacagcagttgcaacagcagcagcaacaacagcagca
catgcagcagcagcacatgcaacagcagcagatgcagcCCAACCAAATGGGCCAGATGCCAATGAACATG
CCGGATGAACATGCAGCAGTTTGCACCCAATCCGAACAACATGATGCAGCAGAATGCCATGTTACAAcag

cagcagcagcagcagatgcagcaaatgggcaacaacccgatgcagcagcagcTGAATGTGGGCGGTGGGA
ACGGGCAGCCAAATCCCCAGATGAACTTCATGCAGCAGGGACCAGGGGGCGGTGGAGCCGGACCGCAGGG
TATGCCTGGCCAGCAGCAACAGTGGCACAATGCGCCGCAGCAGCAGCAGCCGCCACAACCGTACCACAAC
CAATACGCTCCGCATCAGCAGAACATGCAGTGTAAGTCGAGTGTTTGATATTATATACGGTTAAAGATTG
TAATCCGTGTCACTTACCATTGCAGCAAATCGCATCGAGCGCCCGCCGTTGAATGCCAACTCCAAGCAGG
CGCTATCACAGATGCTACGACAACGACAACCATTCCAGCAGCAAGCGCAACAGGGTCCGGGGG
>dme_circ_0005203_junction_seq
CAGCAGCAAGCGCAACAGGGTCCGGGGGGTACAAGGAAAACTTACTGGTTTCGCTC
>dme_circ_0005204
CAAAATAGGCTGTGGGGCATGCACTCTCTCTTTCCAAAGCACCGGCTTACCTTTCCGGCCTCATCTGGCA
GCAGTTCGTGCACCTGGTTGTCCCCGCTTCCGTTTCCGCTGTAAGTGCTTACAATAGGATTGTAATTGCT
GTTGCTGTTACATTGCGACACATCCGTGCCCACCTCCGCCTCGCCTTCGACATCCAGGCCCGAAACG
>dme_circ_0005204_junction_seq
CTCGCCTTCGACATCCAGGCCCGAAACGCAAAATAGGCTGTGGGGCATGCACTCTC
>dme_circ_0005205
GCTAAAGAATGATGCAGAAATGACGATGGCAACTAACGAAGTTGTTAGCGAATTGGACGAGATTGATGCA
CCCAGTAAGGAGGTTAAGGAAGAACTACCCAATCAAACGGGGTCCACAAAGGCTACGGCGTCGCCACCGC
CCACAGGGAATATTCTGCGCCAGAGCAAGACCAAAAATGCT
>dme_circ_0005205_junction_seq
TCTGCGCCAGAGCAAGACCAAAAATGCTGCTAAAGAATGATGCAGAAATGACGATG
>dme_circ_0005206
GGAACTTGTTTTTGATTTCCAGAAAGTTGCCCTTACCAACGTCCATCTTGAAAGGTTCGTTTATGATCGC
GAAACGGATATCGTTTTGTATATCCTGCCAACGGCCATAGCCATGGGTCACAATTCCGGCCAACAGCCAG
TAGTCGTGGCGACGGTGCCAGATCTCGTATTCTCGCCCAGGAACGGCAGCCTTCTCCTCGTTGAGCCACA
AAGTGTGCAGTTCGGTGAACCCGCCGTCGGCGATGTTGAACATAAATTTGCGCTTTAGCACCTCCAAGCT
GTCCTCCGCTCCCTTGCCAGTGGCTCCAGTTGCAGCAGACGTAGCAGCGGCGACTGCCTTTTGATCCTTG
GGTGAACTGGCACTGGGCTTTTCTAGCTCACCGTCCTCCTTGACAATCATCACATCGTCATCGTCGTCGT
CAATTACTGTCGTCGGTGGTATTGGTTTCTTGTCATCCACCTTTTCTTTTTCCTTCTCTTCGGTCTTGGG
CTCCTCCTTTACCTCCGGGTCCTTG
>dme_circ_0005206_junction_seq
GGGCTCCTCCTTTACCTCCGGGTCCTTGGGAACTTGTTTTTGATTTCCAGAAAGTT
>dme_circ_0005207
GTGAAGCAAGAACAAGAGGCTGAAGAGGACAAGAAGCCTGGGGATGTCAAGCAGGAGAACCCTGTGGAAG
AAGCTGCCGGCGACACAAAGCCCAGCGACGCGGAGGTCAAAACCGAGGTGGCTAAAACAGAACCCAAGGA
AGAGACCAAGGACCCGGAGGTAAAGGAGGAGCCCAAGACCGAAGAGAAGGAAAAAGAAAAGGTGGATGAC
AAGAAACCAATACCACCGACGACAGTAATTGACGACGACGATGACGATGTGATGATTGTCAAGGAGGACG
G
>dme_circ_0005207_junction_seq
ACGATGTGATGATTGTCAAGGAGGACGGGTGAAGCAAGAACAAGAGGCTGAAGAGG
>dme_circ_0005208
GTCAAAACCGAGGTGGCTAAAACAGAACCCAAGGAAGAGACCAAGGACCCGGAGGTAAAGGAGGAGCCCA
AGACCGAAGAGAAGGAAAAAGAAAAGGTGGATGACAAGAAACCAATACCACCGACGACAGTAATTGACGA
CGACGATGACGATGTGATGATTG
>dme_circ_0005208_junction_seq

GACGACGACGATGACGATGTGATGATTGGTCAAAACCGAGGTGGCTAAAACAGAAC
>dme_circ_0005209
GTGAAGCAAGAACAAGAGGCTGAAGAGGACAAGAAGCCTGGGGATGTCAAGCAGGAGAACCCTGTGGAAG
AAGCTGCCGGCGACACAAAGCCCAGCGACGCGGAGGTCAAAACCGAGGTGGCTAAAACAGAACCCAAGGA
AGAGACCAAGGACCCGGAGGTAAAGGAGGAGCCCAAGACCGAAGAGAAGGAAAAAGAAAAGG
>dme_circ_0005209_junction_seq
AAGACCGAAGAGAAGGAAAAAGAAAAGGGTGAAGCAAGAACAAGAGGCTGAAGAGG
>dme_circ_0005210
GGCTTCTTGTCCTCTTCAGCCTCTTGTTCTTGCTTCACCTCGCTCTTCTCCGCCGAGGTCTTATCCTTCT
CCTTTTCGGAGTCCTTGTCTTTGTCTTCACCTTTTTCAGAGGCTGGCGCTGGACTGGGAGCCGCACTGGT
GGCTGGAGTGACGCTATTGCTAGTAGTAGCGCTCTTGTCGACATTGCCACCGGTAGGCGGAGCTTCAAGT
GCCGCCACATCCTGTTTCAGGGCTGATCGTACGGGCTCGCACGGCTTGAGAATGAGCTCCGGCATGCTGT
AGTATCC
>dme_circ_0005210_junction_seq
GAATGAGCTCCGGCATGCTGTAGTATCCGGCTTCTTGTCCTCTTCAGCCTCTTGTT
>dme_circ_0005211
ATTATTAAACAGGATGCCGAGAACTCTGATCCGGCGTACTGGGTGAAGCTGCTGCGGCATCACTATGAAC
AGCACCAGGAAGATGTGGGTCGCAGCCTGGGCAAGGGCAAGCGCGTCCGCAAACAGGTCAACTACACGGA
TGGCGGCGTTGTGGCGGCTGATACCACGCGGGATGATTCCAACTGGCAGGACAACGGCAGCGAGTATAAC
TCCGAGTATTCAGCTGGCTCCGATGAGGATGGCGGTGACGATGACTTCGATGACCAGAATGGCGCAGAGA
GAAAGGCCAAGCGGCGATTGGAGCGTCGCGACGATCGTCCATTGCCTCCACTG
>dme_circ_0005211_junction_seq
TCGCGACGATCGTCCATTGCCTCCACTGATTATTAAACAGGATGCCGAGAACTCTG
>dme_circ_0005212
ATGCCAAGCGGAGGCCTTACCTCCTCCAACTCCAACTCCTCATCGTCCTCCAGGAGTGCCAATAGCAAGT
CTTCGGGCGGCAACAAGATGTCCGATGTGTCCAGGCGCAATATACGCGAACGGCTTATCCATCTGCTGGC
CCTCAAGGCCTTCAAGAAGCCTGAGCTCTTTGCGCGCCTGAAGAACGAAGGCATTCGGGATCGGGAGCG
>dme_circ_0005212_junction_seq
AGAACGAAGGCATTCGGGATCGGGAGCGATGCCAAGCGGAGGCCTTACCTCCTCCA
>dme_circ_0005213
GGATCCGAAGGCCAAGAAGGATGAAAAGAAGGACGGCAAGGACAAGGAGCAGCAGCCGGAGCTCTCCGAC
GAGGACCAGCAGCTGCAGGAGGAGCTGGAGCTGCTGGTGCAGCACTTGCAGGAGCCGGATGCCAAGCTCT
ACCAGCCGACGCTGGAGAGTATGGCCAAATTGATCAGAGCCTCCACGACGTCGATGACCTCG
>dme_circ_0005213_junction_seq
CAGAGCCTCCACGACGTCGATGACCTCGGGATCCGAAGGCCAAGAAGGATGAAAAG
>dme_circ_0005214
GTCGCATTTGGACAACTCGCGCTCCCGCTTTGCATCCATCCAGGTAGACTCTGCCAAGCAAAATCTGGCC
GCTAGCTTTGTAAATGGTTTTGTTAACGCCGGTTTCGGTGTGGACAAGTTGCTGTCGGAAGACGGAAACA
AATG
>dme_circ_0005214_junction_seq
AGTTGCTGTCGGAAGACGGAAACAAATGGTCGCATTTGGACAACTCGCGCTCCCGC
>dme_circ_0005215
GGACTGGGCCTAATTGgcacaccgaacagcacacaacaaacacacacgacacacaacttacaacatacaT
CATACGCTTTTTGTTTTCCCTCAAACATGCCTTGTAAATGAGTTTAGTTTTGTTCCAAAAGT

>dme_circ_0005215_junction_seq
TAAATGAGTTTAGTTTTGTTCCAAAAGTGGACTGGGCCTAATTGgcacaccgaaca
>dme_circ_0005216
GTTGGATTTCTCCATTTCCGGGAGGAAGGAAGGAAGTCGGCAACAAGTGTTGGATCCCCAGGAGCAGAAG
TCGCAAGGACTTGCGTCCAGGGTGAGGCAATCAGGACGCGCAGCTGGCGGCTGTGAGGAGTGCCACTAGC
TACACGCCACCGCTTTTCGCCATGGTAAATCACAATGCGGAAACTGCGAAAACGGGCAATGGCACAAATG
CAACAGCGAATCTGATTGTTAAGGCCGACGGAAATGCAACGCAGCCGAAGGCGATGACGTCATCGGCGGC
CAGGATGAATGACGCCCTTTCGGCATCTTTGGCCGATTTGAGCGAGCAGGAAAATGGAACCACGG
>dme_circ_0005216_junction_seq
TTGAGCGAGCAGGAAAATGGAACCACGGGTTGGATTTCTCCATTTCCGGGAGGAAG
>dme_circ_0005217
GACACGTGCGTGAGGCGACCGGCATAAATGTCGACATGCGAATTGGCATCCACACTGGCAACGTACTCTG
CGGCGTCCTTGGCCTACGCAAGTGGCAATTTGATGTTTGGAGCGACGACGTTACATTGGCCAACCACATG
GAGAGCGGCGGTGTCGCTGGGGTAAgtgtgtgtgtgagtgtgtgtgtgtATACACTGGAGAAAATGTTTA
CAAGCATATATAAATTTTAATTATACCTCTTATCACCAATTTGAAATGCTTTattatttatatttaaatt
atttttatcttaaaAGCCAATCTAGAGAAGTGTCTAATTATTGAATAACTATAACAATCCTCTATCAAGC
AGATGTTATTAAAATCCTGCAGATGGAAAAGAACCAATTGGTTAAGCTTCTCACATAATTATGAATTATA
TCAAAGTGTAACTTAGACTTTTTGAGCAGTGAGTGCGAGCGCTTCTTTCATTCCGCCAAGGTGCTTGGTA
CGAATGACAAAGCTGATGAGCGAGGAAAACAAAAATAAATAAACAAAACGCGGAGCCCGCGGGTGGTGAG
AATGGGCTGGAAAAGTGGGCGGGAGGCGCCTCTAATGGCGCTCGGTGGCATTGCAAAGCGAGTCAAGTAC
TCGTAATGGCAATTTAAATTAGCCTCGTCGTGGTCCCAATTAAACGGGTTTCAGTTCAAGTCTGCACAAT
ATTCGAGTGCTGCAGTTCGAAAAAGAACCAAAAAGTTAGCAAGGAATTTTTTTATTAAATAATACCTACA
CATGTCCAATTTTAATTAAATGTTTGTACCGCACATGAAGTTGAAGAATTTCAAATGACTTTCATTGTTC
TGCCCAAATTTCAGTACATTTTCGCCTTAATGCGGTGGTAATTGGTTTTTATAATTTTTGTGAAAAAGAA
ATGGATGACGTCGCATTAAAATATTTTTAATACAAAAATTTCACCGTCCATTTAAATCCAATTTAACAGC
CATGAACATTTCAAGCATTTGCGTGTAATTAAATTTAAAATATGTTCCAACGATGCTTAATTCTTTTGCC
AGGATATTCCGTATGTTTGCTTCGCAAATACCAAAGTCTAAAGCTCTTAGCCCGCTTTCACCAGTTTACT
TTCATACTTTCACAAACAAACATACAAGCAAGTTTCTTTTGGTTTATTCATTTTGCGTAGCTAAGCCGCA
CTCAAGTCCTTCGGATGAATGCTAGCTGAAAATGCTGAAATGTTTTCTTTTATCTTACCTGCGGTCACAC
ACGCAGACTTCAATTATTCGGAATTGAATTGAATTGAAATGCTACAGTCAATGAGCCGGAAAACAGTCAG
ACGCCTCAATTCGTTCTTCATGCACGAATTGCTCGTCCGGAGCAAAAATATAAACAAATTTGCGTAGCAT
CCTAGGATAAATGGCAGTAGGCAGTGCCAGGATAAATTATTTGCCTAAAACTGTCTATGTGTGTACACTG
AAGAAACAATATTGTATATAAATAAAATGTGTTGGATATTCAAGCTTGAATATATTCTGATTTCATCTTA
TTGTAAAATATTTCAAAGGATGAATACCTTGGGTTTTTCCAATTGAAAATATAAAATATAATGAACAGGC
ATACATATAATATGAGGTGGTTCGtttttgtattactcttttttttttgtttacttatttttgtgtaGTTA
TGTTAACACCTGTGTCTTGCGCATACAAGTAGATTGCTCATACGCCCTGTAAGCCTGCCAGCATTGAACA
TCATTTTTCATTTGTGCGTTTATCCTGCAGGGCGAGTCCACATCACGAAGCAAACTTTGGACTTCCTGGG
CGACAAGTTCGAGGTGGAGCAGGGCGAGGGTGGCAATCGAGATGCGTATCTGGCGGATCACAAGGTTGAA
TCGTATCTCATTGTGCCACCGAAACCAGCATACACCTACAGCGTTCCACGCGTGGTGGAATGCATTGAGC
AGAACGATCCCAGTCCCACCaccgaggagaccaaggagatcaaggagacGGATCAATCCCATGAAGCCAC
CGATGTCGCCGATGTCCTTCTTCCGGTAACTGTGGCTCCACCGCCTGCAATCGTTGACGAGAAGATGTCA
CCCACCTCGATCAATAGCCAGGAGGCACCTTTGCATGCCCCTCTCGCCTCAGCCGCCTCCATGTCCATTA
AGGAGCTGTCCGAGGAGGAGGACGAGGCTGATGAGGCCACTGCCGTCACCGAGCCGCTGATGCACAGGGA
TCAGGATGGCAAGAATGACAAGGAGCCGAAGGCAAATGGTGGCCATCGCGGCAGTGGTGATTCCGCCGCC

TCCGAGTCGGTGGCCAAGTCCGCTGCCCTTTCCCTGCCCGCCGATGATCTGCTCAGTATGAGTGGGTCAG
AGAGCGGCATCTCCAACAGCGGAGCCCAGGCGCAATCCTCGAATCCGGCGAGTGTCACACCCACCGCAGC
CGCTCCAGCCGGAGGAGCCGCCTCCAACAGCTTGACGGTGGCCGAGGCCCCGGAGAGATCTCGTCGGAAG
TTGTCCGTTCAAGGGTGAGCATGTGGAGGGGTGGTGCAAGAATAAATTCAACATTACAATTTACTTACTT
ACCTTAGCAACATTTCACTTTATAATAACTTTTCATTGTTTATTGAAATGAATATATTAACATTTAATCT
ATTTAATTTCATGCCTGATCTATCCTATAATTGCCTTTATTTTTAAATTCAGTTATTCTGCTCACACTCA
TTCATTCATCCATCCTGCCATCAATCAAAGGCCGTAATGGGCATTAACACTTCAGTTCTGCTGGCGATAG
CTCTTATTTATGTGTCCGCTTTCACACCTCCGTTTCGCCCAACCAGGGTAAGTAGCCCACCACCCACCAC
CCACTGCTCAGTTCACCCAGTTCGCTGGCAATATAAAACGCAACAGTTGAAAATCACTTTCACAAATGAG
CTCAGGTTATGCATAATTTTATCGCTGGCAGGGCAACTTTTGCATCATAAATTTATCACAGGCTGCATTT
GTTTGCGGATTGGCCTTTGTGAAAACTTTATCCATGATGGCCATGGAACATGTAGAATGGACCATGGACC
GCGAACCATGGAGCAGGGCAATTAAGCTTCTTTGACGGACCAGCTGGGCTGATTTTTCTCCTTTTTGGTG
GTATATAGTACCTATGAAATGGCGGCTGGGGCAGGAACGAGCTGCACGTGAGGCACCATGGAGAACCATG
AGGTCCTGTGCTGGTTTATGAGTTTTAATTTGCCAAGGACATCGAGTGCGGCTTGTGGCAAAGTCACTTT
GATGGCATTTTTAATGCGAATGGGTGACTTTTAATGAGCTTGTCTGGCTGGCCACCCAATCCCCAAAGCG
ACTAGCTGAACTGTAAAAACTGTACTGCTTTTGAGGTGTAATAATGCCAGCGATTTGGGGTAGATTTTTC
ATTATTAATGGGATCATGGTGGACCGAAATGTTGTCGTAAAATATTGGCTACATTTGGTGAGCTCACTAA
AAACTATCACAATGTCACGTAAGTTTACACAATGTTAAACTTTTTGTAAAACGTTTTTGAAGCTATTTGG
CAAGTGGTTAGGGTCCATTTATATTGGTTTACATAGTTAACGAatatatatatatatatatataCCAGGC
AGGAAAGAATCTTTAATTTTACCATGAACTCTTTCAGGTCATGTCACACGCAATCGTCCTTCATCGAAGA
TGACCAAATATGTGGAGTGCTGGGGGGCAGATCGACCCTTTGCCAATATTGCCGAATCCAAGCTGGTGAA
GAACATTGGACTGGCG
>dme_circ_0005217_junction_seq
CAAGCTGGTGAAGAACATTGGACTGGCGGACACGTGCGTGAGGCGACCGGCATAAA
>dme_circ_0005218
GGCGAGTCCACATCACGAAGCAAACTTTGGACTTCCTGGGCGACAAGTTCGAGGTGGAGCAGGGCGAGGG
TGGCAATCGAGATGCGTATCTGGCGGATCACAAGGTTGAATCGTATCTCATTGTGCCACCGAAACCAGCA
TACACCTACAGCGTTCCACGCGTGGTGGAATGCATTGAGCAGAACGATCCCAGTCCCACCaccgaggaga
ccaaggagatcaaggagacGGATCAATCCCATGAAGCCACCGATGTCGCCGATGTCCTTCTTCCGGTAAC
TGTGGCTCCACCGCCTGCAATCGTTGACGAGAAGATGTCACCCACCTCGATCAATAGCCAGGAGGCACCT
TTGCATGCCCCTCTCGCCTCAGCCGCCTCCATGTCCATTAAGGAGCTGTCCGAGGAGGAGGACGAGGCTG
ATGAGGCCACTGCCGTCACCGAGCCGCTGATGCACAGGGATCAGGATGGCAAGAATGACAAGGAGCCGAA
GGCAAATGGTGGCCATCGCGGCAGTGGTGATTCCGCCGCCTCCGAGTCGGTGGCCAAGTCCGCTGCCCTT
TCCCTGCCCGCCGATGATCTGCTCAGTATGAGTGGGTCAGAGAGCGGCATCTCCAACAGCGGAGCCCAGG
CGCAATCCTCGAATCCGGCGAGTGTCACACCCACCGCAGCCGCTCCAGCCGGAGGAGCCGCCTCCAACAG
CTTGACGGTGGCCGAGGCCCCGGAGAGATCTCGTCGGAAGTTGTCCGTTCAAGGGTGAGCATGTGGAGGG
GTGGTGCAAGAATAAATTCAACATTACAATTTACTTACTTACCTTAGCAACATTTCACTTTATAATAACT
TTTCATTGTTTATTGAAATGAATATATTAACATTTAATCTATTTAATTTCATGCCTGATCTATCCTATAA
TTGCCTTTATTTTTAAATTCAGTTATTCTGCTCACACTCATTCATTCATCCATCCTGCCATCAATCAAAG
GCCGTAATGGGCATTAACACTTCAGTTCTGCTGGCGATAGCTCTTATTTATGTGTCCGCTTTCACACCTC
CGTTTCGCCCAACCAGGTCTGATGTCCTTCGCCGACAGACGACGCTCCTCCGGAGCCTTCATCGAAGGAC
GAAAGCTGTCCATCCACAGCGGAGAAAGTTTCCGCAGTCATGCGG
>dme_circ_0005218_junction_seq
AGCGGAGAAAGTTTCCGCAGTCATGCGGGGCGAGTCCACATCACGAAGCAAACTTT

>dme_circ_0005219
GAGATTCGTGTAGCTCATATTCGAGTTTTCAGCTTATTTAACCTAATTTAAATATGTTAACTAAGATAGA
GTGTTTAAAGTGCACCCTACCAATTGAAACCCAAAACAATTAGGGTAAAACAAAAGAT
>dme_circ_0005219_junction_seq
CCAAAACAATTAGGGTAAAACAAAAGATGAGATTCGTGTAGCTCATATTCGAGTTT
>dme_circ_0005220
TCAGTAAGCGTTTGCATATCTGCGCATCTCAAAACTCTGTATCCTGAATCTGCCTTATGGAGTGGAGACC
TTTCAAGAAAACGAATATTGTATATTTTTTCTTACATGCGTAATTTCTGTTAATATATTGCCATATGTAC
TTATTCATATATATTAACACAACTTACCTCTCAATACATGGTATTTTTAAGG
>dme_circ_0005220_junction_seq
TACCTCTCAATACATGGTATTTTTAAGGTCAGTAAGCGTTTGCATATCTGCGCATC
>dme_circ_0005221
GTTCTGAAGAACGGTTTGCTGAGCCTGGAGAAGGAATTCCCGCATATCTTGAACTCCACCCGCGGTCGTG
GAACCTTCTTGGCAGTCAACTGCACCAACACCAAGGTGCGAGATCAAATTATCGGCGCACTCAAGTTGCA
CGGCATTCAGACCGGTGGCTGTGGGGAAATTTCCATTAGATTCCGCCCAGCTTTGATATTTAAGGAATAC
CATGCCAACATCGTCCTCGATCGCTTCCGCAAGG
>dme_circ_0005221_junction_seq
AACATCGTCCTCGATCGCTTCCGCAAGGGTTCTGAAGAACGGTTTGCTGAGCCTGG
>dme_circ_0005222
GTATAACTTGACTTCTTTTCGGCATCCAGTTGCTTTGCTGTGTACAACATTCCCGAATCGGGATTAATTC
GGAACTCGTCGCCCTCATTTCCACCCACGATTTCCAAGTAGACCAATGCATTTTTGCCCAAATCTGGATC
GCTTACTTTGAGCCTTATCACAGGCATGTTAATGGGTGCCGTTTCTGGAATGGATACTTCGTAAATCTCC
TGTGTGAATATGGGTATATTTTTGCTCTCGGGCTTGATTTGAATCGGAACGGATTTGTAGGCGAATCGAC
GGGGAGTTCCGAGGTCCTCCGCCAGTAGGGTTAAGTTGTAAATGAAATGTTGCTGGTTAAGATGGGCAAA
CTGATTCAATTCGATG
>dme_circ_0005222_junction_seq
AAGATGGGCAAACTGATTCAATTCGATGGTATAACTTGACTTCTTTTCGGCATCCA
>dme_circ_0005223
GGTGAAAATCAGCGTACAGGATATGAACGACAATGATCCCATTTTTGAGAATGTCAATAAGGTCATTAGT
ATCAATGAGAACAACTTGGCTGGCTCGTTTGTTGTGAAGCTTACTGCCAAGGACAGGGATTCTG
>dme_circ_0005223_junction_seq
AAGCTTACTGCCAAGGACAGGGATTCTGGGTGAAAATCAGCGTACAGGATATGAAC
>dme_circ_0005224
GCGTACAGGATATGAACGACAATGATCCCATTTTTGAGAATGTCAATAAGGTCATTAGTATCAATGAGAA
CAACTTGGCTGGCTCGTTTGTTGTGAAGCTTACTGCCAAGGACAGGGATTCTGGTGAAAATTCATACATA
TCGTATAGTATTGCCAATCTAAATGCG
>dme_circ_0005224_junction_seq
ATCGTATAGTATTGCCAATCTAAATGCGGCGTACAGGATATGAACGACAATGATCC
>dme_circ_0005225
GTTGTTTTCTTCAGGTCGCAGGAAATAGACAGGGAACCCGATGTGGGGTCCAAGTTAAAGCAGCCATCCT
CGTTGCCGTCGCTCAACCTATAGGATATTATATCGCCTGCATCAAAGTCAATGGCTGAGGTAACGAATAC
CTCGGTGCCCATCGGCGCCGATTTGGTCACTTTGCCATAGCAGTTCACACGTTCAAACTGCGGCCGATTA
TCGTTGATATCCTTGACGACGATGGACAGTTTGATTTCCGTCTGTCTTCTGTACGGCAATCCCCAATCGG
ATGCACGGATTATCAGCTCATAGTTACGCTTCATTGTTTCAAAG

>dme_circ_0005225_junction_seq
CTCATAGTTACGCTTCATTGTTTCAAAGGTTGTTTTCTTCAGGTCGCAGGAAATAG
>dme_circ_0005226
TTCCTCATCCTGGGTCTCCTCTTTCAAAGAGAATTGGATCTCGGCATTGGGTCCAATATCTTCGTCCACC
GCCTCGATTACGGCCAACACTGTTCCACGGGGCAGATCTTCGCGCACCTTAAATATGTACTCTTGCACTC
CGAAAACGGGCGCGTTGTCGTTGACATCGTCGACCAGGACGTAAACCAATGCCTCCGAGGATAGCACGGG
ACTGCCACCATCCTTGGCAGTTATGTGAAGAGCGTAGTTATCCTGCTTCTCGCGATCCAGTGGTTTGTTC
AGACGAAGACATCCCGTAGTAGCATTTACTGTGAAATCGCTGCACTCAACCGACAGGGCATATGTCACCT
GAGCATTGATTCCAGAATCCGCATCCGTGGCATGAAGGCAGTGTACCACAGTTCCTATCCTGGCGCTCTC
AGTCAGCCGGAAGGTGGCCAACGTCTTCTGAATAACCGGGCGATTATCGTTCACGTCGAGGATCGTTATT
GGCAACATTTTTGACGTCGATTTGGTCGGGTTACCCAGATCGTAGACGGTGATGTTGAGAACATATTCAT
TTTGACGCTCTCTATCCAAATATCCAATAATCTGCAGTTCACCGCGGTCTGGATCAATACGAAAAACCGA
ATCGTAGTCCCCGTCTGAAATTGCAAATACCAGCTTTCCATTGTAGCCCAAATCGCGATCTTTGGCCTCT
ATCCAAGCAACTGTTTCGCCCAATTGGACACTTTCGTTAATGGACAGCTCCTGAGGGAAGTTCACGAACT
CTGGTCTATGCACATTTTGGCCATATCGACTGGGTGTTAGAGACAAGTCACTGAAAACGGATGGCGATGC
ACTCTTTACATTGTTTTTTTCGGCCAACGATAATGTTTCCGCCAATCTCCTGGCCACGCCGGTTTCCCGG
CACTCAAAGGATCCAAAACCATGTAGAATACTGGAATCTCCACCCAAATCTTCGGGCATTAGGTGTACAT
TGATGATCAAGTCATCGGAAAAG
>dme_circ_0005226_junction_seq
TACATTGATGATCAAGTCATCGGAAAAGTTCCTCATCCTGGGTCTCCTCTTTCAAA
>dme_circ_0005227
GGACTCAAGGATATCTGGACTATGAGAACAAACAAGTGCACAACCTTATTGTCAGTGCCATCGATGGCGG
AGATCCCTCTCTAACTTCGGACATGTCCATCGTAATAATGATCATCGACGTCAACGAGAACCGATTTGCG
CCCGAATTCGACGACTTTGTGTACGAGGGAAAG
>dme_circ_0005227_junction_seq
ATTCGACGACTTTGTGTACGAGGGAAAGGGACTCAAGGATATCTGGACTATGAGAA
>dme_circ_0005228
CTACCTTTGTGTTATTTTCAAATGCAGAAAATCTGTACGATTCACGTTGGAATGCGAAATTCGAATCGAT
GGCGGACAAAACCTTAATTTTAACTATTAAAATTGCAGAGAAAATTCCGTCGGAGGCACGGACGTGCAAG
GCATAGTCCCGATCATTTTCGTTATTTAG
>dme_circ_0005228_junction_seq
CATAGTCCCGATCATTTTCGTTATTTAGCTACCTTTGTGTTATTTTCAAATGCAGA
>dme_circ_0005229
GTTATTTATTGCCAAACCATTAGATCGCGAACAACTCATGTTCTACAACTTAAGCATAATAGCCACTGAT
GGAAAATTCACTGCCAAAGCCAATGTGGAAATAGATGTAAAAGACATAAACGACAATACGCCTTACTGCC
TAAAACCCCGCTATCATATCTCCACTAATGAATCAATCTCGATTGGAACTACACTCGTTGAGGTCAAGGC
GATTGACTTTGATTTTCAAAGCAAACTGCGCTTCTATCTTTCGGGCAAAGGTGCGGACGACTTCAGTATA
GGAAAGGAAAGTGGCATCCTGAAGGTGGCAAGCGCACTGGATCGGGAGACAACCCCCAAGTACAAATTGG
TCGCACATGTACAGGATGGCAAGGACTTTACGCAAGAGTGTTTCTCGGAAATAATCATCACGGTCAATGA
CATAAATGACAATATGCCCATTTTCTCAATGGCTCAATATAGAGTGAGTGTACCCGAGGATGCACAACTG
AACACATTGATCACGAAAGTGCACGCGATGGATAAGGATTTCGGGGTAAATAGACAAATCAAATACTCGC
TAATGGGTGAAAACCATGATTATTTCAAAATATCAAAATCGACTGGTATCATAAGGCTGCACAAAAGTCT
CGATCGTGAAACAATTTCATTGTTTAATCTCACTGTGAAGGCGGAGGACTGTGGCGTTCCAAAACTACAC
TCCATTGCAACAGTTGCTGTGAACATATTGGACATTAATGACAATCCACCCGAGTTCAGTATGCGTCAGT

ATTCGTGCAAAATTCTGGAAAACGCCACACACGGCACAGAAGTGTGCAAAGTTTATGCCACTTCGATAGA
TATTGGGGTAAATGCGGATATTCACTACTTCATAATGAGTGGCAACGAGCAGGGGAAGTTCAAAATGGAT
TCCACGACGGGCGACTTGGTGCTAAATGCAACCTTGGACTATGAAATGTCCAAGTTTTACTTCTTGACCA
TTCAAGCAATCGATGGCGGCACTCCACCGCTTAGCAACAATGCATATGTGAACATCTCTATTCTGGACAT
TAATGACAACAGTCCCACGTTTCTGCAAAACCTGTACCGCATTAATGTCAATGAAGATATTTTCGTGGGC
TCCAAGATTCTGGACGTCAAAGCCACGGACGAAGATTCAGATGTAAATGGTCTTGTAACTTACAACATTG
AAAGAGGCGACAATATAGGCCAGTTTTCAATAGATCCGAAAAACGGAACAATTAGCGTTTCGAGGCCATT
AGATCGTGAGACTATTTCGCACTACACTCTTGAAATTCAAGCCTGTGATCAGGGAGATCCTCAGAGATGC
AACAGTGTTCCAATCAATATAAACATTTTGGACACTAACGATAATGCACCCATATTTTCCAGCTCTAACT
ACAGTGTAGTACTTCAAGAAAACCGACTTCTGGGCTATGTATTCCTTACCTTCAAGATATCAGACGCAGA
CGAAACACCCAATACCACGCCATACACCTTCGATATTAGGTCTGGAAATGAGGGTGGGCTTTTCCGGCTG
GAGCAAGATGGTTCCTTGAGAACGGCCTCGCGATTTAATCACAATCTGCAGGACGAATTCGTGATTCAAG
TTCGAGTTTTCGACAACGGCACACCTCCATTATATTCCGATGCCTGGGTGGTTGTGAAAATAATTGAAGA
AAGCCAATACCCGCCCATTGTCACACCCCTAGAAGTAACCATAAATTCATTCGAGGACGATTTTTCGGGC
GCATTCATTGGCAAAGTTCATGCCTCGGATCAGGACAAGTATGATGAATTGAACTTTAGTTTGGTGTCCG
GTCCCGATGACATGTATCAGAGCTCGAAGCTGTTCAACATTTCCAACAACACGGGAAAGATCTATGCCAT
ATCCAACCTGGATATTGGTCT
>dme_circ_0005229_junction_seq
ATGCCATATCCAACCTGGATATTGGTCTGTTATTTATTGCCAAACCATTAGATCGC
>dme_circ_0005230
GATATTGGGGTAAATGCGGATATTCACTACTTCATAATGAGTGGCAACGAGCAGGGGAAGTTCAAAATGG
ATTCCACGACGGGCGACTTGGTGCTAAATGCAACCTTGGACTATGAAATGTCCAAGTTTTACTTCTTGAC
CATTCAAGCAATCGATGGCGGCACTCCACCGCTTAGCAACAATGCATATGTGAACATCTCTATTCTGGAC
ATTAATGACAACAGTCCCACGTTTCTGCAAAACCTGTACCGCATTAATGTCAATGAAGATATTTTCGTGG
GCTCCAAGATTCTGGACGTCAAAGCCACGGACGAAGATTCAGAT
>dme_circ_0005230_junction_seq
CGTCAAAGCCACGGACGAAGATTCAGATGATATTGGGGTAAATGCGGATATTCACT
>dme_circ_0005231
GATATTGGGGTAAATGCGGATATTCACTACTTCATAATGAGTGGCAACGAGCAGGGGAAGTTCAAAATGG
ATTCCACGACGGGCGACTTGGTGCTAAATGCAACCTTGGACTATGAAATGTCCAAGTTTTACTTCTTGAC
CATTCAAGCAATCGATGGCGGCACTCCACCGCTTAGCAACAATGCATATGTGAACATCTCTATTCTGGAC
ATTAATGACAACAGTCCCACGTTTCTGCAAAACCTGTACCGCATTAATGTCAATGAAGATATTTTCGTGG
GCTCCAAGATTCTGGACGTCAAAGCCACGGACGAAGATTCAGATGTAAATGGTCTT
>dme_circ_0005231_junction_seq
GGACGAAGATTCAGATGTAAATGGTCTTGATATTGGGGTAAATGCGGATATTCACT
>dme_circ_0005232
GTAAGGAATACATAGCCCAGAAGTCGGTTTTCTTGAAGTACTACACTGTAGTTAGAGCTGGAAAATATGG
GTGCATTATCGTTAGTGTCCAAAATGTTTATATTGATTGGAACACTGTTGCATCTCTGAGGATCTCCCTG
ATCACAGGCTTGAATTTCAAGAGTGTAGTGCGAAATAG
>dme_circ_0005232_junction_seq
TGAATTTCAAGAGTGTAGTGCGAAATAGGTAAGGAATACATAGCCCAGAAGTCGGT
>dme_circ_0005233
GCGCCGGACTCTACCTCGGCCACCCAGGCCTATATGCAGGTGATGACCGCCGGATCAACGGGACCGGAGA
TGCCCTATGTGCGGGTGTACTGCGAGGAGAACTGGCCATCGGAGCAGTACCGGAAGGTGTTCGGTGCCAT

CACAACCACTCTGCAGTTTGTGCTGCCCTTCTTCATCATCTCGATTTGCTACGTGTGGATATCGGTGAAG
CTAAACCAGCGGGCCAGGGCCAAGCCGGGATCGAAATCCTCGAGACGGGAGGAGGCGGATCGGGATCGCA
AGAAGCGCACCAACCGCATGCTCATCGCCATGGTGGCGGTATTCGGACTCAGCTGGCTGCCCATCAATGT
GGTCAACATATTCGATGACTTCGATGACAAGTCCAACGAGTGGCGCTTCTACATCCTATTCTTCTTTGTG
GCCCACTCTATTGCCATGAGCTCCACCTGCTACAATCCCTTCCTGTACGCCTGGCTGAACGAGAACTTCC
GCAAGGAGTTCAAGCACGTGCTGCCCTGCTTTAATCCCTCGAACAACAACATCATCAACATCACCAGGGG
CTATAATCGGAGTGATCGGAACACCTGTGGTCCGCGACTGCATCATGGCAAGGGGGATGGTGGCATGGGC
GGTGGCAGTCTGGACGCCGACGACCAGGACGAGAACGGCATCACCCAGGAGACCTGTCTGCCCAAGGAGA
AGCTGCTGATTATCCCCAGGGAGCCGACTTACGGCAATGGCACGGGTGCCGTGTCGCCAATCCTTAGCGG
GCGCGGCATTAACGCCGCCCTGGTGCACGGTGGCGACCATCAGATGCACCAGCTGCAGCCGTCACACCAT
CAACAGGTGGAGCTGACGAGGCGAATCCGCCGGCGGACAGACGAGACGGACGGGGATTACCTGGACTCCG
GCGACGAGCAGACCGTGGAGGTGCGCTTCAGCGAGACGCCGTTCGTCAGCACGGATAATACCACCGGGAT
CAGCATTCTGGAGACGAGTACGAGTCACTGCCAGGACTCGGATGTGATGGTCGAGCTGGGCGAGGCAATC
GGCGCCGGTGGTGGGGCAGAGCTGGGGAGGCGAATCAACTGAGATAGGGTAAG
>dme_circ_0005233_junction_seq
GGAGGCGAATCAACTGAGATAGGGTAAGGCGCCGGACTCTACCTCGGCCACCCAGG
>dme_circ_0005234
AACTCAACGGCTCAGTCAATTAGCGATGAGCGGAATATCGCACACGGCTCTCTAAGCAGCCAAGTGCTTT
TCGGCCAGGCCATCCATCGGGTAAGGAGCTCAGTTCCGCCGGCGACGGAAATGGCCGCTTAAGTGGTGCG
CATCCGAACCGGAACCACCGGCAGCAAGTCGAGCACACAACCACCACTCAACTCATTAGCCCCGGGAGCC
GTAAAGATGGCCAACTTAAGCTGGCTGAGCACCATCACCACCACCTCCTCCTCCATCAGCACCAGCCAGC
TGCCATTGGTCAGCACAACCAACTGGAGCCTAACGTCGCCGGGAACTACTAGCGCTATCTTGGCGGATGT
GGCTGCATCGGATGAGGATAGGAGCGGCGGGATCATTCACAACCAGTTCGTGCAAATCTTCTTCTACGTC
CTGTACGCCACGGTCTTTGTCCTGGGTGTCTTCGGAAATGTCCTGGTTTGCTACGTAGTTCTGAGGAATC
GGGCCATGCAGACTGTGACCAATATATTCATCACGAATCTGGCCCTGTCGGACATATTGCTCTGCGTCCT
GGCGGTGCCATTTACTCCGCTTTACACGTTCATGGGTCGCTGGGCCTTCGGCAGGAGTCTGTGCCATCTG
GTGTCCTTTGCCCAGGGATGCAGCATCTACATATCCACGCTGACCCTCACCTCGATTGCCATCGATCGGT
ACTTCGTTATCATATACCCCTTCCATCCGCGCATGAAGCTCTCCACCTGCATCGGGATCATAGTGAGCAT
CTGGGTGATAGCCCTGCTGGCCACCGTTCCCTACGGCATGTACATGAAGATGACCAACGAGCTGGTGAAC
GGAACGCAGACAGGCAACGAGACCCTGGTGGAGGCCACTCTAATGCTAAACGGAAGCTTTGTGGCCCAGG
GATCAGGATTCATCGAGGCGCCGGACTCTACCTCGGCCACCCAGGCCTATATGCAGGTGATGACCGCCGG
ATCAACGGGACCGGAGATGCCCTATGTGCGGGTGTACTGCGAGGAGAACTGGCCATCGGAGCAGTACCGG
AAGGTGTTCGGTGCCATCACAACCACTCTGCAGTTTGTGCTGCCCTTCTTCATCATCTCGATTTGCTACG
TGTGGATATCGGTGAAGCTAAACCAGCGGGCCAGGGCCAAGCCGGGATCGAAATCCTCGAGACGGGAGGA
GGCGGATCGGGATCGCAAGAAGCGCACCAACCGCATGCTCATCGCCATGGTGGCGGTATTCGGACTCAGC
TGGCTGCCCATCAATGTGGTCAACATATTCGATGACTTCGATGACAAGTCCAACGAGTGGCGCTTCTACA
TCCTATTCTTCTTTGTGGCCCACTCTATTGCCATGAGCTCCACCTGCTACAATCCCTTCCTGTACGCCTG
GCTGAACGAGAACTTCCGCAAGGAGTTCAAGCACGTGCTGCCCTGCTTTAATCCCTCGAACAACAACATC
ATCAACATCACCAGGGGCTATAATCGGAGTGATCGGAACACCTGTGGTCCGCGACTGCATCATGGCAAGG
GGGATGGTGGCATGGGCGGTGGCAGTCTGGACGCCGACGACCAGGACGAGAACGGCATCACCCAGGAGAC
CTGTCTGCCCAAGGAGAAGCTGCTGATTATCCCCAGGGAGCCGACTTACGGCAATGGCACGGGTGCCGTG
TCGCCAATCCTTAGCGGGCGCGGCATTAACGCCGCCCTGGTGCACGGTGGCGACCATCAGATGCACCAGC
TGCAGCCGTCACACCATCAACAGGTGGAGCTGACGAGGCGAATCCGCCGGCGGACAGACGAGACGGACGG
GGATTACCTGGACTCCGGCGACGAGCAGACCGTGGAGGTGCGCTTCAGCGAGACGCCGTTCGTCAGCACG

GATAATACCACCGGGATCAGCATTCTGGAGACGAGTACGAGTCACTGCCAGGACTCGGATGTGATGGTCG
AGCTGGGCGAGGCAATCGGCGCCGGTGGTGGGGCAGAGCTGGGGAGGCGAATCAACTGAGATAGGG
>dme_circ_0005234_junction_seq
CTGGGGAGGCGAATCAACTGAGATAGGGAACTCAACGGCTCAGTCAATTAGCGATG
>dme_circ_0005235
CCAAGTTGTACTCAGCAGGCGGTATATCCTCGATTCTGAAGGAGCTGGCCGATGACATAGCTCCGGCCGA
TGGCGAGGAGATCTACAACCCATTCACGCCGGTCGTCTCGCCCACGGACGATGCGCCCGCGCACATCGAC
AAGATGTTCCTGCAGACGTCATCCGGATATCGACCCGTGGAGCACAGCTACTACAAGCGCTCCTTCCTGG
GGGCGGCTGTTCGTGGATCCGCCGGAATTTCGGCATCCGGCGGCGGAGGTGGATGTGCAGGCGGTGGCGG
AGGTCGCCTCGATGGCATCTCGGCCGCGGAGCTTGGAGGCGCCGGTCGCCTCTTCCGCTCCAGCGGCAGC
CACGCCCACGGCGGCGACAGAATGGGCGGAAATGGAAGTGGGCGTGGCAGCTACGGTGATACCCGCTGTT
TGCTGGATGCCGACTTTAATCGGGTGAGTTGCGGAAAATAGAAATGATACCTCAGACGTCAGTTATGCAT
ATGAAACTGTCCAAATCTAAGGTTATCACAAAACTTTTCCTAATATTCTGCTGAAAATACTCATTTAAAT
TATTTGTTTTTAACTATTTGAGAATTTAAATATATTTAAACATTCGCACAATAGTGTAGTGTAGATGCCA
GTGATGTCACTCTAATCCAGCAATTTATTTAATTTTACTAGCCAATGGTATCACCAGCAACATACAACTA
GCCTGTGTAGTTCAACGAATCTGGCTGCTCATCTCGAACATATGCCACGGCTTGCTAGCCGGTCTAGCTC
TGGCCCATCTACTCTTCGTGCTGAGCAGCCACCCCATGGACTGGGCCAAGGTTATCAATGGCATGAGTCT
TGCGGGCGAGACGGTGTCATCAACGGCCCAACCCCCAGCCGCATCGACACCATCGGCGATAGACATCGAT
TCCGGCGATCGGGGAACGGAGTCGGGCGAATCCTCATCCCCATCCGCAGCAGCATCCGGACTGGCCCTGA
TAAGTGACTACGCCGGATTTGCGGAGATCTACTTAAATACCTTCTACTGCTTGGCCATAATCTGTCTGGT
TTCCGTTTTCGATCGG
>dme_circ_0005235_junction_seq
AATCTGTCTGGTTTCCGTTTTCGATCGGCCAAGTTGTACTCAGCAGGCGGTATATC
>dme_circ_0005236
CTGAAATCGAAGAGTCTCACGCACTTGGATGGCCTCGACTCGGGTGTGGTGGCCTGCCAGCAAGGTCCTC
CGGGAATGGCCACCAGCACCGCCAGCACCACAGCCACCACAGCCGGCGGCACCGGCGCGACCACCGGATT
GTTCGCCACCACTGGAGCCCCAGGATCGCTGGGCCGGCAGGCCAATGTGGGTAAGTGCAAGTGGTCAGGG
AACCGATGGCGAGGTGGAGGGCACATGCTAGTCTATCGGCCCACTGCTAGGACTTGGCGGCCACTTAGTC
AGCACATCCATCTTGCCTGGACAGCGTTCACTGCGAGAAAAACTATGATTTCAAATCAAGAATTCAAATT
GTCGAAAGTAGATCAATTAAAAATAATAATAAGATAAGTACTCTCTACATTGGATATTAGGTTTATGGGA
AAGTTTTGTGTAGATATGTCAAATAGGAACTTGGATTTGAAATTTCATaaattaagcaaatttaaatata
aaaaaatgtttctgagaattgaaaatattgaaaaatatataCATATAGTGTATATTGTAAAATTGTAAAA
CGGGAAATATTAAATTAATTCTTGTTAACTGAATGAGGATCTTCTAATTCCAATAATATCATAATACATT
TAAAATCTTGATTTTAGATTACAGTTATTCCTCGTGTAGTGTGTAGGTTGTACAGTGTAGTTTGTTTTCA
CTGTATGTTCACTATTCCTGGGTTTTAGTCCCTCGAGTGAAAGTCCTTTCGCAAATTGATAACAAATTAC
TTTGCTTGCTGCTTTGGTCTGTCATGGATTTGTGTTTATTTTGATTTCTTTTAACTATTTCTCTTCTTTG
TGTTGCTGTCATTGATTTAAGCACAGTCAGTTGTTTTTTACTTTGCACTAACTTGTTGTACTTGTTAGTT
ACTGGATTTTTGTTGTCTACTTAAACCGTACGTGACTTTCTGCGCTGTTCGCCCTTTTTCTTTGAATTTT
ACGTTTAATTTATGTTTCAAAGTTGGAATGAACTCTAGGCGTTGATATTAAAAGATTTAAAAACTGTTAC
AAGCGTAGCTCATGTAAGATCTGACTTACGAATTTATTTTGTAGCGCAAATAACTCTCTAATATGATACA
ATTTTCTGCATTATAAGGATATTATTAATGTTACTATTGCACTGTTGGTAATAGCTTTTGTAGCTTAGTA
TTTCCTAACAATTGGCCAAACTTGGTAGATTTGCACTGTAGTAGTACATATGGAGTACATTGAGAATTAA
CGACCAAACAAAATATGCTCACAATATCTGCcttctcctctctgttcttctctctctttctctatctctc
tctccctctctcCCCCGCTGTTTCTATATAATACCTAAATGGGCTAAATAAACTTTGGTTTTTATTTGTT

TGCTGACCGCTAAAACATCCCGCTGATACCCAAAAACAAAATCTCAGACTGAGAAGGGTGAACGAACGGG
CGTTTGCCTGTCACCCTCGGCACCTCAGCCCTCCTCGCACGCTTCACCGCACCACCCACCCACAATTTAC
ACGCAGGCACCGCAGGTCGTGCGGCGGATGCCTTCGCTGATGCTCTCACCCACTCTCGATGAAACCACGA
CGACGGCCAGTTCGTCCTGCTTTGGTACCGGTCACCCCAGTGCCGGATCCTTtcaccagcaccagcacca
tcaccatcatcatcaACCGACTGGTAGCGTCAGCACCGCCAGCTGCACCGCCACCTCCACCACGTCTACT
CATGCTGTACCCTCCTCCAAACACTTTCAGTCTGTTGGCGAGGCGGCGGCCACATCATCGTCCTCGACGC
TGAGTCCGCACAGTGCTACATCCGCGACCTTAGGCGGCGGCGGAGGTGGTGGCGGTGCAGGCGGTGGTGG
TCTAACCTCCCCGGTTGGCTGCTCCACGTCCTCCGGTTCGACGGTGAAAAAGAAATCCTCGTTCATGAAG
CGCAAGAAGCCACTGCTGACGCGCAGCGAGGTGAGTGGGTATCAAATGAATCAACTGAAGCCCTTGATTC
ACCTGGTCAAAAGACAAACAGATCTCGAATGGCTGCACAATTAGAAGGTCCTGTCACCTCAGCGAGAAAT
AAGCCTGCAGTTAAGGATGGTGAATTATAATTGGCGATGGTGACAACTGTTGGATGGGGCGGGGGAATGT
ACATTTTTAATTTCTTTATTTGCCTTTGTCGCCGTAATCAGCAAAAGTTGTTCTAGAGCTTTTCCAACTA
TCCtttcctttttcctttttccatttttttttttcctttttttggttttCCACTGGCCTCCAACGCTTTTCT
GTCACTTGACGAGCAAGAATTCCGCCTCGTTTCCGACCAACACAAACACGAAGAATATTCCTTGATGTTT
GGTTTTTTATTTGCAGTAGTTTTTGTGGGCAGATGGGATGCAGAATCACCTACATCCCATTCCCACGCGG
CCTCCAGATGCAATCTTCACACTTCGGAGGGGCCATCAAAGAGAGTGCTACGTGGCTTCGTCGCTGGTAA
TTTTTGCCAGTGCATCTCTTTTTGGTCTTTTCGAAGGCTAGATTGAAGGGTCCTTCATTTTGGGTTCGGT
CCATGAATTAACAGATTGGGTTCTGCTTCAATAAGATAGCTCTTATTCAACCACTTAACGCTCGACCATT
CATTTGTTTAGGGTAAGTTTAGAAACGGGATGGACGTCATGCTTTGTGGGAACACCGTCGTAACTTAACT
GACCTGGGATCAAATAAAGTTAAAGCTAATGAAAAGGGTCCTGCCATTTAATAATCTTAATATCCGCTGG
GCTAAAAATGTCGCTTTGAGCTTATGAATTTGAATGCATTTCAATTTTCCCTAAAAGAATGTGACAACAA
ATACTACTAAAGTAGATAAACACATAACATGTATAATACGGTTAAATCTCGTCAACCACAGGGGTTCCCG
ACAACCACCGCCCCGACTCCTGCCCTCCGTCTCCACCGAGGAGACGAGCGAGTCGGGAGTGGCGCCTGGA
AAGCAGATCAAGCAGCGGTGGTCCTCCATATTCGGAATCAAGAATCCCCAGCAGAGCCAGCTGTGCGAGC
TGCTGAACAGCTATGCCAAGAACGGAGTGCCCCAGCGGCCGGACAGCATGAGCTTTGATCATCCGGACCT
GGTCAACTCTTTGGCCTATCTGCAGTATATGCACAAATCGTGGCGGGACTTTGTTAATAGCGATTCGATG
AGCGAGTCGGAGGTGAAGATCCAAACGGCCATATGGGAGCTGGTTACCACCGAGGTGTACTACATTCACG
CCCTGCAAACAGTGACTGATGTTGTTCCTAGCCTGTTTGGAGGCCGTGCAGGAGGAGGGTCTGCTGATCG
ACGTGGACCAGGCTCGACTGTTCTCCAATGTGAGGGCTGTGTGCGAGGCGAACATCAAGTTCTGGACACT
GTGGCTCTATCCCATGGTGGCACACAGTGCCATCACCCACGAGCCACTGAGATGCGCTTTTTTTCAGGAG
GGGTTCCTTTCCTTCGCCTCCATTTTTGCGCCATATAAGGATATACTGTGCGGAGCAGAGCACCTGCCAG
TTCTACTGCAAGGAGCTAAACCATAACAATGCCTTATTCACCTCCTACTTGGCGTGGTGCGAGTCGCAGA
AGATGTGCAACCGCCTGCGACTCGCGGACATTGTGGTGCGACCCATGCAGCGACTGACCAAGTACAGCCT
CCTCCTGGCCGCTATCAAGAAGCACATGAGCGACGTAGAGGAGATCGAGGCCATCGACGTCATGATGCAC
AGCGTCGAGAACTTTGTGGGCAGTGTGAACAACCACTTGACGATGCGGCAGGAGAACGAGCGACTCAAGG
GCGTGATGGTGCGGATCGAGTCCTACGATGTGGTGGGACACCAACAACGAGGTGCTGGACAAGATGATCA
AGCAGAACAGCCAAATGTTTGACCTCTGTGCTCCGATGCGCGGATGCCCGGCCTACCATGTGCGCCACCT
GTTCATGGAGGGCGATCACAAGTTCAAGGACAACCTCGGCAAGTCCGATGTGCACTGCTTCCTGCTCACG
GATCTCCTCCTGGTGTGCAAGACGATCGCCAAGCGGGGACTGGGCGCTCTAAAGGTCATCCGGCAGCCGT
ACCTGACCGACCAGCTAATTGTTCAGCTGGCGCCGAACAATACACTGAACTGTGTGTACCTCAATGAGTT
CCAGGTGGCCACCACGGCGTTTACGCTGCAGTGCACCGAGGCCAAGAACTGGTACGACGCCCTGTGGCGG
GCCAAGACGATCTACCAAAGACTGAAGCGCGGAGGCGGTGGCGGCGGCAGCGGAAGTGGAACTGTGGGCG
GCGGCGATAGCTTCCGTTTCGGTGGAAGTGCCACCAGCGGCGGCACCGCCGACTCCCTGGGCGTACGCAA
GTCGCCGATGAACTCCTCCATCTGTAGCCACGTCTCTAGTGCGAACAACAGCCACAGCGGCAGCGTCGAG

TGGAACGACTCCCGCAACATATCCGTCGACTTTGAAAAGACAAACTCGGTGTCCAGCGACGAGGGCTCCA
GCATAATGACAGGGCAACCACGGAGTGAACCTAAAGGGCAAGCAGCAATTGATCAGTGGTCTGCATAAGA
CCAAGATGGGCATTATCGGCCAGATGGGCTCCAAATCGGCGAACACGCTCTCCGTGCAGCCAATGAACCA
TCTGGGCCAGAGCCTGCCCAACCTGAACATGCATCACAGCCACACG
>dme_circ_0005236_junction_seq
CAACCTGAACATGCATCACAGCCACACGCTGAAATCGAAGAGTCTCACGCACTTGG
>dme_circ_0005237
GATGGAGGAGTTCATCGGCGACTTGCGTACGCCCAGGGAGTCGGCGGTGCCGCCGCTGGTGGCACTTCCA
CCGAAACGGAAGCTATCGCCGCCGCCCACAGTTCCACTTCCGCTGCCGCCGCCACCGCCTCCGCGCTTCA
GTCTTTG
>dme_circ_0005237_junction_seq
CGCCACCGCCTCCGCGCTTCAGTCTTTGGATGGAGGAGTTCATCGGCGACTTGCGT
>dme_circ_0005238
GTAACGACTCGAATCCAGATGATATATTGCACCGATGCCTCTCGTGCAGGCGCATTAAGTTCGCTTATTG
CCGGCAATAAACTCATTTACGTACTCGACCCGAGCACCTTGCTCCTTGTGGCCGGAGCCACCTCGGTTGC
GAAGCTGGGGAGGAGTACGGGCTCCTTCTACTCGTCAGTTGCAGGAGTAAACACAGATACGCACATAGCt
acagatacagttacagttacagatacagatacagatacagatacggttagggttaggaatagggatacga
atacggatacTGTGCCCAAGTACTTTACGCGAGTGTTTAGGTGTGTGGGCTGAGCTAACCAACCGAGCGG
GaaacaaacaaccaacagcaaggaaagaaagaaagaaagaaacaaacaaacaaaccaaccaacCGAGAGG
CAAACAAATTGCTAAAATAAATGTACGGTGGGGGCCAGAAGAAACAGCAGcaacaaagactcaacaaaag
gaaaaggacgcgaaaaaagaagaaacaaaaatcggcatcaaaatcggaatcagaatcaaaatcagaatcg
aagagGTCCCATTAATGAACCAACCCAGG
>dme_circ_0005238_junction_seq
agagGTCCCATTAATGAACCAACCCAGGGTAACGACTCGAATCCAGATGATATATT
>dme_circ_0005239
GGGTTTAAAGTCCGTTGGCCTGGGAATGTATTCCAGCAAGGAGACAAAGAGCGCGGCaaaagcgctaaaa
aagacgaaaaaagttaacaaaaagcAGCAGGAATCGTTGTCAAATGGTAACAAAGTGAAATCAACGCCGA
ATGCCAAGAATATCcaaccgaataataacaacaaacaaaccaacttcaacaacaataatagcagtaacaa
taacaacaacaacaacaacaacaacaacaacaacaacaacaacaacaacaacaacaacaacaataatagc
agtatagtgtccaataacaatagcaagaataataCGTTGCCAATTAACGTGGCCCAGAAAACCAAACAAC
ACTTTGAGGCGGAAAAGAGGATCCAGAGTATATCAATGGGCGCCACCACATTGCCGGGCTACGAAAATGT
GCCCAAAATGGAGCGATCCAATAGCTTCTTACGCACCAAGCTGAATAAACTGATTAACCACCTGACGGGC
AGCAAGGAGAATCTGTCGAGGACCGACAGCGATGGGGACTCCAGCCGCACACCGTACATATTCACCCGAA
GTCGTTCGATGATCCAGATGAGACGACCGAATCGCCGCAGTTTCATCGAGCCCCAGCTGGAGCAACTGAG
CGAGGAGACGGAGAAATCGGGGGATCTCACCTCACCGACATCGCCGCGGAAGACATCGGTGGCCGATCTG
CAGGCCAGTTCGCCGGTGCTCCTGCGAAGGAGTGAGCTCAGCTCATCGGTGAGGACTCCAAAACGTCGAC
AATCCGCACAGCCCTTCACGTCGACTCCTTTGGAGGAGGTGCAGTCGCCGGCCCAACGGAAGGGCTCACT
GATGTCGCTCAATCAAACGGAACCCGCCAATCCCCAGCTTAATTTCCAAAAGAGACGATCCAACACGCTG
ATTGCCTCCTTCAAGAGCACCTTCAGTGGATTCACTGGCAGTGGGAGCGGAGCCGAGAAAAAGGAGAAGA
TGAATCCCAAATGGAGTGCCTCGTTGCAATCCCTGCAGGCTATCGATAATATGGTTAGCTACGCCAACAT
GTCCTTCATCGACTACGACAAGTTCAATGGCTACGAGAAGCAACTGGAGCGCCAGCAATCGCTGATGAGT
CTGGCGGAACAGCCGGTGACCATCGCCACCCAGCTGCCGCTGCCGCCTTCGGCGCTGGCCCACTCGCATT
CGCCCTCTCCGCTGGGCGACAACTCGCGCACCTCCGTCCACCTTGGCTCATCTATGGATGAGTCGGTCAG
GACGGTGGTGATGCGCCGCAGGaggagcaagacctcgttggagggcagaagcaagacctcgctggagggt

aggagcaGCATGGCCAGCCTGAACAGTGCCTTCAATTTGGACACCGCCTACGAGCACAATCTGGACCGCG
TACATAATGTCTATCGAGAGAGCTTGGACTCGCGAACCCTGGATCTGCTGAATCAGCGCACGCGGAACTC
CTTCATCCATGATCAGGCGGTGCTCTTCGATGCCCTCAATCTGGAGGACGGCTCGGTGGACCTGCGGTGC
CTCTTCCGTATGACGGGCGTTTGGAGGAGCAGAAGCATGCGGCAGCAGCAGCAGCAGCGGGAACGGGATG
CAGTGGACGGGCGGGGGAAGCCAGGAAAACAGG
>dme_circ_0005239_junction_seq
GACGGGCGGGGGAAGCCAGGAAAACAGGGGGTTTAAAGTCCGTTGGCCTGGGAATG
>dme_circ_0005240
GCTCCTCCGGCTGCATCCACAGACGAGGGCTTGGGTGAGCTTCTGGGAGGCACCGCCTCCGAAGGCGATC
TGCTCTCTGCTGGCGGCACGGGTGGCCTCGAATCCTCCACTGGCAGTTTTGAGGTCATTGGTGGCGAGTC
CAATGAACCCGTGGGCATATCCG
>dme_circ_0005240_junction_seq
GAGTCCAATGAACCCGTGGGCATATCCGGCTCCTCCGGCTGCATCCACAGACGAGG
>dme_circ_0005241
GCCAAGAGAGATAGATAGCGATTCAAGACGCGGATTTGCACAATTGAAATATGAGGAGCGCAATCAGCAG
CAGCGAAATGAATAAATTACGCGAAAATAAAATCAATTTGCATTAGGGCAACTGGAGACGAGTGACTAGT
GACTAGCCGTCAGACAACTTGGACCCCCGAAGACCTCTCGAGAACCCGAGACCCTCCAAATACCATCTCT
CATTCACCAGCTGACAGCCATCTGAAGGACGAACAAGGCGCTGGCCCATGAGCTGCCGGGCGGGCAAATT
ATATTACCTTGTCAGGCGAGTGACATAAGGCGGACCTCTTGGCcccacacccacacccacactcacatcc
acatccacgcccgcgcccacTCAGCCCATGTGTGGATCCCAGTGAGGTGGCAAGTGGGCATAGGAGGAGC
TGGAGGAGGTAGCGGAGGTACTGGGCTCACGTCTTGGGTTACCAAATTGCCGCAATGCTACGCAGCTGCG
TGTGCCTGCGCACCGATGACGACGGGAAGCGGCGCAGCTCCTCCGTGGAAGACGCCTCCTCGTGCCGCTC
GTCGGAAATAGAGCGCGTGGGCGCCAGCATGCAGACACTGGGCCCAAATGGACAGCCGCAGTCATCCGCC
GCGCAGACGAGCACCAGGGCCTCCAGTCTGCTGCAGCTCTTCCAGCGGCGAGGTTGGTGCAAGCACTTCC
GCAAGCCGCTCCGGGGCATGAGCGCCTCCCGAGCCGAAAAGA
>dme_circ_0005241_junction_seq
GGCATGAGCGCCTCCCGAGCCGAAAAGAGCCAAGAGAGATAGATAGCGATTCAAGA
>dme_circ_0005242
GCTATCCGAGCGGCAATCTTCATCCAGAAATGGTATCGCAGGCATCAGGCCCGTCGCGAAATGCAGAGGC
GTTGCAACTGGCAGATCTTCCAGAACCTGGAGTACGCCAGCGAACAGGACCAGGCTGAGGTGAGTCCAGG
ATGCACATATAGTTAGTGATGGGAGGAGGCTTAAGACAGCCGTCGAAGAAAAGCCAAAGATGAACCCTCG
ACGACGACGACGCCAGTCGAGTGCATTTGTAGAGTTTCAAAAGTCGATAGCAATCAACTCAGCCGTGAAA
CTTTGTCGCAAGTGCATTGTTGACCCACTCATAGCTAATTTATTTCTATTCCCTCTGTTTGCTTGCCCCC
AGGGGCAACCGATTGCATCCCAAGTATGTGGCCCTGATTCTACGCGAAGCTGCCAAATCGCTAAAGCAGC
TGCCCAATATTTCTCCGGTGTCCACGGCGGTTTCCCAACAAGTTACGGTTTGCGGGGATCTACATGGAAA
ATTGGACGATCTTCTTGTGGTGCTGCATAAGGAACGGTCTTCCCTCATCTTCCAATCCCTACGTGTTCAA
TGGAGACTTCGTGGACAGGGGCAAGCGTGGCTTGGAGGTCCTGCTCCTGCTGCTCTCCCTTTACTTGGCT
TTTCCCAACGCAGTTTTCCTCAACCGCGGAAACCACGAGGATAGTGTTATGAATGCGCGATATGGATTTA
TCCGGGAGGTGGAAAGCAAGTATCCCGCGGAATCACAAGCGAATCCTGGCCTTCATCGACGAGGTCTACC
GATGGCTTCCCCTGGGCTCCGTTCTCAATAGTAGAGTCCTAATCGTGCATGGAGGCTTCTCGGACAGCAC
AAGCCTGGATCTAATAAAGAGTATCGATAGGGGAAAGGATCTTCGACATAATGTGGAGTGACCCGCAGGC
CACCATGGGATGTGTTCCGAACACTTTGCGAGGAGCTGGCGTGTGGTTTGGACCGGATGTGACTGACAAC
TTTCTGCAAAGACATCGACTCAGCTACGTCATTCGATCGCACGAATGCAAGCCCAATGGCCATGAGTTTA
TGCATGACAACAAG

>dme_circ_0005242_junction_seq
TGGCCATGAGTTTATGCATGACAACAAGGCTATCCGAGCGGCAATCTTCATCCAGA
>dme_circ_0005243
GAGTTGTCCACAGTGCTAGCCATGGTTTCGATCTGCCCCGAGTTTTGAATAAGGTTCATGTTGTCGCGCA
GCCAGTTGAATGCTGCACCGGCAATGGACACGCTTCCCTCAAGGGCGTAAAATGGGACTGCCTTCCGTCC
TAGTTGGTAGCCAACAGTCGTTAGTAATCCGTGCGTTGAGT
>dme_circ_0005243_junction_seq
ACAGTCGTTAGTAATCCGTGCGTTGAGTGAGTTGTCCACAGTGCTAGCCATGGTTT
>dme_circ_0005244
GCCATGGTTTCGATCTGCCCCGAGTTTTGAATAAGGTTCATGTTGTCGCGCAGCCAGTTGAATGCTGCAC
CGGCAATGGACACGCTTCCCTCAAGGGCGTAAAATGGGACTGCCTTCCGTCCTAGTTGGTAGCCAACAGT
CGTTAGTAATCCGTGCGTTGAGT
>dme_circ_0005244_junction_seq
ACAGTCGTTAGTAATCCGTGCGTTGAGTGCCATGGTTTCGATCTGCCCCGAGTTTT
>dme_circ_0005245
GGTGGAGGACGCCATCGAGTGCTACCAGCGGGCGGGCAACATGTTTAAGATGTCCAAAAACTGGACAAAG
GCTGGGGAGTGCTTCTGCGAGGCGGCAACTCTACACGCGCGGGCTGGAAGTCGTCACGATGCCGGAACCT
GCTACGTAGACGCTTCCAACTGCTACAAGAAGGTGGAC
>dme_circ_0005245_junction_seq
CGCTTCCAACTGCTACAAGAAGGTGGACGGTGGAGGACGCCATCGAGTGCTACCAG
>dme_circ_0005246
AACCACCTGGAAAAAGTGCTGCTTCTCCGGTGATGGGGAGTACATTTGCGCTGGCAGTGCTCGCCAGCAT
GCTCTTTACATATGGGAAAAGTCTATTGGAAACCTGGTGAAGATCCTGCATGGCACCAAGGGTGAGCTGC
TCTTGGACGTGGTGTGGCATCCTGTGCGGCCTATTATTGCCAGCATTAGCTCCGGACTGGTATCCATTTG
GGCCCAAAATCAAGTGGAAAACTGGTCTGCCTTTGCACCAGATTTCAAGGAATTAGACGAAAACGTAGAG
TACGAAGAGCGGGAGTCAGAGTTCGACATTGCGGACGAAGACAAGTCTGTGGATCTGAATGCAGACGCGC
AACAGGACGAGGAAATTGAGGTAGACGTGCAGAAAGTGGAGCCGGTGGCTGCCTTTTGCTCCTCCGACGA
AGAGGGCGAGGACGAGAATGCGCTGCAGTTTTTGCCAATGGCACCAGAGGTGGAGGATCCCGAGGATGGC
TGGACCGGTCAGGACGGACTGGAGCCCAGTGCAGTTATGCTTGGCCACATGGAACCGCATGACTATGAGG
ACGACATAATGGCTTCCAAGCGACGACGCATGCAGCTCTATGATGTCAGCTTACCTGATGCACCCACTGA
TGGAAACGCATCCGCTCATTTCTAGCAAGGCCAGCAAAGATAAACAACAGCCCGTGGGTGGCAAAAAGGC
CGCCGGACGTACCAAGAAATAAACATCATTTGGCATTGCCTAATCGTTTTCTTTTTAGTATGTAAATAAT
CCGCTAATCCGCCTTGGGATTCCGACCTTGACTGGCGAAAAGCACCAGGCCGGATTCCATCTCCAAAATC
AAGTACAGGAAGGGAAAACTCGCCATTCTGCTGCTCATCAAAGACATTGTCACAGCTGCGACAGTGGTTC
CCTCTTCATCGGCAATTATTTGGTAGTTTGTTTGCAGGAGTACATTTCCGGTGCCATTCGGTCCAGTTTG
CCCTTTTTTATTGAACAAATATCGGACGCCCATATATGTACATATATATTTTATAGTGGATCACAATTGC
CTATTAAAATGAAAATCATGAGAAACTAAGCTAGCTTAGGATTCCTTTTAGCGAAAGGTCAGTAGTTGGT
TCAAGCTTGGGAATACAGACTCAAGTCTTCGTGAGCCCCAGATGTTCTTTGGAATAGGACAACCAATATT
CCAATCCAATGGTTTTCAAGTGATTGGTGACGCTGTTTAATGCAATTGCAGATTTGAGCAATATCACGAC
CATCGACAAGTGTTGCGTTCAATAGTTTACATAGTTGGTACTGGCGAAACTAGCTAACTGAGCAAAACTG
CCCCCTTCTCATTGAAGTCTACTCTGACCACTGAAGTAGAAGGTGGGGAGCAGAAACGTTTTGGATCCGG
TCAAATCATCTTGCTGCACAGAGTTCTTTATGAGACCTCGGGTGCTGTGACTAAAGTCCTCGTTGATCTG
ACGCACTGTGACCATCTCTGGATGGCATCCTAGTAGCTCCTCTTGATCGAATTCACATTGTCGGTGTAGG
GACTAAAGGGAACCATGTTGACATTGAGAGATCCTTATGCGGTGGAGGAGAGAATATGACAATGAAGCAT

AACAGCGGGAAGAACCATAATTTCACATTATTTCACGGTTATGTTTCAGATTAGAACACGAATTTAGTGG
AGCTCTGTTTTTCTTTGCCCATCTTGTATATATTGACTACTTATGTACTTTCTACTGGCTTATTTGAATG
CAAAACCTATTCTTTGTTCAATATTCCAAGCCATGCTCTCCGAACTGCAAACAGCAAGACCTACAGCTCC
ACATTTTAAAGCTGATTTTCGTGCACTTGACTTTAATATTTTAATTTGAGTATCGTGGTTTGCATTATTG
ATTATATTATTCGAATTGTATTATCTTCCTGTGCATGCGTTCGATTCTACGAGATATCGGTTGAATATGA
ATATATATGAATGAGATCTTGGTTGAGATATatttaaaaaattaaacaattttaaacaaaattaaacaaa
ttctggaatttcggtagaaattaggggaaaaaaataatgaaaaaagtttaagaatttttaaaaatggtcg
gcgtaacatgtttttagaggaatcgatataaatttacaagactaaaaaaaatgagaaaaaaaatatgaaa
acatttttcaaaagtgtggTCAGTTTAATTTTGAAACAAAACTTGGGCACTAGGCGCACTATGCCTCTGG
AATCTTcatgtccatatcgactcgaatattgatcctgatcaggaatatatacacctttccaaaaacactt
tcttttacctattacatacttttcaacgtatctaataACATTTAAATTTACACTAGTTGTAACAAATATT
TTTGATAAGTACCACTATTTCTTAAGATTTCACTGTGCACATCGTTGTACTttgccacacccactcaaac
gcccacaaatcacacaaaatttatacgccgactcttttaaatagtttttggctttatttatgttgtcata
tttttattattaattattgcttattattgtctattatatACGAACAACATACAAGTTTTTAGGGTGATTT
GTCATAGAAAAGACCAATGTATTTTCTGCTTATCTTCATTACTGGATCTCACCTTCCCGAAAGACTCCGG
AGTGAATGAGCAATTTGTGTGTTCTTTGTAGGACAAGGTATGCAAACGGCCTGTTTATATGGAACTTGAT
GTTATTTGTTCCAGAAGATTTCTGCGGAACAGCATTAGGCAAGCCCTCTTCATCTACGACGATCCTGGCG
AATTGGTGGTAGAGGGAGAGGTACGCACCTCTGTCCCCGGTAGGAATCAGCATGCGACCTAGATCTGCGA
ACTCATTTCTCAGATCCGTTAGTCCAGCCTTTGGAAGATGTTCAATATGACGAAAGCTTTGTATTTTTTA
GAGACTTTTACGTACCTCATATACGGTATCTTCGAGTGAAAGTACGGATCGCGTATCGAACTTGGGAAGC
TTGACTTCTACATCAGATTCATTCTTGGAGGCCTCGAGTTCCTCAAGGAGGCGATGTAGTCCCAGGTTCT
TCAATTGTTTGAGCACCAGGCTTACTCTCTGGGAAGGCTTCGGCAGTATGACGATCATTACTAGCTCATG
TTCCCCAAAAGGTAGCTCCAGGACATCCGCTTGCAGACCCTTTACATTATTGACGTAGGCATACTTCCCC
GTTTGCACCATCATCTCCACCTTACCTGCCGGCGAGCCGCCGGCGTTGTAAAACTGTTCCACTTTCGTCT
GGGACTTATCGAATGGATACTGGAGTCATGATATCAGATATATTAAATACAATACAAGCGAGGATGAATC
TGGCTCTGGATCTTACCTTCCATTTGGCCTTAAAGTAGCTAACAGTAATCCCCAGCACAGTTACTGTACT
ATTGAACGAACGACGGCTATAAGAACTGAAGATTTTCGCATGAGAGGCTTTGTCGATATCGTCGTTGATA
ATTATACTCGCCCCCTGGTCGAAGTCCACACCCCTTGGCCGACCCTCTCGAAGAACCACATTTCCTTCTC
CTACTCCTTCTAGGACTACACTGTTGTACCCTTTCACGAGTTCTAAATCTCCTACATCGTCGTAGTAAGC
GTAACGCAGCGAAAACAGTTTTGTGTTTTCCGAATTTAAACTAGAATTTAAAGAAAAAGACATTGAAAAT
TCTAATAGATGTAGTTTCTGAGTAATGTGGTACTTACTAGTGATAAGCTCGGACATCCTCGTACCACCTG
CGCAGTGTTGGATAATCGACATAGATCGCCAGGACATCACGTATCTGCTTCAAGGTAGTCCCCTCTGCTC
CCTCGTAGAACATGATCATAAGTGACCACGTGGAAAATGGCGTCATCGCAAAGTTTTCCAGGTTTAAATG
CGTGGATTCCATTTGCAGCAGGTTGAGGGCAAATTGAAGCCTTGCGTCCGTCATTTTTTGTAATAGACTC
TTCGTATCAACAGTATTTGCACATATATGGGCCCGCAAACTGAGCACCATGCCAAAAAGGCCCAGAAATC
CGAGCTTCATATTGTCAAACAAATTTACTTAGCTAGACTAGGCTAGACCAATGAGAAATATTTCAGCTAA
AGGAAAAATATCATTTACTTTTCTTACCTCTTTGTGCACCCGAACACTACTCAAAACTGGTTTTTTATTT
ACGAAAAACTATATTTATATGATTTATTTTCGTTAATTTGTGGAGTTCAATATTCCATGCCATGTAGACT
TTATTTTTATGTTCCCGTTTTTTTGCACGTGCCTTGCAATTTTTATATCAGTTACTCGTAGGTTTGTTCA
AAAGTAAGTAAAGTATATACGAATATATTTTTATATATGTAATAACGAATCCACGGTAATTGATTAAGAA
ACATACATATATAATCTTTATTCAAATAAAATTGCAAGTGTGGAAGCGGTCACaattcacgtcttatttg
tagagcgaaagaattaaatctgattgtaaggtgcaaagcaaacgcttagcaaacccttggctTTTcagcg
gatgttaatgcttaggacattaggacaagctgaatttttttattgtcaccaaaattgggattcttatGCTA
TGTAGTAAtgtatgtatgtgtgtgtgttgtttgtgtgtgGTACatatgctatatgctatatggtatatAG

GCGAATAATTTGGGATACAAAATATTACATTATTGTTTACAATATTTGGTGGTTCGGTAGGAACACACAC
GCttttttttAACTTCATAACTTCattttaattttcttaggctatattcttatatttataattataataa
aatattattatgacaactaataatattataatattaattgtaattatTTGAAATGTATTACTTGTTTTAT
TATGGATTTCAAAAATATCTTTAGCTTGTATATACTCTATATATTCTTATATAGAAAGATCttcccattt
agttattttcttattgacataaacggtttgtgtgtaatccaacattgtatttgatattgagatgtcatca
atttgtatgaagcaattaaaagctttcaatatattatctccctctattattatatctctatttatgcaat
tatatatatatatagtgacatatccataagaccctaagacttaagcatatgcctatatactaatacactt
acaacatatacaccccaatacaacatacaccactccggatgtacccaacagataccagataagaataaga
ttgttatatgatcctcgagaataaaaaaaaaccccaattctagataagtcacccactggtagactaaaca
tccgtcccctaatttaaacaattccttgcttaagcctcaccccatcgtcacattcccacgttcaaagctc
ggagccgcaatcccaaaaaaaaaagtatcgatttcaataaacaaattataagaatctaagagcacttgtat
ccaagagcaaatgcacttgaatccaagagaaacgcaaagctttttctcttcacgatcagaatcctaaagt
ctaaagtccatattagaaaagctcgataccgaggcttgaacgtcaatcaaatcagaataattatcagagt
tcagtttgagacctaattgtaacaggtcggtgttcttctcaaataaaaagttttgtaatcatttagtgaa
ataaaaactataattttttttcacttataaatattgcaagaatttaattGGCGCAGCCGGTAGGATCCAAT
AAAATAAAAGAGTCCTTTTAGTACTAGCTATCCAAGATCAGCGAATTAAAATAGTGATTCGAAAATATTT
TTTAGAGATCCGCAAAAGAATCTACGTGAAAGTAGTATTCAAAATAAAATCCCGTGCGGTCGGAAACAAA
AATTAATTAAAATTTTTTTAATTCCGAAACTTAAAACCAAGTCTAACGaaaacttaaaaccaaaataaac
tccagaaaaaacaaaagACATCATGGCAGTCCCACAACTCTCAGAAACACATCTAAACCAACTGCTAAAC
CAAATCAAAGAATTAAACTACTACGATGGCGCACCTGGCAAATTATCTGGATTCGTCAACCAAGTGGAAC
AATTGCTCAGTTTATACCCAACACAGGAAGCAAGGCAGGCACACGTCATATATGGAGGAGTGaagcggtt
attagtggattcagccttagaagtcgtaacccaggaaagagctaacacatggttggacataaagaaagca
ctggcaatggcattcaaagaccatagaccttatgtaacactcatcagacaattagaagacacatcatacc
caggaagtatctgtaaatttatagaaaaactagaaacaatactggattatgtttgataagttagaattag
aaagtgaccctgttgataaatcgaattataccgaaatgttaaacaaaactgttaaatcagtaatagatcg
aaaactgccggatagaatttatatgtctttggcacgtaaagatattgatacaatttataaattaaaacaa
gcatcaatggaatttggcctttatgatgctattccagaaaatcaccgttctaatagaacagaaatgaaca
aacgtaggaacaggggaaactataatcaaaataataatcaaatatattacaataatagaaatcacaacta
cagtaattattatcctagcacgaatcagaatcataatacacaaccacctcaaaattcgactcaacctatg
acaaatcaaaaccagtattcaccgcgtttcataccaaataatcaaagagggaattattatgcatttagac
gagacttaacacaagctcagcagaacaacccacttaataacacccttaatttccaaccttcgccatcaaa
taatattaacagacaagggccagtaaaaagacaacgcgagagtcagagtgaccaaagcaggatggatgta
aattttcatcaagctgcctcggacactcaaatgatagagaaggacattcaagtccctatgtaaaaatatt
tcatcataataaaaattataagggaatgatcgatacaggatcatcaattaacatcataagagaaaatttt
gagaacttagaagaaaaggaagaaaacctaacagtatacactattaaaggaccaataacactaaataata
ataaaaccaaccaacagtatgtccgtctgctcaaaaattctacattcacaaattttctgataactatgat
GAACTATTACCTTgcctctggaaaacaaaaattcaggttggtagtcgattacaggaatctaaatgagata
actgttaacgacaaatttcccattccccgaatggatgagatatagatattggacaaattaggtagatgcc
aatactttacaactatagatctagccaagggttttcaccaaatccaaatggatgaaaattctattgcaaa
aaaagctttttcaactaagcatgggcattatgaatatactcgtatgccttttggtttaaaaaacgctcca
gctacttttcagagatgtatgaataatcttctggaagatttaatctacaaagactgtttagtctatttag
acgatattattgtttattccacttcattggaagaacacattttatccctaaagaaagtctttgaaaaact
gagagacgctaatttaaagttgcaactagataaatgtgaattcatgaagaaagaaactgaattcctagga
cacatcgtcacaacaaatggcatcaaaccaaatccaaataaaactaaagcaattacaaattttcgattac

ccaagacacctaagcaaataaaatcatttttgggattatgtggattctatcgcaagtttattcctaactt
tgccaaaaaagttaaacccatgaccctcaaattcaggaaaggtgctataatagacaacaaatgtaaagaa
tacatcgaatcatttgaaaaattaaaagttttgataacttcagacccgatattaatctatcctgattttt
caaaaccctttctctgacaattgatgccagcaatgtagctattggggcagtgttatcacaaaatcacaa
gccagtttgttatgccagtagaacgctaaacgaacatgaaatcaactatgctacgattgaaaaagaattg
ttagctatagtttgggctacaaaatatttcaggtcatacttatttggcaggccatttgaaatattaagtg
atcacaagccactggtatggctcaacaacattaaagaaccaaacatgaaattgcaaagatggaaaataaa
actcaatgaatttgattacaaattcaaatacttccgggcaaagaaaatcatgtcgcggatgctctttccc
gcacgaaaatagaagaagttatggttggcgaggtcgcaaacagcgcagacacaactatacacagtgccat
taaagataatctaaattacatatccataacagaaagaccaataaataacttctctagacaaatagagata
gaaaaaggcgataacgatacaacaagtgtacaacatttctttcaaaagttaaagattaaaatagtctata
aagaaatgacacctgaactcgccaaaaacctcattagggaatatgtgtgtaccaaaaagagtgcaattta
tttccctaatgacgaagattttctgatcttccagggagcgtttaccgaaattataagccctaacaatttc
acaaaactcttgagatgtaccacaaagttaattgatatactaacgtatgcagaattcaaagatttaatct
taatgaaacattaggaacttttacccggatgtatagaaaaaacaatcaatttatttaaagaagaatatta
ctatcctgatagacaaaagcttattcaaaacattatcaatgaatgtgaaatttgtaatctagcaaaaaca
gagaaatcgaaacacgaaattgacatatgagacaacaccagaaatatttaacacaagagaaaaatacgtg
atagatttttttctcacaggaaaccagatcttcttatcttgcattgatatctattcgaaatttgcatcac
tagttgaagtaaaaagtagagattggctagaagcaaaaagagccattactaaaatattcaatgatatggg
aaaaccgtaagaaattaaagcagacaaagactcagcttttatgtgtatagccttacaaaattggttaaga
tctgaaggtgtacaaatttctataacgactagctaaaatggcatatctgatatagagagattccacaaga
ccgtaaacgaaaagctaagaatcattggtagcgagcaaaatattgaagataggtgcacaaaattcgaaac
aattctatacatatacaatcataaaactaaacataatagtaccaaaagatttccagcagacattttccaa
tatgcaggcagcccagactttaacgtacaactaaataaaatcgataagatagaatacctcaataagaata
ggcacgattttgaagttgatatataatatagacaggccccacttgtaaaaagtaaaataaccaatccatt
caaagagacaggaaggattgaacaagtagatgataaacatttcgaagaaacaaaccgtggcaggaaaatc
gtttagccgccgtttacagaagttaatattctaatacaatttcacgtgtctcttttattttgatctgtcc
tttagcttggttcaacagcgactgactgccgcgctctctgcagagtggcgagacgattagtcgataagtt
tctcgataatattggtatgttaaggatgggtgtatgagtgtataagcttatgactagattggctatgtat
aattaggtatgtatgactaggaatgtatgactaagagtaaaggaagagggtagaaggagcagcctactac
aatcgttcactataagtcaaaatttaagaaacagaaaaaatgcaataagagcaaatatgataattccaga
ccaaccaaagaagcacaaagtacacaacatacttctaataatgcttagttgcatactaccacttatcacc
atggtcaagtgcaacaattagaagtaaacccaataagcgcgaaaaatggatatcttatattccaaacagg
aacaatggagattccaaccagctatgaataccattatttaagcataaacataacaaagacaatgctcatg
ttcgaagatatagtaaatgaagcaacaaatgaacaactatcctaatataccacaaatacaatatttagtc
gacaaattaaaacgagaaataaatgggttaagaattattagtcgatgtaaaagaggtcttttaaacgtag
taggaaaagcatacaaatacttattcggcacattagatgaggatgacagagaagagttagaagaaaaaat
aaacaacatgtcagaagactctgaaaaaacccatgacctaaacacgattctagatgcaatcaatagtggt
atagatataattaataagctcaaagtagataaagaacaacaccaacaaattgcggtaccaatatttaacc
tagagcaatttacagaatatatagaagacatagaattgggtatgcaattaactagactaggaattttcaa
tccaaagttacaaaagcatgactatttaaaacatgtaaattcagaaaaaatgctaaagataaaaacgtca
acttggcttaaaacagacacgaacgaaattttgattatttcccatgttcctagcgaagttactaaagttc
caatattccaaattgttccgtacccagatgaacataattatattctaaccgagcaaatattcgataaatt
ctacatatttgataaccaagtattccataaagataccaatagggtagtattcgacaaatgtattattgga

atcatcaaacaagagcaaactcaatgcaaatatattaaaacacataaaaattaccaaataaattatatag
aaccaaatatactattaatatggaatattcctgaaacagctgttaaccaagactgtacaaacaataaaat
attaatatcaggaagcaacatcattaaaatttaaaattgtaccatgcaaatagatgaattcttaatctct
aataatctagcagactttacacaaacaatttatatcaccaacaatgtaacccgtctagaaccaataaatc
acttacaaacgagagaaatgatagaatcccatgtaaaacactataactttttccaaattatatgcattac
aacgttcgtcataatgataattagtttgactctgtatgtagcatataagtttaaaaatatacctaagaaa
attattgtcaaatatcgtaccttgaaaataatgtcaatgagaatattcaacaaggaaataatattacctt
atacccaaatttaacgacctgaggacaggccaaattcaaaggttgggggagtgacatatccataagtccc
taagacttaagcatatgcctatatactaatacacttacaacatatacaccccaatacaacatacaccact
ccggatgtacccaacagataccagataagaataagattgttatatgatcctcgagaataaaaaaaaaccc
caattctagataagtcacccactggtagactaaacatccgtcccctaatttaaacaattccttgcttaag
cctcaccccatcgtcacattcccacgttcaaagctcggagccgcaatcccaaaaaaaaaagtattgattt
caataaacaaattataagaatctaagagcacttgtatccaagagcaaatgcacttgaaaacgcaaagctt
tttctcttcacgatcagaatcctaaagtctaaagtctatattagaaaagctcgataccgaggcttgaacg
tcaatcaaatcagaataattatcagagttcagtttgagacctaattgtaacaggtcggtgttcttctcaa
ataaaaagttttgtaatcatttagtgaaataaaaattatattttttcacttataaatattgcaagaatt
taattatatatatataatatatataataaagttataataatatatataGtggcatattcacatacaaaac
cacataacgtagagtaaacatattgaaaagccgcatccagatcataacgtaaacaataagtgaccaccat
gctaatgtggatcaaataacaaaaatatccactctgcattttggtttaattatattgacacccccatact
gtatgccatctgcgcagtatgcattctaataaacaaattctttgacagcggcacttagccattcttgtaa
acaaatcttaaagtctgcctgctctctctgaggcttctcctccacttaagaatccaagagcaatgctctc
tcaaaatacttaagcatctaagagcaatgctctcccaaaaacactaacatattctttaagcaagTTTTAA
TTAAActttaagtttttaattaaacctcgacaataaagtgaaaccgaaaaatcacaatttgcctaaacaa
acctgaatttattatcaggaagacgctattgaatttgtgagaggctgtaaatccaattggttacctcaaa
gacccacgaaaaagctatagtgcaacccttgtgaaaatcaaaacctatcttaaaaaaaaaaaaaaaatgt
aaaaataagaataataaattaataagcgaaaattaaaacgtattaaaaataagaataataaataaataag
tgaaaattctatttgataaaaattaaaaataagaataataaataaaaagacaacattttaaattaaacaa
tattaaaaaaaaaatacacgataataataatataataactaactaataataataaaaaaaaaaaatatata
tattataaaaataaataaataatccaaaaatcaaaaatggctcaagaaccagcaattgtgccaccactat
cagacagcaacatgacccaggttgcctaccagattggcaatgtggagaaattcaacggtgatccaggctc
actatacacctttgtgagtcgaattgattacatactggctctttatgctaccggagatgaacgccaacag
cagatcatatttgggcatattgaacgcagtatcagcggagaagttatgcgctgcattggagcctatgaca
tgtacacctggcagcagcttagaagacaattggtactcaactataaaccccagacccctaaccacgttct
tttagaagagtttcgaggcaatgtacgagcattcctggaagaagcagaaagccgcagacaaacacttact
agtaagcttgaattagagcaagatcttgaagaaaagactttttaattgaaattaataaaatccagtatag
aatcactaattgaaaaattacctacacacatttatttaagaataaataaccacaacataccagatttgcg
atcacttataaaccttttacaagagaagggcatgtacgaacaaataaatcatacaagtacacatgtccaa
aaacaaaatttctctgataagccacaaaagtcctttaatcaaaatactaatcagtctaacaatatcagaa
aatatccaacacctttcctacattataattcaccaataccatatcaagctccacaaatttatccaacacc
accaactaataacccactttatcgtcatccaataccctaccaccctaatccaaacaatgttattcaacca
agccaacaaaacaatgtttttcaaccaagccaacaaaacaatgcttttcaaccaaatcaacgtacaaact
ttacacctcgaccaatttttaacaccaatagaaacaatgcattcgatcagaataggttcggacaacaacc
ccaatatcaaaatcaacaatcaacacaaaattcaagttcctatgtacccaatcgaccaataaaacgatta
agaccagctaatagtggacagactgggatgagtgttgacgaaacattatatcaagaggacgctttttatc

agcagtgtgttccatatgactatttttattatccaacttacgaccattcagactattatccagaaaatca
atatcaaattgacgaaaacaaccaaaatttacaaagaacacaacagttacagcagattaatacagacgag
acaaacaatgacaaccaagaacccaatgttgaacaggccgaaaattttcagccacaagccttggaaaacc
ccaatatataacaattaaatacaaagaaaataatttgaaatgccttattgataccggatcaacagttaac
atgacatctaaaaatatatttgacttaccaatccagaacactaatactcttattcataccagcaatggac
cgctcattgtcaacaaaagtataaccataccttcaaagattttgttcccaacaacaaatgaatttttatt
gcaccctttctctgagaattacgatcttttattaggaagaaaacttttagcagaagcaaaagcaacaata
agttaccgcgatcaagaggtaactctttacaacaacaaatacaaattaatagaaggaatagcaacacatg
aacagagtcattttcaaaatgtaaatatgataAAATTATAAAttatgaacaggaggtcgaaagccaaata
caagatatgctaaatcaaggtattatacgtaccagtaattcaccttacaatagccccatctgggtggttc
caaagaaacaagatgcatcaggcaaacagaaatttagaattgtaatagactaccgaaagttaaatgaaat
aacagtaggagacagacacccaatcccaaacatggacgaaatcttgggaaaattgggcagatgtaattac
ttcacaactatagacttggcaaagggtttccaccagatcgaaatggatccagaatcagtttctaccaagc
acggtcattatgaatatttgcgcatgccattcggattaaaaaacgcgccagccacctttcaacggtgcat
gaatgatattttaagaccactcttaaacaaacactgtcttgtgtatttggacgacataattgtattctcg
acatcccttgatgaacacctgcaatcgctcggactagttttcgaaaaattagcaaaagccaaccttaaat
tacagcttgacaaatgtgagtttctcaagcaagaaaccacatttttaggacatgttctaacaccagatgg
aataaaaccaaaccctgaaaaaatttaagccattcaaaaatatccaattcccactaaaccaaaagaaata
aaagcttttcttggactgacaggatattatcgtaaatttattccaaactttgcagacatagccaaaccca
tgactaagtgtttaaaaaagaacatgaaaattgacactaccaacccagaatatgactctgcatttaaaaa
attaaaatatctaatatcagaagacccaattcttaaagtacccgactttacaaagaaattcactttaacc
acagacgcaagtgatgtcgctttgggggcagtactgtcacaagatggacacccacttagctacattagcc
gaacacttaatgaacacgaaataaattacagcacaattgaaaaagaactcttagcaattgtatgggcgac
aaagacttttcgacactacctacttggaagacactttgaaatatccagtgaccatcaaccattgagctgg
ttgtaccgtatgaaagactcaaattcaaaactgacccgatggagagtaaaattatccgaattcgattttg
atataaaatatataaaaggaaaagaaaattgcgtggcggatgctctgtccagaataaaacttgaggagac
atatttgagcgaacaaacccaacatagtgcagaagaggacaatagtgatttaatttttattacagaaaga
cctctaaatacatttaacagacaagttatattttcaaaaggaccaccagacattaaagttacgaaatatt
tcaaaaaacacatcacccaaatattttacgacattatgaccagggaaaaagccgaacaatatttgatgga
ccatttttgtggtaagaaaagtgcgttgtatattgagagtgacgctgatttcgaagtcattcaggccgca
cataaattagccataaacaccaaatatacaaaaatcctgcgtagcacgattttgttaaaaacataacca
cttatgcggaatttaaggaattgatcttgactgctcatgaaaaacttctacacccaggcatacagaaaac
tactaaacttttcggagaaacttactatttccctaatagccagctacttattcagaatataataaatgag
tgcagtattcgcaatctggcaaaaacagagcaccgaaatacagacatgccaatgaaaaccacacccaaac
cagaacattgccgcgaaaaattcatgatagacatttactcatccgaaggcaaacattacgttagttgcat
agacatttattcgaaatttgccacattagaagaaataaaaacaaaagactggatagaatgcaaaaacgcg
cttatgcgcatattcaaccagcttggcaagccaaagttactaaaggcggacagagacggcgcattttcca
gtttagccctcaagagatggctggagagtgaggaagtcgaattgcagcttaacacaacaaaaactggtgt
ggcggttattgtacataaaacaattaatgaaaagattcgcataatcaaaacatccgatgacgaagaaacc
aaattgagcaaaatggaaacagtacttaacatatacaatcataaaaccaaacacgacaccactggacaga
cccctgcacacatatttctctacgctggacaaccaatattagctacccaacaaaacaaagaaaacaaaat
aaataatgacagagtggagtacgaagtcgacacaagatacagaaaaggtccactacagaaaggcaaatta
gaaaatccttttaagccaacaaaaaatgtggagcagactgactctgatcattataaaattactaatagaa
atagaattactcactactacaaaacacaattcaaaaaacgaaagaaaaataatcagctctcaattttaca

ggcacctggcacttgataacattgttgccgatgctgatcacaacagttcatggacaacaaattgaaatta
ataatattgacacaaaccacggatatctcatttttctgataaaccagtccagataccatcatcctttga
acatcattgcttgagaatcaatttaactgaaatagacaccatagctgattattttgagcaaagactacgt
accgactaccatgcaccccaggtcaaatttttatacaacaaaatgagaagagaactagctggaatagcct
tgcgacatagaaataaacggggacttattaacattgtaggttcagtttttaaatacctatttggcacact
tgacgaaaatgatcgagtggatatacagaggaaacttgaaacaaacgcccataactcggtaaatttacat
gaactcaatgacgctattcaattaataaatgacggaatgcaaaagatacagaattatgaaaacgacagca
acatcattaacagtcttttatatgaactcatgcagtttacagaatacatagaagatgtggaaatgggaat
gcagctttccagactcggtctatttaatcccaaactactaaactacgataaacttgagaatgtaaacagc
caaaatattttaaacattaaaacatccacttggattaattacaatgataaccaattattaatcatatctc
acatacctattaacttttcattaataaatacagtaaaaataatcccttacccagactcgaacggctatca
gctagaatacacagacacacaatcatattttgaaagagaaaataaagtttacaataacgaaaataaagaa
ataaacaatgagtgtgtcaccaacattattaaacatttaaaaccaatttgtaattttgagtcaatccaca
cagatgaaataataaaatacatagaaccaaacacaattgtaacctggaatttaacccaaacaagtctcaa
acaaaattgtcaaaatttaattaataatataaaaataaaaggaaacaaaatgataaaagtaacccaatgt
aaaatagaaatcaaaagcataattttaagtgaaaatctctttaaaccagaaatagatttgacaccattat
accaaagacactagaataggtctttgattatacacaccacttaacataacaaaaataaaaactgttaaac
acaacgacattaatgaaatgatttcacaaaacaatattacactttacatatttatgactactgtcatcat
tatacttattttattgtacttatatttaagatacgtatcatttaacccattcatgatgctgtatgcaaaa
ctaaaattaagaaaaaaatcaaaatcaaaacacagcacaacaaatagaaatggaagacgttccattacccc
tactatatccatcaatcccagcccaagtataggcttctctttaagggaagggaagtgacatattcacata
caaaaccacataacgtagagtaaacatattgaaaagccgcatccagatcataacgtaaacaataagtgac
caccatgctaatgtggatcaaataacaaaaatatccactctgcattttggtttaattatattgacacccc
catactgtatgccatctgcgcagtatgcattctaataaacaaattctttgacagcggcacttagccattc
ttgtaaacaaatcttaaagtctgcctgctctctctgaggcttctcctccacttaagaatccaagagcaat
gctctctcaaaatacttaagcatctaagagcaatgctctcccaaaaacactaacatattctttaagcaca
gaggcttctcctcattttcactttcatttgatttttagtcttaagctgaacgttaatcaataaacaacac
aatcgatcccgaaattttgattcgttttattttggcaaaacttaattttcagcgttggtcttagttcata
ttcggaacggtccatttaatagactcaaaactatttattgcaaccatttatttgcaATtatatatataat
atagcgaatatattatatattctatattatatatattatattaggttcgacataactgatttcaaatttc
ttatgagtttttacatatctacattctgtattgaattgattaagtatgtcatttatttattatcaaagtt
tttaaagatgctaaataactttgattattttgaacataaaattaatttttttcattttctattaatgtttt
gatcactatcaggatatggaaatctagaaaagataggggttttacttatttcttttggaatatgggaaat
tataagaatttcatttgtatctgtttttaaccaagtggaagttttaatatgtaAAATATGAATTGGGTTT
ACTTCAAGGCTTTGTACTTGGGCAAATACAATACAAATATatttcgatttatagtgagtaatttttcctc
ctctattcttttctttataatatttatcatatatttgtgtaatttctcctgttttcccatatgggtttgt
tgtcttagtttttactaataatgcttgtctatatctagtatatatttcgaattcgtttcgtgatttattT
AAGTAAAGtattgctgaatagttaagttcatgttcgttaagagtaacggctagcgttataagcaggtttg
tgatcctgtggtaatacagcacctagaccaacattgctggcgtttttttttcgaaatttcgataaattaa
tattggttcggatataattaaaaattttagtttttcgaaggctgatatataattggtatctttagaattt
atatgaaacctttttttagtttttttgtgttatgggtttagcaatttttgcgaagttaggaataaatttacg
gtgaaaacagcaaagtcctagaaataattttatttctatactagatttaggtaatgggaaTTTTTGTgta
gtgaaatattgagacctaccgatcttatctagtatttcatccataactagaattggttatttgtcatcaa
tagtaatttcgtttagttagcgatagtcgattactatcccaaactttgTTTAAACTttaactggagaatt

aaaaatgtttgctcttatgatgtttattgtagacccagtgtatataatacatcggagctgtatattgttt
aaaacaatatttatgaaggaattgtgtgttcttcttcgtccgacgcatgtagatgaaaactttcagacgt
atccatttttgaatggcccctggtgctggctctttggctttttattccctgattcggagcagttgaagaa
acgggattgtgttgatagtttacaggattttgtgcctgtgcttggtcgagtttttgcttaaattcTTGAA
ATAGGCGTTCGAAAGATTGGTCATGGCTGTTATtatttttgttgttatattgatttctattttggttata
gtattgatttctattatgattattTGAAGCGTTGTTGTAGTTTGGACTGTACTGTTTTTTATTTAGGGTA
TTTTGAGTGCTTCGGGATCTTGGGCAATCGGACCATGTATTGTCATAACAACACTCTTGCTGGACAATTT
GCTTTAATTTACTAACtgttgttatgtcaaatctagctaattgcatgtagagtctgtctggaagttgttt
attaattacatattttactgttttgtcaattgcgtttgtataaatatgattatgttgttcaatattttct
aaggctagcttatttgttattataaaacattttgcttccagttcttctatgaacttacgaactgatccat
tgaattgtgtgttatatagtcgtcttaaaagttctttaaacggtgtttgggtcttgaactcagcgattag
ggcttccttcaattccggccatgtgttagctgcactgagctgggatactcgttgggcgtcaccaccaatt
tgcattttaatcgcactgaagagaacgtgtaactgacggatgtcagttgttgggtaaagttgtagtatga
gctcgatccttcttatgaacgagcttagttctagtgtcgtaccggtaaatgttggtatttgtcgaatcag
accaagagcctggttcagatgATTCTTGGAGATTAGTGGCACttgttgttgttgtggttgttccattgtt
gttttttttgtttttttgttgatgttgttgttgttgtcttgtttttgttgttCACTCGCACAGCAAAATTT
GCACTGGGGTTATAtttttttttttttttCACTCCTCGTAGCGGTCAAACTTTTTATTTTGCTGGCTTCA
CACTGTCTTTGTTTAATATACAAAACCTGGGGCTTCAGTTACTCTTTAATGGTCTTGTCCTTGCACTAAT
GTTATTTTTATCACTTCTAGTAGCTTCTAGTCACACCTCTATGGTGGGCTACCAAGTTGAAACGATTTTA
ATTAGAAAGCACTGACTGTCGGATTAATCACTTCTTGTAGCACTGATTAGCCACAAAGTTGAAGTTAAAA
CACACACGAAGTTACTTTTGCTGATCATTGACTGCGGCAATAACGAATCCACGGTAATTTATTAAGAAAA
AAGTATAATCCTTATTCTCAAATAAAATTACAAGTGTGGAAGCGGTCACaattcacgtcttatttgtaga
gcgaaagaattaaatctgattgtaagttgcaaagcaagcgcttagcaaacgcttggctgcgacttattta
catctttatatgtatattgagcgtaaataggctctcttttgtatttatatgaattgggcgccctcttgag
tggatcgcggcgcatggatactgatcagccgagaatttgagctgcgtaagtggttttggccctttggtgg
gagtcgcgaatgcgtctgtggatgtttatgtttacattagggcaagctggatttgttattaccaccaaaa
ttggggttcttatggagtacttgaatttatgatataaggcattctataatttggtgtatgactgttgcta
ggtaataatgtgtgtgtgtgtgtgtgtgtgtgtattgtttgtgtgcgtaagtatgtatgcatgtatgata
catatgtatgctactatatatgtatatatatataaagtatatataCGAATTTTTCTTGGTCATTAGAATA
GACTGTTATAAAAGAATAATATAAGAATTGCGTCCAGgagcgtccagggcactatttcctttccggaagc
catgttgctgacttcctaggacgttgctgctctcggtgattgcctctattcgagtatactggatacgttc
gaacagttttccaactatatccagtgggtcgggttcgcaaagctacccgtcgggtggtttctgtccgcct
tgctgctgcagataatgcattttggtgatgcaacccctttgccttgtTCtgcgtcccatcccgaatcctg
cgaaggcccgtgacatcctattgatttaactataggccctggaattgggcgaacatgcagctgcggagct
tctctgcggtcgtagcatcgtccagtccgctgcaggatagtgctattccttgagcttccgttcgcacaga
ggccaaatcaatgagcgccgcttccagatcgctcttatacttgggtacgctttccgaggctttttcatct
acttcgagcaacagctcaccttgctgagttctctgaatttttcgtacgtatcttctcagtttgcttaggc
tcgggtccgatcttagtttccggagcatctctgcgtacgaagactcaccgtcttcttcacgaccgtgcta
gcattcttggcgacatatgcgtagctgcctggcttggactcgtttgttctggatgtcacctttccttctt
cgttgctaccagtcgtgtgctttgggcacttgcccctttttggtatgcttttgtacctcagtctgggagg
caatgctcatcaagcggaggtcgctagtgacccctacgtacaactctatcgccctgttgttaagggctga
gagttcagaagctacgcccttggtccctcttctgacgtgccgttgagtttcaagtgtggaaagcagctta
tcttgcttatcctatcttatgtttatcttgccatacatcctggtgagtatgcacttcaggtccttgttct
gaagagccgtggtcaggtcccgatcgatgaggggatccagctaggcaattcactcctgccttatgcggtg

cactgctgcatctggttaccgactcctctagtgtgggacctcggaagcctggtgctcctcctgagcaggt
cgttgcgatcagggggacttccccccactgcgctatccatcaactataagatggcctacggttacctatg
tgaagcaccagctttagtaagggctctcacgtaggccacggatttaaccctggcttttaccgttggaggt
atgttccgcctcgaatcggacacaccagagccactgcttaggcaccagcacggtgagaggatgatTAAAT
TAAATATAACGGTTCACATTTacgtctcactctcaagatcccccactgcgccaaatgggcagcgctgcca
gctgttttatGAGCTGCAGAATATCGCAAACTTATAGTTTTGCTATGTAACGCCGTCTGCACGGCGATGT
AGACTTGGTGTCCCCACTTTTGGTGTGCCCCATGCGATACGCCGCCGTTCTCGTAGAGGTAGGCGGAACT
CACCGCTGCCTGCCCTTGGATTCCGATGTGAGTTTGCACATTGCTTTTTCGCAGCTTGATTGTTAATTGA
AATCCTTGTTTCTCACCAGGGCGACTTGAACATCGTGGCTTCTTTCGATCGCAGAGGCAAACACATCTAC
ACAGGCAACGCCAAGGGAAAAATACTCGTACTGGATGCTGAAACCTTTGAGGTGGTCGCCAGTTTTCGCA
TTATTGTAGGCACCTCAAGTGCAACGGCTGTAAAAAGCATCGAGTTTGCGAGGCGTGGAGAGTGGGTACT
AATAAGTAATCGGGATTTCATAACGTAACAAATTTATCTTTCAGCGCATTTCTAATCAACACCTCAGATC
GAGTTATTCGTGTTTACGACTCAAAAGAAATAATCATTCTAGGCAAAGACGGCGAACCGGAACCTATACA
AAAGCTACAGGACTTGGTCAATAAG
>dme_circ_0005246_junction_seq
ACAAAAGCTACAGGACTTGGTCAATAAGAACCACCTGGAAAAAGTGCTGCTTCTCC
>dme_circ_0005247
GGCGACTTGAACATCGTGGCTTCTTTCGATCGCAGAGGCAAACACATCTACACAGGCAACGCCAAGGGAA
AAATACTCGTACTGGATGTTGAAACCTTTGAGGTGGTCGCCAGTTTTCGCATTATTGTAGGCACCTCAAG
TGCAACGGCTGTAAAAAGCATCGAGTTTGCGAGGCGTGGAGAGAACCACCTGGAAAAAGTGCTGCTTCTC
CGGTGATGGGGAGTACATTTGCGCTGGCAGTGCTCGCCAGCATGCTCTTTACATATGGGAAAAGTCTATT
GGAAACCTGGTGAAGATCCTGCATGGCACCAAGGGTGAGCTGCTCTTGGACGTGGTGTGGCATCCTGTGC
GGCCTATTATTGCCAGCATTAGCTCCGGACTGGTATCCATTTGGGCCCAAAATCAAGTGGAAAACTGGTC
TGCCTTTGCACCAGATTTCAAGGAATTAGACGAAAACGTAGAGTACGAAGAGCGGGAGTCAGAGTTCGAC
ATTGCGGACGAAGACAAGTCTGTGGATCTGAATGCAGACGCGCAACAGGACGAGGAAATTGAGGTAGACG
TGCAGAAAGTGGAGCCGGTGGCTGCCTTTTGCTCCTCCGACGAAGAGGGCGAGGACGAGAATGCGCTGCA
GTTTTTGCCAATGGCACCAGAGGTGGAGGATCCCGAGGATGGCTGGACCGGTCAGGACGGACTGGAGCCC
AGTGCAGTTATGCTTGGCCACATGGAACCGCATGACTATGAGGACGACATAATGGCTTCCAAGCGACGAC
GCATGCAGCTCTATGATGTCAGCTTACCTGATGCACCCACTGATGGAAACGCATCCGCTCATTTCTAGCA
AGGCCAGCAAAGATAAACAACAGCCCGTGGGTGGCAAAAAGGCCGCCGGACGTACCAAGAAATAAACATC
ATTTGGCATTGCCTAATCGTTTTCTTTTTAGTATGTAAATAATCCGCTAATCCGCCTTGGGATTCCGACC
TTGACTGGCGAAAAGCACCAGGCCGGATTCCATCTCCAAAATCAAGTACAGGAAGGGAAAACTCGCCATT
CTGCTGCTCATCAAAGACATTGTCACAGCTGCGACAGTGGTTCCCTCTTCATCGGCAATTATTTGGTAGT
TTGTTTGCAGGAGTACATTTCCGGTGCCATTCGGTCCAGTTTGCCCTTTTTTATTGAACAAATATCGGAC
GCCCATATATGTACATATATATTTTATAGTGGATCACAATTGCCTATTAAAATGAAAATCATGAGAAACT
AAGCTAGCTTAGGATTCCTTTTAGCGAAAGGTCAGTAGTTGGTTCAAGCTTGGGAATACAGACTCAAGTC
TTCGTGAGCCCCAGATGTTCTTTGGAATAGGACAACCAATATTCCAATCCAATGGTTTTCAAGTGATTGG
TGACGCTGTTTAATGCAATTGCAGATTTGAGCAATATCACGACCATCGACAAGTGTTGCGTTCAATAGTT
TACATAGTTGGTACTGGCGAAACTAGCTAACTGAGCAAAACTGCCCCCTTCTCATTGAAGTCTACTCTGA
CCACTGAAGTAGAAGGTGGGGAGCAGAAACGTTTTGGATCCGGTCAAATCATCTTGCTGCACAGAGTTCT
TTATGAGACCTCGGGTGCTGTGACTAAAGTCCTCGTTGATCTGACGCACTGTGACCATCTCTGGATGGCA
TCCTAGTAGCTCCTCTTGATCGAATTCACATTGTCGGTGTAGGGACTAAAGGGAACCATGTTGACATTGA
GAGATCCTTATGCGGTGGAGGAGAGAATATGACAATGAAGCATAACAGCGGGAAGAACCATAATTTCACA
TTATTTCACGGTTATGTTTCAGATTAGAACACGAATTTAGTGGAGCTCTGTTTTTCTTTGCCCATCTTGT

ATATATTGACTACTTATGTACTTTCTACTGGCTTATTTGAATGCAAAACCTATTCTTTGTTCAATATTCC
AAGCCATGCTCTCCGAACTGCAAACAGCAAGACCTACAGCTCCACATTTTAAAGCTGATTTTCGTGCACT
TGACTTTAATATTTTAATTTGAGTATCGTGGTTTGCATTATTGATTATATTATTCGAATTGTATTATCTT
CCTGTGCATGCGTTCGATTCTACGAGATATCGGTTGAATATGAATATATATGAATGAGATCTTGGTTGAG
ATATatttaaaaaattaaacaattttaaacaaaattaaacaaattctggaatttcggtagaaattagggg
aaaaaaataatgaaaaaagtttaagaatttttaaaaatggtcggcgtaacatgtttttagaggaatcgat
ataaatttacaagactaaaaaaaatgagaaaaaaaatatgaaaacatttttcaaaagtgtggTCAGTTTA
ATTTTGAAACAAAACTTGGGCACTAGGCGCACTATGCCTCTGGAATCTTcatgtccatatcgactcgaat
attgatcctgatcaggaatatatacacctttccaaaaacactttcttttacctattacatacttttcaac
gtatctaataACATTTAAATTTACACTAGTTGTAACAAATATTTTTGATAAGTACCACTATTTCTTAAGA
TTTCACTGTGCACATCGTTGTACTttgccacacccactcaaacgcccacaaatcacacaaaatttatacg
ccgactcttttaaatagtttttggctttatttatgttgtcatatttttattattaattattgcttattat
tgtctattatatACGAACAACATACAAGTTTTTAGGGTGATTTGTCATAGAAAAGACCAATGTATTTTCT
GCTTATCTTCATTACTGGATCTCACCTTCCCGAAAGACTCCGGAGTGAATGAGCAATTTGTGTGTTCTTT
GTAGGACAAGGTATGCAAACGGCCTGTTTATATGGAACTTGATGTTATTTGTTCCAGAAGATTTCTGCGG
AACAGCATTAGGCAAGCCCTCTTCATCTACGACGATCCTGGCGAATTGGTGGTAGAGGGAGAGGTACGCA
CCTCTGTCCCCGGTAGGAATCAGCATGCGACCTAGATCTGCGAACTCATTTCTCAGATCCGTTAGTCCAG
CCTTTGGAAGATGTTCAATATGACGAAAGCTTTGTATTTTTTAGAGACTTTTACGTACCTCATATACGGT
ATCTTCGAGTGAAAGTACGGATCGCGTATCGAACTTGGGAAGCTTGACTTCTACATCAGATTCATTCTTG
GAGGCCTCGAGTTCCTCAAGGAGGCGATGTAGTCCCAGGTTCTTCAATTGTTTGAGCACCAGGCTTACTC
TCTGGGAAGGCTTCGGCAGTATGACGATCATTACTAGCTCATGTTCCCCAAAAGGTAGCTCCAGGACATC
CGCTTGCAGACCCTTTACATTATTGACGTAGGCATACTTCCCCGTTTGCACCATCATCTCCACCTTACCT
GCCGGCGAGCCGCCGGCGTTGTAAAACTGTTCCACTTTCGTCTGGGACTTATCGAATGGATACTGGAGTC
ATGATATCAGATATATTAAATACAATACAAGCGAGGATGAATCTGGCTCTGGATCTTACCTTCCATTTGG
CCTTAAAGTAGCTAACAGTAATCCCCAGCACAGTTACTGTACTATTGAACGAACGACGGCTATAAGAACT
GAAGATTTTCGCATGAGAGGCTTTGTCGATATCGTCGTTGATAATTATACTCGCCCCCTGGTCGAAGTCC
ACACCCCTTGGCCGACCCTCTCGAAGAACCACATTTCCTTCTCCTACTCCTTCTAGGACTACACTGTTGT
ACCCTTTCACGAGTTCTAAATCTCCTACATCGTCGTAGTAAGCGTAACGCAGCGAAAACAGTTTTGTGTT
TTCCGAATTTAAACTAGAATTTAAAGAAAAAGACATTGAAAATTCTAATAGATGTAGTTTCTGAGTAATG
TGGTACTTACTAGTGATAAGCTCGGACATCCTCGTACCACCTGCGCAGTGTTGGATAATCGACATAGATC
GCCAGGACATCACGTATCTGCTTCAAGGTAGTCCCCTCTGCTCCCTCGTAGAACATGATCATAAGTGACC
ACGTGGAAAATGGCGTCATCGCAAAGTTTTCCAGGTTTAAATGCGTGGATTCCATTTGCAGCAGGTTGAG
GGCAAATTGAAGCCTTGCGTCCGTCATTTTTTGTAATAGACTCTTCGTATCAACAGTATTTGCACATATA
TGGGCCCGCAAACTGAGCACCATGCCAAAAAGGCCCAGAAATCCGAGCTTCATATTGTCAAACAAATTTA
CTTAGCTAGACTAGGCTAGACCAATGAGAAATATTTCAGCTAAAGGAAAAATATCATTTACTTTTCTTAC
CTCTTTGTGCACCCGAACACTACTCAAAACTGGTTTTTTATTTACGAAAAACTATATTTATATGATTTAT
TTTCGTTAATTTGTGGAGTTCAATATTCCATGCCATGTAGACTTTATTTTTATGTTCCCGTTTTTTTGCA
CGTGCCTTGCAATTTTTATATCAGTTACTCGTAGGTTTGTTCAAAAGTAAGTAAAGTATATACGAATATA
TTTTTATATATGTAATAACGAATCCACGGTAATTGATTAAGAAACATACATATATAATCTTTATTCAAAT
AAAATTGCAAGTGTGGAAGCGGTCACaattcacgtcttatttgtagagcgaaagaattaaatctgattgt
aaggtgcaaagcaaacgcttagcaaacccttggctTTTcagcggatgttaatgcttaggacattaggaca
agctgaatttttattgtcaccaaaattgggattcttatGCTATGTAGTAAtgtatgtatgtgtgtgtgt
tgtttgtgtgtgGTACatatgctatatgctatatggtatatAGGCGAATAATTTGGGATACAAAATATTA
CATTATTGTTTACAATATTTGGTGGTTCGGTAGGAACACACACGCttttttttAACTTCATAACTTCatt

ttaattttcttaggctatattcttatatttataattataataaaatattattatgacaactaataatatt
ataatattaattgtaattatTTGAAATGTATTACTTGTTTTATTATGGATTTCAAAAATATCTTTAGCTT
GTATATACTCTATATATTCTTATATAGAAAGATCttcccatttagttattttcttattgacataaacggt
ttgtgtgtaatccaacattgtatttgatattgagatgtcatcaatttgtatgaagcaattaaaagctttc
aatatattatctccctctattattatatctctatttatgcaattatatatatatatagtgacatatccat
aagaccctaagacttaagcatatgcctatatactaatacacttacaacatatacaccccaatacaacata
caccactccggatgtacccaacagataccagataagaataagattgttatatgatcctcgagaataaaaa
aaaaccccaattctagataagtcacccactggtagactaaacatccgtcccctaatttaaacaattcctt
gcttaagcctcaccccatcgtcacattcccacgttcaaagctcggagccgcaatcccaaaaaaaaagtat
cgatttcaataaacaaattataagaatctaagagcacttgtatccaagagcaaatgcacttgaatccaag
agaaacgcaaagctttttctcttcacgatcagaatcctaaagtctaaagtccatattagaaaagctcgat
accgaggcttgaacgtcaatcaaatcagaataattatcagagttcagtttgagacctaattgtaacaggt
cggtgttcttctcaaataaaaagttttgtaatcatttagtgaaataaaaactataatttttttcacttat
aaatattgcaagaatttaattGGCGCAGCCGGTAGGATCCAATAAAATAAAAGAGTCCTTTTAGTACTAG
CTATCCAAGATCAGCGAATTAAAATAGTGATTCGAAAATATTTTTTAGAGATCCGCAAAAGAATCTACGT
GAAAGTAGTATTCAAAATAAAATCCCGTGCGGTCGGAAACAAAAATTAATTAAAATTTTTTTAATTCCGA
AACTTAAAACCAAGTCTAACGaaaacttaaaaccaaaataaactccagaaaaaacaaaagACATCATGGC
AGTCCCACAACTCTCAGAAACACATCTAAACCAACTGCTAAACCAAATCAAAGAATTAAACTACTACGAT
GGCGCACCTGGCAAATTATCTGGATTCGTCAACCAAGTGGAACAATTGCTCAGTTTATACCCAACACAGG
AAGCAAGGCAGGCACACGTCATATATGGAGGAGTGaagcggttattagtggattcagccttagaagtcgt
aacccaggaaagagctaacacatggttggacataaagaaagcactggcaatggcattcaaagaccataga
ccttatgtaacactcatcagacaattagaagacacatcatacccaggaagtatctgtaaatttatagaaa
aactagaaacaatactggattatgtttgataagttagaattagaaagtgaccctgttgataaatcgaatt
ataccgaaatgttaaacaaaactgttaaatcagtaatagatcgaaaactgccggatagaatttatatgtc
tttggcacgtaaagatattgatacaatttataaattaaaacaagcatcaatggaatttggcctttatgat
gctattccagaaaatcaccgttctaatagaacagaaatgaacaaacgtaggaacaggggaaactataatc
aaaataataatcaaatatattacaataatagaaatcacaactacagtaattattatcctagcacgaatca
gaatcataatacacaaccacctcaaaattcgactcaacctatgacaaatcaaaaccagtattcaccgcgt
ttcataccaaataatcaaagagggaattattatgcatttagacgagacttaacacaagctcagcagaaca
acccacttaataacacccttaatttccaaccttcgccatcaaataatattaacagacaagggccagtaaa
aagacaacgcgagagtcagagtgaccaaagcaggatggatgtaaattttcatcaagctgcctcggacact
caaatgatagagaaggacattcaagtccctatgtaaaaatatttcatcataataaaaattataagggaat
gatcgatacaggatcatcaattaacatcataagagaaaatttgagaacttagaagaaaaggaagaaaac
ctaacagtatacactattaaaggaccaataacactaaataataataaaaccaaccaacagtatgtccgtc
tgctcaaaaattctacattcacaaattttctgataactatgatGAACTATTACCTTgcctctggaaaaca
aaaattcaggttggtagtcgattacaggaatctaaatgagataactgttaacgacaaatttcccattccc
cgaatggatgagatatagatattggacaaattaggtagatgccaatactttacaactatagatctagcca
agggttttcaccaaatccaaatggatgaaaattctattgcaaaaaaagctttttcaactaagcatgggca
ttatgaatatactcgtatgccttttggtttaaaaaacgctccagctacttttcagagatgtatgaataat
cttctggaagatttaatctacaaagactgtttagtctatttagacgatattattgtttattccacttcat
tggaagaacacattttatccctaaagaaagtctttgaaaaactgagagacgctaatttaaagttgcaact
agataaatgtgaattcatgaagaaagaaactgaattcctaggacacatcgtcacaacaaatggcatcaaa
ccaaatccaaataaaactaaagcaattacaaattttcgattacccaagacacctaagcaaataaaatcat
ttttgggattatgtggattctatcgcaagtttattcctaactttgccaaaaaagttaaacccatgaccct

caaattcaggaaaggtgctataatagacaacaaatgtaaagaatacatcgaatcatttgaaaaattaaaa
gttttgataacttcagacccgatattaatctatcctgatttttcaaaaccctttctctgacaattgatg
ccagcaatgtagctattggggcagtgttatcacaaaatcacaagccagtttgttatgccagtagaacgct
aaacgaacatgaaatcaactatgctacgattgaaaaagaattgttagctatagtttgggctacaaaatat
ttcaggtcatacttatttggcaggccatttgaaatattaagtgatcacaagccactggtatggctcaaca
acattaaagaaccaaacatgaaattgcaaagatggaaaataaaactcaatgaatttgattacaaattcaa
atacttccgggcaaagaaaatcatgtcgcggatgctctttcccgcacgaaaatagaagaagttatggttg
gcgaggtcgcaaacagcgcagacacaactatacacagtgccattaaagataatctaaattacatatccat
aacagaaagaccaataaataacttctctagacaaatagagatagaaaaaggcgataacgatacaacaagt
gtacaacatttctttcaaaagttaaagattaaaatagtctataaagaaatgacacctgaactcgccaaaa
acctcattagggaatatgtgtgtaccaaaaagagtgcaatttatttccctaatgacgaagattttctgat
cttccagggagcgtttaccgaaattataagccctaacaatttcacaaaactcttgagatgtaccacaaag
ttaattgatatactaacgtatgcagaattcaaagatttaatcttaatgaaacattaggaacttttacccg
gatgtatagaaaaaacaatcaatttatttaaagaagaatattactatcctgatagacaaaagcttattca
aaacattatcaatgaatgtgaaatttgtaatctagcaaaaacagagaaatcgaaacacgaaattgacata
tgagacaacaccagaaatatttaacacaagagaaaaatacgtgatagattttttctcacaggaaaccag
atcttcttatcttgcattgatatctattcgaaatttgcatcactagttgaagtaaaaagtagagattggc
tagaagcaaaaagagccattactaaaatattcaatgatatgggaaaaccgtaagaaattaaagcagacaa
agactcagcttttatgtgtatagccttacaaaattggttaagatctgaaggtgtacaaatttctataacg
actagctaaaatggcatatctgatatagagagattccacaagaccgtaaacgaaaagctaagaatcattg
gtagcgagcaaaatattgaagataggtgcacaaaattcgaaacaattctatacatatacaatcataaaac
taaacataatagtaccaaaagatttccagcagacattttccaatatgcaggcagcccagactttaacgta
caactaaataaaatcgataagatagaatacctcaataagaataggcacgattttgaagttgatatataat
atagacaggccccacttgtaaaaagtaaaataaccaatccattcaaagagacaggaaggattgaacaagt
agatgataaacatttcgaagaaacaaaccgtggcaggaaaatcgtttagccgccgtttacagaagttaat
attctaatacaatttcacgtgtctcttttattttgatctgtcctttagcttggttcaacagcgactgact
gccgcgctctctgcagagtggcgagacgattagtcgataagtttctcgataatattggtatgttaaggat
gggtgtatgagtgtataagcttatgactagattggctatgtataattaggtatgtatgactaggaatgta
tgactaagagtaaaggaagagggtagaaggagcagcctactacaatcgttcactataagtcaaaatttaa
gaaacagaaaaaatgcaataagagcaaatatgataattccagaccaaccaaagaagcacaaagtacacaa
catacttctaataatgcttagttgcatactaccacttatcaccatggtcaagtgcaacaattagaagtaa
acccaataagcgcgaaaaatggatatcttatattccaaacaggaacaatggagattccaaccagctatga
ataccattatttaagcataaacataacaaagacaatgctcatgttcgaagatatagtaaatgaagcaaca
aatgaacaactatcctaatataccacaaatacaatatttagtcgacaaattaaaacgagaaataaatggg
ttaagaattattagtcgatgtaaaagaggtcttttaaacgtagtaggaaaagcatacaaatacttattcg
gcacattagatgaggatgacagagaagagttagaagaaaaaataaacaacatgtcagaagactctgaaaa
aacccatgacctaaacacgattctagatgcaatcaatagtggtatagatataattaataagctcaaagta
gataaagaacaacaccaacaaattgcggtaccaatatttaacctagagcaatttacagaatatatagaag
acatagaattgggtatgcaattaactagactaggaattttcaatccaaagttacaaaagcatgactattt
aaaacatgtaaattcagaaaaaatgctaaagataaaaacgtcaacttggcttaaaacagacacgaacgaa
attttgattatttcccatgttcctagcgaagttactaaagttccaatattccaaattgttccgtacccag
atgaacataattatattctaaccgagcaaatattcgataaattctacatatttgataaccaagtattcca
taaagataccaatagggtagtattcgacaaatgtattattggaatcatcaaacaagagcaaactcaatgc
aaatatattaaaacacataaaaattaccaaataaattatatagaaccaaatatactattaatatggaata

ttcctgaaacagctgttaaccaagactgtacaaacaataaaatattaatatcaggaagcaacatcattaa
aatttaaaattgtaccatgcaaatagatgaattcttaatctctaataatctagcagactttacacaaaca
atttatatcaccaacaatgtaacccgtctagaaccaataaatcacttacaaacgagagaaatgatagaat
cccatgtaaaacactataactttttccaaattatatgcattacaacgttcgtcataatgataattagttt
gactctgtatgtagcatataagtttaaaaatatacctaagaaaattattgtcaaatatcgtaccttgaaa
ataatgtcaatgagaatattcaacaaggaaataatattaccttatacccaaatttaacgacctgaggaca
ggccaaattcaaaggttgggggagtgacatatccataagtccctaagacttaagcatatgcctatatact
aatacacttacaacatatacaccccaatacaacatacaccactccggatgtacccaacagataccagata
agaataagattgttatatgatcctcgagaataaaaaaaaaccccaattctagataagtcacccactggta
gactaaacatccgtcccctaatttaaacaattccttgcttaagcctcaccccatcgtcacattcccacgt
tcaaagctcggagccgcaatcccaaaaaaaaaagtattgatttcaataaacaaattataagaatctaaga
gcacttgtatccaagagcaaatgcacttgaaaacgcaaagctttttctcttcacgatcagaatcctaaag
tctaaagtctatattagaaaagctcgataccgaggcttgaacgtcaatcaaatcagaataattatcagag
ttcagtttgagacctaattgtaacaggtcggtgttcttctcaaataaaaagttttgtaatcatttagtga
aataaaaattatattttttcacttataaatattgcaagaatttaattatatatatataatatatataat
aaagttataataatatatataGtggcatattcacatacaaaaccacataacgtagagtaaacatattgaa
aagccgcatccagatcataacgtaaacaataagtgaccaccatgctaatgtggatcaaataacaaaaata
tccactctgcattttggtttaattatattgacacccccatactgtatgccatctgcgcagtatgcattct
aataaacaaattctttgacagcggcacttagccattcttgtaaacaaatcttaaagtctgcctgctctct
ctgaggcttctcctccacttaagaatccaagagcaatgctctctcaaaatacttaagcatctaagagcaa
tgctctcccaaaaacactaacatattctttaagcaagTTTTAATTAAActttaagtttttaattaaacct
cgacaataaagtgaaaccgaaaaatcacaatttgcctaaacaaacctgaatttattatcaggaagacgct
attgaatttgtgagaggctgtaaatccaattggttacctcaaagacccacgaaaaagctatagtgcaacc
cttgtgaaaatcaaaacctatcttaaaaaaaaaaaaaaaatgtaaaaataagaataataaattaataagc
gaaaattaaaacgtattaaaaataagaataataaataaataagtgaaaattctatttgataaaaattaaa
aataagaataataaataaaaagacaacattttaaattaaacaatattaaaaaaaaaatacacgataataa
taatataataactaactaataataataaaaaaaaaaatatatatattataaaaataaataaataatccaa
aaatcaaaaatggctcaagaaccagcaattgtgccaccactatcagacagcaacatgacccaggttgcct
accagattggcaatgtggagaaattcaacggtgatccaggctcactatacacctttgtgagtcgaattga
ttacatactggctctttatgctaccggagatgaacgccaacagcagatcatatttgggcatattgaacgc
agtatcagcggagaagttatgcgctgcattggagcctatgacatgtacacctggcagcagcttagaagac
aattggtactcaactataaaccccagacccctaaccacgttcttttagaagagtttcgaggcaatgtacg
agcattcctggaagaagcagaaagccgcagacaaacacttactagtaagcttgaattagagcaagatctt
gaagaaaagactttttaattgaaattaataaaatccagtatagaatcactaattgaaaaattacctacac
acatttatttaagaataaataaccacaacataccagatttgcgatcacttataaaccttttacaagagaa
gggcatgtacgaacaaataaatcatacaagtacacatgtccaaaaacaaaatttctctgataagccacaa
aagtcctttaatcaaaatactaatcagtctaacaatatcagaaaatatccaacacctttcctacattata
attcaccaataccatatcaagctccacaaatttatccaacaccaccaactaataacccactttatcgtca
tccaataccctaccaccctaatccaaacaatgttattcaaccaagccaacaaaacaatgtttttcaacca
agccaacaaaacaatgcttttcaaccaaatcaacgtacaaactttacacctcgaccaatttttaacacca
atagaaacaatgcattcgatcagaataggttcggacaacaaccccaatatcaaaatcaacaatcaacaca
aaattcaagttcctatgtacccaatcgaccaataaaacgattaagaccagctaatagtggacagactggg
atgagtgttgacgaaacattatatcaagaggacgctttttatcagcagtgtgttccatatgactattttt
attatccaacttacgaccattcagactattatccagaaaatcaatatcaaattgacgaaaacaaccaaaa

tttacaaagaacacaacagttacagcagattaatacagacgagacaaacaatgacaaccaagaacccaat
gttgaacaggccgaaaattttcagccacaagccttggaaaaccccaatatataacaattaaatacaaaga
aaataatttgaaatgccttattgataccggatcaacagttaacatgacatctaaaaatatatttgactta
ccaatccagaacactaatactcttattcataccagcaatggaccgctcattgtcaacaaaagtataacca
taccttcaaagattttgttcccaacaacaaatgaatttttattgcaccctttctctgagaattacgatct
tttattaggaagaaaacttttagcagaagcaaaagcaacaataagttaccgcgatcaagaggtaactctt
tacaacaacaaatacaaattaatagaaggaatagcaacacatgaacagagtcattttcaaaatgtaaata
tgataAAATTATAAAttatgaacaggaggtcgaaagccaaatacaagatatgctaaatcaaggtattata
cgtaccagtaattcaccttacaatagccccatctgggtggttccaaagaaacaagatgcatcaggcaaac
agaaatttagaattgtaatagactaccgaaagttaaatgaaataacagtaggagacagacacccaatccc
aaacatggacgaaatcttgggaaaattgggcagatgtaattacttcacaactatagacttggcaaagggt
ttccaccagatcgaaatggatccagaatcagtttctaccaagcacggtcattatgaatatttgcgcatgc
cattcggattaaaaaacgcgccagccacctttcaacggtgcatgaatgatattttaagaccactcttaaa
caaacactgtcttgtgtatttggacgacataattgtattctcgacatcccttgatgaacacctgcaatcg
ctcggactagttttcgaaaaattagcaaaagccaaccttaaattacagcttgacaaatgtgagtttctca
agcaagaaaccacatttttaggacatgttctaacaccagatggaataaaaccaaaccctgaaaaaattta
agccattcaaaaatatccaattcccactaaaccaaaagaaataaaagcttttcttggactgacaggatat
tatcgtaaatttattccaaactttgcagacatagccaaacccatgactaagtgtttaaaaaagaacatga
aaattgacactaccaacccagaatatgactctgcatttaaaaaattaaaatatctaatatcagaagaccc
aattcttaaagtacccgactttacaaagaaattcactttaaccacagacgcaagtgatgtcgctttgggg
gcagtactgtcacaagatggacacccacttagctacattagccgaacacttaatgaacacgaaataaatt
acagcacaattgaaaaagaactcttagcaattgtatgggcgacaaagacttttcgacactacctacttgg
aagacactttgaaatatccagtgaccatcaaccattgagctggttgtaccgtatgaaagactcaaattca
aaactgacccgatggagagtaaaattatccgaattcgattttgatataaaatatataaaggaaaagaaa
attgcgtggcggatgctctgtccagaataaaacttgaggagacatatttgagcgaacaaacccaacatag
tgcagaagaggacaatagtgatttaatttttattacagaaagacctctaaatacatttaacagacaagtt
atattttcaaaaggaccaccagacattaaagttacgaaatatttcaaaaaacacatcacccaaatatttt
acgacattatgaccagggaaaaagccgaacaatatttgatggaccatttttgtggtaagaaaagtgcgtt
gtatattgagagtgacgctgatttcgaagtcattcaggccgcacataaattagccataaacaccaaatat
acaaaaatcctgcgtagcacgattttgttaaaaaacataaccacttatgcggaatttaaggaattgatct
tgactgctcatgaaaaacttctacacccaggcatacagaaaactactaaacttttcggagaaacttacta
tttccctaatagccagctacttattcagaatataataaatgagtgcagtattcgcaatctggcaaaaaca
gagcaccgaaatacagacatgccaatgaaaaccacacccaaaccagaacattgccgcgaaaaattcatga
tagacatttactcatccgaaggcaaacattacgttagttgcatagacatttattcgaaatttgccacatt
agaagaaataaaaacaaaagactggatagaatgcaaaaacgcgcttatgcgcatattcaaccagcttggc
aagccaaagttactaaaggcggacagagacggcgcattttccagtttagccctcaagagatggctggaga
gtgaggaagtcgaattgcagcttaacacaacaaaaactggtgtggcggttattgtacataaaacaattaa
tgaaaagattcgcataatcaaaacatccgatgacgaagaaaccaaattgagcaaaatggaaacagtactt
aacatatacaatcataaaaccaaacacgacaccactggacagacccctgcacacatatttctctacgctg
gacaaccaatattagctacccaacaaaacaaagaaaacaaaataaataatgacagagtggagtacgaagt
cgacacaagatacagaaaaggtccactacagaaaggcaaattagaaaatccttttaagccaacaaaaaat
gtggagcagactgactctgatcattataaaattactaatagaaatagaattactcactactacaaaacac
aattcaaaaaacgaaagaaaaataatcagctctcaattttacaggcacctggcacttgataacattgttg
ccgatgctgatcacaacagttcatggacaacaaattgaaattaatattgacacaaaccacggatatc

tcatttttctgataaaccagtccagataccatcatcctttgaacatcattgcttgagaatcaatttaac
tgaaatagacaccatagctgattattttgagcaaagactacgtaccgactaccatgcaccccaggtcaaa
tttttatacaacaaaatgagaagagaactagctggaatagccttgcgacatagaaataaacggggactta
ttaacattgtaggttcagtttttaaatacctatttggcacacttgacgaaaatgatcgagtggatataca
gaggaaacttgaaacaaacgcccataactcggtaaatttacatgaactcaatgacgctattcaattaata
aatgacggaatgcaaaagatacagaattatgaaaacgacagcaacatcattaacagtcttttatatgaac
tcatgcagtttacagaatacatagaagatgtggaaatgggaatgcagctttccagactcggtctatttaa
tcccaaactactaaactacgataaacttgagaatgtaaacagccaaaatattttaaacattaaaacatcc
acttggattaattacaatgataaccaattattaatcatatctcacatacctattaacttttcattaataa
atacagtaaaaataatcccttacccagactcgaacggctatcagctagaatacacagacacacaatcata
ttttgaaagagaaaataaagtttacaataacgaaaataaagaaataaacaatgagtgtgtcaccaacatt
attaaacatttaaaaccaatttgtaattttgagtcaatccacacagatgaaataataaaatacatagaac
caaacacaattgtaacctggaatttaacccaaacaagtctcaaacaaaattgtcaaaatttaattaataa
tataaaaataaaaggaaacaaaatgataaaagtaacccaatgtaaaatagaaatcaaaagcataattta
agtgaaaatctctttaaaccagaaatagatttgacaccattataccaaagacactagaataggtctttga
ttatacacaccacttaacataacaaaaataaaaactgttaaacacaacgacattaatgaaatgatttcac
aaaacaatattacactttacatatttatgactactgtcatcattatacttattttattgtacttatattt
aagatacgtatcatttaacccattcatgatgctgtatgcaaaactaaaattaagaaaaaaatcaaaatcaa
aacacagcacaacaaatagaaatggaagacgttccattacccctactatatccatcaatcccagcccaag
tataggcttctctttaagggaagggaagtgacatattcacatacaaaaccacataacgtagagtaaacat
attgaaaagccgcatccagatcataacgtaaacaataagtgaccaccatgctaatgtggatcaaataaca
aaaatatccactctgcattttggtttaattatattgacacccccatactgtatgccatctgcgcagtatg
cattctaataaacaaattctttgacagcggcacttagccattcttgtaaacaaatcttaaagtctgcctg
ctctctctgaggcttctcctccacttaagaatccaagagcaatgctctctcaaaatacttaagcatctaa
gagcaatgctctcccaaaaacactaacatattctttaagcacagaggcttctcctcattttcactttcat
ttgatttttagtcttaagctgaacgttaatcaataaacaacacaatcgatcccgaaattttgattcgttt
tattttggcaaaacttaattttcagcgttggtcttagttcatattcggaacggtccatttaatagactca
aaactatttattgcaaccatttatttgcaATtatatatataatatagcgaatatattatatattctatat
tatatatattatattaggttcgacataactgatttcaaatttcttatgagtttttacatatctacattct
gtattgaattgattaagtatgtcatttatttattatcaaagtttttaaagatgctaaataactttgatta
ttttgaacataaaattaatttttttcattttctattaatgttttgatcactatcaggatatggaaatctag
aaaagataggggttttacttatttcttttggaatatgggaaattataagaatttcatttgtatctgtttt
taaccaagtggaagttttaatatgtaAAATATGAATTGGGTTTACTTCAAGGCTTTGTACTTGGGCAAAT
ACAATACAAATATatttcgatttatagtgagtaatttttcctcctctattcttttctttataatatttat
catatatttgtgtaatttctcctgttttcccatatgggtttgttgtcttagtttttactaataatgcttg
tctatatctagtatatatttcgaattcgtttcgtgatttattTAAGTAAAGtattgctgaatagttaagt
tcatgttcgttaagagtaacggctagcgttataagcaggtttgtgatcctgtggtaatacagcacctaga
ccaacattgctggcgttttttttttcgaaatttcgataaattaatattggttcggatataattaaaaattt
tagtttttcgaaggctgatatataattggtatctttagaatttatatgaaacctttttttagtttttttgtg
ttatgggtttagcaatttttgcgaagttaggaataaatttacggtgaaaacagcaaagtcctagaaataa
ttttatttctatactagatttaggtaatgggaaTTTTTGTgtagtgaaatattgagacctaccgatctta
tctagtatttcatccataactagaattggttatttgtcatcaatagtaatttcgtttagttagcgatagt
cgattactatcccaaactttgTTTAAACTttaactggagaattaaaaatgtttgctcttatgatgtttat
tgtagacccagtgtatataatacatcggagctgtatattgtttaaaacaatatttatgaaggaattgtgt

gttcttcttcgtccgacgcatgtagatgaaaactttcagacgtatccatttttgaatggcccctggtgct
ggctctttggctttttattccctgattcggagcagttgaagaaacgggattgtgttgatagtttacagga
ttttgtgcctgtgcttggtcgagtttttgcttaaattcTTGAAATAGGCGTTCGAAAGATTGGTCATGGC
TGTTATtatttttgttgttatattgatttctattttggttatagtattgatttctattatgattattTGA
AGCGTTGTTGTAGTTTGGACTGTACTGTTTTTATTTAGGGTATTTTGAGTGCTTCGGGATCTTGGGCAA
TCGGACCATGTATTGTCATAACAACACTCTTGCTGGACAATTTGCTTTAATTTACTAACtgttgttatgt
caaatctagctaattgcatgtagagtctgtctggaagttgtttattaattacatattttactgttttgtc
aattgcgtttgtataaatatgattatgttgttcaatattttctaaggctagcttatttgttattataaaa
cattttgcttccagttcttctatgaacttacgaactgatccattgaattgtgtgttatatagtcgtctta
aaagttctttaaacggtgtttgggtcttgaactcagcgattagggcttccttcaattccggccatgtgtt
agctgcactgagctgggatactcgttgggcgtcaccaccaatttgcattttaatcgcactgaagagaacg
tgtaactgacggatgtcagttgttgggtaaagttgtagtatgagctcgatccttcttatgaacgagctta
gttctagtgtcgtaccggtaaatgttggtatttgtcgaatcagaccaagagcctggttcagatgATTCTT
GGAGATTAGTGGCACttgttgttgttgtggttgttccattgttgtttttttttgtttttttgttgatgttgt
tgttgttgtcttgtttttgttgttCACTCGCACAGCAAAATTTGCACTGGGGTTATAttttttttttttt
ttCACTCCTCGTAGCGGTCAAACTTTTTATTTTGCTGGCTTCACACTGTCTTTGTTTAATATACAAAACC
TGGGGCTTCAGTTACTCTTTAATGGTCTTGTCCTTGCACTAATGTTATTTTTATCACTTCTAGTAGCTTC
TAGTCACACCTCTATGGTGGGCTACCAAGTTGAAACGATTTTAATTAGAAAGCACTGACTGTCGGATTAA
TCACTTCTTGTAGCACTGATTAGCCACAAAGTTGAAGTTAAAACACACACGAAGTTACTTTTGCTGATCA
TTGACTGCGGCAATAACGAATCCACGGTAATTTATTAAGAAAAAAGTATAATCCTTATTCTCAAATAAAA
TTACAAGTGTGGAAGCGGTCACaattcacgtcttatttgtagagcgaaagaattaaatctgattgtaagt
tgcaaagcaagcgcttagcaaacgcttggctgcgacttatttacatctttatatgtatattgagcgtaaa
taggctctcttttgtatttatatgaattgggcgccctcttgagtggatcgcggcgcatggatactgatca
gccgagaatttgagctgcgtaagtggttttggccctttggtgggagtcgcgaatgcgtctgtggatgttt
atgtttacattagggcaagctggatttgttattaccaccaaaattggggttcttatggagtacttgaatt
tatgatataaggcattctataatttggtgtatgactgttgctaggtaataatgtgtgtgtgtgtgtgtgt
gtgtgtattgtttgtgtgcgtaagtatgtatgcatgtatgatacatatgtatgctactatatatgtatat
atatataaagtatatataCGAATTTTTCTTGGTCATTAGAATAGACTGTTATAAAAGAATAATATAAGAA
TTGCGTCCAGgagcgtccagggcactatttcctttccggaagccatgttgctgacttcctaggacgttgc
tgctctcggtgattgcctctattcgagtatactggatacgttcgaacagttttccaactatatccagtgg
gtcgggttcgcaaagctacccgtcgggtggtttctgtccgccttgctgctgcagataatgcattttggtg
atgcaacccctttgccttgtTCtgcgtcccatcccgaatcctgcgaaggcccgtgacatcctattgattt
aactataggccctggaattgggcgaacatgcagctgcggagcttctctgcggtcgtagcatcgtccagtc
cgctgcaggatagtgctattccttgagcttccgttcgcacagaggccaaatcaatgagcgccgcttccag
atcgctcttatacttgggtacgctttccgaggctttttcatctacttcgagcaacagctcaccttgctga
gttctctgaatttttcgtacgtatcttctcagtttgcttaggctcgggtccgatcttagtttccggagca
tctctgcgtacgaagactcaccgtcttcttcacgaccgtgctagcattcttggcgacatatgcgtagctg
cctggcttggactcgtttgttctggatgtcacctttccttcttcgttgctaccagtcgtgtgctttgggc
acttgccccttttttggtatgcttttgtacctcagtctgggaggcaatgctcatcaagcggaggtcgctag
tgaccccttcgtacaactctatcgccctgttgttaagggctgagagttcagaagctacgcccttggtccc
tcttctgacgtgccgttgagtttcaagtgtggaaagcagcttatcttgcttatcctatcttatgtttatc
ttgccatacatcctggtgagtatgcacttcaggtccttgttctgaagagccgtggtcaggtcccgatcga
tgaggggatccagctaggcaattcactcctgccttatgcggtgcactgctgcatctggttaccgactcct
ctagtgtgggacctcggaagcctggtgctcctcctgagcaggtcgttgcgatcaggggggacttcccccca

ctgcgctatccatcaactataagatggcctacggttacctatgtgaagcaccagctttagtaagggctct
cacgtaggccacggatttaaccctggcttttaccgttggaggtatgttccgcctcgaatcggacacacca
gagccactgcttaggcaccagcacggtgagaggatgatTAAATTAAATATAACGGTTCACATTTacgtct
cactctcaagatcccccactgcgccaaatgggcagcgctgccagctgttttatGAGCTGCAGAATATCGC
AAACTTATAGTTTTGCTATGTAACGCCGTCTGCACGGCGATGTAGACTTGGTGTCCCCACTTTTGGTGTG
CCCCATGCGATACGCCGCCGTTCTCGTAGAGGTAGGCGGAACTCACCGCTGCCTGCCCTTGGATTCCGAT
G
>dme_circ_0005247_junction_seq
CACCGCTGCCTGCCCTTGGATTCCGATGGGCGACTTGAACATCGTGGCTTCTTTCG
>dme_circ_0005248
GTGTATTCATCGCCAGCCACGTTAAAAGGAAGAACGTGTATGTGAATACGACCATCAGAGGATCTTAGCG
AAGGATTCAGGAGCCCAAATTCTTAATCCCCATCCCACACACACACATATACTCGCACGTAGGCACGCTC
TCTGTGAGAGGAAGGGTAAATAAAAGAAGCAACGAGCAAAATGTCTGGCGAGGAAACGTCTGCCGATCGC
AATGTCGAGATCTGGAAAATCAAGAAGCTCATCAAGAGCCTGGAAATGGCCCGCGG
>dme_circ_0005248_junction_seq
TCATCAAGAGCCTGGAAATGGCCCGCGGGTGTATTCATCGCCAGCCACGTTAAAAG
>dme_circ_0005249
GTGTATTCATCGCCAGCCACGTTAAAAGGAAGAACGTGTATGTGAATACGACCATCAGAGGATCTTAGCG
AAGGATTCAGGAGCCCAAATTCTTAATCCCCATCCCACACACACACATATACTCGCACGTAGGCACGCTC
TCTGTGAGAGGAAGGGTAAATAAAAGAAGCAACGAGCAAAATGTCTGGCGAGGAAACGTCTGCCGATCGC
AATGTCGAGATCTGGAAAATCAAGAAGCTCATCAAGAGCCTGGAAATGGCCCGCGGGGTGAGTCTTATCA
ATGATATACATGTCCTTTAACGTCCCCCCCAACGACTGTTTTGCGTACGTGTGTGGGGTATACACAATGC
ATGGCACGAAAACAACAACACTGGGGTGGAAGTGGGCGTACTGGACGTGGGTGTGGGTGGGGACTTGAGG
GAGCGGCGAGTACAACCACCTTTCCTTGTTGTCGCCGTGTGAATCTCCTTTGCATTGTTTCCAATCTTGG
CAAGATCCCCCGCCTCACTCTATCTCTCTTTTTTGCATACGAGCAACACGTTTTTGTCGAGTTTTTATCA
GTTCAAACTAGACTGATtgagagacggagagagagagagagagagaggagtgagtgagtgagtgagTAAT
CGCCTCGTGGCTGTTGTGCGTATGCGTGCATGTGTGTGTGTGTTTTTCCCCCAAAATGGGCCAAGCTTTG
TGGACCCCTCGCTCTCACTCATCCTGCCTCGCTCACTCCCTTGACCCGGTCCTTACCGCTTCACACCGCT
TTAGAGTGGGTAACAAGGTCAGCAAATCGAGTGACCCCCACAGGAGCATATCTGCTATGTATATACATAT
ATATGTATATTGTTGTCAATATGCTCCACAATTGGAGCTAACATTACACCTCTTCCAATTGGAGTTGGCC
ACTGTTGCCAATTGGGATTCGCTTTGACAGATATCTTCCTATTGGTTTTCAGCCATATGCGAAATATATA
CACACAtatgtatttgtttttctttttttaagttctgtgctattttttgttgtattttGCATTCCAACAT
TTTGTACAAATTACGATTGGCACTCCTCTGTATTATAACGAACGAGAAAATGATTTGCATCAGAGAAACA
AACGTTTGGAAGTTACAGAAACGTATTTGTTTCAATAAATTAATCAAAGTTTAAATTTAGTCCAATAACC
GAATGTCAAGTTTAAAAGACATATGCCACGAAATTTCACGGCTAATTAAATAACTTTAAAGTAGGTTGCT
ATTATGTGCATCTGACGCGCAAATATACGATTGAATAGCACTGTTGCTATGGGAGAAAGAGCCATTTCCG
CGACTTATGTTCTCCCTTGTTTGGGTTTCTTGCAGGCAATGGAACCAGCATGATTTCTTTGATTATTCCG
CCAAAGGATCAAATCTCGCGCGTCAGCAAGATGTTGGCCGATGAGTTTGGAACGGCGTCGAACATCAAGT
CGCGTGTAAATCGGTTGTCCGTCCTCGGTGCCATTACGTCGGTACAGCACAGACTCAAATTATACACCAA
AGGTGCCTCCCAACGGTTTGGTCATCTACTGCGGCACAATAGTCACAGAGGAGGGCAAGGAGAAGAAGGT
GAACATAGACTTTGAGCCATTCAAGCCCATAAACACGTCGCTCTACCTCTGCGACAACAAGTTCCACACG
GAGGCCCTCACTGCCCTGCTCGCCGACGACAACAAATTTGGATTCATCGTGATGGATGGTAACGGAGCGC
TATTCGGTACCCTTCAGGGCAACACGCGCGAGGTGCTCCACAAGTTCACCGTCGATCTGCCGAAGAAGCA
CGGTCGTGGTGGTCAGTCCGCCCTTCGTTTCGCCCGTCTGCGTATGGAGAAGCGCCACAACTACGTGCGG

AAGGTCGCCGAGGTGGCCACCCAGCTCTTCATCACGAACGACAAGCCCAACATTGCCGGACTCATCCTGG
CTGGTAGTGCGGATTTCAAGACTGAGCTTAGTCAGTCTGATATGTTCGATCCTGCGTTTGCAATCAAAAG
TCATCAAGCTGGTGGACGTGTCGTATGGTGGGGAAAACGGTTTTAACCAGGCCATTGAACTGGCGGCCGA
ATCATTGCAGAACGTTAAATTCATACAGGAGAAGAAACTCATTGGTCGCTACTTTGATGAAATTTCTCAG
GGATACTGGCAAATACTGTTTTGGAGTGGAGGACACTTTGCGGGCACTGGAACTTGGCTCTGTAGAGACT
CTCATTTGTTGGGAGAACCTGGATATTCAACGTTATGTTCTCAAGAATCATGCCAACTCGACGTCAACGA
CAGTATTACATTTGACGCCCGAGCAGGAAAAGGACAAGTCGCACTTCACTGACAAGGAGGAGCGGGGTAG
AAATGGAGCTGATTGAGTCTCAGCCGCTGCTGGAATGGCTGGCAAACAACTACAAAATGTTCGGCGCCAC
ACTGGAGATTATCACGGATAAGTCCCAGGAAGGAAGTCAGTTCGTGCGAGGTTTCGGTGGAATCGGCG
>dme_circ_0005249_junction_seq
TTCGTGCGAGGTTTCGGTGGAATCGGCGGTGTATTCATCGCCAGCCACGTTAAAAG
>dme_circ_0005250
CTTCTTGATTTTCCAGATCTCGACATTGCGATCGGCAGACGTTTCCTCGCCAGACATTTTGCTCGTTGCT
TCTTTTATTTACCCTTCCTCTCACAGAGAGCGTGCCTACGTGCGAGTATATGTGTGTGTGTGGGATGGGG
ATTAAGAATTTGGGCTCCTGAATCCTTCGCTAAGATCCTCTGATGGTCGTATTCACATACACG
>dme_circ_0005250_junction_seq
TCCTCTGATGGTCGTATTCACATACACGCTTCTTGATTTTCCAGATCTCGACATTG
>dme_circ_0005251
GCAATGGAACCAGCATGATTTCTTTGATTATTCCGCCAAAGGATCAAATCTCGCGCGTCAGCAAGATGTT
GGCCGATGAGTTTGGAACGGCGTCGAACATCAAGTCGCGTGTAAATCGGTTGTCCGTCCTCGGTGCCATT
ACGTCGGTACAGCACAGACTCAAATTATACACCAAAGGTGCCTCCCAACGGTTTGGTCATCTACTGCGGC
ACAATAGTCACAGAGGAGGGCAAGGAGAAGAAGGTGAACATAGACTTTGAGCCATTCAAGCCCATAAACA
CGTCGCTCTACCTCTGCGACAACAAGTTCCACACGGAGGCCCTCACTGCCCTGCTCGCCGACGACAACAA
ATTTGGATTCATCGTGATGGATGGTAACGGAGCGCTATTCGGTACCCTTCAGGGCAACACGCGCGAGGTG
CTCCACAAGTTCACCGTCGATCTGCCGAAGAAGCACGGTCGTGGTGGTCAGTCCGCCCTTCGTTTCGCCC
GTCTGCGTATGGAGAAGCGCCACAACTACGTGCGGAAGGTCGCCGAGGTGGCCACCCAGCTCTTCATCAC
GAACGACAAGCCCAACATTGCCGGACTCATCCTGGCTGGTAGTGCGGATTTCAAGACTGAGCTTAGTCAG
TCTGATATGTTCGATCCTGCGTTTGCAATCAAAAGTCATCAAGCTGGTGGACGTGTCGTATGGTGGGGAA
AACGGTTTTAACCAGGCCATTGAACTGGCGGCCGAATCATTGCAGAACGTTAAATTCATACAGGAGAAGA
AACTCATTGGTCGCTACTTTGATGAAATTTCTCAGGGATACTGGCAAATACTGTTTTGGAGTGGAGGACA
CTTTGCGGGCACTGGAACTTGGCTCTGTAGAGACTCTCATTTGTTGGGAGAACCTGGATATTCAACGTTA
TGTTCTCAAGAATCATGCCAACTCGACGTCAACGACAGTATTACATTTGACGCCCGAGCAGGAAAAGGAC
AAGTCGCACTTCACTGACAAGGAG
>dme_circ_0005251_junction_seq
GGACAAGTCGCACTTCACTGACAAGGAGGCAATGGAACCAGCATGATTTCTTTGAT
>dme_circ_0005252
GCAATGGAACCAGCATGATTTCTTTGATTATTCCGCCAAAGGATCAAATCTCGCGCGTCAGCAAGATGTT
GGCCGATGAGTTTGGAACGGCGTCGAACATCAAGTCGCGTGTAAATCGGTTGTCCGTCCTCGGTGCCATT
ACGTCGGTACAGCACAGACTCAAATTATACACCAAAGGTGCCTCCCAACGGTTTGGTCATCTACTGCGGC
ACAATAGTCACAGAGGAGGGCAAGGAGAAGAAGGTGAACATAGACTTTGAGCCATTCAAGCCCATAAACA
CGTCGCTCTACCTCTGCGACAACAAGTTCCACACGGAGGCCCTCACTGCCCTGCTCGCCGACGACAACAA
ATTTGGATTCATCGTGATGGATGGTAACGGAGCGCTATTCGGTACCCTTCAGGGCAACACGCGCGAGGTG
CTCCACAAGTTCACCGTCGATCTGCCGAAGAAGCACGGTCGTGGTGGTCAGTCCGCCCTTCGTTTCGCCC
GTCTGCGTATGGAGAAGCGCCACAACTACGTGCGGAAGGTCGCCGAGGTGGCCACCCAGCTCTTCATCAC

GAACGACAAGCCCAACATTGCCGGACTCATCCTGGCTGGTAGTGCGGATTTCAAGACTGAGCTTAGTCAG
TCTGATATGTTCGATCCTGCGTTTGCAATCAAAAGTCATCAAGCTGGTGGACGTGTCGTATGGTGGGGAA
AACGGTTTTAACCAGGCCATTGAACTGGCGGCCGAATCATTGCAGAACGTTAAATTCATACAGGAGAAGA
AACTCATTGGTCGCTACTTTGATGAAATTTCTCAGGGATACTGGCAAATACTGTTTTGGAGTGGAGGACA
CTTTGCGGGCACTGGAACTTGGCTCTGTAGAGACTCTCATTTGTTGGGAGAACCTGGATATTCAACGTTA
TGTTCTCAAGAATCATGCCAACTCGACGTCAACGACAGTATTACATTTGACGCCCGAGCAGGAAAAGGAC
AAGTCGCACTTCACTGACAAGGAGGAGCGGGGTAGAAATGGAGCTGATTGAGTCTCAGCCGCTGCTGGAA
TGGCTGGCAAACAACTACAAAATGTTCGGCGCCACACTGGAGATTATCACGGATAAGTCCCAGGAAGGAA
GTCAGTTCGTGCGAGGTTTCGGTGGAATCGGCG
>dme_circ_0005252_junction_seq
TTCGTGCGAGGTTTCGGTGGAATCGGCGGCAATGGAACCAGCATGATTTCTTTGAT
>dme_circ_0005253
GATCGAACATATCAGACTGACTAAGCTCAGTCTTGAAATCCGCACTACCAGCCAGGATGAGTCCGGCAAT
GTTGGGCTTGTCGTTCGTGATGAAGAGCTGGGTGGCCACCTCGGCGACCTTCCGCACGTAGTTGTGGCGC
TTCTCCATACGCAGACGGGCGAAACGAAGGGCGGACTGACCACCACGACCGTGCTTCTTCGGCAGATCGA
CGGTGAACTTGTGGAGCACCTCGCGCGTGTTGCCCTGAAGGGTACCGAATAGCGCTCCGTTACCATCCAT
CACGATGAATCCAAATTTGTTG
>dme_circ_0005253_junction_seq
ATCCATCACGATGAATCCAAATTTGTTGGATCGAACATATCAGACTGACTAAGCTC
>dme_circ_0005254
TGTGGCGCCGAACATTTTGTAGTTGTTTGCCAGCCATTCCAGCAGCGGCTGAGACTCAATCAGCTCCATT
TCTACCCCGCTCTATACGATTGAATTCCCAGTTAGCGTGCGGATTAGTATCATTACTCAGTTTAGCTCTC
ACCTCCTTGTCAGTGAAGTGCGACTTGTCCTTTTCCTGCTCGGGCGTCAAATGTAATACTGTCGTTGACG
TCGAGTTGGCATGATTCTTGAGAACATAACGTTGAATATCCAGG
>dme_circ_0005254_junction_seq
CTTGAGAACATAACGTTGAATATCCAGGTGTGGCGCCGAACATTTTGTAGTTGTTT
>dme_circ_0005255
TTTGTCTGGCTGCAAAAAGGGGACTATCTTCTGCTTGTCCCATTTCTGGGGATCCACGCAGAAGGTATCC
TCCTTCCGGAGAAGGTGGAATACGTTCTCCAGGATCTTCCAGTCGTCCATGAGGCCATCGCGAATCTGGT
CAACAGTCTCTGCTCCATCCACTGATTCAGCCATACTACACTCGGTCGG
>dme_circ_0005255_junction_seq
CTGATTCAGCCATACTACACTCGGTCGGTTTGTCTGGCTGCAAAAAGGGGACTATC
>dme_circ_0005256
GTGAACGTACGTATTCCTCCTTAAGGATGTCAAAACGTTTCTCGTCGATGGAGAAGTCAAAAAGGTGATC
TAACAGCTTCTCCAACAGCACCACCTGTTTGTCACTGAAGCCCCGAATGGTGAACTGCAAGAGAGAAAAC
ATTTTGTAATTTGAAAGTTCAATGAATAAGAGTTGTACATTCTCACATCGATGCCGCACGACTTTCCCAT
CACACTGAGCTTTAAGCTGGCTAGCTCCGCATCGTACAAGTACTCATTGAGCTGATCCTTCAGCAGCATC
ACCATCATATGGTTCAGATTGCAGTTCAGGGGATCCAGATAAGCAATCGGATTGGACATGTCGAAAGTCA
TGCATGCCTTTGGCTTATTGAACTGATTGTCCTGCTTATGCCACACCCTCAAAATGGGGGTGTCCAAAAT
AATCGTGGGATGTTTGGGAGCATCAGCTGGTACATCGGATATATCAAAGTTTGTTGGTATGAAGCTGTTT
GGCAGCGCCAGTTTCAGGTTCTC
>dme_circ_0005256_junction_seq
TGTTTGGCAGCGCCAGTTTCAGGTTCTCGTGAACGTACGTATTCCTCCTTAAGGAT
>dme_circ_0005257

GGAAATTCGAGAAGCTGCCGATCTATTAGAACTGCCGCAACTGACTCAGCTACTTTCCAGGCCTCAAACC
GTTATGGAGAACTCCAGCGATGAGCCAAATCCGCACATTTGCCTGGTGAGCGCATTGATGTCATGATATC
TATTTTATGATCCTAACCATTTTCTGTTACATACATTAGCGCATCAAAGAAAGCATGGAACGGCATTGCA
TTGGTGACGGGTGCTTCAGTGATGTCACCTTTGAATTGGACGATGGCTTAATGAAGGTACATACATATGT
CTGTTGAATTACTATATGAAGCACAATCGTTTACACTTACCTTTGCTGCATTGCAGGCACACCGCGCTGT
GTTGGTTGGTCGTTGTGATGTCATGCGCGCAATGTTGTTAGGCGACTTTCGCGAGGCTCATTCCAATGTG
GTAGGAACGGGCACAATACCCCTTAACTAAGGACAGTTGTCATAAATTTCCTTTACCTTAGATCGTATTC
CCTGGCGTTACCATTTACACATTCCACAAGCTGCTGTGCTATTTGTACACAGACCAGATTCCGCCAATCT
CGGCCGTCAAGTGCCTCAATCTGCTGGAGCTGGCCAATAGACTCTGCCTGCCGCGGCTGCTCAACCTGGT
TGAATGCCGCGTTATTGAAGATCTCACTCTGATTTCTCAGAACGAAACCAATGAAACGGTGGATCACTGT
CTAAAGCTGCTCGAGCCAGTGAAGGCTGCACAACGCACATCAATTGGCCGAGTGGTGCATGTCATACCTG
TGCGTCAATTACAACCTTATTTGTAAGTTTTCACTCAAGGGCCTAAAGGCGCTACACCAGGATAATCAGG
AGTACTTGCGCGAGCACCGATGGCCTCCAGTCTGGTATCTGAAAGACTATGATTACTATCAGCGATGCCT
GAACGAGCTGAACAAGGAGCTTAAGCTAAAGACTTCGAGGCGGGAATCGCCTAGCGATGACGAGGGCTGT
CTGTGCTTTACGGGCG
>dme_circ_0005257_junction_seq
GACGAGGGCTGTCTGTGCTTTACGGGCGGGAAATTCGAGAAGCTGCCGATCTATTA
>dme_circ_0005258
GGTATTTGATCCGATCTTGTAGGCATCCAGAACAACGCCACTGTTCTGGGCCTCATCCACACTGGACAGA
AGAGATTCGATGTTGTGCAGGGCAAGACTGCGACGTTCTAATTAGAGAATTTTCAAAGAACATTATTCAC
AAAAGCCAGATAAGGGGTGTCTACAATAAAGATTACCATGATTCTTTTCCAGGAGATGCCTTTTTCGTAG
ATAGGTTTTTGCCATTTGCCGTTTGTTCTCCTTCATGTACTGGCGCACTTTGTCATCGTTCACCTTGATC
TCCTCCTCTAGGTCCTCCAACTGCTTGAGTAACTGAGCTTGGGTATTCTGAAGATTGTGGACGGCCTGGT
CTTCTTGGCTTATATTAAGATCGTCGCCAGGTTTTTCTATAACAAAAATTATAGTTTAATAATACATACT
AACTACTTGAGAACCTTTTAAACTTGCTTGGTATTTTGATCAAGTGAATTTGGCGTATGCCTTTTTCTAC
TTTAAATTCTAGGCCCAAGATTTGACGAACATTTAAGGCCAACAGGCAAGCGCATAGATCTTTGTCTGAG
TAAGTGGGAACTCCGTGAGTCTTACAAAGCGTTTTCAAAGCATTAAAGTGCA
>dme_circ_0005258_junction_seq
CAAAGCGTTTTCAAAGCATTAAAGTGCAGGTATTTGATCCGATCTTGTAGGCATCC
>dme_circ_0005259
GCTCAATGATCCTCGACGGCGAGGACGATACTCTGAGCGACAAGATCGGTTGCCCACTGTACACCGCTCC
AGAACTGCTGTGCCCCCAGCAAACGTACAAGGGCAAACCGGCGGACATGTGGTCGCTGGGCGTGATCCTG
TACACCATGTTGGTGGGTCAGTACCCG
>dme_circ_0005259_junction_seq
GTACACCATGTTGGTGGGTCAGTACCCGGCTCAATGATCCTCGACGGCGAGGACGA
>dme_circ_0005260
GAAGCGGTGGAACTGCAGGATTCTCTCGTACCAGGAGGCCACGCTCTCCATCTGCAGATCCGGCTGGTTG
CCCACCACGTGATCAATGAAGTTCAGCTTGGTGGAGGGCAATCCGTTTAGAAGGACATCTTTGGGCGACC
GCTGGAATCCAGGCAGGAAATCTCCAGCGTACCCATTGCGCTCCACGAAAGTGTGGGTA
>dme_circ_0005260_junction_seq
CCCATTGCGCTCCACGAAAGTGTGGGTAGAAGCGGTGGAACTGCAGGATTCTCTCG
>dme_circ_0005261
GGCATTGAAAACAGCCGGGAGCCTGGGATACAATCTAATCATGCCTTGATCTCCCTTAAAGGGTAATGTA
ATCCTCCGCACCATTTGAGTACAAATTATTTTAATTCCCAAATTGATGACAACTTCATACCCAAATCAGA

GAAATAATGTTGAAATCTTTTAACATGCATCACCATCTGAATAAAATATTGAAACTTAGTTTATGTATA
ATAGTATTTGTATAATTAAACAGTCTTTGTGTATTTTACAGAAATATTTCCAGATATTGGGATTCCTATT
CAAAACTGAAATTTGACTGAACCAGTGATAAG
>dme_circ_0005261_junction_seq
ACTGAAATTTGACTGAACCAGTGATAAGGGCATTGAAAACAGCCGGGAGCCTGGGA
>dme_circ_0005262
TTTATTCTTTGACTTGTCGCCGACTTGGACATTTGAATCCCCCCAATAAATTCGGGTACGTAGCCCGAAA
ACTATGTGTTAAAATCGGTGCATTACGGATTCTTTTATTGGCCGATTTCTAGCGACCTTCGTAGGAG
>dme_circ_0005262_junction_seq
GGCCGATTTCTAGCGACCTTCGTAGGAGTTTATTCTTTGACTTGTCGCCGACTTGG
>dme_circ_0005263
GGATAACGAGGCCAGGATGATGAACACAATCAGCATGGTCAAAATATCGATCCTGGCCCAACCAAATGTA
TTCCGAAGCTTCTGCTCCCGACACTCCCTTTTGGCCCGTGTTTGGGCATCCGTGTCGGCCAGGCTTATAG
CTACTCCGGAGCCATTTATCTTGGTCAGGGCGGGGGAAGGATTCCTAGCCTCTGGCGCTT
>dme_circ_0005263_junction_seq
GGGGAAGGATTCCTAGCCTCTGGCGCTTGGATAACGAGGCCAGGATGATGAACACA
>dme_circ_0005264
AAGCGATCCTCGGATCCGTAGATATCCGCAGGGCGGATGATCGTGGCGTTGGGAAAGGCGTCGCGTACGC
GAAGCTCACCCTCGTACTTGCTTTTCAGCCACTCGCTGCCGCCCTTGACGTACAGATCCTTGGGATTGGC
CTCCACGTTCAAAGAGGACAGGTGGATTAAGCGCTCCACGCCAGCCTCGCGCGCAATCCTGAAGAAATAA
AAGTGGTTTACGTCATGCAAAGTTAGAATGAAGAAGTGGGCAGGCAAATAATGCTGGCTATGATGTTCAC
CTAGCGATCCTCTCCGCTCCGTTGACATGGACATCCTTGAACTTGAAGTTCTTGGTCTCGAAGTCCCTGC
CCACCAGGTTGATGACCACGTTCGAGTGCTTGACTGCGTCACGAATGGAGGCGGGATCCTCCAGGTTG
>dme_circ_0005264_junction_seq
ACGAATGGAGGCGGGATCCTCCAGGTTGAAGCGATCCTCGGATCCGTAGATATCCG
>dme_circ_0005265
GTACGAGGGTGAGCTTCGCGTACGCGACGCCTTTCCCAACGCCACGATCATCCGCCCTGCGGATATCTAC
GGATCCGAGGATCGCTTCTTGCGCTACTACGCCCACATCTGGCGCCGTCAGTTCCGCTCGATGCCGCTGT
GGCACAAGGGAGAGAAGACC
>dme_circ_0005265_junction_seq
GCCGCTGTGGCACAAGGGAGAGAAGACCGTACGAGGGTGAGCTTCGCGTACGCGAC
>dme_circ_0005266
AATCTTGGATCGAACTCAGCACAACAGCTGCGATGGCTGGCATCAAGAGCCCCGACAGAATCACTCCGTT
GCCATTCAACAATGGCGAGGAGTACCTGAGACTTCTGCGCGAGGCCCAGCGCGAGTCGAACCAGTCGAGC
CGTGTCGTCTCCTTGGCCAGCTCCCGTCGTGATACGCCCCGTGATAGCCCCAAGAGTCCACCGAACAGTC
CCCAGTCGGAGCTCTGTCCGGATGATGAGCTCAGAAACGTGTACATCAACTACTGGACCAAGGTAGCTGG
AACATTGAGGATTCAAGTCGGGATGCCGGCGACGAGGGCGAGAAAAAGAAGACTAACACAGGCTACTCCA
TCCGTTTGAAGCGACTGGGCTCCAACTCGTTGTTCTCCCGCGAAATTCTGTACTCGCTGCTGGTGACCAA
CGTCCTGTCCCTGCTGCTGGGAGCTGGATTCGGG
>dme_circ_0005266_junction_seq
GTCCCTGCTGCTGGGAGCTGGATTCGGGAATCTTGGATCGAACTCAGCACAACAGC
>dme_circ_0005267
GGTGTCCAGTAGCGCGTCGCCCGACGAGGTCGGGGTGCCACGTGAATTAGTGCGCTTCTTCATCGCCTAA
CTACAAATTCGATTATCAAATGTAGGCGGGTGGCGTGATGTGGTTGTTGTTAGGTGGTTG

>dme_circ_0005267_junction_seq
GCGTGATGTGGTTGTTGTTAGGTGGTTGGGTGTCCAGTAGCGCGTCGCCCGACGAG
>dme_circ_0005268
GTAGCGTGTTTGAGCGGTAATCAGAGTGTACTTGGCAACTTGGTTACAACTGCGTGGTAAGAGTGGTAAG
AGCCTTCAAATCTCAGACAGGAGACAGAAAAACGGAAGATTAGATAAAAGTGCATTTCAGGAACAAAGTG
CGATGGGTTACGCTGCAAGTGGTTGAGGTAGAAATGATGCAATTTCAGACTTAGG
>dme_circ_0005268_junction_seq
GTAGAAATGATGCAATTTCAGACTTAGGGTAGCGTGTTTGAGCGGTAATCAGAGTG
>dme_circ_0005269
GTGAACTCTGGACACCACGTaaaaaaaaTGGATTCTCCCACAACCGAAACGACACCGCCTCCACCCACGC
TATCTGGTCCATTCGTTGGGGCTATTGACGAGGGAACCACTTCCGCGCGATTTATTATATTTCGTGCGGG
CACTGACGAAATTGTGTGCTTTCATCAGATTGAAGTGGAGTCTATTTTCCAGAAGGAGGGATGGTGCGAG
CAGGATCCCATGGCCATAG
>dme_circ_0005269_junction_seq
TGGTGCGAGCAGGATCCCATGGCCATAGGTGAACTCTGGACACCACGTaaaaaaaa
>dme_circ_0005270
GGTTTCTTTTATTTCAATGAGATACTCTTTAGGGCCTTCCTTTTTCTTAAGTTTTTTCGTTGTCTTAACT
TGTTTCACTTCCTCTCCTATTTCGTTTACAATATTTTCTATGACCGTTTCAGGAGCCATTTCTTCAATAA
GGATAACATTTTCTGGTGCTTTCACAATCGGCTTCTTGGGCTTCTTCGTTTTTGTTTTAGTTTCATGTGC
CTCTCCCTCAGTAGGCTCAGGGCTCTCAAAGCTTTGTGGCTCACAAAACTCTTCTAAAACATTAACGACA
TAGCTTTCGACGTCTTCAGGCTTCAATTTAATTGTTTTCTTGATTG
>dme_circ_0005270_junction_seq
GGCTTCAATTTAATTGTTTTCTTGATTGGGTTTCTTTTATTTCAATGAGATACTCT
>dme_circ_0005271
GTGGAAATTAAGAAAAAGAAGGTATCCCGCAAACATGGTCCAAAGGAACAAGTCTTTGAAATCACCGAAA
CAAAAGCTATCGATGAACCTTTGTCTGAAGTTACTGTTGTGGAGATTACAGACGAACAGCCTCAAGAAGA
AGTCTTACCAGCTCAAGAAAAGAAACCTATTAAAAAACAAAAGAAACTAAAGCCCGAAGATGTCAATACT
TATGTTGTCAAAGTCCTTGAAGAGTTAACTGAACCCACTCAATTCGAAACAATTCCGGAAGACGCAGATG
ACAAGCCACAACCAGTAATTGAAGATATATCGGAAAATGTGCAAGTTGTGCAAATAATTGAAGAAGACGG
CACACCAAAACAGG
>dme_circ_0005271_junction_seq
ATTGAAGAAGACGGCACACCAAAACAGGGTGGAAATTAAGAAAAAGAAGGTATCCC
>dme_circ_0005272
GCAAGTGCAGACGCCTTACAAAAACCAACTAAAGACAAGACACCAAAACAGAAAAAGACACTAGAAACTC
CAATTGAGGAAGTTGACGAGACTGTTATCATTGATGAGGGTACAGGGGAACAAACTGACCAGATAGCTAT
AAAACGAAAGCCAAGAAAGGTACAGGGTAACGTTCAGG
>dme_circ_0005272_junction_seq
CCAAGAAAGGTACAGGGTAACGTTCAGGGCAAGTGCAGACGCCTTACAAAAACCAA
>dme_circ_0005273
GCTTCTTCTACAGTAATCTGTACATATGGAATCCCACCTTCCTCGCTGTAAGCAAGGTTTGTGGATGATT
TTGTAACTGGCATTGCTTTTAGCTCCTGGGGTTCGACCGTTGCAAGGCTACCCAAAGATGGTACCGATGT
CACCTCTTGTACTACCGAAACCACTAACTCAGAATTCAAATTTGAATTGGAAGTCAGGTTTACAGTTCCC
GGCTTAAAATCACCATGCCCTTCAATGTTTTCATAAATATTAACAGTTGCATTTTCGGCTGGCTTAAGAA
AT

>dme_circ_0005273_junction_seq
AGTTGCATTTTCGGCTGGCTTAAGAAATGCTTCTTCTACAGTAATCTGTACATATG
>dme_circ_0005274
ATATTTTTGAAAATGCCGAAATAGAGAATTTTTGTAAACCAATTACCGAAGGCACTCAGCTGGAAACAGT
TGTTATTGAAGTGGTGCCAATAGATAATGTGGGTGGTATTCATTTAGCCCCACAGCCATCTACATTATTA
GCCACTTTAACGTCTACCGATATAGTCAATCAATCGCACG
>dme_circ_0005274_junction_seq
TCTACCGATATAGTCAATCAATCGCACGATATTTTTGAAAATGCCGAAATAGAGAA
>dme_circ_0005275
GGACGCAAAGAGCGGGAGAGAACTGTGCGAAGTACCCACAAGCAAAACCGTCACGACCGAGTCATTGAGC
TGCTGGACAGTCCTGAGCAGGAGCACCACCATCAGCACCAGCACAAATCGCATCGCAGCAAGTGGCGCGA
GGAGGTTGAGGTTATCAGACGAAAGGTGCCCGAGGACTTGGAGCTGTTGGCTCGCAGGGAAAAACTTCTT
GCCGCTGAACGGGAAAGCCGCCAGCGAAAACAGACGGCTCGTGAGGAACTGGAAGCTCGGCGGGAGTTGC
TCCGCGAGCGAAACGAGCACAGTGATGCACTTAGTCCAACAACAGTGGCTGCAAGTGTAACCGCAGGTCT
GAACATTCATGTGAAGAGGAAGTCCAAGCCGGATAACTATGAGAAGGAAATCAAACTCAAGAAGCGGCGG
GAAGATGATATCGAGGTTATCCGAGATGATGACGATGAGGAAAGCGAGGAGAGCGACTCAAACGAGGAG
>dme_circ_0005275_junction_seq
AAGCGAGGAGAGCGACTCAAACGAGGAGGGACGCAAAGAGCGGGAGAGAACTGTGC
>dme_circ_0005276
ACTCTGTTGCGCTTCCTTCGCTGTCTTGCTCAGGAACCTCCTCGTTTGAGTCGCTCTCCTCGCTTTCCTC
ATCGTCATCATCTCGGATAACCTCGATATCATCTTCCCGCCGCTTCTTGAGTTTGATTTCCTTCTCATAG
TTATCCGGCTTGGACTTCCTCTTCACATGAATGTTCAGACCTGCGGTTACACTTGCAGCCACTGTTGTTG
GACTAAGTGCATCACTGTGCTCGTTTCGCTCGCGGAGCAACTCCCGCCGAGCTTCCAG
>dme_circ_0005276_junction_seq
CGCGGAGCAACTCCCGCCGAGCTTCCAGACTCTGTTGCGCTTCCTTCGCTGTCTTG
>dme_circ_0005277
GTCCAGTCGAAGCAGTCGGTCCAGATCCCGTTCTAGAAGTCAAAGCAGCCTAGAGGATGAGGTAGATCGT
CAAGACGTGGGGGCAGATGCCTCACCCAGCAGCAGCACACGCAGCGAGGAACGTGGAATGACCCAAGAGC
AGCCGGAAGAGAAGCCCGAGGAGAAACTCAAAGAGAAACAGAAGTCTCTGGAGGAGCAAATTCCGTGCGA
TGACAAGGGCATTCCCCTGCCCAACTATTATCCCGGTGTGCAGGGCTGCCGGTCTGTGGAGGAATTCCAG
TGTCTAAACCGCATCGAAGAGGGAACCTATGGAGTG
>dme_circ_0005277_junction_seq
CCGCATCGAAGAGGGAACCTATGGAGTGGTCCAGTCGAAGCAGTCGGTCCAGATCC
>dme_circ_0005278
GTCACGATGCAATATCCAATTGTCATGGAGGTGGGCCACCGCCCGCAGAAGCTGCTGCGTCAGACATTTG
ACTTCTCCGGGGAAAAAGCTCTGCTTACGATTTTTCATCGTTTCCATCAGGGATTTCAGG
>dme_circ_0005278_junction_seq
TTTCATCGTTTCCATCAGGGATTTCAGGGTCACGATGCAATATCCAATTGTCATGG
>dme_circ_0005279
GGTGGATCACCGAATTCTCCTCGGAATTGCGTGCCTTTTCGATGATCCGGTCGTTGTAGAACACCAGCAT
GTTTGACAGTGTATATACCAGCTCACTGACCACATTTGATGAGGAAATGCGACCTGGAAGCCGGCCAATG
AGATGAGTCTGTAGGCTCTATGTAAGCAAGGAGTTCACTTTACCTGCTATAAAGTAGGATACCCACTTGG
CTGTGGTCTCAAAATCCCCCACCACGTCGGGGTTTTTGCCCACCCACGCCTCGTACGCCTTCAGCATGCC
CTTCAGAGTGTCCATTATATCCGGAAAATATTGTCTGTTCCTCCAAATAATAAAAATAATGAGGTGTTTC

GGTCTACAG
>dme_circ_0005279_junction_seq
AAAAATAATGAGGTGTTTCGGTCTACAGGGTGGATCACCGAATTCTCCTCGGAATT
>dme_circ_0005280
GTTATCGTGGCAACCTGTCATGCAAAACGGTCCTGCAGCTGGACAAGTTCGCACCCTACGAGGGTGTGGA
GAAGGATCACCTGCTGGAGCGGCGGTTTCAGGACATCACCAATGACTACGACAAGTCGCCGCCGCCCACC
GCCTCGACCACGCCCACGCACTATCCCAGCCTGAACAGCATAATATTTGAGAATGGCAGCAGTGGCAACC
TGGGCGATCTCAACGGGAACACGAAGACGGATCTATGCGCCGGCCTGCAGAGAAGTGGCGGCGGATTGGG
GGGCAATGCCGGCAGTGGTGGCCACCTCATATCCAATCTCACAGCCGCCCACAACATGTCCGCCGTGAGC
AGCTTTCCTATCGATGCCAAGATGCTGCAGTTCTCCACGGATCAGGATCCAGTGCATGTGCGAGGCACTG
CAGCAGAAGGGCGACATCGAGAAGCTGACCACATTCCTCTGCAGCCTGCCGCCCAGCGAGTTCTTCAAGA
CCAACGAGAGTGTCCTGCGGGCCCGCGCCATGGTGGCCTACAATCTGGGCCAGTTCCACGAGCTGTACAA
CCTGCTGGAGACGCACTGCTTTTCGATCAAGTACCACGTGGACCTGCAGAACCTCTGGTTCAAGGCCCAC
TACAAGGAGGCCGAGAAGGTGCGTGGCCGACCACTGGGCGCCGTGGACAAGTACCGTCTGCGCAAGAAGT
ATCCCCTGCCCAAGACGATCTGGGATGGCGAGGAGACGGTCTACTGCTTCAAGGAGAAGTCGCGCAACGC
TCTCAAGGACTGCTACCTGACCAACCGATATCCCACCCCCGACGAGAAGAAGACGCTGGCCAAGAAGACC
GGGCTCACGCTCACCCAGGTCTCCAACTGGTTCAAGAATCGTCGACAGCGGGATCGCACGCCCCAGCAAA
GACCG
>dme_circ_0005280_junction_seq
GCGGGATCGCACGCCCCAGCAAAGACCGGTTATCGTGGCAACCTGTCATGCAAAAC
>dme_circ_0005281
CATTTGAATATATCCCCGATTCTGAAATCACTGGACTCACAGGATCTGAGGAGGATCTACTTGATAGAGT
AAGCGTAACTGTTGTACTTGAGAACTTAAACCAAGGATTTTTCGAAAACCATTTTAGACCCTCTCAATCG
ACGAACAAGCTCCGAAAATTATTAAAAAACTACCGGAGAAAATCGAGCCAAAAGAAGGCGAGCAAGCGAA
GCTGGAGGTTAAGGTCGTTGGAAAACCGAAACCGAAGGTCAAATGGCTGCGCGACGACGAGCAGATATTC
GCCTCCGAGGAATATCAGATCGAAAATTTCGAAGATGGCACCTCAGTGCTGGTCATTAACCACGTCTATC
CCGATGATTTGGGTACAATTAGTTTCGAGGCATACAATCCACTCGGTGTGGCAGTGACTACGGCGCTCTT
TGCGGTCGAAGGTACTAATATGGTTTATTCGGCATATGATGCAATCTTTAATATGAATCAACTGTTATTT
GGCTTTGTTAGTAAAATTCTTTTTCAGATGTCTGGAGCTGCATTATTTATGTTTCCCAAACTCTCTCGAG
TCCAGTCTCTCTTAATTTCCCTAAACCCCCCATAACACTTTAGTTTTAAACACCTGCAGAATTATACACC
AAACAAACTTCAAATAGGCAATACTTTTCTAGTTTAAGGAATACTGAACTAAATTTACAAATTTCTTTTT
TCAAGAACACGTTTAATTGAATTTGTTTTTATTACTTCCACTATGTTTCATCTATACCGAAAAAGGCAT
CGTGGGTTCGAAGGATTACCGCAAGCCGGAATGGGTGTCTCAAATGGAGGAAATGCAGGTGGCTCTCAAA
GG
>dme_circ_0005281_junction_seq
TGGAGGAAATGCAGGTGGCTCTCAAAGGCATTTGAATATATCCCCGATTCTGAAAT
>dme_circ_0005282
GTTCGGATAATAATAGCGTGTGCCTCGATTTTGATCGCTGCTGCTCGGTTGATTTATCTATTGTTTTTTG
CGACACTATTGACTTATTCTTTGGCGGTGGAAGACACATTTCCTCCTCTAAAGGTTCTGACAAAAGGAGA
GTTTGTCTGGATTTAGCGATCTTTCTTATGTGG
>dme_circ_0005282_junction_seq
TCTGGATTTAGCGATCTTTCTTATGTGGGTTCGGATAATAATAGCGTGTGCCTCGA
>dme_circ_0005283
GAAGGAGGAGGAAGAAGAGCAGAAGCAAAAACAGCATCTCTTACAACCTGAAATCTGCGAGGCTCAACTG

AATTCAATGGTAAAAGCTGGCAGCGAAGGCTCCTGCAAAAAGTGCAGGAATTGCAATCATACTTTGAGCG
AAACAAGGCTAAGTAGCGAGTCATTTGTCCTGCCACCTCGGAAATTGTGGGACTTTTCAAGGCTACAGCA
ACGGTTGCGCAATATAAGGCAGAGGCCGTCGTGGAAGGAAGTGAACACTTACTGGGAGATCGAGGAGGAG
GCTCGTCTGTCCAGGAACCAAGACTCCGACGATTCAATGGAGCAGACCAGG
>dme_circ_0005283_junction_seq
CTCCGACGATTCAATGGAGCAGACCAGGGAAGGAGGAGGAAGAAGAGCAGAAGCAA
>dme_circ_0005284
GGGCGAAGGGCAGTTCGAAATCATGGAAATTGACGACGACGAGGGGGAGTCATCGAGTCGAAAAGCCAGC
CCCAAGGTACCTGCGTCCAAGCCCATCAAATCTAACTCCTTGCCTTCTATATCCAAGCATAAGCTCTCCC
CGGAACAAGCCAGAGCTGTTGCTCTGGAGCAGATGGCCGGTCTAAAGCCAAAGTCGCGTCGTAAGGAGCC
GCCTGCGCCGCCTTCTGTGGTCGCGCCGATAGATATTGTCAGCTCGTTGAACGATGATTGGGACGACTAC
GACTCTGAGGAAGACAAGTCGGCTTCCGCGTTGGTGACAGAAGTTATAACTCTGCCTTCAGCACACGTAG
TCACTAAGAAGCCACCTCTGCCTGTGAAAAAATTGTCATCCCCAGTTAAGCACAGCCCGAACTCGGCAAT
TTTGCTAAACAACAAGATCAGCGGAGTAAAAGTAATGTTAAAGACCGTGAGCCAAAAGCAACTTGCAGCC
TCAACAGGGGCAGCAGCGGAAAAAACCAAGCCGACTGTCGTTGTGTCACCTCCCAAGGAAACCGAAGAGC
ATACTACGGGGGTTCAAGCGTATGCGTGTAATTAAAAGGAAGATAATCTGGGACCCCGATGTACCAGAGA
CGAAAAAATCGTTTGCGCAGTATGCTAGCACGAAGGCGGCATGTCCAGCACCCACCACACCAAAAACAAC
TGTCAAAAGCATTTCGCCCACCACGACGACTAAGAAGGAGGTGGCACCCAAAAAACGGTCAGCTACACCT
ACTGCTCGGTCTACTAAAGCTGGCACTCCG
>dme_circ_0005284_junction_seq
TGCTCGGTCTACTAAAGCTGGCACTCCGGGGCGAAGGGCAGTTCGAAATCATGGAA
>dme_circ_0005285
TTCGCTACCGTTGTTCGCCTTCATATCGTTAGCCAGCGTATGCGAGGAGGTATCTTCACCGCCCCTTTGG
CTCGACTTGCTTACGGGCTTTACAAACTTAAATGACGGGTCCGAGCCCTCGCCTGCATCATCAGACAGAT
TGGCTGCTCCGGGCCTATTGTCCAGCGAATTTCGACTTACGGAAGCGTTGCTGGAG
>dme_circ_0005285_junction_seq
ATTTCGACTTACGGAAGCGTTGCTGGAGTTCGCTACCGTTGTTCGCCTTCATATCG
>dme_circ_0005286
AGGCAACCCAGCCCGAAGAAGTGCAACCAGTTGAGGAAATTCCGGAGGAGCCACAAGTAAAGGAAGTTGC
TGACGAACGTAAAACGGCACCTAAGCCAAAGCCTAGAAAGGAAGAAATCATCGAGAAGG
>dme_circ_0005286_junction_seq
CCTAGAAAGGAAGAAATCATCGAGAAGGAGGCAACCCAGCCCGAAGAAGTGCAACC
>dme_circ_0005287
ATGAAATGATTGTCGAAGAAAAACGCCGAATCAAGAAAACCAAAAGGCCCAAGTCGACGAAAGAAGTTAC
CGAGGAGTTGTTTGAAGAACAGCCGGAAGAGGAAATATCTCCAGAAGAGGAAGTGCCGCAGAAAGAAGTT
ATTGAAGAAATTGAGGAAATTGTTGAAGAAAAACGTAGGCTTAAGAAAACAAAGAAGCCCAAGCTTACAC
AGCAGGTGACCGAAGAAGAAACTCCCCATGAAGAGATAATCAAAGAGTCTgaagaagttgttcaagaaca
ggaagagatagttgaagaaaagaagaagGTCAAAAAGG
>dme_circ_0005287_junction_seq
gttgaagaaaagaagaagGTCAAAAAGGATGAAATGATTGTCGAAGAAAAACGCCG
>dme_circ_0005288
GCTGCAATCGCACCTACTACGGAGACATTGGACAGACCTACGCCCTGGAGCTGCACCGCCCCAAACAGGA
TCGCGTGCCCTTCGTGTGCGTGCTCACCTTCACGGCGGCAGGTGGACAACACGGCGATGTGGTGCAGGTG
AGTCAGATCCCACTATGGCTTCAAAGTGACCCATAAATACTTTTAATCAATAGGTCATATGGAACAGTTC

TTAAAGCTTTCCTTGTTACTAGTGAATATGTGTCGATGCATGCTTATAAATCGTTTAATATGAAAATATC
ACATAACTTTGAAAGTATTGAAAATATTTTGATTATTCGATCGAAAAAGCAACCACACTTTGTCGTCACA
CATAAACAAAATCATGATAAATTTAATGTTATTTGAATTTCTCTCAGTGCATCGAGGGTTGCTGAGGTAA
AGGGGAAGGCTCTTCTGCATTCGCCATTGCCTTTCACATTTCATTTTGTAACGGGATAAACTACGCCATT
AAAAGATAAGCACTAACAAAGGCGCTTGTAGGAGTGGGTTGGTTTGGATGGGGCCTACAATTGGGTGTTG
GGCAGTGGGTTGCAGAGGCAATATTGATTTCAAAGCCACTCGCCGGAGCGTAGCAAATCCGGCACAACAT
CCCTATTTGGGAGCAGGGGGCGGCGGTGGAGAGCAAGTGCCGGTGCCTGTTAGTTATTACGCCACTTCAC
ACCTCCAATTGGAATGAAGGATGCTGCAGGAGCCGATGGAGTGGGAGGAGCAGCGAAGGCAACGAAGAGA
AGCACCCGGCATCCAGCCAAAGCCTTTGGCATGCTCGAGTGCGTCCCAAGAGCATTCGGCACTGATATCG
ACTGAGCTACGAGCACTGACCGAAACTTTATTACTTACACCCTCCCTTCGGCACACCGGGCGAATATTTT
ATGCCCAGCAGAGCGACTATATAGACTAGATTCGGATGACTGTATGCTATAGGAAGTTCTGGAAACGTTC
TTCTTCCCAAGTTTTTTCTTCACCAGAAAACCTAAAAATCAATATCAATAGTTTGTTATTTTATATGAAA
CCATTCCAAAAAGTATAAATCATAATAATTTACATGATAAATCTGGCTGCATGTCTCGTATATGTTCATC
ATTTGGTGTTGACACTGTGATTAACTGTTATAACTGTTATTATAATCAAGCAAGTCAGTTAGTACCTTTA
CCCAAATGGTCATTTGGGCACCACAAAGTAGAGACCCACCCCACAACACGGCTCTCACGTTTATTGTGCG
AATCGCCCTAGGGTCACCCTGGACAGCTTCACGATTGGCAGGTTCACCTCGTACGTCCATGAGGGTTGTC
CGGATGGATACATGCAGATAGCGGAGTCGGCACGGACTCCGATCGGTGGCATGTGGTGCGGCAGCTCCTG
GGGACCAGTGCTCTTCTACAGCGAGACCCGCTCCCTGATATTCACCATCCGGCTGAACAGGAGCTGGCCC
AGTGGCacaaccgcagtagctttctcaatcagcaacagcaacagcaacagcaacagcaacagcagcagca
gcaacaacaaGGCGCTCCCGGTATCCTGGAGGATTTTACCAACTCCAGTGTAGCCCGTTCGGACCGCTAT
GGCCAGGACTTTAGCCTTTTCAGCGCTTATGCCAACAACAAGACCTTCATGGCCTCACTGGAGAAATCCT
TGGCCAAGGACGAGCAGAACTACACGGAGCCCAAGTACTACCTGGGGTGACCTCATACCCGGAACGTACT
GCTCCAGGATCTTCAGCGACTGCGACAAGAAGCCGTGTCGCCTGCAGTCGCCCAACTTTCCGGGCATCTA
TCCGCGCAACCTCACCTGCTACTATGCAGTGCGACAGG
>dme_circ_0005288_junction_seq
CTCACCTGCTACTATGCAGTGCGACAGGGCTGCAATCGCACCTACTACGGAGACAT
>dme_circ_0005289
GTCACCCTGGACAGCTTCACGATTGGCAGGTTCACCTCGTACGTCCATGAGGGTTGTCCGGATGGATACA
TGCAGATAGCGGAGTCGGCACGGACTCCGATCGGTGGCATGTGGTGCGGCAGCTCCTGGGGACCAGTGCT
CTTCTACAGCGAGACCCGCTCCCTGATATTCACCATCCGGCTGAACAGGAGCTGGCCCAGTGGCacaacc
gcagtagctttctcaatcagcaacagcaacagcaacagcaacagcaacagcagcagcagcaacaacaaGG
CGCTCCCGGTATCCTGGAGGATTTTACCAACTCCAGTGTAGCCCGTTCGGACCGCTATGGCCAGGACTTT
AGCCTTTTCAGCGCTTATGCCAACAACAAGACCTTCATGGCCTCACTGGAGAAATCCTTGGCCAAGGACG
AGCAGAACTACACGGAGCCCAAGTACTACCTGGG
>dme_circ_0005289_junction_seq
ACTACACGGAGCCCAAGTACTACCTGGGGTCACCCTGGACAGCTTCACGATTGGCA
>dme_circ_0005290
GTGCCGCACAGCCAGGTGGATGCCCTTGCCAGCGAAGCCGTGACCACACATCGCGACCAGCTGCTCCAGG
ACTACGTGCAGTCCTCCACGCCCACCCAGCCGGGGGCTGGAGCTCCGGCCGCCTCCCCCACCACCGTCAT
AATCCGCAAGGATATACGCAGCTTCAATTTCAGCGACATCGAGGTCAGTGAGCGGCCCACGGCCACGCTG
CTAACAGAATTGGCCAGAAGGAGCCGGAATGGGGAACTGCTCCGCGATCTGTCCCAAAGAGCGGTGACGG
CGACACCACAGCCGCCGGTCACCGAGTTGGATGACATTTTCATCAGCGTAAAGACGACGAAGAACTATCA
CGACACCCGGCTGGCGTTGATCATCAAGACCTGGTTCCAATTGGCCCGCGATCAGG
>dme_circ_0005290_junction_seq

ACCTGGTTCCAATTGGCCCGCGATCAGGGTGCCGCACAGCCAGGTGGATGCCCTTG
>dme_circ_0005291
TTAACAGAATCGAAAAACAACAACAGAACAACCAGAGCAATAGCAGCAGCAGTTAACGCAACGCACAGAT
TACTTAGCATAATCAACAGGAAAACTAAACATAAACCGATGAACCGATGAAAGCGGCGGATAAAACATTA
TCGTCTACCGATAAGTCTTGAACAGCAACAGCATTACTATTTATTAGTCGTCGAGAATTTGTAAAAGTGT
CAAGTGCAAGAATTG
>dme_circ_0005291_junction_seq
TTTGTAAAAGTGTCAAGTGCAAGAATTGTTAACAGAATCGAAAAACAACAACAGAA
>dme_circ_0005292
CTATTTCCGGGAACTAAGCAGCACCTACGAGATGTGCAAGCTGGGCAAACACACACCCATCCGAGGATGG
GATGGTTTCCTTCAAGTAGGCGCTGCTCGCAACAACGTGCCTGCGGATCGGGAAACAACTCCCTTGGATG
ATTGGCTGCG
>dme_circ_0005292_junction_seq
AAACAACTCCCTTGGATGATTGGCTGCGCTATTTCCGGGAACTAAGCAGCACCTAC
>dme_circ_0005293
GTTCCAAGGATGCCAGTTCGTACAATACGGTGAGTGGACCGCTAACCTGGCGCCAATTCCATCGACTGGC
AGGCCGTGCATCCGGACAATGTGAGCCGCAACCGATACCATCCGTGGTGGTGGGCTACGAGAAGGATTGG
ATCTCGGTGGCACCGCACTCCATCCACTATTGGGATAAGTTTCTCCTGGAACCGTACTCCTATGCCAGGG
ATGTTGTATACGTGGTAGTGTGTCCGGACAACGAGCATGTGGTAAATTGTACTCGCAGCTATTTCCGGGA
ACTAAGCAGCACCTACGAGATGTGCAAGCTGGGCAAACACACACCCATCCGAGGATGGGATGGTTTCCTT
CAAG
>dme_circ_0005293_junction_seq
ATCCGAGGATGGGATGGTTTCCTTCAAGGTTCCAAGGATGCCAGTTCGTACAATAC
>dme_circ_0005294
GTTCCAAGGATGCCAGTTCGTACAATACGGTGAGTGGACCGCTAACCTGGCGCCAATTCCATCGACTGGC
AGGCCGTGCATCCGGACAATGTGAGCCGCAACCGATACCATCCGTGGTGGTGGGCTACGAGAAGGATTGG
ATCTCGGTGGCACCGCACTCCATCCACTATTGGGATAAGTTTCTCCTGGAACCGTACTCCTATGCCAGGG
ATG
>dme_circ_0005294_junction_seq
CTGGAACCGTACTCCTATGCCAGGGATGGTTCCAAGGATGCCAGTTCGTACAATAC
>dme_circ_0005295
ACTGATTTATGTGGAATAAAGTGGCGGAAGTTCGTTAACGGGGAGCGACCGAACGCATCGAGCGATCCTT
TGGCGGACCCGATCCTGCGCTCATATTCGCGATGCATCCAGGCGGACATGCTGTGCGTGTGGAGGAGGGT
CCAATCCACGAAGACGGACCACGCCGACCCGAATGCCTTAACCTTCGAGATGACCACGTCGACCAAGGTC
CACCCGCCCCTTTCGCTGGCCGCCGCCAAGGAGCTATGGATCTTCTGGTACGGCGAGGAGCCCGATCTCA
GTGAACTGGTCGATGCCGAGCTGCTCCGAGTAGCGGGGTAAGTTTCTAGACCGAAGACGGTTCGAATAAA
ATGTGAAAGCTAAATATGAAAGCTAGTTACCGGTACTAGCCACGAGGTATACAATATGACATTTTCATAG
AAGTTTCTAAATAGGTAAACCTATTTACCACTTAAAATTCGATGTGTTACATTTAAGATTGATTGTATTA
TTTTCTTGCTCTCCCTTTCTCTCCcacacacacacaggcacacacaTACCCGTTCTCACTCACACATCCA
AGTGGGTGATGAATTTAATAAAGCTTCAAGGCCACATCCATCGCATGCAATTGCAACAACAAATTTTGCC
ATCTCTTTGACGGTTATGTGTGTGTGGGTGTATCTGTGTGAGAAAAGCCAAAACAAATGCGACACACAAA
CAAACAACAGAGAACTCGCGAAATACATTTCGATTGCGGCAGATGATTATTATTATTGCTCTCCTGATTC
TCTCCACTTTTTTCTCCATCCCCCGTTGTTTCGCAATCTTTCTCCCTCGCCTTTTCATTTTCCTTAGGCG
TCATTCAACTCACATGGCGTCGTATTGAACCGTCAGCAACAGAAAAGAGAAAGAACTAAAGGAAAAAAGC

AACAACTGTCAATCGTAGGCGGTTGTCTTCTCTCTTCCGCCGAATTTCACACGAAAAAAGAAAAAGTTCT
TCCTATATGTTATACACATGCAttttttttCGGAACATTGTAACGCAAAGGTTCAAATTAAATATGCAAT
TGATCCAAAACCGAGTTCTAATCGAAAGTGTCTTATAAACATGAATGTATATATAGTTCCTAGCTAGATT
AAATTATTATGTTAAAAATATTTTAACGTGGCTTACGAATTTACATTACAAAACCCCTAAAAAGTCCTTA
AATCCTATTCCTTTTTTCCTTGTGCATAAAAGAAGGAAAGAGGAAGCAGTAGTGCCGAAACGTCACCTGT
CATTTGCAATGTTTTCGCTAGCTTTCTCTCTCTTTCGCTCTCCGTCGCTCTCGGGTAATTATGCAAACGA
AGAAGAAAGGTGCTCTCGCGCTCTCCCCCTGCTCAGCGTCCCTCTCGCTCGCTCCTTCGAGGGTTGTTCA
ATATCAATACACACTACTGGCCTTCGAAAGAAGCTCGTTCTCTTTCCCACACCCTATCTCCATTTCTTTC
TTCTCTTATCAGCCATTGCGTGCGGCTACTATTTATTCAATATTTTTGTTATTGCCTCATTATCTCTGAC
CACTAACTACGTACTACACTACGTACATTGTTCATAGTAGAATTTGAATTTAAGTTTCGGCTTTTCCACG
GCAAATTCCATTACCCACCAAGCAACCGCAGGTCGCCCGAGATCCGTAATCCCGTTATCTGCAGACCCAT
TTAATAGATATTTCAAAACTTTAATTAAATATGATTGGTAAAAAGTTCTCAATGTTTCAAAGATTCAAAG
ATGGTTTCTTATTAACCAGTGATTGAAACAATAAAGAACAACAAAAATGTGAAGGCTTTTACTTTAGATC
ATAAAGTGGAACGCCAGATAAGGAGCTCCCACCAATTATGTTTTTAACTGAGAAGACCCTCTTCTTTAAA
TATTATTAAATTAAATATTCAATTAAAACATTTTTGTTTAATTAAATTGCCTATCTAAACTAAAAGTATC
TTACAATACGCTTCTTATCCATTTCATAAAGCATAATTATAAATAAATAGTACAGCGTACATACGTAAGA
GATCATAATTATCTTAATAGTTCATTAGTTTCCATTAGAACTACAAATTTTGCATCCGTGTATAGCTACA
GAAGTTCTCCTTTACCCACCGTTTCTCTTTTGAAGTTCTCCGACCGAGATACGAGATAAGGAAGTTTGAC
TGTATCTTTTGTTATCTGCCTACGTCATTGTGGCCCAATAAGTTTACAGTTTGCTTGTTCCGCCGTCATG
CATTAACCCCTTTTTGAGGCGCGCTTTTTTTACCGTCTGGAGTTGAGAGTTGTATGCACGTCGCTTCCAT
CGCTGATGAGATTTCGCGTTGTTTTTCGTTTTAGCCTTTGTCATTGCGTTATTTTATTTATTGCACTTTT
AATGACAAGGACATTGACTAATTGCATTGACATTACTTTGTCTGCGTTTTTTGGTCGAATTTTCGTATGC
TTTTTATAGCTTTTTCGGCCATAAACAACACAACTGGCGAAAAAAATTTTCGTCTTGTTGGTGTGTGTGT
GTGCGTGCTCGCAAGTGCGTGTGAATGAAATTCTGAAAGGGTGAGAAGAGTGGCGAAGAGAGAGCGCCTT
GTAGTTACTGTTACTGTCTCTCTGAGTTGCTGTACATGAAAACGGCAACGCCGCAAATCTCAGCCAGTCA
GCACAGTGGCACAAAAATAACATTTACATGACATAAAATAGGTGCTGTAGAATATAAGATCCCAAATTAC
AATGTATTCTAGCACTGTTTCGAAACaaaaaaaaaaaCAACACACACACACGACTTGACAATGACAAGTA
TTCAAACAGTTTGTTGTACTTCGATAGCATACGATTTTTACCTTAAACAGCTGTCACTTTTGCGTGTGTG
AGTGCTGGGGCAGCTGTGTGCGTGTGCTCCAAAATTGGAGCCAATAATTATGCGGTGCTCTCTGAGAATA
ATAGAGAAAGATTCGAAGAGATATTCTCTCTTTTTCGATTCTTGGCTCTCAATGAGCTTAAACTGGTTTG
CAAGCTTGGGCTTAATTACAATGCGCTTTCTTGCTCATTTATTATGTTATTTTTAAGTCTTTTGCATTCG
TGTGGTTTTACCTGCTTTTTGGTTTCTCGATTTGTTATTGCGCTTACTAAACATTTTTAAATGTGTGTGC
GCTCAGATTATTGACCTTGATCCAACATTTGTTGCTGCTTGTTGTCATGTGTGTTCGTTTTTTGTTTTCA
TTATGTGTCACGCAAATGCAAAACTGCTAAATAACGCTCTCTTAATTACACGTTACAGTTGTTGTGAAAT
TCTCACGCACATTCTTAGAAAGAGAGAGCGGTAAAGAAAAGATCTGCTGCGATAGCAAAATGTTAACCTA
AAATAGCAATTTTTTCGGGCCACAAACAAGAAGAGCAGCTAGTGAGTGTGTGGGTGAACAAGAACGAGTG
CACGCCTAGCGGCACTTTGTCTCCCAAACAACCACTCTCTCTCTCTTACTCGCTACTTTGCACAGAGCGA
CTTTCCACCTCTCTCTTTAAAAACGTCTGAGTGGGAGAGAGAGAGGAGCAGCAACATGTGCACAAACCAC
ACGTGCTAAAAAAATACACCCACACAGACGGCTTCAACGTGTTTGTGGAATTTGCATTGAGCAGTCGCCG
TcgattccgattccccgattccgatttacccgattacaattccTGTTCCAAAGCCAAGAAATTGGAAAAA
GAGGGAGATCAGGATATTTAGCAGCTGTGATGGGGAAATAAAATTAATTCAACAATAATTTACGAACACA
ATTGTACCTTTGAAGCCAGATAATTAAATGGAATTGGATTATTATTGGAAACTATTTGAATTAATCAAAG
GTTATAGGTATAAATCCCGTTTACTTTCCATAGGATACAATGTTGCATATCAtttcaaaaaaataatttt
gtgctttttgaaattcttaaaatctttttttaaattttttacCGCCCAAAATCTTATGTGACAACATTGTC

TTTTGTTTGCTATTGCTGGAATTCAGTCTTTTGCTTCTTTATTTTATTTTTGTTTGTCGTCCCGTTTTGC
TTCACTCGACTCTTGATTTTCTTTTGTTTGCTTGTTTGCCGAGAGAGCCGCTAAAGTTGTGGTTGTTGTT
GCTTGTGTTTTGCCTGGCCTACCCTTGCGGACATTTATTTATAATTTTGGCGAGCTTTGAACTTTTCGCC
GTTGGGTAgtgtgtcaatatgtgtgtgtgtgtttgtggtgtgtgACCTGTGAGAAGAAGAGAAACGAAGA
AGAGCCGGCTTGCAGGATGAATATTGGATGTGTGGGCATGGGAACGTGTAGATGACGGAATAATAATAGT
GGTGTGGAAGAGGAAGAGGACGAGGATGCAGAGAGGAAGCAGACTCTCACGCACTCACACACACACTGTC
ATAATCGTACATTGCCCTTTTATGCCGACGTGTGCGTGTGAGTTTTTTAAGCGTCGTTGTaaaaaaaaCT
CACGCTGATCTCATTGAAGGGAAATAGTAGTAGCCATGAAATACAACAAAAATAGTTATTATGTATACTG
CTAAGCCATTTTTGCTTTGTTTGTTATTTGTCCGTGTTGTTTACTTTTACGTAATTATCTGTGCAACATT
GTTGACCAACGAAGACAAAAAGAAGAAGCAGTGGAAATCAATGCAGGCGTGGGAATCGAAGAGAAAGAGC
GAAAGAGAGATTTACCCCATACATATGTATATATATTTGGAAGTGAGTGTGTGTGTGTCCGTAAGTGGTG
GGGGTGGATGCTTGGAATTTTCCAAAAATTTCTAGACTTTCCATTTTCTTTCACAAATACTCTCCCTCCT
CTTTTCCCTCCCTCTTATTCTTTTACGTACTGCCTTTTCTTTATGTTTCGAGTGTTAGCACTTCCGCTTT
GGATTTTTGTTTTGTTGCTTGGCGGCTTTTTGTCTCCACCTCTCTTCACTTCTCACGCTGCATTTTTACA
TATTGCGCTCTACACATATTTTCATCCTTATCCACCGTttctttatgcttctttatttctggccttctct
gtgcctctcgctttctggcgttctACCCATCTCGCTCGAAGTTTGTTTGTCATTTTTCTACAAGAACAAT
TCAAATGCAAAACGAAAACAAAACAATAACAAATATGAATAGCCTTAAAAGTTTTGTTTTTCCTAAGGCG
TGCAAGAAAGAGAGAAGAAAAGTGGATGGGAAAGAGAGGGCAAAAATGTGAGATTGATTGTTTTACTTTG
TAGACGCCTCTTCCATTGTTTCTATTATTACTATAAATTCTTGTTATTTCTTCCACTTGGTTTTGGGTAT
TTTCCTCGTTTACATTTTGATGAAACATCGAAATCAAAGATGTTGACAAAATAGAGAAACACACCCGCCT
CTTGAAGATTCATTGCGATCTAGAGCTTAGAGAACGTTTTACATAAAGGATTATAGGGATTTTCATACTC
CAACGCAAGTTGAGTAGACTTTAATACACTGACTTCTAACTTTAAAGATGTACTTACATACATATATATA
TGGAATATATTGGATTATAAGGCTTCCATTCACAAGCTACATACATTTTTTGATTCGCCAAATACTTTTA
CCAATTACAAAGATGTTGAAACTATTTTAATCGAGAATGTTCAAATATTTAAAACACCTAAAAGAAGGAC
AATTCTTTAAGGATTTTATGAAAATAAATAAATCATAATTTTATTTTATTTTTCAGCGTATGTGACCGTA
ATGAAGAGTTTAGACAGAGACTGTTCGGATATATGCCTTAACTTTTGTGAAAATTCTGAGAATTGTTTCC
AATTCACAATATGGCAATATCCTAGAATAATGAACAGGAACTCTGTGCAATGGCATTGGAGCTTGTGGCA
TTGCATCGTTCTTCCTGCGACATTCCTAAACTATTTGATTCTATTATAACAAATTCAATTGCGCATAATT
GTTCAGCTTATTCGTTGTATTGAATTTTCGATTATCATCATTAATGCTCCGTTTTCTAATAATCTAATGA
AACTTATTTTTGAAACTTGAAAATATAGGGCTTGTAGGTTGATCTTACTCCACAATTGCTAATCATTTAT
ATTCTCTTTGCAGCCTCCCAGCACTTGTCCTTCTCATTTACGTTCTTTGTGCACGGCGACACCGTTTGTG
CCTCCATAGATTTGCGCGAGCATCCCGCCGTGCGTCCGCTGACCAAGGAGCACTTGACCGAAGCGGCGGC
AGCCTTTGCAGCGGCCAGTTCTCCGCCAGGATCAAATGGATCTGCGGCGTCAGCAGGGGGAGCTGTGCCT
AATCCTGGTCAGGATCCAAATGGAGCCAGCATGGATGGACTGGACGGCGGAGAGGGAGCGGCCAAGGCAG
CGCCACCTCCGCACGCGCGAAAGGTTATGCTGGCGCCCTTTGGAATTGCTGGCATACTTACCGGCAATAG
CTATAAGGCCAGCGATCCCATAGCCGAGAAGATCCTTGAGGATTGGGCCTCGTTTTTTCCGCTGTGCAAT
AAGGATAACACGGATGTGCCACCCGTGGTGGAAGTGGTGTCGGGGTGGTCATAAGATGTATCATCCCACG
AACTACGTACTAGTCACAGACCTGGACGACATGGAGCACATGGAATTCGTCGAGATGCAGAAGATGCAAA
GCTCAGTGGCCGGAGCGGCGGCCGAGTCAGCTGTTCTGGCCTCGCTTTCCTGCCCACCGGGAGCAGCATC
TGCTCCTTCATCGATGGGAGCAGCTCCATCTGCAACAGCGGTTCCCTTGGGTCCAGCTTCCCTCAATGCG
CCAGCTTTAGCAGGAGCAGGTGTCGGAGCCACCGCATCTTCCGCCGCCAAAGAGATATCCCGCAAGGCAG
CTCCACAAGCGGTTAGCGCCCTGGAACGTCTCGCCTTCCAGCCCTACTACGATCAACGGCCCACCTCGGG
CTTCACCTTCAATACCAACAATACTCACATACCCGCCTCGGCAGCCGTGGAAATGCCGGAACGAACCTGG
CAGGATTGCGTGATGAACACGCTCCATGTGGATGCAGCGGCGGCGGCAGCAGCAGTTGCTTCGTCTACGC

CCGCCTCGGGAACTGGGTCATTATCCGCGGATGGGGACGAGAATGAGCAGAATAAACCGCCACAGGACTC
CAAGCAACTGGTGCAGCAGCAGATCCAGCAGCAACAGCAACGCCAAAAGCTCTGGAACTTTGTGGATCCC
ATGCAAAAGGCACCCTGCATATGCACCAAG
>dme_circ_0005295_junction_seq
GCAAAAGGCACCCTGCATATGCACCAAGACTGATTTATGTGGAATAAAGTGGCGGA
>dme_circ_0005296
GGAAACGGGGAGCCTGGGTGATGTAGGTCTCCTCCTCCTCGCGACGTCCGTAGCGCGACGAGTCCTCGAG
ATGCTGGATCTTCTGCAGACCACCTGGGTGTTGCGACTCCGAAACGATTTCGTTCTTGGAGATAACCGTA
AGCTGAGCCACGGTGGTATCCTGACCCATCGCATTGTAGGCGCGGCAGGTGTAGGTGCCGGC
>dme_circ_0005296_junction_seq
TGTAGGCGCGGCAGGTGTAGGTGCCGGCGGAAACGGGGAGCCTGGGTGATGTAGGT
>dme_circ_0005297
GCTTTCCCCAGAAGAGCCTGGAGCGAAGTGGTTCCACCCAGTATGAGCTGGCTGGAGCGCAACAGCCAGG
ATCAGCCAACGCCTCCACCTGCACAGATAGCGGCAGTGTTGGCGGCTATGTTTACCTGCAGAATCACTAC
GCCCCCGGCGCCCACAGCTCCTCAGCCGCCATCAACTACCCTGCGCAACAGCATCCCCAGATGGTCTACC
AAATACAGCAGTATCCCACGTGCcatcagcagcagcaacagcagcacctccagcagcagcagcatttgca
tcagACCGGCGCAGGTCACTACATGCAGGTGACGGCGACGGGTGGTGGCCAGTATCATCACCATCACATG
CTCCATGGTCACGGGCATCATGCCCACCATCACGGTGGGGCGGTGGTCATCGCCGGGAGTGGTGTGGGAA
CTGGCCTGGGATCTGGAGCCACCAGCGTGATCATgcagcaccagcaacagcagcaacagcaacagAATAT
GCACAAGAAGAACTCCATCCGGAACGGCGGAGATGTCCTCAAGCGAACGCGAGCTCAGTCGGCCGTAAGT
ACTTTCTCCCGGGTGATGTTGATAGTTTGGGGTGTGGATTCAGCTGGGAATTATGTGCCGACAGAAAGTA
AATATTTCCAGGGCCCAAAGGAGATTTCCGGGAACAGTTTCTTATCGAAATGTAAAATTTGGGTGGATTG
TTCAGGAGGTTTTTCAAATACAATTTTATCAAATGATAAACAGGTGGCTTTCTAAATCGGTAACGGACTA
TTATAGCATACCTTTAGGACTATCATTAGATAAGTGAAATCAAGAAGACTTTATTTTTAGACCTGAATAT
GCGTAATAGTAACTTATCTTGTTCTAGAAAAGTGACTGCGTTTAATAACTGAAATCAATTAATTTAAGTT
TATTAAATCACAGATGTGCACTTATCAGTTTTCAGAGATATGAGCAGAAAATCAGCCATAAACTATTCAT
GGCCTGAGCTTATTCACAGTCTGGTTGCCTATCAGCTATAAAATATTTGCCATGATTAAAAATTCATTAT
CTTGAGAATGTGTACTGTATACAAACAAAAAACGAACGGCAATGCTTAATTTGGTTTTAAGCATTTAAA
TATTTCTAATTTGTACTCCCTTTTAAAGTGACAGGTTATTTTTTGTTTGGGCATTAATTGCAGAATTAAA
CCATTTCACCATTTTTGTGTAAACAATCCATCAATCCTGAGGGTTGGCAACAGAAGCTCGTCTGTTTATC
TAATTAGTTATCTGTAAAAAGTAAAAGTGTGCCTTCGGGGGATTGACCATTCGTCAGCTGAATGAGTTTG
AACTTTGTCCGCACGCTGAGAACTTGAAAAGTCATAATTGGATATACAGGTGGTATCTTAGATTAGACGC
CGTCAAAAGATAGCGACTTCTATGCAAATATTTGTGCTACTGCAGTGCATTTTCTGATGTTGGCCATGGA
CAGGTCGCTATGCAGCTGATTCAGAAAGTTATAGTGCTCGATAGGTTACAATTCAGGGAATGTCCCAGAA
TATGTTTTCAAAGGTATCTTTATTGCGGCATGATGAGTAATTTACCGATCGAAAGTAACGATTGCTTGGG
GCACGAGGCACATAGCAATAAGCAATATTATTGCCGTCTCTCGAGAGGCGAATGTTAATCAGGTCGATAG
CCCAGAGGGGCGACAGTGACGACGCCAGAGTGAATTGTATTTCAAAGATACTCGTGCGCTATCTTATCGG
GCGGGGCAACAAAACATTCGGTCTCCGGCGTAGACTTTTGTCAGTTATTTGCAAATTTCCAGCCAACCAA
GCACACCACACATCGCACCTTCCATTCCCACACACCGGATGCCTCCAAATACTTATCGGATAAATCTTTT
CTGATCTCCTCCCAATAGGCTACGAACTCTCACAAGATCTGCTTGAGAAACAGATCGAGCTGCTGGAGCG
CAAGTACGGCGGAGTGCGAGCCCGTAACGCAGCGGTCACTATTCAGCGCGCCTTCCGTCACTACATGATG
GTCAAGAAGTTCGCCTCGATCACGGCAATGGCCAAAGCTGAGAAGCGTCTAAGCCGGCGGATGGTGGTTA
CAGCCAGCAGTCTTGGATTGGCGGAGGAGGGTGCCTCCTCCTCGTCAGCCTACGGAAGTGCCACGGAATC
TCAGCTCACCGAGCAGCAACAGCAACAACAGGCGCAGCAGCAACAGCAGCCACGTGTCACCATCATGGCG

GGTCCGGCGGGAGCAGCTTCTCCGGGCTTATCCCGGACGCCACCTACGCGATCGCTTTCCATGCGGGAGC
GACGTCAGCTGGACTGCAGTCCCATACCGCGTAGTCAGTCAG
>dme_circ_0005297_junction_seq
TGCAGTCCCATACCGCGTAGTCAGTCAGGCTTTCCCCAGAAGAGCCTGGAGCGAAG
>dme_circ_0005298
GCTGGGAGTCCTGCTCGTAGCCAGATCGACGGGTGATGGCCTCACGGAAGGAAACCTCGCGTAACAGCCT
GTACTCGAACGTGTCCACACGGAACTCCTCCTCAATGGAGGTGTACGAGTATCCGTTCGA
>dme_circ_0005298_junction_seq
CAATGGAGGTGTACGAGTATCCGTTCGAGCTGGGAGTCCTGCTCGTAGCCAGATCG
>dme_circ_0005299
GTTACCATGGATCACCGCACGACTTGAGCTATGCCAGTTCGGCGGACACCTCGCTAAATGCCTCGTGGGT
TAACACGAGCGGCCACTCCCCGCACACGCCCTACTATTCGGCGGCCCAGATCTATATGCGACCCAAGGGC
GGCAGCACCACGCCCACGCCCAGTTGCAGTGGCAGCACAGGTAGCGGCAGCGGAGGCAGCGGGAGTGGCA
GTAGCAAGAAGGTGCCACCAGAGGTGCCCAAACGCACCA
>dme_circ_0005299_junction_seq
GTGCCACCAGAGGTGCCCAAACGCACCAGTTACCATGGATCACCGCACGACTTGAG
>dme_circ_0005300
GCTTCTTTTGCTGCAGCGCCAGACACCGCCGCCTCCTTCGCTGCTAAGGACCAATGGCCTGTGCAAAACC
GCCGAGAACGGCAGTCTGACCTCCGTGCAGAGTTCCGGATCGGATTCTAGTGTTACCTCTGCGGAACGCA
ATCTAAACAGCGATTTGGGCTCGGATCGCA
>dme_circ_0005300_junction_seq
CTAAACAGCGATTTGGGCTCGGATCGCAGCTTCTTTTGCTGCAGCGCCAGACACCG
>dme_circ_0005301
ATGACTTAGACCTCCGTCGACCGTCCGTTCTCATCCGTCCGCGAAGCCTTTCGCTCGCGTCCCGGTATTC
GGCCAGTAAGCGGctgcgcctgcgcctgcgcctgagcctgggcttgagcttgggcctgagcttgggcttg
agcctgagcctgagcttgCATTTGTGCCTGCTGCTGGAGCATTTGCTGTTGCTGCAACATGTAGGCGGCC
TG
>dme_circ_0005301_junction_seq
GCTGTTGCTGCAACATGTAGGCGGCCTGATGACTTAGACCTCCGTCGACCGTCCGT
>dme_circ_0005302
TTACACAGAGAAAATGTAGGGTAAACAAAATATATAATATACATAGCGCGCACTTATCGGGATATAGTTT
TTAGTATATGCTAATGAAACTACTTTCGCCAATCGCTTTTGTAATTCGTAAGGGGAGGGGTAGATCAAAA
CGGGTATGGGCATAGAAGGTTGCCCAGGGGTAAATTGG
>dme_circ_0005302_junction_seq
CATAGAAGGTTGCCCAGGGGTAAATTGGTTACACAGAGAAAATGTAGGGTAAACAA
>dme_circ_0005303
ATCATAAGGCGAACAATCAGTCAACCAAGCAGAATGGCACGGCTCCAGCTCCAGCAAACCCCACGGCTGC
ACCAGCCCCAGTTCCGGCCACAAATGGGTCTACGGGCGGCGGTGCTGCCTCCAAAACCTGGACCAAGGAG
GAGCAAGCCCTGCTCGAACAGGCCATTAAAACCTATCCAACGACGACACCCGATCGCTGGGATTGCATCG
CCGCCTGCATACCGAATCGCAGTAAAAAGGACTGCTTACGTCGCGTCAAAGAGCTGGTCGAGCTGGTTAA
CTCCAAGAAGGAGGCACAGGCGGCGGTCAAATGAGTGCGGCTCCTGCTCCACTTTACCTGCCTCTCGTCC
GCCTCATCCACTTCTAGCTGCTGTG
>dme_circ_0005303_junction_seq
TCCGCCTCATCCACTTCTAGCTGCTGTGATCATAAGGCGAACAATCAGTCAACCAA

>dme_circ_0005304
GTGGACATCAGCTACCTAAAGTCGCTGGATCCTAAGGAGTGGAAGGACCAGGATCATTACGCCGTTCTTG
GCTTGGGCAAGCTCCGGTTAGTGCGACGGCAGCACGTGGTGTTCTTCCCATGTGTTCGTTTCTATCAATA
TTTAATGGGACTTCCCCAACTAATACTGTATTGTCTGACTAAATCACCCTTTAGATACGAGGCCAGCGAG
GATGATGTTCGACGGGCTTACAGGCGCATGGTTCTGCTGCACCATCCCGATAAGCGGAAAGCCAAGGGCG
AGGAAG
>dme_circ_0005304_junction_seq
GATAAGCGGAAAGCCAAGGGCGAGGAAGGTGGACATCAGCTACCTAAAGTCGCTGG
>dme_circ_0005305
GTGCAGCTGCCGCTCAAGGTCGTTCGCCGTAAAATCGAGCGCGTTGGCTTCGCTTACTTTGCGCAACGGC
GCCAATTTCTAGCTCCCGGCGGCGTGGAGCGCAGCGAAAGCGATGAGAAATTGGAGGGCGTGGGCGAGGA
GG
>dme_circ_0005305_junction_seq
GAGAAATTGGAGGGCGTGGGCGAGGAGGGTGCAGCTGCCGCTCAAGGTCGTTCGCC
>dme_circ_0005306
TTCGATTTATTACCTCTAGTGTAGCTGGAGTCGAAAGATCGTTCTGAACACACAGAACTGGCCTCGGAGT
CGATGTCATCAGACTCATCACGTCCCATGAGCTTCCTACCCATCCGCATTCCGCCGCTGCGCATGTAGTC
CCCGGAAACATAGTCTGGCTGTCCATCATCTCCGACGCCTAATGTATGTTCTGCTTCACGGGATTGCGCC
AGCAGTCGTGCCGCCGCCGATGGTCTTACTGGGTTGTTCCTCTCGGGCGTACGTCGAGACCCCGCACCTG
TAGAGTACATACTGCGTTTCATCAAGCCACCACCTGACCTAGTTGGACTCGTTTCCCTAGAGCTGGCTGT
GCTTCGGGGTATTCCGGAGGCCTTTTTGGGTATGGCGCCAGTGGCCCCACGATAGTAATTACCAATACCA
CCGTACTGATCCCTCAGTTTTGTGCTTGGCGAGGTGGATCTCGATCCTGGCTGTGACTGAGATACTCCTG
CCCGTCCCCGGGGCCGTGGTGTAACCGATCCCGGCGTTGTAGCTGGTGTGCCACCGCTATTGGAATTCAG
GCGAGGTCTGGGCAGTGATCCACTGGCTGCTGCTCCTGTCATCGATGCCTGGTTGGAATTATTAGGTCCT
AAAGGTTTCCTTTGCCTGGAATATAGTGTATATTGCGCTCTAACTTTGGCTCGTTGAGCAGCCGCAGTGT
CCACCGCTGAAATGGATCTCATACTAGGCGTGGGCTTTTGCAGGGTTCCAGGTGTTCGGCCAATGCGGGA
TACAGTGCGTCTCGTTTCAGGTGCAGTCCCAGTCCCAGTACCAGTTCCTCCTCCTCCGCCGCCCTCTCGT
TCCCTTTCTAATGCGCGCTGGGCAGCTATG
>dme_circ_0005306_junction_seq
CCTTTCTAATGCGCGCTGGGCAGCTATGTTCGATTTATTACCTCTAGTGTAGCTGG
>dme_circ_0005307
GGTAATAAATCGAACTACTCACTTAGCGGGAGCCACACCCGCTTGGACTGGAGCACGCAGCGGGCGCCAT
TTGACGACATCGAGACGATTATTCAGTTCTGCGCATCGACGCATTGGTCTGAAAGGAAGGATGGCCTGAT
CAGCCTCACACA
>dme_circ_0005307_junction_seq
GGAAGGATGGCCTGATCAGCCTCACACAGGTAATAAATCGAACTACTCACTTAGCG
>dme_circ_0005308
TGTTGGCCGACGATTGCGTCTTGGAGTTGGAAGACAGGGATGCACACGAATCCTGTTGGCCCTGATCGTG
ATACTGCAAGTGTCCATTGAAGCCATTGGGCGCCAGCGCCGTCTGGTACTGACCGCCGAAGCAGTGCCGG
ATCTCCTCCGACGTCTTCTGAATG
>dme_circ_0005308_junction_seq
CCGGATCTCCTCCGACGTCTTCTGAATGTGTTGGCCGACGATTGCGTCTTGGAGTT
>dme_circ_0005309
GGAGCAACAAAATGCGTCACAACTGGATGCACTTTCTAGAGCTGATTTTGCTGAAGATCATCCAGTGCTA

TCAACACAGCAAGGAGGCTTTGCGGGATATCGACTCGATGATACCAAGGATAGCACCATCCTTGCCTCTG
GATCTGTCCATCAACATTGTCAATCCG
>dme_circ_0005309_junction_seq
GGATCTGTCCATCAACATTGTCAATCCGGGAGCAACAAAATGCGTCACAACTGGAT
>dme_circ_0005310
GAGGACAATAAGAAAACTGCAAGTGTTGCGAAAATTGATTCGAACATAAATAAAGGGCAGCCAAGCGTGG
CCAGACAAGGACAACAAAGGCAAGGGAAGCCGAAGGATTTGTTATTGGCCGAAAAGGCAAAAATCAATAA
AAACGCCACATGTAAGAGGACACCGGACACAAGCGACATGGTGTCCAATCGGCAGGGCATGGGGCTCACG
ACACCTAGACTCCAGTTGCTGTGGTGGAATAACTAACGAGTAGTCCCAGGGAGTAACATGTCGGATCGAT
ATCCAGCCAGCTTCTACGATCAGCATCATAGTCATCATCATCCGCAACATCTTACGCCGACAATGTCACA
CTATCCGCCGCAACCCTACAGATCGGGTTACACCGGCTACCATACCTATGGCTACGGCTATGGGTCTTCT
TCTAGTGCTTCTTACATGCCTAACTACTATCGCTATGCTCCGTACGCCTCGCCGCACTATAGCCAGCACC
ATCAGGGGATGTTCACGCCGGCTGGCCAGCAGATAATCCCCTCCAGCGTACTCCACTATCCGCCCAGACC
GCATCCCTACCAGCAGCTACCGCAGCAGCAGCAGCCACATCCTCAGCAATCTCAGCCTGGCTACGAGCCA
CCACCACCCTTGCCATACAGTTCTTCCGCTTCTTATCAATCACGTGGTTTCGGTGCCTTTGCGGGCCCAA
CGCCCCACTATGCGCCACCAGGCAGATCCTCCACGCCCGCTCCAAATCGCACCGTGTTGCCACCGAGCTA
TTTGGAACCGCTGCGCAGCTACAGCGCCGagcagcaacacctaagtcagcagcagcatctgagccaacag
cagcTGCAACATCACCCGCTGCCTGGAACCCCAAAGCTCGAGGATCCGGATGTCGAGGTGGAGGCGGTGG
CCGAGTCCCCCGCTCAGCGGTTGACCACTTCGCCGCCCATGATCAGTGCCACCGGAACGGACAGCGGCAT
CAGCAATTGCAGCACGCGGGCGAGGAGCGACAGTAGTCAGAGCACGCCCGTTTACAGTGGAGAATATCCG
GTTAACATGTCTGTGGAGTCCGCCTCCTGTGTGCCCTATCACTGTCCGGCGGATGCGCGGGACTACGAGG
AGGAGGAGCACCAGCCACCGGTGACGGCGGAAGTCAGAAGGGCGGACACTCCGCTCGATATTACCGG
>dme_circ_0005310_junction_seq
GGGCGGACACTCCGCTCGATATTACCGGGAGGACAATAAGAAAACTGCAAGTGTTG
>dme_circ_0005311
GATAGTATATGAATCGGGCATTGTGCTCCAGAACGGCACAGTTGCCTCTGGAAACACTTCTTTGCCGTAG
TTCATCCACATCCCCGTAGATCAGGGCAAACTGCCAGGCGAGAGTTCCCCGTTCCGCCGTAAAGGATGAC
TCTACGGCATGGGCCAAGGAATGTGTCTGCGGTGCGTGGAATATGTCCAGCTTGGACTCCACTATCTTCA
CGTTCGCCATGGCGCACTAGCTCTCTACAGA
>dme_circ_0005311_junction_seq
TCGCCATGGCGCACTAGCTCTCTACAGAGATAGTATATGAATCGGGCATTGTGCTC
>dme_circ_0005312
GTATATGAATCGGGCATTGTGCTCCAGAACGGCACAGTTGCCTCTGGAAACACTTCTTTGCCGTAGTTCA
TCCACATCCCCGTAGATCAGGGCAAACTGCCAGGCGAGAGTTCCCCGTTCCGCCGTAAAGGATGACTCTA
CGGCATGGGCCAAGGAAT
>dme_circ_0005312_junction_seq
GATGACTCTACGGCATGGGCCAAGGAATGTATATGAATCGGGCATTGTGCTCCAGA
>dme_circ_0005313
GTATATGAATCGGGCATTGTGCTCCAGAACGGCACAGTTGCCTCTGGAAACACTTCTTTGCCGTAGTTCA
TCCACATCCCCGTAGATCAGGGCAAACTGCCAGGCGAGAGTTCCCCGTTCCGCCGTAAAGGATGACTCTA
CGGCATGGGCCAAGGAATGTGTCTGCGGTGCGTGGAATATGTCCAGCTTGGACTCCACTATCTTCACGTT
CGCCATGGCGCACTAGCTCTCTACAGAGTATTATATATCCAACTGCAATTAAGCTGAAATTATTCTGATT
ACCAGCCGAAACAGAAGCTCTCAATGCCAAAAATGATTTTCGTACTTACGTGCACGCAGCGCTGGAAACT
TTCACGTCGAGGTCAAAACTCGCGATAAAAACTTTTTCGACTTCGGAATGGTTTTGACTGTGTGTTTTTG

GGTTCTGCACAGAAACCTGCACAAAACCAGAATGAAAACAAAGCTATTAACGGATTCTGAACAGCAAGCG
GCTAGTTGGTAGCTTGTTTATTTTGGCGATTCGCCGATTTCCCACTCCCAATTTACAATGCCGTTTGGAG
GTTACTATTTAATCGACTGCCTCGGCCGGCGGTCTGCAGCGGAATGCGCCATCGGAGCTCGATGTGGATG
TGGGCatgggtatggtggtatgggttatgggttatgggtGAAGCGACGTGCCGGGCGAAAGCCCAGCGAG
AATCTTCGAGCCTCAGTGACAACATTTCGCCCAGAGGCGCCGCCGCGGGGCTCCAAATGTTAATGATTGC
ACTGTAAAAAACACATCGTTTTCCACTTAATACTATACGACTTGAGTTATTATTTTATTTTATATATGAT
TATTAATCTATTGTGTTTATCGTGGTGTTAGAAAATTCATTTTGTCATTTGTTTGCGGAGTAAATTGCCA
AGCACAGATGGATTTGGGTTTTCAAAATGGATCATGTTTTCAGACCTAGAAAGTTAAAGCGGGCGAAAGT
TACTGAACTCGttttgaatcttttttttgaaattttatttttAAATCACAAGTATGCTGTTTTTAAGCTAA
ATTaaaaaaaaaaTGCACTAATCATATATAACCATTTCTAGTTTTTTGAAAGATGTACTTCCTAATTTTT
AAAAGTTACCTGATCAAGTACTGAGAATACCGAAAATGTACTGAAATTGCATATTGGTCAtttttttttt
tAATTTAACTGAACTTTTTGGGAGAAAAATCCACCAGCAGGCATGACAATCCACTGAATCATAACGACGA
TAGTGATGATGATTGGTGGCGATGATGATGACATACATGTGCTAGCAAGTGTGAGTTGATACCGCGTAgt
ttgtttgtttgtttgtttTCAGCGACAAGTTGACTTGGCATTGCCCGAAATCGTCAAAGCCGCTGTAACA
GCCAATTAAGAACTTGTGCAGGCTTTTGATAGCGTTTTATAGATTAATAACGACGGATCGTTCCGATCGA
AGCACCTGTATGTGAATGTGTACCTGTGCGTGGGCCGCTGTCAAAGGTAATCGGCGCTCAAGCGCTAAAT
AGGGATTGTATACCGACTAAAGTGCATGTATAAATATACATATGACTTAAATTATTCGTTAATTAAATGG
CAGGTGAAATAAAATATACCAACACAAACAACGAACAAATAACCATATAAAGTGACAACTTGTATTCTAA
AACAAACAAAACAGGCAGCAGAGCGGCAGCAGCTTTTCGATTTAGTATGAGGTAAAAACTTAAGATTTTT
GTCGATTGTAGACTCGGATGACGAAAAAATATTTTATTTACTAGTTAATATAGGAAAAACTTAGTAGAAA
ATAGAAATGCTAAAAGAATTCAGTGATATCCATTTTTAACTATCAATTGATCATGTATTATAAATAAAAC
GAGAATGTTGCTTCACATTTGCTTTTCAAAACAAACGTCGAAGAAAAAGCATCACTTGCAACTcattta
taaatttttatataaactgacaatttttttggtttttAATTAAAAATATGTTTGTGTCGTAGACTATAAA
AACAAATTTATGATTTATATTGCCCTATGACTATATCATAGAGTATATTCTTTTATGCTACACAAACGG
ATATTCCAATTGGAATGACTAGACGCCCAATTGCGTGGGTGGTTGTCTCGGCTAATGTCTTCTACTTCAT
CCTAGACCGCCACCATTTATTTTCAGCTTTTCGCCAAAAAATAGCATCAAAAACAAACCAATTGCTTATG
CTTTTCCTTAAATATCCTAACAATAGCACTGATTTTTAAAGTAGTTAAATGAATATTTTTGTAAATCATA
ACACTTTGCCTCCATATAGCTTTACACATTTACGCATGTCAAGCCGCAGCTTTGAACGAATTGTTGCATT
GAAATTTAGACTTCGCTTTTCCTCTCGATGGGGCACCTTGTGAGGAAAAGCGATAGCTGCGAACGATGCC
GCCAGTTCACGGCAGTTCAGCTTGTCGCCTCGAACAATAAAGACGACCGATCCGGGAGCCataatatata
tataatataGCTTTATAGCACGGCACTGTTTGGATTTTGGCGTGCCAATTGGCGCTAgttgctgccgttg
ctgttgttgttgttgctttgtttgttgtGCTAATTACGTAAATTGCGTGTTTATTTGTTTGTTAATTTAA
ATACTTTACGTAGTAGTGAATTTGTTAGTTGACGACCCGCCAGGTTCGttttttttCTGGGCAATTAATC
TGCGCGCGAGTAAATCTCCCGGAGAAAACAAGAGAAGCGTT
>dme_circ_0005313_junction_seq
ATCTCCCGGAGAAAACAAGAGAAGCGTTGTATATGAATCGGGCATTGTGCTCCAGA
>dme_circ_0005314
GCCACAGCTGACAAAAGCGAAGAAAATTTGAAAGACCGTGAATCCTTTTGAAACGCCCTCTCCGGATTCC
TCATTAAGTGCAAAAGATATAACATCGCAGAGATTTCCCATAAAAATGCTGATCAGGCGCCCTCGCAGGT
TGCCAACGTCGATTTCCGCCAGCAGGACGATGATGAAGATGATGGATGCCCATCTCACCGATTCGATCCG
AGCAACATGGATGTATACCAAATAGAGCTGGAGGAACAGGCACAAATCCGCTCCAAACTGCTGGTCGAAA
CCTGTGTGAAGCACTCGTCTTCGGAGCAGCAGCAGCTCCAAGTTAAGCAGGAGGACCTCATCAAGGATTT
CACTCGggacgaggaggaacagccaagcgaagaggaggcggaggaagaggacaacgaagaggacgaggaa
gaagaaggcgaagaagaagaggaggacgaggacgaggaagCCCTGCTGCCGGTAGTCAATTTTAATGCAA

ATTCAGACTTTAATTTGCATTTCTTTGACACACCGGAGGACTCGTCCACCCAAGGGGCCTACAGTGAGGC
CAATAGCTTGGAATCCGAGCAGGAAGAGGAGAAGCAAACAcagcagcatcagcagcagaagcagcatcaC
CGGGATTTGGAGGATTGCCTAAGTGCCATTGAAGCTGATCCATTGCAGTTGTTGCATTGCGACGACTTCT
ATAGAACATCAGCCCTAGCAGAGAGTGTTGCAGCCAGTCTAAGCCCAcagcagcagcagcaacggcagca
cacccaccagcaacaacagcaacagcagcagcagcagcaacaccctggacagcagcaacatcagctcaac
tgcacgctgagcaatggtggaggtgctttgtacaccatcagcagtgtgcatcagttcggtccggccagca
accacaacaccagcagcagcTCCCCCTCCTCCAGCGCCGCCCACTCTTCGCCGGACAGCGGCTGCTCGTC
GGcctcctcctccggatcttcgcgatcctgcggatcctcctctgcatcctcctcctcGTCAGCGGTCAGC
AGCACCATCAGCAGCGGCCGCAGCAGCAACAACAGCGTCGTCAACCCCGCAGCAACATCTTCATCTGTTG
CGCATCTGAACAAAGGGTACGTGCAATTTGCAACTGACACTGCGACTCTTCCGCGTTGTCGTCTCCTCTC
CTCTGCTGCGGACCATTGTTGTCGGTCTTTGAAATTGTGTTCAATAAATTTGCATTCATTGCATAATAGC
TGCAACAGCAATTGAAAGTCGTTGGGTCTTCGGTTTCTTCTCCTTTTGAGGAAACTATTAAAGCGCAGCA
TGGAAAATGTGAAAATGAATATAATTCGCTGTTATTGGCCAATGGCTGTTGCTGTTTCAGTTTTCTCTTT
AGTGCGAGGAAATTGCTTTTCATGTTGCACGTCTGTCAATGTGCTTGCATTTGCCGCTGCATTTGCTTTT
CCTTTTGGGTGCAGTGCAATTGAAAGCCATGGAAAAGATGTTTTAATTTTCATAAATCGCCTTGAATTGG
TCTCGGCTCTCTTTTGCCTTGGATGATGGTATTTGAAGGGGCTAAGCCCATTCTACTCCTCCGGGAGTAG
TTGGTGGCTCGAACGAATGGGTAGTTGGAACCGGAAATGCAACAAAACATAGGCTTAATAACTACTACGG
TCAGCTGCTGCTTACACAGAGACTTTCAAAAAAAGAAACCAACATATATATTTTAAACAAttttttttAT
AAATATCTAAATCCAATACATTTTTATTGCTGAATGATTTACCGCCACTAATAGCCAAGAAAATGATTAT
TTAACTTTAAATATAAATTTGTTTATTTTCAATAAATTTTATAATATTTTGAACTACAATTCGACATTAT
AAATACTATTCGGCTTGGCTGAACTTGCAAATTGCCTGGATTTTTTGGCGAAGATTTCATTTCGAGTCCT
GGAATGGCAACATTTAATTGCGTCAATTAGTGCGAATGAAAATTAAATCTGTTAGGAAAGGCCTTGAAAT
CAAGCTGGGAATTAGAGGAAAAGTCAAAATCTCGACTGCTTTTTCCACGGCATTCAAATTGATATTCCTC
TGCCGGGGCAAAAATTGACTGCAATCGCTCGTTGTCCACTCCACTCAATTAGTTGTCTTCAACCAGATAA
ATGCATGAAATGAAAATGGAAATGGGAAAAAGTCGAGGAAATATGAAGTCTTTGTCGTCTATCGTCCATG
CCTCTGACTTGTCTGACAACGCGGAAATgccaagcagttttccaccagccagtcagccagccagccagcc
agccagtcaaccCACAACCCGCACCCCTTTTTCTTCGGCTGGATAAACAATGGCGTTTCAAATTGTCAAC
TTGCAAAAGCCACCAAATGATTTGCATTTGAACGCGGCGCGAAAATGAAAAGCAGCCACTTGAGTTTCCT
TTGCGTTTTCTTTTCTTTTTCATTCCTGCCCGGGCCGACGACATTTTCCAGACGCCGTTGTTGTAGTTGT
AATTGTTGTTGCACTCCAATTGTAAAAATTCAATTTGCAATTGCACAAGCGCGTAACGCGCCATCCACTG
TAAGAAAAAACAAATGATGTAAACATGCATCACCAAAGTTGATCACCAAATAAATATTAACAAATTTAAA
CGGTTTCGGAAGTGATGTGTATTAGATTAATATTTGTTACAAAATTTGTAAAGGACAGTTTCTTGGACCA
AGCACAAGGACATTTTATTACAAGAAATAAATTGAAGTTCTGTCTCCATATTTCCGTTCCTGTCGACAGT
TTTTCGGTGCGTGTATCCACATTAATTTCATTTTGTTTCGCATTTCTGAGGTGTGGCAACGCCAATGTGC
ACAGCGGCAACTAGCAACACCAACACCAACAACATCGCGACAACAACAATAGAAGGCTGTTCAAACTCCG
CGCTTAGTTCATTTGCCTCTTCCTTTTGCATTAGAAAAGCGAAAAATAGCAGCAAGACATGCAGTCAAAT
TGAATTCGAAATGAAATGCCAAGAAGCAAACATTGCTGCCTGGCTCCAgtttttctttgcttgtttttct
tttcttctttttccctaatttgcaatgcaaattttttttCGGCGCTCGCACTGTGAAAATTGTGCGGCTGC
AGCTTTCCTCGTGACTGCCGTTTTTACAGGCAATTTATTGTAGGCGGTGGCGTATACGTGATTTTTTGGC
ATTTCTTCTTATCGCACATCTCCTGGAAGTTGAATGCACTCTTTGGCCATTGGATTTAACTTTGATCACC
AGCCCACTGGAGGATGATTAAGATGGGAGCCTTAAAGCTGTCGGAAAAAAATAATTTAGCCATTAGGTGG
CTTAAATCAATTACTGGTGACACTTGTCGATTGATTGCGCTTGTGATCGTGATTGTGATTGTGGCTGTCC
CGTGTTGCAAGAAGGTGACATTTTGGACGATGGCGAGTGGGTGACATGGCGGCAGTGACAGCAAACTCAT
TTAACAAATTGCACTGCGACATGTTGCACACGCGGCGTATGAGCAATGTGCCATTGCATAAGCGACCAGC

TGGACAACTTTCCAGCGGAGGAAATGTGTAAACCAATGCAATTTTCCCTTgcagagcaacagcagcagcc
actgccgacgacacagctgcaacagcagcagcagcaccagcagcagttgcaacacccgcagcagcagcaa
tcttttggcctagcagacagcagcagcagcaacggcagcagcaacaacaacaacggTGTCTCCTCGAAAT
CATTTGTGCCCTGCAAAGTCTGTGGCGACAAGGCATCGGGATACCACTATGGTGTAACCTCCTGCGAGGG
TTGCAAGGTGAGTTTGTGCAGGTCATGCAGTATATATATTTTCAATGGGTTTTTCCTGGCAGGGATTCT
TTCGTCGCAGTATCCAGAAGCAAATCGAATATCGCTGTTTGCGGGACGGCAAGTGCCTGGTCATCAGACT
GAACCGCAATCGCTGCCAGTACTGCCGCTTCAAGAAATGCCTTTCCGCTGGCATGAGCCGCGATT
>dme_circ_0005314_junction_seq
TGCCTTTCCGCTGGCATGAGCCGCGATTGCCACAGCTGACAAAAGCGAAGAAAATT
>dme_circ_0005315
GGTGGCAGACATAACCATAGATGATAATGCTGATGGTGTTGATGAAGAAAAAGTAGCACGCTAGATATCC
GTTTAACAAGTGCATTTAATGCATTAGCACTACAAGAAAATTTCTAGTAAATTTACAACAAAACATAAAT
GCAAGCTTCTAACGAAATGATTTAAATGTTGTCCGGTTTGAAATCCTTCAAAACCATTATCCGGAAATAC
AGGAAAAGCTTATAAATTTGAAAATTTGAGCTCGTATTTATAAAAG
>dme_circ_0005315_junction_seq
TGAAAATTTGAGCTCGTATTTATAAAAGGGTGGCAGACATAACCATAGATGATAAT
>dme_circ_0005316
ACCACCACCTCCTGTTCTACCCACCCGATTGGGAGTCTACGAAAGCGACTATGAAAGTGATCGGTACAAG
TATAGTGGCAGTGAATCGGATGTTGAGCCCGGTATTCGCAAGCAGCCACAACTGACCACCATGGAGACTT
CCTTTAAGTCCAGTGGCTATACAGCTGATACAGAGGAGCATTCTAGCTACCGCAAATCGGAGTCAAGTTT
TTACGAAACCAAG
>dme_circ_0005316_junction_seq
ATCGGAGTCAAGTTTTTACGAAACCAAGACCACCACCTCCTGTTCTACCCACCCGA
>dme_circ_0005317
GGGATAGATGACACCAGCATTGATACTATTGGCATTATACCTCAGAGCCTCCTTGTGGAAACGTCCGTAG
TCCACGCCGAAGATCACTCCAGAAGCTACATCCATGCAGCATATGGTGGCCACGAAGAAGGCCCAGATGT
AGATGGCAGTCACG
>dme_circ_0005317_junction_seq
GAAGGCCCAGATGTAGATGGCAGTCACGGGGATAGATGACACCAGCATTGATACTA
>dme_circ_0005318
GCAAAGCAGGTCAAGAGAAAGCCAAGGCCAAGGCTGGAGAAAGCGCCAAGTCAAAGGAAGCCGCCGCTGC
TGAGGAACCACAACCATCTGACGAAGGAGACGTAGTTGCTAAGCGTGAGGCTACAGATACGCCCACAACT
GAGTCGGAGGCCAATGCCACGACCGCTGAAGCCGGTGAATCTGGCGAAGGCGAGGTTACTGCCAGTACTG
GAGCCGAGGGCGAGGCGGGTGAAGCTGCAGGTGGCGAGCATGTTGCCATGGAACTTGACGAAGCCATGCA
GGCACAGGTGGAAGGTGGCCAGATCAAGTTCATTGTTAACGAGAACGGCCATCTCCTCCAGCTGGACAAC
CACATCCTGACGGACGCCGAAGGCAACCAAATAATTGTGCAAGATCCCGAGCAAATACAGCAGCTGCTCC
AAAGTGTAGGAGTTCTGCAGTCCGGCGAGGGACTTGAGGGCGAAACCCTCCAGATGATGACCGATGGCAG
TGGCCAGATGGTACTT
>dme_circ_0005318_junction_seq
GACCGATGGCAGTGGCCAGATGGTACTTGCAAAGCAGGTCAAGAGAAAGCCAAGGC
>dme_circ_0005319
CTACACCTACGATGAACTTATTGAGAGGGTCTATATCGGCACCTTCCATTCGGTGAACAAGGAGCACTGC
AAGCATTTAATGGCTCGCAACATCGGCAAGTCGGAGAAGTACCCGGACAAGTACTTCTTCTGCCGCGATA
GGCGTCTCAAG

>dme_circ_0005319_junction_seq
CTTCTTCTGCCGCGATAGGCGTCTCAAGCTACACCTACGATGAACTTATTGAGAGG
>dme_circ_0005320
GCAGCCATCAGGCAGTCGGGACGATAGCCCGTGACCAGGAAGATCGATCGCGAGGAATGCCACGTCCGGC
GACAGCAGCGGCATCAGGCGATCAAAGGTTCCCGCATTATCCTGCCACCCGTGCAGTCCTAAAATTGGTT
GGACGTTCTGCGGACCGTACCACTTGCCGGATAT
>dme_circ_0005320_junction_seq
TCTGCGGACCGTACCACTTGCCGGATATGCAGCCATCAGGCAGTCGGGACGATAGC
>dme_circ_0005321
GCTAATTGCCCTGGATTACCTTAAGGTTTGCGACACCACCCTGCTTTTGACCACCGCAGCCTTTGGCGAT
GACGAGATCTTCGACCGCTGGGGTCAACGGATCTTTAACATGATGTCAGCCCAGGGAATACCCACTCCG
>dme_circ_0005321_junction_seq
GATGTCAGCCCAGGGAATACCCACTCCGGCTAATTGCCCTGGATTACCTTAAGGTT
>dme_circ_0005322
GGCTGCCATCAACGACGAAAAGTACGATCAGCAAATGGACTTCCAGGAGGAGCGTGAGACCCTGAAGAAG
CTGCAGCAAGCACGCACGGATCAGCTTTGGCCCGATGAGATAGACACTCCTTTGGATGTGCCCGCTCGCG
AGAG
>dme_circ_0005322_junction_seq
CTCCTTTGGATGTGCCCGCTCGCGAGAGGGCTGCCATCAACGACGAAAAGTACGAT
>dme_circ_0005323
GTTTCATACTTTTAGAGGTTTAAATGTAACAACAATAATATAAACTCATTTCAGAAGTTATTCGCACTGT
CCATCGATCACTTATTTATCCATCCATTTGCACACACCTCTACTCTTCCATGGCCACTTGCTGCGAGCTC
CTCCTGTTTGCCGACGCATGTTGTCGCTCGTGCTCCGCCGTTCGCACCAGACACTCTTCGTAGG
>dme_circ_0005323_junction_seq
GCCGTTCGCACCAGACACTCTTCGTAGGGTTTCATACTTTTAGAGGTTTAAATGTA
>dme_circ_0005324
GATTTATCACGTCCGTTGTGCGGAACATTCCAATGCCCCGTCCGAAGTCCCGGAAATGCGCCTCCCAGCG
CACCATCTTTTCCTCCTGCTTCTGTATGATCTCTGCGCTGAACTGTGTCTTGAATGTGTTCATCAGAGCG
GTCTGCTTGCTCCACGAAATGTCGTACTTAGCGCGTTCGCGACTCAAAGGACTGCGTTAAATAATATGGT
TAGGATCAATATATACATGTAAGTATAGCAACATAGATTTTTAATCTAACACTTACACATGAACTCGAGC
CAGAAGGTCTGTGATCTTGGAGATCAACACAGCTCTGTCCACGATATGAGCAAACATGAAAGGCACATTC
TCCGATGTTATAATTACAATTTG
>dme_circ_0005324_junction_seq
CATTCTCCGATGTTATAATTACAATTTGGATTTATCACGTCCGTTGTGCGGAACAT
>dme_circ_0005325
TGGACATGATTATAAAGGCCTGTCTGTTGTTCTTGCGGTTAACCCGGATTTCCTCTTCTACAATGCGCCC
ATAACCCGCCGCAACTAGTCTCTGCGCATGCGCTTCCAGGTCGGAGAAGTTACCTTTGAAAATCAAGTCT
TCCGACAACTGGTGATCCATACCCACGTGACGTTGGACAATCTTGTTTATGTACGACCGCATGGCCTCTA
GTTCGGTTTTTTGCGATTGGGTCCCATTGCTACCGGATCCATCGTTTTTCTTGCTATTGCGTTGCCTTCG
TCCTAGTTTCGGTTGCTGCTGTACATGACTTCCATTGTTTGTAGCAGAGACCTCAGAATCGTGGCGATGC
CGCCCCTGGTAACCAACCTGTTGCGGATGTGCTTCTGACCTGG
>dme_circ_0005325_junction_seq
ACCTGTTGCGGATGTGCTTCTGACCTGGTGGACATGATTATAAAGGCCTGTCTGTT
>dme_circ_0005326

ATTGTGGATCCATATCTCGAGACCAAATCCTCGGCACAGGCGGTCCAAGAGACGCTGAACGACTTCAGTA
AGCGGCAGGTCGACGTGACGAGCAGTTTGGAGAATTGGACAACGAGCATTGCGGAGAAGCGAGAAGTCGA
ATACCTACTCGAGAAAGTCATGAGCGACAACGAGGAGGACCGTGGCGAAGAGCACCCAGGTGGACACACA
GTTGTATCCCGTATTCACGTCCCAGAGCGTGGACTCCAAGCAGCTGCTGATCAGCACCCGCGAGAAGCTG
ACCAACGTGATCCAGGATATCGAAAGGGCCCAAGATGAGATACAGCAGCGCATCCAGACCACGCTCGGCA
TCCAGACCAAGGATCAGCCATCGCTGGCCAAGATCGAGCAGGTGATCAACAACCTGCGCATGCTGAAGGC
CAAGTTGGACGGCATCAAGTACGACTATCGCACTTTGGTGGAGAGCGTAATTCAGTTCCTGGAGAACATA
GTGCAGCTGCGGCGCGAAATCGACGACTACTTCGCCAGGCAACAGAAGGAACCCGCATCCGGCGCGGATC
GCAGCATCGCGGAGCACGAGAAATTCCGCGATCAGTGCATGGATAAATTTAGATCGCTAATCACACAATC
CGAACTGCTGATCGATCGGGTGCGCGTACTGGAACCGCCGGGAGCCCGCGAAATCGACACCGACCGCATA
CTGAAGCTGCTCGAGAACCTGCGCCTGCACTTCGAGTCCAACAGCAGTGCACGCATGTCCACCCTGGAGC
GTCTGGAGAAGATCGAGCAGTTCCGCTCCGATCTCGAGGACATCGATCGCAGCCTGGACAGCGTCAGCCA
GCAGCTGCACGAGATCAACAACCAGAGCGTCGACAGTCTGGCGGCGGCCAAGACCACGTCGCTGGCGTTT
GAGTATTTTGAACGGACCATAGAGG
>dme_circ_0005326_junction_seq
TTTGAGTATTTTGAACGGACCATAGAGGATTGTGGATCCATATCTCGAGACCAAAT
>dme_circ_0005327
GGTAAGTTCCCAGGGCTGCCGGCTAATCTTGGTAAATATTTCCAGCTGACCAACCGTCATGAATACTGCC
TCCAATTTCGTGCAACTTTTCCTTGTAACCAAAAATAAAGTATGAAATGCAACATTCGATATGCAAGCAC
AACAAATTGTGTTCAGTTGGCAGAAATATTTCAGCTTTTAGCATGTTGATTTTCGAGAAAACAGCGAACT
GCGAACAGCGAGCCGTACACCAATGCGTATGTGCCATGCATTGCGGCGAGCACGAGGCGCATAACGCATA
CGCCGCGTATTGCACGCCACGCGCAATTAAGCCAAACAAAATGCGATCGCAAAGCGGTAATAAAAATAAA
CATCGCAACAGCGAGAGGTGAAAAACGCGAAATAAGAATTAACACCGAGCATAAAATATTCCCAATTTAA
AGACGGCGTGTGCATGATAAATTTACTTGCAACGAGCTCGATAATTAATAAATATTTAGGCGAGGGGCAG
CAAACACTATGCAGTCAACCACTTGACCATAATCAGTCAATCAGCCGGCAAAACGGGGACGTTGTTGTTT
CACCCATTAATGAAGTTGTTGTGTTTACTTGCAAGCCAGCCCACGCATATAAATATGTGCGAATCACACA
GACATACAGCTATATAACGCGATACGGCGATACATAAATGCAATTGCCGCTGGCAATGGTTTCGATCCAA
GCCAATTAGGCGCATCAATTTGGCTCGCTTTGCTTGTTTATCGAAGTGCAAAGCGACGGACACGAGCCAC
ATGTCGATGTCGATGGCTAATCAGACACTTTGGGGTCATTTCCAATGCAGATTTCGGTGCGGAGTTGCCT
CGGAGTTGTCGCTCATTCTCCCCCAGGGGCGAAAGCATGTCCGCAATTGTTCAATTCCCCATTAAATCCA
TACCCAGTTGGGACTTCAGTATCCCCTCCACTTGTGGGCCACACGGTGCCTTCAGTTATCACTGCCGGAA
CAACTACGCACTTTCAATTCAGATCATCTCGCGATGCGAGTTTTCACTGCAAATACCCATTTCGTTTCAC
CCTTATCCATTCATACTCGTATGTGCATGTGGAGCAGGGTGATTCGATATGATGTTCTGTGGAGAAAAGA
AACAAAAGATACACCTTTGACCAACAAAGCATATAAGATCTTTAGCCCTTACAATAAACTACTTTCCATG
ATAATCGCCATCAAACTAGCTTCTGTTTGTGGCTTTGAATGTATTGTGAATATAAACAATCAAAAACCAA
GTCAATTATGTAAATATAAACTGACACCCTGTTGTATTTGGTGGCTCTATCGAAAAGGGGTCACCTGAAT
GCGTAAGATTGGCCCAGTTGAGTGGTGCACTGCGTACATATGTGGGTGGCACTGGTCGGCCCAGCAGGTC
AACAATGGAGTGCCACTCAATATACAAAAACACGACTATTCGCCGATGAATACAAACAACAGTGGCAACA
AAGACTTCTCAACGGCTAACAATCGGCAAAAATGTACAGGCTGGCTGGAAAGAAAAACAACAAGAACGTT
TTTAGAAATGGCCAGAGAGAACGTGAGGAAGGACCAAGAGGCAAATTGCTGAAATCCTTGCAGACAGAAC
GGCGGCACTGGCCGGAAAGGATACGCTTGTTAGCAGCGGAAGTGGCACCCAGGATGAGAAAGAGACAGCA
CCGCACAATTAGACAGAGACAGGTGGAGAGGCAGTGG
>dme_circ_0005327_junction_seq
TAGACAGAGACAGGTGGAGAGGCAGTGGGGTAAGTTCCCAGGGCTGCCGGCTAATC

>dme_circ_0005328
GCTCCATTCTGGCTTGCTGGAGAGCTGCCTCCAGGTCGACTAATTCTCTGCCCAGCAAAGCCAATGCCAG
ACACTGCTCCATTTGGATTTTCCTCGTCTGCCAGGCGAGTTCCAGGGAGTTTCTGCGGTCATGCAGGGCC
TCCAACCACTGCTGAACTTGGTGAACTGCCCTCGTAGCATCTCGTTTAATGTGCTCCGGTCTTGTGTCGA
GGGTCTCCAGCTTTTCCA
>dme_circ_0005328_junction_seq
CTTGTGTCGAGGGTCTCCAGCTTTTCCAGCTCCATTCTGGCTTGCTGGAGAGCTGC
>dme_circ_0005329
AACGCAAAATGAGCGACGAAGGTGGCTGTAAATCGGAGCAGTTAGAGAAATCCGAAGAAGCGGAGGAAAA
AAAGGGGGACGCAGAAGGACAGGAAGCGAAAGCTCCAATTCTCAACCCCATTTCAGTCCCAGAAGTTGAT
GACAAGCCGACGGAAAATAAACCCCAATCGGATAATCACGCGGACAAAACGGACGAAACTCCATCTCAGG
ACTTTGCCGCCTACGAGGAGCACATGACCTATGGAGCGGACGGCGGGGCTATATACACGGATCCCAGCAC
GAAGCAGAAATACAAGTGGTGCGCCACCGGAAACAATTGGCAACCCTTAGGCGTTGATGAAGATGTTGAT
GGACAGGCTGAAGATCCCTACGAAAACGAGCACTACAAGTGGTGTCCCAAATCCCAACAGTGGTTGCCAA
AAAAACAGGAAACTGAAACGGAGCACTACAAGTGGGATGACGAGCAAAAGAAGTGGGTGCCCAAGCACCC
GAACCCTGGCCAGGAAGGGGTTTGCGGTGTGGATGAGCATGGCGAGCGCACTTACACCGACAAGGATGGC
GTCGAGTTCTTTTGGGATGCAACCAAGAGTGCTTGGTTCCCCAAGATCGATGATGATTTCATGGCTCGCT
ACCAAATGAACTATGGATTTATTGACAACACATCAGCGGGGGAGAAAGAGAAGGCTGAAAAGGAGGCGGC
TGAGGCCAAGAGAAAGGAGGAGGAACTCAAGCGTATGACGGCAGAGGCGGAAGCTGCTATGTCTAGAGAC
AATCCAGCATCCAGCGCTGCAGTGCCCACTGGCAAGCGAAAGGCCCAGGAGCCCCCAAGAATGGTTCGAA
ATGGATCCATTGCAGAACACCAAGGTGTATGTCTCCAATTTGCCCCTGGACATTACTATGGATGAGTTTG
CCGATCTGATGGGCAAGTGTGGCATGGTAATGAGAGATCCGCAGACGCAGAAATTCAAGCTGAAACTATA
TGCCGAAAAGGATGGACAAATCAAGGGCGATGGCTTGTGTGATTACATCAAGG
>dme_circ_0005329_junction_seq
GGCGATGGCTTGTGTGATTACATCAAGGAACGCAAAATGAGCGACGAAGGTGGCTG
>dme_circ_0005330
GTTATTTTATAAGTGAGTAGAGAGGGGTTTAATCTATTTCCGGGGCTGAGAGGGTTGTGAATGTGAGATG
GGGTGCGCTGCTATAGAGACAACTCTCTGCAAAGGTCTTTCATCTGGtttttagtttagctttactatta
ttttagtatttttgtttctgtttttgtttacggtttgtg
>dme_circ_0005330_junction_seq
ttgtttctgtttttgtttacggtttgtgGTTATTTTATAAGTGAGTAGAGAGGGGT
>dme_circ_0005331
CGCATGAGCACCAAAGTAATTACAGGTCGGGAAAAGGTGAACCTGCCGGTGGCCAGTATCAATATGGCCA
AGATGTGTGTGCGCTATGGCGAGGATGTGAGACAAATGAGAGTTTTGCGACAACTCCTTCAAAACGAAGT
TTACAAGGAGG
>dme_circ_0005331_junction_seq
CTCCTTCAAAACGAAGTTTACAAGGAGGCGCATGAGCACCAAAGTAATTACAGGTC
>dme_circ_0005332
GTGTATTCAAAAAGCGAAAGTTATAGACATTCTAAAGGCCACGGCCTCAGAATCCTTTTCGCCGATTGTT
GGAGACCTTCACGGCATCCTCCCACAACAGCCCAGTGAGCTGGTGAAAAGCTATCGCAGACAGATTCCTA
TTCTGACAGGATTCACTCAACACGATGGTTCCTTTGTGCTAGCAA
>dme_circ_0005332_junction_seq
CAACACGATGGTTCCTTTGTGCTAGCAAGTGTATTCAAAAAGCGAAAGTTATAGAC
>dme_circ_0005333

TTAACAGATCACCGCCGCATGCTGATGACCGACTACCAGCTGTCACCGGAGCTACTCAACGACTGTGCGG
ATGACATACCCAAGTTCTGTCCGGACGAGCACAAGGCGCAGCTGGTGAATGGCATGACAAGTACAGGTGG
CGAGATCATCCACTGCCTGTTAGAGCACGTTAAGGCTAGGCGTCCGCAAAGACGGGTGACGGCACAATGT
CAGCGAGGACTGGAGACACTGATTAAGGCCAGCGATGCTGGAGAGGATTGGCGAGTCGATCCGGTGCTTA
GACGCGCATGCAAACCAGTGGTGGATGTGGTCTGCAGGGATGTGCAGGGCGGTGAAGCACGAGTCATGGG
TTGTTTGATGGAGCACATTGGCACTCCGGTTATGCTGCCCGATTGCGAGCAGGCCCTTCTCATTATTGAG
TATTTCGTTGCCAGAGATTTCAAGTTGGATCCCCAGCTGTACAAACACTGCCGCGATGACGCTGTGAAG
>dme_circ_0005333_junction_seq
CAAACACTGCCGCGATGACGCTGTGAAGTTAACAGATCACCGCCGCATGCTGATGA
>dme_circ_0005334
TTAACAGATCACCGCCGCATGCTGATGACCGACTACCAGCTGTCACCGGAGCTACTCAACGACTGTGCGG
ATGACATACCCAAGTTCTGTCCGGACGAGCACAAGGCGCAGCTGGTGAATGGCATGACAAGTACAGGTGG
CGAGATCATCCACTGCCTGTTAGAGCACGTTAAGGCTAGGCGTCCGCAAAGACGGGTGACGGCACAATGT
CAGCGAGGACTGGAGACACTGATTAAGGCCAGCGATGCTGGAGAGGATTGGCGAGTCGATCCGGTGCTTA
GACGCGCATGCAAACCAGTGGTGGATGTGGTCTGCAGGGATGTGCAGGGCGGTGAAGCACGAGTCATGGG
TTGTTTGATGGAGCACATTGGCACTCCGGTTATGCTGCCCGATTGCGAGCAGGCCCTTCTCATTATTGAG
>dme_circ_0005334_junction_seq
TTGCGAGCAGGCCCTTCTCATTATTGAGTTAACAGATCACCGCCGCATGCTGATGA
>dme_circ_0005335
GGGCCTGCTCGCAATCGGGCAGCATAACCGGAGTGCCAATGTGCTCCATCAAACAACCCATGACTCGTGC
TTCACCGCCCTGCACATCCCTGCAGACCACATCCACCACTGGTTTGCATGCGCGTCTAAGCACCGGATCG
ACTCGCCAATCCTCTCCAGCATCGCTGGCCTTAATCAGTGTCTCCAGTCCTCGCTGACATTGTGCCGTCA
CCCGTCTTTGCGGACGCCTAGCCTTAACGTGCTCTAACAGGCAGTGGATGATCTCGCCACCTGTACTTGT
CATGCCATTCACCAGCTGCGCCTTGTGCTCGTCCGGACAGAACTTGGGTATGTCATCCGCACAGTCGTTG
AGTAGCTCCGGTGACAGCTGGTAGTCG
>dme_circ_0005335_junction_seq
GAGTAGCTCCGGTGACAGCTGGTAGTCGGGGCCTGCTCGCAATCGGGCAGCATAAC
>dme_circ_0005336
TTAACAGATCACCGCCGCATGCTGATGACCGACTACCAGCTGTCACCGGAGCTACTCAACGACTGTGCGG
ATGACATACCCAAGTTCTGTCCGGACGAGCACAAGGCGCAGCTGGTGAATGGCATGACAAGTACAGGTGG
CGAGATCATCCACTGCCTGTTAGAGCACGTTAAGGCTAGGCGTCCGCAAAGACGGGTGACGGCACAATGT
CAGCGAGGACTGGAGACACTGATTAAGGCCAGCGATGCTGGAGAGGATTGGCGAGTCGATCCGGTGCTTA
GACGCGCATGCAAACCAGTGGTGGATGTGGTCTGCAGGGATGTGCAGGGCGGTGAAGCACGAGTCATGGG
TTG
>dme_circ_0005336_junction_seq
AGGGCGGTGAAGCACGAGTCATGGGTTGTTAACAGATCACCGCCGCATGCTGATGA
>dme_circ_0005337
AACCACCCCTCCATGTCTTCCCAGTGCTCCGCACGAATCACTTTGAGGGACCAGCAAATAGGCCGCGACT
ACCGCGTGTCCCACGGTCTGGCTAAGGCCTGTAAGGACGACATTAAGTTGTACCACTGCCGGCGCGGCGT
CTCTGAAGACAAGCACGTGCGGTTGGCTCAGATCCTGCTATGCCTGGAATCAGTGTCCAAAAACGGGACT
AAACTGGCGCCCCCTTGTCTCACGGAGTTAACAGATCACCGCCGCATGCTGATGACCGACTACCAGCTGT
CACCGGAGCTACTCAACGACTGTGCGGATGACATACCCAAGTTCTGTCCGGACGAGCACAAGGCGCAGCT
GGTGAATGGCATGACAAGTACAGGTGGCGAGATCATCCACTGCCTGTTAGAGCACGTTAAGGCTAGGCGT
CCGCAAAGACGGGTGACGGCACAATG

>dme_circ_0005337_junction_seq
GTCCGCAAAGACGGGTGACGGCACAATGAACCACCCCTCCATGTCTTCCCAGTGCT
>dme_circ_0005338
TTAACAGATCACCGCCGCATGCTGATGACCGACTACCAGCTGTCACCGGAGCTACTCAACGACTGTGCGG
ATGACATACCCAAGTTCTGTCCGGACGAGCACAAGGCGCAGCTGGTGAATGGCATGACAAGTACAGGTGG
CGAGATCATCCACTGCCTG
>dme_circ_0005338_junction_seq
TACAGGTGGCGAGATCATCCACTGCCTGTTAACAGATCACCGCCGCATGCTGATGA
>dme_circ_0005339
GCTCCACAGGTGCACTGCGTTCAGGCAGCTCTCCAGCTTGGGCCGCTCGGCGGGACAATAAGCGGGCAGG
AAAGTGCTCTCTAACCAAGCGGGCAACTGCAACTGGCGCTGCTGCATCCACAGAGCGTTCTGGCAACCCT
CCGGGAGATCCATCAACTGGCTAGAGCTGAATGTCAGGGCGCACTCCAGCATGGACAGATTGTCGGTGGA
CTTGTGGTGCGCGCAGAGTTTCTTTAGATCCTGGCACTCGGCCAAGTCGATAATGCTGTGCAT
>dme_circ_0005339_junction_seq
ACTCGGCCAAGTCGATAATGCTGTGCATGCTCCACAGGTGCACTGCGTTCAGGCAG
>dme_circ_0005340
TACTTGGATCACATATTAAGCAGCCAGAAAAGCCGTACAAGAGATGACCAGGACGTGAGACCGGCGAACC
GGCCCACCATTTTCCACGTACTTGTTGTTGTTGCTCGGCACTGCACATTGAGACCCGACGGAATATACAT
ATGTTTTCTGG
>dme_circ_0005340_junction_seq
CCCGACGGAATATACATATGTTTTCTGGTACTTGGATCACATATTAAGCAGCCAGA
>dme_circ_0005341
ACAGGCACATCAGGTAGTGGACCAGACTGAGCAGCACCAAAAGGGTGGACAGAGTGGCCAATATAAACAT
CACCTCGGCGTTCTCAACACCGTCTTTGCAGAATGCCTTTATCTCGTACTTTTCGCAGACCTTCATGTCC
TCCAGTTTCGTGTTTGGCGGGAACATAAAATCTGTGGGATCGAAGTAGTATTAGATGGCTTATCTTTATG
TATATTCAAGGGCTTACGGAATTGCGTCAGATCAATGCAGCTTTGAGAGTGCTCAACACTGGTGCACATG
TTCCAGAACATGGTGTAGATGAAGGTAACCACTACCAGGAAACAAAGGATCAGACTCCACACAAAGTTTA
GCAGG
>dme_circ_0005341_junction_seq
GATCAGACTCCACACAAAGTTTAGCAGGACAGGCACATCAGGTAGTGGACCAGACT
>dme_circ_0005342
GGTCGCCCAGAACAAGTTCGCGGTACATGGAGTCATCACCGCCATCGCCAGTCGGAGCTTCCGGTTCGCA
TCACTATCATCATCGCCGTTCGCCAAGGATGCGCCAGAGGACCAGAGGCGACAGTCGTCGCCG
>dme_circ_0005342_junction_seq
AGAGGACCAGAGGCGACAGTCGTCGCCGGGTCGCCCAGAACAAGTTCGCGGTACAT
>dme_circ_0005343
GtttttttatttttGAATAAACTGGCAATCTGAAGACAACGTGCATGATCGAGAATAAAAACTTCGCGTA
ACGAAATTGTAAACTGTTAACACAGTACGTATTCGCAACAGCAAAGGCGATTTGTAAGTGACCATGTTTC
AAAGCTACGAAATAAGCATATATAACGAGCAGTTAAAGATTAGCATTTTAATTATAAAGAACTTGTTAAA
TAATAATAGACTACAGATAGTGTATTGACTAGTTTAGTTTATTGTTAAGTATGTAAGAAAGGTGTAAATT
ATAAATGAATTTATCAAGTAAATATGTACTATGTATTTACCCAAGCACAGGCATTTGATAA
>dme_circ_0005343_junction_seq
TATTTACCCAAGCACAGGCATTTGATAAGtttttttatttttGAATAAACTGGCAA
>dme_circ_0005344

TCATATTCCAGGAGGACGAAGACGAGCTAGAAGAGCAATCGTCGCTCTCAGTCGTTGCGCTGCTGCCGTT
GGGCTTCGGTCCCCAGATTCTCACGGTGGCGTCATCGCTTGCGCTGGCCAGCAGGGACGGGTACACGGGA
TTCCAGGACACGCAGTTCACCGTCTTAGTGTGCCCGGCCAGCTTGGCCAGCGGCTCCTCGCGCTTGATGT
GCCAAATGTAGACAACCTTGTCCTCACTGCCGCTGGCAACGAAGCTCTCGTTAACGCCGCCGAAGCAGGA
GTGGATGGCGAAGTTGCTCTGACGTATTCCCTGGAAACGGCGCACAAGGCACTTGTCCTCGATGTCCCAA
AGGTGAAGGCCTTGGTTCGAGACGTTCAGCAAGGCCAAGCGGTCGGCGGAGTTGATACTAAAGGTCATTA
TGGGATGTGGCTCCCTCAGAATATCAAAG
>dme_circ_0005344_junction_seq
GGGATGTGGCTCCCTCAGAATATCAAAGTCATATTCCAGGAGGACGAAGACGAGCT
>dme_circ_0005345
GCTATTTTGCAAATCTTATTGGACAAACTCAAAATTTCACAAAAATGTAGTTTACTACACTTACATAGCT
AACATTACGCCTTCTCATTATGCGATTAGGTTACATCCAGCGAATTCCTACGAGGGTCCTCAATGTGGTA
CATGACATCGGAAGAAAGGAAGATTAGAACAAAGGCATTGCGTTGCAGCTTTTGCGATAGCAT
>dme_circ_0005345_junction_seq
CATTGCGTTGCAGCTTTTGCGATAGCATGCTATTTTGCAAATCTTATTGGACAAAC
>dme_circ_0005346
GATATATTTTGTTTGTCAACTAGAATCACATACAAATGGGTTCGAATCGCTGAACCCGATTCTATGCAAT
TATTGAGATTTTTAAGTTATTGTTACATTAATTGTAGATTTTGGATTTTAAACATTTTGGGTTCATATGC
TAGTTCCCTTTGTATATTTAATTGTTGCTTTTCCCATGGACAACACATTACATTTGTATCAGTAAAAATA
AAACATTTTAAAGAG
>dme_circ_0005346_junction_seq
ATCAGTAAAAATAAAACATTTTAAAGAGGATATATTTTGTTTGTCAACTAGAATCA
>dme_circ_0005347
GGTTAGACGGCTGTTGCACGTGGGTGCCATTTTGATTGACCACTTGCGGCACTACAATGCACTGAGTCCC
TGCTCCACTTGTAGTAGTTGATGTTCCACCTGGCAACTGGTGTATCTGCTGGGGCTGTTG
>dme_circ_0005347_junction_seq
GCAACTGGTGTATCTGCTGGGGCTGTTGGGTTAGACGGCTGTTGCACGTGGGTGCC
>dme_circ_0005348
GGTTGCTGCTGTGGTGGTTGTTGCTGAACCACGtgtgtttgctgtgtctggggctgtggttgctgattct
ggggctgtggttgctgttgctggggctgttgcaccaaaggttgctgatgttggaccgaaggctgctgatg
ctgaaccagcg
>dme_circ_0005348_junction_seq
accgaaggctgctgatgctgaaccagcgGGTTGCTGCTGTGGTGGTTGTTGCTGAA
>dme_circ_0005349
ggcggctgctgttgtaccaaaggcagttgttgctgttgcaccaagggctgttgctgctgttggctcatgg
cctgttgttgctgGGCTGGCTGGCTATGCAGCAATTGCTGCAATTGTTGTTCCAAAGGCATTGATAGCTG
AATGGGTTGCTGTGTGGGCTGTTGTATAAATGGCGCTGCCTGTTGTGGTACGGCCGAGAATCCAGGATGC
TGTTGCTGTTGGGCATCACTGAGCGTTACTTGTCCGCCCTGCTGATTGTAAGTGTGTTGTATTGGAGGAG
CCACGGACGTCGGTGGAACTTGCATCTGCACTGGGATCTGAATAGGTTGCTGATGTTGATGCGGTGCCAT
GACTTGCGATCCCGTAACGAgttgctgat
>dme_circ_0005349_junction_seq
ACTTGCGATCCCGTAACGAgttgctgatggcggctgctgttgtaccaaaggcagtt
>dme_circ_0005350
GATCCCAACGTGCGCGACATTATCGAACGGTGCATTGAACTTAAAAAGGAGGATCGCCCCAGCTGCAATG

AACTGCTAGAATCGGAGTTCTTTGACGAAGATATCGGTATACGAGTGGAGCCAACTGCTTCGGAGCAGTT
CCTTTCTGATCCGAGCATCAGCATCATCGAGTTCCGACTGCGGTTTATGGATCCCAAGAAGCGCTCGTCC
CGTCATAAGGAAAACGAGGCAATTCAGTTTGAGTACAACATCAGGCATGACGAG
>dme_circ_0005350_junction_seq
GTTTGAGTACAACATCAGGCATGACGAGGATCCCAACGTGCGCGACATTATCGAAC
>dme_circ_0005351
ATAACAATTATTTTTGGTGGCATATTTATAACCGGGGTCGTTGGTAATCTCTTAGTGTGCATTGTGATTA
TTCGCCACTCGGCAATGCACACGGCGACCAATTACTATCTGTTCAGCCTAGCTGTTTCAGATCTGCTGTA
CCTACTATTTGGATGCACCTATGTCTCCGTCTTTACCATCGTCGCATTTTCCATGGAGAGATTCCTGGCC
ATTTGCCATCCATTGCACCTGTATGCTATGGTTGGCTTCAAGCGCGCGATTCGGATAATAACAGCTCTTT
GGATTGTTAGTTTTATAAGCGCCATTCCATTTGGACTGCTCTCGGATATTCAATACCTCAACTACCCATT
AGG
>dme_circ_0005351_junction_seq
ATATTCAATACCTCAACTACCCATTAGGATAACAATTATTTTTGGTGGCATATTTA
>dme_circ_0005352
AGTAACTACGTTCATCATATGCTGAAATATTTCAATGACCACAGCTCGCATGTATATGCCCTGCTTGTTG
TGAACATATCGTTTGAAGTACAAGTTACCAGAGTCCTCTCCGGCCTTTTTCGCATTG
>dme_circ_0005352_junction_seq
AGAGTCCTCTCCGGCCTTTTTCGCATTGAGTAACTACGTTCATCATATGCTGAAAT
>dme_circ_0005353
GACACACACGGCGTACCCAATTACATTCTTATTGCCGAGCGCAACTTAAAGCAGTCACCTAGGAAGTTGC
AGCTGCTTTCCGTGGTCCCCATATGTCTGATCATTTGGGACCAAAAACTAACTGGATTTTTAAAGATGGA
AGAACAAATTTAGGAAAAAAAGGAGCAGACGAGCAGATGTAATTCTTCGCATAGGAACAACCCAAAGCAA
CTGCACCGTATTTTTTCAATAAGCCAAAAAAGTTTATAACTACACACAGAGGGAACATCAATTTTGTTTA
AGAGATTCCTTCTGTTTCAGTGTCGTTAAATGaaaaatcaaaaaaaatcgaaaaaTACTTGTTAGTCCAAA
CTCACTTTTTTGAACCACATTGTGGGTCCGAGCTTTATGCTTGACAAAAATTGACTCTGTGATGCTTCCT
TTTTCGGTTTGCATATCATAATAAGCTT
>dme_circ_0005353_junction_seq
TTTTCGGTTTGCATATCATAATAAGCTTGACACACACGGCGTACCCAATTACATTC
>dme_circ_0005354
AGAGAGCACCATTACCCCTTAGGATATCGCAAAGTATTATAAAATCTCGTTCACAGGACAATAATTGTGA
ACAATTTAAGTGGCTTGCACCGACGAAGCAAAATAACGCCGAGGAAAGGAAATCTTCTATAATAGACG
>dme_circ_0005354_junction_seq
GAGGAAAGGAAATCTTCTATAATAGACGAGAGAGCACCATTACCCCTTAGGATATC
>dme_circ_0005355
GTAAACGCTGCACGTGGGCTGACCATCCTCAAGCAGAAAATCCACAGTGGCCAGCGACTCTTCGTACGGG
TGCTGCAGCTCAACCGTGATTTGATTAAATGAAACGGCGGTTACCTCCAGTATCACAGTGCTGTCCATAT
CTGGGACGAATGAGCATaaaaaaaaTATTATTATCATAAAACAGGAATGTGGGGGGAAGTGGAGCACCCA
ACTCACCATTATACTGCTTCTGCTGCTCACGAACACAGCTTGCGAGCAGGGTGTAGAAGAGAGCTTGTTT
CTTGAGGAACGCCACGATTCGCGGAACCTGGGCGGGTTCAGTGAAGGGCAGTGATGACACCAGAGTTCCC
TGCAGTTTGCGGTTGTCGGTAAAGAAATAGCAGTGCGTCTCTTTAGGTAAATTCACATACAGGCCGCGCT
GTGTGTTCTTAATGGCCTGCTTCGATGCAGTTTGTACTATGAGATTAAGCACTGTGGTGGGCGGACCAGG
AGGAGTGGCAACTGA
>dme_circ_0005355_junction_seq

TGGGCGGACCAGGAGGAGTGGCAACTGAGTAAACGCTGCACGTGGGCTGACCATCC
>dme_circ_0005356
GTAAGCCTCATAGGATGGCCACCGCGACGGGGCAGCACAAGACCCACCGATGAACTTGTCATAAGTGAGT
AGCTGTCTCCGGAGTGACCTTGCAGGTGCAGTTGGTATAGGCTCTGGATATCCGTCTCCATGGCCTGAAG
CGCTACAAACGCCTTCTTTTCACCTTGGGCTCGGCATTCAGCTGGTAAATGGAGGACAGACCCTCGAGT
TGAACCGTAAAGTCTGCAAAGTCGCCAGACTTGAGGCAGGCCACTAGCTCGCTGGACTGTTGCTCGATCT
TGCACTCGTGGTGCACCTTGACGTCCGACAGACTGCCGGCGGCGTCCAGCAGGATCTCTACGTAGAACAT
GTCCGTTGATATGAAGAGAAGCTGCTGGTCCTCCATGAACTTGAGGCCCAACTGGCGCGAGAGGCTCTCC
AAACGCTCCACAAGGCCGTGGCGTGACGTGACCTTGATGTAGTGCTGCAACTTGTCCAGCGCCGACTGCA
GCGTGGCCTTGCACAGGGGGTCCAGAGGGTAGTGCTTCTCGATAAA
>dme_circ_0005356_junction_seq
GGTCCAGAGGGTAGTGCTTCTCGATAAAGTAAGCCTCATAGGATGGCCACCGCGAC
>dme_circ_0005357
GGTTTGCCCAAGGACGCTATAATGCTCGATTGCAACTGGTCCAGCCACACCTTGCGCATCTCCTGCGAAC
GGGTGCTTACCAGAAAGGATTTTTCCGGCGTGGTCAAGCGCAGGCTGTCGCCTTCGGTCCACACCCACAG
GGTAGTTAAGGGATACGAGTACAGCGAGTTGCCGCTTACCTGGCAGAGGAA
>dme_circ_0005357_junction_seq
GCGAGTTGCCGCTTACCTGGCAGAGGAAGGTTTGCCCAAGGACGCTATAATGCTCG
>dme_circ_0005358
GATTATGCAGCGCAGTGTGATGAGCTGAGAAAAGCGTTGGCCGAAGCAGAGTCCCGTATCGATGTCCAAA
TCAAAAAGACAGAGGGCTGGCAAGTGCAGTTGAAACACAACCAAGCGACCCAAACGGACCTCGAAGATCC
GATTTTAGTTCAACGCATTGAGACTTTGGAACAACAACTAGCGGAT
>dme_circ_0005358_junction_seq
TGAGACTTTGGAACAACAACTAGCGGATGATTATGCAGCGCAGTGTGATGAGCTGA
>dme_circ_0005359
GGTTCCCAGATTGTCCAGCCATCCGTGAATCGCCAGGATCGGTCGCTCCGTTCGATTACCATACCAACGA
CCGGAGATGTGACCCCAAGGTGCCGGTATTCTAACCTCCTTGAACTACAAAATAGCAATACTGGTTACTA
TGTTCATTAAAATCAGTATGAATTAAAGttttttttttACTATACACTTTCAGTTTCTGCACTGTAAAC
AAAATTTGCCGGTACACGGATTAAGGTTGCTTATCTTCGTATTTCGTCTATAATATTATTTCTGGCATAC
TCACATCGCTTAGAGACATAGTTCCCATATCGATTAGTGGTTTGCTGACCTTTGCACGTCTGTAAATTGG
TCCGAAATCCAGAAATGAAGGCACCGCGTTATCTTAAGTATCTAAACACCTGCAAATAACTCGGCGATTC
GGCGATTCGCACCTCTTTCGGCTACAACAGCTGATCAACGACTAATTTCTAAAATTGTTGTTTCACTTTT
GAAAGCGAACGAAAGAGAAAAAACGCCGATAGAAAGAGTAGCTGTTTTTGGCGCGGAAACAAATCACCAA
CTATAAGAGAGCGAAGAATTTGTTTTTCAAATAGCTGATTAAAAGTATTACCAATTTTTAGCATTGTTTA
CATTCGCGTTTGTCTATTTAAATGTTGATATATTTTTTGTGTATTAATACGAGCTCGTTGAATACCTATT
CTTTCGAAATTCGTAAAAATATTTTAAAATTGTAGCTGGCACTATGATAATAGCCAAAGTTCACTGAAAA
AGCATAGCGAGGGTGGTATGGTCCCATTGTTCTTATCTCCTATTGTTATGATTTTATTTATTTGCTTACT
CATTCGCCTAATGACACAGATCTCAAAAGACGATAGCCCTTTGCACTTTGATTCTTAGACTGGGTCCAGT
CCACTTCCAAAATTATCTTAAGTATCAAAAACATATTGTTTAATATGATAGTTTAAATTTAATACTTGTC
GTGTGTTCACGTTCTCAAACTTTAAAACCTTTACAACTAAAGTTACAGTGTATTATTATCACAACTTGCT
GCGCTTTTTGTTGGTCCATGTGAAAAATCGCTTTGGTTTTCTCGATCTATCTTCCTTTCCGGCCACAGTC
CAGGAGGTCATAGCAGGAGGTCTGTGATGTTGGATGAAGGGCACAATGTAACCGGCGCATTCCTCGGCGG
CATGGAGATGCAGATGATGGGTGCCGTTTTCCACCTCGTAGAACTCAAAGTGCGGATTATCCTTGGCCAA
AATCGATATAGCCTCGTTGTTGCGGACGGAGAGATATGGTGACAAGGAGCCCTTGATGATTAGATATGGC

TTTTTAATGATCCGCCTCGCCATTTCTGCCCCCAGACTATCGTCGATATCTATGTAGTGGTAGTACTTTA
CTCGAATATCCCTAGTGAAATAGAACCGTTCGGGATACAGTTTCGACTTGGCCAACTGGCGGTGGAGGAG
GTGCTTAGCCAGTTCGGGGGATACGGAATTAAAGCTTCCCGCAGCCAGCACCTTTCCCAATTGGTTGTGC
GTGTAGGAGGGCGGCTCAATGTAATTTCCCAGCTTCTGTCGCTCAACATTCACTAGTTGTTTCTCTATAC
TAAGGTCCATGTAGTCTATGTCGTTTCTCAAGGGGAGCAGTATGTCCAGGGAGACGATCATATCGACGGT
GTGAGGAGCAAGAGAGGCATAAATGAAGCTCAGGACTCCGCCCAGTGAGTGTCCAATGAGAGAGACCTTT
GACCATCCGTACTCCTTCATGACCAGTGGAATAGTGAACACGTACTCGTACACACTGTAGTACATTCCCG
GTGGCAAATGCGAGGATCTTCCATGTCCTGGCAGATCGATGCAGAGTACTCCTAGATAGTCAGGAAGCAG
GGGAATGAGCCGATCGAAGGTTCCCAGGTTGTCCAGCCATCCGTGAATCGCCAAGATGGGTCGCTCGTTC
CGATTTCCGTACCAACGTCCAGAAATGTGACCCCAGGGTACGGGAATCTTCACAGCTTCGAACTGAAAGA
TGCATAAACTGTATTAGTCACTTTTAGCAGATGGTCACACAACAGATGGTTGACTTACACTTAAGATTAC
AAATTAATAATTGGTGGGTAAAGGTTGTCTATTAATCTATTAATGTACTTACATCGCTCAGTGACAAAGT
TCCCATTGCTCTTAATTCTCGATTTAAGTAGTACTGGTTTTTGCTTATCTTAATTTGCTCTAAATCATTT
GTATTCCGTTTGATTGTCTACTCGTCTTTCTGCTTTAACATGTTGGCGCAAACTAACATATTGTATTAAG
TTGGGTTTGGTATTACTCAAACTGCAAACAAAAAGAGATCTTGCTATTCTCTTAATTAACTCTTAATTAA
TAATTAAGAGAGCGAGATAAGATTGGGTTCTAAGTTGTATTCTCTCACGAGCTCTTATTACAACTTCCAA
TTACAGTGTATAAAATGCATAGAATGATCTTCGTGTTCTTAATCACATCAAGTTGTTGTTAACTATTGTT
TATGTACATTAGTTTATATTTCATTTATTAAAATACAAATATCTAGAAATTCGCGAGCGCTTAAGTTGAC
ATAACAAAAACATGAATAAGCAAGCTTTCGATGAGAGAAAAAGCTTTCGAGTGTTTCGGGGCGAATGTCA
ATGTGAGATCTGTTAAATTTAAATATTACTTTGTATCCTAAAAGAAGATCCAAATATTTGGATTTAGTTT
ATAATCCGAATTTCAAGTAGCAAATGAAATTGATACACTGGTCTGTATCAAATTTAAGTCAAAAGCATGT
AATCAAAGTCATCTGCTGAAATTATGAACACCACCGCATAAACCAAAAAGCCTACAAAAGCACGTTAAGT
GCTTGTAGATTGCTAACTTAAAAATACCGTCTATTTATAAACAAAGTAGCTCCGCCTTTATTACCTTTTG
AAATGAAAAACGCGTAACAACAACAAAATCATTCATAAATACTGCCAAGTACTAATGACTCTAATTTTAA
CACTGATTATTGCACATTCTTAGATTAAACTCGCGATCGCAAGATCGGAATTTATGCTCTTGCTATTAAA
ACTAAATTAATTGCGATTGGATTTGACTATAACTTTCCTTGGAACAGAGTTGCTCCCATCGGTGGTCGAT
GGTACTGAATAAAGGGACACATGTACTTCGCACACTCCTCGGGGAATTCCAGGTGAACGTGATGGGTACC
GTTTTCCACTTCGTAGAACTCAAAGTGTGGATTATCCTGGGCCAAAATGGAAACGGCTTCGTCACTGTGA
GCGCCCAGAAAGGGCGACATAGATCCCTTGATAATGAGAAAGGGCTTCTTCCCAACGCCCCCGGCCATAT
CGGCAGCCAGTTCCGCACCAATGTCCCAGTAGTTGTAGAACTTGATCCGCCTATCTATGGATAAGTAAAA
CGATTCCGGATTTAGTCCTGACTCCTTCCTTGGGCCAACTCGGGTGTCACCGAATTCAGCGTGCCATTGG
CGACAGCCATTCCCAATTTCGGCAGCTTGTAACTGGGTGGCTCCCGCAGAGATCCGCTCGCCAGGATCTC
ATCCTGCGCCATGTGCTGGTCCAGGTTTCTGCGGAAGCTTTCGAGAGCAATGTCACTGTCGAACTTCGGC
ATCAGCACACAGTCTATGCAAATGACCATGTCCACTGTATCGGGTGCCATTGTCGCGTAAATAAAACTGT
AGAAGGCGCCCAATGAGTGTCCCATGAGCGAAACCTTCTCCCAACCAAAGTGCTTCATAATCCGCGGAAT
AATGAACACGTCGTTGTACACATCATATCGAATTCCCAGTGGCAGAGGCGAGGATCTTCCATGTCCTGGT
AAATCGATGCAGAGCACTCCCACGTGACCGGGTAGCAGTGGAACCAGGCGATCGAAGGTGCCCAGGTTGT
CCAGCCATCCGTGTAGTGCCAGGATGGGGCGTTCCGTTCGATTTCCATACCATCTTCCCGAAATGTAGCC
CCAAGGAGCAGGAATTTTCAGTTCCTTGCACTGTAGGCATAATGAAATTTCTATGAAATTCTATACGGCA
TCTGTTCCTTACTTACTTCGGTAACTGGAAGTTGCTGCAACATGGTTTTTAGTTTATGCAGCAAACGGCG
TTTCAAACGTCTTTTTGCTTTGACGATCAGTCAACGACTTCTTTAAATTTTTATGTTGCTGCTGCTTTTT
GGCGCTAATGTTTACAATATCAGTAATCGAGATACGCTAAAAAAAGCTTCCCACATAAAAGCTTTTTTGA
TTACAAAACGCACAATATAATCTTAAAGCAGGGGAACCTAATTGTACTCTGCATCTTTCCTCAATTTCTA
GTTTATTTTACTATTTCCAAATATGTGTTTCCTAAAACTTAAATAGCACATTAAAGTTTGCTGCTTCTTG

GAGGTCTGTGATTTCGGATGAAGGGAACGATATAGCGGGCACACTCCTCGGCGGCATGCAGGTGGACATG
ATGCTTGCCGCCCTCCACTTCGTAGAACTCAAAGTTGGGATTATCATGTGACAGAATGGACATTGTTTCG
TTGCATCGAGGTCCCACAAATGGTGAAAGGGATCCCTTAATTACCAGATAGGGTTTCTTCTCAATACGCC
TCGCCATTTCAAGAGCAAGTCCGTTTTCAATGTCGAATATGTGATAGAACTTTACACGTCCATCTCTGGA
GAAGAATACCTTTTCCGGGTACATGTTTGATTTGGCCACCTGGCGGTGGAGCATGTGATCGGCCAGGTGT
TGCGGCACCGAGTTGTTGCTGTTCCTGGCGAGCGTCTCACGCAGCTTACTCAAGGTGAACGATGGCGGCT
CATGTTCGGTGCCATCCGCCTGTCGCCTCTCCTCCAGCAAATAACCTTCTATGTCCTTGGTCAACTTGCT
GGGATCCTCGATTCTCCGCGGCAGCAGTACATCCAATGATATAATCATATCGACTGTGCTTGGTGCCATG
GCCGCGTACATGAAGCTCATCACACCTCCGAGGGAATGACCCATTAAGGAGACCTTGGACCAGCCGAACT
CCTTCATCACCCTCGGTATGATGAACACATAGTCGTAGACGTTGTAGGGCACACCCGGCGGAAGCCTAGA
AGATCTACCATGTCCGGGTAGGTCGATGCACAGCACGCCTATGTAGTCGGGAAGCAGGGGAATCAGCCGA
TCGAAG
>dme_circ_0005359_junction_seq
GGGAAGCAGGGGAATCAGCCGATCGAAGGGTTCCCAGATTGTCCAGCCATCCGTGA
>dme_circ_0005360
GGTTCCCAGGTTGTCCAGCCATCCGTGAATCGCCAAGATGGGTCGCTCGTTCCGATTTCCGTACCAACGT
CCAGAAATGTGACCCCAGGGTACGGGAATCTTCACAGCTTCGAACTGAAAGATGCATAAACTGTATTAGT
CACTTTTAGCAGATGGTCACACAACAGATGGTTGACTTACACTTAAGATTACAAATTAATAATTGGTGGG
TAAAGGTTGTCTATTAATCTATTAATGTACTTACATCGCTCAGTGACAAAGTTCCCATTGCTCTTAATTC
TCGATTTAAGTAGTACTGGTTTTTGCTTATCTTAATTTGCTCTAAATCATTTGTATTCCGTTTGATTGTC
TACTCGTCTTTCTGCTTTAACATGTTGGCGCAAACTAACATATTGTATTAAGTTGGGTTTGGTATTACTC
AAACTGCAAACAAAAAGAGATCTTGCTATTCTCTTAATTAACTCTTAATTAATAATTAAGAGAGCGAGAT
AAGATTGGGTTCTAAGTTGTATTCTCTCACGAGCTCTTATTACAACTTCCAATTACAGTGTATAAAATGC
ATAGAATGATCTTCGTGTTCTTAATCACATCAAGTTGTTGTTAACTATTGTTTATGTACATTAGTTTATA
TTTCATTTATTAAAATACAAATATCTAGAAATTCGCGAGCGCTTAAGTTGACATAACAAAAACATGAATA
AGCAAGCTTTCGATGAGAGAAAAAGCTTTCGAGTGTTTCGGGGCGAATGTCAATGTGAGATCTGTTAAAT
TTAAATATTACTTTGTATCCTAAAAGAAGATCCAAATATTTGGATTTAGTTTATAATCCGAATTTCAAGT
AGCAAATGAAATTGATACACTGGTCTGTATCAAATTTAAGTCAAAAGCATGTAATCAAAGTCATCTGCTG
AAATTATGAACACCACCGCATAAACCAAAAAGCCTACAAAAGCACGTTAAGTGCTTGTAGATTGCTAACT
TAAAAATACCGTCTATTTATAAACAAAGTAGCTCCGCCTTTATTACCTTTTGAAATGAAAAACGCGTAAC
AACAACAAAATCATTCATAAATACTGCCAAGTACTAATGACTCTAATTTTAACACTGATTATTGCACATT
CTTAGATTAAACTCGCGATCGCAAGATCGGAATTTATGCTCTTGCTATTAAAACTAAATTAATTGCGATT
GGATTTGACTATAACTTTCCTTGGAACAGAGTTGCTCCCATCGGTGGTCGATGGTACTGAATAAAGGGAC
ACATGTACTTCGCACACTCCTCGGGGAATTCCAGGTGAACGTGATGGGTACCGTTTTCCACTTCGTAGAA
CTCAAAGTGTGGATTATCCTGGGCCAAAATGGAAACGGCTTCGTCACTGTGAGCGCCCAGAAAGGGCGAC
ATAGATCCCTTGATAATGAGAAAGGGCTTCTTCCCAACGCCCCCGGCCATATCGGCAGCCAGTTCCGCAC
CAATGTCCCAGTAGTTGTAGAACTTGATCCGCCTATCTATGGATAAGTAAAACGATTCCGGATTTAGTCC
TGACTCCTTCCTTGGGCCAACTCGGGTGTCACCGAATTCAGCGTGCCATTGGCGACAGCCATTCCCAATT
TCGGCAGCTTGTAACTGGGTGGCTCCCGCAGAGATCCGCTCGCCAGGATCTCATCCTGCGCCATGTGCTG
GTCCAGGTTTCTGCGGAAGCTTTCGAGAGCAATGTCACTGTCGAACTTCGGCATCAGCACACAGTCTATG
CAAATGACCATGTCCACTGTATCGGGTGCCATTGTCGCGTAAATAAAACTGTAGAAGGCGCCCAATGAGT
GTCCCATGAGCGAAACCTTCTCCCAACCAAAGTGCTTCATAATCCGCGGAATAATGAACACGTCGTTGTA
CACATCATATCGAATTCCCAGTGGCAGAGGCGAGGATCTTCCATGTCCTGGTAAATCGATGCAGAGCACT
CCCACGTGACCGGGTAGCAGTGGAACCAGGCGATCGAAGGTGCCCAGGTTGTCCAGCCATCCGTGTAGTG

CCAGGATGGGGCGTTCCGTTCGATTTCCATACCATCTTCCCGAAATGTAGCCCCAAGGAGCAGGAATTTT
CAGTTCCTTGCACTGTAGGCATAATGAAATTTCTATGAAATTCTATACGGCATCTGTTCCTTACTTACTT
CGGTAACTGGAAGTTGCTGCAACATGGTTTTTAGTTTATGCAGCAAACGGCGTTTCAAACGTCTTTTTGC
TTTGACGATCAGTCAACGACTTCTTTAAATTTTTATGTTGCTGCTGCTTTTTGGCGCTAATGTTTACAAT
ATCAGTAATCGAGATACGCTAAAAAAAGCTTCCCACATAAAAGCTTTTTTGATTACAAAACGCACAATAT
AATCTTAAAGCAGGGGAACCTAATTGTACTCTGCATCTTTCCTCAATTTCTAGTTTATTTTACTATTTCC
AAATATGTGTTTCCTAAAACTTAAATAGCACATTAAAGTTTGCTGCTTCTTGGAGGTCTGTGATTTCGGA
TGAAGGGAACGATATAGCGGGCACACTCCTCGGCGGCATGCAGGTGGACATGATGCTTGCCGCCCTCCAC
TTCGTAGAACTCAAAGTTGGGATTATCATGTGACAGAATGGACATTGTTTCGTTGCATCGAGGTCCCACA
AATGGTGAAAGGGATCCCTTAATTACCAGATAGGGTTTCTTCTCAATACGCCTCGCCATTTCAAGAGCAA
GTCCGTTTTCAATGTCGAATATGTGATAGAACTTTACACGTCCATCTCTGGAGAAGAATACCTTTTCCGG
GTACATGTTTGATTTGGCCACCTGGCGGTGGAGCATGTGATCGGCCAGGTGTTGCGGCACCGAGTTGTTG
CTGTTCCTGGCGAGCGTCTCACGCAGCTTACTCAAGGTGAACGATGGCGGCTCATGTTCGGTGCCATCCG
CCTGTCGCCTCTCCTCCAGCAAATAACCTTCTATGTCCTTGGTCAACTTGCTGGGATCCTCGATTCTCCG
CGGCAGCAGTACATCCAATGATATAATCATATCGACTGTGCTTGGTGCCATGGCCGCGTACATGAAGCTC
ATCACACCTCCGAGGGAATGACCCATTAAGGAGACCTTGGACCAGCCGAACTCCTTCATCACCCTCGGTA
TGATGAACACATAGTCGTAGACGTTGTAGGGCACACCCGGCGGAAGCCTAGAAGATCTACCATGTCCGGG
TAGGTCGATGCACAGCACGCCTATGTAGTCGGGAAGCAGGGGAATCAGCCGATCGAAG
>dme_circ_0005360_junction_seq
GGGAAGCAGGGGAATCAGCCGATCGAAGGGTTCCCAGGTTGTCCAGCCATCCGTGA
>dme_circ_0005361
ACCTTTTCGGCGACTGCCATGTAGAATCACATAACAACAAAGTGCATTTAAAGTCAGAAAGGAATCATGG
TGGAATCCTCGAGTCTGGGGAGGACCCCGCCGGCGGCCCAGCAGCCCATTCCGCAGCAGCACTCCCCTTC
GCCATCAGGACTGCGATGCGGCAACATGGAGACGCGTGTTCGCGGCGCCTGGTACCGCGTCCTGGTCACG
CTGGAAACGGACTTCCTGGCCGTCAGCCTGGACGAGTCCTGTGAGGCGGCGCAGCCATCGAACGATGGAC
AGTCTACTACCTTAAATGGAACCTTGGGGGTAAGTTGCGGTCTATTAGGATCAAAAAATCATGGAGCACG
ATTATTTCAAAAACATGTTTTTTAGGTTTTTAAGGTTATTGCTATTGCTTTCAGGACAAAAGGTTTTCAT
TTAACTTAAAAAATTCCTCGACTACCCGTCATTTAACCAAAAGGAGTGCGTAGGAAATGCAGATATGCAA
TTAGGAAACTATTATCGAGTACCTGCTTTCGTCTTTAGAAACATTCTAATATTTATGTTTGAGCTGCCAG
CACTTCTGTTTCTTATAAAAGATGTTGTAATAGTTGTGGCCACGCAGTAGATTGTAGGTCAATATAATAA
TATTCAACAAAATGAATTATATCCTTAAGACTGACAAAGTTATTGGTGACTGCCTATTAACCATTACATA
CAATACCAATCTGCGATAAGCCTAAGATGCAATTATTATTTTTTCCCACAGAAGCAAGCAATCACAgtgg
cggaggaggcggtggcgcaggcggtggtggcacaggtggaggtggcCAGAACGGCACCCTGCCCAATTCC
GCGTCGCTGCAGGGCATGAATATCCAGGATACAGAGCTAGACGGCTCCGCTAGCAACGACAATGGGGACC
GCGATCCTTGCCTGAACAACAACAACAATGCTGGCGACGGTGGCGGAATGGATATGTGCGACGTACCTGA
CCATGTGGCCAACCAGAAGCGGCATGTGCGGATCATCAAGTCGGACAACAACGGCCTGGGTATCTCCATC
AAGGGCGGCCGGGAGAACCGCATGCCCATCCTCATCTCGAAGATCTTCCGGGGCATGGCCGCCGACCAGG
CCAAGGGACTCTACGTCGGCGACGCCATCCTGACCGTCAACGGAGAGGAGTTACGTGACGCCACCCACGA
CGAGGCGGTGAGGGCCCTCAAGCGGTCCGGTCGAGTGGTAGATCTGGAAGG
>dme_circ_0005361_junction_seq
GGTCCGGTCGAGTGGTAGATCTGGAAGGACCTTTTCGGCGACTGCCATGTAGAATC
>dme_circ_0005362
TTACCGGGACCCAGCGACAGCATACCGCCGCGACTGCCTCCTATCTTGCGCGATGTGGTGGCGAATCCCT
TCTCCGCTCCCTCGCCGAGGTCATAGAAGAGTGCCTCGCCGCCGACTACCAAAGCGAAGATGGACTCGTC

CGCCGACCAGGAGGGCTGCCAGTCCGCTTGGTTCTTCTGGACGATGGCAAACACTTCGACACCGGTGGCC
ACTTCGTAGACGAGCAGATTGGGTGACCCCTCCGGCCGGTCCTTAGTGATGGCATAGTGCTCCCAGGTGC
ACAGGTAGCTGCCACGTGGCGAGAATTGGAGGTAGAAGGCTTTCGGCCTGGGCAGCACGCATATCACTGG
CCAGCTGGCTGCGCTGTCCCGGCTGGCCTGGAGCACCTTGACTTCCTGCCCGTTGGAGTAGGCGAAG
>dme_circ_0005362_junction_seq
GACTTCCTGCCCGTTGGAGTAGGCGAAGTTACCGGGACCCAGCGACAGCATACCGC
>dme_circ_0005363
GTTAGCCAGCTTCTCGCGCTTGGACACATCCCACACCTCGACGGCTCCAGGCAGATTGCCGAAGCCGGCG
AGAGCTATGAGGTTGCCAAATGGATTGAAGTAGGCACAGTTGCGTGGACCCTCACCGAAGTCGAAGACTA
CGTCGCACTTGAGG
>dme_circ_0005363_junction_seq
GAAGTCGAAGACTACGTCGCACTTGAGGGTTAGCCAGCTTCTCGCGCTTGGACACA
>dme_circ_0005364
GTGAGTAGAGTGGAGTGGAGCTGAGAAGAGGTGCTGTATCACAATCACAAGAGCAATTACTCCTCGCATC
CCTCAGCCTTCTCGCCGCCGTCGTCATCGTCAACGCCATTAACGTCCTCCTAGGCAGACTGCTTGAGAGC
CTTGAGCTCATCCAGGTACTGTGCTTCCATCTTAATCTTGTTCAGTTGGTTCAGCTCCAGATTCTCGCCC
TGGCTGCGTCGGACCTTCAGCTTCTTAATGTCCGACAGCTTCTTGGCGACGCTGCGAATCTTGCGCTCCT
TATCGCTGATGTGCTGGCCATTGGGATGACCCTGTCCATACGAATTCGGTGGCTGATCGGCAGCGTTATC
TGGCTGGTACTTCTGTCTGGGAGTATGCTGCGGCTTCCGCTCCTCTGCAGCCACGAAAACGGGCGACTGG
CGAGCAACCGGAGCAGCTTGATCGACCACCTCAGTCGGGGCAGAATCCGCATCGCCACCATTTACAACGT
TCACATCCTTCCTAGCGCTTCGCTGTTTGTTCTTGTTATTCCTCTTGTTTCCGTTGACACCACCTGCAGG
TAAATAGAAATGCAAACAGCAATTAGCAAACACACAGCCGAGCCTACTTACGACCGCGCAATCCCTAATC
CTAATGGCATGCTAATACAAAATGTGTGTGAAAGAGG
>dme_circ_0005364_junction_seq
TGCTAATACAAAATGTGTGTGAAAGAGGGTGAGTAGAGTGGAGTGGAGCTGAGAAG
>dme_circ_0005365
GTACTGTGCTTCCATCTTAATCTTGTTCAGTTGGTTCAGCTCCAGATTCTCGCCCTGGCTGCGTCGGACC
TTCAGCTTCTTAATGTCCGACAGCTTCTTGGCGACGCTGCGAATCTTGCGCTCCTTATCGCTGATGTGCT
GGCCATTGGGATGACCCTGTCCATACGAATTCGGTGGCTGATCGGCAGCGTTATCTGGCTGGTACTTCTG
TCTGGGAGTATGCTGCGGCTTCCGCTCCTCTGCAGCCACGAAAACGGGCGACTGGCGAGCAACCGGAGCA
GCTTGATCGACCACCTCAGTCGGGGCAGAATCCGCATCGCCACCATTTACAACGTTCACATCCTTCCTAG
CGCTTCGCTGTTTGTTCTTG
>dme_circ_0005365_junction_seq
CTTCCTAGCGCTTCGCTGTTTGTTCTTGGTACTGTGCTTCCATCTTAATCTTGTTC
>dme_circ_0005366
GCATACTCCCAGACAGAAGTACCAGCCAGATAACGCTGCCGATCAGCCACCGAATTCGTATGGACAGGGT
CATCCCAATGGCCAGCACATCAGCGATAAGGAGCGCAAGATTCGCAGCGTCGCCAAGAAGCTGTCGGACA
TTAAGAAGCTGAAG
>dme_circ_0005366_junction_seq
GAAGCTGTCGGACATTAAGAAGCTGAAGGCATACTCCCAGACAGAAGTACCAGCCA
>dme_circ_0005367
GATTAAGATGGAAGCACAGTACCTGGATGAGCTCAAGGCTCTCAAGCAGTCTGCCTAGGAGGACGTTAAT
GGCGTTGACGATGACGACGGCGGCGAGAAGGCTGAGGGATGCGAGGAGTAATTGCTCTTGTGATTGTGAT
ACAGCACCTCTTCTCAGCTCCACTCCACTCTACTCACCTCCTGTTCGTCCCCTTCCTGCT

>dme_circ_0005367_junction_seq
CTCACCTCCTGTTCGTCCCCTTCCTGCTGATTAAGATGGAAGCACAGTACCTGGAT
>dme_circ_0005368
GATTAAGATGGAAGCACAGTACCTGGATGAGCTCAAGGCTCTCAAGCAGTCTGCCTAGGAGGACGTTAAT
GGCGTTGACGATGACGACGGCGGCGAGAAGGCTGAGGGATGCGAGGAGTAATTGCTCTTGTGATTGTGAT
ACAGCACCTCTTCTCAGCTCCACTCCACTCTACTCACCTCCTGTTCGTCCCCTTCCTGCTGTTTTACGCT
CAGTTTGTGGCCCATTTGCCGCATAA
>dme_circ_0005368_junction_seq
CTCAGTTTGTGGCCCATTTGCCGCATAAGATTAAGATGGAAGCACAGTACCTGGAT
>dme_circ_0005369
GGGGGCTTCCTCTAGTGATGATAGTTCCTCTGAAGACGAGGCACCCAAAAAGGCAGCAACTCTCGCAAAG
CCCATTTCTAAGGCTGCTCCCACCAAAAAGGCCGACAGTTCCACTGAGGATAGTTCTtcggaagacgatg
ctcctaagaag
>dme_circ_0005369_junction_seq
TTCTtcggaagacgatgctcctaagaagGGGGGCTTCCTCTAGTGATGATAGTTCC
>dme_circ_0005370
GGAAGCTCCCAAGAAACCAGCCGCGAAGGCCGTTGCAAAGGCTGCTTCTTCGGAAGATAGCGATAGCTCA
GAAGACGAAAAGCCAGCAAAGGCTGCTCCCAAGGCTCTGGCCAAGTCTGCAAAGGCTGCCTCCTCAGACA
GTGACGATTCCAGCGATGAAGAAACGCCGGCAGTGAAGCCAGCTGTCAAGAAGACTGCTGCTCCTGCGAA
GAAGGCTGACAGCAGCAGCGACGAGAGTGACTCTGGTGAAGAGTCCGGCGAG
>dme_circ_0005370_junction_seq
GAGTGACTCTGGTGAAGAGTCCGGCGAGGGAAGCTCCCAAGAAACCAGCCGCGAAG
>dme_circ_0005371
GCTTAGGCACCCAACAGATATACATCTCTAAATGCATCAGCTAAGCAACAGTATTCTTGCTCAACAATAA
ATATACGAAAACTCTGAACGCGTAAATACAAGAAATATTTCCGTAGCCGTGATTATTTGTTGCATTTGAA
TAAAAACTTCGAAGATACGGGCAGCAGAGTTAACGCAGCGAGCTTAAGCCAACAACGTAGTACGACTAGC
AAAAGAAACCACCAATTTAATTTTATCTAGAGAAACATACAATACGCATACGCAAGCAAAGTAGCCAAAA
TATCTATATGTTTAGTACGTGTATCGCTCATTTATGATTGATATACTCGCATAGCAAAGTTATATTTACA
AATCCCACAACATAATCGCAAGTGAGCCGCAAAAGACAACTGCTGCGATACAGACCGCCGAGTTCGGTCA
GATCCACATACACTTTCATCCAAACCGCGTTAAGACAAAGCCAGCAAAATAGATACCATGGAAGACAATA
ACACGAGCAGCAGTGCGGGCGGTGCGTCCATCAAACACGAGGATCCATCGGTGACACTCACAATAAGGCT
GATTATGCAAGGAAAG
>dme_circ_0005371_junction_seq
CACAATAAGGCTGATTATGCAAGGAAAGGCTTAGGCACCCAACAGATATACATCTC
>dme_circ_0005372
GGTCGATGCACAGCACTCCCAGGTGCTTCGGTAGCAGGGGCAGCAATTTGTCCCACGTGCCCAGGTTGTC
CAGCCAGCCATGCAGCGCCAGGATGGGTCGTACCTGCCGATTGCCGTACCACTTGCCCACCACATAACCC
CACGGCAT
>dme_circ_0005372_junction_seq
ACTTGCCCACCACATAACCCCACGGCATGGTCGATGCACAGCACTCCCAGGTGCTT
>dme_circ_0005373
GCACTCCCAGGTGCTTCGGTAGCAGGGGCAGCAATTTGTCCCACGTGCCCAGGTTGTCCAGCCAGCCATG
CAGCGCCAGGATGGGTCGTACCTGCCGATTGCCGTACCACTTGCCCACCACATAACCCCACGGCATGTCG
ATGCGCAC

>dme_circ_0005373_junction_seq
CATAACCCCACGGCATGTCGATGCGCACGCACTCCCAGGTGCTTCGGTAGCAGGGG
>dme_circ_0005374
GGAGGTTGGTAGTATTATTGGTAAAAAGGGTGAAATTGTCAACAGATTTCGTGAAGAGGTAAGTCACTCA
TGATAAAAGCTTTCAGAAGTCAGCAACTAATTCGGTATTCTTTACTAGTCTGGTGCCAAAATCAACATTT
CGGATGGCTCATGCCCGGAACGTATTGTGACTGTGTCTGGTACAACTAATGCAATCTTTTCGGCATTCAC
GCTCATTACAAAGAAGTTCGAAGAGGTATGTGTGCGGTACAACTGGCCATTCCCTATCATAGTCGTCCCA
TGCCATAGTATTTTTGGTTCACATTACTAACTTCTGACCCGTTTCAGTGGTGCTCGCAGTTCAATGATGT
AGGCAAAGTTGGTAAAACTCAAATACCCATTCGATTGATTGTGCCCGCCAGTCAATGTGGATCGTTAATT
GGTAAGCGGATTGAAAATATCAATAGTCCCCATCCCACTAATGATATTAATTCACACTCGGGACAGGCAA
GAGTGGTTCAAAGATCAAGGAAATTCGTCAGACCACGGGCTGCTCCATCCAAGTGGCCAGTGAAATGCTA
CCCAATTCCACAGAGCGGGCGGTTACCTTGAGCGGCAGCGCCGAGCAGATCACCCAGTGCATCTATCAGA
TATGTCTTGTCATGTTGGAGGTGAGTTTGCTATACACTATTCGGCAAACTACTTTGACTAGATTTTTAGT
CGACAAAGTTTATTGGAATTCCCCTATTGTGTCCGAAATTTGATAATTCCTTTCTAAACAGTCCCCGCCA
CGCGGTGCCACCATCCCGTACCGACCAAAACCACAGGTTACTGGCCCCGTCATCCTGGCCAATGGACAGG
CTTTCACCATCCAAGGAAACTACGCGGTGCCCACACAAGAG
>dme_circ_0005374_junction_seq
AGGAAACTACGCGGTGCCCACACAAGAGGGAGGTTGGTAGTATTATTGGTAAAAAG
>dme_circ_0005375
GGTGGCCAAGAACCCGCTGGCCAGTCTGGCCGCCTTGGGCCTGGCTGGGATGAATCCGGCCAGCACTGGG
GGCATCAACCACACAGGTGAGTTGAGCGCTTCTGCGGCCAGATGCCAGACAGATTTCCAATCTAATCTAG
GCTCTGCCCCAGCAGCCTTGGCTGCACTGGCCGGGTCGCAACTGCGTACAGCGAATCCCGCGAACCGAGC
CCAGCAGCAGCAGCACGAGATGACCGTCTCAAACGACCTGATTGGCTGCATCATCGGCAAGGGTGGCACC
AAGATCGCCGAGATTCGCCAAATATCCGGCGCCATGATCCGGATCTCCAACTGCGAGGAGCGCGAGGGCG
GCAATACCGACCGCACCATCACCATCAGCGGCAATCCGGACTCGGTGGCCCTGGCCCAATACTTAATTAA
TATGAG
>dme_circ_0005375_junction_seq
CCCTGGCCCAATACTTAATTAATATGAGGGTGGCCAAGAACCCGCTGGCCAGTCTG
>dme_circ_0005376
GTGAACTGAAGTTTGTCCCGGGACGTGCGCAAAAGAGCACTCCTCTTACACAGCTGAATGAGGTTAGTGG
GCGCACTTCGCAGTAGGCGGGAGAAAAATAGGCTTTCCAAGCGTGACACCAGCTCCGCTTGGCGCACGAG
ACGATCGAGCGTGGCGCTCACTTGCAATCCTTGTATGTCGCTTACGGCCAATCCCACCATGAGATTG
>dme_circ_0005376_junction_seq
GCTTACGGCCAATCCCACCATGAGATTGGTGAACTGAAGTTTGTCCCGGGACGTGC
>dme_circ_0005377
GAAATATGATGTGAGCGGTGACAGGGAATTTGACGGCATAGTCCCCATAGAATATGTCTTCAAACTCCAG
TTCCCCAGACATCATCGTGATGGACTTCAAAAAGGACCATGTGATGTTCTCAAAGGCAGGATAATCATTG
AAGAGCACGGCAAAGCTGAGGCCAAAAGCCACCAGCAGGCATAT
>dme_circ_0005377_junction_seq
TGAGGCCAAAAGCCACCAGCAGGCATATGAAATATGATGTGAGCGGTGACAGGGAA
>dme_circ_0005378
GCTTTCCATGGTGGGCACTATAAACAATGTGGGCCAATTCGTGGGCATACCCCTAGGAGGATACTTTGCG
GATCGGTAAGTCCCTGTCCCTGTTTGGTAAGAGTATTAGCAGTCTAACAGCATCTTCATCTACCCCAGAT
ACGGACGACGTACCATGCTAGCTGTAGCCGGATCCGTGAGTGCTCTAATGGGCGTGATCCGTTCGCTGTC

GAGCAACTACTCCATGTTCCTGGTGTTCGA
>dme_circ_0005378_junction_seq
GCAACTACTCCATGTTCCTGGTGTTCGAGCTTTCCATGGTGGGCACTATAAACAAT
>dme_circ_0005379
TTAAACACCAGATTGCTGTAGTCGCTGTGCTGCTTGTCCCGCGTCTCGTGAAGCCGAAAGATCGTCTGCC
GTCGCACCATGCTCTCCCTGTGCTCCCCTTCTAAAGAATCCATCAACACCTCGTCGTAGTGGGGGTCCCC
GTCCTCGTCGTCGTCGAAGAGCCTCTTGGGTCGCTGCAACTTCCGGGCTATACTGACAGCACTGCCCTTC
AGCTCGGTGGCGGCATCCTCGTCAGTAAACTGACCTCCTG
>dme_circ_0005379_junction_seq
GCATCCTCGTCAGTAAACTGACCTCCTGTTAAACACCAGATTGCTGTAGTCGCTGT
>dme_circ_0005380
GCTGGTTTCGCCGGAGCGGCGCTTGGAAAAGGCGCATCCAACCTTCACGGAAAGGAGTCAGTTGAAGTAC
GCCCGGAGGCTGGTGGTCAAGTTGGGCAGTGCAGTCATTACTCGAGAGGACAACCATGGACTAGCCCTCG
GCCGTCTGGCCTCTATTGTGGAGCAGGGTTGCCGAGTGCCATTTGGAAGGACGTGAAGTGATGATGGTCA
CCAGCGGAGCGGTGGCCTTTGGAAAGCAGAAGCTCGCCCAGGAGCTGCTCATGTCGCTGTCCATGCGCGA
GACACTAAACCCAAAAGATAGCAAAGAGGGTGCTCGTGACCAAGCCGGACTTTTACAACGAGGAAACACG
CAACAATCTCTTCTGCACGCTCTCCGAATTAATTAGCCTGAATATTGTGCCAATCATAAATACTAACGAC
GCCGTCTCCCCGCCAATGTTCATACGTGACGACGAGCCAGCCGGCGGAGCCCGGAGGGGGTATTCCCATT
AAGGACAACGACAGCTTGTCTGCCATGCTGGCCGCCGAGGTGCAGGCGGATCTGCTAATCCTCATGTCGG
ACGTCGATGGCATCTACAATAAGCCGCCGTGGGAGGACGGGGCCAAGCTGATGCACACATACACCAGCGA
CGACAGCAACAGCATCGAGTTTGGCAAAAAGTCAAAGGGTGGGTACTGGCGGCATGGACTCCAAGGTGAA
GGCAGCCACCTGGGCCCTGGACCGGGGCGTCAGCGTGGTCATCTGCAACGGAATGCAGGAGAAGGCCATC
AAAACCATAATTGGCGGCCGGAAGGTGGGCACCTTCTTCACGGAGGCCACCGAGAGCGCCAATGCAGTGC
CAGTCGAGGTGATGGCCGAGAACG
>dme_circ_0005380_junction_seq
GTGCCAGTCGAGGTGATGGCCGAGAACGGCTGGTTTCGCCGGAGCGGCGCTTGGAA
>dme_circ_0005381
GCTGGTTTCGCCGGAGCGGCGCTTGGAAAAGGCGCATCCAACCTTCACGGAAAGGAGTCAGTTGAAGTAC
GCCCGGAGGCTGGTGGTCAAGTTGGGCAGTGCAGTCATTACTCGAGAGGACAACCATGGACTAGCCCTCG
GCCGTCTGGCCTCTATTGTGGAGCAGGGTTGCCGAGTGCCATTTGGAAGGACGTGAAGTGATGATGGTCA
CCAGCGGAGCGGTGGCCTTTGGAAAGCAGAAGCTCGCCCAGGAGCTGCTCATGTCGCTGTCCATGCGCGA
GACACTAAACCCAAAAGATAGCAAAGAGGGTGCTCGTGACCAAGCCGGACTTTTACAACGAGGAAACACG
CAACAATCTCTTCTGCACGCTCTCCGAATTAATTAGCCTGAATATTGTGCCAATCATAAATACTAACGAC
GCCGTCTCCCCGCCAATGTTCATACGTGACGACGAGCCAGCCGGCGGAGCCCGGAGGGGGTATTCCCATT
AAGGACAACGACAGCTTGTCTGCCATGCTGGCCGCCGAGGTGCAGGCGGATCTGCTAATCCTCATGTCGG
ACGTCGATGGCATCTACAATAAGCCGCCGTGGGAGGACGGGGCCAAGCTGATGCACACATACACCAGCGA
CGACAGCAACAGCATCGAGTTTGGCAAAAAGTCAAAGGGTGGGTACTGGCGGCATGGACTCCAAGGTGAA
GGCAGCCACCTGGGCCCTGGACCGGGGCGTCAGCGTGGTCATCTGCAACGGAATGCAGGAGAAGGCCATC
AAAACCATAATTGGCGGCCGGAAGGTGGGCACCTTCTTCACGGAGGCCACCGAGAGCGCCAATGCAGTGC
CAGTCGAGGTGATGGCCGAGAACGGGTGAGTGGACCGCAGGGCAAATGGCTTATAGGATGACAGGATAAT
GACGGAGAATGATGTGGCCAGACAATGAAAAGAGGTTTAGTAAGGGCTTTCAGCTCTCAAGTAGGGCGCA
TAATTAGCGGTTTTGGAAACGAGCTTCCAAGCACATCGATGGCCAATCAACCGGAGCGGGGTCCTAAGCC
GGGGTCCAGCTAGTACAGATAGTAACATTTAGTGACGCCCAACTTGCAAGATGGTAACGGAGAATGACAA
GGCAACTGGTCGTTAGAAGGCCAGCAGGAGGATGCGGCAAGGGGTGGCAAAAGTAGAAAGTGGGTCAGCT

AGAACTGGGTCTGCTGAAGAATCCTAGGTGGTGGTGCGGTTCTGTTGCTATTTCGAGTGGGCGAAAATAA
TTTAATTGTTATTAAGGCTAACTGCAGCAGCTGGGCGAAAGTGCCTGTTAATTAGGCGGATGCAACTTGT
TGTCCCAGACTTACCCTCTGTATTCCCCTTTTTCAGGCGCGAACCGGGAGTCGCCAGATGCAGGCCCTC
ACGCCCGCTCAGAGGGCCAGTGCTGTCAACACCCTGGCCGATCTGTTGGTGAGCCGCGAGAAGTTTATCC
TGGACGCCAATGCCAAGGACCTGGCCGAGGCGCAGAAGAGCGGACTGGCCAAGCCGTTGCTGTCGCGGCT
CTCGCTCAACCCGGCCAAGCTAAAGAACCTCTCCGTTGGCCTGAAGCAAATCGCCGAGGACAGTCACAAG
>dme_circ_0005381_junction_seq
GAAGCAAATCGCCGAGGACAGTCACAAGGCTGGTTTCGCCGGAGCGGCGCTTGGAA
>dme_circ_0005382
GCTGGTTTCGCCGGAGCGGCGCTTGGAAAAGGCGCATCCAACCTTCACGGAAAGGAGTCAGTTGAAGTAC
GCCCGGAGGCTGGTGGTCAAGTTGGGCAGTGCAGTCATTACTCGAGAGGACAACCATGGACTAGCCCTCG
GCCGTCTGGCCTCTATTGTGGAGCAGGGTTGCCGAGTGCCATTTGGAAGGACGTGAAGTGATGATGGTCA
CCAGCGGAGCGGTGGCCTTTGGAAAGCAGAAGCTCGCCCAGGAGCTGCTCATGTCGCTGTCCATGCGCGA
GACACTAAACCCAAAAGATAGCAAAGAGGGTGCTCGTGACCAAGCCGGACTTTTACAACGAGGAAACACG
CAACAATCTCTTCTGCACGCTCTCCGAATTAATTAGCCTGAATATTGTGCCAATCATAAATACTAACGAC
GCCGTCTCCCCGCCAATGTTCATACGTGACGACGAGCCAGCCGGCGGAGCCCGGAGGGGGTATTCCCATT
AAGGACAACGACAGCTTGTCTGCCATGCTGGCCGCCGAGGTGCAGGCGGATCTGCTAATCCTCATGTCGG
ACGTCGATGGCATCTACAATAAGCCGCCGTGGGAGGACGGGGCCAAGCTGATGCACACATACACCAGCGA
CGACAGCAACAGCATCGAGTTTGGCAAAAAGTCAAAGGGTGGGTACTGGCGGCATGGACTCCAAGGTGAA
GGCAGCCACCTGGGCCCTGGACCGGGGCGTCAGCGTGGTCATCTGCAACGGAATGCAGGAGAAGGCCATC
AAAACCATAATTGGCGGCCGGAAGGTGGGCACCTTCTTCACGGAGGCCACCGAGAGCGCCAATGCAGTGC
CAGTCGAGGTGATGGCCGAGAACGGGTGAGTGGACCGCAGGGCAAATGGCTTATAGGATGACAGGATAAT
GACGGAGAATGATGTGGCCAGACAATGAAAAGAGGTTTAGTAAGGGCTTTCAGCTCTCAAGTAGGGCGCA
TAATTAGCGGTTTTGGAAACGAGCTTCCAAGCACATCGATGGCCAATCAACCGGAGCGGGGTCCTAAGCC
GGGGTCCAGCTAGTACAGATAGTAACATTTAGTGACGCCCAACTTGCAAGATGGTAACGGAGAATGACAA
GGCAACTGGTCGTTAGAAGGCCAGCAGGAGGATGCGGCAAGGGGTGGCAAAAGTAGAAAGTGGGTCAGCT
AGAACTGGGTCTGCTGAAGAATCCTAGGTGGTGGTGCGGTTCTGTTGCTATTTCGAGTGGGCGAAAATAA
TTTAATTGTTATTAAGGCTAACTGCAGCAGCTGGGCGAAAGTGCCTGTTAATTAGGCGGATGCAACTTGT
TGTCCCAGACTTACCCTCTGTATTCCCCTTTTTCAGGCGCGAACCGGGAGTCGCCAGATGCAGGCCCTC
ACGCCCGCTCAGAGGGCCAGTGCTGTCAACACCCTGGCCGATCTGTTGGTGAGCCGCGAGAAGTTTATCC
TGGACGCCAATGCCAAGGACCTGGCCGAGGCGCAGAAGAGCGGACTGGCCAAGCCGTTGCTGTCGCGGCT
CTCGCTCAACCCGGCCAAGCTAAAGAACCTCTCCGTTGGCCTGAAGCAAATCGCCGAGGACAGTCACAAG
GGTAGGAGAACTTAATTATTGGCCACTTTCCAGAGTAGTGGCCAAGGGACCTGCCCTGGACCAGGGCCTG
GACATGGACCTGAGTTCGAGCAAATTGCATTTGTTGCAGTGTCGAAAAGTCAGCGCCAAATTGAGTTAGC
TCCGGGCTGGGATTTGTCTGCCTGCATGatggcaaaatggcaaactggcaaaatggcCATTTCATGAGCT
GCAACAAAGGCGGACTTGGAGAGCCATATGCAGTTTCCAAACCTTAACAGCATTATGAAATCTTCGAGCA
ATTTTACACTTCAACAAAAATTGACACCCCAGTCAGAAGGGATGCTCGACTTGCTGGTGACCTAACCAAT
CCACCCGGGAAGTAGCCACTGAAGCAGCCTTAAATGCCCGGAAGTTTTCGGGATGGGGCTGGAGCTAAGC
AAGTGGAAGATAAAGGACACTTTCTTGTATGCGCACTCGTTTGGTGTCTTGCCAGTGACATTAGCCCGCA
tcctggtcctgtttctggtcctggccctggccctCACCCTGCCCTTGTCCCATTTTAGAGCAGACTAAGT
TGATCGAATATCGCTTGGGAGGTATGGGGGTTGGAGGTCCGGGGGCTTGAGTTTGGTCCTCCCAAGCCAA
CCGTTTTATTGGATGTGATTGTGCGGAAACGCAGAAAGTTTTCCTTCATGTTTTTCTCTAGAAAAAAACT
TTTTCGTTGGGTTCAGGGGCGGGTCGTGGAGCTGGGACATGAGTTTATTGTAAAAATGGCAAGCGAAATT
GTGACACAAAATGGCGGCAGCGTGAAAAATCATAAAAAGGCGAGCGCGCACACACTCATAAAGAAGCGGG

CTGGGAATACTGAAAAACCATAACGCACTCAATAAATGTCGCTCGGGTGGCGAAAAGtgttgtttgtttt
ttgtccctctttttgtcctttttATCCTGCGACAAACAAATGCCACGAACATGGTTTGGCCTCTGGCCCA
CAGAAGCCTTCGTAAGTTGGATAGGTGAGTGTCCTGGCAGTGCTTCGGTTTTCAATTACCCGGGCTACTT
ACGCATCGCACGAAAAGTTTTAATACACAATTAATGCTGAATAATTCAATAGTGTTAGGCCGGGGCACTT
TGGAGATACACTGAGCTTGCTTAACGAAATTGCTTTTCACTCTTTTTTCCATTTGCAGTGCGGACTTCTC
ATAAAGCTATGTATTCCCAACATATTTCAGTTCCCCATAACCTCATCCAACTTTTCCTTGGATACTTGTA
CATAAGCTCGGCTCCACTACTTGCTCTGACTTATCCAATTGTCCTTTGCAAATATAATTTAAAGTTTGAC
GTCCTGCAATGCCGGAGTCAATAATAATGGCCATATAACCAAATTTACCCAGCGCCCCGACTCGAGTTTAA
TGGCTAAGCAAGAGCAGGAGGACCACTCCATCCCCATTCACATCCATTCCATCCATACTATGTAAACAAG
GCCAGCCAACTGCTGTCAGCAGACGTCCAGCCCAGACCAACCCGCAATCTCTTTATGTTATCTGCAGCCA
ATTTTCTAGAGATTCTTCACGACGCaggaggaggaggaggaggaAGGGCCCTCTCAAGGGGCAGGTCAGC
AGAAGTGCGGGGGCGTTGGACAGCAGGCCATTAAAAATAAATAAAGATTACGACGCGCTGAGCGACTTGA
TTTATGTAACGCCTCAAAATGGACGCCGGGGAATCGAGATGCGGTGGAAGGAGAAGCCTTCCGATTCTCC
TTGCGAATGAAACTGTCGCATAAATATCGAGTTATTTTTATGTCCCGGTCCCACATGGCGTATGCATTAT
GCGATTTATGCTGACTTGTGAATTGACTTTCCCTGCCCATGCTCCACCCCAATAATAATATACAATATAT
CCCTCATTCCGTATCTCATCCGGACTTTCTTTTCTTGCAGGAATGTTGGTCGTGTCCTGAGACGCACCCG
CCTTGCGGATCAGCTGGAGCTCAAGCAGGTCACTGTGCCCATAGGCGTCCTACTGGTCATCTTCGAATCC
CGCCCAGACTCGCTGCCTCAAGTGGCCGCCTTGGCAATGGCCTCGGCCAACGGACTGCTCCTCAAGGGGG
GCAAGGAGGCGGCACACAGCAACAAGGCCCTTATGGAGCTGGTCAAGGAGGCGCTGGCCACCGTGGGAGC
GGAGCACGCCGTGTCACTGGGTTTCCACGCGCGAGGAGATCAGCGATCTGCTGTCCATGGAGAACCACAT
CGACCTGATCATTCCGCGCGGCTCCTCCGACCTGGTGCGCAGCATCCAGCAGCAGTCGCTGCACATCCCC
GTGCTGGGCCACGCCGAAGGCGTCTGCCACGTCTATATCGACAGGGATGCGGATCTGGAGAAGGCCCTGC
GCATTGCACGCGACGCCAAGTGCGATTATCCGGCTGGCTTGCAACGCCATGGAGACCCTGCTGATCCACG
AGGACCTGATGAGTGGCGCAATCTTCGGCGATGTCTGCAACATGCTGAAACGTGAGGGCGTCAAAATCTA
TGCCGGACCCCGGCTGAACCAGCAGCTGACATTTGGCCCGCCGGCGGCCAAGAGCCTGAAGCACGAGTAC
GGCGCCCTCGAGTGCTGCATCGAGGTTGTGCCCAGCCTGGACGAGGCCATTAATCACATCCATACGTACG
GCAGCAGCCACACGGATGTCATTGTTACGGAAAATG
>dme_circ_0005382_junction_seq
CACACGGATGTCATTGTTACGGAAAATGGCTGGTTTCGCCGGAGCGGCGCTTGGAA
>dme_circ_0005383
GAATGTTGGTCGTGTCCTGAGACGCACCCGCCTTGCGGATCAGCTGGAGCTCAAGCAGGTCACTGTGCCC
ATAGGCGTCCTACTGGTCATCTTCGAATCCCGCCCAGACTCGCTGCCTCAAGTGGCCGCCTTGGCAATGG
CCTCGGCCAACGGACTGCTCCTCAAGGGGGGCAAGGAGGCGGCACACAGCAACAAGGCCCTTATGGAGCT
GGTCAAGGAGGCGCTGGCCACCGTGGGAGCGGAGCACGCCGTGTCACTGGGTTTCCACGCGCGAGGAGAT
CAGCGATCTGCTGTCCATGGAGAACCACATCGACCTGATCATTCCGCGCGGCTCCTCCGACCTGGTGCGC
AGCATCCAGCAGCAGTCGCTGCACATCCCCGTGCTGGGCCACGCCGAAGGCGTCTGCCACGTCTATATCG
ACAGGGATGCGGATCTGGAGAAGGCCCTGCGCATTGCACGCGACGCCAAGTGCGATTATCCGGCTGGCTT
GCAACGCCATGGAGACCCTGCTGATCCACGAGGACCTGATGAGTGGCGCAATCTTCGGCGATGTCTGCAA
CATGCTGAAACGTGAGGGCGTCAAAATCTATGCCGGACCCCGGCTGAACCAGCAGCTGACATTTGGCCCG
CCGGCGGCCAAGAGCCTGAAGCACGAGTACGGCGCCCTCGAGTGCTGCATCGAGGTTGTGCCCAGCCTGG
ACGAGGCCATTAATCACATCCATACGTACGGCAGCAGCCACACGGATGTCATTGTTACGGAAAATG
>dme_circ_0005383_junction_seq
CACACGGATGTCATTGTTACGGAAAATGGAATGTTGGTCGTGTCCTGAGACGCACC
>dme_circ_0005384

CTACTACTTTCCCTTATAGAAGTGGGACAGAAGCGCATTCTCATGCACCCGCTTTGCGAAACTTTCCTCT
TTCTTAAGTGGCGTCGCATTCGGAAATTCTTCCTTATGAGCCTTGCGTATCATACGCTTTTCGTGATCCT
TTTCACTTTCTATGTAATTTGGGTGTATGTCCGCTGCTGCAAGAAGGAGGAGTTGTGCGTGGCTCCAGGT
TACG
>dme_circ_0005384_junction_seq
GAGGAGTTGTGCGTGGCTCCAGGTTACGCTACTACTTTCCCTTATAGAAGTGGGAC
>dme_circ_0005385
GAAATAAGTCAAAGAAGTCGGGGAAATCGCACTCAACGTCCGCCTTTCCACCAAGACGCATGTAAACGCA
ACCGGAGCCCAAAGAAGGCAAGTGGCAGGGCAGGGAAAGATGCCATCGGCAGATCAGATCCTGTTTGTAA
ATGTCACCACAACGGTGGCGGCGGCGGCTCTAACCGCTGCGGCCGCCGTCAGCACCACAAAGTCCGGAAG
CGGCAACGCCGCACGGGGCTACACGGATTCGGATGACGATGCGGGCATGGGAACGGAGGCGGTGGCTAAC
ATATCCGGCTCGCTGGTGGAGGGCCTGACCACCGTTACCGCGGCATTGAGTACGGCTCAGGCGGACAAGG
ACTCAGCGGGAGAATGCGAAGGAGCTGTGGAGGAGCTGCATGCCAGCATCCTGGGCCTCCAGCTGGCTGT
GCCGGAGTGGGAGGGCCCTTCTCACCGCCCTGGTTCTCTCGGTCATTATCGTGCTGACCATCATCGGGAA
CATCCTGGTGATTCTGAGTGTGTTCACCTACAAGCCGCTGCGCATCGTCCAGAACTTCTTCATAGTTTCG
CTGGCGGTGGCCGATCTCACGGTGGCCCTTCTGGTGCTGCCCTTCAACGTGGCTTACTCGATCCTGGGGC
GCTGGGAGTTCGGCATCCACCTGTGCAAGCTGTGGCTCACCTGCGACGTGCTGTGCTGCACTAGCTCCAT
CCTGAACCTGTGTGCCATAGCCCTCGACCGGTACTGGGCCATTACGGACCCCATCAACTATGCCCAGAAG
AGGACCGTTGGTCGCGTCCTGCTCCTCATCTCCGGGGTGTGGCTACTTTCGCTGCTGATAAGTAGTCCGC
CGTTGATCGGCTGGAACGACTGGCCGGACGAGTTCACAAGCGCCACGCCCTGCGAGCTGACCTCGCAGCG
AGGCTACGTGATCTACTCCTCGCTGGGCTCCTTCTTTATTCCGCTGGCCATCATGACGATCGTCTACATC
GAGATCTTCGTGGCCACGCGGCGCCGCCTAAGGGAGCGAGCCAGGGCCAACAAGCTTAACACGATCGCTC
TGAAGTCCACTGAGCTCGAGCCGATGGCAAACTCCTCGCCCGTCGCCGCCTCCAACTCCGGCTCCAAGTC
GCGTCTCCTAGCCAGCTGGCTTTGCTGCGGCCGGGATCGGGCCCAGTTCGCCACGCCTATGATCCAGAAC
GACCAGGAGAGCATCAGCAGTGAAACCCACCAGCCGCAGGATTCCTCCAAAGCGGGTCCCCATGGCAACA
GCGATCCCCAACAGCAGCACGTGGTCGTGCTGGTCAAGAAGTCGCGTCGCGCCAAGACCAAGGACTCCAT
TAAGCACGGCAAGACCCGTGGTGGCCGCAAGTCGCAGTCCTCGTCCACATGCGAGCCCCACGGCGAGCAA
CAGCTCTTACCCGCCGGCGGGGATGGCGGTAGCTGCCAGCCCGGCGGAGGCCACTCTGGAGGCGGAAAGT
CGGACGCCGAGATCAGCACGGAGAGCGGGAGCGATCCCAAAGGTTGCATACAG
>dme_circ_0005385_junction_seq
CGGGAGCGATCCCAAAGGTTGCATACAGGAAATAAGTCAAAGAAGTCGGGGAAATC
>dme_circ_0005386
TTAATCCAGGGTTGCTGCGCGTCAAGGTGTTTTCAGCATCGCGTCATTCAAAGAACTTCTGATTGGATGC
GAATCGCACGACAGGACTTGCTGGACAAAATGCGTTTCACACTGCCATATAGAAAAAAAGCAAGTGTTAT
TTAACTTTTGTAATTGCCTACAAAATTGTAAAGTTAAGTAATCATTTTCCCCTGTCCTGGTCGCTG
>dme_circ_0005386_junction_seq
TAATCATTTTCCCCTGTCCTGGTCGCTGTTAATCCAGGGTTGCTGCGCGTCAAGGT
>dme_circ_0005387
CGCCGGGACGGGCGGGGGCGCGCACCTTGGACTCCAGCAGCTTGTCGCGCACCTCGGCGAGATCGCCCTT
GGTGAACACGAATCCCACGTTGCCCTTGATGTGGGGTAGCAGCTTCTCCAGCTGCGGGTTGTTCTCCAGA
TGACCGCGGATGGCCTTGCGCATCATGGTGTTCTTGCCCATAAGCACGACGGCCAGTCCACGCAGGCTGG
TACGGATG
>dme_circ_0005387_junction_seq
GGCCAGTCCACGCAGGCTGGTACGGATGCGCCGGGACGGGCGGGGGCGCGCACCTT

>dme_circ_0005388
GGGCAATAGCGCCGGGACGGGCGGGGGCGCGCACCTTGGACTCCAGCAGCTTGTCGCGCACCTCGGCGAG
ATCGCCCTTGGTGAACACGAATCCCACGTTGCCCTTGATGTGGGGTAGCAGCTTCTCCAGCTGCGGGTTG
TTCTCCAGATGACCGCGGATGGCCTTGCGCATCATGGTGTTCTTGCCCATAAGCACGACGGCCAGTCCAC
GCAGGCTGGTACGGATG
>dme_circ_0005388_junction_seq
GGCCAGTCCACGCAGGCTGGTACGGATGGGGCAATAGCGCCGGGACGGGCGGGGGC
>dme_circ_0005389
GCCTGCGTGGACTGGCCGTCGTGCTTATGGGCAAGAACACCATGATGCGCAAGGCCATCCGCGGTCATCT
GGAGAACAACCCGCAGCTGGAGAAGCTGCTACCCCACATCAAGGGCAACGTGGGATTCGTGTTCACCAAG
GGCGATCTCGCCGAGGTGCGCGACAAGCTGCTGGAGTCCAAGGTGCGCGCCCCCGCCCGTCCCGGCGCTA
TTGCCCCTCTGCAC
>dme_circ_0005389_junction_seq
CCGTCCCGGCGCTATTGCCCCTCTGCACGCCTGCGTGGACTGGCCGTCGTGCTTAT
>dme_circ_0005390
GCCTGCGTGGACTGGCCGTCGTGCTTATGGGCAAGAACACCATGATGCGCAAGGCCATCCGCGGTCATCT
GGAGAACAACCCGCAGCTGGAGAAGCTGCTACCCCACATCAAGGGCAACGTGGGATTCGTGTTCACCAAG
GGCGATCTCGCCGAGGTGCGCGACAAGCTGCTGGAGTCCAAGGTGCGCGCCCCCGCCCGTCCCGGCGCTA
TTGCCCCTCTGCACGTCATCATCCCGGCGCAGAACACCGGCTTGGGACCCGAGAAGACCAGTTTCTTCCA
GGCCCT
>dme_circ_0005390_junction_seq
CCGAGAAGACCAGTTTCTTCCAGGCCCTGCCTGCGTGGACTGGCCGTCGTGCTTAT
>dme_circ_0005391
GAACACCATGATGCGCAAGGCCATCCGCGGTCATCTGGAGAACAACCCGCAGCTGGAGAAGCTGCTACCC
CACATCAAGGGCAACGTGGGATTCGTGTTCACCAAGGGCGATCTCGCCGAGGTGCGCGACAAGCTGCTGG
AGTCCAAG
>dme_circ_0005391_junction_seq
GGTGCGCGACAAGCTGCTGGAGTCCAAGGAACACCATGATGCGCAAGGCCATCCGC
>dme_circ_0005392
GCTGCTGGAGTCCAAGGTGCGCGCCCCCGCCCGTCCCGGCGCTATTGCCCCTCTGCACGTCATCATCCCG
GCGCAGAACACCGGCTTGGGACCCGAGAAGACCAGTTTCTTCCAGGCCCTGTCCATCCCGACCAAAATTT
CCAAGGGAACAATTGAAATCATCAACGATGTGCCCATCCTGAAGCCTGGCGACAAG
>dme_circ_0005392_junction_seq
TGTGCCCATCCTGAAGCCTGGCGACAAGGCTGCTGGAGTCCAAGGTGCGCGCCCCC
>dme_circ_0005393
GGTGCGCGCCCCCGCCCGTCCCGGCGCTATTGCCCCTCTGCACGTCATCATCCCGGCGCAGAACACCGGC
TTGGGACCCGAGAAGACCAGTTTCTTCCAGGCCCTGTCCATCCCGACCAAAATTTCCAAGGGAACAATTG
AAATCATCAACGATGTGCCCATCCTGAAGCCTGGCGACAAG
>dme_circ_0005393_junction_seq
TGTGCCCATCCTGAAGCCTGGCGACAAGGGTGCGCGCCCCCGCCCGTCCCGGCGCT
>dme_circ_0005394
GAAGACCAGTTTCTTCCAGGCCCTGTCCATCCCGACCAAAATTTCCAAGGGAACAATTGAAATCATCAAC
GATGTGCCCATCCTGAAGCCTGGCGACAAGGTCGGCGCCTCCGAGGCGACACTGCTCAACATGTTGAACA
TCTCGCCCTTCTCGTACGGTCTGATTGTCAACCAGGTCTACGACTCCGGCTCGATCTTTTCGCCGGAGAT

CCTGGACATCAAGCCCGAGGATCTGCGCGCCAAGTTCCAACAGGGA
>dme_circ_0005394_junction_seq
GGATCTGCGCGCCAAGTTCCAACAGGGAGAAGACCAGTTTCTTCCAGGCCCTGTCC
>dme_circ_0005395
GTTCCAACAGGGAGTGGCCAACTTGGCCGCCGTTTGTTTGTCCGTGGGCTACCCCACCATCGCCTCGGCC
CCGCACAGCATTGCCAACGGATTCAAGAATCTGCTGGCCATTGCTGCCACCACCGAGGTGGAGTTCAAGG
AGGCGACCACCATCAAGGA
>dme_circ_0005395_junction_seq
AGTTCAAGGAGGCGACCACCATCAAGGAGTTCCAACAGGGAGTGGCCAACTTGGCC
>dme_circ_0005396
GGAGTACATCAAGGACCCCAGCAAGTTCGCCGCAGCTGCTTCGGCTTCGGCTGCCCCCGCGGCCGGCGGA
GCTACCGAGAAGAAGGAGGAGGCCAAGAAGCCCGAGTCCGAATCAGAGGAGGAGGACGATGATATGGGTT
TCGGTCTGTTCGACTAAGCTGGATCCCGATTGCAGAATGCCCTCTGCGGCGCCCGCGAACCATCGCTTCC
GCTTTCGGC
>dme_circ_0005396_junction_seq
CCCGCGAACCATCGCTTCCGCTTTCGGCGGAGTACATCAAGGACCCCAGCAAGTTC
>dme_circ_0005397
GGAGTACATCAAGGACCCCAGCAAGTTCGCCGCAGCTGCTTCGGCTTCGGCTGCCCCCGCGGCCGGCGGA
GCTACCGAGAAGAAGGAGGAGGCCAAGAAGCCCGAGTCCGAATCAGAGGAGGAGGACGATGATATGGGTT
TCGGTCTGTTCGACTAAGCTGGATCCCGATTGCAGAATGCCCTCTGCGGCGCCCGCGAACCATCGCTTCC
GCTTTCGGCGTTTACCCACTAAGACCCTTTGTTAT
>dme_circ_0005397_junction_seq
GCGTTTACCCACTAAGACCCTTTGTTATGGAGTACATCAAGGACCCCAGCAAGTTC
>dme_circ_0005398
AAAACATAACAAAGGGTCTTAGTGGGTAAACGCCGAAAGCGGAAGCGATGGTTCGCGGGCGCCGCAGAGG
GCATTCTGCAATCGGGATCCAGCTTAGTCGAACAGACCGAAACCCATATCATCGTCCTCCTCCTCTGATT
CGGACTCGGGCTTCTTGGCCTCCTCCTTCTTCTCGGTAGCTCCGCCGGCCGCGGGGGCAGCCGAAGCCGA
AGCAGCTGCGGCGAACTTGCTGGGGTCCTTGATG
>dme_circ_0005398_junction_seq
TGCGGCGAACTTGCTGGGGTCCTTGATGAAAACATAACAAAGGGTCTTAGTGGGTA
>dme_circ_0005399
GGACCCCAGCAAGTTCGCCGCAGCTGCTTCGGCTTCGGCTGCCCCCGCGGCCGGCGGAGCTACCGAGAAG
AAGGAGGAGGCCAAGAAGCCCGAGTCCGAATCAGAGGAGGAGGACGATGATATGGGTTTCGGTCTGTTCG
ACTAAGCTGGATCCCGATTGCAGAATGCCCTCTGCGGCGCCCGCGAACCATCGCTTCCGCTTTCGGCGTT
TACCCACTAAGACCCTTTGTTATGTTTTCTAT
>dme_circ_0005399_junction_seq
CACTAAGACCCTTTGTTATGTTTTCTATGGACCCCAGCAAGTTCGCCGCAGCTGCT
>dme_circ_0005400
GCTACCGAGAAGAAGGAGGAGGCCAAGAAGCCCGAGTCCGAATCAGAGGAGGAGGACGATGATATGGGTT
TCGGTCTGTTCGACTAAGCTGGATCCCGATTGCAGAATGCCCTCTGCGGCGCCCGCGAACCATCGCTTCC
GCTTTCGGCGTTTACCCACTAAGACCCTTTGTTAT
>dme_circ_0005400_junction_seq
GCGTTTACCCACTAAGACCCTTTGTTATGCTACCGAGAAGAAGGAGGAGGCCAAGA
>dme_circ_0005401

GAACGACACGATGCGCCTGCTGTACATGTACCACGCCCAGGATCCGCCGCACGGCTCGGTGCGTCCTGGC
ACCCTTCCGGATCCGGCCCGCGCCTTCCGCCCCTACCGCCCCATGGTCCTCATGCAGCGCGCCCAGCTGC
CGATGCCCTCACCCACGCACGACGAGCGCGTGCGAGTCCTCGAGCTGCGCAACGAGGATGTGGAGCTGCC
CGCCGGCGACCTACCGCTCTTCTGGTGCAAGATGTTCAAGCTGGAGGACATCAATCGCAAGCACCACCTG
ATCCGGGGTAAGAATATTCGATTCAAGGCCCCGTCTTGATACTCTGTAATCGAGGGTCtaattaataaat
taattaataaaattaataaatattaatattaattttgaatatttaataGTCTAACTAGTACTAATAATCT
GACTAGCAATTTCGCCATCGAACTACAGCCATAGAACGATATATTAAAGTCGCAACTCCTGCAGCTCCTT
TAAGCATTTTTTGTCGAGTGCAGCAAGACTAAACGATATTAAATACAATGCATAATTAAGGCCAAGTGCA
GTGACGCGTTCTGGCATTCCGCTTTTGTCTGGCAGTCACTGCTCCGGCTCTCCGCCGCGGCGGCTGTATT
CCGCATTGTGAGTCGTCGTTGTCTCTTAGCCTCGGCATAACCAAATTGAAATATTAAAAATTTATCCCCA
GCCTCGCGCGCCCTATCTTTCCCACAATGCACATCACATCACTAACCGTTGTCTCCCGTCCTCCGGTTCA
TTTTCTTTTCATGGCTGCAGGTACGAGCCGATTTATGATTCATCGTCCAGCGTGCACTACTTGCAGCACA
TAACGCTGCACGAGTGCCAGGGTGCCCACGCGGAGCTGGAGGAGATGGCACGCGAGCAGGGCCGCCCGTG
CCTCGGAGCCCGATCCATTCCGCTGGCGTGCAACGCCATCGTGGCCTCCTGGTCGCGCGGCAGCGAG
>dme_circ_0005401_junction_seq
CGTGGCCTCCTGGTCGCGCGGCAGCGAGGAACGACACGATGCGCCTGCTGTACATG
>dme_circ_0005402
AGGCGTAATCCGTACTCCACATACTCAGCGCAAAGACGCAGATACGTACTTTTTTTGTAGGTCCGTGGGG
TGAAGCGTTTTGTTGCTGTAGCTCGTTGTTAAAATTTTTTGTTAATTGTAAGCCGGCGTACACACTCACA
CAGGG
>dme_circ_0005402_junction_seq
GTAAGCCGGCGTACACACTCACACAGGGAGGCGTAATCCGTACTCCACATACTCAG
>dme_circ_0005403
CTTTTTATGCTAAAACTGCATCCGGAAAAAGACAAAAGTAATGTGCTTCAGCTGCAGTTTGTGAAGAGCG
CTCTGAGTATGAATCCCTGCATGACGACTAGTCCGCACATCCTGCTGCAGGGTGACGACTGCCTGTACCT
TAACCAGGGCAATAATCTGGAGCTAACGTATGCCGGAAGCCATGGAACGTTTCCATCAAGCGGCCTGGGC
TCTGACGAAGACATTTCCGGTGATGGCGACTGTCTGGAGTTAAAACAAAGCCCGCACACAGGCAGCATTC
TTACGGAGAGTAAGTATTGGACAGTGTTGCAGCTGCCGCTCAACTACGCGGCCACAAACTGGCCAATCCG
ATATGCTGCTATCGATCCCGATGGACTCCACTTGGCGGTGGCTGGTCGCACTGGACTGGCGCACTACTCC
CTAGTGACCCGGCGCTGGAAGCTTTTTGGCAATGAGTCGCAGGAGAAGGACTTCGTTGTCTCTGGCGGCC
TACTGTGGTGGCATGGCTTTGTAGTCATGGGCTGCTATTCGCTTCTAGACCGCACGGATGAGTTGCGTTG
CTATCCGGCAGATTGCAAACTCGACAACCAATACGGGCACAAGCTGCAGGTCCGAGCACCAGTTATTAGC
CTGAACTCATTCCGACACCAGTTGATTGTACTCACTGCCGATGGAATAGTGAGCCTGTTCAATATGTCGA
AAAACTCAGCGTACGCCCTGGATATTGAATGCGCCTATGAATTGGACGTGAAGAGCATTTGCATCCATCC
TGCCTGCATTGTTAGTCTGACTGTGACTAACCTCAAGAATGAGCTAAAGCCCCAGGGACAGCTGGGTGGA
GATCAGGCAGAGACTATCATCGTGAATGTATGCGGCCGCATATTGATGATACAGCGCGATGCCGGCGAAC
AGGGTATGTTTGAAAAATATTAATTGTCGAAATGCTTAAAGTTGGAGAGATGCAAATCATTTATACATTT
CATATGTGAATTTACCGTTATCTCAAAGTAAAGCGAACGTCAGCATGTCTTCTATTTGGAAATGTCTTTG
GTTATCGAGCCAACCATAACAAGGAATTTGAGTATGCATCTTTTCAACTTCGCGGACAATTATTACGATA
TATCATATAGCAACTAAAGTCAAAATCTATGTTTACCAAATAGGGTCCCAAACACCTTGCTGGCCACCTG
CCTGGCTAGCTGCGTTGAGGTCTTCTGGCTGTCACACTCCCTGGAACGCTGTGCGATGCGTGACTGCCTT
TGGCTTTACTCCGGTGCCCACGGAATGCGCGTATGGCTTCCAATACTGCCGCCGGGAAGGGAGCGTCGCG
AAGGCGAGCAGGGCGGCGCCCAACGTCTGCACAGTTTTATGTCAAAGCGAATCATGCTGAGCTTTCCGCT
GAAACTTTATCCGCTCGTCGTGCTCTTTGACAATGTGATCGTTCTGGGGGTGGAGAACGAAAGCACGCTT

TACGCCAATGAGCAGGTCTCCCACTTCTCTCTGCCGTTCGCCGTTATGGAACGCAAGTCGCAGATCTATC
TGCACAAGGTACTTCGCCAACTGATCAAGCGGAATTTGGGCTACTCTGCCTGGGAAATGGCACAGTCGTG
TTGCTCGCTACCATATTTTCCGCACGCTCTGGAACTGCTTCTACACGAGGTTCTCGAGGAAGAGGCTACT
AGCAAACAGCCCATCCCGGATGCACAGCTGCCCAGTATTCTGGACTTTATAAGAGAGTTTCCCGTCTACT
TGGAAACAATTGTTCAATGTGCCCGCAAAACAGAGATCGCCCTGTGGCCGTACCTATTTTCGATGGCTGG
TAAACCGAAGGATTTATTCCAGATGTGTCTGCAGTCGGAGCAGCTGGACACGGCTGCGAGCTACTTGATA
ATCCTGCAGAATCTAGAACCCTCTGTTGTTAGCAAGCAATATGCCACCATGCTGCTGGACATTGCTCTAC
AGCAGCGAAAGTGGGAGCTGGCTAAGGACTTGATCCGGTTTTTAAAGGCTATAGATCCAAATGAGATCGA
CTCACCGCGGTCGTCGATGGTGGTGAATGTGAAAATTGCTCCTCCTCCCCAGGTGAATACACAGCAGCAA
GTGAACCAGAATGCGGATGCCTTTAACATGGTTCTGGGTCCCATTGCCAGAGAGAGAAGCTTCTCCACCA
CGGTGACTAGCAATCTGCCCAAAGATAAGCAGGCATCTGGGACTCCTGGGGTTGCACCTGTGACCGAAAG
CAGCAGTGCAGGAGCACCCTCGGTGGTGCGCCGGCGGTCGACTAAGCAGCGAGAGACGTTCTGCATCGAT
CTGATACTGCAGCGCCATGCTCGACAACTACTTCAGAACCACAAACTAATGGATTTAGGCTACATGTGTG
CTTATCTGGACTTTCACCTTGTCAGCTGGCTTTCGCAGGAATCAGAGCGGGCAGCCAAGTTGGATGATTT
CGCCGGCGCTCTGCAGGCGCTACACGAAGAACTAGATCTCCCCATTCCATTTCCGACTGCGGCAAAGGAC
GACTTTGCTCAGATACGCGGCAGTCTTCGACAGACAGGAGGCGGTGGATCTTCGCAAACGTCAGAGTCTG
GCTATTTTAGCTTGGCCACGCCCAATGGAGCCGCAACACAGTCGCCACAGCTGCAGCCAAGCATTAGGGA
AGAGGAGGAAGAACTGCAGCAGCCTAGCTCCCTACCAATACTTAAGACGCGGTCTGGATCGCAACTGTCG
TTCGATAACTTTCGCTACCGCCGGCTGTACTCGCTACCGACATCAGAGGACGACCTAGCCGTGGATATTC
TGCCTCAGAAGCTGTCCATTAAGCTGCGTTACTTGTTACAGCTGTTTATCGAGGCGAACTGCACGGACTA
CGCGCTGGTTCTTTCCATTTTGCTCCAGGACGCGGCTTCCATCTCGCGGATTGTAAACGGAATAATCCGA
AGCGAATCGGTTCACACCTGTCGACGTACGGAATCTGCACTCAAACAGTTAAGTCAGAGCACCTTTGAAC
ACAGCGGATCTTTGTACCGGGGGTTCGTCCTCACCTTGCAACCGCATGTTTACCTGCTAGAACAGTACAT
ACAGTCTCTGGGGGATGCTCCCTGTTCGCAGCTCCAAGATGCCGGCCCGGGTACCGAGCAGGGCGTGGAC
GTCAGCACGGGACTCCAGGGACTGCAGAACGAAGTGGGCGAATTTGTTCCAAATTCCCAGCAGGCTAACG
GAAACCAATGGACTGTGGCGGACCTTAACCCGAATCACCAGCGGCTCACGCGGCACGCCAGTCTAGAGTC
TAACGGGAACGCAGTGGTGGCCAGCGGTTCCTCTGCCCATTCAACGCCCACCCAGCGCCAACTGTCACGG
CAGAACAGCCGCGAGCGGGAGGGCTGTCGCCTCATGTAAGCCGTTAGCCGTAATCCGTAATGCGAGAGGC
GTAATCCGTACTCCACATACTCAGCGCAAAGACGCAGATACGTACTTTTTTTGTAGGTCCGTGGGGTGAA
GCGTTTTGTTGCTGTAGCTCGTTG
>dme_circ_0005403_junction_seq
TGAAGCGTTTTGTTGCTGTAGCTCGTTGCTTTTTATGCTAAAACTGCATCCGGAAA
>dme_circ_0005404
CTTTTTGGCAATGAGTCGCAGGAGAAGGACTTCGTTGTCTCTGGCGGCCTACTGTGGTGGCATGGCTTTG
TAGTCATGGGCTGCTATTCGCTTCTAGACCGCACGGATGAGTTGCGTTGCTATCCGGCAGATTGCAAACT
CGACAACCAATACGGGCACAAGCTGCAGGTCCGAGCACCAG
>dme_circ_0005404_junction_seq
GGGCACAAGCTGCAGGTCCGAGCACCAGCTTTTTGGCAATGAGTCGCAGGAGAAGG
>dme_circ_0005405
GGGCACAAGAGATTTTGAGTGTCCGCACATATTGGCCTGGGGATAATTATTTGCGTGTCATGACTTATTA
GCGATCGCAGCCTATCTCCGCGAATAAGTGTAAGTAGTCGACCCCAACAAGAAGGGTGGGCCGACCCGAA
CAGCAGCCGGAGCCAGCTGAACAGTCGCCCGACTCGGCCGCATCCAAAAGTTTATTGTTGTTTGG
>dme_circ_0005405_junction_seq
CCGCATCCAAAAGTTTATTGTTGTTTGGGGGCACAAGAGATTTTGAGTGTCCGCAC

>dme_circ_0005406
GTGTAGTTGCTAATGTGGGGTCTACCTTGCATAGCAGACCCGCTGCTGAGGCCATCTCGGAAGCAACCCT
AATTTGGTCGCCTCCGGGCAAATGTTCCTGAAAGTCCTTCACTGAGCTAAGCAGGAAGGGTATGCGCTTG
TCCAGCACATCAACGAGGGCTTCATGCACCAGGTTGCGGAAACATATGATCACACCAATGATCGTCATGC
GTTGCAGCACGCGGTCCACGTCCTGTAAGCGCTTAAATTGCTCCTTCATCACTTCAGGTTTGTCAAAACT
GGTTCGCAGTGTAATGAGCACTTCCTTGTTGGTGCTCACCAGCGACTTCAACTCTTGCACCTGGTTTGCT
AT
>dme_circ_0005406_junction_seq
ACTTCAACTCTTGCACCTGGTTTGCTATGTGTAGTTGCTAATGTGGGGTCTACCTT
>dme_circ_0005407
ATAGCCAAGCCCTCGGAGCTGCTGGCCAGCGTTCGAGCCTACATGAACGTGCTGCAGACAGTAGAGAACT
ACGTGCACATAGACATAACACGAGTTTTCAACAACTGCCTGTTGCAGCAAACACAGGCTTTAGACTCTCA
TGGCGAGAAAACCATTGCCGCTTTGTACAACACCTGGTACAGTGAGGTTCTGCTAAGGCGTGTCTCCGCT
GGCAACATTGTG
>dme_circ_0005407_junction_seq
AAGGCGTGTCTCCGCTGGCAACATTGTGATAGCCAAGCCCTCGGAGCTGCTGGCCA
>dme_circ_0005408
GAATGTGGTGACGCGCGTGGGTCGCTGGATTGACTTCAAGAACGATTACAAGACGTTGTACCCATGGTAC
ATGGAGTCCATTTGGTGGATCTTCAAGCAACTGTTCGACAAGGGTCTGGTCTACCAGGGCGTTAAGGTGA
TGCCCTATTCCACCGCCTGCACAACGTCCCTTTCGAACTTCGAGGCCAACCAAAACTACAAGGAGGTTGT
GGACCCTTGCGTAGTA
>dme_circ_0005408_junction_seq
CAAGGAGGTTGTGGACCCTTGCGTAGTAGAATGTGGTGACGCGCGTGGGTCGCTGG
>dme_circ_0005409
TATATTGTCCATTTCCTGCCAAATCCTCCTTCTCATATCGAACGATATTTTGTAGTAAGAATCGGTATGC
ATTGTACCACGGTAGGAACACATCTTTGATTATGTCGCGCACACCTTCTTCCTTAAAACGCAGACTCTCG
GCCCTTACGACGGGCGAATTGATAAGGTACAGTCGGAGAGCATCCGCTCCATACTTATGGACCACCTCCA
TTGGATCGGGATAGTTCTTCTTGCGCTTCGACATTTTTTGACCATCAGCAGCTAAGACCAGTCCACTTGC
AATGAGGTTTTTAAATGGTGCCTTATTAAATAGCGCCGTCGAAATCACAAGGAGTGTGTAAAACCAGCCT
CGGGTCTGATCGATACCCTCTGCTATAAAGTCAGCCGGGAAATTGTTCATAAAG
>dme_circ_0005409_junction_seq
AAAGTCAGCCGGGAAATTGTTCATAAAGTATATTGTCCATTTCCTGCCAAATCCTC
>dme_circ_0005410
GCTGATCAGTGCATTCAATCACTTGACACGCTTTACGATGTTCTATACACAATGGTTAAAATGATGGCTC
CTTTCACGCCTTACCTTACGGAGTACATATTCCAGCGGCTGGTATTGTTCCAGCCAGCCGGAACCCTAGA
GCATGCTGACTCAGTTCACTACCAGATGATGCCGGTCAGTCAGAAAAAATTTATTCGAAACGATATTGAG
CGCTCGGTGGCTTTGATGCAATCAGTTGTCGAGCTGGGTCGTGTAATGCGGGATCGAAGAACCTTGCCTG
TTAAGTATCCTGTTTCGGAGATCATAGCAATTCACAAGGACTCGCAAATCCTGGAGGCAATCAAGACCTT
GCAGGACTTCATTCTTAGCGAACTCAATGTTCGAAAACTTACGCTAAGCTCTGATAAGGAGAAATATGGT
GTAACATTGAGAGCAGAGCCTGACCACAAGGGCGCTAGGTCAGCGGCTTAAGGGCAATTTCAAAGCGGTT
ATGGCTGCTATCAAGGCTCTCAGGGATGATGAAATTCAGAAGCAAGTTTCGCAGGGTTACTTTGACATTC
TGGATCAAAGAATTGAACTTGATGAAGTTCGCATCATTTACTGCACATCTGAGCAGGTTGGAGGAAACTT
TGAGGCTCACAGCGATAATGAGGTTTTG
>dme_circ_0005410_junction_seq

TGAGGCTCACAGCGATAATGAGGTTTTGGCTGATCAGTGCATTCAATCACTTGACA
>dme_circ_0005411
GATATATTTAACCAAGGCAAATTAGGAAGCTGTAATTCCTCAGTCGAACAAATAGTCAACTTTAAGGGAA
CTCCTTTAAGATCGACAAGCTCACTAGCTATTAAACGCTTCTTGGACGCCTGCTCAGATGAATAGGGAGC
AAACTCCGACTTAATGGCCGTCTTGATTATGCTGGCATAG
>dme_circ_0005411_junction_seq
AATGGCCGTCTTGATTATGCTGGCATAGGATATATTTAACCAAGGCAAATTAGGAA
>dme_circ_0005412
GTTCCCTTAAAGTTGACTATTTGTTCGACTGAGGAATTACAGCTTCCTAATTTGCCTTGGTTAAATATAT
CCTTGGCAGAAGATCTGGTGCCACGATTTGGAAATAGCAGCAAAGCGTCTCTATTTCTGCAGCACAACGT
TAGCAAGGAAATTATTTCACTGCCTACACTTCGCTCTGAGTTGGAGCACTTGTTTGGATT
>dme_circ_0005412_junction_seq
GCTCTGAGTTGGAGCACTTGTTTGGATTGTTCCCTTAAAGTTGACTATTTGTTCGA
>dme_circ_0005413
GGAATTACAGCTTCCTAATTTGCCTTGGTTAAATATATCCTTGGCAGAAGATCTGGTGCCACGATTTGGA
AATAGCAGCAAAGCGTCTCTATTTCTGCAGCACAACGTTAGCAAGGAAATTATTTCACTGCCTACACTTC
GCTCTGAGTTGGAGCACTTGTTTGGATTGTATGGA
>dme_circ_0005413_junction_seq
GTTGGAGCACTTGTTTGGATTGTATGGAGGAATTACAGCTTCCTAATTTGCCTTGG
>dme_circ_0005414
GTTAACTTTAATATTTACGTTGTTGATCACCAAAAAAGGACTACTGCACTAAAGTCTATCGATGAAAACC
TAAGCGGAAAATTGCTTGTTCTAACTCGAAGCCAAGATGCACCGAAACTATCAGCAGGCTATGAACTATC
CCCCGCTCCTTACTCCAAATTCATCAACCAGCATTCAGGAAAATCTATCTTCACGGAAAACCCCTTGGGA
CGCGCCTTAT
>dme_circ_0005414_junction_seq
ACGGAAAACCCCTTGGGACGCGCCTTATGTTAACTTTAATATTTACGTTGTTGATC
>dme_circ_0005415
ATAGATTTTCCTGAATGCTGGTTGATGAATTTGGAGTAAGGAGCGGGGGATAGTTCATAGCCTGCTGATA
GTTTCGGTGCATCTTGGCTTCGAGTTAGAACAAGCAATTTTCCGCTTAGGTTTTCATCGATAGACTTTAG
TGCAGTAGTCCTTTTTTGGTGATCAACAACG
>dme_circ_0005415_junction_seq
AGTAGTCCTTTTTTGGTGATCAACAACGATAGATTTTCCTGAATGCTGGTTGATGA
>dme_circ_0005416
GAATTTTTATCAGTCCCCTTTCAATGCAATACAATACTCTAACGACTCTTTTCACTCATTTTATAATGTT
AGCTGTTGAATTTATTCAAAGGAAAACGCATTAAAAACTTTTTACAAATTGTGATATATATTTCTAAACA
TTTCATTTGACAACTTGAAGTGTAGCGAAGAAAAACTCGAATTAACTCAAATCGAACCGAAAGTAATAGC
AAACAACGCATGTACTTTACACATTTTTTAAACATAGTCTCTTACGATTAGTTTTATGCTGCAATATT
TAGTTCTTACCGTTTGCCATGTTAATTGTAAAAACAATAATGTTTTTTGTAATGATCCCACCCGGGAATG
TCACATAGTGTTTAGTTTCTTTTAATTACTCATTTTTAAAAACACTCAAGCAAGGAAGATAAAATTTCTG
TACTCCCTTAAATTTGGCTGATATTTTGTCGGTATTTTGTGTTTAAAATTATTATtaaattatttataat
tgaaactaaactattaactattgaaaTATTACCTAGCATAAAATATAGGACTTGCGCAGAACTTAAACAT
ACCCCTATAACCATAAAAACACACCTTG
>dme_circ_0005416_junction_seq
ACCCCTATAACCATAAAAACACACCTTGGAATTTTTATCAGTCCCCTTTCAATGCA

>dme_circ_0005417
GGGGACTGATAAAAATTCCTTTTGATTCTTGGCTATCCAAATTAGGTAGACGCGTCTATTAAATTTAAAT
TCGAGCATTCGGCTTAGAGACCTATTTCGCTAAAATGAGTGTTAGGATAAAGGATACGCGCTTTTTCGTC
AGTGGAGGCTTGAAAAGTATTCGTTCGTATATACACAGAGCAATTATGAAGAA
>dme_circ_0005417_junction_seq
CGTATATACACAGAGCAATTATGAAGAAGGGGACTGATAAAAATTCCTTTTGATTC
>dme_circ_0005418
GGCTTGAAAAGTATTCGTTCGTATATACACAGAGCAATTATGAAGAAGTAGGTTAAGCAGCGATCAACAT
TTCTGTTGCCATTTGAGAGTGGGCTGTGTTGGAAAGAGTCCAGGTTTGGACCAAAGACTAGGGAGCTACT
ATGCAGAACTTTAATCTTTTTACATTTATTCATTTCGTAGTTTAACATCTGTACTTATCGTATGCATAAT
TTGCTGTGTAATACTCATC
>dme_circ_0005418_junction_seq
ATGCATAATTTGCTGTGTAATACTCATCGGCTTGAAAAGTATTCGTTCGTATATAC
>dme_circ_0005419
ATTTTGTAATTAGGTGAAATGCGCAACAGCGACCACAAGAAAAACAACAGCACAACTCTCGATGACACAA
CACAAAATAAGACAACAACAAAAACAAAGGAAAAGCAAGCATTGAACACACTAATTAATATAAACTTTAA
TCATGCTTAAAGTTTAGTATTAGCCACAACGCAACCCTCGAATAACCGAAGAAACTTCTTATGTG
>dme_circ_0005419_junction_seq
TCGAATAACCGAAGAAACTTCTTATGTGATTTTGTAATTAGGTGAAATGCGCAACA
>dme_circ_0005420
GTTTCTTCGGTTATTCGAGGGTTGCGTTGTGGCTAATACTAAACTTTAAGCATGATTAAAGTTTATATTA
ATTAGTGTGTTCAATGCTTGCTTTTCCTTTGTTTTTGTTGTTGTCTTATTTTGTGTTGTGTCATCGAGAG
TTGTGCTGTTGTTTTTCTTGTGGTCGCTGTTGCGCATTTCACCTAATTACAAAATCTGCTCGTCAA
>dme_circ_0005420_junction_seq
TCACCTAATTACAAAATCTGCTCGTCAAGTTTCTTCGGTTATTCGAGGGTTGCGTT
>dme_circ_0005421
GCCATCGCCGTACTTGATCTCGTTCAGACGGCCATTGCGGTCGAAAGAGTAGGCTTCTTGCAGGACACCC
CACTTCCAACTGACCAGACGTCCGAACCGATCGTACTCCAAGTCCACGTCCGCGTAGTCGCCGGACTGCG
GTCGGAAGCTGATGGGGCGGGAAGTGCGATCGTAGGTCACGTTAAGCAGCTCCTGCTTGTCGTCCACCAT
AACGACCACCGACTGGGTCTCGCGGTCGTACTCCAAAGTCAGCACGTTGTCTCCGTTCACACGCAGCTTA
CGTCCAACTTCGGTTACGGGACGTGGCGGTCCCTTGCTCTGTTTTCCCGCTTGCAGCGGCTGCTGCCGGC
GCACAAA
>dme_circ_0005421_junction_seq
CTTGCAGCGGCTGCTGCCGGCGCACAAAGCCATCGCCGTACTTGATCTCGTTCAGA
>dme_circ_0005422
GTGTTCCGCTTCGAAGCAGTCGCAACCGCGCTCCAGGCAATTGCACTTGGATTCGGCACCAGCAAACGGA
GCGATCCTTCCGTCCGTGCCGATCACGCGCACCCGGTTTATTCGTTGAGAGTCGCTTTCCGCCACGTAAA
GCTCACCGAGTGGACCGAACGCGATAGACTGCGGCATCACCAGGGTAGCGTGGGTAGCCAGATCCGTATC
GTAGGCAGTGGAGGCT
>dme_circ_0005422_junction_seq
CAGATCCGTATCGTAGGCAGTGGAGGCTGTGTTCCGCTTCGAAGCAGTCGCAACCG
>dme_circ_0005423
GTACAAGATGTTGTCGCTCGAAATCGCAATGCCTTTCGGATAAGCCAATTTGGCATCCTTTGCCAGTGCC
CCATCGCCGCAGTGCGCTTCGTCACCAGGAAGACAGCGCTCCCCGGAGCCCACAACCGCTTCCCAGTTTA

GTTCCGGTTGCGAGTAGTCGTTGGTGTCGCGTACCCGAATGATTTGGTGTGATTCTGGATCCGAAACATA
AAGAGTTCCGTCGAGCGGGCTGAGGGCCATGTGGTAGCG
>dme_circ_0005423_junction_seq
CGAGCGGGCTGAGGGCCATGTGGTAGCGGTACAAGATGTTGTCGCTCGAAATCGCA
>dme_circ_0005424
GCGGTACGAGACGCGAGTCGCGTTCAGCTTGACCACAGTGCGGATGCTGCCGTCGGTCATGATGCGGCGG
ATGTAATTAAAATCACCGACGAAAAGACTGCCGTCAGGAGCGGCGGCCAGTGCGACGGGTGCCAGAAGTC
GCTGCTTTGTCGCCTGGCCGTCGCAATCGGGACACTCCAGAGGCCGCTGGTGGCCATCTCCCATTGTGGT
CAGGATGATTCGCGGCTTATTGCGCAGATATAT
>dme_circ_0005424_junction_seq
TGATTCGCGGCTTATTGCGCAGATATATGCGGTACGAGACGCGAGTCGCGTTCAGC
>dme_circ_0005425
GGGCATGCAAACGGCACACCACCAGCACCAACAGCGGGTTCAGTAAAGGCGGCAATGTGGTTATCGATCT
CAGTGCTAGGGAGGTTAGTAGCCGTGCTTGAAGAAGGTGTTTGCTCCTCATCTCTAACCCCGTCTTCGTC
ATCCTCCTCCTCGCTATGGAAGGGGTTAATAGGTGCCGAGATCGTGCTCGTC
>dme_circ_0005425_junction_seq
GTTAATAGGTGCCGAGATCGTGCTCGTCGGGCATGCAAACGGCACACCACCAGCAC
>dme_circ_0005426
GATATCGATATAGTAAAACTAGATGGATTGACGATCTCCCACGAGGAGGAGATTTACAAGACGGCAGATA
AGGAAAATATGGCACCAAAAAATCAACCATCTCAGCATATAGATAGAAGTCAAAACGAGGTACTGAAAGG
CCATCAGCAGGGTGACGAAAAACAACCCCAACTAGAGCCCCTTAAACCGTATGTGAG
>dme_circ_0005426_junction_seq
AACTAGAGCCCCTTAAACCGTATGTGAGGATATCGATATAGTAAAACTAGATGGAT
>dme_circ_0005427
GGAGCAAATCCAACGAATAGCAGGACTCGCCTGGTCCCCCAATCAGCAGAGGTTGGCGATAGCCACCGCA
GATCGTCATATTCTACTGTACGATGATGCTGGCGAAAGACGCGATAAGTTCAGCACCAAGCCAGCGAATC
CCGCGAATGGTAAGAACTCCTATGTCATCCGGGGACTGGCCTTCAGTCCGGACTCCACCAAACTGGCGGT
TGGCCAGAGCGACAGCATCGTCTATGTGTACAAGTTGGGGGAGTCGTGGAACGACAAGAAGGTCATATGC
AACAAGTTTCCTCAGGCCAGTGCAGTGACTGCTCTGATTTGGCTCACCTCGGGATCCATAATAGCAGGTA
AATCACCGTTTCCAAATGAAATGAACATATTTCATCAGGTATTTACCACAGGACTCGAGGACGGCAAAGT
GCGTGCCCTGCACAGCAAGAGCAATAAATCGCAGAGTCTCTACGGCGGCGACAGCATCTGCATATCCCTG
GCGGCCAATACCAAGGGAACGGGTTTTCTGAGCGGCCACAACGATGGCACCATCATACGCTACTTCATGA
CGGATGAGGCCACCGAACCGCTGGGCAGAGTAGTGCAACATCCAGTTCCACCGTTTGCCTTAGCCTGGCC
ACAGGGTGGCTTCTGTGCCGGTGGCTGTGATCAGAGAATAGTGTTCTATGACAGTATGGGGTCGTCAACT
ACGCACCTTTGATCATTCCCGGACGGAAGGCGAACGGGAGTTCACGGTGGCCGCTTGTAGTCCGAATGGA
CAGGCGGTGGCCTTTGGCAGCTTCGACAGGATACGCATCTTCGCGTGGAGTCCTCGTCAAGGAGCTTGGA
GCGAAAGCGCAACCAAGGAGGTGGCCTGCCTGTACACGCTAAGCAGCCTCCTCTGGCGAAGAGATGGAGC
CCGACTCGCCCTGGGATCCGTGAGTGGAGCTGTGCTCCTCTTTGAGTCCGTACTTCGACGCACCGTGTGG
CAGGACAAGTTCGAGCTGATCTTCGTGGCGCCCAGTCAGCTCTTGGTCCGATCCTTGACAGAGCCCTCGC
AAGCTCTCACTATTGAATCGCAGCTGGGTCTAGAGATCGATGACGTTAGGATTATGGGTCGGGATAATTA
CCTGGTAGCCCGAACGGAGGAGTCTCTTATCCTTTGTGATTTGACCAGAAATTTGAGCAGTGAGGTTCCC
TGGACTGCCTCTGGTCACCATGAACGCTTCTACTTCGAGAATCCAACCGTTTGCCTGATCTTCAATGTGG
GCGAATTGAGCCTGGTGGAGTACGGTGAGAACTCCATCCTGGGATCGGTACGCACGGAATTCGTGAATCC
CCACGTGATCTCAGTGCGTCTGAACGAGCGAGGAAATGCAAAGGAAAACAAGAAACTGGCCTTCCTCTTG

GATGCCAAGACCATATGTGTGGTGGATCTGATTAGCAGGATGACCAGTGGACAGATCAGTCATGAGACGA
AAATCGACTGGCTGGAATTGAGTGAGACTGCCCATAAGCTCCTGTTCCGCGATAAGAAACTGCGCTTGAT
CCTAGTGGATAATTACACTGGCAAGAAGCAGACCTTGCTCAGCAACATATCCTTTGTACAGTGGGTCCCC
CAGAGCGATGTTGTGGTGGCCCAGAGCAACTCAAACCTGGCCATCTGGTACAACATTGATCTTCCGGAGC
ATGTGACCATGCAAAGCGTGCGAGGTGAAGCCATCGAGGTTCTCCGTGAAAATGGGCCGCACAGTGGTTC
GCTCCCAAGACGGACCCAGCGAGCACAACTACCAGCTGGATGAGGGGCTAGTGGAGTTCGGCACTGCCGT
AAATGACAGTGATTTCGGAAGAGCCGTGCACTTCCTGGAGTCCCTGGGTGACAAACCGGCAGCAAAAGCC
ATGTGGCACAATCTGGCTTTAATCGCCTTGGAAGATGGAAACCTTCGCGTGGCCCAGCGCTGCTATGCTG
CATTGGGTAACGTATCAAAAGCGTACTACCTGGCTGGAATGATCCAGCAGGCAGATGAGTTCGAGGAATC
CTCTGGCTCTCCTGGCATCCTCTGCCCGGAAGTCCGCGCCAAGCTGGCTCTGCTCGGCTCGGACTTGCGC
ACTGCTGAGCGTATATATCTGGAGCAAGGTGACATAGAGTCAGCTCTGAAGATGTACCAACAGCTGGGCA
TGTGGGATGAGGCAGTGGCTCTGGCCGAGAGGCGTGGATATAACAGGATCGCCGAGCTGAAGCAACAGCA
CATGGACTACTTGCTGAGTAGCGAGCAGCAGGAGAAGGCTGGTCAGGTGCTCGAGGAGCAAGGTGATCTC
CAGCAGGCGATGTCCCTGTACATGAAGGCCAACAAACCGGCGAGGGCTGCTCGTTTGGCTCTGAAGACAC
CGCACATCCTGCAGGATGAGCAGGTAATGCTGCAAGTGACTGAGGGACTTGTTCGCTCCGAGCTCTATGA
ACTGGCTGGAGACATAGCCCATCGGCTATCCCGGCCCGAAGCGGCACTAGCTCTTTACAGAAAGGGCGGA
GCATATGCCAGAGCACTGGAAATAGGACGAGTGGTGGCCCCACAGGAGGTAACGGCGCTGGAGGAGGAGT
GGGGCGACTGGTTGGTGAGCCGCAAACAACTGGATGCCTCGATAAACCACTACATCGAGGCGGGAGCCAC
GCAAAAGGCCTTGGAAGCCGCCGTGGGCGCGAAGCAATGGCGCAAAGCAGTCCAGATAGCCAAAGTCCTG
GATGAACCGGAGCTAATACAGCGTTATGCTGTGGATCTAGCCAAGCATCTGGCCTTCGCGGGAGATCTGG
ATGGAGCAGAGGATATGTTGGTGCGAGCCAACCTGCACAAGGATGCCATAGAGCTACTCAATCGTCATGG
CAAATGGGAGCGTGCCTATTTGATTGGCGAGAAACACCTTAAGGCGGAGCAAGTCCGTGAGCTCTTCGTT
CAACTGGCTGGAACGCTGGAGGAGCAGGGCAAGTTTAGGGATGCCGAGAAGGTCCTGATAGCGGTCAACG
AACCCGACCTGGCCATAGCTATGTACAAGCGGCGGGAATTGTACGACTCCATGATCCGATTGGTGGAGCG
TTATCACAAGGATCTGCTGGATAGCACCCACTTGCATTTGGCTCGCCAACTGGAATCCCGTGGTAAGCTG
AAGAACGCCGAGATGCATTTCGTAGCCTCCGGGGACTGGAAGTCCGCCGTGCATATGTACTGCTCCTCTG
GTCGCTGGGAGGATGGCTACAGGGTGGCAAAGCTGAAGGGCACCGAGGGAGCCAGCCAGCAAGTGGCCTA
CATGTGGGCCAAGTCAATGCCCACAGAGGGTGCAGTTAGGTTGCTGAGTAAACTGGGTCTGCTGGACACA
GCAGTGGGATTCGCCTGTGATTCCGGACAATTCGAATTCGCCATGGAGCTGTGCAAGTTCGCTGGGAAGC
CAACGGATGAGGTGCACCTGAAGATCGCCATGTCGTTGGAGGATGAGGGGAAGTTTGAGGCGGCGGAGGC
GGAATTCCTGAAGGCCAATAAGCCCAGGGAGGCTATTTTGATGTACCAGCATGCAGGCGATTGGCAGGCG
GCCCTAAATGTGGCCGAAAATCATCTGCCCGATGCTGTGGGAGAAGTACTGATAGGACAAGCTTCGGCTG
CCCTGGAAACCAGTAACTATAAGGACTACGAAGCTCTCCTTTTGAGGGCCCATCGTCCGGACTTAATCAT
AGAGCACTACAAGCAGGAATCCCTCTACGAAGATGCCCTTCGCATCGCCGAGGAACATTATCCATCGGCA
CTGAACGACCTGCGGCGTCTTCAAGCCCAACTTCAACGGGGACAGGCGCAGGCACAAGCTGGCGAGGATG
CCGCCTCCATTTCGCGATCTTATCTGCAAAAGGCAGCAGAGTTTGCCAAGAAGGAGCAGTTTCGTAAGGC
GGCCGAGTGCCTCATGCAGATAGATTCCTCCAATGCCGAGGATGCATCCACGTTGGAAAGAGCTCTGCTA
CGAGCCGCTGAGATTTGCAATCAGTTTTTGGAGGGTCAGGATGCCCAGGAGTTGGCCCAGTCGCTGGGAC
CGCGGTTACTGGCCATAAAGCAGATAGGACCTGCAGCTCAGCTTTACTTGGCGGCGGATATGCCCAAGCA
GGCGGTGGACGTGTTCATCAAGACGGAGCAATGGAGCAAGGCTCGTCGGCTGGCCAAGGAGATCGATCCA
GATCTACAGCTTCTGGCCTATGTGGAGCAGCAGCAGAAGGCTAGCCTCAAGCACGAGGGCAACATTGAGC
AGCTGGCGGACATAGACATAGTGTCCGCCCTAGATCTCCTGGCGGAGCATGGTGAGTGGCAGCGCTGCTT
GGAGAAGGCCAAAGGGCTGAATCCCGCTCTGCTGCAGAAATACGTGGCCGTCTATGCAGCCCAACTTATT
CGGGAAGGCAACTGCACCACAGCCCTGGGGCTCTACTTAAGCTACGGAGCGCCACCGATCGAAGCTCACT

TTAATATATACACCAGGATAGCACTGGACTGTTTGGCTCTGAGGGAGGAGCAAACGGAGAGAGGCACCAC
TTGGCGCCAGTTGAGGGACTTTCTCTTCCGCTTGCTGCAGTCTCTTAAGGCCACTCCGGCGCAGTCCCAG
ACGAAATTTGCAGCCAGCATGGAGCAGTTCCTGTTAATAGCCCACTACTACGCCACCAGAGCCGCATGCA
AGGAGGTACAGGCGCTTCAACCGGTGGCTCTGCGCTTATCCTTGGCTCTGCTTCGCCACACGGATCTGCT
GCCGGTGGACAAGGGTTTCTACGAAGCTGGGATGGATCTCCGCCAGGCAGGTCGCGAAGCCGAGGCCTTC
GTTATGCTCAACCACTATCTGGACGTGTGCGAGGCCATTGAGGAGGGTTCCGGTCAGTTGGTAGACCACT
CCGACCTGGCTAGCACCGATTTTCCCAGTTCCGTGCCCTTGCCGGAGGATATCCACCTGAAGAACGATCC
CAGTCTTCACGAGGAGGTGCGTGAATGGGTTCTCGCCGTGAGCATGGACCAGCAAGTGGACCAACAGCTG
CCCACCGATGATCGCGGCTTGTACGAGTCCAGTTTGGGGCCCAACGATCTGCCCTGCATGCTGAGTGGAT
TCCCGGTAAGAGGCCGTCAACCGGTGACCTTTCAAGGATCGAGCAACCAGGTAAACCGCGACGTGTGGAG
CAAGTTCTCGGTCGCCTTGAAGATGTCACCGGGCAGCGGAATTGCCGACATCATTTCGTTCACCGAAAAA
TGGCAGGGAGCAGCCAACTACGTGATGCATTGAAGATGTACAGAAGAAGAAGTCCAAGAAT
>dme_circ_0005427_junction_seq
AGATGTACAGAAGAAGAAGTCCAAGAATGGAGCAAATCCAACGAATAGCAGGACTC
>dme_circ_0005428
GGTTTGTGGACATCGTGAAGCCTTTTCCAAAACATACTTTGGCCAATGCCACTTATAAGATCATTCGCTT
CTTTTACCCACCCTTTGCCCTGGAAACCGTCGATGTCACCCACGTGGTCAGCGATCCCGAGCTGAATAGT
TTAAACGTGGGGCT
>dme_circ_0005428_junction_seq
CCGAGCTGAATAGTTTAAACGTGGGGCTGGTTTGTGGACATCGTGAAGCCTTTTCC
>dme_circ_0005429
GCTTCCTCCTGGAAGGAGTCACGCCAACGGCACCACCGGATGTTCCGCCGCGTAATCCGACAATGAGTCG
CATGCAAAATGGTCGTTTAACCGTTAACAATCCCAATGACGCTGACTTTGAGCCATCTTGTTTGGTGCGA
ACGCCATCGGGCAACGTTTACATTCCTAGTGGAAATCTAAG
>dme_circ_0005429_junction_seq
ACGTTTACATTCCTAGTGGAAATCTAAGGCTTCCTCCTGGAAGGAGTCACGCCAAC
>dme_circ_0005430
GTATGTTTCTCGATGAGTTTCTCACTGGCTATAAATTTCCCCAACGTGAGTTTAAGCTATGGCCAACTGG
GCAATCTACCCCCCACCACAGCAAGTTTCGAAATAATTAATGGATGAAGCAACACAACACACTAGTTTTG
ATTCCCCTCACTGTTTGTCGGCCAGCTCACTTTCGTTTTAAAGCTGCCAGTTGAAGCCAATAAGCCAAAG
TAACTGGGTAAACACATGTACCAGCAGCAGCACCCAAATGGCCAACAATTTTTAAACAAGATAGAAAACA
CCCGGGAACACGTGTACACAAAGATACACAAAGATACGCCACATACACCCGCAGTCGCACCCGCTTAAAC
TCTGTGAAAATAGTTTCAATAAAGGCAGCAGCAGAGGCAGCAGGAGCAGCAGAGCCAACAAGCTTGGTAC
ACGTGCTGGTGGCTGGCCACGCTCACTAACCTACCGCCCACCGTCTCCTGCCATTGTTATTATTTATGCG
AGCCAAACAGCAGCTTCCGTTTGTGCCACATGCGGCTGGCGGAGGCATGCAACACACGGTGGACAGGAGC
GGAAGAGAAGAGCATTTAAAAAATGTATGCAGCGAAGGCCTTGACTGCAAAATGTTGCCTAATGGCGCAC
TAGCTAGCTGCATTTTTATAGAttttttttACCAGGTCAGCGGATCAGACAAGTGGGGGCAGCAGGGCGC
TTGGTCGTTGGCTTGTTTAACATTTTCCACAATTTCCTTAATCACAGGCTGCTGGCCTAGGAAACACAGC
TGGTACGCGCACTACCCACTACCCTATACCCCCTTGCATGCCATTAAAAGTCAACTGTTTGTTTGCCAGC
GAGTTAGTGTGCGAGAGTGTCAGTGCGCGCCTCAAGTTATGCTACATTTGGTGGCGGTTCGTGGAGCCTC
TGCAACATTTTAATTGCTTTACATAATGCAGCACCAGCATGGCTATGCATAAGCGGGAGGTTGGTCAATG
GTGCCTTCTTTAATATTGCTCTTTTGCAGTTTTTCATTATTTTTAATTGTTTGTGCAGCCATGTGTGCTG
GCACTAAAAGTGCGGCACAGCCAAATGGCTAAATTAATTTGaaaaaaaaTGCAATCAAAGCCGGCTGAGG
CTGGTCATTGAGTGTTGCGATTGGGCTGGCAAATTAGAGAGAAATATTCTTCCAGCATCCAACATCATTA

TTCACATGCCTCACAGCCAACTTGGCTGCTCCCCACGCAGCATAATTACCAGGCGGAAACTTTTCGCATG
CAGTGGCTCCTGTTTTCCCCCTGGCCACCCCACAAACAGGATCAGCGGAAACAACAGCGGCAGGCTGAAA
TAAACTTGAAAAATTCTGCGCAGGAGCAAAAGTTCCTTAAGACGTTCAGGGCTCAGAAACAGAAGCAGGA
ACATTAATAAAACCGCAAGTTTCCACCCCTTGAAGGCAGGAAAAAAGAAAACGAAAGTTTGGCCACTTTT
CAATTTTGGGAGCACTTCAATTTCCACTTCCACCAGGGCTATATCCAGTCGAAACTGAAGCTGGGAGGGA
ACTTCAAAAATTTAACGACTCTCCAAAGCCCACTAAAAGCCAGCGAGAATGGAATCCGGAGAAAGAGCAG
TGCAGGAAATTTAAATTATATTAGGAAAAATGAAGGGTCTTCCAGCAGACCCTCTGTCCGTCTGGACTTT
TGCAATCGGAGCGAGTAATAAATAAACAAAACGACAAAGCTCAAAGCTGAAAATCTACACAAAGAGCCAC
TTGACCAGGGCAGAAGCAATTGAGAGTAGAAAGGCGACATGGAAATAGAATGAAAACTTTGCTTTCAAGT
CACTCCAATTAAAGTTGCTCAGATGGAGACCGAGATGGAGATAAGGGAACGGATCCATCCCGACCCCCAC
CCCACCTTTCGACTATGTTATTTTCCCACATTCGTAACGCGAAACTTGTAATGTATACATGGCAGACATT
CCATCaaaaaaaaataaaaaataaataaaGTACACCTACCACGCCCACTTTTCACCGCCTCACCCGAAAAC
TTTTCTATACACTCGCATTATTTTCTGGCTCGCCCCACGTGACGAGAAGTTTTCCACTCTCTGATGTCAT
GTTGTTGTTGTACCCCAATAATGATTTGTTCTTGTTGCCCAGGTCGTTCATGTTTCGCGTTTGCATAGCA
ACTTTTGTCAGCTGGCAAATTGAATGAGATTGCTGCCAAAGATGGAATCCAATTAGAATTTGAATCAAAA
TAGAAAAGTTCCTTTGAACAAGGAGCGGAAAAAAATACGAGCACGAAAGCATTAAAATCGTTATAATTGCT
TCAACCAGTTGGGAATTGTAAAGGCACCTAGAACTGAAAGCTTGTGCCTAGTACCTCCCCCATTTCGCAC
ATTTCCCCCTTCTAAGCAGTCATTGTTTGACGCGAAACAAAGGAAAGAGCTGGCCAAGCAGCCATAGAGG
ACCATATCTATCCGCAAACTAAACCCTCGAG
>dme_circ_0005430_junction_seq
ATATCTATCCGCAAACTAAACCCTCGAGGTATGTTTCTCGATGAGTTTCTCACTGG
>dme_circ_0005431
GTATGTTTCTCGATGAGTTTCTCACTGGCTATAAATTTCCCCAACGTGAGTTTAAGCTATGGCCAACTGG
GCAATCTACCCCCCACCACAGCAAGTTTCGAAATAATTAATGGATGAAGCAACACAACACACTAGTTTTG
ATTCCCCTCACTGTTTGTCGGCCAGCTCACTTTCGTTTTAAAGCTGCCAGTTGAAGCCAATAAGCCAAAG
TAACTGGGTAAACACATGTACCAGCAGCAGCACCCAAATGGCCAACAATTTTTAAACAAGATAGAAAACA
CCCGGGAACACGTGTACACAAAGATACACAAAGATACGCCACATACACCCGCAGTCGCACCCGCTTAAAC
TCTGTGAAAATAGTTTCAATAAAGGCAGCAGCAGAGGCAGCAGGAGCAGCAGAGCCAACAAGCTTGGTAC
ACGTGCTGGTGGCTGGCCACGCTCACTAACCTACCGCCCACCGTCTCCTGCCATTGTTATTATTTATGCG
AGCCAAACAGCAGCTTCCGTTTGTGCCACATGCGGCTGGCGGAGGCATGCAACACACGGTGGACAGGAGC
GGAAGAGAAGAGCATTTAAAAAATGTATGCAGCGAAGGCCTTGACTGCAAAATGTTGCCTAATGGCGCAC
TAGCTAGCTGCATTTTTATAGAttttttttACCAGGTCAGCGGATCAGACAAGTGGGGGCAGCAGGGCGC
TTGGTCGTTGGCTTGTTTAACATTTTCCACAATTTCCTTAATCACAGGCTGCTGGCCTAGGAAACACAGC
TGG
>dme_circ_0005431_junction_seq
AGGCTGCTGGCCTAGGAAACACAGCTGGGTATGTTTCTCGATGAGTTTCTCACTGG
>dme_circ_0005432
GTATAGGCCGGCAGAGCGGGCGGCTGACCCGCTCCCGACGTGGATCCTCCCGCCTGCGACTTGGTGGAGC
TCTTCGGCGAGACCTGCGGCTTGCGCAGCGTGTTGGCCGAGTAGCCAGTCTTCGGCCACCGCTTGCCGGC
AAACAAGTCGGCCACATAGGCACTCGTCAGCCAAGCGAACACCACGCCCAATGCCGCCCCCGCCAGGACG
TCGCTCCAGTGGTGG
>dme_circ_0005432_junction_seq
CCCCGCCAGGACGTCGCTCCAGTGGTGGGTATAGGCCGGCAGAGCGGGCGGCTGAC
>dme_circ_0005433

GTGTTTCTGGCTCTTGTCTAATCTGGAAAAATGAAGAGCCGCAACAATAATTACATCCCCGCAAACGGCT
AACTACCATAAAATAGGCCATAAACACTCTGCTCTGACTCAGAGAATTTAAAAATAAGAGAGCACTGGGG
AGCCAAAAACAATGCCGAATGTCGGGAGAGTGCCGCCTATTAAATGAGCGTTAAATTAAAGAACGTTACC
CAAACGACGCCAACTCAGTCAGCCACTGACAACGAACCCGACGAAACGATCGACGCGGCGGGAAAACAAG
CAAACGAATCGAAAAGCTAATTACCAGCAGGCAGAAAGGCCATTTGGAATATTAACTAATTAAGCCAGTG
AAATGCGCGACGCACCAGAAGATCAGTCCGTAACGGTTTCCATGCCGGCATCTGCATCCGGCTCTGCCGC
CGGCTCCACGGGACCGTCATCGGATCGCCGGATGACGCAGCGCCTGCTCGTGGAGCTCCTGGTGGTGGTG
GTGCTCGTCATTCCCATCTGCGTGTACGAGTTCGCGGTGGATCCCGTGCGACGCGGCTTTTTCTGCGACG
ACGAGAGCATCAGCTATCCATTCCAGGACAACACCATAACGCCGGTAATGCTCGGCCTGATCGTGGGCCT
GCTTCCGGCCCTGGTCATGGTGGTGGTGGAGTACGTTAGCCACCTGCGCGCCGGAGACATCTCGGCCACG
GTGGATCTGCTTGGCTGGAGGGTATCCACATGGTACGTGGAGCTGGGCCGGCAGTCCACCTACTTCTGCT
TTGGCCTGCTGCTCACCTTTGACGCTACCGAGGTGGGCAAGTACACCATCGGACGCCTGAGACCGCACTT
CCTGGCCGTTTGCCAGCCGCAGATTGCGGATGGGAGCATGTGCTCCGATCCGGTCAACCTGCACCGCTAC
ATGGAGAACTACGACTGCGCCGGCGAGGGCTTCACCGTGGAGGACGTGCGACAGGCCCGCCTCAGCTTTC
CCAGCGGTCACTCTAGTTTGGCCTTCTATGCCATGATCTACGTGGCACTCTACCTCCAGCGGAAGATCAC
CTGGCGGGGATCGAAGCTGAGCCGGCACTTTGTGCAGTTCGCAGTGGTGATGGTAGCCTGGTATACGGCC
CTCAGCCGTGTAATGGACCATTGGCACCACTGGTCCGACGTCCTTTCCGGATCGCTACTTGGCGTGGCTG
GCGCTCTAATCACCGCCCACTATATAGCAAGGATGTTCGACGACGGGGCAAGCAATATTCTAAGCGGTGG
CCTGAGGAGGGAAAACACAGCGGCCACTCTGCAGGAAGAGGTCTGCCCCACCACTCCCCCGCCGTACTCC
GTGAACAACAGCTTCAGCGAGGATCAGTACTGCTCTAAGGTTTAGTGAGACGGATAAGATGCTTTCGTAG
CGATGTCAATTCAAAACATTCCAAAG
>dme_circ_0005433_junction_seq
AGCGATGTCAATTCAAAACATTCCAAAGGTGTTTCTGGCTCTTGTCTAATCTGGAA
>dme_circ_0005434
GAACGAATCCAAAGAGTCCGAAGTAGATGTGCAGCGGCATCATGGCGATCCTGTAGTTCTCCCTCAACCC
CGGCGCCAAAAAGGCCACAAATCCGGCCACATACTGCAGCGAGAAGACGATCACCGCGGAGAGCCCCAGC
CAAGAGTGCAGGGA
>dme_circ_0005434_junction_seq
CGGAGAGCCCCAGCCAAGAGTGCAGGGAGAACGAATCCAAAGAGTCCGAAGTAGAT
>dme_circ_0005435
GTAAGTGGCACCTATCCCCAGAAGGGTGAGTACCGTTTATTCGCCTTATCTCGGCGACTAGGGAGATTTT
AAATAAAGAAATTAATATCAGAAAATTCTCCAACAATGAAACTTTGCCATGGAAACGCGGAAAAACTCTC
GGAATTGATGCGATAAAAGTTTAATTCCAAGTATGGCTGCCCACCACTCCTCTTCGACAACTTCGTAGGC
CTAATGTGCTGACAATCGTTGGATTCCAGACCACTCAGACCTCAGACTCCCCCCTAGTATAATTTAAACT
TTAAAATGACCGGGGACGATCCTAGGCAACAACAGAAAAGGCAGAATGCAATCGGGGGGTTGGAAATGTA
CGACGTAAACTTCTACAAAAGTTGTAAAAAATGAAAAAGTTCGCCAATAAACATTAGAAAAACTTTTGCG
CCTCATCTGACCTTACCATTTTGTGATTTGAATCGCTCTACATGCCCACAAAATCAGTAGGCATATTGCG
AATCCCTTTCTGCGATGGCACCTGCCTAGCGACAAGTTTATCAAATGTTTTCAAACTCAGCCAAATTAAG
CCCAATCCCGAAATATCATCTAAAGTTATCATTATGCTTCTTCTGTCTGTCGCCAGGCAAATGACGAGGG
CCACTTAAGGTTTGAGAAGGTCTGGAGCGATTTTGGCCGAGCCGTCATGGTAATAAGACAAACTGGTaaa
aaaaaCATCAAATATTAATTAAGTTTGCGCCTAATTAGTTTGATTCAGATCCAAAATGCTGCCTTTTCTC
CTTTGCCCAGGGTTAGACATCAAAAAGGTGTTGGCTTTGGAACTCAGGCTCAATTTTGGCAAAAACGCCA
ATGAATTACACTCGCCTGAGATGGTTACAATTGCTCAATTAAAGTCTAATTACAGAGGAACCATGGAGCA
AGGCGGACGAATCGGGTCAAAAGTTAAACAATGTGGCAAACAAAATATTTGACAAAACGGGAACGGCCAA

AGCCAGAACTGGTGCGAAGGATGTCTAAG
>dme_circ_0005435_junction_seq
GCCAGAACTGGTGCGAAGGATGTCTAAGGTAAGTGGCACCTATCCCCAGAAGGGTG
>dme_circ_0005436
GTTTTTATCTTTCTGTCTTCGCTCGGTACACTTTTCTGGACTTCTCTTTCACCGTTTGTGATTTTGGCAA
GCGATTATTGGACCCCTTTCCATTCAAACTCCCCATCTTCCTAATCAATAGGCTAGATAAGGCCGCTTAA
ATCTTTGTAGCTGCATGTCACAGTTGATCGGTTCCTGTGGGTGCTTTTGTTTGATGGGATTGCTTTGCGG
TCTTCAAGTATTCACGGTCTTGAGGGCTCCAGACGAGGATCTTTCCAGCAAATCTATGCGCAAGCAGGCG
TCAAAGAGTTTGTAGACTAGGCACGACCATCACATTTCGttttttttttttttttATAGCGTTGCATCGC
AGTTGATCGGTGCCTGCGGGCATGTATAAATGGAATCTAGCGAGGCGGAAGATTGAGGTCTTCAATTCTA
TGGCCCCAAACAACGCTCCTGCGACATCAATCCCTCAATTAGAATATGTGCAAAGAAGCATAACTAATCA
ACTGAACAAGCGTAAAAGCTAAGATATATGCAACCGATATCTTTTCTTTCGTATTTTGTAGAAATACATT
TTCATGGACTGATAATCTACAGAGAATACCAACTCGGCGCTATGGGCCCGCCCAATACATGTAAATTTGT
GATTTTATGTCCAAAATATTTTTTGTCTTGTAGCATTATGGGTGCAGTTGAATGTACCTGCCCATAATTA
AAGCCTGCAAATCAAGAGTTATTGTTAAACGCAAGAGGGAACATTTTGAACAGACTCAAATCTGTACACG
GCCTCTGTAAGGAATGTGCAATCGATACCCTTTTCTCTCGTATTTTATAGAAATACATGTTGTGGAACAG
TTTAGATGCTATGGAATTGTACCACGTAGTTGGACCACCACACAAATTTAATTAAAGTATGGAGAAAAAC
TTTTTTCTGTTTTCAAGCTGTTCCCGTTGAATGTGCCTGCCCACATCTCATCTATAGCTTCTGAGTAAAT
CCAACAGCGAACCGTCTATTCTTGGGACTTTAACAGCGTTTGAGAAATGGTTGGATCTTTACATTGCTCA
TTTAATACTAAAGTTAATCTTCTTTCCCAATACCTAGACGCTTATTTATGAATTCATTGCAGTGTTTATA
CAGAGACATCGGTTTCAAGCAACCAGTCTTATCAATCACATAGTTGACATACCAGTGCACATTTGCCTGT
ATTAAAACCAACCGAGAACATGTAGCAAAATTTCTTGAGGCAATGGGAATCAAATGCCAACTTGAAAGCA
ATAACACCAACACTAAGTCGTCGCAGTCCAACATCCATACTACACGGCGAATTCGAATTGAAGACGCTGG
TCAGGTCTGAACCCAACTAGAACCCGCTTACACCTGTTTTTCAGCAATTTTTCTGGTCAGGATGTGGATT
ATCTGTCACAGAAACTTCGTTTGTCTTGAGCGTCGCTAAGATTGGCCGTTTGACCCTCCCAAAAGTGAGA
TTCTAAGCAGCCTTAAATTTATTTTACAATTCTTACAGCGGTGCCGCACAGTTGAGTGTGCCTGTGGGCA
TCGACTAAGCAATATTTGAACCGAAGGGCAGGGCAGTGGACCGTTCCGTTGATGATGATGTGCACCGCTC
CGAGCTGGCTAAGTCTACTGCTGTGCTGCTGCAAATCGTTAAAGGGAACCCAAAACGATGCATCAAGTCC
ATCCAGTTAGGCTTCTCTTCCAAAGCACAGCTCACCTGCGCTACAACGACAACCAATTCAATCGCACCTG
CTTCTGCAAGCGATCACAATACGTCAAATGAGCATCGAGCACAGAAAGCAAGCAAACAACCAGGTTAATG
GCTTCCTCTGCTGACTATGAAACTATACGCCTTTACATTGCAGCCAAGAAGCCTCTTAGTGCATATTCAA
CTACCAGCACAGAGCTTCTCAATGACTGGAGTAAGCATTGTTCTGACCGCATCGGACCCTGACCGAGTCC
GACTTGGGTGCCTCTGGTCGCAAGATCTACATTAGGACAGAGCCAGAATAGGAGCGCTTATCTAGCGCCA
TGATGTGCTGAGCTGAACCTAAGCACGTACTGCATTGCACCGCACAGATATGTGCGTTCGGTCCACGCTA
AAGGGAATCAAATGCGTTTGGACCGAGAGCCGATACACCGCACCATCACCAGTATTCGCTGTGAAGTGAG
CTGATGTGTTGTCGTTGTTACAGGGCTGCTGCTGCAACTGCTCCACAGGTATCCTCGCCTAGCGTCGGTA
ACTATCATTTGTCAATTTTAGCGGCCGGCAGTGTACATCAAACATTGAAGTCCGGAGTCCGGAGTGGCAT
GTAATTAACGGCAGATGTGAAACCAGCAAATGGTGCTCAAGCATTCCATTTTAAATCTGAAGATTGTCAG
CAGTAACAGTTAAAGACTATCGACGCAGACTTGCAGAACGAAGCGCAGGAAGGACAAAGCACTTCGATCA
CCAGCGGGTTGTCGTTTTACTTGCACTTTCCTTCCGTAGGTAGCCCAGAATTCGAGAAAGCCCATATCCA
TCGCAAAGGGAAACAAATGCGATTAGTGTTTGAAGACCAAGCTGGGTGTCCAATGTGTCGCACCACGATT
AGCCAGTTGAGCCCATAACTCCTGGCCCGCCGATCATCGTACCGCAAAAGGGAATCAAATGCGAGGTGGT
ACTACAGCAGTCGAGCTCCTCGTTGAGCTCCTGGTTTAAGTCGAACTCCCAACTGAGCTCCTGGTCGAGT
TCTTCGCTTCAGCGTTTTTATCGTAAAGGGAATCCAACGCGATGTACAGCGACAGCGGCAGGCAGGGTCA

CCCAATACACAAAGGTATACCAATATGATCAGAGTTCCAGTCCGTGCAATTACGCTTTTCAGAACCTCTC
AAAAACTAATCTGCACGTGTCTAAATCTGGCTGCCATGCCAGGCGCGACATCAATCATATTCGCCATGTT
GGTCTCAAAGGGAATCCAAGAGACACGCGCCCCGCGGTACAACCAACTGAAGACCAAATAGATTTTGTTA
TGCATTTATAACCAAGCATTTTAGAAGTCATATCCTCATAATTTTTTCCTTTAGTGTTTCCAAATCTATG
TATTCTAAACGAATCGTTTCATTATCTTGTAGGCTTTTATAAACCTTTAATTTATTACTTGTAAACCTTT
CCTCTAAGAGTCAATGCAGACATCCGACTTCGCTGACGATTGCATCGGGACTGCATTACCCTAGGATAGA
CGGTCCGGTCTAGAAGTAGCAGCAAGAGTCAGTTAAATATCAGTACCGCTATTAGCAGAAGATTCATGGC
AGTAAGATGATGTGGATGCACCGTACATAGACGTTTCAAACTGCAGAGGATCGTTTGACCCCCGAACCAA
GGCAGGGAGACCATACATACACAGGCGGAGGCAGTGCTGACCCTCAATACATTCGAGTATTTCAACACTC
TACCTTTTACCTATATTTACTGGGCATTCAATCACCAAATTCGCATCCTGGGTCTATTTGGAGCATATTA
AGAACGAACACTACACGGCGTAGGAGCACCACACGATTTGGTCATTTTTATTATGAGAAGTCCTAAAATC
CTCCTCGGATCATTCCTCATTATCTGCATTTGGAGCAACAAGTAGGCTGGAGGTATCATTTCTTCACTAA
GCACCATCGATTTCACACTACTGGCTTGGGATAGCACCTAATTCTTTACGAGTTTCGGCACTGATGACGA
CTTCAATCCTTGTTTCAACCCGTACCCCTTCGAACCAATATAAAAGTTGGATTGCATACAGCTTTCGCTT
CGCTACACACTGGTCGATTCTTTTTCTTTGTGAGGACCCTTCGTGAGGATATTAGATTTGTTGAAGAATG
ATTCTCTAACTTGGACTGCGGCGTGGGATTCAGTCTAATTTAACCATCGACAGCGGCCTAGGAGTAAATT
TGGACAGCGGCGTGGGATTTTCGGCTCTCAAAGACGACGGCGGCGGACGTACGGATAGTTCAGACTGCGG
CGAGGGATATCAA
>dme_circ_0005436_junction_seq
TAGTTCAGACTGCGGCGAGGGATATCAAGTTTTTATCTTTCTGTCTTCGCTCGGTA
>dme_circ_0005437
GGTGCCGCCGTCGGCGCCCAGCAGCACCGCCTGTTTGCCATAGGGCTTGGGGCCCACGTCGATGATGGAG
CCCGCTCCCTGGCCAACGCGCAGGTACTCGCCCTTGATGGAGTACAGACGGGGGGAGCTCAGGTAGCTGA
AGAACAGGGTGGAG
>dme_circ_0005437_junction_seq
GCTCAGGTAGCTGAAGAACAGGGTGGAGGGTGCCGCCGTCGGCGCCCAGCAGCACC
>dme_circ_0005438
GACACTACGCGTATTGTACAGCTGGCTCTACCGCTTCAATTTGTTTTAGATTCTTCTGTTTCTGCTATT
TGTGTATACAAGAACGATATGAGGCATGTGAAGTGGTTGCTCCTCTGTAACAAGACTACGTGGCTATCGG
CTTGGAGTCCTTGAAAACTTTCTGCATAATCGCGACAGCTAATATATAAAAAC
>dme_circ_0005438_junction_seq
ATAATCGCGACAGCTAATATATAAAAACGACACTACGCGTATTGTACAGCTGGCTC
>dme_circ_0005439
GTCATTGCTGCGAGAAAACATGGACGTGATATTGCTGTGGCTACCCCCAATAGGCTCAATTGTCTCGTAG
CCCAGCGCATCAAGTTCAAACTTATCAAGCTTGCTTGTTCCGCCAACTGAGCTCTCCCGCTTTTCGCTAG
ATCCTCCTCCTCCTCCACCGCTGCTACTATTTCCGTATAGGGAAAAGTCAGTGAAAAAATTCTCGCTTTC
CAATAAGGAGAGTTTTGCTTTAGGGGCGGCCGACTGCTGAATAGTCTCCATATCGCCGAGAGCGGAGTGG
GCCATGTCGGAGCCTCGCAGGTTGAAGCCCATGCCCAGCCGCTCCATCTGTTTAGCCTTAGCAGGGTCCA
TGGTCTTAAGCTTGGCCTCCTCGCGGGTCTTCTGCATGGAGAGTTCTTGGTATGCCAATCGCATGCTGGC
CACCGTCTCTAGTTCGTCCTCGGCCGTCTGTGGCTTGACCACAGGTGCTGCTGCAGCTGATGTCTTCATC
TCGTTAGCCGCGTTGGCACGGGCCTCGATGTCTGCGAAGTTAGTCTTAACTTTCGTGGCTCCAAGACCGC
[truncated: 467,092 more chars]
