# Supplementary material for: Age-related ceRNA networks in adult Drosophila ageing
Source: Front Genet. 2023 Feb 28;14:1096902. doi: 10.3389/fgene.2023.1096902 (PMC10012872; doi:10.3389/fgene.2023.1096902)
Supplement: Supplementary file 11 [file Presentation3.pdf]

CAGGCCATTTTCCACGCAAATATGATACGGCTCAATTGGTCGACTTAATGGAGCAGAAATCGATGTATTC
ATCTCCATTCTGTGCCATAAAATAGAATCACCATAAATTTCTAGCTTTGGATAAAACCTTGTTTTTCCAA
CATACATTTAGGTGGTGTTATTTTCTGCTCACTCCGATTTTAAAACATAAAGATGTTTATGCAAATGTAA
TGTAGTTAATGGGTTGATAATAATATAAATTTATTTGCACTTCACTAGTAATTTGTAGTTTATACCGAAA
TAGTTTTTAAATTATTGAAAATGTTGCTTGCCAAATGCAAAAGGTAATAGTTTCGGCAGTCACAGAGTGG
GAGATGCGTTCAGTCCAAGTATTATAACTATAACCCTTACAATTTATTTTAGAGCACCCACGAGGATGCC
ATGTACGGCACCAAGTTGGACACCATCCGACGGATTCACACCGAGGGCAAGATGGCCATATTGGATGTGG
AGCCGCAGGCACTAAAAATACTCCGCACCGCCGAGTTCACGCCCTATGTCGTGTTTATAGCGGCGCCCTC
ACTGCAGAACATAGCAGATGTACGACGGCAGCCTGGAGCGACTGGCCAAGGAGTCGGAGATGCTGCGCCA
GCTATACGGTCACTTCTTCGATCTGACGATTGTGAACAACGATATTAGCGAGACGATTGCCACGCTGGAG
ACGGCCATCGACCGGGTGCATACCACCCCGCAGTGGGTGCCCGTATCCTGGCTGTACTGACAAGACAGCG
AACTGGAATGCCCCGACTGCCTCGAGGGCTGTGAATGCGAGTGGGAAGCCTACACACAGGCCTCCAACGT
AGCCCTATACTGAGGCACAGATGCAGATGCGCACGACGATGCAGATACACGCCACATCACCACACCACAC
ACACCATTAAAGTATTACCAAGGCACAAGCAACAGCAACTGAAGAAGCAACACAGCAACCACTACGAGAG
ATGACAGTCAGGACTGGAGGATAGAAGGCGCCTGGCGCCGAGCTATATAATTTAAGAATAAATGGGTATT
ATGGGAACTGCGGTCGCAACGCAATGACAAAAG
>dme_circ_0007200_junction_seq
AACTGCGGTCGCAACGCAATGACAAAAGTTAACTGCTCCATCTTCGGGCGCAAGAA
>dme_circ_0007201
TTAACTGCTCCATCTTCGGGCGCAAGAAGAAGCAATGCCGGGACAAATATCTGGCCAAGCACAATGCCAT
CTTCGACACGCTCGATGTGGTCACGTACGAGGAGGTTGTCAAGGTGCCAGGTGGGTTTCATTGAGCTTCC
TGCTCCCGGCCACAGGTGAACTTTTGTTTATCCATGTCCTTCCTGTGCTTTCAGTTGGCGACCCGAACTT
TCAGCGCAAAACGTTGGTGCTCTTGGGTGCTCATGGGGTGGGCAGGCGGCACATTAAGAACACCTTGATA
TCGAAGTACCCCGACAAGTACGCCTATCCCATACCACG
>dme_circ_0007201_junction_seq
CCGACAAGTACGCCTATCCCATACCACGTTAACTGCTCCATCTTCGGGCGCAAGAA
>dme_circ_0007202
ATATTCGTGCGCGCCCAATTCGATTATAATCCGCTGGATGATGAGCTTATACCCTGCGCCCAGGCGGGCA
TATCGTTCCAAGTGGGCGATATACTGCAGGTAGGAAGGATGGCACTCACTACTTTAAAGTATTTTCCGAT
AATAATCGCGTAATTACTGCGATCTTGTTGCAGATTATTAGCAAGGACGATCATCACTGGTGGCAGGCGC
GACTGGACACGGTGGGCGGATCGGCCGGACTGATACCGTCGCCGGAGCTGCAGGAGTGGCGGATCGCCTG
CCAGACGGTCGACAAGACCAAGCAGGAGCAGGGGTGAGTGACCGCGACGATCCTGGCCAGCGGCAGGCGT
CTCGAAATCAAACTATCCGTGTTCTCTATGTGTAATTGGATGCTCGATATAATCAAACAATCGAATGCTA
TATGAATAATTGTACTTATATTGGAAGTGTATGACGAACTCGTAGTTAACCGAAAAGGGAAAGCGCTCTC
TCTTTATGTATCTCTCTATCACACACATGTACCCACCCAGAAACACATACGCGCCACCCACAGAAGACAG
CCACATCTGTACACTAACACTTACAGAGAGTTGCATGCCCCAAAAAGTAGTCGAAGGAAAACCTTTTCAA
ATTTACCAACCGAAATGGGGAAAGCCCCTCCCGCTGCCAAATTTCTACTTGAAAACTCAGACCCAAAAAT
CCTAAGTCCCAAAGACACCGACATATGCACACCTGTACAAAACTCTAATACCTAGTTTGCTAACCATGAA
TCTCGCATCTCGAACCAAGCCAAAGAATTTCTATAAACTATGTATGTATGATAATATAATGCAGGAGAAC
CGGGTGCTGGATGTTCCGCTCACGCAGATGGGTGTGATGGATCAGCAGGTAGTTACCATATAaccaaaaa
ccaccacacaccaccacaccacaAAAATCAAACGAATCTCCGAATGGAGAGCTGCCCAAAATCGTTTTGT
TATGCCACAAAAACCAGACAACACAAACAAACTACTACTCGTTCTATACAACAACAGAATATCTATCGAT
AGCCATTTTGTATTAAGAATTACCCCCACACCCACGCCCGTTACCAGTCAACCACCCAACCACCCGACTG
CCCGAAAATTGTAGCTTATGTAGACGGCACCACCGACCCACCCAGTTCCAGATCCAAGGAATCGCGTAGA

AGCGCCCCTCATGCTCACGATCGCCATCTGAAACAGTAGATGCACCTCAGTCATCGTTGTTTATAAACCC
ATAGTATTGAACTACCCGCTTACAATCCTCAAAAAGTAATACCCCACCAAAAAACTTTAATGATCCTAAA
ATACATTAACACATGTTCACAACACTGCACGTTAGGCTGGCTGAAAGAACACCGCATATCCAATTTGAAG
AAGTTCTAGTTCAGGAAGGGTAAAAAGGACCAAGGGATGGAATTAATATAAAATTAGTATATCATCCAAA
ATGATATCATGATCTCATCTATCATAACTAAGCAAAATCGTGCTTGCTTCATGCTCGAACTTATCCAATG
CCTGATGTACAGCATGTTTTATGCCTGCTCTATAAGCATGCTGCTCTCAACTTACCCAGCTCTATCGTCC
ATCTAATGAAACAATTCAATAACATTTGATTCTCCATTATAACCAACAGTTAACTGCTCCATCTTCGGGC
GCAAGAAGAAGCAATGCCGGGACAAATATCTGGCCAAGCACAATGCCATCTTCGACACGCTCGATGTGGT
CACGTACGAGGAGGTTGTCAAGGTGCCAGGTGGGTTTCATTGAGCTTCCTGCTCCCGGCCACAGGTGAAC
TTTTGTTTATCCATGTCCTTCCTGTGCTTTCAGTTGGCGACCCGAACTTTCAGCGCAAAACGTTGGTGCT
CTTGGGTGCTCATGGGGTGGGCAGGCGGCACATTAAGAACACCTTGATATCGAAGTACCCCGACAAGTAC
GCCTATCCCATACCACG
>dme_circ_0007202_junction_seq
CCGACAAGTACGCCTATCCCATACCACGATATTCGTGCGCGCCCAATTCGATTATA
>dme_circ_0007203
ATATTCGTGCGCGCCCAATTCGATTATAATCCGCTGGATGATGAGCTTATACCCTGCGCCCAGGCGGGCA
TATCGTTCCAAGTGGGCGATATACTGCAGGTAGGAAGGATGGCACTCACTACTTTAAAGTATTTTCCGAT
AATAATCGCGTAATTACTGCGATCTTGTTGCAGATTATTAGCAAGGACGATCATCACTGGTGGCAGGCGC
GACTGGACACGGTGGGCGGATCGGCCGGACTGATACCGTCGCCGGAGCTGCAGGAGTGGCGGATCGCCTG
CCAGACGGTCGACAAGACCAAGCAGGAGCAGGG
>dme_circ_0007203_junction_seq
CGGTCGACAAGACCAAGCAGGAGCAGGGATATTCGTGCGCGCCCAATTCGATTATA
>dme_circ_0007204
CGCAAAATGCATTCTTaacatcagcagctacgaaaacagcaacagcattagcggcaacaacgaatcaacg
acaccaataacaataccattaacaacaaatcatcagAAGAAACCGAAGGAAAACGCTCAAAGTTTTGGAG
TTGCTCACTGGCCGCTTAGtttttgtttatttttatgttcgtttttgGAGGGCAGTAGGCGACGACGG
GCAAAGGAAGCGGGAGAAGGAAAAGCGACGGACACGAAAAACAGGACACAACTCATCAATATTCGAAAAA
GCAATTGAGTCGAGGCATCAATTCATCAATTATTCAAAATGACCGAAGACGAAATTCTCTTTGACGACGT
CTACGAGCTGTGCGAGGTCATTGGCAAGGTAAGTTAGCGCCCCACCGGCTATATCTTTAGCCGATTCTGC
TGCTGACAGCTGGCATCCATGGCCATTATCTCATTATGTATTATGTATCCGCCCCGTATGCCCATCCCAC
CCACCCAGAAGCCGTAAATCATGCAACTATTGGCCTGGGACGAAGCCAGCTCCCCTCTATAAAAAACTTT
CCCAAACGCCCAACTTTTGCATTATTCAAAAATGTATGACCAAACATTTTCTGTGCTAGTTACTTTTGCT
GTGATCATCATAGTTTAGCCAGAAGTTCATTCACAAAGCTGTCAATGTTATTAAGTTGAACTTTGTTTAA
TTTCGTTGGAAAAATATTTTTCACTGATGTTGAAAATGTTCCATTAACACACTTCTAAAGTTTATTGAAA
ATGAAGTGTGAGTCACTAAATAGCACGttttttaattttttGCAATTTATAATCTATAAACTATAAGCAA
AATGACGACCTTGAATCGCAATAGTTTTCCAAATTCAGCTTGGTGACATAGTTCAATAAAAATTAATCAC
GCTCATAAAACAAGATGATAGGAAATCGTTGCTTGACTTTAAATGAGTTATTGAATAGGAATTTGTAAGA
AAATTAAAATTTATTTGTAAAATAATCATCAGTAATGTTATGGCAAGCTTTTGTATTAAAACCCCACGTT
TGTAACTACAAAGAGAATACATAGTTGATTAAATTTCCTCTTTCTCAAGTATCTTGTTTGCTTTTAGACG
CTCGGAAAATATAAATATTTTGCTTTTGTCGAAGCAAGATAAAATTTTATTCGCTCCCATTTAGACACAA
TCAACTTTCAATTGAAGTTTAATCCGAATTTACTTTATTccccccccATCCGGGATTGTTTCTTACAGAG
TGAGGATTATATACACAATACACCTATATATATATGTATACACTCCGCTTTTATTTTTGACGGCACAATG
AGACGACCTTGTTCGATTTGGCCCCGGGCTACCGATTCCTGACTCCTCCAGAGCGCTGTGTGAATAAATT
ATAATTAAGCTGCTCAATTATTCAAAATTATGCCGCTGCCACACGTGTAGCACGCCCCCAGGGCTGCCCG

TTTGCCAGCCCAAGACGCCAATTAAAAAGAAATTGCTCAAGTGCAAGGGCGCCAGGACCTGAGCCTGAAT
CAGGACATGGACACGAACCCGGACGTGGATGTGAATGTGGACGAGGAGCAGCCATCCGACCATTGACTAA
ATGATACATGGCAGGCACATAAGTCAGCGCTAAGTCCATTAGTGCCACTGCCAGCTGGTGTAGTGGGTGG
TACTCCCCACGTGCCCCATTCCAATGTCAACTAATTGCCGCTGTTGGCCCCAACCTCGTTCAATCTGAAC
TCACGCCCCCATTCTCTGCATAttttttttcttttttGCAGAGTGAGAATCGGGCAGAATTGCCACAATGCC
ACAATCTGCGTTTGTTTTGGCCCAAAAGGGAGTAAATTATTAATTATAATTAATTAAAAGACGTGCCCGG
CAGTAATTTGATTTGAGCAAACCCAATTTAGTTGTCTATTCGAGAGATTAACATTCAAGATAATTTATAT
GAGTAATTAAACGTGTGCTATTTAAACACAAGTAACGAGTAATGGAATAAATCAAATTAAACGATGCTAC
TTCTATTTTATACTTATATATGGCTTCTGTATATAAGTATTCCCAATTAAGTAGACGCCCAAAACAAGCG
CAAATTAAATTAAATGAGAAACACTAAATATTAACCCCTTCCTCTTTTCTAGTTCACTATATGCGACAAA
TCCTGGAGGCACTGCGCTATTGTCACGAGAACGACATCCTGCACAGGGATGTTCGTCCCGCCTGCGCTCT
CCTCGCCACCGTCGACAACTCGGCGCCGGTGAAACTTGGCGGCTTCGGATCGGCCATTCAGTTGCCCGGC
ACCCGGGAAACCATAGAAACACACGGACGGGTAGGATGTCCGCATTATATGGCGCCAGAGGTGGTGACCA
GGCGGCTTTACGGCAAAGGCTGCGATGTGTGGGGTGCCGGAGTGATGCTGCACGTCCTGCTCTCGGGCAG
ATTGCCCTTCCTGGGATCCGGAGTGAGGTTGCAGCAGTCAGTAGCTCGCGGTCGGTTATCGGTTTGAAGC
ACCGGAGTGGAAATCAATCTCGGCGAACGCCAAGGACTTAGTCATGAAAATGCTGGCCGCCAATCCACAT
CACAGACTCTCCATAACCGAGGTGCTGGATCATCCATGGATACGGGATCGGGACAAACTACAGCGCACTC
ACCTAGCGGACACGGTGGAGGAATTGAAACGCTACAATGCCCGGCGCAAGCTAAAGGGTGCTGTTCAGGC
CATCGCAGGAGGCACCAACATGGATCCACTATACGCCACCGATGCGGACAGGTTAGTATTTATTTGTAAA
GTCCCATATTTGCTTCAAACCAATCGGAATTTACTCATTTTACTTCGGTCACTGAATCGTACAGTTTTTA
GTATCTATAGAAATTCTATACTGCATTAAATTCCTTCTCGTTTGCTCTTGGCTCTCGTGAAATTCTATAA
ATTCGATGTTGCAAATTGTTTTCAATTCTGCCTTATATATTCGAAGTTTAACTGCAAAACAAATAATCAC
ATCAATTTATTTAATGTGTGAAGCTGAATATTCAATTTGGTTATAAAACTGCAGACGTCATCATGCAATA
AGCCTATAGATTATACACATTCAATTATGTCGGAATCTGTTCATATGTTTCATTGACTTTCTTTGCAGTT
GCCCATAACCGGCGCCACGGACGAATGGGCCGACGAGGAGGCGGGCATTGAGGCAGTGCAGCGCATCTTG
GACTGTCTGGATGACATATATTCGCTGCAGGACGCACACGTTGACGCCGATGTGCTGCGGGATATGCTGC
GCGACAACAGGCTGCACCAGTTTCTGCAGCTGTTCGACAGGATCGCGGCGACAGTGGTGACCAGCAATGG
TAGAGCACCGGCTGCCGAGGCCGTAGGCCGATGTCGCGATGTCCTGGAGCAGCTCTCATCGACGTCCGGC
GGCAACTCACTGGGCGGCAAGTATGCCAAGGAGGAGCTGATGCGGCTACTGGCCGCCCCCCACATGCAGG
>dme_circ_0007204_junction_seq
CGGCTACTGGCCGCCCCCCACATGCAGGCGCAAAATGCATTCTTaacatcagcagc
>dme_circ_0007205
AGGACCTTTCTCCATCGTAAGGCGATGCATACACAGGGAGTCCAATCAGCAGTTTGCCGTCAAAATCGTT
GATGTGGCCAAGTTCACAGCGAGTCCTGGACTGAGCACAGCGGGTGAGGATTATATACACAATACACCTA
TATATATATGTATACACTCCGCTTTTATTTTTGACGGCACAATGAGACGACCTTGTTCGATTTGGCCCCG
GGCTACCGATTCCTGACTCCTCCAGAGCGCTGTGTGAATAAATTATAATTAAGCTGCTCAATTATTCAAA
ATTATGCCGCTGCCACACGTGTAGCACGCCCCCAGGGCTGCCCGTTTGCCAGCCCAAGACGCCAATTAAA
AAGAAATTGCTCAAGTGCAAGGGCGCCAGGACCTGAGCCTGAATCAGGACATGGACACGAACCCGGACGT
GGATGTGAATGTGGACGAGGAGCAGCCATCCGACCATTGACTAAATGATACATGGCAGGCACATAAGTCA
GCGCTAAGTCCATTAGTGCCACTGCCAGCTGGTGTAGTGGGTGGTACTCCCCACGTGCCCCATTCCAATG
TCAACTAATTGCCGCTGTTGGCCCCAACCTCGTTCAATCTGAACTCACGCCCCCATTCTCTGCATAtttt
tttctttttGCAGATCTGAAGCGCGAGGCCACAATATGTCACATGTTAAAGCATCCGCACATTGTAGAGC
TGCTGGAGACGTACAGTTCCGAGGGAATGCTCTACATGGTCTTCGAGTTGTAAGTTGTCAGGTTCGGTGG
TGTCCTTGTGCCCATCGCTAATGTCCTTTCGCTTTGCACTCTTGCAGCATGGAGGGCTCCGACTTGTGCT

TCGAGGTTGTGCGGCGTGCTGTTGCCGGTTTTGTCTACAGCGAGGCGGTTGCTTGG
>dme_circ_0007205_junction_seq
TTTTGTCTACAGCGAGGCGGTTGCTTGGAGGACCTTTCTCCATCGTAAGGCGATGC
>dme_circ_0007206
AAAACTAAAATCGGAAAAACAATGCAACAATTGGAGAGCTCCGCACACCTCAGTGCAACGcagcggcagc
aacatcaacggcagcaaaatcagcagcagcGCGAATGTTGTGCCCCGGGCAAAAACTAAAAATGAAACAA
CTTAATTAGTAGCTGTCGGAGTCGAGAGCAAAAAGCCGCGCACTCCCAGAATTCTGAATCAGCCCAGCTG
GAGCTGGAGGAAGTGGAGGAGGAAGCCGGAGGTTCGCCGAAATTTAGGGAATACTTCGTCGAAGGAGCTA
GTTGCTCCGATAAAAGTGCTTGCGTGTGAGCGCACTTTTTCGAGAAAAATTAAATTGAAAACAGCATCAG
TGCAGAGGCGGTAAACACTCTGAAATACGATTCGAGCCCGCATATAAATTTCTACACTTTACACAATGGA
TTTTCCAGCGAAAAGGACTTCCACAATGCCTCTAACAACTTCCTACAACGCCAACATCAGACGAAGCTGC
CTCGCTTTGTTCGCGCTCTTCCTGTTCGCGGCACACATCAGCCTAACTTCCGGTCAAATCGATTGGGACG
ACGACGATGATCCAGG
>dme_circ_0007206_junction_seq
TCGATTGGGACGACGACGATGATCCAGGAAAACTAAAATCGGAAAAACAATGCAAC
>dme_circ_0007207
GTTGACATGCCAGGCCACTaaaaacgaaaaaaaaaaataaaaaagtaaaaaGAGCTGAAAATGCAAAAGT
GCTGTGACAACCACAAATAAATGATAGAAGGAAAAAAGTTTAATACATTAAAAACAAATACCACAAACAA
AGTCAACGTTTTCATCGTACTGCAAAAAAACCACAAATTTTTTACATATTTACATTAATGAGCTTGTAAA
TATTGGGACGCATTGCCACGAAAAAGTGAGTAAGAATTCTTTTTTAATCATTCCATTTATGTACATACAT
CAGAATGGGGAAAAAATTCATGAGCCAGCACTTTTGGGTTCTTTGTAAACATTACAAAAATTAATTAAAT
CATGTATGTTTCAATTAAATGTATTTTTTGTATTATAAAAATTGCATTTTGTCCTCActctctctctctc
tctctTAACGGCTTACTAATTATCCTCAGTGTAGATATTTTCTTATCAATTCGGGTAGCGTATTTTCTGG
GATTTCCCTGAATTATGTAGCTCCATTTGCATTATAATTAAATAACTAAGTTTACTAGAACTAACTAATG
GGTGATTTATGCAAAAAATCAAATAACAAAATACTTGAGTGCTAAGTGTATAATTTAAATTACCCTGCAT
TAGTTTACAAAGAACCTTTCCGGATTTTCCCCAACTGCAACAAGTTTTTGAGCTGTATAGTTATTATTAT
ACCTATGCCGTATCATGCATTTTCCAATTGTTTTGGGATCGGTTCCGTTGACTAAATCGGATGACTCTAA
ATGCCTGTGATTCATCTTAAATGATTGCACTGATTTGCTCACTTTCAAGATGTTCGTTGCTCATAAGAAA
TAATAATAGATCATTCTAGAATAGGGAATAATTTGATAAGAACTTATATGCGCCTTCGCTAAGTAATATT
GAAAGAGGTAATTAAAGTCTTGTTACTCAAATGTATAGCAATTCGCGAAAATCAATCGAGTGTTTATGAA
AAGCTGAAAAACTTTCCATCGTCTCAAGAAATCAACACAATTGCATTCGGAAGCCCAACAAGTGCCTCTA
ATGGGTGTATAGATACCACTGCCCCCTTCCAGCACCATCGTTATATTTGCTTTTCCAAACAATCTGTCAC
CTACAATGGACTTGAATGGGTTTTCCTTTATGAGGCACaaaaaaaaaTACGCGGAAATCTTAACGACCAA
TTAATGCGCAAATATAAATGTCAACTtgaaatttattgaatttgaatgaaaaataatatgtatatataac
aattaaaaaataaaataGAGAAAGACAAATGAAGTGGATGCGAATTAAGTGCGAGCGAATAATAAAAATT
TCTAATTAATGCGCTTCTAATTTCAAGTTGACAGACGGAAAGCACTGCGCTTGATGTTTTTATTATTATT
GTTTTTATATATGTAGATATTTCGCCAAGTGATTCGCTTTGTGCACAATTGCATTTGATTTCGAATAATT
CATTTGCGGCGATTTATGACACAGTCTGACTGCCACATAGACGGAAAAAAATAAGACAATGAATCAGTCG
ATGGTATAAGGGGGATATAAAAGTGGGGGCAGACTGATGTGTCGCCCACGCGTCGCAATTTTCTTTTCAC
TCGCCGTTGTCCTTGAGGCTTTTCCGCATTTCACGACGGCGACAAGTTTTTAGCTTTAAATTGCCGCCGA
GGCGACAGAAGCCAAGATGGATTGAAATTTAATTTCATGTTCTCAAATTGATTTGTGCGGATATCCATTA
GTCATCCCGGCTTCTAGCCGCTGGCTTCTGGCCAGGATTAGCATTAGACGGGAGTGCAACAGGCGGGCAA
CATGCAACACACAGTCATCTGACTACCAGTCTGCCTTTCTTGgtgtgtgtgtatgtgtgtgtgCTGCACG
TAAGAAACGAGTTATTGGAACAGGTTCATGACCTTGACCATCAACACAAGGCAGCAGCAACAACACGAGC

AACACGGGCAACATCAGCGGCACAACGGCAATTGCAACACGGCCTCCTCATCTCGCCTCCTCCGGCAGAC
AAAAGGAAGTGGTTGCATCTGCATTTGCATTCGCCATTGTTCGCCTTGGATTGGATTAGATGTTgcaaca
acaacagcagcagcaaaagcaacagcaacaAGTTGCTGCCACTCCGGGAAGTCGGGCAGTAGTCGCCATG
CATTTATCGCTCACTGAGTGAGTGACTCACACTCCAAAAGCCAAAAGGGTCTAGTGGGTCTATGTACATC
TACGCTCAGGGAAATGCGTGATTCACTTTTATGGAAACTAAAAAGCCTGCACTTCTGTGAGTTGCCATTA
TATATATATATCAACTTTTATCGAAATTATGCATGCGTTTTGAGTGAATTTACCGCAACTACGCTTACTT
TGAAATAATTAGGAATAAATTCAAAAAGACTACTTATATATTCAGCTTAGTTGTGGTGCTAATGTAATAT
GCATAATTATATCTCCTCATTTAGTAGAGAAATAATAGGTACAACTACATCAGTACTTTAAATAACCCTG
ATACTTTTGATTAATTAAGTAACTACACACTTAAAATGCTATATTTTGCCTTAAAAATTGGCTTAATTCT
AAAATCAACTTAAAATTGTACGCAAATTGCTAACAACGAAATCCGACAGAGCTTAGTGTGATTTCAGTAT
TACCCCAATCCTCGTGTGGCTGGGAAAATGTTGTGAAAAAGAAACTGTTGCTCTGCTCGAAAGGACGCAG
GATGGGTGTGTGGATGGGGGGATGGGGTATGGCTCCTGCAATTGGGAGGCTTTGGAtttttttgtgctta
tttttcgtatttcgcttctttctcgcagttttttttCGCATCTTCCTCATGCCACGCCACTATATAGTTT
ATATAGTTTGGTTGGATGGTGGGGCCACTGGAAGTGCACTGGAAGTACATAGCTGGTGCAACAGGCAGTT
GTTGTTTCTAGCTGGCCGAGGAGATTGGGCTATGCTCCACATAGAAGGCGTGTCCCAAACGAGGATTCGG
ATTGGGAAGAATGaggaacagaggagcaaaggagcaaggagcaaCGTGCAGAGGGGCAAGTGGGCAGGGG
GAGGAAATGTTTTTAATTTAACTTTGGCCATGTCAAATCCCCTACGCAAAGAAAAGCTGAAAAACACTGG
GCAACAGGTGGGGAAGACAATGGGAGTTGGTCAATTATGATTATGGCTTCCTCATTCCGCAGCCGCGACT
GTAAAGTTTAAACAGCGCGGCGACCTTAATAGAGCCAATTGTGTGACGGCCATTGCGTTTGGCATTCCCC
GCATTGTTGTCCAAAAACAAAATCGATATTTTCCATATGCATAAACACAATTGAACAAACAATTTTTAGG
CGAGTGCAGCAACAGCGCACCGGCAATGCCAAACTGCCATATTGTCCTTGAGCTTATCACCTTTTGCACA
GCAACGaaaaaaattccaaacaaaaaaCTAGTCGGCAGCAACaaaaaaaaaaaaaaaaaaTAACTAGCCA
ACAACATTCGCAAATATCAATTCAACTGTTTTCGCaaaaaaaaaagaaatacaaaaaatttgcgaaaaat
agaaaatggaaatacaaaataaaCCTGCATACCAgttttttcatattttccagcgtttttttCGCAAATT
ATTTGTGGCATCATTTACTGAAAACAAACACGTTTGCCTCTATTTTTTAGATATTCCATAGAAAACTGAG
CCATTTTATGATATTTTTCTTCTCAATATGGTTTGCATTGATTGTGTTTAAATTTCATAATTTCTTTTTT
CAAGTTGTTATCTTAATTAATATTTTAAATGGCAAATAGGTTGTATTGAAGAAATAATAGAGAAATGAAT
TTTAAATAATCTGTAAAATAGGAATAACTAATATATACCACTTTACTAATCGAACCTTTGTTAAAGAGTT
TCTAATTTGACTCAAAATCGACTTATTTAAAATTCTTATTCTTTGCTTATTTTTTGGGTTTTATGTTTGT
ATTTTGCCGTTGTCGATGCAACAATTAAATTCATAGAACTCTGCCCCTTTTCAGCTTACAAATAGTTTCA
ATTGCGGTTGAATTTTGACGATACTCACACGGACACGAATAACCCATACCTCAACAATTAGACAAGGACG
GCTGGCGGCGGCAGAAAGAGAGGACAACACGCACGAACACTTGTATTGACGTTTGGTTGGCGACAATTGA
TTTTTGTTACGTTTTCGTTTATTTTCATTCCGCCATCGCTGttttttttttttttttgcatttttttCAGCA
ATTTAGTTCGTTTGCTTTCTGGCTTCGTTATTTAGACAACTGCTGACaaaaaaaaTATTTTCCTATTCCT
CCGGTCCGCAATCGCGCCCTCTCTTTTCCTCCACCAGCGAGGCCACACACGCGCTGCACTTTGCTGCACC
GCTCATAATTCCACttttttttgttgctcgtttttttgtcacatttttgtggcagtttttttattttCGCGA
ATTGCTTGAAAAACTTCTGGTCGCTTTGTCGGCCTGCGACTCAATTTCGTCTTATCTAATTTTTGtttct
atttcgcttctttagtttacaacgcctctctttcttcttttttttcagttttgcagttttccatttgttt
tttttttgtttgttttttGTGTCGAAATCAGGGCAAATTCATCATCATCAACAACATCGTCGCCTTGTTGT
TTTCCTTCttttttttGTGTAACAAAGATAAGGTTTTTTGCTGGCAAACGAGCGAGTGTAGtgctgctgc
tgctgctgctgccgctTTTTATTCTTTATACAATTTCGAGACGAGGACTTTTCCGAGGGGTCGGACATTT
TCGTGTTTGTCGAGTTAATCATTTCGGGCGAAATTTGATAATTTTGAAGGTGACCTCAATTTGTAAGCGG
CGCTGCGAATCGCGAATCGGGGGACCCAAAGTCttttttttCGCAAATTGATAAGGCGGGCAAGAGTAAT
TGCAGCGATGCAACGGCGAGAAAAGCGACAGCCAATAAATAATCAAACCGAAAAAAATGGTGGAAGTCAT

AAACCCATAAAAACTGTAGTTTAACTAGCAACGGAAGTGAAGCGATAGTCATCAGCACTAACAAATATTA
GCTCACTTAAACCAGAATACAATAAAGCTCTCAAAGAAACCGTGCCAAGAAAAGTTTTACAATTAACaaa
aaaaaaaaGAGGTATATAAAGTGTAGAATGTCTAATGAAAATTAAAATAAAATTTAAGTATAACCGAAAG
AACTTCTTTAATATTATAAAATGCACTCACGATGACAAATATGGCGATATCAAAAGGCGGTACTAAGGCG
ACCGATTTTAATATATATAAAAACTCAAATGTGATGCATTTGGCCTGCTCACATATTTATGAAAAAAGTG
CATTTGCATCGCCGGTTAGCCAGGTGGCAATCCAAAGGTCGAAGTTAAGGGGAGGGAGTGGTGGCCAGGC
AGGCAGGCAGCCAAAGAAGTCATCGCATATATCTTCAGGCAGTTTTAATAAAACCCGAAACTGACATCAT
AAAACAAGCAGCAGCAGATATGACACTCGACGTGGCACAGGTCGTATACGTAACCATACCAGCATACGGG
GCGGATGGGTGCTTTGCTTCGGTTTGGTTTTGTCTTCTATGTGAGGGTGTTTAGTTGCTGGTGTTGGTAA
ACAAGCAGCCACACCCATGGTACACACAGACAAAGCAACAAGTCGTTGTACCCAAATATATAGTGCAAAA
TAAAAAAGTTGTGTGTCGGGGTATGGGAAATGTGGAGCGAATGGGATGGAATGGGTTGGGTGGTGGTGAA
AACTAAATAAAATGAAAACGAAGACAGACTTCCAGAGAGCTGGGTGTTTAGGTGAGAGGAGGAACCCACA
CCACACCCGAACCCACACCCAACCATCCAATGGCAGCAGCCAAAAAACAAGCAAAGTCAGAGCAAAAAAC
AAAGTCCTGATGGGTTTGGGGTGGCTTTTTTGTGGGtgctgctgctgtattggtagctgccattcctgct
gtagatgttgctgctgctgctgttcctactgctgttgctgctgctgctgctacctcctgttgcGCATATT
GCAACGAAAAAGTTTCGTAACGTTATTTTCACATTTCCTGCAGATTGTAACGGGCAACGAGGAGCAGCCA
GATAGTGTCGCTGCTGCTTTTGCTATTTCTGCTGCCAAAATGCTCAACAAAACATGTCGAAAATGTGCCA
CATGCCAGTagcaggagcagcaggagctgtaggagcagttggtgcagctggggcagGGACAATGCTCCTG
TTGCTCATCGGCATGCAACAGTTATGTACAGACACACATACACACCGAAGTAGATAGGGACAATAAGCAC
CGACACACATAATGCCGGCAGCCAAATAGTTCCATACAAATGCACAGACAACACATTTCGACATGGCACA
TTTAGCATCCTTGAGCGGCATTTAACTTCATTTCACCCACAGAGACACGGATGCCAGGCCACTTTGTTGT
TTTGCCGTTGAagctgcagcagcagcagcagcagcagcagcagcaATTCTCCTCTTGTTTCATTATTTGA
TTTAAAAACATCTCCGGGGGGCGTTCTCTACCCGTTTATGCAATCAGTGTCAACGGCAAACTTTGCCAAC
TTCTGTGCCTTTTTGTGCCCCTCTCGTTGGCAGGAAATTAAGTTTTTGGCACTTTCGGCTACGGCTTGAA
TACCTCAACAGCAGCACTTCATTGAGGTCAAGTGCAATCTACTGCCCATATGCACAATAAATCAGCCAAT
GGCCAACGCCACCAAGTCAAGCCAAGAGCAAGTCCAATGGCAAGACTTGGTGAGCCGCCTCGAAACTTGT
CTTTCCTCGGTTGTTGCCGGGtttttatttatttagttttttttttttttgtacttttttCTTGGCCGTTT
TTGTAGGTGAGCTGGGCCATGAACGAGGGGCAGGCTCGACCTTACTAACCAGGCTCAATGGAGTGCTTGG
GTGTGGAGTGGAGTCGTGGGGCTCTGGCCCGCAGATCGCAGATCAACGTTAGAAATGGAAATGCGCTTGA
CTGCATACAATGACTGCAACGAGGCAACAGGGAATGTGTGTTACTATAACTGAGCCAGCAGCCTGTTAGC
CAGTTAGTTGGCCAGTTAACTAACCAACAACTTGACTTTGGTTTCGTTTTCGATCTTCCTGTCATTGTGT
GGCCGCTGTGGCGCGTGCTCATCTCGCCTTAGTTAGTCGATGTTGCTGCCTGCACTTGTTGCACACGCCC
GCAGCAAACTTGCAACAGGTGAGAGTTGTCATTGCTGGTAGTCAGCAGATATCAACTTCAGGGTCAGCGG
ATATTTTGAAGCTAGATAAGTAAGATTTATAGCCACTGCCGGCAAATATTACTCATACGACAGGGGATCC
AAAACGAATTAGTTTTAGTTTAGCCAGCTTTTCCTGCACTTAATAGATACAAGTATAAGGAAGTGTAAGT
CATTATTTCGAAGACTAAAAATAAAATGTTGTTCTTAAGTCGTTGCAGGGAAACACCGGAAAATAAGCAA
AAATTAAATAGAATTTTCCCTTCGCTCCCTAAATGAGTTAAACCCCTTTAAATATTTCACTTACGACAAG
CCCAGCTTCCTCATGCTTTTTGCACGCTGATTAACATATTATTCCCAATCAAATTAAGCTAAATTAACAT
ATTTGCACAATTTAAAGCACTTTAATAAATACGTTTTACTATGCGAAGGTCGATCACAACATCACAATAA
AAAGATCATGCAATCAGCCACCATGCAATCCACAAAACAAATTTTCTAGAAAGTAAATCTCAAAACAAAC
AGCGCATAATTAATAAAATTATGAGAACACTTTGGGGGCGATaaaaaaaaaTATAGCAAATTGTAAGCGA
TTATTTCCGCCTTTTTCCGGGCAGCCACAAAAACGATAAATTAGTAATAAATTCATCGATTGGATATATG
AATATGAATAGCGAAAAGAGTGTAAAAAATTGCTATGCAAATTGAGTATTTAGAATAACAAAGAGCGAAG
GCTTTCAGGCATGCAAAGCATCTCCAGCAATTGGAACAATAGAAATAATAGTACTAAAATTACAAAACGA

TTTCAAGGCCTTAACATTGGCAGAAATTTGGTCCAAGTCTTGGGGCCAATTCAGCACCTCAACTTCAACT
TTGAGCTAATGAGAATCGATTGCAGTCGGTCCAAACTGGTTAGCCAAAATGCACTTTGTGCCCAGATCAC
ATCCAATCCAATCCAATCCCAGCCGAACCGAGCTGAACCAAAACCAATTCAATTGATATTAACTCGTTTT
GTTCTAGGCAACATTTTGTGATCGTTCGTGGCTtagaacagaacacaacagtatagaacagaatagagca
gaaCCTTTGGCCAGCCAATCAATTGCTGCCAtttttttttttttttttGAGATTTCGTATTCCATATAT
TCCAAAGCATTGCAGTACATATTATATGGCTACGAGTTTCTAGCTTCTAGCAGGGCGGCCTTGATATTTT
GTTTTATTTATGAAATCAAATGCAGGCGAGCGAGATACGAATACCGACAGCCACCAGATACTTGTGGCAT
TTATTATAAAAAGCTTTCGACTGAATGAATTCCCCTTAGCTTTTGTATCTGTAACACGtatatctacata
tatatgcatatatatatatatatataaatatatagataGTTATAGCTACCCCATTCCACCGACTGTCTTG
GGCCTGTCTCCGATTCTCAGCTCGATGCGAAAGGCAGGAAGCCGCAGAGCTCAAAATAAGCATTGACTTT
GCATATTTGTGTGTGACAGCCCAGCTGTATCATCAACGGATATGGCAGGGAATGTTTGGCATTTCCTTCG
AGGAAAACTCGATTTAGTTTTAATTAGAAAATTACTTAAAATAATACCAAAAAGAAGGCGTTGGGCTGGC
CCTATGAATCGCTCAATCTACCATCTCGCTTGCTCGCTTCGTGCACCCGAAGAAAAGATGCATTTCGTTA
GCATGGATAATGCATGGGTTTCCCTTTCTTGTAGTTACATAATAATGGTTTTATTTAATTGCATGCTAAA
GCATACGATGGCAATTAGTTTTATATTAATAAAAGCATAACTTTGCATATTTCTTAGGTTATTAATTTGA
ATTCGAGGATGTAGAACATCCATGAGATCTTTTTGTAGTTCATAATGTATCTCTTTAGTTGTTATGAACA
GTTGGTTTAGATATAGTTCCTAACACTTGACCATTTGTGATGATATTTTTTCTCGCTGTGCTGCTCCTTC
CTGGCAAATTGCTGGCAGGCTCTTCTGCCGGTTTTCGAAGCTTCTGTTCATCTTCTTCGACTGCTTGGGC
TGCTGCTCCTGGTTGTTCTTCTTGGTAGGCCGGCAACGTCCATCATAATCAACGTCCATCATCAGTTGGC
TGCTGTTCGGCCGCGTGCTCCGTTGACACGCCCACAAAGCTACTGCTCGTGCTACTGCACGCCAACCTGC
TGCTGTTGTTGGGACCTGGAATTGGTTGGTCTGTTGGTTTGCCGGGCCGTTGGGTTGCTGGGTTTGCTGG
GCTGATGGGCCTAACTTTGGCATTGCCCGCCTTTGGCCATGGCAGCTGGAGTAATTTTTCATCAGCGCAA
ACATCTTGAAAACGCTCTTTGGGCTACCAGTTTTACTACTGGGCCTCGCAGATTGCAGCTCAAATGGCAG
CTGCTTTCGGGCCGGTCATGTAAGCCAACATCAGCATATCTGCTATCTGATATTAGAATCTCAATTACTT
TTTCCAGCTCTGCGGCTTTGCTATTGTAATTTCCTTGCGAAGGGGCTGGAAAAACTAAGCCGGttttttt
tCGGTTTTCGGGTAGGCAAATATCCATCGAGGTAAACATGAAAATTTAGTGGCTGTCATAGAGATAGCAC
AGGCCAAAAGGAGAATAGGAAAAACCTGAGGAAAATCGGCGGGGAGAACCTCAAATTGCATCCTATCAGA
TTTAATCATTTATCAGCCAACAGGCAATTACAGGTTGGGGAATTTTCATTCATGGGCCTCCAAAATGGAA
TCTGGATATGAAAAACCCCTTTGCTTGGCCTGAAATTACCCGAAATGGGAATGCAGTCCATAAAAGTTCC
GATGCATTTCATTAGATGCAGGCGGACAGACATTAAGTGGGACTCATTGGGTTAGCCAAACTCATAGCGA
AATCCCAAACACGTTTCGCAATTTACGACTTTTTAATAATGAAACAAAACTAATTTAATGCAAAGCGACA
TCAACGACAACGACATTTGAATAAATTAATGTAATAGACAGCTTAAACAGCAAGAAATATTGCTGTCTAT
TTGTTCAATTGTGCAGTGAGTCTCCAAACATTATTGCGAATCAAAATGGAAAATGTTTACCAGGGtattt
ttttaaattatattaaaaataaataaaatatatttaatctttaatttaattaaataatttgattaattta
attCATAGATAAACATTATCAATTTCTCTGCTTTTTCCAAAATTTAGAGAAGTTTGTTTTCTGGGCGAAA
TCAAATCGATCAAATTAAAACATTTTGAACACATAAAACCTATTTTTTTAAATATTCTTTTTAATACTAT
CGCCAATATCTAAGCACCACTGTAATTGTAACTAGGAAATATCAAAAACTGGTAGCTGCCCGAATAGTAA
AAGCCCCACCAATACTCTGaaaaaaaaaCCACAAAATACAACAACATCGGCAACTAGAACTGCAACAACA
ACTACCACAGACTGGTGGCTTAGCAAGAATTCAACAACAAAATCAAGAAAAATAAATAAATTTTCAGCAT
ATGAAATAATATGACGAAAACAACAACAAAAAATCTAAAGTGCAACAGGTTTTGTGTGTTTTCGCGATAA
CCTTGAAACAAAACTCGTTTAATCATTTCGTAGCGTTCGAAAATGCGGCAACAACAACAACAGTAAATGA
AATTTTATATGGCGCATTATAGATTTAATAAATTAACACTCATTTCCATTGGGGAAatttttcgtatttt
tttttcgtattttGTGTCTGTCGTTTCCAGCCAATTGTGTTGACTTTTCGCTTTTGTTTATTTCAGGGTA
CAGTTGCGATCTAACAATATCGCCAATATATCCTCAAAAAGAAAGCTATGGCCGATTGTTCATATGTGAG

ATGTCAGCAAGAGCGTCGGCTCATCAAAAAGAAGCTATTGAAATGGTCCACAGATATGCTGCATATTGTG
G
>dme_circ_0007207_junction_seq
TGGTCCACAGATATGCTGCATATTGTGGGTTGACATGCCAGGCCACTaaaaacgaa
>dme_circ_0007208
GGTACAGTTGCGATCTAACAATATCGCCAATATATCCTCAAAAAGAAAGCTATGGCCGATTGTTCATATG
TGAGATGTCAGCAAGAGCGTCGGCTCATCAAAAAGAAGCTATTGAAATGGTCCACAGATATGCTGCATAT
TGTGGGGTGAGTTTATCATTTGTATTATTTCCCAAAATATATCTTTGAAACAAGGAAAATAAATGTTTAT
TGTTTTGGAAAAACTGTACAGCTCTTAAATCTCAATTTCCTGTTGCGTCAACAGCTAAATCGACTTTAGT
TTTCCATTCTAATTTTATTATTATCCTTTTAATCCTTTCTCTTCTGTATTGAACTCTATCCACCTTTCAT
TCTATCCAATTAAAGTCCGTTAAAAGCCAAAGGCGCTGATGAAAAGCACCAAAGCCAATAAGCAGAATAA
TTTTTATGCTAGGTGGCTTAAGCATCGCCGCACCCCCGGATATTATACTATCTATATACAAGGTGCTTTC
AAGCTGCAGATTTTCATTTTCCCTGCGATTTTTCAAACCCTTCGACGGCCTCATCGTTCATATTAGGGCC
GCAAATGGGATTTTGCCCGTGAACACGGCAAATGAAGTGCCTGCAAGTCGCTTTTGCCTGAAATTGACTT
TAGCCAGACCTAATCAACATTGACTGGGCTCAAGCCCTTACCATATGTTCGCGTTAAGAAAAAACACCTA
GGAGTCTGGCCAAACACAGAGGAAAATTAACATAAATTGCAGAGCCTTTTTTGCTGGttttttttttttt
ttttGGTGTCGCCCTTGGATATCTATTCCTTTGGCTCCTGGCGGCTGCTTCAGCCTTATCCTTTTGCCTG
AAAACACTCATTTCGGTTTTTGAGCACGTTTAGCACGTTGCCAGCGATCCAAGCTAAAACCCAAAGCCCA
AACCCCATTAAAATGTTAAATGCTCTTTGTTGTTGTTGGGCGAGTGGGCGGCTTTACATGTACATATATA
TGTACAAATGCCGCTCTCCATGTACAAAACGCACACatatatatatatatttatatatataGGTATAGGT
GTAAGTGTATTTTTTTCCTCGCCAGCCAACACACAAACACATCGTCCTTGCCTTGCGTCCCTGTCTCGTG
CACTCAATTACTTCCTCAATGCGCTCCATAATGCTTATCGTCCTTGTTTCTAGTTGACATTCCCGCACAA
ACACAGAGACAGCAGCACCACTTCCAGTGGTTTCCCCCTGCCACCTTTGCGACCCACTAGGAAAACCCTT
AGCACATTTTTCCAGCGACAATTTTTGCTCGGCTCTCCGTCGACGACGACTTCGTTGTCCTCGCTGCACG
AAGCTGCAGCAAAATGTGAAAGAAGCCAGGACGAAAATGGGAGGaaaaaaaaaTATAACGGATATATAT
GCACGGGTGTCTGGCGTTTTGGGGGGTCTGGGTTTCGGGTTAGTTCTAGCCAACATGGGTTAAGAGGGGC
CCCCAAAACGAAGTGCTCTGCTGGAGATAACGAAGCGCACTTAAgcacacgcacactcgcacactcgcac
acacacaTGCGCAGGATGGAAGGTGGTGGGTGGTTGGGTGAGGCGTTATCCCTACGAAATATATTGCACA
ATATAAAAATTGCAGACGATGAGTGCCGAATGCGTTTGCCGAAAAGGCAGCCCAGCACTATAGAGCTTTG
GGGGTTGGGGGATAACTACTAATACTAGCACAAAAACTACTACAACAATTAGGAAAACGAACGAAGCCGA
ACCGAAGGACGAGCCAACGAGGAGCGAGCGTTTTCCTCGACTGTTTTTGTGGCCGATTTTCGTCGCCAAC
GAAttttcctgcatttctttttctgactttttttcctagatttttttttcAACACTCTCCCCCCCCGACCC
ACCACCACCAATGCCACCCAGAAAAACGATGCTGATCGAAGCTGAAGAGGAAGACAAAGACGTCGTTGGT
TAGCGTGGATTGGGTGGGGTGGCTGCAGTTTTCTGGTGGCCCAGTGGGTGTCTCGGTTGGGTTGGCTGTT
TTGGGGGCATAGTGTTGATGGGAAATCTCGCCTGTTGCATTTCCCATTTCCCCACCCAACTGTTGTTGGC
AATGGCTCAATCAGTGAATCAGTGGTGGAGTTTCATTTGATTGTAATGGGAGCCCATCCACTTGTGGATT
CAGGTGTGTGTCCGTGCCCGGTTGCCCAGCCATCTGTATGCCAAACCAAGCGCCGGCATAAAGAAGATTA
CACTAACCAAATTATATAAGCTGCATAAAGGTAATTTTCGACGGTAACGGTTATGGGCGGAAATTTGTCT
GGGGCTCAGGGGGCTTTAGGCGAGTTTTATTAGATATCTCTACAAAAGTAAATGGCACAACTTAGTGTTA
AGTTTGAAATATATGTATTGGGAATGATTATTACCGAGGCTCTTAAGAAACAAACTTAAAATGGGTCTGT
TTATATTTAAATATAGACTCAAATAGATGGACAGCTGTTAACTATTTAATTATATATTTGAAAGACAAAA
ATATTATTATATACATAAACCAACTTGAGCTGCACCAGTGTTTCCTTTGGTCCCAGCCACACCTCGATTG
CTTCCGTAAAAGATTTCCGTGGGATAATGCAATCTACCTGAATATAGCAATAAAGAGCTCAGGCAAATAT
TTTGCTAAGCTTATATTTCCTTGCGAAAAAGTTATCCAGCAGCAGAAAACAAACCCCAACAGGGATTTAA

CTTGTTGCACAGTCGCCGCCTTCGCCCGCTCCACATTTCTCAGCCGAATCCTTTCCATTTAGTTTGTTTG
CCTGGAAAAGTACCCCACCTCCGCCCGATGGGGTGTAAACATATTAAAAAGCCAAACTTTTCCTTTGCCT
TTATTTACaaaaaaatacgaaaaaataaactaaaataaatagaggaaaaaaTCTCGAATATATAGAAGAG
AAAGAGATGCATTATAATGGTGGGGTGTGGGTGTAGTGGTGGCCGAAAGCAACAAGCTTCTCATACACAC
ACatatatatatatatatatataAATTTTTTGCAGCAGAAAGAGAGCGCAGAAAGTTTCCGggtgtgtgt
gtgtgtgagtgttggtgtATTAAGAAGCAGCAGGTGTTGGGCAATATTGTGGGCCATTCCCACACCCCCT
CGCCGTGTCTCTATGTAGTGGTCGGGTCTTGGCCCAAACCACTCTCCACCCACAGCCGCCCACTTTGCCA
AACGCATCTTTCGATTACGCCAACAACAAGCAACAATAAAGAGAACTCCTTCTAGAATAGCGTTGCAGGC
GCCGCTGTTGGCGCTGGCTTTTCCAACCATTCCCCATTTTCCACCCACACCCGCCGCACCATTCGGATTT
TCCACCCACCGTCTTTGGCTGTAAGAAGGCATTTTTATGCCAGAAGGATGCAACAAGCGACGAGGACAAC
GTCGCGTCTGCCTGTGTGTGGGTGGGTGGCTGTGGGTGGGTGGGCAATCCAATCCTGGCCAGCTTTTCAT
GGACTACGCAATAAGCACTCCACCTACTTTCCAATGTCGGGCGAACTATTTTTCTCATCCTCCTTTTATT
TTCTGCGCTTTGCCTTTGCCTTGTTGAATTTTTTCCGGCGGTTTTTAGGTTACATTTTTACGAGTTTTCt
ccacttccactgccacatccacagccacagccacatcctcatccactTGTTGTTGTATTTTGCGAGGATG
ACGCTGCAAGCGCCGAAGGAGTCGCCGGGCGACGCCAtcgtccttcgtccttggtccttcgtGTGAGGCA
GCCAAAATCGTATTGgtgtgtgtgtgtgtgtgcgagtgcttgtgtgtgAGGAAGCGCCACAAGTGGGTGA
AACACTGCGCGAGCGAGAGGGCAATATGGCCAGAAAGAGACGGAGAGGAGGAAGAGAGGAAGGGAGATAG
CTCGACCATAGTTCGGTGCATTTGAGCCACCCATCGCATCCATTCCCAGCCCGATTTTAGCCACCCAACA
TCGGTTTTCTGGGTGCACAGAAATTGGAAAATAGATTTTTTGGTTGGTTGCCTTTGGCCGGTTTCCTTCT
TTGCTTCGCCAGTTTTTTCATTCATTTGAGGGTTTCTTTTTTTGCGAATGTGGGATGGTAAGTGCACAGA
GAGAATACTATATATAAAAATATGATTATTCTAGTCCGAATCTAGAAATAGCCGAAAATGTTaaaaaaaa
CACTTAACACAGAATATATTTCAAGCTCTTAGAATTGATGATCATAATTTGTTTGTAAATTAAAAGAAAA
CATAAAAGTTATATTAATATGATTTCAATTTTCATTTTGTGTCATTGTGTAGCATATTGAATTCAATCTT
TGTTTCTTAGTGTGTTAGATTATGTAGGTCAAATTAAAGTTGCCAGAGTATACAAATGCTAAATGAATTA
AGAAGTTATTAACAACCCACACAACTGTAGTTTTTCGAAAAGAAAATTCAGATGATTTTCTCGCCGTGCA
AATGGGTGTTGATGCTGGTCAACCGGCCGGTTCTCGCCAGGAAGTCAGTAAGCTTGAGGTGGACTTTGGG
TCCTTTCGTGGGATGGGGATTGATTGAGAATTTGCCTTGGGTTCAAGGTATGGCCAGGACAATGTCCCAC
CCCCCTCGCAGGACCAACGCCTCCTCCGCCCAACGCTTTGTGGACAAGACGCAGGCCAacattttttccc
gctgcatttttccaagcttgggtttcattctttttttttCGCCAGAGAGGAAAAAATGAAGCGGAAGCACG
AAAAAGCACAAAGTTAATGCGAAATGGGAGGGTTTTCAAGGGGGTTTTTGGGGAGCTAGGAGGAGCTGGA
GCGCGCCAATATGGCGCTAAGGACATTGGGCAAAATGCACTGCAGAAAAAATTGCACATTATATGTAGGC
AAgtgtgtgtgtgtgtgtgtgtgAGTTAGGGGTTGGGGAGGAGTAGCGAAGgcaaaacagcaaaacacac
aaaaacaaaatggcacaaaaaagagagaacaaaaaaaaaTGTAGTCAACTGAGCTGGCTGACTGTGGAGG
AGGTGGGCGGTGGGTGGCTAAAGCAGTGGGGTGGGTTGGCTAGGGGGCGGTGTTTTTTAGGTGGCGGGAA
AGTGGGAAATTGGGTGAGCTTTGAGTCTGAGTTTCGGCGTCGACTGCGCTGCTGCCTCGTTATTTTGCTG
CTGCATTTGATTTTCGTGCTGAAAAAGTTTCGTGTGCTCGAAAAAAGTTTCGTCGCTTCGTTGACGTCGT
CGCCATTGAGCCACCCAGCCATCCAGCCCACCACCCACTGCACCACCCCCTTTTACGTACTATCTAtgtc
tgtatgagtgtgtgtgtgtgtgtgtgtgtgtgggtgtgtagtgtAGTTTGAATGGCATTTGGTGGGCGGC
GGTGGTGGGGACAACTTGGGTTTGGGTCCTTGCAGCAACATTTGGCTGCACTCGGTTGGCTGGCCTGGCT
CCGTCTCGTAGTTTGGTGGGTGTATCGGGCATTTTTGTCGAATTTGTTGCTGCGAAAAGGAAAACTGGGA
AGGCAGTGGCTGCGCAGCCAGCGCTAGCAGCGACTGCACAACTATTTTCCCGTGCATTTGGCGTCGGCAT
TCGGTTGGCATTGTGCAACAAGTGAGTTTGGCAGCAGCCACCAGCAGGACGACAACTTTGAATTCGGCAG
CAAAGGCCTCCGAAAAATCCAATCTTTGTCAGGTGCACAATTGCACATTTGAAAGCGACTGAAGTCAGGC
AGCGTTTCAACTTGATTTCGACTGATTGAAGCAACAATTGGCGCCAACTGTTGCTGGCCGCCATTTCGCG

ATTTTCTTGATTGACTCGACTTACCACCACTTGCAACCTCAGAAAGTGTTGTGTGCTTTTGTGGGTCGCC
AATTGTGCGGGCTCgtgtgtgtgtgtgtgtgtgtttgtgGAAATTTAATTAAAGACTGTCGTCGAGTGCA
ACGAGATAATTTATGTGTAGGCATTTAATTAAAGCGATCACACAGCCATTTTGCATACACCAAGTCGAAG
GCACAGACAGAGACAGCACGAGGCCGTTAGACAGAGACGGACCGAATGATAAGTGTGGCATATGTGTGTT
AACGCTTAATTGAATTGACTGAATGAAGCAGCCAAGTTATTACCTTTTTTTGGTGCTAATTGCATGTTCG
AGATGTCGAGCAATGAGCAGGTGAGTTGGGAAACATATTTTACAGAGTTCCATTCATTCGCCATTCAAAA
AATTTCTGTTTGAATGGACTCAGTGCTTACAGCTAGAAAACTAATCTGACGAAAGTTAAATGTATGGAAA
ACAATATAGTTAACATCTAAACAAACTCAGTGACTATGAGTATAAGTACACTTTTTAAAAACATTTTACA
TTTATTTACCATGGGAACCATTAGAACattatataagtcttaaaatattagttagatatttatatttata
taatatattatattatataaGTCTAAAATATTACTTGTATATCATTAATAGTCAATCTGTTACCAAGCCT
TTatttatagtattatagtattatagttttatttttttcaatttattatagtaatttACTCAAGTGAACTG
ACCAAATTCTGCAAAATATTTTAAAAGCCTTTGCAAACTTTACTCAACCGATTTTCCATAATTCAATTAT
CACACTCATATAAACAATTTAGTCACAGCTAACGTGATGCAACAACAATTTGCCCGAAAACTGTCCCAGA
GGGTATAGTTTACCTTTCAAAACGTCTCAACACATATGCAAATCTATTAATTCTCTCCAAAGTTTCGTTG
AAAACTTGCAAACCATAAAAGCCATTTAGTTTGCAATTTACACAACAAACGACACATTGAAAGGGGCAAC
GAAAAATCCTGGCACCCAAAAAGGAAAACCCTCAAGAGGAAAACTTACACAACTCAACTCGTCGACAGAT
TGTGGAAAAACACTTGCCAGAACACTCTTAAAATATGCACAATACATCCAAAAAACTTCAAGACAAGGTG
TATAAGGAATTCTTGTACGCTTGTGCTTGTACGAGAAGTTTTTTTGAGAAGAAAACCCAAACCTCTTCGA
AGGAGAGAAAATCCACCGAAAAACATAGACAAACACATGTTCGAGGCAAAAGTCAAGGGAGCGAGCGAAA
CAAAACCAAAACATGCTTCTACGACACTGATGACGACGTGGCAGGCGTTTGTGTGTTGACTTTGTAACAT
CGTTATACAACAACAAACTAGCGCACTTGTTGCATAAGTCGCAGTACACACCCACAAGGGCACACAACAA
GTGGCAGGAGCGAAAGGAGCAGCAGCATCGCCAACAAATGCCGGCAACACAACGCACAAAAGTATGCAAA
GAAAGCACTTCACACGTTCTAACAACGCAGTCGCCGCCGGGTTTTTTCGGTTTCGGTTCATGTATATATA
TGTATGTATGTTTTCCAGTGGGCGGGGAGAGGGGGGTGGTATAGGGAGTTTTCCGGCTGCTGCGCCAGTC
GCGGCACTTTCGGCGATTTTCGACGAAAACCCATGTGTTCTGTGGTGGGGAGTTTACAATCCAACCACCC
GCGATTTTCGCCAAACACGCGAAGCAACAGTTGTCATCTGTCGACAACTTGTTGTTAGGAAAGCTATTCC
CTCTCCCTGTTCTACCATTTAAGCAGTTTTCCTGGAATATGTGCATATTGCAAAGCTCCTTAAATCTTAG
ACAACCAATTTAATTTGGGTGACACAGATTTTATCCCTAGGCATAATCCAACCGAAAAAATATACCTACA
TGAAAAACATTTTACTTTTCTACACCGAAACCTCATCCTTTGTTTCTTCTATGCCCGAAAAAAGGCATGA
CGTCTGCAtttttttttGTATATCCATTTTCAACTCAGCAGGAAGAGCAAATAGAAAGTGCTTGGATTTT
TCCTTGTGGCACTTGTGGCGTTTTTAGCTCCTTGGCAATTGCTTTTGTGCGGACACTACAAACTGTACTT
CCGTTTTATCCTCTGCGTCTTTGTCTGCAGCAGACAAGGGATGACTAGCAAAAGGGGGCTGGCTGATGAC
GAGGGGTTATTGGGCAGAGACGACATCTAATCGATAAATATTCAGCACACTGCTTGTAAAAATACATTGA
AAATGCATTAGACTTTGGCTCAATGGCATGAATTTTAATACGTTTGCGTATTATTTCCCCATACTTTCGC
ACTCGACTTTATTCCCACAtttttttctctttttttttttttGTTAGTTTAATTAATCTGGACTCTGTGTT
CTGGCAGCTGTAATGTGCAAATCGTGTGCCATAAATTTGCAGTTTAAATGCGAATTCGATTGACAAGCCA
GCCAAGTGCACAATTTCTAACTCCGCTCTCCCAAGGTTGACAATACGAAATATATACTTGATATAATAGT
AGAAAAATCCAGTTAACTATTTGCAAAACGCAATCGAAACGCTGCTGCAAGTCAACTGCCATTTGCCGGC
CACTAATCAAACGTAACAAAATTGCCAGCATAAACACGCTTAAAGAATGAATGAGACTGGAGAGTCGAGA
CAAGTTCGCCTTGAGGTCTTACCAAAATGGCGTTAAAACGGTAAAAGGCGTCCGCCGGCGGTAAGGGGTT
AAGAGGCTAGCGGGTAGTTGTGGGTTAAGCGGCTGACGTCTATATGGCAACAACGGCAACAACTTGTTGT
GCGACATTTTATGCGGAAGCAAAAAGTGCCAAGAATACAACGAATTCAAGTTGAGAGTGCAATTGCCAAC
TAGCACCACAATGCGTATACGTGATATGTCGCATTCGTTACGCGATCTACTGTCGATTTTGGCGGCGTAA
AAAATACTTTCTAAAGCTTTTCAAGCGTCTTTAAATATGAGCAGTGAGCTAATTGATTAGCCTTAAAAAC

GTTTAAAAATATTCATAGATTTTATTATACATCTTAACCTTTCGTACGAAATATTGTCAAGAGTTTCAAT
TTTAGTAACATGATTATTTCATATATGTATTTAATGTTTCCCAAAGTATCCCGAGGGGAACAATAAAATT
TACTGGAAAGTAAGCATTATCTCAGAATTTTCGTCACAATTCTCGATTTTGATCGAAGGCAATTCGAATA
AATCCCGCTTGCCACATAATTCCCAACAACAAAGCAAAATAACACTCCCCGGCTGAATCATTTGCTCCAA
TTGCAGTCACTTGCGGCAATGCAATGCGAACTGATAACCGATTATCGATTATGCAACAAATTGCAATAGC
AAAATCTAAGAAATTTTCAATATACTTCGATACTTGTATATACGTATATAACGCGATTGCCCTCCCATAA
ATACGTGAAAACAAAAACGAGTATAATTGATTAAGGAGCATTATCAACAAACTGGCAGTATTACAAAAAT
GTATACTATACGCTTGACATTTTATATATAGAAGCTGATTTGATTGAAGATGTCGTAAAAGCAGAATCAA
ATCGCATGTCCCAATAACTTTGGTGTGGCAACGAAAAACGAGTTGGGCCTGCTGTTGCTTAAATGTAAAC
AAAAGCCACACGATAAGCGAAAAAAGTGTTCATCTACTATCTTTTCAAGCGTATATACAGTTTATATAAA
CAAAGAAATTTCTACGTAACCACAGAGAAAGTCGCCAAGTGCTTACCTTTAAAAATAAATGACATTTAGT
TTCACAGgtttataattattatattttattattaataatataataactataataatataataatatgtat
tattatatttactatatttattataGTCAGAACCTTTAACAAAATCCATTGGCAGTatttataaaatcta
tatttgtatctatatctatttattttatatttaatttattaCTGATACATCGGCGATCGAAAGATGCGTT
TAGAGGGCGGAGAAAGACGAGGGAGTCAATGTGCCTCGGTTGCAGCATTCGCCACATGACTTTGCAACTG
TTGCGCTCtcattgttgtcactgaattcactgttgttgttgtactgtgcgttgttgtgttgtgtcagttg
GATCCGCTGCTTGTGTTGGTGGACAAGGGCTCTTATGGCATGCGCTCATACtgtgtatgtgtgtgtgtgt
gGATATGCCTTCTCGTTCGCTCCCTCGCTTGCCATATGAGATAATACCTTGAACGTGCGTCAGTCAGGTC
GTTGGCGTTTCACAACTGAACTACAATTGTCATATTCATTGTTGTTGTCGTAGTTGTACTAGTAGTACTG
GACATTAACTTAAAGTGAAATGAAAAGTGTGTGCGGAGATTTTCACACTATCCGAATATATAATAAAGTC
AAGTGCGCAGTTGGGGAATAGTTCTATATCTCTATCTGCCATTTAGCATTAGTCATCTCAAAACTGAGCC
GCTACGAATGGCACGTTCCACCATTTTGGACAATCTAACTGCGCACTTCCGAAGAGACACCCGAAAAATT
TAGTCAAAGAGAGTGACTTATCACTTAAAACTAAATTTTTGACGCAAACAAAGCCAACACAAAGTTCACA
CTGGCCATAAGTACAATACATGCAACTaaaaaaaaaaaaaaaccaaacaaaaaaTAGGGGAAACAAAAGT
AAGAAAGCATTTAAGCTCATTTACATGCAACTCACTGAGCGACAGAGAAAGAGCAACGTTCTTGAGTGGC
GCGCTTGAGTTGAGTTGCGGAAGGAGAAACTCACCACAGGAGAGCCACTCTTAATGAGCAGGAAATGCAG
TTTCTCAAGCCGAATATCGACGAGTTAATGCGTTTGAGTGAGTTTTGTAAGTTTATTTCGGACCTTTCTT
GGATTTTTTAATGCAGCCAGAATTTACATTATTCATTAGGACTTATATAAATAGGAAATTATAATCCAGA
TAATTTTGTAACATATTTTAAACATTTTTACATTGTTGAAACCATAATATAAAAACTTAATTCAATTTGT
TGCCGTAATATCTGTAGGTTAGCGTTAAATATATCCTTTTTCTCGTCTTACTATTCCCCCTGTTAATTCA
CCCAAAAAAGGCAGCTAATTGCTGTGACGTCGGCAGCGCAGCGGCAGAAGTGCATTCCTTTTACTTGTGT
GCGTGCGTTAAAAAAGCGACACAAAAAATATGGTGAATTAATGGGAGTGTGTTTTGGTGTGCATTGTTCT
AGGCGTGCATTTGTGTATGCTTGTGTGTGTAAGTGCTCGAATTTGAGTGACTTTTCAGCGAGAGCGTTTT
TAGTAGTGAGCCGCATTTCTTGGGAGGACTCAAATTTTGGCAGCTCGTCGCGCAGCTTGGCAATTTTTTT
ATCAACGCGCGCTGCACTCGAAGTGCAATGCAATTATTGCAGTGCACTCGGATTCTCTCCTTCTCTCTCC
CTCTCTTTGCCTTGTTAACCAAATGCGTGTGTGTGTGTGAGTGCGGCTTACCCCGTTACTCCTAAAGTTC
TCGCGACTGCGCTCTGTGGAAATAACTCAATGAGCGGAGAGGACTCGCTCTCGCATAAAAGCATTCTAGT
TTTGCAGTATGCAgttttgctgtgtgtgtgtgttttccgtggtgtttttcttgttttttGCACaaaaaaaa
aaaCGGCGACGTCGACGTCAGCTCGGCACTGTGCTGGAGTCTTCGTGTTGTTGTGATATAATTTTGCAGT
CGGCTTCGGCAGTCGCAGAAATACAACGCATCCGATATGCAACGGAACGCATTTTCGGACGCGGCCCGAG
TCGTTCCGAGGTGTAGCGtccgattccgattcagattcagattccgaCGATCGCGTATATCAATCGTAAA
GCATTAAGCGCATTGCCCATTTACACGCAGTCCATAGGCGAAAATCGTTTGCCATTGATAAACACACGAC
AATTGCCGAAAATTAATATCCAAATAAATGGCCAAGCAACACAAACAAAGTGAATAAATTAGCTAACAGT
GAGAATGAATAGAACCCTGCCTTACAAAATGCCGACAACACGAATTCTTAATAAAGTGCATCAGCTAAAT

GACAACAAAAATCGAATTCATTGACaaatagttataaataatataaaagaaaaacagtgaacttaaaaaa
tgacaaaataTCAACGTCCTTCAAAAAATGTAGATGAAGAGTATACTCTGCAACCAAAACTCAAATACCC
ACAACAATTTTAAATAGTGAATAGAACAAATATTCGAATCACAATttataaaaattaaaaaatgttgtaa
aaatcctaattgaaatataaaaGGAATTTTATTTAATTTGTACTATTCATCTTAAAAACAGTGACACTTG
TTCTCTCAAAAAGAACTGTTAACCCCAAACAAAGCAATAGAATCAATTTTTTCCATATTAAGTATACGCC
GTGTGTCCTTTTTTTGTACTTTTCACGCATATTCATCGGCGGCATCAATCGCCCGCGTTTTGTTTGATTT
TTTCGGCATTAATATAAATTGCAATCGCCGCTTGTCTCTTGTTTGCTCTGTGTTTACGTGTGTGTGTGCG
AAAacgccgacgttgccgtcgacgcagacgtagctgccgccgttgacgtcgccgtcgacgtcgctgtcgc
cgCTGTCATGCAAAATGCGGAAAAGTTTTTTTCGCGCACTCGCAAATTTTTATAGAATGCATTTTTATTT
CGGTCTTTTGGTTGAATAGTTGCATTGCCCAGCTCAAGTGCGTGTCTCCGGTTGATTGACTATGCTCGCC
tgtgtgtgtgtgcctgcgatcgtgtgcgtgtgtgtgtgtgtgtgAGCCTGACTTAATAAGGCGGCCCATT
GTTGAATAACAATTTCGCGTTTTTTATTAATATTTACCCGAGTGAAATCTTAGTGAAATGTGACTCAATA
AAAATGATGTCCAAAATAATTTAAAGAATTACCACAAGTTTCAATTGACGAAATCCCATAAAAACATCAA
TCAATGCATTTCATAGCTCAATATTTCATAAATGCAATCTTAAAAGGACACAATTAATTTAAAATCAGCA
AGAACTCTCAAAAATTTGCCAGATACGCCAATATTATGAACATTTTTAATAGACAAAGAAGTCATTAGAT
TTCGCTTAAAAAATCATTTACGCGCTGCATTTCATATTTAAATGCCGTCTTCGATTCTCGTTCACTGCAC
CCAAAAAACTGTCTTGTAAAAACACTCCTAGAATCTTGaaaaaaattctaaaaaatagattgaaaggttc
agagaaatggggaaaaaagatgagagaaaaaaaCCACAAAGGCAGCAGCATACAAAATGAACTGTAGCCA
AACTTCTGAAGCTGCAGCAGtaaaacaaaacaaacaaagaagccgaaaaaaaaTGAAATGAAAGAGAATT
GGAGGAGCAGCAAAGAAGTAGCTGCAAGTACACAGTAATAAAGTGTGTATTTTTTCGGGTAGTTCGTCGT
CGCCAACTTTACTGTGCAGGCAAATGTAACAAATTTTTGTCAGTTTCAATTTTCGTCGGGCGCTTGAGGA
CCACCCCCCTcatttctaccatttccaccagtttcaccaattccaccGCCCATGACAATGGCCAGGTCGG
TCGCCAGTCTCCAGTTGCTAGCATTTACTTTGCCGTCGGGCTTGGCGCACAGCATAATGAACAAAAACTA
AGAGCGAACAGaaaacaaaaaaaaaataatacaaaaaaTGTAGAAAACGAGGTGGGGGTGAGCTGAGAAA
AATCAACTCCCAGATATGCTAGACaaaaaaaatggcaaattaaatagccgaaaaaaatGTAGCTGCAGCT
GCGCAACATCAATTGGAACCCCTCCCCCGCCCGAGAGCTTAACCCTCACCTTGCCCATCCAGTTGACCAT
TGGATTTTCCTTACCTTCCGTTCGGtttttcctttttttttttCGCCCAGCTTCTACTTTATTATTTTATT
TTACGTTTTTCGGCAACATCGTCCGCCCGATACCCCTTCATACTCACGAAACTTAAATTACTATTTACAA
TGGtatttttttggctgcattttgttcctatttttttgcaatttttttttttttttttgtttttttATTCT
GTTCAGAACTTGCCAGGCGAATTTTtatatatatatatatGTATACATTTTTTATTCGCATTTCTCGCTG
GCATAAAGTTTTTTATATTTTGGAAGGGGGTTACCCAAGTTGATGCTCTGCTCCGCCAGCTAATTTCATT
TTGGGCGAGTTCAGTTCATTTTTTTATGCAGCTGCCTTCGGTGGATTCCGCTGGAAATCAAATCATCTTC
AGACGGCTATCGAAATATATAATCGCCATGTTAATCAGATTCCAATAGCATACTTTAAATAGCAACCAAG
TTGGTCAGCGATTTTTTATCAAAGCCAGAAAAGTGCAGTGAATGAGATCGTTTGGGGCACACGTCTCGCAT
TTCGCTTCTCTTATCAACGATTTCCATATTTTACAATTTTTAGATATTAAACAAAAACAACAACTGCAAC
AACAACGGCTACACTTTTGTTAGTAAATTTCTGTTGAATtaaaatacgaaacaaaacatataaaaaagtg
caatccaaaaaaaacaaaTATTAACGTTAAACGAACTATAAAGCAAAATAATTAAAAAAGTAGCCATTAC
TTTTGAATTGTTTTcaaatgcaaaaaagaaaaaggttcgtaagcaacaaaaaaaaaGTTAAATGCTAAAG
CATTTTGATAATGCAACATATCAAAGAGCCAGAATTTAAACACAGCAAAAAATAAATAAAATAATTAATA
TACAAACACGCGGGGCAACTGTCAAAAAATCTACAACAACACAAAGTGCAACTTTCAACAACAACAAAAT
AAGTGCAGCAGTACAACAACAAAAAGCATTACACAATTGAACAAACAACATACaaaaaaaaaatattgcc
accaaataaaaaaaaaCCCGAAAGAAAATTCGCGCTTCATTTTCACGCGCTGCATATAATTTACTCTGTG
CTCCACatatatatatatatatatatataATAAATAAAAACCAACCAATAGCCCaaaaaaaaaGAAATGA
AAAGTCCGCATCAACAATAAAAATCTGCCTGCATTTTTGCCTTTTGTGTGAGCTGCCCACCAGAACGAGA

GAAGCACTTTTATTGTATATAAAAATTATATACATCGCCGGGAGGAGCTGCAGCAACCCACTCCAAGCCA
GGGTTGCCACGTCCTGAGCTGCTGTAAGCTCTCcgcagcagctgcagcagcatcagcatcgcagcagcat
cagcagcagcgcagcaaccgccgcagcatcaATTTGGCTTTTGGGCAGAGATAATTTAAGACAAATATAT
GTGATGCTATGCACATCAGCAGCTATGAAATATGTACGTATGCCAAATTGCtttttttttCAAATAATCG
CAAATAAACTAAAAATATATTTAATGATTAATTGTAATTAATGAGTCATAAATAATTAACGAGTTCGTGA
TTAGCTTGGTCATAGATAAAGCGATCCTCAGTTCCGATGCGCATTAATCAATTAGTAAACAAAACATATT
TAATCGTTTATCCACTTCATAATTAACACTGCCAATATATGTTTCATTGCCTACTTTTTGTTTAAGATTT
TTGATAAATGATAAGTTGAGAAAATTATGTAGCCATAATTGTTTATAATCTAGCCTAGATACATTTTTAC
TTGTCTAAGATACATATTCTGATCCATTGCATTTTATATAAGAGTCAGTAGAATTATTAGTATTAGTCAG
CATTAATAATTGGTGAATATTGGCAGATCTTTAAACCCTTTCTCCTTCAAGCGGTGTTCGGGTTCTACGA
TCGCTTATCGTTCGGCAAACCCCGAAAATTACATGAATGTACACAGAAATAAGTTCATAATAATATGTGC
AATGCCTCATTGACATTGTCCTTCAGCGACAGTGCGATGTACGAAAGCGAAATGTGTAAACAAAGATACA
CAATGGGTGTTGGTGAGAGGGGTTTTCATGGCGGTGGGGAGGGGGGTGCGGGTCGAACTGGGAAGTGTGG
GGCTATCAGAGGCGTCACAATGCGATCGCCGCAAGTACAATTGCATTGAGCAATACTTAAGGACAGAAGT
TCAAGCAACTGTCGAGTGTAGACCCATAGATACACAGCTACAGATACTTTGTGTATTCGTGGTATGTGAG
TAGATATCCGTGCTGTATCTGTAGAACTCCATTCAGAAATTCATaaaaaaaaaTATATATCTACTAGTTG
ATATCTACGAGTATAACCGGAAGCCGTGCGAACCAAACGTTTCTCTTAATTTCTCTACGTTTCATCGGTT
TTCGCCAAGATTGCCAATTTGATTCACAACTATGTGCCAGCACTATCTCTGCCCAATCTGAGCAATCTGA
ATCTGCGAAGCGGCGGTTCTTATCAGTGGGGCCCTGACGCAGCCGTTTCTGACCCAAATTACGACAGCTT
TGGCTCGCCCCATCGAGCCTGATACGTGGCGAAATCTGAAAAATGCAACAACAATATAAATTTAAGCAAG
AAATTATTGAACAAAAAATGTATATGTACTCGTACTTTCTTATAAGTTGTCATGCAATCGCGcaaacaaa
acaaaaaaaaaagagagcaacaaaaacaaaaagcggaaacaTTTTGCGCTTTCTTATCAAGGCATACGAA
ATTGATTTAATTTTTTGCTGAAAATTTGCGCCACGAACGAAAATTTTTACTGGCTGCCAAGTGTGGAAAT
TTATATTCCCCATATATATATGTATATTCTTTACAACAATATAGTACAAATAATATGTACTTGTATATAT
TGATAGTTGTAGTTTACGATTCGAGTCGAGTCAACGCATGGTTCAAATTATCGCAATTTCAGtttttttt
attatatatttcatttattttCACAAAATAGTTACAGCCGATAACAAGGCTGCACTCTATATAGGGTTGT
aaaaaaaaaTAACATGATTTCCCCTGAAATTATaaaacaaaaaaaaTCATGCAACGAACTTTGCAACAGA
CCATATTCAACGAAATGAAATTATGCCCGTTTATTCTCTTTGTGATTAATATCTGGTTACGTTATTTTTT
CGGTGATTTATTTGTATACATTTTCGACAGGTTTTTCAAATAAAGTTCAGTCGAAGCTTAGGTAATTTTA
TTGCCGAACCAATTTTCAAGGCTGATCAAGTTTCTTTCAACGGACTGCAGCTGTTTTGGGCCtttttttt
ttttttGTTATATTAGATAATGGACACTCAAAAAGTGTTTACCATCTGGTCGAAAGATAACACACATTGC
CATAACGACTATAAAAATAATAAAAGAGGTGTTGTAAATACAAATGCAATGCTGCGTAAACAGAAACGAA
AACAACAACGTGACAAAAGTGTGAACATTAAATACGTTTATTAACaaaaacgaaagagtgtgaaaaaaac
ctaaaataaacgaaataaggcaaaaaaCCCGCTGACTTGACTAGAAACTGCTGGCGGAAAAGCGGAGAGG
AAAATGTTTAGTCGATCACCTGGCGCTGACCACATACGTGGATCGtttttttttttCACTTAATTACAGT
AGGTTTCATGGGCTTAATATAAGAATTTTATTTATTATTGTAATTTACACCGTGTAAGAAAATAGCCTCG
AGAATATTTATTAGGTGCATTTCACTTCACCATTGCATTTAGAAAAATGCCGCTGTAAATGAATTTTCTG
GTTAATAAATAAATATTCGTTAGGCCCGAGGTTCCCCTTTCACTTAAGGTACTGCCATTTTTTATTCCCC
CATTTAATCGAACCGAAATGCATTGCCTGCATACACATACATATATGTATATCTAGACCATAATTAGCTG
ACGTGCATAAGCCGGGAACAAGACACATTTAATGCCACAACAAATTCAGAACCATCAATGTCGACGGTGG
CAATCTCTTGCCAATCCGGCAAACATTTAACAAACGGAAACAGCTGTCTCGTTCTCGTTCTTACTCTTAT
TCTTATTCTTTTTTCCTGGCCCATGGGCCTTTGGGCCCTGGCAGGAGGCATCTGCATATGCCAAAAGGAA
GCTGCTACCAAACACTCCTCGCTGTGGCAGCAATGCAGCTGGTCAGAGGTACCTGCAGCTAAAGTAGAGC
CTGGCCACATGCGACGGCGGCGAGGTTGTGCCACAGTTCTCTAGCCTAGCCAACAAATAGTGGCAACTGC

CTGCATAAAACTTGGCTAACGACTTTTTTCCGTTGCCGCTCTTGACTTTTGCATTGAAAATCGTGCAATT
CACctgctgctgcttctgctgcagccgcgactgctgctgctgctgctgctgcggcagtaactgcAACAAC
AATGACGTTGAATGCATCTGCATCTCGGCAGCGGCGGCAGCAACAACAACATCATCGTCTGGTTGCATGT
TAATGTTACAGCTGCACTTGCACTTGCAACtcgtcgtcgtcgtcgtcgtcATGAATGAGTCGCGACTTGG
ACTCGAAACCCGGCTCTTTGGCTCTTTCCTAGCTGCAGCAACCCTTCTTCTTCTTTTTTTCCGCGCCCCC
TTGCAACTTCCACATGCAATTATTTTTCTAGGCTGCAAATTGCACGCTCCCtctctgtatctctgcatct
ctgtatctacgtatctTGCGCTCGCCGTGTATCTAACCATCTCTATCTTTGCGTCTGCTTTTGCTGCTGC
TGCTGTTGCACTGCTGCAGTTGCTCCTTGGGTCTTGACTGCCTTTTTGTTGATTTTACAAGGTGTGGAAT
GACTTTGGTAGCGCCTCATCGTCAGACTTTTGTCACAGTTGAGGTTACTACAATAGATCGAAAAGGTATT
CTTAAGTATTGCACTCGGCCATTTTAGCTTAAAAAATGGTTTGTTATTCATGTTATTTAGTTTGCCACAT
GACGTATTTTCGTAAAAAACATAGTCATACTTCTAAAGTTACGTTAGTAGCAAGATCCTTGCCGAAACAG
CATCTTCTCGTCGTTTGTAGGAAGCATATATATAACTATTCTTCTGACCATCATTGTAAGCATTTTCCTG
TGGCTTTGGCTTTGGCATTGGATTCGCTTTTGGCCACGGCTTGGTTTAAGCCTGGTCCGAGACTTGGCCG
CACGTTTATGGTCGCGTTTTAATGTAATTGTTGTTGTATTACAGCAAATTTGTAGCCATTGGATTGTTGT
TGGTGAGGCCTTTGCCGTTGGTGTTGTAATTTGCCATTCATGTTGTCGGCCAAAGTGGCGATTTGTTGAT
TGTCGTTAGAAATTCAAGCTGGCCAACACCTTCGGCTGATTTGCCAGTCGTTTTGTCGTGTGCCGCCGAT
TGTTGGCTTAATCGTTTTGGCCACTTTTTCCTCCGATTAGCCGCATCTAATGATTGAAATTAGACGAATT
CGAAGTTGGTAGATAATCTCTGGCAGAAAACCGGTAGCCAGGTGCCAATTTCTTAACGCGCTATTATCAA
ACTTTGCCCGCAATGGCCGTTAATTTAGGGGTTTAATACCGCTGAAAGGCTTCCTCGCCCACCGCGAATT
TATTATGACACTGATGGAAGGGATGTGCAGCAGGGCACTCCAGTGgcccaatgagtgattgactcgagtg
aatgactgcccgaatgagtgactgactaactgactaactgactgactgactgagtgagtaactCAGAGGG
TCAACGAGCCACAGCCGTGTTATTAGTTAAATGGCGCATAATGGATGAATAACTTGACAAGATAGCGAGC
GAAACAAAAGGTGTAGCCAATGAAAAACGTTAACAAGAGCTTCAAGACAGTTAACAACTGCTCAAATACC
CTCAGAATATTTTATATGAAAATACCAAATTTCCACGATTCTATTTTATAAAAGTAAATTAAGAAATAAC
TTGTAAAAGCCATCAACTTCCCCTTCTATAGATTTCGATATTTAAGGGGTAATATTTTCTTATAAACCTA
TGTACATCCAAAACTCGACAACCTCTCAAGAGTAAAAAGACAATAAACAAAACCGAAGTCAAAGACAAAG
AGTGTGCTATTGTGGGTGTTTGATAGTCCGGCAACGACGACTGCTGCAACCGAACCACACCTTAATAATT
ACTTAAAAGGAAAATAATTTACGAAATTGCATTCATAGTTCAACATTGCAGCGTTTTGCAACAAAAGCCA
GCCATAACAAACAATGAATGCCATCCACaaaaaaaaacaaaaaaaaaacaaacaacatgaaaacaaaaac
acccgcacaagaaaaacaaaaaattgtgcgaaaaacgaggaaaaGCATTGAAAAGCCAGCGTGTGGTCAT
ATTGAGTTTCCTACCGCGGCAAACATTTCTACGACAATTCCCTTGACCCCTGACCCCCCGTGGACAATTG
TGGAATTTTTCCGGGGCTAAGTCAAAGATAAACCCCGCGCGAGGCATCTCAATTTAACCTAATTGCTGTC
CCTTATCCTTGCGACCAATAACATGGTATTTCAATTGTCCGGCATAAAGAGAATTCCTTTGCCATACGGG
GCGTATGAGTAATGTCTGAGAATATTTGTCACGCTGCGGTCAAGCAAATAGAAAAACAACCTGTTCGTCC
AAGCGCACACACACTCCAAATTTGACACATTTGCTCAGTGCAGGCCCGAAAAGCCTTAAAAACTAACTGA
AAATGCATTCGCGCATGGAAAATGGAAAATGTGCTAAGCAAACGCAAATGAATTTTGCAAATTCGAAATT
CAAACGTTAAGCGACAACATAGCAGAATGCATAAATTGAAAACTCATGTGGAAACGAATCGAAGAAAAGC
GGGTGCaaaaaaaaaTGATCGAAAGAATTCTCTTTTGGAGAACTGTCATCAAGAATTTGCTTTTTGTATG
CAAAACAAGTTCTGACTTTTAGATTTTGTTCGAAACCTTGGTTTGCTCAGAAAATGCGCTTAAAATTTGC
TGAAATATATAAAACGCGCAGTTAGCCTTTATCGGAAAATAAACAGATTCAATTTTGCGTCAATTAACAT
TACAAATTGGGAATATAATTCTTTTTACATAAAAAGAATGAAATAAATAGCAAGAATCGAAAATAAATGT
TATTAGAAAACGTAATATTTTGCTGtttttttttGACATTTTGCTTAATGATGCTGCATTAAATGACAAT
CAGCCAAGGCGAGGCTTCAACCTCCAACACTTTTTCGCTTTTCCCGTCCAAAAGATGGAAAGCCTGGCCA
GACGCAAACAGTTTACTCAGACGGATCAGGCGGACAGACACTGTGTAACCTTCGACCCGCTTGGTAACAA

GAAGCCACTGGAAAGCCGGGCGAGGAATATGTATACAAAAGTAAATATGGGAAATTGTTTCCATTTTTTG
TTGCTGTATTCCTATCATTGTTTGCCAAAGTCTGGCAGGTCGGTCACTCAATACCAATTGAAAAGTGCTG
CAAAAAAGTGCAAAAAATAATGTCTCAATTTCATTTAAATCGATTTATATACATCCAAGCTAACAATTTAG
GCATGCAACAGTTAGGAAAATTTGGTTTTTAAAAGCAATAGAATAGTATGTTTAAAACTTAGAAATCGCT
AAATGGCAAATGAAAAATTATATAAAAAGCGGCAAACTGAGAGATCTTGAAATGCGGGAGTGGCAAAAAG
CACGACAGCACTCACGTGTATCTGTATCTAAGCGCCAACGAAGGCAGCGATCAGATACGATCCATTGTTG
CCACCGCTCACACACTCGAAAACCATGAACTGGAAAACCTTGAAAAACTCAACTGCAATTTAGTTTCAAT
GCAATGTGtttttatttttcttatttttttatgcatttttttCCCCCTCTTCGCCAATTTGTACCATCTGA
TTTTCTTGCTGCGCTCGATTTGTTTCTGCGCGATTTCCTAATGAATTCGATTTGCGTGGAAAACCTGACA
AACCTAAGGCCCTAATGGCCCCACTGAATTTCATTAGCCATCCAATCAACGCAATCTGTTTATGCGTGGC
TCTTCAGTTCGTTTTGGTCTGCGCGTGTGTTCTTTGGGCttttcttttccttattatttttcatttcgca
ttttcttttttttttttttGTTGTATCATGCTCCTTTTGCACTGTGCACTCAGTTGTAATTGTGTTAGA
GCAGAGGCCTATGCCACTGCCACGCATGCCGCAAATGCATTATTATTATAATTTCCAGCTCATTCCTCGT
TGTATACTTTTTTCTGGTTCTTTGACACTGGCTTGGGGTTGTGTTATTGTTTTATAGAGAATTCGATTTG
GTTAAGTGCGCTTCCAGGTTTCCATAATTAGTCTAGCAGCGGCAAACAGTGGCAGCAGCCCCCTGCTCAT
TTCTATTTCTATTTCGTTATTTTTAAACTAAATTATTATTAACCAAGTAGGCTGGGCTGGCTTTGAGCGG
CTTTCGCGCTTGACAATTGGTTACAAGTGGGTGCATTGTGCTGAAAGCTTTTATTAATTAGTCCTAATTT
GCCTTTGACTATGCTAGCGGAATATTGCTGttttttttttttttttttttgtttGCTTGCTACCAATGCT
GGCAATCAATTAGACAACGCAACTGGCAGCCAGTCAAGGCTAAAAGCCCGATTCAGTCGGCCTACCAAAA
TGTCAGGCTTGGTGGACAAGCCCCAAATCGGCAGCAATGGCAATaaaaaaaaCAACAACCATGTGGGTGT
TGCTTTGGCGGCTCCAAATTTGGCTAGGCAAAATTTACAAAACAAATTGCATTCATAAATAAAATCGAAT
TCAAAGGCAACAAACAAAATTTGAATAAACTAAACAAATTATGTGGAGCTACGCCGAAGTTGGAGGCAAT
TTATAAATCGTTATAATCGAATGAACACAAGAGAGTCTCATTTTCTCCTGGTGCGCTTCGTCACTACGTG
GAATTCATTCCACGCTGATTAGTTAGTTAAGTTTTCCAATAGAATAGTCATATTTATTTGACCTTCCTAA
TAATGCCAGATCTGAAATCAAAGCTAAAGCCCCAAATTGGCCGACAAAGCTTTGCTTAGTTTTCCATCCA
GTAGAATGCACATTTTTGTCTAAGCCGTTTGTTCAGTTTCTCTGGCAAAGTGCAACACAATTAACACCCC
GCCGAAATGCATACACaaaaaaaaaagggaaaaaaaaCAACGCGGATTTTTTAACAGTAAAATTTGTTTGT
TTAGCGATTTGGTATGGCATAGAGAGAATCTACTGTTGGACGAAACATATTCGATAGTGCAGCGTTAATG
AAAATGAGCAACAAAGCCAAGCGGTTGCACTCACACAGCCAATGTCAGAAACTTAGAACcaaaaccacat
gcaaaatagatagaaaaaaaatgcaagaaaaaaattcaaaCGAGttttttttttttgtggaattttttt
tCAGCATGAACGAAAATGCGCAAAATACACTAAATAAGTGGAAACGTCAATAAGCACCGAAGGAGAATAA
ATGAGCACACCAAACGAAACCAAATTCACATGGCAAAGTCGACTGAGATAGATAGGTAGACATGTAGATA
TAAGTAGGAGCTAGGGAAAATAGAAAAGTAGAAGGCAGAGCTTAGTGAAAAGCTGACGAAATAGGCGCAT
GTGGTGTAATCAAATATGCGaaaaaaaaaaaTATGTATAGGAAAATTATGTAGATGCAAATGATATGGAA
ACGCGCAGATGAATGGTCGAGAGAAATGTGAGGAAAATGAGAAAAAAGGGACGGGCAAGGGAGAAGGAAC
GAAGAAAACGATTCTATCTGTGCAAAGTGCAAAGAAAACGGGCAAAAATTAATTTTTGTAATGCAATCAG
CAAGTTTAATTATGCATAATTGGGAGAACGAAAAGAGGCGGCAGAAGAAACCATAAAATTTTAACAACCA
AAGTTTGCTCGGGTGGCGAAGCTATGATCGCGGATCTTATGAATCATAAACAAATAAAAACTGTACCAAC
ACTGAATTATTAAAAAATAGATAGCCATATACCAAATCGTATTTCGACCAGTTTAAAAGTTCTAGCAAAT
TATCTAGCCTAGATAAAAAGTTTATTTACTGATTTTCCTTAATAAAGAATGTTAGCTTTAAATTTCATAA
TGTTTTTTGCACACTATTGTTTTTCCATATTTAAAGTCTTGCAAGCCATAAAAAAAGTGATGTGAAAGTA
TCAGGAAGCAAGCTTGTGCTTTTATTAGATGATTTTTATGGCCTTTCCAAAACGTGAAATCCAAATGATC
TGAATCCATTCTGTTTGGTTTTCGGTCTGCCGTAAAATCTCCAAGATCGCTCTCAACTAGAAATCGTGGC
ACTTTTTTATGACAAACAAAATGCATTTCGATTCGAGAACTAGCGGCTCAACACACCCAATTGACTTTTG

AGGCACAGCCATATCGCAGTAGtatatatatatctatatatatatatgtatatCTCCGTGTTAATACAAA
TCTGTGCAGTTGCAACAGTAATTTCATATTTCACACGCCGCTCTTTGCTGCAACATTCGTTAATTCCATA
CGCCTTGATATGCAATAATATTCCATTTTTTATTGCTCCGCTAAGTTCAAGATCAGCGGAATGTCGAATT
AAAAAATGGTAGTTGCTTGGATGTCAGATGGATTTATATGATTTTGCTATACCTGTGTAAACTGACAGTC
AGTTAAGATCTTTTAATTTAACTTCTATTATTTTTTAAAGATTTTATGGCGTTCCAGAAACTTATAAAAA
ATGTTTAAAATAACACACTTCCGCAAACACAATTACGATCGCACAAGGCTTGACTCATCATTAGCCCAGT
GAATGAGTTATGAAATTGGCAACAAAAAGGCAACCATTTAAACGTAGTTTTCCGCAAATCAATTAGACAT
AAATTAATTGCCATTAATTAATAAGCAGCAGTTGTAATGGCTAAACTCAACCGCTGGCGACTCAAGGGAT
TGCAATTTGAATAAAAGCCGTCGCAGAGCGGCACAATTCATTTGTAATTGCCAATTGTGAGTCGCTGCAA
AACCGAAAAGTAAATAAATTAAATATTTAGCTAGAAATTTATAAGGCGAATGCGACACGTTGCCAAGCCC
CAATCCGATCCACACCCACAATACAGTGGGGAAAGGGGATCCCATCTGGTGCGGTCTACGTATGCGGAAT
TAAGCGTTCAAATTGTAACCAAATATGGCGCAGCAAGTTCAATTCTAATTTATTTCGGCGAACACAGAAT
GCGCGCTCTATAGTTTTTCTTGCTCGCTTTTTGCGTTCTGGATCAATTGATTCAAGGTTTTCTATTGCTG
CTTTTGATTTAAGCTGTCTCTCCCTCTCTTTAGTTGtatatatacatatataaatatatatatatTGACG
ATCGTATATGTAAGCTGTACATATGTGTGGTGTGTAAAGGCAAATCGTGCGCTTTCATTGGAAGTGACAA
CAAATGCATTCTTAAGTGATTGAAATTTAACACGACACACAGCAAACGAAAATTGAAAACGAATGACGGG
CCACATAAAGTTGACTTTGATTGAACATTAGAGAAAACTATTCAAACATATCTGGAATTTGCAGATTTTC
AGATGTTAAGTTATAACTTTAAATTCTTAGATTAGATCTTTAGATGTCGTTAATAGGTTTTGGGAAAAAC
GGAAAACTCATGCAGTCATTGTATTAAACTATTTTGTTGTATTTACAGCAATTCTTAAATATAGATGAAA
AGTCCTCCTCTTCGTAAGCTGCCACTTTATTGCCATTCATTCTGACTCGTTCAGTTTATGGTCTTCAAAT
ACGATTTATTCGAGCTGCCTAAATATAGAGACATCAATCGGCGAATCTCTGTTTCCTGTCCAACTCGTTT
TCCCTGAAGCCTTTTGACTGCACTGCCCGCCACAAATCACTGGCAATCACAGGCAGCCAGCGGCTTTTGG
CCATGGCCAACAAGCGCGAAAAAAATGGCATTTTCGCGCAAAAAATGCGCAGCTCACACACTGCTCcctg
tgtcctgtatcctgtatcctgcagcctgcatcgtgcatcctTTGGGCCGCCCACACCCATGCGATGTGAG
GTGTTGCTGTTGAGGCTGCGGAGAAAAAAATGCGAAAAGAATTGACAGAAGCCCCGTTTGACTATGGAAT
TTTCGGTTCTTTGTTTCGTCCTCTTTTTACGCCACAAACGGCGGGCAATGCAATCATGTGGATGagttgg
ggggcagttggtgttggttggttggtggctgttgtgtcggtttgttgtgttTGCTCTGGGCAGGGTGCAA
CTGGTGCAACACCCATGAATGTGCCGGAGCACAGGCCTAAAAGGATTAAGGCGATCTGCGTGCAATCCGC
GCAATTATCCTTTCAGCTCCCTCCACCGACTCCGCAATTAGTTGCATCACTCAACACTTCCCTCGTGCTC
CGtttttttcgctgctttattttttttggctcttctcctttttttcgggttgacttttAGCTACATATGGTGA
AGTGGCAAGGAGCATCCTGCCACGTCCTGCCACTTCACATACTCGTTATCGTTTTCGCTGTTTTTCTGTG
TGCCCGCTTTGTTCCACGCTGCGTATACGTAACGTCATCATTTCGCTTGCCATTCATTgggggggggCAG
AAACGAAAATCTGCAGCACTGCACTTTTTCGGTGGCCATTCCCAAAGTGAAGGACACTTAAGTGGCCCCC
CAAATTGGTTTCACACTCGCCCATCAAGTGGAGAGCATACCCTTTGGCGTTTTGCCATTTTGATGCCGCC
CGGCTGCTGCGGAAATTGTCTATTTAGAGCCTCGACTTCGGTTAACATCAGCTACTCGACTTCAACTTGG
CTGTCAAAAACAAAGTTCCTGTTAACAACTGGCTAACTGACGGCGGCCAGTGAAGATGAGTTGAGTGTGT
TCTAGACGAAGATGGGAGCTACTGCTGCTGCTCATTCATGGCCACTGGTGTTGGCAACACAACTGTTGAG
TGCCACAACACCTTGCGCAGCACTGAGAACAAATATTCGGTTTTGGAATTATACGAATTTATTTATAAAT
TATAGATTTACATTTAAAATTTGACATAGCTTTTCTGTTTATTTTATTTTTTGTTAACAAATATATACAA
ATTTTTAGACTTGAAGTGTTCATCTCTACTCTATTTTCTCCGAGTGATCATTTAAGCGAGCGCTTTCCTT
CTTTCACTTGTTTTCCACAGCTTGGCTGCCACTTGTTGGCGTTTGTTGTTGCTGAAGAGCATTTAAATTG
AAATGCCAATGCCGCTGTTTCTGTTGTCGTAGTTGTTGTTTCAACTGCTGCTGCTGCTGTGTATTCTATG
TCATCATTTGCTCTTTTTCATGGCtgtctgaacgtctggatgtcggaatgtctgaatgtcCGCCAGTCTA
ACTAAGCCGAAAACCCATTCATTGCAAATTCAACTAAAACCATGAATAAAATGTGCCACCCACTCTGGGC

CACGTTTTATTGCACATCGTTGTGTAGTTAACAATTGTGGCTCCTCGCCTCTCTCTTTCTCGCGCATCCA
TTTGCTGGCTCTTTCTAACCAAATTGAATTTCCGAATTCAATGAATGAATGAGCGCCTGAATTGGTTTAG
TTTTAGTTGTTTAGCCGGTTTGTTTTCCATGTCTGTGACTGTGTGCTTATGTGTGCGTTGCATACTTTTT
GGCTGCCGCCTGTTGCACGCCAGCCTGCTCCACTTACTGTACACGGATATATATCCGTAGCTTTCCGCAC
ACAATATCCTCACATATATCCGCCTGCATTTGTTTGTTTGTGCCCCATTTGTATTATGCCATCAGTTTAA
TGGTTCGGTCCACGGCCGTCGCctgttgctgctgttgctgctgcgatgttgcatgcagcacttgcagcgc
atctattctctttgccactgttgctgccgttttggttgatgctgctaatgctgcttccaccggcagctgG
AACTTTAATGTCCTCTATCCGCGCCCTTGCCACTCTCATATTACTTGCTGcctttttttatttaggttccc
ctcctttttttaatatttgtatggcaatttcctttttttttATTTGTGTTGTGGCCCTCGGCAATTGTCTA
GGTTGTTCTTGTTGCTGCTGCTGTTGTGCTGCAGTTACTTGTGCGGAAATTGCATTTCTCGGCATTTTAC
ATTTCAATAAATTTCGTCTGATGCCTCAGCCTTTGGGTTTTTATGTCCCTATTTGGCCCGAACTTTATTT
ATATTAAAAACATTTTTTCGTGGCCTTCTTCTTAGCTTAGAATAGTTATTGTACTGCGTTGTCTGAGGGT
TTTATGGGGTCATAACTTTTTCGGTCAGATCAGACTAAAAACATGGCCAATCGTCGAAAATATAAAGTTG
GAAATTTGTTAACAAATGCAATTTATCAGAAATCAGTATTAAAGGGCTTTataataattgaaaaaattct
ttgaacacacgaaaataccagctgatattactgataatattcttaagtaaaaattgtaatttaaacaatg
ctttgaacacacgaaaataccagctaatatatagataatattcttaagtacaaataatatatttaTAATT
TATAAATGCTGTATTTCTTTAACCGAAACAAGAAGATTTCGGAAAATGAAACAGCTTTCAAGGATATTGA
TTaaaaaaaaaCTAGGAGCATAATAAATTTAAAAGCATAATTTCTACTCTGGGATAATATCAATTATTAT
TATATTTTTTAATTCTTCTTTATGACCAGATTTCTTTTTATTGGGTATTTTGAACGGTAGCTGCTTTAAA
AATAAAATGTATTGAATTATGAATCGGATACAAGCTTTAACAAATACAAAGCAATTGATTGATACATAAT
CGATAATTCTTTGAATGCTAAAACATCAATAACTCCCCACACTTTCGCTTAATAATATATTGATTGAGAA
CCGCACTCAAACGAATGGATATGAGAACAGCAATCAAACCGAAAAAAATTATACCCTCCATTTTGCACCT
ACGACCTCTTGAAATAACCCTTCAATCGGCAATTCGACACCTGCAATTAGCAATTTGCAATGTTGCAGCA
CTTAGCACATCAATTATGGCAACATTTCTTTTCGGCCAGCCCAATTTCCTTCTGAGCCTTCTCGGCCAAT
TGCAGTCGAATAGCAGCAAACTGCAGCCTAAACAAACATGCAATCAAATGCAATCGAAACGAACAAAATT
TCGTGTTTCCCGAGTTTTCCGAGtttttaggcttcattctttttttctcgtcccgtttttttttGTTGGCCG
AAAAATGTTGCGGCCAATCAAATTGCATGCAGGCCGAAAAGTGCATACGAAAGCGCATTAGAGCGAGACA
ACGCTAGCACGAACATGTGGGGACGTTTGGGGGGTTAATGTGCTGGTCGGTTGGGGTTAAGGGGGTGTAC
GGTTGTGGATGCACCCACGCCGAAAAAGCCGTACAACAACAAGATGAGCCGAATCGAGACGAGCCAAGCC
GAATGAGGCAATCGAATTTATTTTGCTTCTGCCTCTCTAATTTTCGCCCATTTTGCCCCACAACGAAGCT
GAAAAACCGACGACAGCGACTAAATTTCTATGCCAAAGTGACACACAAAAAGCCTAAAGTacacacagat
gcacacacgcacacaaacacacacacacacacacacacacacCAACATGTGCATATGTGTATTGATGTTG
TCAGTATGTAAATTGCCATATGCGACAAGTTTGCCGAGTTTCTTTTCATTTTTTGGGGTAGGTTGCGTTT
TTAGTTTGTGCAAGGGTTGCTAATGGCTCAAATGGATGGAATGGGTGGATGTGGTGCGGAAGGGGAGGTC
CTGTCAACGCTGCCTGCTTAGAATTGTCCTACTCATCTAAAACCCTGCATTCGTTTGTATGTGTGCTGCG
TATGTCCTTGCATTTAGTGCAATTCATTTACATTTCAGCGCTGCCACTGCCCCTTTCTTAAACCCTTTTT
AGCCATACAAGTTGGCCACAATTTTTGGACCCAGGCACGCAGGACGTCGCATGTCCTTTCCACCGGAGAG
CCTCAAAAAACGGAACCTAAAAATATGCACCTGGAAAAAAACAATTCAATACTTATTTCTATTCCTATAA
ACCTTCATAAATTAATAAAACTTTTTGAAAATGTTGAGAAAGAATGTAGTTATGTTTCCATAAGTTCAAC
ATTGGAATAAAATATCTTATAAGTATCATTAAAGATCAACTTATTTTCTCAGTGCCAGCCACGCTTTTTC
CCCTTATTTTTCCACGCATTGCCGCTGCCACTTTTTTGTGATTTAGCCAAACTCATTTCTTAGCTGCACT
TTTGGCTTTTCCCTTTTGTCCTTGCCGCTCCGCTTCAACCAATTTGTTGTTGCGGCAGGACATGTGACGC
CGGCCGGCTGGCAAGCTGGCTTTTCTTTTCCTCCCCCGCCTTTTGGCCGAGGAAAACCAGTTGGTCCCAC
CCCCGGACATAGATGGGAGTACGTTGACATTAGACATTACTCAAAATGTTATGCAAAATACGATGGCGTG

TCGCGGCCGCAGAAATAACCACAATATGTATGTTGAATTTCTAATTAAAGAATTTTGGTGCACTTCTGGT
TAAAATTTTTTGGGTGTGTTTAAAAAGCATGGAAGTCATAGGTCCGAAAGTCTGAATGCCATGCAGCTTT
AACCCCAATGTACATTTTATTATTGCATAGAGTGGATGTAATTTTCATTGAAATGCGATACGAGCCAATG
AATTTATGTTAAGAGTAAATTTGCATTGATTTTTAATTACAAATTTATTCCAGTTACATACACCTGGCGC
ATTTCAATTTAATCGTATGTTAAGTAAACTTCAAGAGTTCGTAAATTCCGATAAAAGAAATGAGATCATA
AATAAGAGGATTTTACAAGTCCTTACAAGTCCAAACGGCTTAAACTAAAAATAATTATAGACTTTGTAAT
AGAAGTAATAACTCCAAGGATTTTATTTTATAGGCCAATCAAACTTTATCCACGTGCGTTTAGAATCTAA
TTATCTGTGAACTGCGCCATGTGCCCAACACCTTTGTGCGGCTCAGCTAATGCAGAATTTATGGGTCGAA
AATTACACTGCGTATACGTAATATTACGTATGCGCCCCCAAGGCTGGCGGTAACTTTTGACATTTGATTA
CGGCACGCATTACGCCAAAAAGCTGAAAACTGAAACGAGGCAAACTGCGGGAAAATGTCAAATTGCCGTT
GTTTCTGTCTTGTGTGCAATGTTCTTGGCAGGGGTAATGCAGCACATGGCTCGAAAAAATACTACATATA
AATCTATGGATACATATTTATATACATCTTTTAATGCAAATGACAACTTCAACAATGAGACATTACATTA
TGTACTGTCATTACTGTAATTTGCTTATGTAAACGACTTGACAGCCCCCCGGTGGCCAACCGAAAATAAA
ATTTAAGGATGAGAATTGAATAAAACTCAATGAGCCAGCCCGTCGAGGTTGAAATaaaaaaaaaaCATTT
TTTTAATGACTTCTGGCCGAGCATACCTATGGATGGATGCCTGTACATAAATACCATGTGTACATAAGGG
CTTATCTACTTGCAGGCCTCACCATACATACATGTATACATGGGCCATATAATGTGGTACATTAATCGAT
AATTGCAttttttcgtttctttttttttCGTCGTTGTTGTTGCAGTTGCAGTTGTTGCTGTTGCCATTTT
GCCCATTTCTTTTGCCACTTTTCATGCGTTGTCTCTCTCATGGCGCATGCCATTTGCATTTCGTTAAGCG
TATGTGTGTGTAAATGAGTTTCCGTGTTTAATTGCTTCAATTTTTTCGGTTAGTGTGGGTTAAAGGTACC
AACCGACCAAGCAACAACGACAGCAGGGCaaaacaaaaaaaaaaaTGCCACACAAAATGCAAATAAATCA
TACGCCCCGCTGCGGCGGGTCACAATTAGTTTAGTCATTTGGCTTTAATAGCTAACTTTGATTAAACGCA
CTTTCGTTTTTGTTTGTCAGTCGATTGTTGTAGCTTTTAGGCCACCGCACCGCAGCGACGACTCTAGAGC
AAACAAACAGACAGACAGGACGACAGCCCAACTGACAGGATGGGCAGACGAAAGGTGAACGGCAGCTCCT
AGCTCGATCCGCCGATCCGCCCACCTCCCATCCACCTTGCCACCTAGCGTGTCACGTTGCGTTGCCTTGA
ATGGCAAACTCTCTCGCACACACATACTGTCTAAACACCTATTCAAACACTCACACACATGCACTCAGTC
ATAGGTCAGACCACCCATCCATCGACATCGTCTAAACCAACGACACAAGCCCACTTTTCTTCCATTTTTA
TGTACAGTGATCGCTGACATAACGCAGACTCATAGGGCTTAGCAATTAAGAACCACAGTTCCTCAGAACC
ACAATTTAAAGGTATCCAATTTTAGGTATCCCTCCTATAAGAACTATATGTAGATAGTTTAGAGGCTTAG
GAGATCTTTTCTCGAGGTATCACTGTAAGTTCGCGCCTAGTGAATTCTAGAATTCTATTTAGCATACTTT
CGGGCGCCGTTTGCTTGGCATAACGTGAACGCGGGCAGACAGCAATGATGGCGCTAGCACCACCAGAACC
CAGAACTCAGCAGAACTGCCCTGCCCATAACCAGAAGCACTAGCAAAAGCAGAAGCGTAGCCAGAAACTG
AAACATAACCCGAACCCAAACCCAAGCCCAAAACAGCATTGAAATCCCGCCTTGCGGCCGTCGACGTCGT
CATCTTCATACGCAATGTGTGTCGCACCGACCACATGCACAAAACAAACGCACACTCGTGCAGCCAGAGA
CAAAACACGCCAGACACTGACTGTGTGCGTGAGTGCATTGCGTGCGTGCACCGAATATGTTCGTATGTGT
GTGCATCTGGCAGAGCCTGTAGATGAAGAAGAAGAAGAGTCACCAGCTGAGGAACCCCTTGCATTTTGCC
TGTGCGTAGACTTTCCTTCGCTaaaaaaaaCACACGCACCCTGTGTCCGCAGCTGGCCGACTGCAATTTG
TAGCTCGGTTTGTAATGAATGGGAATTACAGAAATTTAACGAGGTGGGAGTGGATAAGCCACAACTAATT
GGTACAAATCTATTGATTGGTGGGACGTGGTTTTGTGCCGAAGGTATCGGAATTAGATGGGAAAACCAAA
AGGTTTTTCTAAATATGGTTATTAACTCACATTACAATTCCAACATCTTTAATATAAGACCTTAAGTGCA
AAATAAGGAATGTTTTTATTTCCAGTTAATTTGATAAATCAGAATTAAAACCAATTTTTCAATCAATCTT
AAATCCATATAGGTCTCGTTTGCCTGCAGAGCATTTGGCTATCGTGCAGATCCCTTTTCGTGCATTCTGC
ATTTTTTAACGACAAAATGCAGGCGTGTGTCTGTGGACTGGTTGCATTTTAAAGTTTGAGTTTTGTACGT
CCCGTGCGTGCATTCACCTCGTTTTTTCGCCTTTTTTCGGTGCGTGCGGCAGGTGGAAAGTCGAGGAAAA
GCGGTAGATGGCGGGAGTGGTAGCGGTGGCGCAACGTTGACGTCGCAACCCCCGAAGCGCTAAAACAACG

ACGACGATGGCGACGACGACCCTGTTTATTTGAGCGCTTAAAAAACTGCAGTTGCCGCTGCAGCTGGGAA
AGCAATGCTATTTTTACACTGCTAAAAGTTTGAACATGCCGCTCCACTTTTGCGCCGGAATGagcagcag
cagcagcagcagcagTTGGGGATGTTTCCCCAATCCCCATGCCCAATCCAGCTCCTCCTGCTGCTGCTGC
TCCACTTGAGCCCCGAGTTGCCATTCCCCAAACGCACGCACCGAACACACGCACTTGTGCGCAACCAGGA
CGATGGGGACCACGGCAAGGACATGGGGGTGCTAGAATTTCTGCACGCACCTTCATTCCAACTTCCGGCT
GGGAATGCTAGCCTAGGCTATTTAACTACACTTTTTTGGCACTAAAAATGCTACAAAATGCCGGTGATAG
CCCACGTATGCAATGTCTAACatatatatgctatatatatgtataAATAATTTTTTCTTCGGCCGGCTCT
CCTTTAATTCGCATTTCGTGTGAGTTGACGCGTAGCCTTTTGCCATATGCGCAAAAGTTTTGCTTTTGAG
CCACGCATGCCTGCCCCTTCAAGTGGTGGGGGTTAGGCTCCAAAACCACATATTCTCGCACTCCGTGGCC
AAATGCATTTTGCATTAAATTGCATAACTTTTGTGCGGCAATTAGAGTTCTCTGGCTGGCCAAGACGGTT
GGGAAGTCCGGGAAATGCAGCCACAATGCAAACATACAAATCTATCAATTTTCCACTCGCACACATTTCG
TATTCAATAATTACGCATACGCAGCGTGTGCCCCCTTTAAGTATAGTGCAACCGTCACAattttccattt
ctttttcgtttcagtcttctttttttcttgcatgcatttttCTCCTATACTTAGTGGCATTGCATTTCAA
TTAAGTAATTTCTGTCTGCTAAAATTCCCAGGAGCAATACACAAATAGGGTTTGGGTCAACAGAAGAAGT
AAAAAAGTGGTTTATTAATTGCCACCACTAGGCAAATTATAGTTTATTAAGCCATGCAACTGGCGTAGCA
TTCTTGAACTGCCCAGAAACtttttttttGAATAATTAATGTAGTGGAGTTTAGTCTTTGATGTGTTTCG
TTTGCTATATTTTCAGATATTTCTGCTCTGTTTTGGTTAGTTTAATGTTTTGTACTAAGATCTAATGATC
AGTGACCTATATTAACATTTATATTTTCTTCATTTTAGGCCCTAGAACGCGTTGCTGAAGAATGTATGGG
TCGCAGGCAATGGAAACATTATCAAGACAAACTGACGTGCAGCCACTTGAATATCGAGGAGCAACAGCCC
ATAGCAATAGCCGGTTCCGAGGACGAGCCATCGCAATACAACCACAGCAGCAAGGAGATCAGCCAGAGCA
ATCCCAACCACTGTAAGACAGAGAACCACCGTCTGGAGCAGCAACACAACGGCAGCCAGCTATTGGAAGA
AGAAGATTCTGGAGAACAACCAAACATCACACGATTCATCACGTACACCAACACCGGGAGCCACCAGTAC
ACCATCACCACCGCCAGAACCCATCGATTGGAGACCGTCGGCCAAGTGCAACTTCTGTGTTAACGGTCGC
CTGCTAACGGTTAACGCCCAGGGCAAGTTGGTGGCCGAGTCAGCAGCAACTGCCACTAGTAGTAGCACTA
GTAATAGTCACATTCATCAGgcacgacagtgacagcaactcgagtgcatcactgccccaccacatcagca
gcagcagcagcagcaacaacaatagcagtggcaacagggcacgccacattgctgctgcaagtgcaagagc
aacaccagcagcggcCACACCCGCCAACTCCCTTGAACTCTACAAGCTGCTGACCCAGCGGGCAGCCAAA
ATGACATCGATGGACTCGATGGCCGCCCAGCTGGCGCAATTCTCACTGCTGGCCGACTTCAATCTGATCA
ACTCGCTGGCCAGCCAAcagcagcagcagcagcagcaacagATCGCTAGTGCGGTAACGCCAACTACCTC
AGAAGTATCTGCAGCCGCAATCAGTCCCGCACTCAAAGATACACCCAGTCCCAGTGTGGATGCACCGCTC
GATCTTAGCAGCAAACCATCGCCGAACTCATCGATTAGCGGCGATGTGAAGTCCGTCAG
>dme_circ_0007208_junction_seq
CGATTAGCGGCGATGTGAAGTCCGTCAGGGTACAGTTGCGATCTAACAATATCGCC
>dme_circ_0007209
GATATTAAACAAAAACAACAACTGCAACAACAACGGCTACACTTTTGTTAGTAAATTTCTGTTGAATtaa
aatacgaaacaaaacatataaaaaagtgcaatccaaaaaaaacaaaTATTAACGTTAAACGAACTATAAA
GCAAAATAATTAAAAAAGTAGCCATTACTTTTGAATTGTTTTcaaatgcaaaaaagaaaaaggttcgtaa
gcaacaaaaaaaaaGTTAAATGCTAAAGCATTTTGATAATGCAACATATCAAAGAGCCAGAATTTAAACA
CAGCAAAAAATAAATAAAATAATTAATATACAAACACGCGGGGCAACTGTCAAAAAATCTACAACAACAC
AAAGTGCAACTTTCAACAACAACAAAATAAGTGCAGCAGTACAACAACAAAAAGCATTACACAATTGAAC
AAACAACATACaaaaaaaaaatattgccaccaaataaaaaaaaaCCCGAAAGAAAATTCGCGCTTCATTT
TCACGCGCTGCATATAATTTACTCTGTGCTCCACatatatatatatatatatatataATAAATAAAAACC
AACCAATAGCCCaaaaaaaaaGAAATGAAAAGTCCGCATCAACAATAAAAATCTGCCTGCATTTTTGCCT
TTTGTGTGAGCTGCCCACCAGAACGAGAGAAGCACTTTTATTGTATATAAAAATTATATACATCGCCGGG

AGGAGCTGCAGCAACCCACTCCAAGCCAGGGTTGCCACGTCCTGAGCTGCTGTAAGCTCTCcgcagcagc
tgcagcagcatcagcatcgcagcagcatcagcagcagcgcagcaaccgccgcagcatcaATTTGGCTTTT
GGGCAGAGATAATTTAAGACAAATATATGTGATGCTATGCACATCAGCAGCTATGAAATAT
>dme_circ_0007209_junction_seq
GCTATGCACATCAGCAGCTATGAAATATGATATTAAACAAAAACAACAACTGCAAC
>dme_circ_0007210
GATTTTGTTCGAAACCTTGGTTTGCTCAGAAAATGCGCTTAAAATTTGCTGAAATATATAAAACGCGCAG
TTAGCCTTTATCGGAAAATAAACAGATTCAATTTTGCGTCAATTAACATTACAAATTGGGAATATAATTC
TTTTTACATAAAAAGAATGAAATAAATAGCAAGAATCGAAAATAAATGTTATTAGAAAACGTAATATTTT
GCTGtttttttttGACATTTTGCTTAATGATGCTGCATTAAATGACAATCAGCCAAGGCGAGGCTTCAAC
CTCCAACACTTTTTCGCTTTTCCCGTCCAAAAGATGGAAAGCCTGGCCAGACGCAAACAGTTTACTCAGA
CGGATCAGGCGGACAGACACTGTGTAACCTTCGACCCGCTTGGTAACAAGAAGCCACTGGAAAGCCGGGC
GAGGAATATGTATACAAAAGTAAATATGGGAAATTGTTTCCATTTTTTGTTGCTGTATTCCTATCATTGT
TTGCCAAAGTCTGGCAGGTCGGTCACTCAATACCAATTGAAAAGTGCTGCAAAAAAGTGCAAAAATAAT
>dme_circ_0007210_junction_seq
AAGTGCTGCAAAAAAGTGCAAAAATAATGATTTTGTTCGAAACCTTGGTTTGCTCA
>dme_circ_0007211
GCCCTAGAACGCGTTGCTGAAGAATGTATGGGTCGCAGGCAATGGAAACATTATCAAGACAAACTGACGT
GCAGCCACTTGAATATCGAGGAGCAACAGCCCATAGCAATAGCCGGTTCCGAGGACGAGCCATCGCAATA
CAACCACAGCAGCAAGGAGATCAGCCAGAGCAATCCCAACCACTGTAAGACAGAGAACCACCGTCTGGAG
CAGCAACACAACGGCAGCCAGCTATTGGAAGAAGAAGATTCTGGAGAACAACCAAACATCACACGATTCA
TCACGTACACCAACACCGGGAGCCACCAGTACACCATCACCACCGCCAGAACCCATCGATTGGAGACCGT
CGGCCAAGTGCAACTTCTGTGTTAACGGTCGCCTGCTAACGGTTAACGCCCAGGGCAAGTTGGTGGCCGA
GTCAGCAGCAACTGCCACTAGTAGTAGCACTAGTAATAGTCACATTCATCAG
>dme_circ_0007211_junction_seq
TAGCACTAGTAATAGTCACATTCATCAGGCCCTAGAACGCGTTGCTGAAGAATGTA
>dme_circ_0007212
GCCCTAGAACGCGTTGCTGAAGAATGTATGGGTCGCAGGCAATGGAAACATTATCAAGACAAACTGACGT
GCAGCCACTTGAATATCGAGGAGCAACAGCCCATAGCAATAGCCGGTTCCGAGGACGAGCCATCGCAATA
CAACCACAGCAGCAAGGAGATCAGCCAGAGCAATCCCAACCACTGTAAGACAGAGAACCACCGTCTGGAG
CAGCAACACAACGGCAGCCAGCTATTGGAAGAAGAAGATTCTGGAGAACAACCAAACATCACACGATTCA
TCACGTACACCAACACCGGGAGCCACCAGTACACCATCACCACCGCCAGAACCCATCGATTGGAGACCGT
CGGCCAAGTGCAACTTCTGTGTTAACGGTCGCCTGCTAACGGTTAACGCCCAGGGCAAGTTGGTGGCCGA
GTCAGCAGCAACTGCCACTAGTAGTAGCACTAGTAATAGTCACATTCATCAGGGTGGGTCAACTATCAAC
TCAAATCCCTCAAGCATGTAACACAATaaaaaaaaaGTGCAGCCTTTTTTGGAAAATCAATCAATTCATT
AACATAGTGCAGTTAATGCAATGTTCAATATTTTTAAAGAATTTTTTAAATACTTCTGATCTTAATAAAA
ATAATATTTATTTAAGAAAGTCAAGCCACTAGAGTAACATTTTTTAAAGATAATTTTGGCTAATATTTTC
TTCGAGTGCAAGTAAACTTTTATTAACCCTCAACTTTGTCCATgtaggcacgacagtgacagcaactcga
gtgcatcactgccccaccacatcagcagcagcagcagcagcaacaacaatagcagtggcaacagggcacg
ccacattgctgctgcaagtgcaagagcaacaccagcagcggcCACACCCGCCAACTCCCTTGAACTCTAC
AAGCTGCTGACCCAGCGGGCAGCCAAAATGACATCGATGGACTCGATGGCCGCCCAGCTGGCGCAATTCT
CACTGCTGGCCGACTTCAATCTGATCAACTCGCTGGCCAGCCAAcagcagcagcagcagcagcaacagAT
CGCTAGTGCGGTAACGCCAACTACCTCAGAAGTATCTGCAGCCGCAATCAGTCCCGCACTCAAAGATACA
CCCAGTCCCAGTGTGGATGCACCGCTCGATCTTAGCAGCAAACCATCGCCGAACTCATCGATTAGCGGCG

ATGTGAAGTCCGTCAG
>dme_circ_0007212_junction_seq
CGATTAGCGGCGATGTGAAGTCCGTCAGGCCCTAGAACGCGTTGCTGAAGAATGTA
>dme_circ_0007213
GGTCAGCGGTCCGGCCAGCAGCAGCAGCCTGTCGCCGGGCGGACTGGTTGGTGGTCACCACCATCCGCTg
aacaacaacaacagcctcagcatcagcaacaacagcaacccacagcagcaacagcCATCGGAACGGCAGCA
ATCGCAGCCCGCATTCCGCATCGCCCATGCTGGCCGCGGCCGTGGCCCAAGGTGGCTACTCCGCCGGCAA
CAGTTTGCTGACCTCATCCTCGTCTAGCATACAGAAGATGATGGCCAGCAATATCCAGCGCCAGATCAAC
GAACAGAGTGGCCAGGAGA
>dme_circ_0007213_junction_seq
CAGATCAACGAACAGAGTGGCCAGGAGAGGTCAGCGGTCCGGCCAGCAGCAGCAGC
>dme_circ_0007214
GTCTCAGGAACGGAAATGTTAGCGATTGCAGCAGCAACAATGGCGGCTCCTCCTCGCTGGGATACAAGAA
GCCGAGCATTTCGGTGGCCAAGATCATTGGCGGAACGGACACCTCACGGTTCGGAGCCTCGCCCAATCTG
CTGTCCCAACAGCACCATTCGGCTCACCACCTGACCCACCAGCAACAGCAGCAACAGCTGAGCGCCCAGG
AGGCATTGGGCAAGGGAACGCGACCAAAGAGGGGCAA
>dme_circ_0007214_junction_seq
GCAAGGGAACGCGACCAAAGAGGGGCAAGTCTCAGGAACGGAAATGTTAGCGATTG
>dme_circ_0007215
TCATTGTTCCGATGGTTGGTGAACGGCAAGGGGGTCTTCTTCACCAGCAGCTGATCGAGCAGGGCGTGTC
CATGGCCATGCCCGTGCCCATGCCCGTTGCCATTGCTACCGTTGCTGCCATTACCCGCCGCACTGCCATT
GCCCTCGCTCGCCTGCAGCGTCTTCCTGATGATGCCATCGTACACG
>dme_circ_0007215_junction_seq
CGTCTTCCTGATGATGCCATCGTACACGTCATTGTTCCGATGGTTGGTGAACGGCA
>dme_circ_0007216
TTATGAACTTCCGTTAAGGTTTTAATCGCTGATCCCAAAATTTGGTTTACAGATTTTGGATCCGATTACC
CTATCAATATGAAGTAGGGCCAAAGTGCGTTTCAGTTCTCCATGGGCAATGGAATGGTAGTATTCGATTG
CCCGTATTGCGGGTTATGAAACGCACTGTACTTTGCTGTCTTGGGTTTTCTTGTAAATGGTTCTTGTCAT
TATCAGTTGCCTTGGAATTTG
>dme_circ_0007216_junction_seq
TTGTCATTATCAGTTGCCTTGGAATTTGTTATGAACTTCCGTTAAGGTTTTAATCG
>dme_circ_0007217
GGCAAAACCCCAGTATATTAATAATGGGAAGAAAAACATAATGAATAAAACCAATTTTTATTTCAATTAT
TAACTTATaaacacaacaacaacaaccacagctccacacagttacagttacaacaagaacaacaacAATA
GCCCTAGAAAATAATATTCATAATTAATCAATCAATCAATCCGTAAAACCAACAACTTCAATAGTTATAA
GCAATTGGCGTCaaaaaaaaaaGCGAAAACAACCAAAATCTTAGCCAGGCAAAAGTTTTGAGCATATTTT
TCATTATTTTTACAACAAACAAACAAACTGATGTAAGTACAATTCATAAAATTAGACTTTCGGTAAACTA
TAATAAAGAAAATAGAGCAGAAAATAACAttttttttttGCATTACATAATTGCTACAAAATTCAAAAAC
AAAGAACGATTTTTTAGTGGAAAACAAAAGCCAATAAGCAAATAAAAAGAATTCGCATTATTATTAGCAA
TAGCCCATAAGTACAGCAAAGTGTATTAGCAATAAAGCTaaaaaaaaaacacaaaagcagagattttaaa
aagaaacataaaagtgaagaaaaacttaaaactgcaagaaaaatgtgaaacCAATTTGTTCTGCGTTGAA
AAAGAAATCCAAAATAAATAT
>dme_circ_0007217_junction_seq
CGTTGAAAAAGAAATCCAAAATAAATATGGCAAAACCCCAGTATATTAATAATGGG

>dme_circ_0007218
AGGCAATCAGCTGGTTTGCGTGGTCGATTTGGTCCTATTGAAATTTTTCGGTTTGGCACCGGAGCAAAAA
TCACATCGCAATCAAAAATTTCGCGTGGCCAAAGGACACACTTTTGAGTTTGGATCAACAGCCGGGAAGA
TCTAGCTAACCGAATAGCAAAATGTCGATGCTGGCGAGAACAGTTGGACGGACG
>dme_circ_0007218_junction_seq
GATGCTGGCGAGAACAGTTGGACGGACGAGGCAATCAGCTGGTTTGCGTGGTCGAT
>dme_circ_0007219
GTAAGTGTATCATCTTGTTTCTGAAGGAAGGGGACACGGGACGTAGTCTACAAATACAATAGTCCGCTGG
CAGCGGCGTGAGCCATCAAGGTGTTGGCCGCCTGCTGAGTCTCCAGCATGAGGCGAGCCTCCTTCATCCA
CTCGCCGGCAATCTTGCGCGATGCACCCTGCAGCAGATTCATGTACTTGAGCGCCTGCAGAAAGTCGCTG
CGATCCACATG
>dme_circ_0007219_junction_seq
CCTGCAGAAAGTCGCTGCGATCCACATGGTAAGTGTATCATCTTGTTTCTGAAGGA
>dme_circ_0007220
CTGGACGATGTGATCAATCGTCTGTCGCAGACCATTACCCTGCTGCCCGGCGACATCATAGTCACCGGAA
CGCCCAAGGGCGTGGGCATGCATCGCACTCCGCCGGAATTCCTTCAGCCCGGTGATGTCGTGCAGTCGGA
GATTGTTGGATTGGGCAAGCTGTGCAACAAGATTGTGGCTCCCTAAGATTGTCAACTCATTTGGCTTG
>dme_circ_0007220_junction_seq
CCCTAAGATTGTCAACTCATTTGGCTTGCTGGACGATGTGATCAATCGTCTGTCGC
>dme_circ_0007221
GTTGACAATCTTAGGGAGCCACAATCTTGTTGCACAGCTTGCCCAATCCAACAATCTCCGACTGCACGAC
ATCACCGGGCTGAAGGAATTCCGGCGGAGTGCGATGCATGCCCACGCCCTTGGGCGTTCCGGTGACTATG
ATGTCGCCGGGCAGCAGGGTAATGGTCTGCGACAGACGATTGATCACATCGTCCAGCTTGAAGATCAAGT
TGCCG
>dme_circ_0007221_junction_seq
ATCGTCCAGCTTGAAGATCAAGTTGCCGGTTGACAATCTTAGGGAGCCACAATCTT
>dme_circ_0007222
GCTGCTGATGAAGAAGATCGTTAAGCGGGAGAAGATGAAGAAGGAGCTCTCGCAGAAGAAGGGAAACAAG
AACGCCCAAAAGCAGGAGCCACCCAAACAAAATGGCAATAAACCCAGTAAAAAACCGGAGAAACTCAGTA
AAAAGCATGTGGCCAAGGATGAGGGATGACGACTTAGAGGAGGATTTCCAGGAGGCGCCGCTGCCCAAGA
AGAAGCAACAAAAACAGCCTCCAAAAAAGCAACAAATTCAGGTGGCCAACTCGGATTCGGAATCAGATGA
CGATGAGCAGGAGGATGAGGCAGATGAGGATAGCGATTTGGACGAAGTAGCCGAAGTTGATGAGGAGGAT
GTTGACAGCGGGAGTGAAGATGATGACCAGCAGGAAG
>dme_circ_0007222_junction_seq
GGGAGTGAAGATGATGACCAGCAGGAAGGCTGCTGATGAAGAAGATCGTTAAGCGG
>dme_circ_0007223
GATCGTTAAGCGGGAGAAGATGAAGAAGGAGCTCTCGCAGAAGAAGGGAAACAAGAACGCCCAAAAGCAG
GAGCCACCCAAACAAAATGGCAATAAACCCAGTAAAAAACCGGAGAAACTCAGTAAAAAGCATGTGGCCA
AGGATGAGGGATGACGACTTAGAGGAGGATTTCCAGGAGGCGCCGCTGCCCAAGAAGAAGCAACAAAAAC
AGCCTCCAAAAAAGCAACAAATTCAGGTGGCCAACTCGGATTCGGAATCAGATGACGATGAGCAGGAGGA
TGAGGCAGATGAGGATAGCGATTTGGACGAAGTAGCCGAAGTTGATGAGGAGGATGTTGACAGCGGGAGT
GAAGATGATGACCAGCAGGAAG
>dme_circ_0007223_junction_seq
GGGAGTGAAGATGATGACCAGCAGGAAGGATCGTTAAGCGGGAGAAGATGAAGAAG

>dme_circ_0007224

GTTCAACTTTGTCGGTCGCATTTTGGGACCCCGTGGCATGACCGCCAAGCAATTGGAACAGGAGACCGGC
TGCAAGATTATGGTCCGAGGCAAGGGTTCCATGCGCGACAAGAAGAAGGTAAGTGCATCGTTCGATCAAG
TGGATTACGGATTCCAAACGATTTGACAGGAAACGTGGCATTACTGCAAGCAGATACACATTTTGGTCTT
ACGGCCATTAGCCGAGCACTTAAATTTCATTTTTAATTATCATAAATAGTAGCCGCGTATATGGTACGCT
TGGATATGAAAATTCAGTCCGTGCCAAATGGGAATTAGTCTTCGTTATGAGGCGAAAAATATGTCCCTCT
TTTGTGTCTAGAGGTTTTTAGTTCTGGCTATGATAACCACATGGTTTTCTTTTGTAAAGTAATAAAAAAA
TTTAattatagattttatacattttatacatttataattaaataatataaatttttcattttttttAACT
AAGCTAGCTTAAAAATCTTTAAAGTTCACTTTAAATCCAAACATCAGATAGCAAATTCTCCAAATTATTT
TCTTATACCCACACTTATTTGGTTGTCTTGTCACTCAAAATGCTCTGATGATTTAATTTCCCCCGACGAA
AAGAGCCATTCAAATGCGGGGAAAAGAATGAAAAATGAATCAAGTCTCTCGAGACGATTAAATGTATTTT
TGTAATTCATAAATGTGGCGGACCAAATGAGCGGCAGGCAGACAGGAGAGTTTTTCAAATTCGCAATTTT
CATGCAATATTTTGATAATTTAGCAGAAATGAATGAGAGGACACCCACACAAAATGTCTGACTCATAGCG
AACGGAAAGATATTGGCCTGCGACACAAGTGAAAAGCGAAAGAATAGAAATGTTCTGTTTACTTGCATTT
TTGGCGGATAGAAATAGACCTGACAGCCAACaaaaaaaaaaaaaaaaaaTTAGTCACAGTGCATGGTTTT
GTTGAGGTGAGACCGACACCGACCCCTCTTTATATTTGCTCAGGTTTTCTGCGCTGCTTTTGCTTGGCCA
CTCATCCGAAGTGGCCAACAACGAAAAGGTCGTTCTTCTCGATTCCTGATGACAAAACTTTGTCCGGCAC
TCGTACAGTGGCCAACTTGACAGTTGTTAATTTGACGTGGAATTAGCACTGGACGACTTCAAAAGAATTT
CTATCCAATTTGAGCCTAATTGATTATAAATTGTGGGTCACAATGTGAGTTGAAGGAGGAATAGCAAAAG
AATTTATTATAGTTCAAGCACGTTCGCCAAAAACTCTTCTTGCCACAAGTAATAACAGCGGTGATATGAA
AAATGGCAATAATGTTTTAAGTGTGTCGGAACTCGCCCACTCGTAATTGTTCCGCGCCCATTAGCACAGT
TTCGAATAAATTCGCATTTCGCGGCTCGCATTTTTGAATATTTTGCATATTGAAAAGAGCAAGAAGAAGG
TGATGCGATTGAATTATTAAGGAAGCCAAGGAATGATTTTAAATAACACAATTTTGCATTTACATTGTTT
GCTAAACTAAATGTGAAAAATGAAGATACTTTGCAAACAGCATTTATTTTAAGAAGGCCATCAGCTTTTA
GGTACTTAGGTAAAAATGTAGCAGACTTTTATATAAGTATTTCAAAAATGTGTGTACATTCTAAAACTTC
TCGTTCCCCGTTTTCAGGGAGGACGCCAACCGTGGCAAGCCTAACTGGGAGCATCTGTCCGATGACCTGC
ATGTCCTGATAACCGTCGAGGACACCGAGAACCGTGCCACAGTGAAGTTGGCCCAGGCCGTCGCCGAAGT
ACAGAAGTTGCTCGTGCCGGGTAAGTGTTTTTCGAGAGGTTTTTCATTAACGAGAGCCCTTCAGAGAAGC
CAAACGTAAACTGATATTTGCCTAATTTAAAGTTCATAATGCGCCCGCAAAAGAGCTTAAAGTAAACGAA
AAAATAAACAAGAAACAATTAAAAACCAATGCGTGTTGCCTATCCCAACTCCTTTTTCTATGTTAAATCA
ATAATTAATTAAATGATTTCTCAGCTAAACTCAATTGAATGTTAACCAATATAATTAATAATGAATTTTC
TTGTATTATTTTTCATACTCTCAACCAACAAAACCTAATCTGCTTTAATGTATTCCGTTTAATACATTAA
ACCCCCAAATCaaaaacaaaaacaaaaaaacacaaaCATACACTCGTACTAACAGGGTATGTCAACCAAA
GCCTCTCTTTATAAAGTATCCATCTAATTAAAACCAACCAAATAACCTAACCCAATCTTCACTCAGACAG
AGCTaaaaaaaaaaaaataaaaataaaaacaaaaGATAACAACGTTAACCAGCAGCTAAAATCGAATGAG
CGAGGATCTAACCATTAACGATTTTGCAAACTGAACCGTTAACGAAACCAAACTGATTCAAATTCGATTT
ACATTTCTTTATTTTGTATTTGATTTATGTGTTCGACGACCAGCTTTCTCATGCGTTGGCTCTGCTTCTT
ATCTGTATCCCGCAGGTGTGCGATGAGGAGTGGCGCCGCCTGGTTGCCGCCTCTGATAGCCGCCTGCTGA
CATCCACCGGCCTGCCCGGCCTTGCCGCCCAGATCCGTGCACCCGCCGCCGCCCCGCTTGGCGCCCCATT
GATCCTGAATCCCCGGATGACCGTCCCCACAACGGCGGCCAGCATATTGTCCGCCCAGGCCGCTCCGACA
GCCGCCTTCGACCAGACCGGCCATGGAATGATCTTCGCACCGTACGATTATGCGAACTACGCCGCCCTAG
CCGGCAATCCTCTGCTCACGGAATATGCTGATCATAGCG

>dme_circ_0007224_junction_seq

CTGCTCACGGAATATGCTGATCATAGCGGTTCAACTTTGTCGGTCGCATTTTGGGA

>dme_circ_0007225

TTATAAAGTTGCTTTCGCCGATATGGTGTGATTTTAGAGTGTTACTTTCTGAGTTATAGTGCTTCGAATT

TCTCGATTCACTATGCATGGTATTTACTGTATCTCAATGAAACCCGCCGGCTTGGCTGGCACTCCGACCG

TTGCTCGCTGATAGGGATGCTCGCGGTTAGTCGCCAAACGACGCTGCTGCTTAATGGCGCCTACTAAAAT

ATACGATACGATACATGTAGATATTGGTTTGCATAGGTTTACATATCGGTTATATGTATAAATAGATGTG

AACATCATCAGCTACAGGGTACAACTTAGGTCTAATAGATATTGCATACATCCTTACTTATCTACCATTC

GCTTGCTCAAACTGAAGCTGAAGGCAGCAGCTGGAAGCCAAATTGCCAACTGAGGGGGAATTATCGACCT

GTTTTTCGTATTCTGTATTCTCTTTCACTTTGTAACATTTAAATTCTCTGCTTCTTTGGAATGAGTCGTT

TATATATGCGCTTGTGTATTCGATAGATTTGATCTGATTTTTAGATTTGGCTGCAAAATAAGTGATTTTC

AAGCCGCTAAGCCTAGGTAAGGTAATTCGTCTCCAGTTCATTGCTAGATATTTTCTAACAATGATTATTA

TTCTGTCGAATCATATATTGCATTTTGTTCCTCCAGCTTGCTGACTAGTCGTGCGTTCAATTGATTCTGG

ATAATGGTTTCTGTTCTTGGACGGCTGCCTTTGAGCCTCGACTAAACTAAGAACTAACTGAAGTTATTAT

TTAAGCACCAACTTGATTCAAGGCTCCTTTCCACTAAGAGTtatgtaaatatatatgtgtatatatatat

atatatatatatataAAAGCTTTTCTGCAATTCTGTTCAAATTATACGCTTTTGACGAGAAATGGCACTG

GCTGGAAAAAATTGAAACGGTTTCCTTTTCGCTCTTCGTTAGGTACATTTGTGCATAAATACTGTCCCTCC

ATCGCAAAACTCACTACATAAACTACATAAAAAACAGACACTTAACTAGATTGCACTTAAGACTCGACCC

TCATACCaaaaaaaaaCTAAAATAGGGTTATTATTATTCAGAAGAGAAAAATTATGAAGTTTAGCCCTCT

TTAATTTGAATACTCATGGGTTCCAAAAACGAAAATCAACAACTTGCATTGGGTGCCGCTGTTGTTGTTG

TTCTCAACTAGAATATACTCATATAAAAAATGTTTCGGGTGGAAAATCATATATGTATGTAGGTTGGTTA

CAGTTAAAATTAAAGCAACTACAATTTAAGGGCCTAAAAATCACTTATTTTTGCCAATATTGCATATTGC

AGAAATG

>dme_circ_0007225_junction_seq

TTTGCCAATATTGCATATTGCAGAAATGTTATAAAGTTGCTTTCGCCGATATGGTG

>dme_circ_0007226

ATATTGGTTTGCATAGGTTTACATATCGGTTATATGTATAAATAGATGTGAACATCATCAGCTACAGGGT

ACAACTTAGGTCTAATAGATATTGCATACATCCTTACTTATCTACCATTCGCTTGCTCAAACTGAAGCTG

AAGGCAGCAGCTGGAAGCCAAATTGCCAACTGAGGGGGAATTATCGACCTGTTTTTCGTATTCTGTATTC

TCTTTCACTTTGTAACATTTAAATTCTCTGCTTCTTTGGAATGAGTCGTTTATATATGCGCTTGTG

>dme_circ_0007226_junction_seq

GAATGAGTCGTTTATATATGCGCTTGTGATATTGGTTTGCATAGGTTTACATATCG

>dme_circ_0007227

GAATACAGAATACGAAAAACAGGTCGATAATTCCCCCTCAGTTGGCAATTTGGCTTCCAGCTGCTGCCTT

CAGCTTCAGTTTGAGCAAGCGAATGGTAGATAAGTAAGGATGTATGCAATATCTATTAGACCTAAGTTGT

ACCCTGTAGCTGATGAT

>dme_circ_0007227_junction_seq

ACCTAAGTTGTACCCTGTAGCTGATGATGAATACAGAATACGAAAAACAGGTCGAT

>dme_circ_0007228

GGTCGATAATTCCCCCTCAGTTGGCAATTTGGCTTCCAGCTGCTGCCTTCAGCTTCAGTTTGAGCAAGCG

AATGGTAGATAAGTAAGGATGTATGCAATATCTATTAGACCTAAGTTGTACCCTGTAGCTGATGATGTTC

ACATCTATTTATACATATAACCGATATGTAAACCTATGCAAACCAATATCTACATGTATCGTATCGTATA

TTTTAGTAGGCGCCATTAAGCAGCAGCGTCGTTTGGCGACTAACCGCGAGCATCCCTATCAGCGAGCAAC

G

>dme_circ_0007228_junction_seq

CCGCGAGCATCCCTATCAGCGAGCAACGGGTCGATAATTCCCCCTCAGTTGGCAAT

>dme_circ_0007229
GTAAGGATGTATGCAATATCTATTAGACCTAAGTTGTACCCTGTAGCTGATGATGTTCACATCTATTTAT
ACATATAACCGATATGTAAACCTATGCAAACCAATATCTACATGTATCGTATCGTATATTTTAGTAGGTA
GGCGCCATTAAGCAGCAGCGTCGTTTGGCGACTAACCGCGAGCATCCCTATCAGCGAGCAACGGTCGGAG
TGCCAGCCAAGCCGGCGGGTTTCATTGAGATACAGTAAATACCATGCATAGTGAATCGAGAAATTCGAAG
CACTATAACTCAGAAAGTAACACTCTAAAATCACACCATATCGGCGAAAGCAACTTTATAACTTTAAATC
AATAAT
>dme_circ_0007229_junction_seq
AGCAACTTTATAACTTTAAATCAATAATGTAAGGATGTATGCAATATCTATTAGAC
>dme_circ_0007230
ATAACACATAAAGAAAATAATAGTTTGATAAAATAAATAAAAGAGTAGAAACACAACTAACCTGTGGAGG
CGGTGCGCTTGAAAATTCTTTTTCAGAACCCTGATCAGGGCAGAGCGGCCAGGCCAGGCCAACGATTCCG
GTCAGGTTTCATCGAACATACAGTGTGGATTGGAATTGGGGATGGTCAGTTATGAGCAGCATTTG
>dme_circ_0007230_junction_seq
GGGGATGGTCAGTTATGAGCAGCATTTGATAACACATAAAGAAAATAATAGTTTGA
>dme_circ_0007231
TTTAGCGTTTAGTTGTACATTATGAATGCTTGCTGCTATTATCATAATATATGTTTGTTTCATTAACTAA
ATATGGCTGTTAAATGTGTTTTAATATGTTGTTTCTGTTTCGCCCCCACTTACTCATCACACGATCGATT
TGAAGTGGGGTACGAAATCTATACATAAAATATAGATATGCAATAACATAATCAAAAAATACTTCATTAA
CAAGAATTAGCGTCAGTTTAACAAAAATAGATTATGCTTAATCGAAGGGTGATAGTCTATATCCGACACA
ATATGGATTTAAATAGACTTCGTGCACAATCCAATGAAAAGTGTAAAGATTCATCTTTGCAATTTGAGTT
AAAAGTGCAGATTTTTAAAAAAATTAAAACAGTTTTGGTTGGTTGAGTAGAGTTTCTTGGATGAGATTCC
G
>dme_circ_0007231_junction_seq
TGAGTAGAGTTTCTTGGATGAGATTCCGTTTAGCGTTTAGTTGTACATTATGAATG
>dme_circ_0007232
GCAGCTTATATACGATGCACTCATCGATTCCGATTCCGATACTGAATTTGAATTCGAATTCAAACCCAAA
CAGACCTTATCGCTGCAAAGAACTATTGCTGTGCAAACAAACCACTACGTATGTATTAAGTATTAATCTC
AGGGAATTGCCCGCGGATGGGTTTCAAAAACAATCTGTAGCGCATCAGTTGGTCAGTCGTATTGCATTCA
AGATACTCTACGTTACCTTTTTAACATAACAAATACACAAAATCTGTAAAATCTATAATAA
>dme_circ_0007232_junction_seq
TACACAAAATCTGTAAAATCTATAATAAGCAGCTTATATACGATGCACTCATCGAT
>dme_circ_0007233
GGCCGATCGCGAGTACCAGAATGCCATGTGCCGCGAGGAGGATCGCCGTGTCGAGCTGGAGGAACGACTC
CAGAAGGAGTTCGAGGCGGAGAAGGCCGAAATGGAGGAGAAGTTCCGCGAACGTCTCGGTCAA
>dme_circ_0007233_junction_seq
GGAGAAGTTCCGCGAACGTCTCGGTCAAGGCCGATCGCGAGTACCAGAATGCCATG
>dme_circ_0007234
GGAGCAATGCGGCGAAGCGGTGGCGCCAAACAAAATGTGAGGATAAACCAGCGTTGGAGCCTTCTGGGAT
TATGCTAAAAATGCTCAAAGTGCAATATAAATTTTTATGAGTGTACGCGCTGAATTTTTACGACTGTCTG
GGGGCGGGGAAAATAAATTAGAGAAAATGCCACAGGGAGGACGATTTAAAACAATACAATAGCTTAAAGT
GGCAAACGGAAATTGCATGCAACCAGGAGGCGTGGCACTGTACGCCCCCCGAGCACGTGACGCGCACACC
AAGCCAACAGCGAAACTAATATAAGCCATTTTGTAGCGCTAATTCGCAGGACACACGTAAACGCAGCACA
GCTACGTCTTACGTAAAAACACCACCCATCTGACACTTGGAAATCAAGCAGAAACCATACACTCACCTCA

TCATCATCATCATCAACATGGCCGTTAGCAATATTGTCTGCGAGTGGCTAAGAGCTTTGGGCCTGGCCCA
ATACGCCGAGAGCTTCCTGGACAATGGCTACGACGACCTGGAGATCTGTAAACAGGTCGGTGATCCCGAT
CTAGATGCCATCGGCGTCGAGAATCCTGCCCATAGACACAAGCTGCTCAAGAGCATCCGATCGCTGAGGG
AAAAGGGCGCCGCATCCGTTTACTTTATGCTCAACGATCCCAACTCGCTCTCGGGCAGCATGGAGATCCT
CTGCGAAACACCACCCAACAACGAACTGGAGCTCGTCCTGCGGGAGCAGCTAGAGACGGACGGAGTCCGG
CTGACCGCGCATCCATACTCAACACCG
>dme_circ_0007234_junction_seq
GCTGACCGCGCATCCATACTCAACACCGGGAGCAATGCGGCGAAGCGGTGGCGCCA
>dme_circ_0007235
GGATGGACAACGCGGCCATTTAGAGGGCCTGGCATCCGTCTATTGCGAATTGCTAATGGCTCCCTTCGGG
GACATTTTGGCAACTATCGAGCGGGCGCGCCAGGCGGCCTGGGCGGAGCGGAGTCCGCTGCACAGTGCCG
CCCAGGTGGTGGGCGGATCAGGTGGGGCTGGCGGCAGCACGGGCagcggcagcggtggggcggccagcgg
tggcagcggcggcagcagcggcgTACTGCACCACCGCCAGCAGCATGGCCATCGTGGCCATTCGATGCAC
GGCGCCGGTCTGCCCAATAGTCATAGCCAGCCCATTTATGTGCCCGGAAAGTACTCG
>dme_circ_0007235_junction_seq
GCCCATTTATGTGCCCGGAAAGTACTCGGGATGGACAACGCGGCCATTTAGAGGGC
>dme_circ_0007236
GCCCTCGAGTTGTCTGTCCGACAAGGAGGAGGATGAGATTTACGGATTCGGCTATGGCGTCTTCGCACCC
CGCGTGGCCAGAGGAGGGTTAACAcaacagcaacagctgctgcagcagcagacgctgcaaacgcaacaga
gcatacagcaacagcagcagcagatgcaacagcagcagcaacagttaccaattgtgccaggacaacagca
gcaaggacctcatcagcatcagACCCTGCCACCGAATGTCGCGCATTTGAACTTTGTGCAGCAAAAGGTA
AGTTGAAAAGAGTTGAAAAGTCCAGAGAAGTAAAATTCTTATCCTGGTAGACGATTTCTAAAGCTTTTGT
GTAAAGGTGATAAGCTTTGTACTTAACGTTTAAAGGAAATCTTTAGACTTTAGTTTTTAATTTATTTAT
GAAAGGGCCGCTCAGAAATTGCAGTAAATGTAGAGCACAATTGTAATGAGGTCAGAATTTTAAAGAAGAA
CTTTAATGAATTTTCATAGATAAATAGCAGAAGTAGCATGTAATCACTATCCCAGCAGAGTTTGAATGCT
TTCTGTTTTATCGTAGTTATTTACTTGACTTAAAATAATCTTTTAAACTATAAAACTCTTAGGAAAGTTA
TATCTCCCTACCAGGGTTTGAAAATAAAACAAAAATGATGTACATCAGCCGCCGACTTGAGTTCCTTACA
CTAATTCAATTTCTGTCCTTTTTATGCCGATTTTTCCCCGCGACTTTCAGAGGGCCGCGAGACAAAGAAA
CGCACCACACTGGCCCGGCTGCTGAAAGGCTTGAAGACTGTCAATCGACGGGATCGGAACAATCAACAGA
ATGGCGCCCAGGCCAGG
>dme_circ_0007236_junction_seq
CAATCAACAGAATGGCGCCCAGGCCAGGGCCCTCGAGTTGTCTGTCCGACAAGGAG
>dme_circ_0007237
GGGTGGCCGTGTGCGCTTCTCGAACAACCGCGAGAGCACAGGCGTAATTTCATTAAGATCCGCCGGAGAT
ATTTCATTGCCGCAACGTGGACCGCCGCGTAGAGGTCTTATCGTGCCGCAGCAGCCGCCAAATCCACCGA
CAATAATACCCTTAACGCACGCCAGGGTGAGTGTTGCCCCCGGGGGGAAAGAAGGAAGTTCCGTTCGGTT
CAGTTGGGTCGGGGCATTGGCCGGTTGTTGATTGCCATTATTATTTGCAGTTGAGTTTATGGCCGAAGGG
CCGACACGTCGAAGGTGGCGCATTAATAAGTAAACCGGTGCCGCCGCTTTAGTGTGGGCAGTTATGATAT
GGCAAATACAAAGGTCGAACCCTTCCATGCCGCACTTCAAAAGGGCAAATTTTTGGAAAGGAATAGGGAT
CATTCAACCATAAAACTGTGCCACAAACGAGCCCAGTCTGGCGTGAAAAGGACAAGCTTTCGAGCCAGCT
TTTCGACCAGCAAACACTAATAGCTGACCAAGGCGAAAGCAATCAAACAGCTCAAGTCTGGCATCTCGTA
TTTATTTGTCAATCGAAAAAGGTGCACCCCAAACCCTACAGACACACGGACAGACACAAAAAATCGGTGA
AAAAGGGTGGACTTCACTTCATTAGTGCGGTTATCAACGGGTCTGCAGCCAGGCCGAGCGATTgaatgag
cgaatgaacgaatgaatgaatgGGGACTTGAATGAGGGAATTAGTGAGTGACGAACGAGAGAGGAATGGG

TAGGAACACCAGCTTGACTGGCAGACGGCACACATATCGAAACGCATCGAACCGAAAGTCATCAATTTCA
TATTGTTACGCATGCCACACTCTTGatatatatatatatatatatggattctggatgctggattcgggatgc
cggatCCAATAAAAGCAAGACATCGACAGGCCGGGACACTTGAATGAAATGGCATTATTGCTACTTCAAC
GCCAATCCACAAGTCAGAGCGCCAGCTCCTCAATCAGCCAGCTAGGTAGACAGTAGCCCTAGAATGGATG
CTGGATGGGGAATGGTGAAAAGGAGGAAACATCCAGAAGGACGAGAACCTGCGTTTTCCACACGTACGCA
TACAAAAGGACTTGCTGAAATAACTTATACGCCTCGTGCGCCGCTGGGGCCACAAACGAGCCAAACGAAA
AGAAAACTTTTTCTTCTCACGAATTGCGAGCGAAAGCCAAAGAGAATGGGGAAACGAAGGTGGAAAACAA
ACAGCAAAGGCAACCAAAAGTGAAAATCTCAACAGTGTTGAAGCAAAATGGTAAAAGGAAAATCTGCCAA
GTAAAAGAAATACGAAAAAGATATCAAGTATTTATTTATATGACCTTTGTTTGCCACTCGACCTCGGGTC
GTGTATACATCCCAGCCTCTCTGGCCGACACCTTCGACTTTCCTTTGCTTATTGTTTGCCATCAAAATGT
TTGCCGTTTTCGATTTTCAAGttttttttttGGCCATTATAAGGGGCACTTTAAGGTGAAATGGATATCA
TTTGTGTTGAAAGGCAGGCAACCTTTTGCTGGACCAAAGATTAAAAAGTTCTCTGAAATTGTTTGACAAT
ATATTTTGCTTGGGAATTCCTTTTGTTCTACAGATTGTTAGATTATTCATTTGCTGATAATTACCATATT
TTCAGGGTAAGTGCAGAAACCAAGTTCCACTTGGATCGGGCTCATTTAACTTGCAGCTATGGTCGCCTCT
CCAACTCCTTCGACATTTCCGACTCCTCCAAGTCCAGATACGCTGCATCTGAATGCGTCTAAATTGATTA
AGTGCTCTTTGTGACAAACAAACTTCGCATTTGGCCAATGTCGTCTGGTCCGAAAATGGACGATGGCCGA
AATGCACGTAGATAGAGGGAAATAAAAACGAGCGGGCGACCGAGGAACGCACACAAACTTGAAAAGCTTT
TTAGCCAAGTTTTTCCCCGGCTCCCCGTGCCGGTTTTAGTATTTTTTCCTTGTTTTCCCGGAGTCGGAGT
TTGGCACTTAAGCCAGCTAAACGTTGTCGGAATACGAACGGCAAATGCTATTGGTTTGGATATGTGCTGG
TTGGGGGAGTTGAGGTTCGGCGACCATTTCATTTGCAAAGTTGACTTGCCCTAACTTGGCTAATATGAAA
ACGCACTCAATCCGCTTTACGCCCCTTTTCAGGATTGGCACCAACAACTGCACGGCCCGCTATCGAACCC
TAAGCGGTGGAATTGGGGAATCACCATCGCTGTCGCCCAGTCCATCGTCGGATTACGAGGACATCGGAGT
GACCAGGGGCCACGGATGTCTACCGCCCAGCCTGCTGGCCGCTAAGGCCAAAAAGAACGGACTGCCGCAC
GGCAAGGCGAACACCATTTGCCAAAAGGCCACGGTGCATCATTGTAAGTTAATTAAACGCTAATGCCAAA
AGGAAAACAGATTTGCAGAAGGTGGAAGAGGACACCAACTGCATCGTCGATGCAGCGACGGGGATTTTGT
TGACACATTATGTGGCCTGCGAAATGTAATTAGCCCCTTGGGGGCTTGCATATGCAATTGGCTTCATCGA
GGCCTTAGCCAACAGTaaagctaaagccaaagccaaagccgaagctaaagccTCATAATGGCCCGACTGG
ACCACAACTCTTTGGCGCGACATGCACTCTAAATGCAAATGCCAGTTTAATGAGTTTTTATGGCCGCTTG
CACGGTTTTAGCGTGTTGGCAAAAGTGAAAGTTCATCTCGGCCAAACTTTCGAGGCGAGTCGAGGCCTGC
CaaaaaaaaaTATGAGCAAAGTTTTGGTGTCCAGCATTGCAGCTTAAGGATCAGAGAAACTAATGCAGTC
TGCGCCCAAAGCGCAATATTCAGCACGAAAACATATTTGATTTTATTCAAAAAAGGCACAAAACTAAAAC
TCACTATAAGTTATTAGCATGTGTTAACGCAAACTATTTATTTCAAATGCAATGGCATACACATGTGTTA
ACATTTATTCTCCGTGTAGACAAAGTCAAGACTCTAATTCCTTCTTCTCTGCCCGAACGTATTGGTATGT
TCGTTTTAAACTTTTTAATGATTTAACTAACAAAACTTTCCTGTTCTTGTTCTTGTTTTTTGCTGCTCTC
CCCTTCAACATGCAATTTATTGCACAACGATGACGACGACGTCGACGACGCAACCAAAACACACGGAAAA
AATACGGATAACTTTCACTTCAATCCGTCTGGGCTTGACTGGGGGATTGGTATGGATTGGATTGTTAGGT
CGGGCGAGATGCGTAGCTCGGCAAAGGAAATTGGCGCATTTAATGAGAATGGACGAAACTTTGTTGCCAC
AAAGGATACGTCGAGGGATTTCAGCAATTCCCAAGATAATACCGACCGTGGCAGCATGAGCGATCAGGCG
TTCGCCTGTTCGGCCAGCTCCGTGGAAAGTTTGCCCAGCGCATCCGGTTCGA
>dme_circ_0007237_junction_seq
GAAAGTTTGCCCAGCGCATCCGGTTCGAGGGTGGCCGTGTGCGCTTCTCGAACAAC
>dme_circ_0007238
GAGGGCAAACGAGGCTACCTGGAGGGCCTGGCCGCCCACTACAGCAAACAGATCAACATGCCGTACGACG
ACGTGCTGGACGCCATCGAGCAGGTGCGCCTGTCCAAGTGGAAGGAGCGCCACGTGCGCATCTCCACGGG

GCACACGCTGTCCCGCCGGATGGCTGGGCCCAGCTGCAGCGGAACGGGTCTGGTCAGCTCCTCGGTGATG
CCCACCAGCCACAGCCAGCCGCTCTATGTGCCCGGCAAGTACTTGGTAAGCATATGCTTCGACTTCGAAC
CGTGACTTTTACGGCCGCAAAGGCTTTTACGACATCCAGCTAATCAGACTCTAATTCATTTCCGATCCCT
TCCCTTTGGTTTATTATGCATGCTCATGTGTGTTCTTTTATGTGTGCATGCACTGAAAGAAATTTACTGG
GGACTACTTGGGATTACTTTGTGAAAATGACAGAGGACAATGGGCTAAGTTGGGCACGCACATCATTATT
GTTTCAATCCTTCTTCATATGAAATACCAATGAAATCGCTTTTAAAATATTGAATATTTATGACTGAGGT
CTTTAAAAAAACGATAAACCTAGTTTTTATTGATCTATATATATTATATATTTTCCGTGCTTGTGCAGCCC
CTCCAGTTGCCTATCAAATCGCGAGGAGAACGAAATCTACAGCTATACGCAGAATGATTTGGCCAATCGC
ATGGCCCGGAACGCCATCGACTCCAAGTCGGCTCTGAATCTGAACGGGATGCAGCACAGTTATCCGGGAG
CACGGACCAATTTCTTTTACAACTTTTCGGCAACAGGTGAGGAGCGTCTAAAATCGGAGGAGGAGGCACC
GGAAGCTGTGGTTAGAGACAGTTTGGCGGCTGTTTGGTTTTTATGGCTTTTAATTCAATTAAATTGGTTT
GCGCTTAAACTTTGGCTGTCTGGTTAATCAAATGAATGTCTGTTTGATTCTCGGTTTGATTGTTCGCCTG
ACTGCCTCCGCATttggatttggaactggatctggattggattggattggTTAGAGGGCCGGAACAAGCA
CAAGCAGCGGAGAACGGTCTTTCACAGGTTTTTGCAGGGCCTCCGGCAAAGTGGAGATGAGCTGAACGCC
ACGGAGGGAATGCAGTTGAAGG
>dme_circ_0007238_junction_seq
AACGCCACGGAGGGAATGCAGTTGAAGGGAGGGCAAACGAGGCTACCTGGAGGGCC
>dme_circ_0007239
GGTGATTTATTTAAGAGCATGTGCTTTCAACGAACGCTGTTTCGCCAATAAAATGTAGCCACCTACCTTT
ATTTCAAGTTTATGAATTGTAAATGTCGCTATTGATTTCCTAAATAGTTTACTTTGGCCGCAAAACATTA
TGTACATCCCTATAAGGATTCGACCTCCGATAAATG
>dme_circ_0007239_junction_seq
CCTATAAGGATTCGACCTCCGATAAATGGGTGATTTATTTAAGAGCATGTGCTTTC
>dme_circ_0007240
GCAGCGTGGAGATGCTGGAGAAGATGATGGCCACCGGCATGAACATCGCGCGCATGAACTTCTCTCACGG
ATCGCACGAGTACCATGCCGCCACCGTGGCCAATGTGCGCCAGGCGGTGAAGAACTACTCGGCCAAGCTG
GGCTACGAACACCCCGTGGCCATTGCCCTGGACACCAAGGGGCCCGAGATCCGTACCGGTCTGATCGGAG
GCAGCGGCACCGCCGAGATTGAGCTGAAGAAGGGCGAGAAGATCAAGCTGACCACCAACAAGGAATTCCT
GGAGAAGGGCTCTCTGGAGATTGT
>dme_circ_0007240_junction_seq
TCCTGGAGAAGGGCTCTCTGGAGATTGTGCAGCGTGGAGATGCTGGAGAAGATGAT
>dme_circ_0007241
GGCGGTGAAGAACTACTCGGCCAAGCTGGGCTACGAACACCCCGTGGCCATTGCCCTGGACACCAAGGGG
CCCGAGATCCGTACCGGTCTGATCGGAGGCAGCGGCACCGCCGAGATTGAGCTGAAGAAGGGCGAGAAGA
TCAAGCTGACCACCAACAAGGAATTCCTGGAGAAGGGCTCTCTGGAGATTGTGTACGTGGACTACGAGAA
CATTGTCAAT
>dme_circ_0007241_junction_seq
GTACGTGGACTACGAGAACATTGTCAATGGCGGTGAAGAACTACTCGGCCAAGCTG
>dme_circ_0007242
GGGCAAGAACATCAAGATCATTTCCAAGATCGAGAACCAGCAGGGCATGCACAACCTGGACGAGATCATC
GAGGCCGGTGATGGCATTATGGTGGCCCGTGGAGATCTGGGTATTGAGATTCCCGCCGAGAAG
>dme_circ_0007242_junction_seq
TCTGGGTATTGAGATTCCCGCCGAGAAGGGGCAAGAACATCAAGATCATTTCCAAG
>dme_circ_0007243

GATCGAGAACCAGCAGGGCATGCACAACCTGGACGAGATCATCGAGGCCGGTGATGGCATTATGGTGGCC
CGTGGAGATCTGGGTATTGAGATTCCCGCCGAGAAGGTGTTCCTCGCCCAGAAGGCCATGATTGCCCGCT
GCAACAAGGCTGGCAAGCCTGTGATCTGCGCCACTCAGATGTTGGAGTCAATG
>dme_circ_0007243_junction_seq
CTGCGCCACTCAGATGTTGGAGTCAATGGATCGAGAACCAGCAGGGCATGCACAAC
>dme_circ_0007244
GATCGAGAACCAGCAGGGCATGCACAACCTGGACGAGATCATCGAGGCCGGTGATGGCATTATGGTGGCC
CGTGGAGATCTGGGTATTGAGATTCCCGCCGAGAAGGTGTTCCTCGCCCAGAAGGCCATGATTGCCCGCT
GCAACAAGGCTGGCAAGCCTGTGATCTGCGCCACTCAGATGTTGGAGTCAATGGTGAAGAAGCCACGTCC
CACTCGCGCTGAGATCTCTGATGTGGCCAACGCTGTGCTCGATG
>dme_circ_0007244_junction_seq
TCTGATGTGGCCAACGCTGTGCTCGATGGATCGAGAACCAGCAGGGCATGCACAAC
>dme_circ_0007245
GAACCAGCAGGGCATGCACAACCTGGACGAGATCATCGAGGCCGGTGATGGCATTATGGTGGCCCGTGGA
GATCTGGGTATTGAGATTCCCGCCGAGAAGGTGTTCCTCGCCCAGAAGGCCATGATTGCCCGCTGCAACA
AGGCTGGCAAGCCTGTGATCTGCGCCACTCAGAT
>dme_circ_0007245_junction_seq
GCAAGCCTGTGATCTGCGCCACTCAGATGAACCAGCAGGGCATGCACAACCTGGAC
>dme_circ_0007246
GAACCAGCAGGGCATGCACAACCTGGACGAGATCATCGAGGCCGGTGATGGCATTATGGTGGCCCGTGGA
GATCTGGGTATTGAGATTCCCGCCGAGAAGGTGTTCCTCGCCCAGAAGGCCATGATTGCCCGCTGCAACA
AGGCTGGCAAGCCTGTGATCTGCGCCACTCAGATGTTGGAGTCAATGGTGAAGAAGCCAC
>dme_circ_0007246_junction_seq
ATGTTGGAGTCAATGGTGAAGAAGCCACGAACCAGCAGGGCATGCACAACCTGGAC
>dme_circ_0007247
GCCTTGGCCTTGGTGGCAGCCTCAACGGCAGCGATGGCAGCCGCGTGAGAGGCATCGATGGTACCAGCGC
CGCGAACCAAGTCGTTAAAGAGGTTCTGGTGCCAGAGGGCAGCCTCGGCCTCCTTGCAGG
>dme_circ_0007247_junction_seq
CAGAGGGCAGCCTCGGCCTCCTTGCAGGGCCTTGGCCTTGGTGGCAGCCTCAACGG
>dme_circ_0007248
AAATATTTTCGTATTTTACGTAAAGCATCTGACGGGTGCGCTGTCCCCTCACCAAACTGAAGAAAAATGT
ACCGGCAACGGGGTGAAATGGGTTGGCCTTCTCCATTTGATCTCCAGCTTTGGGCTTGCTGAAATATCTC
TTCGTCTTGGCCCGCAGCCGGTCGCGGATGATCCTTTGCGCAGCGGCCAGACGAGCGGCCGGACTCTCCT
CCTCCTGTTGAAATTCGAACTTTCGGGGATGCTTGGCTGCCGGTGG
>dme_circ_0007248_junction_seq
ACTTTCGGGGATGCTTGGCTGCCGGTGGAAATATTTTCGTATTTTACGTAAAGCAT
>dme_circ_0007249
GTGCGGCAATGGCCGTCTTGAAAGGTGCAAATCCAGCCAGCTTAACAATTGAGTTGAACTTGGCGAGGTC
CGTCACCGAGGACTCCACCTGGGGCAGGAACATGGACACCTCTTCGAACTCCTTGACGGAGAAGAGCGAG
AAGCCCGCCGCATGCTCGTACAGCACATACAGGTTCGACTGGAGGCGGGGAAATGAAAAATTATCAGTCT
GATGCGGTGGCAGACACATGTGGTTTTGGGTGCTCCGCGTGTGTAGGTTAGTTTTCGCAGGGAAAACATT
TTCATTTCACGGGCAACCCGCAGAAAATTCAAATATATGATTATTGCACTGAAATATTTACCATGTCTAT
GCCGAATATATGCCGATTTAATTAAGAAATATATATTTTTATACACCCACGATGCTGCTACAGTTGTTTT
CCCAC

>dme_circ_0007249_junction_seq
CACGATGCTGCTACAGTTGTTTTCCCACGTGCGGCAATGGCCGTCTTGAAAGGTGC
>dme_circ_0007250
gttaattttccaaaagtaatttGAAGAATTTATTTATCAAACAAAGGTCGCTCGTCAGGTCAGCCTTTGT
TCATTGTCATCAAAACGGGACAGACGGGAATGATCCTTACTTCCTCCTTTTCCTCCACTACATCAACGTA
CTCCTCGCGCTACGATTTCCTTTGCTTTTAGTTTAGTGATACGTTGC
>dme_circ_0007250_junction_seq
CTTTGCTTTTAGTTTAGTGATACGTTGCgttaattttccaaaagtaatttGAAGAA
>dme_circ_0007251
gtaatttGAAGAATTTATTTATCAAACAAAGGTCGCTCGTCAGGTCAGCCTTTGTTCATTGTCATCAAAA
CGGGACAGACGGGAATGATCCTTACTTCCTCCTTTTCCTCCACTACATCAACGTACTCCTCGCGCTACGA
TTTCCTTTGCTTTTAGTTTAGTGATACGTTGCGTTAAATTAAGAAATTATCACATGATATTTGAAAATTG
GTTTTTGCGTTTAAGTAAAGTGTTTTTGATGTTCTCAATTGGATTGTGTTGTACCGACCTCGCGGCCAGC
GTCAAATAACTTACAAATCATTTAAACATAAATTCGCTATGTTCAAATAGCTCGCATTTTGTAGTGTTTC
ATTAATCAGGGAGTCTGAATTTAAGTCTAAAAATAACCGTCAGCGGTTTGGTGAAAGTA
>dme_circ_0007251_junction_seq
AATAACCGTCAGCGGTTTGGTGAAAGTAgtaatttGAAGAATTTATTTATCAAACA
>dme_circ_0007252
ACTTAAATTCAGACTCCCTGATTAATGAAACACTACAAAATGCGAGCTATTTGAACATAGCGAATTTATG
TTTAAATGATTTGTAAGTTATTTGACGCTGGCCGCGAGGTCGGTACAACACAATCCAATTGAGAACATCA
AAAACACTTTACTTAAACGCAAAAACCAATTTTCAAATATCATG
>dme_circ_0007252_junction_seq
ACGCAAAAACCAATTTTCAAATATCATGACTTAAATTCAGACTCCCTGATTAATGA
>dme_circ_0007253
TAGCTAAACTAAACCGCACTAGGTCCTAAGTTAAATTAAGTTCATAGCTTAAGTTAAGATTTCGTACTAC
TTTCACCAAACCGCTGACGGTTATTTTTAGACTTAAATTCAGACTCCCTGATTAATGAAACACTACAAAA
TGCGAGCTATTTGAACATAGCGAATTTATGTTTAAATGATTTGTAAGTTATTTGACGCTGGCCGCGAGGT
CGGTACAACACAATCCAATTGAGAACATCAAAAACACTTTACTTAAACGCAAAAACCAATTTTCAAATAT
CATG
>dme_circ_0007253_junction_seq
ACGCAAAAACCAATTTTCAAATATCATGTAGCTAAACTAAACCGCACTAGGTCCTA
>dme_circ_0007254
GCTTAACCTAAGCAAAAACAATAAAAAATCGCTACTCCGTTGGTTCCACCAGCAGCTTGGTTTCCCCGTC
ACTCTCCGATTTATGCGCTTGCGCCCAGTCAATGACATATTCCCGCAGACTGGACAACGCGAGGACGGCA
GGAGTCTCCAGCTTTTGATCCTTGGCCAGCCAGTCGCAGTATTCGGAACGCGAACAGGCGCCAGCGCTGC
CAAAGCTCCGTACATAACGCACACTCGCCAGCTGATCGGGCCATTGGCGTGAAACGCACTCCAGCAGGGT
ATCCATTTGGGTGGCGGACACGGCACGATGCTGGCCACAGGCCGAGCCAGTTTTCCACAGAAC
>dme_circ_0007254_junction_seq
CACAGGCCGAGCCAGTTTTCCACAGAACGCTTAACCTAAGCAAAAACAATAAAAAA
>dme_circ_0007255
GGTCTAAACTTATCCAAGCCCACGTCAAAGCGTTCGTAGTGCTCCAGCAGTGCTAATCCATATCGATCCG
CAGGTGACAGTTTTGCATGATAGGAGCCAAGGATTACGGGTATGTGATCAGATTGCAGGGCACTAGGAGC
CTTTCTGGCCAACACCTCCATTAG
>dme_circ_0007255_junction_seq

GAGCCTTTCTGGCCAACACCTCCATTAGGGTCTAAACTTATCCAAGCCCACGTCAA
>dme_circ_0007256
ATTATCATATTCGTGGAGACAAAGCGACGCGTGGACAACCTGGTGCGCTTCATCCGCAGCTTCGGAGTCC
GTTGTGGAGCTATTCACGGTGACAAGTCGCAATCAGAACGAGACTTTGTGCTCCGTGAGTTCCGCTCGGG
CAAGTCCAACATTCTGGTGGCCACCGATGTGGCGGCCCGTGGACTAGGTAAGTTTGCTCTCGCAGCGGCG
TAGAATATACACCGTAGTTTGCGGCCGAAAGATTTGGATGGGCCTGTTTAGCGATAAGGTCACACTTGCT
AGGGCCATTGCTTACAACATACCATCACTACCAGAGGACACCGCACCTAACGATCACCCAGCATCATTAC
ACGCACCCACTACATGCGAGCAGAACCTAATCCTACCCGTAAACCAACCTACCCACCATCCGAACTTCCT
AACCACACCTAATGATATATCCCGGTATCTACCCACATCTAGCGAGCGCGCCAACTCAGAAACAGttttt
tttAGACATACGCCAAATGCCGAAATCGAATAATCAACTTGAACTTTTACCCCacccccctgaccctgacc
tgcaacccgcccccacctgcaacacccggcccaacccctgctAAAATAATGTATGAAATGCGTAATGATC
GTATATGATCCATATGCACTGCGGTGTACCTGTTACTACGCAACTTTTGTCTTTGTGCGTGtctctgcct
ctttctctctctcccccccccctctctctctctctTTCTTGTTTCTTTCTCGTTTACAGAGGCCGATT
TTGGTAGTATGATGGGGAGGATAATGAATGAGCGGCGACCACTGCGAACACTCGTGACTTGAGCGACTTT
TGCCAACATCAGAATCAGATCTGTTATGATATATGATGATAGCACATCCTGTGTGATATCTTGCGCAAAA
GGACCGATGAAAGTTCTCAGATTCAGACGGTCGAGAGACACGAATATCGAATATCCCCAACATCATGCAT
CATATGTGGCCTCTGCAGTCCCCGACTTTTTGTTGAGAAAGAGTGAAATAATATGATATGAGACGTTTTT
GCGACCAAAGACTACCATCTACCAACCAAAAACCATCTACCAATCAACCAAATAAGATCCAACAACAACA
ACTGAACCGAACCATCAGAGAGCCAGCTTTTTCCTCAGCAGTGGATGGCGGAAGGCGGCGTGCAAAGTAT
TGACGGGGTGGCCTGGCCCTTGAGAAAGCACTAGAATTGGACATGCACCAAGTCGACagcaaaagccaaa
gcaaagcaagcaagcaaaaacaagcaCATTGACACCACCACCCCCACAACACAAACCACACTCACAAATA
AGAACACCTGACATTTGCTTTAACCAGAGTGAGGACGAGAAGATCCATCCGGAGATGGATGATGTGTGTG
CTTTCGAGGATCCAGACGAAGATGCTGCTGCTGGGTGCTGCGTCCTCCCAAAAGCTCACTACCACCACCC
ACTGACCGCGTTTGCCGCCGCATCAGTTAAGCCCACTGCTCCTGGACGAAAAGGCGGCCCGCTTGTTGTT
GTTAACCCTCCCAAAAAGTTCAATCTTTATATAATGGTCGTGTCTCCTCTCGGTGGTCACCTCCAAAATG
GTCCATGTGAGAAGAGAGCTTAGTCAGAGGCGCACGCGGCTCAATGCAACAAACTCTCCGCTTCGCCGTG
CGATCGAGATCGCTACCTTGCTGTTTCTGGGGAGAGTTTGGACCACGAGCTGCGCGCCGCCAATCAGAGA
TTTTGATTGAGAGAAAATTGAGTGAAACAATTTATAGCACCAAGAAGGGTAAGTTTGCTCTCGCAGCGGC
GTAGAATATACACCGTAGTTTGCGGCCGAAAGATTTGGATGGGCCTGTTTAGCGATAAGGTCACACTTGC
TAGGGCCATTGCTTACAACATACCATCACTACCAGAGGACACCGCACCTAACGATCACCCAGCATCATTA
CACGCACCCACTACATGCGAGCAGAACCTAATCCTACCCGTAAACCAACCTACCCACCATCCGAACTTCC
TAACCACACCTAATGATATATCCCGGTATCTACCCACATCTAGCGAGCGCGCCAACTCAGAAACAGtttt
ttttAGACATACGCCAAATGCCGAAATCGAATAATCAACTTGAACTTTTACCCCacccccctgaccctgac
ctgcaacccgcccccacctgcaacacccggcccaacccctgctAAAATAATGTATGAAATGCGTAATGAT
CGTATATGATCCATATGCACTGCGGTGTACCTGTTACTACGCAACTTTTGTCTTTGTGCGTGtctctgcc
tctttctctctctcccccccccctctctctctctctTTCTTGTTTCTTTCTCGTTTACAGAGGCCGAT
TTTGGTAGTATGATGGGGAGGATAATGAATGAGCGGCGACCACTGCGAACACTCGTGACTTGAGCGACTT
TTGCCAACATCAGAATCAGATCTGTTATGATATATGATGATAGCACATCCTGTGTGATATCTTGCGCAAA
AGGACCGATGAAAGTTCTCAGATTCAGACGGTCGAGAGACACGAATATCGAATATCCCCAACATCATGCA
TCATATGTGGCCTCTGCAGTCCCCGACTTTTTGTTGAGAAAGAGTGAAATAATATGATATGAGACGTTTT
TGCGACCAAAGACTACCATCTACCAACCAAAAACCATCTACCAATCAACCAAATAAGATCCAACAACAAC
AACTGAACCGAACCATCAGAGAGCCAGCTTTTTCCTCAGCAGTGGATGGCGGAAGGCGGCGTGCAAAGTA
TTGACGGGGTGGCCTGGCCCTTGAGAAAGCACTAGAATTGGACATGCACCAAGTCGACagcaaaagccaa
agcaaagcaagcaagcaaaaacaagcaCATTGACACCACCACCCCCACAACACAAACCACACTCACAAAT

AAGAACACCTGACATTTGCTTTAACCAGAGTGAGGACGAGAAGATCCATCCGGAGATGGATGATGTGTGT
GCTTTCGAGGATCCAGACGAAGATGCTGCTGCTGGGTGCTGCGTCCTCCCAAAAGCTCACTACCACCACC
CACTGACCGCGTTTGCCGCCGCATCAGTTAAGCCCACTGCTCCTGGACGAAAAGGCGGCCCGCTTGTTGT
TGTTAACCCTCCCAAAAAGTTCAATCTTTATATAATGGTCGTGTCTCCTCTCGGTGGTCACCTCCAAAAT
GGTCCATGTGAGAAGAGAGCTTAGTCAGAGGCGCACGCGGCTCAATGCAACAAACTCTCCGCTTCGCCGT
GCGATCGAGATCGCTACCTTGCTGTTTCTGGGGAGAGTTTGGACCACGAGCTGCGCGCCGCCAATCAGAG
ATTTTGATTGAGAGAAAATTGAGTGAAACAATTTATAGCACCAAGAAGGTAACGTACATTTTGAAACATT
TTCTTTTTTTCTCTTaaaaaaaaaTACAAACAACCCACAAATCAAAATACCATAGACTCAATAGTATGCa
aaagtataaaaaaaaaaaatttaaaaaaaaaaaaagaaaaGACAAGTGTGTTCAAAGTGTTCAAGACATCTC
GGAAAAACGCTAAAAATTGTTGCAAGCGAGCGCGCATAGCGGAAAAATCATTTGGCAATTTGAAAACTGT
TCGTCAAATAAATAATACACCCCAATTAAGTAAGAACACAGCCAGGAGCTCGAAACTAATCTATGCGCCT
TCTCTTTTCCCCGGACACCTGTAGAACGTGGACGGCATCAAGTATGTCATCAACTTTGACTACCCGCAAA
ACAGCGAGGACTACATCCATCGCATCGGTCGCACAGGACGATCCAACACAAAGGGCACCTCTTTCGCCTT
CTTCACCAAGAACAATGCCAAGCAAGCCAAGGCGCTGGTCGACGTACTCCGAGAAGCTAATCAGGGTGAG
TTCATAGCGTGGTATCAGGTTACAACACCATTTGGGCAGTCAAATCTAGTTTGGGTAAATCTACATCTAG
GTCTTATATTCCAAATCTTAGAATTAGCATGCAGATCCCTTAACCACATCGATTTACTGCTCTTTTTGAT
ACTGACCCTTAGAGGTAAACACAATTCTGATGGTTTCTTATTTCTTTATCAGGGAAATCAATCCTGCCCT
GGAAAATCTGGCCCGCAACTCGCGCTACGACGGTGGCGGCGGACGCTCCCGTTATGGAggcggtggcggc
ggcggtcgcttcggcggcggcggctTCAAGAAGGGCAGCCTGAGCAACGGACGAGGCTTCGGAGGAggcg
gcggcggcggcggcgAAGGCAGACACTCGCGCTTCGACTAGATCGCGGACAATTAGATGAGCAGTAAAAG
AAGCTATAGGAACAGGAGATTTCAGTAGCTAAACTAAACCGCACTAGGTCCTAAGTTAAATTAAG
>dme_circ_0007256_junction_seq
ACCGCACTAGGTCCTAAGTTAAATTAAGATTATCATATTCGTGGAGACAAAGCGAC
>dme_circ_0007257
GGCAAAGAACTCACTGTCGCACTCATCCATGTGATGATGGTAATTGAGCAAAAACTCCAAGACGGCCAAC
TCAAATGGTTCCAGTTGCTGTTCATTCAGCTCGCCGAAGTCCTTGTAGAAGGTCAGGATTAATTTAAATG
ACTTCCCGCAATCAATGGGCTCCTTTAACGT
>dme_circ_0007257_junction_seq
TCCCGCAATCAATGGGCTCCTTTAACGTGGCAAAGAACTCACTGTCGCACTCATCC
>dme_circ_0007258
GAGATTAATGGAGCATTGCCGCAGCTTGCCTCGATTCGAGACCTATACTGCCAACTATCGCTTGCACTTG
ATTAAGACCCTGGATATTGCTTTGGATACACCGGTGGACCAGTTGGGAAATCAGCAGCAGCTCGACGAGT
GTGTGAAATTGGTGGATTCATTTGAATTCAATTCTGATCAATGCTCTCAAATGCTGGACTTGCTAACAAC
CAAAGTGCAAGCAAAGGACTGCATTCCGAATGGTAAACCAAATCATTACTTTGATCTACTTCTACGTTTG
CTGGGTCGATTGAACACGTTAAAGGAGCCCATTGATTGCGGGAAG
>dme_circ_0007258_junction_seq
GTTAAAGGAGCCCATTGATTGCGGGAAGGAGATTAATGGAGCATTGCCGCAGCTTG
>dme_circ_0007259
GTAATACTCAAGGATGTGAGTTTCAACATTATGATGGAGCTACTGGAGTTTATGTACCAAGGAGTGGTTA
ATGTTAAGCACACGGAGTTGCAGTCGTTCATGAAAATTGGACAGCTGCTGCAGATCAAGGGCTTGGCTAC
CAATTCGAATTCCTCGCCAGGATCGAGTGTCTCGGAGAAGTCCTCTAGTCAGCCACCGGCAGAAGAATCA
AGCAACACCAACAGCACCAATCATCACAACTCTAGCACTAATAGCAATAACAATAGCAAATCGGAAACGG
ATCACAATGAGTCCAAAGGCCAGAGCAATTCAAGAACCACTTCGCCAGGTGGTGCCAGCAGAGCCTCCAA
CGATGCCAGCCACCTGTACACATCGAACAAACGTCCGATGCCGTCGGATTTTGGCAGCGATTCCCTGTCC

ATTTACTCCGGCAAGCAGTTGAGACGCTCCCTCAAGGATCACGGTTCCGGCGGAAGCGAAGGCGGCGGAG
GCGATCACGCGGATACCGCAGCCGGATTGGACAACAGCATGAATTCAGAGGAGTTCTTCTTGCCACCCAT
TCCACAAATCACGATGGGAGAACAGCGCTACGATCTCGGAGGTCTGAAACGGGAATCGGATGGTCACCAC
CACGGTCCCTTGTCGGCTGGCGGAGGAAATTCGGGCAGCGTTGGCAGCCTCACCTCCTCGGCCTCACCGT
CTGCACCCATCCGTAATCCCTTTGCCCCGAACTTCATGGACAGCTTCAACTACTACAAGGGCGGCAACAG
CAGTGGTCCCAGCGGTAGTTCCAATAACTCGGTTGGCCCAGGTGGCGTCAATAACAATGGCTCCGTGGGC
CTTGGCTCCGGCGCCGAGTACCCCAACGAACTGTATATGCCCAACGATTACTCCAAGAGCTTTGCCAACC
ACATGGACATTCCCTCAA
>dme_circ_0007259_junction_seq
TTTGCCAACCACATGGACATTCCCTCAAGTAATACTCAAGGATGTGAGTTTCAACA
>dme_circ_0007260
GCTAACATGGACACCGCCGGCATTATTCCCGACATCATCGACGTCAAGCCCGCCTCCAAGGCCACCATCA
CCTATCCTTCCGGCGTTCAGGTTGAGCTGGGCAAGGAGCTGACTCCCACTCAGGTGAAGGACCAGCCCAC
TGTCGTCTTCGATGCCGAGCCCAACTCCCTGTACACCATCCTGTTGGTGGACCCCGACGCACCCAGCCGC
GAGGACCCCAAGTTCCGTGAGCTGCTCCACTGGCTGGTGATCAACATTCCCGGCAACAAGGTGTCCGAGG
GCCAGACCATCGCCGAGTACATCGGTGCTGGACCCCGCGAGGGAACCGGCCTGCACCGCTACGTCTTCCT
GGTGTTCAAGCAGAACGACAAGATCACCACCGAGAAGTTC
>dme_circ_0007260_junction_seq
GAACGACAAGATCACCACCGAGAAGTTCGCTAACATGGACACCGCCGGCATTATTC
>dme_circ_0007261
GAAGACGTAGCGGTGCAGGCCGGTTCCCTCGCGGGGTCCAGCACCGATGTACTCGGCGATGGTCTGGCCC
TCGGACACCTTGTTGCCGGGAATGTTGATCACCAGCCAGTGGAGCAGCTCACGGAACTTGGGGTCCTCGC
GGCTGGGTGCGTCGGGGTCCACCAACAGGATGGTGTACAGGGAGTTGGGCTCGGCATCGAAGACGACAGT
GGGCTGG
>dme_circ_0007261_junction_seq
CTCGGCATCGAAGACGACAGTGGGCTGGGAAGACGTAGCGGTGCAGGCCGGTTCCC
>dme_circ_0007262
GGCACCACACGATCGCGACTTTGGTCACAGTGGACGTGGCGGTCgcggaggcgatcgaggcggcgatgat
cggcgaggaggcggcggcggaggcaATCGCTTCGGTGGCGGCGGTGGCGGTGGCGATTACCACGGCATAA
GGAATGGACGCGTCGAGAAGCGACGCGATGACCGCGGAGGCGGCAACCGATTTGGAGGTGGTGGTGGATT
CGGGGATCGCCGAGGAGGAGGTGGCGGCGGCAGCCAAGACCTCCCCATGCGTCCGGTCGACTTCTCCAAC
CTGGCTCCCTTCAAGAAGAACTTTTACCAGGAGCACCCTAACGTAGCAAACCGATCGCCCTACGAAGTTC
AGAGGTATCGCGAAGAGCAGGAGATCACCGTGCGCGGACAGGTGCCGAACCCCATCCAGGACTTCTCCGA
GGTCCATCTGCCCGACTACGTCATGAAGGAGATCCGCCGACAGGGCTACAAGGCCCCCACCGCTATCCAA
GCGCAGGGCTGGCCTATCGCCATGAGTGGCTCGAACTTCGTCGGCATTGCCAAGACGGGATCCGGAAAGA
CCCTGGGCTACATCCTGCCCGCTATTGTCCACATCAACAACCAGCAGCCGCTGCAGAGGGGCGACGGACC
TATCGCCCTCGTGCTGGCCCCCACTCGGGAGCTGGCCCAACAGATCCAGCAGGTGGCCACCGAATTCGGT
TCCTCGTCGTATGTTCGCAACACCTGCGTCTTCGGCGGCGCCCCGAAGGGCGGCCAAATGCGGGATCTGC
AGCGCGGCTGCGAGATCGTCATTGCCACACCCGGACGTCTTATTGATTTCCTCTCCGCCGGCTCTACTAA
CCTGAAGCGTTGCACCTACTTGGTGCTGGACGAGGCCGATCGCATGTTAGACATGGGCTTCGAGCCCCAG
ATCAGAAAGATCGTCTCACAGATCCGGCCCGACCGACAGACGCTCATGTGGAGCGCTACCTGGCCCAAGG
AGGTCAAGCAGCTTGCCGAGGACTTTTTGGGCAACTACATCCAGATCAACATTGGATCGCTGGAGCTGTC
CGCCAATCACAACATCCGCCAGGTGGTGGACGTTTGTGACGAGTTCAGCAAGGAGGAGAAGATTGAAGAC
CCTCCTGTCAGACATCTACGACACCAGCGAGAGCCCCGGCAAGATTATCATATTCGTGGAGACAAAGCGA

CGCGTGGACAACCTGGTGCGCTTCATCCGCAGCTTCGGAGTCCGTTGTGGAGCTATTCACGGTGACAAGT
CGCAATCAGAACGAGACTTTGTGCTCCGTGAGTTCCGCTCGGGCAAGTCCAACATTCTGGTGGCCACCGA
TGTGGCGGCCCGTGGACTAGG
>dme_circ_0007262_junction_seq
CCACCGATGTGGCGGCCCGTGGACTAGGGGCACCACACGATCGCGACTTTGGTCAC
>dme_circ_0007263
CAGCGGCACTTTCGCTTCTTCTTCTTCGCTCTGCACCGAACAACGACAACAGTTCCACGGGAGTCGCAGG
AATCGTGAGACAATACTGTTCCCAAGCACATATAGTAGTCTCCAAGCTCAGTCGCAGCGTGCGTTTCGAG
ACAGCTCGAAACCAGATTCAGACGACTACGTAGATTCCATCCCGAAAGCAGAGCAGCGGACGAGGACGAG
GAAATCGCTTTTCAACGATCCCGACGAAAGAACTGAGGAAATCAAAATTGAAGGAGTAATGGTGAGTACG
CCGCAAGATTTCATATGAAATGCGTTGGTTTTTCCACTCACTGTCGCCATTTTATCACCCTTCGTGCCA
TTCCTCtttttttttttttttttCTGTACACCTACGAAGCGAGGTGATGAGTAATTAGTGTGAGCGAAAG
AGAGAGTTTGGGGAGAGAGAGAAAAACACATGGTTTGCTAAATTTTGTCAGTCGGAGGTGAGGTTTTCAG
CCATTTGTTGTTTTTTTCTTTGTGTTTGCCCCCGCACATCCGCAAAGGAGTAGCAAAAGCAACAAGAACG
ACAGCGCACGTCATAAAAAGAATTTGCCTGATTTGTGAAACGTGTTCTTGCTTCGTTTGCTATTTTATAG
CGTTTTAATTTGTCTGTATGCCCCGAACATGAACACGCGCACTCACACAGCGGCACATACGGACTTGCTT
GTATGCGTGAATGCCTGTTGACGGCGGCATTTTCTCGTGCTCGTAGAACAGTTCGAACAGTGTGTGCGAG
CGTATAACAGTCGGCGGCTGTCACTGACTAGCGCCGTCCTGCTCACTCGCCAGCATGCCTTTCCATCACA
TTCTTTCCATGGCTCACACGAGGTTTTGTAGAGATATCGCGATGTTTTTGGTATTGGCAATTTTATAATT
TGATGTAACCATCCCGACATAGATTTATACTTACTATGCTTAATTTGGGAAGTGAATGGGGTTAATGGTT
TGCTTCGTTTGGTTATCTAATGCGTACATTGAAATATATAATTTCAGTATAATTCTGTATACTGAGGACA
ATTTTAGGGAAAAGTAGGGTTATTCTCTTAAAATTTTCTCTACACTATAAGTAAAGGTTCCATGCAAACA
CATTTAACTTACAGATTATCAAAAATGCATTTTATCGATCATTTTATCTATTTTGCTACGCTCTTTTTGT
GTTTACTTATCATGAACCATGCTTGTTATGCAATAAAAATTATTTATTAGAAAGTTATACTATAGTATTG
ATAAAAACTCAAGTAACAACCAAATATTAAATGGTAATAGTAACCCAGTTCGTGAACTACGCCATCACAT
AAACGGCCCCATTAGTCTAAAATGTCAAATGACGCCAATTGATTATGAGCTCCCCCTTTGGCGAGCGCGC
GATTCGTCTTTTCGCATGTCAAAAAGCGACAGGTAGACACACAGAAAGTCAAAGGTGCCCCCAGGGACTG
TCCCCTCCTCCCCTCGGGTGTTTGCCCACCGTTGAAACTAGGGGCAAGACATTTGGCTGTCTCATAAATG
TTCAGAGCGTTCCGCTTCGCTAGTTGGGCACAAACGTTAGCGTGCCAGTGGGTCCTACAAATAGACGTTT
AGGGCGGTACGGTGTTCCAAATTGACAGAGTAATAGACaaaaaaaaaCGTGGGCCTGGATCGTGGGTGAT
TTGCATGGTGCACCCCATAGTGATGCCCACTAGGATCACGAAAATGGAGCTTACATACGAAGTAAAGCGA
ATATAAAGTGGAAATTTATAGAAGTTAGTTCGTAACACAATAAACATAATTTATGTTGTAAATTTTGTTT
TATACAACTTTACCATCCAACTTAATCTTGCACTATATTTATTACCACATTGTATATACACTCCGCAGAG
TTAATATTATTTTTCCCTAATTATTGCAAAGTGCTAACATATATAGGTCGGTACCTTCGCCAGTTCTGAC
TGGAACTAGAACTGCGCCAGGTTGATTAATGCCAGAGCAATAAACGCAGGGCTTTGTACATGGGGCTGTT
TAGTTTAGTTGAGCATTTAGGGGCTGATAAACTGGGGTGAAAAATTGGTGGGAGGGTGGGCTGTGAATAG
AAACCCTGCTCGAATTGCATGTTGTTGTCAATCAAATTTGCCTTACTGTTTGCATTATTCGAAATTTATT
TAATTTCCCTAACACGCAATTAGTTTTTATTTAAGTTTATTGCGGTTGTAGCACAGCACAACTCCCATGT
CAGCTGTTTAGCAGCGTCGAAAGAAATCGAAAAGCGAAGACTTACCTTCCAAGCCAAGTCGCAGTTGAAA
TCCAAGTCCCAACTGGCAGACGTCTAGAAACGGCAGCATGCGAGCAAAGTAAGCCAAGTCACTCGTTTCT
TTTTTTCCTCAATTTTCTGTCAAAGAAATACTTAAGAAAAGAAAGTGCAGCTGTCTACGCCGAGCCGGAA
AAAATGATTCCAGTTAAGCATGGTTTAATTCTCGAAGGTCCCTTTTATCAAGGGAAAATTTATGACACAC
AGTTTCTGGGTAATGTGTGTGCAGCGCAAAATTATTTTAGATGGCAGATAAGGAGGAATTGATTATACAA
ATGTACAACGCGAAATTAAAAAGTTTGTGAGAAAACTATCAAACTATATCAACTTCAGCGAAATTCGGTT

ACAAAGCTCAAAGTGGGATATTTTCTTAAAGATTCCACTGAATTCACGGCAGCGAAATAACAAGTCGTGA
AGTCGTTCAATTTCCATGAATAAGGAAAAGGCCCTTGATACTTGACACTAAGTAAACTTCGGCAATGCAA
GATAAGCATATTAGAATTAAATTACTTTAATCTCATTTGCCTTTACCAAAGCCAATTACCATCGCAACCA
TTAATTTTGCCAAAGTTATCTAGAATAAATTACAATACAAAAAGGCACACAGGGTACGCGTGAATTTTTA
ATTTATCAAGTTAATGGGAAAAACACTTGTAATGCCACTAATTTAAttttttttATCTCGAATAAAACAG
CATCAAGATAAATTGGTGGTCAAAGATCAAACCAGTACAAGAAACTGCAAACTACTACCTTAAGTTCAAA
TAAGCTGCAAGATGATGATGATGTGAGATTTCTTTTCTTGAGCATTCCGTTTCTATTTTGCATACTTAGA
TACTCATCGATCTTCCTTTATTATTTCCCAGGGGCACCACACGATCGCGACTTTGGTCACAGTGGACGTG
GCGGTCgcggaggcgatcgaggcggcgatgatcggcgaggaggcggcggcggaggcaATCGCTTCGGTGG
CGGCGGTGGCGGTGGCGATTACCACGGCATAAGGAATGGACGCGTCGAGAAGCGACGCGATGACCGCGGA
GGCGGCAACCGATTTGGAGGTGGTGGTGGATTCGGGGATCGCCGAGGAGGAGGTGGCGGCGGCAGCCAAG
ACCTCCCCATGCGTCCGGTCGACTTCTCCAACCTGGCTCCCTTCAAGAAGAACTTTTACCAGGAGCACCC
TAACGTAGCAAACCGATCGCCCTACGAAGTTCAGAGGTATCGCGAAGAGCAGGAGATCACCGTGCGCGGA
CAGGTGCCGAACCCCATCCAGGACTTCTCCGAGGTCCATCTGCCCGACTACGTCATGAAGGAGATCCGCC
GACAGGGCTACAAGGCCCCCACCGCTATCCAAGCGCAGGGCTGGCCTATCGCCATGAGTGGCTCGAACTT
CGTCGGCATTGCCAAGACGGGATCCGGAAAGACCCTGGGCTACATCCTGCCCGCTATTGTCCACATCAAC
AACCAGCAGCCGCTGCAGAGGGGCGACGGACCTATCGCCCTCGTGCTGGCCCCCACTCGGGAGCTGGCCC
AACAGATCCAGCAGGTGGCCACCGAATTCGGTTCCTCGTCGTATGTTCGCAACACCTGCGTCTTCGGCGG
CGCCCCGAAGGGCGGCCAAATGCGGGATCTGCAGCGCGGCTGCGAGATCGTCATTGCCACACCCGGACGT
CTTATTGATTTCCTCTCCGCCGGCTCTACTAACCTGAAGCGTTGCACCTACTTGGTGCTGGACGAGGCCG
ATCGCATGTTAGACATGGGCTTCGAGCCCCAGATCAGAAAGATCGTCTCACAGATCCGGCCCGACCGACA
GACGCTCATGTGGAGCGCTACCTGGCCCAAGGAGGTCAAGCAGCTTGCCGAGGACTTTTTGGGCAACTAC
ATCCAGATCAACATTGGATCGCTGGAGCTGTCCGCCAATCACAACATCCGCCAGGTGGTGGACGTTTGTG
ACGAGTTCAGCAAGGAGGAGAAG
>dme_circ_0007263_junction_seq
TTGTGACGAGTTCAGCAAGGAGGAGAAGCAGCGGCACTTTCGCTTCTTCTTCTTCG
>dme_circ_0007264
GGTAGCGCTCCACATGAGCGTCTGTCGGTCGGGCCGGATCTGTGAGACGATCTTTCTGATCTGGGGCTCG
AAGCCCATGTCTAACATGCGATCGGCCTCGTCCAGCACCAAGTAGGTGCAACGCTTCAGGTTA
>dme_circ_0007264_junction_seq
CACCAAGTAGGTGCAACGCTTCAGGTTAGGTAGCGCTCCACATGAGCGTCTGTCGG
>dme_circ_0007265
GCCGAAACGATTCTGGGTCGCCACAGTACCATGGAGATTCGCTCGGTTCACCTGGGTGGTCTCTTCCACG
CCGAGGAGGAGATCCAGATGACCTCCACCATGCCGAACTTTGTGGGTCAGATGCAGGGCTTGGTGTTCAA
TGGCCAGCGGCTATTTGGATATTGTCAAGAGTCTGGGACCGGAGCTCTCGGCTCTGCCCAGTGCCACCTT
CAAGTTGACTGCCCGCTTCGTGAACTCTCCGGCCGGACAGCCCTACCATGCAGCCACGTTCCGCTCCAAG
CACTCATACGTGGGTCTGGCCATGTTGAAGGCCTACAACAGCATTTCCATTGATTTCCGCTTCAAGACCG
TGGAACCCAATGGTCTGCTCGTCTTCAATGGCGGACGACGCAACGATTTCGTGGCAGTGGAACTGGTCAA
TGGTCACATCCACTACACTTTCGATCTGGGAGATGGTCCAGTGACGATGAGGGACAAGTCGCGCATCCAC
ATGAACGACAACCGCTGGCATCAGGTCAGCATCAGGCGCCCGGGACCCAAGACGCATACCCTAACCGTGG
ATGACTCCTTCGAGATTATCAGTCTGACTGGAAACAATATGCACTTGGAGCTGGCGGGTATCCTGTACAT
CGGAGGCGTCTTCAAGGACATGTACTCGAAGCTGCCGGCCTCGATCTCGTCGAGGTCCGGATTCGAAGGC
TGTCTGGCATCCCTGGACCTGGGCGATGCCTCTCCCTCGCTGACCAGCGATGCAGTGGTGCCCAGCTCAC
TGGTGGTCAGTGGCTGCGAGGGACCCACCAAGTGTAGCCAGAATGCGTGCGCCAACCGAGGAAACTGCGT

GCAGCAATGGAATGCCTATGCCTGCGAATGTGACATGACTTCCTACACAGGACCCACTTGCTATGATGGA
ATCGATTGCCTACGAATTTGGCAACAACAAGGGCATGGTGCAATACACATTCCCGGAAAACGCACAAGCT
GACACGGAGGAGGATAATATAGCACTGGGCTTCATAACCACCAGGCCGGATGCAGTGCTACTGCGGGTGG
AGAGTGCCACCACGCAGGACTATATGGAGCTGGAGATAGTGGAGGGCAACATCTTCATGGTGTACAACAT
TGGAAGCGTCGATTTGCCGCTGGGCGAGATCGGAACGAAGGTCAACGACAACGCCTACCATGTGGTGCGG
TTCCAGCGCAAGGGAGGCAATGCCACGCTCCAACTGGACGACTACAATGTGCAGGCACTGACGCCACAGA
GGCCACCACTCCACCGTGTTCAATACCATGTCCAACGTTCAGGTCGGCGGCAAGTTCAGCCGGAACGGTC
GCAATCGAATCGAGCGGCCGTTCGCCGGCGTGATTGCCGGACTTTCGGTGAACAAGTTACGCATCTTGGA
CTTGGCCGTCGAACGCGACCCCCATATCACCATCCGTGGAGATGTTCAATTGGTAACTGGAGTATTAGAT
AGAAATGATCTGCAGAGAATGCAGCAGGGTTAGGGAATTTATTTGAAATTTTATTTGAATTCCTCTCAAC
TTTCAACTTTCCAAGTATTTTTCTCATCGTAAACCAAGTTTATTCGAAATTCTTTCTTTGATTaaaaacc
aaaacaaaaaaaaaaagaaaccaaaaTGAAATCTAACCAAAATTGTTGTATTAATCCGCTTCATGAGTTGT
GGAACCCCTTTTCCCAAATTGATTTGACATGATTTGATTGCACCACTGAAATTCGAAAACCCATTCGAAT
ACTCGTAATGTACAGTTCCCGAGCACTATCCTGATATTTGAAATGCATATCCGTGTTAATCGTATCGAAT
GTAACCGCATCTGTACATTGAAGTCATCGTCATCACAATCAAATCCGGGAAATTCGGTTCGACATTCAGT
ACAAACGTTAACCGTTCCAACTAAACCAAAGTATTAGCTAACTAACTGTATAATCTAGTGTAAATGCTGC
ATTtgaatttatgaatttatgaacttatgaatttataatttaCCTCGGGTGCCTTCGTTTTTTAATGTAC
CGCTTTGAACGCACCAACCAAATAACAAAAGCCAATTAAGATTTGCCAGATTTTTCACTAACCAGCCATT
TGAGTTTCCTCGATTTGTTTCCATTGCTGCACAGCACATTAACCAATAACTGTGAATATATATAATCAGC
TATATAAACTGATACGAATTTACTATGCACTAGAATTCGTCGCACTTATGCTGCATGACTCTAATCAAAG
CTCACACTGAAACAAAAGCAAGTGATAGCCAGTGGAGAAAGAAAAGTTTGAAGAAAGAACCAATGAAAGA
ACCTATGGACCTACTCATAAAATTAAATAAATAATAATCTGAAAGAAGCTTCTCCATTGGCTTAATGTGC
CCACACCCATCCACACATATATACTTAATCGAACAGCACCTAACAAACTTACTAATCCAGTTAACAAACC
CCATACAATAACCATCGCTTGGCCCGGCTGCCGCCAGATCAACTGAAGTAAAATTAGACTTTGAACTCTA
CTCGACAGGACGCCGGCGAGTGGTTATCCGGGTGCGCTGGACGATCTGATCTTCTCGGGAGCCGGATCCG
GATGTCGCGGCGACGACGAGGACGAGTGCACGCCGCCATTCGAATCGGGTAGCGGGGATGATCTCATAAC
ACCGGTCTACGTGCCGCCCACCAAGCAGACAACCACGTCCCAGCAGGGGAATAGCTTGAGTACCGGCGGG
AGCAGCAGCGGCGGTGTCATCACCAATGGCACGGAGCGTGCCTGCGACGACGAGGATTGCGTCCATGGAT
CCGGGGATTACGGCGAGACCACGGAACAGTTCACCTCGACGAGCACCGCCCGCGGTTCAG
>dme_circ_0007265_junction_seq
ACCTCGACGAGCACCGCCCGCGGTTCAGGCCGAAACGATTCTGGGTCGCCACAGTA
>dme_circ_0007266
gcggcggcggaggcaATCGCTTCGGTGGCGGCGGTGGCGGTGGCGATTACCACGGCATAAGGAATGGACG
CGTCGAGAAGCGACGCGATGACCGCGGAGGCGGCAACCGATTTGGAGGTGGTGGTGGATTCGGGGATCGC
CGAGGAGGAGG
>dme_circ_0007266_junction_seq
GTGGATTCGGGGATCGCCGAGGAGGAGGgcggcggcggaggcaATCGCTTCGGTGG
>dme_circ_0007267
GACTTTGAACTCTACTCGACAGACGCCGGCGAGTGGTTATCCGGGTGCGCTGGACGATCTGATCTTCTCG
GGAGCCGGATCCGGATGTCGCGGCGACGACGAGGACGAGTGCACGCCGCCATTCGAATCGGGTAGCGGGG
ATGATCTCATAACACCGGTCTACGTGCCGCCCACCAAGCAGACAACCACGTCCCAGCAGGGGAATAGCTT
GAGTACCGGCGGGAGCAGCAGCGGCGGTGTCATCACCAATGGCACGGAGCGTGCCTGCGACGACGAGGAT
TGCGTCCATGGATCCGGGGATTACGGCGAGACCACGGAACAGTTCACCTCGACGAGCACCGCCCGCGGTT
CAG

>dme_circ_0007267_junction_seq
ACCTCGACGAGCACCGCCCGCGGTTCAGGACTTTGAACTCTACTCGACAGACGCCG
>dme_circ_0007268
GACGCCGGCGAGTGGTTATCCGGGTGCGCTGGACGATCTGATCTTCTCGGGAGCCGGATCCGGATGTCGC
GGCGACGACGAGGACGAGTGCACGCCGCCATTCGAATCGGGTAGCGGGGATGATCTCATAACACCGGTCT
ACGTGCCGCCCACCAAGCAGACAACCACGTCCCAGCAGGGGAATAGCTTGAGTACCGGCGGGAGCAGCAG
CGGCGGTGTCATCACCAATGGCACGGAGCGTGCCTGCGACGACGAGGATTGCGTCCATGGATCCGGGGAT
TACGGCGAGACCACGGAACAGTTCACCTCGACGAGCACCGCCCGCGGTTCAG
>dme_circ_0007268_junction_seq
ACCTCGACGAGCACCGCCCGCGGTTCAGGACGCCGGCGAGTGGTTATCCGGGTGCG
>dme_circ_0007269
CTAGGTAGGTTCGCTTCAGTGTCTTCGAATTTTGCGTGTGTGTTAGAGTGTTTCGTGTGTAAGTGTGCTA
GTGTGTGTGTCTTGTCTTTTCCTTGATATGTCTGAGTTCATATTGCACGCAGATCGGTGACAATATTTAG
TCTCAAATACATATAAAAAGTATATGTGTGGGGCTCGGGAGTGGAAGGGACTAACTCAAAGTAGCATCGA
GTTGCTGCACGCAAACGAAAAAG
>dme_circ_0007269_junction_seq
ATCGAGTTGCTGCACGCAAACGAAAAAGCTAGGTAGGTTCGCTTCAGTGTCTTCGA
>dme_circ_0007270
ATTGTGAACGCCAAGATTGCCTGCCTAGACTTCTCGCTGCAGAAGACCAAGATGAAGATGGGTGTCCAGG
TCTTGATCAATGATCCCGACAAGCTGGAGGCTATTCGTGCCCGTGAGCTGGACATTACCAAGGAGCGCAT
CAACATGATCCTCGGAACCGGTGTCAATGTAGTACTGGTTAGCGGCGGCGTTGATGATTTGTGCATGAAG
TACTTCGTTGAGGCTGGAGCCATGGCTGTGCGTCGTGTGAAGAAGAGCGATCTGAAGATCATTGCTAAGG
CTACCGGCGCCGCCTTTATTACCTCGCTGACCAACATGGACGGTGAGGAGAGCTTCGATGCCTCAATGGT
GGGAGAGGCCGCAGAGGTGGCCCAGGAGCGCATCTGCGATGATGAACTTATCCTCATCAAGGGCACTAAG
GCACGGTAAGTGCAGATTATATCATGCAGTAACATAGTTTGCTTTATCGTATCCCTATGCCTTTTTAGTG
CTGCCGCATCCATTATCTTGCGTGGTCCCAATGACTTCTACTGTGATGAGATGGAGCGCTCCGTCCACGA
CGCTCTGTGCGTGGTGAAGCGTGTGCTGGAGAGCAAGAAGG
>dme_circ_0007270_junction_seq
GTGAAGCGTGTGCTGGAGAGCAAGAAGGATTGTGAACGCCAAGATTGCCTGCCTAG
>dme_circ_0007271
GTACTTCATGCACAAATCATCAACGCCGCCGCTAACCAGTACTACATTGACACCGGTTCCGAGGATCATG
TTGATGCGCTCCTTGGTAATGTCCAGCTCACGGGCACGAATAGCCTCCAGCTTGTCGGGATCATTGATCA
AGACCTGGACACCCATCTTCATCTTGGTCTTCTGCAGCGAGAAGTCTAGGCAGGCAATCTTGGCGTTCAC
AATCTTCTTGGGCATCTGCTGGGAGGCAATTGTGCAGTTCAAGGCATAGCCGGGAATCAGCACCGATTCC
CTGGCAGACTTGCCATGGGCCTTCAGGACATTGATGGCTTTAATCGAATAGGCTGCCTGCCCACGGGGAT
CTGTGATTTTCACCGACTGGGCAGCGTCCACAACCATGGCTGAGAAGAACTCTGCGTCAGCACCAATGAT
CTTGGAGCTCATCGAGGTCTTGGCAATGTTAATCAGGGAATCGCGGCCCAGCTCATCAACGGGAGCGGTC
AGATGCTCCGAGATGTACTTGCAGGCCTCCTTGCAGGCAATGCGGTAGCCGGAGATTATGGAGGTAGGAT
GAATCTTCTGCTTGACCAGCTCGTCGGCGTTTTTCAGCAGCTCAGCGGCCAGGATAACCACCGAGGTAGT
GCCATCGCCGACCTCCTCGTCCTGCAGCTGAGCCAGCTCAACCAGGACCCTTAAAATTTAGAAATGAGTA
CTTCTTTGAAAGATTTCCCACATCAACAACTCACTTGGCTGCCGGGTGCTCCACCTCCAGCAGTCGCAGA
ATGGTGGCTCCATCGTTGGTCACTGTGACATCGCCGATGTCGTCCACCAGCATCTTGTCCAGACCCACGG
GTCCCAAGGAGCTTTTCACGATGTTCGATATGGACAGAGCGGCCATAACTGAAAGCATATAAAAGCCGAG
TTGTAGTCATAACCTCACTTCGCATAGGCTTGTCCACAGGATCTTGTAGCTCCTTGGATTCAATGAGTTT

TTTTGTTAATCCAATTAAAATAAGATCGCCCTCCCCCGGGTTGGTTCCGGAACATTCTTCGTGGAGAGCG
TGACCCGCGATGAGTAAGAAACGTGCTCCGCCGGCAGACGCACCGCAGATGTTGACAAAACCCCGGGATT
CTCACCGTTCTGTGTGCGCACCGATGCGCCGGATTGGCGGGTGCCAGCAATGGACAGAGGAGAGGCCAGG
GTCGACATTTTCCTAA
>dme_circ_0007271_junction_seq
GGAGAGGCCAGGGTCGACATTTTCCTAAGTACTTCATGCACAAATCATCAACGCCG
>dme_circ_0007272
CCACCATTCTGCGACTGCTGGAGGTGGAGCACCCGGCAGCCAAGTGAGTTGTTGATGTGGGAAATCTTTC
AAAGAAGTACTCATTTCTAAATTTTAAGGGTCCTGGTTGAGCTGGCTCAGCTGCAGGACGAGGAGGTCGG
CGATGGCACTACCTCGGTGGTTATCCTGGCCGCTGAGCTGCTGAAAAACGCCGACGAGCTGGTCAAGCAG
AAGATTCATCCTACCTCCATAATCTCCGGCTACCGCATTGCCTGCAAGGAGGCCTGCAAGTACATCTCGG
AGCATCTGACCGCTCCCGTTGATGAGCTGGGCCGCGATTCCCTGATTAACATTGCCAAGACCTCGATGAG
CTCCAAGATCATTGGTGCTGACGCAGAGTTCTTCTCAGCCATGGTTGTGGACGCTGCCCAGTCGGTGAAA
ATCACAGATCCCCGTGGGCAGGCAGCCTATTCGATTAAAGCCATCAATGTCCTGAAGGCCCATGGCAAGT
CTGCCAGGGAATCGGTGCTGATTCCCGGCTATGCCTTGAACTGCACAATTGCCTCCCAGCAGATGCCCAA
GAAGATTGTGAACGCCAAGATTGCCTGCCTAGACTTCTCGCTGCAGAAGACCAAGATGAAGATGGGTGTC
CAGGTCTTGATCAATGATCCCGACAAGCTGGAGGCTATTCGTGCCCGTGAGCTGGACATTACCAAGGAGC
GCATCAACATGATCCTCGGAACCGGTGTCAATGTAGTACTGGTTAGCGGCGGCGTTGATGATTTGTGCAT
GAAG
>dme_circ_0007272_junction_seq
CGGCGGCGTTGATGATTTGTGCATGAAGCCACCATTCTGCGACTGCTGGAGGTGGA
>dme_circ_0007273
CAGAAGATTCATCCTACCTCCATAATCTCCGGCTACCGCATTGCCTGCAAGGAGGCCTGCAAGTACATCT
CGGAGCATCTGACCGCTCCCGTTGATGAGCTGGGCCGCGATTCCCTGATTAACATTGCCAAGACCTCGAT
GAGCTCCAAGATCATTGGTGCTGACGCAGAGTTCTTCTCAGCCATGGTTGTGGACGCTGCCCAGTCGGTG
AAAATCACAGATCCCCGTGGGCAGGCAGCCTATTCGATTAAAGCCATCAATGTCCTGAAGGCCCATGGCA
AGTCTGCCAGGGAATCGGTGCTGATTCCCGGCTATGCCTTGAACTGCACAATTGCCTCCCAGCAGATGCC
CAAGAAGATTGTGAACGCCAAGATTGCCTGCCTAGACTTCTCGCTGCAGAAGACCAAGATGAAGATGGGT
GTCCAGGTCTTGATCAATGATCCCGACAAGCTGGAGGCTATTCGTGCCCGTGAGCTGGACATTACCAAGG
AGCGCATCAACATGATCCTCGGAACCGGTGTCAATG
>dme_circ_0007273_junction_seq
AACATGATCCTCGGAACCGGTGTCAATGCAGAAGATTCATCCTACCTCCATAATCT
>dme_circ_0007274
GGTCTTGGCAATGTTAATCAGGGAATCGCGGCCCAGCTCATCAACGGGAGCGGTCAGATGCTCCGAGATG
TACTTGCAGGCCTCCTTGCAGGCAATGCGGTAGCCGGAGATTATGGAGGTAGGATGAATCTTCTGCTTGA
CCAGCTCGTCGGC
>dme_circ_0007274_junction_seq
GAATCTTCTGCTTGACCAGCTCGTCGGCGGTCTTGGCAATGTTAATCAGGGAATCG
>dme_circ_0007275
GCATCATTACCAACTACTTTGTGGTCTCTCTGGCCGTCGCCGACATGCTGGTGGCCCTCTGTGCGATGAC
ATTTAATGCTTCCGTCATGATCTCGGGCAAGTGGATGTTTGGTTCCGTGATGTGCGACATGTGGAACAGC
TTCGACGTCTACTTCTCCACCGCCAGCATCATGCACCTCTGTTGCATATCGGTCGACAGGGTGAGTCCCG
GGTTTACTTTGTCATTGTGGAATAATTGAAGTTCCGAGTGCTACTGGGATGTCTTTTATACCTTCCTTTA
AATGCCCATCAAATTGAATAGTTGCTTGACAGTTTCTAGTGACAATCTTTTTTCCCCTTTACCCGTAGGA

TACTACGCCATTGTGCAGCCACTGGACTATCCACTAATCATGACACAGCGACGCGTGTTCATCATGCTAT
TGATGGTGTGGCTATCGCCGGCGCTCCTCTCGTTCCTGCCCATCTGCTCGGGATGGTACACAACGACCGA
GAACTACAAGTATCTCAAATCGAATCCGCAT
>dme_circ_0007275_junction_seq
CTACAAGTATCTCAAATCGAATCCGCATGCATCATTACCAACTACTTTGTGGTCTC
>dme_circ_0007276
GCTGGAATCACACCCGACAGGCCAAAGCTCATAAATACGCCAGCGCGCAAGGGACGAAGTGCCGGCTCCG
AAAACTTGTCCCATAGCGAGACCACGATGGAGAGGATGCCCAGAATGCTAACAACGGATAGGTATATCAC
CTTTGGCTGGTAATGACA
>dme_circ_0007276_junction_seq
GGTATATCACCTTTGGCTGGTAATGACAGCTGGAATCACACCCGACAGGCCAAAGC
>dme_circ_0007277
AAAAACATTAACACATACTCAGTCACACAAACATGGCCACATTCACGCACTTAGGGTCCTAGCTACTTTT
TGGATTTGAATCATCATCCGCCTGCCCGGCATATAAACATTATACAAGTTATTCTCCGCAGACTACAGAC
TACTGATAGTTGGTAATGGTAAAGG
>dme_circ_0007277_junction_seq
GACTACTGATAGTTGGTAATGGTAAAGGAAAAACATTAACACATACTCAGTCACAC
>dme_circ_0007278
GCACCCAATGTTCACCTTTGGCATGGTGGCCGTCTACTTATCCTGGGTCCTGGTCATTGGACCATTGTTC
ATGCGCGATCGCAAGCCTTTCCAGCTGCGCCGCACCCTGGTCGTCTACAATGCCTTCCAGGTGGCCCTCA
GTGGGTATATGTTCTACGAG
>dme_circ_0007278_junction_seq
GGCCCTCAGTGGGTATATGTTCTACGAGGCACCCAATGTTCACCTTTGGCATGGTG
>dme_circ_0007279
AAGAACACAGTGCTCAACGAGCAGTCACTGTGCGTTGGACTGGCCCGTGTTGGCCAGGAGTCGCAGCAGA
TTGCCGTTGGCACGCTGCTTGAGATGCGCTCGACCGATTTGGGCGGACCGCTGCACTCGCTCATCATTCC
CGCCAAGGAGATGCATCCGCTGGAGG
>dme_circ_0007279_junction_seq
CCCGCCAAGGAGATGCATCCGCTGGAGGAAGAACACAGTGCTCAACGAGCAGTCAC
>dme_circ_0007280
GGTTGAGGCCGGGGATGTCATCCTCAAAGTCAACGGAACGGATGTCCATCGCTATACAACGAAGGAAGGT
GAGTGCCTGCTGGGAATAATGGAATGCTGTCGGAGAAAGCTGTGAATAATTAACTGAAACATATTTTTGA
ACAGTTCTCAAATGCCTGCGCCTGTCGGAACAGCTGGTGACCTTGGAGCTGAAGCGAGGTAGGTGGAAAT
GTATATCATTCCTCGAGGATGcaaaccggtcttgaaccaaagcgaaccgaaccaaactgaactgaaccaa
actgCTTTTCTAATTACCATGATGCAATGCTAAGCGTCTTGGGTTTTTTTCAAGGTTTCTCAAATGATTA
TTGATGTGTACAGTACAACCATACATACATATATAAATTTTAAATCGGTTTGCTTCTCGAAGAATAAACT
TGAAGAATGGAAAACTAACTAACCTTTTATCCTTAGGATCCCAAGCTCAAGGCGCGTATCAAAGAGCAGC
TGGCCAACACCCAGAGTCCGCACTATGTGGACATTGAGTCGCCGAACATCTACGATTACCACAGCAGCAG
CACCAACTCCTCGCCGAATCACCGGCCGAACGCTGGTGGCAAGGGGGCGGCGACCACGCCCTCGCAGACC
GGACTGCGCTACAAGTCACCGACGCACTTGCCCTCGCTGCGCCAGAATTCGTCGCCACTGCTGGCGAGTG
GATCCACCACGACCACAACCACCGCCACGCATACGCACAGCCACAGTCGAAACTCCTCGGCCAGCTCCAC
CAAGATTAAGGTGGTGGAGACAAGCATCACCACCTCGACCACGAACGTAGTGGGCCTCACATCGCCCACC
GGTAGTGTTGGGGGTGGTGTTGGTGGGGAGGCCACTTCGCCCACCTTCCGCCCCTCACGCATTCCACAGG
CGCTCACCAAATGTGCCGTACCCAAGCCCGTGCCCGTTCTCCATTCGCCGCAGAATAAGCGGCCACGCCC

ATCGCAGATTCCGACAAAGGCGGCGAACGGAAACGGGAACGGACATACCGCCCATCTGCCACCGCAATCG
TTGCAGCATTCCAACAGCTACAGCGGCAGTCCGGTGACTCGGCAGCGGTTTGCCGATAGGGAGCCGGAGC
GGGAACCGGAACCGAATTCGGCGCCACCGCAGCCGGCGAAGGCGCCACGCTTTGAGGCTTACATGATGAC
CGGTGACCTTATTCTCAATCTGTCCCGGACACCGCAGACCAGCAATCCGCTTCCCGCCCAGGCCAAAAAG
GATTGACAGTCTTCGCGATTCACCCAGTCGTTTGGTAAATCCGCGTATCAACGGCGCACTAGCACCGCGT
GCCTCTGGTGAATCCTCGCCCACGTCCTCCTCTTCGGTAGACTCGCCCACCAACACCAGCTCAGATTCCG
Tgaagcgcgaggcaaagctgctgcagaaacaacagcagcagcagcaacaaacctaccaacaacaacagca
gcgggacagcatcaacaacagctacaATCGTAAGGATTCGCTGACCAACGATACGCTGCTCATGTGCGAG
GAGTTGGAGCCGGACGAGGAGGGCGAGTATGTCCTGGAAGAGGACAACAAACAGCAGCGGCAGCGCCAAC
AGCAACAACGATACCGCCAGCAGCAAAATCAGCAACGCTACGAATACTACCAAAACGAggacgagctgga
ggagcaggaggaggttgaggaggagcgcgaggaggaTCAAACACACTACGACATCACCAATATTGAAACC
TACCAGAGCGGTGTGGGCCGGGGTgacgacgatgacagtgatcggcagtgtctggtagacgacgacgatg
acgatgatgcctacgatgatgaggagaacgatgcgggcgacgaggactacTCCACCAACTCGCTGGGCTC
CGGTTCAGCCAAGCAACGATTGCGGGCGTTGAAACAGCGCACTGCCACCCGCCAGCAGCAGCGAAATCGG
GATGCCGTCGACTGTGCAGGACGCTCCGGATCGGGATCATCATCTACCACGGTCAAGAGCGAGGCTGGCG
GCCTGGGCCTAGATGAAACCTCCTTCTCAGTGCCAACCTCTCCGATCTCGCTGTCGACGCCGCTGATCGA
CAAGGAGACGGCCAACTCGGTGCCCACGAGCCCGGAGCCCAGTTCGCTTGTTCCGGAGTCgagcagtggc
gctggcgccggagccgtggtggtgcgccggCATAACGGACATGTGGTGCGGAAGTGTGATGCCGCCGGTT
TCCGCACCAGCAAGTCTGAGGACCATTTGCAGCAGATCCAGCGCGAGGGCATCGCCGCTGTGATACCCAT
CGACATTGATGAGGATGTGAATAGTTCGCTGAACACGCTGCTGGACACGCGTCAGGACTCCGAGGACTCG
CAG
>dme_circ_0007280_junction_seq
CACGCGTCAGGACTCCGAGGACTCGCAGGGTTGAGGCCGGGGATGTCATCCTCAAA
>dme_circ_0007281
GGCATCGGATCGCGATCGGATCGTGTGGACTTATAATGCGCCACTCCAGCCGCATCAGTTGGCGGCTCTt
cagcgacagcagcaacagcaagagcagcaattccaacagcagcagcagcagcttcaccagcaacatctgc
agcaacagcagcagcttcagcagcaacatcagcagcagcaacaacagcagcagcagcaacagcagcagca
acTCTACGGTCAGCAATCGCATTCAAATTCACATTCAAGTTCCATTAGTTCGTCGCCCCAGCACTCGGCT
GTGGGCAGTCCGGCCTCGCCCACGTCGGTTTCCTCGTCGGTGATGTCCTCGTCGGGCTCCAAGGGCGCCC
TGGGCCTGGGCAGCAGCAGCAATGGTCCCATGGCCGCCGTGCAGCAACAACAGCTGAGAGAGAGGGAGCA
GGGCGGCCAGGTCGCGCAACCGCCCAGCGGGATTCCTGGCTTGCTTAGCTGCCCAggtggtgggtgcgga
aacaatggtggtggtggaggcattggtggtggtggCAACAACGATCAGAGCGTCTCAGAGGCCATTTCGA
ATATATCTAGTCCCGACTACCAAGACGACGATAATTTATTGAGTTCTCGCGATATTCTAGGCGGCATGGT
ACTTAGCGATCCCAGTGATTCGGACTCCACCATCCTCGTCTCGGACGCGGCCGCCCATCAGCGCCAGCAG
CTCAAGCAGCAGTTGCGCGCCCAGCAGCAACAGCAGAGAGAAAGGGAACGGGATAGGGATCGAGACAGGG
AACAGTCCGAGCACAAAGTGGTCATACAAGTGCGCGGTCTGGACAGTAACAGCAGCGGCGGCAACGGAAC
TAACGGCCGCTCCGAGGAGGATGTGGTCACGCTGACGGACGAGCCGCTGGGCACGATGACCGTTGGCATG
CGGGACGCCTCGCCGCCAGTCTCTGACGATGGCAGCGATGTGGAGTCCCTGCACTCGTATCACTACTCGC
CCAAGGCCGTGGACATGCCCTCGGCCATACGCCTGGCCAAAAGACTGCACTCCCTCGACGGTTTCAAGAA
GAGCGATGTGTCGCGACACCTCAGCAAGAAGCAATGACTTTAGTCGAGCGGTGGCCGATGAGTATCTCAA
ACATTTTACCTTTGAGAAGAAGTCACTTGACCAAGCGCTGCGTGAGTTCTTGCAGCAATTCTCGCTGTCC
GGCGAAACGCAGGAACGGGAACGAGTGCTGGTGCACTTTTCCAAGCGCTTCCTCGACTGCAATCCTGGCA
CCTTTAACTCGCAGGACGCCGTGCACACGCTGACCTGTGCCATAATGTTGCTGAATACGGACTTGCACGG
CCAGAACATAAATCGTAAGATGAGCTGTGCGGAATTCGTCGACAACCTGGCAGATCTCAACGATGGCGAG

AACTTTCCCAAGGATGTGCTCAAGTCGCTTTACCAGGCCATTAAGACCAAGCCGCTTGAGTGGGCACTGA
GATGAAGAGGCAGGTGATCTGCAGCAGCAAAGAGCCAACAATAGTGCCCTGGGCAATGTGGGTCTCAATC
CCTTCCTCGATCCCCCAGAACTGGCCACAGCTGTGGAATACAAAAAAGGCTATGTGATGCGTAAATGCTG
CTATGACAGCAGCTTTAAGAAGAGATGTCCGACAATCTGCACAATGCAATACGCATACATCACGCACTGG
CCACCAAGGCCAACGACTACACCAAGAAGCAACATGTGTTCCGGCTGCAGACGGCCGACCAGGCCGAGTA
TCTGTTCCAGACCAGCGACTCCAAGGAGCTGCAGTCGTGGGTGGAGACGATAAATTACGTGTGCGCCGCT
ATATCAGCGCCTCCGCTCGAGGGAGGGGTGGGCAGTCAGAAACGATTCCAACGTCCGCTCCTGCCCAGCA
AACAATCCAAGCTGATGCTGGAAAGAGCAGTTGGACTCGCACGAGGTGCAGCTGGCGCAGTTGGATCAGG
AGCTTAACGAGCACAAGAAGGGTCCTATTCCCAGCAAGGGCTTGGCTCTGCAGAACTACAAGGAAAAGGA
GAGCTACTTGCAGTATGAAGCTTCGTCGCTACCGCACTTATGTGAGCATTTTGAGCGCCAAGATGCTGGC
TGATCAGCAGCAGCTGGAGCTGCAGGCGCAGCAGCCCTCTCCAGCGTCGCACGAGGAAGAGGCCGACACA
TTCCCAGTGGGCACCACCGCCTGCACGCCACCCACGCCGCAAAGTATTAACCAGAAGGATCAGCAGAAGG
AGCAGCAGCAGCAACAGCCAACGAACAG

>dme_circ_0007281_junction_seq

AGCAGCAGCAGCAACAGCCAACGAACAGGGCATCGGATCGCGATCGGATCGTGTGG

>dme_circ_0007282

GGCGGCATGGTACTTAGCGATCCCAGTGATTCGGACTCCACCATCCTCGTCTCGGACGCGGCCGCCCATC
AGCGCCAGCAGCTCAAGCAGCAGTTGCGCGCCCAGCAGCAACAGCAGAGAGAAAGGGAACGGGATAGGGA
TCGAGACAGGGAACAGTCCGAGCACAAAGTGGTCATACAAGTGCGCGGTCTGGACAGTAACAGCAGCGGC
GGCAACGGAACTAACGGCCGCTCCGAGGAGGATGTGGTCACGCTGACGGACGAGCCGCTGGGCACGATGA
CCGTTGGCATGCGGGACGCCTCGCCGCCAGTCTCTGACGATGGCAGCGATGTGGAGTCCCTGCACTCGTA
TCACTACTCGCCCAAGGCCGTGGACATGCCCTCGGCCATACGCCTGGCCAAAAGACTGCACTCCCTCGAC
GGTTTCAAGAAGAGCGATGTGTCGCGACACCTCAGCAAGAAGCAATGACTTTAGTCGAGCGGTGGCCGAT
GAGTATCTCAAACATTTTACCTTTGAGAAGAAGTCACTTGACCAAGCGCTGCGTGAGTTCTTGCAGCAAT
TCTCGCTGTCCGGCGAAACGCAGGAACGGGAACGAGTGCTGGTGCACTTTTCCAAGCGCTTCCTCGACTG
CAATCCTGGCACCTTTAACTCGCAGGACGCCGTGCACACGCTGACCTGTGCCATAATGTTGCTGAATACG
GACTTGCACGGCCAGAACATAAATCGTAAGATGAGCTGTGCGGAATTCGTCGACAACCTGGCAGATCTCA
ACGATGGCGAGAACTTTCCCAAGGATGTGCTCAAGTCGCTTTACCAGGCCATTAAGACCAAGCCGCTTGA
GTGGGCACTGAGATGAAGAGGCAGGTGATCTGCAGCAGCAAAGAGCCAACAATAGTGCCCTGGGCAATGT
GGGTCTCAATCCCTTCCTCGATCCCCCAGAACTGGCCACAGCTGTGGAATACAAAAAAGGCTATGTGATG
CGTAAATGCTGCTATGACAGCAGCTTTAAGAAGAGATGTCCGACAATCTGCACAATGCAATACGCATACA
TCACGCACTGGCCACCAAGGCCAACGACTACACCAAGAAGCAACATGTGTTCCGGCTGCAGACGGCCGAC
CAGGCCGAGTATCTGTTCCAGACCAGCGACTCCAAGGAGCTGCAGTCGTGGGTGGAGACGATAAATTACG
TGTGCGCCGCTATATCAGCGCCTCCGCTCGAGGGAGGGGTGGGCAGTCAGAAACGATTCCAACGTCCGCT
CCTGCCCAGCAAACAATCCAAGCTGATGCTGGAAAGAGCAGTTGGACTCGCACGAGGTGCAGCTGGCGCA
GTTGGATCAGGAGCTTAACGAGCACAAGAAGGGTCCTATTCCCAGCAAGGGCTTGGCTCTGCAGAACTAC
AAGGAAAAGGAGAGCTACTTGCAGTATGAAGCTTCGTCGCTACCGCACTTATGTGAGCATTTTGAGCGCC
AAGATGCTGGCTGATCAGCAGCAGCTGGAGCTGCAGGCGCAGCAGCCCTCTCCAGCGTCGCACGAGGAAG
AGGCCGACACATTCCCAGTGGGCACCACCGCCTGCACGCCACCCACGCCGCAAAGTATTAACCAGAAGGA
TCAGCAGAAGGAGCAGCAGCAGCAACAGCCAACGAACAG

>dme_circ_0007282_junction_seq

AGCAGCAGCAGCAACAGCCAACGAACAGGGCGGCATGGTACTTAGCGATCCCAGTG

>dme_circ_0007283

CTCTTCGTCGGAGAGCGAGAATCGATTCTTTGGTCTGGTCACTAGCGCCGTGCACAACACACAGATCGAG

GAGGAGTACGAGCCGAGCGCTGGCAGCGCAGCAGCTGCTGGTCACATCAGTATCTCGCACAGCTGCCATG
>dme_circ_0007283_junction_seq
CACATCAGTATCTCGCACAGCTGCCATGCTCTTCGTCGGAGAGCGAGAATCGATTC
>dme_circ_0007284
GAGCCCAGCAAAGTGCCGCCGCAgcggcgagtggtgccggcggtgcgggtggtgccggtggtggtggcgg
tggcTCCGGGTCACAATCGCGACCAGGCAGCCGGAAGCTGCCGCCGCCATTGTTACGGTCGCGCACACTG
CCCGCCATCATTGTGCCAGGACTTCCGGTCGTCTCGCTGCACACGGATAAGCAAACATTTCAGCTGGGGT
GCGTACTATACGTTTTTATTTCGAAGTGGGTTAATTTATAAAAAATAAAGCTTATTCTTTGCACTTTTCA
CACTGCAATTAATCAAAAATTCCATACGATAAGTTAATGTTGCATAAATAATAGTGACATAAGGTTGTTC
AATACTTGCGGAATAATTTATTTATTTTTTATTGAATACAATGCACACAAGTTGGCCATTTACATGCAGT
CTTAAAATTGTTTAAACAACAATAGTTAAATTGAAAGCTGAACAATGCTAGATTATTTTATATAATTCAC
TTCAGCACAAAGTATAAATCGGCTTAATTAAGATCGATTTTCGAGATTTCGAATTAGGTCAAGGGCTTGC
ACATAATTTTCAGCTGATTTGAGAGCAGCTTTTAAAGGCATGTAGGTATGCTATAAAATGCCAGCTCTCG
AAACCCTACACTTTCTGGTCATTGAATGCATGAGTGCATTTTCATGGCAACATGACCATGGCCAGATTCG
ATTAACTAATTGAGAAGAGTGAAGCCAATTTAAATACAATTTCCGCGCTGTAAAGTGTGCTACAGAAAAT
GCATACATACACAAACGACAAAAGCTGATCTAGACAGAAGAATTTTAATATATATCTTATTTCAGAAATA
AATACGATTAACTCAAAAGTCAAAGAAGTAGAGATTTTAATTTGAGTTCTTTCCAGAAATTGTATTTTCC
GGAAGACCTTAAATCCAACTGGTTGAGTGCAAAATAAAGCAGGATATTAGTGAAAACGTTTTGAAACGTT
TTTATCTGGCATTGGCAAAGTGCCACACTTGGCACGTTGCACCAATAGAATAAGTAATTAAAAACATTTG
ACAGGTTATCAGCACAAGTTGATATTTGTGCATATATAGATGTAATATCCACATATTTACCCTGATTTAT
TTTTATCCGATTTTCCAATTTCAATTCAGGACGAACGTTTGTTGGgcagcaaaagccgcagtggcagcgc
cagtggccatcgatggtcgctgctcacacgtgccaccaacagcagcagcatgtgcagcaacaacaacccg
gccagcagtagcagcagcagcagcaacaacaataacaATTTACACATGGCCAACAACAATAACACACTGA
TGGTGAAATCATTGAATTTGCCCAAAGACGATAGCAATTTGGTCTATCGCCGCAAATCCTCGGgcagcac
gccccaccaacatcagaatgcgcaccatcatcatcaacagcatcatcacctgcagcagcagcagcaacag
cagcagcagcatcagcagcagcaactgcaacagcagcagatgctgcagcaTCTGGGGGACAGCTCGCTGT
TTCAGCTGTCCGACGATTTCGAGTGCCACGCGGATGGTTCCCTGCTCCTCAGGAATTCCAGCTCCTCATG
TGGTCGCCGCACGAGCCTATCGACTGGCAGCTCGAAAGCGAGCTGCCGCCTCCTCGGAGGCCTCCACACC
GATGACCCGTGAGCACACGCTCCACGAGATGCCGCTGGCGGAGTCGGCCTGCTCCGGATCTGCCACTGGT
TCGGGCACGACCTTCACGCGTCTGGCCAAATTGCTGCATCAATCAAGCACGCTGGCGGGCAGGTCCAATC
TGCAGCCACCGCCCATGGCTGACAATGCGGTCCAGAGCCTGGGCAGAGGATTGGGCCTGGGACTGGGCGA
GGAGGCGCCACGTCGTCTGTCCTGGGAGAG
>dme_circ_0007284_junction_seq
AGGCGCCACGTCGTCTGTCCTGGGAGAGGAGCCCAGCAAAGTGCCGCCGCAgcggc
>dme_circ_0007285
GGAGCCGGTGGCCAGTACTTCAAAGGCCTCTTCGATGAAGAGCTTGTCGCGGCAGAGCAGCGAAGAGGAT
TCCGCCAGCTGTTCCAGTCTCAACGCAGAGCAGACATCGGGGATCATTCAAGTCTATACCGCCTACAGTA
CGGGTCTGGCCAGTGGAACCAGCTTAAAGATTCACGTAACTCCGAAGACGACTGCCCGCGAAGTGATCAA
CCTGGTG
>dme_circ_0007285_junction_seq
GACTGCCCGCGAAGTGATCAACCTGGTGGGAGCCGGTGGCCAGTACTTCAAAGGCC
>dme_circ_0007286
GTTCCTTCAGCTCTGCCACTCCTTCCTTTCCATTGCGCTGGCTCCAGTTTGTAGCCCTGCTGACGTTGGA
ATCCTCGGAACAGGGCGGTTTGGTTGTGGCCACCGGCGTTGCGGTGGCAACCGCATTCGGGTGGTAGGCC

AACACCACATTGTCCACGTGC
>dme_circ_0007286_junction_seq
GTAGGCCAACACCACATTGTCCACGTGCGTTCCTTCAGCTCTGCCACTCCTTCCTT
>dme_circ_0007287
GGATTCAACAAAGCGTTCAACAAATACTTGATGCCTCTCATCAAATCTTGGAGGCGTGCATCCATGGGTG
AAGCGGCCACCACTCGCTCGATGCTGGCCGTACCCAAATCCCGCGACCGGCACTCCACACACTCGATGGT
TTTCAGCTTCACCTGCACGTTGTCAGCCAAAAGCCGAGAATTGTGCAGGATTCCATAGAATA
>dme_circ_0007287_junction_seq
CGAGAATTGTGCAGGATTCCATAGAATAGGATTCAACAAAGCGTTCAACAAATACT
>dme_circ_0007288
GGTCAAGAAAGCGCCAGCGCGTAGACACAACCTCGTCCATTTCGTACGGATATTTGGCAAGAACATTTAC
GGCAAAGGAAACAATCACTAGCCAGTCAGGCAGTTGGTGGGAGGAGGCGTACATGGACACGCACATGGTG
ATGAAG
>dme_circ_0007288_junction_seq
GTACATGGACACGCACATGGTGATGAAGGGTCAAGAAAGCGCCAGCGCGTAGACAC
>dme_circ_0007289
GACTTTCTGGAGAGCGTGGAGCTCGGCGATTTGCCGCAGCAGAAACCGTTTCAGTATCTGTTGAAACTGA
CCTATGAGTATGTGGCCGACATGTTCAAACAGCCAGACGACATGGAACTCTAATCAAGCACCTCACATGT
CTTTCTATTAAA
>dme_circ_0007289_junction_seq
CAAGCACCTCACATGTCTTTCTATTAAAGACTTTCTGGAGAGCGTGGAGCTCGGCG
>dme_circ_0007290
AGAGAGAACAAGGAACTAAGCAACAACTACTAAATTAATGTGTAGTTACGAGAAGCGCAAGCAATTTTAA
GTTTAATTTTGTaaaaacaaaacaacaaaacaaaaaaGGCGCTTCGATGTCGAAAATGTTGATTCACTTC
AAGTTCACCGAAAGTTTACAAACGAAATGTGTGCTTCTACATCCCTTGGACCCTTTTGACCCTTTGGaca
caacacaccaaacataacacacccaacataacacaagcaacatacaaaccaaccaaCAAACAGAACAAAC
TCTTGGATCAGAAGCTATAGCTACAGGATATGGGATACACTTGTTAATTGGCGTGTATACCTACATGTTT
CAGATGAAATTTAGAACGTGTTTTAAAGATTTTAAGCAGGCAACATGCAGCAACTACTACTATAAATGTA
ACAATTCAATGTGATAGCAAAACAAAACCAGATGTTGATGTGTACAGAGGAGAGAATAAGTATAGATGAT
AAATAAGAGATCAAGCAGAATGTGTGTGTTGAATATTTACAG
>dme_circ_0007290_junction_seq
GCAGAATGTGTGTGTTGAATATTTACAGAGAGAGAACAAGGAACTAAGCAACAACT
>dme_circ_0007291
GTTGCCGCCAGCGATGTGGTGTTATCGCTACTCATCCCCAGGCTATGATGGCTGTGCGAATCCTCGCAGC
CGGGCACGGTCAAGTGGTCCTGAGTGACTCCGCCAGGTCCACTTTCACCCCCGCGCAGAGCGCTTCGCTT
CTGCTTGCCTACTGAGTAGAGGGGATTATCGTTGTACTCT
>dme_circ_0007291_junction_seq
TGAGTAGAGGGGATTATCGTTGTACTCTGTTGCCGCCAGCGATGTGGTGTTATCGC
>dme_circ_0007292
GACGGAACCCTGCGATTGCCCAAAGGCATAACAGGACTTGGCCTCGAAAATGGGCGGCGTCGCAGCTGTC
GTTCCCAGGGCCAGCGACGTGGGAGTGGGCGTTCGCGACGACGGCGTAGCAAACAGACTGCTGCCCTGCA
CCACCTCCTCCTGGATGCGTTGCTCAGCGAGTGCACACAGCAGCTTGAGACCTCCAAGTGGTTCGCGTTC
TGAA
>dme_circ_0007292_junction_seq

GAGACCTCCAAGTGGTTCGCGTTCTGAAGACGGAACCCTGCGATTGCCCAAAGGCA
>dme_circ_0007293
GGTGTTCCTTCGCTCGCATACCGTTCCGCCGCATCGAAGATGTCCATCTCCTCGCCACTGGGCACGTGCA
GTGTGCTGGGTCCCGTCTTGGATGCCAGTTTATTGACCAGACGGATGTTGGCAAAG
>dme_circ_0007293_junction_seq
TTTATTGACCAGACGGATGTTGGCAAAGGGTGTTCCTTCGCTCGCATACCGTTCCG
>dme_circ_0007294
GATAGTAGGTAACCACGCCAGATCCTGGTGACAATGAGGTCTTGATATACGGCAAAATACTCAAGCCCTT
CTGCACGGCATTCTTGGCCAGCAGACCAGCGCCCAACATCACCGAGGGATTCGAAGTGTTCGTGCACGAG
GTGATGGCGGCAATCACAACAGATCCATGACCTATCTTATAGCTCTTCCCATCGTCCCATTGGAATTCAC
CACTTGCAGCCAATGCACTCG
>dme_circ_0007294_junction_seq
AATTCACCACTTGCAGCCAATGCACTCGGATAGTAGGTAACCACGCCAGATCCTGG
>dme_circ_0007295
GGTGAATCGTTCCTTGTTGCGCTGGAACTCTAGTGACTCGTTCTTGGTCAATGCGTCCGAGGAGCGAACG
AAATCCACCTGAACGGAATGGTCGATGACCAGGTCGGCAGGGCAGATGGGATTGATTTTCTCAGGATTGC
CGCCCAGCTCGCGGACAGCATCCCGCATGGCAGCAAAATCCACGACGGCGGGAACGCCA
>dme_circ_0007295_junction_seq
AGCAAAATCCACGACGGCGGGAACGCCAGGTGAATCGTTCCTTGTTGCGCTGGAAC
>dme_circ_0007296
GATGTTGAACGGGCTGCTTCCGCCAGCAATTTGAAATTGAAAACGCATTGGATAAACCTTAGCGCGCAAA
CCAAAAGCAATAAAACGCCAATTGAAGCATTTATATTAGATTGTTAAAGCCAACCGGCGTAGCATTTACT
TGCCTGCCCCaaaaaagtaaaaaaaaacaaaaagaaaagaaaagaaaCCCTTCCAACATCCCACTAAGCA
AAACTAACCAAGAAACGCATTGAAACGCATTCCAATTAGTAGCTGCGGGCAGCACATTGCCTGCATATAC
ATAGTCGATAAGGGTGATTGCATTTATCTGCGGCGTCATCGTGGTGGCCCTCATGATTATGGCTCTGGCG
TCCACAGATTGGTTAATGGCCTCAGACTGGCGGCAAGGTCTCTTTGTGCACTGCATCGAGGATGATTCGG
TGCCGCCGCTGCCATTCAATATCCAGGATCCGCCAGGATGCTACTGGACCCGCGATGTCGGGCTACATTA
AGGCCACAGCCGCTCTGTGCATCATCACGCTGATAACGGACGTCATTGCCACAGTACTGACAGGTCTGGG
CCTCCGGACGCAGAATCATAATCTAAAGTATAAGTTCTATCGTATAGCAGTATTGGTGATGCTTGTATCC
TGCCAATCGACGAGTCTGGGAATTTGGCTGGGCCTATGGCGTCGGCTGGGGTGCGGCCATCTTTCTATTT
GGGGCCGTTGTCCTGCTGCTCTGCGACAAGGAGTCGGAGGAGATCTACTATAAGGAAAGGAAAATCGTAC
ATGAAAACCAAATGCGCGCCTAGGCAAGGCCGCACGACCTGCCTGACGTCGTTCTGTAGAATAA
>dme_circ_0007296_junction_seq
ACCTGCCTGACGTCGTTCTGTAGAATAAGATGTTGAACGGGCTGCTTCCGCCAGCA
>dme_circ_0007297
CATTCTCCAGGCGCATTAACGGATTTGCGGTCAGCACTGGTGAATAATACAATATTCGCCTCCCTGGCTG
TTCGCCATGGCTTCCACAAGTTCTTCCGGCACCTCTCGCCGGGCCTTAACGATGTGATTGACCGTTTTGT
GCGGATCCAGCAGGAGAATGGACACTGCATCAGTGAGGAGGTG
>dme_circ_0007297_junction_seq
GAATGGACACTGCATCAGTGAGGAGGTGCATTCTCCAGGCGCATTAACGGATTTGC
>dme_circ_0007298
GTTAGAATCACGCGTTCCTTAGCTAACTCCAGGGAAACCTTCACCTCACCGGAACGCGTGAATATCGAGA
AAGCACTCAGCTTGGGTATCCGTTTGGTGGTTAGAATGCCGAATCCCTGCTGCGCATCTTCGGGCGGATA
AATCTTGCGTCCCCGCGTGTTTTGCTCTTCGGGAATCGGACATTGGAGCGTCAGTTGGATAAAGTACAAA

TAGCATGGCGCTCCGGCAACGGGACGGCAATCGCAAAATTCGGATGCAATGCGTTTGTAATAGTACTGAC
GACGCTTCGTTGTTCCCGGACGTGGTTCATCGCTTAGCTGCACAATCTGTTCGTCCTCTGGCTCCAGTTC
AAAGCACTCCCAGTCCGGCTCCAGGGCACGAAATCCCTCCTTGCCGATAGGCTGCAACTGATCGTCTAAC
TCACCGATCCTGTGCAGTTCCACGCAAGCCTGCAAGGCAGCCAGTCGGCGGGCCAATGTTTGAGTTGGCA
TCGGAAGACCCTAAAGAAGAGTATTTAACAAATCTTAAATAATTCGTATTACAAGTACTTACCACAATGT
CATGCTTCAATGGCGAGTTGATGGGCAGACGGAGTGTGTACTGAAACAGGGTCACTCCAGCCCTTTCGTT
TCGGGTGCAGCGCCACAACGCCGTCAACTTGGTGAACGTGTCGCTTGGCAGTCGGGCGCAGTACTTGTTG
ACCAAAGCTATAGCAGAACCCAGATCCACGGAGGCGCCTGTTAGCAGGTGCGGCTTGGGTCGGTATGCGG
CCAGGCAGGCACTAAAACGTTCCGCCTCACTCTGCTCCTGCTCCGGCGGCTCTGTGTTGGCGCACTTGGA
TAGCAGCATCTGCTCGATTTCGCGATACTGTGCCATTTGATGCACCAAGTCCTTCGTGGTGCTGGCTAAA
CTATGAGCAGTATCGATTTCGGCTGTA
>dme_circ_0007298_junction_seq
ACTATGAGCAGTATCGATTTCGGCTGTAGTTAGAATCACGCGTTCCTTAGCTAACT
>dme_circ_0007299
GTGCACACCCAAGCGGACGGACTGCGCAGAATGCGGCATCAGGTGGATCAGGCGGACTTCAATCGGTTAT
CTCATACGCTGGAAAGCAAGTGCCGAATGGTGGATCAAATGGACCAACCGCCGACGGAGACACGAGCCCT
GGTGGCCACTCTTGAGCAGATTCTGCACACGACAGAGGACAGGCAGACGAACAGAAGCGCCGCTCGGGTG
ACTCCTACTCCTACTCCCGCTCATGCGAAGCCGAAACCTAGCTCTGGTGCCAACACTGCACAACCACGAA
CTCGTAGACGTGTGTACACCAGGCGCCACCACCGGGATCACAATGATGGCAGCGACACGCTCTGCGCACT
GATTTACTGCAACCAGAACCACACGGCTCGCGTGCTCTTTGAGCTTCTAGCGGAGATTAGCAGACGTGAT
CCCGATCTCAAGTTCCTACGCTGCCAGTACACCACGGACCGGGTGGCAGATCCCACCACGGAGCCCAAAG
AGGCTGAGTTGGAGCACCGGCGGCAGGAAGAGG
>dme_circ_0007299_junction_seq
GAGTTGGAGCACCGGCGGCAGGAAGAGGGTGCACACCCAAGCGGACGGACTGCGCA
>dme_circ_0007300
GCCAGCCAATAGCCAAATTACAAACGAAAACGACGATTTGCAATTTGCAAATTATGCGTGCGGCACCTAG
TGCAATCGAAACCACGTAAACCCAACGAACGCGAGTGTTTAGACCACCTGCAACCTGAAACAACCACCA
TTGTACATAATTTACACGAACAATTTTGCAACGACAACAACAAAGGACGCCGCGCTGAAAGAGAGAAGTT
GACATAACTGCAAAAA
>dme_circ_0007300_junction_seq
AAGAGAGAAGTTGACATAACTGCAAAAAGCCAGCCAATAGCCAAATTACAAACGAA
>dme_circ_0007301
GAATCTTGCCTGCAGGAAAGTAATTGGCACTTGGATGCGATTCTTCTGGATCTCGTAGTGCGGCAGCTTG
TGTACTTCATCTTCGGCCACCGGTTCGGTCATCAACTTGGCCAAACCGCGTTGTGTCTTCCACTGGAGAA
CACTGACCAAGTTCGGATAAAAGCGATCCATGTTCAGAATAAGTCCGTGCATCTGGCGAATCAATTTGTT
>dme_circ_0007301_junction_seq
GTCCGTGCATCTGGCGAATCAATTTGTTGAATCTTGCCTGCAGGAAAGTAATTGGC
>dme_circ_0007302
GCGACTGTTTGTACCGCCCAAGCTCTTGCAATAGGCAGGCAGATGCGAGAGCAGGGAATGTATGTCCTCC
GCCCGAAAGCTCTCCATGTCCACCTCGGCCAGATTGGCGTCCAGATTCTTGGCCAGATCCTCATAGTAGA
TCAGATCCCCCATCGCATTGTACGATGCAGAGAGGGAACTCAAAACGCTGCGCTGCATTCCGCCGCAGCC
ATCTCGATAGCCGTAGTCCAGGAACCACTGGTAATCGGAATTGTCGTGAGTAAAGTCGTGCGACGAGGCC
AACGAGCAGTTGAGATTGTTGCCACCGCCATTAGTAG
>dme_circ_0007302_junction_seq

TTGAGATTGTTGCCACCGCCATTAGTAGGCGACTGTTTGTACCGCCCAAGCTCTTG
>dme_circ_0007303
GCTAACCCAGGTGCCCCGGCGGGCAGAAGCGGGTTGCGGAACCAGTACGCGTCCGTTGTCGTGTGCCTCC
AGCTCCGAAATGATCACGCGCTCCATGCAGACCTCCTGCGGATCACTGAGCACCACCAGGAGCAGTCTGA
GCGATCGCTATCGCTGTGTGCCGCCATCGTTCCTCGAGAAACTCAATAACTTGGGTGAGGAGCGACAGGC
GCCCATTTAC
>dme_circ_0007303_junction_seq
GGGTGAGGAGCGACAGGCGCCCATTTACGCTAACCCAGGTGCCCCGGCGGGCAGAA
>dme_circ_0007304
ATTACGTAAATGGGCGCCTGTCGCTCCTCACCCAAGTTATTGAGTTTCTCGAGGAACGATGGCGGCACAC
AGCGATAGCGATCGCTCAGACTGCTCCTGGTGGTGCTCAGTGATCCGCAGGAGGTCTGCATGGAGCGCGT
GATCATTTCGGAGCTGGAGGCACACGACAACGGACGCGTACTGGTTCCGCAACCCGCTTCTGCCCGCCGG
GGCACCTGGG
>dme_circ_0007304_junction_seq
CCCGCTTCTGCCCGCCGGGGCACCTGGGATTACGTAAATGGGCGCCTGTCGCTCCT
>dme_circ_0007305
GTGATATTTAGATGGGAGATGATTCGATCAGGGATCACGGAGCGAGAACTGATAGCGAGATGTGCTGGTA
TCTTTGATTGATTTAGTTGTCCTTATTTGCGGCAGAGCAGCGACCGACTGCGAGAGGAAGTAGCTAAATT
TTCTTTTTTTACAAA
>dme_circ_0007305_junction_seq
AAGTAGCTAAATTTTCTTTTTTTACAAAGTGATATTTAGATGGGAGATGATTCGAT
>dme_circ_0007306
GGCAATTTCGTCCTTCTGTGGCGACGGGGAACAAATGTGCTCACTGCCTCCAACATTATGGTTACAAGGG
ATGAGCGCGTAAGACTGATAGATGGTTATAATTTAGAGATATCCGATTTGGAGCCTCAAGACGCCGGGGA
TTATGTTTGTCAAATCAGTGACAAAATAAATCGTGATCAAGTGCACACAGTTGAGATATTGGGTGAGTAC
ACGTCGACATGTTTGCACAGCATTAGCTCAAAGATTTGCATTTGACAAGTGCTTCTTAAGGCGCACACAT
CAATTAGCCACTGATTTACTGAACTGTTACCAACGTCCACCCCTTTCTGCCTGCTTTCCACCCGCAGTTC
CGCCCAGTGTACGGGCCATCCCAACATCCGGACAACTGCAGGCCCGCAAAGGAGGACCCATTACGTTGGA
GTGCAAGGGATCTGGTAATCCGGTGCCATCCATTTACTGGACCAAGAAG
>dme_circ_0007306_junction_seq
GGTGCCATCCATTTACTGGACCAAGAAGGGCAATTTCGTCCTTCTGTGGCGACGGG
>dme_circ_0007307
CAACTGCAAGCCGTGTCCTGTGGCCAAGCCGACGTTCCTGTGCGGAGCCGACAACAGGACCTACTCATCA
CTATGCAGATTGGATTATCACAATTGCATACATTCGACGTCCATACGGATCGCCTGCAAAGGATTCTGTC
CCTGCAAGGGTGAGTCTATCCCATATCCTCCATTCCACGACCATAACAAACGGACTAAGACAAATAGTAC
GGCCTTCCTGGGAGTGGAATCAAATGCTCCGCATAATGAGCTCCAGCCTGGCAGTCGGCCAGAGTCGCAG
GACGACGAAAAACGAAGGACCTCCCACGTCCCTTGACTACAAAATAAATTGCATGCATATTTTGTGACAG
CCTTACTCGTCGCCAGTTGTTTGCCCATGAAAGTATGCTGCAAAAACTGAGCCAACAGCTAAATGCGATT
TGTTCGAGAGGATTTAGCATTCTTCCCTCCTCCTCCTCATCCTCTTGGCAATTAAAAATTCCAATTACCA
AACAATGGTTTTTGGCTTTAAATATATTTCTGCCCCGGCACATCACTTGTTAAATATTTAATAGGCCGGC
TAACAAGTAGCCGACGCCATTGTTCCATCGCCCATTGTACAAAAGGGGGCCCGCAAAACCACTCAGTCAG
TCAGCCAGCCACGTTAATTTTCAATTTTCAGTTGGCCAGAAAATAAAGCAAAAGTATGCCTTGTTTGGGG
CTGCAAAAAGTTTGTCTGCTTCAACGATTTTCTGACCGCCATTTTGCCAACGCTTTTCGCAGAGATTGTC
GATGGAAagcggctgcagcgcatctcgggctacaacaacaagtacaacaagaagatcagcctggaccagc

agcagcagcagcagcaacaacaacaacagcagcagcagcagcagcaggcctacaaggacagcaacaacaa
caacatcatgatgaacagcggcaacataatgggcggcaacaacaacgacttcaacacgatcatgaatgta
agttaatgtctaacatacacatactacatacatacatacagacatGCAACAAAGTTAAATTTTTGGTATT
GGTATTGCAGGACAAAGAGGACAATAACCGGCACAACAACCACATCAATGCCCAGTACACTTTCACGCCC
GAGGAGATCAAATATGACAACAAGCACTACAAGTACCTCAAGTACACGGCCTACAAGAAGGTGAGTGGGT
TAACCAGAATATCCAGTGGCCTCTTCTCACATATCAAATTATTTGGCAATTGGCAGGACTCCAAGTACCA
GGAGGACAAGCACAAGATGCGCAACTATAACGAGGTGGTCGAGAAGCAGCAGCAGAAGTTCAATAAGAAC
AGCAACTTGAATGG
>dme_circ_0007307_junction_seq
AGTTCAATAAGAACAGCAACTTGAATGGCAACTGCAAGCCGTGTCCTGTGGCCAAG
>dme_circ_0007308
ATAAACAAAAACACGCAGAGCAACAAGAATCTGGGCGAGCACAAGCGCTGGATACACGATCCCTCGAGTG
AGTAGTAGagcaaagcaaagccaagcaaagcaaagGATTTGCGCACCATTTGGCCCCTGGCTTCGGTCAA
TTAAGTTGTCACTGGAGAACtatatatatatataCATACGTGTATATGCACAATATGGTATGACCTATTA
ATAAATTATATTCGCACGCCTGCAGGTGATGAGATAATCACCAAGGCCAAGTATTTGGAGGAGGAGGCCA
AGCGGCGTGTCAACCAGCAGGACAATCAGGACAGCCAGGACGCGGAGGATATCAACAACGACGACGAGGA
TAACAGCAGCGATGGCGGCAGCAGCAACTCCAGCCCCACAGGCACCAACAATGCCCAGGCCAGTGTCCAG
GGAAATGAGGAGACCGACGACGAAGATAAGAGCCTAAGTCTGGGCGACGACGACGAGTCCAAGGAGGATG
ATGTCTTCTACGAGAACAGTATCGCCGCCGGCGATAAGCAGCAGCAGCAACAGCAGCTATCCCAGCCTGC
TGCAGGACCTTCCGTTATTCAGCAGGACGACGACGAGGAGTTGGAG
>dme_circ_0007308_junction_seq
TCAGCAGGACGACGACGAGGAGTTGGAGATAAACAAAAACACGCAGAGCAACAAGA
>dme_circ_0007309
atgcagatacagatacagatgctgatgcagGCTCAGTGGCTTTGAGCTCTTTGAACGCACCATCGCCATT
TCATCCAAAAGTGCAAGTGACTAAAATGCAAATGGCGAGTGTGTGCCCGTGTGGGTGTGCAATTGTCTGA
CTCTGTGTGGGTGTGAGTGTGGATGGGGG
>dme_circ_0007309_junction_seq
TCTGTGTGGGTGTGAGTGTGGATGGGGGatgcagatacagatacagatgctgatgc
>dme_circ_0007310
GATACCGGGCAACGCATATTGTTGCGATTGCAGGAGCCCCGAACCACGCTGGGCCTCCATTAACTTGGGC
ATCACCCTGTGCATCGAGTGCTCCGGAGTGCACAGAAGCCTGGGTGTGCACTACAGCAAGGTGCGTTCCC
TGACGCTGGATGCCTGGGAGTCGGAGAACGTGAAG
>dme_circ_0007310_junction_seq
GGATGCCTGGGAGTCGGAGAACGTGAAGGATACCGGGCAACGCATATTGTTGCGAT
>dme_circ_0007311
TTTCTTCACCGCCCACTTTCGCGTGCCGCCCAGCGACAGAGTAGCTCGCTTAGCGATACCACTGCCTCCA
CTCTCGCCGTCCTCTACCACGCCGCCACTGTCTATGCTCAGCACTTCGGCTGTCTCGCTGGCCAGCAGCT
CCTGCGGTTTGGGCATACCGCAGACGAACCGCCGCTCCACGTACTTCGCCTTGATCCAAGCCTCGCGCAC
TCCGATCTCGCACTGCTCCGTGGGCTTTTTGAGCCCACAATCATCGCCGATGCGTGCCTCATAGATGCGG
>dme_circ_0007311_junction_seq
ATCGCCGATGCGTGCCTCATAGATGCGGTTTCTTCACCGCCCACTTTCGCGTGCCG
>dme_circ_0007312
GAGAACAACGTGGCCGCCAGCAAAGAACCATCCATGATTGGCCCGCACTCTTCCAGATTGCAAAAGAAAC
GCGCCCTCAACAGCTATTCAAATTTTCCCTTATAACGAATAATTCTCCCCAAATTGGCGACTAATCGAGA

TATATACCAAAGATATCGCAGTGCAGTCAGTAAGTTTAAACTTTTAGCCGTAACTAGTCAATGCCAAAAA
TGATAGTGGATAAATCGGAACTGGTGGGCGAGAATGCCTGGGCAAAACTACTGCCCGATGAGAAGGATAA
GGCCATCATTGTGAAGATGAACAAAAGTAGTAGCAATAACAACGATTCCAAGGAGACGACGCCCCTCAAT
GTGGATCAACTGGTGGCAGCCAGCAATCCAGCAGTTGCCGGCGAGCAAAGTGCCGTCTGCCTGGAGGATA
CCTACAAGCGTACCTCAACTTATGCCTCAAACATCAATTCAACGGGCGGCGAGTCTAGCGGCAGTGGAAC
TCAACTTTCCCGCAAGGAGTCCATCTTGGGCAGCTTTCATCGGCAAATCCGGCGATCCATAAAAGCGGTT
AGCGAATCGCCCGGCCCACTTACGAG
>dme_circ_0007312_junction_seq
TTAGCGAATCGCCCGGCCCACTTACGAGGAGAACAACGTGGCCGCCAGCAAAGAAC
>dme_circ_0007313
TCCAGCGATTCGTCGCTAGCCGGATCCAGATGATCCACCGAGGCGGCGGAGGCAATACGCTGGAACAAGC
TGGCCGAGAGCGTGCGATCCGCAAAGGGTGCATTCACACACTTGGCGCTCTTCGAGTACTCCGACTTAAG
CTCGTCCAGCTGCTTGGCGCTGCACAGCGGAGTGTCCACGTCGTAGGTGTTG
>dme_circ_0007313_junction_seq
CAGCGGAGTGTCCACGTCGTAGGTGTTGTCCAGCGATTCGTCGCTAGCCGGATCCA
>dme_circ_0007314
CGCTTGTCGAAGTACGACCTTAGCGAGAACCAGTTGTCGCTGATTCGCGACATTCGTCGGCGTGGAAAGA
ACAAGGTCGCTGCCCAGAATTGCAGGAAACGCAAATTGGACCAGATCCTGACTCTAGAGGATGAGGTGAA
CGCGG
>dme_circ_0007314_junction_seq
CTGACTCTAGAGGATGAGGTGAACGCGGCGCTTGTCGAAGTACGACCTTAGCGAGA
>dme_circ_0007315
TTCAACGAGCGCTTGTCGAAGTACGACCTTAGCGAGAACCAGTTGTCGCTGATTCGCGACATTCGTCGGC
GTGGAAAGAACAAGGTCGCTGCCCAGAATTGCAGGAAACGCAAATTGGACCAGATCCTGACTCTAGAGGA
TGAGG
>dme_circ_0007315_junction_seq
GACCAGATCCTGACTCTAGAGGATGAGGTTCAACGAGCGCTTGTCGAAGTACGACC
>dme_circ_0007316
GATGCTCTTCCTCCAAGTTGCTGCTGTTTCCGCCAACACTGCTGCTGTTGCCGGCACTCGCTCCCTTCGA
TGCGGTTTTAGTGGCGACCAGGGGCTTCTTATCGCGTGCCTGGGGTCTGGGAAGGGATCCACTTCCCTGG
GGTAGCGAGTAGGTATGATTTAGTTGCACCAAATCCTGCGAAGTCCTTGGCGATGGTCGTACT
>dme_circ_0007316_junction_seq
CTGCGAAGTCCTTGGCGATGGTCGTACTGATGCTCTTCCTCCAAGTTGCTGCTGTT
>dme_circ_0007317
GGCCGCATGCCGCGTCTGAATCGCAGCGTTTCGATGGAGCGTCTTCAGGACTTTGCCACCTACTTCAGTC
CCATTCCGAGCATGGTGGGCGGGGTATCCGATATGTCACCGTATCCGCATCACTATCCCGGCTACTCCTA
TCAGGCGAGTCCCTCGAACGGAGCACCCGGCACTCCTGGCCAGCATGGTCAGTATGGCAGTGGCGCCAAT
GCTACATTGCAGCCACCACCACCACCGCCGCCGCCGCATCACGCTGCCATGTTGCATCACCCGAATGCCG
CCTTGGGCGACATCTGCCCCACCGGGCAGCCCCACTACGGGCACAATCTGGGATCGGCTGTCACCTCCAG
CATGCATCTGACCAACTCCAGTCACGAGGCCGATGGAGCCGCCGCTGCTGCTGCCGCCTACAAAGTGGAG
CACGATTTAATGTACTACGGGGTAAGTTGGATCAACCGCATGTGGCAATGCAGCTTTTATAAACGATGAT
ACCCTTAGAACACCTCATCGGACATTAACCAGACGGATGGCTTTATTAACTCCATTTTTACCGACGAGGA
TCTGCACTTGATGGACATGAATGAGAG
>dme_circ_0007317_junction_seq

ATCTGCACTTGATGGACATGAATGAGAGGGCCGCATGCCGCGTCTGAATCGCAGCG
>dme_circ_0007318
GTGAATACATACGCTTGCCCCTGGATGAGTTGTTGAACGACGTACTCAAACTTTCTGAATTTCCGCTTCA
AGACGATGTAAGTTCGATGTCCCATTTAGAAAAATAGAAAATTCCTTATCGACTTCTTCTCAACCACCTA
GTTATCCAACGATCCGGTGGCCTCCACTTCGCAGGCAGCTGCCGCTTTTAACGAGAACCAAGCTCAACGG
ATTGTTTCGGAAACCGGTGAGGATTTACTCAGTGGCGAAGGAATTTCCAGCAAGCAAAATAGAAACGAAG
CGAAGAATAAGGACAACGATCCGGAGAAAGCTGACGGCGATAGCTTCTCGGTGAGCGATTTCGAGGAACT
TCAAAATTCTGTGGGCTCGCCCCTGTTTGACTTAGATGAGGACGCCAAGAAGGAACTAGACGAGATGTTG
CAATCAGCGGTTCCGTCATaccaccatccccatccgcatcacggccaccctcatgctcatccccatagcc
accaccatgcGTCGATGCACCATGCCCATGCCCACCATGCTGCCGCTGCAGCTGCCGCCCACCAGCGTGC
GGTGCAGCAGGCCAACTATGGTGGtggagtcggcgtgggcgttggcgtcggtgtgggcgtgggcagtggC
ACGGGCAGCGCCTTCCAGAGGCAGCCAGCTGCCGGCGGATTCCATCATGGCCATCATCAGG
>dme_circ_0007318_junction_seq
GGCGGATTCCATCATGGCCATCATCAGGGTGAATACATACGCTTGCCCCTGGATGA
>dme_circ_0007319
GAGAGTGAGATCGCTGAGGTGCTCTACAAGCAGGATGTGGATTTGGGCTTCAGTTTGGACCAGGAGGCGA
TCATCAATGCCTCCTATGCCAGCGGCAATAGTGCGGCTACCAATGTCAAGTCGAAGCCGGAGGATGAGAC
CAAGTCCTCGGATCCATCGATTTCGGAATCCAGCGGCTTCAAGGACACCGATGTGAATGCCGAGAACGAG
GCCAGTGCCGCCTCTGTCGACGATATTGAGAAGTTGAAAGCTCTGGAGGAGCTTCAACAAGATAAGGGTA
AAGTAAAGCAAAGTTTCGACCACGATTTACATACGGAATTTCACAATATTTACAAAGAGATAAAGCTAAA
GTCGAATCGGTGATGGTTATCTAACAGCCATCAAATGTTGTTGGTCTAGCTCTATACCCTATACCCATAT
CCAGTTTCATTTCAAACTAATGGTGTTTTTCTTCCTCCCAACAGGGTGAGTAGCCGCTTCAATAAACTAG
TACATCTACGATCTTGTGTTCTGGTTACATTTGGTCCGTCCGTATCTTATCTAGGATAATATAATTTCAC
TTTCAAAAATAACGAGAGATAAGCTCTACAGAAGCAGGAGATCTTTTTGGGGGACAAGAAGTTGTTATAG
ATATGTATTAAATGGCACGTTTCGTTGCTAGATAAAATTAAATTGGTCGATTAAGTTGGCGTACTTTAAA
TGTCTGGTGGCCTGTTTATTTAACTTATCCATCTGATTGTAATTGATTGAAAGGAATTGCTAATTAGCCG
TAATTTAACATAATTTAATTTTGTTATATTTTGTAATAATACTAAAACCTATCAAAGTTATTTGCTGATA
ACAAAAGGAAATAACGAAATAAGTCAAAAGAAAATGGCCTGCCAAAAAGATGGCGGCTGCTCTAATATAA
ACGAAAATTGATTATCCTGGATTTGTTGGGGTTGGGAGTTTTTGGGTCCTGTGGATGGGCGGTGGCATAC
TAAAACATATTGTTGGCATTGGTGTGCGTGTATTTTCGAGACGTTTATAAATGAATGTAGGCATTTTGAG
TTGACAGGCACGAAAATTAAAAGAAAAATGAGCtttttttttCCGCTTGAGTTGTGTGGCAGCAAAACAG
CCGCCCCACCAGAGCCACCCACTAGCAGTGCCACCGCCACAAAGGCCAGAAACGATGGCTGCTGCTAATC
TGCCACAAACCGCGCCCCGCTTCTCGTTTCCCATTACCCAGGCATCAGCAAGCGGCAGCCTTTCAAACAA
ACTTTGACACGCACACGAACTTTTATTATTAACCGCGGGGGAGGAGGTGGGGAAGTCATGGCATTGAggg
gggggggCAAGCTCGTCTGGGCCTCTGAGACTGGGTCGTGCCGCCTTTGAGACCACGACTACTGAGCCTT
TGGGCTCGAAAGAGGAGGCAGGGCATGGCAGGGCAGATACGATACCGAATCCAAATCCAAGGCGGTGGCA
GTGGCAGTAAAAGTAGCAGCCACCGCGCGTCGCGTCGCCTCGCATGCAACATTTTTCTTTTGAAAAATGA
ACGAAACATGATTCGCCAAAATAGCAAGAGCTAAACATACACACGAACTCACataaatatatatatatat
atatatatatatagaaataatatatgtatagAGAAAGAAGTGCTGCTATGTACACCATATGGTATGGAAC
TTAAGTTCCGGTGGGGCAGCAAACGCGGAATGGTACAAGCTGCAAGGTCTCAACTTTGGCCTATCAAAAG
ATCttttttttttGCTAAGCGAAAACAGTGAAGTAGTCGGTAGAGGGATTTGGGTATGTTATCAATTaaa
aaaaaTAACGACATTTTTAGAACCGAAAAAAATATCGATATTTAGGAGCATACCATTTTTAGTCTAATAT
TTGAGACGGCCATACTAAAGTGACTAATCAGAAATTGTACTTGGTATCGATACCCATTTTATTATTCGAA
GCGGAGTTGTTGAAAAGAAAAGTTTTGCAATATAATTGCCTGCCAGTTAACGAGAAAAACCCTGTGAATA

TTTAAAATAGTTGTACTTCCGAAGTGTGCTATTAGAAACTTTGATAGTGGATTGGTGTATTCAAAACAAT
TGGCTATGGAACTGTATGATTGTTCTCGAACCCTCAATAGAATACCAAATTGTATGTTAACttgaaattg
attaaattgattgaaattTTCTAATTTAAGGGTTTAGATTGTTACCCCCTTGCGACTGGGCCGCTGTGTT
TGCCCTTCGGGTCTCATCCCCTGTTGAACTGGCTCATTGAGCTGGGGAATCGAAGTCGCAGGGGTCGGGA
AAAAGTAGCTTCTGTATGGGATTTCATCCGTCCTTCCCCTCGAGAACGAAAAGTCGCATATCGCTGGCCA
CCATTTTGTGTGCCATGTGGAATAAGGGATAGAAAGAAGAAGTAGTGGTGTGTGTGTGTGGCAAGGGgca
gcagcagcagcacagcagcCACCATATTGGATATATGTTCCTATTCACGAGCATTTATGCTGCTCATATT
GATTGCTTCGTTTGGCCATTTATTAAAAACCCATTGATGCGCACCCAAGTCGCATAGATAGATTCATATC
GCATTCACACGCACATTCGGCTTTGTTTGAGTTTTGGGTTTGGGCCTCGATCTCAGATTTGTGCGTATCC
AAATTCCCCACGGATCGAACCCAACGATTTTCTAAAGTCCGCAGCCTTTGGCGGCATTATCATCCATTGT
TCGCGCCCTACGAAATAAAATGACTAAAATGGAAAGTCATTTTTCGGTTGCTGTTTCAGTAAATGGTATT
TCATTGCCCATACACTATTGATGTGAGTTTTTAAGAAAAATTCATAAATTCCATCTTCTGCACACGTTCA
GGTGAGTTTAGACCCGTTGTTCCTCGCTTTTCACGAGTTTTTATCATCGTTAATTTTGAGCCTTCGAAAT
GAAGTCGTAACCATTCGGTGACATTTTCCGGCCATTGTCCTGTCATGGTTGCCTTGTCATGGGTGCGCAT
ATGTTACTCATTGCCCTTCTAGGATCTGAAATCATGGCAATTAAGTTGCAAGTAGCGCTCGGTTATTCTT
TCGTCTCGCTCTTTGTCGCCTGTCCTCCAATCAGCTTCGTTTGTTTTCAACAGTTTTGATTTTCTATATC
TATCTGTATGTTATATATACATCTACCAATGAATCACAAATCTGATTACATTTGCTGCCTTTTCAATTCG
TTTGGGTTTGTTCTTTTTCCAACtttttttttttGTATTTAGCTTCTCAGATCGTTGACAACTTTTGGAT
CATGACTTGGCCAGAAGTTCTGCGTGGTATCGCCGAGAAATGCTCTTATTCTCAATTATGTTATAGATAT
TTCGGTTTTGGTTTCTTTTCTCGGTGGCTTAAATTCCATTATGCAATCAGTTAGGCCGCTCTTGTTGCTG
CCAAGCTACGCAAGTTTATTGACCACTGGTTTTGTGGAGAGGGGATTTTTGGTGGGAACATGAAACTGAA
TTCTTTTAGGTTTTCGAAAGGCTCTAGAAACACGTTCGATGAATTCTTGCGTTGATAGTTTTATAGACTC
TGGAAAGAAACCGTTAAGGAACTAGGGCTTAAAAATTTCCAATCTAAAGTGTTTTTCTATCTGATTTAAG
AATATGGATTAATTAATTGAAGTTAGCTCAGTCCTTTGCCTGTTCTGCTACATAAGCCATAGAAACTAGA
GTTATTATTTAATTTCCTGCCCATCACATCTGGTCACATCTGGTGCAGCGGTGCACCCTATTGAAGATCT
CTATTCCACATCTTTTTCAACTGTCTGGTGGCCTTCCTTGACCCGCCTCCACCACTCTGCGCCCTGCTGT
TGTCTGGAAAAGCGAGGGTGTGCTGGTGAAGAGTGGCCAGCGGAGACGGTGCGGACTGTAGAAATTGTTC
AAATTCTACGTGGTCAGCATTACGCTACAAATTTACAAAATGAGAAGaaaaaaaaaaaaaaaaaaaaaTG
AAAATTTACATGCTGACAAAATAACAAACAGACAGGGAAAAGAGGGGCATCGTTATAGGTGCGAGGGTCT
GCAGTCCTGACCGGCTTTATTTTTGGTCTGGTCTAGTCTGGCCTGGTCAACGATTTTTTGCTGCATAATC
GTATCACTTTCAAGTGCTTTTCGTCTTGCGCAACCTCAGCCGGCACCTTTCTATGTCTCAGTATTGCCAA
AACTAACTGAGAATTGCATAATGACAACAGCAATAAGTTCGACATTTAATTAGCCGTCGTTGTGGCTAAA
ACTATACTTGACCCATTGAGTACTTATGTATGTGGCTTTAAGGATTATGAAAGTTACTCGTTGTCAACGC
TGGTTTCCCTACTCCAATTTAATTTCCAATGTTGTGGTATTCAAATGAGGCTATTCAAATGAGCTGCCAG
CGTACTCATTCATAAGGTTCATTCAGCTTCTTCGGATTTTGTGGCAACTCATTAACAACCAAAAGTGGCG
TAAAGTTCAGATAATATTGCCCACCGCCATCTATACACATGTCTGTTAACAATAGTGTGTGCCCCCCTTC
TGCTCTCTAAAATCTGACTTGGCTTCTTTTAGCAATTTCATTGTTGACACTATCCGATGACGCGGATCTG
TCCTGTCATAATTGTTGCTCACTGGTTTTCTGTAACAGCTCTTTTGCTCTTCTTTCGAGGATAGTGAAAA
GTCATTGGTGAATTTGAATTCAATACCAGTAAGTTGGCGCAAATGCGCGATGCATTCGTGTAAACTGTAC
TTGTGCTGTTGATTGAAGTGTTGGAAAATGGTTTTTAAAGCGAGTCAAATGAAGAAATGATTAGGCCCGT
TTACTTGTAGCAAGGCTTCCATCTAGGGAAAGCAATTTAAATTTAAAATTTAAATATGCTTTGTTACCAA
AAAATGTTTCGGAAACGACTTTTGATTGTCTTTCACCGGATACAGCCCTGCTTCCCACTAAACCCCTCAA
ATCTCCCATCATTATTTTCTCAATCAGCATCGGCTTCTTTATTATTGTGCCGGCAACAATTTAAACTAAT
TAAGTTGTAATAAAGCACTCAAGaaaacgctcaaaaaaacattgaagagaagaaaaaaaaaTGATTAACC

CGGCGAGTTTTGCTTGTTCAATATTTCTGTGCTCTTTTTTTCCTACTGTTTTCTAGCAGCGATGaaaaaa
aaaTTATACAAGCATCCAATCGAAATGCCCCCTCCTCAACTTGTTGATGATGCTGCTAATTTTTCAGCCC
GAATCGAGCATCGACCAACCAACCAATTTGACTTTTTTCTATTTGCCGTATTTTTGGTAGTTTTTGAAGT
TCACCAAAAGCGTTTTTGGGCCCTATGGTCTTGGCCCGGTCTATTAGTTCAGTGGCCCATTAATGTGCAT
ATTGCCTGCTCTTTTTCCGACTCTCTTTTGGGTGATTCCATGATTGCGGTAATTGTGTTAAttttttttt
ttttGGGTAGATAAGTGTGTATTTTTATTGGGCTGCGAGCTGGAAATCGTTAGGCTTTAAAGGCTGTGCA
TTTAGTTGAAAGGTATTGAGGTCAGTTCAAAATTGCAGCTAAATTGTGCGAAATTATTCAGGAATCGAAT
GTACTGATTTAACACAATTTGCATGCCCATTGAACTAAGTTATTATCTTTGTTATGTAGTTATTAAATGA
CATTTGGTAAACACAAATCTAGATATCATTGTGAAACATTTACAGCAGCTTCTTTTTTTCAAAGCAACTT
ATTGTTTAATGTGTCACAACCCCTCAAAAAGCGAGCAAAACAACATCTACAGTTGCCTATATTTCATAAA
TAAATTTGTTAACATTTTTGTAATATCTAATCATAATGGCGGAATCTGACTCCGGAGAAAGTACGTCGTC
GGTGTCCTCGTTTATATCCTCATCGTCGTCTTCGCGATTAAGTGAGTTTGTGGACGCAAAGACAGAACTG
CAGGATATATATCACGATTTGAGTAATTACCTGTCCAATTTCCTAACCATTTTGGAGGAGACTGTCCTGT
TAAAAGATCGACAAATGCTGGAGCACCTGTGCGCCTTCTCCAGCAGGGTGGAGGCCATTGCAAAGGTTCT
TTCACGTGATCGAATGAAGGTGGCATTTTTTGGACGCACCTCAAATGGAAAAAGTGCCGTGATCAATGCA
CTTCTGCATGAAAAAATCCTGCCCAGCGCCATGGGCCATACCACCAGCTGTTTTTGTCAAGTGCAAGCTA
ATGGCTCGAATGAAACCGAGCACGTAAAGGTCGAGCAGGAGGATGAGCATATGGAACTGAGTGCCCTAAG
CCAACTGGCCAGTGCACATTCTCCTGGGGCCCTAAAACCCTCAACTCTGCTGCAGGTCAATATGGCCAAG
AACCGTTGCTCGATATTGGATTACGATGTGGTTTTGATGGATACACCTGGAGTGGATGTAACAGCGCAAC
TGGACGATTGCCTAGATAGCTACTGCATGGATGCGGATGTTTTCATTCTAGTTCTCAACGCCGAGTCCAC
TGTTTCGCGCGTGGAAAGGCAGTTCTTCAAGGACGTGGCATCCAAACTCTCGCGTCCAAATCTCTTTATA
CTCAACAATCGATGGGATAAGGCCAGCAGTCTGGAGCCGGAAATGGAGCAGAAGGTAAAGGATCAGCATA
TGGAACGTTGCGTTAACCTGCTCGTGGATGAATTAGGTGTTTATTCAACTGCACAGGAAGCGTGGGAAAG
GATCTATCATGTTTCAGCACTGGAGGCATTGCATATAAGGAATGGTCAGATTACGAATCCCTCGGGACAA
ACCCAACAGCGATATCAGGAGTTTCTGCGTTTCGAAAATGATTTTTCGAATTGCCTCGCGGTGTCAGCGT
TAAAAACCAAATTTGGTCCACACTTGCTAAGTGCGCAGAAGATTTTAAACCAGTTAAAATCAACTCTGAT
ATGCCCTTTCATAGAGAAAGTAAGTCGTCTTATCGATGAGAATAAGGAGAGAAGAGCTAACTTGAATGCC
GAAATAGAGGACTGGTTAATACTAATGCAAGAGGATAGAGAAGCGCTTCAATATTGTTTCGAAGAACTGA
CTGAAATGACACAAAGAGTAGGTCGGTGCGTTTTGAACGACCAGATAAAAACGTTAATACCCTCGTCTGT
GCTATCATTCTCGCAACCATTTCACCCGGAATTCCCAGCACAAATAGGCCAGTACCAACGCTCGTTATGT
GCCCATTTGGATAAACTTCTTGAAGATCGTGTCCTTCAATGTCTCTCCATACCCCTACAAAGAAAAATAT
TAGATATAGAGAAAGAAATTGGGCTTCCGATCGCCGAGAACTCTTGCGATTGGCAACTAATCTACGGCCT
GGATTGCCAATCCTATATGAGTGACTTTCAGCCAGATCTTAGGTTTCGATTTTCTTTGGGTTTTACTGCC
CTGTGGCATCGTCTTGAAGGCAACCTACCGTTGCACGCAAGTCCATTTCGAATTCAAAAGTTACAAAATG
GTCACAAGAAATGTTCGCCCCTGCCACCTTTAGTTAACGGAAACCATTGGCAGATGCTGGAATCTTTGGT
GAAGTCTAAAGGTAGCTTGGGCACCGTTTTACTGAGCGCCATGGCCATCCGTTCGTTCAACTGGCCAATT
GTATTGATCCTTGGTGGGCTCGTCGGATCCTTTTACATCTACGAGTACGCCGCTTGGACAACTGCCGCCC
AAGAGCGAAGTTTCAAGAGCCAGTACGCCAGGCTCTTGCAACAACGTCTGCGGTCGGATGTGCAGCAAAC
TGTTAGCGGTTTTGAGCTCCAGTTGCGACAGCACCTGGCAACGGTCCGAAATTGCTGGGAAGCCCAGTCC
AATGAGACACTGAATGACCTGAACGTAAGGACCGCGGAGCTGACCAAACAAATACAATCGATGGAGGTGT
TGCAGCTCAGCCTGAAGAAGTTTCGGGACAAGGGACAGCTGCTGGCCAGTCGGTTGGGAGACTTTCAAGA
GACCTACTTGACCAAGAGCTGACAATTATGAGGGGGGTTACAACAAATTCTAAATGTTCTTAATAGTTTT
AATTTATTTTTGGTTACCTAATTAAGTATTGTAATTCCGTTATGTTACTTAGAAATTTTGTATGTATTTG
GTTGAATATTTTAAAATATTAAACGATTGGTCTTCACTACTTTAAAGTTACTTCGTACCACGAGAATCTA

AGCCATGCAAATTTAAGCAGTTTTTATTGCTGTTGACTGTTTAAATTGCTTGATTAGATTGTTTTTCCTT
CCTCCTAATGGCTcacacacacacacacacacaGTTTCTGGCTGTGGAACATGCACATGCTGGGAATAAG
AACAAGAGAACCAGttttggcttttttttttgttttATTGTAGAAGACATCGGGCTTAAACAAAGCGTCCA
TATTGAAGCGGCCAAAAGCAAAAGTACAACAGATACAAACAGGCCAAGCTGAACTCTTAGCTCCGCCTCT
TGCTcacacacacacacacgcacacaaacacacacgcGCGAGCAGTGCGAGTGCGCATGAACAAATCCAG
CTCAAAACGGGGAGGAAGTGCATATGGTTGTCCCACTCGCACTCATTGTCTGAGCACGAGCTGAGAGCGC
CATTTCTCTTGCGCTACTGCCTACGTCGACGTCGACAGCGATGGCGGAGCAGCGACCGCGGCAGAGGCTT
TGAGCAGCGTTTTAGAATCGTATTTGAATTTTTATAAACGCTCGAGAAACGTTGGACGCTTGACGCGCGC
GCAGAAAATCGCATAAGGGTAAAAATATATGTGAATGGACAATAACGCCAGCAAATTCGTGGGCGTGGAC
AAGCAAAAAGGACTCTGCTCCGGCGAAAATTCGTTAACAACTCTGTGAGCCCGGCAAGGTGACGCGGCAT
TCCGAATCGACAGTTCAAATCGCTCGTTCTGGCCAAGTCAAGTGTTTCGGCTCTTTTTATGCTATGCCAA
TTAGATGATTTGGAAAAATTCTGTTCGACCGTAAAAAAATAATCACTAAACTGTGTCAAACATTAACAAA
TTTGTAAACTATTACGCGTCTGCATTTGTGCAAAAGCTATACaaaaaaaaaaaacaacaaaaaaGTGAAA
GTTACCAAATCAAAAATACATACCAAGCTCCCCCATACTCAAGTGACCATTGTGCGATTCTaaaaaaaaa
aaGCCCCCTACAATAACCAAAGGCATTGCAAAATAACTTAAACCCATTTTCCACACACACAATCGAATTG
TGCAAAGCCCaaaaaaaaaaaatcaaaaaTAAAGACAACACAATACAAAGTCTGTCTGCAACGGAAACGG
CAATATCGAAGTTGCGATTGGATTGCTTTTGCTTTGGCTGCTTTTAGTCGGCACGCCATTGTTGATTCTG
CAACATTTTGGCTGTCAAAACGTTACATAATTGTCAAAGTGTTTGGCCAAATATCAACAACAATCAGCGC
TTTATTGCAACGATTTGTATTCAATCTATTCACAAAAAGCCAACTACAACGATCTTTACGACAATCAACG
CGaaaaaaaaaCTAAAACAGTCAAGACACATTTGACTTTGGCTCTATAGACGTTTGTATTTCTTGGATTA
TAAACACGAATTGTTCACAGATATCAACAGTGTCATCATTGCAGCATCCCTTGAACTAATAAAGGTTCGT
AATACTTTACTCTTTGTTCGATCATATTTCCGAATGTAGGGTTTTAGTATGGGCGTATAACACAGCAACT
TGGCGCCCAACGTGGGGCATAATGATTATGGTTATTGAGTAAAAATTTAGTATTAAATCGTTCTTTCCGA
TTAAAAAAAGTTGATGAGAGAATTCGAGTGCGGTAAGACATTTTTAAAAAAGTTTAAAATGATGGCATGA
TATTAGATCACTCTAGCCTTCCTTGTTTTTATTTCGTTCTATATCAAACAATCTTTATCTCTTCAATCTC
TACTATAAGCACAAGTCAAGCCTTATTTACCTCTTCGATTAGGCTTATAATCTGTACAAACATAGAAAAG
TGTACCGCCCAATGCCACCCCAAAATTACTTCATTGTCGTCACCCCTGTGGAAATATCAAGTAGCCGTCG
GTGTTTAGTGCTCAATCAACCCCATTAGCGGTTCCATTACGAAACGCTCCCACAGTATTTCATACTTCCT
TCCGAGCGAACTTCCTGAAAGGTAACCGAAAATTGAAGTACACCCACTCCCATACCAATATGATCAAACA
ATGGTTGGCCCCATCAACGCCTTCTTTGAGCCTTGTCGCCTAATTGCTGGTGACAGTTGACAGTTGCTCC
TGACCAACCGGACGACAAAACAAAGGCAAGCACACGAACCCGAATCTACTAATATTTTCGTTGTCGTTTC
GGCCACAATGCAAGCTGAAAACCCACGAAACGAGAGGCAAACAATCAGCCCAAAGACCAAAAACGATAAG
TAAACAATTCGAAACCCACACGAACGCATTCCAAAATTAACGGACCACGAATTGTTACCaaaaaaaaaaa
aaaaaaaaaaaaaGTTAAGCAAAGAAGGGAGAACCCTAAAATAACAGGCTATAAGCCAGCAAGCCGaaaa
aaaaaCATGGCCAGAACAGAAGACGAACAGAACGGGCCCAAAATGGTTGCGATGGAAATGGGAAAAGCTG
GCGGCGGGGGAATGAGTTGTTTTGTAACCAATCGTAAGAATAACAAAGCATTTGGTCGAATTGTGTTGGC
CAGGCCCCAGAGAACCACCAAACACTTCCATCTGTTATGTTTATTCTAATTTTTTGACCCGACTCAAGTG
CCACGGTAAAGGTCGTCGTCGTCGTCACACCACTTGAGTCTCTTCTCTCCTGTCTTTCGACCAAGTTGCG
CCCTCGttttagcctttatttttttttcgggtttttggttttGGCGTTATCTAGATGTTTGCTGCCCACCT
TCGGGTTCTCTAGGTTTCCCTGTTCGTGGAACTCAAGTCGTTGTGTTAAGTAGTAATTTTCTGATAATAT
CCTCCAATACCCCCACTCAGTTCTGGGACTTGGTTTTTCCCAAGAATTTCCAATGCAAATATTTGGTCGC
CATCAATATTTGACCAGGAATCAATTGTCTGCTAGTCAATTGTAAGCGATACACTTGAGGCAAGCTTTCC
TATTGTTGTGCCTCGAGTTTAGTTTGTGTTTAGTTGCGATATCTGGGAGCTGTCAAAACTGATCATTAAT
TAAGTGAAGATGTGGGTGAGACCGCAGGGAGCTTGGGAAATCGTTCATTTATTGCAATATTTTTCAGCTG

CTTCCGTCAGACGGGGTTTGAGTGAAGGAGATCTCTCAAAGATCACGTTTTTATGATTATGATTAATGAT
CACCGACGTTCGAGTGACTCTAGTTACTGTCCACTCTGTGATCTCTTCGCTGATCTGACGAACTCCCACA
GGAAAGTGCTACTGTTTTGGGACGCTCCTTATTTACCTACTTTCCTGCTcccccccccGCCACAGATATG
AACCTTCCAACTGTTTTTGTAGCAGAAAATTGATCACTCTCTGGGGAATTTCGAGGCAAAGTGGCCGAGT
ATAATAAGAGATTCAGCTATTTCATTCAAATGATCAAAATATTTTCCTATATTTTCCATGCTTTGATACA
GTCACAAAGATATTGACTCTGGGGAAAGGTTTTTACTCCAAAGGGAATACAGTTTATTTATTATGTATAC
AGTGGGCGATCAAAAGGTTGTATTCTTCCAATTCTATAATGTAGTTTTATAAACCCATTTTTAAACAGAA
CTAAGAGCAAACCGCAAGGATTGGAtttttggatttttttttaataaatgcctttttctttttCATTCAG
GGTTCGTAATACTTTACTCTTTGTTCGATCATATTTCCGAATGTAGGGTTTTAGTATGGGCGTATAACAC
AGCAACTTGGCGCCCAACGTGGGGCATAATGATTATGGTTATTGAGTAAAAATTTAGTATTAAATCGTTC
TTTCCGATTAAAAAAAGTTGATGAGAGAATTCGAGTGCGGTAAGACATTTTTAAAAAAGTTTAAAATGAT
GGCATGATATTAGATCACTCTAGCCTTCCTTGTTTTTATTTCGTTCTATATCAAACAATCTTTATCTCTT
CAATCTCTACTATAAGCACAAGTCAAGCCTTATTTACCTCTTCGATTAGGCTTATAATCTGTACAAACAT
AGAAAAGTGTACCGCCCAATGCCACCCCAAAATTACTTCATTGTCGTCACCCCTGTGGAAATATCAAGTA
GCCGTCGGTGTTTAGTGCTCAATCAACCCCATTAGCGGTTCCATTACGAAACGCTCCCACAGTATTTCAT
ACTTCCTTCCGAGCGAACTTCCTGAAAGGTAACCGAAAATTGAAGTACACCCACTCCCATACCAATATGA
TCAAACAATGGTTGGCCCCATCAACGCCTTCTTTGAGCCTTGTCGCCTAATTGCTGGTGACAGTTGACAG
TTGCTCCTGACCAACCGGACGACAAAACAAAGGCAAGCACACGAACCCGAATCTACTAATATTTTCGTTG
TCGTTTCGGCCACAATGCAAGCTGAAAACCCACGAAACGAGAGGCAAACAATCAGCCCAAAGACCAAAAA
CGATAAGTAAACAATTCGAAACCCACACGAACGCATTCCAAAATTAACGGACCACGAATTGTTACCaaaa
aaaaaaaaaaaaaaaaaaaaGTTAAGCAAAGAAGGGAGAACCCTAAAATAACAGGCTATAAGCCAGCAAG
CCGaaaaaaaaaCATGGCCAGAACAGAAGACGAACAGAACGGGCCCAAAATGGTTGCGATGGAAATGGGA
AAAGCTGGCGGCGGGGGAATGAGTTGTTTTGTAACCAATCGTAAGAATAACAAAGCATTTGGTCGAATTG
TGTTGGCCAGGCCCCAGAGAACCACCAAACACTTCCATCTGTTATGTTTATTCTAATTTTTTGACCCGAC
TCAAGTGCCACGGTAAAGGTCGTCGTCGTCGTCACACCACTTGAGTCTCTTCTCTCCTGTCTTTCGACCA
AGTTGCGCCCTCGttttagcctttattttttttcgggtttttggttttGGCGTTATCTAGATGTTTGCTG
CCCACCTTCGGGTTCTCTAGGTTTCCCTGTTCGTGGAACTCAAGTCGTTGTGTTAAGTAGTAATTTTCTG
ATAATATCCTCCAATACCCCCACTCAGTTCTGGGACTTGGTTTTTCCCAAGAATTTCCAATGCAAATATT
TGGTCGCCATCAATATTTGACCAGGAATCAATTGTCTGCTAGTCAATTGTAAGCGATACACTTGAGGCAA
GCTTTCCTATTGTTGTGCCTCGAGTTTAGTTTGTGTTTAGTTGCGATATCTGGGAGCTGTCAAAACTGAT
CATTAATTAAGTGAAGATGTGGGTGAGACCGCAGGGAGCTTGGGAAATCGTTCATTTATTGCAATATTTT
TCAGCTGCTTCCGTCAGACGGGGTTTGAGTGAAGGAGATCTCTCAAAGATCACGTTTTTATGATTATGAT
TAATGATCACCGACGTTCGAGTGACTCTAGTTACTGTCCACTCTGTGATCTCTTCGCTGATCTGACGAAC
TCCCACAGGAAAGTGCTACTGTTTTGGGACGCTCCTTATTTACCTACTTTCCTGCTcccccccccGCCAC
AGATATGAACCTTCCAACTGTTTTTGTAGCAGAAAATTGATCACTCTCTGGGGAATTTCGAGGCAAAGTG
GCCGAGTATAATAAGAGATTCAGCTATTTCATTCAAATGATCAAAATATTTTCCTATATTTTCCATGCTT
TGATACAGTCACAAAGATATTGACTCTGGGGAAAGGTTTTTACTCCAAAGGGAATACAGTTTATTTATTA
TGTATACAGTGGGCGATCAAAAGGTTGTATTCTTCCAATTCTATAATGTAGTTTTATAAACCCATTTTTA
AACAGAACTAAGAGCAAACCGCAAGGATTGGAtttttggatttttttttaataaatgcctttttcttttt
CATTCAGGTGAATACATACGCTTGCCCCTGGATGAGTTGTTGAACGACGTACTCAAACTTTCTGAATTTC
CGCTTCAAGACGATGTAAGTTCGATGTCCCATTTAGAAAAATAGAAAATTCCTTATCGACTTCTTCTCAA
CCACCTAGTTATCCAACGATCCGGTGGCCTCCACTTCGCAGGCAGCTGCCGCTTTTAACGAGAACCAAGC
TCAACGGATTGTTTCGGAAACCGGTGAGGATTTACTCAGTGGCGAAGGAATTTCCAGCAAGCAAAATAGA
AACGAAGCGAAGAATAAGGACAACGATCCGGAGAAAGCTGACGGCGATAGCTTCTCGGTGAGCGATTTCG

AGGAACTTCAAAATTCTGTGGGCTCGCCCCTGTTTGACTTAGATGAGGACGCCAAGAAGGAACTAGACGA
GATGTTGCAATCAGCGGTTCCGTCATaccaccatccccatccgcatcacggccaccctcatgctcatccc
catagccaccaccatgcGTCGATGCACCATGCCCATGCCCACCATGCTGCCGCTGCAGCTGCCGCCCACC
AGCGTGCGGTGCAGCAGGCCAACTATGGTGGtggagtcggcgtgggcgttggcgtcggtgtgggcgtggg
cagtggCACGGGCAGCGCCTTCCAGAGGCAGCCAGCTGCCGGCGGATTCCATCATGGCCATCATCAGG
>dme_circ_0007319_junction_seq
GGCGGATTCCATCATGGCCATCATCAGGGAGAGTGAGATCGCTGAGGTGCTCTACA
>dme_circ_0007320
GAGAGTGAGATCGCTGAGGTGCTCTACAAGCAGGATGTGGATTTGGGCTTCAGTTTGGACCAGGAGGCGA
TCATCAATGCCTCCTATGCCAGCGGCAATAGTGCGGCTACCAATGTCAAGTCGAAGCCGGAGGATGAGAC
CAAGTCCTCGGATCCATCGATTTCGGAATCCAGCGGCTTCAAGGACACCGATGTGAATGCCGAGAACGAG
GCCAGTGCCGCCTCTGTCGACGATATTGAGAAGTTGAAAGCTCTGGAGGAGCTTCAACAAGATAAGGGTA
AAGTAAAGCAAAGTTTCGACCACGATTTACATACGGAATTTCACAATATTTACAAAGAGATAAAGCTAAA
GTCGAATCGGTGATGGTTATCTAACAGCCATCAAATGTTGTTGGTCTAGCTCTATACCCTATACCCATAT
CCAGTTTCATTTCAAACTAATGGTGTTTTTCTTCCTCCCAACAGGACAAGAACAATGAGAATCAACTGGA
GGACATTACCAATGAATGGAACGGAATACCCTTTACCATCGATAACGAAACTGG
>dme_circ_0007320_junction_seq
TACCCTTTACCATCGATAACGAAACTGGGAGAGTGAGATCGCTGAGGTGCTCTACA
>dme_circ_0007321
TTGACATATTGACTCATCACCATTTGTGCCAGCTGATAAGCAATAATGATCGACTTAGAGGATCTGCCTC
GGCTGCAATCCTTGTCCCCGGCGAAGGACATCGAATATACCTACCACCAGACCCAATTGCAGCTGAATCC
GAAGAAGAAGGTCACCGTCCGGGTGCCCATTGGACCACCCTCGAAGTTGAGTAAGCTGATTAAGCCCACG
GTGGCTTCCACGCCCGCTGTACTGGCCAAGCCAACGCCATCCATCACCCAATCGACGACGCCAGGTAACG
CCAAGACCAACATTTGGAAAGGTGGCTTCCATTTTACCAATCTGGGTAAGTATTGAAAGGCTTCGGTGTT
AATTACTTTATCCAAATGCCAGCACAAATGTTCCCAGTTCAGAACGCAATATATCTATAAATTACCCCCA
TCAATCTAAATTAATTTTTATGAGCAGGTCTTTTAGATAGATTGATAGTCTTCTAGCTATATTTTGAATT
ACACGTTCTGAATAGTGTGGCTATCTGTTTTATTGAGCAAGTCTAATACAAATGTATCCATTTGTGATAT
CAATAGCTAACGGAAGTTATTGTTAAGATATTTGTATTTTTGCATGCATGTAGAATATCAATATCAATAT
CATGaaaaaaaattattttaaattaatattaaggaaatataaaactatatatatcCCTTTATTTTTAATT
TTAATACAAATTTCAACTTTGTTATGTAGAACTAGTTAAAAAAATGACTAATGCAAATTAACTGAAAAAT
TTGCATGGACACGTGTCAAAAGCTTATAAGCTTACACCATATCTACATAAAATTGCAATAGCCACCATGT
CTACATAAGTTACTCTTAGGGAAAATATAAATATGTTCTTTATATTCTTAAGATATTCTTTCTAAAAACT
AAATCTAGAAATAAAAGGTAGGTAATAAAGATACATTTGTAACCGAAATCTACATTTCTAGCTCTCCTTT
AAAAAGCTCTTTCTGTCACTGTTAGTTTAAGCTACATTTAACATTTCTGTTAAAAGCACAATGTATTATG
GTGAGACCGCACTTGGCGGCCCCGAAAAAAACCGCAAAAGCACAAAACTCAACAAAATCGTCTGCAAAGA
GCCTCAACTGGGCACACTTGATAAAAGCACCGCGCTGGACAGACGTTTTTTGCCTCAAATGTTTTGAGGC
CGCAATCGAAAATCGCGACTGATTTCGACGGCGGAGAAATTGCCAAACGCGGCGTAACAAACAAAACACG
TTTTCAAGCTCACCACGTAGGTACGGCGGTTGGCGAAAGAGAGTAGCAAAGCGGACGCGACAATAATTTG
CCAAATAACAGTGAAAGAAGACGCGTAAAGGTGCGAAGTACACGCCGCTTAAATtgtgtgtgtgtgtgtg
tgcgtgtCTGGCTGCGTGTGCTTGTGTGCCGGAACAACAAATGAAAAAGTTTCGGTTGTGCGTTTAAGAA
CTACAGTTGCGAAATGTTGCATGCCCCACTAAATAAGTGCAACTCATTGTGTGGTGGTGGTACTGTCGGT
GGGGGTGGTGGGTTAAAGGCAATCAAAACAACAACGACCAACAGGCTAGCAACAACAGTTATTCAGCAAT
CGCCCCCTctcgctctcgcgctcagtctccctctctcgctccctctccaccgctcagctGACAGAATGCA
ATCGAGAAAAACATCGAGTTAGAAAGAATAGGGAACGAAAAGGAACTAGCAATTTGATAAATAATTTGTT

GATGCAAAGTGCAGAGTGAATGTTGAAATGCTGCCGTTGCCGGTTATTCTCTGCTCATTCTCGAAGCTTC

CATATTTCATTTTTCCTATTTGTTTTGCTCTCCGCGATTCTCAGCAAAAAATTAAATCGTTTTGCATTTA

ATTAATTGCATTAATTGTGGGCACAGACCGAATTGGAATTGGCAAGAAAAAACAGCAGTTGTATGCTTCA

ACGTGTGCGTGTCtttttttttGGTGTGTGAGTCAGAGAGAGACGAGAATCGGGCGATAAAAGAAAACAA

AGAAATACAAAGCAATGTGGAGGCAAAACACATGCAAATATTAGGCAGTGTAAAAACAACAAACGAAGCG

AAAATATTATTGTTTTAACCCTTGATGGACTTGACAATTTTTTGTACCTTAACGTAGTTAAATAATTTTA

CAGCTGTCTTGAAAATCTTAACATACAAATGACTTCAAATTCTAATTTGACGTAGGTCCAATATAGGGTT

AACAAATAAGTGACGATTTCACCACGTAAAACGGCACCACCACCACACACACTCTCACACAAAAGTGCGT

ACCAGACGGCGAGAAAGAAGCACCAGGTGGCACATAATCAAATATCAATTGTTATTAATATTTAATGGCA

CTTGAATGCAAATTGTCTGCAGAAGAGAAAGTTCAAGGACGCAGACAAGCGAAATATATATTTAAAGTTG

TTAAATAAATAAAACGCGTGTTTTCCAGCAATTAAAAGTAAAAATCGTAGAAAGTGAAACATAAACAAAT

AAGGTCTTGCAGAGCAAAAGAAAAGTGAACGGTAAACAATAAACCATTGTGTGTGCTGCAGGGAAGAAGA

AGCAGCGAAGAGGAAAAGAAGATCAAGCAACGGCAACAACAACAAGGAGAACATTTACTGCGCCGCAGCT

TTTTTACCGTTGTAGTCGGGTCTCATCATCCaaaaaaaaTTTGAGGTGAGTGCACTGCAATACAGCCACA

ATCACACGCACACCCACACAAGAACCCTCTCTTGCACGCACATGCACACGTCATTTCCCTACCCCTCTCT

TTCTCTCTCTCGCGCACATCTTTTCATTGAGAATTGAGCCAAATAAAGTTTGGATTTTTGTTAAAATGGC

TTTAAACAAAGAAAGTTTATATCGAACCTTGAAAGAATTTTGCACACAATTTTTTCGTCCGTCCTTGAAT

CGGGTGCTTCACTTCGCATTGCGCACATTTCAAATGCTCGCGAGAGACCTTGAACAAGTTCAAGTTCAAA

GCCATTTCGTCTGGGTCTGGGTCATTCCTTCCGCCTCACGCCCCCCTTTTTCGGGTGGGCGGTTGTCTGT

TAACCTTGTTTAAggggggggggggTGTGTGTGTGGCTGGACAGGATGGGCATATTAAATGCGTTGTGGA

TATCAAACAGAATTCGGACAAAACAACAACCAAAGAAGGATACATGCATATATGTATTTATGTATTATAC

ACACATATTTCGGAAAATTCGATTCTTGACCTGCTTAATTGTCTCTGAATATGAAATTAACTATGCTGAA

ATGCGCTAGGTCATCATCATAACAGCCTTTCTAGGCTATCCACTTTGGTTTGGTATGAAAACAAACTATG

TGTTGTGTTTCTGTAGCAAAACCTTGTTAGTGTTTCTGCACATTTATAATTTCAAGTTTAAATAATAGAG

ATAAGGGATGTTTCCTTACGACAATCTTTCATTTATTTTAATATCTCTCGAAAGTATTACTTATGTACCT

TAACTACATAATTGCGAACAGAAAAACATAATTGTGTTCGTTTTTGCTAAATAAATATGAATCCTAATAT

AATCGCGATGTGGAAATTAGATGTGTTTTTAATAAAAGACGTTACCGACCCGGAAGTTGAACTTTCTTTG

GTGCACTTCCTCGTGCTTAGCAATTTATAAAATCGACTGGCAAACAAAACAAAGGCTGCACCACTCGAAA

CAAAAGCATTTTCATTTAACATTCAAACTGCATATATGGCCATGCGGCAATGTATGAATATTACACTACA

TATCTACATACTTGCATATGTACATATAGTTAGCAAATCGTTATGCTTACTAATTCCATTCTATTTGTTG

TCACATCTTGATTTGCTTACAGAACAAAACCGAGGACAGCCATCAATTAAAAGCAAAGAGCATTGGAAAA

TCGGGGAGATAGGCCGTGGAAACTGCGAATTTTCAGCTTTTTGCGCAGATTTCGGAACGCCGAaggagga

ggaggaggaggaggCCGGAAGGCGGGGCAAGGCGACGTCATTGAACGCATAGATCCCGGACGACGCAGCC

GTAGAGCTGTTGTCCGGGACGGAGAACAACAAGAACAACAGCAGCggaggaggaggaggaggaggaggag

gCGGCGGGCGGAGGTGGACTACGGACTACAAGAGTCACCCACGCACCCATTCGCTCACGTATCGAAACCT

CTGAAAGCAACAAAATGATCTCAAACAAAAAATCGTACGCTATGAAAATGCTGCAGCTGGCTTTGGCCTT

GAGTCTGCTGCATTATAATCCCGATTACCTGCTCCATCGCTGGGACTCGCAGCTGGAATTGGGCACCCAT

GGCGATGGATGGGAGCTGGAGATGCTGCGGACGGTACACCGCCTGGATATGGATCACAACCCGTATGGAA

ATCGTAAAGGACTGAGTCCCCGCATTGAGGATCTCCTAAACTTCGACGATCCTTCCTTGGGGGGAATGGC

CAATGGCATAGGGGGCTGCAAGCTTCCGCCAAGATTCAACGGCAGCACTTTTGTAATGAATCTCCACAAC

ACCACCGGGAATAGCAGTGTCCAGACGGCAGCTCTGCAGGATGTGCAGAGCACTAGTGCTGCCGCCACTG

GCGGTACAATGGTGGTTGGTACTGGTGGTGCACCCACCTCGGGTGGTCAAACCAGTGGTTCTGCACTGGG

TGAAATCCACATTGATACCGCCTCGCTGGACCCTGGAAATGCCAACCATTCGCCGCTGCATCCAACATCA

GAATTGGACACTTTCCTCACGCCACATGCGCTGCAGGATCAGCGTTCCATTTGGGAACAGAATCTAGCGG

ATCTCTATGACTACAATGATTTGAGTCTGCAAACCAGTCCCTATGCAAATCTGCCTCTTAAGGATGGCCA
GCCTCAGCCATCAAACAGTTCGCATCTGGATCTCAGCTTGGCGGCGCTTCTACATGGTTTCACCGGCGGC
AGTGGAGCACCCCTCAGCACCGCCGCCCTCAATGATAGCACTCCACATCCCAGGAATCTGGGTAGTGTCA
CAAATAACTCGGCTGGAAGGAGCGATGATGGCGAGGAGAGTCTCTATTTGGGTCGACTGTTCGGAGAGGA
CGAGGATGAAGATTACGAGGGCGAGCTGATCGGTGGAGTGGCCAACGCCTGCGAGGTGGAGGGTCTGACC
ACCGATGAGCCCTTTGGCAGTAACTGCTTCGCCAACGAGGTGGAAATCGGAGATGACGAGGAGGTAACGC
CAATACACTTGaaaaaaaaaTGAATCAAAGTTTATTTTATACGCAGAGTGTCCAAATCAAAGGTGTGCTC
TAGATGTAAGCTTTACATCTTGTTAACAGATTCAATTGACTGACGTTTTCTTCGAGTGTAGTAGTAAATG
ACTTTGATTTGACTGATTAGAAGTCACTCAAATGGCACCACCAGCAGACCTGTGCTTCGGTGTGTGCTTG
TGCCCATTATCAAGCAGTGTTGTTATaaaaaagaaaaaaGCATTTTGCATTGCTAATGAAGATTTGCGAA
CCCATCGCTTGACCCATTCCACAACCATAGTTCTCCCCTTGGCTGCCTCTCCATTTCTGACCGTTCAGCT
TTCATTATAATTATAGTTTGTTAGTTTCCCCGCCGTCAATCGATATATTCAAATGTGGCTTTCTGTTTGG
ATTGGTCACCTTGCATGCGAGATACTTTTTCACCTTATCAACAGATAAGGGTTTCAACTTTGTTTACACA
TCAGCATGACTATTGTGTCACGGTTATCCACTCATACGCTGTCGATCTGTAAGATTGTAATTGTTATCTG
TGGAGGGTTTCTAAAAGGGTTGATTCATCTAATTGGGAGATTAATAGAAATCAACAAAAGTTGATTTTTA
GTTTAGTTTAGTTTGATTTATTTCTGATATGTTGCCGAAATCCCAAGTCGTAGCATTTTAAAGTTCTGAT
AACATGCGTTAATCTTTAATAAGTATTTTTCATAATACAATAATTATCTTTTGTTTGGCTAACAtttttt
ttAAACCGTGCGCAACAACTTTCACCATCTTTCGTGCCCAAAGTTTACATATTATATGCAAACACACAAA
ACAAGCGGCAATTAGGTCGTTAAACGCTTCGACTGCGCAAAAAACATTTTAACTGCGAAAAAATAAAACA
AAGATGATAAATTCGAGTAATGTGTAAAGGGATTTTTGTCCCGAAATTGAATATTTAAATCAATAAGTTA
ATGGGCAATTTATTAAATTTTTAAATGGGCATTAACGAGTATCTGATAAATCAGATTTTAAATTCGGGAT
ACTTTTTTCATTTAGGGGATAAAACATTTGTTAAATCGCTTTAATGGCAATCCAATCGCCATCTATGTGC
ATGCGACTTTATATAGATATGAATCAGATTGTTTGCGCTCGATTTGTATTTATTTTTAAAAATCGAATTG
AATGTCGGCGACAAGTGCGCGGCGATTTCATTATTTGttttgttttttttttttGCACTCCGGTGTGTGC
TGGTTGTGCGTTTTGCGTATTGCAATTTAAATATGCATAAATATTTATACTTGGCCTGGCCAAGAGCGAC
AGATCGGGGGAGATATGGCCGAGACAGGAGCGTGATGACTAACTCGCTGTTATTTTCGATATATTCAGGC
AGCTCTTTGTTGTTGTTTGTTGCTCACTCGAGTTGCCTGCAATTAATTTGTTTGAtttttttcttatttt
atttttttCCAACATGTCACGAATGTTTTCCATTTCGACCGATGACGGTTTTATTTTTTCACTCCGACTG
AATGTATGTTCTTGAGATGCAGCATATAGAAGACATAAGTATGTGCATTTCGTATGATCTAATTAGCTGC
ATTCGCATTGCATAATCATCGTTGAGCGCTTCATTTTTTTCCTTTGTTCTTTAGCTGCGATTAAAACGAT
TGAGTGGCTGGCACAGGAACTTCCGTCATTTTATTATAATTTATTATGCGATATTTTATTGATCAGGTGT
GGAAAGATACTTTTAGCTCATTTCAGGAAAGTTTCTCTTCTCAATTTGATTAAAGTGCCTTGGAAAGTTT
CTAACAACAATGAAAACTGCGCAAATACTGAGTATCTCTTGGTTTTAAGGTATATCAATTGCATCATTTC
TCGTTTGGTAATTGCAACTATAGTTCCAGCTGTTCGCCATTATTCCATTAACGAATATGGTAACTAGCTT
GGTGTTTACCCCCAACAAAGAACCCTCCATCTTGGACAGGTGTATCAGTTAATGTGTCACATTCTCTCTC
CGGAGAGATCAGGTAGATTTCTACCTGGTCTTTATGTTCTCTCGGGTGAGAGAGGTCGCCACATTGCGTA
CATATGTGTGACCTCAACACGCAGACTGGCGCAGCAAAAAAATGAAGCTCAAAACACAAAACTCCACTTT
GTTGAATGTCGTCTACCTCGCTGTCTTTCCACTAATTGCAAATCTACCGAGCTTGGTAGCTTTTTACGTT
TCGCTTTAACTCATTCGTTTATAGTATATGTACATATGTATGTATACACTCGGTCAGCGAtttttttgtt
gttgttttgggttgggtgttattttttGGGGTCTCGGTCTCGCATTTTGATTGAGATTCGGAGGAATGAA
ATAAAACTCGTCGAAAGCGAGCACAAAGATTCGTTGAACTTCGGTTCTGTTGCCCTGCCAGTGGGTGTCA
TGGAGTTATGATGCAGTATATCCATTCGCGATCCTATAAACTATATAGTACTGTCTCGATCTATGCAATG
CGTAATAATACACATATAATCTACAATTTGTGTATAGAATGTTTTAGAGCATAATTATCGAAATGAAATA
ACTCTTTCGACTGAGTCATGGCATCTCAGGTGTCGTGCCTCCGACTCTTGCTGTCCCTTTCGCTGTCTAT

TAACAATTTTCCATTATTAAGTCATTCGGTAATGCACTTCTAATCTCAGTGTCGTTGTGTTGATTGTAAA
AGGGGTGGCAAGGAAAACTTGAAAACAAAAGCAAAGAGGAAAGCAAACAGCTGATTTGCCGGCATTTTTT
ACACACCACACTTTTTGCATTTCCTTTTACATTCAGCTTGTTCTCTCTCTTCACCACAACTATTAGTCAG
CAAAATTCACCGCTGAGAAAATTCACCACTGACTCGAAAGCTACTCAGCGGAGACATCAACCAAAGATTA
GTTGAGTTACCAACCAAGTGCTTTGCACACCAATATCAACTAATGCTGCAATCGAGAATTTTCAATCGAA
ATTTGAAAATTGATAATGTAGCAGTTAGGCTGAGAAATTAATACTTAAATTACTGATAAGGTCGAATAGG
TAAACCACTTTTAAGGTTGTACTTGTTGTCTACACCACTTGGTGTCGGTTGCTTAGGGCTTATCAGTCTC
TGATTGCTGGTCCGAACTGTTCGAAATTCGTGCAAAACAAACTTTATCAATTGAAATTAATCAAAAGAAA
AAATATATATTTTTATGATCCCCTTCAAACTGCTAATCAGCAGACATTCTCGAGCGCAGCCAAACGAAAG
TTTCAGTCTGCGTTATGCCGATTTACAGTGAATACGTTAACCCGAATCGTGTGTATCGCCCTACCAGCAA
AATCGTATCGAGCGGAAATCAATTTTCAATCGTCTACCAAAATGTGTCTCAACGAGGAGTACAAATATAT
GCACATTTACCTTGCGGTCCGTTTCCAACTTCCTATATCTAGCCATTAATCTGTGTTTACTGTGAATAAA
CTTTCTATTCACATGGCCGGCCGCAGTGCAAAGTGTTGGCaaaaaaagaaataataatacaaaaaaaaaa
aaaaaaaaaaaaaaaataaagaaaaaagcacatatatgtaaaggctgcaaattgaaaaataatttgcctg
taattacgttgcattttccCACTTAATTACCCTGAGCCAAAGAAGCAATGAAGAAGCATTCACTTGGATA
CAAGAGTTTCTACATATACATTTTGTTTCTCCTATTTTTGCCTAAAATTTTATTTCCCACCAATTTTGTT
GTTTAACTGCCATAATTAAATACAGTTAATTATGGTTCGCGAACTTCTGACAAGTGCCGTGCCATACACT
TTTTCAAAAAGAACAACCTAGAAACCAATGAAGGAAACTATCAAAGAAGTTTATGTTATGCGGTTTATTC
GAGTGTGAATTCAGCGAGCAAAAAGTTCAGATGTCAAATTGATTATAATGTGAACAGAGCGGCCAAATCG
AGCTGAGTTTCAATTGAAATTGATAATTGCAGGGAAAACGGTTTGGATGACACAAATGAACTGTTATTAA
GGCCTAATAGCTCGCCATTGTGGGGTTGTTGAAAACGCAAACCGCAAAACAGGACACTCGAAACGGGGCT
TAAGCAATCCGTCAGATACAAGTGCAACACATGCATGTGTAGGATACATGGATACACCGGTCGGCATTGT
TGGTAGTATACAATCCACAATCCGGCCAAGTCTACTGAGGGCTTGACGAGCTCATGATTAATATGCGCCA
CACTTGCTGGCCGCATTGGGATCTCAAATCTACTTACTTCtcttgtatcttgcatcttgtatctCGGGAT
TGTATCCCACAGATGCGATGCTGAAGTGATCGCTCCGTTCGTGCTGTGCTGTCAGTTCAGGTGTCATCAG
AAAAATCTTTTCGCCGAGGCTTTATTAATGACTCTTGCTAACCCGAGCCTTTCCTTTCCCTTCGTGCTGG
CTTCCTTTTTGTGGACCACCACCGTCCCTGTTCTTTGCGCTCGCTTACTTTCTTTTGCTTTCACTCGACT
TTCATCATTTGCCGCCCCTGCAGGATCAGAAACTCATTTATCTTGCTATAGCATTTTGTCCATTCTGCGT
CGGCGTTCTGTGGTCAAGTTAATGTTAAAAATCGGCTCAGTTAAGCGGACTAATTGCCGCCACAAGCGGT
TACAAGTGTCAATATTTTGATGCTACATGGGGGGGCACAACAGCAACTTGATTGATTGTCACTGATCGCG
CTGGTTGCTGCCTCGAATTAAAGCATTACTCCGTTTTATAGAGAACTAAATAGCTCTGGTATATGTGCTT
AGAATTCCCGCCTCTTATCTGTCCACTTATCAATGCTTGAATTCGTCAGTCGCCCGCTTTGGTTGCTCAT
TTAAATGGCTGTCTCGAGAGCGGCCCACGTGTCGCTGGTGGAACTTAAAGGTTCGCCGAACACTACAAGG
TCGTGGAGAGACTTTTCCCGGCACAAGTGATAAGAATTGGCCCATAAATAAGTCGGTTGATATATGTGGA
TTGGAGGCATTGGAGTAGTTTCAAAATCTATGAAATTGTTTCGCGTTTAGTAGGTGTTGTGGTTTATTGA
ACCCTGCGATGATGCTACCAAGTCCTCATCTAGTTTGGTTTGAAACGCTCATTTTCCTTATTATCTCATT
TCGGGAATTTCCAAGTTATATATCTTTATATTCGCTTGTGCTTCTCGAACTGTTAAAATGTCACGTATTT
GCGCGAAATTCTATAAGTACAAGATAAGGTCGTATTTTCGACCCTAACCGATAAGAACTGTAACTGGCTA
CACTGTAAGAAGTCGGATATATGGAATTTCTTTTTTTGTTTACCTTTTGCCCTGGTAAAGAAAAGGAAAC
TCAAATCAGCTTTGTTTTAAAGTTGAAAACACTATTCAACTTTGCTTTAAAGTTTAATTAAAAACGAAGT
TTCCAATCAACAGCTATAAATAGCTATGtatatatatatatatatatTGTATGTGTGTGTTTTAAACAAC
CCTCCGAAATGAAGaaaaaaaaaaTGTAATAATGAAATGCCAGCAGGGGAGGATTCGGCTTTTTGGATTT
TTGTTGGGATCCCGAGACCTTCTTCTTTGCTCCGCTCAATTTAATTGCTCATTTCTACCGAACATGCGCA
ACGTACAGCAGCACACTTTCAACTACAGTGAAACGTCTAGAGGCTGAACTAGAGAAATTGTTAATATATG

TAAAACTTTGAAACTTTAAATCATTTTAAAATTAACCTATTTCAATTGAAAATATACACTTTTAAATCGT
GCATGGTTGCCTTTAACTTTATTTTACCTTTCTTTCTATTCGGTAGGCTTTGTATATTTGATATTTTCTG
AATCGGATGATTTGATTTGTAGTTGCTTTACTGTATTTACACACACGCACTTGGTCCCTGGGGAATTGGC
TCTGCTGCCGCTCCGCCAGCTGCTCAGTCGTCGTCGCTGTCGGCGTTGGCGCGGCAGCAATTGATTGGGC
TCAACATAGTGGCCCTTAATTTTTCCGACGGTTCTCAGGGGTTAAGGAGAGCAAGGGAGATGGCAGCAGA
GCGGAGACCAATGATGCCGCCGCCATATTCTGCTGACTAATCCAGGAGATAGGCGTGGCCACAATGATTG
ATTACTTTTTGTTGCCACTGTGGAAAAGTCCAAGAAAGCCTCACGAAGGGCCGACGAGCATGAAATGGGa
aaaagaaaaaaaTGTACGCTACGGGAAAATGTCACACGaaaacaaaaacctcaaaaataaaaaaGTAGAG
GCCAAAAGATTTCAATTAAAACATCAGAAAGTTGTATGCTCGAATTGCAATTGTACTGCTAATGTCGACC
ATTATCCTGTGATTATCCCTCCAGTCCAATGGATATTATGCCTCACCATAGAACACTTTGACTTTTACCT
GTTTTGTTTGAAACGAAAAACGGTTTTGCCCGGGCTTAAGTGTGGGCGACAACGCCCCCGTTCCCTGGCC
AAGTCCGCCCGACTTGACTTGGCCAGAACAAAAAAGAAAAGGATTAAAGTGGTTTCGGCCAGCAGCGTTA
ATAATGCATCATTAACGCTATAATTTGAGCTCGAATTCCACCGCATGAATTGGCAGAATTCGACCAAACA
TCTTTATTGGCAAACCGACAAGCGATCTGCAAATATTTCGTTTCCGATTCTCTTTTGAAAAGTTTTCGCC
AAGTGTATGATCCGCTCTTAGGTGGTAAATTTATAGCCACAGAATATTGTTTACAGTGTGGCGAAGCTGG
CGGCGGTTGATAAGAGATCCGGTCTGGCGTTCGTTAGCTACGGCGTAAACAAGTCTCGGGCCACAAAGCA
CGCCGTAAGAGAGTGCTCCGGCCCCCCTTCCAAGTTCCGCATACGCCCCCCCTGCAATCGGCCTAATATA
TACTATATAACCCAGCGTTGTGCCCACGGACTTGGCTCTCTGTGGCGTTTCATTGATAAGATTCGCCACT
ATCGATCAACGCTGTTCACACAGAGCGAGCACTCTTCGAGAGGTTTCCGCGGGTTAAGGGAGCGCTTGAT
GTTCTATGAGTTATGGCACGGAGGCGATAAGAATTGTGAAATAAGAGAATCTATATGTAGGGGGGCACTT
ATTACTGGCCAGCCAATAATAGTACGTACGTACACCTCGGCTTATGTTTAGTAGCCAAATTTACAGCACT
ATCAGTCAGTCAGGTATAATCTTTTAAGTGGCATTATGTCTTACCAATTGGCCAAGGGTGGTATCTTAAG
AGAGCTCGGTAAGAATTGGATTATAACTCTTAGAAAACTAGCTACCCTTCTACGACAGTCCTTCAACTTT
CAGATCCAAAGTTGCTAACAGCACGATGACTTTACAATAGACTAATTACTACTATATAAGCACTTTAATC
AAACGAACTTTAAACATTTATTAATATAATTTAAAAATGAAAGCCTATATTTAAGTCCGTGTGATATATT
AAATTCATAGAGTATCCAACATTCTTGGCTATCCATAAAGCTCTTACGTAGTTTTATCTATTCAGAAACG
TTTTTATCAGCTGGCGCGAAATGTAAATCGACATTATCATATTAAGAAAAGCGGCACGAAGTGAGGAAGA
GCAAAGAAGAGCGGGCGAGGTGCCCATTATGGACTATGACTCGTCCGGCTCTTGGAGCCTTCAGTCTCTC
TTTGGAAACTAGCAAATGCGGATGACGTCGTGATCGAGATCGTGAATGATATAAACAAAACAACAGATAC
AACAAGACTGAAAGTGCAACTGATATAATCCAGTATAGTGCAGATCTATTGATGAATCGAATTGTAATCC
TTGCAAAGGAGTTGTGAATATGAAAGTGTTCATCCATAGGAACATAATGGTCAGGCATTTGTGCTAGCAA
TTGATAAAATTATATATTATATTATCTATGGAGATATCAATGTGCTACCTTATTGACTGGAATGTCTTTC
CCTTGCAGGGAGAGTGAGATCGCTGAGGTGCTCTACAAGCAGGATGTGGATTTGGGCTTCAGTTTGGACC
AGGAGGCGATCATCAATGCCTCCTATGCCAGCGGCAATAGTGCGGCTACCAATGTCAAGTCGAAGCCGGA
GGATGAGACCAAGTCCTCGGATCCATCGATTTCGGAATCCAGCGGCTTCAAGGACACCGATGTGAATGCC
GAGAACGAGGCCAGTGCCGCCTCTGTCGACGATATTGAGAAGTTGAAAGCTCTGGAGGAGCTTCAACAAG
ATAAGGGTAAAGTAAAGCAAAGTTTCGACCACGATTTACATACGGAATTTCACAATATTTACAAAGAGAT
AAAGCTAAAGTCGAATCGGTGATGGTTATCTAACAGCCATCAAATGTTGTTGGTCTAGCTCTATACCCTA
TACCCATATCCAGTTTCATTTCAAACTAATGGTGTTTTTCTTCCTCCCAACAGGACAAGAACAATGAGAA
TCAACTGGAGGACATTACCAATGAATGGAACGGAATACCCTTTACCATCGATAACGAAACTGG
>dme_circ_0007321_junction_seq
TACCCTTTACCATCGATAACGAAACTGGTTGACATATTGACTCATCACCATTTGTG
>dme_circ_0007322
AACAAAACCGAGGACAGCCATCAATTAAAAGCAAAGAGCATTGGAAAATCGGGGAGATAGGCCGTGGAAA

CTGCGAATTTTCAGCTTTTTGCGCAGATTTCGGAACGCCGAaggaggaggaggaggaggaggCCGGAAGG
CGGGGCAAGGCGACGTCATTGAACGCATAGATCCCGGACGACGCAGCCGTAGAGCTGTTGTCCGGGACGG
AGAACAACAAGAACAACAGCAGCggaggaggaggaggaggaggaggCGGCGGGCGGAGGTGGACTAC
GGACTACAAGAGTCACCCACGCACCCATTCGCTCACGTATCGAAACCTCTGAAAGCAACAAAATGATCTC
AAACAAAAAATCGTACGCTATGAAAATGCTGCAGCTGGCTTTGGCCTTGAGTCTGCTGCATTATAATCCC
GATTACCTGCTCCATCGCTGGGACTCGCAGCTGGAATTGGGCACCCATGGCGATGGATGGGAGCTGGAGA
TGCTGCGGACGGTACACCGCCTGGATATGGATCACAACCCGTATGGAAATCGTAAAGGACTGAGTCCCCG
CATTGAGGATCTCCTAAACTTCGACGATCCTTCCTTGGGGGGAATGGCCAATGGCATAGGGGGCTGCAAG
CTTCCGCCAAGATTCAACGGCAGCACTTTTGTAATGAATCTCCACAACACCACCGGGAATAGCAGTGTCC
AGACGGCAGCTCTGCAGGATGTGCAGAGCACTAGTGCTGCCGCCACTGGCGGTACAATGGTGGTTGGTAC
TGGTGGTGCACCCACCTCGGGTGGTCAAACCAGTGGTTCTGCACTGGGTGAAATCCACATTGATACCGCC
TCGCTGGACCCTGGAAATGCCAACCATTCGCCGCTGCATCCAACATCAGAATTGGACACTTTCCTCACGC
CACATGCGCTGCAGGATCAGCGTTCCATTTGGGAACAGAATCTAGCGGATCTCTATGACTACAATGATTT
GAGTCTGCAAACCAGTCCCTATGCAAATCTGCCTCTTAAGGATGGCCAGCCTCAGCCATCAAACAGTTCG
CATCTGGATCTCAGCTTGGCGGCGCTTCTACATGGTTTCACCGGCGGCAGTGGAGCACCCCTCAGCACCG
CCGCCCTCAATGATAGCACTCCACATCCCAGGAATCTGGGTAGTGTCACAAATAACTCGGCTGGAAGGAG
CGATGATGGCGAGGAGAGTCTCTATTTGGGTCGACTGTTCGGAGAGGACGAGGATGAAGATTACGAGGGC
GAGCTGATCGGTGGAGTGGCCAACGCCTGCGAGGTGGAGGGTCTGACCACCGATGAGCCCTTTGGCAGTA
ACTGCTTCGCCAACGAGGTGGAAATCGGAGATGACGAGGAGG
>dme_circ_0007322_junction_seq
GAGGTGGAAATCGGAGATGACGAGGAGGAACAAAACCGAGGACAGCCATCAATTAA
>dme_circ_0007323
TTGACATATTGACTCATCACCATTTGTGCCAGCTGATAAGCAATAATGATCGACTTAGAGGATCTGCCTC
GGCTGCAATCCTTGTCCCCGGCGAAGGACATCGAATATACCTACCACCAGACCCAATTGCAGCTGAATCC
GAAGAAGAAGGTCACCGTCCGGGTGCCCATTGGACCACCCTCGAAGTTGAGTAAGCTGATTAAGCCCACG
GTGGCTTCCACGCCCGCTGTACTGGCCAAGCCAACGCCATCCATCACCCAATCGACGACGCCAGGTAACG
CCAAGACCAACATTTGGAAAGGTGGCTTCCATTTTACCAATCTGGGTAAGTATTGAAAGGCTTCGGTGTT
AATTACTTTATCCAAATGCCAGCACAAATGTTCCCAGTTCAGAACGCAATATATCTATAAATTACCCCCA
TCAATCTAAATTAATTTTTATGAGCAGGTCTTTTAGATAGATTGATAGTCTTCTAGCTATATTTTGAATT
ACACGTTCTGAATAGTGTGGCTATCTGTTTTATTGAGCAAGTCTAATACAAATGTATCCATTTGTGATAT
CAATAGCTAACGGAAGTTATTGTTAAGATATTTGTATTTTTGCATGCATGTAGAATATCAATATCAATAT
CATGaaaaaaaattattttaaattaatattaaggaaatataaaactatatatatcCCTTTATTTTTAATT
TTAATACAAATTTCAACTTTGTTATGTAGAACTAGTTAAAAAAATGACTAATGCAAATTAACTGAAAAAT
TTGCATGGACACGTGTCAAAAGCTTATAAGCTTACACCATATCTACATAAAATTGCAATAGCCACCATGT
CTACATAAGTTACTCTTAGGGAAAATATAAATATGTTCTTTATATTCTTAAGATATTCTTTCTAAAAACT
AAATCTAGAAATAAAAGGTAGGTAATAAAGATACATTTGTAACCGAAATCTACATTTCTAGCTCTCCTTT
AAAAAGCTCTTTCTGTCACTGTTAGTTTAAGCTACATTTAACATTTCTGTTAAAAGCACAATGTATTATG
GTGAGACCGCACTTGGCGGCCCCGAAAAAAACCGCAAAAGCACAAAACTCAACAAAATCGTCTGCAAAGA
GCCTCAACTGGGCACACTTGATAAAAGCACCGCGCTGGACAGACGTTTTTTGCCTCAAATGTTTTGAGGC
CGCAATCGAAAATCGCGACTGATTTCGACGGCGGAGAAATTGCCAAACGCGGCGTAACAAACAAAACACG
TTTTCAAGCTCACCACGTAGGTACGGCGGTTGGCGAAAGAGAGTAGCAAAGCGGACGCGACAATAATTTG
CCAAATAACAGTGAAAGAAGACGCGTAAAGGTGCGAAGTACACGCCGCTTAAATtgtgtgtgtgtgtgtg
tgcgtgtCTGGCTGCGTGTGCTTGTGTGCCGGAACAACAAATGAAAAAGTTTCGGTTGTGCGTTTAAGAA
CTACAGTTGCGAAATGTTGCATGCCCCACTAAATAAGTGCAACTCATTGTGTGGTGGTGGTACTGTCGGT

GGGGGTGGTGGGTTAAAGGCAATCAAAACAACAACGACCAACAGGCTAGCAACAACAGTTATTCAGCAAT
CGCCCCCTctcgctctcgcgctcagtctccctctctcgctccctctccaccgctcagctGACAGAATGCA
ATCGAGAAAAACATCGAGTTAGAAAGAATAGGGAACGAAAAGGAACTAGCAATTTGATAAATAATTTGTT
GATGCAAAGTGCAGAGTGAATGTTGAAATGCTGCCGTTGCCGGTTATTCTCTGCTCATTCTCGAAGCTTC
CATATTTCATTTTTCCTATTTGTTTTGCTCTCCGCGATTCTCAGCAAAAAATTAAATCGTTTTGCATTTA
ATTAATTGCATTAATTGTGGGCACAGACCGAATTGGAATTGGCAAGAAAAAACAGCAGTTGTATGCTTCA
ACGTGTGCGTGTCtttttttttGGTGTGTGAGTCAGAGAGAGACGAGAATCGGGCGATAAAAGAAAACAA
AGAAATACAAAGCAATGTGGAGGCAAAACACATGCAAATATTAGGCAGTGTAAAAACAACAAACGAAGCG
AAAATATTATTGTTTTAACCCTTGATGGACTTGACAATTTTTTGTACCTTAACGTAGTTAAATAATTTTA
CAGCTGTCTTGAAAATCTTAACATACAAATGACTTCAAATTCTAATTTGACGTAGGTCCAATATAGGGTT
AACAAATAAGTGACGATTTCACCACGTAAAACGGCACCACCACCACACACACTCTCACACAAAAGTGCGT
ACCAGACGGCGAGAAAGAAGCACCAGGTGGCACATAATCAAATATCAATTGTTATTAATATTTAATGGCA
CTTGAATGCAAATTGTCTGCAGAAGAGAAAGTTCAAGGACGCAGACAAGCGAAATATATATTTAAAGTTG
TTAAATAAATAAAACGCGTGTTTTCCAGCAATTAAAAGTAAAAATCGTAGAAAGTGAAACATAAACAAAT
AAGGTCTTGCAGAGCAAAAGAAAAGTGAACGGTAAACAATAAACCATTGTGTGTGCTGCAGGGAAGAAGA
AGCAGCGAAGAGGAAAAGAAGATCAAGCAACGGCAACAACAACAAGGAGAACATTTACTGCGCCGCAGCT
TTTTTACCGTTGTAGTCGGGTCTCATCATCCaaaaaaaaTTTGAGGTGAGTGCACTGCAATACAGCCACA
ATCACACGCACACCCACACAAGAACCCTCTCTTGCACGCACATGCACACGTCATTTCCCTACCCCTCTCT
TTCTCTCTCTCGCGCACATCTTTTCATTGAGAATTGAGCCAAATAAAGTTTGGATTTTTGTTAAAATGGC
TTTAAACAAAGAAAGTTTATATCGAACCTTGAAAGAATTTTGCACACAATTTTTTCGTCCGTCCTTGAAT
CGGGTGCTTCACTTCGCATTGCGCACATTTCAAATGCTCGCGAGAGACCTTGAACAAGTTCAAGTTCAAA
GCCATTTCGTCTGGGTCTGGGTCATTCCTTCCGCCTCACGCCCCCCTTTTTCGGGTGGGCGGTTGTCTGT
TAACCTTGTTTAAggggggggggggTGTGTGTGTGGCTGGACAGGATGGGCATATTAAATGCGTTGTGGA
TATCAAACAGAATTCGGACAAAACAACAACCAAAGAAGGATACATGCATATATGTATTTATGTATTATAC
ACACATATTTCGGAAAATTCGATTCTTGACCTGCTTAATTGTCTCTGAATATGAAATTAACTATGCTGAA
ATGCGCTAGGTCATCATCATAACAGCCTTTCTAGGCTATCCACTTTGGTTTGGTATGAAAACAAACTATG
TGTTGTGTTTCTGTAGCAAAACCTTGTTAGTGTTTCTGCACATTTATAATTTCAAGTTTAAATAATAGAG
ATAAGGGATGTTTCCTTACGACAATCTTTCATTTATTTTAATATCTCTCGAAAGTATTACTTATGTACCT
TAACTACATAATTGCGAACAGAAAAACATAATTGTGTTCGTTTTTGCTAAATAAATATGAATCCTAATAT
AATCGCGATGTGGAAATTAGATGTGTTTTTAATAAAAGACGTTACCGACCCGGAAGTTGAACTTTCTTTG
GTGCACTTCCTCGTGCTTAGCAATTTATAAAATCGACTGGCAAACAAAACAAAGGCTGCACCACTCGAAA
CAAAAGCATTTTCATTTAACATTCAAACTGCATATATGGCCATGCGGCAATGTATGAATATTACACTACA
TATCTACATACTTGCATATGTACATATAGTTAGCAAATCGTTATGCTTACTAATTCCATTCTATTTGTTG
TCACATCTTGATTTGCTTACAGAACAAAACCGAGGACAGCCATCAATTAAAAGCAAAGAGCATTGGAAAA
TCGGGGAGATAGGCCGTGGAAACTGCGAATTTTCAGCTTTTTGCGCAGATTTCGGAACGCCGAaggagga
ggaggaggaggaggCCGGAAGGCGGGGCAAGGCGACGTCATTGAACGCATAGATCCCGGACGACGCAGCC
GTAGAGCTGTTGTCCGGGACGGAGAACAACAAGAACAACAGCAGCggaggaggaggaggaggaggaggag
gCGGCGGGCGGAGGTGGACTACGGACTACAAGAGTCACCCACGCACCCATTCGCTCACGTATCGAAACCT
CTGAAAGCAACAAAATGATCTCAAACAAAAAATCGTACGCTATGAAAATGCTGCAGCTGGCTTTGGCCTT
GAGTCTGCTGCATTATAATCCCGATTACCTGCTCCATCGCTGGGACTCGCAGCTGGAATTGGGCACCCAT
GGCGATGGATGGGAGCTGGAGATGCTGCGGACGGTACACCGCCTGGATATGGATCACAACCCGTATGGAA
ATCGTAAAGGACTGAGTCCCCGCATTGAGGATCTCCTAAACTTCGACGATCCTTCCTTGGGGGGAATGGC
CAATGGCATAGGGGGCTGCAAGCTTCCGCCAAGATTCAACGGCAGCACTTTTGTAATGAATCTCCACAAC
ACCACCGGGAATAGCAGTGTCCAGACGGCAGCTCTGCAGGATGTGCAGAGCACTAGTGCTGCCGCCACTG

GCGGTACAATGGTGGTTGGTACTGGTGGTGCACCCACCTCGGGTGGTCAAACCAGTGGTTCTGCACTGGG
TGAAATCCACATTGATACCGCCTCGCTGGACCCTGGAAATGCCAACCATTCGCCGCTGCATCCAACATCA
GAATTGGACACTTTCCTCACGCCACATGCGCTGCAGGATCAGCGTTCCATTTGGGAACAGAATCTAGCGG
ATCTCTATGACTACAATGATTTGAGTCTGCAAACCAGTCCCTATGCAAATCTGCCTCTTAAGGATGGCCA
GCCTCAGCCATCAAACAGTTCGCATCTGGATCTCAGCTTGGCGGCGCTTCTACATGGTTTCACCGGCGGC
AGTGGAGCACCCCTCAGCACCGCCGCCCTCAATGATAGCACTCCACATCCCAGGAATCTGGGTAGTGTCA
CAAATAACTCGGCTGGAAGGAGCGATGATGGCGAGGAGAGTCTCTATTTGGGTCGACTGTTCGGAGAGGA
CGAGGATGAAGATTACGAGGGCGAGCTGATCGGTGGAGTGGCCAACGCCTGCGAGGTGGAGGGTCTGACC
ACCGATGAGCCCTTTGGCAGTAACTGCTTCGCCAACGAGGTGGAAATCGGAGATGACGAGGAGG
>dme_circ_0007323_junction_seq
GAGGTGGAAATCGGAGATGACGAGGAGGTTGACATATTGACTCATCACCATTTGTG
>dme_circ_0007324
ATTTGCGAGAATAAGCAATTTTTTAATTAACCGATACGAGAACTGTGGATTGATTGCGGGTCTAGGGTAA
ACATGGATGCCTCTTAGTGTAACGGTTTCTGTTGTAGCGCCTACCTAGCTTAGTTTTCTTTGGCCTGGCC
AGGCCAGAAAATCCCCGAGAAACTCTGGCCAGCAGCTGGTCGGTGTATAAGTAGCAATATACCCAAACCC
ACAAGTGCTGGACAAG
>dme_circ_0007324_junction_seq
ATACCCAAACCCACAAGTGCTGGACAAGATTTGCGAGAATAAGCAATTTTTTAATT
>dme_circ_0007325
AAGTTCCCACCGCCTTGCCACCCCTCACGCCGGGCACCAATCGCAAGGTCAACGAGGTACTGAAGGCATC
CTTTGCCTCCTGGGAAAAGGAGGTCCAAAAATGCAACATAACCAAAGGTAAGAAAATGCGTAGAAAGTGA
TTTGATTAGAGGACTATGACTATAGATATGCGCAAAAAACCTAAAGAAGAGTTATCCCTGCTGACCGCTG
AAACTTCCAATTTGAAGAAGATAATCTTAATTATCCTAACTAACCGTGAACTATCAATTGGAGTTCCGGC
CCGAGTACCAATGAATATTAATAGTTTCCCACAGCAATAACAAAGTGCGCTCCCACGCCTAATTAAGTCG
CCACCGAGCTAGAGGCATTAGAAAACGATTAATTTCCATCGCTATTTATATTTATGTATATTTCTATTCC
TATTTACACTCCACCCACAATGTTTTCTTTCTATTATTTCCCATGCAGAATCCGCGTGAATGGACGGAGG
AGCATGTCATCTACTGGCTCAATTGGGCTAAAAATGAGTTCTCATTGGTCTCCATGAACCTGGACCCCTT
CTACAAGATGAAGGGACGCGCCATGGTGGATCTGGGCAAGGAGAAGTTCCTGGCCATCACGCCGCCCTTC
ACCGGCGACATACTGTGGGAGCACTTGGACATCCTGCAAAAAGGGTGAGTACGTGAGCCGAATGCCAGTG
CGCCGACGGCAAAAGAGTAAGCCATAAACATGGACACGACTGGCGCCAGTAACGAAATTTACCTGCCCCA
AAGAGAACCAAAGCCAACCGTGGCAAACCCAAAACGGAAGCCTGTCTAACAAAGTCAAGGAGTCCGTCAT
GATGGTTGGGTGGTTGGTGGCTCTATTGGGTCGGAATGGGATGTGAACCGGTTCTCTGACCTGTTGAGCA
GTCCCATACCCTTTATATTAAACtttttttttCCACTCCTGAAGCTTCCTGTCCAGGCTGAAATTTGATT
TGAAGTAGAGGTCGCAGTTAGACCAAGCACTTGAAAAAAACGAATGTTTTCAGTGAACTCGAATAGAAAA
AAGAAAACTTTTCTTGGTTTTGCACCAAGATAAACAGATTTATAAAAGAGATTGTAGAAATGCAACTCAA
TAAATTTGAATCAATTTATTTGTACACTATATAGTTAGCGGTTGAAACTTAAGTGTACGTACGTACCTGT
AAAAAATAAACTCATTGGGACAGTTTCATATTTTTATAATTTATTGAGTTTGGCTAGCTCTTAGTTTGGG
aaaaaaaaaaCCCTATTAGTATTTTTATGCAACTAATTCAATTTGTTGCTCACCTTTTTCACCGAACCCC
TTTTCGGCATTTCGACATTTTCAGAATTGCGAGAAACCAAATGAGGATATTGTGCACGGCAATTCCTTTG
AGTCGGCCACCACGGCCTCGGTCTGTGGATCGGACCACCAGGTGGCCAATTACCCCAACGAGACGCACGC
CAACAGCAACAACAGCAGCATAAACAGCCGCCTGTCCATGGATTATGTGACGTCGGCGGGGAATAGTGAT
AATAAAAACTTCCACCGCGCCACGCCCCACTCCCACAATGG
>dme_circ_0007325_junction_seq
ACCGCGCCACGCCCCACTCCCACAATGGAAGTTCCCACCGCCTTGCCACCCCTCAC

>dme_circ_0007326
AAGTTCCCACCGCCTTGCCACCCCTCACGCCGGGCACCAATCGCAAGGTCAACGAGGTACTGAAGGCATC
CTTTGCCTCCTGGGAAAAGGAGGTCCAAAAATGCAACATAACCAAAGGTAAGAAAATGCGTAGAAAGTGA
TTTGATTAGAGGACTATGACTATAGATATGCGCAAAAAACCTAAAGAAGAGTTATCCCTGCTGACCGCTG
AAACTTCCAATTTGAAGAAGATAATCTTAATTATCCTAACTAACCGTGAACTATCAATTGGAGTTCCGGC
CCGAGTACCAATGAATATTAATAGTTTCCCACAGCAATAACAAAGTGCGCTCCCACGCCTAATTAAGTCG
CCACCGAGCTAGAGGCATTAGAAAACGATTAATTTCCATCGCTATTTATATTTATGTATATTTCTATTCC
TATTTACACTCCACCCACAATGTTTTCTTTCTATTATTTCCCATGCAGAATCCGCGTGAATGGACGGAGG
AGCATGTCATCTACTGGCTCAATTGGGCTAAAAATGAGTTCTCATTGGTCTCCATGAACCTGGACCCCTT
CTACAAGATGAAGGGACGCGCCATGGTGGATCTGGGCAAGGAGAAGTTCCTGGCCATCACGCCGCCCTTC
ACCGGCGACATACTGTGGGAGCACTTGGACATCCTGCAAAAAGG
>dme_circ_0007326_junction_seq
GGGAGCACTTGGACATCCTGCAAAAAGGAAGTTCCCACCGCCTTGCCACCCCTCAC
>dme_circ_0007327
ATGCCTGCGCAATATGGAATTGGCGATTTGTAAAACAGATCTGTCTGCCACGAAATTTATGCTGCCGCCC
GCTTTGCCCTCCTCAGCGGCCATTGGTTCCTCGTCGGCGGTCGCCTCCACGGCCTCGCATTTCCTGGATA
AGGCGGCCCACGAGCTCTTTCAACTGAACGCCATCAACGGACACCTGTTCAAGTCGCCGGCCAGCAGCCA
CCTCAATTCGGTCGGCAGTCCGTCGATACTTAGCCAATTGAACGGCATCGGCAACAGCGGCAACCATTCG
GGCCAAGTTAGCACCATGAGGCTCAAGAAGAACCGCAAAGTCACCTTTCTGTCCAGCCTAGTTGAGGGTG
AGTTAAGGAATTTCCCAAACGTATTAAATTACTTTGTTATCTGAGCAATTTTCATGGCGACCAACAAGTG
GGAGTAAAAAACTTTCACAGTTTATGATTGCTTTAATTATAATTATATTTCAAATACTTAATCTAGGTCA
TCTTAATGATCACTAGAAGCCGCTTAACTTAGTTTAGGCATAAGCTCAACACTTGGCTTATTCAAAACTA
GGTAGCAGTCATTGCACGTCGTTGATCGATGGCTGCATAAATCTATAATTTCTATATATAACTACGAATA
AGTGAATTTGCTGATCAAGTGATCGATGATAGGCTGTCCATCGTTTTCTATGCCAGTTTGACAGGCACTG
CAGTGGGAGCCCCAATGACAGAGTGATACCCATTGCTAATGCAGAACACGTAGAACGCATTCTAAATTTG
GTAGAGCAAACAAAACTCAATTATTATGTATTATGCTTTGTTGCTGGGCTTTAATTTaaaaaaaaaCACC
CTGCTTTTTCGGATGAGACTTTGCAATTAAGTATATTTTATGGAATAATCTGATCATACGGTATATAACC
AAAGTAAGGGTGTGCCAAATTCGTGTGCATTCCATTGGTATCTTTTGTGTAAGCCATCTTTTGTTTATTC
GCCATAAAAGCCATAAGCGTGCCACTTGGCTTATGACGCATCTGCATAGACAAAAGGCCTGATCAAAATT
TGGATTTGCTTGGTAACGCCTTAAAATGTAGGGACCTCAATGCGAAGCCGACCAAAGAAAGTGAAACAGG
CAACAATGGGATCCAAGATCGTAGGGCTTCCCTTTATTTCAGCCAGCTTGTTCTTAAAAATAAGAATAGC
CTTTGTGATAGATACCCTTCACACTCATGACATATTAAACTGGTTCTTTATAGATTATTTTGAAGTAATT
TAAGTTTTTTGCGTCTTAAGTAGGATCAGTTATTGTATGCCCAACCAACTTTGTCTTAATAGAGCTGCCT
CCTCAAATAACAAGAGTATCCAAATCTTCGGCTATGCAAAGCAGTCATTCTCTTATTTTCCCACTCCCTT
GGATTCTTTGTGTCCCCAAGAAATTGACGACCGAGTGAAGGCATTTGACACATCGCCTGCGGTAAGCATA
TCCGTTGGCCCGCCGAGCCGTTGACCACTTTCATCCGGTCTTTCGGGGCCAACAGCCTGACCCCCAGCTG
ATCCTTACCCCGCTGCCCTGCCAGTGACCACTccccccccccaaaaaaaaagaaaaaGGACATGCGCAT
GCGCAGTCGCTTGGAAAATGCAGCCAGGTAATCCAATCCGAAGTGTCTGGCCTTTAGAAGGAGCCAAGTT
TTGGGTTTCCATCAGTCTGAACAGTAATTGAAGCCTTGTGGATCGTAGAGCTCCAAAAATTGGATTCCAA
TTGAGTATGTGAATTCAGCTGGTATTCGCTCAGCAATTGATTACGATATTTACGGAGCATTTCGTTTGAC
TACTCTGACCTCCATTATCATTAATGGCATTCCCGCAGACAAAGTTCCCTCGTTTGAGCAAGTCAAACGT
ACAATTCAATTTATTAGAACGCGTTCAACACGATCCGATCCGGACCCATGGGACAACATTGGGGCAGTCC
GCCGCAGCCAGCCACTGTCAATTGCCGTAGCAATCTATGCCACGACTCTGCGCTATTGTATCTCTGATTA
TAATGCCCGTCGAGCCACAGTTTTACGTTGCACTCGcacacacacacacacGCATTTTGTAATTGTAATT

GGGTAAAAGTGGCTTGTTTATAAATATGTACAACACACAATCATGGATGCCTCTTGTGTTGACTGTTGCT
CATTGTGCTGCAAATGGGGGTGGCAGGTGGAGCACACCTCTTGGGGAAAGATaaaaaaaaaCATGCAAAT
GTCAGCGGGGGAATTGGTGTTTTGGCCAGTGAAAACAAACAAAGCTGATGGATTGATCATGCCTAATTTA
TTGCCTTCTAATTAAGAACCAATGTCAATCAAAGCAGCAGATTCTTACTGCATTTAAATTTAAATTAGCT
CAGAAAAGTAATGCGAATGGAATGCAAACATTGGAATTGACTTCTAATATGACACAATAATGTTCTACTT
GTATCTTAAAACAAATTAGTCAGCAAATGAATCAGCCTTGAGATGTTAGTTAAAACCCTAAACTAATAAT
ACAAATTTCATCTTGCTTGCAGTCGAAAAATATTTTCATCAAGGAGGAGCCGATCCATGGATGCAAGGAT
CTGTGCAGTTTGTCGGATATTTCAGATCATGAGGCATCATTAGG
>dme_circ_0007327_junction_seq
ATATTTCAGATCATGAGGCATCATTAGGATGCCTGCGCAATATGGAATTGGCGATT
>dme_circ_0007328
GAGCGATCTCTACTGTGGGGCCTGGGTGATGGGATCGACCTTAACATCGGGGAACTGGAATTCGGGGAAC
TTCAGCATGTCGGTCTTGCCATCGCTGCCAAACTGCTTGGCCACACGGTCCAGTTCGGTCTTCAGCTCCC
GCTCAATATCGGGATTGGA
>dme_circ_0007328_junction_seq
TCAGCTCCCGCTCAATATCGGGATTGGAGAGCGATCTCTACTGTGGGGCCTGGGTG
>dme_circ_0007329
GATTTGGCGGGAGGACAACGACCGCTGGGTGGAGGAGCACCGACTGGAGGCGCACTCGGATTGGGTGCGC
GACGTGGCCTGGGCGCCGTCGATCGGATTGCCCCGCTCGCAGATTGCCACGGCTTCGCAGGATCGCCATG
TGATTGTGTGGAGCAGCAACGCAGATCTATCCGAGTGGACCTCGACTGTGCTGCACACATTCGACGATGC
CGTGTGGAGCATCTCGTGGTCAACCACCGGCAACATTCTTGCCGTCACCGGTGGGGACAACAAT
>dme_circ_0007329_junction_seq
TCTTGCCGTCACCGGTGGGGACAACAATGATTTGGCGGGAGGACAACGACCGCTGG
>dme_circ_0007330
CTTGTACCTCAGCGACTCAGCCTGGGCAATGGCGCACAGGCCACGAGCGGCCACCTTCTCGGCGTACAAC
TCAATGCGTCCGGTCTCGAAGTTGAAACGCTTCTGCACCATGGCGGTCAGCTCCCGAATGCGACGGCCCT
TCTCGCCCAGCACCTGCTGGGTCTTGG
>dme_circ_0007330_junction_seq
TTCTCGCCCAGCACCTGCTGGGTCTTGGCTTGTACCTCAGCGACTCAGCCTGGGCA
>dme_circ_0007331
GTTTACAAAACTTTCGCCTCGGACAGATCGTCCAGTGGCTTGCTGGGCGGGGGGATCTTGTACTCGGTCT
CGGGCGTCTCGTAGATCTTCTCCTCCTTGGGCTCCACAACGGACACATTGTCGGGCAGCGGCTTCTTGGG
GCCGATCTTG
>dme_circ_0007331_junction_seq
GGGCAGCGGCTTCTTGGGGCCGATCTTGGTTTACAAAACTTTCGCCTCGGACAGAT
>dme_circ_0007332
GATCGGCCCCAAGAAGCCGCTGCCCGACAATGTGTCCGTTGTGGAGCCCAAGGAGGAGAAGATCTACGAG
ACGCCCGAGACCGAGTACAAGATCCCCCCGCCCAGCAAGCCACTGGACGATCTGTCCGAGGCGAAAGTTT
T
>dme_circ_0007332_junction_seq
TGGACGATCTGTCCGAGGCGAAAGTTTTGATCGGCCCCAAGAAGCCGCTGCCCGAC
>dme_circ_0007333
CTGTGGCCCAAAGTAGCTTTAAGTGTCCCGATGACTTTGGATTCTATCCACATGACACGTCCTGCGACAA
GTACTGGAAGTGCGACAACGGCGTTTCCGAGCTGAAGACCTGTGGAAACGGTCTGGCTTTCGATGCCACA

GACTCCAAGTACCTAACCGAAAACTGCGACTATCTACACAACGTGGATTGCGGCGATCGCACAGAGCTGG
GAGCCCCCAATTACCACCCCCCACTGCTCTCGCCTGTACGGCATCTTCCCCGATGAGAACAAGTGCGACG
TGTTCTGGAACTGCTGGAACGGCGAGCCCTCCAGATACCAGTGCTCCCCCGGATTGGCTTACGATCGCGA
TGCTCGCGTGTGCATGTGGGCTGACCAGGTGCCTGAGTGCAAGAACGAAGGAGGTGGCCAACGGATTCTC
TTGCCCGGCGGCTGGTGAGCTGGCCAACGCCGGATCCTTCTCGCGCCACGCCCATCCCGAGGACTGCCGC
AAGTACTACATCTGCCTGGAGGGTGTGGCACGCGAGTACGGATGCCCCATCGGCACAGTGTTCAAGATTG
GCGACAGTGATGGCACTGGCAACTGCGAGGATCCCGAGGATGTTCCCGGGTGG
>dme_circ_0007333_junction_seq
CGAGGATCCCGAGGATGTTCCCGGGTGGCTGTGGCCCAAAGTAGCTTTAAGTGTCC
>dme_circ_0007334
GCGGAGACCTAAAAGGAGCCCCCAATCCCATTCGTAGTAAAGAGGAACATCCTCCGTGTTAAGGCGCATA
TGTGAGGCGTCCTTATTCCGCAGGGCGTATTCCGTCCACCAAACTGCTGTGTCCAAAGGACTCATGGGTC
GATCCCTGAAGGATTGCGACATTTTCTTGGCTTTCAGAGCATATCTTGGGTTCTCCAGCAGCTTCCGAAT
A
>dme_circ_0007334_junction_seq
TCTTGGGTTCTCCAGCAGCTTCCGAATAGCGGAGACCTAAAAGGAGCCCCCAATCC
>dme_circ_0007335
GAGCCTTTGCCAATGGACGCACAACTAGATTGTGAGAGCTGAAGTGGTAGGAAAATATGCCTAGGATGTT
GGCAGCCTGCACCGAGTCCTGGTACAGATCCTTTTGCAGGAACAGGCAGAACAAGACAGCCAGCCAATAA
AACTCGAGGTGCATTTCCACAGTTCAGTCCGCTCATGTCGGAGCTCTGTCCACAAATGAACACAATCTAA
TCTAAGGTGTCGGCAGAAAGCTTAACTGATATC
>dme_circ_0007335_junction_seq
GGTGTCGGCAGAAAGCTTAACTGATATCGAGCCTTTGCCAATGGACGCACAACTAG
>dme_circ_0007336
TGATCCTGGCGTTCAAAGGATGCTGCATGACAAAAGCGAAAGTTTTGACTTGATCATCATGGAACCATCT
AGTCTGGACGCCCTTTATGGCCTGGTTGAGTTCTACAACGCCACGCTGATGGGATTGTCCAGTATACGGA
TTAACTGGCACACATACGAATTGGCAGGAAATCCAGCTCCATCAATATACGAACCCATTTCTCCGG
>dme_circ_0007336_junction_seq
CCATCAATATACGAACCCATTTCTCCGGTGATCCTGGCGTTCAAAGGATGCTGCAT
>dme_circ_0007337
CTGTGGCGGCATCCCTGGCGACGGTCCTACCACAAAAGGAAGAAGGCCCAATGGGAGACAGATGCCAATT
ATTGTGCCCTGGTATGTATACCCCAAGTGCGGCCCGTGCTCGTGCTGCGGTTTCTTTTATTTAATTTGCA
TTTAAACTCGATTGAAATGCAAATGTTTTTATTGTGGTCATAGCGGGAGGGCGGCGCTTCTGACTGGAGC
TGCTGACTTAATTAAGACATCGCTGGCCGAGCTGCAGCTTAATTTTGCATTTGATTTTCCCATTTTCTCT
CCTCCACCAACTCGCTTCTGTGCACGATGCCCACTTAGGGTGAGTAGTATTGGAGTATGGGTGGTATTGG
CTAAATAGGGCTAAGCCAAGCGGAGGGGGCCACACGTCTGTCTATGTCTGCGTCTGGGTGTGCAGTGTGT
ATCTATGTCCTTGGCATGGTAACTAATGCGATCGGTTTATTTCCGCACACATGGCTTTGTTGGCTTCCTG
CCTTCATCGGTTCCTCGGTCTGACTTTGGCACGTTACGTATGCGCCCTGTGTGCGCCTAAGCGCCTGCAT
TTGCCAGGGATATGCCAGCACCTGCATGCCAAGCAGCCGCCAATGCCGATGCCGTGGCCACAGATGCAGA
TGCAGCAGCTGGAAAGATATTTTCAGACCGCCTGCAAAAGGTTGCAAAACATCTCCAGGTTGTAAGCCGC
CACACCAACGCCACACACATCATCGATGCGCCGGGTCTTTGGAGACCAGGTGCCAGGTGACTTGGCTGCG
CTGCTGCTGCTAAATTGTTTGTTATGCAAAAGCGTTTGTTGGGCTTACGGCGATGTCATCGCTGGCACCC
AGCTAACACCTGGCCATCCTGGTTCATTCCTGACTTTCCATGCCACTCGGCCACCTTGCCACCCACTTCT
GCTAGGCAATTATCCCCTCCGCCAATCGCGTCCAGGAAGGGCCTTCGAAGGACCTGCCCTGGCTGATTGC

CGAATACACTCTAATTTGGCCCGCCTTTCGTTTGCATTCCAGGGGTCTACGGCAACGAGACTTTCCCCCT
GCATTTGGACCACGGCCGTGGTTTCGGGCGGCCCTTCCACGACGAGCTCTCCATCCTGGCGCCGGTGCTG
CAGTGCTGCCTGATACGCAAGTCGACGCTCGTCAAGCTGCTAGAG
>dme_circ_0007337_junction_seq
CAAGTCGACGCTCGTCAAGCTGCTAGAGCTGTGGCGGCATCCCTGGCGACGGTCCT
>dme_circ_0007338
TGCAAAAGGAGGGCGGCACCCAGCTGAAATTGATCATCGAGTACCCAAACGATATCAAGGCCTTAATGAA
GCCGATGCGGTGAGTTTGGCCAACGGATGGACAAAGATTTCCCGGTCTGTCGCCGCTTAATGCATTTGTC
CCTTGGATTTCCGCGTGCGTAATGAATGCAACTTTCGCCCACTTGTCGACAACACTTTTCGCACAGTCGA
GTAAATAAAATTGCGCTTTTTATTCGCTGACTTTCCTGCGGCCTGTCGATGGAAATTCCACGTCCATTGC
CGTCGTTTTGCGGTGGCACTTCAAGCTGCTCGCAAATTTAAAACATTCATGCACTTTTTAATGAGATTTA
AACGCATTGGCTTGGTCAGCGTGCTGGTTTTCCATTCCTCCATCTCTGCCATCGCAGCTGCCAAAGTGCA
AAGTGTCCTTATAATGCCATTCATTTGCAATTTAAATGAAAGCCCTTCTCCTTCTCTGCTCTTCtttttt
ttttttttaaataaatatataggaacatttcgttttttttttttttttgtttttAAATAGCACTCCCTTT
CGTGTAAGTAATTAAGCTGGCACATAGCACGTAGCAATGCAGGTTCAACGTGGGAAAATTCAAAGTGGCC
CCACGCAAATGCATTAAGCTGGTGACAGAAGCCCGCAGTTTCTATTCATTTCATTATGTCGCATCCCCAC
CTCTGATCCCGCTCATTTGCAATTTGCATTTCCACAGGTTTCCGCGGGAGCAGCAAACGTTGCCCAACCA
CTTCTACTTCACGGACTATGAGCGCCACAACGCCGAGATTGCCGCCTTCCACCTGGACAGGTGAGTTCCG
TCGACTCCATTCCATTCCATTCCGATTCGACGCCATTCTGAACTTTCACCGGGTGCATTCGAGTGCACAA
GAATTCCGTTGTGAAATCGTTCGATCGAATTAAATCTGCCCAGGCATCGTGTAAATGTCTGGCATACTCT
TATTTATTTTTAATTTAATTTTCAATTATACGTAAATATTTATGGTGAGCATTGAACGGATTCCCACCGC
AGCACAAAGGCGGGGAAATCGGGAAAATATTGTGCAAAATCAATTTGAAATATTCAATTAGGCAAACCAG
ACTGTTCTCTGCAGACTGGCTGAAAATACCTGAATTTAATTTTAAGCGATTAAAAAAGGGATTGTTCCGA
AAGCAACGAAAAAAATAGTTATATTTTTTTGATCTTTTTATCATAGCATAGTAAATGGAGCATTCACTTG
CACTAAAACTATTATTATTAAAATCAATTTATTTTTAGAATAATAAACCTATTGATTGCATTTTCGGTTC
ATGCCTCTTAATTAAGCCTTTGTTTGAATTTTTTTATTATAGTGTCCCTAAAAGGGTATCATACTCTAGG
TGTTCCCTACAAATAATAGTCATTTCCTTGGTCAATACTCGTAATAGTAAAATTCCGGGTGTCAGTGCTC
AAATTATTTTCGCTACCACATTTCAACTTTATGGCCCCTTGATAGTTATTTCTTTTTTTAGTGCATGACA
AAGCAATCAAATTAACCAATTTGTCTGCCATGTGAAACGGTCCCTTGGCCAACATTCGTCCAGGCCAGCA
TTCAAATTTATAACGCTTGGGATGCCTGACCCTCGGCGATTAGAATCCCAATAATCCTGacacacacaca
cacacacaTACTGAGGTCAGACCGCAGTCAATTTATGTTCATTATTTATGATGGCCGTGCCGAATAAAAT
AAAAAATGTCCATACATTTACCCGTTCGGGGTGGGCAAGGCCAAACGGCGCCATTAAATGATGTTTAAGA
ATATATTTTTTAAAAAAGGCGAAAAAGCTGTGGTGCCAACTCATGTAAATGATGTGGCCACATAAACCCA
ATGCCTGCCAGTCTGGCTATCTGCGCCCCTTTCATGGCTAATTAACTTGGCCAAGTGGCCTGACATGTCCT
GCCGTACTTTAGACTGAAATTGTCTGCATAGTTAGCAGCTGCTTGCCTTTGACAAAACCTTGACGGCCAG
GTTAATTGCCGGGGGATTTTTGAATTTAGATAAGGCGTCATAACTTGTGTAATTGTTGTGGCTGCCAACC
ACACGCACATGCCGCACACATCCTCTAATCGAGTTGGCTCCACTCTCCTGTCGATTGGCTGGCCAAAACT
CCGGAGGATTAGACGGACCAACTTCTGGCAACTCGAGCTGACAGCCCTCTAATGGAGTCGCATGTGTGTG
ATGGAGTAGGGCCGTGTGGGTGATGGAAATTTACGGCATTTTCCCATTTTGACCTCAGTGGCTGCCAATG
CCCGACGATGGCGCCCAGACAATGAAACATGCTTCATGTTGCCAAATAATTTCGATAATATTTTCTGGTC
AGTCTGGCCACCAACATTACCAACCACCATAAATCCCATGCGAACCCTTTAGGATCGAAAGAGATTAACC
GTGACTAGGCGAAAAGATTGATGAAAACTTCATTTATTAGATTCGATATGAGGTCTGCTTTTTACGCTGC
TACCATCGTAATTTGATTGATGAAAATAAAGCTAAAGAGACTCGGATTTCGCTAAATTAGCACAATTGTT
GTGCTTGGTCTTGATTTCCTTAAATAAACAAGGAATTATTTTCCTTATTATATTACTCCCTACCGTTTCC

CTGACAACAGCCCACAAATATTTTTCTCGTTCTTGATTACGAATTCCGGTTCACACGCAGTCGTTTTGCT
TCGCCCCCACAAACCATTaaaaaaaaGTTTACCCACAGAGCTCTTATTTCTCGTAGGATGTTTCAGTTAA
TACAGGGTACTCATAGATCATACGGGGTATATTCGACATGTAACTCAATTCAACAAAGGGAATAGAAAGA
TTTTCACTTGTATGTGATGATGttttttattttaatattatttagattttttttGTGGTAAGAAACATATA
GCTCATATTTATAAGTACTTTGCTTACAGGATATCTTCAATTCACCAACCTTCGCCTTATTTTCGCCCGC
CTGTATTTTTTTATTTTGGCATTTTGTTAACCGCTTTTCTGCTCTCCTCTCCGGTTTCAGGGATCCTGGG
ATTCCGGCGTGCCATGCCCGTTGCCGGTCGTACGCTGAACATTACGACCGAAATTTATCAACTGGCCGAG
GAGAATTTATTGAAGACGTTCTTTGTTTCGCCATCGCTGAATTTATGCTTCCATGGCAAGTGCTCGTACT
ATTGTGACACCTCGCATGCCATTTGCGGCAATCCGGACATGTTGGAGGGCTCCTTCGCCGCCTTTCTGCC
CAACTTTGAGTCGGGCAATCGCAAGGGTATGTATCGTATCCTAGACACGCTGGTGTATCAGCGTACACAT
CGCACAGCTACCTCATCCAGCATCCAGCACGCATGACAGtggattgaaatgggatgggatgggatgggat
ggtataggatgacatggtatgggTATCATGTCTGCGGGCCCGGCTAAGTGGTTTTTGATTAATGCACGGC
CAGCTTTAACACTGCTCCAGATTACAATTTATGCTCTGCCCGCTTTCATTACCACATTCGTGCGCCTGGC
CGTTGGGGCCAAATAACAAATCACTTTATTTACTTAGTTCGTAAGCTGTTTATTTGTTTGGGTGTCGCAA
CAGGTGAGctatggactacggactatggactatggacgatggactatggCTAGGTGTTGGTCAGTTCTTA
GAGATACAACTTACTTAAGGCTTAGAGGGAATTTTTAATAGCTGGCTGCCTGGCTGGCTGGCCCAAAAAG
CAAAAGAACTCACTTTTAATTTGCATAATTTTTGTTGTTCGTATGCCTTTTATCCTTTGTTTGTGCGAAT
GCCGGACTCGTTTACCTTTCACCCAACCCAGCGAGCCACTTCATTGCCATGCCCAATTCATTCATTCGTT
TGGCCGTTGAATTAAGAGCAGAAATATTGTATCAGTTCTTCAGCGACTGGTAAAGCGTAAAATGGAATAT
AAAATGCAGGGACCCAGGACTCAGTCGTTGGCGTTAATAAATTCTAGAGTCCACTCCATCAACGTAAAAA
AACACGAGCGGTGGGCGAATaaaaaaaaaaGGGAAAATGGCAACAGCTTTTAAATGCAAATTACCGAAAA
ATTAAAGCCCAGTCCTGTCCACTTCAGGACTTCGGGACTGTCTCGGTTGGGGAGCTGTGGCCATAAAAAG
GGTTCAGCTCAGCTGGACTCATCCCTCAGATTTGCCGCAGCACAGGCATTTTATTTTTAGTCTATTTTGA
ACGGAGatatatatatatatatgtacgtatatatatatttttttttAGAATCCCTGCCCCGAAGCCTCAAAG
GAATAATTCTCTTTATTTCCCCCtttttttttGCGGCTCGCTCTCCAAACAGTTTACAAGTAGAAAATGG
TTAAGTGTATACTTGAGATTTGGCGTAAGGCAAAACTGTAAATTATGCACAAGAGATCATTTTTCTTTCT
CCTCTTGCACAACCCTATTTCTTCCTCCtttttttttgtcccccctccctttgagctattcaggtttttcct
actttccttttccgGCCTGGCTAAGTTTTGTTTCCCCTTCGTTGTAAACAACGAGGAAATTTTTGTAACT
GAACAGAATTTTAATTAAGTTGCAAATTTGATTTGCACGCTTGTTAAATGCTTTGGGAATTCATTTAAGG
GGGATCTACGGGTAACTAAATTGCAAGGACCGAAAGAAAAGGGAAAAATAATCGTGATAACTAAACAATT
GATTAGATTATAGCTGGGATAAAGTGGAGCTCAAGCATTTATAAATGAATTCTGTCTAATTGAGGAGAGG
AGTTGACTTTAATCACAGAAATTGGCTTAAATACAGGTGCTTCAATAGCATCTAACTAAGTTTTTCAGGG
AATTTATTATTTCTACTTATCTTATTTTCGCCTGGAACTTCATCTGCATACATTTCAAATACATCTTACA
TGAACTTAAATTATAACAAAAACTTTGTGGGCATTTCATTTCATCCAAATGAGTCAAATTTTAAATTAAA
TTCTTTTATTATTCGGTTAATAAAATAGGACTGTGATAATTGCATTCCATGCAAGTCCGCTGACTTTGGG
CCATCATTTCCATAAAAGTCATTGCATCTTAAAGCTCTTCTGCATGGCGTTCACAAACTTATCAAGTCCA
TTTAACGCGAAGCTAACAAAAATTGCAAATTGCTTGCCTCACATTCTATGTTTTTTTGCGCGTTTCCCAT
TTCCCGCTGCTCTTATAAAATTTAATTTTGCTTGATTTATGCATAtttttttttCTACCTTTTGGCTAAG
CTAAGGTGAACAGTGGAGCTAAAGAGCGCTATGAGAATCCCCGCTCCGCTGTCACCATGGCAGCGTTAAC
TTACCTGGGtttttcgctttatgtttggcttttttcacatattttttcTCGTAGCAGTTTGTTGCTTTTC
CCCAAGTTTGCATTTTCCGCTTTCTGTTTGCCTTGCTTTGCTTTGCGCGCCGCCTGCTGCATTCTTCCCC
GCGAAAAGCGGCGGGAAAACCCCGACCCCAAAATGGGCGCCCCTATTTTGGTCCCCGCTTCCGGTCATTA
CACATTCTCTGCCGATGCCACGCCCACTCAGCATAAAATGCCCAACGCGCCGAGCTTTTATGATGCATTT
TTGTGCGCTCTGTTCCCCTTCTTATTTTTAATTCTTTTATATACATATATATTTCATATATAATTTGTTG

TTTTGCTGCTCCCGCTCCTGTTTCTGCCGCTCCTGTTCCCGATCCTGCTCCTTGTCGCtgttgctgtttc
tattctgttgttgtcgttgttgttgtcgctattgttgACCCCCTGACCCCGCAACACAGGGACGCAAGGT
AAGTTGCGGTGGCCCAGAGGAAACGCAGGGGTCGAGACTAAAGTTTATTACCCACCTTCAGTTCGGTGC
GAATAATTTAATTTAAGCAAAATTAATTTGGAGTTGCAGTTGGAGGAATTCGCAAGAAAGACGGCAAAG
CAGCCATAAACCAAAATTATTAGTTTTACACTCGTGGAAAAACTGAAATTTTGTGTTCTTAAAGTTaaaa
accaaaaaaaatttataaatgaaaaaCTTTTAATTGAATTTAAGGGTTACATAGAGAAACAGAAAAGTAG
CGTTTAAAAGCCACATAAACTTTTCATTTTTAGAAAGTGCGATATCTTTAGGCATTTAAATGAGACATTC
AGAACTTGAAGATGTTACTTTAAAATATTAGAAAACTATATTTCTTGCCGTGCAATTAGAACCAAATGCC
TTTCTCTAAGACAGATATTACTCGGGCGATTGCGTAAGCGGTTTACTGTCGGCCTGCAAGAATGCGAATT
GATTTCCGGTCGTCCTTTGCttttttttttACCCAACAGCGTATGTATACCCCAAGTGCGGCCCGTGCTC
GTGCTGCGGTTTCTTTTATTTAATTTGCATTTAAACTCGATTGAAATGCAAATGTTTTTATTGTGGTCAT
AGCGGGAGGGCGGCGCTTCTGACTGGAGCTGCTGACTTAATTAAGACATCGCTGGCCGAGCTGCAGCTTA
ATTTTGCATTTGATTTTCCCATTTTCTCTCCTCCACCAACTCGCTTCTGTGCACGATGCCCACTTAGGTC
CGTGATATTCCGCCCTACGACGATGGAAGGCGACTGTACGACCTTATGGACATGGCCGTCTTTGATTTTC
TCACTGGCAACATGGATCGTCATCATTACGAGACATTCAAG
>dme_circ_0007338_junction_seq
GGATCGTCATCATTACGAGACATTCAAGTGCAAAAGGAGGGCGGCACCCAGCTGAA
>dme_circ_0007339
GTTTCCGCGGGAGCAGCAAACGTTGCCCAACCACTTCTACTTCACGGACTATGAGCGCCACAACGCCGAG
ATTGCCGCCTTCCACCTGGACAGGTGAGTTCCGTCGACTCCATTCCATTCCATTCCGATTCGACGCCATT
CTGAACTTTCACCGGGTGCATTCGAGTGCACAAGAATTCCGTTGTGAAATCGTTCGATCGAATTAAATCT
GCCCAGGCATCGTGTAAATGTCTGGCATACTCTTATTTATTTTTAATTTAATTTTCAATTATACGTAAAT
ATTTATGGTGAGCATTGAACGGATTCCCACCGCAGCACAAAGGCGGGGAAATCGGGAAAATATTGTGCAA
AATCAATTTGAAATATTCAATTAGGCAAACCAGACTGTTCTCTGCAGACTGGCTGAAAATACCTGAATTT
AATTTTAAGCGATTAAAAAAGGGATTGTTCCGAAAGCAACGAAAAAAATAGTTATATTTTTTTGATCTTT
TTATCATAGCATAGTAAATGGAGCATTCACTTGCACTAAAACTATTATTATTAAAATCAATTTATTTTTA
GAATAATAAACCTATTGATTGCATTTTCGGTTCATGCCTCTTAATTAAGCCTTTGTTTGAATTTTTTTAT
TATAGTGTCCCTAAAAGGGTATCATACTCTAGGTGTTCCCTACAAATAATAGTCATTTCCTTGGTCAATA
CTCGTAATAGTAAAATTCCGGGTGTCAGTGCTCAAATTATTTTCGCTACCACATTTCAACTTTATGGCCC
CTTGATAGTTATTTCTTTTTTTAGTGCATGACAAAGCAATCAAATTAACCAATTTGTCTGCCATGTGAAA
CGGTCCCTTGGCCAACATTCGTCCAGGCCAGCATTCAAATTTATAACGCTTGGGATGCCTGACCCTCGGC
GATTAGAATCCCAATAATCCTGacacacacacacacacacaTACTGAGGTCAGACCGCAGTCAATTTATG
TTCATTATTTATGATGGCCGTGCCGAATAAAATAAAAAATGTCCATACATTTACCCGTTCGGGGTGGGCA
AGGCCAAACGGCGCCATTAAATGATGTTTAAGAATATATTTTTTAAAAAAGGCGAAAAAGCTGTGGTGCC
AACTCATGTAAATGATGTGGCCACATAAACCCAATGCCTGCCAGTCTGGCTATCTGCGCCCTTTCATGGC
TAATTAACTTGGCCAAGTGGCCTGACATGTCCTGCCGTACTTTAGACTGAAATTGTCTGCATAGTTAGCA
GCTGCTTGCCTTTGACAAAACCTTGACGGCCAGGTTAATTGCCGGGGGATTTTTGAATTTAGATAAGGCG
TCATAACTTGTGTAATTGTTGTGGCTGCCAACCACACGCACATGCCGCACACATCCTCTAATCGAGTTGG
CTCCACTCTCCTGTCGATTGGCTGGCCAAAACTCCGGAGGATTAGACGGACCAACTTCTGGCAACTCGAG
CTGACAGCCCTCTAATGGAGTCGCATGTGTGTGATGGAGTAGGGCCGTGTGGGTGATGGAAATTTACGGC
ATTTTCCCATTTTGACCTCAGTGGCTGCCAATGCCCGACGATGGCGCCCAGACAATGAAACATGCTTCAT
GTTGCCAAATAATTTCGATAATATTTTCTGGTCAGTCTGGCCACCAACATTACCAACCACCATAAATCCC
ATGCGAACCCTTTAGGATCGAAAGAGATTAACCGTGACTAGGCGAAAAGATTGATGAAAACTTCATTTAT
TAGATTCGATATGAGGTCTGCTTTTTACGCTGCTACCATCGTAATTTGATTGATGAAAATAAAGCTAAAG

AGACTCGGATTTCGCTAAATTAGCACAATTGTTGTGCTTGGTCTTGATTTCCTTAAATAAACAAGGAATT
ATTTTCCTTATTATATTACTCCCTACCGTTTCCCTGACAACAGCCCACAAATATTTTTCTCGTTCTTGAT
TACGAATTCCGGTTCACACGCAGTCGTTTTGCTTCGCCCCCACAAACCATTaaaaaaaaGTTTACCCACA
GAGCTCTTATTTCTCGTAGGATGTTTCAGTTAATACAGGGTACTCATAGATCATACGGGGTATATTCGAC
ATGTAACTCAATTCAACAAAGGGAATAGAAAGATTTTCACTTGTATGTGATGATGtttttttattttaata
ttatttagatttttttGTGGTAAGAAACATATAGCTCATATTTATAAGTACTTTGCTTACAGGATATCTT
CAATTCACCAACCTTCGCCTTATTTTCGCCCGCCTGTATTTTTTTATTTTGGCATTTTGTTAACCGCTTT
TCTGCTCTCCTCTCCGGTTTCAGGGATCCTGGGATTCCGGCGTGCCATGCCCGTTGCCGGTCGTACGCTG
AACATTACGACCGAAATTTATCAACTGGCCGAGGAGAATTTATTGAAGACGTTCTTTGTTTCGCCATCGC
TGAATTTATGCTTCCATGGCAAGTGCTCGTACTATTGTGACACCTCGCATGCCATTTGCGGCAATCCGGA
CATGTTGGAGGGCTCCTTCGCCGCCTTTCTGCCCAACTTTGAGTCGGGCAATCGCAAGG
>dme_circ_0007339_junction_seq
CCCAACTTTGAGTCGGGCAATCGCAAGGGTTTCCGCGGGAGCAGCAAACGTTGCCC
>dme_circ_0007340
TGCAAATGCCAGCAATTTGGAGCGCTTCCAATTGCGCATCACGAAGAAGGAGCTGTACGGCGAGCAGGAC
ACTCTGGTGGATGCGGTGTTACGTGACATGATCAAGCTGCCAATACAGCATGTTGGTAAGACAGTCTGCT
TCCGTTCTATTCAGCCGTTCGGCAATTGTGGGATTGTAAAGTCATAAATACTGGCCAAGTCTGTTGGCCA
CCTCACCCAACGAACTTTCGAACCTTAGTGGGGTTTACACGAGGCCAAAAAATGTAAGCGGGCCAAAAGA
GACTCAGCTGCACATTTAACTCAATTATGTAGAGCTTGGCGGCGGCTGGCATTATTGAGGTGCCACTGCG
AAACTATTTTCATAGCGAAGTTTTTCCAGCGCATTCATTAAACTCATAATTTATTAATAATAAACTTTTA
ACAGTGGCTGCACGGAGCTGCGAAAAGAAAACTCATTTGCAGTTAAGGGGAAACCAAGGGATAGACAAGA
GTTGGGCGGGCCGAGCGCAACAATGACACTGTGGCAGCATAATTGCTGCAACTTTAGCTTTGTTCTGCCT
GGCATTTATCACGTATACGCCGCGTATACACAGGCGCATACTAACACAGACAAACAAATGCAGCCCTTTG
GCGTTAGAACAACTTTAGAGAGATAATTCATAAGTAATATAGGACCAATTTTCATTTGTTTATTTTTAGA
ATAAATTCCAATGAAATGTGGCGTGTGTGCGGCAGGAGTTTCGGTTTTATTTTCCCTTACGGTCAGTCAG
CAGCTTGACTGTAAAGCCCAAACTACAATGAGCCAAGTTTTTGGTTCACTACACACACTGAATTGCAAAA
GCAAGCCAAGGCGGTTTTTATGTCGGGCTTCTTTGTGCGGTTGCGTTTACGCAATATTTGTGTGCTCTTT
GAATGCATTTTAATTGCTATTTAGTCGAGTGTAATTTGTTTATGCAGTCCGCTCGGTACGGTACGGTTTG
CAATTTTAAAAGCAGTCTGACTGCAGTCTCCGTGATCGGTGATGTGAGAATTGCATGAATTTTTGGTTTT
TGCTTCAATTAATTCCGAGTGCTCGATTAATTTACATTCATTCCTCTGTCTTTTCTCCGTTCGCAGTGTG
AGTTCCGTCGACTCCATTCCATTCCATTCCGATTCGACGCCATTCTGAACTTTCACCGGGTGCATTCGAG
TGCACAAGAATTCCGTTGTGAAATCGTTCGATCGAATTAAATCTGCCCAGGCATCGTGTAAATGTCTGGC
ATACTCTTATTTATTTTTAATTTAATTTTCAATTATACGTAAATATTTATGGTGAGCATTGAACGGATTC
CCACCGCAGCACAAAGGCGGGGAAATCGGAAAATATTGTGCAAAATCAATTTGAAATATTCAATTAGGC
AAACCAGACTGTTCTCTGCAGACTGGCTGAAAATACCTGAATTTAATTTTAAGCGATTAAAAAAGGGATT
GTTCCGAAAGCAACGAAAAAAATAGTTATATTTTTTTGATCTTTTTATCATAGCATAGTAAATGGAGCAT
TCACTTGCACTAAAACTATTATTATTAAAATCAATTTATTTTTAGAATAATAAACCTATTGATTGCATTT
TCGGTTCATGCCTCTTAATTAAGCCTTTGTTTGAATTTTTTTATTATAGTGTCCCTAAAAGGGTATCATA
CTCTAGGTGTTCCCTACAAATAATAGTCATTTCCTTGGTCAATACTCGTAATAGTAAAATTCCGGGTGTC
AGTGCTCAAATTATTTTCGCTACCACATTTCAACTTTATGGCCCCTTGATAGTTATTTCTTTTTTTAGTG
CATGACAAAGCAATCAAATTAACCAATTTGTCTGCCATGTGAAACGGTCCCTTGGCCAACATTCGTCCAG
GCCAGCATTCAAATTTATAACGCTTGGGATGCCTGACCCTCGGCGATTAGAATCCCAATAATCCTGacac
acacacacacacacaTACTGAGGTCAGACCGCAGTCAATTTATGTTCATTATTTATGATGGCCGTGCCGA
ATAAAATAAAAAATGTCCATACATTTACCCGTTCGGGGTGGGCAAGGCCAAACGGCGCCATTAAATGATG

TTTAAGAATATATTTTTTAAAAAAGGCGAAAAAGCTGTGGTGCCAACTCATGTAAATGATGTGGCCACAT
AAACCCAATGCCTGCCAGTCTGGCTATCTGCGCCCTTTCATGGCTAATTAACTTGGCCAAGTGGCCTGAC
ATGTCCTGCCGTACTTTAGACTGAAATTGTCTGCATAGTTAGCAGCTGCTTGCCTTTGACAAAACCTTGA
CGGCCAGGTTAATTGCCGGGGGATTTTTGAATTTAGATAAGGCGTCATAACTTGTGTAATTGTTGTGGCT
GCCAACCACACGCACATGCCGCACACATCCTCTAATCGAGTTGGCTCCACTCTCCTGTCGATTGGCTGGC
CAAAACTCCGGAGGATTAGACGGACCAACTTCTGGCAACTCGAGCTGACAGCCCTCTAATGGAGTCGCAT
GTGTGTGATGGAGTAGGGCCGTGTGGGTGATGGAAATTTACGGCATTTTCCCATTTTGACCTCAGTGGCT
GCCAATGCCCGACGATGGCGCCCAGACAATGAAACATGCTTCATGTTGCCAAATAATTTCGATAATATTT
TCTGGTCAGTCTGGCCACCAACATTACCAACCACCATAAATCCCATGCGAACCCTTTAGGATCGAAAGAG
ATTAACCGTGACTAGGCGAAAAGATTGATGAAAACTTCATTTATTAGATTCGATATGAGGTCTGCTTTTT
ACGCTGCTACCATCGTAATTTGATTGATGAAAATAAAGCTAAAGAGACTCGGATTTCGCTAAATTAGCAC
AATTGTTGTGCTTGGTCTTGATTTCCTTAAATAAACAAGGAATTATTTTCCTTATTATATTACTCCCTAC
CGTTTCCCTGACAACAGCCCACAAATATTTTTCTCGTTCTTGATTACGAATTCCGGTTCACACGCAGTCG
TTTTGCTTCGCCCCCACAAACCATTaaaaaaaaGTTTACCCACAGAGCTCTTATTTCTCGTAGGATGTTT
CAGTTAATACAGGGTACTCATAGATCATACGGGGTATATTCGACATGTAACTCAATTCAACAAAGGGAAT
AGAAAGATTTTCACTTGTATGTGATGATGttttttattttaatattatttagatttttttGTGGTAAGAA
ACATATAGCTCATATTTATAAGTACTTTGCTTACAGGATATCTTCAATTCACCAACCTTCGCCTTATTTT
CGCCCGCCTGTATTTTTTTATTTTGGCATTTTGTTAACCGCTTTTCTGCTCTCCTCTCCGGTTTCAGGGA
TCCTGGGATTCCGGCGTGCCATGCCCGTTGCCGGTCGTACGCTGAACATTACGACCGAAATTTATCAACT
GGCCGAGGAGAATTTATTGAAGACGTTCTTTGTTTCGCCATCGCTGAATTTATGCTTCCATGGCAAGTGC
TCGTACTATTGTGACACCTCGCATGCCATTTGCGGCAATCCGGACATGTTGGAGGGCTCCTTCGCCGCCT
TTCTGCCCAACTTTGAGTCGGGCAATCGCAAGG
>dme_circ_0007340_junction_seq
CCCAACTTTGAGTCGGGCAATCGCAAGGTGCAAATGCCAGCAATTTGGAGCGCTTC
>dme_circ_0007341
CTATCGACCGGCGGTCGCAGCGGCAGCTCAATCGACGCCGTTGGCACAGCGCCTTCAGCGCTTTCGATAT
CGCcagcaattgcagttgcagcaagagccgcatcagcaacagcaacagcaacagcaacagcagcagcaac
aacaatcgacTGCTGATATCGACGTTTACGATAGCCCCTTTAACGGCCAGTTATCAGCTGAACGAATAGG
CGTAGCCAATTGGCGGAGGGGGAACGTCGAAGGGCCCATTGGCGGGAGAGCTCCAGTTGAGGCTGAGTAC
TCAGCGGAGCAGGAGGAGCTGCCGCCGGATCGAGTGCTATATCCAGACTCGGAACCGGAGGAAGAGGAGG
CAGCGCCACGACGCCGTGTTGTGATCAGGCGCCGCATAGTGCGCACCAGTCGCACCGCTTCACAAGATCC
ACAGACTCAAACTGCAGAGGTTGTTCCAAAATCCTCCAACGATAGCTCAACTCCTGCCGAGATCAACGGC
CGCAATCTTGGCTGGCTGACCAGACTGCGCAGCTTTGGATCGATCAATAGAAAACACCAGGAGACCGCCG
CACCACCAAAAGGCGGCCAGCAAAAAGCCATTTCTGCTGCAAGCAACAACAGTTGCAACACCAATCCCAT
CATGGCCGTCCTGCGTACTATGAAATTAAAGGAGCGACTGGTCATCAGTCTGGGTGCCACCCTCGTCCTG
CTCACCCTTCTGCTTATCGTCGATGTGCAGATGGACTTCGGTGTGGCCAATCGCCACCTCCTTCAGCAAC
AGCACCAAAAGATCCGACTGGGCAACGACTACGATGGTGGCACGGGTGGCGGTGGAATGCTGCACGAGTT
CAAGCGCAAATTCCTGCAGAAGAGGGTAAGTCCGCATTCCCGGTGTCCGTCTTGTACACGAAATCTGACT
CATCTATTGCTCCAGTCCCGTCATTAATCAGCGGATACCTTTCGAGTTCGCTTGGAATGTGGGCTTGGAT
GGATGGCAAGCTAGGATGTTGTAATGATTTCCTGCGAGTATTGTAATGAAAACTGACCCTCATTGAATTA
ATGAGGCATTAGTGCATGCATAAGGTAGCTGGAATTCTGTTATCAGTTATCAGAGCTTGAAACATGGGGG
GAGCATGCAAGATGGGATTTCTTGTTTGTACAGTACTCAAAGTTTGCTATTAGGTAAGTGGAAAGTAAGT
CGTCTGGCAGAAAATTGTTATCATTGCAATATTTTAGAATGCGGCAAGACCATAAACCATATACCCCTAG
TTCGATTCGTTTCCTTCTTTTTCATTTCAAACTCCCAGGTAGCAAAAAGCAGCGTGTTGAAACTTAGTAT

TCCCCAGACTGGCCAAAGCAAACGGGCCACCGCTGGCCATTCCGGGGGATTAAAAATTATGCACAAATCG
CAAACGGGGTCGGGCCATTGACTCTGACTCAAACACCCCACTGTTCCCTCGGGGCGACTGAACACGGCAG
GGTACATAATTCAATTTAGCTGAAATGCAACAAAAGTTGCAATAAAACTTGGTTGGCCCCGGCATTAAAA
CGTAATTTGCTACAAAACATAAACCCGAGGCAGGGCCAACTTCTTGGGAACTACAAGGAATACAAGAAAG
CGGAACAAGCACAGGAGCGCCATGTTAATGCCTTTGCCATGGAGAATATATACAGACGGACGGGGCCGAG
TCGCTTTACCTTTGAACACTTCATTTTTTCATGCTGGCCACTTTTTGCGTTGCGTGCGGTGGGCGAAAGT
GGGTGGACTAGAAGGGTCCGCCGCCATTTTGCTGTGGCTGGCGGAAGTCGAGTAATAAAACATCAAAATG
ACGCAGCAAGGCGCAAGGGAAACTGTTAAGACCATCCTTCCCAGCTCCCAGGACTTCCACTTCCTCTCAG
GTGCAGTAAACGACTCCGGTTCGCATCGCGCATCCCGTGCTGCTTCGGTTTTGTTTCGTTCTGTTCCGTT
CAAGCTTTCGCTACCGCTCCCTCTCCGGCAACATAAAACGCCAATGTTTAGCCAGTAAATATTTAATTAA
TTCATAAGCCGCCTGCCACCAGCCCGTAAAATGGATGCCTTCGGAAACCGTTCCTTCGTATTCGCTGCGT
CTGAGGGATACCTCTTCCCCGAAAGATCGGCAGCATCAGGGCGGCCAGAAACGCCGGGCTTGAAGCTCCT
TGCTATCTACTTATGCGCCAGGGTAAATGCAAAATTATGATGCCTCCATGCACTGAGTCGTGCAACGCGG
CTGTGAAGCGTAATAAATTCTATTTAAGCATCAAATGTTTTGCCCGTCTCCGTCTTCAGACGCTCCGAAA
AGCCCGTGGAGGAAGCCGCTCCGCTTCCTGATTTCCTGTCTCGTCCCTCCCCCCTCCCCACTTCCCTTCG
CCCATCAGCCTTATAATTTTTTAATTATTTCATTAAAAACTGAAAACTGAGGCAGCAACTTGGCAATGCT
GCAAATGGTGTTTGCCGTCCACGAAGCAGAGGGAAAATTCATTTTCAGTGATGATTCTCATTTTTTGGGC
TGTTATCAAtctttattctatttttcttgttttctttttatcttcgggcttttcttaaccctttTAATTA
AAGTTGCTGTAGCTGACAGAGATTTTAATTCGTTTCTTCTAATACTTTTTAATTGGATTAAATCAAATAA
AAATGTGTTAGGATTTAACTTAACTTAACTATTAACTTAAACGATTTCGTAATCTTTATTGGATTCTAAT
ATGATTAGATTTTAGATATATATAAGGAACTCTGAGCAATTTGAACGTAACAAGTTATAGTTATATCCAT
GAACTAGACAAGATGGCATTTTCCATGGAAGGCAGGCGGAAATGCCGCACTGAAATCCAATTAGGTCTCC
CTTAAATCCATCCATTAACCCCTGACAGTGTCACAGCTTTCGACTTGCATAAACTTTCACCTATCACCTA
TGATCCTGTGGCTGCGCGTTGTCGCTGCATTTGTTGCAGGGCCGTCTATTGCCATTACAGTTGCAGTTGA
CTGGTTAATTAACCGCAGCACATGGCCAGCTTATGTGGCCAGCGTTACGCACACCACGTGCCACTTTAAT
GAGCAAAGTTTCGCTATTGCCAAAGTGTAATTAAGTGCACGCCACATCGATCAGTTCAGTCGAAAACAAA
GAGGGTTACCACCACTACCCTTTTAGAGGCATATCCCCCATAGCGCAAGCCATTTTGAGCGAAAGCCATA
AATATGAGGACCCTCTTCCTGCAAGTGGTTGCACAAGGGGCGACTTAAATACTTATTAGCACATAAAGTG
GCATTATGCGGCTGCCAATAAAATGCGCGAAACGCGCGAAATCCGCACAGGCTCATTGCGCTGGAAAATT
CTTAATAAGCCCCCGAGCCGTGGAAAAGGCTCTTTGTCGCCCAGATATTGTCTCATCCGAGATGGGGAAA
ATGGCAAATGTTGACAGCTTCTCGGCTGTTGGCCTATCTGCTGTCTCGGCCCAGACATTCTCTGGCAACT
GTTAATTGAAAAAGTTTTGCATTCTCACCCGGCAAACAtgtgtgtgtgtgtgtgtgtgcgtgtAGGTTTG
TATGCAACACACCTGTCTGCTTGACGGATAAGGCAAGGCGCCGACAGACAGACCGACAGACAAAAGACTT
TCGCCCCAAAAACTTGGGATACTTGCACAATTAAAAAAATATATATAAGTATTTTCAGAAAACGACAATT
TACGTAAAACTCAAATTTCAAAATTCTGGCCTAATGATGTGCACTCCCCACAGCCTGTGCACACGAGTGC
TGTCATTTGGCTTAATGGTCACACAACTGGTGCATAATTCTCGGTTTCCCGGTACATACATATGCTAACG
AGGTGGAGATGGAAAATGGGCCGGAAACGGGGCACCCGGCCAGGCCAGGTTAGATCTCAAGCATTTCGCC
GAGAGCTATTAAGAGTCAAGGCTAGGAAAATGGATGCAACAAATTCAAAAACTTTAATTCATTTTTCAAG
CACTCTTTTTGTCTGAAGCAGGCGCTGTCTAAACGGCTGCTGATTACCAGCACAAGCACAGTGGAATAGG
CTGGCAAAATGGTGTGCAAACTTTGAGAGAAATGTGAATACTAAATGAATACGAATTTCTGTCATCTAAA
CCGCATTTGAATGGACTAAAATGCAATTTATATGTCAGAGAAAGCAAACTTATAAATTGTAAAGAAATTT
GTCTCCCCGTCTTTGTTCAGATTGTCTAAAGAAACTTCTGCAAATAATGTTCGCCAAAGTTTGCTGTATT
TACCTGATATTAGAGGCATGTCCCCTAGGTGTCGCCACTGTTCCAACTTGTATGGGTTGCGGTTTCGCCC
CCTTGAAGTGCCTGCAATTTCAGTTTATTGCAGTCGAGTGAAGAGTCCCATGTGTGTCTAAGCTtgtggg

tgtggatggggatctggatgtggatgtggatgtggatgtggatgtgggtgtgttCGTCTTAGGTAAACGC
AACTAAAATCCTGGCTGACAAAGTTTCCAGCTTACCGGCAGCAAGTGCGTGTCTTTGGGAGCCTGGCCAG
GGGGCACTTAGCTGTCTAGGCTGCTTCAGTTCAGTGTAGCTCGATGGCTGCCACTAAAAGGCAGTGGGGC
ACTTGGCAGTTGCAATTAGCAACGAGAGCCACCCGGACCATTGTCAAGAGCACCCGGCTTTTGGGTCTCC
GCAAATTATGGCAAttttttgccctgttatttttgtttttgtttttCTTGTACATTTAACTCACTTTTAT
GCAGCTCCTTTTTAACAGAGGTACTCTCGCTTTCATTTTGCTTTTATTCCAGCCAACGCCTCGGGCTCCA
AGGAGGCATCCACGCAGGCGGGGGCGAGCCAGAGTGGCGGGGCGACCAGTGGTCAGGATGCGGCTGCCGG
AGCTTCGGGCGGAGCAGCCGGACCCGGCACATCAAGGAGCACATCCACGAGGAAGCCCACACCACACGAT
CGCTATGCGGATCTGCAAAAGCACCTGCTTAGCGATGAGTACTCGCATGTGATTGTGGACAATGCGCCGG
ATGTGAGCAGGGACAATCCCACGCTGGCCGAGATGCTGCACCGCAAGGCCAGGGTGAGTGGCACATAAAA
ACCAGTAGCGAAAAAATTCAATTTCCCTTGAGCGGAAGGACATAATGCTTACAttttttttttttttACT
TCGGGCCCCCAAGCCACTTTATCCGGCTACGTGGTAATTGATAGTGAAGGTGGGGGAGTTTTACGGCTCT
AGGATGGAAAATGGATAGTAAAAGGAGCGTAGGGTTGCGGCAAGCAGTTACGGCACTTTTCGGGCTTCAA
CGGAATTGAAATGATTTTTATTGAAATTTACGCTCCATTTACGTGACACATTTACGTTTTAGACATTTAG
CCTGTACACTTGAGAAAAACTTAACTATGCCTAAGAAAATGAGATAACCTTGTTTTCGAAGTATAGAATG
GTACCAATTTTATGGATACAATCTTCGCAACGAACTAGATAGAATGTTATTACAAGACTATAGTGCATTG
CATTTAGGATTTGTTTTCCAACTCTATATATATGTTTCGCTGAGTGTATGCCCCCCAGAAAGTTCTCTGT
GCCCACATCTTTACGGGCATGTCTCTATCTGTCGCTGGTAAGTGTTGCCTGATTGTTGTTGCCGATGGCA
ACTGTTCCACTGCTCCTTCCCGATTCCGACCACCGATAACGTTGATGAGAGTTTGCCTTGTCTCAACATG
GCGTATGCGTAATTTCATGCGAGCACAATCAAATAAAAGTGCAATAAAGAAATCAATGTGGATTTGCACC
ACCACCCACCCATCCATCGGTTATCTGCGTCACCGTTGGTGGCATCTCTTCCGTTTCCCAACTCGGTTTT
CCACCCGCACACTTGAGACAGCAATGAAATTTATTTCGACAAGTTTTCATTTGATTTTCTCTTTCGGTAA
TCAGCTTAGTTTTCTCCATCTTCCGACCGGTCCTTTTCAGCGATTTTCCTTTGCTTTTACAATGCGTTTG
GCGAATCGTTTTGCTAGTTTTTATCAAAATTGAATATGTAGCGTATGAGTAATTTCATTTGCTCCAATAG
TTGCCCAACATTTTGTCCCAGCTGAAGTTGCACAAAAATTCCGGGAGTTGCGGCGAAAGTGAAGCAAATC
GAAAATGGGTAAAATGCAAAGTGGCAAAAACTTGTCAAGTTGTTGTTGCTCTTTTTGTTGTTTTAGCAGC
ggtgtgtgtgtgggtggtgggtgggtgtTGTCTGGCTTGGCTAAGTGTGGGCGTCGGCATTAGCATAAAA
TGCTTGATTTTCTAATTTATAGCACATCACCGAAAAGCAAGCCGGAAAGTTCGCTACTTCtttttttttt
tGGTGTGTCGCACGTAGCAAAATGCAAATTTCATTAACAAGCGAAATTGCTGCCAGTAAATGTGCAGGGT
AAAAAAAGTTGGCAGAAGGTTTAGTTTTACCTGGCTTAGTCGGGCCAAGAAGTTCGGATGCTCTTGCAAG
CAAAGTGCAAATTTTATGAAAGTGATTCTATTAAATTACTTGCTTACTACACAATGGTACTTAGTTTTAA
GATGCTCTGTAAGTTTGGGCTTTACAAAATTACGATAAATTTGTTGCGCTGTCGACTAGCTAAAGTTAAT
CCCATCAAACCAATTTGGGATTGTTCAGTCTTAAAACCAGTTAAGTATCATATGTATTTGAGCGCAATCT
CCTGCTTACTAGTCCCAATTGCAGTGAAAAGCTAATCCTTACAAGTCAGCCTCCTACAACTAATCAACCT
AAAAAGATAGAAGGATCTGAGTTAGCGGAAACAGAATCAAGGCGTGGCCCAGATTCTGTGGCTCCATCTC
CTCACGATTCCCCCATTGCTGCTGTCGCCTGTCGCTCGTCTCCTGGCGATATCTCATTAAAATTCTGTCG
CCGAGCAGCTCCCCACATGCTCGACGTTCTACCATATCTCATTCCGTTTAGCTGGCGGCTTAGTCAGCAG
TTTTTTGGCCCAGAGCTCCGGTAACAATGCAGCCAAGAAAAGACTGCAGGTGGGGTAAAACAAAAAAGAA
TGCAATGTGACAAAATGCAAATGTGCCTAAAATTACTAGATGAGTTATTACCAGCGGCAGCTGAAGAGTC
AGGTACAAGCCTGTCCGGCATTAGCCGGTTATATAATTACGACAAAGTTGCCGCTGAATCAGCCCAAGTC
CCCGGGTCGCGTTTAATTAGTATGACAAATTTTTAATTAGCTTGCAATTGCGAGCGCCGAGCGGTGGCAG
AAATCTTATTTCGAGGGAATTACACCCGGCTAAAAAGAAGAAACAGAAGAAGTTTTACCAGCTGTCGGTC
AGGATGCGAATCCTGAGAATCCTGCCAACGCCGGCTAATAAAAGACTATGCTCAATGTCGACGAGTGTGT
AAAACTCCGAGAGCTTGCTGACATTTCGGATGGCgaagtggaaatggaagtggaagtggaagtTTTCCAA

CCTCGCCTTACTCCACACCCATTCGCTACTCGCGGATCCCAGCTGCAGTTTGGTCTGCTGTCATTGGTTT
TAATGTTCGCGCTCTTGCCGCCGCTGAAGTTTTATCCCCGGCTACAATATAAGTTTAATTTCACTTTCCT
TTTGGCCCAGCTCCCTCTCACTGCAAACTCGGCTCAAAAGTGTCCGTGCTGCAGTCCCGGGTCCGTCCAT
TTCGATGGCTCCTCGTTGGTCTTGGCGGCCGCAAAATTTCACACGCACAAACTTTTCCTTCTCTCCATCT
AAGCAGCACTTTTGCGGCTATGTGGCTGCGGCCAGGACGCAGCTGCTCCATGCTGGGTAGTCGGGAATGT
TGGGGAAATCGGGTAGAAAACTGGAGGACTGTGCGCGTTTGCGAAACTTTACTGACATTTTCTTATGGCA
AAATAATTGCGGACTTGCGGGGCGGCTGGGGTTTGGTGGGACTTAAGCAAAGGCCAGGACATGCCGGCTG
ACAGCTGTTATTATAGTACCCACTTCGGCCGGCGGTATAACACCGAGGATAACAGCACCAACAATGGCAG
TAGAAAACCAGTCATTCGGCCAGTCATAATCCCGGGTAAAAAGTTTACCTTAGACGCGAGACTCCCATGG
GCGGAGCTCCAATCCGGTGAAAAATGAAATTTAATATTTGGTTAATGAGTAATTGTTATCGAAGCAGCGA
GTAGATACTAGCACATCCAACAGGTATTCCAGTTGTGaaaaaaaaGTAACCATTCCTTTTGTATGCTACA
TTTTTTTAAGCTTCATAACTATCAGTTACCTGTAACATAGTTTTCGTTAAACAAAATCCAGGCTTATGGC
AACTTTCCACCGCTTTTCACGTTTCTCTTTAGCGTAAATTCGCGAAATAATATTAAGCTGTAAGCAAAGT
TTATTCGTGGCCATTGCCATGGCAGCGATGTTTTGCAGGACTTGGCCAGCGGGATTCAGGACTCCGGCAC
TTGGGAATTTAGGAGACCGCCTGCCCTGTCAAATCGTCAAACCGAGCGCAATTAAGCTGATTAGTGAGCG
TCATTCAAAGTGAAGCCCGTCCACGTTCGTCGTCCCATCACTGACATAAATAAATTTAATTTCCGCTAAA
CATAAACTCAAAGTAAACTTTTGACGCGAGTAACCGCCAGGAACGTGCAACTGTCGAGGGTTGCGGGGTA
CTACCAATTATATGAAAATCTCtttttcaattatttttccttctttttttttcgtgcatttcgcagcgttt
ctgttttttGCGCTGCGGTACGTTGTTGTCATCGTCGTTGAGCTGTCAACGCTTTACATAACAAAGTTTTT
CGCGGGCTTAGACCACTGGAATGGTTgggggggtgggggTTTGGCGGTTGAAGGACAGTGGGCGGGTGGG
GACGGCCATGGGGCCGTTTTTAGGGGGGCTGGTGGCCGACTGTCACTGCGGTTGTCAGCGTTTGTAAGAC
CATGTTTGCCGGGATATGTGCTTGTGGGCCACGTGTCACGTGCGCTTTTGGCGTGCTTTTAGCTCCTCCC
AGTAGCAGCCCCCCATTTGGACCCACCTTCTTCAGCACTTTCTCTTGTAATGCGATTTCGGGTTTTGTTG
ACTGCCGGCCGCTGCAACTGATTTACGTGACTCAATTACCCAAGGGATTTGAATTCGATTCGGCTGGTGC
TCTTTCTTTGTTTCAGTGTTGTTTCGGCCCCGAATATGCAACAGCTGGCCATTCGGACCAAGCCGCAGAC
TCGCAGAAACATATGGCCACTCTGCGGACAGTGGCCAGTGCTTAGTCAGTGTCCGGATCGGGCGGCTTCA
TCAATTACGTAGCTTCTGCTCCGGATTCCATTGGCCAACTCTAGTCCATGGGCTAGAGCTGCTCCACCGC
AGTTGACAAATGAAGCATGGGCTGAAATTTGAATGTGCCAAGCTAACACGTTGCTTAGCATACTTTGGGG
CACACTGCCAAAAGAAGCTCACGAATGAGATATTAAGTCCCTATTAAAGAGAAATATATAAGAAACGCAA
AAAACTCCAAATAACATATACCCCAGGAATTCTTATATAATTTGATCATATATTCTTTCACTTGTGCATT
TCTTTCAATGTGTCTTAATTTATATGGAACatatatatatgctatattattatatattattattatatat
tatgtattgatattagtctattCTTGGACCTGCAAAAAACCGCATTTTAAGTTTTTAGAAGCAATTATAT
TTAAATGTTCAACCCCTTGGCTCAGCTTTAATTCTGCAGACTCGAACCTAATGGGACCTTCGTCACTTCT
TTTGGCCCCTTCCTGACCTGCGAATCCTGGTCATTGTCACCGGATTTCGAGCACTTTTACGCCGCCAGGT
GTAAGGGGTTGTTCTGCTCTCGGAAGTTGTCACCATTTCCATAGAACGCTGATATATTGAAAGTCATTCG
GGGCACGAGAAGGTTGTCTGCCAGACATTTGTTTCCTTCCTGCGCACAAAGACCATCTTAGGCGGCGAAA
AAAGGGTTAGACGTTGAAGTATAACAGACGTCGGTGGGCAAATTATGAATGGGCTGGGAGTGGGTGGTGG
AGCAAGTAGATGTAGGAGGACAATGAGTTATGTAGACACCATTGAGAGCAGCATTTGGGGGAGCGCGAAA
CGGATATGACTTCATTATCATGATAAGCTTGCTGCACAATGGAGCTGACAACGCCGCCCGTTTCCCACTT
TTTCACTGACCAACGAAGCGAGTACAGGGTTTTGCATTTCAATTTGTCGTAACTGACTGTGACTACTGGT
ATAAATTGTTGTGCATTGTGAATTGCAAACGGGATAGCTAATGCCGATGAAGAGCACTTTACAGCTTAAA
TTCTGGAACGGGGCTGCAGTGCTTAACCAGCACAAATTGGACTAAATCTGAAAGCGTAACTGCCAGGGCT
TAAGACAAAAGCATTGGCCATGTGAGCGGCACATGTCAAACGTGGAGTGCAAAGCATAATCGGAGAGGAA
AAGATATTTTCCGACAATTGCCAGTCTGTCTGGCAGCAACAGGAAAAATCGACCACCAAAGATTACGGCT

GGGTGATGTGGGGGTGTCTTGGCATTTGCAAACCAAATTGTCATGACAGCAGCGGCAAATAAAGGAAAAA
GGAGCACAAGGAGCAGGGCATTGCTTTGACAAATCAATGTGAGAAATCTTGATATGCAGCCCCAAAGCAT
AGCATACTTATGAGGGAGCCCCGAGAACTGGCTACACTGCGCAAAAAGTCCACTACTCCCTTAACGAGTA
ATACGAGATAAGATCTATCCATTGTTACTTTAATAAAATATGTATCTTGATATCACACTTCTACGCATAT
ACTTTGGCATAATCATAAATAAGGCAGAAAAGGAGTTTAATGGTTTTGTGCCAACAGAAGAAAGGTATTT
TAACTTCAATTGTTAAAGGAAGttttttttATTATTATGTATTACATTTACTTCGAGTGTACGGCTCCAG
CTTGATTGTCCTTATGACTTCCTTTTGCCTGCAAACGACAGCTGGCTCTCTTGGCTGCGATGCTCGATTT
TTACTTACATTTCTGCCATTTTGTTTCCCTTTTGCCCGCACTATCTCTGAACCCGAACAACTTTCTCATC
TCTCTTTGCAGTGTAAGACAGTCTGCTTCCGTTCTATTCAGCCGTTCGGCAATTGTGGGATTGTAAAGTC
ATAAATACTGGCCAAGTCTGTTGGCCACCTCACCCAACGAACTTTCGAACCTTAGTGGGGTTTACACGAG
GCCAAAAAATGTAAGCGGGCCAAAAGAGACTCAGCTGCACATTTAACTCAATTATGTAGAGCTTGGCGGC
GGCTGGCATTATTGAGGTGCCACTGCGAAACTATTTTCATAGCGAAGTTTTTCCAGCGCATTCATTAAAC
TCATAATTTATTAATAATAAACTTTTAACAGTGGCTGCACGGAGCTGCGAAAAGAAAACTCATTTGCAGT
TAAGGGGAAACCAAGGGATAGACAAGAGTTGGGCGGGCCGAGCGCAACAATGACACTGTGGCAGCATAAT
TGCTGCAACTTTAGCTTTGTTCTGCCTGGCATTTATCACGTATACGCCGCGTATACACAGGCGCATACTA
ACACAGACAAACAAATGCAGCCCTTTGGCGTTAGAACAACTTTAGAGAGATAATTCATAAGTAATATAGG
ACCAATTTTCATTTGTTTATTTTTAGAATAAATTCCAATGAAATGTGGCGTGTGTGCGGCAGGAGTTTCG
GTTTTATTTTCCCTTACGGTCAGTCAGCAGCTTGACTGTAAAGCCCAAACTACAATGAGCCAAGTTTTTG
GTTCACTACACACACTGAATTGCAAAAGCAAGCCAAGGCGGTTTTTATGTCGGGCTTCTTTGTGCGGTTG
CGTTTACGCAATATTTGTGTGCTCTTTGAATGCATTTTAATTGCTATTTAGTCGAGTGTAATTTGTTTAT
GCAGTCCGCTCGGTACGGTACGGTTTGCAATTTTAAAAGCAGTCTGACTGCAGTCTCCGTGATCGGTGAT
GTGAGAATTGCATGAATTTTTGGTTTTTGCTTCAATTAATTCCGAGTGCTCGATTAATTTACATTCATTC
CTCTGTCTTTTCTCCGTTCGCAGTGTGAGTTTGGCCAACGGATGGACAAAGATTTCCCGGTCTGTCGCCG
CTTAATGCATTTGTCCCTTGGATTTCCGCGTGCGTAATGAATGCAACTTTCGCCCACTTGTCGACAACAC
TTTTCGCACAGTCGAGTAAATAAAATTGCGCTTTTTATTCGCTGACTTTCCTGCGGCCTGTCGATGGAAA
TTCCACGTCCATTGCCGTCGTTTTGCGGTGGCACTTCAAGCTGCTCGCAAATTTAAAACATTCATGCACT
TTTTAATGAGATTTAAACGCATTGGCTTGGTCAGCGTGCTGGTTTTCCATTCCTCCATCTCTGCCATCGC
AGCTGCCAAAGTGCAAAGTGTCCTTATAATGCCATTCATTTGCAATTTAAATGAAAGCCCTTCTCCTTCT
CTGCTCTTCttttttttttttttaaataaatatataggaacatttcgttttttttttttttttgttttttA
AATAGCACTCCCTTTCGTGTAAGTAATTAAGCTGGCACATAGCACGTAGCAATGCAGGTTCAACGTGGGA
AAATTCAAAGTGGCCCCACGCAAATGCATTAAGCTGGTGACAGAAGCCCGCAGTTTCTATTCATTTCATT
ATGTCGCATCCCCACCTCTGATCCCGCTCATTTGCAATTTGCATTTCCACAGGGTGAGTTCCGTCGACTC
CATTCCATTCCATTCCGATTCGACGCCATTCTGAACTTTCACCGGGTGCATTCGAGTGCACAAGAATTCC
GTTGTGAAATCGTTCGATCGAATTAAATCTGCCCAGGCATCGTGTAAATGTCTGGCATACTCTTATTTAT
TTTTAATTTAATTTTCAATTATACGTAAATATTTATGGTGAGCATTGAACGGATTCCCACCGCAGCACAA
AGGCGGGGAAATCGGGAAAATATTGTGCAAAATCAATTTGAAATATTCAATTAGGCAAACCAGACTGTTC
TCTGCAGACTGGCTGAAAATACCTGAATTTAATTTTAAGCGATTAAAAAAGGGATTGTTCCGAAAGCAAC
GAAAAAAATAGTTATATTTTTTTGATCTTTTTATCATAGCATAGTAAATGGAGCATTCACTTGCACTAAA
ACTATTATTATTAAAATCAATTTATTTTTAGAATAATAAACCTATTGATTGCATTTTCGGTTCATGCCTC
TTAATTAAGCCTTTGTTTGAATTTTTTTATTATAGTGTCCCTAAAAGGGTATCATACTCTAGGTGTTCCC
TACAAATAATAGTCATTTCCTTGGTCAATACTCGTAATAGTAAAATTCCGGGTGTCAGTGCTCAAATTAT
TTTCGCTACCACATTTCAACTTTATGGCCCCTTGATAGTTATTTCTTTTTTTAGTGCATGACAAAGCAAT
CAAATTAACCAATTTGTCTGCCATGTGAAACGGTCCCTTGGCCAACATTCGTCCAGGCCAGCATTCAAAT
TTATAACGCTTGGGATGCCTGACCCTCGGCGATTAGAATCCCAATAATCCTGacacacacacacacacac

aTACTGAGGTCAGACCGCAGTCAATTTATGTTCATTATTTATGATGGCCGTGCCGAATAAAATAAAAAAT
GTCCATACATTTACCCGTTCGGGGTGGGCAAGGCCAAACGGCGCCATTAAATGATGTTTAAGAATATATT
TTTTAAAAAAGGCGAAAAAGCTGTGGTGCCAACTCATGTAAATGATGTGGCCACATAAACCCAATGCCTG
CCAGTCTGGCTATCTGCGCCCTTTCATGGCTAATTAACTTGGCCAAGTGGCCTGACATGTCCTGCCGTAC
TTTAGACTGAAATTGTCTGCATAGTTAGCAGCTGCTTGCCTTTGACAAAACCTTGACGGCCAGGTTAATT
GCCGGGGGATTTTTGAATTTAGATAAGGCGTCATAACTTGTGTAATTGTTGTGGCTGCCAACCACACGCA
CATGCCGCACACATCCTCTAATCGAGTTGGCTCCACTCTCCTGTCGATTGGCTGGCCAAAACTCCGGAGG
ATTAGACGGACCAACTTCTGGCAACTCGAGCTGACAGCCCTCTAATGGAGTCGCATGTGTGTGATGGAGT
AGGGCCGTGTGGGTGATGGAAATTTACGGCATTTTCCCATTTTGACCTCAGTGGCTGCCAATGCCCGACG
ATGGCGCCCAGACAATGAAACATGCTTCATGTTGCCAAATAATTTCGATAATATTTTCTGGTCAGTCTGG
CCACCAACATTACCAACCACCATAAATCCCATGCGAACCCTTTAGGATCGAAAGAGATTAACCGTGACTA
GGCGAAAAGATTGATGAAAACTTCATTTATTAGATTCGATATGAGGTCTGCTTTTTACGCTGCTACCATC
GTAATTTGATTGATGAAAATAAAGCTAAAGAGACTCGGATTTCGCTAAATTAGCACAATTGTTGTGCTTG
GTCTTGATTTCCTTAAATAAACAAGGAATTATTTTCCTTATTATATTACTCCCTACCGTTTCCCTGACAA
CAGCCCACAAATATTTTTCTCGTTCTTGATTACGAATTCCGGTTCACACGCAGTCGTTTTGCTTCGCCCC
CACAAACCATTaaaaaaaaGTTTACCCACAGAGCTCTTATTTCTCGTAGGATGTTTCAGTTAATACAGGG
TACTCATAGATCATACGGGGTATATTCGACATGTAACTCAATTCAACAAAGGGAATAGAAAGATTTTCAC
TTGTATGTGATGATGttttttattttaatattatttagatttttttGTGGTAAGAAACATATAGCTCATA
TTTATAAGTACTTTGCTTACAGGATATCTTCAATTCACCAACCTTCGCCTTATTTTCGCCCGCCTGTATT
TTTTTATTTTGGCATTTTGTTAACCGCTTTTCTGCTCTCCTCTCCGGTTTCAGGGATCCTGGGATTCCGG
CGTGCCATGCCCGTTGCCGGTCGTACGCTGAACATTACGACCGAAATTTATCAACTGGCCGAGGAGAATT
TATTGAAGACGTTCTTTGTTTCGCCATCGCTGAATTTATGCTTCCATGGCAAGTGCTCGTACTATTGTGA
CACCTCGCATGCCATTTGCGGCAATCCGGACATGTTGGAGGGCTCCTTCGCCGCCTTTCTGCCCAACTTT
GAGTCGGGCAATCGCAAGG
>dme_circ_0007341_junction_seq
CCCAACTTTGAGTCGGGCAATCGCAAGGCTATCGACCGGCGGTCGCAGCGGCAGCT
>dme_circ_0007342
TGCAAAAGGAGGGCGGCACCCAGCTGAAATTGATCATCGAGTACCCAAACGATATCAAGGCCTTAATGAA
GCCGATGCGGTGAGTTTGGCCAACGGATGGACAAAGATTTCCCGGTCTGTCGCCGCTTAATGCATTTGTC
CCTTGGATTTCCGCGTGCGTAATGAATGCAACTTTCGCCCACTTGTCGACAACACTTTTCGCACAGTCGA
GTAAATAAAATTGCGCTTTTTATTCGCTGACTTTCCTGCGGCCTGTCGATGGAAATTCCACGTCCATTGC
CGTCGTTTTGCGGTGGCACTTCAAGCTGCTCGCAAATTTAAAACATTCATGCACTTTTTAATGAGATTTA
AACGCATTGGCTTGGTCAGCGTGCTGGTTTTCCATTCCTCCATCTCTGCCATCGCAGCTGCCAAAGTGCA
AAGTGTCCTTATAATGCCATTCATTTGCAATTTAAATGAAAGCCCTTCTCCTTCTCTGCTCTTCtttttt
ttttttttaaataaatatataggaacatttcgttttttttttttttttgtttttAAATAGCACTCCCTTT
CGTGTAAGTAATTAAGCTGGCACATAGCACGTAGCAATGCAGGTTCAACGTGGGAAAATTCAAAGTGGCC
CCACGCAAATGCATTAAGCTGGTGACAGAAGCCCGCAGTTTCTATTCATTTCATTATGTCGCATCCCCAC
CTCTGATCCCGCTCATTTGCAATTTGCATTTCCACAGGTTTCCGCGGGAGCAGCAAACGTTGCCCAACCA
CTTCTACTTCACGGACTATGAGCGCCACAACGCCGAGATTGCCGCCTTCCACCTGGACAGG
>dme_circ_0007342_junction_seq
CGAGATTGCCGCCTTCCACCTGGACAGGTGCAAAAGGAGGGCGGCACCCAGCTGAA
>dme_circ_0007343
TGCAAATGCCAGCAATTTGGAGCGCTTCCAATTGCGCATCACGAAGAAGGAGCTGTACGGCGAGCAGGAC
ACTCTGGTGGATGCGGTGTTACGTGACATGATCAAGCTGCCAATACAGCATGTTGGTAAGACAGTCTGCT

TCCGTTCTATTCAGCCGTTCGGCAATTGTGGGATTGTAAAGTCATAAATACTGGCCAAGTCTGTTGGCCA
CCTCACCCAACGAACTTTCGAACCTTAGTGGGGTTTACACGAGGCCAAAAAATGTAAGCGGGCCAAAAGA
GACTCAGCTGCACATTTAACTCAATTATGTAGAGCTTGGCGGCGGCTGGCATTATTGAGGTGCCACTGCG
AAACTATTTTCATAGCGAAGTTTTTCCAGCGCATTCATTAAACTCATAATTTATTAATAATAAACTTTTA
ACAGTGGCTGCACGGAGCTGCGAAAAGAAAACTCATTTGCAGTTAAGGGGAAACCAAGGGATAGACAAGA
GTTGGGCGGGCCGAGCGCAACAATGACACTGTGGCAGCATAATTGCTGCAACTTTAGCTTTGTTCTGCCT
GGCATTTATCACGTATACGCCGCGTATACACAGGCGCATACTAACACAGACAAACAAATGCAGCCCTTTG
GCGTTAGAACAACTTTAGAGAGATAATTCATAAGTAATATAGGACCAATTTTCATTTGTTTATTTTTAGA
ATAAATTCCAATGAAATGTGGCGTGTGTGCGGCAGGAGTTTCGGTTTTATTTTCCCTTACGGTCAGTCAG
CAGCTTGACTGTAAAGCCCAAACTACAATGAGCCAAGTTTTTGGTTCACTACACACACTGAATTGCAAAA
GCAAGCCAAGGCGGTTTTTATGTCGGGCTTCTTTGTGCGGTTGCGTTTACGCAATATTTGTGTGCTCTTT
GAATGCATTTTAATTGCTATTTAGTCGAGTGTAATTTGTTTATGCAGTCCGCTCGGTACGGTACGGTTTG
CAATTTTAAAAGCAGTCTGACTGCAGTCTCCGTGATCGGTGATGTGAGAATTGCATGAATTTTTGGTTTT
TGCTTCAATTAATTCCGAGTGCTCGATTAATTTACATTCATTCCTCTGTCTTTTCTCCGTTCGCAGTGTG
AGTTTGGCCAACGGATGGACAAAGATTTCCCGGTCTGTCGCCGCTTAATGCATTTGTCCCTTGGATTTCC
GCGTGCGTAATGAATGCAACTTTCGCCCACTTGTCGACAACACTTTTCGCACAGTCGAGTAAATAAAATT
GCGCTTTTTATTCGCTGACTTTCCTGCGGCCTGTCGATGGAAATTCCACGTCCATTGCCGTCGTTTTGCG
GTGGCACTTCAAGCTGCTCGCAAATTTAAAACATTCATGCACTTTTTAATGAGATTTAAACGCATTGGCT
TGGTCAGCGTGCTGGTTTTCCATTCCTCCATCTCTGCCATCGCAGCTGCCAAAGTGCAAAGTGTCCTTAT
AATGCCATTCATTTGCAATTTAAATGAAAGCCCTTCTCCTTCTCTGCTCTTCttttttttttttttaaat
aaatatataggaacatttcgttttttttttttttttgtttttAAATAGCACTCCCTTTCGTGTAAGTAAT
TAAGCTGGCACATAGCACGTAGCAATGCAGGTTCAACGTGGGAAAATTCAAAGTGGCCCCACGCAAATGC
ATTAAGCTGGTGACAGAAGCCCGCAGTTTCTATTCATTTCATTATGTCGCATCCCCACCTCTGATCCCGC
TCATTTGCAATTTGCATTTCCACAGGTTTCCGCGGGAGCAGCAAACGTTGCCCAACCACTTCTACTTCAC
GGACTATGAGCGCCACAACGCCGAGATTGCCGCCTTCCACCTGGACAGG
>dme_circ_0007343_junction_seq
CGAGATTGCCGCCTTCCACCTGGACAGGTGCAAATGCCAGCAATTTGGAGCGCTTC
>dme_circ_0007344
CTATCGACCGGCGGTCGCAGCGGCAGCTCAATCGACGCCGTTGGCACAGCGCCTTCAGCGCTTTCGATAT
CGCcagcaattgcagttgcagcaagagccgcatcagcaacagcaacagcaacagcaacagcagcagcaac
aacaatcgacTGCTGATATCGACGTTTACGATAGCCCCTTTAACGGCCAGTTATCAGCTGAACGAATAGG
CGTAGCCAATTGGCGGAGGGGGAACGTCGAAGGGCCCATTGGCGGGAGAGCTCCAGTTGAGGCTGAGTAC
TCAGCGGAGCAGGAGGAGCTGCCGCCGGATCGAGTGCTATATCCAGACTCGGAACCGGAGGAAGAGGAGG
CAGCGCCACGACGCCGTGTTGTGATCAGGCGCCGCATAGTGCGCACCAGTCGCACCGCTTCACAAGATCC
ACAGACTCAAACTGCAGAGGTTGTTCCAAAATCCTCCAACGATAGCTCAACTCCTGCCGAGATCAACGGC
CGCAATCTTGGCTGGCTGACCAGACTGCGCAGCTTTGGATCGATCAATAGAAAACACCAGGAGACCGCCG
CACCACCAAAAGGCGGCCAGCAAAAAGCCATTTCTGCTGCAAGCAACAACAGTTGCAACACCAATCCCAT
CATGGCCGTCCTGCGTACTATGAAATTAAAGGAGCGACTGGTCATCAGTCTGGGTGCCACCCTCGTCCTG
CTCACCCTTCTGCTTATCGTCGATGTGCAGATGGACTTCGGTGTGGCCAATCGCCACCTCCTTCAGCAAC
AGCACCAAAAGATCCGACTGGGCAACGACTACGATGGTGGCACGGGTGGCGGTGGAATGCTGCACGAGTT
CAAGCGCAAATTCCTGCAGAAGAGGGTAAGTCCGCATTCCCGGTGTCCGTCTTGTACACGAAATCTGACT
CATCTATTGCTCCAGTCCCGTCATTAATCAGCGGATACCTTTCGAGTTCGCTTGGAATGTGGGCTTGGAT
GGATGGCAAGCTAGGATGTTGTAATGATTTCCTGCGAGTATTGTAATGAAAACTGACCCTCATTGAATTA
ATGAGGCATTAGTGCATGCATAAGGTAGCTGGAATTCTGTTATCAGTTATCAGAGCTTGAAACATGGGGG

GAGCATGCAAGATGGGATTTCTTGTTTGTACAGTACTCAAAGTTTGCTATTAGGTAAGTGGAAAGTAAGT
CGTCTGGCAGAAAATTGTTATCATTGCAATATTTTAGAATGCGGCAAGACCATAAACCATATACCCCTAG
TTCGATTCGTTTCCTTCTTTTTCATTTCAAACTCCCAGGTAGCAAAAAGCAGCGTGTTGAAACTTAGTAT
TCCCCAGACTGGCCAAAGCAAACGGGCCACCGCTGGCCATTCCGGGGGATTAAAAATTATGCACAAATCG
CAAACGGGGTCGGGCCATTGACTCTGACTCAAACACCCCACTGTTCCCTCGGGGCGACTGAACACGGCAG
GGTACATAATTCAATTTAGCTGAAATGCAACAAAAGTTGCAATAAAACTTGGTTGGCCCCGGCATTAAAA
CGTAATTTGCTACAAAACATAAACCCGAGGCAGGGCCAACTTCTTGGGAACTACAAGGAATACAAGAAAG
CGGAACAAGCACAGGAGCGCCATGTTAATGCCTTTGCCATGGAGAATATATACAGACGGACGGGGCCGAG
TCGCTTTACCTTTGAACACTTCATTTTTTCATGCTGGCCACTTTTTGCGTTGCGTGCGGTGGGCGAAAGT
GGGTGGACTAGAAGGGTCCGCCGCCATTTTGCTGTGGCTGGCGGAAGTCGAGTAATAAAACATCAAAATG
ACGCAGCAAGGCGCAAGGGAAACTGTTAAGACCATCCTTCCCAGCTCCCAGGACTTCCACTTCCTCTCAG
GTGCAGTAAACGACTCCGGTTCGCATCGCGCATCCCGTGCTGCTTCGGTTTTGTTTCGTTCTGTTCCGTT
CAAGCTTTCGCTACCGCTCCCTCTCCGGCAACATAAAACGCCAATGTTTAGCCAGTAAATATTTAATTAA
TTCATAAGCCGCCTGCCACCAGCCCGTAAAATGGATGCCTTCGGAAACCGTTCCTTCGTATTCGCTGCGT
CTGAGGGATACCTCTTCCCCGAAAGATCGGCAGCATCAGGGCGGCCAGAAACGCCGGGCTTGAAGCTCCT
TGCTATCTACTTATGCGCCAGGGTAAATGCAAAATTATGATGCCTCCATGCACTGAGTCGTGCAACGCGG
CTGTGAAGCGTAATAAATTCTATTTAAGCATCAAATGTTTTGCCCGTCTCCGTCTTCAGACGCTCCGAAA
AGCCCGTGGAGGAAGCCGCTCCGCTTCCTGATTTCCTGTCTCGTCCCTCCCCCCTCCCCACTTCCCTTCG
CCCATCAGCCTTATAATTTTTTAATTATTTCATTAAAAACTGAAAACTGAGGCAGCAACTTGGCAATGCT
GCAAATGGTGTTTGCCGTCCACGAAGCAGAGGGAAAATTCATTTTCAGTGATGATTCTCATTTTTTGGGC
TGTTATCAAtctttattctatttttcttgttttctttttatcttcgggcttttcttaaccctttTAATTA
AAGTTGCTGTAGCTGACAGAGATTTTAATTCGTTTCTTCTAATACTTTTTAATTGGATTAAATCAAATAA
AAATGTGTTAGGATTTAACTTAACTTAACTATTAACTTAAACGATTTCGTAATCTTTATTGGATTCTAAT
ATGATTAGATTTTAGATATATATAAGGAACTCTGAGCAATTTGAACGTAACAAGTTATAGTTATATCCAT
GAACTAGACAAGATGGCATTTTCCATGGAAGGCAGGCGGAAATGCCGCACTGAAATCCAATTAGGTCTCC
CTTAAATCCATCCATTAACCCCTGACAGTGTCACAGCTTTCGACTTGCATAAACTTTCACCTATCACCTA
TGATCCTGTGGCTGCGCGTTGTCGCTGCATTTGTTGCAGGGCCGTCTATTGCCATTACAGTTGCAGTTGA
CTGGTTAATTAACCGCAGCACATGGCCAGCTTATGTGGCCAGCGTTACGCACACCACGTGCCACTTTAAT
GAGCAAAGTTTCGCTATTGCCAAAGTGTAATTAAGTGCACGCCACATCGATCAGTTCAGTCGAAAACAAA
GAGGGTTACCACCACTACCCTTTTAGAGGCATATCCCCCATAGCGCAAGCCATTTTGAGCGAAAGCCATA
AATATGAGGACCCTCTTCCTGCAAGTGGTTGCACAAGGGGCGACTTAAATACTTATTAGCACATAAAGTG
GCATTATGCGGCTGCCAATAAAATGCGCGAAACGCGCGAAATCCGCACAGGCTCATTGCGCTGGAAAATT
CTTAATAAGCCCCCGAGCCGTGGAAAAGGCTCTTTGTCGCCCAGATATTGTCTCATCCGAGATGGGGAAA
ATGGCAAATGTTGACAGCTTCTCGGCTGTTGGCCTATCTGCTGTCTCGGCCCAGACATTCTCTGGCAACT
GTTAATTGAAAAAGTTTTGCATTCTCACCCGGCAAACAtgtgtgtgtgtgtgtgtgtgcgtgtAGGTTTG
TATGCAACACACCTGTCTGCTTGACGGATAAGGCAAGGCGCCGACAGACAGACCGACAGACAAAAGACTT
TCGCCCCAAAAACTTGGGATACTTGCACAATTAAAAAAATATATATAAGTATTTTCAGAAAACGACAATT
TACGTAAAACTCAAATTTCAAAATTCTGGCCTAATGATGTGCACTCCCCACAGCCTGTGCACACGAGTGC
TGTCATTTGGCTTAATGGTCACACAACTGGTGCATAATTCTCGGTTTCCCGGTACATACATATGCTAACG
AGGTGGAGATGGAAAATGGGCCGGAAACGGGGCACCCGGCCAGGCCAGGTTAGATCTCAAGCATTTCGCC
GAGAGCTATTAAGAGTCAAGGCTAGGAAAATGGATGCAACAAATTCAAAAACTTTAATTCATTTTTCAAG
CACTCTTTTTGTCTGAAGCAGGCGCTGTCTAAACGGCTGCTGATTACCAGCACAAGCACAGTGGAATAGG
CTGGCAAAATGGTGTGCAAACTTTGAGAGAAATGTGAATACTAAATGAATACGAATTTCTGTCATCTAAA
CCGCATTTGAATGGACTAAAATGCAATTTATATGTCAGAGAAAGCAAACTTATAAATTGTAAAGAAATTT

GTCTCCCCGTCTTTGTTCAGATTGTCTAAAGAAACTTCTGCAAATAATGTTCGCCAAAGTTTGCTGTATT
TACCTGATATTAGAGGCATGTCCCCTAGGTGTCGCCACTGTTCCAACTTGTATGGGTTGCGGTTTCGCCC
CCTTGAAGTGCCTGCAATTTCAGTTTATTGCAGTCGAGTGAAGAGTCCCATGTGTGTCTAAGCTtgtggg
tgtggatggggatctggatgtggatgtggatgtggatgtggatgtgggtgtgttCGTCTTAGGTAAACGC
AACTAAAATCCTGGCTGACAAAGTTTCCAGCTTACCGGCAGCAAGTGCGTGTCTTTGGGAGCCTGGCCAG
GGGGCACTTAGCTGTCTAGGCTGCTTCAGTTCAGTGTAGCTCGATGGCTGCCACTAAAAGGCAGTGGGGC
ACTTGGCAGTTGCAATTAGCAACGAGAGCCACCCGGACCATTGTCAAGAGCACCCGGCTTTTGGGTCTCC
GCAAATTATGGCAAttttttgccctgttatttttgtttttgtttttCTTGTACATTTAACTCACTTTTAT
GCAGCTCCTTTTTAACAGAGGTACTCTCGCTTTCATTTTGCTTTTATTCCAGCCAACGCCTCGGGCTCCA
AGGAGGCATCCACGCAGGCGGGGGCGAGCCAGAGTGGCGGGGCGACCAGTGGTCAGGATGCGGCTGCCGG
AGCTTCGGGCGGAGCAGCCGGACCCGGCACATCAAGGAGCACATCCACGAGGAAGCCCACACCACACGAT
CGCTATGCGGATCTGCAAAAGCACCTGCTTAGCGATGAGTACTCGCATGTGATTGTGGACAATGCGCCGG
ATGTGAGCAGGGACAATCCCACGCTGGCCGAGATGCTGCACCGCAAGGCCAGGGTGAGTGGCACATAAAA
ACCAGTAGCGAAAAAATTCAATTTCCCTTGAGCGGAAGGACATAATGCTTACAttttttttttttttACT
TCGGGCCCCCAAGCCACTTTATCCGGCTACGTGGTAATTGATAGTGAAGGTGGGGGAGTTTTACGGCTCT
AGGATGGAAAATGGATAGTAAAAGGAGCGTAGGGTTGCGGCAAGCAGTTACGGCACTTTTCGGGCTTCAA
CGGAATTGAAATGATTTTTATTGAAATTTACGCTCCATTTACGTGACACATTTACGTTTTAGACATTTAG
CCTGTACACTTGAGAAAAACTTAACTATGCCTAAGAAAATGAGATAACCTTGTTTTCGAAGTATAGAATG
GTACCAATTTTATGGATACAATCTTCGCAACGAACTAGATAGAATGTTATTACAAGACTATAGTGCATTG
CATTTAGGATTTGTTTTCCAACTCTATATATATGTTTCGCTGAGTGTATGCCCCCCAGAAAGTTCTCTGT
GCCCACATCTTTACGGGCATGTCTCTATCTGTCGCTGGTAAGTGTTGCCTGATTGTTGTTGCCGATGGCA
ACTGTTCCACTGCTCCTTCCCGATTCCGACCACCGATAACGTTGATGAGAGTTTGCCTTGTCTCAACATG
GCGTATGCGTAATTTCATGCGAGCACAATCAAATAAAAGTGCAATAAAGAAATCAATGTGGATTTGCACC
ACCACCCACCCATCCATCGGTTATCTGCGTCACCGTTGGTGGCATCTCTTCCGTTTCCCAACTCGGTTTT
CCACCCGCACACTTGAGACAGCAATGAAATTTATTTCGACAAGTTTTCATTTGATTTTCTCTTTCGGTAA
TCAGCTTAGTTTTCTCCATCTTCCGACCGGTCCTTTTCAGCGATTTTCCTTTGCTTTTACAATGCGTTTG
GCGAATCGTTTTGCTAGTTTTTATCAAAATTGAATATGTAGCGTATGAGTAATTTCATTTGCTCCAATAG
TTGCCCAACATTTTGTCCCAGCTGAAGTTGCACAAAAATTCCGGGAGTTGCGGCGAAAGTGAAGCAAATC
GAAAATGGGTAAAATGCAAAGTGGCAAAAACTTGTCAAGTTGTTGTTGCTCTTTTTGTTGTTTTAGCAGC
ggtgtgtgtgtgggtggtgggtgggtgtTGTCTGGCTTGGCTAAGTGTGGGCGTCGGCATTAGCATAAAA
TGCTTGATTTTCTAATTTATAGCACATCACCGAAAAGCAAGCCGGAAAGTTCGCTACTTCtttttttttt
tGGTGTGTCGCACGTAGCAAAATGCAAATTTCATTAACAAGCGAAATTGCTGCCAGTAAATGTGCAGGGT
AAAAAAAGTTGGCAGAAGGTTTAGTTTTACCTGGCTTAGTCGGGCCAAGAAGTTCGGATGCTCTTGCAAG
CAAAGTGCAAATTTTATGAAAGTGATTCTATTAAATTACTTGCTTACTACACAATGGTACTTAGTTTTAA
GATGCTCTGTAAGTTTGGGCTTTACAAAATTACGATAAATTTGTTGCGCTGTCGACTAGCTAAAGTTAAT
CCCATCAAACCAATTTGGGATTGTTCAGTCTTAAAACCAGTTAAGTATCATATGTATTTGAGCGCAATCT
CCTGCTTACTAGTCCCAATTGCAGTGAAAAGCTAATCCTTACAAGTCAGCCTCCTACAACTAATCAACCT
AAAAAGATAGAAGGATCTGAGTTAGCGGAAACAGAATCAAGGCGTGGCCCAGATTCTGTGGCTCCATCTC
CTCACGATTCCCCCATTGCTGCTGTCGCCTGTCGCTCGTCTCCTGGCGATATCTCATTAAAATTCTGTCG
CCGAGCAGCTCCCCACATGCTCGACGTTCTACCATATCTCATTCCGTTTAGCTGGCGGCTTAGTCAGCAG
TTTTTTGGCCCAGAGCTCCGGTAACAATGCAGCCAAGAAAAGACTGCAGGTGGGGTAAAACAAAAAAGAA
TGCAATGTGACAAAATGCAAATGTGCCTAAAATTACTAGATGAGTTATTACCAGCGGCAGCTGAAGAGTC
AGGTACAAGCCTGTCCGGCATTAGCCGGTTATATAATTACGACAAAGTTGCCGCTGAATCAGCCCAAGTC
CCCGGGTCGCGTTTAATTAGTATGACAAATTTTTAATTAGCTTGCAATTGCGAGCGCCGAGCGGTGGCAG

AAATCTTATTTCGAGGGAATTACACCCGGCTAAAAAGAAGAAACAGAAGAAGTTTTACCAGCTGTCGGTC
AGGATGCGAATCCTGAGAATCCTGCCAACGCCGGCTAATAAAAGACTATGCTCAATGTCGACGAGTGTGT
AAAACTCCGAGAGCTTGCTGACATTTCGGATGGCgaagtggaaatggaagtggaagtggaagtTTTCCAA
CCTCGCCTTACTCCACACCCATTCGCTACTCGCGGATCCCAGCTGCAGTTTGGTCTGCTGTCATTGGTTT
TAATGTTCGCGCTCTTGCCGCCGCTGAAGTTTTATCCCCGGCTACAATATAAGTTTAATTTCACTTTCCT
TTTGGCCCAGCTCCCTCTCACTGCAAACTCGGCTCAAAAGTGTCCGTGCTGCAGTCCCGGGTCCGTCCAT
TTCGATGGCTCCTCGTTGGTCTTGGCGGCCGCAAAATTTCACACGCACAAACTTTTCCTTCTCTCCATCT
AAGCAGCACTTTTGCGGCTATGTGGCTGCGGCCAGGACGCAGCTGCTCCATGCTGGGTAGTCGGGAATGT
TGGGGAAATCGGGTAGAAAACTGGAGGACTGTGCGCGTTTGCGAAACTTTACTGACATTTTCTTATGGCA
AAATAATTGCGGACTTGCGGGGCGGCTGGGGTTTGGTGGGACTTAAGCAAAGGCCAGGACATGCCGGCTG
ACAGCTGTTATTATAGTACCCACTTCGGCCGGCGGTATAACACCGAGGATAACAGCACCAACAATGGCAG
TAGAAAACCAGTCATTCGGCCAGTCATAATCCCGGGTAAAAAGTTTACCTTAGACGCGAGACTCCCATGG
GCGGAGCTCCAATCCGGTGAAAAATGAAATTTAATATTTGGTTAATGAGTAATTGTTATCGAAGCAGCGA
GTAGATACTAGCACATCCAACAGGTATTCCAGTTGTGaaaaaaaaGTAACCATTCCTTTTGTATGCTACA
TTTTTTTAAGCTTCATAACTATCAGTTACCTGTAACATAGTTTTCGTTAAACAAAATCCAGGCTTATGGC
AACTTTCCACCGCTTTTCACGTTTCTCTTTAGCGTAAATTCGCGAAATAATATTAAGCTGTAAGCAAAGT
TTATTCGTGGCCATTGCCATGGCAGCGATGTTTTGCAGGACTTGGCCAGCGGGATTCAGGACTCCGGCAC
TTGGGAATTTAGGAGACCGCCTGCCCTGTCAAATCGTCAAACCGAGCGCAATTAAGCTGATTAGTGAGCG
TCATTCAAAGTGAAGCCCGTCCACGTTCGTCGTCCCATCACTGACATAAATAAATTTAATTTCCGCTAAA
CATAAACTCAAAGTAAACTTTTGACGCGAGTAACCGCCAGGAACGTGCAACTGTCGAGGGTTGCGGGGTA
CTACCAATTATATGAAAATCTCtttttcaattatttttccttctttttttttcgtgcatttcgcagcgttt
ctgtttttGCGCTGCGGTACGTTGTTGTCATCGTCGTTGAGCTGTCAACGCTTTACATAACAAAGTTTTT
CGCGGGCTTAGACCACTGGAATGGTTgggggggtgggggTTTGGCGGTTGAAGGACAGTGGGCGGGTGGG
GACGGCCATGGGGCCGTTTTTAGGGGGGCTGGTGGCCGACTGTCACTGCGGTTGTCAGCGTTTGTAAGAC
CATGTTTGCCGGGATATGTGCTTGTGGGCCACGTGTCACGTGCGCTTTTGGCGTGCTTTTAGCTCCTCCC
AGTAGCAGCCCCCCATTTGGACCCACCTTCTTCAGCACTTTCTCTTGTAATGCGATTTCGGGTTTTGTTG
ACTGCCGGCCGCTGCAACTGATTTACGTGACTCAATTACCCAAGGGATTTGAATTCGATTCGGCTGGTGC
TCTTTCTTTGTTTCAGTGTTGTTTCGGCCCCGAATATGCAACAGCTGGCCATTCGGACCAAGCCGCAGAC
TCGCAGAAACATATGGCCACTCTGCGGACAGTGGCCAGTGCTTAGTCAGTGTCCGGATCGGGCGGCTTCA
TCAATTACGTAGCTTCTGCTCCGGATTCCATTGGCCAACTCTAGTCCATGGGCTAGAGCTGCTCCACCGC
AGTTGACAAATGAAGCATGGGCTGAAATTTGAATGTGCCAAGCTAACACGTTGCTTAGCATACTTTGGGG
CACACTGCCAAAAGAAGCTCACGAATGAGATATTAAGTCCCTATTAAAGAGAAATATATAAGAAACGCAA
AAAACTCCAAATAACATATACCCCAGGAATTCTTATATAATTTGATCATATATTCTTTCACTTGTGCATT
TCTTTCAATGTGTCTTAATTTATATGGAACatatatatatgctatattattatatattattattatatat
tatgtattgatattagtctattCTTGGACCTGCAAAAAACCGCATTTTAAGTTTTTAGAAGCAATTATAT
TTAAATGTTCAACCCCTTGGCTCAGCTTTAATTCTGCAGACTCGAACCTAATGGGACCTTCGTCACTTCT
TTTGGCCCCTTCCTGACCTGCGAATCCTGGTCATTGTCACCGGATTTCGAGCACTTTTACGCCGCCAGGT
GTAAGGGGTTGTTCTGCTCTCGGAAGTTGTCACCATTTCCATAGAACGCTGATATATTGAAAGTCATTCG
GGGCACGAGAAGGTTGTCTGCCAGACATTTGTTTCCTTCCTGCGCACAAAGACCATCTTAGGCGGCGAAA
AAAGGGTTAGACGTTGAAGTATAACAGACGTCGGTGGGCAAATTATGAATGGGCTGGGAGTGGGTGGTGG
AGCAAGTAGATGTAGGAGGACAATGAGTTATGTAGACACCATTGAGAGCAGCATTTGGGGGAGCGCGAAA
CGGATATGACTTCATTATCATGATAAGCTTGCTGCACAATGGAGCTGACAACGCCGCCCGTTTCCCACTT
TTTCACTGACCAACGAAGCGAGTACAGGGTTTTGCATTTCAATTTGTCGTAACTGACTGTGACTACTGGT
ATAAATTGTTGTGCATTGTGAATTGCAAACGGGATAGCTAATGCCGATGAAGAGCACTTTACAGCTTAAA

TTCTGGAACGGGGCTGCAGTGCTTAACCAGCACAAATTGGACTAAATCTGAAAGCGTAACTGCCAGGGCT
TAAGACAAAAGCATTGGCCATGTGAGCGGCACATGTCAAACGTGGAGTGCAAAGCATAATCGGAGAGGAA
AAGATATTTTCCGACAATTGCCAGTCTGTCTGGCAGCAACAGGAAAAATCGACCACCAAAGATTACGGCT
GGGTGATGTGGGGGTGTCTTGGCATTTGCAAACCAAATTGTCATGACAGCAGCGGCAAATAAAGGAAAAA
GGAGCACAAGGAGCAGGGCATTGCTTTGACAAATCAATGTGAGAAATCTTGATATGCAGCCCCAAAGCAT
AGCATACTTATGAGGGAGCCCCGAGAACTGGCTACACTGCGCAAAAAGTCCACTACTCCCTTAACGAGTA
ATACGAGATAAGATCTATCCATTGTTACTTTAATAAAATATGTATCTTGATATCACACTTCTACGCATAT
ACTTTGGCATAATCATAAATAAGGCAGAAAAGGAGTTTAATGGTTTTGTGCCAACAGAAGAAAGGTATTT
TAACTTCAATTGTTAAAGGAAGttttttttATTATTATGTATTACATTTACTTCGAGTGTACGGCTCCAG
CTTGATTGTCCTTATGACTTCCTTTTGCCTGCAAACGACAGCTGGCTCTCTTGGCTGCGATGCTCGATTT
TTACTTACATTTCTGCCATTTTGTTTCCCTTTTGCCCGCACTATCTCTGAACCCGAACAACTTTCTCATC
TCTCTTTGCAGTTGCAAATGCCAGCAATTTGGAGCGCTTCCAATTGCGCATCACGAAGAAGGAGCTGTAC
GGCGAGCAGGACACTCTGGTGGATGCGGTGTTACGTGACATGATCAAGCTGCCAATACAGCATGTTGG
>dme_circ_0007344_junction_seq
TGATCAAGCTGCCAATACAGCATGTTGGCTATCGACCGGCGGTCGCAGCGGCAGCT
>dme_circ_0007345
CTATCGACCGGCGGTCGCAGCGGCAGCTCAATCGACGCCGTTGGCACAGCGCCTTCAGCGCTTTCGATAT
CGCcagcaattgcagttgcagcaagagccgcatcagcaacagcaacagcaacagcaacagcagcagcaac
aacaatcgacTGCTGATATCGACGTTTACGATAGCCCCTTTAACGGCCAGTTATCAGCTGAACGAATAGG
CGTAGCCAATTGGCGGAGGGGGAACGTCGAAGGGCCCATTGGCGGGAGAGCTCCAGTTGAGGCTGAGTAC
TCAGCGGAGCAGGAGGAGCTGCCGCCGGATCGAGTGCTATATCCAGACTCGGAACCGGAGGAAGAGGAGG
CAGCGCCACGACGCCGTGTTGTGATCAGGCGCCGCATAGTGCGCACCAGTCGCACCGCTTCACAAGATCC
ACAGACTCAAACTGCAGAGGTTGTTCCAAAATCCTCCAACGATAGCTCAACTCCTGCCGAGATCAACGGC
CGCAATCTTGGCTGGCTGACCAGACTGCGCAGCTTTGGATCGATCAATAGAAAACACCAGGAGACCGCCG
CACCACCAAAAGGCGGCCAGCAAAAAGCCATTTCTGCTGCAAGCAACAACAGTTGCAACACCAATCCCAT
CATGGCCGTCCTGCGTACTATGAAATTAAAGGAGCGACTGGTCATCAGTCTGGGTGCCACCCTCGTCCTG
CTCACCCTTCTGCTTATCGTCGATGTGCAGATGGACTTCGGTGTGGCCAATCGCCACCTCCTTCAGCAAC
AGCACCAAAAGATCCGACTGGGCAACGACTACGATGGTGGCACGGGTGGCGGTGGAATGCTGCACGAGTT
CAAGCGCAAATTCCTGCAGAAGAGGGTAAGTCCGCATTCCCGGTGTCCGTCTTGTACACGAAATCTGACT
CATCTATTGCTCCAGTCCCGTCATTAATCAGCGGATACCTTTCGAGTTCGCTTGGAATGTGGGCTTGGAT
GGATGGCAAGCTAGGATGTTGTAATGATTTCCTGCGAGTATTGTAATGAAAACTGACCCTCATTGAATTA
ATGAGGCATTAGTGCATGCATAAGGTAGCTGGAATTCTGTTATCAGTTATCAGAGCTTGAAACATGGGGG
GAGCATGCAAGATGGGATTTCTTGTTTGTACAGTACTCAAAGTTTGCTATTAGGTAAGTGGAAAGTAAGT
CGTCTGGCAGAAAATTGTTATCATTGCAATATTTTAGAATGCGGCAAGACCATAAACCATATACCCCTAG
TTCGATTCGTTTCCTTCTTTTTCATTTCAAACTCCCAGGTAGCAAAAAGCAGCGTGTTGAAACTTAGTAT
TCCCCAGACTGGCCAAAGCAAACGGGCCACCGCTGGCCATTCCGGGGGATTAAAAATTATGCACAAATCG
CAAACGGGGTCGGGCCATTGACTCTGACTCAAACACCCCACTGTTCCCTCGGGGCGACTGAACACGGCAG
GGTACATAATTCAATTTAGCTGAAATGCAACAAAAGTTGCAATAAAACTTGGTTGGCCCCGGCATTAAAA
CGTAATTTGCTACAAAACATAAACCCGAGGCAGGGCCAACTTCTTGGGAACTACAAGGAATACAAGAAAG
CGGAACAAGCACAGGAGCGCCATGTTAATGCCTTTGCCATGGAGAATATATACAGACGGACGGGGCCGAG
TCGCTTTACCTTTGAACACTTCATTTTTTCATGCTGGCCACTTTTTGCGTTGCGTGCGGTGGGCGAAAGT
GGGTGGACTAGAAGGGTCCGCCGCCATTTTGCTGTGGCTGGCGGAAGTCGAGTAATAAAACATCAAAATG
ACGCAGCAAGGCGCAAGGAAACTGTTAAGACCATCCTTCCCAGCTCCCAGGACTTCCACTTCCTCTCAG
GTGCAGTAAACGACTCCGGTTCGCATCGCGCATCCCGTGCTGCTTCGGTTTTGTTTCGTTCTGTTCCGTT

CAAGCTTTCGCTACCGCTCCCTCTCCGGCAACATAAAACGCCAATGTTTAGCCAGTAAATATTTAATTAA
TTCATAAGCCGCCTGCCACCAGCCCGTAAAATGGATGCCTTCGGAAACCGTTCCTTCGTATTCGCTGCGT
CTGAGGGATACCTCTTCCCCGAAAGATCGGCAGCATCAGGGCGGCCAGAAACGCCGGGCTTGAAGCTCCT
TGCTATCTACTTATGCGCCAGGGTAAATGCAAAATTATGATGCCTCCATGCACTGAGTCGTGCAACGCGG
CTGTGAAGCGTAATAAATTCTATTTAAGCATCAAATGTTTTGCCCGTCTCCGTCTTCAGACGCTCCGAAA
AGCCCGTGGAGGAAGCCGCTCCGCTTCCTGATTTCCTGTCTCGTCCCTCCCCCCTCCCCACTTCCCTTCG
CCCATCAGCCTTATAATTTTTTAATTATTTCATTAAAAACTGAAAACTGAGGCAGCAACTTGGCAATGCT
GCAAATGGTGTTTGCCGTCCACGAAGCAGAGGGAAAATTCATTTTCAGTGATGATTCTCATTTTTTGGGC
TGTTATCAAtctttattctatttttcttgttttctttttatcttcgggcttttcttaaccctttTAATTA
AAGTTGCTGTAGCTGACAGAGATTTTAATTCGTTTCTTCTAATACTTTTTAATTGGATTAAATCAAATAA
AAATGTGTTAGGATTTAACTTAACTTAACTATTAACTTAAACGATTTCGTAATCTTTATTGGATTCTAAT
ATGATTAGATTTTAGATATATATAAGGAACTCTGAGCAATTTGAACGTAACAAGTTATAGTTATATCCAT
GAACTAGACAAGATGGCATTTTCCATGGAAGGCAGGCGGAAATGCCGCACTGAAATCCAATTAGGTCTCC
CTTAAATCCATCCATTAACCCCTGACAGTGTCACAGCTTTCGACTTGCATAAACTTTCACCTATCACCTA
TGATCCTGTGGCTGCGCGTTGTCGCTGCATTTGTTGCAGGGCCGTCTATTGCCATTACAGTTGCAGTTGA
CTGGTTAATTAACCGCAGCACATGGCCAGCTTATGTGGCCAGCGTTACGCACACCACGTGCCACTTTAAT
GAGCAAAGTTTCGCTATTGCCAAAGTGTAATTAAGTGCACGCCACATCGATCAGTTCAGTCGAAAACAAA
GAGGGTTACCACCACTACCCTTTTAGAGGCATATCCCCCATAGCGCAAGCCATTTTGAGCGAAAGCCATA
AATATGAGGACCCTCTTCCTGCAAGTGGTTGCACAAGGGGCGACTTAAATACTTATTAGCACATAAAGTG
GCATTATGCGGCTGCCAATAAAATGCGCGAAACGCGCGAAATCCGCACAGGCTCATTGCGCTGGAAAATT
CTTAATAAGCCCCCGAGCCGTGGAAAAGGCTCTTTGTCGCCCAGATATTGTCTCATCCGAGATGGGGAAA
ATGGCAAATGTTGACAGCTTCTCGGCTGTTGGCCTATCTGCTGTCTCGGCCCAGACATTCTCTGGCAACT
GTTAATTGAAAAAGTTTTGCATTCTCACCCGGCAAACAtgtgtgtgtgtgtgtgtgtgcgtgtAGGTTTG
TATGCAACACACCTGTCTGCTTGACGGATAAGGCAAGGCGCCGACAGACAGACCGACAGACAAAAGACTT
TCGCCCCAAAAACTTGGGATACTTGCACAATTAAAAAAATATATATAAGTATTTTCAGAAAACGACAATT
TACGTAAAACTCAAATTTCAAAATTCTGGCCTAATGATGTGCACTCCCCACAGCCTGTGCACACGAGTGC
TGTCATTTGGCTTAATGGTCACACAACTGGTGCATAATTCTCGGTTTCCCGGTACATACATATGCTAACG
AGGTGGAGATGGAAAATGGGCCGGAAACGGGGCACCCGGCCAGGCCAGGTTAGATCTCAAGCATTTCGCC
GAGAGCTATTAAGAGTCAAGGCTAGGAAAATGGATGCAACAAATTCAAAAACTTTAATTCATTTTTCAAG
CACTCTTTTTGTCTGAAGCAGGCGCTGTCTAAACGGCTGCTGATTACCAGCACAAGCACAGTGGAATAGG
CTGGCAAAATGGTGTGCAAACTTTGAGAGAAATGTGAATACTAAATGAATACGAATTTCTGTCATCTAAA
CCGCATTTGAATGGACTAAAATGCAATTTATATGTCAGAGAAAGCAAACTTATAAATTGTAAAGAAATTT
GTCTCCCCGTCTTTGTTCAGATTGTCTAAAGAAACTTCTGCAAATAATGTTCGCCAAAGTTTGCTGTATT
TACCTGATATTAGAGGCATGTCCCCTAGGTGTCGCCACTGTTCCAACTTGTATGGGTTGCGGTTTCGCCC
CCTTGAAGTGCCTGCAATTTCAGTTTATTGCAGTCGAGTGAAGAGTCCCATGTGTGTCTAAGCTtgtggg
tgtggatggggatctggatgtggatgtggatgtggatgtggatgtgggtgtgttCGTCTTAGGTAAACGC
AACTAAAATCCTGGCTGACAAAGTTTCCAGCTTACCGGCAGCAAGTGCGTGTCTTTGGGAGCCTGGCCAG
GGGGCACTTAGCTGTCTAGGCTGCTTCAGTTCAGTGTAGCTCGATGGCTGCCACTAAAAGGCAGTGGGGC
ACTTGGCAGTTGCAATTAGCAACGAGAGCCACCCGGACCATTGTCAAGAGCACCCGGCTTTTGGGTCTCC
GCAAATTATGGCAAttttttgccctgttatttttgtttttgtttttCTTGTACATTTAACTCACTTTTAT
GCAGCTCCTTTTTAACAGAGGTACTCTCGCTTTCATTTTGCTTTTATTCCAGCCAACGCCTCGGGCTCCA
AGGAGGCATCCACGCAGGCGGGGGCGAGCCAGAGTGGCGGGGCGACCAGTGGTCAGGATGCGGCTGCCGG
AGCTTCGGGCGGAGCAGCCGGACCCGGCACATCAAGGAGCACATCCACGAGGAAGCCCACACCACACGAT
CGCTATGCGGATCTGCAAAAGCACCTGCTTAGCGATGAGTACTCGCATGTGATTGTGGACAATGCGCCGG

ATGTGAGCAGGGACAATCCCACGCTGGCCGAGATGCTGCACCGCAAGGCCAGG

>dme_circ_0007345_junction_seq

GGCCGAGATGCTGCACCGCAAGGCCAGGCTATCGACCGGCGGTCGCAGCGGCAGCT

>dme_circ_0007346

GTTAAGTCAGGCGGCAGCGAAGTGGACTTAAGGCAACGGAATGCCAGCATCCGGAACCTCTTCGCCCCCT
CGTCGGCTGAGTTGGCCaaaaaaaaGGAAGCTCGTCAGAGGAGCCGCAGCCAGAGCAGCTTTTCGCTCCA
GAGGGCCAGCAGCCAGGATGACGGGATTGGTAATGGTATGGGAATGGGGCTTGGGAGCAAGATGGGCAGC
CAGATGGGCCTGCCCAACTCCAAGAGCTTCGATGATTACGCTCTGGGCATAAATCAATTGCCAATGACTG
CGAATCAATCGGCAGTGAGACAATACAATGGCCAGGACCATAACGTGCTGGGCTCAAGTCCCTATGCACC
GGCTTCTACAAGGGCAACGGCATTGGTAATTCCCAGGACATCGCACAATGGCTGCCATCAGAGTGCATTT
GAATTGGCAATGAGCCGGACTCCATCGGAGCACAAAAACTGGAAGTCGACGAGCCAGCTGGAAAATTTTA
TTAACAGCAACGAGAAGCTgcagcaggagcagtggcagcagcaccagcagcaggACACTTCTGTTGACTT
TGGTAATGAAATGACCTGGGGCAAATCTGCTGAGAAATCGCTGCCATTGAATCACCTGGTGCAAGAAGAA
GGATTTTCCCGAGACGACCAGTGGGATTTGGACTTTCAGACGGAGCAGCGCGAAAGTTTGCAGAGCCAAA
GGTAAATTGACAAAGGGCCCAAAGCACAGGGAATGTGATAGAGAACAGAGAGGTGAAGAAATAGCCAGAA
AGGCAACTAACCCAAACTGCGTTTAGCTTTTCTATTTTCCATTTTCCAAACCCTTGCACCCGCCGCAATT
TGCTGAAGTTTAGCGGATGCTTCAATGGTCATACGGAGAAGAAGGGAGCCATGTAAATGAGCCAAACGTG
TAAGCCAAACTAACCTTGACACTCCTCCACTCGACTCGATCGAATTGGCAAAACGATGGCTGTAACAGTT
TGGCCACCatttgcatatcgaatggcatcgcgattgattggccgttcgattgggtcgataaggtggcggc
tccgtaatggcaacagtagttgtaagcagctcaagtgcaaaccgctcaaatagtgtggcaacaaatcgat
caataagtcgggtcttttgtggcatatttgcatatcgaatggcatcgcgattgattggccgttcgattgg
gtcgataaggtggcggctccgtaatggcaacagtagttgtaagcagctcaagtgcaaaccgctcaaatag
tgtggcaacaaatcgatcaataagtcgggtcttttgtggcatCGGCACCGCCATCGCTATCGCCAAcgga
atcggattcggattcggattcgTATCCCGGACAGTGGAATTCTCGACTGCCATCGATGCGTGGCCAAGTC
ACGCGGGGCAACAGGgtggatatagatatagatgtggatgtggatgtggatgCACTGAGCGTGTCACTTC
AAGCTGTTGCACAATCGCTGATTTGATTCGAATCGAGCTGAGTTTCAAGCGGGTTACGCATTGCATTTGT
TAACAACAAAATTACAAGGGTATCCCCTCCACTGCAGTGCAATCATGCAGAGCAAATgggggggggggggg
ggAAACTGGATAAAGTGGGGGTCTCGCAGCTGCGATTCAAGTGCGGCACGACTACTATATAATATCAAGT
ACAGAGCAAGAGTCCACGGAATGCAGTTCGTTGACCGGCGAAGGCTAATTGAGCTGGAGAAAGGTAGCGG
AAAGGCATTTGTCAAGTTTGATTGAAAGGGAGTTTGATTTATTGCATTGCGATACGATAACCCAGTTTAA
CTCAGAGTGCTCCAATTCGGTTTAAGATTGATTTTTGGGGTCGATTGTCGGAGTGTTTTGTACTCTTTGT
ATTGTTCACAATATGTTACTTCATATTGGAAAAGCTTATAATTGAAATTGATTGGATAGATCGATTAGGA
AGGCCATAGCAACTTTTCACGATATTTTCTTATGAGCCCCTCCTTTGGTAGTGAAATGATTAATTTTGCG
GAATATTAACATATCCCTCTCTCAACACAACGCGATATTGTGCTCTAAATTGTTTTGCACTTTATTGCCC
CCAGCTCATTTAAGTACATCTCGCCTCATTTCGCTTTCTTAACTAttttctttttttgcatttttttttA
CTTGCGCGGCTATATGTCTGATTAATAAAAACAAAGCGCAAATTGGAAAGGAAATTGCTGTCACTCACTG
GCGATGTTGTTGCTGCTGTCAGCAATTGCATTAAAACGATTGGTGTTGCTTTTGCTGTTGCTATTTTTAC
TCGACTCTTAGTTGACTCCAGAGCCTCGACCCTCGACGATGCGGCATTTAAATACACCGCATGAGTTTAA
TTTAATGCAACGCAGCAGTTTTATCAAGCGACAACAAAATGCGACGACAGCAATTGCTGTTAACTGTGTT
CGCTCcactcgcactcgaactcgaactcacactcgcactTGGCATGTCACTGCCACAGCGCTTATCGCCC
TCACCGGCTGGCTGATAGGGAAAACTCGTGTGGATGACGCTTctccagtcctccagtcctctagccctcc
agcccATCAGTCCACCGTCAGCACCCACCCACTTTTCCTGGGATTACGTATGTGCTTTGCATTTGACTGC
CCATTGATGTTGCCAACGCGCTGGTGACGGCGAGAGATGTGCCATTGTGTTGCCAGGCCAAGTCTCTTCA
GCTGAGTTCCTTGTACACGTACTTCATGTACCTAAACCCACCAATCCATGACCCCTAAAAACCCTTCCGC

TCTTGTCTGGCCTTAACCCCTTGGCCACGCCCACTCTGCAACTGTCAAATCATCTCGTCATGGATATGAG
CAATTGCCAGGATCTCATACTGCTGTACAGAGCTCTTTGGGGGCGAGCCTGCATAGAAAATTGGCATGAT
ATGTGATTTGATTGACATGGCACTGCGATCCTGCTGCTGCTTCTTCGCAGCCATTTTCGGCCGTTCTTTG
TTTGCCAATTTAGTGCCGCCTCTGTCATTTTATTTCTATCAGACCTGCTTGTAATCAGCATTTTTGCCAA
CGATTTTGACGTTTAATTTGTGAGCCGAGTTACGTCAGCAACAAATCGGTAGGCCTGTCAATCAAAGGGT
TTCGCATTCAATTTGCAGGCCATACAGTAATGCACTCAAGAAAATATTGAAACTGAAATTAAACCAAATG
TAGCCAATATTTTGCTTTCAGCAACAAGCATACACTTTACGAAAATTAAAGTATTTTAAACACAAATGTG
TACAGACCAATTGTAAACTTTTATGATGTTTTAAAATTAGCTTTTATAGGCGAAGGAAAATTCTGTTTGA
TACCTTGACAATTTCTTGGTTTACTTTCCACTTAAGCCCTAttttttttCGAGTGGATGTGGAGGACTGG
AATATTTTGGTCACATTTAATGCAGTTACGGCATTTGTTGCCCCGGGCAATTCAACATTTTGCCCACAAT
TTTCGGTTGTCGAACGACCAGGGCCGTGTTGACTTTGCATGTGGGCAGAAAACAAGGCAGCAACAAGCTT
CGTTAGCTTACCACCACCCGACGGCCAAACAGACAACGGCCACTTTGCAGGTTCTATTCCAGATCCAGAT
CCTGGCACCGACCAACTCTTCCTGTTGTAGTTACAATTCAAATTCCGACTGAAACCCGCCACGAGCTGGC
TGCCTGCAAAATATGCTTCATGCTACACGGGCCAGCAAAAACATGAACAATAAAAATGGAGACAATAAAA
TGGAACTACAAAAACTACAGCAACTTGATGGCACCGACAAAAAGCACTGGCAATAGCAACAACACGCTAG
AGCAGCGATAACGGTGGCCGGCGaaaaaaaaaaaaTGGCTGCTGTCGCATTTCCGGTGGCAGTCAACATG
GCGGATGCCGTGACGCCGCTTTATGAATAACCAGACACAAAATACGAACTGCAAAATGGCCATAGGGTCA
TATTTTTGAAGCTTGATATGGTTAGGGATAGACCAACTGATATGTGGAGTGGGTGTTATGAATTAGCCAA
TTGAAAATAAATACAGAGCTTATCACCAAAATAAACAATATCAATACGATGGGGACCTTGAAAAATGCAA
ATTTAGTCATTGAAATTAAAGAAATAAATGGTTTTAATGAAAAATCAATTCGTGATATCATTTCAAATTG
GCAAAGCGAAAATAATATATTATTCGTTGATAATTGATCTTTTATTTAGCGTCATTTTTGCAGATGTTGG
TAAATTTTTTATTGTGGCTATCACAATCTAGTGCACACTGTTGATTTTTAGAAAATGGTCGAAACTTTAA
GAAGGTTATTCCACTACATTTTACATTTACACCAAATAAATTTGTTTTATTAGCAAAATAAATATTTTAT
AACCTTTGTTATTTTGTTACTCTCCTGTGACTGTAATGTTTTTATTGGATGCATATATAAAGCATTTATT
TATATGCTTATTTTTCATAAAAAGTTGTTCCCAAAATAAAACGTATCTCCTTTGCTTTAGTGCTATTGGC
GGATAGGATTTGCTGGCATTTCACTTGTGACTTCTTTGTATTCCCTTTTCGACCCTTCAAGTCATGAAAA
CTTAATAGAAATTTGATTGCCTATCACCAGAACTACACATCGCACCAGCCGGCACCCTTGAATTCGCATT
GGTTTTTAATGAAATTTCCCGCttttttttttGCAGCCTATCGACCGGCGGTCGCAGCGGCAGCTCAATC
GACGCCGTTGGCACAGCGCCTTCAGCGCTTTCGATATCGCcagcaattgcagttgcagcaagagccgcat
cagcaacagcaacagcaacagcaacagcagcagcaacaacaatcgacTGCTGATATCGACGTTTACGATA
GCCCCTTTAACGGCCAGTTATCAGCTGAACGAATAGGCGTAGCCAATTGGCGGAGGGGGAACGTCGAAGG
GCCCATTGGCGGGAGAGCTCCAGTTGAGGCTGAGTACTCAGCGGAGCAGGAGGAGCTGCCGCCGGATCGA
GTGCTATATCCAGACTCGGAACCGGAGGAAGAGGAGGCAGCGCCACGACGCCGTGTTGTGATCAGGCGCC
GCATAGTGCGCACCAGTCGCACCGCTTCACAAGATCCACAGACTCAAACTGCAGAGGTTGTTCCAAAATC
CTCCAACGATAGCTCAACTCCTGCCGAGATCAACGGCCGCAATCTTGGCTGGCTGACCAGACTGCGCAGC
TTTGGATCGATCAATAGAAAACACCAGGAGACCGCCGCACCACCAAAAGGCGGCCAGCAAAAAGCCATTT
CTGCTGCAAGCAACAACAGTTGCAACACCAATCCCATCATGGCCGTCCTGCGTACTATGAAATTAAAGGA
GCGACTGGTCATCAGTCTGGGTGCCACCCTCGTCCTGCTCACCCTTCTGCTTATCGTCGATGTGCAGATG
GACTTCGGTGTGGCCAATCGCCACCTCCTTCAGCAACAGCACCAAAAGATCCGACTGGGCAACGACTACG
ATGGTGGCACGGGTGGCGGTGGAATGCTGCACGAGTTCAAGCGCAAATTCCTGCAGAAGAGGGTAAGTCC
GCATTCCCGGTGTCCGTCTTGTACACGAAATCTGACTCATCTATTGCTCCAGTCCCGTCATTAATCAGCG
GATACCTTTCGAGTTCGCTTGGAATGTGGGCTTGGATGGATGGCAAGCTAGGATGTTGTAATGATTTCCT
GCGAGTATTGTAATGAAAACTGACCCTCATTGAATTAATGAGGCATTAGTGCATGCATAAGGTAGCTGGA
ATTCTGTTATCAGTTATCAGAGCTTGAAACATGGGGGGAGCATGCAAGATGGGATTTCTTGTTTGTACAG

TACTCAAAGTTTGCTATTAGGTAAGTGGAAAGTAAGTCGTCTGGCAGAAAATTGTTATCATTGCAATATT
TTAGAATGCGGCAAGACCATAAACCATATACCCCTAGTTCGATTCGTTTCCTTCTTTTTCATTTCAAACT
CCCAGGTAGCAAAAAGCAGCGTGTTGAAACTTAGTATTCCCCAGACTGGCCAAAGCAAACGGGCCACCGC
TGGCCATTCCGGGGGATTAAAAATTATGCACAAATCGCAAACGGGGTCGGGCCATTGACTCTGACTCAAA
CACCCCACTGTTCCCTCGGGGCGACTGAACACGGCAGGGTACATAATTCAATTTAGCTGAAATGCAACAA
AAGTTGCAATAAAACTTGGTTGGCCCCGGCATTAAAACGTAATTTGCTACAAAACATAAACCCGAGGCAG
GGCCAACTTCTTGGGAACTACAAGGAATACAAGAAAGCGGAACAAGCACAGGAGCGCCATGTTAATGCCT
TTGCCATGGAGAATATATACAGACGGACGGGGCCGAGTCGCTTTACCTTTGAACACTTCATTTTTTCATG
CTGGCCACTTTTTGCGTTGCGTGCGGTGGGCGAAAGTGGGTGGACTAGAAGGGTCCGCCGCCATTTTGCT
GTGGCTGGCGGAAGTCGAGTAATAAAACATCAAAATGACGCAGCAAGGCGCAAGGGAAACTGTTAAGACC
ATCCTTCCCAGCTCCCAGGACTTCCACTTCCTCTCAGGTGCAGTAAACGACTCCGGTTCGCATCGCGCAT
CCCGTGCTGCTTCGGTTTTGTTTCGTTCTGTTCCGTTCAAGCTTTCGCTACCGCTCCCTCTCCGGCAACA
TAAAACGCCAATGTTTAGCCAGTAAATATTTAATTAATTCATAAGCCGCCTGCCACCAGCCCGTAAAATG
GATGCCTTCGGAAACCGTTCCTTCGTATTCGCTGCGTCTGAGGGATACCTCTTCCCCGAAAGATCGGCAG
CATCAGGGCGGCCAGAAACGCCGGGCTTGAAGCTCCTTGCTATCTACTTATGCGCCAGGGTAAATGCAAA
ATTATGATGCCTCCATGCACTGAGTCGTGCAACGCGGCTGTGAAGCGTAATAAATTCTATTTAAGCATCA
AATGTTTTGCCCGTCTCCGTCTTCAGACGCTCCGAAAAGCCCGTGGAGGAAGCCGCTCCGCTTCCTGATT
TCCTGTCTCGTCCCTCCCCCCTCCCCACTTCCCTTCGCCCATCAGCCTTATAATTTTTAATTATTTCAT
TAAAAACTGAAAACTGAGGCAGCAACTTGGCAATGCTGCAAATGGTGTTTGCCGTCCACGAAGCAGAGGG
AAAATTCATTTTCAGTGATGATTCTCATTTTTTGGGCTGTTATCAAtctttattctatttttcttgtttt
ctttttatcttcgggcttttcttaaccctttTAATTAAAGTTGCTGTAGCTGACAGAGATTTTAATTCGT
TTCTTCTAATACTTTTTAATTGGATTAAATCAAATAAAAATGTGTTAGGATTTAACTTAACTTAACTATT
AACTTAAACGATTTCGTAATCTTTATTGGATTCTAATATGATTAGATTTTAGATATATATAAGGAACTCT
GAGCAATTTGAACGTAACAAGTTATAGTTATATCCATGAACTAGACAAGATGGCATTTTCCATGGAAGGC
AGGCGGAAATGCCGCACTGAAATCCAATTAGGTCTCCCTTAAATCCATCCATTAACCCCTGACAGTGTCA
CAGCTTTCGACTTGCATAAACTTTCACCTATCACCTATGATCCTGTGGCTGCGCGTTGTCGCTGCATTTG
TTGCAGGGCCGTCTATTGCCATTACAGTTGCAGTTGACTGGTTAATTAACCGCAGCACATGGCCAGCTTA
TGTGGCCAGCGTTACGCACACCACGTGCCACTTTAATGAGCAAAGTTTCGCTATTGCCAAAGTGTAATTA
AGTGCACGCCACATCGATCAGTTCAGTCGAAAACAAAGAGGGTTACCACCACTACCCTTTTAGAGGCATA
TCCCCCATAGCGCAAGCCATTTTGAGCGAAAGCCATAAATATGAGGACCCTCTTCCTGCAAGTGGTTGCA
CAAGGGGCGACTTAAATACTTATTAGCACATAAAGTGGCATTATGCGGCTGCCAATAAAATGCGCGAAAC
GCGCGAAATCCGCACAGGCTCATTGCGCTGGAAAATTCTTAATAAGCCCCCGAGCCGTGGAAAAGGCTCT
TTGTCGCCCAGATATTGTCTCATCCGAGATGGGGAAAATGGCAAATGTTGACAGCTTCTCGGCTGTTGGC
CTATCTGCTGTCTCGGCCCAGACATTCTCTGGCAACTGTTAATTGAAAAAGTTTTGCATTCTCACCCGGC
AAACAtgtgtgtgtgtgtgtgtgtgcgtgtAGGTTTGTATGCAACACACCTGTCTGCTTGACGGATAAGG
CAAGGCGCCGACAGACAGACCGACAGACAAAAGACTTTCGCCCCAAAAACTTGGGATACTTGCACAATTA
AAAAAATATATATAAGTATTTTCAGAAAACGACAATTTACGTAAAACTCAAATTTCAAAATTCTGGCCTA
ATGATGTGCACTCCCCACAGCCTGTGCACACGAGTGCTGTCATTTGGCTTAATGGTCACACAACTGGTGC
ATAATTCTCGGTTTCCCGGTACATACATATGCTAACGAGGTGGAGATGGAAAATGGGCCGGAAACGGGGC
ACCCGGCCAGGCCAGGTTAGATCTCAAGCATTTCGCCGAGAGCTATTAAGAGTCAAGGCTAGGAAAATGG
ATGCAACAAATTCAAAAACTTTAATTCATTTTTCAAGCACTCTTTTTGTCTGAAGCAGGCGCTGTCTAAA
CGGCTGCTGATTACCAGCACAAGCACAGTGGAATAGGCTGGCAAAATGGTGTGCAAACTTTGAGAGAAAT
GTGAATACTAAATGAATACGAATTTCTGTCATCTAAACCGCATTTGAATGGACTAAAATGCAATTTATAT
GTCAGAGAAAGCAAACTTATAAATTGTAAAGAAATTTGTCTCCCCGTCTTTGTTCAGATTGTCTAAAGAA

ACTTCTGCAAATAATGTTCGCCAAAGTTTGCTGTATTTACCTGATATTAGAGGCATGTCCCCTAGGTGTC
GCCACTGTTCCAACTTGTATGGGTTGCGGTTTCGCCCCCTTGAAGTGCCTGCAATTTCAGTTTATTGCAG
TCGAGTGAAGAGTCCCATGTGTGTCTAAGCTtgtgggtgtggatggggatctggatgtggatgtggatgt
ggatgtggatgtgggtgtgttCGTCTTAGGTAAACGCAACTAAAATCCTGGCTGACAAAGTTTCCAGCTT
ACCGGCAGCAAGTGCGTGTCTTTGGGAGCCTGGCCAGGGGGCACTTAGCTGTCTAGGCTGCTTCAGTTCA
GTGTAGCTCGATGGCTGCCACTAAAAGGCAGTGGGGCACTTGGCAGTTGCAATTAGCAACGAGAGCCACC
CGGACCATTGTCAAGAGCACCCGGCTTTTGGGTCTCCGCAAATTATGGCAAttttttgccctgttatttt
tgtttttgtttttCTTGTACATTTAACTCACTTTTATGCAGCTCCTTTTTAACAGAGGTACTCTCGCTTT
CATTTTGCTTTTATTCCAGCCAACGCCTCGGGCTCCAAGGAGGCATCCACGCAGGCGGGGGCGAGCCAGA
GTGGCGGGGCGACCAGTGGTCAGGATGCGGCTGCCGGAGCTTCGGGCGGAGCAGCCGGACCCGGCACATC
AAGGAGCACATCCACGAGGAAGCCCACACCACACGATCGCTATGCGGATCTGCAAAAGCACCTGCTTAGC
GATGAGTACTCGCATGTGATTGTGGACAATGCGCCGGATGTGAGCAGGGACAATCCCACGCTGGCCGAGA
TGCTGCACCGCAAGGCCAGG
>dme_circ_0007346_junction_seq
GGCCGAGATGCTGCACCGCAAGGCCAGGGTTAAGTCAGGCGGCAGCGAAGTGGACT
>dme_circ_0007347
GAGCGACTGGTCATCAGTCTGGGTGCCACCCTCGTCCTGCTCACCCTTCTGCTTATCGTCGATGTGCAGA
TGGACTTCGGTGTGGCCAATCGCCACCTCCTTCAGCAACAGCACCAAAAGATCCGACTGGGCAACGACTA
CGATGGTGGCACGGGTGGCGGTGGAATGCTGCACGAGTTCAAGCGCAAATTCCTGCAGAAGAGGGTAAGT
CCGCATTCCCGGTGTCCGTCTTGTACACGAAATCTGACTCATCTATTGCTCCAGTCCCGTCATTAATCAG
CGGATACCTTTCGAGTTCGCTTGGAATGTGGGCTTGGATGGATGGCAAGCTAGGATGTTGTAATGATTTC
CTGCGAGTATTGTAATGAAAACTGACCCTCATTGAATTAATGAGGCATTAGTGCATGCATAAGGTAGCTG
GAATTCTGTTATCAGTTATCAGAGCTTGAAACATGGGGGGAGCATGCAAGATGGGATTTCTTGTTTGTAC
AGTACTCAAAGTTTGCTATTAGGTAAGTGGAAAGTAAGTCGTCTGGCAGAAAATTGTTATCATTGCAATA
TTTTAGAATGCGGCAAGACCATAAACCATATACCCCTAGTTCGATTCGTTTCCTTCTTTTTCATTTCAAA
CTCCCAGGTAGCAAAAAGCAGCGTGTTGAAACTTAGTATTCCCCAGACTGGCCAAAGCAAACGGGCCACC
GCTGGCCATTCCGGGGGATTAAAAATTATGCACAAATCGCAAACGGGGTCGGGCCATTGACTCTGACTCA
AACACCCCACTGTTCCCTCGGGGCGACTGAACACGGCAGGGTACATAATTCAATTTAGCTGAAATGCAAC
AAAAGTTGCAATAAAACTTGGTTGGCCCCGGCATTAAAACGTAATTTGCTACAAAACATAAACCCGAGGC
AGGGCCAACTTCTTGGGAACTACAAGGAATACAAGAAAGCGGAACAAGCACAGGAGCGCCATGTTAATGC
CTTTGCCATGGAGAATATATACAGACGGACGGGGCCGAGTCGCTTTACCTTTGAACACTTCATTTTTTCA
TGCTGGCCACTTTTTGCGTTGCGTGCGGTGGGCGAAAGTGGGTGGACTAGAAGGGTCCGCCGCCATTTTG
CTGTGGCTGGCGGAAGTCGAGTAATAAAACATCAAAATGACGCAGCAAGGCGCAAGGGAAACTGTTAAGA
CCATCCTTCCCAGCTCCCAGGACTTCCACTTCCTCTCAGGTGCAGTAAACGACTCCGGTTCGCATCGCGC
ATCCCGTGCTGCTTCGGTTTTGTTTCGTTCTGTTCCGTTCAAGCTTTCGCTACCGCTCCCTCTCCGGCAA
CATAAAACGCCAATGTTTAGCCAGTAAATATTTAATTAATTCATAAGCCGCCTGCCACCAGCCCGTAAAA
TGGATGCCTTCGGAAACCGTTCCTTCGTATTCGCTGCGTCTGAGGGATACCTCTTCCCCGAAAGATCGGC
AGCATCAGGGCGGCCAGAAACGCCGGGCTTGAAGCTCCTTGCTATCTACTTATGCGCCAGGGTAAATGCA
AAATTATGATGCCTCCATGCACTGAGTCGTGCAACGCGGCTGTGAAGCGTAATAAATTCTATTTAAGCAT
CAAATGTTTTGCCCGTCTCCGTCTTCAGACGCTCCGAAAAGCCCGTGGAGGAAGCCGCTCCGCTTCCTGA
TTTCCTGTCTCGTCCCTCCCCCCTCCCCACTTCCCTTCGCCCATCAGCCTTATAATTTTTAATTATTTC
ATTAAAAACTGAAAACTGAGGCAGCAACTTGGCAATGCTGCAAATGGTGTTTGCCGTCCACGAAGCAGAG
GGAAAATTCATTTTCAGTGATGATTCTCATTTTTTGGGCTGTTATCAAtctttattctatttttcttgtt
ttctttttatcttcgggcttttcttaaccctttTAATTAAAGTTGCTGTAGCTGACAGAGATTTTAATTC

GTTTCTTCTAATACTTTTTAATTGGATTAAATCAAATAAAAATGTGTTAGGATTTAACTTAACTTAACTA
TTAACTTAAACGATTTCGTAATCTTTATTGGATTCTAATATGATTAGATTTTAGATATATATAAGGAACT
CTGAGCAATTTGAACGTAACAAGTTATAGTTATATCCATGAACTAGACAAGATGGCATTTTCCATGGAAG
GCAGGCGGAAATGCCGCACTGAAATCCAATTAGGTCTCCCTTAAATCCATCCATTAACCCCTGACAGTGT
CACAGCTTTCGACTTGCATAAACTTTCACCTATCACCTATGATCCTGTGGCTGCGCGTTGTCGCTGCATT
TGTTGCAGGGCCGTCTATTGCCATTACAGTTGCAGTTGACTGGTTAATTAACCGCAGCACATGGCCAGCT
TATGTGGCCAGCGTTACGCACACCACGTGCCACTTTAATGAGCAAAGTTTCGCTATTGCCAAAGTGTAAT
TAAGTGCACGCCACATCGATCAGTTCAGTCGAAAACAAAGAGGGTTACCACCACTACCCTTTTAGAGGCA
TATCCCCCATAGCGCAAGCCATTTTGAGCGAAAGCCATAAATATGAGGACCCTCTTCCTGCAAGTGGTTG
CACAAGGGGCGACTTAAATACTTATTAGCACATAAAGTGGCATTATGCGGCTGCCAATAAAATGCGCGAA
ACGCGCGAAATCCGCACAGGCTCATTGCGCTGGAAAATTCTTAATAAGCCCCCGAGCCGTGGAAAAGGCT
CTTTGTCGCCCAGATATTGTCTCATCCGAGATGGGGAAAATGGCAAATGTTGACAGCTTCTCGGCTGTTG
GCCTATCTGCTGTCTCGGCCCAGACATTCTCTGGCAACTGTTAATTGAAAAAGTTTTGCATTCTCACCCG
GCAAACAtgtgtgtgtgtgtgtgtgtgtgcgtgtAGGTTTGTATGCAACACACCTGTCTGCTTGACGGATAA
GGCAAGGCGCCGACAGACAGACCGACAGACAAAAGACTTTCGCCCCAAAAACTTGGGATACTTGCACAAT
TAAAAAAATATATATAAGTATTTTCAGAAAACGACAATTTACGTAAAACTCAAATTTCAAAATTCTGGCC
TAATGATGTGCACTCCCCACAGCCTGTGCACACGAGTGCTGTCATTTGGCTTAATGGTCACACAACTGGT
GCATAATTCTCGGTTTCCCGGTACATACATATGCTAACGAGGTGGAGATGGAAAATGGGCCGGAAACGGG
GCACCCGGCCAGGCCAGGTTAGATCTCAAGCATTTCGCCGAGAGCTATTAAGAGTCAAGGCTAGGAAAAT
GGATGCAACAAATTCAAAAACTTTAATTCATTTTTCAAGCACTCTTTTTGTCTGAAGCAGGCGCTGTCTA
AACGGCTGCTGATTACCAGCACAAGCACAGTGGAATAGGCTGGCAAAATGGTGTGCAAACTTTGAGAGAA
ATGTGAATACTAAATGAATACGAATTTCTGTCATCTAAACCGCATTTGAATGGACTAAAATGCAATTTAT
ATGTCAGAGAAAGCAAACTTATAAATTGTAAAGAAATTTGTCTCCCCGTCTTTGTTCAGATTGTCTAAAG
AAACTTCTGCAAATAATGTTCGCCAAAGTTTGCTGTATTTACCTGATATTAGAGGCATGTCCCCTAGGTG
TCGCCACTGTTCCAACTTGTATGGGTTGCGGTTTCGCCCCCTTGAAGTGCCTGCAATTTCAGTTTATTGC
AGTCGAGTGAAGAGTCCCATGTGTGTCTAAGCTtgtgggtgtggatggggatctggatgtggatgtggat
gtggatgtggatgtgggtgtgttCGTCTTAGGTAAACGCAACTAAAATCCTGGCTGACAAAGTTTCCAGC
TTACCGGCAGCAAGTGCGTGTCTTTGGGAGCCTGGCCAGGGGGCACTTAGCTGTCTAGGCTGCTTCAGTT
CAGTGTAGCTCGATGGCTGCCACTAAAAGGCAGTGGGGCACTTGGCAGTTGCAATTAGCAACGAGAGCCA
CCCGGACCATTGTCAAGAGCACCCGGCTTTTGGGTCTCCGCAAATTATGGCAAtttttttgccctgttatt
tttgtttttgtttttCTTGTACATTTAACTCACTTTTATGCAGCTCCTTTTTAACAGAGGTACTCTCGCT
TTCATTTTGCTTTTATTCCAGCCAACGCCTCGGGCTCCAAGGAGGCATCCACGCAGGCGGGGGCGAGCCA
GAGTGGCGGGGCGACCAGTGGTCAGGATGCGGCTGCCGGAGCTTCGGGCGGAGCAGCCGGACCCGGCACA
TCAAGGAGCACATCCACGAGGAAGCCCACACCACACGATCGCTATGCGGATCTGCAAAAGCACCTGCTTA
GCGATGAGTACTCGCATG
>dme_circ_0007347_junction_seq
CACCTGCTTAGCGATGAGTACTCGCATGGAGCGACTGGTCATCAGTCTGGGTGCCA
>dme_circ_0007348
CTATCGACCGGCGGTCGCAGCGGCAGCTCAATCGACGCCGTTGGCACAGCGCCTTCAGCGCTTTCGATAT
CGCcagcaattgcagttgcagcaagagccgcatcagcaacagcaacagcaacagcaacagcagcagcaac
aacaatcgacTGCTGATATCGACGTTTACGATAGCCCCTTTAACGGCCAGTTATCAGCTGAACGAATAGG
CGTAGCCAATTGGCGGAGGGGGAACGTCGAAGGGCCCATTGGCGGGAGAGCTCCAGTTGAGGCTGAGTAC
TCAGCGGAGCAGGAGGAGCTGCCGCCGGATCGAGTGCTATATCCAGACTCGGAACCGGAGGAAGAGGAGG
CAGCGCCACGACGCCGTGTTGTGATCAGGCGCCGCATAGTGCGCACCAGTCGCACCGCTTCACAAGATCC

ACAGACTCAAACTGCAGAGGTTGTTCCAAAATCCTCCAACGATAGCTCAACTCCTGCCGAGATCAACGGC
CGCAATCTTGGCTGGCTGACCAGACTGCGCAGCTTTGGATCGATCAATAGAAAACACCAGGAGACCGCCG
CACCACCAAAAGGCGGCCAGCAAAAAGCCATTTCTGCTGCAAGCAACAACAGTTGCAACACCAATCCCAT
CATGGCCGTCCTGCGTACTATGAAATTAAAGGAGCGACTGGTCATCAGTCTGGGTGCCACCCTCGTCCTG
CTCACCCTTCTGCTTATCGTCGATGTGCAGATGGACTTCGGTGTGGCCAATCGCCACCTCCTTCAGCAAC
AGCACCAAAAGATCCGACTGGGCAACGACTACGATGGTGGCACGGGTGGCGGTGGAATGCTGCACGAGTT
CAAGCGCAAATTCCTGCAGAAGAGGGTAAGTCCGCATTCCCGGTGTCCGTCTTGTACACGAAATCTGACT
CATCTATTGCTCCAGTCCCGTCATTAATCAGCGGATACCTTTCGAGTTCGCTTGGAATGTGGGCTTGGAT
GGATGGCAAGCTAGGATGTTGTAATGATTTCCTGCGAGTATTGTAATGAAAACTGACCCTCATTGAATTA
ATGAGGCATTAGTGCATGCATAAGGTAGCTGGAATTCTGTTATCAGTTATCAGAGCTTGAAACATGGGGG
GAGCATGCAAGATGGGATTTCTTGTTTGTACAGTACTCAAAGTTTGCTATTAGG
>dme_circ_0007348_junction_seq
TGTACAGTACTCAAAGTTTGCTATTAGGCTATCGACCGGCGGTCGCAGCGGCAGCT
>dme_circ_0007349
CTATCGACCGGCGGTCGCAGCGGCAGCTCAATCGACGCCGTTGGCACAGCGCCTTCAGCGCTTTCGATAT
CGCcagcaattgcagttgcagcaagagccgcatcagcaacagcaacagcaacagcaacagcagcagcaac
aacaatcgacTGCTGATATCGACGTTTACGATAGCCCCTTTAACGGCCAGTTATCAGCTGAACGAATAGG
CGTAGCCAATTGGCGGAGGGGGAACGTCGAAGGGCCCATTGGCGGGAGAGCTCCAGTTGAGGCTGAGTAC
TCAGCGGAGCAGGAGGAGCTGCCGCCGGATCGAGTGCTATATCCAGACTCGGAACCGGAGGAAGAGGAGG
CAGCGCCACGACGCCGTGTTGTGATCAGGCGCCGCATAGTGCGCACCAGTCGCACCGCTTCACAAGATCC
ACAGACTCAAACTGCAGAGGTTGTTCCAAAATCCTCCAACGATAGCTCAACTCCTGCCGAGATCAACGGC
CGCAATCTTGGCTGGCTGACCAGACTGCGCAGCTTTGGATCGATCAATAGAAAACACCAGGAGACCGCCG
CACCACCAAAAGGCGGCCAGCAAAAAGCCATTTCTGCTGCAAGCAACAACAGTTGCAACACCAATCCCAT
CATGGCCGTCCTGCGTACTATGAAATTAAAGGAGCGACTGGTCATCAGTCTGGGTGCCACCCTCGTCCTG
CTCACCCTTCTGCTTATCGTCGATGTGCAGATGGACTTCGGTGTGGCCAATCGCCACCTCCTTCAGCAAC
AGCACCAAAAGATCCGACTGGGCAACGACTACGATGGTGGCACGGGTGGCGGTGGAATGCTGCACGAGTT
CAAGCGCAAATTCCTGCAGAAGAGG
>dme_circ_0007349_junction_seq
GTTCAAGCGCAAATTCCTGCAGAAGAGGCTATCGACCGGCGGTCGCAGCGGCAGCT
>dme_circ_0007350
GCACCTTTGTGATGGAACAAAGTCCATTAAGTCGCCGTACATAGGTGAGTATTAGCCCATATTCCGAGTG
CTCTGCCACATCTTTCATTCGCGTGACTTGTCAATTTTCTGACAGACAATGGCTGTGGCAAACAACTGGC
ATTGATGCGACAGGGCGAAGCACCAAAAGGCAGCTCAGTACCCAACAATTAGATTGGCCGGAGAAAATGC
TATCAGTGGTGACAGTGGCAGGGAAAAAGGGCTGGGGAAATGGAAATGGCAACAATTGCTGTCACCATGG
CGATAAACGATAACAAATTCATTAATTAAAATTTAAGTGCACAACAACAAAAGAAAGATTGCCATGCAAA
TGGCTTTATCGCTGCTCTGCGCATTCCGGAATTGATTTGCATTGCCAAACaaaaaaaccaaaaaaaaaaa
GAGTGGACTTTAATATGCACAAAAAGAAACAATTAAATGTCGTGTAAATTACTCACAAATGTTCATCAAT
AAATTCGCATAGCGCTGAAGGGTTAAAATACCACTGTCAAGAGATGACTAAAGCACTTGAAAGTTATCCA
CCAATTATGGCAAATGGTAAATATTAATAATTTTTCAATTTGTTTTACCGGTAATTTTATGTTGTTCCAC
TTTTTTCTACCCACTTTTTTCATCAAGCCGTTTATCATAATCACTTTATGGGCTGACTTTGTTTTCACTT
TAATTAAATTTTATTAACATCAGCAATTTATGACACGTTTTTCCGTTTCAGCAAAGGTAACCACAAAAAC
GCCAAACCATAAATTGATTACATTCAAAAAATTATTTAGAGAAATAAAAGCCAAAGGAGAATGGAATTTT
TACCAGAGTATTTGTG
>dme_circ_0007350_junction_seq

GAATGGAATTTTTACCAGAGTATTTGTGGCACCTTTGTGATGGAACAAAGTCCATT
>dme_circ_0007351
GAATAGTTCGATCGCGTAGAGCACATGATGGTGGCCAGGATGGCGTCCGTGGCGAAGACATTGCCGACGG
TCTTAGACAAACGCCGGATGACGGGATCGTCTGTGGTGGTCACCGTATGCACGATGCGATCGATCTTCTG
CAGCGGCTTCTCGTTCTTCACATTGATGCGGTCGTAGGTCTTGTCGTAG
>dme_circ_0007351_junction_seq
ATTGATGCGGTCGTAGGTCTTGTCGTAGGAATAGTTCGATCGCGTAGAGCACATGA
>dme_circ_0007352
GCGCGGTGCTGTGCTCGCCAACGAGCTGAGGAACAACGCATGCAAGCTGGCCAAGTGGACTGTGCAGGCC
GTCCTGGCCGGGTCCGATCAGCTAAAGCTCGGCTATGTGTCGCGTATCAATCCGAGAGATCACTCGC
>dme_circ_0007352_junction_seq
TCGCGTATCAATCCGAGAGATCACTCGCGCGCGGTGCTGTGCTCGCCAACGAGCTG
>dme_circ_0007353
AGGACATCAGTTGTGGTTATTCTTAGCGGGTTGTGGCAAAAGAGCCCAAGACTGGAAGGGATGCAGGAGT
AGGGGGGCGTGGCGTCTAAATCTTGTTGTGCGCGTAGTTGTAGACCTGCTGGAAACCTTCGTCATCGCCC
TCGCCATCGTCCGAG
>dme_circ_0007353_junction_seq
TTCGTCATCGCCCTCGCCATCGTCCGAGAGGACATCAGTTGTGGTTATTCTTAGCG
>dme_circ_0007354
AATCGTCCAGTTTCACCTTGAAGCTGGAGTACTTTTCCTGACAACTCGTTAGGTTCCAAACGTTGACGGT
GATCTTCAGCTTGATCGCACTTGGCTGCATGTTCTCGGTAAGTCCCCAGCCGGCCACATCCATGCCGTAG
TCCACAAAGTTGTTTTGGACTAAACCATCCGTGGGAAGGCATATTGGACGCACGTAGTCGGTGTAGCTG
>dme_circ_0007354_junction_seq
TATTGGACGCACGTAGTCGGTGTAGCTGAATCGTCCAGTTTCACCTTGAAGCTGGA
>dme_circ_0007355
GATATTCCCGCTGCACAGCTCGCTGATACTGCGCCTTGTTGGCCGGGGTAATCAGGTTGCTAATAAAATT
ATGTGCAGCTGGTGCATTGGGCAGCACTTGGAAACAATTCTCGTCCACGGAGGGCGTGTCTGGAATTATT
GACCAAGATGGTGAGGAGCAGGCCATGGGACCTGTCTGGTCAATAGCCGGCAGTGACTCGGAAAGCGCGT
TGAGATTCAATGGCTCTTCCATAGCTTCGTCCTCAGCCATTGTATTGCACTCGGTAGCACCATCATTGGC
TA
>dme_circ_0007355_junction_seq
TTGCACTCGGTAGCACCATCATTGGCTAGATATTCCCGCTGCACAGCTCGCTGATA
>dme_circ_0007356
TCCAGGCTCCCAGAATAACGATGCGATCTGCTAAGAGGCGACAAGTAAAGAAAAAAATCCGCTCTGTCAA
GAAAACAATTTATTCGCCTTTTAATAAAAAGGAAACCACTCCCAAGGATGGCGATGAAATCGTAGTGGAA
ACGCTGGTCGTTTACAGCTCGGTGACGGTCGTCTGGCAGGATGGCACGGTGGAG
>dme_circ_0007356_junction_seq
GGTCGTCTGGCAGGATGGCACGGTGGAGTCCAGGCTCCCAGAATAACGATGCGATC
>dme_circ_0007357
GAGCAGGAACAGTCTTTGGCGGAGGCTGCTCCGCGTACACCGACGCTTGCGAATCCTCCTCCATCCTCAC
AGGAGCTGGGTCGGACCCCCAACCAGGAGAGCCGCCATAACGCAAATTCCCACTCCCGTCGATCGGGTGG
TGGCCAGGTGCCATCGAAAGCCCAGGG
>dme_circ_0007357_junction_seq
GTGGCCAGGTGCCATCGAAAGCCCAGGGGAGCAGGAACAGTCTTTGGCGGAGGCTG

>dme_circ_0007358
GATTACTTGCTTAACATTAAGCTGGGCTTCCATGGCTGGATTGACTGGAACATTTGTCTGGACGAGATTG
GCGGTCCTAACTACGTGGACAACACGGTGGATGCCCCAGTGATTGTGAATACAACAAGTGGGATATTTTA
ATACTTCCATTTATTCTGTTTGTGATTAGTGATATGATTTTCTTGCAGCATTCGAGGAGTTTTACAAGCA
GCCCATGTTCTATGCCATTGGTCACTTCTCCAAGTGGGTTCCAGAGGGATCCGTTCGCATAGATGCAGTA
CCTAGTAATGTTAATCTGGATTCCGTTGCATTCCTGCGACCGGACAACAAAATCACCGCC
>dme_circ_0007358_junction_seq
CCTGCGACCGGACAACAAAATCACCGCCGATTACTTGCTTAACATTAAGCTGGGCT
>dme_circ_0007359
ATTAACATTACTAGGTACTGCATCTATGCGAACGGATCCCTCTGGAACCCACTTGGAGAAGTGACCAATG
GCATAGAACATGGGCTGCTTGTAAAACTCCTCGAATGCTGCAAGAAAATCATATCACTAATCACAAACAG
AATAAATGGAAGTATTAAAATATCCCACTTGTTGTATTCACAATCACTGGGGCATCCACCGTGTTGTCCA
CGTAGTTAGGACCGCCAATCTCGTCCAGACAAATGTTCCAGTCAATCCAGCCATGGAAGCCCAGCTTAAT
G
>dme_circ_0007359_junction_seq
AATCCAGCCATGGAAGCCCAGCTTAATGATTAACATTACTAGGTACTGCATCTATG
>dme_circ_0007360
GTACCTAGTAATGTTAATCTGGATTCCGTTGCATTCCTGCGACCGGACAACAAAATCACCGCCGTTCTCT
TCAATAGTGGTCGCGCCGATCTGGACATCACTTTGGTGGATAGTATTCGGGGTCAGTTCGTTGTCAATGT
TCCGGCCAAATCCATTCACACTCTGCTGTACA
>dme_circ_0007360_junction_seq
GCCAAATCCATTCACACTCTGCTGTACAGTACCTAGTAATGTTAATCTGGATTCCG
>dme_circ_0007361
CGGTCGTTGTGGTCGCTAACTAGTTCTTCTCAGTTTGTCAACTTTCTGCGAACAAATCCACATAGTTTCT
TGCACGACGCGTTGTTTGGCTTACATTTATATAACCACTAATTGCGGCACTTTCGTTCGCGAAATTCGAT
TGCTACTTTATTTGGCTAACAAATAGTTAAATGTGCAAAACACACGACAGCTTTACttttttttAGTTAG
TTCAGGGCGCACACTCGAGTTGTTGTATTTGGTTTTGTTGGCAG
>dme_circ_0007361_junction_seq
GAGTTGTTGTATTTGGTTTTGTTGGCAGCGGTCGTTGTGGTCGCTAACTAGTTCTT
>dme_circ_0007362
TCCTGCTCACAATTTCATCGGTGGACATACGCATCACCTCCTCGCCCAGGGACTCCTCCCCGTCCTCCCA
GATTGACTTATCCTCCAGAGTCTGAGCCATGTCTAATACGATTGTTGATTAGCCGTTGCTGAATGTAATT
CTTTGTTAAAATGCTATTATTGCCGATCTTTTTGGGCAAACACTCTGTCGATGACTTG
>dme_circ_0007362_junction_seq
TTTGGGCAAACACTCTGTCGATGACTTGTCCTGCTCACAATTTCATCGGTGGACAT
>dme_circ_0007363
GCTTCTTAATCAGCTCGATGGCTTCAGTTCCACTGCAGATATTAAGGTGATTGCGGCCACCAATCGCGTA
GACATTCTGGATCCTGCTCTGCTGCGCTCTGGTCGTCTGGATCGTAAGATCGAGTTCCCACATCCCAACG
AGGAAGCCCGTGCCC
>dme_circ_0007363_junction_seq
CCACATCCCAACGAGGAAGCCCGTGCCCGCTTCTTAATCAGCTCGATGGCTTCAGT
>dme_circ_0007364
CTCCAAGGGCCCAACATTGATGGTACGGGCAGCAGTTTGGGTTCGGCCACAGGTACGGGTACGGGAACGG
GATTGGGCACGGGAACAGCCACGCCAACGCTGCCCAATGAGCCCTCCACCCAGGCGGAGGCGGAGGAGG

>dme_circ_0007364_junction_seq
CCCTCCACCCAGGCGGAGGCGGAGGAGGCTCCAAGGGCCCAACATTGATGGTACGG
>dme_circ_0007365
GAGCTCCAAGGGCCCAACATTGATGGTACGGGCAGCAGTTTGGGTTCGGCCACAGGTACGGGTACGGGAA
CGGGATTGGGCACGGGAACAGCCACGCCAACGCTGCCCAATGAGCCCTCCACCCAGGCGGAGGCGGAGGA
GG
>dme_circ_0007365_junction_seq
CCCTCCACCCAGGCGGAGGCGGAGGAGGGAGCTCCAAGGGCCCAACATTGATGGTA
>dme_circ_0007366
GGTGAAGAACAACAATAGGCTATTCTCATGGTATTTGAGACGGCGGAGGCCACCATTTCGGCCGCCTAC
GAAACCATCCAGCCGGCGGTCAAGCTCTTCGAGCCATCCATTCAGCGCCTGGACAAC
>dme_circ_0007366_junction_seq
CGAGCCATCCATTCAGCGCCTGGACAACGGTGAAGAACAACAATAGGCTATTCTCA
>dme_circ_0007367
GCCATCCATTCAGCGCCTGGACAACGTGATGTGCAAAAGCCTGGACATTCTGGAGCAGCGCATACCACTG
GTCTATCTGCCGCCCGAAATGGTAAGTAGTCGGCTAGTAACTCCCCCCCATGCGCTGACAAAAGGGCATA
ATTCTCTGCTTGAAGATGTACTGGAACACCAAGGAGTACATGTCCGATCACCTGGTGCGTCCGGTTCTGA
AACGCGCCGATTCTGTCAAGCAAATCGGTAATGCTGTCCTCGAGAGTCCACTTACCACTTATGCAGCCGA
GCGCATTGATGGCGCTTTCACAGTCGGCGATAAGTTCGTGGACAAGTACCTGGTGCCCATCCAAACAGAT
CAGGATCAGACCGATGGGTGAGTCCTGGTATCTCACATCTGGGTTAATCTTCAAACGCATGTTGGGGAAT
CTATTACATTTCATTTTATTTTAAACTGAAGTTCTTATATTTTGGCACTTTTACTAAACTAACTATTTAT
AATTCAAAATTGTTGTAGTGAAAAAATTGGTTTCGGCCCTGAATGATAACTTTTCGCCGATTCGACAACA
ATTAAACCGAATGTTTGGCCACAAAAGTTAGTATGCAAAATTACATATTTCTCCATATAACTAGAGATAA
TCAAAATCATATGAAAGATAGTTTAGTTTGCCAAAAGTTTTACATAAAAAGTAAGCGAAAGCAAGTGATT
TGAAAAGTCAGCATCCAGGCAAATCACCTTAAGGTGCACTCATCTCATAGTTTATTTATAAATGCCCACG
TTGTCGGGAAGGTTATCTCTGGGGAACGGCGGATTCATTGAAACCTGAAGCCAGAGAAAGTTGCGATAAG
CTGAGCTCTGGCCGGAATTTGCTCTCTCATGCTCaaaaaaaaTTGTATAAGACACTTATTCCGAGTATCC
GCCTTGCAGGGCCCACAGGAGGATGATAACGAGGCCGTGCCGGACGAGAGGGGCGCCATCAAGGCGATCC
ATCATGGTCAACGCTTCTCCCGCAAACTGAAGCGCCGCCTTACCCAAAGAACCATCGCAGAGGCTCGCGC
CCTCAAAAAGCAGAGCAAGGAGGCGATCCACGTGCTCTTCTATGCCGCAGAATTGGATTGCCACCGATCC
CAAGCAGGCCGTTCAAAAGGCCAAGGAACTGTGGGTCTATCTCAGTGCAGATGAACCCGAAAATCAAGCG
AGGCCCGCCACTCTGGAGCAGTTGATTGTGCTACTGACCCGCGAATCGGCTAGGCGTGTGGTTCATCTAG
TGAACTTCAGTGCTCATGTGGCAGCCAACATACCCAGGAAACCTGGCGCACACGACAACAGAGGTTGCCC
ACCATATTATCTATATCAACCACCGCATCATCACAATCTCACGGCTGGACAAGGTTAAAACCATCTCCAA
AGAGGAAGCTGAATCTCTTTTCAAGCGCATGCTGGCGTTCTATGGTAGCCTTCAGGGCCTGACCAATGCC
TACCTGGGAGCGCGTGGCCAGCTTTCTATCCGGACGCATGGAGGCCGAGAAGGTGACGGGCAGCGATGGC
GGCAATTCCAACCACCGGTCATCGCGGAGGAGGCAGGATCCGAACCATTATTCAGCCACCCACAATAACA
TCAACGGCGTCTACTAGCATTGAATTCTTTATTGCTATC
>dme_circ_0007367_junction_seq
TACTAGCATTGAATTCTTTATTGCTATCGCCATCCATTCAGCGCCTGGACAACGTG
>dme_circ_0007368
GTACTTGTCCACGAACTTATCGCCGACTGTGAAAGCGCCATCAATGCGCTCGGCTGCATAAGTGGTAAGT
GGACTCTCGAGGACAGCATTACCGATTTGCTTGACAGAATCGGCGCGTTTCAGAACCGGACGCACCAGG
>dme_circ_0007368_junction_seq

GGCGCGTTTCAGAACCGGACGCACCAGGGTACTTGTCCACGAACTTATCGCCGACT
>dme_circ_0007369
TTACTGGACCATCCCTGCCCGTCTCCATGGTATTTGCGCCGCGCTCGGCCACCGATTTGTCGCCCAGTGT
AATGCAGTTGGTGGAGTTTAACATGGTCAAAGGCAACGAGGGTTTTCGAGAAAGTATCATTCCCCATCTC
GAAGTTCAGCTTAACGACTGCCTCTCAGTTAATGTGGAGGGTAGTGAGTGCCCGTG
>dme_circ_0007369_junction_seq
TTAATGTGGAGGGTAGTGAGTGCCCGTGTTACTGGACCATCCCTGCCCGTCTCCAT
>dme_circ_0007370
TTATCAAACCCAAAGTAAAAAGTGTAGAGGGTGGAAATCCCTCTAGCTCTATTGGCAGTGCTCCCAACAC
GCCCCAATCTCGACCCAAAGGCGCAACTCCCAACAAAGAACGCGAGTCGTTTAGCGGAGCCATTCCAAGT
GAGCCGCTACCACCAGCTGGAAACTCGCCTGGAGCAACGAATGGACGGGAAAGTCAGGATAACGGCCGCC
GTGAGTCATGGCTGCATCCTAACAACCTTGAGAAGGTACGCCAGCACAACATTCAGACTGGAATGGATCA
GGGATCGCCCCATAACAGCACCCTCAACGAGCTTGTGCCACG
>dme_circ_0007370_junction_seq
ACAGCACCCTCAACGAGCTTGTGCCACGTTATCAAACCCAAAGTAAAAAGTGTAGA
>dme_circ_0007371
GTTTTGGAGCCACCAGCGCAGCACCACTTAATTTCAACGCAGGCAACACGGGCGGATCACTTTTCGGAAA
TACAGCCAAGCCGGGAGGAGGTCTTTTTGGAGGAGGTACTACAACCTTGGGTGGCACGGGTGCTGCCCCA
ACGGGAGGACTGTTTGGAGGAGGAACTACCAGTTTCGGAGG
>dme_circ_0007371_junction_seq
TTGGAGGAGGAACTACCAGTTTCGGAGGGTTTTGGAGCCACCAGCGCAGCACCACT
>dme_circ_0007372
GCCTTTTTAACTCGGGATTGAACAAGCCAGCAACGTCCGGATTCGGAGGTTTTGGAGCCACCAGCGCAGC
ACCACTTAATTTCAACGCAGGCAACACGGGCGGATCACTTTTCGGAAATACAGCCAAGCCGGGAGGAGGT
CTTTTTGGAGGAGGTACTACAACCTTGGGTGGCACGGGTGCTGCCCCAACGGGAGGACTGTTTGGAGGAG
GAACTACCAGTTTCGGAGG
>dme_circ_0007372_junction_seq
TTGGAGGAGGAACTACCAGTTTCGGAGGGCCTTTTTAACTCGGGATTGAACAAGCC
>dme_circ_0007373
GAGGCCTTTTTAACTCGGGATTGAACAAGCCAGCAACGTCCGGATTCGGAGGTTTTGGAGCCACCAGCGC
AGCACCACTTAATTTCAACGCAGGCAACACGGGCGGATCACTTTTCGGAAATACAGCCAAGCCGGGAGGA
GGTCTTTTTGGAGGAGGTACTACAACCTTGGGTGGCACGGGTGCTGCCCCAACGGGAGGACTGTTTGGAG
GAGGAACTACCAGTTTCGGAGG
>dme_circ_0007373_junction_seq
TTGGAGGAGGAACTACCAGTTTCGGAGGGAGGCCTTTTTAACTCGGGATTGAACAA
>dme_circ_0007374
GCCTTTTTAACTCGGGATTGAACAAGCCAGCAACGTCCGGATTCGGAGGTTTTGGAGCCACCAGCGCAGC
ACCACTTAATTTCAACGCAGGCAACACGGGCGGATCACTTTTCGGAAATACAGCCAAGCCGGGAGGAGGT
CTTTTTGGAGGAGG
>dme_circ_0007374_junction_seq
AGCCGGGAGGAGGTCTTTTTGGAGGAGGGCCTTTTTAACTCGGGATTGAACAAGCC
>dme_circ_0007375
GAGGCCTTTTTAACTCGGGATTGAACAAGCCAGCAACGTCCGGATTCGGAGGTTTTGGAGCCACCAGCGC
AGCACCACTTAATTTCAACGCAGGCAACACGGGCGGATCACTTTTCGGAAATACAGCCAAGCCGGGAGGA

GGTCTTTTTGGAGGAGG
>dme_circ_0007375_junction_seq
AGCCGGGAGGAGGTCTTTTTGGAGGAGGGAGGCCTTTTTAACTCGGGATTGAACAA
>dme_circ_0007376
GCCTTTTTAACTCGGGATTGAACAAGCCAGCAACGTCCGGATTCGGAGGTTTTGGAGCCACCAGCGCAGC
ACCACTTAATTTCAACGCAGGCAACACGGGCGGATCACTTTTCGGAAATACAGCCAAGCCGGGAGGAGG
>dme_circ_0007376_junction_seq
TCGGAAATACAGCCAAGCCGGGAGGAGGGCCTTTTTAACTCGGGATTGAACAAGCC
>dme_circ_0007377
GGCGCCGACGTCCCAAAGCCTCCGAATCCTGTTGTACTGGTGGCGGCAGCCGGTTGGCCAAACAAAGAGC
CCGTGGGCTGAGCAGCAGCTGTTTGCCCAAAGGCCGTCATAGTCGGCTTGGCGGCTCCAAAGGCGGGTAG
CGTCGATTGTCCAAAGAGGGAGGTGCTTGCCGCCGTCTGGGTGGATCCAAATAT
>dme_circ_0007377_junction_seq
TTGCCGCCGTCTGGGTGGATCCAAATATGGCGCCGACGTCCCAAAGCCTCCGAATC
>dme_circ_0007378
GAGCAAAGGAAATACGCGGCATATTTCCCAAGAGCTATATACACTTATGCGAATATAATATCGTGAATGG
GGAGTACTGCATCCAGCGCACGGATATTGTCGAGGAGATCACTAAAGTTCTTCTCGAGTGGGGCTCCATA
GCCAAGGATTACTTTCTGGACCACAAATCCCAGCTTTCCCAAAATTCGACGAAAAATGAACGAATTAAAT
AATAATAGGGCGGCTTTGATCTCCGGAAACCTGCCATTGGACGAGGTGCGAAAGGTGAAGCTGCTGGCCA
CCAATCAGATCGATACTGGCAACAAGCTCCTTGGCCTGGACATGGTGGTTCGCGACGAGAGCGGAGACAT
CTTGGACACCAATGCCATCTGCACCACCGAGCTCTACGAGCAACATATGCACGCTGTTCAGCGCATCGAC
AAGGCCAAT
>dme_circ_0007378_junction_seq
CGCTGTTCAGCGCATCGACAAGGCCAATGAGCAAAGGAAATACGCGGCATATTTCC
>dme_circ_0007379
GAGCAAAGGAAATACGCGGCATATTTCCCAAGAGCTATATACACTTATGCGAATATAATATCGTGAATGG
GGAGTACTGCATCCAGCGCACGGATATTGTCGAGGAGATCACTAAAGTTCTTCTCGAGTGGGGCTCCATA
GCCAAGGATTACTTTCTGGACCACAAATCCCAGCTTTCCCAAAATTCGACGAAAAATGAACGAATTAAAT
AATAATAGGGCGGCTTTGATCTCCGGAAACCTGCCATTGGACGAGGTGCGAAAGGTGAAGCTGCTGGCCA
CCAATCAGATCGATACTGGCAACAAGCTCCTTGGCCTGGACATGGTGGTTCGCGACGAGAGCGGAGACAT
CTTGGACACCAATGCCATCTGCACCACCGAGCTCTACGAGCAACATATGCACGCTGTTCAGCGCATCGAC
AAGGCCAATTGTGAGTATCTAAAAGTGCTGCGGGAAAAACTATATCTGTCAGCTATAAATTCAAGTTCAG
GTTATAAACAAATTGTATAAATGAGTTCGCTTCTTGATATTTCTATAAATATAATTATAACGATTATACA
TTAAGTATGTGTTCAGTTAAATAAGTTCAGTTAAAACTATTCTTCTTCTTCTTATATATTATTCATATTT
CATCAAATGTGTCACGTGCACTATGTGCACTTGCTGAGAAAGTTTAATCAGCTTAATGCTATTTTATTTT
TACGAATTATGATTCATGAGAGATTTGACTCATTAATTCGTATATGTATAGTGAAAACCCATGCTTTAAC
TAATCCTAAttttttttATCCAACCGTTAGGCGGCTGTCAAGCGAGCGGGGAACTACAAGAACACCTAAC
AAGTACTCTCACAACATCCTGCTACACGTGAACGCCTTCGTTTGCAAGTTCCAGGAGGACTCTGACCTGC
TCTTCACGCTTTTCGACGGCGAAACACACAAACCCATTAGCGAGAACTATGTGGTCAAGTGGTCACGGAC
AGGTATTGCGCGTGATACTGACCAGATCGACAACAACCGCGTGCTGTTCACGGATCTCAGTAAGAGCGAC
CTAGCCATTGCCAAAATGTACCTGGTGTGCTATGCCATCCGAATCGGTTCCATGGAGTTTAAGGATTCTG
CCGAGTCGAAACGGACCAGCATGAGCATTGCCAACAGCATGCTGAACGCCTCCAGTCGCAAGGCCTCCCA
GCTATCGGTGAGCTCCAGTGGATCGTCCAGCAGCAATGGGGAATACATCATTCGACGTCCGTTTGGTGTG
GCCTGCAAGGACCTCACGCCCTTCATCAATAAGTCGGACGACTTCCGAGGCAACATAGACCTGCCCTTCA

TCATGTGCGAAAAGGAAACGCTGGACGGAACGCTGCGGAAGCTAATCGCGAATAAGGATATTGGCAAGAT
TGACTCCAAGATGGCTGTAACTATCGAGGTGCTTCGGGGCGACATCAAGCAGATAAAGGAAGAATTTCCA
CGCTTGATGCACACAAATGTGCCAGTTGCCCGAAAAATGGGATTTCCTGAGGTCATTTTGCCAGGCGATG
TGCGAAATGATTTGTACCTTACCATCTGCAGTGGAGAATTCGCACGCATTGCCAAGACCTCGGAGAAGAA
CGTTGAGGTGTCGGTGTGTGTGGCCAATGAGCAGGGATACCTTATGCCCGGTGTGCTAAGCATTGGAGCT
GGACATCAACCCATTGATGAGTACAAATCGGTTGTATACTACCACGATGACAAGCCTAAGTGGCAGGAGA
CCTTCAAGATCCACGTGCCCATTGAAGACTTTAAACAATGTCATTTGCGCTTTGTGCTCAAACATCGTAG
CAGTAATGAGCAGAAGGATCGAACTGAAAAACCCTTCGGTCTCGCGTATGTGCGTCTAATGCAGGCCAAC
GGGACAACCATCACGCAGGGTCAACATATCCTGGCCGTTTACAAAATTGATCACAAAAAGTACGACAAAA
CAGTAGCCAATTGTTATTTAGAACTTCCAGCCACGGTTGCAGAGCTGCAAGGTGCAAAGCCTTCTATCGG
TGGACTTACTCTCTTGCCAAAGGATCAGCTATCGATTGGGGTTAACCTGTGCAGCACCAAGCTCACGCAG
AGCGGTGAGCTTGCTGGGACTTCTGAATTGGTCGGCGCACAAGGAGACGCTGGAACAGTCGCTAAACGCC
TTGTCCACAGTGCCTGGAGAGGAGGTGGTGAAGTTCCTGCAGGACATACTCGATGCCTTGTTTAATATAC
TGGTGGAGAATGATCATCCTGAGAAATATGACCAGCTCGTCTTTATGAGCATTATACATTTGATTGAAAC
GGTGTCCGATCTCAAGTACCAACACTTTCTCAGTGTTCTCGATGTGTACATTAATGAAAGCTTCTCGTTT
ACACTTGCCTACACGCAAATTGATGGATGTGCTGCAGAAGAATATTAGTGAAGCGATCTCACCAAAAGAA
AAGTCTGCTGATGGCAATGATCTGGAGGAGAGTCCAGAGGTGCGCCGCCTGTACAAGACAACTCGTTACC
TTCACTATGTGATGAAGTTCGTGATTCGATCGCGTGTGCTCTACGCCGAGATGAACTGCAACACGGACTA
TATGGACTTTGCTACCCGGCTGCAGGAGCTTCTTCGCATGTTCATCGACATGATTGGCTGTCCGAGCAAT
CTGCTTAAATCGGAGGGAGCGCTGCTCAAGAATCTGCACATCATCGCCACAGATTTAATGCAGGTCTTTG
AACATGTGCGCTTAAGGCATATCCATTGTGGAAATCCTTGAGAAATTCCCGCCGCGACGTCTTACTCAGT
CTAAGATGGGATGCATTAAAGATTTTGTGGAGACAAAACTGTTTACGTTGCCCAAGTGTCGGGCCATCCT
ACTGCCCGTGTTCTGCAAACACATCAAGGATCACCTCGAGAGCAAGGAGGAG
>dme_circ_0007379_junction_seq
CAAGGATCACCTCGAGAGCAAGGAGGAGGAGCAAAGGAAATACGCGGCATATTTCC
>dme_circ_0007380
GAGCAAAGGAAATACGCGGCATATTTCCCAAGAGCTATATACACTTATGCGAATATAATATCGTGAATGG
GGAGTACTGCATCCAGCGCACGGATATTGTCGAGGAGATCACTAAAGTTCTTCTCGAGTGGGGCTCCATA
GCCAAGGATTACTTTCTGGACCACAAATCCCAGCTTTCCCAAAATTCGACGAAAAATGAACGAATTAAAT
AATAATAGGGCGGCTTTGATCTCCGGAAACCTGCCATTGGACGAGGTGCGAAAGGTGAAGCTGCTGGCCA
CCAATCAGATCGATACTGGCAACAAGCTCCTTGGCCTGGACATGGTGGTTCGCGACGAGAGCGGAGACAT
CTTGGACACCAATGCCATCTGCACCACCGAGCTCTACGAGCAACATATGCACGCTGTTCAGCGCATCGAC
AAGGCCAATTGTGAGTATCTAAAAGTGCTGCGGGAAAAACTATATCTGTCAGCTATAAATTCAAGTTCAG
GTTATAAACAAATTGTATAAATGAGTTCGCTTCTTGATATTTCTATAAATATAATTATAACGATTATACA
TTAAGTATGTGTTCAGTTAAATAAGTTCAGTTAAAACTATTCTTCTTCTTCTTATATATTATTCATATTT
CATCAAATGTGTCACGTGCACTATGTGCACTTGCTGAGAAAGTTTAATCAGCTTAATGCTATTTTATTTT
TACGAATTATGATTCATGAGAGATTTGACTCATTAATTCGTATATGTATAGTGAAAACCCATGCTTTAAC
TAATCCTAAttttttttATCCAACCGTTAGGCGGCTGTCAAGCGAGCGGGGAACTACAAGAACACCTAAC
AAGTACTCTCACAACATCCTGCTACACGTGAACGCCTTCGTTTGCAAGTTCCAGGAGGACTCTGACCTGC
TCTTCACGCTTTTCGACGGCGAAACACACAAACCCATTAGCGAGAACTATGTGGTCAAGTGGTCACGGAC
AGGTATTGCGCGTGATACTGACCAGATCGACAACAACCGCGTGCTGTTCACGGATCTCAGTAAGAGCGAC
CTAGCCATTGCCAAAATGTACCTGGTGTGCTATGCCATCCGAATCGGTTCCATGGAGTTTAAGGATTCTG
CCGAGTCGAAACGGACCAGCATGAGCATTGCCAACAGCATGCTGAACGCCTCCAGTCGCAAGGCCTCCCA
GCTATCGGTGAGCTCCAGTGGATCGTCCAGCAGCAATGGGGAATACATCATTCGACGTCCGTTTGGTGTG

GCCTGCAAGGACCTCACGCCCTTCATCAATAAGTCGGACGACTTCCGAGGCAACATAGACCTGCCCTTCA
TCATGTGCGAAAAGGAAACGCTGGACGGAACGCTGCGGAAGCTAATCGCGAATAAGGATATTGGCAAGAT
TGACTCCAAGATGGCTGTAACTATCGAGGTGCTTCGGGGCGACATCAAGCAGATAAAGGAAGAATTTCCA
CGCTTGATGCACACAAATGTGCCAGTTGCCCGAAAAATGGGATTTCCTGAGGTCATTTTGCCAGGCGATG
TGCGAAATGATTTGTACCTTACCATCTGCAGTGGAGAATTCGCACGCATTGCCAAGACCTCGGAGAAGAA
CGTTGAGGTGTCGGTGTGTGTGGCCAATGAGCAGGGATACCTTATGCCCGGTGTGCTAAGCATTGGAGCT
GGACATCAACCCATTGATGAGTACAAATCGGTTGTATACTACCACGATGACAAGCCTAAGTGGCAGGAGA
CCTTCAAGATCCACGTGCCCATTGAAGACTTTAAACAATGTCATTTGCGCTTTGTGCTCAAACATCGTAG
CAGTAATGAGCAGAAGGATCGAACTGAAAAACCCTTCGGTCTCGCGTATGTGCGTCTAATGCAGGCCAAC
GGGACAACCATCACGCAGGGTCAACATATCCTGGCCGTTTACAAAATTGATCACAAAAAGTACGACAAAA
CAGTAGCCAATTGTTATTTAGAACTTCCAGCCACGGTTGCAGAGCTGCAAGGTGCAAAGCCTTCTATCGG
TGGACTTACTCTCTTGCCAAAGGATCAGCTATCGATTGGGGTTAACCTGTGCAGCACCAAGCTCACGCAG
AGCGGTGAGCTTGCTGGGACTTCTGAATTGGTCGGCGCACAAGGAGACGCTGGAACAGTCGCTAAACGCC
TTGTCCACAGTGCCTGGAGAGGAGGTGGTGAAGTTCCTGCAGGACATACTCGATGCCTTGTTTAATATAC
TGGTGGAGAATGATCATCCTGAGAAATATGACCAGCTCGTCTTTATGAGCATTATACATTTGATTGAAAC
GGTGTCCGATCTCAAGTACCAACACTTTCTCAGTGTTCTCGATGTGTACATTAATGAAAGCTTCTCGTTT
ACACTTGCCTACACGCAAATTGATGGATGTGCTGCAGAAGAATATTAGTGAAGCGATCTCACCAAAAGAA
AAGTCTGCTGATGGCAATGATCTGGAGGAGAGTCCAGAGGTGCGCCGCCTGTACAAGACAACTCGTTACC
TTCACTATGTGATGAAGTTCGTGATTCGATCGCGTGTGCTCTACGCCGAGATGAACTGCAACACGGACTA
TATGGACTTTGCTACCCGGCTGCAGGAGCTTCTTCGCATGTTCATCGACATGATTGGCTGTCCGAGCAAT
CTGCTTAAATCGGAGGGAGCGCTGCTCAAGAATCTGCACATCATCGCCACAGATTTAATGCAGGTCTTTG
AACATGTGCGCTTAAGGCATATCCATTGTGGAAATCCTTGAGAAATTCCCGCCGCGACGTCTTACTCAGT
CTAAGATGGGATGCATTAAAGATTTTGTGGAGACAAAACTGTTTACGTTGCCCAAGTGTCGGGCCATCCT
ACTGCCCGTGTTCTGCAAACACATCAAGGATCACCTCGAGAGCAAGGAGGAGGGTATGTGCTTGAGCAAT
AACTAAATTGCGTGTGCCACTAATTTATCATTTGGGAACTGCATTTTGTTCTTAATCCAATGCATCGTCC
TTGAAATTTGTCTTGTGAATGCACCGACAAGACGCAAAATTAAAACATAACTCAACACATGAACAAGTTA
ATTTGGCTCCTTAAGTAACTAACTAAAAACAAGCTTTTAAAATTATTTTTTCCTCACATAATTGCTTTAT
AAAACTATTTGCAAAATGAAGACCATCCATTTGCGATGCCTTTAACCTTTAATTACTTTGAACCATTTAA
TGAATAAATACAATTCCATTACTCGCATTCATAATTCTTGAGGCTATTAAACACTTGGTCAATTGCTTTA
AGCAATATTTTCTCCTTGACATGTGATTTGTCTTAATTTTGCTCAGTTCCAGTTCGAGCTATTTAGAAAT
TAGTTAAGAACGTAATCTTTGCAAGCTATGTTTAGTTAGTGTTAAGCATGTTGCATTTGCTTTTTATTTT
AAGAACGTTAGCATGTGATTTGGCAGCTTAAACGATTTGTTGCCAACTGTTTTTATGTGTCTTGTGTCCT
AGTTATAGATCAATGTTCGTCCATATCCTAGTGCTAAGCATCAGAAACGATTGTACAACGTAGTGAAATA
GAAATGTTATTTCCACGATAGATCCGTGTTAGTTAGCGATCTTTCTGTGTGTGTCTATAGTGAGATTTCC
TTTGTATCTACCATGGGGCGTAGCGAACTCATGTAAGTGTTTAGCTTGGCCATTAGTACTGCACCCGAAC
ACCCGTCATCGTCATAGTTAGAAAGCAATTTGCATTTGGCAGCTCATCAAAGCACCTTACAACAAACCAC
ATCAACCACCTGGGAATAGTAAACTATaaacacacctcaaaacacacaccgaaacacacactgaacacac
ccacCCAAATATGAATGTGCGATCTCTCCTCACTGGCACCCAATTATTCAGCTATACCTATCTGTCTGTA
ACTCTTCTCTAATTCCACAATCATTATATGTTTTTCGTACAATATTTCAAAAATCAAAACGAATCTTTAT
TCACGCACAACCCAACCGATGTGTTGTTATCGTTTCTCTGTTGTTGCCACCGCAATTGCCTTGTACAAAA
AAACTAGGGAGACTCGAAAACCGATATCTGGCAGCAAGAAAAGAATCTTTCGAAAGCCGCCAAAGTGCTT
GGACAGAAAAAATCCCAACTGCATACATGTGATACGACTGCCAACAAAAAGGTAAAGGAAATAAGTCTCT
CATCTCCAAATCTCAGTCAGATCAGCTGCAGTTCCTCCTGTGATCAGCGTCAATCCCTTGATTTGTAACC
ACAAATGTTTTGTATTATTATGATGTACCTGCTAAGATCTTTATTATGTACCGCTGTTAAGCTTGCAGCT

AACTGGGCAGGTTATCCGCGCCCACTTTGTGCAATTGGCTTTCCAAGCTTCACGCTGTCGATGGCTAGAC
AATCATGAGACTATTTTCCCAGCTACTTGGTTTTCCCGCCATCGACAACCGCTAAGTTAGGCTTAAGTTG
TTCCAGCTTTTTGCAATGTAATGTTCTATGGTTTCGTGTTTTTTTAAATCTGCAATGTTAATTTATATTT
AATTGAACACTAACGTTAACTTTTTCCCGTGAAACCCAATCAGATAGTCAATGGATCTCTGATAGCTCAA
GAAGCACATAGATTTAAGCCGCATTACCTATTAATATTTCCAATCTTTGGCAATTTGGTAATGAAACAAA
TCTTGTATGAATTTTCTAAAGGCATTCCCAAATTGCTTGGATTGAAAATAATAATTTGGCTTACATTTAA
TATATCAACTTTCATCGTCAGGATTGCCGAGTGCATCAACATCATGAACAACATACTGAAACTTCTGTTC
CGATCGGATGTGGGTTCCACGCACAATGACATTCGAGACATTATGATCATCCTGTTTCGCACTGTGATGA
AGGCAGCTCACGCCCTGGACAGGGACACGGGGTTGGTGGGCAAATTCTTTGCCATTATGCTGGGTATACT
GCAGCGCATGGACGCCCAGCACTACGAGTACTTTGTGAAGGACCTGCACCAGAGCGGCGAGTTGAAACAC
TTTGTCATAGAGATCCTTCTGGTATTTGAGGAGCTGGTGTCTCCGCACCAGAAAGCAGTGTTTCCGCGGG
ACTGGATGGACATGATTATGCACCAGAATACTGTGATCCTTGGTGCTCTGAAGCACCTCACCGTGGTCAT
TACGGACTACTTCTTGTGCCCGTTCGAGAAACAGATCTGGTCGAACTTCTTCCAGTGCTCTATTGCCTTT
CTGGTGCAATCCCCGCTTCAATTGAATGATTTCAACGATAATAAACGGCAAATTGTGTTCGCAAGATATC
GTGACATTCGCAAGGATACGGCCATGGAGATCAGGAAGATGTGGTTCCAGCTGGGGCAGCACAAACCCAA
GTTTGTGCCGCAGTTGGTGGAGCCAATACTGGAAATGAGCATGATACCGGAGAAGGAGCTGCGCCAGGAG
ACCATTCCTATCTTCTTCGACATGATGCAATGCGAGTACTATAGCTCGCGATTGGAACACGAAAGTTACG
GCGACACCAAATTCAACAATGCCCACCACAAGGGTAACTTTTCCGACTTCAAGACAGCGATGATTGAGAA
GTTGGACATCCTGATTGGAGCTGGTAAAGGCGACGCTGAATACAAGCATCTGTTCGAGACAATTATGCTA
GAGCGCTGCGCTGCCCACAACACTCTTAACGTGGATGGCACTGCCTTTGTGCAGATGGTAACCCGGCTGA
TGGACAAGTTGTTGGAGTATCGCTTTATAATCCAGGATGAGAGCAAGGAGAATCGCATGGCTTGCACGTT
CTCTCTGTTGCAGTTCTATTCGGAGGTGGATCTCAAGGAGATGTACATACGTTATGTCAACAAACTGTGC
GCTCTGCACATGGAGTTCGAGAACTACACGGAGGCGGCGTTCACGCTTAAGCTGCACACGGAACTGCTGC
GTTGGACGGATACGGAGCTGTCGCACCAACTGCGCAGCTATCGGCATAATAACTGCCGTACTCACCGGCA
ACTGAAGGAGGCTCTTTACTTCGAGATCATGGAGTACTTCGACAAGGGCAAACAGTGGGAGTGCGCCATC
GATATGTGCAGGGTGCTGGCCCGCCAGTACGAGGAGGAGATCTTCGACTATCTCAAGCTGGCGGAACTTC
TGAATCGCATGGCATTGTTCTATGAGAAGATCATCAAGGAGCTGCGCCACAATTCTGAATATTTCCGCGT
GTGCTTCTATGGGCGTGGATTCCCGCGGTTTCTTCAGAACCGTGTCTACATCTTCCGTGGCAAGGAGTAC
GAGCGGCACAGTGACTTCTGCGCCCGTATGCTAGTACAGCATCCACAGGCTGAGCTCATGCAAACGCTGG
AGGCTCCGGGCGACGACATCACCAACAGTGATGGTCAGTACATACAGGTGAACAAGGTGGAGCCGATAAT
GGGTCAGGCGTTCAACAAGTTCAACGATAAGATTATCAACAACGAAATCGTGAAATACTTCACCGCCAAC
AACGTGCAAAAGTTTCAGTTTTCACGTCCCTTCCGGGACAGCACGAACGGTGGCGATAGGGACGATGTAC
GAAATCTGTGGCTGGAGCGCACAGAGCTGCGTATCAGCTACCCACTTCCGGGTATTCTGCGCTGGTTCCC
CGTCGTTGAGACCAATACTTTTAAAATCTCTCCCCTAGAGCGGGCGGTCGAAATTATGAAAGATACGAAC
CGTGACATTCGACAACTGGTGATCCTGCACAAGAGCGACGAAACTCTGCACATTAATCCGCTGAGTATGA
AACTTAATGGCATTGTGGATCCGGCTGTAATGGGTGGCTTTGCCAAGTACGAGGAAGCCTTCCTAACCGA
CGACTACTTGGAGCAGAATCCGGACGACAAGGAGTTGGTAGAAGAGCTCAAAGAACTGATTGCGAACCAG
ATTCCACTGCTCGATTTAGCAATTCAGCTTCATCGCTTGCGGGCACCAGATAGTTTGAAAGCGCTGCAGG
AACACCTGGAGCGTTGCTTCGCCGACATGCAGCAGCATGTGGAGCAGCGCTATGGTCGCAAGTCCTGCGA
CCTCAAAATCGAACGGGATTCCGTGGTGATGCGACGTCCAAACTCCTTTCTGCCTTCGCTTTTCGATGGC
AGCAACAATCGACACTCCGAAACCAGCATGGGATCTTCAGAGGTGAGTGCTTTCGATGATTTGGAGTTCT
TTGGAATGCTTGCCCTAACCAAAACTCTTTTTAACTCTTTCTTCATGCTAACCGCTGGCTTTTAGTTTGA
GACTTTAACATTATTTTGATTTGGTTTGATTTTTAAATTGCATTTTCTTTCGTTCTGTTTAATTATTTAC
GTTCTGTTGCTTATCGTCTACGCTTATTTTGCTATCCCAACATGTCCAGGGAGCGTGAAGCCCATAGCCT

GTCCAGCAGTCAGTGGTACACACCGCCATTGTCCACAATAACATCGACGCCGGAGAAGGAGATCAACACA
TCCATAGCCTCGCTAGCGAGCACATCAAATAGTTCTCTAAGTGGTCCCAAGACGCCAGATCCTCATGTCC
TGACAGAGGAGGCTAACCCCTAAGCGACCTCTGCGTTCGGAAATGGAGAAGGAACGTCGACTCTCTCGTC
CTGCTAGCATCGCTACGCCAACCGCCAGTATCAAGAATTTCCCTGACACGCGCTCCCTGTCGGAAAGCAG
CAACCGCAACTCTGTTGGAAACCACCGATTCCACTTCAGAGGAGGACATTCGACCTCCGCCTTTGCCCGC
CAAGGCACGTGACTCAACTGATTTTACCAGCCTGTCGCAAAACATGGATTGGACCCCGAATGGTTATGCT
ATGCTCAGCACTATTAGCAATACCAGCAGCATGAGCACCACCTCAACCCTGACGAAGACCAGTATTACCA
ATACCACGTACGAGTATTTGGAGACCACGAACTTCAGTTTGGTGGGTGCCATCGATGGAAACAAACCGCG
TCCGCCGACGCCGCCACCGAAGCCATCGCGCCACAGCAAACACATTCCATAGGGTAGCGAGGGGCATCTT
TGAACCCCAGAAGCTTCAGTGCAGCGCCATATATGTTCAAACGTTGTCGAGTGCCAAATATTCCTGTTAC
TATATATATAGCCGGCAATCGAGCCTGTATCCACATCCACAAAATCAGCAACGACCTTTGCCTCAACCTT
TGACCGCCTAACAAATCAAGTGTCTTAAAAACGATCGCATTAGCATGTAAAATTCAGATTTCCGATAACT
ATGTTTAGGTGTTTGATTACGTTTAAATGATATTATGTTGAACATGTCATTTTTGTTTATTTAAGGTTTT
TGTCGTGTAATTTGTTTTAGAACGAACCCAAATGCTTGTCTTCCACAAGTACTAATTAATGTTGTACACA
TATATGTTGTACTTTAGATAACTACGATAAGATGTAGGCTATGTAATGTTGTCGAAAAACGTTGTACGTC
TCTTATGCAAACAAGTGCCACAGTCTTAATGCAATAGCAACCATATCTTCGATTTTACGAACGAGCCACG
TGGTTTTAGCACAAATTTACTAACGAGTCAATTACGAAATACTAATGTAAAACGAATTCTTGTATGCTAC
AATTTTACACAATTTTTTAAAGAAAGCATAACTATTTACACTGTTTTTATACGATTTCGTATAGTCATAA
AGTAGAGCAACTAAATATTGAGATTTATTCGTTAAGAATATTATATGTATGAACAACTTGCACTGAAACC
ATAACGCACAATTATTTAAACAAAAGTTTGAATATTTAATTTTAGCCAAAATTACTGTTTAATTATGTTT
TAAATGGATTCTGTGTAATTTTCGATCTGTGTATCGGCCAAAGATTAAGCAATAATAAAATAAAAGTAAG
CATGTATTATCTACTATTCCTTATAGCTTGACAACTGAGTCTGAAAATTAGATTTTAAAGATAAAACAAA
TTCtttttttttttGGTTAAAAAAGTTTATTAAATTTTTTGTTTGGGGTTGTTTTGATTAGTAAAACGCG
AGACCTGTCAAAAATGAACACATTAGCCAGGCCGGTTAAAAGTTGGTTATAAATAAATATTGTCAAGTAT
ATAAAATATAAGATGTCAGATTTGTATAGAAAAAATCAATCCTGAAGTTGGAGCTTAAGTTTAGTAAATT
TTAAAATGGCTTCAGTTTCATCCTTGAAAAGGCACACAATAATACGGTTTGAAAATGCACGGAATGTTCG
TTGTTCAGGGACAGCAGGGTCCTGAGATTTGAGACCAAGGTGTAAGAGAGTGGGCGTGAAAGGTGTCAGG
AACAGGATCAAACATCAACTCCACGGGACGAGTGCAGATCCACGCATATTTTAGAGTTGCGATTCTGCTT
CTGACTTATCTCTAGTACTTGGAACATCTCCTTGAGAGCCTCGCTGGTGCCCGTCTTGCGGCGATTGCGT
CTTCGCAGGGTCACACCTCCCGACTTAAACTCACGCATCAGAGCGTCAAGACCTGAAACAAAAAAGGTGA
AAGTGAATGAGACCCAGGCTTTGCGGCGAAATTACAATAAGTCGCTACCAAAGCCGAACGAACATGCGGT
TAGATGTTAGCATAGGATTTGACATAATAGTGTCCCGAGGAGCAGTCAATACTCACCTACGTAATCGAGC
AATCTTGGGACTTTAAAGGACTTTGGCTAGGTGGCAATTGGACATAAATCACGGACAGAGTCAAACGGGT
TGATGCGAGCACCATGTCGGGTACTCAGCAACTGTCACCGCCGACAAATGAAGTTGGACCCTGATACAAG
TCAATGGAAATATACGGTCTCCCACTCCTGCTCAAGAtgtgtgtgtgtgtgtTTTCAGTGTGGTTTTC
AGCACGAGAGCTCAGGACTGGCAGCAGCAGAAGCAGGAGCAGGAAAAGTGGCGCAATAAAACACGGAAAT
ATAAAACGCACAAATAATAATAATAAGCCATGCCCGGCCGCCCAGTAACAACAATGCGGCTTGCGGTTCT
GGAACGGTGCTATGGCCATGGCCCTTCCTCGTAAAAATAATAAATTGCGTCCTCTCCGCCATCGTCATCA
TCGTCGTCGAATTTGATACGGTCCTAGCTTGACAGTTTTGGGCGGGGATGGAGGAAATGGGCCAGGAACC
AACATAAAGCAAGGCAAGCGTAAAGTGTAGGAAAAATGCGAGAGTTTCGTTTAAAGCCAAAATTCAGGCA
GGTTTCCTTGGTGCCGGGCCAAGGAAGCCAGTCAAAAGCCTTTTGCTCCAGTTCGCCGGAATCTCGGCAT
GGCGGCTCTGTGGAGAAACTTAAATCAAATTTAAGTTTTCAATGTATCGCATGAAACCGGAAATGGCACT
CAGAGAGACGAGCAGAGAGCTTTAGTGTCGAGGAGGATATTGATAAATGTGTGCGCCCCCACTCCCAAAG
ACAATTCGTTTAAACCGTACTCTAGCGCACACACATAAGCCCGTAGATTCGGCAAAAAGGCAGGTCATGG

AGGTCGGTCGCTCGTAGAAAGTGAAGCTGGTAGCAGGTATCTGGCGGCCATGGCAGCCACACCAGCAATG
CCAGGCAGCAACTTGTTGTTATGTATACACACAACACAGCCAGGCTTGGACAATATCAGCATTTCCAttc
acttcgcttcgcttcgcctcgcttcgctccatctcgttccggtgtggaacgcttcatttGTGTTTGCCTC
CCCGCCGAGCAGGACGACGAGCTTGACTTGACGAGTGCGGCCAGGACACGAGCAGCGGCTGATGCCTAGC
CGTAAAGCTGATGTACGGCTGGTTGCCAGTTCAAACGCTTCACGTGCCTCAAGAGACGCGCGCGCGTTCA
CCGCTCCACTCCGCTCGCAAGGCGCTACGCTACTCAGTTGCATATAGCCAAGTTTTCtatacatatggaa
tatatatattggaatatttatatatacgtatatTTACGTATAAACCGTCACATTCGTGGCTGCGTTTTTC
TGGCTTCCAACAATAAGCGCGTTGATTGATTTCGATTTGAATGTGCGCTCGTTACGCATACGACTCGAGG
TGCGCGCCAAAAAGTAGTCAACAGAAATGAAACCAGCGCCAAAACAAAACGGAAACGGAAGTGCTTagtg
acagtgtgcagtgtcagtgaaagtgtgggagtgagcgagtgtgtgtgCCTAGCATGGCCATGACATTGCT
GCCTTGATCTGAAGTTTAAACCCCAA
>dme_circ_0007380_junction_seq
CTGCCTTGATCTGAAGTTTAAACCCCAAGAGCAAAGGAAATACGCGGCATATTTCC
>dme_circ_0007381
TATATTAAACAAGGCATCGAGTATGTCCTGCAGGAACTTCACCACCTCCTCTCCAGGCACTGTGGACAAG
GCGTTTAGCGACTGTTCCAGCGTCTCCTTGTGCGCCGACCAATTCAGAAGTCCCAGCAAGCTCACTGTAT
GGAAATGAAATATTTAAGATGTTTGTAAAGTATTTAATTATAAGTCCCCCACTTACCGCTCTGCGTGAGC
TTGGTGCTGCACAGGTTAACCCCAATCGATAGCTGATCCTTTGGCAAGAGAGTAAGTCCACCGATAGAAG
GCTTTGCACCTTGCAGCTCTGCAACCGTGGCTGGAAGTTCTAAATAACAATTGGCTACTGTTTTGTCGTA
CTTTTTGTGATCAATTTTG
>dme_circ_0007381_junction_seq
TTTGTCGTACTTTTTGTGATCAATTTTGTATATTAAACAAGGCATCGAGTATGTCC
>dme_circ_0007382
GAACTTCACCACCTCCTCTCCAGGCACTGTGGACAAGGCGTTTAGCGACTGTTCCAGCGTCTCCTTGTGC
GCCGACCAATTCAGAAGTCCCAGCAAGCTCACTGTATGGAAATGAAATATTTAAGATGTTTGTAAAGTAT
TTAATTATAAGTCCCCCACTTACCGCTCTGCGTGAGCTTGGTGCTGCACAGGTTAACCCCAATCGATAGC
TGATCCTTTGGCAAGAGAGTAAGTCCACCGATAGAAGGCTTTGCACCTTGCAGCTCTGCAACCGTGGCTG
GAAGTTCTAAATAACAATTGGCTACTGTTTTGTCG
>dme_circ_0007382_junction_seq
TAAATAACAATTGGCTACTGTTTTGTCGGAACTTCACCACCTCCTCTCCAGGCACT
>dme_circ_0007383
GCAATAAATCTTCAGGACTATGGAGCGACGCGGTCGCATTCCGGTGGCCAAGTTCCGCACCCAGGAGGCC
AAGACACGCAGTAGGAGTCGCGTCAGGGAGCCGGACTACAACGAGCAGGACCCAGAGCACGATATGTTTA
CCTTGCTGGAGGATAACATTGCCCTCCGGGAAGAGAACAAGGCTCTTAAGCTGGAAATCGAGACCCGATA
AAACCACACAGGAAGAGCAGCAGGCTCAGCATGAGAAGATGTGTGCTACTCTGGAATCCCTGCAGCAGCA
GCAGACCAACTTCGAGACCCAAATTAAGGAGCTAAGCGCCAAGTTCAACAAGGCTCTGAACGAGCGAAAC
GCTTTAGATAAGCTGCACAAAATGAAGGTCGATCAAATTCACAAGTCTCACCAGCGAGCTGGAGCATATT
CGCGAAAAACAGCAGGATCAGGCACCTCCAATAGCTCCCCGTTCGGTGCTCGGTTCTGTTGAACTTAAGC
GAAAGCTCTTCAAGATCCTGCAGTCGGGCAGCGCAGACAGTGCTGACAGTATCGGAACTCAGGAGCTGGA
CAGTCTGGTTGATGCCAATTCGGAAGGAGTGCCCATTACCAGCGGTCTGGTGGAGCGTCTGGCCAATGAG
TTTTTGACTCTCAAAAATTTCACAAATTCGGTGGAGCTGCAGTTGTACGAGGCCAACGAAAAGATGGCCG
AGCTGTTGGAGCAGGGTATGATGTGGGATTGTTAAACGAGCTTATTAATATATTGTGATTCTTATaaaaa
aaaTTACACTTATGAATTCGAGATAAGTAAGTGTTCGTTAGAAAAAACACAACCCCTTGCATGGAAGGAA
TAGATGACTCATTTTAATAGTCCATGACTATAGGTTTTAATTAttttttttttCTCATTCTCGTTTTAGC

AATGGAAGCCGCTCTTATCAAGTTGAAGCAGCGCAATGATGAGCTCACTGCGAAGACTCGAGATCTTACT
GATGGCCAGCCAGGATCCAGGACCAGTACGTCGTCCAGCGTACTGAACGAGCAGGTGGAGTTCGAGCAGA
TTCAGGACCAGGTGCAGCAGCAGGCCAGAGAGCACAACGAGCGCATTGTGGAAATGGGTTAGTCCCAAGT
TCTGGTTACACTTTGATTTTGCACTTGATCTATGGCTATCAATCTATCTATTCTGTCCATCTGTCAATCC
ATTAGCAAACACGCAGCGTGTTTGACATTTTCAAGCCGTTCTCGAATATCTTCAAGAGCAACTGAATCAC
ATCAAGATCAGCTGGTGGAGACGATTCCCCCAATGCACATATATAATACGATCTGAAAAATGCATGGAAT
GTGGCCAGCTGGGCGTGTTGTTGTTCCCCATTCTTGGTGCCGGGTCAACCTTTCTTCTTTCCTCTACCaa
aaaaaaaaaCCAGCCGAGACACGTAAGCAACTCGCTTGCCGTGCGCCGCTTTCCCCCACTCACGATCCCC
TGGCTTTCGATGCACTAACTTGCCCCCTATATGCTTTGATAGATCTTCTAATTTTCTTGCACTACCACCA
ATCAACCCAATCTCTTCCTGCTCCTGCTCGTCCGCCCCGTTCTACCCTTGTTCCTGTTTCTTGCATTTGT
AGCCAAAATCTAATGGATGCGGCTATAGCCAAAACTACTAACGATGAACTAAAAAAATTACAACTTAAGT
TGGAGATACTCGAGGAGCAGCTGCGCGAGGCAGTCACGCGTGCTGATCGTGCCGAGGAGCAACTGGCGCA
CTACCAGCAGTCTGAGCAAAAGTCGCTGGTCGctccgacaccgccgcctcctccaccgccgccccctcct
ccAATGCCAGGAAAGCTTTTTCCACCCGGGGGATTGGTGGCAGCTGGCGGTGGCAGCCTGAGTGACCATA
TTGCCGGACATAGCTTGCGCAAGGGCAGCGGCGACAAGATCATCGAGAATGTGAAACATCAGGTACAGGC
CGAGGCTGCAACAGG
>dme_circ_0007383_junction_seq
ATCAGGTACAGGCCGAGGCTGCAACAGGGCAATAAATCTTCAGGACTATGGAGCGA
>dme_circ_0007384
ATAAAACCACACAGGAAGAGCAGCAGGCTCAGCATGAGAAGATGTGTGCTACTCTGGAATCCCTGCAGCA
GCAGCAGACCAACTTCGAGACCCAAATTAAGGAGCTAAGCGCCAAGTTCAACAAGGCTCTGAACGAGCGA
AACGCTTTAGATAAGCTGCACAAAATGAAGGTCGATCAAATTCACAAGTCTCACCAGCGAGCTGGAGCAT
ATTCGCGAAAAACAGCAGGATCAGGCACCTCCAATAGCTCCCCGTTCGGTGCTCGGTTCTGTTGAACTTA
AGCGAAAGCTCTTCAAGATCCTGCAGTCGGGCAGCGCAGACAGTGCTGACAGTATCGGAACTCAGGAGCT
GGACAGTCTGGTTGATGCCAATTCGGAAGGAGTGCCCATTACCAGCGGTCTGGTGGAGCGTCTGGCCAAT
GAGTTTTTGACTCTCAAAAATTTCACAAATTCGGTGGAGCTGCAGTTGTACGAGGCCAACGAAAAGATGG
CCGAGCTGTTGGAGCAGGAATGGAAGCCGCTCTTATCAAGTTGAAGCAGCGCAATGATGAGCTCACTGCG
AAGACTCGAGATCTTACTGATGGCCAGCCAGGATCCAGGACCAGTACGTCGTCCAGCGTACTGAACGAGC
AGGTGGAGTTCGAGCAGATTCAGGACCAGGTGCAGCAGCAGGCCAGAGAGCACAACGAGCGCATTGTGGA
AATGG
>dme_circ_0007384_junction_seq
GAGCACAACGAGCGCATTGTGGAAATGGATAAAACCACACAGGAAGAGCAGCAGGC
>dme_circ_0007385
GCAATAAATCTTCAGGACTATGGAGCGACGCGGTCGCATTCCGGTGGCCAAGTTCCGCACCCAGGAGGCC
AAGACACGCAGTAGGAGTCGCGTCAGGGAGCCGGACTACAACGAGCAGGACCCAGAGCACGATATGTTTA
CCTTGCTGGAGGATAACATTGCCCTCCGGGAAGAGAACAAGGCTCTTAAGCTGGAAATCGAGACCCGATA
AAACCACACAGGAAGAGCAGCAGGCTCAGCATGAGAAGATGTGTGCTACTCTGGAATCCCTGCAGCAGCA
GCAGACCAACTTCGAGACCCAAATTAAGGAGCTAAGCGCCAAGTTCAACAAGGCTCTGAACGAGCGAAAC
GCTTTAGATAAGCTGCACAAAATGAAGGTCGATCAAATTCACAAGTCTCACCAGCGAGCTGGAGCATATT
CGCGAAAAACAGCAGGATCAGGCACCTCCAATAGCTCCCCGTTCGGTGCTCGGTTCTGTTGAACTTAAGC
GAAAGCTCTTCAAGATCCTGCAGTCGGGCAGCGCAGACAGTGCTGACAGTATCGGAACTCAGGAGCTGGA
CAGTCTGGTTGATGCCAATTCGGAAGGAGTGCCCATTACCAGCGGTCTGGTGGAGCGTCTGGCCAATGAG
TTTTTGACTCTCAAAAATTTCACAAATTCGGTGGAGCTGCAGTTGTACGAGGCCAACGAAAAGATGGCCG
AGCTGTTGGAGCAGGGTATGATGTGGGATTGTTAAACGAGCTTATTAATATATTGTGATTCTTATaaaaa

aaaTTACACTTATGAATTCGAGATAAGTAAGTGTTCGTTAGAAAAAACACAACCCCTTGCATGGAAGGAA
TAGATGACTCATTTTAATAGTCCATGACTATAGGTTTTAATTAttttttttttCTCATTCTCGTTTTAGC
AATGGAAGCCGCTCTTATCAAGTTGAAGCAGCGCAATGATGAGCTCACTGCGAAGACTCGAGATCTTACT
GATGGCCAGCCAGGATCCAGGACCAGTACGTCGTCCAGCGTACTGAACGAGCAGGTGGAGTTCGAGCAGA
TTCAGGACCAGGTGCAGCAGCAGGCCAGAGAGCACAACGAGCGCATTGTGGAAATGG
>dme_circ_0007385_junction_seq
GAGCACAACGAGCGCATTGTGGAAATGGGCAATAAATCTTCAGGACTATGGAGCGA
>dme_circ_0007386
ATCGGTTTTTATAAAAAAGGACTCAAACTCGACACTAACACAGCACACAGATCTGGACACAGCTCTGGAT
GACAATAGTACCTTGTGCCTGTACCAGGCGCCCACTCTGATGGACTCTGGCTCGGGGTCATCGTTATTGT
CTGGCGCAATGGTAGATCAGCAGATGCAGACTGTGAAGCCGGCGAGTTCCCTTAAGCAGTCCGTTTCCTT
GGAAAAGG
>dme_circ_0007386_junction_seq
CTTAAGCAGTCCGTTTCCTTGGAAAAGGATCGGTTTTTATAAAAAAGGACTCAAAC
>dme_circ_0007387
GCAGCCGGACGAGCTCCACCGGAACCCTACTACGGTCGCTATATTGGAGATTTTACCAACTTTGCCCATG
GCATTAAGGTGAGTTTTATACTTCATATAAATACTGCCTTCTTGGGCAACATAAGCTTTGCGAAATTTTG
CGTAATAAGTAATGCTATTATTTTACGTTTTAAGAAGAAAGTATAGTACTATATTTATAAACCATATAAT
CCTTTTCCGCAGGGTCAAATCTACGCGGTGGACGAGTCGACGCTGTTCGTCAAATCCTTTGCCTACGACG
GCACCGGACCGGACGCCTTCTTCTGGGTGGGCAAGACGCCAAGGCCCAGTCCCGATGGCTACATCATTCC
CTATCCGGAGGAGTACACGGGCATGTGAGTATCGCGCAGTGTCAATAATGCACTCCATACAATAGTTTTC
ACACACTGGCACTCACATTTTTGGGGCTAGCCATTGATGGTCGGAGTGGACCTGTGTCCAAGATGGAAAT
GGAGAGATGGAGATGGTGATGGAGATGTGCTGGCTACTCGAGCGCAGCAGGCCATTTCCTCAGAACGTGA
GCGAATGTTTTGGGCAAATGTATGCCCTGCCGCCGTATCCTTGCAGACTTTACGTTACGCACGGCTGACA
ATGAAACCACTTTCAAAAATTAAAAAACATTTTTCATCTCGGCTGCCATTTGTGCTCGTTGTCGAGTATT
CCTTTTGCCATCCGGCGCCTTTGTGGCCCAGCAAGTTGCCGTTGGTGGTGTCGCTTCGTTGCCTCCTAAT
GTGAAAAACTCATTTATTTCTTCACGTTAGATTTGCGGTCACATGTGTGTGGGGCAGCAGGACGCCAACA
CCCCTCCCCCAACCCTGTGAGTATCGCGCAGTGTCAATAATGCACTCCATACAATAGTTTTCACACACTG
GCACTCACATTTTTGGGGCTAGCCATTGATGGTCGGAGTGGACCTGTGTCCAAGATGGAAATGGAGAGAT
GGAGATGGTGATGGAGATGTGCTGGCTACTCGAGCGCAGCAGGCCATTTCCTCAGAACGTGAGCGAATGT
TTTGGGCAAATGTATGCCCTGCCGCCGTATCCTTGCAGACTTTACGTTACGCACGGCTGACAATGAAACC
ACTTTCAAAAATTAAAAAACATTTTTCATCTCGGCTGCCATTTGTGCTCGTTGTCGAGTATTCCTTTTGC
CATCCGGCGCCTTTGTGGCCCAGCAAGTTGCCGTTGGTGGTGTCGCTTCGTTGCCTCCTAATGTGAAAAA
CTCATTTATTTCTTCACGTTAGATTTGCGGTCACATGTGTGTGGGGCAGCAGGACGCCAACACCCCTCCC
CCAACCCaaaaaaaaataaataaataaaaaaaCAGTCACAACAACAGTAAACAGCGAACAAAGAAAGCCG
CAGGGCGGGCAAAGAAATTGGGGGCGTTGAAAAGAGATAGCGAAGTTTCAGCCTGTGGTGCGCTACTATT
TACTTTGAGTCGCCACAAGGTTAAGCCGCTCAACTCAACTATTTACCTTGCCAGTCGTCTCCTTGCGCCG
CCACTTGAACGCACTAAGCTGGTATTAGCCAGGTCCCCGCCCACTAAAAATCTGCACCACCCACTTTTGC
CGCTACTTTTCGGCCTAATCCTGCGTTGCAGCCAAGACACGCACACACAATTTGACGTCAGTTGTCCGCT
TAGGACATTGCATATTTCATTATCACAAACAATGCAGCCCCAAACTAATCCCGCCGCCCCATCATAATAC
CCAGATCCCTAGGCATATGTCTATTCCTAACCACTTCGCACCACCTGTATTCCAAGGCAAAGGTAAAGTG
GCTTTCCACTTGGCATTGACATTACATCTGCTGAATGCCATATTTCATATTCATGATTTAACTGGGCAAA
AGGCTGGAGCATCCCGTTGAATGTATTTTTTGTTTCGAAGTGCATCCAAAACACGTATCATATCCTTATG
TTAACCCATTTTTGCATATCCAATCTTTGGAAAAATATTTTCGCATGCCCAAGAATAACAGAGCGCATCG

TGCGAGGCATAAGGATAAGCGATTCCCGAAAAGCGGATTCCCATCTCCCCTCGTATTCCCAGTGCGCAGT
CTCCATTTCGAATCCCAAATCACGCTCCCAAAACGAAAGCGGCAAAGAAAAACCTGAGGATGTTTACGCA
CTGGATTTCCCCATTCGAAAAATGTGAAATACTCGGCGCGGTTTCGCGCTTGAGAAATCGTTTGATGATG
TGTGTgttgcttggttggttggttggttggCGGGCGGATTGGTACCGATGCAGAAGAAACAACCAAGGCG
AATCCATGGCCCAAACCACGGGCATTCATATTGTAATCGACTTTAATGCGTTTCATTGTTGATGCCACGT
GCCGGGCTTTATTAGTTCAATGTACAACGATGCCATCGCCTCCATTTCCATTTCCCCATCTCTCATCTTT
AGTCCTTCGTCCTTCCTGTCCTTCTGACAAAGAGCAACATTATGCCAGGAGAGGGGATATGAAGGTGGAG
GTGGAAGCCCGGAACGCCGTAGCGATTGCACGCTTTTACGCTTTCAGACACATTACCCATACCACCCGTT
TGTCCATGCACCTTCTGAAATGCGTGCTTGGTATGGATagggtggtgctgaatggtgggtgggtggctga
aggtcgacggtgggttggaatgtggtgtggggCGGACTGGTGTTAGCACGATGATGATTGCTGTATTATG
TGGCGCACCGCTGCTATCGCAATAGCATGCATTTGAGCCAACAAATTGCGCGCGTTTATTATGTCTAATG
ATTTTcatgtatgtatgtatgtacgtatgtatgtacgtatgtgtgtGCCATGACGTCGCCAAGCGATAAT
TGATAAATGCAACTGATTGGCAGTGGGGCAGGTTCTGCTGTTGGAAACCGAAGCCCCACAAACGCAGCTC
CGCATCCTCTTGTTGGTTGACGAACGGCAAGTGACAGCCATAAAACTGACTTCCTGCTAAGCTTTTCGGG
CAGGCCAATCAGCGTTAATTATCCCAACTGCTACTGGCAGGCTTCGTTGAAGGGCTGCCAATCCTGCTGC
ATCACTAGATTTTCCAGCACACGGAAAAATTACAAGGGATATATCCTTAAAATTTAGATTAGAAGTTCAT
TTCCACTTAGAATTAACCAAACATTGCCCACTTAATATACAAATTAAGACGTTAGATTTCCAAATTATAA
ACAATAATCTGATTGCTTATCTCATAGCTTCCGTTATTTTCTTCAAACACGTTTTTTAAGTTTCTAAGAA
ATAACCTTGTAACGTGAACCTATatttttttgagtgccttgtgccttgcttttcatgctgctcactttttt
tttATGTTCACGCTCGTTTTACCTCCCCGCTGAAGTGTGTGGCGCGGTGGAGTGAAGTGGGTGGTTTGGT
TGGGGGGCATGTGTCACAATGGATGCATCGAGCATAAGTAATTAAATTAGAATTTTTTGAGAAGCATAAA
AGCGACGCCGCGCTGATTAACGCCCGTTGCCATGACAATGAGGATGATGCTGTTGCCTTCGCCTGCCGCA
TACCACCACCCATCGCCCACAACAAGCCCCACCCCCTGGCTGTTGCTCATTTGCATCTGCAGTACAAAAA
CCTCGCTGTTTCCTCCTCCACCTTGGCGTTTGCCTTCTCGCTTAGTGCTGATTTATGCAGATTTCATTAA
AATTTGCTGCATGCAAAGTAGCAAAATGAAGTTTTAGTTTTGTCCTTCATTGTCGAGCGGGCAACCCATT
CTTTAACTCCGCTCTCCGCCATCCACTACCCGGATCATACTTCATTGTGTTAATCAACGTTGACAGCGCA
GCCGCAACACGAACAACTGTTTTGGCTGTCGACTGGCTGTTCGCCCCTGGCTGATGTGTCAACTGCCTTT
AACTCTATTAAGCACCAGAAAGTTGGCGGATTCATTTGGCATCCAACCTAACAGACACATCCCATTCCAT
TACCCGCCTATACATGTTTTCAGAACTCAAGTAGTTGCGCCTGTTGCTGTCACTTCACCCTCGGTGCGCT
CAGAAGTAGGCAATGCTTTTTATCCTCACACCTTGCGCGGTTTGCATTGTCTACGATCTTTTCGCAGTAC
TTTCATTTAATTTCGTTAAAAATTAAAATTTTTCAAAATATAATATACACGTTTAATTTAGAAGTGGGCA
GAACTATCTCCCTGTAATATAATTTTCTGCTAACTAGATATTTAGCAGCTCCTTAAGGAACCCCCATCTG
TGCTTATTCATCAAGCCGCTCTTCGCTCCTAAAATACCAAAGCCGTACTAATTATTATTGTTGTTGTCGT
GATTAATGCCCCCGACCACGGCACTTTAATACAAATTTAATTGAGTAGTCCGGGGTACCCATCCGTCTGC
GGTGTGTCAGTGACAAAAATGCGGGGGCGTTGGCTTGGCTGCGACGCTTCGTCACGGAAACATGAAATAT
TCATAATAATTTTAGAATATTTTCGGTGGTGGTTTCCCAGTGGGCTTTGTGCTCAGCTTTGGAGAGGGGA
GTAGAGTTGGCGGGTTAATGGGGTTGAATGCtggatggatgattggatggttggatggctggatggttgg
GCTGTCTGCTGAGTTGGCGCGTTTTATTGTCTGCGAAATGGCCCCGATTGTTGTGCGTTTTGTTTATCGA
GCGGGAATGCAAAGTGCCAGTCAGCAGGCGCAACAAGTTGGCCAGTTTAGTCTTAGTCAATGGGCCAGCA
TACATGAAAAGACGCCTGTGCGTGGCTCTTTGCACGAACCTACAACTGAATTCTAAACTAATTGGGTGAA
AGTGCACACAGGGACTTATGTAAATATCATATATGTGACTGTTCAACGAGCTGAACGAGAGCAATAAAAT
AAAATCGATGCTTGTAGCGGGCCAGCCAGCCAACCAGCCCCAAAGTAATCCGAACCCAAAACCCAGCCTG
AGTGCATCAATAAATGTCGAACGGAGAGGTCCTTGCCAAGTGGGAGGACAAGCAAATCAAATAAAGCAAC
AAACATATTTGCCAGGCGGAAACTGTAATGGAACTAGGAATCGAAAGCAAACAGAATTCGAGCAACTGAA

GctggaggactggaggagtggaggactgaaggacCGAACTACTGGAGACAAAGGACCAATCGCTTGGGGT
TGCTGGCATCAGGAGCGGGACATATATAGCGTTTGTGTATCCAGCAGATACATTTGATGCACTGTTGACA
CAGAGCCATTCAATTTGTTTTGGCTCATTAGAGCGGTGGTGCAACTGTTGCTGCTTGCCGGTATCTGGTG
TTGTGGTCCCAATTGTGGACCAAATCTGGACACGACCTCGAAATGGGAACAAGGATCTTGGACCTCTGTC
ATTGTGTCAGTTCAAGGGTGTTGGCTAAGTGCTTAGCCGTGTCTAATGCGGATTGCGTGGCCTCCGTCGA
GATTCATTAGACGTCGACCTTTCCACTTCAGCGACTGACTAACCGCCCAACTGACTGATTGACTCGCCAC
TTGGCCCAGTTGCGATAAATCGATCCTGCGCCGCCCTCAAGCTCAAGCTCTTCCTCCACCCTCCTCCAAG
GATGTGGCTCTGCCATTCGCGACATCAGATACTCATTCATCTGGGCAAACAGTCGCTGACTGTTCAGACC
CCTTGGGATTGGCATACTTAGACTTCAAGTTGATTATTAATATACCCCGGGGCTGCTGCTCCGCCCCGCA
AGTGAAATGAGATGCAGTAAGTGCAGTAATGGATATGTAATCATCACAAAATCCAAATGATTACAAAAGC
CATCGCGCTGCACTTGGGTAAGCCAACGAAGGGATTATTTTCATTCCAATGCCAAGGTATCATATAGGTT
TCCAAACTAAGTGGCTTAAGTATAAATCTTCTACATATATATACCAGCAGAGCTTGGATTCGCGGCCTTC
ATCTGTTCTATTAACTTAAATAAATAACATAACTAtgttcacacatgaacacgaatatatttaaagactt
acaattttgggctccgttcatatcttatgtaaatgaatcgagagcgataaattatatttaggattttgtt
atctaaggcgacatgggtgcattgctcaaaaacatgtaatttaagtgcacactacatgagtcagtcactt
gagatcgttccccgcctcctaaaatagtcccttagtgggagaccacagataaggtcctcgccgctcaaga
taggcagatgtgcccgagcgtgggacctcgataaggcggggactatttacttaggcctctgcgtaggcca
tttactttaagatgcgattctcatgtcacctatttaaaccgaagatatttccaaataaaatcagtttctt
acaaaaactcaacgagtaaagtcttcttatttgggattttacatttggtcaatcgagcctttaatcgact
ctgcagtttcccccctaccaaaggtaaggaactcagagaaaggccagctcctttaagcatcttacagctaa
aggtagcaaaaataagtgactcttgtttcccccctaccaaaggtaaggaacagagtataaatataaaaagc
aaaagatacaaaagaatcttttatgttttaaaacaagcaccttatagtctatagctaaaggttgctttgt
gtaccattataaattgtggtaaggcgtgcttgaggccatacatcagcaattgtgaaattaaaaagtgcat
aacaaaagtgccttataaatgctctaatagcattaaatcagctcataaatagagtgcagtgtatatgcca
taagagcataaattaaataaaaagtgcctgaaaacagtgccttataaatgctctaatagcattaaatcag
ctcataaatagagcgcagtgtatatgccaaaagagcataaatgccgaaataaatggctaaaaaacaaaaa
atctgactggactacaaaaataataaaacgtgccaaaaaaaaaaaaaaaatcatctttaaacatcgacgg
agccttaaagaagagaaggaagtcaaattcaaaggagcctctaccagcagcagaagcagcaacaacagca
gcagcagaagcagcaacagcagtagcaacagcagcaacaacagcagcaacagcagcagcaacaacaacga
catcagctaagtcaaaacaagaattttctgtttatccaaacacacatatatatataaatacatataaaat
acatatacacgtactatatatattaagaaattacaaaaaattttcaaaatgatgtcagaaaagactattc
aattccttaagaagcagtccgaaattattttggaaattagaaagttggaagtaaaaccaacattaacaga
tgtagaaattctaaaattaaatgagcttcaaaaatgtttcattgctaatcatagcaatttgttaaagatc
ggcgttgtcgatcatgaatattttaacgcgaagcagtatgatttaataatgatggtgttagaaaaaatta
aaaataaaaatgaaaaaattaagggcgagtcggtagaaaacactttccctaaatcaaacactgtccctaa
atcaaaccctccccctacattaaaccttgaaatgcgtggtcaccctgaaaaagagggtatagcacaaaac
aacgctttaaaagtagagcaggcatttcgtaataatgttggccaatttcgagtatatctagaagatacgt
ctaaactaatagacagtagtccagatttccttaaaataaggaaaaataaaattgaatttttatggcataa
aatagataacctgattgaacaggtgaatagtcattttgagagttcgctattcgaagaagaaattagcgaa
cttgaatttgacaaacaaaatattcttacagccattaatagtcgactcagtggcacaataaataaagctg
aaatgtcgacggttgttaaggcggaggagttaccaaccctgcctaaaatacagattcccactttctttgg
tgattccaaagaatgggatctttttaatgaactctttacagagctcatacatgtgagagaggatctcagt
ccttctctcaaatttaattatctaaagtcagcattaaaaggagaagccagaaatgtggttactcatttac
tgctcggctctggagaaaattatgaagccacttgggagtttttgaccaagcgatatgagaataaaagaaa

catattctcagatcatatgaataggcttatggatatgccaaatttaaatttagaatccaataagcaaata
aagacatttattgacacgattaacgagtcaatttatattataaaattaaaggcacaattaccagaagatg
tggatgcaattttcgctcacataattcttcggaaattcaataaagaatcactcaatttatatgaaagcca
tgttaaaaagacaaaagaaatacaggcactttctgatgtcatggactttttagagcaaaggctcaattct
atatcatcattctcacaggaagtaaaacctgtaaagaaaatgattaataataacaagaataaaaattata
gtgacaattgtgcatattgcaaactaccagggcattatttaattcaatgccataaatttaaaataatgaa
tccagcagaacggtctgactgggtaagaaaaaatgggatttgcctaagatgtctgaggcatccgtttggt
aaaaaatgtataagcgagcagctttgttcgacttgtcgtaaacctcaccacacgttacttcactttgcag
gtcataatccagaaaaagtgaatacgtgtagaacaacaggtcaagccttgttggccacggccttgattca
agtaaagtcgaggtatggaggctttgaacaattaagagcattgattgatagtggctctcaaagcacaatt
atttcagaagagtctgcacagattctaaaattgaaaaaatttcggtctcatactgaaataagtggagtat
cttccacaggaacgtgcatctccaagcacaaagcggttatttcgataagaaattctccgaaaaatttaga
aattgaagcaattattctcccaaaacttatgaaggcacttccagtcaacacgattaatgttgatcagaaa
aaatggaagaactttaaattagccgaccccgattttaataaaccgggtcgcattgatttaatcattggag
cagacgtatatactcacattctgcaaaatggagttataaaaatagacggtctccttgggcaaaaaactga
tttcgggtggatagtttctggatgtaaaaaatccaaaggaaaagaaaccattgtagccacaacaatagaa
ataaaagagttagatcgctactgggaagtggaagaagaagaaaaagatgatatcgagtctgaaatctgtg
aaaataaatttatcaaaacgacaaaaaaagattcagatgggcgatacattgtgtcaattccattcaagga
ggatgtcaccttaggagattcaaagaaacaagcgatagctcgttacatgaatctggagaaaaaactaaaa
aatgaaaaacttaaggttgactacactaaattcatgaatgaatacatggatttaggacacatgattgaag
tgagtgatgaaggcaaatatttttttaccgcaccaggcagtgattagagattcaagccttacgaccaaatt
gagagtagtttttgatgcttcagcaaaaactacgaataacaaaagtttgaacgacataatgtgggttggg
ccacgagttcaaaaagatatttttgacattattattaaatggagaaaatgggaatttgttgtttcggcag
acattgaaaagatgtaccgacaaattaaaatagataataatgatcaaaaatatcaatatattttatggag
aaattctccaaaagaaaaaattaaaacatataaattaaccacagtcacttacggaactgcatctgcacca
tatttggctaccagggttctggtagatattgcagataaatgtaaaaaccaagttattagtgcaataatta
ggaatgatttctatatggatgacctaatgactggagctgattcggtagaagaagctaataaattaataac
attaattccccatgaattgcagaaagttggattcaacttaaggaaatggatttccaacaattccaaaata
ttaaccactgtggaggacacaggggacaataaggttctcaatattatcgaaaatgaatgtgttaaaactt
taggactaaaatgggaacctcaaaaggatttatttaagttcagcgtaaattgtaatgatgaatcaaaaaa
tataaataagcgcgttgtgttatcaacgctagcaaaaatatttgatccgttaggatggttggcaccagtc
acggtttcaggaaaactttttattcaaaaactttggataaataaaagtgaatgggatcaggaattatcca
tagaagataaaaattattgggaaaaatataaagaaaatttattattgttagagaatattcgaatcccaag
gtggattaattcaaacagttcttcagtcattcagattcacggatttgcggacgcctccgaaaaagcatat
gctgcagtagtctatgctaaagtaggacctcatgttaatataatagctagcaaaagtagagtcaacccta
taaaaaataggaagacaattcccaaactcgagctgtgtgcagctcacctgcttagtgaattaatccaaag
actaaaaggatcaattgacaatataatggagatctatgcttggagtgattccacgattaccttagcatgg
attaacagtggtcaaagtaagatcaaatttataaaaagaagaacggatgacattcggaaattaaaaaata
ctgaatggaatcatgttaagtcagaggataatccagcagatttagcatccaggggagtggattctaacca
gttgatcaactgtgatttttggtggaaaggtccgaaatggctagcagacccaaaagaactttggcctcgg
cagcagtctgtagaagaacctgtcttaataaatacggtattaaatgacaaaatagatgatcctatttacg
aattaatagaaaggtattccagtatagaaaaacttatacgtataatagcatacataaatagattcgtgca
gatgaaaacaagaaataaagcctattcatcaattatttcagtaaaggagataagaatagcggaaacagtt
gttattaagaaacaacaagaataccagtttaggcaagagataaagtgccttaaaatcaaaaaggaaatca

agacaaataataaaatattgtcattgaatccatttttggacaaggatggggttctaagagttggaggaag
attgcaaaattccaatgcagaatttaatgttaaacatccaatcattttagaaaaatgccacctaacaagc
ttattaataaaaaatgctcataaggaaacattgcatggagggataaacctaatgcgaaactatatccaaa
gaaagtattggattttcgggttgaaaaattcgttgaaaaagtatttaagagaatgtgtaacgtgtgcaag
gtataaacaaaatacagctcagcaaataatgggtaacttgccaaaatatagagtgacgatgacattcccg
tttcttaatactggaatagattacgcaggtccttattatgttaaatgttcaaaaaatcgtggccaaaaaa
catttaaaggatacgttgctgtattcgtttgcatggccaccaaagccatacacttagaaatggtaagcga
tctaacttcagacgcatttttagcagcactcagaagatttattgctagacggggaaaatgttccaatatc
tattcagacaacggaacaaattttgtaggagctgcaagaaaattagatcaagagttatttaatgcaatac
aagaaaatataacgattgcagcgcagcttgaaaaggacaggattgattggcattttattcccccggcagg
acctcacttcggaggtatttgggaagctggagttaagtcaatgaaataccatttaaagcgtataatcgga
gacactattttgacttacgaagaaatgtcaactcttttatgtcaaatagaagcatgcttaaattcaaggc
cattatacactatagttagtgagaaggaccaacaagaagttttaacaccaggtcatttttttaattggaag
accacctttagaaatagtcgaaccaatggaagatgaaaaaatcggaaatttggataggtggagacttatc
caaaaaatgaagaaagatttctgggttaagtggaaaagtgaatatttgcatacgctccagcaaaggaata
aatggaaaaaggaaattcctaatatagaagaagggcaaatagttttattaaaggatgagaattgtcatcc
tgcaagatggcctttaggaaaggtggaaaaggtgcataaggggaatgatgataaggtccgagtggctaaa
gtaaagatgcaggaaggatatatcactagacccattactaaaatttgtcccttggaaggaataaagtctg
ttgacaaaaatgaggctgaccaagagccaaaaagacgaactagagcgacatcgggaatgtccaagatcgg
aatcattatggcaatgttgttgtttgtgttaagttgtcaagtttctagcgcattacctaaagatatagca
ccaagatattctatagacaaaataaataaaacctcagcaatatatctagacccgctaggagatgttgaga
ttgtgagtacttcttggaatttggttatctattataaaatggatccatattttaaaatgttaacaaaggg
taatgcgcttatacaaagtatgaggaaagtttgcgaaagacttcatagctttgaagagcaatgtagtcta
gtcttagataatatgcaaagtcagttatcggaacttgaagaaaacaataaattgtttatgatacagtcta
gatctagaagcaagcgtgctcctttcgaatttatgggttccttgtatcatattttatttggtataatgga
tgaagatgatagagagcaattagaagaaaatatgaagaatttgttagataaccagaacaaccttgataaa
ctaattcaaaaacaaacatctgtggttgattcaacttctaatctattaaagagaacaacagaagatgtta
actccaattttagaagtatgcaaataagaattgagaacatgacagaagttcttaaagaaaattattatgt
ttataaggaatcaataaaattctttatgattacgaaacagctacactcattgattgaagaaggcgaaaaa
attcaagcaggcactataagcctgttgattgatattaatcacggtaggctaaatacaaatattctcaggc
caaatcagcttaaaaaagaaattgccaaaattcagcagagtctttcggagaacctagtaattccaggaaa
acggtcaggtacggaacttaaggaggtgtatacactgttaacagccaggggtttattcatcgacgataaa
ttgatcattagtgcaaaagtgcctctgtttagcaggcatccatccaaattgttcaggcttattccggtgc
caattcgaaatgaagatcggataataatggtgcatacaacgtccgaatatttaatttataattttgagat
agattcctatcacataatgacggaagccacattaaatcaatgtcagaaatggcaactaaataagagaata
tgcaaaggaagttggccctggaattcagcgaatgataatgcatgtgagattcagcctctaaagccagata
aagcggcgaactgcatctataaaacagtagtcgactctaaaagttactgggtagagttagaaaagaaaag
tagttggttgtttaaggttcctgcgaattcaaaagtccgtctgcaatgtactggctctcaaattgaattg
tttgatttgcctcagcaaggagttttaagcattgcgccatattgtacggcaagaaccgacgataaaattc
tagttgcccaccataacattcagtccgaaagtgaagaattattatcaacaccttatataggagaagttag
tgaagcgccgaagattatttgggatccgctgaaactatcaatattaaatcatactgaggaatttgaacga
ttgaataatgaaattaaatttatgaaagagaaccatcaaaaagatttacatttccatcatatttccggac
atgctggattaattattgctttaatactaatgatagtattaataatatatttcatacggaaatgtgctgt
gcaacaaagaatgcaagcaataacctttgcaggtccgttgccagtactataaatatcaatagtaaataaa

caataaaataatataacaaataaaaatatacagtccactatatcgttgtttaatagaaaatgtacttcta
catagaaaaagcaaaatgtttaaaataagttaattgagtacaaattgttgaattaaaaataatataaacc
ataattgtaatccaataaaattaaaagccagaaaaactaggcccattgaaatcttagttgcaaaataaat
gaacatatatcaaataaatacagtccactactgttataaatgcaactaatatactaatgtacatctcagc
ttgctggccctttggcagaatgttcacacatgaacacgaatatatttaaagacttacaattttgggctcc
gttcatatcttatgtaaatgaatcgagagcgataaattatatttaggattttgttatctaaggcgacatg
ggtgcattgctcaaaaacatgtaatttaagtgcacactacatgagtcagtcacttgagatcgttccccgc
ctcctaaaatagtcccttagtgggagaccacagataaggtcctcgccgctcaagataggcagatgtgccc
gagcgtgggacctcgataaggcggggactatttacttaggcctctgcgtaggccatttactttaagatgc
gattctcatgtcacctatttaaaccgaagatatttccaaataaaatcagtttcttacaaaaactcaacga
gtaaagtcttcttatttgggattttacaACTATGTATGAAGAAGATGAATGAAGATTTAAATTGCATTAA
CACAATGCACTTACACTATTGCATGATGAATGCTGAAAACGAAGCCCaaaaaaaaaaaaGCGCTAATTGG
CTAAAGCTTAAAGCTGGCTGGCAAAAAGTTGGAAAATCTAATAGTAGTATTGTAGTACAATAACAATTCA
TGCATTGGAAAAATGAAAATCACATCGGGAGGAGGGCAAATTGTGGGGGCATGAGACGTGTGTAATGCCT
CGGTTCGCTGCATTAGCCATAAATCTTCATCAGCGCTCGCCTCATAAATAATTCGAATATGCGAAAATGA
GAAATGTTTTTGGTTTCGTTGTCGCTCGCCTGGTTTTTGCTTTAATTTTAATGACATCAAGTCGCAGTGT
AACATGCATACAAATACCATTCAGGCGACCACAATCGAGGCCAAAGTCCGATCTATTGCATTTTTCTGGC
TGGTTACACTTCAATCCCCATCCACATACTGACAATAAACGTGCAAATCTGATGACATTCTGTGTGTCCT
CTTTGCCTTTTTAGCCATTTAATTTTCAGGGTTTCAAAAGATTTCTTAAATTCCTAATATTTCTGTACAT
TTAATTGGCAATGGAATATGGCTATGGTTTTGTGTTTGCTTTTTCTAATAAGCTGTAAATATGGTTGGGC
AGCACACACATTCGGCTTCCCTTGTACTTGTAGTTTACTTTAGTATACCCTGGACCCCATTTAACTCTGT
ATCTCTGCATCTCTCTCGTTTCCGGCATCCCGGTCTCGCTTTCGGAACCACCCACAGGGATCCACCCATT
CTGCAAGCGCACAACCGAACGGACATCATACTGCGCTTACCCATGGGCAAAAGGATTAAGGACATTCGAT
GGCTGTCAGTGTGGTGCAGACGCTTCACG
>dme_circ_0007387_junction_seq
GCTGTCAGTGTGGTGCAGACGCTTCACGGCAGCCGGACGAGCTCCACCGGAACCCT
>dme_circ_0007388
CTACAATGGTTACTTGCCACAGGGAGATCGCGGTCGACGACGCTCCAAGTTTGTGCTCCACAAGCGGACG
GAGGCGAGTGGCGTCAAGCGTTCCAAGCACTACATCGTCCAATCGCCACAGACTTCGAAGGCCATTCTGG
ATGCCAATCAGCATTCAATCTCGTACACACTCTCACGCAACCAGGCGGTAATCGTGGAGTACAAGGAGGA
CACGGAAACGGACATGTTTCAGGGTGAGACACCCCAAAGGTTTCTGGGCCGCCCGGCGGTCTCAAGAGTT
TCGGTTTCACGGTCTGACTTTGATGAATGTTGTTTGCCTAATTAGAGCCGCCAAGGCCCAGCAATAGTGC
CCCATTCCTGGAGGTCAGAGGTCTGCTCTGTTGACTTCTGACCTTAGCTGGTCTCACAAAGCGACATTCA
ATATTCATATCAGTCTGGCCAGCAAAGTATCTCTTCCTGATTGACATCACAAGTTACACTTGTGATTTAT
TTACTTTGTGATATATTGCCTAATCATTTTCTCATCCCCCATATGGCTCACACTCGACTAATCATGATTG
CTGTTCTCCGCCAGGGTGGGACGCTCATCGGAGTCACCCATTGACTTTGTGGTGATGGATACGCTGCCAG
GCGATAAAAAGGATGCCAAGGTTATGCAGAGCACAATTTCTCGGTTCGCCTGCCGCATTCTGGTCAACCG
CTGTGAGCCTGCCAAGGCGAGAATATTCGCTGCCGGCTTTGATTCGAGCAGAAATATATTCCTTGGGGGA
GAAGGCCACCAAGTGGCAGGACAATGTGGAAATCGATGGCCTGACCACCAACGGTGTGCTGATTATGCAC
CCCAAAGGATCCTTCTGTGGCGGCAATGCCAAGTGCGGACTATGGCGCGAGTGCTCCGTGGGCGGCGACG
TTTTCAGCCTTCGCGAATCGCGTTCAGCCCAGCAAAAGGGGCAGCCGGACCAAACACGATTTGGAGAAAC
TTATCGATGCCATTAATGCCGGTCGTCCACAGTGCCCGGTGGGCCTGAACACGCTGGTTATACCACGCAA
AGTTAACATTGGGGATCAGGTGAACCAGCCTTACGTGTACTTAAACTGTGGCCATGTCCAGGGGCCATCA
CGATTGGGGTCAGGATGAGAACACTGGCGCCCGCCGCTGTCCGATGTGCCTGGAACTCGGTCCGGTGGTC

ACCCTGTGCATGGGCTTGGAACCAGCCTTCTATGTGGACGTGGGCGCCCCGACGTACGCGTTCAATCCAT
GCGGACACATGGCGACTGAAAAAACTGTCAAGTGAGCGATTGCAGCCAGTTGTGGCTATGAAATGACTCT
ACTAATTGTGGGTTTATCCTTGAATCTTGTAGATACTGGGCCAATGTGGAGATACCTCACGGTACCAACG
GATTTCAGGCCGTCTGTCCCTTCTGTGCGACGCCCTTGGATGGAGCGACTGGCTACATAAAG
>dme_circ_0007388_junction_seq
CTTGGATGGAGCGACTGGCTACATAAAGCTACAATGGTTACTTGCCACAGGGAGAT
>dme_circ_0007389
GCTGGAAAACTAAACACCTACCGCTTCTGCGACAATGTCTGGACTCTCATGCTTAACGATGTGGAGTTCC
GCGAAGTGCACGAGATCGTCAAGGTGGACAAGGTGAAGATCGTGGCCTGCGACGGCAAGAGCGGCGAGTT
CTGAACACCACCGACCCGATCTGaacacccaatgtaaccccactaaacacaccatgtaaccccacaaaac
acaccatTATAACCATTACAAATAGCTGTAAGATTCGTAGGCGATAAGCTGGG
>dme_circ_0007389_junction_seq
CTGTAAGATTCGTAGGCGATAAGCTGGGGCTGGAAAACTAAACACCTACCGCTTCT
>dme_circ_0007390
GGTGGTGCAGGCGATCATGCAGTCGCCAAACGGGTGGTGTTGTGTTGGTGGGTTAGCGTGTGGATGCAAT
TAGTGCTGGTCAGCGATGGGTCCTTACCCTGATAGTCAAAGTTAACTACTTTGTGGCTATGAAGGAAGGT
GTTTGGATGGCTATTGGAGATGTTTGGGATGTGATGGTGCACGGCCTGCTGGCGGTGCATAAGCTGGCGT
TGACCAGCCAGAGCAGTTGTGGTTAGTG
>dme_circ_0007390_junction_seq
TGACCAGCCAGAGCAGTTGTGGTTAGTGGGTGGTGCAGGCGATCATGCAGTCGCCA
>dme_circ_0007391
GGTCCAGATGCGGCCATGAAGCTGCCCCAGGTCACGCCCCACACCCGCTGCCGCCTTAAGCTGCTCAAGC
TGGAGCGCATCAAGGATTACCTGATGATGGAGGACGAGTTCATCCGCAACCAGGAGCGCCTGAAGCCCCA
GGACGAGAAGAACGAGGAGGAGCGCTCTAAGG
>dme_circ_0007391_junction_seq
GAGAAGAACGAGGAGGAGCGCTCTAAGGGGTCCAGATGCGGCCATGAAGCTGCCCC
>dme_circ_0007392
GGCGGTCGGGAATCTGGTGCTCGGTGGCATAGCCGCGGCTCGACTGCATCACAGCGGTGGGCAGTGCCTT
GGCCAGCGTGGCCAGCTCCTGCTGGCGGCGGGCGCCCTGGCGGGCCAGACTCTTCAG
>dme_circ_0007392_junction_seq
GGGCGCCCTGGCGGGCCAGACTCTTCAGGGCGGTCGGGAATCTGGTGCTCGGTGGC
>dme_circ_0007393
ATCTTTGGCAAGCTGCTGGAGAAGAATCCCGGCATCGTGGACAAGGTGCGCGTGGTAAAGGGCGATCTGC
TGGAGCCGGACCTTGGCCTGAGCGCCAATGACACCAACACACTCGCCTCCAACGTCGAGGTGG
>dme_circ_0007393_junction_seq
AACACACTCGCCTCCAACGTCGAGGTGGATCTTTGGCAAGCTGCTGGAGAAGAATC
>dme_circ_0007394
GTTGTTGGGCCTTCAGGTCCTTGGGACCCACTTCCAAGCGCAGCGGCACACCCTTTAGTTCCCAGTGATT
AAACTTCCAGCCGGGAGAGTAGTTGTCTCGGTAATCACCCTCGCAACGAACTCCACCGCCGACAAGGCGC
TTCTCAAGGGCCTTGCAAGCGTCAAGCAGCTGCGCCCGCTCGTCATCCTTCGTGTTAACTGTGATGCCGC
AGGGCACCACAATGGCCTGAATGCAGGCAACATGCGGGGGCAACACCAGGCCCTGGTTGTCGGCATGCAC
CATGATCATCACGCCAATAGTGCGAGTTGTAATGCCCCAGGAGTTCTGGTAAACGTACTTCTTCTGCTGC
GTTTCAGGGTCCTCGTAAACGATTTCGAACATCTTAGAGAAATTCTGGCCCAAATGATGACTGGTAGCAC
CTTGAATGGCACGTCCTGAAGCCGAGATGAATGCCTCCACGGTGGTGGTGTAGTCACCACCAGCGAACTT

CTCCTTCTCAGTCTTGCGGCCCTTGACCACGGGAATGGCCAGCAGATGGGTGTACACCAGGGCATAAAGA
TCGAGAAT
>dme_circ_0007394_junction_seq
TGTACACCAGGGCATAAAGATCGAGAATGTTGTTGGGCCTTCAGGTCCTTGGGACC
>dme_circ_0007395
GTGATAAAATACGTCAATTgaaatcggcgaaatcggagaaatcCCTTGTTGAAGCCGAGGTTAAACTTTT
GCTGGCTTTGAAAACGGACTACAAATCTCTAACTGGTCAAGAGTGGAAACCAGGTACTGTGGCACCCGCT
CCCACCACTGTAAATGTGATCGATCTTACTGGTGGAGATTCAGGCAGTGACGTAGGCAGTGTATTGAGCA
AGATTCAGGCCCAAGGTGATAAGATCAGGAAATTGAAATCAGAGAAGGCAGCCAAGAACGTAATCGATCC
TGAGGTTAAGACTCTGCTTGCTCTTAAAGG
>dme_circ_0007395_junction_seq
AGGTTAAGACTCTGCTTGCTCTTAAAGGGTGATAAAATACGTCAATTgaaatcggc
>dme_circ_0007396
GTACCACAGCTCCAGCTCCAGCAGCAGCTCCTGTAAAAGTCAAGCAGGAGAAGAATCCGGACCCAGCTTC
TGTCCTTACTGTAAACACACTGCTCAATAAGATCGCCCAACAAGGTGATAAAATACGTCAATTgaaatcg
gcgaaatcggagaaatcCCTTGTTGAAGCCGAGGTTAAACTTTTGCTGGCTTTGAAAACGGACTACAAAT
CTCTAACTGGTCAAGAGTGGAAACCAGG
>dme_circ_0007396_junction_seq
CTCTAACTGGTCAAGAGTGGAAACCAGGGTACCACAGCTCCAGCTCCAGCAGCAGC
>dme_circ_0007397
GGAAGGAGAGAGCCCAGGATATGTCATCCTCGCAGGTCAGTGAGAAAGACAGCCAGTGATCAATCTGTGT
CCGTTCGATGGCCGTTTCCCCGTACAGCTTGTAGTCCGGAGCAGCGCGAGCGAGGGCGCGCAGCACATCG
TT
>dme_circ_0007397_junction_seq
CGCGAGCGAGGGCGCGCAGCACATCGTTGGAAGGAGAGAGCCCAGGATATGTCATC
>dme_circ_0007398
GTGGTACATGGCCTATCCGGATAAGGTGAAAAAGAAGATCACCCGGGAACTGGTCACTACGATACTGGCC
CGCAAGCCCAAAATGTGCTCCTTTCTGGAGTGGAAGGACTGCAAAATTGTCTACAAAAG
>dme_circ_0007398_junction_seq
GGAAGGACTGCAAAATTGTCTACAAAAGGTGGTACATGGCCTATCCGGATAAGGTG
>dme_circ_0007399
GCACGCAGCATATCCGAGGAGTTCAGCAGCGGCGACACACCTCGTTTCAAGGACGAGGAGTCGGCCAGCA
AGGCCGAATCGGGACATGGACCCAGCAGTGGTGGTGCAGGTGGTGGAGGCGGAAGTGGCGGTGCTGGAGG
CTCATCCGCAGCTGGTGCATCGGCATCGGCTGCCGGTGGCTCCTCCGGGCACTATAGACCCGATTCCGGT
TCCGGTCACAAATCGGATAAATCggagaaggatcgcgagaagaaggagaaggagcgcgaggaaaaggaTA
TAGGAGATGATCAAGGTGTTCGATGGCAACAACTCATTCCGACGCCAGCAGTACCGCGTGATCATCGTGC
AGCGCACCTATACGCTGGAGCAGCTGCTGACCACCGCGCTGCGGGCATTCCACATTACGCGCGACCCGCA
GGCCTTCTATCTGACCGATCTGTATGCACCCGCCGGCATGGAGGATACTCCGATGCTCGATCCCACGCCC
GTGCTAAACCTGGTGCACCTGGAGGGCAAGCGACCGGCCATATACCTCCGGTTCCACGACCGTGACCGTG
GCCATGTGCGCGTCTACCCCGGCAAGCTGCAGTGCTCGATGCTGGAGGATCCCTATGTCAGTGTTCCTGT
AGATAATAGCACAGTTATTAAGGATTTGATACGCGATGCCCTGGACAAGTTCGGGCTGCAGGATAACCAG
ATACAGGACTACAGGGTGCTCGGAAGTGTTGCTCGATCGCGGCGTGACCGAACGCATTTTGTCGTGGAAC
GAAAGGCCCTGGGATATTATGAAGCAGCTGGGCAAGGACTCCATTCGACAGATGGAGCTCATGCGGTTCT
ACATGCAGCACAAGCAGGATCCGCATGGTCCCAATATCGCGCTGTTCGTGGGCAATCTGCCCACCGGTCT

CTCGCAGCGCAACTACGAGCAGATCCTCAACAAGTACGTAACCGACGAAAATAAGTTCATCAGCATCGGA
CCCATCTACTACGAGTATGGCTCAGTGGTGCTCACCTTTGAGGACTCCATGAAGGCGGGTTCGCGCCTTC
TACAATCTCCGCGAGACCATTATCGAGGACAAGAAACTGCTGGTTCTGCTCCTGCCGAACATCGAGCCCA
GCATGGTGCCCTCGGATGTGAGACCACTGCTGGTCTTTGTCAATGTTAAGTCCGGTGGTTGCCAGGGATT
GGAGCTGATCTCCAGCTTCAGGAAACTGCTGAATCCCTACCAGGTATTCGATCTGGATAATGGCGGTCCT
TTGCCTGGGTACGTTCAACCTATAACCGTGTTTGTGATTCGCCCACTTATCTTTGATTCTATCATTAGTT
TGTATGTATTCCGGCAAATAACCAACTACAAGATTCTGGTTTGCGGCGGAGATGGCACCATTGGTTGGGT
GCTGCAGTGCCTGGACAATGTAGGCCAGGACTCGGAGTGCTCCAGCCCACCTTGCGCCATTGTTCCACTG
GGAACAGGGCAACGACTTGGCTCGCGTTTTGTGCTGGGGCTCCGGCTACACCGGCGGTGAGGATCCGTTG
AATCTGCTGCGCGATGTTATCGAGGCGGAGGAGATACGGCTGGACCGCTGGACGGTTGTCTTCCATCCAG
AGGACAAGCCCGAAGAGCCGGCTATGAAGGCGCCCTCGCAAACAACCG
>dme_circ_0007399_junction_seq
GCTATGAAGGCGCCCTCGCAAACAACCGGCACGCAGCATATCCGAGGAGTTCAGCA
>dme_circ_0007400
GCACGCAGCATATCCGAGGAGTTCAGCAGCGGCGACACACCTCGTTTCAAGGACGAGGAGTCGGCCAGCA
AGGCCGAATCGGGACATGGACCCAGCAGTGGTGGTGCAGGTGGTGGAGGCGGAAGTGGCGGTGCTGGAGG
CTCATCCGCAGCTGGTGCATCGGCATCGGCTGCCGGTGGCTCCTCCGGGCACTATAGACCCGATTCCGGT
TCCGGTCACAAATCGGATAAATCggagaaggatcgcgagaagaaggagaaggagcgcgaggaaaaggaTA
TAGGAGATGATCAAGGTGTTCGATGGCAACAACTCATTCCGACGCCAGCAGTACCGCGTGATCATCGTGC
AGCGCACCTATACGCTGGAGCAGCTGCTGACCACCGCGCTGCGGGCATTCCACATTACGCGCGACCCGCA
GGCCTTCTATCTGACCGATCTGTATGCACCCGCCGGCATGGAGGATACTCCGATGCTCGATCCCACGCCC
GTGCTAAACCTGGTGCACCTGGAGGGCAAGCGACCGGCCATATACCTCCGGTTCCACGACCGTGACCGTG
GCCATGTGCGCGTCTACCCCGGCAAGCTGCAGTGCTCGATGCTGGAGGATCCCTATGTCAGTGTTCCTGT
AGATAATAGCACAGTTATTAAGGATTTGATACGCGATGCCCTGGACAAGTTCGGGCTGCAGGATAACCAG
ATACAGGACTACAGGGTGCTCGGAAGTGTTGCTCGATCGCGGCGTGACCGAACGCATTTTGTCGTGGAAC
GAAAGGCCCTGGGATATTATGAAGCAGCTGGGCAAGGACTCCATTCGACAGATGGAGCTCATGCGGTTCT
ACATGCAGCACAAGCAGGATCCGCATGGTCCCAATATCGCGCTGTTCGTGGGCAATCTGCCCACCGGTCT
CTCGCAGCGCAACTACGAGCAGATCCTCAACAAGTACGTAACCGACGAAAATAAGTTCATCAGCATCGGA
CCCATCTACTACGAGTATGGCTCAGTGGTGCTCACCTTTGAGGACTCCATGAAGGCGGGTTCGCGCCTTC
TACAATCTCCGCGAGACCATTATCGAGGACAAGAAACTGCTGGTTCTGCTCCTGCCGAACATCGAGCCCA
GCATGGTGCCCTCGGATGTGAGACCACTGCTGGTCTTTGTCAATGTTAAGTCCGGTGGTTGCCAGGGATT
GGAGCTGATCTCCAGCTTCAGGAAACTGCTGAATCCCTACCAGGTATTCGATCTGGATAATGGCGGTCCT
TTGCCTGGGTACGTTCAACCTATAACCGTGTTTGTGATTCGCCCACTTATCTTTGATTCTATCATTAGTT
TGTATGTATTCCGGCAAATAACCAACTACAAGATTCTGGTTTGCGGCGGAGATGGCACCATTGGTTGGGT
GCTGCAGTGCCTGGACAATGTAGGCCAGGACTCGGAGTGCTCCAGCCCACCTTGCGCCATTGTTCCACTG
GGAACAGGGCAACGACTTGGCTCGCGTTTTGTGCTGGGGCTCCGGCTACACCGGCGGTGAGGATCCGTTG
AATCTGCTGCGCGATGTTATCGAGGCGGAGGAGATACGGCTGGACCGCTGGACGGTTGTCTTCCATCCAG
AGGACAAGCCCGAAGAGCCGGCTATGAAGGCGCCCTCGCAAACAACCGGTAAGAAGAAGAAGGCACACCA
AGCACACCTATCGCAATCGCAACAAACCAATCAGCATCATCAATTACCGGCTCTGACATCGAGTGATATA
TCAGGGTAAGAAGAAGAAGGCACACCAAGCACACCTATCGCAATCGCAACAAACCAATCAGCATCATCAA
TTACCGGCTCTGACATCGAGTGATATATCAGGTCATTTGGTGTTTTCAAAACTTCGTTCGTGTGTTCTTT
CTCTACGTTACATACTCGTAAACCCGTCTAGTGTCTAACCCTTGAAGAATCCGTTCCAGTACCTGTCCCA
GTCCTTGACCACGCCCGAACCTTATATTTTGTGTTGTGATTAAACCTGTGCTTTGACTAAATACCGGATT
CGGCTGTAATATTCCGCCAATTACACGTAAATCCAAAATATCAAGACTGTACAAATGTAGCATATGTAAA

TTTTTAAAGGCTATCTTTAACTAACTTGtttttttttGATAAATGTATCCAAACTTTATCTGGTAATACT
TTGTAGTTACAGGAACAAAGATCCGCTATAGAAATTTAATAGGAAATACTTTTGTTAACTCTCAAGATAT
AGAAGTCGTACGTCTTTACAGTGCACAAAATTAAAGTCTCTCCCTAAGATACCCCAAAAGAAACCCCTTA
TGTAACCACAAGCCTAGTTTGGTATCTGATGAGAAATGCTGTTTAGTTTCGATGTGTAAAATATCCTTGT
AGGGATTTGCATGTCACAAACTTCCGGAAAGGTAAGCTAAACACCGATCAGCTCAGACACTACAAGGTTC
AACCATTAATTAGGTAAATTAAGCCATGGATATTATACACCTCGATGTTGTCAGTTAAATATTTGGTGTC
CTGTGTCGATGTTTAATCCGAGTAATATGATAATTGGAGATGACTAAACGATCTTGGCGTTCCATTCAGG
GCGGTGCCCAAAACGAGGATAACTCTCAGATCTTCGTGATGAACAACTACTTTGGCATTGGAATAGATGC
GGACCTCTGCCTGGACTTCCACAATGCGCGTGAAGAGAATCCCAATCAGTTTAATTCGCGTCTGCGCAAC
AAGGGCTACTACGTCAAGATGGGACTGCGCAAGATCGTGGGTCGCAAGGCTGTCAAGGATCTGCAAAAGG
AACTGCGCCTGGAGGTCGATGGCAAGATTGTCGAACTGCCGCCCGTCGATGGCATCATCATCTTGAACAT
TTTAAGGCTGGGGCAGCGGTGCCAATCCCTGGGGTCCCGACAAGGACGACCAGTTCTCAACGCCCAACCA
TTACGATGGAATGTTGGAGGTGGTCGGTGTTACGGGCGTGGTTCACTTGGGTCAAATCCAATCCGGAATT
CGTACGGCCATGCGCATAGCTCAGGTAAGCACAAAACCTTTGCTTGGGATTAGATACCACATCATTAATT
TATTATCATCCAGGGTGGTCACATCAAGATACACTTGAATACCGATATGCCCGTCCAGGTGGACGGTGAG
CCCTGGATTCAGAGTCCCGGCGATGTGGTCGTCCTAAAGTCGGCCCTCAAG
>dme_circ_0007400_junction_seq
TGTGGTCGTCCTAAAGTCGGCCCTCAAGGCACGCAGCATATCCGAGGAGTTCAGCA
>dme_circ_0007401
GTTTTTCTTAATGGTCAAATATAAAAATTGGCTCTGGCGCATGTTTCGTTCAATCCATTGCATAGCCCAC
GACTGGTGGCGTCGGGCGCGCCCTCCGATCTCTGCCGCATTTGGCTAGAGACGTATGTCACGGAAATCGG
TCGAGTGTGCTCCGATTAACCCCTGGCACTGAGCCCCTTAATCGCAGCCGGAGGTGATTTGCCGAACGCA
AAGGGTGGTGGTGTTTGGTTTGGTTTCGTGTTTGTGTTATGATCAAACTCCCACCCAGCCGCCCCACACC
CCACCCCCACAAATCCGCAAGCGGATTCGCAAGGGGCGGGACTAGGGACGGATTGGGCTTGGATTTGGAT
TTTGGTGAACACTGCCATGCGATTTCCGTGACCTTTCCATGGCAGTGCGTGTCTGATGGATTGCGGGAAG
AACTCTGGTCTGTTCGTGGACGTGTGTCTCTCGGTGTGACCTTGTTTGGGCGGCGTACCTACAGCCGAGT
GCGTCCCGCGCTCAATGG
>dme_circ_0007401_junction_seq
ACAGCCGAGTGCGTCCCGCGCTCAATGGGTTTTTCTTAATGGTCAAATATAAAAAT
>dme_circ_0007402
TTTCATTTGTTTTTGTTGAAATCAAAAGCAAAATGGCTTTGGCCAACGTGCTGCAGCTAAGCCAGATAGC
TGGCCATGTCATCCTGTTTATCCTGTCGCTGTGCATCGTCGTGCCATTGTTTATAAACCTCGACCAATTC
TGGTGGCCACTGCCTGCTCTTTACAACTGGCAAATGGCGGGAGGAGGACGGCATGTTCGATGTCCAGTGG
GCTTCCAATGGCTTCTGCAACTTTCCCCTGATTACCGGGTTCTTCCTGCTAATTATTTCGGTGGTGCAGA
TTTATAGGTATGTATCTTGTGGGTATTATTCGGGGTTTTTGACATCTAGAAAGTGAATTTCTTTGCCAAA
TAGATACGCCCGCATGAAGGAGGAGGCCTCGTTTATAGCTCTGTTCATTGACGTCGTTCTGGGCATTTGG
ATGCTGGCTATG
>dme_circ_0007402_junction_seq
CGTTCTGGGCATTTGGATGCTGGCTATGTTTCATTTGTTTTTGTTGAAATCAAAAG
>dme_circ_0007403
CACTGCACCTTGCCGAACTGGGCGCGACGCTCCTGGTACTCGCGGTACTCCTTGTAGTGCATCTGGATCT
CCTGCGAACGGTAGCGCGTTAGCTTTATGGACTTCCTCGTCTCCGGAGTTATGGCAGCCCGATAGCCGCC
CTTCCGTAGGAGTGAGTCAATGGTCTGCAGCTGGTCCCAGCCCTGTTCGGTGGCCACCTGCGGCAAATAG
GTGGCTGTGCGCTTGCAGCCGCGTTCTGTGAGGAACTCGATGCGGATGCCATGGACGCCCAGTTGCCAGT

CCAGGTGGCCCTGAGCCTCCTCGAAGTTCTGCAGAATCGAGACCGAGACAGTTAGCCGTGGCAACTCATC
TCTAGAGATAGGTGCAAATCTAGAGTCCTTAAAGGCGCTGGTCAAGGCGTACTCCCGAAGACCATGGTGC
AGTTCCATTGCACTAAAAG
>dme_circ_0007403_junction_seq
CCATGGTGCAGTTCCATTGCACTAAAAGCACTGCACCTTGCCGAACTGGGCGCGAC
>dme_circ_0007404
GTAAAACAATTTCAGCAAAACAGCATAGTAATTAATTAACTGATAGGATGCAAAGTTCAGATTCAGATCA
ACATCAGCTGAGTCTTAACGAGCCTCTGGCAAAGGCTTAACCAGTATACCCACTTTCCTACGACCTAAGT
AACCGTTCAATGAATAAGTATGCTCTGTGTACTCGTATTTAATAATTCATGTTTCTGACCGACCGCCCGC
CTATCTCCCTAGTACTATTTTTGCCATTGTATTAGTCAAATTTTGCACGCAACCGCTGCTGGCTTTTGCA
ACAGAACAGCAACATTGAGAATACAACATCAGAGTATCTTCTAAACAAACAAAAAATCTTGTGATT
>dme_circ_0007404_junction_seq
TTCTAAACAAACAAAAAATCTTGTGATTGTAAAACAATTTCAGCAAAACAGCATAG
>dme_circ_0007405
GCAAAACAGCATAGTAATTAATTAACTGATAGGATGCAAAGTTCAGATTCAGATCAACATCAGCTGAGTC
TTAACGAGCCTCTGGCAAAGGCTTAACCAGTATACCCACTTTCCTACGACCTAAGTAACCGTTCAATGAA
TAAGTATGCTCTGTGTACTCGTATTTAATAATTCAT
>dme_circ_0007405_junction_seq
CTCTGTGTACTCGTATTTAATAATTCATGCAAAACAGCATAGTAATTAATTAACTG
>dme_circ_0007406
GGTGTACGTAAAGATGCGCAACGAGCGGGAACTGCGAGGACGTCTTCACGTGAGTTGATGCCTCTTTTTA
CTGTTGCCCAGCAATCTTTCAATATACACCTTTATCTAGGCCTTTGATCAGCATTTGAACATGGTGCTCG
GCGATGCGGAGGAGACGGTGACCACTGTGGAGATCGACGAGGAGACCTATGAGGAGGTGTACAAGACCGC
CAAGCGCACTATTCCCATGCTATTCGTTAGAGGCGATGGAGTCATCCTGGTTTCGCCACCCATGCGGGTG
GGCTAGCGG
>dme_circ_0007406_junction_seq
TTCGCCACCCATGCGGGTGGGCTAGCGGGGTGTACGTAAAGATGCGCAACGAGCGG
>dme_circ_0007407
ATCGCGTTGCTGAGTACTCCGACGAGGACGAGGAGGCCGAGTTCAGTTCGGGCGAATTCAACGACGAGGC
CAACGAACTGGGTCTGATCCGGGGCTACGATCCCAAGGATCCGAGGGACTACAACCATCCGGCCAAGCAT
GATATGCGCAAGTATCGCAACAAGCTGCAGCGCTCCGGAATCGAGGACTGCGCCTTAGTGGGTCACCATC
CCGGCAGCATTCAGCACCATCATCCCGGCGTGGTCGTCGATAGCGATAGCGAGTTCGAGATGAAGGACAA
GAGTTCGTCACGGGCCAAGTTTAGCCGGGGTGAGTCAATTGAATCACATCGAATCATATCGTGTAATCCC
GCCCAGCTGTTCCGCGTGGCACCCaaaaaaaaaaaaaaaaaagaacaaaaaaaaaaacacaaaaaTTGTCT
CTTACCCAAAAGTATTTTCATTACCGCTCGATATAATTGCGTGGCAACAAGTCAGCAGGCGGCGGAGGTC
TTCTATTTGGGGGGCTCTAAAATTCATTATGCAAAGTGAATAAATATCACTTAGTGGCTATCTATATAGA
TTACAAGCACACATTCGCACATACAGCAAATGAAAAGGGGCAGAACCCATTCACTAAAAAACTAGAAACG
CAAAAACAGAAACGTTCTTCTTTTTTTAAACCTTTTAATCAGATGGTAAAGAATGTGAAGTAGATAAAAT
GGTATGGCATGATTTAAATATTCACTAAACAAAACCATGTAAATAAAGATTTGGATTATCGAATATTTGC
ATAGATTCTGTCAGATATCTCTTAGGATTAACTTGTTCTAATTTCTTCTTCCCATGCCTATTCAGGAATT
TCCCAGAAATTAGCATAAGCTAATCTATAAGGCATTTCCATATTATTCACATTTAGCCCATTCTAGTCAT
CGAAACTGTGCTTAGCCTTGTCTAACATTGTTAGTACCTTGTTGCTACCTCCTCATTCTTGCTAAGGCAT
TTGCCAATTGCGAAATATTGTCAGTTCCTTGCTGCTAACCCAGATCCGCAGGCACAGATACAAGATACAA
AAAGCCGACCCAAAACAAAGACCCACTACCACCAACCCGGCGGCCAAATCAAATGAAATATGTATCAAAT

ATGTATCCCCATAGTATAGAAGAAAAAGAGTGCATATACATACATTTGCACAGATACTTAAGTTCGGGGA
AAAGCATGCCCCAAACGGGGTGGCCAAGAATTTCTGTGGCAAATAGTTGACAATTGTTGGGGCGGCGGCC
TTGGCGTGTCGCTCACTCGACTCAATTTGCTTAGATTCGCCCGGTGGCATACTTTCGAATCTATGGCTAT
ATCTCAGGGCTATATATCGGGGTCGTGGATTTCCGCATAAGAATATATCCAAGTTGGACTAGATATAAAG
GGGGATTTGTACTACGAAAAAGGATTATTAAATGGGATTATTTTCCCCACTTTCTTTTCAAAGCGAACAG
CATATTTAGAAATTCCATGCTTTATTTATGCCTTTTTCCCCTTAAAAGCATTCGTACTAATCACAACAAA
GACAATTTTCTTAAGCTGCCGCTTAAATATTTAAACAGCGTTTGGCTTTGACTCACTCGGCTCAGCCGTT
TGGGCCAATTATGGACTTTTGCCTCGTCCTGGTGCCCGCCCATTGCCTCCCTGCGCCCCTTTTTATCTAC
GCACCTGGCCAACATCCGTAGCACAGGTGTAAAATTAGCCTGCGGCTTTTATTGTCATGGCTTCTGCTGT
TTAttttttttttGTTGTGGCTGGCTTTTTATCGCAGGATCATTTTGAATGGCTGGCGTAGACAAAAAGT
CGACTAATGACAGGCCGTAGCACaaaaaaaaaacctaaaaaataaaacaaacaaacaaaaagaaacCGAT
GAGCGCTGGGCAATTAAAAAAGAACGATGCATAAATTTTAACAGCTCCCCACAAGTCGTCAACGCTGTCG
CAGTCCTTGAGATTTCGTTTAATGACCCATAAGCAACTCTAGATTGATCCAGCGGCAATTAAGGGGTATT
AATGAGGAAAGGCAAGAGCAGGGAAAACAATATTTTCAACTTAATATAAAGATCTTGTTTCCTTATGATC
AAGATCTTGTAGCTTCTAGTGGACAACTACTGGTGCAGTTTAATATTTTTAGCCCTTTCCCTTAATAGTT
ACAAAATTGAAGCAATAAAAGCAAATATTAATAATAATTTTTATGGTCTTTCCTCCTTTTAGAACGATCT
CGCTGCAGCAGCGAAATTTCAAGCAAAAGATCGTGGCACTTTTGAAGCGGTTCAAGGTGAGCGAGGAGCT
GGAGGGAGAGTCCGGTCATCGAGGCACGGCGGCGCTGCGCGGAGAACGCGACCTGGATGCGCTCTTCCAG
GAGCTGGAGTCGCTGTCCTGTTGCGAGGGCGACGACTCCGGACCGGACATGGACAGCATATCGGTGGGCT
CCACGCCGAAGCCATCGCTGCGTCCCTTTTTCACCAACTCGCGGATTATGTTGCACGACAACATCACCGG
AAACGGCGGAGATCTGAGCCAATTGGTGGGCGGTGGCCTTGGACCCGGAACCGGAACAGCTGG
>dme_circ_0007407_junction_seq
GCCTTGGACCCGGAACCGGAACAGCTGGATCGCGTTGCTGAGTACTCCGACGAGGA
>dme_circ_0007408
ACTCTGTTCGCTGACAATAACACGTCTGTCCATCCTGACTCCGCTGCCCGGTGACACCACCTCCTTATCG
CTCGCGGTGAAGATGCAGAGCTCCAAGAGGACGCTGCGCAGCCACGAGATTCCCATCAACGGCCTGGCCC
TGACTTCATCCGCCTCCCTGCAGGGCAACTCGCCGATGTCGGTGGCCGGAGGAGTTGGAGGTGGTGGTGT
TGGCACTCCGGGTAGTGTGGGCACTGGAGGCGGACTGGGCGTGGCTGTGCCCTTGACAGAAACCGAGCTG
GACTTGCACTTCAGTCTGCAGTATCCGCACTTTATCAAGAGAGATGGCAATAGGACTGGTTATTTTACTG
CAACGCCGCAAAAAATACAAATCACGCACCATTTTGGGCTATAAAACGCTGGCCGAGGGCATCATTCGCA
TGGACGCGGTGCTCCAGAAGTCGATGGACATGATCATCGAGCTCACCGCTTCCGGGAAGAATGGCAGACC
TGGCACCGTGGTGGCCTGTCTGCGAGCAGAGCGCGTTTCTTCCATTCCAGTTGACCATGACAATAAGAAC
AACAACAGCGTCCTGCTGGCAGG
>dme_circ_0007408_junction_seq
AGAACAACAACAGCGTCCTGCTGGCAGGACTCTGTTCGCTGACAATAACACGTCTG
>dme_circ_0007409
CCACAAGAAGTCAATTGCGATGTTGCCTGCCTAGTCTACGACTCATCTAATCCCCGTTCCTTTGAGTACG
TGGCCCGCATCTACATCAAGTATTATGCGGAGAGCAAGATTCCAGTGATGATAGTCGGCACCAAGTGCGA
CATGGACGAACGCCGGCAGGACTATCTAATGCAGCCCTCTGAATTCTGTGACAAG
>dme_circ_0007409_junction_seq
AATGCAGCCCTCTGAATTCTGTGACAAGCCACAAGAAGTCAATTGCGATGTTGCCT
>dme_circ_0007410
CAACGATTCCAGGGGTCAGCTTCGAAACCCGAAAAGTACACTCAGCCTGCGAACTTTCACGCTGTTTAAC
TTAGCTAACTAATTTTTGTACAAGAGCAAGAATAAGTAGTCGCAACCAAGTATTCATATGTACATTTATA

CAATCCTGTATATTGGAACGGACAACTATTTATAATCACAACAACAACATCCGGATCATCGAAGAAGGAT
CGAGCAATTCTGAAATACCAAGTAGTGTACACCAAGAAACCCCGAATCCAAATCCCAGGCCCCCAGGAAA
GGAAGTCAACCGATTATAGACATATATATTAGGCGACTAATCGTATATTCCAAAACACTCCATTAGCGTT
TAGTTAGGACAGATATATTTATACGTATACATATAGATGTTTAACGAAATTTTCAATTAGGCGCCAGATT
AGGAACAAAATAAAGCAATAAACGACGAACCTCAGCGTTAAGCAAATAGATCAACCTACCTAGATTGAGC
ATATAAAGTTTATATAATCCAAAAAAATCAAGCCAAGCCGAGAGTAAGTCGAAAGTAGTAGAAAATCAAA
GTATTCAAATCTCTCTTGGAGTTTCCTGTTAGCAGCTAGCACCAATTAGATTGCCGCAAATACACCTGTT
TAATTAGCAGACACTTTAGATTTTGTACGTAGCTAGGAACTTAATCTGCAAATTTTTGATACATCCCCTT
CCGATTAATGCGGCCAGGTGCAGTAGTTGCAATTCCGGTTTTAGTGTAGAGTCAGTAGATTGTCGCTGTA
GCCTAGTGTATAATCAGATCAAAAAAGCCACAGTCCCGTGACATCCGACAGCCATGGACGAGTCGCAACC
GAAGACCCTATACGTGGGCAACCTGGATAGCTCAGTGTCCGAGGACCTGCTAATTGCCCTCTTCAGCACC
ATGGGGCCCGTCAAAAGCTGCAAAATCATTCGGGAACCGGGCAACGATCCATATGCCTTCATCGAATATT
CCAACTACCAGGCAGCCACAACAGCTCTGACCGCCATGAATAAACGCCTATTCCTCGAAAAGGAAATCAA
GG
>dme_circ_0007410_junction_seq
CGCCTATTCCTCGAAAAGGAAATCAAGGCAACGATTCCAGGGGTCAGCTTCGAAAC
>dme_circ_0007411
CAGCAACGATTCCAGGGGTCAGCTTCGAAACCCGAAAAGTACACTCAGCCTGCGAACTTTCACGCTGTTT
AACTTAGCTAACTAATTTTTGTACAAGAGCAAGAATAAGTAGTCGCAACCAAGTATTCATATGTACATTT
ATACAATCCTGTATATTGGAACGGACAACTATTTATAATCACAACAACAACATCCGGATCATCGAAGAAG
GATCGAGCAATTCTGAAATACCAAGTAGTGTACACCAAGAAACCCCGAATCCAAATCCCAGGCCCCCAGG
AAAGGAAGTCAACCGATTATAGACATATATATTAGGCGACTAATCGTATATTCCAAAACACTCCATTAGC
GTTTAGTTAGGACAGATATATTTATACGTATACATATAGATGTTTAACGAAATTTTCAATTAGGCGCCAG
ATTAGGAACAAAATAAAGCAATAAACGACGAACCTCAGCGTTAAGCAAATAGATCAACCTACCTAGATTG
AGCATATAAAGTTTATATAATCCAAAAAAATCAAGCCAAGCCGAGAGTAAGTCGAAAGTAGTAGAAAATC
AAAGTATTCAAATCTCTCTTGGAGTTTCCTGTTAGCAGCTAGCACCAATTAGATTGCCGCAAATACACCT
GTTTAATTAGCAGACACTTTAGATTTTGTACGTAGCTAGGAACTTAATCTGCAAATTTTTGATACATCCC
CTTCCGATTAATGCGGCCAGGTGCAGTAGTTGCAATTCCGGTTTTAGTGTAGAGTCAGTAGATTGTCGCT
GTAGCCTAGTGTATAATCAGATCAAAAAAGCCACAGTCCCGTGACATCCGACAGCCATGGACGAGTCGCA
ACCGAAGACCCTATACGTGGGCAACCTGGATAGCTCAGTGTCCGAGGACCTGCTAATTGCCCTCTTCAGC
ACCATGGGGCCCGTCAAAAGCTGCAAAATCATTCGGGAACCGGGCAACGATCCATATGCCTTCATCGAAT
ATTCCAACTACCAGGCAGCCACAACAGCTCTGACCGCCATGAATAAACGCCTATTCCTCGAAAAGGAAAT
CAAGG
>dme_circ_0007411_junction_seq
CGCCTATTCCTCGAAAAGGAAATCAAGGCAGCAACGATTCCAGGGGTCAGCTTCGA
>dme_circ_0007412
AAAATCAAAGTATTCAAATCTCTCTTGGAGTTTCCTGTTAGCAGCTAGCACCAATTAGATTGCCGCAAAT
ACACCTGTTTAATTAGCAGACACTTTAGATTTTGTACGTAGCTAGGAACTTAATCTGCAAATTTTTGATA
CATCCCCTTCCGATTAATGCGGCCAGGTGCAGTAGTTGCAATTCCGGTTTTAGTGTAGAG
>dme_circ_0007412_junction_seq
TAGTTGCAATTCCGGTTTTAGTGTAGAGAAAATCAAAGTATTCAAATCTCTCTTGG
>dme_circ_0007413
GTAAAATTTTAGCGACGCGTTCAACGTACATTTGCAGCCCCTAACAACTTTGAAAGTTTCGCAAAATTAA
TACAAACCGGTAGCGGCTAAACTTGACCGTCAAATGAAGTGTGCGAACAGAGTGCAGAGCAAG

>dme_circ_0007413_junction_seq
GAAGTGTGCGAACAGAGTGCAGAGCAAGGTAAAATTTTAGCGACGCGTTCAACGTA
>dme_circ_0007414
GCCGAATTTATCGCGCCAGATTAAACAAACAGAAACAAAAAACAGAAGCCGAGACCAGTTCCACATCGTG
CCATTTTTATCTGGGCATATAGGCAGACGTGATTACGTGATTAGTGCGGCTGGGGCTGCCGTTTATCAAT
AGCGAGAGCAACAATGGTTTCCTACTTCGTACCTC
>dme_circ_0007414_junction_seq
GCAACAATGGTTTCCTACTTCGTACCTCGCCGAATTTATCGCGCCAGATTAAACAA
>dme_circ_0007415
GAGGAGTACGAGTACAAACTGAAGGAACTGGAACAGTTCTGCAGCCCCATCATGACCAAGATGCACAAGG
GAGGTGGAGATGGCCAGCAGGCTCCAAACTTTGGACAGCAAGCTGGCGGTTATAAGGGTCCCACCGTCGA
GGAGG
>dme_circ_0007415_junction_seq
GGTTATAAGGGTCCCACCGTCGAGGAGGGAGGAGTACGAGTACAAACTGAAGGAAC
>dme_circ_0007416
GCTTGCTGTCCAAAGTTTGGAGCCTGCTGGCCATCTCCACCTCCCTTGTGCATCTTGGTCATGATGGGGC
TGCAGAACTGTTCCAGTTCCTTCAGTTTGTACTCGTACTCCTCCTTCTCGGCGGTGGT
>dme_circ_0007416_junction_seq
ACTCGTACTCCTCCTTCTCGGCGGTGGTGCTTGCTGTCCAAAGTTTGGAGCCTGCT
>dme_circ_0007417
GACAATGGCAGAACCTTTTTTTCGGGTCAATTGCCATCCCATATTCATGAAAGGAGCGAATTATGTACCA
GCCCACACACTTCCGGAGTTATCGGCCGATGCCGATGCGGGTAAGTATATATACCTAATGGCAGCAGAAA
TAAATTAGCTTATTATACGAAATCCAATTAAGTCGCACATTTGCTTAAGTCCGCAAACGAGGCCAATATG
AACATGATACGCGTTTGGGGTGGCGGCCTTTATGAGTCGGATACCTTCTACAACTTAGCGGACTTTTACG
GACTATTGGTGTGGCAGGACCTGGCGTTTTCTCAAGCAGCATACCCGCTAGCAAATGACTTTGTAGCTTC
TGTGTGCGTTGAGACAGTACAGAATGCACAGCGTCTTTCTTATCATCCAAGTTTAGCCCTAATAGTCACC
AACAATGAGATTGAGCTATTCCTGTCAAGCAACAAATCTGATTTTGGAGAAAACGCCACTAGAATGGATA
GTGATTACAAAGCCTTGTTTATTGAAACATTAATTCAAGAGTTAAACGTTATCTCTCGTAAAGATTTTAG
TCCTCGCCCTGACCCCATGGCCTCTACGCCTTCATTAGGTGTTCAGGAGTCTGGCAAAAACCTCTCCAAC
AACCCGCAAAGCTTACAGGTGATGGTGCACTTTTGGGACGACAAAGATGGATTCAGCCCTGAAACGTATC
CAAACGCTTTGTTTCGGAATTCGGTTACTCTAGCCTTCCAATGAAATCAACGTGGCAAAGGGCACTAGCT
GATTCTGCAGATAATAGCGACCTTGAAATTGCGAGACTTATTCGAAGCCGTCAGCACGACCCTAAGGGAT
TCATACCGATACTCCAATTGATTGCCTATCAGCTTCCGTTTGTTCCCCAAAATTGGGACGAAAATATCGA
TGTTTTTATATTTTAGCCAAGTGACACAAGCCATGGCAACTAAAACCGCTTTGGACGTGTTTCGTAGTCT
ACGCACAGGAAACCATACAATGGGAGCTCTTATATGGCAGTTAAACGATGTTTGGGTGGCTCCAACTTGG
TCCTGCATCGACTTCTACGGAAACCCTAAGCTATTATACTACTGGACCAAGGAACTTCTTGCCCCAACGA
GTGTTATAGCCCTATACGAAAAAACCTCAATATCACGCTTACAAGAGAGGACTTCTCCGAGCATAGAGAC
TCGCGGTTATACCATGTACTGGTGAACACCTATCTATGGACGGGCTCGATTCCTAAGAAGACTATTGCTC
GCCCTTTTGGACTGGGGCTCCAATAAACTTGAGATGCGGCAAATACCTTTGGAATATTTGCTGTACATAA
ACATTTGAAGGAAGAACTGTTTTTGGAAATCATTTTGGAGGACGAATACGGCAACTCTGTGACTAGAAAC
TTTTTTTACCCCGTACCCTTTAAAAAAATTATGGGCATTAAAGATTCAGAACTAACAGTAAGTGTGCCCC
TCTTTTTAAAACGCCTAATATTTGCTATTTTCTTACTTGTATGTAAGTCAAACAGCTGGAGGTCGAAGGT
CAGGACTGCAAAGCGGCAACATCCCCTTATGCAAATAGTTTTAGTTTGCGCATTACTGTCAGATATCCGG
CTCTACTTGTATACCTCGAGCTATTGCATCCTTATTATGTCAAGGAGCGGCACAAGTTCTCGACAAATGG

CTTTACTCAAACCCAGCCAAAAAAGACGATACACCTAGTGATCGAAAGCGACTCG
>dme_circ_0007417_junction_seq
GATACACCTAGTGATCGAAAGCGACTCGGACAATGGCAGAACCTTTTTTTCGGGTC
>dme_circ_0007418
GTGCTGGAATCGTTGCTGGTCGTTGGATCAGTCGTTTCTCCGAAATCCGTCGTGGAGTCTTCACTGACCG
TTTCCGCCGAGTCAGAGGCAACGTCAACACTGGTGGGCAATTCAGTAGTAGAAGGTTCGGTGATGTTGGG
CGGTGTAGTGG
>dme_circ_0007418_junction_seq
GGTTCGGTGATGTTGGGCGGTGTAGTGGGTGCTGGAATCGTTGCTGGTCGTTGGAT
>dme_circ_0007419
GATTAAGGATGCGGCGGTCATTGGAAAGCCAGACGAGGAGGCTGGCGAGCTGCCGCTGGCATTTGTGGTC
AAACAGGCTAATGTTCAACTGACCGAGAACGAAGTCATTCAGTTTGTCAACGACAACGCCTCGCCCGCCA
AGCGTCTAAGGGGTGGCGTGATCTTTGTTGACGAAATTCCAAAGAACCCCAGTGGCAAGATTCTGCGTCG
CATTCTGCGGGAAATGCTTAAGAAGCAAAAATCCAA
>dme_circ_0007419_junction_seq
GGGAAATGCTTAAGAAGCAAAAATCCAAGATTAAGGATGCGGCGGTCATTGGAAAG
>dme_circ_0007420
TTATGGCGAGCCGCGCACGTGGTGGTCATGGCCAACAGGTCCTTCTCCCTTGCTAGAAAGAACTGGAAAA
CCATGCGCAGTGTTTCGATACGCTCGGCAACCTGATTTAGCTTCTCCTGAGCCGCCAGAGTGTCCTGGTA
GCTTTCCTCGCTGCCATCAAAGTTTGCG
>dme_circ_0007420_junction_seq
GCTTTCCTCGCTGCCATCAAAGTTTGCGTTATGGCGAGCCGCGCACGTGGTGGTCA
>dme_circ_0007421
GGTTGATATGACCGCTTGGAATTGTCTGTGGGAAGCCAAAAAGAGGTTTGAAGCAAAAGGACGTTTTGTG
AACAAGTCTGAGAGATTCATCAACCGAATGTATATGAAAGCGGTGCGCAGAAAGATGGTCCAACCGTATC
CGGAGGA
>dme_circ_0007421_junction_seq
GAAAGATGGTCCAACCGTATCCGGAGGAGGTTGATATGACCGCTTGGAATTGTCTG
>dme_circ_0007422
GATGTGATCCAATTAATTTAGGAAGGATGCGGACTGGATGTATAAATTCGCAAGCAAATAACAGTTTCCG
GAATACGGAGTCTGATCCGGACTACTATATGTTTGGCACCCAATTGAGCACATTAGTACGACCCACGTCG
ACTCAAGAGCCTGACGATCAGGTAAACTGTCCAGAGACAGAGATGAATGAGAGCTGGGTAAG
>dme_circ_0007422_junction_seq
AGACAGAGATGAATGAGAGCTGGGTAAGGATGTGATCCAATTAATTTAGGAAGGAT
>dme_circ_0007423
GTTCAGCAGTGCCGGCAACGGATCCTCCAACATCGTGGCAATGGGAACTGGCGACTTTTTGGATGCCGGA
GAACTCCAACTTACTGCTACTGCGGGAATGAGTGCGGGAGCCGGAACCAGTACCACGGGCAGTGAGACTG
GTATGGAGGAGTATGGCGAA
>dme_circ_0007423_junction_seq
TGAGACTGGTATGGAGGAGTATGGCGAAGTTCAGCAGTGCCGGCAACGGATCCTCC
>dme_circ_0007424
TGGCCATTTTGCGCTGTTTCAATGGCAACGAACTGGCGCCGCTTTTGGAATTGTTATTATTGTTGCCAGT
TCCTTGGTCGCTATCAGGCTCGTCCAAGAAGAACTCGTCATCATCTGCATCATCTTCTTCTTCTTCGCCT
TCTCCACCACCTTCGTCTTCGTCGATGTAG

>dme_circ_0007424_junction_seq
TCCACCACCTTCGTCTTCGTCGATGTAGTGGCCATTTTGCGCTGTTTCAATGGCAA
>dme_circ_0007425
GGGCGTTGGAGAGCCTCTTACCGAACAGCAACAGCAGCAGTTACACGCTGAGCAGGCGCATCTCTTTCAG
CATCGAACTGGCGGTCAGCGTCCGCCCACCCAGAGTGAGTTAGAGCAGGTGGCTCAAGAGCTATTGCTTC
AGCGAAGCGGCCAG
>dme_circ_0007425_junction_seq
AGAGCTATTGCTTCAGCGAAGCGGCCAGGGGCGTTGGAGAGCCTCTTACCGAACAG
>dme_circ_0007426
GTGGTGGTGCGGATGATGTTGCACACTGGTGGCCGAACTGGTGGCTGTGACAGGCAGTGGGGCTCCACTC
GCTCCCACCGCTGTGGTCTGCTGGGCGCTACTTTTGCGGACTACTTCCTTCCAGCCATCGACTTCGGTGC
GCTTTGCAGGATTCAAAG
>dme_circ_0007426_junction_seq
ACTTCGGTGCGCTTTGCAGGATTCAAAGGTGGTGGTGCGGATGATGTTGCACACTG
>dme_circ_0007427
GGCAGCGGCTATTGGACAGTCCTCGCCCAAAAAGGCTGAGGGTGGTGCTACATCTGCGGTGGTCACCTCT
GCCGGTGGACGCAGCAGTGGCGTGGTGGCTCCATTTGGACGTGGCAAGCCTGTAGCTGGCCAAGGAGGAC
CTGCAGCAACGGCGGCTTCCAAC
>dme_circ_0007427_junction_seq
AGGACCTGCAGCAACGGCGGCTTCCAACGGCAGCGGCTATTGGACAGTCCTCGCCC
>dme_circ_0007428
TGCCGCCATACCAACGCTGTGCAGCTGCTCCTCCTGGCTGTCTGGCACCCGTTCCAGGTGGTTGTCTGCC
TGGATTTCCTCCAAACACATTCGACTGCGTTCCACCCTGCGGCGGTGGCTGCGGTGGCTTGGTG
>dme_circ_0007428_junction_seq
CTGCGGCGGTGGCTGCGGTGGCTTGGTGTGCCGCCATACCAACGCTGTGCAGCTGC
>dme_circ_0007429
AGTGCCGCCATACCAACGCTGTGCAGCTGCTCCTCCTGGCTGTCTGGCACCCGTTCCAGGTGGTTGTCTG
CCTGGATTTCCTCCAAACACATTCGACTGCGTTCCACCCTGCGGCGGTGGCTGCGGTGGCTTGG
>dme_circ_0007429_junction_seq
CCCTGCGGCGGTGGCTGCGGTGGCTTGGAGTGCCGCCATACCAACGCTGTGCAGCT
>dme_circ_0007430
TACTTCGGTTCGTGGAAAGCTTAACTTTATATCCGTGGGATCTAAGTTCAAGACTCAGCTGGGCGAGCTA
ATGGAGAAACTGGAGCAGAACGTAGGCAACGCAATGGACTTTTAACCAAAATCGAAATTTAATTAATGTT
ACCTTTCAGGGCACCAACTTCATACGCTGCATCAAGCCCAACAGCAAGATGATCGACCGACAGTTTGAGG
GCAGCCTGGCGCTGGCCCAACTGAAGTGTTCGGGCACAATTTCGGTGCTGGAACTCATGGAGCACGGTTA
TCCATCGCGTGTTCTCTTTGCCGATCTCTACAGCATGTACAAATCGGTGCTGCCGCCGGAACTTGTTTCA
CTGCCGGCGCGCACCTTCTGCGAAGCCATGTTCCAGTCCCTCAATCTGAGCGCCAAGGACTTCAAGTTCG
GTATCACCAAGGTCTTCTTCCGGCCTGG
>dme_circ_0007430_junction_seq
GTATCACCAAGGTCTTCTTCCGGCCTGGTACTTCGGTTCGTGGAAAGCTTAACTTT
>dme_circ_0007431
GCTACGTTTACGATCGGAGCAAGTATCTCAATAGTGCGGTGACCGAGTGGAACCTGGTCTGCAGTCGAAG
CCTGCTAAGTGCCACCAGTGATTCGTTATTCATGCTGGGCGTGCTCCTGGGCAGCTTAATCTTTGGCCAG
ATGTCCGACAAGCTGGGACGCAAGCCCACCTTCTTCGCCTCACTGGTGCTTCAGCTAATCTTCGGCGTTT

TGGCTGCCGTGGCACCCGAATACTTTAGCTACACGATTTCCCGTATGATTGTGGGCGCCACCACTTCGGG
AGTCTTCCTG
>dme_circ_0007431_junction_seq
GGGCGCCACCACTTCGGGAGTCTTCCTGGCTACGTTTACGATCGGAGCAAGTATCT
>dme_circ_0007432
GATGTCGCCTCAGTGGCGGTGCCGACGAAGGAGGCGTACTTTAATGGCTCCACTTACCTCCGCCTCACCA
CGCCGATGCCCATTTGGGATCACTCGGCGATTAGTTTCCGCTCGTGCCGCGGCGGCGAGATCCTCGCCCA
GCAGTACAACAAGAACTCCATTGTAATCTCAGTGCTCAATGACTTTCTGCAAATCTCACTGGCTGGACCC
GCCGTCCATGGGCCCAACAACCGGCTGGATGTCAAGCTGCCCTACCAACTGCTGGACAACCGCTGGCATA
CGCTGCAGTTCAAGTACGAGTACGGAAATCTCTACCTGCATGTGGATCGCGCGGCAAGCATATTTGGGTA
AGTAACCATGCCGTTCTGGCTGCCCCAAATTGGATTTCAGGCTCTATCGCCCACTCATTCGGGTCAGTCG
TGGACAATCGTGCCATGCAGCAAAGCACATGGAAGTGAATCACATCACGAGGGTTAAGTAATGCATGTTA
AGTAATTGTGCTGGCTAACTGAATAGAGGTAGAAAATGGGTAGAGATGAACTCATTCGAAAAATCAATTA
AACGATTGTATAGAATACGTGCGAGAAATGATTAAATAGAGGAAACCAACCAAGAAAATAATAATTACTA
GAAATGAATATTAACGAAAACAATTTGCTAATCTTAATAATGAATTTATCTTTCTTCTCCAGGCCAACTC
CACGTACAACAGTCAGTTCCTGACTAACCAGGACATTGGCTACAAGGACGCCATCTTGATACTGGGAAAC
TCCTTCTCCGGTTGCCTTCTGGATGGACCAGGTCTGCAATTTGTGAACAACTCGACGGTGCAGAATGTGG
TCTTTGGTCACTGTCCCTTGACCCCGGGTCCCTGCAGCGATCACGATCTTTTCACTCGACTGCCAGACAA
CTTTTGTCTGAATGATCCCTGTATGGGCCATGGAACCTGCTCGTCCAGTCCAGAGGGATACGAATGTCGG
TGCACGGCACGTTACTCGGGAAAAAATTGCCAGAAGGACAATGGCTCGCCGTGTGCCAAAAATCCATGTG
AGAACGGTGGTTCCTGCCTGGAGAATTCCCGAGGGGATTACCAGTGCTTCTGTGATCCCAACCACAGTGG
TCAGCACTGCGAGACGGAGGTGAACATCCACCCACTCTGCCAGACGAACCCCTGCCTGAACAATGGAGCA
TGTGTGGTGATTGGCGGCAGTGGAGCACTCACCTGCGAGTGTCCCAAAGGATACGCTGGTGCCAGGTGCG
AGGTGGACACGGATGAGTGCGCCTCACAGCCGTGTCAGAACAACGGAAGCTGCATAGACCGTATCAATGG
ATTCAGCTGCGATTGCAGCGGCACCGGTTACACGGGTGCGTTCTGCCAGACGAATGTGGATGAGTGCGAC
AAGAATCCGTGCCTGAACGGCGGCAGATGCTTCGATACCTACGGATGGTACACCTGCCAGTGTCTGGATG
GTTGGGGCGGCGAGATTTGTGATCGACCCATGACATGCCAGACGCAGCAGTGTTTGAACGGTGGCACCTG
CCTGGACAAGCCCATCGGCTTCCAGTGCCTCTGTCCGCCGGAGTACACTGGTGAACTGTGCCAAATCGCA
CCCAGTTGCGCACAGCAGTGCCCCATCGATTCGGAGTGCGTAGGCGGCAAATGCGTTTGTAAGCCAGGCT
CATCGG
>dme_circ_0007432_junction_seq
AAATGCGTTTGTAAGCCAGGCTCATCGGGATGTCGCCTCAGTGGCGGTGCCGACGA
>dme_circ_0007433
ttgcagTAGGCTCCGCAATAGGGTTTCTGGCAGATGCACTGGAAGGATCCGGGCTTGTTGAAACACCGGC
CCAATCCGCCGCAGTAATCTCCCTCCATGTTGCACTCGTCGATGTCATTTTCACAGCGAATGCCCTCGAA
CCCGGTGCCCTGGCAATCGCACTCGTACCTGCCGACGAGGTCCTTACACTGACCACCGTTCTGGCATGGA
TTCGATTCGCACTCGTTAATG
>dme_circ_0007433_junction_seq
GCATGGATTCGATTCGCACTCGTTAATGttgcagTAGGCTCCGCAATAGGGTTTCT
>dme_circ_0007434
CTGGGCTGCACCTTCTATCATCCCAAGCCCTGCCGCTTCGGCAAGAACTGCGTAAACAAGCTGGAGTGCA
TCTTCTACCATCCTGAGATGCAGAGCAAATTCAAGTGGGTGGCATCTTTGGGCTAATG
>dme_circ_0007434_junction_seq
TCAAGTGGGTGGCATCTTTGGGCTAATGCTGGGCTGCACCTTCTATCATCCCAAGC

>dme_circ_0007435
ATCGTTCGCAGCGCAACCAAAAGGAGCTCTATGTGCCGACCCATCGGCGACGGAGCGAACCCGAGGCCAC
CAAAGAGCGCAGTCAAAGAGAGCGCACCCGAGAACGACGCTCCAGCCGCGATACCTCTAGGGACACTAAC
CGTAATCAGCGCCAGCG
>dme_circ_0007435_junction_seq
GGGACACTAACCGTAATCAGCGCCAGCGATCGTTCGCAGCGCAACCAAAAGGAGCT
>dme_circ_0007436
GCTTACACAGATGCCGTGGACTCGGTGCAGTGGTCGCATCGTGGACTGAGATTTATATCGGGGAGCAAGG
ACGGCACAGCACACATCTGGACGTTTGAGTCGCAGCAGTGGAAAAGTTCCAAACTATGCATGACGGAGC
>dme_circ_0007436_junction_seq
AAAAGTTCCAAACTATGCATGACGGAGCGCTTACACAGATGCCGTGGACTCGGTGC
>dme_circ_0007437
TTCTATCTGGCACCCAATGTCCGCTACGATGTGGGTCAGTCGTTGAAGTGCCGCGTCCTAGAAGTTAATG
CCGAGCGAAAAATCTGCTACGTGAGCAATCGTGCCGAGTATTTGGGCAAGGGAATTAAAATTCTGACTGA
TTATGCGTCCGCTCACGTTGGAAACGTCTATATGGGCACAGTAGTCCGATGTGAGGACACTTACGTCCTA
GTGAAGTTCGGCAACGGCATCAAAGGAGTTTTGCACAGGCAGAATCTAAAGGAAAATAGCTCATTTTTCG
AGGGACAAACAACTAAGTTCCGCATCCTAACACGCAACAAGGACCAAATCACTTTGACTTTGCCGGAGGA
CAAG
>dme_circ_0007437_junction_seq
AATCACTTTGACTTTGCCGGAGGACAAGTTCTATCTGGCACCCAATGTCCGCTACG
>dme_circ_0007438
GATCGGCAAGGTCAACGGAATACTGGAACAGTTCTATCTGGCACCCAATGTCCGCTACGATGTGGGTCAG
TCGTTGAAGTGCCGCGTCCTAGAAGTTAATGCCGAGCGAAAAATCTGCTACGTGAGCAATCGTGCCGAGT
ATTTGGGCAAGGGAATTAAAATTCTGACTGATTATGCGTCCGCTCACGTTGGAAACGTCTATATGGGCAC
AGTAGTCCGATGTGAGGACACTTACGTCCTAGTGAAG
>dme_circ_0007438_junction_seq
ATGTGAGGACACTTACGTCCTAGTGAAGGATCGGCAAGGTCAACGGAATACTGGAA
>dme_circ_0007439
GGATGCCGGGCAAGGTGGGGGTGAAGATTAAGGCGGCGCGCAACCTGCCCGTGATGGACAAAAGCAGCGA
AACCACGGACGCCTTCGTGGAGATCAAACTGGCCTCGGTGACCCACAAGACGGATGTCTTCCGCAAGAGT
TTGAATCCCACCTGGAATACCGATTGGGTTCCGTTTTGAGGTGGACGACGCTGAGCTCCAGGATGAGCCC
CTGCAAATCCGCCTGATGGACTACGACACGTACTCTGCGAACGATGCGATTGGAAAGGTCAACATCAGTT
TGAATCCTCTGTGCCTGGAGAGCTCTAGTCAAGCGGTGCACGGCAAGGGTACCGTGTTATCCGGCTGGAT
TCCTGTTTTCGACACTATGCACGGTATTCGCGGCGAGATCAATGTGATTGTGAAGGTTGACCTGTTCTCG
GACGTCAACAAGTTCCGTCAGAGCTCCTGCGGCATCCCCTTCTTTCACTGCTCAGTGTGTGCCATTTGGC
TACCGCGCCCAAGTGATTCATGGCTTTGTCGAGGAATTGGTGGTGAATGACGATCCGGAGTATCAGTGGA
TCGACAAAATACGCACACCGCGTGCTTCCAACGAGGCTCGCCAGGTGGTGTTTCTCAA
>dme_circ_0007439_junction_seq
ACGAGGCTCGCCAGGTGGTGTTTCTCAAGGATGCCGGGCAAGGTGGGGGTGAAGAT
>dme_circ_0007440
GCTACCATGCCGATGACTGATATAAAAACTTGCGGGGCATATAGCGCATGTTTTACGCGAATCGTCCCAA
AAGCGTATACTCTCTATTTTCTTCCGCTTAGCTAGAACTATCAGCTCTGCCCAGCAACTGGAGTTAAACT
GAAGTTAAACTAATTCACGTTTACTTAACAATTTTACAGCATCTGTAGATGGATTCTTTATGCAATATTT
GAACTGTGAGGAATCGTTCTAATCCTAACTTAGTAGTGCTAATTTTTTCGGTCTTGTTACTTGTTTGCTG

GATGTTTTTAGCGTTAAATCAATTGCAATTTCTGTACATAGTTGAT
>dme_circ_0007440_junction_seq
TCAATTGCAATTTCTGTACATAGTTGATGCTACCATGCCGATGACTGATATAAAAA
>dme_circ_0007441
TAACAAGACCGAAAAAATTAGCACTACTAAGTTAGGATTAGAACGATTCCTCACAGTTCAAATATTGCAT
AAAGAATCCATCTACAGATGCTGTAAAATTGTTAAGTAAACGTGAATTAGTTTAACTTCAGTTTAACTCC
AGTTGCTGGGCAGAGCTGATAGTTCTAGCTAAGCGGAAGAAAATAGAGAGTATACGCTTTTGGGACGATT
CGCGTAAAACATGCGCTATATGCCCCGCAAGTTTTTATATCAGTCATCGGCATGG
>dme_circ_0007441_junction_seq
CAAGTTTTTATATCAGTCATCGGCATGGTAACAAGACCGAAAAAATTAGCACTACT
>dme_circ_0007442
CGACAACAACATTCAAGACTGCTAACTGGAATTGCTTCGAACCATCACCATTAAAAAACGAAGAAACAAC
CACAACAAAGTGCTGCCAGCAACAAAAACGTAATCAGGAATTTAAGCAAAACCAAAAAGAAACATCAGGG
ACAGGCAGGTAGACCCAGGGAGCATGTTTATGGAATTGTCATCGTTAGCGAGCAACTGACGCAACAGCTC
AAGGCATGTGgcaacagcagcagcaacagcagcagcagcaaCACTGAGCGGCAGGAGCAGAAACACCTGG
CAAGAGAAGCAACTAGGAGCAGGACAACCTCCGCGGACAGGTGGTGTGCTGCTATAAAGCAGCACCATAG
CTGCCATTAAAATAGACgccgcggcgcaacagcaacgctgccgtcgtcataatcaacgccagcgacaaca
ccacccgcatcatcgccatcgcagacgacgacggcgcagcagcaacatcagcagcaacatcagcagcaac
agcaactgcaacatcagcaactgcagcagcagcGGGCTGTTGGGCGCAGTGGGCGTGGCCGGCAGCTGCC
CATTGCTCGCCACACAGCAACTAATCGAGGAGCCTGGCAGCAGCAACAACACAGGCGACGACGACGGCGG
CGCAACAACGCAGCAGCAACACTGCAGCCTGCAACATCAGCAATCCGGTCGACGGCGACACTACGACGGC
CTGTCATCTGTAGGCAATGCAACGCGCTGTGCAGCAACGG
>dme_circ_0007442_junction_seq
GGCAATGCAACGCGCTGTGCAGCAACGGCGACAACAACATTCAAGACTGCTAACTG
>dme_circ_0007443
GCTGCGGGCACAAGCGACGGTGCTAAAGAAGGCGGTGCTGGAGGAGCAGTCCAAGGAGGCCAGTCTGCGG
GAGCAACTGCAGCAGTACGCCACCTCGTTGCGCCGTACGGAGCAGGAGGTGGACTCGCTGGGCTTTCGCA
ACAAGCAGCTGGAGTCGCGAGTTTCGCAGCTGCAGCAGGAAATCTCGGTGCACGAGCAGCCCAAAAAGAA
GGACAAGGATTCTGGCGGCAGGCGGGGCGTGCAGAGCAATAGCCGCCCAGATGCCGATCCATCATTGGAT
GCCGCTCAGGAAGCCCTCATCTTCGAGGAACTGCAGAAGAAAATCATGGAGAATGCGCAGCTGACGTCAT
TG
>dme_circ_0007443_junction_seq
CATGGAGAATGCGCAGCTGACGTCATTGGCTGCGGGCACAAGCGACGGTGCTAAAG
>dme_circ_0007444
ATTTGCATGCATGCCTCCAAGGTGCTGCACGATCGCATGTTTGCCTGCATCCTGCACGCCACCATGAGAT
TCTTTGACACCACTCCGTCTGGAAGGATTCTGAATCGCTTCTCCAAGGATATGGGCGCCATTGATGAGTT
GCTGCCGCGGGCCATGATGGATTTTATACAGATCGCTCTGGTGATGTTTGGAATTCTGATTG
>dme_circ_0007444_junction_seq
GCTCTGGTGATGTTTGGAATTCTGATTGATTTGCATGCATGCCTCCAAGGTGCTGC
>dme_circ_0007445
GATAAAGCAAATGAGGCCATTGATGATCGTGGGTCCTAGGAAACAGCGGAAAAGTGCGTTTCTGAGATGC
GGCTTCTGTTTCTTGCGGTGAGATCGCTCTAGTTCCTTAAACCATTGACTAAAAATTGATCACAATTGAC
AAATGTTTAATACGACTTTGTAGCAAGTCAAACTTCCTTAGTGTTTGTTGTCAGTTAAAGAGGATGTTAG
ATCTCAGTACTTACTCCTCCAAACGGTCGCCCAACTGCTCGGATCGATCTTCCTTCAGACATTGTGTCAG

GTCATCCGTATTAAGGCCTCTGCGGGAACCTCGATATA
>dme_circ_0007445_junction_seq
TTAAGGCCTCTGCGGGAACCTCGATATAGATAAAGCAAATGAGGCCATTGATGATC
>dme_circ_0007446
GCAAATGAGGCCATTGATGATCGTGGGTCCTAGGAAACAGCGGAAAAGTGCGTTTCTGAGATGCGGCTTC
TGTTTCTTGCGGTGAGATCGCTCTAGTTCCTTAAACCATTGACTAAAAATTGATCACAATTGACAAATGT
TTAATACGACTTTGTAGCAAGTCAAACTTCCTTAGTGTTTGTTGTCAGTTAAAGAGGATGTTAGATCTCA
GTACTTACTCCTCCAAACGGTCGCCCAACTGCTCGGATCGATCTTCCTTCAGACATTGTGTCAGGTCATC
CGTATTAAGGCCTCTGCGGGAACCTCGATATAGTAATG
>dme_circ_0007446_junction_seq
CCTCTGCGGGAACCTCGATATAGTAATGGCAAATGAGGCCATTGATGATCGTGGGT
>dme_circ_0007447
GGTCTACAACGTGGTCGACGAGTCGTGGTCAAAACGAAAGCAACGTCATTGTAAGAGAGAGAACAAATCG
GCGAAAAAAACCCTATAAACAAAACACAATTTCGTGGGTGTCTCGCATCGTGTGAGAACGAAAAGCTGGCG
CTACAAATAATAAACTAAATTAATTAAACTAAGTAACTGCGAGAGAGCAACTAAAACAGAATGAGTGCCA
ACCGAAGAAGACGCAGTGCATCAGCTGCGAGTTCAATTgcagcagaaacagcaacagcagctacagcagc
aacagcagaagcgatatcaactgcaCCCTCCAAAGAGCAACTTTCTCCACCGATTGCTGGTGGCAGTGCT
TCAGCTGATGAGGATTTACTGGAAATGCCTCGCATTTGCCGATGCTGTTGCAAACGGGATTTGAAATTAC
TCGGCTTATTCGAGGCCAGCCAACCAACACTGGCCAACACAACAGTGgcatcagcatcagcagcagcatc
agTGGCATCGAACAAATCATCGGGAACATTAGCAAAAACATGTGCGACAGAAGCAGAAACCGAAACTCCA
CAGACATTGTCCGCAAATCCAGTAAATCCCGGGACTAAAGCTACATCATCTCCATCAGAAGCAGCTACAA
CGTTGTCCACATCCACACGCCCCGCGTCCGCCTCATTCTCATCCACCTCCTTTTCCACATCCACGTCTCC
CATGAGGCGTCGCACCACTAACACCACCGCTCCATCCGCTTCGCCCGCTCCACGTGGTCGTAGCAGCGAT
AATGCCGTCGACTACACCCTCAGCGGGGCCCACAGCAGCATGGACATTGTCCTCGAGGAGATGACCATTT
GGATGCTCAAT
>dme_circ_0007447_junction_seq
CGAGGAGATGACCATTTGGATGCTCAATGGTCTACAACGTGGTCGACGAGTCGTGG
>dme_circ_0007448
GGCAAAAATAGCCGGTAAAACAGAGACAGCAAACGGAATAGCAGACAAGGATTCCAAACACCATAATGGG
CGGATCGGCAGTGCAGGTGATCAACGCCTCCGAGGAGCACACATTTGTGCTCAACGAGGATGCGCTGAGT
GAGGTCCTAATGCGGGATGAG
>dme_circ_0007448_junction_seq
GCTGAGTGAGGTCCTAATGCGGGATGAGGGCAAAAATAGCCGGTAAAACAGAGACA
>dme_circ_0007449
GGAAAGCTCCAGTCCCGGACGAGGAACTGCAGACGCTGGAACGGCTTTTTGCCGGTGTCGGCCAGCGCGA
GGCGACCATACTCAGTGAAGAGCTGCAGGTGCTGCAGGTCGTCCTCCTGGATGTTCTGTGACAGGTTGTA
TATCTGCACCGAGGACAGCATTGTGCTCAACGCAAAAACGGTGGCACAATCGCGCACCGTGCTCTGGCTG
>dme_circ_0007449_junction_seq
GGCACAATCGCGCACCGTGCTCTGGCTGGGAAAGCTCCAGTCCCGGACGAGGAACT
>dme_circ_0007450
GCTTTTCCTGTTCACTAGCATCCGGATGACGGGTGGCGAACAGAGCCATCAACTTGAAAAGCTCGTCCAC
GGCGGCCGCGTACTGCGTGGGATGCGGTGTGATGTTTTTAAATGCCCACTGAAGATTCTGATGCTGGGAC
AATTGGCGTGTGAGTGCACGGCTCTGCTCGCAGCACAGACGAA
>dme_circ_0007450_junction_seq

GCACGGCTCTGCTCGCAGCACAGACGAAGCTTTTCCTGTTCACTAGCATCCGGATG
>dme_circ_0007451
GATCCCGAACCGGCGGTACGGCGTGAGATATGCGCTGGTCTTTATCGCATTTGCTTAGGAAACGCACATA
GCTATCGCCTCTTACTAGCACCACTGCTGCACAAGCTCATCGCCCTGTTGCCATTGGCCGAGCAGATGAG
CTCTGGCAACCAGCACACGCAGTTTTTGCTCTCGGAAGAGGGCAAGGATCCCTACGGACCCGCCTGCCGT
GACTACTTTTGGCTGCTGGCCAGATTGGTGGATACTCTCTCCCCGGAAATGGTGGCCGAGGAGCACATCG
ACATTGAAATGCTGTGCGAAAGCATCTCGCAGAGCATTCTCACCCGAGAGTATTATGAACTGCGCCATGG
TTACCAGGACGATGGCTTGGTGGGTCTCCTAAATCTCATGTCCAATTTGATCAAGTACGACACCACATTC
AAGTACACACCCAAGGCG
>dme_circ_0007451_junction_seq
CACCACATTCAAGTACACACCCAAGGCGGATCCCGAACCGGCGGTACGGCGTGAGA
>dme_circ_0007452
GGTTTTCCATACACTGTGAAACGAACTCCTTCTCCAGGCGCAATTGTTGACCCATGTAGTACATGTTGAT
GTACTGTATGGCCGCCAAGGAGACATCGTTGCTCTGGGCACGCAACGCTATCTTCCAAAGATGGTACATG
CCAACGGCACTGGCGCTCGCCGAGATCTGGTCGCTATGATTGTCGTACTCGGCCATGGCGATGCGGGCGA
AGCTACAGAGCT
>dme_circ_0007452_junction_seq
ATGGCGATGCGGGCGAAGCTACAGAGCTGGTTTTCCATACACTGTGAAACGAACTC
>dme_circ_0007453
GTGTGTTTCCGGGCTGGCAGACATCATTTAGCTTGAACGAGGGCTGCACCGGTGTACTCCCGGACGTAGA
GCTGCCCTGGCTCGCCTTTGGTGAATGGTGCTGCTTTCCGCCGGAAACCACATCCATCAGATCGACATCT
GAGTCCGATTTTGCCTTGGCCGCAGCAACCACGGCGGCAGCTGCAGCAACGCTGGCCTGCCGGTTCATTG
CCACCAGCGTTTGCTGTGCAGTTCCGGTAGCCGCTGCAGCAGCGGCCACAGCAGCGGCAGCAGCAGCCAC
ATTCGACTCTCCAGACTTCTGAAGGGCCATCGCCGACTGGGCCACCATCATGGCGGCGGCAGCGGCTGCA
AAGGCTGGTGGAACGTCCGAACAATTGGCGCGGGAGCTGACCAGCTGGGCCAGCTCCTCCTCGCAAGGCG
ACACGTCCTCGTCATCGAAGTCAGCCAGGTTCTTTTCAGATTTGTTACTCATCGGGGATGAGTAACTGCC
ATCGTTCTCGGCACCACTGAGCAGCTCATTGATATTGGTCTCGATGGCCCTGATAAAAG
>dme_circ_0007453_junction_seq
ATATTGGTCTCGATGGCCCTGATAAAAGGTGTGTTTCCGGGCTGGCAGACATCATT
>dme_circ_0007454
GATTTGTTACTCATCGGGGATGAGTAACTGCCATCGTTCTCGGCACCACTGAGCAGCTCATTGATATTGG
TCTCGATGGCCCTGATAAAAGGTCCCTCTCCCTGCAGATGCTTTAGCACGGCGTCGGGTACCAGCTGACT
CGTAGCACCATCGCTGCACTCATCGCTGTCCGCTAAAAGTTCGCCCTCGCCATCACCATCGGCTTCGCCA
TCAGCCTCGTCACTGCTGGGCACATCTTCAACTCCGTCCCAAATCTCGACCAGCTCATGGGGCAGAATGA
TCTGTTTGTTGGTTTTTTGTCGCCGTCGCTTGTTGCTCGTCTTTTTGCGGCTGCGATGCAGTTGCAGCTC
CAGTGCGGCCCGCGATTGCAGCTCCTCTTCACTGCTTGTGTTGTCGATAAT
>dme_circ_0007454_junction_seq
CCTCTTCACTGCTTGTGTTGTCGATAATGATTTGTTACTCATCGGGGATGAGTAAC
>dme_circ_0007455
GGCGGAACGGAATCTGGCCAAAAAGAAGCAGAAGACGGGAGGAGCCACAATTGGCTACTCCTACGAAGAT
ACCGGCGATGGAGCGACTATTGGACCTCAGCCTTTTGGAGCCGCCAGTTCAACTGTCATTG
>dme_circ_0007455_junction_seq
TTTGGAGCCGCCAGTTCAACTGTCATTGGGCGGAACGGAATCTGGCCAAAAAGAAG
>dme_circ_0007456

GGAGAAGATGCTGCTGAGCGGGCGCAAGTCAAGACGCGAGCGTCGGGCGCAAAAGGAGAGAAGGATCGCC
AATAGACCATTCAGTCCGCCAAGCTATGCTGCCAAGGAGGATAAGGACAAAAAAGGTGGTGGTAAGGGCG
GTGAGAAAGAAAAGGAGGAGGACAGCGAATCGCGTTCGCCGTCGCCCGCGGAAGCAG
>dme_circ_0007456_junction_seq
TCGCGTTCGCCGTCGCCCGCGGAAGCAGGGAGAAGATGCTGCTGAGCGGGCGCAAG
>dme_circ_0007457
GGGCTCCGGACATCCGAGTTCTGGCGGTGGCGGACGGCCCAATCTGCGCGAGCGGCTTAAGAGAAAGATG
CAGAACCTTTTGAACCGGCAATGTAAGCCAACTGGAGTGTAGTGTGATGTTCACCAAGCGCCTTCTGAAA
TCCCTTGAATTAGGTAACTAAAACGGAAATATCTTGCAGATAAAGCCGATAAGCGTGCGGAAATCGAAAA
GACCGAGCGTGAACGACTGCAACAGCAGGAACGCGAGGATGAGATGCGCGAACTGGCCCTCAAGCTGCGC
AGAAG
>dme_circ_0007457_junction_seq
GCGAACTGGCCCTCAAGCTGCGCAGAAGGGGCTCCGGACATCCGAGTTCTGGCGGT
>dme_circ_0007458
GGTCGTTCTCGTATTTATACTCAGCATTGCATCCCTCATTATATACTTTGTTGATGCATCTAGCGAAGAA
GTCGAAAGATGCCAAAAGTGGAGTAACAACATTACTCAACAGATCGATCTCGCATTCAATATATTTTTA
TGGTTTACTTTTTTATACGAGGTAAGCGAAACAATCCCTTTTAAATTAACCTATCACAATGAAAATTCCA
AAAATGTTATATGCATACAAATCGGTGTTGCTCAATCAATTGGAAATCAATCAGATTCCCAAATGTATTT
AGATATCCTTTACACACATACACTGTCACACATAACAGCCACACATGAAGACCCaaaaaaaaaaCAGTGT
CCTGATCGATTTGTATGCATGCAATGTGCCTTACTCGACATGAAAACATACGCCTGTATGCAATTGTGTA
TATACCTTGTGTATTATCGTGAACAGGGTCTTCGATTTCTTCGAGCGCTACGTCTCATGACTGTTCCAGA
TATTTTACAATATTTAAACGTACTAAAAACATCGAGCTCCATACGCTTGGCTCAACTAGTATCAATTTTT
ATATCCGTGTGGTTAACAGCAGCGGGCATTATACATCTGGGTATGTAGAAacacacacacacacacaaac
actcgacacacatacacTGACGCGCGATCGATTGTGCCTGTGTGTGGATGACTCTGTGATGGGTTTGCAG
TGTGTGTGAGAAGCCAGTCCCGAATATTACGCATACGCCACGTTGCAGCCTACTACAGAGCCCCAAAGCC
AGGCACCTTCGATACGCCACAAGATTTACGAACATATATGTATCTGTTTCGATGTGTTTATGAAGTGACC
AACCGAAAAGGACTTTAGATGACCTGATCCGCTGATGCGGCTGATAATTGCATTTGCATTTACTTAAAAT
TTAGGCTGGAGAACTCTGGCGATCCGCTGGATTTTAATAATGCTCATCGTTTATCGTATTGGACCTGTGT
CTATTTCCTAATTGTGACCATGTCAACGGTAGGATATGGTGACGTTTACTGTGAGACTGTCCTGGGAAGA
ACATTTCTCGTGTTCTTTCTGCTCGTCGGCTTG
>dme_circ_0007458_junction_seq
TCTCGTGTTCTTTCTGCTCGTCGGCTTGGGTCGTTCTCGTATTTATACTCAGCATT
>dme_circ_0007459
GGCAATGTTTGCCAGCAGTATACCGGAGATAATTGAACTCGTCGGTAGTGGCAATAAGTATGGCGGTGAA
CTGAAAAGAGAACACGGAAAGAGGTAGTCTATAAAAACTATAATTAAATCCTATaaaaaaaaaaaCATAA
TTATCGCATTCGATGTGAAAAACACAAAGCCACATTCTAAAGATAATCCACAAAAAGTTATAAATCGCAA
TAAATCTAAAAGTATAACATAAAAAGATATATATAAACTTTACCAAGACAGCAAAATGCTTTTAGTTAGG
AACTACCATCTGATGAGGACTATTATCTTAAGATATCaaaaaaaaaaaaaaaaaaaaaaaTTCTAACAGT
TAGGATCGGTTGAAGTACAAAAGAAAACTTTTGTAAAAAACACACATTCTTAGTATTTTGAGCCCATCTA
GGGTGAAGTAAAACTCCAATTAGATAAATCACTAAGTCTTAATATTGATCAAAGTCGATAAATTAAATAG
TCTGTTACCCTTTCACTTTGTTTCCCTAATTTCATTGTATCTCTAAGATCGCATATACAGTTGACTTACC
CGCATCAAAATGTAGTTCAGCCGACTCTTAAGTCGTCCGTTTTAAACTATTTTGTTCCTTGATAAATATG
TATTTCGAAAACCGAAACAGATATGACATTTGATAAATCATCAAACAAGTAATAAAAGTAAAGTACAATT
TTATCATTCGATTCTTACTTTGTTTCGTCTTTATTTATTATAATTTTTAAACATTTTTGTTGCCTCGCAG

TCAGAGACATTTCAGCAGATTCGAAAATACATCGTTCTATTTGTACAAGCCATTAAACATTTTAATGTTC
CATTTACTTAGGTTTGGTTTTCCAATTTTATTTGTTTATTTTCCTTGGTACTTGCGTTCCTCCTAATTTG
TTTCGTTTCGTTAAACATTTTTATTGAACTCTTTGGCAATGTCTATCATATATAAATCACTTTGTAGGCA
AGCGTGAGTTCATTTTGGCCATTTTACTTTGGATTTTGTCTGAAGAGTAAACATGTTTTATTCAAGATAT
TATCGTTTGACACTTATACTTTGCCATGCTTTAAACGTTTCGTATTAACACCAGAGTTATGATACAAGAT
ATGCGTTTATCAATTTGCAATAGTTCAAGGTTCATATATATATTTATTTCGATATCCCAAAGCATTTGTG
CAATCTACTTAGGCGCATTGTTGTACCACATCCCACACAGATTTATAGAGCTCCCACTTATAAACATTTA
ATCTACAACACTAGTCCCCATAAAAAAGCATCGGTCACGCATTGAATAAAGTAAAACTAAACTATCCAAA
GTCATGACACGTAATTCAAATCGTATTTTCTTGAAGAGAGCAAACTCCAGCCACTTTGCAAGATTTGTAT
TATTCAATTCATATTTTCCCACCTTATTCCTACTTAATATGTTACTTCATTTGTCAGTCTCGCTGGTCCA
TTTACTTAAAGTAAATTCGGTGAACCGAAGAATTGCTTTCTCATCATCCTACTACTTATTCAATCCGAAG
ATTCGGAAGCGTACCTTAATCCCTTAATACTTCATAGTAAAATGTAATTAGGATTGTACAAAGTACGGTA
TTTGAATAGCGTAAAGACTAGCGCGCACTACCAATAATATTTTCAGATGACTTATAGGCGGGATCTTGCT
CCCCACGAACATCTCGACTCCAAAATTTGTCTCTGTCTAGGCCCCAGCACTGTCGTGTATTGTAAACTAC
ATATGTAAATTATTAGTTGTAACCAAGTAAGAGTACAGTAAATAGATGCGAACGAATTGAGAAATCGTGG
CGAGAAATCGTGTAAAGTAATGTACAATTAGTCTTAAAGTATGTGTATCTGTATCTGATGGTGTGCGTGC
CGAGTATGTGAATCGGTGTAAATAAATGAGTGAGTGCAATTTGAGTGAATAGAGTGAGTGTGAGTACTGC
TGTGAGTGTGAATCAGAGCTTAATTGCGGGTTGAGCGGTTCCCATTCTGCACTGTTCTTCGTCGTCAGCG
TCAACGTCGTTAAAATATAATAATTTTGTCTATTTCTTTTAAAGCCACACGAACACTAACACTCCATAGA
TCAAATTTGTTAGCCCAGAACAGAACAAAACCAACCAGTTAAAGACaaaaaaaaaaaaaaaaaTAACAAA
CTGAACTGAACTGAATCTTCTTTTTTGGCACCTCTCAATCAATCTAAATGTGTATGTGTGTTGTTGAATT
TAGGCCGTTTTCGCTAGTTGGATACCAGAAATCACTGAACTGGCTGCCCAAAGAAGCAAATATGGTGGAA
CATATAGCAAGGATCCTAGAAAAAGGTACACGAACACGTTGCTGACTGTACCGCTAAGAGAAATTACCAA
AACTATACAACACGAAAATATCAAGATAAACAAGAAATGATTATAAATGCAAACAAATACCaaaaaaaaC
ATCGCAGCAATCGAATAGTCTTGAATTCTATTTGTATTACAACATTTTCTTTACCTAACTTCAGTCAATG
AACCAACCAGCTTACTTATTATTTCATTTGATTTCTTATTTTTCACCTTGTTGTTGAACATTTTTATCGA
CGTTACCTGGAATTGCATAAAAATGACAAACTCTGACGGCAACAAATAAGTGACGAAAATTGTTGCCAAA
TTAAAAACAAAATATTTATATTGTTTTGTTATTTTCATAAAGCTAAACCaaaaaaaaaaGAAAGAATTAA
GAAAAATCAATTAGGACTTGAAAGGCATTGGTTTCTTGGCGGCATGTGTCTATATTTAGTTAAACAATCA
ATTCTGAAAAAGGAAACACATTCAAAGAATCATGGCAAAAATTACACCAAAATCCAGGGAATAATCGATT
CCATACAAAAGATATAATTTATGTAATTTACTTGTCAAATCCTTCTGTAATTCTCGTTGTCGTTTACACA
CATTATACTTATTGTTTGAAACTATGGCTTCATTTTTGCGTGCGTTTCATTATTAGGCTTATTGTGTTAG
CTAGACAGATAAATTGTAAAAGTTCATAAATTGCTTAAACCATCAAAGAATTATCGTTAAACAAAAATGT
TTCTCTTGTTTGGCGCCTGTGGGTATGCAACACTTTTTGTCGTCTAAAATCTCTGGGCCTTCTTACCCCT
ATTCGTCTCACTAATCTTTACTGTACAATAATTGCAGACCTTTTGCTAATTAACCAAAGTTTTATCGATG
GGCTCCCCGCATTTGTTGTGTTCAATATTTAAAGCATGCACATTTCAAACTTTTGCTAGACAACAAAAAA
ACCTACATAAACAATATTCAATAACCACACAACACAATACATTTTTCAGATAATAGTTTACAGAAGTATT
TGAAAAGGGCTCCCCAGGCGAAATGGACTTATGTTCCAATGATTTTTTAGAAAAGGGCTAGGCTCGCTGC
ATTTGAAAAGTGTAATTTTTGAGTTCGTCTTTGGTTTCTGTTTAGTTTTGACTTTGAATTTTGATTATTA
CCGTACATACTATATAGAGCAGAGCCACTATTGCGTGTTTTTATGCAACTTCAGCTGCGTCATTGTTTTT
GAGTTCGAAGTGTTAAGATCAAAAACTAGCGCTGAATCAATATACATATGTAAGAATATATATACATATA
TGTTCTCTTAACTGGCTTCTTATTTAATTACCGAAATGCATTTTTATTGTCTCGTTTGTTTGTGCGTTGG
TTTCATTATATTTCGATTTTCCAATTTCGGTTGAAACACACTTACACCCAGTTGCTGCTTTAGTTCCCGC
ATAGAGTTTTGAGTTTCCAATATGAATATGATTTCCATATGTTTTTCACTTGTTCCTATATGCGATACAT

ACATAAATAAAGTTGCAGTAGTTACGATTTTTGCAATTTCAACTTATCCAACGTACTATATCGATTACTT
GTAATTTATTTACCATACCAATACGAAACTAATTCCACACCTTACCTAATCGAACAATGTAATCATCTAT
ATTTCAAAGTCGTTAATTTGCTTGCACCCACTTTGTATGCCGCAGCCACGCATAAAAATATAAAACTAGA
ACTTaaaacaaaaaacaaaaaaaaaaaaacgaaaCGAAACCAGTACGAAACCGGCGCCTTTTTGGCGTTGA
AATATGTTTTTATGACTATCATCATTTAGATAAAAATCAATTATGTTTGTATATCAGTAAAGAGTTGCAG
TATCCGTATCCTTTGATCGTATTCAGTATCAGCATCATGACCTCACATTATCTGTAACTCTTTCCCGTTC
CGAACCGATTCCCACCCGATACGATACCCAACACTCACACACATACGTGCGGTTTAGTCTGTTAGAATGT
TAGCATCACCCGATTTGAGTAGTCCTAGTCTAGAGTTTAGGCACTGAAACCCACCACAAAGATGAAGTCG
AAAGTCAAATGGCCGTCGATGTGTGTGCGTGTGTACTTGGCACCGAGTTACTAGGCGTTACTCCGCCACC
TAGTCTTTGAATATGTTTACTGTATATACTATATGTACGTATGTTCCGTAATCTATCCACACAACACGTA
ACCCCAAGAAACCATTAGAGCCATAAGAAACTAGCTGCAAGCCGATGAAACGGCCCATCGATTTATGAAG
ATAATAAGACCGATTTGTTACCATACCTTTTGTTTCGTTGATATCATTTTGATTTTTCTTGTTTACGATC
GAAGACATAACAATAACTTCTAGTCTGGTTCAACGAAGCCGCAACAGCCAGAGGCACAAGAACAACAAGA
AACACCAAGAGGAGCTCCGTATGAGATATATCCGAAATTATCCACTTGACTGCGTTTATCATGTACTTAT
ATAGGAGTCCTTCTTCGAGTCTGCCGAACCCATTCCCCACATCATACTCTCTGTTTCTCTAACTCTCTGC
TGATCCTTCGGGCCACTTTCTCTGTCTCTTGTATCCTGTGTCAAACGAAATTTGTGTAATGCTTTTTTAT
TTATGTTAAACAACAATCaaaaaaaaaaaaaaaaaaCAAAACCGAATAGATCAAAAATACTTCACTGTCA
TCATTATCTTTCATACTGTAAACAGGGTAGTCTATAAAAACTATAATTAAATCCTATaaaaaaaaaaaCA
TAATTATCGCATTCGATGTGAAAAACACAAAGCCACATTCTAAAGATAATCCACAAAAAGTTATAAATCG
CAATAAATCTAAAAGTATAACATAAAAAGATATATATAAACTTTACCAAGACAGCAAAATGCTTTTAGTT
AGGAACTACCATCTGATGAGGACTATTATCTTAAGATATCaaaaaaaaaaaaaaaaaaaaaaaTTCTAAC
AGTTAGGATCGGTTGAAGTACAAAAGAAAACTTTTGTAAAAAACACACATTCTTAGTATTTTGAGCCCAT
CTAGGGTGAAGTAAAACTCCAATTAGATAAATCACTAAGTCTTAATATTGATCAAAGTCGATAAATTAAA
TAGTCTGTTACCCTTTCACTTTGTTTCCCTAATTTCATTGTATCTCTAAGATCGCATATACAGTTGACTT
ACCCGCATCAAAATGTAGTTCAGCCGACTCTTAAGTCGTCCGTTTTAAACTATTTTGTTCCTTGATAAAT
ATGTATTTCGAAAACCGAAACAGATATGACATTTGATAAATCATCAAACAAGTAATAAAAGTAAAGTACA
ATTTTATCATTCGATTCTTACTTTGTTTCGTCTTTATTTATTATAATTTTTAAACATTTTTGTTGCCTCG
CAGTCAGAGACATTTCAGCAGATTCGAAAATACATCGTTCTATTTGTACAAGCCATTAAACATTTTAATG
TTCCATTTACTTAGGTTTGGTTTTCCAATTTTATTTGTTTATTTTCCTTGGTACTTGCGTTCCTCCTAAT
TTGTTTCGTTTCGTTAAACATTTTTATTGAACTCTTTGGCAATGTCTATCATATATAAATCACTTTGTAG
GCAAGCGTGAGTTCATTTTGGCCATTTTACTTTGGATTTTGTCTGAAGAGTAAACATGTTTTATTCAAGA
TATTATCGTTTGACACTTATACTTTGCCATGCTTTAAACGTTTCGTATTAACACCAGAGTTATGATACAA
GATATGCGTTTATCAATTTGCAATAGTTCAAGGTTCATATATATATTTATTTCGATATCCCAAAGCATTT
GTGCAATCTACTTAGGCGCATTGTTGTACCACATCCCACACAGATTTATAGAGCTCCCACTTATAAACAT
TTAATCTACAACACTAGTCCCCATAAAAAAGCATCGGTCACGCATTGAATAAAGTAAAACTAAACTATCC
AAAGTCATGACACGTAATTCAAATCGTATTTTCTTGAAGAGAGCAAACTCCAGCCACTTTGCAAGATTTG
TATTATTCAATTCATATTTTCCCACCTTATTCCTACTTAATATGTTACTTCATTTGTCAGTCTCGCTGGT
CCATTTACTTAAAGTAAATTCGGTGAACCGAAGAATTGCTTTCTCATCATCCTACTACTTATTCAATCCG
AAGATTCGGAAGCGTACCTTAATCCCTTAATACTTCATAGTAAAATGTAATTAGGATTGTACAAAGTACG
GTATTTGAATAGCGTAAAGACTAGCGCGCACTACCAATAATATTTTCAGATGACTTATAGGCGGGATCTT
GCTCCCCACGAACATCTCGACTCCAAAATTTGTCTCTGTCTAGGCCCCAGCACTGTCGTGTATTGTAAAC
TACATATGTAAATTATTAGTTGTAACCAAGTAAGAGTACAGTAAATAGATGCGAACGAATTGAGAAATCG
TGGCGAGAAATCGTGTAAAGTAATGTACAATTAGTCTTAAAGTATGTGTATCTGTATCTGATGGTGTGCG
TGCCGAGTATGTGAATCGGTGTAAATAAATGAGTGAGTGCAATTTGAGTGAATAGAGTGAGTGTGAGTAC

TGCTGTGAGTGTGAATCAGAGCTTAATTGCGGGTTGAGCGGTTCCCATTCTGCACTGTTCTTCGTCGTCA
GCGTCAACGTCGTTAAAATATAATAATTTTGTCTATTTCTTTTAAAGCCACACGAACACTAACACTCCAT
AGATCAAATTTGTTAGCCCAGAACAGAACAAAACCAACCAGTTAAAGACaaaaaaaaaaaaaaaaaTAAC
AAACTGAACTGAACTGAATCTTCTTTTTTGGCACCTCTCAATCAATCTAAATGTGTATGTGTGTTGTTGA
ATTTAGGCCGTTTTCGCTAGTTGGATACCAGAAATCACTGAACTGGCTGCCCAAAGAAGCAAATATGGTG
GAACATATAGCAAGGATCCTAGAAAAAGGTACACGAACACGTTGCTGACTGTACCGCTAAGAGAAATTAC
CAAAACTATACAACACGAAAATATCAAGATAAACAAGAAATGATTATAAATGCAAACAAATACCaaaaaa
aaCATCGCAGCAATCGAATAGTCTTGAATTCTATTTGTATTACAACATTTTCTTTACCTAACTTCAGTCA
ATGAACCAACCAGCTTACTTATTATTTCATTTGATTTCTTATTTTTCACCTTGTTGTTGAACATTTTTAT
CGACGTTACCTGGAATTGCATAAAAATGACAAACTCTGACGGCAACAAATAAGTGACGAAAATTGTTGCC
AAATTAAAAACAAAATATTTATATTGTTTTGTTATTTTCATAAAGCTAAACCaaaaaaaaaaGAAAGAAT
TAAGAAAAATCAATTAGGACTTGAAAGGCATTGGTTTCTTGGCGGCATGTGTCTATATTTAGTTAAACAA
TCAATTCTGAAAAAGGAAACACATTCAAAGAATCATGGCAAAAATTACACCAAAATCCAGGGAATAATCG
ATTCCATACAAAAGATATAATTTATGTAATTTACTTGTCAAATCCTTCTGTAATTCTCGTTGTCGTTTAC
ACACATTATACTTATTGTTTGAAACTATGGCTTCATTTTTGCGTGCGTTTCATTATTAGGCTTATTGTGT
TAGCTAGACAGATAAATTGTAAAAGTTCATAAATTGCTTAAACCATCAAAGAATTATCGTTAAACAAAAA
TGTTTCTCTTGTTTGGCGCCTGTGGGTATGCAACACTTTTTGTCGTCTAAAATCTCTGGGCCTTCTTACC
CCTATTCGTCTCACTAATCTTTACTGTACAATAATTGCAGACCTTTTGCTAATTAACCAAAGTTTTATCG
ATGGGCTCCCCGCATTTGTTGTGTTCAATATTTAAAGCATGCACATTTCAAACTTTTGCTAGACAACAAA
AAAACCTACATAAACAATATTCAATAACCACACAACACAATACATTTTTCAGATAATAGTTTACAGAAGT
ATTTGAAAAGGGCTCCCCAGGCGAAATGGACTTATGTTCCAATGATTTTTTAGAAAAGGGCTAGGCTCGC
TGCATTTGAAAAGTGTAATTTTTGAGTTCGTCTTTGGTTTCTGTTTAGTTTTGACTTTGAATTTTGATTA
TTACCGTACATACTATATAGAGCAGAGCCACTATTGCGTGTTTTTATGCAACTTCAGCTGCGTCATTGTT
TTTGAGTTCGAAGTGTTAAGATCAAAAACTAGCGCTGAATCAATATACATATGTAAGAATATATATACAT
ATATGTTCTCTTAACTGGCTTCTTATTTAATTACCGAAATGCATTTTTATTGTCTCGTTTGTTTGTGCGT
TGGTTTCATTATATTTCGATTTTCCAATTTCGGTTGAAACACACTTACACCCAGTTGCTGCTTTAGTTCC
CGCATAGAGTTTTGAGTTTCCAATATGAATATGATTTCCATATGTTTTTCACTTGTTCCTATATGCGATA
CATACATAAATAAAGTTGCAGTAGTTACGATTTTTGCAATTTCAACTTATCCAACGTACTATATCGATTA
CTTGTAATTTATTTACCATACCAATACGAAACTAATTCCACACCTTACCTAATCGAACAATGTAATCATC
TATATTTCAAAGTCGTTAATTTGCTTGCACCCACTTTGTATGCCGCAGCCACGCATAAAAATATAAAACT
AGAACTTaaaacaaaaaacaaaaaaaaaaaacgaaaCGAAACCAGTACGAAACCGGCGCCTTTTTGGCGT
TGAAATATGTTTTTATGACTATCATCATTTAGATAAAAATCAATTATGTTTGTATATCAGTAAAGAGTTG
CAGTATCCGTATCCTTTGATCGTATTCAGTATCAGCATCATGACCTCACATTATCTGTAACTCTTTCCCG
TTCCGAACCGATTCCCACCCGATACGATACCCAACACTCACACACATACGTGCGGTTTAGTCTGTTAGAA
TGTTAGCATCACCCGATTTGAGTAGTCCTAGTCTAGAGTTTAGGCACTGAAACCCACCACAAAGATGAAG
TCGAAAGTCAAATGGCCGTCGATGTGTGTGCGTGTGTACTTGGCACCGAGTTACTAGGCGTTACTCCGCC
ACCTAGTCTTTGAATATGTTTACTGTATATACTATATGTACGTATGTTCCGTAATCTATCCACACAACAC
GTAACCCCAAGAAACCATTAGAGCCATAAGAAACTAGCTGCAAGCCGATGAAACGGCCCATCGATTTATG
AAGATAATAAGACCGATTTGTTACCATACCTTTTGTTTCGTTGATATCATTTTGATTTTTCTTGTTTACG
ATCGAAGACATAACAATAACTTCTAGTCTGGTTCAACGAAGCCGCAACAGCCAGAGGCACAAGAACAACA
AGAAACACCAAGAGGAGCTCCGTATGAGATATATCCGAAATTATCCACTTGACTGCGTTTATCATGTACT
TATATAGGAGTCCTTCTTCGAGTCTGCCGAACCCATTCCCCACATCATACTCTCTGTTTCTCTAACTCTC
TGCTGATCCTTCGGGCCACTTTCTCTGTCTCTTGTATCCTGTGTCAAACGAAATTTGTGTAATGCTTTTT
TATTTATGTTAAACAACAATCaaaaaaaaaaaaaaaaaaCAAAACCGAATAGATCAAAAATACTTCACTG

TCATCATTATCTTTCATACTGTAAACAGGCGATTTTTGCAAGTTGTATACCTGAGATTATAGACTTGATT
GGAACAAGAGCCAAATATGGGGGCACATTAAAAAATGAAAAAGGGCGAAGGTGAGACAAACCAAATTTTC
TTACAATCAATCGTAAACAATTAATCAACAAACTGATTAAACCAAAAATTATATAAATAAACTACTACTA
CAACAACAACTACAATAAGACTACTACCACAAAAATAAAATACCAATCATAATTTTTAAGCCAACAACAA
ATTTTCAAAACTAACCAACAACGAGCATTTATGTTTCCAAAAGTAAACACCAAATCAAAATGAAATTTCT
GCCAAAAGCAGTCGAATGAACAGTGAAACGATATCAATTGAAAGAATTTACTTTTAGTTTGCTCTTCATT
CATGCCAAACGATTTGCGAGTGTGGAAAAGTAGCTGAAATTTGATTTTGATTCTACCAATAGATACACAC
CAGCCTAACACCAGCCATCCACACTAAAAAGCCACCACAAACATGGCCAATCCAAtccaattcaaccaac
cagccaaccacgaacccaccaataccaataccaatccaatCACTGTAATCATCATCAGCAACTCAAGCGA
CACATACAATATATTACTCATACGCCCAGTGGGACCGCTTGGAAAGCTCAGGACGGCACATAAACAACAC
AGTGACAACACAGCATTTGTACATTCCAACTATCCAGGCCatcaatcaaaatcagccaatcaatcaatca
atcaCCCAGACCCGAATTCAAAACATTTAATCGATCAAACACTCACCACCACCGcaacaacaccaacaac
aacaacaaacaGCCACGGAATATAAAACACCCACACAGCAATTGaaaaaaaaaaGCCGCAATCAACAAGA
AAATTTTAACTAAAATACTCATATTGCTTATATGAAAAAAGAACAACAAGTGTATGTTTTAGTTCTCGAA
CAACTCATTTTGTGTTGGTTGTACTTCAGTTGATTTCCTTTTAGTTTTGCGACCTAGACGTCTCTACATA
TGTATGTAGTTaaaaaaaaaCAAATATAAAAGGTAACTTAGGACGTAACCCCCTTGGAAATGGGGGGCTG
TGCCGGGTTATGCATTAGTTTCATTACTTTTACCTAAGTTTCGCCCATCGATTTGCGTTTTCATTTGCCA
CTTTGGATTTTTGTGTTCTTTGAATTGGCATTTTGCAGATTTGATCGTATTTGAGAATCCGTACCACATA
CAATCAATTGTTTCGTACTTAAAATACTGTTCTGAGTGTATCTACTATATCATGTGCGTACTCTTAACTA
CCAAATGCTATCTTGTTGTGAGTTTCCATACTACATCCTCAATTTGAACGCCAAATTATCAACTTACTCT
TCTGCTACTTTCTACTCTCTATATCGTACTCGTACATCCTGATCTTGAACCTAAATTCTTTAGCTCCTAA
CTTTTCGTTTGATTTGCACGTTCGTCTTTAGTTCCGAATAAACGCGGCTTAATCGTGTTCGATTTACAAT
TTTGGGTATATTGTTTAAATATTATTCTTTGGTTGATCTAGAAACGAATTGGATACGTGCAATTTGCAGG
TCGACCCAGCGAGTGTGAAGTCAGAGCTGATGATTGGCATTAATCGAAATTCAATATGCGTATACTCTTA
AGTTTTTCTCTTTTTCTGTATGCCATATTCATCCTAGCCTCGTTTTGCATTTGTATTAGCCCTTGAAGTG
AATCAGTGATTGGTGTTTATTATTGTGTTACATTATCGTAATTGTCTATATAGTCCTTGCTATCGAAGTC
GTAACTCGtatatatatatctatatacataaataaatatacatgcgtatatgtatatCCACCTAAATGAT
TTAACATTGAACgataataatgcatactaaatacaataataataataataataataataataTTGAT
TCGATTGTATACGTAAATGTTAGAATCCATACAATTCAAAATGTACACACACACATGCATAGAGTCGAAC
TCTCTATAGACTTTTTCTCCCCAAGTACAGACAGCAATTTGTAAATACCAAGCAATTTCCACGGAAGACT
TCAATTTGCTCAATATTTTTTGGCTAGAAAATGTACAATATTAGAACACACAGCTTGTGAACTGGCTTTG
ACATCTTGACCAAAATCAGCAGGCAGCTGCCATCTGAGAAACGGATTAAACATCGATGTGTGTGTAAAGC
GAATCAATGAAACCTATCAATCAATAATACCCATATATCTTTCCCGCATACTATACTCTTACTCTCTGCC
TCTCTATATATTCATATCTTTCTCTCTTCAAACCTTCAATCAGTTATCAGATACTATCAGTGTTGTAGTT
CTTGGAAAACTTGTATACACATACCCCATGCTATAAACATTCACACGCATCTACTCTAATACTCTTATGG
TTTTGATACTTTCCTCTTCTGTTCACAAACTTTCGTTTTAACGAACATTCGAATACAACGGTATGATCAA
GTAATAAAGATATAAACAATCATAACCTACAGcacacacacacacacacaGATTATACAATGCGAATCGG
ATGAAACTGTAACTCCACTCCAACCCACTTTTGTCGTAAACTCAGTGTTGTGTATGGATGCGCTCGGCGA
TTTCATTTGATTTCCTTTGCCCACCTTGTTCGGATCAGGATTAAGATCGAAAGCAAATTCCCAATCAAAa
tcaggatcaggatgaggatcaggTAGGAGGTCCAGGACGATTAGATATGCGGTTATGATGCGCGGCCCCT
GTTCTCTAGCTAAGGAACCAACCCCCTCCGATGAGGACAGTTCAAATTCTGATTCTGAGTGTAACATCCG
TATCCGTAATCGCAACCGAACCCGACTCCACCATTGCatccgtatccgtaaaccgtaaccgtatccgtat
ccgAGCCCCTCCAaatctctacataaacacatctcctgtctctctcttcaactctgtctctctttcaaga
tctctcCTATCCACAATTTTTGATTTCCTCAAAAGACCCACTCACATTCATTGCACATTTGTGAGCAATC

GATTTGGCGCATTGAAATCGACTTATGTTTTCTTATTCTGAGCAGCAATTCTGTATATGAAAATAAAACA
ATGAAAATGCGTGGCCAGGCATCAAATATGTTAAACCTTCTCACTCTATATTATATCATTTCAAGaaaaa
aaaaCGAAACATTTAATTCCAAACGTCACCATTCGTTTCTTTAGCTGCAGCAATGAAAAGAAATACATGC
AACACCAGAACTAATCGAATGTAGTGTCTTTACTTAGTTTCTTTGTTCTTTAAATGCAAATTGAAGTTCT
TCTTTCGTTTTAGCGAACTAGTCGAGAACAATTTTTTATTGCCACCGTAGAGGTAATGAGTTGCCTTGCA
TCCGGATTTGATCTGCAAAAATGGATTGAACATACAACAGAGCACAATCATGTCATCGATCATTCAATTG
TTGATCACCTTTAGATTGTACTGACTTTTTGTTAAATATTTCTACGAACGAAGACTTATATCGTTTTCCT
GAAGGACTATTATCCTTTGACTTTCTTCGACATTGTAGCTACAACCGTGGGTGTATAGTAATCGTGAACT
GAACGATATGGTTCGCTGATTCTCAACGCACATCCACAAACTCACAAAATCACAAACATCCAACACTCGA
AAAAATAATGTAGAAGTATGCATGTGTAGTAGTGGCACTGACACTCACTTTTTACTTGGTATGGGAAGAA
TGCCTGCCCGGTTTGCCTGACTGCCTGATTGCATGACTGTGACTACAGTAAATACCCAAAAACAAATAAT
CTATTGTATAATTAGCACCCGTAGAAGTAATTTATATACCTTGAATGAGCGTAGTGTATTATACAATGTG
TATATAGAAAACAAATGCGTTGGCCAGTTGCTGACTACTTTTGATGCTTAACTTGATGCATGTAAGTTGA
TTGCAACCGAAAATGGTGTGCGTAGCTAAGCCTTTATAGTACCTAAAACAATATTGATTTTCGATAATCG
TTTATATATACTTATCATTTCGATTGGCGAACTGCAGCGGCAGAGTGCCGAAAATGCGGCGACAAGATCC
TCATTTTGTGACTTTTGATTTATTGCCATTGATTGATTTGTGTACAAGCCCCCGGGGTAGCAGACTAAAT
TCATGCATTTTGCATTTATCATTTAAGTTTTTAGTTTCAATTATGAGTTCGTGTATTTTGTATAATTTTG
ATAAATGTTTGTTTCCTAAGAAAATGCAAAGTAATTATACTTTACCACCAAATAATACTGACAAGCCATA
AAGATAATCATAACCAGGCAACGATAAGCAGTTGATAAATAATACAAACAGATCAAAGCATAATCAATAT
AAAGAAATATACAGAAATACAAACAATGAATATACCCGAATCTATAACCTCATCGAAGAACATgcactca
ttcgaacactcattcaaagcactcaAAcacacgcacacgcacacgacacactgacaacacacacagacac
acCTATGTACTATATTATGGACTTGGCTTCATGGCAGCTCAATGGAATGTGATTAAATCTGAGATAATAA
TCTAATAATTCGGCTAAGAAAGTGAATTTAATTTCATGACTTGAACTAAATTCATTTTACTTTTTTTCCG
CTTTAATCGCACACTAACACTGAAACACCGCAAAacacacacacacacacacacatacatgctcatttga
aacacacacatacgtcacacatGTACAGAGCTTAAGTGCATTCCACACTAGATTTAAGTCAAATTGTTGC
ATTAATCGTTTGAATCGCTGCCAATTCAAGGAACTAATCTATTTTTTCTATCTACCCCCCACCTTCAACA
ACACAAGGGTAAGTCCATCCAGAATCCAATTAGCCTTTAAGCAGAAGAATAACCTGTTTACAGTCGTATT
GCTAATTTGTTGTGTCGTTGTTTTATTTCCATTATTATTTGATAAATGTATTTTCAATTCAGTTTTAAAA
CAGCAGCAAGAGAATAATTAGCGCAGTATCTAAGCAGTTAAAAAGAATGTGGATTAGCGGTTAGCAGTGG
CGCGGAAAAGTAGGCAATAGTTATTTGCACTGAAAAGAGATCAAACCATTTGCATAGTAATATTATTTAA
AATTGATTTAGATCTGTGCAGCTGCTGGCCGCTTAATCGATAGTTGGGAGTTAAGTTAGTTCAAGGCACT
ATTTTAAGTATTTGCATTAATCTAGCGACTAGACACAGTCAGTGAAAGTTATGTAATCTTTATGATTTGA
AAATACCTCTTACGTTCACTATGTAACTAATCAATGCCTAAACTCAACTATATATATGCCATATCATGTA
CTGTAATGTAAACCAAATAGCAAAGAGCCTGATCTGGAGCTAGAGGGACTGCTGAAGCGTCACTATACCA
CAGTGGCCTTTTTTCAGGGCACTATGATGAACGCAGTCGACCTGGAGCGAGTCAAGGTacacaaacacac
accgtgacacagacaccaagccacggccacacccacacccacacccacacgcacacgcacaccaacagac
acaaacacgaacacctacagacacacagagacacacaTATGAGAACCAGTGAAGACACGCAGAAACTTAA
CGATTTAAACGATTTAATCGAGCCCCCGATTGTCAGGTGTCCCAGCAAAAGAACAAAATCAAACTCCAAT
AAAATCAAGAACATGAACAACAACAGCGAATCTTACGTGCAACTCTCATACTTAGATCCTGTGATTTGTT
TTTGTGCCCGCAACTTAGTTCTTCAACAGCTAGGATGCAAATTTCACTCTACTTGCCCTCCGATTCGAAT
AATTATCAAGAATTTGAATTCCATTCGCTGCTGACCTTTCACAGcaaaaatagaaaacaaaacaaaaatg
aaaCAAGACACGCCCCCTTCCTCGTTCTATTTTTTATGCCATAAATCTATGCTCGCTCTCACTTTGAACT
TGAACAATTCaattatttttatttttattgcaattttatagctttattacggcattattattaATGGTTT
CCGGTTGCTGCTGATGAGGCTTCCGAGAAGAGCATTTTGCCTAACAATTCCTCTCAGAGCTTCGTGATAA

ATGATGTTTATAATATTTTAGATAGACTTTAGTTAAGAATCCCGTTCACAATATACAAttttttttgtt
tcatcgttttctttttttATCTTGGCTTAAAGTATTTTTACCCCGCTTAATGGAATGACTTTTTTAGCT
GAATTTTTTTCATATATCCTTTTAATCCAAATTTAAGCTCAAGCTTACAAATACTTCACAATTTATTATA
TTTTATATTTATTAATTCGTAGTAAGCAAAAGTTGAATTCCAATATTCCTCTTGTATCATATCCGTACAG
TTTTTATACTCCCATAGAATTTACTTAGTCTTGAGAGCCTAGTTAAGTAATGTTAATTAGATATTTAAGA
CGAGTTTTTTGTCTAGAGCATAGACTTAATTTAAGTAAGGAGGTTACAATATTTCTACTTTAGTTCAGTT
TACTTTTAATGAATCACAGTTTTTGTTGCACCTTCGGCTTGGAGATTTTCTTTACAACAATTCTGTCTGC
ATTGTTTGTTTTTCTTCTTTTTCATTTGCATATTTGCGGACTACGAAAATCGCACCATAATCATAAATAT
CTGTATATTGTTTGTTGTGTTTCGAACACCAACCCACACACATGCAATGATATGATATTCTTCTGTTCCT
TTGAATACAATTCAATCTACGTGAACTCTGTGTACATTGGAATCATTGAAAACTCTAAAAACTTTGTATT
ATTTTCCCACAACAACCACGATAAACCGaaaaaaaaaacaaaaCAATCGATTTTAAATGAAAAATTATAA
AGGGTacacaaacacacaccgtgacacagacaccaagccacggccacacccacacccacacccacacgca
cacgcacaccaacagacacaaacacgaacacctacagacacacagagacacacaTATGAGAACCAGTGAA
GACACGCAGAAACTTAACGATTTAAACGATTTAATCGAGCCCCCGATTGTCAGGTGTCCCAGCAAAAGAA
CAAAATCAAACTCCAATAAAATCAAGAACATGAACAACAACAGCGAATCTTACGTGCAACTCTCATACTT
AGATCCTGTGATTTGTTTTTGTGCCCGCAACTTAGTTCTTCAACAGCTAGGATGCAAATTTCACTCTACT
TGCCCTCCGATTCGAATAATTATCAAGAATTTGAATTCCATTCGCTGCTGACCTTTCACAGcaaaaatag
aaaacaaaacaaaaatgaaaCAAGACACGCCCCCTTCCTCGTTCTATTTTTTATGCCATAAATCTATGCT
CGCTCTCACTTTGAACTTGAACAATTCaattatttttatttttattgcaattttatagctttattacggc
attattattaATGGTTTCCGGTTGCTGCTGATGAGGCTTCCGAGAAGAGCATTTTGCCTAACAATTCCTC
TCAGAGCTTCGTGATAAATGATGTTTATAATATTTTAGATAGACTTTAGTTAAGAATCCCGTTCACAATA
TACAAttttttttgtttcatcgttttctttttttATCTTGGCTTAAAGTATTTTTACCCCGCTTAATG
GAATGACTTTTTTAGCTGAATTTTTTTCATATATCCTTTTAATCCAAATTTAAGCTCAAGCTTACAAATA
CTTCACAATTTATTATATTTTATATTTATTAATTCGTAGTAAGCAAAAGTTGAATTCCAATATTCCTCTT
GTATCATATCCGTACAGTTTTTATACTCCCATAGAATTTACTTAGTCTTGAGAGCCTAGTTAAGTAATGT
TAATTAGATATTTAAGACGAGTTTTTTGTCTAGAGCATAGACTTAATTTAAGTAAGGAGGTTACAATATT
TCTACTTTAGTTCAGTTTACTTTTAATGAATCACAGTTTTTGTTGCACCTTCGGCTTGGAGATTTTCTTT
ACAACAATTCTGTCTGCATTGTTTGTTTTTCTTCTTTTTCATTTGCATATTTGCGGACTACGAAAATCGC
ACCATAATCATAAATATCTGTATATTGTTTGTTGTGTTTCGAACACCAACCCACACACATGCAATGATAT
GATATTCTTCTGTTCCTTTGAATACAATTCAATCTACGTGAACTCTGTGTACATTGGAATCATTGAAAAC
TCTAAAAACTTTGTATTATTTTCCCACAACAACCACGATAAACCGaaaaaaaaaacaaaaCAATCGATTT
TAAATGAAAAATTATAAAGTAAACCACCCGATTTGGAACTTGAGGGTTTGTTCAAACGTCATTTTACCAC
CGTGGAGTTCTTCCAAGGAACCATTATGAATCCGATCGATCTTCAGAGGGTTAAGGTAAAACAAAAATGT
ATTTTCATTTTATCCGTTCTATATGTTTGTTCGTTTGCTTTTGTTACGTTGTTCATTTAACTATATTCCT
TTCCGTGTCTATTTTCGTCTCGACCTGTGAGCTTTAATTTCATTATGTATTAACTATTAACCGAAGTCCC
AGAATAAGCGTACTTCTACTTCTTACGTACTTATTTATGTAACGAACGCTCAAATGTAATTACAACCCGC
CTATTAATATACTAATCTATGTATTTACCtgtgtgtgtgtgtgtgtAATGTCTGTTTAACTGTACAAGCG
ATATTCGTGTAATACATGCGTGATGTATGCGACCATACTGCATTTTCTGGACCTAGTTTAGTTTACAACA
CCACACCACACATCCATCGGTACATGTATCCTTTGAATTTAAGCACTCAACTAACATCTGATCATGAATC
AAACGAATCGAACGTGAATTCACGAAACAAACACTCGTTGTACACAGAAACACCGTAAATTGATGTTGAA
ATTGTTGTGTAAAATAATAACGATATCATCTTGATTTATCTTTATTTTAATCTGCATTTATTGCAACGAT
TAACGTATACGTAATTTCCTATTTATGTATGTGCTAAGTATATTACTGTACATGTTAAGTTAAGTGTTAA
TTGTATAATATGGATTACATAGGATTTGAGGGTTTTTAGTGTTAATTCTTTAATATGGATTTTTTGAAGA
AATTGAGAATATACATATTAAATTGGTAGGTAAGCATATTTTAAACAAGTTGAAACaaaaaaaaaaCTGT

TAATATTATTTGCAAATATTTCTAAAAATTCAAGATCATAGGCTTAGGTATTCTAAAATATGTAAAATAA
CATTTTTTATTAGAACAGGTTTATTACATATATTCCACGTAAAGAAAGATATTTATACAGTTATACAGTA
AGTCATATTTTGAGCAGTAAATGTTTTTGTGAGTCTGCCCAATTCATTTCTTTTAATTTTGAGGGAAATG
TTTAATTATCGATAGTAAGCCATTATTAAGTACGTGCCCTAATAGTTTGTATATATGTATATTTATCAGC
TTAATCGATATCCTTTGAATGGACACTCATGAGCAGCCTTGGCTTTGTATAATGCCATATTGCGATGCAG
ATAGTTACTTTCCACAGTATCTGAGCTTATCTTTTGCATGCGAGCATTCTGCATGTCTATAATCGGATAC
AGATGCTTGTAGCTAGTGCCAATCTATTGTCTAAACGCCCACTTGAGTTGCTCTTGAGTTTTGTGAGGCT
TTGGTATTTGGTATTTGATTAAGTTTAAGTTCCCCCACGCCAAAGCCAATCACCCCGACACCTCTGTGTA
CTTGTTCTTTGTGAAAACACCCCACCCGTGCACCGAGCGTTCGCCTTATGATTTCTTAATGTGTCCTTAG
TTTGGAGTTGAAACCGAAAGACTGTGTTATCCAGCAACCCAACCAACCAAACACCCACCCACCCAGCCAC
TGTACAACCGCTTACTATATCCCCAACCGACACCCTGTGGGCAATGCTAATGAATACTACTTGTACCCCC
GCCCaaaaaaaaaaCCCTACTCAGTACGTACGCAATGCATATGCAAGATATTTATATATATATTGATATG
ATATGGACTCGATCGCAGCCAGATCAATCGAGCCAACCAAAAACAAACACTCACCTGTGGCATTTTAAGT
ACTCATACTCGTACCATAGTTGTCCAGTCGATGTGCGTGTAAACCAAAACAAAAATCGCTTACTAATTGT
TTATATTGTGGACAATGCTCTAAACGTGACACCACATCTGCAATATCTaatcacaataacagtaacaata
acaatCACTGAACCATAACAAACTACTTGGAACAGTTCAACATAACAAAACTAACTCACATGAGACAAAC
ATCAAATGCCCATAGACATAGTCTGACCATAAACAGCTATACTAGATAGCAATTATAACTATACTATACT
AATCTGTAACCATTAATTTCAATCCTTCTATCCATATGGATATGTCGAATGGAATAGGGTTCATGAAGCC
GACGCCTGCCTCGTGCTAGCTAACAAATATTGCCAAGATCCCGACGCAGAAGATGCTGCCAACATCATGA
GAGTCATCTCGATCAAGAACTACAGCGACGACATTCGAGTCATCATCCAGCTGATGCAGTACCACAATAA
GGGCATACTTGCTGAACATACCATCATGGGATTGGAAACAGGGCGACGATGTCATCTGCCTGGCCGAACT
GAAGCTGGGCTTCATTGCCCAGAGTTGTTTGGCGCCCGGGTTCTCGACCATGATGGCCAATCTGTTCGCC
ATGAGATCGTTCAAGACG
>dme_circ_0007459_junction_seq
TCTGTTCGCCATGAGATCGTTCAAGACGGGCAATGTTTGCCAGCAGTATACCGGAG
>dme_circ_0007460
GACATATTGTGGTATGCGGTCATATAACATACGAATCCGTGTCGCATTTCCTGAAGGACTTCCTCCACGA
AGATCGGGAGGATGTCGATGTCGAAGTGGTCTTTCTCCATCGGTAAGTCCATCCAGAATCCAATTAGCCT
TTAAGCAGAAGAATAACCTGTTTACAGTCGTATTGCTAATTTGTTGTGTCGTTGTTTTATTTCCATTATT
ATTTGATAAATGTATTTTCAATTCAGTTTTAAAACAGCAGCAAGAGAATAATTAGCGCAGTATCTAAGCA
GTTAAAAAGAATGTGGATTAGCGGTTAGCAGTGGCGCGGAAAAGTAGGCAATAGTTATTTGCACTGAAAA
GAGATCAAACCATTTGCATAGTAATATTATTTAAAATTGATTTAGATCTGTGCAGCTGCTGGCCGCTTAA
TCGATAGTTGGGAGTTAAGTTAGTTCAAGGCACTATTTTAAGTATTTGCATTAATCTAGCGACTAGACAC
AGTCAGTGAAAGTTATGTAATCTTTATGATTTGAAAATACCTCTTACGTTCACTATGTAACTAATCAATG
CCTAAACTCAACTATATATATGCCATATCATGTACTGTAATGTAAACCAAATAGCAAAGAGCCTGATCTG
GAGCTAGAGGGACTGCTGAAGCGTCACTATACCACAGTGGCCTTTTTTCAGGGCACTATGATGAACGCAG
TCGACCTGGAGCGAGTCAAG
>dme_circ_0007460_junction_seq
GAACGCAGTCGACCTGGAGCGAGTCAAGGACATATTGTGGTATGCGGTCATATAAC
>dme_circ_0007461
GACATATTGTGGTATGCGGTCATATAACATACGAATCCGTGTCGCATTTCCTGAAGGACTTCCTCCACGA
AGATCGGGAGGATGTCGATGTCGAAGTGGTCTTTCTCCATCGGTAAGTCCATCCAGAATCCAATTAGCCT
TTAAGCAGAAGAATAACCTGTTTACAGTCGTATTGCTAATTTGTTGTGTCGTTGTTTTATTTCCATTATT
ATTTGATAAATGTATTTTCAATTCAGTTTTAAAACAGCAGCAAGAGAATAATTAGCGCAGTATCTAAGCA

GTTAAAAAGAATGTGGATTAGCGGTTAGCAGTGGCGCGGAAAAGTAGGCAATAGTTATTTGCACTGAAAA
GAGATCAAACCATTTGCATAGTAATATTATTTAAAATTGATTTAGATCTGTGCAGCTGCTGGCCGCTTAA
TCGATAGTTGGGAGTTAAGTTAGTTCAAGGCACTATTTTAAGTATTTGCATTAATCTAGCGACTAGACAC
AGTCAGTGAAAGTTATGTAATCTTTATGATTTGAAAATACCTCTTACGTTCACTATGTAACTAATCAATG
CCTAAACTCAACTATATATATGCCATATCATGTACTGTAATGTAAACCAAATAGCAAAGAGCCTGATCTG
GAGCTAGAGGGACTGCTGAAGCGTCACTATACCACAGTGGCCTTTTTTCAGGGCACTATGATGAACGCAG
TCGACCTGGAGCGAGTCAAGGTacacaaacacacaccgtgacacagacaccaagccacggccacacccac
acccacacccacacgcacacgcacaccaacagacacaaacacgaacacctacagacacacagagacacac
aTATGAGAACCAGTGAAGACACGCAGAAACTTAACGATTTAAACGATTTAATCGAGCCCCCGATTGTCAG
GTGTCCCAGCAAAAGAACAAAATCAAACTCCAATAAAATCAAGAACATGAACAACAACAGCGAATCTTAC
GTGCAACTCTCATACTTAGATCCTGTGATTTGTTTTTGTGCCCGCAACTTAGTTCTTCAACAGCTAGGAT
GCAAATTTCACTCTACTTGCCCTCCGATTCGAATAATTATCAAGAATTTGAATTCCATTCGCTGCTGACC
TTTCACAGcaaaaatagaaaacaaaacaaaaatgaaaCAAGACACGCCCCCTTCCTCGTTCTATTTTTTA
TGCCATAAATCTATGCTCGCTCTCACTTTGAACTTGAACAATTCaattatttttatttttattgcaattt
tatagctttattacggcattattattaATGGTTTCCGGTTGCTGCTGATGAGGCTTCCGAGAAGAGCATT
TTGCCTAACAATTCCTCTCAGAGCTTCGTGATAAATGATGTTTATAATATTTTAGATAGACTTTAGTTAA
GAATCCCGTTCACAATATACAAttttttttttgtttcatcgttttcttttttttATCTTGGCTTAAAGTAT
TTTTACCCCGCTTAATGGAATGACTTTTTTAGCTGAATTTTTTTCATATATCCTTTTAATCCAAATTTAA
GCTCAAGCTTACAAATACTTCACAATTTATTATATTTTATATTTATTAATTCGTAGTAAGCAAAAGTTGA
ATTCCAATATTCCTCTTGTATCATATCCGTACAGTTTTTATACTCCCATAGAATTTACTTAGTCTTGAGA
GCCTAGTTAAGTAATGTTAATTAGATATTTAAGACGAGTTTTTTGTCTAGAGCATAGACTTAATTTAAGT
AAGGAGGTTACAATATTTCTACTTTAGTTCAGTTTACTTTTAATGAATCACAGTTTTTGTTGCACCTTCG
GCTTGGAGATTTTCTTTACAACAATTCTGTCTGCATTGTTTGTTTTTCTTCTTTTTCATTTGCATATTTG
CGGACTACGAAAATCGCACCATAATCATAAATATCTGTATATTGTTTGTTGTGTTTCGAACACCAACCCA
CACACATGCAATGATATGATATTCTTCTGTTCCTTTGAATACAATTCAATCTACGTGAACTCTGTGTACA
TTGGAATCATTGAAAACTCTAAAAACTTTGTATTATTTTCCCACAACAACCACGATAAACCGaaaaaaaa
aacaaaaCAATCGATTTTAAATGAAAAATTATAAAGGGTacacaaacacacaccgtgacacagacaccaa
gccacggccacacccacacccacacccacacgcacacgcacaccaacagacacaaacacgaacacctaca
gacacacagagacacacaTATGAGAACCAGTGAAGACACGCAGAAACTTAACGATTTAAACGATTTAATC
GAGCCCCCGATTGTCAGGTGTCCCAGCAAAAGAACAAAATCAAACTCCAATAAAATCAAGAACATGAACA
ACAACAGCGAATCTTACGTGCAACTCTCATACTTAGATCCTGTGATTTGTTTTTGTGCCCGCAACTTAGT
TCTTCAACAGCTAGGATGCAAATTTCACTCTACTTGCCCTCCGATTCGAATAATTATCAAGAATTTGAAT
TCCATTCGCTGCTGACCTTTCACAGcaaaaatagaaaacaaaacaaaaatgaaaCAAGACACGCCCCCTT
CCTCGTTCTATTTTTTATGCCATAAATCTATGCTCGCTCTCACTTTGAACTTGAACAATTCaattatttt
tatttttattgcaattttatagctttattacggcattattattaATGGTTTCCGGTTGCTGCTGATGAGG
CTTCCGAGAAGAGCATTTTGCCTAACAATTCCTCTCAGAGCTTCGTGATAAATGATGTTTATAATATTTT
AGATAGACTTTAGTTAAGAATCCCGTTCACAATATACAAttttttttttgtttcatcgttttcttttttttt
ATCTTGGCTTAAAGTATTTTTACCCCGCTTAATGGAATGACTTTTTTAGCTGAATTTTTTTCATATATCC
TTTTAATCCAAATTTAAGCTCAAGCTTACAAATACTTCACAATTTATTATATTTTATATTTATTAATTCG
TAGTAAGCAAAAGTTGAATTCCAATATTCCTCTTGTATCATATCCGTACAGTTTTTATACTCCCATAGAA
TTTACTTAGTCTTGAGAGCCTAGTTAAGTAATGTTAATTAGATATTTAAGACGAGTTTTTTGTCTAGAGC
ATAGACTTAATTTAAGTAAGGAGGTTACAATATTTCTACTTTAGTTCAGTTTACTTTTAATGAATCACAG
TTTTTGTTGCACCTTCGGCTTGGAGATTTTCTTTACAACAATTCTGTCTGCATTGTTTGTTTTTCTTCTT
TTTCATTTGCATATTTGCGGACTACGAAAATCGCACCATAATCATAAATATCTGTATATTGTTTGTTGTG

TTTCGAACACCAACCCACACACATGCAATGATATGATATTCTTCTGTTCCTTTGAATACAATTCAATCTA
CGTGAACTCTGTGTACATTGGAATCATTGAAAACTCTAAAAACTTTGTATTATTTTCCCACAACAACCAC
GATAAACCGaaaaaaaaacaaaaCAATCGATTTTAAATGAAAAATTATAAAGTAAACCACCCGATTTGG
AACTTGAGGGTTTGTTCAAACGTCATTTTACCACCGTGGAGTTCTTCCAAGGAACCATTATGAATCCGAT
CGATCTTCAGAGGGTTAAG
>dme_circ_0007461_junction_seq
GAATCCGATCGATCTTCAGAGGGTTAAGGACATATTGTGGTATGCGGTCATATAAC
>dme_circ_0007462
GACATATTGTGGTATGCGGTCATATAACATACGAATCCGTGTCGCATTTCCTGAAGGACTTCCTCCACGA
AGATCGGGAGGATGTCGATGTCGAAGTGGTCTTTCTCCATCGGTAAGTCCATCCAGAATCCAATTAGCCT
TTAAGCAGAAGAATAACCTGTTTACAGTCGTATTGCTAATTTGTTGTGTCGTTGTTTTATTTCCATTATT
ATTTGATAAATGTATTTTCAATTCAGTTTTAAAACAGCAGCAAGAGAATAATTAGCGCAGTATCTAAGCA
GTTAAAAAGAATGTGGATTAGCGGTTAGCAGTGGCGCGGAAAAGTAGGCAATAGTTATTTGCACTGAAAA
GAGATCAAACCATTTGCATAGTAATATTATTTAAAATTGATTTAGATCTGTGCAGCTGCTGGCCGCTTAA
TCGATAGTTGGGAGTTAAGTTAGTTCAAGGCACTATTTTAAGTATTTGCATTAATCTAGCGACTAGACAC
AGTCAGTGAAAGTTATGTAATCTTTATGATTTGAAAATACCTCTTACGTTCACTATGTAACTAATCAATG
CCTAAACTCAACTATATATATGCCATATCATGTACTGTAATGTAAACCAAATAGCAAAGAGCCTGATCTG
GAGCTAGAGGGACTGCTGAAGCGTCACTATACCACAGTGGCCTTTTTTCAGGGCACTATGATGAACGCAG
TCGACCTGGAGCGAGTCAAGGTacacaaacacacaccgtgacacagacaccaagccacggccacacccac
acccacacccacacgcacacgcacaccaacagacacaaacacgaacacctacagacacacagagacacac
aTATGAGAACCAGTGAAGACACGCAGAAACTTAACGATTTAAACGATTTAATCGAGCCCCCGATTGTCAG
GTGTCCCAGCAAAAGAACAAAATCAAACTCCAATAAAATCAAGAACATGAACAACAACAGCGAATCTTAC
GTGCAACTCTCATACTTAGATCCTGTGATTTGTTTTTGTGCCCGCAACTTAGTTCTTCAACAGCTAGGAT
GCAAATTTCACTCTACTTGCCCTCCGATTCGAATAATTATCAAGAATTTGAATTCCATTCGCTGCTGACC
TTTCACAGcaaaaatagaaaacaaaacaaaaatgaaaCAAGACACGCCCCCTTCCTCGTTCTATTTTTTA
TGCCATAAATCTATGCTCGCTCTCACTTTGAACTTGAACAATTCaattatttttatttttattgcaattt
tatagctttattacggcattattattaATGGTTTCCGGTTGCTGCTGATGAGGCTTCCGAGAAGAGCATT
TTGCCTAACAATTCCTCTCAGAGCTTCGTGATAAATGATGTTTATAATATTTTAGATAGACTTTAGTTAA
GAATCCCGTTCACAATATACAAtttttttttgtttcatcgttttctttttttttATCTTGGCTTAAAGTAT
TTTTACCCCGCTTAATGGAATGACTTTTTTAGCTGAATTTTTTTCATATATCCTTTTAATCCAAATTTAA
GCTCAAGCTTACAAATACTTCACAATTTATTATATTTTATATTTATTAATTCGTAGTAAGCAAAAGTTGA
ATTCCAATATTCCTCTTGTATCATATCCGTACAGTTTTTATACTCCCATAGAATTTACTTAGTCTTGAGA
GCCTAGTTAAGTAATGTTAATTAGATATTTAAGACGAGTTTTTTGTCTAGAGCATAGACTTAATTTAAGT
AAGGAGGTTACAATATTTCTACTTTAGTTCAGTTTACTTTTAATGAATCACAGTTTTTGTTGCACCTTCG
GCTTGGAGATTTTCTTTACAACAATTCTGTCTGCATTGTTTGTTTTTCTTCTTTTTCATTTGCATATTTG
CGGACTACGAAAATCGCACCATAATCATAAATATCTGTATATTGTTTGTTGTGTTTCGAACACCAACCCA
CACACATGCAATGATATGATATTCTTCTGTTCCTTTGAATACAATTCAATCTACGTGAACTCTGTGTACA
TTGGAATCATTGAAAACTCTAAAAACTTTGTATTATTTTCCCACAACAACCACGATAAACCGaaaaaaa
aacaaaaCAATCGATTTTAAATGAAAAATTATAAAGGGTacacaaacacacaccgtgacacagacaccaa
gccacggccacacccacacccacacccacacgcacacgcacaccaacagacacaaacacgaacacctaca
gacacacagagacacacaTATGAGAACCAGTGAAGACACGCAGAAACTTAACGATTTAAACGATTTAATC
GAGCCCCCGATTGTCAGGTGTCCCAGCAAAAGAACAAAATCAAACTCCAATAAAATCAAGAACATGAACA
ACAACAGCGAATCTTACGTGCAACTCTCATACTTAGATCCTGTGATTTGTTTTTGTGCCCGCAACTTAGT
TCTTCAACAGCTAGGATGCAAATTTCACTCTACTTGCCCTCCGATTCGAATAATTATCAAGAATTTGAAT

TCCATTCGCTGCTGACCTTTCACAGcaaaaatagaaaacaaaacaaaaatgaaaCAAGACACGCCCCCTT
CCTCGTTCTATTTTTTATGCCATAAATCTATGCTCGCTCTCACTTTGAACTTGAACAATTCaattatttt
tatttttattgcaattttatagctttattacggcattattattaATGGTTTCCGGTTGCTGCTGATGAGG
CTTCCGAGAAGAGCATTTTGCCTAACAATTCCTCTCAGAGCTTCGTGATAAATGATGTTTATAATATTTT
AGATAGACTTTAGTTAAGAATCCCGTTCACAATATACAAttttttttttgtttcatcgttttctttttttt
ATCTTGGCTTAAAGTATTTTTACCCCGCTTAATGGAATGACTTTTTTAGCTGAATTTTTTTCATATATCC
TTTTAATCCAAATTTAAGCTCAAGCTTACAAATACTTCACAATTTATTATATTTTATATTTATTAATTCG
TAGTAAGCAAAAGTTGAATTCCAATATTCCTCTTGTATCATATCCGTACAGTTTTTATACTCCCATAGAA
TTTACTTAGTCTTGAGAGCCTAGTTAAGTAATGTTAATTAGATATTTAAGACGAGTTTTTTGTCTAGAGC
ATAGACTTAATTTAAGTAAGGAGGTTACAATATTTCTACTTTAGTTCAGTTTACTTTTAATGAATCACAG
TTTTTGTTGCACCTTCGGCTTGGAGATTTTCTTTACAACAATTCTGTCTGCATTGTTTGTTTTTCTTCTT
TTTCATTTGCATATTTGCGGACTACGAAAATCGCACCATAATCATAAATATCTGTATATTGTTTGTTGTG
TTTCGAACACCAACCCACACACATGCAATGATATGATATTCTTCTGTTCCTTTGAATACAATTCAATCTA
CGTGAACTCTGTGTACATTGGAATCATTGAAAACTCTAAAAACTTTGTATTATTTTCCCACAACAACCAC
GATAAACCGaaaaaaaaaacaaaaCAATCGATTTTAAATGAAAAATTATAAAGTAAACCACCCGATTTGG
AACTTGAGGGTTTGTTCAAACGTCATTTTACCACCGTGGAGTTCTTCCAAGGAACCATTATGAATCCGAT
CGATCTTCAGAGGGTTAAGGTAAAACAAAAATGTATTTTCATTTTATCCGTTCTATATGTTTGTTCGTTT
GCTTTTGTTACGTTGTTCATTTAACTATATTCCTTTCCGTGTCTATTTTCGTCTCGACCTGTGAGCTTTA
ATTTCATTATGTATTAACTATTAACCGAAGTCCCAGAATAAGCGTACTTCTACTTCTTACGTACTTATTT
ATGTAACGAACGCTCAAATGTAATTACAACCCGCCTATTAATATACTAATCTATGTATTTACCtgtgtgt
gtgtgtgtgtAATGTCTGTTTAACTGTACAAGCGATATTCGTGTAATACATGCGTGATGTATGCGACCAT
ACTGCATTTTCTGGACCTAGTTTAGTTTACAACACCACACCACACATCCATCGGTACATGTATCCTTTGA
ATTTAAGCACTCAACTAACATCTGATCATGAATCAAACGAATCGAACGTGAATTCACGAAACAAACACTC
GTTGTACACAGAAACACCGTAAATTGATGTTGAAATTGTTGTGTAAAATAATAACGATATCATCTTGATT
TATCTTTATTTTAATCTGCATTTATTGCAACGATTAACGTATACGTAATTTCCTATTTATGTATGTGCTA
AGTATATTACTGTACATGTTAAGTTAAGTGTTAATTGTATAATATGGATTACATAGGATTTGAGGGTTTT
TAGTGTTAATTCTTTAATATGGATTTTTTGAAGAAATTGAGAATATACATATTAAATTGGTAGGTAAGCA
TATTTTAAACAAGTTGAAACaaaaaaaaaaCTGTTAATATTATTTGCAAATATTTCTAAAAATTCAAGAT
CATAGGCTTAGGTATTCTAAAATATGTAAAATAACATTTTTTATTAGAACAGGTTTATTACATATATTCC
ACGTAAAGAAAGATATTTATACAGTTATACAGTAAGTCATATTTTGAGCAGTAAATGTTTTTGTGAGTCT
GCCCAATTCATTTCTTTTAATTTTGAGGGAAATGTTTAATTATCGATAGTAAGCCATTATTAAGTACGTG
CCCTAATAGTTTGTATATATGTATATTTATCAGCTTAATCGATATCCTTTGAATGGACACTCATGAGCAG
CCTTGGCTTTGTATAATGCCATATTGCGATGCAGATAGTTACTTTCCACAGTATCTGAGCTTATCTTTTG
CATGCGAGCATTCTGCATGTCTATAATCGGATACAGATGCTTGTAGCTAGTGCCAATCTATTGTCTAAAC
GCCCACTTGAGTTGCTCTTGAGTTTTGTGAGGCTTTGGTATTTGGTATTTGATTAAGTTTAAGTTCCCCC
ACGCCAAAGCCAATCACCCCGACACCTCTGTGTACTTGTTCTTTGTGAAAACACCCCACCCGTGCACCGA
GCGTTCGCCTTATGATTTCTTAATGTGTCCTTAGTTTGGAGTTGAAACCGAAAGACTGTGTTATCCAGCA
ACCCAACCAACCAAACACCCACCCACCCAGCCACTGTACAACCGCTTACTATATCCCCAACCGACACCCT
GTGGGCAATGCTAATGAATACTACTTGTACCCCCGCCCaaaaaaaaaaCCCTACTCAGTACGTACGCAAT
GCATATGCAAGATATTTATATATATATTGATATGATATGGACTCGATCGCAGCCAGATCAATCGAGCCAA
CCAAAAACAAACACTCACCTGTGGCATTTTAAGTACTCATACTCGTACCATAGTTGTCCAGTCGATGTGC
GTGTAAACCAAAACAAAAATCGCTTACTAATTGTTTATATTGTGGACAATGCTCTAAACGTGACACCACA
TCTGCAATATCTaatcacaataacagtaacaataacaatCACTGAACCATAACAAACTACTTGGAACAGT
TCAACATAACAAAACTAACTCACATGAGACAAACATCAAATGCCCATAGACATAGTCTGACCATAAACAG

CTATACTAGATAGCAATTATAACTATACTATACTAATCTGTAACCATTAATTTCAATCCTTCTATCCATA
TGGATATGTCGAATGGAATAGGGTTCATGAAGCCGACGCCTGCCTCGTGCTAGCTAACAAATATTGCCAA
GATCCCGACGCAGAAGATGCTGCCAACATCATGAGAGTCATCTCGATCAAGAACTACAGCGACGACATTC
GAGTCATCATCCAGCTGATGCAGTACCACAATAAGGGCATACTTGCTGAACATACCATCATGGGATTGGA
AACAGGGCGACGATGTCATCTGCCTGGCCGAACTGAAGCTGGGCTTCATTGCCCAGAGTTGTTTGGCGCC
CGGGTTCTCGACCATGATGGCCAATCTGTTCGCCATGAGATCGTTCAAGACG
>dme_circ_0007462_junction_seq
TCTGTTCGCCATGAGATCGTTCAAGACGGACATATTGTGGTATGCGGTCATATAAC
>dme_circ_0007463
GACATATTGTGGTATGCGGTCATATAACATACGAATCCGTGTCGCATTTCCTGAAGGACTTCCTCCACGA
AGATCGGGAGGATGTCGATGTCGAAGTGGTCTTTCTCCATCGGTAAGTCCATCCAGAATCCAATTAGCCT
TTAAGCAGAAGAATAACCTGTTTACAGTCGTATTGCTAATTTGTTGTGTCGTTGTTTTATTTCCATTATT
ATTTGATAAATGTATTTTCAATTCAGTTTTAAAACAGCAGCAAGAGAATAATTAGCGCAGTATCTAAGCA
GTTAAAAAGAATGTGGATTAGCGGTTAGCAGTGGCGCGGAAAAGTAGGCAATAGTTATTTGCACTGAAAA
GAGATCAAACCATTTGCATAGTAATATTATTTAAAATTGATTTAGATCTGTGCAGCTGCTGGCCGCTTAA
TCGATAGTTGGGAGTTAAGTTAGTTCAAGGCACTATTTTAAGTATTTGCATTAATCTAGCGACTAGACAC
AGTCAGTGAAAGTTATGTAATCTTTATGATTTGAAAATACCTCTTACGTTCACTATGTAACTAATCAATG
CCTAAACTCAACTATATATATGCCATATCATGTACTGTAATGTAAACCAAATAGCAAAGAGCCTGATCTG
GAGCTAGAGGGACTGCTGAAGCGTCACTATACCACAGTGGCCTTTTTTCAGGGCACTATGATGAACGCAG
TCGACCTGGAGCGAGTCAAGGTacacaaacacacaccgtgacacagacaccaagccacggccacacccac
acccacacccacacgcacacgcacaccaacagacacaaacacgaacacctacagacacacagagacacac
aTATGAGAACCAGTGAAGACACGCAGAAACTTAACGATTTAAACGATTTAATCGAGCCCCCGATTGTCAG
GTGTCCCAGCAAAAGAACAAAATCAAACTCCAATAAAATCAAGAACATGAACAACAACAGCGAATCTTAC
GTGCAACTCTCATACTTAGATCCTGTGATTTGTTTTTGTGCCCGCAACTTAGTTCTTCAACAGCTAGGAT
GCAAATTTCACTCTACTTGCCCTCCGATTCGAATAATTATCAAGAATTTGAATTCCATTCGCTGCTGACC
TTTCACAGcaaaaatagaaaacaaaacaaaaatgaaaCAAGACACGCCCCCTTCCTCGTTCTATTTTTA
TGCCATAAATCTATGCTCGCTCTCACTTTGAACTTGAACAATTCaattatttttatttttattgcaattt
tatagctttattacggcattattattaATGGTTTCCGGTTGCTGCTGATGAGGCTTCCGAGAAGAGCATT
TTGCCTAACAATTCCTCTCAGAGCTTCGTGATAAATGATGTTTATAATATTTTAGATAGACTTTAGTTAA
GAATCCCGTTCACAATATACAAtttttttttgtttcatcgttttctttttttATCTTGGCTTAAAGTAT
TTTTACCCCGCTTAATGGAATGACTTTTTTAGCTGAATTTTTTTCATATATCCTTTTAATCCAAATTTAA
GCTCAAGCTTACAAATACTTCACAATTTATTATATTTTATATTTATTAATTCGTAGTAAGCAAAAGTTGA
ATTCCAATATTCCTCTTGTATCATATCCGTACAGTTTTTATACTCCCATAGAATTTACTTAGTCTTGAGA
GCCTAGTTAAGTAATGTTAATTAGATATTTAAGACGAGTTTTTTGTCTAGAGCATAGACTTAATTTAAGT
AAGGAGGTTACAATATTTCTACTTTAGTTCAGTTTACTTTTAATGAATCACAGTTTTTGTTGCACCTTCG
GCTTGGAGATTTTCTTTACAACAATTCTGTCTGCATTGTTTGTTTTTCTTCTTTTTCATTTGCATATTTG
CGGACTACGAAAATCGCACCATAATCATAAATATCTGTATATTGTTTGTTGTGTTTCGAACACCAACCCA
CACACATGCAATGATATGATATTCTTCTGTTCCTTTGAATACAATTCAATCTACGTGAACTCTGTGTACA
TTGGAATCATTGAAAACTCTAAAAACTTTGTATTATTTTCCCACAACAACCACGATAAACCGaaaaaaaa
aacaaaaCAATCGATTTTAAATGAAAAATTATAAAGGGTacacaaacacacaccgtgacacagacaccaa
gccacggccacacccacacccacacccacacgcacacgcacaccaacagacacaaacacgaacacctaca
gacacacagagacacacaTATGAGAACCAGTGAAGACACGCAGAAACTTAACGATTTAAACGATTTAATC
GAGCCCCCGATTGTCAGGTGTCCCAGCAAAAGAACAAAATCAAACTCCAATAAAATCAAGAACATGAACA
ACAACAGCGAATCTTACGTGCAACTCTCATACTTAGATCCTGTGATTTGTTTTTGTGCCCGCAACTTAGT

TCTTCAACAGCTAGGATGCAAATTTCACTCTACTTGCCCTCCGATTCGAATAATTATCAAGAATTTGAAT
TCCATTCGCTGCTGACCTTTCACAGcaaaaatagaaaacaaaacaaaaatgaaaCAAGACACGCCCCCTT
CCTCGTTCTATTTTTTATGCCATAAATCTATGCTCGCTCTCACTTTGAACTTGAACAATTCaattatttt
tatttttattgcaattttatagctttattacggcattattattaATGGTTTCCGGTTGCTGCTGATGAGG
CTTCCGAGAAGAGCATTTTGCCTAACAATTCCTCTCAGAGCTTCGTGATAAATGATGTTTATAATATTTT
AGATAGACTTTAGTTAAGAATCCCGTTCACAATATACAAtttttttttgtttcatcgttttcttttttt
ATCTTGGCTTAAAGTATTTTTACCCCGCTTAATGGAATGACTTTTTTAGCTGAATTTTTTTCATATATCC
TTTTAATCCAAATTTAAGCTCAAGCTTACAAATACTTCACAATTTATTATATTTTATATTTATTAATTCG
TAGTAAGCAAAAGTTGAATTCCAATATTCCTCTTGTATCATATCCGTACAGTTTTTATACTCCCATAGAA
TTTACTTAGTCTTGAGAGCCTAGTTAAGTAATGTTAATTAGATATTTAAGACGAGTTTTTTGTCTAGAGC
ATAGACTTAATTTAAGTAAGGAGGTTACAATATTTCTACTTTAGTTCAGTTTACTTTTAATGAATCACAG
TTTTTGTTGCACCTTCGGCTTGGAGATTTTCTTTACAACAATTCTGTCTGCATTGTTTGTTTTTCTTCTT
TTTCATTTGCATATTTGCGGACTACGAAAATCGCACCATAATCATAAATATCTGTATATTGTTTGTTGTG
TTTCGAACACCAACCCACACACATGCAATGATATGATATTCTTCTGTTCCTTTGAATACAATTCAATCTA
CGTGAACTCTGTGTACATTGGAATCATTGAAAACTCTAAAAACTTTGTATTATTTTCCCACAACAACCAC
GATAAACCGaaaaaaaaaaacaaaaCAATCGATTTTAAATGAAAAATTATAAAGTAAACCACCCGATTTGG
AACTTGAGGGTTTGTTCAAACGTCATTTTACCACCGTGGAGTTCTTCCAAGGAACCATTATGAATCCGAT
CGATCTTCAGAGGGTTAAGGTAAAACAAAAATGTATTTTCATTTTATCCGTTCTATATGTTTGTTCGTTT
GCTTTTGTTACGTTGTTCATTTAACTATATTCCTTTCCGTGTCTATTTTCGTCTCGACCTGTGAGCTTTA
ATTTCATTATGTATTAACTATTAACCGAAGTCCCAGAATAAGCGTACTTCTACTTCTTACGTACTTATTT
ATGTAACGAACGCTCAAATGTAATTACAACCCGCCTATTAATATACTAATCTATGTATTTACCtgtgtgt
gtgtgtgtgtAATGTCTGTTTAACTGTACAAGCGATATTCGTGTAATACATGCGTGATGTATGCGACCAT
ACTGCATTTTCTGGACCTAGTTTAGTTTACAACACCACACCACACATCCATCGGTACATGTATCCTTTGA
ATTTAAGCACTCAACTAACATCTGATCATGAATCAAACGAATCGAACGTGAATTCACGAAACAAACACTC
GTTGTACACAGAAACACCGTAAATTGATGTTGAAATTGTTGTGTAAAATAATAACGATATCATCTTGATT
TATCTTTATTTTAATCTGCATTTATTGCAACGATTAACGTATACGTAATTTCCTATTTATGTATGTGCTA
AGTATATTACTGTACATGTTAAGTTAAGTGTTAATTGTATAATATGGATTACATAGGATTTGAGGGTTTT
TAGTGTTAATTCTTTAATATGGATTTTTTGAAGAAATTGAGAATATACATATTAAATTGGTAGGTAAGCA
TATTTTAAACAAGTTGAAACaaaaaaaaaaCTGTTAATATTATTTGCAAATATTTCTAAAAATTCAAGAT
CATAGGCTTAGGTATTCTAAAATATGTAAAATAACATTTTTTATTAGAACAGGTTTATTACATATATTCC
ACGTAAAGAAAGATATTTATACAGTTATACAGTAAGTCATATTTTGAGCAGTAAATGTTTTTGTGAGTCT
GCCCAATTCATTTCTTTTAATTTTGAGGGAAATGTTTAATTATCGATAGTAAGCCATTATTAAGTACGTG
CCCTAATAGTTTGTATATATGTATATTTATCAGCTTAATCGATATCCTTTGAATGGACACTCATGAGCAG
CCTTGGCTTTGTATAATGCCATATTGCGATGCAGATAGTTACTTTCCACAGTATCTGAGCTTATCTTTTG
CATGCGAGCATTCTGCATGTCTATAATCGGATACAGATGCTTGTAGCTAGTGCCAATCTATTGTCTAAAC
GCCCACTTGAGTTGCTCTTGAGTTTTGTGAGGCTTTGGTATTTGGTATTTGATTAAGTTTAAGTTCCCCC
ACGCCAAAGCCAATCACCCCGACACCTCTGTGTACTTGTTCTTTGTGAAAACACCCCACCCGTGCACCGA
GCGTTCGCCTTATGATTTCTTAATGTGTCCTTAGTTTGGAGTTGAAACCGAAAGACTGTGTTATCCAGCA
ACCCAACCAACCAAACACCCACCCACCCAGCCACTGTACAACCGCTTACTATATCCCCAACCGACACCCT
GTGGGCAATGCTAATGAATACTACTTGTACCCCCGCCCaaaaaaaaaaCCCTACTCAGTACGTACGCAAT
GCATATGCAAGATATTTATATATATATTGATATGATATGGACTCGATCGCAGCCAGATCAATCGAGCCAA
CCAAAAACAAACACTCACCTGTGGCATTTTAAGTACTCATACTCGTACCATAGTTGTCCAGTCGATGTGC
GTGTAAACCAAAACAAAAATCGCTTACTAATTGTTTATATTGTGGACAATGCTCTAAACGTGACACCACA
TCTGCAATATCTaatcacaataacagtaacaataacaatCACTGAACCATAACAAACTACTTGGAACAGT

TCAACATAACAAAACTAACTCACATGAGACAAACATCAAATGCCCATAGACATAGTCTGACCATAAACAG
CTATACTAGATAGCAATTATAACTATACTATACTAATCTGTAACCATTAATTTCAATCCTTCTATCCATA
TGGATATGTCGAATGGAATAGGGTTCATGAAGCCGACGCCTGCCTCGTGCTAGCTAACAAATATTGCCAA
GATCCCGACGCAGAAGATGCTGCCAACATCATGAGAGTCATCTCGATCAAGAACTACAGCGACGACATTC
GAGTCATCATCCAGCTGATGCAGTACCACAATAAGGGCATACTTGCTGAACATACCATCATGGGATTGGA
AACAGGGCGACGATGTCATCTGCCTGGCCGAACTGAAGCTGGGCTTCATTGCCCAGAGTTGTTTGGCGCC
CGGGTTCTCGACCATGATGGCCAATCTGTTCGCCATGAGATCGTTCAAGACGGGTAAGTGTGGGGTTGCA
GTCCCAGTCCAGAGATCAGGCCACTCACCTAAGAAATACCAGACACACTCACACTACTGGCGCCCAGTGA
AAGCAGGCCGAAGAAGTTAGATATTTTTTTCAGATCTACCGATTACAGATTACAAATTCGCGGACAATGT
AACGCCCCCggggggggTGCAAGTAATTAACTATATGTGGGGTGTACTGTACCGTATATACGTATGAAAT
CAAAAATATGCAAATGTCTCTAAATTACGTCTCTAACCCATAGTCGCCAGACATGCAGTCTTGGACAAAT
GATTACCTGCGCGGCACTGGCATGGAAATGTACACTGAAACATTGAGTCCCACATTTATTGGAATACCAT
TTGCTCAGGCCACTGA
>dme_circ_0007463_junction_seq
TTGGAATACCATTTGCTCAGGCCACTGAGACATATTGTGGTATGCGGTCATATAAC
>dme_circ_0007464
GGTTCATGAAGCCGACGCCTGCCTCGTGCTAGCTAACAAATATTGCCAAGATCCCGACGCAGAAGATGCT
GCCAACATCATGAGAGTCATCTCGATCAAGAACTACAGCGACGACATTCGAGTCATCATCCAGCTGATGC
AGTACCACAATAAGGGCATACTTGCTGAACATACCATCATGGGATTGGAAACAGGGCGACGATGTCATCT
GCCTGGCCGAACTGAAGCTGGGCTTCATTGCCCAGAGTTGTTTGGCGCCCGGGTTCTCGACCATGATGGC
CAATCTGTTCGCCATGAGATCGTTCAAGACG
>dme_circ_0007464_junction_seq
TCTGTTCGCCATGAGATCGTTCAAGACGGGTTCATGAAGCCGACGCCTGCCTCGTG
>dme_circ_0007465
GGTTCATGAAGCCGACGCCTGCCTCGTGCTAGCTAACAAATATTGCCAAGATCCCGACGCAGAAGATGCT
GCCAACATCATGAGAGTCATCTCGATCAAGAACTACAGCGACGACATTCGAGTCATCATCCAGCTGATGC
AGTACCACAATAAGGGCATACTTGCTGAACATACCATCATGGGATTGGAAACAGGGCGACGATGTCATCT
GCCTGGCCGAACTGAAGCTGGGCTTCATTGCCCAGAGTTGTTTGGCGCCCGGGTTCTCGACCATGATGGC
CAATCTGTTCGCCATGAGATCGTTCAAGACGGGTAAGTGTGGGGTTGCAGTCCCAGTCCAGAGATCAGGC
CACTCACCTAAGAAATACCAGACACACTCACACTACTGGCGCCCAGTGAAAGCAGGCCGAAGAAGTTAGA
TATTTTTTTCAGATCTACCGATTACAGATTACAAATTCGCGGACAATGTAACGCCCCCggggggggTGCA
AGTAATTAACTATATGTGGGGTGTACTGTACCGTATATACGTATGAAATCAAAAATATGCAAATGTCTCT
AAATTACGTCTCTAACCCATAGTCGCCAGACATGCAGTCTTGGACAAATGATTACCTGCGCGGCACTGGC
ATGGAAATGTACACTGAAACATTGAGTCCCACATTTATTGGAATACCATTTGCTCAGGCCACTGA
>dme_circ_0007465_junction_seq
TTGGAATACCATTTGCTCAGGCCACTGAGGTTCATGAAGCCGACGCCTGCCTCGTG
>dme_circ_0007466
GGCAATCATGAGCCCTCTCCCACGAGTCAACATCGACGAGCCGAAATATGATCAGAACTCCTACTTGGGC
AGAGCCAAGCACTTCTTCCTGCTGACCAACCCGCTTAATGTGCTGGCCTCCGCCTCGAAGTTGGAGGAGG
CCCGCCAGATTGTGATAAAGTACCGGGCTGGGCAAGGATGTTCCCGAGTGCAAGACCATCGACGACGTGT
GGCGCGCTAAGTACCTGTACGATTCGGCGTTCCATCCAGAGACCGGGGAGAAGCAGATAGTCATCGGGCG
CATGGCTGCGCAGATGCCCATGAACACAATCATCACCGGCG
>dme_circ_0007466_junction_seq
ATGCCCATGAACACAATCATCACCGGCGGGCAATCATGAGCCCTCTCCCACGAGTC

>dme_circ_0007467
GTCACCAGACACACAGGCCTGGCAAAATGATTATCTTCAAGGTACAGGGTGTGAGATGTACACCGAAACC
CTATCACCTTCATTTACCGGCATGACATTCCCACAAGCCAGCGAGTAAGACTCaaaaatgagaaaataga
gaaaaaacaaaaactaaaacaaaaaaaaaaaaTTTATATGCAAACTGTATAAAAAAtactactacttcta
ctactactactactacaactTTTTGCATTGTACAAAATAAGTTTGTTTAACAATTCTTTTTCCTATTATT
TAACAACTTGATTCGATTCCGTTTGATTTACTCTCCTTCTCCTCATATTAACAACAACTAAACAACTGTT
TGTCGCCTCTCCCCACTGTCCCACCTCTGGCCATGTATCTTTATATACATATATCAAAAACCCATACTCT
AGAACCATAGCCACTGAAAGATTCAACTTAGCCATAGCCACCAGTACTCGAGAACCCGAGATCAAATCTT
TCGGGGGCGAACGCATTGAACAACCTGTATATTAAACCCACACTCATTTCGATTGGCTCAAACTGCATAT
ACCGTATCCGTATACCTGATAGTATCCGTATCCGTACTCTTATTCGTATCCGTATTCGCAACTGCAATCG
TAATCCGCATCTGATCGCCACAATCGCTTGCTAACCTTGAACTCCGCTCGCTGCGATTTGTAGAGCGTCA
GACACGTACACTGCAAATAATTCTAGAGACCCCACCCACAGAACAGCTCCACTTGTGACTCCACTTCCGA
AGTCCCGAACTCATTGCATGTGCAGAATGTTTTTTAGAGTGTACCCTAGTTGGCAATGGATGATGATGAT
TCACTGTCGGTAGttttgtttttttttttcaaacaataatttttttctagcatttttttCCACTAAGCAT
TTTGCTACGCATGCCCTTTTTTTCTTGATGGTTCATTGATTTTTAAAAGTGTACGCTTTCAAAGCACCCA
CCAATACAACGacacatacacacacactcacaaacaccccactcacacacacacaATATGCATGCCCTGT
TTTTGAGAGAGTTCGACTACACTTTTGTAGCATACTAAAACTAGGTTCAATTACTTGTAAAGCAACGAAC
TAGACTAAGTTGAAGCGATATCGGGCGATCGGGAACTGATTAACTTTCCCTCTTCCCCGCGTCTAGGACT
GTGTTTCTCCAAGCTGAAGCTTCTGCTGCTGGCCATCGAGATCAAGGGCGCAGAAGAGGGGGCGGACAGC
AAGATTTCCATCAACCCTCGGGGGGCAAAAATCCAGGCCAACACCCAGGGTTTCTTCATAGCACAAAGTG
CCGACGAGGTGAAGCGTGTAAGCAAACCCTTGTACCAAGTAGTAGCCACAGCTATCTTTTTAGAGTCTTT
ATGTATCTATGGATTTAATTTAACTTTTTCCGTTTTGATTTTAATATGTATTTGATTTGTTATTTCGTTT
GTGAATCGAATCGAACGTTTCGAAGACAGCCAGAATCGAAAAAGCCAAGAGAAACGATAGGAAATAACAA
TTGATTTTAGTTTGTAAGAAGGAACTAGCGAGTGTTGAGTGAGGCAGTTTCTAGGTATAAGGCGCTATCT
TTCTAATCTTTATGAATATCTAAATATTTTACCTCTCCACCATGAAACTATTCGTATATTCTTACATATT
TTTATACATAAATATCCATGTTTTTATTATATATTTATTATGTATGTGTGTTGGCGCGTGTGGCCTCGCT
CTCAATGAAATCAATACCAAAAAACTGCAACAACCAATCACAACAACTAACTATAACTATAACTATGTAT
AATCGAACCGAATCAACAATACCAAAAACCCATTGGCATGGCTGCAACTGTTCGAACACACCTCGCACAT
ATCCgtgtgtgtgtgcgactgtgtgtgCCGCACTTGCAACGTGACATAACCAAATACAACCAATTAAACC
GAAATCGAATCGAATTTAATACCAAATAATACAATCCAATGCCATAACTTTGTCCCACAGTGACTGTTCA
GCCCAGGAGCAAATTCGATGACTTAGGTAAATCAAGCTACAAGCCGAATTATCCAAAGAACAACAAGAAA
ACACACTCTAATCTTGCCCACATTAAAAACCAAAACACTTGAAGCCACACAAACACTTCTCAGTTTTCAA
GTTTTACCCGataccaataccaataccaataccaaCCGTTACCGATTCCAACTTTTGTATAAAGTACATT
TTGCATACCCCCGTTACCTTTTGTTTGTACATTGAATAAAGCAAAGCGAAGGTCCCGATTCCCGAAAACG
ATCCCCAGTTGATTTTCCTATCAGCACCTCACCTCAACCTCAAGAGCACAACAAAATCAAGAATTTTCCT
TTGATTTTGATTGTTTGTCTTTAGTTTTTGAAACGATTTTGCAAGGTGAACCATTGGACCCACCCCTTTGT
GCTCAGCCCCTAACCATAAAACCGACGTTTCCGATATCATAACCATCCAGCATATACTCTTATGTATACT
TATATATATCTGAACAACAAGCCATTTCGTCCTGCCCGATTACCTTGCCCATGTGCAGTTCCATTTTGGT
CACTGTTTGTTTTGACATGCGCCTCTTTGTAACCCACGAAGTAAGAGTTAAATACACCCACACCTATAAC
GTTTAACTATTTATGCCCATACAATTATCCAATGAGGATAAAACCCTGAAGCAAACAAAACATTACGTAA
CATTGACCTTGTAAATATGTTTGTGCGATATGCAACCCCAGTTCTTTGATTACCTATACTACAGCCACAG
TCAACCAGCAAGAAATGTTAACCAAAACTCTTCGCTTTTCCTTTAACCATCACCAAAAAAGAAATCCATT
AGAGTACCACCATGATAGTTGACACCCACCATTTTGAGTTCAAAAGTGAATTTTGCGTTTTACGGGGCGT
ATGGGTAATATGCCAGCCTGCAGAAGTGAAATACAAAGGCGGAACTGCCATATTCGAGCAGAGAACAGGC

GCCACGCAAATTATTCAAATTTATTCTAATATTTTTCTATTTTTAAAACTCAAGCCTAGTTTGCAACTCG
ATTACAGCTTTGTATTTCATTAAAAGGCATGAGAACCTCTTTACCATCTGTATTTCATTATGAACTTATA
ACTCACAACCCATTGATAGCCCTCTTTTGATAATCGATTTTTGTTCCAATTCTGAAACTGCACAtttttt
ttAAAAGTCTGTCTCGCTAATCCTCGCTCTTGCTATCTCTGTCTCTGTAATCCCCTCTTAATCTTTGTAC
TTTATACTTATGTGTTGAGAATAAGCCGAGATCAAATTAAGTGCATGGAAATGAGCACAACAATCAATTA
ACATTCTATCACACCATCACCATACGAAGAACCTTTAATATAAAAAACTATACTCTATTTTCGATCAATT
TTGACTCACTTTTGCAGCATCCAGCTCTAATTTAAATACTCTTCCTAATACTTATGACTAAATTATCTTT
AATACTTACCGGTAGACCTAAGTTCTTGAAATGCTGCGCCGTCCTCACGAATCCCCTAACACTTTTCGTT
GATTTGTGTTCTTGGGCGTGGCACTCACCTCACTCACCGATCTCTATTTACATTTTAAAGTATAAAGTTT
ATACATATATATATCTAACGCGATTGTATATAACCGCGATCGTAGCTGCAAAGCCAAAGTGTAAATAACA
TTAGATCAGGTTACGCGGCAGAAGTTAACATGGCGTTTTCAGAGAATAACCAAAGTTTATGTATTTGCTT
CTATGTAGCATAATACTAGAACAATCTCAACCTATACTCTGTAAACACCCAACTTAGCTGCAAATGTTCT
TAAGTAGTTAGTACGTTTGCCGAGTTTGCGTTGCCTGCGTTTTGGCTTTGAAACCAGCGAAAAATCAATT
TCGGGATCCAATGTGTTGCAGCTGAATATGTGAAACGCAGTTCGTAGTGCCTAGAGCTTAAGTTTCTGAA
CCAATCCCAGATACCGAACCCAGATATATACACCTAACTGAACTCTATATACATATATGTGCATTTGTTT
GTGTGTATGAGCGAGTGTCTATCTGAGTATCTACCAAATACCAACCAAAAACGTAACCATCTTGCAGATG
TGGGCATGATTATGATGCAGACGGGCATGGTCAACCAAGGCATCACGTCGGTGATGAATACAATGGAGGG
TACGCTTTCAAATCCATTTCCACAAACGAATCCCCAAACCAATTATTTGCGATTTGCGGCGCTCTATCCA
GTATTAATATCCGTTTATATATATACTACCAAGCCAAAAGAATTATTTGTTGCTGCTAATCCCGAACCTA
ATCAAATCGATATAAAAGATAAAAGGAGCGACAACCAAAGTACAAACTATTGTAAATATAAGTAAAAGTC
CGTTGATCCGATATACTTTGACATCTAATTAAAGAAATTCCTAATTTGCATCTTCTTTAGTAATTTTAAA
GACTTTGGTTAGCGCTCCTGAGATTTGGCTTGCCAAATTCATAGAGAAAGGACTATCCTTTGAAAACTAA
TTGAACTTGGAAACTAAATCAAAATTACTAAATTGATGTACAACTGTCAGCGTTTGTGTGCCTTAGTTAA
TGCTAAAAACCATGAAAAGCTCTTAGCTGCTAAATGCCAAGAATCCCCGCCAAGAACCGAAACCCGCATC
AGTTGGCTGGAAAACTGGGAAATCAGATTGTGAAGAAATGGGTATATAGAATCGATAATAGCAACCAGGG
AACTCCGAGTAAATAAGTTTTAGACTAAGAGTTGAGTTCAGTTCGTACTTCTACTTCTTCTATCGCTCTA
TCGTTCTCTCTATCGTACTCAGTATAAATATAACATACACCTATTATAGAACTAATTTAGTTTTGCTACG
ATTTCTGGTTTGGTTCGCCCAAGAGTCGCGTATAGTGTCATAATAAATCGAAAGCATATCTAGTATATAT
TCCTTATATACATTGCATACATATATCTATTTATGATCATAAACTGCATACCACATATGAATATATCTAC
ATAAATATAATATATATTATTTACGAACGTTCAAGTACAACGTGTGCCGTGTTTTTTACCTATGATTTTC
GTATAGCGTATTTAATTTCCTATGATTTATTCATCATTTTCGTTGAATGATGTTCCAATGATAAGCAGGC
GCAATATTGCGAGCAGATGAAAATGAAGAAAAACTAAATTATATTGAAAGCAACAATCATTACCTTGCCA
TCATTTACCTATTCACACGATCCTATAAAATTCTGTTTTCATGAAACAAAAACAGGGTAAATCAAGCTAC
AAGCCGAATTATCCAAAGAACAACAAGAAAACACACTCTAATCTTGCCCACATTAAAAACCAAAACACTT
GAAGCCACACAAACACTTCTCAGTTTTCAAGTTTTACCCGataccaataccaataccaataccaaCCGTT
ACCGATTCCAACTTTTGTATAAAGTACATTTTGCATACCCCCGTTACCTTTTGTTTGTACATTGAATAAA
GCAAAGCGAAGGTCCCGATTCCCGAAAACGATCCCCAGTTGATTTTCCTATCAGCACCTCACCTCAACCT
CAAGAGCACAACAAAATCAAGAATTTTCCTTTGATTTTGATTGTTTGTCTTTAGTTTTTGAAACGATTTT
GCAAGGTGAACCATTGGACCCACCCTTTGTGCTCAGCCCCTAACCATAAAACCGACGTTTCCGATATCAT
AACCATCCAGCATATACTCTTATGTATACTTATATATATCTGAACAACAAGCCATTTCGTCCTGCCCGAT
TACCTTGCCCATGTGCAGTTCCATTTTGGTCACTGTTTGTTTTGACATGCGCCTCTTTGTAACCCACGAA
GTAAGAGTTAAATACACCCACACCTATAACGTTTAACTATTTATGCCCATACAATTATCCAATGAGGATA
AAACCCTGAAGCAAACAAAACATTACGTAACATTGACCTTGTAAATATGTTTGTGCGATATGCAACCCCA
GTTCTTTGATTACCTATACTACAGCCACAGTCAACCAGCAAGAAATGTTAACCAAAACTCTTCGCTTTTC

CTTTAACCATCACCAAAAAAGAAATCCATTAGAGTACCACCATGATAGTTGACACCCACCATTTTGAGTT
CAAAAGTGAATTTTGCGTTTTACGGGGCGTATGGGTAATATGCCAGCCTGCAGAAGTGAAATACAAAGGC
GGAACTGCCATATTCGAGCAGAGAACAGGCGCCACGCAAATTATTCAAATTTATTCTAATATTTTTCTAT
TTTTAAAACTCAAGCCTAGTTTGCAACTCGATTACAGCTTTGTATTTCATTAAAAGGCATGAGAACCTCT
TTACCATCTGTATTTCATTATGAACTTATAACTCACAACCCATTGATAGCCCTCTTTTGATAATCGATTT
TTGTTCCAATTCTGAAACTGCACAttttttttAAAAGTCTGTCTCGCTAATCCTCGCTCTTGCTATCTCT
GTCTCTGTAATCCCCTCTTAATCTTTGTACTTTATACTTATGTGTTGAGAATAAGCCGAGATCAAATTAA
GTGCATGGAAATGAGCACAACAATCAATTAACATTCTATCACACCATCACCATACGAAGAACCTTTAATA
TAAAAAACTATACTCTATTTTCGATCAATTTTGACTCACTTTTGCAGCATCCAGCTCTAATTTAAATACT
CTTCCTAATACTTATGACTAAATTATCTTTAATACTTACCGGTAGACCTAAGTTCTTGAAATGCTGCGCC
GTCCTCACGAATCCCCTAACACTTTTCGTTGATTTGTGTTCTTGGGCGTGGCACTCACCTCACTCACCGA
TCTCTATTTACATTTTAAAGTATAAAGTTTATACATATATATATCTAACGCGATTGTATATAACCGCGAT
CGTAGCTGCAAAGCCAAAGTGTAAATAACATTAGATCAGGTTACGCGGCAGAAGTTAACATGGCGTTTTC
AGAGAATAACCAAAGTTTATGTATTTGCTTCTATGTAGCATAATACTAGAACAATCTCAACCTATACTCT
GTAAACACCCAACTTAGCTGCAAATGTTCTTAAGTAGTTAGTACGTTTGCCGAGTTTGCGTTGCCTGCGT
TTTGGCTTTGAAACCAGCGAAAAATCAATTTCGGGATCCAATGTGTTGCAGCTGAATATGTGAAACGCAG
TTCGTAGTGCCTAGAGCTTAAGTTTCTGAACCAATCCCAGATACCGAACCCAGATATATACACCTAACTG
AACTCTATATACATATATGTGCATTTGTTTGTGTGTATGAGCGAGTGTCTATCTGAGTATCTACCAAATA
CCAACCAAAAACGTAACCATCTTGCAGATGTGGGCATGATTATGATGCAGACGGGCATGGTCAACCAAGG
CATCACGTCGGTGATGAATACAATGGAGGGTACGCTTTCAAATCCATTTCCACAAACGAATCCCCAAACC
AATTATTTGCGATTTGCGGCGCTCTATCCAGTATTAATATCCGTTTATATATATACTACCAAGCCAAAAG
AATTATTTGTTGCTGCTAATCCCGAACCTAATCAAATCGATATAAAAGATAAAAGGAGCGACAACCAAAG
TACAAACTATTGTAAATATAAGTAAAAGTCCGTTGATCCGATATACTTTGACATCTAATTAAAGAAATTC
CTAATTTGCATCTTCTTTAGTAATTTTAAAGACTTTGGTTAGCGCTCCTGAGATTTGGCTTGCCAAATTC
ATAGAGAAAGGACTATCCTTTGAAAACTAATTGAACTTGGAAACTAAATCAAAATTACTAAATTGATGTA
CAACTGTCAGCGTTTGTGTGCCTTAGTTAATGCTAAAAACCATGAAAAGCTCTTAGCTGCTAAATGCCAA
GAATCCCCGCCAAGAACCGAAACCCGCATCAGTTGGCTGGAAAACTGGGAAATCAGATTGTGAAGAAATG
GGTATATAGAATCGATAATAGCAACCAGGGAACTCCGAGTAAATAAGTTTAGACTAAGAGTTGAGTTCA
GTTCGTACTTCTACTTCTTCTATCGCTCTATCGTTCTCTCTATCGTACTCAGTATAAATATAACATACAC
CTATTATAGAACTAATTTAGTTTTGCTACGATTTCTGGTTTGGTTCGCCCAAGAGTCGCGTATAGTGTCA
TAATAAATCGAAAGCATATCTAGTATATATTCCTTATATACATTGCATACATATATCTATTTATGATCAT
AAACTGCATACCACATATGAATATATCTACATAAATATAATATATATTATTTACGAACGTTCAAGTACAA
CGTGTGCCGTGTTTTTTACCTATGATTTTCGTATAGCGTATTTAATTTCCTATGATTTATTCATCATTTT
CGTTGAATGATGTTCCAATGATAAGCAGGCGCAATATTGCGAGCAGATGAAAATGAAGAAAAACTAAATT
ATATTGAAAGCAACAATCATTACCTTGCCATCATTTACCTATTCACACGATCCTATAAAATTCTGTTTTC
ATGAAACAAAAACAGTGGCCACCTTCCGGAAAGGCGTCCGAGCCGTACAAATGGTTGGGCGTGCAAGTAC
GTATAACTTACTCTACctctctctctctctctctctctctctctctgcctctctctctctatctatctcc
tgtctctgtctccactactctactgtTACCAATAAGCATACGAAACATACATCTCATAACCAGAAACCAC
GAAACCCAACATGATTTCCAATTGGCAAACCAAAATTTATCATCACATTCGCCCTATAAACTCTCCTGCT
AATCAAGTTAAAACGCTCAATTACATGTTTATAGCGCCAAACTAACTGTCATCTAACTACAAACTAACTA
ACCAACTCTAATTGCTATTGTTCAGCTGTATATGCCATTTGCCATTCAGCAAACCAATCGAAAACTAAGC
CAATCAATCAGGCAGCCAAACAATCGAAAATCAATCAATCTTTGTCGGAGCTCAGAATTcagaactcaaa
acacagaacacaaaacaccaacacaaGCACACAAAGTCCCTGGACCCGCCCCCCAAATAACCAACCGCCC
CCTTTTCCAACACTAATGCACTACACAACACACACATATGTACATGTTTCGTTTCTACTCGTTTGTTCTA

GTAATTGTGTACTGTTTCGTATTCAACTTTAACCCTAGTGTGCGTGTGCGTGTGTAGTAAAGTCAAGAGA
AAACCAAACCGAACTTCGCTTTGAGTCAGTTAATATTGGAAATCAACTGTTTGGATACTAAATATTCAGT
AACCATAATCGATCGATTCAAGCGTTGTGTTTTCGTATGAGCTCCATACTATCCATACTATTCGTTTTTG
TTATCAAGTCGAGTCATATTCTACATAATAatcgtactcctactatatcgtaaagtatcttgtacaaacg
ttacctacatattgtattgtatctatcgtatcgcattatatcatgtcgtaAAGTAATTCGTTAATTCGTG
ATACAAATTCTAACCGTTCACGTATCTTTGGACAACTATGTCATATCCAAACAAATACTTCAACTTTCCT
ATTTATATGATATACTAAGTGGTTACACCAACACTCCAATGAACACAACACTTCGGGTTCCACTGACCTG
CAATCCTGAAACACAATATCACACTCTACACTCATTTTCTGTAATATATATATATACAAATTCGATCTTC
TACGCCCGGACCTGTGTTCCTTTCTATATAATATATCAAATATGTAAACCTATCATCTTTCTATTGTACC
TCCCTACCCACACATTCGTGTTCCGTATCAACTACTACAACTACAATAAATACTCCTATTTATATGTCTC
TGTGTGTGTGTGTAGAAGACGATGAATACTCGTTGTCAAGTAAGTCAAACTAACTCAAAATGTTGTTTAT
CCATACCAGAAATTAAGAACATCCATTTCAATATCGCATTTGTACTTGTCGAAAACATATCGTTATCATT
TGAATTACTAGAGTCCTTTAAAAATTTCCCTTCAAAATATAGTGATTTGTATTGTTAGTTTTGTTTCGGT
TATTACTTGTTTTCGTTTTGAAGAGAGAACCCCATTATGTTAGAGACATTCTCAGTTTTAGTTTTAAACC
ATACATAAATCAACATGAATAAATAAAATGCAAATGACTTGAGAAAGCAAAGCAACTGAGCCAATGATTA
GAACCAATATTCGATCACAACCACCCAAAGCAGTAAAATGAACATAAAAAGCAAGCAGCTCCTCCTCATC
CCCCCGAACACTGCACCAATCCCAATCGTCCGGGGAACAGGAGATTCCAATTAGAGAGCTATTAAATTCC
AAATTCCGTGCAGTTAAATTGCTGACCATCAATGATCGTTTCAATCAGCTCCCAAAATCAGAAGCTGGGG
ATAAACTAGATAGTGCAACTCGTAGTTGGAGAGATCGTTTTTGAAGTTCTTCTTTATGTGGGTGTTAGCA
GTTCTAGCAGCCTAAGTGTTAAGTGCTTGACAATCGGATACTAAAGTCCAGTGAGCCAAAAAACCAGCTA
TTGATGACTATATAGAGTATTACCTATCTTGCTACTCAGCTTGTAATCCGCACTTAGTCAGCGTGTTGCT
TAAATAATGCTTCAAGCTTTAACCAATTTGGTTTGTATCCCTATGTACCAGAATACCAATATATATCACT
GCTTGCTTAGCTCTATTTATACCTATACTCTTTTTACCTATATACCTCTTTATATGTATTATGTAAACGT
TTGTATAATTGTAAGATGACACCGTCACCAGTTCTGCTCCAACTCATATTTATTTGCATGTTTTGTTCGA
TATTCTATAAAGCAAATACCCTAAAATGAATTCCCAAAAACCATCCCCCGACCCCATCTTGAACACTTTT
CGTTCAGTTTCGGCCGACGGCCACTTTTCATTTGATTTGAGTTCGTCAGGTTTTCCTGGGTTTTCCCAAG
AGTCAGACTAATTTCTCTATGATTTTCTCTCGAATTTCGTGTTTATTTTGAACACAAACCAATACTCTTA
CCGCTTCAGTTCTTacaaaaaccaaacacaatccccccctaaaaaattaaaaaacagaaaaaGAATTTGAT
GTATATTCAAAAGCAGTTTTGAGCTTCACATaaaaaaaaGCTTGACCATATAAATAACACCaaaaaaaaa
aTTAAATTGCATACACACCGAATCTGATTTACCGAGCTGTGTGATAACAAACCACCGTATTTTACAGATG
AACACCATCCTGCACCCACATTTACTCCTCCAGAGCTACCCAAGCGGGTGCATGTGCGTGGATCTGTATC
GGGTAAAGTCTCGTCTTCCAGTCGAACGAACGAACACTCAATTTGGTATCAGTCAGAGATCACATTCACA
GAAAGGTCATTTTGAATCGGAGGTGTTTAGGTGGATAGCACAGTAATCCAGCCAAACAGCTACCCACCCA
GTCTCACATAACACACACACACATCCACGCTCAAGATCAAGATAACATTCttatcagttatcagttatca
gttTGCCCCACGATTTCGTTTGCCTTGGTTCGCTTGTTCCTCAGTTGAAAACGTAAGAGATAAAAATGTA
TCCAATCCAATCGATAACCCATTGATAATTGTTTAGATATTCGTTTGCATAACTCTGTTTTTGCGTTGAT
TCTTGAACAGAACTCGTTCCGCTCTGCTAATGTAACTCGTTGATATACTCTCTAATCTAAAGAAACGATA
TACTATATATCTTCCGTATTATCATATCCACATATCGATGTCTATTTACCTTAACATACTTATTTGTACA
CATCACTCGGTGTGGCTTTGTTTTCAATTGCATAAACAATCTGCTTGTTACCAATTTTGTTTATTACTGT
CGCAACCACACACAGCATCACATAATGACAAGAACTAAGAACTAAGAAACCAGTAACCTAAACCTAAACC
AAGTGCAACATGCAACACGCGGCTCAAAAATACCTTTCTCGATTAACTAATGACTAATACTTAAAGCCAA
TGAGTATCTCTTCACACTCTCTGATCAGCGTGACGAAGCTCTTGCCAAGATTACTAATCGATTGATCTGC
TCTCTATCTCTGATCTCGTCAAAAACAACAACGCTTCTGCCTTGCCAAATGCCAAATGATACAAAACAA
AAACTGTCAATCTGCCACAGGTGATATCACTCGTGACAGAGAAGATACGAATCGTAAGTTTCGATGGTAT

TGATTACATATCATTTTCATGTGCGCAACCATCTCTTGTGATTACAGGTACTCAATCGCAATGTGCGCCG
GCCGAATGGCACTGGCAACGGTACGGGTGGCATGCACCACATGAACAACAcggcagcggcggctgcggca
gctgcggcggcggGAAAACAGGTGAACAAGGTGAAGCCAACGGTGAATGTGAGCCGGCAGGTGGAGGGCC
AAGTGATATCGCCATCGCAGTACAACAGGGTAAGCCAATCGCACTTATCCTGCGGGGACTGTACatatat
atatatatatttataatatatatTTAACATGGGGTGGAACAGCAGGCCATCTACTTAACTATAGAGTTTG
ATCACATTAAACAGCACTCTGTTATCAAATCAAATATATATAGCAAAGCAAAGTCGGTAATCGAGCAAGA
GCAAGCAGCAAGATAACTCAATGAATACACCCACTAGGAAAACTCTGTTTGCAGCTGCATCAAGTTTTGA
TTTTCCACTTTTCCGTTGCGTTACAATCGAACaaaaaaaaaaaaaaaaaaCGAGAGATTATGTTACACTC
AATTATGGTTACTGTGTTTGCCACACTCGTTTTATTTGACACAACAAAATGGTCAGGATAAGGATAAGGA
TGAATAGGTATCTCGTACATATGTAAATGCTCTTTAACCAACTAATCGAAACACCTCAACTAACTGTCTC
TCACTCACCTGCTCAACCACTCAACCACTCGCTCACTCTCGAAAAAAACAAACAAACAAATCAACCAATT
ATTAACTTTTAACCAGTCTAAATGTATAAAAACGAACAACATCTTGTTTCAGGCCACCAGAGAATGATGC
TAACCCTTATGCGGGCTATCAACTTGCTTACGAAGTTAAAAAGCTCATGTATGCGTCCATAATTTCCTAG
ATTTTTAACTTACAAACTCTATCGAAACTAAAAAAGCCAAAAGCCTTTTCTTTAACCATACCTTAGTATT
CGTTAATACTTATCTAACTACATCTTATGCGCATTTTGTTTGACACACATTGATATTGATTGATCGAAAG
CTATGCCATATTCCATCAAAATCGCGTACTAAACATAATATGAATTTTAACAAATTTGTGCGtttttttt
tGTCATGTCGCTGTCTTCTGTGTCTGTGTGCGTATGTGTTTGTGCGTTTGTATATGTTGGTGTGTATGTG
TGAGCGCCCCATGATTATTTTGCCATCTAAACTATCCCCAGCATAACTAACCGCATTCCATTCTGAAGCA
TTCTGTCTACTAACATCTATCTGCCACCCAAAGTAAACTAACAAGTAACAACTTTACTTTGGGTTTAATG
TTTAAGTAAAAAGGGTTCTAATTCTAAATAATCTTTGAAGCAGCTTTTTTTAAGAACTTCTTAAACTCTC
CTTTTGCTGATTTATTTGCCGTGCTAATGTCTTGTGTATATTTTAACTGTACGCAAATATCTATGTAAGA
AAAGTGTTTTACAATATGGATTTCCATTAGGGAAACTTAAGATATTTTTACCAATCTTGAGAAAAGGATC
AATCAAATATTGTAGCGATCTTTTGGCGCAGACTACGAAACTTCTaaaaaaaaaTATTTCAACTTCTTCT
TCGAAACTCATAAAACTAAGTTGGTTCTAGACCACAAAAGCATACATTAAGCTCAATAATCGCCCATTTT
TCTCCTGCATTCTATATCTATTTCTCTGAATCATTTGAACTTGAATTGCAATCTAAGTGTTGTATGTGTT
TTTCTGTGTTCATCTTTGTATCAAGACTTGCACCTGTGTACCATATAttttatatttttacgattttttt
tttAATTAAGAAGACACAACATTAAGTATTGTAGTTGAAAATGCCAAGTTCGGCGGATCGGAAGGGTTAT
GGATTAACAAAGGCCGCTCCCTCGCGCATGCCGAGCACATGAATCACACTGAAACACCTGGAGGGAGTTA
GCCGACCACCTAATACACTAGTTTTTGATCCAACCATCCCAACCGCCGCCGGATGGAGCTGTTGAGCATT
TTGCGTAGTGACACATTGATTTATTCTCAAATACTTTTTCGCATTCATTTGGAGACTTTTGTTATTAGCA
CATGATCTTCTCTAACTCTCTATCTATCTTACTATCCGTGTATTTATCTTTGTAAACGTAAAACTCTTCT
TAACTATCTTTAACTATTCGTATATTGAGATCACATTTTAATTCAATATGCCCCAAGTACTCGTACTCGT
ATTGAAGTATTCACATCATTTATTGAACGAAACACGTACTCGTAGCGTTTCCGATGAACACTTTGCATTT
GGATGTAGCGTTTGTTTTTGAATTGAATTGGTTGATAAACTGCATATGCAAGTCAAAAGTTGAATCGAAG
TGAAACCCATTATGatcgaatcgaatcgaatcgaatcCAGCTACAGCACAGACCGCCCGATGGCTAACTC
CTGTCTCTATCCTATCGATCTACTTGTGTCTAGGGCCGACGAGTCGCAGTTCCGGCACGGGCACACAGAA
TCAAAATGGCGGCGTATCCTTGCCCGCCGGCATTGCGGACGACCAGTCGAAGGACTTTGATTTCGAGAAG
ACCGAAATGAAGTACGACTCGACGGGCATGTTCCACTGGAGTCCGGCAAAGAGCTTAGAAGACTGCATAC
TGGGATCGCAACCAGGCGGCCATGACCGTGCTGAACGGCCACGTGGTCGTGTGCCTGTTTGCCGATCCCG
ATTCGCCGCTGATTGGGCTGCGGAATCTGGTGATGCCGCTGCGGGCATCCAACTTCCACTACCACGAGCT
GAAACACGTGGTCATCGTGGGCTCGGTGGACTACATAAGACGCGAGTGGAAGATGCTGCAGAACCTGCCC
AAGATCTCGGTGCTCAACGGATCGCCGCTGAGCCGTGCCGACCTGCGGGCCGTTAACGTCAATCTGTGTG
ATATGTGCTGCATCCTGTCGGCGAAAGTTCCTAGCAACGACGATCCCACGCTGGCCGACAAGGAGGCGAT
CCTCGCCTCGCTGAACATCAAGGCGATGACCTTTGACGACACGATCGGTGTGCTCAGCCAGCGCGGCCCG

GAGTTCGACAATCTGAGCGCCACCGCCGGCAGTCCCATTGTGCTGCAGCGGAGGGGCTCGGTCTATGGCG
CCAATGTGCCCATGATAACAG
>dme_circ_0007467_junction_seq
TATGGCGCCAATGTGCCCATGATAACAGGTCACCAGACACACAGGCCTGGCAAAAT
>dme_circ_0007468
GACTGTGTTTCTCCAAGCTGAAGCTTCTGCTGCTGGCCATCGAGATCAAGGGCGCAGAAGAGGGGGCGGA
CAGCAAGATTTCCATCAACCCTCGGGGGGCAAAAATCCAGGCCAACACCCAGGGTTTCTTCATAGCACAA
AGTGCCGACGAGGTGAAGCGTGTAAGCAAACCCTTGTACCAAGTAGTAGCCACAGCTATCTTTTTAGAGT
CTTTATGTATCTATGGATTTAATTTAACTTTTTCCGTTTTGATTTTAATATGTATTTGATTTGTTATTTC
GTTTGTGAATCGAATCGAACGTTTCGAAGACAGCCAGAATCGAAAAAGCCAAGAGAAACGATAGGAAATA
ACAATTGATTTAGTTTGTAAGAAGGAACTAGCGAGTGTTGAGTGAGGCAGTTTCTAGGTATAAGGCGCT
ATCTTTCTAATCTTTATGAATATCTAAATATTTTACCTCTCCACCATGAAACTATTCGTATATTCTTACA
TATTTTTATACATAAATATCCATGTTTTTATTATATATTTATTATGTATGTGTGTTGGCGCGTGTGGCCT
CGCTCTCAATGAAATCAATACCAAAAAACTGCAACAACCAATCACAACAACTAACTATAACTATAACTAT
GTATAATCGAACCGAATCAACAATACCAAAAACCCATTGGCATGGCTGCAACTGTTCGAACACACCTCGC
ACATATCCgtgtgtgtgtgcgactgtgtgtgCCGCACTTGCAACGTGACATAACCAAATACAACCAATTA
AACCGAAATCGAATCGAATTTAATACCAAATAATACAATCCAATGCCATAACTTTGTCCCACAGTGACTG
TTCAGCCCAGGAGCAAATTCGATGACTTAGGTAAATCAAGCTACAAGCCGAATTATCCAAAGAACAACAA
GAAAACACACTCTAATCTTGCCCACATTAAAAACCAAAACACTTGAAGCCACACAAACACTTCTCAGTTT
TCAAGTTTTACCCGataccaataccaataccaataccaaCCGTTACCGATTCCAACTTTTGTATAAAGTA
CATTTTGCATACCCCCGTTACCTTTTGTTTGTACATTGAATAAAGCAAAGCGAAGGTCCCGATTCCCGAA
AACGATCCCCAGTTGATTTTCCTATCAGCACCTCACCTCAACCTCAAGAGCACAACAAAATCAAGAATTT
TCCTTTGATTTTGATTGTTTGTCTTTAGTTTTTGAAACGATTTTGCAAGGTGAACCATTGGACCCACCCT
TTGTGCTCAGCCCCTAACCATAAAACCGACGTTTCCGATATCATAACCATCCAGCATATACTCTTATGTA
TACTTATATATATCTGAACAACAAGCCATTTCGTCCTGCCCGATTACCTTGCCCATGTGCAGTTCCATTT
TGGTCACTGTTTGTTTTGACATGCGCCTCTTTGTAACCCACGAAGTAAGAGTTAAATACACCCACACCTA
TAACGTTTAACTATTTATGCCCATACAATTATCCAATGAGGATAAAACCCTGAAGCAAACAAAACATTAC
GTAACATTGACCTTGTAAATATGTTTGTGCGATATGCAACCCCAGTTCTTTGATTACCTATACTACAGCC
ACAGTCAACCAGCAAGAAATGTTAACCAAAACTCTTCGCTTTTCCTTTAACCATCACCAAAAAAGAAATC
CATTAGAGTACCACCATGATAGTTGACACCCACCATTTTGAGTTCAAAAGTGAATTTTGCGTTTTACGGG
GCGTATGGGTAATATGCCAGCCTGCAGAAGTGAAATACAAAGGCGGAACTGCCATATTCGAGCAGAGAAC
AGGCGCCACGCAAATTATTCAAATTTATTCTAATATTTTTCTATTTTTAAAACTCAAGCCTAGTTTGCAA
CTCGATTACAGCTTTGTATTTCATTAAAAGGCATGAGAACCTCTTTACCATCTGTATTTCATTATGAACT
TATAACTCACAACCCATTGATAGCCCTCTTTTGATAATCGATTTTTGTTCCAATTCTGAAACTGCACAtt
ttttttAAAAGTCTGTCTCGCTAATCCTCGCTCTTGCTATCTCTGTCTCTGTAATCCCCTCTTAATCTTT
GTACTTTATACTTATGTGTTGAGAATAAGCCGAGATCAAATTAAGTGCATGGAAATGAGCACAACAATCA
ATTAACATTCTATCACACCATCACCATACGAAGAACCTTTAATATAAAAAACTATACTCTATTTTCGATC
AATTTTGACTCACTTTTGCAGCATCCAGCTCTAATTTAAATACTCTTCCTAATACTTATGACTAAATTAT
CTTTAATACTTACCGGTAGACCTAAGTTCTTGAAATGCTGCGCCGTCCTCACGAATCCCCTAACACTTTT
CGTTGATTTGTGTTCTTGGGCGTGGCACTCACCTCACTCACCGATCTCTATTTACATTTTAAAGTATAAA
GTTTATACATATATATATCTAACGCGATTGTATATAACCGCGATCGTAGCTGCAAAGCCAAAGTGTAAAT
AACATTAGATCAGGTTACGCGGCAGAAGTTAACATGGCGTTTTCAGAGAATAACCAAAGTTTATGTATTT
GCTTCTATGTAGCATAATACTAGAACAATCTCAACCTATACTCTGTAAACACCCAACTTAGCTGCAAATG
TTCTTAAGTAGTTAGTACGTTTGCCGAGTTTGCGTTGCCTGCGTTTTGGCTTTGAAACCAGCGAAAAATC

AATTTCGGGATCCAATGTGTTGCAGCTGAATATGTGAAACGCAGTTCGTAGTGCCTAGAGCTTAAGTTTC
TGAACCAATCCCAGATACCGAACCCAGATATATACACCTAACTGAACTCTATATACATATATGTGCATTT
GTTTGTGTGTATGAGCGAGTGTCTATCTGAGTATCTACCAAATACCAACCAAAAACGTAACCATCTTGCA
GATGTGGGCATGATTATGATGCAGACGGGCATGGTCAACCAAGGCATCACGTCGGTGATGAATACAATGG
AGGGTACGCTTTCAAATCCATTTCCACAAACGAATCCCCAAACCAATTATTTGCGATTTGCGGCGCTCTA
TCCAGTATTAATATCCGTTTATATATATACTACCAAGCCAAAAGAATTATTTGTTGCTGCTAATCCCGAA
CCTAATCAAATCGATATAAAAGATAAAAGGAGCGACAACCAAAGTACAAACTATTGTAAATATAAGTAAA
AGTCCGTTGATCCGATATACTTTGACATCTAATTAAAGAAATTCCTAATTTGCATCTTCTTTAGTAATTT
TAAAGACTTTGGTTAGCGCTCCTGAGATTTGGCTTGCCAAATTCATAGAGAAAGGACTATCCTTTGAAAA
CTAATTGAACTTGGAAACTAAATCAAAATTACTAAATTGATGTACAACTGTCAGCGTTTGTGTGCCTTAG
TTAATGCTAAAAACCATGAAAAGCTCTTAGCTGCTAAATGCCAAGAATCCCCGCCAAGAACCGAAACCCG
CATCAGTTGGCTGGAAAACTGGGAAATCAGATTGTGAAGAAATGGGTATATAGAATCGATAATAGCAACC
AGGGAACTCCGAGTAAATAAGTTTTAGACTAAGAGTTGAGTTCAGTTCGTACTTCTACTTCTTCTATCGC
TCTATCGTTCTCTCTATCGTACTCAGTATAAATATAACATACACCTATTATAGAACTAATTTAGTTTTGC
TACGATTTCTGGTTTGGTTCGCCCAAGAGTCGCGTATAGTGTCATAATAAATCGAAAGCATATCTAGTAT
ATATTCCTTATATACATTGCATACATATATCTATTTATGATCATAAACTGCATACCACATATGAATATAT
CTACATAAATATAATATATATTATTTACGAACGTTCAAGTACAACGTGTGCCGTGTTTTTTACCTATGAT
TTTCGTATAGCGTATTTAATTTCCTATGATTTATTCATCATTTTCGTTGAATGATGTTCCAATGATAAGC
AGGCGCAATATTGCGAGCAGATGAAAATGAAGAAAAACTAAATTATATTGAAAGCAACAATCATTACCTT
GCCATCATTTACCTATTCACACGATCCTATAAAATTCTGTTTTCATGAAACAAAAACAGGGTAAATCAAG
CTACAAGCCGAATTATCCAAAGAACAACAAGAAAACACACTCTAATCTTGCCCACATTAAAAACCAAAAC
ACTTGAAGCCACACAAACACTTCTCAGTTTTCAAGTTTTACCCGataccaataccaataccaataccaaC
CGTTACCGATTCCAACTTTTGTATAAAGTACATTTTGCATACCCCCGTTACCTTTTGTTTGTACATTGAA
TAAAGCAAAGCGAAGGTCCCGATTCCCGAAAACGATCCCCAGTTGATTTTCCTATCAGCACCTCACCTCA
ACCTCAAGAGCACAACAAAATCAAGAATTTTCCTTTGATTTTGATTGTTTGTCTTTAGTTTTTGAAACGA
TTTTGCAAGGTGAACCATTGGACCCACCCTTTGTGCTCAGCCCCTAACCATAAAACCGACGTTTCCGATA
TCATAACCATCCAGCATATACTCTTATGTATACTTATATATATCTGAACAACAAGCCATTTCGTCCTGCC
CGATTACCTTGCCCATGTGCAGTTCCATTTTGGTCACTGTTTGTTTTGACATGCGCCTCTTTGTAACCCA
CGAAGTAAGAGTTAAATACACCCACACCTATAACGTTTAACTATTTATGCCCATACAATTATCCAATGAG
GATAAAACCCTGAAGCAAACAAAACATTACGTAACATTGACCTTGTAAATATGTTTGTGCGATATGCAAC
CCCAGTTCTTTGATTACCTATACTACAGCCACAGTCAACCAGCAAGAAATGTTAACCAAAACTCTTCGCT
TTTCCTTTAACCATCACCAAAAAAGAAATCCATTAGAGTACCACCATGATAGTTGACACCCACCATTTTG
AGTTCAAAAGTGAATTTTGCGTTTTACGGGGCGTATGGGTAATATGCCAGCCTGCAGAAGTGAAATACAA
AGGCGGAACTGCCATATTCGAGCAGAGAACAGGCGCCACGCAAATTATTCAAATTTATTCTAATATTTTT
CTATTTTTAAAACTCAAGCCTAGTTTGCAACTCGATTACAGCTTTGTATTTCATTAAAAGGCATGAGAAC
CTCTTTACCATCTGTATTTCATTATGAACTTATAACTCACAACCCATTGATAGCCCTCTTTTGATAATCG
ATTTTTGTTCCAATTCTGAAACTGCACAttttttttAAAAGTCTGTCTCGCTAATCCTCGCTCTTGCTAT
CTCTGTCTCTGTAATCCCCTCTTAATCTTTGTACTTTATACTTATGTGTTGAGAATAAGCCGAGATCAAA
TTAAGTGCATGGAAATGAGCACAACAATCAATTAACATTCTATCACACCATCACCATACGAAGAACCTTT
AATATAAAAAACTATACTCTATTTTCGATCAATTTTGACTCACTTTTGCAGCATCCAGCTCTAATTTAAA
TACTCTTCCTAATACTTATGACTAAATTATCTTTAATACTTACCGGTAGACCTAAGTTCTTGAAATGCTG
CGCCGTCCTCACGAATCCCCTAACACTTTTCGTTGATTTGTGTTCTTGGGCGTGGCACTCACCTCACTCA
CCGATCTCTATTTACATTTTAAAGTATAAAGTTTATACATATATATATCTAACGCGATTGTATATAACCG
CGATCGTAGCTGCAAAGCCAAAGTGTAAATAACATTAGATCAGGTTACGCGGCAGAAGTTAACATGGCGT

TTTCAGAGAATAACCAAAGTTTATGTATTTGCTTCTATGTAGCATAATACTAGAACAATCTCAACCTATA
CTCTGTAAACACCCAACTTAGCTGCAAATGTTCTTAAGTAGTTAGTACGTTTGCCGAGTTTGCGTTGCCT
GCGTTTTGGCTTTGAAACCAGCGAAAAATCAATTTCGGGATCCAATGTGTTGCAGCTGAATATGTGAAAC
GCAGTTCGTAGTGCCTAGAGCTTAAGTTTCTGAACCAATCCCAGATACCGAACCCAGATATATACACCTA
ACTGAACTCTATATACATATATGTGCATTTGTTTGTGTGTATGAGCGAGTGTCTATCTGAGTATCTACCA
AATACCAACCAAAAACGTAACCATCTTGCAGATGTGGGCATGATTATGATGCAGACGGGCATGGTCAACC
AAGGCATCACGTCGGTGATGAATACAATGGAGGGTACGCTTTCAAATCCATTTCCACAAACGAATCCCCA
AACCAATTATTTGCGATTTGCGGCGCTCTATCCAGTATTAATATCCGTTTATATATATACTACCAAGCCA
AAAGAATTATTTGTTGCTGCTAATCCCGAACCTAATCAAATCGATATAAAAGATAAAAGGAGCGACAACC
AAAGTACAAACTATTGTAAATATAAGTAAAAGTCCGTTGATCCGATATACTTTGACATCTAATTAAAGAA
ATTCCTAATTTGCATCTTCTTTAGTAATTTTAAAGACTTTGGTTAGCGCTCCTGAGATTTGGCTTGCCAA
ATTCATAGAGAAAGGACTATCCTTTGAAAACTAATTGAACTTGGAAACTAAATCAAAATTACTAAATTGA
TGTACAACTGTCAGCGTTTGTGTGCCTTAGTTAATGCTAAAAACCATGAAAAGCTCTTAGCTGCTAAATG
CCAAGAATCCCCGCCAAGAACCGAAACCCGCATCAGTTGGCTGGAAAACTGGGAAATCAGATTGTGAAGA
AATGGGTATATAGAATCGATAATAGCAACCAGGGAACTCCGAGTAAATAAGTTTTAGACTAAGAGTTGAG
TTCAGTTCGTACTTCTACTTCTTCTATCGCTCTATCGTTCTCTCTATCGTACTCAGTATAAATATAACAT
ACACCTATTATAGAACTAATTTAGTTTTGCTACGATTTCTGGTTTGGTTCGCCCAAGAGTCGCGTATAGT
GTCATAATAAATCGAAAGCATATCTAGTATATATTCCTTATATACATTGCATACATATATCTATTTATGA
TCATAAACTGCATACCACATATGAATATATCTACATAAATATAATATATATTATTTACGAACGTTCAAGT
ACAACGTGTGCCGTGTTTTTTACCTATGATTTTCGTATAGCGTATTTAATTTCCTATGATTTATTCATCA
TTTTCGTTGAATGATGTTCCAATGATAAGCAGGCGCAATATTGCGAGCAGATGAAAATGAAGAAAAACTA
AATTATATTGAAAGCAACAATCATTACCTTGCCATCATTTACCTATTCACACGATCCTATAAAATTCTGT
TTTCATGAAACAAAAACAGTGGCCACCTTCCGGAAAGGCGTCCGAGCCGTACAAATGGTTGGGCGTGCAA
>dme_circ_0007468_junction_seq
CGAGCCGTACAAATGGTTGGGCGTGCAAGACTGTGTTTCTCCAAGCTGAAGCTTCT
>dme_circ_0007469
GACTGTGTTTCTCCAAGCTGAAGCTTCTGCTGCTGGCCATCGAGATCAAGGGCGCAGAAGAGGGGGCGGA
CAGCAAGATTTCCATCAACCCTCGGGGGGCAAAAATCCAGGCCAACACCCAGGGTTTCTTCATAGCACAA
AGTGCCGACGAGGTGAAGCGTGTAAGCAAACCCTTGTACCAAGTAGTAGCCACAGCTATCTTTTTAGAGT
CTTTATGTATCTATGGATTTAATTTAACTTTTTCCGTTTTGATTTTAATATGTATTTGATTTGTTATTTC
GTTTGTGAATCGAATCGAACGTTTCGAAGACAGCCAGAATCGAAAAAGCCAAGAGAAACGATAGGAAATA
ACAATTGATTTTAGTTTGTAAGAAGGAACTAGCGAGTGTTGAGTGAGGCAGTTTCTAGGTATAAGGCGCT
ATCTTTCTAATCTTTATGAATATCTAAATATTTTACCTCTCCACCATGAAACTATTCGTATATTCTTACA
TATTTTTATACATAAATATCCATGTTTTTATTATATATTTATTATGTATGTGTGTTGGCGCGTGTGGCCT
CGCTCTCAATGAAATCAATACCAAAAAACTGCAACAACCAATCACAACAACTAACTATAACTATAACTAT
GTATAATCGAACCGAATCAACAATACCAAAAACCCATTGGCATGGCTGCAACTGTTCGAACACACCTCGC
ACATATCCgtgtgtgtgtgcgactgtgtgtgCCGCACTTGCAACGTGACATAACCAAATACAACCAATTA
AACCGAAATCGAATCGAATTTAATACCAAATAATACAATCCAATGCCATAACTTTGTCCCACAGTGACTG
TTCAGCCCAGGAGCAAATTCGATGACTTAGGTAAATCAAGCTACAAGCCGAATTATCCAAAGAACAACAA
GAAAACACACTCTAATCTTGCCCACATTAAAAACCAAAACACTTGAAGCCACACAAACACTTCTCAGTTT
TCAAGTTTTACCCGataccaataccaataccaataccaaCCGTTACCGATTCCAACTTTTGTATAAAGTA
CATTTTGCATACCCCCGTTACCTTTTGTTTGTACATTGAATAAAGCAAAGCGAAGGTCCCGATTCCCGAA
AACGATCCCCAGTTGATTTTCCTATCAGCACCTCACCTCAACCTCAAGAGCACAACAAAATCAAGAATTT
TCCTTTGATTTTGATTGTTTGTCTTTAGTTTTTGAAACGATTTTGCAAGGTGAACCATTGGACCCACCCT

TTGTGCTCAGCCCCTAACCATAAAACCGACGTTTCCGATATCATAACCATCCAGCATATACTCTTATGTA
TACTTATATATATCTGAACAACAAGCCATTTCGTCCTGCCCGATTACCTTGCCCATGTGCAGTTCCATTT
TGGTCACTGTTTGTTTTGACATGCGCCTCTTTGTAACCCACGAAGTAAGAGTTAAATACACCCACACCTA
TAACGTTTAACTATTTATGCCCATACAATTATCCAATGAGGATAAAACCCTGAAGCAAACAAAACATTAC
GTAACATTGACCTTGTAAATATGTTTGTGCGATATGCAACCCCAGTTCTTTGATTACCTATACTACAGCC
ACAGTCAACCAGCAAGAAATGTTAACCAAAACTCTTCGCTTTTCCTTTAACCATCACCAAAAAAGAAATC
CATTAGAGTACCACCATGATAGTTGACACCCACCATTTTGAGTTCAAAAGTGAATTTTGCGTTTTACGGG
GCGTATGGGTAATATGCCAGCCTGCAGAAGTGAAATACAAAGGCGGAACTGCCATATTCGAGCAGAGAAC
AGGCGCCACGCAAATTATTCAAATTTATTCTAATATTTTTCTATTTTTAAAACTCAAGCCTAGTTTGCAA
CTCGATTACAGCTTTGTATTTCATTAAAAGGCATGAGAACCTCTTTACCATCTGTATTTCATTATGAACT
TATAACTCACAACCCATTGATAGCCCTCTTTTGATAATCGATTTTTGTTCCAATTCTGAAACTGCACAtt
ttttttAAAAGTCTGTCTCGCTAATCCTCGCTCTTGCTATCTCTGTCTCTGTAATCCCCTCTTAATCTTT
GTACTTTATACTTATGTGTTGAGAATAAGCCGAGATCAAATTAAGTGCATGGAAATGAGCACAACAATCA
ATTAACATTCTATCACACCATCACCATACGAAGAACCTTTAATATAAAAAACTATACTCTATTTTCGATC
AATTTTGACTCACTTTTGCAGCATCCAGCTCTAATTTAAATACTCTTCCTAATACTTATGACTAAATTAT
CTTTAATACTTACCGGTAGACCTAAGTTCTTGAAATGCTGCGCCGTCCTCACGAATCCCCTAACACTTTT
CGTTGATTTGTGTTCTTGGGCGTGGCACTCACCTCACTCACCGATCTCTATTTACATTTTAAAGTATAAA
GTTTATACATATATATATCTAACGCGATTGTATATAACCGCGATCGTAGCTGCAAAGCCAAAGTGTAAAT
AACATTAGATCAGGTTACGCGGCAGAAGTTAACATGGCGTTTTCAGAGAATAACCAAAGTTTATGTATTT
GCTTCTATGTAGCATAATACTAGAACAATCTCAACCTATACTCTGTAAACACCCAACTTAGCTGCAAATG
TTCTTAAGTAGTTAGTACGTTTGCCGAGTTTGCGTTGCCTGCGTTTTGGCTTTGAAACCAGCGAAAAATC
AATTTCGGGATCCAATGTGTTGCAGCTGAATATGTGAAACGCAGTTCGTAGTGCCTAGAGCTTAAGTTTC
TGAACCAATCCCAGATACCGAACCCAGATATATACACCTAACTGAACTCTATATACATATATGTGCATTT
GTTTGTGTGTATGAGCGAGTGTCTATCTGAGTATCTACCAAATACCAACCAAAAACGTAACCATCTTGCA
GATGTGGGCATGATTATGATGCAGACGGGCATGGTCAACCAAGGCATCACGTCGGTGATGAATACAATGG
AGGGTACGCTTTCAAATCCATTTCCACAAACGAATCCCCAAACCAATTATTTGCGATTTGCGGCGCTCTA
TCCAGTATTAATATCCGTTTATATATATACTACCAAGCCAAAAGAATTATTTGTTGCTGCTAATCCCGAA
CCTAATCAAATCGATATAAAAGATAAAAGGAGCGACAACCAAAGTACAAACTATTGTAAATATAAGTAAA
AGTCCGTTGATCCGATATACTTTGACATCTAATTAAAGAAATTCCTAATTTGCATCTTCTTTAGTAATTT
TAAAGACTTTGGTTAGCGCTCCTGAGATTTGGCTTGCCAAATTCATAGAGAAAGGACTATCCTTTGAAAA
CTAATTGAACTTGGAAACTAAATCAAAATTACTAAATTGATGTACAACTGTCAGCGTTTGTGTGCCTTAG
TTAATGCTAAAAACCATGAAAAGCTCTTAGCTGCTAAATGCCAAGAATCCCCGCCAAGAACCGAAACCCG
CATCAGTTGGCTGGAAAACTGGGAAATCAGATTGTGAAGAAATGGGTATATAGAATCGATAATAGCAACC
AGGGAACTCCGAGTAAATAAGTTTTAGACTAAGAGTTGAGTTCAGTTCGTACTTCTACTTCTTCTATCGC
TCTATCGTTCTCTCTATCGTACTCAGTATAAATATAACATACACCTATTATAGAACTAATTTAGTTTTGC
TACGATTTCTGGTTTGGTTCGCCCAAGAGTCGCGTATAGTGTCATAATAAATCGAAAGCATATCTAGTAT
ATATTCCTTATATACATTGCATACATATATCTATTTATGATCATAAACTGCATACCACATATGAATATAT
CTACATAAATATAATATATATTATTTACGAACGTTCAAGTACAACGTGTGCCGTGTTTTTTACCTATGAT
TTTCGTATAGCGTATTTAATTTCCTATGATTTATTCATCATTTTCGTTGAATGATGTTCCAATGATAAGC
AGGCGCAATATTGCGAGCAGATGAAAATGAAGAAAAACTAAATTATATTGAAAGCAACAATCATTACCTT
GCCATCATTTACCTATTCACACGATCCTATAAAATTCTGTTTTCATGAAACAAAAACAGGGTAAATCAAG
CTACAAGCCGAATTATCCAAAGAACAACAAGAAAACACACTCTAATCTTGCCCACATTAAAAACCAAAAC
ACTTGAAGCCACACAAACACTTCTCAGTTTTCAAGTTTTACCCGataccaataccaataccaataccaaC
CGTTACCGATTCCAACTTTTGTATAAAGTACATTTTGCATACCCCCGTTACCTTTTGTTTGTACATTGAA

TAAAGCAAAGCGAAGGTCCCGATTCCCGAAAACGATCCCCAGTTGATTTTCCTATCAGCACCTCACCTCA
ACCTCAAGAGCACAACAAAATCAAGAATTTTCCTTTGATTTTGATTGTTTGTCTTTAGTTTTTGAAACGA
TTTTGCAAGGTGAACCATTGGACCCACCCTTTGTGCTCAGCCCCTAACCATAAAACCGACGTTTCCGATA
TCATAACCATCCAGCATATACTCTTATGTATACTTATATATATCTGAACAACAAGCCATTTCGTCCTGCC
CGATTACCTTGCCCATGTGCAGTTCCATTTTGGTCACTGTTTGTTTTGACATGCGCCTCTTTGTAACCCA
CGAAGTAAGAGTTAAATACACCCACACCTATAACGTTTAACTATTTATGCCCATACAATTATCCAATGAG
GATAAAACCCTGAAGCAAACAAAACATTACGTAACATTGACCTTGTAAATATGTTTGTGCGATATGCAAC
CCCAGTTCTTTGATTACCTATACTACAGCCACAGTCAACCAGCAAGAAATGTTAACCAAAACTCTTCGCT
TTTCCTTTAACCATCACCAAAAAAGAAATCCATTAGAGTACCACCATGATAGTTGACACCCACCATTTTG
AGTTCAAAAGTGAATTTTGCGTTTTACGGGGCGTATGGGTAATATGCCAGCCTGCAGAAGTGAAATACAA
AGGCGGAACTGCCATATTCGAGCAGAGAACAGGCGCCACGCAAATTATTCAAATTTATTCTAATATTTTT
CTATTTTTAAAACTCAAGCCTAGTTTGCAACTCGATTACAGCTTTGTATTTCATTAAAAGGCATGAGAAC
CTCTTTACCATCTGTATTTCATTATGAACTTATAACTCACAACCCATTGATAGCCCTCTTTTGATAATCG
ATTTTTGTTCCAATTCTGAAACTGCACAttttttttAAAAGTCTGTCTCGCTAATCCTCGCTCTTGCTAT
CTCTGTCTCTGTAATCCCCTCTTAATCTTTGTACTTTATACTTATGTGTTGAGAATAAGCCGAGATCAAA
TTAAGTGCATGGAAATGAGCACAACAATCAATTAACATTCTATCACACCATCACCATACGAAGAACCTTT
AATATAAAAAACTATACTCTATTTTCGATCAATTTTGACTCACTTTTGCAGCATCCAGCTCTAATTTAAA
TACTCTTCCTAATACTTATGACTAAATTATCTTTAATACTTACCGGTAGACCTAAGTTCTTGAAATGCTG
CGCCGTCCTCACGAATCCCCTAACACTTTTCGTTGATTTGTGTTCTTGGGCGTGGCACTCACCTCACTCA
CCGATCTCTATTTACATTTTAAAGTATAAAGTTTATACATATATATATCTAACGCGATTGTATATAACCG
CGATCGTAGCTGCAAAGCCAAAGTGTAAATAACATTAGATCAGGTTACGCGGCAGAAGTTAACATGGCGT
TTTCAGAGAATAACCAAAGTTTATGTATTTGCTTCTATGTAGCATAATACTAGAACAATCTCAACCTATA
CTCTGTAAACACCCAACTTAGCTGCAAATGTTCTTAAGTAGTTAGTACGTTTGCCGAGTTTGCGTTGCCT
GCGTTTTGGCTTTGAAACCAGCGAAAAATCAATTTCGGGATCCAATGTGTTGCAGCTGAATATGTGAAAC
GCAGTTCGTAGTGCCTAGAGCTTAAGTTTCTGAACCAATCCCAGATACCGAACCCAGATATATACACCTA
ACTGAACTCTATATACATATATGTGCATTTGTTTGTGTGTATGAGCGAGTGTCTATCTGAGTATCTACCA
AATACCAACCAAAAACGTAACCATCTTGCAGATGTGGGCATGATTATGATGCAGACGGGCATGGTCAACC
AAGGCATCACGTCGGTGATGAATACAATGGAGGGTACGCTTTCAAATCCATTTCCACAAACGAATCCCCA
AACCAATTATTTGCGATTTGCGGCGCTCTATCCAGTATTAATATCCGTTTATATATATACTACCAAGCCA
AAAGAATTATTTGTTGCTGCTAATCCCGAACCTAATCAAATCGATATAAAAGATAAAAGGAGCGACAACC
AAAGTACAAACTATTGTAAATATAAGTAAAAGTCCGTTGATCCGATATACTTTGACATCTAATTAAAGAA
ATTCCTAATTTGCATCTTCTTTAGTAATTTTAAAGACTTTGGTTAGCGCTCCTGAGATTTGGCTTGCCAA
ATTCATAGAGAAAGGACTATCCTTTGAAAACTAATTGAACTTGGAAACTAAATCAAAATTACTAAATTGA
TGTACAACTGTCAGCGTTTGTGTGCCTTAGTTAATGCTAAAAACCATGAAAAGCTCTTAGCTGCTAAATG
CCAAGAATCCCCGCCAAGAACCGAAACCCGCATCAGTTGGCTGGAAAACTGGGAAATCAGATTGTGAAGA
AATGGGTATATAGAATCGATAATAGCAACCAGGGAACTCCGAGTAAATAAGTTTAGACTAAGAGTTGAG
TTCAGTTCGTACTTCTACTTCTTCTATCGCTCTATCGTTCTCTCTATCGTACTCAGTATAAATATAACAT
ACACCTATTATAGAACTAATTTAGTTTTGCTACGATTTCTGGTTTGGTTCGCCCAAGAGTCGCGTATAGT
GTCATAATAAATCGAAAGCATATCTAGTATATATTCCTTATATACATTGCATACATATATCTATTTATGA
TCATAAACTGCATACCACATATGAATATATCTACATAAATATAATATATATTATTTACGAACGTTCAAGT
ACAACGTGTGCCGTGTTTTTTACCTATGATTTTCGTATAGCGTATTTAATTTCCTATGATTTATTCATCA
TTTTCGTTGAATGATGTTCCAATGATAAGCAGGCGCAATATTGCGAGCAGATGAAAATGAAGAAAAACTA
AATTATATTGAAAGCAACAATCATTACCTTGCCATCATTTACCTATTCACACGATCCTATAAAATTCTGT
TTTCATGAAACAAAAACAGTGGCCACCTTCCGGAAAGGCGTCCGAGCCGTACAAATGGTTGGGCGTGCAA

GTACGTATAACTTACTCTACctctctctctctctctctctctctctctctgcctctctctctctatctat
ctcctgtctctgtctccactactctactgtTACCAATAAGCATACGAAACATACATCTCATAACCAGAAA
CCACGAAACCCAACATGATTTCCAATTGGCAAACCAAAATTTATCATCACATTCGCCCTATAAACTCTCC
TGCTAATCAAGTTAAAACGCTCAATTACATGTTTATAGCGCCAAACTAACTGTCATCTAACTACAAACTA
ACTAACCAACTCTAATTGCTATTGTTCAGCTGTATATGCCATTTGCCATTCAGCAAACCAATCGAAAACT
AAGCCAATCAATCAGGCAGCCAAACAATCGAAAATCAATCAATCTTTGTCGGAGCTCAGAATTcagaact
caaaacacagaacacaaaacaccaacacaaGCACACAAAGTCCCTGGACCCGCCCCCCAAATAACCAACC
GCCCCCTTTTCCAACACTAATGCACTACACAACACACACATATGTACATGTTTCGTTTCTACTCGTTTGT
TCTAGTAATTGTGTACTGTTTCGTATTCAACTTTAACCCTAGTGTGCGTGTGCGTGTGTAGTAAAGTCAA
GAGAAAACCAAACCGAACTTCGCTTTGAGTCAGTTAATATTGGAAATCAACTGTTTGGATACTAAATATT
CAGTAACCATAATCGATCGATTCAAGCGTTGTGTTTTCGTATGAGCTCCATACTATCCATACTATTCGTT
TTTGTTATCAAGTCGAGTCATATTCTACATAATAatcgtactcctactatatcgtaaagtatcttgtaca
aacgttacctacatattgtattgtatctatcgtatcgcattatatcatgtcgtaAAGTAATTCGTTAATT
CGTGATACAAATTCTAACCGTTCACGTATCTTTGGACAACTATGTCATATCCAAACAAATACTTCAACTT
TCCTATTTATATGATATACTAAGTGGTTACACCAACACTCCAATGAACACAACACTTCGGGTTCCACTGA
CCTGCAATCCTGAAACACAATATCACACTCTACACTCATTTTCTGTAATATATATATATACAAATTCGAT
CTTCTACGCCCGGACCTGTGTTCCTTTCTATATAATATATCAAATATGTAAACCTATCATCTTTCTATTG
TACCTCCCTACCCACACATTCGTGTTCCGTATCAACTACTACAACTACAATAAATACTCCTATTTATATG
TCTCTGTGTGTGTGTGTAGAAGACGATGAATACTCGTTGTCAAGTAAGTCAAACTAACTCAAAATGTTGT
TTATCCATACCAGAAATTAAGAACATCCATTTCAATATCGCATTTGTACTTGTCGAAAACATATCGTTAT
CATTTGAATTACTAGAGTCCTTTAAAAATTTCCCTTCAAAATATAGTGATTTGTATTGTTAGTTTTGTTT
CGGTTATTACTTGTTTTCGTTTTGAAGAGAGAACCCCATTATGTTAGAGACATTCTCAGTTTTAGTTTTA
AACCATACATAAATCAACATGAATAAATAAAATGCAAATGACTTGAGAAAGCAAAGCAACTGAGCCAATG
ATTAGAACCAATATTCGATCACAACCACCCAAAGCAGTAAAATGAACATAAAAAGCAAGCAGCTCCTCCT
CATCCCCCCGAACACTGCACCAATCCCAATCGTCCGGGGAACAGGAGATTCCAATTAGAGAGCTATTAAA
TTCCAAATTCCGTGCAGTTAAATTGCTGACCATCAATGATCGTTTCAATCAGCTCCCAAAATCAGAAGCT
GGGGATAAACTAGATAGTGCAACTCGTAGTTGGAGAGATCGTTTTTGAAGTTCTTCTTTATGTGGGTGTT
AGCAGTTCTAGCAGCCTAAGTGTTAAGTGCTTGACAATCGGATACTAAAGTCCAGTGAGCCAAAAAACCA
GCTATTGATGACTATATAGAGTATTACCTATCTTGCTACTCAGCTTGTAATCCGCACTTAGTCAGCGTGT
TGCTTAAATAATGCTTCAAGCTTTAACCAATTTGGTTTGTATCCCTATGTACCAGAATACCAATATATAT
CACTGCTTGCTTAGCTCTATTTATACCTATACTCTTTTTACCTATATACCTCTTTATATGTATTATGTAA
ACGTTTGTATAATTGTAAGATGACACCGTCACCAGTTCTGCTCCAACTCATATTTATTTGCATGTTTTGT
TCGATATTCTATAAAGCAAATACCCTAAAATGAATTCCCAAAAACCATCCCCCGACCCCATCTTGAACAC
TTTTCGTTCAGTTTCGGCCGACGGCCACTTTTCATTTGATTTGAGTTCGTCAGGTTTTCCTGGGTTTTCC
CAAGAGTCAGACTAATTTCTCTATGATTTTCTCTCGAATTTCGTGTTTATTTTGAACACAAACCAATACT
CTTACCGCTTCAGTTCTTacaaaaaccaaacacaatccccccctaaaaaattaaaaaacagaaaaaGAATT
TGATGTATATTCAAAAGCAGTTTTGAGCTTCACATaaaaaaaaGCTTGACCATATAAATAACACCaaaaa
aaaaaTTAAATTGCATACACACCGAATCTGATTTACCGAGCTGTGTGATAACAAACCACCGTATTTTACA
GATGAACACCATCCTGCACCCACATTTACTCCTCCAGAGCTACCCAAGCGGGTGCATGTGCGTGGATCTG
TATCGGGTAAAGTCTCGTCTTCCAGTCGAACGAACGAACACTCAATTTGGTATCAGTCAGAGATCACATT
CACAGAAAGGTCATTTTGAATCGGAGGTGTTTAGGTGGATAGCACAGTAATCCAGCCAAACAGCTACCCA
CCCAGTCTCACATAACACACACACACATCCACGCTCAAGATCAAGATAACATTCttatcagttatcagtt
atcagttTGCCCCACGATTTCGTTTGCCTTGGTTCGCTTGTTCCTCAGTTGAAAACGTAAGAGATAAAAA
TGTATCCAATCCAATCGATAACCCATTGATAATTGTTTAGATATTCGTTTGCATAACTCTGTTTTTGCGT

TGATTCTTGAACAGAACTCGTTCCGCTCTGCTAATGTAACTCGTTGATATACTCTCTAATCTAAAGAAAC
GATATACTATATATCTTCCGTATTATCATATCCACATATCGATGTCTATTTACCTTAACATACTTATTTG
TACACATCACTCGGTGTGGCTTTGTTTTCAATTGCATAAACAATCTGCTTGTTACCAATTTTGTTTATTA
CTGTCGCAACCACACACAGCATCACATAATGACAAGAACTAAGAACTAAGAAACCAGTAACCTAAACCTA
AACCAAGTGCAACATGCAACACGCGGCTCAAAAATACCTTTCTCGATTAACTAATGACTAATACTTAAAG
CCAATGAGTATCTCTTCACACTCTCTGATCAGCGTGACGAAGCTCTTGCCAAGATTACTAATCGATTGAT
CTGCTCTCTATCTCTGATCTCGTCAAAAAACAACAACGCTTCTGCCTTGCCAAATGCCAAATGATACAAA
ACAAAAACTGTCAATCTGCCACAGGTGATATCACTCGTGACAGAGAAGATACGAATCGTAAGTTTCGATG
GTATTGATTACATATCATTTTCATGTGCGCAACCATCTCTTGTGATTACAGGTACTCAATCGCAATGTGC
GCCGGCCGAATGGCACTGGCAACGGTACGGGTGGCATGCACCACATGAACAACAcggcagcggcggctgc
ggcagctgcggcggcggGAAAACAGGTGAACAAGGTGAAGCCAACGGTGAATGTGAGCCGGCAGGTGGAG
GGCCAAGTGATATCGCCATCGCAGTACAACAG
>dme_circ_0007469_junction_seq
AAGTGATATCGCCATCGCAGTACAACAGGACTGTGTTTCTCCAAGCTGAAGCTTCT
>dme_circ_0007470
GATGAACACCATCCTGCACCCACATTTACTCCTCCAGAGCTACCCAAGCGGGTGCATGTGCGTGGATCTG
TATCGGGTAAAGTCTCGTCTTCCAGTCGAACGAACGAACACTCAATTTGGTATCAGTCAGAGATCACATT
CACAGAAAGGTCATTTTGAATCGGAGGTGTTTAGGTGGATAGCACAGTAATCCAGCCAAACAGCTACCCA
CCCAGTCTCACATAACACACACACACATCCACGCTCAAGATCAAGATAACATTCttatcagttatcagtt
atcagttTGCCCCACGATTTCGTTTGCCTTGGTTCGCTTGTTCCTCAGTTGAAAACGTAAGAGATAAAAA
TGTATCCAATCCAATCGATAACCCATTGATAATTGTTTAGATATTCGTTTGCATAACTCTGTTTTTGCGT
TGATTCTTGAACAGAACTCGTTCCGCTCTGCTAATGTAACTCGTTGATATACTCTCTAATCTAAAGAAAC
GATATACTATATATCTTCCGTATTATCATATCCACATATCGATGTCTATTTACCTTAACATACTTATTTG
TACACATCACTCGGTGTGGCTTTGTTTTCAATTGCATAAACAATCTGCTTGTTACCAATTTTGTTTATTA
CTGTCGCAACCACACACAGCATCACATAATGACAAGAACTAAGAACTAAGAAACCAGTAACCTAAACCTA
AACCAAGTGCAACATGCAACACGCGGCTCAAAAATACCTTTCTCGATTAACTAATGACTAATACTTAAAG
CCAATGAGTATCTCTTCACACTCTCTGATCAGCGTGACGAAGCTCTTGCCAAGATTACTAATCGATTGAT
CTGCTCTCTATCTCTGATCTCGTCAAAAAACAACAACGCTTCTGCCTTGCCAAATGCCAAATGATACAAA
ACAAAAACTGTCAATCTGCCACAGGTGATATCACTCGTGACAGAGAAGATACGAATCGTAAGTTTCGATG
GTATTGATTACATATCATTTTCATGTGCGCAACCATCTCTTGTGATTACAGGTACTCAATCGCAATGTGC
GCCGGCCGAATGGCACTGGCAACGGTACGGGTGGCATGCACCACATGAACAACAcggcagcggcggctgc
ggcagctgcggcggcggGAAAACAGGTGAACAAGGTGAAGCCAACGGTGAATGTGAGCCGGCAGGTGGAG
GGCCAAGTGATATCGCCATCGCAGTACAACAG
>dme_circ_0007470_junction_seq
AAGTGATATCGCCATCGCAGTACAACAGGATGAACACCATCCTGCACCCACATTTA
>dme_circ_0007471
GTACTCAATCGCAATGTGCGCCGGCCGAATGGCACTGGCAACGGTACGGGTGGCATGCACCACATGAACA
ACAcggcagcggcggctgcggcagctgcggcggcggGAAAACAGGTGAACAAGGTGAAGCCAACGGTGAA
TGTGAGCCGGCAGGTGGAGGGCCAAGTGATATCGCCATCGCAGTACAACAGGGTAAGCCAATCGCACTTA
TCCTGCGGGGACTGTACatatatatatatatatttataatatatatTTAACATGGGGTGGAACAGCAGGC
CATCTACTTAACTATAGAGTTTGATCACATTAAACAGCACTCTGTTATCAAATCAAATATATATAGCAAA
GCAAAGTCGGTAATCGAGCAAGAGCAAGCAGCAAGATAACTCAATGAATACACCCACTAGGAAAACTCTG
TTTGCAGCTGCATCAAGTTTTGATTTTCCACTTTTCCGTTGCGTTACAATCGAACaaaaaaaaaaaaaa
aaaCGAGAGATTATGTTACACTCAATTATGGTTACTGTGTTTGCCACACTCGTTTTATTTGACACAACAA

AATGGTCAGGATAAGGATAAGGATGAATAGGTATCTCGTACATATGTAAATGCTCTTTAACCAACTAATC
GAAACACCTCAACTAACTGTCTCTCACTCACCTGCTCAACCACTCAACCACTCGCTCACTCTCGAAAAAA
ACAAACAAACAAATCAACCAATTATTAACTTTTAACCAGTCTAAATGTATAAAAACGAACAACATCTTGT
TTCAGGCCACCAGAGAATGATGCTAACCCTTATGCGGGCTATCAACTTGCTTACGAAGTTAAAAAGCTCA
TGTATGCGTCCATAATTTCCTAGATTTTTAACTTACAAACTCTATCGAAACTAAAAAAGCCAAAAGCCTT
TTCTTTAACCATACCTTAGTATTCGTTAATACTTATCTAACTACATCTTATGCGCATTTTGTTTGACACA
CATTGATATTGATTGATCGAAAGCTATGCCATATTCCATCAAAATCGCGTACTAAACATAATATGAATTT
TAACAAATTTGTGCGtttttttttGTCATGTCGCTGTCTTCTGTGTCTGTGTGCGTATGTGTTTGTGCGT
TTGTATATGTTGGTGTGTATGTGTGAGCGCCCCATGATTATTTTGCCATCTAAACTATCCCCAGCATAAC
TAACCGCATTCCATTCTGAAGCATTCTGTCTACTAACATCTATCTGCCACCCAAAGTAAACTAACAAGTA
ACAACTTTACTTTGGGTTTAATGTTTAAGTAAAAAGGGTTCTAATTCTAAATAATCTTTGAAGCAGCTTT
TTTTAAGAACTTCTTAAACTCTCCTTTTGCTGATTTATTTGCCGTGCTAATGTCTTGTGTATATTTTAAC
TGTACGCAAATATCTATGTAAGAAAAGTGTTTTACAATATGGATTTCCATTAGGGAAACTTAAGATATTT
TTACCAATCTTGAGAAAAGGATCAATCAAATATTGTAGCGATCTTTTGGCGCAGACTACGAAACTTCTaa
aaaaaaaTATTTCAACTTCTTCTTCGAAACTCATAAAACTAAGTTGGTTCTAGACCACAAAAGCATACAT
TAAGCTCAATAATCGCCCATTTTTCTCCTGCATTCTATATCTATTTCTCTGAATCATTTGAACTTGAATT
GCAATCTAAGTGTTGTATGTGTTTTTCTGTGTTCATCTTTGTATCAAGACTTGCACCTGTGTACCATATA
ttttatatttttacgattttttttttAATTAAGAAGACACAACATTAAGTATTGTAGTTGAAAATGCCAA
GTTCGGCGGATCGGAAGGGTTATGGATTAACAAAGGCCGCTCCCTCGCGCATGCCGAGCACATGAATCAC
ACTGAAACACCTGGAGGGAGTTAGCCGACCACCTAATACACTAGTTTTTGATCCAACCATCCCAACCGCC
GCCGGATGGAGCTGTTGAGCATTTTGCGTAGTGACACATTGATTTATTCTCAAATACTTTTTCGCATTCA
TTTGGAGACTTTTGTTATTAGCACATGATCTTCTCTAACTCTCTATCTATCTTACTATCCGTGTATTTAT
CTTTGTAAACGTAAAACTCTTCTTAACTATCTTTAACTATTCGTATATTGAGATCACATTTTAATTCAAT
ATGCCCCAAGTACTCGTACTCGTATTGAAGTATTCACATCATTTATTGAACGAAACACGTACTCGTAGCG
TTTCCGATGAACACTTTGCATTTGGATGTAGCGTTTGTTTTTGAATTGAATTGGTTGATAAACTGCATAT
GCAAGTCAAAAGTTGAATCGAAGTGAAACCCATTATGatcgaatcgaatcgaatcgaatcCAGCTACAGC
ACAGACCGCCCGATGGCTAACTCCTGTCTCTATCCTATCGATCTACTTGTGTCTAGGGCCGACGAGTCGC
AGTTCCGGCACGGGCACACAGAATCAAAATGGCGGCGTATCCTTGCCCGCCGGCATTGCGGACGACCAGT
CGAAGGACTTTGATTTCGAGAAGACCGAAATGAAGTACGACTCGACGGGCATGTTCCACTGGAGTCCGGC
AAAGAGCTTAGAAGACTGCATACTGGGATCGCAACCAGGCGGCCATGACCGTGCTGAACGGCCACGTGGT
CGTGTGCCTGTTTGCCGATCCCGATTCGCCGCTGATTGGGCTGCGGAATCTGGTGATGCCGCTGCGGGCA
TCCAACTTCCACTACCACGAGCTGAAACACGTGGTCATCGTGGGCTCGGTGGACTACATAAGACGCGAGT
GGAAGATGCTGCAGAACCTGCCCAAGATCTCGGTGCTCAACGGATCGCCGCTGAGCCGTGCCGACCTGCG
GGCCGTTAACGTCAATCTGTGTGATATGTGCTGCATCCTGTCGGCGAAAGTTCCTAGCAACGACGATCCC
ACGCTGGCCGACAAGGAGGCGATCCTCGCCTCGCTGAACATCAAGGCGATGACCTTTGACGACACGATCG
GTGTGCTCAGCCAGCGCGGCCCGGAGTTCGACAATCTGAGCGCCACCGCCGGCAGTCCCATTGTGCTGCA
GCGGAGGGGCTCGGTCTATGGCGCCAATGTGCCCATGATAACAGGGTAAACTGCATCGCACTAAGTTGGC
CGAGTTTGCGAACGACCGAACGATTGTGAGGCTGGCCAGGATTTGGACGTGGGTGTTGGCATgggggggg
gggggggTTGAACTGTAGGGGTGCTGCTGGGTTGGTGGGTACATCCTATGTCCTGGCCAGGCTCACCCGC
TTTCTGCTCCGAATGCGGTTCAAGTGTGTGCTCTACTAATGCTTCACATGTTGGTTGTCGTTTAAAGGAC
TTACTTCAATCAAAACGCCTTAACGCTGATCCGCTCACTGATAACGGGCGGCGCCACGCCCGAGCTGGAA
CTGATCCTGGCCGAGGGAGCTGGCCTCCGGGGAGGCTACAGCACTGTGGAGAGTCTGAGCAATCGAGACA
GGATGTCGAGTGGGTCAAATCTCACTGTACGACGGTCCGTTGGCTCAATTTGGGGAGTGCGGCAAGTATG
GGGACCTATTTGTGGCAGCCCTCAAGTCGTACGGAATGCTGTGCATCGGATTATACCGATTCAGGGACAC

CAGCTCCAGTTGTGATGCGAGCAGCAAACGTTATGTAATAACCAACCCACCCGATGACTTTTCACTACTG
CCAACAGATCAGGTGAGCTTCGTTATAGGTATACATTATTATTCCAATATACAATGTGCATTATATAGGT
ATTCGTTTTAATGCAATTCGATCCGGGCCTGGAGTACAAGCCGCCAGCGGTACGAGCACCTGCAGGCGGA
CGTGGCACCAACACACAAG
>dme_circ_0007471_junction_seq
GCAGGCGGACGTGGCACCAACACACAAGGTACTCAATCGCAATGTGCGCCGGCCGA
>dme_circ_0007472
GAGTCAAGAAAATCCGACCACTTTCTTCAGTGCGCACTTCCAATGCGAGTCGTCACTCCATTCCAGTGGA
ACTCCAGCGGATGGGCACATGGTTTTTGTCCGCCACCAGCGACCAACTAACAGTCAGTTGTCTTCGCGAC
TTGAACAGGTGCGGACGTCGCTGCTCCTCGAATTTATATTCGAAACTCTTTACAAATCTTTTGTGCGTGC
AGTTTAAGATCGAAGAAAGCGCCGCGGCGCTCGTTGACATGAAGTTAATTGACGTGATTATATACCTAAT
TGCCATTTTGATCTTCATCTATTTGCTGCTGGAGTAGCAGAAGAAGCCAAAAGTTGCTCGACATTTGCCC
CAGAGCTTTCTACAGTCTACCAGATCCATCAGCCAGCTACCCGACAATAAAACAACGAAAATCTCTAGCA
AATCCGAAAGAAGATGAGG
>dme_circ_0007472_junction_seq
TCTCTAGCAAATCCGAAAGAAGATGAGGGAGTCAAGAAAATCCGACCACTTTCTTC
>dme_circ_0007473
GGCCCGGCACGTGCTAATCGAAAATGCAGAAATTATACTAAGAGCAGATAGGTAGGCACATCTCAGTCCA
AACGTGGTCAGTGGTCACGAAACGAGGAAAGAGCAACAAAACCTAAAATCACAAACCGAAGACTTCAAAG
TACACAAGAATTTGAAGCCACTGCAATGCGTAGGAGGCGTGGCACGCCCCTGGGCGTCAGCTGGACGAAC
TGCGTCCTGCTCCTGGCCACCGGTCTGCTGGTGGTGCTCATTTCGGTGCACGCCAAGAACCCCAGGCCGC
ATCGAGGACTACCCGCCCCGGCTCCTGCACCAGTTCCTGCATCCGTAGATCAGGTGGCAACTGAAAATGC
CCACAGGCGGGAACCTACCGTCGATGGGAGTGTACTTCAACGGCTATCGGGTTCAGATAGGGCTGTTGAC
gaggatgaggatgtcgatgaggatgaggatgaggGGCTAAAGCACCAGCACCTCGAGCATCGGCAACAAT
TTGACGCATATCTGGGCACAATCCGACGGCTGCGTCGTCCATACCGCAGCAAATACACCATCGAGCAGCT
GATGCGACTGCATGTGACACCAAAGGTATCCGGAGACATTGACATGGATCCCTGCAAGGCGG
>dme_circ_0007473_junction_seq
GACATTGACATGGATCCCTGCAAGGCGGGGCCCGGCACGTGCTAATCGAAAATGCA
>dme_circ_0007474
GGCGGTTTCATGGGCGACATTGCCCTGCCGGAGGGTGATTCCGGCCCGGTGGTTGCCCACACGCCCATCG
ACGACGAGGAGCAGGAGCGCCAGGAGGTGGAGCAGCTGCTGGCTGACTCGCCCAGCCCGGTTCACGACCC
CCCTTCCGTTTCCGGTGCGGCCAACTCAGGGCCGACCAAGTTCAATGCCACCTTCCAGCTGGATCTGGAG
AAGCTCAAGCAGGAGGTCTACCACGAGGGCCTACAGGTGGAGGAGGAGGGCCTCACCGACATCATCCGCA
AGAAGACCAAGCTGCCCAGCTCAGGTCCGGTCAACCTGAAGGCAGGACAAAGCCAGTCACAGAGTCAGGA
GTGCCAATGAACCCGTGAAGCCCGGTTCGGAATCGCCCTCCGATTACGCGCAAGCATCCAGCTACAAGAA
TGAGGTACTGCCTCCGTCGGAGCAGCGAATCCATACGGACAAGCCGGCACGGTTGACGCTGGCCGAGATT
GGACCCAAGTTCAGCAACAAGACGCAGTCGCTGCACCGTAATGTGGACACACGCAAGATTCAGTCGGACA
ACAAGGACATCGAGGACGATTCGGGCAACAATTTTGTGGCCGAGAGGAGGCCGTCACAGATTGTGCCCAC
TTCCTTGCCGGAGCAGCGATCCACTCCGGCTCACGTGTACAGCAATGCCGGAGGCAATGGCATGCAGTTG
GACGACGAGGTGGACTTCTTGCATTCCCGTGGAGTCGTGAGGAGGCGTCACAGGCGAGGACCAAAACAGC
GGAAGGAGCAACCAGCACTTGGAGCACGAGGTCAAGCTGCAGAGATTGAGGGAGGAGCTCAGCCGTCCGG
TCATCGAGGCCCACCATCACCAGTCGCACCACCAGAAGCAGGCGCAGCACCGCCAGCACCACCAGAGCCA
CCATCGAAAGAAGCACCGTCGACAGCAGCAGCACCATCGTCGACGTGGCCAGAGCACCACGCAATATGTG
AACCATTCCCCGCCAGGATTCAATCAATCGCAGCAGGCCCAACAGCAGTCAGTGCCTGAAATGGATTTGT

TGAAAGCGGA
>dme_circ_0007474_junction_seq
TGCCTGAAATGGATTTGTTGAAAGCGGAGGCGGTTTCATGGGCGACATTGCCCTGC
>dme_circ_0007475
GCTGGGCAGGGAACACGGCGTGAAGTTTATCTTTTGGCTCTTATACCGTCCACTTGTCATTGGCGGCGGC
TTGCGTGCATTTTGCTTCATCTTTTCCTTGCGGGCCACGAGCTTCTTAGTTTCGCGCACCATGTAAATGG
CCTCTGGG
>dme_circ_0007475_junction_seq
TTCGCGCACCATGTAAATGGCCTCTGGGGCTGGGCAGGGAACACGGCGTGAAGTTT
>dme_circ_0007476
GTTACGAGACGATCAAGCGCATTGCGGTGGACGAGCAGTACAAGCATCAGATCCGCTGGTAAAGAACGCA
CAATCCCCAGCCAAACGGAAACCAACTCAAAACTGCATTTACGCGACGTACTGAGAACCACCAGAACTGG
AGGAGATTCGCCCCCGATTGTAAATAACAAGAGCTAGAGAATGAATTAGAGGAGCACAAG
>dme_circ_0007476_junction_seq
GCTAGAGAATGAATTAGAGGAGCACAAGGTTACGAGACGATCAAGCGCATTGCGGT
>dme_circ_0007477
ATTAAGAGTGAGGAGGCCAAGCTCTTCGATGCAGAGACCGGAGCACCCACCGACCTGCACCTGCAGTTGA
TCCACTGGGCATACAGTCCGCAGCCGGACAAGCTGAAGCAGTACATCGAAAAGCTGGCCAAGAAGACGCC
CGAAAAGCGCAAGCAGGAGAGCAGCAGCAGTGCCAGCGATTCCAGTGCCACCAGCCAGGATTCCGATGGC
GAGGATAAGCCCAAAAGGAAGAAGAAGAGGGAGGAGTGAGG
>dme_circ_0007477_junction_seq
AAAGGAAGAAGAAGAGGGAGGAGTGAGGATTAAGAGTGAGGAGGCCAAGCTCTTCG
>dme_circ_0007478
GCTGTGCAGAAGAGCAGCAGCAGCATGGCCAGCGGATAGACGAATGTGCGTTGGAAGGTGCTCGATGTGC
GCAGCTTGTCTAGTTCCTTGCGCTCGGAGTCCAGTTCTCTTAGTCTATCGTTGAGCTCGTCCACGTCACT
GGCCATCGCCTTGCGCTGGTATAGATGAGCCTGGGA
>dme_circ_0007478_junction_seq
CCTTGCGCTGGTATAGATGAGCCTGGGAGCTGTGCAGAAGAGCAGCAGCAGCATGG
>dme_circ_0007479
GGTGCTCGATGTGCGCAGCTTGTCTAGTTCCTTGCGCTCGGAGTCCAGTTCTCTTAGTCTATCGTTGAGC
TCGTCCACGTCACTGGCCATCGCCTTGCGCTGGTATAGATGAGCCTGGGAGTGTTGCAGCAGTGGCTGGG
GATAGAAG
>dme_circ_0007479_junction_seq
GTGTTGCAGCAGTGGCTGGGGATAGAAGGGTGCTCGATGTGCGCAGCTTGTCTAGT
>dme_circ_0007480
GGGCTCGACCCGGCCACCCACTTGATTGTGCCGGGCGCGAAGGCCAAGAAGCCGTTTGCCATGCCCATGA
AAGATCTCCAGTCGGAGGTGGTGATCAAGGAAGAGCCCATCGATGCCAATGAGGAGGAGGTGAAG
>dme_circ_0007480_junction_seq
CATCGATGCCAATGAGGAGGAGGTGAAGGGGCTCGACCCGGCCACCCACTTGATTG
>dme_circ_0007481
GGTGGTGATCAAGGAAGAGCCCATCGATGCCAATGAGGAGGAGGTGAAGGTGGAACAGCAAGATGATTAC
CAGAATGGCAACGACTACTCGCATCATGCCGACGACGATGGGCATGAGATTGACCAGGTGGGAGATGTGG
ACGATGA
>dme_circ_0007481_junction_seq

TTGACCAGGTGGGAGATGTGGACGATGAGGTGGTGATCAAGGAAGAGCCCATCGAT
>dme_circ_0007482
GGTGAAGGTGGAACAGCAAGATGATTACCAGAATGGCAACGACTACTCGCATCATGCCGACGACGATGGG
CATGAGATTGACCAGGTGGGAGATGTGGACGATGAGTGTGTGATCCTGGATGACGACGATGAAGAGGATG
AGGATCATGTGTACGACGATGAGCCCCAGAGCGGTGATGGCGATGCCGAGGGGAAAGCTCAGGACGATGG
AGCCGAGAACAATGGCGACGACAAGGCTATTATCCATG
>dme_circ_0007482_junction_seq
AATGGCGACGACAAGGCTATTATCCATGGGTGAAGGTGGAACAGCAAGATGATTAC
>dme_circ_0007483
GAGGGCGTCGAATCCAGTGCCGCTTACATCACAAAGACCTTTTCGACCTTGGGGTTGGAGAACTTCGTAT
TGGTCACCGGACAGGTGACGGTACCGTCGTCTTTGGTGATGTCCGGCAGAGCCATCTGACCAAAGATCTG
TCCGTTGGGCAGCATCATCGGCTGATT
>dme_circ_0007483_junction_seq
GTCCGTTGGGCAGCATCATCGGCTGATTGAGGGCGTCGAATCCAGTGCCGCTTACA
>dme_circ_0007484
GCCGAGGAGAGCATCAATGATGAGCTGAGCGTCACACAGATCTGCAAGCGCAAGCTGGACCACCTAAAGG
GCATTACACCGCCAAGCAGCGTTACAGGCGACCTGTGGCAGGGTTCCGTGGACCAATGGAAGCGGATCCG
ACTAGACCGCCTGGTCATCGAGCACCTGCTGCGCATGGGATACTATGAAACGGCCGAGGAGCTGGCCGCC
AAGTCCGATGTGCGCCACCTGACCAACCTGGACATTTTTCAGACGTCGCGCGAGG
>dme_circ_0007484_junction_seq
CTGGACATTTTTCAGACGTCGCGCGAGGGCCGAGGAGAGCATCAATGATGAGCTGA
>dme_circ_0007485
GATATCATAAAATTAGCAACTCTTTTCGGGATCCCATAATGGCAATTAGCATGTGTCGTTGGTTTTGAGA
GACTCATAAACAAATTAGTAATCGTTTAGTGATAAATGTTAATCGACCGTTTAGACTGCGTCAATGGATT
TGGTTTGAAAGAGG
>dme_circ_0007485_junction_seq
TGCGTCAATGGATTTGGTTTGAAAGAGGGATATCATAAAATTAGCAACTCTTTTCG
>dme_circ_0007486
GGATTTGCACCACCCAACCGTCAGCAACCGACGCCTCCTGGCTGGCCACCCGGCTGGCAGTGGGGAGCCG
GTGGAAATCAGAATCAACCCGGATTTGACTGGGTTCAAACAGGATTTGCACCAGGCTGGAATGGAGGTGG
AGGGCAAGGACCAGGATGGAATGGTCCAGGATGGAACGGAGGCGGTGGAAGAGGacctccaccaagacca
g
>dme_circ_0007486_junction_seq
GGTGGAAGAGGacctccaccaagaccagGGATTTGCACCACCCAACCGTCAGCAAC
>dme_circ_0007487
GTTGTCTGCGAAGCAGTGGATTATGATCGTGTCCTGTCGGAGTTGAAGGCTTCGGGGAATACCACCGTGG
AGACGCGTCAGGCTCTGGCTCTCAAGGCATTCACGCACACCGCCACCTACGATGATGCCATCTCAGATTA
CTTCAGGAAACAGTATGGATCTGGGGTGTCCCAGCTACCGTTGCGCTATGGCATGAATCCCCACCAGAAG
CCGGCTCAGCTCTACACGCAGCTGGCCAAGCTTCCACTGACGGTCCTCAATGCCTCGCCGGGATTTATCA
ACCTGTGCGACGCCCTCAACGGTTGGCAGTTGGTTAGGGAACTGAAGAAGGCTCTGCAGCTGCCGGCGGC
CACCAGCTTTAAGCACGTTTCTCCAGCGGGTGCGGCTGTTGGAGTGCCTCTAAATCCGGCACAGGCCAAG
CTGTGCATGGTGGATGATCTGTACGAGCAGTTGACACCTCTGGCAACGGCCTATGCTCGTGCCCGTGGAG
CCGACCGCATGAGCTCCTTCGGCGACTTTGTGGCCCTCTCTGATGTGTGCGATGTTGTGACGGCCAGGAT
AATCTCGCGAGAGGTGTCCGATGGTATTATTGCCGCGGGTTATGAGCCCGAAGCTCTGGAGATCCTGAAG

AAAAAGAAGAATGGCGGCTACTGTATACTGCAGGATGGACCCCAACTACGAACCCTCCGCGGTGGAGCGC
AAGACTATTTTTGGTCTGACGCTGGAGCAGAAGCGCAACGATGCTGTCATCGACGCCTCGCTCTTTTCCA
ATGTGGTGAGCAAGCGTGGTCCTCTGCCCGAGGTGGCTGTGCGGGATCTGATCGTGGCCACCATTGCCTT
GAAGTACACTCAGAGCAACTCCGTGTGCTACGCCCGCGATGGCCAGGTCGTGGGCATCG
>dme_circ_0007487_junction_seq
GCCCGCGATGGCCAGGTCGTGGGCATCGGTTGTCTGCGAAGCAGTGGATTATGATC
>dme_circ_0007488
GTGCTTCCCGTACTGCTGTTGATGCTGCTGCATCTGATGGTGGTGCTGACCCACCGCGATGCTTGCTCGG
CTGTTTGCCTGCCGCAGGGCAGCCATCGGGACACCGCCTCCTCCGATCTGAGCCCGCGACTCAGGACGCG
AGTGCGGCATGCCGCCGATGGCACGGAAGTCGGACTCGCTGCGATACTTCCGGAGATTGGGCGGCACCCG
ACTGCGGGTCGTCTCGTTGTCGCTGCTATCGTAGCCACTGCTCCCGGCTCCAGCCGGTGCACCCGAGCTA
TCCCGAAAGGCGCGGTTCACTCGTCCTCGCACTCCGCCCAGGGCACTGGAACCACCATGGCCATgacccg
gtcccgcacccggacccggaccgggacGATGCGATCGCTGCGATCCAGCATTTGGCGGAAAGACGCGCGG
CAGTCCGAACTTCAAGTATGT
>dme_circ_0007488_junction_seq
CGCGCGGCAGTCCGAACTTCAAGTATGTGTGCTTCCCGTACTGCTGTTGATGCTGC
>dme_circ_0007489
GGGCGTTGTCCACCGGCGTGATAGTAAGCAGAACGGAATCGTCCTCTTCCTCGTCATCGTAGTCCGATCG
CCTTCCGTCCGTCGGTGCTTTTGCTGTTGGCATTTCGGCGTCGGGTGCGAGGTATGTGATCACCGCTGCT
CCTTCTGCCGCGTTCAG
>dme_circ_0007489_junction_seq
TCACCGCTGCTCCTTCTGCCGCGTTCAGGGGCGTTGTCCACCGGCGTGATAGTAAG
>dme_circ_0007490
GAATCCCAAATTCCGGCGCAAACTGTCCGGAATTGTACATATCTGGATTAACGACTTCACCTAGGACATG
CTAAATCTCCTACGAAGGCGCCCAACGTCGGTGCCCCTCATCCGGGCCGCGCTTAGCGCTCCGCTGCACT
CAACGGAAAACCGGCCGGGTTACATAGTGCTGGGTACCGGAGATCGGTGAGGAGGGACCGCTAGGCGAGG
GCGTGCTCAAGCCGGACGGACTTCCCGCCTTCAGTGACATCACCATCGAGATGTGCTTGGGCGCCATTGG
TCAGCAGGCATCGGCGGTGGAGAACTCGGTGAAGGCGCTGGAGAGCGACCTGCACAGTGGCAAGCAGCTA
AATGCCGCCAGCATCTTTCGGGAGCTGGACACCGTGACGG
>dme_circ_0007490_junction_seq
ATCTTTCGGGAGCTGGACACCGTGACGGGAATCCCAAATTCCGGCGCAAACTGTCC
>dme_circ_0007491
GGTGTGGCATCCGGCAGTTAAGTTCTACGATGTGTTCGATGCTGATTCCGTCAATAGCTCCACCCCGGTG
GGTGGCTTTTATGTGGACTGCTATTCCAAGGAGCACAAGTTCGGCAGAAACAATGGTTGGATG
>dme_circ_0007491_junction_seq
CAAGTTCGGCAGAAACAATGGTTGGATGGGTGTGGCATCCGGCAGTTAAGTTCTAC
>dme_circ_0007492
TGCCTCGGCCGACGGGTCGCGACCCTGGAAGCGACGGAACACTTCCGCCGTGGGCACAGATCCGCCGCAG
CTGAGGAAGGTCTGCTTGTAGCGCCGGCCAACGACCGCGTAGTTCTCTTCGCTTCGCTGCTCAGCAAAGC
TGCTCGAAATGTCGGCGGCTATGAGGCGGGAATACAAGTGACTGAACTGGGCGGCCGCCCAATCTCCGGC
AAAAATGTCGGTCAGGGAGCACGGATGTGCGTCCTTCTTGTCCAGCGGCATGCATTGATAAACCGGCCAC
AGCTTGCGAACCACCTCCAGCCAGAAGGCATTCGAGCGGTGCAGCTCCACGTCGAGATCCGCCAGGTAGA
GGTCCTGGCACAGATTATAGCCAGCCAACTGAGTCTTCAGCAATCGCATCTTCTGTGAAAGCTCAGCCTC
CAAGGGCTCACCGCTGCTAAAGTGTCCGGATAGGCTTCGAATCACTGTGGGATCGTCGAGGAAGTTGCTC

ATCACATGACCGGACACTTGAGAGGCATCCCACTCGATGTTTGAGAG
>dme_circ_0007492_junction_seq
GAGAGGCATCCCACTCGATGTTTGAGAGTGCCTCGGCCGACGGGTCGCGACCCTGG
>dme_circ_0007493
GCCGCCGACATTTCGAGCAGCTTTGCTGAGCAGCGAAGCGAAGAGAACTACGCGGTCGTTGGCCGGCGCT
ACAAGCAGACCTTCCTCAGCTGCGGCGGATCTGTGCCCACGGCGGAAGTGTTCCGTCGCTTCCAGGGTCG
CGACCCGTCGGCCGAGGCACTTCTAAAGTCGCTGGACATATTTGAACCCTCCCAGACCACAGATAACAAC
TGAGGGTCGCCCTCTGTTCCGCTAAAAC
>dme_circ_0007493_junction_seq
TGAGGGTCGCCCTCTGTTCCGCTAAAACGCCGCCGACATTTCGAGCAGCTTTGCTG
>dme_circ_0007494
TTTAAGCTGGCCTGGCGCCGTCGTCCTGCTCCTCCGGATTCGGACAAGTTTTATAACTTCACTACACTAG
CCTGCCAGCTCAACGAAGAGGAGGAGGGCGTGGCGCCGACGGACTCACGCCTTCGTCCCGACCAAAGGCT
GATGGAGCAGGGCGACTGGGACGAG
>dme_circ_0007494_junction_seq
GCTGATGGAGCAGGGCGACTGGGACGAGTTTAAGCTGGCCTGGCGCCGTCGTCCTG
>dme_circ_0007495
GGGTCGTACTGTGGGCCGAGTCCGCCGAGGTTGTTTCCAGGCCCGACTACATTGCCGCCAAGGCCCCCAC
CTCCTGCCCTGCCATCATAGTACCCCTTGTAGGTATTAGGGCTCTGTCCGTCGACCAGCGTATTGTCGAA
CTGGTTGGGGTCAGCGCTTTGCTGTCCAGTATACAGGGGGTTGTATCGCGGAGAGTAATCCTTGTTGTAG
CTAGGCTGATAGTTCTGAGGCTGCTGTCCCGACTGCGGATCCGGATCCGGCAGGTTTTCGGAAAATTGGG
GCGTGATCTGGCCAACTAAGTTGAGCACAAGAATACTCGCCAGAAGGCCGATTTTTGCTAGCATAGTTGG
TCCTTAGCTTTAATAGCGTGGGTTCTGGTTTAGATG
>dme_circ_0007495_junction_seq
TTTAATAGCGTGGGTTCTGGTTTAGATGGGGTCGTACTGTGGGCCGAGTCCGCCGA
>dme_circ_0007496
GGCTAAAAAGATCTGCAAGGCACACAATGCCGACCTGATAAACGTGGATAATGTAGAGAAGCACTCGTTT
ATACTCAAGAATCTCATCCTGCAGAATCAACGGCAGAACCGATTCTTCATCTCCGCCCGGCAAACGGGCC
CGTTGAATTGGGTCAACGATGACAACACCCAGTTAGTCCAGATCGAAGACTCCTTTTCCATGGACGAGCA
AGTGCCCTTGGAAAACGAAGATCTGCACGACAATAGATTCCTTGTGCAAAACGACCTCAACAACCAAAAT
ATCAATAATCCAAATCAGTTTTACAACTCTCTTCCGGGGACT
>dme_circ_0007496_junction_seq
TCAGTTTTACAACTCTCTTCCGGGGACTGGCTAAAAAGATCTGCAAGGCACACAAT
>dme_circ_0007497
TCGAAAGGGAACACGAGAACAAAATGATATATCGCTGATAATTGGGACGCTGCCGAAACGCCTGCAGAAC
AAAATCGCGGCCAGCTGCAGCCACGCAGCAGCAGCGGCAACAACAACAAAGGCGTTGGAGACGGAAAACG
CTGAGAATGCGGAGCCCGTGGCTACGACAGCAAATGCTTCAAGGCCAGG
>dme_circ_0007497_junction_seq
CTACGACAGCAAATGCTTCAAGGCCAGGTCGAAAGGGAACACGAGAACAAAATGAT
>dme_circ_0007498
GTTGTATAAATTGTTACAGTGCAACTACAAGGAATGGCTGAAGCAGACTCCTTGGTGTTTATAAATGTTG
TATATATATCGGCAAAATCGATCATTAATTTTTTTATGAACTGTTCAATGTGAGCGTCAATAGATTTATA
TAAATATTGTAATAAATAATTACGTAAAAAT
>dme_circ_0007498_junction_seq

ATATTGTAATAAATAATTACGTAAAAATGTTGTATAAATTGTTACAGTGCAACTAC
>dme_circ_0007499
GTGTCCCTCAGCGAACTGAACGTGTTGGTCACCAGGATGTGGGTGCAGCCCATCTTGCGCATGGCCCACA
CATTCGCCCTATAGTTAATGTTGCTGGGCATGATGTCGTGGTTGCGTCCATTTCGTGATAGCAGGCACAC
ATTGACGCCTTCTATCTGGCCATCGATGATCACATCGGATGGTTTTCCAAACGGCGTGCATACTGCATAC
TCCATGCGCTCTGCCAAGTAGATGGGCTTGTCCAAATTGGCCTCTCCAATTATGCCAATTTTGATGGGGA
TGG
>dme_circ_0007499_junction_seq
CCAATTATGCCAATTTTGATGGGGATGGGTGTCCCTCAGCGAACTGAACGTGTTGG
>dme_circ_0007500
GCTGTCGCCGAACACCCAGAAGTGCCTCAAGGAAGACATCCAGGCGAACCAACTGGTTATGGGCGAGTTC
GAAGTTTCCGACGTACCCGGCCAGATCATCGACTACATTGTGAGTTTTCCGCTCGGTAcacgtacttaac
ttactccacgcacttaacgaactcAATTTGCGTCCAACAGGCACGCGACACCAAGGGACACATCCTGTCG
CAGAAGGAGCACATCACTAAGGGCAAGTTCAGCTTCATGTCCGAGGTGTACGATACGTACGAAATCTGCT
TTATATCCAAAGTTCCTGCACGTAAGTTATAGTTTGCAATAAACTCCCATAGCGTATATATATAAACGTT
TCGATTTCTGTGCAGATCAACGCGGCGTTATACAGGAAGTATCGCTCTTGACCAAAAAGGGAGTGGAGAC
CAAGAGCTACGAAGGCGTAAGTACACTTATGTCCTTTTTGCATGTTTTACACTTGACTTGCAATCCTTTA
ATCATATGCAGATTGGCGAAGCATCCAAACTGAAGCCCCTTGAAGTTGATCTTAAGCGTTTGGAGGATCT
CTCCGACTCCATTGTGCGTGATTTCGTTCTCATGCGCAAGCGGGAGGAGGAAATGCGCGATACCAATGGA
GAAAACCAACAGCCGCGTGCTGTTCTTCAGCATCTTCAGCATGTGCTGCCTGCTCGGCTTGGCGACGTGG
CAAGTTCTGTACCTCCGCAGGTACTTCAAAGCCAAAAAACTCATCGAATAGTCGTCTAGCCGTAGTCTTA
ATGCGTGCGTGGAACTGTGTTTAAATGCCTG
>dme_circ_0007500_junction_seq
CGTGCGTGGAACTGTGTTTAAATGCCTGGCTGTCGCCGAACACCCAGAAGTGCCTC
>dme_circ_0007501
GCATTTAAACACAGTTCCACGCACGCATTAAGACTACGGCTAGACGACTATTCGATGAGTTTTTTGGCTT
TGAAGTACCTGCGGAGGTACAGAACTTGCCACGTCGCCAAGCCGAGCAGGCAGCACATGCTGAAGATGCT
GAAGAACAGCACGCGGCTGTTGG
>dme_circ_0007501_junction_seq
ATGCTGAAGAACAGCACGCGGCTGTTGGGCATTTAAACACAGTTCCACGCACGCAT
>dme_circ_0007502
GATTCCAGGTACATCATTAACTGGTCCATGGAGTTGGAGTCCTTGGCGAGCACCTCAGCACCTTCGGCCA
AGTATCGTCTAATTGGCGGTGCCATTTTCCTGGCCACAATCTCTATGAGCTCAACAATCTGATTGCGTTC
GGTGTCCAATGCATTTTCAATGACAGTTTTGATGGTGCTTGCGCATCTCTCAGCCTCTAAGTTAG
>dme_circ_0007502_junction_seq
CTTGCGCATCTCTCAGCCTCTAAGTTAGGATTCCAGGTACATCATTAACTGGTCCA
>dme_circ_0007503
ATTGAATTCCATAATGAAACCGAAGTTTAAATGCGCGCTTTCGGATCGAATTCGCCCAAATTTGTTTTCT
GCCACACAATGATACGCGCCCTGGTCCAGCGCTTGCTTCGGCTCGTAGATAATCAGGTTGCCTCCTGATA
TGGTGTATCGATCCTGCGCCAGGGGATCAATCTTTTGGTACTCGAGTCTGTCGTCTACGTATACCTCTCG
GTACCATG
>dme_circ_0007503_junction_seq
TCGTCTACGTATACCTCTCGGTACCATGATTGAATTCCATAATGAAACCGAAGTTT
>dme_circ_0007504

ATCGGTTATAATTTTCGAGTTACTGGCTGGAATTGGGACATGAATCGGACGCCGAAGATGCTGAACAGCA
AGGTGGCTGGCTACAGCAGCCTAATCACCTTCGCCATCCTGCTAATCGCCTGGCTGGCCGGATTTTCCTC
TCGCCTCTTCGCCGTCATCCGTTTCGAGTCGATTATCCATGAGTTTGATCCGTGGTTCAACTACCGGGCC
ACCGCCTACATGGTGCAGAATGGTTGGTACAACTTCCTCAACTGGTTCGACGAGCGCGCATGGTATCCGC
TCGGCAGGATTGTGGGCGGTACCGTCTATCCCGGCCTGATGATTACGTCCGGCGGAATCCATTGGCTGCT
GCACGTACTCAACATACCGGTCCATATTCGTGACATCTGCGTGTTCCTGGCGCCGATCTTCAGTGGCCTG
ACCTCCATCTCCACCTACCTGCTGACCAAGGAGCTGTGGTCCGCGGGCGCCGGCCTCTTCGCCGCCAGCT
TCATCGCCATCGTGCCTGGCTACATCAGTAGGTCGGTGGCTGGATCGTACGATAACGAGGGCATTGCCAT
ATTCGCCCTGCAGTTCACCTACTTCCTGTGGGTGCGCTCAGTGAAGACTGGATCCGTGTTCTGGTCGGCC
GCAGCCGCTTTGTCCTACTTCTACATGGTGTCCGCCTGGGGTGGCTACGTGTTCATCATCAACCTGATAC
CCCTGCACGTCTTCGTACTGCTCATTATGGGCAGGTACTCGCCGCGTCTGCTGACCAGCTACAGCACCTT
CTACATCCTGGGACTGCTGTTCTCCATGCAGATCCCCTTCGTGGGATTCCAACCGATACGCACCAGTGAA
CACATGGCTGCGCTGGGAGTGTTTGTGCTCCTTATGGCCGTGGCCACCTTGCGCCATTTGCAGTCCGTGC
TGTCGCGCAACGAGTTCCGGAAGCTGTTCATCGTCGGCGGATTGCTGGTGGGCGTTGGCGTCTTTGTGGC
CGTCGTGGTGCTCACCATGCTGGGCGTTGTGGCCCCGTGGAGTGGACGCTTCTACTCGCTGTGGGATACT
GGCTACGCCAAGATCCACATTCCCATCATTGCATCCGTGTCGGAGCATCAGCCCACCACTTGGTTCTCGT
TCTTCTTTGATCTGCACATCCTGGTGTGCGCCTTCCCAGTGGGAGTGTGGTACTGCATCAAGCAGATCAA
CGACGAGCGCGTTTTCGTGGTGCTGTACGCCATCAGTGCGGTTTACTTCGCTGGTGTGATGGTGCGTTTG
ATG
>dme_circ_0007504_junction_seq
CTTCGCTGGTGTGATGGTGCGTTTGATGATCGGTTATAATTTTCGAGTTACTGGCT
>dme_circ_0007505
GCGTCCACTCCACGGGGCCACAACGCCCAGCATGGTGAGCACCACGACGGCCACAAAGACGCCAACGCCC
ACCAGCAATCCGCCGACGATGAACAGCTTCCGGAACTCGTTGCGCGACAGCACGGACTGCAAATGGCGCA
AGGTGGCCACGGCCATAAGGAGCACAAACACTCCCAGCGCAGCCATGTGTTCACTGGTGCGTATCGGTTG
GAATCCCACGAAGGGGATCTGCATGGAGAACAGCAGTCCCAGGATGTAGAAG
>dme_circ_0007505_junction_seq
GGAGAACAGCAGTCCCAGGATGTAGAAGGCGTCCACTCCACGGGGCCACAACGCCC
>dme_circ_0007506
GGTGGAGAAACCCGTCACCACTACTGCACCCAAGGATCCTACGGGTCCGTCCACCAAGACTTCAGCTCCT
GCCAAAAACTCCAGCACACTGCCAGATAAAACTCCACAGAGCAGCCAGGAAAAGCATCCTCCCCTCATTC
AAGAGAAAACTGCCCAGGGAAATGCGAGCAGCAAGCCTGAGAATGAAACTACT
>dme_circ_0007506_junction_seq
GAGCAGCAAGCCTGAGAATGAAACTACTGGTGGAGAAACCCGTCACCACTACTGCA
>dme_circ_0007507
GTGTCTTAAGCTTGGAATATTCCAAAGGTTTCAATGAGTCGGACGCTATTAACACAATTCACACGGGCCA
CAATAAACGAACCAGTAAATTTCTATTTGACACAATTTTTCGCATAAGTTCGGGAGTATCGAATGCCTTT
GGACTTTCGGATACCGAAGACGAGGTCGAGTATACCGAAAATTCCAGCCTAAAAAACTGTGACTGTGGAT
TGTGGCTTTTCGAACGAAGAAATCCGGATTGTTGGGGGAAAGCCCACGGGTGTTAATCAATATCCTTGGA
TGGCACGAATCGTGTACGACGGCAAGTTCCATTGCGGAGGATCCTTACTGACGAAGGATTACGTTCTATC
GGCCGCTCACTGCGTGAAGAAACTGCGAAAATCCAAAATCAGAGTCATATTCGGCGACCACGATCAAGAG
ATCACCTCGGAGTCACAGGCCATCCAACGTGCGGTTACGGCTGTGATAAAGCACAAGAGCTTTGACCCCG
ATACATACAACAACGACATTGCCCTGTTGCGACTTCGCAAGCCTATATCGTTTTCTAAAATTATAAAGCC
AATCTGCCTACCGCGCTATAATTACGACCCCGCAGGGTGCCAATCATGTCCATCACGGAATGCCGAAATC

AAAGATACAAAAGTACTCGAATTACCTCATCCATGTTGTGTGCGGGTAGGCCTTCCATGGACTCATGCCA
GGGCGACAGTGGAGGACCGCTGTTGCTATCCAATGGCGTTAAGTATTTCATCGTCGGCATTGTGTCCTGG
GGCGTTGGTTGCGGCCGCGAAGGATATCCGGGTGTTTACTCCAGA
>dme_circ_0007507_junction_seq
CGAAGGATATCCGGGTGTTTACTCCAGAGTGTCTTAAGCTTGGAATATTCCAAAGG
>dme_circ_0007508
GAAAGTCACCAAGAAGTTTCGCCACCATTGCGGATCGGGCTTTGCTGGCCACCTTGATGGTTTGCATATT
CCGGAACATGACTGGCGGCTTTACAGATCCGCCGAACTTGTTCAAAAACTTAAGTATTATTTTCTCGTGA
TTGTCTTTCGGATCTTCGGTAAGCCGCCGAAGAACCGATTCCAA
>dme_circ_0007508_junction_seq
CGGTAAGCCGCCGAAGAACCGATTCCAAGAAAGTCACCAAGAAGTTTCGCCACCAT
>dme_circ_0007509
TGAAGGTGAAGAGCCTGCAAATAGTAGTGAAAACAAATCGCGTGTACTTAAAACCAGTCAGAATGCCATT
AATATTAAACTAGCTACCGACTTATCGAATGAGAGTGGATACGAATCCATAGCACCTGCCAAAACAATTT
CAAATACTGAAGTAATCGAAGAAGATCAGGAGGAGACGAGCGAGGACGATGCCGAGTGTGGGATAAACAT
GCCTGCCTCCAAACTCAAAATTACCTACAAATTCCATCAAACGGAAACCAAGTTGCTTCGAAAGCTCTTT
AATGTCCATGGCCTTACTGAAGTTCAGGGTGAAAATAATAACTTTAATTTACTCTGGACGGGTGTTCATA
TGAAATTAGATATTGTTAGAAATTTGGCGCCGTATCAGAGGGTTAATCATTTCCCTAGGGTCCTACGAAA
TGACACGAAAGGATCGACTATATAAAAACATCGAACGCATGCAACATCTTCGGGGTATGAAGCATTTTGA
TATTGTGCCACAAACATTCGTTCTGCCAATCGAATCCCGAGATTTAGTAGTAGCCCACAACAAGCATCGC
GGACCATGGATAGTTAAGCCAGCGGCATCCAGTCGAGGAAGGGGAATATTTATTGTTAATTCGGCCAGAT
CAAATACCTCAAGATGAGCAGGCGGTGGTGTCGAAATATATAGTGGATCCGCTGTGCATTGATGGTCACA
AATGTGATTTGAGGGTATACGTTCTAGTCACTTCCTTCGATCCCCTAATTATTTATTTATATGAAGAGGG
CATTGTGAGATTGGCCACAGTCAAGTATGACAGGCATGCCGATAACCTGTGGAACCCGTGCATGCATCTC
TGCAATTACAGCATTAACAAATACCATTCTGATTACATAAGGAGCTCCGATGCTCAGGATGAGGATGTAG
GCCATAAGTGGACGTTGTCCGCTCTTTTAAGGCACCTGAAACTCCAGAGTTGTGATACACGTCAGCTAAT
GCTGAATATTGAGGATTTAATTATTAAAGCTGTGCTAGCCTGTGCTCAATCTATTATTTCGGCCTGCAGG
ATGTTCGTTCCCAATGGAAATAACTGCTTTGAGCTGTATGGATTCGATATATTAATAGACAACGCACTTA
AGCCATGGCTACTGGAAATCAATCTATCGCCGTCAATGGGTGTGGATAGTCCTTTGGACACTAAGGTGAA
GTCATGTCTGATGGCAGACCTATTGACGTGTGTGGGCATTCCAGCCTATAGTCCCGAAATGAAATCTCAT
TATGACCAGAAATGGTCGCGTTTCCGCAGCTCAAGCTGCCAAAGGATAGCAACTTTTCCCAGTACCTCGC
AAAAGACAAAGAAGTCGAAGAAAAAAGGAGCTGCCATTAATCTTAATCTAACCGGCGAAGAGCAGCGCAT
ATTGCGCAATGCACGCCTACAGTATTCTCGTCGTGGAGGATTCGTGCGCATCTTTCCCACAGATGACTCA
ATGCAGCGGTATGGTAATTTCCTTGATTCTGCTAATGGCATTCCGATCTCCACACCAAATGTACAGAGTC
AGACATTCCAAACGCCGATTATACAGCACAACTACAACCAGATGCTTCACCAAAATTTATACTCCAAGGA
CGGCCGGCAAAAGCAGGACGATAATAATGAAGCTGATCGGATTTGGCAATACGAAAGGGCTTTGGAAACC
GATTCGGAAATTCCATTTGTCAAGAAGCCCACAGTTGAGAAGTGCGAAGTGGAGGGGAGGCGGTTGCGGA
AGATCATGCTCAAGAAAATATCCAATGGGTCAGAGCTGACGCCTTTTCAAGCGCGTCAGACGTTCAGCAT
GTACTTGGAATCGGTTCTTCGGCGGCTTACCGAAGATCCGAAAGACAATCACGAGAAAATAATACTTAAG
TTTTTGAACAAGTTCGGCGGATCTGTAAAGCCGCCAGTCATGTTCCGGAATATGCAAACCATCAAGGTGG
CCAGCAAAGCCCGATCCGCAATGGTGGCGAAACTTCTTGG
>dme_circ_0007509_junction_seq
GATCCGCAATGGTGGCGAAACTTCTTGGTGAAGGTGAAGAGCCTGCAAATAGTAGT
>dme_circ_0007510

GCCATAAGTGGACGTTGTCCGCTCTTTTAAGGCACCTGAAACTCCAGAGTTGTGATACACGTCAGCTAAT
GCTGAATATTGAGGATTTAATTATTAAAGCTGTGCTAGCCTGTGCTCAATCTATTATTTCGGCCTGCAGG
ATGTTCG
>dme_circ_0007510_junction_seq
TCTATTATTTCGGCCTGCAGGATGTTCGGCCATAAGTGGACGTTGTCCGCTCTTTT
>dme_circ_0007511
CTAACCCCTTCACTTCGAACGCGTATCCGACTGATCGTGGATTTGGCGGATCCGGCGGATATAATAGTCA
ATTCGGCTCTAAACTACCGGCAATCAACCCAAGCAGCGTTTTGCGAAACGATCTGACAACGTCGTCACTC
TTCAACTTCAACACCAATTTGAACCCATCTTATAACAACCTTAACCGGAGGAATAATAATTTTTAATAGA
TAGTTCG
>dme_circ_0007511_junction_seq
GGAATAATAATTTTTAATAGATAGTTCGCTAACCCCTTCACTTCGAACGCGTATCC
>dme_circ_0007512
ATCTCAAGGTGCGTGAGCTAAACGTGTCCTTCGACACATACAAATCTGATCCCGAACAGCATCCGAGTTA
TCAAGCTGAGTGGCAAATGTTTTGGAAGCGCCGCAAGGATGAGCTTATACTGGCTGGCATTAATCATCGC
ACGTACAACTTCCAAAACGAATGGATTAATTTCTTTAATGCTCGTATCGAAGAATTG
>dme_circ_0007512_junction_seq
TTTCTTTAATGCTCGTATCGAAGAATTGATCTCAAGGTGCGTGAGCTAAACGTGTC
>dme_circ_0007513
GTCGCGGCGCCCAAGAAGCCGGAACCGGCAAAGGCGCCAAAGGCACCAAAGAGCAAGTCGGAGAAGGAGA
ACAAGCCGGTTGTGGCTGCCCGCAAGGCCAACGCTCCGGTGGCTAAAAACGCTAGTCCAGTGAAAGGCGG
CAAGGGTCCCGCTGGCGGGGAT
>dme_circ_0007513_junction_seq
AGGCGGCAAGGGTCCCGCTGGCGGGGATGTCGCGGCGCCCAAGAAGCCGGAACCGG
>dme_circ_0007514
GGAGTGCATCATGGACGACAAGTGCAGTACCAATTTCAAGCAGATGACACGACTCTTCGTGGAACACCAC
AAGGCGGCAATGGAGCAGCATGTAGCGGTGGAGAACTGCAACACTTCGGTGGTCTTCTCGGTGTCCTCCA
GTCCGTGTGTGGCCGGAAGATGTGGACGATTTGGAAG
>dme_circ_0007514_junction_seq
TGGCCGGAAGATGTGGACGATTTGGAAGGGAGTGCATCATGGACGACAAGTGCAGT
>dme_circ_0007515
GAGGAAAATCCATACAACAAGTTTTGAGCTGCGATGAATTTACACTGAAGTCCGAGGATCCAGTGGGCGA
AAACTTGTTGGGCGAAAGCCAGGGATGTGGTATATTATCCAAAATTTGCAGGGCCACTATTCCACTTTTC
CACAAGCCACATGGATTTAATTTTATTCTTTGCAACTTGGCATTATTCGGAATGTTTTTCAG
>dme_circ_0007515_junction_seq
ACTTGGCATTATTCGGAATGTTTTTCAGGAGGAAAATCCATACAACAAGTTTTGAG
>dme_circ_0007516
GTGAAGAAGAATCTAACGCGAAGCACATTTTTGCAAGGTCTCGTCCACTGCAAATTGGACGTGTTTAAAA
AAGTACAGATATGTTTACAACGCGAAAAGAAGTGGACGCACACGTCCACAAAATGTTGGGAAAACTTCAG
CCGGGAAGAGAGGTAAATAAACGGCCAAAGTGTGCATCGGAATTTCCAAAAAGAAAATCATTGCGAAATC
TTCAGGCGCCGCCATTGAAAGCACACCGTTGTGTGCGTGCGGCGTCTGCTTGTGTTTGTGTGTGTGAGTC
GGCGTCACTTGGCCGTCGTTTTTGAGATTGAATAAGAAATTGCGCGCTTTTCTTTCAACGGCTGCATTTC
ATATACTTGACAAATAGAACGCTTTTTTCGCAGCTGCATGGCTTTCGCCTTCTCTGTATAAATCAAATTA
GATATTTGAGGTTATGTGTACCATATCAATCTGCTAGAGTGAGCGCTGAATTTGTAAGACCGCCAAAAAT

AATTCGAATTTCCATTGTGGATTTGAATGATTTTTTGCGGTCTAACGTAGCTCTATCTAAATAATAAGCA
CACAATAAGTCAAATCTTACAAAGTTATTCTTATCCAGCTCACAGTGATTGTCTAAAAATTTAACAGACT
TTTTTGAACCTAACGTACTAATCAATAAAATGTATATTGTAGCGCGATATCAAAGGCTTGGCTGTAGCTC
GATTGTACATGAAAGTTCAGGAATACCCAAAAGCCATCGA
>dme_circ_0007516_junction_seq
AAGTTCAGGAATACCCAAAAGCCATCGAGTGAAGAAGAATCTAACGCGAAGCACAT
>dme_circ_0007517
GGTATTTTGCTCGCCGTCTCCGCCACTGGTGTCATCTCTTTCGCCGTTACTGTCCGCAAGATTTACACGC
ATTCGCAGGTTGAAGACTTGCTTGTTGCCGCTTAAATCAAGGCTATTAGCCTGATCCAACCAGTACCTGG
CGCACTCCGTGATCGATGCGTTG
>dme_circ_0007517_junction_seq
CCTGGCGCACTCCGTGATCGATGCGTTGGGTATTTTGCTCGCCGTCTCCGCCACTG
>dme_circ_0007518
TTAAGTCGCACCACCAGATCCCACCAGTTACTTTGCTCCGGTGCGCCTAGTTGACCCACTAGATTGGCAA
ATGGCATCATATGTGGCCGGGCGGCAGCATTGCGGTTTCCCAAATTGGAGGAGTCATACACCTCACGCTG
GATTTGCAATTTACGTCTGGTCTGAATGGTTGTGGCATAAAGG
>dme_circ_0007518_junction_seq
TCTGGTCTGAATGGTTGTGGCATAAAGGTTAAGTCGCACCACCAGATCCCACCAGT
>dme_circ_0007519
ATCCCACCAGTTACTTTGCTCCGGTGCGCCTAGTTGACCCACTAGATTGGCAAATGGCATCATATGTGGC
CGGGCGGCAGCATTGCGGTTTCCCAAATTGGAGGAGTCATACACCTCACGCTGGATTTGCAATTTACGTC
TGG
>dme_circ_0007519_junction_seq
TCACGCTGGATTTGCAATTTACGTCTGGATCCCACCAGTTACTTTGCTCCGGTGCG
>dme_circ_0007520
TGTTGTTCTCCTCCTCCTCCACCTCTTGCGAATCGTTATCTTCATCCTCCTTAGCTGCTGTACTATTTGG
CTGCTGCTGGTCGGGCTTGGGCTTGGTCATGGAGAAGGTGAAGCTGCTGGATCCAATTGAGGTAGTCGGA
GCAACAGCTGCGGGCATCGCAGCGAAGTTGAACGTGGCTCCATCGCCAGCCTGATCTTTGTCACCAGCCG
CTGCGGCTCCGAAACCAAAGCTAAACTTGGAGGTAGGTGGCAGATTAAGTCCCGATCCTAGTGACTTCTC
CTTCTGTGGTACCGTGTCATTCTTGGGTGCATCACAGGCGCTGCAGTAAAGAGACTCTCCGGGATTGTTC
ACATAGCATGCGCTACATGACCAACTACCGACTGCAGGTTTGAAGGCGTCCCCGAATCCAGAAGTCATTG
AAGACTTTCCAAATCCAAGGGCTGCCGTTTGAACTGACGTAGGTGCCGAAGCCGAGGCGGTAACAACTGC
CACTGCTGGCTTCTCCTTTGGCTGTGTGGCGCCACCGAAACTAAATCCACCTGTGGCTGGAAGAGTCGCT
GAGGAGGCAAAGCCAAACGAAAACTTGTTTGAGGAGCTAGTGGTAAGATTTAACGCGTTCCCTTGGTCTA
ACCCCGATTGCTTGGGGGGAACAGTGGCATCTTTGGGCTCCTGGCAGGCCAAGCAGTAGAGCTGATCCTG
GCCATTGTTGGTATAGCAGGCTTGGCAGTTCCAACTGCCTGCCTTAGGCTTAAAGGCATCTCCAAAGCCT
TTAATTGGCTTCTCCTTTTCTCCAGCGGCAGAAGGGACCTCCTCCTTTTTGGGTTCATTTCCAATACTTT
GTTGAGCCTTTTGCACGGCCTCGAGGAATTGCTGACAGATGTCTTGCGACTTAAATCGCACAGCGAGCAA
TGCGGTGGTTAACTCCTCATCGGAATAATCCTGAGCACCCCATGTGACGGCCGCTTTACAGTTCGTTGCA
TAAGTGAACTTGGTCTCAGGCAACAGTCGCTGATTGCAGCACAGCTTATGGACCTGCTCGCGCCGCATAA
GCAGGCGAACCTTGTTGGGATCAGTGCGATCCCGAAGGAGCTTCATATTACCAAGGCCGCGCTCCTTCCA
CTCATTCGCTTCCTTATCCCAACGCAGAAGCTTGGCACGGTGCTCGAAGAGCACATCCTCATTCTCTTCG
CCGGTGACCACCTCCACTATATCGGGCAGGGCAATAACAGGCACGAACTGAGCGGTGGGCACATACTCCT
CCTCAGCGTCCGAGGAGTTTGACTTGTTTAATTGTGACTCCTGCTGAAGCGTTTTGCCTTGAACTGTATT

GTCCTTGATCAAAGCCGTGGTGAATAAAGTAGTGTTGTTGCCGCTGCCCACCGCAGAAGCGGTACCAAAA
GAAAGGTTTCCGAACGGAGAGGCGGTGGAAGTTCCAAATCCTGATGACCCATTCGGCGTGAAGCTAAAGC
TGGCAAATGGTGTGGCAGAGGCTGGCGCAGATGTTGCAGGAGCAGCACTGACTGACAGGGTTCCAAACAG
TGACTTAGCTGCTGTTGCTGGTGGGTCCGGATTAGGCTGAGTTTTTGTCTGCTCCTGAGGCTTATTGATT
AGCGAATTGGCTGCTGGGGTGCTGGTTACAAAGCTCTTGAATGCCGCTGGAGCAGTCGCAGTTGATCCTT
TCTCCGCTGTGTTTACCGTCGGTTTCACGGTTTCCTTCGACTTGGCGGCCTCGCTTGCCTTTGTAAAAGC
CACCCTGAACTCTTCAGCCAACTCCCCTGTTTTGAAACGCACCAGGAATCTCTCCAATGTAACTTGCTCA
TCCGCAAAATCATTTGCCGCCCACAGAAGGGATTTCTTATCCTTGTCCTGGTTGGCAACATTTATCGTAA
TGTCTGCAGTGATTGTGTGATTAGCACACACTTTGTGAGTTTGATCGCGACGCATCAAGACTCGCGAAAC
GCCGGTGGCTTTGTCGCATAAAATTTTGATCACCCCTGTCCCGCGCTCCTTCCACTCCTTGTCCACATAT
CGAAAGAGCTTGGCTCGGCTGGTAAACTTGATATCCTCGCCCTCCTCCCCTGTTCGAACCTCCACTTCAT
CGGGTAGTGGAATGATGGGCTTGAAATCGGGACGAGGATCGTAATCATCTGCTGAAGTATCTTGTGGCAG
AGTTTTGTTGGGATCAGTAGCCCCACTTTGGTTTTGCTCAGCTTCCTCTTGTTCCTTTTGTTTTTCGGCT
GCTGCCTTAGCCACCTGTGTCTTGAATGAAAAAGGACTTTCGGAAGACTTACTGCCAAAGTTAAAGCTGA
GTGATCCTACTCCTGACACCGATGGCtgtgccggttgtgccgattgtgccggctgttccggtGCTTGAAC
CAAACTTGGCTTGATATGCTGTGCTGGAATGGTCACACTTAAAGTGGGTTGGACACTGGCAGTCGTGGGT
TTCGGCAACGGATCTGAACTGGTTATGACCACATTGGCTGGCGGCTCCTTTTCCACGGGCTGATTGTTCA
ACGCACGGTTAAACATAGACGGCACACTAACAGGGGCGGGTGCGGGTGGCTGTGCAGGAACAGAAGGAGC
CACTTGAGGTATATGAACAGGAGGCGGAACAGCAGTTGTTGCCGGTGGATTCACAGTTCCAGCAATTGGT
GGAGCAGGAGCAGTACTAGGTATTGGCACCGTTGGAACAGTTAATGGTTTCGACTGCGGAACTTGAATGG
GCGAAGCTCCAAAGACCGGTGTTGTCGTATTGAAGAATCCTGGAGTTGGCAGTGCAACCACAGGCTTGGA
CTCAAGAATATTCAAGGGAGCCGGAGGAGCTGCTGGCTGAGCGGGAGGAGGTATCAAACTTGGGGGAGGC
TGGGTAAAGTTGCCCATGGCGTCAATG
>dme_circ_0007520_junction_seq
CTGGGTAAAGTTGCCCATGGCGTCAATGTGTTGTTCTCCTCCTCCTCCACCTCTTG
>dme_circ_0007521
GATTCCTGGTGCGTTTCAAAACAGGGGAGTTGGCTGAAGAGTTCAGGGTGGCTTTTACAAAGGCAAGCGA
GGCCGCCAAGTCGAAGGAAACCGTGAAACCGACGGTAAACACAGCGGAGAAAGGATCAACTGCGACTGCT
CCAGCGGCATTCAAGAGCTTTGTAACCAGCACCCCAGCAGCCAATTCGCTAATCAATAAGCCTCAGGAGC
AGACAAAAACTCAGCCTAATCCGGACCCACCAGCAACAGCAGCTAAGTCACTGTTTGGAACCCTGTCAGT
CAGTGCTGCTCCTGCAACATCTGCGCCAGCCTCTGCCACACCATTTGCCAGCTTTAGCTTCACGCCGAAT
GGGTCATCAGGATTTGGAACTTCCACCGCCTCTCCGTTCGGAAACCTTTCTTTTGGTACCGCTTCTGCGG
TGGGCAGCGGCAACAACACTACTTTATTCACCACGGCTTTGATCAAGGACAATACAGTTCAAGGCAAAAC
GCTTCAGCAGGAGTCACAATTAAACAAGTCAAACTCCTCGGACGCTGAGGAGGAGTATGTGCCCACCGCT
CAGTTCGTGCCTGTTATTGCCCTGCCCGATATAGTGGAGGTGGTCACCGGCGAAGAGAATGAGGATGTGC
TCTTCGAGCACCGTGCCAAGCTTCTGCGTTGGGATAAGGAAGCGAATGAGTGGAAGGAGCGCGGCCTTGG
TAATATGAAGCTCCTTCGGGATCGCACTGATCCCAACAAGGTTCGCCTGCTTATGCGGCGCGAGCAGGTC
CATAAGCTGTGCTGCAATCAGCGACTGTTGCCTGAGACCAAGTTCACTTATGCAACGAACTGTAAAGCGG
CCGTCACATGGGGTGCTCAGGATTATTCCGATGAGGAGTTAACCACCGCATTGCTCGCTGTGCGATTTAA
GTCGCAAGACATCTGTCAGCAATTCCTCGAGGCCGTGCAAAAGGCTCAACAAAGTATTGGAAATGAACCC
AAAAAGGAGGAGGTCCCTTCTGCCGCTGGAGAAAAGGAGAAGCCAATTAAAGGCTTTGGAGATGCCTTTA
AGCCTAAGGCAGGCAGTTGGAACTGCCAAGCCTGCTATACCAACAATGGCCAGGATCAGCTCTACTGCTT
GGCCTGCCAGGAGCCCAAAGATGCCACTGTTCCCCCCAAGCAATCGGGGTTAGACCAAGGGAACGCGTTA
AATCTTACCACTAGCTCCTCAAACAAGTTTTCGTTTGGCTTTGCCTCCTCAGCGACTCTTCCAGCCACAG

GTGGATTTAGTTTCGGTGGCGCCACACAGCCAAAGGAGAAGCCAGCAGTGGCAGTTGTTACCGCCTCGGC
TTCGGCACCTACGTCAGTTCAAACGGCAGCCCTTGGATTTGGAAAGTCTTCAATGACTTCTGGATTCGGG
GACGCCTTCAAACCTGCAGTCGGTAGTTGGTCATGTAGCGCATGCTATGTGAACAATCCCGGAGAGTCTC
TTTACTGCAGCGCCTGTGATGCACCCAAGAATGACACGGTACCACAGAAGGAGAAGTCACTAGGATCGGG
ACTTAATCTGCCACCTACCTCCAA
>dme_circ_0007521_junction_seq
CGGGACTTAATCTGCCACCTACCTCCAAGATTCCTGGTGCGTTTCAAAACAGGGGA
>dme_circ_0007522
GGACAATACAGTTCAAGGCAAAACGCTTCAGCAGGAGTCACAATTAAACAAGTCAAACTCCTCGGACGCT
GAGGAGGAGTATGTGCCCACCGCTCAGTTCGTGCCTGTTATTGCCCTGCCCGATATAGTGGAGGTGGTCA
CCGGCGAAGAGAATGAGGATGTGCTCTTCGAGCACCGTGCCAAGCTTCTGCGTTGGGATAAGGAAGCGAA
TGAGTGGAAGGAGCGCGGCCTTGGTAATATGAAGCTCCTTCGGGATCGCACTGATCCCAACAAGGTTCGC
CTGCTTATGCGGCGCGAGCAG
>dme_circ_0007522_junction_seq
GGTTCGCCTGCTTATGCGGCGCGAGCAGGGACAATACAGTTCAAGGCAAAACGCTT
>dme_circ_0007523
TGACTTCTCCTTCTGTGGTACCGTGTCATTCTTGGGTGCATCACAGGCGCTGCAGTAAAGAGACTCTCCG
GGATTGTTCACATAGCATGCGCTACATGACCAACTACCGACTGCAGGTTTGAAGGCGTCCCCGAATCCAG
AAGTCATTGAAGACTTTCCAAATCCAAGGGCTGCCGTTTGAACTGACGTAGGTGCCGAAGCCGAGGCGGT
AACAACTGCCACTGCTGGCTTCTCCTTTGGCTGTGTGGCGCCACCGAAACTAAATCCACCTGTGGCTGGA
AGAGTCGCTGAGGAGGCAAAGCCAAACGAAAACTTGTTTGAGGAGCTAGTGG
>dme_circ_0007523_junction_seq
AACGAAAACTTGTTTGAGGAGCTAGTGGTGACTTCTCCTTCTGTGGTACCGTGTCA
>dme_circ_0007524
TGACTTCTCCTTCTGTGGTACCGTGTCATTCTTGGGTGCATCACAGGCGCTGCAGTAAAGAGACTCTCCG
GGATTGTTCACATAGCATGCGCTACATGACCAACTACCGACTGCAGGTTTGAAGGCGTCCCCGAATCCAG
AAGTCATTGAAGACTTTCCAAATCCAAGGGCTGCCGTTTGAACTGACGTAGGTGCCGAAGCCGAGGCGG
>dme_circ_0007524_junction_seq
ACTGACGTAGGTGCCGAAGCCGAGGCGGTGACTTCTCCTTCTGTGGTACCGTGTCA
>dme_circ_0007525
GTGAAGCTGCTGGATCCAATTGAGGTAGTCGGAGCAACAGCTGCGGGCATCGCAGCGAAGTTGAACGTGG
CTCCATCGCCAGCCTGATCTTTGTCACCAGCCGCTGCGGCTCCGAAACCAAAGCTAAACTTGGAGGTAGG
>dme_circ_0007525_junction_seq
CGAAACCAAAGCTAAACTTGGAGGTAGGGTGAAGCTGCTGGATCCAATTGAGGTAG
>dme_circ_0007526
GCTATTGTATGTGCATAAGGCCAAGTTGTACCGCCTAAACGAGAGCGATTGGAAAGAGCGCGGCTTGGGG
GATGTGAAGATCCTGCGCCATAGGCAGACCAAAAAGCTGCGCGTGGTCATGCGTCGGGAGCAGGTGTTCA
AGATCTGTCTTAACCATGTGCTTAACGAGAAT
>dme_circ_0007526_junction_seq
CTGTCTTAACCATGTGCTTAACGAGAATGCTATTGTATGTGCATAAGGCCAAGTTG
>dme_circ_0007527
GTTATGTCCTTTTCCTTTTCATTTTTTGCAGCTGTAAATGAGAAGGTTGCCATTTGTCCAAAAGCGGGTG
CACTGCCAAACACAAAGTCTTTAGCAGGTGACTGGGTTTCAATTTTGGGTATTCCAAAGGCGTTGGACCC
TCCAAATAACAATGGGGTTCCGCCAATGATAGATTTTGGTTCTTCCTTGCCATCTGATTTTTGCAAATTA

ACTTTGTGGCCTCCAAAAGGATTGGCGTCTCCGAACACAGGTGCGGCAATTCCACCGAAGGCCAACTTCG
GTGCTTCTTGGGTCGCTGGCGATTGGGAATCCGCTTTATTGCCTCCGAAGACAGACTCTGAGGAATCTTT
CTTCTGTTGCTCAGCCGCTTTATTTCCACCCCCGAAAAAGAACTTGACAGAACCGTCAGACGAATTTTCG
TTTGACGAGCCGCTACGGAATATAGATCCCAAAACGTTTCCTTG
>dme_circ_0007527_junction_seq
GGAATATAGATCCCAAAACGTTTCCTTGGTTATGTCCTTTTCCTTTTCATTTTTTG
>dme_circ_0007528
GGAATCGGACTCTGTCTGGCGGTGGAGTGCGCCATGAAAGGCGCCAATGTGACGGTGATAGCGCGCGACG
AGAAAATGCTGAGTGAGTAATGTTCTGGCTAGGAAACCCCACCCAAAATACTCTTATCTTTGATATACGA
TCTTTTTTGCAGGCGGAGCAGTGGCTCTGATGGAAGTGATTCGCCAGCGGCCAGATCAAAAGTTCCAGTA
CAGAAGTCTGGACATAAGCGGCGATTATGACCAGG
>dme_circ_0007528_junction_seq
CTGGACATAAGCGGCGATTATGACCAGGGGAATCGGACTCTGTCTGGCGGTGGAGT
>dme_circ_0007529
TTCTTTTTGGCCATCAAGGAGATGGTGGAATGGCTGGGCTACAAACCATTCAAGATCACCTACTCCTCGG
ACAACTTCCAGCAGCTGTACGAGTGGGCTGTTGTGCTGATTAACAAAGGATTGGCCTACGTGTG
>dme_circ_0007529_junction_seq
TGATTAACAAAGGATTGGCCTACGTGTGTTCTTTTTGGCCATCAAGGAGATGGTGG
>dme_circ_0007530
GTAACAGACTCCATCGTGGGCAGCCGCGTATCCAAAGTTAATGTTAATAGCCTTTGCGTGACCGATGTGC
AGGATGCCATTCGGCTCTGGCGGAAATCGTGTGTGCACTTTGCCTCCAGTTCTCGCCAGGTGCTCCTTTA
GTAGCCGCTCCGTGTGCTCGGTGACCACGTAGCCATCTGCCTTGAAGTTCTCTCCGGGAGCATGGAAGTG
TACCTTG
>dme_circ_0007530_junction_seq
CTCTCCGGGAGCATGGAAGTGTACCTTGGTAACAGACTCCATCGTGGGCAGCCGCG
>dme_circ_0007531
GGAAACTACGGCAGCGTGATGACCCGCATCAATGTGGAGTACACGACCAAGGTATCGAAAGGGAAACAGT
CCACCACATTTCTGGTTAAGACCACATTCGCTGACCGGGATCCAGCCGGCGATGTGCTCATCCACTATGG
TGTCTACACCCGTGAAATGGATATATACGAGCACATCCTTCCCCAGCTGGCGGATATGGTGAGGAAGGAG
CTTAAAGACTCCAGGAAGCTCTTTGCGGCCACCATGAATGTGGACCGCGAAAGGGACTCGATCATCTTCG
AGGACAT
>dme_circ_0007531_junction_seq
AAAGGGACTCGATCATCTTCGAGGACATGGAAACTACGGCAGCGTGATGACCCGCA
>dme_circ_0007532
GAACTCCGCCGTAGTTTTCACCAGGCCCCAGGGCGGGATTTATCTGCAGCTGAGTTATGGTTAGTCCTTC
ATCCTTGTAGTGGCAGCGAAGTGCATGCTCGATGTATTCGTGGGTAAGCCACTCGGGTGCCGCTGCCATC
TTGTTTAGTTGCTTCAATTTTTTCGACTGAACC
>dme_circ_0007532_junction_seq
TAGTTGCTTCAATTTTTTCGACTGAACCGAACTCCGCCGTAGTTTTCACCAGGCCC
>dme_circ_0007533
CTTGTAGTCGTTGCATGTACTTATCTTTTATCTTTTCGAAATGAGGCTTATACTTTCTTAGCTCTGGTAA
TCTGTCCAGCATTTCAATGAAGCTCTGCATGCCAGTTGTAATAAAAGGGTCGGTCTTTACAGTGGGCATC
TCGAAGAGGCCATACTTAAACTCCTTTAGGAAGTCAGG
>dme_circ_0007533_junction_seq

CATACTTAAACTCCTTTAGGAAGTCAGGCTTGTAGTCGTTGCATGTACTTATCTTT
>dme_circ_0007534
GTTCAGTAGCTTGAGGCATTCCGCCAGACGCTGGTGATAGATCTGAATAAAGTGATCGAATTCCTTGATC
TTGATATCCAGCGATGCCGATGTTGTGATCAGGTACCAAAGGTCTTGAGCCGGGCTGCCCCACTTGCCAA
CCTGCAGGTCCACCAATATGGTTCTATCGATCTCGCCGTTATCCTTGTAGTTGAACATGATGTTGTTGGA
CCAACAGTCGCCGTGGTTCAGCACATTGAACTCGTCCTCGTCCACCTGGTTGACTTGCACTGCTTCCTCG
AACACCTCCAAGGGGTCCCACATCCGGGCGAT
>dme_circ_0007534_junction_seq
CCTCCAAGGGGTCCCACATCCGGGCGATGTTCAGTAGCTTGAGGCATTCCGCCAGA
>dme_circ_0007535
TCTCTGGGTAAGCAGCGAGAGGAGCAATTCCTCAAGGCCATGCGCAACTGGGACCTCGAGAATGCCGAAA
GCTACATCGCCCGGATGTGGGACCCCTTGGAGGTGTTCGAGGAAGCAGTGCAAGTCAACCAGGTGGACGA
GGACGAG
>dme_circ_0007535_junction_seq
GCAAGTCAACCAGGTGGACGAGGACGAGTCTCTGGGTAAGCAGCGAGAGGAGCAAT
>dme_circ_0007536
GTTTTCGAATATCCAAGCAATTTCAGATGCTTTACCAATTCGGAGTGGTAAAACCATATGAAGTAATCAA
ACTTTGATGTTTTTATGTCCAAGCTTGTAGAGGACAAAAGAAAGTAGTATAGATCCTGGGTCACTGATCC
CCACTTAGGGACTTGCAGATCGACAAAGTAGGTATCCTGTATCTCGCCTTTCGTATTGTACTGGGACATA
ATGTTGTTGGCCCAACCATCGCCATG
>dme_circ_0007536_junction_seq
TAATGTTGTTGGCCCAACCATCGCCATGGTTTTCGAATATCCAAGCAATTTCAGAT
>dme_circ_0007537
GTTTATCCTCCAGCTTTGTGGATGAGAGCAAGAAGTAGAGCAGATCCTGTGCCACGGTTCCGTACTTGGG
CAGCTGGTAGTCGACCAAATACACCTCCTTGATCTTTCCAAAGGCATCATACTGAAACATTATATTGTTT
GACCACGAATCGCCGTGGTTCAGAACGTTGAACTCCGTTGGCTCGACCTTGGCGAACTCAAAAATCTTAT
CTATAGCAACAGGCTGTAACGCTTTCTGATAGAGGAAAGAACAATTAAATATTTTCCGCGACTAATTGGG
CGTTTGAAGTATTTACTACTTACGACTTTGTCTAAATAAGCCTCATGACCTTCGTAGGTGGCGCAGCTTT
TAACGAAATTCGCGCCCATTCCTTTTATCATTTCAGACATAACGGGCCTGCTTTCCTCCTTAAAGAAGCC
TTGAAGTAGGATTTTGGGATAGGGTCCTTTGGTGGCCACTCGTACAGCTGATGCAGCATGCCATTGGGAT
AGCTTTCGCAGAACCCTCTCCGTGTGCTCTTGATCCAGCCCTTCGAGGCGGTTGGCATTCTTGAACCCTT
TCAACCCCAAATCCTCCAGGGCCACGTAGTCTGTCTTGGCATTCTTGAGATCATAGTTTTTGGCGCCGAA
TGTGATGTCCACTCCTACTGCCTT
>dme_circ_0007537_junction_seq
CGAATGTGATGTCCACTCCTACTGCCTTGTTTATCCTCCAGCTTTGTGGATGAGAG
>dme_circ_0007538
GAGGAAAGCAGGCCCGTTATGTCTGAAATGATAAAAGGAATGGGCGCGAATTTCGTTAAAAGCTGCGCCA
CCTACGAAGGTCATGAGGCTTATTTAGACAAAGTCGTAAGTAGTAAATACTTCAAACGCCCAATTAGTCG
CGGAAAATATTTAATTGTTCTTTCCTCTATCAGAAAGCGTTACAGCCTGTTGCTATAGATAAGATTTTTG
AGTTCGCCAAGGTCGAGCCAACGGAGTTCAACGTTCTGAACCACGGCGATTCGTGGTCAAACAATATAAT
GTTTCAGTATGATGCCTTTGGAAAGATCAAGGAGGTGTATTTGG
>dme_circ_0007538_junction_seq
TTTGGAAAGATCAAGGAGGTGTATTTGGGAGGAAAGCAGGCCCGTTATGTCTGAAA
>dme_circ_0007539

GGTTCAAGAATGCCAACCGCCTCGAAGGGCTGGATCAAGAGCACACGGAGAGGGTTCTGCGAAAGCTATC
CCAATGGCATGCTGCATCAGCTGTACGAGTGGCCACCAAAGGACCCTATCCCAAAATCCTACTTCAAGGC
TTCTTTAAGGAGGAAAGCAGGCCCG
>dme_circ_0007539_junction_seq
GGCTTCTTTAAGGAGGAAAGCAGGCCCGGGTTCAAGAATGCCAACCGCCTCGAAGG
>dme_circ_0007540
AAATATTTGAAGATTTGCTTAAGGCAAATGTGGATGGATACTCAAAAATTAAGAACTTTAAGGCGGACAT
TGGATCCGCTGCAGGTGAAAATTATGCCACCATTATGCTTCGAGTCAAAATCGAAGTTGAGCTGCAGGGT
AAGTTACTGCGCCTGATGTCTAAGAAAACTGTCTCATTTGCAAACCTTAGATGGCAAATCGAAATCGGTA
TCCTACATGGTTAAGTTGCCCCACCAAGTGGAGGCTATTCAGGAGATGATGAAACGAACCAACATCTTCG
AAATCGAGCGTACCATG
>dme_circ_0007540_junction_seq
CAACATCTTCGAAATCGAGCGTACCATGAAATATTTGAAGATTTGCTTAAGGCAAA
>dme_circ_0007541
GAGGAAAACAGGCCGATGATGAACGATATGATGAATGGCATGGGACAGGTGTTTGTCAAATGCTGTAGCA
CCTACGAAGGTAACGAGGCCTATATAGAAAAAGTCGTAAGTTTTATCCCGAAGTTTGCTATAAAAATGTA
ATTTACGAACTTTCTTATATCATATCTCACTAGAAAGCTCTTAAGGACGTGATGATAGACGAGTTGTTTA
AGATGTGCGTAGTCGATCCCACGGAGTTCAACGTCCTGAATCACGGTGATTCTTGG
>dme_circ_0007541_junction_seq
CAACGTCCTGAATCACGGTGATTCTTGGGAGGAAAACAGGCCGATGATGAACGATA
>dme_circ_0007542
TTCCTTTTGGGCGAAGACATCATCGCTGCTCCCGTTGTGGTCGAGGGCGCCACCAAGCGAGACATTTACC
TGCCAGAGGGCGAGTGGCAGGATGGAAACAGCGACCAGGTGTACACTGGACCCACCTGGGTGATGGACTA
CGCCGCTCCCTTGGATACCCTACCCTACTTTTTGCGCGTGGGATTCACACTGGAGTAGAGCGGAAACCG
>dme_circ_0007542_junction_seq
GATTCACACTGGAGTAGAGCGGAAACCGTTCCTTTTGGGCGAAGACATCATCGCTG
>dme_circ_0007543
AGTTCCTTTTGGGCGAAGACATCATCGCTGCTCCCGTTGTGGTCGAGGGCGCCACCAAGCGAGACATTTA
CCTGCCAGAGGGCGAGTGGCAGGATGGAAACAGCGACCAGGTGTACACTGGACCCACCTGGGTGATGGAC
TACGCCGCTCCCTTGGATACCCTACCCTACTTTTTGCGCGTGGGATTCACACTGGAGTAGAGCGGAAACC
G
>dme_circ_0007543_junction_seq
GATTCACACTGGAGTAGAGCGGAAACCGAGTTCCTTTTGGGCGAAGACATCATCGC
>dme_circ_0007544
TTTCCCAATAGCAATGTCTACGTGCGACTTAGGAGAGGTGATAAGTTGCAGTATGAGCTAATGAAGGGCG
AGAAGTCCCTGCAGACGGTGACCTTCGACAACTTTGACAGCAGCGCCAATCTGGTGGAGACGAGCAGTGG
TAGCTACCAGCTTACCGATGGCCAATCCACCGTGGAGTTCACCCTGGTCAAAAGCGGAGTGGTTGCCGGC
ACAGGCAGTGAGATAACCCATGTGAAAGTATCCCGAGATCAGGTGCTGCAAGGCACCCAACCCAGGGATT
GCGTCCCCCTGAAGACCGGATCGACGCACTGGTTCAGTGGTCCGGAGCAGAAGCAACACTACTGGCCAGT
GGAGCGACAGAGGCATAGTAACTATTCCTACCTGCCAAAGGAGCTGGATAACTTTGGTGTGGGTGAGCGA
TATTGGTTGAACGGCGACGGAGTCTTTTTGTACGTGGAGAGCAACACTCCTCTGTTTATTGATCAGAATA
GTGCTGCCTATCCTGACCAGCTGTGTCTGAGTGCTATTAGTGCTCTACCCTATGATCCTCGGGTGACCTC
TGCCAAATTCGTTTACCACGTAGCTGTGGCCAAGGATGCCAAGGCGGCGCACCGGTATGCCGTTAAAACG
TTCCTCGGCCAACCCACAGGCTATCCCGATGAGCGAATGGTCATGCATCCTGTGTGGTCCACATGGGCTC

TCTACAAGGCTGAGGTATCCGACGATGTGGTTCGTGACTTCGCCAAGCAGATTCTGGATAATGGGTTCAA
CAACAGCCAGCTGGAAATCGACGATGATTGGGAGGATTGCTATGGAGCCATGACCTTCAGGAAGAACAAG
TTCCCCGATAGCAAGACCCTGACTGATGACCTCAAGGCCTTGGGTTTCCGTGTGACCCTGTGGATTCATC
CATTCATTAACAACAACTGCACAGCCATTTACGATGAAGCTAAGTCTAAGGGTTACCTGGTGCTCGATCA
CGAGGGAAGCTCCGACACGCAATGGTGGAACAGCAAGCCCAAGGATGCAGCCATTTTGGACTTTACCAAA
ACTGAGGTTCAGGAGTGGTTCAGCAAGCGTTTAAAGCGCCTTCAGGATGAGGATGGCATTGATAGCTTCA
AGTTTGATGCCGGAGAGACCAGTTGGCTACCCAGTGATCCTGTACTCCAGGCTAGCAGTTCCATGGTGAC
ACCACTGCAGGCGGTGGGCGACTACGTGCGTACTGTAGCGGCATTCGGAGACATGGTGGAGGTTCGATCA
GCCCAGAACACTCAAGATCAGCCAATCTTCGTGCGCATCGTGGACAAGGACTCCGAATGGGGATGGAACA
ACGGCCTGCTCACACTGATCTCTTCGATGCTGCAAATGAACCTCAACGGTTATCCTTTCGTCCTTCCCGA
TATGATTGGTGGTAATGGCTACTACGAAAAGCCACCAACCAAGGAGCTTTTCCTCCGTTGGCTGCAGGCC
AACGTCTTTATGCCCGCCCTGCAGTTCTCCTTCGTTCCCTGGAACTTTGACGACGAGGCCATTGCGATCA
GCAAGAACTTCACCGCCCTCCATGCCACCTATACACCTTACATTATGAAGCTCTTTAAGCGCGCTGTGGA
TTCTGGTGAACCTGTGAATG
>dme_circ_0007544_junction_seq
GCTGTGGATTCTGGTGAACCTGTGAATGTTTCCCAATAGCAATGTCTACGTGCGAC
>dme_circ_0007545
GCCAACGTCTTTATGCCCGCCCTGCAGTTCTCCTTCGTTCCCTGGAACTTTGACGACGAGGCCATTGCGA
TCAGCAAGAACTTCACCGCCCTCCATGCCACCTATACACCTTACATTATGAAGCTCTTTAAGCGCGCTGT
GGATTCTGG
>dme_circ_0007545_junction_seq
AGCTCTTTAAGCGCGCTGTGGATTCTGGGCCAACGTCTTTATGCCCGCCCTGCAGT
>dme_circ_0007546
GTTCTTGCTGATCGCAATGGCCTCGTCGTCAAAGTTCCAGGGAACGAAGGAGAACTGCAGGGCGGGCATA
AAGACGTTGGCCTGCAGCCAACGGAGGAAAAGCTCCTTGGTTGGTGGCTTTTCGTAGTAGCCATTACCAC
CAATCATATCGGGAAGGACGAAAGGATAACCGTTGAGGTTCATTTGCAGCATCGAAGAGATCAGTGTGAG
CAGGCCGTTGTTCCATCCCCATTCGGAGTCCTTGTCCACGATGCGCACGAAGATTGGCTGATCTTGAGTG
TTCTGGGCTGATCGAACCTCCACCATGTCTCCGAATGCCGCTACAGTACGCACGTAGTCGCCCACCGCCT
GCAGTGGTGTCACCATGGAACTGCTAGCCTGGAGTACAGGATCACTGGGTAGCCAACTGGTCTCTCCGGC
ATCAAACTTGAAGCTATCAATGCCATCCTCATCCTGAAGGCGCTTTAAACGCTTGCTGAACCACTCCTGA
ACCTCAGTTTTG
>dme_circ_0007546_junction_seq
GCTGAACCACTCCTGAACCTCAGTTTTGGTTCTTGCTGATCGCAATGGCCTCGTCG
>dme_circ_0007547
GGGAACGAAGGAGAACTGCAGGGCGGGCATAAAGACGTTGGCCTGCAGCCAACGGAGGAAAAGCTCCTTG
GTTGGTGGCTTTTCGTAGTAGCCATTACCACCAATCATATCGGGAAGGACGAAAGGATAACCGTTGAGGT
TCATTTGCAGCATCGAAGAGATCAGT
>dme_circ_0007547_junction_seq
GTTCATTTGCAGCATCGAAGAGATCAGTGGGAACGAAGGAGAACTGCAGGGCGGGC
>dme_circ_0007548
TTCCATGGTGACACCACTGCAGGCGGTGGGCGACTACGTGCGTACTGTAGCGGCATTCGGAGACATGGTG
GAGGTTCGATCAGCCCAGAACACTCAAGATCAGCCAATCTTCGTGCGCATCGTGGACAAGGACTCCGAAT
GGGGATGGAACAACGGCCTGCTCACACTGATCTCTTCGATGCTGCAAATGAACCTCAACGGTTATCCTTT
CGTCCTTCCCGATATGATTGGTGGTAATGGCTACTACGAAAAGCCACCAACCAAGGAGCTTTTCCTCCGT

TGGCTGCAGGCCAACGTCTTTATGCCCGCCCTGCAGTTCTCCTTCG
>dme_circ_0007548_junction_seq
TTTATGCCCGCCCTGCAGTTCTCCTTCGTTCCATGGTGACACCACTGCAGGCGGTG
>dme_circ_0007549
GGAAAAGCTCCTTGGTTGGTGGCTTTTCGTAGTAGCCATTACCACCAATCATATCGGGAAGGACGAAAGG
ATAACCGTTGAGGTTCATTTGCAGCATCGAAGAGATCAGTGTGAGCAGGCCGTTGTTCCATCCCCATTCG
GAGTCCTTGTCCACGATGCGCACGAAGATTGGCTGATCTTGAGTGTTCTGGGCTGATCGAACCTCCACCA
TGTCTCCGAATGCCGCTACA
>dme_circ_0007549_junction_seq
CTCCACCATGTCTCCGAATGCCGCTACAGGAAAAGCTCCTTGGTTGGTGGCTTTTC
>dme_circ_0007550
TTCCATGGTGACACCACTGCAGGCGGTGGGCGACTACGTGCGTACTGTAGCGGCATTCGGAGACATGGTG
GAGGTTCGATCAGCCCAGAACACTCAAGATCAGCCAATCTTCGTGCGCATCGTGGACAAGGACTCCGAAT
GGGGATGGAACAACGGCCTGCTCACACTGATCTCTTCGATGCTGCAAATGAACCTCAACGGTTATCCTTT
CG
>dme_circ_0007550_junction_seq
CAAATGAACCTCAACGGTTATCCTTTCGTTCCATGGTGACACCACTGCAGGCGGTG
>dme_circ_0007551
GGATAACCGTTGAGGTTCATTTGCAGCATCGAAGAGATCAGTGTGAGCAGGCCGTTGTTCCATCCCCATT
CGGAGTCCTTGTCCACGATGCGCACGAAGATTGGCTGATCTTGAGTGTTCTGGGCTGATCGAACCTCCAC
CATGTCTCCGAATGCCGCTACAGTACGCACGTAGTCGCCCACCGCCTGCAGTGGTGTCACCATGGAACTG
CTAGCCTGGAGTACAGGATCACTGGGTAGCCAACTGGTCTCTCCGGCATCAAACTTGAAGCTATCAATGC
CATCCTCATCCTGAAGGCGCTTTAAACGCTTGCTGAACCACTCCTGAACCTCAGTTTTGGTAAAGTCCAA
AATGGCTGCATCCTTGGGCTTGCTGTTCCACCATTGCGTGTCGGAGCTTCCCTCGTGATCGAGCACCAGG
TAACCCTTAGACTTAGCTTCATCGTAAATGGCTGTGCAGTTGTTGTTAATGAATGGATGAATCCACAGGG
TCACACGGAAACCCAAGGCCTTGAGGTCATCAGTCAGGGTCTTGCTATCGGGGAACTTGTTCTTCCTGAA
GGTCATGGCTCCATAGCAATCCTCCCAATCATCGTCGATTTCCAGCTGGCTGTTGTTGAACCCATTATCC
AGAATCTGCTTGGCGAAGTCACGAACCACATCGTCGGATACCTCAGCCTTGTAGAGAGCCCATGTGGACC
ACACAGGATGCATGACCATTCGCTCATCGGGATAGCCTGTGGGTTGGCCGAGGAACGTTTTAACGGCATA
CCGGTGCGCCGCCTTGGCATCCTTGGCCACAGCTACGTGGTAAACGAATTTGGCAGAGGTCACCCGAGGA
TCATAGGGTAGAGCACTAATAGCACTCAGACACAGCTGGTCAGGATAGGCAGCACTATTCTGATCAATAA
ACAGAGGAGTGTTGCTCTCCAC
>dme_circ_0007551_junction_seq
CAATAAACAGAGGAGTGTTGCTCTCCACGGATAACCGTTGAGGTTCATTTGCAGCA
>dme_circ_0007552
GTGTGAGCAGGCCGTTGTTCCATCCCCATTCGGAGTCCTTGTCCACGATGCGCACGAAGATTGGCTGATC
TTGAGTGTTCTGGGCTGATCGAACCTCCACCATGTCTCCGAATGCCGCTACAGTACGCACGTAGTCGCCC
ACCGCCTGCAGTGGTGTCACCATGGAACTGCTAGCCTGGAGTACAGGATCACTGGGTAGCCAACTGGTCT
CTCCGGCATCAAACTTGAAGCTATCAATGCCATCCTCATCCTGAAGGCGCTTTAAACGCTTGCTGAACCA
CTCCTGAACCTCAGTTTTGGTAAAGTCCAAAATGGCTGCATCCTTGGGCTTGCTGTTCCACCATTGCGTG
TCGGAGCTTCCCTCGTGATCGAGCACCAGGTAACCCTTAGACTTAGCTTCATCGTAAATGGCTGTGCAGT
TGTTGTTAATGAATGGATGAATCCACAGGGTCACACGGAAACCCAAGGCCTTGAGGTCATCAGTCAGGGT
CTTGCTATCGGGGAACTT
>dme_circ_0007552_junction_seq

CAGTCAGGGTCTTGCTATCGGGGAACTTGTGTGAGCAGGCCGTTGTTCCATCCCCA

>dme_circ_0007553

GGTTACCTGGTGCTCGATCACGAGGGAAGCTCCGACACGCAATGGTGGAACAGCAAGCCCAAGGATGCAG
CCATTTTGGACTTTACCAAAACTGAGGTTCAGGAGTGGTTCAGCAAGCGTTTAAAGCGCCTTCAGGATGA
GGATGGCATTGATAGCTTCAAGTTTGATGCCGGAGAGACCAGTTGGCTACCCAGTGATCCTGTACTCCAG
GCTAGCAGTTCCATGG

>dme_circ_0007553_junction_seq

CCTGTACTCCAGGCTAGCAGTTCCATGGGGTTACCTGGTGCTCGATCACGAGGGAA

>dme_circ_0007554

GCCAACTGGTCTCTCCGGCATCAAACTTGAAGCTATCAATGCCATCCTCATCCTGAAGGCGCTTTAAACG
CTTGCTGAACCACTCCTGAACCTCAGTTTTGGTAAAGTCCAAAATGGCTGCATCCTTGGGCTTGCTGTTC
CACCATTGCGTGTCGGAGCTTCCCTCGTGATCGAGCACCAGGTAACCCTTAGACTTAGCTTCATCGTAAA
TGGCTGTGCAGTTGTTGTTAATGAATGGATGAATCCACAGGGTCACACGGAAACCCAAGGCCTTGAGGTC
ATCAGTCAGGGTCTTGCTATCGGGGAACTTGTTCTTCCTGAAGGTCATGGCTCCATAGCAATCCTCCCAA
TCATCGTCGATTTCCAGCTGGCTGTTGTTGAACCCATTATCCAGAATCTGCTTGGCGAAGTCACGAACCA
CATCGTCGGATACCTCAGCCTTGTAGAGAGCCCATGTGGACCACACAGGATGCATGACCATTCGCTCATC
GGGATAGCCTGTGGGTTGGCCGAGGAAC

>dme_circ_0007554_junction_seq

GGGATAGCCTGTGGGTTGGCCGAGGAACGCCAACTGGTCTCTCCGGCATCAAACTT

>dme_circ_0007555

ATTCTGGATAATGGGTTCAACAACAGCCAGCTGGAAATCGACGATGATTGGGAGGATTGCTATGGAGCCA
TGACCTTCAGGAAGAACAAGTTCCCCGATAGCAAGACCCTGACTGATGACCTCAAGGCCTTGGG

>dme_circ_0007555_junction_seq

CCCTGACTGATGACCTCAAGGCCTTGGGATTCTGGATAATGGGTTCAACAACAGCC

>dme_circ_0007556

GGTCATCAGTCAGGGTCTTGCTATCGGGGAACTTGTTCTTCCTGAAGGTCATGGCTCCATAGCAATCCTC
CCAATCATCGTCGATTTCCAGCTGGCTGTTGTTGAACCCATTATCCAGAATCTGCTTGGCGAAGTCACGA
ACCACATCGTCGGATACCTCAGCCTTGTAGAGAGCCCATGTGGACCACACAGGATGCATGACCATTCGCT
CATCGGGATAGCCTGTGGGTTGGCCGAGGAACGTTTTAACGGCATACCGGTGCGCCGCCTTGGCATCCTT
GGCCACAGCTACGTGGTAAACGAATTTGGCAGAG

>dme_circ_0007556_junction_seq

AGCTACGTGGTAAACGAATTTGGCAGAGGGTCATCAGTCAGGGTCTTGCTATCGGG

>dme_circ_0007557

GTATCCGACGATGTGGTTCGTGACTTCGCCAAGCAGATTCTGGATAATGGGTTCAACAACAGCCAGCTGG
AAATCGACGATGATTGGGAGGATTGCTATGGAGCCATGACCTTCAGGAAGAACAAG

>dme_circ_0007557_junction_seq

TGGAGCCATGACCTTCAGGAAGAACAAGGTATCCGACGATGTGGTTCGTGACTTCG

>dme_circ_0007558

GAGAGCCCATGTGGACCACACAGGATGCATGACCATTCGCTCATCGGGATAGCCTGTGGGTTGGCCGAGG
AACGTTTTAACGGCATACCGGTGCGCCGCCTTGGCATCCTTGGCCACAGCTACGTG

>dme_circ_0007558_junction_seq

CCTTGGCATCCTTGGCCACAGCTACGTGGAGAGCCCATGTGGACCACACAGGATGC

>dme_circ_0007559

GGATGCATGACCATTCGCTCATCGGGATAGCCTGTGGGTTGGCCGAGGAACGTTTTAACGGCATACCGGT

GCGCCGCCTTGGCATCCTTGGCCACAGCTACGTGGTAAACGAATTTGGCAGAGGTCACCCGAGGATCATA
GGGTAGAGCACTAATAGCACTCAGACACAGCTGGTCAGGATAGGCAGCACTATTCTGATCAATAAACAGA
GGAGTGTTGCTCTCCAC
>dme_circ_0007559_junction_seq
CAATAAACAGAGGAGTGTTGCTCTCCACGGATGCATGACCATTCGCTCATCGGGAT
>dme_circ_0007560
GAGGTCACCCGAGGATCATAGGGTAGAGCACTAATAGCACTCAGACACAGCTGGTCAGGATAGGCAGCAC
TATTCTGATCAATAAACAGAGGAGTGTTGCTCTCCACGTACAAAAAGACTCCGTCGCCGTTCAACCAATA
TCGCTCACCCACACCAAAGTTATCCAGCTCCTTTGGCAG
>dme_circ_0007560_junction_seq
CACCAAAGTTATCCAGCTCCTTTGGCAGGAGGTCACCCGAGGATCATAGGGTAGAG
>dme_circ_0007561
ACGAGCAGTGGTAGCTACCAGCTTACCGATGGCCAATCCACCGTGGAGTTCACCCTGGTCAAAAGCGGAG
TGGTTGCCGGCACAGGCAGTGAGATAACCCATGTGAAAGTATCCCGAGATCAGGTGCTGCAAGGCACCCA
ACCCAGGGATTGCGTCCCCCTGAAGACCGGATCGACGCACTGGTTCAGTGGTCCGGAGCAGAAGCAACAC
TACTGGCCAGTGGAGCGACAGAGGCATAGTAACTATTCCTACCTGCCAAAGGAGCTGGATAACTTTGGTG
TGGG
>dme_circ_0007561_junction_seq
CAAAGGAGCTGGATAACTTTGGTGTGGGACGAGCAGTGGTAGCTACCAGCTTACCG
>dme_circ_0007562
GTTACTATGCCTCTGTCGCTCCACTGGCCAGTAGTGTTGCTTCTGCTCCGGACCACTGAACCAGTGCGTC
GATCCGGTCTTCAGGGGGACGCAATCCCTGGGTTGGGTGCCTTGCAGCACCTGATCTCGGGATACTTTCA
CATGGGTTATCTCACTGCCTGTGCCGGCAACCACTCCGCTTTTGACCAGGGTGAACTCCACGGTGGATTG
GCCATCG
>dme_circ_0007562_junction_seq
GGTGAACTCCACGGTGGATTGGCCATCGGTTACTATGCCTCTGTCGCTCCACTGGC
>dme_circ_0007563
TTAAATTTAAATGTAAGCCTAGTTGTacacaacgacacaagcaacacacacacacactcacacaaacaaa
cacacgcacacgTATTAGGGGGCGTGGCAGGGCAAAAAAACAAAGGTTGTTCTCCATTGGATATCCTTTA
CTTTTTGTTTGTCGAGAAATGGGACGCACAAAGCAAACTTTTG
>dme_circ_0007563_junction_seq
GAAATGGGACGCACAAAGCAAACTTTTGTTAAATTTAAATGTAAGCCTAGTTGTac
>dme_circ_0007564
ATCTTCGATGATCACTACCACTTCGCACATCACAAGGTCTCGAAGCGGTCGCTCTCCCCCGCCACGCATC
ACCAGACTCGCCTGGATGACGACGATCGCGTCCACTGGGCGAAGCAGCAGCGGGCCAAGTCGCGATCCAA
ACGGGACTTTATCCGCATGCGACCCTCAAGGACCTCCTCGCGAGCCATGTCGATGGTGGACGCCATGTCC
TTTAACGACTCCAAGTGGCCGCAGATGTGGTATCTGGAATCGTGGTGGTGGCCTGGACATGAATGTGATA
CCCGCCTGGAAGATGGGCATAACCGGCAAGGGCGTGGTGGTGACAATTCTGGATGATGGCCTGGAATCCG
ATCATCCGGACATACAGGATAACTACGGATCCCAAAGCCTCGTACGATGTGAATAGCCACGACGACGATC
CGATGCCGCATTACGATATGACGGACTCGAACCGCCATGGAACTCGCTGTGCCGGCGAGGTGGCAGCCAC
CGCCAACAATTCGTTCTGCGCGGTGGGTATTGCCTACGGCGCCAGTGTGGGCGGAGTCAGGATGCTGGAC
GGAGACGTCACGGATGCGGTTGAGGCACGGTCGCTGTCGCTGAATCCGCAGCACATTGACATATACAGTG
CCTCCTGGGGACCCGATGACGATGGCAAGACGGTGGACGGACCCGGCGAACTGGCATCGCGCGCCTTTAT
CGAGGGCACAACTAAGGGTCGCGGCGGCAAGGGCAGCATCTTCATATGGGCATCGGGCAATGGTGGGCGG

GAGCAGGATAACTGCAACTGCGACGGCTACACGAACTCCATCTGGACGCTGTCCATCTCCAGTGCCACGG
AGGAGGGCCATGTGCCCTGGTACTCGGAGAAGTGCAGCTCCACGCTGGCCACCACCTACAGCAGCGGCGG
GCAGGGCGAGAAGCAGGTGGTCACCACGGACCTGCACCACTCGTGCACTGTCTCCCACACGGGCACCTCG
GCGTCGGCCCCGCTCGCCGCTGGCATAGCCGCCCTGGTGCTGCAGTCCAACCAGAATCTCACCTGGCGCG
ATCTGCAGCACATTGTTGTGCGCACCGCCAAGCCGGCGAACCTTAAGGACCCCAGCTGGTCACGCAATGG
GGTGGGGCGGCGGGTGAGCCACTCCTTTGGCTACGGATTGATGGACGCCGCCGAGATGGTGCGCGTGGCC
CGCAACTGGAAGGCGGTGCCGGAGCAGCAGCGGTGCGAGATTAACGCTCCCCATGTCGACAAGGGTCATT
CCACCTCGTACCCATATCACCCTGCAACTGACGGTTAATCACTGTCGATCGGTCAATTACCTGGAGCACG
TCCAGGCCAAGATTACGCTAACGTCGCAGAGACGAGGAGACATTCAGCTCTTTTTGAGGTCTCCCGCAAA
CACCAGTGTCACGCTCCTAACGCCTAGGGTAAGTCTAAGAACTTTTTCCAAGATACCAAACATTGTGCCG
TCAGATCGATGCTTTAAACCTGTACCGTTTAACATGCTGCGCTTTTGTGGATCTCACCCACTAAACACCC
CATGAACCCACCCATTTACCCACCGGCTTGTAGGCCATGCTCTGCTCAGGGAATGGTCGCTGATCTTTTA
TGGCACCACTCAGAGCATCGGTCCCAACGATCCGATCTCGGTGCCCAAGCCAAGTGGTTCGGAGGCAACC
ACCCCGAATAGCAGCAGCACCACCAGCAATCTGCATCAAGCCTATTCACCCCAGTATCCCCGCATTCCAC
CCAATAACTTTGGTAGCTCACCCTCTGGCGGTTCCAAGTTGCCCCTGGGCAAAGTGCCTCCACCCAATAA
ATCCAGCTATGTGACAAACAATCCGCTGCTGAATTCCGCACCACCCAAACAGGGTTACCAACAGATTTCG
GCCACCTACGGTGTGATTTTGGGCAAAGCCAATGGAAAGTCTAACAATAACAGCAAGGAGAAGACCAACA
ACAAGGGAAACAAGAGCAATAATGGTAACAAGGGAAAATCAGGCGGATCGAGTGGTAACCGCAAGGAGCA
AACCACTCAGAGCACCATCATTCAGACAAGCACCAGCAAGAATAAGTACTACCGCATCTCGCAGCAACAG
CAGCAAAAGAACAATAAGCAGGACAGGAATGGAGTGCAGACACAGAGACCCAAGGCCAACTCCGGCGAGA
AATCCTATGACGAGAAGAGTCGCAAAGTGGTGGGTGAGATCACGACGAATTCCGGCAACGGATCCATCAA
AGCAGCCAAGCAGGTGAAGGAGAGTACCACCACCTCGTCGAATTCACGGATACCCAAGCTATTCGAGCGC
TACGAGAAGATCCAGGCAATTTTTCCCGAACTAGAACCCTATGAAAACAGTTCTCCCAAAGGAAAACCCA
AGCAGGCCAAGCAGGGAAAACAATTCGAGGTGGATCTGTTTAAGCCCACCAATGGGGGAAATAGTCGCCA
GGGCAATACGAAGAAGTCACCATCGGTGCCGCCGCCCTCGCAAACGATGGCTACCCTCTCCATTTTGCCC
ATTTTACCCGCCGGCGGTAGTAGCTTCCTGCCCGATCAGAAGATTCTAAAGAAACAGCAACTGCTGATGG
CCGCGGCCGGAGTGATGGCCCCCGCTCAGGTGGAGGTGGAAATGGAGGAGGTACACGCGACTCCGGATTA
CGAGGCACGGAAAGACCAACGAAAGGAGGTCAACGGACCAAATGGTAGCCAACCAATTTTTGGAACCACA
TATTTTGCTTTTTAGTTTCGTACAAAAGCATTATTTGTAGTATTTTCACTGAGCCACCTTATCATTTATT
AGTATCAATTTGTACTTGTTTAGTAGATTTCGTCATGATCGTTTTCTGTGTCGAAAGTATCCATATTTTG
CTTTCTATTTTATGATTTTCGACCCGTGGCCGTCAATGAAAGTTTGACAGCCACTGAGTGGCACTTTCTT
GTTACTTTCATCAATCCAAGTGCAACTTTAAAATGttttttttttCGGTTTGTTGGCCATTGAGCTGACG
TTGCCATGGCTGCTGTCAGTGGAGCAATTTTTCATTTTTATCCTTTTTGTCGAATTGTGATGAAAGTTGC
GATTAGCTTTCGGCACTTGATCTGCGCCATGTACATGTACATATAGTACGTATTTCGTTCATATGGTAGA
TTTATTGGCCCTTCACTTGACTTGTTTGCCCGCCTGCAATCTGCAATATATCATACATTTTATAACATTT
TGCGTTTGTCGTTCGCTGTTGATTTTGTATAGATGTTTCGATTTTATTTGGATTATTGAGCCCTCGGCTA
GTCAGCAATAACTAAAAGCATTTGATTTCTCTACTCTGCGTTGCAGCCACAAATCACACAATGGGATATG
ATATTCTACGGCACCGAAACGCCCGCCCAACCCGATGACGTGGCCAATCCCAGCCAGTCGAACCAGTTCA
ATCTGTACGGCAACGATATGGCCCACAATGACGTCGAGTACGATTCCACCGGCCAGTGGAGGAATATGCA
GCAGG

>dme_circ_0007564_junction_seq

ACCGGCCAGTGGAGGAATATGCAGCAGGATCTTCGATGATCACTACCACTTCGCAC

>dme_circ_0007565

GAAACTGATCGAGTACAAGACGTACACTCAAGCACTGCCCTATTTCGATCGTCTGGACTATGTGTCCTGC

ATGGCCAATGAGTTGGCCTACTGCTTGGCGGTGGAGAAGTTGCTGAATGTGGAGGTTCCACGCCGGGCCA
AGTACATACGCACCATGTTTTCGGAGATAATGAGGCTGACGAATCACACGATGGCCATTGGTTCGTCGGT
ACTGGACTGTGGAGCTATAACTCCACTCTTTTGGCTGTTCGAGGAACGTGAGAAGTT
>dme_circ_0007565_junction_seq
TTTGGCTGTTCGAGGAACGTGAGAAGTTGAAACTGATCGAGTACAAGACGTACACT
>dme_circ_0007566
AGCCGTATTGATATTGTGGCCATGTCGGGTATTTGCGCCGTTGCGCGTGGTCTTGCCCATCTGGAAGTGT
AAAGTTGGACTGGACATGCGACCGTCTCCTCCATTGCTGTATGCGAGGACTCGCATGTTGTAACTCTTCC
CTGGCGTCAGATTGTTGATGTACGACTCCAGTTTCTGTCCGATTGGCACTATGGTATTGTTGGCCGTAAT
CATGTTCTGG
>dme_circ_0007566_junction_seq
GGTATTGTTGGCCGTAATCATGTTCTGGAGCCGTATTGATATTGTGGCCATGTCGG
>dme_circ_0007567
CATCTCAGCCTCACAACCAATATCCCGAGCAACGTGCACAAAAACTATTGGAAATATCCTAACCCACAAT
GAAAAACGACGTCGTGCGATGGAGCAGGCAGCCAACTAGCAACACCACTAACAGCAGCAGCAGCAGTAGA
AGCGATAGCAACAGCACTCATAAGCACAGAAGCAAAAGCAACAAATTAAATGCTCGACAATTAGGGTCAA
ATGCTGCCAGAAGTTGTCAGCAAAGATCCTCTGTGGCAACAACACTAGAAGATGAACAACAAACAATCAT
TGAATGTGATATAGGAAATTTCAATTTCGATTGCAATTTATTCAAAACTAGTTTTTAACGCAACACAAA
CAGAAACGTAGTGGCAAGAGCAGCAGCAAAAGTAAGAGCAACAGAAGTAGACCCCTAGCGAAAACGAAAG
CGGTGTTTCTATTAGCTCTGCAATTTAGTGCCGTAGTTTTTTATGTAATATTAATGTCGGTTTCGTGGC
CGGAAGTGTGGCAACTGCGGCATCATCGGCAGGCGGCTCATCGCCGGCAGCTCCATCATCTGCGCCCTCA
TCCCCGCCCACAGTTGCTGTACCACCGCCGCCGCCACCTTCGTCGGCACTCAAAGTGGATCCAAATGGTC
AGTCACCAGTGCTGCCGCCCTACGTTCTCGATTATGAGACGGGGGGCAAGGCCAAGCTAACGCCAAACAA
TGGCAAGTTCGGCCAATCGGGCAGTTCCGGGAGCAATAACAACCACATCGTCGGACACTATACCCACACC
TGGGCGGTGCACATACCAAACGGCGATAATGGCATGGCCGATGCGGTTGCCAAGGATCACGGATTCGTCA
ATTTGGGCAAGG
>dme_circ_0007567_junction_seq
GATCACGGATTCGTCAATTTGGGCAAGGCATCTCAGCCTCACAACCAATATCCCGA
>dme_circ_0007568
ATAAGAGCCGTATTGATATTGTGGCCATGTCGGGTATTTGCGCCGTTGCGCGTGGTCTTGCCCATCTGGA
AGTGTAAAGTTGGACTGGACATGCGACCGTCTCCTCCATTGCTGTATGCGAGGACTCGCATGTTGTAACT
CTTCCCTGGCGTCAGATTGTTGATGTACGACTCCAGTTTCTGTCCGATTGGCACTATGG
>dme_circ_0007568_junction_seq
TCCAGTTTCTGTCCGATTGGCACTATGGATAAGAGCCGTATTGATATTGTGGCCAT
>dme_circ_0007569
GTACGAGCGTTTCATTTTTGCCATACGAATCTGACTAGCACTGCGTAAGCCCAACAATAGTTAACTACTC
TCTGCCATTTGGGCAAATAAATAATAAAATTTTATATTTGAGTTAAATTCGACTGGGAAGACAGCAACAA
AAGACAGCGCTTGAATATGCAAACAAAAGTGTACAAAAATATTTTGCCCGCTTCGATATAAGTTTGTATG
TGAATGCGAGTTGAATAAATAGCATTCGCTGTGTGTGCAAAGTGTGCTGGGTTGGGAAATATGCTTACTT
TCTGTGTATTTCGTATTTGACTTTTGATAGCCAGCCAATCGTCGCATTGTGAACTTGACACACAATCGAA
AGCGCCACACTTGTTCATTATGAAATTGCAGCAGAATTTCTTATAAACACCCTTACCAAAGG
>dme_circ_0007569_junction_seq
AATTTCTTATAAACACCCTTACCAAAGGGTACGAGCGTTTCATTTTTGCCATACGA
>dme_circ_0007570

TTTGCCAAGAGTCTTCGCCATTTGCATTAGCTGCGACGAGTGGTGGCCAAAGCTCAGAATCAAAGACGGT
TGTCCGCAAATCATCTGAAGCAATCGCAGGCTCTGGGCCACCGCAGGCAATGGAGATGTGTTCTTCTGCA
GGTAGAGACACTCGTAAAGCTGAATAATAAGAATATCCAGTATAAAAGACTCTTTTCTGAATGTTCAATG
TACATCTACTCACATTGGGCAGCTCCAAGCTGAGAACTCCCTGATCAAAATAGATGAAGTCCCAGTTAAA
GTGATGAAGTTGCACCAGTCCGTACAAACCAGCCTGTTCCAGCAGGGTTTGAAAATAGGAGTAACAACTG
GGCACACAGACTATGTGATACATTTTCATGGAGATGTCGGGCATTTCCTCTGAGGCCACGGGTTGCAATT
GCTTGAGAAGTGTCTGAAATGTACCCAAAACACTGCGGATCATGTAGATG
>dme_circ_0007570_junction_seq
ACCCAAAACACTGCGGATCATGTAGATGTTTGCCAAGAGTCTTCGCCATTTGCATT
>dme_circ_0007571
GGATAAACCACATTTGTAACTTTAGTCCACAGGAAATATCGACAAAGTCGACGAATTACCTACGATTAAA
TTAAAGGGTGTAACCACGGCATTGGTTCGATTCGGGTATTTTAGTCGATCTCGTATACCTTATCCGGTTC
AAAGCCCGGAGCAGGTGCTGCCGGTGGCCCATGTGCCACCGATCCGCCCGCCATTGGCGGAACACCACCA
GTGCCCTGGTTGTAGCCAGCGGCATTGGGATTCTGGTTGGCGTAGAGCAGGGGATTCATGTACATCCCGC
TGGGATGGACAATATTTGAGTGCAGGTTGAGGAGATCCTTGTACATGGTCGGATCGCCGTT
>dme_circ_0007571_junction_seq
GATCCTTGTACATGGTCGGATCGCCGTTGGATAAACCACATTTGTAACTTTAGTCC
>dme_circ_0007572
GCTTTGGGGAATGGTGTACATTGTTCAATTCTCGTTCTTCGACAAAGGGTTTCCACCATTCCGAGGGGTC
GTCCTTTTGGACACTGGATCCTCCACCCAATCCGCCCAGCATCTCCAGATCGGAATCGCTATCACCGCCA
CCAGCGTTGCCATTACGAGTCTTGCGCTTTTTCACCTTTCCCGAACTGGCCGCCAATCCCGACATACTGG
CGTCCGACTTGAAG
>dme_circ_0007572_junction_seq
TCCCGACATACTGGCGTCCGACTTGAAGGCTTTGGGGAATGGTGTACATTGTTCAA
>dme_circ_0007573
GTGGACAAGGGACTGCTGAAGAAGCTGAAGCCCCATCAGGTGGCTGGCGTTAAGTTCATGTGGGACGCGT
GCTTCGAGACGCTCAAGGAGAGCCAGGAGAAGCCCGGTTCCGGTTGCATTCTAGCCCATTGCATGGGTCT
GGGCAAGACTCTGCAGG
>dme_circ_0007573_junction_seq
TGCATGGGTCTGGGCAAGACTCTGCAGGGTGGACAAGGGACTGCTGAAGAAGCTGA
>dme_circ_0007574
GATGACAGAAGGAAGCGTATCGAAGATCGTCAGAAGCTCTATAACCGCATCTTTGTGAAATCCGAGAGCG
TGGAGATCAATGAACTGGTGCTAGACTTTGACGAGGAGTCGAAAAAGGCCCTGTTGCAGG
>dme_circ_0007574_junction_seq
GAGGAGTCGAAAAAGGCCCTGTTGCAGGGATGACAGAAGGAAGCGTATCGAAGATC
>dme_circ_0007575
GCGGCCAAAGAGGAGGATGACAGAAGGAAGCGTATCGAAGATCGTCAGAAGCTCTATAACCGCATCTTTG
TGAAATCCGAGAGCGTGGAGATCAATGAACTGGTGCTAGACTTTGACGAGGAGTCGAAAAAGGCCCTGTT
GCAGG
>dme_circ_0007575_junction_seq
GAGGAGTCGAAAAAGGCCCTGTTGCAGGGCGGCCAAAGAGGAGGATGACAGAAGGA
>dme_circ_0007576
GTGATGCCCCAAAGGAAGCGGCGTCGAAAGAAGAGCGAATCCGACAAGGGCTCCTCCGATTTTGAGCCCG
AGGAGAAGCAGAAAAAGAAAGGTCGAAAACGCATCAAGAAGACCTCAAGCGGCGAGAGCGATGG

>dme_circ_0007576_junction_seq
AGAAGACCTCAAGCGGCGAGAGCGATGGGTGATGCCCCAAAGGAAGCGGCGTCGAA
>dme_circ_0007577
CTTGGCTTTCAACGATGGCTCCACCAGAACCTGCATCATGGGCAGTAGATCCCGCGCAGAACGACTCATT
GGACCGGCACATACCATCGTATCCTTTTCCTTGCCCGTTCTGAACGTACATCCGGCCATATTGACCGCAC
CGGAAGTGGGTTTGTGCCCGAAGATGCCGCAATTGAAGGCCGGTATGCGTATGGAACCACCAATGTCTGT
TCCCAAACCAAAACCCGTGCAGCAGGCAGCGATCAGAGCGGCTTCACCACCCGAAGAACCGCCGACGGAA
CGCCGCAGATCGTAGGGATTGTTGGTGCAGCCGATTAGCATGTTCCTCGACTCAATCCACTTGTTCACCT
CCGGCACATTGCTGGTGGCGATGATAATGGCGCCACTTTCCTTCATCAGGCGCACGCATTCCGCATCCGT
GGTCGAACG
>dme_circ_0007577_junction_seq
GCACGCATTCCGCATCCGTGGTCGAACGCTTGGCTTTCAACGATGGCTCCACCAGA
>dme_circ_0007578
GACATTGGTGGTTCCATACGCATACCGGCCTTCAATTGCGGCATCTTCGGGCACAAACCCACTTCCGGTG
CGGTCAATATGGCCGGATGTACGTTCAGAACGGGCAAGGAAAAGGATACGATGGTATGTGCCGGTCCAAT
GAGTCGTTCTGCGCGGGATCTACTGCCCATGATGCAGGTTCTGGTGGAGCCATCGTTGAAAGCCAAGCTA
AAACTGGATCAGAAGGTGGATCTGAAGCGGCTGCGTTACTTCTATGTGTCCTCCAATGGCATGGCTCAGT
GCAATCCTATTAACCGGGAAACCGAACGGGTTATGTACAAGATTCGCAAACATTTCGAGGCAGTGAGTGG
AAAGGACGTGCGGCACGCTGACTTGCCCTATACCAAGCTGACCGGAAAGATGTGGCGCTATTGGATGACC
CAGGAACCGGCCAACTTCAATCTGCTGTTGGGCAATGGCGCCGAGCTCAATCCATTTGTGGAGCTCTTCA
AGAAGATACTGGGGCAAA
>dme_circ_0007578_junction_seq
GAGCTCTTCAAGAAGATACTGGGGCAAAGACATTGGTGGTTCCATACGCATACCGG
>dme_circ_0007579
AACCTGCATCATGGGCAGTAGATCCCGCGCAGAACGACTCATTGGACCGGCACATACCATCGTATCCTTT
TCCTTGCCCGTTCTGAACGTACATCCGGCCATATTGACCGCACCGGAAGTGGGTTTGTGCCCGAAGATGC
CGCAATTGAAGGCCGGTATGCGTATGGAACCACCAATG
>dme_circ_0007579_junction_seq
GGCCGGTATGCGTATGGAACCACCAATGAACCTGCATCATGGGCAGTAGATCCCGC
>dme_circ_0007580
TATCTTCTTGAAGAGCTCCACAAATGGATTGAGCTCGGCGCCATTGCCCAACAGCAGATTGAAGTTGGCC
GGTTCCTGGGTCATCCAATAGCGCCACATCTTTCCGGTCAGCTTGGTATAGGGCAAGTCAGCGTGCCGCA
CGTCCTTTCCACTCACTGCCTCGAAATGTTTGCGAATCTTG
>dme_circ_0007580_junction_seq
CACTGCCTCGAAATGTTTGCGAATCTTGTATCTTCTTGAAGAGCTCCACAAATGGA
>dme_circ_0007581
TAGTGGAATGGTGCAGTCCTGGGAGAACTGTGAAAGAACAGCACACCATCGTCGCCGAGCAAGTCCTGGA
CGGACTTCTTGCACTTGGCCGTGGCTTCCCTCATCAGCTTCTCCTTTTCCTTGGGCAGCACACTGTCGAT
CAGACCATAAATGGCCGCCATGCTGTAGTCACTTTGCCCCAG
>dme_circ_0007581_junction_seq
CCGCCATGCTGTAGTCACTTTGCCCCAGTAGTGGAATGGTGCAGTCCTGGGAGAAC
>dme_circ_0007582
AGGCTGAAGTACGCGAAGTCATTGAACTTGACCAGAGGATAGTAGTGGAATGGTGCAGTCCTGGGAGAAC
TGTGAAAGAACAGCACACCATCGTCGCCGAGCAAGTCCTGGACGGACTTCTTGCACTTGGCCGTGGCTTC

CCTCATCAGCTTCTCCTTTTCCTTGGGCAGCACACTGTCGATCAGACCATAAATGGCCGCCATGCTGTAG
>dme_circ_0007582_junction_seq
CAGACCATAAATGGCCGCCATGCTGTAGAGGCTGAAGTACGCGAAGTCATTGAACT
>dme_circ_0007583
GCTATGTGGATGCTATTGATGCCTGTGGCACTGCCATTCCCAAGGATGTGGCCAAGATTGTGGACTCCGT
TAAGGAGATTATCAAAATCTGCGACGATATTTGCATCTGCATTCGAAACTTTGTGCCACGGATAAGTCC
GTGGGCTC
>dme_circ_0007583_junction_seq
TTTGTGCCACGGATAAGTCCGTGGGCTCGCTATGTGGATGCTATTGATGCCTGTGG
>dme_circ_0007584
TATTTTGGCAAGCCGGTAAATGTCATTGTCCTCAACGCGTCGCAGCTCCGTGTTGCGAGAATACTTCGAG
GATAAACCTATGGCGCCGTTGCTTCCGTTCACCTGCACGCCCATTCCGCATCCGTTCCAGCCATACGCCA
TGGTGTTCCCTTAGCTACCCAGGATAACCTTACGGGAAAGCCTTTGCCTGTGGCAGGTCGATGGTTCAAG
TTCCCAGATGTCCAGTGCGATTGTATGAGATTTCCGCTATAAAACCATGTTTGCATCTATCTTCTCTCTC
TATG
>dme_circ_0007584_junction_seq
ATGTTTGCATCTATCTTCTCTCTCTATGTATTTTGGCAAGCCGGTAAATGTCATTG
>dme_circ_0007585
AGTTCGAAATGAGTATAGACAGATCGGGGATGTTCATCGGTCGTGGCTGCAGCACTGGCTCCTGAATTTG
TTCGCTGGGATTGAGAATTGTCAGATTGGGCACATTCGGGATGGTCGTCGACGTAGTTCCGGTGCCTGTC
AATGTTGCAAAGTTGGAGTTAGTAGTACTCCTTTGCTGTCGTCGAGGTGGCGCTATGGGAGGGGTGTTTC
CTGACGGCTGGGGCACACTCTTGTCCCGCCTCACAATGTCTTTTTCAAAGTTGTCCAGGTTCAGGCCAAC
GCTACCTTGAGCGTCTGCCCCAAGAGTGGCAGTGCTTGACTGGTATTTGAGGCTCAGCCCCTCGTTGTCC
ACTTCCATTTCCTCTTCCAGCAGCTCAAGGCTTATGAGCGCACCGGGACCGTCAACAGGCCGGGCAGGGG
TGGACTCTAGAGAAGTCAGTGGATACACTTTTTTTAGTTTTGTCAGTTTAAGGCAAACATAGTCATACTT
GCCATTTAGGAAGTCTAGTGCCACAAAGTCTGCCGCACTGAAGAGCTCTGCCTGCACCAGAAGTTGGAGC
AATACCCCAACCGTGGGCCGCTCGTTGAGCTTGCCAGAGGTCTTCCACTCGTCGATCATCATCTGCGACT
TGCTTTGGTCCGGCGGTAGTCGGTTTGCAGCCTCGTCAATCTGGAACACGTCCTGCGCAGTG
>dme_circ_0007585_junction_seq
GTCAATCTGGAACACGTCCTGCGCAGTGAGTTCGAAATGAGTATAGACAGATCGGG
>dme_circ_0007586
GGTCAGTATCGGAGATGAACGCCACTTCTCTATATGCCGGGTAAGCCGTGGGACTTACGCTTAATTATGC
TCATATATTATTTAATAATAGTAGGAAGAAAAGGGTCCGCCACAGAGGCTTTAAGCCCTGATCATATTAT
ACTTGCATTGTGATTGACTCACTGCCATAATGTATCCTTTCACACACACGGATTAAATTCATTATCGATT
CATTATGTGGGCTGTGGGGAAACTTTAAAAGCAAACTTTACGAGCAAAAATTCAAATACAAATTTAATCA
AACTTTGGCCGTGTGGCTTCCGAAAATCCCAGACCCCGCTTTAACTTGACTCTCGCCTTTGGCAGCTCGT
ACGTCCTTTATAATTGGCATACAAAATAAAAACCGAAACAAAAGTTTAGCCAAACTCCCCAGGGAAAGTG
GAAACATTGAAGTTGGTTTCAACTTTGTTTAAAATTGGAGTGGTCATTACTATTCTAATGTTTAAATGTG
TCGCTTTAATGAATTCTTTTATATTATAAGCTGAAAAGATAGTTTTTCTAGTCTTACTACACttttttt
tCCAACTTGCTGGATTATCAGCCaaaaaaaaagaaaaaCGCGTCGAGCTTTTAAAATATTTCAACGATTAA
AGTGCCAGCAATTAAACTACCATTTCATGGTGAAAACAATCAAACAATTAGTGTAAATCGATGGGTTTAT
TTGCTTACTTCGCGCATATGTTTTTTGCAAGCTGCCCGCTTTAAATTGCTTAATTTTTTTCCCCAAGTGC
TTTGTTCGTTACCGTAGTATGCGTATGATACGAAATGAAATACAAACTCATGTATAAAAATCAGAGTAAA
TAAAAAAGTGACAGAGAAAGCCAGTGAGTGAGACTGGGGAAGTAAAATGCCAACAAACGACAAAAATTGC

GGTGGGGAATGTTTGTGGAGCTCGGGAAAACTCACAGCAGCCCTCTGGCATATGCACATTTTGCACAATG
TTTGCTCTCAGAGGGGGCAGGTGGAAATGGGGAGGAAGGGGCATTGTGGGGTCACCGAATGTCATTGTTA
AAAGTAATGGGGGTTCCCTTCCCTCCATTTCCTCCATTTTTACATCACTTTACTCAACTCTCCGGGGAAT
TGTAGCTCAGTTGAGGAATGCCAAAAATCGCGTTGCTCGCTCAATGAATTATAATGTAGAATCGTTCAAT
ACGTATTGTGTCAGGCGATGGGCGGAAAATCCAGTGGGTTGATAGCCAGGATCTTTGCTTAACACAAAAG
GAAACGAGCCGAGCACGTCTGGCACATGTTAGTGTAAGAGTGCCCTGAATCGACTGTCAAGCTTAATGGC
AAGTAAAGTAACTTTTCCGACTTTTGTGTGGGTTTTTCTGGGGCGATTTATATATTTTTATTGGGATATC
GAAAGTTTTCGTCCGGTAAGCAATCCTCTTGTATATTTTTACATTCGTTTTTAATACTGGGAGTCATAGT
ACTAACACATTTTCTTCAATAACCATAAATATTTAAATTGTGCCATTTTGAAAGTTTCCTTACTTTTGAC
CATTCAAAAGCCCTCACCCATGTCACACATGGCGTATGCTTGATTAATTTTCATTACCGGGAAAAGCATG
AGAAGGGTGCTCTGGAAAATGTAAACAAAGGGCCCAATTTAATTACCTCACTTTTCCAGAACTGTTCAAG
TTTACCCAACTTTCAAGGCGCTTCGCATTAAGGTTATGCTTCCGTTAAAGCCTTTGAAGTTGACTGTTAG
CAAACTTTTTTATGGCCAACTTCAAGGCGTCGGCAGGGCCAATTAAAATGCCAAGATTTTTGCTGACTTT
GTTGTTGGCAACGATGCGCCTGTGTTTGTGTGTATGTGGGTTAATTTATTTAGCTACCAATTGGCACGTA
AGGGGTTAATTGTCAGCCGTCAGCAAAATACGCTCCGCTCGAGTTTATCGATTGCACATAACTCGCACAT
GTTGTTCCTCAGCCACCCAGTGGCCCAAACTTTTGTGATTCACACTTTGCTCGGACGCCCATTTTGCGCC
ATTTTGCCACTAGTTTGTTGATTTTGGAGTTCTTCTTAGCTGCGGGCTGCCTTGAGTTTCATTAGAGCAA
GGATGTATGTGTTACTTGCTTGCTGCCTTTTGAGTTATTCCTTTCGTTTGATTTAAATTTAAATTAAAAA
TCACTCATACGCACCATCTGACAGGCAAACTGACAGCTGCGGGCCGCTCAATGGCGAACAAGAAATTAAA
AGCAAAATAAATAAGAGCACCGTCCTCCACATATTGCACAAATGTCAAACACCTGGCCAAAGCCACTATA
TAAAATTCATTTTAAAAGCAAGCAATCAAAGTGCCCAGACATCCcacacacactcacacacacacacaAA
ACACCCAGGTTCCACGTTCAATAAAAGCTTAGAAAAGAGTTGGGCAGAATTTTAAAAGCCCCTTCCACCA
GTTGGCCAGCAATTAAGCCAGTTTGTAACAATAAATTTCAGTTGCTGGCAGCACGAGGTGTTTGCCAACT
GTTCACTATATTGACTTTCAATTTGGGCGCCGCTCAGTTGGTTGGACTTTATTTTTAGGCACCCTTATGG
GGCAACAATGCAGTAGCTTAAAGTCGTATGGGGTATAGTTAGTTATCAGTTAGCATCAGTTATCGAAATA
ATTAGTATTAACCCTTGAAAACCTTAGAAAATCGATAGAAAAGTTTCTGCAATAATAAATCCAATTACAA
ATTTCGATTTTCCAGCCAGCAAAGATGCCGCCCATCCATAATAGATTACATTTCTGCTCTCTATAGACTG
GCAAACATTTGCCAATTGGCAAACAAATGCCAAATAAATCAAAATCAAACAAGAGAACTGCAACCCCTAC
GTGCAAAAAAAGAAACCGAACCCCAAAGGTGCCCTTAAAAACTTGTTCCCTTGGCCGCTTTACAATCCAA
CCTCAGCCCACCTTGTACCCAGCCCCCTTCCACCTGAAACTCACCTGCTTTTCCGCACCTCAGCCCCTTT
GGTCCCACCGACAACCCCCGTGAGCGATATTCCACTGCGCTTGTACAAACACGTAATCGAATAATCCAAT
TTGTTTGCGCAAAAACTGAAACTTTTTCATAGGGCTTCAGTTGACGACGCCGCGTTGTGAGTCACAAACA
CCCAAATCCCCACCACACCTATCGACCTCAACCTCACCTTTTGAGCTAATTGTTTGAGCGAGTTTTGGAT
ATAAGTGCAGCGTCCTGGCCCCACGAAAAGCCAAATGTCCTTGTTCCCTACGGCATTGTCTGCAAAGTGA
TGTAATTGTTACCCGCTACTTTGATTTGATAGTTAGCCCAATTTGGGTGCCAAGTGGttttttttAGAAA
TGTTCAAGAGAACATAAGTTGACCCAGTTTATAAGTGCAGCTATTTTAAGTGAATACTGAAACTGTTTCC
TTTTCATTAGCTGGCTTACACCATTTCTACGTATTTTGTCATCTTGCTGGCTATATCTGGACTCACTGAA
TTTAGTACACTCAAGTATTTCCACATCCATTGAAATCCTTTCCAGCAACTAATCCTATTCCACCTACCAT
TGTGTTCTTCACTTTGCCTTTAATCAACCTTTTGTTTCGTTCGCCGTTGTGGTTCTCCACCCCAAAACGG
TTTAGGCAACTGTATCCTACCGACTGACCTTTCAATTAAATAAACGTTCCGTTGGTCACCTAGCCAAGAG
GACCACCAACTGTGTGTGTTGACAACGGAGCTCTACCTCCACCTCTCAAGGCCACCTGCTGTTGCTGTTG
TCGGGCATCTTTTCACACCCACATGGATGCGGGGTTTTCCACTTTTTTTGGCCACAATCTTGTCAAACAT
TGGTTGCGCCCCATTCAGCCGGACGTTTCCCCCTAGTTTCACTTGCAGCTTTTCAGGAGCGAGTTACAAA
GCAAGGTTAACTCTTGGGGTTAAAACAATAGCAAAAATGTTTCACAATTTAATAACTTGAAGAGCTATGC

ATATGAGGATGCGAGTGTTTAAACTTTTAAACTTTACTTACTAAAGAGAGCGCACAGTGATAAGCAGAAT
AAGTGATTTAAATAGTTAAGTTGGAGGATGGCGGGCATTACTGGGCTCAGACGTATTAGAGGCCTACACT
TGAGATTTCCCAAGTGTTCCGACGGTGGCTTTGTGTACTAAGCTGCCAATACAGGACAGATTCCCAGGAC
GCGAAAGCTCCTGGTTCTTCCCCCCACCCCCTGAGATGAGCAGATGAATAGCGCGGCTAATCCGCCTGCT
TATTACTGAGAGCAGTTCTGCTTGTGGGCAAAGTAGTATGCGCTTTCTGCCGGAGACAAACAGCTGGCAA
ACTTATCCGTCCGTTTGGGTGCGTGCGCCGGAAGCCAAGGCGGGTTAAGGGGTGAGCTTCAGCTGGCAGG
TGGGTATGTGGGTGGTGGTAAGTGGGAGGCAACCGGAACGGAAACACGGAACGCGACACTTTATGACTGC
TTCTTGGGGACCTGCACTCAGTTCGGTTTGATGTGCTGCCAGTTCCAAAAAGGAACAAGCATAAAACTCT
TACTACACGGAAAACATTTTAAATTAAAAATGAAAAGGTCTTAAGTCATTTGGAAAATAAACGAAGTTGT
AAAGCATGTGTGTGATAATCATCATACATTGCTTGTATTATTCATACTATATATTCAATAAGTTTTTATA
ACTATTCTTTTACTTTCTGTGCACTGGTGGGCGTGGCTCCCAAACCCATCTCCCGTCAATTTCCCACCCT
CTGGCAGGAATTAGGAATGCATTGTCTGAGCGCTCAGCTCAGTTTTCCGTTCGGGTTTTCAGCTCAGTGT
TGCTGCAGTTTGTGCGTCAACTCTTTGGCTCAAGTGCGGGCCACATTTCATAGGCTTCCCAGAGTAACCT
CCACCTCCTCTGACACCCCACCTCAAATCACCCACGATCCAACCACTTCCCCCCCGATTTACAACCCTTC
GCTGGAAGACGCGACACAAATTAGCCATcttttttttgccttcttttttttttGTAGTTTTTCCATTTGGCT
GAGAACATTTTCAGTTTGCTTTGCAGTGCACAACTGGCAACAGTCGGCGGGTTTTATTCGATTCGTTTTG
CACTCGATTCTTTTGTCTGCCCTGAAGGAAATAATCAGAGTAGTTAACCAAGGAAAAAAGTTGAATTCCC
CAGTCTTAAAGTGAATATCTTGGCTTCTCTCGAGCTAATTTAATTATTAGTGCTGCTGCAAAGGAAACGA
ATGATTGCTAATTCGGTTTGGGCAGCCAGTGGAAGGAAAAATATTAAATTCAGTGCAAAGATTCTCAACT
AAGTTAAAAGTAATCCGAGTTATCTGAATTGAAATGAATTATTTAGAAACTATTTTGTGGTAAGAAAATA
TAGAATTAGTACAGATAAATAATTTGAAAATATGTTTACGTCTTTTACAATGGAAAAATGCGGTTAAGAA
AGAAATCTCCTGGAAAATGCATCAAACTTTTCACATACATTTCAATTTGTGAAGTTGTGCCAGCTTTATA
CAATTTATTTAAATATTTTCATAGACTTTTTCTCAGTCATGTCAAGTCAGTGAAGTTTCAGCGAGCACAT
TTTATATATGCAATTTCTCGGAAAACGAATTCAAATACACTTAACATATTACAAAAATGTTCAAAATAAA
TACTCTTGCGGTTCCTCTCATTCGTTTTATCAACCCAAGGGAATGTTGTGCGCCATATTTGTCTTGCTTT
TGCCAATATTTGTTCTCGTAATTTTCTATGGCATCCATCGCCAGCTGCCAAGTTTTCTACGTTTTTGTGC
CCTTTGGCCAATGGACTGCTGCTCACTCAAATGATGCGTAAAAATCACATTAGCGGCAGCCGTGCGACAA
TTTGGCCAGAACCAAGGTCTCAATGCCCCAAAGTGTGCTATATGTTTTGCAGGAGCGTGTTACCTTGGCC
ATTTAAGCCTTGACTTGCCGGCTAAGCCAAGTTTTTGAATTTATAATGCCAGCATGCAGGCTGAAACATT
AATGGGGGACTCACGAGAAGTTTTTCATTGTTTTTACAAAGCTTTTTTACTCAGCTTGGTGTTGTTCGCA
ACTTTTATATTAGGCTTGTTTATTTAACACTTCAAACTTAACTTAGATACAGCTTGATCAGTACAAAAAT
GGCTTGCTACATTTTCGTTAAATTTTATTATATGTGGTAGTTACAAAATACATTTAATACAAGTTTTAGG
GAATGCAATTTTTAATTTTCCGAGCTGTCTGGCAGACAAGATTTTTCGGTTCCTATTTTTTTGATTTTCC
AGACTGAACTACACTCGGTTTTGTGTTCTTCCTTCTATTTCCTGCATGGATGTTTTCATAAATTTTACTC
CCttttttttttGCTATTTTTACTGCCTGCGACTGGCATGAAAAGCATTTTACACATGGCAGCATTTTTTA
ATTTTGCCAGCCGGCTTTTAGAAATGGGACAGCACAAATACACACACGTGAGTGTGTATAGTACACATGC
ACTTACTAATTAGAAATAAAAACGCGAACTGCACTTGCATTTTGCTCATTACGTTCTCTCAGCCATTTCG
CTCTTTATTTCTTGTTCTCTTGTATGTGTGCCGCCCTTTTCGAGCTTGCATTTTGCGCTAAAACATAGGG
TGGCTGGTCGTTTGTTTGGAAATAATAGACACCCTGTCTTTGGGCCAAGAACTCCGCCGACACGTTGGCC
AAATCCCCGCCCATGGTCTGCATAACTCCGGTGTCCTTTGGGCAGCGATGGAATCGGGTCAACTGCTGCC
ATTCAGCAGCTGGACAGCCCTGGGATAAGAAGCCCGTTGCCTGATCCCTTGCCAGACTCTCGGCAAACAG
CATAAATGCCCTCCAATGGCTGCATTCATGCCAAAAGCAACCTGGTTGCTGGCTGCCCCAGTTGGCATAG
AAATCCACATGACCCACGGGTCGATCAAATCCATAGCTGCCCACACTGGTGTGCAGCACCTCCACATAGT
CGGCATCCTCGGCGGTCAGCCGCTCCTTTGGTTTGGCATACCGAAAGAAGGGTAGAGCCGGATCCAAAGC

TCGGATCATCCGAAGGCGACCGGTCTGCAAATGTTTGCCTGCCAGTCCCGCCACATGTGCGCCCATGCTG
AATCCGACGAGCTGAAGATCCTCGAAACGCATTCCCGCCTCCTGATGCAGGAAGTCCACGAACTTGGCCA
AAACCTGACCCACCGGCTTGACCCTATAGCTGGCCGTTATGTAGTCCGCTATGGCTCCACGTCCCCAGTC
CACAGTGAAGATGTTGTAGTTGCCATTTCTCAGATCAAAGTAAGCTGGCAATAGCTCGTTGTACATGTTA
GCATTTTCATTACCCAACCAGCCGTGAATGAGGATTCTGAAAAGTATGATATATTATTTTGAACTTTGAA
TGAAATATCTTAATCATATAATTCTTGAGTGCAACCCCAATACGTTTGTATACAATAAAACAAGTTTCCA
TTGGCCGTAGGGCCTTTAAAACCGAAATTGGCTATCGAAATGGCTCTCAAAACTCACAGATAATGGTCGG
GTAGCTTTCATCCGGCTGCACTCACCTGGTGGGATTGAAGGGACTGAAGCGAGACCGGCGCAACGAGGCG
GCATCGTAGAGATTCACCGGTTCGCTGGTGAACTGCTGGTTCTGTTCCTGGTCCCCCTTGTTCATCACGT
TTAGGCCAAAGGCGGCGGTCAGCGTGTTGCCCACAACCTGCTGGAATAGCCGGCGATGTGGCGATTCCTT
CTCGGATTTCAATGGTCCTGTCTGGCGGTTGGCATTGTCCTGCGGGACCAGGGTATGCAGGTGGAAGTAG
GTGTGGTGCTCGGCACTGTACAGCAATTTGTAGTCCGGCTGCAGGCGGAGTAATTGCTGTTCAACCGAAC
TGGGCCCGTGGGCCAAGTAATGCCTCTCCAATTGCTCCTGCCGCCCATGCTGCTCATCCAATATTGGCAG
CTGGCCGGGACTCATTAGGCCAGACGACAGCAGTGGCATTTGTGTGTCAAAGTAGTTTGCCTGCAACCGC
TTACCGCCCCGATTTCTCATCCGCATCATCATTGTTCCCATTCTGAGGGCACTTTCCACTTCATTTGTCA
CTTTTACTTGCACTGCGGGTCTCGGGCTCGGATTCGTCTTCGCCGGAGTCATCGCCATCGCCATAGTCAT
CACCTTCTCCTCCTGCTGCAATTGATTGTTGTCTGCAGCGACATTATAGTCCTCAAAACCAGGCCTGTAA
TCAGACGCTTTCAGCTTTGCTGCAGCGAGCCACAAACAAACTGGTGTCAACGAAATGAAAATGCATTAGT
CTCGAAACTGTGCAACTAGACCAATCCGCAAATGAGTAAGGCACACAAAGAAAAACCAGAAATCATTGAT
TTTTAAAAAGGTGttttttttatgatttttcacattttCATAGCACTGTTTAATTAAGTCTTAAAAAAGA
TTTTATTATGCATAAACTTTCTCAAAAGTGACACAACACTTAGTTTTTGATCAATTGACAACAACATTTT
CTGATATTTTCTGTCCGTGTACATTACTGTTGCTCCCGCAGTTCATGATGCTGATGATAGTCACCTGCCT
GCAAAACGACTGCCAAATGTCCAACATTTCCCATATTTTCAAAATCTGATCTTTCCTCGACGAACTGGAA
TTCCGAACTGAATTGTACTGAACGAAAGCCAGGCATTTACCACAATATTTTGACCATTTTCATTTTTCGG
CTTATTAATATGCAGAACTTCACACTTTTTCTCACTCTCATCTTCGATGATTTCTCTTAACGAGAAGTAA
TTGTTAATGCCTGAGTTGACGGGAATTACTGGAAGAGTGCCCTCCCATTAGCTGTTAATCTGTTGGGTCA
ACAAAAGTTCTGGTAGTAAGTGGTCATGGGAAGGGGAAGCGCAAGAGAGGAATCTGTGCCACTGACCAAG
TTTTTGTACCATTTAGCACTCGATGACGGGAGTTCAATCTTTTTTGGGATAGGTCAATATACTGGCCTAA
AATATTTCTGAATGTATAGTTAAATAACACAAATACTCGTTCATTACGGACGGAAATAACAAAAGTATTT
AAAATAAATAAAATAAATCGTTTTAACACGTGCTACCAACTTTTATGAAACGCTATTGAACTGAATGCAA
GAACATTTCAACAAGCCACAATTCGATTTAACATTCTCTTTATATTTAAAACACTATGTATCTCAATCTT
GTGATTGTGTTTTCCCCACAAAAAAATATTTTCTTAAGCCTGCGGCATAAACATACTAATCGAAAATTTG
CACATTTCTTCTTGTTTACTTTCCAGGTAAGTTCACATATTTCCGCGAATTCCGCCAACCGGAAGTGGCG
GTCGCCATTTTAACGACATCCGGCGCGTGCATTTCTTGCCTGAAAATAAGAACGACGACGAAGGACCTGC
TGCAGGACATAGCTGTTTTCCATTGGGTTTATCGCCGATCGCAAAACGTGACGAAAACAACGAGAACAGC
GTGATGTAGGGTAGAAATAAAAGCCTCAGGAGCAGGGAAAGATAAACGAAG
>dme_circ_0007586_junction_seq
CCTCAGGAGCAGGGAAAGATAAACGAAGGGTCAGTATCGGAGATGAACGCCACTTC
>dme_circ_0007587
GCGCAGTCGCGGCTTTGGTTTCATCACATTCCAAGAACCCTGCACCGTGGAGAAGGTTCTCAAGGTGCCC
ATTCACACACTCGATGGCAAGAAGATCGATCCCAAGCATGCCACCCCAAAGAATCGACCTCGTCAGGCCA
ACAAGACCAAGAAGATCTTCGTGGGCGGCGTCTCACAGGATACTTCGGCCGAGGAGGTTAAGGCTTACTT
CAGCCAGTTCGGACCCGTCGAGGAGACGGTTATGTTGATGGACCAGCAGACAAAGCGCCACCGTGGCTTC
GGATTTGTCACCTTTGAGAATGAGGATGTGGTCGATCGCGTATGTGAGATTCACTTCCACACCATCAAGA

ACAAGAAGGTCGAGTGCAAGAAGGCACAGCCCAAGGAAGCAGTCACACCGGCTGCTCAGCTTCTCCAGAA
GCGCATTATGTTGGGCACCCTCGGCGTCCAGCTGCCCACAGCTCCTGGCCAGCTGATTGGAGCCCGTGGT
GCCGGCGTGGCCACCATGAACCCACTGGCCATGCTTCAAAATCCCACACAGCTACTGCAATCCCCGGCAG
CAGCCGCTGCCGCCCAGCAGGCCGCCCTCATATCACAGAACCCATTTCAAGTACAAAACGCCGCTGCGGC
AGCCTCGATTGCCAATCAGGCTGGCTTCGGCAAGCTGTTGACCACATATCCGCAGACTGCGCTGCATAGC
GTCAGGATATGCACCCTACTCGATCCCCGCCAGCGCCGCCACTGCCAACGCCGCCTTGATGCAGGCTCAT
CAGGCGCAAAGCGTGGCCGCCGCTGCCCATCATCACcagcagcagcaacagcagcagcatcatcaccagc
agcagACCCACAATGCCCATGTGGCCGCCGCCCAGCAGCAGCAGCAGAGCCACCACAACGCCGTCTCGAA
TCCCGCTTCGCAGGCGCACTCGGCAGCAGCGGCTGCCGCCCTGGCGGCCAATGCCGCGAATGGGGCAGGT
GCCGCTGGAGCCCACAGCCTGGCCGCTGCCGCTCAGCAAGCTGGCTTAATGGCCGGCAATCCCCTGAAtg
ccgctgctgccgccgccgccgctgctgccAATCCGGCGGCTGCCTACTCCAACTATGCGCTGGCCAACGT
GGACATGTCCAGCTTCCAGGGCGTCGATTGGAGCACCATGTACGGCATGGGCATGTACGTCTAAGACGCG
AGTTTTGTTTG
>dme_circ_0007587_junction_seq
TGTACGTCTAAGACGCGAGTTTTGTTTGGCGCAGTCGCGGCTTTGGTTTCATCACA
>dme_circ_0007588
CTAATTCGCGTTCAGTCGGCGGAGGGCATCAAACGCATCGAGATCTCGCCCAAGTCAAATCTAAAGCATC
TGTACGACAGCGTTCAGAATGCCCTGAAGGTGGATGGATTTGGTCTCTTCAAGGAGCGCAACTTCCTCAC
AGAGCTGCAGGCAAGTGGCTCCCAGCTGGTGGGGACTTCCTTGAGGCATGGCGACATGGTCTACTTAAAA
CAGATGGCTGGCACATCATCGCGCGCGCACTAGCACCACTGTCCTGGACAGTCAGGCGTTCAAGACTAGC
ACCATTAGCAATCCAAACAGCGCTCGTCCATCCTTCAATGTGATCGAAGATGACGTGGACCAGGCTCTGA
GCAAGGCTGATGGCACCATTAAGCGCGAACGTGATAGCAAGCTGGTGCCACCACAATGCCAACGGCCGTT
GCGTTCACTGCTCCGCACTGGAGCCCTATGATGAGTCCTATCTGAAGGAGCACAATATCAAGCACTTGTC
CTTCCACTCGTACATACGAAAGCAGACATCCGGCATGGATCAGGGCAAGTACTTTGTCTTCGACGACATC
AATTGCCGCATCAAGCCGGGATGTCGCGAGCATCCACCATGGCCCAAGGGCATCTGCTCCAAGTGCCAGC
CGTCGGCCATTACGCTGAATCGTCAGACATATCGCCACGTGGACAACGTCATGTTCGAGAACACTAAGAT
CGTGGAGCGCTTCCTCAACTACTGGCGCACCACAGGACACCAGCGCATGGGATATCTGTATGGGACGTAC
GAGCAGCACACTGATGTTCCGTTGGGCATTCGGGCCAAGGTGGCTGCCATTTATGAGCCGCCACAAGAGT
CTACCAGGGATTCGATCAATATTCAGCCGGATGAGTTCGCCGACGATGTGGATGCCGTAGCCAGTGCGCT
GGGACTGAAGAAGGATTGGTTGGATTTTCACTGATCTCATCACGGACGATGCGAGTATCGGCACTGTCAA
ACAGATCCGTGGCATCGAGTCGCACTTTATTACGGCCCAGGAGTGCATCACAGCTGGCGAGCTGCAGAAT
CGACATCCAAATCCGTGTAAATATGCCTCCAATGGCGTCTTCGGCTCTAAGTTTGTGACCATTTGTGTAA
CAGGGAGATAAAACCAAACAAGTGCACATGGAAGGCTATGCGGTTTCGGCTCAGTGCATGGCTCTGGTGC
GCGATAATTGTCTGATACCCACTAAGGATGCGCCCGAGCTGGGCTACGTGCGTGAATCCACGGACAAGCA
GTATGTTCCCGACGTTTTCTATAAGG
>dme_circ_0007588_junction_seq
CAGTATGTTCCCGACGTTTTCTATAAGGCTAATTCGCGTTCAGTCGGCGGAGGGCA
>dme_circ_0007589
GTTAATGAGTCCCATATTCACATGCCCTAATCTTAATTTTGTGTGCTATACTTTTGGTTAATTTTTGCTG
CTAGTCTACGTGTTAACAAAAACCCTTTCCAATCTCTACAATTCGCCATTAGTTGTTAACTTGACAATGA
ATTTTAATTGTTGCAATAATTCGAGAGTATCGTAATTTAAAAACGCCTTTCTAACGAATAAGTAGTGCCC
TTAGTGATTTGTCTCCTACAGAATTATTCATTGTAGAATTCACAATTCGAATTACACAAAACTTATTGTA
CTTAGCAATTAGCGCAGCTTTAAAAAAACAAATTAGCAATGCTTTGTTGAAAAGTTTAACTTTTAAAGTG
CTTAGCCTTAGCCACCGGATATCGAAACAAAAGACCGAAGCATTAACAATTGCAAAG

>dme_circ_0007589_junction_seq
AAAGACCGAAGCATTAACAATTGCAAAGGTTAATGAGTCCCATATTCACATGCCCT
>dme_circ_0007590
GTGGTGATAACAGCTTTGAGCTAACCAACGACTCCAGCTCCACCTCAAACGACGATTACGCTTGCAACAT
TCCAGACTTGAGTGAGCTGCAGCAATAGATAAAAGCACCGACCATCGGCGGAGATGACATTCCGTTCGAT
AACTTATGCAACCCAGATGTATTCATTTGGCAGCTGG
>dme_circ_0007590_junction_seq
AACCCAGATGTATTCATTTGGCAGCTGGGTGGTGATAACAGCTTTGAGCTAACCAA
>dme_circ_0007591
GTAACCACAAGTAGCGAATGGGCAGCTTAGAAACGCAGCTTCTGGAAGAACCACTTGTTCTTGCCCTCCT
TGTAGACGGACTCGAACTTGACGCGGGTCTGGAAGCGGTGCGTCTTGCGCTTTACGGGATCCTTCAGGTC
CTTGGGCGACAGCTTCTCAAAGCTGATGTCGTGCGCCGT
>dme_circ_0007591_junction_seq
GCTTCTCAAAGCTGATGTCGTGCGCCGTGTAACCACAAGTAGCGAATGGGCAGCTT
>dme_circ_0007592
GAAACGCAGCTTCTGGAAGAACCACTTGTTCTTGCCCTCCTTGTAGACGGACTCGAACTTGACGCGGGTC
TGGAAGCGGTGCGTCTTGCGCTTTACGGGATCCTTCAGGTCCTTGGGCGACAGCTTCTCAAAGCTGATGT
CGTGCGCCGT
>dme_circ_0007592_junction_seq
GCTTCTCAAAGCTGATGTCGTGCGCCGTGAAACGCAGCTTCTGGAAGAACCACTTG
>dme_circ_0007593
GGGCTTGACCTTGGACTTCTTCTTCAGCTTGTTCTTGCCCATCTTCTTGGTCACCTTGCGCGGGTAGCGA
TCGATACCGGCGACGAGGGCGTGTCCGAAGGGCTTCTCCGGGGTTCCATCGTCGTGG
>dme_circ_0007593_junction_seq
GGGCTTCTCCGGGGTTCCATCGTCGTGGGGGCTTGACCTTGGACTTCTTCTTCAGC
>dme_circ_0007594
ATGAGGAAAATCATGAAGCAGGGCAAGATCGTAATCGTCCTTAGCGGACGTTACGCCGGTCGCAAGGCCA
TCATCGTCAAGACCCACGACGATGGAACCCCGGAGAAGCCCTTCGGACACGCCCTCGTCGCCGGTATCGA
TCGCTACCCGCGCAAGG
>dme_circ_0007594_junction_seq
GCCGGTATCGATCGCTACCCGCGCAAGGATGAGGAAAATCATGAAGCAGGGCAAGA
>dme_circ_0007595
ATACAGCAAACACTTTTATCACTACTGATGGATTAATCAATTCTGTATCATCCCATTCCAGATCACCCAG
CTTTGCAGGGACTTTGGTATCTGACTGGCCTGACTACGGCTGCCACTGCCCTCAGCTACGTCATGAATCG
GCATAATACCTTTAAGATTATCCAAAAAAAGACGTAGGGCCGGGCATTTCCGCTAAATATCTGAGTACTA
TGAAGGAGTAGTATCTCTCCTTAAGCAATGATTAG
>dme_circ_0007595_junction_seq
GTAGTATCTCTCCTTAAGCAATGATTAGATACAGCAAACACTTTTATCACTACTGA
>dme_circ_0007596
GCACCGACCATCGGCGGAGATGACATTCCGTTCGATAACTTATGCAACCCAGATGTATTCATTTGGCAGC
TGGGTGTGGACCTTCTTATGTTATGCATGAGGTCCAACTAGAATCTAAGATTCAGTGCACGATTTGTTCA
TATCACGTCTGTAATCGTGACAGTACATGACAA
>dme_circ_0007596_junction_seq
CGTCTGTAATCGTGACAGTACATGACAAGCACCGACCATCGGCGGAGATGACATTC

>dme_circ_0007597

ATAGGACATGTCGCTCCGACGAGTTCACCTGCGGCAATGGACGTTGCATTCAGAATCGCTTCAAGTGCGA
CGATGACGACGATTGTGGTGACGGATCGGATGAGAAGAACTGCGGGGAAAAGGCGAAATGCGGATCCAAT
TTCTTCGCTTGCAAAAGTGGACCGTGCATTCCCAATCAATGGGTTTGCGATGGAGATTCAGACTGCAGAA
ATGGCGAAGATGAAATGGTGAGTTAACTAAAATAAAACACAAGTTGATAACAAGGGAAAACACAGAAACC
GTGCAAGCAAGATATATTTATATTTTTGCCGTGTAAAGTCCGGAAATCTTTTCCCCTACCTTCAACTTGC
AATGAAAATTGATATGAGCACATTATCAAACCACATACACACAAACATGCACACATACAAATGCCAAGTT
GTTTAACAGCCCTCGCATATAAATATGTATTTATATGGAGCAAGCTAGGAAGCATAAATTATGCAAGACG
CGAGCATTATTCAAAACTGGCAACGACGACGAGGACCATGTCGGCAACTGTTTCCCATAAACATTTATAT
GTATGACGCATGGCTTTTGTTGTCCGTAGGGTGGTGCCCGAAATTGCATGGCTCCGAACAGCAATAAAAA
CAGTGCAGAAACTATCCGAACAGTTCGCAAAAAATACTCTTTTACGAATTTACTGAGAAAATATTTTATA
GTAATTCTTTAAAGCAAAAAATCGTATTAAAATTCTTAAACTGTTTGATTGCCAACaaaaaaaaaGGTTA
AAATATTTATTATCTTGCATCACAAAAACATCACACAATATCGAAACACAATATAAAAACCAACTTAAAA
ATTTCACCTTTAAAAATCCGATTAATTAAATTCGTAAAAATAAACGGGCTATTTACACACAAGTACTCCT
CTTTACAAAGCGTATAGAATGTGGAAAATGTTTTCTGGCATCTCCTAAAAGTATGCAGTACAAAAAATGC
AGGCAATGGCCACAGGGACCTCTATATAAGTGTGCATGTACGTATGCTCTCGTGTGTATGTGTGCGTGCT
GGTGCATAAGGGCTTTGCGTGTGCGAGAGTGTGTTTGTGAACCGGGCTTAGCTGCCTGAATATTTGGTTG
GCACTTAACAAGAAAAGCAAACTGTTGTAGCAGCAGTAAAGTTAACTTCCTTTATCCTCGCCACGGGTAG
AGATTCATCTTTTAAAAGACGACCTTGTGAAAAACTTAAGCATTAAGCTCTGACTTTTAAGCAAACTTAA
TTTTAAAAGTACCCATTTTATCTATATAATTCATTCACTTTTAGCAAAACTGCACCGTCAGTCTATTGAA
CTTCTGCCAAGCGGGTGAGTTCCAGTGCTCGGATAGAATCACCTGCCTGCACAAATCGTGGGTTTGCGAT
GGCGAGGCCGATTGTCCCGATGGCGAGGACGAATCGCAGTCCAACTGCCTAAAAGTGTCCTGTCGTCCGG
ATCAGTTCCAATGCAACGATCAAAGCTGCATCGCTGGCCATCTGACCTGCAACGGGAAGAGGGACTGTGC
GGATGGCAGTGATGAAATTATGTGCGACATTAGTGCGACGCCAAGGACTTGCAATGCAACCACTGAATTC
GATTGCGGCGGTGGTCTGTGCATCCCGAATGCCAAGGTCTGCAACCGCAGGAAGGATTGCCCGAATGGCG
AGGATGAGCCGGCCGGAAAGTGTGGCATCAACGAGTGCTCCTCGAAGAATGGCGGCTGTATGCACCAGTG
TGTGAATCTGGAGGTGGGTTACCGCTGCGAGTGCCACGATGGCTATAAACTGGGCGCCGATCAACACACA
TGTGTGGACATCGATGAATGTGAGACTCCTGGCATTTGCTCCCAGGTGTGCGTCAACGAGATCGGAGCCT
TTAAGTGCGAGTGCGAGGCGGGCTACATGAGACTCCTGAAGAATCACACCAGGTGTAAGGCCAGCGAAGG
ACATGCCTCGCTACTTCTGGCCAGGCGCCATGACATCCGGAAGATAGCACTGGATCGACCGGAAATGACG
TCAATAGTGAATAGTACAAAGGCAGCCACGGCTCTGGACTTCGTGTTCCGCACGGGAATGATCTTCTGGA
GCGATGTTGCAACCCAGAGTATTTACAAAGCGCCCATTGATGAGGGTAACGATAGAACGGTGGTGCTGAC
AAAGTCCTCGGTGACCTCGGATGGCCTGGCTGTCGATTGGATCTATAACCATGTCTACTTTACGGACACC
CACAAGTGCACCATCGAACTGACCAACTTCGAGGGCAGCATGGGCAAGGTCCTGGTAAAGGACTCCCTGG
ACATTCCTCGTTCCATTGCTTTGGATCCCATCGAGGGCTGGATGTACTGGTCCGACTGGGGCGCCTCTCC
GCGCATCGAAAGGGCGGGTATGGATGGCACCCACCGCACTGCCATCATCACCTACGATGTTAAGTGGCCA
AATGGCATCACATTGGACCTGGTTCAAAAACGCCTTTACTGGGTGGATGGCAAGTTGAACACCATCTCGT
CGTCAAACTACGATGGCTCGCAAAGACATCAGGTGCTCTACTCCGGCGAATACCTGCGACATCCCTTCTC
GATCACCACCTTCGAGGACTACGTCTACTGGACCGACTGGGATAAGCAAGCCGTCTTCAAGGCCAACAAG
TTCAATGGAATGGATGTGGAGCCTGTTACGGCGACGCATATGGTAAGTGTATTTCCCAAATTGTTTCTTT
TTAAGATTACATATATCGAGAATTTTGCAATCGTAACTGCTTGCGATGATAGCACACTTCCTATTAAATT
TAATACATAACATATCCGGAGGAATGTGACCTTAAGTACAAGTGTCTGATTGCCTGGTATGTAAGTCGTC
GCTTGGGAATTATGGTAGATAATTGTGAAACGTTTGAGCTTCTTATCTAAGCTACTCATTCCAAGAAACG
AGAAATTAAATACATTTAAAAGAATATATTTGTAAGGATAGATAGAAAAAGTATTTTTATAACCAAAAAA

CTTTTACCATTTCCAATTCTAGTTGGAACATCCCATGGTGGTGCACGTGTACCATCCTTATCGCCAGCCA
GATGGCGTGAATCACTGCCAGTCGGTGAATGGCCACTGTTCTCATCTTTGCCTGCCAGCTCCCAGGATTA
ACGAGCGGAGTCCTCGCATATCCTGCGCCTGTCCGACAGGCCTGAAATTAATGCCCGACGGGCTAATGTG
CGTCGAAGATCGTAAGTAGATTTTACTGTGTCCTTTGGGTATTTTACGGCGGATTTCGTAATTGCATTTG
AACATTCCGCCGCGATGGCACCGCTTTGTTGTCCATCTGACCAACGGTGGAAAATCGATGCGTGGAGATG
CAGAACGCTTTCCCGCTGCGGTTGCGGCCATTTAAGTGGCCATCTGCACTGCGCTTTTCATTGCATTGCA
TTTCCAACTTTTCGAATTTCATTATACCTATGAAATAACCCTTTGTTTGTAGTGGGTATCCCAACTGTGC
GTCCCACTGCACCGATGCACCGGACAATCGAACAGTCGTCCAACTCAACCACAAAACTACCTCAAGATGG
GTACAGTGAAACGCCGGG
>dme_circ_0007597_junction_seq
CTCAAGATGGGTACAGTGAAACGCCGGGATAGGACATGTCGCTCCGACGAGTTCAC
>dme_circ_0007598
GAAGCCACACATGTTCGCCCGAGGAGTTTGCCTGCAAGTCGGGCGAGGGCGAGTGCATTCCCTTGTCCTG
GATGTGCGATCAGAACAAAGATTGTCGCGATGGAAGCGATGAGGCGCAGTGCAGTAAGCCATAACCGAAT
ATTTCCTAGCTCTCTGTTACCTGCTTTGCTTTGCTTGGTGTTGGTTGCCGTAATAATTGAAATTATCGTA
GATTTTTCCCTGCTTTCCTTTGGTGTTGGTTGCTTATTTATTTCTAGATACTGTAGTGCACTATTCCCCG
TACTTGACTGTGCCATGAACACATGTCCCTGAGACTTGTGTTGTGACATCGTGAAGCTCGTATGCAATAA
TGTACAAATATTTACGCACACACAGAGGGAACACTTTGGTTTTCTGTGTGTGCGCTGTTTCTGTTGTTGT
CACTTTCACATCTCGCCCTTCTCTATATGCTTTCTTTGTTTGTTGGCAATTTTTGTAATGGTGTTGGCTT
TTCCCAAGCCTTAAAGAAATGGTCTTTGCGGCTTTAGCTTTTCCTCATCCCATCCATGCTCACACTAATC
ACAATCATCAAATCCTCCCGCCGATAACCCATAGTGAAATTAAACTAATCATTGGAATGTACTTCGGCAG
ATAGGACATGTCGCTCCGACGAGTTCACCTGCGGCAATGGACGTTGCATTCAGAATCGCTTCAAGTGCGA
CGATGACGACGATTGTGGTGACGGATCGGATGAGAAGAACTGCGGGGAAAAGGCGAAATGCGGATCCAAT
TTCTTCGCTTGCAAAAGTGGACCGTGCATTCCCAATCAATGGGTTTGCGATGGAGATTCAGACTGCAGAA
ATGGCGAAGATGAAATGGTGAGTTAACTAAAATAAAACACAAGTTGATAACAAGGGAAAACACAGAAACC
GTGCAAGCAAGATATATTTATATTTTTGCCGTGTAAAGTCCGGAAATCTTTTCCCCTACCTTCAACTTGC
AATGAAAATTGATATGAGCACATTATCAAACCACATACACACAAACATGCACACATACAAATGCCAAGTT
GTTTAACAGCCCTCGCATATAAATATGTATTTATATGGAGCAAGCTAGGAAGCATAAATTATGCAAGACG
CGAGCATTATTCAAAACTGGCAACGACGACGAGGACCATGTCGGCAACTGTTTCCCATAAACATTTATAT
GTATGACGCATGGCTTTTGTTGTCCGTAGGGTGGTGCCCGAAATTGCATGGCTCCGAACAGCAATAAAAA
CAGTGCAGAAACTATCCGAACAGTTCGCAAAAAATACTCTTTTACGAATTTACTGAGAAAATATTTTATA
GTAATTCTTTAAAGCAAAAAATCGTATTAAAATTCTTAAACTGTTTGATTGCCAACaaaaaaaaaGGTTA
AAATATTTATTATCTTGCATCACAAAAACATCACACAATATCGAAACACAATATAAAAACCAACTTAAAA
ATTTCACCTTTAAAAATCCGATTAATTAAATTCGTAAAAATAAACGGGCTATTTACACACAAGTACTCCT
CTTTACAAAGCGTATAGAATGTGGAAAATGTTTTCTGGCATCTCCTAAAAGTATGCAGTACAAAAAATGC
AGGCAATGGCCACAGGGACCTCTATATAAGTGTGCATGTACGTATGCTCTCGTGTGTATGTGTGCGTGCT
GGTGCATAAGGGCTTTGCGTGTGCGAGAGTGTGTTTGTGAACCGGGCTTAGCTGCCTGAATATTTGGTTG
GCACTTAACAAGAAAAGCAAACTGTTGTAGCAGCAGTAAAGTTAACTTCCTTTATCCTCGCCACGGGTAG
AGATTCATCTTTTAAAAGACGACCTTGTGAAAAACTTAAGCATTAAGCTCTGACTTTTAAGCAAACTTAA
TTTTAAAAGTACCCATTTTATCTATATAATTCATTCACTTTTAGCAAAACTGCACCGTCAGTCTATTGAA
CTTCTGCCAAGCGGGTGAGTTCCAGTGCTCGGATAGAATCACCTGCCTGCACAAATCGTGGGTTTGCGAT
GGCGAGGCCGATTGTCCCGATGGCGAGGACGAATCGCAGTCCAACTGCCTAAAAGTGTCCTGTCGTCCGG
ATCAGTTCCAATGCAACGATCAAAGCTGCATCGCTGGCCATCTGACCTGCAACGGGAAGAGGGACTGTGC
GGATGGCAGTGATGAAATTATGTGCGACATTAGTGCGACGCCAAGGACTTGCAATGCAACCACTGAATTC

GATTGCGGCGGTGGTCTGTGCATCCCGAATGCCAAGGTCTGCAACCGCAGGAAGGATTGCCCGAATGGCG
AGGATGAGCCGGCCGGAAAGTGTGGCATCAACGAGTGCTCCTCGAAGAATGGCGGCTGTATGCACCAGTG
TGTGAATCTGGAGGTGGGTTACCGCTGCGAGTGCCACGATGGCTATAAACTGGGCGCCGATCAACACACA
TGTGTGGACATCGATGAATGTGAGACTCCTGGCATTTGCTCCCAGGTGTGCGTCAACGAGATCGGAGCCT
TTAAGTGCGAGTGCGAGGCGGGCTACATGAGACTCCTGAAGAATCACACCAGGTGTAAGGCCAGCGAAGG
ACATGCCTCGCTACTTCTGGCCAGGCGCCATGACATCCGGAAGATAGCACTGGATCGACCGGAAATGACG
TCAATAGTGAATAGTACAAAGGCAGCCACGGCTCTGGACTTCGTGTTCCGCACGGGAATGATCTTCTGGA
GCGATGTTGCAACCCAGAGTATTTACAAAGCGCCCATTGATGAGGGTAACGATAGAACGGTGGTGCTGAC
AAAGTCCTCGGTGACCTCGGATGGCCTGGCTGTCGATTGGATCTATAACCATGTCTACTTTACGGACACC
CACAAGTGCACCATCGAACTGACCAACTTCGAGGGCAGCATGGGCAAGGTCCTGGTAAAGGACTCCCTGG
ACATTCCTCGTTCCATTGCTTTGGATCCCATCGAGGGCTGGATGTACTGGTCCGACTGGGGCGCCTCTCC
GCGCATCGAAAGGGCGGGTATGGATGGCACCCACCGCACTGCCATCATCACCTACGATGTTAAGTGGCCA
AATGGCATCACATTGGACCTGGTTCAAAAACGCCTTTACTGGGTGGATGGCAAGTTGAACACCATCTCGT
CGTCAAACTACGATGGCTCGCAAAGACATCAGGTGCTCTACTCCGGCGAATACCTGCGACATCCCTTCTC
GATCACCACCTTCGAGGACTACGTCTACTGGACCGACTGGGATAAGCAAGCCGTCTTCAAGGCCAACAAG
TTCAATGGAATGGATGTGGAGCCTGTTACGGCGACGCATATGGTAAGTGTATTTCCCAAATTGTTTCTTT
TTAAGATTACATATATCGAGAATTTTGCAATCGTAACTGCTTGCGATGATAGCACACTTCCTATTAAATT
TAATACATAACATATCCGGAGGAATGTGACCTTAAGTACAAGTGTCTGATTGCCTGGTATGTAAGTCGTC
GCTTGGGAATTATGGTAGATAATTGTGAAACGTTTGAGCTTCTTATCTAAGCTACTCATTCCAAGAAACG
AGAAATTAAATACATTTAAAAGAATATATTTGTAAGGATAGATAGAAAAAGTATTTTTATAACCAAAAAA
CTTTTACCATTTCCAATTCTAGTTGGAACATCCCATGGTGGTGCACGTGTACCATCCTTATCGCCAGCCA
GATGGCGTGAATCACTGCCAGTCGGTGAATGGCCACTGTTCTCATCTTTGCCTGCCAGCTCCCAGGATTA
ACGAGCGGAGTCCTCGCATATCCTGCGCCTGTCCGACAGGCCTGAAATTAATGCCCGACGGGCTAATGTG
CGTCGAAGATCGTAAGTAGATTTTACTGTGTCCTTTGGGTATTTTACGGCGGATTTCGTAATTGCATTTG
AACATTCCGCCGCGATGGCACCGCTTTGTTGTCCATCTGACCAACGGTGGAAAATCGATGCGTGGAGATG
CAGAACGCTTTCCCGCTGCGGTTGCGGCCATTTAAGTGGCCATCTGCACTGCGCTTTTCATTGCATTGCA
TTTCCAACTTTTCGAATTTCATTATACCTATGAAATAACCCTTTGTTTGTAGTGGGTATCCCAACTGTGC
GTCCCACTGCACCGATGCACCGGACAATCGAACAGTCGTCCAACTCAACCACAAAACTACCTCAAGATGG
GTACAGTGAAACGCCGGG
>dme_circ_0007598_junction_seq
CTCAAGATGGGTACAGTGAAACGCCGGGGAAGCCACACATGTTCGCCCGAGGAGTT
>dme_circ_0007599
CAAAACTGCACCGTCAGTCTATTGAACTTCTGCCAAGCGGGTGAGTTCCAGTGCTCGGATAGAATCACCT
GCCTGCACAAATCGTGGGTTTGCGATGGCGAGGCCGATTGTCCCGATGGCGAGGACGAATCGCAGTCCAA
CTGCCTAAAAGTGTCCTGTCGTCCGGATCAGTTCCAATGCAACGATCAAAGCTGCATCGCTGGCCATCTG
ACCTGCAACGGGAAGAGGGACTGTGCGGATGGCAGTGATGAAATTATGTGCGACATTAGTGCGACGCCAA
GGACTTGCAATGCAACCACTGAATTCGATTGCGGCGGTGGTCTGTGCATCCCGAATGCCAAGGTCTGCAA
CCGCAGGAAGGATTGCCCGAATGGCGAGGATGAGCCGGCCGGAAAGTGTGGCATCAACGAGTGCTCCTCG
AAGAATGGCGGCTGTATGCACCAGTGTGTGAATCTGGAGGTGGGTTACCGCTGCGAGTGCCACGATGGCT
ATAAACTGGGCGCCGATCAACACACATGTGTGGACATCGATGAATGTGAGACTCCTGGCATTTGCTCCCA
GGTGTGCGTCAACGAGATCGGAGCCTTTAAGTGCGAGTGCGAGGCGGGCTACATGAGACTCCTGAAGAAT
CACACCAGGTGTAAGGCCAGCGAAGGACATGCCTCGCTACTTCTGGCCAGGCGCCATGACATCCGGAAGA
TAGCACTGGATCGACCGGAAATGACGTCAATAGTGAATAGTACAAAGGCAGCCACGGCTCTGGACTTCGT
GTTCCGCACGGGAATGATCTTCTGGAGCGATGTTGCAACCCAGAGTATTTACAAAGCGCCCATTGATGAG

GGTAACGATAGAACGGTGGTGCTGACAAAGTCCTCGGTGACCTCGGATGGCCTGGCTGTCGATTGGATCT
ATAACCATGTCTACTTTACGGACACCCACAAGTGCACCATCGAACTGACCAACTTCGAGGGCAGCATGGG
CAAGGTCCTGGTAAAGGACTCCCTGGACATTCCTCGTTCCATTGCTTTGGATCCCATCGAGGGCTGGATG
TACTGGTCCGACTGGGGCGCCTCTCCGCGCATCGAAAGGGCGGGTATGGATGGCACCCACCGCACTGCCA
TCATCACCTACGATGTTAAGTGGCCAAATGGCATCACATTGGACCTGGTTCAAAAACGCCTTTACTGGGT
GGATGGCAAGTTGAACACCATCTCGTCGTCAAACTACGATGGCTCGCAAAGACATCAGGTGCTCTACTCC
GGCGAATACCTGCGACATCCCTTCTCGATCACCACCTTCGAGGACTACGTCTACTGGACCGACTGGGATA
AGCAAGCCGTCTTCAAGGCCAACAAGTTCAATGGAATGGATGTGGAGCCTGTTACGGCGACGCATATGGT
AAGTGTATTTCCCAAATTGTTTCTTTTTAAGATTACATATATCGAGAATTTTGCAATCGTAACTGCTTGC
GATGATAGCACACTTCCTATTAAATTTAATACATAACATATCCGGAGGAATGTGACCTTAAGTACAAGTG
TCTGATTGCCTGGTATGTAAGTCGTCGCTTGGGAATTATGGTAGATAATTGTGAAACGTTTGAGCTTCTT
ATCTAAGCTACTCATTCCAAGAAACGAGAAATTAAATACATTTAAAAGAATATATTTGTAAGGATAGATA
GAAAAAGTATTTTTATAACCAAAAAACTTTTACCATTTCCAATTCTAGTTGGAACATCCCATGGTGGTGC
ACGTGTACCATCCTTATCGCCAGCCAGATGGCGTGAATCACTGCCAGTCGGTGAATGGCCACTGTTCTCA
TCTTTGCCTGCCAGCTCCCAGGATTAACGAGCGGAGTCCTCGCATATCCTGCGCCTGTCCGACAGGCCTG
AAATTAATGCCCGACGGGCTAATGTGCGTCGAAGATCG
>dme_circ_0007599_junction_seq
CCGACGGGCTAATGTGCGTCGAAGATCGCAAAACTGCACCGTCAGTCTATTGAACT
>dme_circ_0007600
ATAGGACATGTCGCTCCGACGAGTTCACCTGCGGCAATGGACGTTGCATTCAGAATCGCTTCAAGTGCGA
CGATGACGACGATTGTGGTGACGGATCGGATGAGAAGAACTGCGGGGAAAAGGCGAAATGCGGATCCAAT
TTCTTCGCTTGCAAAAGTGGACCGTGCATTCCCAATCAATGGGTTTGCGATGGAGATTCAGACTGCAGAA
ATGGCGAAGATGAAATGGTGAGTTAACTAAAATAAAACACAAGTTGATAACAAGGGAAAACACAGAAACC
GTGCAAGCAAGATATATTTATATTTTTGCCGTGTAAAGTCCGGAAATCTTTTCCCCTACCTTCAACTTGC
AATGAAAATTGATATGAGCACATTATCAAACCACATACACACAAACATGCACACATACAAATGCCAAGTT
GTTTAACAGCCCTCGCATATAAATATGTATTTATATGGAGCAAGCTAGGAAGCATAAATTATGCAAGACG
CGAGCATTATTCAAAACTGGCAACGACGACGAGGACCATGTCGGCAACTGTTTCCCATAAACATTTATAT
GTATGACGCATGGCTTTTGTTGTCCGTAGGGTGGTGCCCGAAATTGCATGGCTCCGAACAGCAATAAAAA
CAGTGCAGAAACTATCCGAACAGTTCGCAAAAAATACTCTTTTACGAATTTACTGAGAAAATATTTTATA
GTAATTCTTTAAAGCAAAAAATCGTATTAAAATTCTTAAACTGTTTGATTGCCAACaaaaaaaaaGGTTA
AAATATTTATTATCTTGCATCACAAAAACATCACACAATATCGAAACACAATATAAAAACCAACTTAAAA
ATTTCACCTTTAAAAATCCGATTAATTAAATTCGTAAAAATAAACGGGCTATTTACACACAAGTACTCCT
CTTTACAAAGCGTATAGAATGTGGAAAATGTTTTCTGGCATCTCCTAAAAGTATGCAGTACAAAAAATGC
AGGCAATGGCCACAGGGACCTCTATATAAGTGTGCATGTACGTATGCTCTCGTGTGTATGTGTGCGTGCT
GGTGCATAAGGGCTTTGCGTGTGCGAGAGTGTGTTTGTGAACCGGGCTTAGCTGCCTGAATATTTGGTTG
GCACTTAACAAGAAAAGCAAACTGTTGTAGCAGCAGTAAAGTTAACTTCCTTTATCCTCGCCACGGGTAG
AGATTCATCTTTTAAAAGACGACCTTGTGAAAAACTTAAGCATTAAGCTCTGACTTTTAAGCAAACTTAA
TTTTAAAAGTACCCATTTTATCTATATAATTCATTCACTTTTAGCAAAACTGCACCGTCAGTCTATTGAA
CTTCTGCCAAGCGGGTGAGTTCCAGTGCTCGGATAGAATCACCTGCCTGCACAAATCGTGGGTTTGCGAT
GGCGAGGCCGATTGTCCCGATGGCGAGGACGAATCGCAGTCCAACTGCCTAAAAGTGTCCTGTCGTCCGG
ATCAGTTCCAATGCAACGATCAAAGCTGCATCGCTGGCCATCTGACCTGCAACGGGAAGAGGGACTGTGC
GGATGGCAGTGATGAAATTATGTGCGACATTAGTGCGACGCCAAGGACTTGCAATGCAACCACTGAATTC
GATTGCGGCGGTGGTCTGTGCATCCCGAATGCCAAGGTCTGCAACCGCAGGAAGGATTGCCCGAATGGCG
AGGATGAGCCGGCCGGAAAGTGTGGCATCAACGAGTGCTCCTCGAAGAATGGCGGCTGTATGCACCAGTG

TGTGAATCTGGAGGTGGGTTACCGCTGCGAGTGCCACGATGGCTATAAACTGGGCGCCGATCAACACACA
TGTGTGGACATCGATGAATGTGAGACTCCTGGCATTTGCTCCCAGGTGTGCGTCAACGAGATCGGAGCCT
TTAAGTGCGAGTGCGAGGCGGGCTACATGAGACTCCTGAAGAATCACACCAGGTGTAAGGCCAGCGAAGG
ACATGCCTCGCTACTTCTGGCCAGGCGCCATGACATCCGGAAGATAGCACTGGATCGACCGGAAATGACG
TCAATAGTGAATAGTACAAAGGCAGCCACGGCTCTGGACTTCGTGTTCCGCACGGGAATGATCTTCTGGA
GCGATGTTGCAACCCAGAGTATTTACAAAGCGCCCATTGATGAGGGTAACGATAGAACGGTGGTGCTGAC
AAAGTCCTCGGTGACCTCGGATGGCCTGGCTGTCGATTGGATCTATAACCATGTCTACTTTACGGACACC
CACAAGTGCACCATCGAACTGACCAACTTCGAGGGCAGCATGGGCAAGGTCCTGGTAAAGGACTCCCTGG
ACATTCCTCGTTCCATTGCTTTGGATCCCATCGAGGGCTGGATGTACTGGTCCGACTGGGGCGCCTCTCC
GCGCATCGAAAGGGCGGGTATGGATGGCACCCACCGCACTGCCATCATCACCTACGATGTTAAGTGGCCA
AATGGCATCACATTGGACCTGGTTCAAAAACGCCTTTACTGGGTGGATGGCAAGTTGAACACCATCTCGT
CGTCAAACTACGATGGCTCGCAAAGACATCAGGTGCTCTACTCCGGCGAATACCTGCGACATCCCTTCTC
GATCACCACCTTCGAGGACTACGTCTACTGGACCGACTGGGATAAGCAAGCCGTCTTCAAGGCCAACAAG
TTCAATGGAATGGATGTGGAGCCTGTTACGGCGACGCATATGGTAAGTGTATTTCCCAAATTGTTTCTTT
TTAAGATTACATATATCGAGAATTTTGCAATCGTAACTGCTTGCGATGATAGCACACTTCCTATTAAATT
TAATACATAACATATCCGGAGGAATGTGACCTTAAGTACAAGTGTCTGATTGCCTGGTATGTAAGTCGTC
GCTTGGGAATTATGGTAGATAATTGTGAAACGTTTGAGCTTCTTATCTAAGCTACTCATTCCAAGAAACG
AGAAATTAAATACATTTAAAAGAATATATTTGTAAGGATAGATAGAAAAAGTATTTTTATAACCAAAAAA
CTTTTACCATTTCCAATTCTAGTTGGAACATCCCATGGTGGTGCACGTGTACCATCCTTATCGCCAGCCA
GATGGCGTGAATCACTGCCAGTCGGTGAATGGCCACTGTTCTCATCTTTGCCTGCCAGCTCCCAGGATTA
ACGAGCGGAGTCCTCGCATATCCTGCGCCTGTCCGACAGGCCTGAAATTAATGCCCGACGGGCTAATGTG
CGTCGAAGATCG
>dme_circ_0007600_junction_seq
CCGACGGGCTAATGTGCGTCGAAGATCGATAGGACATGTCGCTCCGACGAGTTCAC
>dme_circ_0007601
GAAGCCACACATGTTCGCCCGAGGAGTTTGCCTGCAAGTCGGGCGAGGGCGAGTGCATTCCCTTGTCCTG
GATGTGCGATCAGAACAAAGATTGTCGCGATGGAAGCGATGAGGCGCAGTGCAGTAAGCCATAACCGAAT
ATTTCCTAGCTCTCTGTTACCTGCTTTGCTTTGCTTGGTGTTGGTTGCCGTAATAATTGAAATTATCGTA
GATTTTTCCCTGCTTTCCTTTGGTGTTGGTTGCTTATTTATTTCTAGATACTGTAGTGCACTATTCCCCG
TACTTGACTGTGCCATGAACACATGTCCCTGAGACTTGTGTTGTGACATCGTGAAGCTCGTATGCAATAA
TGTACAAATATTTACGCACACACAGAGGGAACACTTTGGTTTTCTGTGTGTGCGCTGTTTCTGTTGTTGT
CACTTTCACATCTCGCCCTTCTCTATATGCTTTCTTTGTTTGTTGGCAATTTTTGTAATGGTGTTGGCTT
TTCCCAAGCCTTAAAGAAATGGTCTTTGCGGCTTTAGCTTTTCCTCATCCCATCCATGCTCACACTAATC
ACAATCATCAAATCCTCCCGCCGATAACCCATAGTGAAATTAAACTAATCATTGGAATGTACTTCGGCAG
ATAGGACATGTCGCTCCGACGAGTTCACCTGCGGCAATGGACGTTGCATTCAGAATCGCTTCAAGTGCGA
CGATGACGACGATTGTGGTGACGGATCGGATGAGAAGAACTGCGGGGAAAAGGCGAAATGCGGATCCAAT
TTCTTCGCTTGCAAAAGTGGACCGTGCATTCCCAATCAATGGGTTTGCGATGGAGATTCAGACTGCAGAA
ATGGCGAAGATGAAATGGTGAGTTAACTAAAATAAAACACAAGTTGATAACAAGGGAAAACACAGAAACC
GTGCAAGCAAGATATATTTATATTTTTGCCGTGTAAAGTCCGGAAATCTTTTCCCCTACCTTCAACTTGC
AATGAAAATTGATATGAGCACATTATCAAACCACATACACACAAACATGCACACATACAAATGCCAAGTT
GTTTAACAGCCCTCGCATATAAATATGTATTTATATGGAGCAAGCTAGGAAGCATAAATTATGCAAGACG
CGAGCATTATTCAAAACTGGCAACGACGACGAGGACCATGTCGGCAACTGTTTCCCATAAACATTTATAT
GTATGACGCATGGCTTTTGTTGTCCGTAGGGTGGTGCCCGAAATTGCATGGCTCCGAACAGCAATAAAAA
CAGTGCAGAAACTATCCGAACAGTTCGCAAAAAATACTCTTTTACGAATTTACTGAGAAAATATTTTATA

GTAATTCTTTAAAGCAAAAAATCGTATTAAAATTCTTAAACTGTTTGATTGCCAACaaaaaaaaaGGTTA
AAATATTTATTATCTTGCATCACAAAAACATCACACAATATCGAAACACAATATAAAAACCAACTTAAAA
ATTTCACCTTTAAAAATCCGATTAATTAAATTCGTAAAAATAAACGGGCTATTTACACACAAGTACTCCT
CTTTACAAAGCGTATAGAATGTGGAAAATGTTTTCTGGCATCTCCTAAAAGTATGCAGTACAAAAAATGC
AGGCAATGGCCACAGGGACCTCTATATAAGTGTGCATGTACGTATGCTCTCGTGTGTATGTGTGCGTGCT
GGTGCATAAGGGCTTTGCGTGTGCGAGAGTGTGTTTGTGAACCGGGCTTAGCTGCCTGAATATTTGGTTG
GCACTTAACAAGAAAAGCAAACTGTTGTAGCAGCAGTAAAGTTAACTTCCTTTATCCTCGCCACGGGTAG
AGATTCATCTTTTAAAAGACGACCTTGTGAAAAACTTAAGCATTAAGCTCTGACTTTTAAGCAAACTTAA
TTTTAAAAGTACCCATTTTATCTATATAATTCATTCACTTTTAGCAAAACTGCACCGTCAGTCTATTGAA
CTTCTGCCAAGCGGGTGAGTTCCAGTGCTCGGATAGAATCACCTGCCTGCACAAATCGTGGGTTTGCGAT
GGCGAGGCCGATTGTCCCGATGGCGAGGACGAATCGCAGTCCAACTGCCTAAAAGTGTCCTGTCGTCCGG
ATCAGTTCCAATGCAACGATCAAAGCTGCATCGCTGGCCATCTGACCTGCAACGGGAAGAGGGACTGTGC
GGATGGCAGTGATGAAATTATGTGCGACATTAGTGCGACGCCAAGGACTTGCAATGCAACCACTGAATTC
GATTGCGGCGGTGGTCTGTGCATCCCGAATGCCAAGGTCTGCAACCGCAGGAAGGATTGCCCGAATGGCG
AGGATGAGCCGGCCGGAAAGTGTGGCATCAACGAGTGCTCCTCGAAGAATGGCGGCTGTATGCACCAGTG
TGTGAATCTGGAGGTGGGTTACCGCTGCGAGTGCCACGATGGCTATAAACTGGGCGCCGATCAACACACA
TGTGTGGACATCGATGAATGTGAGACTCCTGGCATTTGCTCCCAGGTGTGCGTCAACGAGATCGGAGCCT
TTAAGTGCGAGTGCGAGGCGGGCTACATGAGACTCCTGAAGAATCACACCAGGTGTAAGGCCAGCGAAGG
ACATGCCTCGCTACTTCTGGCCAGGCGCCATGACATCCGGAAGATAGCACTGGATCGACCGGAAATGACG
TCAATAGTGAATAGTACAAAGGCAGCCACGGCTCTGGACTTCGTGTTCCGCACGGGAATGATCTTCTGGA
GCGATGTTGCAACCCAGAGTATTTACAAAGCGCCCATTGATGAGGGTAACGATAGAACGGTGGTGCTGAC
AAAGTCCTCGGTGACCTCGGATGGCCTGGCTGTCGATTGGATCTATAACCATGTCTACTTTACGGACACC
CACAAGTGCACCATCGAACTGACCAACTTCGAGGGCAGCATGGGCAAGGTCCTGGTAAAGGACTCCCTGG
ACATTCCTCGTTCCATTGCTTTGGATCCCATCGAGGGCTGGATGTACTGGTCCGACTGGGGCGCCTCTCC
GCGCATCGAAAGGGCGGGTATGGATGGCACCCACCGCACTGCCATCATCACCTACGATGTTAAGTGGCCA
AATGGCATCACATTGGACCTGGTTCAAAAACGCCTTTACTGGGTGGATGGCAAGTTGAACACCATCTCGT
CGTCAAACTACGATGGCTCGCAAAGACATCAGGTGCTCTACTCCGGCGAATACCTGCGACATCCCTTCTC
GATCACCACCTTCGAGGACTACGTCTACTGGACCGACTGGGATAAGCAAGCCGTCTTCAAGGCCAACAAG
TTCAATGGAATGGATGTGGAGCCTGTTACGGCGACGCATATGGTAAGTGTATTTCCCAAATTGTTTCTTT
TTAAGATTACATATATCGAGAATTTTGCAATCGTAACTGCTTGCGATGATAGCACACTTCCTATTAAATT
TAATACATAACATATCCGGAGGAATGTGACCTTAAGTACAAGTGTCTGATTGCCTGGTATGTAAGTCGTC
GCTTGGGAATTATGGTAGATAATTGTGAAACGTTTGAGCTTCTTATCTAAGCTACTCATTCCAAGAAACG
AGAAATTAAATACATTTAAAAGAATATATTTGTAAGGATAGATAGAAAAAGTATTTTTATAACCAAAAAA
CTTTTACCATTTCCAATTCTAGTTGGAACATCCCATGGTGGTGCACGTGTACCATCCTTATCGCCAGCCA
GATGGCGTGAATCACTGCCAGTCGGTGAATGGCCACTGTTCTCATCTTTGCCTGCCAGCTCCCAGGATTA
ACGAGCGGAGTCCTCGCATATCCTGCGCCTGTCCGACAGGCCTGAAATTAATGCCCGACGGGCTAATGTG
CGTCGAAGATCG
>dme_circ_0007601_junction_seq
CCGACGGGCTAATGTGCGTCGAAGATCGGAAGCCACACATGTTCGCCCGAGGAGTT
>dme_circ_0007602
GAAGCCACACATGTTCGCCCGAGGAGTTTGCCTGCAAGTCGGGCGAGGGCGAGTGCATTCCCTTGTCCTG
GATGTGCGATCAGAACAAAGATTGTCGCGATGGAAGCGATGAGGCGCAGTGCAGTAAGCCATAACCGAAT
ATTTCCTAGCTCTCTGTTACCTGCTTTGCTTTGCTTGGTGTTGGTTGCCGTAATAATTGAAATTATCGTA
GATTTTTCCCTGCTTTCCTTTGGTGTTGGTTGCTTATTTATTTCTAGATACTGTAGTGCACTATTCCCCG

TACTTGACTGTGCCATGAACACATGTCCCTGAGACTTGTGTTGTGACATCGTGAAGCTCGTATGCAATAA
TGTACAAATATTTACGCACACACAGAGGGAACACTTTGGTTTTCTGTGTGTGCGCTGTTTCTGTTGTTGT
CACTTTCACATCTCGCCCTTCTCTATATGCTTTCTTTGTTTGTTGGCAATTTTTGTAATGGTGTTGGCTT
TTCCCAAGCCTTAAAGAAATGGTCTTTGCGGCTTTAGCTTTTCCTCATCCCATCCATGCTCACACTAATC
ACAATCATCAAATCCTCCCGCCGATAACCCATAGTGAAATTAAACTAATCATTGGAATGTACTTCGGCAG
ATAGGACATGTCGCTCCGACGAGTTCACCTGCGGCAATGGACGTTGCATTCAGAATCGCTTCAAGTGCGA
CGATGACGACGATTGTGGTGACGGATCGGATGAGAAGAACTGCGGGGAAAAGGCGAAATGCGGATCCAAT
TTCTTCGCTTGCAAAAGTGGACCGTGCATTCCCAATCAATGGGTTTGCGATGGAGATTCAGACTGCAGAA
ATGGCGAAGATGAAATGGTGAGTTAACTAAAATAAAACACAAGTTGATAACAAGGGAAAACACAGAAACC
GTGCAAGCAAGATATATTTATATTTTTGCCGTGTAAAGTCCGGAAATCTTTTCCCCTACCTTCAACTTGC
AATGAAAATTGATATGAGCACATTATCAAACCACATACACACAAACATGCACACATACAAATGCCAAGTT
GTTTAACAGCCCTCGCATATAAATATGTATTTATATGGAGCAAGCTAGGAAGCATAAATTATGCAAGACG
CGAGCATTATTCAAAACTGGCAACGACGACGAGGACCATGTCGGCAACTGTTTCCCATAAACATTTATAT
GTATGACGCATGGCTTTTGTTGTCCGTAGGGTGGTGCCCGAAATTGCATGGCTCCGAACAGCAATAAAAA
CAGTGCAGAAACTATCCGAACAGTTCGCAAAAAATACTCTTTTACGAATTTACTGAGAAAATATTTTATA
GTAATTCTTTAAAGCAAAAAATCGTATTAAAATTCTTAAACTGTTTGATTGCCAACaaaaaaaaaGGTTA
AAATATTTATTATCTTGCATCACAAAAACATCACACAATATCGAAACACAATATAAAAACCAACTTAAAA
ATTTCACCTTTAAAAATCCGATTAATTAAATTCGTAAAAATAAACGGGCTATTTACACACAAGTACTCCT
CTTTACAAAGCGTATAGAATGTGGAAAATGTTTTCTGGCATCTCCTAAAAGTATGCAGTACAAAAAATGC
AGGCAATGGCCACAGGGACCTCTATATAAGTGTGCATGTACGTATGCTCTCGTGTGTATGTGTGCGTGCT
GGTGCATAAGGGCTTTGCGTGTGCGAGAGTGTGTTTGTGAACCGGGCTTAGCTGCCTGAATATTTGGTTG
GCACTTAACAAGAAAAGCAAACTGTTGTAGCAGCAGTAAAGTTAACTTCCTTTATCCTCGCCACGGGTAG
AGATTCATCTTTTAAAAGACGACCTTGTGAAAAACTTAAGCATTAAGCTCTGACTTTTAAGCAAACTTAA
TTTTAAAAGTACCCATTTTATCTATATAATTCATTCACTTTTAGCAAAACTGCACCGTCAGTCTATTGAA
CTTCTGCCAAGCGGGTGAGTTCCAGTGCTCGGATAGAATCACCTGCCTGCACAAATCGTGGGTTTGCGAT
GGCGAGGCCGATTGTCCCGATGGCGAGGACGAATCGCAGTCCAACTGCCTAAAAGTGTCCTGTCGTCCGG
ATCAGTTCCAATGCAACGATCAAAGCTGCATCGCTGGCCATCTGACCTGCAACGGGAAGAGGGACTGTGC
GGATGGCAGTGATGAAATTATGTGCGACATTAGTGCGACGCCAAGGACTTGCAATGCAACCACTGAATTC
GATTGCGGCGGTGGTCTGTGCATCCCGAATGCCAAGGTCTGCAACCGCAGGAAGGATTGCCCGAATGGCG
AGGATGAGCCGGCCGGAAAGTGTGGCATCAACGAGTGCTCCTCGAAGAATGGCGGCTGTATGCACCAGTG
TGTGAATCTGGAGGTGGGTTACCGCTGCGAGTGCCACGATGGCTATAAACTGGGCGCCGATCAACACACA
TGTGTGGACATCGATGAATGTGAGACTCCTGGCATTTGCTCCCAGGTGTGCGTCAACGAGATCGGAGCCT
TTAAGTGCGAGTGCGAGGCGGGCTACATGAGACTCCTGAAGAATCACACCAGGTGTAAGGCCAGCGAAGG
ACATGCCTCGCTACTTCTGGCCAGGCGCCATGACATCCGGAAGATAGCACTGGATCGACCGGAAATGACG
TCAATAGTGAATAGTACAAAGGCAGCCACGGCTCTGGACTTCGTGTTCCGCACGGGAATGATCTTCTGGA
GCGATGTTGCAACCCAGAGTATTTACAAAGCGCCCATTGATGAGGGTAACGATAGAACGGTGGTGCTGAC
AAAGTCCTCGGTGACCTCGGATGGCCTGGCTGTCGATTGGATCTATAACCATGTCTACTTTACGGACACC
CACAAGTGCACCATCGAACTGACCAACTTCGAGGGCAGCATGGGCAAGGTCCTGGTAAAGGACTCCCTGG
ACATTCCTCGTTCCATTGCTTTGGATCCCATCGAGGGCTGGATGTACTGGTCCGACTGGGGCGCCTCTCC
GCGCATCGAAAGGGCGGGTATGGATGGCACCCACCGCACTGCCATCATCACCTACGATGTTAAGTGGCCA
AATGGCATCACATTGGACCTGGTTCAAAAACGCCTTTACTGGGTGGATGGCAAGTTGAACACCATCTCGT
CGTCAAACTACGATGGCTCGCAAAGACATCAGGTGCTCTACTCCGGCGAATACCTGCGACATCCCTTCTC
GATCACCACCTTCGAGGACTACGTCTACTGGACCGACTGGGATAAGCAAGCCGTCTTCAAGGCCAACAAG
TTCAATGGAATGGATGTGGAGCCTGTTACGGCGACGCATATGG

>dme_circ_0007602_junction_seq
GTGGAGCCTGTTACGGCGACGCATATGGGAAGCCACACATGTTCGCCCGAGGAGTT
>dme_circ_0007603
GTAGAGCACCTGATGTCTTTGCGAGCCATCGTAGTTTGACGACGAGATGGTGTTCAACTTGCCATCCACC
CAGTAAAGGCGTTTTTGAACCAGGTCCAATGTGATGCCATTTGGCCACTTAACATCGTAGGTGATGATGG
CAGTGCGGTGGGTGCCATCCATACCCGCCCTTTCGATGCGCGGAGAGGCGCCCCAGTCGGACCA
>dme_circ_0007603_junction_seq
TGCGCGGAGAGGCGCCCCAGTCGGACCAGTAGAGCACCTGATGTCTTTGCGAGCCA
>dme_circ_0007604
GTTTATAGCCATCGTGGCACTCGCAGCGGTAACCCACCTCCAGATTCACACACTGGTGCATACAGCCGCC
ATTCTTCGAGGAGCACTCGTTGATGCCACACTTTCCGGCCGGCTCATCCTCGCCATTCGGGCAATCCTTC
CTGCGGTTGCAGACCTTGGCATTCGGGATGCACAGACCACCGCCGCAATCGAATTCAGTGGTTGCATTGC
AAGTCCTTGGCGTCGCACTAATGTCGCACATAATTTCATCACTGCCATCCGCACAGTCCCTCTTCCCGTT
GCAGGTCAGATGGCCAGCGATGCAGCTTTGATCGTTGCATTGGAACTGATCCGGACGACAGGACACTTTT
AGGCAGTTGGACTGCGATTCGTCCTCGCCATCGGGACAATCGGCCTCGCCATCGCAAACCCACGATTTGT
GCAGGCAG
>dme_circ_0007604_junction_seq
ATCGCAAACCCACGATTTGTGCAGGCAGGTTTATAGCCATCGTGGCACTCGCAGCG
>dme_circ_0007605
ATAGGACATGTCGCTCCGACGAGTTCACCTGCGGCAATGGACGTTGCATTCAGAATCGCTTCAAGTGCGA
CGATGACGACGATTGTGGTGACGGATCGGATGAGAAGAACTGCGGGGAAAAGGCGAAATGCGGATCCAAT
TTCTTCGCTTGCAAAAGTGGACCGTGCATTCCCAATCAATGGGTTTGCGATGGAGATTCAGACTGCAGAA
ATGGCGAAGATGAAATGG
>dme_circ_0007605_junction_seq
GACTGCAGAAATGGCGAAGATGAAATGGATAGGACATGTCGCTCCGACGAGTTCAC
>dme_circ_0007606
GAAGCCACACATGTTCGCCCGAGGAGTTTGCCTGCAAGTCGGGCGAGGGCGAGTGCATTCCCTTGTCCTG
GATGTGCGATCAGAACAAAGATTGTCGCGATGGAAGCGATGAGGCGCAGTGCAGTAAGCCATAACCGAAT
ATTTCCTAGCTCTCTGTTACCTGCTTTGCTTTGCTTGGTGTTGGTTGCCGTAATAATTGAAATTATCGTA
GATTTTTCCCTGCTTTCCTTTGGTGTTGGTTGCTTATTTATTTCTAGATACTGTAGTGCACTATTCCCCG
TACTTGACTGTGCCATGAACACATGTCCCTGAGACTTGTGTTGTGACATCGTGAAGCTCGTATGCAATAA
TGTACAAATATTTACGCACACACAGAGGGAACACTTTGGTTTTCTGTGTGTGCGCTGTTTCTGTTGTTGT
CACTTTCACATCTCGCCCTTCTCTATATGCTTTCTTTGTTTGTTGGCAATTTTTGTAATGGTGTTGGCTT
TTCCCAAGCCTTAAAGAAATGGTCTTTGCGGCTTTAGCTTTTCCTCATCCCATCCATGCTCACACTAATC
ACAATCATCAAATCCTCCCGCCGATAACCCATAGTGAAATTAAACTAATCATTGGAATGTACTTCGGCAG
ATAGGACATGTCGCTCCGACGAGTTCACCTGCGGCAATGGACGTTGCATTCAGAATCGCTTCAAGTGCGA
CGATGACGACGATTGTGGTGACGGATCGGATGAGAAGAACTGCGGGGAAAAGGCGAAATGCGGATCCAAT
TTCTTCGCTTGCAAAAGTGGACCGTGCATTCCCAATCAATGGGTTTGCGATGGAGATTCAGACTGCAGAA
ATGGCGAAGATGAAATGG
>dme_circ_0007606_junction_seq
GACTGCAGAAATGGCGAAGATGAAATGGGAAGCCACACATGTTCGCCCGAGGAGTT
>dme_circ_0007607
GACATCCCTCGCTTGCCTTTAAGGATCGTTTCCTATCTCATCAAGACCAAGTCCCATTAGCTAGGCAGTT
AGTGGTTTTTTTCCGGCACCCTTTGTGATTATAGCCTTGAAGTAGGTGCTCGGGATTTATTAAATTTTT

TACCCGTTGTGGGTAATATTTGCAAAACAAGACTCTATATACCATTATTTGATATATTTAATAAAACAAA
AAGGATTTTTCAAAATGCAATCGAAAATTTTCTTCTTCTAGGAATTACTGAATTATGAACAAAGCACACT
ATAGATTGCCTCTCGATGTATCTTTAAATTACGTATTTCCATGATCTTGATCTTTTTAGGAAGCCACACA
TGTTCGCCCGAGGAGTTTGCCTGCAAGTCGGGCGAGGGCGAGTGCATTCCCTTGTCCTGGATGTGCGATC
AGAACAAAGATTGTCGCGATGGAAGCGATGAGGCGCAGTGCAGTAAGCCATAACCGAATATTTCCTAGCT
CTCTGTTACCTGCTTTGCTTTGCTTGGTGTTGGTTGCCGTAATAATTGAAATTATCGTAGATTTTTCCCT
GCTTTCCTTTGGTGTTGGTTGCTTATTTATTTCTAGATACTGTAGTGCACTATTCCCCGTACTTGACTGT
GCCATGAACACATGTCCCTGAGACTTGTGTTGTGACATCGTGAAGCTCGTATGCAATAATGTACAAATAT
TTACGCACACACAGAGGGAACACTTTGGTTTTCTGTGTGTGCGCTGTTTCTGTTGTTGTCACTTTCACAT
CTCGCCCTTCTCTATATGCTTTCTTTGTTTGTTGGCAATTTTTGTAATGGTGTTGGCTTTTCCCAAGCCT
TAAAGAAATGGTCTTTGCGGCTTTAGCTTTTCCTCATCCCATCCATGCTCACACTAATCACAATCATCAA
ATCCTCCCGCCGATAACCCATAGTGAAATTAAACTAATCATTGGAATGTACTTCGGCAGATAGGACATGT
CGCTCCGACGAGTTCACCTGCGGCAATGGACGTTGCATTCAGAATCGCTTCAAGTGCGACGATGACGACG
ATTGTGGTGACGGATCGGATGAGAAGAACTGCGGGGAAAAGGCGAAATGCGGATCCAATTTCTTCGCTTG
CAAAAGTGGACCGTGCATTCCCAATCAATGGGTTTGCGATGGAGATTCAGACTGCAGAAATGGCGAAGAT
GAAATGG
>dme_circ_0007607_junction_seq
GACTGCAGAAATGGCGAAGATGAAATGGGACATCCCTCGCTTGCCTTTAAGGATCG
>dme_circ_0007608
AATCAACTTGCAGCCAGGAACAATTCCGCTGCGGCAACGGAAAGTGCATTCCGCGAAGATGGGTCTGCGA
TCGGGAAAACGACTGCGCCGATGGAAGCGATGAATCGACGTCCCAGTGCCGTAAGTGTCGGTTAATATCT
TTGATATAAGCAGTAGATATCGGACTATATGCGATTGAAAACGTAATTGAATCGAGCAAGTGTGGCTAAC
CAAAAGGAATCAAATTTTAAATGGATTTAATTATGTCTAAGAGttttttttttttttGAAATCCTAATAA
CCCAAAAAAATGATAATTTGCTCAGGGGGCCTGTGTTCCTCCTTGATGTTCATGTGTAAAAATGGTGAGC
AGTGTATACACCGGGAATTCATGTGCGATGGCGACCAGGACTGTCGCGACGGATCCGACGAATTGGAATG
TGGCAGTAAGCTAAAAAGAATGAATTATTCCATCTCACATTTGCATACTAACTGGAGGATTTGTCCATCA
CTAAAACGAGCGAAAAAGCAGAGTCACTTAATCGAAATTTAAATGCATGGAAGACAGTTGCCGGCCTCTG
AGATCTCGGGAGGACTTTATGACTTGCCAGGGACTGACCAGGACATAAATTAATGTCACCTCTTGTGGAC
AATATCGCGACGTGACCATCGGCGGAACTAATTTGTATTTATGCATTAGGCTCACTTCGGCTCACTTTTC
CGTTTTGGGTGTAGTTTCCGCTTTTTACACACTTTATTCCTCGCATTTCTCTTGCTGCCGAGCAACTTGT
TTAGTGGTTTATTGTCTGTGACACAATTTTCCAACTATATAAAGAGGAGAGTGCCTGGCGGAGAAGAGCA
GGGAAAATGTTTTTATGTCGGACGGAATTTCAGCGCTGTAATTGAAAAAATGATGCGATTTTTATGATGT
TATCCTTGATGAAAATAAAAACCGGTAATCAAAAGCCGAACGCAGCGCCAAATTGAGCCTGTGGTTTATA
CGCGGGGAGTCGTGGCTCAGTGGTCGGCTACCAGGATGTACAGTGGTCGCCAAAATACTAGGGCTTCAAA
ATGTATCTAATATTTGGAGTGTTTTCATTTATATTTCTAAAAGTAAATTGCACATTTACATACATTGCAA
ATATAATTTTAGATCCTTATAACAACATTTTAAATTCAAGTATATATTGTGTATATTTCCATGTTCACAA
CTGTATGTTGTACGAGCATAAAGCTACGTCTGCTCATGTGAATGGATATGTTCGCCTTTTTATGACTTTT
ATGTGCTTTAAAATTGTCATCAAACCTTGTTGACATTTTCGCTCTTACTCAGTTACCCTTGTGAATCTTA
ATTTAACTTGttttttttttCGGTAAAAAAAAGTTCAGCTCACTGCTGTGGATTTCTCGGTATAAAAGTG
CTATTTTCAGTAATGGTGCCCGCCCCGTTTATTTAATTCCATTTAACACTCCACTCGGACTCTTCATATC
TTTGTTTACCATTATGGGAAAAGCTCGGCTGGCCATCTCACAATGCGACCAAGGGTGGATAGTTGAATAA
TAAACAAAACGCAAACTGCCCCACAAAGGCATTAAGCTTCATTGGCTTTCAAGTGCCCGAGAGTTGGAGG
CGGAGACGGGCGGCGTGGCCACTGCACTCGACCAGGACATGTCTTGTACATGGTCTAAAAAGCATTTTAA
ATAAAATTAAAATCAAATTGCAAGTATATGGGTTTTGGCGTCAGATAACAAGAAAATGCTATTGAATACA

ATGCCAATGTTCTGCCAGGATGTGTGTGCGTTGAGGAAGGAGAGCCAGAGCCGAGCATCCTCGGCGGTCC
ATTGACTGTGACCCCACGATAGCAAATTGATTCTGCCAGGACTCACAAAAGCCGAAGCCGAGATGGAAGC
TGTGTCCCTATGGACACACAAGAATGAAACGTCCATTCAGCCAAGTAGTTACCTTCGAATGGATAAAATG
GAAAGTTATATGTATATATACATACATATAGTTAGTTATATGGAAGGACCCCAGGCGAATATTTTCCTCA
CCAGCGATGGAGAATTTAATGAAGCCATAAATCATGGTCGGCGAAATGATATTGGATAATTGAATTTATA
TCAGGTGCATGGTGTGCGGAGCAAACGAAATAATCGAAAAGTATTTTCAACTGAATATGAACGATGCTTA
ACAGAAAATGATTTTAATGCTGGAGCTGCCATTTAATTATGAAAAATGTAAATGAAAGTCAACTGATCCA
ATAATTTTGAGGAAACCATATTCCCTGAAATGTAATTCACTGCAGTGCACTGGACACCCAATGGAGCAAT
TATAAAATAAAGCTAGAACTGCTACTATAGATTTAATGGACATTTATGAAAGATATTCAAATATAGGTGT
AGCGTATAAATAAACCAATTAATTGATTAAATTTACTAGACAAATTGCTTTGAACTTATACTTACAATTT
AAATTGCATTTTCGGGGCTGAAAAAGCGTCATTTTATTTATTTATAAAATAGCTAAATATAAAAATAAGT
TACCTAATATTTTGTTAAATATTTCCGAAGCATCCTAGCAATTTCAGTAAATATTCAGCAGGCGAACGAC
GAATAATTTCTGTAAGCTGGTCACAGTAAATTCCAATTACAGGCATATCACGCATACGACCCGTTGTACA
TGCTAAGCAACCGCCAAAATGGCGAATACCAAGAAGAGAATAATAATAAATTAATTTATTTGCAACGATG
TCAGAAATTTCCGCCGTACGCCGTGCACGTGTAAACGGTAAACAAATAGACGGAATGAAGCAAGTATGAA
AAACAGAGGAGAGGAAAGGCAGGCGGAGATAGACAAAACGCCAAAAAGCATATTCAAATGTGTGGCTCAA
GCATAAGATTTTATTGCTAGcacgcacacacacacacacacacaGAGAGTCACCGCGGCAATGTATGTAT
GTACATAATCATATCAGGGAAAATCACTCCCACACGCACTCACGGCGTGAAACAAAGGGCCGTGTGGTAG
GCAATGAAATGGAGAAAGAAAGCGAAAGAAAGCCCAAAAGCGAGGCGCCAAGAACTGGCATACTAATACG
CATTAAGGGTTAAACACAAGCGTTTTAATGCGCCAGTGTGTGTGCgtgtctgtgagtctatgtgtctatg
tgtctatgtgtggttgtgttgtcagctttgtttaatgtccGTTCCTTTGGCAAGGATAACTTCAATGGAA
CTTCCCGAGTGTGAGTGTGTGCGATAATTGCTTTAATGCATACGCATGTGTGCGTTTTAACTTTATGGAG
TGTTGGCTGATAATGTCTTAATTACTGCCTAGATAAAATGCCAACTGTTTAGCCCCCGAATGGTTGTTGG
TTGTGCCAgtgtggtgtgtgtgtgtgtgtTGGCATGACAACTATGTTTATTACTTGGAGAACGGTTGCTG
TCCCACTCCCTCTTATATGGCAGCTGCTTATAAACTTAATTACAGGACATCCCTCGCTTGCCTTTAAGGA
TCGTTTCCTATCTCATCAAGACCAAGTCCCATTAGCTAGGCAGTTAGTGGTTTTTTTCCGGCACCCTTTG
TGATTATAGCCTTGAAGTAGGTGCTCGGGATTTTATTAAATTTTTTACCCGTTGTGGGTAATATTTGCAA
AACAAGACTCTATATACCATTATTTGATATATTTAATAAAACAAAAAGGATTTTTCAAAATGCAATCGAA
AATTTTCTTCTTCTAGGAATTACTGAATTATGAACAAAGCACACTATAGATTGCCTCTCGATGTATCTTT
AAATTACGTATTTCCATGATCTTGATCTTTTTAGGAAGCCACACATGTTCGCCCGAGGAGTTTGCCTGCA
AGTCGGGCGAGGGCGAGTGCATTCCCTTGTCCTGGATGTGCGATCAGAACAAAGATTGTCGCGATGGAAG
CGATGAGGCGCAGTGCAGTAAGCCATAACCGAATATTTCCTAGCTCTCTGTTACCTGCTTTGCTTTGCTT
GGTGTTGGTTGCCGTAATAATTGAAATTATCGTAGATTTTTCCCTGCTTTCCTTTGGTGTTGGTTGCTTA
TTTATTTCTAGATACTGTAGTGCACTATTCCCCGTACTTGACTGTGCCATGAACACATGTCCCTGAGACT
TGTGTTGTGACATCGTGAAGCTCGTATGCAATAATGTACAAATATTTACGCACACACAGAGGGAACACTT
TGGTTTTCTGTGTGTGCGCTGTTTCTGTTGTTGTCACTTTCACATCTCGCCCTTCTCTATATGCTTTCTT
TGTTTGTTGGCAATTTTTGTAATGGTGTTGGCTTTTCCCAAGCCTTAAAGAAATGGTCTTTGCGGCTTTA
GCTTTTCCTCATCCCATCCATGCTCACACTAATCACAATCATCAAATCCTCCCGCCGATAACCCATAGTG
AAATTAAACTAATCATTGGAATGTACTTCGGCAGATAGGACATGTCGCTCCGACGAGTTCACCTGCGGCA
ATGGACGTTGCATTCAGAATCGCTTCAAGTGCGACGATGACGACGATTGTGGTGACGGATCGGATGAGAA
GAACTGCGGGGAAAAGGCGAAATGCGGATCCAATTTCTTCGCTTGCAAAAGTGGACCGTGCATTCCCAAT
CAATGGGTTTGCGATGGAGATTCAGACTGCAGAAATGGCGAAGATGAAATGG
>dme_circ_0007608_junction_seq
GACTGCAGAAATGGCGAAGATGAAATGGAATCAACTTGCAGCCAGGAACAATTCCG

>dme_circ_0007609
CAGCTGCAAAAGATGGCCACAACCTGCATGCCGCATTTCACGGAATATTTTCAAACCACCACCCAGTCAG
AGTTAACAACAAATGAAAGTGCCAAcagcagaagcagcagcagcagcagTGGCTGTAGAGAACCGTCCAA
AACCAGTGGCTTCTTCTACAGCGCCAGCACACATTACCAGAGCAAAGTGCAGCAGCTAATCAGCTTCAGC
TTCACCTCATCGTTCCACCTGCTCTTCATCACCTGCATACTGAGCATCAGCAAATTCTGCAATGCGGCGG
CGGTAAATGCCGCCCATGTGACCGCTGCCACGCCCACTGCCAGCGAGATAATGCACAATCTGAGCGGATC
GGCGCTGCTAGAGCGTCTGAAAGCCAGTACTCTGGGACCCACCCTCGATCAGCTGAAGCATCTGGCTCCT
GCGGCAGCCAACAGCATTAGTCCTGGTACAGGATCTGGCATGGGAATCGGGATGGGACACTTTGGCAGTT
CCATTAATTCGGTGCTAAACGGAAATCCTTTAAAGTTCTTGAATGTGTCAGGAAAAATCGGCACTCCTTT
GGACATATGGTAAGTTTTTACAAGCCCATAAAAATGGTTTATTCCTAGAAGTTCTAAATGCTAGAGGTAT
TTGTAGTTATAAATCTTTTAGTCACTCTTTTTATCAACTTGTATTTATTTTTATGATTCATGAAAGGCTT
AAACATTCATACATATTGCCGGCGAATTATTAGAATTAGTCAACTATTAACATCACGTCACAAATTAAGC
TTTCCATCATATCAAAACTAATCATTTTTGTAAGAGTTGCCGTATTTTAAATATCTATTAAAAAGCTATG
AATATAAAATtcaaaactaaagtgcagaataatcaaaatttaaactgaaaaataatTTAAATCACAATAT
ACTTTGCAGCCTTAATTGAGAGTTGTAAACACTTAAGGCTCGTAATCATGGTTCGACTTTGTCGGATCTT
CTTCGATTTCTCCGAGTGTAAGGGGATGCGGTGTTTATAGTTGGTGGCTCCAACTCCAACTCTCTCATTT
CTGGCGATCATGCCACCAGCCATGTGTACATACGTATGCATACCCCGATTTCCAGTCCGACTCCGATTTT
AGCTAAGGCTCCGACTCCGACTGACTCGGCTCAAGTTTTATTTATGTGAATTTTGTTGCCAGCCGAAAGC
CAAAGCGTCTGGCAATGCCAGTAAACTGCACTTCCATTTCCATGTCCCAACATTCCACATCGTCATACGA
TTTCCAGACTGCAGAGCTCTTTCCCTTTTCTCCCCATGCTCCGTGCTCTTTTTTGTCGGCAGAAACCTTT
TACTTTCCTCTACAACTCGCCTCTGCTTACACTGTAATAAAAGTTCCACTAAAACTCATGCTTAAAGTGA
CTAAAGTACCGGGATAAAATCGAGTGTGGCATACTTTTTGGCGTGCTATATATGACATTaaaaaaaaGGG
TTCTTTAAAGTTCAGAATTTATGCATTTTCTTTCGGTGTACACTCTTTGCTTGTTCTGCTTCCCCTTATA
ATTATTCCATTTTATGCTTATTTTTTGTACCGGCCGGCATCGCTACATGAAGCGAATGGAGCGCCACTTG
AAGACTTAAACTCCGGCGGCTCTTGTGAAATGTACagagagaataccgagaaagagagaagaggagagaA
ACCGTGGTAGACGGGGGAGCAGAGAACAAAAAGAGCAACAAGTGGCCGATTCTTGGAGTTTCTTGGCCTT
GCACATTGGTCAACGTTTTTTTGTAAATCGATCCGAGCACGTGGCAGATATATGTACATTCTACTCCAAC
ATAAACTGGAAGTAGCTCGACTTATTTATAAACATTTGTTGTTACCAAGCTGATCTCGATATCTATTTTG
ATTATCCCCCACCCAGAAGTAATTCAAGCACTTTAAAAAATATGGCTTAGCTTTAAAAAAACATAAACAG
AAGATTGACTAACGAAGTAATTTTAGCTTCAAACGCGGCTGTTTGTAAATGACATTGAATAAGCAGGCAG
TTTACATAAAGGCCTACGTaaaacattcaaagaacattcaaaacattcaaacattcaaaTTGGTAAGAAA
ATTGCTGAATAATCAATTATTTGCGCAAATTAAAAGTACAGTGTAGAACCAAAAATACAATAGACCCTGA
TTAAATATCAGGGCATAGTATCGACCATTTCACCATTGTTAGTCGCTTTTAATCTGAATATTTTAGTCCA
TCATATCGAAATATGAATAGTTTTTGTTATGTTCGCAGTAAGGGAATAACAAATGGTTGGACTCCAAGCA
AGTTTGTTTTTAATCTTCAACGAAACCTTCAACTTGGCTAAAAGCGGAGGGTTAAAACGCTTTGGATTTA
AGCCAACGAATGTCAAAGGTAAAAACAAAATTAATAACGCTGGGTCATGATAATAGCCGTAGCTTTAAGG
TTAAAAGCAGATGATTGAAAGTGGGACTAGCGAATTATAAACCGTTGCATCAAAAGATAGTTTGCCATCA
ATCTATGTCAAATTTATTTTCGTGCTTTTAAGTGGTTTCTGGTCGAGAATTATCAACTCCTGTGAGTAAA
TGCATTTCCGCTGTACTTTTGACAGTTTTAATATAAACCAATAGCTCAGGTGCCAAATAAATCGTGAGAC
AGGATTTAATACGATTTATAGGTCATGGCATGACAGTAATTTCCGCCCAACTAAACTTGTAGTAAGCCAA
AACGAAATCATTGCAAACTACTCGGGAGGACAAAATTGACAACTCTCATTTGGCAGCAAGTTCTGGGCAC
GTCCTTGACAGCTTTAATGACTGCTTTTAAACTGCGACAACGCAAGTTTCCCAGGCATCGACTTCCTGGC
CGAGCTGATTTATTTCAAAATCGAATTTAAACAAGTTTGCAGGCGACGATGGCGAGGAAAAAATCACCCG
GCCAGTCCCAACTTGGCCGGGACATTTTTAATTAACTTGGCTAATTACGCCTGGAATTTGCACGTGCTCT

CTGGATCCCACTGTCCCCGATACAAACTTGGTCCCTTAACTGGAGTTGGCTGCATTACAGACCGTCTGCC
TTAATTACAAAAACATTTGCCATTATGTATAATTATGAGCAACGaaaaaaaaaTTATTTTCATTTGTGCT
TTTGTTGAAAACTGGTTTCCAACACTAATGGAAGCCAACAAAATGCACTGCAATTGTTCGTGCTCGAGTG
ACAAAATTCATAAGCGATGCATGCAAAACACAATCTAACTGTGTTAACTGTATCTCAATGCTGGCCTGAA
ATGAAATGAAATCTGCCAACAAAGTGCGAGGGTCAGACCTTCGTTAGCCTTCTATTTCCCACACAGCCCA
GCGAATTTCTCTTCTCAAATTGGTTTTCTATTTTCGGGCATTCAATTAAAATATTACGCATACGCAGCGT
TGGCGTGGCGCATTCAATTGCAAAGCAAAAAAGGTGGCGTGATTTGTTGGCTAGCATTAGCTGGGCTGTC
AAAAAATTCCCACAATAGATACTAGGAAAGTGCATTCATTAGAAAATCAGCAGAGCATATTCTCGCCCGG
CGCTATATCTCACAACAAACAGATGATGACGTTGGCGAAATTCCGAGCGCCTGTAAGGAACATCATTCAT
TATTAGCAGATAGTACTGAGTCATCTTAATGCCATTTTCGACGCTCATGCGACTTTGGCCAACAGATGTG
TGAATAATTGTTATTGTTGTTAATAATGTTGTTTGACTGTTTTGGTCGGCTATGACGTTACCGATCAGGC
CACCTTTTTTTCCACATTCAACAAAACTGAAGCCAAATAAGCAGCTACAATAACAAGACACTCGTACTGA
AGCATATCGAACGAATTCACTTCTCTTGCCAACATTTAAGAGCCGTTTCAATAGTTTCTCTTATGttttt
ttttAACTTTAAAGAGCTCATGTGCTGGTCGGTTTTTTGTTCTTAAGTTCAGAGTTGACGATCCCCTTCG
GAGAGGGCAAACTGTGCTGGTCGGTATCCAAAGCGCAGTGCTCACATGATTATACAATTATTATTTGTTG
ATTATTTTTTATCGTCGTACCAAAGGTGCCCATTTTTATCAGCCCATATGTTATCTCCACAGCAGTGGaa
aaaaaaTCACACGAAATTATTAAAATTATGAGATAATACGTTATGAGATAAAAGACCTAAGCTACTATGT
AATTGCATAGAGTAATTCTGACTCATCAAATCTATTTCATGCACCTTATGAATGACTTGAAAATTTAAAA
TTAAAACCAACTACGTAGTTATGACTTGCTTTCAGTGATTATAAGATAACAAAATTTATTAATCTTCCGC
TTACCTAGGAACTGAATTTATGAGTAAGTCGAAATCTATTTTTGCAATAAAAACTAGTAGGTACCAAACA
TTGATAAGGCGTCTGCAAAATCGTAGTATTCGTATCTTTTGATAGTGCTGTTTAGTTTTGATTCTGCTCA
CCCAGAAGGTTATCAGTCTTATTTGAAGTGCCTAGCAGGCGCTTACAGATAGTCAGATAACAAATCTACA
CGAAATAGTTGTGATTACAAATCGAATACGTATTCAATTCAAAAGTTCTAATATGAAGTTTCTACAACCA
TTTGGTTGCCATTACAGTtgttcacacatgaacacgaatatatttaaagacttacaattttgggctccgt
tcatatcttatgtaaatgaatcgagagcgataaattatatttaggattttgttatctaaggcgccatggg
tgcattgctcaaaaacatgtaatttaagtgcacactacatgagtcagtcacttgagatcgttccccgcct
cctaaaatagtcccttagtgggagaccacagataaggtcctcgccgctcaagataggcagatgtgcccga
gcgtgggacctcgataaggcggggactatttacttaggcctctgcgtaggccatttactttaagatgcga
ttctcatgtcacctatttaaaccgaagatatttccaaataaaatcagtttcttacaaaaactcaacgagt
aaagtcttcttatttgggattttacatttggtcaatcgagcctttaatcgactctgcagtttccccctac
caaaggtaaggaactcagagaaaggccagctcctttaagcatcttacagctaaaggtagcaaaaataagt
gactcttgtttccccctaccaaaggtaaggaacagagtataaatataaaaagcaaaagatacaaaagaat
cttttatgttttaaaacaagcaccttatagtctatagctaaaggttgctttgtgtaccattataaattgt
ggtaaggcgtgcttgaggccatacatcagcaattgtgaaattaaaaagtgcataacaaaagtgccttata
aatgctctaatagcattaaatcagctcataaatagagtgcagtgtatatgccataagagcataaattaaa
taaaaagtgcctgaaaacagtgccttataaatgctctaatagcattaaatcagctcataaatagagtgca
gtgtatatgccaaaagagcataaatgccgaaataaatggctaaaaaacaaaaaatctgactggactacaa
aaataataaaacgtgccaaacaaaaaaaaaaaatcatctttaaacatcgacggagccttaaagaagagaa
ggaagtcaaattcaaaggagcctctaccagcagcagaagcagcaacaacagcagcagcagaagcagcaac
agcagtagcaacagcagcaacaacagcagcaacagcagcagcaacaacaacgacatcagctaagtcaaaa
caagaattttctgtttatccaaacacacatatatatataaatacatataaaatacatatacacgtactat
atatattaagaaattacaaaaaattttcaaaatgatgtcagaaaagactattcaattccttaagaagcag
tccgaaattattttggaaattagaaagttggaagtaaaaccaacattaacagatgtagaaattctaaaat
taaatgagcttcaaaaatgtttcattgctaatcatagcaatttgttaaagatcggcgttgtcgatcatga

atattttaacgcgaagcagtatgatttaataatgatggtgttagaaaaaattaaaaataaaaatgaaaaa
attaagggcgagtcggtagaaaacactttccctaaatcaaacactgtccctaaatcaaaccctcccccta
cattaaaccttgaaatgcgtggtcaccctgaaaaagagggtatagcacaaaacaacgctttaaaagtaga
gcaggcatttcgtaataatgttggccaatttcgagtatatctagaagatacgtctaaactaatagacagt
agtccagatttccttaaaataaggaaaaataaaattgaatttttatggcataaaatagataacctgattg
aacaggtgaatagtcattttgagagttcgctattcgaagaagaaattagcgaacttgaatttgacaaaca
aaatattcttacagccattaatagtcgactcagtggcacaataaataaagctgaaatgtcgacggttgtt
aaggcggaggagttaccaaccctgcctaaaatacagattcccaccttctttggtgattccaaagaatggg
atctttttaatgaactctttacagagctcatacatgtgagagaggatctcagtccttctctcaaatttaa
ttatctaaagtcagcattaaaaggagaagccagaaatgtggttactcatttactgctcggctctggagaa
aattatgaagccacttgggagtttttgaccaagcgatatgagaataaaagaaacatattctcagatcata
tgaataggcttatggatatgccaaatttaaatttagaatccaataagcaaataaagacatttattgacac
gattaacgagtcaatttatattataaaattaaaggcacaattaccagaagatgtggatgcaattttcgct
cacataattcttcggaaattcaataaagaatcactcaatttatatgaaagccatgttaaaaagacaaaag
aaatacaggcactttctgatgtcatggactttttagagcaaaggctcaattctatatcatcattctcaca
ggaagtaaaacctgtaaagaaaatgattaataataacaagaataaaaattatagtgacaattgtgcatat
tgcaaactaccagggcattatttaattcaatgccataaatttaaaataatgaatccagcagaacggtctg
actgggtaagaaaaaatgggatttgcctaagatgtctgaggcatccgtttggtaaaaaatgtataagcga
gcagctttgttcgacttgtcgtaaacctcaccacacgttacttcactttgcaggtcataatccagaaaaa
gtgaatacgtgtagaacaacaggtcaagccttgttggccacggccttgattcaagtaaagtcgaggtatg
gaggctttgaacaattaagagcattgattgatagtggctctcaaagcacaattatttcagaagagtctgc
acagattctaaaattgaaaaaatttcggtctcatactgaaataagtggagtatcttccacaggaacgtgc
atctccaagcacaaagcggttatttcgataagaaattctccgaaaaatttagaaattgaagcaattattc
tcccaaaacttatgaaggcacttccagtcaacacgattaatgttgatcagaaaaaatggaagaactttaa
attagccgaccccgattttaataaaccgggtcgcattgatttaatcattggagcagacgtatatactcac
attctgcaaaatggagttataaaaatagacggtctccttgggcaaaaaactgatttcgggtggatagttt
ctggatgtaaaaaatccaaaggaaaagaaaccattgtagccacaacaatagaaataaaagagttagatcg
ctactgggaagtggaagaagaagaaaaagatgatatcgagtctgaaatctgtgaaaataaatttatcaaa
acgacaaaaaaagattcagatgggcgatacattgtgtcaattccattcaaggaggatgtcaccttaggag
attcaaagaaacaagcgatagctcgttacatgaatctggagaaaaaactaaaaagaaatgaaaaacttaa
ggttgactacactaaattcatgaatgaatacatggatttaggacacatgattgaagtgagtgatgaaggc
aaatatttttttaccgcaccaggcagtgattagagattcaagccttacgaccaaattgagagtagtttttg
atgcttcagcaaaaactacgaataacaaaagtttgaacgacataatgtgggttgggccacgagttcaaaa
agatatttttgacattattattaaatggagaaaatgggaatttgttgtttcggcagacattgaaaagatg
taccgacaaattaaaatagataataatgatcaaaaatatcaatatattttatggagaaattctccaaaag
aaaaaattaaaacatataaattaaccacagtcacttacggaactgcatctgcaccatatttggctaccag
ggttctggtagatattgcagataaatgtaaaaaccaagttattagtgcaataattaggaatgatttctat
atggatgacctaatgactggagctgattcggtagaagaagctaataaattaataacattaattccccatg
aattgcagaaagttggattcaacttaaggaaatggatttccaacaattccaaaatattaaccactgtgga
ggacacaggggacaataaggttctcaatattatcgaaaatgaatgtgttaaaactttaggactaaaatgg
gaacctcaaaaggatttatttaagttcagcgtaaattgtaatgatgaatcaaaaaatataaataagcgcg
ttgtgttatcaacgctagcaaaaatatttgatccgttaggatggttggcaccagtcacggtttcaggaaa
actttttattcaaaaactttggataaataaaagtgaatgggatcaggaattatccatagaagataaaaat
tattgggaaaaatataaagaaaatttattattgttagagaatattcgaatcccaaggtggattaattcaa

acagttcttcagtcattcagattcacggatttgcggacgcctccgaaaaagcatatgctgcagtagtcta
tgctaaagtaggacctcatgttaatataatagctagcaaaagtagagtcaaccctataaaaaataggaag
acaattcccaaactcgagctgtgtgcagctcacctgcttagtgaattaatccaaagactaaaaggatcaa
ttgacaatataatggagatctatgcttggagtgattccacgattaccttagcatggattaacagtggtca
aagtaagatcaaatttataaaaagaagaacggatgacattcggaaattaaaaaatactgaatggaatcat
gttaagtcagaggataatccagcagatttagcatccaggggagtggattctaaccagttgatcaactgtg
atttttggtggaaaggtccgaaatggctagcagacccaaaagaactttggcctcggcagcagtctgtaga
agaacctgtcttaataaatacggtattaaatgacaaaatagatgatcctatttacgaattaatagaaagg
tattccagtatagaaaaacttatacgtataatagcatacataaatagattcgtgcagatgaaaacaagaa
ataaagcctattcatcaattatttcagtaaaggagataagaatagcggaaacagttgttattaagaaaca
acaagaataccagtttaggcaagagataaagtgccttaaaatcaaaaaggaaatcaagacaaataataaa
atattgtcattgaatccatttttggacaaggatggggttctaagagttggaggaagattgcaaaattcca
atgcagaatttaatgttaaacatccaatcattttagaaaaatgccacctaacaagcttattaataaaaaa
tgctcataaggaaacattgcatggagggataaacctaatgcgaaactatatccaaagaaagtattggatt
ttcgggttgaaaaattcgttgaaaaagtatttaagagaatgtgtaacgtgtgcaaggtataaacaaaata
cagctcagcaaataatgggtaacttgccaaaatatagagtgacgatgacattcccgtttcttaatactgg
aatagattacgcaggtccttattatgttaaatgttcaaaaaatcgtggccaaaaaacatttaaaggatac
gttgctgtattcgtttgcatggccaccaaagccatacacttagaaatggtaagcgatctaacttcagacg
catttttagcagcactcagaagatttattgctagacggggaaaatgttccaatatctattcagacaacgg
aacaaattttgtaggagctgcaagaaaattagatcaagagttatttaatgcaatacaagaaaatataacg
attgcagcgcagcttgaaaaggacaggattgattggcattttattcccccggcaggacctcacttcggag
gtatttgggaagctggagttaagtcaatgaaataccatttaaagcgtataatcggcgacactattttgac
ttacgaagaaatgtcaactcttttatgtcaaatagaagcatgcttaaattcaaggccattatacactata
gttagtgagaaggaccaacaagaagttttaacaccaggtcatttttttaattggaagaccacctttagaaa
tagtcgaaccaatggaagatgaaaaaatcggaaatttggataggtggagacttatccaaaaaatgaagaa
agatttctgggttaagtggaaaagtgaatatttgcatacgctccagcaaaggaataaatggaaaaaggaa
attcctaatatagaagaagggcaaatagttttattaaaggatgagaattgtcatcctgcaagatggcctt
taggaaaggtggaaaaggtgcataaggggaatgatgataaggtccgagtggctaaagtaaagatgcagga
aggatatatcactagacccattactaaaatttgtcccttggaaggaataaagtctgttgacaaaaatgag
gctgaccaagagccaaaaagacgaactagagcgacatcgggaatgtccaagatcggaatcattatggcaa
tgttgttgtttgtgttaagttgtcaagtttctagcgcattacctaaagatatagcaccaagatattctat
agacaaaataaataaaacctcagcaatatatctagacccgctaggagatgttgagattgtgagtacttct
tggaatttggttatctattataaaatggatccatattttaaaatgttaacaaagggtaatgcgcttatac
aaagtatgaggaaagtttgcgaaagacttcatagctttgaagagcaatgtagtctagtcttagataatat
gcaaagtcagttatcggaacttgaagaaaacaataaattgtttatgatacagtctagatctagaagcaag
cgtgctcctttcgaatttatgggttccttgtatcatattttatttggtataatggatgaagatgatagag
agcaattagaagaaaatatgaagaatttgttagataaccagaacaaccttgataaactaattcaaaaaca
aacatctgtggttgattcaacttctaatctattaaagagaacaacagaagatgttaactccaattttaga
agtatgcaaataagaattgagaacatgacagaagttcttaaagaaaattattatgtttataaggaatcaa
taaaattctttatgattacgaaacagctacactcattgattgaagaaggcgaaaaaattcaagcaggcat
tataagcctgttgattgatattaatcacggtaggctaaatacaaatattctcaggccaaatcagcttaaa
aaagaaattgccaaaattcagcagagtctttcggagaacctagtaattccaggaaaacggtcaggtacgg
aacttaaggaggtgtatacactgttaacagccaggggtttattcatcgacgataaattgatcattagtgc
aaaagtgcctctgtttagcaggcatccatccaaattgttcaggcttattccggtgccaattcgaaatgaa

gatcggataataatggtgcatacaacgtccgaatatttaatttataattttgagatagattcctatcaca
taatgacggaagccacattaaatcaatgtcagaaatggcaactaaataagagaatatgcaaaggaagttg
gccctggaattcagcgaatgataatgcatgtgagattcagcctctaaagccagataaagcggcgaactgc
atctataaaacagtagtcgactctaaaagttactgggtagagttagaaaagaaaagtagttggttgttta
aggttcctgcgaattcaaaagtccgtctgcaatgtactggctctcaaattgaattgtttgatttgcctca
gcaaggagttttaagcattgcgccatattgtacggcaagaaccgacgataaaattctagttgcccaccat
aacattcagtccgaaagtgaagaattattatcaacaccttatataggagaagttagtgaagcgccgaaga
ttatttgggatccgctgaaactatcaatattaaatcatactgaggaatttgaacgattgaataatgaaat
taaatttatgaaagagaaccatcaaaaattgaaagatttacatttccatcatatttccggacatgctgga
ttaattattgctttaatactaatgatagtattaataatatatttcatacggaaatgtgctgtgcaacaaa
gaatgcaagcaataacctttgcaggtccgttgccagtactataaatatcaatagtaaataaacaataaaa
taatataacaaataaaaatatacagtccactatatcgttgtttaatagaaaatgtacttctacatagaaa
aagcaaaatgtttaaaataagttaattgagtacaaattgttgaattaaaaataatataaaccataattgt
aatccaataaaattaaaagccagaaaaactaggcccattgaaatcttagttgcaaaataaatgaacatat
atcaaataaatacagtccactactgttataaatgcaactaatatactaatgtacatctcagcttgctggc
cctttggcagaatgttcacacatgaacacgaatatatttaaagacttacaattttgggctccgttcatat
cttatgtaaatgaatcgagagcgataaattatatttaggattttgttatctaaggcgccatgggtgcatt
gctcaaaaacatgtaatttaagtgcacactacatgagtcagtcacttgagatcgttccccgcctcctaaa
atagtcccttagtgggagaccacagataaggtcctcgccgctcaagataggcagatgtgcccgagcgtgg
gacctcgataaggcggggactatttacttaggcctctgcgtaggccatttactttaagatgcgattctca
tgtcacctatttaaaccgaagatatttccaaataaaatcagtttcttacaaaaactcaacgagtaaagtc
ttcttatttgggattttacaACAGTAGTAAAAAGAACGTTAAATTAAATCTATTTAAAATACTTGGAAAA
ATAATTAAAACCTATTTGTCACTTTTGACCCGAAACAACAGCTTAGAAACAGTTAATAAAATTAGGAATT
ACAGATAGGAAATCTAAGTGATTTGTTAGCACCTAACTAAGCAGCTGACCCCTATTAATGAACTTAAAGT
AGTAAGATTAAATTAACTACATGAATTTCAACAAACAATGTTTTAAGACACAATTAAGTAGGCACGAGGC
ATAATAAAAACTTACATTTACGCAATTATAAGCAATTGAATTTGGTTACCCAGTCTGCCCATCCTTCAAT
ACCTTTTACTAGTCAGTAAAAACCGATTGAACTTTTTGAACCAATGCGTAAAACAGTGTTATCACTTTGA
ACTGCTGTTTAGGTGCAAAGTTCTCGCTATCAAAATTGGCTGGTTTCACATTTTATCTAAAGTGGAAATA
TTTCGGTTAATTTGCGTAATGCCATACTGTTATTGCGGTGTAGTTGACCATGGTCGAATCGGTGATTAAT
CACCGACTATTAATAGGATTGCAGTTGTAATCAAATCGGATGGTCGACGGGGGTGGGTTAGGCACAATCG
CCGACAACCTTTCGGGCTATTAATAAAACATTTATATTGATTTGAACTCTGATTGACTTTTGCGCTGATT
TCTGTTTATTATTTCGCCCTCTTGGCTAACCAAGACAGCCCTCCTGTCATGCTGCCTTTGCTGTCATTGA
ATTAATGGTGCGAGATTTTCATGTGAAATATACTCTTGACCAGCATTTTCTTTCCTGATTGATTCCTGCT
CCAAGTAAAATAAAACTGTTCAATGAACTTCGCATTAAGCATTTAATTGGCCAGTTGCCTCTGCGTTGGA
CGGACATTCGTGATTCTGTCAATCAAATTGGCGGCTATTGCCCACGGAGGCAGCTGCTCCATGTACGCAG
TCTTTGTGGAGCACACCGCAAATTATTGCCCCACTGATTTAATTTGTATTAAATAGATGGCTGTGAGAAT
TGGGTGAAATGCCCTGGAAATAAATGCCTGGAGAGGATAACTATAGGATTTTTCCGAAAAACATTAAATT
AAATATCAAAGAAATTCGTTTTGAGCATGTACTGTTATTCAATGTTCAACAGAGAGGAGAAAATATCAAG
TTACATCTGTACTATGTTTTTCTATGATTTCTTTGAGTATTTAAAACGTAATCAAAAATTCCGTTAGCAG
AATGATTTTTCATTTTATTAAGAAAGTCGATTTAAATGCATTAAGCTGTACAAAAGTATTCGCTTGCTTA
CCTTTGTGCATAAAAATAAGCTGGCTTCACAGTTGAAAACGATGAGAATAAATTTGAGAAAATAATTTGT
GGGTGTCCATTAACCAATTGCCAGCTATAAAATGCATTTGCTAGAAATGCAAAATAAATGGCTTGGAGAT
GTATACATTTCTTTTTGGTTTATAATCCAAGCTCTGTCAAATGAATTGGATGGGGCATTCTCTTTTAATA
TTTATTTCAATTAAGCTACTAAACGACTCACTTAAAATGCAAATTGCATTGCACATTTACATACAGATTT

GTCTACAAAAGCAATTGCAATTTCTGAGCACCAAACAAGTGCAACCCTacacacacacacacacacacac
aaacaaacacGTACGACAGGAGCAAACAAAACTCTTTTCATAAATTCAAAATGTGTGTGCACATTTTTAA
TAATTTACGGGGACTAATAAAAAATATTCATCCAGCAAAAGAATGAAAAGCTGCAGCTAAAGGCAGGAAG
AAAATGAAGAGTTGAATACATTTTTTTGGCAAATAAATACACATGCTTGCATAAGGAAAAGGCAAAATGC
ACTGGCAAGCTTAAAGCAAGAGCCAAAAATACAATCAGCGAAAAATTTGAGGCGATGGAAGTTGGAATTT
GAAGCCAGCATGTCTTGAAATTCCCATGATAATGAACCACTAACACACTCGTCAAATGCGTGTCACTCGG
GTTCTCTCTCtgtgtgtgtgtgtgtgagcgtgtgtgtTAGTCCTTTTGGACATGTGTGCGTGCCGAGGGC
AATGCATGGCAGGTCTGATTTCGTAATTAAATGCGTGTGTGTCAAAAGGAGTGGACCAATGTGAGCTAAA
TTGGAAATTCTAAACAAAATGTGTCCTATATTTTCACCAGCTAACCAAAAAACTACATACAGAAAAGCTT
TCAACACAATTTTCATAAAAATAACTCATACGACCAGTCGACCAGTTCAACATTCTGTTCTACGCATAAG
TTTTCTAATAATGCTGAATGTTGTTAGATAAATCTGGGAAAAGTGAAAAACCCGAGCAATTGTTCTGAAG
AAATAAGTAGCTTGCATACAGAATTCACATTTGCGGAAATTCTTTATACATATATAATATTTGTTAATAC
AATAATATATCCAACTGGATCCTATGTCTTTTGCCTACAGTTTAGGTCCACTCTTAGGAGATTCTGTGCG
TGTTCGTGACTTTTTGGGTGAGCTCGTGCGTGATTGTGACGGTGCCTGAGCAATTGTCAATTTGTGTTAA
ATGCGCGCAATTAAACGGTAAATGCTACGCGAATAGACCGTCTcacacacacaagcgcacacacacCGAG
ACACTTTAACATAATGACGTCACATTAACGGAAGTCGGTTGGCTTTAGCATATTTGCTATGCTCGATACT
AGCATACTAGCATATATACGTGCATATGCTAATCGGTCCAAAGACACAGAAAATGAAAGACAGATTAGGG
CGGCCGAGTAAAATGTCAAGTGGCCCGTAAAACGATTGATGAAGCCATCACATCCGAAACTTTCCAACTA
GCTGCCATCAAATTCTCGCTTATTAATTTCCCCCGAAAACTTTTCGCCTTTCATTTGGGGTCCTTTTGCG
GTCCGCCACAAGAACATAGACCGCAGAAACACCCACTTGGCCACTTTTATATACATAGACATGTGTAAGC
CTCGCCCTTCGGCTGGATTTCATTATTTACAAGTTTTTGTTTATTGTCAAGTCTCGGCTTTCATTAAAAA
GGGTTTTAAGGCTTGTCAACAAACGCCTTTTCTCCACAATTTCCCCCTCCCTTTTCGACCGAGCATCGCT
ATCTCGCTTCCTCAGTGTGTGCTTAACGAGCCAAAGTTTTCAACTTAAATTTTAGTTATGAATTTTATTA
GGCCCTCCCATTGGGACGTACAAACTCCAAGCACTGGTCGCCGACGTCTCCCGTTTCCGTTTAATTATAG
TCTGCGGCTAACCCGAGGATTTCAACTTAATTAATTAATATGCATTTGATTTTGCAGCCTCTCGGTCTGA
GCAACTACTGCGATGAGAAGCAGTTCCAGTGCTCCACCGGCGAATGTATCCCCATACGGTTCGTGTGCGA
TGGCTCCTCGGATTGCCCGGACCACAGTGATGAGCGGCTGGAGGAGTGCAAGTTTACAGGGTGAGTGCAC
AGCACGAAAAATCCCATTAAAATGCAGGAATAAATTACTCTGATTTGACGCGAAATGGATTTGTTATAAT
TGTAAATTAGCGTTTAAGCACTGACTGGCATTCGATTGTTTTAAAAGCTTATCTAATTTCGAAATGTGTA
TCGCTGAATTGAAAGTATTTGTTCGAGTGTGTAAATCAATAGCACTGACATTTTCCATCTGTAAATGAAA
TAACCTGCAAGTTTCAGTTGAACTTTGAttttttttGTTAGCAACTCGACCCTGCGAGTTCATCATGGTG
CTTCCTGCATTTGTGCACAAATAAATCACCCGAAATTCTATAGAGCTCTTCTGCCCTTGCCATTCTCCAT
TTCCATTCCTTACAATTTTTATCTTTCCGGCTCTGATTAGGCCTTCACTTTAGCTGTTGTCTTATTCAAA
TACACTCGAAAGGTTGCACAAAACCTCGCACTTGAAAGAAATCAACAGCCATAGCCGCAAAATGTATTTG
CAGCCCGGttttttttttttGGCTGTATCTTTTGCTGCTGGAGCCGTTTTAAAGGAAAGAAGAAAGTTTTG
CCCCAAGCTGATGTTGCTTTTTCATCTTATTTATTTTGCTATATATGTGTGGCCATGGGCAAGAACACGG
ACACGAAGTATCTACAAAGTCAAGCGCTAGCTCCGCAATTGGGTTTGGTTAAGGGATCGGGATTTAATGG
ATGGACGTAGGTCCTGGTCCTGAAAGCCCAGCTGTTGCCTGCGAACTACGTGCGCTCTCGCTCTAATCTG
TTGTCAGTCATTTGTAGTTTTTCAATTTAGAAATGTCACAGAACTAACCATTTACTTCGCTCTGCGTGTG
CTGTTTGCCTCGTATTGGttttggtgtttttgtgtttttcagcttttgtgtgttggccgtttgtTTGGCT
TCGCTGTTGAGTAAGTAACAACTGGCTGTTTATTTAGTGTCATGTAGGCAGGACTAGAGCTTCCCCAAAT
CCCCATGACTCACCGAGCTTATTTTCATTTAATTTTTACACTCTGCGTGTATGTGTGGTATTTTTTATTA
GCCTCTCTACATTTAATTTAAGTTTGGTTAGCAGACCAGTCTGTTTCGATCCACGGTTCGTTGGTTTTGT
TTGGCCTGGGGCTTCCCGAAAATTTTAGCATTGCCTACTTTCGGGCAGCAGTGCTTAAAACACTGCCAGC

CGTAAATATTGGGCACGGACTTGCGTTTGGGGCTTAGTCTTTAAGCACAGAAACCAGCTGAGCCGCAATT
ATATATAGTATAGAGGGTCTTAAGCACACGCCTGATAAATTACCACCAACCCAGTTCCAGTTGCATTCGA
GTTGAGTCTAATTGGAGGCACTGCAAGCAAGTCAAGTGCAATTTAACTAAACCATTGCATCAGCTTTTGG
GTGATAAACTGAACTGCATTCAAACTGCTCAAACCAAATGGATGTAATCACTTAAAGAATGAAAAGCTTA
TTGAAACTTGATTGCTCCAAGTATTCAATAAGACTTGTGGTACGAAATCTTCATCCCCATCACCAACACA
AATATGTAAATATTCTACAAATATTCTGATGAACTTAACTTTTTAACTCATTTGTCAAATTTTTAAAGAT
AATGTACATGGAAGTACAATGTATAAATGTACGTTTCTATATTTGCAGTTTTCGATTATAATAATGTTAA
TAATCAAATCACTGCCAAATTAAGCATTTTTTCAGATATATATATTTATACTTTGaaaaaaaaTTGTTT
GTTTCAATTAAAGGTCGTTTATATGCGCCTTAAACTTTTTATCAACCGAACGAACAGTCGAGAAACAACA
AATAATGCGTATCCCACAACAAGGAAATCCAGATTAAACTTGACCTAAAACTTATATTGAAGTATAAGTT
CTACACAAAGCCAGTTCAGCCGGATCTCTAGCTAATAATCTAGGAGAGTGCAGTAGACATACCTTATCAG
TGAACTTATAGTTCTAGTGAACTAGCAAGGCGTCACTGAACACATTTGAGATTATTTATTAGTGGCATAG
GGTCATTAGGTTTGGGTATTTTTCTTGTTTTGACAAATTACAATTAAATTGCGCACAAAAATATAGATTC
ACTTGCTAAGCTAATTCAATTGAAATGCCTGCGAGTCCTAAACTCATTCGCTTGAGTGAGAGTTAATTGT
GAGTGGAAGCTTTGAGTGGAACTCTTATCAATCCGAGTGCAAGCTTCTGTCGCGCGGAGTCTGCGAAATA
CTGGATGCCAGTACATCTGTTCCGCATCCGACGAGGACCAAACGCGATTCAGTTTGCTTCGAGCTCTGCA
AAGAAACGGACGTGTGTGCTCTCCGGCCAGGATAACTGAGATTTCAGGACTCCGAGGCGGCAAACATAAA
ATCAGTTTCGTAAAAGTCAAGGCGAGAGGCATATAAAAAATAAAGTGCAAAATTCTTCAAGTACACAGTG
ATTTGTAATTAAATATTTTAAGAAAATAAATACATTTCAACAAATTCATTAAAATAAATTTAAATAAAAG
CAGTTATCGGCCAGCAAAAATCAATGGCAAGGCCAAATTGTGCCGACAAACGCTGGCAGACAGATTGAAA
ACAGTTAGCTGGCGTCTCTTTGGCGGTAACATCAAAAGGCAGCAACAGCAGCTTTTCCCCCCAAACGCAA
GCTTTTCCGCAGGATAACTCTCTCtgtgtgtgtgcgtgtgtttttccttttccgtttttttgtgttttttt
ttCACGCTTCTGCGCGCCACTTTTTATTACATTTTCATTTAGACCTCTCTTTTTGGCAGAATCTCTTACA
AACGCTCTTCAGGGTCTGGCATAATTCTGTTGAGATTCAGCAAAGATTTGGCCTTTGTTTTGCATTGCAT
TGCATGAACTTGACACCGAATCCGAATGCAAACACTTTAAAATGCAGAGGAATCTGGAATTGCTGTCCAG
CTTAATCATAATTAATCTTCACACATTACTCGCACTTTAATCTGTTATAATTGGTTTTAATATTTATTAA
TTTTCATTTGGAATTCCCGGTTGTGTGAGAGTTTGTGAGCGTCATAATTCCTGTCGGCATGTCAAAGTAA
AAAATTGCTATTAAATGTAAACACTCAACTCGGCTTAACTCAACTCCCCTTAAAATACaaaaaaaaCTGC
TTCATGCGAGGATTCGGTGAAGTTCTGCAAACGCATCCGCATTAATAATAATTCAACAAAAATATGACAA
GCGTTAAATACGTGGAAAACTGTGAAAGGGGGCTGCATAAAGCATAATTAAGAACTTTACCATGTGAAAA
CATTTAAGTACATAAAGTAAACACATGAAGTGCAACATTTACTACACTTTCATAAAATTCAACTATTCAT
CTTTAAAATGCGTGGCTAAACAAGTAAACAAAGTATCTGTTTTTAAAATAGCAGAAAACCTTACAAAACA
AGCGCAATCCATTAAAATAGTTATAAACCAAACAATTAACCATTGGAATTGCACACAATATTTACAAATA
TTTTAAAGCAAGTCCATTGAAAAAAAGACAGTAAATAAATTCAGTTTCCACGCTTGAGAGGAAAAACTTT
AGTGACTTCGTTGAATATGATACCAATTAAAATGACATTTTCGCCAGAATAAATTCGCATATTATTTGTG
AGCGGAAAACAGTTGACTACTTTTGAGTTACTGCCACTGCTGTTAACCGTTCTGATGCTGTGATGATGTT
GCCTGTTGCCATTTTTGCTGGCAGACCACTTGACTGCCATTTAAAATAATTATGATTACACGTTCGCAAC
AGAGAGCCCAGTGCAAACATTCGCGCGACGACAAACCCGAATAAACAGTAATTTAATTTAATAATTCAGC
GGAGCACTTAGGCAAGAACATTATCACACTGCCGTGCAGTGTACAGTAGAGCCTCCAAAGAACCAACATG
GGACCAATATGCCGCATCCTGGGCTGCTCCTTTCTGCTGCTCATCGCCGGATTAGTGCCGCACCATGCCT
CCAGAGTCGTCGGATTGGGTATGATGGGGAAATCTGTTATGGTTGTGGAATGATAATTCCCGCAAATCAG
ATTAGCAACCAGATTAAAATAGGGAATACGCTGGTTTTCGCTTAATATAGTTTTTAATTGCCGATCTCGA
ACAATGGGTAATCTTCTGTTTCAAACAATACGCCTAAATAAATCGACATTCGTAACCTTTTTTATAATAA
TGAAATTAATAAAACGTACATACAATATTTTTAAGAACGATACGCCTACCACATACGATTATACACTTAA

GCCTGACATCATTGCAAGTATTCCAAGTACAGTAAATGTCACTTGTCCATTATCTTAATAATGTAAATTC
CCATGAAACAGAAAAAAGCCCAATCAGGAAATGTTCGGTCAAAGTTTTTCGCTATCAAGGCGCGGACCCA
TTAACCGCTTCAAGTGAATTTCAATTTACTGTTATTGTCTGTGCGACGTAATCGCTGAGTGGGTGTTATC
CCTTATGCATGGGCGTGGCACAGTAGGTGGGCAAAAATCAATGGCAATTCCACAGAGATGACAAAAAATA
ATGTCTTAGGAGGCAGTAAATAAATAAATAACTTATAGGCAACTAAACTGGGGATGGCCATTAACAACAG
AGCTGATCGAAGACATCGAAGAGGAGCGCTAAGTGCCCCGCTTCCTTGGCGTTGCTCATACGCCGTGTTG
CCAGCATACACCGCTTTACCCATACGCCGCGTAAACAGCCAGAGACAAAGGACGAAAGACGCCGTAGACG
CTTCCCGACTTTTAAACAGCTTTTATACCATGTGGTGCTGGATGCCATACATATTGGTCATGGGTATGCT
AGTGCTCGATATCGAGGGCATAAATATTCATGCAGCACATTTGGAAAAAGCAGACAGCTGATAAGCCTGC
CACCCACCCAAGGAATAAAAATCGAGAAAAATATGTGACGAAGCAGGAGCTGAATCCTCTATATATGTGT
GTATTTTCTGTGCGCCGAGTGCGGTCTTTAGATAACTTCAGTCACATATCCTTGGCCTTGTTCCTTGCCC
CCCAATCCGTGTCCTTTCCGGAAAGCCATAAATTATGTATTCCGCACATTTTTAAGCGCAGCAATCCATC
GAATTGGCTGTATCCTAAGTTGGCCAGGAATTCACAGAAATGGGAATTACTTTTCGAATACTTCACAGAG
CCATCTATTCTTCTTTTTCCATTCCGCGAATCAATTTGTTTGCGTCGCTTCCCGGAACAAGTTTATTTAA
CATATAAATGGGCAAACATTTAAAGTGCGATTACACTGTCCAAAATTTAGCAACAGTTGCCAAGCCATAT
GTAACACAAAACTTTGCCTCATTAGAGCGACTCGCGTCTGTTCTGGGAATGATTTATGATGCCGACATTG
TCTCATGTACACCAGACCAAAAACAAACCCTTAGCCGCCCCATTTGCCTGTTCCtttttttttATTGAAA
TACTTGAGGCGGAGTACAACTTCAATAAAGTGGAAAAAGGTGCCATAAACAAGGAGCGGCGAGCCttttt
tttACGACACAACCCGCTGTGTGCGGAAGCAGATACTTGTCTAAACAAAGGCCGaaaataaataaataaa
aaaaaCCGGATGGGGAAAACAATTGTAAACTGTGAAAAAGGAAAATCCCCTTAAATGTGGCACAAATTTT
TCATTTATTTATTGAGACGAGACAAGCGGACAATGGGCAGTGAGAGATGAGGGTTAGGTGGAGTTTGGGG
AGAAGTGAAATTCACTTTCGCATTTCTCGTATACTTTTTGTTTTCGCTTGCCTTTCGCCGTTTAAATGAA
TCGGACAATTCAATTTATGCTCTGCCAGACAGACAAACTTCATTAGGACATCTTTTTGCCCCGGAACCCA
AGAATCAATAAAAGTAATTCCATGTTATATTTCTATCTGTGCGTCTGTGTCTTTGTTTTTCTATAAATGT
GAATATTAGATTTGAAAAGTGTTTAGACAACTTAATTCATGCAGTAATTTATAGAATATATTTGAACAAA
GGGCACTCGAATGGATATTGGAAAACGGCAAATATCAAAAAACTTTTAATGCGCCAAATCGATGAATGAC
CGCAAAAACTTTTCCTCCATTTTCCGTGGACACTTCCCAAGTACAAGGCAACTTTTCTAGCACTAAGCTT
TTGGCCTGTAAATGAGCTTGGGGACTGTTGTCCTGGAGGAGGAGGAGCAGAAGAAGGACCTGCATTGTTA
CCAAACTTTGTCGAGGTTAGTTCCCGGTGTCTGTATGGAAAGTTTTCGATGTATTTGCTTTGGCTTAAGT
CGGCTGACTTTGTCACTTTTGTTGTCGCCAGTTGGGAACCTCAATTTGTGATAGCCCCCACACTGGAGTT
TAATAAATATTGGCGAGCATTCAAATAGACGCCACCAGGTGTGTCATTCATTATCGCACGACGTGTCGTC
AGATTTTCTCCCCTCGGCGAAAGGACTTTAAATGAATTGCACAATCTGCAGTGGGGGTGTCGGTTTCCCG
CGTACACCAATGCTAAGGGGAATTAAGAGCCTGTCTGGCAGTAACACCACACTTTCCCGCCCACTTGAAA
CACCCAGAATATCCCATATCTTTTGCTTCATGTTGTCGCGACGCGGCAGACTGTAAATAATGAAGTTTGC
ATCTGGGTGGCAAGTGGCAAAGCAGCTGCAAAAAGTGCTTTTAGGCACGGGAACAAAAAATGCATACATT
TATTTAGATATATGCGTAATCCAACTTATATTCAACACACATATATAATCTTTTGGCGAAAGGTCAACAG
CGAAAATTTGTGAATATGGTATTCAGGCATAAAAAATGACCATTCCAAAAATGTTGAAAGCATAAAATAA
CGATTTAGGTCCCTTACGAATTATTTGCTTTCTTCTTTTGTAAATACAAATCATTAATAAATCAATAAGA
TTATGTTCTAGGTTTATTACttttttttattttatttatcatcaattttCTCACCGTGTAATAAAGCAAA
ACAAATTGCTTGTAAAAAATGTCTATTTGTGCATTAGCTCAGAATAATGTCCTTAATTCTGTGTGTAATA
TTCTGGCGATGACAGACTATTTATGGTTTCATAAATAAATCATTCATATATTTACTTTTAGCATAAAAGA
ATTGTAGAAAAATTGGAAATGCACTTGAATGAACACTACACCAAGCCACAATTCGACTATAAATACTTAA
TCATTCCCTTTCGAATTCCGTTGATGCGTTAGCTCATTAAGCCCAGGCTAAATCAAACCGAAACCAGACT
ATAACCCACACCAGACCCCGGGAAACTAATCCCCCGACTAAACCCAATTTTAATGTTCTTTTTTGGGCGC

TAAATGCCGCTGTCTAGGTAAATACGAACCAGAACGTTTTCGCCATTCAAGCTCACTGTTTCCATTTCCC
AGTTTTCCCAGTTTCGCCGTTTTCCCGGATCCCCAAACGCAAAACGCAAAACGTGATGCGCATTTTTCGC
ACTGCGGCGGCTGTATGACTGAGTCCCCTGGGTTTTGGCTTCCACTCCGGGTGGCTGTTTCGGTTTGAGG
TTACCGGGAACTACCGGAGTTTGGGATTGCCACAAAAGTTGCGCTTAAGTTTATGGCAAACGACTCCCGG
CCGTGTGGGGGTAAAACTTCACCCAGCGGAGGAGCCAAGGATCCCCTTAAAGTGATGTCAAACCGAATGC
AGATGTCATTCAGAGGAATTCGTTTGAATTAAGCGCCAAATTGTCTGCACTCTTGTACAAGCTCTCAAAA
GGTGCAAAGTAAACACTTAGGAAAAAATTCACAAATCTGAAAACATTCTACATAAATTACAATTAAAAAT
CCTTAGACTACATGTTTGAAGTTTTGCATAGATATAAGCTAATTATATAAAATGTTATTCCAATTTTACA
GCCTTAATTGCCATTTTACGGCTGTTAAGAAATAGATAACCACTTCAAACAGTAGCATTCATCACATTCC
CCTGTCAATTGGCTAACAAGCCAAAGCTAACGTGATCAGGAATTTCCTTTTATCTCGTAATTGCATTACC
ATCACTCAGCCAGCCAAATAAATTGTTTTCCTTGCAACGAAGCATCCTGAAATAATAGGAATATCCTTTA
GTCCTTTAAAAGAAACATCTAAGCGAGTAAAGTCATAAAATAAAGTAAGCATAAAATGGCAGAAAAACTA
TTTCAACTAAAGCATAAAACAAAAGCTAAAGCGGAAGCCGCAGGAACATAAAAATCACATTTATTTTTGA
CACACTTAACCATTCGAGCATTAAAAGTTTGAAAGCCATGCTCTGCATAAATGTGTGCATATATATGTAC
GTTTAGAAACATTTATATCTAGTTATggggtgggggaggggggggggAATATTCTATCGAAGCCATTATC
TTCGACAGTTATGTAAAATTGATTTCGAACATTTACTCTTGCAGAGTAAGTGTCGGTTAATATCTTTGAT
ATAAGCAGTAGATATCGGACTATATGCGATTGAAAACGTAATTGAATCGAGCAAGTGTGGCTAACCAAAA
GGAATCAAATTTTAAATGGATTTAATTATGTCTAAGAGtttttttttttttGAAATCCTAATAACCCAA
AAAAATGATAATTTGCTCAGGGGGCCTGTGTTCCTCCTTGATGTTCATGTGTAAAAATGGTGAGCAGTGT
ATACACCGGGAATTCATGTGCGATGGCGACCAGGACTGTCGCGACGGATCCGACGAATTGGAATGTGGCA
GTAAGCTAAAAAGAATGAATTATTCCATCTCACATTTGCATACTAACTGGAGGATTTGTCCATCACTAAA
ACGAGCGAAAAAGCAGAGTCACTTAATCGAAATTTAAATGCATGGAAGACAGTTGCCGGCCTCTGAGATC
TCGGGAGGACTTTATGACTTGCCAGGGACTGACCAGGACATAAATTAATGTCACCTCTTGTGGACAATAT
CGCGACGTGACCATCGGCGGAACTAATTTGTATTTATGCATTAGGCTCACTTCGGCTCACTTTTCCGTTT
TGGGTGTAGTTTCCGCTTTTTACACACTTTATTCCTCGCATTTCTCTTGCTGCCGAGCAACTTGTTTAGT
GGTTTATTGTCTGTGACACAATTTTCCAACTATATAAAGAGGAGAGTGCCTGGCGGAGAAGAGCAGGGAA
AATGTTTTTATGTCGGACGGAATTTCAGCGCTGTAATTGAAAAAATGATGCGATTTTTATGATGTTATCC
TTGATGAAAATAAAAACCGGTAATCAAAAGCCGAACGCAGCGCCAAATTGAGCCTGTGGTTTATACGCGG
GGAGTCGTGGCTCAGTGGTCGGCTACCAGGATGTACAGTGGTCGCCAAAATACTAGGGCTTCAAAATGTA
TCTAATATTTGGAGTGTTTTCATTTATATTTCTAAAAGTAAATTGCACATTTACATACATTGCAAATATA
ATTTTAGATCCTTATAACAACATTTTAAATTCAAGTATATATTGTGTATATTTCCATGTTCACAACTGTA
TGTTGTACGAGCATAAAGCTACGTCTGCTCATGTGAATGGATATGTTCGCCTTTTTATGACTTTTATGTG
CTTTAAAATTGTCATCAAACCTTGTTGACATTTTCGCTCTTACTCAGTTACCCTTGTGAATCTTAATTTA
ACTTGttttttttttCGGTAAAAAAAAGTTCAGCTCACTGCTGTGGATTTCTCGGTATAAAAGTGCTATT
TTCAGTAATGGTGCCCGCCCCGTTTATTTAATTCCATTTAACACTCCACTCGGACTCTTCATATCTTTGT
TTACCATTATGGGAAAAGCTCGGCTGGCCATCTCACAATGCGACCAAGGGTGGATAGTTGAATAATAAAC
AAAACGCAAACTGCCCCACAAAGGCATTAAGCTTCATTGGCTTTCAAGTGCCCGAGAGTTGGAGGCGGAG
ACGGGCGGCGTGGCCACTGCACTCGACCAGGACATGTCTTGTACATGGTCTAAAAAGCATTTTAAATAAA
ATTAAAATCAAATTGCAAGTATATGGGTTTTGGCGTCAGATAACAAGAAAATGCTATTGAATACAATGCC
AATGTTCTGCCAGGATGTGTGTGCGTTGAGGAAGGAGAGCCAGAGCCGAGCATCCTCGGCGGTCCATTGA
CTGTGACCCCACGATAGCAAATTGATTCTGCCAGGACTCACAAAAGCCGAAGCCGAGATGGAAGCTGTGT
CCCTATGGACACACAAGAATGAAACGTCCATTCAGCCAAGTAGTTACCTTCGAATGGATAAAATGGAAAG
TTATATGTATATATACATACATATAGTTAGTTATATGGAAGGACCCCAGGCGAATATTTTCCTCACCAGC
GATGGAGAATTTAATGAAGCCATAAATCATGGTCGGCGAAATGATATTGGATAATTGAATTTATATCAGG

TGCATGGTGTGCGGAGCAAACGAAATAATCGAAAAGTATTTTCAACTGAATATGAACGATGCTTAACAGA
AAATGATTTTAATGCTGGAGCTGCCATTTAATTATGAAAAATGTAAATGAAAGTCAACTGATCCAATAAT
TTTGAGGAAACCATATTCCCTGAAATGTAATTCACTGCAGTGCACTGGACACCCAATGGAGCAATTATAA
AATAAAGCTAGAACTGCTACTATAGATTTAATGGACATTTATGAAAGATATTCAAATATAGGTGTAGCGT
ATAAATAAACCAATTAATTGATTAAATTTACTAGACAAATTGCTTTGAACTTATACTTACAATTTAAATT
GCATTTTCGGGGCTGAAAAAGCGTCATTTTATTTATTTATAAAATAGCTAAATATAAAAATAAGTTACCT
AATATTTTGTTAAATATTTCCGAAGCATCCTAGCAATTTCAGTAAATATTCAGCAGGCGAACGACGAATA
ATTTCTGTAAGCTGGTCACAGTAAATTCCAATTACAGGCATATCACGCATACGACCCGTTGTACATGCTA
AGCAACCGCCAAAATGGCGAATACCAAGAAGAGAATAATAATAAATTAATTTATTTGCAACGATGTCAGA
AATTTCCGCCGTACGCCGTGCACGTGTAAACGGTAAACAAATAGACGGAATGAAGCAAGTATGAAAAACA
GAGGAGAGGAAAGGCAGGCGGAGATAGACAAAACGCCAAAAAGCATATTCAAATGTGTGGCTCAAGCATA
AGATTTTATTGCTAGcacgcacacacacacacacacacaGAGAGTCACCGCGGCAATGTATGTATGTACA
TAATCATATCAGGGAAAATCACTCCCACACGCACTCACGGCGTGAAACAAAGGGCCGTGTGGTAGGCAAT
GAAATGGAGAAAGAAAGCGAAAGAAAGCCCAAAAGCGAGGCGCCAAGAACTGGCATACTAATACGCATTA
AGGGTTAAACACAAGCGTTTTAATGCGCCAGTGTGTGTGCgtgtctgtgagtctatgtgtctatgtgtct
atgtgtggttgtgttgtcagctttgtttaatgtccGTTCCTTTGGCAAGGATAACTTCAATGGAACTTCC
CGAGTGTGAGTGTGTGCGATAATTGCTTTAATGCATACGCATGTGTGCGTTTTAACTTTATGGAGTGTTG
GCTGATAATGTCTTAATTACTGCCTAGATAAAATGCCAACTGTTTAGCCCCCGAATGGTTGTTGGTTGTG
CCAgtgtggtgtgtgtgtgtgtgtTGGCATGACAACTATGTTTATTACTTGGAGAACGGTTGCTGTCCCA
CTCCCTCTTATATGGCAGCTGCTTATAAACTTAATTACAGGACATCCCTCGCTTGCCTTTAAGGATCGTT
TCCTATCTCATCAAGACCAAGTCCCATTAGCTAGGCAGTTAGTGGTTTTTTTCCGGCACCCTTTGTGATT
ATAGCCTTGAAGTAGGTGCTCGGGATTTTATTAAATTTTTTACCCGTTGTGGGTAATATTTGCAAAACAA
GACTCTATATACCATTATTTGATATATTTAATAAAACAAAAAGGATTTTTCAAAATGCAATCGAAAATTT
TCTTCTTCTAGGAATTACTGAATTATGAACAAAGCACACTATAGATTGCCTCTCGATGTATCTTTAAATT
ACGTATTTCCATGATCTTGATCTTTTTAGGAAGCCACACATGTTCGCCCGAGGAGTTTGCCTGCAAGTCG
GGCGAGGGCGAGTGCATTCCCTTGTCCTGGATGTGCGATCAGAACAAAGATTGTCGCGATGGAAGCGATG
AGGCGCAGTGCAG
>dme_circ_0007609_junction_seq
GCGATGGAAGCGATGAGGCGCAGTGCAGCAGCTGCAAAAGATGGCCACAACCTGCA
>dme_circ_0007610
AATCAACTTGCAGCCAGGAACAATTCCGCTGCGGCAACGGAAAGTGCATTCCGCGAAGATGGGTCTGCGA
TCGGGAAAACGACTGCGCCGATGGAAGCGATGAATCGACGTCCCAGTGCCGTAAGTGTCGGTTAATATCT
TTGATATAAGCAGTAGATATCGGACTATATGCGATTGAAAACGTAATTGAATCGAGCAAGTGTGGCTAAC
CAAAAGGAATCAAATTTTAAATGGATTTAATTATGTCTAAGAGttttttttttttttGAAATCCTAATAA
CCCAAAAAAATGATAATTTGCTCAGGGGGCCTGTGTTCCTCCTTGATGTTCATGTGTAAAAATGGTGAGC
AGTGTATACACCGGGAATTCATGTGCGATGGCGACCAGGACTGTCGCGACGGATCCGACGAATTGGAATG
TGGCAG
>dme_circ_0007610_junction_seq
ACGGATCCGACGAATTGGAATGTGGCAGAATCAACTTGCAGCCAGGAACAATTCCG
>dme_circ_0007611
CAGCTGCAAAAGATGGCCACAACCTGCATGCCGCATTTCACGGAATATTTTCAAACCACCACCCAGTCAG
AGTTAACAACAAATGAAAGTGCCAAcagcagaagcagcagcagcagcagTGGCTGTAGAGAACCGTCCAA
AACCAGTGGCTTCTTCTACAGCGCCAGCACACATTACCAGAGCAAAGTGCAGCAGCTAATCAGCTTCAGC
TTCACCTCATCGTTCCACCTGCTCTTCATCACCTGCATACTGAGCATCAGCAAATTCTGCAATGCGGCGG

CGGTAAATGCCGCCCATGTGACCGCTGCCACGCCCACTGCCAGCGAGATAATGCACAATCTGAGCGGATC
GGCGCTGCTAGAGCGTCTGAAAGCCAGTACTCTGGGACCCACCCTCGATCAGCTGAAGCATCTGGCTCCT
GCGGCAGCCAACAGCATTAGTCCTGGTACAGGATCTGGCATGGGAATCGGGATGGGACACTTTGGCAGTT
CCATTAATTCGGTGCTAAACGGAAATCCTTTAAAGTTCTTGAATGTGTCAGGAAAAATCGGCACTCCTTT
GGACATATG
>dme_circ_0007611_junction_seq
GAAAAATCGGCACTCCTTTGGACATATGCAGCTGCAAAAGATGGCCACAACCTGCA
>dme_circ_0007612
ACCAGACCTGTCGTGCCGACGAGTTCACCTGCGGCAATGGACGGTGCATCCAGAAGCGATGGAAGTGCGA
CCACGACGACGACTGCGGCGATGGATCCGACGAGAAGGAGTGCCCAGTGGTGCCCTGCGACTCCGTGGCG
GAGCACACCTGCACGAATGGAGCCTGCATCGCCAAGCGGTGGGTCTGCGACGGTGATCCGGACTGCTCCG
ATGGCTCCGATGAGCGGGGTGAGTGAATAATGCCAGCCATAAATTTCCATTCCTTGCTAGTCCGCAGAGA
AACTTACCCTTATCCTTACTCAAACCGAGACCCAGACACAAAACCCTGCCCCTGCCTTTGAAACATCCTT
TCGTTTTTTGGTGCTCGCCTAAGCCACATGAATTATGGCCATGTTGGGCATAACTCAGCGCCCTGCCAGC
TTTGGCAGCTTTTGTTGTCAACGCATGGCACGCATAAAATTGCATTTTTGCATGCTCAGGGGGTGCATAA
TTTCCCCTGTGTGCGGCCATTTccccccccccccccccTTGCATACTTTTCCGCAGGAGAACTTGCCAAA
CTGAGGGCCGGAAACTTTGCCTGGTTCCGCAGCCTAACCCTCGTTTTATTTTGCACCTTTCCCACAAAAG
GAAAATGTCAGCAATTTAAGCTATTTAAGTTGAAGTGCATCCTGGACTCCTCAGCAGTATTGTTCGAATA
CTTTTGCGCAAATTACTCTTGAATAAAATAAATTAAAGTGATATCAGCACATTCAATAACTTACACGAAG
AAGTATTTAAATTAATGGAATGTTTCATATTGAACAACACTTATAAAAACTTCTCGTTTCTTTTTATTTC
AGTTCCTGTGCGAATGTGACCAAGACGACCACGCCCTGCTTATCGCACGAATACCAGTGCAAGGATCGCA
TCACCTGCCTGCATCACAGCTGGCTCTGCGATGGTGACCGCGACTGTCCCGATGGCGACGATGAGCACAC
AGCCAACTGCAAGAACGTCACCTGCCGGGCGGACCAGTTCCAGTGCGGCGATCGCAGCTGCATTCCAGGA
CATCTCACCTGCAACGGCGACAAGGACTGCGCCGATGGCAGCGACGAACGGGATTGCGGATTGAGTCTGA
GTCTGGGGATCAACCAAGGAGGATGCAACGCAACCAGCGAATTCGACTGCGGTGGAGGTCAGTGCGTTCC
ACTCTCGAAGGTCTGCGACAAGAGGAAGGATTGTCCGGATGGCGAGGACGAGCCGGCAGGAAAGTGCGGG
ATAAACGAGTGCGCCTCGAAGAACGGAGGCTGCATGCACCAGTGCATCGATCTGAAGGTGGGCCACCACT
GCGAATGCCACGAGGGTTACAAGTTGTCTCCGGACAAGCGGAACTGCCAGGATATCAATGAGTGCGAAGT
TCCCGGAAAGTGCTCTCAGATATGCGTGAACGAAATAGGAGGCTTCAAGTGTGAGTGCGAAGCGGGCTAC
ATGAGGGATCCCAAAAATCACACCAGATGCAAGGCCAGCGAGGGACACGCCTCCCTGCTCCTGGCCAGAC
GCCATGACATCCGCAAGATAGCACTCGACCACATGGAAATGACCTCCATTGTGAATAGTACAAAGGCAGC
CACTGCCCTGGACTTTGTGTTCCGCACGGGTATGATCTTCTGGAGCGATGTGACCACCCAGAGTATATAT
AAAGCACCGATTGATGAGGGAAACGAAAAGACGGTAGTGTTGACTAAATCCTCGGTGACCTCGGATGGCC
TGGCTGTCGATTGGATCTATAACCATGTCTACTTTACGGACACCCACAAGTGCACCATCGAACTGACCAA
CTTCGAGGGCAGCATGGGTAAGGTCCTGGTAAAGGACTCCCTGGACATTCCTCGTTCAATTGCTTTGGAT
CCCATTGAGGGCTGGATGTACTGGTCCGACTGGGGCGCCTCTCCGCGCATCGAAAGGGCGGGTATGGATG
GCTCCCACCGCACCACCATCATCAGCTATGACGTCAAGTGGCCCAATGGTATCACCTTAGACCTGGTTAA
GAAACGCATCTACTGGGTAGATGGCAAGCTTAACGTTATCTCGTCGGCGAACTATGATGGCTCGCAAAGG
AGCCAGGTGCTCTACTCCGGCGAATACCTGCGACATCCCTTCTCGATCACCACCTTCGAGGACAATGTCT
ACTGGACCGACTGGGACAAACAGGCCGTCTTCAAGGCGAACAAGTTCACCGGGGAGGATGTGGAGCCCGT
TACAGCAATGCATATGGCTCCAGCATCCAATGGTGGTGCACGTGTACCATCCTTACCGCCAGCCGGATGG
CGTGAATCACTGCCAGTCGGTGAATGGCCACTGTTCTCATCTCTGCCTGCCAGCTCCCAGGATCAATGAG
AGGAGTCCTCGCATATCCTGCGCCTGTCCCACCGGTCTGAAGCTGATGGTCGATGGCCTCATGTGCGTCG
AGGATCGGTAAGTACTCGAGAACTTTTAGTAACTCTTTAGCCATTTTGCTTTTAACTTAACCAGCATGGC

TTTGATTATGATTTTATGCTTACTAATTAACCCCCAAACCCGATTACCCAATCCGGCATGACTTGCATCC
GCATGTAGCACTTTATTTGCCTCCAGTCACGAGACCCCCGCGAGCCCGCAAAACGAAACCGAGGCCAAAA
TCCCCGAGGAGGCGGCTGCCCACGGGTGTCCAGGTGGGACATGCTGACATTCGGATTGAAATTAACGATG
ATCTCCAGCTGAGCACCAGGCTACCCCTGTTGCCCACGACTTTCGG
>dme_circ_0007612_junction_seq
GGCTACCCCTGTTGCCCACGACTTTCGGACCAGACCTGTCGTGCCGACGAGTTCAC
>dme_circ_0007613
TCCTGTGCGAATGTGACCAAGACGACCACGCCCTGCTTATCGCACGAATACCAGTGCAAGGATCGCATCA
CCTGCCTGCATCACAGCTGGCTCTGCGATGGTGACCGCGACTGTCCCGATGGCGACGATGAGCACACAGC
CAACTGCAAGAACGTCACCTGCCGGGCGGACCAGTTCCAGTGCGGCGATCGCAGCTGCATTCCAGGACAT
CTCACCTGCAACGGCGACAAGGACTGCGCCGATGGCAGCGACGAACGGGATTGCGGATTGAGTCTGAGTC
TGGGGATCAACCAAGGAGGATGCAACGCAACCAGCGAATTCGACTGCGGTGGAGGTCAGTGCGTTCCACT
CTCGAAGGTCTGCGACAAGAGGAAGGATTGTCCGGATGGCGAGGACGAGCCGGCAGGAAAGTGCGGGATA
AACGAGTGCGCCTCGAAGAACGGAGGCTGCATGCACCAGTGCATCGATCTGAAGGTGGGCCACCACTGCG
AATGCCACGAGGGTTACAAGTTGTCTCCGGACAAGCGGAACTGCCAGGATATCAATGAGTGCGAAGTTCC
CGGAAAGTGCTCTCAGATATGCGTGAACGAAATAGGAGGCTTCAAGTGTGAGTGCGAAGCGGGCTACATG
AGGGATCCCAAAAATCACACCAGATGCAAGGCCAGCGAGGGACACGCCTCCCTGCTCCTGGCCAGACGCC
ATGACATCCGCAAGATAGCACTCGACCACATGGAAATGACCTCCATTGTGAATAGTACAAAGGCAGCCAC
TGCCCTGGACTTTGTGTTCCGCACGGGTATGATCTTCTGGAGCGATGTGACCACCCAGAGTATATATAAA
GCACCGATTGATGAGGGAAACGAAAAGACGGTAGTGTTGACTAAATCCTCGGTGACCTCGGATGGCCTGG
CTGTCGATTGGATCTATAACCATGTCTACTTTACGGACACCCACAAGTGCACCATCGAACTGACCAACTT
CGAGGGCAGCATGGGTAAGGTCCTGGTAAAGGACTCCCTGGACATTCCTCGTTCAATTGCTTTGGATCCC
ATTGAGGGCTGGATGTACTGGTCCGACTGGGGCGCCTCTCCGCGCATCGAAAGGGCGGGTATGGATGGCT
CCCACCGCACCACCATCATCAGCTATGACGTCAAGTGGCCCAATGGTATCACCTTAGACCTGGTTAAGAA
ACGCATCTACTGGGTAGATGGCAAGCTTAACGTTATCTCGTCGGCGAACTATGATGGCTCGCAAAGGAGC
CAGGTGCTCTACTCCGGCGAATACCTGCGACATCCCTTCTCGATCACCACCTTCGAGGACAATGTCTACT
GGACCGACTGGGACAAACAGGCCGTCTTCAAGGCGAACAAGTTCACCGGGGAGGATGTGGAGCCCGTTAC
AGCAATGCATATGGCTCCAGCATCCAATGGTGGTGCACGTGTACCATCCTTACCGCCAGCCGGATGGCGT
GAATCACTGCCAGTCGGTGAATGGCCACTGTTCTCATCTCTGCCTGCCAGCTCCCAGGATCAATGAGAGG
AGTCCTCGCATATCCTGCGCCTGTCCCACCGGTCTGAAGCTGATGGTCGATGGCCTCATGTGCGTCGAGG
ATCG
>dme_circ_0007613_junction_seq
TCGATGGCCTCATGTGCGTCGAGGATCGTCCTGTGCGAATGTGACCAAGACGACCA
>dme_circ_0007614
ACCAGACCTGTCGTGCCGACGAGTTCACCTGCGGCAATGGACGGTGCATCCAGAAGCGATGGAAGTGCGA
CCACGACGACGACTGCGGCGATGGATCCGACGAGAAGGAGTGCCCAGTGGTGCCCTGCGACTCCGTGGCG
GAGCACACCTGCACGAATGGAGCCTGCATCGCCAAGCGGTGGGTCTGCGACGGTGATCCGGACTGCTCCG
ATGGCTCCGATGAGCGGGGTGAGTGAATAATGCCAGCCATAAATTTCCATTCCTTGCTAGTCCGCAGAGA
AACTTACCCTTATCCTTACTCAAACCGAGACCCAGACACAAAACCCTGCCCCTGCCTTTGAAACATCCTT
TCGTTTTTTGGTGCTCGCCTAAGCCACATGAATTATGGCCATGTTGGGCATAACTCAGCGCCCTGCCAGC
TTTGGCAGCTTTTGTTGTCAACGCATGGCACGCATAAAATTGCATTTTTGCATGCTCAGGGGGTGCATAA
TTTCCCCTGTGTGCGGCCATTTccccccccccccccccTTGCATACTTTTCCGCAGGAGAACTTGCCAAA
CTGAGGGCCGGAAACTTTGCCTGGTTCCGCAGCCTAACCCTCGTTTTATTTTGCACCTTTCCCACAAAAG
GAAAATGTCAGCAATTTAAGCTATTTAAGTTGAAGTGCATCCTGGACTCCTCAGCAGTATTGTTCGAATA

CTTTTGCGCAAATTACTCTTGAATAAAATAAATTAAAGTGATATCAGCACATTCAATAACTTACACGAAG
AAGTATTTAAATTAATGGAATGTTTCATATTGAACAACACTTATAAAAACTTCTCGTTTCTTTTTATTTC
AGTTCCTGTGCGAATGTGACCAAGACGACCACGCCCTGCTTATCGCACGAATACCAGTGCAAGGATCGCA
TCACCTGCCTGCATCACAGCTGGCTCTGCGATGGTGACCGCGACTGTCCCGATGGCGACGATGAGCACAC
AGCCAACTGCAAGAACGTCACCTGCCGGGCGGACCAGTTCCAGTGCGGCGATCGCAGCTGCATTCCAGGA
CATCTCACCTGCAACGGCGACAAGGACTGCGCCGATGGCAGCGACGAACGGGATTGCGGATTGAGTCTGA
GTCTGGGGATCAACCAAGGAGGATGCAACGCAACCAGCGAATTCGACTGCGGTGGAGGTCAGTGCGTTCC
ACTCTCGAAGGTCTGCGACAAGAGGAAGGATTGTCCGGATGGCGAGGACGAGCCGGCAGGAAAGTGCGGG
ATAAACGAGTGCGCCTCGAAGAACGGAGGCTGCATGCACCAGTGCATCGATCTGAAGGTGGGCCACCACT
GCGAATGCCACGAGGGTTACAAGTTGTCTCCGGACAAGCGGAACTGCCAGGATATCAATGAGTGCGAAGT
TCCCGGAAAGTGCTCTCAGATATGCGTGAACGAAATAGGAGGCTTCAAGTGTGAGTGCGAAGCGGGCTAC
ATGAGGGATCCCAAAAATCACACCAGATGCAAGGCCAGCGAGGGACACGCCTCCCTGCTCCTGGCCAGAC
GCCATGACATCCGCAAGATAGCACTCGACCACATGGAAATGACCTCCATTGTGAATAGTACAAAGGCAGC
CACTGCCCTGGACTTTGTGTTCCGCACGGGTATGATCTTCTGGAGCGATGTGACCACCCAGAGTATATAT
AAAGCACCGATTGATGAGGGAAACGAAAAGACGGTAGTGTTGACTAAATCCTCGGTGACCTCGGATGGCC
TGGCTGTCGATTGGATCTATAACCATGTCTACTTTACGGACACCCACAAGTGCACCATCGAACTGACCAA
CTTCGAGGGCAGCATGGGTAAGGTCCTGGTAAAGGACTCCCTGGACATTCCTCGTTCAATTGCTTTGGAT
CCCATTGAGGGCTGGATGTACTGGTCCGACTGGGGCGCCTCTCCGCGCATCGAAAGGGCGGGTATGGATG
GCTCCCACCGCACCACCATCATCAGCTATGACGTCAAGTGGCCCAATGGTATCACCTTAGACCTGGTTAA
GAAACGCATCTACTGGGTAGATGGCAAGCTTAACGTTATCTCGTCGGCGAACTATGATGGCTCGCAAAGG
AGCCAGGTGCTCTACTCCGGCGAATACCTGCGACATCCCTTCTCGATCACCACCTTCGAGGACAATGTCT
ACTGGACCGACTGGGACAAACAGGCCGTCTTCAAGGCGAACAAGTTCACCGGGGAGGATGTGGAGCCCGT
TACAGCAATGCATATGGCTCCAGCATCCAATGGTGGTGCACGTGTACCATCCTTACCGCCAGCCGGATGG
CGTGAATCACTGCCAGTCGGTGAATGGCCACTGTTCTCATCTCTGCCTGCCAGCTCCCAGGATCAATGAG
AGGAGTCCTCGCATATCCTGCGCCTGTCCCACCGGTCTGAAGCTGATGGTCGATGGCCTCATGTGCGTCG
AGGATCG
>dme_circ_0007614_junction_seq
TCGATGGCCTCATGTGCGTCGAGGATCGACCAGACCTGTCGTGCCGACGAGTTCAC
>dme_circ_0007615
CTCCTCAAGGAAAGCACATTGTGAAAGTCGCGGGAAAAAACTACGGAAAATGGGGCGCATTTGGTGTTTA
CTCTTCGTTAGCAGTTTGCTGCACACTCAGAGCCAAAGTTTTCGCGTTCTGGCTATAAATGGTATGAAAA
TTATGTTGATTATTTCGTTTGGTCCGAATTTAATGGTCAAACATAATATTTGCGTTGACACTATTATAAA
TTAAGTGAACTGTTTATTTATGGACATTAAATAACTAAAATACCTAAAATAATCGCAGAAATATACGCCA
TACTAGAGTTATTTAAATAGAAACTATACGCATAGACGAAACATAATTTATGCATATCATGCATAATTCC
AGCTTATTTGCCAAAATGAGTAGTGCTAAATAAATAAACTAAGAGCTCGATGGATTTTAAAATCCAAACA
ATTTAATTTCATATATTCCAGTATATGTACTGTCACTACGACTAAATATTAAGTACTCTTTAAAAGTCTG
TAGTGTTGCTATCATGTCCTTTTATCAGCCTTCTTTAAGAGACCCAAAACGAAACTGTTGAAAAGCTGAA
GAACACTCGGTTTAACTAGTCCAAGTTCGGCGGGAAACTTTTAACTATTATCAGGTGGAAAACTTTTCGA
CACCTTTTAACTTAAATTAAACACACCAAAATCCCAATCATCTGGATTTACAGAAATCCCGAGTTCTCCT
CTGCAGttttttttGCCAGTTTTCCGCATTTCCCGGAGCCCGTATCCTTTATACAGGATTCCGGCTATGC
CTCGCATGCAACAGAATATTTTTGCAAGCGGCGCTTAACATTTGCATACAAATTGGCTGAGAGCAGAGAG
ACTGAGCGGGAAAATACACGGAAACATCAACTGCCAAAGGCCAAAGTTTTTTAATCAACTGAAAAGCCGT
TTAAACTTAGTTAAAAAATAAATTATGCCGCAAAATGCTGCATGACAGCGCAACTTTCCTATTTCTTCCA
ttttttttatttatgccaattttgtcttttttttggggggggggATGGCCTGTTAAAATGCAATCAATGA

AGGATTCGCCCTGTCATGTCAGACATGATGCAAAAAGTGCTGTAATTTGATTTTTAATTGGAGACCGAGG
CGACATCCCAGTTTAATATGAGCGGACCAGTAGATATTACCAATTTACCCGCTGGGCACAATTCCCAGAA
AATAATGTCACATATTAGCAAAAACGAAACGCAAAGAATAACAACATAAAACCGAATCGACAATCATTGA
ACCAAAAGTGGGCGTGGGGAAAATGTTTTTCACTCCCCTGCACTTGTTTCCTTTCATTCGATTTTATACT
TTTTATTCTCGCTCGGGTCACACATGTGGCCAATTTTTATTGTTCACTGAATGTGTAtttatatttgcct
tttttgttttgccatttttttatttttatttttttttttttttAACAAATATTGGCGCACATCAAAGTTTTGTT
TTGCGCCAGTTGCCTGCGCACACAAACAATTTTTTTGTTGCCACATTTCGTTGGAATTCATAAAAAGTTT
AGAAAAGTAATCATATAAAATGTTATTGATATGGCCAACACAGGCCTGACAAGCATAAAAATTGATTAAA
CTTTACAGGCCCAAATAAACGCATTTCCTTTGGTGCTGGCCAAGATTTGCATAATCAACAGCAATTCTCA
AAGGCCACAAGGCATTTTCCAATCATTTACACCAAAAATATTCCCAATTCCATTTTATAGTGATCGTAAA
AATAGCCCAAAAGTGCCCGAAGAATTTGTTAAATGCCAGGACTTTCTCCTTCCAAGAAAAAGTTTTGTGG
CAAGGAAAATTTCTAATTGCCATGGCTGTTTGGCGAAAGTATGCAATAGAATTTTGTGACTGCAGAGCGG
TGGAAAAAAATCGCTGCGTTTCATAGCAACAAGTCAGCGGAATCTAAAACCGAATAATCCGATTACAAGCC
ATACACGTACTCCatatatatgtatatatatatatatatatatatatgtttatacatatatgtatctgtg
tacaCGCGCGGTGTAAAATAAAAATGACACATTTTTGCTGCCCCTTGGAGTGGCATTGAAAAGCCATTGC
AGCGCTCTTTTTGTTTGTGCTTGTGGGGTATTTTCCCTGCTCAGCACGTGCTCAAGTGCGTGTAAATAAA
TAtgtgtgtggcagtgtgtgtgtTTATAAACAGGGCGTGTGGGCGTGGCCACACATACATACATATACTC
ACAAACACGCGCATACTTCTGAGTGGCCCCCATTTCCTCTGCTGTTTGTTTGGCTTTTGTTTTTGATACG
GGTCGCCTGCAGAGTTCAAAGCCAGGCAAACATACAACTGCGGTCGAAGTAAAACATACTTCTCGCAACA
AAAATTCTTCAAATTTCAAATGTTTTTGTGTGTCAATTATGCCTATTTAAATAGGGCTATTCAAAGTCAA
TCAAGTCAAACCGCCATTTGACTTTATATGGTTAATTGCTGAATTAAAACAAAAAGGCGCTTTTGCCAGC
TCTGTCAGAGTTTGAACAGTTAATTTTTGCAAAGTTTTTTGTTTAATATCCTACTAAACCTACTTTTAGA
AAATATGTATATGTAGAAAAAGCTCTGCCAACTCAGCTTAAGTTTAAGTTTGGTTTTAATCCACTCACCA
ACTACTTACTAAACCAAATTAAGTTAGTTTTACATTGATGAGCAGTATATAAAGTACCTCAAAATAAATC
ACTTTAGTCTATTTATTTCAACTGCAACATCAGAGTAACCATGATTTAATCGGATTTTTCTGCTTTTCGC
AGAGTAAGTATTGCGCCGCTACCTGGCGATGAAGTCCTTTGAATCCACGTATACGGATACGGGGAATCCC
GTGGAGAATTTCGCCAGTCATTTAGCTAACCGACGACTATTTCGATTGTGTGTGTTTAGTGAACGCCTGT
CCCAACAACGAGTTCAAATGCCAAACGGTCGACCAGTGCATTCCGCGCAGCTGGCTCTGCGATGGCAGCA
ACGATTGCCGCGACAAGTCCGATGAGGCGCACTGCAG
>dme_circ_0007615_junction_seq
GCGACAAGTCCGATGAGGCGCACTGCAGCTCCTCAAGGAAAGCACATTGTGAAAGT
>dme_circ_0007616
GCCAATCCCTGGAGGCCAAGTGCGATGAGAAGCAGTTCCAGTGCCACAGCGGCGACTGCATACCCATTCG
ATTCGTTTGCGACGGCGATGCGGACTGCAAGGATCACAGCGATGAGCAGATTAAGGAGTGCAAGTTTATA
GG
>dme_circ_0007616_junction_seq
AGCAGATTAAGGAGTGCAAGTTTATAGGGCCAATCCCTGGAGGCCAAGTGCGATGA
>dme_circ_0007617
GCGCTTTTGAAACGGACTCCAGCTGGGTAACTCGTGTGGCAAACTGAACCGGAACCGGCAGAGGCAGCGG
CAGCAGGAGCAGGAGGAGGACCTGGACCAGCGGAACTGGCAACCACCTGGCCAAATGGGTGGCAATAGCA
CAGGAGCGAATACGAGCGCCTTCAGCGCTGGCGGTTACATTGGGCCCACGTCGGCCAGCAGTCATCACAG
CCTGGGAACTTCATCGGCGGCAGCGGCAGCAGCAGCAGGAAGTGACCTGATACCCGCACCAATTGGCACG
GGCAACGCCATGGGAGTGTCTTCGTATGCATACGGTGGAACCAGTTGGGAGGAGTTCTGCGAACGACACG
CCAGAGTGGCTGCCTCGGATTTCGCCAAGGCGTGCATCACGTACATTAATGGCAATCTGCCGCCGGAGGA

GGCGAGGAACATCCAGCATCGCAGCTTTGCTCAGAAATTTGTGGAATCCTTTTCGGCGCACTACGACACG
GAGTTCTTCAAGCGGAGGAGCACTCTTAAATCAGGTGCGGGCTCGCTGGACTTCGAGGAGGAGCACGAGG
TGCCAAAACTGCTCTCAAAGTCTCTATTAAGACGACTATCATTCAAAGGACTGCGCAAGGGCAAG
>dme_circ_0007617_junction_seq
ATCATTCAAAGGACTGCGCAAGGGCAAGGCGCTTTTGAAACGGACTCCAGCTGGGT
>dme_circ_0007618
TACAAGTCGACACTTCTCCCACTTTTGGGAGCCCGTCGGTTGATCCAGACTTTCCGGCGTCAGGTTGTTC
ACTGTGCCCTCCTTCCGGCACTCCACAACGATCTTGGCCAGCTTCGTCTTGCTCTGCTTGCCGCTGCCAC
CGCTGCCATCCAAG
>dme_circ_0007618_junction_seq
CTTGCCGCTGCCACCGCTGCCATCCAAGTACAAGTCGACACTTCTCCCACTTTTGG
>dme_circ_0007619
GTTGGCAGTGGCCAAGGATCAATTGGTTCCCTTGCTCGTGGTGCAATCCATGAGCTCATTTCCTGGAATT
GCCGGCATGTTTGTGGCCGGAGTGTTTAGTGCCGCCCTCAGTTCTTTGTCCACGGGTATGAATTCCCTAT
CTGCTGTTTTCCTGGAGGATTACATTAAGCCCCTTGCGAAGAAACCCCTTACGGAACATCAGACCGGTGT
CACCGTGCGACTTTGTACGGTAATAATTGGCATTATCAGTGTGGCCCTGGTCTTTGTGGTCGAGCGGATG
AGCTCCCATGTGTTGCAACTCGGCATCGCCATTGGTTCAGTGGTCCAAGGTCCGCTTCTTGGTCTGTTTT
CGATGGGTCTGCTGTGCCCAAGCATCAACTCAAAAGTGAGTTTTCCCTCCTCCTTCAAAGTTGTTAGGAT
GAAAGTTAACAAATAATCATTTTGTTTAAGAGCGCAATCACGGGATCAATTTTTGGCTTCTTCTTCATGG
GTTGGCTTAGTATTTCGGCCCAAATTGCCATTAACTCTGGGGAAATTCATCCCGAGGAAAAGCCAGTCAG
CGTGGAAGGCTGCGATTATGAGTTCGACCGTTCTCTGCTGACATCTGCAAATGCCACTCTTCATAGTGGA
GAATCCGCGTAAGTTTGCGATATCCTTAATCGCCAAAATCTTTTCTATTCGGTTCTCATCATAGAAGCCT
AATCCCACAGGTCCCTTAGTTTCGCCGAGCAGTTATACCACATATCCTTCATGTTTTATACTGTAATGGG
GGGAATATTGAGTGTGATTGTGGGCCATTTAGCCGGCTTCATATTTGGTCGCAATAAGCCCGGCGACGTG
GAAGACGATTTGCTATCACCCTGCATACGCAGATTCCACAAGTCGAAATATTCCAGCGTAAATTTAAATG
AGAACTCCTTAAGGAGTTCTGATGAGAAACCATAGAGCATTCTTTGACTAACCTATGTTTAATTTACTAA
TCCATTCAATTTTCTTATATTTCGATTCCGTTTCACTCTAAAAACTTAAGTTTTAAAAGGTACTTAAATG
GTTAAAAAGAAATACTTAATTGCCACTTAATTTCTTGACTTACCCATAAGATTGATCAAATGTAAATACC
AGACCTTTCGTTTAGGAAAGTTAGCCATTCAATTACGTTCTTAAAAATAAAGCTTCAATGACTTACACTA
CCATAGCCTAAGTATTTTGGTTTGAAGTTAATTATTTTTGTCTTTGGGTTGCTTTAAGTTTTATGGGTTC
TTGACAAGTCATATATGTAAATGTTAAAAGTTCGTTGCAACTGCATTCTTTATTGTTTTGAATATAGTTT
AAGCCCACTTTATACCCAGAACATAGAGGCTCCGTCAAATCGAGCGCCACGTGCTGACTTTTGTGCACTT
TTCCAGTAACTGGAATAAATATACATGTCATCACGCCAATCGTGTGCCTCGTCTGCATATTCTACACAGC
AGCCGGCGGACTGAAGGCCGTTGTCTGGACAGATGTGATCCAAACCGTGATTATGGTGGGAGCCATTTTC
TTCGTCATCATCAAGGGCACCGTCGATTTGGGCGGATTGGGCGTGGTTATTCAGCGGAATTTCGACACCG
GACGCATGGAGTGGCCTGAGTGGGTGAAAACTGTTTACTAGGGCAGGTACCACACATGGCATGTAATCCT
GCCAGGATGTGTGGTGCATGGGCTCCGCTCGCTGGTCTATATGGTATAATATGGTACATTTTCGAAACAG
ATTGACGCTGGATCCCAAGGTGCGAATGTCCATGTTGGCAATGATGGTGGGCAACGTGGCACACAACTCG
TATAACCTCGGATGCAATCAGATCATCACCCAGCGATACCTGTCACTGCCCGGATTAAAGCAGATGGCCC
AGTGCTCGTTCCTCTTCATTTCCGGATTGGTTCTTCTGATGGCCATCTGCATGTACAACGGCCTCCTGCT
ATCCGCAACCTACTACGACTGTGATCCATTGACCACGAAG
>dme_circ_0007619_junction_seq
CTACGACTGTGATCCATTGACCACGAAGGTTGGCAGTGGCCAAGGATCAATTGGTT
>dme_circ_0007620

GAGCGCAATCACGGGATCAATTTTTGGCTTCTTCTTCATGGGTTGGCTTAGTATTTCGGCCCAAATTGCC
ATTAACTCTGGGGAAATTCATCCCGAGGAAAAGCCAGTCAGCGTGGAAGGCTGCGATTATGAGTTCGACC
GTTCTCTGCTGACATCTGCAAATGCCACTCTTCATAGTGGAGAATCCGCGTAAGTTTGCGATATCCTTAA
TCGCCAAAATCTTTTCTATTCGGTTCTCATCATAGAAGCCTAATCCCACAGGTCCCTTAGTTTCGCCGAG
CAGTTATACCACATATCCTTCATGTTTTATACTGTAATGGGGGGAATATTGAGTGTGATTGTGGGCCATT
TAGCCGGCTTCATATTTGGTCGCAATAAGCCCGGCGACGTGGAAGACGATTTGCTATCACCCTGCATACG
CAGATTCCACAAGTCGAAATATTCCAGCGTAAATTTAAATGAGAACTCCTTAAGGAGTTCTGATGAGAAA
CCATAGAGCATTCTTTGACTAACCTATGTTTAATTTACTAATCCATTCAATTTTCTTATATTTCGATTCC
GTTTCACTCTAAAAACTTAAGTTTTAAAAGGTACTTAAATGGTTAAAAAGAAATACTTAATTGCCACTTA
ATTTCTTGACTTACCCATAAGATTGATCAAATGTAAATACCAGACCTTTCGTTTAGGAAAGTTAGCCATT
CAATTACGTTCTTAAAAATAAAGCTTCAATGACTTACACTACCATAGCCTAAGTATTTTGGTTTGAAGTT
AATTATTTTTGTCTTTGGGTTGCTTTAAGTTTTATGGGTTCTTGACAAGTCATATATGTAAATGTTAAAA
GTTCGTTGCAACTGCATTCTTTATTGTTTTGAATATAGTTTAAGCCCACTTTATACCCAGAACATAGAGG
CTCCGTCAAATCGAGCGCCACGTGCTGACTTTTGTGCACTTTTCCAGTAACTGGAATAAATATACATGTC
ATCACGCCAATCGTGTGCCTCGTCTGCATATTCTACACAGCAGCCGGCGGACTGAAGGCCGTTGTCTGGA
CAGATGTGATCCAAACCGTGATTATGGTGGGAGCCATTTTCTTCGTCATCATCAAGGGCACCGTCGATTT
GGGCGGATTGGGCGTGGTTATTCAGCGGAATTTCGACACCGGACGCATGGAGTGGCCTGAGTGGGTGAAA
ACTGTTTACTAGGGCAGGTACCACACATGGCATGTAATCCTGCCAGGATGTGTGGTGCATGGGCTCCGCT
CGCTGGTCTATATGGTATAATATGGTACATTTTCGAAACAGATTGACGCTGGATCCCAAGGTGCGAATGT
CCATGTTGGCAATGATGGTGGGCAACGTGGCACACAACTCGTATAACCTCGGATGCAATCAGATCATCAC
CCAGCGATACCTGTCACTGCCCGGATTAAAGCAGATGGCCCAGTGCTCGTTCCTCTTCATTTCCGGATTG
GTTCTTCTGATGGCCATCTGCATGTACAACGGCCTCCTGCTATCCGCAACCTACTACGACTGTGATCCAT
TGACCACGAAGGTGGGTATTTATTAGAAAACCATGCATATTGTTACTAATCCTGAAAGTTACTTACAGTT
GGCGGTGGCCAAGGATCAATTGGTTCCCCTGCTCGTGGTGCAATCCATGAGCGCATTTCCCGGAGTTCCC
GGCATGTTTGTGGCCGGAGTGTTTAGTGCCGCCCTCAGTTCTCTGTCCACGGGCATGAATTCCCTAGCCG
CTGTTTTCCTAGAGGATTACATCAGGCCCTTGACGAGGAAGCCGCTTACGGAACACCAAACTGCTGTTAT
CATGCGTGTTTGTACGGTTTTAATTGGAGTCATGAGTGTGGCCCTGGTTTTTGTTGTGGAGCAGATGGGC
TCCCATGTAATGCAGCTTTCCATGACCGTTGGATCGGTGGTACAGGGTCCCTTGCTGGCTCTTTTCGTTA
TGGGCCTTCTGTGCCCAAGCATCAACTCAAAG
>dme_circ_0007620_junction_seq
CCTTCTGTGCCCAAGCATCAACTCAAAGGAGCGCAATCACGGGATCAATTTTTGGC
>dme_circ_0007621
GCTGTTTTCTGGGCGTAGAACAGCTGTGCATAACGGGGCGCTAGACGAAGCGGAGGAGGAGGAAGCAGGA
GCTAACCAGAAGAGCCAGAAGCACACAAAGACGAATCCATGTCCAGCGAGGAAGACAAACCGCCGGCGCC
GCCTGTGCGTCTCACCAGCAACCGGGGCGGCAATGAACGATCGGGAGGAGGAGTGGGCGTCGGTGGCGGA
GGACTGGGAGGTGGCGGTATGGGAGATGTACCGCCCGACATGCGTCCGCTGCCCAAGGGAACCCGACGAC
TCCGATCGGAAGAAGAAAACGCTGAAGAGCAAGATCAAAGGATCGAAGCCCTCGCACACGGACTCCAAGC
CGAACATCTCCTATCCCACGAACTTTGAGCACACGGTCCATGTGGGATTCGATGCTGTCACCGGCGAGTT
TACC
>dme_circ_0007621_junction_seq
ATTCGATGCTGTCACCGGCGAGTTTACCGCTGTTTTCTGGGCGTAGAACAGCTGTG
>dme_circ_0007622
GCTGATCGATTTGAGCAACTGGTCCAACTTGTTACAATGATCAGCGACAAAGGAGAACTTCCCATAGCAA
TGGCTTTGGCTAATGTGGTGACTACATCCCAAATGGACGAGCTGGCTAGGGTTCTGGTCACCCTATTCGA

CGCCAAACACCTGTTATCGCCACTCCTATGGAATATGTTTTATCGCGAG
>dme_circ_0007622_junction_seq
ACTCCTATGGAATATGTTTTATCGCGAGGCTGATCGATTTGAGCAACTGGTCCAAC
>dme_circ_0007623
GCAACTGGTCCAACTTGTTACAATGATCAGCGACAAAGGAGAACTTCCCATAGCAATGGCTTTGGCTAAT
GTGGTGACTACATCCCAAATGGACGAGCTGGCTAGGGTTCTGGTCACCCTATTCGACGCCAAACACCTGT
TATCGCCACTCCTATGGAATAT
>dme_circ_0007623_junction_seq
ACCTGTTATCGCCACTCCTATGGAATATGCAACTGGTCCAACTTGTTACAATGATC
>dme_circ_0007624
TTATCCAAAACCATGTCGATCAGCTCGGGGATCTGTCCAATTGATCCCCAGATCTTGGCCTGCACTGAGG
GATACATTTCCTTTTGGTCAATAGTTAAATTGATGAGCTTGTCCAGAATTTGGGAGACCTTAAGCTTTTT
GGAGTCATCGTTGGACTTGCAAAATTTGACTAGGTTCTTTAACCATGGCGTCATGTACTCCAAACACAAA
TG
>dme_circ_0007624_junction_seq
ATGGCGTCATGTACTCCAAACACAAATGTTATCCAAAACCATGTCGATCAGCTCGG
>dme_circ_0007625
GGGCATGCCGGAGGCATGGGCGCGACTCCTGATGAACTCCAACATCAGCAAGCAGGAGCAGAAGAAGAAT
CCCCAGGCCGTGCTGGATGTGCTCAAGTGGTTCGACAACACTACCAAACAGAGGCCAAGTTCCAAGTACA
TGACAAATGCCATTACGACGCATTCAGGGTTCATCGCTCAGCCGCGTCAGCAGCAGCAGTCCGAGCAGCA
CGCCAACAGACTCGGAGCTGCACGGCTCCAACAGCGGAGGTAACCTGATTGGCGTCCAGTTGGGCAGTAT
GACTCTGGGTCCCAACGCCAACAACGTGGCCGTTGCTGGGCAAATTCTGGGCAACCACTACCAAcagcag
cagcagcacctccttcagcagcagcagcCACTTTTGCATCAGAACCACAACCAGCATCACATGGGCATAA
GTCAATCGCACTCGTACAACTTTGTCGGACACACGGTCTCCTCATCCACATCGCAACATTCATCCGCTAA
CGAGGACGATATGCTGGGACCACAGCACCCGCAGCAACAGCCGCCACCTCCACCGGTGGCCTCTAGGCCT
GAGCGCACGAAATCCATCTACACGCGGCCAATCGAGGATCTACAGCCAGCCATCATCCCAATGCCAGTGG
CGCCAGCAACCACGCCTGCAACGCCGCTGCAAAATCATAGGACGCCAGGCGGAATATCAGCACCAGCGGC
ATCGCCAATGATGCACAACAATGCCACGACGACGCTGGACAAGAATAAAAACAATGCAA
>dme_circ_0007625_junction_seq
ACGCTGGACAAGAATAAAAACAATGCAAGGGCATGCCGGAGGCATGGGCGCGACTC
>dme_circ_0007626
GCAACAGATCCACGCCCAGCAGGTGCCAGGTGGACCACCTCAGCCGATGGGCGCACTGAATCCGTTTGGC
GCCCTGGGCGCCACCATGGGCCTGCCACACGGACCGCAAGGTCTGCTGAACAAACCGCCCGAACACCACA
GGCCGGACATCAAGCCGACGGGTCTAGAGGGGCCAGCAGCCGCCGAGGAGCGATTGGTGAGTACCACGCC
CTAGATCAAAATTCTGCATATTTCAAGCTAATTTTCATGCTGATCTTGCAGCGCAATTCGGTTTCGCCGG
CCGATCGTGAGAAGTATCGCACACGCTCGCCGCTGGACATCGAGAACGACTCGAAGCGTCGAAAAGATGA
GAAACTGCAA
>dme_circ_0007626_junction_seq
GAAGCGTCGAAAAGATGAGAAACTGCAAGCAACAGATCCACGCCCAGCAGGTGCCA
>dme_circ_0007627
GATCCACGCCCAGCAGGTGCCAGGTGGACCACCTCAGCCGATGGGCGCACTGAATCCGTTTGGCGCCCTG
GGCGCCACCATGGGCCTGCCACACGGACCGCAAGGTCTGCTGAACAAACCGCCCGAACACCACAGGCCGG
ACATCAAGCCGACGGGTCTAGAGGGGCCAGCAGCCGCCGAGGAGCGATTGGTGAGTACCACGCCCTAGAT
CAAAATTCTGCATATTTCAAGCTAATTTTCATGCTGATCTTGCAGCGCAATTCGGTTTCGCCGGCCGATC

GTGAGAAGTATCGCACACGCTCGCCGCTGGACATCGAGAACGACTCGAAGCGTCGAAAAGATGAGAAACT
GCAA
>dme_circ_0007627_junction_seq
GAAGCGTCGAAAAGATGAGAAACTGCAAGATCCACGCCCAGCAGGTGCCAGGTGGA
>dme_circ_0007628
ATCCCAGGAGCGCACCGTGTTGTCTAAGCCGCCCGTCCACAGCCTGGAGCCATCCGGACTGATGTCGATG
CATGAAGCGCCGTCGGTATGGCCCTGGAACTGGCGCACCAGGATCTCGTTGTGCAGG
>dme_circ_0007628_junction_seq
CTGGCGCACCAGGATCTCGTTGTGCAGGATCCCAGGAGCGCACCGTGTTGTCTAAG
>dme_circ_0007629
GTGCAGCGAACCCTGGCCGGTCATTTGAGGGATCGGGACAAGGAGCGCGAGCATCACATGCGCGAGGAGT
GCATTGGTCACTTTACCGCTTTGCTGACAGATCTGGTGCGAACGCCGGACTTCACGTGGAAGGAGG
>dme_circ_0007629_junction_seq
CGAACGCCGGACTTCACGTGGAAGGAGGGTGCAGCGAACCCTGGCCGGTCATTTGA
>dme_circ_0007630
GGAGTGGCTGCGTCCGGATGTTAAGATCACCTACACACCCATCGATGGGGACTCCTCATCCGATGAGGAC
ATGGACGACAGCGACGAGGAGGACGATAATGAAATGGGCCAGGGCGACGAGGAAACTCCCAGCGGCAGTG
>dme_circ_0007630_junction_seq
GGCGACGAGGAAACTCCCAGCGGCAGTGGGAGTGGCTGCGTCCGGATGTTAAGATC
>dme_circ_0007631
AACCCCACTGTGCCACGCTGTGGTCAGTGCAAGGAGAAACTCCACGGGATCACCCCCTCCCGGCCCAGCG
AGCGTCCCAGGATGTCCAAGCGTTTGAAGACCGTCTCCAGGACCTACGGAGGTGTCCTGTGCCACAGCTG
CCTGCGAGAGAGAATCGTCCGCGCTTTCCTCATCGAAGAGCAGAAGATCGTCAAGGCTCTGAAGAGCCAG
CGCGAGGCTTTGGTCAAGCCCGTCAAGAAGG
>dme_circ_0007631_junction_seq
GAGGCTTTGGTCAAGCCCGTCAAGAAGGAACCCCACTGTGCCACGCTGTGGTCAGT
>dme_circ_0007632
GTATGGTAGTAAAGTGGAGCTCTCCAAGGGCTGGGCCCATGTCCTTCAGCCGACGCCGGAACTCTGGACA
CAAACACTGCCCCACCGCACACAGATTATTTACACGCCGGATATTAGTATGATTATCCACCAGCTGGAGG
TCCGACCCGGTGCCGTGGTG
>dme_circ_0007632_junction_seq
GCTGGAGGTCCGACCCGGTGCCGTGGTGGTATGGTAGTAAAGTGGAGCTCTCCAAG
>dme_circ_0007633
TTTTCAAACGTCACAATGCGATGCATCATCTCCATCATCAGACAGCAACACCAAATATTGGCTTCTCAAA
GGTAAGCATTTAAGTTTTAGTTCATTAATACTCTAGTATAGTTTTCTGCCTTATTTCGTTTTCACTTTCT
CGGAAATATTGCCCACAATTCATATTCAATCGGAGCGTAATTAGGAAAGCGATCAGCGTGATATGCGTTC
ATTTACCATATAACCTCGGCAATTTTCAATCCCGATGACATTTCGATTGGGCTCCCAGGGAACCGAGAGA
AACAACTCAATTTAAGCCAACGCGGTGACAACTCAACCGAAAGTTCCCGGGAAAACAACTCTATTAATGG
CACAAAAGCTGGCCATATCATAAGTCAGAAATCGTTTTGCTCGCCGCCACTTGCTGCGCCAAAAGCGAGA
AGAGGAAATCAGCCAAAAGCCAGCGGGCCGAACAATAAATGCCCGGGAAAAGCCACAGATTTACAGGAGC
AGCCACCAGACaaaaccaaaaaagcaaaaataaaaaacaaaaaaCACAGAAGACAGAAGACAGTAGTCAG
TCAAGCTGCCTGGCACTGACTGCTCCAGTCGGGCGAGATTGATCTACATACCTGTGCACACTCGTGACTG
AATTAAATACTATAACCACGGTAGGAGTACTACTCATACTCCCCCTTTTCGGCTCTCATCGGGAGCTCTG
CTGCGGTTGTCAACGTGGGTTTAGGCCAGGCTTTTGGGCTAACAATTTATTAGTGCTCAGACGCGAAAAT

GAATTTGCATTTCCGACACAAGATTCAATTTATGCTTTTTCAACTAGGGGGTGTTAGAAAAGAGGTAGCG
TGCTAGCATGATAGGGTGTTTGAATGAACATAGGTGCATTTTAAAGTAGGTTGTTATTATTAAAACGAAA
ATAGAATCCTCTTTATTTTAAGTAAGTTAATATAGATATATTTGGATTAAGAATATTTTGTATTCATTAC
CAATCACCCTGTATTCACATATACGTATTTGTCTTACAGGCACTCTCCGGCTTCTTGATGATGCTCACAC
AAAATGGCAAATTGCTTTATATATCGGATAATGCTGCGGAGTACTTGGGCCATTCCATGG
>dme_circ_0007633_junction_seq
GCTGCGGAGTACTTGGGCCATTCCATGGTTTTCAAACGTCACAATGCGATGCATCA
>dme_circ_0007634
TGTGGGCGGCCGCGCATGCGGGTACATTGCATAGGATACCAGCAGCTAAGGCGTTCAGGTAAAGCTGCGC
GTGACTTTCTGTGGGAGCCACAAGAAGCTCAGGTAGTATTGTACTGCTGGCCAGGACGTTCCACGAGCTG
GAGTTGTTCTCAGACACCACCAATACGAG
>dme_circ_0007634_junction_seq
AGTTGTTCTCAGACACCACCAATACGAGTGTGGGCGGCCGCGCATGCGGGTACATT
>dme_circ_0007635
TTAACGCGAATATTTATATCTGTGCACTTGCTAGCGCCCTCGATAATCATGTTGAGATCATTCTCGGTCA
TCTGGCCGAAAAGATCCTTGCGAGACTTGAGCAAACTCAGCGAGGAGCGCATTAGTGACGTAATGGCCTC
CATAACGGATACATCTTCACTTCCCCTAAATGCTTGTTGACCCAAGATCATCCACATGTTATAGAGGTTG
CTGCAGCCCCCCAAATCTTCAGGGGGCAGCACATTGCATAGGTTCTGGAGGCAAATCAAGTATGATACTC
TTAATTTTTCCACTTTCTCCTTGATATTCTCTTGCTGGGACACAAGATTTTCCACAGCAGGTTGCAGTGG
TTGAGCCTTTTGCCATAGTTTCTCGATCACTTTGTTTTGTTTGATAGTCTCCAAGAAGTTGGCGGGCAGT
CCCCCCGATCCACTGTCAGCTGTCTCGTCCTCCATGTCGTAATCGGCCACGCTCTCAGACTCTGAATGCT
CGCTATCTGTATCCCAGTTGCTCTGTTCATCGCTGGAGCCCAGGTTGG
>dme_circ_0007635_junction_seq
CTCTGTTCATCGCTGGAGCCCAGGTTGGTTAACGCGAATATTTATATCTGTGCACT
>dme_circ_0007636
GCTGCAGCGCCATCTGAACAAAGCTGTCCGGACCCAGACGCTGTTTCTTTATGAAATCCTTTCCAAAGCC
GTTGAATTTCAGAACCTTCATTTGGAGAGCATCGGCAAGTTTATCCACGTTTTCCTGGGCGAC
>dme_circ_0007636_junction_seq
CAAGTTTATCCACGTTTTCCTGGGCGACGCTGCAGCGCCATCTGAACAAAGCTGTC
>dme_circ_0007637
TTGAAGCCGCTGAGTACCTCGTAGTTCTCCAGCTCCACCTCCTGGTTGGCATACGCCACGTAGGCCTTGC
CCTTGTTGGGGATAATCTTGGCCGGCAGCATGTCGTTGCAGTAGAAGGCGCGGCCAATGAAGATGGTGTC
GCCATCGGAATCGTGACCAGCCACAACGGCTGCCTGGGGGATTGCTCCGTTGCTGAAG
>dme_circ_0007637_junction_seq
TGCCTGGGGGATTGCTCCGTTGCTGAAGTTGAAGCCGCTGAGTACCTCGTAGTTCT
>dme_circ_0007638
GGCAGCCGTTGTGGCTGGTCACGATTCCGATGGCGACACCATCTTCATTGGCCGCGCCTTCTACTGCAAC
GACATGCTGCCGGCCAAGATTATCCCCAACAAGGGCAAGGCCTACGTGGCGTATGCCAACCAGGAGGTGG
AGCTGGAGAACTACGAG
>dme_circ_0007638_junction_seq
CCAGGAGGTGGAGCTGGAGAACTACGAGGGCAGCCGTTGTGGCTGGTCACGATTCC
>dme_circ_0007639
GGTGGAGCTGGAGAACTACGAGGTACTCAGCGGCTTCAACTACGAGTGGCTGCCGGCCGAGAACGGAGAG
GTGCCTCCCGGCGCCGTCAAGGTCGGCCAGAATGTCGACGGAGAGACCTTGTACGCCGGAAGGGGCTACC

ATGCCGGCAGTTTGACC
>dme_circ_0007639_junction_seq
AAGGGGCTACCATGCCGGCAGTTTGACCGGTGGAGCTGGAGAACTACGAGGTACTC
>dme_circ_0007640
GGAATGTACAGGCAGCCGTGGGACGGATGCACCTTGCCCACGGTCAAACTGCCGGCATGGTAGCCCCTTC
CGGCGTACAAGGTCTCTCCGTCGACATTCTGGCCGACCTTGACGGCGCCGGGAGGCACCTCTCCGTTCTC
GGCCGGCAGCCACTCGTAGTTGAAGCCGCTGAGTACCTCG
>dme_circ_0007640_junction_seq
CTCGTAGTTGAAGCCGCTGAGTACCTCGGGAATGTACAGGCAGCCGTGGGACGGAT
>dme_circ_0007641
GTCGCGGCTGGGCGTCGTAGAGATGCGTCGACGCTTAGTCGGTTGACTTATTGGGTCCGACTTCATTTCC
TTCTTCAGCTGGGCCACTTCCTCCTTGCCGAGTGCATCGATCTCGATGATGACCACCTCCTGATTTCCTT
GCTGCAG
>dme_circ_0007641_junction_seq
TGACCACCTCCTGATTTCCTTGCTGCAGGTCGCGGCTGGGCGTCGTAGAGATGCGT
>dme_circ_0007642
AAAGAGGTGGTGCCGAAGTATGACGACGAGTTGGATGCGCTCTGCAAGGAGTTTTTTGATGATGGCCCCT
CCGCCGGCAAAGGAGAAGCTAATGAGGAGCAGCAGCAAGACGATGCGCACCTGCAGCAAGGAAATCAGGA
GGTGGTCATCATCGAGATCGATGCACTCGGCAAGGAGGAAG
>dme_circ_0007642_junction_seq
GAGATCGATGCACTCGGCAAGGAGGAAGAAAGAGGTGGTGCCGAAGTATGACGACG
>dme_circ_0007643
TTATCATAACCCCCCGTTTAGACCTCAGTGGGCAAACTCTGGCTCTGGCAGCCCGCCTGCAGTTTCTTGG
AGCTGCGGAAGAACATGTCGAAGAGCCTGGCGCGCCGATTCTGGAACTTTTGGGGCATGGTTGGTGTTAT
AGCTATG
>dme_circ_0007643_junction_seq
TTGGGGCATGGTTGGTGTTATAGCTATGTTATCATAACCCCCCGTTTAGACCTCAG
>dme_circ_0007644
GTTCCAGAATCGGCGCGCCAGGCTCTTCGACATGTTCTTCCGCAGCTCCAAGAAACTGCAGGCGGGCTGC
CAGAGCCAGAGTTTGCCCACTGAGGTCTAAACGGGGGGTTATGATAACTATACATATAGGAAG
>dme_circ_0007644_junction_seq
GGGTTATGATAACTATACATATAGGAAGGTTCCAGAATCGGCGCGCCAGGCTCTTC
>dme_circ_0007645
GGATAAGGGCAAGGACGAGGTATCCAAGGACAAAAAGAAGAAGAAACCCACAAAGGACGACAAGAAGGGC
AAGTCGAATGGCAGCGGTGACTCGTCCACAGACTTCAGCTCTGACAGCACAGACTCCGAGGACGACCTTA
GCAACGGACCGCCCAAGAAGAAG
>dme_circ_0007645_junction_seq
CCTTAGCAACGGACCGCCCAAGAAGAAGGGATAAGGGCAAGGACGAGGTATCCAAG
>dme_circ_0007646
GGGCAAGGACGAGGTATCCAAGGACAAAAAGAAGAAGAAACCCACAAAGGACGACAAGAAGGGCAAGTCG
AATGGCAGCGGTGACTCGTCCACAGACTTCAGCTCTGACAGCACAGACTCCGAGGACGACCTTAGCAACG
GACCGCCCAAGAAGAAGGTCGTC
>dme_circ_0007646_junction_seq
CAACGGACCGCCCAAGAAGAAGGTCGTCGGGCAAGGACGAGGTATCCAAGGACAAA

>dme_circ_0007647
ATTTCAATGAAAACAAAAGGATATGTCGTAAATCGCTAGGCTAAAGCACATTTTATGGTGAAAACAACAG
CAATCACTATCAGCAACACCTAAATGTATGAGGCTTGTAACATTGAAGTCGCACAACGttttttgaattt
ttttaatg
>dme_circ_0007647_junction_seq
GCACAACGttttttgaatttttttaatgATTTCAATGAAAACAAAAGGATATGTCG
>dme_circ_0007648
GATAAaattgtttgaattgttgaattgtttCTCCATCGAACATATTCAATCCTTTCTTCTGCACTCATCA
TTTAGTTGTTCTCTAAAATAATGTTATAATATAATAAACTTGCAAAAATGTCACGTACTACATATATATA
ACATGTTTTGAAAGTGTAATCGTTCATCtttttttttttggttatttttttGTAATGCTTGCTTTGCTTAG
CTCTCGAAACTAAG
>dme_circ_0007648_junction_seq
CTTGCTTTGCTTAGCTCTCGAAACTAAGGATAAaattgtttgaattgttgaattgt
>dme_circ_0007649
GCCACCGTCAACCTGAGTGCCATCAACAACGTTCCCTTGATCCGCCCATGGGCCCTCACCTTCTCGTACG
GTCGTGCCCTGCAGGCCTCCGTCCTGCGTGCCTGGGCTGGCAAGAAGGAGAACATTGCTGCCGGCCAGAA
CGAGCTGCTTAAGCGCGCCAAGG
>dme_circ_0007649_junction_seq
CAGAACGAGCTGCTTAAGCGCGCCAAGGGCCACCGTCAACCTGAGTGCCATCAACA
>dme_circ_0007650
GGCGTAGCGGGCCAGGACATTGGCGTTCTCCAGGATCGACTGGTAGGATGGGGTGTTCTTGCCGATCTTC
AGGACGCAACGCCACTTGGCGAAGTCGCAACCGTCCTTCTTGTACTGGGCGCAACGGGCGGCCAGGTCAT
CCAGACCCTGGGTGGTGACCTCATCCTCAGAGCCGAACA
>dme_circ_0007650_junction_seq
GTGGTGACCTCATCCTCAGAGCCGAACAGGCGTAGCGGGCCAGGACATTGGCGTTC
>dme_circ_0007651
GGTTTTTACGAAGGGCGAAGCGCGTGCAAGTCGGAGTCGACAGCCTTCTTTGGATAATGTCACGGATACG
GATGACGACACAACGCCGCTGCTGCAGCAACAGCAGTCCAATGGTAGGCAGGTGGGTCAAGTGAGCTCGG
CTTCTTCGGCTGGTGGAGCAGCTGGGAGCAGCTCAAGCGTTGCGGCGGCGGCTGTCGCTGGAACCACGCG
ACATGGAACCTTCCGCCGTGGTCACGCCGGTCGAAATCCGTTCGAGGAGTCCCAAAGCTCTGACGATGAG
AACGGCTCTGCTGGCCTCTACTGTGCGCCCGGCCACGTCTTCTGGAGCACATGAACGCATCAATCCCTTT
GATCGTGCTCCGAATCTACCGGCCCACCTAGCCGAACAGTTAACTGAGAGTCGTCGCTCGGTCTGGTCGA
GAATAAATTTTGCGCTCGTTCTTCCGCCGGCAGCCAGCTGTTATTAGTGTGGCGGCGCCACCCTATCTGG
AGCGCGTTGAGTCCGGTACCAGTGCCATGGGTCTGCCGGTGACGGGGACAATTGCTGTAGCCTCGCCCGC
ATCGAACAACATCCTCAATCCGAATTTAAGCGGCTCCTTTAAGGACGAAGACGATATGCCACCGCATCGT
TCGATCTACGAACCGATAGCAATCAGCACGCCTGCAGCTGACTCAGCAACAGTCGACGATTCGGCGTTTC
TGCAAACGCCCACGCAAGGCGGCATAGGCGTGGCCGCCCTGCCGCACAGCGCATCCGATCGCCAGTTTCT
CATATGAGGCGAGCTAGTTCGCCGCGAGTCCTTAGCCTGTAGGTTGTAGGTGGTCGTCGATCCTTGAGAG
ATGAGACATGAGCCCAGCCTGGGTACGAGTTTTTCGTTTGGGTTCGCGATCAGCGCTGTGGCTGCCAGCT
TCTTT
>dme_circ_0007651_junction_seq
GATCAGCGCTGTGGCTGCCAGCTTCTTTGGTTTTTACGAAGGGCGAAGCGCGTGCA
>dme_circ_0007652
GAACTGCCGCGAAATGGACGCCGCTGCCGACGGAGTACCGCCAACGGCGCCAGCTGCGACGGCGACGACG

GCCAACAGTGATCTGGACTTGGCGGCACGTCGGCGTCTCTTCATCTCACAGCCCTCGACGCTGGCGACGC
GCCGCCAGCAGGCGGTTTGTGTGCGGCGGGCCCACAAAATGTATGGCAGCAGCAAGAACCCCAACGTTGT
CCTCGACGGCCTGAACATGACAGTGCCCAAGGGTTCCAT
>dme_circ_0007652_junction_seq
TGAACATGACAGTGCCCAAGGGTTCCATGAACTGCCGCGAAATGGACGCCGCTGCC
>dme_circ_0007653
ATAATGCCGCACAACATAACGATGGGCAAGAACGAGCCCATCGCCACATAAGTCGCAGTTCGCTCCGTGT
CCACAGCACAGGAAATAACGAATCCGAAGCTCATGCCACACAGTCCATTCAGGATGCAGAGTGCCACCAC
CAGCCATATGTTACCCACCAAGG
>dme_circ_0007653_junction_seq
ACCACCAGCCATATGTTACCCACCAAGGATAATGCCGCACAACATAACGATGGGCA
>dme_circ_0007654
AAATAGGCACAGGAATTAATGCAAATTTAGAGGTCATGATAAGTCCCTTAATATATGCCAAAAGCAATCA
CTACTTTAAGTTGGGATAGACAGATAGATTTCAGCTTCCAACATAACATCACATTTTCATCCTGTGTCGT
GTAGACCCAAGAGAAGTTCTTATCCCTTCTTGAACTTGAGCAGCAGGATGG
>dme_circ_0007654_junction_seq
CCCTTCTTGAACTTGAGCAGCAGGATGGAAATAGGCACAGGAATTAATGCAAATTT
>dme_circ_0007655
TAATTAAATCCTTAACAAATAGGTATAAAACATCGATTATTTAATTGTCTGGAAATTCAAATATGTATCA
ATTGTGTCTTCGTTGGTAACATTTTACAGCGGTGTGTGTTTCGATTAATAGGTAGATAGATTCATAGAAA
CTAGCAAACTCTAAG
>dme_circ_0007655_junction_seq
AGATTCATAGAAACTAGCAAACTCTAAGTAATTAAATCCTTAACAAATAGGTATAA
>dme_circ_0007656
AAACAACTCTTGGGCTACAGCTTGCACTCATAAAGGTTTCTTTTATTTTCCTCAGTGCAAGTTCCAACTT
TGACCCCATGTCTTGTCCCCTGTGCGTGCAATGCAAATTGCCAAAGAAAATTTATTCCAACTACTGAATG
CGAATTTTCTAGAGCGCCAAAAGTTAACCAAATATGTCAATTGAATGCTTTGTGTGGCAGTGATAAAGGG
GATTCGAGTTACTGGCGATGGTAAAGGGGAAGCGTGTGGGACGTGGCATGTGGCAAACAACAAACAAAGC
GCACAGGACCAACTAACCAAGAACGGAGCTTGTAGGTTAAAAAGCAATTTATCTTTGACTTTTGCCAAGT
AAATTCCCGCCATTGTCTAAAGTGGTTCGAATGCCCCATGCCTCGTCCTCGAAGTTTTTG
>dme_circ_0007656_junction_seq
GCCCCATGCCTCGTCCTCGAAGTTTTTGAAACAACTCTTGGGCTACAGCTTGCACT
>dme_circ_0007657
TAAAGTGCAGCAACTGGCAGCGCAGACAAGTCGAAAGGGCAGCCAATTAAATGTAAGAAGCGACGTCCGC
TGCTTTACAACAAGTATCGCTGATTGTCCAGCCGAGTAACAGTATTTTTCACGAGGTGCGCAGAGCGAAA
GGAAAATCAAAGAGGAAAATCTAATACACAAAAAG
>dme_circ_0007657_junction_seq
CAAAGAGGAAAATCTAATACACAAAAAGTAAAGTGCAGCAACTGGCAGCGCAGACA
>dme_circ_0007658
GCGAAGAGCAGACGGAGGCGTCAAGTCAAACAAAAGTAAAGTGCAGCAACTGGCAGCGCAGACAAGTCGA
AAGGGCAGCCAATTAAATGTAAGAAGCGACGTCCGCTGCTTTACAACAAGTATCGCTGATTGTCCAGCCG
AGTAACAGTATTTTTCACGAGGTGCGCAGAGCGAAAGGAAAATCAAAGAGGAAAATCTAATACACAAAAA
G
>dme_circ_0007658_junction_seq

CAAAGAGGAAAATCTAATACACAAAAAGGCGAAGAGCAGACGGAGGCGTCAAGTCA

>dme_circ_0007659

GCGAGCTCTTCACGCTCTTCCAGTCAATACGATTAGCAAGAGCATTTTCTCATTTCTCTGTTCACCGCGA
TGTTTGGCTTCGGAAATAAATGAAAATCGGGGGAGAAATCAAATCACCGAATTTTATTCACTAATCTAAT
GcacacacccatgcacacaaacacacacacacgcccacgcacacaAAAGTATGCTAATACATTTGGGCAC
AGGCATAAATATTTTACGCGTGCAACAGTGCGTATACTCAATTTGTTAAATAGTCCGCACAAACATGCCG
CCAACACGATGGCCCGGCGCCAATGTCATCAGTTTGTCACCGCTCAAATCGGAAAATTGATGAGGTGGTG
GGGCATTAACCCGTGGCTTGGGGTGGAAAACAATCCCAAGGGACAGTGAAAATACATACCAAGATTGGAA
AAAGCCATATTCAAAATTAACAAATTTTCATTTCTTGTTCATCTTTTGTTCGCGTCGCTTTTTTATCAAA
TTAAAAGAAGCTCTGTATGAGAAACTAAAAGTTTCAAGTTATTATTAATATTACTGATTTACTTGATTAC
ACTTTTAAAGCATAAAGTTTTGAGAAAGTTTTTCCAAAAGCCAACCGAAGTTTTTTCCCCTTGGTTTCTA
TTGTTTTACGAACTGCAATTATTTCCGTTCTAAACTTTTTTTGTTCGCCAGTTAGATATTATATTCTGTT
ATTACACCAAGAACTATAAGTTCTTAGCAATGAGTATAGAATTGCATTGAAAATTACCTTTTTTAACATA
GATATTTCTGCTGCTATCAGTTAGCATAATAATATCAGCATTAACAAAGGTATCCTTCATCCTGCGTTTT
TCCCCTCTATCCTTTCTTTTTACTTCCTCTGACATCCTTTTATCGGCCCTAAATTTGTACTGGCTTTTTG
TGTGTGCTCGGATTTAAGCTGCGATAAATTCGCAGATTCTACGGCTCAGATTCTTCATCTGCCATTACGC
ACGCATCTCCataataatgatagtagtcataataataataatGATGATGATGGCGGTGATGATGCCA
CATAAAAGGCGGCGGAGCCCATTTCAATTTCAATTCGACAAAAGGCGCGTGTATCTGCAAGATACATTTC
TATCTCAAGAGCGCAAAATTGCGCGGCAAACCAAAGCTCGCAAAAAGCAAAAAATCGCATATATGAATGC
TAAAATATATTTCTGGCTTACTATTATATTGTTGATGCCTTTGCTGTTCGAACTATGAAAGGCTTTGCAA
AAAGTAACGCACATCGCAGACAAACTTTTATCCGTACGACAATTGaaaaaaaacaattcgataaatcaaa
agaaaaatgaaaaaaaaCGCTTGTGCGAATTTGAGCTACGGTTTTTTGTTTTGAATACACAGAAAGTAAT
TGGATTGCGtttaaatttgtaatagcatttagatttctaattgaatttTATTAAAAATTCGTTTGTTTTA
TTAACCAAGCAAGCAAATTAAACGAATAAAATAAAAAATGCTTCTGGTTGAAAAAATATTGGAAATACTA
GCTTATTTCTTCTGTGCATTGTGGCGTTCATTTGTTTACAGCCCTGCGATATAAAACTTTGTCTCGTTTA
CATCTTTTTCGAAATATATTGTATAATATTCGGTTTTGGTTTAAGATAAAACCCAAAACTGGAAGATGAG
CAAAAAGCCGGCTGAGCAAAGGAAATCACATTGGGGTAGTTGTTGTTATATAAAACAAATTGTACGCGAA
TTTTTGcacaaaaacacacagatacacgacacacacacacaAAGAGCCGCAAAATCCGGCGCTCAGGAAA
ACAGAGAGCGAAAGAGCggatgaagaggacgaaggggaggaagatgaggaaACAAACGAATTTCAGCAAC
GACGCAAATTTCGATTTCCTCGAAATTTCTACCTTCTATTATGCTCGTTCCTCTCCCGACTGCTGCTTCT
TCTTGCGCTTCTTGTTTGGGCATCATATACTTTTTTGTTTGCAAAAATAAAAAATAAGGAGAAGAAAACA
GTGAAGAAGAAGCAACAGCAGCGGCGGCAGCAGCAGCGCAGCTTACTAAGCTTTTGAGATGCCGCCGTTT
TGCTCCttgtttttttgtttttgctacgctcttgctgctttgcctttgctttttcgggtattttgcctttg
ggtttttttgctttttttgcgtttttgccttttgcgtGAACAAGAAGCAGCAGCAACTACTGTACTTGGAG
TTTTTCCTTTGCTACATACATTCGTATATCCTTTTTGCCGCTTTGTTTGTTTGGAATTTCTCAATTTTCA
AATACTGATGCCACCCACACGCACACACACAAACGTACACGCACTCCCACTCGCACACATACACGCACTT
TACGCTTTTTTGTGCTTTGCTGCGTCTCCAGTTTCTTGTTATTTCGCCCTTTGTTGTATTATGTCATAAT
TTATTTGTCGCCCAGGACTAAACAATGCCGGAATTTTGCACAATTGATAAAGCCAATGAATCACATTTCG
TTTTCATTAGCCCCCACTCGTGCTGTCTTGCTCTTTCTGGCTGCACCGGCTCCTCTcttttactttttac
tttttacttttctgctacctttgttgtctttttttgcatttccgctttacgtttgtgtttCGCTCGCACTC
ACGCACTCAAACACACGCAAACACAGACGCACACATAGCTGCAATTACTGTTGTTGCCCAGCTTGTTTAA
CTTTTGTTTGTTGTTTGAAACACACACAAGCACGCAGCGTCGTCGAAAATATCGCGAATTTACAAGCGCC
GGCAAAAATTTACTAGAGAAATCCGCCCGCAATTACACAGAGCGACGCAGCAAACACGTCCAAACTGTTC
GCCGAGCTTTGGCGCACTGCTTAACTTATGCTTATGAATGGAACGGCGGCGACAAGCAGCCCGTTCGGCG

AAAGAACGAGAAAGCGATAGTGATACTCCCCCAATTTCTCTTTCTCCTCGGCGCTATGTTAACCGATTCG
GGAAAGGCGCCGATTTGAATGTAATACGATTGCGATCAGATGGAAAATTCGCATCTGTTTTAAAACGTAA
AAGATAAAAACTACCAGCTTCTACTTCTTCTTTACGATGTTGTCTTGCATTGCAAAAATCGAACTCCACA
GCTTTAATCATTTAGGCTTCACTTAAACTTAGCACTAAAAATAATGCTCAATATCTTCTTAAACACCAAA
TAGACATGAAGGAATATCAAATACACATTCCTATGACaaaaaaaaaTGTTAAACAATTTCAGCTCTTTTT
AACATCAAGATACAAAGAACAAAACCTTTGGGTTTAAATCGGTTAACATTGCTTAACATTAAGCGGTTGT
AAGAGAGCAGCAACTTAAGAGAATTTGTACACTCTTTTATCTTGCTCAATTTGAGCTTTGAAGAGAGCCG
GCAACTTTTGTTTTTGTTTGCGACGCCAAAATTTCAATGTCGACGTCGCAGTCGGCAGCGTAAGCTTTTC
GAGCAGCGAAAAACAAACAAAACGGAATGCGAGTaaagcaaaaaagagacacaaaaaaaaTGCAGTTTGT
TGAATCTAGTACTAAGATTTAAATTTATTACAGACAAACGTAACTTTTATTTTGCACTAGAAAAatatta
catattatattattatcttgtgttatattatattataattaCTTACCGAACACCAAGTACTTTACAGTAT
TCAAGTTATTTTCCTTTTTTAACGTAATTAATGAAAATATTCATTATCTTTTAATCTTAATTTAAAATAT
TAACTTAAACTTCATCTCAAATAGGAAGGCGCCGATTAAAAATCGGAAATAGAGATGGCAAATAAAACGA
ATGGTTGTTGTCTTTACTAAAGGTGAGTTGCGCAGTTGCTAGTAGTGTGACCAGACGGCGATTCACAAAA
TTTCTCCAAAGTGTGAACGTATGCGAAGATACTTCGTTGATGGAAGAGGAGTGTCACCTTTTTCTTGGTT
TTTCCAGAGCGTCATACTGCAAAGAGTTGTTGTTGCGCGCGGGCGGACGACGAGAAAAAATTGTAAACGC
GGATGAAAACAACGGTTGATTTTCACCCAACGCTCGTGATTTTCGACCGCTTTTGAGTGTGGAAGCGAAA
AAACCGCCTGTTTTTTAACTGCATGa
>dme_circ_0007659_junction_seq
AAAAACCGCCTGTTTTTTAACTGCATGaGCGAGCTCTTCACGCTCTTCCAGTCAAT
>dme_circ_0007660
GTGCGTATACTCAATTTGTTAAATAGTCCGCACAAACATGCCGCCAACACGATGGCCCGGCGCCAATGTC
ATCAGTTTGTCACCGCTCAAATCGGAAAATTGATGAGGTGGTGGGGCATTAACCCGTGGCTTGGGGTGGA
AAACAATCCCAAGGGACAGTGAAAATACATACCAAGATTGGAAAAAGCCATATTCAAAATTAACAAATTT
TCATTTCTTGTTCATCTTTTGTTCGCGTCGCTTTTTTATCAAATTAAAAGAAGCTCTGTATGAGAAACTA
AAAGTTTCAAGTTATTATTAATATTACTGATTTACTTGATTACACTTTTAAAGCATAAAGTTTTGAGAAA
GTTTTTCCAAAAGCCAACCGAAGTTTTTTCCCCTTGGTTTCTATTGTTTTACGAACTGCAATTATTTCCG
TTCTAAACTTTTTTTGTTCGCCAGTTAGATATTATATTCTGTTATTACACCAAGAACTATAAGTTCTTAG
CAATGAGTATAGAATTGCATTGAAAATTACCTTTTTTAACATAGATATTTCTGCTGCTATCAGTTAGCAT
AATAATATCAGCATTAACAAAGGTATCCTTCATCCTGCGTTTTTCCCCTCTATCCTTTCTTTTTACTTCC
TCTGACATCCTTTTATCGGCCCTAAATTTGTACTGGCTTTTTGTGTGTGCTCGGATTTAAGCTGCGATAA
ATTCGCAGATTCTACGGCTCAGATTCTTCATCTGCCATTACGCACGCATCTCCataataatgatagtagt
cataataataataataatGATGATGATGGCGGTGATGATGCCACATAAAAGGCGGCGGAGCCCATTTCAA
TTTCAATTCGACAAAAGGCGCGTGTATCTGCAAGATACATTTCTATCTCAAGAGCGCAAAATTGCGCGGC
AAACCAAAGCTCGCAAAAAGCAAAAAATCGCATATATGAATGCTAAAATATATTTCTGGCTTACTATTAT
ATTGTTGATGCCTTTGCTGTTCGAACTATGAAAGGCTTTGCAAAAAGTAACGCACATCGCAGACAAACTT
TTATCCGTACGACAATTGaaaaaaaacaattcgataaatcaaaagaaaaatgaaaaaaaaCGCTTGTGCG
AATTTGAGCTACGGTTTTTTGTTTTGAATACACAGAAAGTAATTGGATTGCGtttaaatttgtaatagca
tttagatttctaattgaatttTATTAAAAATTCGTTTGTTTTATTAACCAAGCAAGCAAATTAAACGAAT
AAAATAAAAAATGCTTCTGGTTGAAAAAATATTGGAAATACTAGCTTATTTCTTCTGTGCATTGTGGCGT
TCATTTGTTTACAGCCCTGCGATATAAAACTTTGTCTCGTTTACATCTTTTTCGAAATATATTGTATAAT
ATTCGGTTTTGGTTTAAGATAAAACCCAAAACTGGAAGATGAGCAAAAAGCCGGCTGAGCAAAGGAAATC
ACATTGGGGTAGTTGTTGTTATATAAAACAAATTGTACGCGAATTTTTGcacaaaaacacacagatacac
gacacacacacacaAAGAGCCGCAAAATCCGGCGCTCAGGAAAACAGAGAGCGAAAGAGCggatgaagag

gacgaaggggaggaagatgaggaaACAAACGAATTTCAGCAACGACGCAAATTTCGATTTCCTCGAAATT
TCTACCTTCTATTATGCTCGTTCCTCTCCCGACTGCTGCTTCTTCTTGCGCTTCTTGTTTGGGCATCATA
TACTTTTTTGTTTGCAAAAATAAAAAATAAGGAGAAGAAAACAGTGAAGAAGAAGCAACAGCAGCGGCGG
CAGCAGCAGCGCAGCTTACTAAGCTTTTGAGATGCCGCCGTTTTGCTCCttgtttttttgtttttgctacg
ctcttgctgctttgcctttgctttttcgggtattttgcctttgggtttttttgctttttttgcgtttttgc
cttttgcgtGAACAAGAAGCAGCAGCAACTACTGTACTTGGAGTTTTTCCTTTGCTACATACATTCGTAT
ATCCTTTTTGCCGCTTTGTTTGTTTGGAATTTCTCAATTTTCAAATACTGATGCCACCCACACGCACACA
CACAAACGTACACGCACTCCCACTCGCACACATACACGCACTTTACGCTTTTTTGTGCTTTGCTGCGTCT
CCAGTTTCTTGTTATTTCGCCCTTTGTTGTATTATGTCATAATTTATTTGTCGCCCAGGACTAAACAATG
CCGGAATTTTGCACAATTGATAAAGCCAATGAATCACATTTCGTTTTCATTAGCCCCCACTCGTGCTGTC
TTGCTCTTTCTGGCTGCACCGGCTCCTCTcttttactttttactttttacttttctgctacctttgttgt
ctttttttgcatttccgctttacgtttgtgtttCGCTCGCACTCACGCACTCAAACACACGCAAACACAGA
CGCACACATAGCTGCAATTACTGTTGTTGCCCAGCTTGTTTAACTTTTGTTTGTTGTTTGAAACACACAC
AAGCACGCAGCGTCGTCGAAAATATCGCGAATTTACAAGCGCCGGCAAAAATTTACTAGAGAAATCCGCC
CGCAATTACACAGAGCGACGCAGCAAACACGTCCAAACTGTTCGCCGAGCTTTGGCGCACTGCTTAACTT
ATGCTTATGAATGGAACGGCGGCGACAAGCAGCCCGTTCGGCGAAAGAACGAGAAAGCGATAGTGATACT
CCCCCAATTTCTCTTTCTCCTCGGCGCTATGTTAACCGATTCGGGAAAGGCGCCGATTTGAATGTAATAC
GATTGCGATCAGATGGAAAATTCGCATCTGTTTTAAAACGTAAAAGATAAAAACTACCAGCTTCTACTTC
TTCTTTACGATGTTGTCTTGCATTGCAAAAATCGAACTCCACAGCTTTAATCATTTAGGCTTCACTTAAA
CTTAGCACTAAAAATAATGCTCAATATCTTCTTAAACACCAAATAGACATGAAGGAATATCAAATACACA
TTCCTATGACaaaaaaaaaTGTTAAACAATTTCAGCTCTTTTTAACATCAAGATACAAAGAACAAAACCT
TTGGGTTTAAATCGGTTAACATTGCTTAACATTAAGCGGTTGTAAGAGAGCAGCAACTTAAGAGAATTTG
TACACTCTTTTATCTTGCTCAATTTGAGCTTTGAAGAGAGCCGGCAACTTTTGTTTTTGTTTGCGACGCC
AAAATTTCAATGTCGACGTCGCAGTCGGCAGCGTAAGCTTTTCGAGCAGCGAAAAACAAACAAAACGGAA
TGCGAGTaaagcaaaaaagagacacaaaaaaaaTGCAGTTTGTTGAATCTAGTACTAAGATTTAAATTTA
TTACAGACAAACGTAACTTTTATTTTGCACTAGAAAAatattacatattatattattatcttgtgttata
ttatattataattaCTTACCGAACACCAAGTACTTTACAGTATTCAAGTTATTTTCCTTTTTTAACGTAA
TTAATGAAAATATTCATTATCTTTTAATCTTAATTTAAAATATTAACTTAAACTTCATCTCAAATAGGAA
GGCGCCGATTAAAAATCGGAAATAGAGATGGCAAATAAAACGAATGGTTGTTGTCTTTACTAAAGGTGAG
TTGCGCAGTTGCTAGTAGTGTGACCAGACGGCGATTCACAAAATTTCTCCAAAGTGTGAACGTATGCGAA
GATACTTCGTTGATGGAAGAGGAGTGTCACCTTTTTCTTGGTTTTTCCAGAGCGTCATACTGCAAAGAGT
TGTTGTTGCGCGCGGGCGGACGACGAGAAAAAATTGTAAACGCGGATGAAAACAACGGTTGATTTTCACC
CAACGCTCGTGATTTTCGACCGCTTTTGAGTGTGGAAGCGAAAAAACCGCCTGTTTTTTAACTGCATGag
tgcgtgcgagtgtgtgtgtgtgcgtgcgtgtacgtgtTCGTAATTTAGACGTGCTGCTCGCTGCGATTTT
CGTTGTTCTGTGTGGCCCCTGTAACTACATGTAAACGCGGATGAAAACAACGGTTGATTTTCACCCAACG
CTCGTGATTTTCGACCGCTTTTGAGTGTGGAAGCGAAAAAACCGCCTGTTTTTTAACTGCATGagtgcgt
gcgagtgtgtgtgtgtgcgtgcgtgtacgtgtTCGTAATTTAGACGTGCTGCTCGCTGCGATTTTCGTTG
TTCTGTGTGGCCCCTGTAACTACAGTAAATACTCGTGCAAATAACGCGTCAAAGCGTGCGAAAAACGCAG
GGAGAACGCCAGCTAGCGCCTTCTCTTTCACTTTTTCGCTGATTCCTCGCCGCCTTAGACTAGCATTTTG
TTGTTGCGCAGCCTGCGCGTATAtgtgtgcgttcgtccgtgtgtgtgcgcttgtcctgcgtgtgcgtgtg
tgtgtAAATTCCAAAACATACGCCGCTGCACAGTGGCACAAAAACGAGAGAAACGTAACGTAACGAAATA
GCTATTTGGATTCCCGGCTTTCGGGCTTTCCCTGTGTGCGTGTGTCCGTTTGGGTGTGTCGCATTAATGT
TAATTTTCCGCTGAAGTTTTTCACTATTCCACCATTCCACGCAGCTTCGCTGTGGAGAGGAAGCAACAAA
TGCGATGCGTTGCGTTGCGTTTCTTGTTTCTTTTAACTAACTACGTGATGCAATGCGTTTTATGGCATTC

CAACCAAGTGCCAATTAGAATTGATTATTATTCATTATTTCGCTTATTACCCACACAGGCGCGTGTTTGT
GCGTTTGTGTGTAAGCGGCTCTCTTTTGGCGCTCTCTCGCTCGTCCTCTCTCTTTCAAACAAACTCCAGT
GAGCCGCAACAACAAAATGGCAAGGCCAGGCAAACACaaaaaagaaaaaaGTCTATCTTACGTTTTTCCC
TTGCCGTTTCACGTAGCGCAACGCCTAACGTAATCGCAATGCGAGAAGCaatcaaataaataaatataaa
taaaatgtaaaatttaattacaggtgaaaaatGTGGGCGAAACGTGTGCGATAAATAAGAGCCCGAGATA
GCGAAAGAAAGTGTCCAACTATCGGTCTGCTACACCGGCACTGTCGATGTCCGCCAGCTAGCCAAGTGCT
GCAGAGACATCCTACGATCGAAACGAGCTAACGTTACAATAGAACACCCCCTCCTTTTCCCCCCTCAAAC
AATCGAAAAGCCGGCAAGACGACGTCCAAAAAGAAGCAGTCACAGCCGACGCGGCCGAAGACGAGCATGT
TCAAGTGCATTCCCATCTTCAAGGGCTGCAACCGGCAGGTGGAGTTCGTGGACAAGCGCCACTGCTCCCT
GCCGCAGGTGCCCGAGGAGATCCTGCGGTACTCCCGTACCCTGGAGGAGCTCTTCCTCGACGCCAACCAC
ATCCGGGACTTGCCCAAG
>dme_circ_0007660_junction_seq
CGCCAACCACATCCGGGACTTGCCCAAGGTGCGTATACTCAATTTGTTAAATAGTC
>dme_circ_0007661
gtgaaaaatGTGGGCGAAACGTGTGCGATAAATAAGAGCCCGAGATAGCGAAAGAAAGTGTCCAACTATC
GGTCTGCTACACCGGCACTGTCGATGTCCGCCAGCTAGCCAAGTGCTGCAGAGACATCCTACGATCGAAA
CGAGCTAACGTTACAATAGAACACCCCCTCCTTTTCCCCCCTCAAACAATCGAAAAGCCGGCAAGACGAC
GTCCAAAAAGAAGCAGTCACAGCCGACGCGGCCGAAGACGAGCATGTTCAAGTGCATTCCCATCTTCAAG
GGCTGCAACCGGCAGGTGGAGTTCGTGGACAAGCGCCACTGCTCCCTGCCGCAGGTGCCCGAGGAGATCC
TGCGGTACTCCCGTACCCTGGAGGAGCTCTTCCTCGACGCCAACCACATCCGGGACTTGCCCAAG
>dme_circ_0007661_junction_seq
CGCCAACCACATCCGGGACTTGCCCAAGgtgaaaaatGTGGGCGAAACGTGTGCGA
>dme_circ_0007662
gtgaaaaatGTGGGCGAAACGTGTGCGATAAATAAGAGCCCGAGATAGCGAAAGAAAGTGTCCAACTATC
GGTCTGCTACACCGGCACTGTCGATGTCCGCCAGCTAGCCAAGTGCTGCAGAGACATCCTACGATCGAAA
CGAGCTAACGTTACAATAGAACACCCCCTCCTTTTCCCCCCTCAAACAATCGAAAAGCCGGCAAGACGAC
GTCCAAAAAGAAGCAGTCACAGCCGACGCGGCCGAAGACGAGCATGTTCAAGTGCATTCCCATCTTCAAG
GGCTGCAACCGGCAGGTGGAGTTCGTGGACAAGCGCCACTGCTCCCTGCCGCAGGTGCCCGAGGAGATCC
TGCGGTACTCCCGTACCCTGGAGGAGCTCTTCCTCGACGCCAACCACATCCGGGACTTGCCCAAGGGTGA
GTGCGTAGCTTAGGAATCCTTGAAAATGAACTGGACCAAACGACCAAGGACACTGACATTGGGTTAAAAG
CAGTTATTTCATTTCATATTATTGTGAAGAATCCGTTCATGATGCGTATTTGGGTTTCTTGCTAACCAGA
TCTTAAAACATGAGCACATTTAACCACAAATCGAATGTTTACTTCTGCAATGAATAACAGAAAAGTTTAT
TAGGTCTTGAAAAATTAATGATTCATGGCGAAAAATAGTCATTCGAAAAGCAAAATAATTGCCAATCATA
AAAAATGAAATCGACGATAGATAAAGATACATTATTTTGTATATTATTGTTTAGGATTTTGCCAGTAGTG
AGTTGGGAGAGAAAAATTAGAAGTTGGTATCAGAAAAAGTTGTACTGTACATATGGCGTGGTTCCGGAAA
ATCAACCTGCGCTGACTGACTGACTTCAATGAATCTCAACTTTTGGCGCAAAACCGTAAATGTCATAGTT
CAAAGTGCGTGGGAAATTGAATAAACGCCGTCGAGTCAGGTTCGCTTGAAAAAAGTAAAACCTAACCTAA
GTGGGCGGCAAATGAAAATTAAATATTAATAGATGCAGTGTTTGTTTGTGCTAAAGTATAGAGATACATA
CATACAGTGTGCAAAAGCCTCACGTACGAGTATACCTTTTGTTTTATTTGAAATATTCAAATTACAATGC
AAACAAGTCCACAATCCAGATCAATTTCCAAGCAACATGACGGTCGCCTAGAAATATGCTCATAAATGCC
AATGGACCAATAGGTTGAACATTCCGATAGGATTGTTTGATCTGCTCAAACCACTCACTTTTATCTGTTT
AACAACTTTGAACAACGGCCTTGTCTAGCggcgatgacgatggcgatgccgatggcTTTACGTTGGCAAA
CTTGTCCCACCGAATGGCCTGGTCCTAAGCTCAAGGGGTTGATGGCTGGGCACGTAATTTGCATCCCCGG
GTGCGTTGACCCCGCCCAGGGCCATTCCCCCATCTCCTGCACCCCCTGTTCCTCCTTATGGCGTCTTGTT

TAGCttttttttttcttttttGTATTTTCCCCGTGAAAAGCCCGCAGTCAGGGCCATTCTCCTTTGGTCA
GCTATCTGCTGGAGCATCTCGCCGTTTACCATTTACCAAGACAACAATTACGACAAAAGAAGAAGTTTCC
CTCGGCCGAGAGCGGAAGTCTTTAATTAATGGCGGGGGGAAAAAAGGGGGGAATGAATCGCCAAGTGGGC
AGCACTCACTGGCTTTTTCTTTGACAGCAGTGCAGCCATCATAATGGAGAGCATAGCTGCATAATTGAAT
CTGCAATTACGCAACTGGCGATCAAATCTCTCCGCCAAGTGCTGCATTCTAATTAGTGGCCAAGTGCCCC
CGATAATAGTCACATAAATTGGGTGTGGTCGCTTTTCCAAATAATGCTTCATCTTCTTTTGTTTCGTTGG
CCGGTGAATAATTCTGTCGCTGTTGTTTATTTGGTTTTGCCAGCGGTCTTCCGCCACGATTGCAGCTGGT
AAATAAACAAGTAAACAAGCCAGCAGCAATCGGACATAAACATTAGTGCTGGCTGGCTCGTTGACATAGC
AACAAGTTGTGGGCGAAGGCAAATAGATAGCTCGCTGCCTGCAGTTGCAACTGCAATTCATTTGCGGTCA
GCTCGCGGAGGTCTTCTTGGAGTTTTCCCACTGATTTATCCCCAGCATTTGCATCAACCGCACTCACTAA
TGAACTTCTAATGCAAATGCAGAGCGCTCTTCCAGCTTTCATTGGGGAAGCCACATTTTGCAACATGTGG
CAGCAACTACATGTTGCTCTGGGGCAGCAACTACAATGGTCTAGTCTGCCATATAATGATGATGTTCTGG
CGGCGTGCTCGTTCGAATGCTAAGCAAAATGACAGCAGCGGATGTGATTGTTGCTTTAGTTTAGCTTCTG
CTGATGGAATCGCTATGCTCAACCCTTTCTGGATTGTTGAGAGCGGTACACAATTTCCGCGTTGTCATGG
CATCGACAATTGGGGAGGGTACACGGCGAAAAAGGGATATTAAATCGAGATGAATGGCCCATAACTCGAT
TGAAATAGGTTCCAGGCAGTTTTGAAAGCTTGTTTAGTGTGTCGTAACTAGTTGTAAACATTACCAACGT
CTTAATCCCAACTATAACTAACTGATAATCTATTTAGGAAGATATATCTTCTTTCAATTTAATATACTTG
ATTTTAGTAGTGTACTCGCAAACTGTCTTCCTCAATTGAATGGTTGTTAACTCGGCGACAATCTGCCTCA
ATCTGCGACCATTCGGTTTGCCCTCATCGGATGGAGTTGGCTGGCTTGCAATTGAATGGGAAAATGCTGT
TTTGCGTTTAGCATTTTGCACAATCATTTAATGGTGCTCACTCCGCTCATCGCTGCTTCTATGCTTCCTC
ACTATAATTGGTACTTTGCATTATGCAAGTGCTCGATTTTTGGCTCTTCCCCGCTGCGATACCACCGTGT
AGCGTGTGGAACCTACCCCAATGAAACGACTCGGCCAAGAAATTGGCTTCTTCAGTGCATACTAGCCACC
CAAAATCCGCACCCATTCACAGTCACCGGCAACCGCTGGCCCCATTCATTTGGGCCAATTAGTCACGCTC
GAAGATAAGATACCGAGTAATCACCTTATCGCCTGGCATTGCCCCAATGGGCCTGAGGTTCAACCTGCTT
AGCAGACCGACCGCCCGCCCGCCTACCACCAAGCTACTTACCTTCACCACCCACACCTCCTTCTTTTCGT
TGGCTTTTGCTGGCTTTCGAAACCTGAAGCGGAGCAGGTGCCTTTTATTGTTGTGGGTCAGCGGCGGGAG
AGTGAAGGGGCTCTTGTGAAAACTTAACCGGCAGTCTGGCTATTGAACACACACAAAGAAAAATGCCAGA
GCAGTGGCAATGACATCATTTGGCAGTAATCGCGCGTACCTGACACAGCGAACTCGACAACGGCTACGAA
AACAGTCAGCAAGAATCTAAAATGGCGGTCGGCTTTTAGTGAGTTTGGCTAAGTGAATGCTACTCACTAC
TCGCCGCACAACTTGGCTCAACACCTGCTGGCCATCCCCAGAAAATGAAAAAGATGTATATAGCAAAACA
ACGATTTGGCAACATTTTCGGTCATCAGTATGTCTAAAGATACTGTTAAACTATACATTACGTTTAGTTT
GTTGAGGGGGTTAAAGAGGCCTAGCTGAAATATTGTCAAGGAACTGAGATATGTATAGATGTGCTTATAA
AAGGAAGATACAAAAAGGTGCTGTATATTTTGTGGCTCTTATTATTCTTATTATTAGAAAATACCACAGC
ATAACACTATCATGACTTTAAAAACAAATCTAAGATCTAAGATGAAAAGCACAAGGAATATAAACTTAGT
TTAACTCCTATTCCAAAGAATTTAATCAACTAATGCCAACTTAGATAAAGCCATTCTAAGCAAGACAATC
ATTCTGCCACCTTATTCGATGAAGTGCAGTTCCGATTTAACTGCAGTTATCTTTTAGCCCCGAGACGAAC
TGTACCTCACTTGTGGAATTTTTCATTCGATTTATTCCGAGTTTTTCATTTCGTTTTCGTTACACTCAGC
CAACTCAACGCAACGCTCGTCTCTCGCATTCCCCAGCGTCCACAGCTTACCACTTTTATTCTAGCCACTA
CTGACTGAGCTTGAGCAAACCGGTTTGCCTATAAATAAATAAATGTTTTCCACTTAATTGTTTGTGAAGC
CTTTTGTATCAAACATTGCGTCTGACCGACCGACAGACTGAAGAGACTGTCGAGAATTTCGGCTTGCAGC
TGTTTAAAAAGTTATTTAGGCAAAACGCATTTATATAGTGCGGTGATTGTAGTCCATGATTTGGCTAAGA
TGCAGCTGAGATTAATGGCGCTTGATACGCTTATTGCAATACGCTGTATTTGCATCTAAAACAGATACTA
AATTTCCCAAGCAGCTGTTTGGGAAACGAGGAGCGGTTAAGTGCTTTTCCGCTGACTAGCAGTTGGCACT
GCTGTTGTTAttttttttttttttATTCGGTGGCTGCATCTTCTGCCGTTGGCCATAAAGAAAGTCGTcc

gtttgcccgtttgccagtttgcTGGCTTGTTGGCATTGGCATGACAAATGCACCGGGGCGCACACAAACA
CTCACTCTACGGGTCTGCAAATCGTGGATGGTTGGATAGAAAAATGAAGATGTACAGATTGGAACAGGCG
CCCAGATACACGAAGCTAGACACTACAACGATTCCAATTTCGATTCCGATTCCTTCTGCGGATGCCACTG
GAGAAGCTCGCAAGCTCGTGTCTTGTTTGTTCGACGATTACACATATTTGTATATTTTTCGACTCCCCTG
TTGATGTTGCAGTGGCAAAGGCATCGGCAGCCATTTGTATCCGCTGGCAGAGaaaaaaaaGAAGTCCATA
ACTTCAGGTTTAATTCTGAGCTAAATCTACTTGGGGATATTCATAAAATACAATTTACAACTTTTGAACT
CATTAACTACCAAGTAAAGTAGTTTGTTCAAATCTTTTGTTAACTTGCTAAGTGATTTTCCCTGTGTATT
TTGTATCTCCAGTGCTGCGTGAAATACTAAACGTAGTCGCAGTCTTCAGTTCCAGCTCTGCACATAATTG
CCGCTGTCTGTCTGTCCCTCCAGTCGGTGTTTTTGTATTGCTGGCTGTTCTAGCTCAGCTGCTTTCGGTG
GCTGGTAGATGCGTCCGTTTAGATATATATCTACGCCGAAAAATGTATCTAGAACCCGTTTCGTCGCGAT
TGTACGCCGTTTTCTGGGCGCCTAGTCATTTGCAACATGCACACACCACTTGCCAACTGCGACTATACGA
CCATGTGGATATATGGATATATGTACATATGTATGGATATATGGCAGTATGTGCGCTGTGTGAGTGGGCC
AGATACTTTTGGCTTTTCAGATTACACTTGCAGATACTGGGGCACATTAAAAAATTGCTGGCGGGCAGCG
AAGCGCACATTTCCATACCCACACATAGGTCATGGATCAGTGCAGTTGCACTGGAGTCTGGACCCTGGAT
TTCATTTTCCGGATTCCGAGAGTCATCAGGGTCTATTAGGTTTGCTTTTAGGCAGCGCAAATTGGTTTGC
CGACCGGGCTTTTAACAATGGCAAATTGAATTGTTTTTGTTACAGCCGGTCGGCGCTGCGCGTAGGGGCT
GAGATATGTAGTGGCCGGTCCAGAAATCGAATTATGCACATGACGATGATGATGCAGCTCAAACAGccag
caaccagcaaccagcaacTTGTCTGCTCTCTGTGATGCAACCTCGGAACCTCAGTTGCGAATTGCTGGCT
CTGTTTTGGCCTGGCTAAAAGCCAGCCACCTACCGCCTGGCACTTGCTGTGCGCCTGCTCCGACTAGATA
CAATAGTAAAAGTGTGCTGCCCTGCCgagtgagtgagtgagtgagtCTACCATATCTGTATCTATCTGTA
TGTGCGCTTGAGTGGCAGACAAACTGGTAGTACTCCATGGCTGGCACTCTGTACTCTGCACTCGTACTCA
TGGCTATATATGCCGGCCGGCAGACCGGCCGGTAATTTATGGCCAAGATACATACAACAAGCCCGAGTTG
CAGCGTGGGCTTTTgttgcttggtaggttgcttgataggttggttggGTGGCaaaaaaaacaaaaaaaaa
aGTATCTTTTGTGACCAGCACTTGGCGAAAAGTTTACAATTCGCATTCATTTTTATTGTCGTCATGCCTT
GTTCTCGTTTTTGTCTCTCGGCCATTCACCACCACCCCCTTTTTGGCCCCTTTTGTGGGCCATTTTCATA
TGCAAGTTACGttttttttgtattcttttttttGGCCTGCCCGGACGGACGGACTGCCTGCCTGCCTGTC
TTCCTGCAtttttttttttGTTGCCCCTTTGTCGCTGACAGTTGCAACTTTTTGACGCATGATTTGTTGA
AAGGCTTTTCGCTTTTTCTGCTTTTCTGTGCATAAACAGCAAACGGAATCTGGCCATTGTAGGTGGCCAA
AAGCAATGCAGCTGACACTGATGGAGTCTAAGCCGCTTTGTCTTTAAAATAAAAGCCCCTTATGCATTGT
TGCATCGGTTGCTGGTTTCTCTGACATTGACTTCCACGTAACCATGTTGAGTGTAATATCTTAAGTGTGT
TGTAGTGTAATAAAAATTAACTACTAAAATAGATAATAGCACTATAATTATCGGTTCAAAATACCATTCA
TATTATGACATATGTCAGACCTTCTGGTACAAATTAAAAAGTTATGGTTCTATTAATCATTTTGTTGTCT
CTTAATTAAAATGGATTTATGGGTTGCCGTTCCCAAAATTCCTTTCGATTTGTATGACCGTAAATGCCCA
AAGCTAGTGTTTCTTGGCCGAGGTACGGAATTCGCTCGTTCAGTGCCCCAAAAAACTGGGTCAACAACTC
CAGCACTTGGCCAGCACACCACGAGCACTTCTATTGTCATATGGGGcaaacaaagaacgcaggaaaaaat
cagaaaaaaagaaggaaaatagaaagaaTTTAATAAACTTTACGGCCAGAACTACGAATTATTTTCATTA
TTCTGGTGGCGACTGCTCTTTGGGGTTCGGCCCAAAGTAAACTCAAGTAAAGAACCCAGCAACAATGGCC
GATAAAAATCGCGCTGGCAGAACAAAAGCAGCGGCTGTTAACCTTGAGGTCTATGACTCAGTGAGTCAGT
AGGGGGCGCAACTTTCTTTCCAAAGATGAGTACTACTCCACATTGCAGGAAGGTCAGGGTATGGGTATTT
TGTGTTTCATATTGGATACTGGGCCTCTTCCCTTCACTTTGGAAAGTTTCCGAGTGGAGTGCCAACATAA
TTTATTTCGAAATTCGAATCGAAGTCGTGATTTTCATAAATGAATTGTTGGCGACATGTCTTCTGTCTGT
CTGACTGCCTGCCTAGCTAGATGGCCGGGTCGAAAGGAAAGCTGCAAGGCTGGCCAAAATGGAGGATTCT
AATGGTGAACTGACTGTTTGGCCGGGTCACGGCACTAACAAATGACAACCCCCTAAAGATTTATGACCAC
GAAAACGAACTAACTGAGTTGCAATCCATTGGCTTTTGTTTGCAAAAGGCTCCCGAGTACGAGTCGTGCT

GAGGTCTAAATCTTGttttttttttCTCTTCTCCGTGCTTGCAGGGTAAGTGCCAAGTATCCGCCTTCAA
AAAACGATGAAAAACTGCCCCATAAACATGCAACAAGTGTGCCCATGATGCTCTAAATGACATTGATGGA
CTATAGATTTGAAAAAAGTGTAGAttttgtttttttttCGGTTAGATGGACAGCACGTGTCCGTtgattg
cagtgattgaatgattgagtgaCGGAAAGGGGGCGGCAGATCAATATATTTATATATCCAATGGAGTGCC
ACATGCCATACCACTGATGTGGCAACAATTGCATACAATAATTGCATCCGTGTCACTGATTGGAATGGGT
TTTTATCTTTTATTTGCGGTTGGGATTGATGAGCCTGCAACTGAATCAGCATATCAAATGACGCATTCAG
CTTTCCCCCATGTTGTGGCCTGATCAACCTAATTATGGAAAATGTGGAGCTGTCGACCGAAGGTTTATTC
ATGATAGCTCTTCTTGTAAAACTAATTATATAAACTGTGCCAGATAGATTTAATCAAATGAAGAAGGAAA
ACATTAAAAGCGGCATAGCATATTAAATAATGAGAACTAACAACATAAATGGGTAGAATTTAAGATCAAT
CACACTTACAGATTGAGTTAAACGTTAAATTATTAAATCAGTAACAGGAATCCCCGATCTTTTCAATTAC
CTAGTACCtttttttttCAATTTAAGTGCTCTTCCTGTTCTTTTATCTTGTTAAATTGCTCTGTATTACA
GACTTTCAGCGACTTTAAACTCAATTTAAGTGCAAACAATTGCATTGTCTTATCTCTCTTGGCGCATGCA
ACATCCGTATTGCCACTGCTCTTTTCATGTTTTGCAACTACAAGTTGTTCCTCAAACAAAGAAGATGCAG
CTGGGCTTTTTAATAACTTTTCCCGAGCGAAACTTCTGCCGCATATATGCGGACATAAACGCGCACCACC
CGCAGAACCACCGACTCAATCAGCGGCATAGCCCCACATATACATACTCGTATATGCATTTTCCGCTCAA
GCGAGCATAAATCCGTAAACAGAAACCCGTAATTGGAGTCAAGTGCAGCTTTTCAAGCGATGCGACGCAT
ACGCATTATAATAAATGGAAACAAAatatatatatatatTCAGAATATAACTTGAATAAGTAAATCAACT
GCAGAGCAGGCCAACCCCCCTTTCGAACTCAAATTCCCAGTCCCCGCCACTTGACAATATGCATATCCAT
GGCAACGCAACTGCTTACGAATACCGAACGACGAATCATTCTCATTCATTCACAGCCCCTGAAACAACAT
TATAGACAATGACGTCGAAGGGGCGCTTCCCATTTCCGTCCAAAGGTTTCAGTTTCATTTGATCGCAAAA
CGAAATTCAGCAACTTTTCGAGGCTATCGCATTAAAACACAGagaaaaatatttaaaacattatttttaa
ttgaattccattaaaaaaaaaCGAAGGAAACTCATTTTATAATGATCCAAAGtatatattatattatata
tatatTGATTTTTGTGTGCACTTGGCCAGGTTGACTGCACTTGTTAGCTGATTATTCGCTGATTTTAATA
TGACTTTGTCGGGCTGTGTTTGGTTTACTACTTACTTTTAGCTTGTTGTTTTGCTGATTGTGCAGTTTGC
CGTTTATACACATGACCCACAAATGACCACAAACTTATGCCCGGCTCGGTTCGGCTCGGCCCAATCTCCA
CCAAGTGAATGGGTTCGAAGTGGTTCCAGTTTGGCATTGCTGTTGAAATCTGAGCTCGATTTTCCTGGCT
CTGGGTGAAAGGCCGCCCACGTTAGGATCGCTTTAGATTATCCGCCAGGCAGGAATCGTTGATTAATGGT
CGTTAAAGAGCGATAAGTTGATCAGCGAAATATTAAAAGATCTCTTTCAGTGAGCATCATGCTTTCTATT
TGCCAAGCGAGCATGTGGTTTATCTAATGTGTGGGTGGGAGGGTTGCGACTCCCTCGGGGAAAGCCTTTC
ACATTCAATTCCTGCGGCGTCTAAGTGCACATTTTATTGAACCCCATGGGCCATGGGCTGCGGCACAGGT
GATGTAAAAGTTTGCGTACCTTTCTATGTAATTTCCCAAGCAAATTTTCTTATTGCCAACCGCAGGAAAA
TTGTATTTCTCAGCTTTGTGTGGGAGTTGCACATTTTCCATTTTCATGTAGCACTTTTTCTATGCTGGCT
CAAATTAGCCAGCTCGATTTTGTATGCGGCTGCTTTGCCAGCTGCAGTTTGCCACAACAATGGGCGTTCT
GGCCGCAAATTTTGCAAGTGCAACAGTGCCACGGcaacaccaacaataacaacacaacagcaatagcaac
aaacaaaaccaaaaccagctgcaacaacaaacACCCAAAACACTCGAGACCAACTGCACTTGACAATAGA
ATTTTGTACGCGACACTGGCATCGTGCATTTGCCACCCGTGCCTGCATTTTGTATGCAAGCGACGGCAAT
AATAACAATTCTAATAAAAACACAAATTCCCCTGGCTCTAGTGGATTTCTGTGGGTGTACTTGTGTTTGC
GTTTGTGGGTGCGTTTCTATGGACACAGCTGAGCGCAGAACAATAGCATCCGATCCGCACAGTGGCAGAA
TAAACAAAAACATAGTATCTTGAAATATCTTTCAAAGACTGAAAATATAAAACTAACAATTTATTTCTTG
TTTATAAGAAATGTAAAGTAAACTACTAGTGGTTAGATTTATAAATAGTTGATTAACCACTGTAAATAAA
GATTAACTGAATCATTTTAATTGATCAGCTGGAGAGGCGGTGCTATAAATTCGCCCACCACTGTGCACTC
AAGTACAGGTGTCATTGTTGGTAGTCGCTAGTTTTGCACGTAAATCATTATGACAAGTTGACCCGCTTAC
GAAAGGTAAAGCTAAAAGGGCTCAGTCGACGCCGCCGCCTGCTGATATACTATTTTTCACCttttttttt
ttGCAACAACCTGCTAATTGTTTGTTTATTTGCTTATTGCGTTTTCTATTTCAACGCCATATATCTGGCA

TATATATTCGCTATTCGCTTGCACTGCAGTTTTTTCCGTTTATGGAGAGCTGCTTTCATGCCGTGTTTTG
TTTATTAAGTAACCAGACCAAAATTCATGTTTTCTCGAAGCCCCCAAATGCGGGGCCGTTGCCAAGCCGG
GTTTATTTGTGTGAATTCGTTAAAtttttttttGGCTAGCAGCGAAACAAACAAACAATAGAATTGTGAG
AATTTGTCGCTGGACTCTAATTTTTGCAATGGAATGTCGCCGCGATTGGCTTCGCTTGGTTCAGGCGTAC
AAATTGCAGTGCATTGCCAGGGGCCTGCTAAAATATAAAATGTGAATTTTTCCGATTTATTCAGCTGGAA
TTTCACCCAAACATTTGTTTTCTACTTAGTTATTAGAGTGGAAGTACAGACTTTTACTGCCTGCTTTTTA
ATCAGTTTGTGTTGTCAGCTTTTTAGCTGATTTTCCCTGCTGGGCAACACTATTTGCCCAAAAGCTGATT
GGAAACCATTGTTGGAATGCCCGAAAGTCGAGCGCCCCAAAACTCAAACATAAACTCAATTAAATGCTAT
TTGAAAGTCATGCCCTTAGGCCCCGTATGACACTTGTTTCCCCCGTTTCTTGCATTTCTCCCACAAAATG
TGTGTCATCTTTCCTGCTCTTGCTGTTTCTTACACTATGTTGCAATTGGGGCGTCAACTGGGTCAAAGTT
AAAGTGCGCGCaaaacagaaaaaaataaataaaaaaCAAATCGCGGCGCCTCTGTTCGAGTGGCGTGCTC
ATTTTGTTGTTCTTGGAGACTGCCGGCGCTTTGGTCCCCAGGTTCCAAGTGCAGTCACTTTCACACACAC
AGTTTGGGCACAAGTAGGAGAAAAATAAGTCGGAGACAAGGCATCAAAAGAACGTGGTGCACTGGAAGAA
AAACCCATTCCAAACATAAGAAATATACTCCAACTACTACTACAATTTGCTGGCATATTAGCACGCTCTT
CATAAATTAATATTACTCATACCttttttttttcaattttaccaattttcatatttttttttttttGTAA
GCGTAAGCTGAAAGAATTAGCAGAAATGGCAAGCGATGGCTGGAGGGGGGAGAGACGAATCGGGGCAGCA
TTCGAACAAAGGCAAAAATCAAAGCTTTGTCGTGGTCTTGGACGAGCAGCCGACCATTAGAGTCCATGAC
TTTGTAGGATGGTCGCCAAACAGCGATTGTTGTTGGCCCAAAGTCAAAGCGGGCCGCGAGCGACCGCCAA
GTGATGGGAACATTTCAATATACTCTAAACAAACAAACAAATTGAAGAGTTTCGGACTGACTGACTGACG
GACAGTCCGTCGGAGGAGGAGAAGAAATTGTATAATTAATAGCATTAAACAAAAACTAAATCAAAATGTC
AGAAGGGGAAAAATCAGCGCAAATTGAAAAATCATTTCTTCTCACGTCGGCCCATTTTATTCAATAGTGT
CGATTTGCTCGCTGCCCTCTGCTCCATCCCATCCCATGCCCATTCCCATGCCCAGACCCAATCCGATTTA
TTTCGATCGTGTCGGTCTCTGGAGCCCATTGACGTCACGAGGGGATGAGCAATTAAAAACTACTCGGCCA
GCAGGCAGCCATCCAACGAGCTTGTACAAACAACAAATCAAAAGTGAAGAATCCCCTCGCCTTGGCAACA
GCGATCGGCCAACAGAAATGAGTTGGTGAAAAGCATAAAACAACAAACATAAATGTGGCAAGCAAATCTG
TACATATATATATGTACATACATGCTGTTCAAATGTAAATGCATTCATGTTTACCATACAAATAAAGCAT
ACATATGTATAAAATACAAACAAATAAAGATTACCATGTTAATTTGTTAATAATTATCCATTAACGTTTG
CCAAACCAATAAAGCTTATCTAAAGTAGTTTAACAAATGCATTAAATGCACGCATTAATTGGCAATTAGT
AAACAGATAATCCTCCATATAATTATGTTGCCAAATGCACTTACGTTTGCCAAGCaaaaaaaaaGACttt
ttttttACTAGTTCAAGAGCTAATTGCACGGATTACGCAGGCAATTAGTTAATTGAAATAATATTCACCG
AGTGTCGCAGGTGGCCAAAGGTTATGGCAGGATTGTGATTAAATTAGGAGCAGCGGAAAATTGATTACCA
AGTGACTTGGTGCTTGGGAAACCCAGCGCAGGCTTAAAATGTCAATTGCAGATGTGCTCAATCTGTGGAG
TGAATGttttttttttttttttttGAGGGGCGGACGCATCTGTGTTCGTCTTGTCGGGGGCTTTTCCTA
AACTTGTTGCCTGCCTAACGAGCGTAAAACAGTCATTAAATTCACCCAGCCAGCCACCCACAGTTCAAAT
ACCTGAGACGAGTCTCTTTGTTTGCTTGCTAATTGAATCTGAATTAAAGGCCTTAGTCAAATACACTTCA
TATGCGCTCGTTAATTCCGTTTTTAATGCACTTTAAAAGCACCTGTTGTGCTTAGAAATCGTTGTTGCCC
TCTTTCCCATCATCATCATTATAGTCGCATCTTTTGCCACAATTAAGAGTCATTTACTTATCACAATCTG
AACAGCGCCACCTCCTCTTAGCCCGCTTCATTTCATCTCATCTGATCCGAGCTCAGTGCAGTTCAGCTTC
TGCAGGCGATGTGGCAATCATCCCGGGGTGCCATCAGTGATGAAAACTTGCAGATTACTTAAAAGTTTAG
CTAGCAAATTTAATAGGTACTAGCTTAACTAGTTCTAACAAGCATGTATTTATGGTAACATTGATGTATT
GCTGGTAATTCAAGTTACTCATAACAGGAAGTGGCTTTTTCTTGAAAGTATTCCTTTCGAAAAGGTTATG
TTACTAACTTTTTACTGATTTTGAACCTGCGGGAAGATACAAGTTGCATAATCTTAGATGCAATCATTTA
ACTCAGACATCTTTAACTAGCCAATGGGGACTATAAAATAGCTGAAATGGCTGCACAGAAGTGGCGATGG
CTTTACCCTTGGCTCTGCCACCAAAGACGACGTTCAGGTTGAACGAGAATTGTGGTGGCGGAAAGCCATT

TTCCCTGTTGACGCTTAGGGATTTGCTCTCAACAATGGCAGCCATAAAATTGCAGGGAAGTCGGGATGGC
GATGGCGGTGACAACAAGGACTTGGCATGGTGCCCCAACTAACAGGTCCTGGAGAAAGGGAACCTTTTCA
TCATTTTGGATGCATGCAATAAGCGAAATGGCAACGTGCCATGGCAACTCATAACGACGGATGCCGAGTG
CTGGGGATTAAAACGTAACCGAGCTATTGAATCGGGATTCCACTTTTTCCCAATGATGCAAGGCAAATTG
AAAGGCCCTGGGATGGGGATTTTTCTTCTTATTTTCTTTTTCTCCTCGTCGCTTTCTAGGGCTTGTGAAA
TATGCATCCTCGACATCATCTTCATTAAGTGCAGTTCGCATTTGAGGAAGATGATGTATGGCGGTGACCT
CGATAGCAACACCTCCCACTTTTCTGTCTGGGTCGCCTTGATATACTCTGACAAATCATTTCACATTTCA
GATTCACATTAAACACAGCGTGCTCTTAAAATTTTTGCTCGTTCAATGACATTGAAAAGAATTTTCATAT
GTGTGTTCttttttttttttttttGTGCCAACCCGATTTGTGATGTCGTCGTTGTTTGCTGACGTTTCGC
ATGGCTTGttttatttttgatgcctttatttcggtgatttttctttttGTTATATATGTATGTAGGTATA
CGTTCCTGGCACAGTTCTTCTTCATTGATCTTGTCTGGTTTGTTGAAAATTATAAGCGCTTTAAAATGCA
AAAATCGCTACCGCATTGttcccgaatcccgaattcccgaattTTTGGAGCTCTCTCACTTCATAACGAT
AAAATATGATAAACAAATTAAATACATTTTGGGGGCATTTTCCGTTTCTTTGAAGGGCGCCGCCGCGCCT
TTATTGCATAATTGCACAAATTGATTACAATTCCGGACGCTATTTTCCAACTGTGCCGGCACACCCAGCA
TTTCCTCTAATTTCCCGTCTCCAAATGAATTTCCAAATGTGGCACCCAAAAGTGTGTCCCAATTCTTGTT
TCCATGTTCCATGttttttttgttccaactgttgtgagtgtgtctgtgtgtgtgAAAGGTCAACGAATTG
TTTGTCACCGCCGTTGATCTAATGACCGCCAACTGTGCGGCGCACAGCTGAGCATCTTGTGCTGTTTTGT
GTGGCATAGCCATATGTTTCCAATTGCCTTCGTGTGGATGTGGATTTTTTCTGGGAGATGGGCGAGGTAG
GAAAAGCGCCTTGCTGCGTCCATTTGTCTGCAAGTGGCGGGATCCAATTTTGAGTGTTTTGCTTGATGGA
TTGACGGATGTGTGATGTGTGGGGCTGCTGCTGTTTTTGCTGCGCCAAAAGCTTTTAGGCCTTCGAAAGA
TCGCGAGCAGATTGCCAAGGATCGTGGTGCAAAATGGCAAAGACGGTTGAACTTCAATAACTGGGACTGC
ATAACTTTGTAGAACCCCTTTTCATTTTTAACTCCAATAAATTTGATTTGAAAATGAATCTTTGAAACAA
GTTGATAAAGCGAAAAGACAATGCTTTCAAAGACGGTTGAACTTCAATAACTGCAAATGCTAATTAGCCC
AACTGTATAACTTTCTAGAACCCATTTCATGTTTAATTATAATAAATTTGATTTGAAAATTAATCTTTGA
AACAAGTTGATAAAGCGAAAACACAATGCTTTCCTACACATCAGTTGGTGTTTCAAGTTGTTCAACTGTA
GTATCTATAATTGGCCAGCACATTCAAGGCCATGAACAAGGCCTTCTGCCATTGCACACAATGCAGCACA
ATGCATACGGACTGCAAAAACATAAAGATAAATGTCGGTTTATATATGTTTATATAATTTTTAATTAAAG
ACTGTCGCAGCAGCAGCTCATGCTCGGGCTGCAGCAATCTAATTGTGTGGCTGTGTGGCAATATGGCAAG
TAGCAATGCAGTTGCAAATAAATAGCTTATCGGCAACCTGCCACAACTGGTGACATTCCCAGTAAAATGG
GGGTCTATGCCTGGCCACATCCGCTGATTGCCACGAGGCCACAGCCTCGCTTCTCCTTCCATTGTGTGCG
GGCCACACAACTCGAGCATGTCCTGCCTGcaggacaccagcacaccagcacacATTTCCCCCCCTCATTT
CCGTCATGTTGTTGCGCTGGCTGGCCTGTCGCGGCGAGTTCGCGTTGCGGGTCAGTCCCACGTGTCGTAT
TAGTTGGATTGAATGTGGACACATGGGGCAAGTCAGCCCGCGTTGACCGATGACGAtgtttcgtttggtt
tggtttcgtttcgtatcgtatcgtatcgttgctgtttcgttcttttcTTCTGGCTTTTCCGGGGGTGTAC
AAGTGTTTTGCCCCATGTATCTGGGTCAATTTAAACAGTGCTGAAAAGGCTTGAGGCCATCCCACACCTA
CTGGCACCTGTCGTTTTGCTTTTGCTGCCAGTTATGTGGCTTTTATACGAGGCTCTCGTGGAAAAGCTTT
TGTGGGCGTGCTGGTAAACAGCGCACACATACATGTTTACATTCTGTTTTCCCATTTCCCGGGTCCTTTG
CGTGCGCATTTCCGCTTTGGCAGCTCGAAAAATTTATTCGATCAACAAGTTTCGTTTGTCATTTTACGCT
TCGTCTCGCTCTATCAGCGCGGGGACAAACAATAATAACGAAAACCTTGGCCAAAAGAGGTGGAAACTCC
GCTCTATATACCGAATATGCAAAACTTAATAGTTTTTAAAACAAAACGGCCCGACTGCGCAATGAAAAT
CGTTCGCTTTAAAAGCGAAAAACTGAATTGATAGACAGTGACAAGGCCATATGCGGAAATCTCTCGTTCA
GTGGGTCTCATGGAATATTTATAGCAAATTTATTAATATATTAACGCGGCCCTTTGGAGCAATTCATGCG
TGAGCAAGTCAATCAGCCGAGCAGATAGAAATGTGTAACCCGATAACCAACCAGCTTGGCTTTGGCTCTG
GCTCATTGAAAAGCCATAACAGCAATCAGTAAACAATATGTTTTCCCTTCTTCACtttttttttATTCCA

GGATATACCCGACATTCCCGACGATATCAAGCATCTGCAGAGCCTGCAGGTGGCTGATTTAGTTCGAAC
CCGATACCCAAGTTGCCTTCGGGCTTCTCCCAGCTAAAGAACCTGACCGTCCTGGGACTCAACGACATGT
CGCTCACCACATTGCCAGCGGATTTTGGCAGCCTGACTCAATTGGAATCCCTGGAGCTTCGCGAGAATCT
GCTCAAGCATCTGCCCGAGACAATAAGCCAGCTGACGAAGCTGAAGCGTCTGGATCTGGGCGACAACGAG
ATCGAGGATCTGCCACCCTATTTGGGTTATCTGCCGGGTCTGCACGAGCTTTGGTTGGATCACAATCAGC
TGCAGCGATTGCCGCCGGAGCTGGGACTCCTGACCAAACTGACCTACCTCGATGTCTCCGAAAATCGACT
GGAGGAGCTGCCCAACGAGATCAGCGGTCTGGTCAGCCTGACGGACCTCGATCTGGCCCAGAATCTACTC
GAGGCACTGCCCGATGGCATTGCCAAGCTGAGCAGACTGACTATCCTGAAGTTGGATCAGAATCGATTGC
AGCGGTTGAACGATACGCTAGGAAAAGTGAGTAAAATAGTTTTTGATTTTAAATATAGTAAAAGCCAAAT
TAAATCGTGTAATTGGAAGTTTTTGAAATGCAAAGTCCACACAGTTCAGTTTTCCAGCTCATAACGTTTG
CTTATCTTATCGTCGTTATACCACAATATTTGTCAGGCCCAAACAAAATACTTGTTTATATTTAAAAATA
GAAGCGTTTCTTCGATACAGGttttttttGCAAACAAATATTTTAACTTAATTATGTATCTTACATTCTA
TATAACTAAACTATCTTATTTATTCATACTTTGCTATAGGCTGCGAAAACATGCAGGAGCTGATTCTGAC
CGAGAACTTTCTCTCCGAGCTGCCCGCCTCCATTGGCCAAATGACCAAGTTGAACAATCTGAACGTGGAT
CGCAATGCCCTGGAGTACCTGCCCCTGGAGATTGGCCAGTGTGCCAATCTGGGCGTGCTGAGTCTGCGGG
ACAACAAGCTGAAGAAGCTGCCGCCAGAGTTGGGCAACTGCACCGTGCTGCATGTGCTCGATGTGAGCGG
CAATCAGTTGCTGTATCTGCCCTACTCGCTGGTCAATTTGCAGCTGAAGGCCGTCTGGCTGTCGGAGAAT
CAGTCGCAGCCGCTGCTCACCTTTCAACCGGACACGGATGCGGAAACGGGCGAACAGGTGTTGTCTTGCT
ATCTGCTGCCCCAGCAGGAATACCAGCCAATTACCCCAG

>dme_circ_0007662_junction_seq

CAGCAGGAATACCAGCCAATTACCCCAGgtgaaaaatGTGGGCGAAACGTGTGCGA

>dme_circ_0007663

GATATACCCGACATTCCCGACGATATCAAGCATCTGCAGAGCCTGCAGGTGGCTGATTTAGTTCGAACC
CGATACCCAAGTTGCCTTCGGGCTTCTCCCAGCTAAAGAACCTGACCGTCCTGGGACTCAACGACATGTC
GCTCACCACATTGCCAGCGGATTTTGGCAGCCTGACTCAATTGGAATCCCTGGAGCTTCGCGAGAATCTG
CTCAAGCATCTGCCCGAGACAATAAGCCAGCTGACGAAGCTGAAGCGTCTGGATCTGGGCGACAACGAGA
TCGAGGATCTGCCACCCTATTTGGGTTATCTGCCGGGTCTGCACGAGCTTTGGTTGGATCACAATCAGCT
GCAGCGATTGCCGCCGGAGCTGGGACTCCTGACCAAACTGACCTACCTCGATGTCTCCGAAAATCGACTG
GAGGAGCTGCCCAACGAGATCAGCGGTCTGGTCAGCCTGACGGACCTCGATCTGGCCCAGAATCTACTCG
AGGCACTGCCCGATGGCATTGCCAAGCTGAGCAGACTGACTATCCTGAAGTTGGATCAGAATCGATTGCA
GCGGTTGAACGATACGCTAGGAAAAGTGAGTAAAATAGTTTTTGATTTTAAATATAGTAAAAGCCAAATT
AAATCGTGTAATTGGAAGTTTTTGAAATGCAAAGTCCACACAGTTCAGTTTTCCAGCTCATAACGTTTGC
TTATCTTATCGTCGTTATACCACAATATTTGTCAGGCCCAAACAAAATACTTGTTTATATTTAAAAATAG
AAGCGTTTCTTCGATACAGGttttttttGCAAACAAATATTTTAACTTAATTATGTATCTTACATTCTAT
ATAACTAAACTATCTTATTTATTCATACTTTGCTATAGGCTGCGAAAACATGCAGGAGCTGATTCTGACC
GAGAACTTTCTCTCCGAGCTGCCCGCCTCCATTGGCCAAATGACCAAGTTGAACAATCTGAACGTGGATC
GCAATGCCCTGGAGTACCTGCCCCTGGAGATTGGCCAGTGTGCCAATCTGGGCGTGCTGAGTCTGCGGGA
CAACAAGCTGAAGAAGCTGCCGCCAGAGTTGGGCAACTGCACCGTGCTGCATGTGCTCGATGTGAGCGGC
AATCAGTTGCTGTATCTGCCCTACTCGCTGGTCAATTTGCAGCTGAAGGCCGTCTGGCTGTCGGAGAATC
AGTCGCAGCCGCTGCTCACCTTTCAACCGGACACGGATGCGGAAACGGGCGAACAGGTGTTGTCTTGCTA
TCTGCTGCCCCAGCAGGAATACCAGCCAATTACCCCAG

>dme_circ_0007663_junction_seq

CAGCAGGAATACCAGCCAATTACCCCAGGATATACCCGACATTCCCGACGATATCA

>dme_circ_0007664

GCCCGTGACCTGGAATCCGATTCTGAGCCGTTCGAGGAGCGCGAACCGTCGCGCACCGTGGTCAAGTTCT
CGGAGGAGGCCACCCAGGAGAAGGAGACGCCCTTCGTGCGCCAGAACACGCCGCATCCCAAGGATCTCAA
GGCCAAGGCGCAGAAGCTGAAGGTGGAGCGCAGTCGAAACGAGGAGCATGCCAATCTGGTCACCCTTCCG
GAGGAGGGTGAGTTGTGGTCCTAGATTCGATTCAAATGCCAAGCTTCTGCTGTGTTTCCATCGATAGGGT
AACTCTAAGCATTTTCTAGCGCAAATATACGATTGCTTTTATACGTTGTCATCCTTTTTTGGCTGTATAC
TAATCCTACACTTAAGACTAGAGGCTAAGTAGCTTGATTATTAATAGACCAAAATTAACTGGGTTATACA
TTTTTAAACAATTAAGATTAGATTGTAACTTACATTACAATTTACATTTTGCTTTATGGAGAATTTTATT
CTACTCACTTTGGTCGAGGAGCTGTAGAGTGCCATGTAAAGTCAAGTaaaaaaaaaTATCCCTTTTCTTC
TCCAGGAATGGCACCAAGTTAGCTGAGACCCCAACCGAGACCCGAACCATTGCAAACAATCACCAACAGC
AGCCACACCCAGTGCAGCAACCAATCGTTGGTGTAAATTCCAAGCAACCAGTGGTTGTGGGTGTAGTCAC
TCCAACTACGACGACAGTCGCCCCAACCGGCGTACAGGGTGGCTCAGAGGGAGCCAGCTCCACGGCCAAC
AATGTAAAGGCAGCAACAGCAGCTGTGGTGGCTGAGTTAGCAGCCACTGTTGGTGGAAGTGATGAGGTCC
AGGATGATGACGAGCAGGAGGATGAGTTTGGGATACTCCGCATCACCTGAAGAATAAGCGTGTCCAGCAT
TTAACCGACAAGCAGGCCAGTGAAATCTTGGCAAATGCCCTAGCCTCACAGGAGCGGAACGATACCACAC
CGCAGCACTCGCTTTCCGGCAAAGTGACATCACCAATTGAGGAGGAGGAGCAGCTGGAGGTGGaacagga
gcagcaacagcaacagcagcagcaTCCCTTCGACTCCTCGCTCTCGCCCATTTCGGCTGGCAAAACAGCA
GAGGCTAGCACCGACCCAG
>dme_circ_0007664_junction_seq
AAAACAGCAGAGGCTAGCACCGACCCAGGCCCGTGACCTGGAATCCGATTCTGAGC
>dme_circ_0007665
GCCCGTGACCTGGAATCCGATTCTGAGCCGTTCGAGGAGCGCGAACCGTCGCGCACCGTGGTCAAGTTCT
CGGAGGAGGCCACCCAGGAGAAGGAGACGCCCTTCGTGCGCCAGAACACGCCGCATCCCAAGGATCTCAA
GGCCAAGGCGCAGAAGCTGAAGGTGGAGCGCAGTCGAAACGAGGAGCATGCCAATCTGGTCACCCTTCCG
GAGGAGGGTGAGTTGTGGTCCTAGATTCGATTCAAATGCCAAGCTTCTGCTGTGTTTCCATCGATAGGGT
AACTCTAAGCATTTTCTAGCGCAAATATACGATTGCTTTTATACGTTGTCATCCTTTTTTGGCTGTATAC
TAATCCTACACTTAAGACTAGAGGCTAAGTAGCTTGATTATTAATAGACCAAAATTAACTGGGTTATACA
TTTTTAAACAATTAAGATTAGATTGTAACTTACATTACAATTTACATTTTGCTTTATGGAGAATTTTATT
CTACTCACTTTGGTCGAGGAGCTGTAGAGTGCCATGTAAAGTCAAGTaaaaaaaaaTATCCCTTTTCTTC
TCCAGGAATGGCACCAAGTTAGCTGAGACCCCAACCGAGACCCGAACCATTGCAAACAATCACCAACAGC
AGCCACACCCAGTGCAGCAACCAATCGTTGGTGTAAATTCCAAGCAACCAGTGGTTGTGGGTGTAGTCAC
TCCAACTACGACGACAGTCGCCCCAACCGGCGTACAGGGTGGCTCAGAGGGAGCCAGCTCCACGGCCAAC
AATGTAAAGGCAGCAACAGCAGCTGTGGTGGCTGAGTTAGCAGCCACTGTTGGTGGAAGTGATGAGGTCC
AGGATGATGACGAGCAGGAGGATGAGTTTGGGATACTCCGCATCACCTGAAGAATAAGCGTGTCCAGCAT
TTAACCGACAAGCAGGCCAGTGAAATCTTGGCAAATGCCCTAGCCTCACAGGAGCGGAACGATACCACAC
CGCAGCACTCGCTTTCCGGCAAAGTGACATCACCAATTGAGGAGGAGGAGCAGCTGGAGGTGGaacagga
gcagcaacagcaacagcagcagcaTCCCTTCGACTCCTCGCTCTCGCCCATTTCGGCTGGCAAAACAGCA
GAGGCTAGCACCGACCCAGGGTAAATACCGAAATTGTTTCAATTAATTACATTTATATTGCTCTATCTCT
GAGAACTGTGTCCAAAACAGCCAAAGTGCTTTGGATGCAGCTTGAGATGGCCAGGCATTGATGGGAAATC
GATATGAAAGTGGGCGGGCACTGGTAATTTTCACTCATTTGCCAACTGGGTGACCCGTAAGAGAGGACAC
TGAAGTGGAAACTTAATTGGCATCCTCTCGGACCACCCATAGCCCCTCTTAATTGTTTCAGCTCTCCACA
CGCCAACCAAACATTCATACACTCCGCTCttttctttttttttttttttgttttGGGGTCTAGGCCTCAT
CAATGGGGCAGTACTTCCGACATAATTATATCAGCaaaaaaaaaaaGAGAGGTTAATAGCAAGAGAGAGC
CAAGTCGATTAAAAGCGCGTTATGCCAGCGGAATACTACAGGTGTAGAAACCGAGTTGGGCTTGTGAAAT
ACACGCAACAGTTGAGCGTTAACATGGCCTATAAATAGGCAAGGCACGTAGTGTTTGCTTGCTTATCAGG

TAACGATATCAGCTTGCAAAAGCTGATaaaaaaaaTGAGCAGAGATAGGTGGAAAAACTGTGCAAACCTC
ACTCAAAAAACAGAGGGTAAACAAGCAAAGAGAGCGCCGAATGGCGATAAAAAATGTGGTGCAAATGAAA
CTGAAATGAGTTGCCTCATTATACACATATGCGGCTAGGTCTATATTGGTATGGCTACCTGGGAAAACTG
TGAGTGAGAAGAGAGCAGCTACGGAGAGACTAGAGACCACATCGAGTGCAATTGCCAGACTTTGCGACCG
GGTCTGGGCGTTTGGCCCACTCGCCGCCTGCTTTAGTCATTCTTCAGTTACGCTTGCGTTTTGTCAACGT
CGCGAGAGACGCGCTACATTCGTCAGCAGCTCAACGTATATTTGCACTTGTTATTATACGGTTTTTTATT
ATTTTTGTTTGCTTCTCAATTGGCATTCAAAACAGAGAGCGAGTTATAGCCAAGACTACCGAGATCTTGG
CCAATAACAATAGCAGAAGCTTCTCACGCAAATATCAACGCCTTGACTTAGTCATAAAAGCGGAGGAAAA
CAACATTTCATTGTTAAATACTTCTCCGGAGGAGAGTTTGAAATGTATTTTCTTCGGTTTTACttttttt
ttttttGCTTACGTGTTTTGTTGTCCCGAGCGAATATTGTTATCGATTTTTGTCTGCTGTCGGTCATAAT
TCCTTTATGGAGGGCGTTGAGAAGGAGGCAGCGGTTCATTCGTCTGGAAAGTTTCTCACGTTGCGCATAC
GCAGCGTATGATGGCCCGAGGCCGTAATTGAATTTCAGCTGCAGTACTCCGTCGATTCAAGTGTTCAAGG
TTGGGAAGCGGACCGGAAGGAAGGGAAACCCCTTGGGCCCTATTTGCATATCGGCTGCAGTGCATATCCC
CGAAAATGAGCTACTTGAAAGTAAATTTAGTCTTGTTTATATAGCAAGCGTTGCTGGGTTTTCTGTTGGA
TATTTGCAGATGGCAAACATGAAAAGTGTGCTTggttcggttcggttcggttcgCTTGTGTGCATATCCG
GTTTCCCGGAATTTCCTTCGCCTTTGCACAGATTTTTCTGGCTAGATATTGCAACCGCTTCGGCAATACT
ATCAACATTTGCACAATTCCTTTGACATTTCTACGCTCATGTCCTCTATTTTTATAAATTTTTTGCTGGA
TTCCGGCAAACAATGAGCTGCATTTGCTGCACATTTCCTGCAGGATTCTTAAAGCACGGTTACCTTCTCT
TTCGAAATAGAATGCCGTGGTACCGTCCGAGTCGACCAATGTTGGCCAATAAATAAACCGTTGATAAGCT
CAAGGCAATGGCATTCATTATTTATGTATTGTATCGTTGATTGCATGGGATTCTTATTGAATTTTTCCCG
CAGTATGTTGCGTGTTCGTCAATGTGCTTGTACATATATTTATATATGATTCTTTGTCGGTTTTCCGGTT
TGTTTGTTCGGTGCGCCCTTTTCATCACTGATGTAACAAAGTATGCATTATTATTTGCTGTTCTCTTGCA
GCTCTTGTGCAAACAGCAATTTAAACTATTTGCAGTTGGCATTCCTTCTATCATGGGCATCAAACAAACA
CGATTTCGATTTGGATTTTGGCTTGCCGAATGTTTAAGTTTTGCGAATGCAATCATAGCCCTGAACTCTC
AGGCCTTGTGTTTTTCACAGTTGCATGCTAAGTTTATCGTTTAAACCGCTACTCCCCAGTGGTAGAATTT
TCCCAACCCCCTGCCATCAGTTGTGACAGTTTTCTCTCCCCTTAAAATAAGAGGaaaaaaataggaaaaa
aaaaCCATCTTCCCATTTTCGCGCTAGAAAACAAACAAAAATGCAATAAACGCCAGCTGCAGCGGAGAAG
AGAGGAGCCTGTTCGGGTCAAAGAATTTCACTGCGTATTTTCCAATATTTTCGCCGCCATGTTGATAGCA
TGGAAATGTGGAAAATTCGAGTAATTGTCACTGAGGTGGCTGTCTGTGTCCCAGCAAGCCGAACCAAACC
AAACCAAGCGAACATTGGTTTGTCAACAAACATGGATGCGGGAATGTGACGACCTGAATTGTTGCCCTCA
AAATGCAATCAAAATGAACACACACCGAGCTGTTTTCATTACCAAAAGCGCTCTTCGCGCCCATGATATC
ATCCGTTTCGTTTTAAGCACATTAATCAGTACGCAAACCTCTGCTGCCCTACTGATATCGAGAAATAACG
CATACGACAGGTGGACCAAGGTCATTACCTACCACTTGACTTTGCTGTACACTGGGCTGGGTTGGTCCCC
CCTTTTGTAATGTTCCTACGTATGTGTCCAAAAATTTCGTAATGATTCAGAGCTGCCTTATCGCCAGTTT
GTTTTACTACATTTTCCCCCCTTTTTGGGCCTTCTTGTTTTGCTTTATCACATTGGCTTAGGCATTCCAA
AGTTTGATTAGATAGCAGTGTTTTGAACGCACACCCCTTTCaaaaaaaaaaaaaaaaaaCATCGCTTCAT
TGGAGTGAAAATCGAAAAAGAGGAGACAGTTTTGATGGAGGAAGCTTAGTGTGGCTTTGTTGTCGGAATA
TTCCTGGGTGATTTTATGATGCTCCCATAAAAACTTTGTTCATTAATTTTTGTCGCCACATTTAAGCGGA
CGTCGCACTGTCTTTTTTGTTGATTTCCAAAGCTTAACACTCGCAATTTGTCAGAGGCTGCAGCATCGCT
ACCCGCCGTTTAACCTGGTCGACAATTAATCATGAAACCTGCCAGCACTCCACAACTATGGCATTTTCCA
TAAGCGCCGGAAAACAAAATAAGACGACAAAAAATGCAGATCTCGTCGATTCCAAAAGGGCTTTAAGTCG
TTTTAAGCGCACTCGAAGCATTGATCTCTTGCCATTGATAATGTCGGGGTAATTAAAATCGATTGATTCG
ATGCGGTCATTGGCTTAAATTCCATCAGAGTGGAAAGCGAAATTTGCATGGACGGGGAAAAGTTGTGGCC
CAAGTTGTTCTTTTTTTGTTTCCCCTTGTCATAACAAATGTGACAAGTTGAAGCTCCTGGAGAGTGCCGT

CCACATCTTGGCCACAGAATGCCGTGAGAAAGAAATAGTCGAGTGAAATATGATTTCTGCCTTTTGGCAT
GTGTCCATGTTGTGGGCTAATTTCCCAGAGACAGACACGACGACTTCTGCCACATGGTCATTTACATATA
AATGACAAAACAAAAAGAGAGCAGCGAATTGGAGAAGCTGCAGGATTCCCGACCCGTGCCATTGATTTGG
ACTTTGGCATTTGTCAGCTGGCAGCAGATTCCGATTTGCGATTGCCATTGCGATTCATAACCAGCGTGCA
AATGCTAATGCATTCGGGGAATCATCATTTCTTATTGCCACTTTCTCATTTTACGAGCTGCTCTcgtttc
gtcttttggtgtttttaaatttttatatgtttatgtttatgttatttaTTTTACGGGTGACGAAGGACAA
AAACGATGGCGTTACTTCGTTCGGGGCTCACCTCATTGAGCTGCACGCGTGATGTCATCGTAAAATGCTT
AAATGCCCGCTTAAGTGGGTGCCGCAAAAAGTTCGCAGGCACTCTGGCAATCAGTAAAATAAGTCTGATA
AGAAATATGAGGAAAACAAATGGTCTTATGCGATCCTTCTTGTGCAGTTCCTGTTTCAGTTCGTGTATTA
AAACTTCACTTTCCTTGCCTTGCCCCCCAGACAGATTGCCTACATTCCGGCGTCCTCAGGAACCGGAATG
AAATCACACACAGCACGTGTCATTTGCTCTCCTCCCCGGGAAAAAATCTTATTTTGTTTTATGGCTTTTT
TCTCTGGTTATTGTTGTCTCGGACCGTTTTTCTCCCACTCATCTGGGTGGAAAGCATGAAAAGATGGCTT
TATTTCGAACGTATGCATGTGGGGCCGAAATATACATATGTATATATATCCATAAAAATATCTTTCGCTT
ACGCATTTGTCCGGCAATTCAGAGAGTCAAATTGAGAATGAAGGAGATGAGTTGTTGGCATTTAAAACAT
TTAAACAGCATATTTTGCTGCCACAACACCTTCGCTTAAGCTTCTACCCCATGGAAATTGTTGAATTATG
GAGCACAAACAAATTTGGGTCTGTAAGCAAAGAAATTCAAATTTTCTGAATACCCCACTCAACTTACCAA
ATTCAATAATGGTACATTGAGTGAATGGCAATGTTGGTTTGTAGGTGTAGTAGACTTAAATCCGGCAATA
TTAATGTTGACTTGACAGGCAGACAGGAGTCGATGGAGGTGAGGCTCAAGCTGAGTGCCCCTGTCTTTTC
ATTTATGCATTGATTAACATGAAAACTCGCATACAATTACAGAGTGAAATTGTCTCAAGAGCCGACTCCC
TGTTTTCTCCGTCCCGCCATCCTGGCGAATCCTTGACAGTCGCTGGATGTGGAGATGGGGAAAAGAAAAT
GATTTTGTTGGCCAAAGTGCCGACGGATTTATTGAGTGCAAATCAGCAATAGCGAAAATGAAAGCAATAA
ATGTACTTCTGAGAAGGGAAAAGTTACTGGTACTCATCTTTCTTACACCACCTGATTCCCAGCCTCtttt
ttttCGACCTGAGGGACGTCTGAGAGTTTTCTTGGCAGACTGTCTGTTTATTTATTTAATAATTATACAA
AGTCGTATTCTCAGCTGGGGGACTTGAGGGGTTTCATTCAAGGTCGTTGCTTTAACGCCTGATTCTAGTG
AGGGGAAGCGGTTCATTGACTGCTGTTTATGACCAAGTAACAGACGCTGTTGGCCAACTTTTGGTCGTTC
AATTTAAATGTCATCATGGCAAAATTTATGCTAAACACGAATCCTTTTATTTATTTGTTTCGTGGGAGGT
GTTTCAAATTTGATTAAATCATCGCAATAAATGTGTAATCTCACTGATGCTCTATCGCATTGATGGGGTT
TTCCGTTCCAATGCACTTGAATGGATGCATATGGTTTACCCACTTAGCCACCAGTCCGTGCTTTTATCTA
TTCCTCTGGCAGACACAGATCGATTGCGCTCCTTTAGCTCGTCGGCAGCGTTAAAAGTTAGCTTCGCATA
TTAAAAAGTGCAGTCAAATAAAAAATAGGTTGCCTCACAACATACACACGGTGTCTGGACAAGATGCCTG
GCAGCTGCTTATATTAAAAAGTGCACTCAGATAAAAAGCAAATCAAGCCATGTTCAATGTCAAAATCAAA
GTCAAAGAGGGCAACAACACCCTGCCCCGAAATGGCCAGCGCAGCAACGGCAACTTTAGCTACTCCGGAG
CTGGAAGTGGTCGAAATAGTCCGGCGATGAGCAGCCTGCGCAGCTCACTGGCCTCCTCCAATCGGAGTCC
CAGTGGATCACTGCAGCGAAAGGGCAAAAGAGTACGGATTGTGACCAGCTATGATCCGATTGATCGGGAT
TACGAAAGGCAGAGGCAGCATTATGGTAAAAATCCGGGGATTTAGAAATATTAAAATATATATCCACAAA
AAACTCTGATATTAAGCAAATATTTTTCATTCTTCCAGGACAACTTGGATGGCGTTACGGAGCTGCGTTT
GGAGCAGTACGAAATCCACATCGAGCGCACTGCAGCCGGATTGGGTCTGAGTATTGCCGGTGGCAAGGGC
TCAACACCCTTTAAGGGTGACGATGATGGCATCTTCATATCAAGGGTCACCGAGGCGGGACCCGCTGATC
TGGCTGGTCTTAAAGTGGGCGACAAAGTTATCAAAGTCAACGGCATTGTTGTCGTGGATGCGGATCACTA
CCAGGCGGTGCAGGTGTTAAAAGCCTGCGGTGCCGTACTTGTTTTGGTGGTACAGCGTGAGGTGACTCGC
CTGATTGGCCACCCCGTGTTTAGCGAGGATGGTAGTGTGTCCCAAATCTCGGTCGAGACGCGACCACTGG
TGGCGGATGCCCCTCCAGCTGCATCCATTAGCCACGAGCGTTACATCCCGGCACCCATTGAAATTGTGCC
CCAACAGCAGCATCTACAGCAACAGCAACAGCAGCCCATTCAGCAGGTGGCCCCAACGCATAGCTATTCG
GGCAACGTGTTTGCCACACCCACTGCCGCACAAACGGTGCAGCCTGCTGTGTCTGCTGCGCCCAATGGAT

TGCTACTGAATGGCAGGGGAGGCGCCACTGAGCTACATCCAGCTGCACACAACGCTCATCCGCGATCAGA
TTGGCCAAGGACTGGGCTTCAGCATTGCGGGCGGAAAGGGGTCGCCGCCGTTCAAGGACGACTGCGATGG
CATTTTCATATCCCGCATCACGGAGGGCGGACTAGCGTACCGCGATGGGAAAATTATGGTGGGCGACCGG
GTGATGGCGGATTAACGGCAACGATATGACTGAAGCCCATCATGACGCAGCCGTCGCCTGTTTGACTGAA
CCGCAGCGATTTGTGCGTTTGGTGTTGCAGCGCGAGTACAGAGGTCCACTGGAACCACCGACGAGTCCGC
GAAGTCCGGCAGTGCTCAACTCTTTGAGCCCCTCTGGATATTTGGCCAACCGACCAGGCTAATTTCAGTC
GATCGGTGGTGGAAGTTGAGCAGCCGTACAAGTACAACACCCTGGCTACGACCACGCCCACACCGAAGCC
CACTGTGCCAGCTAGTATTAGCAACAATAATAACACTCTGCCCAGCAGCAAAACCAATGGATTTGCCACT
GCTGCTGCTGCCACGATAGACAGTTCCACGGGTCAACCGGTGCCCGCTCCCCGTCGTACCAACTCTGTTC
CAATGGGAGATGGAGACATTGGGGCGGGTTCCACCACCAGTGGGGATTCTGGCGAGGCACAGGGTAAGGA
AGTTGGATAACTAACTTTAGTAGCTGTGTAGACTATAGTCTAGTTTCCTGTTTAGCCAGTTGCACGTCAT
TTTAATGTTGCCCATAAGCTCGTTACCTTAACTTTTTTGTTTTCTACAATCATGATCAGTCGGTAGGATT
AAGATAAAACCAAAATGTATATGCTTGCGCTGCAGTATCTTACATATATTTCTAATCAGCCTAAAAGGTA
AGTAGCTAAAACTTTAATGTTATTTTAACAAAAAACTGCAGCGCAAGTCGTCCAAGCAAATTGTTGTTGA
TATTTGGCGTCCAAAAACCAAAATTCAAAACTTAAACATGAACACACTAAATAGTCATAAGATACCTAAT
ATGTGCTAATCCATTTCTCCGCCAGAATATCAATTACTCATAACCCTAGCTGCTAGATCTATATATTTGC
TCATCTACTCGTATATATAACGAAATTGTTTGTGTTTGTTTTGCATGTGCCCTGTGCGCCTTGTCTGTCC
TCCCAATATATGCTATATGCTCATTTTGCATACATAGTCGAATTATATATAATTATCGACCGATTAGACG
CTAAGTCTGCACCGATTTATGTCCCAGCTTTTAAGCACCAATCGAGAAAAAGAGTCAAAATGCTAGGAGG
CGTTTGCGGAATTGTATCTAAATTAATGATTAATGATTCATAGTTAGGCACGTTAGCTTAGTTCTAGATC
TAACGATGGCTTCATAGCCCATCATCAAAAGTGAAACCAAAATTTACAAAAACCAACATATTTTTGTTTC
TTTTTCTCTGATCAACCACCACCCCCTCCAAAAACCAAAAAACTTGCTTAAAACCGATATTTCCGAATAC
TTTGCTAAATCCAAAAACCACGTAACAACCAACGTACATACCCAACCACGACCGTGAGCAGCCCTCCTCG
TTGCGACCGCTGACCAGCGATGATTTTCAGGCGATGATCCCGGCACACTTCCTCAGCGGCGGCAGTCAGC
ATCAGGTGCATGTGGCCCGGCCCAACGAGGTGGGCGTGAGTGCCGTCACGGTGAATGTGAATAAGCCTCA
GCCGGATCTGCCCATGTTCCCGGCGGCACCTACCGAACTTGGCCGCGTCACCGAGACGATCACCAAGTCC
ACGTTCACGGAGACGGTGATGACGCGCATCACCGACAATCAGTTGGCGGAGCCGCTTATCAGTGAGGTGA
CTATCTCCACATCCCGCATACACTAGAAGCGCGTACAGTCGCTGCCCAGAGTCCAAGAGTTAATGAATCG
TTGTGTAAAAGTCACAAGCAAAGTTATCCGGACACGGGCACACATTATCCTGTAGCTCAGATGACCTGAA
AATATGTaaaaaacaaaaaaaaaaaaaaaTCTATATAGTGTATCCAGGCACGGAATTCGAGCTTTTCTCG
TTACCGCACATAAACACGCACAATCCACCCGTACATGATGCACAACAACACACACCAAAATTGATTTAAT
TTAATTCTAAATTAATTTATGATTTATAGTCCCTACAATTGGCAAACTTTGACCTATTGGCTGCGCTTCC
CATCTTTAATTTGAATTTGCTAAATAAATATATGTGCGTTTTGCAAACGATTTGATTTAACTATTGGTCA
GAGGCCCGCCCCGTTTGAGACGCTGAGTTCATCTATGTTTCATGCCAAGCATTATGCAAAACATGCACAT
TTACAAGAGTTGTGATTGCTCCGACTAATTGAGTAGCAGTTGAGATGGAATTGGATTAAGTTGGTTATGG
CACGGAGCTGGAAACTACTATTAAGCGATTAATCAATTCAAATTTAATTCAATTAAATGTGGAAGAACTT
TGGCATGCCACTCGTTTCTGTACTAAGTTAACTTTCCCTCTACTAACACTGGATAAATATAATTTTCCAA
CTGTTCCACAAAAATCCTGGCTTATGTTTGTTTTGCAAATAACTTGAAACCAACAAAAAATATCCATTTA
AGTGGGTATTAGCCTTAATGCTTTCGAGGGGTTTTGAAATGttttttttttttttttATTTTGGGCTGGT
ACAATTTTTGATAGCCTTGGCCAACACTTGAATATTTAGTGTCCTTTGGAAGTAACCGAATCTATTGAAG
TGAGCGGCAAAGCGCTTTCGTTTTGAACGACAGAATGGTTTACTCACTCACTTTTCGTAGAGCCACTCGA
GTGTCATTGCCGACGTGATGCAAATCCCAGCAAGAAATCCCAGAGACTCTGTGCATGTGGCAAGCTTTGT
CACTTGCCACTTGCAGCATAAATAACTAAACCGGCAAACTATGCTACATGCACACTATATCCTTAAATAA
ATAATAATAAAGTGAGCACATGCCGAAAAAAGATTTAGAAACTTTAAATGACTCACAAGAGTTCCCAACA

GCGTTTATTTCGTGTGTAGCAGAGTGTGGCCATAAGTAAACTTTAAATATTTCTGTTTGCCTTCGCAATC
TTCATCCGTGTGTCTGTCTCCGGGGCACTGATAAATAAAACATTTAATTGTTTTTAATTTACTTTCCCCT
TCTGCGTTTGCATTCTGCTTTGGAGCGAGATATCCGTTGGCTTCGAACTCGAAAGAATTCAATTTCTTCG
AGGTAATTGGAAAGAGTTAGTAGCAGTTAGCCGAGTACATGTGTTTATTTGGCATTTTGACACGAATGAG
CCTTTGTGGATGTCAGCTCTCAGGAGATAGTTCCGAGTTTCCCGGCAATGAATATAAAACAGAAATATTA
TGCTTTGATGTTGCAATATGCCAGGACTTGAGCTAAACGATGCTCACCTCAACCACAGGAGCCGCAAAAG
CTTTGTTTAAATCTGTGTGAAAATGCGTGAGCCAGGAAAATTCAGCATAAAGTGGGCGTGGTTTCACCCA
CTCTTTTTGCATGTTGTTTAATCTAAAATTAAGTTATTCATAAGAACAGCACAAAACGTATTCATTATTT
ATTGAATGTGAATCACTCTTGGTACTCGGTTTTCATGTTTAATGAATGCTTCGACTTAAGTGCATGactt
tgtttttttggtgtttggcttggcttgtcttggtttttgtgtCTCACCAAGGGAAACCCCCTTAATTTGT
ATGTACACCTTAATATAAATATCACCAACAATTTTGCACCACCATTGAACGCCAGCACATGAGCACCCTT
CATCCATGGACTCACCCAATACACGGCAGACTCAGTAGGATTCTTAGCGTTAGGACCATCGATCTGTGTT
AATTTTGTGATGTGCTAACACGATTTCCCATTATCTGTCCTGTAGCGTAAGTACAGACGGATAGATTAAC
TAACTAACAATTACCTGCAGCAGCAGCTGGCAAAAAAGTTTTCGAATTAAAACACTCGAGTGGCCGCAAC
TTGCGAATGCAAATGAGCTGCTGCTGCAGAACAATTGTTCAGAATCCAGTGGCTCGTAGGATGGCTTTAT
TATAAGCAGCCAACTAAGCCAGCTGCCTGCTCGAAAGTTTCAAGTCTTTTGGCCATTTATGAGTATAACA
ATTACCCCTGGGGTTTCTCCTCCTTAAATCCGTCAGGATTGTGCCCGGCGGGATTGCCTCCAAGTGCGGC
AAGCTGCGCATGGGCGATCGCATATTGAAAGTCAACGAGGCGGATGTTTCCAAGGCCACACATCAGGATG
CGGTTCTGGAGCTGTTGAAGCCAGGTGATGAAATTAAGCTGACCATTCAACACGACCCATTGCCGCCAGG
ATTTCAGGGTAAGTTTTGTGGACTGGCAAAGTCCTTATTAGCCCATCTTAGACTGCTTTTGTACTGTAAA
TATGTATATACGCTTCGGATCTACTTCTTACTCCTCCTCTATAATCAATTTTTGTATACTCTGCCACTGC
TCACAAACAAATTCAATACGCACAGAGCATAGAAGTAGTTTATGTAAAAACAGAGGTAAGCTCCTCTTGG
CTTTCAGTGGGGAAATATTTGCAATTAGTCAAATGCCTTTTCCTTAACTTTTTGTTTGCTTTGTTTAGAC
CTGAAGAGAGTCCCGATTGTTATTTGTCAGCGTTTAAGCCGTGTCCATGCTCATTTCCATTCTTTCCTAA
TTGTTAATTGTAAATCGCAGGGAAGTGCTACTCAGCAAGGCGGAGGGCGAACGTTTGGGCATGCACATCA
AGGGCGGCTTGAATGGCCAGCGTGGTAATCCCGCCGATCCCTCCGACGAAGGCGTCTTCGTGTCCAAAAT
TAACTCGGTCGGTGCGGCCAGACGGGACGGAAGGCTTAAGGGTGGGCATGCGTCTGCTCGAGGTTAATGG
ACACTCGCTGCTGGGCGCCTCGCACCAGGATGCGGTCAATGTGCTGCGCAACGCTGGGAACGAGATTCAA
TTGGTCGTCTGCAAAGGCTACGACAAGTCCAATTTAATTCATTCCATTGGCCAGGCCGGCGGCATGAGCA
CTGGCTTCAACTCGTCTGCATCCTGCAGCGGTGGCAGTCGCCAAGGGACTTCGATGTCTTTGCCTCGCAG
AAGCCAGATGCACAACAGCCAACTGGCCCATCTGTCCTGGCAGCTGCTGCTGCCATGGTGCATGGCGCCT
CGTCGCCCACACCACCTGCTGCCACCTCCAATATAACTCCCTTACCCACAGCCGCGGCAGTAGCATCTGC
TGATTTAACAGCACCGGACACGCCAGCAACCCAGACCGTGGCGCTCATTCACGCAGAACAGCAGGCCCAT
CAACAACAACAGCAGACGCAGCTGGCGCCTTTGGGTCAGGAGAAGAGTACCCAGGAGAAGGGTGCTGGAA
ATCGTACGCGCGGCTGATGCCTTCACCACCGTGCCACCAAAGTCGCCATCGGAACACCACGAGCAGGATA
AGATACAGAAGACGACGACGGTTGTCATATCGAAGCATACGCTCGACACGAACCCAACCACCCCCACAAC
GCCGGCGGCGCCTCTTTCCATCGCTGGAGCAGAGTCAGCGAACTCGGCTGGTGCTCCTTCTCCAGCTGTT
CCTGCATCCACGCCCGGCTCAGCGCCAGTTCTTCCTGCCGTGGCCGTGCAGACACAGACGCAAACGACCT
CCACGGAGAAGGATGAAGAGGAGGAGTCGCAACTACAG
>dme_circ_0007665_junction_seq
GGATGAAGAGGAGGAGTCGCAACTACAGGCCCGTGACCTGGAATCCGATTCTGAGC
>dme_circ_0007666
GAATGGCACCAAGTTAGCTGAGACCCCAACCGAGACCCGAACCATTGCAAACAATCACCAACAGCAGCCA
CACCCAGTGCAGCAACCAATCGTTGGTGTAAATTCCAAGCAACCAGTGGTTGTGGGTGTAGTCACTCCAA

CTACGACGACAGTCGCCCCAACCGGCGTACAGGGTGGCTCAGAGGGAGCCAGCTCCACGGCCAACAATGT
AAAGGCAGCAACAGCAGCTGTGGTGGCTGAGTTAGCAGCCACTGTTGGTGGAAGTGATGAGGTCCAGGAT
GATGACGAGCAGGAGGATGAGTTTGGGATACTCCGCATCACCTGAAGAATAAGCGTGTCCAGCATTTAAC
CGACAAGCAGGCCAGTGAAATCTTGGCAAATGCCCTAGCCTCACAGGAGCGGAACGATACCACACCGCAG
CACTCGCTTTCCGGCAAAGTGACATCACCAATTGAGGAGGAGGAGCAGCTGGAGGTGGaacaggagcagc
aacagcaacagcagcagcaTCCCTTCGACTCCTCGCTCTCGCCCATTTCGGCTGGCAAAACAGCAGAGGC
TAGCACCGACCCAGGGTAAATACCGAAATTGTTTCAATTAATTACATTTATATTGCTCTATCTCTGAGAA
CTGTGTCCAAAACAGCCAAAGTGCTTTGGATGCAGCTTGAGATGGCCAGGCATTGATGGGAAATCGATAT
GAAAGTGGGCGGGCACTGGTAATTTTCACTCATTTGCCAACTGGGTGACCCGTAAGAGAGGACACTGAAG
TGGAAACTTAATTGGCATCCTCTCGGACCACCCATAGCCCCTCTTAATTGTTTCAGCTCTCCACACGCCA
ACCAAACATTCATACACTCCGCTCttttcttttttttttttttgttttGGGGTCTAGGCCTCATCAATG
GGGCAGTACTTCCGACATAATTATATCAGCaaaaaaaaaaaGAGAGGTTAATAGCAAGAGAGAGCCAAGT
CGATTAAAAGCGCGTTATGCCAGCGGAATACTACAGGTGTAGAAACCGAGTTGGGCTTGTGAAATACACG
CAACAGTTGAGCGTTAACATGGCCTATAAATAGGCAAGGCACGTAGTGTTTGCTTGCTTATCAGGTAACG
ATATCAGCTTGCAAAAGCTGATaaaaaaaaTGAGCAGAGATAGGTGGAAAAACTGTGCAAACCTCACTCA
AAAAACAGAGGGTAAACAAGCAAAGAGAGCGCCGAATGGCGATAAAAAATGTGGTGCAAATGAAACTGAA
ATGAGTTGCCTCATTATACACATATGCGGCTAGGTCTATATTGGTATGGCTACCTGGGAAAACTGTGAGT
GAGAAGAGAGCAGCTACGGAGAGACTAGAGACCACATCGAGTGCAATTGCCAGACTTTGCGACCGGGTCT
GGGCGTTTGGCCCACTCGCCGCCTGCTTTAGTCATTCTTCAGTTACGCTTGCGTTTTGTCAACGTCGCGA
GAGACGCGCTACATTCGTCAGCAGCTCAACGTATATTTGCACTTGTTATTATACGGTTTTTTATTATTTT
TGTTTGCTTCTCAATTGGCATTCAAAACAGAGAGCGAGTTATAGCCAAGACTACCGAGATCTTGGCCAAT
AACAATAGCAGAAGCTTCTCACGCAAATATCAACGCCTTGACTTAGTCATAAAAGCGGAGGAAAACAACA
TTTCATTGTTAAATACTTCTCCGGAGGAGAGTTTGAAATGTATTTTCTTCGGTTTTACttttttttttt
tGCTTACGTGTTTTGTTGTCCCGAGCGAATATTGTTATCGATTTTTGTCTGCTGTCGGTCATAATTCCTT
TATGGAGGGCGTTGAGAAGGAGGCAGCGGTTCATTCGTCTGGAAAGTTTCTCACGTTGCGCATACGCAGC
GTATGATGGCCCGAGGCCGTAATTGAATTTCAGCTGCAGTACTCCGTCGATTCAAGTGTTCAAGGTTGGG
AAGCGGACCGGAAGGAAGGGAAACCCCTTGGGCCCTATTTGCATATCGGCTGCAGTGCATATCCCCGAAA
ATGAGCTACTTGAAAGTAAATTTAGTCTTGTTTATATAGCAAGCGTTGCTGGGTTTTCTGTTGGATATTT
GCAGATGGCAAACATGAAAAGTGTGCTTggttcggttcggttcggttcgCTTGTGTGCATATCCGGTTTC
CCGGAATTTCCTTCGCCTTTGCACAGATTTTTCTGGCTAGATATTGCAACCGCTTCGGCAATACTATCAA
CATTTGCACAATTCCTTTGACATTTCTACGCTCATGTCCTCTATTTTTATAAATTTTTTGCTGGATTCCG
GCAAACAATGAGCTGCATTTGCTGCACATTTCCTGCAGGATTCTTAAAGCACGGTTACCTTCTCTTTCGA
AATAGAATGCCGTGGTACCGTCCGAGTCGACCAATGTTGGCCAATAAATAAACCGTTGATAAGCTCAAGG
CAATGGCATTCATTATTTATGTATTGTATCGTTGATTGCATGGGATTCTTATTGAATTTTTCCCGCAGTA
TGTTGCGTGTTCGTCAATGTGCTTGTACATATATTTATATATGATTCTTTGTCGGTTTTCCGGTTTGTTT
GTTCGGTGCGCCCTTTTCATCACTGATGTAACAAAGTATGCATTATTATTTGCTGTTCTCTTGCAGCTCT
TGTGCAAACAGCAATTTAAACTATTTGCAGTTGGCATTCCTTCTATCATGGGCATCAAACAAACACGATT
TCGATTTGGATTTTGGCTTGCCGAATGTTTAAGTTTTGCGAATGCAATCATAGCCCTGAACTCTCAGGCC
TTGTGTTTTTCACAGTTGCATGCTAAGTTTATCGTTTAAACCGCTACTCCCCAGTGGTAGAATTTTCCCA
ACCCCCTGCCATCAGTTGTGACAGTTTTCTCTCCCCTTAAAATAAGAGGaaaaaaataggaaaaaaaaaC
CATCTTCCCATTTTCGCGCTAGAAAACAAACAAAAATGCAATAAACGCCAGCTGCAGCGGAGAAGAGAGG
AGCCTGTTCGGGTCAAAGAATTTCACTGCGTATTTTCCAATATTTTCGCCGCCATGTTGATAGCATGGAA
ATGTGGAAAATTCGAGTAATTGTCACTGAGGTGGCTGTCTGTGTCCCAGCAAGCCGAACCAAACCAAACC
AAGCGAACATTGGTTTGTCAACAAACATGGATGCGGGAATGTGACGACCTGAATTGTTGCCCTCAAAATG

CAATCAAAATGAACACACACCGAGCTGTTTTCATTACCAAAAGCGCTCTTCGCGCCCATGATATCATCCG
TTTCGTTTTAAGCACATTAATCAGTACGCAAACCTCTGCTGCCCTACTGATATCGAGAAATAACGCATAC
GACAGGTGGACCAAGGTCATTACCTACCACTTGACTTTGCTGTACACTGGGCTGGGTTGGTCCCCCCTTT
TGTAATGTTCCTACGTATGTGTCCAAAAATTTCGTAATGATTCAGAGCTGCCTTATCGCCAGTTTGTTTT
ACTACATTTTCCCCCCTTTTTGGGCCTTCTTGTTTTGCTTTATCACATTGGCTTAGGCATTCCAAAGTTT
GATTAGATAGCAGTGTTTTGAACGCACACCCCTTTCaaaaaaaaaaaaaaaaaaCATCGCTTCATTGGAG
TGAAAATCGAAAAAGAGGAGACAGTTTTGATGGAGGAAGCTTAGTGTGGCTTTGTTGTCGGAATATTCCT
GGGTGATTTTATGATGCTCCCATAAAAACTTTGTTCATTAATTTTTGTCGCCACATTTAAGCGGACGTCG
CACTGTCTTTTTTGTTGATTTCCAAAGCTTAACACTCGCAATTTGTCAGAGGCTGCAGCATCGCTACCCG
CCGTTTAACCTGGTCGACAATTAATCATGAAACCTGCCAGCACTCCACAACTATGGCATTTTCCATAAGC
GCCGGAAAACAAAATAAGACGACAAAAAATGCAGATCTCGTCGATTCCAAAAGGGCTTTAAGTCGTTTTA
AGCGCACTCGAAGCATTGATCTCTTGCCATTGATAATGTCGGGGTAATTAAAATCGATTGATTCGATGCG
GTCATTGGCTTAAATTCCATCAGAGTGGAAAGCGAAATTTGCATGGACGGGGAAAAGTTGTGGCCCAAGT
TGTTCTTTTTTTGTTTCCCCTTGTCATAACAAATGTGACAAGTTGAAGCTCCTGGAGAGTGCCGTCCACA
TCTTGGCCACAGAATGCCGTGAGAAAGAAATAGTCGAGTGAAATATGATTTCTGCCTTTTGGCATGTGTC
CATGTTGTGGGCTAATTTCCCAGAGACAGACACGACGACTTCTGCCACATGGTCATTTACATATAAATGA
CAAAACAAAAAGAGAGCAGCGAATTGGAGAAGCTGCAGGATTCCCGACCCGTGCCATTGATTTGGACTTT
GGCATTTGTCAGCTGGCAGCAGATTCCGATTTGCGATTGCCATTGCGATTCATAACCAGCGTGCAAATGC
TAATGCATTCGGGGAATCATCATTTCTTATTGCCACTTTCTCATTTTACGAGCTGCTCTcgtttcgtctt
ttggtgtttttaaatttttatatgtttatgtttatgttatttaTTTTACGGGTGACGAAGGACAAAAACG
ATGGCGTTACTTCGTTCGGGGCTCACCTCATTGAGCTGCACGCGTGATGTCATCGTAAAATGCTTAAATG
CCCGCTTAAGTGGGTGCCGCAAAAAGTTCGCAGGCACTCTGGCAATCAGTAAAATAAGTCTGATAAGAAA
TATGAGGAAAACAAATGGTCTTATGCGATCCTTCTTGTGCAGTTCCTGTTTCAGTTCGTGTATTAAAACT
TCACTTTCCTTGCCTTGCCCCCCAGACAGATTGCCTACATTCCGGCGTCCTCAGGAACCGGAATGAAATC
ACACACAGCACGTGTCATTTGCTCTCCTCCCCGGGAAAAAATCTTATTTTGTTTTATGGCTTTTTTCTCT
GGTTATTGTTGTCTCGGACCGTTTTTCTCCCACTCATCTGGGTGGAAAGCATGAAAAGATGGCTTTATTT
CGAACGTATGCATGTGGGGCCGAAATATACATATGTATATATATCCATAAAAATATCTTTCGCTTACGCA
TTTGTCCGGCAATTCAGAGAGTCAAATTGAGAATGAAGGAGATGAGTTGTTGGCATTTAAAACATTTAAA
CAGCATATTTTGCTGCCACAACACCTTCGCTTAAGCTTCTACCCCATGGAAATTGTTGAATTATGGAGCA
CAAACAAATTTGGGTCTGTAAGCAAAGAAATTCAAATTTTCTGAATACCCCACTCAACTTACCAAATTCA
ATAATGGTACATTGAGTGAATGGCAATGTTGGTTTGTAGGTGTAGTAGACTTAAATCCGGCAATATTAAT
GTTGACTTGACAGGCAGACAGGAGTCGATGGAGGTGAGGCTCAAGCTGAGTGCCCCTGTCTTTTCATTTA
TGCATTGATTAACATGAAAACTCGCATACAATTACAGAGTGAAATTGTCTCAAGAGCCGACTCCCTGTTT
TCTCCGTCCCGCCATCCTGGCGAATCCTTGACAGTCGCTGGATGTGGAGATGGGGAAAAGAAAATGATTT
TGTTGGCCAAAGTGCCGACGGATTTATTGAGTGCAAATCAGCAATAGCGAAAATGAAAGCAATAAATGTA
CTTCTGAGAAGGGAAAAGTTACTGGTACTCATCTTTCTTACACCACCTGATTCCCAGCCTCttttttttC
GACCTGAGGGACGTCTGAGAGTTTTCTTGGCAGACTGTCTGTTTATTTATTTAATAATTATACAAAGTCG
TATTCTCAGCTGGGGGACTTGAGGGGTTTCATTCAAGGTCGTTGCTTTAACGCCTGATTCTAGTGAGGGG
AAGCGGTTCATTGACTGCTGTTTATGACCAAGTAACAGACGCTGTTGGCCAACTTTTGGTCGTTCAATTT
AAATGTCATCATGGCAAAATTTATGCTAAACACGAATCCTTTTATTTATTTGTTTCGTGGGAGGTGTTTC
AAATTTGATTAAATCATCGCAATAAATGTGTAATCTCACTGATGCTCTATCGCATTGATGGGGTTTTCCG
TTCCAATGCACTTGAATGGATGCATATGGTTTACCCACTTAGCCACCAGTCCGTGCTTTTATCTATTCCT
CTGGCAGACACAGATCGATTGCGCTCCTTTAGCTCGTCGGCAGCGTTAAAAGTTAGCTTCGCATATTAAA
AAGTGCAGTCAAATAAAAAATAGGTTGCCTCACAACATACACACGGTGTCTGGACAAGATGCCTGGCAGC

TGCTTATATTAAAAAGTGCACTCAGATAAAAAGCAAATCAAGCCATGTTCAATGTCAAAATCAAAGTCAA
AGAGGGCAACAACACCCTGCCCCGAAATGGCCAGCGCAGCAACGGCAACTTTAGCTACTCCGGAGCTGGA
AGTGGTCGAAATAGTCCGGCGATGAGCAGCCTGCGCAGCTCACTGGCCTCCTCCAATCGGAGTCCCAGTG
GATCACTGCAGCGAAAGGGCAAAAGAGTACGGATTGTGACCAGCTATGATCCGATTGATCGGGATTACGA
AAGGCAGAGGCAGCATTATGGTAAAAATCCGGGGATTTAGAAATATTAAAATATATATCCACAAAAAACT
CTGATATTAAGCAAATATTTTTCATTCTTCCAGGACAACTTGGATGGCGTTACGGAGCTGCGTTTGGAGC
AGTACGAAATCCACATCGAGCGCACTGCAGCCGGATTGGGTCTGAGTATTGCCGGTGGCAAGGGCTCAAC
ACCCTTTAAGGGTGACGATGATGGCATCTTCATATCAAGGGTCACCGAGGCGGGACCCGCTGATCTGGCT
GGTCTTAAAGTGGGCGACAAAGTTATCAAAGTCAACGGCATTGTTGTCGTGGATGCGGATCACTACCAGG
CGGTGCAGGTGTTAAAAGCCTGCGGTGCCGTACTTGTTTTGGTGGTACAGCGTGAGGTGACTCGCCTGAT
TGGCCACCCCGTGTTTAGCGAGGATGGTAGTGTGTCCCAAATCTCGGTCGAGACGCGACCACTGGTGGCG
GATGCCCCTCCAGCTGCATCCATTAGCCACGAGCGTTACATCCCGGCACCCATTGAAATTGTGCCCCAAC
AGCAGCATCTACAGCAACAGCAACAGCAGCCCATTCAGCAGGTGGCCCCAACGCATAGCTATTCGGGCAA
CGTGTTTGCCACACCCACTGCCGCACAAACGGTGCAGCCTGCTGTGTCTGCTGCGCCCAATGGATTGCTA
CTGAATGGCAGGGGAGGCGCCACTGAGCTACATCCAGCTGCACACAACGCTCATCCGCGATCAGATTGGC
CAAGGACTGGGCTTCAGCATTGCGGGCGGAAAGGGGTCGCCGCCGTTCAAGGACGACTGCGATGGCATTT
TCATATCCCGCATCACGGAGGGCGGACTAGCGTACCGCGATGGGAAAATTATGGTGGGCGACCGGGTGAT
GGCGGATTAACGGCAACGATATGACTGAAGCCCATCATGACGCAGCCGTCGCCTGTTTGACTGAACCGCA
GCGATTTGTGCGTTTGGTGTTGCAGCGCGAGTACAGAGGTCCACTGGAACCACCGACGAGTCCGCGAAGT
CCGGCAGTGCTCAACTCTTTGAGCCCCTCTGGATATTTGGCCAACCGACCAGGCTAATTTCAGTCGATCG
GTGGTGGAAGTTGAGCAGCCGTACAAGTACAACACCCTGGCTACGACCACGCCCACACCGAAGCCCACTG
TGCCAGCTAGTATTAGCAACAATAATAACACTCTGCCCAGCAGCAAAACCAATGGATTTGCCACTGCTGC
TGCTGCCACGATAGACAGTTCCACGGGTCAACCGGTGCCCGCTCCCCGTCGTACCAACTCTGTTCCAATG
GGAGATGGAGACATTGGGGCGGGTTCCACCACCAGTGGGGATTCTGGCGAGGCACAG
>dme_circ_0007666_junction_seq
CACCAGTGGGGATTCTGGCGAGGCACAGGAATGGCACCAAGTTAGCTGAGACCCCA
>dme_circ_0007667
GGAGGTTGTGCTGCCCAAGAACCAGGGCTCCCTGGGATTCAGCATCATCGGCGGCACGGATCATTCCTGT
GTGCCCTTCGGCACTCGAGAGCCTGGCATATTCATATCGCACGTAAGTACAGACGGATAGATTAACTAAC
TAACAATTACCTGCAGCAGCAGCTGGCAAAAAAGTTTTCGAATTAAAACACTCGAGTGGCCGCAACTTGC
GAATGCAAATGAGCTGCTGCTGCAGAACAATTGTTCAGAATCCAGTGGCTCGTAGGATGGCTTTATTATA
AGCAGCCAACTAAGCCAGCTGCCTGCTCGAAAGTTTCAAGTCTTTTGGCCATTTATGAGTATAACAATTA
CCCCTGGGGTTTCTCCTCCTTAAATCCGTCAGGATTGTGCCCGGCGGGATTGCCTCCAAGTGCGGCAAGC
TGCGCATGGGCGATCGCATATTGAAAGTCAACGAGGCGGATGTTTCCAAGGCCACACATCAGGATGCGGT
TCTGGAGCTGTTGAAGCCAGGTGATGAAATTAAGCTGACCATTCAACACGACCCATTGCCGCCAGGATTT
CAG
>dme_circ_0007667_junction_seq
ACACGACCCATTGCCGCCAGGATTTCAGGGAGGTTGTGCTGCCCAAGAACCAGGGC
>dme_circ_0007668
GGAGGTTGTGCTGCCCAAGAACCAGGGCTCCCTGGGATTCAGCATCATCGGCGGCACGGATCATTCCTGT
GTGCCCTTCGGCACTCGAGAGCCTGGCATATTCATATCGCACGTAAGTACAGACGGATAGATTAACTAAC
TAACAATTACCTGCAGCAGCAGCTGGCAAAAAAGTTTTCGAATTAAAACACTCGAGTGGCCGCAACTTGC
GAATGCAAATGAGCTGCTGCTGCAGAACAATTGTTCAGAATCCAGTGGCTCGTAGGATGGCTTTATTATA
AGCAGCCAACTAAGCCAGCTGCCTGCTCGAAAGTTTCAAGTCTTTTGGCCATTTATGAGTATAACAATTA

CCCCTGGGGTTTCTCCTCCTTAAATCCGTCAGGATTGTGCCCGGCGGGATTGCCTCCAAGTGCGGCAAGC
TGCGCATGGGCGATCGCATATTGAAAGTCAACGAGGCGGATGTTTCCAAGGCCACACATCAGGATGCGGT
TCTGGAGCTGTTGAAGCCAGGTGATGAAATTAAGCTGACCATTCAACACGACCCATTGCCGCCAGGATTT
CAGGGTAAGTTTTGTGGACTGGCAAAGTCCTTATTAGCCCATCTTAGACTGCTTTTGTACTGTAAATATG
TATATACGCTTCGGATCTACTTCTTACTCCTCCTCTATAATCAATTTTTGTATACTCTGCCACTGCTCAC
AAACAAATTCAATACGCACAGAGCATAGAAGTAGTTTATGTAAAAACAGAGGTAAGCTCCTCTTGGCTTT
CAGTGGGGAAATATTTGCAATTAGTCAAATGCCTTTTCCTTAACTTTTTGTTTGCTTTGTTTAGACCTGA
AGAGAGTCCCGATTGTTATTTGTCAGCGTTTAAGCCGTGTCCATGCTCATTTCCATTCTTTCCTAATTGT
TAATTGTAAATCGCAGGGAAGTGCTACTCAGCAAGGCGGAGGGCGAACGTTTGGGCATGCACATCAAGGG
CGGCTTGAATGGCCAGCGTGGTAATCCCGCCGATCCCTCCGACGAAGGCGTCTTCGTGTCCAAAATTAAC
TCGGTCGGTGCGGCCAGACGGGACGGAAGGCTTAAGGGTGGGCATGCGTCTGCTCGAGGTTAATGGACAC
TCGCTGCTGGGCGCCTCGCACCAGGATGCGGTCAATGTGCTGCGCAACGCTGGGAACGAGATTCAATTGG
TCGTCTGCAAAGGCTACGACAAGTCCAATTTAATTCATTCCATTGGCCAGGCCGGCGGCATGAGCACTGG
CTTCAACTCGTCTGCATCCTGCAGCGGTGGCAGTCGCCAAGGGACTTCGATGTCTTTGCCTCGCAGAAGC
CAGATGCACAACAGCCAACTGGCCCATCTGTCCTGGCAGCTGCTGCTGCCATGGTGCATGGCGCCTCGTC
GCCCACACCACCTGCTGCCACCTCCAATATAACTCCCTTACCCACAGCCGCGGCAGTAGCATCTGCTGAT
TTAACAGCACCGGACACGCCAGCAACCCAGACCGTGGCGCTCATTCACGCAGAACAGCAGGCCCATCAAC
AACAACAGCAGACGCAGCTGGCGCCTTTGGGTCAGGAGAAGAGTACCCAGGAGAAGGGTGCTGGAAATCG
TACGCGCGGCTGATGCCTTCACCACCGTGCCACCAAAGTCGCCATCGGAACACCACGAGCAGGATAAGAT
ACAGAAGACGACGACGGTTGTCATATCGAAGCATACGCTCGACACGAACCCAACCACCCCCACAACGCCG
GCGGCGCCTCTTTCCATCGCTGGAGCAGAGTCAGCGAACTCGGCTGGTGCTCCTTCTCCAGCTGTTCCTG
CATCCACGCCCGGCTCAGCGCCAGTTCTTCCTGCCGTGGCCGTGCAGACACAGACGCAAACGACCTCCAC
GGAGAAGGATGAAGAGGAGGAGTCGCAACTACAG
>dme_circ_0007668_junction_seq
GGATGAAGAGGAGGAGTCGCAACTACAGGGAGGTTGTGCTGCCCAAGAACCAGGGC
>dme_circ_0007669
GGAGGTTGTGCTGCCCAAGAACCAGGGCTCCCTGGGATTCAGCATCATCGGCGGCACGGATCATTCCTGT
GTGCCCTTCGGCACTCGAGAGCCTGGCATATTCATATCGCACGTAAGTACAGACGGATAGATTAACTAAC
TAACAATTACCTGCAGCAGCAGCTGGCAAAAAAGTTTTCGAATTAAAACACTCGAGTGGCCGCAACTTGC
GAATGCAAATGAGCTGCTGCTGCAGAACAATTGTTCAGAATCCAGTGGCTCGTAGGATGGCTTTATTATA
AGCAGCCAACTAAGCCAGCTGCCTGCTCGAAAGTTTCAAGTCTTTTGGCCATTTATGAGTATAACAATTA
CCCCTGGGGTTTCTCCTCCTTAAATCCGTCAGGATTGTGCCCGGCGGGATTGCCTCCAAGTGCGGCAAGC
TGCGCATGGGCGATCGCATATTGAAAGTCAACGAGGCGGATGTTTCCAAGGCCACACATCAGGATGCGGT
TCTGGAGCTGTTGAAGCCAGGTGATGAAATTAAGCTGACCATTCAACACGACCCATTGCCGCCAGGATTT
CAGGGTAAGTTTTGTGGACTGGCAAAGTCCTTATTAGCCCATCTTAGACTGCTTTTGTACTGTAAATATG
TATATACGCTTCGGATCTACTTCTTACTCCTCCTCTATAATCAATTTTTGTATACTCTGCCACTGCTCAC
AAACAAATTCAATACGCACAGAGCATAGAAGTAGTTTATGTAAAAACAGAGGTAAGCTCCTCTTGGCTTT
CAGTGGGGAAATATTTGCAATTAGTCAAATGCCTTTTCCTTAACTTTTTGTTTGCTTTGTTTAGACCTGA
AGAGAGTCCCGATTGTTATTTGTCAGCGTTTAAGCCGTGTCCATGCTCATTTCCATTCTTTCCTAATTGT
TAATTGTAAATCGCAGGGAAGTGCTACTCAGCAAGGCGGAGGGCGAACGTTTGGGCATGCACATCAAGGG
CGGCTTGAATGGCCAGCGTGGTAATCCCGCCGATCCCTCCGACGAAGGCGTCTTCGTGTCCAAAATTAAC
TCGGTCGGTGCGGCCAGACGGGACGGAAGGCTTAAGGGTGGGCATGCGTCTGCTCGAGGTTAATGGACAC
TCGCTGCTGGGCGCCTCGCACCAGGATGCGGTCAATGTGCTGCGCAACGCTGGGAACGAGATTCAATTGG
TCGTCTGCAAAGGCTACGACAAGTCCAATTTAATTCATTCCATTGGCCAGGCCGGCGGCATGAGCACTGG

CTTCAACTCGTCTGCATCCTGCAGCGGTGGCAGTCGCCAAGGGACTTCGATGTCTTTGCCTCGCAGAAGC
CAGATGCACAACAGCCAACTGGCCCATCTGTCCTGGCAGCTGCTGCTGCCATGGTGCATGGCGCCTCGTC
GCCCACACCACCTGCTGCCACCTCCAATATAACTCCCTTACCCACAGCCGCGGCAGTAGCATCTGCTGAT
TTAACAGCACCGGACACGCCAGCAACCCAGACCGTGGCGCTCATTCACGCAGAACAGCAGGCCCATCAAC
AACAACAGCAGACGCAGCTGGCGCCTTTGGGTCAGGAGAAGAGTACCCAGGAGAAGGGTGCTGGAAATCG
TACGCGCGGCTGATGCCTTCACCACCGTGCCACCAAAGTCGCCATCGGAACACCACGAGCAGGATAAGAT
ACAGAAGACGACGACGGTTGTCATATCGAAGCATACGCTCGACACGAACCCAACCACCCCCACAACGCCG
GCGGCGCCTCTTTCCATCGCTGGAGCAGAGTCAGCGAACTCGGCTGGTGCTCCTTCTCCAGCTGTTCCTG
CATCCACGCCCGGCTCAGCGCCAGTTCTTCCTGCCGTGGCCGTGCAGACACAGACGCAAACGACCTCCAC
GGAGAAGGATGAAGAGGAGGAGTCGCAACTACAGGTATGTACCCACCTGACCGTGGATGCACGTCTCTTG
TCTTGGCTTTAATATCTGTGTGTGTGTGTACGTCTTTCAATATTTGTGCGTGTACCATTCGTTTGTGTAA
AATACCTGTCTGTGTGCGAGTTTGGGTACCCAGATCCAGATCCAAATACAGATACACCCATGTAACAGCT
AACACGAACCGACAGTCGCCACCCCAACAGAAAACTCGACTCTAAACCCGACCAATCTCTTAACTTGATT
TTTGTTTCGTATTTCGCTTGTTATGTGTTTGTGTAACTTCACTAATGCCTTTGTCCTGTCACTGACATTT
CGTGTCCTGTGCTTGGAGTGCGTGCGTGTGTGTGTCAAAAATTTTAAAATGTATACAAAGAAATAAGATG
ATAAACCAAAAACACCATACTACTACCATCCATATTCACAAACCACAAAGGTCACAAAACAGTTGTTGTG
ATAAATTTGGCATGTGATGCCAGTTGaaaaaaagggaagGGTATGTACCCACCTGACCGTGGATGCACGT
CTCTTGTCTTGGCTTTAATATCTGTGTGTGTGTGTACGTCTTTCAATATTTGTGCGTGTACCATTCGTTT
GTGTAAAATACCTGTCTGTGTGCGAGTTTGGGTACCCAGATCCAGATCCAAATACAGATACACCCATGTA
ACAGCTAACACGAACCGACAGTCGCCACCCCAACAGAAAACTCGACTCTAAACCCGACCAATCTCTTAAC
TTGATTTTTGTTTCGTATTTCGCTTGTTATGTGTTTGTGTAACTTCACTAATGCCTTTGTCCTGTCACTG
ACATTTCGTGTCCTGTGCTTGGAGTGCGTGCGTGTGTGTGTCAAAAATTTTAAAATGTATACAAAGAAAT
AAGATGATAAACCAAAAACACCATACTACTACCATCCATATTCACAAACCACAAAGGTCACAAAACAGTT
GTTGTGATAAATTTGGCATGTGATGCCAGTTGaaaaaaagggaagaacaaaaaaagaaaaaaaaaaaaaC
ACCAAATAGAATGGGAATCATAGCTATATATTTTCAACTGAATTCACAGGTACCAGCATGATAGCATCAG
ACCATTAAACCTACTGACTGTAACTAAAACAAAATAATCATTGCATCCACTCGTTCGAAAGCACTTTCAT
TCTCTCATGTAAACACTAGCGGCAACGTTATTCACATCCATTTCGTGTTTTACGTACTTTTGTTGCTGCT
TTAAGGCGATTTTTTTGTTCAGTTGCATTTGAATTAAAAATGAATAAAAAATCATAGCCTATCTATGTGG
GCCGATTGTACCAGGGGGCGTATGAGTTATGTATTTGTTCTGCATGCGTGTCGGGCTTGCGCTTAGCTGC
TCTCAGCTCCTCTGAGCTGCTCCTTTGCTCCTTTGGCAGAGGAGTGGAGTAGATGGCCCCACATTTATTG
ATATGGCAGCAGACAATTTATTATTTATTAGCATATTGGCTGCTGTTGCTGCTGGCTTTCTGAGAACGCC
TTTCTTGGTCTGGCCCACTCTGATCAttgttcttgttgagtttcgttgttgttgtggttattgttcg
cgttgctcttgctgttgttgttgttATTTATGCTACTTTCATATTATATTCATTTCAGTTTGCGTCTTTA
CTGTTACCGTTCGTTGGTACCTTTGCCCCCACTGCGTAGGCGGCATTGTTATTATTGTTAACGTTGATGT
GGTTCTTTTTGTTGTTGGCTTTAAAGCATGCCCCATTTGAATTTGCATTGAAAGCAGTAGGGAACAAACG
ATATAACAAAAAGGGAAAGAAATCGGGCACACAttacatatatatatgtatatatacaaacatatactta
ctatataGATGGCACGTATAATAAATGTAAATGTAAATAATTCAAAAACTGCAGAAAGAAAATTTCCATT
AAAACGtctctcttatcttcttctcgtccttgttcttctttcattcttctgaattcttGCAATAATGTGC
GCGCTTTTCAACAAATTGTTGAtttttttattctttttgtttttgttttatttataacatttgttattgt
ttctttcaaacattttgCAATCTGTTCCGTTGGTTTTCTGTTGTTTTCCGCCCGCCATCGATATCATTGC
AGCTTACTGCTGAGCAGCGCATTGCTTAACAAGCGGATGTTTATCACCATAAGACTGAATATTTAGTTTA
AACTGACAGAATATAAATGATAGCAGCCAAAATAAAAGGACAGGAGGCAACAGCTTGGGTGTGCAGGCTC
TACTATTGTATTTGTACCGATGGAGTTGCAATATATACAAAATaaaaaaaaaaaaaaaaaaCTAGGATTT
GAGGAAGCGTGTTCAAAGGGCCAAGAGTTACTACTTATTGCGCTTTCGTCCCGTAATCCCCCACTCGAAA

TTCAATCAAAATCCGGACGCTTTCAACTCAGCAAATGGTTTCAATGTCTTCTCACTAGGACTCTGTCTCC
ACCTCCCATTCGATTGTTAAAAAGTGTGTCCTTATCAACACGAACAGCAACAAATTTACACTCCAAACTC
CACACATCCCAGACAATTGTTGAGATGAAAGGAACTCAGTACCGAAAATGTGTAAATGTATTTATCAACG
CGGAATGCTCTAATGTTGTCGCCATTTTGCGGCATATAAAGTATTTCACTTATACATATAGTCTTTCGAT
GTTTATACTATGTGAAACGAAAACTGTTTACTACATAACACGCAGAAGACACCCACACTATATATTACTA
AATGTGTAACAACGTGTGGCAAGCACACACAAAAGGTCCAATTATGCAACTGAATAATACTCAAGAGAAG
AATAGAATCTTTCCGACAAAAACTAAAGCCGATGACAAAGGGGCATCTTGTTATAACCTTTTGAAAGAGC
CAAGCTATACCTATACTAGGGATATTTTGTTACAGTATCTACTAAACTGTTACCACATAGTTAAAACCGA
ATTCACACCGTATATTGATTGAATACCTTAATGCAAATGGTTAGGTTTGAGTTTTTCACTTTCGTTGTTA
CCTAATGATAGTTAAATTAAAAGCAAGCTAAATGGAATTTAAAGTGTGAATTTTTGATGTCAATAAATCT
TAATGTATTTGTCGTATAAAAACCTCAAAAAATGGGTCAAACTTTTAATTGCATTTTGGTTTTTGGGAAG
ACCAAAGATTGGATTCTCGGAACTTCCTTAGTTTTCGGCCAAAAAGTTTAATTCGGGAAAGTTTTCGTTT
GATTTCGTTATTTTCCTTTTGCCATGTGAATCGTCTTTGTCTCCGTATCTGCTATCTCTCTGTATTTTCG
TATCGCTGCTGTTCGCTATCGTCATCGTGTGCCGAGTTCATCAACCCAAAATCATCACCCAGCAAATTGG
CCAGAAGAGTCCCAGTCGCAGTCGCAGTCACTCACCTCATCATCAAGCAGAAAATCACTGGTAATTGGCT
AATGGACGGAGGTTTCCAAGGAGCACCAAAAACAAAACCAAACAGAATAGTACAACTTTTGTGTGTGTCG
TTCGTACCAAAGCATCACCAACTAAATAAAGCGCACCCGCATTCATCCCCACttttttttACCGAGAATA
CTTCACCAGCATTTCACCCACTCACCATTAACGTTGCACACTGCAATTTGTGTTTCAAGCTTGTGTGCGA
TGACGTTGCACTCGAAAAATACCAAACCAAAACCCAAACTAAAACCAATCTATACCGAACTAAATGAGCA
ATCCACCAACAATTCAAACTCTAATAGCAACGAGGCACCCAGACTCCAGATCGCCTGGCACCCGGAGCAC
GTTACTCCTACCAGCAGATGCTGGAGTCGCAGGAGGAAAATCGAACTTCTGGCAAAGCCACCCAGGCGAA
GCCCAAGAAGGCGGAGCCCGTATACATCATAGATgatgaggacgatgaggatgatgatgacgCAGGTTCC
CAGCGGCTGGACGACATTGACGCTATCCAAGAGGAGGAAGAAGAGGAGGACACGGAGAGCTCTCCGCCCT
TGGAAGTGCAGTCACCACCTTCCTATGCAGACACTGATTCCGATACCTTCGACCGTCGGCGTGGTCGATA
TACAAGCGACTCGGAAAGCGACTGCCGGCCGTACAAACCATCGCGATCGGGACGCAGTACGCCTGAATAC
GGTGGTCATCGCAAGTCTGTAAGCTTCGATCTAAGTGGAGACGAGCGATCCAGGTCGGACTACGAGCGAT
CCCGCTCCCGCACTCCAGATGGTTATACTAGGGCCTACGATTCAGAGCACGATACTCGAGGATCTGGTCG
TAATCGTCTGCCGCGCAAAGGCATCTTGCGGGCCACCAGCCCGTGCGCCACGCTGGAAAAATCGGAACCC
ATTGTGCCAGCAGTGGCACGCATCCGTGATGTGGAATCGCCGCAACTTCAGCGCGTCTCACGGGCCACTT
CTCCGGATCGACCCGGCGAACTGGACCGGGAAAATCCTTTCCGTCATCCCACTTCCGAGGATGATGAATA
CACGCGGATTGCAAAGGAGGTCAGCAAATCGCCTCGGTCGCCGCGCTCCACCCGCGAGCAATTCTTTTTT
GGTTCTTCGAGATCCAGTGAGGATCTGCTGGAGCAATCCCAGTCACTATCCAGCGGTCGCAGTACCCCCA
GCACAGTGATCTCCAAGTCGACGGAGAACCTTACCGGCACGCGGCCAAAGATACCTCCACCCACCGCTCC
AAAGCCGCAATTAAAGAAGGCGAACCTGGTGCGAAATGTAGCACTGGAAACATTCCAGGCCAGCGATGTG
GGACAAGGTGATTTTACTGTATTCGAGCACGATGCCAGCACGAATACGATCCATCCCGTGGAGGCCAAAG
TTCCCGTCAGGGTGTCATCCAAGACGAAACCGAGTCGGCCGAGGGAATCACCACCACCGCCGCCAGTGAA
TTTCGCCACCCTGCCGAAGTTGCCAAGCACGCCAACTTTGCGAGAGGCGGAGGAGTCCATCTACATGGAG
GCACTGCCGCCGACGCCCTCGCAGCTTCGGCCCCACGAATTCGTGCACGAGAACTCACCGGACAATATTC
TGGTCTCGCCGCAGGAGCACCGCGAGTATTTGCTGCGTGAGAACGAGCTGAGGAATCAGTTGAGGGCCCC
CTATGAACCCGCTCCACCTGTACCCATTGTCGGCGGCGGATATTCGGTAACGAATCCCTTTCTAGATAGT
TTCGACACCGACATTGACAGCCTGCCATTGCCACCACCGCCCACACCGCCTGCCCAACACCAACATACAC
GTCATTTGCATGAGCCGCCGCAGTTTCCGCAGTTTGCGCCACCACTAACCGCTAACGCATCCACGTCTCC
ATCTTGTTCTTCCAATGCTGCTTCTGCTGGTGGCGCCACTAACCGCTTGATGGATTCGCCGCAACAACAG
CTGCTGGTCGCCGGGTCGCCCACCCAGATCTTTCCCACGGCCCAGATACTGCCCGTGCAGTATGCCAGTC

TGCCGCAACCGCAGCAGCCCAACACGCTCACCCTTAAGACCGTTTCCCCCACTTCGTCCCAAGACCCTAA
ACAGGCCCTTTTTAACCTGGTACTCCTGCCCGGCGACCAGGTGGCCAACAAATCCTCGCCAGCAATGCTC
TACCTGCAAACGGTGAGTGAATCGAGTGCCAAGCCCCGGCGGGGACCACCACCTCCTCCGCCCGTTACCG
TGACCTCACCCACGCCCTCGTATGCCGCCATGAAGTTCCCGACCTTCTCGGAAATAGTTGTCAGTCCCGA
TGCGAATAGCAATTACCAAAATATGACCACTGCTCGCGTTGGCGTCTCGAATTCCTCGCTGGCCCACAGC
ACCAGTTCCTTTTCAATCGGCTCCTCCAGCTCTTCTCTTCTTATGACCAAAAATTCGTCATTGGGAGAAG
GTAGTGTACCCTCCAGTTACTACGAGACGGCCGAGGAGATATACTCCACTATCGAGGAGGAGGCCATCTA
CAGCAACACCTCCTTCTTTGATTCGCGTCGAAGTAGTGACACTTCGGTATCCTGTCGCGCCTCCAAGTCG
CCCACCCCTCCTCCTCTGCCTCCCAAGCGACCTTTAAAGCCCACCAAGTTGCGTACCGCCCCCGCTCCGC
CGCCAGTGGCCCAAAAGCCTCATGGCCTGAGGGGCATGGCCAGTTTCAAGTTGCGCTCCCAATCCCAGCC
GCAGCTGGATGGGGCGTGGCAACAGCCCAACCTACCTCGAACGCCAACGACGCCCACTGGCAAGGAGACA
ACAGTGTAGTTTAGGGTCGGAATGAAATCTATGAATAAATCTAAAGTATTTCCAAAGAAACATTATCCAG
TTAATACTACTAACTCAAACTCTTTTTAAAAGGCATTGAAAATTGTaaaaaaaaaaaaaaTAACAGCCCA
TTCCGCTACAAATATTTCAGTACAAAATCTTCTTAAGTGAGTTCACCAATAACATAAAAATAGTGTCAAG
AAATACCCATAACAATAAATACGAATTGCAAAACTACTTTAGCAATAACTCAATATAAAATCCAAGAGCT
TTATTGGTCAGCGAACTCAAGGTTTTAAAACTAAAATGGTTTTTAAAGCGACAAAAATTTATTCGCCATT
TAAGTGAAGAGCTTAGAAATGTCGAGGAATATCCGACCGCCAAATGCGATTTTAGATTTATTCCTCTTCA
AAAATCGATCCACCTTGGGTAGACCGTCATGCAACCAATTGCCATCGGTGGCTTTTAACAATGGCGATGC
ATTTAATTGCTCGTGAGCTGACCGCAGGACCTTTCCACTTTTCCCGTTGCCACCACCACCACCTCGTTCT
CTCTTTTCGTCCACGTCTCTGCTCATACATTTTAATTAAATTCTAACACCTTTTTGCCATCAATTAAATT
GCCCAGCCCAGCCCCTTGCATTTTTCTTGCTCTCTTTGTTTCTGGCATCGACTGCAATTTTCAATATTTT
CATTCCACTCGCTCGCCATTTAAATTGATTGATTTGTGGGTAGTGTCTGAGCTCCGAACCCGCTGAACTT
gcaggcaggcaggcatgcaggcagATTGGGTGGCACACCTTTCCTGCTAATTAGATGCAACTTTCTGTTG
ACTTTCGAGACGCCCTCGACCCCCATCGAGCGTGTACTACCTACTTTGCCACAAAATTGCAGTTGAAATT
GAGCAGCAAGCGGTTGAAGTTTAATGGGCGCCACCTCGTCCTCCTGCTCGTGCTCGTGCTGTCACCCGCT
CACCTGGCAACTCCTCCCGAGTCTCGGGACAAGTTTAATTTGACGTTGATTGAAACAGTTGGCTGCTTAT
GGTCGCCCACCTGCAGTTACCACGCCCACCGGGCCATTAATTTTTGCTACTGCTGATGCCGCCGCTGCCC
ATTTTTATTATTGCCTTTTGTTTTGGgaaaacaaaatacgaaaaacgaaaatacgaagaaaaaccaacga
ataaataTGTAGTATAACGAAGAGAAATACCTGAGTATAAAACGAAGTAACAGAAAAACAGACAGAAAAC
ACAATTTTACAATTTATAAAGTTAATTTCGCCCTAGCGTTGCTAAGTTGATTTTAATGCTAATTTATTTT
ATCAAGACAAAGAAGCAAGCTGAACATTTTTAATTGCATACCTTAAGGCAAATAAACATTGCGTATTATT
CTTATTTGCGTTTCGTGGGCTCTTTTCATTTTGTCTTGTCCCCCTGGCCATGTATTGTGTAAACAAGGTT
TTTAGTACTCGAAAAACGATTGTTTAAATGGGAGCGATAACCCAGTACCCAAGTGGTGTGGCGGCCAGTG
ACCTTGACTCACTTAGCATTTGTATCTAATTGCGTCGTGACCCCAATTGGTCAAGAGGCAGGCACTGACT
GCCTGGCGACCTGCCGCTTTCGCACCTGGCCCTGCACTGATTTTGCTGTTGACTGGTTGCCCCTTTTTAA
TGTTCCATACCTCGGCTGGGTTCTCTTTGTTGCAATCTTATCGCAAAGCACTCGTAATTGCTTAGCGATA
AAAAAGAGTTGGTTACCCCTTATCAACGCCTTATCTTTCACTGCTCGCATGGTCTGAATAATTCAATGGC
TTATTGGCTTTTAATGCGCTGGCCATTTTGGCTCTCACACACTTATACACAATGGGACATCTCTGGGATC
GGTTACcacacacatacacacacacacacacaTTCTCAACACTTTCATGGCTCAATAAATTCCTATTAGC
TGGACTGCTGAGCAGGGCAAAAAGCAGCTCCTCCAACACTGGAGACTGATGCTGATAAACCAGCTCCATA
GCTGCCCTACTCCTCCATGTTGTTGGCTCTTCGCATTGACTTTAATTGAAAATCGAAAAATCCGATACGA
AATTAATTTCTCTTCTAAAAATACAGCTCAGTTAAGCTGGCACTCCTGCTGCTTTCACTTTTTCCCCATG
CACTTGTCCTGCCCTCCACTCTTCCACGCCATCGCCATCGCCCTTGGTTCTACGGCTTGGTTTATTTATG
AGCTCTTGGACTGGTGTGGTCTGATGGTCTGGTTGCTTCTTACTGCTCCTGGATGGAGGGTCGAACCCCA

CTAGTCTGGTATAAGGTTGCAATTTGTGCAGTTTAACGCTGGCCTTTGCCCTGTTTGCCTTTGTTTCGTC
TGGGTCTTCCTCCTTCTGCCCGCTGTCCTTTCCCACCCTACTGCTGCCATTTAATGACTTTGTCGTCGTA
GTCGCAGTCATCGTTGCTCTGCAGGCACTAACTATCCAGCCCGGGTTGGCTTTATCTGGCCACTGTGTCT
CATGTTGCCCAGCACGAGGAATTGCATGAAAGCATAAAACCGACAGTACACACGACTTTGGGTGATAAAT
GGCCAGCTCCGGTTTGGTTCGTTCCGTTCTAACCCACGCAGCTGTAATTTGCGCCCTCCTCTCCAGTTCA
ATCCGAACTCAAATCGCAGCCCAAACTCAAACCCACACCCTACCGTAAATTATATGTCCCGAGTGCAGGA
CATAGCAGCACAGACCCAAAAATGTCGGCTTGGTCACTTTATGATTGAGCCAGCCTATGGCTGATCCTGC
TAACGCCCTTTCTGGGCGTAACCAGTTCTGGACAAACAGACAGAAGACTGTCGGACTGCCGCCTGCCTGC
CTGACTTTTTAATAAACTTGTGTAGTGCCATCAATTTTCTTTATGGCCATTTCGGATTTTTCAGCGCTCG
CTTTGAAAATTGCAGTTTTTCCCAGTCATTTAATTGAAAATATGGCTGAGTGCTGCCAGCGTTTCAGCTT
TATCTGTTTATTGATTATTTTCGTCGTTAAATAAATCCTTGAGATATCttttttttttGTGTGTGTGCGT
TGAGTTGCTAACAAAAACGTTTTGCCATTTTGCAGGTCAACACCTGCCAGCAGGGATGGAGCAGAGGAGC
AGCAGGAAGAGGTTAGAGCCAAACCCACTCCGACTAAAGTGCCTAAATCGGTTTCCGATAAAAAACGCTT
CTTTGAGTCAGCCATGGAGGATCAACACAAGCCCACACAAAAGACAGGGTGAGTCAATGGTTAGTGCGAA
AAGTTGAATGAGTGGCTCAATGATGTCGCTGTGCAGTTTGCCCAAGCTGGTGGTCCTGCCACTTAAATCT
CGCTTCTTGGCCCAAGTTGTCAATCAAACCACTTATCTGCGGTTGCCAAGCCACCACAACGGTGGTCGTC
ACCTTTCGCTTCTCGGCTGGCGTAATTAGAAAAGGGTGGAGGAAAGCGCGGTTGCCTGCGCCATTTGCAT
GACAAAGTCTAGAGCTGTAGTGGTGGAATCCACTTAGGCAGCTAAATGGCTAAGCTGCATCGGATCGCTA
TTTATTTAATTTCCCAACTTAATAGCGGGACGATGTCCTTAGCTTACACTGATTTTATGCACTCTCTTTT
TTGCTTGCAGGACAAAGTTTTCTCATTCCTGTCAAAGGATGAAGTGGAAAAGCTGCGACAGGAGGAGGAG
AGAAAAATTGCAACATTGCGCCGTGATAAGAATTCCAGATTACTGGATGCCGCCAACGACAACATTGACA
AGGATGCCGCACAGCAGCGGACCAAGAGCAACAGCAACAGCAGCAGCGGCGACGATAATGACGACAGCGA
CCAAGAGGAGGGCATCGCTCGCGGGGATTCCGTTGACAACGCCGCTCTGGGGCATTTCGATGATGCGGAG
GACATGAG
>dme_circ_0007669_junction_seq
GGCATTTCGATGATGCGGAGGACATGAGGGAGGTTGTGCTGCCCAAGAACCAGGGC
>dme_circ_0007670
GGAGGTTGTGCTGCCCAAGAACCAGGGCTCCCTGGGATTCAGCATCATCGGCGGCACGGATCATTCCTGT
GTGCCCTTCGGCACTCGAGAGCCTGGCATATTCATATCGCACGTAAGTACAGACGGATAGATTAACTAAC
TAACAATTACCTGCAGCAGCAGCTGGCAAAAAAGTTTTCGAATTAAAACACTCGAGTGGCCGCAACTTGC
GAATGCAAATGAGCTGCTGCTGCAGAACAATTGTTCAGAATCCAGTGGCTCGTAGGATGGCTTTATTATA
AGCAGCCAACTAAGCCAGCTGCCTGCTCGAAAGTTTCAAGTCTTTTGGCCATTTATGAGTATAACAATTA
CCCCTGGGGTTTCTCCTCCTTAAATCCGTCAGGATTGTGCCCGGCGGGATTGCCTCCAAGTGCGGCAAGC
TGCGCATGGGCGATCGCATATTGAAAGTCAACGAGGCGGATGTTTCCAAGGCCACACATCAGGATGCGGT
TCTGGAGCTGTTGAAGCCAGGTGATGAAATTAAGCTGACCATTCAACACGACCCATTGCCGCCAGGATTT
CAGGGTAAGTTTTGTGGACTGGCAAAGTCCTTATTAGCCCATCTTAGACTGCTTTTGTACTGTAAATATG
TATATACGCTTCGGATCTACTTCTTACTCCTCCTCTATAATCAATTTTTGTATACTCTGCCACTGCTCAC
AAACAAATTCAATACGCACAGAGCATAGAAGTAGTTTATGTAAAAACAGAGGTAAGCTCCTCTTGGCTTT
CAGTGGGGAAATATTTGCAATTAGTCAAATGCCTTTTCCTTAACTTTTTGTTTGCTTTGTTTAGACCTGA
AGAGAGTCCCGATTGTTATTTGTCAGCGTTTAAGCCGTGTCCATGCTCATTTCCATTCTTTCCTAATTGT
TAATTGTAAATCGCAGGGAAGTGCTACTCAGCAAGGCGGAGGGCGAACGTTTGGGCATGCACATCAAGGG
CGGCTTGAATGGCCAGCGTGGTAATCCCGCCGATCCCTCCGACGAAGGCGTCTTCGTGTCCAAAATTAAC
TCGGTCGGTGCGGCCAGACGGACGGAAGGCTTAAGGGTGGGCATGCGTCTGCTCGAGGTTAATGGACAC
TCGCTGCTGGGCGCCTCGCACCAGGATGCGGTCAATGTGCTGCGCAACGCTGGGAACGAGATTCAATTGG

TCGTCTGCAAAGGCTACGACAAGTCCAATTTAATTCATTCCATTGGCCAGGCCGGCGGCATGAGCACTGG
CTTCAACTCGTCTGCATCCTGCAGCGGTGGCAGTCGCCAAGGGACTTCGATGTCTTTGCCTCGCAGAAGC
CAGATGCACAACAGCCAACTGGCCCATCTGTCCTGGCAGCTGCTGCTGCCATGGTGCATGGCGCCTCGTC
GCCCACACCACCTGCTGCCACCTCCAATATAACTCCCTTACCCACAGCCGCGGCAGTAGCATCTGCTGAT
TTAACAGCACCGGACACGCCAGCAACCCAGACCGTGGCGCTCATTCACGCAGAACAGCAGGCCCATCAAC
AACAACAGCAGACGCAGCTGGCGCCTTTGGGTCAGGAGAAGAGTACCCAGGAGAAGGGTGCTGGAAATCG
TACGCGCGGCTGATGCCTTCACCACCGTGCCACCAAAGTCGCCATCGGAACACCACGAGCAGGATAAGAT
ACAGAAGACGACGACGGTTGTCATATCGAAGCATACGCTCGACACGAACCCAACCACCCCCACAACGCCG
GCGGCGCCTCTTTCCATCGCTGGAGCAGAGTCAGCGAACTCGGCTGGTGCTCCTTCTCCAGCTGTTCCTG
CATCCACGCCCGGCTCAGCGCCAGTTCTTCCTGCCGTGGCCGTGCAGACACAGACGCAAACGACCTCCAC
GGAGAAGGATGAAGAGGAGGAGTCGCAACTACAGGTATGTACCCACCTGACCGTGGATGCACGTCTCTTG
TCTTGGCTTTAATATCTGTGTGTGTGTGTACGTCTTTCAATATTTGTGCGTGTACCATTCGTTTGTGTAA
AATACCTGTCTGTGTGCGAGTTTGGGTACCCAGATCCAGATCCAAATACAGATACACCCATGTAACAGCT
AACACGAACCGACAGTCGCCACCCCAACAGAAAACTCGACTCTAAACCCGACCAATCTCTTAACTTGATT
TTTGTTTCGTATTTCGCTTGTTATGTGTTTGTGTAACTTCACTAATGCCTTTGTCCTGTCACTGACATTT
CGTGTCCTGTGCTTGGAGTGCGTGCGTGTGTGTGTCAAAAATTTTAAAATGTATACAAAGAAATAAGATG
ATAAACCAAAAACACCATACTACTACCATCCATATTCACAAACCACAAAGGTCACAAAACAGTTGTTGTG
ATAAATTTGGCATGTGATGCCAGTTGaaaaaaagggaagGGTATGTACCCACCTGACCGTGGATGCACGT
CTCTTGTCTTGGCTTTAATATCTGTGTGTGTGTGTACGTCTTTCAATATTTGTGCGTGTACCATTCGTTT
GTGTAAAATACCTGTCTGTGTGCGAGTTTGGGTACCCAGATCCAGATCCAAATACAGATACACCCATGTA
ACAGCTAACACGAACCGACAGTCGCCACCCCAACAGAAAACTCGACTCTAAACCCGACCAATCTCTTAAC
TTGATTTTTGTTTCGTATTTCGCTTGTTATGTGTTTGTGTAACTTCACTAATGCCTTTGTCCTGTCACTG
ACATTTCGTGTCCTGTGCTTGGAGTGCGTGCGTGTGTGTGTCAAAAATTTTAAAATGTATACAAAGAAAT
AAGATGATAAACCAAAAACACCATACTACTACCATCCATATTCACAAACCACAAAGGTCACAAAACAGTT
GTTGTGATAAATTTGGCATGTGATGCCAGTTGaaaaaaagggaagaacaaaaaaagaaaaaaaaaaaaaC
ACCAAATAGAATGGGAATCATAGCTATATATTTTCAACTGAATTCACAGGTACCAGCATGATAGCATCAG
ACCATTAAACCTACTGACTGTAACTAAAACAAAATAATCATTGCATCCACTCGTTCGAAAGCACTTTCAT
TCTCTCATGTAAACACTAGCGGCAACGTTATTCACATCCATTTCGTGTTTTACGTACTTTTGTTGCTGCT
TTAAGGCGATTTTTTTGTTCAGTTGCATTTGAATTAAAAATGAATAAAAAATCATAGCCTATCTATGTGG
GCCGATTGTACCAGGGGGCGTATGAGTTATGTATTTGTTCTGCATGCGTGTCGGGCTTGCGCTTAGCTGC
TCTCAGCTCCTCTGAGCTGCTCCTTTGCTCCTTTGGCAGAGGAGTGGAGTAGATGGCCCCACATTTATTG
ATATGGCAGCAGACAATTTATTATTTATTAGCATATTGGCTGCTGTTGCTGCTGGCTTTCTGAGAACGCC
TTTCTTGGTCTGGCCCACTCTGATCAttgttcttgttgagtttcgttgttgttgttgtggttattgttcg
cgttgctcttgctgttgttgttgttATTTATGCTACTTTCATATTATATTCATTTCAGTTTGCGTCTTTA
CTGTTACCGTTCGTTGGTACCTTTGCCCCCACTGCGTAGGCGGCATTGTTATTATTGTTAACGTTGATGT
GGTTCTTTTTGTTGTTGGCTTTAAAGCATGCCCCATTTGAATTTGCATTGAAAGCAGTAGGGAACAAACG
ATATAACAAAAAGGGAAAGAAATCGGGCACACAttacatatatatatgtatatatacaaacatatactta
ctatataGATGGCACGTATAATAAATGTAAATGTAAATAATTCAAAAACTGCAGAAAGAAAATTTCCATT
AAAACGtctctcttatcttcttctcgtccttgttcttctttcattcttctgaattcttGCAATAATGTGC
GCGCTTTTCAACAAATTGTTGAtttttttattctttttgtttttgttttatttataacatttgttattgt
ttctttcaaacattttgCAATCTGTTCCGTTGGTTTTCTGTTGTTTTCCGCCCGCCATCGATATCATTGC
AGCTTACTGCTGAGCAGCGCATTGCTTAACAAGCGGATGTTTATCACCATAAGACTGAATATTTAGTTTA
AACTGACAGAATATAAATGATAGCAGCCAAAATAAAAGGACAGGAGGCAACAGCTTGGGTGTGCAGGCTC
TACTATTGTATTTGTACCGATGGAGTTGCAATATATACAAAATaaaaaaaaaaaaaaaaaaCTAGGATTT

GAGGAAGCGTGTTCAAAGGGCCAAGAGTTACTACTTATTGCGCTTTCGTCCCGTAATCCCCCACTCGAAA
TTCAATCAAAATCCGGACGCTTTCAACTCAGCAAATGGTTTCAATGTCTTCTCACTAGGACTCTGTCTCC
ACCTCCCATTCGATTGTTAAAAAGTGTGTCCTTATCAACACGAACAGCAACAAATTTACACTCCAAACTC
CACACATCCCAGACAATTGTTGAGATGAAAGGAACTCAGTACCGAAAATGTGTAAATGTATTTATCAACG
CGGAATGCTCTAATGTTGTCGCCATTTTGCGGCATATAAAGTATTTCACTTATACATATAGTCTTTCGAT
GTTTATACTATGTGAAACGAAAACTGTTTACTACATAACACGCAGAAGACACCCACACTATATATTACTA
AATGTGTAACAACGTGTGGCAAGCACACACAAAAGGTCCAATTATGCAACTGAATAATACTCAAGAGAAG
AATAGAATCTTTCCGACAAAAACTAAAGCCGATGACAAAGGGGCATCTTGTTATAACCTTTTGAAAGAGC
CAAGCTATACCTATACTAGGGATATTTTGTTACAGTATCTACTAAACTGTTACCACATAGTTAAAACCGA
ATTCACACCGTATATTGATTGAATACCTTAATGCAAATGGTTAGGTTTGAGTTTTTCACTTTCGTTGTTA
CCTAATGATAGTTAAATTAAAAGCAAGCTAAATGGAATTTAAAGTGTGAATTTTTGATGTCAATAAATCT
TAATGTATTTGTCGTATAAAAACCTCAAAAAATGGGTCAAACTTTTAATTGCATTTTGGTTTTTGGGAAG
ACCAAAGATTGGATTCTCGGAACTTCCTTAGTTTTCGGCCAAAAAGTTTAATTCGGGAAAGTTTTCGTTT
GATTTCGTTATTTTCCTTTTGCCATGTGAATCGTCTTTGTCTCCGTATCTGCTATCTCTCTGTATTTTCG
TATCGCTGCTGTTCGCTATCGTCATCGTGTGCCGAGTTCATCAACCCAAAATCATCACCCAGCAAATTGG
CCAGAAGAGTCCCAGTCGCAGTCGCAGTCACTCACCTCATCATCAAGCAGAAAATCACTGGTAATTGGCT
AATGGACGGAGGTTTCCAAGGAGCACCAAAAACAAAACCAAACAGAATAGTACAACTTTTGTGTGTGTCG
TTCGTACCAAAGCATCACCAACTAAATAAAGCGCACCCGCATTCATCCCCACttttttttACCGAGAATA
CTTCACCAGCATTTCACCCACTCACCATTAACGTTGCACACTGCAATTTGTGTTTCAAGCTTGTGTGCGA
TGACGTTGCACTCGAAAAATACCAAACCAAAACCCAAACTAAAACCAATCTATACCGAACTAAATGAGCA
ATCCACCAACAATTCAAACTCTAATAGCAACGAGGCACCCAGACTCCAGATCGCCTGGCACCCGGAGCAC
GTTACTCCTACCAGCAGATGCTGGAGTCGCAGGAGGAAAATCGAACTTCTGGCAAAGCCACCCAGGCGAA
GCCCAAGAAGGCGGAGCCCGTATACATCATAGATgatgaggacgatgaggatgatgatgacgCAGGTTCC
CAGCGGCTGGACGACATTGACGCTATCCAAGAGGAGGAAGAAGAGGAGGACACGGAGAGCTCTCCGCCCT
TGGAAGTGCAGTCACCACCTTCCTATGCAGACACTGATTCCGATACCTTCGACCGTCGGCGTGGTCGATA
TACAAGCGACTCGGAAAGCGACTGCCGGCCGTACAAACCATCGCGATCGGGACGCAGTACGCCTGAATAC
GGTGGTCATCGCAAGTCTGTAAGCTTCGATCTAAGTGGAGACGAGCGATCCAGGTCGGACTACGAGCGAT
CCCGCTCCCGCACTCCAGATGGTTATACTAGGGCCTACGATTCAGAGCACGATACTCGAGGATCTGGTCG
TAATCGTCTGCCGCGCAAAGGCATCTTGCGGGCCACCAGCCCGTGCGCCACGCTGGAAAAATCGGAACCC
ATTGTGCCAGCAGTGGCACGCATCCGTGATGTGGAATCGCCGCAACTTCAGCGCGTCTCACGGGCCACTT
CTCCGGATCGACCCGGCGAACTGGACCGGGAAAATCCTTTCCGTCATCCCACTTCCGAGGATGATGAATA
CACGCGGATTGCAAAGGAGGTCAGCAAATCGCCTCGGTCGCCGCGCTCCACCCGCGAGCAATTCTTTTTT
GGTTCTTCGAGATCCAGTGAGGATCTGCTGGAGCAATCCCAGTCACTATCCAGCGGTCGCAGTACCCCCA
GCACAGTGATCTCCAAGTCGACGGAGAACCTTACCGGCACGCGGCCAAAGATACCTCCACCCACCGCTCC
AAAGCCGCAATTAAAGAAGGCGAACCTGGTGCGAAATGTAGCACTGGAAACATTCCAGGCCAGCGATGTG
GGACAAGGTGATTTTACTGTATTCGAGCACGATGCCAGCACGAATACGATCCATCCCGTGGAGGCCAAAG
TTCCCGTCAGGGTGTCATCCAAGACGAAACCGAGTCGGCCGAGGGAATCACCACCACCGCCGCCAGTGAA
TTTCGCCACCCTGCCGAAGTTGCCAAGCACGCCAACTTTGCGAGAGGCGGAGGAGTCCATCTACATGGAG
GCACTGCCGCCGACGCCCTCGCAGCTTCGGCCCCACGAATTCGTGCACGAGAACTCACCGGACAATATTC
TGGTCTCGCCGCAGGAGCACCGCGAGTATTTGCTGCGTGAGAACGAGCTGAGGAATCAGTTGAGGGCCCC
CTATGAACCCGCTCCACCTGTACCCATTGTCGGCGGCGGATATTCGGTAACGAATCCCTTTCTAGATAGT
TTCGACACCGACATTGACAGCCTGCCATTGCCACCACCGCCCACACCGCCTGCCCAACACCAACATACAC
GTCATTTGCATGAGCCGCCGCAGTTTCCGCAGTTTGCGCCACCACTAACCGCTAACGCATCCACGTCTCC
ATCTTGTTCTTCCAATGCTGCTTCTGCTGGTGGCGCCACTAACCGCTTGATGGATTCGCCGCAACAACAG

CTGCTGGTCGCCGGGTCGCCCACCCAGATCTTTCCCACGGCCCAGATACTGCCCGTGCAGTATGCCAGTC
TGCCGCAACCGCAGCAGCCCAACACGCTCACCCTTAAGACCGTTTCCCCCACTTCGTCCCAAGACCCTAA
ACAGGCCCTTTTTAACCTGGTACTCCTGCCCGGCGACCAGGTGGCCAACAAATCCTCGCCAGCAATGCTC
TACCTGCAAACGGTGAGTGAATCGAGTGCCAAGCCCCGGCGGGGACCACCACCTCCTCCGCCCGTTACCG
TGACCTCACCCACGCCCTCGTATGCCGCCATGAAGTTCCCGACCTTCTCGGAAATAGTTGTCAGTCCCGA
TGCGAATAGCAATTACCAAAATATGACCACTGCTCGCGTTGGCGTCTCGAATTCCTCGCTGGCCCACAGC
ACCAGTTCCTTTTCAATCGGCTCCTCCAGCTCTTCTCTTCTTATGACCAAAAATTCGTCATTGGGAGAAG
GTAGTGTACCCTCCAGTTACTACGAGACGGCCGAGGAGATATACTCCACTATCGAGGAGGAGGCCATCTA
CAGCAACACCTCCTTCTTTGATTCGCGTCGAAGTAGTGACACTTCGGTATCCTGTCGCGCCTCCAAGTCG
CCCACCCCTCCTCCTCTGCCTCCCAAGCGACCTTTAAAGCCCACCAAGTTGCGTACCGCCCCCGCTCCGC
CGCCAGTGGCCCAAAAGCCTCATGGCCTGAGGGGCATGGCCAGTTTCAAGTTGCGCTCCCAATCCCAGCC
GCAGCTGGATGGGGCGTGGCAACAGCCCAACCTACCTCGAACGCCAACGACGCCCACTGGCAAGGAGACA
ACAGTGTAGTTTAGGGTCGGAATGAAATCTATGAATAAATCTAAAGTATTTCCAAAGAAACATTATCCAG
TTAATACTACTAACTCAAACTCTTTTTAAAAGGCATTGAAAATTGTaaaaaaaaaaaaaaTAACAGCCCA
TTCCGCTACAAATATTTCAGTACAAAATCTTCTTAAGTGAGTTCACCAATAACATAAAAATAGTGTCAAG
AAATACCCATAACAATAAATACGAATTGCAAAACTACTTTAGCAATAACTCAATATAAAATCCAAGAGCT
TTATTGGTCAGCGAACTCAAGGTTTTAAAACTAAAATGGTTTTTAAAGCGACAAAAATTTATTCGCCATT
TAAGTGAAGAGCTTAGAAATGTCGAGGAATATCCGACCGCCAAATGCGATTTTAGATTTATTCCTCTTCA
AAAATCGATCCACCTTGGGTAGACCGTCATGCAACCAATTGCCATCGGTGGCTTTTAACAATGGCGATGC
ATTTAATTGCTCGTGAGCTGACCGCAGGACCTTTCCACTTTTCCCGTTGCCACCACCACCACCTCGTTCT
CTCTTTTCGTCCACGTCTCTGCTCATACATTTTAATTAAATTCTAACACCTTTTTGCCATCAATTAAATT
GCCCAGCCCAGCCCCTTGCATTTTTCTTGCTCTCTTTGTTTCTGGCATCGACTGCAATTTTCAATATTTT
CATTCCACTCGCTCGCCATTTAAATTGATTGATTTGTGGGTAGTGTCTGAGCTCCGAACCCGCTGAACTT
gcaggcaggcaggcatgcaggcagATTGGGTGGCACACCTTTCCTGCTAATTAGATGCAACTTTCTGTTG
ACTTTCGAGACGCCCTCGACCCCCATCGAGCGTGTACTACCTACTTTGCCACAAAATTGCAGTTGAAATT
GAGCAGCAAGCGGTTGAAGTTTAATGGGCGCCACCTCGTCCTCCTGCTCGTGCTCGTGCTGTCACCCGCT
CACCTGGCAACTCCTCCCGAGTCTCGGGACAAGTTTAATTTGACGTTGATTGAAACAGTTGGCTGCTTAT
GGTCGCCCACCTGCAGTTACCACGCCCACCGGGCCATTAATTTTTGCTACTGCTGATGCCGCCGCTGCCC
ATTTTTATTATTGCCTTTTGTTTTGGgaaaacaaaatacgaaaaacgaaaatacgaagaaaaaccaacga
ataaataTGTAGTATAACGAAGAGAAATACCTGAGTATAAAACGAAGTAACAGAAAAACAGACAGAAAAC
ACAATTTTACAATTTATAAAGTTAATTTCGCCCTAGCGTTGCTAAGTTGATTTTAATGCTAATTTATTTT
ATCAAGACAAAGAAGCAAGCTGAACATTTTTAATTGCATACCTTAAGGCAAATAAACATTGCGTATTATT
CTTATTTGCGTTTCGTGGGCTCTTTTCATTTTGTCTTGTCCCCCTGGCCATGTATTGTGTAAACAAGGTT
TTTAGTACTCGAAAAACGATTGTTTAAATGGGAGCGATAACCCAGTACCCAAGTGGTGTGGCGGCCAGTG
ACCTTGACTCACTTAGCATTTGTATCTAATTGCGTCGTGACCCCAATTGGTCAAGAGGCAGGCACTGACT
GCCTGGCGACCTGCCGCTTTCGCACCTGGCCCTGCACTGATTTTGCTGTTGACTGGTTGCCCCTTTTTAA
TGTTCCATACCTCGGCTGGGTTCTCTTTGTTGCAATCTTATCGCAAAGCACTCGTAATTGCTTAGCGATA
AAAAAGAGTTGGTTACCCCTTATCAACGCCTTATCTTTCACTGCTCGCATGGTCTGAATAATTCAATGGC
TTATTGGCTTTTAATGCGCTGGCCATTTTGGCTCTCACACACTTATACACAATGGGACATCTCTGGGATC
GGTTACcacacacatacacacacacacacacaTTCTCAACACTTTCATGGCTCAATAAATTCCTATTAGC
TGGACTGCTGAGCAGGGCAAAAAGCAGCTCCTCCAACACTGGAGACTGATGCTGATAAACCAGCTCCATA
GCTGCCCTACTCCTCCATGTTGTTGGCTCTTCGCATTGACTTTAATTGAAAATCGAAAAATCCGATACGA
AATTAATTTCTCTTCTAAAAATACAGCTCAGTTAAGCTGGCACTCCTGCTGCTTTCACTTTTTCCCCATG
CACTTGTCCTGCCCTCCACTCTTCCACGCCATCGCCATCGCCCTTGGTTCTACGGCTTGGTTTATTTATG

AGCTCTTGGACTGGTGTGGTCTGATGGTCTGGTTGCTTCTTACTGCTCCTGGATGGAGGGTCGAACCCCA
CTAGTCTGGTATAAGGTTGCAATTTGTGCAGTTTAACGCTGGCCTTTGCCCTGTTTGCCTTTGTTTCGTC
TGGGTCTTCCTCCTTCTGCCCGCTGTCCTTTCCCACCCTACTGCTGCCATTTAATGACTTTGTCGTCGTA
GTCGCAGTCATCGTTGCTCTGCAGGCACTAACTATCCAGCCCGGGTTGGCTTTATCTGGCCACTGTGTCT
CATGTTGCCCAGCACGAGGAATTGCATGAAAGCATAAAACCGACAGTACACACGACTTTGGGTGATAAAT
GGCCAGCTCCGGTTTGGTTCGTTCCGTTCTAACCCACGCAGCTGTAATTTGCGCCCTCCTCTCCAGTTCA
ATCCGAACTCAAATCGCAGCCCAAACTCAAACCCACACCCTACCGTAAATTATATGTCCCGAGTGCAGGA
CATAGCAGCACAGACCCAAAAATGTCGGCTTGGTCACTTTATGATTGAGCCAGCCTATGGCTGATCCTGC
TAACGCCCTTTCTGGGCGTAACCAGTTCTGGACAAACAGACAGAAGACTGTCGGACTGCCGCCTGCCTGC
CTGACTTTTTAATAAACTTGTGTAGTGCCATCAATTTTCTTTATGGCCATTTCGGATTTTTCAGCGCTCG
CTTTGAAAATTGCAGTTTTTCCCAGTCATTTAATTGAAAATATGGCTGAGTGCTGCCAGCGTTTCAGCTT
TATCTGTTTATTGATTATTTTCGTCGTTAAATAAATCCTTGAGATATCttttttttttGTGTGTGTGCGT
TGAGTTGCTAACAAAAACGTTTTGCCATTTTGCAGGTCAACACCTGCCAGCAGGGATGGAGCAGAGGAGC
AGCAGGAAGAGGTTAGAGCCAAACCCACTCCGACTAAAGTGCCTAAATCGGTTTCCGATAAAAAACGCTT
CTTTGAGTCAGCCATGGAGGATCAACACAAGCCCACACAAAAGACAGGGTGAGTCAATGGTTAGTGCGAA
AAGTTGAATGAGTGGCTCAATGATGTCGCTGTGCAGTTTGCCCAAGCTGGTGGTCCTGCCACTTAAATCT
CGCTTCTTGGCCCAAGTTGTCAATCAAACCACTTATCTGCGGTTGCCAAGCCACCACAACGGTGGTCGTC
ACCTTTCGCTTCTCGGCTGGCGTAATTAGAAAAGGGTGGAGGAAAGCGCGGTTGCCTGCGCCATTTGCAT
GACAAAGTCTAGAGCTGTAGTGGTGGAATCCACTTAGGCAGCTAAATGGCTAAGCTGCATCGGATCGCTA
TTTATTTAATTTCCCAACTTAATAGCGGGACGATGTCCTTAGCTTACACTGATTTTATGCACTCTCTTTT
TTGCTTGCAGGACAAAGTTTTCTCATTCCTGTCAAAGGATGAAGTGGAAAAGCTGCGACAGGAGGAGGAG
AGAAAAATTGCAACATTGCGCCGTGATAAGAATTCCAGATTACTGGATGCCGCCAACGACAACATTGACA
AGGATGCCGCACAGCAGCGGACCAAGAGCAACAGCAACAGCAGCAGCGGCGACGATAATGACGACAGCGA
CCAAGAGGAGGGCATCGCTCGCGGGGATTCCGTTGACAACGCCGCTCTGGGGCATTTCGATGATGCGGAG
GACATGAGGGTGAGTGCGGATGTGCCAAGCTCTCATTTCGGATTATTGGTGCTTGCATTATGCGCTTTTT
ATGTTACATCTGAATGCACAGACACACTATACACACACTTCACACCAATCCACCCAATCAATCAATCAAT
CACCCTTCACCCCATGCGATATTATGATAAccatacacccactcacccacacacacacacacgaaactca
cacaACTTTCACGGTGTTTGTGCATTTGCTTAGTATAATCTCTTATGCAAAGTTAAATTTTTTAGTTTTA
TTGGTGAAGTTTTTCCGCTGCCTTTTGGTTTGGTTCTGGTGCCGGTTCTCTTACAGCTGCTTTTAAATAT
GGGTTGCTCTGCTCTTAAGTTTAGATGCCCTCAATCGTTCACATTGCaaaaaaaaaaaaaaaaaaaaaa
aCACCCCACAAAACACCACCACTTTGTTTACTATCCATGCAGTCAACTGACGGAGCTGCTAATCATCATT
GAGACTTAGTGTGTTATTAAATAAGGATAGCGGTCTATGGCTGAGTGTTCTTTAATAGTAAGTACACTTC
TCACCACACAATATCTCCACATACGCACTCACACACACTTGCATACGATTATAAGCGTCTAAGCCCTAAG
CAGTTTAAGTTTTATGTAGCACTTAAGCGTTGTATGGACTCGTAAATCGCAGCCAGACTCCCAGCCCTTA
GTCTACTTAAAATGTAAGGaaaaaaaaaaCAGTGAGGAACTTGAGAAGGAGGTGGAGTGTAAATGCCGGA
AAAACATGTTATGGTTCTGGCGTTGAAATCTTTTCACATAGTACATAGACCACGCCCGGTGGGCTTCGAA
TCTCATCTCGTGTATATGATAATCTAGTCGAAGCGGCAGAACTCAAAAGTGTAAGTATATCCGCTGCTGT
TTTACTTAAtttttttttGCTGGCATTGCGTATACGCAATGTGTGCCTCCGGGCCTGCAACCTTGCTCTT
CCTTTTGCTCCTTTTCCTTCCGCGTGGGCGTCCTactccactccattccactccactcGAATTCCCTGCG
GCACAATGCCAGCTGTTTTTACAATTGAATAACTTCATTTGGGCATTTCGCAAATTGCGTTTTGTGCGTT
TGCTCACTCAACTGGGCGGTGCCAGTTCAGATTTGGATTTTGGATTCGCTACGGCTCCCCGGCAATGGAT
CGGCAAACGAGCCACCAGAAGAGCATCACCCAACACGTACGCATAATTTAGTATCCGTTGGGAATCGATG
ATTTTATGCTAAGTGTGTTGCAGTGAATTAAGTCGCGGCAGTCGGAGACTCGGAAAACGGGCACTCGGAT
TTGTTTGGCTCAAATGATATTTTATACCCAGCTCGAAACGGGCAATTATAATTAGCGTCCGTTGAAGTGG

TGGGTGGACCCCGCTAACTTGATGGGTCCTCCAAGCACTGTCGATGGCATGCAACACGTAGCAACCACAA
CCATTGTGTCAAAGGGCAACTAGTATTTCCTCTGTGCGTGGAAAAGGTGCCAACCAGCGGCGAACAAACA
AAAACTGAACAATGAAAGAAACTAAAAGCTTATGCCAGGTGGTACCACCCAGAACATGCTATGGTTGCTC
CATCCACCAAAAGCACCACCCATACATTCGCCCACCATGGCAGCCATTTCGTACGCGGAGCTGGCGCTTA
TCAGCCAACTTTCACACATATGTGCTCCGCTTGGTTGAAGGACTCATAGTCGTCGCCCCCACGCGATGTT
CCAGTGGCTGGCTCTAACAACATTATATAATGAGCAACAACAAAGAAATTTAATACAATTTCGCCCCAAA
GCTGTTGCAGGAAAACATTAAAAAGAGCGCAACCGCCAAGCGTCAGCAAAAGTCGCCACTGAAATACCCT
TTTCAGGTCATTAGCCTAACGATCAGTTGACCAGTTGCTTGACCTCTGCCATAAATAGGAACACACGAGC
TGATGTTAATGTTCTGCTTTTAATTTATAACAACTTATCTGACTGATTAAAAACTGCAACCTGTTTCCGA
CCAAAAGGGTATTCACTGCGGCAAAATAGTaaaaaaaaaaaaaaaaCAATAGAGTGAAAAAAAGGCAGAG
ACGTATGACAACAGCAGATAGATAGCTTGACAACGGGTTCATGACTTTTTTAGTCCTTTGTGTTTGTGGA
TAGAATTTACTCTATGAATAAAGCTACGAAAATAACGCAAAATGTAAAGAAAATTCAATTCACTTTACGC
GTTTATGTACAAAGCATTACAAAGCGTTCTATGGAATTTCAAAGTAATGATGCGTCCCTTAACTCTGTAG
ATATGGGATGCTTTCATTTATCTCTGACGATCTACAGCAGCATTTGTTTTTCTAACCTTGCGCATAAATC
AAGCCGCAAGAACTAATCATTAAATGGTTACAAATTTGGGAATAAATATCTTATGACTTCACAGTCCCCC
GGAGCGGAGAATTCGGCTCAATTTTCCCAGCAGTCGCCATAGTCTGGTTGTCAACATAGCCGAAAAGACG
AGAAGGTGGCCTTGAGGTGGAATTGGTTGGGGCACAGCTGCCAAAAGTTCGGGAACGAAACGAATGGAGA
TCCGTCCACCCTCCTCCGTTCCGaaaaaaaaCCCGCCTGCCAGCTGGCTTTACAACTTTACATGCATAAT
ATAATAAATTGAAATGTGTGTTATTATGTTGCTCGGTCCGTAGCCTTGGAATTCATAACGAGGAAAAGAA
GATAAACATGTTAATAACCTTGTAATAAATTTCTAGCAAGAATTTCTACTAGTTTCGTTCCTCTTTCCTG
TTCTTGTTTATATATATTCTTTCCATGCATAAATTTTGTaaaaaccgaaaaaaaaaaaaTGAAGAGAAGC
ACGGAGAAGTAGAAGTAGAATATATGCTGAAAAACCCCATATCAAATTAGCCGGCAAGGCAGTGTTCTGC
AAACTGTTTGTATTTATATTCAAATTAAACTCGTCTAAATCTATAAAAGTTGTGGAACACAAAAGGCTCG
CCAGATCCGTGTTTATGAATTTTATTTGCGACATCAAATTGGCTTCCGTGACGACAAAAACCTAGTCTCG
ATGTCGGCAAAACGCAATCGAATTCTTAGTCGTGCATTAATCGTTTAATTTATTGTTTGATAATTTATGT
ACTATTATTAGTTCGTCTGTTCCGCCGAGCATTCTACTATACAATTTTTCTGCTCCACTCCGTTGATGGC
TGATCGCCGAAGATACCGCCAGATTCTACAGATACATACATATGCAGACACAGATACACAGATACAGATC
GAGATACTCCCTCGCTCTCGCTTTCAATTGGTCTGCGTTTGCGTTGCTGTTGACCCTGAAAAATATTGGC
AGCGACAAGACAATTGGTTGATTTTTAAATTTTTTGTGGTCAGCTGAGATGGCGGCGCGGTGGCGGtttt
tatttttttttttttcttttGTGTCTCTTCTGTCGGTTTTTATATTAAATTTTTTACATTTAAATGTGTAT
GTTTGCGGCCGTTTGCTTCTTGGACGTTTTTGTGTTTTTATGCAGATGAAAATATTTATGTTTTGTTGAT
TGCGCATAAATTAATTTATATTTTATGGCGTTGGCGCTTCGTTCGTTCCTCAAATGGATCGAGCCGCGGC
ATATGCAAATCGCACTGTCGTTGTCTGTGTGTCTGATATAAAAGCATAATTTGACCTTAAGCTAAAATGA
GGGCGTTAATGATGTTTTTTGTTTTCCTTTTCTTAGTCTATTTTATACATTTTTTTGGTTCGCTCGTCGG
TAGTATGCGtttttttttttcggctgattgttttgtttgggctgttgcttttttttggTCACATTAATAT
TAATTACAACGTCGTGGAAATCCCAGCTACATTTCGCCATAGACAGAATAAAATCCCCGTTGAAAAAGAG
AACCAAACCAAACGAAAAAGCGGCGGAGATTGCTTTGCCGAAGGAGCATTGTGTGTTTATTATTTTACAA
TTAGCTTGATTCTCTGCACCTTTTTAATTGTACACCACAAAAACTTTCTGTTTCTGCTTTCTAAACCACT
TTTCCAGGAATCCACTCGATGAAATTGAAGCAGTATTTAGAAGTTAAACTAAACTAAGCTCTGACACCCT
CACTACGACTTGTGCGAATGTGACACGTTCATTAACCATAGCTTTAAGTGTAACCTCCATATATAATACT
ATGTATGTAAAGTGTTTATTTCGAACGAGACTTTTTTCGAGACTAAGCTACACCTACTGAAGCGAGCAGT
AAACCAACTAATCTGGAATATAAAGTCATTTTTATACAGCGACACGAAAACTTTTTATACCCGTATACCC
CGTAACATGTTAACGCTGAAACGTGAAGTTTATAGAGTGACAACTACCACGAACCCCGCCCCCACAGGCA
CGCCCACTTTCACCTGATATGGATAAAGGCATGGTGTTAATGCCACATGCTGTCTCTCCCTTTTGCATTT

TATTAGCCTAATTATTTCTCTCTTTTCAAATTGCCCAACTTTCCGAACAACAACTGCTAGTCTGTCTGAG
TTGGCTACACTTGAAATTTTTCAGTACACATCATGCACACTCACACACCTGCAGCACTTCTAGGACCTTC
ACACGCACACACGTACACGCAGAtatactacaacacacgtatactaaacacccgtaCACAAACGAACTCC
AGTGCCTTGTGTGTTGCTTGCATAATATATATGCGTTTTGCATTTTTGAAATGTTTGTTATTAAAAATAA
TGATTTTTAGAGCAGGACAAGAAGTCCAATTAGTTGTCTGTGTTCTTTTCGTAATGTCACCTTTGCCCCC
CCGGGCACTTCGTTAATTGCCATAGATTAATGTGTGTAGATAAAGTCACGATTTCGAAGTCAGACACGAA
GGTATTGTTGGGTTAATGTCGACTTATCGCATTAGCGATTTTAAATCCTCGGATTACGATGCTACGATGC
ATTTACCACGATTGTTATTCGTCAAACATACCAGAATCTTGAATTTAATTTATGAATACTCAACTAATTA
ACAGGGACTCGACGAGGTGTTCTATTTGTATGCCCATACTAAGAAGTTAGCGTTAATAGTTCCTGAATTT
TATTTCGCAAACAAGCTTTCAGCAAAGCAAATGTATTCCTAAAGTCTCCAGCTGGTTAAGTTTTACGATA
GTCAAACGGTGTTATAACTTTCGATATATTTTATAAATATTTATATGTTAATATCTGTGAACAAAGCAAC
CACATAAATAAGAACTGCTTAACGATGTATTAAAATAACTACTGACTACGGTTATGGAATCACACCACTt
tatcattatcattaccattaccattactattaTTAATCATCCACATATGTACGTATTTGTCATCGATTTG
TGCCATACATATTTTATGTAACTCTGTTATTTTTGCATACAAAAGAAGTACAATTTATGATACTAGAAAT
AAAGGCGAATGATTTAAATTAGCTCTAGTGTAATTTATTTATCCCACCAATCACCACATTTCCATTATTG
TTAAACTTTAACCAATACTCGAGCACTCTTCGGTTGTGGTTTTTGCATTATTTACCTCACCTACTTTTTA
CCACTGACTTTTATTGGGGTGTTTTAATTATTCTGGCTTTCATCATAATGAGCAATTAGCAATTGAAAGA
TGAAAAACAATCATTTAGAGCTGCGAATGGTTGGGGGAGGGGAGAGGAAGTTTCATTGGGCATTGCATAA
ATCTGTCAATTGCAAAAGTTGAACGCGCCGGGATACGTTTGTGGGACCCAGGGAACATGGCTTGGATTAT
TGTCGGAGGCGAGCGGTCGAGTGCCGGGGCAAAGTTCATTTAAAAGTCGAGAGGACAAATGAATGAATAA
AAGGAGCAAGGAGGCAACGAAATGAGCAAGAAAAGAATCCACTAGAATATTATAAGTGCAATTCCCTGGG
TCAGGAGCTCGGCGCACTTTGGCTACTTCACGTAGCCTTAGTTGGCTCCTTCATTCCGGATCCTGGCTCC
TCCAGATTCCTCGGGCATGACATTTGCGTAGGTAGCGAGAGTGTTTCAACGAAAAGTTTCTCTCTCCCTT
ACCGCCGCTGGTGCTCCAAGGACCAAGGACTAAGGAGCCCGGCGGCGTTCAAATTGAAAAACATAAATCA
CTCAAGCAGAAGTTGCTGCCAGCGACttagtagtagtagaagtcgtagtagtatgtagtagtagCCCCGG
CTTTGTGTGAATAATTTCGTATTAAAGTTAAATTGAAAGTGTGTAGCAATTTGTTAAAGACAAAAGTCAG
TTTAGCTGCAAGGAGGGCATAAAAACGTCGAGCGAGCGGATAGGCCACAAAGTTCAGACAGGAGGAAGGA
ATCCAGCCAAAATCCCATTGCAAATAGTTCTAAGGCGCTTTATTTTAAAATTAATTTTGCATACCACTCT
ATGCTGCTGCCATGCTGAAGCTCTGTGAGCATATACGACCATAGTTATATAGTGCGAGACGGCTCTTGCC
AAGTTTTACGACTCCACAGACAGAGGCTCCTCGACTGCTCGGCTCCACGCTAATCTCCAGCTGCCATGTT
ATTGCACTTGTTGGCAAAGTTTTTCAAAAAGTGATTTTGTCAGGGGTTTGGCGGCTGGGGTCGCTTTCTC
CTCTCGGGCTAAAAACTTTGCAGTATGTTTCTTGGTTTTTAATTACAACAAATTCGACTTGTTATGCCAA
AAAGTTAGCAGGGATTCCAGGGCACGTACGCAAGTGTGACTAAAAGAAATTAACGTCATTATGTTTTGTA
ACTGCTCATATCACGTGCTAACCGCATCTCACCGATCTCCCCTTTGGTTTTGACAAGCAAATAATTTGAT
TAAATTTACATATATGTGTGTGTTGTTTTACTTTTGCACAATTCGAACACGAAAACCAACTCTCAAAAAT
TATGACAGAACCGCACAAAGTCTGGCGTCGACAATGCCACATGCCCCCAGCAAAATACCCAAACAGAAGT
TGAAAGCGAAACAGATTGAGTCGTCTCTTCCTACTCCCACGGCACTTGATCCGCCCAAGCCCTCGCTGCT
GCCCAAGCCAAAGGTCAAGGTTGTCATTCCACCAGCACTGCAACAGCGTCTTaagcagcagcaggtgcag
cagcagcagcaACAACACCAACAATCCCAGCCCGAAGAGCCCTCAATTCCGGAGGAGGAATATCACCAGC
AGCCGGAGCCGGATTCGCCATCCGCCGCAGGAGGCAGTCGCATACCCGTAAGAACCCTAAAAGCCGAGCG
CCGCGCCTTGACCTTGGCAGCCCGCCAATCCGGACTTAGTGTAGAGGAGTATCTGAAGCGACTGGAGACC
CAGGATGATAAAGAGTCCCCGGCTTCGCCCACAACGCCCACTGGCAAAAGCCGTTCCATGATTAAGTAAG
TTGAATGCGTGACGGTCAACGAGTTGACATGCGTGCAGAAGGTCCAGGTCCATGCCCAGGCCCCCGAAAG
GTCACAGGTCAAGTGTGGTCCAAATGTGAGCGTGCGAAATGCCAGAGACTAGCCCCTGGTCTAGGTTTGA

GTATATAGAACACCCACACCAAATGCACCCACTCACGCGAGGCAATACGAACAGCTCTCAATGTTTCCGT
ATATGTATAAATCATTGTATAAATCTATATGTCGATTGGAACACCTACTTTTACATCTTTGTTTTTAGTT
AGCGGTGTCTGTCTTAGCCACACCCACTTTTTCCTTCTCTGTCTGTATATACAATAGTCATATCTTGTAA
TTAGTTTTGCTTAAGCCGGAGTTCAGATTGAGTATCAGTTGCCCATACATCTTAAGTGGATGATATATAT
GCTAACCAATTGATTCTCGCCCATTTAGGTGCTGAGAAGCGCGCCTCCTGGCGGGCAGCTCGTCTTCGAT
CACTGGAACAGGGTGCCGTGGAGGCACAAGATGTGATCAAGAATATGCGCAAGATGACCGACGATCTGAT
CACCCACGAATCCAGGGCCAGCGAG
>dme_circ_0007670_junction_seq
GATCACCCACGAATCCAGGGCCAGCGAGGGAGGTTGTGCTGCCCAAGAACCAGGGC
>dme_circ_0007671
GGAAGTGCTACTCAGCAAGGCGGAGGGCGAACGTTTGGGCATGCACATCAAGGGCGGCTTGAATGGCCAG
CGTGGTAATCCCGCCGATCCCTCCGACGAAGGCGTCTTCGTGTCCAAAATTAACTCGGTCGGTGCGGCCA
GACGGGACGGAAGGCTTAAGGGTGGGCATGCGTCTGCTCGAGGTTAATGGACACTCGCTGCTGGGCGCCT
CGCACCAGGATGCGGTCAATGTGCTGCGCAACGCTGGGAACGAGATTCAATTGGTCGTCTGCAAAGGCTA
CGACAAGTCCAATTTAATTCATTCCATTGGCCAGGCCGGCGGCATGAGCACTGGCTTCAACTCGTCTGCA
TCCTGCAGCGGTGGCAGTCGCCAAGGGACTTCGATGTCTTTGCCTCGCAGAAGCCAGATGCACAACAGCC
AACTGGCCCATCTGTCCTGGCAGCTGCTGCTGCCATGGTGCATGGCGCCTCGTCGCCCACACCACCTGCT
GCCACCTCCAATATAACTCCCTTACCCACAGCCGCGGCAGTAGCATCTGCTGATTTAACAGCACCGGACA
CGCCAGCAACCCAGACCGTGGCGCTCATTCACGCAGAACAGCAGGCCCATCAACAACAACAGCAGACGCA
GCTGGCGCCTTTGGGTCAGGAGAAGAGTACCCAGGAGAAGGGTGCTGGAAATCGTACGCGCGGCTGATGC
CTTCACCACCGTGCCACCAAAGTCGCCATCGGAACACCACGAGCAGGATAAGATACAGAAGACGACGACG
GTTGTCATATCGAAGCATACGCTCGACACGAACCCAACCACCCCCACAACGCCGGCGGCGCCTCTTTCCA
TCGCTGGAGCAGAGTCAGCGAACTCGGCTGGTGCTCCTTCTCCAGCTGTTCCTGCATCCACGCCCGGCTC
AGCGCCAGTTCTTCCTGCCGTGGCCGTGCAGACACAGACGCAAACGACCTCCACGGAGAAGGATGAAGAG
GAGGAGTCGCAACTACAG
>dme_circ_0007671_junction_seq
GGATGAAGAGGAGGAGTCGCAACTACAGGGAAGTGCTACTCAGCAAGGCGGAGGGC
>dme_circ_0007672
GCAACGAGGCACCCAGACTCCAGATCGCCTGGCACCCGGAGCACGTTACTCCTACCAGCAGATGCTGGAG
TCGCAGGAGGAAAATCGAACTTCTGGCAAAGCCACCCAGGCGAAGCCCAAGAAGGCGGAGCCCGTATACA
TCATAGATgatgaggacgatgaggatgatgatgacgCAGGTTCCCAGCGGCTGGACGACATTGACGCTAT
CCAAGAGGAGGAAGAAGAGGAGGACACGGAGAGCTCTCCGCCCTTGGAAGTGCAGTCACCACCTTCCTAT
GCAGACACTGATTCCGATACCTTCGACCGTCGGCGTGGTCGATATACAAGCGACTCGGAAAGCGACTGCC
GGCCGTACAAACCATCGCGATCGGGACGCAGTACGCCTGAATACGGTGGTCATCGCAAGTCTGTAAGCTT
CGATCTAAGTGGAGACGAGCGATCCAGGTCGGACTACGAGCGATCCCGCTCCCGCACTCCAGATGGTTAT
ACTAGGGCCTACGATTCAGAGCACGATACTCGAGGATCTGGTCGTAATCGTCTGCCGCGCAAAGGCATCT
TGCGGGCCACCAGCCCGTGCGCCACGCTGGAAAAATCGGAACCCATTGTGCCAGCAGTGGCACGCATCCG
TGATGTGGAATCGCCGCAACTTCAGCGCGTCTCACGGGCCACTTCTCCGGATCGACCCGGCGAACTGGAC
CGGGAAAATCCTTTCCGTCATCCCACTTCCGAGGATGATGAATACACGCGGATTGCAAAGGAGGTCAGCA
AATCGCCTCGGTCGCCGCGCTCCACCCGCGAGCAATTCTTTTTTGGTTCTTCGAGATCCAGTGAGGATCT
GCTGGAGCAATCCCAGTCACTATCCAGCGGTCGCAGTACCCCCAGCACAGTGATCTCCAAGTCGACGGAG
AACCTTACCGGCACGCGGCCAAAGATACCTCCACCCACCGCTCCAAAGCCGCAATTAAAGAAGGCGAACC
TGGTGCGAAATGTAGCACTGGAAACATTCCAGGCCAGCGATGTGGGACAAGGTGATTTTACTGTATTCGA
GCACGATGCCAGCACGAATACGATCCATCCCGTGGAGGCCAAAGTTCCCGTCAGGGTGTCATCCAAGACG

AAACCGAGTCGGCCGAGGGAATCACCACCACCGCCGCCAGTGAATTTCGCCACCCTGCCGAAGTTGCCAA
GCACGCCAACTTTGCGAGAGGCGGAGGAGTCCATCTACATGGAGGCACTGCCGCCGACGCCCTCGCAGCT
TCGGCCCCACGAATTCGTGCACGAGAACTCACCGGACAATATTCTGGTCTCGCCGCAGGAGCACCGCGAG
TATTTGCTGCGTGAGAACGAGCTGAGGAATCAGTTGAGGGCCCCCTATGAACCCGCTCCACCTGTACCCA
TTGTCGGCGGCGGATATTCG
>dme_circ_0007672_junction_seq
TGTACCCATTGTCGGCGGCGGATATTCGGCAACGAGGCACCCAGACTCCAGATCGC
>dme_circ_0007673
GCAACGAGGCACCCAGACTCCAGATCGCCTGGCACCCGGAGCACGTTACTCCTACCAGCAGATGCTGGAG
TCGCAGGAGGAAAATCGAACTTCTGGCAAAGCCACCCAGGCGAAGCCCAAGAAGGCGGAGCCCGTATACA
TCATAGATgatgaggacgatgaggatgatgatgacgCAGGTTCCCAGCGGCTGGACGACATTGACGCTAT
CCAAGAGGAGGAAGAAGAGGAGGACACGGAGAGCTCTCCGCCCTTGGAAGTGCAGTCACCACCTTCCTAT
GCAGACACTGATTCCGATACCTTCGACCGTCGGCGTGGTCGATATACAAGCGACTCGGAAAGCGACTGCC
GGCCGTACAAACCATCGCGATCGGGACGCAGTACGCCTGAATACGGTGGTCATCGCAAGTCTGTAAGCTT
CGATCTAAGTGGAGACGAGCGATCCAGGTCGGACTACGAGCGATCCCGCTCCCGCACTCCAGATGGTTAT
ACTAGGGCCTACGATTCAGAGCACGATACTCGAGGATCTGGTCGTAATCGTCTGCCGCGCAAAGGCATCT
TGCGGGCCACCAGCCCGTGCGCCACGCTGGAAAAATCGGAACCCATTGTGCCAGCAGTGGCACGCATCCG
TGATGTGGAATCGCCGCAACTTCAGCGCGTCTCACGGGCCACTTCTCCGGATCGACCCGGCGAACTGGAC
CGGGAAAATCCTTTCCGTCATCCCACTTCCGAGGATGATGAATACACGCGGATTGCAAAGGAGGTCAGCA
AATCGCCTCGGTCGCCGCGCTCCACCCGCGAGCAATTCTTTTTGGTTCTTCGAGATCCAGTGAGGATCT
GCTGGAGCAATCCCAGTCACTATCCAGCGGTCGCAGTACCCCCAGCACAGTGATCTCCAAGTCGACGGAG
AACCTTACCGGCACGCGGCCAAAGATACCTCCACCCACCGCTCCAAAGCCGCAATTAAAGAAGGCGAACC
TGGTGCGAAATGTAGCACTGGAAACATTCCAGGCCAGCGATGTGGGACAAGGTGATTTTACTGTATTCGA
GCACGATGCCAGCACGAATACGATCCATCCCGTGGAGGCCAAAGTTCCCGTCAGGGTGTCATCCAAGACG
AAACCGAGTCGGCCGAGGGAATCACCACCACCGCCGCCAGTGAATTTCGCCACCCTGCCGAAGTTGCCAA
GCACGCCAACTTTGCGAGAGGCGGAGGAGTCCATCTACATGGAGGCACTGCCGCCGACGCCCTCGCAGCT
TCGGCCCCACGAATTCGTGCACGAGAACTCACCGGACAATATTCTGGTCTCGCCGCAGGAGCACCGCGAG
TATTTGCTGCGTGAGAACGAGCTGAGGAATCAGTTGAGGGCCCCCTATGAACCCGCTCCACCTGTACCCA
TTGTCGGCGGCGGATATTCGGTAACGAATCCCTTTCTAGATAGTTTCGACACCGACATTGACAGCCTGCC
ATTGCCACCACCGCCCACACCGCCTGCCCAACACCAACATACACGTCATTTGCATGAGCCGCCGCAGTTT
CCGCAGTTTGCGCCACCACTAACCGCTAACGCATCCACGTCTCCATCTTGTTCTTCCAATGCTGCTTCTG
CTGGTGGCGCCACTAACCGCTTGATGGATTCGCCGCAACAACAGCTGCTGGTCGCCGGGTCGCCCACCCA
GATCTTTCCCACGGCCCAGATACTGCCCGTGCAGTATGCCAGTCTGCCGCAACCGCAGCAGCCCAACACG
CTCACCCTTAAGACCGTTTCCCCCACTTCGTCCCAAGACCCTAAACAGGCCCTTTTTAACCTGGTACTCC
TGCCCGGCGACCAGGTGGCCAACAAATCCTCGCCAGCAATGCTCTACCTGCAAACGGTGAGTGAATCGAG
TGCCAAGCCCCGGCGGGGACCACCACCTCCTCCGCCCGTTACCGTGACCTCACCCACGCCCTCGTATGCC
GCCATGAAGTTCCCGACCTTCTCGGAAATAGTTGTCAGTCCCGATGCGAATAGCAATTACCAAAATATGA
CCACTGCTCGCGTTGGCGTCTCGAATTCCTCGCTGGCCCACAGCACCAGTTCCTTTTCAATCGGCTCCTC
CAGCTCTTCTCTTCTTATGACCAAAAATTCGTCATTGGGAGAAGGTAGTGTACCCTCCAGTTACTACGAG
ACGGCCGAGGAGATATACTCCACTATCGAGGAGGAGGCCATCTACAGCAACACCTCCTTCTTTGATTCGC
GTCGAAGTAGTGACACTTCGGTATCCTGTCGCGCCTCCAAGTCGCCCACCCCTCCTCCTCTGCCTCCCAA
GCGACCTTTAAAGCCCACCAAGTTGCGTACCGCCCCCGCTCCGCCGCCAGTGGCCCAAAAGCCTCATGGC
CTGAGGGGCATGGCCAGTTTCAAGTTGCGCTCCCAATCCCAGCCGCAGCTGGATGGGGCGTGGCAACAGC
CCAACCTACCTCGAACGCCAACGACGCCCACTGGCAAGGAGACAACAGTGTAGTTTAGGGTCGGAATGAA

ATCTATGAATAAATCTAAAGTATTTCCAAAGAAACATTATCCAGTTAATACTACTAACTCAAACTCTTTT
TAAAAGGCATTGAAAATTGTaaaaaaaaaaaaaaTAACAGCCCATTCCGCTACAAATATTTCAGTACAAA
ATCTTCTTAAGTGAGTTCACCAATAACATAAAAATAGTGTCAAGAAATACCCATAACAATAAATACGAAT
TGCAAAACTACTTTAGCAATAACTCAATATAAAATCCAAGAGCTTTATTGGTCAGCGAACTCAAGGTTTT
AAAACTAAAATGGTTTTTAAAGCGACAAAAATTTATTCGCCATTTAAGTGAAGAGCTTAGAAATGTCGAG
GAATATCCGACCGCCAAATGCGATTTTAGATTTATTCCTCTTCAAAAATCGATCCACCTTGGGTAGACCG
TCATGCAACCAATTGCCATCGGTGGCTTTTAACAATGGCGATGCATTTAATTGCTCGTGAGCTGACCGCA
GGACCTTTCCACTTTTCCCGTTGCCACCACCACCACCTCGTTCTCTCTTTTCGTCCACGTCTCTGCTCAT
ACATTTTAATTAAATTCTAACACCTTTTTGCCATCAATTAAATTGCCCAGCCCAGCCCCTTGCATTTTTC
TTGCTCTCTTTGTTTCTGGCATCGACTGCAATTTTCAATATTTTCATTCCACTCGCTCGCCATTTAAATT
GATTGATTTGTGGGTAGTGTCTGAGCTCCGAACCCGCTGAACTTgcaggcaggcaggcatgcaggcagAT
TGGGTGGCACACCTTTCCTGCTAATTAGATGCAACTTTCTGTTGACTTTCGAGACGCCCTCGACCCCCAT
CGAGCGTGTACTACCTACTTTGCCACAAAATTGCAGTTGAAATTGAGCAGCAAGCGGTTGAAGTTTAATG
GGCGCCACCTCGTCCTCCTGCTCGTGCTCGTGCTGTCACCCGCTCACCTGGCAACTCCTCCCGAGTCTCG
GGACAAGTTTAATTTGACGTTGATTGAAACAGTTGGCTGCTTATGGTCGCCCACCTGCAGTTACCACGCC
CACCGGGCCATTAATTTTTGCTACTGCTGATGCCGCCGCTGCCCATTTTTATTATTGCCTTTTGTTTTGG
gaaaacaaaatacgaaaaacgaaaatacgaagaaaaaccaacgaataaataTGTAGTATAACGAAGAGAA
ATACCTGAGTATAAAACGAAGTAACAGAAAAACAGACAGAAAACACAATTTTACAATTTATAAAGTTAAT
TTCGCCCTAGCGTTGCTAAGTTGATTTTAATGCTAATTTATTTTATCAAGACAAAGAAGCAAGCTGAACA
TTTTTAATTGCATACCTTAAGGCAAATAAACATTGCGTATTATTCTTATTTGCGTTTCGTGGGCTCTTTT
CATTTTGTCTTGTCCCCCTGGCCATGTATTGTGTAAACAAGGTTTTTAGTACTCGAAAAACGATTGTTTA
AATGGGAGCGATAACCCAGTACCCAAGTGGTGTGGCGGCCAGTGACCTTGACTCACTTAGCATTTGTATC
TAATTGCGTCGTGACCCCAATTGGTCAAGAGGCAGGCACTGACTGCCTGGCGACCTGCCGCTTTCGCACC
TGGCCCTGCACTGATTTTGCTGTTGACTGGTTGCCCCTTTTTAATGTTCCATACCTCGGCTGGGTTCTCT
TTGTTGCAATCTTATCGCAAAGCACTCGTAATTGCTTAGCGATAAAAAAGAGTTGGTTACCCCTTATCAA
CGCCTTATCTTTCACTGCTCGCATGGTCTGAATAATTCAATGGCTTATTGGCTTTTAATGCGCTGGCCAT
TTTGGCTCTCACACACTTATACACAATGGGACATCTCTGGGATCGGTTACcacacacatacacacacaca
cacacaTTCTCAACACTTTCATGGCTCAATAAATTCCTATTAGCTGGACTGCTGAGCAGGGCAAAAAGCA
GCTCCTCCAACACTGGAGACTGATGCTGATAAACCAGCTCCATAGCTGCCCTACTCCTCCATGTTGTTGG
CTCTTCGCATTGACTTTAATTGAAAATCGAAAAATCCGATACGAAATTAATTTCTCTTCTAAAAATACAG
CTCAGTTAAGCTGGCACTCCTGCTGCTTTCACTTTTTCCCCATGCACTTGTCCTGCCCTCCACTCTTCCA
CGCCATCGCCATCGCCCTTGGTTCTACGGCTTGGTTTATTTATGAGCTCTTGGACTGGTGTGGTCTGATG
GTCTGGTTGCTTCTTACTGCTCCTGGATGGAGGGTCGAACCCCACTAGTCTGGTATAAGGTTGCAATTTG
TGCAGTTTAACGCTGGCCTTTGCCCTGTTTGCCTTTGTTTCGTCTGGGTCTTCCTCCTTCTGCCCGCTGT
CCTTTCCCACCCTACTGCTGCCATTTAATGACTTTGTCGTCGTAGTCGCAGTCATCGTTGCTCTGCAGGC
ACTAACTATCCAGCCCGGGTTGGCTTTATCTGGCCACTGTGTCTCATGTTGCCCAGCACGAGGAATTGCA
TGAAAGCATAAAACCGACAGTACACACGACTTTGGGTGATAAATGGCCAGCTCCGGTTTGGTTCGTTCCG
TTCTAACCCACGCAGCTGTAATTTGCGCCCTCCTCTCCAGTTCAATCCGAACTCAAATCGCAGCCCAAAC
TCAAACCCACACCCTACCGTAAATTATATGTCCCGAGTGCAGGACATAGCAGCACAGACCCAAAAATGTC
GGCTTGGTCACTTTATGATTGAGCCAGCCTATGGCTGATCCTGCTAACGCCCTTTCTGGGCGTAACCAGT
TCTGGACAAACAGACAGAAGACTGTCGGACTGCCGCCTGCCTGCCTGACTTTTTAATAAACTTGTGTAGT
GCCATCAATTTTCTTTATGGCCATTTCGGATTTTTCAGCGCTCGCTTTGAAAATTGCAGTTTTTCCCAGT
CATTTAATTGAAAATATGGCTGAGTGCTGCCAGCGTTTCAGCTTTATCTGTTTATTGATTATTTTCGTCG
TTAAATAAATCCTTGAGATATCttttttttttGTGTGTGTGCGTTGAGTTGCTAACAAAAACGTTTTGCC

ATTTTGCAGGTCAACACCTGCCAGCAGGGATGGAGCAGAGGAGCAGCAGGAAGAGGTTAGAGCCAAACCC
ACTCCGACTAAAGTGCCTAAATCGGTTTCCGATAAAAAACGCTTCTTTGAGTCAGCCATGGAGGATCAAC
ACAAGCCCACACAAAAGACAGGGTGAGTCAATGGTTAGTGCGAAAAGTTGAATGAGTGGCTCAATGATGT
CGCTGTGCAGTTTGCCCAAGCTGGTGGTCCTGCCACTTAAATCTCGCTTCTTGGCCCAAGTTGTCAATCA
AACCACTTATCTGCGGTTGCCAAGCCACCACAACGGTGGTCGTCACCTTTCGCTTCTCGGCTGGCGTAAT
TAGAAAAGGGTGGAGGAAAGCGCGGTTGCCTGCGCCATTTGCATGACAAAGTCTAGAGCTGTAGTGGTGG
AATCCACTTAGGCAGCTAAATGGCTAAGCTGCATCGGATCGCTATTTATTTAATTTCCCAACTTAATAGC
GGGACGATGTCCTTAGCTTACACTGATTTTATGCACTCTCTTTTTTGCTTGCAGGACAAAGTTTTCTCAT
TCCTGTCAAAGGATGAAGTGGAAAAGCTGCGACAGGAGGAGGAGAGAAAAATTGCAACATTGCGCCGTGA
TAAGAATTCCAGATTACTGGATGCCGCCAACGACAACATTGACAAGGATGCCGCACAGCAGCGGACCAAG
AGCAACAGCAACAGCAGCAGCGGCGACGATAATGACGACAGCGACCAAGAGGAGGGCATCGCTCGCGGGG
ATTCCGTTGACAACGCCGCTCTGGGGCATTTCGATGATGCGGAGGACATGAG
>dme_circ_0007673_junction_seq
GGCATTTCGATGATGCGGAGGACATGAGGCAACGAGGCACCCAGACTCCAGATCGC
>dme_circ_0007674
AATATTGTCCGGTGAGTTCTCGTGCACGAATTCGTGGGGCCGAAGCTGCGAGGGCGTCGGCGGCAGTGCC
TCCATGTAGATGGACTCCTCCGCCTCTCGCAAAGTTGGCGTGCTTGGCAACTTCGGCAGGGTGGCGAAAT
TCACTGGCGGCGGTGGTGGTGATTCCCTCGGCCGACTCGGTTTCGTCTTGGATGACACCCTGACGGGAAC
TTTGGCCTCCACGGGATGGATCGTATTCGTGCTGGCATCGTGCTCGAATACAG
>dme_circ_0007674_junction_seq
TTCGTGCTGGCATCGTGCTCGAATACAGAATATTGTCCGGTGAGTTCTCGTGCACG
>dme_circ_0007675
GGCGTCGGCGGCAGTGCCTCCATGTAGATGGACTCCTCCGCCTCTCGCAAAGTTGGCGTGCTTGGCAACT
TCGGCAGGGTGGCGAAATTCACTGGCGGCGGTGGTGGTGATTCCCTCGGCCGACTCGGTTTCG
>dme_circ_0007675_junction_seq
GGTGATTCCCTCGGCCGACTCGGTTTCGGGCGTCGGCGGCAGTGCCTCCATGTAGA
>dme_circ_0007676
GTCACGGTAACGGGCGGAGGAGGTGGTGGTCCCCGCCGGGGCTTGGCACTCGATTCACTCACCGTTTGCA
GGTAGAGCATTGCTGGCGAGGATTTGTTGGCCACCTGGTCGCCGGGCAGGAGTACCAGG
>dme_circ_0007676_junction_seq
CACCTGGTCGCCGGGCAGGAGTACCAGGGTCACGGTAACGGGCGGAGGAGGTGGTG
>dme_circ_0007677
GTCAACACCTGCCAGCAGGGATGGAGCAGAGGAGCAGCAGGAAGAGGTTAGAGCCAAACCCACTCCGACT
AAAGTGCCTAAATCGGTTTCCGATAAAAAACGCTTCTTTGAGTCAGCCATGGAGGATCAACACAAGCCCA
CACAAAAGACAGGGTGAGTCAATGGTTAGTGCGAAAAGTTGAATGAGTGGCTCAATGATGTCGCTGTGCA
GTTTGCCCAAGCTGGTGGTCCTGCCACTTAAATCTCGCTTCTTGGCCCAAGTTGTCAATCAAACCACTTA
TCTGCGGTTGCCAAGCCACCACAACGGTGGTCGTCACCTTTCGCTTCTCGGCTGGCGTAATTAGAAAAGG
GTGGAGGAAAGCGCGGTTGCCTGCGCCATTTGCATGACAAAGTCTAGAGCTGTAGTGGTGGAATCCACTT
AGGCAGCTAAATGGCTAAGCTGCATCGGATCGCTATTTATTTAATTTCCCAACTTAATAGCGGGACGATG
TCCTTAGCTTACACTGATTTTATGCACTCTCTTTTTTGCTTGCAGGACAAAGTTTTCTCATTCCTGTCAA
AGGATGAAGTGGAAAAGCTGCGACAGGAGGAGGAGAGAAAAATTGCAACATTGCGCCGTGATAAGAATTC
CAGATTACTGGATGCCGCCAACGACAACATTGACAAGGATGCCGCACAGCAGCGGACCAAGAGCAACAGC
AACAGCAGCAGCGGCGACGATAATGACGACAGCGACCAAGAGGAGGGCATCGCTCGCGGGGATTCCGTTG
ACAACGCCGCTCTGGGGCATTTCGATGATGCGGAGGACATGAG

>dme_circ_0007677_junction_seq
GGCATTTCGATGATGCGGAGGACATGAGGTCAACACCTGCCAGCAGGGATGGAGCA
>dme_circ_0007678
GTCAACACCTGCCAGCAGGGATGGAGCAGAGGAGCAGCAGGAAGAGGTTAGAGCCAAACCCACTCCGACT
AAAGTGCCTAAATCGGTTTCCGATAAAAAACGCTTCTTTGAGTCAGCCATGGAGGATCAACACAAGCCCA
CACAAAAGACAGGGTGAGTCAATGGTTAGTGCGAAAAGTTGAATGAGTGGCTCAATGATGTCGCTGTGCA
GTTTGCCCAAGCTGGTGGTCCTGCCACTTAAATCTCGCTTCTTGGCCCAAGTTGTCAATCAAACCACTTA
TCTGCGGTTGCCAAGCCACCACAACGGTGGTCGTCACCTTTCGCTTCTCGGCTGGCGTAATTAGAAAAGG
GTGGAGGAAAGCGCGGTTGCCTGCGCCATTTGCATGACAAAGTCTAGAGCTGTAGTGGTGGAATCCACTT
AGGCAGCTAAATGGCTAAGCTGCATCGGATCGCTATTTATTTAATTTCCCAACTTAATAGCGGGACGATG
TCCTTAGCTTACACTGATTTTATGCACTCTCTTTTTTGCTTGCAGGACAAAGTTTTCTCATTCCTGTCAA
AGGATGAAGTGGAAAAGCTGCGACAGGAGGAGGAGAGAAAAATTGCAACATTGCGCCGTGATAAGAATTC
CAGATTACTGGATGCCGCCAACGACAACATTGACAAGGATGCCGCACAGCAGCGGACCAAGAGCAACAGC
AACAGCAGCAGCGGCGACGATAATGACGACAGCGACCAAGAGGAGGGCATCGCTCGCGGGGATTCCGTTG
ACAACGCCGCTCTGGGGCATTTCGATGATGCGGAGGACATGAGGGTGAGTGCGGATGTGCCAAGCTCTCA
TTTCGGATTATTGGTGCTTGCATTATGCGCTTTTTATGTTACATCTGAATGCACAGACACACTATACACA
CACTTCACACCAATCCACCCAATCAATCAATCAATCACCCTTCACCCCATGCGATATTATGATAAccata
cacccactcacccacacacacacacacgaaactcacacaACTTTCACGGTGTTTGTGCATTTGCTTAGTA
TAATCTCTTATGCAAAGTTAAATTTTTTAGTTTTATTGGTGAAGTTTTTCCGCTGCCTTTTGGTTTGGTT
CTGGTGCCGGTTCTCTTACAGCTGCTTTTAAATATGGGTTGCTCTGCTCTTAAGTTTAGATGCCCTCAAT
CGTTCACATTGCaaaaaaaaaaaaaaaaaaaaaaaaCACCCCACAAAACACCACCACTTTGTTTACTATC
CATGCAGTCAACTGACGGAGCTGCTAATCATCATTGAGACTTAGTGTGTTATTAAATAAGGATAGCGGTC
TATGGCTGAGTGTTCTTTAATAGTAAGTACACTTCTCACCACACAATATCTCCACATACGCACTCACACA
CACTTGCATACGATTATAAGCGTCTAAGCCCTAAGCAGTTTAAGTTTTATGTAGCACTTAAGCGTTGTAT
GGACTCGTAAATCGCAGCCAGACTCCCAGCCCTTAGTCTACTTAAAATGTAAGGaaaaaaaaaaCAGTGA
GGAACTTGAGAAGGAGGTGGAGTGTAAATGCCGGAAAAACATGTTATGGTTCTGGCGTTGAAATCTTTTC
ACATAGTACATAGACCACGCCCGGTGGGCTTCGAATCTCATCTCGTGTATATGATAATCTAGTCGAAGCG
GCAGAACTCAAAAGTGTAAGTATATCCGCTGCTGTTTTACTTAAttttttttttGCTGGCATTGCGTATAC
GCAATGTGTGCCTCCGGGCCTGCAACCTTGCTCTTCCTTTTGCTCCTTTTCCTTCCGCGTGGGCGTCCTa
ctccactccattccactccactcGAATTCCCTGCGGCACAATGCCAGCTGTTTTTACAATTGAATAACTT
CATTTGGGCATTTCGCAAATTGCGTTTTGTGCGTTTGCTCACTCAACTGGGCGGTGCCAGTTCAGATTTG
GATTTTGGATTCGCTACGGCTCCCCGGCAATGGATCGGCAAACGAGCCACCAGAAGAGCATCACCCAACA
CGTACGCATAATTTAGTATCCGTTGGGAATCGATGATTTTATGCTAAGTGTGTTGCAGTGAATTAAGTCG
CGGCAGTCGGAGACTCGGAAAACGGGCACTCGGATTTGTTTGGCTCAAATGATATTTTATACCCAGCTCG
AAACGGGCAATTATAATTAGCGTCCGTTGAAGTGGTGGGTGGACCCCGCTAACTTGATGGGTCCTCCAAG
CACTGTCGATGGCATGCAACACGTAGCAACCACAACCATTGTGTCAAAGGGCAACTAGTATTTCCTCTGT
GCGTGGAAAAGGTGCCAACCAGCGGCGAACAAACAAAAACTGAACAATGAAAGAAACTAAAAGCTTATGC
CAGGTGGTACCACCCAGAACATGCTATGGTTGCTCCATCCACCAAAAGCACCACCCATACATTCGCCCAC
CATGGCAGCCATTTCGTACGCGGAGCTGGCGCTTATCAGCCAACTTTCACACATATGTGCTCCGCTTGGT
TGAAGGACTCATAGTCGTCGCCCCCACGCGATGTTCCAGTGGCTGGCTCTAACAACATTATATAATGAGC
AACAACAAAGAAATTTAATACAATTTCGCCCCAAAGCTGTTGCAGGAAAACATTAAAAAGAGCGCAACCG
CCAAGCGTCAGCAAAAGTCGCCACTGAAATACCCTTTTCAGGTCATTAGCCTAACGATCAGTTGACCAGT
TGCTTGACCTCTGCCATAAATAGGAACACACGAGCTGATGTTAATGTTCTGCTTTTAATTTATAACAACT
TATCTGACTGATTAAAAACTGCAACCTGTTTCCGACCAAAAGGGTATTCACTGCGGCAAAATAGTaaaaa

aaaaaaaaaaaCAATAGAGTGAAAAAAAGGCAGAGACGTATGACAACAGCAGATAGATAGCTTGACAACG
GGTTCATGACTTTTTTAGTCCTTTGTGTTTGTGGATAGAATTTACTCTATGAATAAAGCTACGAAAATAA
CGCAAAATGTAAAGAAAATTCAATTCACTTTACGCGTTTATGTACAAAGCATTACAAAGCGTTCTATGGA
ATTTCAAAGTAATGATGCGTCCCTTAACTCTGTAGATATGGGATGCTTTCATTTATCTCTGACGATCTAC
AGCAGCATTTGTTTTTCTAACCTTGCGCATAAATCAAGCCGCAAGAACTAATCATTAAATGGTTACAAAT
TTGGGAATAAATATCTTATGACTTCACAGTCCCCCGGAGCGGAGAATTCGGCTCAATTTTCCCAGCAGTC
GCCATAGTCTGGTTGTCAACATAGCCGAAAAGACGAGAAGGTGGCCTTGAGGTGGAATTGGTTGGGGCAC
AGCTGCCAAAAGTTCGGGAACGAAACGAATGGAGATCCGTCCACCCTCCTCCGTTCCGaaaaaaaaCCCG
CCTGCCAGCTGGCTTTACAACTTTACATGCATAATATAATAAATTGAAATGTGTGTTATTATGTTGCTCG
GTCCGTAGCCTTGGAATTCATAACGAGGAAAAGAAGATAAACATGTTAATAACCTTGTAATAAATTTCTA
GCAAGAATTTCTACTAGTTTCGTTCCTCTTTCCTGTTCTTGTTTATATATATTCTTTCCATGCATAAATT
TTGTaaaaaccgaaaaaaaaaaaaTGAAGAGAAGCACGGAGAAGTAGAAGTAGAATATATGCTGAAAAAC
CCCATATCAAATTAGCCGGCAAGGCAGTGTTCTGCAAACTGTTTGTATTTATATTCAAATTAAACTCGTC
TAAATCTATAAAAGTTGTGGAACACAAAAGGCTCGCCAGATCCGTGTTTATGAATTTTATTTGCGACATC
AAATTGGCTTCCGTGACGACAAAAACCTAGTCTCGATGTCGGCAAAACGCAATCGAATTCTTAGTCGTGC
ATTAATCGTTTAATTTATTGTTTGATAATTTATGTACTATTATTAGTTCGTCTGTTCCGCCGAGCATTCT
ACTATACAATTTTTCTGCTCCACTCCGTTGATGGCTGATCGCCGAAGATACCGCCAGATTCTACAGATAC
ATACATATGCAGACACAGATACACAGATACAGATCGAGATACTCCCTCGCTCTCGCTTTCAATTGGTCTG
CGTTTGCGTTGCTGTTGACCCTGAAAAATATTGGCAGCGACAAGACAATTGGTTGATTTTTAAATTTTTT
GTGGTCAGCTGAGATGGCGGCGCGGTGGCGGttttatttttttttttttctttttGTGTCTCTTCTGTCGG
TTTTTATATTAAATTTTTTACATTTAAATGTGTATGTTTGCGGCCGTTTGCTTCTTGGACGTTTTTGTGT
TTTTATGCAGATGAAAATATTTATGTTTTGTTGATTGCGCATAAATTAATTTATATTTTATGGCGTTGGC
GCTTCGTTCGTTCCTCAAATGGATCGAGCCGCGGCATATGCAAATCGCACTGTCGTTGTCTGTGTGTCTG
ATATAAAAGCATAATTTGACCTTAAGCTAAAATGAGGGCGTTAATGATGTTTTTTGTTTTCCTTTTCTTA
GTCTATTTTATACATTTTTTTGGTTCGCTCGTCGGTAGTATGCGtttttttttttcggctgattgttttg
tttgggctgttgcttttttttggTCACATTAATATTAATTACAACGTCGTGGAAATCCCAGCTACATTTC
GCCATAGACAGAATAAAATCCCCGTTGAAAAAGAGAACCAAACCAAACGAAAAAGCGGCGGAGATTGCTT
TGCCGAAGGAGCATTGTGTGTTTATTATTTTACAATTAGCTTGATTCTCTGCACCTTTTTAATTGTACAC
CACAAAAACTTTCTGTTTCTGCTTTCTAAACCACTTTTCCAGGAATCCACTCGATGAAATTGAAGCAGTA
TTTAGAAGTTAAACTAAACTAAGCTCTGACACCCTCACTACGACTTGTGCGAATGTGACACGTTCATTAA
CCATAGCTTTAAGTGTAACCTCCATATATAATACTATGTATGTAAAGTGTTTATTTCGAACGAGACTTTT
TTCGAGACTAAGCTACACCTACTGAAGCGAGCAGTAAACCAACTAATCTGGAATATAAAGTCATTTTTAT
ACAGCGACACGAAAACTTTTTATACCCGTATACCCCGTAACATGTTAACGCTGAAACGTGAAGTTTATAG
AGTGACAACTACCACGAACCCCGCCCCCACAGGCACGCCCACTTTCACCTGATATGGATAAAGGCATGGT
GTTAATGCCACATGCTGTCTCTCCCTTTTGCATTTTATTAGCCTAATTATTTCTCTCTTTTCAAATTGCC
CAACTTTCCGAACAACAACTGCTAGTCTGTCTGAGTTGGCTACACTTGAAATTTTTCAGTACACATCATG
CACACTCACACACCTGCAGCACTTCTAGGACCTTCACACGCACACACGTACACGCAGAtatactacaaca
cacgtatactaaacacccgtaCACAAACGAACTCCAGTGCCTTGTGTGTTGCTTGCATAATATATATGCG
TTTTGCATTTTTGAAATGTTTGTTATTAAAAATAATGATTTTTAGAGCAGGACAAGAAGTCCAATTAGTT
GTCTGTGTTCTTTTCGTAATGTCACCTTTGCCCCCCCGGGCACTTCGTTAATTGCCATAGATTAATGTGT
GTAGATAAAGTCACGATTTCGAAGTCAGACACGAAGGTATTGTTGGGTTAATGTCGACTTATCGCATTAG
CGATTTTAAATCCTCGGATTACGATGCTACGATGCATTTACCACGATTGTTATTCGTCAAACATACCAGA
ATCTTGAATTTAATTTATGAATACTCAACTAATTAACAGGGACTCGACGAGGTGTTCTATTTGTATGCCC
ATACTAAGAAGTTAGCGTTAATAGTTCCTGAATTTTATTTCGCAAACAAGCTTTCAGCAAAGCAAATGTA

TTCCTAAAGTCTCCAGCTGGTTAAGTTTTACGATAGTCAAACGGTGTTATAACTTTCGATATATTTTATA
AATATTTATATGTTAATATCTGTGAACAAAGCAACCACATAAATAAGAACTGCTTAACGATGTATTAAAA
TAACTACTGACTACGGTTATGGAATCACACCACTttatcattatcattaccattaccattactattaTTA
ATCATCCACATATGTACGTATTTGTCATCGATTTGTGCCATACATATTTTATGTAACTCTGTTATTTTTG
CATACAAAAGAAGTACAATTTATGATACTAGAAATAAAGGCGAATGATTTAAATTAGCTCTAGTGTAATT
TATTTATCCCACCAATCACCACATTTCCATTATTGTTAAACTTTAACCAATACTCGAGCACTCTTCGGTT
GTGGTTTTTGCATTATTTACCTCACCTACTTTTTACCACTGACTTTTATTGGGGTGTTTTAATTATTCTG
GCTTTCATCATAATGAGCAATTAGCAATTGAAAGATGAAAAACAATCATTTAGAGCTGCGAATGGTTGGG
GGAGGGGAGAGGAAGTTTCATTGGGCATTGCATAAATCTGTCAATTGCAAAAGTTGAACGCGCCGGGATA
CGTTTGTGGGACCCAGGGAACATGGCTTGGATTATTGTCGGAGGCGAGCGGTCGAGTGCCGGGGCAAAGT
TCATTTAAAAGTCGAGAGGACAAATGAATGAATAAAAGGAGCAAGGAGGCAACGAAATGAGCAAGAAAAG
AATCCACTAGAATATTATAAGTGCAATTCCCTGGGTCAGGAGCTCGGCGCACTTTGGCTACTTCACGTAG
CCTTAGTTGGCTCCTTCATTCCGGATCCTGGCTCCTCCAGATTCCTCGGGCATGACATTTGCGTAGGTAG
CGAGAGTGTTTCAACGAAAAGTTTCTCTCTCCCTTACCGCCGCTGGTGCTCCAAGGACCAAGGACTAAGG
AGCCCGGCGGCGTTCAAATTGAAAAACATAAATCACTCAAGCAGAAGTTGCTGCCAGCGACttagtagta
gtagaagtcgtagtagtatgtagtagtagCCCCGGCTTTGTGTGAATAATTTCGTATTAAAGTTAAATTG
AAAGTGTGTAGCAATTTGTTAAAGACAAAAGTCAGTTTAGCTGCAAGGAGGGCATAAAAACGTCGAGCGA
GCGGATAGGCCACAAAGTTCAGACAGGAGGAAGGAATCCAGCCAAAATCCCATTGCAAATAGTTCTAAGG
CGCTTTATTTTAAAATTAATTTTGCATACCACTCTATGCTGCTGCCATGCTGAAGCTCTGTGAGCATATA
CGACCATAGTTATATAGTGCGAGACGGCTCTTGCCAAGTTTTACGACTCCACAGACAGAGGCTCCTCGAC
TGCTCGGCTCCACGCTAATCTCCAGCTGCCATGTTATTGCACTTGTTGGCAAAGTTTTTCAAAAAGTGAT
TTTGTCAGGGGTTTGGCGGCTGGGGTCGCTTTCTCCTCTCGGGCTAAAAACTTTGCAGTATGTTTCTTGG
TTTTTAATTACAACAAATTCGACTTGTTATGCCAAAAAGTTAGCAGGGATTCCAGGGCACGTACGCAAGT
GTGACTAAAAGAAATTAACGTCATTATGTTTTGTAACTGCTCATATCACGTGCTAACCGCATCTCACCGA
TCTCCCCTTTGGTTTTGACAAGCAAATAATTTGATTAAATTTACATATATGTGTGTGTTGTTTTACTTTT
GCACAATTCGAACACGAAAACCAACTCTCAAAAATTATGACAGAACCGCACAAAGTCTGGCGTCGACAAT
GCCACATGCCCCCAGCAAAATACCCAAACAGAAGTTGAAAGCGAAACAGATTGAGTCGTCTCTTCCTACT
CCCACGGCACTTGATCCGCCCAAGCCCTCGCTGCTGCCCAAGCCAAAGGTCAAGGTTGTCATTCCACCAG
CACTGCAACAGCGTCTTaagcagcagcaggtgcagcagcagcagcaACAACACCAACAATCCCAGCCCGA
AGAGCCCTCAATTCCGGAGGAGGAATATCACCAGCAGCCGGAGCCGGATTCGCCATCCGCCGCAGGAGGC
AGTCGCATACCCGTAAGAACCCTAAAAGCCGAGCGCCGCGCCTTGACCTTGGCAGCCCGCCAATCCGGAC
TTAGTGTAGAGGAGTATCTGAAGCGACTGGAGACCCAGGATGATAAAGAGTCCCCGGCTTCGCCCACAAC
GCCCACTGGCAAAAGCCGTTCCATGATTAAGTAAGTTGAATGCGTGACGGTCAACGAGTTGACATGCGTG
CAGAAGGTCCAGGTCCATGCCCAGGCCCCCGAAAGGTCACAGGTCAAGTGTGGTCCAAATGTGAGCGTGC
GAAATGCCAGAGACTAGCCCCTGGTCTAGGTTTGAGTATATAGAACACCCACACCAAATGCACCCACTCA
CGCGAGGCAATACGAACAGCTCTCAATGTTTCCGTATATGTATAAATCATTGTATAAATCTATATGTCGA
TTGGAACACCTACTTTTACATCTTTGTTTTTAGTTAGCGGTGTCTGTCTTAGCCACACCCACTTTTTCCT
TCTCTGTCTGTATATACAATAGTCATATCTTGTAATTAGTTTTGCTTAAGCCGGAGTTCAGATTGAGTAT
CAGTTGCCCATACATCTTAAGTGGATGATATATATGCTAACCAATTGATTCTCGCCCATTTAGGTGCTGA
GAAGCGCGCCTCCTGGCGGGCAGCTCGTCTTCGATCACTGGAACAGGGTGCCGTGGAGGCACAAGATGTG
ATCAAGAATATGCGCAAGATGACCGACGATCTGATCACCCACGAATCCAGGGCCAGCGAG
>dme_circ_0007678_junction_seq
GATCACCCACGAATCCAGGGCCAGCGAGGTCAACACCTGCCAGCAGGGATGGAGCA
>dme_circ_0007679

GAACCGCACAAAGTCTGGCGTCGACAATGCCACATGCCCCCAGCAAAATACCCAAACAGAAGTTGAAAGC
GAAACAGATTGAGTCGTCTCTTCCTACTCCCACGGCACTTGATCCGCCCAAGCCCTCGCTGCTGCCCAAG
CCAAAGGTCAAGGTTGTCATTCCACCAGCACTGCAACAGCGTCTTaagcagcagcaggtgcagcagcagc
agcaACAACACCAACAATCCCAGCCCGAAGAGCCCTCAATTCCGGAGGAGGAATATCACCAGCAGCCGGA
GCCGGATTCGCCATCCGCCGCAGGAGGCAGTCGCATACCCGTAAGAACCCTAAAAGCCGAGCGCCGCGCC
TTGACCTTGGCAGCCCGCCAATCCGGACTTAGTGTAGAGGAGTATCTGAAGCGACTGGAGACCCAGGATG
ATAAAGAGTCCCCGGCTTCGCCCACAACGCCCACTGGCAAAAGCCGTTCCATGATTAAGTAAGTTGAATG
CGTGACGGTCAACGAGTTGACATGCGTGCAGAAGGTCCAGGTCCATGCCCAGGCCCCCGAAAGGTCACAG
GTCAAGTGTGGTCCAAATGTGAGCGTGCGAAATGCCAGAGACTAGCCCCTGGTCTAGGTTTGAGTATATA
GAACACCCACACCAAATGCACCCACTCACGCGAGGCAATACGAACAGCTCTCAATGTTTCCGTATATGTA
TAAATCATTGTATAAATCTATATGTCGATTGGAACACCTACTTTTACATCTTTGTTTTTAGTTAGCGGTG
TCTGTCTTAGCCACACCCACTTTTTCCTTCTCTGTCTGTATATACAATAGTCATATCTTGTAATTAGTTT
TGCTTAAGCCGGAGTTCAGATTGAGTATCAGTTGCCCATACATCTTAAGTGGATGATATATATGCTAACC
AATTGATTCTCGCCCATTTAGGTGCTGAGAAGCGCGCCTCCTGGCGGGCAGCTCGTCTTCGATCACTGGA
ACAGGGTGCCGTGGAGGCACAAGATGTGATCAAGAATATGCGCAAGATGACCGACGATCTGATCACCCAC
GAATCCAGGGCCAGCGAG
>dme_circ_0007679_junction_seq
GATCACCCACGAATCCAGGGCCAGCGAGGAACCGCACAAAGTCTGGCGTCGACAAT
>dme_circ_0007680
GTGCTGAGAAGCGCGCCTCCTGGCGGGCAGCTCGTCTTCGATCACTGGAACAGGGTGCCGTGGAGGCACA
AGATGTGATCAAGAATATGCGCAAGATGACCGACGATCTGATCACCCACGAATCCAGGGCCAGCGAGGGT
AAGTTCTCGAGCCGGGGTGAACACCTCGAAAACTAACCAAACCCATAGCCAAATCCAAAAACCATACACA
TTAACTTGCGCCCGATGAGATGGAGGAATGGCCCGAAATTTGCCTGCGAACATAACCAAAAACGAGCAAA
TGAACCCACATAGCTACATGGTTTGCTTCACTAACTAACTGCAAGAAATACTAATATCCTTCAGCCTCTC
GATTGTAACTAATGAAGATGCAAAATTCAAATTTAAATCGCAATCGAGATATCGGGATCGGCAAATGTGT
ATCCCTAAGGGAATGAAAATTGGAAACCCCTGAAATTGAAATCTATACCCCTACCTAATACGTTGCTCAC
CCATTTCTCTATCTCTCTTATTTATTGTCTACCCACTAATCAGGCTCAGCCGAAAATAGTCTTTCCTAAA
ATTGCCATAAAATCGAGCGACGGTCCAGTCATTGTGCGCGAACGCGAGAAAATCCTCGATGAGAAGATTG
TGCGCCGCACTGAGGAGGTGGCATGCCCCATTACGGGGCGACCTCAACTGCGCACCGTCGAGTACATTGA
GAAAATCATCGAGACGGAG
>dme_circ_0007680_junction_seq
GTACATTGAGAAAATCATCGAGACGGAGGTGCTGAGAAGCGCGCCTCCTGGCGGGC
>dme_circ_0007681
GGATATCCGGCAGCAGCTGTAAGGAAATGGGCTGAAGTGTTGAGGTTGCATATACGCCGTGTGGTGGGTA
TGGGTTCGAGTATGAGTATGTGATCCGAGTGAATCCTTACACGCACAACCTTCCGCACAAACACTCACTA
CCCACCTACCCACACACACAGAAACACACATATCAGCGTGTTAGAGGGCTCGTTGTCATCTCTCTATATT
AGAACTACTAATTGTATACATATGTATATTATGTATTTACGATATTCGCATATGTATTGATAATTTTAAT
TAATTGTGCAAATAATGTGTCCCCTGCATTTACAAAATCCCATTTGTTACTAATCATCAAAGTGTACGCA
TTAAAACAAAATATTTTCATTGCAAATTAAAAACAAATCGAATTGCCAAATGAGATATTGCAAGAATTTA
ACTGAAAATTTAATGTTGTGCAGCAACCGCAAAGAAACGACAACAACACAAATCGCCACACTGAGCCCAT
CAGAATTAATACGACAACATCATCGGACaaaaaaaaaaGGGAAACAAAAATCTTATTTAATTGAATTAAA
TAATTTCATTTTGTTTGCATTTACTATCATTTGCATACTGCATCTGGTTTTTCTCCCTCATTGTGTTTTT
CCAAATGGGCCTTGCTTATTTTCCACAAATTAAATTTACGTTTTATGTTCTAGGGTGGAGACCTGCAAGG
AGCGAATTATATCCTTGGAGCTGCAGGTGCCCGAAACGGAGGACGAGGGCAAGCTAGATGACACGCAACC

GCCGCTGGAACTCGAGGCGgatgacttccaggacgacgaagatgatgacgacgacgatgatgatgaagat
gacttcgaggatgaagatgaggaggaggacaccgCCTCCATTGTGACCGTGCAGGCGAACAATGAGAAGC
TATTCCTGCGCCACGATTCTGATACCTATGGTCCGTCGTCCATTGAAAGTGTTGCATCCGGCAAGGTGCG
CATCAAGCCCACGCCGTTGTCCCTTCACCAAACTCTGGCCAAGGAGACCACAATTGTGGAGGTGCTGAAT
CCCGTCATCGGAGGCGATGGCAGTGCCCGCACCATTCACGTGAGCGGTGGCCTGCCCTTCGATGAGGACG
CTTTCGAGCAGGGCGCCGTGGCCGTGCTGCGCAGCGAAACTTTCGTCTTGCCAGGAGCCGCCGGCGGTGG
TAGGTCTGAGTTGTCATTAAACGCTAAAATGAAAACCGTGCTCGAGGAGCTGCTGGAGAACGAGCGCGTC
AAATTGAATTTGCAAAAGAGCCTCGAGGGTGAGGAGGGGGAGGACGACGACGATGAGGATGAGAATGGTA
GTGCTTATGGCGACGCGCGGGACGAAGAGGAGAGCGCCTATGGCGATGCCATTGACGATGTAGTTGACTT
TGGCAGCGCCACGGTCACGAGCCGCGCAGCTAATGATAAGGACGATGTCAAGAACGGGAATCAGCAGCTC
CTCGAGGAGCTGATTAAGGACAGCAACCGCAACACAATCATTGCTAAATTGGCGGGCCAATTGTCAGACG
ATGATGAGGAGGATGACGACGAGGAGGAGAGCAGTGACTCTTCGGACGACGATAGCGATGACTCCGACGA
AGAGGACAACGCACAGCAGCAGCTGAATGTCACCTATGTGCAGGGCGCTCAGGTAATCGAGAATCTTAAT
ACACTGGCCACGGGCGGAAAGCTACAAAAGTCGCAGACCTACACGGCCATCAAGCAAGCAGAGGAGCCAA
AGGAGGAGGACACGAAACTGGAGGAGGATCCATCCGCGGAGAAGCCTTGCGGAGCTGATACCCTGGCCAA
GCTGCAGGCCGAGCAACGCGCCCTGGCCGAGATCTCAAAGCAGTTGCGCAAGTCTGTGGAGGATTTGCTC
AGCGCCGATGGCGAGACGGTGGTGGAAACTCAGAGGACTTACACTTTGCCCGCTCCCAGCGATAGTTCAG
CGGTGGTCACGGTCACCGAGGTGGTCAAGAAGCAGAAGAAAAAGAAGAGCGAAACTGGGGAGGAGCTTTT
CAATCGCCTGCTAAACGCCGCAGAGTCGGAACGTCAGGTTTTCGACCGACAGCAGGGTGAGACCATCACA
ACCACCACCACGACCACAGAGGCTGTGAGCTCGGAGGACAACGAGCCGAGTACCAGTGTGGTGACCACCA
CGCGGACTGTGTCCAATATTGTGACCAGCGGCATTCccaggccagaaaccagcaagcagaacaaccgcag
tagcagcagcagcaacagcaacagcagcaataataaccgcaacaagaacaAGCGAAAGGGCAAAAAGAAG
TAGAGGATATCCGGCAGCAGCTGTAAGGAAATGGGCTGAAGTGTTGAGGTTGCATATACGCCGTGTGGTG
GGTATGGGTTCGAGTATGAGTATGTGATCCGAGTGAATCCTTACACGCACAACCTTCCGCACAAACACTC
ACTACCCACCTACCCACACACACAGAAACACACATATCAGCGTGTTAGAGGGCTCGTTGTCATCTCTCTA
TATTAGAACTACTAATTGTATACATATGTATATTATGTATTTACGATATTCGCATATGTATTGATAATTT
TAATTAATTGTGCAAATAATGTGTCCCCTGCATTTACAAAATCCCATTTGTTACTAATCATCAAAGTGTA
CGCATTAAAACAAAATATTTTCATTGCAAATTAAAAACAAATCGAATTGCCAAATGAGATATTGCAAGAA
TTTAACTGAAAATTTAATGTTGTGCAGCAACCGCAAAGAAACGACAACAACACAAATCGCCACACTGAGC
CCATCAGAATTAATACGACAACATCATCGGACaaaaaaaaaaGGGAAACAAAAATCTTATTTAATTGAAT
TAAATAATTTCATTTTGTTTGCATTTACTATCATTTGCATACTGCATCTGGTTTTTCTCCCTCATTGTGT
TTTTCCAAATGGGCCTTGCTTATTTTCCACAAATTAAATTTACGTTTTATGTTCTAGCACAAAAAGTAGG
AGCAATCCCAAATCCGTGCCCAGCAGCCGGCCGAAGTATCCACGATGATTCTTCACGAGAACCATTTATA
AACTTTAAGCATTCCCGACTGCCCGAGATTTTCGGGCACACTGTTTTTAGTTTTAGTCAATAAGCAAACT
AAACCCGCAACTAAATCAAACTAGATTCGAAACGAAACAGCGAGAGCCGAGAACataaatcaaataatcg
taaataataataataataGTTAGCAGTGTGTAAGAGCGTCCAGTTGTAATCCGTAGTAT
>dme_circ_0007681_junction_seq
AAGAGCGTCCAGTTGTAATCCGTAGTATGGATATCCGGCAGCAGCTGTAAGGAAAT
>dme_circ_0007682
GTGGAGGACAGGTACGTGCGCACCCACCACGTGTTCTCCTTCAATGGGAagcaccagcaccagcgtcgca
cgcagcagcagcagcagacgcagctgagtcagcagcagctgcagcagcagtatttccagcagcagtactt
caaccagtaccatcagcagtatcaCATCCCCGTTGGCCAATTCATGGATCGCTACGACAAAAGCCTACGA
TGGCCAGCAGGAGCACGAGCGGGTGAGTAGCCATTGTCCACGGAGAACAGTAGACTGCCAGAAAAAAGTC
AGGAAACGGGACAGCAAATTATAAGATATCAGTATCAAATGCAGTAATTATTTTGATATTAATTGCAAGA

AATGTTTGAACACTAGTAGTTCAAGTAATTGTTAGATAATCTGGCTCATTTTTTATGCCTAAAAAACAAA
AATAAACTATTCTCTCCGTGTATCTGCAATTGGCAAGACAATAAGCTGTGAATCACCACTGGCTAATGCG
GCATTTCTGTGCTGTTTGAGCTCCAAACGAGGGTCTGCAGCGTTTACGTTAATTAGTTTGATTGCTGACC
TGTGCCTCTTTTGCCAATCTATTCCTCGGCATTGCAGATGAGTTCCCGCACTTTTTGTCGCACCACCATG
AGGGGCACGGTCACCACGGTAGCCTGTACAACAATCCGTTTGGATCGCTGGCCAACTACCATGACGTGGG
CGAGTTCCACGAGGTGCATCAGCGCAACTACGACAGCAACAAGAAGAACATGGAGCTGAAGAAGCAGCAG
CAGATGGAGATGAAGAAGCACAGG

>dme_circ_0007682_junction_seq

GCAGCAGATGGAGATGAAGAAGCACAGGGTGGAGGACAGGTACGTGCGCACCCACC

>dme_circ_0007683

GTTACATGGCTGAAGGTTGACAAGGGCAAGTTCTTTGAATTCATAAACGGACGAAATCCGCCATTCCGTA
ACTCAACCATCGAGGGAGCCGAAATCGACGTAAGTACCAAATGATGCCAGTGTTTCGGCCAACTATTTAC
TTAATCGTCATTACCGCTGTCACTTCGCGATTTTGGTAATCATTCTACAATTTGACATGTTTAAATAAGT
CCGTAAATTGAAGCTAATTGGAAGGCACTTGCTGCACATGCGAGATGTCCTGCTGATCAATTGATTTGTT
AATGTGCTTTTTGTGTCCCTGCACTTTTCTAATTTGTCCATTCCACGCCCCCTTCAATTAACAGTGGGAC
AATTCCAACGAGCAGCAGGTGACACTGAAGGATGTCCAGTTCGATCTATCCGGCCAATTCTACTGCGAGG
TCTCCACGGACACCCCCATTTTTACCAAGGCCAGCGCCGATGAGCTGATGAGCGTGTTCTGGTGCGTATG
CGTGATATTTGAAGCAGCCAATTCGACGATTGCGCAATTCAATTGTTTGGCGTAATCAGAGTCCAATTGC
AATGGATTATTTACGGCAAGGCAGCTATGTGCGAAAAAATGCCGCCGGGCGGGAGGTTAATCAGTGGCAAA
CGACTGCCAGGGGATGTGAATTTGGATGTGGATGTGGCATGCAGTGCATTAAGATTAACATCAGTTTAAG
CCATTTTATAATTATGGCCTACATGGGCGGCCAGGACAGCTTTCATTTTAATTGCACATGCCCGTTGGCC
AGCTGCTGTTGGCGGCGAGAGGAAATGAAATTTATATAAATTCATTATGGCTGCCCGGGCAACTCTAAAA
TGAAATTTATTCATAAACGCGCGCCGCTGGCTATGGGAAATGGCAACAACTGCAGCACTGTGTGGCCAGC
AGATGAGCAGATGTGGGACTAGCCCTTCGGGGCCTGATCCATATTTAATTTAAGCCGGACGCTCGTGTCC
AGATGTGCCACTGGAATGGGCAATGGGATTTGACATTCAAGTGACTTCTGTGCGCAGCCAAATGTAATTG
TTATTGTTGAGCTGGCCGCGTCATGTGCTGTCCATCCATACAATTCACTCGCCCACTGACTCATCGCCCT
TTCGGGCTCCTTTGTGTATCGACAGGGTGGAGGACAGGTACGTGCGCACCCACCACGTGTTCTCCTTCAA
TGGGAagcaccagcaccagcgtcgcacgcagcagcagcagcagacgcagctgagtcagcagcagctgcag
cagcagtatttccagcagcagtacttcaaccagtaccatcagcagtatcaCATCCCCGTTGGCCAATTCA
TGGATCGCTACGACAAAAGCCTACGATGGCCAGCAGGAGCACGAGCGGGATGAGTTCCCGCACTTTTTGT
CGCACCACCATGAGGGGCACGGTCACCACGGTAGCCTGTACAACAATCCGTTTGGATCGCTGGCCAACTA
CCATGACGTGGGCGAGTTCCACGAGGTGCATCAGCGCAACTACGACAGCAACAAGAAGAACATGGAGCTG
AAGAAGCAGCAGCAGATGGAGATGAAGAAGCACAGG

>dme_circ_0007683_junction_seq

GCAGCAGATGGAGATGAAGAAGCACAGGGTTACATGGCTGAAGGTTGACAAGGGCA

>dme_circ_0007684

GTCAACTATTTGGAACACGTGCAGGCGGTGATCTCGGCAAATGCCTCACGACGCGGGGATCTTGAACTTT
TCCTTACATCGCCAATGGGCACAAAGTAAGTGAAATAATATTACTTTGAAAAATTCCTAACTATATTTTG
GTTTTGTGAATTCattaataatatcaataatattaataTTACTCCATAGATCCATGATCCTGTCGAGGCG
CGCCAACGATGATGACCACCGCGATGGCTTCACCAAATGGCCCTTTATGACCACTCACTCGTGGGGCGAG
TATCCGCAGGGGACCTGGAAATTGGAGGTAGTATATAAATTACCTATGTAGTCCGGCCTTTCTGAACGAA
TTCGCCCACAGGCTCGCTTCAACTCGCCCCAAACACGACACGGCAATCTGCTGGAATGGTCCTTGGTCCT
GCATGGCACCAAGGAGGCGCCCTATCGCACCCTGCACCCCTCGTCGCCGCACTCCAAGCTGGCCATTGTG
AAGAAGGCGCACGAGGACAAGAAGATGAAGTAGGGCGGGTCGCGCAGAGGGTCAGAGG

>dme_circ_0007684_junction_seq
TAGGGCGGGTCGCGCAGAGGGTCAGAGGGTCAACTATTTGGAACACGTGCAGGCGG
>dme_circ_0007685
GGTGGCGGGAACGCCCTGGGTGGAGCACACCCAGTAGGAGCTCTTCAGGTAGAAGTGGGCATAGAATGCC
ACCTCCAGCTCCTCGGCCGTTTGGCAACCGGGTTGACCAGTGGGCTTGGCCACAGCAGCTCCGCTGCAGA
AGGAGGAGTAGCCGAGGATTAGGAAAAAGGCGAACAGAGGCAGGAACTTCATCTTGAACTAGGGG
>dme_circ_0007685_junction_seq
AGGCAGGAACTTCATCTTGAACTAGGGGGGTGGCGGGAACGCCCTGGGTGGAGCAC
>dme_circ_0007686
GAGAACCATAGCCATAAACAGAAGCAGAAGCCAGGGCATAAAAGACGACTAGTCGACAGTCGTCTGTGTT
AATAAACCACCGCCCCATGACCACCAAGAGCGGCACCATCCCGTCATCAGTTCCACGTACTCTTCTGCCC
AATGCGAACCCAAACCACCCGCATTCCCAACTCTTACTACTTGCATGCCCTTCGAATGGCGGCTGCCACA
TAAAATTAAATTTAATTTAGTTAATTGAGGCTTTTATTAATCAGGGGAA
>dme_circ_0007686_junction_seq
TAATTGAGGCTTTTATTAATCAGGGGAAGAGAACCATAGCCATAAACAGAAGCAGA
>dme_circ_0007687
GACTCAAACTAACAAAACACTTTTAAACTGTACATAAATTACGGCGCGCGTGTTGTAAAGGCGTAAAGAT
AGCAGATTCCACTGAAACACTTGCCAATACTGGACAGAGTTGCGTGCAATATGTGCAACGGTTTTCGGCA
ACGAGCGTGCGCCACGAAACGCACTGAATCGAAACGGAACGGTGGGAATCTATAATGCAACAGCAACAGG
AAGAATTCGATACAGAAACAGAAACTGTAGGGGGAAAGGAAAACATTTGGAAAATGTTGTTCCCGAAAAC
TGAAGAAAATGCATCGTTTTCTTAACATCCAGCAAAATATCAAACTTCGCAACTATCATCAGTTACTTTT
GTATTTTGTCGCTGCTGCTGGAGGAAGAGCGGAAGTGAGAAGAGGAAGACGGACCGGAACCGGAAAGAGA
AGCCTTCGCAAAGGAAGCAGAAAAGGAAGCAGAGCAGCAGCAGCAGTAAAAGTAACAGTTAAAGTAACGT
TTAGAGCAACGTCGAGAGTAGTTGAAGCATAATATTATTCTATTAGCCCAATACGTTAACCCAACGCTGC
CACTGTTCCTAAGACACTAAATTTGACACAAAAAAGAAGAAGAAACCACTTGAAATAAACGTAGACCATA
GTCGTAACCGTAAAACGAAACGCCAACGCCTCTACACAATATACGCTACTGAAACACGAATCCTATGCCA
ATTTTTGATCATCAAATCGCTTAGCACCTATCATAGACTCCCATAAACGCCTCATCAAACGTCAAAATCT
CAAACGACAGTGTCGCAACTAGACGTTGTTTTCGACAAATACCCAAAACCCCCACAAAAACAACGCCGAT
CTCGCCCCTTTGTCACGCACAccaaaaacacaacaaaatagacaaaaataataaaaaaaaaGGCTGGCTA
AGGCTAAGGAAAGCGGAAAAATATGGCCACACCCCAAGTCAAGGACTTGGTGTTGCGCTCGCCCGCTGGC
TCCTCCGATGTCATTTCCTTCGCCTGGCCCCTGCAGATCGGACACGGACAGGATAAGCACGACAATGGCA
TCGACATCATCGACACCATTAAGTTCGTCTGCGATGAACTGCCATCGATGTCATCGGCCTTCGAGGAAAC
CAATCTCCACCAAATTGACACTGCCTGCTATAAGACTATGACCGGTCTGGTCGATCGCTTCAACAAGGCC
GTCGACAGTATCGTTGCATTGGGTGAGTTGATTTACATTGTTGTGGTATCGAAAGCaaaaaaaaaaTTAG
TCATGAAAGGGAAATCAAAGCAAAGGCCACTAGATTTTAAAGAttttttttcttttAAAGATTTAAATTA
ATATCATAATGCAATATGCGGTCTGAAGATATAATTTATTGTAGTACTTGATATTTTTCTTTGCATCAAA
TACCCGCTAATATTAGCACACATGAACTTGTTCCTAATGTGTTCTCTTTGCTCTTAGGGAAAAAGGAACT
TCACTGCCCGCCGAGCGCCTGAACAAGTTCGCTCACCCTAGCCTTCTAAGACACATATTGCAATTAGTTT
ACAATGCCGCCGTACTCGATCCGGATAAACTCAACCAGTACGAGCCCTTCTCGCCAGAGGTTTACGGCGA
GACGAGCTATGAGCTGGTGCAGCAGATGCTTAAGCACGTCACTGTCAGCAAGGAGGACACGTTTATCGAT
CTCGGCTCGGGGGTGGGCCAGGTGGTTCTGCAGATGGCCGGATCGTTTCCCCTGAAAACCTGCATCGGCA
TCGAGAAGGCCGACACGCCGGCGCGATATGCGGAACGCATGGATGTTATCTTCCGCCAGTATATGGGATG
GTTTGGAAAACGCTTTTGCGAATACAAGCTGATAAAGGGTGACTTCCTGGTCGATGAGCATCGTGAAAAC
ATCACCTCATCGACGCTGGTTTTTGTAAATAACTTCGCCTTTGGGCCAACTGTGGACCATCAGCTCAAGG

AACGGTTCGCAGATCTTCGCGACGGAGCACGGGTCGTGTCCTCCAAGTCGTTTTGCCCGCTGAACTTTCG
GATCACAGACAGGAAACCTCAGTGACATTGGCACCATTATGCACGTCAGCGAAATTCCGCCCCTCAAGGG
GTCCGTTTCCTGGACCTGCAAGCCCGTTTCGTATTATCTGCATGTGATCGATCGCACCATATTGGAGCGA
TACTTTCAGCGCCTGAAAACTAAGGGCGGCAACGATCACGAGAGCGTGG
>dme_circ_0007687_junction_seq
AAGGGCGGCAACGATCACGAGAGCGTGGGACTCAAACTAACAAAACACTTTTAAAC
>dme_circ_0007688
GGTAATTAACACGCTGCACAACAGCAACCACAACTCAAAACACCTAGAGCGTCAGCAGCAAGAACAATGC
ATAAATCTACTTTCCTGGTTCAATGCGCCGGCTGCGGTGAGCAACAACAGACGTGATTAGAAAAGAAAAG
AAAAACCCAATGCGAGGGAGCCACCGCACAAGAACTAGTTTTCTTTTTCGTTTTCCTACCTTTAGCATAA
TGGGAAATCGAACCCGCAACAAACGGTAAAAAAAGAAGGGGGGAAAAGAGCCGCAACGGTATAAAGAACT
TCAGCTAACCATGTTGCCTAGAATTGGTAAAAAGGCGACCGAGGAAGCTTAGAAAGTGCTGTGGAAACCA
AAGCAGGCCAATGAATCGTTTCGGCCACATTTAAAAGGCTTTTCCAACTAAGCACAGTAGACTGGCTAAG
TGCATCCGAGCGCGAATTATGCCCATGAAACATGTGTTGTAAAAGATACACAAGAGGGAGTTGCAATTGC
AATTGAAGTAATTACAAATGCACAACCATTCGGCGGCGGCattctatattctatattctaCATACTAAAT
GTGCCGAACAAAGGCTGCAAATTGCTGAAAAACTCAAACAAAAGAAGAGAGCCGGCGAGTTGCATGAATG
CAAGACCCAAATGCATACACAAATAAATTAGCAACTAAACAAAAAACAAAGACGAGCTCCAGCAGCTGCT
GCGAT
>dme_circ_0007688_junction_seq
AAAGACGAGCTCCAGCAGCTGCTGCGATGGTAATTAACACGCTGCACAACAGCAAC
>dme_circ_0007689
GTGCCTATGTGCCTCAAGTTCAGAAGCAGATTGCCCAAGAGCAGGAGCGGATGGCCAGGCTGAAAAACAG
AGCCAGCCAGTTGGATAAGCAGATAAAAGTGCTGATCGATGACAGTGTGGCTTTACTTAAAGTGCGAATG
AACGAGCTGGGCATCCATGTAAACTCACCAAACGATTTGATCGCCCAGGCAAAGGAGATTGTGGGTAGAC
ATAAGGATCTACAGCACACTGCAAGTCGAATGCGAAACGAAGTAACCTTCTATGAGGGCGAACAGAAGCT
GCTCCTTAACAAACAACTAAAAAATCTACCCGAGTATCAAAAACTCTGTGGTACTGTTAATGGAAAGGTC
AAGCTGGAGGTGCCCCCGGAACTTTCCGAGACCACGGCCCAGGAGCTGGTGCTAAAGGAGATCGCCAACA
CGCTAAGTCAACGAAAGAAACTTTACGCCCAGGTGTCCACCATTGAACAGGAGACTTCGGTTTTGCAGAA
GACAGCAGAGGAAAGGTCCACGGCAGCGACGCTCTTGGCACAAGGAACAAATATGATTGTGTCGACCGGA
TCATCTTCCTCCTCCTCTACCACTGTTTGTGCAAGTGCAGTTACGGCGCAGAGCAACAAGTTGAACTCCG
TGAAGAACTCTCGTCGCAATCGCGAGCACCGAGCGCGTTCCCAGGAGTGGCCAGAGGTTCCCGAAGTGGG
TAAAATCCAGGAAAGCAATCCCGAAGTGCTGGCACAGAAAATAGTCGAGACCTGCCGCCAAATCGAAGCT
GGCAAGTTCCAAGGAGCAGGTGCGCCCTCATCGCAAGTGAATGGCAAGAATAAAGCAATTATAGAGGTCC
CACCACCTCCGGCCACTGCTCCAGTGAGCATTAAATCCTCGCCTGGGCACCACTATAAAGACACAACACT
GATGCCAGCACCCAAACAACAACAGCAACAACAGATGACGCTTTCCCAGCTTCCGAAGTGTGAGCTGCCC
GGACTATCGACTAGTCGCAAACAGGAATCTCCCAAGGTTGCCAACTTTGAGGATCGGTTAAAGAGTATCA
TCACCACGGCCTTAAACGAAGATCAGGAGCAGCGCAGCAAGGCAGTGGAGTCGTCACCCTCTCCCTCACC
TCTTCACAGTCCAGCACCCAAGCGATCAAAGCAGCATCCGGCTGGAGCGATAAACCCTGCCCAGTCGCTG
CCCAACAATCTGCACAACATCATCACCGTCTCCACGCAGGGTTTGATGCACCTTAATGCCAATACAACAA
TATCCCCAATAACACCACCACTGCCGGGTCCAGGTGCAGGAGCAACCGCATCCACTGCGCCGCCTCCCCC
AGCGAATCTTCCTTATGGGGCTTACGGCGGGGCAGTGGCAAAGACAACGATATCGGGGAAGTACCAGGCA
GCTAAGGAACCGAAGTATTCGCCAGTTAGACAGGCACCATTACCTCCTCCCCCCTCCCACATGGCATCTT
TGTATCCAGCTGGACAGCAGACTACTCCGGCGGACCTAGGTTACCAGCGGCGACGCAGCTCTGTTAGTGC
CACTAGCTACGAACACTATATGGTACAGCAGCAGCAGCAACTCCAGCAACAACAACTCATGCTAGCAGCC

GCTGCTCATGCGGCCCAGCGTCAACAGATGCGCGTAGAAGAACAACAGCAGCAGCAGCAGcaccaacacc
accaccaccaccaccaccaccatccccaacaccGTCTGCCGCAGCATGTGcagcatcagcatccgcatca
gcatcaTCCCAATGAGTTCAAGGCACCGCCGGCTGATAGTCATCTCCAGCGATCGAGCAGTCGTGAGCAA
TTAATAGTGGAGCCCCCGCAGACGCAACCGCTGGAGCTGCTGCCTCGAGCAAGTTCCGCCAATTCGGATT
ACAGCGGATATCGCATCCGTCCGCCAAGCAGGCCAAGCTCCAACTCCTCGCAACCAGATTACACGCAAGT
TTCACCCGCAAAGATGGCTCTGCGGCGGCATCTGTCGCAGGAGAAGCTGAGCCAACATGTCACGCCCCAG
GCCACACCGCCGCTTCCTGGTCACGGAGGAGCACCCACATCAGGGAAGACCATTGGCGATTTGGTTAACG
GGGAAATCGAGCGAACGCTTGAGATTTCGCATCAGAGCATCATTAATGCGGCTGTGAATATGAGCACTAG
TGGCGCTAGCTTCATGGAGCGGGCCTTCCTTAATGAGCGCTCCAACGATCGTTTGCTGATCAACTTGAAT
GCCCAACGACCAGAGAGAGTTCATGTTCGTCCGCTCTCCGAAGAATCGCAGGATCCACAGCCCACCAGTT
ATGCTCAGGAGAGAGGACCTGGTCTAGGTGCTGGTGGAGCAGCAGCTGGAGGTAACAGCAATCTGGCCAC
TTTGGCACATGTAGCCTACGCCCAAAAGGCACAGGGCGGCGCTCGAGCCAACGCAGGAACGGCACCACCC
GCAACCCACTCCTCCTCTGCTCGTTCTGGACGTGACTATCAGCCTGTGGCTTTGCCCCGTGCTGAGCTTA
AGGGCAGCATTGAGGCCTATTTCCACGAGGAGCAGCAGCAGAAGCAGTCGAAGGGCGCAGGATCAGCAGG
ATCGTCCTCTTTGCGGGGACCGCGTCTTAATGGTGCCAATCCTCCATTGGAAGGGTAAATTGCCGCTCGT
TTTTGTCCGACGAGCTTGGACCTTCTAGGCGAATCAAATTTTATACGTTAAAAATATTAGCCTTTTTGAA
ATTTCTAGAAATCTGTGCTCCAGTAAATTTTAAATATGTTTAAAATAAGAGTGCTCTTCTCAACTTTTAT
TGAATGCAATGTTTCTTCTTCGAACTGGTTTACCAAACATATTTTGTATATCTGTAGGGTCTAGCAGCAT
CGCTGCAGGATCATGTCCGTGCCAGAAAGTACAAGGAGGAGACCGAGGAGCGTCAACGACGTGCAGCAGC
CGCTGCCTCGTCTTCGGCGGGACCGCCTGCTGGCATGGAACTGCCAACGCACTATGCTCATCAGGCGCCT
CCAGCCCACAGCTACCACCACCATGGTGCAAGCATCAATGGAACACCGCACAAAGTAGAACGTGGGAATA
AAACGCAGCTCACCGCTGGCGCCGCATCAGCAACCGCCACGTCCCTCGAAATTGGCACACTACGAGCCGC
CAACGACGCAGCAGCAGCATGCCCATGCCCACCTGTACGCCAACGGACAAGTGCTACCTCCGCCTCCAGC
TCATGATGCGACCACGCCCTCCCCGACGCCCTCGTCATCTTCATCGTCCTGCGGACGTCGAAGCAACAGC
AACAATGGAAAGTTGCTCGTGGATCCACCTTTGCTAATGAGTCCCGAGATCAATTCACTGTTGGGCGACG
AAAGGCCTTTgcagctgtcgcaccaccaacagcagcagcagcaaatgctgcatcaccatcaatcgcagca
acagcagcatctgcagctgacccaacagcaactgcGTGTAGCTCATCTTGGACATGGTCTTAGTCACGGT
CACAGCACCATGCCCACTTTGGGCGGACAACGCAATGGCAATGGCAATGCCGCCGACGATGGGTAAGCTA
TACGTAGACCAAGCAAAAAACTCGACCAGGGTATCTGTACCCAAATATGCACCACAACAAGCACCACCAC
CTGCACACGTAGCATTCCCGCTAGAGCCACATATTCTTCACCAGCACAAATACCAACGACTAGCCACCCA
AACGGTGGCAGAAGATCTTACCATGAAGCGAAAAGAGCAGGAATCGGATTCAGAACTCGAGCCGGAAGTG
GTTGCTGCGTTTGTCGAAGATGGAGGTTCCCATAGCGGATCCTCATCGTCGAGTTCCAGCTCGCACTCAG
GATCTGATTCCAGTTCTGACGTGCCATGAATTGCTTTTAAAACGCTTtatacatacacacacacacatac
atctgaacataattatacatttatacatTTTGTAAATACAACATGCACAAAAAATGATCTTTTCTCACAT
CTCGTTCATTTTAATTGTTGTTTGTTCTATCTAGTTTGTTTTTTGCCCGTAACACTTGTAGTCCTCAATG
TAATTGTTGCTTGtttttgttttagtagatttgctttttttttattttgttatgatttttATTAATGCTT
CTTGTATATATTTTATGCCATCCACATTGAAATTTGTAAGCTTCAAAGGGTGAAATCTGACGGCGATCAT
CAATGAATTTTACCAAATGTAAATGAATAAATCTTATAGTGTATCGCGAGTGAAATTTGGTTACATCTTC
TAAAGCTCTTAGTTGGGCTTTACGTACATTTTCCACAAACCTCAACAAACATTATAGTACAATATAAAAA
CGTAAACGACCATAAAATCAAAGCCAACAACATTCAACATGCTGAGATAAATCGAGCTCCACTATTATTC
TAAGCGTCATGCCAAATCAATCATCTATCTATACCCACTGCATATAATAGTCACTCAGAGATATCAGCAA
TCACACAAAAAACCCGTGTAAAGCGTAAAGGCTCTCTTGTAAAAACCATATATAAGAATACAacacacac
acacacacCCCCATAAGCAAACATTTATTTCAAAATTAACCCAAAAAAGCAACTCCTGGAATGTTATTAA
TGCATAATTAATTTTCAATTATACTAAAATTCGAAGAAATCTGCAAATGCAAATTGGTAATGCTAAGAAC

CAAACCAAACAAATCAAACAAAAATATGccccccccccccccTCATCTTATCCAGCCTTCTTTATTATCC
CTTCTATTATGCTCTACTCCATACAGTTAACGATTTGGCAACGCAACGTACTATAACTAATTACGATCCC
AGACGTCGCCTTCGCACGACTTTGTCTGGCCCAACGAAACTATCAGCTGCCCATAGTAATCAAAATCTGA
ACGGATATGTTATGGCCGATAGTAGTAGTAGTTGCCCCACAATACCCCAATAACCAATGATCgattgcat
tgaattgacttgcattgaactgaattgaattgaattgaattgattgattgattgacttgcatACCTCAAA
ATTCCTCAAACAAACTAAACAAAAAGCAAAACCGAAAACATGTTTGTTGTTGGGCGAAGATACGATCTTG
TAATAACCATAAATAATAAGCAGAGTACAAAAAACCTGATGAAAAACAAAGATTTCAAATCAGAAACAAC
CTACAAAAGACAACTTTTAATTTTTGCGTTTAATAATTTACCTACTAAAAGTACAGAATTTATATGCCaa
aaaaaaaaaaaaaTATATATATATATATACTTAAAAATCCCGAACAAAATTTATGAAAATTTAACGAGCA
TGAGGTAAGTTCTGTTGCGCCTAATTGGTTTCTTTCTTTTAATTATGATTTATATATTTTACTTTCATTA
CGTAACTCCTTTGTTTCAACATTCAATCACCACACTAAACAAATAAAACACAAAAACACCCATTAATACA
AATGAACACAAGAACATACCCTCCTGCACGCAACGCGCAATGCAGCTGTACCAAAAAGATAATGAAATAT
GAAAAAAGCTTAGGACAACACGCGTTATCGCCAGTGTATCGTTATCACCCTTTCCAATTGACTTTTCCAA
ATATCCACTCAAACTCACAATATCACCATCACCCAAACGAAATCTAAAAATCGCACTAGTATTGGTCGCG
TTAGCGAGATAAAACTAATATTTTATCCTAATTCTTCACAGGACGATGTTATTGCCGCTGATGATGAGAC
CCACTGGCAGCATCGAGTCAGCTCTGGTTTCGACCGCCTCGTGGCCTTCGCCAGCACTGAATTGGATAAG
ACCAGGCGAAGCATCGATGGCGATACCGTGCCGGCCTCGATTAGTTGCAACACGTCTCCGGACTCGGGCA
TTACGCACAGCAGCAGCAACTCAGATGCAGTGCGCACATTTTTGTCCACCTCGTCCAGCTCCTCGCAGCT
GGAGCTGCCCATAGGAagcggtggtagtagctgcaacagcctctacaaccatcatgcccagcacaacaac
attggcattggcggcagcggaggcagccacccacatcagcaacagcaacagttgcaggcgcagcaccaac
agcagcagcaccaTCTTTCCGATCACATGAGCGACAGTAGCATGAAATCCTCGGCATCCTCCTCGCTGCA
TGCTGGTAGCTCCCTTAAGACCGTGTCGCCAGTGGATCCCAGTGCCATCGAGTCGCCGCCACTCTCGGAC
GTTGGATTGCCCAGAACTCCCAGTCCCAGTGCAGCCAGTGTGGCCACTCCACCACTGGCCAGTGGACCAC
CCGTGTCCGGGGACTTAGGTGGTATACCTCTGAAGTACCAGCGCAAGTCCAAGACCAGTTCGGAAAAGCA
CTACAAAAAGAAGTTCTGCGAAAGAAACTGGGAGTACGACTGTGATGAACTGCTGGCCTACTCCAACTCC
AGTGCACCGCCGACGGCAGCAGATGCAGCGGGCAATGGGCCACTTAACTTGGATGCTCCCAATACTGTGG
GCGGAGCACCTGCACCGCTCTTGGGAAAATCGGGAAAGTCACCCAAGGAAAAGTCTTCACCGCACCACAC
GGGCTTAAACCATCAGCAGCAGCACCATCACAAGTCATCCAAATTTCGGCCAAAGGGCAAGGACTGGGAC
TGGTCAATGGACAGTCGGAGCCAAACGGGCGAGCTGCTGGCCGCCAGTAACAACATGAACATTAACAACA
ACTCAGCCACCACAACGAGTAATTAATTTAGTAAACTAATTTAGTTCAATTTGCTGAATGGAGCGTGACA
GAGAGAAATCCAGAAAAGTATGCAAAGCTTTCATGCTTTTAGCCAGATTATTTGTAAATGGGTCCAGACG
GATCGGATCCCGGATCCTCGGAATTGTAATGTATTTGATAAAAGATTTTTT
>dme_circ_0007689_junction_seq
TTGTAATGTATTTGATAAAAGATTTTTTGTGCCTATGTGCCTCAAGTTCAGAAGCA
>dme_circ_0007690
GATGAGCATAGACGGACTGTGTCAGTGTGCACCCATAATGTCGGAATACGAAATCATCTGCCCGGCGAAT
GCGGAGAATCCCACGTTTAGGCTAACCATCCAGCCCAAAGACTACGTGCAGATCATGTGCAATCTGACGG
ATACCACGGACTACCAGCAGCTGCCGAAAAAGTTGCGGATCGGTGAG
>dme_circ_0007690_junction_seq
GCTGCCGAAAAAGTTGCGGATCGGTGAGGATGAGCATAGACGGACTGTGTCAGTGT
>dme_circ_0007691
GATAAAAGTGCTGATCGATGACAGTGTGGCTTTACTTAAAGTGCGAATGAACGAGCTGGGCATCCATGTA
AACTCACCAAACGATTTGATCGCCCAGGCAAAGGAGATTGTGGGTAGACATAAGGATCTACAGCACACTG
CAAGTCGAATGCGAAACGAAGTAACCTTCTATGAGGGCGAACAGAAGCTGCTCCTTAACAAACAACTAAA

AAATCTACCCGAGTATCAAAAACTCTGTGGTACT
>dme_circ_0007691_junction_seq
ACCCGAGTATCAAAAACTCTGTGGTACTGATAAAAGTGCTGATCGATGACAGTGTG
>dme_circ_0007692
GTGTTTGTTGTGGTCGAATAGGCCCTGCGGCAGGGAACGGAAGAGATTGGCGCTCAGATTGATATCCGTG
ACATTCGTGAGATGGGCAAAGACGTCGTGCGGCAACTGTTCGATGCCATTATCGTGAATATCTATGCCCA
GCACCGAGGTGGCACCTTCGAAGTCG
>dme_circ_0007692_junction_seq
CAGCACCGAGGTGGCACCTTCGAAGTCGGTGTTTGTTGTGGTCGAATAGGCCCTGC
>dme_circ_0007693
TTTATGTACAGAGACGGGGTGTGTATGCAATGCAATCGATTATGAATCCTTGGGGTCTATATATCTAGCA
TACGTACATACATACATACAAAGAAGGCAATTTGTATTTCATTTCGGCTGCTTTTGTTGCGTTTGTTCAC
ACAATTACAGAGTCTATACTACTATTTGGATACGTCGTATGTGTATTGCAAGTGG
>dme_circ_0007693_junction_seq
GGATACGTCGTATGTGTATTGCAAGTGGTTTATGTACAGAGACGGGGTGTGTATGC
>dme_circ_0007694
GGATTCATAATCGATTGCATTGCATACACACCCCGTCTCTGTACATAAACTGGATATTGATTTCTGTACT
TTTCCAAAACTAGTATGTGTATCGCACAGCAGATTCAAGGACCTTACACACAACTATCACTAGTATCAAC
TGTAATCTCACGCCCATTTAAATGACGCTATATAAATAAGTACATAACACGCGAATACATAATTCAGTGG
ACACACATTATCATGTCATCTACCAAACAATTAGGCTAGCTCTTCATCCATACAAAAACCAGAATGAATA
TCGTCTATGATACGTAAATGTAATTAAGCTAAACTATGTGTATATCGTCTAAGCTATAGATTGTCTCTGA
TATGTAATACCTAAG
>dme_circ_0007694_junction_seq
AGATTGTCTCTGATATGTAATACCTAAGGGATTCATAATCGATTGCATTGCATACA
>dme_circ_0007695
CTTAATTACATTTACGTATCATAGACGATATTCATTCTGGTTTTTGTATGGATGAAGAGCTAGCCTAATT
GTTTGGTAGATGACATGATAATGTGTGTCCACTGAATTATGTATTCGCGTGTTATGTACTTATTTATATA
GCGTCATTTAAATGGGCGTGAGATTACAGTTGATACTAGTGATAGTTGTGTGTAAGG
>dme_circ_0007695_junction_seq
TTGATACTAGTGATAGTTGTGTGTAAGGCTTAATTACATTTACGTATCATAGACGA
>dme_circ_0007696
AGCATGGGCTTTCCTCTGGTGTCACTGACGCAACGGAATCGCACCTTCGCCGAGCTGGCTTACTTGAAGG
GAGACCCCTGTCCCACAGACAACACCTCGGAGCTCTCAACGCACATCCTTTTCAACTGTAATATGCGTGC
CGGTCGGGGTAAGTACACTGTCAGAAAAACGCATTATATGCTGCAACGTTCGCCTTAAATTATACATAAA
TTAGTAGCTACTAAAGTCGTTTTCTTTTCTTTCAATTTGATATGTTTTGGAATTACTGATTCCAAGAGAT
ATATAACATAGTCGGTAGTTTGTTTCAAAATTTTATTTGTGTACTCCGTTACGGAAAATTGCACATTCAG
GACTTTTTGTAGAAGGAATCGCCATTTGGCAGGACTTTCTGTGGACATTAACTGACTGCGTGGCAGCATA
TATACGATTATTTCATGGCCAGTGGACCCATTTCTGTAGCAAATTGCTGCATGCACCTAATGGCTCCGCC
TCCTTTCTCTTCTTCCttttttttttttAGGGGACAGCCCGTGCTACGTTCGGTTGAGGACTGTGCCTAT
CGGTTCGAGTGGGAGACCAATGTCTTCTGTCCGCCGCACGAGTGCACCTTCAGTGCGGACACCTGCGATC
TGGTGCACGACGAGCTGGGTCGTCGTTTCAACTTCAAGAGTGCGCCGTTCACCAAGGATGGCAAGATTGA
GGATTGACTACAACGCCACGAAGATGTCGGTAAACATTTGCGGAGCGCATCGCAAGGCAATGACGGACTA
TTCGCAGGCGCTGGTCAACATATTCTTCACACACGAGTCGCCTAATTGCGGTCGGGAAGGGTGAGTTCGA
GTGTGTGCATTGGGTTCTTTTTATGCAGTCCGGCTAATTCGATTTACATAATCTGGTGCTCTACGGCCGG

CTCAAGGAGCGGGAGAATCCGCGTTGTCGCTAGTcgcttccgtttccggtcccggttccgcttcccttc
cgctGGCGAAACACGCGCGCATTGCCGCCATTTTGTTTGCCTTGCGCTCTCCCTTCATTTTCCCTTGTTT
TTGGTATGCTTTTGGCTGTTTTGTGTGAAAACGTAGGAATCGCATTAGATTTGGCGTACAGCCCAATGCA
CTTTTCGTGCCGGGCACTTCTTGCTGCTCCTCCATCACAGCACCACTCCAGTTGGATCCTCGCGGCATTT
TATCGCGTAATTTTTGTCGCTGGCACTCGCGCttttttttttCACAGCGGCGCCGTTCTTGCAACAGCAA
CATTTTTGGGCAGCAGTTTGCCTTCCGACTAAGATTCTTTTTGTTCGTCTGCAAGCAAAGTGCTTGAAAT
TATACAAGAACAACGAATTATACaaaaaaaatcaagcgaacgaaaaaaacTGTCTGCGAGATAATGCGGT
AAAATAATCTTGCTGCACTTGGGAAAATCTCTCtttttctctggggcttttcctgtttttttctatacctt
tttttgtgtttgGTTGATGTGATACTACTGCATGAATATTTAATGCATGCAGAGCGCAGACAGTTGGATC
GCACTGGAGGTGGCTGTTGGGGCttttttttttCTCTGTGGGGGCTTTAGAGGCATGAGGCTTGCTCTGC
CGAGATTTCCCCCGAGCCATCGGGGTTAGCTGGCTTGTGTTCGCACAATGATTTTTATTCGAGCCATTGC
ATGGCACTTAAAACTTTGCTATTAAAACAAAAAGTTTTTATTTTGACAAATAAAACTGACGACGATTTAA
TTATGACACATTTCCATTGTCTGCAGCCCTGCCCGTAAAATGGTATTATTTTAAAACACTGTGTGCGGCA
CACAAACTGTTTCGCCATTGTGGGACGACAATGCGCAATCTGCAAACGATCAGACAATTTTAATATGCCC
AGTGACAGGCGTTGGCAGATACCAGAAATTTGGGTGGCTGCCGGAGGTGGAGATGGTCCCTGGCTAGCCC
AAATCCCAACGAGTTGTAGATTTTGTGTGTCTTTGCGACCGAAGCTGAAGGCTTTGATTACCCCGCTCTA
TCCACACTCAAATATATACCCATTTGACCGACAACAGTAGCAGCCTGACCCTCCAGGACTCCAGGACAAC
CGGCTGGAGCGAAAGCAGGACCAACAGCAGCAGCAGCAGCTAAAGCTGTGTTGTCGTCTGTGGTGATAAT
TATTTTTGGCTTTTATGCGATAACTTTGCGGTGTCCAGGCCCAATCCACTTTGCCTAACCCGCTTGTGCT
CTATGCTGTATCATTTCAAATTTTGATAGGGATACTCGTATTACATGTGTCTCTGTGTGTGTGAGGATGC
CTGCCTTTGTCTGGGCCATAAAAGTGGTGAGGCTGACTTTCTGGATCCCTGCTGTGCCGCTGCTTTTGTT
CGCCTTTATTAGGGCTAAGATTGTTTTGAATTGAGGGCTACATTTGAACTAATTAAAGTGCTCTCGAAAC
TAATGTCCTATTATGCTTATACTCCTTCCAGGCACAATGAACGTCCAGATCCGGCTGATATGCAGCGACC
AGACCGAGAGCAGCAGTACCATCTCGAGCG
>dme_circ_0007696_junction_seq
ACCGAGAGCAGCAGTACCATCTCGAGCGAGCATGGGCTTTCCTCTGGTGTCACTGA
>dme_circ_0007697
AGCATGGGCTTTCCTCTGGTGTCACTGACGCAACGGAATCGCACCTTCGCCGAGCTGGCTTACTTGAAGG
GAGACCCCTGTCCCACAGACAACACCTCGGAGCTCTCAACGCACATCCTTTTCAACTGTAATATGCGTGC
CGGTCGGGGTAAGTACACTGTCAGAAAAACGCATTATATGCTGCAACGTTCGCCTTAAATTATACATAAA
TTAGTAGCTACTAAAGTCGTTTTCTTTTCTTTCAATTTGATATGTTTTGGAATTACTGATTCCAAGAGAT
ATATAACATAGTCGGTAGTTTGTTTCAAAATTTTATTTGTGTACTCCGTTACGGAAAATTGCACATTCAG
GACTTTTTGTAGAAGGAATCGCCATTTGGCAGGACTTTCTGTGGACATTAACTGACTGCGTGGCAGCATA
TATACGATTATTTCATGGCCAGTGGACCCATTTCTGTAGCAAATTGCTGCATGCACCTAATGGCTCCGCC
TCCTTTCTCTTCTTCCttttttttttttAGGGGACAGCCCGTGCTACGTTCGGTTGAGGACTGTGCCTAT
CGGTTCGAGTGGGAGACCAATGTCTTCTGTCCGCCGCACGAGTGCACCTTCAGTGCGGACACCTGCGATC
TGGTGCACGACGAGCTGGGTCGTCGTTTCAACTTCAAGAGTGCGCCGTTCACCAAGGATGGCAAGATTGA
GGATTGACTACAACGCCACGAAGATGTCGGTAAACATTTGCGGAGCGCATCGCAAGGCAATGACGGACTA
TTCGCAGGCGCTGGTCAACATATTCTTCACACACGAGTCGCCTAATTGCGGTCGGGAAGG
>dme_circ_0007697_junction_seq
ACGAGTCGCCTAATTGCGGTCGGGAAGGAGCATGGGCTTTCCTCTGGTGTCACTGA
>dme_circ_0007698
GCGCTTGTTACGAGCAGAATTCCACGACCATCAGCCTGGGCAACTCCAACTCGCAGCTGCGTTTCAATCA
GACCGGTTCGCTGTATTTGCTGTACGAGGATGGTGCCGAGTGCTCCACAGCAACGGGCATGCGGCGCTGG

TCGACAAAAATCGAGTTTGTCTGTGCGAACAATGCGACGAAGGACAATGGAGCCAGCACTGCTGGCGGTA
GCGATTCTTTGAAAATAATTGAGGACTCCAATTGTCAGCTGTTGATCCAATATCAGACGCCGCTTGCCTG
CCGGGAGCCGATTAGATGCAAGGCAACCACCTATGTGGACCACACAAACGATGGACTGGGCAGCAGCGGC
GACGAGCTGATCGACCTAACGCCATTGATTAGCGCCACCGACAACTACGAGGCAAGAGTGGAGCTGCCGG
CCAGCATGGAGCACCTGGTGCCCAAGACGACCAAGGTGAGTCGTTGTAGAACCTGATTTCCCACAGGCAA
AAATGACCAAACACCATGCCAATTCAGATCGTAATTTGCTGGCTTTTGTCTTTGCCACTCAACTCAAACC
CGGACCGACTCCTCCGTTCCAATTCCGTTTCACTCTCTTATTTCTTTGCAGTTCTTTTTGAATGTGTGCC
GTCCATTAGTGCCCAAATACCAATTAGGCTGTGCCGGCGGATCTGCTGCCTGCATGGCCAAAGTGACTGC
CGCCGGCGCCCCCGAGGAGGAGCGTG
>dme_circ_0007698_junction_seq
GCCGCCGGCGCCCCCGAGGAGGAGCGTGGCGCTTGTTACGAGCAGAATTCCACGAC
>dme_circ_0007699
GATTACACGCAAGTTTCACCCGCAAAGATGGCTCTGCGGCGGCATCTGTCGCAGGAGAAGCTGAGCCAAC
ATGTCACGCCCCAGGCCACACCGCCGCTTCCTGGTCACGGAGGAGCACCCACATCAGGGAAGACCATTGG
CGATTTGGTTAACGGGGAAATCGAGCGAACGCTTGAGATTTCGCATCAGAGCATCATTAATGCGGCT
>dme_circ_0007699_junction_seq
TTCGCATCAGAGCATCATTAATGCGGCTGATTACACGCAAGTTTCACCCGCAAAGA
>dme_circ_0007700
AAATTTTCCACCACGGAATGTAAACTCAAGGAGCCCATTTACGGCAGCACCTTCGACTTCAGTGGTCTCC
ACTCGGATTTGGCCCATGTGGTGAAGAGCATGAACATTGGAGGCGACCAATTCGAGTTCAATATATGCGG
AAATCTTTCAAGAACCTGCAATGGGGAAAGTAATGTGGCAGCTTGTCTCAAAAGACAGGGAAAGGAGTAT
ATTTTGGGGTCGTCAGCACGAGTTGTTCTACAACAATGGAAATATGTTTCTGAAGTACAAAAGCGGCGCA
AAATGCGACAATGGCACTGCCGACAAGCCCAACTACCAGCTGCATGTAATGTTCTCCTGCGACTATACTT
TGGATGCCCAGCCTATGCACGTAACGCCTTACGGCCAACGAAGTGTGTTCTTTTTACATCTCATATAGGA
CGCCTTTGGCATGCCTATCCATTCCCGAGGGTCTACAATCCAATAGTTGCAGAGTGGGCGACACAAAGTC
AAATGGAACCTTCGATCTGATGCCGCTGAGTGATAGTAACTATCATACGAGCAATCGTCAAGGCGCCTTC
TTTGTGATCAATGTCTGTAAGCCGGTGCTTTATGGAGAGAACAGCATGTGTCCGGCAGGCAGTAGCGTTT
GTCTATTTGATAGTAAAGCCACAAATCCAAAAGAACGGATTTATCAATTTCGGCAATGTACAAACGCATC
CGGTGGTGGAGAAAGGGCAGCTACTGCTCAGGCATGAGTCACCAACGCCATGTGCCAAGAACTCCAGTGC
AAACTACACCAGCGTGATTTACTTTAGCTGTGACAAGTTCATAAGGGTGCGTAGCTTAGGGTTTTTAGTT
TATGGTGATGCTAATCTCATATCTTTCTTTTAGAATGCCCATCCGGAGTTCGCGGGTCTGGGAGCCGATT
CCTGCACCTATCAGTTCAACTTTGCAACGCCTTTGGCCTGTAATGATCTCAAACCGTGCACGGCCTTTAC
CTCGACAAATGAGCTGTGAGTTGTCTATACACTTAAGTATAAGGAATCCGTGTTAAGTACTTGTTGTGCC
CTCTTAACAGGTTGGATCTGAGTTCGCTTAGCTCTAAGCCAGCTCGCACTTTGCTTAAGGATGGCAAGAA
CTATACAATTGCTGTGTGCGCACATGCTGGGGCGCCCTGCCAGGAAAATGGCGG
>dme_circ_0007700_junction_seq
CTGGGGCGCCCTGCCAGGAAAATGGCGGAAATTTTCCACCACGGAATGTAAACTCA
>dme_circ_0007701
GAGAAGTCACATCGCAAGAAGACAGCCAAAAAGGTCACCCCATCAGCCCGAAACGCCAAGGTATCGCCCT
TGAAGCGAGTCGCAGATAGTTCACCACCCAGCCAGAAGCGCGTTAAGACTGAGCCCAAGTCAACACCCAG
GGAGAGAGCTACGCCCAAGGCCAGTCCCAAGCCAAGGAGCACGCCGAAGGCCAGCCCCAAGCCACAGACA
CCAACAGCAGCGCGGCTCCGTACTCCCAATGCCAAGATCAATTTCAGCCCGTCTATTTCGCTGCGTGGAC
GTCCCGGTGGCAAAACCGTGATCAACGATGACCTCACTCCACAGCCGCGTTCCAAGAAGACCTATGAGTT
TAACTTCGAGCTCGACGTTAGCATGGATGCCAACGTAATTG

>dme_circ_0007701_junction_seq
GACGTTAGCATGGATGCCAACGTAATTGGAGAAGTCACATCGCAAGAAGACAGCCA
>dme_circ_0007702
GTGAAGGAGGGCACAGTTTTCACCGATTTAGCCAAAGGCCAGTGGCGCATTGGACCCTCGATCGGAGTTG
GAGGATTCGGTGAGATCTACGCGGCTTGCAAAGTGGGTGAGAAGAACTACGATGCTGTGGTCAAATGCGG
AGCCACATGGCAATGGTCCTCTGTTCGTGGAGATGCACTTCTACCTGCGCAACGCCAAGCTGGAGGACAT
CAAACAGTTCATGCAGAAGCACGGTCTCAAGTCCCTGGGCATGCCCTACATACTGGCTAATGGTTCCGTG
GAGGTCAATGG
>dme_circ_0007702_junction_seq
TGGCTAATGGTTCCGTGGAGGTCAATGGGTGAAGGAGGGCACAGTTTTCACCGATT
>dme_circ_0007703
GTCGATGTGCATGTGCGCGTTGTCCGGATTGGTGCAGCCGGGCTCGCCGCTGTCGCACCAGCAGCGCTGC
TGATACACAGAGCAGTCCGACGGCCAGGTCTCGTTGGTATTGGTGCAGACCAGGCGGCGAGTGTCCACCT
CCTCCGGGCACTCCGGAATGCACACGCATTTGGGCTTCTCGTCGTGCATCTGGCAGATGCGACCGGCTCC
GCAGCTCATCGTTTCGCACAGATCCACCTCGACAACGGGATTGA
>dme_circ_0007703_junction_seq
CACAGATCCACCTCGACAACGGGATTGAGTCGATGTGCATGTGCGCGTTGTCCGGA
>dme_circ_0007704
GATCAATTCACTGTTGGGCGACGAAAGGCCTTTgcagctgtcgcaccaccaacagcagcagcagcaaatg
ctgcatcaccatcaatcgcagcaacagcagcatctgcagctgacccaacagcaactgcGTGTAGCTCATC
TTGGACATGGTCTTA
>dme_circ_0007704_junction_seq
cGTGTAGCTCATCTTGGACATGGTCTTAGATCAATTCACTGTTGGGCGACGAAAGG
>dme_circ_0007705
GTGCCAAAAACGTCTGACACACTTTAGTGACTTGGACGGACGATCGTTTCACTAATTGCCGACACACATC
ATTTGCCTAATTGGCGGTGGAAAAGAGTAACAAGTGCGAGACGAGAGCAAACGTCGAGCGCAAATTGAAT
TTACGGTGCCCCATCAAAGTCAAGTCGGCCGACTCCAACTAATTTAATGATCAAAAGTAACACATTTTTT
TCGCATTCGGCGGCAGCCGCTGAGCGGTGTTAATTGAAAGTGTCACGCTTCGTCACAATAATAAATATAA
TTCCCATCGACTACTGGCTTTCGTCTATGTGAAGTGAGTGCCCGAGTGAAATGCGGTCGGTGGAAGTGGC
CCAAAGCGGCCCATCTGCCATCTTCTGGCTCGTCCAAGTGCTAATCGTGGGGGCGGCACAGGCTGCCACG
CCCTTCGCGAAAGAGTACCGCAACGCGGGACTCTACAATGGCAACACACTCGTCGATCTGGACGGTTCCA
CCACCCTGGACATGGGCCAGGAGAAGCCCACCAGCAGCAGCAGCGGTCTCAGTGGAGGCTTCAAGTCGGA
GTCGTCGGTGGTGCTGCCCGGCATGCTGCAAAATTCAG
>dme_circ_0007705_junction_seq
GTGCTGCCCGGCATGCTGCAAAATTCAGGTGCCAAAAACGTCTGACACACTTTAGT
>dme_circ_0007706
GCTAACTTATTGTAAGGCCCAGCTCTCCAATTGCCTTGTTTTATTTGATAATTACTATCCAACAGCTCCC
CTTTCACCAGTGTGTTTTTATTTCATTAATAATTCTAATTAGTCTAGGGACGTTTTTCGTAATACTTTAT
AACGTTGAACCGGAAAAACGCAACTGCGCTGATGAACAATGTTACTGATTTCTTTGTACCGATTACCAAT
TACTATGATTAT
>dme_circ_0007706_junction_seq
TGTACCGATTACCAATTACTATGATTATGCTAACTTATTGTAAGGCCCAGCTCTCC
>dme_circ_0007707
GGCCAAAGAAGACAACATCGCTATTAGGAGAAGACCAAGCCCAAAAAGTGAGGACAATACCCATGAAAAT

TCTGGTGAAATCTCTTGACTGGAGTCGCAGCTGAAGTTGCAGCTCGCAGATTCAACTGGCAGATAGATTG
CTCGCGGAGCACATTTCCGCGCACTCGGTGGGACAGTGGGACTAGACTGTTTATATACACTGCATCCATC
CAGCCGAGATCCGATCCACACAATGCCCGGCCACTTGCACGCCATAGATCTTTGAGGCCAGGCCTGCAAT
ATTTGGAGAGCATTGCTGTTGGTGCCGCAATCTGTGGATAAATTGTAATAAATAGCCAGACAAACGAATA
CTTGGCACAAAAATAAATAAGAATCGAAGAACGCGGAACGAAAAGCACTGCGCACGCATTGCAACACAGA
TTTCTGGATTCGTAGCCATTCGAGGTGTGTGTTGAGTGCATTGGCCAGATCGCTTGGGTCATAATCCCAA
CCCCATCCATATCACCTTCTGGACTTCTGACTTCACATAGACGTAGGTCGCGGCCAAAAAGTGTTCCAGG
CCAGAGGCAATCGCAACCGGAGCGAGGTGTACACCTTTGGCACCATGGAGGGCGGTTACGTCAACGAAG
>dme_circ_0007707_junction_seq
ACCATGGAGGGCGGTTACGTCAACGAAGGGCCAAAGAAGACAACATCGCTATTAGG
>dme_circ_0007708
TATTCGTTTGTCTGGCTATTTATTACAATTTATCCACAGATTGCGGCACCAACAGCAATGCTCTCCAAAT
ATTGCAGGCCTGGCCTCAAAGATCTATGGCGTGCAAGTGGCCGGGCATTGTGTGGATCGGATCTCGGCTG
GATGGATGCAGTGTATATAAACAGTCTAGTCCCACTGTCCCACCGAGTGCGCGGAAATGTGCTCCGCGAG
CAATCTATCTGCCAGTTGAATCTGCGAGCTGCAACTTCAGCTGCGACTCCAGTCAAGAGATTTCACCAGA
ATTTTCATGGG
>dme_circ_0007708_junction_seq
CAAGAGATTTCACCAGAATTTTCATGGGTATTCGTTTGTCTGGCTATTTATTACAA
>dme_circ_0007709
GATTTCTGGATTCGTAGCCATTCGAGGTGTGTGTTGAGTGCATTGGCCAGATCGCTTGGGTCATAATCCC
AACCCCATCCATATCACCTTCTGGACTTCTGACTTCACATAGACGTAGGTCGCGGCCAAAAAGTGTTCCA
GGCCAGAGGCAATCGCAACCGGAGCGAGGTGTACACCTTTGGCACCATGGAGGGCG
>dme_circ_0007709_junction_seq
GTGTACACCTTTGGCACCATGGAGGGCGGATTTCTGGATTCGTAGCCATTCGAGGT
>dme_circ_0007710
GAAACGACATTTCAACAAGAAATTTAGGCATTAGAAGACTCGAATTACACATGTGCGAAATTCATTTACA
GGACAACTAAAGTACTAAGCGCAGATCGGACCGACCAACGCGAATTAAACAAATACAGTAAACAGTTATA
ACAAACAAACACTTACAACTAATAGAAGAATTAAATGTTTTCAAGATTGACAGATAAAGATATAGT
>dme_circ_0007710_junction_seq
TTTCAAGATTGACAGATAAAGATATAGTGAAACGACATTTCAACAAGAAATTTAGG
>dme_circ_0007711
GAATTAAATGTTTTCAAGATTGACAGATAAAGATATAGTGTAACAAATAGAATATGTCGATCGGAGTTGG
ATAAGAAAAATCAAAGGACTACACCTAGGAGCCCAACCAGTCGATGAACAATTGACGAAGCTCGTTATTC
TCGATACCTTACAACACCATACAAACACACATTTAAATTTGACAATTTTGAACTGAGTCTAGCTCCCTGC
GATCCACTTTAAAATCCATGTGGAAATCCCTCTAGAAatatatagatatatatacttatatatGTACAAC
CGAGGATCGCAGGAAGACAAACTCGTCGAGCGCCGAGGTTTGGTTCTATAGTTCTAGATGTACATTCGTT
TACAGATACATTTTGTAAATTTGTTTCGTTTTTAAAACCGCTGCACACGTTTCCTTCATTCAGACTTTAA
GTTCGTTTCATTTGTAATGTTGTTCAACAATAAAACTATGCACTACATACAGCAGGGGCAGCAACGGACT
GGCTGCCCCACCATCGTCATCAGCGTGTTAATGTTATTGTTTAGTAATTATAACAATATGCCAATTGCAT
ACATACAACAAGGCCTGAGGGTAACGTTAGATTAAAGAAGTCGGCTCAAGCTCACACCTCTAATGATGTT
GGACACTTTTGAAACCAATAAGAGGCACAGTTAAGCTGTTGATTA
>dme_circ_0007711_junction_seq
ATAAGAGGCACAGTTAAGCTGTTGATTAGAATTAAATGTTTTCAAGATTGACAGAT
>dme_circ_0007712

AAACCCTCAACAAAATCCAATCTTCCCTCTGTTTAAACTAATCAACAGCTTAACTGTGCCTCTTATTGGT
TTCAAAAGTGTCCAACATCATTAGAGGTGTGAGCTTGAGCCGACTTCTTTAATCTAACGTTACCCTCAGG
CCTTGTTGTATGTATGCAATTGGCATATTGTTATAATTACTAAACAATAACATTAACACGCTGATGACGA
TGGTGGGGCAGCCAGTCCGTTGCTGCCCCTGCTGTATGTAGTGCATAGTTTTATTGTTGAACAACATTAC
AAATGAAACGAACTTAAAG
>dme_circ_0007712_junction_seq
CAACATTACAAATGAAACGAACTTAAAGAAACCCTCAACAAAATCCAATCTTCCCT
>dme_circ_0007713
TTTAACAGCATAACCCAGCAAACAGCAACAACCTGCAGCTCCTCGGCGGGCTCATCTTCCGGGTACAGTA
GTGGCGGCTCTGCGGGAATTGTTTCGCGATCAGTGCCCTCAACTCCACTGCCACATCAACAGCAAAATAG
CCTCACATCGTTGAATGGAGGCGGTGGTGCAGCGTCCAGCGGAGGAGG
>dme_circ_0007713_junction_seq
GCGGTGGTGCAGCGTCCAGCGGAGGAGGTTTAACAGCATAACCCAGCAAACAGCAA
>dme_circ_0007714
TCCCGGGTTGACTCGGATGAAGCAAAGACCCAACTTGTTGAGCAAGCAGCAATCACTTGACTGCGACAAC
CGGGATCCCATGATAGGGGCTCATCGCAAGGGTCGAGGATATGTGTACAGCTACCCTACAAGCACGTCTG
CCTCCGCACCACCGAG
>dme_circ_0007714_junction_seq
CAAGCACGTCTGCCTCCGCACCACCGAGTCCCGGGTTGACTCGGATGAAGCAAAGA
>dme_circ_0007715
TTTGAATCGTCTTATTCCATTCAATAATGAGCTCCTCGCGGTGCGTCAAAGATTGCAGGACATTTAAAAA
CTTTTCCCACAGCAGCACGTTGACAGGCACTTTGGTGTGCGCTCGTATCCATGTGACAATGAGAGTTTGG
AATATAGCTGACCCTAGGATCCCGCCCAAAGTGGCACTCTTGTTGGTGCCACTTGGCAATGTATGCTGAT
TTTGGTG
>dme_circ_0007715_junction_seq
CACTTGGCAATGTATGCTGATTTTGGTGTTTGAATCGTCTTATTCCATTCAATAAT
>dme_circ_0007716
GGTGCTACAAGTGCTGCTCCAGGCGTTGCAGTTTAAGCATCACACCATTGCCATTGTAGCGGTGGATTCC
TTAAAGTTGTGTGCCGAGCGTGGACGCCAACTGGCGGCTATTGAAAGAGTTCCCCAGCTAATCATCACTG
CCATCTGCAAAGCTTTGGAAATTCAAAGCGTAACCAAGCCTAAGGATTCCGACAAAGTGGTGCTGACTTC
TCTGATGTTGTGCCTGGGCGAATTCTGTATGGCTATTCCGGCTCCTATTATGCTGACGCCCTTCAACGAA
CAAGGTGATACGCTAGTGCTCCAG
>dme_circ_0007716_junction_seq
CGAACAAGGTGATACGCTAGTGCTCCAGGGTGCTACAAGTGCTGCTCCAGGCGTTG
>dme_circ_0007717
GAATCGGAGGAAAGGGACACGTTTTATTGAAGCGTCGCTGCGGCAATGTTGGGCTCTCGGGCGGCGTGT
CACCGCCAGCTCCTGCGCCGCCTCCCACGGCGATATGCTGCAGCGAATGCTTTTTGGTCACGCTCTTGAA
CGGAGCCCCCAGTTTTCGAGGGCCACGCGGCGAAGCGGAGGACATTCCGGTGGCTGCCTCCATGGCCGCG
GTGGATCCAGCTGAAGCCGATGGCGCAATCCCCATTATGGGCCCCGAATCAATCGAGGCTTG
>dme_circ_0007717_junction_seq
TTATGGGCCCCGAATCAATCGAGGCTTGGAATCGGAGGAAAGGGACACGTTTTATT
>dme_circ_0007718
GCAGCACCGGCAGTGGGACCAAATCCGTTGACCACATTGATCACACCGGCTGGGAAGCCTGCCTCCTTCG
CCAAAGCAGCCATGTGAAGGGCAGTCAGAGGAGTTTGCTCCGCGGGTTTCATGATGATGGTGCATCCGAC

GGCCAGAGCGGGGCCCCACTTCCAGGCCAGCATCAGCAGGGGATA
>dme_circ_0007718_junction_seq
ACTTCCAGGCCAGCATCAGCAGGGGATAGCAGCACCGGCAGTGGGACCAAATCCGT
>dme_circ_0007719
GTAATCTCCAGCGACCATGTAGGCATTGGTGGTGGCTCCCATGCGGACGGAGCTGTAGGAGCTACCGCAC
TCCTTGGCCACAGCCTCCTCGTAGTCGCCGCAGCGCATGGTGGGGAAGTTGTTCTCGGTCACGGACTCGG
CGTAGTAGATCACTGAGCGGCTGTGGGCGCAGGATCCGGTGAGATCAACACCACATCCGGGCTGGCTCTT
TCCTCCGTTGGGGTAGAAGGCTCCCTTGCCGATGGGCTTCAGGAATCCCAGGGTTCCTCCGTTGGTCTGG
ATGGACTCCAC
>dme_circ_0007719_junction_seq
TTCCTCCGTTGGTCTGGATGGACTCCACGTAATCTCCAGCGACCATGTAGGCATTG
>dme_circ_0007720
GCTGAAAAGGGGCAGGGCGGGGTCCAGACCAATGATGGTGTGCAGCTGGCCGTTCTTCACATTCTTGCCA
GCATAGCCCGAGACATGGGCGCCCAGGCTGTGACCAATCACCATGGTGTTGTCCAGGTTCAGGCCGTGAT
TGCTGCGCATAAAGTTGATCAGGGTAGCCACCTGCTCGCCGACTCCGGGAACAGCCAGAACGGAGGAGGC
GTAGTCCACGGAACGAGCACGTCCCCAGTCGACGGCAATCAT
>dme_circ_0007720_junction_seq
GAGCACGTCCCCAGTCGACGGCAATCATGCTGAAAAGGGGCAGGGCGGGGTCCAGA
>dme_circ_0007721
GTTGCCAAGATGATTAACTTCCTGAAGGACAACCACGGTCTGAATCTTAATGACGTATATGTGATTGGTC
ACAGCTTGGGCGCCCATGTGGCTGGATATGCCGGCAAGAACACCGACGGCCAGGTGCACACCATCATTGG
TCTGGACCCCGCCCTGCCCCTCTTCAGCTACAACAAGCCCAACAAGCGTCTGAATTCCGACGATGCCTGG
TATGTGGAGTCTATCCAGACCAACGGTGGCACTCTGGGATTCCTGAAGCCCATCGGCAAGGGAGCTTTCT
ATCCCAACGGAGGAAAGACCCAGCCCGGCTGCCCTCTGGATGTGACTGGAGCCTGCTCCCACGGACGCTC
AACAACCTACTACGCCGAGGCCGTCTCCGAGGACAACTTCGGAACCATGAAGTGCGGAGACTACGAGGAA
GCCGTTGCCAAGGAGTGCGGAAGCACCTACTCCAGCGTTCGCATGGGAGCTGATACCAACGCCTACATGG
TCGAGGGTGACTTCTATGTGCCCGTGAACAGCAAGGCTCCCTTCGGCATGATTAACTAATTGCTGTGCAA
TAAAAACGATGCTTACCTCGGCTATTCCGATCAAGGAATCTGAGCGCGTCCATGGGGAGAACGGCTGGTA
CGTTCCCCAGGAGGATGGCACTTCCGAGTGGGTGGACATGGACGTTGCCGAACAGTGGATGGAAGCCCAA
GAGTTGCTGGAAAGTCGTGGCTTGACCACCGTTCCCGTAAAGTTCTACCTTTACACCTCCTCCAACCCCA
CCAAGGGCAAGAAGATCACCGCCTCCACCAAGTCCATTGATGCCTCCAGCTTTAACTCCGCCCATCCAAC
TCGCTTTGTGATCCACGGCTGGACCCAGAGCTACACCGCCAGCATGAACAAGGACATCCGCAGTGCCTGG
CTCTCCAGAGGAGACTACAACGTGATCGTCGTCGACTGGGCCCGTGCTCGTTCCGTGGATTACGCGACTT
CTGTTTTGGCTGTCGCTGCCACCGGAAAGAAGG
>dme_circ_0007721_junction_seq
TTGGCTGTCGCTGCCACCGGAAAGAAGGGTTGCCAAGATGATTAACTTCCTGAAGG
>dme_circ_0007722
AGGACGGACACGGAGGTGCAATCCGAACAGCCGATACCCCCCCGACATCCATCGGATAGTAAGTACTAAG
TATTTTAGCCTATATATGCTCAGTTACATTCGATACCCAAACATGCCTTACAACTAACTACTAAATCGAT
CCCATACTCACTGGCCAACTGCCTTTGGCGCTTGCCTAATTTTAGCCTTTGTCTTCCCCGATTCCCCCAT
AGCCAAGTATCGGCCACCACAATCCCCAGCTCGTCCTTTGAGGAATGACACTAAAGAGCACAATCCCTGC
GCCAAGGATGAAAGCCAACACCTTAGAAACTTCTGCACGAATGTGGACGACTATCCGGACCTTTCAGGCC
TCACACATAAGCTAAAAAACAACTTTGCCAAGTTCTTTAGCAACGATCTTCAGCCCACGGATGTGAGCTC
TCGCGTTGGTGGCTCAGACGAGCGATTCCTTTGCAGGAGCATCAGGAAGCTGGTGTACCCAAAAAAGGGC

TTGAGGGCGGACGACACCTGGCAGTTAATTGTCAATAACGATGAGTACAAACAGGCCATCCAGATCGAAG
AGTGCGAGAGGAGCGGATCAACCCTGTGACTTCGCCGCCAACTTTCCGCAGAGCTATAATCCAATCTGCA
AGCAGCACTACACACAGCAGACCCTGGCCAGCATCAAAAGTGATGGCGAACTGGACGTGGTGCAGAATTC
CTTCAAGATCCCTTCCTGCTGCAAGTGCGCGTTGAAGACTGGGTGATTCTGGAAAATGGGATTCTCCAGT
CGATGGCTGCGACAATGTGATATTGAGG
>dme_circ_0007722_junction_seq
CGATGGCTGCGACAATGTGATATTGAGGAGGACGGACACGGAGGTGCAATCCGAAC
>dme_circ_0007723
CGCCAGCTTTAAGTTCGGCTCGCATTTTATAAATATCACATTATGTTTCTGTTCTTCGTTTTGTTTCGG
GACGTAGGGAAGCACCTTTTAGAGTGGACAAGAGTTTTTTATCCGTAAGCTTTAACTTTGCAAGATCCTT
TGTCACTAGCCAAGGCCTTACTAAATGGCAGTTAGAGGTG
>dme_circ_0007723_junction_seq
AGGCCTTACTAAATGGCAGTTAGAGGTGCGCCAGCTTTAAGTTCGGCTCGCATTTT
>dme_circ_0007724
TTTGGGCAATGTGCGCGTCCTATTGAGGGATCAGTGTTTGAAGAACAATGTGCCGAACAGCAAGACACTG
CATCTGGACTTGAACGATTCCACCACAGAGCAGCTATACGAGAGTCTGAAGATCTTCGACCGGCTGTTCG
CCGAGTTCGAG
>dme_circ_0007724_junction_seq
CTTCGACCGGCTGTTCGCCGAGTTCGAGTTTGGGCAATGTGCGCGTCCTATTGAGG
>dme_circ_0007725
GACAACAAGAAAgagcagcggaagaggagaagacgaggagcaggagcaggaggggaggaggcagagcaAC
AACTGTGGAGCTGCAGCTGGAAAGAAACAACCTCCTGCGATGGACACGTTTAAACGCTGGCTAAACAAAC
CCAAGG
>dme_circ_0007725_junction_seq
TTTAAACGCTGGCTAAACAAACCCAAGGGACAACAAGAAAgagcagcggaagagga
>dme_circ_0007726
GAATGGAATTGCGAAGCCAATCGGGTGTTTAAGTTACAGCAAACCATAAACATAGCATAGATTAAATACA
GATAGGATAGTGATAGGATAGAATAGAATACTGCGTAATTGCTTTTTCGAAGGCGTAGTCACCGTCACAG
TCGTCATGTCAATGTCGTCATTTAGTATCGTACGTTTCGG
>dme_circ_0007726_junction_seq
TGTCGTCATTTAGTATCGTACGTTTCGGGAATGGAATTGCGAAGCCAATCGGGTGT
>dme_circ_0007727
GCTAACTGGTGAGaacaacccaaacaagcaaacaaccaaacaactaaacagccaaccatccaacCGATTC
CACATGTAAAACAACTTAACTCGCTGCCGTTAACCACCACCAACTATCTAAGTTAGAACATTATGCTTTC
TATCGATTAGTTCACTTATATCTGTAGATATATTCGAGCGACGATTATGTTTGTT
>dme_circ_0007727_junction_seq
ATATATTCGAGCGACGATTATGTTTGTTGCTAACTGGTGAGaacaacccaaacaag
>dme_circ_0007728
GCTAACTGGTGAGaacaacccaaacaagcaaacaaccaaacaactaaacagccaaccatccaacCGATTC
CACATGTAAAACAACTTAACTCGCTGCCGTTAACCACCACCAACTATCTAAGTTAGAACATTATGCTTTC
TATCGATTAGTTCACTTATATCTGTAGATATATTCGAGCGACGATTATGTTTGTTGTTTAGCCATATCTG
ATATTTATTAACGCAATTAAGCAGCTTTGTTATAATTTATT
>dme_circ_0007728_junction_seq
CAATTAAGCAGCTTTGTTATAATTTATTGCTAACTGGTGAGaacaacccaaacaag

>dme_circ_0007729
atgataagatgataagatgCGTCCGCATCCGCAGCGTTGCCTTATACACATCTTTTAGTCAACACCAACC
ATTTCGAATGCTTTCCAACCGAGCGCATTGTGAGTGCGCCTAGCGCACACGATATAATACTGAAATTCCC
GTGCCTGTCGAGGCTTACCTTATGATATGTATACCGTG
>dme_circ_0007729_junction_seq
AGGCTTACCTTATGATATGTATACCGTGatgataagatgataagatgCGTCCGCAT
>dme_circ_0007730
CCCGGGACACGGCGGAAATTGAGCGCAGTAATCTGGTGAATATCTGCAAGCTGGTGGTCAAGGAGCTGTT
GGAGCAGTCGCTGCGTTATGGTCGCATGCTGGACTCGGATCACCTGCCGTTGCAGCATTTCTTCATCGTC
ATCGAGCATGTCCTGGGGCATGGATTGCGCCCGAAGAAGGGTCTGCTGGGACCGAGGAAGGAATTGTGGG
ATCTGCTGCAGAGCGTCGAGCATTACTGCCCGGAGGCGCAGGACATTACGGCGAGTGTAAGGGATCTGCC
CACCGTTCGTACCCACATTGGCCGCGCTCGAGCCTGGCTGAGGATCGCTTTGATGCAAAAGAAGCTATCG
GATTACCTGCAGGCACTCATCGAGCACCGGGAGGATTCACTCTTCGACTACTATGAGCCACATGCTCTGA
TGATGAGCGATGAGGATCGTTGTGATAATGGGCATTTTGGTGGGCTTAAACGTGATCGATTGCAATCTGT
GCGTCAAGGAGGAGGATCTGGACTCACAGCAGGGCGTCATCGACTTCTCGTTGTACCTGCGTTCAAGCTC
GAGAAACGCCGATGCGGCTCCAGAAGACAGTGCTCCGCCTGCTCTGCTGGATGCCACTGGGCAGGGCAAC
ATGATTGCGGTGCTGGACCAGAAGAACTACATTGAGGAGCTCAACAGGCATCTAAAGCGCCACTGTGGGC
AATCTGCAGGCCAAAGTAGAGTCCTTAACGACCACAAATGCCCTGATGAAAGAGGATTTGGCAATCGCTC
GGAATAGCTTGTTGGCCTTGCAGGCCGAGAACCAGGCCATGCGACAGTCCACGTCGGCAGGGGACAACAA
TTCAACTGGTTCGGGTGGTTCATCCGACAAGGACAAGGAGAAGGCGTCCGAAGAGCTGGTTGAGGAGCGT
CGCAAGAACAGCGAGCTGGAGAAGGAGCTCAAATTGCAGGTGTCCCTCAAGGCGGAATCGGACATGGCCA
TGAAACTGCTGGAGAAGGATATTCACGAAAAGCAGGACACGATAGTCTCACTGCGCCGCCAGCTGGACGA
TATCAAGCAGATCAACCTGGAGATGTACCGAAAGCTGCAGG
>dme_circ_0007730_junction_seq
AACCTGGAGATGTACCGAAAGCTGCAGGCCCGGGACACGGCGGAAATTGAGCGCAG
>dme_circ_0007731
ACAAGCGCTTTGGCACCCTGATTATTTCTGTGGGTGTTGCAATGATATCAACTGGCGTATCGTACTTCTG
GAATAGATTCGGGGGCAGTGAGTCCACCACCTGCATGTCGTGCACGATTGTGACAATAATTGTCTTCGGT
GTAATGGCTCCCAGTTCAATAAGGAGGCCGATGTCCAGATCAGCGAAACCATTTCCGCGTCCAATGCGGT
AACCCTCGCGTGACACTACAACAGAGCCAATAACCACGATGTCCAGCTTCAGACCGCTG
>dme_circ_0007731_junction_seq
AACCACGATGTCCAGCTTCAGACCGCTGACAAGCGCTTTGGCACCCTGATTATTTC
>dme_circ_0007732
GGTGTTAAGGCAGCCGGCGCAGCAGAACTGCCGCACATCGAAACCGAATCGCACGCAGTACTTGCCAAGC
GCTATGGAGCTCACCTCCTGCTTGCAGGTCTCGCAGGTGGAGCCAATGGTGCGCTGATAGCTGCCCAGGC
ACACCTCATTGCAGAAGTCCATGGTCTCCCACATGAGCTGCTTCTCGTCC
>dme_circ_0007732_junction_seq
GGTCTCCCACATGAGCTGCTTCTCGTCCGGTGTTAAGGCAGCCGGCGCAGCAGAAC
>dme_circ_0007733
GCTCGGCAAAAGCCTGCTGCTCCCAGTGGGTTCGAGTACATCTGCGATACCGCCTGTGTCGACGCCCTGC
TAGAAGATCAACCCGGCAAGTACTTTGTACGCCGCAAGAAGTTCCTGGTCGAGGAAG
>dme_circ_0007733_junction_seq
CGCCGCAAGAAGTTCCTGGTCGAGGAAGGCTCGGCAAAAGCCTGCTGCTCCCAGTG
>dme_circ_0007734

gtggatgcagaaggtgaagctgagcaggaagcagaagACGACGCAGCTCTGGATCGAGAGGCGACAGATG
ATGTAGATATGCAGTCCGTGGGCGCAGATTCACACCAGGCGGAGGATGCAGAGCCACCGGAGGGCCGAGA
TGAGGTGGACACAAGCTTAGAGGAGCAGGAAAATGGCCAAGATCCTGATGAACGGGAGAAAGATGCGGCC
GCCGATGAAGATGATCCGGATGCAGAAGGAGATGCGGAATCGGAGCAGG
>dme_circ_0007734_junction_seq
GCAGAAGGAGATGCGGAATCGGAGCAGGgtggatgcagaaggtgaagctgagcagg
>dme_circ_0007735
gtggatgcagaaggtgaagctgagcaggaagcagaagACGACGCAGCTCTGGATCGAGAGGCGACAGATG
ATGTAGATATGCAGTCCGTGGGCGCAGATTCACACCAGGCGGAGGATGCAGAGCCACCGGAGGGCCGAGA
TGAGG
>dme_circ_0007735_junction_seq
GCAGAGCCACCGGAGGGCCGAGATGAGGgtggatgcagaaggtgaagctgagcagg
>dme_circ_0007736
GCGACTACGACGAGAACATGGCCAATGACAGCGACCTGGAAATGTCCAATCTAGCCATGTCCGAGGGCTC
CAACAATGCCGAAATCATTAGCGGAGGGCACTAGATGGCTATCTCCTAACTTCCTAACACATCGCCAAGT
ATTACCCCAAAAAGACAAACACCATTG
>dme_circ_0007736_junction_seq
TATTACCCCAAAAAGACAAACACCATTGGCGACTACGACGAGAACATGGCCAATGA
>dme_circ_0007737
AAAGCGAAAGATCAACAGCTGCGCCTCCGTCTCCATCTGGCTGCAGTTGGTCAACAGCTCGGTGCTGATC
TTAAAGAAGTGCAGCTTGGAGGTAGCATATATCTCCCTGGCGAACATGCTGAAGATCAGAATCAGACGTA
ATTGATCGCTCGGCGACCTTTCCTCCTCGGTGGCCAGGAACTCCACAATCTGAGGATTTGCTTGCAGCAG
CTCAAACAACTCTTCCCGCTGTTGGATGCGCAGGAACCACTGCGAATGAAAG
>dme_circ_0007737_junction_seq
GATGCGCAGGAACCACTGCGAATGAAAGAAAGCGAAAGATCAACAGCTGCGCCTCC
>dme_circ_0007738
GTGCCTGAAAAGGCTTACCGGTAAATTAGATCCCTTCTTCGAAGATCCTTTGCAGACCGCCAAGAGCGGA
GGACATCTCTTTGGCTATCTTTGCGCCTTGGACGAGGTGGTCAAGGCTGGCTTGGCAGTGGAGACGCCTC
TGGAGGATTTGTTGCCATTGCTAGAGAGAATTCTCAGCGGCATTCTCAAGTTCCTCAATGTGGCCAACGC
CCGGCGACAGGAAGAGGCAGCCACAGCAGCCAGTTTCCAGGACATGGACGAGAGTCTCCAGCTGCTGGTC
AGCGAAAGCTCCTTCAAGTGTGATGGGGAGGAAGAGGCTTGTCGAAAGTACTTACTGATGAGCTTCTGGT
TAACGTTGAAGGGGCTGTTGCGATCTGGCCACCAGCATTGGGTGTTACCAATTGCAAACGTCTGCCAAAA
CAGTTAACACCGAGACTCTGCGTCGTTGCCTCAATATTAATGTTTCCGTTTTGACGCTTTGCCGGCACAA
AGGAGCCATAGAGGCGGCCGGCTTGAGCATTGGCAGACTTACCCGTGCCATCACCAGTAGCTTAGACAGC
TGCGATTCGGGATTCCAAATGCTTCACGACTGCCTAGAGCGAGAGCTGCTGACTGAAAGCCGTCAAGTGA
GCACCACACGACGTGGTGCCGGCTTCGCCATAATGTTCCTTCAC
>dme_circ_0007738_junction_seq
TGCCGGCTTCGCCATAATGTTCCTTCACGTGCCTGAAAAGGCTTACCGGTAAATTA
>dme_circ_0007739
AAGTTCTCCTCCTCCAGATTCGCGGCCAACTTGGCGCTGGCCAGCAGCGCCGCCGGCAGATCGCAGCGGA
ATTCGTGCGACCGCACAAAGCAGGTGGCCGCCAGTTGGCGGACCTGATCACATTTGTGCCGCAACAAATG
CCAACTGAGTCGGCGGAAGCGAATGAGCAGGGGATTTGGCTCTTTCCCGCCCGACG
>dme_circ_0007739_junction_seq
AGGGGATTTGGCTCTTTCCCGCCCGACGAAGTTCTCCTCCTCCAGATTCGCGGCCA

>dme_circ_0007740
GGGTTAGCAAAGCTGGCAAAGACGAGGTCTTCGATGGTGAAGTCCGACGAGGAGTCCGTCCAGTAGACGT
CGCCCTCTTTGCTCACGGTCACGCCGTTGAAAATCTTGGCAGGGCGGTTGATGGACTTGCCGGCAAGCTC
CTGGGCGGGCGAGACCAGCAGAGTCTTCTTATTGGTGCCCAGGTCCACCTGCCACAGGCCGTAGTAGGCG
TCGGCGATGATGAGGTTGTTGCCCTGCG
>dme_circ_0007740_junction_seq
TCGGCGATGATGAGGTTGTTGCCCTGCGGGGTTAGCAAAGCTGGCAAAGACGAGGT
>dme_circ_0007741
GAGCGCGGATAGCGAGCATCCCAACGGCTTCACCCTGTTCACTCGCTTCCCCAGCGTGCGCCTGTTCCTG
GCCCGCATGCTGGCTTTGTTCGAGCTGCCCTTCCGCTACTTGAACAGCGTATATCCGAACAA
>dme_circ_0007741_junction_seq
GCTACTTGAACAGCGTATATCCGAACAAGAGCGCGGATAGCGAGCATCCCAACGGC
>dme_circ_0007742
GCGTTGCAGTCTCAGCTGACTCTGCTTTCTCACCGCCCTCCCCTGCGGCGGCAGCTGGGGCTGTCTCGCC
ACCAGTGGTAATGGTGAGCGAGAGATCGTTGAGAGACTTCAACACCTTGTCCATGTCGTCCTGGG
>dme_circ_0007742_junction_seq
TTCAACACCTTGTCCATGTCGTCCTGGGGCGTTGCAGTCTCAGCTGACTCTGCTTT
>dme_circ_0007743
GGGCGGTGAGAAAGCAGAGTCAGCTGAGACTGCAACGCCTGTGCAGACCAAGACTATTCTCGTCAACGTA
GAACTGATCCAATTTGGCGAGAAGAAAAGTGCTAGCTCCACTGGTGCCAATGCCGGTGCAGGCGAGGATG
GAAAAGCAGACGCGCAAAACGGAGGGGAGGCCGTAACTGTGGCAGGAGGCGAC
>dme_circ_0007743_junction_seq
GGAGGCCGTAACTGTGGCAGGAGGCGACGGGCGGTGAGAAAGCAGAGTCAGCTGAG
>dme_circ_0007744
GGGCGGTGAGAAAGCAGAGTCAGCTGAGACTGCAACGCCTGTGCAGACCAAGACTATTCTCGTCAACGTA
GAACTGATCCAATTTGGCGAGAAGAAAAGTGCTAGCTCCACTGGTGCCAATGCCGGTGCAGGCGAGGATG
GAAAAGCAGACGCGCAAAACGGAGGGGAGGCCGTAACTGTGGCAGGAGGCGACGTGGGTG
>dme_circ_0007744_junction_seq
GTAACTGTGGCAGGAGGCGACGTGGGTGGGGCGGTGAGAAAGCAGAGTCAGCTGAG
>dme_circ_0007745
GCAGACGCGCAAAACGGAGGGGAGGCCGTAACTGTGGCAGGAGGCGACGTGGGTGGTGCACGCATCGAAG
CTGTCGACACCACCACAGTATAGTATGACTTTGCGCTTGGCGCAGCCAATGAACGGTCGCGGCGGACTTC
CGCCGACAGCGCAATGTTTGGAGTTCAAcgaaaaaaa
>dme_circ_0007745_junction_seq
CGCAATGTTTGGAGTTCAAcgaaaaaaaGCAGACGCGCAAAACGGAGGGGAGGCCG
>dme_circ_0007746
TGTAATTGCCAGCGTGTATTTAGTGAGTGTGTATTTTGAGTGTACtttgtgtttgttcggagttcgcttt
ctatttgttttttgtttggtatttcccgattttttctaaacaaaatgttactttttttcgTTGAACTCCA
AACATTGCGCTGTCGGCGGAAGTCCGCCGCGACCG
>dme_circ_0007746_junction_seq
CGCTGTCGGCGGAAGTCCGCCGCGACCGTGTAATTGCCAGCGTGTATTTAGTGAGT
>dme_circ_0007747
AAAGAGTTGCGTTTGATTTTCTTGTTGGTTAGAATAATGACGGAGAAGAAGAAACTACAAGCTGCAAAAC
ATGATATTCAGTGTATGCACAAGTGTAATTGCCAGCGTGTATTTAGTGAGTGTGTATTTTGAGTGTACtt

tgtgtttgttcggagttcgctttctatttgtttttttgtttgg
>dme_circ_0007747_junction_seq
gttcgctttctatttgtttttttgtttggAAAGAGTTGCGTTTGATTTTCTTGTTGG
>dme_circ_0007748
AAATGGTGTTCCACTTTGCATTTGTGGAACGAGTATTTCTGCAATCGTTTTAATTTTCTTAGGCAAAAAA
CAAATGCAATTGTTTAGAAAAAGCGTCAATGATTTGTGTTCTCGTTAGAAAGAGTTGCGTTTGATTTTCT
TGTTGGTTAGAATAATGACGGAGAAGAAGAAACTACAAGCTGCAAAACATGATATTCAGTGTATGCACAA
GTGTAATTGCCAGCGTGTATTTAGTGAGTGTGTATTTTGAGTGTACtttgtgtttgttcggagttcgctt
tctatttgtttttttgtttgg
>dme_circ_0007748_junction_seq
gttcgctttctatttgtttttttgtttggAAATGGTGTTCCACTTTGCATTTGTGGA
>dme_circ_0007749
TCTCCCCGCGCTGTAAATCGGGCACCGAACAAACCTCCATTGGAGCCGTCAGCCTTCATTCGATACTGGT
CAAATGCGAGGTGGATGCCGATCCGCCGGACTCCGTGCGCTTCAGTTGGACCTACAACAATACCCGGAAC
GTGTCGCCGGTAAGTCAGCTCACAAAAACTACTTTCTCTTGGGACACAGTCGAAAAAATCAATGTTTAAA
CTAAAGATATTACCTATAAATATAAGTCAAAATCAGCTATCGTTTTGTCTAACCCCTTTTTCAAAGAGTC
ATGTTATCAATTGGTATAACAATTGAAATTTAATTGATTTCGTTAAATACATTGATTGTTGTTCGGAATC
AAGTTCAAAGCAGCTATTTCTCTGTGCTTCGTACTGGGGCAAAGGTACATGCCTCGTCCGGAATCCAAAC
CGAACTTATTGTGTCTGTAATTATGCCGGGAAACGTGCTGCAGCCGGAGCCTTTGTCGCAATTACTCGGA
ATTccagccagccagccagccagTCGGTCCGGAAGAATAGAAGGGTGTAGGAACAGATAGGTGGCATGGG
TGGTATGGTGCCAAGTTCGAAGTCGGGTGCTATTAGCAGAACTTGTGCTAAATACTCGAGGGTTGTCTGG
GATTTTCGTTCGACAATTCAGAGCAGACGAACAATGGTTGAGGTAGGGAATCGGGCGCCGTACGTGACTG
AATTAAAATTTAATCAAAATGTATAAATTCGGAATGGTGGGGCAAACATAGCGACATTCGAGGGAATTTA
AAATGCAAACCGTTTAGGTGCAAAACCGAGGACTGCAGAACTAGCAGAAAATGGCaaaaaaaaaaaaaTG
GGGAGAAAGACAGATGGAGGAGGAAAAATACCAGAATGGCTGACCAAACGAACCGAAAGACTTGCATATG
TGAGTCGACTTTCCTTGAAATGTCGGTTCTGGGACAGCTGTGCACAATAAATAAAGTAATTCATTTACCG
CAAAAGTTATTTGAAAATCTGCACGAAACCCGATGGCCGGCTGGCCACAGGATGACTTATTCCGTCTCCA
GCTCGATTCGTTTGGCTTGCAATAAAAACTTATTTGCCTTCAAATATTTATGCCCACCCGAGCTGGGATT
CGTTTTTGGTTGAATGCGGATGCTCTGGGTTTGCTTTGATTTCAATTCGATTTGGTTCGCTGCGAATCGA
GTGGGTTAGTGCATCTGGTCCTTCTGCCTACGTTCGCACAATGAAGCCGCCCTAATCCCTGTCTATTTAC
CCTACATTACAGGGTAAGTGAAACTGGAAGTGGAGGAATCCTGCCCCTCCTACTCAAGTCGAGTCGAGGA
AGTTGTTGCCACAAGATGCCGGCACTTACCTCCATCTTCGCCGCAATTTAAACAGTACTGGTGTCGGCAT
TGCCACCAATGTCGGCGTCGGAGACGTAGCCGTAGCCGGAGACCTCCGTCCTCCGTCCGCCTTCCTACAC
CCGGACTATCTCTTATGCACACATAAGGATCTACTTACATAATTGGCGAGTGTGTGCCGGGTGCAGGACG
GGTTGGACTGAAAGGACCGGGTGGCATTGCCGTCGCCGGCACCGGTGTAGTTTGGCAAAATGTTGCCAAA
TAATCCGCAGGTTGAACTGCCAGCCGTTGCCTCAGGACTTTGCCAATTTCGATGGTTATTAATGCAAAGT
ACACAGAAGCATGGTGATGCACTCAGGCAAAGATTTTCGAAACGTTTCAGAAACAAATATATAACACTTA
AATAATGTGCTAATTCTTAAGTATATATTCGCTTGGTTACAATTATATCATCACTACTGCAATACAAATC
AACATTGGATCGTTGGAAAAATATGTTTCTTCGTTATTTTAAAGAAACCTTCTTTTACTTCACCCTAAGT
TTGTTAAGCTTTTAAAGACAGCATAGAATTATAGACCAATTGAGTTACACCCACTTTGTGCATCGCACCC
TTGGGCCAAAATTAAAGCTGACTTAATAATTTTAGGGGCGTAGTCGCCAGTCCTTATTTTTGTTTTAACG
GGGGCAACGCCTTCATTTGCGGCAAAGTGGCGCTTTAAGGCGACAGGAGCTGCGTTTAAATGCAATTACG
GATGGGGTGGCCGTTGGGCCATTAAGCCCCAGCTTGTCTATGACTATGACTAACTGCTATCAGGATACCC
TCTCATACCGCCCTTTTCATTGGAACCTTTTCCGCAGAACACTCCGGAGGCACCGAAGGCCTGCGAATTG

CGCAACGACACCGTCCTGGAGGTGGTTTGTGTGGCGGGCAGTGACGGAGGACTTTCGCAATACTTCATGC
TGGAGGTGGTGGGCGGAGACCCACTCTACTCTGGAGATCCGGGTGGATCGGCGGGCCAGGGACGAGGCCA
GTTCAGCGAGGCCATCGGCCTGGGCGACAACGAGATATCCACGCTGAATGATCAGGGTGAGTGGCGCTGC
ATCTGGATTCGCCCGAATCCAGTTAGTCTAAGTTGATAAGTTCCGGCAGGGTAAACGCAAATTGCTCAAT
TAAATTAAAATTTACTCACGGTTCGGTAAACAGCCGGCAGCTAAAAGCgaaacagaatcaaaatcagaag
cagatacagaatcagcttcagatttagaatcTGGGTGAATGTAGGATAGAGCTGAGAGTGTGGAGCCTAG
AGAGTGTGCAATAATTCACTTGAAATCCATATGGTCAATTAGTGTGCATGTCCTTTGACTTTTCTGCTTG
AATTATTAATAAGTATTTTGGGAACTGCCATGGGGGTGTATGACCGGGAGTTTTCTTCAGCTCCACTCGA
GTGTAAAAGGGACAACATAATTAATGCTATGGGTCCAGTCCCCAGGTTCAATGATTTTACCCTTTGTCCA
GCCACTCGGGGAGTGCATGCTGAGTTATTAAATTATCTCTGTCACTCCTAGATGGGAATATCATATGAAA
ATTTCATTTCATTTCGTTTGGCTGCTCCTCTCAACAGCCTTGATGAAGCGCTGAAGCTGGCCGGGCGAGT
TTATTAGCAAAGTAGCCAAGCGCAAAGCTCATCACGAGGATGGTCatgaaaatgaaaatgaaaatgGTAC
GACGATGATGACCGTGGTGATGATGATGGCCCGACATCATCTCCGGACTCTGACCCACGCCCTGCTTGGT
CCTTTGGCTGGCAGCTTTAAAGTTTCATGCTAGATAAAGGACACACACACTCGCACTTCGGTGTTGGTgg
gggggggggggggggggAGTGAGGTTGGAAAAGTGCCGCAAGGACAACATAAATTACTTACGCTTACTTG
AGTGGGAATTTGTGCGTAACCAACGAAGCTCACTCGATTGTGTCCCATTACGGCAAACGGATATTCAGTT
TTAAATTTTAGACAGTGTCCTGTTTCTGTTTCTGTGTCAGTTTCTATTGCTGTTTCTCCGTGGGCTTCAG
TACAGTTGTGCCTCCGTATTTTTCAGGCATGAGCAATTATTTATGCGCTGGGTCTGCGAATGGGACAGAA
AAGTTAAAAATACATGGCATACTCTGCAGGGTGGGGCGAGTGAATTATAAATGCGCCTCTTGTCATGCCC
GCATCCCAAATCAGCCAAAACCTCTGtatataaatatatatatatatatatatattgctatataACATAA
ATGTCTATAATTCCAGGGCGACATCTGCACCCATCTTCCGCATGCAGGAGAACAGTCCACAGTTTCGTTT
GAATAATTTAGAACCGGGACGTGAATATCAATTTTTAGTTTACGCCGTCAACGCCAAAGGACGAAGCGAA
CCGCCAGTGGTGATTGAGAATGTACGCGTGGCCGCCCAACTGGGACCATATGGGTGAGTGCCCCAATCCC
CCAGTGTGATCCCGTCCTGTCCTGTCTTCTGTCTTCTGTCCTGTTTTTCCACTaaaaaaaaGCGACTCGA
TACTTCACGGTTGAGAGGCGAAAGCTCAAAAACAAAAAAGGCAGAACTCTCCTGCTAATCACATAAAGAG
CACAAGTTATGGCCACCCGAGTTGCTAGTTTGTGGGTCACTTCTTTACCATGGCCAATAAAATAGCCAAA
AACCTGAACATGGAACTGAAACCGAAACCGAAACTGAACTCAAAGTGGAACCCTTGAACAGCTGAGGACG
GAACTGTCTAATTCAACGCTTGACTGCATTTTCAAATCAACGCAAGTTCGGGGAAATCGTCTTCAATCAG
CCGCCGCGAAAAGTTCGCACATGGCGTCACAGGGACATTGGGATACAGAGATGTCCTTAGAGCGCAAGTA
GATGCGAACCTGAAGACTGCCTAACTGCCTCTCCAGCTGGGATGCCAGTGCCACCCCAAAGGACGAGACG
CCCTTTTCTAATCCACTGACTGAGTTATGCAGTGGAGCGGAAAGCATTTCAGCATTTTCCATCAATTCTT
TGAGTGGGGCACACGCATATTACGTATACGCAGGGGCGTGTCGCAACAGCCTCTTCATTTTGCTCCTGCC
TAACGACATAATGCGACTGCCGCCTGTCGCAGTGTGGCGTACATGTGAGGTCCTGTGTGTGCGCGCGTGT
GCGTATGTGTTTGTGTATGCCAAGCGTAAGCGGAATTGCTTCAATTTTCGTTTTGCTTAACGGCCACCGT
AGACACTTTTCTTTTATTGTTCCTTTCGCCATTCTCAGCTCTCTCAGTTCGCACACAACTACGATGAGTA
CGACAACAATGTTGTCATTATTTTCATGCTGATTTTTATTTTTATCTGcatttccattaccatttccact
tccatttTCCCTCCTCTGGCCATTTTCATTTCGCTTTTCATGCGCTTCCTTCGCCCAGTTAGCCATAAAT
CAGTCATATTGGCCGATTTAATGGGGCTAATTGCATTTGATACGCAAATGTTCGATTAAGCGCACTCACT
AGATTTTCGGAGCGCACTCAATTCGCTGGCGAAACCTATTTACATGAAACAACAGCTGCCCGGCTTTTCT
CGTCGAGTTACTTATGTATGTTAATGGGCTGCCGCAGACTCGTAAATTCGGCCTCTGTGATTACACAACC
GAGACCATGGACTCGGCACTTCCTATTTCGGTGGCTGGGGATCCTTCTAATTTATGCGGAATTTTAACAG
CCACCTACTAGCCCACGCAGGTTTTTGATTCTCGCCGGCATATGTGAGCCGATAAATTGCAAAGCCGTGG
GTCGTTGCCACCAAACCGAATTCAAGAATATAAATAAAATGAAGCCAAATCAGCTGACGCTGATTTAAAT
CAGTTTACGCAGAACTCATCAAGAGAATTTATTACACTTTGCGCCGGGTAAGGAAAACGTGAAAAAAATA

TCAggcaaagtggcaatgtggcaaagtaacaatgtTGGGAAAGGCAAAACGAAGGAGCAAACTGATTAAA
AAACCGGCTGAGATGCCGTGAACCATGGCATCAGAAGCCAGCCGATTCCAGTCGATCCAGATCCAGTGCA
AAAGGCCGGGCAATAACAAAAATTAAATTGAGCCGCAGGTGTAGGCAATTTCCACTCAAACGCTCACTTC
AAAAGCCACAACGATGGGACGGAGCAAGGAGCAGGTCAtgaaaatctgaaaaccagaaaacctgaaGGTA
AGGAGCTGGTCCTGGCAATGTTTCAATTCTGAGGCCGTTGCTTTGACGGCCCGCAAATGGAGCGGAAACG
CTTGGATGCCAACGAAAATGGAGGGGCTGGCATTAACGAATGTCTGGCATAAATCTTGCCCGGGGATCAT
CTTACAATCAGTTTGGCCCGGGCTCTGGGATCTGGGCCCTGGGGCTTGGCTCGCACATTATGTTGCAATT
TCCGCTGGCTGGAAATCTAATAAATATAAACAATGCCTCTCACATGTTGCTCATAATTGCCGGGGCTTTG
CGGCAAGTGCCATCAGCTCTAATCGCTGGTAATAAGTGGCCAAAATGGCCTTTGATTTTCATAATTTCTC
CTCCGCAGCAGCCACTATTTATCATACCACCAGATCTCCCATAACAGAGCACCCAGCGATTTGGCTAATT
TCGATTTTCAACCCAATTGGCCCGCTCACAAGGGTCGGAATGGGAAAACACAATAAGCAAACAGTCAAAA
GCAATCAGTTAGGCAAAAGAACACGGCAGAGCACAGCAAAACAAATAAATAAATAAACGGCAGCGAGGCG
AGGCAAACAGAAATCAGCAGGAGCATCAAACTAATTCCGAGCAAATAACAAATCATTGCTAGGAGCCCTC
AAACTATCAGATGCTCCACCTACGATCCATGTTGGTGCACTGGAAAAAGTAAAGGCGGATATGGGACTCT
TAAGAAACGTTGCAAGCATGTTAAATATTAAAATGTATAAATAGTATTAACGTTTAGTGCCTAACTTTTG
GTTGTATTATTTTTGTTCAGTGTAGTAATAGTATCCCTCTTCTCTCCTTCCAGAACGAATCCATTCTGTC
CGAGGACCCAACGCCCGCAGAAACAACGCATCATGGAGGCGGCGGCGTGGGCGGCAGCGGGAGCTCCAGC
GGCCTGGGCACCGGCGCCAGCACGGGCAGCGGTCCGGAGAAGCAATCGACGCTGCTGATACTCGCCGCCG
TCGTGGTGATAGGCGCCGTCGTGGTGACGTCAATTATCGTGGCCGGCATCGTCGTGGTCTGCCGGCACAG
GCCGCGGCCACCTGAGCCGCAGGAGGTGCGCAAGAGCATGCGG
>dme_circ_0007749_junction_seq
GCCGCAGGAGGTGCGCAAGAGCATGCGGTCTCCCCGCGCTGTAAATCGGGCACCGA
>dme_circ_0007750
GTGCTCAACTCGAGGATACAGTCGAACGGACTGGCCAGCACGGTGACGTACCTGCCCCAAACGGATTCCG
AGTTAATAACGCTGGCCTGCTGGGCCAGCAATGCTGTGGGACGCCAAACGACGCCATGTCTGGTGCACAT
CCTGCCGGCAAGGTAAGTGAAACTGGAAGTGGAGGAATCCTGCCCCTCCTACTCAAGTCGAGTCGAGGAA
GTTGTTGCCACAAGATGCCGGCACTTACCTCCATCTTCGCCGCAATTTAAACAGTACTGGTGTCGGCATT
GCCACCAATGTCGGCGTCGGAGACGTAGCCGTAGCCGGAGACCTCCGTCCTCCGTCCGCCTTCCTACACC
CGGACTATCTCTTATGCACACATAAGGATCTACTTACATAATTGGCGAGTGTGTGCCGGGTGCAGGACGG
GTTGGACTGAAAGGACCGGGTGGCATTGCCGTCGCCGGCACCGGTGTAGTTTGGCAAAATGTTGCCAAAT
AATCCGCAGGTTGAACTGCCAGCCGTTGCCTCAGGACTTTGCCAATTTCGATGGTTATTAATGCAAAGTA
CACAGAAGCATGGTGATGCACTCAGGCAAAGATTTTCGAAACGTTTCAGAAACAAATATATAACACTTAA
ATAATGTGCTAATTCTTAAGTATATATTCGCTTGGTTACAATTATATCATCACTACTGCAATACAAATCA
ACATTGGATCGTTGGAAAAATATGTTTCTTCGTTATTTTAAAGAAACCTTCTTTTACTTCACCCTAAGTT
TGTTAAGCTTTTAAAGACAGCATAGAATTATAGACCAATTGAGTTACACCCACTTTGTGCATCGCACCCT
TGGGCCAAAATTAAAGCTGACTTAATAATTTTAGGGGCGTAGTCGCCAGTCCTTATTTTTGTTTTAACGG
GGGCAACGCCTTCATTTGCGGCAAAGTGGCGCTTTAAGGCGACAGGAGCTGCGTTTAAATGCAATTACGG
ATGGGGTGGCCGTTGGGCCATTAAGCCCCAGCTTGTCTATGACTATGACTAACTGCTATCAGGATACCCT
CTCATACCGCCCTTTTCATTGGAACCTTTTCCGCAGAACACTCCGGAGGCACCGAAGGCCTGCGAATTGC
GCAACGACACCGTCCTGGAGGTGGTTTGTGTGGCGGGCAGTGACGGAGGACTTTCGCAATACTTCATGCT
GGAGGTGGTGGGCGGAGACCCACTCTACTCTGGAGATCCGGGTGGATCGGCGGGCCAGGGACGAGGCCAG
TTCAGCGAGGCCATCGGCCTGGGCGACAACGAGATATCCACGCTGAATGATCAGG
>dme_circ_0007750_junction_seq
AACGAGATATCCACGCTGAATGATCAGGGTGCTCAACTCGAGGATACAGTCGAACG

>dme_circ_0007751
TCTCCCCGCGCTGTAAATCGGGCACCGAACAAACCTCCATTGGAGCCGTCAGCCTTCATTCGATACTGGT
CAAATGCGAGGTGGATGCCGATCCGCCGGACTCCGTGCGCTTCAGTTGGACCTACAACAATACCCGGAAC
GTGTCGCCGGTAAGTCAGCTCACAAAAACTACTTTCTCTTGGGACACAGTCGAAAAAATCAATGTTTAAA
CTAAAGATATTACCTATAAATATAAGTCAAAATCAGCTATCGTTTTGTCTAACCCCTTTTTCAAAGAGTC
ATGTTATCAATTGGTATAACAATTGAAATTTAATTGATTTCGTTAAATACATTGATTGTTGTTCGGAATC
AAGTTCAAAGCAGCTATTTCTCTGTGCTTCGTACTGGGGCAAAGGTACATGCCTCGTCCGGAATCCAAAC
CGAACTTATTGTGTCTGTAATTATGCCGGGAAACGTGCTGCAGCCGGAGCCTTTGTCGCAATTACTCGGA
ATTccagccagccagccagccagTCGGTCCGGAAGAATAGAAGGGTGTAGGAACAGATAGGTGGCATGGG
TGGTATGGTGCCAAGTTCGAAGTCGGGTGCTATTAGCAGAACTTGTGCTAAATACTCGAGGGTTGTCTGG
GATTTTCGTTCGACAATTCAGAGCAGACGAACAATGGTTGAGGTAGGGAATCGGGCGCCGTACGTGACTG
AATTAAAATTTAATCAAAATGTATAAATTCGGAATGGTGGGGCAAACATAGCGACATTCGAGGGAATTTA
AAATGCAAACCGTTTAGGTGCAAAACCGAGGACTGCAGAACTAGCAGAAAATGGCaaaaaaaaaaaaaTG
GGGAGAAAGACAGATGGAGGAGGAAAAATACCAGAATGGCTGACCAAACGAACCGAAAGACTTGCATATG
TGAGTCGACTTTCCTTGAAATGTCGGTTCTGGGACAGCTGTGCACAATAAATAAAGTAATTCATTTACCG
CAAAAGTTATTTGAAAATCTGCACGAAACCCGATGGCCGGCTGGCCACAGGATGACTTATTCCGTCTCCA
GCTCGATTCGTTTGGCTTGCAATAAAAACTTATTTGCCTTCAAATATTTATGCCCACCCGAGCTGGGATT
CGTTTTTGGTTGAATGCGGATGCTCTGGGTTTGCTTTGATTTCAATTCGATTTGGTTCGCTGCGAATCGA
GTGGGTTAGTGCATCTGGTCCTTCTGCCTACGTTCGCACAATGAAGCCGCCCTAATCCCTGTCTATTTAC
CCTACATTACAGGGTGCTCAACTCGAGGATACAGTCGAACGGACTGGCCAGCACGGTGACGTACCTGCCC
CAAACGGATTCCGAGTTAATAACGCTGGCCTGCTGGGCCAGCAATGCTGTGGGACGCCAAACGACGCCAT
GTCTGGTGCACATCCTGCCGGCAAGGTAAGTGAAACTGGAAGTGGAGGAATCCTGCCCCTCCTACTCAAG
TCGAGTCGAGGAAGTTGTTGCCACAAGATGCCGGCACTTACCTCCATCTTCGCCGCAATTTAAACAGTAC
TGGTGTCGGCATTGCCACCAATGTCGGCGTCGGAGACGTAGCCGTAGCCGGAGACCTCCGTCCTCCGTCC
GCCTTCCTACACCCGGACTATCTCTTATGCACACATAAGGATCTACTTACATAATTGGCGAGTGTGTGCC
GGGTGCAGGACGGGTTGGACTGAAAGGACCGGGTGGCATTGCCGTCGCCGGCACCGGTGTAGTTTGGCAA
AATGTTGCCAAATAATCCGCAGGTTGAACTGCCAGCCGTTGCCTCAGGACTTTGCCAATTTCGATGGTTA
TTAATGCAAAGTACACAGAAGCATGGTGATGCACTCAGGCAAAGATTTTCGAAACGTTTCAGAAACAAAT
ATATAACACTTAAATAATGTGCTAATTCTTAAGTATATATTCGCTTGGTTACAATTATATCATCACTACT
GCAATACAAATCAACATTGGATCGTTGGAAAAATATGTTTCTTCGTTATTTTAAAGAAACCTTCTTTTAC
TTCACCCTAAGTTTGTTAAGCTTTTAAAGACAGCATAGAATTATAGACCAATTGAGTTACACCCACTTTG
TGCATCGCACCCTTGGGCCAAAATTAAAGCTGACTTAATAATTTTAGGGGCGTAGTCGCCAGTCCTTATT
TTTGTTTTAACGGGGGCAACGCCTTCATTTGCGGCAAAGTGGCGCTTTAAGGCGACAGGAGCTGCGTTTA
AATGCAATTACGGATGGGGTGGCCGTTGGGCCATTAAGCCCCAGCTTGTCTATGACTATGACTAACTGCT
ATCAGGATACCCTCTCATACCGCCCTTTTCATTGGAACCTTTTCCGCAGAACACTCCGGAGGCACCGAAG
GCCTGCGAATTGCGCAACGACACCGTCCTGGAGGTGGTTTGTGTGGCGGGCAGTGACGGAGGACTTTCGC
AATACTTCATGCTGGAGGTGGTGGGCGGAGACCCACTCTACTCTGGAGATCCGGGTGGATCGGCGGGCCA
GGGACGAGGCCAGTTCAGCGAGGCCATCGGCCTGGGCGACAACGAGATATCCACGCTGAATGATCAGG
>dme_circ_0007751_junction_seq
AACGAGATATCCACGCTGAATGATCAGGTCTCCCCGCGCTGTAAATCGGGCACCGA
>dme_circ_0007752
TCTGGACTCGCGGGGCGGAAACGTGAAGTTGGCGCCACATGCGGCCATAGCTAGCGATCTGGGGCAACGT
CTGTTCTTCAGCATCGGCGATAATCCCAAGGATTCGCGCCTGCAAATCAACGATGTGAAGCCGGAGGACG
GAGGCGTCTATCGGTGTCGCGTCGACTTCTTCAACTCACCAACGCGTAACTTCAGGCACAACCTGACCCT

AGTTGGGACGACCCCCGCCCAAAGTCACCTGGTGGCGCGACGACACCGAGCTAATCGGCACCAGCCACAC
ATCCGTGGAGGAGGGCGCCACCGTGATGGTTAATCAGCTGCTGATTGGAACCACCACGCGGGACTTCTAC
GGAATACGCATCGAGTGCCGGGCGCAGGGAACTCGACTGGTGGATCCGGTCCGCAAGGATGTCACCGTGC
AGGTTTACTGTGAAACCGGTGCGCGTAAAGATTGCAACTCCCAACGAACTGCTGACCGCTGGCCAACCGA
TGCCCATTCGCTGCGAGTCCTGGGGCTCATATCCTGCGGCCAAGATCACCTGGCTGCTCGACGGCGAGCC
CATCCGCAATGCCGAGGTCACCGTTCACAGCGACAAGGAGGGTGAGTAAATGTCCTGGCCAAATTGCCCG
AAAATGTCTGACTATTTTATGGAGCATGGAGTGCATGGAGTGGGTAGCTGGGTGCGGAGAAAGTTTTCGT
TCTTGTTGTCGTTCCCAGGACATCGCAACTTTGTGCCCGGAAAATTTGCTTCGAGCGTTTGACATTTGAT
AGCTGGCCGCAGTGCAGCAGGCACTTCTACAACGTTCACATCCCATATAACACTGTGCGGCACTTAATAG
CTCCAGATTCTCGGGGCAGCAACAAAGGCAGCCGAGGCGACCACTGGGTGACCCTAAATGCGAGCCGGAC
AAACTTTTGCCGCCAGCCGCAGAGGGATAGACAAAAGGACGAAGGGACAAGTTGACTCTCCACCAGCCGC
CGCTACTTTGATGAACTGCAATGGCGAAAAGTTTTCGCCAAACTGCACAAATTTGCACTCCATGCGGCCA
AAGGCCAATGATTCGGCTAATGTGCGAATTTTTATTGGAATTCATCGTCCTTTGCGCTCGCATCTGCTGG
CAAATGGTAGCGATTTTTTTCCCACCAAATCGCATTTATCAGATCCTTTCGCCTACCCACTGAAGGTATC
GGAGATGTCTCAGTTTTCGGCATCCATTAGTTGCCTTTTTTGTGGAGCCACTGGCTTTTTCGCTTTCTAA
TTATGCCATGTAAATGGATAGTTGCTGCCAAACGTTGCCAGGCATTAGTCAACTCGTGCATAACCGACTC
GACTTCCAATTGAGGAACAGATTTCCACACACGAAGAGTCCTGCGCTTGTTAGTTTGCCTGGCCCGGCAT
CGATTGTTGCCAAAACGTGGCTCGACTTTGGGGCTTGGAGCTGGTCGATGTCGTAACTTAATACCCAGTT
TGCCATGAGATCCTCTGCTCCGCTTCCACATCGCCTAACGAAgggctaacgaaggccggggctaacgaag
gccgCCTATCAGACAGGGACATATGGCAGATCTAAGGGACTTTAAGTTTCAACTAAAAATCCGAAACATA
TCACAGTAACATGATATATCGATATACTGCTTATTATGCTATATTTTAATATAAAGAATATGGAATTCCT
GTCATCAATGAGCTCTTCTCTGTTGACAAAAGCCGCTTAAGTATTAAGAACTAATATTTAAGATTACATT
TTTACCAACGTATCCTTTAGCTAATACGCCATCCTTTTGCCTCCCGTTTACTTTCCGCAGGGACGGGAAT
ATCACAACGAGTATCCTGACATTGAAGGTAACATCGGAGAACGATAATGCCGAGCTGACGTGTCGCGCCA
CGAATCCCTGGTTCTCCGGTGGCGCCATCGAGGACAAGCGCATCATTCGCGTGGCATG
>dme_circ_0007752_junction_seq
AGGACAAGCGCATCATTCGCGTGGCATGTCTGGACTCGCGGGGCGGAAACGTGAAG
>dme_circ_0007753
AAGTTCTAATTCAGAATTGTAACAAGTCACAAGAAGTTAGTAAAAGGATTATTTAGAAGAAATGGTGTTC
CACTTTGCATTTGTGGAACGAGTATTTCTGCAATCGTTTTAATTTTCTTAGGCAAAAAACAAATGCAATT
GTTTAGAAAAAGCGTCAATGATTTGTGTTCTCGTTAGAAAGAGTTGCGTTTGATTTTCTTG
>dme_circ_0007753_junction_seq
TTAGAAAGAGTTGCGTTTGATTTTCTTGAAGTTCTAATTCAGAATTGTAACAAGTC
>dme_circ_0007754
TTATTATACACATATATGTATACATATATTGCAAACaaaaacgaaaaatgcaaaaaaaaaacttaaaaaa
aaTCTAAAGGAAACGTAAAAGTTAGCGTTATTATTAAATATGTTTGTAAAACGTCAACAATATGCAAAAA
ATGAAATTAAATGAAATGAAAAGCGAGCGAAAGGCGCATTGAGGGCATCTAACTAAAGATTGGTCGTATA
TTTCGAAACACAAATTGAACCCATGGCCTAAATACTAAGCGACGTGTGTGTAAGAAAGTTTTGCATCTTT
ATATTAGCGTGTAATTAAACAAATGAAATAGCTAAACGAAGCCCAAGCCTAAACAGCAAACTACAACTAG
AGCTACTACAAACTACCCACGAGTATCATCCCTCCCTGCCTCCCCTCATTAGCCCACAATAGCGATCAGA
TCGAATCCTATAGCCCCCGAGCGTTAAGTCCTACTGCCTTTCAAAAGATTCCAAGAAGAACACCAAATAT
AATGAAACACGAAGAAATGTTAAAACTCGAACAGGCAGCACTTATCGACTCGACCTGTTTGTTATGCCCA
TCTAGTAACTAACACCAATCTCAGATCCTAACTAGCAGATCCGAAAAACGAATAGGACCCATCTCTAGTT
AAAGTTGATGTTTGCCTAGGATGCCAATCAGTAAAACAAAAAAGAAGAACATATTCAAATAACGCAAGTG

>dme_circ_0007754_junction_seq
AGAAGAACATATTCAAATAACGCAAGTGTTATTATACACATATATGTATACATATA
>dme_circ_0007755
TTATTATACACATATATGTATACATATATTGCAAACaaaaacgaaaaatgcaaaaaaaaaacttaaaaaa
aaTCTAAAGGAAACGTAAAAGTTAGCGTTATTATTAAATATGTTTGTAAAACGTCAACAATATGCAAAAA
ATGAAATTAAATGAAATGAAAAGCGAGCGAAAGGCGCATTGAGGGCATCTAACTAAAGATTGGTCGTATA
TTTCGAAACACAAATTGAACCCATGGCCTAAATACTAAGCGACGTGTGTGTAAGAAAG
>dme_circ_0007755_junction_seq
AATACTAAGCGACGTGTGTGTAAGAAAGTTATTATACACATATATGTATACATATA
>dme_circ_0007756
AGTGGGTTCGATGGACTTAAAACTTAATATTTAGCTGAATGTGGCTACGAGAATGTAAGATCGTTGATCG
TAGAGAAGACTTGGTTCCGTCTATTGAGCAAATTGGATTAGTACATTAGTATTATCCGGCTTAAACTACT
TGCACATGCAATTAAAACGTTAATATCGTAAGACTCGAGTGCATATCGTTCAATATCATGGCCAGCTCAT
GGTGGAGTATGTAATGAGTCATGAGTGGATGAGTGTGTGCATCCGTTGGAGG
>dme_circ_0007756_junction_seq
GTGGATGAGTGTGTGCATCCGTTGGAGGAGTGGGTTCGATGGACTTAAAACTTAAT
>dme_circ_0007757
GGTACTCCTCGGAGATGGGGCACGAATCCAGATGCTTGGCATTTAGCGGCGGACTAGCCGTGGACACTGC
CATTTGTGTGGACGACTCGAAGAAGACCGAAGCCATTGAGGTGGTTTCACGACACCGCTCCTTGCTGCAC
ACTCCGTTTCCGCAGTATGTGCAACCAAAGGCGGTGGACACGCACGATTGACAGCTGGCCAGTTCCTGGC
AACGGTGCACATCGTGGAGTAACTCTGAAGTGAGGGTAAGACGGCTCTTCGACGGGCAAGCCACATAGTC
GTATTGCTCGCGTCCGTAGATCGCCGATCTTTGAACCTGG
>dme_circ_0007757_junction_seq
TCCGTAGATCGCCGATCTTTGAACCTGGGGTACTCCTCGGAGATGGGGCACGAATC
>dme_circ_0007758
GATAACAACCATGTACTGGTAGTTGTTTTTGTCCCCGTAACCGGGAACCAAGGTAGCTGTGTGACCCACC
ACATGCAGGGGTCCGCACATAGCGGTGGTTCCTCCAGTGGCGTTGCACGAGGGATCCGCTCTTACCGAGA
TGTTCGCCCACGTTCGCGCCGAGACGTCGAAGGCCCACAGTTCGTTGGAGATGCCGTGGCCCTTGACCAC
TCCACCGTACATGAAGATCTTGTCGCC
>dme_circ_0007758_junction_seq
CTCCACCGTACATGAAGATCTTGTCGCCGATAACAACCATGTACTGGTAGTTGTTT
>dme_circ_0007759
GGCGGCCGCCCGATGGAGTCCGCTGAagcagccgtggaagaggagcagcgtgaggaggagcagGCATTTT
CGCCTGTACTTGGCGTTGAATGTGGGTGAGGTGCTGCTCCGGTTCGGTTGCTTCTGCGGTGGCTCCGCCG
CCAGGCACATGATTTGC
>dme_circ_0007759_junction_seq
TGGCTCCGCCGCCAGGCACATGATTTGCGGCGGCCGCCCGATGGAGTCCGCTGAag
>dme_circ_0007760
GGCTTTCGTTTTATATATAACAACAAAGTATTGCAACTAACCAGAAGAACAGAAGTTTTGTATATATCAG
CTAACTACATATGTCATGTCTTTTGCTTTTGTTTTTCGATCTCGATTTCGGCCTTTTCCCAATCTGGAAT
G
>dme_circ_0007760_junction_seq
GATTTCGGCCTTTTCCCAATCTGGAATGGGCTTTCGTTTTATATATAACAACAAAG
>dme_circ_0007761

ATAGTTACAACAAAGTTGCAGTGGAACGCCAAGTCCAAAATCGGTTCGCTGGAGAATGCCGCCCACAAGC
CGGGAGGCGGGGACAAGAAGATCGAGACCCTGAAGATGGACTTCAAGGACAAGGCCAAGCCGAAGGTGGG
CTCGACGGCCAATGTGAAACATCAGCCGGGTGGCGGAGACATCAAGGGTATGTGGCAAACCCCTTTCGTG
ACCTTTCACCCCACGCCTCCGGGCGATTTCATGTTTGAGTTTGACCCAGAGAGACGAGAACGAGTGTTCA
TACATAAGTATTGATTTTTGTAGCGGTTAAGCATGATTTCCGATTGCTGTTGGCTTATGATGGttttttt
ttGGATGCTCCACCTGAGTTACCAACTGGCATGGGCATGGGCCGCATCTATTCTATTCTATACTTATTAT
CCGTTCGTTTCTCTCAGTGCATGAGCTGCAATGTTCTCTTCAGTTTTAAACAGCATGCTGCCACTTCTAA
CCACCTTTCAAGGGTTATTACTATTAAGAAAGACTACATTTAGAACTAACACCCAGAACACCAAAAAATA
CCAAAACCTTGAACCACTTTCCAGAGCTACGATAGGAAGATGAACATCATCTATGGCGAGGGCTCCAAAA
TAGTCAGCCATTGCGCCAATAACAAACGTTTGGATCAGAATTTGTATAATGAGGAGGAGTAGTGtttttt
ttttAGTTGGGTAGGTAACACCCCCCAAAAATGGTGCTAAAACGAACCACCCACACAGACACCTAGTTTA
GTTTTGGGTTAAGCATTGTACATACATATTTACTATATAATCAGATTGGAGGGAAATTCATTATGTTTAG
TTCCTAACCATACCGACCGAATCACTATGAATAATTGAGAATAACTTATTGTTTCTCCATCCTTATTTTA
ACGAACCGAAACATGTTTAAACCATATTTTGGAAACTGTATTTTACTTTTAACTAAACCATTAAACAACG
AATTCGAAGGATAACAACCCGAAGGATGTAAGTAGTCGTGATAACTCTCTTTTCCCAGTGTAATAAGTGT
GCTTTTGTATAGAAACCTAGATGCATATCGTTTCTTTCATATTTTTGGTATATTTTGAAAACACCTAAGC
AACACTTTTCGAGATCCCAAAATCACAGCACCATGTAGCGTATTTTTGTACCATTAAATTTAGACTTAAt
ttttttttGCCATCCATTTGTTACGTTCTGTGCTCTGCCGTTTCTGTGTGTGAAAATTTCCAAATTTCAG
TTATGTCCCCGATTGGCCGTTTGTCGTGTGTCCATAATACGAGTAATAATCCCACCTGCaaaatcgaaaa
caaaaaaacccggaaaatggaaataTACTCCATTTGCCAAATTCAATCTGAATCTATGGTCTTGCTAATT
GATTTCTGTACGTTGCCGGTGCCGGTGCTACTTTTCAGAATCCAAACGCAAAAACTAGAAATTAAGGCAC
AAAGCAAAGTTGGCTCTTTGGATAATGTAAAGCACAAGCCCGGTGGCGGTGAGAAGAAGATTTTCGATGA
TAAGGATTATTTGAAAAATGTTGAACACTCTGTTGCACTGACAACACCACCAACACAGG
>dme_circ_0007761_junction_seq
GTTGCACTGACAACACCACCAACACAGGATAGTTACAACAAAGTTGCAGTGGAACG
>dme_circ_0007762
GATACGTTGAGCCACCCAAGGCCCCAAGGCCATCGCAGGCCACCTGTCCGCGTTCCACGGCCTTCTATCC
CCGGAATCCCATCATTACGGTTACGGAGCACACGCCCACACCTTCGCCCGATTACATGAAGCGTCAGGTG
AGCCATTATCATTGTTATGATTACGACTTGGTCTACAAAGTTTGTCCTTCGCAAAATGAGTTCCCACTCA
AGATATGATAATGAAGTACGACTAAAAACCGAATAATTTTCCTACAGGGCTCCATCGACTCCCAGCTGGA
TGCCTTGAGTAATGGCGGCAGTATTGCTGGTATGACTGGAAACGGAGGTGGTAATGGTAGCACTGGCATG
GGCAGCTCCACCACCCGTTACCGTGGTCAAATGCTGCGCTCCCACACGGACTCCCACATCGATTACACGG
GCGTGGATGAGTCGGAGGCGCCGGGCTCCTCCTTTTATATAACACGCGATGGCGGCATTGACTACGAGAT
TATCCTGCTGGCCATTAGCAATGTTTTCAAGCGGGATCCGGCACAGGTTTGCTCACTCCGAGTCTTGGAA
GCGGGTCTGAACATCTGCGAGTTGCTGATTGAAATGGGTGTGCTCAAACTGGGTGAGCATGCCCACGAGA
TCTCGATGAGTATTACGAGGAGAGCTCTGCAGGTCTTGGGGTGTCCACACGGATGCAATGATGGGTGTTC
GAGGGCCACCTGCTGACTTTCTGCGCAACCAGTGCCAGAAGATCCTGTCGAGAATGTTGCGGCAGGCTGG
TCAAAGGACGAAGCGCTTTATGCAGGAGATGGTGAAGACTTCACCGCTGCCGGAACTCATCGACTATTTC
CATGCCTTCCTGGCCTTCTGCGTGGATCCCAGCTCCCTGCTGTCGCCACTGA
>dme_circ_0007762_junction_seq
GATCCCAGCTCCCTGCTGTCGCCACTGAGATACGTTGAGCCACCCAAGGCCCCAAG
>dme_circ_0007763
GTACGGCAGGCACACCTTGGACACCAGAAAGGTGCCTCGAGTATTGATGTTGTGCATCAGGTCGTAGCGC
TTCATGTCCGTATCCGGGGTATTGGTCAGCGAGATGGCGCTGGCGTTATTGATCACAATGTCGATGCCAC

CGAACTTGGCCACCGCGGCCTCAACGGCGCTGCGCACCTGTTGCTCATCCCGCACATCCACAACGCAGGG
ATAGGCCTTTCCGCCGGCCTTCTCGATCTCCTCCGCAGCCGAGTAGATGGTGCCCGGCAGCTTGGGATGC
GG
>dme_circ_0007763_junction_seq
AGATGGTGCCCGGCAGCTTGGGATGCGGGTACGGCAGGCACACCTTGGACACCAGA
>dme_circ_0007764
GCTACAGCGCCATTTACCAGCCAGGTGGCTTCTGGTTGGGATATCCACCCTGCTGGTTGGCATAACTGCC
TGGGTATGTTCCGTAGTGGCCGGGTGGCGGTCCCTGCGGGAATCCCTGGTACTGATAGTCTGCAGAAGAT
GATCAGCATTATTGAAGATTCATTTTAAGCGGTGACGTTAACTTACTACCAGGATACGGCAAGGTGGCGT
ATGGATTGAAGCTCTGCGGCATCGGAGCGGGCACATAGGCGGGGTAGGGCACACCTGGCTGGGCTCCATA
TGGAATAGGCATGCCTTGCACTTGGGCCGGATACGGAAT
>dme_circ_0007764_junction_seq
TGCCTTGCACTTGGGCCGGATACGGAATGCTACAGCGCCATTTACCAGCCAGGTGG
>dme_circ_0007765
GATGCCTCACAGTCCGCAGCTGAAGCCACATCAGTTCCAGGCCCACACGCCGACGGATCAGTACTCATCC
TATGCGGACAACTTCGACCTGTCGCTGCTGCCGCAGGAGTCCGCATCCATTCCCACCACAGTCTTCCAGT
ACAACGCCCCCCAGATCAAGGTCGAGTGCGCCTGGGATAGCCAGCCCATCCAGCAGCAACAGCAGCCCAC
TGCGCCCTATACCAACCCCTCCAGCCACCAGCTGATTCCTCCGCCCGCCTATCCCCACAGTGCCTATCCC
TCTCCGCAATCCAGTCCGCTGCAGTCGGAGTTCGCTGCCTATGGGCTTGGCAGATTTGGCGGTAGCTACG
ACAGTCTGAACAGCCCATCGCCCTCGCTGGAGGCGGTTAGCATCAAGCAGGAGCTGCACATCCTGCCACC
CTCTCCGCCGGAGTCCAACTGCGAGACGCCATCACCCAGATCCTCCTGCGGGGAGAGCATCAAGGCCGAG
CCCTTGGATGCGGACATCGAGAGCCTCATCGATCTGAACTCTCTGCTGCAGCAGCAGAGTCTACAAAGCC
CACAGAATCTGCAGGACACCAAGCCCGATCACCAGCTACTGCGGGAGTGCCTCGAAGACACCAGCTTCCA
GAAGCGCCACAATCTGAAGCCTCTGGCTCTGGAATCCTTCATCGGAGGCTTGGCCGAAGTGCGCGGCGAC
TTTGAGCCCGTCATTTCACTGGCCCTAGAGCATGCCAAACGAGAGGCGGACGCCATCTGCGCGGAGCTGC
AAATATCGCAGGGATCCCAATGGCTGGTCGCCCGCCCAGGTACACGCATGGCTCCGATCCACCTTGGCCC
AGTTCAGATTGCCGCCGGTGGCCGATTTGGAGCTGCACTTCTGCGAAAACGGAGCCGCACTGGCATTACT
CAGCGAGGAGGAATTCGTGCGCCGACTACCAGAG
>dme_circ_0007765_junction_seq
GGAGGAATTCGTGCGCCGACTACCAGAGGATGCCTCACAGTCCGCAGCTGAAGCCA
>dme_circ_0007766
ATAACGAGATGAGAGATCTGAGCGTCACCTTCTACGGCAGCAGCGCCATCAACCGACAGAAACTGGGCCT
TCTGGCCCTGAGTTCCGCCACTTTGGCTCTGAGCCTGCTCGTGGCCTAATTGCTTTCCAATTCGACCTGA
GTTACGAATTTCACCGCCAATAGCACAGTAAAGTGAATCGATTGACTTTGATCTGGGATAGTCGAACGGA
GATTGGACAGcaaggatacccaaggatacccaaggataTCGCGGTCCTGCACAACACTCTTAGTCGTTTA
TGATAGCCTAAGCGATGATCCACATAGGCATAAGCATTTCTCAATGTTCGACTCGAATTTCTCAAGCAAA
TTTAGTGAATTAATCAAGAAACGCAAAACGGAACCATTCATTATACATGTAACAATCAATGTAAAAGAGC
ATGCAACTGTTCAAACTAGGACCCATAAAACATTCACTACACCCACTCACAGCCACATTTGCCGACCATT
CCAGCACTCAAAAATAAGTAAAGAAAACCCACTGTACATAGATTTTATTGCATACTCCTAGGTATAAACT
GCACCAAAGAAATAGTCACTCGACGACCGaaaaaaaaTACATTTAAATATAACTAGTATGTACTGGATGA
TGGCCACAGATGAGCCGGGGGTTAAGCTACCGGCGTCACGGCGGTGTGTGGAGTACTTTGTAGCATTTTG
CGGATAGTTAATTTAATTAATACATAAATGCATAAACTAATTATTGGATACGGCACTGCCACTGCGCGCC
ACACCATGCGTTTTAGTCAAATTCAAAGTCCCCCAGTACTCCAACTAATTTGCAGCATTCGAGACGGAGT
GCATTTGAATGGCTTACAGCAGACAGCGTTTTCTAGCTTTTTGCAATTTGTATGTATGTAGCGCTAAGTA

GCTTAATTGACCGCATAAATGCAATTAAATTAGTCAACAGACCTATTGATTTGGTATGTAAATTAATCGA
CACACGAAAATGGCAGCGTATAGAAGCTTCCACATGGCTATTGGATTACAATTAGTGCG
>dme_circ_0007766_junction_seq
ACATGGCTATTGGATTACAATTAGTGCGATAACGAGATGAGAGATCTGAGCGTCAC
>dme_circ_0007767
GCGAGGAGAAGCTCCACTCGCACGTCCTCTCCATCGAGAATGTGGATCGGCACAAGGGCGGCGTCTACAT
ATGCACGGCCAACAATCGCGTGGGCCAGCCCGCTTCCAGCCAGGTCGTGCTCCATGTCCTGTGTAAGTAT
GCCACCCACAACCATATTTAAACACTGaaaaaaaaaaCCATCAACAATATAAATATTTTCGAAACAAGCA
GTGTACACATCCATTTGTTAAACGGAATAAAAACATCTCTTTTAGTTTGAAACAATTTTGTaaaaaaaaa
aCTACTGTAGGTAGGATCTTTTTCTCATAGTAAATACATTCGATATTCGAATTGGttttttttttCCAA
GTGTAGGTACACAATGGCAGCGAAACGAATTAATTTTTTCTGTGCGCGACCGTGCCACGGAGTCCGCCTG
TCAGTCCAATTTATGTCAAATCTGATTTCGATTTCGAACGGGTATTGACTTTTAACGCTCTCGCGGCACA
TGCATTCGCGTAGATGATGGAGTCATGGAAGGGCAGAGGTCACGATTCAGTGGTCAGGGGTTGTGGGTTG
TGGGCTGAGCGATGCCCAACTGAAATGGGGATTGCTCTTATTACATTTACACACTTACCGACATTTATTG
GTTGGCTGGCTGGCAATTATTGAAATGCCTGGCGGGGACTCCCGCTCGTTCTGACATTTATTAAGAATTT
ATATGTCCGTATAATTTTGATGCGAGTTCGTTACCGATCTTGTTGCCCCCTCTGGTCATTACATGTGCTA
ATTACATGGCTCCCATCCCAATTTACACTTTACTGATACCAATGTCTAGGGGGGTCGCCATTGGGTCATT
GATTTTTCGCGTTCGCTACCAGTCGCTGCCATTCCGTGAGACAGGACAATGTTCATGTTATTAACTAATT
TCTTAACATTCTCTTCGTTATTTTGCGCCCGCAGTTTCGCCGGAAATCTCCGTGGAGCGTCCTGTGGTCT
TCTCCGGCGAAGGGCACGAGGCCACGCTGGTCTGCATCGTCCACGGCGAAACACAGCCTGAGG
>dme_circ_0007767_junction_seq
ATCGTCCACGGCGAAACACAGCCTGAGGGCGAGGAGAAGCTCCACTCGCACGTCCT
>dme_circ_0007768
GTTTCGCAACGACAGTCGCTCCAGTTTATTGTCTGGCGAGCGATTGATGTTCCGGATCCAGACAACGTCC
GGTTGCACCGGTCGATCAGCTCGGCCAAAGCTATACATCGCCCGATCGGTGACATAGTATTGAGGTGTTG
TCTGACAGGAAGGCGGACGTGGGCGCACTCGTCGAGCATA
>dme_circ_0007768_junction_seq
GCGGACGTGGGCGCACTCGTCGAGCATAGTTTCGCAACGACAGTCGCTCCAGTTTA
>dme_circ_0007769
CGAATAGATCCTACGAAGCCTGGACGCCGTGAGCCTTTTTCAGTCCCTGGAACAGCATCAGTTTCTCCCG
GTTAACTTCCTTAAGCGTCTGCACCGCCAGCTCCAGTTGCACCTTCTTCAGCGCATGTTCGCTGGCGATG
>dme_circ_0007769_junction_seq
CTTCTTCAGCGCATGTTCGCTGGCGATGCGAATAGATCCTACGAAGCCTGGACGCC
>dme_circ_0007770
GGTTCACCAGCTGCTCCGCCGCAACAACCGACAGGCCTACGCCGTCAGCGAGCTGGCTGGTGAGTAAACT
GTTTATAACTCCTCTTTTGTGTGAATCTAACCCGTTTGAATTCCTGTTTGCAGGTCACCAGACCTCCGCC
GAGTCCTGGGGTACCGGACGTGCTGTGGCCCGTATTCCCCGTGTGCGTGGTGGCGGTACCCACCGTTCCG
GCCAGGGAGCCTTCGGCAACATGTGCCGTGGTGGACGCATGTTCGCCCCCACCAAGACGTTCCGTCGCTG
GCACCGCAAGGTGAATGTCAACCAGCGTCGCTACGCCCTGGTGTCGGCCATCGCCGCCTCAGGAGTGCCA
GCTCTGGTCCA
>dme_circ_0007770_junction_seq
CCGCCTCAGGAGTGCCAGCTCTGGTCCAGGTTCACCAGCTGCTCCGCCGCAACAAC
>dme_circ_0007771
CTGTTGCTCTTCCGTTACCACGATGGAAGCCGTGAATCAAGTTTCAATCGTAACGCCAAGAAGAGCATTT

TATCTGGCAACGCAGGAGATCCCTGCAAGCATCGGACAACCAGAAGAGTAGCCAGTTGCTTGCACGCTGC
CAAG
>dme_circ_0007771_junction_seq
AGTAGCCAGTTGCTTGCACGCTGCCAAGCTGTTGCTCTTCCGTTACCACGATGGAA
>dme_circ_0007772
GGGTTAAGCTTGATCAGCTGGCGCACGTTGGTCAGGGGGTTCAGGCGGCGCACGCTGCGGAACACACGCT
TGCGGGGATCGCGCAACACCTTGCGGATCTCCTCAGACTTGAGCAGACGCGACAGATCGGTGTTGGCCAT
CTTGGGCTGGGGCAGGTTGTAGCCCTTCTTCAAGG
>dme_circ_0007772_junction_seq
TGGGGCAGGTTGTAGCCCTTCTTCAAGGGGGTTAAGCTTGATCAGCTGGCGCACGT
>dme_circ_0007773
GGTCCTCAAGCGTCGTGCCGCCCTCGCCGCCGAGAAGCGCACCGTGGCCAAGGTGCTGGCCAAGGCCAAG
AAGCAGAACGTCGAGCTGGCCAAGTCGCACTTCGCCAATGTGGCCACCAAGGCTGCGGCCAATCGCGCCA
AGCTGCTGGCCGCCCGCAAGAAGAAGGTCGCCGCCAAGAAGCCAGCGGCCAAGAA
>dme_circ_0007773_junction_seq
TCGCCGCCAAGAAGCCAGCGGCCAAGAAGGTCCTCAAGCGTCGTGCCGCCCTCGCC
>dme_circ_0007774
GTTGCTGCCCTGCTTCCTCTCCGGAGTCGTTACGAACACGAAGCAGATCGAGATGCTCAAAACAGCCACA
GAGCGTTCGCCGGGCACTCCGCTGATATTTGTTCCGCTGCATCGCAGCCACTTGGACTACATCATG
>dme_circ_0007774_junction_seq
GCATCGCAGCCACTTGGACTACATCATGGTTGCTGCCCTGCTTCCTCTCCGGAGTC
>dme_circ_0007775
GCTAACGCATTGGTGGACATCACAGAGGTGGCTGCTGCGCAGTCAAAGACCACCTGGCGCGAGATGCTTT
CGATGAGATTGCGGTGCTCCTCGCACACAACATCGGTGCCGTACAAGGAGGAGGTGGACTGATTGTGCTG
TAGGACCCTGAAATAAAAGAGTAATAAATATATATTTATATTCAGAACCATATTCAATCACATAAGAAGC
ATATCTTTGAACATTAGAACATACGACTACTCATATCAAATCATCTTCAGCAGCCTACCTGGCAGATGGC
TTGTAGACCTTGGCAATGTTTCCATCTTCTCGGGCTATCTTGTTGTACGAATTGACCAGCTCACGTATGG
AGTAGGGCTCGTTAAAGTCGATGCGCATGAGGCCGTAGTTTGATTTCAGGGCCTTCCAAATGCCCGAAAT
GGCCTTGCCGAATGATTCGGGTATCTTCTTCTCGCCCTTTTGTTCGCGCACAAAGTTTCCGTCGACGAGA
CGCTCGTAGTTTACGGAAACGGGTACCAGCAGTGCGTCCGG
>dme_circ_0007775_junction_seq
CGGAAACGGGTACCAGCAGTGCGTCCGGGCTAACGCATTGGTGGACATCACAGAGG
>dme_circ_0007776
GCTGATAGACACCGCGCTGGAAAACTGCCAGATCTATCGCTACGAATTTATTCTGAACAAACCGACGCAG
GTGCTGGAAAATTTGCTGTACCAGCAGTTGGACGACCTCTTGATTAGTGGTTGCGTTTTGACCGAAAAG
>dme_circ_0007776_junction_seq
GATTAGTGGTTGCGTTTTGACCGAAAAGGCTGATAGACACCGCGCTGGAAAACTGC
>dme_circ_0007777
GCTCCATGACGATTTGCCGAATGGCGCTGAGGGCAGGCGCTTGGCCAACGTCCTGGCAGAGTGCCTGGAC
GAGGACGGCTACGAAGACGTGCGCGATGGCGAGGCGGATGAGCCCAAACTGCTGTTCGCCAGCGAAACCC
CGTCGCAGCAGCGCTACATTTGCGAGGTGCTGGCGCCCTTCGCCTGGACGTATGTGACGGTGGCGCA
>dme_circ_0007777_junction_seq
TCGCCTGGACGTATGTGACGGTGGCGCAGCTCCATGACGATTTGCCGAATGGCGCT
>dme_circ_0007778

GCGAGAGCAAAGAAAAGTATAAGCAGCGTCGGGGTTCCGACTCAAACGACGAGGGGAAGCCTCCAAGTTC
TGGTGGTCCGGCCAAGAACTCCCAGCCGGAGGATTCAGCAGCTACAATGAAATCAGATGCGCCCGTGGAG
AATGCTAACGGCACGAATGGAAACTCCAACGGCAGCACCAATGGTGCTTGCGACAATGTCAGCGGTGTTG
TCATCGTGAGCGACATCCTGCAGCAATCGACGAGCAGTTTT
>dme_circ_0007778_junction_seq
CATCCTGCAGCAATCGACGAGCAGTTTTGCGAGAGCAAAGAAAAGTATAAGCAGCG
>dme_circ_0007779
GGACACCGTGCGCGAGCTCATCCGCAAGTACTCGCAGTTCATCAACTTCCCCATCCGCATGTGGTCCAGC
AAGACCGTCGAGGAGGAGGTGCCCGTGGAGGAGGAGGCCAAGCCCGAGAAGAGCGAGGATGACGTGGAGG
ATGAGGACGCCAAGGTCGAGGAGGCCGAAGATGAGAAGCCCAAGACCAAAAAGGTTTCGAAGACCACCTG
GGACTGGACCCTGATCAACGACAGCAAGCCCATCTGGACGCGCAAGCCCGCCGAA
>dme_circ_0007779_junction_seq
GCCCATCTGGACGCGCAAGCCCGCCGAAGGACACCGTGCGCGAGCTCATCCGCAAG
>dme_circ_0007780
GGTGACCTTCAAGAGTTTGCTCTACGTGCCCAAGGTGCAGCCATCGGAGTCCTTCAACCGCTACGGCACC
AAGTCCGACAACATTAAGCTGTACGTGCGCCGTGTTTTCATCACCGACGAGTTCAACGACATGATGCCCA
ACTACCTGAGCTTCATTCGTGGCGTCGTCGACTCCGACGATCTGCCCCTGAACGTTTCCCGCGAAACTCT
GCAGCAGCACAAGCTCATCAAGGTGATCAAGAAGAAGCTGGTCCGCAAGGTTCTCGATATGCTGAAGAAG
ATCGACAAGGAGGCGTATGAGAAGTTCTGGAAGGAGTTCTCCACCAAAGTAAGTGTCGCTTTCTAGGAAT
TAAGAAACGTTCTTTGCCTGTTCAAATAATTAGTTATTTATGATAAGCATTTCCGCAGAATCCATTATAA
TATGGAGAATAGAATGACGTGTATTATTTTTTGCCAACAAATTAATATATTTAGTTTGTTGCTTTAAAGA
ATTCCGCGCTGATAAGATAAGGAAATCCACGTAGTATTATAATATTGGCACTGGTGACGTGTAGAGTTTA
TACTTAAATATTTTATTTTAGTTAAATAAGCATTTCCATTAGCTATAACCTCGAATTCTTGGATTTTCGT
TGCTTATCAAGACTTAATTGACTCATTTCAGGCATCAAATTGGGCGTGATGGAGGATCCCAGCAACCGAT
CGCGTCTCGCCAAGCTGCTCCGCTTCCAGACCTCCAACGGCAAGGGCGTCACCTCGCTGGCGGAGTACAA
GGAGCGCATGAAGGCCAAGCAGGAGCACATCTACTACATCGCTGGTGCCAACCGCGCTGAGGTCGAGAAG
TCGCCGTTCGTCGAGCGTCTGCTGAGCAAGGGATACGAG
>dme_circ_0007780_junction_seq
CGAGCGTCTGCTGAGCAAGGGATACGAGGGTGACCTTCAAGAGTTTGCTCTACGTG
>dme_circ_0007781
GTATTGAGCAGATGATGCGCCAGACCTTGGGTGTCTCCCAAGACGAGCAGGTGGAGTTCGACGAGGAGGA
GGAGGATGATGCCGAGGAGACCGCCACCGGCAGCCAGGAGAGCGGCAACGCCGATGATGAGGAGGAGGAG
CAGCAGCACGACGAGCTGTAAACTACTTCACCTCACCCACTGTCCAAATAGATT
>dme_circ_0007781_junction_seq
TTCACCTCACCCACTGTCCAAATAGATTGTATTGAGCAGATGATGCGCCAGACCTT
>dme_circ_0007782
GATGATGCGCCAGACCTTGGGTGTCTCCCAAGACGAGCAGGTGGAGTTCGACGAGGAGGAGGAGGATGAT
GCCGAGGAGACCGCCACCGGCAGCCAGGAGAGCGGCAACGCCGATGATGAGGAGGAGGAGCAGCAGCACG
ACGAGCTGTAAACTACTTCACCTCACCCACTGTCCAAATAGATT
>dme_circ_0007782_junction_seq
TTCACCTCACCCACTGTCCAAATAGATTGATGATGCGCCAGACCTTGGGTGTCTCC
>dme_circ_0007783
GGTGGAGTTCGACGAGGAGGAGGAGGATGATGCCGAGGAGACCGCCACCGGCAGCCAGGAGAGCGGCAAC
GCCGATGATGAGGAGGAGGAGCAGCAGCACGACGAGCTGTAAACTACTTCACCTCACCCACTGTCCAAAT

AGATTGTACATAAGAGT
>dme_circ_0007783_junction_seq
ACTGTCCAAATAGATTGTACATAAGAGTGGTGGAGTTCGACGAGGAGGAGGAGGAT
>dme_circ_0007784
GATGTATCCCAACGACGACTGTACCTTCTTCCAAGTGCTGGCGAGGATTGTCTCTGGTACTCTACACGCT
GGCCAGGAGGTGCGTGTCCTGGGAGAGAACTACACCTTGCAGGATGAGGAGGACTCTCGCATTCTGCAAG
TCGGTCGTCTGTGGGTATTTGAGTCTCGTTACAAG
>dme_circ_0007784_junction_seq
TCTGTGGGTATTTGAGTCTCGTTACAAGGATGTATCCCAACGACGACTGTACCTTC
>dme_circ_0007785
GATATTTGGACCCGTGGAGTCGGGACCGAAGGCCCAAATGGAACGCGCCGCAAGCAGATCCCAATCGTAG
TTGACTTGGAAGAACTCGCCGATGCGCTTTTTGTTCCAGTTTATGCAGACGGTTCCGTTCTCAATGTCCT
CGGCCAGTCCCTTCTCCAGTGGCTCCGAGATCATGG
>dme_circ_0007785_junction_seq
CCCTTCTCCAGTGGCTCCGAGATCATGGGATATTTGGACCCGTGGAGTCGGGACCG
>dme_circ_0007786
GTATCCGACACTGTCGTGGAGCCGTACAATGCCACGCTGTCCATCCACCAGCTGGTGGAGAACACCGACG
AGACATTCTGCATTGACAACGAGGCTCTCTACGACATCTGCTTCCGCACGCTGAAGCTATCGTCGCCCAC
CTACGGCGATCTGAACCATCTCGTCTCCGGTGAGTACCCCTAGTCAATCCATCTAATGCATCCTCTGTTT
GTCTGTCCCATTTTCCCGTTTCCTGCTCCTACTCCTTCTGCTCTCCAATCGAAATCAATGGCGGAAAGGT
GTCTGAGGTGGTGGTGGAGCCATATAACGCCACGTTGTCGCTGCACCAGCTAATTGTGGACACCGATGAG
ACGTTCTGCATCGACAACGAGGCTTTGTATGACATCTGCTATCAGAGCTTGCGCATCTGCTCGCCCACCT
ACCAGGATCTCAACCACCTGGTGTCAGTGAGTAGCCAAGACATTAGCATTAACGTGAAGCAAAGATGAAG
ATGAGGATAATGATGACCAGATGTCCACACCAGGAGCAATAAGTCCACCCTACCTTTCCCACAATCAAAT
CAAAAGAACAGAATTTGAAGTCCATGTAATCTGTAATTTTTTTCTGACAGTAATGTAGCTTTTCCTCACC
CAACCAGGGTCACAATGTCCGGAGTGACCACCTGCCTGCGCTTCCCTGGCCAGCTGAACGCTGATCTTCG
CAAGCTGGCGGTGAACATGGTGCCCTTCCCGCGTCTGCACTTCTTCATGCCCGGATTCGCTCCGCTGACC
GCCAAGGGATCCCAGCAATATCGCGCCCTCACGGTGGCCGAGCTGACCCAGCAAATGTTCGATGCCAAAA
ACATGATGACCGCCTGTGATCCCCGGCACGGACGCTACCTCACCGTGGCCTGCATCTTCCGGGG
>dme_circ_0007786_junction_seq
ACCTCACCGTGGCCTGCATCTTCCGGGGGTATCCGACACTGTCGTGGAGCCGTACA
>dme_circ_0007787
TTCTGGGAAATCATCTCCGACGAGCACGGCATCGATCCCAATGGTTACTACCACGGCGAGTCCGCCCTGC
AGCACGAGCGGATCGATGTTTACTACAACGAGGCCAGCAGCGGGAAGTACGTGCCGCGAGCGGTGCTCAT
CGACCTGGAGCCGGGCACCATGGACTCCGTCCGCCAGTCGCCGGTGGGCCAGCTTTTCCGGCCAGATAAC
TTCGTTTACGGACAGTCGGGAGCCGGGTGAGTACAAATCCAGTTGCAGATAATCGCTGGTGTTCCAAACC
ACAAGAGATAAGCTCTTGTGCGTCAACGTGACACGCCATAGATGTTTGCGATCTATTTTTGATTGATATT
AGATATGAGCAACATGTTTTGTTTCATCATTTCCTTATCAATGTATTGACTATTGAATAACAAGCTAGAA
CTTCTTAATGGCGCTTAGCACCAAAAATGTTCTAACTCCGTTGAAAACAGTAGCATTTCGATGTCCTCCT
GTTTCTGTTTCGTAGCCAGCCTCTTTGGGCATTTTCCTAATGCATTTCCGTTGCACTCGTTAATGGGCGA
ATCTGGTGTGGTGTTCATGTGCTAGGACATAGCTTTTGTTTTTCTGCCGCTTTTCGGCATGCTTTTGTTT
CGGCCAAAGTCCGCTAGCGCACTATCATCGATGGTGCTGTGAGTGTCCTTTTGCCACGGCAGTTATTGCA
GCTGCGCCAGCCTTGTCGCAACATCGAAAAcaggagcagcagcgggagcaggagcagcagcaACATCCGT
GACAACAGCAGCCACTGAGGGCGGCCTTATCCTTGCTTTGATAAGTGCCAACCAATTGACAAGATGAGCA

CGGCATTGTTATCACAGGCGAGCTTATCAGCTAACAGCCGCCGGTGCGATAGTCCTGCCGCGGTCAGGAC
CTCCCGGCCGGCCCACTTTATGCTCAGCTGCCATGAGGATAATGACCATGATGGCTATGATGATTGCAAT
TATAAAATGGCCTGCGGATGACGATGAAAATTAATTTTGTACATGTCAGACGCTGTTGCTGTGGCCTCAA
TAATGACCCACAAACGCCGCGAACTTCGATGAGTTTTGGCGGATGAGTAGCACACAGCTATTATATGGTC
TAATTTAAGCTTAACATATATATGAATAGTTTAGTATATATTACATTGTAATAACATCTTGTTTATATCA
CAGGGCAACAACTGGGCCAAGGGCCATTACACAGAGGGCGCCGAGCTGATCGATTCCGTGCTGGAGGTGC
TGCGCAAGGAATCCGAGGGCTGCGACTGCCTGCAGGGCTTCCAGCTGGCTCACTCGCTTGGCGGAGGCAC
TGGCTCCGGCCTGGGCACGCTGCTCATCTCGAAGATTCGCGAGGAGTACCCCGACCGCATCATGAACTCC
TTCTCCGTGGTGCCATCGCCCAAGG
>dme_circ_0007787_junction_seq
TCCTTCTCCGTGGTGCCATCGCCCAAGGTTCTGGGAAATCATCTCCGACGAGCACG
>dme_circ_0007788
GAACAATTCCACATCTTTGTCGGCGACTTGAGCTCAGAAATTGAAACTCAGCAATTGCGCGAAGCATTCA
CACCATTCGGCGAAATCTCGTAAGTATTAAAATGCAAATTTTCCATTTGCCAGTTAACTCGGAATTGCCT
ttttcccattttccccaattctgcagcttttatttcgcctttttccccttttttttttGCGAAAATTTTCC
TGTCACACATTCTCTGATTATTGACAGGCCCGTGTGGAGAAGGctcgcaactcgcaactcgctactcCAT
AAAAAATTCATAACTGAACGGCAAAAAATGAATTTATGCAAATGCCACTTTGGCCGAACTCTTTTCGCTG
TCTGTAAGTGTCGCCAGAAGCGAGGGAGGGTGAGGCAAAAAGACGGGGCATTCAAATATTTAAGTTGCTA
TTTTTCGGCATGAAATGGCAAAGTCACTAGCCACCCACAACCACTTACCACCCACGAGCCACCTCCTCTC
CCGTGGCAACGACAGCATCAAAAACTTGGCTTAATTAACTTCTGTTTAATCGCAAAGTTTCGAGTCAAAA
TGTTAGTCCGTCTCCTGGGAGGAGCTGCTGCTCCTCCACCTCCTCCGCCCACAAAAAGAGGCCCTGCGGC
GGCTTATCAAAAATTACGCCAAAGTATTTGATTTTCGGATTTGCCGCTTGTTTGAACCACGAAACGGAGG
ACTCAGTCAGACTCAGTCTATCAGCAAATCTGCTCTGCTCATCTTCCGGCCGATGGCCAATTTAAATTTT
AATAATTTGAAAATCATTCTAATGACTCTGGCATGATTGGGTTAAAGGGTTCTCTGATTCGCTAATGAGC
TCATGTCAGGTCATGTCTTTCATCAAAAGCGGTTGACAGTGTTGTGTTTGCATAACTTGAATATGAAATT
TAGGGAAGTGTTTAAAAGTCTGCAATTCCAATTCAGAATAAGCCCGAGTAAGCCCGATTGTTGCCTACTC
TTAGGCACCTTTTAATAAAACACGTTTACAAGTTAACCTCAGCCAGGAATAGAAAAACGATACCACTCCC
AATCACTGCCTGTTGACAACCGTATCGATGGCTACGCATTTCAGTCCCATTGAACAATCGTTTGGCACCT
TTGTGATAGCTATCGCTGACATCATTATCGCCGATACTCCCTGAAAACCCATCCCACATCGCCAGCTGAT
TATCTTCGAAGGATGTTGCAGGTTGGGTGGTCGATGGTGGGTAGGGAGAGGGACGGGGTTGGTATGGGAC
CGGCTTAAAGAACTTTATTTCTTATGGCTTTTCCTTGGGAGCCGAGTCGATCTGCTAGCCAACATGTCAA
AACGCTTAACGCACAAAGTCGCCGGGATTCGGTCGTGCGGCTCCCAAGGATCAAGATGTCCCCTCTCCGG
CTGTCAAAACTTTTATGCGTGCCTGGCGTTGTTTGCCCttttttttggctttgtttttctgctttttttC
GGGGCATTGTTGCCCGGCTTGGTGTTGCCTGGTTTTGGCATTATATATATATATATAtttttttttttCGT
CGGAGGTCCCAGCGCATTTTGTTTCGTTTTGTTAAATGCGCGGCGCGTGTGAATATTTAATAAGACCTGG
CAACAAGCCCCAGAAGCCAAAGCCCAAAACAAAGTCAAAGCCTGGCCGAAAGACCCGATGGGTTCGCTAG
CTCGGCGTTTTACTTATAATAATCATaaaaaaaaGTTGCCGGCCAGCGTCGCCAACTTCGCCGCCGCTGT
GGTTTATGCTTCATAAATGAAAAGTAAACTGCCGGACCGGGGGTAATTGCCAAAGTTTCCTGGCAGCCCG
CTTTAGTGCGGTGTCGAGGCCCCAATTGTCCAAATGAGAAGTCGTACAGCCGCTGGGGGAAATCAATTTG
GGCCCCCGAGTCGGAAGTTGGCCAATTGGCACAAGTGCCGGGTCACTAGTGTAAGTGACCAAAACGAGCT
GGTCCTTTTATCCCGCAGCCGTAATGAGCGCAGTTCGATTTGGTAGAAGGGAATGTATCAAGATGGGGGC
AAATCACGGTTCGGATGGCAGAAATTCATCAAAAACTTTATCGTTATCTTAAAGCCCAGCGATGAGATGA
CCGTACTGGCTTCTTGGCAGCTCAAATCCGAAAGCTTTCCTTGGCAAACTGCAAGATAAATAAAAAGTTT
GCGGCATGAGGTCAGATTTCTAATTTCCGAATTACAAGGGGCCACGCCCACTTCCACGCCCAAGCCGGCA

AACACAGACTGAATATATTTGTATTTGTACTCGAACGGAAATATTTGAACACGTCGGCCAGACGAAGACG
CAGGCGGAGTCCTCGAGAGATGTCTTTATTAGGAATTTGCATAAATTTTAAAGTTTGCCAGGACTTCCGT
TCGGCTGAAAGGCAGGGTACAAAATATATATATTTAAGACTTAGTGAACTCTGTTCGGGACTTTAATGTT
TACATTTCGTGTTATTTTCTCTCATTTTCTTGCAGCGACTGTCGCGTGGTGCGCGATCCACAAACCTTGA
AGTCGAAGGGCTATGGCTTTGTGTCCTTCATCAAGAAATCGGTAAGAGCCGCCAATGGCATTAGCATATT
TAAGTGCATATTTATGCAGTCGTGCACAGGCTCTTTCTTTGAATTTGGCAAAGCACGCACAAAAATATCA
AAAAATTTGGTTTTACTAAAAATAACATTTTGAATAATGCTGAAGAAATCTCATATAGATTGTATATATC
ATTTAATTTTTATTACCTTGTAAAACCTTATTTATTTGAGATGACTTTATTCAAGTGATATTTTTCACCG
TGTCCTTCTTACCACGATTCTGCTTCTGACCGTCTCTCTGTGTTCATTATCGCCCCGCAGGAAGCCGAAA
GCGCCATCACGGCCATGAATGGCCAATGGCTTGGCTCCCGCTCAATTCGCACGAATTGGGCCACACGGAA
ACCGCCGGCGAGTAAAG
>dme_circ_0007788_junction_seq
GCCACACGGAAACCGCCGGCGAGTAAAGGAACAATTCCACATCTTTGTCGGCGACT
>dme_circ_0007789
GCGACTGTCGCGTGGTGCGCGATCCACAAACCTTGAAGTCGAAGGGCTATGGCTTTGTGTCCTTCATCAA
GAAATCGGTAAGAGCCGCCAATGGCATTAGCATATTTAAGTGCATATTTATGCAGTCGTGCACAGGCTCT
TTCTTTGAATTTGGCAAAGCACGCACAAAAATATCAAAAAATTTGGTTTTACTAAAAATAACATTTTGAA
TAATGCTGAAGAAATCTCATATAGATTGTATATATCATTTAATTTTTATTACCTTGTAAAACCTTATTTA
TTTGAGATGACTTTATTCAAGTGATATTTTTCACCGTGTCCTTCTTACCACGATTCTGCTTCTGACCGTC
TCTCTGTGTTCATTATCGCCCCGCAGGAAGCCGAAAGCGCCATCACGGCCATGAATGGCCAATGGCTTGG
CTCCCGCTCAATTCGCACGAATTGGGCCACACGGAAACCGCCGGCGAGTAAAG
>dme_circ_0007789_junction_seq
GCCACACGGAAACCGCCGGCGAGTAAAGGCGACTGTCGCGTGGTGCGCGATCCACA
>dme_circ_0007790
ATAAATGCGCTAAACAAACAAAAACCGAAAACGACCAAAATCCAATGTGAATCAAAAGGGGCAGTGGGAT
TTAACCCCAGGGCCAGGACAGATGTTGCTGTTCCCTTGCGAACGGGAAAATAGTGAACATGGCCCATAGC
CCATGGTTATTGATGGTCCGAGTAGAGGCAATTACTGTTGAAGCACCGAAAAACCGAGACGCAAAGAAAT
ATTAAACAAATTATGAGCATCAGAGCGATTGTGGCGCAATAAAATTGGCCATAAAAAAACTGTTCATACC
GCGCAGCAAGCAATAAAAGAGGAAAACTAATATTTGACAAACTGTCAGCGGAGTCACTCGAAAGTAAAAT
TGTGAACATTTGCAGAAAGTTGCTGGGTAGCACTACCCAAAGAAAACCCCGAGCGTTTGCACTCGTTGCG
TATACGCCGTGTCGGCAGTTCTGCATTTTTACAGGAGGTGTTAAATGCGTTCGCTAGTCTCAGCTGGAGC
GGCAGCTCCACCGACCACAGTCCCCCTCGGATCCCCCGCTGGAAAGTGCCAGTGAAAGCCAGTGAAAGTA
CCAAGCTGTGCACTATTTCCGCGTAACTGAACATTATCGCTCTCGCTCCCTCGCGGGAGGGGATGCGCTC
CTGGAAAAAATCGCAGTCGATCCGAAATGCAAACACTCGAAACGAGAGTGCGGCCCCTGCAGGGCGCTCAG
CTAACTCAGCTGCTCCTCGGTGGCATCCTCTGCCTCACAATCGG
>dme_circ_0007790_junction_seq
TCGGTGGCATCCTCTGCCTCACAATCGGATAAATGCGCTAAACAAACAAAAACCGA
>dme_circ_0007791
TCTTCGAATCCGACTCCAAAGTAAATAACTGGAGGACCTACACGGCAACAACATCAGAAACCCACTTGAT
CCCATTTAAGCAAGAGCGAGCTTAGGGAGGCTGGCTGGAACCCATAATCGACTTCAATTGAATACATTAA
TGTAGCACCATGGGCGCCAATACATCGAGCAGATCCGACGCGAGTCTGCTCGCCGATGACGATGGTAAGT
ACAGAAAGGAGGTCTATTGACCCTTGACAATAAATATAATATTTTTTACAATGCCTACTACGCGCCGAGC
AACATAACttttatttttttatttatttttattCAAACTGAATAGTTTAAACAAGTGCTGTCAAAATAAAA
TTGCCCACAAATTAAGTAAATTTTCTGAGAATGTGCGCTAAAATCGTCCAATAGTTTTTCACAATGGGAT

CGAAAAACGCAGTGGGCTTAGGGCTTAAACATCTAACGCTAAGTTTGGGGAGCACATACACCCGGGGCGA
ACCGGAGGCAATGGAGTTTGTAGACGATCTGTGCCTGGCGAGGATATTAGGACTCCTGGAACTGCCCGAT
CAGATTGCTATGATGCAGAGCTACGAGCGCTGTCGTCCTCTGCTGGGAAGTATATGGCGTCACCGACTGA
CCAGGATCTCTCTAAACCTGCTCGAGATACCCCTTCTCGGCGATGACTTCAGGTTCCTCTTGGCTAGCGC
CTGCCAGCAGCTGCATGAGCTGAGGATCAGCTTCTTAGGCAGGGAGCACTTCCAGGCGCTCATCATGCAT
TGTTTTCCCAATTTGTGGCTTTTGCAAGTCGATGTTCTACCCCCGTTTTTCCTGTGCCCACGGCAAAGAC
TTCAGTTGATTCAGATTATCCCGAAATTCCATCCCGAGGACCACTCGCTCGCAATGGGCCACATGCTGTG
CGTAAAATGCCAAAGCCTAATTCCGAGTTAGAAGCCATCGGGTGTGTGCAAATAAAAGTTCATATATATA
TTTGTTACGAATATTTATGCACTGTGGACGAATAGGCAACACACACTATTGCCTTAATACTCATATACAC
TTTCCCTATCTTACGTTCCAGTGTGTGCATTGTCCAAAAGCGGGATACCGGCATCCTGTACGCGATGAAA
TATGTAAGTCGGTCCGCCTGCGAAATGCGCGGAGCTCTTGGTGGCGTCATCAAGGAGGTGGAACTGCTAT
CGTCGTTGGAGCACCCGTTTCTCGTCAATTTGTGGTTCTCTTTTCAGGG
>dme_circ_0007791_junction_seq
TCGTCAATTTGTGGTTCTCTTTTCAGGGTCTTCGAATCCGACTCCAAAGTAAATAA
>dme_circ_0007792
GGAGGCGGAAACCGCCATCACTGCAATGAATGGCCAATGGCTGGGCTCGCGCTCGATACGCACAAATTGG
GCCACACGAAAGCCGCCGGCAACCAAGGCAGACAGTGAGTATCCCATCCCATCAGAGACACCAATCCAAA
CCACCAAGCCATCCCGCATCTCAATGTCACATCTCGCTCTTCCACAGTGAACGCCAAGCCGCTGACCTTT
GACGAGGTCTACAACCAGAGTAGCCCCACCAATTGCACTGTCTACTGTGGCGGCATCAACGGAGCCCTCT
CTGGCTTCCTCAACGAGGAGATTCTGCAGAAGACCTTTTCGCCGTATGGCACAATACAGGAGATACGGGT
CTTCAAGGACAAAGGATACGCATTCGTGCG
>dme_circ_0007792_junction_seq
TCAAGGACAAAGGATACGCATTCGTGCGGGAGGCGGAAACCGCCATCACTGCAATG
>dme_circ_0007793
GTTTAAATTATTCATATAAAAGTTGCAAACTTAAAGTGCACATAAAAGTCAGTGATCCAAGTGTGGTTGG
TTGTGTGTTATCATAAACTAAAGTCAACACTTCATGTGAAAAACAAAATAGTTGTGGCAGAACTACATAT
GTATAATTACACAACAGCCGATTGAATTGTCTCGTCGGACATCTCTCTCATTTTGTAATATCTATAATAT
TGATAGATCGGATCGTTAAGCTCATACAGATAGAGAAGCGTCTGGCCAATGCGGATGTGATTGTTTTTAT
GTGTGGATAAAATTGTATCAATTGATAAGGCGACATTTTAAAAAGAAGCTGGGAAATATATTTACAGATA
TCTAATGATAACAAAATAATATTTAATGAAAC
>dme_circ_0007793_junction_seq
ATGATAACAAAATAATATTTAATGAAACGTTTAAATTATTCATATAAAAGTTGCAA
>dme_circ_0007794
TTGGAGGTGCAGCCCAAGCTGGCCGAGTACCTGAAGAGCTTCAAGAACATTGAGGACTTCCGCGTGGTGG
AGCCGGAGATCAACGAGATGCTGCAAGGAGTGGGCAGCCTGAATCCAGGCCGCCAGCTCAATCGCCACCG
CAGTGTGTCGGAGAGCGATGGCACAGCTGTAATTGCGGCCGGCGGTCGTCGCCTCAACACGACcatcacc
aaccgcagcgactacg
>dme_circ_0007794_junction_seq
ACGACcatcaccaaccgcagcgactacgTTGGAGGTGCAGCCCAAGCTGGCCGAGT
>dme_circ_0007795
ggtggttgtggttttggtggttgctttggtggCTGAGGAAGCATTGTTCGTGGTTACACACACAGTGGCT
GATGATGAGGCGTTGGATGGGGCGTTGTTGGACGAGGACTTGGACTTGGACGAGGTGTTGTTCGTTTTGA
CATTATCGTTATTGTTGTTGAGCGTTTGTGAAAGCGGTGGCT
>dme_circ_0007795_junction_seq

TTGTTGAGCGTTTGTGAAAGCGGTGGCTggtggttgtggttttggtggttgctttg
>dme_circ_0007796
ggtggttgtggttttggtggttgctttggtggCTGAGGAAGCATTGTTCGTGGTTACACACACAGTGGCT
GATGATGAGGCGTTGGATGGGGCGTTGTTGGACGAGGACTTGGACTTGGACGAGGTGTTGTTCGTTTTGA
CATTATCGTTATTGTTGTTGAGCGTTTGTGAAAGCGGTGGCTGTT
>dme_circ_0007796_junction_seq
TTGAGCGTTTGTGAAAGCGGTGGCTGTTggtggttgtggttttggtggttgctttg
>dme_circ_0007797
GGAAGCATTGTTCGTGGTTACACACACAGTGGCTGATGATGAGGCGTTGGATGGGGCGTTGTTGGACGAG
GACTTGGACTTGGACGAGGTGTTGTTCGTTTTGACATTATCGTTATTGTTGTTGAGCGTTTGTGAAAGCG
GTGGCTGTT
>dme_circ_0007797_junction_seq
TTGAGCGTTTGTGAAAGCGGTGGCTGTTGGAAGCATTGTTCGTGGTTACACACACA
>dme_circ_0007798
GAATTGGCCGCTGGCTCAGCGTCTCGCTGGGCTTTGGCATCATGACTCGTGCTGGCAACAGGTGCAGTTG
TGGCTTGGCTGTTGGTAGTGGTGTTTGtggtggtggtgctgctgctgggattgctgttgctgctgctg
>dme_circ_0007798_junction_seq
gctgctgggattgctgttgctgctgctgGAATTGGCCGCTGGCTCAGCGTCTCGCT
>dme_circ_0007799
TTTGGAAGCCTATCTCTGGAGCCACCACATCCCGTTGAGCAGGCAAGGAAGCAGACAAGAGTTTCCCCAA
CCCGACAACGAGGATCAGCCGCTGACGTAGCCGGAGCAGGAGCAGCGAGTGTGCTGCCCACTCTGGAGAG
GAAACCGGAGTCAAAGAAACAGCAGGAGTAGCAGCCGCAACTGAGGAGCATGTCCAGCAAGGAGGAGACG
CCATCCACGGGAGGTGCCGCTCCTGTGGCCACTGTCGCCGCCTCGGGCAGCGCCACCACTTCCTACGCCA
ATGTGGTGCAGAACCTGGAGACCAAGAAGTCCGGCACCAGCAACACCACCACCACCACCGCCAGAGCCAC
CGTGGACAACAAAGAGAACCAGCCCAATCTGAAGAGCTTCAAGTCGTGGAGCGAGGAAGCCGCTGCCGCC
GAGGCAGCCGGCGAATCGCCAATCGTCGGCAGCGTTAGCCTAGCCCAGACGGGTGAGAAGCGTGCCGCTG
CCTCCGGTGGAGATAACACCGCCGAGAACTCATCGGAATTGGACGACAACAACGACTTCGTGCCGGTACT
ATCGCATCACCGCCGCGATCGCAAAAAGGCGCGCAAAGAGAAGCCGCGCGATCAGCAGCCGGCCGGAGGA
CGTGGCGATGGTCAGAAACCCCAGCAGGCTGGTGGCCGCCGCGTCCCCGGATCCTCCGACACCGAACGCA
AGTCAGTGCGCCCACGTGCGCCGCGCGGCGGTAGTCCCCGCAAATCGGCAGCTGGAGCTGCTGGCGCAGC
CGGATCCAATGCTGGAGCTGTGTCCTCCGCCGTTCCTGGTGGACCGAAAGTACACAAGGATCGCAAGGAT
ACCTCGCCCAGTGCCAGCATCGAGGGAGCCGGCAGCAGCGGCAACGAGGCCAAGTCCGCCGACGGGGATC
AGCAGGCGGGCGAGAAAGGCGCCGCTGGAGCAGCTCCGCCGCCCAAGCGATTCATTGCCGCCCCACCGCC
CAAGGTCAACGCATGGAAGG
>dme_circ_0007799_junction_seq
CCACCGCCCAAGGTCAACGCATGGAAGGTTTGGAAGCCTATCTCTGGAGCCACCAC
>dme_circ_0007800
ggattcaccggccagacgacgccaggaatcttctggcagaaggcgccaggactcCGATGGCAGAAGACGC
CGTGAATCGTCCGGCAACAGGCGCAGTCGCAGCCGTGGACGTTGGCACAGTTCCTCGCGCAGTCGCA
>dme_circ_0007800_junction_seq
CGTTGGCACAGTTCCTCGCGCAGTCGCAggattcaccggccagacgacgccaggaa
>dme_circ_0007801
GCAGCAACAGGAGCAGCAGCAGATATTGCGACTATAGCGGCAACAAACAGACATGGCCAGCCAAAGGATA
CGGAGCCGGCCAGAAGAAGGACGAAAAGGACGAAGAATGAGCAGCGCCTGCCGACCGGGAGCCTTGCATA

CGGCATTCAATATGCTGGCGTTGAGCTGGATTATAATCTCAGAGTTATTGCTCAGTGGTAAGTGTTCAAA
CGACCCACATATAAATGGCATATTGCAGGGCTTCCTCTTCCTCATCCCCATCCGTTTCCGAATTCCCCAC
CACTTGAGGCAGTCTGTCATGTTAAACAAGCTGAATTTATTATGCCAACTGCGTGTTGCGGGCCGTGGTT
AAAATTGATTATTTAATCTACCAAATAACTGTTTATACACATATATTATGGACTGAACCACCCATTCATC
CACCCATTGACCAAGCAAAAAACCAACTCTTCCGCTGAACGGCGGTGAAGTAGTCAATTTATTAAATGGC
CCGGGAATGGGCGAGCATTGGCCTTTGATTAATATTGGTTTGTTCAATTAAAGTTGCCATAATTTGGCCA
ACCACATGCCTGTCACTCCACTTGTTTCCGTCGCCATCCCGCCGATCCCACAAATCGATGAAAACTTTTC
GAATGACATGTGTATGAACCGTTGCGAGGACAGGTCATTTATTGAGTACACTACCCTTCCAGACCGAAAC
TACATATTTATCATGGGCTTTTATGAGTCAGGATAAGAAGACAATGAATATTAAAACATTTTAATATATT
TTCATTCGCTCTGAGCCGGCACTTAATCAAGTGCTCGGTTTGATTAACAAATTGTGTTTCCCGCGATCTC
GAAATATACATTTAGGTCCGAACTTTTACTCATTTAGTTGAATTTATCTTTATTCATTTAAATAAATGTA
TAGATCACATTAAGTATATAAGTGGGTAAGAAGAACACCCCGAAATTTAATTAGGGTACCTCACCTTGTA
ATTGAATAATGTTCCAAGTAAAATTAATAAGATCATTCCACATGCAGATATTTGCTATACCTAAAATGAT
GTGTGGAAATTCTTTTAAGGCCATATCTGTAAATGTGTACGATTTCATCATTGGCAATTAAAGGAGCATT
TAAACGTATTAAAACCTTAAAAAAATTATTTAACTTTAATTGCATATCTCAATGGCAAATAAAACATTTT
CTGCTCTTCACGTAAATTAATTTAACGTTTTGTGCTTATGGAATAATCATATTGGCACATCTCTTTATTG
GAATTTATTTTTAAATTTCCAATTGATTCGAATGAGCCATGTTTAATGTTGATAGTTGCGGCAACTCGCG
TTGAATTGCGCTATTCAGCCGGTTCAAACGAAAATTGCAAATCGAAATCGAAACGAAATGGAATAAAATA
AATAAATTGCATTTAATGCAAGTTAAATTTTTGGGCCGTACCCTCGACATTGTCATAAATTAACATGGAA
GTGGAGTTGCCGGTAGTTCATGTCTGATACCATTCCTTTGCCGCCCGGTTGGGTAATTGGGTTTTTCGGT
ATTTCttttttttttATGCCCAGTGTAGACCGCCTTTCCGTTTCCCGTTGGCCCCCGAAAATCGAATTCG
TACGGTTGCGTATACGACATGTGGGACAAGTCATTCGGGAGATTCTACGGTTAAACGACTTTTGGGACTG
TCTTTAATATTAATGCCACCGCACTCTTTCAGGGTAAGCGACACTTCCTCCAGGTATACTCGCGGGGAAC
AGCAGCCAAGTCAACAATACGGAGGATATTGTCGTCGGCGGCATGCTTAATTATTATAATTACTTGCACT
TTGTCTTGCCGTCGACGCCGTCGCTCCAGCAGTCTTCTGCCTCCTGCCGCCCGTCGCCTATTGTCCTGGC
TGCTCCTTCCGCTCGGCTCCTTCGGCTGCCACAGCTCCTCGACTATTCACCACTCAAGCTTTTCATTAAC
GCATTTTCCTTCCGTTCCTTCGCAGGCGGCGCTTTACGCGGTACGCTGGTACTTCGGACAGGAGGAGTTC
TACCGCTACGTTCCACGCGAGGCCAAGCCCACATTTGTCTTCGCCGTTGCCGGCATTAATGTTGAT
>dme_circ_0007801_junction_seq
CTTCGCCGTTGCCGGCATTAATGTTGATGCAGCAACAGGAGCAGCAGCAGATATTG
>dme_circ_0007802
GCAACAGGAGCAGCAGCAGATATTGCGACTATAGCGGCAACAAACAGACATGGCCAGCCAAAGGATACGG
AGCCGGCCAGAAGAAGGACGAAAAGGACGAAGAATGAGCAGCGCCTGCCGACCGGGAGCCTTGCATACGG
CATTCAATATGCTGGCGTTGAGCTGGATTATAATCTCAGAGTTATTGCTCAGTGGGTAAGTGTTCAAACG
ACCCACATATAAATGGCATATTGCAGGGCTTCCTCTTCCTCATCCCCATCCGTTTCCGAATTCCCCACCA
CTTGAGGCAGTCTGTCATGTTAAACAAGCTGAATTTATTATGCCAACTGCGTGTTGCGGGCCGTGGTTAA
AATTGATTATTTAATCTACCAAATAACTGTTTATACACATATATTATGGACTGAACCACCCATTCATCCA
CCCATTGACCAAGCAAAAAACCAACTCTTCCGCTGAACGGCGGTGAAGTAGTCAATTTATTAAATGGCCC
GGGAATGGGCGAGCATTGGCCTTTGATTAATATTGGTTTGTTCAATTAAAGTTGCCATAATTTGGCCAAC
CACATGCCTGTCACTCCACTTGTTTCCGTCGCCATCCCGCCGATCCCACAAATCGATGAAAACTTTTCGA
ATGACATGTGTATGAACCGTTGCGAGGACAGGTCATTTATTGAGTACACTACCCTTCCAGACCGAAACTA
CATATTTATCATGGGCTTTTATGAGTCAGGATAAGAAGACAATGAATATTAAAACATTTTAATATATTTT
CATTCGCTCTGAGCCGGCACTTAATCAAGTGCTCGGTTTGATTAACAAATTGTGTTTCCCGCGATCTCGA
AATATACATTTAGGTCCGAACTTTTACTCATTTAGTTGAATTTATCTTTATTCATTTAAATAAATGTATA

GATCACATTAAGTATATAAGTGGGTAAGAAGAACACCCCGAAATTTAATTAGGGTACCTCACCTTGTAAT
TGAATAATGTTCCAAGTAAAATTAATAAGATCATTCCACATGCAGATATTTGCTATACCTAAAATGATGT
GTGGAAATTCTTTTAAGGCCATATCTGTAAATGTGTACGATTTCATCATTGGCAATTAAAGGAGCATTTA
AACGTATTAAAACCTTAAAAAAATTATTTAACTTTAATTGCATATCTCAATGGCAAATAAAACATTTTCT
GCTCTTCACGTAAATTAATTTAACGTTTTGTGCTTATGGAATAATCATATTGGCACATCTCTTTATTGGA
ATTTATTTTTAAATTTCCAATTGATTCGAATGAGCCATGTTTAATGTTGATAGTTGCGGCAACTCGCGTT
GAATTGCGCTATTCAGCCGGTTCAAACGAAAATTGCAAATCGAAATCGAAACGAAATGGAATAAAATAAA
TAAATTGCATTTAATGCAAGTTAAATTTTTGGGCCGTACCCTCGACATTGTCATAAATTAACATGGAAGT
GGAGTTGCCGGTAGTTCATGTCTGATACCATTCCTTTGCCGCCCGGTTGGGTAATTGGGTTTTTCGGTAT
TTCttttttttttATGCCCAGTGTAGACCGCCTTTCCGTTTCCCGTTGGCCCCCGAAAATCGAATTCGTA
CGGTTGCGTATACGACATGTGGGACAAGTCATTCGGGAGATTCTACGGTTAAACGACTTTTGGGACTGTC
TTTAATATTAATGCCACCGCACTCTTTCAGCTCACTGCCTGAAGGACCTGAAGATATTTGTACCAGAGGC
CGTATTAATGGGAAATGCAGCGACATTGTCTTGTCAATACGATTTGGAGCAGGTAAGCGACACTTCCTCC
AGGTATACTCGCGGGGAACAGCAGCCAAGTCAACAATACGGAGGATATTGTCGTCGGCGGCATGCTTAAT
TATTATAATTACTTGCACTTTGTCTTGCCGTCGACGCCGTCGCTCCAGCAGTCTTCTGCCTCCTGCCGCC
CGTCGCCTATTGTCCTGGCTGCTCCTTCCGCTCGGCTCCTTCGGCTGCCACAGCTCCTCGACTATTCACC
ACTCAAGCTTTTCATTAACGCATTTTCCTTCCGTTCCTTCGCAGGCGGCGCTTTACGCGGTACGCTGGTA
CTTCGGACAGGAGGAGTTCTACCGCTACGTTCCACGCGAGGCCAAGCCCACATTTGTCTTCGCCGTTGCC
GGCATTAATGTTGAT

>dme_circ_0007802_junction_seq

CTTCGCCGTTGCCGGCATTAATGTTGATGCAACAGGAGCAGCAGCAGATATTGCGA

>dme_circ_0007803

GCTGGGTCGCTGGGTCACCGGACGGGTGGCCGGATTGAGCACATACTGCATGTATTGCTCCTCCTCGGGA
CTCAGAGGCCGGACAGGACCCACCGCCTTCAGAGTAACGCGCAGCGAGACCGGACGCCTCTGATTGTACA
CCTCGCAG

>dme_circ_0007803_junction_seq

CGGACGCCTCTGATTGTACACCTCGCAGGCTGGGTCGCTGGGTCACCGGACGGGTG

>dme_circ_0007804

TGTGTCGCGATATTTATAAGCCAGGAGAGTGCCCGGCTCTTTCAGCGAATGCCAGTGGATGTGCCCGGGA
ATGCTACACCGATGCCGACTGTCGTGGAGACAACAAGTGCTGCAGCGACGGCTGCGGACAGCTGTGTGTG
CATCCTGCTCGTCCAACTCAGCCGCCCAGGACTCAGGCTCCAGTGGTCTCCTATCCGGGCGATGCCAGAG
CCGCTCTGGAACCCAAGGAGGCGCATGAGCTTGATGTCCAGACAGCCATCGGCGGCATAGCCGTGCTGCG
CTGCTTCGCCACCGGCAATCCAGCCCCGAATATCACCTGGTCGCTCAAGAACTTGGTGGATCAACACGAA
CAAGGGACGATATGTGCTGACCGCCAACGGTGATCTGACCATTGTCCAGGTGCGCCAAACAGACGATGGC
ACCTACGTCTGCGTGGCCAGCAATGGTCTGGGAGAACCCGTGCGTCGTGAGGTCGCCCTCCAGG

>dme_circ_0007804_junction_seq

CCCGTGCGTCGTGAGGTCGCCCTCCAGGTGTGTCGCGATATTTATAAGCCAGGAGA

>dme_circ_0007805

GGTGATATTCGGGGCTGGATTGCCGGTGGCGAAGCAGCGCAGCACGGCTATGCCGCCGATGGCTGTCTGG
ACATCAAGCTCATGCGCCTCCTTGGGTTCCAGAGCGGCTCTGGCATCGCCCGGATAGGAGACCACTGGAG
CCTGAGTCCTGGGCGGCTGAGTTGGACGAGCAGGATGCACACACAGCTGTCCGCAGCCGTCGCTGCAGCA
CTTGTTGTCTCCACGACAGTCGGCATCG

>dme_circ_0007805_junction_seq

CTTGTTGTCTCCACGACAGTCGGCATCGGGTGATATTCGGGGCTGGATTGCCGGTG

>dme_circ_0007806
GTCCCAGCCAGAGTGCACGCAGTGCATCTGCGAGAATCCGTGCGAGGGCTACAGTTGCCCGGAGGGCCAA
CAGTGCGCCATCGATGTGGCCAGTTCCGACGATCGCCAGTTTGCTCCAGTGTGTCGCGATATTTATAAGC
CAGGAGAGTGCCCGGCTCTTTCAGCGAATGCCAGTGGATGTGCCCGGGAATGCTACACCGATGCCGACTG
TCGTGGAGACAACAAG
>dme_circ_0007806_junction_seq
CGATGCCGACTGTCGTGGAGACAACAAGGTCCCAGCCAGAGTGCACGCAGTGCATC
>dme_circ_0007807
gctccactgaaagcacaaccgaaggatccacagacagcacCCAATCCACCGATCTGGACAGCACCACCAG
TGATATCTGGAGCACCAGTGATAAGGATGACGAATCGGAGAGCAGTACTCCTTACTCCTTTGATTCTGAA
GTTACCAAGAGCAAGCCCCGTAAATGTAAGCCTAAGAAGAGTACCTGCGCCAAATCCGAGTACGGTTGCT
GTCCGGATGGTAAGTCCACTCCGAAGGGACCGTTTGACGAGGGCTGTCCCATTGCAAAGACCTGTGCGGA
TACAAAGTACGGTTGTTGCTTGGACGGAGTGTCTCCGGCTAAGGGCAAGAATAACAAGGGTTGTCCCAAG
TCCCAGTGCGCTGAGACTCTCTTTGGCTGCTGTCCCGATAAATTCACCGCCGCCGATGGTGAGAATGATG
AGGGATGTCCAGAGACCACTACTGTACCACCTACGACTACCACAGAGGAGACACAGCCAGAGACTACTAC
TGAGATCGAAGGATCAGGTCAAGACTCGACGACTTCTGAGCCAGACACCAAGAAGTCCTGCTCCTTCTCC
GAATTTGGTTGCTGTCCCGATGCGGAAACATCCGCCAAGGGCCCTGACTTCGAGGGATGTGGTCTTGCGT
CACCGGTTGCCAAGGGCTGCGCAGAGTCTGAGAACGGTTGCTGTCCGGATGGACAAACACCTGCAAGCGG
TCCGAATGGCGAAGGTTGCTCTGGATGCACACGTGAGCGCTTTGGATGTTGTCCGGACTCCCAAACCCCG
GCCCATGGTCccaacaaggagggctgctgcttggacactcagtttggctgctgccccgacaacatcttgg
ccgcgcgtggacccaacaacgagggctgcgaatgccactacactccgtatggctgctgcccggacaataa
gtcggccgcaactggctacaaccaggagggatgcgcctgcgagacgacccagtatggctgctgtcccgac
aagatcactgccgccaagggacccaagcacgagggctgcccttgcgagaccacccagttcggatgctgtc
cagatgggctcaccttcgccaagggaccGCACCACCACGGATGCCACTGCACTCAGACGGAGTTCAAGTG
CTGCGACGACGAGAAGACCCCGGCCAAGGGCCCCAACGGCGATGGCTGCACGTGTGTGGAGAGCAAGTTC
GGTTGCTGTCCCGATGGAGTCACCAAGGCGACGGACGAGAAGTTCGGTGGATGCGAGAATGTCCAGGAGC
CACCGCAAAAGGCCTGCGGCCTGCCCAAGGAGACGGGCACCTGCAATAACTACAGCGTCAAGTACTACTT
CGACACCTCCTACGGAGGATGCGCTCGCTTTTGGTACGGTGGCTGCGATGGCAACGACAACCGCTTTGAG
AGCGAGGCCGAGTGCAAGGACACCTGCCAGGATTACACCGGCAAGCACGTATGCCTGCTGCCCAAGAGCG
CAGGTCCCTGCACTGGCTTCACCAAGAAATGGTACTTCGATGTCGACCGCAACCGGTGCGAGGAGTTCCA
GTACGGCGGATGCTACGGTACCAATAATCGCTTCGATAGCTTGGAACAGTGCCAGGGCACTTGTGCTGCC
AGTGAAAATCTGCGCTACCTGTGAACAACCTGTGGAGAGCGGACCATGTGCCGGAAACTTCGAGCGTTGG
TACTACGACAACGAGACGGACATCTGCCGACCCTTCACCTATGGTGGATGCAAGGGCAACAAGAACAACT
ATCCCACGGAACATGCCTGCAACTACAACTGCCGCCAGCCCGGCGTGCTTAAAGGGTTTGAGATCAGATT
TAGTATGCTATGGTGCCTTGAGTTGGGTAATGAGACTAGTTGTATGCAATATTTTGCTTGGGTTAATGAG
TTATTTTAAGCTTAAACCTCCTGGTTTTTCCTGGCTAACGGTATGTTCTTCCCAAACGTTCCTTCTCCCC
CAAAAACTGCTTGGTTACTATATTGTATGTGCATGATTGCTTGTTACTTGCTATGCCGACTTTCCGAATC
TGCACCCGCATCCGAATCTCCTCCTGCACCAAACACCTTCACCATCCCACAACCCCAGACCGCTGTGCGC
TCCCTAAGCAAACTGGTGATTGCAGTGAAAAACTGGCCAAGTGGCACTTCTCCGAGAGTGAGAAGCGCTG
CGTGCCCTTCTACTACAGCGGTTGTGGTGGCAACAAGAACAACTTCCCGACCCTGGAGTCCTGCGAGGAC
CACTGCCCACGCCAAGTTGGTAAGCTTTAAATGTCGCCTGCTCCGGAGGCTGCTTTCAATGTGTTCCTGT
TTTCCGCTACCCAAACCCCCGCCCTACTGACACTCTTTCAATCCCTCAACCCAACTAGCTAAGGACATCT
GTGAGATTCCGGCCGAGGTGGGCGAGTGTGCCAACTACGTTACCAGCTGGTACTACGACACTCAGGATCA
GGCCTGTCGCCAGTTCTACTACGGCGGCTGTGGGGGCAATGAGAACCGCTTCCCCACCGAGGAGTCCTGT

TTGGCGCGTTGCGACCGCAAGCCCGAACCCACAACCACCACCCCCGCCACCAGGCCACAACCCAGTAGGC
AGGACGTCTGTGACGAGGAACCCGCTCCAGGAGAATGTAGTACCTGGGTCCTCAAGTGGCACTTCGATCG
GAAGATTGGGGCTTGCCGTCAGTTCTACTATGGCAACTGTGGCGGCAATGGCAATCGCTTCGAGACAGAG
AACGATTGTCAGCAGCGTTGTCTTAGCCAAGAGCCTCCTGCCCCGACTCCTCCACGAGCACCTGCTCCAA
CCAGGCAGCCAGATCCGGCTCCTACTGTCGCTCAATGCAGTCAGCCTGCTGATCCAGGACAGTGCGACAA
ATGGGCGCTCCACTGGAACTACAACGAGACCGAGGGTCGCTGTCAGTCGTTCTACTACGGCGGCTGTGGC
GGCAATGATAACCGCTTTGCCACCGAGGAGGAGTGCTCTGCTCGCTGCTCTGTCAACATAGACATCCGCA
TTGGTGCCGATCCCGTGGAGCACGACACGTCCAAGTGCTTCTTGGCCTTCGAACCGGGTAACTGCTACAA
CAACGTGACCCGCTGGTTCTACAACAGCGCGGAGGGACTCTGCGACGAGTTCGTCTACACGGGCTGCGGC
GGTAATGCCAACAACTATGCCACCGAGGAGGAGTGCCAGAACGAGTGCAACGACGCCCAGACCACATGTG
CACTGCCTCCGGTCCGTGGACGATGCAGTGACCTCTCCCGGCGCTGGTACTTCGATGAGCGCAGCGGAGA
ATGCCACGAGTTCGAGTTCACTGGATGCCGCGGTAATCGAAACAACTTCGTGTCGCAGAGCGATTGCCTA
AACTTCTGCATTGGGGAGCCTGTAGTGGAGCCTTCAGCGCCGGTTAGATTGTTCTTCTTTTCATGTGTCT
TAATCTTTATGCGAATATTATCTTACAGACCTATTCGGTGTGCGCCGAGCCTCCGGAGGCCGGCGAATGC
GACAACCGTACCACCGCGTGGTTCTACGACAGCGAGAACATGGCCTGCACGGCCTTCACCTACACGGGAT
GCGGAGGCAATGGAAACCGCTTCGAGACCCGCGACCAGTGCGAACGGCAGTGCGGCGAGTTCAAGGGAGT
GGGTAAGTTTGGGGCAGCCGGGCTGGGTACTTGTTTCAGATGCTATAATCTCTTGTGTCAGACGTTTGCA
ATGAACCGGTGACCACGGGTCCGTGCACCGACTGGCAGACGAAGTACTACTTCAACACAGCGAGCCAGGC
TTGCGAACCGTTCACCTATGGTGGATGTGATGGCACCGGCAACCG
>dme_circ_0007807_junction_seq
ATGGTGGATGTGATGGCACCGGCAACCGgctccactgaaagcacaaccgaaggatc
>dme_circ_0007808
GTAGTACTTCGTCTGCCAGTCGGTGCACGGACCCGTGGTCACCGGTTCATTGCAAACGTCTGACACAAGA
GATTATAGCATCTGAAACAAGTACCCAGCCCGGCTGCCCCAAACTTACCCACTCCCTTGAACTCGCCGCA
CTGCCGTTCGCACTGGTCGCGGGTCTCGAAGCGGTTTCCATTGCCTCCGCATCCCGTGTAGGTGAAGGCC
GTGCAGGCCATGTTCTCGCTGTCGTAGAACCACGCGGTGGTACGGTTGTCGCATTCGCCGGCCTCCGGAG
GCTCGGCGCACACCGAATAGGTCTGTAAGATAATATTCGCATAAAGATTAAGACACATGAAAAGAAGAAC
AATCTAACCGGCGCTGAAGGCTCCACTACAGGCTCCCCAATGCAGAAGTTTAGGCAATCGCTCTGCGACA
CGAAGTTGTTTCGATTACCGCGGCATCCAGTGAACTCGAACTCGTGGCATTCTCCGCTGCGCTCATCGAA
GTACCAGCGCCGGGAGAGGTCACTGCATCGTCCACGGACCGGAGGCAGTGCACATGTGGTCTGGGCGTCG
TTGCACTCGTTCTGGCACTCCTCCTCGGTGGCATAGTTGTTGGCATTACCGCCGCAGCCCGTGTAGACGA
ACTCGTCGCAGAGTCCCTCCGCGCTGTTGTAGAACCAGCGGGTCACGTTGTTGTAGCAGTTACCCGGTTC
GAAGGCCAAGAAGCACTTGGACGTGTCGTGCTCCACGGGATCGGCACCAATGCGGAT
>dme_circ_0007808_junction_seq
GCTCCACGGGATCGGCACCAATGCGGATGTAGTACTTCGTCTGCCAGTCGGTGCAC
>dme_circ_0007809
TTCACTGGATGCCGCGGTAATCGAAACAACTTCGTGTCGCAGAGCGATTGCCTAAACTTCTGCATTGGGG
AGCCTGTAGTGGAGCCTTCAGCGCCGGTTAGATTGTTCTTCTTTTCATGTGTCTTAATCTTTATGCGAAT
ATTATCTTACAGACCTATTCGGTGTGCGCCGAGCCTCCGGAGGCCGGCGAATGCGACAACCGTACCACCG
CGTGGTTCTACGACAGCGAGAACATGGCCTGCACGGCCTTCACCTACACGGGATGCGGAGGCAATGGAAA
CCGCTTCGAGACCCGCGACCAGTGCGAACGGCAGTGCGGCGAG
>dme_circ_0007809_junction_seq
CGACCAGTGCGAACGGCAGTGCGGCGAGTTCACTGGATGCCGCGGTAATCGAAACA
>dme_circ_0007810

GGCAATCGCTCTGCGACACGAAGTTGTTTCGATTACCGCGGCATCCAGTGAACTCGAACTCGTGGCATTC
TCCGCTGCGCTCATCGAAGTACCAGCGCCGGGAGAGGTCACTGCATCGTCCACGGACCGGAGGCAGTGCA
CATGTGGTCTGGGCGTCGTTGCACTCGTTCTGGCACTCCTCCTCGGTGGCATA
>dme_circ_0007810_junction_seq
CGTTCTGGCACTCCTCCTCGGTGGCATAGGCAATCGCTCTGCGACACGAAGTTGTT
>dme_circ_0007811
GTAGAACTGACGGCAAGCCCCAATCTTCCGATCGAAGTGCCACTTGAGGACCCAGGTACTACATTCTCCT
GGAGCGGGTTCCTCGTCACAGACGTCCTGCCTACTGGGTTGTGGCCTGGTGGCGGGGGTGGTGGTTGTGG
GTTCGGGCTTGCGGTCGCAACGCGCCAAACAGGACTCCTCGGTGGGGAAGCGGTTCTCATTGCCCCCACA
GCCGCCGTAGTAGAACTGGCGACAGGCCTGATCCTGAGTGTCGTA
>dme_circ_0007811_junction_seq
GGCGACAGGCCTGATCCTGAGTGTCGTAGTAGAACTGACGGCAAGCCCCAATCTTC
>dme_circ_0007812
GAACTGACGGCAAGCCCCAATCTTCCGATCGAAGTGCCACTTGAGGACCCAGGTACTACATTCTCCTGGA
GCGGGTTCCTCGTCACAGACGTCCTGCCTACTGGGTTGTGGCCTGGTGGCGGGGGTGGTGGTTGTGGGTT
CGGGCTTGCGGTCGCAACGCGCCAAACAGGACTCCTCGGTGGGGAAGCGGTTCTCATTGCCCCCACAGCC
GCCGTAGTAGAACTGGCGACAGGCCTGATCCTGAGTGTCGTAGTACCAGCTGGTAAC
>dme_circ_0007812_junction_seq
CCTGAGTGTCGTAGTACCAGCTGGTAACGAACTGACGGCAAGCCCCAATCTTCCGA
>dme_circ_0007813
GGCATGTTCCGTGGGATAGTTGTTCTTGTTGCCCTTGCATCCACCATAGGTGAAGGGTCGGCAGATGTCC
GTCTCGTTGTCGTAGTACCAACGCTCGAAGTTTCCGGCACATGGTCCGCTCTCCACAG
>dme_circ_0007813_junction_seq
TTTCCGGCACATGGTCCGCTCTCCACAGGGCATGTTCCGTGGGATAGTTGTTCTTG
>dme_circ_0007814
GTTGTTCTTGTTGCCCTTGCATCCACCATAGGTGAAGGGTCGGCAGATGTCCGTCTCGTTGTCGTAGTAC
CAACGCTCGAAGTTTCCGGCACATGGTCCGCTCTCCACAGGTTGTTCACAGGTAGCTACATTGGAAGAAT
GCCGATAATCGTGGCTCTGGAATAAGGAACAGATGCCCACTTACGCAGATTTTCACTGGCAGCACAAGTG
CCCTGGCACTGTTCCAAGCTATCGAAGCGATTATTGGTACC
>dme_circ_0007814_junction_seq
CCAAGCTATCGAAGCGATTATTGGTACCGTTGTTCTTGTTGCCCTTGCATCCACCA
>dme_circ_0007815
GTTATTGCAGGTGCCCGTCTCCTTGGGCAGGCCGCAGGCCTTTTGCGGTGGCTCCTGGACATTCTCGCAT
CCACCGAACTTCTCGTCCGTCGCCTTGGTGACTCCATCGGGACAGCAACCGAACTTGCTCTCCACACACG
TGCAGCCATCGCCGTTGGGGCCCTTGGCCGGGGTCTTCTCGTCGTCGCAGCACTTGAACTCCGTCTGAGT
GCAGTGGCATCCGTGGTG
>dme_circ_0007815_junction_seq
CCGTCTGAGTGCAGTGGCATCCGTGGTGGTTATTGCAGGTGCCCGTCTCCTTGGGC
>dme_circ_0007816
tcggccgcaactggctacaaccaggagggatgcgcctgcgagacgacccagtatggctgctgtcccgaca
agatcactgccgccaagggacccaagcacgagggctgcccttgcgagaccacccagttcggatgctgtcc
agatgggctcaccttcgccaagggaccGCACCACCACGGATGCCACTGCACTCAGACGGAGTTCAAGTGC
TGCGACGACGAGAAGACCCCGGCCAAGGGCCCCAACGGCGATGGCTGCACGTGTGTGGAGAGCAAGTTCG
G

>dme_circ_0007816_junction_seq
GCTGCACGTGTGTGGAGAGCAAGTTCGGtcggccgcaactggctacaaccaggagg
>dme_circ_0007817
GTGGCATCCGTGGTGGTGCggtcccttggcgaaggtgagcccatctggacagcatccgaactgggtggtc
tcgcaagggcagccctcgtgcttgggtcccttggcggcagtgatcttgtcgggacagcagccatactggg
tcgtctcgcaggcgcatccctcctggttgtagccagttgcggccgacttattgtccgggcagcagccata
cggagt
>dme_circ_0007817_junction_seq
tattgtccgggcagcagccatacggagtGTGGCATCCGTGGTGGTGCggtcccttg
>dme_circ_0007818
gatgttgtcggggcagcagccaaactgagtgtccaagcagcagccctccttgttggGACCATGGGCCGGG
GTTTGGGAGTCCGGACAACATCCAAAGCGCTCACGTGTGCATCCAGAGCAACCTTCGCCATTCGGACCGC
TTGCAGGTGTTTGTCCATCCGGACAGCAACC
>dme_circ_0007818_junction_seq
CAGGTGTTTGTCCATCCGGACAGCAACCgatgttgtcggggcagcagccaaactga
>dme_circ_0007819
GCGCTCACGTGTGCATCCAGAGCAACCTTCGCCATTCGGACCGCTTGCAGGTGTTTGTCCATCCGGACAG
CAACCGTTCTCAGACTCTGCGCAGCCCTTGGCAACCGGTGACGCAAGACCACATCCCTCGAAGTCAGGGC
CCTTGGCGGATGTTTCCGCATCGGGACAGCAACCAAATTCGGAGAAGGAGCAGGACTTCTTGGT
>dme_circ_0007819_junction_seq
ATTCGGAGAAGGAGCAGGACTTCTTGGTGCGCTCACGTGTGCATCCAGAGCAACCT
>dme_circ_0007820
GCGCTCACGTGTGCATCCAGAGCAACCTTCGCCATTCGGACCGCTTGCAGGTGTTTGTCCATCCGGACAG
CAACCGTTCTCAGACTCTGCGCAGCCCTTGGCAACCGGTGACGCAAGACCACATCCCTCGAAGTCAGGGC
CCTTGGCGGATGTTTCCGCATCGGGACAGCAACCAAATTCGGAGAAGGAGCAGGACTTCTTGGTGTCTGG
CTCAGAAGTCGTCGA
>dme_circ_0007820_junction_seq
TCTTGGTGTCTGGCTCAGAAGTCGTCGAGCGCTCACGTGTGCATCCAGAGCAACCT
>dme_circ_0007821
GGTGGTACAGTAGTGGTCTCTGGACATCCCTCATCATTCTCACCATCGGCGGCGGTGAATTTATCGGGAC
AGCAGCCAAAGAGAGTCTCAGCGCACTGGGACTTGGGACAACCCTTGTTATTCTTGCCCTTAGCCGGAGA
CACTCCGTCCAAGCAACAACCGTACTTTGTATCCGCACAGGTCTTTGCAATGGGACAGCCCTCGTCAAAC
GGTCCCTTCGGA
>dme_circ_0007821_junction_seq
ACAGCCCTCGTCAAACGGTCCCTTCGGAGGTGGTACAGTAGTGGTCTCTGGACATC
>dme_circ_0007822
TACCTGCGCCAAATCCGAGTACGGTTGCTGTCCGGATGGTAAGTCCACTCCGAAGGGACCGTTTGACGAG
GGCTGTCCCATTGCAAAGACCTGTGCGGATACAAAGTACGGTTGTTGCTTGGACGGAGTGTCTCCGGCTA
AGGGCAAGAATAACAAGGGTTGTCCCAAGTCCCAGTGCGCTGAGACTCTCTTTGGCTGCTG
>dme_circ_0007822_junction_seq
AGTGCGCTGAGACTCTCTTTGGCTGCTGTACCTGCGCCAAATCCGAGTACGGTTGC
>dme_circ_0007823
gctccactgaaagcacaaccgaaggatccacagacagcacCCAATCCACCGATCTGGACAGCACCACCAG
TGATATCTGGAGCACCAGTGATAAGGATGACGAATCGGAGAGCAGTACTCCTTACTCCTTTGATTCTGAA

G
>dme_circ_0007823_junction_seq
AGTACTCCTTACTCCTTTGATTCTGAAGgctccactgaaagcacaaccgaaggatc
>dme_circ_0007824
GAATCAAAGGAGTAAGGAGTACTGCTCTCCGATTCGTCATCCTTATCACTGGTGCTCCAGATATCACTGG
TGGTGCTGTCCAGATCGGTGGATTGGgtgctgtctgtggatccttcggttgtgctttcagtggagccttc
ggtagtactttcCTCCGTTGGACCGCTTTCCGTA
>dme_circ_0007824_junction_seq
actttcCTCCGTTGGACCGCTTTCCGTAGAATCAAAGGAGTAAGGAGTACTGCTCT
>dme_circ_0007825
ggaaccatctgtggaAGAGGATGAGTCTGTGGAACCAGATACTTCAGTGGAAGGTGAGGAGTCGGTGATG
GTAGAATCAGATGTAACATCAGAGCTTGTGGAACTGGACAGGTCCTCGACGGTACTTCCCTCGGTGGTCG
AACCATCACTGGCGATGGCGTCGGTGCTTTCGGTTGATCCGGAGGACTCCGTGGAGATTGTGGTGTCCTC
GCTTGCgccttca
>dme_circ_0007825_junction_seq
GATTGTGGTGTCCTCGCTTGCgccttcaggaaccatctgtggaAGAGGATGAGTCT
>dme_circ_0007826
GTCCTGTGTCTTTCCAATGGAACCAAGTCAGTCAACTGCGATGAAGAAAAGGTCGAACCTCTATCTGAAA
AGTGTAACTCTGAAGCCTGCACTGAGGATGAGATTTTACCTTTGACCAGTACTGATAAGCCCATCGAGGA
TGACGAGGAGGATTGCGATGAAGATGG
>dme_circ_0007826_junction_seq
ATGACGAGGAGGATTGCGATGAAGATGGGTCCTGTGTCTTTCCAATGGAACCAAGT
>dme_circ_0007827
GTTGACTGACTTGGTTCCATTGGAAAGACACAGGACCTCACGGACTCGCTCACCCCCACCACAAGGCTTG
CTGCACTTGCCCCAAGGTCCAGTGAACCATTCGCCGGGGCAAACCTTCTCCTCGCCCTCACAATCCT
>dme_circ_0007827_junction_seq
CAAACCTTCTCCTCGCCCTCACAATCCTGTTGACTGACTTGGTTCCATTGGAAAGA
>dme_circ_0007828
GTGTATCCGGAGGAATTCTGTGAACCAGAGGTACCCACTCTATCCCGGCCGTGCAAATCCCCCAAGTGCG
AGGCGCAATGGTTCTCCTCGGAGTGGAGCAAGTGCTCTGCTCCTTGCGGAAAAGGTGTCAAATCTCGCAT
TGTCATTTGCGGGGAGTTCGATGGCAAGACGGTAACTCCAGCAGATGATGACTCTAAG
>dme_circ_0007828_junction_seq
GGTAACTCCAGCAGATGATGACTCTAAGGTGTATCCGGAGGAATTCTGTGAACCAG
>dme_circ_0007829
GTAAGGTGTATCCGGAGGAATTCTGTGAACCAGAGGTACCCACTCTATCCCGGCCGTGCAAATCCCCCAA
GTGCGAGGCGCAATGGTTCTCCTCGGAGTGGAGCAAGTGCTCTGCTCCTTGCGGAAAAGGTGTCAAATCT
CGCATTGTCATTTGCGGGGAGTTCGATGGCAAGACGGTAACTCCAGCAGATGATGACTCTAAG
>dme_circ_0007829_junction_seq
GGTAACTCCAGCAGATGATGACTCTAAGGTAAGGTGTATCCGGAGGAATTCTGTGA
>dme_circ_0007830
GTCACATGCAACAATCGCATCACCTTGGCGGAAGTCAATCCATCGCTGTGCGACCAAAAGTCCAAGCCAG
TGGAGGAACAGGCTTGCGGCACGGAACCGTGTGCACCACACTGGGTTGAGGGAGAGTGGTCCAAG
>dme_circ_0007830_junction_seq
ACACTGGGTTGAGGGAGAGTGGTCCAAGGTCACATGCAACAATCGCATCACCTTGG

>dme_circ_0007831
GAGCCACCGCCGCAGGAAGCACTGCAGGCATTGAACTGGTGATGCGTCCATGTGTGCGTATCCTGCTGCG
AGTGACTAAGGCTCTCCGGAATGCTGTACTCGTAGTCGAGGCTGATGTTCTTCTCCTGCACCAACATAAC
TATGAAGAGGCTCTCCGAGATGGGACCGCTGCAGGTCAGTTGGTCGGGAGCGGCGAAGCCCATTGGCTTT
CGTTGGTAGTTCCACCACGAGTTCGCGAAGAACATGGGGCGTGGGAAGTCGATGCGCCAATCGCCGTTCA
GGTAGTA
>dme_circ_0007831_junction_seq
CGATGCGCCAATCGCCGTTCAGGTAGTAGAGCCACCGCCGCAGGAAGCACTGCAGG
>dme_circ_0007832
GGCTCTCCGGAATGCTGTACTCGTAGTCGAGGCTGATGTTCTTCTCCTGCACCAACATAACTATGAAGAG
GCTCTCCGAGATGGGACCGCTGCAGGTCAGTTGGTCGGGAGCGGCGAAGCCCATTGGCTTTCGTTG
>dme_circ_0007832_junction_seq
GAGCGGCGAAGCCCATTGGCTTTCGTTGGGCTCTCCGGAATGCTGTACTCGTAGTC
>dme_circ_0007833
GTCGAGGCTGATGTTCTTCTCCTGCACCAACATAACTATGAAGAGGCTCTCCGAGATGGGACCGCTGCAG
GTCAGTTGGTCGGGAGCGGCGAAGCCCATTGGCTTTCGTTGGTAGTTCCACCACGAGTTCGCGAAGAACA
TGGGGCGTGGGAAGTCGATGCGCCAATCGCCGTTCAGGTAGTA
>dme_circ_0007833_junction_seq
CGATGCGCCAATCGCCGTTCAGGTAGTAGTCGAGGCTGATGTTCTTCTCCTGCACC
>dme_circ_0007834
GTGGCTCCCTCTGGAAGGAGCAGCAAGTCATTGTATCCAGGAGCCAGATCCTTCGTGGTGATGGTGTTGC
GAATGGTCTTGCAGGTGCTGCCATCACCACCACACTTTCGGCACTTGTCCTCCTTGGCATCGCTACCCAG
CATCAT
>dme_circ_0007834_junction_seq
CCTCCTTGGCATCGCTACCCAGCATCATGTGGCTCCCTCTGGAAGGAGCAGCAAGT
>dme_circ_0007835
GACTCCATCAAACTGCTGCCTGTCGAAGCGGGAGCACTGCTGCGCCCGGAAGTCGGATTCTTCCTCCGGA
CAGTCTTGGGTGTTGCAGGAGAAGTAACGGCGACTGCCTCCACTGCAGACTGCCTCGCCACGATCACTGT
GGAGTGGTAAGAGATGTAAGAGATTTAAAACTATTGGCAATCTAGAGGTCTCTTTCCTAATATAACATTC
GGCTATGATAGGGATGGAATAGGGTTGTTTTCTTTTACGGCAGGTCTTGTATACTCACTCTCTTCGCAGG
CACTCCCGAGTCTGGTAGGACACTCCACCGCCGCAGGTCCTGGAACAGTCCGAGGGTGAGCTCCAAGGTG
TCCACTCATCGGGATC
>dme_circ_0007835_junction_seq
AGCTCCAAGGTGTCCACTCATCGGGATCGACTCCATCAAACTGCTGCCTGTCGAAG
>dme_circ_0007836
GACTCCATCAAACTGCTGCCTGTCGAAGCGGGAGCACTGCTGCGCCCGGAAGTCGGATTCTTCCTCCGGA
CAGTCTTGGGTGTTGCAGGAGAAGTAACGGCGACTGCCTCCACTGCAGACTGCCTCGCCACGATCACTGT
GGAGTGGTAAGAGATGTAAGAGATTTAAAACTATTGGCAATCTAGAGGTCTCTTTCCTAATATAACATTC
GGCTATGATAGGGATGGAATAGGGTTGTTTTCTTTTACGGCAGGTCTTGTATACTCACTCTCTTCGCAGG
CACTCCCGAGTCTGGTAGGACACTCCACCGCCGCAGGTCCTGGAACAGTCCGAGGGTGAGCTCCAAGGTG
TCCACTCATCGGGATCGTTACCCTCGCCGCCGGGCGTCACCGAGCTCTCCGGCAAGTACAT
>dme_circ_0007836_junction_seq
GCGTCACCGAGCTCTCCGGCAAGTACATGACTCCATCAAACTGCTGCCTGTCGAAG
>dme_circ_0007837

AATCAGAATCAGAATCGCAAACGGAATCAGAACCTTCCAGCCAACAAGTGACAAGTGCCGAAGTGCTCTC
GATAAAATGCCAGATATATGCAAACCGATTGAAGCAGTGATAGTGCACTGAAATCACAGTTGATATACAA
TATATCAGAGTAAAGCCACGTTTTAAAGACCTCCTTTTTGATTTTCG
>dme_circ_0007837_junction_seq
GTTTTAAAGACCTCCTTTTTGATTTTCGAATCAGAATCAGAATCGCAAACGGAATC
>dme_circ_0007838
CAACAAGGAGGCAACAGCGACGACGAGGAGGGATACGGTGGATACTATGGAGCCGGTGGCAGTGGCTATT
ATGGTGGCGCCGCCAGTGGGGGCTACTCCTACGGGggtgatgacgatgaagatgag
>dme_circ_0007838_junction_seq
CTACGGGggtgatgacgatgaagatgagCAACAAGGAGGCAACAGCGACGACGAGG
>dme_circ_0007839
GTGCAAAAGCAGCATCAGCTGAATGGCAAAATGGTGGACGTAAAGAAGGCCTTGCCCAAGCAGAATGACC
AACAGGGAGGTGGCGGAGGACGCGGTGGTCCGGGTGGTCGTGCCGGTGGAAACCGCGGAAACATGGGCGG
TGGAAACTACGGCAACCAGAATGGTGGCGGCAACTGGGAACAACGGTGGcaacaactggggcaacaaccg
cgggggtaacgacaactggggcaacaacaGCTTCGGTGGTGGCGGCGGCGGCGGTGGTGGTTATGGCGGT
GGCAACAACAGCTGGGGCAATAACAATCCGTGGGACAATGGCAATGGAGGCGGCAACTTTGGAGGCGGCG
GCAACAATTGGAACAATGGTGGCAATGATTTTGGAGGCTACCAGCAGAACTATGGCGGCGGTCCGCAGCG
AGGTGGCGGCAACTTCAACAACAATCGCATGCAGCCCTACCAAGGAGGTGGTGGATTCAAAGCAG
>dme_circ_0007839_junction_seq
TACCAAGGAGGTGGTGGATTCAAAGCAGGTGCAAAAGCAGCATCAGCTGAATGGCA
>dme_circ_0007840
GGCGGCGGCAACAATTGGAACAATGGTGGCAATGATTTTGGAGGCTACCAGCAGAACTATGGCGGCGGTC
CGCAGCGAGGTGGCGGCAACTTCAACAACAATCGCATGCAGCCCTACCAAGGAGGTGGTGGATTCAAAGC
AG
>dme_circ_0007840_junction_seq
TACCAAGGAGGTGGTGGATTCAAAGCAGGGCGGCGGCAACAATTGGAACAATGGTG
>dme_circ_0007841
GCAAGACACAGTACCCTGACACCTAACTGTTAGAGCGTCGATAGAGTTTGGCAGTTAGCTTATTAAACGC
TGTGAGATCAACTGACGATGGCCTTCGCTATGTTATGCGTATGTAAGATAATCCCAGTACGACGTAACAC
GCAGCCAC
>dme_circ_0007841_junction_seq
ATCCCAGTACGACGTAACACGCAGCCACGCAAGACACAGTACCCTGACACCTAACT
>dme_circ_0007842
GAGCAGAAAACACAAAATATATAAAAACATGTATTACATTTTACAACTTTACTAGGCTAAGTTCTCATAG
AATTAAATCGAGTAGAGCGTGCGGAGCGACTTCTGCATCCATCCGCACGCTACCCTAGTCTTAATAATTT
TACTATTCAAGTTCTAAGCGAACCTGACC
>dme_circ_0007842_junction_seq
ACTATTCAAGTTCTAAGCGAACCTGACCGAGCAGAAAACACAAAATATATAAAAAC
>dme_circ_0007843
GAGCTCCTGCTGACGGATCAGCAAGCGAACAGCGTGCTAGGAGCCTTGGGGCTGCTGCGCCTCCTGCTGC
CACAGCTTATACGCGGCTACCCCGGCGATAGCCACGAGGATTCCGAGTCCTTGGCCGGCAAGAAGCAGCA
ACAGCAGCAGACGACGACCTCCGACTGTCGGCAAATTATTGAAATTTATGATTATTGTTTGCACCTGCTG
AGCACGCAGCACACCGCGAATCACGCCATCATAAATGCCACCCTGGAGGTCATCAACGGCATACTCCAGG
CTGTGGACGCCGCATCCGATGGCCAATGTAGCCAGAGTCTGGGCCAAAGTTTGCGCCAACTGTTGTGCAA

TCAACAACTGCAGCACAATGAGTATTTGCGGCGAAGGAAATCATTAAAGAATCAAATATTCCAATTGAAA
AATTACGAAGTGGCGACAAGTCAACACCAGCTCGAGGATGAGGATGAGAACGAGGATGTGGATGAGCTGG
TGGTTGGGGCCACTGCAATGCAAATGAAAAAGAATTCAAATGCAAAACTGCAGCAGGCGAAGTGTCGAGa
gcagcagcagcatcagcatcagcagcaACTCGAGGTTGACAACAGCTCGCTGGGAATTAATGCTGGGGAA
GATGCCCCGACCGAGGCTCCAAGTTCCGTCGCTGATGAAGGGTATGGCAAATAAGGCAGAATGCAAGTCG
AGATGGTTTGTGGGTTTGCGTTTGGGAGTGTGTGCGCGCAGTCATGTGGAAATGTGACATGCTGACGGAG
GATCAGAGGAACTGGGGGAAAAGACGGGCGTGGCAGGACAGTGAGGGCCGTGAAGACAGACGGGCAGGCG
GACGGACGGGCGGACGGAACCAACAGACAGACCATGCCGAAATCAAGAAAATTGAGCAATTGCACAGGCT
GATGGAGACGCCCACACACAATGCACATATGAAAATTATTAGTGCATGCTTTTAGGCGGCGGCACATGCA
GGAACGAGGTCAGGCCAAATTTGCATTTGCATTTGCCGCCAGCGGAATCCTGCACAAATATTTACACAAA
TTGTAGGGCTAACCCATACCCGAACTCATGCCCACACCATTTTTGCATTTTCCAGGGGGGAGAGCCGGAG
TCAACGAAGCTGCGCTGCCACATTAGGAATGCCGCACGCAGCATCAGCGAATGCGTTGCCTCGGATGAGG
ACAAGCAGGGGCAAGGACACCGGCAGCAGCGGGACGAGGATGGTGTCGTAGTGGCGgaggatgatgatga
tgatgatgatgatgatgacgacgacgacgaCATGGAGCTGCTGAGTGCCGAGTGTGATGACTTTACAACC
CTGTCGCAACTAAATGAACAACAACAGGCGCTGTCAGCGGCATTGAAATTGCCCACAACAACAGCAGCAT
CATCAGGCGGAGCAGCAACATCGCAGGACGATAAACTGATTGATGTTGATGCCGATGTTGGGGGGCTGCC
TAAACCGCAGCATCAGTCATCTTTGCAGAATCTGCTCGCCGGCAGCGATGACAAATCCCAGCATTTGAGT
GACATCGACAATGAATCTTTCAACAGCATCGATTTCGATGCCGAAATCACAATTGCCGGCAGCAAAGAGC
AGCAGCAGCAACATCCACCGGCGGATGATTCGGTGGAATCCGGCGATGCAACAGCAATTGGCACATTCTT
CAACAATCTGCTGTCGCATTCCAATGCAG
>dme_circ_0007843_junction_seq
AACAATCTGCTGTCGCATTCCAATGCAGGAGCTCCTGCTGACGGATCAGCAAGCGA
>dme_circ_0007844
GGGGGAGAGCCGGAGTCAACGAAGCTGCGCTGCCACATTAGGAATGCCGCACGCAGCATCAGCGAATGCG
TTGCCTCGGATGAGGACAAGCAGGGGCAAGGACACCGGCAGCAGCGGGACGAGGATGGTGTCGTAGTGGC
GgaggatgatgatgatgatgatgatgatgatgacgacgacgacgaCATGGAGCTGCTGAGTGCCGAGTGT
GATGACTTTACAACCCTGTCGCAACTAAATGAACAACAACAGGCGCTGTCAGCGGCATTGAAATTGCCCA
CAACAACAGCAGCATCATCAGGCGGAGCAGCAACATCGCAGGACGATAAACTGATTGATGTTGATGCCGA
TGTTGGGGGGCTGCCTAAACCGCAGCATCAGTCATCTTTGCAGAATCTGCTCGCCGGCAGCGATGACAAA
TCCCAGCATTTGAGTGACATCGACAATGAATCTTTCAACAGCATCGATTTCGATGCCGAAATCACAATTG
CCGGCAGCAAAGAGCAGCAGCAGCAACATCCACCGGCGGATGATTCGGTGGAATCCGGCGATGCAACAGC
AATTGGCACATTCTTCAACAATCTGCTGTCGCATTCCAATGCAG
>dme_circ_0007844_junction_seq
AACAATCTGCTGTCGCATTCCAATGCAGGGGGGAGAGCCGGAGTCAACGAAGCTGC
>dme_circ_0007845
GCTTCGGAGTCGGTCAGTAAGCTCTTTCGTCAATCAAGCGGCTCTAAGTCCACGCCCTCCAAGTCCGCTT
CCACTCCGGCTCCCGCCGACAAATCGGATGCCATCTCGGCCGCCTCGCTTACGCTTTCGCTTACCTCCTT
GGCCAGCAGTAACCTGGAGCCGCCGGAACGGCAGCCCTTGATTGCGGAGACGCCCACGCCAGTGGAAGAT
TCCTGCTCAATAACCGCCTCGCACACGGCCTCCACAGCCCTCATGATGGATGCTCCAGCCGTGGAAGTGG
CAGCCTCTAAGCCCGAAACACCGCAGCTGCGTGGCACGCCCAATGGCTAATCCCTTTCTAGTGGAGAACT
CGCCGCTCCGACAGACAGTTGTCGGTCGTGCCCTGATCACCGTGAAAATTGGCAGCATCCTGGAGCAGTC
GTTGGTCTACTATACGGCTCGCTTGGTGGCAGCTCGGTTTCTGCTCAGTGGACAAGCGGCGGGATTGCAG
CCCGATAGCATCAGCAGAGTGTCTATCAAGAGCCTCTCCTTGGCCGTGATTGCCCAATGTGTGCGTTTGG
CTCCGAAGATTCTTCAACTCAGCCTGGAGATTAGCGAGCAGGAACTGCAGCTGCTGGAGGAGGCCACCTC

TCAGATTGGAAGTGGTGATAGCACTCAAGTTTCGAGTCCCCAGAGCAGCGACAATTCCCAGGTGGGCGGA
GAGAAGCCACCCCTGGATAGTAGTCTCGTTCCGACTAGCCTGGAAGAGAACCTGCTTCTGCTAGACATTA
AGGATGATCATTTCGGGCCGAGCACATGTCCAGCATACTTGCAGTCAGCGACACCCACCCTAAGCCGCAG
TGCAGACGCTTCAGTTCTCCTACTTGAAGGCGGCACTACTTCATCGCGGTCTGCCAAGAAATCGGAAGAG
ATGCTCTCCAAGTCCGAGATCATTGAAAGTTCGTACAGGCCCACAGTGGCAGTTGAGGATGTGCCTCCAT
TGAGCATGCCTCCACGTCCACCCAAGCGCACAAAGTCAACGCGTAGTCGAGTAGGAGTTCTTGGAACCTC
CTCTACCACTGAGAGTTCTTCACCTCAAAGCCGTCAGAAGCTTTCGGATATCCTGCTCTTCCATGACCAC
TGCGATCCCATCTTGCGGGGTGGCGTCCAGCAGGTTGTGGGTAACTTCCTACAATCTAGTGGGGCTGGAC
TGTTTCTGGATCTGCAACGTGGTCTTGGTCTGCAACACTTGTTGGCCATTTTGCTGAAG
>dme_circ_0007845_junction_seq
GCAACACTTGTTGGCCATTTTGCTGAAGGCTTCGGAGTCGGTCAGTAAGCTCTTTC
>dme_circ_0007846
GGGCTTTGAGGACGAGATCCACACAGTGGTTATACAAGCGTTGAATGCATTCGATAAGATATTTCCGAAT
GTGGTGTCCAAATATTTGACAGAGCCGCCATGTCATTATCATGCTCATcagcagcagcagcagcagcaga
aggagcaacagcaacaagagcaggacaaccagaagctggagcaggacctgcaacgacatTCAAGTGGACA
ACAAAAGCGTTCGGGCCAGGCGCAAACATTTGGCCAACAAACTTTTGCAAAGGACCAGGATAATGCCTTA
AGCAGTCAACGGCAGCAACAAAGGCGTCCTAACGATGCCGGGACATGTGCCAACTCCTCGGCCACTGATA
ATGATGAGCTACTGGCTGCTCTTTTGAATGATTTCCAATTGCAATCGACTGGCATGCGGCAGCAGCCAAA
GAATAATAGCACCGACACCGGGCAGTCGGGTAATGAGCCCGATCTCGAACCCAATCCCAATGCCGCAGTG
GAGCCTTTCTGTGTGTTTGCCATCTCACCGAAATTGCTCTTGAGCAAACTCCGGCTCTGCCATCACAATA
AATATTGGCTGGTCCAGAATAAATACGCCGAAGTTATTTCGAATTTAAATTACGTGCTGCTGCGGTCGTA
CTACGCAAATTTTCGTTGTGCCATCGACAACAAAAACAGCGGCGCCAGGAAGCAGGACTCCAAGTGGCCA
CCGATGGATGCCAGCAGTGTCTGCCACTCTGTACGGGACGCGGAGGGCGAGGACATTGTCTGCACTTATG
AG
>dme_circ_0007846_junction_seq
GGGCGAGGACATTGTCTGCACTTATGAGGGGCTTTGAGGACGAGATCCACACAGTG
>dme_circ_0007847
GGGCTTTGAGGACGAGATCCACACAGTGGTTATACAAGCGTTGAATGCATTCGATAAGATATTTCCGAAT
GTGGTGTCCAAATATTTGACAGAGCCGCCATGTCATTATCATGCTCATcagcagcagcagcagcagcaga
aggagcaacagcaacaagagcaggacaaccagaagctggagcaggacctgcaacgacatTCAAGTGGACA
ACAAAAGCGTTCGGGCCAGGCGCAAACATTTGGCCAACAAACTTTTGCAAAGGACCAGGATAATGCCTTA
AGCAGTCAACGGCAGCAACAAAGGCGTCCTAACGATGCCGGGACATGTGCCAACTCCTCGGCCACTGATA
ATGATGAGCTACTGGCTGCTCTTTTGAATGATTTCCAATTGCAATCGACTGGCATGCGGCAGCAGCCAAA
GAATAATAGCACCGACACCGGGCAGTCGGGTAATGAGCCCGATCTCGAACCCAATCCCAATGCCGCAGTG
GAGCCTTTCTGTGTGTTTGCCATCTCACCGAAATTGCTCTTGAGCAAACTCCGGCTCTGCCATCACAATA
AATATTGGCTGGTCCAGAATAAATACGCCGAAGTTATTTCGAATTTAAATTACGTGCTGCTGCGGTCGTA
CTACGCAAATTTTCGTTGTGCCATCGACAACAAAAACAGCGGCGCCAGGAAGCAGGACTCCAAGTGGCCA
CCGATGGATGCCAGCAGTGTCTGCCACTCTGTACGGGACGCGGAGGGCGAGGACATTGTCTGCACTTATG
AGGGTAAGCAGCTAAATCGTCTTATTACAGCCGCTCCCGCTGGCAGTCCACCATCCGCCGCTCGCCAGCT
TGTTTATTGTGCAGATTATTTTACGAGTGTGTTTTCGCCCAGCTCTCTGGAGCGTTTGGAGCAAGTGCGC
CAGAGCATCTGGAGCATCCGAGGCAATTCGAGTGCCGGGATGGGGCACTCCTCTGACAACTCTTATTGAA
CATTTGTGCACTCAAGTGTGTGGCCGGGCTCTTTGTGCATTCTGCCGGAGACTGCGAGCGCATCCtgccg
agtgccgagtgccgaATCCAGAATGACGGGTTCCACTGACAGCCTGACGGACTGGCTGACAGAGCTTGGG
GGTTTTCCTTTTCGTGGCACCAGCTCGCACATATATTTGTTTATTTATTATTTTGACAAAGCTAAGCAAT

TTACTAAATGGCACTGAGAGGGAAAACGTACATCAACTCGAGAAGAGTCGGCAAACAATTGCGCCAAAGG
ACGACAGACGGATCAAACTGAATGCCGAGAAATTGTAATAGCATGAGGAGAGCTTAAATGTGTGCCTACA
ATCCTATCTATTGATGTGGAGCATTGAATTTCGTATTCCAAACGTAGTTGCAATTATGGTTCACTGTGTT
TTCGCAGAGTTCAAAACTTGGTTGCCCGCATTTTTTGATGGGCGCTCGCGTTATAAACACTTTACTTCAA
AAACCATTTTGCCAGAGtaaaaacttaattataaattattatatattatattaaaaataaCGTAGTAAGA
ATCAACAAttttttttttAATATTCAATATAACAACAAAGGTCATTTTCAAATCAAAGGTTGGCTTTTCA
TGCTGATGGCCATTTTCTTCAGTGTTCTTTCTACCTTGTGGGCCAAATCGACTGGGCAAACCGCATCTGC
ACGGCCATTCGAAACTGCCAAACTGCATAATGAAAACTTGGCTCACTTTTTGATTTACAACGTGCCGTCA
ACTGACTCAAAAGCACTTTGCCCCCTTGCAGGGCGCAGTTTCTGGCTGAACTTTTACATCTGCTCGGCGA
CGATGATGCGCGGGTGCGGGAACATGCCGCCTGCTGTCTTTGCCGCTTTATAATGCAAACGGCGCGCCAG
GACCCATCGCAGGACCAAGGAGCAGGAGGCGGAGGTGGGGACGATATCGAAGGCAATGGCAACGTCAACG
TTGAAACTCAACAAACTAATTTCAATTTGCTGTGGGATTTTTTCGACTATCGCATATTTGGCAGCATGTC
CGTGACGTTGCGTAATTTATTCCGGGCCAGTTCGACGATTGTGCCGCCTCTGGCTGAGCTGGATGCATTG
GCCACCTCGAATTCCGCGCCATCTTACCCCGACACTGGCAGCACGTCCGGCTCCTCCACATCGACATCTG
CCTCATCCGGAGGATCCGCAGCGGCAGTCTCCGCGGCGTCGGCCTACTTCGAGGCAAGTTACGGTATCGG
CATCGCCGAGGGGCATGTTTTTGCGCTGGCATCCGCGTCCCAACGTCAAATCGCGCAGGAGGAAAAAGTT
TTGGCCAAAGTTTTGTATCGCTTGACAAATAAATTGATGACGCTGAATGATAAAAATGTGCAGGTTTGGC
ATAATTTACGCGCTGCGCCTCCTGCTGAGGCACTTCAATTTCGTGGACTATCAACAGGTCTGGCTGGAAT
TCAATTTCGTTGAAATTTGCATTAGCTATGCCTACTACAACAATGCAACAGCTGCGGACCTCGGCTGCCA
GAACGATTTGATTGATGTGATGGGCAAATTAATGGCAG
>dme_circ_0007847_junction_seq
ATTGATGTGATGGGCAAATTAATGGCAGGGGCTTTGAGGACGAGATCCACACAGTG
>dme_circ_0007848
GGTGCCATGCTGAGCTCAGGTGAGCCAAACACCGCCCACCTGGACTTTCTGCTGCGTCACAGCGTCAAGA
TGCTGAACATCTACTATCACCTGGTCACCAATCAGCGCCCACCCACCGCGGGATCACAGTCGGGAAGCAG
CAGCTCCAAGCAGCCCAAGAGCGAGCTCTTTGCCCGCGAACAGCCAGCGGCAACCCTTCAGGCTCTGGGT
TACTTTGCCGGCGATTACGTTTACATGAAACTTTACAACATTTTAAGAGGTGCTAATGACAGCTACAAGG
GTAAGTCAGACCACGGACTCGCAGATTCCGAGCCGGGAACCGCCATTAAGTCGCTGCTGGCGCCGAAATG
GCAAAAGTTGCGCGGCACGTGCACTGAGATTTCCCAGCATCCAGCATCACAGGATTCGGTTCgagatgga
gctcctgatggagatggagctgcagctggaTGCACGTACAGCGCAATGCGCCGTTTTATATGCAACACTT
TCCAACGCTGCCGCAGCAATGGCAATGAGTTGTAAAAATTCAGCCCGCGACGAAATGCAGCCAACGTAGC
ATCCACACTTGACTTTTGCCACTCGCAGCTCAAACAGAAAACTTCTCCACTCGGAGTGACTTAATGTGCG
GGTTGCATTAGGTCGCCAGCACATTGCATCCGCCACTAACTCCTCGTTCTCCTTCTCCTGGATAAGTAAC
TCGAGTTTTTCTATTTGTCCTCCCCAGGATAACCATAAATCAGGAGGCCGGCAGCTTATTAATTTGTCTC
CTGAAAACCTGCCTCCACGCAGTGAGCCTCTGCCTGGAGGGCATGGCGAGTGCTTCGCCCCCGGAGCTGA
AATTAATCGAAGAAATACTCCACTATTTGACCAGGCTCATAAATTATGCGCCCGCCGAGTGCGTTGCATG
TCTGCGGCAACTGTTGAAATATTTATTCGCCCAAAATTATGCCAGCCAAGTACGGCTGCAGCCATCGGCC
ATCGGTGGAAATGGCAGTGAAATTGGCCACCATGCCGCCTTCATGCGTCCCTACTTTGCGGCCAAGGGCA
GGGGTCACGGCGCCAGCTCCACATTGTTGCCAACAATAAATTCAAAACCAGCGGTGGCGGTGGGATCCCA
GCGAGGGGCACCAACCGACGCTCGCCAGCCAATTGATGCCGGCCCACTCCAGGACATGGGCATGCTCTTT
GTGCACGGCCTGCAGCCACCCACTCCGCCGGCGGGCGACTGTGTCCGGCTTATAAAGCTATTCGAGCCGA
TGGTCATATACTGTCTGACGGGTAAGTACACTGCGAGAAGCTTATCTTTCATTTAGGCTACATTTTAAAT
GTATATATGGGTAGGTAAACTGGTTTCCAAAGGTACATTTTTATTTTATATAAATGTCCAAAATGTAATA
CCCATTTTTCCCACTGCATAAACGCAACGTTGGCCAGCGATTGCTAATTAATTTAGTTGTAGCCAATTT

GTTTGATTAAAAATGCACAGACACGAATACCGACCATCCTGGGGAGCAGTCCTTAATCTCTTACGTGTGT
GAGTGTGTGCGCGGATGGGTCAGAGTGCAACCATTGATATTTTATTAATGGCAAAAATTTGCAGAACTCT
AGTATTTCTCCATGTAACATGTACCTTTCCCTTGCGTTCCCTTTTCAGGCTCTTCATGAAATCCAATGCG
CTGGTACAAGCGCCTATTTTGCGATTACTTTCCCAACTGCTGGACTTGAATGTGACCTATAGCATACTGG
ACTCGAAGAATGTAATATTTGATCAGGTACTGAGCAATATGGATCTGATTGAAGGCGGAATAGATAGGAA
ATGCGTTTATCATGGTGCCGCCCATGCTGAGATTCCTGGTGCAGCTGACGCACAAGAGCGACCGGCAGCT
GATCACGATACCCAAGATCATCAGCATCACCAACAATCTGCTGGCCAACGGATCGGTGCGCGTGGTGGCT
CTGCTGGCCCTGAAGACCCTAAGCTACGAGCTGTTTTTATGCACAGCCAGCTGGAGGAGGCGCTGGATA
CCGAGGGCCATAACTCAGGGCGAGACGCGTGCCAGAGTCCTTTGAGTGCAGCCCCCACACCGGAAACCAG
AGAGGCCTTGCTGGCGCAGCGACGCGAACTGGACACCCAACGAGAAGTGGTGCTGGGCATGCTGGAGAAG
TTCATCGAAGCACGGCCGAGTCAACAGGTCCTCGCCCTACTCCTGCTGTTCGAGCGGAGTGTGCAGCAAC
TGGATACGCCACCGTATCGCAGTGCCCAGGATGCGGACGCCGTGTACGGGACGCTTTGTCGAGGGCTGTG
CTCCCGCCAATGGCGTCTCCACAACGCTGGGGATCTGCGGCTCCTGGAGAGCTGTTTCCGCAATAACGGC
AACCATGTGCTGGCCGATAGCAAGAGATTCCTGCAGCTGTTGCAGCTTTTCATCGAGCAGGGTGGGTTAA
ATATGCAGTGGCCTAAGCTTGACAAGGCACTCAGCCAATTCACAGCTGCCCATCAAGCCCTCAATTACGG
CGTGCATTACGACTCCGGCAGTCCTGTGCCTGCACTGCTTCCGCCCATCAGTCCGTCCGCCCTCGACTGA
ACTCATAAATATGCGAGAAGCCTGCTTAAATATAGATGCCCACACACACTTGGAGGCGTCAAGCATATTG
TAATATTTATGAATGCAGACAGAAACTTAAAACCCAGCCCGACATGCAAATGAACGTAAAACTGTCTATC
AGCCGCCGGTTTGGCAGGCTGCCAATGCAAATGACATATGAATGTCCTGAGTAGGTGTCATCGGGCGTGC
CTGGCATCCACTTAATAATATAATTGAAGTAATGCCACAAATTACAACACTCTCAGTTTGGCAACGATGG
CCGTGCGACTTAACTGGTACTTAGGGCCAACGCCTGCAATTGCATTAATTGAACTCGATGACATTAAACT
GCCGACTGGCAGATGTTCGATTCCTAAGATGACGGCCCATTGTGGCCATGTCTCATCACTTTCCATTCAC
TTCTCGGGTATCCATCGAAGATTCAATGCTCACAGGGCTTTGTCTGCTGAGTTTAAAAGCTTTTTCCACC
CAATATATACAAAGCCGGTAGCCAAACACCTGTAGGCCCTCCTCGACCCTCGATACCCGATGCTCATCAA
CGATTTGTCATTGccgccttcccgtccgtccatctctccgtccgtccgtccgtccgtccgttcaaccctc
cgCATTTACATACACACGAATGCACTTAGTTGCAGTTACGGGGCAATTTTTTATTTTGCCctgctgttgc
tggtgctgctggtgttgttgctgttgccactgctgttgctgatccatgtggcactgtagttAATGTGCCA
CATGTCGTATACGTGACATTTTTAATACCTACGCTTCAACGCCGCACATTTTCAACTATTAATGCGCATT
TAAGCTACACATGCATTAACTGTCGGGTCTGTCTTCCATGCCCGTGCAGCCCGTGCCGTTAGGGCCAGCC
ACGCCCCCATTCGCTCATGCCATGCCCCCGCCGCCCATTCCGAACCCCCTTCCTATAGACCAGTCAAGCG
TTATGCTACAGTTAAAACCAACTTAATTACAGAGTCTCACTAGCACAGCTGGGCGTGTGTTGGTTCAGCG
AGTGTTCTGGGGATCCAAGTGGAGTACTTGTCGTAGGCAGAGTCAATTGCATGTGAGAACTACTCCACTC
CACTACTCGACTCCGGTGCATCCGCGTGTCTAACGGCTTTGGCCGTTGGTCACCCAACTCCCATCCCAAA
TCGCCCGTATTAATGGCGAACTGACACTTTACTGCCGACGGCAATTCAACTTCATTTAGCTTGTAAGTTA
TGCGCAGAGCTTAAAAACTTTAACTAATTATCGTTTATTTAATTTGGACATGCATAAAAAAGATGAGGCG
ACGGTTGGGCGAATTAACAGGGTTTACGCAGTCTTTGCCGCTCAGGTGCCATGAGTAATTAAGTCTGGCC
TCCCACTCGTTTTCGCCCGGGTGTTTATGTGGGATGCAGGTGAATAGTTTATGGCTGCAGCATTTTAATT
GAAATTGAAATTGCACCGCAACTTAAATAACATTCTGCGTGTAAACATTGACTCGAGGAGGCACGCGAGC
TGTTTAGCAAAAAATATTCTGTTTAAAATGGGTAGCACAGCCAATGGGAACTGCATTCGCCACATGTGCG
GAATGGGCAGGATGGTTCTGTGTCCTAATTACTGCCGGAAAATATGTTGATGTTGGCACTGGCAACTTTG
TCACACAAATTTATGCAAACTTTCACACTTTACTGCACTGGCGGTCAACAACGAAAGGAGTCATCAGCCA
GTGTACTTACGTCTCTTCTCAAACGAATCTCTTTTTACATTTTCTTTGCAGGGGCGTTGGGAACTTTGGT
GATTTAGCTCTGGCCATGGTAATGCTGTCCAACGTAATCCTTAAAACGGAGGAGATTTATCTAGTGAATC
ACATTAAACTTTATTTGAAAAATAATCCAACGGCCGAGCGTCGTCTGCAGGCCCTGATGCCCAGCTCCCC

ATCGGCAGCTCCTCATTGGCAGGACGAGGCACCATCGACGTCTTCGGCGGCGGCAGCTGCCAGAGCGGCC
GCCGCATCCTTTTCCGCCGGCCGTTCCTCCATCAGTGAAATTAACTATTTCGCCAAAGTTCTCTGCGAGA
AGCTGCTCGCCTGTTTGGAGGTCCTCCTGGGCTTGGAACCGAGCTCATCCAGCCACGCCTACTGCCAGCT
GACCGGGCGCTTCATGGATGCCCTGCTCAACGTCTGCTGTCGCTCACGGCACAAGGATGCACTTCAATCA
GTTTTCCGTTTGGTTTTGGCCGAGTCTGAATTTCTCTGCAAATACTATAGCCTGCTGTTGATGAGTGCCG
CTGGCCTGGTCGGATCCTACTTACTGGATGCCGTGTTGCTGGCCTGCCTGAGGTTGGTGCTCGCCATGCG
GCTGGAGGAACCGGTGGCTCTTTTGGAACAGGCCGCCCAGCTGCCGCTGAAGACCAACTTGCAGAGGGCG
TTGCTACGCGAAGTTTGTCGTGCAAGTGCGGGTAAGTGAACTAAGACAAAAATCGATGTTAGAATTATAA
ATTAACTAATAAGTAATGGTTAAACACAGAATTATTATGCCTGGTGTTATTAATACACATGGTTTAAAAT
ATTTTTCTGACACTTGAGGCTTTGTCTCTCAACTTTTTTGCGAACAAATTTCTCCCAGTGTAGCTAAGTA
AAGAGACGGCAGCGGAGTAATGGCGAATCAGACACATTAGTTTAATTTGAATTTGGCAGACGACAGCGTT
GCCGGGCCGCCAACACCCCCAATCCCCACCGCCCGTCCTCCGGATGCCCCAACCATATACGACAATGCGA
ATTTTTCCAGCTGACAGTTGTCGCACTCGCAATTTTGCGGTTCACCGCATCGGGTTTTTGGTTTCGGCTT
TCGGTTCACCGTCCCGCCGGCGACAGTGAGCCTTTAAACGGTTGGCTTACTTTAAGCACAGTTCATTGGC
GGGCATAATTTTAAATGATTGCCACTCGAGCATGCGCCACTTGTTGGATAATGGCACAATCCCAATTCCC
ATCCCCATCCCCAGGACAATCCCCATTATTTGCCTGTGACCCGAACACATCACGATTGTCCACTAATTGC
AGGGGCTGCGATTGGAGTGCGCAACAAGTGCGCCGACTGTTCGAAGGCAGGTATCTGAATTTCCTAATTG
CTGACCACTTGGAATTTATTTGCGAGCTATGCCAAGAGCGTCCCGAGTGCGGCTCATTACTGCAGGTGGC
CTTGTTCCGGAATGCCCATCGATTGAGCAGGGCAATCCGTTCGCATCGTCCTTCGGCTATTGGGACGTCT
TTGCGAGCCCGCTGAACGGGAAGCCTCCGGAGACGTTGGCAGCGATGCGGATCCAACCCTGCAGGCCATG
CAACTCCTGAGTCGCCTGCACCAGCTTTATGAAGGAGAACGCTCGCTGCAGTTGCCCATCGAGCGGATGG
CCCGGCGGCTGAGCGCCCAGAGTGGGCAGCCGGCCAGGAACGTCATCTACGAGCGTCTGGTGGAAGGAGA
CCTGGCCGGCGGAGAGGACCAGGACGCCCTGCGAACCCTTCT
>dme_circ_0007848_junction_seq
AGGACCAGGACGCCCTGCGAACCCTTCTGGTGCCATGCTGAGCTCAGGTGAGCCAA
>dme_circ_0007849
GCCCCCTGCCGTTCAGCGTGGAGCACACGAAGCTGGTCTGGACGCTCAATTACAGTCTGATCGAGTGGAC
ATCGAAATTCGTGCCGCAGTGTCATCTGTTGTCAAATACAATAATTGCGGCCAACAATTTCCTGAAGACG
ACCGCCGACGAGGAGCTCTATCTGTGCGTTCTGCATTGTGAGTACAAACTGGAATGGAGATGGGAGGATG
GGATAGGATAGCCGCGTGGAAAGGACTTAGCATGCCACTTAAATGGTTGTCAGCCTTGTCTAGCCGCCAT
CAGGCCACTTACTAACTGTCAGCCGACTAGCGGGCCAGCAAATCTGACTGCCATTGACCGGGGCAAACGT
TCCCTGGGCTGTGATGGAAACCTCGATCCCTTGACTGGTGTGGCTTAGTCCTGGGCAGCCCACGTTGCGC
TTAATTGGTTTTTTGATTGACAATTTTCAAACGGCAACGGAACTGTCAGGCGGACGGGTGAAAAGCCACG
CGAAAGGACCGCAGCCAGTGCTGTCATAACTGACGcaggagctggaacaggaacaggagcaggagcagga
aGAGCAGCCAATGGCAGTAGCCAAATGTTTATTGGCCTTGGCGGAACACTCACACATGGCCCGTTGGTCA
ATGGGAGCCCGTTTGCAAATTGAGCGTAATAAAAAAAGAGTAGAGCATTTTGCATTTTGTTTAAGCGTTG
TACACATACGTCTGCTACATAAAGAAATTCCTCAGTTGGCGAACGTAATTTGCAAGCATTTAATATAGAG
TTGAGATTTTAATTGCAGTTTACGAGTACATGGCGAGCGGCAAGTAATTAAAAACTGTTTTTGCACTGCA
TATTTCCGGGCGACGTTCGGTCGAAAATTAAATTCGACTTTTTAAGCAATTTATGAAATTTGCACGCAAT
TCAATTGTCCCATTTTGTTGCTTTTGCTGGCCATGATGTCCTTGTTGTTGCCGCTGCCTTTTGCTTTGAT
GGCCGCCACTCGGAATCCAAATCGGCATTGGAATCAGAATCATCATCATCGTAGCGAAGAGAAAGCGGGA
GAAAGCGCAATGGGGCGCAGCGACAACAGCGGCCATAACAATAACAATTTGCCACTCAATTAGCGTGGCC
ATTACTGAAAAACCCACTTAAGTATGCAATGATAAAAATTCAAAAGCGAGTCGAGAGCCGCCGCATTGCT
ATTTCTATGAGCGCTGTCCTTTTTCATTGCCTTTATTTTGCTACTTTTTATATACGTGTATATGTTGCTT

ATTGTATTTATTTATCTCTTTGTCATTGACAGGGGCCTGGAGCGCATGGTGGTCAATAGTGGAGTGCCGC
CTCCTGGCATTCAGCCTACTGGAAAGGATGCCGCTGCTGGAGAACCGGGCGCCGAAGGATCCAAGGCGGG
AGTTGGCGTCGTGGTCACACCGCAGATGCGACATAAAATTGAAAAACTTGCACTCGAATTGCTGAAAATG
GAAAATGAGAAATTTTCGATTCCGGCATTGAAATTGCTTTTGTCCTGCATGTATGTGG
>dme_circ_0007849_junction_seq
AAATTGCTTTTGTCCTGCATGTATGTGGGCCCCCTGCCGTTCAGCGTGGAGCACAC
>dme_circ_0007850
GTACAAAGAACAAGGGCATTGCCGGCAGTCTGGCCATCAAGTATGCCCGTTTCCTCAATAAAATCTGTCA
TGATCTCGACGCCGGCTTGGCCGCCCTGCAGCAGGCGCTGGAACGTGATCCAGCCAACACTCGTGTGGCC
CTGCAAATGATCGATTTGTGCCTACAGCGCCCAAAAGTCGATGAGCAGGAGGTAGTCGAAATTATGGATA
A
>dme_circ_0007850_junction_seq
AGCAGGAGGTAGTCGAAATTATGGATAAGTACAAAGAACAAGGGCATTGCCGGCAG
>dme_circ_0007851
GGTTTTGTTTGCCCAGCGCAAAGTGGAGTTCCTTGAGGATTTCGGCAGCACGGCCAGGGGTCTACAAGAC
GCACAGCGCGCTCTGCAGCAGGCACTCACTAAAGCCAAGGAAGCGCAAAAGAAAAGGTAAGAAATCCATG
AAGATGTATCCACACTCATTCATTAATCATGGACATTCCAATAGTGATGGCAGTCCTAGTCGCAAAAACT
CATCAAGCAGCAAAGAAG
>dme_circ_0007851_junction_seq
CGCAAAAACTCATCAAGCAGCAAAGAAGGGTTTTGTTTGCCCAGCGCAAAGTGGAG
>dme_circ_0007852
GCCATGGAGCATTGACTTGCACCACTGAGACCCCGAATCCCTAGTCCCCGATTCCCGCCAGGCCATGTTG
GAGCCAACGCTGACGCCCGATCTGGAGGAGGGCATCGCCGAGCTCAGTCTGGAGGACGTGGATGTGGACT
CGGTGCCAGAGCGTAAGCCGCACTTCCTCGACTACGACGCAGTGCCCCCGCCAGACTATGAAGAAGGCTT
TGGGGCCAGTTCCGGGGAGCAGCTAAAGATGGACAAGAAGTACGAGCGGGATGGCGAAGTGAGCGACTAC
GAACTGGCCGCCAGTGGCTATACCAAGAAGGAGTTCACCCAGGACGACAACACGCTGCACTTCACCCAGA
GCGCAGTCGGACTGGGATCCGGAGCCGTTGGTAAAAAACCAGCAGCCAAGCATTTTCTGGACGAGAACCC
CATTCCGCCGGACTACATGTTGGCCCAGGAGCTGCGGGAGATGAGAGAACATCGCAGCCTGGACAGGAAC
TTCGAGCGACAGTCGGCGCAGCAGCAGCAGCTGGATGAATTGCCTCCGAGGAATGGAGGCGGCTCTCCAG
CCAGCGCAGGTCGTCCATCGCGCAGCAAGGAACCATCTTACACGCTCTCCCGATTTCTGGACGGAGATGC
ACCTGCCCCTGCTCCAAGGCTGCCCAAGGGAGCTGCCTGGACGACCAGCTTCGATGAGCGATACACCTCC
TCGGCAGTGGAAGCCACCTTGGGATCCAAGGTGGAGTGTGTTTACTCACTGCTCTCCATGTTGGGCTCAA
ATGATCCTCTCGAAATGGCCAAAAAGTTCCTGGAGCTGTCCGGAAATGCTCAAAGTTGTGCAACTCTTAG
ACGCTCTGGTTGCATGCCGCTTCTGGTGCAGATGATGCATGCACCGGACAACGACCAGGAGGTGAGGAAG
TGTGCTGGACAGGCGCTGCACAATGTGGTGCACAGCCATCCGGACGAGAAGGCTGGCAGAAGGGAAGCCA
AGGTGCTCCGACTGCTTGACCAGATCGTTGACTACTGCTCCTTCCTGAAGACGCTGCTCCAGAGTGGCGG
AGAGGCTATTGCAGATGACTCCGACCGTCATCCGCTGGCCGCCATCTCTTCACTGATGAAGGTGAGCTTC
GATGAGGAGCACCGACACGCTATGTGCGAGCTGGGGGCATTGCACGCGATCCCCAATCTGGTTCATTTGG
ATCACGCCGTTCATGGACCCAAACCGGAGGATCAGTGCTGCAATTCTCTGCGAAGATACGCTTTGATGGC
CCTGACCAATCTGACCTTTGGGGATGAGAACAACAAGGCGCTGCTGTGTGGTCAAAAGCAGTTCATGGAG
GCCCTGGTGGCCCAATTGGACTCGGCCCCGGACGATCTGCTCCAGGTGACGGCCAGCGTGCTGCGCAATC
TCTCCTGGCGAGCGGACAGCAACATGAAGGCAGTGCTGAATGAGATTGGAACAGTGACAGCTCTGGCCCT
GGCGGCCATGCGGAATAGGAGTGAGAACACCCTGAAGGCCATACTCTCGGCCCTGTGGAATCTCTCGGCG
CACTGCAGCACCAACAAGGCGGAGTTCTGTGCCGTAGATGGGGCCTTGGCATTCCTAGTAGGAATGCTAA

GTTACGAAGGTCCCAGCAAAACATTGAAGATCATTGAGAATGCGGGAGGGATCCTGCGGAACGTGTCCAG
CCACATTGCTGTTTGTGAACCTTATCGGCAGATCCTGCGCCAGCACAACTGCCTGGCTATTCTGCTGCAG
CAATTGAAGTCCGAAAGCCTTACCGTGGTCAGCAACTCCTGTGGAACTCTATGGAACCTGTCAGCTCGCT
CTGCCGAGGATCAGAAGTTCCTGTGGGACAATGGAGCAGTGCCCATGCTCCGTTCGTTGATCCACTCCAA
ACACGCCATGATCTCAGAGGGCAGCTCTTCGGCCTTGAAGAACCTTTTGAACTTCCGACCAGCTGTTCAA
AATCATCACCAGCTGGATCCAATTGCCCGCTCCATGGGCCTCAAAGCATTGCCCACTCTGGAGGCACGAA
AAGCCAAGGCATTGCAACAGGAGCTGGGTGAGCGTCACACGGCCGAGACGTGCGATAACTTGGACACGGG
CGGAAAACTGGATAAAGAACGAGCCTCCAGTTCATCCAGACGTCATCCTGCGCCACGCTTAACCCGATCG
GCAATGCTAACGAAGAGCGAGAGCAGGGATTCGGTGTACTCGGCCAAATCCGATTGCGCTTACGATCATC
TAATTCGCTCAGCCTCTGCTTCAGATGCCCATCGAAAGGTGAAGCCCAAGATCACGGACTTCGATCTGGA
AATGGAGCAGGACACGGAGGCAACCGAGGAACAACCCATTGACTACTCGGTGAAGTACAGCGAGAATGCC
ACCAAGACCTCAACATATCAGGAAACGGACCTGGATCAGCCCACGGACTTCAGCCTTCGCTACGCTGAGA
ACCAAATTGAATCCGACCTGGACATATCAGGACCAGCAGGCGGACAAAAGAGCACTATCACTCCTCCTGC
TGAAACAGTTCCCGAGAAATCCGAGGGCCAGGAAATATTGTTGATCCTAGACGACAGTGTTAAGTGCTAC
CAGACGGAGGATACCCCTTATGTTATATCCAATGCGGCTTCAGTCACAGATTTACGAGTCGCTGCTAAAG
CGGATGCAGAAGCGGAAGTCAAGCCAGAAGTTCGCGAGGTGACTTCCAAAGAAGGAGCTCCCAAAAAGCT
TCCCAAGCTTTCGCAGTGTGGCTCCGGTTCGTACACTCCAGAGAAACCCATTAACTACTGCGAGGAGGGA
ACTCCTGGGTACTTTAGTCGCTACGATTCACTGAGCAGCTTGGATGAATCCGGTAAGGCTAACCAGGCAA
TAGTAGGAACGGATGCGGATATCAAGCCCAAATTAGAGAAGCAGGAGGAACAGGAATCCCAGCCAGCAGA
GCAGGTCCTGACCAAACCTCCCACCCAGGCAAACTCGGCATTGGAAACCCCGTTGATGTTCTCGCGACGC
AGTTCCATGGACTCCCTAGTCCACGATCCGGATGTCGATGTGGCCAACTGCGATGACAAGAGCTCGGTGG
TTAGTGACTTCAGTCGCTTGGCCAGTGGGGTAATATCCCCATCCGAGATCCCAGATTCCCCCACACAAAG
TATGCCCCAATCTCCACGACGAAATAGTGTCGCTGGTTCGGGGCAGAATGTCGATTCTCCACCGGTTGTA
ATCCCGGCCAGCCTACAACCATTACGCAGCGTTTTCGAGGACGACCTGAGTAGCTTCAACGTGGAGCATA
CACCTGCTCAGTTCTCCACGGCCACCAGTTTGAGCAACCTGAGCATAGTGGACGATGAAAAGGCACCGGC
CAGTGTGGCCGAGGAAGACAACGAAGATGAGCTCCTTTTGGCCAACTGCATCAACATGGGTATGCAGCGG
AAGCCAACTGAGGCTGTGAAATCGACAGTTGTGAACTCAGAAGTGGACGTGGCTGAGGAGACCATTCGTA
GCTACTGCACCGAAGATACGCCAGCATTGCTCTCCAAAGTACCAAGTAACACTAACCTTTCGGTTATCTC
CATGAGCTCCACCGATCCCAAGGATGCCACGGCTGGTCAGGCACAGATGTACGCTCATCAGCTATCCGAC
GATGTATCTTCCAATGCATCGGACTGCGGTGGAGCCTCTGGTCACCTGCTACAGCAGTGCATCCGGGATG
GCATGAAGAAACCCCTCGGTGAAGCCACTTCAGATCCTATAGCCATGCTTCGACGAGGCGGAAACGAATT
GCCTGGCTATCTGCCCTCTGCCGATGAAATGAACAAGTTTTTGGTGGAGGACAGTCCCTGTAACTTCTCA
GTGGTCTCTGGTTTATCTAATCTCACCGTGGGATCCAGTCTGGTTGGGCCAGCCGTACAGCTCAAGGAGA
CCGAACCGGTGAGTAGTGATCGCATTTAAACTAAATAGCAAAGTAGGCTTTAAAAATAACAGCATTGCAT
TGAAATTCAGTTGTGATATCAATATCATATCATTACTATGATTATTGCAATTGTATAATGTTTTGCTTCA
ATATTAATTGGTGGTTCAAGTTAGAAAATCATATCTCAACTATTTTAACTGACTATATTAAATCAATCGA
TTTCCCAATAGAATCCAGTGCGGATCAGAATCCTGAGATGAAGGCGAAGCCTGGCAAACAGGAGCAAGTC
CGAAGACCGCCGCATTGGCAAGATGATTCGCTGAGTTCTCTATCCATTGACTCGGAAGATGACACCAACT
TACTGAGTCAGGGCTATTGCCGCTGGTTGCAATAGACCGAAATCGAATCTTGGCTTCAGCTCGAACGGCA
AGCGCTCCAGCTCGCTGAGCTCCTCGCAGCCAATAGCCATCAATGCGGCCACCTCGGCCAGCTCCCTGAA
TTCGGCGATGACAGTACGCAAGAGCCAACAGCAGGAGTCCTACAGTTCCGTGGACTCTAGCGATTCTAAC
GACAACCAGTCAAAGTCGCTCTTCGAGCTGTGCATCCTCAAGGGCATGTACAAGACGAAGGAACCCGGCG
CCCGGGCCCAGCAGATGCAGGAGCAACCTATAGTGGGTTCATCCTCCGTCCAGTCGAATCCCAGTCTAAA
GCAGTTCGATTCGCTGCCGGTGCAGCTGCCATCTAGTGGACAGGTCAAGCGGCAGCGCcatcatcatcat

caccatcatcatAGGGAGCGAGAGCGAGAACGCAAGGACGAGAAGCTGCTGCAGGAGTGCATCAATACGG
GCATCTCGAAGAAGATCAACGCCGTGCCAAAAAACGTCCTGGCTACGTCTGCGGCTGCATTGGAACCGTG
TCACCCAATGGCAGCTACTACATCAGCAAGTGCCCTCAGCACAGCAGCTCCAGACGTAGAGCAGAAAGCG
CACGCCACCAGCAATCCGCAGCAGCAGTCGTCCACGCACCCGAGCAGCCACATCCTCCCGAATCCTATCG
ACGCCATCGCCACCGTCACCGACACAGTAAGAAGTCCAGCAGCTCCAAATCAAGGGAACGGGAACGCGAG
TCAGAACGGTCTCGAGACCGCTACTGGGTCTAAGGACTTGGATTCGGAAGATCGATCCAGCGATGAATCA
AATCAGTCCTTCATCATGGAAACCATGGTCAGGTTGGACAGTGCTCTCAACGAAACGTGCATCTCCGGAG
CAAGCGAGAAGCACAAGGATCCCGACCTGATGCTGAAGTCTGTGGAGAGACTGACCATGGAGTTTGTTAC
GTCGGCGGagcagttgcgcagcagcagccacaaccacagcagcagcaacagccacaagaacaacagtagc
aacaacaCCTGGAACGAGAGCACCTGTCCCAATGATGTGAGTTTCCCCAGCGTCAGTCAAACGGCACCGG
TTCTGGCCTCCTTGAGCTTGGATGAAGATGCCACGGAGGCGAGATCCCTGCACGAGTTGATTGAGATAAC
GCCCACGAATGAACAGCAACCGGAATCGCTCGAAGGGGAAACTGATACCCTGGTTAATGGTCATGCTGAT
AGCTACTCAGGATCGTCTGGAGGTCTTAACTTCCAGTTGGGTGGGCAGGTGCAGAATGCTGGCGTTAGAT
TGGAGCCGCAAAGACTATTATTTAATGGAACTAGTGCCTCCATTATGACAAACTCGACCATGATTGCGTT
CGAAGCGCGAGCTCTGGCGGAAAATCTACTCCAGCCCGCTGCCACTGACGATGACACCACGGAGATGACC
TTCAGCTTGAACAGTTTGGATCTGGATAATATTCGTCCGCCGTCGGGAATGGAATCGCTGAACAGCTGCT
ACCAGGATCACTCCCAGCCTAGTTCTCTGCGCCAGGCGATGCCCAGCAAGAGTCCACGCTTCGCGCGAAA
AATGTTTCCAGCCAATTTGGTAGCCAGACGAGCACTGGGTCACTTAGCTGGCAGTGCGGAGAGCGTGAAC
TCCAGCTGCAACCTGCTGGACAACATCAAGCCTCCGTCACTGATGGATGAGCTGCTGGACTCGATGATCA
GTGTGGACAGCATTCAATCTGAGGTGGCCGATGGCGAGCAGGACTGCAGCATGGCCACCACCATTTCCGT
GTCCAACTATGAGACGGCTGCTTGCGATGATCAGACGATGACCGTGCTCCAAAGTTGTTTCGACGAGGAT
GAGGATGCCACAATGAATGACTACAGCTCGGCCGAGTCCACACCCAAGCACGGATCTACTCCATCACCCA
ATCGTCGTTCCCTCACACCGAAAGACAAGCGACGTCTCACAAAGGATCGCTTCAAGACCTACACCATAGC
CACCAGCTGCGAGATGGAAGCACCAGAGGCCAATGAAACTTTGCAGATAGAAATTGTGGAAGCTGCCGTT
CCTGTGGCCACGCCATCGCCACGCGCCAATGGAAGGAGAAGGGGCAGTGCGGAGCGGTACAAGACCCAAC
TGATTGAATGTCCCCTGGCTTTGATACAACCCCAACCCGATGATTGCCCAAGCGAACAGCTCTCCTCAAT
TCGAGCAATGATGCAACAGTTCACTTTCATCACGGACATTAACATTGGACACAGTCAGGAGACTTGCGAG
AGTACTGATCATCCAGAAGACGCAGGGGAATCCCCGGAGTGCGATCAGAACTCTGAGACGGAGTCGTGCG
ATGGTCAGGAACCGGATCAGCTGCCTCCTCCTCCGTCAATTGTGGACCTTAGAACATCAGTCGTTAAGCC
CACAACCTTGGAGCCCGCCACGGCCGTAAAACTGGTGCGAGGTCGCAAGAAACCAGCCTATGTCTCACCC
TACAGCATGCAAAGTCAACGGAATAGCAACAATGCCGCCCCGTCCAAGAAGAAAACCCTATCTCCAACAA
TTGCCAAACGAAGTCTGGTGCCCGGCGGTTCAGGAGTTAGGTTACCAGCGAAAAAGAAGCCGACGCCGCC
GCCTGAGCCGGCACCCGCACGCTTGGAACGCCAGGGAACCTTTGTCAAGGACGAGCCCACTAACTCCAAT
GTTCAAGTGCCCGTGGTGGAGACAAAACCGGCTCAAACCAGTCCCACTCACCGCGCCTCGAAGTTACCCA
CAAAGAAAGGCACGGCCAGCGGTGGGTCACCATCGAAAGCGGGCTCTCCCAAGAGGATTCCCTTGGCGCC
GGCCCGCCGAATGACGCCTCAAAGGGCAAACACCAGTCTGCGACTAGCTGCGGGCAAATCTCACGCTGCC
TCACGTGTGGTCTCCGGCCGCGTGTCGTCCACTACTCCACCATCCCGCAGCAACTCCAACCTAAATGGAA
GCAGTGCAGCCGCAGCAGCTGCGGCCAAGATCAACCATGCGCAAAGTCGTATCGCCAACATCTGGAAGAG
AGTGGACGAGGCCAAAACCAAGCAATCGTCATCCAACCTGAGGACGCAGAAAACCAAATCTTCCAACATG
CTCAACGCAAATGGAACCAAGCCGACGCTCCTGCGCAGCTCCACGTTTGACAATACCCCCAGCACCGCTG
GAGGAGTCAAGTCCAAGCTGCCAGTGGTGGGCGCGCGGAAATAGAGGGTTAATTTGTTCAGGATAGTATG
GATCACAAATTAGCCAAGTTAAAGCGAAAACGGAAGTGGAGATTGTAAGAGGAACAGGACTGTATTTGTC
AAGCATTTTTTAGGAACCGAACACGCACAGGTCCACATCCACAAGCCGAGCTAGAGTCAGAGTCAAAATC
AAAGAGTTTTTAGGATAGGAGTTAATATACATACGCTATGTAATCCATATTTTACTCTCCTTTTACTTTA

TACATTATACGTAGACCCTAAAACTTTGTAAGCAACCAGTTTAGCATAGAGTTAGCGCAAACTCCTAGTA
ACTTCAACTTATCCAAATCACAGTCAGAGAAGGAAGCTCCCGATGCTCCCGATCGTGTGTAGCCAAATGT
ATTGCATATATATACACAAAATCAGGCCTTTCAACATGTGTACAACTACCCAATATATACAAAATGTATA
CTGGCATATATATAAATGCTATATATTCGATTGTCGCATTAACTAGGAAAGGTTTACCAAGTCGAATGTC
TGTTCTGATTTAACTTTTTTAACACTTAGTTAGATGAGCTGTCCTCCAACTGGTGAGCTACTGAAATCAA
TTACAATCATATAGTTGCACATCTTTATTGAGAATATACTCTAAGTTTAAAATATGCCCCTGGCTTCCTT
ATGCAAACACCAAATATCTGAAGAGAGAAGAAATTCACATTTTTTAGTCACTGCTTTTTAAGTATTCTTT
TATATAATTTAGCAAGTTAAGGGTAACTGTTTTAGGAAACAGCTTATGCATTAAGCTCATCATTTGCGAC
TTAGTTTTTACCGATTCAATGCGATTAACCATTTTCAATATGAATAAATCGAAATATGCTTTTTGTCAGC
AAAAAAATTAAACCAATGTGAATTTACTCGATTATAATTTATATCAAAATAAAACATATTTTGAAAGCCAG
ATCGCCTTTTTCGGTCGGTCACACTGGCAGCAATTGAATTGGAGTGaaaaaaaaTAAATGAATAAACAAA
CTAGCATTGCGTCCGGGAACAGACGAATAATCAACCGAGGAACACTCGTTTTCAACGCCATGCACTCGAT
GACGCGGACGCGCGCACAGTAGAGCCTCCCGATAAGAGTCCCGGAATCCGCAAGGCCAGAGACTACACAG
ATTTtgtgggtgtgttgtgttgtgtgtgGGAAAAAAACGAAAGAGTGAAGAGCAGTGCACGAAAATAAAC
GCAAAAAAATCAATGGGCGACCCCCCCAGCAGAGCAGCTCACAAGGGAAATTCGAGACCTTGAGTAGGGCA
GCACATCATCCCATCCCATCCAAGCCGAACCAACCCAACCCATGAACTTACTCCCAATCCGCAGCAGCGT
CTGTCTATTTACATTTGCATTTTCACCCCACCTGGCGCGACGACGGACAGGTGTTGTCACAAATCTTGGA
GCTACGGAGTGGCGCACAGTGGGGTGGATTGGCTGGTGGCTTGTGGCGAGTGCCGAATGTTGAGTAACCC
AGACCACTGTGCATGTATCACACATAGATACATGCGCACACCTAATCAGTGCTTTTCGCCAAAGATAAGG
GCTCTTTTCGAAGAGTTTGCCACTGGCCTGGCTTTTGCCCACATTCAAACGCAGCTGGCTGGCTTGCTGG
CTGACTGTTTCTGTTTCTGCTTCTGATTTCCGACTCTCGGGCACCGTGACTCAGACCAGGCGATCCGCTC
GCAGCCCCGCCGACACCATAACCAGCTACATCGTCATAGCCAGCGCAGGTCTCGGTCTCTCTCGCTCGCG
CTCTTGGCCGTAATTCGGGCTGAAAACCATTCAAAACCGTAAAGACAATACTGTCAATCGATTCAGCAGT
CCCAAAACCATAATTCTTCGATCCCAAATCGGGTTATTCGCAACACACTTCGGACTCCGTTGCTCAAACT
CTCTGTAGATCGGAGTTTTTACAAGCTGTACACTGTACAAAAAAATAGGCAGCCTATCCAAGTGTAGTTA
TCTTTTTATATTCGAAGGCGTTGACTGCAATAATTCATTTAAACGTTAAATATTATACAGTTTCTGAACT
CTTTAGTATATTTATGGGAGCTCAAACTTTTCATATTGTATCGTCAACCCTACGATCCCAAACTTTTGTT
CACTCTATCGAAGTTCAACTGTTACTCCGTGTGCCTTTATTGTTATGAACTTTTTACTTTTTCGGGGTCC
CCAGCGTTTTGTGATATTAAATTAATACATTTAGCTTTTGTTCATTTGCGTATTGTTTTCGCCACACAAT
AAAATGCGTATTGTGTCCGTATTGTGCAGGGGAAACCAGCCCAGATCCCCATATCCAGCACCAGACCCAG
ATCCAACGAAACCGGCCAGATGAGCAGCGGAATGAGGTCGCAGGACGGTCCACGGGCGAAGGTCAATGCC
GCCAGTACGCTGCTCACCCAGGAGGAGAATGAGGCCGTCTTCAAGCTGCTGGGCAAGAAGTGCCAGG
>dme_circ_0007852_junction_seq
TTCAAGCTGCTGGGCAAGAAGTGCCAGGGCCATGGAGCATTGACTTGCACCACTGA
>dme_circ_0007853
GTTCCAAAGAAGTGTTCGTTCTGCGCTCCCTGCTCTGCTCCACGAAGCTGACCCAGAACGCTGACCTTCT
CTCGCTGCTGCAGTGGCGCACATATCCCGAAACGATCCAGGACTCGCTGACGGGAGTCCTTCGGCTCAAC
GATGAAGAGCTGGTCAAGTTCCTGCAGGACGTGCTGGACGCCCTGTTTGCCAT
>dme_circ_0007853_junction_seq
AGGACGTGCTGGACGCCCTGTTTGCCATGTTCCAAAGAAGTGTTCGTTCTGCGCTC
>dme_circ_0007854
GGTATGTCTACGATGGCATTCAGTTGAAGGAGTCAACCGCGCAGTCGGAGAACATTGCGGACAACGGCGG
AATGCGACTGGCCTACACCGCCTACCGGAAATGGTACGAAAACCAGTTGACACTTCCTAATGGGGCTCAG
GATATGACCAAGGAGACCCTGCCCAACCTGCGATACACCGCCAAACAACTATTCTTCATTAGCTTTGCTC

AGAGCTGGTGCAACGATGTACATCCCAAGGTGAAGGCCCTTCAGGTGTCAACCGACGACCACATGCCAGG
AAAGTTCCGCGTTATTGGAACTTTATCTAACTTTGAGGAGTTTTCAAAGGAGTTCAACTGTCACGCCGGA
TCCGCCATGAATCCCATCGAAAAATGCATACTTTACTAGCATGGGAATTAAAGCGAAGCACAACGCGAAG
CAGTTGCTCTGCATTTGCATTGCTGGCCACGTCCTAAGTGCGATGGTCGCGGCTGCTCCTACCAGCGAGG
AAGATCTCAAACAGGACACGCCCTACATGAAGGAACTATTGCGTCAGGCGAAGACTGCTGAGATAGAGAG
CTTTATGGACCAGAAGGCGGATCCGTGCAACGATTTCTACGCATTCTCCTGTGGCAATTATAAGCGCATC
AACTCGGCTTTGAGCATGCAGGTGGTCACCACTGGAGTATTCGAGACTCTGACCAAAGGACTTAACCGAA
AGATCCTGAAGATGCTCAACACACCCCACGATTCCCACGACACTCCGGAAGACATTAAGGTGAAGCATTT
CTACGAGTCCTGTCTCCAGATCAAGGAGCTCAACTCGACGTACAGCGAAAAGCTGAAGCGGCTCATTGCA
GAATTTGGAACGATGCCGCTGCTCGAAGGATCCTCCTGGCAGGAGGACGACTTCGACTGGCTGAATACTA
CTGCTCGCATGGCCTACCGATACGGTATAACTCCCATTTTCGGCATTGAGGTGAACAAAGACTTGGCCAG
CAATACCCGGAATCGGATATACTTGGGTCAGCAGGACTTCCCGCTGGAGGCGCGGTCCATGTACGTCGAT
GATGCAACAGCAGTATATCGTCAGAAATATCGTAATAATATCCAGCGCATCTTGCAGCGTTACCTGGGCG
TGAAGATGGATTTGGCCAAAAAAACGGCCAAGGAACTGATTGACTTTGAGGTGGACTTGGCTCAAGGACT
GGTGGACGAGAGTGAAGGACTAGATGTGGGTGAATTGACCCAGTTACTCACAGTGGACGAAATCCAGAGA
AGGTACTCCCCCACCTTGGATATCGACCGATTGCTATTTGTGAGCATGGGAGAGCGCATTTCAGACCAGA
TCTACGAATATAACAATCGTTATCAGCAAAACTTGGTCGAAGTCATCAAGCGTACGCCGAAGCGCACTGT
AGCCAACTATATATTTTTCCGGCTGATCTGGGAGTTTGTGGAGACGCCTTCAGATTCGCCGGAGAAGCAG
ATGAAGGCATGCGTGGACCTTACCAAAAAGTACTTTGCCAAGAATCTTGACAACATGTTCTATCGTCGCT
ACAACAACGAGAAATCCTCTAGAGAAATCGACAACATGTGGCGTCAATTGAAGTCTACCTTTAACGAGAC
CCTCCGTTCCAGTCCCGCCTTGAATTGGATTGAGCGACCCACCCGGAATCTTGCTATGGCGAAGCTACAG
GCAATGACGCTGGAGGTCAACAACTACGCAGACGACAACTTCACCGAGGAGTTTGCCGAACTCAATCTGC
AAAGTGACGACTACGTGGAGAATGTGCGATATACAAGCCTACTTGGGGCCAAACAGATGCGGGAGATGCT
TCACAAGCCGGCCAAACCCTTTGAGGCGGGCTCCCAACTCAGTTACACCCCGGCCAACATCCTGATAGAG
AACACCATCAAGGTTCCGGTGGCCCTGCTACAACCCTTCTACATTTGGTCCGATGTCTACCCCAATGCGA
TTATGTTCGGAACCCTGGCCTCCCTAATTGGACACGAGTTAATCCACGGATTCGACGACAGCGGGCGGAA
GTTCGATGAGAAGGGAAACTCGAAGGATTGGTGGGATGAGAAGTCCAGCAGCAACTTCCTGAAACGAAGG
GACTGCTTCACCAAGCAGTATGGCAG
>dme_circ_0007854_junction_seq
GGGACTGCTTCACCAAGCAGTATGGCAGGGTATGTCTACGATGGCATTCAGTTGAA
>dme_circ_0007855
GGACTGGTGGACGAGAGTGAAGGACTAGATGTGGGTGAATTGACCCAGTTACTCACAGTGGACGAAATCC
AGAGAAGGTACTCCCCCACCTTGGATATCGACCGATTGCTATTTGTGAGCATGGGAGAGCGCATTTCAGA
CCAGATCTACGAATATAACAATCGTTATCAGCAAAACTTGGTCGAA
>dme_circ_0007855_junction_seq
CAATCGTTATCAGCAAAACTTGGTCGAAGGACTGGTGGACGAGAGTGAAGGACTAG
>dme_circ_0007856
ATTCCGGGTGGGTCGCTCAATCCAATTCAAGGCGGGACTGGAACGGAGGGTCTCGTTAAAGGTAGACTTC
AATTGACGCCACATGTTGTCGATTTCTCTAGAGGATTTCTCGTTGTTGTAGCGACGATAGAACATGTTGT
CAAGATTCTTGGCAAAGTACTTTTTGGTAAGG
>dme_circ_0007856_junction_seq
ATTCTTGGCAAAGTACTTTTTGGTAAGGATTCCGGGTGGGTCGCTCAATCCAATTC
>dme_circ_0007857
GTGTTTGTTCTGTAATGATAACCAAATCGTGTATATTATTGAAAGTGCGGTGCGTCTTCCCGACACCAGA

ACCTAATTGTAAATGTCTTGCATTGTGCACACCCCAAGGTGGAGGAGACTAATTGAACCGACTGCAAACC
GATAATTGGAATATACAAATGTAGTTGTAAAATAAGAAAGTCGCTTAAGCGCTAGTTGGAATGCATCCCC
ATGTCTACACCTGTCACTGAACCGGGAGCCGACTAATTTGTAATTAATGCACCATTTCGAGAGCGTGAAC
TGAAAGCTAATGCAAGCCATTATGTACACCGCCCACGAATGTACGGATTATTGTAAATGTAAAGAGCAAT
TGTTATGCCACCATGTGTACACTTCAGCGATCGCAGCGCATTTTGTGTAAATTCAGTAGCAGCCCCAGAG
TCATCTCTCTTAGAATACGTTTTCAAATGTGTAGAACCGTTGCCGGCCT
>dme_circ_0007857_junction_seq
TTCAAATGTGTAGAACCGTTGCCGGCCTGTGTTTGTTCTGTAATGATAACCAAATC
>dme_circ_0007858
TTATCATCGAGAAATTGCTGCGCGTCACGGAGGTGGGACAAATCTATTTGCTGATACGCACCAAGAAGGG
CAAGGATGCCTTCGCCAGGATCGAGGATCTGTTTAACGATCCGGTAAGTGATGGAGACCTGCTAATTGTT
TTCTCTCCCGCCTCCCTGGCTAACTAATTTAAATTTTTAACTGCGCACAGGTCTTTGCCAAGATGAAGCA
GGTGAATCCCAAGTACCGCTGCCAGATCACCATCATCAGTGGCGACTGCTCGCTGCCTGGACTGGGAATC
AGTGCCGACGAGCGGGAGACCATCATGGAGAACG
>dme_circ_0007858_junction_seq
GACGAGCGGGAGACCATCATGGAGAACGTTATCATCGAGAAATTGCTGCGCGTCAC
>dme_circ_0007859
GATCAGGATCATCAGCCAGCCGCACACCACCACAAGCCGAGTGGTCCACCGCTGCTCCCCAAACAGACAC
GCAAGGACATCGCACGGAGGACCGAGGAGCAGCCGGATCACGAACAGGATCACGACCAGCAATAGGATCT
AGTGCAGCCGAACGGGACATTCACCGATCGCACGACCATGTTCACGGGCAAGCTGCAGATCAAGGTGTGC
GAGGCGAGTGGCCTGCGGCCCACAGACTTCCAGAAGCGCCACAATCTCACCTTCGGCAAACTGGCCGACG
AGCAGCTCATCGACCCCTATGTCTCCATAGATGTCGACGAGAGTCACTTCGGGTGAGTGTCCTCCCACTT
CGATTTCAGCTCAAATATTCAAATATGGTTGGGAAACAAAAGTCAGTTAGATAGTTACAAATGTTAGTTA
CCTAGCTAAAGTGTTGCATACTTAAATGGGATTTGGCAGCTAACTTTATTTATTATTTCATTATCTGGTT
ATTTTGTTACGCACAGGATCGAGCCACCACGCGGCCAAAGACATTCGATCCTGTTTGGAACGAGCAGTTC
GTCCACGACGTGACCAATGTGAGCAACATCAACTTGACCGTCTTTCATGATGCTGCTCTGCCGCCGGATG
ACTTTGTGGCCAACTGCATCATCTCCTTCGAGGACCTCATGCAGAGCGAGACGGCTGTGCAGGATCTATG
GGTGGGTTTCGAACATATAGTAGCTAAATCTCTAGGAATACTTATACTTACTTTTACTTCTTGAAACCTC
TCAGGTTAACTTGGAGCCGCAAGGCAAGATTCACGTCATCATTGAGCTAAAGAATCGCACGGGTACGTAG
CGAAATAAAGATTTGAACAATGTTCAGCGAATGCTGATCAATTCCATCTGATTTCAGATAAAGCCAAAGC
CGAGGCCGTGGTGGAGCACACCGTGGCCGTCAACAAGGAGTTCAAAGAGCGCGCCGGATTCAACCGACGT
CGCGGTGCCATGCGTCGTCGGGTCCATCAGGTTGGTGCCCATTTAACCAGTTGAGAATGAGCGTAACTAA
TATGTTTCCTTTGCTTGAAACCAGGTGAATGGGCACAAGTTCATGGCCACGTTTTTGCGTCAACCCACCT
TCTGTTCGCATTGCCGGGAGTTTATCTG
>dme_circ_0007859_junction_seq
TCTGTTCGCATTGCCGGGAGTTTATCTGGATCAGGATCATCAGCCAGCCGCACACC
>dme_circ_0007860
GATTATTACAAAGCTTTTATGAGTGTGTTTATTTCTATTGATCTTCTTTTAACTTACGTGCTTTAAATGA
CTTTGTATGCATTACGCGGTTATCACAATTTCATTTTCTTTTCAATTCTTGTTGTTAGGTTTCCTTCACG
>dme_circ_0007860_junction_seq
CAATTCTTGTTGTTAGGTTTCCTTCACGGATTATTACAAAGCTTTTATGAGTGTGT
>dme_circ_0007861
GGCCTTCACCACTACCACTGAGGAAACGCAAGACTCGACTATCTCCAGCGAGAGCTGTAGCTTTAACTAC
AGTGATCTTCCCATCGGCAAAGGACCCCGCATTCGCACACCCATCAAAAATGGTCGAAGAGTTAAGACTA

ACCTGGAGCTCCGACAGAAATTCAAGACGCTCAATGGCCAGGACGAGGAAATTAAGGTGGAGCATGTGAA
AACAAATGGGGAG
>dme_circ_0007861_junction_seq
GGTGGAGCATGTGAAAACAAATGGGGAGGGCCTTCACCACTACCACTGAGGAAACG
>dme_circ_0007862
GGTAGAAGTACTTCAGGGTCTCGCTCATCCAAAAGCTTTCCATCAGATCGCGCAGACGCGTGCTCTGTGT
GTTCTTTACATTTCCCATCGATGTGTAGCCTGCATTTACTTTAGCGTGAGTCTCAAACGCCTGGAAGATT
TTCCATCCCATGTCCTGGTATGTCCGATTGCCAGTTATTGA
>dme_circ_0007862_junction_seq
CCTGGTATGTCCGATTGCCAGTTATTGAGGTAGAAGTACTTCAGGGTCTCGCTCAT
>dme_circ_0007863
GTAGTAGTTGGACCAACCTTGGTTAGTTAGCTCCGATTCCTCCGCGGCCAGCGGCGCACAGCAATCGATT
TTTATTCGAGGCGAATGCTGTGGAATTTCCCATCCAACTTTCCCACACTCTGAATTCCGGCGGCGGCATT
TAGTGAAACACTATGAAGGACGGCGG
>dme_circ_0007863_junction_seq
TTTAGTGAAACACTATGAAGGACGGCGGGTAGTAGTTGGACCAACCTTGGTTAGTT
>dme_circ_0007864
GCTTTCCGAGCTGGACGAGACCATGCGCTTGCGTGATCGCTCTGGCATTTTGATGATGGCATCTGTTCGA
GTGGTGTAGGACTTCTTGGTGAGGTTCTTGAACTCCAGTGCGCCACAGTTGATGTAGAAGCCGCCCTCCA
GA
>dme_circ_0007864_junction_seq
ACAGTTGATGTAGAAGCCGCCCTCCAGAGCTTTCCGAGCTGGACGAGACCATGCGC
>dme_circ_0007865
TTATAATTTGCACTGCTGTCTCCTACTTTCCGCGGCGCTGACGCGGCGGCAGATTGTTCTTCTTGCCAAG
AATCTTCATTAGCTGCTTGGACATGAAGAACGGCTTCTCCGGTGAGCCGTCGTTGCGCTCGCAGTCCTCC
AGGAAGCACAGAAACAGTGTGTCCACTGCCGTCGAGTACACACCGAAAAACACGCTCGTTATGAGAAAGG
CGGCAATTACCACCACCGTGGTGGGCACTGCTATGTAATTTAGCCGAATAATGCTCGGATTATTGTCCAG
GAAGTAGTAGGTGGACGCCCCCGCCCCTGCGGTTAGCAGCAG
>dme_circ_0007865_junction_seq
ACGCCCCCGCCCCTGCGGTTAGCAGCAGTTATAATTTGCACTGCTGTCTCCTACTT
>dme_circ_0007866
GCAAGAAGGCGGCGTAAACCAAACGACCGAGTCGATGACCCGCGCCTTGATCACGGACGAGGCGCGCACC
GCTGGTGCCTCCATGGGTCCCGTACAGCTCGGGTACGTATGCACCAGGACAGCTTCGAAAATGGGAAAGA
CGCACGCTCAGGCGTGGGCCGCAGTTCCGTCGGCAGGCAGATACTCTTTGGGCGAATAAGAGTGTACATA
AAGGGATAAATTGTAATTTTAAATGGTGTACTTTTGGTTATCTTAACCCATTGGGTCTTACTTTTGTATA
TGTAATCTACTCTTTGTTCGATTTGTTTATACATATGTACATACCCAAAGATCCTATCTTTAGAAGTATC
ACATAAAGTAATCAGATTAGTGCCGCCCAGGGAGCTTTTGCCTTGCCCAAttttttttAGCTCTTAACCA
ACGAAATGCCACACAAAATTATTGCTTACTGCCGCCAACGCCAAACTAATCAAACTCAGAACGCCAATGC
AGCTGCGCCGTCACAGAGAAAGATTAACCGAACCATGTCCATTTATACCGTCCAGCTTGGAGTCCCCTGG
AATTTCGAGCAATAATTTTCTGTGCAAGATCCCAAAATGACACATCGAATGCCACTAAATTAAACGTCGT
TCTCTTCTTATGCTCCTCTCTTCCCCACACAAAAATGTCCAACCGAAAAATAATGTTGAAAATATAGTTA
CATGGAGAAATCAAACGAACGTGAGGATTATCTTCCATGGTTGGTACCCTGTCCCTTCaaaaaaaaaaaa
gaagaaaaaaaCATGACTAGGTCAAACAATCAGTCAAACTCGTATCCATTAGCCAACGTACAGCGCTCAT
CAAtcgtatttgtatccgtattcgtattcgATCGGTCAATCAGCTGTATATAGAAACATGTCATTAGCTT

CGctcgcctcgcctcgcctcgccttgcctcCCAGCACCGCCCCGCATCCCGTCCAGAACTCAGAGTGCCA
CCTAGAGTGCTAATGTGTCACCCATTCAACCGATCCCACCCAGTGCCTCATTCATCCCGATCCTCGCTCT
CGTAACGCCTCGCGCGTCCATTGGTAACTTGTGCTTGAGAGTTTAGTTTTCCTTGTTTGTCGTAAATAAC
CGGAATGACATTGTTGGATAGCCCGTAGCGGAGATCTCTCACTAATAGCTGCTTCGTAATCCCCTCTAGG
CTTCGTGGCTGGCGTCATCCTGAGGGAGCGACTGCCGGCCTTCGAGCGGATGTTGTGGCGCGCCTGCAGG
GGTAACGTCTTCCTGCGACAGGCGATGATTGAGACGCCGCTGGAGGATCCCACCAATGGGCGACCAGGTG
CACAAGTCCGTGTTTATAATCTTCTTCCAGGGTGACCAGCTGAAGACGCGCGTCAAGAAGATCTGCGAGG
GCTTCCGGGCCACGCTCTATCCTTGCCCTGAAGCTCCAGCCGATCGGCGAGAGATGGCCATGGGTGTGAT
GACTCGCATCGAAGATCTGAACACCGTGCTCGGCCAGACGCAGGACCATCGCCATCGTGTGCTGGTCGCT
GCGGCCAAGAATCTTAAGAACTGGTTCGTCAAGGTGCGCAAGATAAAGGCCATCTACCATACGCTGAATC
TCTTCAATCTGGACGTGACCCAGAAGTGTCTGATCGCCGAGTGCTGGGTACCGCTGCTGGACATTGAAAC
CATCCAGCTGGCCTTGCGCCGCGGAACCGAAAGATCGGGATCGTCGGTGCCGCCAATTCTCAACCGGATG
CAGACGTTCGAGAATCCACCGACCTACAATCGAACGAATAAGTTTACCAAGGCCTTTCAGGCATTGATCG
ATGCCTATGGCGTGGCCAGCTATCGGGAGATGAATCCGGCACCCTACACGATCATCACCTTCCCCTTCCT
GTTCGCCGTGATGTTTGGAGATTTGGGCCACGGAGCTATCATGGCGCTCTTTGGTCTCTGGATGATTCGA
AAGGAGAAGGGACTGGCGGCTCAGAAGACGGACAACGAGATCTGGAACATATTCTTTGGCGGACGGTACA
TCATCTTCCTCATGGGCGTCTTCTCCATGTACACGGGTCTTATATACAACGATATATTCTCCAAGTCGCT
GAATATCTTTGGATCGCATTGGCACTTATCCTACAACAAGTCCACCGTGATGGAGAACAAGTTCCTGCAG
TTGAGCCCGAAAGGCGACTACGAGGGCGCTCCGTATCCGTTCGGCATGGATCCCATTTGGCAGGTGGCGG
GAGCCAATAAGATCATCTTCCACAACGCTTACAAGATGAAGATCTCGATCATTTTCGGTGTTATTCACAT
GATCTTCGGTGTGGTGATGAGCTGGCACAATCATACGTATTTCAGGAACAGGATCTCCCTGCTCTACGAG
TTCATTCCCCAGCTGGTCTTCCTGCTGCTGCTTTTCTTCTACATGGTTTTGTTGATGTTCATCAAGTGGA
TCAAGTTTGCCGCCACCAATGATAGAGCCCTACTCCGAAGCCTGTGCGCCTTCAATCCTGATCACCTTTA
TCGACATGGTGCTTTTTAACACGCCCAAGCCACCTCCGGAGAACTGTGAAACCTATATGTTCATGGGCCA
GCACTTTATCCAGGTGTTATTCGTCCTGGTTGCCGTCGGCTGCATTCCCGTAATGTTGCTGGCCAAGCCG
CTGCTCATCATGCAGGCCCGCAAGCAGGCGAAC
>dme_circ_0007866_junction_seq
CATCATGCAGGCCCGCAAGCAGGCGAACGCAAGAAGGCGGCGTAAACCAAACGACC
>dme_circ_0007867
GCTTCGTGGCTGGCGTCATCCTGAGGGAGCGACTGCCGGCCTTCGAGCGGATGTTGTGGCGCGCCTGCAG
GGGTAACGTCTTCCTGCGACAGGCGATGATTGAGACGCCGCTGGAGGATCCCACCAATGGGCGACCAGGT
GCACAAGTCCGTGTTTATAATCTTCTTCCAGGGTGACCAGCTGAAGACGCGCGTCAAGAAGATCTGCGAG
GGCTTCCGGGCCACGCTCTATCCTTGCCCTGAAGCTCCAGCCGATCGGCGAGAGATGGCCATGGGTGTGA
TGACTCGCATCGAAGATCTGAACACCGTGCTCGGCCAGACGCAGGACCATCGCCATCGTGTGCTGGTCGC
TGCGGCCAAGAATCTTAAGAACTGGTTCGTCAAGGTGCGCAAGATAAAGGCCATCTACCATACGCTGAAT
CTCTTCAATCTGGACGTGACCCAGAAGTGTCTGATCGCCGAGTGCTGGGTACCGCTGCTGGACATTGAAA
CCATCCAGCTGGCCTTGCGCCGCGGAACCGAAAGATCGGGATCGTCGGTGCCGCCAATTCTCAACCGGAT
GCAGACGTTCGAGAATCCACCGACCTACAATCGAACGAATAAGTTTACCAAGGCCTTTCAGGCATTGATC
GATGCCTATGGCGTGGCCAGCTATCGGGAGATGAATCCGGCACCCTACACGATCATCACCTTCCCCTTCC
TGTTCGCCGTGATGTTTGGAGATTTGGGCCACGGAGCTATCATGGCGCTCTTTGGTCTCTGGATGATTCG
AAAGGAGAAGGGACTGGCGGCTCAGAAGACGGACAACGAGATCTGGAACATATTCTTTGGCGGACGGTAC
ATCATCTTCCTCATGGGCGTCTTCTCCATGTACACGGGTCTTATATACAACGATATATTCTCCAAGTCGC
TGAATATCTTTGGATCGCATTGGCACTTATCCTACAACAAGTCCACCGTGATGGAGAACAAGTTCCTGCA
GTTGAGCCCGAAAGGCGACTACGAGGGCGCTCCGTATCCGTTCGGCATGGATCCCATTTGGCAGGTGGCG

GGAGCCAATAAGATCATCTTCCACAACGCTTACAAGATGAAGATCTCGATCATTTTCGGTGTTATTCACA
TGATCTTCGGTGTGGTGATGAGCTGGCACAATCATACGTATTTCAGGAACAGGATCTCCCTGCTCTACGA
GTTCATTCCCCAGCTGGTCTTCCTGCTGCTGCTTTTCTTCTACATGGTTTTGTTGATGTTCATCAAGTGG
ATCAAGTTTGCCGCCACCAATGATAGAGCCCTACTCCGAAGCCTGTGCGCCTTCAATCCTGATCACCTTT
ATCGACATGGTGCTTTTTAACACGCCCAAGCCACCTCCGGAGAACTGTGAAACCTATATGTTCATGGGCC
AGCACTTTATCCAGGTGTTATTCGTCCTGGTTGCCGTCGGCTGCATTCCCGTAATGTTGCTGGCCAAGCC
GCTGCTCATCATGCAGGCCCGCAAGCAGGCGAAC
>dme_circ_0007867_junction_seq
CATCATGCAGGCCCGCAAGCAGGCGAACGCTTCGTGGCTGGCGTCATCCTGAGGGA
>dme_circ_0007868
CTCATCACCACACCGAAGATCATGTGAATAACACCGAAAATGATCGAGATCTTCATCTTGTAAGCGTTGT
GGAAGATGATCTTATTGGCTCCCGCCACCTGCCAAATGGGATCCATGCCGAACGGATACGGAGCGCCCCTC
GTAGTCGCCTTTCGGGCTCAACTGCAGGAACTTGTTCTCCATCACGGTGGACTTGTTGTAGGATAAGTGC
CAATGCGATCCAAAGATATTCAGCGACTTGGAGAATATATCGTTG
>dme_circ_0007868_junction_seq
ATTCAGCGACTTGGAGAATATATCGTTGCTCATCACCACACCGAAGATCATGTGAA
>dme_circ_0007869
CGCCCACAATCGCAGATAGGAAGCGGTGTGGGATACCGAACCTAGGACGTACTCGATGGTGTGGATGCTC
TGGTGAATGAAGATCTCAGAGAGCTCCTCTTCCTCCTCATGGCCACCGCCACCACCGTGTGAGCCGCTAT
TGGACACGCCGCCGGCTTCTGCATCTGAAGTTGCTCCTGCAATGGGCTGTACCTGCAAGTGAAGTATATT
ATTAACTGCGTTAAAGACGAACATTTGGGGGCACTAAAGTCGAACTTCTCTAAACGAAAAAGTATTGACA
GCATATTATACTATTTAAATTTAATTATGCGATTAGAGAAGTTCGATTTAACATCGACAATAAACGACAC
GACTAACCTCTTCCTACGGTTTGTGGATTGAAGTGTTTTAGTTTATTCGGTGTGGATTTGGTTGGGGTTA
TAATTTTGTTAATCGGGAGCACAAATAAACGAGAAGCAGTTAGTATAAGGTGGAAAGTTTGGAGTGCGAG
GTGCTTGGATATGTTGTGATGTAGATGGAACGCCGTTCTATGAAATTAATGGGGTGCATCGAACTAGCGT
CTACTTACGTTCGCCTGCTTGCGGGCCTGCATGATGAGCAGCGGCTTGGCCAGCAACATTACGGGAATGC
AGCCGACGGCAACCAGGACGAATAACACCTGGATAAAG
>dme_circ_0007869_junction_seq
AACCAGGACGAATAACACCTGGATAAAGCGCCCACAATCGCAGATAGGAAGCGGTG
>dme_circ_0007870
GGTGACGCTGGCCAGCATCCTGAAGGCCAGTGGCCACAAGGAGATCCCCATCCAGAAGACTTGGCGCCTG
AACGAGCGCCACTACGGTGGACTCACTGGCCTGAACAAGGCCGAGACCGCCGCCAAGTACGGCGAGGCCC
AG
>dme_circ_0007870_junction_seq
GACCGCCGCCAAGTACGGCGAGGCCCAGGGTGACGCTGGCCAGCATCCTGAAGGCC
>dme_circ_0007871
CAAGGGAGTGGATAAAATCACATCTGTGGACCTGGAGCACCCCGAAGTGGTGAACTTTATAACCGATGTC
AAGGCAAGCGAAGCTAATGGAGCGGCGTCCGAGACGCCCCTGTTCCACTCCGAGCTGAAGGAGATG
>dme_circ_0007871_junction_seq
CCTGTTCCACTCCGAGCTGAAGGAGATGCAAGGGAGTGGATAAAATCACATCTGTG
>dme_circ_0007872
GGCTTCCTCCACGGCTTCTTCGTCGGACTCGGGAGCTTCCTGGCTTTCCAAGGTCAGAGCAGCAGTTTCC
GTTTCCAGTTCCTCGGTTTGGGCCACCGTAGCGAAGGTCACAGGAGCGGGAGTAGCTGGGCTGGCGGTCT
TTCTCCTTTCCGTCTCAGACCCAAGAGCAGGGAAATCCTCGCCAGACAAGGCCTTTGTCTTGACCAGCGG

GATTTGCTGCACCAGACTGGGCTCATGCGACCAGAGCTTGTCCCCAAAGAGGTGGAGCATCTTGATGGCC
ACACCGTGACCTCCGCACATGTACAGTTCATCGCTGGAGCGAGCCAACTGGCCCACGCCCACCGCAGATT
TGTTGTTTGTGAG
>dme_circ_0007872_junction_seq
CGCCCACCGCAGATTTGTTGTTTGTGAGGGCTTCCTCCACGGCTTCTTCGTCGGAC
>dme_circ_0007873
CTTGTTTTCGGGGAAGCTGCGATCCTCGTGCAGCATCTCGATCTTCTCGATGGACTCCGAGATCTCCGGC
CAGAACTCATCCACGATGTTGTCCAGCTTTTTGAGGGCGAACACCTTAAGATCCGGCATTGGCTCATCGA
GTAGGGAAATTATCCCCGCGGCGGACGTAAGACTCATTTTCCAGCTGCG
>dme_circ_0007873_junction_seq
CGGACGTAAGACTCATTTTCCAGCTGCGCTTGTTTTCGGGGAAGCTGCGATCCTCG
>dme_circ_0007874
AACTTGGACCGGCCTCCTTTGGCAGATAGCTCTGCATGAGAGCCAGGGAATCCTTCTCGTGACCCCGATG
AATTACTCCAAGCGAAGCGGTTGCCGTCAGCTTGGCCCAATTCGTGGCCCTGGCCAGCCAGTCGAGGTTG
TCGCGCAGGAATTGGTCGGAGGTGGTGCCGCTGTGCATGAAGGCGTTGGCAATAACAGTGGCTGTGTGGC
AGATAGACACGCGGACCGCTTCCTTTGTGCCTCGAAGGACCTGCAAATCGGCGTGGTTGCTGCGGATGAG
GAATTGGAGCTGTAGGTCGATGGAAACCTCGCCGGAGAGGATCGATATGAGCTTTTCAATGTTCTTCTGA
TGCAACTTTTCAACCTCGTTAAGGGAATCGATTGTGCGCTCTACTTTATCATCAGCTAGGTGAATAAAAT
GCGATTTATAAGTATTTTAAGGGACTGGATTAGAAAACCGGGTTTACCTGGCGTCTCCTCTGTGATATCC
TCGTCAGACTTGGACTTGTCTCCCTCCGACTTGGCACCATCCTCGGAGGTGGTACCCTGGGGCTTAAATG
TGCTGGGGAGGGCGGTTGGGATAGGAGCGGTGTTTTTGAGGTGCTGCAGCACATTGCCCAGAAACTCTTG
CGTGGCCGACTCG
>dme_circ_0007874_junction_seq
GCCCAGAAACTCTTGCGTGGCCGACTCGAACTTGGACCGGCCTCCTTTGGCAGATA
>dme_circ_0007875
GCTAACATTATAGACTACCTGCTGCAACAGCTGAAGGATGCCCAAAACGAGAATGTGCGTCATGGCGGCT
GCCTGGGACTAGGTCTCGCTGGCATGGGCACCCATCGCCAGGATCTGTATGAGCAGCTGAAGTTTAATTT
GTATCAGGATGACGCG
>dme_circ_0007875_junction_seq
GAAGTTTAATTTGTATCAGGATGACGCGGCTAACATTATAGACTACCTGCTGCAAC
>dme_circ_0007876
GGAGAAGAAGAGCGAGGAGCGCGAGAAGGTGGCCACCGCAGTTCTGTCCATTGCCGCCCGCCAGAAGAGA
CGCGAGAATGCCGACAAAAAAGAGGATGAGAAGATGGATGTGGACGAGGACAGCAAGGAGGGTGCTGCT
>dme_circ_0007876_junction_seq
GGACGAGGACAGCAAGGAGGGTGCTGCTGGAGAAGAAGAGCGAGGAGCGCGAGAAG
>dme_circ_0007877
GGGGGGAGTGGAGCGGAGAAAGCCAGTGAATCAGTGCCGCAACGTAACGGTAAACCCCCGATCCCGGCCA
CCTTAGAACCAGTACCCATCCCAAAGTTATGAACCGCACCAGGAGGAAGGTGGTGCGCCCAAAGAATCCC
TTCACCAAAGCCAACTTCTTCTCACAATGGAGCTTCTGGGTGAGTTTAACTACAAACGTGTGGATATGTA
GAATTAATCAAAACATGAAAACAGACTTGGAGTCACTCCTGTATTAGTCATGAGAATATCACTTACGGTA
AATCAAATGTACTATTCTGTAGCACGTGTTCTCACCATATTGTAGTATCAGGTTCGTATCAGGGCAGCAC
CTATCGTATAATTAGTTTAGTTGTATTAGCCTTTTTGGACCGATAAGTTTGGTGTGCTTGATAAGGTTCC
CGTTATAATGAGAATGGCAATTCGATGGCACAGTTTTATTAAATGTCACCGCGGGCCACAAGGTTACATC
TTTATCTGGCGTCGACAATGAACGCTTGCCAATTGCAACCAGATGGATCTGGCACCAGGGGTTTATTACG

ATTTCCTCCAATAAACAAAGCGTTCATAGTCTGACTAGAGCTGATAAGGCTGAAATTTTAATTCACTATT
CAATGTCACATTTACTACGAATATTCAAATGTTCGCTCTACGGCTAGACAATTTACAATGTATGTACACA
ATAAGACTGAATTGTAATTTATGAAAAAGTCTTAGCGATTCCAGGCAGGGCCGTCACATGAGCGCGGTTT
TAATTTAAGATCGTGGAGTTCGATTGCCCGTTTTCTCATGATTTTTCAGTAGGTACATAAAGAAAAGTAA
TTCGTCAGAAGACTGAATCAATTGGTCTTAGTTTTGCGTACCCTGAGCGTACGATTCCGATCCGAGTTTA
TGGTGATTTTAAGACAACAAAGTCTGTAAACCAATTTGGATTTTTATAACCTGTATTTTGCCTCGATAGA
ATCGAGTCGCCCATAGATCCAATATTCAAATGATTAACGCGAATCAATTAACGCCCATTTGGTGTTGATG
TTCACAAATACTACATGTCTACATTCATAATTTTATTGTTAGTTAATTTGGACGATAATCTGCTGACATG
GAATAAGCAACATTACTGAACATTCAAATTAACATTTATACTGTCTTACTACCCAGTTTCCACACTATAC
ATGCATATATAGATATGTGCAAGTGTCTACACACGTACAAGCGTTTTATTAAAATGTAATCAAACAATGC
CTCCGGGGGTTTTCGCAAATCTTGGACTGACGAAATTCCCAAGAAAATGATGTCGCTTGCAGGCCTTGTC
TATGTTTTATCAGAGGAGTTCCCTTAAGCAAAATAGCGCCTACTGTATCTAAATTGGGGTCTTCGTTACA
TAGACTTTACTTAATCAAACACCAATAAAGAGTAGGAAATCGTATAAAATCTTAGGGCCTAACAACCGAA
TATGACCTTGTCTCACTCATTATCAGAACATTTAAAGGTCTTCTGTAGTACTACTGTATTGCCATCGTAA
GCCTTAACATGTGCGCCTTTAGTTTAATTGCAATTAAACTAAGATAAGGATATCAGGATTTAAGTGCTTT
AAAGTAGTTATACTGTAGGTTTCTGTGTTACTACTCTGACCTTTTAATCTTTCAGGTGGATGCGAGACCT
GTTCAAGCGAGGCCTCCAGGGACCGCTGACGGACGAGGAGCTGTATCAACATAGAAAGACCCTGGATAGT
GAAAGAGTGACGAACAAATTTGCGGAACT
>dme_circ_0007877_junction_seq
AAAGAGTGACGAACAAATTTGCGGAACTGGGGGGAGTGGAGCGGAGAAAGCCAGTG
>dme_circ_0007878
ATAGCGGCGATATCATGGAGGTGCAACCAAGAGCGGCATGGATGTAGGCAGTGCCTCGAATGGCCTTCAC
CTCCTTGAGGCGAACTTCGGCGATGACCCGGGCAAAGCTTTTCTCCCACGCATACATCTTGATCACCTGA
ATGCCCTGGATGATCTCGTTCATCAGCTTGACACGCATATCCGTTCGCTCTGCCGTCATTTGGCGATAGT
AGGCGGCTTTCTTGGCTGCCCACGCTTGCAGTGGGATAAAGCTGAGCATAAAGGACACACCGATCACAGC
CGATATACCGATTTCACGGTACATGAGAAAGCCAATCAGTATCGACTCCATCGGCCCTTTCCATG
>dme_circ_0007878_junction_seq
AGTATCGACTCCATCGGCCCTTTCCATGATAGCGGCGATATCATGGAGGTGCAACC
>dme_circ_0007879
CACCACACATTTTTTCGTTGCCTCCGGATCATAATCCCTTAGACGATTGTGGTGGACTTGGCCATTGGAC
AGCAAGGAACCGTTTAACTCTACGTTCttttgtttgtcctttttgctgattttgcgcttgtttttgcctg
tttgttgatttgcTTCCAAGTTTATGGGCACCTCCGCTTTGTCTCCGTCTAGTAGAAAATCTTTGCAACG
TTTCGCCGAGACCAAGGCCTCGGCAATAAACGTGATAGACATAGGCCAAAAGGTTACCATAGAATCG
>dme_circ_0007879_junction_seq
CATAGGCCAAAAGGTTACCATAGAATCGCACCACACATTTTTTCGTTGCCTCCGGA
>dme_circ_0007880
GGTGCCCATAAACTTGGAAgcaaatcaacaaacaggcaaaaacaagcgcaaaatcagcaaaaaggacaaa
caaaaGAACGTAGAGTTAAACGGTTCCTTGCTGTCCAATGGCCAAGTCCACCACAATCGTCTAAGGGATT
ATGATCCGGAGGCAACGAAAAAATGTGTG
>dme_circ_0007880_junction_seq
TGATCCGGAGGCAACGAAAAAATGTGTGGGTGCCCATAAACTTGGAAgcaaatcaa
>dme_circ_0007881
GTAGCGTATCATGCCCAGAATAACACCAATGATGGCAGCTGGAATCAGGAGCCAATAGTTTGCAATCGCC
ACAATAATGACAACGCCAGAAACATCGATAATGAAGGCGAGGCAGTCCAGCAGTGTGTGCGGCAGATCCG

TATCCACTGTCCTTATATCCTTGGAGAAGCGATTCAGAATTCGTCCTGAGGAGTTCGTATTGAAGAAG
>dme_circ_0007881_junction_seq
TCGTCCTGAGGAGTTCGTATTGAAGAAGGTAGCGTATCATGCCCAGAATAACACCA
>dme_circ_0007882
GCAGATCCTGGGAGATGATACGGCCGCCGAGGACTTGAACATCACCCTTGCGATGGAGGAGAAGACCCAG
TGACCGAGACGCACACTCATCTACTTGTAGATTTGCTTAAGGTTTAGATCATAAGAACTGAAAGTATTAT
GTACATAGCAAAGTTGTACTTAAAGCTAACGAAATACCTATTGCCTTGTACTTACGGAATATTTGTAAGC
TTGATTATCGCCACGAATTTATGTATGTATATGTTCCATAGCGTACTGA
>dme_circ_0007882_junction_seq
TGTATGTATATGTTCCATAGCGTACTGAGCAGATCCTGGGAGATGATACGGCCGCC
>dme_circ_0007883
GTGGATGTCAATGTGCGTTCGCGCATGAATGTGCCCTTCCGTATTGGCAGTGCCAGTGGAGATGATGCCC
TGGAGAAGGAGTTCCTGAGCAAAGCCGAGGCCGAGGGCATGATTCAGTTGAAGGGTCATCGGTCGGTGGG
CGGCATTCGAGCCTCCCTCTACAATGCTGTAACGCTGGCGGAGACCCAACAGTTGGCCAATCTGATGCTG
GCCTTTTACAAGAACAATAAAAATTGAACAG
>dme_circ_0007883_junction_seq
TTTTACAAGAACAATAAAAATTGAACAGGTGGATGTCAATGTGCGTTCGCGCATGA
>dme_circ_0007884
GGTGCCCGCCTTTGAGACCGCCGAAGGACAGTACTTGAGCGAGTCCAATGCCATCGCCTACCTGCTGGCC
AACGAGCAGCTGCGCGGCGGAAAGTGCCCCTTCGTGCAGGCCCAGGTCCAGCAGTGGATCTCCTTCGCCG
ACAACGAGATT
>dme_circ_0007884_junction_seq
GTGGATCTCCTTCGCCGACAACGAGATTGGTGCCCGCCTTTGAGACCGCCGAAGGA
>dme_circ_0007885
GGTGCCCGCCTTTGAGACCGCCGAAGGACAGTACTTGAGCGAGTCCAATGCCATCGCCTACCTGCTGGCC
AACGAGCAGCTGCGCGGCGGAAAGTGCCCCTTCGTGCAGGCCCAGGTCCAGCAGTGGATCTCCTTCGCCG
ACAACGAGATTGTGCCTGCCTCCT
>dme_circ_0007885_junction_seq
GCCGACAACGAGATTGTGCCTGCCTCCTGGTGCCCGCCTTTGAGACCGCCGAAGGA
>dme_circ_0007886
GTGGAGCAGACTGCTGAAGACCACAATGTCGGCCAATGTGATCCTCTCGCCGGCCAGGAAGGTGGCGTCC
TGCAGCTTCTGGTTGAGCTGCTGCAGCACAGCCTCGGCCTCTTGCTTGGCAGTGCTGTTCTTCTGCTGCG
GCAGAATGCCCAGCAGGGGGAAGACCCAGGCACAGGAGGCAGGCACAATCTCGTTGTCGGCGAAGGAGAT
CCACTGCTGGACCTGGGCCTGCACGAAGGGGCACTTTCCGCCGCGCAGCTGCTCG
>dme_circ_0007886_junction_seq
GGGGCACTTTCCGCCGCGCAGCTGCTCGGTGGAGCAGACTGCTGAAGACCACAATG
>dme_circ_0007887
GCACTGCCAAGCAAGAGGCCGAGGCTGTGCTGCAGCAGCTCAACCAGAAGCTGCAGGACGCCACCTTCCT
GGCCGGCGAGAGGATCACATTGGCCGACATTGTGGTCTTCAGCAGTCTGCTCCACCTGTACGA
>dme_circ_0007887_junction_seq
TCTTCAGCAGTCTGCTCCACCTGTACGAGCACTGCCAAGCAAGAGGCCGAGGCTGT
>dme_circ_0007888
GAGGATCACATTGGCCGACATTGTGGTCTTCAGCAGTCTGCTCCACCTGTACGAGTACGTCCTGGAGCCC
AGTGTGCGCAGTGCCTTCGGCAACGTGAACCGCTGGTTCGTCACCATCCTCAACCAGAAGCAG

>dme_circ_0007888_junction_seq
GTTCGTCACCATCCTCAACCAGAAGCAGGAGGATCACATTGGCCGACATTGTGGTC
>dme_circ_0007889
GTTCCAGGCCAAGACCGGAGCCGCCAAGCCCCAGCAGCAGGCTCAGCAGCAGAAGCAGGAGAAGAAGCCC
AAGGAAAAGAAGGAGGCGCCCAAGAAGGCTGCCGAGCCCGCCGAGGAGTTGGACGCCGCCGATGAGGCCC
TGGCCGCCGAGCCCAAGTCCAAGGACCCCTTCGATGCGCTGCCCAAGGGCACCTTCAACTTCGATGACTT
CAAGCGCGTGTACTCCAACGAGGACGAGGCCAAGTCCATTCCCTACTTCTTCGATAAGTTCGATGCCGAG
AACTACTCGATCTGGTTTGGCGA
>dme_circ_0007889_junction_seq
CCGAGAACTACTCGATCTGGTTTGGCGAGTTCCAGGCCAAGACCGGAGCCGCCAAG
>dme_circ_0007890
GGACCCCTTCGATGCGCTGCCCAAGGGCACCTTCAACTTCGATGACTTCAAGCGCGTGTACTCCAACGAG
GACGAGGCCAAGTCCATTCCCTACTTCTTCGATAAGTTCGATGCCGAGAACTACTCGATCTGGTTTGGCG
AGTACAAATACAACGAGGAGCTGTCCAAG
>dme_circ_0007890_junction_seq
GTACAAATACAACGAGGAGCTGTCCAAGGGACCCCTTCGATGCGCTGCCCAAGGGC
>dme_circ_0007891
GGGCACCTTCAACTTCGATGACTTCAAGCGCGTGTACTCCAACGAGGACGAGGCCAAGTCCATTCCCTAC
TTCTTCGATAAGTTCGATGCCGAGAACTACTCGATCTGGTTTGGCGAGTACAAATACAACGAGGAGCTGT
CCAAG
>dme_circ_0007891_junction_seq
GTACAAATACAACGAGGAGCTGTCCAAGGGGCACCTTCAACTTCGATGACTTCAAG
>dme_circ_0007892
GTAACAATAAAGTTTGAGATTTAAAATGCAGGAAGAGCACAATGCCCATTTCTTAAGTTCCAACTGATAA
GTACACTAAAGATATCCAATATCTGTCGTGCTGCCTCCCACGTTTGGCGGAATCGTGTGTCTCGCCTCCT
GCATTTTGTACTGGAGAATTTGTTTGTAACCGCCTAAGCATAACACAGCATGATATTGTCAACGGAACAG
CCGCCTGCAGTCAGAAAATATTTATAAAAAATAAAAG
>dme_circ_0007892_junction_seq
GTCAGAAAATATTTATAAAAAATAAAAGGTAACAATAAAGTTTGAGATTTAAAATG
>dme_circ_0007893
GAACAATTTAAATCGGAGTTTTGGAACAGAATTGAAGAGGCTCAAGAGCCAAAGCCTGTTGGAAAGAAGC
GACGAACCCCTGGAGGACTCAAAAGAACGCGGGGCAACAAGCTTCTAAAAGAGCTCGAGATCCAATCGCC
GGAAACGGCGGCCGCTATTGCGGCAGCACAGGCGGAACTCAACGCCGCAGGAAAGTCGGGAGCAGCAGGA
GCACGGTCCAGCCGCAAGAGGACCAACACGATGTCTCTATACGATCGGCGATTCCAGACGACCACCGAGG
AGCGCCGCGTGGCTAACGGATACGGTGGCAATGCTGCGCGATCGGTCACTGGCTCCTCGTCAAACGAGAT
CGAGTCCCAGGAGCCGCAGAGTCATCAGTATTACGGTCCCAATGGCGGCAGCAATGCCGCGGGAGCAGGC
GCAGGCTTGAACGGAAGTGCGGCGGCCCTGAACCATGCCCCTTCCATGGGCACACTGTGCAACATTGGCA
ACAGTTGCTACTTGAATTCTGTGGTTTACACCCTGCGCTTTGCCCCGCACTTTTTGCACAACCTACACCA
CCTGATACAGGACTTGAATGTTGTGCAGCAGACGATAGTTCGCCAACAAACGGCCAGGTCTGCGTCCCTC
GGCAAAAAATGTGAGTGCCGCTCAGTTGGAGCACGCCCGCAGCTGGAGCAGCAAGGATCTGGCCACCAGTA
CGGATCAGTACAGTGGCCAGAATGGCGGAGTTAATAGCGGAAACGGTGGTAGCGGCTCGAGCAAGTCGAC
TCATCAGTCGGTAACCGAAAAACTCCATGAACTGTATAACAACCTGCACGGCAACGAGATGGCAGACTCC
ACGGAGCCCTACCACGCGGACACGCTGCTGCACGCCATTCAGGACGTAAACGCCACCTTTGAAGGTAACC
AGCAGCAGGACGCACACGAATTCCTAATGTGCGTGCTTAATTGCATCAGGGAAACGAATCAGTCTTTGAT

CAAGGCCATTGGCGAGTGTCCTGAGGTCATCGCAAATGGGATACATAGCCAACCCGGATGAGGTCGACAC
CGGAGAGGGACAGGATCGCACTGATTCGACTGCGTCGCAGAACCTGAACGCTGGCAATGGATCGCTGGCA
ACTAGTCAAACAACCACGACCAAAACGTCGTTCTTCTCTCGCAAATCAAAAAGAAAAGACGAGGTCAAGC
CATCCAAGTCGACGCGCGTTCAGTCGCCACTCAAGGAGAATAGTCCCACGGCGGGCGGGATAACCGGTGC
GGGCACAGCCCATGCCACCGCCAACAGCCTGTTCTACCTGAACACAGTTGATCTCAGCGGCGCCAGCAGC
ACCAGCGGTAGTGCCAGTACGAGTGCCAGTGGCGTTGTTTCCACAAGTGCAGCGTTGCCCACACCCCCGC
AGGCTACAAAATACTCCAGTGACGACGAGATGAATTCCGCCACCGTGCTGAAGGACAAAATGCGACTGGA
GGAGCGAATACGGGAGCTCAATCTAAACTTCTTCAGCTCGGACTTCGAAGGCATTGTGGTGCTGACCACC
AAGTGCCTCAGTTGCGAGACGATAACGCGGCAAAAGCAGGGCATGCTTGATATTTCTGTGCCGGTGCCGA
TCTCGGGGTACGACAATGCTGATCTCCAAGACAAACCGAGCACCTACATACAGGAATTCCTGCATTACGA
AAGAGTATTTCCGCGGGGAGAATAAGTATAGTTGCAACCAGTGCACCGGCTACACCGAAGCCATCCGCTC
CATCTCGTACGAGGTGCTGCCGCGGTTACTGGTCATTCAGCTGAATCGCTTCTCTGGCGGCATGGAGAAA
>dme_circ_0007893_junction_seq
GAATCGCTTCTCTGGCGGCATGGAGAAAGAACAATTTAAATCGGAGTTTTGGAACA
>dme_circ_0007894
GATGTTGTACAGCAAAACTGATGACTTTAAGAGAGAGCGAGGGGAGGCCTTGCTTAATTGATTGGGAGGT
ATTGTTATGTCTGTATTGCAACTAGCTGAAACTATTATAATCTCCTCATAGTCCGGGTGGAGTTTGTTGC
TGAGTGGCGCTATCTCTGTGCCAGGATCCTCAGTTTCCATGCAGTAGGTGTCCTTTACAATATCCTTAAG
TGGCGGCAGCTTGGTTACGCGTGGCACCTTGGCTGATACGCGGGAATCGATTATTTTTAGCTGTTGGGCA
CGTTTCATCTCTAGCATACGCAGCACCTTGTTCTTGGCCATGGTAATGGGATCCATGGGCAAAGAAGATT
GTTCTGTGCGAGGACATTTAAGGCTGTTTACAATATTGACTATTTCCGCTTTTTTTGGTGAAG
>dme_circ_0007894_junction_seq
TTGACTATTTCCGCTTTTTTTGGTGAAGGATGTTGTACAGCAAAACTGATGACTTT
>dme_circ_0007895
GGAAATCCCAATCCTCGTCACAGCCATGTTTGATCGCCGTGCAGTACACAACGCCCCGAATGCCCAGCGG
CACTGGGTTAACCTCGTCAGGATTAGCCTCTGATCGCCAGCTACGGTAGAAGCTCAAGGCCTGG
>dme_circ_0007895_junction_seq
CCAGCTACGGTAGAAGCTCAAGGCCTGGGGAAATCCCAATCCTCGTCACAGCCATG
>dme_circ_0007896
TACTCCTCGCAGGAGGCCAAGCAAGAGACTGCGAATATAGTGGCTCATGAGTTGGCCCACCAGTGGTTTG
GCAACCTGGTCACGATGAAGTGGTGGACGGACCTTTGGCTGAACGAGGGATTTGCCACTTATGTGGCCAG
TTTGTGTGTGGAGGACATCCATCCACAGTGGCG
>dme_circ_0007896_junction_seq
GTGTGGAGGACATCCATCCACAGTGGCGTACTCCTCGCAGGAGGCCAAGCAAGAGA
>dme_circ_0007897
TGAAACCCTCGCCGACTACATATGGTGCGAGTTCCAGGAGTCGGTGCCCATGTCCACCTACCTGGTTGCC
TACTCCGTGAACGATTTCTCGCACAAGCCCTCCACCCTGCCAAATAGCGCTCTATTCCGAACTTGGGCCA
GACCCAATGCCATCGATCAGTGCGACTATGCCGCTCAGTTCGGACCCAAAGTGTTGCAATACTATGAGCA
GTTCTTCGGCATCAAGTTCCCGCTACCCAAGATCGATCAGATTGCAGTGCCCGATTTCAGTGCCGGCGCC
ATGGAGAACTGGGGTCTGGTGACCTACAGGGAGATTGCCCTGCTCTACTCGGCGGCACACTCCTCGCTGG
CGGATAAGCAACGAGTGGCCAGCGTGGTGGCTCATGAGTTGGCGCACCAGTGGTTTGGTAACCTGGTCAC
CATGAAGTGGTGGACCGACCTGTGGCTGAACGAGGGATTCGCCACCTATGTGGCCAGTTTGGGTGTGGAG
AATATCAATCCGGAATGGCGTTCCATGGAGCAGGAGTCGCTGTCCAATCTGCTGACCATTTTCCGCAGAG
ACGCCCTGGAGAGCAGCCATCCCATCTCGAGACCCATCCAAATGGTGTCGGAGATCTCGGAGAGCTTCGA

TCAGATCTCATACCAGAAGGGCTCCACGGTGCTGCGCATGATGCACTTGTTCCTGGGCGAGGAGTCCTTC
CGCTCCGGTCTGCAGGCCTATCTGCAGAAGTTCTCCTACAAGAATGCTGAGCAGGACAATCTCTGGGAGT
CGCTCACCCAGGCAGCTCACAAGTATCGATCTCTGCCCAAGAGCTATGACATCAAGAGCATCATGGACTC
ATGGACCCTGCAAACTGGGCTACCCTGTGATCAACGTGACGCGAGACTACGCTGCCAGGACGGCCAAGCT
CAACCAGGAGCGCTATCTCCTGAACACCCAGGTAGCCCGAGCTTATCGCGGCGGTTGCTGGTGGGTGCCA
CTGAGTTACACCACACAGGCGGTGCAGGACTTCAACAACACGGCACCCAAGGCCTGGATGGAGTGCGGCA
AGAATGGCGAGAGCCTGCCCAAGACCATTCAGGACCTGCCAGGACCCGATCAGTGGGTGATCTTTAACAC
GCAGCTCTCGACGCTGTACAAGGTCAACTACGATGCCCAGAACTGGAAGCTTCTTATTGAGACCTTGACC
AATGGAGATTTCGAACGCATTCACGTGATCAACCGAGCTCAGCTGATCGACGATGCCCTATATCTGGCCT
GGACCGGCGAACAGGACTACGAGATCGCCATGAGACTGATCGAGTATCTGCAGCGGGAGCGGGAGTATTT
GCCCTGGAAGTCGGCCTTCGAGAATCTGAAGCGTGTGGGCCGCATTGTCCGCCAGACACCCGATTTCGAG
TTCTTCAAGGGTGGGTTCTGGTCTCTTAAAGTATCCTAATGCTCTTCTACCACGTGCTCCCCGTATAATC
TCATTCTCATTTGTATTTCGAAACAATCCTTTCAAAATTGCTCACACCTAACTTCATCTATACACCTAAC
TTGTCATGGGCAACCCACCGACAGTTGTTCTTTGTCGTAAGTCTGACTAGATAGCACTAGATATTTTATA
ATTTGACAAATGCCGGGTATATGAACTTTTATTAAAGTCCTTTTAAATGAATTTATTCATCCTCAATGCA
GCCGCTACATGAAGAAGCTGATTCTGCCGATCTACGAGCACCTGAATGGCATCAACGACACATTCAGCGC
GATTCCGCAGCAGGATCAGGTGCTGCTGAAGACAATGGTGGTCAACTGGGCGTGTCAGTACCAGGTGGGC
GATTGTGTGCCCCAGGCCCTGGCCTACTACCGCAACTGGCGAGCGGAGGCTAATCCCGACGAGAAGAATC
CGGTTCCGATCAACGTTCGTAGCACTGTGTACTGCACCTCCATAAAGCACGGATCCGATTCGGATTGGGA
ATTCCTGTGGACGCGTTACAAGAAGTCGAATGTGGCGGCTGAGAAGCGCACCATTCTGACTGCTTTGGGT
TGTTCGAGGGAAGTGTGGCTGCTGCAGCGTTATCTGGAGCTGACCTTTGACCCCAAGGAGGCCATTCGCA
AGCAGGACTCGATGTGGGCCTTCCAAGCGGTGGCCTTCAATGAGGTGGGCTTCCTGCTGGCCAAAAAGTA
TTTCATGGACAACGTCGACTTCATCTACAAGTTGCTACCATCCGCTCACCAAGGACATGTCCCGCCTACT
CTCACCGCTTTCCGAGCAGGTCATCACGCTGAGCGACTTCAACGAGTTCAAGGACTTCGTGAACAACTCC
CGTCAGTCGCTGAAGGGATTGGAACAGGCCATCCAACAGACACTGGAGATCATGCTCACCAATGTTCAGT
GGAAGGAGCGAAACTACCATCAGATGTCCCGCTCCATCCAGCAACTGCTCTAGATTACCCAGCTCCCAGT
CCAGCCTCTCATCAGCTCACTAAGAGAAGCAACTCTTGATTTCAGCAGGAATGTTATGAATGTTTAAAGT
CGCTTAAATAAACTATTTAATACTCTAGTATTCGTGTATCTCAAGATTTCGGCTATGAAGTGGTTTCTCT
TGTTCGTTCTGGCTATTGGCCTGGATTTATCTGTGGCCAACTCGATTTCTAGGTATAACCACTACCGACT
GCCAACTGCCTTAAGACCCCAAAGGTATTACTTACGTATCCTGACACTTTTGGAAAGTCCTGATGATCTC
CGTTTCGCTGGAAATGTGCAAATCGTGATTAAAGCACTGCAAAATACCAGGAACATAACGCTGCACTCGA
AGAACCTGACCATCGATGAGTCCCGGATCACGCTGCGCCAAATTAGTGGAGCGGGAAATATGGATAATTG
TGTATCCTCCACATCCGTTAATCCCACCCACGACTATTACATCTTTCACACCTGCAAGGAACTTTTGGCC
GGCAATGTATACAAACTGTTCCTTCCATTCTCGGCTGATCTAACACCTCAACTTTTTGGTTACTACCGCA
GTTCGTACAAGGATCCTGTGACCAACAAGACACGGCTGGCTGTCTGCGACGCAATTCGAACCATCCTCCG
CTCGAAAGGCATTTCCCTGCTTCGATGAACCCGGTTTTAAGGCATCCTTCGTGGTCACTTTGGGCTATCA
CAAGCAGTTCAATGCACTGAGCAATATGCCCGTAAGGGAAATCAGGCCACAGCGAGTCCCTTGCAAACTA
CATATGGTGCGAGTTCCAGGAGTCGGTGCCAATGTCCACCTATCTGGTGGCATATTCGGTTAATGATTTC
TCCTTTAAACCCTCCACTCTGCCAAATGGAGCTCTATTCCGGACTTGGGCCAGACCCAATGCCATAGATC
AGTGCGATTATGCCGCGGAGTTTGGACCCAAAGTGCTGCAATACTATGAGGAGTTCTTCGGCATTAGGTA
TCCGCTTCCCAAGATCGATCAGATGGCTGTTCCGGATTTCAGCGCTGGTGCCATGGAGAACTGGGGTCTG
GTCAAATACAGAGAATCCACACTTCTCTACTCGCCAACCCACTCATCACTGGCGGATAAACAGGATCTGG
CCAATGTAATAGCTCATGAGTTGGCCCACCAATGGTTCGGTAATCTGGTGACGATGAAGTGGTGGACCGA
CCTCTGGCTAAACGAGGGATTCGCCACATATGTGGCCGGCTTGGGAGTACAGGAAATCTATCCGGAGTGG

CACTCAAGGGATAAGGGCTCGCTGACAGCTCTGATGACCGCCTTTCGCCTGGACTCACTGGTTAGCAGCC
ATCCCATATCGAGGCCCATTCAAATGGTGACGGAGATCGAGGAAAGCTTTGATGCCATCTCTTACCAAAA
GGGATCCGCAGTGTTACGAATGATGCACTTGTTCATGGGCGAGGAGTCCTTCCGTACGGGGCTAAGGGAG
TATCTTAAGCTTTACGCCTACAAGAATGCCGAACAGAATAATCTATGGGAGTCACTGACCACAGCAGCTC
ATCAAAATGGAGCTCTACCCGGGCACTATGACATAAATACCATTATGGACTCGTGGACTCTGCAAACCGG
GATTTCCTGTGCTTAATATTACCCGTGACTATTCGACTGGAACCGCTGAGATTACCCAGGAACGTTACTT
GCGCAATTCCCAGATACCACAAGCCGACCGCGTTGGCTGCTGGTGGGTGCCACTGAGCTATACCACCCAG
GATGAGAATGACTTCAATAACACGTCACCCAAGGCGTGGATGGAGTGCAGCAGTACGGACGAAGGTGTGC
CTACGACCATTGATCATTCAGCTGGACCCGAAGAATGGCTGATCCTCAACATACAGTTGTCGACTCCTTA
CAAGGCCAACTATGATGCCAGAAACTGGAAGCTGTTGATTGATACTTTGAACAGCAAAGATTTCCAGAGC
ATTCACGTGATCAACAGAGCCCAGCTTATAGACGATGTTCTGTACTTCGCCTGGACGGGGGAACAGGACT
ACGAAACCGCACTGCAAGTGACCAACTATTTGCGGAGGGAACGAGACCTTATTCCTTGGAAATCAGCCCT
GGACAATTTGAAGTTGTTGAACCGCATTCTGCGACAAACTCCCAACTTTGGGTCATTTAAAGCGCTACAT
GCAGAAGCTTCTTACTCCGATCTACGAGCACCTGCACGGCATGAACGACACCTTTAGTTTGATGACGCAG
CAGGATGAGGTCCTTTTGAAGACCACGGTGGTCAATGTGGCGTGCCAGTACGATGTTTCCGATTGTGTGA
CCCAGGCGCAGGCCTATTTTCGCCGATGGCGGGCAGAGACCAATCCTGACGAGAACCATCCGGTTCCATT
AAACCTTCGCAGCACAGTGTACTGCACCGCAATAAGTCAAGGCACAGAGGAGGATTGGAACTTCCTGTGG
TCGCGGTACCGGAAATCAAATGTGGCATCCGAGAGGCAGACCATCTTGAGTACCCTGGGCTGCTCGAAGG
AGGTTTGGATACTGCAGCGTTACATGGAAAAGTCCTTCCACCCCAAGAGCGCCATTCGCAAGCAGGACTC
ATCGCTGTGCTTTCAGGCGGTGGCCTCCAGAGAGGTGGGCTTCCTCCTGGCCAAGCAGTACTTCATAGAG
AATGTTGAGCTTATCCACAAATAGCTATTATCCGCAGACGAGAGTCATGTCCCGACTGTTGACGCCGTTA
TCCGAGCAAGTTAGCACAATAAATGATCTGAACAAGTTCAGGTCCTTTGCCAACGATTCCCGACATTTCC
TGAAGGGCATTCGTCAGGCGATGCAGCAGAGCCTGGAGACGATGCTCACGAATGTGCAGTGGATGGATTG
GAACTATCATAAATTCTCACGGACTATACGCCACCATTTGTAATGCTCACGAATGAGTTTAGATCCTCCC
AGGAAGCCTACTTTATTTTATAGTATTCTAAGTGAAGATTGAAAATACAAAACAAGATTACCCGTTTCTC
AGATAGTGACGATAGAACGACGAAGAAATTTCGAAATTCTTTTTGCTAATTTTTTCCAAAAAAATAATAA
ATATATTTATTAATCTGCATGCTTAGTACTATCAAGTATTCTCGGAGTACTCCTAGATTAAATCGTAGTT
TTGGGAAACAAAAGTCAGCGATTAAATCACAAAGACATTGGTTAGATCATTTTTAAAACAGTTTTGATGT
TAAAACTAGGAAAACGATTCGTTTATTAAGTTATGCAATTATGAATGGAAGTAGTTTTGATGGACAATTT
CTTCCGGTTTTGTAACAAATAATAATAAACGCAGCACGGaaaaaaaacttaaaaaGATGCAAATGCGAAA
GTGGAGATTTACTTATACCAGAGCCTCTTTCAGGAGTTAACTAATTCAAGTGCGGGATTCACTTTTTGCA
GAAACCCAGTATGAAGTGGCTTGTGTTATTTGTAATAACCCTTGGCTTAGGATTAGCCACACCCAATTCG
ACCTACAATCATTATCGCCTGCCGACGGCACTTCGACCTATCAAGTATGACCTGCACGTGCTAACCCAGC
TGGAATATGCTGATGATTTTAGTTTTAACGGAAGTGTGAAAATCCAAATCCAAGTCTTGGAAAACACCAA
CAACATAACTTTGCATTCGAAGGAACTGACGATCGATGAGACGGCAACCACTCTTCGCCAAATTACCGGA
GAGGATCTAAAGAATAACTGCGTGTCCTCCACAGAGGTAAATCCCGAACACGACTTCTATATCCTTCGCA
CCTGCAGACAACTTTTGGCTGGCCAAGTCTATGAGCTCAGTTTGCTATTTTCGTCCACATTGTCAGACCG
ATTGCGGGGATACTACCGCAGCTCCTACGTGGATCCTGTGGCCAATGAGACCAGGTGTGGACTGGCGATT
ACTCGAAGAAGAAAACAGATATTTAATCCTTGTATGTTCACCGGACTTAGCAATATGCCCGTGAAGCATA
CCGGCAAAGAGTTTTGTTCACAACATCATTTTAAAACTTGACGATGAAGTGCGTTTTCCTGATACTGGCT
GCACTTGGATTAAGTTTTGCGGGGGAAGGTAGCACCTATGACCATTTTCGCTTGCCAACGGCTCTTCGAC
CACAAAGTTACGATGTTCGCATCCTGACACAATTGGAAAATCCGGACGATTTTCACTTCAACGGAACTGT
GAAAATTCAAATAGAAGTTTTGCAAAACACACACAATATAACTTTGCACTCAAAGGATCTGACCATCGAT
GATACGGAAATCACTCTCAGCCAAATTGGCGGAGAAGAGACAACGGAGAACTGCATAACGTCTACGGCAG

TGAATCCCACACACGACTTCTACATTCTCAACACGTGTAAGGAGCTATTGGCTGGACAATTTTATGAGCT
GAGTTTGCCCTTTTCGGCCAAGTTACAAGATCAACTGGCGGGCTACTACCGAAGTTCCTATGTCAATACA
GTGGCCAATGAAACGAGGATGGATCTCCGTTACTCAGTTTGAGCCAGCTGCGGCTCGACTGGCATTTCCC
TGCTTCGATGAACCTGGCTACAAGGCATCCTTTGCCATCACCTTGGGTTATCACAAGAAGTACACCGGTC
TCAGCAACATGCCCGTGAATGAAACAAGGCCACAG
>dme_circ_0007897_junction_seq
CATGCCCGTGAATGAAACAAGGCCACAGTGAAACCCTCGCCGACTACATATGGTGC
>dme_circ_0007898
GTATCCGCTTCCCAAGATCGATCAGATGGCTGTTCCGGATTTCAGCGCTGGTGCCATGGAGAACTGGGGT
CTGGTCAAATACAGAGAATCCACACTTCTCTACTCGCCAACCCACTCATCACTGGCGGATAAACAGGATC
TGGCCAATGTAATAGCTCATGAGTTGGCCCACCAATGGTTCGG
>dme_circ_0007898_junction_seq
CTCATGAGTTGGCCCACCAATGGTTCGGGTATCCGCTTCCCAAGATCGATCAGATG
>dme_circ_0007899
GTCGCGAGCGCGACTTCTTTAGCCGCCCGGTGGCACCGCCCAGGCCATCGCCGTTCCAGGACTTGGACTT
GGCGCGAAAGTGGCGATAGGCCTCGTTGTAGGCGGGATCCAGCATGGTGCGGTAGGCATGTG
>dme_circ_0007899_junction_seq
GGATCCAGCATGGTGCGGTAGGCATGTGGTCGCGAGCGCGACTTCTTTAGCCGCCC
>dme_circ_0007900
TTACTTAACTTTTGACGCAAAATGGATTCACGAATGCTAAATGTGTTCCAAGGAGATACCTTTGAGGCAA
ATTATACCAAATACGAGTCTGAGCCGATATTCTGGTCATCTAATGATGAGTGAGTGATTGTCATCAGAAA
AATGTACTAAAAACTAATGCAAACCCTTTCAGACTGCAACTGACAGATACACTGAGCGGCATCCTACCCA
TCGCGCCCACAGACGAATCACTGCTCAACCATAACCGGGAGGAAGATCTGCAAAACAGAAAGAATCTGGT
AGGTTTTGGCTCGAGGGGCATACGAGCGAATCACATTTCAGTATTAATTCGATTAACTCTTTAAATAGCT
GATTTACAACGACTTTCTGAAACAGGAGCTCAGCAACCATGATGCCAATGAGCTAAATGCCCAGGTCTAT
CGGTCCATCGCCGACAGCGGGGGCGAGATTGCCTATGAGCAGCAGCGCCAGATCAGCGCCATTGTGGAGT
ACGACAAAGAAGATATTAAAATCGACGGCCTTACGGAGGAGGAGGACATACAAAGCCAGTCCACATTTCG
CACCCGCACAGACAGCTCTTCGATTGGTGACTATGAATCGCACTCTAGCGACTATGATGATGAATTTGAC
ATTGTCAACGAAGAAAAAAGTCCAAGGCTGCAGGTTGTCCATAACAATATCGGCAACTTGCAGAGCTCCT
GTGAGCGCAAGGGACGGACGCTCAGTACTAACACTATTATCTCCGAAGTAGACGCATTGGGGCTAAGTAT
TGATGTTAATAACTTTGACCTAGCTGATTTTATTACCAAAGACGATTTTGCTGAGAATTTAAACGCCTGT
CGCAAAAAGGAATCTCTGCAAGCACAGGCGCAAATTAAATCTAATACTTTAGCAGATGTTCCCATACTTC
TTAATCCACCGGTTGCCACAATTGTGGCAAAACCCGGGGTCCCATCCTCTAAACCTGGCGATTCTGATTA
TGACTCTGACTCGATTATCGACGTTGAAACTGTCGATGTACTCGATATGAATATGGCCAATATCTGGTCA
ATAGATAAGCCGGCAAAAATGGGCCAAACTCCCGATGAATTACGCTATGTGGACAATGTAAAGGCCGATC
CCTCTTGGAGTCCGAGATCCGCAAAAAAGACCACACCTACTATCAAAGAGGTAAGTCCTATGACGACATA
CTAGTTAAGAAATTCTTTATAACAGTAACTGTCATACTCAAAACAGAATAAGCCGGAACAACTGGGGAAC
CATCAGCTCGCGGTCCCAGCATCAAACAAACCGAAGCATAAGAATATTTGCCTGGTGCCTAATAAAAAAC
AGTGCGATTTTCTTAAACGTAAGG
>dme_circ_0007900_junction_seq
AAACAGTGCGATTTTCTTAAACGTAAGGTTACTTAACTTTTGACGCAAAATGGATT
>dme_circ_0007901
GGTAAGTCGATGCGATGCGTGCTGCAATTTATGCCCGGGTATGTCAATATATCAGCCAATAATTTGCCTA
TGACTTGTCAGTGGTTTAATTAAAATGAACTGCAGCATGAAGAGTGCACAcatatatatgcatatatata

tatccatatccataCTACCACGATGGCAATCAAATAGAGAGTTTCGCCAACCGGACAGCCAATGGCGTGT
AAATAAAACTAATCCAGAGTTGAGCGACCGCATAAATCAGCCGCAGATCGAAATGTTGCAAG
>dme_circ_0007901_junction_seq
AATCAGCCGCAGATCGAAATGTTGCAAGGGTAAGTCGATGCGATGCGTGCTGCAAT
>dme_circ_0007902
AACACACCTATTGCTGGAGAAATCTTTATGTAAGTCAAAATCAAACCCTATTTAATTTAAAAATTGAATC
AGTTTTCTATGGGCATTGTTGCCTTGTTTAATTTTGGTATGTTTGTCGCAAAGTCGAGCATATCCTTTCT
GAATCAAACGAAAACGAAGAGAGAGGGCATGTTGTGGCGGTTATAAAAATCATAACCACAACTGTGGCAG
AAACTCGGTTCATTTTACATGGCCCGACATTGCCTTTGGCATTTGTGCCTCCTCCCAGCGACTGAATTGG
AGACCTTTATAACTTGTATTGTGTATATGTGTGTGTGCTAGCCACTTAGTAAATATTTTATGAATTATCT
GTTTTCTATTAAGATGTTTTAACAGAGCAACCAAAACGTGGGCCGAGCGACAACCGCAGGACCATCGAGC
TGCGACGAAATGGGCGCTATAAAGCCCAATAAACATAAGTCTGTTGTCCTTCGCAAATGCACAAAAGTGT
ATGTGTGG
>dme_circ_0007902_junction_seq
TCGCAAATGCACAAAAGTGTATGTGTGGAACACACCTATTGCTGGAGAAATCTTTA
>dme_circ_0007903
GTGCCAAGCAAACCGCAGAATCTAACCATCTTGGATGTTTCGGCCAACAGCATCACGATGTCCTGGCATC
CGCCAAAGAATCAAAACGGAGCCATCGCCGGTTACCATGTGTTCCACATACACGACAACCAAACTGGCGT
GGAGATCGTCAAGAACTCTCGCAATTCGGTGGAAACCCTCATTCACTTCGAACTGCAGAACTTACGTGAG
TTTAACTACGTTTTCATTTGGGGCTTATTTGGCGTAAAAGCGTGAGTTTAACTACGTTTTCATTTGGGGC
TTATTTGGCGTAAAAGGTTTTCAAACTTTTAATTTATTCACTCGATAAATTTGCGAAAGTCCCGGTCGAT
CAAAGGGTTTTCCCCGCCTATTCTCGCCAATTGGTGACCCCCGTCAAGTTCCGAACTCGAACAGTTTAAT
ACTGTCAACAGTCGAACGCCCACGTTGTGAAAAGCGGCACACACTTTTCCGCGTCGCCCCCGCCGCGAAA
TGGAAAATGGGAATGGAAAACCGCTTGAGCCTGCCGAAGGACCTACCTATTATTAGCCATAATTGAGGCT
CTAGCCAAATTGTTTGCCAGCTGCTTTGTAATTAAATAGTTGCAAGTGCTCGCTTGCCACTTTTTGTTTG
CCAACAACAACAACTTTTGACGCTTATAATTTGCACACCCCAAAAGTCGTCTAATTTTCCTGATTAAGGC
GTGTGGGCCAGAGAATACATCCGCAGGATATGCCGCATCAGGAGCAGCTACTTAGAAGGATCCTGGTAGA
AAAGTGGCTCGGTTTTTTGGAGGTTTTTAGGCGCCTCCGGGGCCACCATGCAATTAACTTGCTACAAAGT
TATGCGCCGCATTCCACACTCTCATGCAGCTACAGCCACATTGTTTAATTTAATTACACGTCTTCGGATG
CAGGATGCACCTGCTAACTTCTGCTCGCAAATTTCGTGGCAAAGGAGCCTCCTTTTTGCGGCATGCTGGG
CCGCCATGGCAACGTTTAATTATTTCCAAATTTAATCCAAGTAGATGTCAGCCCGTTTCTCTTCTTAATT
AAATCCAATTTAAGCTGACAGCGCCGCTTGAAGCAATACCCTTTTGCTTTCTGTTTTTTCTCGCCATGAA
AAATCAATTCACTGTTATTGTTGCCACTGTCCCTTGCATTTACCCCGCGTAAAATTTAATTATAAAGCCG
CACAGCAGCCCCCTttccttccattccattccattcccttcTCCTGCAACCAGTGACATGCGGCGACGCA
TCTCATAATAATGAGCAGCGAAAAGATGCCCACCGCTCAATTTAAGGCGGCTGGTGGCAGGTGGCATGTG
GCAAGCGGCGTGGCAGGTGCTCCACATACTCGCGGGAAAAACAACTGACATTCCCTGGAAGTATTTACGA
CCAATAAATGCCTCAACTGTCGCATCCAAATGCATTCGACTGCAAATGAAGCAAATTAAAGGCTTACGCA
GGTCCTTGAATAATTATGATTTCTTTTGAATTTCCACTACTATAGTTAATAAAGAAGGTACTTATATATG
TAATACGTATTTTCAGGGACCCTACACCGACTACCGTGTGATAGTCAAGGCATTCACCACCAAGAACGAG
GGCGAACCCTCCGATCAGATTGCCCAGCGTACGGATGTCGGAGGACCCAGTGCCCCGGCGATTGTGAACC
TCACTTGTCACTCCCAGGAGTCCATCACGATTCGATGGCGAAGACCCTATGAGTTCTACAACACCATCGA
CTTTTACATCATCAAGACGCGTTTGGCTGGCCAGGATACGCATCGGGATATCAGGATTAATGCCTCGGCC
AAGGAACTGGAAACTGCG
>dme_circ_0007903_junction_seq

TGCCTCGGCCAAGGAACTGGAAACTGCGGTGCCAAGCAAACCGCAGAATCTAACCA

>dme_circ_0007904

GTGCCAAGCAAACCGCAGAATCTAACCATCTTGGATGTTTCGGCCAACAGCATCACGATGTCCTGGCATC
CGCCAAAGAATCAAAACGGAGCCATCGCCGGTTACCATGTGTTCCACATACACGACAACCAAACTGGCGT
GGAGATCGTCAAGAACTCTCGCAATTCGGTGGAAACCCTCATTCACTTCGAACTGCAGAACTTACGTGAG
TTTAACTACGTTTTCATTTGGGGCTTATTTGGCGTAAAAGCGTGAGTTTAACTACGTTTTCATTTGGGGC
TTATTTGGCGTAAAAGGTTTTCAAACTTTTAATTTATTCACTCGATAAATTTGCGAAAGTCCCGGTCGAT
CAAAGGGTTTTCCCCGCCTATTCTCGCCAATTGGTGACCCCCGTCAAGTTCCGAACTCGAACAGTTTAAT
ACTGTCAACAGTCGAACGCCCACGTTGTGAAAAGCGGCACACACTTTTCCGCGTCGCCCCCGCCGCGAAA
TGGAAAATGGGAATGGAAAACCGCTTGAGCCTGCCGAAGGACCTACCTATTATTAGCCATAATTGAGGCT
CTAGCCAAATTGTTTGCCAGCTGCTTTGTAATTAAATAGTTGCAAGTGCTCGCTTGCCACTTTTTGTTTG
CCAACAACAACAACTTTTGACGCTTATAATTTGCACACCCCAAAAGTCGTCTAATTTTCCTGATTAAGGC
GTGTGGGCCAGAGAATACATCCGCAGGATATGCCGCATCAGGAGCAGCTACTTAGAAGGATCCTGGTAGA
AAAGTGGCTCGGTTTTTTGGAGGTTTTTAGGCGCCTCCGGGGCCACCATGCAATTAACTTGCTACAAAGT
TATGCGCCGCATTCCACACTCTCATGCAGCTACAGCCACATTGTTTAATTTAATTACACGTCTTCGGATG
CAGGATGCACCTGCTAACTTCTGCTCGCAAATTTCGTGGCAAAGGAGCCTCCTTTTTGCGGCATGCTGGG
CCGCCATGGCAACGTTTAATTATTTCCAAATTTAATCCAAGTAGATGTCAGCCCGTTTCTCTTCTTAATT
AAATCCAATTTAAGCTGACAGCGCCGCTTGAAGCAATACCCTTTTGCTTTCTGTTTTTTCTCGCCATGAA
AAATCAATTCACTGTTATTGTTGCCACTGTCCCTTGCATTTACCCCGCGTAAAATTTAATTATAAAGCCG
CACAGCAGCCCCCTttccttccattccattccattcccttcTCCTGCAACCAGTGACATGCGGCGACGCA
TCTCATAATAATGAGCAGCGAAAAGATGCCCACCGCTCAATTTAAGGCGGCTGGTGGCAGGTGGCATGTG
GCAAGCGGCGTGGCAGGTGCTCCACATACTCGCGGGAAAAACAACTGACATTCCCTGGAAGTATTTACGA
CCAATAAATGCCTCAACTGTCGCATCCAAATGCATTCGACTGCAAATGAAGCAAATTAAAGGCTTACGCA
GGTCCTTGAATAATTATGATTTCTTTTGAATTTCCACTACTATAGTTAATAAAGAAGGTACTTATATATG
TAATACGTATTTTCAGGGACCCTACACCGACTACCGTGTGATAGTCAAGGCATTCACCACCAAGAACGAG
GGCGAACCCTCCGATCAGATTGCCCAGCGTACGGATGTCGGAGGACCCAGTGCCCCGGCGATTGTGAACC
TCACTTGTCACTCCCAGGAGTCCATCACGATTCGATGGCGAAGACCCTATGAGTTCTACAACACCATCGA
CTTTTACATCATCAAGACGCGTTTGGCTGGCCAGGATACGCATCGGGATATCAGGATTAATGCCTCGGCC
AAGGAACTGGAAACTGCGGGTAGGTTTTTATATTTAAACTATAGTTTTGTTCAGATATTGTGCAACTTTA
AAGATATGTTTGAATTTACTAACAAACGCCAGAACAATGTATTTCTGTATAGAATGGCGAAATAAATGTA
AGTGAATTAGTCCCTTTGGAGTCTGGCGCTAATATTTCATGCGCAAGTTTTCGTGAAAATAAAAGAGTTT
TCCCGCGCTGTTAGCCAACATTTTGTTGGCCTGCCAAGTGGGCAACAATGTAGACCTCACCTACCACCCC
CCTGCGTTATGTCAATCGAGTCGGCTTATCGCGGCCATTTGGCCCGTTCTGCGGCCCATaaaaaaaaaaG
GAGAAGTTCTATGATAAACGGCCTAATACAGACAGATATTCAAGGGGGAGCGGCAGTAATAATTGCATTT
TTATGCAGCAAACAACAAACGGCATAGAATAAAGCCCACCTGCCACGGCACCTGTAAATATTTCATAAAA
AATTCTACATGCAAAAAATGCAacattcacacacacattcacacacacGCGTTGCAAACCGCCCACCTGC
GAAAATAAATTAAATTTATGTTGTTTTTCCTGTCCGCCTTTGTGCCGCGTCTTCCCTTTTTATTTAGCGC
CTGAAATGGATGGAGGaaaaaataaaaaataataaaaaaaatatacgccaaagtaaataaataaaaacga
aaaGTTTGTCGTGGGGAATGTGGCGAGGCAAACAAATTTCGCAACAATAAAATGTTATCATACTCATATC
TGACTTTTTCCATATCTGAAAACTAGTTGGATCCTTGTTTAGTCCTGTGTTTTACCCTTCCGCCCCAGGA
TCTCCTGTCCTCTGTCGCCCGGCTTTTGTTTTTGGCCAGTTAAAAATATTTTTGTAATTGAAATATTCTG
CAGCGGCAAACAAGCTGGCTGTTGAACAACTAAACAACAGAAATGGCAATAATTCTACCTTTGTTTTTCA
TTGTTACTATAGTTTGCGTATATAAATGCCAAATATTTGGCTTTTCCGTATGAAGGAGACAGAATTCGAA
GTTAGATGGAAACTAAATTAGAAAATTGGCTCGTACGCAAGGAAAATTCTGATTTGGATAAACTAAATTA

GTATGCATTTTAGAAATTTGTTGACGCGTTTTCATTAACTGCAGAAATTTTCAAGGGGTCAAACATCAAA
CATTGAATATTTGTTGTATTTCGAAATGAAATAATATATCCTTTAGTTTATCCTTTCAAATTTACATTTG
CATTAATAAAACACGATACTAATACTACTCTTTTGCTTATTTCCTTGCAGATGATTCTTCAAAACCTAAC
AACCAACTCGTATTACGAGGTCAAAGTGGCAGCGGCAACGTTCAGTGTCATAAATCCAAAGAAAATTGTG
CTGGGCAAATTCTCGGAGTCAAGGATT
>dme_circ_0007904_junction_seq
GCTGGGCAAATTCTCGGAGTCAAGGATTGTGCCAAGCAAACCGCAGAATCTAACCA
>dme_circ_0007905
ACTAATCCAAGGCGGCTATGCCCTGGCCACGACGGAATCGGATGAGCCCCTGGATCTAATGACGGCCACA
CAGAAGCTGCTGGTCGACGGCTGTGAGACTATGACGGCGGTGCTGGGCATGACGGGCGTCATCTCGATGT
TCTGCCACTACATCGGACGCGGCTTCCAGTGGTACCTGCTCACCTACGACGACGAGGAGAAGTCGCTGGG
CACTGTCTCCGCAGTGCTCTTCTACATCCTCGCTCTGCAGACGGGCCTGACCTCACTGTCGCCGGACAAA
CGCTTCATCAGGCTGTGCCGCAATCTGTGCCTGCTGATGACCGCTCTGCTGCACTTCCTGCACAACATCG
TGTCGCCCATCTTGATGTCGCTCAGTGCGGCACGGAATCCCTCGCGGAAGCGCCATGTGCGCGCCCTCTC
CGTCTGCGCGTTCCTCGTGGTGTTGTCAGTGTCTCTGCTCTACCACCTCTGGTCGCAGCAATCCATCTCC
ACCTGGCTGCTAGCGGTTACCGCCTTCTCTGTGGAGGTCGTCGTCAAGGGTTCTCGTTTCGCTGGCCACC
TACACGCTCTTCCTGCTGGACGCGCGTCGGCAGTTCTTCTGGGAGAAGCTGGACGACTACCTGTACTATG
TGCGCGCGTTCGGCAACTCAGTGGAGTTCTGCTTCGGCATCCTGCTGTTCATCAACGGTGCTTGGATTCT
GATCTTCGAGTCTGG
>dme_circ_0007905_junction_seq
GTGCTTGGATTCTGATCTTCGAGTCTGGACTAATCCAAGGCGGCTATGCCCTGGCC
>dme_circ_0007906
GACGAGATAGAGTCTAGCAAGATAACCGACTACGTGGATCTGCTGAAGTGTCTGCAGAACAGAGACGACA
TCGAAGGTCTTCCACCCGGAGGAGGTCTACAGTCCAAGTGAGTGTTTCATGCCCGTAAATAGCGTCCGGC
GATTGCTAATCAGTGGTGTTTTTTTGTTTACCTTTGCAGATATCACGTTATCGAGCGCACATTCAAGCAA
GAGTTTCCGGATCGACCATGTGAA
>dme_circ_0007906_junction_seq
GCAAGAGTTTCCGGATCGACCATGTGAAGACGAGATAGAGTCTAGCAAGATAACCG
>dme_circ_0007907
GACGAGATAGAGTCTAGCAAGATAACCGACTACGTGGATCTGCTGAAGTGTCTGCAGAACAGAGACGACA
TCGAAGGTCTTCCACCCGGAGGAGGTCTACAGTCCAAGTGAGTGTTTCATGCCCGTAAATAGCGTCCGGC
GATTGCTAATCAGTGGTGTTTTTTTGTTTACCTTTGCAGATATCACGTTATCGAGCGCACATTCAAGCAA
GAGTTTCCGGATCGACCATGTGAAGTACGAAATCTGTTCTATAATCTGTAACTCAGTTCGCTGCGCTTTC
GCCAATGCTAACACATATATTTTATCGCTTGCATAGCACCGTTTTTGGCCATTATAGCCGTAGCCTTTGA
AATTGCCTTGTTTTTTTCTGATTCAATCAGTAGATCGACACCAGAGGATTTGGTAATGATAGAGCTATCA
CAGCAGTTCATTCGATCGTCACCATTCACCATTGTTCCATGTTTAGTTGGGTTCCCCTCTCGGCCCTTAA
TCGTAACCTCTAATCATGGCTGCCTATTCTCTTTCTTTTTTTCTAGCTGTATGCATATGATGATATATGA
ATTTTGTAATGCTAATTGTTCATTTTTAaaccaaccaaccaaccaaccaacaaccaaccagcaaGAGAAC
AAAGATAACTAAACACCCTATGCTTCACATACTCTAAAT
>dme_circ_0007907_junction_seq
AACACCCTATGCTTCACATACTCTAAATGACGAGATAGAGTCTAGCAAGATAACCG
>dme_circ_0007908
TTCCTCCGCTCGGCCACCGGCAACCTGCCGTCGGAAGTCATGCGCACCAGGGCCACCTCCGCGGGCGTGC
CAATAAAGGCGCCACAGGCTCCGGCGATCGTGCCCATGGCCATGCTGTCCGTGATGCCGGGACTCCTCTG

GAACTTCTCCCTGAACAGGTCGTTCAGATACGTGTACATGCCCAATCTTCCCGTGGTGTAGG
>dme_circ_0007908_junction_seq
TACATGCCCAATCTTCCCGTGGTGTAGGTTCCTCCGCTCGGCCACCGGCAACCTGC
>dme_circ_0007909
GAAGCTCTCGGTGCTGCGCACAGTGCTCCTGGTTGTGGACGTGGTCGGGTACTTCCGCGGCGACTCCTCG
CTCTTGTCCTCTTCGGAGGCCAGGACGCGAAAATCATCGTTGTACGGCACCTCCAAGTCCAGCGGCAGGG
GCGGATTACGATTG
>dme_circ_0007909_junction_seq
GTCCAGCGGCAGGGGCGGATTACGATTGGAAGCTCTCGGTGCTGCGCACAGTGCTC
>dme_circ_0007910
TAGAAGGTACAAAGTGCGGTGAGTGCGGCTGCGGTTTggattgcggtggcatcagtgggtggggattggg
tgccctagtgggtgTCGATGCATTCGAGGTGCTGGGGCGAGCCTTGGTCGGGGATTAATGGGCGTGTGGA
ATGCGGTGGAGTGTCTGTTCGTGTGAGTGGGCGTGGTATTTCACATTTTCAGCAGAAACGGCAAGTTTAA
GGCAGTGTTATGCAAG
>dme_circ_0007910_junction_seq
CGGCAAGTTTAAGGCAGTGTTATGCAAGTAGAAGGTACAAAGTGCGGTGAGTGCGG
>dme_circ_0007911
GAGTTCTGGGAAGCAACCGAGAAGACACGCATCGAGAATGCCATTGCCGAACGGAAGGATGCCGTCGCCC
AGCAGGAGCGTCTCAAGCGCATGTATCCGGACCGGGATGAATTCCTTGAGGCCCTTAAAAAAGAACGTGC
CTCCCTCTACGTCGAAAAGCTCAAAAAGTTTGAGGCTGCTCTGGAGGCAGAGCGCAAAAAGCGCTTAGCC
GACCGCATTATTCGACGCCGCGAGGAGCGCCGACAGGCCTTCCTTCGCGAGAAAGAGGAGGAGCGTCTTC
GCAAGGAGGAAGAGATTCGCTTGGCACAGGCTGCCGAAGAGCGCGCCGCAGCAGAAGCCCGCCGTTTGGA
GCGTGAGGCTGAGGACGAAAAGCGTCGTGCCCAATACGAAAAGCAGCGTGCCAAGGAGGAGGAAGCTGAG
CGTAAAATCAAGGAAGATCGAGACCGCCTGTCCAGGGAGTTGGCCTCGGAGCGCGAACGCACTGAAAAGG
ACCGTGACACATGGCGTCCTCGAGGCGGTGACCGTCCAAGCGCATCTAACGGCGGATCCAGCGAGTGGCG
TCGTGCTGCACCTGCGGCCAGTgaacgcaacgaccgtggtggcgagcgtatcgagcgcggaggagaacgg
atcgagcgcggtggagaacgcctcgagcgtggtggagagcgcatTGAACGAGGCGGTGACCGTGACCGCA
AGGACAACGAGGGAGCGGACTCCTCTTGGCGTGTGCGACGAGAGCCAGATTCACAGCGTGCAGCTGCTCC
CAAAGACAGTGGCGCTCCGCAATCGCGCGATGACAAGTGGCGGCGTGGCGGAGAACGTGACCGCGATTTC
CGCATtgatggggctcgtcgcgacagggatgatgggcctcggcgtgaccgtgacgatggtcctcgccggg
atcgtgacgatgAGCGCGGTGGATTCCGCCGCGCCGACGGGGCGCGTCGTACCGACGAGCCTCAGCGGGA
AACTGGAGGGAACTGGCGCGACGCTCCACGCCACGCTGATCGGGAAACCCGTCGCCCAGCTGAGAGGCGC
GACCGAGATGTGCGCGAGACTCGTGGCGATCAACGCGGCTCTGCTCCTAAGGAGGCAGCCAGTGGCGGAG
TAGGCGGAAACTGGCGTACTGCTCCTGCGACCCGTGAGGAAAAACCAGCAGCTAAGCGCGATCAAGCCCA
GGAAAAGGG
>dme_circ_0007911_junction_seq
CTAAGCGCGATCAAGCCCAGGAAAAGGGGAGTTCTGGGAAGCAACCGAGAAGACAC
>dme_circ_0007912
GTTCGATAAACAACGGTGTGTGGAGCGGGGCGTGGTTCGCCCGACTTTAATCTACAGAATTGACTGGAAA
AGGCCAAAAAGGCGAAAAGAGAgtgtgtgtgtgtgtgctcgtgtgctggtgtttgattgtgtgtgtgAGT
GCCAGCTGCACATGTAAGTTTTGTTTATAATCGCCAACTGGAGAGCAGCAACAATGAAAGTCAAAGTGGA
GCGCACAACGAAAAAGCCCGCCATCAGAAAGCCCGAGGATCCAGATCCGGCGGAAGAGGACAGGGTCAAG
ATGGTGCAGGATGACCCAGAGGACCAGGAGAACCAGGCGGTGGATGAGGAGGAGCTGGACTTTCTGCCCG
CCGATCTAAGCGCTGCGATATCGACGGCGACAACGAAAATAGCAACACCGACGCGCAATCTTATCGTAAG

TGCGATACTCTATTTGTGTACCCTACATACGCGGGAGTATTATCAGTCGGAGAACGGGTTGACTTTACAA
CTGTTACTTTCTTATAGGTCACATGCACCTTGGTAGCTCGGTCATATGCTAAAAGAATCTAAAGAAACTT
GACCAATAAGCCCAAAATTATTGTGCTACCAATAGCGAATGGTTGCCTTGAAATTCCCTTGCAGATAGTT
GAATCTAGAAAATAAGATGATTATTCACTTATGAATTCGCACACTGAAAGGGTATTCGCGCTTCGTTGCC
ATCTGGTTTTATTGCTATTCACTCGGATTAATAAAGAGGAAGTGGGATAGAAGAGGATAGGTACGGTCGG
GCGGTACAAACTGGTACTGCATAGAACGCAATAATTCCGTACTCCCCCCGGTTTTGGATATGAATCTCAA
CATATCTCACAGAAAGCGTCTGAATGCTAAGGCTGACTTTGTCTGTGAGGCGAAAACATATAGAAAACGG
CACACATACGGGTCTGGAAACTTGTTTAAAAAATATATTGAACTTTTTGTTTTTTGTTGTTGCCTTGTAT
ACTCGTCTGCCtttttttttATTATGATTATTCGCCGAATTGTGTGGTGCGCATTTTGTTGTAGCTTTCC
ATTGTTATTGTTGCCGCTGTCTCGTTTTGGTTTTTATTTCGTATTTACTTTGTCGACATCTTTGGAATTT
CAATTTACACTTTTGTTGTTGTTTCGCGCTGTCACTGTGCCGCTCTCTCACGCGCACAAGGCAGTTAAAT
GGCagagagagagagagagagagagagagccagagagCGCGAGCAGCTCACACAGATACTTTTATCGCATGC
GAGAGAGGCAGACGTGAGTGGGAGAGCGCGACGAGTGCGAGTGAGTGGCTTATTGTGCATTCCCTCGTTG
CACTTGATTCGTAGTCGCAGCCAAGGTTGCCCTCTTTGCATTTGTTTTTCTATTGTTCCACTATCTCTCT
GTTTCTATTTCGTTTGGTTTTCGGACATTCCCCTCACCATCCATCGCCATCCCCTGTGTATCTTGTATCT
GTCAGATATTTGCACCCTGCATCTGTTTCTTTTTTTGTGGCGTAGGTGCGGTCTTTATTGCTGCTTGATT
CTCGTTCGCAACACGTGCATCGCCAATTTCATATTGCTGCACTGTGGCCAATAAGCCAAGAAACCGCTCG
GAGGTCAAGGCTTTCGCTTCCGTTTCTAATTGGATTCCACTTGGGACTGCGGCAAGCTATGAAATACTGG
ATAGCATATCTATCTGAAATTTGTATTTGCCGCATTTTGGTGTGATTTTAAATTTAACTTTGACGAAGCT
TATTGATTTTCAAAGATATGTTTTTAAATGTCTATGCTAACTGTCTATATCCTTAGGTGGACATATATAT
TTTTTATTTTATTAGCAATAATGACTGCCACTTTCGGCTGCCTTTAGAATTCAAAGGGAAATCTTGAATG
CGAAAATAGACTTGTCTTCTGTTCGGCACGCAAATCCCCCGGCTAATACCAGACTTGACAAGCGAAATAT
ATATATGTACATATATATCTTTCCACCCCGATTTCCCATTCGGCTGACATTCGAAATTATGCTGCAGTTG
ATTTTGCCATAGAATTAAATCCAATGCCAAGGTCAAGTGGCACTCAGATGCTGCGGCTCacccccgttct
tcccccccgaatacctcgaatccccccTCGAATCTCGCTGGTATTCCACTTAGAATGTCAAGCGACTCA
CGCGTCGAAATGTTTGAGATGCAATGTTGGGTATGGTGATAGGATGGGGATATGGCGATCGGGATGGTGG
GCTTGGGGTTGTATTTTGCGATTATATGTATACTGAATATGGTGCTGTGCACTTGAACAACTCAAATTGC
CTTTTTCGCATTGCGCTGCATAAAACATGCAGCGTGCTACATAAATATTCCATGCATTAAAAGCAAACGC
ATTAAAAGTCACGAGCAAATCGGCTAACGAGCAGCGAACTCTCCTCGGAGATAACCTTTTTACGGATTTT
TATGTTTGCACACTCGGGGTCAAGAGAATAGGGTTTAATTAATTGCAAAACACAAGCTGGAGATCACATG
AGATCACTATAGGTATAGGATAGGATTAATCCTAGAGATTGTGTTATTAGGTGATCACAACCACTTTCTG
CCAATAATAATTGCCTACATTTATATTTGATTCGTTTTAGTATTGATTTaaaaaaaaaGCCCTTTCGTTG
CGACACCAACTGTACAGAACAGAACCGCAGATTCGttttttttAGCGCTTTATAATCACGATCGGATATC
CAGCAGAAATTGCCAATTTAATGTGCGCATCAACTAAAAATAAATAAATATAAAATTGCCAATGCTTCGG
CCGCCGCCTTTGTTGCCATCGACATCAGCGTGCTCACTTAGCCagcaaataaataaataaatgcacacac
atcacaacaaaacacaacaaaataaaCAATTAATTATTGTTGTTCTTGTCTTGTTGTAATTTCGTCTTCG
GTTCCTACGTTTTTTATGGCGAATCTTTTACGACATTTCGCCTTTGCTGTCCTCGCACTCGTTTTCTCAT
TTCTTATATCTTATTTGCTTTTATTATTATGGCTTTCGATTGTTCCAGGAAAATTTCCGGGGATTTCGAA
AATTCAACCGCGTTGCTTCACGCACGTTCATAGATTAAAATCCATATAAATAGATTGTGTAGATAGCAGA
ATGCACTTGTTACCCAAAAGCGATTTCTTTGATTAATTTGTGGGTTTCGGGGGAAATGTGCAATGTGCAC
CTTGCCAGAATTGATAAGAAAAAAGGTAAATATAACTCTTAGTGCCCAGTGAAAACTGGGAAATGATCAA
TTGGTTTTACTTATCAACTTTATGTGTTAGCTTTCCTTCTTCGAAATAATAACAATTTTATTTAAAATAA
TATAGCTTACGAATGCTAGCTAACTCCAGCTGTAAGAATAGAAACTTTATAACAAGATTATTTATAGGAT
GAGCTGGCCATTTTTTATCAGATCTTTACTAAGACGGCTTAGCTTATCTCTGGATCTGTGTTCTTTCGCT

AGATTTGCTATTTCAATTAGTTAAAATCATCGGTATGAAAGATACTTTGTACATACATGGCTATCTGCAC
TTCAAAGAGCGTTTAGCTTGTGCATTTTGGCGCTACGCTAATGAAATCGTCTCATGACGTCAATGGCTTG
AACTTAGCATAAGTTGAGGCCTTTGAAAAGAAAACAACTTTAGAGTTGAGAAGCCGGTTTTCGGGCCGCG
ATTCATTTGAGAGCCAAATTTGGTCAACACGGTTACTCCGCGCCATGACGTCACAAGTTTGGGGGCCAGA
GGAAAGCGATCGCTTTTCCATACCATTAGAAAATAAGACCGAGAGCATTATGTCATCACCCAATCGAGCA
TTTCGAACTAACGAGCTGGTaaaaaaaaaaaaTATGTAAGCAGCCAAAGGAAAATGTCAGAAGTTGCGTG
TTTTCGCACTAGCTTTCGATTTTCTCGGAATCAAAATGGGGCCGTCATGGGTAGCAAAAGTGGGAGAAAT
AAATACGTTTATCAGGACCGCTGAGAGGGGGGTGGGGGTTAGATGGGGCACTGATAGGGCTTATCTCCTA
CATACATAGACACCGCAAAAATTCACAAGAGTCACGCTTTCATCTGAAATTTAAATAGATATAGTGAATA
CAAACCACAATCTATCGTAAATGAGCAGTATAATAGAAATTTATTAATTAATCTGCTGTTTTATGCAAAT
GTATTTATTCCCTTTTATATTTTTGTGAGTTTACTAAACTCTCGCAATTAGAATGTGGCTACTTAATGCT
AAGTGCCAAAGAATAATACCCACTATTCGACTAATTGAGTTATACGTCGGCAAAACAACTAATTCCAGGC
GAAATGCCGGCGGAAATGGCACATGCCGCCAAATGGGATTGGGAAATGGGTGTGGATTCGGAATTGGAAT
TGGCCGGATTGCCCGCCGCAATTCGAGCACATCTGGAATGGAGTGGAGTGTGGCAGAAAGTGCACTCGCC
AGTGAAAGATTTACACGATCCTGTGTGTTCCCCCGGCTTACCAAATGCACTTTGTGCCGCGTCATCCAGT
GCGGAATTCCAAGAAGAGGCGCAGCATCGGGCGCTGGAATGCCAGGGGGTCGGTCAGGAGGGATCGTCGT
CGTCGAAGGGACTGCACCATCGCCAGCGGGTTCGATACGCCATGGCGTTGAAAAGTGCGAAACGAAAAAG
TGCGAGGAGAAGATAAGTCCGGAGGAAATTATCGCGAACTGACGCCCGACCAACAGCGTACAACACTCCC
CAGATTGTGTCAACTCCTTGAAGTGCAACTGAAATATGGGGGTATGCTGGATTCACGAACGAAATTGACA
AATGCATCCATTAAGTCGTTAAAATTTACCTGCCTAATCTGGTGTTTTTCGATGTGATTCCATCTTATCC
GAGTTCATAGGCTTCTGGAATGTTCGAATGATTGTATATACGTCCTATTTACTAACTAGAAAGTTAGATG
AGAAATCGATTGAGACAAAATGACTAGTAAATATTTGCATATAATGTAATGGCTATCATTTGGCTATCAT
TATCCTTGTGTGCCTGTTAAATTTCCTATCCGCGTTTTCCGAGGTATCTCCTCGTCGACCTCCTTGTTTC
TTATCGAATCCCTTATGTTCGGGGGGAATTTCGCAATGCTGCATTGGGATTTCCAGCAGCAGCACCATAG
CCATGCCATAGCCTCGCATCGCCTGATTTGGCCTGGCTTTTAGCCTGGCTTCCTCTCTTTTCGAGCTGAG
TGGACCAGTTTTGGGGAGCACTCATTTTGAGCTCCACTCGAAGTGAGTGGAACTCAGAGTGCGAGTGAAA
CGGGTTCTGGTCGCTTCGGCATTCGGCTGGCAAGTGGGTGCCAAGTGTGGAGCTGAATGCCCTAGGCCGT
GGCACGTCCCTCTCTCCCACACCAAGTGGGCCAAAATGAAAAACAGCAAAACAAAACGCACGAATTCTGG
TTAAAACGGCGATGAGTCATCCGCACTGCGAGGTGAtgtatgtgtgtgtgtgtgtgtgtgtAGAATCGGT
TGTAGCCCGTCCATTCATAATCGCGTTTGCTGAATGAACGAACATCTATATGGGCACACACGAATCGGCG
TCTTTATAAACAAATATATAGTACAGCTGCGGTCGCGATAGTAGAAAAGCAATTACGGAATTTCACAATA
GCTGAATGCATAATAAGTTAATACTAGTGTTACCTGTTGTACGCCGTAATTAGCCGTTTAAATAGTTAAT
TTTTAATTATTTTGTTTAATATTACGATGATATCACGACTTCTGATTAGGGGGAATATTAAGGCTAAACT
TAAAGGCAACACTGCGTATGCTTAATATGCTAATAAAAATGCCAAAATGAGAATTTGTCACTGGAGGTTT
CTATAAATATGCCAGTTTATGTATGTATTTCAAAACCTATATCTGTATAAACAAAGTATATTTGGGTTTC
TATCGCCAACTGCTGATGGACACGGACTAATATTTGCCGCAGACACCCGACATGACTAGGCATTTGGCAC
CTCGACGCTGGACAGGATTAGGGTGATGTCATCGGGTTTCCCGCCGCCGGAGTATGAAACATTGTGTTGC
CTGGCCTTGATGGCGAATGGTGATTGGAAACTGGCATTCATGGACAGTTCCCTGGCCTTCTCCACCACCC
GGCTGGCGCCCACAAGTAGATCGTGCTCTCCACGCTCCTTTAGGCCATTCAATATACTGAGCAACATGCT
CTCGGGCATGTTATCGAACAAGCCATCGGTGGCCAGGAGCACCAGGTCACCGGGTAGTAGGGAGTGCCTC
GTGGACACGGCCATTTCCGGCTTGTCGCAGTAGTAGCTCTCCTTCCGATCCTCCGGCGGCACTGTCAGCT
GATAGGGCGTGTTGAAGTCGTGGGTTTGCTCCACTGAGCGGTGCAGCACCCGTCCATTTCGCACCACCAA
GAAGCCGCTGTCCCCCAAATTGGCGGTGTACAGGGTGCAGTCCTTCCGATGCATCGTCGCCAGGCAGGCA
GTGCTGCTACCCACAACAGGATGCTCCCGGTGGGACAACTCCTGGAAGCCGGCGATGAGCATGTTCCGGG

GACTGCGTCCATCGAAATCCGATAGCTGCGTCTGCCCGGAACAGCAGCTCATCAGCTCCTTGGCGAAACG
ACCCGCATCCACGCCCAAGTCCCGCCAACCGCCCACGCCGTCGGCCACGCCCATCACCTCGGCCAGCGGC
GTGGAGCTGACGAACCAGCTATCCTCGCCGAAACGCTGATTGGACCGCTCTCCCGGAAATCGCGGCTTCT
TCGATCTTCCCTGCACCACCGTGACGAGATAGGGATCCCGCGAGGATTTACCGGATTGAGGCGGACGCTC
GAAGCGGCCGGACAGTTGGGTGAACTGGTGGATCTGGTGGACCGAGAAGCGTTTCAGCAGGAGCGGCCAG
TTTCTCAAACAGGTGATCATTTCTCTTCGCTCTTAGGCACTGGAATTTGAAAAAAACTAAGTTTTGGGAA
ATTTACTTCAACGAGTTGGGTTTGAATATTCACTTTACTTATGTATGAAAGTTCCAAAAATGTAACAGCT
ACAGTTAAACAGCCTGCTTACATGTATATTATATTTCAATAAATATTTTGCTATAGCCACCCATCGACTA
GAATAATACCATGAAATCGTACACTTTCCACGTAATTGGAGCAAAAGGGCACTTTGTAGTTATAAAAATT
ATTAATCACAACGCAATTGCCAACTTGGGTTTCTGTTTACACCATAAACATTGGTTAATCGTAAGGCCCA
GATACGAAAACCATACACTCTAGATGGGCTGGCACGGTCTGTTTTTCCTGGCATTTGTCATAGGTAGATT
TGATTTCTTTGTTCAATCTTATCTGGATGGAGCCTACGACACTCTGGACTCCGAATGGAAATCGGAATCG
AACCCCCATCATCACCGGAAGTTTTGATCACGCTACGGAACTGCAAATGTTATCGCCGTGGATTTTCTTA
TCGGCCCAAAGGAAAACAATACGCAATGATAACTTTATTTTAAACCCCTGATGTAAACGCCTTTCAGATA
CGGCGTCTTATCATGTTATACCCAAACGCATTCGTTGGAATCGCTCATTATTCATTATTTGATTTGtttt
tttttttCTTCGTTCGTTGTGTTTGCCAAATTGTTAAATTGTCGGTAACTCTAAGGTCCGACTTCCTGTC
CAATGCTCGATACGTGATTCTGGCCAGCAAGAGCTGATCAGACTACTCTATCAGCTCTAGCTTCCGAGCT
GGAAGAGCTCGGAATTCGTATCTGAAACTGGAAGCGGAGACGTCAGAAGGTCTTATCACCTTAGTTCCtt
tttttttGTGACGTTATGaaaaaaaaaaaTGCGAAAAGTATTTCCTGCAGCGGCTACTGTTCCGGAATAT
GTTTTATCGTGGTTAAAATGATATTACTAGCAACAATTATTGGACATTATGCTACCGAAATCGGGGTTTT
ACCCAGCATTCAAATTTGTTCTATCGCTAGTAGAGCTCTTTAAGCACAGGAAACTCAGACAAAAACAACA
CACGATTTTGTTTTCATTTCATGTGTAGAAATTTTCTGAATCACTAAGATTCAGGCCAGGCTTAACTTAA
AGCGAATCTTGAATTGGAAACCGAGCTTCTGATTCATGGCGATAAGGGTTGCGTATCAAGCGAGGCTTTA
GATTTCGTGGGTTCCTATAGATTTGGGTCATTTATGTAGTTCTTGGCCTGATCCTGCACACTAATTGACT
GTGAATTTCGCTCTCTCGTTGCAGGTAAGTAGAGCAGAGACCACAGAGCTAAGTGGAGCCACAAATGTCC
AGTGTCACATGACCCACATTTGGGTGCACAACCCCTTCTCCTGCCCCCCCTCATGCCATTTACTTTACCA
AATGGTTTTGATTTCGGTTTGGATTCTCATTCCTCATGTAAGTGGCGGCACAAGGTCAGTGTGGCCtttt
ttttttttACATGGCCGTCGGCCGTTGGGGCCCCATCAATATTTATTGAAAGCTGGCATATCAATAGATA
AATGCCTAGAAACAACACCTGCAACAACGGAgaatcgaattgaatggaatggaatggaGCGGAGTGGAGC
CACCTGAGCCTCCGGCGGCGACATCCGATCCGCCTGGCTATGGCGACCCATTTCGGAAAGCCATTCCCCG
ATAAGAGGAGCAAAGCCACAAAGGCGCACCATTCATTTGATGGCGTTGCTGCATTCGGTGGGTCGGTGGG
GTTTACCGGGCGCTTTAAATTTAGTTGCTTATGTCATAACTAAATTGTCTGGCCGGGGCGATAAGATAAA
TGTGGCTATGGCATCGGCAATCGGGTGCCCAAGAAACGGGTAAACAAAAAGCGTAGTCAAAGAAAAACTG
GGCACGTGAGAGACCGAAAACCGCCTCTATCAACTAAACaaaaaaaagcacaaaaaaaaTATCTCGAAAA
GTATCCAAAAGTCAGCGAGCAGAGCGGGTTTAACCCACGAGATACACGTATTTATGTGAGCACTATTCAC
TTTATTTTGCATTTTGTCGAGCTTGCTCAGGAAATGCCAGAGCGACCGAGCTTTCAACTCACTCATTCGA
TTCACTCGCTCTCTTTGCGCTCTCTGAGTGCGATaaaaaaaaCACTCATTCGCCATGGCCCGGGTTTTTC
GAATAAAAGGCGAGCCATCGGCGATGACGCATTTAGTCTAAACAAGTTGGCCGAGCCAAACGTTTCGGGA
ATAGAGTGATAGTAGTGATAGTTCGAATCTGAAGTCGTGCGAATTACGTGAAAAGTTTTGTAGTGTGTGC
TAAATGCAAACAGTGCGACTCTCTCAGCATGAAAATTCAACAAAAACAGAGCGAGAATTCTAGGAAATAT
TGAAAACAGACAGCCCAAATAACAAATAATTCGCACGATCGCGCGCCACTCGCGGAACGcagatacagat
acagatacaaatacaTTTAGCCTGCGGTACAGATACTTTATAGTGCCAGTGCTTCGGTGTGCGGAATACA
AAGGCGAATTTCGCCAGAAATCGCTTGAGTCACTTGGCAGACTTTGTGCGGCCATACGAATCAAAAGCGA
ATCAAATATATTCAAAACACCTTTGGTTATTCAATAAAAATCAGTCAAAACGGAAGACCTACTGATCTAC

GACAAGATGACGCTGGACAGCTACAACATCTTCAACGATGAGTACTTGTTCAACATGCCGCTGTCGCCGC
TGCCAAAAGTGGTAAGAAATGGGCCTACGTCTATGCCATTTGAGTTAGCCAGGGTAGCAGGGTCCCGCCC
TTTTTGGGTTAAATTGGTTAAATGCCAGCGTTATCGGTGTTTGCCATGAAATTTCTAGAATTCCTCAGAG
TGGACTGATACACATATGTATGTACATATATGAACGAGTGTGCATTCAAATGGCTGTTATCGTTTGCGAA
TTGGTTTCTTGGAAGCCGGAGATCCCCTTTTTCTCAATGCCTTTCCAAAGCCCCATTTTTATGGGCCTGT
ATGCTATAGTTTATATTGTGTTTGCCCTGCGATAAGAAATTTCAACCTATTCCGATACGCTCGATCGGCC
GATTTGCGCTCATTATTCGAAAAATGAATTGTTTATTCTTTGCTAGCGATAAGGGAATTATTGTTGGCT
GTGGCAAGTGTGTGTGttttttttatgatttatttttGGGGGCTAGTGCGGGACATCCATCATTGGTTTC
GTAATTATTGAAAATCATTATTTTGCCTTCGAGTGCTATGTATGTACAAATGTGTGTTTTTATTCGAAAT
AATTCAGTTCAAATCAACTTAGATTGTGTTATATTCTTTTGGGCAACTCTTGCTCGCACCCTTGTTAATT
TATTAACTTTTGCGTGTGCGGATTCATTAAATCGAGCAAGTGCACCCTGCTGCATAAAAAAAAGCATAAAA
AGTTATTGCCAAAAATCACCCTCGATTGCGAGGCACGTTGCGATTTCATGCCACCATTAACAGTTAACAT
TTTCCGGCTTAAAGTCGGCTTCTTGCAGTTTTGTCTGCTGTCTTTTGGCCATATCTACCCATTTTTAGCC
ATTTTTGCCCACTGCACACTTCACAGTCGAGTGATCCGGTCTGGGTTTTGCGACTGCGTCTGGGTTTTGG
GCTTGGGTTTGGttttctttttttttGGGTCTGGCTTGGTTTGGTGGAACATGGAACAGCCGGCGCCATT
TGCTGATATCGCACACGTGCGATTTTCGTGACAATTTATCAAGTTTTCTGTGCATACAATATTGTTTTTC
ACTTTCCGCCGCGAAAGACGGCAAAAAATGAggggggggtggggggCTTAATTTGATGGTAGAAAGTGAAC
AGAGGGTCCGATTGAGTGACCCTTTGACTCGTGGACCTCAAACACACTCGCGTGACACTTGATACTCGAC
TTTTACAGCtgtgtgtgtgtgtgtgtgtgtgtgtgtTTTTTCATTTTAATCCCATATATATTTTATTTATAT
GTCTTACAATTGGGGCGGTTCAAAGTGACGGACTGCAAGTCAAACCACTTGCTCTTAGCCCCCCAAATGG
TTCCATTAAACAGCACATGTAGCGCCCCCACATTATTATCCCCAAAGCCGCAGAAAGAGCCAAAGTCCAA
CTTTCTTTTAATTAACCGAATGGCCGCCGTATATGGTCCGCTCTTGAAGCAATGTTTTTGTGCTTTTTCG
AGGGGCTTTACGTGGGGGGTTTACTTTCGCAATTGttttttttCGGTGGCTCAGTTGTGGGCGACTTCGA
TTGGTTTTGAGTTGGAGTTTTTAGGCATTGAGATGAATGAAATCACTTGAAGGCGCTGCTTTCGAGGGCA
GACAGTAACTTTCCTTTGTCTAGAGCTGCCCACCAGTCCACTGGCACCTAGAACTGGCCTAAAAACGCAC
ATTTTTGGCCAACCAACCACACACACACATATGAACAGCGATGATGTGAAGTTGGAGCGATCGTCATGCT
TGCGGAGCGTGCAACTGCTGGAGTGCATTCAATTAGGTTCGAATTAAGCATTAAATGTGTCTGCGCACTT
AATTGCATTTACAGTCGATTTAATGGGCTCTCTTCTCGGCGCAATCAAATGCATTTGGCATTCGGCGTCA
TCTACTTTAGCTGCCAGCAAAGTTTGCTGCAGATAATTTGACAGCGAGCGGTTGTCCTGTAATTGAAGGA
CTCTGttttttttCCTTGCCTTTTATTTCGAGCTGCCATCGTTGGTTTTGCGTTGGCAAATGTCCTGGCG
TTTGATGGCTGCGGTCACGTCCTTCTTGGAATTGGCCAAGCGTtggaaatggaaatggaaaaatggaaaa
ggaaaaCACCAATAGCCCGTCGAATGGAAAGTGAAAACAACTTTTACTGCTGTCTGCTGGCTTGCACTTC
GTCCTGTTCGTCctttttgtgctttttgcgctttttgcTGGGTGTTGTCCTTTGTGGCGCCCGAAAGTCG
CGCCGTTTGTTTGCCATTTCCTCGAATGCATACTTCGCCATAAATTCAGGGGCTTCCCCGCTTCTGCGTT
TGCTGTCGCGCCGAAATGCATTTAAATAAATTATTCCCAGACCATTTTGTCGCGTCATTTTATTAGCTGC
ATGTGCTGCTAGCTAGTGGCCACTGTAGTTGGCCATATCGGCTTAATTAAGCCATCTCAACGAGCGAAGT
GGGCTATCGATTGATAAGTAGTATTCAAATACAAATTACGACTGGGTTCATTGCCCTGCTTAAAACGCAG
AACAGAAAATATTTTCACCCTTTTTTCATTCTGAatatatatatatatatgtgtatatatCTCTGGCAAA
TTTATTTCTTGCCTTGTCCACGCCCTCGGAACAAAAGTCTGATTAATTTGACACCAAACTCCAAGCCGGC
GGACTTCGAGTCCTGCAAATTAATTTAACTCCGTTGCCTTTGCTTTGCGCTTTTTGGCAAATTTTGAATT
ATGGCCGAATGTTAAACTTTAATTCAATTTCGGGTCGTGTGGGCGTTTCGAAAATGGACAAACGTCAAGT
GATTTTAATGACGGCGCTGCTTTCTGTTCATGCAAAAAATCTACTTaatatatatatatgaatatattgt
atatCTGTGTGTGTGTACTTTGCATAAATGTTGCTTATATAAAGGGGGGGCTCCTCGGTGTTTTGTTTTG
CGTGCCTAAAAAAGGCCGGCCAGCCGTGTGGATGCGACTTTTTCGCCGGCCGTTGACTCTGGGCCACTCC

AAAATTCTTTGAAGTCAGGGAAGAAACCAGAggggggggggTGGTGAATTGGGGACTGGGGGAATGGGGC
TTTATCAGCCGAGCAACGAAAGGCAACTGAGACTGCAGACAGCAGACAGGCGGCACTTAGCGAAGTCAAC
ACTACACGACAATCGGCCATTTCGCTTGGGCATTGCGGTCACGTTCTCCTTTTGCCTTTGCCAGCAAGCT
aaaaaacaaaaacaaaaaaataagcgaaaataaatgaaattaaataaaacagaaaatATGATTGCCGCCC
CTCCCTGCGTTAATTGTTTTATTCGCAGAATTTGCAATTCGTACGAGAGTCCGTCAAAGGTTCCTATCTG
AAAATTATGCTTATCGTCAAGGGAAGTGCGATTGCACTTACAGAGTTGAAGGTGCACTGAGAATAAAATT
GGTGTTAAATGTGAGCGGAATGTATTAACTAAGTAGAAATGGTAATATTTGAGTTGCAAATGGTATCATA
ATTTAAGTTTGCATCTACTAAACAAATTTGCCAATTAACACTTGCACGCCATTTCTCGCAGTGCATTTTG
CAACATGCCGCACATGAACTTGTCAACTTTTCAAGTCTCttttgtttttttttATTTAGTTTCGCTGTGAC
ATTGCTGCAACTTGGAGCAAACAATGCTGAGTAGACAATTTACTCAGTCTTAGCCGATTGAAAATCAAAA
TTGTATTATTTAGCCCGGATAATTATGCGCATTTCTAACTAAACAAGCGGCGACCTTCGGCATGCAATTT
CTGTATTATTATTTCTGTATTTAACAAGCTTGGCAACAAATGTATGCCACAGCCACAGCAAGTAAAGCGT
TGAAACAAAAGCAAAATGCGCGCTTGGAGTTGCCAACTTTAAGTTAACATTTGGCCAAAACGGCGGCCTA
TGCTAACGCGCTCTGTTAGCCTTTGTTTCGGCCGCTCCAAACGCTTATTAGCCGCTTTGATATAAACAAT
GCAAATTGTCGTCGCTGTTTTCGTTTTTGCCAACGCTTGTTTTTGCCTTCGATGATGCCAAGttttttgt
ttttttttGTGCCGTCATTACGTCATTGTCAGGCTAAAATTGGTCTAAAAATAGCCCAACACGTCAGCTG
AAAGCAGAAGGCAAAGTCTCGGGCCCGAGAATTTCTTAGCCACTCAACTTGTTGCATCTTCACCTTCCTC
CTCTGCCCAGCGTTTAAAAACATGTGAATGTGTTTTTGAACTAATTAGAGCCAAGGTCAAAGTTGATGGT
CTAATCAAAATGATAGCGCGGATTTACAGGCAATTGATAAGTTTGAGTTTTCAGGGAATTCCTTTGGGAA
GTGGCAATTTTTAGGTTTTGAAATATTTTTGTAAGTTGCAGTCACCATACATTTATTTAGGGGCCACTTT
GGTTTGGCTTGAACCGCCCGCAAATCAAATTTGCAGCAGTCGCGTGTATTAGTCAGCAATTTGTTGCTTT
TCGCTACGTCAGTGttttactttttcttttttttttttACTCTCCACTGCTGGCTGTTCGATGTTCGCTG
CTCGTTTCATTTGGTTCATAATTGACCCCCAGATGTGAACTCGGTGTCCGGAGATATGACTCTGTCCAAC
GTTCCGATAGGAGAATCGAGATCGCCATCCGAGCTGGCTGGCAAGCAATTTAGACAATTCAATTCGCCGC
GCTTGATTGCCAGCATTCTATATCATCACTTGTTTTGCATACGTACATTTTCCAAATTGACTGATTCATC
ATCGGCTAAATGCGGTTCATGTTCGCGAGGGACAAGTTCAAATGGCAAAAAAGTGGGTTAAAACGAAAGT
AGCCAGCCAAACAAAGGTGGCCAAACAGCCAAGGCGGCCGAGTTGAAAGTAAATTTCGCATACGAAATTA
AAAAGTAAATATATCACAAAGAATAAAATCGATTGTTTGCTGTCAATAAGGGAACGGCCTTTGGGATTGG
GAAACACGGCTGCGATAAGATCGTCAAATAAATAAGCACACAAGCCGCTCGAACTCGCTGCACGGAGAGA
AAAGAAGGCATACTACTTCAAAAATGTAGCCCCAGTGCAACTGGTAtgtgtgtgtgtgtgtgtTGCTCTT
AGCCCACAACAAAAGCCAGCAATTAGCGTATTGGCCAACTTGCTGGCGACGGAAATAAACGCTCTTGGCG
AGACAGGGGGTctgaactgaactgaactgaactgaaAACATAGTTAATGTGGATGTGCCACACGCACACA
CCTGTTCGTGGGACATCTGTCGAATCGGACATGTGTATATATGTATGTAGCTTATGTTTACTCGGAATCA
TCGCTTTTGGCCAAAACATTGGGCCTGATTTATGGGCAACTGAAAGGCTTTTCCGTGGAAGCGAAGAATG
AGCCTCTGGCAAGTTCGGCAAATCGGAAGCAGAAGCATATACATATGTACATAAGCCACCACCACATTCT
ACTATAGCTAACTAAATGTACATATTTCGCTTTGAATGCGGCAAAAGCGAGCGTGGTCGTCGTGTGGGCA
GCCGCTCAATTAAGAGCAGCCAGCGTTTAGGATCATTTGATTTCCCAGCGGCTGTCTTCTAATTTGAGCA
GCCAGCTCGCACAACACACGCCCAGCAGACAGCAGACAGCTCTCCGGATTCGGATGCGGGTTCGGGTTCT
CTAGTAGTTCTCAAGatatataaatatatatatgatatgtatatatatacatatatatGCATACATCTGC
GGCGATCTCTCGATCGCGATCGTTGCCAATTTCGCTCGCCGGAGATTCTGATGCGATTCAGTCGCAAACG
AACAACGAACCATCGAAGTTTAGAAAATATCTAACAATTAAAAGCGATCGCGCGCGCTTTTCCTCGAA
TCGCATCGCATCGAATCGAAACGCGAAACTCAAACAACGAAACGAAAATAAATTTGGAAAACTGCAATTG
TCGTTGGGTTTTCGGTGCGCGTGCATTGCCAAAACGAATCAGGAGCaaaaaaaaCGAATCAGTCGGCTAT
ATGTACATATATACATATAGACGCAAAACAACGCACATTTCTCGGCTGCAAGCGCGTGGATCGAAatata

tatgccatacgaatatacatatatatatatatatatatatgtaaatataGCCAAACCCAGAAATAAAAGCGC
AAAGGGAGGCATAACACTCGCTGTGTTGAGAGGCGTTGCCCATGGCCGAGGAATTTCTGAATACAACAAA
TGCAGCATTAATTGAACTAGCTCAAGGCTCCATGTCCGTGTAAATCTCTATAATTATATAATAATCGAAT
TTGCGGCTCACACAGTGGAGCGACTTAAACTGGACACGTGTCGCTGGATTTTGGGCCTCGCACACTTACG
ATAAACTGCGTCGATTATGAGCGCTTTCGCCGAGCGGAATGTGCGTTTCAATCCTTTGCGAAATCAGCAA
ACGCTTATCGTTAAGCGGCTGAGACCGCCGGAGAGCCAAAACTTCAAGAACTGAGCAATCGAACAACGAA
CATATACGAAATCTGCTCACGGCTCGCAGCTAGCAAGAAGTCAAATCAAACGAACCCTTTTTCTTTATTA
GGAAAGAGCCAACGCAGAAAGCAAACGAAATACACAATACACACaaaaaaaaGAAGCTATGAAACAAAGC
CAAAAACGAACGAATTTCTAAGCAACTAGAACAACACTGCGACAACAAACAGCATAGGAGCATCCAAACG
AACGGAATTATAAAGACAAGAAAGGATCATCAGCACATTGTTAGGAAACTTTGGGAACAGAGTCCCAATT
TGTACATATACTCTAGTTTAGCTAGTAACTAGATTCTTAGATTGTTGAATTGTTTTTGTTGATTTTGAAT
ACAATTCGCATATATCATTGTTGTCCCAATTGTTGTGTTAGCTTAGCTAGGCTAAGAACCCCAACGAATA
CTAGTCAGCCGCCCAACTGTGCGTATACGCAACGCGAGTGCGTATACTTAATATTTAGGTTTAGATCGAT
AAGCAGCGAACAAATGATGAAAAATCTCAACGGTAGGACGCACAATGCGTGCTACCATCCCTACTACCAC
CAATCGCTGCATTTTGCTcagcagcagcagcagcagcagcagcatcatctgcagcaacagcagcaacata
tgcagcagcagcagcagcaacagcaagcgccgcagcaacagttgcgtcatcagcaacggcaactgcccac
gcagccagcctaccagcaatcgcaATCGGTTGCCCACAATGCTTTTCCACTGCGCAGCAGCAGCAACAAT
TATGGCCATGTTGCTAGCAGCGCCTACGCTGCCAGCAGCGGCAGTCACAACAGCAACAATGCGGCTGCCA
TGGCCGCCGTCTGCCAAATGCAGAATTTTTTCAACcagcaacagcagcagcagcagcaacTGGAGTTCAA
CAACAATTGCATGCCCATTAATTACTACCAGCAGCAGCAGCAGCAACATTACCCTTCCGAGTCCCAGTCC
TCCGCCTCCGGCTGGAATCCCGAAACCCCTGGGCAGGCGCAACTCGCCTTGACGGCCAcaacatgcaaca
ccactgcagctgcaacatgcaacaccactgcagctgcaacaacgagcacaactgcaACATCTGCTGCCGC
TGGCAGCGACAACAACCACAGCGACAACTTTGCAATGGACGCCAGTGAGATAGCCACTTTCCTCGCCAAC
GAGCTTTTCCTACAGCAGGTACCACAGATCGATCGATGATGATGCCTCCGATTCTGGTCGAGATTGGGAT
AGATTTGGGCGCGAAACAATTGGCAAGCGGCATGCGTACTTAATCATCGCCGAAAATAGAACAAATGCGG
AACAACAAAGGGGTGGGCAAATATAAACAAAGTGATGGATTGGCCGCCTCTCTTTCGGTTTCTTCATCTC
TCTTCGCCTCtttttttttcgttttttGTTTGGCCAAAAACACGTTCGCTAATTCGCTAGAAATGTACGG
ATAAGCAAGAGGTCTTACAGACTTAGCAATAGCTTTTGATGTTGACTGATATCTGAACTCGACCCAAATT
ACTCATGTGATAAGAATAAATTCCCAAACATCCTTAAGCTATTCGCGGGCAACACTGCGTATGCGTGATA
TATGTATACTGATATGGCATTGTAGAGGGTTTCGTGCTTGTCATGAGCTTTAATGAGCGTTAATAGTAAT
CCATCTTCTCAGATACTTGCACTCATAGATGTCTTTACTAATCATGCACCTTTTCTGCTCTTGCAGGCTC
GGCAACTTTGAGACCGGCCAGAGTGTTCTCACACTGACGACGCCCACGTTGACGCCGACCACCACGCGCA
ACATCGAGGACACACTGGGCCACTTGCTCTCGGACACGCAGACCGATCGTGTGGCTGGTTGCGCGGGATT
TGCAGTGCCAAAGGTGCTACCCAATGCCATTGATGTCCTGGGCATGGGTATTCCCACCGGTGTTTCGTCG
CTCCCACTTCAGCAGACATTCGATTTGAGCCTGGGGCAGGGCAGCGAGTCCGAGGACTCCAACGCTTCGT
ACAACGATACGCAGATGAACGAGGAGCAGGACACGACCGATACTTGCAAGTGCCCATACGGACAGCACCT
CGTACCAAGCTGGCCACATCATGGCGGGCAGCGTGAACGGCGGCGGTGTCAACAACTTCTCCAATGTCCT
GGCCGCCGTGAGCTCTAGCCGCGGATCGGCGTCGGTGGGCAGCAGCAACGCGAATACCTCAAATACGCCG
GCTCGTCGTGGCGGTGGCAGACGCCCCAACCGGTCGACGAACATGACCCCGGAGGAGGAGCAGAAGCGGG
CCGTGCGCCGGGAGCGGAACAAACAGGCGGCGGCCCGTTGCCGCAAGAGGCGCGTGGACCAGACCAACGA
GCTCACCGAGGAGGTGGAGCAGCTGGAGAAGCGGGGCGAGAGCATGCGCAAGGAGATCGAGGTGCTGACG
AATAGCAAGAATCAGCTGGAGTACCTTCTGGCCACCCACCGGGCCACCTGCCAAAAGATTCGCTCCGACA
TGCTGAGCGTGGTCACCTGCAACGGTCTGATTGCCCCGGCCGGACTCCTGAGTGCAGGGAGCAGCGGCAG
CGGCGCGAGCAGCCATCACAACCACAACAGCAACGACAGCAGCAACGGCACGATTACGGGCATGGACGCC

ACGCTGAACTCCACCGGGAGGAGCAATTCACCCTTGGATCTCAAGCCGGCGGCGAACATCGATAGCCTGC
TGATGCACATCAAGGACGAGCCACTCGATGGCGCCATCGACTCAGGATCCAGCCTGGACCAGGACGGTCC
GCCGCCCAGCAAGCGCATCACCTTGCCGCCCATGTCCACGATGCCGCACGTTCACTT
>dme_circ_0007912_junction_seq
CCATGTCCACGATGCCGCACGTTCACTTGTTCGATAAACAACGGTGTGTGGAGCGG
>dme_circ_0007913
GTTCGAATCTGAAGTCGTGCGAATTACGTGAAAAGTTTTGTAGTGTGTGCTAAATGCAAACAGTGCGACT
CTCTCAGCATGAAAATTCAACAAAAACAGAGCGAGAATTCTAGGAAATATTGAAAACAGACAGCCCAAAT
AACAAATAATTCGCACGATCGCGCGCCACTCGCGGAACGcagatacagatacagatacaaatacaTTTAG
CCTGCGGTACAGATACTTTATAGTGCCAGTGCTTCGGTGTGCGGAATACAAAGGCGAATTTCGCCAGAAA
TCGCTTGAGTCACTTGGCAGACTTTGTGCGGCCATACGAATCAAAAGCGAATCAAATATATTCAAAACAC
CTTTGGTTATTCAATAAAAATCAGTCAAAACGGAAGACCTACTGATCTACGACAAGATGACGCTGGACAG
CTACAACATCTTCAACGATGAGTACTTGTTCAACATGCCGCTGTCGCCGCTGCCAAAAGTGGTAAGAAAT
GGGCCTACGTCTATGCCATTTGAGTTAGCCAGGGTAGCAGGGTCCCGCCCTTTTTGGGTTAAATTGGTTA
AATGCCAGCGTTATCGGTGTTTGCCATGAAATTTCTAGAATTCCTCAGAGTGGACTGATACACATATGTA
TGTACATATATGAACGAGTGTGCATTCAAATGGCTGTTATCGTTTGCGAATTGGTTTCTTGGAAGCCGGA
GATCCCCTTTTTCTCAATGCCTTTCCAAAGCCCCATTTTTATGGGCCTGTATGCTATAGTTTATATTGTG
TTTGCCCTGCGATAAGAAATTTCAACCTATTCCGATACGCTCGATCGGCCGATTTGCGCTCATTATTCGA
AAAATGAATTGTTTTATTCTTTGCTAGCGATAAGGGAATTATTGTTGGCTGTGGCAAGTGTGTGTGtttt
ttttatgatttatttttGGGGGCTAGTGCGGGACATCCATCATTGGTTTCGTAATTATTGAAAATCATTA
TTTTGCCTTCGAGTGCTATGTATGTACAAATGTGTGTTTTTATTCGAAATAATTCAGTTCAAATCAACTT
AGATTGTGTTATATTCTTTTGGGCAACTCTTGCTCGCACCCTTGTTAATTTATTAACTTTTGCGTGTGCG
GATTCATTAAATCGAGCAAGTGCACCCTGCTGCATAAAAAAAGCATAAAAAGTTATTGCCAAAAATCACC
CTCGATTGCGAGGCACGTTGCGATTTCATGCCACCATTAACAGTTAACATTTTCCGGCTTAAAGTCGGCT
TCTTGCAGTTTTGTCTGCTGTCTTTTGGCCATATCTACCCATTTTTAGCCATTTTTGCCCACTGCACACT
TCACAGTCGAGTGATCCGGTCTGGGTTTTGCGACTGCGTCTGGGTTTTGGGCTTGGGTTTGGttttcttt
ttttttGGGTCTGGCTTGGTTTGGTGGAACATGGAACAGCCGGCGCCATTTGCTGATATCGCACACGTGC
GATTTTCGTGACAATTTATCAAGTTTTCTGTGCATACAATATTGTTTTTCACTTTCCGCCGCGAAAGACG
GCAAAAAAATGAggggggggtgggggggCTTAATTTGATGGTAGAAAGTGAACAGAGGGTCCGATTGAGTGAC
CCTTTGACTCGTGGACCTCAAACACACTCGCGTGACACTTGATACTCGACTTTTACAGCtgtgtgtgtgt
gtgtgtgtgtgtgtTTTTTCATTTTAATCCCATATATATTTTATTTATATGTCTTACAATTGGGGCGGTT
CAAAGTGACGGACTGCAAGTCAAACCACTTGCTCTTAGCCCCCCAAATGGTTCCATTAAACAGCACATGT
AGCGCCCCCACATTATTATCCCCAAAGCCGCAGAAAGAGCCAAAGTCCAACTTTCTTTTAATTAACCGAA
TGGCCGCCGTATATGGTCCGCTCTTGAAGCAATGTTTTTGTGCTTTTTCGAGGGGCTTTACGTGGGGGGT
TTACTTTCGCAATTGttttttttCGGTGGCTCAGTTGTGGGCGACTTCGATTGGTTTTGAGTTGGAGTTT
TTAGGCATTGAGATGAATGAAATCACTTGAAGGCGCTGCTTTCGAGGGCAGACAGTAACTTTCCTTTGTC
TAGAGCTGCCCACCAGTCCACTGGCACCTAGAACTGGCCTAAAAACGCACATTTTTGGCCAACCAACCAC
ACACACACATATGAACAGCGATGATGTGAAGTTGGAGCGATCGTCATGCTTGCGGAGCGTGCAACTGCTG
GAGTGCATTCAATTAGGTTCGAATTAAGCATTAAATGTGTCTGCGCACTTAATTGCATTTACAGTCGATT
TAATGGGCTCTCTTCTCGGCGCAATCAAATGCATTTGGCATTCGGCGTCATCTACTTTAGCTGCCAGCAA
AGTTTGCTGCAGATAATTTGACAGCGAGCGGTTGTCCTGTAATTGAAGGACTCTGttttttttCCTTGCC
TTTTATTTCGAGCTGCCATCGTTGGTTTTGCGTTGGCAAATGTCCTGGCGTTTGATGGCTGCGGTCACGT
CCTTCTTGGAATTGGCCAAGCGTtggaaatggaaatggaaaaatggaaaaggaaaaCACCAATAGCCCGT
CGAATGGAAAGTGAAAACAACTTTTACTGCTGTCTGCTGGCTTGCACTTCGTCCTGTTCGTCctttttgt

gctttttgcgctttttgcTGGGTGTTGTCCTTTGTGGCGCCCGAAAGTCGCGCCGTTTGTTTGCCATTTC
CTCGAATGCATACTTCGCCATAAATTCAGGGGCTTCCCCGCTTCTGCGTTTGCTGTCGCGCCGAAATGCA
TTTAAATAAATTATTCCCAGACCATTTTGTCGCGTCATTTTATTAGCTGCATGTGCTGCTAGCTAGTGGC
CACTGTAGTTGGCCATATCGGCTTAATTAAGCCATCTCAACGAGCGAAGTGGGCTATCGATTGATAAGTA
GTATTCAAATACAAATTACGACTGGGTTCATTGCCCTGCTTAAAACGCAGAACAGAAAATATTTTCACCC
TTTTTTCATTCTGAatatatatatatatatgtgtatatatCTCTGGCAAATTTATTTCTTGCCTTGTCCA
CGCCCTCGGAACAAAAGTCTGATTAATTTGACACCAAACTCCAAGCCGGCGGACTTCGAGTCCTGCAAAT
TAATTTAACTCCGTTGCCTTTGCTTTGCGCTTTTTGGCAAATTTTGAATTATGGCCGAATGTTAAACTTT
AATTCAATTTCGGGTCGTGTGGGCGTTTCGAAAATGGACAAACGTCAAGTGATTTTAATGACGGCGCTGC
TTTCTGTTCATGCAAAAAATCTACTTaatatatatatatgaatatattgtatatCTGTGTGTGTGTACTT
TGCATAAATGTTGCTTATATAAAGGGGGGGCTCCTCGGTGTTTTGTTTTGCGTGCCTAAAAAAGGCCGGC
CAGCCGTGTGGATGCGACTTTTTCGCCGGCCGTTGACTCTGGGCCACTCCAAAATTCTTTGAAGTCAGGG
AAGAAACCAGAggggggggggTGGTGAATTGGGGACTGGGGGAATGGGGCTTTATCAGCCGAGCAACGAA
AGGCAACTGAGACTGCAGACAGCAGACAGGCGGCACTTAGCGAAGTCAACACTACACGACAATCGGCCAT
TTCGCTTGGGCATTGCGGTCACGTTCTCCTTTTGCCTTTGCCAGCAAGCTaaaaaacaaaaacaaaaaaa
taagcgaaaataaatgaaattaaataaaacagaaaatATGATTGCCGCCCCTCCCTGCGTTAATTGTTTT
ATTCGCAGAATTTGCAATTCGTACGAGAGTCCGTCAAAGGTTCCTATCTGAAAATTATGCTTATCGTCAA
GGGAAGTGCGATTGCACTTACAGAGTTGAAGGTGCACTGAGAATAAAATTGGTGTTAAATGTGAGCGGAA
TGTATTAACTAAGTAGAAATGGTAATATTTGAGTTGCAAATGGTATCATAATTTAAGTTTGCATCTACTA
AACAAATTTGCCAATTAACACTTGCACGCCATTTCTCGCAGTGCATTTTGCAACATGCCGCACATGAACT
TGTCAACTTTTCAAGTCTCttttgttttttttATTTAGTTTCGCTGTGACATTGCTGCAACTTGGAGCAA
ACAATGCTGAGTAGACAATTTACTCAGTCTTAGCCGATTGAAAATCAAAATTGTATTATTTAGCCCGGAT
AATTATGCGCATTTCTAACTAAACAAGCGGCGACCTTCGGCATGCAATTTCTGTATTATTATTTCTGTAT
TTAACAAGCTTGGCAACAAATGTATGCCACAGCCACAGCAAGTAAAGCGTTGAAACAAAAGCAAAATGCG
CGCTTGGAGTTGCCAACTTTAAGTTAACATTTGGCCAAAACGGCGGCCTATGCTAACGCGCTCTGTTAGC
CTTTGTTTCGGCCGCTCCAAACGCTTATTAGCCGCTTTGATATAAACAATGCAAATTGTCGTCGCTGTTT
TCGTTTTTGCCAACGCTTGTTTTTGCCTTCGATGATGCCAAGttttttgtttttttttGTGCCGTCATTA
CGTCATTGTCAGGCTAAAATTGGTCTAAAAATAGCCCAACACGTCAGCTGAAAGCAGAAGGCAAAGTCTC
GGGCCCGAGAATTTCTTAGCCACTCAACTTGTTGCATCTTCACCTTCCTCCTCTGCCCAGCGTTTAAAAA
CATGTGAATGTGTTTTTGAACTAATTAGAGCCAAGGTCAAAGTTGATGGTCTAATCAAAATGATAGCGCG
GATTTACAGGCAATTGATAAGTTTGAGTTTTCAGGGAATTCCTTTGGGAAGTGGCAATTTTTAGGTTTTG
AAATATTTTTGTAAGTTGCAGTCACCATACATTTATTTAGGGGCCACTTTGGTTTGGCTTGAACCGCCCG
CAAATCAAATTTGCAGCAGTCGCGTGTATTAGTCAGCAATTTGTTGCTTTTCGCTACGTCAGTGttttac
tttttcttttttttttttACTCTCCACTGCTGGCTGTTCGATGTTCGCTGCTCGTTTCATTTGGTTCATA
ATTGACCCCCAGATGTGAACTCGGTGTCCGGAGATATGACTCTGTCCAACGTTCCGATAGGAGAATCGAG
ATCGCCATCCGAGCTGGCTGGCAAGCAATTTAGACAATTCAATTCGCCGCGCTTGATTGCCAGCATTCTA
TATCATCACTTGTTTTGCATACGTACATTTTCCAAATTGACTGATTCATCATCGGCTAAATGCGGTTCAT
GTTCGCGAGGGACAAGTTCAAATGGCAAAAAAGTGGGTTAAAACGAAAGTAGCCAGCCAAACAAAGGTGG
CCAAACAGCCAAGGCGGCCGAGTTGAAAGTAAATTTCGCATACGAAATTAAAAAGTAAATATATCACAAA
GAATAAAATCGATTGTTTGCTGTCAATAAGGGAACGGCCTTTGGGATTGGGAAACACGGCTGCGATAAGA
TCGTCAAATAAATAAGCACACAAGCCGCTCGAACTCGCTGCACGGAGAGAAAAGAAGGCATACTACTTCA
AAAATGTAGCCCCAGTGCAACTGGTAtgtgtgtgtgtgtgtgtTGCTCTTAGCCCACAACAAAAGCCAGC
AATTAGCGTATTGGCCAACTTGCTGGCGACGGAAATAAACGCTCTTGGCGAGACAGGGGGTctgaactga
actgaactgaactgaaAACATAGTTAATGTGGATGTGCCACACGCACACACCTGTTCGTGGGACATCTGT

CGAATCGGACATGTGTATATATGTATGTAGCTTATGTTTACTCGGAATCATCGCTTTTGGCCAAAACATT
GGGCCTGATTTATGGGCAACTGAAAGGCTTTTCCGTGGAAGCGAAGAATGAGCCTCTGGCAAGTTCGGCA
AATCGGAAGCAGAAGCATATACATATGTACATAAGCCACCACCACATTCTACTATAGCTAACTAAATGTA
CATATTTCGCTTTGAATGCGGCAAAAGCGAGCGTGGTCGTCGTGTGGGCAGCCGCTCAATTAAGAGCAGC
CAGCGTTTAGGATCATTTGATTTCCCAGCGGCTGTCTTCTAATTTGAGCAGCCAGCTCGCACAACACACG
CCCAGCAGACAGCAGACAGCTCTCCGGATTCGGATGCGGGTTCGGGTTCTCTAGTAGTTCTCAAGatata
taaatatatatatgatatgtatatatatacatatatatGCATACATCTGCGGCGATCTCTCGATCGCGAT
CGTTGCCAATTTCGCTCGCCGGAGATTCTGATGCGATTCAGTCGCAAACGAACAACGAACCATCGAAGTT
TAGAAAATATCTAACAATTAAAAGCGATCGCGCGCGCGCTTTTCCTCGAATCGCATCGCATCGAATCGAA
ACGCGAAACTCAAACAACGAAACGAAAATAAATTTGGAAAACTGCAATTGTCGTTGGGTTTTCGGTGCGC
GTGCATTGCCAAAACGAATCAGGAGCaaaaaaaaCGAATCAGTCGGCTATATGTACATATATACATATAG
ACGCAAAACAACGCACATTTCTCGGCTGCAAGCGCGTGGATCGAAatatatatgccatacgaatatacat
atatatatatatatatatgtaaatataGCCAAACCCAGAAATAAAAGCGCAAAGGGAGGCATAACACTCG
CTGTGTTGAGAGGCGTTGCCCATGGCCGAGGAATTTCTGAATACAACAAATGCAGCATTAATTGAACTAG
CTCAAGGCTCCATGTCCGTGTAAATCTCTATAATTATATAATAATCGAATTTGCGGCTCACACAGTGGAG
CGACTTAAACTGGACACGTGTCGCTGGATTTTGGGCCTCGCACACTTACGATAAACTGCGTCGATTATGA
GCGCTTTCGCCGAGCGGAATGTGCGTTTCAATCCTTTGCGAAATCAGCAAACGCTTATCGTTAAGCGGCT
GAGACCGCCGGAGAGCCAAAACTTCAAGAACTGAGCAATCGAACAACGAACATATACGAAATCTGCTCAC
GGCTCGCAGCTAGCAAGAAGTCAAATCAAACGAACCCTTTTTCTTTATTAGGAAAGAGCCAACGCAGAAA
GCAAACGAAATACACAATACACACaaaaaaaaGAAGCTATGAAACAAAGCCAAAAACGAACGAATTTCTA
AGCAACTAGAACAACACTGCGACAACAAACAGCATAGGAGCATCCAAACGAACGGAATTATAAAGACAAG
AAAGGATCATCAGCACATTGTTAGGAAACTTTGGGAACAGAGTCCCAATTTGTACATATACTCTAGTTTA
GCTAGTAACTAGATTCTTAGATTGTTGAATTGTTTTTGTTGATTTTGAATACAATTCGCATATATCATTG
TTGTCCCAATTGTTGTGTTAGCTTAGCTAGGCTAAGAACCCCAACGAATACTAGTCAGCCGCCCAACTGT
GCGTATACGCAACGCGAGTGCGTATACTTAATATTTAGGTTTAGATCGATAAGCAGCGAACAAATGATGA
AAAATCTCAACGGTAGGACGCACAATGCGTGCTACCATCCCTACTACCACCAATCGCTGCATTTTGCTca
gcagcagcagcagcagcagcagcatcatctgcagcaacagcagcaacatatgcagcagcagcagcagcaa
cagcaagcgccgcagcaacagttgcgtcatcagcaacggcaactgcccacgcagccagcctaccagcaat
cgcaATCGGTTGCCCACAATGCTTTTCCACTGCGCAGCAGCAGCAACAATTATGGCCATGTTGCTAGCAG
CGCCTACGCTGCCAGCAGCGGCAGTCACAACAGCAACAATGCGGCTGCCATGGCCGCCGTCTGCCAAATG
CAGAATTTTTTCAACcagcaacagcagcagcagcagcaacTGGAGTTCAACAACAATTGCATGCCCATTA
ATTACTACCAGCAGCAGCAGCAGCAACATTACCCTTCCGAGTCCCAGTCCTCCGCCTCCGGCTGGAATCC
CGAAACCCCTGGGCAGGCGCAACTCGCCTTGACGGCCAcaacatgcaacaccactgcagctgcaacatgc
aacaccactgcagctgcaacaacgagcacaactgcaACATCTGCTGCCGCTGGCAGCGACAACAACCACA
GCGACAACTTTGCAATGGACGCCAGTGAGATAGCCACTTTCCTCGCCAACGAGCTTTTCCTACAGCAGGT
ACCACAGATCGATCGATGATGATGCCTCCGATTCTGGTCGAGATTGGGATAGATTTGGGCGCGAAACAAT
TGGCAAGCGGCATGCGTACTTAATCATCGCCGAAAATAGAACAAATGCGGAACAACAAAGGGGTGGGCAA
ATATAAACAAAGTGATGGATTGGCCGCCTCTCTTTCGGTTTCTTCATCTCTCTTCGCCTCttttttttc
gttttttGTTTGGCCAAAAACACGTTCGCTAATTCGCTAGAAATGTACGGATAAGCAAGAGGTCTTACAG
ACTTAGCAATAGCTTTTGATGTTGACTGATATCTGAACTCGACCCAAATTACTCATGTGATAAGAATAAA
TTCCCAAACATCCTTAAGCTATTCGCGGGCAACACTGCGTATGCGTGATATATGTATACTGATATGGCAT
TGTAGAGGGTTTCGTGCTTGTCATGAGCTTTAATGAGCGTTAATAGTAATCCATCTTCTCAGATACTTGC
ACTCATAGATGTCTTTACTAATCATGCACCTTTTCTGCTCTTGCAGGCTCGGCAACTTTGAGACCGGCCA
GAGTGTTCTCACACTGACGACGCCCACGTTGACGCCGACCACCACGCGCAACATCGAGGACACACTGGGC

CACTTGCTCTCGGACACGCAGACCGATCGTGTGGCTGGTTGCGCGGGATTTGCAGTGCCAAAGGTGCTAC
CCAATGCCATTGATGTCCTGGGCATGGGTATTCCCACCGGTGTTTCGTCGCTCCCACTTCAGCAGACATT
CGATTTGAGCCTGGGGCAGGGCAGCGAGTCCGAGGACTCCAACGCTTCGTACAACGATACGCAGATGAAC
GAGGAGCAGGACACGACCGATACTTGCAAGTGCCCATACGGACAGCACCTCGTACCAAGCTGGCCACATC
ATGGCGGGCAGCGTGAACGGCGGCGGTGTCAACAACTTCTCCAATGTCCTGGCCGCCGTGAGCTCTAGCC
GCGGATCGGCGTCGGTGGGCAGCAGCAACGCGAATACCTCAAATACGCCGGCTCGTCGTGGCGGTGGCAG
ACGCCCCAACCGGTCGACGAACATGACCCCGGAGGAGGAGCAGAAGCGGGCCGTGCGCCGGGAGCGGAAC
AAACAGGCGGCGGCCCGTTGCCGCAAGAGGCGCGTGGACCAGACCAACGAGCTCACCGAGGAGGTGGAGC
AGCTGGAGAAGCGGGGCGAGAGCATGCGCAAGGAGATCGAGGTGCTGACGAATAGCAAGAATCAGCTGGA
GTACCTTCTGGCCACCCACCGGGCCACCTGCCAAAAGATTCGCTCCGACATGCTGAGCGTGGTCACCTGC
AACGGTCTGATTGCCCCGGCCGGACTCCTGAGTGCAGGGAGCAGCGGCAGCGGCGCGAGCAGCCATCACA
ACCACAACAGCAACGACAGCAGCAACGGCACGATTACGGGCATGGACGCCACGCTGAACTCCACCGGGAG
GAGCAATTCACCCTTGGATCTCAAGCCGGCGGCGAACATCGATAGCCTGCTGATGCACATCAAGGACGAG
CCACTCGATGGCGCCATCGACTCAGGATCCAGCCTGGACCAGGACGGTCCGCCGCCCAGCAAGCGCATCA
CCTTGCCGCCCATGTCCACGATGCCGCACGTTCACTTGTCCACGATATTAACGCCCACCGGCGCCTCGTC
GGGATCTCTGCAGACGCCGATCACGAGCACGGCGCCCGGCGGATTCGGCAGCGCCTTCCCGGTGACCTCC
AACGGCAGCAGCATCAACAACATCAACAGCATCGGCAACAACATGAACTCTCCCACGCTGAATGCGCATA
ACAAGGTGCCTAAGGAGCGGCCCAATACGCTGGCTTTCCAGCGTCCTCTGGGCCAGATGCACCTCACCAT
GGCCAACAACAAGGCGGGTGGGCCCACGCAGATCCAGGGCGTGCCCATCCAGACGCCCTCGACCGGCACC
TTCAACTTCGACTCCCTGATGGACGGCGGCACTGGGCTAACGCCCGTCTCCGGACCCCTCGTACCGAACA
GCTCCTCCACGAACAAGCACCCGCTGGAGCTGCCCACGCCCACCGCCGAGCCGTCCAAGCTGGTCAGCTT
ATAACCGGACCGGATCTGGGACGGGGATGGGGGCCGAGTCAGGATTAAGTTAAGTTTTATATAGTTGGCA
GGCAAACAACGGAAGGAAACCGCGAAGCACTTTTCTAGTTGTAGGAGAT
>dme_circ_0007913_junction_seq
GCGAAGCACTTTTCTAGTTGTAGGAGATGTTCGAATCTGAAGTCGTGCGAATTACG
>dme_circ_0007914
TATATTTACGCTTTACGATTCGTTTAAATGTACAATTTTAAAACCGGCTTAGTTCCTTTCATTAGACGCT
CCTGTTTGCTTTCTACTTTGTTTCATCCTCGCATCTTTTGTATTCGTTTTATAATAAATATCACAACGCA
TCCTGCAAATCTACACGCCCTATTATCATCATTATCCTTATCATTCTCATCATGCATCGTCGGTcgttcg
cgttcgcgttcgcgttcTTATATTTTTTGAAAATATACAACGCGCAATGAAGAGGTTTGAAAATTGCACA
AATTACAAG
>dme_circ_0007914_junction_seq
GAGGTTTGAAAATTGCACAAATTACAAGTATATTTACGCTTTACGATTCGTTTAAA
>dme_circ_0007915
GTGATCCGCACCGCCCTGGCCATGGGCGCCGATCGCGGCGTCCACGTAGAGATCCCGGCCGCCGAGTACG
AGCTTCTGCAGCCCATCCACGTGTCGAAGATTCTGGCCAAGTTGGCTCTTGACGAAAAAGCCGACCTGGT
CATCCTTGGCAAGCAGGCCATCGACGATGATGCCAACCAAACGGCCCAGATGACCGCCGCCGTGCTCGAC
TGGCCGCAGGGCACCTTCTGCAACAAGATCGAGAAGACAGACGCCGGTCTGACCATCACGCGTGAAATTG
ATGGTGGCCTGGAGACAATCAAGACCAAGACCCCGGCTGTGCTGAGCGCCGATCTCCGGCTGAACACCCC
TCGCTACGCCACGTTGCCGAACATCATGAAGGCCAAGAAGAAGCCGCTGAAGAAGGTTACGGCCAAGGAT
CTCGGCGTGGACACCTCGCCCCGCATCGAGGTCATCTCCG
>dme_circ_0007915_junction_seq
ACCTCGCCCCGCATCGAGGTCATCTCCGGTGATCCGCACCGCCCTGGCCATGGGCG
>dme_circ_0007916

GAACCTAGAGCAGGCCAGGTTGCAGGAAGGCGAAGAGGAGGAGCCTCTTGGCCACGACCTGCCCGAGCAC
ATGCTGGTGCGAAATAGCGGAACTGAGGATGCAGTGCAGCCACTGCCGCAGCAGCAGCAGACGATCGATA
CCACCGGCAATAGCCAGGTGATGGCAGCCTCTTTCATGGCGTCCAAGACCTGCCCCTATCCCTTGTCCGT
CGTCTGGTGGAAGACCAACA
>dme_circ_0007916_junction_seq
TTGTCCGTCGTCTGGTGGAAGACCAACAGAACCTAGAGCAGGCCAGGTTGCAGGAA
>dme_circ_0007917
GATAACATGGCCGCTAGGAACTCACTCACTCCACAGCAGGGACTGGGCTTCTTTGGGCTGCTAATCCTGC
TCTGCTCGGCGGTTTTGGGCAAGTCGCAGATGTGCGAAGTGGAAACGGGGCAGACGAACATCATCCTGGA
CATCGAGGAGAGCCGGGAGTCTTTGTAAGTACACCACCCTCCTTCTTCACTGGATTATACAATACATATG
AAAATACAATAAACACGGCCGTGTTATCGACTAGCCAGCCCATTTAAGTCGTTTAACATGGCCTTAAACT
GGAAATATGAGTTTCAAGACAACTGCGGCGTCAAATCTATTACGTGGATGCTAAAACATGAATGGCCATA
TGCTTCTCAGCCAAAATGCTCTGCGGCCAACCTCGACTTCAAGATCATAGACTAAAAGCACGAAAGGTGC
AACACCGTAATTATAATTTTAGACGTGTTCCAGCGCATAGGCAGCCCATCACATCCACATCGCATCAAAG
AACGAAAAGAAAAGCACGTCAGAGCAGGGAAACTAAGTGTACTTCGAAGATACATTGTACAATTTACATG
CCGCTGAGGTGCACCTCGGAAAACCTTAATTGGTTTTTAGCCTCATTAAGCTTGCAGCTAGAAAGAATGT
GGGGTCAAGAATAGCCATCAAATATTGCTCATACGACGTGTTGTACATGCGATTAGTGTAGGGAAGCGAT
GTATCTCCCTCTTACAGATACAATTTGAATAAATTCATTACATGCAACTATAAGATATAATAGTGTATAG
TACTTTTAGTTCGTTATGCAACTTCAGAGCTCACTAGTGGCGCCAAATAACAAGAGCACGCGAATGGTAA
AGACTTTCTCGTGCATCTATGATGCACCTACGACGTGTGCCCACATATGTACATATATGTGCACACACTG
AGATACTTCGCAAGTGATCGCTGCAACTTGACCCCAAATGCAAAGAGAAAGGCTGCTGTCTGCAGTTTTC
TCGGCTTGCGCGTTTTCCCGCCGATCCGTTTGCCGGTTTGGCCATACTTGTACCAATAACCCAGCTTGGT
CCATTAGATCCCGGCTCTTACTTTATTATTTATGTGCTTTCCGCAGGTCATCGGACAGCCGACCACGCCG
CCAGAGCTGCCGATCTTCGGCGATCCGGATACGGAGATTGCTCTCAACTTGGTCTTCCCCAAGGGTCAGC
CCATCTTCCAGCTGAACGGCAAGAAGCTGCAGCTGCTGCAGCCCCTGGATCGCGACGAGGAGAACCTCAG
CCACATCGTCTTCCAGGTGTGTAGACCATTCTCCGGAAGCACTTTCCACTGCAAGGTATCATCGACTCTC
CACGTCTGCCCGTCTCGTTTCAGGTCTCCTGCACCACCAGGTCCACGGGCAAGAAGCGCACCATTCCCAT
CATTGTTCGGGTGTCGGACATAAACGACAATGCGCCGCGCTTCATGAACACGCCCTACGAGGTCACCGTT
CCCGAG
>dme_circ_0007917_junction_seq
CACGCCCTACGAGGTCACCGTTCCCGAGGATAACATGGCCGCTAGGAACTCACTCA
>dme_circ_0007918
TTTATACAACTTAAACAGTTTATATTAATGCATGTACATGTTTTCAACAATTCCTAAAGAAAACGACTGC
GTCTGGCAGTTTCGTAAGTGTGTGGGAAGTATGCACTCTGCGTTCGCTTAAATATAATGACTG
>dme_circ_0007918_junction_seq
CTCTGCGTTCGCTTAAATATAATGACTGTTTATACAACTTAAACAGTTTATATTAA
>dme_circ_0007919
GGTTCTGGGCTCTCAAGCAGATCATGATCCGGTTGTGGTCCGTTTGACCCGTGGGCAACCAGATGCGCGC
CTGAGGCGCAAATGGTACATCGCTGACATGGACGAGCTTGATGCGCATGTCATGCCACGAGTTGCTAATC
GCGGCAACACAGTCCGACAGGAATACCTTTGAAAATCCGTCTTCGCACCGGATCACTCGGTGGCCACGAA
CTACGTCTGAGCTATCGAAGGACGGGACGGGACCCCCCGGCATGGCCATGACTCTGTCAGTCACCAT
>dme_circ_0007919_junction_seq
GCATGGCCATGACTCTGTCAGTCACCATGGTTCTGGGCTCTCAAGCAGATCATGAT
>dme_circ_0007920

CCTCGTACCACTGGCGGAAGGGCGTGGCATCGATGACCACGATGCTGTTCTTCACCAAGGTCTTGGTTCG
CACCAGCTCGTTGTTGGAGGCGTTGTACACAACATCGGCGATACGGGTCTTGCGCGCCACTCCCTCGGAG
GCCCAGGCGAAG
>dme_circ_0007920_junction_seq
CGCCACTCCCTCGGAGGCCCAGGCGAAGCCTCGTACCACTGGCGGAAGGGCGTGGC
>dme_circ_0007921
GCATCGTGGTCATCGATGCCACGCCCTTCCGCCAGTGGTACGAGGCTCACTACGTGCTGCCCCTGGGACG
CAAGCGTAACCCCAAGCACGCCCAGAAAGAGGACGAGAACGACGTGCTGACCAAGAAGCGCAGCGAAAAG
GTTATGAAGAA
>dme_circ_0007921_junction_seq
AGAAGCGCAGCGAAAAGGTTATGAAGAAGCATCGTGGTCATCGATGCCACGCCCTT
>dme_circ_0007922
GAGGACGAGAACGACGTGCTGACCAAGAAGCGCAGCGAAAAGGTTATGAAGAAGTACCTGGAGCGCCAGA
AGTACGGCAAGGTCGAGCAGGCCCTCGAGGATCAGTTCACCTCCGGCCGCATCTTGGGGTGTGTATTCTT
TCTAATTCATGATTAATGATTTTTGTTATAGTGATGTAAAAACCCTAGCGATCCAGCTACTTTATTGTGT
TGATCTTGTTTTAAGAGAGACGCAAGCCTGTTGGGTGTGATTCTGCTTGTCATAGTTACTCTGATCAGAC
CACTAAACTTATACTTTTCCATTTACTGGAAGCCCCGCTCTAATTCCCTGTTATTTTTCCTTTCAGCTTG
CATTTCTTCCCGCCCCGGACAGTGCGGTCGCTCCGACGGCTACATTTTGGAAGGCAAGGA
>dme_circ_0007922_junction_seq
CCGACGGCTACATTTTGGAAGGCAAGGAGAGGACGAGAACGACGTGCTGACCAAGA
>dme_circ_0007923
GTTGGGTAGACAGCGCTTTCACGAACTACGTCAGTGCATGCAAAACGATGAAGATGATAATCCAGGAAAT
ACACTCACACCTGATTCTCAGGTATACATTTTTGTAATCTAGCCTGTTTGCTCAGCTTTCCGTTTGCCCA
GCTTACCCACGTGTACTTATGATTTAAATTAAACTTGCAGGAATAGCTCAACGGATGGCATTTTTACCAA
CCGTTCGACGCATCCGTTTAAGGTCATTGAGACAGATGCGATGAGCATACAGAGCATGACATCACTGGGT
CGCGTGGGCCGAATCTTGGCGGGCAGCATTGATCCATCTGGCTATAAGCATAGATCGCGAGTCCCTGGCC
TCGTTGAATGCGCAAcagcagaagcagcaggcgcagcagcaacaacaggcgcagcCTACATCGTCTACCA
GCTCCAATACTCCACCGAGCGTTGGAAACACCTCTTCCGGGATGAGCACGCCCAGGGTGTCGCCCAAGCA
TCAGCAGAGTGGTAAGGAAGTAGCCGCCACCACGACAGGGATTCCTAGTGCCGCATCCTCCGCGCAGAGC
TCTGGTGTTGGCTCGATGGTTTTGCAGGAACCAGATGTGATAGCAAGCACAAAGCTTGCCCAGTCTAAAA
CTACTGATTCCATCACCTCGTCGGGACCCGTGGCGCCACCGCGAAGAAAGAAGAAGTCTCGTAATTGCTC
CATCAGTTCCTCAGATCAGTATAGTCTTCCTCCTGCGGACAACGAAGACACTGATAGTGATACGCGGTCT
GTGAAGAGTGTGCAGGGTCTTCAGCAAAAGCTGCGGGATGACTTTGTCATGGAGTTCGAGAGCTCTCTTA
ACAATGCAACTAGTTCAGCCTCCAATATCACCCTGACCTGCTCCTCCACCAATACAGTTGTTTCTGCATC
GGCTTCGACTTCGGCAGCATCTTCGTCCTTGCCATCAACAATTGCAATTCATATGCGCCCCATGGTCGCT
TCGGTCACCATTGCTGGTCCAAAACTCACTCAATCTCTGGGCAGCAGTTCCACGTCGGTCCACTCCAAGC
CAAGTCCATCAAATTCAGCGAAAAGCAATTCCGCACCAGCGAGGGTTAGGTTCCATTGATCCGAATCAGG
GTCTGAATCTGTTTAAGGCCATCCAGGGAGGTTATGTGGTGAAACCACGCGACGAGGCCAGCAACAAGGC
GCAAGGCCCATCCCAAGATGAAATCGAGCGCATTGAGAAAATGCTGATGGAAAGCGCGAACCGACCAAAG
CTGGCGGGAACGGGATCCAACAGTTTAGGAAGCTCTACACGGCTATCCCGGATTTTATCGCGGTGTTAAC
GATAAGGAGCGCCGGAAGTCCGCTGGCGACGAAGATGTGCTCAAACAGATGAACATTTATGTGCGCACGC
GAACCAGCTCAGGCAAGCAGCTAACCGACTTTGAGATCCTTGAACAAGTTCCTGTTAAAAATTTAGACAC
GGGAGAAAATATGCCTCTATCCGAAATGCGTTCACCACCAGTGCCTGAGGGATGGAATCCGCTCTCGCTA
CACATCCTTAAGCTGACCTCCCACCTGGAGGTGATCCAGAAGGAGTCCGACGAGGAGAGTGTGATTGGCA

TACCGCCTAGCATCGAGGGTCAGCAGCCCGACGAAGAAGAACTGGAAACGGAAGATGGCAGTCGACTGAA
AAAGAAAACGGCCCGGATCAAGAGGTTCTTTGGCAGCACAATGCGAAAGACGGTGGACAAGGCCAAGTCG
ATTGCATCCGAAGTGTCGCATGCGCGACACAAAGAGGATGTGGCTGATATTGTTGATGCAATGAATCCCG
AGCAAAATATCAAAATCAAGGCTTCCTCGTCCAACAAGGGACCCTATGAGTTTACCAAACTGCAGCACGT
CCAGGATTTGAGTGGCGAAGATACCAGTGCCGTGTGGTGCATGAAGTTCAGTTCCTGCGGTCGACTCCTG
GCCACTGCTGGACAGGACAAGGTGTTAAGGATTTGGGTTCTTAAAGATGCCTATCCGTTTTTCCAGGGAC
ATGCGCAACAAATACAATGCTGACCAAAAATCCTCACCTACGCCCTCGCAAGAGTCACTCGTCTCGCAGC
ATTCGGCTGAGGAAGCCATTGCCATGGCTACTGCCGCAGAGAAATGCACGGGCCCCTTCATGCCAAAGCC
CTTCTGCACCTACAATGGACACACCTCCGATTTACTGGACGTCAGCTGGTCAAAGAATTACTTCATACTG
TCAAGCAGCATGGACAAGACTGTGCGGCTCTGGCACATTTCCAGAAAGGAGTGCCTTTGCTGTTTTCAGC
ATATCGATTTTGTCACCGCTATTGCGTTCCATCCACGCGATGATCGCTACTTTTTGAGTGGCAGCCTCGA
TGGCAAACTAAGGTTGTGGAACATACCCGACAAAAAGGTGGCACTGTGGAATGAGGTAGATGGCCAGACA
AAGCTTATAACGGCTGCCAACTTCTGCCAAAATGGACAGTTCGCCGTTGTAGGGAGCTACGATGGTCGCT
GCATATTTTATAACACGGATCAGCTGAAATACCACACGCAGATTCATGTGCGCTCCACCAGGGGTAAAAA
CCGCATTGGCCGCAAGATCAGTGGCATTGAACCGATGCCCGGCGAAGACAAGATTCTAGTCACATCCAAC
GACAGCAGAGTGCGCCTGTACGATTTGAGGGacttgaacttgtcctgcaagtacaagggctacttgaatg
tgtcctcgcaaattaagggctCGTTCAGCCACGATGGAAAGTACATCATAGCCGGGTCCGAGAATCAGTG
CATCTATATTTGGAAAACAAACCACGATTACTCAAAACTCAGTTCTGGTTCGCCGCGATCGCAGCGACTT
TTGGGAAGGCATTAAAGCACACAATGCTACGGTCACTTGTGCCATATTCGCGCCACACCCAGAGGCTATC
ATCAAGCCCGAACCGGATGAGATCTCCAATGAGAAGGTTGGCTCACAATCAGCCGGATCCACTGGTCGAG
CAGCACAAAAAGGGATGTGGTTATGTGTTGGTCAGCGCCGATTTCAATGGCGCCATCAAGGTGTTCATCA
ACAAGACGAAGCCGAAGCACAGCAGTCTACCCTACACGGCCATTGCGGATTAGTCTTTAGATAGAGGATG
GGAGATATAGAAGA
>dme_circ_0007923_junction_seq
TTAGATAGAGGATGGGAGATATAGAAGAGTTGGGTAGACAGCGCTTTCACGAACTA
>dme_circ_0007924
GTCAGCTTAAGGATGTGTAGCGAGAGCGGATTCCATCCCTCAGGCACTGGTGGTGAACGCATTTCGGATA
GAGGCATATTTTCTCCCGTGTCTAAATTTTTAACAGGAACTTGTTCAAGGATCTCAAAGTCGG
>dme_circ_0007924_junction_seq
GGAACTTGTTCAAGGATCTCAAAGTCGGGTCAGCTTAAGGATGTGTAGCGAGAGCG
>dme_circ_0007925
GACTTGTTCCGCATGGCAGCCAGCTCCGAGATGAGCATCTCGTGGAAGGCTTGCTTCTTTTGCGGCGTGT
TGCCCACGCTGATCGAGCTCTTCATGGTGCTCGTCTGGCCGCCGTTAACATTGATCCTCATGCAGTTGCC
CGATGACTGGATGGCCAGGACATCGGACTCCTGCTTCAGGGCCTCGTGCTTGG
>dme_circ_0007925_junction_seq
GACTCCTGCTTCAGGGCCTCGTGCTTGGGACTTGTTCCGCATGGCAGCCAGCTCCG
>dme_circ_0007926
GCTAATGTCATAAGGAGTCTCAAGCGCCGTCTCGCAAAGCCGCTGAAAATGTCCGAGGACCTGGACGAGC
TCTTCCTGACGCCCATTCCGGCGCCCTCGGCCGTGGAGCTGCAGCAGTGCATACTGTTCGGCTTCCGGCG
CCGCCTTCATCCCGCCCAGATATTCGAGGAGTGCCGCAACGCCTTTGGGCCCTTCTGCCCCAGTGCGCAG
TACGTGGCGAAGTGGTACCAGCGGTTCCAGGACGGCTTCTTCAATCTGGACGAGAAGGACGACGAGGAGC
TGAGGATCGGGCATGGCGGCACGCCCACCACCTGCAGTGATTACGATGATGACAGTGATTGCGGGGAGGC
GACCGTTCTGCCCCTGATCCTAAGTGAAATGATCCAACGCAGTGAGGAGGATGACGATGATGATGGTACT
GATGGTTTTGAGAAGGAGAGTGATGATGGTCTAGATGGTCATGTGATTGGTTATTGGCAGGGTTATGGCT

ATTTGGGTGGCAAGCAAAGGTATAGTTGGGCTGGAACTAAACTGTATGTACTTAACCCCAGTTTGTACTC
CATGATATTGATCCGTGTCTTCCAATCAATCTTCTCCTTGCAGGTGGAAACTCGGCTTGCGAGCTGCCCC
CGCTGCCGAAATCTCTCATCGGCTTCAACAAGTTACTGTGCTCCGATTCGGGACTCCTGAGTGATGTGGA
AGGAAACAATAACAGCAGCGATGGCAGCCGAAAACCAAACGACAATCTTGCTGTGGAGACACAGGATACC
AAGGAGCATGCCACCGATAAGTCACTGCCCTCCTCGCCGAACGGAAACACATCCATACCACCAATGCTGG
TGGCCAAATCCGAGGCCAAGTCCCCCAGCTTATCCGCCAAAGGAAACCCCCAGGCGGTCGAGGCGGGCAG
CACTCTGGATGCCCAGATTGCGACGCTGCGGAAGGAAATG
>dme_circ_0007926_junction_seq
CCAGATTGCGACGCTGCGGAAGGAAATGGCTAATGTCATAAGGAGTCTCAAGCGCC
>dme_circ_0007927
ggcttcttgtccttatcatcgctcggcttcttgtccttgtcgtcacccggcttcttgtccttatcgtctc
ccggcttcttgtccttgtcatcggctggcttcttatcattattgtcagcaggtttcttgtctttgtcgtc
gccag
>dme_circ_0007927_junction_seq
gcaggtttcttgtctttgtcgtcgccagggcttcttgtccttatcatcgctcggct
>dme_circ_0007928
GTTGCCGCTGTTGTAGTGGTGGTGCTGGATGATGTCGCCACTGCCCACCCAGTGGGTGTACTGGGGCTGG
GTGCGGATGCTGGCTCCGTAGTTGATGGTCACTCCACTGGCTCCGTTGGTGCAGTGAGCGGCGGTCAGGA
CCCAGGTGTTGCCGATGATCGAGCCACCGCACCACCAGTTTCCG
>dme_circ_0007928_junction_seq
GATCGAGCCACCGCACCACCAGTTTCCGGTTGCCGCTGTTGTAGTGGTGGTGCTGG
>dme_circ_0007929
GTGAAGCCAGCCGGTCCTCCAACCTGGCATCCGTAAGCGGAGACGAAGGAAGTGATTCCGATAAGCTTAT
CACCCTCCTTTGTGACCAGAGGACCACCAGAATCACCCTGGCAGGTGGCCTTTCCGTCCGGAGTTTCCAC
ACAGATGGTGTTCTCGGAGGCGGTATCAGTTCCGTA
>dme_circ_0007929_junction_seq
TGTTCTCGGAGGCGGTATCAGTTCCGTAGTGAAGCCAGCCGGTCCTCCAACCTGGC
>dme_circ_0007930
GCTTACTACGGAACTGATACCGCCTCCGAGAACACCATCTGTGTGGAAACTCCGGACGGAAAGGCCACCT
GCCAGGGTGATTCTGGTGGTCCTCTGGTCACAAAGGAGGGTGATAAGCTTATCGGAATCACTTCCTTCGT
CTCCGCTTACGGATGCCAGG
>dme_circ_0007930_junction_seq
TCCTTCGTCTCCGCTTACGGATGCCAGGGCTTACTACGGAACTGATACCGCCTCCG
>dme_circ_0007931
GTTCCTTGAGCTAGACGCGCCCATCGAAAGCGGTCGCCTCCATCGGCACCTGCTGCCCGTTCAGGCCGTA
AACGTGGGCGAAACTTTCCACATAGTGGAGCACGACGCTCTGCAGCAGTCGGCAGAAGAAGCTCAGGAGG
AGGAGTAGGAGGATAAGCCCTCGGTTGAGCATTAACAAAATCACAATGGAATCCACGTTCGGTAAA
>dme_circ_0007931_junction_seq
AATCACAATGGAATCCACGTTCGGTAAAGTTCCTTGAGCTAGACGCGCCCATCGAA
>dme_circ_0007932
GTTCTTTTATTATGATCCCACAGTGTGGACCATCGAAAATGCTCTGCATAAGCTTGGCAAGTCATCGAAG
CGCAAATGGCCACTGAACGATCCTGAAGAATTGCGGGTTAGCCGCGTGCTAGATTTTGAAAATGCCTTTG
ATGTAAAGTACCAGACTAAGGCGACGAGGGAGTTCTCCTGCTGCCAAGGACTGGACGTGACTGCCTGGGG
CAT

>dme_circ_0007932_junction_seq
AAGGACTGGACGTGACTGCCTGGGGCATGTTCTTTTATTATGATCCCACAGTGTGG
>dme_circ_0007933
GGTCTTCACGGGGCGTCCGCCTTTTTCGCTCAAATAAGCAAACAGATTTCGGTGTTTTTCGGTCAAGGCC
TTGAATTCTGGCGATGATTCCAGGCTGGCCAGCTCATAGTCATAGGCAGGACAAGGTGCCTTAGCGGCCA
GTATGGGATCCTCCCTCTCGGGCGAGGTGTGAATGGGTATGGGTTGCCAGTTGATGTCCGTGTTCCAGAT
>dme_circ_0007933_junction_seq
GTTGCCAGTTGATGTCCGTGTTCCAGATGGTCTTCACGGGGCGTCCGCCTTTTTCG
>dme_circ_0007934
GCGGAGACGAACATAAACGACGATGGCGAGAAGAAAACCTTGAAGATCCCCATTGCCGTGTTGAAGGTCG
GCGAGACCCGTAGCTTAAGACCAAATGTCGAGTTCCCCAATGGATCAGTGACCTTCAAACTGGTGCAGGG
AAGTGGACCCGTACACGTATGCGGCAAGG
>dme_circ_0007934_junction_seq
AGTGGACCCGTACACGTATGCGGCAAGGGCGGAGACGAACATAAACGACGATGGCG
>dme_circ_0007935
GGACTACGCACGCGGCCAGAAGCTGGTCATCAAACAGATCCTCTTGGGCGCCGAGGCCAAGGAGAACGAG
TTCAACGTGGTCGAGGTGAGTACTCCATTCATTGTGTATGCGAAACGGGGCATTCAGTGCATGTAGTAAA
TCCCATAAATTGTGTATGCGCGCGCCGGTTGGTGTCCTCTTCtttttttctttttcatttttttttttcgt
tgcatttttCCGGCCGGCGGGGCAGGGACCGGTTTCCATTTTTCCCCCAGACCAGAACAACCTCGAACTT
TTCGCTCGTTGTGGTGGGCTAGGGTAATTGTGTCTGTACATCTGCAAACGGCCGCCGAGTAACCAGCCCC
GGACTTTCGATTGCCATGGTACATTACCAATGGGATGTGCTAGGTACATAAATTCTTTTTAGTACAAGGT
ACTTGCAAAGAAGTACTAGGTACTTTACAACTGTCCCAGTAAATCCAGGGACTTAGGTGTTTTGTATATT
GTTAGGAACTTTCGAGAATAACTATCAAAGCACATCTTCTTTAAAGAATATAATTTTTATTATAATAAAT
ATTTTTAATTTGTGCTTTAAAATTTCTCATTAGTTGTACCTAACCATCATTATACCTTAACCATCAAGAT
CCTCATAGTTTAAGTACCCCACCTTTCCGAACCAAATTCATTGGCGCAACTGTACGCAGATACACATACG
GCAAGACAAAAGAGCACTCTTTATTGACTCAAGTCAACGGCGATACACGAATCGGCTGCCTCGGCATCCG
CAATTGTTTGCATTAGGTCTGGAAAGCTTTTCGAGAACTTGAACATACTGCGCAGCTTCCTGTCCATCCG
GCAAATCACTCCCGCCGCCGGAATACTCCAATTCCAGCAGATCATGTCGCACTTCAGCAGGGCGAGATCT
TCGCGCAGTGACTTGAGGTTCTTCGCGAGGAATAGAGAAGAGGTTTCGGGTCAAAGGTCATAGTTTGCAG
CAAAGAAAGCTGGCACTCACCAAAGCCACTGTCGTATCAATTGAGGCAATCTCGTGTCCCTTAATGACCA
CCAGTGTGGTGGGGGCACTTCCCTCGCTGTTTCTTTGCGTCACGTGCGACACAAGCGACATTTTCAAGTA
CTCGGCTGCCGCGTAGTCCACGCTGCCCTTGAGATCGGCCATGGTCACCTCAATGCCGTTAAACTAGGTT
GATTGCCAATAGAAATTATTAGGCCCTATATATGCAGTGTGTCAGCTCACCTTCTCGGTTGTCAAGAAAA
ACTGCGGCTTCATGCTCTTGTAGAGCAGGAAAAGCGCATTGAAGACGATGCCCACCACCATTCCGTACTC
CAGGGTCCAGAAGACACAGGTCAGCACGGTAACCAGGAAAGGCAGCATATCGCGCTCTGTAAGATAACCA
GACTTACTTTAACTTCACGTGGGATCCCAAATGCCATGTAGCACTCACTTTTAGCCCGCCAAATCTCGCC
AATGGTCTCGTACTCCACCATAAAGAACATGGCTGCAATGATGATGGCTGCCAGGGTCGCCTTCGGAATG
TAGGCAAATGTGGTGGTGAGGAAGGCCAGGGTCATGAGGACGAGAGCGCCGGTAACCGCACCACCCAGCG
GGGTTTTTACTCCACTGGCGTTGTTGATGGCTGTCCTTGTAAAGGATCCAGTGATAGGCATGGAGGAGAA
GAAGCTGCTGAGGATGTTGCTGACGCCCAGGGCAATCATCTCCTGCGATGCGTCCACTATCTTGCCCTTG
GCTGAAAGTAGAAGAAGCTCAAGTTATGGAATATGAACTGAAGTTATTCCTCCGCACTCACAGAAGGCCT
TGGCCACAGCTATGCTCTCCAATATGGACAACAGTGGTATGGACACCAGGGCAGTGCCTACGTTCGATAC
CATGCCACCGAAGCTAATGGTTTCTCCAGTCTCTGGATCCTCCGTGTGAAAAGGGGGAAGCTTGAAAGGC
GGCAGGCCTGGAGTGATGCTGCCGCTGACTAGAAAGGGCAGTTTGCCATCGTTCTTCAGAAGATAGCACA

GGAGTATGCCAATTAACACGGCCACAGCATTGCGGGCCAGTGAAATATACTTCCACACGCTTTTGAAGCC
GAAAGTTAGGTTTTTCAGTTGCTGAAATTATTTAAACATTTATAGTCTCGCTAATCAAACCTTCTAATTA
AGCAATTTAATTAAGCATTACCCACCCTCATGAGCAGCAGGAGCACAAGTGTGCAGCAGCCGAGGATGGC
ATCATTTCGGCGTGTTTGTGTTATGTGACCGAAGAAGTATATCCAGCTGTTAAGGAACCCACTGGCACTA
CTGCTAATGCCAAAGAGGCTATTAACCTGGCCGGTGGCAATGGTTAGGGCGGCTGCCATCGTAAAACCCG
TGGTCACCGGCACCGATATGAACCGCATGAGCACTCCGAGATTGAGTAAACCCATTAGGGTGATGATGCA
GCCGGACAGAAAGCATATTAGCACCGCATACGCTGGGTTGCCGTCCACATATGGCTGCACCATAAGCGCC
ATGATGGCCGTAGGGCCTGTGGAAGAACGATTTGAATTACTTAGCGCACCACCTCACCAGAGGCTCCCCA
ACTCACCCACTGTGATATCCTTACAAGTTCCAAGCAGTATGTACACAAATCCACCCATAAAAGCGGAGTA
GAGCCCATAGGCTGGTGGCAGATTGGCCACAGCTCCGTAGGCTATGGCCTGTGGCACGGCTGTGAGGCCC
ACGGTGAGACCCGCCACCACATCCATGGCCAGGAAGTTCAACTGGTACTTGGGCAGCCAATCGGTGACGG
GCAGGTATCTGTGGACGGAGCGCATGCTGCAGCAGCTCCTCGCCTTTGAGCTCACGGCACCGCAAACGTT
CGGCAGACGCTCCCGGTAGAGATCATCTGGAAAGCACAAGTGGGCTATTTAGTAGGTGTGCCTCCTGGGG
CGCCTTGAAATCTTGATATGGCCATAGTTAAGTGGTTTATTTGATTGAAAAACGCTCTTTTATGTACACC
CATTCGCAATCTCTGCACATGATCTGAAGTGCCTCGATGATTTCCTGCGGTTCGGTGGTCTTGTGACTTC
TGGAAAAATACCAAAGATGTCTACAAGCTGATAGACAAGTTCATCCAGCAGGCAGCACAATGCCTTGATG
TTAGCACTTTGAACCCTCTGTTTTGGTGTCTCATATTCTAGATAATTAGTGAGCCGCATATAGTATCCTT
GGCTAGTTTGTATCTTATGCATTTATATTATTCAACGGGGGAAATTTACGTTTTGATCCCTTCTTGCTTG
ATGGACACGTTCATCCCCAGGAAGCCAGCTTGTCACTGATAACTCATCTTTCGCAGCTCGTAATAGCACT
GTCATCTTGTGGATTCTCTTGTCTAATTACGGACTCATTACCGGGTATTTACCATAAATAATGTAATGTC
TCCGAAGACCTTTGTTTAGTTTCCTTTAATTGTAGTTTATCTCATCAAGTTTTAGGTAAACTAGTTCTTT
ATTTATCACAGCACAATCAAACAGTGTTTGTTCCTTCACTAATTATAGCCCCATTGAGTAATAGTTTGTG
TTTTCTGATATTCAGTTGGTTGGTTTTAATAGAAGGGGAAGGAGTAGTTTCCTTCGTATCACACTGCTTT
CACTTATCCTCTTGAGGGAACGCGCTTCTCCACTTACCTTCGTTATCGCGCATGTCGACAAGATCTTTGG
AGCACTTTACGATCGGCCAATACTAGTTTTGATTCGAATTACACGTTGCGGATGATTAATTAGCAGTCAC
TTGTTAGATTCGCGCTTGCTGGACTCCGGTTCAGATGAGCCTGGCTACAGTTTAAAGGGAACGAACCGAT
CCACTCCAGTGTGGGCAGACGAGTGGTCAGGCGGCCGCCAGTCGACGGTGTTATTACCGAAAGCGACTGT
GCGAATATGTGAGTGCGTAGGCAGTTATCATCGGACAGTTCAGTTAGTTTTGGGGAAGGTGTTCTGTTCG
GCCGACAACGATAAGAGATAGAGACGAAGACGAAGACTGCAGAGCGATTAAGTGGGCCAGTCGTGATGAA
CTTCGCGCTATCTGCTCGCTGGATCCGAAATTGGTGTAATCTGGGAATTCAACAACTATCTTCGGCCACT
TGAAAGGCATATGACGGGGATCTATATGGGTCAATCTTGGCCAGCAATCTTATGCAGTTAAAAGCAGGAA
AAGATTGATGTTTCAATTGATGTTCCTACCTAAGTGAACAATAGATAACATTCGAAACGTGTCTAACTTC
AATTTATTTATAAGCCACTTGAAAAGTCAATGAATCTATCTAATTTGCGAATCTTCGCCTTGTTTACGCA
GGGTGAACACACCCAAGGACTCCGTGCAAATTCCCATCGCCGTATTGAAGGCCGGAGAGACCCGCGCCGT
CAATCCCGACGTGGAGTTCTACGAGTCGAAGGTGACATTCAAGCTGATCAAGGGCAGCGGACCCGTCTAC
ATCCACGGGCACAACATCAAGGACGATGTGGAGGTGGTCGACATGGAGGAGGATGACGAGGAGGATGATG
TCGCCGAGGACGAGGAGGACGAGCACCCAAAGAAGCGCGCCAAGATCGAGAACGCCGCCGATGGTAAAAA
TGCCAAGAACAACAAGAAGAAGTAAACTGCTGACCGCCAATCATCCATCGAG
>dme_circ_0007935_junction_seq
AACTGCTGACCGCCAATCATCCATCGAGGGACTACGCACGCGGCCAGAAGCTGGTC
>dme_circ_0007936
GGTGAACACACCCAAGGACTCCGTGCAAATTCCCATCGCCGTATTGAAGGCCGGAGAGACCCGCGCCGTC
AATCCCGACGTGGAGTTCTACGAGTCGAAGGTGACATTCAAGCTGATCAAGGGCAGCGGACCCGTCTACA
TCCACGGGCACAACATCAAGGACGATGTGGAGGTGGTCGACATGGAGGAGGATGACGAGGAGGATGATGT

CGCCGAGGACGAGGAGGACGAGCACCCAAAGAAGCGCGCCAAGATCGAGAACGCCGCCGATGGTAAAAAT
GCCAAGAACAACAAGAAGAAGTAAACTGCTGACCGCCAATCATCCATCGAG
>dme_circ_0007936_junction_seq
AACTGCTGACCGCCAATCATCCATCGAGGGTGAACACACCCAAGGACTCCGTGCAA
>dme_circ_0007937
GGTTGTTGCTCTTTCCGTGCTTGGCCATCGCGTTCAGCAGAGCCACGGGCGTGGCGGCTGCTCCTCCGGC
GAAAACCGTGTCACCTGAATGGAATGAAGGCGAGAAACCGCAGGTTTTATTACCAATATTTTTATTACGC
ACACGCTGCTAATCAGTCAATCGGCAGTTATTGTACACTTTAATCTATTCAGCAGGTCTTATCTAAAGAT
TTGCATGACATGCTATCCGAGCGCCTACAAATAATGCTTTTCAATATTGTCTAATTAAATTAGTTATGCT
TTGAGTGTTCAATGATAACGAAATAACCAGGATTACTATCAGTTCTTGAACTTTATGATATCTTAATTTG
AAGAGCGTGGCCTAATCCATTCAATGGAATCCTGAGCTGGCAATTAATGCACTTCGAAATAAAATAAGTT
TATAATGTCGCTAAGAGAAAAACTTGTTTTGAAATTAGAACACTCTCTGATAATTGATAATAAAAAACAT
TCATATATATATTTATATTGTTATTAAAATTGTTATAAATAGTAATATAAACATTCTTTAGAGCTTTAAT
CAGCAAATAATGTTTCTCTAAGTCATTCATGGTTATTTACATATTTTTATTGCGCTGGTCATGCTTTCAT
TGAGACTATAAATTTGTTTCCAAAACGAATTGTTCCGAAATTCGGTAATTTTCTCTCTGATCATTCCACT
TGCTCTCGAAAGCTTAAAAGCTATAGCTATACATACATACATTCATATGTAAAAATGTATATTAGTTAAT
AGGCAGAGTGATAATGCGAATGCGAGGAGTGGGAGCGCTTATTGCCCAGAAACGCGTGATAATGAATGTT
TGTAAATAAATAACACCGGTTTCCCAGTTGGCATTACATGGCAAACATGTCGATTCGAGATTATAGTATA
ATATATAGATTTTGTCATTTGATTAACGAGCCAGAAAGTAGAAGCACATCAATTGATCAAAATGTATCTG
ATTGGGCTGTGTTACGTAAGAGAATTGTAGCTTTTAATCGGAACTTTGGGACGAATGATACTTGTATACT
TGGTCTATAAAAATGTATAAATTTTGTATCTATTTAATACACATCTGAATTGCATCTCTAATGCAGTCTT
AAGCACATGCAACATGTTTGGCCAATCTATTTTGGTCTCCCCCCTGAAGAGGCTGGCATTCCGAAGTTCC
TCTGTATGAGGGAATAGAAAGAACCATGATGTACATTAAGTCGAACGAGCGAGCCCCTTCAACTGGGGTA
ATCATGCGATAAGTGCGATTCTTATCGGTAGACGTCTGAGCCACGTCGACACACACGGTCAAGTTTTCCG
CGAGGCTTCACAATCAATTTATTTATTTATTTACCGTTACTTGAAAAGGCCAATTATGCATTTCTTACGC
ATAGGCTTAGTTAGCTAGTGCCTACTGTACCACTTATTATCTGCCAAAAACGTGCCAAACGAAAAAGTTT
TCCAAATCAAATTACGTCGCAGATTTGGCATGGCTCGTGTTGGTGATTTTGGCTAAAATTAGAGATACCG
ATTTTGGGTGACGTTTCGCTGGGCGCTGCGATGTGCCACTCCCCCTCCAAAGACCCGCCCCCCTCCGCAA
TATCACCAATTTGCAGGGACCTGCTGATGAGATGCCGTAGAAGTGCTAAGAAATTGAGATTTAAGCGGGC
GCCGAGGGCGTGTCCACATATTTGTTTGCATCTGAATCCCAGGAGACTCGCCTCCAAATTGGGACAATTG
AATTTAATCAGAGCAGAGGGTTCCCAAAAATCAGATGACTGGTTTCGTTAAACGAACATATACAAATTAT
CTAACAGATTTATCGTTTTGATAAGAAAGCTTGTTAGGAATTAATGAATATTTTTCGAAGCACTCCATTC
ATGCTTTTGTTTCCATAATTCACCTGCTGATACACGTTGCATAACCTTGCCTTCTTATAGAGGTGCTGCT
ATATACCATAGTATATGGTATATAGGTTCCTACAGAGAGCAGGCCTTATCAGCACCACATCCAGAGGCCG
GCTGACTTGTTTATGAGCTACATACAAGGTTGTTCTTGCCACCAAGCTGAGCTTTGCATCGGACCTTTCT
CTTCTTTTTGCCCGGGGTACAAAGTACAACTTGGCTGGAAACTCATCTATAGCGCCGAGTATACTCGTAT
GCATTATTAAATGTGTATAAATATCTACATATGTACGTGGTAGGGGCACGCACAGCTGGTTTCACGCGAT
TAAATGAATATAACTTACAGTTGTGCTGCTGCAACACCAGGAATTACGAATAAAAACATGGGGTCTACTG
ATAGCCGCCACAAGGAGGATAGCATTTTGATAAGGGCAGCTACTTGAAGTGTGATTTCCCTATTGTATAA
CTCCCCTAACTATTTATCATACTAGAACCCCAATCAACTAAAAACCTATTCCCTATCACAAGGTAATACA
ATACACTAAATATTTGATTCTAACTTGTGTATTATATATCCCCACGAGTGGCTCCTAATCACGGTCAATT
AAATGCCAGCCATCCATATACCCAGCACCCTGTGAGTGCCCTTCCCTATTTACGTACATATGTACGTATG
TACATGCATATGTGCTCCAAAGTAGTTTGGATTGCGGGCACACATGGGCTTACATTTGCATGGCCCCACA
ACCCGGTGTTCTCGTTTTCATTCTGTTTTCATAAGCAAGCCCACTAGAAGGTTACATAACCGAAAGATTG

CAAACAGCGCAAGGCAGCGATAACTGGGAGGCCAGTTCCGCTAGGACGGTGGAAGGGGGGGCGTGGCGGG
CTCCTGGCCAAATGATATGGGGGCCAGTGGGACTCACCGGATTTTACACATGCCACGGCCTCTTCCGGCT
TGACGATTGGAGGCTCTCGAGCGATGGGATGCGACAGCTCCCGCACGTAG
>dme_circ_0007937_junction_seq
GATGGGATGCGACAGCTCCCGCACGTAGGGTTGTTGCTCTTTCCGTGCTTGGCCAT
>dme_circ_0007938
ATGACGAGATGAAGGCGCTCACCCAGTGAAGAGCCAGCAGTTACTCGAATCCGAGGAAGCGAGGAGCGAC
GAGATGTCGAAAATGAGGGGTAATTGTAATTGAATCGAGTTTCCGGCTTAGAAAAGCCAGCCTTGCTAAA
TCCATTAAATGTTTGCCATTGAATAGGTCGCATTCTGATCCCACTGCCCAATACCGGAGCGATGGGTCGC
AAGCGCAACAACTTCTACATGCGGAGTCTGTTCCTGCTGGCCCTGGGCATTTTCGGTCTGCTCCAGTACA
ACAACTTCAATTATCGTGGACTCGCGGGACAATGTGCTGGGCGACGCGGTGACCAACGACTCGGACGACG
CCATCCTGGCCATGGTGCCCGCCACCCTGCACAAGTATCTGACGCCCCACTCGCGCAATCACAGCGCCTC
GGGAGCAGGAGCCCTCAACGGCGCGGGCCCTGCTCCTGAATGCCAGTAGTCCGGGCGCAGCTACTGCCAG
CACCATCAGCTTCGACGTGTACCATCCTCCGAATATCACCGAGATCAAGCGCCAGATCGTCAGGTACAAC
GACATGCAGATGGTGCTCAACGAGGACGTCTTTGGACCGCTGCAGAACGACTCTGTGATAATCGTGGTCC
AGGTGCACACGAGGATCACCTACCTGCGCCACCTGATCGTCAGCCTGGCGCAGGCCCGGGACATTTCGAA
GGTGCTGCTGGTGTTCTCGCACGACTACTACGACGACGACATCAACGACCTGGTGCAGCAGATCGACTTC
TGCAAGGTGATGCAGATCTTCTACCCGTACTCCATTCAGACGCATCCTAACGAGTATCCCGGCGTGGATC
CCAACGACTGTCCGCGAAACATCAAAAAGGAGCAGAGCTCTGATCACCAACTGCAACAATGCGATGTATC
CCGATCTCTATGGCCACTATCGGGAGGCGAAGTTCACGCAGACGAAGCACCATTGGATCTGGAAGGCCAA
CCGGGTGTTCAACGAACTGGAGGTGACCCGCTATCACACAGGGACTGGTGCTGTTCCTCGAGGAGGACCA
CTACGTGGCCGAGGACTTCCTGTACCTGCTGGCTATGATGCAGCAACGCACCAAAGACTTGTGCCCCCAG
TGCAACGTGCTGTCCCTGGGCACCTATCTCAAGACCTTCAACTACTACACGTACCACAGCAAGG
>dme_circ_0007938_junction_seq
TTCAACTACTACACGTACCACAGCAAGGATGACGAGATGAAGGCGCTCACCCAGTG
>dme_circ_0007939
GGTACTGAATGTACTGCATCAACTGTAATATAAGCTACGTAAATAGGAATTTATGCGAATAAGAGTAATT
AAGTGAAGCGTGTAGTTAAACATTTCTACAAACCGGACATAAAAAGATTCAACCATCTTAAAGGACTTGC
AGCGCTATGGATTGATATGTTCTCAAGCGTTGGCCATTTCTCACTTGAGTTTAATCGGGTTAGTGTTCTA
TCACCCTTTATATTCAAAGCATTCTTTAGATTTATTTAGGCGTTTATTGGATGCACCCGTGCGCGAAATA
TTTTCTCCCCCACAAATCCAATATGTATGGCTTATTGAAAAAAAGGAGGCACTTGGGTGGTTGCTACTGA
TTGATTTAAAGTTCGCTGTGTTGAAAACTAATTCGATTTGGGCTCTGAAATGTAAATTTATTTAAGCTGA
GCCAAAAAATAATATTTATACAATTTTATCTCTATTAAAATACCTTCCAATTTTATCTTTGATGCAATATT
TATGACTGGCATATAAAAAagttttttttgtttaatttatttatggtatttgtattatgttttggtttgaa
tgattatttatgAGATGCACGAGACTAAAGATTTTTCTTAAAATTATTTTCATTGTTTTGGACTTACTTT
CTTGGCGAACAAAAATATACCCAATGGCCAAATCTCTTTTCTCCCAATTGATAATTGATAATATCCTTAT
GTATAAAGCTTGCCtattatatatacatattttatatacatttatttatatatatgtatatTTAAAACAG
AGTAAAAGTGTTCATAATTTTTTTAACTATCCCATAATGCTTGGTAAGGCTAGAAACATTAGTCTTCAGA
TAAAACTTTAACTTTAACATATAACATAAATTTATACCAAATGGATAACAATGGCTCTAATCtttttttt
ttCCAAACTCTTAACTATATGTTTACAACCTTAGTCTTTCGAACAAATATTTAGTATTATATATTGTCT
TAGCTTATTTCAAAATGATTTCAATTGATCTAATCTTTTCTTCATTTCACTATTCTTTCAGGAACAACTG
GAACCATTCGGAATACCAATATCTTCCCCTGATTTCATGTTGAATTTTTTATGCAAATTACATAT
>dme_circ_0007939_junction_seq
TGTTGAATTTTTTATGCAAATTACATATGGTACTGAATGTACTGCATCAACTGTAA

>dme_circ_0007940
GGAACAATGAAAAACGCAAGGAAAAGTCTCGCGATGCGGCTAGATGTCGTCGCTCCAAGGAGACAGAGAT
ATTCATGGAACTCTCGGCTGCTTTGCCATTGAAAACCGACGATGTCAACCAACTGGACAAGGCCTCCGTG
ATGAGGATCACCATCGCTTTCCTCAAGATTCGCGAAATGCTGCAGTTCGGTGCCCAGCTTGCGCGACTGC
AATGATGACATCAAGCAGGACATTGAAACTGCAGAGGATCAGCAGGAAGTCAAGCCCAAGCTGGAAGTGG
GCACTGAGGATTGGCTCAACGGAGCTGAGGCCAGAGAGCTCCTCAAACAGACCATGGACGGATTCCTGTT
GGTCTTGTCCCACGAGGGAGACATCACCTACGTGTCCGAAAACGTAGTCGAGTATCTGGGCATCACCAAG
GATTGACACACTTGGCCAACAGATCTGGGAGTACTCGCACCAGTGCGATCACGCCGAGATCAAGGAGGCT
CTCAGCCTAAAGCGTGAACTTGCCCAGAAGGTCAAGGATGAGCCGCAGCAGAACTCTGGAGTGAGCACCC
ATCACCGGGATCTGTTTGTGCGCTTGAAGTGCACCTTGACCAGTCGTGGACGAAGCATCAACATCAAGAG
CGCTTCGTATAAGGGTCATTCACATCACGGGACACTTGGTGGTCAATGCAAAGGGCGAACGTCTGCTGAT
GGCCATTGGCCGACCAATTCCGCATCCTTCGAACATTGAGATTCCGTTGGGAACTAGCACCTTCCTTACC
AAACACTCCCTGGATATGCGTTTCACCTATGTGGATGACAAAGTGAGATACATACAGAATTAAAGACGCA
AATCCCATTTTAAAAACCATAACTGCTTAGGTTTCTTTTTGAGGGATTGACTAATTGCTCTGCCAAAAGT
GGCACTTAACTATCATTTACTTACTCATAATGAACAAGCACAAATTAATAGATTACGTAAACATATTAAG
CAGCCTAGAAAAGCATCAAAATGGGAACCCGTCTAGAGGATAGTAACATTAATTCTCATTTGTTCCCTGC
TTTGATTTCAGGATGCATGACTTACTGGGATATTCGCCCAAGGATCTACTTGACACCTCTTTGTTTTCGT
GCCAGCATGGAGCAGATTCCGAACGCCTGATGGCCACCTTCAAGAGCG
>dme_circ_0007940_junction_seq
GAACGCCTGATGGCCACCTTCAAGAGCGGGAACAATGAAAAACGCAAGGAAAAGTC
>dme_circ_0007941
GTGCTTAAGGAAGAACCTGACGATTTGTCCCACCACCTGGCCTCCACCAATTGCATTCAGCTGGACGAGA
TGACTCCCTTCAGTGACATGTTGGTTGGCCTCATGGGCACCTGCCTGCTGCCCGAGGATATCAATTCCCT
GGACTCGACCACCTGCTCCACGACGGCCAGCGGCCAGCACTACCAGAGCCCcagcagcagcagcacctct
gcacccagcaataccagtagcagcaataatagctatgccaacagtccgctcagcccgcttacacccaatt
ccacggcaaccgctagcaatcccagtcatcagcagcaacagcaacatcacaaccagcagcagcagcagca
acaacagcagcagcatcatccccagcaccacgacaacagcaacagcagcagcaacatcGATCCGCTGTTC
AACTATCGCGAGGAGTCGAATGACACAAGCTGCTCGCAACATCTGCATTCGCCTAGCATCACATCAAAGG
TGAGTTACCTAGACTTTCAAGCATTGATTATCCTTACTGATCGATATGTCCCATCTATTGCAACAGAGTC
CCGAGGACAGCAGCCTCCCATCGTTGTGCAGTCCCAATTCACTAACACAGGAGGATGACTTCTCTTTCGA
GGCCTTTGCCATGCGTGCGCCCTACATCCCGATCGATGATGATATGCCCCTGCTAACGGAGACGGATCTC
ATGTGGTGCCCACCAGAGGATCTGCAGACCATGGTGCCCAAGGAGATCGACGCCATAcagcagcagttgc
aacagttgcagcagcaacatcaccagcaatacgccggcaacactggctaccagcagcagcagcagcagcc
gcaactccagcagcagcaTTTCTCCAACTCCCTGTGCTCCTCGCCAGCTTCAACGGTCTCGTCATTGTCG
CCATCGCCCGTGCAACAGCATCATCAGCAGCAACAGGCAGCGGTCTTCACCAGCGATTCCAGCGAACTGG
CGGCATTGCTGTGCGGATCCGGAAATGGAACACTTTCGATCCTGGCAGGTAGTGGTGTTACGGTTGCGGA
GGAGTgcaacgagcggctgcagcaacatcagcagcagcaacagcagacgagtggcaacgagttcaggact
ttccagcagctgcagcaggagttgcagttgcaggaggaacagcagcagcgtcagcagcagcaacaacagc
agcaacagcagcagcagcaacagcagTTACTCAGCCTAAATATCGAGTGCAAAAAAGAGAAATACGATGT
GCAAATGGGAGGCAGTTTATGTCATCCCATGGAGGATGCATTTGAGAATGACTACAGCAAGGATT
>dme_circ_0007941_junction_seq
GCATTTGAGAATGACTACAGCAAGGATTGTGCTTAAGGAAGAACCTGACGATTTGT
>dme_circ_0007942
GTGCTTAAGGAAGAACCTGACGATTTGTCCCACCACCTGGCCTCCACCAATTGCATTCAGCTGGACGAGA

TGACTCCCTTCAGTGACATGTTGGTTGGCCTCATGGGCACCTGCCTGCTGCCCGAGGATATCAATTCCCT
GGACTCGACCACCTGCTCCACGACGGCCAGCGGCCAGCACTACCAGAGCCCcagcagcagcagcacctct
gcacccagcaataccagtagcagcaataatagctatgccaacagtccgctcagcccgcttacacccaatt
ccacggcaaccgctagcaatcccagtcatcagcagcaacagcaacatcacaaccagcagcagcagcagca
acaacagcagcagcatcatccccagcaccacgacaacagcaacagcagcagcaacatcGATCCGCTGTTC
AACTATCGCGAGGAGTCGAATGACACAAGCTGCTCGCAACATCTGCATTCGCCTAGCATCACATCAAAGG
TGAGTTACCTAGACTTTCAAGCATTGATTATCCTTACTGATCGATATGTCCCATCTATTGCAACAGAGTC
CCGAGGACAGCAGCCTCCCATCGTTGTGCAGTCCCAATTCACTAACACAGGAGGATGACTTCTCTTTCGA
GGCCTTTGCCATGCGTGCGCCCTACATCCCGATCGATGATGATATGCCCCTGCTAACGGAGACGGATCTC
ATGTGGTGCCCACCAGAGGATCTGCAGACCATGGTGCCCAAGGAGATCGACGCCATAcagcagcagttgc
aacagttgcagcagcaacatcaccagcaatacgccggcaacactggctaccagcagcagcagcagcagcc
gcaactccagcagcagcaTTTCTCCAACTCCCTGTGCTCCTCGCCAGCTTCAACGGTCTCGTCATTGTCG
CCATCGCCCGTGCAACAGCATCATCAGCAGCAACAGGCAGCGGTCTTCACCAGCGATTCCAGCGAACTGG
CGGCATTGCTGTGCGGATCCGGAAATGGAACACTTTCGATCCTGGCAGGTAGTGGTGTTACGGTTGCGGA
GGAGTgcaacgagcggctgcagcaacatcagcagcagcaacagcagacgagtggcaacgagttcaggact
ttccagcagctgcagcaggagttgcagttgcaggaggaacagcagcagcgtcagcagcagcaacaacagc
agcaacagcagcagcagcaacagcagTTACTCAGCCTAAATATCGAGTGCAAAAAAGAGAAATACGATGT
GCAAATGGGAGGCAGTTTATGTCATCCCATGGAGGATGCATTTGAGAATGACTACAGCAAGGATTGTAAG
TATCTTGGGTGGCAGTTTTGAGCAGACATGAAATGAAGTATTTTATTAAGAAGTGTCAGATTATGTAGAA
GAACGGTAGAATGGACTGGCAATTGGGGGGTAATTATGGGGTGAACATTTTACGACAATTTTTGTCTTTT
AATAGAATGTGGCATTTGATAGATTTAATAAAGCTGAATAGATTGATTTACGGCCCCTTGGGACCACTCT
CTCTCTCATTCGGCTTTGAAAATATTCCACAATTTCGCCTCCTGCATTTGGAGAGGCCTAATTTTATGGC
AGTTCCGCGGCTAAAAATACCCGTGGAAACTTGCTTTATTTTTTATGATATGTTGCCAACGCACACGCAC
GCACTCCAACGCCTACATGTAAAAATAGAGGAGGCAAACATAAAGAAAAATTCAGAATTTCAGTTGCGTG
TCGCGGAAGATGTCACTTGTGTTAGTTGTGGGTTGGGTTCATTTTCCGTCTGCGGGAACCGCGGGAGAGC
TAATTGATGTGGGTCTGCTCGCCACTCCAGTGGTTTGAATCGATTTGCGTAATATTCACACACTGCTCTC
TTACCTTTGACACATGTCACGGTGCGAGGACTGTGTGTGATTGATGCCTAGGAGCTGACGGAATCACATA
GCCTTGTCAACTGTATTGACTGCAAGTGCTGGGTCTTATGACAGGGTATATCGGGGTATATTGACAGATG
CCTGTAGGCCTCTATGAATTTTCCACTAGCTAATCGTGGCTGATTTATGTCGCACACAGCAGGCGCGAGA
AAACGAAACAGACTGGGGATATGGGCTACTTGTTGTATATGTAGAGTTGTAGATACAAAGATACTGAGTC
AAAGGCAGAACGACATATATATTGTGTAGAATGTGCCCACTTATTTATGTGCAAAATGTTTGAGACAGCC
AAATATTATTGTTTTAAATTAGCACCAATGTCATGGGCAAAGGAATTAATTTTTGGCTGGCATTCGATTC
AATAAATTTGGGGATTAGACGGCATATTTCTAATAAAGATTCTTCGCTTCTTATTAACTTGAATTGCTTC
TATTTTAGCCGCCAATCTGGACTGCTGGGACCTGATTCAAATGCAGGTGGTGGACACGGAACCCGTGAGT
CCCAATGCCGCATCTCCCACTCCCTGCAAGGTGTCTGCCATCCAGTTGCTCcagcaacagcagcaactgc
agcagcagcagcagcagcagcagaacattatcttgaatgcagtgccattgattaccatacagaataacaa
ggaattgatgcaacagcagcagcagcagcagcaacagcaacagcaggagcagctgcagcagcCGGCTATA
AAACTGCTGAATGGAGCCAGCATTGCGCCGGTGAACACCAAGGCCACCATCCGCCTGGTGGAGAGCAAGC
CGCCCACCACGACGCAGTCTCGAATGGCGAAGGTGAACCTCGTGCCGCAGCAGCAGCAGCATGGAAACAA
GCGCCACCTGAACAGCGCGACAGGAGCGGGCAATCCCGTGGAGTCCAAGCGTCTGAAGAGCGGAACCCTC
TGCCTGGACGTGCAGTCCCCTCAGCTCCTGCAGCAGCTAATTGGCAAGGATCCTGCCCAGCAGCAAACCC
AAGCAGCCAAGCGAGCAGGCAGTGAGCGCTGGCAATTGTCGGCGGAGAGTAAACAGCAGAAGCAGCAACA
ACAGCAGTCGAACTCGGTACTAAAGAACCTGCTGGTCAGCGGACGCGAT
>dme_circ_0007942_junction_seq

AAAGAACCTGCTGGTCAGCGGACGCGATGTGCTTAAGGAAGAACCTGACGATTTGT
>dme_circ_0007943
GTTTTTTGCTGGAAGGCTCAAACAAGTTTTTTGGTGATGGTTGTATTCTGGTTTATAACTTTCAAGCAAA
AATGCTGTGACGAGAAAAAAAGTTTTTGAACATGTTTTaaaataaatattaacaaaaaaaaacaagaaaa
caacaaaaaaaTATATAGTAATGTAAAATTAGGTTTAAG
>dme_circ_0007943_junction_seq
TATATAGTAATGTAAAATTAGGTTTAAGGTTTTTTGCTGGAAGGCTCAAACAAGTT
>dme_circ_0007944
GGGGAGATCTCGCCGGTGAATGCGCCCTTGGCCACCTTGTAGGCATTCTGGCCGGCCAGACCCAGCTCGC
AGGGCAGCAGGTTGCGGGCGTACATCAGGTAGATGGCCGGGCAGCCGATGACCACCTCCGTGTTGGGGTC
GAGGGCGGCCGAGCTCAGGGTCTTGGCGATCTCGGCGATGGACTTCTGGTCGCC
>dme_circ_0007944_junction_seq
CGATCTCGGCGATGGACTTCTGGTCGCCGGGGAGATCTCGCCGGTGAATGCGCCCT
>dme_circ_0007945
ATCGGAATCGAAATGAGgatgtatgatgtatgatttatgatgtaGGGAGGACGAAGACGATGTTGATGGG
TAGCCGGGCTGTATTCGTTAGCCCTGCACCCGCGAGTTCAATTTGATTATGAATATGAATTTTAGCCAGT
TTTGTGAGTTACAATGTTTTTCGTTCAAGAAATCTCGCAGGACCAGGAGAGAGAGAGAGG
>dme_circ_0007945_junction_seq
AATCTCGCAGGACCAGGAGAGAGAGAGAGGATCGGAATCGAAATGAGgatgtatgatg
>dme_circ_0007946
CATGTGCCTTTCATCCCCGATTCCCGGCCAGAAACAGGCGACGTCTGTTGGCCACTGCAACGGATCGTGC
AGCCTGGACAGGAATGGCAACCGCAGCGCAGCCATTGTCCCGAATGGAGGAGAGCTGAAGCACAGAGGCC
ATGCCACAGACGCTACATCACACACCCTGGTCAAAGATGACGACAATTTGGGCTACAAGGAGGAGAACGC
GGTCTACAAGGAGCAGCAGCTGCTGCAGCAACAGGACCAGACGCTGAAGGAGGCGGAGCAGGTGGAGGAG
GAAGAGGAGTACCACCCGCAGATCAGATGGCCGGATCTGGGAGCACAGGCATTCCTGCACATCGGTGCCC
TCTACGGACTGTACCAGCTCTTCTACGCGAACTTCTACACATTCCTCTGGGG
>dme_circ_0007946_junction_seq
ACGCGAACTTCTACACATTCCTCTGGGGCATGTGCCTTTCATCCCCGATTCCCGGC
>dme_circ_0007947
ATCATCCTGGTCCGCGAGCTGGAGAAGAAGTTCTCGGGCAAGCACGTCGTCGTGATTGCGGAGCGCAAGA
TCCTGCCCAAGCCCACGCGCAAGGCCCGCAACCCCCTCAAGCAGAAGCGTCCACGCTCCAGGACTCTGAC
CGCTGTGTACGACGCCATCCTTGAGGATCTGGTCTTCCCCGCCGAGATTGTGGGCAAGCGCATCCGCGTC
AAGCTGGACGGCTCCCAGCTGGTCAAGGTGCACCTGGACAAGAACCAGCAGACCACCATTGAACACAAAG
>dme_circ_0007947_junction_seq
AACCAGCAGACCACCATTGAACACAAAGATCATCCTGGTCCGCGAGCTGGAGAAGA
>dme_circ_0007948
TTCTCGGGCAAGCACGTCGTCGTGATTGCGGAGCGCAAGATCCTGCCCAAGCCCACGCGCAAGGCCCGCA
ACCCCCTCAAGCAGAAGCGTCCACGCTCCAGGACTCTGACCGCTGTGTACGACGCCATCCTTGAGGATCT
GGTCTTCCCCGCCGAGATTGTGGGCAAGCGCATCCGCGTCAAGCTGGACGGCTCCCAGCTGG
>dme_circ_0007948_junction_seq
CGCGTCAAGCTGGACGGCTCCCAGCTGGTTCTCGGGCAAGCACGTCGTCGTGATTG
>dme_circ_0007949
GCCGTCCAGCTTGACGCGGATGCGCTTGCCCACAATCTCGGCGGGGAAGACCAGATCCTCAAGGATGGCG
TCGTACACAGCGGTCAGAGTCCTGGAGCGTGGACGCTTCTGCTTGAGGGGGTTGCGGGCCTTGCGCGTGG

GCTTGGGCAGGATCTTGCGCTCCGCAATCACGACGACGTGCTTGCCCGAGAACTTCTTCTCCAGCTCGCG
GACCAGGATGATCTGGATCTTCTGGAACACCTTCTGCTGTGGAATGGGCAC
>dme_circ_0007949_junction_seq
GGAACACCTTCTGCTGTGGAATGGGCACGCCGTCCAGCTTGACGCGGATGCGCTTG
>dme_circ_0007950
GTGTTCCAGAAGATCCAGATCATCCTGGTCCGCGAGCTGGAGAAGAAGTTCTCGGGCAAGCACGTCGTCG
TGATTGCGGAGCGCAAGATCCTGCCCAAGCCCACGCGCAAGGCCCGCAACCCCCTCAAGCAGAAGCG
>dme_circ_0007950_junction_seq
AGGCCCGCAACCCCCTCAAGCAGAAGCGGTGTTCCAGAAGATCCAGATCATCCTGG
>dme_circ_0007951
GGAACGCGTTGGTCAGCACACTCGGGATGCCACAATCCAGGGACTGGGAATCGCTCAACAAGCCGCCAAT
GTCGCCGCAACTGCCCGAGGTTGACCACGATGATTATTTATAATTATTTATTTAAAGATCTATTTATTCT
GTTGCTCCCTGTAAATAAAACAATTTTAAAAATTTAAAGAATTCTATTCAAACTTTGTTTTTTAAAGAGT
TGGAGAAAAGCGAACTCTTGAATTTATACACACATTTTAAATACACTTAAGAGGCATTATTTATACAGGA
TATTACAAATCGCTTCTTTTCCGATTTGGAAAGGCCGAGATTATGTCTTATCTGTTGAAATATAATTCGT
TTCACCTATAAAAGGACCAGTCTTTTAGTTTAAATTATCAGTCGCTTGTCAAATACTGAAACAATTAGAT
TAATTTGTGGATTTTATTTGTCCTCATCCTGACCACTTATTGGCCACAATTGGAAGCTGGCTTCGACGGG
ACATTAGTAAGCTTAGTCATTTTAAAAGATTTCTTTGCATCTAACTATGATTCTAAATCCTCAGAAGGAC
GTTGGTCTATACACCCTAAATGCTACCCTGCAAGTTGCTGAAGTCGCTTCGAAAGCAGCCAATGTGGCAA
TCACTGCCAGGGGATAAACTTAAGTTAGggtattatttataagaaattaaattaatagattttattttat
atattttttgtatattgttattCAAACTGATAATGTAATATACGCTTTTCAAACGATCATTCCAAATCAG
TTGTGGGCTTATCGCAAATGATTTCGTAGTGTTTTTATTTTGATTGATTCAAAGAAGGGGTTTCCTCTCT
GATTCTTAGTCTCCCGCATTGACGAGGTAAAAAATCCCTATGCATATGAAATATGCAAATTTAAAAATCC
CCCAATCCGACAGGTTGGTTTTGATCGGTTTGGATTCCTCTCGTGTACTTTTCAGCCATAAAAATCCCCT
TTCGAGCCTTATCAGGCGCTGAACTTAAGCTGATTCGCCTATAAAAGCTCTCGGCGTTCCTGGTGCAATC
AACAGTCGATCACTTTCCATTGCAACAGCAACATCAGAGCTATAGCTACTCTTGCAAAATCTAAAGTCAA
ATAAAACCACCATGAACTTCTACAACATCTTCGTTTTCGTCGCTCTCATTCTGGCCATCACCATTGGACA
ATCGGAAGCTGGTTGGCTAAAGAAAATTGGCAAGAAAATC
>dme_circ_0007951_junction_seq
TTGGCTAAAGAAAATTGGCAAGAAAATCGGAACGCGTTGGTCAGCACACTCGGGAT
>dme_circ_0007952
GATGTTGCTGAAAGTGGCCAGCGCACTGGTCTGCAGAACGCCGGAGGAGCCGTTGTTGCCACTGCTGCCA
TTGCTGGCGGTGTTGCCGTTGTTGTGGGGCAAACCCGACCCTATGACGCCCACATTGGGATTGGGCGTGA
GTCCGGCGTAGCTGTTGCCGTTG
>dme_circ_0007952_junction_seq
CGTGAGTCCGGCGTAGCTGTTGCCGTTGGATGTTGCTGAAAGTGGCCAGCGCACTG
>dme_circ_0007953
GGGATCGGTACTTTAATAATGCTCTGGAGTCACGCAAAGAGCGTCGAATGCGGAAGGCAGAGCTGCGGGA
ACAGGCTCGTGTGGAGATGAATGCACGGGGAAAGATTCCTACGGATGGGCGCTCCAAAAAGTGTGACCTG
AATAAAGCTCGATTTAT
>dme_circ_0007953_junction_seq
AGTGTGACCTGAATAAAGCTCGATTTATGGGATCGGTACTTTAATAATGCTCTGGA
>dme_circ_0007954
TTATTCGAGGAGGTTTTTCAAATTATTTTTCAAAGAAAATTCCAGACACTGCTGAGAAATATCCGAAATA

ATCTGAAACGAAAGATTACCGCTTATTCTAGGAGCATTCACAAGTGCTTTCGGGGTTGGGGTTTTACATT
TTTCACAAAAATCGTATTCCTTCCGAAGGTCAACACACACAGGGATACAAATACATTTTCAATGTTCACA
GGG
>dme_circ_0007954_junction_seq
TACAAATACATTTTCAATGTTCACAGGGTTATTCGAGGAGGTTTTTCAAATTATTT
>dme_circ_0007955
GATAACAGATAACTCGACTGGCTGATAATTTTGATGCGTGACTGGGCGCTGCGCCTTCTCTATCTCCACC
TCCTTACAGGGTCTTGTCGAACAGGAACTCGCCCAGTTCGCCGTTGGTGTCCATCATCTTCTTGAGAGTG
GTCAGCTTGCCGGCGAGCTCGCGCTGTCCGTGGAGCTGCTCCTCCAGATAGACACCGGTCAGGTAGTCCA
CCAGGTGGTAGTGGTTGTAGGGCTTGTTCTCGCAGGTCTGGATCAGCTTGCGGATGGACTTGGTCACCTT
GATCTCCAGGTCGAGGGCGTCGGACAGGGCGGCGGCACCATCGGTCCACTCCTGCTTGGCCACAGTCTGT
GGGGGATTCGCCAGGATTAGCTGGGCTTTTGGGTTTTCTAGGCGAGTCGTCGACTTACCGGCACATTGAT
CAGATCGCTGACTCCCTCGGTCAGTTGACCGCGCATGGACAGGTACTCCACCAGCTTGGATCCGTGCTCA
CGTTCCTCCTTGGCGGCCTTGAAGAAGTGCTCGGCGAATCCAGGGCGGTTGACGGTGTCGCGGGAGAAGT
AGGCGCCCATGGCCAAGTACTGGTAGGAGGCGTTGATCTCCTCCTGGATCTGATTGCGCATGCCCTTTAT
GCAGGCATCCTTCATGTCCACCCAGTCCTTG
>dme_circ_0007955_junction_seq
GGCATCCTTCATGTCCACCCAGTCCTTGGATAACAGATAACTCGACTGGCTGATAA
>dme_circ_0007956
ATTACCAAGGACTGGGTGGACATGAAGGATGCCTGCATAAAGGGCATGCGCAATCAGATCCAGGAGGAGA
TCAACGCCTCCTACCAGTACTTGGCCATGGGCGCCTACTTCTCCCGCGACACCGTCAACCGCCCTGGATT
CGCCGAGCACTTCTTCAAGGCCGCCAAGGAGGAACGTGAGCACGGATCCAAGCTGGTGGAGTACCTGTCC
ATGCGCGGTCAACTGACCGAGGGAGTCAGCGATCTGATCAATGTGCCGGTAAGTCGACGACTCGCCTAGA
AAACCCAAAAGCCCAGCTAATCCTGGCGAATCCCCCACAGACTGTGGCCAAGCAGGAGTGGACCGATGGT
GCCGCCGCCCTGTCCGACGCCCTCGACCTGGAGATCAAGGTGACCAAGTCCATCCGCAAGCTGATCCAGA
CCTGCGAGAACAAGCCCTACAACCACTACCACCTGGTGGACTACCTGACCGGTGTCTATCTGGAGGAGCA
GCTCCACGGACAGCGCGAGCTCGCCGGCAAGCTGACCACTCTCAAGAAGATGATGGACACCAACGGCGAA
CTGGGCGAGTTCCTGTTCGACAAGACCCTGTAAGGAGGTGGAGATAGAGAAGGCGCAGCGCCCAGTCACG
CATCAAAATTATCAGCCAGTCGAGTTATCTG
>dme_circ_0007956_junction_seq
CAAAATTATCAGCCAGTCGAGTTATCTGATTACCAAGGACTGGGTGGACATGAAGG
>dme_circ_0007957
GATAACTCGACTGGCTGATAATTTTGATGCGTGACTGGGCGCTGCGCCTTCTCTATCTCCACCTCCTTAC
AGGGTCTTGTCGAACAGGAACTCGCCCAGTTCGCCGTTGGTGTCCATCATCTTCTTGAGAGTGGTCAGCT
TGCCGGCGAGCTCGCGCT
>dme_circ_0007957_junction_seq
GTGGTCAGCTTGCCGGCGAGCTCGCGCTGATAACTCGACTGGCTGATAATTTTGAT
>dme_circ_0007958
GTGACCAAGTCCATCCGCAAGCTGATCCAGACCTGCGAGAACAAGCCCTACAACCACTACCACCTGGTGG
ACTACCTGACCGGTGTCTATCTGGAGGAGCAGCTCCACGGACAGCGCGAGCTCGCCGGCAAGCTGACCAC
TCTCAAGAAGATGATGGACACCAACGGCGAACTGGGCGAGTTCCTGTTCGACAAGACCCTG
>dme_circ_0007958_junction_seq
GGGCGAGTTCCTGTTCGACAAGACCCTGGTGACCAAGTCCATCCGCAAGCTGATCC
>dme_circ_0007959

TACTTGGCCATGGGCGCCTACTTCTCCCGCGACACCGTCAACCGCCCTGGATTCGCCGAGCACTTCTTCA
AGGCCGCCAAGGAGGAACGTGAGCACGGATCCAAGCTGGTGGAGTACCTGTCCATGCGCGGTCAACTGAC
CGAGGGAGTCAGCGATCTGATCAATGTGCCGGTAAGTCGACGACTCGCCTAGAAAACCCAAAAGCCCAGC
TAATCCTGGCGAATCCCCCACAGACTGTGGCCAAGCAGGAGTGGACCGATGGTGCCGCCGCCCTGTCCGA
CGCCCTCGACCTGGAGATCAAGGTGACCAAGTCCATCCGCAAGCTGATCCAGACCTGCGAGAACAAGCCC
TACAACCACTACCACCTGGTGGACTACCTGACCGGTG
>dme_circ_0007959_junction_seq
TACCACCTGGTGGACTACCTGACCGGTGTACTTGGCCATGGGCGCCTACTTCTCCC
>dme_circ_0007960
GAAACAAATTTATAAGGGAATTATGAGAGCAATTTTGAGGAGACAGTGTAGGAGCGTGTTAGAAAAAGAA
GGAGGAAAAGGAGGTGGTCTCCACTCCTTACTGCTTCTGCAGATACTCGTCGAACAGGTAGACGGACAGG
GATGGGTCGGGGACCTTCATCAGCTTGGCGAGGTCGTTGGCATAGCCGGACAGCTTGCGCACGCTCTCGG
CCTGCTTGCCCAGGAAGTTCTCCTCGAAGTAGTGGGCCAGCTCGGGATCCCTCTCGGCGTCGGTGGCGTG
GGTGGCACGGGAGTGCACGTGAGTGGCTCCGGTGGCCAGCTGCTTCTCGGTGTCCAGAGCCAGAGCCAGG
GAGTGCAGTTCGTCGACCTCAAGAGTGCCGCGCTTGGTGCTCACGGAGCCGGAAGACTCGTGGCGGGTGT
TGAAGTCAACGATTCCTCCGCGACGGGTCACCTGCTTGATCAGGGCAATGCTGTCCTCGAAGGAGCGGTC
CGAGAGTCCCTGGTACAGCTTCTGGAAGCCGGGGCGG
>dme_circ_0007960_junction_seq
CTGGTACAGCTTCTGGAAGCCGGGGCGGGAAACAAATTTATAAGGGAATTATGAGA
>dme_circ_0007961
TTCGTCGACCTCAAGAGTGCCGCGCTTGGTGCTCACGGAGCCGGAAGACTCGTGGCGGGTGTTGAAGTCA
ACGATTCCTCCGCGACGGGTCACCTGCTTGATCAGGGCAATGCTGTCCTCGAAGGAGCGGTCCGAGAGTC
CCTGG
>dme_circ_0007961_junction_seq
CTCGAAGGAGCGGTCCGAGAGTCCCTGGTTCGTCGACCTCAAGAGTGCCGCGCTTG
>dme_circ_0007962
AGCGACTACAACTCACGGAGCAGCTCCAACTCGGAGCAGAGCGAGAAGGTGTTCTTCTTCAAGTCCGCCC
ACAAGATCCTTGCCAGCACAAACAGCGTCAAGGTGGTGATCCGGGCCACGGAAAGGTGTCCTCCAGTGCT
GAGGAAAGTTCAGATGTGGGGACTGCCCGCTCGTTCGCTGGACAAAGCCGATCGCGAGCTGGTCAAGACC
ATCTGGAGCGAGATAAGCGATCCCTACGGCCAGAGGGACGCGGCCCAAACGCCCACCGGGCAGAGATCAC
CACCGCGGAATGTTCCAGAGCTCAGGGACCAGTCGACCCTCTCTATTCCTGAAGAGTTCCTCGACTCCAT
CACCTGGGAGCTAATGGGTAAATATCAGAAAATCCTAGCTTTTTATGTTTAAGTAGTAGTACAACTGAAT
ACATACCCATGTCAATTCATGAAATCAATCGTTTTGCACGGTTTTATTATTCATAATTCTTTATAATCTT
TATGAATCAATTATCATGTCTTTATCAAAACCAAACAAGGTAAATATCGGAAGTCATGTAAAGAATTCAT
TTTCTTCATAATGAGAATTGCATTATAATGTCCGAAACGCAACAACACTTAAATATAAAGATAATAGGAT
GCTCTGTGAACTGTTTACTTTGTAGCTCTCCTTATCAATTCTCCCATTGCCTCTTCAGAATCTTCCCCAC
CGTGCTGCCATCTGGAAAAGTGGTGGACCAGTCCACCATCGACAAGCACGCCGAGGAGGAGGCCAAGTGG
GGTCGCCAGCCCTCGGATCCGTTTACAGGCCTGGAGTTCAATGCGCAACGCAAGGCGATTCTCCACCTGG
CCCTAAAAGCGCGCATCGAGAAGTTCCTCATGGAGAATTCGGAGCACTTCAAGGCGGTGCCCCGTTCGCT
GGGCACCTCTCGCCAGCGTCGTCGTCACGCCTCGCAGTTCGCCAGCAGCACCTGTCAGCCGGGACAGATT
AGTCCGGCTGCCGGAACCTATTCGGCACTGAGCAACGCCTACAAGCAGCAGCAGCGGAAGAGCAGGGCCA
CTGCCACGGCCACATCCTCATCGGCGGCATCCTTCGGCGGATCTCCGCACTCGCCGCCGGCGGCCAAAAG
AGCAAGGCACAGCCAACACGGTAGCGCCTGTGCCACGGTGACCTCGGCCACAACCTCCATCGATCAGGCC
ATTCAACAGGCGCTGCAGAAGGTGACGCGCTTTAGCCAATTGCCATCGAACTCCAGCGCAGGCTCGGGAT

CCTCAAGTGCTCCGCCCGCCAGCTGCATCAACTGCCGGAGTGCGCAGTTCGGCTACGAAATCCGCACCTG
CCGACATCTGGTCTGCCGGGAGTGCCTGGTGCTCCTCTCGCAGGATCAGCAGTGCGTGTGCAAAGTATTC
TTCCGCTGCGCCGACGTCGAGCGCTACCACAAGCTGGATCCTTAAGAGCTTCCCCG
>dme_circ_0007962_junction_seq
ACAAGCTGGATCCTTAAGAGCTTCCCCGAGCGACTACAACTCACGGAGCAGCTCCA
>dme_circ_0007963
ATGTGGGGACTGCCCGCTCGTTCGCTGGACAAAGCCGATCGCGAGCTGGTCAAGACCATCTGGAGCGAGA
TAAGCGATCCCTACGGCCAGAGGGACGCGGCCCAAACGCCCACCGGGCAGAGATCACCACCGCGGAATGT
TCCAGAGCTCAGGGACCAGTCGACCCTCTCTATTCCTGAAGAGTTCCTCGACTCCATCACCTGGGAGCTA
ATGG
>dme_circ_0007963_junction_seq
CTCGACTCCATCACCTGGGAGCTAATGGATGTGGGGACTGCCCGCTCGTTCGCTGG
>dme_circ_0007964
GTTTGTGGTCCAAGTGGTTGCCCGCCGAGGCGGAGGAGACTGTTCTTCGAGGAGGATATTACACTGCATC
CCCGAGCAAAGGACACAGGATTGTGGCCCTGAACAGCATGGACTGCTACCTGTACAACTGGTGGTT
>dme_circ_0007964_junction_seq
TGGACTGCTACCTGTACAACTGGTGGTTGTTTGTGGTCCAAGTGGTTGCCCGCCGA
>dme_circ_0007965
CTTGGGATCGGAGTTGGTGAACTTGTTCCTCCAGAACTTCCTTAGCAAGTCGGGCTTCTCAGCCATTTCC
AACAGCAAGCGATCGATTCCCGCGGGACTGGTGTCCTCGGTGTACTCCTTGGTGAACTGGTACTCCAGCT
CCCACTTGGGCTGCTCGTCGGGCGTCAGG
>dme_circ_0007965_junction_seq
CCACTTGGGCTGCTCGTCGGGCGTCAGGCTTGGGATCGGAGTTGGTGAACTTGTTC
>dme_circ_0007966
GCAAAATGGACGAGTACCAGAGGGAGCACTCGGATGCCTCCCATGATATAACCGCCTATGTATCCAATCC
AATCAATGCGTACCTGCTGACTAAGCGGCTAACCACCGATTGGCGTCAGGTGGAGAACCTCATGGAGCAC
GACGTGGGCACGGATTTCCTGCAGAACATCACCCAGTATCGCAGCCTCCTGAAGTTCCCCTCCGACGAGG
ACCTCAATGGCGCGGCGGTGGCCTTGCTCCGCCTGCAAGACACCTACCAACTGGACACTTCGAGTGTGGC
GAGGGGCAAGCTGAATGGCATTCAGTACAGGCACGGAAATGTCCTCGGATGACTGCTTCGAGTTGGGTAG
ACAATCGTACGTGAACCACGACTACTACCACACGGTGCTCTGGATGAACGAGGCGATGGCCCGCATGCTG
GAGGAGCCGACCAACCACACCCAGAGCTTCACCAAGGCCGACATCCTCGAGTACCTGGCCTTCTCCACCT
ACAAGGAGG
>dme_circ_0007966_junction_seq
TACCTGGCCTTCTCCACCTACAAGGAGGGCAAAATGGACGAGTACCAGAGGGAGCA
>dme_circ_0007967
GGCAACATAGAGAGCGCGCTGACAATGACCAACGAGCTGCTCCAACTGCTGCCCCACCACGAGAGAGCCA
ATGGAAACAAGCGGTTCTACGAGAAGGAGATTGCCCAGCAGCTGCAGTTGCGGAAAATGAAGGGCGACGA
TGGCACCGATGAAATGCCCAAGTCCGATTTGGCCCGTGGCCAAGAGTGACCCGGCCATCTTTGATATGAC
AGAACGAAGGGCTTACGAGATGTTGTGCAGAGGCGAACTAAAGCCATCACCATCGGATCTGCGATCCCTG
CGATGTCGCTATGTCACCAACAGAGTGCCCTTCCTGCGTCTGGGTCCCCTGAAATTGGAGGAGGTTCACG
CGGATCCGTACATTGTGATCTATCACGATGCCATGTACGACAGCGAGATCGATCTGATCAAGCGGATGGC
CCGCCCGCGATTCCGCAGGGCCACGGTCCAGAACTCCGTGACCGGAGCCTTGGAGACGGCGAACTACAGG
ATCAGCAAGTCCGCCTGGCTAAAGACCCAGGAGGATCGGGTGATTGAGACTGTAGTGCAGCGCACTGCAG
ACATGACTGGCTTGGATATGGACTCCGCCGAGGAGCTGCAGGTGGTAAACTACGGCATTGGAGGTCATTA

CGAGCCGCACTTTGATTTTGCCAGGGTGAGTTTCCGAGTTTCCTCCATTAGTTACATACCTTAAATAAAG
TAGAAAAACCTTATTAAAATGCGTTTTAATCCTTTCTAGAAAGAGGAGCAGCGCGCCTTTGAGGGTCTCA
ATCTGGGCAACCGCATTGCCACCGTTCTATTCTAT
>dme_circ_0007967_junction_seq
CAACCGCATTGCCACCGTTCTATTCTATGGCAACATAGAGAGCGCGCTGACAATGA
>dme_circ_0007968
GTACAAGGCCGCCCCGCCCTCCGAGGCCCGCCGGTGGGCGGGATTGGGGGATGCTGCGGTCAGGCCACCG
CTGCTGCATCCTGCACCGGTTCCAGGTCCCTGGGCAGCTGCTCCACCGGTCTCGGGCAGCATGCGATCCT
TGGTCTTGG
>dme_circ_0007968_junction_seq
TCGGGCAGCATGCGATCCTTGGTCTTGGGTACAAGGCCGCCCCGCCCTCCGAGGCC
>dme_circ_0007969
gaaccaaaaaaGTGCAACACCCCACAGAACATGCAACCCAAATCAAACAAATAATAATTCAACACACGCC
ACAAAAAACAACAACATCGACATCAGTGATACTAGAAACAATTCAATGAAAACAAATGATTTTCAGCGGC
GTTCTCGCCTGCT
>dme_circ_0007969_junction_seq
ATGATTTTCAGCGGCGTTCTCGCCTGCTgaaccaaaaaaGTGCAACACCCCACAGA
>dme_circ_0007970
GACTTCTGCCACCACAAAGACAACCACGCTCACCACCCCCGCCAAACTGTATGGCAAGGATCTGAGTGAG
TACGATGATGTGGATGTGGAGAGCCTGCTGGCCCAGCTCTCCCCCGAGGAGATAACCATACTGGCCAAAG
AAGTGGATCCCGATGGACAACTTCCTGCCGCCAGACCAGCGCAATAGCTATGAGTGCACCAAGGAAGCCA
CCGGGCCTCTCAATCGCAAGCAGCTGATCGAGCACATCAATAAGCAGGCCATCGAGACCCCGGATCAGCC
GGAGTTCGAGCCGTTCGTTCAGGGCAAGGTTCGTGGAAAGAAGTGGGTCCCACCGCCAAGGGATGCCCGC
GACATCGAGGCCGAAGAACAAATCGCCATTGACATGGGCGAGGAGTACGAACACGCCCTTAACGATGCCA
CGCAGGAGGAGATCATCGATCTGGCCGCCATCCTCGGCTTCCACTCGATGATGAATCAGGACCAGTACCA
CGCCTCCTTGCTCAACAAGGGTCAGCCCGTGGGCCTGGGATGGGATGGCATTACGAAATCCACTCAGCAG
AAGCTCTTCCCTATGGATCCGCCCAACAACACAGATGTGGAGGAATCCATCAAGCGGGTCAAGGACGATG
ACTCCAAGCTGATTGACCTCAACTTGAACAACATTAAGGAACATCTCGGACGAGAAACTCGAGCAACTGT
TTGCCGCACTGCCTCAGAACGAACATTTGGAAGTCCTCTCGCTAACAAATGTGGGCCTCACCGACAAGAC
AGCCCTCCTGTTAGCCGCGGCCATAGAGAAGAGCAAAACCCTGAGAGTATTAAATGTCGAGACCAACTTT
ATCAGTCCGCCCGTGATTGTTAAGTTGGTGCAAGCCCTGCTCAAGTGCCACACCATCGAGGAGTTTAGGG
CCTCCAATCAGGCGGTCTGCGGTTCTGGGCAACAAGATCGAGATGGAAATCACCGACCTGGTGGAGAAGA
ACTCGTCGCTGCTGCGCCTTGGCTTGCACCTGGAGTTCAACGACGCCCGCCACCGAGTGGCCGCTCACCT
GCAGCGCAACATCGACAGAA
>dme_circ_0007970_junction_seq
GCTCACCTGCAGCGCAACATCGACAGAAGACTTCTGCCACCACAAAGACAACCACG
>dme_circ_0007971
GATCCTTGTTCAGACCCTCGAAGACCTGCGCCACTCGCGGCTGCAGGTTGTAGAAGAACAGCGGCTGCTC
CCGCAGCTTAAAGTCCATCACCATGGTGGATATCACCTTGGCGGCCGTGAAGTCCGCTCCATAGATGTGG
GTGCAATCGATGACCACCGGCAGTGTGGACTTCTGACCCTGCTT
>dme_circ_0007971_junction_seq
CCGGCAGTGTGGACTTCTGACCCTGCTTGATCCTTGTTCAGACCCTCGAAGACCTG
>dme_circ_0007972
CACTGCTGTCCTTTCAAATCGCTGGAGGCAGTTGGCAGATAGCCACCTTGCTAACCTTCCTCACAGGTCT

CATTGAGATCCTGATGGGAGTTTTCCGGCTGGGATTTCTCATCGATTTCGTTTCTGGTCCCGTGGGAGCG
GGATTCACGAGTGCCGTGTCCTTGATCATCTTCAGCTCCCAGATGAAGGACTTCTTGGGCATCAAAACAA
GTGGCAACACCTTCCTGCAAGTGTGGATTAGCATTGTGAACGATATACACAACATCAGCTGGCCGGACTT
CATCTTGGGAATCGTCTGCATTACTCTGCTCCTGAGCCTGAGAGCCCTGGCCAGTTGTACCCTGGGTCCC
AAGGAGGGAAAAACCACAGCGCAAAAGCTTCTGACCGGCATTTTCTGGACCATCGGAACTGCCCGCAATG
CCCTGCTGGTCTGTGGAACTGCTGGCCTGGGTTACTGGTTGTTTGTCAACGGCAAGGAGAATCTCGTGAA
GACAGTTGGCTTTGTGCCCAAGGGATTGCCCTCCTTTCAGCCACCACCCTTCCACATGGATGCCGTTGTC
AACGAAACCACAGGAGAAGTCCTTCAGGAGGCCCAGAGCTTCTGGGACATGGTCAGCACCCTGGGATCTG
GGCTCATTGTGGTTCCATTGATTGCTCTTCTGGAAACTATGGCCGTTGTTCAGGCTTTCGGGTGAGTTGT
CCGCTTATTTGATATCGTCATTTTCATGAGTTGGATCTCGTTCTTCCCCAACGAGTTCAAGAATCTTCAA
AATTCAAGTAGGATATGAATCATCAGTAATTGGTTTATATTTAAAGCTAATTTTAAATCGGATTGACGAA
AGCAACGAGTTTTTGTttataacccttatacccttataacccttGTTGTCCCAAATTAGTGTCAACTTTT
GAATGACACGTGCAAATATTAAAATAGGTCAGTCGCTTTATTCCTTGGGGAAGAACATATTGTTTTGCTT
TCGAATTTCATTTGACTGGATTGTAATATTTTCCCCGTTTGGTTTTTTCCTTTTAGCCTGATGGCAAACC
CACAGATGCCACGCAGGAGCTGATAGCCTCTGGAGTGTGCAACGTGGCCAACTCCTTTGTCCAGGGTCTT
CGGAGCAATGGCGGCATCGCCAGGGGAGCCATCTTAAATGCCAGTGGAGTGAGGACCCAGCTGTCCAATC
TCTACACCAGCGTGATAGTGATTATTGCCCTG
>dme_circ_0007972_junction_seq
CACCAGCGTGATAGTGATTATTGCCCTGCACTGCTGTCCTTTCAAATCGCTGGAGG
>dme_circ_0007973
GAAACGAAATCGATGAGAAATCCCAGCCGGAAAACTCCCATCAGGATCTCAATGAGACCTGTGAGGAAGG
TTAGCAAGGTGGCTATCTGCCAACTGCCTCCAGCGATTTGAAAGGACAGCAGTGCTGATATGGCCGTAGG
TCCAATAGGCACATCCTTACTGGACCCAATAAATAT
>dme_circ_0007973_junction_seq
GCACATCCTTACTGGACCCAATAAATATGAAACGAAATCGATGAGAAATCCCAGCC
>dme_circ_0007974
ATCGTTGTAACCAATACCATTCCACACGAGATCCAAAAGCTGCAGTGCCATAAGATCAAGACGATCGACA
TCTCCATTCTGATCGCTGAGGCCATTCGCCGCATTCACAACAAGGAGTCCATGTCCTATCTGTTCCGCAA
CGTAACGCTGGAGGACTAGGGGACCCACTCACTCATATAACCACTCACAAGTCACAAAAATAGTGTTGCA
ACCGACCCGTTTTGGG
>dme_circ_0007974_junction_seq
AATAGTGTTGCAACCGACCCGTTTTGGGATCGTTGTAACCAATACCATTCCACACG
>dme_circ_0007975
ATTAGGCAGCGTACGGCCACCAAAGCGTTGCAGGAGCGACATGGATAACACCTCCACCTCGGACATTGTC
ATCATCAATGGCAACTCGCATCCGGATCTGGCCAATATGGTGGCCGAGCGTATGGGAATCAAGAATGGGG
GCTGCTCCGTGTTCCACAAGTCCAATAGGGAGACTATTGTGGAGATCAGTGATTCCGTTAGAGGCAAGGA
CATCTACATCATCCAAACGGGCACAAAGGGATGCCAACAATAATATCATGGAGCTTCTGATTATGGCCTA
CGCCTGCAAGACTTCCTCGGCTCGTTCGATCGTGGGAGTGATTCCATATTTGCCCTATTCCAAGCAGTGC
AAGATGCGCAAGCGCGGCTGCATTGTGTCCAAGCTGCTGGCCAAGATGATGTGCACCTCTGGCCTGACGC
ACATCATCACTATGGATCTGCACCAGAAGGAAATCCAGGGCTTCTTTGACATACCCGTGGACAATCTGAG
GGCGTCGCCCTTTCTGCTCCAATATATTCAAGAGAGCGATTCCCGACTATCGAAATTCGGTGATTGTAGC
TCGCAATCCGGGCGTGGCCAAGAAGGCCAACTCCTACGCCGAGCGACTGCGTCTTGGCCTGGCCGTCATC
CATGGCGAACAGAAGGAAACGGATGCCGATGAGGTCGACGGTCGGTACTCCCCTCCACCGACCAGG
>dme_circ_0007975_junction_seq

CGGTCGGTACTCCCCTCCACCGACCAGGATTAGGCAGCGTACGGCCACCAAAGCGT

>dme_circ_0007976

GAGCATAACAAGTCTGCGTTTGGAAAATGACAAAGCCCGCAGTTCCACGGAGGAGTACTTATCCAATCCC
CTGAACTCCTTTTCCCTCATTCGTCGCATGAATCGCGACTGGATTATTTGGCAACTCTACATGGACGATC
CGGTGGGTATTTCTCAGGTGGAGCGAATCCAGGAGTTGAGGGAACACATGCCCACCCACACGGATGTGGA
GGAGGCTGTCACTGCCTTGGATCGCATTCAAAGCACCTATGGCCTTAAAGTGCCGGAAATCTCCCATGGA
TTCCTCAATGGCAAACAGTACAAGTGTGAGTCTCACTGTATTGGATACCTATGCCATGGGACAAATCTTA
TTTGACCAGAAAAACTATTTGGCAGCTGCTAGCTGGATCTATCAGTCCGTTGTATTGATGGAGGCCTTTT
CGATGGCTGCTCCGCTTGAGATTTCCAAAAATGAAGTGCGGATGGTATATGCTGAAACCCTGTTAAAGCT
TAGACCAGCACGCCGATGCCCTGAAAGTTGTCAACATTGCTCTGACTGATAACCCGCACGATATTAAACT
GTTGCTGAAGAAGTCGGAAATCGAAACGATGATAAGGACGGGCACCAATAATGCGCCTCCGGTAAAGGGT
TCAGGCAACAGGCGTGCCAACTGCCTACCAGATCGGCTGTCGAGGTCAGTTCCCGCCGTCCGCGGATAGC
AAGCTTTACTGTCTGTACAACAGAACCACTTCGCCTTTTCTGATCCTTGCTCCGCTCAAGATGGAACTGG
TGGGCCTGGATCCTTACATGGTGCTCTACCACGATGTGCTCTCACCCAAGGAAATCAAAGAGCTCCAAGG
TATGGCCACTCCTGGTCTAAAAAGGGCCACCGTCTATCAGGCCTCCTCCGGTCGGAACGAGGTGGTGAAG
ACCAGGACCTCCAAAGTGGCCTGGTTCCCCGATGGCTATAACCCGCTCACTGTTCGACTTAATGCCCGCA
TCTCCGACATGACCGGGTTCAATCTCTATGGCTCCGAGATGCTGCAGCTGATGAACTACGGCTTGGGAGG
CCACTACGACCAGCACTACGACTTCTTTAACAAGACTGCTGACGGATGTAGAACAAGGTGGAGCCACCGT
GTTTCCAAACATTAGAAAGGCCGTCTTCCCACAACGCGGATCTGTGGTAATGTGGTACAATCTCAAGGAC
AACGGTCAAATTGATACTCAAACTCTTCATGCCGCTTGCCCCGTTATAGTGGGTTCCAAATGGGGTGTGC
AACAAGTGGATTCGAGAGCGCGAACAGATATTCAGCAGACCTTGCCTCAAAAAGCGAATGTGATTCTCAG
CGAAACTGTTTTTAGACAAGTGATACTATACGACCACTATAAAACTCAATAAAGTCATATTAGAATCGAT
AATGCTGTCAATTAAACATTCCCAGCGATAAGAACAATTGCAAATCAAGTCGCGACATTCCGAAGAGGAA
TAGGTGTTTTCCCCAGAGTCGTTCATCAAAATCAAGAAATGGAAATTCCTGCTAAATATCGTTTAATGGT
CGTGAACCAACCATTAATTGACATTTTGCATTAAAAGCACAAGATAAGAAAGCGGGTTTCTATTTTTAAA
AACATGAAAATAAATGCACATCGATCTAGATCAACTCCTAGTAATACGTGCTGAAAACGAGAAAGGAAGA
AGGAATGCTATATCCTATTTGTCCAATCCGCTGAATGGCTTTTCAATCATCCGACGATTGCATCAGGATT
GGATCAACTGGCGAAAGTACATGGAGCAACCAGTGGGGATATGGCAATTGAAAGCATTCTACAGCTGGAA
GGATGAGCTTCCCACCGAACGTGACTTATGGGATGCCTGCGAGGGCATAGCAAGAATTCAGAGTACATAT
GACCTGAAAGTTGGCGATTTTATTAATGGGAACATTAATGGAAAACAGTACAAGTGACAGCATGAGTACT
GCAGACATTCTTTCGGTAGGAGCTTACCTTTTCATGAAAAATAGACCATCGGATGCTATTCAATAGCTGC
AGGAAGTGCCACAGCGCCTCCAAGAGGAACTTTTAATCCAGCCAAGGCACCTGCCAATCAAAGAAGTGGA
TGCTCTAAGATTACTTGCCGAGGCCCAAATAAAAGATCGAAAACTATTCAGAAGCCTTGCCTCTGTTGCA
CAATTGCTTGAAGCTGCAGCCCCACGATGCGCGTGTACTTCGACTGTGGAAAAAAACAACGGACTTCATT
GAGAATCAACCAGATCAATCCCCTACAGAAAATAAGAAGCGGAATGTTCCAATCGCAAATGCGTTCAAAT
TAAGCTGCAACGGACCACTCGAGAGCTCCACTAGACTGCACTGTTTCTATAACTTTACCACGACTCCATT
CCTTCGCTTAGCTCCACTCAAAACGGAGCAAATCGGATTGGATCCATATGTGGTGCTTTATCACGAAGTG
CTCTCTGCTCGAGAAATATCTATGTTGATAGGCAAGGCTGCTCAGAATATGAAAAACACCAAAATTCATA
AGGAACGAGCAGTTCCAAAAAAGAACAGGGGAAGAACTGCCAAGGGTTTTTGGTTGAAAAAGGAGAGTAA
CGAGCTGACGAAGAGAATTACTAGGCGCATCATGGACATGACTGGCTTTGACCTGGCTGATTCTGAAGGA
TTTCAGGGTGATTAATTACGGCATAGGTGGACACTATTTTTTGCATATGGATTACTTTGATTTTGCGTCT
AGTAATCACACTGATACACGTAGTCGCTACTCCATCGATTTGGGCGATCGTATTGCTACTGTGCTTTTCT
ATGCTGACTGATGTCGAACAAGGTGGAGCCACCGTCTTTGGAGACGTGGGATATTATGTGTCTCCCCAGG
CTGGAACCGCAATCTTCTGGTACAATTTGGATACGGATGGAAATGGTGATCCCCGCACAAGGCACGCCGC

CTGTCCAGTGATTGTGGGTTCCAAATGGGGTGATGACCGAGTGGATACGCGAAAAGCGGCAGATCTTCAT
CAGACCCTGCCTGCCAAAAGCAAAGGGAACCTCTACGAAATCATAGACAAACAAATTTCCGGCATAATCA
CTCTGCACACTTAACAAAAACCCACGGAGCTCAGCTTAAATGCCTTTTTAAAATGCCAACACGGAAAAAT
AAAGTGCTGAGTGGTTGATAGTCCCTGAAAGTCGAGGAGGGGGTACGGAAGGGCATCTGCTTCAGCACCA
AGG
>dme_circ_0007976_junction_seq
CGGAAGGGCATCTGCTTCAGCACCAAGGGAGCATAACAAGTCTGCGTTTGGAAAAT
>dme_circ_0007977
CTCCGACTATTTTGGCTCTGGCAGTTGGCTCCGGCACGTAGACCCACATAGAACTTCGACCAGAAAGGTA
ACCCACGATGCGCTCTTCGAAGTGGCTGCTTCCGGTGAGGGTGCTGCTCACCTACCAGGTGCTGCTTCTA
CTCCTCCGCCAGGCGAAAGGCGAGGAGAGCCACTGCACCTCGGTGGCAGGAATGGTCAAGCTGCTCGATT
TGGAGGCGCAGCTGATCGACAACCTAGAGGATTACGCTGTCGAGCTGGAGAAGAAACTGCAGACCGTAAG
AAGGGAGCATAACAAGTCTGCGTTTGGAAAATGACAAAGCCCGCAGTTCCACGGAGGAGTACTTATCCAA
TCCCCTGAACTCCTTTTCCCTCATTCGTCGCATGAATCGCGACTGGATTATTTGGCAACTCTACATGGAC
GATCCGGTGGGTATTTCTCAGGTGGAGCGAATCCAGGAGTTGAGGGAACACATGCCCACCCACACGGATG
TGGAGGAGGCTGTCACTGCCTTGGATCGCATTCAAAGCACCTATGGCCTTAAAGTGCCGGAAATCTCCCA
TGGATTCCTCAATGGCAAACAGTACAAGTGTGAGTCTCACTGTATTGGATACCTATGCCATGGGACAAAT
CTTATTTGACCAGAAAAACTATTTGGCAGCTGCTAGCTGGATCTATCAGTCCGTTGTATTGATGGAGGCC
TTTTCGATGGCTGCTCCGCTTGAGATTTCCAAAAATGAAGTGCGGATGGTATATGCTGAAACCCTGTTAA
AGCTTAGACCAGCACGCCGATGCCCTGAAAGTTGTCAACATTGCTCTGACTGATAACCCGCACGATATTA
AACTGTTGCTGAAGAAGTCGGAAATCGAAACGATGATAAGGACGGGCACCAATAATGCGCCTCCGGTAAA
GGGTTCAGGCAACAGGCGTGCCAACTGCCTACCAGATCGGCTGTCGAGGTCAGTTCCCGCCGTCCGCGGA
TAGCAAGCTTTACTGTCTGTACAACAGAACCACTTCGCCTTTTCTGATCCTTGCTCCGCTCAAGATGGAA
CTGGTGGGCCTGGATCCTTACATGGTGCTCTACCACGATGTGCTCTCACCCAAGGAAATCAAAGAGCTCC
AAGGTATGGCCACTCCTGGTCTAAAAAGGGCCACCGTCTATCAGGCCTCCTCCGGTCGGAACGAGGTGGT
GAAGACCAGGACCTCCAAAGTGGCCTGGTTCCCCGATGGCTATAACCCGCTCACTGTTCGACTTAATGCC
CGCATCTCCGACATGACCGGGTTCAATCTCTATGGCTCCGAGATGCTGCAGCTGATGAACTACGGCTTGG
GAGGCCACTACGACCAGCACTACGACTTCTTTAACAAGACTGCTGACGGATGTAGAACAAGGTGGAGCCA
CCGTGTTTCCAAACATTAGAAAGGCCGTCTTCCCACAACGCGGATCTGTGGTAATGTGGTACAATCTCAA
GGACAACGGTCAAATTGATACTCAAACTCTTCATGCCGCTTGCCCCGTTATAGTGGGTTCCAAATGGGGT
GTGCAACAAGTGGATTCGAGAGCGCGAACAGATATTCAGCAGACCTTGCCTCAAAAAGCGAATGTGATTC
TCAGCGAAACTGTTTTTAGACAAGTGATACTATACGACCACTATAAAACTCAATAAAGTCATATTAGAAT
CGATAATGCTGTCAATTAAACATTCCCAGCGATAAGAACAATTGCAAATCAAGTCGCGACATTCCGAAGA
GGAATAGGTGTTTTCCCCAGAGTCGTTCATCAAAATCAAGAAATGGAAATTCCTGCTAAATATCGTTTAA
TGGTCGTGAACCAACCATTAATTGACATTTTGCATTAAAAGCACAAGATAAGAAAGCGGGTTTCTATTTT
TAAAAACATGAAAATAAATGCACATCGATCTAGAAGTAAATACTCATAACTACGGCAGTACATGGCATCA
AAGAACAAACAATGGGTTATTTTATCAGGTGCCTTCTGGTGATGCTGTTTCACAGCACCTTGGTCCTCGG
ACACCGTCCGCTTGAAAAGAGTCACTCCTTATCACTGGTGACAATGGTGCCACTTCTCGAGCTGGAAAAG
AAACTGATTGATAACCTCGAAAACTATACAAATGCCCTGGAGCAGAAGCTGGAGATAATTCGCAGGTCAA
CTCCTAGTAATACGTGCTGAAAACGAGAAAGGAAGAAGGAATGCTATATCCTATTTGTCCAATCCGCTGA
ATGGCTTTTCAATCATCCGACGATTGCATCAGGATTGGATCAACTGGCGAAAGTACATGGAGCAACCAGT
GGGGATATGGCAATTGAAAGCATTCTACAGCTGGAAGGATGAGCTTCCCACCGAACGTGACTTATGGGAT
GCCTGCGAGGGCATAGCAAGAATTCAGAGTACATATGACCTGAAAGTTGGCGATTTTATTAATGGGAACA
TTAATGGAAAACAGTACAAGAAAACTATTCAGAAGCCTTGCCTCTGTTGCACAATTGCTTGAAGCTGCAG

CCCCACGATGCGCGTGTACTTCGACTGTGGAAAAAAACAACGGACTTCATTGAGAATCAACCAGATCAAT
CCCCTACAGAAAATAAGAAGCGGAATGTTCCAATCGCAAATGCGTTCAAATTAAGCTGCAACGGACCACT
CGAGAGCTCCACTAGACTGCACTGTTTCTATAACTTTACCACGACTCCATTCCTTCGCTTAGCTCCACTC
AAAACGGAGCAAATCGGATTGGATCCATATGTGGTGCTTTATCACGAAGTGCTCTCTGCTCGAGAAATAT
CTATGTTGATAGGCAAGGCTGCTCAGAATATGAAAAACACCAAAATTCATAAGGAACGAGCAGTTCCAAA
AAAGAACAGGGGAAGAACTGCCAAGGGTTTTTGGTTGAAAAAGGAGAGTAACGAGCTGACGAAGAGAATT
ACTAGGCGCATCATGGACATGACTGGCTTTGACCTGGCTGATTCTGAAGGATTTCAGGGTGATTAATTAC
GGCATAGGTGGACACTATTTTTTGCATATGGATTACTTTGATTTTGCGTCTAGTAATCACACTGATACAC
GTAGTCGCTACTCCATCGATTTGGGCGATCGTATTGCTACTGTGCTTTTCTATGTGATGACCGAGTGGAT
ACGCGAAAAGCGGCAGATCTTCATCAGACCCTGCCTGCCAAAAGCAAAGGGAACCTCTACGAAATCATAG
ACAAACAAATTTCCGGCATAATCACTCTGCACACTTAACAAAAACCCACGGAGCTCAGCTTAAATGCCTT
TTTAAAATGCCAACACGGAAAAATAAAGTGCTGAGTGGTTGATAGTCCCTGAAAGTCGAGGAGGGGGTAC
GGAAGGGCATCTGCTTCAGCACCAAGG
>dme_circ_0007977_junction_seq
CGGAAGGGCATCTGCTTCAGCACCAAGGCTCCGACTATTTTGGCTCTGGCAGTTGG
>dme_circ_0007978
ATAAATATATGTCTATCTAAAAGATAGCGAATTAAGCGATTCACCTGAGATAGCTTTTCCAACCATCGTA
GGTCTTTGGCTGACTCTTCATAAGCAGTTGCTCATTGCACACATTGTATTGGCGGTGGTCAGTGATCTGG
GCGAGAGCACGTTCTTTGATGACGCAGCTCTTTTCCAAATGGCGAGCACGTAGTGCCAACTCAATATTTT
TGTTCCGGTGCTCTCGGGTTAATTAAATCGCTGCGTGCCGCTATCAATGCGCCGCTTATCAGCAGCCGAA
CAAAACGCTATAAACGAACTACGGTTATTATTTATCTCGGCTCGATGTTAACTTGCCAGCTCCGACTATT
TTGGCTCTGGCAGTTGGCTCCGGCACGTAGACCCACATAGAACTTCGACCAGAAAGGTAACCCACGATGC
GCTCTTCGAAGTGGCTGCTTCCGGTGAGGGTGCTGCTCACCTACCAGGTGCTGCTTCTACTCCTCCGCCA
GGCGAAAGGCGAGGAGAGCCACTGCACCTCGGTGGCAGGAATGGTCAAGCTGCTCGATTTGGAGGCGCAG
CTGATCGACAACCTAGAGGATTACGCTGTCGAGCTGGAGAAGAAACTGCAGACCGTAAGAAGGGAGCATA
ACAAGTCTGCGTTTGGAAAATGACAAAGCCCGCAGTTCCACGGAGGAGTACTTATCCAATCCCCTGAACT
CCTTTTCCCTCATTCGTCGCATGAATCGCGACTGGATTATTTGGCAACTCTACATGGACGATCCGGTGGG
TATTTCTCAGGTGGAGCGAATCCAGGAGTTGAGGGAACACATGCCCACCCACACGGATGTGGAGGAGGCT
GTCACTGCCTTGGATCGCATTCAAAGCACCTATGGCCTTAAAGTGCCGGAAATCTCCCATGGATTCCTCA
ATGGCAAACAGTACAAGTGTGAGTCTCACTGTATTGGATACCTATGCCATGGGACAAATCTTATTTGACC
AGAAAAACTATTTGGCAGCTGCTAGCTGGATCTATCAGTCCGTTGTATTGATGGAGGCCTTTTCGATGGC
TGCTCCGCTTGAGATTTCCAAAAATGAAGTGCGGATGGTATATGCTGAAACCCTGTTAAAGCTTAGACCA
GCACGCCGATGCCCTGAAAGTTGTCAACATTGCTCTGACTGATAACCCGCACGATATTAAACTGTTGCTG
AAGAAGTCGGAAATCGAAACGATGATAAGGACGGGCACCAATAATGCGCCTCCGGTAAAGGGTTCAGGCA
ACAGGCGTGCCAACTGCCTACCAGATCGGCTGTCGAGGTCAGTTCCCGCCGTCCGCGGATAGCAAGCTTT
ACTGTCTGTACAACAGAACCACTTCGCCTTTTCTGATCCTTGCTCCGCTCAAGATGGAACTGGTGGGCCT
GGATCCTTACATGGTGCTCTACCACGATGTGCTCTCACCCAAGGAAATCAAAGAGCTCCAAGGTATGGCC
ACTCCTGGTCTAAAAAGGGCCACCGTCTATCAGGCCTCCTCCGGTCGGAACGAGGTGGTGAAGACCAGGA
CCTCCAAAGTGGCCTGGTTCCCCGATGGCTATAACCCGCTCACTGTTCGACTTAATGCCCGCATCTCCGA
CATGACCGGGTTCAATCTCTATGGCTCCGAGATGCTGCAGCTGATGAACTACGGCTTGGGAGGCCACTAC
GACCAGCACTACGACTTCTTTAACAAGACTGCTGACGGATGTAGAACAAGGTGGAGCCACCGTGTTTCCA
AACATTAGAAAGGCCGTCTTCCCACAACGCGGATCTGTGGTAATGTGGTACAATCTCAAGGACAACGGTC
AAATTGATACTCAAACTCTTCATGCCGCTTGCCCCGTTATAGTGGGTTCCAAATGGGGTGTGCAACAAGT
GGATTCGAGAGCGCGAACAGATATTCAGCAGACCTTGCCTCAAAAAGCGAATGTGATTCTCAGCGAAACT

GTTTTTAGACAAGTGATACTATACGACCACTATAAAACTCAATAAAGTCATATTAGAATCGATAATGCTG
TCAATTAAACATTCCCAGCGATAAGAACAATTGCAAATCAAGTCGCGACATTCCGAAGAGGAATAGGTGT
TTTCCCCAGAGTCGTTCATCAAAATCAAGAAATGGAAATTCCTGCTAAATATCGTTTAATGGTCGTGAAC
CAACCATTAATTGACATTTTGCATTAAAAGCACAAGATAAGAAAGCGGGTTTCTATTTTTAAAAACATGA
AAATAAATGCACATCGATCTAGATCAACTCCTAGTAATACGTGCTGAAAACGAGAAAGGAAGAAGGAATG
CTATATCCTATTTGTCCAATCCGCTGAATGGCTTTTCAATCATCCGACGATTGCATCAGGATTGGATCAA
CTGGCGAAAGTACATGGAGCAACCAGTGGGGATATGGCAATTGAAAGCATTCTACAGCTGGAAGGATGAG
CTTCCCACCGAACGTGACTTATGGGATGCCTGCGAGGGCATAGCAAGAATTCAGAGTACATATGACCTGA
AAGTTGGCGATTTTATTAATGGGAACATTAATGGAAAACAGTACAAGAAAACTATTCAGAAGCCTTGCCT
CTGTTGCACAATTGCTTGAAGCTGCAGCCCCACGATGCGCGTGTACTTCGACTGTGGAAAAAAACAACGG
ACTTCATTGAGAATCAACCAGATCAATCCCCTACAGAAAATAAGAAGCGGAATGTTCCAATCGCAAATGC
GTTCAAATTAAGCTGCAACGGACCACTCGAGAGCTCCACTAGACTGCACTGTTTCTATAACTTTACCACG
ACTCCATTCCTTCGCTTAGCTCCACTCAAAACGGAGCAAATCGGATTGGATCCATATGTGGTGCTTTATC
ACGAAGTGCTCTCTGCTCGAGAAATATCTATGTTGATAGGCAAGGCTGCTCAGAATATGAAAAACACCAA
AATTCATAAGGAACGAGCAGTTCCAAAAAAGAACAGGGGAAGAACTGCCAAGGGTTTTTGGTTGAAAAAG
GAGAGTAACGAGCTGACGAAGAGAATTACTAGGCGCATCATGGACATGACTGGCTTTGACCTGGCTGATT
CTGAAGGATTTCAGGTGATGACCGAGTGGATACGCGAAAAGCGGCAGATCTTCATCAGACCCTGCCTGCC
AAAAGCAAAGGGAACCTCTACGAAATCATAGACAAACAAATTTCCGGCATAATCACTCTGCACACTTAAC
AAAAACCCACGGAGCTCAGCTTAAATGCCTTTTTAAAATGCCAACACGGAAAAATAAAGTGCTGAGTGGT
TGATAGTCCCTGAAAGTCGAGGAGGGGGTACGGAAGGGCATCTGCTTCAGCACCAAGG
>dme_circ_0007978_junction_seq
CGGAAGGGCATCTGCTTCAGCACCAAGGATAAATATATGTCTATCTAAAAGATAGC
>dme_circ_0007979
GTTCCCTCGCTGGCCTCCACTTTGCTCCACCACAACCAGTCGCCGAAGCACAGCAATCCGGGATCCAGTG
GGATCCAGGATGCCCATCCCAACCAGCCAGGTGCCGCTGCAGATGCCTTCCTGGTCAAGTGTACCCAGTG
CCACAAGCGATTCCCCGAGTATCAGGTAAGATTTGGATAACCAACAAAAGCCTAGTTACTGAGCAGTACA
AATAATTATTGCTCATTAATTCTTGGTAATTTTTGATTTAAGAGTAATTCAGCTTAGGAAATTAAAGAAC
TTTCGTCAGTAGGATTGAAGTAGGGGAAATGCCAAGTGATTATGATTATAGGGCCAAGTTCATGGGGCTA
AACGCCAGATAAGCGCCATCTTAATCAAGTAATAAATTTAAATCCTTTTTCATTCACTTTCACAGTCCCT
CAGCGAGCACATTGCCAGCGAGCATCCCCACGACAAGCTGAACTGCGGAGCTGCCCAGCCGGAGAGCGAT
GCCGAGGACGAGCAGAGCAACATGAGCGGCAGCAGCAGGCGGTACGCCAAGTCGCCGCTGGCCAGCAACA
ACAACAGCAGCACCGCCAACGCCAACAACAACAGCACCAGCAGCCAGTCCATGAACAACAACAGCGAGCT
GGCCAAGAACCACAACAGTGCTAACAAAATGTCGCCGATGTGCTCACCTGGCTCCCTCACGCCCGGCGAC
CTCTTCGCCCAGCTGCAGCATCCGCCGCCCCAACTGCCGCCCCACCTGCACGCCCAGTTCATGGCCGCCG
CCGCCTCCCTGGCCATGCAGTCCGCCCGCACCgccagctcgcccagccaacagcagcagcagcaactgca
acagcagcagcaactgcagcagcagcagcagcatcaaatggccatgcagcaactgctgccgccgcaactg
cccggcagcaacagcagcgtgggcagcaacTCCGCCTACGATTTGGATCTCAGCGCCCCACGCTCCACAT
CCAGTCCGGGCTCCACCACCGGCGACCTGAGTGGCGCCTATCCATGCATGCAGTGCACCGCCTCCTTCGC
CAGCCGcgagcaactcgagcagcacgagcagctcCACTCGCCATGCGGACCGGCGGCGGTCAGCAATGTC
TCCCAG
>dme_circ_0007979_junction_seq
ACCGGCGGCGGTCAGCAATGTCTCCCAGGTTCCCTCGCTGGCCTCCACTTTGCTCC
>dme_circ_0007980
AAGATCAAGCACGCCTCGCCCATCCCGGAGCGCAAGAAGATCTCCAAGGAGAAGGAGGAGGAGCGCAAGG

AGTTCCGCATCAGGCAGTACTCGCCGCAAGCCTTCAAGTTCTTCATGGAGCAGCACATAGAGAACG
>dme_circ_0007980_junction_seq
TTCTTCATGGAGCAGCACATAGAGAACGAAGATCAAGCACGCCTCGCCCATCCCGG
>dme_circ_0007981
CTCCAAGTTGGCCGCCATCGATTCGTGTGTAGGATCAATTAAGATTCCGAGTGGTCGAGAATCGGCTCAA
ATCAAATTAAAATCAACTAATATTTTGGTATTCAGATATTCAAATGGAATTCATTCATCGCCTGCGACTT
TTATTCGGATCTGCCAACTATTTTTGAATTTGAATTGTGTGTCTGCGGCTGGCGCAGAATCTCTGATAAA
GCAGAGGAATAAAATCGGAAGAACAACAAATACAAATACAAATGAAATGCGGGGAGCAGTATTTACATGC
CAAATGAATGCTGGATAGGCGAAAGGGAGGGGGGTTTCTCTTATaatgcaaatgtgaatgtgaatgcgaa
tgcgaatgcgaGTGGAAGAATTCCCGGCGCGAGTGATAAATAATCCGACGACAAACAAAGCAGAAGCCTA
CACCGCGAGAAAGAGCAGTGCAAACACAATTATCTTTATTGAGAGCAACAATATCAAGATCGAGATAATA
AAGCATCCTAAAACCCGCGCCTTAGTTCGTTGTAGTCTCGCCACAGATATAGATATTCAAAGGCAAAAAG
GTGGTGTCGGCATCGCCAGACAAACAAGTAAAGCATCTATTTCATACAAAACAACCAATtaaataataat
aaaaataataataatCGTAGAGAGGCAGAGCCAAATCAAATTCCCGGCCGCCGATGTGCCCCAgtgtgtg
tgcgtgtgtgtgtgtgtgctgtgctgtgctgtgcgagtgttagtgtgcgGAGCATTTCTGTGATATGA
GTGCTAAATGCCACAGGGCGAAGCAGCAGCATCATGCATCCAGCGGGCGAAAAAAGGGGCGGTCGCCCCA
ATGATAAATACACGGCGGAAGCCCTCGAGAGCATCAAGCAGGACCTAACCCGATTTGAAGTACAAAATAA
CCATAGGAATAATCAGG
>dme_circ_0007981_junction_seq
GTACAAAATAACCATAGGAATAATCAGGCTCCAAGTTGGCCGCCATCGATTCGTGT
>dme_circ_0007982
GTCTGTGCAATAGTCGAATATGAGTACGCGGCCAAGGAGCCGGACGAGCTGGATCTCCAGAAAGGTGCCA
TTATCCATCGGATCAAGCAGATGCCCGGCGGCTGGTGGCAGGGCACCCTGAAGGCCTCCGGCGTAACGGG
GATGTTTCCGGATAACTTTGTGCGCGTGCTGGAGTCCGGAGTCAGTAGCAATGGCAACGGAACCACTGGC
AGTGGCGACCACATCGAGGTGGGCACCGCTGTGCAGCTGAGGGTGCGTACACGCCGGGTAACGGGGGGAA
TGCGGGGTTGCTTTCTACCCCCATTTAACCTATTGGGCCAATAGAAATTGAAATTCGTTGCGCACTGAGA
GAAAGAATCGTCCTAATCTTATTAACAAGTTCCAAAGCTCTTGATTGTTTGGTAACTTTTGGTTTTGACA
TAAAATAACCAACATATAttttttttattttACGATTTAAATGTAAATGTAGATTTGACGCTTGTAAGAA
CATTCGGTATTTTCTCGCAGTGCAGTACATTTGGTTTCAGTCTGTTCATTTCACTCAATTCTGCTTCACT
CTGTTTTCTCATTTAGTCACTCTGTTTTCTCATTTAGGTCGAGGAAGGCTGGTGGCGCGGTCGTCTGCGC
AGCAAAGTCGGCGTCTTCCCATCGAACTTTGTGCAACATATCGAGCCGTCTCCCGTTTTGGCCTCCAAAC
GGCCACCGACAATTGGCGCCACCGTGACCACATCGGCGCTGACGTCCAAGGCggccaatgtggccaatgt
ggcAGCCACAGCCACAGTACCAACCGGCGGAGCAGTGCCTCCTGCGTCCACCAGCATCTCCACCTCCACA
ACTAAGCAGGCAACGAAGAGCAGGGCAACTGCAGCAGCAGCAGCATCTGCTCCAGCGGAGCCGGCGGCCA
TAGTTTCGGGATCGGGAGCAGCAGCTGGTGGAGGAGCAGCGGCAGCAGTGCCCATGTTGCCACCCAAGCC
GCAGCGGGAGTTCTGCCGGGTGGAGTTCCCCTACGCCCCGCAGAACGACGATGAACTGGAGCTGAAGGTG
GACGACATAATCGCAGTTATCAGCACGGAACTGCCCGATAAGGGCTGGTGGAAAGGAGAAATCCGCGGCA
AAGTGGGCGTCTTTCCCGACAATTTTGTCAAAATGCTGGCACCTTCGGAAG
>dme_circ_0007982_junction_seq
TTTGTCAAAATGCTGGCACCTTCGGAAGGTCTGTGCAATAGTCGAATATGAGTACG
>dme_circ_0007983
GTTGCACCGATCAACGAGCCACCGAGTGCCACACAGATTACGCAGAATCAGAGGAAGGTCAGCAACACCG
CCAGCACCACCAGCCACATGACCACAACGAACTCAAGCAATTCCAGCACCACCTCGGTGACCACGCAGCA
GCAGAAGGAGCAGATCGGAGGCAGTTCCTCCACCACATCGAAGGTGTACATCAAGAAGACCGGGAGCAGT

AGCAATAGCACTACCAGCAGCACCACCACAGCTTCCTCTTCGGCGGGTCGCAAGGAGAGCTTTGGTTCCC
GTGACTCCCTGAACGATATCCTTAGTGAAACGGGTCTGCCCACCGGCAATGTGGCTGCCCAAAGGAAATC
CCTGGAGAACAAGAACCTGGACCTGACTAAGCCACCTGGAGTGGGAGCATCAGTGTCCATTGGACAGCAG
CGGAGCAGCACCTCTAGTGCCACCACCTCCTCAGCCACTACAGTAGCCACTGGACTAACCTCATCATCAG
TGAGCAAGTCCATGGAGAACATGCTGGACGAGAAGGTCAAGTCGCCACCACCGCCGGTGCTCAGCAAGAA
GCCCAGTGTGCCGCTCAAGAAAACTCCCACCGGCGGAGTTCCTATGACTGGGTAACCTTTTGGGCGGCAA
GAAAAAGAATACCGAGGGAAAGCTCACCACTGCCGTGTCCCATGATTTGGCGGATGGTCTTGCGGCCAGC
AAGATGCTTTCAGGTGCTAATCAACTACCTGAATCTGGAGCAGCTGGCAATGTGGTGATCCGGAGAGCTG
CCACGATTGCAGTGGAGGATACAGATTTCGATCGCGTGGAGAGGGCTTCCATACTCACGGATATGCGGCA
AGGAAGGGTCAAGGCGCCAAAGAGACGTCCACCATCGGCGGCTATCAATGTGCTGGGCGAAAGTAATAAC
AATTCCGTATATGTAAATGGCAGTGGAATGTCCGGATCTGGTGGAGCAGGAAGTGCCAGTGAGTTGAGTG
AAGATGGCGGTGCTGGAGGGAAGACTTCTGATGACAATTCCACCGGCGAGGAACCACAGTTGGCCAAACC
CAAGCCACGTGAGTGGGAGAAGAAGAAGGCGCCTTGGATGGAGGAGCTGAAGGCCTCGCAGGTCACCAGG
AAAAAGACCTCACCCAGTGTGGAGCCACGAGGAGCTTCGGTGGCCGAAAGGACATCCAAACTCTTTGCGG
CCGAGCAAGCTGTCAGCAGCAATAGCAGCAGTATCACAACGAAAGTCCAATCTTCAGCGGTGACCTCCTC
CATGTTTGTGGAGAACTCAACGACCAGCAGCTCGGTGATCACCAGTAGCTCCCACACCACCACCAACGAT
GCCATCATGTCGCGCAGCTTGTCGGCTGCCAAAGTCCAAACCGCCAGCAGCGACGAGGAGACGAGAGCCA
CAAGGCCAAACAGTCTAGCTATACGAAATCAACGAAGTGTTTCGCCTCTGGTAAAGAGCTCCAATGCCAG
CAGCTCGCAGTCCCAGGAGGAGAAGCCCACCAGCCAGCTGAACAACCACCAGGATGCCAGTTCCTCAAGG
GTGGCCCAATTGGAGCAACGGGTGGATAAGCTGGAGGGGCTGGTGCTAAACCAACAGCGAACCATTGAGG
AGCTGGTAAATGCCCTGAAATCGGAAGCTGAACGCGTCAAGATTTTGAGGAGCGAACTGGACAAATACGC
GCA
>dme_circ_0007983_junction_seq
TGAGGAGCGAACTGGACAAATACGCGCAGTTGCACCGATCAACGAGCCACCGAGTG
>dme_circ_0007984
ACTCCAATCCACGACAACGACCCCTGGCTTTAAAATGGATCCCGACTGTTTTGCGATGTCCTCGTACCAG
TTCGTCAACTCGCTGGCCTCCTGTTATCCGCAGCAGATGAATCCGCAGCAGAATCACCCGGGAGCGGGAA
ATTCGAGTGCAGGCGGCAGCGGTGGAGGGGCGGGCGGTAGCGGGGGCGTGGTGCCCAGCGGCGGAACGAA
CGGCGGTCAGGGCAGTGCGGGGGCGGCCACTCCGGGGGCGAACGACTACTTCCCAGCCGCGGCGGCCTAT
ACGCCTAACCTGTATCCAAACACACCGCAGGCCCACTACGCCAATCAGGCGGCCTACGGCGGACAGGGAA
ATCCGGATATGGTGGACTACACgcagctacagccgcagcgactgctcctgcagcagcagcagcaacagca
acagcagcaacatgcccatgcagcggcagcagttgctgcccagcagcagcagcaactcgcccagcagcaa
cacccgcaacagcagcaacagcagcagcaggcgaacatcagttgcaAGTACGCCAACGATCCAGTGACGC
CGGGCGGAAGTGGGGGCGGGGGTGTTTCCGGTAGCAACAACAATAACAATAGCGCCAACAGCAACAACAA
CAACAGCCAGTCGCTAGCCAGTCCACAGGATCTGTCCACCAGGGACATATCGCCCAAACTGTCGCCCAGC
TCAGTGGTAGAGAGTGTGGCCCGATCGCTGAACAAGGGCGTCCTGGGCGGAAGCTTGGCGGCCGCCGCTG
CCGCCGCCGGCTTGAACAACAACCACAGCGGATCGGGAGTGTCCGGAGGACCTGGAAACGTCAATGTTCC
CATGCACAGTCCGGGGGGCGGGGATTCGGACTCCGAGAGCGATAGCGGCAATGAGGCGGGCAGTAGCCAG
AACAGCGGCAATGGAAAGAAGAACCCGCCGCAGATATATCCGTGGATGAAGCGAGTACATCTCGGGACGA
G
>dme_circ_0007984_junction_seq
GGATGAAGCGAGTACATCTCGGGACGAGACTCCAATCCACGACAACGACCCCTGGC
>dme_circ_0007985
GTGAATTTGTGATGAAACTGAATTTATAAACGCACTCAAACGCCGGCTGAAAAAGTTAACTTAACTAA

ACTAACACTTACACTAAGCAGTACCCTTATCGTAATTTCAACATAAAAATTAGCGAAATTCACGAGCCGC
AAACTTTGTATCGATATCCCCATGTGTCCGAAGTCATTTGAGCAGTGTGTGTAGTTTAAGATATCAAGCA
AAACTGTG
>dme_circ_0007985_junction_seq
GTAGTTTAAGATATCAAGCAAAACTGTGGTGAATTTGTGATGAAACTGAATTTATA
>dme_circ_0007986
GGACCCGATCCCACGCTTTATATATGCATATACCTTTACATATATCTGTTTACATTCTATAACGATTTAC
TGTAAATGAAACATAATTACTTACTTTTCGTTTTGTACCCCGGTTTTTGGCTCTAAATTTGTTAGGTGAA
TTTGTGATGAAACTGAATTTATAAACGCACTCAAACGCCGGCTGAAAAAAGTTAAACTTAACTAAACTAA
CACTTACACTAAGCAG
>dme_circ_0007986_junction_seq
TAACTAAACTAACACTTACACTAAGCAGGGACCCGATCCCACGCTTTATATATGCA
>dme_circ_0007987
GAGCCGCCCACCTGCGTCCTTTGCGAATTTATCATGACCAAGTTGGATGCCGATCTAAAGAATAAGACCG
AACAGGACGACATTAAGCGAGCGATTGAAGCCGTGTGCAATCGCCTGCCAGCCACGGTGCGCAAACAGTG
CGACACCTTCGTCGATGGATATGCCTCTGCGGTCCTGAAATTGCTGAGCGATG
>dme_circ_0007987_junction_seq
TCTGCGGTCCTGAAATTGCTGAGCGATGGAGCCGCCCACCTGCGTCCTTTGCGAAT
>dme_circ_0007988
GAGGCATATCCATCGACGAAGGTGTCGCACTGTTTGCGCACCGTGGCTGGCAGGCGATTGCACACGGCTT
CAATCGCTCGCTTAATGTCGTCCTGTTCGGTCTTATTCTTTAGATCGGCATCCAACTTGGTCATGATAAA
TTCGCAAAGGACGCAG
>dme_circ_0007988_junction_seq
GGTCATGATAAATTCGCAAAGGACGCAGGAGGCATATCCATCGACGAAGGTGTCGC
>dme_circ_0007989
GTTATCGACAAGTACGGCGATAAGATCGCCGATCTGCTGTTAAAGGAAATGGATCCCAAGCTTATCTGCA
CGGAGTTGGGAATGTGCATCCTGGCTGATCTGGATGATCGTGAGTATTAAATTCTAGAGCTACGTTAAGT
ATACGTTTAAACTAATGAATTTCATTTTCGTAATACAGTCGAAGTGGATGAGGCTTTGAAGTACGACGTG
ATTGCTCTGCCGCGCCAGGACAACAAGCTGTCTAGCAGCATCAAGGAGCCGCCCACCTGCGTCCTTTGCG
AATTTATCATGACCAAG
>dme_circ_0007989_junction_seq
CGTCCTTTGCGAATTTATCATGACCAAGGTTATCGACAAGTACGGCGATAAGATCG
>dme_circ_0007990
TACATGCTGCATTTCATTCAGGAGACTCTGGCTACCCCCTCCACGGATGACGAGATCAAGCACACCGTGG
AGAACATCTGCGCCAAACTGCCCTCGGGAGTTGCCGGTCAATGCCGCAACTTCGTGGAGATG
>dme_circ_0007990_junction_seq
CGGTCAATGCCGCAACTTCGTGGAGATGTACATGCTGCATTTCATTCAGGAGACTC
>dme_circ_0007991
CTTTGGTCGAGGGTGGAGAGCTGTGCACCCTGTGTGAGTACATGCTGCATTTCATTCAGGAGACTCTGGC
TACCCCCTCCACGGATGACGAGATCAAGCACACCGTGGAGAACATCTGCGCCAAACTGCCCTCGGGAGTT
GCCGGTCAATGCCGCAACTTCGTGGAGATG
>dme_circ_0007991_junction_seq
CGGTCAATGCCGCAACTTCGTGGAGATGCTTTGGTCGAGGGTGGAGAGCTGTGCAC
>dme_circ_0007992

CTGGAGAAGCACGAGAAGAAGCAACTGGGCGCCAGTGAGCCGAAGTTCAGCCAGCAAGAGATCCTGGACA
TGCAGCTGCCCATTGATCACCTAATGGGTGCAGCTAATCCCGGAGCTTTGGTCGAGGGTGGAGAGCTGTG
CACCCTGTGTGAGTACATGCTGCATTTCATTCAGGAGACTCTGGCTACCCCCTCCACGGATGACGAGATC
AAGCACACCG
>dme_circ_0007992_junction_seq
TCCACGGATGACGAGATCAAGCACACCGCTGGAGAAGCACGAGAAGAAGCAACTGG
>dme_circ_0007993
ATGCCAATGGAGCCTGCTGCATGATCGGCATTTGCCAGAAGAACTCCGCCTCCTCGATGAAGGATGTCCC
AATCATGCCGCTGCTGCCTGTTATTGAGCCGGCTCAGGTAAAGATCACCATCGAGAAGCTGGAGAAGCAC
GAGAAGAAGCAACTGGGCGCCAGTGAGCCGAAGTTCAGCCAGCAAGAGATCCTGGACATGCAGCTGCCCA
TTGATCACCTAATGGGTGCAGCTAATCCCGGAGCTTTGGTCGAGGGTGGAGAGCTGTGCACCCTG
>dme_circ_0007993_junction_seq
GGTCGAGGGTGGAGAGCTGTGCACCCTGATGCCAATGGAGCCTGCTGCATGATCGG
>dme_circ_0007994
CTGGAGAAGCACGAGAAGAAGCAACTGGGCGCCAGTGAGCCGAAGTTCAGCCAGCAAGAGATCCTGGACA
TGCAGCTGCCCATTGATCACCTAATGGGTGCAGCTAATCCCGGAGCTTTGGTCGAGGG
>dme_circ_0007994_junction_seq
CAGCTAATCCCGGAGCTTTGGTCGAGGGCTGGAGAAGCACGAGAAGAAGCAACTGG
>dme_circ_0007995
TCATGCAATTCCACAGCAGATTAGAGCAAAACACAGAGAACATCGCTCTTAAAGACTCAAGTATCCCAAG
GGGCTTCCCCTTCAAAGCGGCTGTGTTGCGATGCTCTTTGTTTTGATGTGGCTTTCCGATTTTTCTACTG
GACTATGTCGATGATAATATGCCTACGTGCAGTCGATCCATGACTGATTTGCATCGGATCCGAATCCAAT
ATCCGAGCATCTCGCATGTCGGTGCTAATAGGCGGTGAGTATCTTTATGCGGAGGGCCGGCCGTCCTCTA
CTGTTTTCAAGATCAAGGCCACAATACGATACGAGTGGTATTCATTCATCTTCCTGCTTAGACAAGTTTT
CAACAGTGCAGAAAAATAAAAAAGAAGGCTGGAATTAGATGACCCTCATTTACATGGCACGTCTTTGGTG
ATAAGTTCTGATAAGTACTACTAATAGTGCATAGACTTAAAATAAGTAAATTTGCTCTTATTCCGCCTTT
TGCGCATTTCCGAACTAAAAAAATAAATTCAGATAGTAATCTTTTACAACTAATTACCAAGTTAACATTA
AAGTTTTTATTTTGATTGTTAAGTGAAATTCTTCATTACATGATTTAATACACTTCCTTATCAGGATGTC
TAATTTGTATTTAGATTTACATAATAATGACCAGTTAACGGTTGTTGACATTTCTACGTGAGTCTGTGGT
TTATTCACTTTTTATCGATTCAAAATAAGCGTGGCAAACGTGTTTGGTTTCCCATAAACAAATCATGAGC
GAAGTCTCACGCAAAGCAGTTCAATGGGTCAAAGTTTTCGAGAATTAGCGCTTCTAACCCAGCTAGACTA
CTGCATCTTTATGGCCAAATCCACACCTGAACTTGATCTGGATGCTGGACGGACTACGTGCGGAAGAAGA
GCAGCAGTCAGTCGAGAAGCCTTCGCTCGAAGTCAGCTATAAAAGAGTTGTAGATCTTAACTATAACTTC
AACTGTTTCTGCTTTGTTATTGTTGGGGGTTTCGTTCGCTTATAGCCATTATAGACAGTATTTGAAGATT
TGACTCACAAATTATGGCCAGCAGTTGTGGCTGAATTGATTAGCTGGCATTTGGCTGTGGCTAGCGGTCT
TTTAGCCCCTCGATAAGGTCAAGTTCATTTCCGCATGGTGAGGCAGTAGTTAGTGAGCTACGTTCTCTGA
TCTCCGAAAAGCGACTACCCCAAAGTAACTGTTTCGGAGTTTCAGGCGGATTTCATGGATAGGGAAAGGG
AAATTGCCCAAGAGGTCAGCCAAAGTGGCCATAAACAATTAGCCATTAGACGGGCCTTATGTGACGCACT
TGGCAATCGCGGCAATTAGAGTTGTTACCAATCTCTCCGCGGGTCACTCAACACAAAATGTATCTCGAGC
CTCGATTTTATCTGCCAGCGTGTGAACTGCCGGAAATTTCACTCTTGTGGATGGATGCATTTAATTCTTT
CCGAACCTGATTTTTGACCAAGTTAATTGGGAGGGAATAAAAGGCGTACTTTGTACTTTGTCCGACTATA
ACTTGGCTTGGCTGAATTCAAAGACCCAAACAAGCAGTCGCTTCTAGAGTAAACAAATTTATGAATGACA
TAAAAAAGCGGATAAAAGCAGAAAATTATCTCGCTCTTGAAACTCCGAAATTAAACGTTAGTCCAATGAT
TTACTGCTTCACTTATAAGCGTGTTAATTGCCTAAATTTCCTTCACTGCTGAACTTGATTACTTGTTCTT

TGGCGGCGGTCCACTCAATTGCCATAATATCAATCTCGCAGCCAGACTTCAAGTGTCAAGTGCAGTGTCT
GGCTGCAATTAACCCTCTACTACCCCATGGTACCTCGCCTAAGGTCACATAAACCTTAAATTCAATTCCT
ATTTAATCGAGTGCAATTTAATATTTCCACAATTTTTGGTTCTAAGTACCAAGTATTTCAGCAAGGTCTC
AGTGGGTTAATCTCCGTCAAAGAACCGAAATGCACGGCAGGCGTTGCAATCCACGGTCAACACGACAGGT
TTCAGGTTTCCTTCTAAGCCCCTGAACAGCCAAATCGTTGGTTTTGACACAGATATAGCCATGTTATGGC
CGTATTGGCCGGATCGGCGCTTGCATTTGGCATTTCGTTGGGCAAACACCACCAGATGACCTCGACTTTC
AATCGCTGTGAGAATGTGTCTGGCATTCTTTGCGAATGTATCTTCAGACGATTCTCACACAGATCTTGAT
ACGCACCTCGGAATCGAGAATTACACGCCGATAGGCATCGCGAGAAAAATCTACTAGCCGGCCATTACGA
GTGAGATGCATTTTCCCCAACATTTATGCCTGCGAGCTTTACATCTGGGCAGGCTCACATCAATTAACCC
GCAATTACAATAGAATTTAATCATGTGCCTGCCAGTTCACGTCGCACAGTTGGCCAAGTTGGCCAAAGGG
GGGTTAAGCGATGGGGCTGGGCAAAAAGGGGCGTATTAATAACCGCGCTAAGTTCAAGAACATCGCTGAT
GGTTATTGTCATAGTTTCGTCGCGGCAATATGTAATTAAATCTGCGGATTGCAATCGATTATAAATACAT
TGTGAGGCATTTGCGGGTGGCGAAGGCTTTGTTTGTTGATTGAGTGAGTGATTTAATAAATTGTTCCGAC
TTTAGGAGCAATAATTTTCTTCTACGATAATTGAAGTTATAATTTGAAGGACAACGTATTCAAGTACTCC
ATGTACTTAACACCCTATAGAAAGGTGGCCCAAATCTAATAGTTTCATATCGGTTACTTAATTGTTTATT
ATACCATTTTAAGTTTAATGATTTGCTGACCCCATTTTATGATAGCTTCCTCGGAGGTTTTTATGGCTTT
TTGAAAGGCAAAAACAGATCTCTGTTATTAACAGAGTGCGGTTCGAGTCCATGCTCCCCCCTGATTGCTA
AGTATACACTGGGTTGGTTTTATGCTAAAAGCATTATCCAAGTCTTGCTCCATAAGTATCGTTAAATAAA
TTTAACAATAATTCAATCGCTGACTGGTCGCACTATTATATCGCTACGGTATATATTTGACGAAACAGAC
GGCGGCCACACTACAGACAAATAAAAAATTGAACCAATCGTTGCAATTGCACTTCAGTTTTCGTTCAGAA
ATAAATACGTAGCTGCAAATAGAGGACTATCTCCGCCGACATTTAAGAAG
>dme_circ_0007995_junction_seq
AGGACTATCTCCGCCGACATTTAAGAAGTCATGCAATTCCACAGCAGATTAGAGCA
>dme_circ_0007996
GCTTTTCGGGCTCTGGCAACGGCTGCATCTTGGATTTGCTTTCACCACAGTTGGAAGATACCGGCCTCAA
GACGACGACTTTCCTGCAGCACTTCAAGGACGTCGTCACGACGAAGTTCACGGGGAGGGACCACCACCTG
CCGCCGCTGcaccccgcacaccccgcccacccggcgcaccAGCAGTCGGCGCTGCACCACCACCACCACC
AGCAGCAGAGCTTCTCCACCATTTTCCCCACGTATCTAGGCATGGACCGCTCCTCCGCCGTCGGCGGTTG
CGGCGGGGGCGGCGGTCTGGGAGTGGGCGTGGTCACCAGCTCGGCCACGGCCTTGGGACCGGGTGGCCTG
ACCAACGGGGGCGGCGGAGTGGTCAGTGGCGCACTCAACGGATTGGAGGCCATGTCGGCCGAGTCCACGG
GTCTGTGCCTGCAGGATCTGGTGAGTGCCGGGACGGCCAACGGAGCAGGCTCCGCAGGATCGGCAGAGAG
TGCCACCACCACCAGCACGGCACTGAGCAGCGGCAGCACCGGAAGCTCCACGGTCAACGGAGGAGGCAGC
AGCACCTCCGGCACGGAGCACCTGCACAGCCACCACAGCCTGCACGACTCCAGCTCGTCGGTCAGCATCT
CACCCGCCATCTCGAGCCTGATGCCCATCAGCAGCCTGAGCCACTTGCACCACTCCGCGGGCCAGGACCT
GGTGGGTGGCTACTCCCAGCACCCGCATCACACGGTGGTGCCGCCGCACACGCCCAAGCACGAGCCGCTC
GAGAAACTCAGAA
>dme_circ_0007996_junction_seq
AAGCACGAGCCGCTCGAGAAACTCAGAAGCTTTTCGGGCTCTGGCAACGGCTGCAT
>dme_circ_0007997
GTTTGGGCCGAAACCGGCGATTTCCGTGATAGTCATAGCTCCATGACCGCCGTAGCAAATAGTTTGGATA
GCACCCACTTGAACAACTTTCAGACGTCATCGACATCCTCGATATCGAACCGGAGTCGGGATCGCAAAGA
TGGGTGAGTAGCAGTAGCTGCCATATCCGTAACCCCCAGCACCACCACACCAAAAGTGCTGTTCCCAGAC
CGTATCCGCTATCTCCGGCGGCCCATCGATAAGGAACATAATTTATAATAATTTCGGAAAGCGTGGCCAA
CGATTCCACCTTTGTCGGCGAAATTTACTCGGCCGGGTCCGTTTAGTTTTCGATTAGACTGCCTTTGATT

TGCGACCTCACGACGACAGCAGAACAGAAAAAAGTGAGAATGGAGCCGAACGAAAATGATATTTCACAGG
GGCAGTAATTTAGTTTATTTGATTAAGTTTTCGGCAGCTACGAATTCGATTTGGTCTCACTTTCTGGCTT
CCCTGGCCCAGAACATACGAAAAAACACAAAAAGCACGCCGCATGATCGATGACATTCGGGAACATACAT
TAACTGGTTATAAATCTGTTTGCCGGCGGTCCGCCTTTGAACTTTTTCGAGGGTGGTGGGTGGTTAATGG
CTGGTGTTTGCTGGCGACGCCTGAAGGTTGCATGGCAATTAGTAACGGTGCTCCGCTGACGCCAGAAATC
GGAGGGCGCTCAGAAGACCTTTCCCCTTCGCGCTTATCTACCCTACACTGGTAACCCGAGCAAACCCAGA
CGGACGGCATAGCTACAGCTTAGCTACTCCAACCGATGGGCAAACAAATTACACAGCCGATTTATATATC
CACATTATATATCCATCCTTAAAAAGTATCTAAGAGTATGAATTGCTTCTCCCACAGGGCAATCGCAGTG
TAAACGAAACCACGATCAAGACAGAAAACATATCAAGTTCCGGGCACGATGAGCCGATGACCACCAGTGG
CGAGGAGCCGAAGAACGACAAGAAGAACAAGAGGCAGCGGCGCCAGAGGACACACTTCACCTCGCAACAG
CTCCAGGAGCTGGAGCACACCTTCAGCCGGAACAGGTATCCGGACATGTCCACCCGCGAAGAGATCGCCA
TGTGGACCAATCTTACAGAGGCCCGCGTCAGG
>dme_circ_0007997_junction_seq
GACCAATCTTACAGAGGCCCGCGTCAGGGTTTGGGCCGAAACCGGCGATTTCCGTG
>dme_circ_0007998
GAGCACAAAGCCGTTGGACTCATCGCCCCTTTCCAAGATTCCTAAGGCCGTCTCCGTCATCTCTTGCAGG
GAAGGCTCTCCCGCCTCCCTGGCTATGGAGTACGTAATGTGATTGTTTCGGAACAGGCCCATCAAATGGT
CCACCTTCTTGACATTCACATTAAGCAGATCCTTACGGTTATGTACCAATGCTGGAGGAACTGTGTCGTT
GGCGTGATGGGCCAGCCACTCGGCAGGAAGGTTTCTGTTATCACCGCGGGTGCAAATTGTTTCCGTGGGT
CCTTCGAAAATAGTAGTCTTCACCTCGGAGGCATTCAGAATGCCCATGGGCGACATTCCTCCGCCCAGGA
TCACATTGAACCGATTTCCCGGAGCATTCTCCACCAACTGACGGGCAATATCGACATGAAAGCCCACCGA
TTCCGCGGGCACTTCCGTGTCGCACTCCCAGTCCCGGTCGTAGATGTGGGCGTAGGTGGCGGCGGGCGTG
GCGTGCGTGATCCTGGTTGTGGTGACCACGCCGGTGCGCTTGCCCTCCTTCTGGGCCCACTCCATGACGC
TCTGGACCCTGCCTTGCTGCCCATTCTTCTTGGAGCGGGTGGCGTCCATTCCAATGGCTCCGTAATGGGT
TTTCGAACCCGAGAAGATCGCAGTGGCAGTGCCCGCCGAATCCGGCACTTGTTTGTCCACGTTGTAGG
>dme_circ_0007998_junction_seq
TCCGGCACTTGTTTGTCCACGTTGTAGGGAGCACAAAGCCGTTGGACTCATCGCCC
>dme_circ_0007999
CTAATCAAGAATGACGAGCTGTGGAAGGAGCGCATGGCGAAGCTGGAGGAGAACCTCAAGAAGACCAACA
CCATCCTGGAGAAGGAGTACGCCAATGCTGTAGAAAATGTGCACAAGCGATTCGTCAGCACCGCGTCGTC
GCACAAAGTGCCTCCCTGCCAGGACCTGAAATCCCAGCTGCTTGCCTGCTACCGCGCGCATCCCGGGGAG
ACCTTGAAGTGCATTGAGGAGGTGGCCCAATTCCGACAGTGCATCGATCTTCATCGCGTCCAGAAGCTGG
ATGCGGAACCAGAGTCGTCGAAAGTGACCTCCAAGGCCACCG
>dme_circ_0007999_junction_seq
TCGTCGAAAGTGACCTCCAAGGCCACCGCTAATCAAGAATGACGAGCTGTGGAAGG
>dme_circ_0008000
GAGTACGCCAATGCTGTAGAAAATGTGCACAAGCGATTCGTCAGCACCGCGTCGTCGCACAAAGTGCCTC
CCTGCCAGGACCTGAAATCCCAGCTGCTTGCCTGCTACCGCGCGCATCCCGGGGAGACCTTGAAGTGCAT
TGAGGAGG
>dme_circ_0008000_junction_seq
GGGGAGACCTTGAAGTGCATTGAGGAGGGAGTACGCCAATGCTGTAGAAAATGTGC
>dme_circ_0008001
GAGAACCTCAAGAAGACCAACACCATCCTGGAGAAGGAGTACGCCAATGCTGTAGAAAATGTGCACAAGC
GATTCGTCAGCACCGCGTCGTCGCACAAAGTGCCTCCCTGCCAGGACCTGAAATCCCAGCTGCTTGCCTG

CTACCGCGCGCATCCCGGGGAGACCTTGAAGTGCATTGAGGAGG
>dme_circ_0008001_junction_seq
GGGGAGACCTTGAAGTGCATTGAGGAGGGAGAACCTCAAGAAGACCAACACCATCC
>dme_circ_0008002
GTACTGTTTGTTGATGAATGGCTTAGGCCTTGCAAAATCGAGTCAGTGGGCATTTGTATAGTTTGCTGAT
TTCTCTTCTTGCTCGAATTGCAACAGAGACCCAACCAGGCCCGCTGGTTCTCTTTGTTAAATACTATAAA
GTAAAGGAATAAAACAAATCCCTGTAAAGTGGCTGTCAGGCAGAATAAATAGGCGAAGATCCTTCCGAAA
TCGAAGTATGTACACAATCCAAAAATCCATGTTATTCCCAAAAGAAAGAAGAGCAACACAAACAGGCCGA
GTTGCTTGAAGATAAGGTCACGTTTGAACAGGGCTGTATAGACACTCCAGGAGAT
>dme_circ_0008002_junction_seq
ACAGGGCTGTATAGACACTCCAGGAGATGTACTGTTTGTTGATGAATGGCTTAGGC
>dme_circ_0008003
GTTATAAAAATCATTAGGAGGCCCAACAGAGAAAGGGTCAAGCCGACATTGGTGATTACATCTAAGGAGT
GATCATCTTCATTGGTGTCCAGACCTGCCTGCATTTTGCTCACTCCCAGTAGAAATGTAAACTGGGTCAG
ATGATCCGCATGGCAGAGGATCACTGGGTGCGATGAGGCTTCCAAGCTGCCACCACCGCTGGTGGATATT
CCATCGCTAAGCCAG
>dme_circ_0008003_junction_seq
GCTGGTGGATATTCCATCGCTAAGCCAGGTTATAAAAATCATTAGGAGGCCCAACA
>dme_circ_0008004
GTAACCACATCCTCCCTCAAATTGAGTTTCATTCGCTTTTGCATTACGCAAGAAAAATGGCAACTTTCCA
GGTAGCTTGGTATCTTACGAAAAGATTTTAGATTAAAACATATATGCTATAATTTTCCCTCAACCCACTC
ACCATTCAATCCTGGTATTGTTATGGATATAACATTACTAGTTGGCCGTCGTTTGATTTCCTCTTCCGTC
TCCACAAAGAGTGAATCTCGA
>dme_circ_0008004_junction_seq
TTCCGTCTCCACAAAGAGTGAATCTCGAGTAACCACATCCTCCCTCAAATTGAGTT
>dme_circ_0008005
GTTGTGATATGCGTTTGAACATATTCGTTATGAAGTGAATTAAATACGCCAGCAGAGCGTTTCAATTAAG
CGAAAACCGGAAGAGAAAAAAAGCTCTAAAATGGTAAGTGGTATTTAATTAAAAAAGCAATCAAAACTGA
TAATAGAGCACATTACAAGCACTCTTAAATGAATACAGAAGCTTAAGAGAGTTCTCGAGCTGACATATGC
AATCTGAATGATTGTGATTTTTGGAGCATTTCCAACAACCTTATTTATCATTTCACTTTACTTGTATCTG
CGTGTGGCATTTGGGACTCTATTCTCCTGTACTGCTACAACATTTCAATGCGCCCAACAAAGGCAATAAA
CATCAAATATCGGTTAGATACATCTTCATCAGACAACCGAACAGAAAAGCTGAGAGCCCGTGTGGAATGa
aaaaaaaaaaaacaaaaaGCAACAGCAGACACAAACAAAAAGCGAAAACACACTGGCGGCCCGAGGATGA
AGCCTCATGTGTGGCCAACTAGACGTATGCGTAATGTATGTAAATATATTTCAAGTGTTTTGCTTGTTTA
GTATTACTTCCGGCGGAAGCGATTGATTGTGCTGCGTATCTGGATGGCAGAGGTATGTCTGCTGCCTCCT
AccccccccccccTTTTATTCCACCTCCTTTGAGGGGTTTGATTTCGCACAACAAATCAAATTGATTGC
TTTTCGGATACATTTAAACGAAATGAAAATGCGCAATCGCTATTGGTTTCGCATATTTCAAATTTATCGA
CTCGAATCAAAGCTTGTGTCCCGTCTGGGAATGTAATTAAAGTCCAAGAGCTTCTGCTTTTACGATACGA
AAGAGACCAATACGGCAACATGGATCATTCAGGCCAGATACACCAGACACTAGAGTGAAAATATTGATGT
TTGTTTAAGAGCAGCCATAAGCATAAATACTAATCGGTTACTGTTTTCCCTTCAATCGCAGAGTAAGTAG
CGTTCAAATGCCAAGTCAACAGTTACACAAACTCATTAGCAAACGACAATTAAATGGTTAAATGGTTATA
AGGAACCCAATCACTCTCACTGGCATATATTCTCATATTACCCAATATATCGTGCGATTCATTTCAAGGC
TGTTTGCACCCACAAACATATTAACAGCCACGCACTTAATTAATAACTAAAATACATAAATCCCCCACAT
TTGTCTGCCACGTACCGCAATTAACTAATTATCCATTTTTCACATCAGACATGGACGTTCAGTCGTTGGG

CGTGGCCCGCCCTCACAGCCATAGTGAGATATACATAAATTAATCGAGGCCGTTTGTTGTTGGTGGCGCA
TAAACAAGCAGGACACGCGTCGCATTAATGCGTACACGGATTTCGCAGGCTCAGAGATCCACGGGCTCGA
GCCTGCGAGTGCAGCCAGAATCTACACTCAAATACAAATACAAATACTTTTCAGATTCAAAGGATTCAAA
CTCAATTAATTTGTAGGACATAAGTCAAGGATATAAGCTAATTCTAAGAGGATTCGGGACACTGGGACAT
GATCGGAGAATCAGAATTGTGTTAATGTTTTTTATGAAACCATGCCTAATAAGTTTTAAGAACAACCCTC
TGTATATTGTTCTTCAGTGTATTCAGTTAGCGGAAATCAACGCCCCGAAACGGCCATTGGCAAGTGTGTG
TGTGAGTTTGTATATGGGAGCGTTAGTGTGACTACAGGCTATATGGTATATGCGATTCGCATTTATCTTA
ATTAAGTTATTGATATTGCTACATGGCTGCACGGGTTAGCCTGAGTCATAGACTAAGGGAGTAGTCCTCT
ACTAATTGGCCGTGATTTATGATTTCTGATTGCTTAACCGCAAGTCACTTGCCATTTGTATTGGCCACGT
TCACAAATTGCTAATGAATTGAGGCAGTCAAGGGGCGAATCGCATCGGGTGTCGGCATCCTGGCCCAGAC
AACCCTTTAATTATGGCGAAGAAAAGGGAAACATGGCGAACTTTTCTCATTAGAAGGGTCTGCTCTTAAA
GGAAAAGGTAACAATAGCAACGTTCACAGGACACTGTCATCTGGCTCCAACTTTGATGTTATAAGTAAGA
GTTAATTCAATTAATTATCGCATTAAAGACAGCTATAAATGGGTTGATATTTTAGAAATCTTGCGACTGG
GGCACTAAGAAATTCTGGAATTCAAATAAAACTGAAAGAATATTCTAATATATATATTTGGCTGCTTACT
TTCTCTCGGTGCATTGGCGGCCCTTCAATTTAAATGCCTGCCAATGGGGTCGGCATTGACTTGACTTTAA
TTACATTGCAGTTAGTTGGCAGCCACAGTCGTAGACATAGTCGCAGAGCCTAGGGAAATAGCTGCAGCGA
GAATCAAACTCATTATCCAGCACTCGAGGCGCCAAGACAAAGACGGGGTCTCAAGTCCTGCgtcttatgt
cttatgccttatgtcCTGTGGCCTCAGCCCGGGGCCACAGTCTCTCTGCTCTGTCAGTCCCCATTCCTCA
GATAGGCCACATCGTTTGTCGCCGTCCTCGCACTTGAAGGCATTGTCAAAGCGATCATTAAAGCGAGGAA
AAAGAATAGAAAAGCCGAGGCCACAATGGAAACGTGGCCCAGGGGCGGGGGTTTGTCTGGTTCTGCCCGC
CTTGTCATCGTGGCATTGGCATTGGCATTTGCCTTGATTGACACCCAGCTGACCGCATTGATAGATAGTT
GGTAGTCAGTAGTCGGTCTACCTCTTTGTCAGCCCATTGCTGATTGCTTTTAAAGCCTCTGCGCAGGACC
TTAAAGCCAGCTCCTGTTCCGCAGCCTCGCTGGTCGTGTTTGCGCTCACTTAATCAGAATCAGTGCGTTA
AGTGCTTTATGCTACTTGGTCCCGAAGCATAATTGCAGCACATCAATCTGCAAATGGGTCTCATTAATTT
GCCCAAACAAGATGCTCGACGAAAGGGGAAAGCGGAGCGGACTCTGAGTTTAAAGCAAAGATGAAACCAA
ACAGATAACTTAAGGTCTTTGCAGCTAAGTACAGCACGGGAAAAAATATGTACTGCATTCGCTTTCTAAT
TTACATCTCATTGAAAAAATAACAACCCAAAATAAGCAGATTATTAAGTTTTTCTGCATTTGTAAGACCC
CAATCATCCAATTATATTGTACTTCACATCAATGCAAGGAAGAGTAGATCATTTTGTTACAAAATTGATT
ATTGAGGCTTTTCCTCGGAGTGGCTGGAGTGTCAGTTGTTCAACTAATACACTCACAGCAACCAGTCGAC
CAGTCGCATTATTGTTGTTTTTCCTGCCTCAGCTTTTCATCTGCTGCTGTTGCAAAAGTCCAGTTATTGT
TATCCTGCTGCCGACGTGCGTGTTTGTGGCACTTTAGACAAAAATCGATTTGCCTATAAAATGCTTTACA
GCAACGACCACAATGCGGCCACAGGCGAGAGCTGAGGTTTGAGCTGCTTGGCTGCGATGGAATTGGATTT
GCCCTTTGTGCGAGTGTGTCAATATGTCAGTCAACTCTATATACACCCCATGGTTATGTGGCTATATGGT
TTGAGCTGTCATATGAGCCGCAGCCGCTGCCGGTCACTCGCCAATTCACGCTAATGGGTTCCGATGCCTC
GACCTCATCCCTTTTTGCCTGGTTTAATGCGTGTGGAATGATATCACTAATTTAGTTAAGTGTTCAGAGC
TTTTAGCTGCCTGAAATCTTATGCCATTTACATGGCGCCCGAACTGGGGCAGGTGAATACCAAGCATAGA
GTTTGATAAATGGCTAGAAAACTTGGTCGAGCACGTCAACGCCGACTTGTCATATTAAATATAAATTTAA
GGAATTTCTGAGGCCAACACGTGCCACTGCCGTTTACCAAACATTTTTGCGACTTATTAGCTTTGCTGCG
GAGAAGGAGTTGAGTTCAAGGGGAGATTCTCGCTGACAAATACTGAAAATCCATCAATCTTCATTGCGCT
TCACTGGAATTTCCATCAACGGCGCTGGGTCGGTGGAGAAGCAATCAGCCAGCATTTTGTCAAGCATCCT
GCTAAGGACATTCGTTTGATTGCAGGCATTTGCGAATCCTTTGCCATTAAAAGCGCATTTGTACACTTAC
ATACATCGTGGTATCTGTGTAAAAGTATTTATATACGGCTTTGATACGCGCTCAGCTTATTTTTTGGTTA
CTTCGGGCCATTAGTCGCCCACACTCCGCCACTCACCCACTCACCCAATGACCTGACTTTTTTGGCCATG
TCGTCtttttttttAGATATTCATAGGAACATGAATTCGCACACACACGCCCGCAGCATTACGTCTTTTT

CCCTGTTGTTTGCCACACTCACATCACATTGCTTAACACACCCCCTCGAACGCACGAAAATCGATCGCAT
TTTCATGGCAGACATTATGGAAATTCGACTCGATTCTCGAGTGTGTGTATCTGGGCTGGGGTTAGGGAAA
ATGGCATTTTGAGGCTTGTAACGAGTTGAGGGTTGCCCGTACCCGTGTCCCCGTGTCCGTGTCGGTGCTA
ATGCGTCAAAAGTGTGCGTTGGCCTGGCCAAAAAAAGTTCTCAGCCAAACTCAAACTCCGCAACTTTTTA
ACTGACAGCTGGTAACATTTGGGGAATGCAAACTGTGCCGCCGGCAGCTCCAAAAACTCAAACACGAAAA
CAAACATCGGGCGAACACAAGAGAAGCACCTGTTTCACCAGTGCTGTCGGCGCCTAGCTTAAGTCAGAAA
AGAAACAAGTCAGCAAAATATTTAGAAACAACAATATTTTTAAAAACAAACAAAAGAGATGCATAGTATA
TTTTAAGAATGCAGTTTTTGCAAACATTTATTAATAGTGCATAAATGTCATGCTAAAAACTAAAACACGT
CTAAAGAAAGACGCACTTTATAGAATTTTATGTTTTCAAATTTTATCCGCCGTTTAAGTCGCAAAGTCAC
TATGGAGTCGTTCAAAAAATCGCAGCACTGATGGTTTTACCCTTCCTCTGGCAGGTGAAGTTCTCCCTCA
ACTTTTTGCATTTACAATATTTTCATATTGCTGCTCCAACTGTCGTCGCCATTTTGTCAACTGTTTTCAG
CGCGCAGCAAATATTGTTACGCTTTATTGACTTCCATGATGCGGGCAAGCTGCTCCATGACAGTTGCAGC
CACGCCTCCACGCAGGAGCATCCTGTTCCGCCCCTGGCCAGGACATTTGACACGTTTTCGCACTTATCGC
TGAGATTACTTTTTGCTTTGATTGCATTTCTTTTGGCTCGACGTCGCGACTCCTTTGCTTCATTTATTGT
CTGTTTGATTGCTTCATATTTTTGGCCAAAACCTGGAATCCTGGCATCCACTACAATCCCCCGGTTTATT
GTTGAAAGTTTCCCGTTGGCGTTTTATGTGCTCTCCACTTTTATCGCTTTATGAATTATGCGTCGCGTGC
GTGCGTCCCAAAGTTGGTGGCGTTGCGCATACGCAATGTTGGACCTGCCGCCTTCAATTCGCTGGCACTC
GAGTATTATCGCCATTAATGGCTTGTCAATAGTGCCAATGACTTGGCCGGGTGTCCTTTCGTGTGTTAAA
GTCAAATGGTGTGCAATAATCTAAATAAGACCGAGTCCAAGGAGGCTAATTATAGTCAAGACAACGAAGC
AGCCACTTGATAACGATTATGACAAGCCATTAAGCAGGCAAAGGCGAATGGAATAGCCATTATTAAGCGA
CTCACTGGGCGGATATTTGGGTAAGGAGAATGGGCGGTATTGGCTATCAAAAAATGTCATTTACATAACA
CGAAACCTTATACTACGACAATTTAATTGATTTTTAATAAAGAGAAAAGGTTATTAACCGATGGACATTT
TGTGTAATATGACATACCCGAATATTCAATACAATGTTTACTATTAATTATTCAACGATATTCGTTTGTG
TTAGGATTGGTTGCACaaaaaaaaCTGAAATAATGTCTTTATTTTTTACTAATTAAGTAATTTTAACTCT
GTGTTCGTTCCAGAACTTCTTATCAAAAGTTCAATTACACGGTAAAAGTAAATATTTGTTTTCCGACCGC
TTGAATACACAGGTTTGGTGTTCCGCGGGCCCTTTCCTCATGTCTCCAGTAAAGCTCCTTATTGGCCTTT
CACGGAGGGTTGAACTAACTTGAGTTCACTTGACGGATCCAACTAATTACATATTCCATGGCTTTGGCGA
AAACCAAACGCCAACGAAAATTCCATTAATTTGTAATAATAATTCCATGATGCCGAAGTCATTAATTATG
TAAACGGCTGGTTGACTGCTGGACATTTTTAATTAAATGCGGCAGTCGAAATATGTTTGCCAACTGGGCG
CCAACTGGTCGCATACGCAATGTCTAGTTAGTAGTGTTGCAACAGCCGGGGCTCTAAAGAATAATTAAAT
AACCCGCCAGCCAGTAGAAGGAGTGACTGGGCACGTATTTCGCGCTCAAATGCATCGCTGAAATTTTTAT
TTATTGCATTTTGCGAATTGCGACACGCGCACTAGTTTTGCTAGGTCCTTTGGGGACGAGAGCAGGACAC
GTCGCTTAAAAGCAATTTATTTTCCCCTTATGAGAGGGTCAGCTGCTGGTTGAAACGCTTTACCACCGTC
CGCTTTTCGCAAGCTCgtgtgtgtgtgtgtgtgtTTTTTAATAAAACTTAAAACCGCACAAGACTCTCCC
TGAGCGCATTTGTAAACATTTCGAAATTGCATTTGGGCTGCTTCCCGGGTGAAATCTGCATATTGAAATC
GTGTCCTCTCCAATGGCAGCAGGCATTTTTCATTTGTTGCTGTTACTTCCGGCCGAGATTTGCCAACGTC
GATGATGCCAAAGGGCAATGCGTCCCGCCCATTTGCATTTTACAGCAAAACAAGACATATCCCGGAGCAA
CTGCTCTGCATCTGCTCGGCATCAGGATGAGCAGCAGAAACATCGTCCTCTAAGCCGGAAGTGTTGCCAT
GTGCCTCCTCCTAGATTTGCGCCGTTTTGTTGGCCTTGCGAGGCTTCAGCTTTGAGTGGAGCTCTGGCCG
GCGGCCATCATTAGGACTAAAATCCTGTAGGACTGTAGGACTGAATGGCAGAGCCACCAGAACAAATTTG
CAGCATAGAAATGGTTCCGGATAGGCGGCCATTGCAAATTGTGTTAGGATGCCAGTTGGCAACTGCCAGT
GGAAATATAAATCTAATTTCCATCTCGATTCGATTGGAAATCCATTTTTCAGTGTGAGTGCATGAAATTG
TTTCGCTGACGTCCCACGAAGAGTATTTTGCTAAATTGTATTTGACGATTAATCGATTTACCATTCTCAT
GAGAACTAACGACGAAGAAAGGATCACTTTATCAGTATTTTTGTCAGAACAAGGCAAAAAATCGTAAAta

agctcctaatgatttttcgaattaactggtctttaaatcttgaaacttttatttttttgccaatttattaa
aatattgatgattataccccttatcaaaaaatcgaaaattgacaaaaaggaatttttttttcaaatatcc
aaaagtgatagaagttttatgtttaggtcataggctacataaaaAAGGCAACTCCAAGCTCATAAGTTAT
TTTATAAGCAAGCTTCATTAAAGAGTTGTGTTTTATTTCGTTTATAATGGAAATCACCCTGATAAATGGA
CAAACCCTGCAATTGAAATAAGCACATCGCCCTTAATTCCCCTTACATTCTCTCATAACATTAAATATTT
AAACCCCATTTTTTCGCTGCATGCGTAAATATCAGCTCTGCATACAATAACGTTAATTTACCCTTAATTC
CGCCTTAGGTCAGCTTTAAAAACTAATTGTGTTAAGGGCGGCTTAATTCTTTGCTTTCCCCTGCGAAGAC
CAAACCTAAATCGGAAAATGGGAAATCCAGGCGCAGTGGGGCGGCTACAACCGCAAATTCCGAAGTGAGA
GATGGGCGCGATGTAGTAGGATTGTTCGGCATTTATCAGAGCTTTTCTTTTGGCGGCATCGACAGTTGCT
AATGATGTCCTAATTTCGAGATGTCACAGTGCATGCGTAGAGATAAAAAAGTAAATGGAAATGGCCGAGG
GCAAGGGCGAGTCAATCGATGGCGGGCGGTAATAAATGACCACGaaaaagacaaaaaaaaaaaGAAATGC
TGGTCGATTGGGCCTGCATATGGACAGATATTAATGGGCCTGGCCAAAAGTGCGATTCATTCGATTTTCC
GATCATCTTGGCTGCTTCTTTCTCCCTTTTCTGATTTTTATGTCTCTAGGTGTGCGACGCACACTTCAAG
CTGCATTAAAGGTTAATCATACGCCCCATGGCCGCCGCCGCAGCTTAACGATGGTAAAAATGCTAATCCT
CAGGAAAATCGACTTGGCGAAGCCAATGCCAATGGCACTGGCCGTGACAATGATGCTGGCGATGTTGATG
ATGATGATGAGATCCTTCATCCTTCATCATTAACAACGTACATCACGTCTGACTGTGAATTGCTTGGCAG
CGGTTTAACGCTAATCGCATACAAATACACACTAGACAGCAGACAGGATACACCCATAGTCACCAAAGTG
GCAAAGTGGGAGCACCAGGGTGGATCTTCCGGTACCAGTAAAACTAGGGAATTGGCTTCCACTGCCTTCT
TCCTGATTTGAATAACACGCCTCAGGGATTACCACAAACACACACAGTCCTAGGCACACAGAGaaaaaaa
aCACACCGAAATGGGTCATCAAATGGTTGTATGAACAACAAACATATCGGAGTGGGGCTCCTTAAGGACA
TAAGATTCGaaaaaaaaaCCCTTGAGATAGGATAAATTTACATTATTATATGTTATTATGATAACATTTA
TAATAATGTTTTAATTTGTGACTCAATATAACCTAAGGCAAATACAATTTTTTATTCATAAAAAAGCCTC
TATAAGACCACTTTACTTAACCGCCAGTTGAGATACGTGGCCCACTATAGATACTCGCTGTAATTTCGAT
GTTAATTTCCCGCCTCTGGCATTCGATTAAAGCGATGCTCTTGGCTAATTCGACTTGACCCTGGCACAAC
TTGGCTGCATCCTGTACGTGCGCGGGTGTGTGTGTTTGTGCACAATAAAACATGTGACTCACTTGAGTTA
TGTGGCACATTAACTTGGCCGAGTCTTAAGTGGCTTTTAACCTTATTTCGTTTCCGTTTCTGGTTTGCAC
TGGCTGTGGTTCTTTTCTTTTTATTTTGGTTCTTTGCAGCTACTTAGGAAATTCCAAATGCAAGTTGGAA
TACATAAAGCTGACTGTGGCATACTCTATTCCGAAAAGTGCAGGCATTTAGCTGGCATCATTAAACCAAG
GCAGGCTTATCACGGTATAGAATCCTTTTCACCTCGCTACAAGGCCAAATTGGAAGAAATTCTGTGACTT
CTCTCTATTTTCTCTTTGGTGCTAAGTGCCTGCCAAGAAAATGCACTAATGTTGGCTAGTTTAAATTCGT
TCATATGTGTGTTAAACATTTCAATGGGCGAATACATAACTACAGACAAACAGTGGAGGTAAAAATAATA
GGACAATGTGTCTTATTTAATTTATACCTATACAAATTGTAAATAATAAGGATTTGACTTTCAATTAACT
AATTTGTACGGAATGAATTGATATTAAAGTAAGCAttttttttttCATTCCCGTTCTAAATGGATGGTCT
CTGCTGTCTGGCGGTTGCATGTGCATCCCACATTCGCATCCAAACTTCTGCGGTGCACGTCAGAAATAGC
TGCGGGCTGATTTGTTAAGTCGACGATGGCGAAACGAGGAACCACAATTAGACGTGACCTCATCTTCTCG
TAACGAACTCATTAAGAATTCTTCACCCGACCGCACGCAAAGTCATCCTCAGACTCGAATTAGCTGATTG
ATTATGGATTTTACTCACTTTGCGGTTTGCCGCGAGGCCCACACTCTGGCGCTGGCTAAGCCAACTTAAA
GTTTCAGCTGCCAAAAGTCAACAAGTAGTTCATAATAAAGCGACTTAATGTCCTGCCATAAAAAGTGAGA
GTCATGTGAGGAATTGCGAGGTAAAATGGAAAATGCGGCGCGGGAAAGCAAGAAACGAGAAACGTGAGTT
AGAGAGCGTGTCAGTGACGCGTAAATTCCTCGGGTTGAGCATTCTGGCCGGGGACTTTTCCTCCATATCC
CATCCACAGAGCAGAGCAGAACGTGGGCTGCGAATGGCTCTGCGATTGGCATATTAAATAATAATATTTA
CGCGCGAGAGCAACCAAAACCAAAGCAGTGGCCTGCTAGGGGATGTGTAAACAGGATATCCACACAGTAT
ATATAAACTTTCGATGCTGAAAAGGCAATGCGCGTGAAACTTAAACCAACAATCGCGAGGCAAAACAAAA
ACAGTTGGGCGTTGGCCTAAGCTACTTAGGCGCGAAATGTTTTCGTATTTCTTAGGCGCTTCTTCGGCTC

CTTGGCCGTTTTCGCAGATTAAGCCACTTAGTTTCGACGGCCTGTCGTTGTCACTGAGTAAACAATATTT
AAATGAGTTTAAAGGGGCTGCGTTGCCCAGCGGAAATTGAATTTGTAATGAAAGCGACGGGAGGCGAAGC
CACTAAGTGTCGCCTTGCATTTGCCAGACCAGCGCGGCGTATACGCAATATCCAGGAAGAGAAGAAGGAA
GAACATGGCATCATAAATTGCCGTCGTGTGTGCGTAATTAATTTTTACAGCTCGTATTAATTGACGCTTA
TGCTGCTGCCGCAAATGTGCTAAATTTAATGGCAAACTTTTTTGCTTGGTGTGTGTGACCGAGGATTTAA
TGACAAATTGCATTAGGAGAGAAGAAACGAAAAAACGTGCCCGAATCCAATGGACTAATTCTGCTTTCTC
CACGCACATTAACTTTTTCAATTAAGTTGTCGATCAAAAGGCGAATCTGCAAAATACACACACAGAGACA
GCTTACACACACTCGCCGACGACAGACACGCACATTTAAATCAATAAAAATCAATCATACGCAGTGTTGG
GCGGCAGGTAAGCTGGGCCAAAACGTGCAGCATAATTTAATTTACGGCACAAAATGCTGCCTAGCTGGCA
CAATAAAAATGATAGACATTTTGCCGCCGGAGTGTTGACTTTATGAAATTATATTGCGCACAGATAGAAA
GAAGCATTAGGCGAAAGTAACCAGCTGCAGATGATGGTAAAAGCATCTCACCTCAGCTCAACTGAGCAAT
TAAATGGAACCAGCATTTATGAAATGAGTGAGCTGCAATCTTCACTTCCAGTTCACTGGCCATTTAAAGG
ACTTTAAGTTGCGTAGTCGTTAAATTTATGTATGCACTTGCCATAAATCTTGGTTTAATTTCGAGCTTCC
CTCACTCAACCGCATGCCATATATTTTATAGAATAATAAATTGCATGTCGAAAATCAATTTCCATTTTGG
TGTGTAATATGCAGGAACTTCCTTGGTTTTCTTGGCTTGAAAGCTGTTTTTCAGGTTCCTGTTTCCTATT
CCTAATGTCCGCTGGCAGTTCAGGAAATTAAAAATCAGCTTCCACAATAAAATAAGTACATGTGTCGGCG
TGTGAGAGGCTTCAATATTAAATGTTTTCTGTTGCAACAACTCAATCTTAGCAGGGGTGCAAGTAGTTCT
CGCATTTCAGCGACAGCTGCTGACACATTCTGCAATCCAACAGCATTTCGCATTTGAAATGAGCAATAAT
GAAATACCGCATTTCGCCTTCGCTCACCTGCGATGCCACGCCCACTTTGTCCCGACAAATCAGAAGTTTT
GCGTAGGGAAAATGGAAAAACTTTCTCACGTACAAAATCTACTTTTCTATTACGTTTTTTCCACCAACTC
ACCCACCCACCTCCCACACGATTTTCATGTTCGATTCTGCAACGTTGGCGTTTTTATTTCACGACTTTCT
CCGGGTTACTTTCACTGCGCCGGCACGTTGTATTCAATTTGTTTTGGTAACAGCTTGGCGCAAGTTCTAT
TAAATAATAAAGTCGATTTGCACGATGTTCACAAAGGCAAATGGCAGCGGGCTTTTCTCTTTCTGTATTA
TTGTTATTCGATGCAAATAGCGACCAACTTTCTAAGAAGAATATGAAATACTTACCAAAAAGCCAGCTAA
CTTTTGAGAGAAACTTAAAGGATTTTGGTGCTTAAAATGGACTTTTCAATCCGCATTATGCTTTATCCCA
AAGCAGAAAAGAAAGTAAAGCATGCAGATGTGTTTAAACTGCTTGCTGTGGTGACACTTAAAAAATATTA
ATAACTGCTTTTTTTATTAACTTAAAAATAACATATCTTACAGAGATATCTTTTTGGGCATAAATGAATT
ATCCAAACTACTTTCTTAGGCCAAAGGTATTATTCCCaaaaaaaaaGTTGTATGAAAATATGACAGTTCT
TCTTAAAATTATTCAAGCTGGCGCCTTCCAATTAACTTTTGATAAAATTTCATCAAAACCCCAAAAAAAG
CGTACAAAGGAAAGCTGAAACCCTCGCCCCATTTTAAGGCTGACCAAAATTTCTCATTTGAAGGGCTTTA
TTTCGTCTTCATTGCTTAATTAAAAACATAAACATGAAAAAGCATATAAAGTGACCGCCATATGGGCAGC
TGCCCGCCAAAAacacacagacagacagacagacagacacacacacacacacaGGGTGGAGGAATGCCAG
CCACTCACTCATAAATTCACGAGCACAGAAGCAATTTAATTAAAATTAATGTATTTGCCACTCTCTTTCT
TTTTTTCCGTCTGTGTGGAAAACCCTTCTCTGCCATTTCACATTTTGCGGTTTCGCCTGACTCGATTTTG
ATGGCTGCTTATTTTTGGCTCGGCTCGGCTCGGAATTCAGGAAAAAGGCCATGGTCAGCATGGAGGGACA
GTTTCTGGTGCGACAAATTTACGACGATGAAATAACATACAATCTAATTGGAGCAGCTGTCGAGATACTC
AGTAAGTAGTTAGCTGGTGCCATTTTGTTATGCAAGCATATGTGGGTCATAAAATACTAACTCACTACTG
TCTCCACTCCCGCACTGTTTGTCCTTGAAGATATTCCCGCAGATGACATCCTGGAGCTGTTCGGTAAGAC
TTTCTTCGAGTTCTGCCAGGACTCGGGCTACGACAAGATCCTGCAGGTCCTGGGAGCCACGCCGCGGGAC
TTCCTTCAG
>dme_circ_0008005_junction_seq
GGGAGCCACGCCGCGGGACTTCCTTCAGGTTGTGATATGCGTTTGAACATATTCGT
>dme_circ_0008006
GAAGAACATGACGGCATGCATGCCAATCCACCAGACGAACGCCTTCGGGTAGTTGCAATCGATGAAGAGC

AGCTGGAAGGCGTGCACCATGATCAAGATGAACTGGACCTGTGGGAGCAGAGGATTAGGCTAGAAACAGG
GTTCACTTCACATGCAGCTTACCATCTGCAGAGTGGTCAGGTACTTCTTCCACCAGAGGTATTTCTGGTA
CTGCGGACCCATGGCGGAGAACATGTAGTAG
>dme_circ_0008006_junction_seq
CGGACCCATGGCGGAGAACATGTAGTAGGAAGAACATGACGGCATGCATGCCAATC
>dme_circ_0008007
GGAAAAAGGCCATGGTCAGCATGGAGGGACAGTTTCTGGTGCGACAAATTTACGACGATGAAATAACATA
CAATCTAATTGGAGCAGCTGTCGAGATACTCAGTAAGTAGTTAGCTGGTGCCATTTTGTTATGCAAGCAT
ATGTGGGTCATAAAATACTAACTCACTACTGTCTCCACTCCCGCACTGTTTGTCCTTGAAGATATTCCCG
CAGATGACATCCTGGAGCTGTTCGGTAAGACTTTCTTCGAGTTCTGCCAGGACTCGGGCTACGACAAGAT
CCTGCAGGTCCTGGGAGCCACGCCGCGGGACTTCCTTCAG
>dme_circ_0008007_junction_seq
GGGAGCCACGCCGCGGGACTTCCTTCAGGGAAAAAGGCCATGGTCAGCATGGAGGG
>dme_circ_0008008
GGAAAAAGGCCATGGTCAGCATGGAGGGACAGTTTCTGGTGCGACAAATTTACGACGATGAAATAACATA
CAATCTAATTGGAGCAGCTGTCGAGATACTCAGTAAGTAGTTAGCTGGTGCCATTTTGTTATGCAAGCAT
ATGTGGGTCATAAAATACTAACTCACTACTGTCTCCACTCCCGCACTGTTTGTCCTTGAAGATATTCCCG
CAGATGACATCCTGGAGCTGTTCGGTAAGACTTTCTTCGAGTTCTGCCAGGACTCGGGCTACGACAAGAT
CCTGCAGGTCCTGGGAGCCACGCCGCGGGACTTCCTTCAGGTACTTTTGCCTTATTTACTCGATATATCT
GGCAGTTAACTTAATTGAAGGCCATTTTACACTCATGCGCCATGCATCTGCGGCACAATTGCATTGGCCA
ATCGACTTTTAAGTCATTTTTCATGCTGCTCCCTTGGCAGTTTCTCGGTTTCGGTCTCAGTCTGCTCGAG
TTCGACATTCGCCATCATTGATAAGCATTAAGTATTTTGCTTGAGCGCACACATCGCCACTCAAACCCAA
ACCCGTAACCCATTTCCCTTGGCGCCTTTTGTTGCACTCACATTCGATTTATTTTCAATTTTCCACATTT
TTCACCGCAAATGCTTCTCGAATCCTTTGTCATAATGAAAAATGAAATTGAATTTTTTCGTCTGCACCAC
CGCAAAATCAACAACAACAGTGCCACACGGAGAACAAATGCCACAAATGCGGGCCATCAATTCACTTTGA
ATTCCGTGGACAGCATGATTTTCATTGAAGAATATTGAAGATATCGATTGACCTTTTATTCACAATGTAT
AATTTTTAAAAACAAACTTCTTGCTTGGAACTCTAATATGTAtttttgttttttttttttttCAATTAG
CTAAGATAAGAAGAAAGTTCCTCTATATTAATTATCCATATTACCTATCCGCTTTCCCTTTTCTCTCTGT
GCAACAATCAGGGCCGTTGCATCCAAGCTGCACGGCGTCGAGGTGGAAATCGACATTGTCAAGCGCAAGG
GAGAGCCCATCGACGAGGCCGAAAAGGAGCGGGCCATCGCCCGGGAGAATCAGCAACTGCTGGAGGACGC
GGTGGCCACGACGACGACGGGATCGGCAACGGTTGTCCTGGCTCCTTCGTCGGATGCCGAGCGAAACAAT
AATCAcaatggcagcaatggcagcaaCAATAATGGAATGGCCAACAACGGCAACACCGTCAACGTGAATA
ACAACAACGATGGCCAACAGATTGCCAGCGAGACAG
>dme_circ_0008008_junction_seq
GATGGCCAACAGATTGCCAGCGAGACAGGGAAAAAGGCCATGGTCAGCATGGAGGG
>dme_circ_0008009
GGAAGGGACTCTACATCTCGGATGTGCCGCTGCACGACGCTGCCAGGGATTTGGTTCTGCTCTCCGAGAA
GTTCGAGGCGGAGTACAAGCTCACCAAGAACCTGGAAATGCTCACCGACAAGCTGCAGCAGACATTCCGC
GATCTGGAGAGCGAGAAGCAGAAGACCGACAGGGTAAGAAAAAGTCAATTTACCCTATCCCTTTGGGAGA
AAGTGCACAGAGAGAAAAACAAATTGTGTTGAGACCATTGTACAGCTTACAAATTGCACTTGTACAACAT
ACTGTGGCATTACTTTTCTCTCTGTGCATCACTCTCACATGCTGGAAAATGCAAGCAATTTGCCATATTT
GTGTCCGTTTATTTATGAGACCATACTTCCTTTGCCATTCGACAGGCTACGACTCCGTGACGTTGATGTT
CTCCGGCATCGTGGGCTTTGGCCAATACTGTGCGGCCAACACAGATCCCGACGGGGCCATGAAAATCGTG
AAAATGCTGAATGAACTCTACACGGTCTTCGATGCTCTGACCGACTCCAAGCGAAACCTGAACGTGTACA

AGGGTTGAAACAGTTGGCGATAAGTACATGGCCGTTTCCGGACTGCCCGATCACTGCGAGGATCATGCCA
AGTGCATGGCACGAGTGGCCCTCGATATGATGGACATGGCCAAGAACGTCAAAATGGGATCCAATCCAGT
GGGTGAGTGTGGAATATAATAAATTCCGAATATATGCACTTATTTGTCACTCTGTTATTTTGGTGTATAA
TGAGAGCAAACTTCACGTTTAGGACTAACACAAGTAAAACTCGCTAAATTTCGCCGCAATTGTATTTGAG
TCTCGGCTTCGCTTTTCATCAAATTTTAGCATATTTTGGCGTCTTTCCTTTTGTGCCACATTTTGTGCAG
CCTTTGCGGAGCAACCAAAAATGCATTCACAACTGACAACTTGTGCTCAAGTTGCTCAAGTGCCCGCGGA
TGAAAAGCGGGGTTGGGGCTGGGAGAATGCTGGGTTTTTCCAGGGGGCCGTCACTCTAATTGTGCGCATT
GAAAGTAGTTGGAAAAATTGTCTCGCTCGTGTTTTTGTTCGTTCGTGTGAGTATGCAACTTTCAGCGTGTT
CGCGCAATTTGTTGCTGAATGcgccattcgccattcgcaattttccatttgccattcgccattttGAGAG
CAACAGCAACTCAATGGCGCATAAATTTCAATGTCTATCCCCTCGCCCCTTGCAATCTCTTAACCCTCTA
CTGGCCGGATTGAGTGGCAAGTGCACGTAAAATATTAACAACTTGTGGCAGCAGGCAAGTGCGAAAAAAG
TTCACTCAACAAAGTTTCACATTCTATGCGCAAGGCGTGGGCGCAAAGTTTTGGATGAAAAACTGCGAAA
ATGGCTAAAATTAATTCAAAATTGATGACACACAATGTCAAAGGACGCGTCTTGTACCATGGTACATGGT
CTCGGATCCTTGCCAGTGGCCACTTGACCGTCAACTGTCACCAGAGACGTTGAAACTTGTCCGAGGATGG
TGACGAGGCCTCCTGCCTGCTGTTACAATCTTTTTTTCGGCTTAAACACTTTTGCTTTGAGTGCTCGGGC
TTAAAATACACTGCACACATGCAGGGCCACAGATAAATGTAGTTATGAAAAGGGGGGGAAGCAGGGACGG
CGGTTCATCCACAGGACGAAGCGAGGACGAGTGTGAGGGCGAGGATGCGGATGCCCCACTTGATGGCATC
CTTCACAATTACAGCATTGTACGTGTGGACAATGCTTGTGGCTTGTCAGTCACTTTTTGCACATGTCTTG
GCATCTGTATGTATATTAGGGTGGCTCACAGCGAAAATGGAAACAAACTatattggaatatttgaatatt
tggaataCCATTATGTAAATCCATATTCTATAAAGCAGAATATGTTTGTGATTTTGGATTCTTAATATAA
TATCATTCAAAGAGCAAATATGAATTTTATTACATTACTATTAgtatattaaataatctaaatataaata
tattaaatatatattaatatatatattataaatatatataaatataaatatattaaatatattatattaa
atataaTTACCGACTCACCCTACTGTAGATATGTCCCAGGATCTGCGTGCGTGTGCCATATACCCACCAA
CACAAACTTAGCAACTCTTGCTTGGGCTCGTGTCACTTGGCTTTATTGTCCGAGTTGTTGTTTTTCTGTT
GCTAAAATTCCATTACCTTATCGTCTTGCCGTTGGCTGTTCTCGTAAGGAAGTCTTCTTTTGGTCCTGGG
ATCATCCATCTGTTCCGCCCACCATAAGCCATAAGCCCCAACTAGTCTCCATTTCCATCTGGCCTCGCCG
CAACTGCAGTCAAGTTTTTGTGGCTGTCAGTATTTCATATTTGACTGATTGCCTTTGGTAATGCAGGGGG
AAACCCCTTTCATGCGGACGAAAAAGAAAACTCGGGCAGGGGAATTCGAGAATCGAGTCAGCGTTTGATA
GATCGCTCGAAATCCAATTAACAATCGCCACGGGCAACTAATGGCAAAAACTAGGACACGACATCCGAGC
CTGTTGCACTGCCCTTCCCCAGGATGCTCTAaacccccaaacccccccttttgcgaccctccccccaacga
acctcccTTTCCGCCACTGGCGTCATTTTGCGCTTCATTAAATCCTGATAAAAAGCAGCAGCAGCAGAAG
CTAGTCATTCCGCCCGGCACCTCCCCCTCCGTTTATGAAGCCAATTATACCGATGttttttttttATGCAA
AAACTTTGCTTCCAGCCCTCTTTTCCCGCTTTTCCTGCCAGTTTTTCTCAACTCAACTCTTTGTTGGCTA
CTTCCTGTGCTTACAGTCCATAAATATTGATTCGGCTCTCAGCAAAAACTTTCTGCAGTTGCTATGTATT
TTATACACTCGAGGGCGAAACCAGGAAAATCATTTTCCTTACTGCCATGCATCTTGGCTGATGGCTAAAA
ATAAACCAGCAATTTTCCAGAAAATAGTTTCGAACTAGAATGGACAGTACGTAATGTCCTTTAGCATTTT
CTGGCAAAAAGTTTGCTTCTCTAATTGGAAAAAAATTGTGAAGAAAACTGTTTTTGTTTTCCATATTACGT
TGACATTTGAAGTAATAATAAATAAAAAACAAAAACATTGTATAGCTCCATCATTTAATCAATAGAAACT
GTTTTAAGCTCGATGTTGAATCATTCATCAGCAGAGCGGTGCACTGGTTCAATTTGATTTTGTTGCGAAT
TTTTTCCCAACTCGACTTTCTGCACTTTTTGCCTTCCAAAAGTTTTGATACTTTTCTTATCGCACTCCAC
CGCCTAGGCGATTAGTGTTATGTAATGTTTTTCTGGTAATTACAACTTTTGTCCTTCGTTTTTATATCTG
AACTGCTGGCACTTTACCCATTTTCACATATTATCTTGGCTCTGCGTTTCTGCGAAACTCTCTCAGCATC
GTCACCTACTAACAAGGACCTATTGTGGTCGGGGTTGAGACTTGCTCTTATTCCCTATGGTCGCCGCTCA
TTTCGTTTTAAATTTAATTAAAGAAAACTTTTTCCTACTGCTGCTGCAAGTTTTTGGAAAGTAATTGTGG

CAACTTTCGGTTGGTTTTTGCAGCTACATTCCGTTGCGGTTGGCAACTTTTCGGGGTGTTGATGATGGGA
CGTTGGCTGGCGAAAGTTTATTAGCGCAATGAAGTGCCTTTCTAATACGCTGCAAGGCCCGATAAATTCT
GTTACGAACTCAGATTATACATTTATTTGCTAGATAAACTCCTTACAAATTCTACAATTCTAAATTCTTT
GTTTTGGTCCTTGTTTTTTCAGGCAAATCACCATAGGCATCCATTCCGGCGAAGTGGTGACTGGAGTGAT
CGGCAACCGAGTGCCACGCTACTGTCTGTTCGGAAATACTGTCAATCTCACCAGCCGGACGGAGACCACT
GGCGTTCCGGGGCGCATCAACGTCAGCGAAGAAACCTATCG
>dme_circ_0008009_junction_seq
GCATCAACGTCAGCGAAGAAACCTATCGGGAAGGGACTCTACATCTCGGATGTGCC
>dme_circ_0008010
TTGCTACAGGCTTTCCAGCGGCTGGTAAGTCAATTAAAAAGCCAATTTGTGTGGGTTCGCTGCTGGTTGC
CATTTCCACGGCATTTCTACGGTTTCCGCCGGGAAACTGAGTCACGGGCACGGCTGCGTCTGCGGGTGCG
GGAACAACTGCGTCGGAATCCTGCCAATAGGGCCAGTCACCAGCGGAACCGGCGAACCCAGCGCCGCCAT
CTGGTCATGGACTACATCTTCGAGTGCTTCTTCGACGACACCTTCGAGCAGATCTCCCGCAACGGACTGC
AGGACCGCCAGTCGCGGCGAGATGTCCTCGACCACCTGAGCTCCATCATCAAGGGCTGCAGCGGCGGCCA
GAACTCGCAGACGGATGAGGTGGCCGCCATCGCGGTGACGGCGGCCATGAGGTACCACCGAATGGCCAAG
GAGCAGAACGGCCAGGGTGAGCTTTATGTACAAATCGAGTGTTGCCAATGTGGCATTTCTGTGTGCCAAA
CTTCAATAGTTAATATGAATAAATATTTACTTAAAAGAGTCCATACAAACTAGCACTGGGCTTTAGCAAA
GCTCAACTTAATTAATAATGATTTGACAATTAGTTTCCGGTACTTAAAAAAACGAAATAAATAATTAGAA
CAAAAGCCAAGTCAGTCTATTTCGTTGTCGTATTAAATAAATGTTAATGTTTAAACCAAGCCTTATGAAT
GGAAACCAATGATTAATTAGCCAAATAACATTGCTTTGCTTTTGAGTCGAATAAAAGTGGAATTTAATAA
AGCTTTCTAGAAAACGGTTAGAAAAGTACTAAAACTATGCATAAATATGTATATCCTGCTGCTGCGATCC
AATTGACCTTTTAGAGTGTAAATAAATATGGAAAGTTGTTTCGGGCTTAATTTCCTTTGGCAGAGTCTGC
TAAACTTAATTTTCTTCCATTTTAAATGCCATCTCTTCATATGGCAATTATTTATAATTTAAAAAGTAGC
ACTTCAGTTGGGATTCAGCCCCTTCACCACACCCTCCATTCCATGCGTATTCCCCAGGGTAAGTGGAGTC
GGGCCAACTAAATAATTTAATTTAAATGCATATGTATGCGTATGTGTGCGTAAGCGTATGCGAGTGAGTG
TGCGTGTGTGTGTGGCAAGACTTTCCTGTTTGCTTTTTGGGCGTGGCTGTGTGCTTTGCATACTTTAATA
AATATCATCCCATCGCATCGGATTATTACGCCCATAAGCAGGCAGTTTTCACTTTAATCACGTGTTTCTG
CTAATTGCGTTAAGAAAGTGTTGCTGATGTCGTTCTGTTGCTCTGTTGTTGCTGCATCGTAAAACTTGTA
AAGTGCCTCAAAGTGTTGCAAGTTATTTGGGGCGTACGCATGTTGGCTGCCTCTAATTAAAAGGCTGCGT
AAACTTTTGCGAGCAGGAAAAGTGCCTGGAATTAATGGCATGCCTCTGTCAGGGAATCTGGACTCGTGTG
CAATAAAACTTGCTCCTTGTTTGTCCTGTCCTCACTCACCAAGTTGCTTTGTTGCTGACTCCAGCCTTTT
GGACCCGCTGGCCTTGGTTTGGCAAACATTGTGGCCGTTTCCGTTTTCCGCTGACTTCATTTCTGGGCTG
TGCCAAAAAGCCAACGAAACCGAAAGCCAAAAAACCAAAGCAGCAACAAGTGCAGCCACGTAATTAGCTT
TTTTATGGCTGCGCTGCTGCGATTTGATGACATTATTTAACCGCTTTCTCCACTACCGACAATTTGCCTG
CATCGCGGGAAAATCGGTCACAATGTGTATACAAATTTGGTTTATGGCATTACCCACTTCGAAATGTTAT
TAAACGTGGCTTGTTTTATATAAATTAACTTTTAACTATTTTCCTGTTCCCATGACTAATTTATCCCCTT
TTTGTGCAATTCAGTGTGAGTCCTCAGCCCGGTCCCCATCCTGTCCTGACATTCTCCCCTTTTCCCCACT
CCCCCCTCTTCACTCTTTCATGTGTCGCGCTCTTTTGCTGCCTCGAGCGATTTTCACACTAGCCGGGTTA
ATTGGATAGTTGCCTGGGTGCCATGCCGAGGTCTTCATTCCCCCAAAGAGTTTTTCATACTAGCTGTATG
CTTGGACATTTATGGAGAGCTCTTACTTTCATAACAGTCACAGTAAGCCTTTCTATACACTTAGCTTATG
GCTATCAGAGCAAATTATAAGTTGCTTACAAAGCAAATCGCCAAGAATTAAAGATTAAACTTTAAAATTC
GGATTATGTGCCGAGATACCGATCAATCTTAATGGCTTGCCAGTTTTCCACACAATAATAATGTTTTCGG
TAACAATAAGATACTATAAATACCCGCTGGGAATGCAAGGAAATGGTGGACGAACGTGAAGGGTAACCAG
AGAAATGAAAGGATTTAATTAAATTAATTCATCAGCCATTGTTGTGGCACCTACACAGAgtgtgtgtgt

gtgtgtgACTGCCATAATTGAGTGAAAGATGGCGACAATTTACAACCACAGACACGACTGCGAATACACT
TTTACAGAGAATGGCTCTCAATCACTGGCGGTGGCTTTCTCTCACTTCTGCTGGTGTCATGTATAATTAA
GATTTTCATTTGCAGCTTGCTTTTgggggggggTTTCCCATTTTCCGCCTTCTTTTTCCGACCCTCCCAC
ATGCACATGAGGTCCGTGTGTGAGTGTCGCTGATGCGATAAGCTGAAAACCAACAGGCAGCGATGTAAAC
CTTTTGTTATTTCGGCTTTGTTCGCCAGCACTTCGGAGGATTAAAGTCAAAAATGCGTGTAATAAGCGCA
CAAAGGCATGCTCAACCCCACCACGCCCACTAATCCACACAGATACAGCCCGCCCCCTTGGATATACCAT
ACCCATACCATTGCCGAGTTGACTGTTTGCGCTTCTTAAACAAACAGAAGGCAAAAAACATACTGACTGG
CAAGCATTAAGACGACATTTATGATGCCCTCTCGCAAAACAGCAGCCAAAGTCATGAATAAGCGTCAATA
GCTGCAAATGTATGTGGGTGTACGATTGGGAGTGTGTGTGTGGAGTGTGCGACCCAAGAGCTGAGAAAAA
CAAGCAATGTGACAATAAACGTTTCGGCCAAATGCCGTTGAAGTTGCATAAATCAGTGCTGGAGCAGCAT
ATTGCCAAAGTTTTCGCAGAACAGTGAACACTCGTGAAAAGGAATTGTGTTGAAGCTAAAAGCTCACAAT
GAGATTTACACAATTTACTTACGATGTTAGGGTTCTAATGCAACAGTAACGGATTTGCAAAGAAAACACC
TGAAAAGGGTGTTTAACAAAACACTAAAACCTAATTGTTTATTGAGAAAAAAACATGATTTAATTCAGTA
ATTGCATGGCTTAAATGGCTTAAAATAGCAAGTTACATTATTTCACTACTGCATTTGCATAACCTGCCGA
CTTAATGGCACTCGGAATAATTTATACCCTCCAAGGCACTGGGAACTTCACAAGCTGAGAACTCATACTC
TTTTCATCGGATTTAATCGAAGTGCACACTGTATAGCAAAGACATTAACATGAAACGCCGGCTAATACTC
GCTAAGCAGACCATTAGACAGAAATTTGATTTGTCAGCCCACTCAGCAGCTGATTAAAGTGTTAATGTCT
GACTTGGGGGCAAATCACTTCCGACTGGAGGAGAATATGCCAGCCACGAGGTGCTATATACGGCTATTGA
GGAACCTGTCATGGGATGCGATTGGGGAATGGCTTGATTCTTGCAGGGACTCACTTTTATCGCCGCCAAT
AAAACGCAAATGAATTTGCCAAGCCAATTTCCAAGCTGTCACTGGATGGTGGCTCAAAGTGCGCTTATCG
TCTGATGTGTGGGTTACTTCTACTTCCGGTTTATTGTGGTTTCTGCTCTTTTATTTGCCTACTTTTATGC
GTAGACATAAAAGCCGTGTGTGTATGTGTGCGCTGTGCCGGAAATGTGCTTTAAGTGCATTGTACATGCT
AATGCCTGAATGGAGACAAACGCCCACCCCTCACACAACACATTACGCACATATACGCAATGTCAAGTTA
GGCACAGAAAAAAATATTGCAGAAATGTCGAAGTTGAATAACTTCTAGGTCTCAGTAATCCGTCTTTAAG
GCGTCTTAAAGTGTGCTGCGAGAAACGAACCTTGGTCTCTTCTTTTAGATTCAAGTAAAAACAAGGAAAG
GCTTTATATTTTTCTCTGTGCTTCTTCTCGTTTTATGAAAGGGCTTTGAGCAACAAGCGAGCAGCTTAAT
TGAGGTCTGGTACCCGCCCAGACTCATTTTCTCAGCTCCAGAGACTCCTGCTAATGTGCGAAGCCCATTT
TGATGCTTGTCGCCCATTTGGGCGTCCACTAAACTCACTCCGAATGTGAGCCTAAAAACTAACTGACTTA
TCCGAATCCGGCTTTTTGTTCTTGTTTTTTTGCAGATTCATTGATGTCCCCATTGAGTCATGCGGCAAGT
CCTCGCCCCTTCGCATTGCCCTACAGGCGAACGCTCCCGAACTGCTGCTCATACTGCTGAGG
>dme_circ_0008010_junction_seq
TCCCGAACTGCTGCTCATACTGCTGAGGTTGCTACAGGCTTTCCAGCGGCTGGTAA
>dme_circ_0008011
TACTACAGTAGACCCCCGTCGAGCGTGAACACCGTGCCGCTCCGTACTGCGCCCACGCCCCTGACGCAGG
ACACTGGTCTGCCTTCGCCTCCCCTGGCCACCTCGCCCACTCCCTCGGTGCAGAAGTCCACGGAGGTCTG
AGGATCGGCACTCAACCCGATCCTTAAGCTGCAAAG
>dme_circ_0008011_junction_seq
CACTCAACCCGATCCTTAAGCTGCAAAGTACTACAGTAGACCCCCGTCGAGCGTGA
>dme_circ_0008012
ATCTCCGTGGGAACCCAGAGGTTCAAGTGGCGCGGAAAGTACTTCGTGGAGACCCCGGCTGCCGCCACCG
AGAAGATCTTCTTCACCACCGATGATGTGCACAAGAAGAACCCCGCCGAGATCCGCATCACCTGGAACCA
GTACAATTTGACCTCGAACGCCAATGCCAACGTGATGATCTCGCTGTGGGGCTACCGGGAGACCAAGATC
GAGCCGCAACTGGAGTACATCGATGTGATCGAGGCCTCCTACTCGAATAGCGGCAGCTACGTTATCACGC
CCTCGAACTACATCAACCGCAACAACATCAACCGGGACATGCAGTTCGGATTCCTCCAGATCAATCTCAC

TCAGCCCGATCAATACTCAGGATTGGCCATCAGTCCGGTAAGTAGATACATTTGTGGCAAGCAACCTCAT
ATAAATCGTTTAAACAGGTTAATACATGGTTTCACTCAGTTGAGTTGCCTTAAATTAAATTCATGGCCAC
ATTCCTTTGTTATTAAGCTTTAATTGATTTCCGGTTGTCTCAATGTGTCATCTCAATATCTCAGTTCCGA
GTGCCTGGCGCTCTCATGGTCTATCCATGTTTTTATTCAATTTGTCGGACAATTTCAATGACAATGCGAA
CGGGCTATGTGTACTTCGGCTGCCACCAGAGCCACCAGAAACCAGATACCCAGTCATCCAACCCATTCCC
TTCGGCTGCAATCAATTTGCTTTGGCTTGGTTGGCTGGAGATTGCTTATTATGACAGCCAGTAATTGCGA
GATATTCTCGAAGCTAATCAATAATGGAAATGTGTTTTTAATGGCCCCGGCCAATGGTAATCGCTGCCAT
GTTTGTTCGCCAGTCTCCTGCATCTAAGCCTTTTTCCTCACGTCCAGCAATTATTTTGCACTTAAACGGG
AATCATCTTCCGAACCATCAAATTTGTGGCCCAAAAATACGAGTCGTGTTTCGTTGGATGGCCAGTTTGA
ACATTACGAATTTGCAAGGGCTTATTTATTGTAGAAACTCTGGCCTACACAAGAAGAAAAGTTTAAGAGA
GTTTATATTTTTGCAAGATACTCATGATAGTGTTGTAAAATATATAGGTATCTATCATTCCCTTTTTTAT
CGCTGTGCCTTTTCCCCCAAGACGCAATATAAGACAATTTCTTTCTTTTTTGGTTTACGGCACCTTGGGG
TGTCGTTAGACGGCCTAAAGTGGCGGATGGGCCCAAATAAATTGTTAGCATTGTTTCAACATTTAATTTC
CATTTGATGGCCGAGTAATTGTGGAACTTAATGAAAAATATTTCCATGGTAGCGCAAGGCGCGAATGGCC
ATTGAAGGATAACTAAGCAACGGCTAATGGCAAAATCGTGTTGCTCGTCAAAAGGAGCAAGTTAAATCGC
GTGGGAGTTCATAAGCTGGCAGTGTGCAACCTGCACGAATGTCAAAGGTCGATTAGTGTCATCCGTTTGG
GCTGCTTGCTTGAAATTAATCTTGGGCATACATAATCTCACACTGCACTTTCCCATTTGCAGGGAACTTT
GCGGCCGACCTGCCACTTTGCCCCTGCACCTTGGACCAGGCTGTCCTGGACAAAGGACGCTTCCGCCCTG
ACCGCGAGTGCGACAAGGACTCGAACCCTAGCTGCCTGCGTCATCGCGGGGCCATCCACTGCGTGGTCAG
TGGAACGCCAGTGTGCCCAAGGAGCTGAACAGCAGTGCTGCTACGATCGTTATGGCTTCCTGATGCTGAC
CTACGACCAAATGTGGGGCTCTCGTCCCCGTCGCGTCCACAACCTGGGCAAGATGCCCTGGAACGAGGCC
AGCAAGGTGCCCTCGCTGTCCATGTGGTTCCACGACATGCGCCCCTTCTACTCCTGCTGCTACTGGCAGG
AGGAGCAGGCGGTGGGCTGCGAAACCTACCGCTTCGAGCGTCGTCCCTCGCAGGATTGTGTGGCCTATCA
GGCGCCTGGAATCGCAGGTGTTTTCGGCGATCCCCACTTTGTGACCTTTGATGGCACGGCTTACACATTC
AACGGACTGGGAGAATTCGTCCTGGCACGCAGCGTGGATGAGAGTAATAAGTTTGAGGTCCAAGGACGAT
TCGAGCAGCTGCCAGTTAATTATTATGGCGAGGTGAAGGCCACCCAGTTGACAGCGGTGGCCATGCGGGG
AAACACCACCACGACGATTGAGGTCCGTCTGCGCCCTCTGCACGCCAGATGGCGCTATCGCCTGGATGTC
CTGGCCGATGGACGAAGGGTTTACTTCGATCGTGAGAGTCTGAAGTTCCAGCACTTCGACGGTGTTACCG
TGTATACCCCGACTTACCTGCTCAATCAGTCGCAGGTGGTGGTGCAATTCGATGCTGGTATCGGAGTGGA
GGTGGTAGAGAACGAAGGATACATGACCGGTCGCGTCTTCCTGCCATGGAAGTTTATAGAACAAAACCGC
GGGACTCTTTGGCAACTGGAGTTTTAATAAACTAGACGACTTTATGCTGCCCAATGGACAAGTGGCCCAA
CTCAATCTTAACGATCTGCGCAGCATTCATACCAACTTTGGAATCAAATGGATGCTCACGGATCGCGAGG
TTCCTGGAGTGGGAGCCGCTCTATTTAAACGAGAATTTGGACGAATGTCTGGTTACTATGCGAATGCCAC
CTTCCAGCCGAATTATGTCCTAGATCCTGCGGACTTCCTGCCAGCGAATCGCTCTTACGATCTTGAAAGA
GCTGAAGAACTTTGTGGAGAGTGTATGCAGTGCCAGTACGATTACGCAATGACTTTGAATAGAGACTTGG
CACACTTTACCAAAAATTACTACGATACTATAG
>dme_circ_0008012_junction_seq
TTTACCAAAAATTACTACGATACTATAGATCTCCGTGGGAACCCAGAGGTTCAAGT
>dme_circ_0008013
GTGAAGGCCACCCAGTTGACAGCGGTGGCCATGCGGGGAAACACCACCACGACGATTGAGGTCCGTCTGC
GCCCTCTGCACGCCAGATGGCGCTATCGCCTGGATGTCCTGGCCGATGGACGAAGGGTTTACTTCGATCG
TGAGAGTCTGAAGTTCCAGCACTTCGACGG
>dme_circ_0008013_junction_seq
AGAGTCTGAAGTTCCAGCACTTCGACGGGTGAAGGCCACCCAGTTGACAGCGGTGG

>dme_circ_0008014
GAACTTTGCGGCCGACCTGCCACTTTGCCCCTGCACCTTGGACCAGGCTGTCCTGGACAAAGGACGCTTC
CGCCCTGACCGCGAGTGCGACAAGGACTCGAACCCTAGCTGCCTGCGTCATCGCGGGGCCATCCACTGCG
TGGTCAGTGGAACGCCAGTGTGCCCAAGGAGCTGAACAGCAGTGCTGCTACGATCGTTATGGCTTCCTGA
TGCTGACCTACGACCAAATGTGGGGCTCTCGTCCCCGTCGCGTCCACAACCTGGGCAAGATGCCCTGGAA
CGAGGCCAGCAAGGTGCCCTCGCTGTCCATGTGGTTCCACGACATGCGCCCCTTCTACTCCTGCTGCTAC
TGGCAGGAGGAGCAGGCGGTGGGCTGCGAAACCTACCGCTTCGAGCGTCGTCCCTCGCAGGATTGTGTGG
CCTATCAGGCGCCTGGAATCGCAGGTG
>dme_circ_0008014_junction_seq
GCCTATCAGGCGCCTGGAATCGCAGGTGGAACTTTGCGGCCGACCTGCCACTTTGC
>dme_circ_0008015
GATTGATCTGGAGGAATCCGAACTGCATGTCCCGGTTGATGTTGTTGCGGTTGATGTAGTTCGAGGGCGT
GATAACGTAGCTGCCGCTATTCGAGTAGGAGGCCTCGATCACATCGATGTACTCCAGTTGCGGCTCGATC
TTG
>dme_circ_0008015_junction_seq
GATGTACTCCAGTTGCGGCTCGATCTTGGATTGATCTGGAGGAATCCGAACTGCAT
>dme_circ_0008016
GCCCCACAGCGAGATCATCACGTTGGCATTGGCGTTCGAGGTCAAATTGTACTGGTTCCAGGTGATGCGG
ATCTCGGCGGGGTTCTTCTTGTGCACATCATCGGTGGTGAAGAAGATCTTCTCGGTGGCGGCAGCCGGGG
TCTCCACGAAGTACTTTCCGCGCCACTTGAACCTCTGGGTTCCCACGGAGATCTGGAATCGGATATAGCC
CTCGGCCTTCAGGTAGGGCTGCACACAGATTACGCGGTTCCGATCCACATAGGTTCCCAGCACGGACTCC
GTATCGAAGTGGCACGTCACCCTGATGGCCGGATCAAAGCAGGGTCCAGTGATATTGACGACTTGACCAC
CCAGCATATTACCAGACTCGGGGGCAAAGGTAAGAGGTAGCAGAGCAGCATCTAAATGAGTAAAAGATGC
ATATAAGATACATGGAAATTGACAGTTCCCAAAACCCAATCAGTTTCTAAGCTTTAGTTGCCACCAATGA
CAGGAACCCCGCAATCAAACGTACCGATATCCTTGTTGCAGGAGCCAATTAGAATCTGCTCGTCCACGCG
GAAAATGTGGCGACCCGGGAATCCGTTGGCCCAACCTCTGTTGGCCAGGTCGCGAATCACCATGTTCTGA
CTGTAGGGATTGTACTCGTACGCCTGGGTTCCATTACCGGCATTGAATCCGACCTGGAAAGGGGGAGCAA
AGTTCGCGTGTCAAGGGTGTGGGAACTTTTCGGGGAAGAAGTGCAATTAAATACATGCATTCGGATTTCC
CGCACATTTAATTAACTGTTCACAAAGTTCGCAATTATGTTTCGGGCTTAGAAAAAGTTTAAATTGTGCG
TTTAATTACTGCAAGTGGCTGGTGGCTGAAGGGCACTCGAGTTCCAAAAGCAAAACGATTTAAGCTAATG
ATAATTGTGAATAAGTGATTCGGCAAAGGAAATTAAGGGTTAACTATTAGGTATTTTGTATTATTTCGAA
ATTTTCTTAGAGAAACTTTATGACTCTCTGTGAATTCTTAATCGAAAAACTTAACTTGCACTTTACTTTC
TTCCTCAGATTGGCGAACAAATCACGAAAGCAAACAAGGCATGGCTAGATCCTGGGAAACTAAAGGCAAA
ACAAACTCTCCACTCGCAATTAGTTGGCAAGTTTTTCTTTGGAGTCAACTGTTTAATTGAGTGCCAACCG
ACAAAGAGTGAGGGGACTAAAAAAGGGATAGTTTGACCACTTACATATGCAGGAACACCACCCTCGCCCT
TCGTCGTATCACCTCCGGCCTCAGTATGAGAAAGCCAGTTCAGAACGGCATAGTTGAAAATAATATAG
>dme_circ_0008016_junction_seq
CAGAACGGCATAGTTGAAAATAATATAGGCCCCACAGCGAGATCATCACGTTGGCA
>dme_circ_0008017
ATCCCAATAACAATGTCGCCCCGCCGACCACATACTCCACGGCAGCCGGAGGATACACCATAACCGCCGC
CCGTCTTGCGGAGCTCAGGAGCAACTTTATGTACTGGTACTTCGACAAGGACATGTACGGAGGACGTGGC
GACTACCAGTTCGACATCCATGCCTCCATGACGCAGCTGCACAAGAATCTGAACTTCCAGCTGCCCTTCT
ACGGCTTCCGATTCAACTACACCAGGGCTTTCCCTCAACGGCTACTTGGAGTTCTCGGATCCGCCAGAGT
ACCTAACTTACCCCCTGGTCTTTCCGATCAAGGACTGGCCCGCCAAACGCGATCCCTCCTTCATGGGCAT

CTTCTTCAGCAAGTGCCGTGTAGGACGAATTTATCCATCGGATATTGACCAGCGGACGCCCGGTGTTTAC
TTCCGG
>dme_circ_0008017_junction_seq
CCAGCGGACGCCCGGTGTTTACTTCCGGATCCCAATAACAATGTCGCCCCGCCGAC
>dme_circ_0008018
ATAGGCATTTCCGGTTGGCTAACTGGACATTATAGCTTCCGATGTCAGCCAGTCGACTATAGTAATAATC
CTAGAACGTTAAGGGTATGTGTTAACCGCCACACGCAGTAAGATAACGAATAACGATAAGCATTTTGGTT
ACGTTTTATTTACGTTTTGGTGTGAGTGCACGAATAGTTGTTATAGATAATCATACACGCACATTTACTC
ATATACACATACCCATGGTAGTACCTAAGAAGATGGGTCATTGTGACACAAACTATAATTTACGGTGTTG
TGGGTTGGGCAGCATTGCTTGCGTCCTTAACAAAACTACTCCTAGCACCTAAGCCCATAGGATGCTATCA
CAGATTTAGCATGTGTAAGCCGTACATAACTGCCTTCTCTTCCACTATCTCTAAACTTAACTCTCGAGAT
TCACAGTACCAACTGTGATTGAGGCCTATCTGTTCCCTAACGATCCAATCTATTCGCATTCCATCTAAAT
TTCTTTACTTTTAATTGAATATTGACCCTTCTTGGAAAGCCATATTAACCCAACTGAATCTCGAATGCGT
GTGCGTATGTGTTTAAATAAATTTATTATTTTTCAATAGTGCTCATGCAAGAGTCAATGATGAGACCTGT
TGCCGGTTGAATCGACACGACTGCACTCCCTATTCCATCTGTTATGACTGCGAGACCCAAATTCTTGCTG
GCTTGCATTTAACTTGCACCTAGCAGTGCTCATTCACTGGATTGAATAGGGATGCGCAGCACTAAGTCCA
AGGGGATGTTAAAAACACGTCCCACATATGTACATATAATAGTTCTAATATAAACACCTCCCTCTCCCTA
TTCCCTGAATCAGATGGTTCATGCCTGCTGGTGGTACTACTTCTCGAAGTTTACAGAATTCATGGATACG
GTGAGTTGCAGATCAACGGATCAAAGAGACCGCGATCGG
>dme_circ_0008018_junction_seq
ATCAACGGATCAAAGAGACCGCGATCGGATAGGCATTTCCGGTTGGCTAACTGGAC
>dme_circ_0008019
TCCAGGTTTTTCAGCTTGCCCTTGCGGAACTTCTTCAGTTTGTCCAGCATGTCCTTCTTCTCGGCTTCGC
GTTGGACCTTGGTCTGTTTGGCCAGCATCTTGCCCATCTTGCGCTGCTCGCGGATCTGCTTGATGCGCTC
GGATTTCGCCTGTCCTTGCTGTTTGGCCATCAGGTTGGCGCGCACCTTCTGCATG
>dme_circ_0008019_junction_seq
CATCAGGTTGGCGCGCACCTTCTGCATGTCCAGGTTTTTCAGCTTGCCCTTGCGGA
>dme_circ_0008020
GCCCTTCTTCTTGCCGCCGAAGCCGAACTTCTTGTTGCGCTTCTTGCGATTCTCGGCTGACTGCTTCTGC
TTGGACTCAAGCGCCTTGGCGTCCTCCAGGAAGTCCAGGTTTTTCAGCTTGCCCTTGCGGAACTTCTTCA
GTTTGTCCAGCATG
>dme_circ_0008020_junction_seq
GCGGAACTTCTTCAGTTTGTCCAGCATGGCCCTTCTTCTTGCCGCCGAAGCCGAAC
>dme_circ_0008021
GCGCAACAAGAAGTTCGGCTTCGGCGGCAAGAAGAAGGGCCTCAAGAGGAACACAAAGTCCTCCTCCGCG
GGATTGGATGGCGACAAGTCCTCCAGGCGCCAGCGGGGCGTAAAGGCAGGTGCTTCG
>dme_circ_0008021_junction_seq
CCAGCGGGGCGTAAAGGCAGGTGCTTCGGCGCAACAAGAAGTTCGGCTTCGGCGGC
>dme_circ_0008022
GTACGCGCCACAATAAAATCGAAAAGTTGCTGAGCTCAACGATCCACCGCATCTCCGCCCAGCCACGCCC
ACACACTCGCCCATCCAAGATGTTAGTCGCAGAGGTGATCTCCACGAGATGCCTGCCGCATGTCCAAGAA
CTTCGGGGAGAGAGAGAGCCTTGGTGGGCGCGCATGGAGATCCTCCGCCGAATGGGCCGAGTGCACGAGG
ATGGCTGGAGTTCGTTCGGGCCATCGATTGGGTTGTGTTTTCGGTGAGCCGGAAGCTTGCTTTAAGTAAA
ATCTAATTTTAATGTCTGTCGTCTGG

>dme_circ_0008022_junction_seq
AAATCTAATTTTAATGTCTGTCGTCTGGGTACGCGCCACAATAAAATCGAAAAGTT
>dme_circ_0008023
GGTAGTCAGTCACAGATTAGTCTGAATTGAGTTCTGCCTTAGAGGCAGCAAGCACATCGCACTAAAAACG
CTGGCCACGAAAGGTTTCAATGCCAACCAGGCGGCGGTCACCGGGGGCAACGGCAACACCTG
>dme_circ_0008023_junction_seq
CGGTCACCGGGGGCAACGGCAACACCTGGGTAGTCAGTCACAGATTAGTCTGAATT
>dme_circ_0008024
GGTAGTCAGTCACAGATTAGTCTGAATTGAGTTCTGCCTTAGAGGCAGCAAGCACATCGCACTAAAAACG
CTGGCCACGAAAGGTTTCAATGCCAACCAGGCGGCGGTCACCGGGGGCAACGGCAACACCTGGTAGCAGG
ATTTCTGGTACTTTCGCCCTGCAATCACACAG
>dme_circ_0008024_junction_seq
CTGGTACTTTCGCCCTGCAATCACACAGGGTAGTCAGTCACAGATTAGTCTGAATT
>dme_circ_0008025
GGTAGTCAGTCACAGATTAGTCTGAATTGAGTTCTGCCTTAGAGGCAGCAAGCACATCGCACTAAAAACG
CTGGCCACGAAAGGTTTCAATGCCAACCAGGCGGCGGTCACCGGGGGCAACGGCAACACCTGGTAGCAGG
ATTTCTGGTACTTTCGCCCTGCAATCACACAGGTTTCCTGCTGGGAAATGAAGCCGCAACAAACAGCTGG
CTCGGCCGATTCTCAGACCTTCCCGGGGCAATGGTTTGTGGA
>dme_circ_0008025_junction_seq
AGACCTTCCCGGGGCAATGGTTTGTGGAGGTAGTCAGTCACAGATTAGTCTGAATT
>dme_circ_0008026
GATTAGTCTGAATTGAGTTCTGCCTTAGAGGCAGCAAGCACATCGCACTAAAAACGCTGGCCACGAAAGG
TTTCAATGCCAACCAGGCGGCGGTCACCGGGGGCAACGGCAACACCTGGTAGCAGGATTTCTGGTACTTT
CGCCCTGCAATCACACAG
>dme_circ_0008026_junction_seq
CTGGTACTTTCGCCCTGCAATCACACAGGATTAGTCTGAATTGAGTTCTGCCTTAG
>dme_circ_0008027
GATTAGTCTGAATTGAGTTCTGCCTTAGAGGCAGCAAGCACATCGCACTAAAAACGCTGGCCACGAAAGG
TTTCAATGCCAACCAGGCGGCGGTCACCGGGGGCAACGGCAACACCTGGTAGCAGGATTTCTGGTACTTT
CGCCCTGCAATCACACAGGTTTCCTGCTGGGAAATGAAGCCGCAACAAACAGCTGGCTCGGCCGATTCTC
AGACCTTCCCGGGGCAATGGTTTGTGGAGTAATCAGATACTC
>dme_circ_0008027_junction_seq
CAATGGTTTGTGGAGTAATCAGATACTCGATTAGTCTGAATTGAGTTCTGCCTTAG
>dme_circ_0008028
ttgctgctgctgctgctgttgcggctgcggctgttgctgctgatgctgttgcGTGGGAAGATTGGTCAGG
GTGACCACATTCCCGGCACTGAGCTGCTGCAGGACATTCCCGTTGCTAGTGAGAATGGCAGTGTTGCTAG
TAGTGCTCCCATTGTTGCCATTATTCCCGTTCGACAGACTGCCGCTGCCTCCATGGCAACTGCTGGCGCT
GCTCGACATGTTGCTGATGCTCGACGGACTCAACGAGCCCAGCGACTGAGACGGCGAGTTGCTGCGCTGA
ATGATGGGCGGCGGGTTCGGATTGGAGGAGG
>dme_circ_0008028_junction_seq
ATGGGCGGCGGGTTCGGATTGGAGGAGGttgctgctgctgctgctgttgcggctgc
>dme_circ_0008029
CAAAGTCCCCACAAGTGCAGCCAATCGCTGCAGTACTCGCAAAGTTCCAAGGCGAATCTGAGGCAGCACA
GCTCGGTCCACACACAACAGCCAGCAGGTCCTGTGAGGGAGTTCCAGATCGATCCCTACATCATACTCGA

TGACGACCTCAAGTACTTCTACGACGATGTGCGATATGCTGCTGAAATCGGGCACATCCCAGCCAGAGTT
GGACACCATTGCCAGCTATTACTTCGATGGCCAGGGCAAGGCTCTGCGACCCATGGTTACCATGCTGATG
GCCAAGGCGATAAACTATCACCTGAACAACGAGTCACAGGTGAGTACTAGTAGATGTGACTAATTAATAT
CTACATGAGAAGAGCTTACAAGCTTAGTAATGGTAGTCGTAAATAAATACTGCCTCTGTTTTTGATTTTA
TTATTTTCAAAAAGAACTCGTGGTTATTACCTAACGTAAAAGTATTCACATTTCTAACGAACTTTCCTGt
ttttttttttAAGTTTAGTAGAGTTTAAAATGTTGCTATTAGTTTCTGTCATTTATCTGTGTAGACGAGT
GTAATTGATTAGCACTGTTTTGCGAAATGTGCGCAGCCCGACCAAATGAAATCACACATACCCCTGCAAA
CACTTACATACATATGTATGTAGGTGTAGTTTTTGGATATTTGCCCTAGTCCTAAATCATGTATCTTCCT
ATCGCCTTGACAGCCCAATTAGTACACAAACAGCGACAGATCGCCCTCTTTTCGGAGATGGTGCACTCGG
CCAGTCTGGTCCACGACGATGTCATCGATCAGTCGGACTTCCGACGCGGCAAGCCCAGCGTGAATGCTCT
GTGGAACCACAAGAAGGGTAAGGATCGATCCGCGTGACATGGGTGATATGGCATGTGGCATCATCGATGC
CACCGATGTTTGCCAGAGCCAAACAAACAAAGCCAAACACACGCCCCGAGTCGCTCGGCAACCGAAACAG
CCGAATAGCAATCATTGGGGCCCAAAGCCTGACTTTGACATCAATTTGTTGCGTATTTCGATTTGAAGTG
TTTATTGACTTTGTCAACATCTATCTTGCATCATTTGAAAGGCCTCAAAATCGAATGGCGCTGCCTCTTG
CCGCTGCGTTCAATAATTCAATAATAGATCCCAAAATAGTTCCCTTTCGACATGTCTTCAAATTAAAATG
CACAAATTAATCCAAACCCCATCTTCACAAGAAGCGGAAATTTCTCGCCTTTGATTGTATTGGCGCTCAT
CTTAGTCACATGTCGGTTGGGTTTTCTTGCCTTTAAGCCAACTCGCTGCTGGCTGTTAACGTTGCAACAC
ATAACTGTCTTACAGCGATTGTGTTGGCCGATCTTTTGTTATGGCCGCTTGTTTCTGACCATGTCTCATC
GCTTTTACAGGTCACAATGGCTGGTGATTACATCTTATCGATTGCCTCGATTATGATAGCTCGTCTGCGC
AGCGATGATGTGACGATCGTGCTGAGTCAGG
>dme_circ_0008029_junction_seq
GATGATGTGACGATCGTGCTGAGTCAGGCAAAGTCCCCACAAGTGCAGCCAATCGC
>dme_circ_0008030
ACTCGAGGACAAAGGGCTGGCCCATGATGTCGTCCCCATTCCCCACACTGGCCGTTTGCCTCACATATGT
CTATCTGGTCAAGGTGAGTGTACGGAGCACAAAATTCCAATTTGCAGGCTGCCCAATTTGGGCTTCTTAT
TAGTAGTTAAACGATAAGAAGGTGGTCTTACTAAATCAATTGTAGGTAGGTTTATGACTACTGTACTGTA
CTGTTTCACATAACAAACTTATCAACTTCGTGATAATAAAATGGGGTAAAATTAATGTTGATACCAACTG
CCGTCCACAGGTGCTGGGACCCCGATTAATGGAGAACCGAAAGCCTCTGAATCTGCAAAACACTCTAGTC
ATGTACAATGCCATACAGGTTGTATTTAGCGCATGGCTGTTTTATGAGGTAGGTATTCCCGAACCACCCA
TCCATCCCATCCCACCCCGCCCCACTTCCCCATCATCCCAGCAGCTTTTCATCGCGTGGCATCGTCGCTG
GACAGGTCCACGCTTCTGCGGACCGTGGAGGACCGTGCTTTCCAATCGAATTTAACCTATTTTTGCTGCT
AATGTCTCTGTTCTAGTGTAGGTATTCCCGAACCACCCATCCATCCCATCCCACCCCGCCCCACTTCCCC
ATCATCCCAGCAGCTTTTCATCGCGTGGCATCGTCGCTGGACAGGTCCACGCTTCTGCGGACCGTGGAGG
ACCGTGCTTTCCAATCGAATTTAACCTATTTTTGCTGCTAATGTCTCTGTTCTAGTGCTTGATGGGCGGC
TGGTGGGGCTCGTACAGCTTCCGGTGCCAGCCGGTGGACTACACAGATAGTCCCACATCCCGGAGAGTAA
GTGTCCCTGGCAAAGCGGCCTTGCGCTAGAGTACCTATAAGTGTGTGCTTATCCGATCCGATTTGACCGA
TTTTACCCCGACCACTTCAGAGAAATAGCTAATACCGTAGTTATTCGCGCCCCATTCTATATCTGCAGTT
CAGGCAATCCAGCGCGGCTTCTGCTTAATCTAATCCTACTATATTGTTATTGATCCACTGGCCACTGACC
TAGCCAACGCTTTTTGGAGTGCTAACCAGCCACGCCTCGCCGTGTAGTCGTTTCATTGTATTAACTGCGT
ATTGTACTCAAATAAACCCCGACTGCCAAAGATGCCTGCGCCAGCTTCGGGCGCCCGGCTTTGACTAATA
CCGACCTAGTGTACCCTAACGCACCTCATGTGCAGGACGCACTTGTCCTGTTCTGACCCCCAGCTGCTCT
GTTTTGTATATAAATGCTAGTCAAACCAACACCAGTGCTACAGATAAGACCGGTGGATAATTTGCTTCTA
TTAATAGAATTATCGCGTTTAAGCCTAAGTTGATACCATCTAAGTGCTCTATACTCACACTAACCCCCAC
ACCGCCAAACTTGCACATCTACAAATAGTTGACAGCTGAGCGAGCAAGCGACCTGAAAGTGACGTCTTTC

AGCCAGTATCCGGTGACGATGCCAACACCAGTACCAGCATATCTCTCACGAATCATCCAAGAAATCGAGA
GCCAAGCGAAGATATATAAGTTACCTTTTGTGCTCTACATTTCCAGAGTAAGTGTCCCTGGCAAAGCGGC
CTTGCGCTAGAGTACCTATAAGTGTGTGCTTATCCGATCCGATTTGACCGATTTTACCCCGACCACTTCA
GAGAAATAGCTAATACCGTAGTTATTCGCGCCCCATTCTATATCTGCAGTTCAGGCAATCCAGCGCGGCT
TCTGCTTAATCTAATCCTACTATATTGTTATTGATCCACTGGCCACTGACCTAGCCAACGCTTTTTGGAG
TGCTAACCAGCCACGCCTCGCCGTGTAGTCGTTTCATTGTATTAACTGCGTATTGTACTCAAATAAACCC
CGACTGCCAAAGATGCCTGCGCCAGCTTCGGGCGCCCGGCTTTGACTAATACCGACCTAGTGTACCCTAA
CGCACCTCATGTGCAGGACGCACTTGTCCTGTTCTGACCCCCAGCTGCTCTGTTTTGTATATAAATGCTA
GTCAAACCAACACCAGTGCTACAGATAAGACCGGTGGATAATTTGCTTCTATTAATAGAATTATCGCGTT
TAAGCCTAAGTTGATACCATCTAAGTGCTCTATACTCACACTAACCCCCACACCGCCAAACTTGCACATC
TACAAATAGTTGACAGCTGAGCGAGCAAGCGACCTGAAAGTGACGTCTTTCAGCCAGTATCCGGTGACGA
TGCCAACACCAGTACCAGCATATCTCTCACGAATCATCCAAGAAATCGAGAGCCAAGCGAAGATATATAA
GTTACCTTTTGTGCTCTACATTTCCAGATAGGCATTTCCGGTTGGCTAACTGGACATTATAGCTTCCGAT
GTCAGCCAGTCGACTATAGTAATAATCCTAGAACGTTAAGGG
>dme_circ_0008030_junction_seq
TATAGTAATAATCCTAGAACGTTAAGGGACTCGAGGACAAAGGGCTGGCCCATGAT
>dme_circ_0008031
GAAAATGCTTAGCTGTCTCGGAGACACCACGGCGTATACTTAATCTTGTCAACGGGCCAAAGCACGGCTA
AGCAAACATGAACTGCCTCTGTCGCCCATCACGGATTATGTCCGTGCGCATTAATTTGCTGGATGAAACC
GAGTTTATACACGAGATCAAG
>dme_circ_0008031_junction_seq
TGAAACCGAGTTTATACACGAGATCAAGGAAAATGCTTAGCTGTCTCGGAGACACC
>dme_circ_0008032
GGATGATTTGCCCGGCCAGGCTCTGCTGGATGTCGTCTTCGCCAGGCTCAATTTAATCGAGACCTCATAC
TTCGGCATACGTTATATAGACGAGGAGAATCAGACGGTAAGTGTGTAGAGCAAAAACGATCAAAATCGAA
AAAAGTGAAGGAGACCGCTTCCGCTGGGCACACATATTGGTTTTGGAAATTCATTTTCGTTTTCATTGAA
ATTCATTCAGGCGCGATGAAAAATGAAAACAAAGAGCCCAGCAGCCAGACTATGGCAACTCTTTGAGAGT
CCTTTGGCTGCGGGGCAAGTTAGATATTTTCGCTTTCGCTGGTCCTGCTGGCTGCGTTGTCCTTCATTTA
CCTTTACACATACCATACACGTTGCTCATACGCCACGTGGTTAGCCCTTAGATTTATCCCTCATTTGAAT
ATTCCCACAGCACTGGCTCGATCCTGCGTCGCGTATTTCTCGCCAACTCAAGCCCAAGTCGGATCCTTAT
GACTTGTATTTCGGTGTGAAGTTCTATGCTGCCGATCCCTGCAAGCTGCTGGAGGAAATAACCAG
>dme_circ_0008032_junction_seq
CCTGCAAGCTGCTGGAGGAAATAACCAGGGATGATTTGCCCGGCCAGGCTCTGCTG
>dme_circ_0008033
GGGCGAGGACAGCGTGGAGTACTTCCTGGGGCTGACTCCGAGCGGGATTGTCGTGCTGCGCAACAAGACG
ACGGTGGCTCATTACTATTGGCCGCGCATTGCCAAAGTTTATTATAAAGGACGCTATTTTATGCTCAGGA
TAAGCGATAAAAATTGTAAATTCTCAAACGCACCCAACGCGACAATGGGAACTGGAACTGGAAAACTGTA
AACTGTTAGCTGGAAACCCGAAACGACAATGGCCACTGTGACGATGTGGATGAGGATCCCCATTTCGTAG
CCCATCCCAGCCATAAATTTGTTTGCCGACAAGTGCTTTTTACAATTTGCAGTTCGTTCCGCGAGTGAAT
GGTCGAAAGTTGGCACTAAAGGGTAGTAGACCTATGACAAAGTCGAGTAGAAAGTATCTGACAGTTGAGA
GAGTTCTAGGCGTGCTAAATAGATGACGTGAAAGAAATGTAGAAAATTCAATACCAATTCACTTTACTTG
ATCCTTAGTCATTTTCAAGGATTTATACGAGTAAGTCATGATATCCTCGAAGTTGAGTACTTTAGTGTAG
TAAACACAGATCGTTCTTTAAAACTTACAGGGTATCCTATAGTCCATAGCTCCCTTTGAGTGCTTTCAAT
TTCTTATTCCCGTTTTGCAGTGTCGCCAATTTTTCTGTGTGCTCCTTATGGCGACAACTTTTGCCTGTCA

GCGGCATCAAGTGGCGCCAGGAGCCAGAAGCGTAGGAGGACTCGTTAGACAACAAGTAGCTAGTAGCTGG
TAGATGGAAGATGGTAGCTTTGGTAGTAGATAGCAGATAGAGGACGCCAAATGGCTGGAGCTGCCGAAAG
TTGCACACTTTCGGGTGTTAACGCTTTGATAATCAAAAAAGTTTCCCAGCCAGTTGACGCGCACAAAAGG
CGGAACTTAAGCATTTTCCTTCGCCTTGATTTTTCCCTTTTCCTACCCAGCttttttttttttttCGGCC
AGTACTTTACAGTTTGGAATGGGAGGGGGCGAGCCCGACAGGGGCACTCATTCAGTCACTCCGCCTCTTT
GTCGGCATGTCGAGTCCCAGTTGAGTTTCCAGTTCCCATCGTGACTCGACTCTTGCGCTTATCACACCTT
TGCGATTCGATTCTGTTCCTGTTTCCCGCAGAACGAGGAACGAGCTGAGCACCTACGGATTCGAGACGCC
TCGCAAGTCGGCCTGCAAGCACCTATGGCGCTGCTGCGTGGAACACCATGCCTTCTTCAGGCAGGTGCGA
GTGGCCCCTCTGCCCAGTTCGCAATCGCATGCCGCCGATTTGTTCCAACTGGGATCGCGCTTCAGGCACA
G
>dme_circ_0008033_junction_seq
TCCAACTGGGATCGCGCTTCAGGCACAGGGGCGAGGACAGCGTGGAGTACTTCCTG
>dme_circ_0008034
GATTGAGGAAATCCATTGACCGACGGTTTTACAGCATTCAATCAAGCGCAAATTGGCTCAACTTGCAAAA
AGGGCACATTTTCGGCCTTGCAAATTCCAATCTTGAAACTAAATGACGCCTGTGTCTGAAACCGAGACCC
TGCTGCCAAATACCGCAACGCTGGACGCCAGACGACGGCCACAAATTAGCCAGCCAGCCGTAGATTTGCA
AGTCTAAGGAGTCGCCCTGCCCACCAGAACGCAGAAGGGGATCCCTGTTCAAAGGAGCCAGCGAAGGCCA
CACACAGGAGAAAGATCAAAAGGAGCACCAGCCAGTGTGACTGCGACTATTTTTCGGGAGCGCCAAAGGA
TTCATTAAAAAACCGCCAACCTGGAACTAATGGAATTAGAGAATATTGTGGCCAATACGGTCTACTTGAA
AGCGAGAGAAG
>dme_circ_0008034_junction_seq
AATACGGTCTACTTGAAAGCGAGAGAAGGATTGAGGAAATCCATTGACCGACGGTT
>dme_circ_0008035
GCATTCAATCAAGCGCAAATTGGCTCAACTTGCAAAAAGGGCACATTTTCGGCCTTGCAAATTCCAATCT
TGAAACTAAATGACGCCTGTGTCTGAAACCGAGACCCTGCTGCCAAATACCGCAACGCTGGACGCCAGAC
GACGGCCACAAATTAGCCAGCCAGCCGTAGATTTGCAAGTCTAAGGA
>dme_circ_0008035_junction_seq
GCCAGCCGTAGATTTGCAAGTCTAAGGAGCATTCAATCAAGCGCAAATTGGCTCAA
>dme_circ_0008036
ATGGACTTGCGGAGCAGACGTGGATTGCTGGTACTGATAACACCGGGTGGTCGGGGGTCGGCATTCTGGT
GGACAGACGGACGAGGCACAATTATTCTCAATCTATGCCAATTTGGCAATTCTCGTTCAAACATTCAAAT
GCCAAACAAACGAAACGACTGCCGCGCTATTGGGTGCAAAACAAATCAATTAAGGCGAGCAACGAGCAAC
GCCTGCGAAATTGTTATAAATTGACACGGCGAATGCTCCTCGGTTCCATTGAATTATTTATGAATGAATT
TTATTAGTTGCCCTCATTGGCGGCTCATCAGTGTCCGCTGACTTGACATTAATTGCAGTCGAGTGGAGTC
GGTTCGCGGCGAGCTGCGACTTACAGCCGAGCAAACAGTCAATTAAATGCTCCACATTTTGCCAAACATT
TGATTTCATTACAGCCCGGTTCAGTACGTTGGTGCTTCGATTGGTAGCCAGCCAGCACTTGCCAATAGAT
AAATGTTTGCTCGGGCCAACAGCTTAACAGCTAAAAACCAACACACATGCCGCATTCCCACATTAAGCGG
CCTTGTTTGGCAGCTCATTTGACAGGCGAACAACATGATCGATGCTTGTTATTTACTCTTGCTCATTTTC
CCATTCGACTGCAGCCCGGTGATTTTCATTTGCATTTAG
>dme_circ_0008036_junction_seq
CAGCCCGGTGATTTTCATTTGCATTTAGATGGACTTGCGGAGCAGACGTGGATTGC
>dme_circ_0008037
GTTAATTAATTGTAATTGTGGTAACAAGAATACCACTTACTATACCACTTTGAGAGCTAAGGCCGTCAGT
TTGTCACAGCACCCAAAAATAATGTTACTTAAATACGAGCGCTATACAATATACACGCGTCGCCAGAAGC

GGGACAACGGCGGATGGGCAGGAACAGCACTACGTAGACGGCTGAGTAATAT
>dme_circ_0008037_junction_seq
CAGCACTACGTAGACGGCTGAGTAATATGTTAATTAATTGTAATTGTGGTAACAAG
>dme_circ_0008038
AGTTTCAAAATCAAAATTGAGGAATACCAACTAGAGGATAAGGCTACTTAAGGATCAAAAAACACCAAGG
AGACGAGATTTTCTACCAAATCGAGAGACGAGGGGCAGGTTAATTTCGTCATTTTTGGCCAAGACAGCAA
ATAGAGGAACAGCAAAGCGAAAATCATTTTATACCTCACACAACAACTACACACTAACTAAGATTAGGCT
ACGCAACTGTACATTGTACTTAAGTGTTCAAAGTATATTTAGGTTTACTTTGTATATAAGAAAAGTAGCT
AAAAGCACGCGGACAGGGAGGCAGGAGCACCACAGTCACTAGCCACTAAGCAGAGTCACAGTCACGATCA
CGTTCACTCCAGGATCAGGACTCGGGGCGGGATCAGCAGACGCTGAGGAAGCTGCCACGATGACGATGAG
TACAAACAACTGCGAGAGCATGACCTCGTACTTCACCAACTCGTACATGGGGGCGGACATGCATCATGGG
CACTACCCGGGCAACGGGGTCACCGACCTGGACGCCCAGCAGATGCACCACTACAGCCAGAACGCGAATC
ACCAGGGCAACATGCCCTACCCGCGCTTTCCACCCTACGACCGCATGCCCTACTACAACGGCCAGGGGAT
GGACCAGCAGCAGCAGCACCAGGTCTACTCCCGCCCGGACAGCCCCTCCAGCCAGGTGGGCGGGGTCATG
CCCCAGGCGCAGACCAACGGTCAGTTGGGTGTTCCccagcagcaacagcagcagcagcaacagccctcgc
agaaccagcagcaacagcaggcgcagcaggccccacagcaactgcagcagcagctgccgcaggtgacgca
acaggtgacacatccgcagcagcaacaacagcagcCCGTCGTCTACGCCAGCTGCAAGTTGCAAGCGGCC
GTTGGTGGACTGGGTATGGTTCCCGAGGGCGGATCGCCTCCGCTGGTGGATCAAATGTCCGGTCACCACA
TGAACGCCCAGATGACGCTGCCCCATCACATGGGACATCCGCAGGCGCAGGGTGAGCATGGATCTTATAT
TGTGGCTTTTATGGTTTGCTGAGTCTTAGATACACGGTTACCAATGGCTTTTGGAAATATTTTTTATTGG
CATAATTAGAATTTATTGACATGATCCTTGTGTAATAAAGAATCAAAATTAATTATAGAAAATAGAAACA
CGTTACTTTTTTCGTTTCCTATAACAAGAGCAAGTCCTTATTTTTTGACAACAAGGGCAATTAACTTTTC
GTTATTGAATCTTCTTATATGGATAATTGTTGTTTATTTTTATTTACTAATTTCCCAAATCGATATCCTT
ACACCTCCCGCATATTTCGCCAACCACTTAACGCTTTTCCTATCTTTTTCACTGCCGCTTTTGCCGCTCG
CACCTGCAGTTGGGCTATACGGACGTTGGAGTTCCCGACGTGACAGAGGTAAGATCGAGTCCACATTGCG
CTTGTCACCTACTTCAAATTCTGCTAAAACCTAACATCTTTTACCGGTTGATGGTTTCAAGATATACCCC
TAAAATTACAATATCACTTATCTAAAAACTTACCCATCACAGGGTCCATCAGAACCATCACAACATGGGC
ATGTACCAGCAGCAGTCGGGAGTTCCGCCGGTGGGTGCCCCACCTCAGGGCATGATGCACCAGGGCCAGG
GTCCTCCACAGATGCACCAGGGACATCCTGGCCAACACACGCCTCCTTCCCAAAACCCGAACTCGCAGTC
CTCGGGGATGCCGTCTCCACTGTATCCCTGGATGCGAAGTCAGTTTGG
>dme_circ_0008038_junction_seq
TGTATCCCTGGATGCGAAGTCAGTTTGGAGTTTCAAAATCAAAATTGAGGAATACC
>dme_circ_0008039
GTGGATCTCGGTGCCTTCAAGGTGCCCGAGCACCTGAACGACGCCTACTTCCGCCGTCTGAAGGCTAAGA
AGGACAAGAAGACCGGCGAGGCCGACATCTTCGCGGCCAAGAAGGAGCGCTTCGTGCCCAACGAGCAGCG
CAAGAAGGACCAGAAGGAGG
>dme_circ_0008039_junction_seq
GAGCAGCGCAAGAAGGACCAGAAGGAGGGTGGATCTCGGTGCCTTCAAGGTGCCCG
>dme_circ_0008040
GCGGGCACGAGTTGAGGGCGAAGGGTCCGGTGACCAGCAGGAGTCCGGAGGCCAAGACCTTCAACAGGAC
GACGCGCTTGCCCTGGTGGCGTCCGGCCAGCAGGATGAGCACGGTTCCGGGGGTCAGGTTGCGGCGCGT
>dme_circ_0008040_junction_seq
CGGTTCCGGGGGTCAGGTTGCGGCGCGTGCGGGCACGAGTTGAGGGCGAAGGGTCC
>dme_circ_0008041

GGGTCCGGTGACCAGCAGGAGTCCGGAGGCCAAGACCTTCAACAGGACGACGCGCTTGCCCTGGTGGCGT
CCGGCCAGCAGGATGAGCACGGTTCCGGGGGTCAGGTTGCGGCGCGTGTTGCGTTTGTGCTCGCTGAAGT
TTGCCTTGGAGGGGCGCTTCTTCACGAAC
>dme_circ_0008041_junction_seq
TGCCTTGGAGGGGCGCTTCTTCACGAACGGGTCCGGTGACCAGCAGGAGTCCGGAG
>dme_circ_0008042
ATCGGAGGACCCAAGAACGGCGGTGAGCGCACGGTCTTCCTGAAGAAGAGCAAGGCCTCCTACCCGACCA
AGACGTTCGTGAAGAAGCGCCCCTCCAAGGCAAACTTCAGCGAGCACAAACGCAACACGCGCCGCAACCT
GACCCCCGGAACCGTGCTCATCCTGCTGGCCGGACGCCACCAGGGCAAGCGCGTCGTCCTGTTGAAGG
>dme_circ_0008042_junction_seq
CAGGGCAAGCGCGTCGTCCTGTTGAAGGATCGGAGGACCCAAGAACGGCGGTGAGC
>dme_circ_0008043
GACAAGAAGAGCCCCGTCGTGGAGAAGGCCAAGGTGCCCATCAAGAAGGTGAAGAAGATCGGAGGACCCA
AGAACGGCGGTGAGCGCACGGTCTTCCTGAAGAAGAGCAAGGCCTCCTACCCGACCAAGACGTTCG
>dme_circ_0008043_junction_seq
AAGGCCTCCTACCCGACCAAGACGTTCGGACAAGAAGAGCCCCGTCGTGGAGAAGG
>dme_circ_0008044
GGCAAGAAGCACCCCGTCAACTCCTACCTGAAGGGCGGTATCCTGCGTTACTCCAAGGCCCAGGTAAATA
TCGCCGGAAACAGTGGGGAAATGGCCTCTGGCCCGAGTGACGCCTCCGCCCGATGAGAAGGCGCAGCAGC
GTGGCTTACTTGTGGTTCTCCGATTCCAAGGGATCCGATACTTGCTCATCTATCCGCGGGATGGACCACA
CACCATCCACCCACTTTGAGTTAGTTTTACTTGAAAACTCAGTATTCGTCAGTGCGGTTCCTGAATCCAA
ATGCATTTATTTTAATTCCCGTGTGTTCAAGTGGCTATGAGTACAAACCATGCATACTTTGCCTAAATTG
TTTACTCTTCTCACAAATAAGTTCAATAATCCTCAATGCCCTTGCTTGTGACTGTGCAACAAGTGGAGCG
TATTAGTGATACACGTAAACAAAATAACCCCCTAGTTCAAAGGCTTGTGGATCAAGGATCAAGCTAACCA
CTTTTCCAACCTATTTCAGAATGTACAAGCGTCGTGCTCTGTACCGCCTGAAGGACAAGAAGAGCCCCGT
CGTGGAGAAGGCCAAGGTGCCCATCAAGAAGGTGAAGAAGATCGGAGGACCCAAGAACGGCGGTGAGCGC
ACGGTCTTCCTGAAGAAGAGCAAGGCCTCCTACCCGACCAAGACGTTCG
>dme_circ_0008044_junction_seq
AAGGCCTCCTACCCGACCAAGACGTTCGGGCAAGAAGCACCCCGTCAACTCCTACC
>dme_circ_0008045
CACCCCGTCAACTCCTACCTGAAGGGCGGTATCCTGCGTTACTCCAAGGCCCAGGTAAATATCGCCGGAA
ACAGTGGGGAAATGGCCTCTGGCCCGAGTGACGCCTCCGCCCGATGAGAAGGCGCAGCAGCGTGGCTTAC
TTGTGGTTCTCCGATTCCAAGGGATCCGATACTTGCTCATCTATCCGCGGGATGGACCACACACCATCCA
CCCACTTTGAGTTAGTTTTACTTGAAAACTCAGTATTCGTCAGTGCGGTTCCTGAATCCAAATGCATTTA
TTTTAATTCCCGTGTGTTCAAGTGGCTATGAGTACAAACCATGCATACTTTGCCTAAATTGTTTACTCTT
CTCACAAATAAGTTCAATAATCCTCAATGCCCTTGCTTGTGACTGTGCAACAAGTGGAGCGTATTAGTGA
TACACGTAAACAAAATAACCCCCTAGTTCAAAGGCTTGTGGATCAAGGATCAAGCTAACCACTTTTCCAA
CCTATTTCAGATGTACAAGCGTCGTGCTCTGTACCGCCTGAAGGACAAGAAGAGCCCCGTCGTGGAGAAG
GCCAAGGTGCCCATCAAGAAGG
>dme_circ_0008045_junction_seq
GAGAAGGCCAAGGTGCCCATCAAGAAGGCACCCCGTCAACTCCTACCTGAAGGGCG
>dme_circ_0008046
GTGGCCAAGTCGGCCAAAAAGGGCAAGAAGCACCCCGTCAACTCCTACCTGAAGGGCGGTATCCTGCGTT
ACTCCAAGGCCCAGGTAAATATCGCCGGAAACAGTGGGGAAATGGCCTCTGGCCCGAGTGACGCCTCCGC

CCGATGAGAAGGCGCAGCAGCGTGGCTTACTTGTGGTTCTCCGATTCCAAGGGATCCGATACTTGCTCAT
CTATCCGCGGGATGGACCACACACCATCCACCCACTTTGAGTTAGTTTTACTTGAAAACTCAGTATTCGT
CAGTGCGGTTCCTGAATCCAAATGCATTTATTTTAATTCCCGTGTGTTCAAGTGGCTATGAGTACAAACC
ATGCATACTTTGCCTAAATTGTTTACTCTTCTCACAAATAAGTTCAATAATCCTCAATGCCCTTGCTTGT
GACTGTGCAACAAGTGGAGCGTATTAGTGATACACGTAAACAAAATAACCCCCTAGTTCAAAGGCTTGTG
GATCAAGGATCAAGCTAACCACTTTTCCAACCTATTTCAGATGTACAAGCGTCGTGCTCTGTACCGCCTG
AAGGACAAGAAGAGCCCCGTCGTGGAGAAGGCCAAGGTGCCCATCAAGAAGG
>dme_circ_0008046_junction_seq
GAGAAGGCCAAGGTGCCCATCAAGAAGGGTGGCCAAGTCGGCCAAAAAGGGCAAGA
>dme_circ_0008047
CTAAAAAGGTGGCCAAGTCGGCCAAAAAGGGCAAGAAGCACCCCGTCAACTCCTACCTGAAGGGCGGTAT
CCTGCGTTACTCCAAGGCCCAGGTAAATATCGCCGGAAACAGTGGGGAAATGGCCTCTGGCCCGAGTGAC
GCCTCCGCCCGATGAGAAGGCGCAGCAGCGTGGCTTACTTGTGGTTCTCCGATTCCAAGGGATCCGATAC
TTGCTCATCTATCCGCGGGATGGACCACACACCATCCACCCACTTTGAGTTAGTTTTACTTGAAAACTCA
GTATTCGTCAGTGCGGTTCCTGAATCCAAATGCATTTATTTTAATTCCCGTGTGTTCAAGTGGCTATGAG
TACAAACCATGCATACTTTGCCTAAATTGTTTACTCTTCTCACAAATAAGTTCAATAATCCTCAATGCCC
TTGCTTGTGACTGTGCAACAAGTGGAGCGTATTAGTGATACACGTAAACAAAATAACCCCCTAGTTCAAA
GGCTTGTGGATCAAGGATCAAGCTAACCACTTTTCCAACCTATTTCAGATGTACAAGCGTCGTGCTCTGT
ACCGCCTGAAGGACAAGAAGAGCCCCGTCGTGGAGAAGGCCAAGGTGCCCATCAAGAAGG
>dme_circ_0008047_junction_seq
GAGAAGGCCAAGGTGCCCATCAAGAAGGCTAAAAAGGTGGCCAAGTCGGCCAAAAA
>dme_circ_0008048
TCGGCCAAAAAGGGCAAGAAGCACCCCGTCAACTCCTACCTGAAGGGCGGTATCCTGCGTTACTCCAAGG
CCCAGGTAAATATCGCCGGAAACAGTGGGGAAATGGCCTCTGGCCCGAGTGACGCCTCCGCCCGATGAGA
AGGCGCAGCAGCGTGGCTTACTTGTGGTTCTCCGATTCCAAGGGATCCGATACTTGCTCATCTATCCGCG
GGATGGACCACACACCATCCACCCACTTTGAGTTAGTTTTACTTGAAAACTCAGTATTCGTCAGTGCGGT
TCCTGAATCCAAATGCATTTATTTTAATTCCCGTGTGTTCAAGTGGCTATGAGTACAAACCATGCATACT
TTGCCTAAATTGTTTACTCTTCTCACAAATAAGTTCAATAATCCTCAATGCCCTTGCTTGTGACTGTGCA
ACAAGTGGAGCGTATTAGTGATACACGTAAACAAAATAACCCCCTAGTTCAAAGGCTTGTGGATCAAGGA
TCAAGCTAACCACTTTTCCAACCTATTTCAGATGTACAAGCGTCGTGCTCTGTACCGCCTGAAGGACAAG
AAGAGCCCCGTCGTGGAGAAGGCCAAGG
>dme_circ_0008048_junction_seq
AAGAGCCCCGTCGTGGAGAAGGCCAAGGTCGGCCAAAAAGGGCAAGAAGCACCCCG
>dme_circ_0008049
ATTTCATAATTTTAACAACTAGCAAATTTTACATTGTATTTAGCGCGGAGTTAGTTAAATATTTGCAAGT
ATCAACATCAAAAACATGAATGAAAATCTCAATTATTGTATATTTAGTTTTTATTTGACGACTACTTTTA
TTCATGATAACAATGATGGTGGGCAATTGCCCATCGTCAAGTTTTGAGCTTATATTCGGTTGAGG
>dme_circ_0008049_junction_seq
CAAGTTTTGAGCTTATATTCGGTTGAGGATTTCATAATTTTAACAACTAGCAAATT
>dme_circ_0008050
CAGAATCCGAATACATTGTTAACACAGTCGAAATGCGAAACAAATAATTATATTTAAGCGACGGAGATCA
TCATGTGTTCGTTTAAGCTTAGTCCACTATAAATAACAGCCAATTGACGATTAGGCGATTAAGAGAAAGG
>dme_circ_0008050_junction_seq
ATTGACGATTAGGCGATTAAGAGAAAGGCAGAATCCGAATACATTGTTAACACAGT

>dme_circ_0008051
GTTTTCTGAATTTTCGAAATTTCAATTGTTTTATGCTCTGCGTTAGTTTGCCTATGCTCTCTAAAACTCT
TCGCTCGACCCTCTCGGTACATCTTGCTAAATGTTGCATCATAATTTCTGCTCAATGCTCGCCGCGCTTC
AATTTGAATTGAATTGAATTTTTGGCCTTGTCTCAACGCTGGTTTTGCTTTAAACTAGGGTTTAATGGGT
AGTTTCATATGGTACAACATAAGAATGTGATTCGGTTTACAATTTGTTTAATAAGTTAAATAATATTACG
TAACAAAAAAAGAAGCTTTAGCATGTGCATCTTATGTGAAGAAGGTATAATATAAAAAAGAGGACTCGAA
CTAAATTTCAATGCCTTCGACTTGATTGATTGATTAATTGGTTGGTTGTTCGAGTGATAAACAATCATAT
AGAAATAACAA
>dme_circ_0008051_junction_seq
GTGATAAACAATCATATAGAAATAACAAGTTTTCTGAATTTTCGAAATTTCAATTG
>dme_circ_0008052
CAAACTTGTTATTTCTATATGATTGTTTATCACTCGAACAACCAACCAATTAATCAATCAATCAAGTCGA
AGGCATTGAAATTTAGTTCGAGTCCTCTTTTTTATATTATACCTTCTTCACATAAGATGCACATGCTAAA
GCTTCTTTTTTTGTTACG
>dme_circ_0008052_junction_seq
ACATGCTAAAGCTTCTTTTTTTGTTACGCAAACTTGTTATTTCTATATGATTGTTT
>dme_circ_0008053
CCGCGCTCAAAAAACACGAGAAAGCCCATAGAAAACTAAAGTATAATTCGCCGCCTGTATTGACTAAGAA
CTCTTAACAATAATATAATAACTTTAAACAAGGTACAATATGTAGTGTGTGTCTAGTAGATTTTTCTCTG
TATAACAAAATCAAAAACCAAACAAAAGCGAACTCAATTTCCGCCCTAGAAAATGTTTCATTATCATACG
CAGCTAAGCAAACTTGTTATTTCTATATGATTGTTTATCACTCGAACAACCAACCAATTAATCAATCAAT
CAAGTCGAAGGCATTGAAATTTAGTTCGAGTCCTCTTTTTTATATTATACCTTCTTCACATAAGATGCAC
ATGCTAAAGCTTCTTTTTTTG
>dme_circ_0008053_junction_seq
GATGCACATGCTAAAGCTTCTTTTTTTGCCGCGCTCAAAAAACACGAGAAAGCCCA
>dme_circ_0008054
GCTTTAGCATGTGCATCTTATGTGAAGAAGGTATAATATAAAAAAGAGGACTCGAACTAAATTTCAATGC
CTTCGACTTGATTGATTGATTAATTGGTTGGTTGTTCGAGTGATAAACAATCATATAGAAATAACAAGTT
TGCTTAGCTGCGTATGATAATGAAACATTTTCTAGGGCGGAAATTGAGTTCGCTTTTGTTTGGTTTTTGA
TTTTGTTATACAGAGAAAAATCTACTAGACACACACTACATATT
>dme_circ_0008054_junction_seq
AAAATCTACTAGACACACACTACATATTGCTTTAGCATGTGCATCTTATGTGAAGA
>dme_circ_0008055
tacaaaaCTTCTTTATGTATAGACCGACCTAAAAGATAAGCTAACACAAAAACACAAACACAAGGTCGGA
TCAGATTAATCAGTAATTTTAGCCGCGCTCAAAAAACACGAGAAAGCCCATAGAAAACTAAAGTATAATT
CGCCGCCTGTATTGACTAAGAACTCTTAACAATAATATAATAACTTTAAACAAGG
>dme_circ_0008055_junction_seq
AACAATAATATAATAACTTTAAACAAGGtacaaaaCTTCTTTATGTATAGACCGAC
>dme_circ_0008056
GTCAATACAGGCGGCGAATTATACTTTAGTTTTCTATGGGCTTTCTCGTGTTTTTTGAGCGCGGCTAAAA
TTACTGATTAATCTGATCCGACCTTGTGTTTGTGTTTTTGTGTTAGCTTATCTTTTAGGTCGGTCTATAC
ATAAAGAAGttttgtactttttttttatattttatgatttttgttgatgtttttGG
>dme_circ_0008056_junction_seq
attttatgatttttgttgatgtttttGGGTCAATACAGGCGGCGAATTATACTTTA

>dme_circ_0008057
GGTCGGAGGAGAAGCGGTTGATCGGGCGCAAGACGCGCAGCGTGCGACTGGACAGCTTCCAGACAAGCCG
CTGCTTGTAGGGCGCCTTGTTCTGCCCGTTGTAATGGGACAGAATACCCGAAACGATGCTGAAGCCGCAG
>dme_circ_0008057_junction_seq
AATACCCGAAACGATGCTGAAGCCGCAGGGTCGGAGGAGAAGCGGTTGATCGGGCG
>dme_circ_0008058
GCGTCGAGGGCGTGATCACCGTGGTCTTCACGTCGCACATCAGGATCTCCAGGCGGTTCTCCAGTTCCTC
CAGCTTGATGAGCTGCTGGTAGAACTTGAAAAAGTCGCCGCCGATCTCCTTCGCCAA
>dme_circ_0008058_junction_seq
AAAAGTCGCCGCCGATCTCCTTCGCCAAGCGTCGAGGGCGTGATCACCGTGGTCTT
>dme_circ_0008059
GATCCTCGGCGGGAATGGGTTTCAAGTCCAGCTGCTTGATGGCCAAGTGGAACGAGGCCACGCTCATGCA
GGCCATGTGCTTCGGCTTGACGGCCATGCGATCCAGGAAGCGGTCCACCAGGCTCATGGCCGAGAACAGG
ACGTCGGAGGGCAGCTCGTACCACATCTTCAGGCAGCGCAGCACGTAGGCGGATCCATCTCGTCCTCCGG
CA
>dme_circ_0008059_junction_seq
GTAGGCGGATCCATCTCGTCCTCCGGCAGATCCTCGGCGGGAATGGGTTTCAAGTC
>dme_circ_0008060
GGAAGCGGTCCACCAGGCTCATGGCCGAGAACAGGACGTCGGAGGGCAGCTCGTACCACATCTTCAGGCA
GCGCAGCACGTAGGCGGATCCATCTCGTCCTCCGGCAGTAACCTCACGCTGCAGAAGGAATACATGTTAT
GTTGGTCTCCATCTTAAAACTAAAGCAGCTCACTACACTCACCCTTGATTCCTTGGGCAACAGCAGCACC
GTGTGGAACTTGTCCTGCAGGACGTCGTACTCCTTGAGGGTTTCGTACAACTCGTCCGAGGTGAGGGCGT
GCAGCTGATCCTCTAGCTTTTTCAAGGACGACGACGATTCGCCGTTGTTGTTGCTGGCTCCACCGACGGC
TCCTGCCTCGGACTCCGCCAGATCCTCCAGTTGTTGTTGTTGTTC
>dme_circ_0008060_junction_seq
CCAGATCCTCCAGTTGTTGTTGTTGTTCGGAAGCGGTCCACCAGGCTCATGGCCGA
>dme_circ_0008061
GCTCGTACCACATCTTCAGGCAGCGCAGCACGTAGGCGGATCCATCTCGTCCTCCGGCAGTAACCTCACG
CTGCAGAAGGAATACATGTTATGTTGGTCTCCATCTTAAAACTAAAGCAGCTCACTACACTCACCCTTGA
TTCCTTGGGCAACAGCAGCACCGTGTGGAACTTGTCCTGCAGGACGTCGTACTCCTTGAGGGTTTCGTAC
AACTCGTCCGAGGTGAGGGCGTGCAGCTGATCCTCTAGCTTTTTCAAGGACGACGACGATTCGCCGTTGT
TGTTGCTGGCTCCACCGACGGCTCCTGCCTCGGACTCCGCCAGATCCTCCAGTT
>dme_circ_0008061_junction_seq
GCCTCGGACTCCGCCAGATCCTCCAGTTGCTCGTACCACATCTTCAGGCAGCGCAG
>dme_circ_0008062
GCACCACCCAATCCCGATCCCGATCCCGCACCAGCAGATCCACCCGCGGATGCGGCAGCAGCGGCGGCAG
CCTGGGCCGAAGTCGAGGGCTGCTCGTCGTCGACCAGAATGGCGTTGATGCTGTGGGCGTCCATGGCGA
>dme_circ_0008062_junction_seq
GCGTTGATGCTGTGGGCGTCCATGGCGAGCACCACCCAATCCCGATCCCGATCCCG
>dme_circ_0008063
GAATAGAAAATAGAAATTGAAAAGGACCAAGTGCAAACGTACAAAGCTTTGCTAAAGCAGAACTaaaaaa
aaTCAGCAGGAGGAAAATGCACAGCTGATGGAGAAAAGTCCCAAGCACCTTCAGCCCAGTGTGTGTGGGA
AAAATCGATCCGCAGACGGGAAAAATTGAGGATAAAATGCCAGCCTAATAAACAGAGTTCCAATTGTGAT
ACAAACGCAGTACAAATTAGTTAACAACTAGCTTAAATGGCCAACGACAAGGTTTTATAGCTAACTATCT

GACGCAATTGAATAAGCGTAAAGCTGCCAGCCAATTTATTCACTTATTTTCGAGTGCAATAGAAGCAAAA
ATTCACAACTAAACAAAGTTCATCTCAACATTCCTGCGCAACACAAAACAACAAAATGGCTGTAAAAACG
>dme_circ_0008063_junction_seq
ACAAAACAACAAAATGGCTGTAAAAACGGAATAGAAAATAGAAATTGAAAAGGACC
>dme_circ_0008064
GTGGGCTGCGTTTTCAGAGCATGCTGCACGTACTCGTGCAGGATGCAGACGAAGCTCTTGCCATTGGGAT
TCATGATCCACTCCTTCCGACCGCGCGGGTTCGTTGATCCTTCGCCAGCATTCGCTAGTATTGGGAACTT
AATAAGCTTAGTGCCATCCGGCAGCGTGGGTCGTTGAATATTCTTGATGTGTTTGCGGTTTTTGGTGAAC
TTGCGGCGAGCATTCCAACTCCGAAATCGCAGCACACGAATCACCTTGAATTTGAAGAGTTTCTGGCAGT
AGG
>dme_circ_0008064_junction_seq
TTGAATTTGAAGAGTTTCTGGCAGTAGGGTGGGCTGCGTTTTCAGAGCATGCTGCA
>dme_circ_0008065
CTAACTGAGCCGCCGAGTCAACCGAAAACCACCAGAGCCTGTGATTTCTACATGCGTGCGTGCTCCCCCT
TCCCCTCACCCTACCCCAGCCTCGCCCAACAAAGTGCGTGAGATCGAGGGGGATGCAAGACCGAAGGGGC
TTTTGGCtttttttttGGTTTCCTCTAGCGATATTTGTGCCGTG
>dme_circ_0008065_junction_seq
GGTTTCCTCTAGCGATATTTGTGCCGTGCTAACTGAGCCGCCGAGTCAACCGAAAA
>dme_circ_0008066
AAGCGTTGAGATGCCATCTTCATTGGGAGGCTGTGGTTGCGTCACTAAGCGATGTCCGAGTGTGTGCGAT
CGCGATCTATGTCCTCATGCAAGTGGGGCATCAAAGGTCCTTTCGTTGTCCTGGGTTCGGATTCTTACTC
CTTGGGGCCTGACAGCTGCTAAAACATATATTCCAGGGCG
>dme_circ_0008066_junction_seq
CAGCTGCTAAAACATATATTCCAGGGCGAAGCGTTGAGATGCCATCTTCATTGGGA
>dme_circ_0008067
GGCTTAAATTATCGCcaaagaaaatgaaaatcaaaaacaaaaacaaaagcaaaagcaaaacaaaatGCAG
TTGAAATCCAACAGTAACAACAGAGCAGTCGCAGCAGACCACAAAATATTGTTGTTAATGCGAAACGATC
TCCAAACAAAATAAAGAACTTGAAAAGTAGACAACAAGTAATTCCTCAGTCAAGTAAAACGTTCAAATTT
GTTGCAATTCGAGAACTAAGAGAAAACTACGAAAAAGATTCAGAAAATTACAACTGCAAAAAACGTACAG
AAAACCAATTGAAACAAAATTAAGCGCAATCAAATAATAAACAAGCTGCGTACGCTAACTTATTAAATAA
TTTT
>dme_circ_0008067_junction_seq
TGCGTACGCTAACTTATTAAATAATTTTGGCTTAAATTATCGCcaaagaaaatgaa
>dme_circ_0008068
GTTTCACATTTATTAAGGTCCGCCAAGGAAGCCCATATGAACATGCTTCGAGTCTGGGGAGGCGGAGTTT
ACGAATCGGACTACTTTTACCAGCTCGCCGACAGCTTGGGTCTTCTAATTTGGCAAGATATGATGTTTGC
TTGTGCCATGTACCCGGTTAACGACGAATTCCTATCGTCAGTGCGTGAGGAGGTTCGCCAGAATGCCATG
CGACTATCACACCATCCAAGCGTAGCCATTTTTGTGACCAATAACGAAAACGAAGCTGCTTTGGTACAGA
ACTGGTACGGCACGCTTTTCGAAAGAGATCGATTTGAGAGCGAGTACCGGGAGCTCTACCTTGCTAATGT
CATCCACGAGTTGAAGCTTGTTTCGCATAGCTCTCGACCTCAGCCACTGGTCTCCTCACCTTCAAATGGC
AAAGCTAGTGAACCGGACAACTATATTTCGAGCAACCCTCAGGATAACCAAAATGGCGATGGTACATTTC
TACGACTACACTAAGGACGGCTGGGATCCAGGAATTTTCCCACGTCCTCGATTCGTCAGTGAGTTCGGCT
TTCAGAGCTTTCCAGGCGCATACGCATGGCAGCGCAGCAAGGGCGAGGACGATGACCTCCTCGGCCTGAT
CACACACCGACAACACCACCCACTGGGAAATGTTCCTGTTATCGCACTAGTGGAGAGACACCTACCGCTG

CCCTTTCCAGAGAATGAGAACTACGCGACAGCACTGATTTACTTCAGCCAGATTGCTCAAGCCATGGCCA
CAAAGGTGGAAACCGAATTATACCGTAGCCTTCGGGACACTCCCCACAGAACGATGGGGGCCCTTTACTG
GCAGCTGAATGATGTATGGGTGGCGCCATCCTGGTCCAGCATTGATTTCTACGGCAACTGGAAG
>dme_circ_0008068_junction_seq
CAGCATTGATTTCTACGGCAACTGGAAGGTTTCACATTTATTAAGGTCCGCCAAGG
>dme_circ_0008069
GTACTGCTACGCCAAGGAAGTGCTGACCGAGCAGGCGCGGTTGGAGAAGCTCAGGGGCGACGGAGCCGAC
GACCACGTACTGCGCAAGCAGGAGGAGGTCATCCAGGAGTGCATCATGATGGTGCCCGACTCCAAGAGAA
G
>dme_circ_0008069_junction_seq
TCATGATGGTGCCCGACTCCAAGAGAAGGTACTGCTACGCCAAGGAAGTGCTGACC
>dme_circ_0008070
GGTGACCAGCTCCTTTTCGTTGAACCACAGGGCGATCTCCTTCTCGGCAGACTCGACGGCATCGGAGCCG
TGGATGATGTTGCGTCCGACCTGAATGCAGAAGTCACCGCGGATGGTGCCGGGCAGCGAGTCGGCGGGGT
TG
>dme_circ_0008070_junction_seq
GGTGCCGGGCAGCGAGTCGGCGGGGTTGGGTGACCAGCTCCTTTTCGTTGAACCAC
>dme_circ_0008071
GACCCTCCCACACCATGGGCACCACGGGGCCGGAGTTCATGTAGTTCACGAGTCCGGGGAAGAAGGGGCG
GGCGGACAGATCAGCGTAGTGCTTCTCCAGCAGCTCCTTGGAGGCCTGGTTACAAAACGGTTCGTTATCA
GTTAATTGCAGTTCTGACCTCTAAACACATGTCATTCCGCGATGACGCCTGCACCCAGCCTTTGAGGTTA
GGTCCGATGGCAACCCCGCGTGGGGCAACCCGTTTCCACGTATTTATATCTATTGATTTCTTCCTAATTC
AATTATCCGCTTACCCAGGTGAACTTCAGGGCGACCAGCTTGAAGCCCTTCTGCTCGAAGCGCTCGATGA
TCTTGCCGACGAGCCCGCGCTGGACGCCATCGGGCTTGACCATGATGAAA
>dme_circ_0008071_junction_seq
GACGCCATCGGGCTTGACCATGATGAAAGACCCTCCCACACCATGGGCACCACGGG
>dme_circ_0008072
GTTCACGAGTCCGGGGAAGAAGGGGCGGGCGGACAGATCAGCGTAGTGCTTCTCCAGCAGCTCCTTGGAG
GCCTGGTTACAAAACGGTTCGTTATCAGTTAATTGCAGTTCTGACCTCTAAACACATGTCATTCCGCGAT
GACGCCTGCACCCAGCCTTTGAGGTTAGGTCCGATGGCAACCCCGCGTGGGGCAACCCGTTTCCACGTAT
TTATATCTATTGATTTCTTCCTAATTCAATTATCCGCTTACCCAGGTGAACTTCAGGGCGACCAGCTTGA
AGCCCTTCTGCTCGAAGCGCTCGATGATCTTGCCGACGAGCCCGCGCTGGACGCCATCGGGCTTGACCAT
GATGAAAGTCCTCTCCTT
>dme_circ_0008072_junction_seq
GCTTGACCATGATGAAAGTCCTCTCCTTGTTCACGAGTCCGGGGAAGAAGGGGCGG
>dme_circ_0008073
GTCCAAGGAGCAAAATGAGCCAGCCAAGGAGCCCACACCAGAACCTATGGAAGTGGACGGAAGTTCGAAG
GGCAGTTCGGACACCGCTCAAGTAGACACACCAGCCATCGAAAACGTTTCTGCCGCTCCAGAAACAGAAG
CAGCAGCAAAGACTACGGAGTCTAGCAATGATGTGGTGGAAGTGGCCACAGAGGGCAAAGAAACAGTTTT
AGAA
>dme_circ_0008073_junction_seq
CACAGAGGGCAAAGAAACAGTTTTAGAAGTCCAAGGAGCAAAATGAGCCAGCCAAG
>dme_circ_0008074
TTGCAACGGCTTCCAAAACTAATATACGCTTTTGCTGTGTGTATTATATTATTTATCATTAGTATTACTA

TTAGAATTTGTGTTTCTCTTCTTTTCATTGAAAAAGTTTCCTCGTACTCTAGCCTGTATTTACGAATTAA
GTTAGGAGAGATCGCCCGGAAGAGACCTTTGCCTAACACAACAGTTTCCAATGAGCTCATTTGGATCTTT
aaaaacttaaatgcttcaaacgaaacgggcttgaaatacttaaaaaaaatgtccaaaaaaggGTGAAAGTTA
TGCATAGACGTATCTTAAAGCCGCAGACCATTTGTATTGTGTTACAATAGTTTAAATATGTCCGCGATCC
GTTTGCTTATTTTTTGAGTGTTGTCCAATTGGTTTTATAATTAAGGATATTAATGGCTTACAATTTAAAA
ACATCTATTTTACATACATTTTCCG
>dme_circ_0008074_junction_seq
AAAACATCTATTTTACATACATTTTCCGTTGCAACGGCTTCCAAAACTAATATACG
>dme_circ_0008075
GGTCCGCCAAGGAAGCCCATATGAACATGCTTCGAGTCTGGGGAGGCGGAGTTTACGAATCGGACTACTT
TTACCAGCTCGCCGACAGCTTGGGTCTTCTAATTTGGCAAGATATGATGTTTGCTTGTGCCATGTACCCG
GTTAACGACGAATTCCTATCGTCAGTGCGTGAGGAGGTTCGCCAGAATGCCATGCGACTATCACACCATC
CAAGCGTAGCCATTTTTGTGACCAATAACGAAAACGAAGCTGCTTTGGTACAGAACTGGTACGGCACGCT
TTTCGAAAGAGATCGATTTGAGAGCGAGTACCGGGAGCTCTACCTTGCTAATGTCATCCACGAGTTGAAG
CTTGTTTCGCATAGCTCTCGACCTCAGCCACTGGTCTCCTCACCTTCAAATGGCAAAGCTAGTGAACCGG
ACAACTATATTTCGAGCAACCCTCAGGATAACCAAAATGGCGATGGTACATTTCTACGACTACACTAAGG
ACGGCTGGGATCCAGGAATTTTCCCACGTCCTCGATTCGTCAGTGAGTTCGGCTTTCAGAGCTTTCCAGG
CGCATACGCATGGCAGCGCAGCAAGGGCGAGGACGATGACCTCCTCGGCCTGATCACACACCGACAACAC
CACCCACTGGGAAATGTTCCTGTTATCGCACTAGTGGAGAGACACCTACCGCTGCCCTTTCCAGAGAATG
AGAACTACGCGACAGCACTGATTTACTTCAGCCAGATTGCTCAAGCCATGGCCACAAAGGTGGAAACCGA
ATTATACCGTAGCCTTCGGGACACTCCCCACAGAACGATGGGGGCCCTTTACTGGCAGCTGAATGATGTA
TGGGTGGCGCCATCCTGGTCCAGCATTGATTTCTACGGCAACTGGAAG
>dme_circ_0008075_junction_seq
CAGCATTGATTTCTACGGCAACTGGAAGGGTCCGCCAAGGAAGCCCATATGAACAT
>dme_circ_0008076
GACCTCTGGCCTCACACTGATCCAGTGAGTTAGGCAGCACCTCGTACTTAATCAGTCCGGACTTGAAGTT
CATCTGGCCATTGGCGATAATCCACACCATGGCGACCACGCTGACGATAATGCTTGTGACAGCCGTTTTG
GCATTGCAGCGCGGCACAAACATTCCCAAGAGGAACACACCCACCACTGCGCCCGTATTTATGCCCGTAA
TG
>dme_circ_0008076_junction_seq
CACTGCGCCCGTATTTATGCCCGTAATGGACCTCTGGCCTCACACTGATCCAGTGA
>dme_circ_0008077
GTCAAGTTCTACTCCAACTATAGGACGCGATTCCACGTCCGCACCGTCGCCTCCTGGTTGGTGAAGATGG
CCTCGGAGGCTAGGCGCAACAATAGTAAAATATAAGAGTGGCATGCCGGACAGATAAGTGGAGCACTAAA
CCCCGGATGATTTACGGGGCAACG
>dme_circ_0008077_junction_seq
TAAACCCCGGATGATTTACGGGGCAACGGTCAAGTTCTACTCCAACTATAGGACGC
>dme_circ_0008078
GTTTCAGAGTTGAAAATCTCGATGGCAATGCAAACAACTTCGTTCGGCTAACAAAACCCGACTAAATTCC
AACGTCAACGAGAGCATAGACGATATTTTCGGTTTCTTCCCAAGTGATACCGATTCCGAGAGCTACGGCC
TTATATGTAGAGTGGGGGCAGGCAGAGATCCCGGCCAAAATTAACATATTCCTGGAGACTATCAGCCAGC
TGGCGGCCCTGGCGACCTCGACCGGCATCATGCAGCTGCTCTTATCGTCCGTTTGCATGGCGCTGACCAG
CGCGGTGATTGGATTTGCCTACTACCAAAAGCAGCAGTTCTATCCTGCGGTGGTCTACATCACCAAATCG
AACGCCTCCATGGGG

>dme_circ_0008078_junction_seq
CATCACCAAATCGAACGCCTCCATGGGGGTTTCAGAGTTGAAAATCTCGATGGCAA
>dme_circ_0008079
GATTTTTGCAAACTGGCCGCTGACCAGGCATTGGGAAAAAGGACAATAAGAGGGAGCCCAGAGCAAGAAG
TCCAACTAACCGACCAACTGTGCCTCAAGAAACAGCAAATCAGCGGGGAACTCCCAAACTGGACAAGCAG
AAGCCCGGATACCTCACATTTGCCATAAGACACCAGTCAAAAAACGACGCGCACTGCAGAACGCATTAGG
ATTAGCCCGGCACTAGAGCTCGTGCATTAACATCCGCTTCCGGTGCTCCGCAGAACGAGTTGGCTGGAGA
GGATACCCCGCCACCATGACGTTCGACACTCGTAGGCACACCACCGGACAGCCGGGAAGCACAGCGCCCA
GCTCAAGCAGCAGCACCACCAGCACAACAACCACCACAACGAGTCCGGCGCAGAGTGCCGGATCAGGATC
GGGCATAGGAACAGGCACAGGAACAGTCGCCAACTCGAGTCTGCCAGGAGGAGGATCAGGTAGCTTGGAC
GGCAACCAGGATCAACAGCCGGCCACAGACAGTCAGAGCTCTGATGACGTGGCCGCTTCCTTGTCAGCGA
ACAGCGTGGACAGCACCATCACAATAGT
>dme_circ_0008079_junction_seq
ACAGCGTGGACAGCACCATCACAATAGTGATTTTTGCAAACTGGCCGCTGACCAGG
>dme_circ_0008080
ATTGTCAAAGCCTCCCAACTGTCCGAAGCGGTTAATAAGATCCACAAGCCAGCCACGCGCATTCTTGGGC
TCCGGCGGCGGACGGGCGTACATTTTGTTGCTGTCCAACTGACCCCAGATGTAGTCCGGCGCTGCGAAGC
ACTCCGGTTGACGGCCTGCGTTAAATGTG
>dme_circ_0008080_junction_seq
CTCCGGTTGACGGCCTGCGTTAAATGTGATTGTCAAAGCCTCCCAACTGTCCGAAG
>dme_circ_0008081
GCTTCTAATGCCTGCCACTATAGCCAAGTACTTTATGCCCACCTGGAACGTTGTGTTAGACCTTCTGGAC
AGCTTCACAGACGAGGAGTTAAAGCGAGAAGTGAAACCTGAGGGACGCAATGACTACATCAATGGGATTG
TAAAGTCAGCccgcttactggccagccgcttgactggccagGAGGAGCTAATCCGAGATCTTGAGAT
>dme_circ_0008081_junction_seq
agGAGGAGCTAATCCGAGATCTTGAGATGCTTCTAATGCCTGCCACTATAGCCAAG
>dme_circ_0008082
GGTGTCCAGCTTCAACGGCAAGATGAATGCCCTTAATGAGATCAACAAGGTGCTCTCCTCGGTCGCATAT
TTCTCCCATCGATCCCAACCTTTGCCCCACTGTATGCCCGAAGACGAAATGGACTGGCTGACGGCGGATC
GCATGGCGCAATGGATAAAGTCGTCAGATGTATTGG
>dme_circ_0008082_junction_seq
CAATGGATAAAGTCGTCAGATGTATTGGGGTGTCCAGCTTCAACGGCAAGATGAAT
>dme_circ_0008083
GGTGATCGGTGCCGAACGCTATTTAGCTCCACACTGCAGTGGTCATGTCCCGACATTTCAACCATTAAGG
CGGTAGTGCAACTGGCCTGGGCCTCGTCTTGCGGAAATCTTCAGGCTCTAGGCAATAGTAGCGGCGACTT
TGAAGACGAG
>dme_circ_0008083_junction_seq
CAATAGTAGCGGCGACTTTGAAGACGAGGGTGATCGGTGCCGAACGCTATTTAGCT
>dme_circ_0008084
GGTTCTCACCATTTCGTTTATTCTGAATCCGAGTGCCAACGAGGCGTTGACTAGCGATCCGAACTGGCCA
AAGTTCATCACCTCCATTGTTCTGAAGAACCCGCTTCGCCACGTGCGTCAGGTGGCCTCTGAGCAGTTGT
TTTTGGCTTCTACCTATTGCGCTGGGGATCGGCGACCGTTC
>dme_circ_0008084_junction_seq
CTATTGCGCTGGGGATCGGCGACCGTTCGGTTCTCACCATTTCGTTTATTCTGAAT

>dme_circ_0008085
AAAGGGTCCGACACAGCACCGAGAAGAACTCAGCGCAAGTTGACTCGTACTGGGGAACCAGCGTCTTCAA
CGCGCCAACCAGCAAGTTAACCATGTATACGAACGGTCGCCGATCCCCAGCGCAATAGG
>dme_circ_0008085_junction_seq
AACGGTCGCCGATCCCCAGCGCAATAGGAAAGGGTCCGACACAGCACCGAGAAGAA
>dme_circ_0008086
AGCTGGCACAAGGCAATGAGGAGATCGCAAGCAGCGGCGATTGTGTGCGGACTGCGACAAACTGGTGGCG
GGACGGTGTCCTGCCGTAGACTGCCATGACGCCGTAAATGCAGAAATTCGCGAGAGGCCGTGAACAGGAA
GTCGTCGATCAGTTCGTGGATGAGCTCGTTCAGCTGCGCCTTTGAATCGGCGCCAAGGAAAAACATTAGC
TCTTTGGCAAGGCAGAGGTGGCCTTCAAGGAGCTCCTCATGCACTTGTGTGTCGCCGGTGGCGTGGACGT
TTTCGCGAATGCGTTGCAGCCACTTAATCTCATCGCCTAACAATCCCTCGCTAATCTGCAGCGGCCAGTT
GTAGATGCATCCATAAGAAAGGGTCCGACACAGCACCGAGAAGAACTCAGCGCAAGTTGACTCGTACTGG
GGAACCAGCGTCTTCAACGCGCCAACCAGCAAGTTAACCATGTATACGAACGGTCGCCGATCCCCAGCGC
AATAGG
>dme_circ_0008086_junction_seq
AACGGTCGCCGATCCCCAGCGCAATAGGAGCTGGCACAAGGCAATGAGGAGATCGC
>dme_circ_0008087
TGTATTGGTGAGTAGCTTCATATTAGGAACACAGAGCTGGCACAAGGCAATGAGGAGATCGCAAGCAGCG
GCGATTGTGTGCGGACTGCGACAAACTGGTGGCGGGACGGTGTCCTGCCGTAGACTGCCATGACGCCGTA
AATGCAGAAATTCGCGAGAGGCCGTGAACAGGAAGTCGTCGATCAGTTCGTGGATGAGCTCGTTCAGCTG
CGCCTTTGAATCGGCGCCAAGGAAAAACATTAGCTCTTTGGCAAGGCAGAGGTGGCCTTCAAGGAGCTCC
TCATGCACTTGTGTGTCGCCGGTGGCGTGGACGTTTTCGCGAATGCGTTGCAGCCACTTAATCTCATCGC
CTAACAATCCCTCGCTAATCTGCAGCGGCCAGTTGTAGATGCATCCATAAGAAAGGGTCCGACACAGCAC
CGAGAAGAACTCAGCGCAAGTTGACTCGTACTGGGGAACCAGCGTCTTCAACGCGCCAACCAGCAAGTTA
ACCATGTATACGAACGGTCGCCGATCCCCAGCGCAATAGG
>dme_circ_0008087_junction_seq
AACGGTCGCCGATCCCCAGCGCAATAGGTGTATTGGTGAGTAGCTTCATATTAGGA
>dme_circ_0008088
GAACACAGAGCTGGCACAAGGCAATGAGGAGATCGCAAGCAGCGGCGATTGTGTGCGGACTGCGACAAAC
TGGTGGCGGGACGGTGTCCTGCCGTAGACTGCCATGACGCCGTAAATGCAGAAATTCGCGAGAGGCCGTG
AACAGGAAGTCGTCGATCAGTTCGTGGATGAGCTCGTTCAGCTGCGCCTTTGAATCGGCGCCAAGGAAAA
ACATTAGCTCTTTGGCAAGGCAGAGGTGGCCTTCAAGGAGCTCCTCATGCACTTGTG
>dme_circ_0008088_junction_seq
CCTTCAAGGAGCTCCTCATGCACTTGTGGAACACAGAGCTGGCACAAGGCAATGAG
>dme_circ_0008089
TAATGTTCGAATTTGTGTGGAGCATCTGCAGCGCAATCGGCGACTTATGGAAAAGTTGCGTCAGGCACTT
GATTATTTGGTACGCCCTCTTCTGGTAGTGCGTCTTCGCCCGCTGTATCGTCTCTAACAGACCCTCACGT
TCCTCCGCCACTCCGTTGAGAGCATTGTGTATGCGATGGTGCTGCCACGAGTCGTCGATCAGCAGGATGT
TCAGAAGCAGATCAGTGTGGTGACGCATGTCGTGACAATAGGCGAACCCACACTGCCACAACAATTCGGT
TAGCACTGCGCGAGAGAAATGTGGATTCTCCCAGCTGCAGTATTGCAACAGCTTCAGACCCTCGTCGCCC
ACGTTAG
>dme_circ_0008089_junction_seq
AGCTTCAGACCCTCGTCGCCCACGTTAGTAATGTTCGAATTTGTGTGGAGCATCTG
>dme_circ_0008090

GTCGATTTTGAGGTGGGACAGCCGAAATTCTTAAGTGCCCAGGGCGATCAATTGGGTGCAGTTGGGTCTC
GCATAGTGGCTCCCATGCCCGGGATTTTAGAGAAGGTGCTGGTAAAGCCCGGTGACAAGG
>dme_circ_0008090_junction_seq
AAGGTGCTGGTAAAGCCCGGTGACAAGGGTCGATTTTGAGGTGGGACAGCCGAAAT
>dme_circ_0008091
GGCGGGTATTCATTTCCATGAAGTGGAAAGAGAGATCTTCTTTGTCTAGAATGAACTCCACGGTGCCAGC
TCCTACGTAGCCCACAGCTTTCGCTGCACGAACAGCAGCCTCGCCCAGCTCACGACGCAAATCTTCCGAC
AAACCTGGCTGTTGTATAAATGAGCATTATTTTTAAAATAGCCGAAGCTCTTGTGCACTTACAGCTGGCG
CCTCTTCAATAATCTTTTGATGACGTCTTTGCACGGAGCAATCTCTTTCCCACAGATAGACAGCATCACC
ATACTGATCCGCAAATACTTGAACCTCTACGTGGCGTGGAGATCGGAC
>dme_circ_0008091_junction_seq
GAACCTCTACGTGGCGTGGAGATCGGACGGCGGGTATTCATTTCCATGAAGTGGAA
>dme_circ_0008092
GCTTCTGCAGACACAAAACCCCTACCAGACAGCGACGATGAGGAGGATCAGCATACGAATGCCGTCTCGA
TTCCTATCGCCGTGCATAACATGGCCTCGGCCCTTCTGCAAGTGGAGCAGGCTATTGGAAAACGGTTTCT
GAAGGAACCCTACGGCATGAAGAAGTGGGACCCCAAGCAGGAGGCTTTAAAACTTGCCTGCGACTCGCGT
TTGCACCAGTGGGAAGTTTCGCTAATGGAAA
>dme_circ_0008092_junction_seq
CACCAGTGGGAAGTTTCGCTAATGGAAAGCTTCTGCAGACACAAAACCCCTACCAG
>dme_circ_0008093
GAAGAAACGCAAGTTTATCGTAGAAGAGGAGGATGATGAAGCTACAGACGAAGAAGAGGAAGAGAAAAAG
GATGACGATATGACCGATGAGGATGCGGAGCACGAGAACGAAAAACACGATGAGGACGTTGAGGACGACG
AAA
>dme_circ_0008093_junction_seq
CACGATGAGGACGTTGAGGACGACGAAAGAAGAAACGCAAGTTTATCGTAGAAGAG
>dme_circ_0008094
GAAAAAGGATGACGATATGACCGATGAGGATGCGGAGCACGAGAACGAAAAACACGATGAGGACGTTGAG
GACGACGAAAGTGTGACCTCTACCCCCTCATCTTCCAGAGTCAATGGGCGAATTTTAAGGCGACCCCGCA
CACGGCCAACAAGCAGAAGACTTACTTCGAAAGAGATCGAAGAACATGCTCAGGAAGAC
>dme_circ_0008094_junction_seq
AGAGATCGAAGAACATGCTCAGGAAGACGAAAAAGGATGACGATATGACCGATGAG
>dme_circ_0008095
CTCGAAGCTAAGAAACCGCAGCTGGCTCGTCGACGTGCTGGCCATCTTCTTTGGCATAGGCACCTGGCTG
GGCGTCAATGGCACCTTCATTCAGCTTCCCCTGCTGGTGGACGAGGCACCAGAGGGCTGGAGTCTGCCCT
CTTACCTTAGCGTTATGG
>dme_circ_0008095_junction_seq
AGTCTGCCCTCTTACCTTAGCGTTATGGCTCGAAGCTAAGAAACCGCAGCTGGCTC
>dme_circ_0008096
AACTCGACCGCCCATAGCGACTACAGCGTCCAGTCCCCGGCGAGCGGATCGCCTCTGCCCCTTAGCGCCG
CTGCCAACGCGACCAGCAACAATGCAAACAGCTCAAGCGACAGCAACAGCGCCATGGGAATCTTACAAAA
CACAAGCGCCGTCAATGCGGGCGGCAACACCAACGCTGCCGGGGGACCCAATACTGTGCTCCGTGTGATT
GTCGAGTCCTTGATGTACCCCGTCTCGCTGGACATACTGCACCAGATCTTCCAGCGCTACGGCAAGGTGC
TGAAGATTGTCACCTTCACAAAGAACAATTCATTCCAGGGCCCTCATCCAATATCCGGACGCCAATTCCG
CCCAGCACGCCAAGTCGCTCCTGGACGGGCAGAATATATACAACGGTTGCTGCACGTTGCGTATTGATAA

CAGCAAGCTGACGGCTCTGAATGTTAAGTACAACAACGACAAATCGCGCGACTTCACTAACCCCGCTCTG
CCACCTGGTGAACCGGGAGTGGATATCATGCCCACCGCCGGCGGACTGATGAACACCAATGATTTGCTTT
TGATCGCCGCCCGTCAGCGACCTTCTCTCTCAGGTGATAAAATAGGGTGATAAAATAGGTAACACAAACG
GAACATTTTCCCAAATTTTTCTTTGCTTTCTTCAGCTTCAAGTTGCTAACCACTCAAACGCCTTACTTTT
CCACCAAACTTTTTCGATTTTCAATCAAGAGATTTTCCGACACAATATTTTATATGACCTATTTCTGTCG
TTCAAAGTAATACGGTTATTCTGTGTAATTAGATTACTAAttttttttGCCGAAGTATTTTGACGTTGCT
TTATCACTTTTTCAAAATTTAAAAATCCAAATTAAAATGTAAAGAGGCTTAACATAAGCTAATTTCTTTT
TGGCATTAGTAAAAATATTGTGTTTTTTGCTTTTACACCTGCTATCGATTCACACAGAGCTTAAGTAGCA
TGCTAACTAATAAAACATTGTCAGCCTGGGTAGATTGCTTACTCTTGTTCAATTCTCTTTCAGTTCAACG
GCCTGGGCGCCCCCGGAGTCCTGCCGCCCTTTGCCTTGGGCCTGGGCACTCCACTGACCGGTGGATACAA
CAACGCACTGCCCAACTTAGCAGCTTTCTCGCTGGCCAACAGTGGCGCCCTGCAGACGACCGCTCCTGCA
ATGCGAGGTTACTCGAATGTCTTGCTTGTCTCGAATTTAAATGAGGAGGGTACGTCTGCACAAACGATTA
CTCTATTAATTGCGAGATCTTTCAACTTTATTTGTTTGTTGTCCGGGCCAAGCTAGAGGATGGCTGTTAG
TGGGAGAGGTGCATTAATGCACTCCTCTGCGGACACATCTTCATTTGCTGTTGACCTCTGTCTCTTTTTT
AATCTGACTCCTTTTCGATCTCTACCCTGGAATAAGGGTGCACCTATTGTCCTGTCTAGCTTTGCCCGGC
TACAACGTTTTCCACAGAAATTTACACTCCTTAAACTATTTATAAGTAGTTCACTTGATTATTCATTGAA
TTTACACTGTACGAATCGAGCGAAGCCAACGCTTATCCTTTACTTCATGTAGCTGCTCTCTGTGTTCTCC
TTTCATGCTTGCTTCCGTTTGGCATTTACGCCATTTACGTGCAATCAGCAAAAGTGCCATGTATTATTTA
TAGCACAGTATTGGATGAGGCACGGGGGTGCGGTGGGCTAACAAAGCAATACTTGACAAAAATGCGGCAC
AAACTGTTTAAAATGCTGAAAAAGCCTTTCCAAGGGTCCGCGGGTTGGAATTTGGTAATTTGGGTATACA
TCGGGCATCTGGCAGTTTGCACGTAAAATGCATTCGCCAGAGATGGGAGATGTGGATCAGAATTATTAAC
TCATAGAGCTCATACTCCTGGACCTCGTGAAAAACTCCATTACTATCGCAAACCAATCGTATTCCAGAGA
TGGTCGAAGGCCATTCTCCTGCAAGAAATGTAAATAAATTTAGCGCATCCCGATCATGATGTAAAACCCA
TTTAAATGGAGAAATGGTCTTTCATATAGCAGTGTCAATATTTATTGTATTGCGAAATGGAATAAACCTA
CTAAAGCTGGCACCAAATGGCTTTCTAATAGCTTTTTGCCCAGAGCAGCAGGGACAAATCCATCAGGAAG
CTTGAAATAGGAATGGAAAATGTTCTTTTGGTATCCAGTCTTCTCCTTCTGCCTTCTGCCATTTATCCTG
TCATAAGTTCTGCTTACAAATTGTAAAACAATGAAAATGAGAATACACTCGACGTCCTGGAAACCCCTAC
GTACATAAATTAAAAACAGAGCAGACGCAACTGGCAACAAGTGAAACCGCACTCTGGCGGGAGCGGTGGT
GCATTGGCAACACCACTACGTGTGCATCCTGTTGCTCCTCCGTTGTCTTCGTGTGCGTTGGTGCGTCTGT
GTGTGTGTGAAATTACATGAATTAAGTGTGCTCGTTGTTGGTTTCCATTGTTGAGGCACAGGACCCAGGT
CCAGAGGGCGAGAGGGTAAGAGAGCGAGCTCCTGTGGGCTGAGAGCTGAAATGCATGCACAGAACACATG
CTTTAACATGTGCTCATCCTTCCGCACACACAGACAAACACACATGAACTCCAGCACCCACTAACACACA
CGCAGCACGAAAGATTGAGTGCGGAAGTGCATAGCTATAAAAATGTCCATTTATTTAGTTATCATTGCGC
TTGTTGCTGTCGCCCAttgctgcttttgctgcttttCTATAGTTTGTGTGCACACGAAGAAAATACGTGG
CTTGTCGTGTCCCTCGGACATTAAAATCCACACACATTCGCCACAATATTTCGAAAGAGGACAACCTCTC
TTATCACAAATGCGAATTGTCGTCTCGAAAGTATAAATATTTTAAATTTAAATATTAACATTTTGTTAAA
CCACTCGACATTCAGCGCTTTCGGACTAAGAAATGGGTTTTTTTCTCTGTGCGTGTGTGTGGTTTTTACC
TAACGCTTTCTCGGTCCTTGTATACATATTTATATACTTTTTATACTACACACAGCCATGATCATTGTGT
AGTTTTTGCTGGCTCTTACAATTGAAATAAAATTTACTTGTGACTTTTTGTAGCTCGTTGGTTCGGTTAG
CCAGTCGGTTGGTTGGTTTCTCAAATGGAAAAGGGGATTGTCTCCAGTCTCCGTGCTGGATTTTCGTCGT
CTACCGTCTGTTTGCCAAATGAATTTAACTAACTTTGTAAAGTTAATGGGGTTTGCTGTTTGAATTGCTC
TTCCAGCTGCCCCCAGCTCCGTGTGTGTTTTATTATTTTATGGGATTCTCAGTTGACTTTTCACTTGCGC
TGCATTTCTCCATTTAGCTTGCCCCATTATGTACTTGATTTAATTACCAAAACACATCGATTGAGCAAGT
TTTTGTCCGGTGCTACGAGAATTCTGCCAAGTGTGGCGCATAATTGCAGCATAATGGATTTTGTTGCCAC

AACACGAGCTGTACCCTTCTCCTCCGCTTTCTCAATACTGGCGATTCAATCTCTGGTGATTTTTTTGCCC
CAATGCGCATGCATGGTATGTGTGTTCAACAACCAGTCTGGCTCTCGACCCTTTGTCTCTGGTGACACAA
AACGTTTGTTTGCGGCCACACACAGGCTTCCCATTTATTTGTATGCAAATGCATTTAATTTGAGTTTAAA
TGCCCAAATATAAACTTCTGCCAAACAAACGCAACCACATGGAGAATGACCGGCAACAATGAGTTAAGCC
GCTTGCTTACCCGGAACTTCACCCTTGTGTGCTGTGCCTTTGATTTATATTTACAAATTGCATCCATTCT
GTCAGCAGTTCGGAGCTTATCGCTCAAAGTAAACAGAAACGTTGCCAAACGTATTAATTGCTTAAAAATT
GTTTCTGTTCTGTCGTGCCGTTGTTCTTGTTGCCGGATTTGTGCAGCGTAAACCACAGAGCACCACAGGG
CAACTTTGAGCCACTGAGCCGGGTAAATCCGATCAGCAGACCGCTTAGCAGACTCCTGCTGCATCTGAAG
TTACAGCTTCTGCTCCTCACCCTCCTTATCTGCCTTCCACTCGTACGCCAGTTGCCTGCCGCCTGCACTG
CTAATAAAGCTTAGATTGCTGATTAAACGCTTCGAAGGGCTCAGCCCAGAGCGAAGTGGAAAAGTTGGAT
ACCAATACGCCCTGTTGCACAGCCGAGATGGAGAGATGTCGATAAGTCGAACGTTAAGCGTATTATTATT
GGGGTGCCAATAGATGTAGATAAACTCGACGGCTTCCCAAGGAGCCGAGCAAGCACCCAGCGTGTAATTG
ACGGCCAGAGTCTCGACGGGCAATCAACCGAATGGCAAGCTAAAATTTTATGTAATATCCTTTGAATTTC
CAAGTAGCTCTAAGCAGACAGCGGACAGACAGGAGCACCACCAGTGACACTTAAAAGAGCTAGGAGGTGC
TCTACAAACAAGTGCACAATAAAAGCCGTCACATTTAAGTGGGGttttttttttAAATATTTTGAGATTC
GATTATTTCACAGACAATATATTAGCGAGTCAAATATCTAATTGTTTCGGCTATTGCTCAGTGAATATCA
TCATCATCATATCATCGTCATGTATAAAGCCTCAATTCAAGCAGTCAGGATCAGGCCCGGTTTTTGATCA
GTGTAGTAGGCAACTGGCAATCGAGTAGGAAACGTCAACCAGCAAATGGCAAACAGTTGCTTTTGTTATG
TTAATAGCAACACGCTAGGACACGTAACAGCGATTTTCACATGAGGTGGGCCAAAGACGCTGCGGGCCAC
TTTTGGTTGGTGAATTTGGCACTTCAAAGGGTTAGAGAAAGGGAGCGACCGAGGGGCGAATGAGTATGCG
GTTCGCAGCCCGCGTTCACCTGAAAGCTGTTTGCATACCAAATAATAAAAGTGGAGAGGTTAACTCTGGA
ACCGGGAAGGACGCACTTTAAGCGCAGGGATTAATCTAGCCGAAAACAGCAACAAGCGGCGCAACAAAAA
GGGCAAAGGAACGGAAAAGTAGGCCCAGAACCCTTTTTGGTGTCAGCCAGTCAGACATTTTATTAGTTTT
GACAGCGCTTTGTTAGCTCTGCCTGCGACTAGGCGAATACCCAATTGAAAGGTCCCAGAGGCCCTCTGGG
GCGTGCTCTTTAGACGCTTGACACATTCCGCTTAGGCTGTCAAGCATTCAATATCGTCCTATACCCCACT
CGGCCGGAAACACAGCCAGCTGGACCAACAAGCCAACAGCCCATATCATCCGATTGCCCACTTTTGGAGG
CTGGGACTTAGTTGCCTTTGCATAACAATAAAAGAAACCTAATCAGTCTGGAAATCGTCTGCGAAGCATG
CTCTGATACCCTAACACTCGCACATATGCTATGTAATCCGCTTCTGTAATTTTAAGAGCGTGATAAAAAT
TTATATTATTGACGAAAATATTGTCGAAACGTGTTTAATTGATTTTTTATGCGTTCAAAGCTCCAGCGTG
TTGTTGTGTGATTTTCCCTCTTTATATGTGCGCCGATTGTTGTTCCTATGAATGCGCGAAATAATAATTT
CATAAACTTTTATGCGATACATGTTGTGGCAGAAATGCAATTTGATAAAGTGCCCGAAACAGGAAAAATT
AAATCCAATGTGCAAATAATTTCAATGAATCGCAACAAATCGCTTTAGCATCTAAACGCAGTATGACAGG
CGTTCCTGGCAATGTGATTAAATGGGAGCTATCGATTTGAATTTGAATTTCCGTTTGGCTTTTGACACCG
GCTATAATGGAGATGGAAAAGAAGATAAGTTCAGAGGGCTGTTTCAACTTTATAGACTCTCAGGCCAGCA
TGCCACCGCCATCCGCATAATTCTTCATCTTCATAGAATATGCTCAAACACGCGACTAATGGGCCGCCGG
GGAATCGGCTTCGCTGTCTGCATACGTTAGTGTCGCGGAGCCACaaaaaaaaGAAACTTTTGGAGGAACA
TTCAGACGATAATAAATTATTATCAAGTCTCTGTCTATTGGACGCGGCTGGGAATACAAATAAAGATTGT
GGCTACCGGGCACACTTTGCACGGCAAACGGCAGCTGTTGCTCATACGCCCCGTTGCACGCCGAGTTATG
CTGGTGACTGGAGAAGAGCCGCATGCCTGGCATTCAATATGCTCCACTGCCAATACCTGCAGATGTGGAT
GTTGATGTCGACGTCTGCTGCTGCTGCTACTTCTGGCCTCGCCTGCAATTGTTACCCCGAATTCCTGGAA
AAAACGAAACGATATTGCTCGGTGTTTGGCATAAGTCAAGTCCATCGGATAACTGGGAACTAAGGCAGTC
AGTCAGGCAGCCAGTCGTCTGTATAAAATGGATTTACTGCCAACTGTTGAACTGCGTTCTCCGTCCTGCG
AACTCCTCCTTGCACCTCACTATTTACTACCTGCATTTCCATTGCTAGTTTTGTAACTTGTTAGCGCAGC
GCTCGCTTGGCTACGCTTCGAGTAGCTGACATAGCCCCTACTTATATAGCCGGCATGGCTTCCGAAGTTC

TTGCACTGGTAAAAAATCTAGAAGATTCATTGACTCTTTAGTATATCTTAATTTCAAAAGACTTGAACTC
CTGCATCACATGGAAGAACTGGCGTGGGCTGCTTAATATTTAAGAAGGGAATAAATTAATCTTAAGTAGC
GAAGTAAAATTGGTCAAAGTGTAAAGCCTCTTCTGTGCGCGTGTGTGTGGCTAAAAGAAGTTGAAAATTT
ACTTTCCGCATTATTCGATTTGTTGTCTTGGGCAGGGCTGCACTTTACATTGTTTGCTTTGGGGTCGGTT
TTGCCATTGGCATTGCCGTTGCCCTTTGGCCACCAAGTCGACCGGCAACTATTTTTTATGCTTGATGGCA
ATTTGCGCCCCGCTTCGAATTTGCGCTGATTGGCTGCACATGGGATGTGAAGCCCGGCAAGGGTCACCCA
ATAGACCGGATCTTGGCCAACTGTCAAAGCAAAAGACACTGGCCATAGGAAAGAGTCAGCAGCTTCCTTA
AATTCTGCTTTCAGCCTTAATGCAGTTCTACAGCTTGGTCTCAGCTCACCTTATATTGCGTAGTTTGTGG
CGTAAGTAAAGCCATAAAATACAATTCAATAACGCGTCCGGTGCCGCCGAATTGTTGCACACCAGCACAC
CTTGTCTGACAGCTGTAAAATCCGTGGCCTTAAAACGAAATGCGGCAAGGAAGCCAAGCCAGCTGGGCCA
ACGCAAAAGCTGTAGCCAAGCAGACGCAGTTCGAATTGAAGCTCGTAAAAAATAGCACAGAAAACTTATT
GAGCCAGTTATGAGTTTCGTACATGATACTAACGGCGTGCCCCCCGGCAATAATGCGAGTGCTTGGGTTG
TAGGCAAAGAAAGCAAGGTGGAAAAATGATAGTGCCCAAGATATCTGGTGTACATGTTATGGCTTTTTGG
TAGGGTGTAAAGTTTTTCCGCGGTCGCCGTAAACCGTTTGGAGATGTCCAAAAATTCAGAAAGCAGTCGA
GGGGAATGAAAGGGAATACTCTGGACACTTCAAAGCTTAAAGCATAAAAAGGTACTACACTTAAAAACAA
GTTAATTTCTTACCGAACCCATTGTTCGACAGTAAGTTTTAGAACTTTTAAATAGTATATACTTTTTTGG
GATTCCACGTGTAAAATTTTATATAAGAGAAAGTTTACCGGAGCAGCAGCTACAAAGTTCTTTAGTTGGC
CCGCTCACCATTCGTGCGGCAATACCCAAATTACTTTACTTACCACTACTCTCACAACCATTTACTCATA
ATTCTCAACTGCTCCAATCCGCAGGTGTATACGGCGATGTGCAACGTGTAAAGATTCTGTACAACAAGAA
GGACTCGGCACTCATACAAATGGCGGAGCCCCAGCAAGCTTATTTGGG
>dme_circ_0008096_junction_seq
TGGCGGAGCCCCAGCAAGCTTATTTGGGAACTCGACCGCCCATAGCGACTACAGCG
>dme_circ_0008097
AACTCGACCGCCCATAGCGACTACAGCGTCCAGTCCCCGGCGAGCGGATCGCCTCTGCCCCTTAGCGCCG
CTGCCAACGCGACCAGCAACAATGCAAACAGCTCAAGCGACAGCAACAGCGCCATGGGAATCTTACAAAA
CACAAGCGCCGTCAATGCGGGCGGCAACACCAACGCTGCCGGGGGACCCAATACTGTGCTCCGTGTGATT
GTCGAGTCCTTGATGTACCCCGTCTCGCTGGACATACTGCACCAGATCTTCCAGCGCTACGGCAAGGTGC
TGAAGATTGTCACCTTCACAAAGAACAATTCATTCCAGGGCCCTCATCCAATATCCGGACGCCAATTCCG
CCCAGCACGCCAAGTCGCTCCTGGACGGGCAGAATATATACAACGGTTGCTGCACGTTGCGTATTGATAA
CAGCAAGCTGACGGCTCTGAATGTTAAGTACAACAACGACAAATCGCGCGACTTCACTAACCCCGCTCTG
CCACCTGGTGAACCGGGAGTGGATATCATGCCCACCGCCGGCGGACTGATGAACACCAATGATTTGCTTT
TGATCGCCGCCCGTCAGCGACCTTCTCTCTCAGGTGATAAAATAGG
>dme_circ_0008097_junction_seq
GACCTTCTCTCTCAGGTGATAAAATAGGAACTCGACCGCCCATAGCGACTACAGCG
>dme_circ_0008098
CGCGGAAGCGACGAACTTTTGAGTCAAGCAGCGGTCATGGCGCCCGCTTCCGACAATAACAATCAGGACC
TGGCCACAAAGAAGGCCAAACTGGAGCCGGGCACTGTGCTGGCCGGCGGAATTGGTAAGTTTCAGCtcta
tcctcttttatctgtgtctgcctatcctctctacctctctctctttccctctccgGCTTACCTTTGCATT
AACCTTGCCTTTCCTTTTGTCTTGGATTAAAACCCTGCCTTTACGTCTCCCGTCCCCTGAGCGACGCTTG
CATTAAGTGCATTTACAGCCATGGTCTTGGGCCCAGTTGGGGTTTGTCTGCCAATTAACCACGCCCACTG
GGACAATATTGTACACTGAAAAACGCGTTTACCATAACTGACCGAAAAACATGCTATATAGAAATAAAAA
GAATTTAGAAATCTTTAAATTTTTCAGAAAAGAAAACATATTGTAAATGGTGAAAGTACTGTCGCCCTCT
TATTTCGGTGGTGTAGTTTCTGTTAAGTTGGCTTGTGGGTGGTCCCAGTGCAGTATTAGGCTTAAGCCTT
CAACATTGGCAACAAAGTCGCCTGGCCACTTGAGTACCCCTATTGAAGGCAAGCGCGCTTTGATGGCTAT

AGCCAATTAACCTACTAATTAGCATGGAAACAAGTCAACAGCGAAAATATTTGGTTCGTCCCTTTTGTGC
TGCCGGAAAATTATTACGGGAAAAATGTTTGGCGTCCGCGCTGACTGGCTACACACTGTGTCGACACATT
AATTAGCCGCCCACACCACCCACACACGCTTAGCCGGTACGAGTATCGCCCTGTATATGCCGCCGCAAAG
TAATAAAGCTAATCAAAAACCGCACACCAAGCCGTCAAAACCATAGTGAACCGAAACCAGCAGAATGAAC
CACACATTAGGCAAATATTTGACGGGTTCGTAGGCACCCCGCCAAAACTGGATTTGAAAGCCAATACCTG
CCCACAAAAATTCAGTGAGCCACAAAATGATTGCCAATGCAAACAAATCAATAGGCAACAATTTTCGCTG
AAATTCAAAAGTAATCGACTGCTCTACCCGCCGATTGCGTTGCTTTTGTGTGAGCGAATTTAATTAACGC
CATATGCAGGTGCTGTGCGTCGATATCCTTTTCGTGGGAGTTTTCAAAATGCTTTTGCAATTTAGCCGTA
ATTAAGAGCGTTCACCGCCGCCAGCAACATCGGTGGGTGAGTATGAGTGGGTGGTTACCACAAAGGGAGC
ACTTGAGCATTAGCCGCCAGACAGCCAAAGGTCACGCCAGGACAAGAGATGGGTGGACGGAGGACGAAAA
AAGGGTTGCGAAAGGATCGGCAGCCTGTAAATGGCAATCGGCTGAGAGCGAAAAAATGTACGCAAAAAGC
GGAAGCATAATATTTATTTATTACTTTACAAAGGACTTTTCTATTAATTAAAAAGCAACAAGCAGACACC
CGCACACGCACAGAGAGGAAGGCAGTTAGCGGTTCGCCTTTCACCCCCTCTACCGTCTGCCATTGTTCGA
CGGTCGATGCAGACGACTACTTTGACCCAACGGCATTTTTGCGGCCATGGCCCGACTACACCTATTGGAC
GCCTCCCACCTGCGGCCATTTTAATTTTCCACGTAAAGTTCTTGTGCAAacacacacacactaacacaca
TGCCGCATTTTGTCCTTCTCCATTAAAGTCGCGCTTAAAGGAAGCAGGGTATTCGGTGAAGCAGGAATGA
GAAAGGCTAGGTATCCAATGAATTCATTTTTGTGCTCACCTCTTAAATCTCTCCTTTCTCACACAAAGAG
GCCATGCCGATGATGCCAAAAATAATAATAAAGGGAATTGCTAAATAAATGTGTAGAGACACTTTTATTA
CACTGCAAGACCCAATTCATGTCTAAGGGCTCGAGATTGACACAGAGCACAACAAAGACCAGACAAGTAA
ATAGCAGAAATCTAAAAAGCAACAATGAGCATTGTATGCCTCTGTGGTCGAGCCTTAGATAAACACACGC
ACTCCTCGGGTCAATTGTATCCAAGCGCCACTAAACCAATACATCGATATCTGGATTCGCACACACGCAC
GCTTCCCGTTTCCGTTACAAGAGCTTAGAAGGACATGCAACAAATTTTTCACGTAAATGTAATAAAAACA
CTTTGGCATGTGCAGCGCGTAGGAGGTGGAGATGCAGATCTTTTAGCAACAAGGGGGGAAAGGCGGGGAC
ACGCCCACCCACTTGATATAAGGGCTCGGCTTTTCGTGTATTGGTGGGTGACTGCGTGTGGCTGGAAAAA
TGTTCACACACACTCATGCTCGCAAGGCAGTACACAGTCTGCAAAAGGAAAACTAGTAGGTCTCGGACCT
TCATGGTCCGAGTTTTGACCGGCTAATAACTAGCTTCGCTTGATTTATTTGATTTTTTATTATTAGGTAT
ATTTTATGCCAGGTACACGAGACCCCATAGAGTTGAACCGTAACGTTTAATTCCTAGAATAACCGGAAGA
AAAAATGTTGACAAGGCAATCAAAATTAGTTCCAATCTTTTTAGAAATAAGATTTCTTGAAAAACTACTT
CAACTTAATTATTTTTTATTACAATACCCATTTTTGTTATCAAGATAATTAAAAACTTTTTAAATTGATA
CGGCCGACTTTTTCTCGTGTAGACCATAGCGTGTCGCGTGTCCGGCTGAAACACCACACCAAACGTGGCT
AGAAAGTGTTTTGCTTTCGTTGTCGCGACACTCGCCCCCTTCCCCGCAATTTCAGTTTGTTGTTTATTGT
TACAGCTCTCACACATGCATCGCCTCCATGCTGTTTGTACAGTCCACCAACATTTGTTTGTTTATTGACC
GATTTTGGGGGCTTAACGGTCAGCTCACCACCTATGGCAGCTAGGACCTACCGCCAAAAAAAGGAGTTTT
GCAGCTGTTGCGTGAGGACCATAttttttttttCTCCAGCTCTTCGGCATTGCTAATTCTCTGACCAGACA
GCCAGCGGCATCGGAAAAATATGCTGAAAAAATATCTAAAAATATCTATCAAGGGTCATCCCTTGAGCCT
TAAATCAGCCCACTAGTGTAGTGTCATCAATTTAAAGCTCAAGGTTTGCTGTCTGCACAGTGGGTCGTTG
CCAATCCCTTCTGTGGTCTGATCGAACACTTAAACGCTTTTATGGCTGTAACCAATAAGACGACAGTTTG
GATACGACAAGTGTCGGTTGGTTCCATGTCCTGCGAATCCTGCGCGTGCCGCTCTGGCTTGGGTATCATT
CGCACGTCCCTGGCTCTGCCTCCTGGTCTACGCGTTGCGTGTAAATATTTTCGCAGTCAATAAAATAATA
TTGTCATTAtttttttttGTTGTTTTCCCTGCCATCTGTATCAATATTGAGACAGCATTGAAAGGAGTGTA
AAAATCCGGCCAGACCAAGGGCAAACAGCAATTGTCAATTGGTTTGACTCGCTGATGACTGGCTTGCGTT
GTGTGCAGCTATTTATGGCAGTCACGAAACGTGAGTGCGGTTAGTGGCAGCGGACTTTCAGGCAGTGCTC
GGCCTGACTTCTGAATTTCCTGCCACTGTATGGGTGACTGTGGGAAACTGCGAAATATTTGTGTGTGCCA
TTTAATTAAGCATACGACCCGTTGGACGGTCGAAGTTTTAATGCACAAATGAAGTGGAAAAAGTTTTGCC

AGGACCAACAAACCTCCTATGTTTGCTCATAACTTACACCATTGTTATACGTTACAGACTGCTAACTAGC
TCTCAGTTGAAATCGAGCACTaaaaaaaaTACTTTGGAGTATAATAGCACAAAAAAACAAAGATCCATAG
AAATATAAACTGCATTGGGAACAGTGTGTTCGATGTGTAGCCAAAAAATATACGCAAATATAATGAAATT
AAACGTAGTTCCTTGATTATTTTGTTGTGAACTATATTTTCTAGTCCTAGCTCTCTGCTGTTTGACTGAA
AGCCACTTGACTGCACACAGACATGAAGTTCGCCATTTCCCAGACTGTATCCTGCAGCCTGCTAAATGCC
AATGCAGTTTCAGACTCCCACTCGGATGTGTGAGCGAAAAAAACTATTTTCCTAATTGAAATGCAATTAA
ATTTATTTAAAGTCGTCGTCATCAACGCTCAGGCTGTTGAAACCCAGCTGCGAACGTTGGCTCCGTGCGT
TTGTATTCACTTGGTGAAAGCTCCAATCTTTCCCACTCCCCTTCCTTTTTTCTGGCAAAATTTTAATGAA
TTACATTTCTTGCGGGTGTTAAGCTCGAGCTGTAAAAAGAAAATCGTTATTTTCAATTATGGGCCAGTTT
TTGTGTCTGGGGATTTTCCAGAAATCCTAAGGCAACGGAACCTCTGGCATTGGGTTTCATTTGCTGCCAA
AAAAATGTGCCATGATTGATTACGCATGGGCAAATGTTTAAAATATATAGACAAGGCCTGGCTGGCTAAG
CAAATATCATTAAGTCGTTGTGCTCACATTGTGGCGGAGGTAGCAGAACGACAGACACGTAAAGGACACA
AGTTTGCCCAACAATTTAAAATTTATGATGTGTGAGCTTGTAAAAATCTAATTTACTGGCGCATAATTAA
AGACGCTCGAACGGCTCCATGTGCGTGAGTTCGGCGTGTATTTATTTAAATTATATATTCGACTGTTGAC
ACACCCTCGGCAAAGTTTGGTGCCGGAACTCAGACAAACACTGGCGTCGCGCTCACATTAAATCATTAAA
TGCCGCCATGTAAGCATAAATTTAATTGTTATTAACAATCAGCACTTATTTCATCCCGAACACCACGCCC
ATCGCCGCTGGGGAAAGCCCttttcttttgttccactcgccttttccattcattttcatttacatttttc
gtttggggtttCTGTTTGCTTTATGAATTTAAATTGTCATTAATTAACTTGGTTCGCATCGGTTAAGAGA
AGGGACGGGGGTAAATTATTACACATACGCCACGCTGGACAGACTGACCATTCATTGCAACCCAACCATC
GACGTCCGAACTCAATTACAGTTTGTAAACTTTTCAATTAGCTATAAATAGTTTATTTATGTATATTTTT
CGTGCAACTCATTGTAAGTGGTGGAGAGGCTGTGTGTTTAATTGAAAATGGCAGGATGGGGCGCGTTCCT
CGCCTTTCGCAAAAAGCGTGTGAGGGTCATTAATACTGCTGAGCTTTGGCCCTACGTCCTTGCAGCTCAT
TTTAACGCGCAGCCGAGCGGATGATTACTATTCATATCATCCATGGGGCATTGACAGTCAACAGGCAGCG
CCAGCGACATCATTATCGAATGCAAATTTTATCGATGCACTTTTTTCAGCCACACCAGTTAATGCATTTC
AGGCTTGTTGGCATAGCATTATCCTAGGGTATATGTGCGCCGGTGAGGTATCAGTCGAGCTGCAACAGCT
GTTTTTAAACTGATGATTAATTTTGATTCTGCAATTGCAATTAATGTTTGCTGCGCAAATATTTGTTCGG
GTTATTCAGTTCGCTGTTAATTAAAAAGCAGCAATACCATAACTGCTAACCAATCTAGCCGCAGGCTACG
TACCCCAAACCGAAAGCGCCTGATTCAAATTGAAATTTTTTGCCAGTAGAAATTGTAGCTGCCGTGCTTT
TGGTTGCGTTTCCGGTTGTCGTCATCGCTGGGCGCCATCCTTCTTTTCAATTACGACGCCTGCCTTTGTT
GGAAACAGTGCTGCAGCCGTAGGCTCCGATGGTTGCAACGCTTGTCGATGAGCTCATTGGATTGATGTTT
CTCTTTTCACACCATCAGGCACTACGATTTAAAACAAATGTAGCTAAGGAGAACCGACAGTGCAGAAAAA
TCGAATCGAGCTCCTGGCAGCGGAACGTaaaaaaaaTCCACAAATTTCGCCAGTTTTTCGCCCGGTTCGC
CGACATCATCATGCATGATAGATACCTTTACATATTTTTGCCGTCGCCTTTTTTTCCAGGCAACTAGCCG
ACGGCACTTGATTTGTTGTTTGTCTTGTGCAGAACATTTCCGGACCGCGTCCATCGACACTTTTATTTAG
TTTTGATTCATAGCAGTCCCCAGTCTTTTTCGATTTCGTCTGTTATTTATGGTGAGTTTGTGGGGCAGTA
GAGTGATGGAGCAGATATTATCGTTCTGCAGTTGCCCATCGATTTTTAATAAATTTTTGAGTTGCCGCTC
GAAAGGCAGAATGCCCAACTATAACGACCGCACAGGTCCTGACCCGTCTGCATAATTTCGTTTCGGGTTC
ATTTCCGTTTCGAACTAATCAAAAAGTGTAATTTCCGATTAGTTGTATTCAACATTGACCGGCAAGCAGG
GAGAGTTTCCACAGTGCACTTCGAAGTGGCTTGACTTTTCGCTTTTACAGGCACAAAGGCTTCTGGAAAG
TGGGTTAGGGAAAGAATCGGTTCAGCCTCTTTGGTGGCTTAGTAGTAGTATTGTGAAGTATATCAGAACA
TTGACCCCAGCGTCTGGGTTTTGATTGAATGTACGGAATTGGCTGGATAATGACCTTTTTACATTAAATG
TTATCGAAGAAGTTTGGGATCGAACCATCGATTTATCAAAGTTCTAGAAACTCTTTCCCCAAACGCTGAA
GTGGCGGGGCAAGGCCTTAGATCTTAAATTTAACAAATTTATGGAACTTTTGAACACGATTGTCCTAATG
CTTTGTTAGACCTTAAATTCATTTAAATTCGGGCACTATTAAAACGGAGATGGACTCTGTCCGGAACTC

GAAACCACGACTATCTACGACCATGTGTGGGCTTGTTTAGCAAACACCAGAGTGTTTGAAAATCGGATAA
GGGCATGAGGGGCGACGGCATTTCATTTGCATATGAACAGCAAACTGTTCTCTTGCAGGTGCGGCAAAAT
ATAAAAAGCGCACAAACCAAAACATTAACAGGTGTTGACTAAGCGCCAGGCAGATTCTAGTCTAGCCCTC
CACTTTTCCTCTTGCAACTGCATAAAGAGTGGCAAATTTGTTTGTCTTTGTTTTAAAAAATTTAAGGCAC
TTCTGTGGTCATGCCCAGGCTCACTTAGCGAAAAATTGTCTTTGAAGATGGTATAGCGATTTATTAGGAG
ACAGCCTATTGCTTTCTTGTTGAATCTGCGACAAGCCACTGGTACAACTAATTTACAttttttttagacg
ggtttttttGATTGAGGGAAGTTAGAACAGATAATATCTCGCAGTGCTGTTGTAGGGATATCCCCGAAAT
CTGGCTGCAAACGCTTTATTTATGCAAAAATAACAAACAAGTATCCGGCGAGGAGAGAAACTGCAAAAAC
GTCGACGAGTGCCTAAAAGCCTCAAACGTTGTCTGTGCGCCGGACATTGTCGCCGTGTTGCTGTTGGGTT
ATTCACAGCCGTTGAAAGTGTGCAATGAATGAATTTATGGCGGTACCCCCGCAGTTGGCTTAACTGTGCA
AAGTGTATACAAATGTGGCTGCGCTTGGCCATGTTATCAACATTTTTATTCGCACCACCCCACTCTACTT
TAGGAACAAAAGCAGCCAGCAAATAAAACCCAAAAACAAACAAATTAAGTGAAGCGCGGAAGCGGAAGTG
CAGACAGCGCTGGCCATTAAGTGAACCGGAGGTGGTCCCCGAAAGAGGCGGGCATGTTGCAAAagagaga
gagagagaCCTATAGGGATTGTCACCTGTTTAATATTTCAACGTTTGCTTATTTTATGCTTTTTTGTTGC
AGCCCAAGGCCTCAAAAGTCATCCACTTGCGCAACATTCCGAACGAGTCCGGCGAGGCAGATGTGATTGC
CCTGGGCATTCCGTTTGGACGTGTGACCAACGTGCTGGTGCTCAAGGGCAAGAACCAGGCTTTCATCGAG
ATGGCCGACGAGATCTCCGCAACGTCAATGGTGTCCTGTTACACAGTAACTCCGCCCCAGATGCGCGGCC
GCATGGTCTACGTGCAGTTTTCTAATCATCGCGAACTAAAGACGGACCAAGGTCACAACG
>dme_circ_0008098_junction_seq
GAACTAAAGACGGACCAAGGTCACAACGCGCGGAAGCGACGAACTTTTGAGTCAAG
>dme_circ_0008099
TGAATAAATTATCAACGTGTGCAAATCAAACTCGAATTTAAACGGATAACAACGAAATCAATTGAGCAAG
CTTCCATACCCGGTTGCTTACCGATTTGTAAGTCCTTTATACTTCACGCATTAAGTTTTACCCTATCTAT
TACCCTCTTCCCCATTGTCCGCCTGTTTCAAAAAGCTTATTTTACCTGCAGCCCACACACTCCATTAATA
CCTCTCTATCCCACATCTCGGTGTTTCCTTTAAAGAAAAATATTATTTTTGTTATGGTACCATCTATGAT
TTATATATGATTTAATGGTTTATTTCGGCAGCTTACTCCTATGACCTTTCATGTTGTTGTCTCTTTCGCA
TAATTTAGCTGACTCATAGGCATGCTCAATGCAATTTTAATAATTTGTTAAACAGCGATATTCTTTAGCA
ACTTTTTGAAACTCTGCTTTGGGGGGTTTGGCTTATTTCCGTGGTCAACTAGAGAAGGGAACTGTAGCCG
TGGATCCAGTAACCAGTGTCGTACAACTAGCGCCGTTATTTTAGTCACAAACGACTCAGTTAAAAATTGT
TCAAAGGGGGAGAGTAGGTAGCAATGATACGGCCGGGTGTCAGGGGATGTACAGGGTGGCACGGCGCCAA
GACGAGCGTAATTTGCAAACAAAAAGCCATTAAAATATCAAATTAGCGTCTGGCTGTGGCTGTGAGTTCT
GGATTTAGTAGAAGTCTCCAGTTCTCTTCGGTGCCAGTTTGCCCGTGCCCCGCCTCTCTTTCGCCTTTGT
CGCCCGTCTGCTACCTTCCCTGATCCCAGACATTTGTTTGTGCTTGCCCACAGCATTGTGCAAAATCGAA
AGTAAGTGAAATGTAGCGTCCGCATGCGCTTCGTTCAGTCACCAATTTGTCAGTCCAGTACTCATTGCCA
CGTATGTACTGCCCTGATCACTGAGCGAAAGAAATGATCAAGAACCATTGTTGTTAATTACATACTGATT
AAAAAGCAAACGAAGTGAGTATCTAAAGGTATATTGGAGTGGGGGTATCATCATTTCAGCCGGCGTAGGC
GAGCTTACAAAAAGATTTCAAAATATAtttttttttCAACTACTTAGTTATCAGACTGTATATTTTAGTT
TCTAGTTTCGCTTATCAGTAATTAACAGAAATGAGTTCTTGCCCGCCACAAATGTCTCGTGTATTTCTTT
TAGATAACAGAATTTACACTCCACATTAAAATCCATTTGTTTCACTGCCACGGCTGAATGCGTGCCAAAA
ATTCTATTAAAATCAAACAGAAACAAAAGCTGAAACACGTTTTACCTGTACTGCACTTCAAACTGAGTCA
CTGGGTTGAAAGAACTTCGTTGGGGATGAAGCAAGCACAAAATGGTTACTTGAATAATACaaaaaaaaaa
aaaaaaaaCAACAACAGCAATCAAATCATCAAGGGAAAATTTATGACGGTCACTCAGACCCCTTGCAAAC
TGCGCTGACAATAAAGCCGTACTGGAAGCTATCTCGATCAATGGATCGGGTCGTTCTGGCAGACCAAGAC
AGAAACTTCTCCACGCTCCACATTTGGACAATCTGCCGTCTCCCTTTGTCCCAAGCGATACGTTCTGGGG

CCACGCTTGCGCGTTTTGCCCTGGGTTTCGATTCGTTTTCGGTCTTGGGCTTGTGTATTTGTACCCTTCC
CTACTCGTATTTTTGCCCAAACGCGTTTCATAAATTATCAAGTTGAATGCCCAGTTTCGAAATTTCAACG
GGCCTGACCTTGAGCACACATTTTCGGAGCTTCGGGTCCCGCTCTCGTGCTTTTAAGTGTTTGCGATTGT
ATCTGTATTCATATGGTTTTGCTACCCACTTTTCACATCGGGTATACCAAACTGCTGACATGACTATAAT
GAGGTCATTTACAAGCTAAGACTTTATTTTCATCTTAAAACTAATACGACGAAAATCTTTGGTAGTTATT
CACGTGTTTCACTCTGTTGATTATTGCCAATGGCTTCTATACAAATTAACTAACGCACATGCGTAACCCA
AAAGAACAGTGCGGAGATGGTCTGCTTCAAATGATAAATGATGCTCATTTTTTCCAACTTAGCTCCCTAA
CAACCGCGAAGATAACGTGCGTGTGAGACGTAACTTTAACCGGTTGGAGCCCTTGAATTTTCAATGCAAA
TACTTAGAAAAACTCACGTTAGTGGGAAGGTCAAGTTAATTAGGATTGATGGAATGGGAGGGTGGGTCTC
AGCATTAGAAGTCCGGGAATATAAAAAGTATATTTGGGTAAATACTAATAGATTTAATTATGTCTGGCAA
CGAATTTTCGTTGATTTTCCAGACGAAACTCGTAAAACATGAAACGCACACGTTTTGAAATTAGATGAAA
AAATAATAAATCTTCGTATCCGAAACATCATTCCCATTTTTGAGTATTTTATTGTGCGTGACCCAAACAC
AAATATTTATTGTTATTTAGTGTGATGGATTATGTTAGAAGTAGCAGGATAGGTGGTAATGCGTCTATTC
GTTAAGGTGTTGATGCGCATACAAATTAACATAGCCCGTGAGTATCGGTGATTCCGGGCTGTCATATTAC
GGAGCATATGTGGCATGTGTGCGGCACGCATCATTGAGAAGATTTGCCGCATAATAGACCGCTCAAACGG
CCCACAAACCGACGAACACATCAATGTTGGTCGGTGCTCATAGCAGGGAAATTTACAAGGAAAAATTATT
ATACTACACAATTACACTACAAATATTATTTGCTTCCGCAACATATTTTAATTTTGTAACGCTTTCTGGG
TTCTCTTATTTCTAAGTGTAAATTTTATTTTTTATCGGACTGCACGGTGTGTCAAAACAAGCTTGACAAC
ATAAATACCGCACACGATTTTTGAATGGATAAGAAATTTGGTGAGCTTTATTAATTGCCGGATTTTGTAT
AATTAGTAGTACAATTTCCTGGTTTTCTTATATCCCGAAATCGACCGAAACCGAGTAGAAGGCGAAGAAG
CGATACACAGATCGTGATTAACGTTTTATTAAAATAACCTAAAATTTGTTGACCTGATTATGTACGACAA
AAAATATTAAAATTTTCTTATACATATTTACTACATTTCCCTGCGCTTGCAGtttttttggtttgtttttt
tATTTATGGGCTTACCCTCACTATATATGCATTTATATTTCTGGTCGTCCCTAAAAACAATTTTATAGCC
CCATTTTCCGTTTCCATCCTATGCACAATCTTTGAATCCATTTTTGTTTTTGGTTGTCTGTTGTCTTCGT
AGCCTGTCTGTTCTGTACTTTCGTGGGTGTGCTCTATTTTAAAATGTTTTCTAAACATTACTACAATAAT
AGGATTTGCCATTTTCACAATAGATTTATATTCTATAAATAACCATAGTACCATAGATAATACGTTTCTT
TACAATCAATGCTAACTTCATTTTTGCAATAAAATGCAAACCAATCCCGTTTTTTCATCTTAACCTCCCT
ATATCTGTGCCTTTTCTTTGCATTTATCTTTTTTCGTACAAGCTTACCCCAAGATCATGCCTTGCATTGT
CAATGTATATGTGTAATATATATTTATATATTAGAATTTTTTGTCTCATATGGGAATTGTTCTGTTTTCT
TTCGATTTTTACAGGCGCTTGTACGCTCCCCTCTCTACTAGTTCAGCTACTGCTACTAGCACCACCAAAC
CAACGCCACCAATCAACAGCACCATCTTCGCGAACGTGTGTCTTGAACAGTTTTAAAGTCTCTCGACTTA
GTCAACTCACCCACAGCGCATATGCCTATACATATGTTTGAAAAGTAATCAGAGAGATATAAAATCTTTG
GTGACATCCGATGAGATAAGATTAACTCCCCGAGCCTTAGGGGAATGTCAAATGCAAATTTATTAGAGAG
CTCTCTGACCCAGAAAATTAATTTCTATAGATATCCTTCAAATCGTCAGCCTCTATTGCTCGATTTTAAA
TTATTTTGTCTTCCCATAAATTATTAGCGACAGAACTCTTTTAAAAATATATACTTATTTTTAGTGTAAG
TCCTTTATACTTCACGCATTAAGTTTTACCCTATCTATTACCCTCTTCCCCATTGTCCGCCTGTTTCAAA
AAGCTTATTTTACCTGCAGCCCACACACTCCATTAATACCTCTCTATCCCACATCTCGGTGTTTCCTTTA
AAGAAAAATATTATTTTTGTTATGGTACCATCTATGATTTATATATGATTTAATGGTTTATTTCGGCAGC
TTACTCCTATGACCTTTCATGTTGTTGTCTCTTTCGCATAATTTAGCTGACTCATAGGCATGCTCAATGC
AATTTTAATAATTTGTTAAACAGCGATATTCTTTAGCAACTTTTTGAAACTCTGCTTTGGGGGGTTTGGC
TTATTTCCGTGGTCAACTAGAGAAGGGAACTGTAGCCGTGGATCCAGTAACCAGTGTCGTACAACTAGCG
CCGTTATTTTAGTCACAAACGACTCAGTTAAAAATTGTTCAAAGGGGGAGAGTAGGTAGCAATGATACGG
CCGGGTGTCAGGGGATGTACAGGGTGGCACGGCGCCAAGACGAGCGTAATTTGCAAACAAAAAGCCATTA
AAATATCAAATTAGCGTCTGGCTGTGGCTGTGAGTTCTGGATTTAGTAGAAGTCTCCAGTTCTCTTCGGT

GCCAGTTTGCCCGTGCCCCGCCTCTCTTTCGCCTTTGTCGCCCGTCTGCTACCTTCCCTGATCCCAGACA
TTTGTTTGTGCTTGCCCACAGCATTGTGCAAAATCGAAAGTAAGTGAAATGTAGCGTCCGCATGCGCTTC
GTTCAGTCACCAATTTGTCAGTCCAGTACTCATTGCCACGTATGTACTGCCCTGATCACTGAGCGAAAGA
AATGATCAAGAACCATTGTTGTTAATTACATACTGATTAAAAAGCAAACGAAGTGAGTATCTAAAGGTAT
ATTGGAGTGGGGGTATCATCATTTCAGCCGGCGTAGGCGAGCTTACAAAAAGATTTCAAAATATAttttt
ttttCAACTACTTAGTTATCAGACTGTATATTTTAGTTTCTAGTTTCGCTTATCAGTAATTAACAGAAAT
GAGTTCTTGCCCGCCACAAATGTCTCGTGTATTTCTTTTAGATAACAGAATTTACACTCCACATTAAAAT
CCATTTGTTTCACTGCCACGGCTGAATGCGTGCCAAAAATTCTATTAAAATCAAACAGAAACAAAAGCTG
AAACACGTTTTACCTGTACTGCACTTCAAACTGAGTCACTGGGTTGAAAGAACTTCGTTGGGGATGAAGC
AAGCACAAAATGGTTACTTGAATAATACaaaaaaaaaaaaaaaaaaCAACAACAGCAATCAAATCATCAA
GGGAAAATTTATGACGGTCACTCAGACCCCTTGCAAACTGCGCTGACAATAAAGCCGTACTGGAAGCTAT
CTCGATCAATGGATCGGGTCGTTCTGGCAGACCAAGACAGAAACTTCTCCACGCTCCACATTTGGACAAT
CTGCCGTCTCCCTTTGTCCCAAGCGATACGTTCTGGGGCCACGCTTGCGCGTTTTGCCCTGGGTTTCGAT
TCGTTTTCGGTCTTGGGCTTGTGTATTTGTACCCTTCCCTACTCGTATTTTTGCCCAAACGCGTTTCATA
AATTATCAAGTTGAATGCCCAGTTTCGAAATTTCAACGGGCCTGACCTTGAGCACACATTTTCGGAGCTT
CGGGTCCCGCTCTCGTGCTTTTAAGTGTTTGCGATTGTATCTGTATTCATATGGTTTTGCTACCCACTTT
TCACATCGGGTATACCAAACTGCTGACATGACTATAATGAGGTCATTTACAAGCTAAGACTTTATTTTCA
TCTTAAAACTAATACGACGAAAATCTTTGGTAGTTATTCACGTGTTTCACTCTGTTGATTATTGCCAATG
GCTTCTATACAAATTAACTAACGCACATGCGTAACCCAAAAGAACAGTGCGGAGATGGTCTGCTTCAAAT
GATAAATGATGCTCATTTTTTCCAACTTAGCTCCCTAACAACCGCGAAGATAACGTGCGTGTGAGACGTA
ACTTTAACCGGTTGGAGCCCTTGAATTTTCAATGCAAATACTTAGAAAAACTCACGTTAGTGGGAAGGTC
AAGTTAATTAGGATTGATGGAATGGGAGGGTGGGTCTCAGCATTAGAAGTCCGGGAATATAAAAAGTATA
TTTGGGTAAATACTAATAGATTTAATTATGTCTGGCAACGAATTTTCGTTGATTTTCCAGACGAAACTCG
TAAAACATGAAACGCACACGTTTTGAAATTAGATGAAAAAATAATAAATCTTCGTATCCGAAACATCATT
CCCATTTTTGAGTATTTTATTGTGCGTGACCCAAACACAAATATTTATTGTTATTTAGTGTGATGGATTA
TGTTAGAAGTAGCAGGATAGGTGGTAATGCGTCTATTCGTTAAGGTGTTGATGCGCATACAAATTAACAT
AGCCCGTGAGTATCGGTGATTCCGGGCTGTCATATTACGGAGCATATGTGGCATGTGTGCGGCACGCATC
ATTGAGAAGATTTGCCGCATAATAGACCGCTCAAACGGCCCACAAACCGACGAACACATCAATGTTGGTC
GGTGCTCATAGCAGGGAAATTTACAAGGAAAAATTATTATACTACACAATTACACTACAAATATTATTTG
CTTCCGCAACATATTTTAATTTTGTAACGCTTTCTGGGTTCTCTTATTTCTAAGTGTAAATTTTATTTTT
TATCGGACTGCACGGTGTGTCAAAACAAGCTTGACAACATAAATACCGCACACGATTTTTGAATGGATAA
GAAATTTGGTGAGCTTTATTAATTGCCGGATTTTGTATAATTAGTAGTACAATTTCCTGGTTTTCTTATA
TCCCGAAATCGACCGAAACCGAGTAGAAGGCGAAGAAGCGATACACAGATCGTGATTAACGTTTTATTAA
AATAACCTAAAATTTGTTGACCTGATTATGTACGACAAAAAATATTAAAATTTTCTTATACATATTTACT
ACATTTCCCTGCGCTTGCAGttttttggtttgtttttttATTTATGGGCTTACCCTCACTATATATGCAT
TTATATTTCTGGTCGTCCCTAAAAACAATTTTATAGCCCCATTTTCCGTTTCCATCCTATGCACAATCTT
TGAATCCATTTTTGTTTTTGGTTGTCTGTTGTCTTCGTAGCCTGTCTGTTCTGTACTTTCGTGGGTGTGC
TCTATTTTAAAATGTTTTCTAAACATTACTACAATAATAGGATTTGCCATTTTCACAATAGATTTATATT
CTATAAATAACCATAGTACCATAGATAATACGTTTCTTTACAATCAATGCTAACTTCATTTTTGCAATAA
AATGCAAACCAATCCCGTTTTTTCATCTTAACCTCCCTATATCTGTGCCTTTTCTTTGCATTTATCTTTT
TTCGTACAAGCTTACCCCAAGATCATGCCTTGCATTGTCAATGTATATGTGTAATATATATTTATATATT
AGAATTTTTTGTCTCATATGGGAATTGTTCTGTTTTCTTTCGATTTTTACAGGCGCTTGTACGCTCCCCT
CTCTACTAGTTCAGCTACTGCTACTAGCACCACCAAACCAACGCCACCAATCAACAGCACCATCTTCGCG
AACGTGTGTCTTGAACAGTTTTAAAGTCTCTCGACTTAGTCAACTCACCCACAGCGCATATGCCTATACA

TATGTTTGAAAAGTAATCAGAGAGATATAAAATCTTTGGTGACATCCGATGAGATAAGATTAACTCCCCG
AGCCTTAGGGGAATGTCAAATGCAAATTTATTAGAGAGCTCTCTGACCCAGAAAATTAATTTCTATAGAT
ATCCTTCAAATCGTCAGCCTCTATTGCTCGATTTTAAATTATTTTGTCTTCCCATAAATTATTAGCGACA
GAACTCTTTTAAAAATATATACTTATTTTTAGTTGTGACCTTTTCGGTTGTTTGTCTCCTATGGCTCTAG
TTCCGCGGTGTAAACATTTTAAATAAACAGAAGGTGCATCGCTGAAAACGACTCCACATGTGTGGGTTTC
TTTAAAGTGGG
>dme_circ_0008099_junction_seq
CCACATGTGTGGGTTTCTTTAAAGTGGGTGAATAAATTATCAACGTGTGCAAATCA
>dme_circ_0008100
CATAGCGTCGCTCACATCATTGTTGAAAGTCAAACTTATGTTTTGCACAACAATTTTCCCTAGGTAAGTT
TTTGATAATGAAAGCCAAATTAAGGATTTCATGAGTTTAAGTTTAACATTTTAATAAAATAAAAAGAAAA
AGATTACTTCTAAGCTCTTAGGAATTTTATAAATTGAAGTTAAATTACAACAAGAATTACAAAATCAATC
AAAATTCTTTTGAAAGGTTCGATATAAGTTCGATAATATCCCAAGTTTAATTTGCTTTTTCCACACCTAT
AAAACGTAATGCCATGGAAATAATACAGGGAATATTATATTGCATGAGTATGTTCAAAAAGTATATAATT
TTTATTTCAAATTAAGTTGAAAAGTTGTATAACTCTTAATTAATACGGTCTAATTTTTTACTTTTCTACC
AACAAAGCTCGTTCCCTTACCAAGAATCCGCGCTGATGTCCGTCGGGTGATTTCGCTTTACAATAGGATA
TTGCAAGCAATTTCCGTTACGCGACCGGATGTAATGG
>dme_circ_0008100_junction_seq
ATTTCCGTTACGCGACCGGATGTAATGGCATAGCGTCGCTCACATCATTGTTGAAA
>dme_circ_0008101
GTTTTTGCAACAGCCGCACGGAGCACAACCCGAATCTCACAACAAGATGCCACTTCCACCCAAAGTAGCC
CTTCATCCGGTGTTTGTTTGCCGGGGCATGCTGCCTGGCATATGCAGAGATGAGTTTTTAAACTTTGTGC
GGCCGCCA
>dme_circ_0008101_junction_seq
TGAGTTTTTAAACTTTGTGCGGCCGCCAGTTTTTGCAACAGCCGCACGGAGCACAA
>dme_circ_0008102
GGATACTGTCTCTGACCTCGCAGTTATTCATTTCCGATACGGCTTAAGAGCTGTAATTCCTTTGCAACTC
CTTTCTATCAAAGTAAACACAAGTGGCAGCACTaaaaaaaaTTAAGCTAATTGTTTATATTTAAAAAAAC
ATAAATTTACTTCATAATTTCCTATTTATTTTAGTTTATATTAATAATTGAATTATATTTTTAAGCGTGA
ATGAGCCTATTTAATTTTTTCTGTGAAAAATAAGTACAACAAGCAACTGAAACTTTTTTTCTGACCTCGA
CTAACATAATACAATTCATTTATTGTTCCGAAGTCGTCTTCGGTTTTTTGCGGAGACGCTCTAACCTTTC
TCTCACTGTTGCGTTGCATGCTGTATGGGTTTTCATTTCCGGCTTCCGTTCCTGTTGAGTTCCTTAAGCG
GATTACACGGACAGAAGCTATAACAGGGGCACGCTCTGTCGAAGGAAGCTGTAGGGGAGAAGATGCCGAA
ATAGTCTATGAGCGGTGTGTTGCCCTCACTTATTTTTTGACTTATCAAG
>dme_circ_0008102_junction_seq
GCCCTCACTTATTTTTTGACTTATCAAGGGATACTGTCTCTGACCTCGCAGTTATT
>dme_circ_0008103
GCCATATATTGTAGTATCTATAGGGTTTAAGCTGAAAGTGTACGAATTTTAAAATGTGAAATTGATAAAC
CAGTCAACACACCAGATCTACACAAAATGCCCACGCATAAGTTAACTACACGAGCGAAGTATTTCGGCCA
CACCTGTAAAAGTTGAAAGAATTCACAAAATTTGATATG
>dme_circ_0008103_junction_seq
GTTGAAAGAATTCACAAAATTTGATATGGCCATATATTGTAGTATCTATAGGGTTT
>dme_circ_0008104
GGAAGAGGAGACAGTAGTCCCCCAATCGCCTTCCAAAAAATCCAGAAAACAGCCTGTTAAAGAAGTGCCG

CAGTTCAGTGAAGAGGACGAGTCTGATGTGGAGGAGCAAAATGACGAGCAGCCAGGGGACGACAGCGACT
TTGAGGGTAAGCTATaaaaaaaaCGTGGTCTTCTGAATGGATATGGCTAACATTACATGCATAGTATGCA
CGTATATATAGATCTGCATGGATAAGAATCCATTTTACGTTTTCTTTGCCACGTGGTTGTAAAGATTTTA
TAATTATAAGACGAGTGCACATAACCCACGATCCTCTGGTTTTATACCGACTCGGTTCATATATCGATaa
aaaaaaaaTTGTCTTTATTGGTGCTCTCCCATCAGCGGTTATAAGAAAATTGTCTACTTTTCAGGACCGA
AGAGGCAGCGGGCTTAATTGACGACGAGGCTGAGGAAGATGAAGAGTACAATAGTGACGATGAGGAAGAT
GATGATGACGATGAGTTGGAGCCTGGTGAGGTTTCCAAAAGTGAAGGCGCGGATGAGGTAGATGAATCCG
ATGATGATGAAGAAGCGCCT
>dme_circ_0008104_junction_seq
TGAATCCGATGATGATGAAGAAGCGCCTGGAAGAGGAGACAGTAGTCCCCCAATCG
>dme_circ_0008105
GATGAAGAGTACAATAGTGACGATGAGGAAGATGATGATGACGATGAGTTGGAGCCTGGTGAGGTTTCCA
AAAGTGAAGGCGCGGATGAGGTAGATGAATCCGATGATGATGAAGAAGCGCCTGTAGAGAAGCCAGTTTC
CAAGAAATCAGAAAAAGCCAACTCTGAAAAGTCCGAAGAAAATAGAGGAATTCCAAAAGTAAAGGTTGGC
AAGATTCCGCTGGGAACGCCAAAAAACCAGATCGTTTTTGTCACAAACCTACCAAACG
>dme_circ_0008105_junction_seq
ATCGTTTTTGTCACAAACCTACCAAACGGATGAAGAGTACAATAGTGACGATGAGG
>dme_circ_0008106
GATGATGATGACGATGAGTTGGAGCCTGGTGAGGTTTCCAAAAGTGAAGGCGCGGATGAGGTAGATGAAT
CCGATGATGATGAAGAAGCGCCTGTAGAGAAGCCAGTTTCCAAGAAATCAGAAAAAGCCAACTCTGAAAA
GTCCGAAGAAAATAGAGGAATTCCAAAAGTAAAGGTTGGCAAGATTCCGCTGGGAACGCCAAAAAACCAG
ATC
>dme_circ_0008106_junction_seq
TCCGCTGGGAACGCCAAAAAACCAGATCGATGATGATGACGATGAGTTGGAGCCTG
>dme_circ_0008107
GATGATGATGACGATGAGTTGGAGCCTGGTGAGGTTTCCAAAAGTGAAGGCGCGGATGAGGTAGATGAAT
CCGATGATGATGAAGAAGCGCCTGTAGAGAAGCCAGTTTCCAAGAAATCAGAAAAAGCCAACTCTGAAAA
GTCCGAAGAAAATAGAGGAATTCCAAAAGTAAAGGTTGGCAAGATTCCGCTGGGAACGCCAAAAAACCAG
ATCGTTTTTGTCACAAACCTACCAAACGGAGTATCTCCACAAGGACTTGGTCGCATTGTTCGCCAAGTTC
GGACGACTTTCCGCTTTGCAGCGTTTCACTAATTTGAATGGCAATAAATCAGTTCTCATTGCCTTTGATA
CGTCAACTGGAGCAGAAGCTGTTCTGCAGGCTAAGCCTAAAGCGTTAACCCTTGGAGACAATGTTTTGTC
TGTGTCCCAACCGAGAAATAAGGAAGAGAACAACGAACGCACGGTGGTGGTTGGCCTGATTGGACCTAAT
ATTACCAAGGACGACTTAAAAACCTTTTTTGAGAAGGTAGCTCCCGTGGAGGCGGTGACTATATCCAGTA
ACCGCCTCATGCCCAGAGCTTTTGTACGTTTGGCGTCGGTCGATGACATACCGAAGGCTCTTAAACTGCA
TAGCACTGAGCTGTTTTCTCGCTTTATAACGGTTCGTCGGATCTCACAAGAATCGATTTCACGTACCAGT
GAACTTACCTTGGTTGTCGAAAATGTGGGCAAACACGAGTCTTACAGCTCCGACGCTCTAGAAAAGATAT
TCAAAAAATTTGGTGATGTTGAAGAAATCGACGTTGTGTGCAGCAAGGCAGTTTTAGCTTTTGTCACGTT
CAAGCAGTCGGATGCCGCCACGAAGGCTTTAGCACAACTCGACGGAAAGACTGTTAATAAGTTTGAGTGG
AAGCTTCACCGATTCGAACGCAGCACCTCGGGGAGGGCCATTCTTGTGACGAACTTGACTTCAGGATGCT
ACTGAAGCCGACCTGCGGAAAGTGTTTAACGATAGCGGCGAAATCGAAAGCATCATAATGTTGGGCCAAA
AAGCGGTAGTAAAGTTCAAGGACGATGAGGGTTTCTGCAAATCTTTCTTGGCTAATGAAAGCATTGTAAA
CAACGCCCCTATATTTATTGAGCCAAACTCTCTGCTGAAGCATAGATT
>dme_circ_0008107_junction_seq
AGCCAAACTCTCTGCTGAAGCATAGATTGATGATGATGACGATGAGTTGGAGCCTG

>dme_circ_0008108
GGCGCGGATGAGGTAGATGAATCCGATGATGATGAAGAAGCGCCTGTAGAGAAGCCAGTTTCCAAGAAAT
CAGAAAAAGCCAACTCTGAAAAGTCCGAAGAAAATAGAGGAATTCCAAAAGTAAAG
>dme_circ_0008108_junction_seq
AGAAAATAGAGGAATTCCAAAAGTAAAGGGCGCGGATGAGGTAGATGAATCCGATG
>dme_circ_0008109
GTTTCCAAGAAATCAGAAAAAGCCAACTCTGAAAAGTCCGAAGAAAATAGAGGAATTCCAAAAGTAAAGG
TTGGCAAGATTCCGCTGGGAACGCCAAAAAACCAGATCGTTTTTGTCACAAACCTACCAAACGGAGTATC
TCCACAAGGACTTGGTCGCATTGTTCGCCAAGTTCGGACGACTTTCCGCTTTGCAGCGTTTCACTAATTT
GAATGGCAATAAATCAGTTCTCATTGCCTTTGATACGTCAACTGGAGCAGAAGCTGTTCTGCAGGCTAAG
CCTAAAGCGTTAACCCTTGGAGACAATGTTTTGTCTGTGTCCCAACCGAGAAATAAGGAAGAGAACAACG
AACGCACGGTGGTGGTTGGCCTGATTGGACCTAATATTACCAAGGACGACTTAAAAACCTTTTTTGAGAA
GGTAGCTCCCGTGGAGGCGGTGACTATATCCAGTAACCGCCTCATGCCCAGAGCTTTTGTACGTTTGGCG
TCGGTCGATGACATACCGAAGGCTCTTAAACTGCATAGCACTGAGCTGTTTTCTCGCTTTATAACGGTTC
GTCGGATCTCACAAGAATCGATTTCACGTACCAGTGAACTTACCTTGGTTGTCGAAAATGTGGGCAAACA
CGAGTCTTACAGCTCCGACGCTCTAGAAAAGATATTCAAAAAATTTG
>dme_circ_0008109_junction_seq
GCTCTAGAAAAGATATTCAAAAAATTTGGTTTCCAAGAAATCAGAAAAAGCCAACT
>dme_circ_0008110
TCTTTCCGTCGAGTTGTGCTAAAGCCTTCGTGGCGGCATCCGACTGCTTGAACGTGACAAAAGCTAAAAC
TGCCTTGCTGCACACAACGTCGATTTCTTCAACATCACCAAATTTTTTGAATATCTTTTCTAGAGCGTCG
GAGCTGTAAGACTCGTGTTTGCCCACATTTTCGACAACCAAGG
>dme_circ_0008110_junction_seq
TGTTTGCCCACATTTTCGACAACCAAGGTCTTTCCGTCGAGTTGTGCTAAAGCCTT
>dme_circ_0008111
GGCTTTAGCACAACTCGACGGAAAGACTGTTAATAAGTTTGAGTGGAAGCTTCACCGATTCGAACGCAGC
ACCTCGGGGAGGGCCATTCTTGTGACGAACTTGACTTCAGGTGGGTTACCCGTTTCGTAAAATTGTTTAA
AATTATTAATCATTATCATGTAAAATTTCGTTGTAGATGCTACTGAAGCCGACCTGCGGAAAGTGTTTAA
CGATAGCGGCGAAATCGAAAGCATCATAATGTTGGGCCAAAAAGCGGTAGTAAAGTTCAAGGACGATGAG
G
>dme_circ_0008111_junction_seq
GCGGTAGTAAAGTTCAAGGACGATGAGGGGCTTTAGCACAACTCGACGGAAAGACT
>dme_circ_0008112
GCATCATAATGTTGGGCCAAAAAGCGGTAGTAAAGTTCAAGGACGATGAGGGTTTCTGCAAATCTTTCTT
GGCTAATGAAAGCATTGTAAACAACGCCCCTATATTTATTGAGCCAAACTCTCTGCTGAAGCATAGATTG
TTAAAGAAACGCTTAGCTATTGGTCAGACCCGCGCCCCGCGGAAGTTCCAAAAGGACACTAAGCCAAACT
TTGGTAAAAAACCATTTAACAAGCGCCCGGCACAAGAGAATG
>dme_circ_0008112_junction_seq
TTTAACAAGCGCCCGGCACAAGAGAATGGCATCATAATGTTGGGCCAAAAAGCGGT
>dme_circ_0008113
CGAAACTTGTAGACTGCTTCTTCAAAAAAATGTCTATACATTGGCCGAATTTTATGTGGTTTTATTGTAC
TACCAAGCACCTTTATTTTATTATTTAACAAATCTGTAAACATATTATATTTCAATGGATTTATAAATTT
AATAATGTGGAAATTGAGCAGCACTATAAACATGTTCAGTACTAATATTAAACGAGGAAAAGCTTTTACC
CAG

>dme_circ_0008113_junction_seq
TATTAAACGAGGAAAAGCTTTTACCCAGCGAAACTTGTAGACTGCTTCTTCAAAAA
>dme_circ_0008114
GAACGTGAGCAAAAAtttttttaaagcatttcaaaagtaaatatttaatttttaaatAACAGAACACTT
GGTGACATTTATCTTTCGTTTTCGTTACAAAATAGCACAAACATTTATTCGCTCTGCGTTCATTACAGTA
GTTCATTATTTTTTGAAATAAAATGATTTAATAATATGTAGCAAGAAATCGAGGGTATAATCGTATATAA
ATAAGTTCAATAAGCAAAAATTCCTAATTTGGTTACCAGGATGCTATTGTATTCATCGTTTTTCTTATTA
C
>dme_circ_0008114_junction_seq
CTATTGTATTCATCGTTTTTCTTATTACGAACGTGAGCAAAAAtttttttaaagc
>dme_circ_0008115
GCTTCCAGGAACCAGCAAACGACGAGATGTACTCCCATTGTTATAGGTTAAGCTAGCTGAATGCGTAAAC
TTTGGAGCTGAGCGAGCTGTGAAAGATTTTGTGATAGTTGCGGTAGCAGTGGAAGTGGTGGTAGAGCTG
>dme_circ_0008115_junction_seq
GGTAGCAGTGGAAGTGGTGGTAGAGCTGGCTTCCAGGAACCAGCAAACGACGAGAT
>dme_circ_0008116
TAATGTAGGATCAACAGTTAAACCACCAACCAAGTTAAGCGGCACACGACCAGCTACAGCTCCAGTGAGC
AAGGTAACTTTAGGGGCCAAGACCATAACTAACAAGCCGACTGCAAGTGGTACTGCATCTGATAATGTAA
CCAGGACAACACTTCGCCCTTTGGTCAG
>dme_circ_0008116_junction_seq
CCAGGACAACACTTCGCCCTTTGGTCAGTAATGTAGGATCAACAGTTAAACCACCA
>dme_circ_0008117
CTACAAAAACTTCGACTACATCTTCACTAACTGGAAATCCCCGAAAGTCACTAAGCAGTAATGTAGGATC
AACAGTTAAACCACCAACCAAGTTAAGCGGCACACGACCAGCTACAGCTCCAGTGAGCAAGGTAACTTTA
GGGGCCAAGACCATAACTAACAAGCCGACTGCAAGTGGTACTGCATCTGATAATG
>dme_circ_0008117_junction_seq
ACTGCAAGTGGTACTGCATCTGATAATGCTACAAAAACTTCGACTACATCTTCACT
>dme_circ_0008118
ATATTTTAGAACCGGAAAAAGATGTCGAAGTTGCGAAAAGTTTGTCTGAAAAAACTTCTGTTGCTACAGT
GGCTGGAGGTGCTGTGGTTGGTGCTACCAAAACCCACTCAGCCACCAAAGCAGGATCTACAGCAGCTAGT
GCAAAATCGAAAACAGAAACCCTTGTAATGAAGAAGACTACAGCGAGTTCTACTTCTGTTTACGGGGCAA
ACAAGTCGGCCGCTCCACGTCCAAGTACCGCCCGCCTTGGAATCAAAAG
>dme_circ_0008118_junction_seq
CAAGTACCGCCCGCCTTGGAATCAAAAGATATTTTAGAACCGGAAAAAGATGTCGA
>dme_circ_0008119
GTCGCTCTTCTTCAACAACGAAGTTCTTTTCAGTGCCAAGATTTTCAACCTGGGCAACAACATCTCCAAC
AGGGTGCAGCAGCTCCTCCTTTACAAAGGCTTGTTGCTCACTCCTATGATCCTCTACTTCATTTGAAGAC
TTACTAGCAACTTTATCACATATAGCAAA
>dme_circ_0008119_junction_seq
TACTAGCAACTTTATCACATATAGCAAAGTCGCTCTTCTTCAACAACGAAGTTCTT
>dme_circ_0008120
TGGAAGTACATACACGAGGTGCGGCAGAGTGAAAAGCTTCAACAGGAAAAGCTTCCACTGACAAAGGAAA
CCGGCAATGGCTTCGGGCCCGGACGGGACTCAGATAATCAAGTTCACGGAAACGGAGCTGCCGCTGTCTT
CAATCTGTACGAAGAGGACG

>dme_circ_0008120_junction_seq
GCTGTCTTCAATCTGTACGAAGAGGACGTGGAAGTACATACACGAGGTGCGGCAGA
>dme_circ_0008121
GATGCCTCAAATCCGCGGGTGTTCTTCACTCAGGCCTACATCCAACGTTGCAAAGCGAAGATTCGGGGAG
CCCTGGCCGCCATTACCAGGCCCATAAATGTGGCTGTGATTCTTCAGAAGATCGGTGTGCAGGAGAAGAT
CTTCTACTCGTTGCTGGACGAAATCGCGCCAGCTGGCCAGG
>dme_circ_0008121_junction_seq
CTGGACGAAATCGCGCCAGCTGGCCAGGGATGCCTCAAATCCGCGGGTGTTCTTCA
>dme_circ_0008122
GTTATCTTAACATCCGCCGCATTGGCAAAACTAACAATTAATGGATAAATGCGCAAGTGGTTGATTGATT
TGATGTCCGATGCTTTCAAAGATCTGCTCCTGGGCGCGGCGTTGTCGATGCGTTTGCATTTATGTACCAT
GCGGGGGGTGTCCATATGGTAGGCTTAAAACTATAGATTGGGCTGCTCTTCTATTCTTGTTAGACTAATT
CAGACTATTCACTATTTAGATCTTCATGTCGTTGATGTATGAGTCCAGTTCGGCGTCCAGCTCCTCAGCA
GTGGGCGGGGCCTTGCGCTCCCGTCGCTGGCCGCCTGCCGCCGGCTTGCCACCGACCGGACGTTTGAAGC
CGCGACGCGCCGTGCCACCAGCTTGGCCACCTAAAAAACAGTTCGGAATGATAGTGTAAGCATTGGAGCA
AGGATGGATCGTCATGGGACCGTATCATGGTCATTATAGAAAG
>dme_circ_0008122_junction_seq
GGGACCGTATCATGGTCATTATAGAAAGGTTATCTTAACATCCGCCGCATTGGCAA
>dme_circ_0008123
ATAGCCTCGTTGTCGACCATGAAGGCGCAGTCGGAATGCTCCAGGGTGGTGTGGGTGGTCAGGATGGAGT
TGTAGGGCTCGACCACGGCAGTGGACACCTGGGGGGCTGGGTAGATGGCGAACTCCAGCTTGGACTTCTT
GCCGTAGTCCACGGAGAGACGCTCCATCAGCAGCGAGGTGAAGCCGGAGCCGGTACCTCCACCGAACGAG
TGGAAGATGAGGAAGCCCTGCAGACCGGTGCACTGATCGGCCAGCTTGCGGATCCTGTCCAGAACCAGAT
CGACGATCTCCTTGCCGATGGTGTAGTGGCCACGGGCGTAGTTGTTGGCCGCATCCTCCTTACCAGTGAT
CAGCTGCTCGGGGTGGAACAGCTGACGGTAGGTTCCGGTACGGACCTCATCGACCACAGTGGGTTCCAGA
TCCACAAACACGGCGCGGGGCACGTGCTTGCCAGCTCCAGTCTCGCTGAAGAAGGTGTTGAACGAGTCAT
CACCTCCGCCCACGGTCTTGTCAGACGGCATCTGGCCATCGGGCTGGATGCCGTGCTCCAAGCAGTAGAG
CTCCCAGCAGGCGTTTCCAATCTGGACACCAGCCTGACCAACATGGATAGAGATACATTCACGCTG
>dme_circ_0008123_junction_seq
CAACATGGATAGAGATACATTCACGCTGATAGCCTCGTTGTCGACCATGAAGGCGC
>dme_circ_0008124
TGATCAGCTGCTCGGGGTGGAACAGCTGACGGTAGGTTCCGGTACGGACCTCATCGACCACAGTGGGTTC
CAGATCCACAAACACGGCGCGGGGCACGTGCTTGCCAGCTCCAGTCTCGCTGAAGAAGGTGTTGAACGAG
TCATCACCTCCGCCCACGGTCTTGTCAGACGGCATCTGGCCATCGGGCTGGATGCCGTGCTCCAAGCAG
>dme_circ_0008124_junction_seq
ATCGGGCTGGATGCCGTGCTCCAAGCAGTGATCAGCTGCTCGGGGTGGAACAGCTG
>dme_circ_0008125
GCCCGATGGCCAGATGCCGTCTGACAAGACCGTGGGCGGAGGTGATGACTCGTTCAACACCTTCTTCAGC
GAGACTGGAGCTGGCAAGCACGTGCCCCGCGCCGTGTTTGTGGATCTGGAACCCACTGTGGTCGATGAGG
TCCGTACCGGAACCTACCGTCAGCTGTTCCACCCCGAGCAGCTGATCACTG
>dme_circ_0008125_junction_seq
CTGTTCCACCCCGAGCAGCTGATCACTGGCCCGATGGCCAGATGCCGTCTGACAAG
>dme_circ_0008126
TTGTAGGGCTCGACCACGGCAGTGGACACCTGGGGGGCTGGGTAGATGGCGAACTCCAGCTTGGACTTCT

TGCCGTAGTCCACGGAGAGACGCTCCATCAGCAGCGAGGTGAAGCCGGAGCCGGTACCTCCACCGAACGA
G
>dme_circ_0008126_junction_seq
GCCGGAGCCGGTACCTCCACCGAACGAGTTGTAGGGCTCGACCACGGCAGTGGACA
>dme_circ_0008127
GGCACCAAGTTGGTCTGGAACTCAGTCAGATCCACGTTAAGGGCACCATCGAATCGCAGAGAGGCGGTAA
TCGAGGACACGATCTGGCCAATCAGACGGTTCAGGTTGGTGTACGTGGGGCGCTCAATGTCCAGATTGCG
GCGGCAGATGTCGTAGATAGCCTCGTTG
>dme_circ_0008127_junction_seq
GCGGCAGATGTCGTAGATAGCCTCGTTGGGCACCAAGTTGGTCTGGAACTCAGTCA
>dme_circ_0008128
GGAAGTGAATACGTGGGTAGGGCACCAAGTTGGTCTGGAACTCAGTCAGATCCACGTTAAGGGCACCATC
GAATCGCAGAGAGGCGGTAATCGAGGACACGATCTGGCCAATCAGACGGTTCAGGTTGGTGTACGTGGGG
CGCTCAATGTCCAGATTGCGGCGGCAGATGTCGTAGATAGCCTCGTTG
>dme_circ_0008128_junction_seq
GCGGCAGATGTCGTAGATAGCCTCGTTGGGAAGTGAATACGTGGGTAGGGCACCAA
>dme_circ_0008129
GTAATATATATGAGCCACACCAAAAAGAGGTTCAATGCAATGCGAAAATAGTTGAAAACTTTTGTTAAGA
TTCATTTACATTGGTCGGCAACCAACTAATAAACTGATTGTCTTCGTCCTTAAGCCGGTTTGCACTTACA
GTATAAAAGCTTTATCTCCATGATCTTTGTCACTCTTTCTTGTCTGACTATAGTGTGATCTTCGGTCCGG
TCGTCCGTTTAGTTGTCACAATTGTCCAGGAAGGCCAAGGGGAGGTCTGGGTCGTGCTGCATTTTGCAAT
TGGCTAGCTAGCTACAATGTATAAATAAGTTTAACTATTAGACCTTTACAAAGTATTGCAACAAAATGCC
GTTCTCTCTCAATATACAAAACAAGCTACAAATAGTATAACGTCTTCTTTGAGTTTAGAGCAGACTGAAG
AGAGCATGTATAGCTTTCATATATCATAGGTTCGGCCGCCGTAACATCTCAATTTGTTATCAAAAATGTT
CATACAAAATTTTACACCTAAAGTGAGTAT
>dme_circ_0008129_junction_seq
TACAAAATTTTACACCTAAAGTGAGTATGTAATATATATGAGCCACACCAAAAAGA
>dme_circ_0008130
GCCACACCAAAAAGAGGTTCAATGCAATGCGAAAATAGTTGAAAACTTTTGTTAAGATTCATTTACATTG
GTCGGCAACCAACTAATAAACTGATTGTCTTCGTCCTTAAGCCGGTTTGCACTTACAGTATAAAAGCTTT
ATCTCCATGATCTTTGTCACTCTTTCTTGTCTGACTATAGTGTGATCTTCGGTCCGGTCGTCCGTTTA
>dme_circ_0008130_junction_seq
TGTGATCTTCGGTCCGGTCGTCCGTTTAGCCACACCAAAAAGAGGTTCAATGCAAT
>dme_circ_0008131
ATAAAGCTTTTATACTGTAAGTGCAAACCGGCTTAAGGACGAAGACAATCAGTTTATTAGTTGGTTGCCG
ACCAATGTAAATGAATCTTAACAAAAGTTTTCAACTATTTTCGCATTGCATTGAACCTCTTTTTGG
>dme_circ_0008131_junction_seq
TTTCGCATTGCATTGAACCTCTTTTTGGATAAAGCTTTTATACTGTAAGTGCAAAC
>dme_circ_0008132
GTACATGGCCTGCTGCATGCTGTACCGCGGTGATGTTGTGCCCAAGGACGTCAACGCCGCTATTGCCACC
ATCAAGACCAAGCGCACCATTCAATTCGTCGACTGGTGCCCCACTGGCTTCAAGGTTGGCATCAACTACC
AGCCACCCACCGTGGTGCCTGGAGGTGATTTGGCCAAGGTGCAGCGTGCCGTGTGCATGTT
>dme_circ_0008132_junction_seq
CCAAGGTGCAGCGTGCCGTGTGCATGTTGTACATGGCCTGCTGCATGCTGTACCGC

>dme_circ_0008133
GCCTCGGCGATGGCCGTGGTGTTGGACAACATGCACACGGCACGCTGCACCTTGGCCAAATCACCTCCAG
GCACCACGGTGGGTGGCTGGTAGTTGATGCCAACCTTGAAGCCAGTGGGGCACCAGTCGACGAATTGAAT
GGTGCGCTTGGTCTTGATGGTGGCAATAGCGGCGTTGACGTCCTTGGGCACAACATCACCGCGG
>dme_circ_0008133_junction_seq
GACGTCCTTGGGCACAACATCACCGCGGGCCTCGGCGATGGCCGTGGTGTTGGACA
>dme_circ_0008134
ACGGGCCCAGGCCTCGGCGATGGCCGTGGTGTTGGACAACATGCACACGGCACGCTGCACCTTGGCCAAA
TCACCTCCAGGCACCACGGTGGGTGGCTGGTAGTTGATGCCAACCTTGAAGCCAGTGGGGCACCAGTCGA
CGAATTGAATGGTGCGCTTGGTCTTGATGGTGGCAATAGCGGCGTTGACGTCCTTGGGCACAACATCACC
GCGG
>dme_circ_0008134_junction_seq
GACGTCCTTGGGCACAACATCACCGCGGACGGGCCCAGGCCTCGGCGATGGCCGTG
>dme_circ_0008135
GTTCGATCTGATGTACGCCAAGCGTGCCTTCGTCCACTGGTACGTTGGTGAGGGTATGGAGGAGGGAGAG
TTCTCCGAGGCCCGTGAGGATTTGGCTGCCCTCGAGAAGGACTACGAGGAGGTCGGCATGGACTCCGGTG
ACGGCGAGGGTGAGGGCGCTGAGGAGTACTAAGCGTCACGCCACTTCAACGCTCGATGGGAGCGTCATTG
GTGGGCGGGGTAACCGTCGAAATCAGT
>dme_circ_0008135_junction_seq
GGTGGGCGGGGTAACCGTCGAAATCAGTGTTCGATCTGATGTACGCCAAGCGTGCC
>dme_circ_0008136
GCTTTATCTCCATGATCTTTGTCACTCTTTCTTGTCTGACTATAGTGTGATCTTCGGTCCGGTCGTCCGT
TTAGTTGTCACAATTGTCCAGGAAGGCCAAGGGGAGGTCTGGGTCGTGCTGCATTTTGCAATTGGCTAGC
TAGCTACAATGTATAAATAAGTTTAACTATTAGACCTTTACAAAGTATTGCAACAAAATGCCGTTCTCTC
TCAATATACAAAACAAGCTACAAATA
>dme_circ_0008136_junction_seq
TCTCAATATACAAAACAAGCTACAAATAGCTTTATCTCCATGATCTTTGTCACTCT
>dme_circ_0008137
GCTTTATCTCCATGATCTTTGTCACTCTTTCTTGTCTGACTATAGTGTGATCTTCGGTCCGGTCGTCCGT
TTAGTTGTCACAATTGTCCAGGAAGGCCAAGGGGAGGTCTGGGTCGTGCTGCATTTTGCAATTGGCTAGC
TAGCTACAATGTATAAATAAGTTTAACTATTAGACCTTTACAAAGTATTGCAACAAAATGCCGTTCTCTC
TCAATATACAAAACAAGCTACAAATAGTATAACGTCTTCTTTGAGTTTAGAGCAGACTGAAGAGAGCAT
>dme_circ_0008137_junction_seq
TGAGTTTAGAGCAGACTGAAGAGAGCATGCTTTATCTCCATGATCTTTGTCACTCT
>dme_circ_0008138
TTATTTTTTGAACGATATTTATAGAGCCATAAAGCCTGTTTAAACCAGCTACAGATACCAATTATAGTGT
AGCTTACCGCATACAAACACCAACAAAGACATACATACATACATACTCACTTTAGGTGTAAAATTTTGTA
TGAACATTTTTGATAACAAATTGAGATGTTACGGCGGCCGAACCTATGATATATGAAAGCTATACATGCT
CTCTTCAGTCTGCTCTAAACTCAAAGAAGACGTTATACTATTTG
>dme_circ_0008138_junction_seq
AAACTCAAAGAAGACGTTATACTATTTGTTATTTTTTGAACGATATTTATAGAGCC
>dme_circ_0008139
TTCCAGCTTTTGTTCCGCGAATGTGATTACCCCAAGGGCTTCATGGTCTGGATCGGATTGCACGGCGTTA
TGTTCCTGTTCCTGTTCTCCGACTTTTACAAAGCGAAATATCTCAACGCAGCTCGACGTCGCCGGCAGGC

GGTCAAGGCCAATGGCTATGCCAACGGCTCGGCTTCGAATGGGCACAGCAAGCATCTTGGAGAGGGTGAC
GCCCTGATTGCAAACGGTTGCAATACTGGGGCTTGCATGGGTAGGTCTTAGTGCCACCGCACACCTAATC
TGGGGCTAATTGGGGGACACAAGGTCATTCATTCCCTGAGTGATAAAAGTGGAGACTGAGCACACATATT
TTCCTTACATACTCAAGGCCCTGACGCCCCGACCTCACCAAGGTGAGCGTCTGGTGCGGGAGGGGCGAGC
CGTCGTGGGCTGTTATATCATCATAATTTTGTTGATCAACTTAAAATTCTAACTAGCTGAGTTAGGCAAA
CAGCACTTGCGAACTCTCAGCATAGGCGGGGGACGTTTCAATTAGCGCTCACATGTGAAGCCGGATGCGA
AGGCCCAAGCCCAGTCCCAGCCCAATCTGCCGCCCATTAGAAAAGCACATGGGCGGCATGTTCGCTGCCA
GCACACGTGCCATCGTCCAGTGGAGTCTGAACCCATTGAACTCATTGAGCACTCTGTGTTTTCTTTTCAG
CCCGGTGATGGAGGACGAGTACGTCAAAAGCAAGGGGCAATCGAATGGTGCCTACAAGGAGGGCTTCTTC
AAGGAGGGCGTGTTATCCAACAACGATGCCATCTTCAACCCGGACAGCAGTAGTTCTAGTCTGCACCAGC
GCAAAGTCAAATAACTGCTGATTAGGTGTACTACGATGATATTCATTATGTTTATGTTAAGTGATTAGCG
TGTAACTGAACCAGATTGAAGAACAAGACGAGCAAGAAACTTGGCAGAAGTAGCAGAAGACGATTGGCAC
GACAGCAGCGAAAAATTAATAGAGCTCGG
>dme_circ_0008139_junction_seq
ACAGCAGCGAAAAATTAATAGAGCTCGGTTCCAGCTTTTGTTCCGCGAATGTGATT
>dme_circ_0008140
CAACGTACCGAACTCAGCCCGCCGACCCACCAAACATCCGCAACCGCCGAAGTAAATGCACCGCTGGCCA
GCTCGACGAGTGCATCCATTGCGGTAACTGCGAGTGCGACGGCGGCATCAGCCCCTCCAGCAACGGGCAC
CACTACCTTAGGCAGCAGCATTAGCGGCAATGCTGGCTCAACCTCCAGCGGTAACAGTTCCGGAGTATCG
GCGACTGGAGGAGCACAATCCGCCGTGGGGTCTGGCGGCTATCCAAAGACAGAATCGAGCAAGTCGAGTG
GCACCGCAAGCgcaggcagtggcagtggcagcagcagcaatacaagtagcaccaagcacggcagcaacat
caaggatattagcagcagtagcaatcagcaggcatcgactgcgagcagcagtagTGCACCCAGCCTTTAT
GTCTCCGTGCCACTGTCCACGGCCAACGTACCTGGAATCAATCTGCCCACCAGCAGCACTAGCAGTAGTA
CCACCAGCGGAATCCCATTCGGCATCGTCGCGCAGCAGTGGTGCACAATCTCAGCATCAGCAGCAGCTAA
GCAATGCTTTGGTTGGTCCGCCCATGGGAGGATCCGCTGGGGCTTTTCATGGCGGAACAACCAGCGCTGG
GTCCTCGTCGGTCATCCAGCAGCAGAGCGGCAAGTCATCGCCGGCTCTGGGCACGCTAGTCAGTGGCAAC
AGTGGTGGTAGCATAATCTCCGCCAGTGGCTTTCCACTACCGAGTGGTAATCTCACTGCCACGACCACTG
AGAGCGGCAACTTGAAGATCAGCTATGAGAAACAGACGACACGAGTGCAACAATTGCAGGAGCAGGAGGC
GCCACCGGCCAGACGCAGCAGG
>dme_circ_0008140_junction_seq
GGAGGCGCCACCGGCCAGACGCAGCAGGCAACGTACCGAACTCAGCCCGCCGACCC
>dme_circ_0008141
CTCGGTTAACACACCAAATACAGGACCGATCCCATTACTTGCAACACAGAAACCACAAACAGAAGCGACA
ACAAAATGGCAATCATACTGCAAGAAGCCCAGAAGTGGTATCGGGACCTAATGGACAATAAAAGCGGATC
CTCGCGTTAACGACTTCTTTCTGCTATCCTCGCCACTGCCCACACTTTGTATGTGCATATTCTATGCCTA
CTTCAGCAAATCCCTGGGGCCCAGGCTGATGGCCAAACGGAAACCGATGGAGTTGCGATCTGTTTTAGTT
GTATACAACGCGATACAAACAATATTTAGCGCGTGGATCTTTTATGAGGATGGTCAACATTTGCTGGTGG
TACTACATATCTAAGTTCACCGAGTTCTTCGACACACTCTTCTTCATTCTGCGAAAAAAGAACGAGCACG
TCTCCACACTACACGTCATCCATCACGGCTGCATGCCGTTCTCCGTCTGGATGGGTCTGAAGTTTGCGCC
AGGGCGGTCATAGCACTTTCTTTGCCCTTCTCAACTCGTTTGTGCACATTGTAATGTATTTCTACTACAT
GATCGCCGCCATGGGTCCAAAGTACCAGAAGTACATCTGGTGGAAGAAGTACCTGACCACCTTCCAAATG
GGTTCAATTCGTGGCCATTTTCACCCACCAGTTCCAGCTTTTGTTCCGCGAATGTGATTACCCCAAGGGC
TTCATGGTCTGGATCGGATTGCACGGCGTTATGTTCCTGTTCCTGTTCTCCGACTTTTACAAAGCGAAAT
ATCTCAACGCAGCTCGACGTCGCCGGCAGGCGGTCAAGGCCAATGGCTATGCCAACGGCTCGGCTTCGAA

TGGGCACAGCAAGCATCTTGGAGAGGGTGACGCCCTGATTGCAAACGGTTGCAATACTGGGGCTTGCATG
G
>dme_circ_0008141_junction_seq
AACGGTTGCAATACTGGGGCTTGCATGGCTCGGTTAACACACCAAATACAGGACCG
>dme_circ_0008142
GGAGTAGCAACATCGGCAACATTAGCAATAGTTTAAACAATAATCTGCCCGGAGGCAGTTCGTCCACCAG
CAGTGCTGGAAACGTGCCTGGTGGAAGTGGGGGAATTATATCAGCTTCGAGCGGTGGTGCTACGCAATCG
ACAAGCAGTCAATCGTCAACAGCACCAGGAACCACCAAATCATCGGCCAGCAGTTCGAGCAG
>dme_circ_0008142_junction_seq
CCAAATCATCGGCCAGCAGTTCGAGCAGGGAGTAGCAACATCGGCAACATTAGCAA
>dme_circ_0008143
GCTAACGTTCACCTGTGGTCCCAGCTTCTGCCTCGGCAGTCCACCTCCTGGGATGTTACCCTGCGCCCCA
ATGGCGTGCAGTACGACAAGGTCATTGCCATCTCCGATCTGCTGCAAGGAGAGTTCACCAGAACTAACGC
>dme_circ_0008143_junction_seq
TGCAAGGAGAGTTCACCAGAACTAACGCGCTAACGTTCACCTGTGGTCCCAGCTTC
>dme_circ_0008144
GGCGATTTTTTTAGTGGCTGGCTGCCTGGGATTTGTTGGTTGGGATTTTTGGTGGTATTTCGTCAGCTCA
CTATTTGCGCGCCTTTTTGCAAACGCCGGCCATTTACCCAAACGCACTGATTTATTGCAGTTTGATGCAC
TAAAAGGGTTTATATTTG
>dme_circ_0008144_junction_seq
TTTGATGCACTAAAAGGGTTTATATTTGGGCGATTTTTTTAGTGGCTGGCTGCCTG
>dme_circ_0008145
GATTTGGTGCACTGCACAAACGAGCCGAATGTGTCGATACCTCATCTGGCCAATTTACTTATCGAGCGTT
CACAGAATGCCAACTGGGTCGTCGTCTACAAGTCGCTGATAACCACACATCATCTGATGGCATATGGCAA
TGAGGGTTTGTATTTGTGTATTTATCGGAATCTAGTGTTTGTGCGAAGCTTAGGGGCGTATTTCAAATAC
ATTTTTACTTTCATTTAAGCGTTCGAAATGAGGTAATCTATTTTGTTTGATTTAATATACAGATGAATAG
GTCACGTACATCATGAGATTCTTATTATTTTCCTTCTTGTACCTTTAATGGCAACTTATTACTGCAGAAT
TACTCTTTTGGGATGACCTCTGACCAAGTATATCGTTGTGTGGTattttactttcagtttttgttcgttc
agttttatttcttagtaatttttttGGTGACATAAAAAGTTGTCTTCTCTGAAAAATAAATAAATAAATG
TTAAATTTTTAACACATCAACTTTTATAGTTCCCTCCATTTAATTGCGATTTATAATTATCTAAATAATA
TTTAACAGCATTCCTATTGTTTTTCCTAAATCTTTTCAGCGATTTATGCAATATCTTGCCTCTAGCAATT
CTACATTCAATCTCAGCTCCTTTCTGGACAAAGGAACTGTACAAG
>dme_circ_0008145_junction_seq
TCCTTTCTGGACAAAGGAACTGTACAAGGATTTGGTGCACTGCACAAACGAGCCGA
>dme_circ_0008146
GATTTGGTGCACTGCACAAACGAGCCGAATGTGTCGATACCTCATCTGGCCAATTTACTTATCGAGCGTT
CACAGAATGCCAACTGGGTCGTCGTCTACAAGTCGCTGATAACCACACATCATCTGATGGCATATGGCAA
TGAGGGTTTGTATTTGTGTATTTATCGGAATCTAGTGTTTGTGCGAAGCTTAGGGGCGTATTTCAAATAC
ATTTTTACTTTCATTTAAGCGTTCGAAATGAGGTAATCTATTTTGTTTGATTTAATATACAGATGAATAG
GTCACGTACATCATGAGATTCTTATTATTTTCCTTCTTGTACCTTTAATGGCAACTTATTACTGCAGAAT
TACTCTTTTGGGATGACCTCTGACCAAGTATATCGTTGTGTGGTattttactttcagtttttgttcgttc
agttttatttcttagtaatttttttGGTGACATAAAAAGTTGTCTTCTCTGAAAAATAAATAAATAAATG
TTAAATTTTTAACACATCAACTTTTATAGTTCCCTCCATTTAATTGCGATTTATAATTATCTAAATAATA
TTTAACAGCATTCCTATTGTTTTTCCTAAATCTTTTCAGGGTATAACGAAGCCTCCCTTTTAAGCCTCCC

AAATTTGTTTCAAACCCATCGACTATTCAATCGATTTACCTATTTCAAATTATTAACTAATACCTACATT
TTGATCATTCCTCTCTCATTCTCTCTTTCAACTCATGCGAATGCGCGCGAACGTTTGGCTTGATCATTTG
TAATTTTGTTTACTTAACAAAAATCTCTAAAACAAAAATATTAATACCGAAATGCGCTGCTTACTGACCT
ACATACCGCACACTACATGCTGGCTAACAACAACTGAAAAATGCAATTATCCAAACTAAATATTCAAAAT
CAAACAATATAAACCTTATATACAGGGTGAGTATTATACAGAAGCTATTAGCAATTGGATACAACTTTCC
TTATTTGGCTATAGGCGTTGAATTCCTTACTGGTTGTTACTAAAATTTGTTTTACTTATGATTTAGAGCG
AAATTAACTAACCAAAAGACCATTCTTCTTAAGTTTCGTATTTGGCATTGTCCAAAATTTGTGTGACCTA
TATTGTTGGAGACCAATACTATAATTTGTTAAATCAACAGGGCGTCATCAATATGAGCTTTATGCTCTTG
TTCCGCGATCTCATACGATTGTTTGCTTGCTACAATGATGGCATCATAAATTTGCTTGAAAAATATTTCG
ATATGAACAAGAAACATGCCCGCGATGCGTTGGATCTTTACAAAAAGTTCTTAGTGCGCATGGATCGTGT
TGGAGAGTTTTTAAAAGTGGCTGAGGAATGTGGGCATAGACAAGGGTGACATACCCGATTTGACCAAGGC
GCCCAGTTCGTTGTTAGATGCCTTGGAGCAGCACTTGGCCACGCTTGAGGGTCGAAAAGTATCCGCTGCT
AATACTCCTACGCAGTCATCAAG
>dme_circ_0008146_junction_seq
CTGCTAATACTCCTACGCAGTCATCAAGGATTTGGTGCACTGCACAAACGAGCCGA
>dme_circ_0008147
GATTTGGTGCACTGCACAAACGAGCCGAATGTGTCGATACCTCATCTGGCCAATTTACTTATCGAGCGTT
CACAGAATGCCAACTGGGTCGTCGTCTACAAGTCGCTGATAACCACACATCATCTGATGGCATATGGCAA
TGAGGGTTTGTATTTGTGTATTTATCGGAATCTAGTGTTTGTGCGAAGCTTAGGGGCGTATTTCAAATAC
ATTTTTACTTTCATTTAAGCGTTCGAAATGAGGTAATCTATTTTGTTTGATTTAATATACAGATGAATAG
GTCACGTACATCATGAGATTCTTATTATTTTCCTTCTTGTACCTTTAATGGCAACTTATTACTGCAGAAT
TACTCTTTTGGGATGACCTCTGACCAAGTATATCGTTGTGTGGTattttactttcagtttttgttcgttc
agttttatttcttagtaatttttttGGTGACATAAAAAGTTGTCTTCTCTGAAAAATAAATAAATAAATG
TTAAATTTTTAACACATCAACTTTTATAGTTCCCTCCATTTAATTGCGATTTATAATTATCTAAATAATA
TTTAACAGCATTCCTATTGTTTTTCCTAAATCTTTTCAGGGTATAACGAAGCCTCCCTTTTAAGCCTCCC
AAATTTGTTTCAAACCCATCGACTATTCAATCGATTTACCTATTTCAAATTATTAACTAATACCTACATT
TTGATCATTCCTCTCTCATTCTCTCTTTCAACTCATGCGAATGCGCGCGAACGTTTGGCTTGATCATTTG
TAATTTTGTTTACTTAACAAAAATCTCTAAAACAAAAATATTAATACCGAAATGCGCTGCTTACTGACCT
ACATACCGCACACTACATGCTGGCTAACAACAACTGAAAAATGCAATTATCCAAACTAAATATTCAAAAT
CAAACAATATAAACCTTATATACAGGGTGAGTATTATACAGAAGCTATTAGCAATTGGATACAACTTTCC
TTATTTGGCTATAGGCGTTGAATTCCTTACTGGTTGTTACTAAAATTTGTTTTACTTATGATTTAGAGCG
AAATTAACTAACCAAAAGACCATTCTTCTTAAGTTTCGTATTTGGCATTGTCCAAAATTTGTGTGACCTA
TATTGTTGGAGACCAATACTATAATTTGTTAAATCAACAGGGCGTCATCAATATGAGCTTTATGCTCTTG
TTCCGCGATCTCATACGATTGTTTGCTTGCTACAATGATGGCATCATAAATTTGCTTGAAAAATATTTCG
ATATGAACAAGAAACATGCCCGCGATGCGTTGGATCTTTACAAAAAGTTCTTAGTGCGCATGGATCGTGT
TGGAGAGTTTTTAAAAGTGGCTGAGGAATGTGGGCATAGACAAGGGTGACATACCCGATTTGACCAAGGC
GCCCAGTTCGTTGTTAGATGCCTTGGAGCAGCACTTGGCCACGCTTGAGGGTCGAAAAGTATCCGCTGCT
AATACTCCTACGCAGTCATCAAGGGTTTGTTAATCTATTTCTAAGCTCCAAAATCTATCTTTTAGATATT
ACTTTTATTTGTTATATGCTAACATAATTTAAACATTGGGTTCCTACTAACAACCACTTGTATTTTGTAC
ATTTTATTCAAATTTTATAATGCAACATAAAATAATAAGATAAATTATGTAAAATTTGGCAATGAATTAT
GGATAGTTTACAAATTATTTAGTTCTTGTATAAATACTTTATTTACTGAACTGTTATCATTAATGTATC
AATGTATTATTTGCGATAtatttttaacattcttttcttttgtttttctatgattttccttggtttttGC
GTTGCACCTGTTTTTGATGGCTGGGAAACATTAGTAATCAGCGTAATGTAAAATCCGCTGTATCTGCCCT
GTCTTCTACCAGGTAATCAGCGTAATGTAAAATCCGCTGTATCTGCCCTGTCTTCTACCAGCTCGGCATT

TGGTACTGCAGCTGCGTCCAGCAAATTTGATACCACCAACGGCATTGACGAGCAGCTTAAGGCCCAGGTT
TTGGCCGAAGAGGAAGCCGCTATGAACCAATACAAGGTCCAAGGTGTCGTCGCCCACTAGCAGCGGTGCT
GCTGGCGCTAGCGCTGCACTAACAAATCCATTTCTATCGTCGCCGCCAGCCGCGCAGGCTGGCCAGCCGA
TAGTTGATCTGTTCGGTGCCGCGTCGGCGCAGCCCGCTGCTGCTGCAGCAGCCACCAAGGCCTCTGACGA
TCTGCTGCAGTTGGGCAATCCTTTCGCGGACATGTTTGACGCCAGCGGTGGGGGAGCAGCTGCTGTAGGA
GCCACAGGTGCTGCACTCAATGCCAATAATTTGTGGATGCATAATAATGGGTGCTGCACTCAATGCCAAT
AATTTGTGGATGCATAATAATGGTAGCATGTTATAATTTTCTTATTATTTCGTCCTAAGTTCTTGTTTAG
CGATTACGATATGAGTTAAATTTATTTTGTGCTGTACATAGGTAACATTCGTTGTGTAAGTGTAGTGATC
TTACGTATTTATGTTGTCGTACTCTAAAAATCAGAGCAATGAGGAGCGCACTTTTTAGGCGCTCTAGACG
CCCCTCACCCTTTGTCTGTCTTCCTATCACTTCAGCTTAGACGTCTATTTGCACAAGTTTTCATGCATCA
TTATCCATAGTTGAAATCATAATGGtatattttgtatccatttaattatttacgataattatttttgtaa
gttttgatttttttttAAATTCTTGTCTAATGTATTGTTACTCTTCATTTTTTCATCACTGTTTTTTTCC
AAACTGAAACTGTAATGACAACATCTTCAACAACGATACCGAAAACAACTCCTCGGTTACGAAAACCAAA
CCAATCGCAAATCTTCCAAACGACCATCGCCCACTGTCCACCGCCCACACTACACCCAACTAAACTGAGA
CAAAACCACCAACAAATTTCAAAATAAAACAAACAAAAATAGGTTTCAATGGCGCATCAGTTTCCTCCGC
TGCTGCTACAAATGCATTCGTTTCCGATAGTAATTTCTCATCCGTATTTGGTAATACGGAACCGGCAGGT
AAGTCACTCAATGGTCTACAGTCTTCATTCATTCCTAACTGCAAGCTACAATCTAATCCTAAGAATACAT
ATTTGAATACTTAATATGCAGTCACTAACAAAATGCTTATGATTTACTAACTATTCCAAATTGAGTAAAA
ATCGGAGTGGTTCGTTTAAGTTTACTAATTGGCAAAGGCAATAACAATGCTAGCAATATCATCCAACTTT
GATGTGTTTGAATTATACTGATATAAATGTAGCATGTAATTTAATATGCCTGATTGATTTCATGTATTAA
ACAAGTTCAACAAAAAGTTAAGAAAATTCCATTTTACGTATATATAGGTCTTTGAAATTCTAAAACTGGA
TTAGAAATATGCGTGATTATGTTAGGAACCTAATCCCTTACCTGTATTCCTCAATGCTAgtttttgaatc
atattcggacttttaataatttctttttatttttttagtatttattttAATGGTTATCCTCTGAATGTTTG
CGTTAAATTTTACACAGCTTCGACGTCGTTGCCAAATCCATTTTTCGATGATATGTCTTTGCCCCAAATC
CCACTCCCTGCTGAATCTGTTGCTGCACCCTTTGgcaacaatataaatttcatggatcagcagctgcagc
aacaacagcaagcggctttatatgtgcaacagcagcagcaacagcagctgcaactacagcagcagcaaca
ccggccgcaactgccgcaacagcaaGCGATATTGACACCTTCCATGTCAGCAGCTCAGTTTCCACCAGGT
GAAGATTTGCAGACCCTCGCCGCAAAACGAATACGAAAACAAACCCATTAAACACAAGCATATGTTCCTC
CGCGTGCTTTGTTTTTGTACAAAGCTTGATCCATCACTGGCTTGTAGTGCTGCAGCGGACACTGACTTTT
CTATCTCAGCATCCAAAATCTCCTGAGCCTTGCGCCCAGTGCATCAGATGCTCATCCACACCAAACAGAA
CACGCTTTTCCCTCTGTTGTCTGCTCGTGTTTTGTAGCTATTTTGTCTGCAATCGACTATAAACTCGTCT
CGTAATTGACAAGAAAGCTGTGTTGTTGTCTAAATAGTTTTAATTTATTTTTTGGAAAAAAGTATTTAAT
GTGTGTTTCATGTGATAACTGTGTGATAACTATTCTTTTGATTTACGTTTTTGAGTTCAGGTCAACTAAT
TCTAGTGTCAGATTCCCTATTTGCTATTTGAACCTGAACTTTTCACATAACTATCAGGACGTATTGTATT
TACGATGATATAATGCTTAAATTACACTaaaaaaaaaCGCACTTATTTCATTAACAAAAAACTATTCTGC
TTTGGTAGCTTTTGTAAATAATCTTACGTCTGTCATGTGTTTGTAAATAACAACCTATTATTAATCCTAC
ATATATCAAAAATCATTAGAGATCGATATATCCCAAATCTTTATATATAAAATGCAatttcatattattt
attttgtaaatgttatttttttctctttttctctctcttatgttcttaTGTACTGAACGTAAAAATCCAAT
CAAAATATCAAAACTAAAAACAATTTCACATTGTATCATGTTAATGTTGGTGGCACAAATACATATTGAA
CACTTAAAATCTCAACAAAACGCTAAAACATATTTGTGTGTTTTATGATTACGCTGGCaaaatgaaaaaa
aaaaaaacttaaaaaCGAATTAAAACCGAAATATCGAATATCTACGTCAATCGAACTCAACGCTAACTCG
ACAAACATCAAAACCAATCCTCTGGGCTCATCGGCTTCATTGTTAATGCATATCTACCatatatatatat
ataCCAATGTGTTTGTCTCTGTGTATAAATGTCTGCTAAAAACGGTGCATTGGCTATGGCTATCCCCAAA
CAACCATGTTGTCTGAATTGTAAAAACCTATCAAGGCTTCGATGCCTTGGGCGACGTCCTCAAGCCGGCC

TCGGCCAGCAACAACAGCAATCAAATGAACGTTGTCGCCGCAGGTTACTCCAGCAATAGCAGTTCGATCC
TAGCGTTAcaacagcagcagcaccagcagcaccagcagcaacaccagcatcaactgaaccagcagcaaca
gcagcagcCACCGGCTGGCACTGGAAAGATAATTACCGGCGACTTGGATAGTTCACTAATGTCACTCGTT
GATAACTTAAATATTAACAAAACGGCAAGTGCAAAGTGAGTAAATCGAGCCAGTAAACTGACGATTGTGT
GTGCGTGCGTTCGAAAGCGAAGCCAAACCAAACCAGAATGTTGATCAGAATCCATGTTGTCACATCCCTT
CCAGTTCATTCGCTGTAGATGGCTGAATCGTGTCGATTTTGATCGACTGAAGATTATGTTCCACATCCGA
AGAAACAGATCCTTTGATGATCCCTTACCATTTATCCAATACCATTCCGAACTGACCCTCCCATATTCAT
GTTCCATACGTGTACATAAAATCCATCAAAAAAATGGTTCGAAATCACGCAGAAATCTATTTAGATACCC
aaaaaaaaaaaCCCAAAATTCAAATGCCGTGTTCATTGACGTCGTCTATATATCATTACTCTCTTACCCC
ATTATTCTCATAGACCCGTGCAATGGAATTCACCTAAAAATACAGCGAAACCAGGTGCTAATTGGACACC
CCAACCAATGGCGGCTACTACTGGTGCTGGCTATCGTCCAATG
>dme_circ_0008147_junction_seq
TACTACTGGTGCTGGCTATCGTCCAATGGATTTGGTGCACTGCACAAACGAGCCGA
>dme_circ_0008148
GGTTACGATATGTCTCCCTTTATTCGGCGCTACGCCAAGTATTTGAATGAGAAATCGCTCTCCTATCGAG
CCATGGCCTTTGACTTTTGCAAAGTCAAACGGGGGTAAGTAACGCCAGCTCACATTTAATTGTCATCTGA
TTGATTTGTTTCGCAATCTATCATTTTAGCAAAGAGGAAGGCTCGCTACGCAGCATGAACGCGGAAAAAC
TTCTAAAGACTTTGCCAGTTTTGCAGGCACAATTGGATGCACTTTTGGAGTTCGACTGCCAATCCAACGA
CTTGTCTAATGGTATGAATATATTTCCAAATTATATTGTAAAACTATAACGACTTTAATATTTTTCAGGC
GTCATCAATATGAGCTTTATGCTCTTGTTCCGCGATCTCATACGATTGTTTGCTTGCTACAATGATGGCA
TCATAAATTTGCTTGAAAAATATTTCGATATGAACAAGAAACATGCCCGCGATGCGTTGGATCTTTACAA
AAAGTTCTTAGTGCGCATGGATCGTGTTGGAGAGTTTTTAAAAGTGGCTGAGGAATGTGGGCATAGACAA
GGGTGACATACCCGATTTGACCAAGGCGCCCAGTTCGTTGTTAGATGCCTTGGAGCAGCACTTGGCCACG
CTTGAGGGTCGAAAAGTATCCGCTGCTAATACTCCTACGCAGTCATCAAG
>dme_circ_0008148_junction_seq
CTGCTAATACTCCTACGCAGTCATCAAGGGTTACGATATGTCTCCCTTTATTCGGC
>dme_circ_0008149
GGTTACGATATGTCTCCCTTTATTCGGCGCTACGCCAAGTATTTGAATGAGAAATCGCTCTCCTATCGAG
CCATGGCCTTTGACTTTTGCAAAGTCAAACGGGGGTAAGTAACGCCAGCTCACATTTAATTGTCATCTGA
TTGATTTGTTTCGCAATCTATCATTTTAGCAAAGAGGAAGGCTCGCTACGCAGCATGAACGCGGAAAAAC
TTCTAAAGACTTTGCCAGTTTTGCAGGCACAATTGGATGCACTTTTGGAGTTCGACTGCCAATCCAACGA
CTTGTCTAATGGTATGAATATATTTCCAAATTATATTGTAAAACTATAACGACTTTAATATTTTTCAGGC
GTCATCAATATGAGCTTTATGCTCTTGTTCCGCGATCTCATACGATTGTTTGCTTGCTACAATGATGGCA
TCATAAATTTGCTTGAAAAATATTTCGATATGAACAAGAAACATGCCCGCGATGCGTTGGATCTTTACAA
AAAGTTCTTAGTGCGCATGGATCGTGTTGGAGAGTTTTTAAAAGTGGCTGAGGAATGTGGGCATAGACAA
GGGTGACATACCCGATTTGACCAAGGCGCCCAGTTCGTTGTTAGATGCCTTGGAGCAGCACTTGGCCACG
CTTGAGGGTCGAAAAGTATCCGCTGCTAATACTCCTACGCAGTCATCAAGGTAATCAGCGTAATGTAAAA
TCCGCTGTATCTGCCCTGTCTTCTACCAGCTCGGCATTTGGTACTGCAGCTGCGTCCAGCAAATTTGATA
CCACCAACGGCATTGACGAGCAGCTTAAGGCCCAGGTTTTGGCCGAAGAGGAAGCCGCTATGAACCAATA
CAAGGTCCAAGGTGTCGTCGCCCACTAGCAGCGGTGCTGCTGGCGCTAGCGCTGCACTAACAAATCCATT
TCTATCGTCGCCGCCAGCCGCGCAGGCTGGCCAGCCGATAGTTGATCTGTTCGGTGCCGCGTCGGCGCAG
CCCGCTGCTGCTGCAGCAGCCACCAAGGCCTCTGACGATCTGCTGCAGTTGGGCAATCCTTTCGCGGACA
TGTTTGACGCCAGCGGTGGGGGAGCAGCTGCTGTAGGAGCCACAG
>dme_circ_0008149_junction_seq

GGGGGAGCAGCTGCTGTAGGAGCCACAGGGTTACGATATGTCTCCCTTTATTCGGC
>dme_circ_0008150
GGTTACGATATGTCTCCCTTTATTCGGCGCTACGCCAAGTATTTGAATGAGAAATCGCTCTCCTATCGAG
CCATGGCCTTTGACTTTTGCAAAGTCAAACGGGGGTAAGTAACGCCAGCTCACATTTAATTGTCATCTGA
TTGATTTGTTTCGCAATCTATCATTTTAGCAAAGAGGAAGGCTCGCTACGCAGCATGAACGCGGAAAAAC
TTCTAAAGACTTTGCCAGTTTTGCAGGCACAATTGGATGCACTTTTGGAGTTCGACTGCCAATCCAACGA
CTTGTCTAATGGTATGAATATATTTCCAAATTATATTGTAAAACTATAACGACTTTAATATTTTTCAGGC
GTCATCAATATGAGCTTTATGCTCTTGTTCCGCGATCTCATACGATTGTTTGCTTGCTACAATGATGGCA
TCATAAATTTGCTTGAAAAATATTTCGATATGAACAAGAAACATGCCCGCGATGCGTTGGATCTTTACAA
AAAGTTCTTAGTGCGCATGGATCGTGTTGGAGAGTTTTTAAAAGTGGCTGAGGAATGTGGGCATAGACAA
GGGTGACATACCCGATTTGACCAAGGCGCCCAGTTCGTTGTTAGATGCCTTGGAGCAGCACTTGGCCACG
CTTGAGGGTCGAAAAGTATCCGCTGCTAATACTCCTACGCAGTCATCAAGGGTTTGTTAATCTATTTCTA
AGCTCCAAAATCTATCTTTTAGATATTACTTTTATTTGTTATATGCTAACATAATTTAAACATTGGGTTC
CTACTAACAACCACTTGTATTTTGTACATTTTATTCAAATTTTATAATGCAACATAAAATAATAAGATAA
ATTATGTAAAATTTGGCAATGAATTATGGATAGTTTACAAATTATTTTAGTTCTTGTATAAATACTTTAT
TTACTGAACTGTTATCATTAATGTATCAATGTATTATTTGCGATAtatttttaacattcttttcttttgt
tttctatgattttccttggtttttGCGTTGCACCTGTTTTTGATGGCTGGGAAACATTAGTAATCAGCG
TAATGTAAAATCCGCTGTATCTGCCCTGTCTTCTACCAGGTAATCAGCGTAATGTAAAATCCGCTGTATC
TGCCCTGTCTTCTACCAGCTCGGCATTTGGTACTGCAGCTGCGTCCAGCAAATTTGATACCACCAACGGC
ATTGACGAGCAGCTTAAGGCCCAGGTTTTGGCCGAAGAGGAAGCCGCTATGAACCAATACAAGGTCCAAG
GTGTCGTCGCCCACTAGCAGCGGTGCTGCTGGCGCTAGCGCTGCACTAACAAATCCATTTCTATCGTCGC
CGCCAGCCGCGCAGGCTGGCCAGCCGATAGTTGATCTGTTCGGTGCCGCGTCGGCGCAGCCCGCTGCTGC
TGCAGCAGCCACCAAGGCCTCTGACGATCTGCTGCAGTTGGGCAATCCTTTCGCGGACATGTTTGACGCC
AGCGGTGGGGGAGCAGCTGCTGTAGGAGCCACAGGTGCTGCACTCAATGCCAATAATTTGTGGATGCATA
ATAATG
>dme_circ_0008150_junction_seq
GCCAATAATTTGTGGATGCATAATAATGGGTTACGATATGTCTCCCTTTATTCGGC
>dme_circ_0008151
GGTTACGATATGTCTCCCTTTATTCGGCGCTACGCCAAGTATTTGAATGAGAAATCGCTCTCCTATCGAG
CCATGGCCTTTGACTTTTGCAAAGTCAAACGGGGGTAAGTAACGCCAGCTCACATTTAATTGTCATCTGA
TTGATTTGTTTCGCAATCTATCATTTTAGCAAAGAGGAAGGCTCGCTACGCAGCATGAACGCGGAAAAAC
TTCTAAAGACTTTGCCAGTTTTGCAGGCACAATTGGATGCACTTTTGGAGTTCGACTGCCAATCCAACGA
CTTGTCTAATGGTATGAATATATTTCCAAATTATATTGTAAAACTATAACGACTTTAATATTTTTCAGGC
GTCATCAATATGAGCTTTATGCTCTTGTTCCGCGATCTCATACGATTGTTTGCTTGCTACAATGATGGCA
TCATAAATTTGCTTGAAAAATATTTCGATATGAACAAGAAACATGCCCGCGATGCGTTGGATCTTTACAA
AAAGTTCTTAGTGCGCATGGATCGTGTTGGAGAGTTTTTAAAAGTGGCTGAGGAATGTGGGCATAGACAA
GGGTGACATACCCGATTTGACCAAGGCGCCCAGTTCGTTGTTAGATGCCTTGGAGCAGCACTTGGCCACG
CTTGAGGGTCGAAAAGTATCCGCTGCTAATACTCCTACGCAGTCATCAAGGGTTTGTTAATCTATTTCTA
AGCTCCAAAATCTATCTTTTAGATATTACTTTTATTTGTTATATGCTAACATAATTTAAACATTGGGTTC
CTACTAACAACCACTTGTATTTTGTACATTTTATTCAAATTTTATAATGCAACATAAAATAATAAGATAA
ATTATGTAAAATTTGGCAATGAATTATGGATAGTTTACAAATTATTTTAGTTCTTGTATAAATACTTTAT
TTACTGAACTGTTATCATTAATGTATCAATGTATTATTTGCGATAtatttttaacattcttttcttttgt
tttctatgattttccttggtttttGCGTTGCACCTGTTTTTGATGGCTGGGAAACATTAGTAATCAGCG
TAATGTAAAATCCGCTGTATCTGCCCTGTCTTCTACCAGGTAATCAGCGTAATGTAAAATCCGCTGTATC

TGCCCTGTCTTCTACCAGCTCGGCATTTGGTACTGCAGCTGCGTCCAGCAAATTTGATACCACCAACGGC
ATTGACGAGCAGCTTAAGGCCCAGGTTTTGGCCGAAGAGGAAGCCGCTATGAACCAATACAAGGTCCAAG
GTGTCGTCGCCCACTAGCAGCGGTGCTGCTGGCGCTAGCGCTGCACTAACAAATCCATTTCTATCGTCGC
CGCCAGCCGCGCAGGCTGGCCAGCCGATAGTTGATCTGTTCGGTGCCGCGTCGGCGCAGCCCGCTGCTGC
TGCAGCAGCCACCAAGGCCTCTGACGATCTGCTGCAGTTGGGCAATCCTTTCGCGGACATGTTTGACGCC
AGCGGTGGGGGAGCAGCTGCTGTAGGAGCCACAGGTGCTGCACTCAATGCCAATAATTTGTGGATGCATA
ATAATGGGTGCTGCACTCAATGCCAATAATTTGTGGATGCATAATAATGGTAGCATGTTATAATTTTCTT
ATTATTTCGTCCTAAGTTCTTGTTTAGCGATTACGATATGAGTTAAATTTATTTTGTGCTGTACATAGGT
AACATTCGTTGTGTAAGTGTAGTGATCTTACGTATTTATGTTGTCGTACTCTAAAAATCAGAGCAATGAG
GAGCGCACTTTTTAGGCGCTCTAGACGCCCCTCACCCTTTGTCTGTCTTCCTATCACTTCAGCTTAGACG
TCTATTTGCACAAGTTTTCATGCATCATTATCCATAGTTGAAATCATAATGGtatattttgtatccattt
aattatttacgataattatttttgtaagttttgatttttttttAAATTCTTGTCTAATGTATTGTTACTC
TTCATTTTTTCATCACTGTTTTTTTCCAAACTGAAACTGTAATGACAACATCTTCAACAACGATACCGAA
AACAACTCCTCGGTTACGAAAACCAAACCAATCGCAAATCTTCCAAACGACCATCGCCCACTGTCCACCG
CCCACACTACACCCAACTAAACTGAGACAAAACCACCAACAAATTTCAAAATAAAACAAACAAAAATAGG
TTTCAATGGCGCATCAGTTTCCTCCGCTGCTGCTACAAATGCATTCGTTTCCGATAGTAATTTCTCATCC
GTATTTGGTAATACGGAACCGGCAG
>dme_circ_0008151_junction_seq
TCCGTATTTGGTAATACGGAACCGGCAGGGTTACGATATGTCTCCCTTTATTCGGC
>dme_circ_0008152
GGTTTCAATGGCGCATCAGTTTCCTCCGCTGCTGCTACAAATGCATTCGTTTCCGATAGTAATTTCTCAT
CCGTATTTGGTAATACGGAACCGGCAGGTAAGTCACTCAATGGTCTACAGTCTTCATTCATTCCTAACTG
CAAGCTACAATCTAATCCTAAGAATACATATTTGAATACTTAATATGCAGTCACTAACAAAATGCTTATG
ATTTACTAACTATTCCAAATTGAGTAAAAATCGGAGTGGTTCGTTTAAGTTTACTAATTGGCAAAGGCAA
TAACAATGCTAGCAATATCATCCAACTTTGATGTGTTTGAATTATACTGATATAAATGTAGCATGTAATT
TAATATGCCTGATTGATTTCATGTATTAAACAAGTTCAACAAAAAGTTAAGAAAATTCCATTTTACGTAT
ATATAGGTCTTTGAAATTCTAAAACTGGATTAGAAATATGCGTGATTATGTTAGGAACCTAATCCCTTAC
CTGTATTCCTCAATGCTAgtttttgaatcatattcggacttttaataatttctttttatttttttagtatt
tattttAATGGTTATCCTCTGAATGTTTGCGTTAAATTTTACACAGCTTCGACGTCGTTGCCAAATCCAT
TTTTCGATGATATGTCTTTGCCCCAAATCCCACTCCCTGCTGAATCTGTTGCTGCACCCTTTGgcaacaa
tataaatttcatggatcagcagctgcagcaacaacagcaagcggctttatatgtgcaacagcagcagcaa
cagcagctgcaactacagcagcagcaacaccggccgcaactgccgcaacagcaaGCGATATTGACACCTT
CCATGTCAGCAGCTCAGTTTCCACCAGGTGAAGATTTGCAGACCCTCGCCGCAAAACGAATACGAAAACA
AACCCATTAAACACAAGCATATGTTCCTCCGCGTGCTTTGTTTTTGTACAAAGCTTGATCCATCACTGGC
TTGTAGTGCTGCAGCGGACACTGACTTTTCTATCTCAGCATCCAAAATCTCCTGAGCCTTGCGCCCAGTG
CATCAGATGCTCATCCACACCAAACAGAACACGCTTTTCCCTCTGTTGTCTGCTCGTGTTTTGTAGCTAT
TTTGTCTGCAATCGACTATAAACTCGTCTCGTAATTGACAAGAAAGCTGTGTTGTTGTCTAAATAGTTTT
AATTTATTTTTTGGAAAAAAGTATTTAATGTGTGTTTCATGTGATAACTGTGTGATAACTATTCTTTTGA
TTTACGTTTTTGAGTTCAGGTCAACTAATTCTAGTGTCAGATTCCCTATTTGCTATTTGAACCTGAACTT
TTCACATAACTATCAGGACGTATTGTATTTACGATGATATAATGCTTAAATTACACTaaaaaaaaaCGCA
CTTATTTCATTAACAAAAAACTATTCTGCTTTGGTAGCTTTTGTAAATAATCTTACGTCTGTCATGTGTT
TGTAAATAACAACCTATTATTAATCCTACATATATCAAAAATCATTAGAGATCGATATATCCCAAATCTT
TATATATAAAATGCAatttcatattatttattttgtaaatgttatttttttctctttttctctctcttatg
ttcttaTGTACTGAACGTAAAAATCCAATCAAAATATCAAAACTAAAAACAATTTCACATTGTATCATGT

TAATGTTGGTGGCACAAATACATATTGAACACTTAAAATCTCAACAAAACGCTAAAACATATTTGTGTGT
TTTATGATTACGCTGGCaaaatgaaaaaaaaaaaaacttaaaaaCGAATTAAAACCGAAATATCGAATAT
CTACGTCAATCGAACTCAACGCTAACTCGACAAACATCAAAACCAATCCTCTGGGCTCATCGGCTTCATT
GTTAATGCATATCTACCatatatatatatataCCAATGTGTTTGTCTCTGTGTATAAATGTCTGCTAAAA
ACGGTGCATTGGCTATGGCTATCCCCAAACAACCATGTTGTCTGAATTGTAAAAACCTATCAAGGCTTCG
ATGCCTTGGGCGACGTCCTCAAGCCGGCCTCGGCCAGCAACAACAGCAATCAAATGAACGTTGTCGCCGC
AGGTTACTCCAGCAATAGCAGTTCGATCCTAGCGTTAcaacagcagcagcaccagcagcaccagcagcaa
caccagcatcaactgaaccagcagcaacagcagcagcCACCGGCTGGCACTGGAAAGATAATTACCGGCG
ACTTGGATAGTTCACTAATGTCACTCGTTGATAACTTAAATATTAACAAAACGGCAAGTGCAAAGTGAGT
AAATCGAGCCAGTAAACTGACGATTGTGTGTGCGTGCGTTCGAAAGCGAAGCCAAACCAAACCAGAATGT
TGATCAGAATCCATGTTGTCACATCCCTTCCAGTTCATTCGCTGTAGATGGCTGAATCGTGTCGATTTTG
ATCGACTGAAGATTATGTTCCACATCCGAAGAAACAGATCCTTTGATGATCCCTTACCATTTATCCAATA
CCATTCCGAACTGACCCTCCCATATTCATGTTCCATACGTGTACATAAAATCCATCAAAAAAATGGTTCG
AAATCACGCAGAAATCTATTTAGATACCCaaaaaaaaaaaCCCAAAATTCAAATGCCGTGTTCATTGACG
TCGTCTATATATCATTACTCTCTTACCCCATTATTCTCATAGACCCGTGCAATGGAATTCACCTAAAAAT
ACAGCGAAACCAGGTGCTAATTGGACACCCCAACCAATGGCGGCTACTACTGGTGCTGGCTATCGTCCAA
TG
>dme_circ_0008152_junction_seq
TACTACTGGTGCTGGCTATCGTCCAATGGGTTTCAATGGCGCATCAGTTTCCTCCG
>dme_circ_0008153
GGTTTCAATGGCGCATCAGTTTCCTCCGCTGCTGCTACAAATGCATTCGTTTCCGATAGTAATTTCTCAT
CCGTATTTGGTAATACGGAACCGGCAGGTAAGTCACTCAATGGTCTACAGTCTTCATTCATTCCTAACTG
CAAGCTACAATCTAATCCTAAGAATACATATTTGAATACTTAATATGCAGTCACTAACAAAATGCTTATG
ATTTACTAACTATTCCAAATTGAGTAAAAATCGGAGTGGTTCGTTTAAGTTTACTAATTGGCAAAGGCAA
TAACAATGCTAGCAATATCATCCAACTTTGATGTGTTTGAATTATACTGATATAAATGTAGCATGTAATT
TAATATGCCTGATTGATTTCATGTATTAAACAAGTTCAACAAAAAGTTAAGAAAATTCCATTTTACGTAT
ATATAGGTCTTTGAAATTCTAAAACTGGATTAGAAATATGCGTGATTATGTTAGGAACCTAATCCCTTAC
CTGTATTCCTCAATGCTAgtttttgaatcatattcggacttttaataatttctttttatttttttagtatt
tattttAATGGTTATCCTCTGAATGTTTGCGTTAAATTTTACACAGCTTCGACGTCGTTGCCAAATCCAT
TTTTCGATGATATGTCTTTGCCCCAAATCCCACTCCCTGCTGAATCTGTTGCTGCACCCTTTGgcaacaa
tataaatttcatggatcagcagctgcagcaacaacagcaagcggctttatatgtgcaacagcagcagcaa
cagcagctgcaactacagcagcagcaacaccggccgcaactgccgcaacagcaaGCGATATTGACACCTT
CCATGTCAGCAGCTCAGTTTCCACCAGGTGAAGATTTGCAGACCCTCGCCGCAAAACGAATACGAAAACA
AACCCATTAAACACAAGCATATGTTCCTCCGCGTGCTTTGTTTTTGTACAAAGCTTGATCCATCACTGGC
TTGTAGTGCTGCAGCGGACACTGACTTTTCTATCTCAGCATCCAAAATCTCCTGAGCCTTGCGCCCAGTG
CATCAGATGCTCATCCACACCAAACAGAACACGCTTTTCCCTCTGTTGTCTGCTCGTGTTTTGTAGCTAT
TTTGTCTGCAATCGACTATAAACTCGTCTCGTAATTGACAAGAAAGCTGTGTTGTTGTCTAAATAGTTTT
AATTTATTTTTTGGAAAAAAGTATTTAATGTGTGTTTCATGTGATAACTGTGTGATAACTATTCTTTTGA
TTTACGTTTTTGAGTTCAGGTCAACTAATTCTAGTGTCAGATTCCCTATTTGCTATTTGAACCTGAACTT
TTCACATAACTATCAGGACGTATTGTATTTACGATGATATAATGCTTAAATTACACTaaaaaaaaaCGCA
CTTATTTCATTAACAAAAAACTATTCTGCTTTGGTAGCTTTTGTAAATAATCTTACGTCTGTCATGTGTT
TGTAAATAACAACCTATTATTAATCCTACATATATCAAAAATCATTAGAGATCGATATATCCCAAATCTT
TATATATAAAATGCAatttcatattatttattttgtaaatgttatttttttctctttttctctctcttatg
ttcttaTGTACTGAACGTAAAAATCCAATCAAAATATCAAAACTAAAAACAATTTCACATTGTATCATGT

TAATGTTGGTGGCACAAATACATATTGAACACTTAAAATCTCAACAAAACGCTAAAACATATTTGTGTGT
TTTATGATTACGCTGGCaaaatgaaaaaaaaaaaaacttaaaaaCGAATTAAAACCGAAATATCGAATAT
CTACGTCAATCGAACTCAACGCTAACTCGACAAACATCAAAACCAATCCTCTGGGCTCATCGGCTTCATT
GTTAATGCATATCTACCatatatatatatataCCAATGTGTTTGTCTCTGTGTATAAATGTCTGCTAAAA
ACGGTGCATTGGCTATGGCTATCCCCAAACAACCATGTTGTCTGAATTGTAAAAACCTATCAAGGCTTCG
ATGCCTTGGGCGACGTCCTCAAGCCGGCCTCGGCCAGCAACAACAGCAATCAAATGAACGTTGTCGCCGC
AGGTTACTCCAGCAATAGCAGTTCGATCCTAGCGTTAcaacagcagcagcaccagcagcaccagcagcaa
caccagcatcaactgaaccagcagcaacagcagcagcCACCGGCTGGCACTGGAAAGATAATTACCGGCG
ACTTGGATAGTTCACTAATGTCACTCGTTGATAACTTAAATATTAACAAAACGGCAAGTGCAAAGTGAGT
AAATCGAGCCAGTAAACTGACGATTGTGTGTGCGTGCGTTCGAAAGCGAAGCCAAACCAAACCAGAATGT
TGATCAGAATCCATGTTGTCACATCCCTTCCAGTTCATTCGCTGTAGATGGCTGAATCGTGTCGATTTTG
ATCGACTGAAGATTATGTTCCACATCCGAAGAAACAGATCCTTTGATGATCCCTTACCATTTATCCAATA
CCATTCCGAACTGACCCTCCCATATTCATGTTCCATACGTGTACATAAAATCCATCAAAAAAATGGTTCG
AAATCACGCAGAAATCTATTTAGATACCCaaaaaaaaaaaCCCAAAATTCAAATGCCGTGTTCATTGACG
TCGTCTATATATCATTACTCTCTTACCCCATTATTCTCATAGACCCGTGCAATGGAATTCACCTAAAAAT
ACAGCGAAACCAGGTGCTAATTGGACACCCCAACCAATGGCGGCTACTACTGGTGCTGGCTATCGTCCAA
TGGTGAGTTTTGCCTACTCTAACTCTTCTGTCTACCGAACTACTCTACTCAGAAACAATGAATACTACTC
GAAAGAATCTACGTTGCCTCGAATTTGTTCAAATTTAAAAAGTGACTAAAACAAAAGATTCGAATACACT
AGAATTTGTGCCGAAGCAGAGACAAAAAATAATAAGAACTATAACAATGCCAGAGAAGAAAACAAACGAC
ACATTTCTTTATTTTTGTTAATGCATTGTTTTTTCCTATCATTTTCTAACAATTTTGTGTCACCCAACAC
TTTAGGCTACACTTCATGCGAAGTACACttttttttACCTGATTTAATGTGAACGATATTATCAAAATTA
ATGTTTATTTACCAATTGCACAATCCGTATAAATTTACAACTTGAGCTACTTGGTTTCCTTGGTTTTCAT
GACATTTTTGATCTGTATGAACTATTTTTTAGCTTGCATAAATTGTCTTGATAAGATTTAAAGTAAATTT
TTATAATTAATATGTCTTCGTCGTGAGAATCCCCTTTAAAATTCTAAGTGTTTTTAAATTTCGTATATCA
TAATTGTAAATTGGATGACCAAAATCAAATCGCAAATCAAGTCTGCGGGTTGTAAGTTTGACTTTATTTT
GTCCACACATATTCTTGGCCCTAGTTTCCACACACCCCACCGTAAACTACAGAATCACAGCAAACAGCAC
TAGATCATAAGCATTAGGGCATCCTGGAGAGAAGGGCAACGACGGTGGGTTTTATGCCTCCACACACACA
AAACACGCAGGCCAAATACGTCAAGACACAtatttccattttatagttttaatttcttttattttaattt
ttaatttgcattttaACGGAGTATATATAAACTATCAACTTCTTTCTTTTCTTTATTTTATGTTTGCTTT
ATATCTCAACTATCCCAACGCATACCGCaaaatcgaaatcgaaatcgaaatcgaaaatcgaaaatcgTTA
ACCGAACCGAATCAAATTGAATCACAACTAAATATATATTGAATCCAATCAAACAAACAACAACAAACGA
ATCACGTATGACCACTCACGCTGCGCCTGTGCCAATGCtgtctgtgtgtgtgtatgtgtgtttgtgatat
atcaatatatattctatcaatatgcctatatatatatataCCAATATGCAATACGTAGGCACATGGCATG
ACTGTAAGTCCTGCGCCAATCACAATCAATCATCCGTATATACATGCTAGTTATCCTGTAATGCCAAATT
ATATGCAGGTAAGATGATGCCAAAACGAACGAAATGAAACATTACAAGAAACCATTACAATTACTCAAAC
AGAATGAAAGATTATAAATAAATATAATTTTGGTTTTGCCCTGAGTTGCCGTTTTCCTTAAATCAAGAAA
TTCTTTCAGTTTTATTTCCTATCATTTCGTTTAACAATCGAACTTCTATCATCACAGCACTATAACGTAT
AAATGTGACCTCTACGCCACTTCCTTCTAACTTGCaaaaaaaaaaaaaaaaaaCATGCCAACAATTCCCA
CTTGGTGACCAGTAACAAGCGTTTAACATGCCTCTCCTCCCTCTATGGTCTCCGTCTCTGAACTATAGTG
CATTACAATCTTGCATTCGTATCTGTTAAATTATTATTTAAATCTACATATTTAAGTTTAGTAGACATTC
AGTTCTTAATGGAGTTACTATTTCACTTGTCTGTAGAGCTAACCACCTTCCGTATGATTCCATGTAGCAA
CTGTTTTCCAAGCTCACGGCGGGCCCCCTCCTTTTAAGCCCAAACGACTAATCTTTTCCTGCCACTGATT
TTGTAGCATTGCGTCTGTCATAGCAGTTAATAAGTATTCCCTCCTCCAGTGCATGAATTTCAATTGAACA
AACATTTTATGTTCGCTCTTAACGCCTTTTTTAACGACTCTTGTAGGGGAATGCCGGTGATGGGACAGCC

AAGTATGATGGGGGGGCAGGCAGCAGCCCCTTTGACTGGGGTGCAACCGACGATGATGGCGGCGCCGCAC
AGTAATGCAACGGGCATCATGCAACCCATCCAGCCAACGCAGAATGGCAGCAATAAAGGCGTCCCACTCG
ACCCATTTG
>dme_circ_0008153_junction_seq
AATAAAGGCGTCCCACTCGACCCATTTGGGTTTCAATGGCGCATCAGTTTCCTCCG
>dme_circ_0008154
GGCTTCGATGCCTTGGGCGACGTCCTCAAGCCGGCCTCGGCCAGCAACAACAGCAATCAAATGAACGTTG
TCGCCGCAGGTTACTCCAGCAATAGCAGTTCGATCCTAGCGTTAcaacagcagcagcaccagcagcacca
gcagcaacaccagcatcaactgaaccagcagcaacagcagcagcCACCGGCTGGCACTGGAAAGATAATT
ACCGGCGACTTGGATAGTTCACTAATGTCACTCGTTGATAACTTAAATATTAACAAAACGGCAAGTGCAA
AGTGAGTAAATCGAGCCAGTAAACTGACGATTGTGTGTGCGTGCGTTCGAAAGCGAAGCCAAACCAAACC
AGAATGTTGATCAGAATCCATGTTGTCACATCCCTTCCAGTTCATTCGCTGTAGATGGCTGAATCGTGTC
GATTTTGATCGACTGAAGATTATGTTCCACATCCGAAGAAACAGATCCTTTGATGATCCCTTACCATTTA
TCCAATACCATTCCGAACTGACCCTCCCATATTCATGTTCCATACGTGTACATAAAATCCATCAAAAAAA
TGGTTCGAAATCACGCAGAAATCTATTTAGATACCCaaaaaaaaaaaCCCAAAATTCAAATGCCGTGTTC
ATTGACGTCGTCTATATATCATTACTCTCTTACCCCATTATTCTCATAGACCCGTGCAATGGAATTCACC
TAAAAATACAGCGAAACCAGGTGCTAATTGGACACCCCAACCAATGGCGGCTACTACTGGTGCTGGCTAT
CGTCCAATG
>dme_circ_0008154_junction_seq
TACTACTGGTGCTGGCTATCGTCCAATGGGCTTCGATGCCTTGGGCGACGTCCTCA
>dme_circ_0008155
GGCTTCGATGCCTTGGGCGACGTCCTCAAGCCGGCCTCGGCCAGCAACAACAGCAATCAAATGAACGTTG
TCGCCGCAGGTTACTCCAGCAATAGCAGTTCGATCCTAGCGTTAcaacagcagcagcaccagcagcacca
gcagcaacaccagcatcaactgaaccagcagcaacagcagcagcCACCGGCTGGCACTGGAAAGATAATT
ACCGGCGACTTGGATAGTTCACTAATGTCACTCGTTGATAACTTAAATATTAACAAAACGGCAAGTGCAA
AGTGAGTAAATCGAGCCAGTAAACTGACGATTGTGTGTGCGTGCGTTCGAAAGCGAAGCCAAACCAAACC
AGAATGTTGATCAGAATCCATGTTGTCACATCCCTTCCAGTTCATTCGCTGTAGATGGCTGAATCGTGTC
GATTTTGATCGACTGAAGATTATGTTCCACATCCGAAGAAACAGATCCTTTGATGATCCCTTACCATTTA
TCCAATACCATTCCGAACTGACCCTCCCATATTCATGTTCCATACGTGTACATAAAATCCATCAAAAAAA
TGGTTCGAAATCACGCAGAAATCTATTTAGATACCCaaaaaaaaaaaCCCAAAATTCAAATGCCGTGTTC
ATTGACGTCGTCTATATATCATTACTCTCTTACCCCATTATTCTCATAGACCCGTGCAATGGAATTCACC
TAAAAATACAGCGAAACCAGGTGCTAATTGGACACCCCAACCAATGGCGGCTACTACTGGTGCTGGCTAT
CGTCCAATGGTGAGTTTTGCCTACTCTAACTCTTCTGTCTACCGAACTACTCTACTCAGAAACAATGAAT
ACTACTCGAAAGAATCTACGTTGCCTCGAATTTGTTCAAATTTAAAAAGTGACTAAAACAAAAGATTCGA
ATACACTAGAATTTGTGCCGAAGCAGAGACAAAAAATAATAAGAACTATAACAATGCCAGAGAAGAAAAC
AAACGACACATTTCTTTATTTTTGTTAATGCATTGTTTTTTCCTATCATTTTCTAACAATTTTGTGTCAC
CCAACACTTTAGGCTACACTTCATGCGAAGTACACttttttttACCTGATTTAATGTGAACGATATTATC
AAAATTAATGTTTATTTACCAATTGCACAATCCGTATAAATTTACAACTTGAGCTACTTGGTTTCCTTGG
TTTTCATGACATTTTTGATCTGTATGAACTATTTTTTAGCTTGCATAAATTGTCTTGATAAGATTTAAAG
TAAATTTTTATAATTAATATGTCTTCGTCGTGAGAATCCCCTTTAAAATTCTAAGTGTTTTTAAATTTCG
TATATCATAATTGTAAATTGGATGACCAAAATCAAATCGCAAATCAAGTCTGCGGGTTGTAAGTTTGACT
TTATTTTGTCCACACATATTCTTGGCCCTAGTTTCCACACACCCCACCGTAAACTACAGAATCACAGCAA
ACAGCACTAGATCATAAGCATTAGGGCATCCTGGAGAGAAGGGCAACGACGGTGGGTTTTATGCCTCCAC
ACACACAAAACACGCAGGCCAAATACGTCAAGACACAtatttccattttatagttttaatttcttttatt

ttaatttttaatttgcattttaACGGAGTATATATAAACTATCAACTTCTTTCTTTTCTTTATTTTATGT
TTGCTTTATATCTCAACTATCCCAACGCATACCGCaaaatcgaaatcgaaatcgaaatcgaaaatcgaaa
atcgTTAACCGAACCGAATCAAATTGAATCACAACTAAATATATATTGAATCCAATCAAACAAACAACAA
CAAACGAATCACGTATGACCACTCACGCTGCGCCTGTGCCAATGCtgtctgtgtgtgtgtatgtgtgttt
gtgatatatcaatatatattctatcaatatgcctatatatatatataCCAATATGCAATACGTAGGCACA
TGGCATGACTGTAAGTCCTGCGCCAATCACAATCAATCATCCGTATATACATGCTAGTTATCCTGTAATG
CCAAATTATATGCAG
>dme_circ_0008155_junction_seq
TTATCCTGTAATGCCAAATTATATGCAGGGCTTCGATGCCTTGGGCGACGTCCTCA
>dme_circ_0008156
GGCACATGGCATGACTGTAAGTCCTGCGCCAATCACAATCAATCATCCGTATATACATGCTAGTTATCCT
GTAATGCCAAATTATATGCAGGTAAGATGATGCCAAAACGAACGAAATGAAACATTACAAGAAACCATTA
CAATTACTCAAACAGAATGAAAGATTATAAATAAATATAATTTTGGTTTTGCCCTGAGTTGCCGTTTTCC
TTAAATCAAGAAATTCTTTCAGTTTTATTTCCTATCATTTCGTTTAACAATCGAACTTCTATCATCACAG
CACTATAACGTATAAATGTGACCTCTACGCCACTTCCTTCTAACTTGCaaaaaaaaaaaaaaaaaaCATG
CCAACAATTCCCACTTGGTGACCAGTAACAAGCGTTTAACATGCCTCTCCTCCCTCTATGGTCTCCGTCT
CTGAACTATAGTGCATTACAATCTTGCATTCGTATCTGTTAAATTATTATTTAAATCTACATATTTAAGT
TTAGTAGACATTCAGTTCTTAATGGAGTTACTATTTCACTTGTCTGTAGAGCTAACCACCTTCCGTATGA
TTCCATGTAGCAACTGTTTTCCAAGCTCACGGCGGGCCCCCTCCTTTTAAGCCCAAACGACTAATCTTTT
CCTGCCACTGATTTTGTAGCATTGCGTCTGTCATAGCAGTTAATAAGTATTCCCTCCTCCAGTGCATGAA
TTTCAATTGAACAAACATTTTATGTTCGCTCTTAACGCCTTTTTTAACGACTCTTGTAGGGGAATGCCGG
TGATGGGACAGCCAAGTATGATGGGGGGGCAGGCAGCAGCCCCTTTGACTGGGGTGCAACCGACGATGAT
GGCGGCGCCGCACAGTAATGCAACGGGCATCATGCAACCCATCCAGCCAACGCAGAATGGCAGCAATAAA
GGCGTCCCACTCGACCCATTTG
>dme_circ_0008156_junction_seq
AATAAAGGCGTCCCACTCGACCCATTTGGGCACATGGCATGACTGTAAGTCCTGCG
>dme_circ_0008157
GCGATCATCACGGCGGGGGCATGGGTGTTTCCCGAGCTGACCTTGGGCATTATGCTGGTGTCCATGACTC
GGAGTCCGCGAATTCCGTGGACCCGCAACTCGTGGTTGACCACCGCCATGGGGTCGTGGCTGGGACCCAT
TTTGCAGGAGCCAGCCTG
>dme_circ_0008157_junction_seq
TGGGACCCATTTTGCAGGAGCCAGCCTGGCGATCATCACGGCGGGGGCATGGGTGT
>dme_circ_0008158
ACGACAATTGGGGCCTGGGACTGGCCATTGCCGCCAACTTGCATGTGCCGTATCGCATCGAAATTGGCAG
GACAATACGTGACCTGCATTCGCAGGATAAGCCGGGCAAAGGTGAGTATCGCATGTGCTGGTCACTGGGC
CTGCTGCACTGAGCCACAAGCAACTCTTAAAATTGGAGCTTAAGAGTGTTGCCATCTGAGAGCAGAAATC
TATTATTGAAAATTATATCCATATTTAATGTTCTTGATAAAGTTTGCAATATAAAAGATAGTATGGCATT
TTAGAAGTCCTAGTTTTAAATTCCTAGATGAATTAATTGACTTGAAAAGATGCCAAGTCACAAATGATGA
TAATGATAATGATAACACGAGTTATATTTCTTTGAAAGAGCAATCTTTGATCCTAATTGTATTAGGATCT
GCGTAGCAGTTACAATATTTATACTTGTGTTGTTCCCGTGCTGAAAACTCATTGCATTCGGTTTCGATTT
TCAGCAATTATTATCACAGCCCCGCTGAACGATGCGAGCACCATCCTCCTGTTGTCCACCATCGAGTTGG
ACTGGACGGAGCAGGCGAGGAAAGACGGTCAGGGTTCAAGGCCCGGGAGGCAGCACACCAAAATCCTGCT
CATCGACATGGCGGCCAGCTCATTGGACACTCAGCATGGGTTCTACAAATATTTGTCTCGGATGGGCGGA
TCACAGAGTGACGCCTTCGCACGCAACACGGGTTCTCCAGCCGAAGTGGTCAGCAGCAATCTTTTGGTGC

TGACTCTGCTAAACGACAGACATCGACTATTTCTGAATGCGGCCGGACTGATGGCAACAATGCAGAATTT
TCGCGCGCCCCTCAACGGCAACTACAAGGATCCACCACCCTCCGGGCATCGCAATGCCAGCCTGCACAAG
CTGTACCCGTACCGCAATCTCCTCCCGCTCTACGATACGATTGTGACCACCACTACCCAATCGGACACTT
CAGAAACCAGTCCCCAATTTGATTTTGTGGTGCTGGACATCGTTAGGGACGCCCCGGTCGCCACTTACAA
GTGGAGACCACTGCTTATTCTGGAGAGCGACCCAGGAAGTCGAGGAGGTGGGTCGGTGGTGGAAGGGGGT
GGCTCCTACATCTCCCACTCCATACACCCTGGATACGACGAGTGGTTGCTGGTCAGCAGTGTCCTCCTGT
GGCAATGTGGTGCCATCTGCTGGACCATCGCGGCGATCTGTGTTTGCCTGTTGGTCATCATGATAGCGGG
CATAGTAGCCGGGGGAATCGCCATGAGG
>dme_circ_0008158_junction_seq
CATAGTAGCCGGGGGAATCGCCATGAGGACGACAATTGGGGCCTGGGACTGGCCAT
>dme_circ_0008159
GTAACTGCATCTGCTTATCAGATCTGTTCAGAGCCGACCCTCCGTCACACCTTTGTGTTTCCCAGTAATT
CTGCCTGGCGTTGCCGTGGCTCCTCGTTCGGATCGGCTTTCCGCTGCCTTCCACTGGATGACGACGGGTT
ATCCGGCGGTCGACGCACGGTCATGCACCCCCGATCCGTCGCCCCCACCACCCCGCGGATTCTGGTCTCG
ACCGGAAGCCGTATTGGGCGGGGACGGGCGGCGGTCCGGTGCTGAAGCCGGCGACAGTTGCCCGAGTCAG
CCACTTTCAAAATTTGTTGGTTAAGTAACTTAGTAGCTTAGCTTCGGATTTTCG
>dme_circ_0008159_junction_seq
AACTTAGTAGCTTAGCTTCGGATTTTCGGTAACTGCATCTGCTTATCAGATCTGTT
>dme_circ_0008160
CCTGCACTTGGGATTGTGTGACGATGTTCCTTCCGCCGAGGCAGACACAGTGGTAACTGAAAGTGAAGAG
CACTACACCGCATCCGATGACTTCGACTATTAGGTGTTACGAACAAATCCCCATTAAG
>dme_circ_0008160_junction_seq
TAGGTGTTACGAACAAATCCCCATTAAGCCTGCACTTGGGATTGTGTGACGATGTT
>dme_circ_0008161
AATGGTCGACTTTAAGGTCCAACAACGACGATACAGGACCAGCGAAAAGACTGTCGTCAGCACCACCTAC
GGACCCATCAAGGGTGTAAAGAGGAAGTCCATCTACGGCCAGTCCTACTTCAGCTTCGAGCGGATCCCCT
TCGCCAAACCACCGGTGGGCGAGCTACGCTACAAGGCTCCCCAGCCCCCGGAGGTCTGGACGGAGGTCAG
GAGCTGCACCTCCCAGGGTCCCAAGCCACTGCAGAAGCACTTCGTGTTCGAGATGACCGATGGCTCCGAG
GACTGCCTCTACCTCAATGTCTACACAAAGAATGGTGAGTGCATAGTAGTAAGCTATCTTTTTTCCTTCA
CGGACAAAACAGCCTTTTTAAGCGTATCTGTGATCTATTCTTCAGTTGCTTTTTATCGAAAAGTGCAATC
AAATTTTATATTATTTTGCTGGCTATCACACTGCATCACTATGGTATATTTAATATATTATTTACTTCAC
AGTTTGTATCCCACCAAACCAATGCCCGTGATGGTCTGGATCTATGGCGGTGGCTTCCAGTTTGGCGAGG
CCTCACGGGAATGTTACAGTCCGGATTATTTGCTGCGTGAGGATGTGGTGGTCATTTCTATCAACTACAG
GTTGGGACCACTGGGTACGAATGATGACACGTGGaaaaaaaaaCATATATTTAACATAAGCCTTCCAGGA
TTTCTGTGCCTGGACGATCCCGAACTCGATGTGCCCGGCAATGCGGGACTCAAGGATCAAGTGTTGGCCC
TGCGCTGGGTCAAGGCCAACTGTTCGCGTTTCGGAGGCGATTCCGCGAATATAACAATTTTTGGGGACAG
TGCGGGAAGTGCCTCGGTTCACTACATGATGATCACAGAGCAGACGCATGGACTTTTCCACAAGGCCATC
TGCATGTCGGGTAATACGCTTTCCCCCTGGGCAGTGACTCCTCAGAGAAACTGGCCATATCGGTTGGCTG
TCCAGGCGGGTTACGCAGGTGAAAACAACACCCGCGATGTCTGGGAGTTCCTGAAGAACGCCAAGGGATC
CGAAATCATCAAAGCCAATGGAGAACTCTGCATTGACGAGGAGAAGAAGGAGCGGATTGGATTCTCTTTT
GGGCCCGTAATCGAACCGTATGTAACCAGCCACTGTGTGGTGCCGAAGAAACCCATCGAAATGATGAGAA
CCGCCTGGAGCAATAATATCCCACTTATCATCGGAGGCGTTTCCAACGAGGGGCTTCTCTTGTACTCAGA
GACCAAGACGAATCCGAAGTGTCTAAACGAGTTGGACGACTGCCGGTTTGTGGTGCCTATCGAATTGAAT
ATGGACAGGGAAAGTGCCCTGTGCCGGGAGTACGGCGATCAGCTGAGGCAGTGCTACTACGGCGATAAGA

CGCCCAGTCTGGACACCCTGCATGAATACCTTCAGG
>dme_circ_0008161_junction_seq
CTGGACACCCTGCATGAATACCTTCAGGAATGGTCGACTTTAAGGTCCAACAACGA
>dme_circ_0008162
GCTCCCCAGCCCCCGGAGGTCTGGACGGAGGTCAGGAGCTGCACCTCCCAGGGTCCCAAGCCACTGCAGA
AGCACTTCGTGTTCGAGATGACCGATGGCTCCGAGGACTGCCTCTACCTCAATGTCTACACAAAGAATGG
TGAGTGCATAGTAGTAAGCTATCTTTTTTCCTTCACGGACAAAACAGCCTTTTTAAGCGTATCTGTGATC
TATTCTTCAGTTGCTTTTTATCGAAAAGTGCAATCAAATTTTATATTATTTTGCTGGCTATCACACTGCA
TCACTATGGTATATTTAATATATTATTTACTTCACAGTTTGTATCCCACCAAACCAATGCCCGTGATGGT
CTGGATCTATGGCGGTGGCTTCCAGTTTGGCGAGGCCTCACGGGAATGTTACAGTCCGGATTATTTGCTG
CGTGAGGATGTGGTGGTCATTTCTATCAACTACAGG
>dme_circ_0008162_junction_seq
TGTGGTGGTCATTTCTATCAACTACAGGGCTCCCCAGCCCCCGGAGGTCTGGACGG
>dme_circ_0008163
TCCTACTTCAGCTTCGAGCGGATCCCCTTCGCCAAACCACCGGTGGGCGAGCTACGCTACAAGGCTCCCC
AGCCCCCGGAGGTCTGGACGGAGGTCAGGAGCTGCACCTCCCAGGGTCCCAAGCCACTGCAGAAGCACTT
CG
>dme_circ_0008163_junction_seq
GGTCCCAAGCCACTGCAGAAGCACTTCGTCCTACTTCAGCTTCGAGCGGATCCCCT
>dme_circ_0008164
ATGTCAAGCGGTGCACACATGTTCGCGCCAAGCCCTGCCAGGTCAACATCGTTCTGAAGCAGGTGCAAGG
CAGCGAGGACTGTCTGTACCTCAATGTCTACACCAGGGAGGTGAGTCAAAGAACCAAACAACAGGTGCAC
GAGGAAATTTTGATCGATTTATTATTGACCGCCTTTTGACTTAAATAAATCATAAGAAGTATTTTACTAT
TTGTTATAAATTATATTTTTTGAATGTTTAATGTAAACTTAAAACGATGATTGTTTTCTCTTATGTAGAA
AGCTAACAAATATATATTCTTATACCAGATTTGATATGAAAACCATAGGTGAAATGGTATTTAGTGAGTC
CACGATCAAATTGTACTCTTAAATACAATTAAGCAACGGTTTACATCATATGCAATTTTCTGATATATAT
TTTAATGCTGGGAGCAAATTAATGTCTTGTTATATAATTTACGACATTTTAATAGACCTGATGATAAAGA
ATTATTGCACAACTCGTATAGTTATTATTAAATACGGGTTTAAGAGTTTTGTTTTCTTCTAAAAAGTATA
CATTTGTCTGGTATATAATATGGATTATTACAttttttttttttAAGTTTACATCCACACAGACCTTTG
CCGGTTCTGGTTTGGATCTATGGCGGTGGATTCCAAATGGGAGAAGCATCGCGGGATCTGTACAGCCCGG
ACTACATTATGATGGAACATGTCGTACTGGTCGTAATATCCTATCGGTTAGGAGCCCTGGGATTCCTTAG
TCTTGCAGACGAAGAACTGGATGTTCCTGGAAACGCCGGACTAAAGGATCAAGTTATGGCCTTGCGGTGG
GTCAAACGGAATTGCCAGTTCTTTGGTGGCGATCCCGATAATATAACCGTTTTTGGTGAGAG
>dme_circ_0008164_junction_seq
CCGATAATATAACCGTTTTTGGTGAGAGATGTCAAGCGGTGCACACATGTTCGCGC
>dme_circ_0008165
TGGCAGTCCATCTACGGCAACAACTACTACAGCTTCGAGGGCATCCCGTTCGCCAAGCCGCCGGTGGGCG
AGCTCCGCTTCAAGGCGCCCGTGGAGCCAGAGCACTGGTCAGATGTCAAGCGGTGCACACATGTTCGCGC
CAAGCCCTGCCAGGTCAACATCGTTCTGAAGCAGGTGCAAGGCAGCGAGGACTGTCTGTACCTCAATGTC
TACACCAGGGAGGGTGAGTCAAAGAACCAAACAACAGGTGCACGAGGAAATTTTGATCGATTTATTATTG
ACCGCCTTTTGACTTAAATAAATCATAAGAAGTATTTTACTATTTGTTATAAATTATATTTTTTGAATGT
TTAATGTAAACTTAAAACGATGATTGTTTTCTCTTATGTAGAAAGCTAACAAATATATATTCTTATACCA
GATTTGATATGAAAACCATAGGTGAAATGGTATTTAGTGAGTCCACGATCAAATTGTACTCTTAAATACA
ATTAAGCAACGGTTTACATCATATGCAATTTTCTGATATATATTTTAATGCTGGGAGCAAATTAATGTCT

TGTTATATAATTTACGACATTTTAATAGACCTGATGATAAAGAATTATTGCACAACTCGTATAGTTATTA
TTAAATACGGGTTTAAGAGTTTTGTTTTCTTCTAAAAAGTATACATTTGTCTGGTATATAATATGGATTA
TTACAtttttttttttttAAGTTACATCCACACAGACCTTTGCCGGTTCTGGTTTGGATCTATGGCGGTG
GATTCCAAATGGGAGAAGCATCGCGGGATCTGTACAGCCCGGACTACATTATGATGGAACATGTCGTACT
GG
>dme_circ_0008165_junction_seq
TACATTATGATGGAACATGTCGTACTGGTGGCAGTCCATCTACGGCAACAACTACT
>dme_circ_0008166
GTGAAGGGGGTGAAGTGGCAGTCCATCTACGGCAACAACTACTACAGCTTCGAGGGCATCCCGTTCGCCA
AGCCGCCGGTGGGCGAGCTCCGCTTCAAGGCGCCCGTGGAGCCAGAGCACTGGTCAGATGTCAAGCGGTG
CACACATGTTCGCGCCAAGCCCTGCCAGGTCAACATCGTTCTGAAGCAGGTGCAAGGCAGCGAGGACTGT
CTGTACCTCAATGTCTACACCAGGGAGGGTGAGTCAAAGAACCAAACAACAGGTGCACGAGGAAATTTTG
ATCGATTTATTATTGACCGCCTTTTGACTTAAATAAATCATAAGAAGTATTTTACTATTTGTTATAAATT
ATATTTTTTGAATGTTTAATGTAAACTTAAAACGATGATTGTTTTCTCTTATGTAGAAAGCTAACAAATA
TATATTCTTATACCAGATTTGATATGAAAACCATAGGTGAAATGGTATTTAGTGAGTCCACGATCAAATT
GTACTCTTAAATACAATTAAGCAACGGTTTACATCATATGCAATTTTCTGATATATATTTTAATGCTGGG
AGCAAATTAATGTCTTGTTATATAATTTACGACATTTTAATAGACCTGATGATAAAGAATTATTGCACAA
CTCGTATAGTTATTATTAAATACGGGTTTAAGAGTTTTGTTTTCTTCTAAAAAGTATACATTTGTCTGGT
ATATAATATGGATTATTACAtttttttttttttAAGTTACATCCACACAGACCTTTGCCGGTTCTGGTTT
GGATCTATGGCGGTGGATTCCAAATGGGAGAAGCATCGCGGGATCTGTACAGCCCGGACTACATTATGAT
GGAACATGTCG
>dme_circ_0008166_junction_seq
CCGGACTACATTATGATGGAACATGTCGGTGAAGGGGGTGAAGTGGCAGTCCATCT
>dme_circ_0008167
GTTGTTTTCCAATATTGAGACACATCTGCGGACCTTTCTCATCTAGTGCCTCCCATGTGGTCGGGCCGAT
CTGTGGACAATTGGGATTGTCGTTTCGGGCGAATGCCACCCACATGCCCACCAGTCTCTGTATGGTTTGG
TACTCCATCGAGGACTTATCCACCTTGTTGGCCAAAATGTGATAGAAAAGGTAGGAGAGATCGTCGGCAT
GACTAACTCCGCGAACGTGTTTTCCGCACATCACCTGCCGGAAGTGATTGAAATGGGGCGAGTCCACATC
GAAGCGGTACAG
>dme_circ_0008167_junction_seq
GGGGCGAGTCCACATCGAAGCGGTACAGGTTGTTTTCCAATATTGAGACACATCTG
>dme_circ_0008168
GAACAGACCTTCAAAGGAGTTGCCGCCCAGGATCAGCGGCAGGTCATTACCCCAAGCTTCCGGCAGCATC
TCCCTGTGGCAACGGGGCAAAACGCAATCCCTGGTGATATATGGTTCCACAACGGGAGTAAAGGGAAACA
GGACGTACTGCCGCCGTTCCTCTTGGGAGACAAGAGTAATACCCTGTGCGGCCATTTCGGAGGCGG
>dme_circ_0008168_junction_seq
ATACCCTGTGCGGCCATTTCGGAGGCGGGAACAGACCTTCAAAGGAGTTGCCGCCC
>dme_circ_0008169
GGCAATCCTCGCTGCCTTCGACGATGCTGAGCACGAAGTGCTTTTGCATCGGCTTGGCCCGCGGATAGGT
GCAATCCCGCACCCCCAACCAGGGATGTGGTGGCTGGGGGGCTCGGAAGCGCAGCTCCCCCACCGGCGGC
TGTGCAAAGGGGATTCCCTCGAAGGCGAAGTACATCTCCTCGTC
>dme_circ_0008169_junction_seq
CCTCGAAGGCGAAGTACATCTCCTCGTCGGCAATCCTCGCTGCCTTCGACGATGCT
>dme_circ_0008170

CATAATCCGAGATGGAAGGACGCCAACCGGAGCGCCAGAAGCAGTCGGCCAGCGTCTTGTGCTCCTCCTC
AGAATCCTGCTCATCGCTGAGTACGTGAACGTCTACGTGGAGAATGTCCGCTTGCATGACAATTGTAGCG
TGCCGGCATTCAGAAAACGCAGCAGCAACAGCAGACGACGCGGCTGAGCGAGCAGTACGTGAAGAGATTC
TCAAGAACGAGCGGGAGTAGGACGAAAAGGACGCGCTGCGGCAGGAGCAAGGACATCCCACATCCAGCAT
CCAACGCACATGCACGACAGTAGGGCTTAGCACCTGCACTCGAGCCGCAGACAGCAGAAGAGCCAACGCA
CGGAATAAGGTAAGTGCCAGCTGAATAAAAATGCCCTTCTTCCACCGAAAAGAATACGGTTACAGGGGAG
AAAAGCGACAGGGATCACGTGCACTGCTGCTTTTCAGCTTATAAGACCGTGAAGCACCTTTCTCAAGTCA
AAAGTAGGAGAGAAAAAAATGTAACCAATTACTCCTCGCTGCTCCTCGTAGAAGAATCGATATCCAGAAA
TGAAGAAATGAAGTTATAAGTAAATATTTCTGAAACCTAAGAACAAAAAAATATGAATTTTATTGAATTG
TAAATGTAACAAATTTTTTACACTTTTAACTATATAAAATATATAGAATAGACTCTTAAACCGATCCGTT
AATAAGCCTTATGCCAGTTTGGTTAATTGACTGCCCACATCCTTCGTTTAAGTTCTTGAATTGTTGATCC
AATGACCAAATGTCAGTCAGGCAATGAGAAGGCTACAGCTTTGTTCCCTGCACCAATAACCTCCACCACC
ACCAACTTCCTAGTCATTCCACGTAGGCATTCTCCTCGTGTTGTCAGGAGTCTCGGTTCTGCCTGCCTGC
CTGCGAAACTGACATGTCTGGCATATGCAAAATGCCAACTGTCCTGGCCACCCAGCCCAGACCACTTCCC
TGCCACGCCACACACAAAACCCCTTCGTTTGGCTGTTGTTTTTGTCAACTACAACAAGGGCAAGGACACT
ACACCAAGTGCAAACAAGGTGATGCTCCAGCTGGCAAACATACCAAAAAACAAGACGAATACGCAGTGGG
CGGGGGATTCTTACCCACCACAAAAAAAGGGCAGAGTGTAAAGAAAGAAATGGTTCAAGGAACACACTTC
ACTGAATTGCCTTGAAGATACCACAAGAGCGGAAGAGAAGAACCGAGAATATAGGGCTAACGAATAGTTA
AATCCGATGTAGCTATCGGATGTGAACTTATAAATAGATATTTATACATATATATTAAGTCCCATTTAAA
TAGCACATCTCTTCATTTATTCTAGAATGAATGAATTGGTGCTCGTGACTTTGTGAATCAATAAACATTT
TCTGAAAGGCAGTAAATTCCATCTAATATTTGTAATATCCCCAAGCGTATAAAATTAAACCAATAACttt
ttttttCATTCCAGTTTCTCTGAAACTGCCATATATTACTTTGCACAAACCCGAAAGAGAAAATTAAAAA
CGGGAAGCAAAAAGTGCGAATACTTGCTGAAACAAGCAGAGCGATAACCAGGCCACGCCACAAACAGCTA
CTTGTGGTCAATTATACAGGAGGGCAAAAAGGGATAACACAGCAATCCGGACATCAAGTTATCGAAAAAA
CAACGACAACATTGCGACGTGGCCATTTTGACCAATAATAATAATGGTTATGCTGCTCTCGAAGCGACAA
CTCCCGCCATCAGCAGCAACGAGAACGCGGCGGAAAGTACCGCCCACGAGGAGGAGGTCGACGTGGGCGT
TGTCATCTCTGCTGGCTCTGGTCGTCCTGGATATATGTATTGCCCGATGCCTGGCTGCGGGCATTAGTTA
TAGCAGCAACAGCGGCAACCTGAGCACTTCGCAAAAGAGTCCCTCGTCGGCGAATAATGGCGGGCTCGTG
CTGATCGGTTCCAGCAGCGCCACTTCCAGTTCCGTGCCCTCATTATCCCTCACTTCATCATCGTCGGCGG
GCTTGTCGTCCCCCGGCAGCAGCTCATCCTCCTCCTCATCTTCCACGAGCACCAGCGGATCCAATAGCAA
CCGCAGTAGTAGACCCACTAGCAATAACAGCAACTCGAACTCGAACTACAGCCCGAACCATGATCAATTA
CCCGCCCAGCTCTCGTCGCGGATTATCAACACACGCAATGGCGCCATCTCAGGCGTAATTGTCCAATTAG
ATGGAAGGCACTTGGATCCGGTGGAGGCGTACCGGGGAATACCCTACGCCTCGCCGCCGGTCGGCAATTT
GAGATTCATGCCGCCCGTCTCCGCGGCCATGTGGTCAGGTGTCAAAAAGGCGGACAGGGTGAGTCGGCTC
TATGCATTTTAGCGCGGGGTAACAGGAGCCATTAAACTGGATTAGCCCAAATTATTTCCGTAGACCGCAG
AAACTGTTTTAAAATATTTATTCAAAGGACTTAGAGAAGCGCAATCATATTGTATTTACATATTGTATAA
TAGTTCCTTATAGCAATTTGTAATTTGGCCACTATAAAACGAAATCTATCTTTATTATGCAACTACCTAT
ATTGTATAGCTTTTTGAGAAATTTTTACATTTGATTTGGTTAAATTGGTCAATATCATGACAACTTTATA
CAGTAATTAATCAAGGAAGAAGACATTTCAAGCAAGCTTCAAAATTGGTCCATCAAATCCTATTAGGGAA
AGCCCGATTCCTCCTCGTCCAAGTAATTTAATAAATTTCCCGAGAAGTCTGCCGACTTCCATCGTCTGAC
AAGAAGCTTTGTCCGTCTTAAAGCACTAAATCATCTCCCCAAAGGGTTGAGTTTGATTTGCCTTTCGAGT
GCATAAAATGATCAAAATGCCATAAGTCAAGTGCGGAAATCGCCAAGTCTCTCAAGAGGGCCCAACAATT
GTCACCTCAATTAACTGACTCATTATTGAGTCAAGTGCGCAGGCGGCGGTCCGAAAATACTGAAAGCCAA
GTGTAACTGAAAGTACTGAATATAATGGAAAATGCTACAACCCGTGCAACCCGGGCCGGCGACAACAATG

GACATAACGATAATGGCTTCTGAAAGTAAGGAATGAAAAAATGGGGAAAACACAAGTCAAAGGTCAACTT
AGTCGGCCGACTTCCTCGCGGAATATCTCGCAAATTAAATATTTACGCCGTCCCCGGCAGCAAACAGCGC
TACGGGAAAGCAGCAGCTACACAGCGGCATCTAATTTACAAATGCTCCCAAGTGGCAAATCATTATTCAC
AAAGATGCCCAGAGATATTCCCTGCTCCCTTACTTCCCATTGTTGTAATaaaaaaaaaaGGGAAGCGAAC
TGGGGCCGCCCAGGAGCAAAAGATTTTGCCCCTTTTCTCAGTCACCAGACCCATAATGCCGATGGAAAAG
GGCCGCGAAATCAGTACGGCACACAGTGCGGCCCGGCAAATAAAATATAATTTTTCAAGTTTAACAAAGA
ACAGCACCTGCGGCCCCGCAGCAGACTGATGattctcgcattctcgcatttctcgaatttcCCCGCCAAT
TTTTGGGTAATTCAAGGCCTCCTCCGTCGCATCGTCTTCGCTTTTCCGGATAGGTAAGAGCGGCTTTATT
TGTCAGCCCGACTTGTTGACAGAGGGGGTCAGCCGCAAAAGGAGCCAATAAAACGGCTTGAGTAATGGTC
GAGAGATTTTTCCAGCACGTCAGCGTAATAAAGTTGTCCACGGACAGTTGGAACAATAGGTCATTAGCCT
GATTTTGGGAAATGGGTACCGCTCAGGATACCAAGTTAATGTTGTTACGTGGCTTGGCTTGAATTTCAAT
TTCAGTTCTGTGGCTTTGCACTGGGTTATCTTTTTTTGAATCCTTGATTTCCTGATTCGAGAAGAAGGCA
GACAAACAAACTCATTTCTTGATTCATTTGTTTACATAATTTTAATGCCACGAACTAGTTTATTCACCTC
TAGCCTGCACTCCTCCtcacctgtcacctgtcacctgtcTGCCCTTTGAACTAAAGTTTGCTCTGAATTT
TTTCTCCCACACGAACTTAATGTTGCGTAGGCCACATACCTTTCCACCACAAGTTACTCGTCAACTTCCA
TCGGCCCCATTGCAAAAATAATTTGCCGCAACCACAAAAAATGGAGGCAGCGCTGAAAGTAAAAAAGAGA
GAAAATTATGTCCACACATATTCCTAGCCCGGCAAAGGCAGCAGCAGCTGTTCATGGCTTTGACAAGCCA
CGCGTGTCACCAACAAACCACCGAACCACCACCACCCGGCCACCCACTCCATCGCCCTCCACTCCCACTT
TGGCAACCTCATCCACGCATTTTCCGCCACAACGTCTTTAATAAAAATGAAATAAATTCCGCCAGCCACT
TTGCACTTTGGTGCTGTTGTTCCAACGGCTGCACATTGGTCCTGGCCGTTGCCTGTGGCGTAAGGATTAT
GAAAAACAAGCGGATTAGTGGCCAACCTCTCCTGACCAGGTTCCAGGAGCCCGGGACCCAGGGTTCCGGG
GTCACAGAGCTCCAGGTCCTGGCTCATAAACGGAGTGTAAAAATCGCAATAAACTGACGAGTTTTATGCA
TGACGCCAAAGTTTTTAATAAAGACGAAAGTAAAAGCTTGACGCAAAAACGCTAAGTGGAGAAACTCAAG
TTAACGATTTTCCTAATAAAAAAGCGCATTAATGAATGGCGGGCAGGAGGTGCGGTAAGGTCCTTCACAA
GGACCAGCAGAATGTGCGTGCAGCAGTTTGTGAGGCATCTCTTTCGCACCAACAAACAGAAGCTTGCACT
GTCATTAAAAACGTTAATTCCAATTTGAGGCTCAAGAGCAAGTGGTAACCGCCAGGATGCGGAGGGTTAA
GGGCCCAGAGCTGAGGTGGCAGATGGGCGGTGGGAAGACTTCTCCAGGACATCAGCCGCATAAATTTCCG
ACTGACTCGACGGCGGCGGCGGCGGCGGTACAAACTGTTGCCCGTTTTAAATGAAATTGCGAAAATTCAT
TTTAAATTGGATTTTATTTAATTTGACTGAGGCAAGAAAGCTTTACGCTCGAAGGAGATTTTCGGAGCAA
GGTGTGAGTTCCAGGGCCTGCGACTCACACTCAGTCTGTGTCGCCAGCCAATCTAATTTGTGTGAACGGT
GGGAGGGATGGAAAAATCAGAATGAGGACGACAACCTTCTGGCCCGTTGGCCACAGGTAAAATTAATCTA
TTTTACTTGACGTGGCCGCAATGTCGCGTATAATTTTATGCGATCCGGTGCCCTTCGTTCATCACCTGCC
CATTCCAATGAGGTGTTGAGAATTCCAAATTGCATGTATTTCGGAATAACGCAAGGGGTGGGGAGGATGA
CGATGAACCATGCCTATAACCTTGTTTACTTTTGACCGCGTGCCTTGGCTACCGAGCAGTTTCTCACTGG
CCGGGGTCACAATCTTCAAATTGTTTGCTCGAAATTCGCAGATATCTGTTTGTGGCAAATAATCTACCCT
GAAAACAGGTGCTGTACGAGAAATGAAATGCGATATTAGTTATTAGTCACCAGGCAAATATGTTTCTAAG
TGAACCAGAAATGTAACAGGCTAACAGAATTTGGCTAGATTTCTCTTAAACCAGTTAACCAAATATTTAG
TACGGCATTTCAAATTGGAAAACTTAAAACGAAGATATTAAAACTACTACCTAGCCGATAACTGAAGTCT
GCACTCGGCATTAATAAAAATAAGTCGACATAATTAAAAAGAAACACCATGTATTTAAATTATATGCATG
GCGATTGCTCAATTTGAAAGTTGACAATATACCAATATAACATATACAATGTAAATTAAATTCAACCCAT
GCCATTTAATAGAGTACGCGGCTTCGCGTTTGATAGCTCTGTTTAATTTCGAAAAGCAATCCAATGGTAT
TTTCCCACCTCTATCACGTAACATCTCAGCGAAATGGAAATAGTAAAGCATAAGATGGTTTTTAAAGTAA
ATTGCCGCACCGGGAAACATGATTATGCGCCCCCACAGCTGCCAGTGGAATCTACAAGTAATTATCAAAT
TTTTAATGCCCTGCCATTTGCAAATGCAGCATCGATGGCGAGAGGAACTCACCCTGACCTTAATTGCGGC

ACCCGCTTTATTAGCGCCACTCAACCACTCCCCTCCAGCAGTCAACATACACATATATATATAACCCACC
TGCACCACCTGTATTGCGTGCCACCACCCGTTCTTATCTGCGAAAGTTCAGCGGTTGCAAAGTGGCATAA
ACAAGATCGCTTGCCACTTGAGAAGTGTGCGCCATTCGTTTGCCAGGGGGGGCAACATTAAAAAGGCAAA
ATGTGGCTTTTATGACCTCGAACTGGGGAGGATGGAGCTGGATTTCGTTACCTGTCCCCTGGAccactgg
cagccactagcagccactggctgccactACAACTACGAGAGAGTTGCTGTAAAAACTTTTCACTTTCATT
ATTTTTAATATTTTCTGCTCATCCAAGCTGTTCCAGCTCCCGTTCATGTGGCTGCAGGGTTCCGAGGTTG
ACACCATGTCCGCTACATTAAAAAAAGGCGAAGGGAGAATGGAGACCGAAAATGAAACTTTTATTACCTG
CCTTATTGTTTTGCCATAAAATTAATGATTATTATGGCAATCGCATGGCCACCACAAGCGTCCATTAACT
GGCGACCCACTGAGGGTCACTTCCAAGTATATTCCCAATTTTCTGCGCGGCGTTTGGAATGCCATAAAAA
CCAAAAATTAAATATACTCGCCATGGTAAACAAAAAGCAGAGTGGGCGGTAATAACAAAGATAAAAGGGC
AAACGGCGAAATAAAGTTAAGGAAATTAAATAAAATAAATTCTCGTTTCGCAATGTCATTAATGTTAATG
CATTTTTGGGCAGCCAGCCGTGGGAATGGAGCAGCAACTCTCTCTTAAATGTCCCTATTGATTTTCGCTT
TATGACAGTTTGGTGGCCAACAGGAGCTGTGTCGGTTAATGAGTTAAGCCGCCTACTTTAAGGGATCCCA
GCACTCAACTGCAAATTAGGAGGAGCTCTGACGGCCACAAAGGACTGATGAATGGAATTGAAAGCTTTCA
CCAGACAAATCGTATCGGCCTTAATTCATTAGTGGCCACAGTTTCCCTTTCGTTCGATTGCCAATCCTTT
AAAAGCCTGTACTGACACAAATCTTCAACTGCCATCACGAAATCGAGCGCAATTTTAACTGGGCATCATG
CCTTTTCCGGGAATGTATGGAACACAGGTGGGCTCAACTTGGCCGTATGTGTATAACTCTTCCACACATA
GATCAGATTAAAAACAAATAATAACACTGCCAGCTAAAAGCAGATGGCAGAATAAAAAGCCTTGAACACT
GGGGAAAAATATTTTTAATTGACATATGTACTTTATAATTTACCAACTTAAGATATTTCAAGGTATAACT
GAAACAATATAGAATACATTTTATAATCAAACCAATTTTAATGTTTATTTCTATATGTAGAAATGATACA
AAGTTATTTTGAAGATGTATTATTCAACAATTTTTCAGAGTTTAATACTTATTATAAGTCATGAGACATA
CAATTAATAAAAGTTACAATTAGTTTAAGTATTGTCCTTCGATAAGCACACTTTTTGTTCCATTCTTAAT
TACCTAAATACGTATTTTTTCTATCGGTGTATTCCGAATCCCATCAGATTCATAAATGAGCGCAAGGCCA
AAAAATGAGTACGCTTCTGGCATTTGCATGGCCCGTTTGTGGGAATAGAGGGAGGAAATCAGTAATTGCA
CAAGTGCACACACAAAGGGAGATTTGCCAATGGGAAAAATCGAACGATAAGAGCCAAATCGAAAACCAAT
TTACCAAGATCGAAAATAAGCCTCGGCCAAAAGGAGAAAAATGCAGTAGCGAAGAAATGAAGAGGAGACG
AATTGACAAAAACAAATTAAAAGGCAAACTTTGCGTTCAAATCTAAAAGTTGTTATGGCCAAAAGACAAT
CACAAAGTTGACTTTGACTCGGGCTCTGTTCAAGTCATTACATGCCCCCCTGAAAAAATCCCTCAAAGAT
TACACAATTCCGGCAAATTAATCTATTTATAACGACATTCGAGGCCGGGCATAAAAATAAGCCTATTTAC
CAAAAACTTTTTTCGCTTGTATTTTTATTTTTCGGGGTTAGGAAACCTTTTTTAACAACGAGCGAAATGC
CATAATGGAAAGTTTCTCCTTTGACAAGCTGGGCACTCAAATCTTTTTCGATTTTGCAGAATTCAGCCCG
GTTTGCCCGCAGAGATTGCCGGACATTCACAACGAGACGGCGGCCCTGGAACGGATGCCCAAGGGCAGAC
TGGAGTATCTGAAGCGATTGCTGCCCTATCTGCAGAATCAATCAGAGGACTGCCTCTATCTAAATATTTA
TGTGCCCATTCAAGG
>dme_circ_0008170_junction_seq
ATCTAAATATTTATGTGCCCATTCAAGGCATAATCCGAGATGGAAGGACGCCAACC
>dme_circ_0008171
CATAATCCGAGATGGAAGGACGCCAACCGGAGCGCCAGAAGCAGTCGGCCAGCGTCTTGTGCTCCTCCTC
AGAATCCTGCTCATCGCTGAGTACGTGAACGTCTACGTGGAGAATGTCCGCTTGCATGACAATTGTAGCG
TGCCGGCATTCAGAAAACGCAGCAGCAACAGCAGACGACGCGGCTGAGCGAGCAGTACGTGAAGAGATTC
TCAAGAACGAGCGGGAGTAGGACGAAAAGGACGCGCTGCGGCAGGAGCAAGGACATCCCACATCCAGCAT
CCAACGCACATGCACGACAGTAGGGCTTAGCACCTGCACTCGAGCCGCAGACAGCAGAAGAGCCAACGCA
CGGAATAAGGTAAGTGCCAGCTGAATAAAAATGCCCTTCTTCCACCGAAAAGAATACGGTTACAGGGGAG
AAAAGCGACAGGGATCACGTGCACTGCTGCTTTTCAGCTTATAAGACCGTGAAGCACCTTTCTCAAGTCA

AAAGTAGGAGAGAAAAAAATGTAACCAATTACTCCTCGCTGCTCCTCGTAGAAGAATCGATATCCAGAAA
TGAAGAAATGAAGTTATAAGTAAATATTTCTGAAACCTAAGAACAAAAAAATATGAATTTTATTGAATTG
TAAATGTAACAAATTTTTTACACTTTTAACTATATAAAATATATAGAATAGACTCTTAAACCGATCCGTT
AATAAGCCTTATGCCAGTTTGGTTAATTGACTGCCCACATCCTTCGTTTAAGTTCTTGAATTGTTGATCC
AATGACCAAATGTCAGTCAGGCAATGAGAAGGCTACAGCTTTGTTCCCTGCACCAATAACCTCCACCACC
ACCAACTTCCTAGTCATTCCACGTAGGCATTCTCCTCGTGTTGTCAGGAGTCTCGGTTCTGCCTGCCTGC
CTGCGAAACTGACATGTCTGGCATATGCAAAATGCCAACTGTCCTGGCCACCCAGCCCAGACCACTTCCC
TGCCACGCCACACACAAAACCCCTTCGTTTGGCTGTTGTTTTTGTCAACTACAACAAGGGCAAGGACACT
ACACCAAGTGCAAACAAGGTGATGCTCCAGCTGGCAAACATACCAAAAAACAAGACGAATACGCAGTGGG
CGGGGGATTCTTACCCACCACAAAAAAAGGGCAGAGTGTAAAGAAAGAAATGGTTCAAGGAACACACTTC
ACTGAATTGCCTTGAAGATACCACAAGAGCGGAAGAGAAGAACCGAGAATATAGGGCTAACGAATAGTTA
AATCCGATGTAGCTATCGGATGTGAACTTATAAATAGATATTTATACATATATATTAAGTCCCATTTAAA
TAGCACATCTCTTCATTTATTCTAGAATGAATGAATTGGTGCTCGTGACTTTGTGAATCAATAAACATTT
TCTGAAAGGCAGTAAATTCCATCTAATATTTGTAATATCCCCAAGCGTATAAAATTAAACCAATAACttt
ttttttCATTCCAGTTTCTCTGAAACTGCCATATATTACTTTGCACAAACCCGAAAGAGAAAATTAAAAA
CGGGAAGCAAAAAGTGCGAATACTTGCTGAAACAAGCAGAGCGATAACCAGGCCACGCCACAAACAGCTA
CTTGTGGTCAATTATACAGGAGGGCAAAAAGGGATAACACAGCAATCCGGACATCAAGTTATCGAAAAAA
CAACGACAACATTGCGACGTGGCCATTTTGACCAATAATAATAATGGTTATGCTGCTCTCGAAGCGACAA
CTCCCGCCATCAGCAGCAACGAGAACGCGGCGGAAAGTACCGCCCACGAGGAGGAGGTCGACGTGGGCGT
TGTCATCTCTGCTGGCTCTGGTCGTCCTGGATATATGTATTGCCCGATGCCTGGCTGCGGGCATTAGTTA
TAGCAGCAACAGCGGCAACCTGAGCACTTCGCAAAAGAGTCCCTCGTCGGCGAATAATGGCGGGCTCGTG
CTGATCGGTTCCAGCAGCGCCACTTCCAGTTCCGTGCCCTCATTATCCCTCACTTCATCATCGTCGGCGG
GCTTGTCGTCCCCCGGCAGCAGCTCATCCTCCTCCTCATCTTCCACGAGCACCAGCGGATCCAATAGCAA
CCGCAGTAGTAGACCCACTAGCAATAACAGCAACTCGAACTCGAACTACAGCCCGAACCATGATCAATTA
CCCGCCCAGCTCTCGTCGCGGATTATCAACACACGCAATGGCGCCATCTCAGGCGTAATTGTCCAATTAG
ATGGAAGGCACTTGGATCCGGTGGAGGCGTACCGGGGAATACCCTACGCCTCGCCGCCGGTCGGCAATTT
GAGATTCATGCCGCCCGTCTCCGCGGCCATGTGGTCAGGTGTCAAAAAGGCGGACAGG
>dme_circ_0008171_junction_seq
GTGGTCAGGTGTCAAAAAGGCGGACAGGCATAATCCGAGATGGAAGGACGCCAACC
>dme_circ_0008172
GGACAAAAATTTTGTTGCAAATGAAATTAAATACAGAAAGAAGCTGCACAAAGGAGCAGGCTAAATACAG
CGAGTAAACAAACTGTCGAAACTATTTAAGGCGCTCAAAGAAAATCCATGTAAATACAGCGTCCAGAAAA
TGTGAGCCATTCGGTTTGACGCAACAAAGAAGGGAAACAGCGAACAGTTGGGGAAAAAGAACAATTAACC
AAACTGGAAAGCAAACACGAGCGGAATGTGAAAAGTTTGGACAGAGCGGGAAGTGTAAGACCGAAATTAA
TATCGAAATATCCCAGAAGCCGGCAGGAAAGACCGGGTCCAGGTCCCGGAGGCGGAAGACAAAGCCATGA
AGTTTCGTTTGGCAGCATTTTGGCTATTTCTGCTGACAGTCGGCGGCAATCACAAGCTATTAAGCCACGT
TTCCCCAATGGGCGTGGCAGCCGAAAAGCAGCCGTTGCACAAAGGAAATGAGCTGGAAAACGCAATGAAA
TTGCGTGAAGCCCCAAAGCAATCCCGGGTTATCGGGGACATCACGACGACAATTCAGCCGGACTCGGATC
CGGGCCGATCAATTGGCCATCAGGCGTTGCGACGTGCCAAGGCCCCGCCGCCCAGCTTGGAGCTGCATTT
CAGGCAGAATCTGAACAAGCTTTTCGCCGGCGATGAAACCACCCACGTGGGCCACACCAATGCCACTGAC
AACACCACGGCCGCAGTTTTGGCCACCGACGAAGGTTCCAGCGAACACGAGCCGTCAACCACTCATCACC
CGGAACGGCGACACGTGGTCCCCGATAAGCTGCAGTACACCCAGGAGATCCAGGTGAAGCAGGGCCGGCT
CATGGGCATCACACGTCGCTTCCAGGTGACCAGCGGGCTGCGCCAGGTGGACCAGTTCCTGGGGCTTCCA
TACGCGGAGGCGCCCACCGGAAATCGACGCTTCATGCCACCGG

>dme_circ_0008172_junction_seq
ACCGGAAATCGACGCTTCATGCCACCGGGGACAAAAATTTTGTTGCAAATGAAATT
>dme_circ_0008173
GGACAAAAATTTTGTTGCAAATGAAATTAAATACAGAAAGAAGCTGCACAAAGGAGCAGGCTAAATACAG
CGAGTAAACAAACTGTCGAAACTATTTAAGGCGCTCAAAGAAAATCCATGTAAATACAGCGTCCAGAAAA
TGTGAGCCATTCGGTTTGACGCAACAAAGAAGGGAAACAGCGAACAGTTGGGGAAAAAGAACAATTAACC
AAACTGGAAAGCAAACACGAGCGGAATGTGAAAAGTTTGGACAGAGCGGGAAGTGTAAGACCGAAATTAA
TATCGAAATATCCCAGAAGCCGGCAGGAAAGACCGGGTCCAGGTCCCGGAGGCGGAAGACAAAGCCATGA
AGTTTCGTTTGGCAGCATTTTGGCTATTTCTGCTGACAGTCGGCGGCAATCACAAGCTATTAAGCCACGT
TTCCCCAATGGGCGTGGCAGCCGAAAAGCAGCCGTTGCACAAAGGAAATGAGCTGGAAAACGCAATGAAA
TTGCGTGAAGCCCCAAAGCAATCCCGGGTTATCGGGGACATCACGACGACAATTCAGCCGGACTCGGATC
CGGGCCGATCAATTGGCCATCAGGCGTTGCGACGTGCCAAGGCCCCGCCGCCCAGCTTGGAGCTGCATTT
CAGGCAGAATCTGAACAAGCTTTTCGCCGGCGATGAAACCACCCACGTGGGCCACACCAATGCCACTGAC
AACACCACGGCCGCAGTTTTGGCCACCGACGAAGGTTCCAGCGAACACGAGCCGTCAACCACTCATCACC
CGGAACGGCGACACGTGGTCCCCGATAAGCTGCAGTACACCCAGGAGATCCAGGTGAAGCAGGGCCGGCT
CATGGGCATCACACGTCGCTTCCAGGTGACCAGCGGGCTGCGCCAGGTGGACCAGTTCCTGGGGCTTCCA
TACGCGGAGGCGCCCACCGGAAATCGACGCTTCATGCCACCGGGGTGAGTGTACAAAACTGTCCTCTAAG
AGTCTTTAACTTTATTTGATACAAGATATATACATAGATAGATAGCTTTTAAGCTAAAACAATTTCAGC
TGTTCTCAAAGACACCTCATGATGCCGATCGGCTTGGGTCTACCGTTTGGCAGATGGAACTTTCAATTTG
AAAGTATACTTTTCTTACATAAGCGAGCAATAGCATTATAAATATTTTCCCTTAATTAATTAAGATTAAA
GAAAATGCTAATGGCAAAGCCAAGTAAGCCAAGTATCATCACAATATCATTGACAAGGGCGACCAAAACC
AATAAATATTATCCCCTGAGTCCGTTTTTGACATTGATTGCAAAATGCTTATTTAAAATATACTGTGTT
AAAGTAAATCGATTTATTTGCATACATCAAGTCTTCGGAAGGGCTAAAATAATTCAATAAAAGTTTGTTA
AGTTGAACGGGCAATATTCTATAATATAAATAACGATATCCTCTGTTTGAACCAACCTGCTGGCAGGGAG
TACCACCTATTTATTTCCCCTTGCCCGACCCGCCGTAGGCAGGCAGCACTATTTGTGACCGCAAATATGA
TGGCAAATTGCCACTTTGCAGCACCAACAGGCAAACCCGAAAACCTCGAAACCCCAAAAACCCCAGAACT
CAAATAGGAGCAGGAGCAGGGGTGTCTTCGGAAGGACCGACCACTCCTCGAACAACCGACATGTGCCGCC
GCACTTCCCCTGCCGACCATGCCAGAACATCCTGTTGGTCCTGGGGACGCTGCTGGGGCACTTAGAATTT
AGCCAGCAATTTAAGCAATTATCGTTGAAGCTGGCCCGGCATTAGGCTTTCAGGACTTGACATGTGCCCT
CTCTAACAGCGATCTGGCAGGATGTTGGTTCCGAAAGCACGTCCCtgtgtgtgtgtgtgtgCTTCCTGTC
TCGAATGGCTATTATTATTGCAGCTTGTTTTTACAAGGATTGCAATGTAAATCCCAGGCGTCCCCGCCCC
TTCGCCCTTTTATTTTGCCATCTGCTGTGCGATTATGTTTCGGACTTTGTACATGAATATTTTGCTTGTT
TAATTAAGAGTGAAAATTGCTGGTATTATATCTATTTAAAGTTTGTTTATCAAAATATGAGCTACGTTGC
CCGTGCCAATTTGTCTGTGAGATTTGAAGCCGCCAAGCTGCCAACACTTCAGCCAACTTCGGGCCTGCAT
CCTGCTGTTCTGGGCACAAACAAGCCGCAGCTTCAATTGTTGCCACCACAACTAGGTCCCTTCCACTTGG
TCATCCGGTTTCATTGGCTATGCACCAAGAATATAATTTATTTTCCATATAATTTTACAAGCTACTACTC
ATATCTAATTAGTGTACCCAAACTAAGAAATACCGTCATTTATTTGAATAGGAAACTAAAACTCTAAAAC
TGGTAGATTAGTCAAAACAAGTTGAGGTGTCCAAAAAATGTTTTCAGTGTAGGCGGCATAACACGCCTTG
GTCCTCCGAATTGCATTACGTGAAGACAATAAATTAACACGCCCCAGAGGCTGTCGCAAGTGTTGGCCGC
GATTTGCGTGTCAGATGTGCTGCTTATGTTGTAGTTTCTAGTTGCAGTTTCCAGAGGCAGTTTTCAGCAT
TCAGTTTGCACTTCGCCGTTTGTCGCGCTGAGTTTATTGAGCACTTCAGCGCCGCGCACTCAATCCGAGC
GGACAGCAGCGCCACTCAGCAGCAACATATTGCATCACCgcagcaacaacaacagcagcagcagcaacaa
caacaggagcCGGAGACGGATGGGGCGAGGAGATATTTGCCAGGGGAAGTTGTTGTGAGTGTGGCATTGC
AAACAGGCTGTCGCTGATACAACTTGCCTTGAAATTCGCCCCGCAAATATAAGCCAATGCAAATATGCTC

ACGCTGCGTATGCGTAATGTGTAGAGATCCTGTCAAAGCGGAGGACATTCAAACCCGTATGCAAACAGAG
TCCTGTGCGTTTGTGTGATAGATTTTGTTTGTGCGCGACAACCAAACAAATACACATCGTTTTCGATTCA
GCTCTGCGTTTTCCATTTCGGCCACATCTTTCTGGAGCCTTTTCCTTTGCCTGTTGCCAAATTTATTAAT
AACAAAATGCAAATATTGCTCACATCCGCATATTGAATCCTCGTTCGGCGTTTGCTCTTTTCTTTGCATT
TTTATGGGCTCAATAACTCGACAAAATGAAATTTGTTCGCTGCGAATGTCAAAGTGTAAATGTTTCtttt
tttttAGTTGCTGTCGAGCTAGTAGCGAAATTGGGATTTGAACCCGCTTTGGGGTATATAAATCCTGAAA
CTGAAACGTTCTTCCATAACAAAGTTGTAATATTAATTTATCATGATTGGGATAATTTGCTGTTTCGAAA
TTCTTTTTTTGATATCCGATAATAACTCTTATTATTCATTTTCCTAATGAAAGAAAGTGTGTTGCCATCA
TTTGTTGCGAATTGTACCCTTAGAGTTTTACGGCAGTAAAAACTTTTAGTAGGATAATTGTAAACAATTT
TTTTACACGTATGTGTATTATGTCCGACCTAAAGCAATTGTGGCAACCATCAGCTATGCTAATATCATTA
TTGAATTGGTTCGAACTTCTAATAATATTTTCATTCCTGGCTGTGCGTGTGATTGAAGCCACTTAATTTG
CGTAAGCCACGTCATTTGCATATCGATCGCTCCTAAATAGCCGCAAAACAATCTGAAGCCGTAACAATGC
GGTGTCAAAATGTGTAAGCGATTTGCCAATTCTCCACCGAGATGGATTGCCCTCATCTGCTCCGGTTTGT
CCCTCAGCATCACCCCCCTAAGCCGCAGAAATTTGGATTTGGATTCGGAAGCAGAGACCGGAGACCAGAA
TCCGAACTAACTTGACCAAATCGTGGTTGTCCACTCACCGACGATTCTCCGCATCTGCGAGCACGGTTTG
ACGTTAACTGCAACACCAAAGCGTAACCGCAGTCAACTTGGCCACGAATCGGATCCGGACAGGACCAAAT
ACGGAGCACCGAGTGTCCTTTGGTCTCCGATGGCACTTGTGTGCAGGATGGGCGAGCTCTATGGTGTGGA
GGCAGTGTCTGTGTTTGCTTGTTTATGGCGTTTTCAAGTGCCTGTTGACCGTGTTACATAAATACGGAAT
TGACTTTTTGGCCACAGCGCTTCGCCAGCAATCGAATGACAGCGAAACAGAGAGGAGAAAAAGCGAAATG
TGTTTGGCCACAATGGGTTAGGATGTCATTCGGTATGCCCTCTTTGTCAGTTCCCTGCTGGGGGATATTG
AATTCTTAAAGATTTGTGTAACGTTCGATAGAGATGTTCGTAAAAACGTCGTGAAAAGCTTTTACATTTC
CTGAAAACTGTTGCTGAGTAATGAAAATACTCTCTAAAATCAAATATGAAAAGAATTAATATCTTTGTCT
ATTTTCTTTGCAAGAAAAGAATATAAGTCTTAAAGTCTTGCAAAACTTTCGAACGTAGTCTATGATCTAG
TAATAGAAAAACTGGGTAATATGTTTCTTATTATTGTCTATGGTTCTAGGGTGCTCCACTACCATGGCAG
GGCTTGAAGATAGCGCGACATCTTCCGCCCGTGTGTCCGCAGAAATTGCCAGATCTTTCGCCCCACGGCA
GTGAAAATATGTCCCGGGCTCGTCATAAACACCTGAGTCGACTGCTCCCTTATCTGCGGACGGAGAGCGA
GGACTGTTTGTATCTCAATCTGTATGTGCCACATGAAGGGTAGGTCGGCCCCATATATCAGAGATCTGAA
AATTTGATAGAGTCGGAGCTTGTTACCGATAAAATGCAATTAATGTGCTGTCACACATTGTGCGTATTAA
TCTCTGCTCATTAACGGGTATCTGACAGTCGAATATAATCAATAATCTTAATTTATATTAAAAGCCTTGC
CATATGTGACAAATCCAAGCTGAAGTGAATTTTGTATCTAAAATTTAGGAGCCTCAAAGCACCCCCAAGC
AATACGCTGTGCTGGTCTATCTGCACGGGGAGTCCTTCGAGTGGAACAGTGGCAATCCCTACGATGGCTC
CGTCTTGTCCAGCTATGGAGAGGTGATTGTGGTCACCGTCAACTACCGGCTGGGAGTGTTGGGGATTCTT
ACGGCCGAGCATCGATGCGCATAATATAGCGAATTACGCCCTGCTCGACCAGATTGCGGCGCTACACTGG
ATCAAGGAAAACATCGAAGCCTTCGGCGGTGACAATTCGCGTGTCACTTTAATGGGGCACAGTACGGGGG
CGGCGTGTGTCAATTACCTAATGGTATCGCCGGTGGCCTCAGGGCCTCTTTCACAGGGCCATCCTCATGT
CCGGATCCGCGATGTCCGACTGGGCCGCCAGCAATCAGTCCCTGCAATTGACGATGCAAATCGCCCATGC
CCTCGAATGTCCACTCAACGAGCACGTGGAGGCAGAGGACGACGATGTCCTGCTCGATTGTTTGCGCCAT
CGTCG
>dme_circ_0008173_junction_seq
TCCTGCTCGATTGTTTGCGCCATCGTCGGGACAAAAATTTTGTTGCAAATGAAATT
>dme_circ_0008174
GGACAAAAATTTTGTTGCAAATGAAATTAAATACAGAAAGAAGCTGCACAAAGGAGCAGGCTAAATACAG
CGAGTAAACAAACTGTCGAAACTATTTAAGGCGCTCAAAGAAAATCCATGTAAATACAGCGTCCAGAAAA
TGTGAGCCATTCGGTTTGACGCAACAAAGAAGGGAAACAGCGAACAGTTGGGGAAAAAGAACAATTAACC

AAACTGGAAAGCAAACACGAGCGGAATGTGAAAAGTTTGGACAGAGCGGGAAGTGTAAGACCGAAATTAA
TATCGAAATATCCCAGAAGCCGGCAGGAAAGACCGGGTCCAGGTCCCGGAGGCGGAAGACAAAGCCATGA
AGTTTCGTTTGGCAGCATTTTGGCTATTTCTGCTGACAGTCGGCGGCAATCACAAGCTATTAAGCCACGT
TTCCCCAATGGGCGTGGCAGCCGAAAAGCAGCCGTTGCACAAAGGAAATGAGCTGGAAAACGCAATGAAA
TTGCGTGAAGCCCCAAAGCAATCCCGGGTTATCGGGGACATCACGACGACAATTCAGCCGGACTCGGATC
CGGGCCGATCAATTGGCCATCAGGCGTTGCGACGTGCCAAGGCCCCGCCGCCCAGCTTGGAGCTGCATTT
CAGGCAGAATCTGAACAAGCTTTTCGCCGGCGATGAAACCACCCACGTGGGCCACACCAATGCCACTGAC
AACACCACGGCCGCAGTTTTGGCCACCGACGAAGGTTCCAGCGAACACGAGCCGTCAACCACTCATCACC
CGGAACGGCGACACGTGGTCCCCGATAAGCTGCAGTACACCCAGGAGATCCAGGTGAAGCAGGGCCGGCT
CATGGGCATCACACGTCGCTTCCAGGTGACCAGCGGGCTGCGCCAGGTGGACCAGTTCCTGGGGCTTCCA
TACGCGGAGGCGCCCACCGGAAATCGACGCTTCATGCCACCGGGGTGAGTGTACAAAACTGTCCTCTAAG
AGTCTTTAACTTTATTTTGATACAAGATATATACATAGATAGATAGCTTTTAAGCTAAAACAATTTCAGC
TGTTCTCAAAGACACCTCATGATGCCGATCGGCTTGGGTCTACCGTTTGGCAGATGGAACTTTCAATTTG
AAAGTATACTTTTCTTACATAAGCGAGCAATAGCATTATAAATATTTTCCCTTAATTAATTAAGATTAAA
GAAAATGCTAATGGCAAAGCCAAGTAAGCCAAGTATCATCACAATATCATTGACAAGGGCGACCAAAACC
AATAAATATTATCCCCTGAGTCCGTTTTTGACATTGATTGCAAAATGCTTATTTTAAAATATACTGTGTT
AAAGTAAATCGATTTATTTGCATACATCAAGTCTTCGGAAGGGCTAAAATAATTCAATAAAAGTTTGTTA
AGTTGAACGGGCAATATTCTATAATATAAATAACGATATCCTCTGTTTGAACCAACCTGCTGGCAGGGAG
TACCACCTATTTATTTCCCCTTGCCCGACCCGCCGTAGGCAGGCAGCACTATTTGTGACCGCAAATATGA
TGGCAAATTGCCACTTTGCAGCACCAACAGGCAAACCCGAAAACCTCGAAACCCCAAAAACCCCAGAACT
CAAATAGGAGCAGGAGCAGGGGTGTCTTCGGAAGGACCGACCACTCCTCGAACAACCGACATGTGCCGCC
GCACTTCCCCTGCCGACCATGCCAGAACATCCTGTTGGTCCTGGGGACGCTGCTGGGGCACTTAGAATTT
AGCCAGCAATTTAAGCAATTATCGTTGAAGCTGGCCCGGCATTAGGCTTTCAGGACTTGACATGTGCCCT
CTCTAACAGCGATCTGGCAGGATGTTGGTTCCGAAAGCACGTCCCtgtgtgtgtgtgtgtgCTTCCTGTC
TCGAATGGCTATTATTATTGCAGCTTGTTTTTACAAGGATTGCAATGTAAATCCCAGGCGTCCCCGCCCC
TTCGCCCTTTTATTTTGCCATCTGCTGTGCGATTATGTTTCGGACTTTGTACATGAATATTTTGCTTGTT
TAATTAAGAGTGAAAATTGCTGGTATTATATCTATTTAAAGTTTGTTTATCAAAATATGAGCTACGTTGC
CCGTGCCAATTTGTCTGTGAGATTTGAAGCCGCCAAGCTGCCAACACTTCAGCCAACTTCGGGCCTGCAT
CCTGCTGTTCTGGGCACAAACAAGCCGCAGCTTCAATTGTTGCCACCACAACTAGGTCCCTTCCACTTGG
TCATCCGGTTTCATTGGCTATGCACCAAGAATATAATTTATTTTCCATATAATTTTACAAGCTACTACTC
ATATCTAATTAGTGTACCCAAACTAAGAAATACCGTCATTTATTTGAATAGGAAACTAAAACTCTAAAAC
TGGTAGATTAGTCAAAACAAGTTGAGGTGTCCAAAAAATGTTTTCAGTGTAGGCGGCATAACACGCCTTG
GTCCTCCGAATTGCATTACGTGAAGACAATAAATTAACACGCCCCAGAGGCTGTCGCAAGTGTTGGCCGC
GATTTGCGTGTCAGATGTGCTGCTTATGTTGTAGTTTCTAGTTGCAGTTTCCAGAGGCAGTTTTCAGCAT
TCAGTTTGCACTTCGCCGTTTGTCGCGCTGAGTTTATTGAGCACTTCAGCGCCGCGCACTCAATCCGAGC
GGACAGCAGCGCCACTCAGCAGCAACATATTGCATCACCgcagcaacaacaacagcagcagcagcaacaa
caacaggagcCGGAGACGGATGGGGCGAGGAGATATTTGCCAGGGGAAGTTGTTGTGAGTGTGGCATTGC
AAACAGGCTGTCGCTGATACAACTTGCCTTGAAATTCGCCCCGCAAATATAAGCCAATGCAAATATGCTC
ACGCTGCGTATGCGTAATGTGTAGAGATCCTGTCAAAGCGGAGGACATTCAAACCCGTATGCAAACAGAG
TCCTGTGCGTTTGTGTGATAGATTTTGTTTGTGCGCGACAACCAAACAAATACACATCGTTTTCGATTCA
GCTCTGCGTTTTCCATTTCGGCCACATCTTTCTGGAGCCTTTTCCTTTGCCTGTTGCCAAATTTATTAAT
AACAAAATGCAAATATTGCTCACATCCGCATATTGAATCCTCGTTCGGCGTTTGCTCTTTTCTTTGCATT
TTTATGGGCTCAATAACTCGACAAAATGAAATTTGTTCGCTGCGAATGTCAAAGTGTAAATGTTTCtttt
tttttAGTTGCTGTCGAGCTAGTAGCGAAATTGGGATTTGAACCCGCTTTGGGGTATATAAATCCTGAAA

CTGAAACGTTCTTCCATAACAAAGTTGTAATATTAATTTATCATGATTGGGATAATTTGCTGTTTCGAAA
TTCTTTTTTTGATATCCGATAATAACTCTTATTATTCATTTTCCTAATGAAAGAAAGTGTGTTGCCATCA
TTTGTTGCGAATTGTACCCTTAGAGTTTTACGGCAGTAAAAACTTTTAGTAGGATAATTGTAAACAATTT
TTTTACACGTATGTGTATTATGTCCGACCTAAAGCAATTGTGGCAACCATCAGCTATGCTAATATCATTA
TTGAATTGGTTCGAACTTCTAATAATATTTTCATTCCTGGCTGTGCGTGTGATTGAAGCCACTTAATTTG
CGTAAGCCACGTCATTTGCATATCGATCGCTCCTAAATAGCCGCAAAACAATCTGAAGCCGTAACAATGC
GGTGTCAAAATGTGTAAGCGATTTGCCAATTCTCCACCGAGATGGATTGCCCTCATCTGCTCCGGTTTGT
CCCTCAGCATCACCCCCCTAAGCCGCAGAAATTTGGATTTGGATTCGGAAGCAGAGACCGGAGACCAGAA
TCCGAACTAACTTGACCAAATCGTGGTTGTCCACTCACCGACGATTCTCCGCATCTGCGAGCACGGTTTG
ACGTTAACTGCAACACCAAAGCGTAACCGCAGTCAACTTGGCCACGAATCGGATCCGGACAGGACCAAAT
ACGGAGCACCGAGTGTCCTTTGGTCTCCGATGGCACTTGTGTGCAGGATGGGCGAGCTCTATGGTGTGGA
GGCAGTGTCTGTGTTTGCTTGTTTATGGCGTTTTCAAGTGCCTGTTGACCGTGTTACATAAATACGGAAT
TGACTTTTTGGCCACAGCGCTTCGCCAGCAATCGAATGACAGCGAAACAGAGAGGAGAAAAAGCGAAATG
TGTTTGGCCACAATGGGTTAGGATGTCATTCGGTATGCCCTCTTTGTCAGTTCCCTGCTGGGGGATATTG
AATTCTTAAAGATTTGTGTAACGTTCGATAGAGATGTTCGTAAAAACGTCGTGAAAAGCTTTTACATTTC
CTGAAAACTGTTGCTGAGTAATGAAAATACTCTCTAAAATCAAATATGAAAAGAATTAATATCTTTGTCT
ATTTTCTTTGCAAGAAAAGAATATAAGTCTTAAAGTCTTGCAAAACTTTCGAACGTAGTCTATGATCTAG
TAATAGAAAAACTGGGTAATATGTTTCTTATTATTGTCTATGGTTCTAGGGTGCTCCACTACCATGGCAG
GGCTTGAAGATAGCGCGACATCTTCCGCCCGTGTGTCCGCAGAAATTGCCAGATCTTTCGCCCCACGGCA
GTGAAAATATGTCCCGGGCTCGTCATAAACACCTGAGTCGACTGCTCCCTTATCTGCGGACGGAGAGCGA
GGACTGTTTGTATCTCAATCTGTATGTGCCACATGAAGGGTAGGTCGGCCCCATATATCAGAGATCTGAA
AATTTGATAGAGTCGGAGCTTGTTACCGATAAAATGCAATTAATGTGCTGTCACACATTGTGCGTATTAA
TCTCTGCTCATTAACGGGTATCTGACAGTCGAATATAATCAATAATCTTAATTTATATTAAAAGCCTTGC
CATATGTGACAAATCCAAGCTGAAGTGAATTTTGTATCTAAAATTTAGGAGCCTCAAAGCACCCCCAAGC
AATACGCTGTGCTGGTCTATCTGCACGGGGAGTCCTTCGAGTGGAACAGTGGCAATCCCTACGATGGCTC
CGTCTTGTCCAGCTATGGAGAGGTGATTGTGGTCACCGTCAACTACCGGCTGGGAGTGTTGGGGATTCTT
ACGGCCGAGCATCGATGCGCATAATATAGCGAATTACGCCCTGCTCGACCAGATTGCGGCGCTACACTGG
ATCAAGGAAAACATCGAAGCCTTCGGCGGTGACAATTCGCGTGTCACTTTAATGGGGCACAGTACGGGGG
CGGCGTGTGTCAATTACCTAATGGTATCGCCGGTGGCCTCAGGGCCTCTTTCACAGGGCCATCCTCATGT
CCGGATCCGCGATGTCCGACTGGGCCGCCAGCAATCAGTCCCTGCAATTGACGATGCAAATCGCCCATGC
CCTCGAATGTCCACTCAACGAGCACGTGGAGGCAGAGGACGACGATGTCCTGCTCGATTGTTTGCGCCAT
CGTCGGGTGAGTAGTCCTCGACCAATTGAATTATTTAGGCGCGCCAACAACCGCATCAGCGATATGCGGA
CCATTATAAACAGCCGTAATGCCTGGTAATGCCTTTCAATGGTCTACACGGAAAGAAATGAGGGTGGAGT
CATTAGTCAAAATTAAAATAGCTTCAACCAACTTTCTTTCCCAATTGGTATCTTTTGAAACGACGAACAA
TGCCCTTTGCATATGATACACTTGTGGTTTGACAGTTCATTTTGTTTTAAAATTTAAAGAGCGTCACTTA
TTTTCGCACAGTGCTTTCTGTGGCGATGCGAGCGCGTCTACTTCATTTGCACTGGCATCTTCATTTGAAT
AGTGTTTTGTGTCCTTTGATTCAATTCGTCAGGCCAGCGGTATTTGGCGGCTTTGTGACCTGCCTAATGA
TTCGTTAAATTTACGAGCTTCCAGCACCACGGTGTCAATAAGGCGTAAAGGCCTTTTGGCTCAACATCCT
CATCAAACTTCGCTTATAGGACCTTGACTAATCAGGAGTAATGACGATGGCTTAAAATTTGCATGGCGAT
TTCCAGGGACGAGGGACCGTAGGTCAGCAAAAGCAAAAAGGACGACTACGATGCGGATGGATGGCAGGGA
TTCAATTTAGTTACATTATAATTGCTCAATTCACATTCAAGTGATTTAATTTACGCTCCTACTTTTGCAG
TTTCTTGCAGTTTTATAATTGCGTATTCATTGCACGCATTCCGGCGGCTTATCCTATCAGTATGCCAGTG
TCCCTTTCCATTAAAGCATGAGCTCTAAACGCTTAAAAATAAACACGACCTAAGAGCACTTTTTATTTAT
CAGCATTTAATGCATGTGTGGGGCTTTGTGCTCTTGTGTGGGAGGAAGTTCATTTGTATGCGCAGAGAGC

ACACAAGAGCGAACTAAAAAACAATTTGCATACGAAATGAAAAAAAGACGCATGTAACTAAATATGTAAA
ACTAAATCAACAACGGCACAATAATAGTAATTATGTTAATTACGCCAGCACACACTCACCCACAAACTCC
CAGCCATTCCGCTCACAAACACTATGTGGCAACAGTCGGTTTTATCCGCGTGTGTGTGCGCCTTTGAAAA
CTTTCTACTCGCTTTCTTGTCCTTAATACAAATTGGCACAGTAACTTGAAAACCCAAGCCAGAACTTTTG
CTGATGTAAATTGTCGATTCTAGTAGTTGGCATACGGCCTTTTTAGGCTCTACTGGGGATACAAACTTGA
AACAGGATAATACAAGAAGAGCACAATTTTAAAAGTGCTAATTTAGGGTTAGGCTACAATTATCTAGAGC
GTTGGAATATTTAATACTAAAACTAACAAATTTTTTTATTCAATATGGATTAAACATAACATATTTTTAA
AGTGATTTGAGTTTTCAGGAGTATTCAAACCTTATTTTGTTGACCTAAACATCTTTAATTAACACAATTT
CGAGTGCGTTTAGAAAGTAGGAATACCTCGCTACTGAAGGGTTCATTTCGAAATGTTTTAATGAATGAGC
TAAGCTTCGGTTTGAGCCTGGGAATTGCAGCCTCGTGTGGCAGCTTACTTAAATTACGGCAGAAGGAGCG
AGTGAATGAGTGAGTGAGTAGAGGTTTAGTAGCATGTGTGCCAGCCAGAGCGAGTGTTAAATGAATGCAA
TGAATTATGCATGAATTACTGAGCAAATGTGCTCATCGCTTTTACTGCAAAGgcacacaactcacacaca
cacacacacccactgaaacacaaacaGGTAACAACTACTAGTGCAAACAAACGGAGAGTCACAGTCGCAT
TTGTTTAACCCACTCTCACAAACAGGCATAAGTCTTTGTCTTTGGGTCTTAGGCTTCCTAAATATTTAAT
AGAACCTCTGCCCGAATCCTGCCCATTGATTTGAATGTGTGCTTTTCCAACTTGTTAATTTATTCAAATT
ATTTACCTATTGCCCCAGCCTAATGTGTTTGCCTGCCTGTGATCTCTATTTACTCTTGGCCGAGCTTAAC
TAAACTATTGCCTGAAATGCATATTTGAATGCCATAAATTCTGATTGGCCACTTGGCGGAAGAAAAGAGC
AATTCGTTCCATTAGATTACACTCCATGCAATAACTTTGTGAAACGAACTATTGTTTGTTGATTTTGGAA
CAATAAATGCCACTTGCTCCTAGGAAAATCGTAATGAGAAAGCTGATGTTTTTGAATAAGATATGTAGTT
TAAATAATCGATGTACAAATGCATAGCCTATGCAATTAAATATTTTTCATGTATTTTACTTCATTATTAT
TTCACCTTATAAATGCAACAACTCAACCATATCACGAATACGCGATGCGGGCCACAAAGTACAAGTTGAG
CGCTGTTAATAGACAAAATAAAAGGCAAGGCATATTAAAAATTCATTAAATGAAAAGCGAGCAGAATCTA
GAACTATAGCCGTAGTAGAGACAAGGCTCAACTGGGGCATAATAAATGTTCGGTTTGCCTGCACAGGACT
CGCTGCAAACGAGTGTGTGTGTGTCTTTCCAGTTGCTACATGCAGCCTTAAGGACATCATCAATCTTCGT
TCCTGCCACCAGCACACTGACAAATTGTGCATTTCGTTTCTAGCAAGCAGTATCCAGTTTCCAGCATCTA
TATTTGTGCCACCGCACACAAAATATATAGAGAGAAATCGCACATATTGTGTGTCTAACGAGTTAATCGC
AGTTGCAGCTCAACTTGTGACAGTTTTGCACCAGCCGAGCTAATAAGGGGCAACAACTGGGCGGAGATAA
ACTCTTACCCATATGAAAATCATAAATCACACTGTTCCATGGCGAACAACTTCGCAGCAGCGACAACTTC
GACGATGAAGTGGTCTTTCGAAGACCCCGTATCTAGGGGCACAACTCGCATTCCCGGGCTGCCAAAATTG
TTTTAGCTTCCACAAATTGTCAAAGTCAGAGGAAAACACAAGCTCACGTAGCTGTGGCCGTGCGGTGCAG
ACTTTAGCCCTAAACTTGGCCAAGTGCCGATAGTCAGGTCCAGGGATGACCACATAAGCCGGGAACATGT
GCTGTGGAACTTTGCCTGCACTAGAAAAACAAAAAATCGTCCTTATATAAAACGAAATAAATCCTAAAGC
ACTTATAAAACACTAACTAACTTGATTCTCAAGTACAGATTTTTTGCTGATTCTGAGAAGTGAGATATAA
CCTTTATAAATCATAATAGGAAATTAAATTAACGCAAATTAAATGTAATTTAATAGTAAGCACACATTAT
ATCATTAGATTATGTgtttacatttttttgaattataaacaaatttttacaacatttttttttataaaaatc
aattgtaagcttaatttttttAAAAGTGTCTCCTCGTTTAACTTCTTTAAAGAGCGGGGAAGCTTTATTT
GGTCTCGAAAGTGTTTGATGGCATTGCATCGATTGCTGCAGTCAGCTCTTGGCTTAAATGGATTACGATA
ATGTCCCTCTTCAATGGGATTCGAAGGTGTTGAGTGAATTAACACACACATCCACCGACAGATGGCAATT
TTCGGTATTTTTTCAGGCTTAGCATGTGAATGGGTGCCAGGCATCCTTGGCATCGCATCCAATTGAATTT
TCCCCCGAGATCTGCTTTCACTTGGGTCTTATTCAAGCGTAGAGGACGGAATGATTAAGAGAGCCTGGGG
TTTTAATTCAGCCGCATTCCCAGAATCATTGGCGAAAGGCGGACCAAATATTACGACTGTGCAAACGGGG
TACAGTCACTTGGTTCGGTCCAAGGTTATTCCGAGTTGAACTGAGCTGAGCATGCAGATTGTTCATTTGA
ATTTCTACTCCGAGCATTTCCACCTCAGCTGGCTGGTTCAGTTCTGGTCTGCATTTTCCAGATAACAATT
GTACAACTCCGGCGGCCAAAATATTCGAAAATTCTCTTCTTCTCCCCCGATTGTTGTGGCTATATTTTTT

GTCGATGTTGTCGGTCCTTTTCTTAATTTCTGCCAATTTCTGTGCCATCGACACTTCAGCTGGAGCTCTG
GCATGTGGATTTTTATGCAAACTATGCCAGAAATTGCACCCAACTGAACCATTTTGCCACTGCAATTTTG
TATCAATACGTGGGTGCGGTGGGTTGGCCCAAACGAAGGCCAAACGAGCCAACAAGCGGGTAAAAAGAGC
AAAAAGCGTAAATATTGCAACGAACGGAAATGCTTTGCAGCACGAGCAACTTCAGACCAGAAATCGCAGG
AGCATTCGGATTGTGAGCTGATTGTATTTCATATCCACGTGGCGTTTTTAGGTCGGAAAAAGGTTGGGTT
CCCTGTATTTGTATTGGCAATACCCACACATAAATACGAGTGTAAATCCGTACTAGTATCTGCGTGGGAA
TTGGTTTGCATTGCGGCTCTATTATGGCTTTGAATGGGTAAAAATACTTTTGGAAAAAACGCCATAACTA
CAGGAAGTAACACAAGTGCCAGTGCCACACGTTGATGGAAGGCATTTGGTTACTTGAAAAGAAAATTTGA
ATGTGTGAAATGGGAAACCTCAGAGCTCCAAACGTTTTTATTGCCTAGGGTTTAGGAGTTATAATTTTAG
ACACGTAATTTAAAAAGACATTTTCGTTCAGAAATTGCAAATTATTCCAAGCGAAAGCAGCCCTTAAATT
CTTGTTGTGTGTTCTCGCACATGATTCTGTTTGGTTGTAGGAATTGATGTTTAGGAATAAAAAACTAAAT
GGATTTTATCAGAAATGTAGTATAACATTGGCATTGAAATCCTGTCCATACTTATATTTTTTAGAGCTCC
ACGCGGAACGCTCTTAATCTTCTGAGTAGTAATTTGGCAACGCCCTTAAGGCCATATGTGTATCTTGTAG
GTCATCTCGTTTGGCTTTATGGGGCCACGTGTGTGCGGTACGTGCAAGTGACCGTCCAGCAACAAATCAC
ACATAAAATTTATTAGAAATTTCAAATCAAAGTGAAAAGTGACCGCTGTCAGCCAGCGGAGCCGCTTCAG
TCCCAGCGGCAGTGCTACATTTATACCCAGCTCGTATGTAAGGCCTTTTAACGCATTTTTTGGCATTCTT
GTTTCTTTACAGCTACCAGGATATCTTGCACATACCAACGGCGCTCACACAATTTTCAACATCATTGGGT
CCAATTGTCGATGGACATGTAATACCAAATCAACCGTACAAGGTCATGGGGCATTATACGGAGCACTTCA
GCCG
>dme_circ_0008174_junction_seq
TGGGGCATTATACGGAGCACTTCAGCCGGGACAAAAATTTTGTTGCAAATGAAATT
>dme_circ_0008175
GGACAAAAATTTTGTTGCAAATGAAATTAAATACAGAAAGAAGCTGCACAAAGGAGCAGGCTAAATACAG
CGAGTAAACAAACTGTCGAAACTATTTAAGGCGCTCAAAGAAAATCCATGTAAATACAGCGTCCAGAAAA
TGTGAGCCATTCGGTTTGACGCAACAAAGAAGGGAAACAGCGAACAGTTGGGGAAAAAGAACAATTAACC
AAACTGGAAAGCAAACACGAGCGGAATGTGAAAAGTTTGGACAGAGCGGGAAGTGTAAGACCGAAATTAA
TATCGAAATATCCCAGAAGCCGGCAGGAAAGACCGGGTCCAGGTCCCGGAGGCGGAAGACAAAGCCATGA
AGTTTCGTTTGGCAGCATTTTGGCTATTTCTGCTGACAGTCGGCGGCAATCACAAGCTATTAAGCCACGT
TTCCCCAATGGGCGTGGCAGCCGAAAAGCAGCCGTTGCACAAAGGAAATGAGCTGGAAAACGCAATGAAA
TTGCGTGAAGCCCCAAAGCAATCCCGGGTTATCGGGGACATCACGACGACAATTCAGCCGGACTCGGATC
CGGGCCGATCAATTGGCCATCAGGCGTTGCGACGTGCCAAGGCCCCGCCGCCCAGCTTGGAGCTGCATTT
CAGGCAGAATCTGAACAAGCTTTTCGCCGGCGATGAAACCACCCACGTGGGCCACACCAATGCCACTGAC
AACACCACGGCCGCAGTTTTGGCCACCGACGAAGGTTCCAGCGAACACGAGCCGTCAACCACTCATCACC
CGGAACGGCGACACGTGGTCCCCGATAAGCTGCAGTACACCCAGGAGATCCAGGTGAAGCAGGGCCGGCT
CATGGGCATCACACGTCGCTTCCAGGTGACCAGCGGGCTGCGCCAGGTGGACCAGTTCCTGGGGCTTCCA
TACGCGGAGGCGCCCACCGGAAATCGACGCTTCATGCCACCGGGGTGAGTGTACAAAACTGTCCTCTAAG
AGTCTTTAACTTTATTTTGATACAAGATATATACATAGATAGATAGCTTTTAAGCTAAAACAATTTCAGC
TGTTCTCAAAGACACCTCATGATGCCGATCGGCTTGGGTCTACCGTTTGGCAGATGGAACTTTCAATTTG
AAAGTATACTTTTCTTACATAAGCGAGCAATAGCATTATAAATATTTTCCCTTAATTAATTAAGATTAAA
GAAAATGCTAATGGCAAAGCCAAGTAAGCCAAGTATCATCACAATATCATTGACAAGGGCGACCAAAACC
AATAAATATTATCCCCTGAGTCCGTTTTTGACATTGATTGCAAAATGCTTATTTAAAATATACTGTGTT
AAAGTAAATCGATTTATTTGCATACATCAAGTCTTCGGAAGGGCTAAAATAATTCAATAAAAGTTTGTTA
AGTTGAACGGGCAATATTCTATAATATAAATAACGATATCCTCTGTTTGAACCAACCTGCTGGCAGGGAG
TACCACCTATTTATTTCCCCTTGCCCGACCCGCCGTAGGCAGGCAGCACTATTTGTGACCGCAAATATGA

TGGCAAATTGCCACTTTGCAGCACCAACAGGCAAACCCGAAAACCTCGAAACCCCAAAAACCCCAGAACT
CAAATAGGAGCAGGAGCAGGGGTGTCTTCGGAAGGACCGACCACTCCTCGAACAACCGACATGTGCCGCC
GCACTTCCCCTGCCGACCATGCCAGAACATCCTGTTGGTCCTGGGGACGCTGCTGGGGCACTTAGAATTT
AGCCAGCAATTTAAGCAATTATCGTTGAAGCTGGCCCGGCATTAGGCTTTCAGGACTTGACATGTGCCCT
CTCTAACAGCGATCTGGCAGGATGTTGGTTCCGAAAGCACGTCCCtgtgtgtgtgtgtgtgCTTCCTGTC
TCGAATGGCTATTATTATTGCAGCTTGTTTTTACAAGGATTGCAATGTAAATCCCAGGCGTCCCCGCCCC
TTCGCCCTTTTATTTTGCCATCTGCTGTGCGATTATGTTTCGGACTTTGTACATGAATATTTTGCTTGTT
TAATTAAGAGTGAAAATTGCTGGTATTATATCTATTTAAAGTTTGTTTATCAAAATATGAGCTACGTTGC
CCGTGCCAATTTGTCTGTGAGATTTGAAGCCGCCAAGCTGCCAACACTTCAGCCAACTTCGGGCCTGCAT
CCTGCTGTTCTGGGCACAAACAAGCCGCAGCTTCAATTGTTGCCACCACAACTAGGTCCCTTCCACTTGG
TCATCCGGTTTCATTGGCTATGCACCAAGAATATAATTTATTTTCCATATAATTTTACAAGCTACTACTC
ATATCTAATTAGTGTACCCAAACTAAGAAATACCGTCATTTATTTGAATAGGAAACTAAAACTCTAAAAC
TGGTAGATTAGTCAAAACAAGTTGAGGTGTCCAAAAAAATGTTTTCAGTGTAGGCGGCATAACACGCCTTG
GTCCTCCGAATTGCATTACGTGAAGACAATAAATTAACACGCCCCAGAGGCTGTCGCAAGTGTTGGCCGC
GATTTGCGTGTCAGATGTGCTGCTTATGTTGTAGTTTCTAGTTGCAGTTTCCAGAGGCAGTTTTCAGCAT
TCAGTTTGCACTTCGCCGTTTGTCGCGCTGAGTTTATTGAGCACTTCAGCGCCGCGCACTCAATCCGAGC
GGACAGCAGCGCCACTCAGCAGCAACATATTGCATCACCgcagcaacaacaacagcagcagcagcaacaa
caacaggagcCGGAGACGGATGGGGCGAGGAGATATTTGCCAGGGGAAGTTGTTGTGAGTGTGGCATTGC
AAACAGGCTGTCGCTGATACAACTTGCCTTGAAATTCGCCCCGCAAATATAAGCCAATGCAAATATGCTC
ACGCTGCGTATGCGTAATGTGTAGAGATCCTGTCAAAGCGGAGGACATTCAAACCCGTATGCAAACAGAG
TCCTGTGCGTTTGTGTGATAGATTTTGTTTGTGCGCGACAACCAAACAAATACACATCGTTTTCGATTCA
GCTCTGCGTTTTCCATTTCGGCCACATCTTTCTGGAGCCTTTTCCTTTGCCTGTTGCCAAATTTATTAAT
AACAAAATGCAAATATTGCTCACATCCGCATATTGAATCCTCGTTCGGCGTTTGCTCTTTTCTTTGCATT
TTTATGGGCTCAATAACTCGACAAAATGAAATTTGTTCGCTGCGAATGTCAAAGTGTAAATGTTTCtttt
tttttAGTTGCTGTCGAGCTAGTAGCGAAATTGGGATTTGAACCCGCTTTGGGGTATATAAATCCTGAAA
CTGAAACGTTCTTCCATAACAAAGTTGTAATATTAATTTATCATGATTGGGATAATTTGCTGTTTCGAAA
TTCTTTTTTTGATATCCGATAATAACTCTTATTATTCATTTTCCTAATGAAAGAAAGTGTGTTGCCATCA
TTTGTTGCGAATTGTACCCTTAGAGTTTTACGGCAGTAAAAACTTTTAGTAGGATAATTGTAAACAATTT
TTTTACACGTATGTGTATTATGTCCGACCTAAAGCAATTGTGGCAACCATCAGCTATGCTAATATCATTA
TTGAATTGGTTCGAACTTCTAATAATATTTTCATTCCTGGCTGTGCGTGTGATTGAAGCCACTTAATTTG
CGTAAGCCACGTCATTTGCATATCGATCGCTCCTAAATAGCCGCAAAACAATCTGAAGCCGTAACAATGC
GGTGTCAAAATGTGTAAGCGATTTGCCAATTCTCCACCGAGATGGATTGCCCTCATCTGCTCCGGTTTGT
CCCTCAGCATCACCCCCCTAAGCCGCAGAAATTTGGATTTGGATTCGGAAGCAGAGACCGGAGACCAGAA
TCCGAACTAACTTGACCAAATCGTGGTTGTCCACTCACCGACGATTCTCCGCATCTGCGAGCACGGTTTG
ACGTTAACTGCAACACCAAAGCGTAACCGCAGTCAACTTGGCCACGAATCGGATCCGGACAGGACCAAAT
ACGGAGCACCGAGTGTCCTTTGGTCTCCGATGGCACTTGTGTGCAGGATGGGCGAGCTCTATGGTGTGGA
GGCAGTGTCTGTGTTTGCTTGTTTATGGCGTTTTCAAGTGCCTGTTGACCGTGTTACATAAATACGGAAT
TGACTTTTTGGCCACAGCGCTTCGCCAGCAATCGAATGACAGCGAAACAGAGAGGAGAAAAAGCGAAATG
TGTTTGGCCACAATGGGTTAGGATGTCATTCGGTATGCCCTCTTTGTCAGTTCCCTGCTGGGGGATATTG
AATTCTTAAAGATTTGTGTAACGTTCGATAGAGATGTTCGTAAAAACGTCGTGAAAAGCTTTTACATTTC
CTGAAAACTGTTGCTGAGTAATGAAAATACTCTCTAAAATCAAATATGAAAAGAATTAATATCTTTGTCT
ATTTTCTTTGCAAGAAAAGAATATAAGTCTTAAAGTCTTGCAAAACTTTCGAACGTAGTCTATGATCTAG
TAATAGAAAAACTGGGTAATATGTTTCTTATTATTGTCTATGGTTCTAGGGTGCTCCACTACCATGGCAG
GGCTTGAAGATAGCGCGACATCTTCCGCCCGTGTGTCCGCAGAAATTGCCAGATCTTTCGCCCCACGGCA

GTGAAAATATGTCCCGGGCTCGTCATAAACACCTGAGTCGACTGCTCCCTTATCTGCGGACGGAGAGCGA
GGACTGTTTGTATCTCAATCTGTATGTGCCACATGAAGGGTAGGTCGGCCCCATATATCAGAGATCTGAA
AATTTGATAGAGTCGGAGCTTGTTACCGATAAAATGCAATTAATGTGCTGTCACACATTGTGCGTATTAA
TCTCTGCTCATTAACGGGTATCTGACAGTCGAATATAATCAATAATCTTAATTTATATTAAAAGCCTTGC
CATATGTGACAAATCCAAGCTGAAGTGAATTTTGTATCTAAAATTTAGGAGCCTCAAAGCACCCCCAAGC
AATACGCTGTGCTGGTCTATCTGCACGGGGAGTCCTTCGAGTGGAACAGTGGCAATCCCTACGATGGCTC
CGTCTTGTCCAGCTATGGAGAGGTGATTGTGGTCACCGTCAACTACCGGCTGGGAGTGTTGGGGATTCTT
ACGGCCGAGCATCGATGCGCATAATATAGCGAATTACGCCCTGCTCGACCAGATTGCGGCGCTACACTGG
ATCAAGGAAAACATCGAAGCCTTCGGCGGTGACAATTCGCGTGTCACTTTAATGGGGCACAGTACGGGGG
CGGCGTGTGTCAATTACCTAATGGTATCGCCGGTGGCCTCAGGGCCTCTTTCACAGGGCCATCCTCATGT
CCGGATCCGCGATGTCCGACTGGGCCGCCAGCAATCAGTCCCTGCAATTGACGATGCAAATCGCCCATGC
CCTCGAATGTCCACTCAACGAGCACGTGGAGGCAGAGGACGACGATGTCCTGCTCGATTGTTTGCGCCAT
CGTCGGGTGAGTAGTCCTCGACCAATTGAATTATTTAGGCGCGCCAACAACCGCATCAGCGATATGCGGA
CCATTATAAACAGCCGTAATGCCTGGTAATGCCTTTCAATGGTCTACACGGAAAGAAATGAGGGTGGAGT
CATTAGTCAAAATTAAAATAGCTTCAACCAACTTTCTTTCCCAATTGGTATCTTTTGAAACGACGAACAA
TGCCCTTTGCATATGATACACTTGTGGTTTGACAGTTCATTTTGTTTTAAAATTTAAAGAGCGTCACTTA
TTTTCGCACAGTGCTTTCTGTGGCGATGCGAGCGCGTCTACTTCATTTGCACTGGCATCTTCATTTGAAT
AGTGTTTTGTGTCCTTTGATTCAATTCGTCAGGCCAGCGGTATTTGGCGGCTTTGTGACCTGCCTAATGA
TTCGTTAAATTTACGAGCTTCCAGCACCACGGTGTCAATAAGGCGTAAAGGCCTTTTGGCTCAACATCCT
CATCAAACTTCGCTTATAGGACCTTGACTAATCAGGAGTAATGACGATGGCTTAAAATTTGCATGGCGAT
TTCCAGGGACGAGGGACCGTAGGTCAGCAAAAGCAAAAAGGACGACTACGATGCGGATGGATGGCAGGGA
TTCAATTTAGTTACATTATAATTGCTCAATTCACATTCAAGTGATTTAATTTACGCTCCTACTTTTGCAG
TTTCTTGCAGTTTTATAATTGCGTATTCATTGCACGCATTCCGGCGGCTTATCCTATCAGTATGCCAGTG
TCCCTTTCCATTAAAGCATGAGCTCTAAACGCTTAAAAATAAACACGACCTAAGAGCACTTTTTATTTAT
CAGCATTTAATGCATGTGTGGGGCTTTGTGCTCTTGTGTGGGAGGAAGTTCATTTGTATGCGCAGAGAGC
ACACAAGAGCGAACTAAAAAACAATTTGCATACGAAATGAAAAAAAGACGCATGTAACTAAATATGTAAA
ACTAAATCAACAACGGCACAATAATAGTAATTATGTTAATTACGCCAGCACACACTCACCCACAAACTCC
CAGCCATTCCGCTCACAAACACTATGTGGCAACAGTCGGTTTTATCCGCGTGTGTGTGCGCCTTTGAAAA
CTTTCTACTCGCTTTCTTGTCCTTAATACAAATTGGCACAGTAACTTGAAAACCCAAGCCAGAACTTTTG
CTGATGTAAATTGTCGATTCTAGTAGTTGGCATACGGCCTTTTTAGGCTCTACTGGGGATACAAACTTGA
AACAGGATAATACAAGAAGAGCACAATTTTAAAAGTGCTAATTTAGGGTTAGGCTACAATTATCTAGAGC
GTTGGAATATTTAATACTAAAACTAACAAATTTTTTTATTCAATATGGATTAAACATAACATATTTTTAA
AGTGATTTGAGTTTTCAGGAGTATTCAAACCTTATTTTGTTGACCTAAACATCTTTAATTAACACAATTT
CGAGTGCGTTTAGAAAGTAGGAATACCTCGCTACTGAAGGGTTCATTTCGAAATGTTTTAATGAATGAGC
TAAGCTTCGGTTTGAGCCTGGGAATTGCAGCCTCGTGTGGCAGCTTACTTAAATTACGGCAGAAGGAGCG
AGTGAATGAGTGAGTGAGTAGAGGTTTAGTAGCATGTGTGCCAGCCAGAGCGAGTGTTAAATGAATGCAA
TGAATTATGCATGAATTACTGAGCAAATGTGCTCATCGCTTTTACTGCAAAGgcacacaactcacacaca
cacacacacccactgaaacacaaacaGGTAACAACTACTAGTGCAAACAAACGGAGAGTCACAGTCGCAT
TTGTTTAACCCACTCTCACAAACAGGCATAAGTCTTTGTCTTTGGGTCTTAGGCTTCCTAAATATTTAAT
AGAACCTCTGCCCGAATCCTGCCCATTGATTTGAATGTGTGCTTTTCCAACTTGTTAATTTATTCAAATT
ATTTACCTATTGCCCCAGCCTAATGTGTTTGCCTGCCTGTGATCTCTATTTACTCTTGGCCGAGCTTAAC
TAAACTATTGCCTGAAATGCATATTTGAATGCCATAAATTCTGATTGGCCACTTGGCGGAAGAAAAGAGC
AATTCGTTCCATTAGATTACACTCCATGCAATAACTTTGTGAAACGAACTATTGTTTGTTGATTTTGGAA
CAATAAATGCCACTTGCTCCTAGGAAAATCGTAATGAGAAAGCTGATGTTTTTGAATAAGATATGTAGTT

TAAATAATCGATGTACAAATGCATAGCCTATGCAATTAAATATTTTTCATGTATTTTACTTCATTATTAT
TTCACCTTATAAATGCAACAACTCAACCATATCACGAATACGCGATGCGGGCCACAAAGTACAAGTTGAG
CGCTGTTAATAGACAAAATAAAAGGCAAGGCATATTAAAAATTCATTAAATGAAAAGCGAGCAGAATCTA
GAACTATAGCCGTAGTAGAGACAAGGCTCAACTGGGGCATAATAAATGTTCGGTTTGCCTGCACAGGACT
CGCTGCAAACGAGTGTGTGTGTGTCTTTCCAGTTGCTACATGCAGCCTTAAGGACATCATCAATCTTCGT
TCCTGCCACCAGCACACTGACAAATTGTGCATTTCGTTTCTAGCAAGCAGTATCCAGTTTCCAGCATCTA
TATTTGTGCCACCGCACACAAAATATATAGAGAGAAATCGCACATATTGTGTGTCTAACGAGTTAATCGC
AGTTGCAGCTCAACTTGTGACAGTTTTGCACCAGCCGAGCTAATAAGGGGCAACAACTGGGCGGAGATAA
ACTCTTACCCATATGAAAATCATAAATCACACTGTTCCATGGCGAACAACTTCGCAGCAGCGACAACTTC
GACGATGAAGTGGTCTTTCGAAGACCCCGTATCTAGGGGCACAACTCGCATTCCCGGGCTGCCAAAATTG
TTTTAGCTTCCACAAATTGTCAAAGTCAGAGGAAAACACAAGCTCACGTAGCTGTGGCCGTGCGGTGCAG
ACTTTAGCCCTAAACTTGGCCAAGTGCCGATAGTCAGGTCCAGGGATGACCACATAAGCCGGGAACATGT
GCTGTGGAACTTTGCCTGCACTAGAAAAACAAAAAATCGTCCTTATATAAAACGAAATAAATCCTAAAGC
ACTTATAAAACACTAACTAACTTGATTCTCAAGTACAGATTTTTTGCTGATTCTGAGAAGTGAGATATAA
CCTTTATAAATCATAATAGGAAATTAAATTAACGCAAATTAAATGTAATTTAATAGTAAGCACACATTAT
ATCATTAGATTATGTgtttacatttttttgaattataaacaaatttttacaacatttttttttataaaaatc
aattgtaagcttaatttttttAAAAGTGTCTCCTCGTTTAACTTCTTTAAAGAGCGGGGAAGCTTTATTT
GGTCTCGAAAGTGTTTGATGGCATTGCATCGATTGCTGCAGTCAGCTCTTGGCTTAAATGGATTACGATA
ATGTCCCTCTTCAATGGGATTCGAAGGTGTTGAGTGAATTAACACACACATCCACCGACAGATGGCAATT
TTCGGTATTTTTTCAGGCTTAGCATGTGAATGGGTGCCAGGCATCCTTGGCATCGCATCCAATTGAATTT
TCCCCCGAGATCTGCTTTCACTTGGGTCTTATTCAAGCGTAGAGGACGGAATGATTAAGAGAGCCTGGGG
TTTTAATTCAGCCGCATTCCCAGAATCATTGGCGAAAGGCGGACCAAATATTACGACTGTGCAAACGGGG
TACAGTCACTTGGTTCGGTCCAAGGTTATTCCGAGTTGAACTGAGCTGAGCATGCAGATTGTTCATTTGA
ATTTCTACTCCGAGCATTTCCACCTCAGCTGGCTGGTTCAGTTCTGGTCTGCATTTTCCAGATAACAATT
GTACAACTCCGGCGGCCAAAATATTCGAAAATTCTCTTCTTCTCCCCCGATTGTTGTGGCTATATTTTTT
GTCGATGTTGTCGGTCCTTTTCTTAATTTCTGCCAATTTCTGTGCCATCGACACTTCAGCTGGAGCTCTG
GCATGTGGATTTTTATGCAAACTATGCCAGAAATTGCACCCAACTGAACCATTTTGCCACTGCAATTTTG
TATCAATACGTGGGTGCGGTGGGTTGGCCCAAACGAAGGCCAAACGAGCCAACAAGCGGGTAAAAAGAGC
AAAAAGCGTAAATATTGCAACGAACGGAAATGCTTTGCAGCACGAGCAACTTCAGACCAGAAATCGCAGG
AGCATTCGGATTGTGAGCTGATTGTATTTCATATCCACGTGGCGTTTTTAGGTCGGAAAAAGGTTGGGTT
CCCTGTATTTGTATTGGCAATACCCACACATAAATACGAGTGTAAATCCGTACTAGTATCTGCGTGGGAA
TTGGTTTGCATTGCGGCTCTATTATGGCTTTGAATGGGTAAAAATACTTTTGGAAAAAACGCCATAACTA
CAGGAAGTAACACAAGTGCCAGTGCCACACGTTGATGGAAGGCATTTGGTTACTTGAAAAGAAAATTTGA
ATGTGTGAAATGGGAAACCTCAGAGCTCCAAACGTTTTTATTGCCTAGGGTTTAGGAGTTATAATTTTAG
ACACGTAATTTAAAAAGACATTTTCGTTCAGAAATTGCAAATTATTCCAAGCGAAAGCAGCCCTTAAATT
CTTGTTGTGTGTTCTCGCACATGATTCTGTTTGGTTGTAGGAATTGATGTTTAGGAATAAAAAACTAAAT
GGATTTTATCAGAAATGTAGTATAACATTGGCATTGAAATCCTGTCCATACTTATATTTTTTAGAGCTCC
ACGCGGAACGCTCTTAATCTTCTGAGTAGTAATTTGGCAACGCCCTTAAGGCCATATGTGTATCTTGTAG
GTCATCTCGTTTGGCTTTATGGGGCCACGTGTGTGCGGTACGTGCAAGTGACCGTCCAGCAACAAATCAC
ACATAAAATTTATTAGAAATTTCAAATCAAAGTGAAAAGTGACCGCTGTCAGCCAGCGGAGCCGCTTCAG
TCCCAGCGGCAGTGCTACATTTATACCCAGCTCGTATGTAAGGCCTTTTAACGCATTTTTTGGCATTCTT
GTTTCTTTACAGGCTACCAGGATATCTTGCACATACCAACGGCGCTCACACAATTTTCAACATCATTGGG
TCCAATTGTCGATGGACATGTAATACCAAATCAACCGTACAAGGTCATGGGGCATTATACGGAGCACTTC
AGCCGGGTTTGTTAAGCAATTCCCTCCCCTTTCCCATTTGTACCCATTTTATCCATTGCTATTGCTTTTC

CCTAACCTGGCCCTAACCATTGCCAATTCCTTTGCGTTTGATATTTACTGGTGCTCCGTGCTTTGGGGAG
CTGTCAAACGCTCCCTTTCATCCGCTTTTGGCCAGGGCAGACAGCTCACCGGACTATTTATTGTGCGTTA
CTTGATTAGGCGTCTGGTATTTCTTTCTTCGGTGCATGTGTTTGCCCTCAGGTGAAGTCAAATCGAGTGT
GAAAATCGGGGCCTGAGGTGAACCATTTAACGCGCCGATTTTGCGTTGGCGTTAGCTCAGTTATCATCAA
GCAAATTACGTCTCGATGTCAATTCTGGGGTGCCAACCGAAAAAACATTCATTTCAACACCCAATGCACC
CTACCTTTGGTCCATTCTAGATGTTTCTCCAGACACTATTTAGGGTCGGGAAATGGGTTTAGCTACGGTC
ATATCGAAGGTGAAACACTGAAGGCCAATAGTTATAGTGATTCTGCGTGGAGCAAAACGGATTTGATTGG
ATTACATGAACCTAAAATGGTAGAAGTAATACCGTTTATGGTACTAGATTTACTCGTCACAAGAATATTC
AAAAAGGATCTTGTTTTATATAGCTATCAGATAACTTGGTGTTGTCTTTAAAAGTACTTTTCGCCGTGCG
TCAAAATCAAATTAAATTAGCCTAATACAAATAATGGCCTGGGTCGCATCAAAGAGCGGCTTTGAGGGAT
TGTGTACCGGCGGGCCCCTTGAAATTACACCCGTCCATCAATCAATTGGGCAACAAGTAATAACTCAACT
CCGCGCAGGACTCCGCACTCCGTACGTATTCCTCGGCCAGGAGTCTCCCCTCGGATCTCTCACATTTCCC
CGAAGGACATTAGGGCACGTGCTGCTCAGCCATTCAGTCAACCTAACACGGTATAATCTAATAAAATGCG
TGATTAATTTGATTTTAATTGTTATCCTTTTGCAAGTCAAACGTGTTTGACACGCAGTGCCGAGAAAAAT
AGAATAATAGCTGCCAGGAAGAGCCATTAATTACTGCAAACAGACAGGGTTCCGGGCGCAGAGGGGTAGG
TCACGGTTCAGTTGAGAAACTGAGGTGGAAGCACGTTTCACTCGACCGGAGATGGCGTCCGGAGCGGTAA
CCCCCAGGTTGGGCTTTTAATCACCTTAATCAGCGATGACCAAGGATTAAAGCCTTCCCAAATGAGTGAG
GTGCCCGGAGGAGTGCAGGAAAAGTGGTCCTTGAGCACGCTCAAGTGTCCAGTTGCCATTCCATTGGCAG
CTCCATTCAATTTAGCAAGTTCTGCAGCAGCTTTGCATTTACTTTTATCGGCGCAAACTTTCCGGCCCAA
CTTGAGTGGAACTTTTAATGCCATGTCCACGGCGCAACAGGAAGACAATGCCCGGTTGGTCGCTCGAGGG
GCTCCCCAGGGTGGAGGCCTGTACGGGATCTGGACTGGAGCTGGGAAAAGAGCACTTTCCATAGACGAGC
ACCAGTCGCACCATTTATCAGGCGGCGTAACAAGTTACGCCACAGGAACGAGGGCTCGTTTTATTTTAGT
GCTTTTCACTGCGTTTTATGGGATTTCATTGGACTGCTGCACTGAGAGATGCAATGGGTTGTAAGCTTAT
GTATTATGTATTAAGtaaatagaaaatagaaataaatagaataatagaaataaatagaaCATTTTTGTTT
TAGGCCTGCTTGTTGTGCAAACAGTTTTGCCTTGATGGGCGAAATGAATATAATATGATTATCCAAGGAA
AAGTTGAGCATAAGTGTTCTAGATTTTGTAATATATTATAAATTTTGAACTTAACTTAACGTTCTTGAAA
ATATCCTTAATGTAGTGCTGTGTACTTTCCACACATCGCATAAGTGAGAAAGTTTGCACTCCTGTTCTTA
GCACTGGCATATATTACTCCCCAAAAGTTTGCCTTCTTAACGTCACCTTTTAATTTGGTAAATCGGCTTA
TATTAATTCCCACTAACACCTGTATTAACACCTTGAGTGGCGAAAATATCACTTACTATTTTACAGTGCA
CAAAAGTAGCCTTCGTCCACCAGCGGCAAACACAAGCTGTGCATGTTGACTGCTCCTCCACAACGCCCCC
AGCGCAACTGGCCTCTCTCTCACACCGTCTCTCGCTGTCTCTTTCAGGTTATGATTTATTATTCGGCATC
ACGGAGTCAGAATCCTATCACACACTGGCCGCATTAGCACTCGAGGAAGGATTACGTGAAAATGAACGCG
ATAATTTATTGCGCTTCTATATGCAGAGCCGCTTCGATATACGCCCCGATTTGGCATTGGCTGCCACGCT
GAAGAA
>dme_circ_0008175_junction_seq
ATTTGGCATTGGCTGCCACGCTGAAGAAGGACAAAAATTTTGTTGCAAATGAAATT
>dme_circ_0008176
GGTGCTCCACTACCATGGCAGGGCTTGAAGATAGCGCGACATCTTCCGCCCGTGTGTCCGCAGAAATTGC
CAGATCTTTCGCCCCACGGCAGTGAAAATATGTCCCGGGCTCGTCATAAACACCTGAGTCGACTGCTCCC
TTATCTGCGGACGGAGAGCGAGGACTGTTTGTATCTCAATCTGTATGTGCCACATGAAGGGTAGGTCGGC
CCCATATATCAGAGATCTGAAAATTTGATAGAGTCGGAGCTTGTTACCGATAAAATGCAATTAATGTGCT
GTCACACATTGTGCGTATTAATCTCTGCTCATTAACGGGTATCTGACAGTCGAATATAATCAATAATCTT
AATTTATATTAAAAGCCTTGCCATATGTGACAAATCCAAGCTGAAGTGAATTTTGTATCTAAAATTTAGG
AGCCTCAAAGCACCCCCAAGCAATACGCTGTGCTGGTCTATCTGCACGGGGAGTCCTTCGAGTGGAACAG

TGGCAATCCCTACGATGGCTCCGTCTTGTCCAGCTATGGAGAGGTGATTGTGGTCACCGTCAACTACCGG
CTGGGAGTGTTGGGGATTCTTACGGCCGAGCATCGATGCGCATAATATAGCGAATTACGCCCTGCTCGAC
CAGATTGCGGCGCTACACTGGATCAAGGAAAACATCGAAGCCTTCGGCGGTGACAATTCGCGTGTCACTT
TAATGGGGCACAGTACGGGGGCGGCGTGTGTCAATTACCTAATGGTATCGCCGGTGGCCTCAGGGCCTCT
TTCACAGGGCCATCCTCATGTCCGGATCCGCGATGTCCGACTGGGCCGCCAGCAATCAGTCCCTGCAATT
GACGATGCAAATCGCCCATGCCCTCGAATGTCCACTCAACGAGCACGTGGAGGCAGAGGACGACGATGTC
CTGCTCGATTGTTTGCGCCATCGTCG
>dme_circ_0008176_junction_seq
TCCTGCTCGATTGTTTGCGCCATCGTCGGGTGCTCCACTACCATGGCAGGGCTTGA
>dme_circ_0008177
GGTGCTCCACTACCATGGCAGGGCTTGAAGATAGCGCGACATCTTCCGCCCGTGTGTCCGCAGAAATTGC
CAGATCTTTCGCCCCACGGCAGTGAAAATATGTCCCGGGCTCGTCATAAACACCTGAGTCGACTGCTCCC
TTATCTGCGGACGGAGAGCGAGGACTGTTTGTATCTCAATCTGTATGTGCCACATGAAGGGTAGGTCGGC
CCCATATATCAGAGATCTGAAAATTTGATAGAGTCGGAGCTTGTTACCGATAAAATGCAATTAATGTGCT
GTCACACATTGTGCGTATTAATCTCTGCTCATTAACGGGTATCTGACAGTCGAATATAATCAATAATCTT
AATTTATATTAAAAGCCTTGCCATATGTGACAAATCCAAGCTGAAGTGAATTTTGTATCTAAAATTTAGG
AGCCTCAAAGCACCCCCAAGCAATACGCTGTGCTGGTCTATCTGCACGGGGAGTCCTTCGAGTGGAACAG
TGGCAATCCCTACGATGGCTCCGTCTTGTCCAGCTATGGAGAGGTGATTGTGGTCACCGTCAACTACCGG
CTGGGAGTGTTGGGGATTCTTACGGCCGAGCATCGATGCGCATAATATAGCGAATTACGCCCTGCTCGAC
CAGATTGCGGCGCTACACTGGATCAAGGAAAACATCGAAGCCTTCGGCGGTGACAATTCGCGTGTCACTT
TAATGGGGCACAGTACGGGGGCGGCGTGTGTCAATTACCTAATGGTATCGCCGGTGGCCTCAGGGCCTCT
TTCACAGGGCCATCCTCATGTCCGGATCCGCGATGTCCGACTGGGCCGCCAGCAATCAGTCCCTGCAATT
GACGATGCAAATCGCCCATGCCCTCGAATGTCCACTCAACGAGCACGTGGAGGCAGAGGACGACGATGTC
CTGCTCGATTGTTTGCGCCATCGTCGGGTGAGTAGTCCTCGACCAATTGAATTATTTAGGCGCGCCAACA
ACCGCATCAGCGATATGCGGACCATTATAAACAGCCGTAATGCCTGGTAATGCCTTTCAATGGTCTACAC
GGAAAGAAATGAGGGTGGAGTCATTAGTCAAAATTAAAATAGCTTCAACCAACTTTCTTTCCCAATTGGT
ATCTTTTGAAACGACGAACAATGCCCTTTGCATATGATACACTTGTGGTTTGACAGTTCATTTTGTTTTA
AAATTTAAAGAGCGTCACTTATTTTCGCACAGTGCTTTCTGTGGCGATGCGAGCGCGTCTACTTCATTTG
CACTGGCATCTTCATTTGAATAGTGTTTTGTGTCCTTTGATTCAATTCGTCAGGCCAGCGGTATTTGGCG
GCTTTGTGACCTGCCTAATGATTCGTTAAATTTACGAGCTTCCAGCACCACGGTGTCAATAAGGCGTAAA
GGCCTTTTGGCTCAACATCCTCATCAAACTTCGCTTATAGGACCTTGACTAATCAGGAGTAATGACGATG
GCTTAAAATTTGCATGGCGATTTCCAGGGACGAGGGACCGTAGGTCAGCAAAAGCAAAAAGGACGACTAC
GATGCGGATGGATGGCAGGGATTCAATTTAGTTACATTATAATTGCTCAATTCACATTCAAGTGATTTAA
TTTACGCTCCTACTTTTGCAGTTTCTTGCAGTTTTATAATTGCGTATTCATTGCACGCATTCCGGCGGCT
TATCCTATCAGTATGCCAGTGTCCCTTTCCATTAAAGCATGAGCTCTAAACGCTTAAAAATAAACACGAC
CTAAGAGCACTTTTTATTTATCAGCATTTAATGCATGTGTGGGGCTTTGTGCTCTTGTGTGGGAGGAAGT
TCATTTGTATGCGCAGAGAGCACACAAGAGCGAACTAAAAAACAATTTGCATACGAAATGAAAAAAAGAC
GCATGTAACTAAATATGTAAAACTAAATCAACAACGGCACAATAATAGTAATTATGTTAATTACGCCAGC
ACACACTCACCCACAAACTCCCAGCCATTCCGCTCACAAACACTATGTGGCAACAGTCGGTTTTATCCGC
GTGTGTGTGCGCCTTTGAAAACTTTCTACTCGCTTTCTTGTCCTTAATACAAATTGGCACAGTAACTTGA
AAACCCAAGCCAGAACTTTTGCTGATGTAAATTGTCGATTCTAGTAGTTGGCATACGGCCTTTTTAGGCT
CTACTGGGGATACAAACTTGAAACAGGATAATACAAGAAGAGCACAATTTTAAAAGTGCTAATTTAGGGT
TAGGCTACAATTATCTAGAGCGTTGGAATATTTAATACTAAAACTAACAAATTTTTTTATTCAATATGGA
TTAAACATAACATATTTTTAAAGTGATTTGAGTTTTCAGGAGTATTCAAACCTTATTTTGTTGACCTAAA

CATCTTTAATTAACACAATTTCGAGTGCGTTTAGAAAGTAGGAATACCTCGCTACTGAAGGGTTCATTTC
GAAATGTTTTAATGAATGAGCTAAGCTTCGGTTTGAGCCTGGGAATTGCAGCCTCGTGTGGCAGCTTACT
TAAATTACGGCAGAAGGAGCGAGTGAATGAGTGAGTGAGTAGAGGTTTAGTAGCATGTGTGCCAGCCAGA
GCGAGTGTTAAATGAATGCAATGAATTATGCATGAATTACTGAGCAAATGTGCTCATCGCTTTTACTGCA
AAGgcacacaactcacacacacacacacacccactgaaacacaaacaGGTAACAACTACTAGTGCAAACA
AACGGAGAGTCACAGTCGCATTTGTTTAACCCACTCTCACAAACAGGCATAAGTCTTTGTCTTTGGGTCT
TAGGCTTCCTAAATATTTAATAGAACCTCTGCCCGAATCCTGCCCATTGATTTGAATGTGTGCTTTTCCA
ACTTGTTAATTTATTCAAATTATTTACCTATTGCCCCAGCCTAATGTGTTTGCCTGCCTGTGATCTCTAT
TTACTCTTGGCCGAGCTTAACTAAACTATTGCCTGAAATGCATATTTGAATGCCATAAATTCTGATTGGC
CACTTGGCGGAAGAAAAGAGCAATTCGTTCCATTAGATTACACTCCATGCAATAACTTTGTGAAACGAAC
TATTGTTTGTTGATTTTGGAACAATAAATGCCACTTGCTCCTAGGAAAATCGTAATGAGAAAGCTGATGT
TTTTGAATAAGATATGTAGTTTAAATAATCGATGTACAAATGCATAGCCTATGCAATTAAATATTTTTCA
TGTATTTTACTTCATTATTATTTCACCTTATAAATGCAACAACTCAACCATATCACGAATACGCGATGCG
GGCCACAAAGTACAAGTTGAGCGCTGTTAATAGACAAAATAAAAGGCAAGGCATATTAAAAATTCATTAA
ATGAAAAGCGAGCAGAATCTAGAACTATAGCCGTAGTAGAGACAAGGCTCAACTGGGGCATAATAAATGT
TCGGTTTGCCTGCACAGGACTCGCTGCAAACGAGTGTGTGTGTGTCTTTCCAGTTGCTACATGCAGCCTT
AAGGACATCATCAATCTTCGTTCCTGCCACCAGCACACTGACAAATTGTGCATTTCGTTTCTAGCAAGCA
GTATCCAGTTTCCAGCATCTATATTTGTGCCACCGCACACAAAATATATAGAGAGAAATCGCACATATTG
TGTGTCTAACGAGTTAATCGCAGTTGCAGCTCAACTTGTGACAGTTTTGCACCAGCCGAGCTAATAAGGG
GCAACAACTGGGCGGAGATAAACTCTTACCCATATGAAAATCATAAATCACACTGTTCCATGGCGAACAA
CTTCGCAGCAGCGACAACTTCGACGATGAAGTGGTCTTTCGAAGACCCCGTATCTAGGGGCACAACTCGC
ATTCCCGGGCTGCCAAAATTGTTTTAGCTTCCACAAATTGTCAAAGTCAGAGGAAAACACAAGCTCACGT
AGCTGTGGCCGTGCGGTGCAGACTTTAGCCCTAAACTTGGCCAAGTGCCGATAGTCAGGTCCAGGGATGA
CCACATAAGCCGGGAACATGTGCTGTGGAACTTTGCCTGCACTAGAAAAACAAAAAATCGTCCTTATATA
AAACGAAATAAATCCTAAAGCACTTATAAAACACTAACTAACTTGATTCTCAAGTACAGATTTTTTGCTG
ATTCTGAGAAGTGAGATATAACCTTTATAAATCATAATAGGAAATTAAATTAACGCAAATTAAATGTAAT
TTAATAGTAAGCACACATTATATCATTAGATTATGTgtttacatttttttgaattataaacaaatttttac
aacatttttttttataaaaatcaattgtaagcttaattttttttAAAAGTGTCTCCTCGTTTAACTTCTTTA
AAGAGCGGGGAAGCTTTATTTGGTCTCGAAAGTGTTTGATGGCATTGCATCGATTGCTGCAGTCAGCTCT
TGGCTTAAATGGATTACGATAATGTCCCTCTTCAATGGGATTCGAAGGTGTTGAGTGAATTAACACACAC
ATCCACCGACAGATGGCAATTTTCGGTATTTTTTCAGGCTTAGCATGTGAATGGGTGCCAGGCATCCTTG
GCATCGCATCCAATTGAATTTTCCCCCGAGATCTGCTTTCACTTGGGTCTTATTCAAGCGTAGAGGACGG
AATGATTAAGAGAGCCTGGGGTTTTAATTCAGCCGCATTCCCAGAATCATTGGCGAAAGGCGGACCAAAT
ATTACGACTGTGCAAACGGGGTACAGTCACTTGGTTCGGTCCAAGGTTATTCCGAGTTGAACTGAGCTGA
GCATGCAGATTGTTCATTTGAATTTCTACTCCGAGCATTTCCACCTCAGCTGGCTGGTTCAGTTCTGGTC
TGCATTTTCCAGATAACAATTGTACAACTCCGGCGGCCAAAATATTCGAAAATTCTCTTCTTCTCCCCCG
ATTGTTGTGGCTATATTTTTTGTCGATGTTGTCGGTCCTTTTCTTAATTTCTGCCAATTTCTGTGCCATC
GACACTTCAGCTGGAGCTCTGGCATGTGGATTTTTATGCAAACTATGCCAGAAATTGCACCCAACTGAAC
CATTTTGCCACTGCAATTTTGTATCAATACGTGGGTGCGGTGGGTTGGCCCAAACGAAGGCCAAACGAGC
CAACAAGCGGGTAAAAAGAGCAAAAAGCGTAAATATTGCAACGAACGGAAATGCTTTGCAGCACGAGCAA
CTTCAGACCAGAAATCGCAGGAGCATTCGGATTGTGAGCTGATTGTATTTCATATCCACGTGGCGTTTTT
AGGTCGGAAAAAGGTTGGGTTCCCTGTATTTGTATTGGCAATACCCACACATAAATACGAGTGTAAATCC
GTACTAGTATCTGCGTGGGAATTGGTTTGCATTGCGGCTCTATTATGGCTTTGAATGGGTAAAAATACTT
TTGGAAAAAACGCCATAACTACAGGAAGTAACACAAGTGCCAGTGCCACACGTTGATGGAAGGCATTTGG

TTACTTGAAAAGAAAATTTGAATGTGTGAAATGGGAAACCTCAGAGCTCCAAACGTTTTTATTGCCTAGG
GTTTAGGAGTTATAATTTTAGACACGTAATTTAAAAAGACATTTTCGTTCAGAAATTGCAAATTATTCCA
AGCGAAAGCAGCCCTTAAATTCTTGTTGTGTGTTCTCGCACATGATTCTGTTTGGTTGTAGGAATTGATG
TTTAGGAATAAAAAACTAAATGGATTTTATCAGAAATGTAGTATAACATTGGCATTGAAATCCTGTCCAT
ACTTATATTTTTTAGAGCTCCACGCGGAACGCTCTTAATCTTCTGAGTAGTAATTTGGCAACGCCCTTAA
GGCCATATGTGTATCTTGTAGGTCATCTCGTTTGGCTTTATGGGGCCACGTGTGTGCGGTACGTGCAAGT
GACCGTCCAGCAACAAATCACACATAAAATTTATTAGAAATTTCAAATCAAAGTGAAAAGTGACCGCTGT
CAGCCAGCGGAGCCGCTTCAGTCCCAGCGGCAGTGCTACATTTATACCCAGCTCGTATGTAAGGCCTTTT
AACGCATTTTTTGGCATTCTTGTTTCTTTACAGGCTACCAGGATATCTTGCACATACCAACGGCGCTCAC
ACAATTTTCAACATCATTGGGTCCAATTGTCGATGGACATGTAATACCAAATCAACCGTACAAGGTCATG
GGGCATTATACGGAGCACTTCAGCCGGGTTTGTTAAGCAATTCCCTCCCCTTTCCCATTTGTACCCATTT
TATCCATTGCTATTGCTTTTCCCTAACCTGGCCCTAACCATTGCCAATTCCTTTGCGTTTGATATTTACT
GGTGCTCCGTGCTTTGGGGAGCTGTCAAACGCTCCCTTTCATCCGCTTTTGGCCAGGGCAGACAGCTCAC
CGGACTATTTATTGTGCGTTACTTGATTAGGCGTCTGGTATTTCTTCTTCGGTGCATGTGTTTGCCCTC
AGGTGAAGTCAAATCGAGTGTGAAAATCGGGGCCTGAGGTGAACCATTTAACGCGCCGATTTTGCGTTGG
CGTTAGCTCAGTTATCATCAAGCAAATTACGTCTCGATGTCAATTCTGGGGTGCCAACCGAAAAAACATT
CATTTCAACACCCAATGCACCCTACCTTTGGTCCATTCTAGATGTTTCTCCAGACACTATTTAGGGTCGG
GAAATGGGTTTAGCTACGGTCATATCGAAGGTGAAACACTGAAGGCCAATAGTTATAGTGATTCTGCGTG
GAGCAAAACGGATTTGATTGGATTACATGAACCTAAAATGGTAGAAGTAATACCGTTTATGGTACTAGAT
TTACTCGTCACAAGAATATTCAAAAAGGATCTTGTTTTATATAGCTATCAGATAACTTGGTGTTGTCTTT
AAAAGTACTTTTCGCCGTGCGTCAAAATCAAATTAAATTAGCCTAATACAAATAATGGCCTGGGTCGCAT
CAAAGAGCGGCTTTGAGGGATTGTGTACCGGCGGGCCCCTTGAAATTACACCCGTCCATCAATCAATTGG
GCAACAAGTAATAACTCAACTCCGCGCAGGACTCCGCACTCCGTACGTATTCCTCGGCCAGGAGTCTCCC
CTCGGATCTCTCACATTTCCCCGAAGGACATTAGGGCACGTGCTGCTCAGCCATTCAGTCAACCTAACAC
GGTATAATCTAATAAAATGCGTGATTAATTTGATTTTAATTGTTATCCTTTTGCAAGTCAAACGTGTTTG
ACACGCAGTGCCGAGAAAAATAGAATAATAGCTGCCAGGAAGAGCCATTAATTACTGCAAACAGACAGGG
TTCCGGGCGCAGAGGGGTAGGTCACGGTTCAGTTGAGAAACTGAGGTGGAAGCACGTTTCACTCGACCGG
AGATGGCGTCCGGAGCGGTAACCCCCAGGTTGGGCTTTTAATCACCTTAATCAGCGATGACCAAGGATTA
AAGCCTTCCCAAATGAGTGAGGTGCCCGGAGGAGTGCAGGAAAAGTGGTCCTTGAGCACGCTCAAGTGTC
CAGTTGCCATTCCATTGGCAGCTCCATTCAATTTAGCAAGTTCTGCAGCAGCTTTGCATTTACTTTTATC
GGCGCAAACTTTCCGGCCCAACTTGAGTGGAACTTTTAATGCCATGTCCACGGCGCAACAGGAAGACAAT
GCCCGGTTGGTCGCTCGAGGGGCTCCCCAGGGTGGAGGCCTGTACGGGATCTGGACTGGAGCTGGGAAAA
GAGCACTTTCCATAGACGAGCACCAGTCGCACCATTTATCAGGCGGCGTAACAAGTTACGCCACAGGAAC
GAGGGCTCGTTTTATTTTAGTGCTTTTCACTGCGTTTTATGGGATTTCATTGGACTGCTGCACTGAGAGA
TGCAATGGGTTGTAAGCTTATGTATTATGTATTAAGtaaatagaaaatagaaataaatagaataatagaa
ataaatagaaCATTTTTGTTTTAGGCCTGCTTGTTGTGCAAACAGTTTTGCCTTGATGGGCGAAATGAAT
ATAATATGATTATCCAAGGAAAAGTTGAGCATAAGTGTTCTAGATTTTGTAATATATTATAAATTTTGAA
CTTAACTTAACGTTCTTGAAAATATCCTTAATGTAGTGCTGTGTACTTTCCACACATCGCATAAGTGAGA
AAGTTTGCACTCCTGTTCTTAGCACTGGCATATATTACTCCCCAAAAGTTTGCCTTCTTAACGTCACCTT
TTAATTTGGTAAATCGGCTTATATTAATTCCCACTAACACCTGTATTAACACCTTGAGTGGCGAAAATAT
CACTTACTATTTTACAGTGCACAAAAGTAGCCTTCGTCCACCAGCGGCAAACACAAGCTGTGCATGTTGA
CTGCTCCTCCACAACGCCCCCAGCGCAACTGGCCTCTCTCTCACACCGTCTCTCGCTGTCTCTTTCAGGT
TATGATTTATTATTCGGCATCACGGAGTCAGAATCCTATCACACACTGGCCGCATTAGCACTCGAGGAAG
GATTACGTGAAAATGAACGCGATAATTTATTGCGCTTCTATATGCAGAGCCGCTTCGATATACGCCCCGA

TTTGGCATTGGCTGCCACGCTGAAGAA
>dme_circ_0008177_junction_seq
ATTTGGCATTGGCTGCCACGCTGAAGAAGGTGCTCCACTACCATGGCAGGGCTTGA
>dme_circ_0008178
GCTACCAGGATATCTTGCACATACCAACGGCGCTCACACAATTTTCAACATCATTGGGTCCAATTGTCGA
TGGACATGTAATACCAAATCAACCGTACAAGGTCATGGGGCATTATACGGAGCACTTCAGCCGGTTTGTT
AAGCAATTCCCTCCCCTTTCCCATTTGTACCCATTTTATCCATTGCTATTGCTTTTCCCTAACCTGGCCC
TAACCATTGCCAATTCCTTTGCGTTTGATATTTACTGGTGCTCCGTGCTTTGGGGAGCTGTCAAACGCTC
CCTTTCATCCGCTTTTGGCCAGGGCAGACAGCTCACCGGACTATTTATTGTGCGTTACTTGATTAGGCGT
CTGGTATTTCTTTCTTCGGTGCATGTGTTTGCCCTCAGGTGAAGTCAAATCGAGTGTGAAAATCGGGGCC
TGAGGTGAACCATTTAACGCGCCGATTTTGCGTTGGCGTTAGCTCAGTTATCATCAAGCAAATTACGTCT
CGATGTCAATTCTGGGGTGCCAACCGAAAAAACATTCATTTCAACACCCAATGCACCCTACCTTTGGTCC
ATTCTAGATGTTTCTCCAGACACTATTTAGGGTCGGGAAATGGGTTTAGCTACGGTCATATCGAAGGTGA
AACACTGAAGGCCAATAGTTATAGTGATTCTGCGTGGAGCAAAACGGATTTGATTGGATTACATGAACCT
AAAATGGTAGAAGTAATACCGTTTATGGTACTAGATTTACTCGTCACAAGAATATTCAAAAAGGATCTTG
TTTTATATAGCTATCAGATAACTTGGTGTTGTCTTTAAAAGTACTTTTCGCCGTGCGTCAAAATCAAATT
AAATTAGCCTAATACAAATAATGGCCTGGGTCGCATCAAAGAGCGGCTTTGAGGGATTGTGTACCGGCGG
GCCCCTTGAAATTACACCCGTCCATCAATCAATTGGGCAACAAGTAATAACTCAACTCCGCGCAGGACTC
CGCACTCCGTACGTATTCCTCGGCCAGGAGTCTCCCCTCGGATCTCTCACATTTCCCCGAAGGACATTAG
GGCACGTGCTGCTCAGCCATTCAGTCAACCTAACACGGTATAATCTAATAAAATGCGTGATTAATTTGAT
TTTAATTGTTATCCTTTTGCAAGTCAAACGTGTTTGACACGCAGTGCCGAGAAAAATAGAATAATAGCTG
CCAGGAAGAGCCATTAATTACTGCAAACAGACAGGGTTCCGGGCGCAGAGGGGTAGGTCACGGTTCAGTT
GAGAAACTGAGGTGGAAGCACGTTTCACTCGACCGGAGATGGCGTCCGGAGCGGTAACCCCCAGGTTGGG
CTTTTAATCACCTTAATCAGCGATGACCAAGGATTAAAGCCTTCCCAAATGAGTGAGGTGCCCGGAGGAG
TGCAGGAAAAGTGGTCCTTGAGCACGCTCAAGTGTCCAGTTGCCATTCCATTGGCAGCTCCATTCAATTT
AGCAAGTTCTGCAGCAGCTTTGCATTTACTTTTATCGGCGCAAACTTTCCGGCCCAACTTGAGTGGAACT
TTTAATGCCATGTCCACGGCGCAACAGGAAGACAATGCCCGGTTGGTCGCTCGAGGGGCTCCCCAGGGTG
GAGGCCTGTACGGGATCTGGACTGGAGCTGGGAAAAGAGCACTTTCCATAGACGAGCACCAGTCGCACCA
TTTATCAGGCGGCGTAACAAGTTACGCCACAGGAACGAGGGCTCGTTTTATTTTAGTGCTTTTCACTGCG
TTTTATGGGATTTCATTGGACTGCTGCACTGAGAGATGCAATGGGTTGTAAGCTTATGTATTATGTATTA
AGtaaatagaaaatagaaataaatagaataatagaaataaatagaaCATTTTTGTTTTAGGCCTGCTTGT
TGTGCAAACAGTTTTGCCTTGATGGGCGAAATGAATATAATATGATTATCCAAGGAAAAGTTGAGCATAA
GTGTTCTAGATTTTGTAATATATTATAAATTTTGAACTTAACTTAACGTTCTTGAAAATATCCTTAATGT
AGTGCTGTGTACTTTCCACACATCGCATAAGTGAGAAAGTTTGCACTCCTGTTCTTAGCACTGGCATATA
TTACTCCCCAAAAGTTTGCCTTCTTAACGTCACCTTTTAATTTGGTAAATCGGCTTATATTAATTCCCAC
TAACACCTGTATTAACACCTTGAGTGGCGAAAATATCACTTACTATTTTACAGTGCACAAAAGTAGCCTT
CGTCCACCAGCGGCAAACACAAGCTGTGCATGTTGACTGCTCCTCCACAACGCCCCCAGCGCAACTGGCC
TCTCTCTCACACCGTCTCTCGCTGTCTCTTTCAGGTTATGATTTATTATTCGGCATCACGGAGTCAGAAT
CCTATCACACACTGGCCGCATTAGCACTCGAGGAAGGATTACGTGAAAATGAACGCGATAATTTATTGCG
CTTCTATATGCAGAGCCGCTTCGATATACGCCCCGATTTGGCATTGGCTGCCACGCTGAAGAA
>dme_circ_0008178_junction_seq
ATTTGGCATTGGCTGCCACGCTGAAGAAGCTACCAGGATATCTTGCACATACCAAC
>dme_circ_0008179
TGTTCCACGGCTTCATTGCTCGCCGGTTCCCGGAGCATCTTGGTCTCCTGGTTCAAGTCCAGCAGCTTTA

GCACAGTGAAGCCCACGTTGTGGGTGCGATTGGAAGCTCCTGGCTTCAAAAAGGTGACCTTGGCCAGATA
CCGGATGTGTCCATAGGCACCCTCGAAGGACGAGGGGCAGTGGCTGGGAATGGGGCAAGCAAAAGTGTAG
G
>dme_circ_0008179_junction_seq
CTGGGAATGGGGCAAGCAAAAGTGTAGGTGTTCCACGGCTTCATTGCTCGCCGGTT
>dme_circ_0008180
GAAGGCACTGTAAATGGCAAGCAATAGAAACACAATTATGTCAAATTCGAAAGTTCGTTAACATCAACAA
TCAATGCAATAACAACAAATGCTGCTGCGACAACGGCATCCGCAGGCCCCTCAAAATGTAATTGGGCCAG
GGCGTGGCAGCTTGGCAGTGGGCAACGGTCAACGGCAACGGCAGCAGCAATAGCAACACCACTGAACCGC
AAAAATCGACAGCAGCAGCATCAGGACACCAAACAAAATACGCAACATAAATGAAGTTAACAACCTGTCA
AATTGCTAAAGTATTTCCGCTGTTGACAGCCTTCTTCTTCCTCAATTCTCCCAGCGCAGCCTCATTATCC
TTCGGTTTGAGTCCCACCTTCGCCTCCGCCTCCGCCTCCAGCtccttgtcctcgtcatcgtcctcgtcct
cCCACTTTCACTCAGAAAAACCCCTCCACTCGGATGTACTCGTCCTGCGAGCGGCCAAGCCATCCAGGGC
CATTGCAACAAGCGCAGATGCGGCTACGCGGTCTGTGCCAGCGTCAGCGGCAGAAACAACACTTGGCAGC
AAGTCAATTTGGCCGACAGCTATACAAACAAAAAGTCCACGTAATGCGCGTGAAGCTGGACGTCACAAAC
GTCAAATGCAAAAGCCCGCAATCcagcagcaaccactgcagcagcagcaacagcgcattaatcccccgca
gcaaaagcgacagcagcagcagcagcagcagcagcaagtgcaacatcagGCTCAACGAGTGGGCGTTTTA
ATACCTCCTTCGCTGCACGTTGATTTGACGCATGTGCAACAGGGCTTTCAGAATTTCCTCGACTTTTTCC
AACTGCATTTATTTAATGTCACCGTCGATTTTCTACGTGATGTGGG
>dme_circ_0008180_junction_seq
TCACCGTCGATTTTCTACGTGATGTGGGGAAGGCACTGTAAATGGCAAGCAATAGA
>dme_circ_0008181
CCACCCTATCCACAACATTCCAATCCCGCATTAAAAGTAGTTCCTTGCCACCGCCCAACGGTAGAGTAGC
CTCAGCGCCGCCAGCCACTGCAGCAGCAGCATCAGCAGCCAAATCAGCATTAAGCGCTAATCAGGCTCAT
GCTAATCAATCACAATCATCACATGCCAAGAGCGGTAGCATCAGCGGCAGAATTTCAAAGCCATCGGCCT
CATCCGCCcaacagcaacaacagcagcaacagcaacagcaaATGCGTAAGAAAGACAAAGATAGATCGAA
TAGTAGTTGCATTGGCAGTGGTAGTCATGTTGGTGCTGGTAGCGGTAGTCAAGCAAATAGTGTCAagcat
ctgcagaataagcaaaagcaacaacaactacagcagcaacagcaacaaaagcaacaacaacaacagcaac
cgcaacagcaacagcaaaagcagcagcaaaaacagcagcagcaactcgatgttgcagcagcagcaacagc
agcagcagcaacatcggcaCCTCCCCAAAACCCAACCCGGCCTGCCACCATCAACATCAAACGGCTCACG
GTACCAGGTCAGGATAGAAAATTGGCTGAAAGTGAGTAAAGTTTCACCGAAAACTTGAACCAATCAAAAA
CCAAACTCTTCAATAAACCCAAGCTAAACCAAAATTAAAGCCCATTTAAAAAGTAAAATACACGGAAAAT
GCTGTAAAATGACATCTGATGCGCGAAATGTTTGTGTTGCAACTTTTGTAAAGCCCTAAACTTaaaacaa
aaaaaaaaaaaaaaaaaaaaaaaacgaaaaGACAATCTCTTTTCACGCTATCAATAATTAAGCATTTTA
CACCTTGGTCAAATCTGTTCGTCCACAATTAGTGAAAAGTGATTAAGCTTATGATTTACCGATTGGCCAC
ATCGATTAGATGGACTTGTGTGTGTGTTTTTGTTTGGGCCAAGCCAGGTGGAATTGTGTATCTGTATTGA
TAGATTGGTCGTAGAAAGTGTACAAAATTGTAGTTTGATTATAGTTATTCCACTGGCAGCCCGattcaaa
aatgaatataaaattcaccgaaaaagtacgaaaatcaaacaaaattccaaattcTTAATTGCTGTTTATT
GTGGCTACACCCAAAGAGCAAAATGCCTTATTAAATGTTATTAAGTGTTAGTTGTTGTTGTAGTCAGTGT
ATGTATTCGTTGTATATCAGCCGTGCGTCTATATAAATGTATCTGTATAAGCAATTTAAATTTAATGCCC
TTGAATGTGCGTCTCATGATTTGTAGATTTTCGTTGTTAAATCACATCTCAGAATTTCAAATATACCTTT
GTTTGTTTGTGTACATATGTAAAGTTTGTAGCATTTCTATCTTATCTCATTTTAGATTATATGTCTATGA
TTATTTCTTTTAAAACAACATAATCTACAAAAAGACACTTAAGCACAGACACTAACAACAATAAGGTAGA
AGTGTTAGTTAGTATTTTGATGTTCGAAATGGAGGTTTGGGTTAGTTAAACAGTTTTAGAAGAAAGTAAA

TCGTTTCTACTAACTACAGTTCACTTTCAAATCAACGTAGGAACGTGTACTTTTGTATCTACTACTTTAA
TTTCAATATTACCAACTGTTAAAATACATGATGAGTGTTCGATAATATCTAAGCGAACTCGAATTGCCAG
GGACTAACTGACAAATCTTTTTTAAGCTGTCGTCGACGCCTTCGTTGTTGCCTGCCaaaaaaaaaGAAAC
ACCAATAAAACATATCCAGCAATGTGCCACACCAGGATTTGTACAGCTATACCAATCTTTTGAAACATAA
AACATATATTAGTTTTATTACTCCTCTTCGGATTTTCATGTTGCGTGTCTGTTTGTGTATTTGTGGCATG
CTGTTTCTCATGTTGTGCGCTTTATTaaataaaacaaaaaataaaaaataaaaaTATTCCTTTGGTTCTA
CTAACAATATGTTGCCTTTTAAGCTTCTGGCTTAATATTCTTCCGTTGACTTCAGTTTCCTTCAGTTTTC
GGTTACCGAACTAAGTTAGAAATATTTATTTGGATTTTATGTTTCAAATACTAACCAGTAAAACTCAAAA
ACACAAAATTTGCTTAACGAACAAAGGACTTATCCaaaaaaaaaTTTATGAAGATCAACAGCTACGGCAG
CATAATGAAATAACAGAAACATAAAATGCGCTTATAATTAAGTTAATTCCAAAACGCATAACTTTCCAAA
CTGAGCAGAACTACCATTTATAAGTATTTTTGAATATTTTCCCAACCAACAGAACGAAGGCGAGGATGGT
CGTGAGACTGCCATTTCCATTTCCAGCTGTAGTCCCCAGCCTCCGGTGCCGGAGGTGCCCGAGGAGCCCC
TGGTGAAGTCGCCACCAGAACCGACCCGGGTCAAATCCCCGGAGCAGATCATAATGCGCTCTCCTGATCC
AGTCAACTGGACGGTGCCCCTGGACACTGGCAAAACCTTCACGGTGACGCAGAATGTCAAAGATGGTAGA
GTAGACACAAGGTTGTTGACCACTATGTAGAGCAGAAGATTGGAGTACAGATTGGAGTAAGATACTAATT
TGAGCGTGACTTTAATTTCTGCCTACGAAGTGCATCACTACCCTGACAAATATGTGCTGCAGCTAAGTAA
ATTCtttttttttttttAGGGGAGAACTACAGCCGACCTCAGAGCGAGATTAAAGCGTCGACACCGGTGG
AGAAGCCCCCACCGCCACCACAATCGGCTCCGCCACAATTAACCGAACAGGCTAAGATGGACGG
>dme_circ_0008181_junction_seq
AATTAACCGAACAGGCTAAGATGGACGGCCACCCTATCCACAACATTCCAATCCCG
>dme_circ_0008182
CTACTCTACATGAGTTGGAGCCCTTGGTTAGCGATACGGATGATAGAAAAACTCACGAGAAGTATGAACA
TTTGATTAACCTAGCCCTCAAATCGACTTGCTCATAGCTTCTGCCATCTCATTCAGTGATGTCCTTCATT
AGCCCACCCGAAAGAGCAGTATCATCCAAAATATAATAATAGCTTTCAGAACCCACATTCATCCGCACCT
GCATGTTGCAAGTGTTGCATGCTGCAGCCATCGAGAAGCACAGCAGTTCCCACTTGAAGCTCAGAATCTC
TCAGGAGCCAGCACTACTTCCCATTGCTAATGACTCTCCCGATTCTTGCAGACAAGTGACCATTATGGAG
CGCAAGACCACCTCGCGTCCCTTCTCGCAGGCCATCGACCAGGAGCGCCTAAACCACTTCATCACGAAAA
AGGAGAACTTTGGCTTCGCCGACGCCGCCGTGGCCGCCGCGGCCCTCAAGGACGAGGTGGACAACCGGCA
GGCCGGCGAATCCGGCCAGGTGGTGGTCATGAACGGCTCTGCCCCGCCGCACTCGAAACCGAATTTGGAT
TTGTGGTTCAAGGAGATGGTGGAGCTGCGCAAAAAAGCCGGCGAATACAAGGACCTATTACAAAGGAGGA
GAAGGAACAGGAGAACAATAAGAAGTCCACGCCATTGCAGAAGGCCCAGAAGCCGCGTGTTCCTGGCCAA
GCCTTTTTGATTGATAATAAGGATGAGATTTCAGCACTGCCAGCACGATTTAGCAATATACGCCATCACC
TTGAACGCACCACAGGTCCGGGATGTGGAAGAGGGAGCTTTGTTGCCCTCGCCAACGCGCGAGAAGCTGA
TGCCGGCTATTACCAAGCGGGAATCGGAATCTCAGCGAGGAAGTCCCAAGAAGACCGCCTTGTCCAGGCA
CGGATCGcctcagaagggcagtcctcaaaagggcagccccaagaaggTCCTTAAAAGGTGAGTAGTTCCC
CGCTTTTTCCCTCACCTTTGAGCAGAGGATACAAGGAAAGAATGCATACGCATATCCGATTTTAATTCCA
AAGTTAACCATATCCGAATAGCAAATTTACTCTTTTGCAACAATGACACAAGTACACAAAATGCACTTAC
CAATAGAGTTACGAGTTTGGGAACAGAACAAAACATTGTACACGCTCCAACAATAAGTATACGCCCCGTT
ACCAATACCTTGATTTGGTTTCCTATGATTTTCGTTTTGCCATAGTTTGCTCAACTGCTTCAACCGTTTG
ATTTGCATTTTCCCCGACAGTCGAAGGAGTCGACTCGTTTTTCATCATTGTCAAGTGCACCGAAGACTTC
TTGGGATATGAGATTTGTGCACAATCCTAATGTAGTTTCTATTTACTTACATTTGCCAGTTTTTATCGAG
GGTTTGTGTTCTGAATTCGGTTGAAAGTTGATTTTCATGTTCTACGTTTAAGCTATGATTTGTAGAGAAC
CTTTTGAGAACATATGAGTCAATCCCTTAAAACCACAACTACTTACATTTATATATTGAGGTGGCCAATA
CTTAGGAAAATCCCACATAATCATTCATTCATATTTCGTATATATAAATCATTTCGGTGTATTGGGGCCA

TGAGTGTTTTAGTGTGCTGGGATGTAAGTAAGTTCGATAATGGCTGTATATTACTTTGCGTTAAGCTAAG
CTTTAAAGACATCTATTTCGCTTTTAATTTATATATATACTTCGATAATTTTAACTAAATAAAAAAGTGT
ATCTTTCGGTGTCTGATTAGTATTCGGATTTTGTCAGGAATTTGCCAACTTAGTCTTAACCTTAGTATAT
AATTGGGAGAGATCTATATTATAATCGTTGTGGAAAGGAAAAAGGTTAAATCGTAGCGCATTGCCATGAA
AACGTGTACAATAATTCACTTGAAACACCCACACAAATATCACTAGTAGAAAGGCTCTCTCACACCGTCC
AACATTTGGAATAAGAATTTCTCTAGTCAATTGCGAATTTAAATTAAAACATACAAATCGTACTTTTGTC
ATCAATCAAACCGCCaccaccaccaaccaaccatacaacaaaatcgcaaataataaaacgaaatgccaaa
acaaaaaccgattccgaaaaccgaaaaccgaaccatCCTCCATCCGTTTCCGTGTCCATGTGCGTGCTCA
GCCCGGTCGCAATCGGCTGGTCCGGGCGTTGCTGAGAATGAGTCACCGAAGCGGCAGATCCGGTCGGCGA
GTCAGGCACCCAGCAGCCGTAAAAAGACGCCGACAACCGGAAGCGGAAGCGAACGCAAGGCACGCCCATG
TAAGTAGCCATTTTGGAGAAGGCAAAAGAAAATCCCCCGCCGAATACCCAGAACTCGACGTGAACGATGC
GATCATCGTTCATTGCCTTATTTATTTGTTTAGTCCTAGTTTGCATATGTCATGTGTTAATTAGATTGCG
TTCCGATTTATCGATTTTTCGTTGCCTTAATTTTGTTTCTTTTGCCAGATTGGTTCCTAGTTTAGTTTAG
TTTAGATTATCTTATCACACCTCCAGTTTGTACGTTCTTCCAAGCCCAAAACGTATTTCGATCGACCATC
TAGTTGAGTGTCTAATGAAACAAGTAATTCATAGTTGAAACAACTTTCATTTTGGGTTTACAGTATTTGT
TAGCTTGGTCTCTGGTCATGCAGTTAAAttttttttagttttttATTCTAATTCCTTACCCATACACTTT
AGTTTCGATCACTGTTCTCTCTGTAATGCAGCGTTTGATTGGTCTTCCGAAATACATAACAATCTATAGT
TTAGCATGTTGAGATTCCGTTACTAACAAGACCTACTAAATAATTAACAAACCAACTAAAACAATAAACG
AACTAAACTTATCTGTAAGTAAGTAAAAGTAATACTAACAATCCAGTTCAGTGTTGTGCCTAACCGTTCG
AGTTTTAATTTGTATTCCGTTATCCTCCTACTAACCATAACTTTGTAACTTTGTTGGCTTCCAACCCGAT
TATCTGTAACATACATTTATATTCTAATTTATGTGGGACAGGGGCAACCAACTACCTGTTTAATATACTG
AAATTCTCCTCTTGCCAAATTTAGGGTCATAAAGCCAACATTGTGTTCTCCCTTCATTAGCAGCCAATTA
ACGAAATACCGATAGTCTGGTATAAGTAGACTAACGAATTTGAATTGTTAACACAACTAACCGAAAGAAA
ACTGGAACGCTACTAATAGCATTTTACGTTTGGTTCATTAAGTTCCTTGTTTTGTTTGAACTTTTGTTTT
AGGACATTTTAAGCTTTTGCTTTGATTGCATTCTCGACTCGTCACCAACCAAATAAAACATTTTTAATGA
AAATACATGTAATAATTTGTTTTTCACCTGCGTTTTGTTTTCGGCaaaaaaatgtaaaaaaaaactatga
caaacaacaacaaaaatcatcgtaaaacaaacgaaaccgaaaaccgaaaaccgTTTACGTTCTTATAGCC
ACCCTATCCACAACATTCCAATCCCGCATTAAAAGTAGTTCCTTGCCACCGCCCAACGGTAGAGTAGCCT
CAGCGCCGCCAGCCACTGCAGCAGCAGCATCAGCAGCCAAATCAGCATTAAGCGCTAATCAGGCTCATGC
TAATCAATCACAATCATCACATGCCAAGAGCGGTAGCATCAGCGGCAGAATTTCAAAGCCATCGGCCTCA
TCCGCCcaacagcaacaacagcagcaacagcaacagcaaATGCGTAAGAAAGACAAAGATAGATCGAATA
GTAGTTGCATTGGCAGTGGTAGTCATGTTGGTGCTGGTAGCGGTAGTCAAGCAAATAGTGTCAagcatct
gcagaataagcaaaagcaacaacaactacagcagcaacagcaacaaaagcaacaacaacaacagcaaccg
caacagcaacagcaaaagcagcagcaaaaacagcagcagcaactcgatgttgcagcagcagcaacagcag
cagcagcaacatcggcaCCTCCCCAAAACCCAACCCGGCCTGCCACCATCAACATCAAACGGCTCACGGT
ACCAGGTCAGGATAGAAAATTGGCTGAAAGTGAGTAAAGTTTCACCGAAAACTTGAACCAATCAAAAACC
AAACTCTTCAATAAACCCAAGCTAAACCAAAATTAAAGCCCATTTAAAAAGTAAAATACACGGAAAATGC
TGTAAAATGACATCTGATGCGCGAAATGTTTGTGTTGCAACTTTTGTAAAGCCCTAAACTTaaaacaaaa
aaaaaaaaaaaaaaaaacgaaaaGACAATCTCTTTTCACGCTATCAATAATTAAGCATTTTACA
CCTTGGTCAAATCTGTTCGTCCACAATTAGTGAAAAGTGATTAAGCTTATGATTTACCGATTGGCCACAT
CGATTAGATGGACTTGTGTGTGTGTTTTTGTTTGGGCCAAGCCAGGTGGAATTGTGTATCTGTATTGATA
GATTGGTCGTAGAAAGTGTACAAAATTGTAGTTTGATTATAGTTATTCCACTGGCAGCCCGattcaaaaa
tgaatataaaattcaccgaaaaagtacgaaaatcaaacaaaattccaaattcTTAATTGCTGTTTATTGT
GGCTACACCCAAAGAGCAAAATGCCTTATTAAATGTTATTAAGTGTTAGTTGTTGTTGTAGTCAGTGTAT

GTATTCGTTGTATATCAGCCGTGCGTCTATATAAATGTATCTGTATAAGCAATTTAAATTTAATGCCCTT
GAATGTGCGTCTCATGATTTGTAGATTTTCGTTGTTAAATCACATCTCAGAATTTCAAATATACCTTTGT
TTGTTTGTGTACATATGTAAAGTTTGTAGCATTTCTATCTTATCTCATTTTAGATTATATGTCTATGATT
ATTTCTTTTAAAACAACATAATCTACAAAAAGACACTTAAGCACAGACACTAACAACAATAAGGTAGAAG
TGTTAGTTAGTATTTTGATGTTCGAAATGGAGGTTTGGGTTAGTTAAACAGTTTTAGAAGAAAGTAAATC
GTTTCTACTAACTACAGTTCACTTTCAAATCAACGTAGGAACGTGTACTTTTGTATCTACTACTTTAATT
TCAATATTACCAACTGTTAAAATACATGATGAGTGTTCGATAATATCTAAGCGAACTCGAATTGCCAGGG
ACTAACTGACAAATCTTTTTTAAGCTGTCGTCGACGCCTTCGTTGTTGCCTGCCaaaaaaaaaGAAACAC
CAATAAAACATATCCAGCAATGTGCCACACCAGGATTTGTACAGCTATACCAATCTTTTGAAACATAAAA
CATATATTAGTTTTATTACTCCTCTTCGGATTTTCATGTTGCGTGTCTGTTTGTGTATTTGTGGCATGCT
GTTTCTCATGTTGTGCGCTTTATTaaataaaacaaaaaataaaaaataaaaaTATTCCTTTGGTTCTACT
AACAATATGTTGCCTTTTAAGCTTCTGGCTTAATATTCTTCCGTTGACTTCAGTTTCCTTCAGTTTTCGG
TTACCGAACTAAGTTAGAAATATTTATTTGGATTTTATGTTTCAAATACTAACCAGTAAAACTCAAAAAC
ACAAAATTTGCTTAACGAACAAAGGACTTATCCaaaaaaaaaTTTATGAAGATCAACAGCTACGGCAGCA
TAATGAAATAACAGAAACATAAAATGCGCTTATAATTAAGTTAATTCCAAAACGCATAACTTTCCAAACT
GAGCAGAACTACCATTTATAAGTATTTTTGAATATTTTCCCAACCAACAGAACGAAGGCGAGGATGGTCG
TGAGACTGCCATTTCCATTTCCAGCTGTAGTCCCCAGCCTCCGGTGCCGGAGGTGCCCGAGGAGCCCCTG
GTGAAGTCGCCACCAGAACCGACCCGGGTCAAATCCCCGGAGCAGATCATAATGCGCTCTCCTGATCCAG
TCAACTGGACGGTGCCCCTGGACACTGGCAAAACCTTCACGGTGACGCAGAATGTCAAAGATGGTAGAGT
AGACACAAGGTTGTTGACCACTATGTAGAGCAGAAGATTGGAGTACAGATTGGAGTAAGATACTAATTTG
AGCGTGACTTTAATTTCTGCCTACGAAGTGCATCACTACCCTGACAAATATGTGCTGCAGCTAAGTAAAT
TCttttttttttttAGGGGAGAACTACAGCCGACCTCAGAGCGAGATTAAAGCGTCGACACCGGTGGAG
AAGCCCCCACCGCCACCACAATCGGCTCCGCCACAATTAACCGAACAGGCTAAGATGGACGG
>dme_circ_0008182_junction_seq
AATTAACCGAACAGGCTAAGATGGACGGCTACTCTACATGAGTTGGAGCCCTTGGT
>dme_circ_0008183
CTACTCTACATGAGTTGGAGCCCTTGGTTAGCGATACGGATGATAGAAAAACTCACGAGAAGTATGAACA
TTTGATTAACCTAGCCCTCAAATCGACTTGCTCATAGCTTCTGCCATCTCATTCAGTGATGTCCTTCATT
AGCCCACCCGAAAGAGCAGTATCATCCAAAATATAATAATAGCTTTCAGAACCCACATTCATCCGCACCT
GCATGTTGCAAGTGTTGCATGCTGCAGCCATCGAGAAGCACAGCAGTTCCCACTTGAAGCTCAGAATCTC
TCAGGAGCCAGCACTACTTCCCATTGCTAATGACTCTCCCGATTCTTGCAGACAAGTGACCATTATGGAG
CGCAAGACCACCTCGCGTCCCTTCTCGCAGGCCATCGACCAGGAGCGCCTAAACCACTTCATCACGAAAA
AGGAGAACTTTGGCTTCGCCGACGCCGCCGTGGCCGCCGCGGCCCTCAAGGACGAGGTGGACAACCGGCA
GGCCGGCGAATCCGGCCAGGTGGTGGTCATGAACGGCTCTGCCCCGCCGCACTCGAAACCGAATTTGGAT
TTGTGGTTCAAGGAGATGGTGGAGCTGCGCAAAAAAGCCGGCGAATACAAGGACCTATTACAAAGGAGGA
GAAGGAACAGGAGAACAATAAGAAGTCCACGCCATTGCAGAAGGCCCAGAAGCCGCGTGTTCCTGGCCAA
GCCTTTTTGATTGATAATAAGGATGAGATTTCAGCACTGCCAGCACGATTTAGCAATATACGCCATCACC
TTGAACGCACCACAGGTCCGGGATGTGGAAGAGGGAGCTTTGTTGCCCTCGCCAACGCGCGAGAAGCTGA
TGCCGGCTATTACCAAGCGGGAATCGGAATCTCAGCGAGGAAGTCCCAAGAAGACCGCCTTGTCCAGGCA
CGGATCGcctcagaagggcagtcctcaaaagggcagccccaagaaggTCCTTAAAAGGTGAGTAGTTCCC
CGCTTTTTCCCTCACCTTTGAGCAGAGGATACAAGGAAAGAATGCATACGCATATCCGATTTTAATTCCA
AAGTTAACCATATCCGAATAGCAAATTTACTCTTTTGCAACAATGACACAAGTACACAAAATGCACTTAC
CAATAGAGTTACGAGTTTGGGAACAGAACAAAACATTGTACACGCTCCAACAATAAGTATACGCCCCGTT
ACCAATACCTTGATTTGGTTTCCTATGATTTTCGTTTTGCCATAGTTTGCTCAACTGCTTCAACCGTTTG

ATTTGCATTTTCCCCGACAGTCGAAGGAGTCGACTCGTTTTTCATCATTGTCAAGTGCACCGAAGACTTC
TTGGGATATGAGATTTGTGCACAATCCTAATGTAGTTTCTATTTACTTACATTTGCCAGTTTTTATCGAG
GGTTTGTGTTCTGAATTCGGTTGAAAGTTGATTTTCATGTTCTACGTTTAAGCTATGATTTGTAGAGAAC
CTTTTGAGAACATATGAGTCAATCCCTTAAAACCACAACTACTTACATTTATATATTGAGGTGGCCAATA
CTTAGGAAAATCCCACATAATCATTCATTCATATTTCGTATATATAAATCATTTCGGTGTATTGGGGCCA
TGAGTGTTTTAGTGTGCTGGGATGTAAGTAAGTTCGATAATGGCTGTATATTACTTTGCGTTAAGCTAAG
CTTTAAAGACATCTATTTCGCTTTTAATTTATATATATACTTCGATAATTTTAACTAAATAAAAAAGTGT
ATCTTTCGGTGTCTGATTAGTATTCGGATTTTGTCAGGAATTTGCCAACTTAGTCTTAACCTTAGTATAT
AATTGGGAGAGATCTATATTATAATCGTTGTGGAAAGGAAAAAGGTTAAATCGTAGCGCATTGCCATGAA
AACGTGTACAATAATTCACTTGAAACACCCACACAAATATCACTAGTAGAAAGGCTCTCTCACACCGTCC
AACATTTGGAATAAGAATTTCTCTAGTCAATTGCGAATTTAAATTAAAACATACAAATCGTACTTTTGTC
ATCAATCAAACCGCCaccaccaccaaccaaccatacaacaaaatcgcaaataataaaacgaaatgccaaa
acaaaaaccgattccgaaaaccgaaaaccgaaccatCCTCCATCCGTTTCCGTGTCCATGTGCGTGCTCA
GCCCGGTCGCAATCGGCTGGTCCGGGCGTTGCTGAGAATGAGTCACCGAAGCGGCAGATCCGGTCGGCGA
GTCAGGCACCCAGCAGCCGTAAAAAGACGCCGACAACCGGAAGCGGAAGCGAACGCAAGGCACGCCCATG
>dme_circ_0008183_junction_seq
GCGGAAGCGAACGCAAGGCACGCCCATGCTACTCTACATGAGTTGGAGCCCTTGGT
>dme_circ_0008184
TGTCGCGGTTGGGGCATAGAAATTGATCCGGAATTGTATAAGAAACAGAAGGATCTTTGGGATCAGGTTT
CAAAGCGCAGCTCACTTTCGGCACTTTCCCTAGCCTCTTCAGTTCATAGGTAAATATAACTCGGGTATTT
CATCCAGTGAGGCTTACCTATTATCATTTTTATGTGGCGCTTACCTTaaaaaaaaaaaGAAATCATAATC
AAAAAGGTTTTTTCCATTCCTGCTAGACCTATTACAAAGGAGGAGAAGGAACAGGAGAACAATAAGAAG
TCCACGCCATTGCAGAAGGCCCAGAAGCCGCGTGTTCCTGGCCAAGCCTTTTTGATTGATAATAAGGATG
AGATTTCAGCACTGCCAGCACGATTTAGCAATATACGCCATCACCTTGAACGCACCACAGGTCCGGGATG
TGGAAGAGGGAGCTTTGTTGCCCTCGCCAACGCGCGAGAAGCTGATGCCGGCTATTACCAAGCGGGAATC
GGAATCTCAGCGAGGAAGTCCCAAGAAGACCGCCTTGTCCAGGCACGGATCGcctcagaagggcagtcct
caaaagggcagccccaagaaggTCCTTAAAAG
>dme_circ_0008184_junction_seq
agggcagccccaagaaggTCCTTAAAAGTGTCGCGGTTGGGGCATAGAAATTGATC
>dme_circ_0008185
CTACTCTACATGAGTTGGAGCCCTTGGTTAGCGATACGGATGATAGAAAAACTCACGAGAAGTATGAACA
TTTGATTAACCTAGCCCTCAAATCGACTTGCTCATAGCTTCTGCCATCTCATTCAGTGATGTCCTTCATT
AGCCCACCCGAAAGAGCAGTATCATCCAAAATATAATAATAGCTTTCAGAACCCACATTCATCCGCACCT
GCATGTTGCAAGTGTTGCATGCTGCAGCCATCGAGAAGCACAGCAGTTCCCACTTGAAGCTCAGAATCTC
TCAGGAGCCAGCACTACTTCCCATTGCTAATGACTCTCCCGATTCTTGCAGACAAGTGACCATTATGGAG
CGCAAGACCACCTCGCGTCCCTTCTCGCAGGCCATCGACCAGGAGCGCCTAAACCACTTCATCACGAAAA
AGGAGAACTTTGGCTTCGCCGACGCCGCCGTGGCCGCCGCGGCCCTCAAGGACGAGGTGGACAACCGGCA
GGCCGGCGAATCCGGCCAGGTGGTGGTCATGAACGGCTCTGCCCCGCCGCACTCGAAACCGAATTTGGAT
TTGTGGTTCAAGGAGATGGTGGAGCTGCGCAAAAAAGCCGGCGAATACAAGGACCTATTACAAAGGAGGA
GAAGGAACAGGAGAACAATAAGAAGTCCACGCCATTGCAGAAGGCCCAGAAGCCGCGTGTTCCTGGCCAA
GCCTTTTTGATTGATAATAAGGATGAGATTTCAGCACTGCCAGCACGATTTAGCAATATACGCCATCACC
TTGAACGCACCACAGGTCCGGGATGTGGAAGAGGGAGCTTTGTTGCCCTCGCCAACGCGCGAGAAGCTGA
TGCCGGCTATTACCAAGCGGGAATCGGAATCTCAGCGAGGAAGTCCCAAGAAGACCGCCTTGTCCAGGCA
CGGATCGcctcagaagggcagtcctcaaaagggcagccccaagaaggTCCTTAAAAG

>dme_circ_0008185_junction_seq
agggcagccccaagaaggTCCTTAAAAGCTACTCTACATGAGTTGGAGCCCTTGGT
>dme_circ_0008186
GCGGGTGAGTCAAATGGGCTATTGAAAAAGACCATCAATAAGTTGAGCACTGAGTACCGCCTGCAATTCG
TTTGGCCCACCGTCCGACGCATAAAGGGCGGCGGCGAGGCGACGTCTAGGGCCGCTGCCGGCGACTATCC
GAGAAAGTCCATATCGCTGGGCGCCCTTCGGTCCGGCGGCCAAGGTCACTGTCACAGTCACACCCAAAAC
CAGAATCAGAGCCAGAGTCAGGGTCTGACCCAAGCCCAGAATGGTCACACACATCACACGATGATGGGTG
GCGGTGCGGGCTTGCCGACGGTGCATAAAAAACGAACAACAAATCAGAAAGAAGGTGCGACAACAAATTG
TCGTCGCTAAGCATTTATTTATCATACTAAAACCAAAACAAAAAACAATAAATTACATATATATGTGAAA
CAGACGAAACATTTCCTACTAGTGCATAGTTTCTTTCAACTACTATATGTAATACAAAAACAAATTTTTA
ACAATCAAAAAGCGCGTGCGTACTAAATGAGTTGACATTTGTTTTTTAATTAAACGTTTCGTTACTAAAT
CAACCTAACAAACTAATTCACGGCAGATTGCGTAATATTTTGTTATATACTAACCTCCGTAACCGTTGCG
ATTTGTTTATTTTTCAAGATTTTTAAATGAAAATTTGTGCCGTGGCAGCAGCATTGAGCaaaaaaaaaaa
aaaaaaaaCCAAAAGATAAAAAGCCCTATAGAAAAGTGTTGAATAGTGTTGGTAAATGAACGTAGTCTAA
ACGATTTTTCTCCTCTCACCGCCGCCATATTAAAAATGCAAACTTGCCACAAAGCAATCAATCGAATTCG
AAATCAAGTTTTTATGTACACGTCGTCGTAACTTTCGCTTTATAATCGTACTCGTACCTATAAATATGTA
TACTACAGTGCTCAAAACTAAGATAGATATTTCCTAGAATAATTATCTTGACCATTTCCGTATGATTTTC
CCCTTCTTTCCGTACGTACAAGCAAATGTTTCGTTTAACGCTTTTGTAATTGAAGTTGTATTATTTCAAG
CAGTTAGCCAACTTAAATCTATCTCATATAGAACGAAATTATGCTAAATTCCTTTCCAAAAACAAAACTA
AACCAGACACTAAAAGGTTAAACACAATCAAGACTATCTTTTTAGCCCAAAGCTGACGTAAGTGTTGAAC
TAAATTTAAACTTTATTAGTGCTAAGCCAATTCAATTTCCTTTACTGCCGAGCGTAAAATTTGCGTTGAT
CAATCGAAAATTGATGGAATTATTACCTGGTCGTGCCCATTCTCATCCAAAATTCAAAAAACCCACCAAC
ACGCAGTGTCAATGCAACTTTACGtgacgaactgatgaattgatgaattgaAAATAGCCAAGTGTAGTAT
GAGCATGTTGTGACCGCAGGTTGTGTGATATATTCCGTTTTCATTTCGATCGAAGCGTAGGAGTTGTTGC
ATAAGTTGCCTTAGTTTCATTGCATGTGTGAAACGACAACCAATCGAAATTACTTTAGTGTCATGCATCA
AACCAGACAACCATCAATCAATCAGTCAACACGGCATCAGCATCAACTCGCTATCAATCGTATATACCAA
TAATACTCATGTATAACAATTCATCGGCTAGACGAATTGGCAAGCTTAGTGTGGGTTAGTTTAGGCTGTG
GGCAGTAAATGTGATTACCACTTTCCAAATACTAACAAAATTTACAGTTCAAGCAAAGCGCATAGGTAAG
GTAAACGGAAACGGAAGTGAACAAACCGCAATCGAATAGAATAAAATGAAACCTAACCCCTCTACCCATA
TGCTAAATCGTTAAGTGCATCTGCATCTAAGTGGCATTTAGTGGCTTCAAGTGCAAAATGCTTTGTCTTG
CTTTAGCCCTTGTGCGACGCCTTTAGCTTCCAGCGATAGTGTACAAAATTAGACGTAGTGCTTACaaaaa
tacaaaaaaaaaaaaatatctaacaattaaaaaatataaaatatGCAACGCTGCATTCAACTCGAATTGTA
ATTACATATTTTGCATAAGAAAGAATACAACAATTGGTTTGAATTTCGTTCTATATTCCAAATAAAAGTT
ACCCGAAATTGTTCAAACATATCCCCAGAAACTACCAGGAAACCATGCCAAACACGCTTCAACCAAACAT
TCAACATTATTTTCGGATTTTCGGTTCTGAACTCAATGCCAAAAACACTAATTGATTTTCATTTGAAGTT
TACATTTAGCATTTCTCTTAGCCGAGTAAGCTGACCCGACACAAACTAACCACCCAATCACAACCACGAC
CTCAAAACCACCCACTTAAGCGCTGCTTCCGGCTCATTTATTTGCTGTACTATAATATAAAATCACCTTC
TCCTTAACCCTGAGTTGAAGTTTAGCGTATCTGAGACACCTTGCAAAAGAAAACCTAACCTTTGTTTCCA
CCTTTTTGGCCTTTAGTTTCCTATTGCATTTTCTACAATTAATTATACTTTCCTTAACTAATCTTAATGT
ACCCACTGATGGCAGACATCAGGCAGAATCTGTATAAAGCTATCGATCGACTTGGATTGCAGCATTAAAT
TTCGTGTCTGTGTGCTTTCAGCACAAGTGACCATTATGGAGCGCAAGACCACCTCGCGTCCCTTCTCGCA
GGCCATCGACCAGGAGCGCCTAAACCACTTCATCACGAAAAAGGAGAACTTTGGCTTCGCCGACGCCGCC
GTGGCCGCCGCGGCCCTCAAGGACGAGGTGGACAACCGGCAGGCCGGCGAATCCGGCCAGGTGGTGGTCA
TGAACGGCTCTGCCCCGCCGCACTCGAAACCGAATTTGGATTTGTGGTTCAAGGAGATGGTGGAGCTGCG

CAAAAAAGCCGGCGAATACAAGGACCTATTACAAAGGAGGAGAAGGAACAGGAGAACAATAAGAAGTCCA
CGCCATTGCAGAAGGCCCAGAAGCCGCGTGTTCCTGGCCAAGCCTTTTTGATTGATAATAAGGATGAGAT
TTCAGCACTGCCAGCACGATTTAGCAATATACGCCATCACCTTGAACGCACCACAGGTCCGGGATGTGGA
AGAGGGAGCTTTGTTGCCCTCGCCAACGCGCGAGAAGCTGATGCCGGCTATTACCAAGCGGGAATCGGAA
TCTCAGCGAGGAAGTCCCAAGAAGACCGCCTTGTCCAGGCACGGATCGcctcagaagggcagtcctcaaa
agggcagccccaagaaggTCCTTAAAAG
>dme_circ_0008186_junction_seq
agggcagccccaagaaggTCCTTAAAAGGCGGGTGAGTCAAATGGGCTATTGAAAA
>dme_circ_0008187
GCGGGTGAGTCAAATGGGCTATTGAAAAAGACCATCAATAAGTTGAGCACTGAGTACCGCCTGCAATTCG
TTTGGCCCACCGTCCGACGCATAAAGGGCGGCGGCGAGGCGACGTCTAGGGCCGCTGCCGGCGACTATCC
GAGAAAGTCCATATCGCTGGGCGCCCTTCGGTCCGGCGGCCAAGGTCACTGTCACAGTCACACCCAAAAC
CAGAATCAGAGCCAGAGTCAGGGTCTGACCCAAGCCCAGAATGGTCACACACATCACACGATGATGGGTG
GCGGTGCGGGCTTGCCGACGGTGCATAAAAAACGAACAACAAATCAGAAAGAAGGTGCGACAACAAATTG
TCGTCGCTAAGCATTTATTTATCATACTAAAACCAAAACAAAAAACAATAAATTACATATATATGTGAAA
CAGACGAAACATTTCCTACTAGTGCATAGTTTCTTTCAACTACTATATGTAATACAAAAACAAATTTTTA
ACAATCAAAAAGCGCGTGCGTACTAAATGAGTTGACATTTGTTTTTTAATTAAACGTTTCGTTACTAAAT
CAACCTAACAAACTAATTCACGGCAGATTGCGTAATATTTTGTTATATACTAACCTCCGTAACCGTTGCG
ATTTGTTTATTTTTCAAGATTTTTAAATGAAAATTTGTGCCGTGGCAGCAGCATTGAGCaaaaaaaaaaa
aaaaaaaaCGTGAGTCAAATGGGCTATTGAAAAAGACCATCAATAAGTTGAGCACTGAGTACCGCCTGCA
ATTCGTTTGGCCCACCGTCCGACGCATAAAGGGCGGCGGCGAGGCGACGTCTAGGGCCGCTGCCGGCGAC
TATCCGAGAAAGTCCATATCGCTGGGCGCCCTTCGGTCCGGCGGCCAAGGTCACTGTCACAGTCACACCC
AAAACCAGAATCAGAGCCAGAGTCAGGGTCTGACCCAAGCCCAGAATGGTCACACACATCACACGATGAT
GGGTGGCGGTGCGGGCTTGCCGACGGTGCATAAAAAACGAACAACAAATCAGAAAGAAGGTGCGACAACA
AATTGTCGTCGCTAAGCATTTATTTATCATACTAAAACCAAAACAAAAAACAATAAATTACATATATATG
TGAAACAGACGAAACATTTCCTACTAGTGCATAGTTTCTTTCAACTACTATATGTAATACAAAAACAAAT
TTTTAACAATCAAAAAGCGCGTGCGTACTAAATGAGTTGACATTTGTTTTTTAATTAAACGTTTCGTTAC
TAAATCAACCTAACAAACTAATTCACGGCAGATTGCGTAATATTTTGTTATATACTAACCTCCGTAACCG
TTGCGATTTGTTTATTTTTCAAGATTTTTAAATGAAAATTTGTGCCGTGGCAGCAGCATTGAGCaaaaaa
aaaaaaaaaaaaaCCAAAAGATAAAAAGCCCTATAGAAAAGTGTTGAATAGTGTTGGTAAATGAACGTAG
TCTAAACGATTTTTCTCCTCTCACCGCCGCCATATTAAAAATGCAAACTTGCCACAAAGCAATCAATCGA
ATTCGAAATCAAGTTTTTATGTACACGTCGTCGTAACTTTCGCTTTATAATCGTACTCGTACCTATAAAT
ATGTATACTACAGTGCTCAAAACTAAGATAGATATTTCCTAGAATAATTATCTTGACCATTTCCGTATGA
TTTTCCCCTTCTTTCCGTACGTACAAGCAAATGTTTCGTTTAACGCTTTTGTAATTGAAGTTGTATTATT
TCAAGCAGTTAGCCAACTTAAATCTATCTCATATAGAACGAAATTATGCTAAATTCCTTTCCAAAAACAA
AACTAAACCAGACACTAAAAGGTTAAACACAATCAAGACTATCTTTTTAGCCCAAAGCTGACGTAAGTGT
TGAACTAAATTTAAACTTTATTAGTGCTAAGCCAATTCAATTTCCTTTACTGCCGAGCGTAAAATTTGCG
TTGATCAATCGAAAATTGATGGAATTATTACCTGGTCGTGCCCATTCTCATCCAAAATTCAAAAAACCCA
CCAACACGCAGTGTCAATGCAACTTTACGtgacgaactgatgaattgatgaattgaAAATAGCCAAGTGT
AGTATGAGCATGTTGTGACCGCAGGTTGTGTGATATATTCCGTTTTCATTTCGATCGAAGCGTAGGAGTT
GTTGCATAAGTTGCCTTAGTTTCATTGCATGTGTGAAACGACAACCAATCGAAATTACTTTAGTGTCATG
CATCAAACCAGACAACCATCAATCAATCAGTCAACACGGCATCAGCATCAACTCGCTATCAATCGTATAT
ACCAATAATACTCATGTATAACAATTCATCGGCTAGACGAATTGGCAAGCTTAGTGTGGGTTAGTTTAGG
CTGTGGGCAGTAAATGTGATTACCACTTTCCAAATACTAACAAAATTTACAGTTCAAGCAAAGCGCATAG

GTAAGGTAAACGGAAACGGAAGTGAACAAACCGCAATCGAATAGAATAAAATGAAACCTAACCCCTCTAC
CCATATGCTAAATCGTTAAGTGCATCTGCATCTAAGTGGCATTTAGTGGCTTCAAGTGCAAAATGCTTTG
TCTTGCTTTAGCCCTTGTGCGACGCCTTTAGCTTCCAGCGATAGTGTACAAAATTAGACGTAGTGCTTAC
aaaaatacaaaaaaaaaaaaatatctaacaattaaaaaatataaaatatGCAACGCTGCATTCAACTCGAA
TTGTAATTACATATTTTGCATAAGAAAGAATACAACAATTGGTTTGAATTTCGTTCTATATTCCAAATAA
AAGTTACCCGAAATTGTTCAAACATATCCCCAGAAACTACCAGGAAACCATGCCAAACACGCTTCAACCA
AACATTCAACATTATTTTCGGATTTTCGGTTCTGAACTCAATGCCAAAAACACTAATTGATTTTCATTTG
AAGTTTACATTTAGCATTTCTCTTAGCCGAGTAAGCTGACCCGACACAAACTAACCACCCAATCACAACC
ACGACCTCAAAACCACCCACTTAAGCGCTGCTTCCGGCTCATTTATTTGCTGTACTATAATATAAAATCA
CCTTCTCCTTAACCCTGAGTTGAAGTTTAGCGTATCTGAGACACCTTGCAAAAGAAAACCTAACCTTTGT
TTCCACCTTTTTGGCCTTTAGTTTCCTATTGCATTTTCTACAATTAATTATACTTTCCTTAACTAATCTT
AATGTACCCACTGATGGCAGACATCAGGCAGAATCTGTATAAAGCTATCGATCGACTTGGATTGCAGCAT
TAAATTTCGTGTCTGTGTGCTTTCAGCGTATGAACATTTGATTAACCTAGCCCTCAAATCGACTTGCTCA
TAGCTTCTGCCATCTCATTCAGTGATGTCCTTCATTAGCCCACCCGAAAGAGCAGTATCATCCAAAATAT
AATAATAGCTTTCAGAACCCACATTCATCCGCACCTGCATGTTGCAAGTGTTGCATGCTGCAGCCATCGA
GAAGCACAGCAGTTCCCACTTGAAGCTCAGAATCTCTCAGGAGCCAGCACTACTTCCCATTGCTAATGAC
TCTCCCGATTCTTGCAGAACAAGTGACCATTATGGAGCGCAAGACCACCTCGCGTCCCTTCTCGCAGGCC
ATCGACCAGGAGCGCCTAAACCACTTCATCACGAAAAAGGAGAACTTTGGCTTCGCCGACGCCGCCGTGG
CCGCCGCGGCCCTCAAGGACGAGGTGGACAACCGGCAGGCCGGCGAATCCGGCCAGGTGGTGGTCATGAA
CGGCTCTGCCCCGCCGCACTCGAAACCGAATTTGGATTTGTGGTTCAAGGAGATGGTGGAGCTGCGCAAA
AAAGCCGGCGAATACAAGG
>dme_circ_0008187_junction_seq
CTGCGCAAAAAAGCCGGCGAATACAAGGGCGGGTGAGTCAAATGGGCTATTGAAAA
>dme_circ_0008188
CTCCACTCGGAGTATCGGAGCACTTATCGCTGGCATGAATTTACGGGCAACTCGCGGCCAGAGGTTGTGC
GACGGGCGCCTGCCCCAAACCCAAGTCAATTTGTTGGTAAGTTGCGAAGGCATTTGTCTTGCGCCCATTG
TCACGGACTATTTATTGATCCCGCTCTGATTCGACTTTCGATTGGATTTTGCGCATCTGCAGGAGCGACA
AATGAGCCGCCATTGCCACGCCGGAAAAAATGTCCAGAATTAGCATATAAATCGCACGAGTTTATTATAG
GATCGGAGTATACAGATGGACGCCGAGATGCCAGTGCACATCGTTTGGCGAGAGGTAAGTGTCGTTTCCC
GAATTTCCAATTTTCCACCTTCATAATGGGCAGCTGTAGCGAGCATTAAATATAGAAAATTGCTATAATA
TGATATATATAAAATAGATTTTATCTCTCATAAGGAATAGGGAAATGAAATGTTCCTTTAGCACAATGaa
aagtaaaaaaataaaaaaTTTCGTATATTACAAATCTATAATATTGGATTTACAAAAAAGTATAATTTTC
TATACTTTCTTATATACTAAAGTCCGAAATGTAATTAAGAACCTTTTTGCCACTTTAAGCCAAACTTCCA
TTTCTGCGCATAAAAAGTTAAGAAGATGTCCGCTTATAAATATGCAAGATTATTGTGCATATTCACGTTC
CATGAACGAGCTAAAAAATGCAAATCCCAGCTAGCTTTCAACATGTTATAGTAGTAGTGTATTCAGTTTA
TGGCCACACAAGCTTTATGCGCTTTTGACATTTGAAGGGGACCATGAAAGAAAAATGAAATGCTCTGAGG
TTTTCCAAAAAGTAGAAAACTTTCCTATTTATATACCCTATGTTTCAGTAAGTCGTCGAGGAGGCGAGGA
AATAGTCAGCCCTCCTTGCACTGCCTAAAATTAAATCGCGCTAAAAGATGAGCATTTAATTGTACACCTA
AATTACGCGTAAATTATTTGTTTTAATTAGGCTTAAAGGGTATAAAGTTTTCATTGAAAGTATACTTTCA
TATACGATTTTTATGGGCTTCAGAAGCCATTACGGGAGGCTTGGCATTAGGGTTAATGCGTTTAAGCCGC
GGCCCTCATAAAAAGTCGACGCTCTCAAATGCCACAGGAAATACACAAAAGCCGTTCAGAAAAGCTCCAT
GTGAGCTTGGTCCTTTCCTAGTTCATTTTGGGCCCCTCATCTCCAACGGTAGACTAATTAATAATGAGGC
ACACAGTGGCAAATGTTGCAAGGACATTAAGATACGTGTGCGAAAATCAGTGGCGTACGCGGATGGAAGG
TTTTGAGGGAGTTTTGTGCCCTTGGGCATTTATTTTGCTTTCCAAAATGTGTCCATTTGTGCTTTGAAAT

GTTGAAAAGCGATTGCTTAAATTGTATTAATAATTAGCTTATCATTCGAGTGACTAAATGTTGTTACAAT
AGAGTCAAAATGTTAATCAATCAAAAGTGCATAAAATGAATGTATAGTATAACTCTCATTAAAATACCCC
GCGGCAATATTCTTAGTACGCCACTGACCCAAATACTGGTTTGTGCGTCGAAATGCCGGGGAGAGTGTGC
TTTTGACATACTATCATGGGCACCCTGACAATACATAATTAATTGACTTTACCTCATGACGCACGTCAAC
AGGGCGGATGCGTATTATTTACGAGCAACTAATTAACCAGACATGCCCGTATAACTTTAATGTCCTTAAT
GCCATCTCTGTCTTTCTGCAGTCGGAGGAGCGCGGTGGCACACCTTCGCGCCGCAGCAAATCGGAGGGAC
CACCCGTTGTGCCCAATGGACGTGCGTATCCCATTGCCACGGAGATCGACGGAACCACAAGAAAACAGG
>dme_circ_0008188_junction_seq
GAGATCGACGGAACCACAAGAAAACAGGCTCCACTCGGAGTATCGGAGCACTTATC
>dme_circ_0008189
GGAACAGAACATGCAATGAAAACCTTTCAACAGCTAATTATAGTTATTTGATGTTTAACGCTATATAATT
AGTTATAAATAATTGCCTAGGCTTAGCGTTGATAAAAGGTTTTATTCGCATTGTGGGATGTAATGCAGCT
ATCAGAATTCTGTTATATATATCAATA
>dme_circ_0008189_junction_seq
TATCAGAATTCTGTTATATATATCAATAGGAACAGAACATGCAATGAAAACCTTTC
>dme_circ_0008190
ATGCTGGTTTTACTGACGCGTGCTGGAAAGATTTTTGGCATTGACAATGTCTCTGGGAAACATCACTGGC
AGCTGCATCTTCCCAATGTTATTGGATTCGCTAACGACGAACAGATGCGTCTGATTGTCCAGCGCTCGGC
CAAACACTTTCCACTTCAGCCGTTGTGCACTATTTTGGGCAAGAATGCTGGTCAGCGGCAATGGTGTGCT
CTATCGTTTTAATCCTATTACCGGCAAAGTAGCCGAAGGTGGCCTTGTTCAGCTGGACTACAGAATTAAG
CAGCTTTCGTTGCTGGGAGAAACGGAAAAGGACTTTCTGAAGGGCATTCTGTTACTGGATGCTTCGAATA
AGGTACATGTTTATCCCGAACATGCGGCCCCACTGGTAAGATATAAAATTCTATGATATATCAATAGAGT
TGAAAATCTTTACATGCACGTACAGGCTGATGGCATGTATCTGTACACCGCCGATTTGAAAACTGCAGAG
CTGGCCGGCTACTTTGTTAAATATGCGGGCGGGGTAAGTAATGCAGCCTTTTAATATGAAAGCACTTAAC
TTATTAAACCATTTCCGTTAGCAACTGTCCAGCACGCACATCTGGAATGCTCGCCTGGGCGGCCACAATT
CCGAGCAGCAGATTATCGGCGTTGCCGGCAAGAATCCCATCGAGCATGTCCACTCCCAGGGACGTG
>dme_circ_0008190_junction_seq
ATCGAGCATGTCCACTCCCAGGGACGTGATGCTGGTTTTACTGACGCGTGCTGGAA
>dme_circ_0008191
GTAGTGACAGCGATACACCCGAAATCGATTTGTTGGCCCAGCTCAATAAACTCGCAATTACGGACACCTC
CACCGACGGTCAGCAATCGGTTGCCAATGCAGGTGACGACGGTCCTCCGCTATTGCCGGATGAAGCCCGC
ATTGCCAAGGCCTCCAAGAATCTGCTGAAACGCAGCAACCCG
>dme_circ_0008191_junction_seq
CAAGAATCTGCTGAAACGCAGCAACCCGGTAGTGACAGCGATACACCCGAAATCGA
>dme_circ_0008192
GTGCTTCTAAAATCAAAAGAAAGCAGCAAAATTTAAAAGTGAGACCTAAAGTCGAAATATTTTCCAATAC
TCTTCGGACTTACCCAATAACTTAAGCTTACCAATAATTTATGCAAGACTAAATTGCAATAAAGGCATAT
AATCCCTAACAATATAACTCTCATTGAAAAAATCTTAAATAATTTAAACCCCAGTGAATTAAACAGCAAA
AGACAGTTAATAGACACAGAAAATGCTAATAAAAATAGCATAATAAAAGCACGGTTACATAAACTTAGAC
GCTCGAACATTTACATAGGATATCAGAAGCTGGAATCATGGTTTCGGAGGAGAACTGGAATAGCGACACG
ATGTCCGACTCGGACATGATCGACTCAAAGAACGACGTCTGTGGCGGAGCCTCCAGTTCCAGCGGCAGCT
CGATTTCGCCGAGGACACCACCGAACTGCGCCCGCTGCCGCAATCATGGCCTAAAGATCACCCTAAAGGG
ACACAAGCGGTACTGCAAGTTCCGCTACTGCACGTGCGAGAAGTGCCGACTGACGGCGGACCGCCAGCGG
GTGATGGCTCTGCAAACGGCCTTGAGGCGAGCCCAGGCGCAGGATGAGCAGCGGGCGCTGCACATGCACG

AGGTGCCGCCTGCCAATCCGGCGGCCACGACCCTCCTGAGTCATCACCATCATGTCGCAGCTCCCGCTCA
TGTCCACGCCCACCATGTGCACGCCCATCACGCACACGGAGGACACCACTCGCATCACGGACATGTCCTG
CACCACCAGCAGGCAGCGGCGGCCGCAGCAGCAGCTCCGTCGGCTCCAGCCTCCCATCTGGGTGGCTCCA
GCACGGCCGCTTCCAGCATCCACGGTCACGCCCACGCGCACCACGTCCACATGGCAGCTGCTGCAGCCGC
CTCGGTGGCTCAGCACCAGcaccaaagccacccacactcgcaccaccaccaccaccagaaccaccaccag
caTCCGCATCAGCAACCGGCCACGCAGACCGCCCTGCGATCTCCGCCGCACAGCGACCATGGTGGCAGTG
TGGGTCCGGCCACCAGCAGCTCCGGCGGTGGAGCACCCAGTTCCAGCAATGCGGCAGCGGCCACCTCGAG
CAACGGATCCAGCGGCGGTGGAGGAGGAGGCGGAGGGGGCAGCTCGGGTGGCGGGGCAGGAGGTGGTAGG
TCATCGGGAACATCGGTGATCACTAGCGCCGATCACCACATGACCACGGTGCCTACGCCCGCCCAATCGC
TGGAGGGGTCCTGCGACTCGTCGTCGCCCTCGCCGTCGTCCACTTCCGGTGCAGCCATTTTGCCGATCTC
AGTTTCCGTCAATCGAAAGAACGGCGCCAATGTGCCCTTGGG
>dme_circ_0008192_junction_seq
GAAAGAACGGCGCCAATGTGCCCTTGGGGTGCTTCTAAAATCAAAAGAAAGCAGCA
>dme_circ_0008193
GCCGATCCACTGGTCGCCTTCTCCAGGTAATTGAAGTTGTTTACTTTCTCGATTGTATTTGTCACTAACT
TTTGTATAATTCGTTAATGCTTGCCACATGAAGGCAGAGGTAAAAACTTAAACCAAGTGTCCCtatatat
tatatattagtattttaattttcgatatttctgaattcatttatattcaATCCTACTATAACCTATGCTT
AATTTATTAGTTGTGTTATTATGTAGTTAAAAGATTTCAGTTATCtttttgtttttttttaaacccatt
ttatgttttttCCACAGGGATCAAGTCTGGAAACCCTGGCTACTTTGTCATCTTCAATCCCACCGAACTG
CCGCAGGCGAGCAACTTTACTATCCCAGACAATCTGCCGGACAAGATGACGGTGTCCTATTTCAGCGAGC
AGTACAACAATAACATGGATAACTCCGGAAAGG
>dme_circ_0008193_junction_seq
AACAATAACATGGATAACTCCGGAAAGGGCCGATCCACTGGTCGCCTTCTCCAGGT
>dme_circ_0008194
GGAAAGTTGTTTGTCTGTGGGAGTAGAAGCCATATTCATTGGGTCCCAGGCTGAAGTCCTTCGGCGATGA
CGAGATCTGCTCATCCTCGAGGCTATCGAATAGCGCAAAGAACTTGGTATTCTTCAGGCGGAAGCCTCGA
ACGCCCAATGCCACCAGATGCTTTAGGACTGCTCCGAACTCGTTCCTAATGATG
>dme_circ_0008194_junction_seq
GACTGCTCCGAACTCGTTCCTAATGATGGGAAAGTTGTTTGTCTGTGGGAGTAGAA
>dme_circ_0008195
CTTAATGCACCCGGCCACTCCGCATCCGGCCAGGAGGAGTTGCCAGTGTTCGCAGAGCCACCCGATCAGC
AACCGCCCGCAATGCTCTGTGGCTTCCAGCGGCGATAGGCAGATCGAAACCAGTACATTGGAGTCGCGCA
CCTCCAAGCGG
>dme_circ_0008195_junction_seq
TACATTGGAGTCGCGCACCTCCAAGCGGCTTAATGCACCCGGCCACTCCGCATCCG
>dme_circ_0008196
GATTGAAACCTACATAGACGACTGGCTGGCAGACGCCGATAAGGTGCACGCCACCAAGTTGAAGGAGCAG
ATCCTCGAAGTGTGGGACCGGCTCATCCAGTATTGGATCGATTTCAATAAGGCCGGTTCATCGTACGGGC
CGCGGGTACCAACGGATGCCCTGAAAAGCACATTTGGCGAAATC
>dme_circ_0008196_junction_seq
TGCCCTGAAAAGCACATTTGGCGAAATCGATTGAAACCTACATAGACGACTGGCTG
>dme_circ_0008197
GATTCTGTTTAAGGCGAGCGGGATTCGTGCTCTTGGGAATGGCGGATACACCAGTGTCGATAATCCAGCG
GAGGAGGACCTGCGCTGGCGTCTTGCCATGGCTTGCGGCAATCTCCTTCACCTCAGGGATGTCCATCAGG

TCCGGCAGATCTCTCACGATGCCCGCTCCAGCATTAAACTTGGCGATTCCCTTGGATCCCAACGGGGAGT
AGGCAGTTACG
>dme_circ_0008197_junction_seq
GGATCCCAACGGGGAGTAGGCAGTTACGGATTCTGTTTAAGGCGAGCGGGATTCGT
>dme_circ_0008198
TTTAGCAACTACCAAGTCAATGGATTCGGGACTCAAAACCTTTTGAAGGCTCCTCCAGCGCCTTCCTCCG
TGCATGCGCTCAGCCAGTCGGAGGACGAGGTAGCCACGAAGTCAAGGAGTGTGCCTGACATCCTGCGGGA
GCTGAAGACGGAGCTGCAGCAGGCGGAGAAGCGTCGTACCCGCCTGCACAGTCACAACGCGGAGCAGCAG
CCAACGGTGGAGTTGGCAGTGGACAGAAACTCCTTTAGGGAGCTGGCTGGTCCGGCACAGAAGTTGGCCC
AACGTATCAGTACTATGGGTTTCCCTCTGGAACGGGTGGCAAAGGTGG
>dme_circ_0008198_junction_seq
TTCCCTCTGGAACGGGTGGCAAAGGTGGTTTAGCAACTACCAAGTCAATGGATTCG
>dme_circ_0008199
GTGAACATGGAAATTGACGCTGTCGAGCGTCGCTTTGGTCTCCGCCAGCGTAGTCATTGCTAGCTCCACC
GGAGCTGGCACTTCATTGCCAGATTGCTCCTCCTTGGAAtgatcctcatgcggcggctcctgctgctgct
gatcctgctgctgttgatcTATGCGCTTCTCCAGCTGTGTCTG
>dme_circ_0008199_junction_seq
gatcTATGCGCTTCTCCAGCTGTGTCTGGTGAACATGGAAATTGACGCTGTCGAGC
>dme_circ_0008200
TATCAACGGCAGCTGCACGACCAGGCGCTGCAGTGGCTACTGAAGATCGGTCCGAAATATCCGCAGGAGT
TTAAGGCCCTGATGGGTCAAACACCGGAACTGCGACAAAAACTAGAAGCGGCCATTCGCAGCCAACAGCA
GTCGATTAACATTGCCCAAAAAGCAAGCGAGGCGCAGAGGAACCTGCTGGCCAAGCCGCAGAAGCCTACG
ATCAAGCTGAAGACGGATTTCAGTAACTTCCAATGAGCGCCTCCCCCGGGAATCGAACAAAATAG
>dme_circ_0008200_junction_seq
CGCCTCCCCCGGGAATCGAACAAAATAGTATCAACGGCAGCTGCACGACCAGGCGC
>dme_circ_0008201
GCGTGGGCAGAGGTTTATATTGTGGCCATGATAGGAAATGGAAAGGCTCCGGCTTCCCTACTGAATCTCC
AATCCCAACAATCCGGCTTTCAATCCTTAACCAATCTGGAAACGGACTCGGATGTTCCTGACAGTAGGGG
TGAAAGTCTTTTAGGATTAGTGCAACCTGAGCTCCACAATCTTTCAACACACTGGCTGAGTGCTATGAAG
GATCACGCCTTGCTGCTTCTACCTGCGGAGTTCCAATCCCAACTACCGCACGACGGAGGAGCCTTTTACA
CCACGGACACCATTAATTCGTCAAAACCACATTACATGACCAGTTGGCCCCCAATCCTCTATGCCTCCGC
ACTATGG
>dme_circ_0008201_junction_seq
CCCAATCCTCTATGCCTCCGCACTATGGGCGTGGGCAGAGGTTTATATTGTGGCCA
>dme_circ_0008202
GGAACATCCGAGTCCGTTTCCAGATTGGTTAAGGATTGAAAGCCGGATTGTTGGGATTGGAGATTCAGTA
GGGAAGCCGGAGCCTTTCCATTTCCTATCATGGCCACAATATAAACCTCTGCCCACGCCTTTAAAATGCT
TAGCTTCTCCAAA
>dme_circ_0008202_junction_seq
CGCCTTTAAAATGCTTAGCTTCTCCAAAGGAACATCCGAGTCCGTTTCCAGATTGG
>dme_circ_0008203
GATCTCCTATGTCGCGAGCCACTCCAGATCCAATCCACGCTGAGCAAACTTCACAAGCAGCAGCTGTCAC
ATGGGAGGGGGTATCCGGCGCGAAAGCAGGGCGCAAGGCAGCTCCCACCTGAGCCTGGAACTGCTCCAAG
AGTAGGTGACCAGGGAACTCCGGTTCCGGAAC

>dme_circ_0008203_junction_seq
GGTGACCAGGGAACTCCGGTTCCGGAACGATCTCCTATGTCGCGAGCCACTCCAGA
>dme_circ_0008204
ATCGTTTATAAACAGCGACCATTTTTCCGCCTCATCGACAAGGGGACGCTCGGAAACTGAGGAGGAGGCT
ATATCCGAAATGGAGCTGGCCCACACTTTGATCCTTAATGAGGATGCTCTCAAGCAGTTGCCCGAACACA
AGCGGCCTGTGTTCGAGTTGGAATGGCTGCGCTACTTGGAGAAGGCCCTGCCACTGGTGTCCAAAGCGGA
GATCAAGGCGAGCCAGAAGAAGCTGGTGCAGCAGCTCTCTGAAAGAATCCAGGGCGCACCAGGTCCGCCC
ATTCGTAAGCTCATTGCTAGCGCGTTGGCCACGCTATTCTCCGTTGGTGACACCTTCATGCTCTTCGACA
CCGTCAACGCCTGCAATGACATCCTGAAGAACAAGGACGACTCGCCCAGCTACTTGCCCACAAAGCTGTG
CCGCCATTTGTGTGCTGGGCTCTATGTATGAGAAGCTGGGCAGGATGATGGGCCGCACCTACGAGGATAC
GGTGCAGATCCTTATTCGAACGCTTCGCAACGCTGAGTCGCAGGCGCGGATTGAGATCATGCACACGCTG
GAGAAGGTGAGCGCTGGCATGGGCACAGCCATTGCCAACGTGCACAAGGATATTTACAAGGCGGCCAAGC
ATTGCCTGTTGGACCGAGTTATGGCTGTTCGAGTGGCTGCAGCCCGCTGCATCCTTAAAATGATATACAG
TGCACCATTCCTGTACCAAACGGAGCTGGAAAGCTTGGGAACATTGTGTTTCCGAGCCTTCGACGGCAGC
AACTATGAAGTGCGATGTGCGGTAGCTCAGCTGCTGGGAACACTTCTAGCCTATACTCAACAACTGGCAG
AAGCGGCAACAAGCAAAAAGAAACAGGGACAAGCGGTGGCTATTCAAGCGGGCAAGGGAGCTACCCAACG
CTTGGTTTCCCTGGATGAAGCTCTGGGCATTCTCATGTCAGGTTTCCTTCGTGGTGGCGCATCTTTTCTC
AAGGGTACCGGAGAGATTATCAAAGGCAGCTCAGGTGTCAACCGTGAGGTGCGAGTGGGTGTAACGCATG
CATACGTGGTTTTCGTGCAATTCATGGGCAGCGTTTGGTTGGAACGACAGTTAAGCACATTTCTGGCACA
CGTTTTGGATCTCGTGGCCAATCCAAAGGCAGCGTGCTCCCACGTAGATGCAGTGTATTCACGGAAATGC
ATTAGCTTTATACTCCGATCAACGATTGGAAAGATGCTAGGCGAGAAGGCGCAGAGCGCTGCCTGCAAGG
AACTGGTCCATCTGGTAGCCAAACAGATGAACTCAATTGACTTCAATCCCGAGAACGCCAAGGATTCTAA
TCAAGAAACACTGTTCAGTCAGCACCTGCTGGTGTGTGCCCTCCAGGAATTGAGTTCTCTACTTATTGGA
TTAGGAACCACGGCCCAGAACTTGTTGGGAGATCAATCCCTGCAAGCGATCGATGCTACCTGTGCCGTAT
TGGTACATCCCTGCGCCGCTGCCCGTTTGGCTGCCGCTTGGTGCTTACGATGCGCTTGTGTGGCGGTTCC
TGGGCAAATCACGCCACTAATTGATCGCTTTGTGGAGGCCATCGAGCAGATGCGATCCTCGCCAGAAGCA
ATGGCCGGATATAGCTGCGCTTTGGCGGCAATTTTGGGAAGCGTTCGGTACTCGCCATTGGGCATACCCC
ACACAAAGGGAAAGGTCGTGTTCAACTGTGCGGAGGAACTGCTGCGATCTGCTTCCCAGAATAGCCGCAT
GTCGCTGCACCGTACCCAGGCCGGCTGGCTATTAATTGGAGCTATAATGACTTTGGGATCGCCGGTAGTC
AAGGGCTTGCTGCCGCGAATGCTGCTACTGTGGCGGAACTCTTTCCCGCGGTCCAACAAAGAGCTTGAAT
CGGAAAAGGCGCGCGGCGATGCCTTTACCTGGCAGGTGACCCTAGAGGGCAGAGCTGGAGCTCTTTCTGT
AATGCACAGCTTCCTGCTTAATTGCCCGGATCTCCTGAACGAAGACATAACCAGACGACTACTCACCCCC
ATCGAAAGCGCCCTGGCCATGTTGGTCAAGCTTGGCATCTGTCCTAAAGAGCTATGGAACCCAGCTAAAG
GCACCGGCAGCTATGGTGCGGCTTCGTCTCTTTGAAACGCTGACGCTGCTGCCTGCTAATGCGCTAGAGG
CCTCCTATACCCATCTTCTTCGCATGCTCGTCTCGGAGTTTACTCTTTCGGATAACGCGGCCAACACTAC
AAACTCATTGTTGCGGACGCTTTGTCATGGTGATGATTCGATCATACTGGGCACCTGGCTGCAAGAAACT
AATCATCGCACCATTGAGGATCAGG
>dme_circ_0008204_junction_seq
ACTAATCATCGCACCATTGAGGATCAGGATCGTTTATAAACAGCGACCATTTTTCC
>dme_circ_0008205
GGAATGTCCACTACACAGCTCTAGAATGGGCCGAACGCCTGGTTGAGATCAGGGGACTGTGCCGTCTGCT
GGATGTGTGCTCCGAACTGGAGGATTATAAGTACGAGAGTGCAATGGATATCACAGGCTCATCATCCACA
ATTGCATCCGTTTGTCTGGCCCGGATATATGAGAACATGTACTACGATGAAGCTAAGGCACGGTTCACGG
ACCAGATCGACGAATACATTAAGGACAAGCTTTTGGCGCCTGATATGGAATCCAAGGTGCGGGTCACCGT

TGCGATTACTGCCCTGCTTAACGGTCCATTGGATGTGGGCAATCAGGTTGTGGCCAGAGAAG
>dme_circ_0008205_junction_seq
GTGGGCAATCAGGTTGTGGCCAGAGAAGGGAATGTCCACTACACAGCTCTAGAATG
>dme_circ_0008206
GTCCTGTCTCTATGGTGTGGTCACTACTTTTGTGAATCTGTGCAATGCGTATGAGAAGCAGGAGATGTTG
CCTGAGATGATAGAACTTGCCAAGTTTGCTAAGCAACACATACCGGAGGAGCACGAGCTGGACGACGTAG
ATTTCATCAATAAGCGTATTACTGTGCTGGCAAATGAGGGCATAACAACTGCTCTTTGTGCACTGGCGAA
AACTGAAAGCCACAATTCACAGGAGCTGATTGCCAGGGTTTTGAATGCG
>dme_circ_0008206_junction_seq
GGAGCTGATTGCCAGGGTTTTGAATGCGGTCCTGTCTCTATGGTGTGGTCACTACT
>dme_circ_0008207
GATTTCATCAATAAGCGTATTACTGTGCTGGCAAATGAGGGCATAACAACTGCTCTTTGTGCACTGGCGA
AAACTGAAAGCCACAATTCACAGGAGCTGATTGCCAGGGTTTTGAATGCGGTTTGTGGACTGAAAGAGTT
GCGAGGAAAAGTGGTTCAAGAAGGCGGCGTTAAGGCGCTGCTTCGTATGGCGCTTGAGGGAACTGAGAAA
GGAAAACGCCATGCTACCCAGGCGTTGGCTAGGATTGGCATAACCATTAATCCGGAAGTATCTTTCAGTG
GCCAGCGATCGCTTGATGTAATCCGTCCTTTGTTGAACCTTTTGCAACAGGACTGCACAGCGCTGGAGAA
TTTTGAGTCGCTAATGGCACTCACCAATCTAGCCAGCATGAACGAGAGCGTGCGCCAGAGGATTATCAAG
GAGCAGGGGGTCTCCAAGATAGAGTATTATCTGATGGAGGACCATCTGTACCTGACTCGTGCAGCTGCCC
AGTGTCTGTGTAATCTG
>dme_circ_0008207_junction_seq
TGCAGCTGCCCAGTGTCTGTGTAATCTGGATTTCATCAATAAGCGTATTACTGTGC
>dme_circ_0008208
GATTAAAGTACAATATGCTCTGGCTTTCGAAGGTATCAATGGATGGATGCATATGGCGGTGGATAAGGAC
ATCACAGGTGCTATTTGCAAAGCCACAACTCATTTGAAGACCCTCGGCTTGAACGTGAAGAAAGTGAAAC
TACCCAATCTCGAGAATTCA
>dme_circ_0008208_junction_seq
AGTGAAACTACCCAATCTCGAGAATTCAGATTAAAGTACAATATGCTCTGGCTTTC
>dme_circ_0008209
GCCAATCAGAAGGACTATCCCAACAAGCGGTCCAGGGAGAATCTGGCATGTGACGAAGTGACATCGACGA
CGTCATCATCAACGGCAATGAACGGCGGCGGACGGACGCCGGCGCTAACCCGCAGCTGCTCCAGTCCGGC
GGTCTACGGGTAAGTGAAAGTCGGCTAAATGGGGAACCCAGCGCAAAGGTATGCGACCCGAAGACGGGTT
TCAAACAATACTCTCTGTGAGTCGGTTAGAAATTTTTGGCAATTATGTGAAAAGTCGTGAATCGTCATGT
ATTTTGGTATGCAAGAAGTTAAAAACCTAATATTTCCACATGCAAACGTAGAGCATTGCATCTTGAAAAC
TGTTAGAGATATGATAAAATAATCAAATAATCAAACACTAATCACTCATAGTAGATTGCATAGATTGCAA
TCTAAAGCTGATCACAATAACTGTGCTAGTTAGGAAGTTACAAAGCATTGCGATCAAGAACGGAACTCAA
GGGGCACCATATCGCCAGCCGATCGGGAAATCGCTCGATTGTTCTACTGTTGCATATTTGCATGTACATA
TGTATGAATAGTGCTGCTTGTGCTTACCATTGCATTTCATTTCCTACTTGGTCCAAGTTCAGCACCCCCT
CTACAAACTGACCGTTCTGATAGTACAACATGGCGATAAATTGTCTCAGATTGTCATACAATCTTTCCGT
CATTTCGCCCCATCTCTTTCTCGCACTTGTGAGCTCTGCTTTAACATTAAAAACAAACAGTTTTGTTTTT
GCTTTGGCACGTGCTGTGAGATAAATCGCTCGCTCTGTAGCGCTCTCTTGCTCGCACATTTAACTCGTAT
TTTTGCACGTTACGAAATGTGGTGTACGTATATGTACATACATGTGTATGAAAAGTATGCGAAAGCAGCA
AAACATATGCAAAACAATAATAACAAAGGTAGCAACAACAACGATTGTCATACAGAGAAATTTTCAATGT
CAAACTTTTGCATATTACTCAGTTACTCACGTTGGGCATCGTACTAAATCTCGGCTTTGAGTGGGGTAAC
CCTCAACTTatacatacttacatatgtatgtatgcatgtacatatgtacatgtatacCGCTTTTCCACCT

GACAAAGGTGTGATCACATCGTGATGGGCTTCAGTATCTTCCATATAGTAATGAATTATTATAGATGAAA
AACTAAAAATAATTTTTAGTTCAATCATAATCTCAGGCCTATACTAATGCTAAAAATCATTTGATTTGAA
TATACATTATCGGTCAATATATTTCCAATGCAACATTTGGAGACAAGTGTTTCTAATGAAACAGTTATTT
AAAATTATTTCACATATCTGTaaaaaaaaaaaGTGAATAAGATATAGGGGTGGCAATGACTCACAATAGG
TCACAGTTATAATCAATCGTTATTCACCACCAATACAAAGGAAACTTCCAAAATGTAATAATATATTTTT
TTACGATATTGGCAATTCCCTGCGCTTACAAATTACCGATCGATTTCTGTTTGGGGATCTATAGACCACC
GTCATTACAATGTTTGTTAGTAAAACACTAGCGATTCCTGTTTATCGAATCGACAGCTGAGTCACTGTGG
AATTATTTATGAAATCTTTTAATTGCATTCTTAATACATTACTGTGTACTTTGTGTATATTTTTGTATAT
GTAGTGCTATCAGAAGGGGCTTGCTGGCGTAAATGCCGCAATGACAATCATCTTTACGTGTAAAGTAAAG
TTCCAACGTGCTGCCGATGACTAATGCAAGTGATTTCCGTGTGACCCACAAAGTGCAGCCAGCCCAAATA
AATAAATATAAATGAAAAACACGAAGTTGCGTTGCCATGGCTCTCAGTGCTATCAATTAACACGCAAGCC
AAATGACAAATAATCGATGGAAGGGAGTCGATGAGGTGGGAAGGGGGAGGGGTGTCTATGGCCAAAACCG
CAGGGCATGCTGGTCCCTGGGACAGCACTTTTATCTAAACATTACATTGGCaaaaaaaaGTTCGAACCAA
ATGTGTGTAATTTCAATTCCAAAGTAACATTTAGAGAAGAAGTTCGATTCTTGCACAGGGGAGTTGTACG
AAAACGCCATCGAAAGAAGTTCCAGGGTAGTTTTTCTCTTAATGGTAGTTTTATAAGGTGAAATTTGGTT
TTTCGGAGTGTAGTTAGACGGTGCACAATTATAGATACACATAGAGAGGGAAGGAAAAACAATTGCGCGG
CTCATTGAGAGTGCTACGAACTCGGGGACGGCCATGAGGCAATGTGAAGCCACCCCTCGGCCCGCTGACC
ACCCTGTCGCTTCCCCCTTGCCCACCCCCACCTTTGGCTTCGCCCACTGCCAATAAAACTGAACCCAACT
GAACTGAGACTATGCCTCTGTGTGTGGCATGACTCATGCAACAGCAACAGAGGCCCCAGCAGACGAATCC
AATCGAAACGAAACGAAAACGCTCCGCGGTGGTGGAGTAGGTGAAAGAGAAGGAGGGTAGGGGTAGAGGG
GTGAACAAAAAATAAACAATTTGTTGTAGCAAAATTTAGACGTTGTGTACAACAATAGATACAGAAAACA
GGGcatgcccatccccatccccatccccatccccatccccatcGCAATGATCATATGCCCAGACTCTTTT
GTTGCATGACATCGCGAATTGGCAAACTTCCATCGCTTTATAGTGCgagtgagagagagagagagagaga
gagaTCCCCGACAATGGTCACTTTTTGTACGGCGTTTGAGGGCAGCAAGAACTTTTCCATTATTTACTCA
TAAAAATTAATGATCCAATCCAATCCGATCCGCATGTATCTTTGCAGGATATAGAAACACACCCCGCCTC
GCCCGTTTTCCCGCATCTATTGCTGGGCAATGGACGCGACGCCGACGATCCCAGCAGCGTGGGCGCCAAC
TGTGTGCTGAACGTTACCTGCCAAAGTCCCAATGAGAGCCATCTGCAGGGCCTCAAGTACATGCAAATAC
CTGCCAGCGATACGCCACATCAGAACATCAAGCAGTACTTCCAGGAGGCCTACGACTTTATCG
>dme_circ_0008209_junction_seq
TACTTCCAGGAGGCCTACGACTTTATCGGCCAATCAGAAGGACTATCCCAACAAGC
>dme_circ_0008210
GATATAGAAACACACCCCGCCTCGCCCGTTTTCCCGCATCTATTGCTGGGCAATGGACGCGACGCCGACG
ATCCCAGCAGCGTGGGCGCCAACTGTGTGCTGAACGTTACCTGCCAAAGTCCCAATGAGAGCCATCTGCA
GGGCCTCAAGTACATGCAAATACCTGCCAGCGATACGCCACATCAGAACATCAAGCAGTACTTCCAGGAG
GCCTACGACTTTATCG
>dme_circ_0008210_junction_seq
TACTTCCAGGAGGCCTACGACTTTATCGGATATAGAAACACACCCCGCCTCGCCCG
>dme_circ_0008211
ATCCCCATTGTCCTTATTGGACTCAGAAGAAGTGGGATTGAACTCGTAGCTATTCCCCTTGTATCTGTCG
TATCCGCCTCCGTCATATCTTTCATTGGAACCGTGGTCCCTATTATCGCGATCCTGTCCCCTATCACCCG
AATTAGTGACCTGCTTGCGAACGTGATTGATAGCATCGATGACGTTGCTCTGGATGCTACCAGTTATCTT
GACTATTAAGTCGCACTTGTCCAATTTGACGCGGACATTGAAGTCGTTCTGGATCCGGGATATATTGGAG
CCCCCTCTTCCAATAACTTTTCCGACCATCTCGGAAGCGATGCTCAAGGATTCGCTAAAGTCTCCTCCTC
CTCCCCCGGCACTGTCGCGACTATCACGATGCCCTGCTCCGTAATTATCCCGATTGTCGCGACGTCCACC

GCCATAATCATTGCGTTTAGCGCGATAGCCTCGTCCTTCTCCGCCGTAG
>dme_circ_0008211_junction_seq
GCGATAGCCTCGTCCTTCTCCGCCGTAGATCCCCATTGTCCTTATTGGACTCAGAA
>dme_circ_0008212
GGTGTGTGTCGGATCGCTCGATCTGGCAGCCACGCACTCGGTGAAACAAATTATTAAATTGATGGAGGAT
GACATGGACAAATTCAACACCATTACATCTTTCGTTAAGAACATGTCCAGTACGGACAAGATCATCATAT
TTTGTGGACGCAAGGTTCGTGCTGACGACCTATCCAGTGAACTTACGCTGGATGGTTTCATGACCCAGTG
CATTCATG
>dme_circ_0008212_junction_seq
GATGGTTTCATGACCCAGTGCATTCATGGGTGTGTGTCGGATCGCTCGATCTGGCA
>dme_circ_0008213
GATATCTCTAAGAGTCCAGTAGGGGTGTATGCCAGTCACTCCCGGCGGATCCATCTTCTGAGCCTCCGCT
TGGCCTTCATTCGGTGGCCAATCGATGTCCACTCGGCTACTCTTGTTTAGGACACGGTTTTGGGGCCTCG
CCTGCAGATGAACTCCTCTATCGGTGAAGTAGAGAAACATCTTGCGG
>dme_circ_0008213_junction_seq
ATCGGTGAAGTAGAGAAACATCTTGCGGGATATCTCTAAGAGTCCAGTAGGGGTGT
>dme_circ_0008214
GTGATGCAGTAGTGGCTCTTCTCCTATAGAACTGATCGCTGGGTTTGAATGTTGTTGGCGAGGAGCGGAA
GGCGCACTCGTCTGATGTGGGACTCTCCGCTGTGGGACTTGTGAGACTCGTATTTGTCAGTGAGG
>dme_circ_0008214_junction_seq
CTTGTGAGACTCGTATTTGTCAGTGAGGGTGATGCAGTAGTGGCTCTTCTCCTATA
>dme_circ_0008215
GTAAAAACTACAAAACTGAAAATCAATTTACTTGTATCAAGTCAAGCTCAATTACAATAAGTGTTGTTCC
TTTGTGTGAGATGTCGGGATTCAGATATTGTGTATTAACTAAGCTTAAGTAGGGGACCCAACCAATTGAA
TATGTATGATATGAATTGTAAATGAAGTAATAAATGTAATAGTGAATGTACAACAAGGACGCGACTTGTT
TCTCGGATCTCACAGCTGGGAGACCGCTTTGGACTGAATGCCGCTAGGATATGTAAGAAG
>dme_circ_0008215_junction_seq
ACTGAATGCCGCTAGGATATGTAAGAAGGTAAAAACTACAAAACTGAAAATCAATT
>dme_circ_0008216
GTAAAAACTACAAAACTGAAAATCAATTTACTTGTATCAAGTCAAGCTCAATTACAATAAGTGTTGTTCC
TTTGTGTGAGATGTCGGGATTCAGATATTGTGTATTAACTAAGCTTAAGTAGGGGACCCAACCAATTGAA
TATGTATGATATGAATTGTAAATGAAGTAATAAATGTAATAGTGAATG
>dme_circ_0008216_junction_seq
AATGAAGTAATAAATGTAATAGTGAATGGTAAAAACTACAAAACTGAAAATCAATT
>dme_circ_0008217
CGGCGGCGAGGCAAGTTCAAATTCAATCGCACCAAGAAACACGCTCCACATCGCCGACCCGACCTGCTCC
GGCGCATTATTGCCCAGCAAAAACTACTTGGATAGCTGTCGGGGATCGTCGGCAGCTACGATGCTGCAGA
CGACGGGAATGGTAATGGGGG
>dme_circ_0008217_junction_seq
CTGCAGACGACGGGAATGGTAATGGGGGCGGCGGCGAGGCAAGTTCAAATTCAATC
>dme_circ_0008218
GAACAGGACTGACAGCTTACGATTGTTTAGCCCTTGGCGAGTTTGGCGTCCAAAATCAAAAGGATGATCT
TGCCGAGGCTTGGTATAACCTTTCTTTGACTCGTTTTGATAATATAATCGAAAAATACCAAGTGCACAAG
GCATGGGCACTTCTCCTGGCGAAGAATAAGCAACTTACTGAAGCCTTTCAACATTTCGAGAACAAGCCTG

AGGGAATAGTCGCCAGTAATGAAGTTATACACTTTGAGGGTGTGTTGGCGACTACGCAAAACTGCACTGC
TGTGGTCCAAAAACCAAGTAAAAAACTCCACTGTCGCTACAACACCTCCACGACTCCCTTCACGCGGATT
GCCCCTCTAAAAATGGAGGAGCTGGGCTTAGATCCGTACATGGTGGTCTTTCACGATGTGATTTACGACA
CCGAAATTGATGGGATGCTAAACTCGAGTGATTTTGGATTATCCGAGTCTGTTTCTGGTCTGAAGTCTGA
GGTGAGAACGTCCAAGGACTCACATATAGTAGATGCTAAAACACTCAATGAGCGCGTAACCGATATGACC
GGACTCAGTATGGAGATGAGCGATCCATTTTCGCTGATTAATTACGGCTTAGGTGGGCACTTCATATTGC
ACCATGACTTTCATGAATATACGAATACGGCTGAGAGAAGTTGACTCGGGTGGCGCCACAGTTTTCCCGA
TGCTTAATATTACGGTTATGCCCAAGAAGGGATCAGCGGTTTTCTGGTACAATCTTCACAACTCAGGAGC
TGTAAATTCGAAAACTTTGCACACAGCTTGTCCAGTGATAAGCGGTTCGAAATACGGTGCTCACGAAGTG
GATAAACGAACTGCCGCAAATGTTTGTCACACCATGTATGAAAGACTCGAACCTACGCCCACCCCAAAAA
GAATAATTCTGTAATCTGTAATGGAATTAAATTTAAATTAAATTTGTTTTCCCTTTTCTGGATGGATCCC
TAATAGATATGAGTAGAGAGAACATGGAAATGAAGAGTGATTATGAATCGTACTTGGGAAATCCATTGAA
CAGCTTTCGACTAATACATCGCCTACACACGAGTTGGAGAAAATGGTATCAATATGCCATTAAGGTCGAG
AATAATGCTTTAGGGGCACATTGAAAATGCCCGACTGATGAGAAAGATGCTACCAACCTCGTCGGATTTG
CAGCAGGCGTGTCGCGGAATCCATGACTTGATGTCCTTTTACGATTTGAAGCCAGAAGAACTGGCAGCTG
GTAACTTAGCAGGGTACTCGCAGCCAGG
>dme_circ_0008218_junction_seq
GTAACTTAGCAGGGTACTCGCAGCCAGGGAACAGGACTGACAGCTTACGATTGTTT
>dme_circ_0008219
GATCCTTTAGTCGCAGAATAGCGCTGCATTCGTGTTTGGTGTATGCTCTAACATGGGAAACAAAAGCGCG
CATGCCTTTGTCATAGACGCCCTTGTCGGCCGCCTGCAGCCGATGGAGTTGATCTAAGACGGCTG
>dme_circ_0008219_junction_seq
AGCCGATGGAGTTGATCTAAGACGGCTGGATCCTTTAGTCGCAGAATAGCGCTGCA
>dme_circ_0008220
TGATGTGATCTATGAGACAAACCAGTTAGTGGTGTCCAATCCCGATTGCAACCCAGCCTGGAGCCTGGAT
GAGCTCAATGAAAATGTGACCCATCAGCTGGGTGGGTGTACGGAAGACTTGGGAAGCCTTTTGGAAGACT
TCCGTCTGGAGAGTCAACTGGCTTTGGCCAGAGTACAGGAATTCGTACAGCAGATTGCCCACTTGCCGGC
CAAGTGCCAATTGCTGGGGGCTTCCCCGCTTAATCCTCTAGCTTCAGCTGGCGGAAGTGTCTGTTTCATT
AACGCGATGGCGGAAATCAATCTGGGGATGGCCCAATCCATGCACAATGCATCCCTAATCCTGGTGCAGA
CCCACCAGACCGCCGAGGATCAAG
>dme_circ_0008220_junction_seq
CAGACCCACCAGACCGCCGAGGATCAAGTGATGTGATCTATGAGACAAACCAGTTA
>dme_circ_0008221
GCTTCTGTTTTAACTAATTGGAGGCAACTGCTTCTTGGGGTTCCGCCGGCTCTGTCTGCTTCTTGGCCCC
ATCCCTTTTCACCTTCGCCGTGCTCTTGTCCCACATCTCTGGAACGTAGACAAAGGGATCGATTTTCAGC
TGCTCACTGCTTGGCGGGCTCTTCAGCAGGACACGTGTAATGAAACGTCCCTCGCGCTTGGGCCTCATAG
TGTTAATATCCTGCAGTAGGAACTTGATGTTCTCCTCAAGCTTCTGGACATCCATGTCCAAAGTGCCCAC
ACTGGCGGTGATCAGGCCAAAGTTTTGCTGGTACTCATCCTTGGCTGCACTGTAACTAATGCCGCTGGAG
AATTTGACGATCATCTCGGCCAGGTTGGTGCCCAGCGTCTCGCTCTTTGGATTTGGGAACTTGCGTTTCA
TCAGGCCTCGCAGGGCCACCAGCTCGGCCAGTATATTCGGATGGGCAATGACGTACTGGTAGTCGGAGAG
CAGCAGCTCGCCGCTGG
>dme_circ_0008221_junction_seq
TAGTCGGAGAGCAGCAGCTCGCCGCTGGGCTTCTGTTTTAACTAATTGGAGGCAAC
>dme_circ_0008222

GCTGAAAATCGATCCCTTTGTCTACGTTCCAGAGATGTGGGACAAGAGCACGGCGAAGGTGAAAAGGGAT
GGGGCCAAGAAGCAGACAGAGCCGGCGGAACCCCAAGAAGCAGTTGCCTCCAATTAGTTAAAACAGAAGC
CTAT
>dme_circ_0008222_junction_seq
CCTCCAATTAGTTAAAACAGAAGCCTATGCTGAAAATCGATCCCTTTGTCTACGTT
>dme_circ_0008223
GTTTTTATTTATGGAAGGGCGGTCAATATGGAGAGAGGCAGGGACAAACTAAAATATCAAGGACGACTTT
AGAAAGATTGCTTGGGAAAGTAAAGGTCTTAAGTTCAGCATTTCTCTCATCCTTTTAAAATCAGTTTTCA
GTTATGTATTTCGATTAGATAAGGAATGTAAGATCAGATTTTACTGCATAATGTTTATATGTCTTTTTAA
AACAACCTACTTTCTTATCACCCAAGCAATGTTGCATAATGATTAAAGAATATTTTCAAAGAAATGGATT
TCAGACCTATACTTTGGCTTGAGCCCTCGCATATAAAT
>dme_circ_0008223_junction_seq
ACTTTGGCTTGAGCCCTCGCATATAAATGTTTTTATTTATGGAAGGGCGGTCAATA
>dme_circ_0008224
TTATTTATGTACATACATGATTTTTTCACTCACAAGCAGGCGAAAGGCACTTGaacaaagaaacaaacaa
acaaacaGGCGACGAGTACGCCCAACTTACTACCATATGCACAAGCTATATAGCCATCTATATGCATTTG
CATGGGTCACTTGCTTATGTATGTATTTGTGTCTAGGG
>dme_circ_0008224_junction_seq
TTGCTTATGTATGTATTTGTGTCTAGGGTTATTTATGTACATACATGATTTTTTCA
>dme_circ_0008225
TCGAGTTCCCAGGCCAGAAGCAGCGGTCACGACGAACTGGGCAACCTACTCTGGCAGCAGGATTCCTGTG
AGTTCCACGAGCACTATCCCTCCGATTCCAGCTGCCCAGTGCCGCCCCTTCGTCACTGCCACGCCCCAGG
GCATGGACATGCCACCAAGACGCTGACCAGACGCAATGCCGATCTATTTAGCGGAGATCTGGGCATACCC
GAGATCAACACCCATGTGGGCAGTGTGGTGCACACGTTCGAAACGCTGGCCCTGCAACGTCGCCTGGCGG
AGGAGGAGGATCGCCATCCGGAGCGGGAGCTGAGTAAGTATGAGGCCAACGGCGGCACCACCACCCAAAG
CAAGTACAGCCAGCTGAAGCGCCGCTCGCAGGAGATGCGCCAGCGGCTGGTGCAGCAATTGCACCGCCAG
CGTTTGCAGCAGCAGCAACTGCAGCAGCAACACGAGCCACACCTGCAACATCAGCAAATCGCACGCTCAC
GAACACAGTCGCAGCCACAAAAGCAGCCGACCATGCACGAGGGGGTTTGACCAACAGTTGCAGCACCCA
AACACTGGCCACCGGCTTTTGGCCATTCCAGCCGAGCCAGCGAGTGGATGACTCCCGGCAGCAACTGCAG
CAGTCCACCTTCGAGGACTATCGCAAGAACTTGTTCGGCACCCTGCGAAGACATGGCAGCCTGGAGGATA
ACGAAGAGCTGCAGGACGAGTCGTTCATAGTGAACGCGGCTTTTGATGAGATCTTCTCCTGCTACGATGA
CGATAACGAGGATGACAAGGGCACGCAGACGCAGACCAATACCGATGATCTCTTCGAGGACGAGCATATG
CTGGTCAGCCTGAGCGAACTTGATGACGAGGTGTTCGCCAGCCGAGCGCAAACGGCCTCGACCACACCCA
CAGCCAGTGCGGGAATGGGAGCTGGACGAACGCCCAAACACAGCACGCCCCAGTTAAACCACAACAAGTA
TACGGCTCCATCGCCACCTCGCTCCCGCACTCTTACCAATCTCTTCGAGCGTTTGAAGTCGCAGGACAAC
AAGACGCTACAGAAGTACGCCGCCAATGGGGCGCTCAACAAGACAGAGGACTTCAATAGAGGCTCCACAG
TTAG
>dme_circ_0008225_junction_seq
AGGACTTCAATAGAGGCTCCACAGTTAGTCGAGTTCCCAGGCCAGAAGCAGCGGTC
>dme_circ_0008226
GTGCCCCGCAGGTCCTGCGTCTGCATCCTTCCCTGCACACCGTCTCCGTGAAGCGAGGAGAGGCGGCCAG
CCTGACAATGGTCGTCTGTGCGGATCCGCGGCCACAGCGAGTCGCCTGGGAATGGGGATCACTGCGTCTG
GAGGCCGGCTCGGGCATCGGTGCGTATACTTGTTTGATAGGCAGTTACCGCTCAGCGCTTACCATCCGCC
CCATTGGTCAGCTAATGGTAATGCAATTAGACGCTGGACTTAGTTTTGCCTACTCGGAATTCAATTAGCG

CCGCTCCACAGGATGCACAACGATGGCTCCGCCCGCTCGAGGGCTCATTAGATTAACTGCCTCGTCGCAG
AAAGGGTTGCGGACGGGGGGAGGGCATTCGCGCAGAATTGTTCCGCAAATCAAGACAAATTACATGACCA
CGCGGAGTCAGAGTCGGACAAGAAACTGGCAGTGTCATGTCCGTTGTTGCAAAGTGGGTAGTGCAAAGAA
TGGTCGGTCGAGATGGGGGTGCCTCGGGCAGACTTCCCACGCCTGTCAGCCGCCGGTAATCCGAGCTTCA
ACAGACAGCCAGCGCACACAACCCAACTCATTTTCCAGCTGAAACTCTTTCGGGATGAACATCCGCAACC
AGATGCGGCTGCATCCCTTCTTAACTTCCGCTACCCTCGCAATTCCATGAACTTGAACTGCATCCTTGCC
AGTGTTGGCAACTATTGATTACCTGATTAAGCTCAACCTGAAAATTGCAGATTTAAATTAAATTACTCCA
AAAAAGAGAATTTTCTCATGTTAGTTCATTTAAAAGTTCAAAGAAAACCAATAATGTGTTCATAAAGTAT
TAGGATATTAAATAAGTTCTGCGGAAATTAAATAATATAAAACACAGACACAGATAAGAATATAATTGAA
TAACTAGTTCAGGTGAGAATTAAATCTGGTGTTCTTTATTCTAGAAAACATCTGACTGGGCAACACTGGT
CCTTGGTCCGCAAGTCGCATTACTCTTCATTTTCAGCTTGTCTGCCCGTGTCCATCCTTGCTCGTCCTTC
GTGCGTGTAATCCTGCGTGCTCTCACTGCCACTGACACAAATCAAACGGGGCGCATTCCCGTCCAATTCG
TTCTCCTTTGCTCCGCGTCCAtttttttttCGCTTGCGATGCTAGTTAGCCCATCGAATCGAATGATTAA
TGTCGGCCGCCGACAAATTGTGTGCAATTCCAGTGCAATTACTTAGCCCACGAAAGCCAATTCGCATTTG
GTAACAACTAAAAAGCACAAGGACTTGCCTGATTTCCCATTTCATATCCCACAGAATCGCTTCCGGGCCG
ATGACATGCAGCCGGACACGCGGGAGGATTGCTACCTGTCCACCCTACACATCCTGCACGCGGACGAGCA
CGACTCCAGGCCCTACTACTTGGTGGTGGAGAACGAGCGGGGCACCGATCGTCATGCCATACACCTGATT
GTGGAGGGTACGTTTGCAGGGTGCATAGACTTTTCTTTGATTTTCTTTACCCCCTCCCCACATACTGCTC
CCCATCACATACATCACGTATTTAAGTTACACTCAATTTTTTCAATTCACTTCATCATCAAGAAATTAAT
GCATAGAATACCATACATTCCTGCAAAACACTTTTATGCTAGCATCGTCACAGTCAAATTCACTTACTCA
TGAACCCATATTATTATTCAGTCTTTATCCAAAGATATAAGTCAAATGTTGAATCTTAGAAACAACAATT
TCAGTAATTCATATCCTTAAAATTTCAATGAAAGTTCACTTTAATTAGCCGAATAGTGATTAATCCTTGC
TTATTGCCACAAGGTAGTGTAAATTCGCTAAAGAACACATGAACTGAGAACCCATTAAACATATCCCACC
CACCCATCATCGCATCGCTGCCCAATGGTTTTAATTAAAATCAATCGCACGTGCAATACATGAATTGCAT
AACATATATTTTGCATATGAAAATGAAAATTCCCCTTAATGCTTGCATAATCAACCCCACCACCCTCCAC
AACATCGAATACGAATACAAATACGAATCCGTGTATCGAATCGAATGGAATTTCCGGACAGAGTAAGTTG
AAAGAAGGAGGGAAAACACCCaaaaaaaaaaaaaGAAGGCGAACAATCATCGGGCCCGTATTAATTGCAC
CGCCAGTGCCGTCAAGGCCGCGACAATTAAAACTTCCTCCTTGATTATGGCGCCTGTTTCACCACCCAGC
AGCCAACACCACCACCCCTTTCTAGCTGGAAACCAGCCCCCCCAAGGCCGCTGTCACACTAATTGCAAAA
ACACAAAAACTTGCCAGCTCACAAAGCATTGAAATGCGAGAAGGACGCGGTCATGGCGGAAAAAATGGCT
GCCAGGATGGATGCGTCTCTTTGGCGCTTTGTGTGTGTGTTGTGTTGGCTCGttttatttttattttatt
ctttttgcagcttttccctttttccCTACACACTGCTTGAAACTCGAAACTCAAATAAATCTCATAAAATA
TTCATTAGCGTCCAACGCCTGCGGTCAGTCTCCGTTTCCTGTTTCCTCCGCTGAATCCTGCCCCCTGTGC
ACACAAAAGGGTTAAGGGTTTTCCAGCCGGCTTTTTGTTAGTTGCGCCAACATTTTCATCTCTTCTCCTT
CGGCGAAGGAAGATGGCTTAAAGCCAGAGTTCGCTGCCTAATTAGAAATCAAAACGCAGACAAAAAGCGG
TGAAACTTTTTACAATTGTAAAACAATTTTCTAAAGGAGCAAGGGCGAAACTTTGCCATTGCTGAAGGAC
ATCTGAGCCGTTCCTGCACACAGTTACCCATCCACATCCACGTGATCTGTAAACACTTTTCAGGCTGTGT
CCCGAGTCCCAGGCACCTAATCGCAATTTCTCAGTTGTCAATCAAGCTGTCAATTTGACTGGCTGACTGA
CTGCTGCAGCTGCCTTCCAGTTGTCAAATGCCGTTTGACAGATCTCTCAAGTGCGACCCGATACTGTGGC
ACATATATCTTAGGCTAAGGGGACCCTTCACTTCACCCCTTCGATATCTATCAAAATTTGCGAACACCTC
GTTGGGAATTTACACGTTCCTCGCAATCAATAAGTCCTTGGCTCATTTATCTCGGCGCTCCAAGGGCTCA
TTTCAAAAATATATTCATATACGTACATACATAGCCCTCTGTCTAGCTGTCTATTTGCAAATTGCCAGCG
GTATTTTCACAGCTAATTTCTACTATTTTGCAGCGAGACACAACCACACCCTTCAAAGAATAATGGGTGG
AATTTATATTCCTGAATATTGCACACTCTAATATGAATCTTCCCCTTTCTCTTTCCTTTCGCCTCTCGCA

TGGCACAGGGTAAGTTGAAGCGTAATACGTacacacccacagacacagacacacacacacaTGCATGTTC
AGGGAGGGGGCCATAGATAAATGACCATTTTGATATATTTTTATGCAGCCGCATCCTGCACCCAACAGGT
GGCGCTGGCGTGCCAATTTATGCGCCCCTACTGGCCGAATAGAGCAGCTCGCCCACCAACACAAATGCAC
ACGTACAGCATTGGAACAATGAAAAATGTTTTCCACTGCACGTACGTGTGTGTGTGAGGGACATAAAATA
TTATACATTTTTCATATATTCAATATATGCATCGGCGAGCAAACATTTTTATTTGGCGCAGAACAAAGTA
GAAATACCCTCACACATGTTGAGAAAGTGGTTCGATAAGACTATTTTGTAGAATAGCTAAGGAAATTTAA
CTAGTGTAAATTATAATTATTTTAGGGCGGAAGGGAAACTTTGATTTAAACTTTTCTTTCTCAATAATTT
TCATTAGAATCATAACTTAGAATATAAACATTTGTTATAACCCAAAACTCCAGAAGGGTAAATAATATTT
TGTTTTCCAATAGCAGCAATTCGAAACAGGGGCAACCTATCGCTCATTGTTGCTGCATGCCATGCAGCCC
CAGTCCTGAAATATGAGTTATGAGAGGTTATTTGGCCATTCACCGCTAACGTCCTTTGTTCCACTTGTTT
AGGAGTTTAGAATAACTACATATATTCTGTTTATACACACACAAGTTCAAAGTGTACCACTGAACAGATC
AATATTAAAAGGAGAACGGAAATGGATTTAGAATTTCCGTATCGAGCACCACACGCTAGAACCACAATTC
CACAAAATTCGTTTTGTGAGCTAAATTTCTGGGCCCATCTCGAGAGTAAATTGATGGGGGCAAGGATGCA
AAGGGGCAAGTGGCAAGGGGCATATCTAATTTCCTAAGCCCCAAGTAAACAATTGCCTGCGGCAAGGCGA
GGCAGCGACAAAAACTAAATAGATATTCAGATATGCGTGAGGGATACAATTTGCCAATCAGCGATGGCAG
CCGATGATGGCACCACCCAACCACTTAACCACCACCCACACAATTGTCACTTGGGCAAACAATTTTCGAT
AACGATTTGCTCCACCCATCTCATTCTCATTCTCATCTCTTTTCCTTTTTGTTTGTTGCCCGCAGCCGAG
AAAGCTGATTTGAAATCACGCTCGGATAGCACGAGTGGCCCTCAAGGTGATTCGATTTACACCACGCCAG
CGGGCTTCcatcatcaccagcagcagcagcagcagcaacaacagcagcagcagcacgcagcagcagcagcagc
cgctgccctacatgccgcatcccagcatccgcagcaacagtaCCCGGGACACGGATCACCGGAGGCAATG
AAGG
>dme_circ_0008226_junction_seq
GGACACGGATCACCGGAGGCAATGAAGGGTGCCCCGCAGGTCCTGCGTCTGCATCC
>dme_circ_0008227
GATCCGAGGGATTTCAGCGATTCTCGGAGCAGCCCAAGTACACGGAGGTGAATCCCGCCGAGGACACGCT
GCTCACATGCAAAGTAATCGACAAGCGCGGCACGTGCTCCTGGCAAAAGGATAACAAGGTAATTATATCA
GCCCACCGAGCCCATCTATCGATGGCTCCAGTTTATAAACATTTTGATTAAACTCATAACTTGGCCAAAA
GTCTCGCCCAGGCGCAAACAGCCGAACTTTGCTCGAACACTAGCACACCGAGacacatagaaatacacac
acacacacatacacacacacacaGGCTCAACGACGTGTCCTGGCTAGAAAGCTCATATCATATGCGTGGC
GCGTCCTGCGGTTCAATTAAGTGTTGTTCTTTTGGCCTCCACATGTGGCGAATGTAATTTACACCCTGGC
CAGGACGAGTCAATAATGAAAGTTGGCCGTGGACGCGGACAAGGGGCCAAGGGTCAGGAGTCACATGACC
TGGGAACAGCACCCCAGACAGCCCTTCATTGTTCGCCGTTGATTAGAACACTGGCCGGCGCAACTTTTAA
TCGAAAGTGCGTGACAACAAAGGACTAAGGGGGCTTAGGGGGGGTTTGGCAGTAGATCCCTCGCCAAATA
AAATATATTTTTTGTTTTTATTAATTTGGCTTATGCTGTCACGGCTGCCATTTTATTTGCTGCATTTTC
GCGCTGATCCCAATTTATTTCAGCCCAGGGCTTTACTTTTTTGTTACATGCAAATTAAGTTTTATAAGCC
ATTAGAAATTTAACGAAGTTTAAGCTAAATGTGTGTGCAACATTTTTTGGTTGTTTGTCGACATCATAGA
CCATTAAACTAATTCTACAAATGGCTTCGTTGTAAGCTCTGCTTTATTGTTCTCAGTGATTAATTGAATT
ACTTTGTAGGACTTGAGCTGCATGGTTGAATTTAAATCCTAATTATTTTCATGGTCAATAAGTGTCTTGT
TTCGAGTGTTTAATATTCTATATAATCGAAAATGTCTGCCGAACCATAGTTCATATTTGTGTTCTGACCA
TTTGTTATGACCATTGCGGGCGGGTTGAGTTGATCTCAAACGACTTCGTTTGCATACCAATCCAAAGTGG
CCTCATTATGGATCAGCCTCCGGCGAAGGGAATATGTTGTTGCCGTTTGTTATTCAGGGCAATTGTGTGC
GTTTGAAGCATAACTTTTATTTGCCACTCCCCGCAGTGACTGCCATTCCCGTGCCCCACTTGTTGTGGTA
GTTGCTTATGCAAATGCACACTGGGACTTTGGACGGCAGTCCGACGCCATTGTATGACAAAAATCAGAAA
GCATTTCAAATGTCCTTTTTCATATGCTGCGCATCGCAAACTCTCGCTCTGCATCACATACTTAAATGTT

TACATGCTTTGTGTCTCCACTCGAACAAGTTAAAGGAACCGAAaggacgaaggacgcaggacgcagcaca
caggacgAGGCAAACAGCATTTGCATGCATCGAGCGTCGCACACACACAAACATGCTGATGTTGCTGTTT
CGGGCTTCATTTAAAATTCTTCTTATTTTTGCATACTTATTCCACTTCCCTCTTTTTCTCCTATGTGTGC
TTAACGTTTCCCAATTTCCTCCATTTGGGTTTATAGTTCTGGCCATACATTCTACGTGTAGTTATCATAA
GAAGCGACGTTCCAAATCGACTGGCCaaaaaaaaaaTAAGAGATGAGGATGCGGCTATGTCCATTGCCGA
GCTGCATTCTAATTTAGCCAAAGGATGTCTTTTGAACATTTTTCAATTCTATCCATGCCTCATCTGTGTT
TTCCTTGCAGTGCACTTCCCGATAATTTCGAAGCACAAGTGGAGAATAGTCGACTATGATATCAACAACC
AGGCTCAGCCATTTGAATAAAAGTAATTTTTGGCCATCGCACATTTTTTCGCAGGCCAACAAATCGAGCT
CAACGATGCGGATGCCCAGGCTAAAACTCCGGAGTTTTGCCTCCAAGTTGTTGCTAGCAGTATCTATATT
GAAGTTGAAATATGCAGAGAATTATGCCGATACGAATATCGATTCGAGAATGTTTTGGCAGCAATGGCAG
CATCAATGGGCGAGAGCCAAATATTTGTTCGGTTCGATTTTTTATCTCCCATGCTGAAATTGTTTTTCAA
GCAGTTTTGGCAGCTCGGTCGCCCATTGAGCTCATATCTTTTGTTACCGCAAAGCCTGCAAAACTTAACT
TAACCCTTTGGTGCCCGGCGACCGGCCATTGATTATTCCCTGTGGCCTAAGGTCAGTGGAGTCAAATGGC
ATGTAGTATCTGCAGACATTTCACATGCATGCCAGCCGCAAGCTGTTTAATAAACCAGAGATACTGTATT
TATGCTGGCTGGCCAGTCAACTCAAGGTGGTCAAATGCATGTCACACATTATTTTATCAGCATGGGACAA
AGAAAATGCATTTGTGGCTATTTGCGCAAATTTAAGCACTAAATATCAAATGGCAGGACAGACGAGATTA
GATTATGCACTTGCATGTGCTTTCCTTTTCTTTCACTGCAGTGAACTGCCTTTGAATGCTCGTTTTAAAC
GATTGTTATGCCATGGCGCACAAGCTCAATGTAGCAATTAATTTAACCCATTATGCCACGGACAAGTCAG
ACAACACTAAAAGCCCAAATGCAATTAGGCAGCTCAGTTCTGGGGGTTTTTGGGGTACGGTCCGTTCCCA
TAAATACTTAAATGCGAACGATGCCACGCTCAGAGATCACTAATTGTAATTTAATTTCCAATTAGCAAAC
AAACTAATGCCAATTTACAGACAGAAGCGACTGAAACGGCGACTCGAAATGGAGTTAAATGACACATAGT
GGTTGGGTGGATAGTAGTGGCGCTGGTGGGCGGACTAGGCGGTGGAGATTCGGGGTTGGAATTCCGGGCG
AGGACACCACCGTGTGTCCTCCTCACCATTGGCTCGCCGATTTGTCCGTGAATGTTTGACCAAATCGGAG
AACAAATTATACGGCTGCCTGCATCGAAAAGTTAAACATGCTAAACATGTCGTAGACGACGCCCAGTTCA
TTTCAACGCCATCATCGTTTTTATCCCCATCCGTATCCAATCTGTATCCTTGTTCAATCGACACCGGAAT
AAATATCTAAATGGAAAAATGTTTATATTGAAACCAAGTTTGATAGGAGCGGAAACATCTAGTCCATTTC
CATATGTACTCCAATCGTAAAAATCTTTAATAATATTACAAATTAGTATAATTGAATTTTTTCTCCGTGT
GTGGAACTAAACCGATTCATTTCCTCACCCCCAGCCCCGTTGGCATCTATACAAAGAAATACGAATGGGC
CTCACGTATGCCGACAAGCGACAATGGAAATGTCCTGCATCTGGATCTCCATCCGCCGCCGGTGCAGCTC
GGTGGCGACTGCTCCCTCTGGATCCGCTCCGCCACACTGGACTTTGACGACGGACTATGGGAGTGCCAAG
TGACGGCCAGTGACTTCACCACGCAGGATGCCCTCACCAGCCAGCCGGTGCGGCTTGTCGTACGTGGGTA
AGTGCACTCCTTGGGAGCACACGCAACGAAATGTTGCCAGTTTATGTGGCGAACACGGAAAAGGGAATTA
TAAATGTTTGCTTTCAATTGCAtttttttttttACAATTAATAGTTTATTTTTTCAGCAACACAAATGAA
TATTCATTGGTATATTTATTTCAATCCAACTGATAGTACAtttttttttttGGGTGCAGCATGCCGCATT
ACCCCAGTGGCTGTTGTTATCCAGCGGCAGTTGTTAATGTTAATTAATTCCCATTTGCTGCTGCTCTCTC
CCCCAGGGCGACCAGGAGATCTCGCCCATCCACCCGCAAATGAATGCCACCGAGCCGGATAATCCACGCA
CCTGGTCGGCCACCTCGGTGGTGCAGGTGTCGGCGATGCGGGAGCGCCACGGAGATATACTGCGATGTGC
CGCCTACCACGAGTCCTACACAGCCAAATCGGTGGTGGTGGAGGCCAGGCTGGACGTGAAATGGTGAGTG
TTGCGATAACGATATGCAAAACTGTGATACAGCACCTGCAGTATGAATGCGGGCCGGACCACTAAAGTGG
CTTCATTCGATTTGGCTTAAAGTCAGGGCAGGGCAATCTAAGCAAGAGCCATGGAGACCGAGTAGAAGTG
GCAGTGGAATCACAATCCAAATCCGAATCGGAGACAGAAAGCTGTAACAATTAAATTAACAACAATTTGC
TGTAATTTCCTGTTTTGCTGAGCAATTCAAGAGTTGACTTGTCCCAAAGGACGTGGCCAGGAGCAGGACT
TGCCCGGCTTGTTGACTCTGTTTGCTCGGCTGCCATTGCTGTTTCTGCAGTTTCAATTGCTGTTTTTGTT
TTGCCTAACCAAGTTGAAcgtctccatctccgtctccgtctccgtTTCTCTGTTTCTATTTTGTGTTCCT

GTGTTCTGTTTTTCTCGCAGAGTGAGCTTAGCTTAGTTGCAGCTAAGGATGCGGATACTACCGCCCAGCT
TGTTTATGTTGTTCCGAAATTGCTAGTGTCGGGTGGAGTCACCCCCTCGCTGGGAATTTATCTGAAGTGT
AGCAGAAATTTGTAATTGTATTAAATTGCCCGCTGATCCAAGCCACCTCCCATCTCCTCTCCTCCTCCAA
TCTCTTGCCCACAGGGTGAGTCCATTGACGTATAAGTACACTGGAAATACACATTCTTGGGTTCAGGATG
GATGCTGAGTTATTGCAGCCTATTTAGTTTTAGCTACCAATACCTTTGTCAGTTGGCTTAAGTTTTTCTC
TGTGTGCAGGTTGGCATCGCTGGAGTTCTTTGGCAGTTTGCGTTGCATAGTTTTGGTGGACTTCTAGGTT
GCGTTAAAAACTTTACTGGTCTGGCTGTGAGCACGTGTCTTTCCCGTTCAAATAACTCGTCTTGGGACCG
CTTTTTGTAGCTGGCTGAATGGTAGAGCTAATTAAACGCAACTCACTCTGCGTATGCGCAATATTTGTCT
TGCCCCAGTAATAAATATGTTGTAGAAATGTTTGCACTTTATGGAATATATAGTTAAATTTAGTACCAGT
ATATGTAGCAAGGATTAGTCCTTATGAACAGCCTTTCCTTCTTACGTAAGCACGTTTCCTGTTGCACATT
TCCTTTATATGAGGAGCCTCGCCCACTTTTCAACAGAACTGATGCTATTTCGCGTAATTTTAATTACCTG
CTCAGCGGAAATTGTTTGAGACAAGGAAAACATCATAGAACACGGATTGTTTTTAAAAACACTGCAGCGG
TTCTACCCTGTAATTCGAAGTACTCGTTTTCTATTCAGAAATCATTAGTATTCTGACAAAATAAAAATAA
AAATAGTCATGCTTCGAGCTTAGAACTTTCTGCATCGCAATACCATTATAGAGTAATTGATATTTATTAA
AGCCTATGCTTATAGTAAAGAGACTTAACGTTCGTATATAGCCATATTAAGCTAGCATAGCAAGCAGCCA
TCTATAATGCCTGCAGGATTAGGCTCCTTCCGTTTCTGCTTTCGTGGTTAATGCCGTCGTGCATTAGTAT
GCAAAACAGGCAGATTGTAGGGAAAGAATCGGAGCACTCCTCATGGCACTCCTGCCTATGGCTCGTCCTT
TCTCTTTGTGCTGCGTATTTGCATCTCTTTCCGAGGCAAGGGCTTTGACGATGCCACGTAAGTACGTGAT
TTTTGCGATTCCATTTGTTTGCACTTGCCCCCAGACAATCGGGGCAGCATAAAACACACATAGCTACACC
AGATCGTGATCGGACAGACATATATGTAGGATATAGGAGAATGGGAAGTTGTTATGCGAAGGTGTCGTCA
TCTAATAACGAATGCCACATGCAACACATTGACGTGTTGACTTAATGAAGCGAATGTGCGCGTGTTTATC
AGAAATTAAATGTCAATTGTTGAAAACTAATTTCCACGCATGTTCTCCAAACACAAGCGCCAACCTACCT
ACTCTGCCTTCCATGTCTGCCCACTGCCACCCACCGAATTAAATGGCGCGTGCCGTGTTGGGTAAACAGC
GGGAGTCGCCCAGTTGGAACCAGTGGGTAATAAATCAATGCCACGAAGTGAGCCGCTGACAGGCCGAGCG
ACGTATGCTCGGCAGCTCCTTAATCTCCTCCCAGGTGGTGTCCTTGTGGCCTTCGGCCGAGCTGCAAGTG
CAACTGCTGCGCGGGCGGCTTGCAAATGAGGTACCCAAGGACACGACACAGGACACGAAATCCTGTCACT
TTGATTTACGTGATTTCACCGCTTTCTCTCACTTGCTGGACCTTCGAGAACTAGAGACGAAAAAAAGAAG
AGCGCTTGCCAAACTTTTTCGACTTGGCAGATTTGCATTTGAATGGCTTTCTAATTTATTGCCTTTAATT
GCCGGTTGCCAGGGACCGCCTTAAAGATCCATGCTCCCACTTAAAGCACCATCGAATGAATGTATTGGCT
GAACTGCTGTTTTTTTCCCATCTTTTTATGCGGCTCCCTCAAAAGAATCTGAGAGCCCAGAGCATTTCGC
AGCTTGAAAGGCATTTCTCATATTAAAAATGTATCAAATATTTCTGTGAGCCAGCTCTTTGGCCCACCCG
TCTGTTAGTCGGAGTCGGGCAGCCTGCACCAAAAAATAACTCATACGCATACGCAATTTTCAATATTGCC
AAGCAAGGATTGCAGCAGCATGAGGACACATGGGAAAAGGCTGGGGTCCGGAGGAGGAATGTGGCATCCT
CCGTTGCATGCATCAGTTTCATTTTGCTCGACTGCGAGCAGCTCAAAGAGCGGCCTGCAAAGTGCGCTCC
ATGCCGAAGTCCGTGACCAGATGGAATCCGTATATTAGTTGCCCATAATCATAATCAATTGCCACACGTA
CAACTGGCTTTTTGCATCGATACATCGAATCCTCCGATGCATCGCCAAAAAGGGGAAAATCAATTAATTT
TTCAATCCATTTAAACTGTTGCCAAGTTAGATTCCATTAAAAGTTGTTAATACTGGGCACCCTACCCTTT
AATATCTTGCTCTCTCGACTTTCCTTTCAGAGTAAGTCGCTATCTGCACTGCTCGTATTTACTGTGTGTG
GTGTGGCCATAAAAACCGCAATTCCACAAAATATGACGACAACACGGCTGGTGCCGCAACCTGCATGTTT
TCCACCTGACACGCATCGAATCCTCGAGGATCTGGAATCCAGGAGAGGCACTGCCAAAGGGATGGCCAGC
CGACCCACCGGAAAGGGTATTGCGGGTTAAGGAACGGACCATGTTTGCGGTCATAAATCTGCATCCCAGC
TCTCTTTGCttttttcgatgtcattttttttccgatctttttttttttGACCGAACTGGAATGGAAACCA
GTGTCATGGAGATGACCAGCATAGCCACAGAGGAGGTGACAACGCAACTGGGaaaaaaaaGGTCGAAACC
TATTCCTCCTCGGGCTACTCATTTAATTCCTCCCCTCGCTGGGCTCCCGTGCAACTAATGCACACTTAAA

ATAACTAACAAAACTCCATTAAAAGCTGCAGCCGGAAAGCGCCTGCGAAACATTTTAAGCGCTTGCGTTG
CCACCTCCTCGTCGCTTTCCCGCTTTTCCACTTTCCCTACATCTTACCCCTTGCCATTTCCCCCCTCCAT
ACAACCGTACAATTTCGCGGTGGACGGCGACTAGCAACTGGACAACGGACTAAACCAAAACGTAACACAA
AATCAAAATGAACTTATGACCGCAGCGCCAGCGGAATAAAAAAATGGAGCTGCAACTGCCGAGGGCGAAA
AACTGGACAGCAAACTCGGAAAAAAGGATAACTCTaaaaaaaaaGAACAGCAAGAAATATTTAAATTTAG
TGCCGAATACCACAGCACGTACATAATTTATGACCAACCAAGGACACGAGCAATTTAGCAGAGCCGGGAA
CaaaaaaaaaaaTGCACAAAAATAAACAAGCGCACGAAAAAAGTCCACAGCACATTTATATGTACATGTC
TATGTGTGTGTAACAAACTAACTAACTAACAGCGACATAGTGCCAAAGTGTCAAAAAAAGGACCTAGTTT
CTCATTACTTGGCAGTAATATTTTAGAAATAGATACTATACATTTAAGATTAAAACAAACTTCATTATCA
TATTTATATGTTTACACTACTTTAATTACTTACATAAAATGCAAGTCAGTATATGTATTCTTAAAAATAT
TCAAGGCTGAAGAACTGTATAGCCTCAAATTACTTGCCTATCCGAGATTCCCAGACCACTAATGCCATAT
TGGAGTGCCCATAGTGGTTGCTTGAACTCCACCCTCTAACCACTCTATATAATTATCTTCGGCAGTGCGG
CTCGGCAAGTGCGTTGCGCCACACCAACTCAATTAAAAAGTCATCTGGGCGGACTAAGCGCGACTCCTTT
ATGCATTTTTCACCATTGTTTCCGTTGTCGTTGCAGGGTGCCCCGCAGGTCCTGCGTCTGCATCCTTCCC
TGCACACCGTCTCCGTGAAGCGAGGAGAGGCGGCCAGCCTGACAATGGTCGTCTGTGCGGATCCGCGGCC
ACAGCGAGTCGCCTGGGAATGGGGATCACTGCGTCTGGAGGCCGGCTCGGGCATCGGGTGCGTATACTTG
TTTGATAGGCAGTTACCGCTCAGCGCTTACCATCCGCCCCATTGGTCAGCTAATGGTAATGCAATTAGAC
GCTGGACTTAGTTTTGCCTACTCGGAATTCAATTAGCGCCGCTCCACAGGATGCACAACGATGGCTCCGC
CCGCTCGAGGGCTCATTAGATTAACTGCCTCGTCGCAGAAAGGGTTGCGGACGGGGGGAGGGCATTCGCG
CAGAATTGTTCCGCAAATCAAGACAAATTACATGACCACGCGGAGTCAGAGTCGGACAAGAAACTGGCAG
TGTCATGTCCGTTGTTGCAAAGTGGGTAGTGCAAAGAATGGTCGGTCGAGATGGGGGTGCCTCGGGCAGA
CTTCCCACGCCTGTCAGCCGCCGGTAATCCGAGCTTCAACAGACAGCCAGCGCACACAACCCAACTCATT
TTCCAGCTGAAACTCTTTCGGGATGAACATCCGCAACCAGATGCGGCTGCATCCCTTCTTAACTTCCGCT
ACCCTCGCAATTCCATGAACTTGAACTGCATCCTTGCCAGTGTTGGCAACTATTGATTACCTGATTAAGC
TCAACCTGAAAATTGCAGATTTAAATTAAATTACTCCAAAAAAGAGAATTTTCTCATGTTAGTTCATTTA
AAAGTTCAAAGAAAACCAATAATGTGTTCATAAAGTATTAGGATATTAAATAAGTTCTGCGGAAATTAAA
TAATATAAAACACAGACACAGATAAGAATATAATTGAATAACTAGTTCAGGTGAGAATTAAATCTGGTGT
TCTTTATTCTAGAAAACATCTGACTGGGCAACACTGGTCCTTGGTCCGCAAGTCGCATTACTCTTCATTT
TCAGCTTGTCTGCCCGTGTCCATCCTTGCTCGTCCTTCGTGCGTGTAATCCTGCGTGCTCTCACTGCCAC
TGACACAAATCAAACGGGGCGCATTCCCGTCCAATTCGTTCTCCTTTGCTCCGCGTCCAtttttttttCG
CTTGCGATGCTAGTTAGCCCATCGAATCGAATGATTAATGTCGGCCGCCGACAAATTGTGTGCAATTCCA
GTGCAATTACTTAGCCCACGAAAGCCAATTCGCATTTGGTAACAACTAAAAAGCACAAGGACTTGCCTGA
TTTCCCATTTCATATCCCACAGAATCGCTTCCGGGCCGATGACATGCAGCCGGACACGCGGGAGGATTGC
TACCTGTCCACCCTACACATCCTGCACGCGGACGAGCACGACTCCAGGCCCTACTACTTGGTGGTGGAGA
ACGAGCGGGGCACCGATCGTCATGCCATACACCTGATTGTGGAGGGTACGTTTGCAGGGTGCATAGACTT
TTCTTTGATTTTCTTTACCCCCTCCCCACATACTGCTCCCCATCACATACATCACGTATTTAAGTTACAC
TCAATTTTTTCAATTCACTTCATCATCAAGAAATTAATGCATAGAATACCATACATTCCTGCAAAACACT
TTTATGCTAGCATCGTCACAGTCAAATTCACTTACTCATGAACCCATATTATTATTCAGTCTTTATCCAA
AGATATAAGTCAAATGTTGAATCTTAGAAACAACAATTTCAGTAATTCATATCCTTAAAATTTCAATGAA
AGTTCACTTTAATTAGCCGAATAGTGATTAATCCTTGCTTATTGCCACAAGGTAGTGTAAATTCGCTAAA
GAACACATGAACTGAGAACCCATTAAACATATCCCACCCACCCATCATCGCATCGCTGCCCAATGGTTTT
AATTAAAATCAATCGCACGTGCAATACATGAATTGCATAACATATATTTTGCATATGAAAATGAAAATTC
CCCTTAATGCTTGCATAATCAACCCCACCACCCTCCACAACATCGAATACGAATACAAATACGAATCCGT
GTATCGAATCGAATGGAATTTCCGGACAGAACCCTACGAGATGAGCTATCTAATGGGCGTGGCCGGGGGC

TGCATGGCCGCCATCCTTTTGCTGGTTTGCCTCTGTATCTATGCGATCAAGAGCAAGAGATGCTGCTTCA
AGGG
>dme_circ_0008227_junction_seq
TCAAGAGCAAGAGATGCTGCTTCAAGGGGATCCGAGGGATTTCAGCGATTCTCGGA
>dme_circ_0008228
ACGCGCCGAGCATCCGTCTAATTGGCTCACCGGAAATCGACTTGGAGGAGGACAAGGATGCCCTGGTCCT
GCGCTGCGTGGCCGATGCCAATCCGCCGGCCAGCATCGTCTGGCGGCGGGCAGGTCGCTCCGAGATAGCC
AGCTTGCAGGGTGAGCTTAGCTTAGTTGCAGCTAAGGATGCGGATACTACCGCCCAGCTTGTTTATGTTG
TTCCGAAATTGCTAGTGTCGGGTGGAGTCACCCCCTCGCTGGGAATTTATCTGAAGTGTAGCAGAAATTT
GTAATTGTATTAAATTGCCCGCTGATCCAAGCCACCTCCCATCTCCTCTCCTCCTCCAATCTCTTGCCCA
CAGGGTGAGTCCATTGACGTATAAGTACACTGGAAATACACATTCTTGGGTTCAGGATGGATGCTGAGTT
ATTGCAGCCTATTTAGTTTTAGCTACCAATACCTTTGTCAGTTGGCTTAAGTTTTTCTCTGTGTGCAGGT
TGGCATCGCTGGAGTTCTTTGGCAGTTTGCGTTGCATAGTTTTGGTGGACTTCTAGGTTGCGTTAAAAAC
TTTACTGGTCTGGCTGTGAGCACGTGTCTTTCCCGTTCAAATAACTCGTCTTGGGACCGCTTTTTGTAGC
TGGCTGAATGGTAGAGCTAATTAAACGCAACTCACTCTGCGTATGCGCAATATTTGTCTTGCCCCAGTAA
TAAATATGTTGTAGAAATGTTTGCACTTTATGGAATATATAGTTAAATTTAGTACCAGTATATGTAGCAA
GGATTAGTCCTTATGAACAGCCTTTCCTTCTTACGTAAGCACGTTTCCTGTTGCACATTTCCTTTATATG
AGGAGCCTCGCCCACTTTTCAACAGAACTGATGCTATTTCGCGTAATTTTAATTACCTGCTCAGCGGAAA
TTGTTTGAGACAAGGAAAACATCATAGAACACGGATTGTTTTTAAAAACACTGCAGCGGTTCTACCCTGT
AATTCGAAGTACTCGTTTTCTATTCAGAAATCATTAGTATTCTGACAAAATAAAAATAAAAATAGTCATG
CTTCGAGCTTAGAACTTTCTGCATCGCAATACCATTATAGAGTAATTGATATTTATTAAAGCCTATGCTT
ATAGTAAAGAGACTTAACGTTCGTATATAGCCATATTAAGCTAGCATAGCAAGCAGCCATCTATAATGCC
TGCAGGATTAGGCTCCTTCCGTTTCTGCTTTCGTGGTTAATGCCGTCGTGCATTAGTATGCAAAACAGGC
AGATTGTAGGGAAAGAATCGGAGCACTCCTCATGGCACTCCTGCCTATGGCTCGTCCTTTCTCTTTGTGC
TGCGTATTTGCATCTCTTTCCGAGGCAAGGGCTTTGACGATGCCACGTAAGTACGTGATTTTTGCGATTC
CATTTGTTTGCACTTGCCCCCAGACAATCGGGGCAGCATAAAACACACATAGCTACACCAGATCGTGATC
GGACAGACATATATGTAGGATATAGGAGAATGGGAAGTTGTTATGCGAAGGTGTCGTCATCTAATAACGA
ATGCCACATGCAACACATTGACGTGTTGACTTAATGAAGCGAATGTGCGCGTGTTTATCAGAAATTAAAT
GTCAATTGTTGAAAACTAATTTCCACGCATGTTCTCCAAACACAAGCGCCAACCTACCTACTCTGCCTTC
CATGTCTGCCCACTGCCACCCACCGAATTAAATGGCGCGTGCCGTGTTGGGTAAACAGCGGGAGTCGCCC
AGTTGGAACCAGTGGGTAATAAATCAATGCCACGAAGTGAGCCGCTGACAGGCCGAGCGACGTATGCTCG
GCAGCTCCTTAATCTCCTCCCAGGTGGTGTCCTTGTGGCCTTCGGCCGAGCTGCAAGTGCAACTGCTGCG
CGGGCGGCTTGCAAATGAGGTACCCAAGGACACGACACAGGACACGAAATCCTGTCACTTTGATTTACGT
GATTTCACCGCTTTCTCTCACTTGCTGGACCTTCGAGAACTAGAGACGAAAAAAAGAAGAGCGCTTGCCA
AACTTTTTCGACTTGGCAGATTTGCATTTGAATGGCTTTCTAATTTATTGCCTTTAATTGCCGGTTGCCA
GGGACCGCCTTAAAGATCCATGCTCCCACTTAAAGCACCATCGAATGAATGTATTGGCTGAACTGCTGTT
TTTTTCCCATCTTTTTATGCGGCTCCCTCAAAAGAATCTGAGAGCCCAGAGCATTTCGCAGCTTGAAAGG
CATTTCTCATATTAAAAATGTATCAAATATTTCTGTGAGCCAGCTCTTTGGCCCACCCGTCTGTTAGTCG
GAGTCGGGCAGCCTGCACCAAAAAATAACTCATACGCATACGCAATTTTCAATATTGCCAAGCAAGGATT
GCAGCAGCATGAGGACACATGGGAAAAGGCTGGGGTCCGGAGGAGGAATGTGGCATCCTCCGTTGCATGC
ATCAGTTTCATTTTGCTCGACTGCGAGCAGCTCAAAGAGCGGCCTGCAAAGTGCGCTCCATGCCGAAGTC
CGTGACCAGATGGAATCCGTATATTAGTTGCCCATAATCATAATCAATTGCCACACGTACAACTGGCTTT
TTGCATCGATACATCGAATCCTCCGATGCATCGCCAAAAAGGGGAAAATCAATTAATTTTTCAATCCATT
TAAACTGTTGCCAAGTTAGATTCCATTAAAAGTTGTTAATACTGGGCACCCTACCCTTTAATATCTTGCT

CTCTCGACTTTCCTTTCAGAGTAAGTCGCTATCTGCACTGCTCGTATTTACTGTGTGTGGTGTGGCCATA
AAAACCGCAATTCCACAAAATATGACGACAACACGGCTGGTGCCGCAACCTGCATGTTTTCCACCTGACA
CGCATCGAATCCTCGAGGATCTGGAATCCAGGAGAGGCACTGCCAAAGGGATGGCCAGCCGACCCACCGG
AAAGGGTATTGCGGGTTAAGGAACGGACCATGTTTGCGGTCATAAATCTGCATCCCAGCTCTCTTTGCtt
ttttcgatgtcatttttttttccgatcttttttttttttGACCGAACTGGAATGGAAACCAGTGTCATGGAG
ATGACCAGCATAGCCACAGAGGAGGTGACAACGCAACTGGGaaaaaaaaaGGTCGAAACCTATTCCTCCTC
GGGCTACTCATTTAATTCCTCCCCTCGCTGGGCTCCCGTGCAACTAATGCACACTTAAAATAACTAACAA
AACTCCATTAAAAGCTGCAGCCGGAAAGCGCCTGCGAAACATTTTAAGCGCTTGCGTTGCCACCTCCTCG
TCGCTTTCCCGCTTTTCCACTTTCCCTACATCTTACCCCTTGCCATTTCCCCCCTCCATACAACCGTACA
ATTTCGCGGTGGACGGCGACTAGCAACTGGACAACGGACTAAACCAAAACGTAACACAAAATCAAAATGA
ACTTATGACCGCAGCGCCAGCGGAATAAAAAAATGGAGCTGCAACTGCCGAGGGCGAAAAACTGGACAGC
AAACTCGGAAAAAAGGATAACTCTaaaaaaaaaGAACAGCAAGAAATATTTAAATTTAGTGCCGAATACC
ACAGCACGTACATAATTTATGACCAACCAAGGACACGAGCAATTTAGCAGAGCCGGGAACaaaaaaaaaa
aTGCACAAAAATAAACAAGCGCACGAAAAAAGTCCACAGCACATTTATATGTACATGTCTATGTGTGTGT
AACAAACTAACTAACTAACAGCGACATAGTGCCAAAGTGTCAAAAAAAGGACCTAGTTTCTCATTACTTG
GCAGTAATATTTTAGAAATAGATACTATACATTTAAGATTAAAACAAACTTCATTATCATATTTATATGT
TTACACTACTTTAATTACTTACATAAAATGCAAGTCAGTATATGTATTCTTAAAAATATTCAAGGCTGAA
GAACTGTATAGCCTCAAATTACTTGCCTATCCGAGATTCCCAGACCACTAATGCCATATTGGAGTGCCCA
TAGTGGTTGCTTGAACTCCACCCTCTAACCACTCTATATAATTATCTTCGGCAGTGCGGCTCGGCAAGTG
CGTTGCGCCACACCAACTCAATTAAAAAGTCATCTGGGCGGACTAAGCGCGACTCCTTTATGCATTTTTC
ACCATTGTTTCCGTTGTCGTTGCAGGTGCCCCGCAGGTCCTGCGTCTGCATCCTTCCCTGCACACCGTCT
CCGTGAAGCGAGGAGAGGCGGCCAGCCTGACAATGGTCGTCTGTGCGGATCCGCGGCCACAGCGAGTCGC
CTGGGAATGGGGATCACTGCGTCTGGAGGCCGGCTCGGGCATCGG
>dme_circ_0008228_junction_seq
TGCGTCTGGAGGCCGGCTCGGGCATCGGACGCGCCGAGCATCCGTCTAATTGGCTC
>dme_circ_0008229
GCTCGCTCATGCGGTAGTGCGGTCTCGCTTATTTAACCATAAATCTAGTACATTTTCCCACAGACCGAGC
AATGAAGATAACTTGCCAAATGGCAGAAATGGTTGTTTTCATAGGAAATTGGATTATGAAGATTGAGTTA
GTTTCTCACCTAAAGTCACCAGAGAGTTTTCCAATTATTTATGCAAATTCAATTTAAACGAAGAGCAACA
ACTACAGATGGAAACATCCCAAAGTTTATGTGGTGCAGTAGTTATAACCGGTCAATGTTAAAAGTGTTGG
TAAGATCTGACAATAGCTGATTCGTGATTGATTGAGCCGGCTGGTTAAAGAGCTGTCAAAGACAATGCAA
TTCCCATGTACAGCATAGTCGTACACGTCGACTACGAGACGCACTAATACTCACTGCATTAAATCCATGC
AAACGATAGGTGATATATTCGCAGATAGACAACCAACAAGCTATATATCCCGAGATACCTATTGCTCCAT
TTGTCAATAACATAAGTCCAGAGCCAAATATATGAGTTATTCAAATCAATATGACATAAACATGGTCAAC
ATGGGTTCAATTCGTCAGCATAAAAGCTTTCACCAATCCATATAAATTAAACAATTATTCAGCCTGCAAA
CATATTCATCAAGCCACAGTtgtgacaaatcgatatgtgacaaatcgatatTGTTTGCTACCCGCATCGC
TTTCACCGATAAATGCAATAGTTATGGGAATAATGCATTTGTAAATCAGTTTCATAATGGTGTTCTAAAT
AAAAGAACCTTTCCGATAAAACTCCGCTAAAATACATTAAATACAAAATTCGTATAGGTTTTATTTTATA
TCCGAAGGGAAAACAAAACTTACATATATATATTCATCAATTTAGCCATGTCCGTCGGTTTATATCAGTT
TCGATGAAACTTTTCAATTTGCTCCCTATTGCAGACAATATATATTACTTAAGGCGATTGGACCACTATA
TAGTGGAAAAAACTATGTTCCTTCGATGTTTTAATTTGTTTAATTTATCAAGAATAATTATATAAAACAG
AACGAAAATTATTTGAAGATAAGAAAAAATAAATCATTTCGTTGTTTGATCAACAAGCATATATCACTGA
AACTAGCAGCCGAAATAACATTTATGCATCTCAATTCCCTCAATCTTGATTATATCTGATTTCTGCAAGG
GTATAATTAGCTTCGCAATGCCTTTAAGAAAATCAAGTTGCATTATTTCAACATGCAGTTTGGTAGAGAC

TAAATGGGAAAGACGAATTGAAATTATTTAATTTTTAAATTGAATTGAAAATATTTGTAAAAAATTGTTT
AAATATTACTAATATTGCCGGATTTTTTCTGTATTAGACTATTTCTAGAAGATCCTTAGCTATCATTAAT
CGCCACCCAAGCATGCGAAAAATCCAACGACAATTTGTCATGGACTCCGCGAATTGGACTCCACACAGTT
CAATGCACAGTGCACTTTTTACTTGTACATAAATAAATATATATAAACATATATAATCCCGAACTTGTTT
GTCTCGGTTTCGCGTTCATTTGTTTCGGCGTGCAACGTGCTTAGAAAGTTTTCTATCTGAAAATAAGGGA
CTGACGAATTTCTCGAAGCTAGTTCATCTTCAGTTCGTTTACATTTCGGGAAAGTCACTTTTATGACCAG
GCATACATCTTCTCCCTATGTTTTTCGTGAGGACCGAAAGGTCGTGTTCTCCATTATTCCATGTCTAATG
GCGGACCTTATTGGAAAAATGCGTGAGTGGCGGTGTGGTCACAAAAAATACCACGAAGTCCGAGGAAATA
TAATAAACGAGTTTCCACAACGGAAGTCTTTTGGCTTTGAACGATTTAATGaactgccacgccctaactt
aaaaaaaggtttcgctgtttttcatttgtatatttgttttgccaattgttatggatatgccaaCGCCTAC
GCGTTCGGTCTTGTTTTAATTTAATAATCCTGCAATAACATTAATGCTGGTATGGTATGCTGCTGATTTT
AAAACTAACTGCAAATTACAGATGATAAGGAATAGGAAGTCGGCGACGGCTTATGCAAATTCCAAACTGG
CGAACACCAAAAttttttttttttACAGTGGCTGCTCATGTAAATAATATTCAAGGATCTTGTTTTGCGA
GCGGTATCAGGTTATCTGTTATAAACGCAAAGTGAAATGTGAAATGAAACCTATACGACACGGCTGCATG
AATATTGCCGACCAAGTCGAGAACAACTGGTGTAATACGCATCAAATGTTGGCTATAAGCACGAGGAAAT
TTAAACATGAAAACAGCCTGAGGTATGGCGAATCGCAAAATGAATCGCCTCGCTTATAATCAATTGTACA
ATGAACCGCAAATTAAGCCACACCCGAGTGAAGACAGCAGCGACCACTGAAACTGACACTGGAAATATAA
AAAACATTGTTAACACAGTTGGTTTCCCGGAGAAATAAATAAGATACACACATATATAACTGGAATAAGT
TATATCTCACTTTTGATCGTACTTGCTCGCATGACTGATAATCAAG
>dme_circ_0008229_junction_seq
GTACTTGCTCGCATGACTGATAATCAAGGCTCGCTCATGCGGTAGTGCGGTCTCGC
>dme_circ_0008230
GATGAGGGCGAAATTGGACGCGGTGCATTTGGGGCAGTCAACAAGATGACCTTTAAGAAATTGGACAAGG
TGATGGCCGTCAAGCGCATTCGATCCACCGTAGACGAGAAGGAACAGAAGCAGCTGCTCATGGATCTCGA
GG
>dme_circ_0008230_junction_seq
CAGAAGCAGCTGCTCATGGATCTCGAGGGATGAGGGCGAAATTGGACGCGGTGCAT
>dme_circ_0008231
GATGGAAATGGAGGGCGGCTTGGGCTGGCTATACATGAATGTAATATACTTTCGCGATGGACTTGCACAG
TTTATTATTAACGCTTGCTCCTGGTTTTTGAGGTTGTGAGAGAGGAGTGAATTGCTAAACGAGGGTGGG
>dme_circ_0008231_junction_seq
GAGGAGTGAATTGCTAAACGAGGGTGGGGATGGAAATGGAGGGCGGCTTGGGCTGG
>dme_circ_0008232
CGCAACACAAATCACGAAAATACAGAAAAGATACACAGATGCACCCTCCACCCCACCCTCGTTTAGCAAT
TCACTCCTCTCTCACAACCTCAAAAACCAGGAGCAAGCGTTAATAATAAACTGTGCAAG
>dme_circ_0008232_junction_seq
AGCAAGCGTTAATAATAAACTGTGCAAGCGCAACACAAATCACGAAAATACAGAAA
>dme_circ_0008233
GACAGTTTCTCTTTCACATTTGCTGTTCCTATATAGTATACGTGTGTACACATATATAGTTGTTATCGAC
TTTGTTTTACTCCGTTTGGTTATTTGCGAAAGTGGCTACGATGCGActgctgatgctgctgctgctgctg
GCCCAG
>dme_circ_0008233_junction_seq
gctgatgctgctgctgctgctgGCCCAGGACAGTTTCTCTTTCACATTTGCTGTTC
>dme_circ_0008234

GCAAACCTTCTTTGTATTGCTCTGTACAACAGACACATCTGCGAGGAGGAAGCCCCACAGCTTTAGAATG
AAATGGAGATGCAAAGCTACTCTGGTCGTGGACCATCGACAGGCAcatcagaaacagcaacagcaacagc
agcagttgcatctgcatctgcatcagacacagcagcaacaTTCACACTGCGACACTTCAATTGTCGGCTG
GAAGAGGCGGATTGGCCTGGGCCTCGCCAGCCGTTTATTGACTGCGAAGGCAGCCACACCAACAGCACAT
CCTACACCCCCAGCAGCCTGCCCACCAGCAAAAACCTCATCGAGGGACCCAACAACACATTCCAGTGTGC
ACGGCAAGCTATCGACGAAGCGGAGCACAACGCATTCTATAGTTGCGATGGGGAAAGCCCATTGTCTGAA
AACTGCACTTTGAGGCTGAAGGCGCGACTGTTGCGATGGCCGTACGAGGCGTATGAGCACATTCGACTGG
CAATCAACGGGGCACTCAGTGATTATGCTAGTGATAAACTTAGTGATAGTTCGAGCTGCGATGAAGGAGG
CATGGTGTTGCAATGTAAGCTGGGCCAGGCCCATGAACTATTGCTTTGTCATTTGCAACAACTCGAGAGG
GAGCCACTGCGGGAGGTGGGAAGCGCGGAGGAGAGCTTCTCCAACGACAGCCTTCTGCAGGCCTTTCAAA
TCGAACTGGGCGCAGAGCGCGATGGACTCTGGTCCTTCCTTAATCTAAGCGAGCTAATGGAGCCCGATCA
CGGATTCCTGGGCATCATGAATGTCACCAACACTAGCTTAGACATGTTTTTGCCGAACCTTACAGCCCTG
AACACGACCTCCACGGCCTCCACTGCAGACATGGCCACCGAGTTCGCGGTAACAAAGAGCTTTCTGGACT
ACAGTCCCCACGGCTACGATTTCCTATTCCTGTTCGTGGTGTTCTTTATCTTCGCGGGTGGCCTGGGTAA
TATCCTTGTGTGCCTAGCCGTTGCCTTGGACCGGAAACTGCAGAATGTGACTAACTACTTCCTGTTTTCC
CTGGCTATAGCCGATCTCCTGGTCAGTTTGTTTGTGATGCCCATGGGCGCGATACCAGCTTTTTTGG
>dme_circ_0008234_junction_seq
CCCATGGGCGCGATACCAGCTTTTTTGGGCAAACCTTCTTTGTATTGCTCTGTACA
>dme_circ_0008235
GAGTACGAGCTGTTGAACGATATATCCGTGATATTTGATGCCATAACTCAGTGCACAGGCAGTGCTTTGG
ATACGCACAGCTCGGGAGGCAGTTCAGCCACCACTGAACGCAGTTTGAGCGGCGGAAGTTCATCGAGAGC
TCCGCCGAAGGGAG
>dme_circ_0008235_junction_seq
AGTTCATCGAGAGCTCCGCCGAAGGGAGGAGTACGAGCTGTTGAACGATATATCCG
>dme_circ_0008236
ATAAAGAACACCACGAACAGGAATAGGAAATCGTAGCCGTGGGGACTGTAGTCCAGAAAGCTCTTTGTTA
CCGCGAACTCGGTGGCCATGTCTGCAGTGGAGGCCGTGGAGGTCGTGTTCAGGGCTGTAAGGTTCGGCAA
AAACATGTCTAAGCTAGTGTTGGTGACATTCATGATGCCCAGGAATCCGTGATCGGGCTCCATTAGCTCG
CTTAGATTAAGGAAGGACCAGAGTCCATCGCGCTCTGCGCCCAG
>dme_circ_0008236_junction_seq
ACCAGAGTCCATCGCGCTCTGCGCCCAGATAAAGAACACCACGAACAGGAATAGGA
>dme_circ_0008237
ATGCTTCACCACTTCGGCGGCCTTGTCGCCCAGTGGCTTGCCGTGCCAGTTGTCCAAATCCTCGATGTTG
GGGAAGTCCCTGCCCTTGAACGTCTTTGCAATGATGGCGGTGGGCTTGTTCTTGGTGATTGCAGCACAAT
GGAAAGCCTTGCTCAATTCCTCGACATCATGACCATCGACAACGACGGCATTAAAGCCGAAGGCCTCCAG
ACGATCACGGTAAACATCCAGCTTATGCTGCAGGGAGGTGGCCTCCGACTGGCCCAAGCGGTTCACATCA
AAGATCACGCAAAGGTTGTCCAGTTTATAGTGGCCGGCAAAGTGCAGCGACTCCCAGATGGAACCCTCGG
CGGACTCACCATCACCCACCACCACGTAGGTGCGGTAGTCGGCCTTGTCGAAGTTCTTGCCCACATAGGC
CATGCCAGCGCCGACGGCGACACCCTGTCCCAGGGATCCAGTGCCCACATCGATGAAGTTCAGACGAGGC
GTCGGGTGACCCTCCAGATCGCTGTCGATCTTGCGCAGGTTGTTCAGATCCGCGATGGGGAAGAGACCAG
CCTCAGCCCAGGCGGCATACAGAATGGGAGCAGCGTGTCCTTTGGACAGGATGAAGCGATCGCTGGAGGG
ATCGCGTGGGTGCTTCAGGTTCAGGCGCAGCTGCTGGAAGAACAGCACGGACATGATCTCAGCGATCGAG
GCACATGATGTGGGATGGCTATAAAGAGGGAAAAGTAGGCAAAATATTAGATTGTGACAAGGCATTTCGT
TTGCATGTAGTTCTAGGTTAAGGTAAGTGCCTAATGAATTATAGAAGTCGACCTCACGGTGGGTAAGTGA

AGGCCGATCAACAAAGGAAAAGTGGAACTGAACACTGTGCATACTTGTTAAAAACGTTGTTTAATAAAAG
AAATCCATTTGTCTATTGAAGCTTTTATTTAAAccccccccccccccTCTGTTCATATTTCAAATGAAGA
TTTCTCGAGTATCCAATTGCAATGTACATATAAATAAGATAAGATCCATTCTTGGAATTCAATGTTCCCA
TGCGATCGTGTTCCaaaaaaaaTTGCTTTGAATCGCAGATCTGGCACAACTCCAACCGCAAAGAAAACAC
GCAACCCCAGACAAAATGGGGGGCTCGTCTTACCCCAGACATATAAATAAACTATAACGAGTACATGCAT
ATGTATTTATTGGCGTTATATGGTGTGATATGTGTGTTTGTCGCGACGTGAGTCCATCGCAATTCCTCAC
CGGTTTCTGGGACCTTGAACTTTTCTGATCACCAAAAAACCTAAATAATAGCCTTAAAAACCAAATTCTT
GGCTTCCACGATTGTGTTGCATGGCGATCTTTGTGAACGCACGTGCTTCTAGATAGTTCCGAAGTGCGTC
CCCGTTTCCTGCAGCCATATCAAGAACTATTGAAGGTGATACGGAGTGTTAGCAGTGACCCGGCGCGCtt
tttttttttttATCAGTTTTCAGTGATTTTCGATTATCATGCGGGGAACACACTTGAGCTGGTCACCTCT
AATCGGTCATATCGTGGTCAATGAGTAACCAAACCGGGCTTATCTCGTCGGCAGAGGTCACATTTAGAGC
AAACAGAGCAGATCTTATCTTGAACCGTCACaaaaaaaaaaaaaaaCGAAACAGAATGGAAAAACTAATG
GAGGCGATAAAAGTGTTTTCCTTATCAGCATAGTAACTTTTTTATTTACTGTTGCAGTAATAATTTGATC
GCGTCTATCCGAGATATAATTACATTAAAAGCCATACAGTCGTAAATCTCAAGTGTAGGGGATCATTCCA
TTACTATTCTCATCCCTCTCTTTCCAATTCCACTCCCGTCACAAGCTGTGCTTTCCAACACTTGTATGTA
CATATGTACATACGTATATTAAATGCCTCTGTTTGATATGTAAGTATGTTTGTTTTTACCCCATGTACAT
ATGTATGTGCTTTCGGCGTTTTGAACGCCACGCACAAAACAAAAGCAAAAGAAATTAAAAGGCAGTAAGA
AAAACCTAACGTTTCAAGGTTGTCTAAGGTCAAAAAATATTAGCTCACAATTTAAGGTAGGTGAACGCGG
CTTATCACTGAGCACGCAGGCAGGGCAACAAATTCGCAAATTCACTAAAGCTACTGGACAACACGTGGGT
TGGCAACTGAATCCCAGGAGGGGTCACTAAAGGAACCGGTCTTCCCATATTTACTTCTCACATTCATAC
AAACATAGATGCATAACTATGTATATGCGTAACTATTTTTGTTTTATTTTCTTTTGTAATGTTTCAAAAC
ACATACATATGTATGTATGTATTTAGCAGCCCCCTCAAAAACTTTTTCGCACCCACCCTGGAAATGGAGG
GGGTCACCTTTGAAGCAACAAGTGCAAGGTGCACAACTTTTAAAATTCACATCTACACAAACAAATCTAA
ATACGCACACTTTAATATGCCTATGGACTTTCAGTTATTGATAAACTTACCCCGATTTGGAGGCCTGGGT
GGCGTTGATAGAGTGAATTCGCAGTTTCTGGGCCAGATCTTTCAGATCCTGGACGGTCTTAGCTTCGGGC
TTGTGGTACGACATCTTTGAAATGCGAATG
>dme_circ_0008237_junction_seq
GTGGTACGACATCTTTGAAATGCGAATGATGCTTCACCACTTCGGCGGCCTTGTCG
>dme_circ_0008238
TTCTTGGTGTCTCCGTCTAGGGCAACAACACGCAGGTTGTTCTGGCCAATCTTGGCCAGAGCTGTTCCAT
AGGCCAAACGGGTGGCAATGGAATCACCCAGCTTGTAGGCGGGTGGCGAGCTCAACTTAATGTTGTTGAT
GTCCACGTCGGGAGCAGCACCAGTCTTGGGCACCGGCTTAGGGGTCAGCTTGACGTTCTTGTTGACAATC
AGTCCCTCCAGATGCTTCACCACTTCGGCGGCCTTGTCGCCCAGTGGCTTGCCGTGCCAGTTGTCCAAAT
CCTCGATGTTGGGGAAGTCCCTGCCCTTGAACG
>dme_circ_0008238_junction_seq
ATGTTGGGGAAGTCCCTGCCCTTGAACGTTCTTGGTGTCTCCGTCTAGGGCAACAA
>dme_circ_0008239
GAATCTTGTGGGAGTGGCCGTTGGAGCCGCCTGCCGTCGCCGCACTGTGGCCTTTGTGTCCACCTTTGCC
ACCTTCTTCACCCGTGCCTTCGATCAGATCCGTATGGGCGCCATCTCGCAGACGAACGTTAACTTTGTGG
GCTCCCACTGCGGCTGCAGTATCGGTGAGGATGGTCCTTCGCAGATGGGTCTGGAGGACATTGCCATGTT
CCGCACCATTCCGGGCAGCACCATCTTCTATCCCTCCGATGCAGTGAGCACGGAGCGTGCCGTCGAACTG
GCCGCCAACACCAAGGGTGTCTGCTTCATCCGTACGTCCCGTCCCAACACCTGTGTGATCTACGACAACG
AGGAACCCTTCACCATTGGCCGCGGCAAGGTGGTGCGCCAAAAGAGCTCCGACGAGGTGCTTCTGATCGG
AGCTGGAATCACTTTGTACGAGTGCCTGGCTGCCGCCGATCAGCTGGAGAAGAACTGCATCACCGTCCGT

GTGATCGATCCGTTCACCGTGAAGCCATTGGACGCCGAGCTGATCATCGAGCACGGCAAGCAGTGCGGAG
GACGCGTAGTCGTCGTGGAGGATCACTACCAGTAAGTGTTCTTTGTAGTCTTTTTATTTGCCTCCTTTGC
TAACTTGAAATTTACTTAATTTCTTATAGGCAAGGAGGCCTGGGTGAAGCTGTGCTGAGTGCCCTGGCTG
GCGAGCGCAACTTCGTTGTCAAGCACTTGTACGTGCCCACCGTGCCACGTTCGGGTCCTCCGTCG
>dme_circ_0008239_junction_seq
CACCGTGCCACGTTCGGGTCCTCCGTCGGAATCTTGTGGGAGTGGCCGTTGGAGCC
>dme_circ_0008240
GGCCGAGCCGCTCGAGGAGGGTGCTCCGGTGCCGGTGAACGACATTAAGGTGGTGCTCCGTCCGCGTCCA
TGGCTGGAGCGCTGGGAACGACAGAATCTGCGTGGTGTGGCCAACATCGATGAGTATCTAAAAGACAAGC
ATCGCCTGTCGGCGGCCAAGGTGCAGAAACCATGGGAGAA
>dme_circ_0008240_junction_seq
CGGCCAAGGTGCAGAAACCATGGGAGAAGGCCGAGCCGCTCGAGGAGGGTGCTCCG
>dme_circ_0008241
GAGGAGGAGCAGGACGATGAGTACGAGCGTAAGTCAGTGGACTCTGCCATGGATCTGTCCCAAGGAACGC
CAGTGAAGGAGGATGAGCAGCAGCAACAACCGCAGCAGCCGCTGGCCATGAATCTCAAGG
>dme_circ_0008241_junction_seq
CAGCAGCCGCTGGCCATGAATCTCAAGGGAGGAGGAGCAGGACGATGAGTACGAGC
>dme_circ_0008242
GGGTATTGGCCACTTGGCTTTACCTGGTGCAACATTTATGTGACGTGTGACGTACTGGCCTGCTCATCCA
GTATATTGCACATGTGCTTCATCAGTTTGGGACGCTATATGGGAATAAGAAATCCATTGGGCTCGAGGCA
TCGATCTACGAAACGATTGACTGGTATAAAGATAGCCATTGTTTGGGTAATGGCCATGATGGTATCCAGT
TCAATAACCGTTCTTGGGTCTGGTCAATGAGAAGAACATAATGCCCGAGCCTAACATATGCGTTATCAAC
AATCGCGCCTTTTTCGTGTTTGGATCTCTGGTAGCTTTTTATATACCCATGCTGATGATGGTTACCACAT
ACGCATTGACGATTCCCCTCCTCCGGAAGAAAGCGCGATTTGCCGCCGAGCACCCGGAAAGTGAACTTTT
TCGCAGGGTAATTTCATACTCGTTATATGGCTCCCAGTTAATTTCCAAGAAAAATCGTAATTGTAAACAT
ACTGAAATAATATAAAAAGAGAGTTTCTCGCCTGTCATATACCCATTACTTAGCCAAAAGTAAACTGGCA
AGAAAAAAATCTGCACACAATCGTTCTGTAGATGACTACTAAATTTCCGGTTAAGAACTCGCTCTTCCAT
CATCTCTCGCTTCTTCTTTTAAACACAGCAATTTGTCCCATTGATTGGCCAATCGAAACTCGACCAAGTG
AACGAATTAGCCCTTACTCAATCAAGCATCGCCTATCCCGCTATCCCTCATGCTCTAAAAATCTGTACAC
TAAAGAAAGTCCCCTAATCGTCAAAAGCTTAACTAATAGATTTTTCAGAAGGCATTATACATTTTTCTGA
GTGCATCCATGATGCTTTTACGTTCATGTTTCCAGGGTTGGGTGGACGCTTCACCCTAAGGCCGCAGCAC
AGCCAGCAGCAGTTGCAGATGTTCAGTAGCTTTTCTGGCAGCAATAACAAATTCCTATCAATGGGCGACG
GCAATCGCAACTTCAACACTGAAGGAGAGATGGAGGAGGGTGGAGCTCCTAGCAGAAGGAGTGGCGTAGA
GCCCGCCGAACGACCTTTGATGCAACAGCGAACGGCGAGCAGCAGGAGCATGGGCACGGTTAGTTTCCGT
AATGTCGTGAACGGGACTAGCGGAGCCGCAGGAAGTGGGCGCAGGACGACTGGCACTGCCCACAGCAGCT
TCCGGTTCTCTGGCGCCGGCATCTTTCGGCACTCGTCGTCGTCTCCCGCCTCGAGCTGCCACTCCACCAG
CACGTCGCGTTCGAGCTCCTTCTGGCGCAAACACGGCGGCTATCCAAACCTAATGGACAG
>dme_circ_0008242_junction_seq
ACGGCGGCTATCCAAACCTAATGGACAGGGGTATTGGCCACTTGGCTTTACCTGGT
>dme_circ_0008243
AAAAGCTACTGAACATCTGCAACTGCTGCTGGCTGTGCTGCGGCCTTAGGGTGAAGCGTCCACCCAACCT
GGAAACATGAACGTAAAAGCATCATGGATGCACTCAGAAAAATGTATAATGCCTTCTGAAAAATCTATTA
GTTAAGCTTTTGACGATTAGGGGACTTTCTTTAGTGTACAGATTTTTAGAGCATGAGGGATAGCGGGATA
GGCGATGCTTGATTGAGTAAGGGCTAATTCGTTCACTTGGTCGAGTTTCGATTGGCCAATCAATGGGACA

AATTGCTGTGTTTAAAAGAAGAAGCGAGAGATGATGGAAGAGCGAGTTCTTAACCGGAAATTTAGTAGTC
ATCTACAGAACGATTGTGTGCAGATTTTTTTCTTGCCAGTTTACTTTTGGCTAAGTAATGGGTATATGAC
AGGCGAGAAACTCTCTTTTTATATTATTTCAGTATGTTTACAATTACGATTTTTCTTGGAAATTAACTGG
GAGCCATATAACGAGTATGAAATTACCTGCGAAAAAGTTCACTTTCCGGGTGCTCGGCGGCAAATCGCGC
TTTCTTCCGGAGGAGGGGAATCGTCAATGCGTATGTGGTAACCATCATCAGCATGGG
>dme_circ_0008243_junction_seq
CGTATGTGGTAACCATCATCAGCATGGGAAAAGCTACTGAACATCTGCAACTGCTG
>dme_circ_0008244
AGAACCGGAAGCTGCTGTGGGCAGTGCCAGTCGTCCTGCGCCCACTTCCTGCGGCTCCGCTAGTCCCGTT
CACGACATTACGGAAACTAACCGTGCCCATGCTCCTGCTGCTCGCCGTTCGCTGTTGCATCAAAGGTCGT
TCGGCGGGCTCTACGCCACTCCTTCTGCTAGGAGCTCCACCCTCCTCCATCTCTCCTTCAGTGTTGAAGT
TGCGATTGCCGTCGCCCATTGATAGGAATTTG
>dme_circ_0008244_junction_seq
ATTGCCGTCGCCCATTGATAGGAATTTGAGAACCGGAAGCTGCTGTGGGCAGTGCC
>dme_circ_0008245
GGAGGAAAAGAAAGACACCAAGGAGGCAGCAAATGGTGCAAGCAGCTCTAACGGCAAGGAACTGGACACC
ATCAAGGAAGGGTCCGATGAGGCAGACTCTACCGGGGAAGCCGAGCAAGCTCAAAGTGATGAAAAGCCCT
CTAAGAAG
>dme_circ_0008245_junction_seq
TCAAAGTGATGAAAAGCCCTCTAAGAAGGGAGGAAAAGAAAGACACCAAGGAGGCA
>dme_circ_0008246
GGCAGACTCTACCGGGGAAGCCGAGCAAGCTCAAAGTGATGAAAAGCCCTCTAAGAAGGTACCGACCGGC
GTGGATGAGGTCAGCAGCAGCAATGGCGGTGGCGGAGCTGCCGTCAACGATGACGAGCGACCGAGCACAT
CGAATGGTGAG
>dme_circ_0008246_junction_seq
CGAGCGACCGAGCACATCGAATGGTGAGGGCAGACTCTACCGGGGAAGCCGAGCAA
>dme_circ_0008247
CTTCAAGCTAAGCAAAAACGTAGTAAATGAACAAACGaaaaaaaaaatagcatcaaaaatcaaaagtcca
aatcaaaacaagacaaacaTAAATGTAAATTGGTATGCAAATAAACTTAACCATAATTGCCGATGcaaca
acaacaacaacaacaacagatcaaacaaatacaacagcaacaataaaaactacaataacaacaacaacag
caacaacaagaacagagttactgcaacaagaaaagcaacaacaaccgCTGCTAGTAGATATATaaaaaaa
aaaCCGTACAACATTTATGTAAAACTTACATACATTTTTTGTCGAGGCACCCCAGCAAAGTAATATTTTG
CAACGTAGACTCTTAGGCGTCGCACACAAAAACAACCCAAGAACacaacagcaacatctgcaacatcagc
aacCAGAGCAAAATACAACATCCATCCGAAATCGCAAACATCCAACAATCCACCATCGCCACCACCACGA
ACCACATGAGCCTGAACAAGATCAAGAAGCGGGACGCGAGCGACTTATGACTGATGCGCTCCAAGGGCAA
CGTCGGCGTCGGCGTTGGGGTCAACGTCGGCGTCGCCGTTGGCAGCGGCAGCCGGTACAAGGTGACGGCC
CGCTGTGTGCCCGAAGTGAGCATCACGTCGCCGACGACAGCAACCGGCTCCATCCAGCCCAAGCACTTTG
CCCTGCTCCGCAGTCGCTCGCAACCTTCGCCGCCGACGTCTAACATCCTTTTCTCCACGCCAGGCGGGGC
CACTCTGGTCGAAGAGGAGGATTTCAGCTTGGCCAGCGGCAGTGGTACAGCGGTTAAGAACGTGCTTCAG
CGCCAGCTGGCAACCACCGTTCAGCAGGAGGATCCGGATCTGGAGCATTCCGCGCAGGAGCAGCACACTA
AGTTGTCCGAGCCGGCGGAACCGCCCAATGAGCTAACGCCGTTGGTGGAGGAGCCCGGCTCTATAGCGCC
AACCACATCGACCCAGCGTTATAGCCACAACTCCAAGACCACTCGCTCCTGGCCAGTCATCTACCGGAAT
CCACATCACTGCGACTACGAGGCCTTGTACGTGCAACAGCAGCAGCAACAGCAGAGCTTCAAGCAAAACT
GCTACTCGAATCAGTTTAAGCAGTCCATCGTCTACTCCAGCAGCAATGAGGAGTTGCCCGGCGGCGAGCC

GGTAGCCTCGAGTTCCGTCGATAACCGCCAGACGACGTGCTACTACTTTGCACCCAGCCGGCACAACAGC
ATTTATGAGCACCCCTCCTCGGTGGTTGGAGGATCCCTGCAGTTTGATAATCCAACGACCACAGCAGCAG
CGGCGGCGGCAGTCGAGAGTCTAAGGCGGAGCAACAGCATCCTGTTGCGACAGAACAGCTGCGCCAAGGT
CATTCAGCGTCGGGGCAGCGGTAGTGGTTCACCCAACTCACTGGGCTCCCAGATGGAGGGTCTGGGAATG
GGCATGGGCGGTAGGGGAGGCGGCAGCACCTGTTGCTCCAGCTGCTGCATGGGCCCGCCGCCGGTGCTCT
TCCTGTTCGTCACTCTGCTGATGACTACAAGCGCCACAGCCATGCTGTGCGCTGCCATCATGACTGATCA
CTGGGAGCACGTTACATGGGACCGCAACAGCCTTGATCGCTACTCCAATCGATCGGGACTGCATCTGGAG
TGGCTTCTCGACGACCAGGTGGCTATGCTCAAGCCGGACAAGAGGGGCCGATCATCGCTTTCGGCGCGAC
TCTGTTTTCCTGGTGCCCATGCACGGCGGCATCTGGACACTGTGCATTGATCTGCCCATTCAGCAGCTGC
AGGAACTGCGGCGCAACCCCAAGTTCCCACGCGGTGCTCCTCCGTGTGTAAACTACCTGGCCGGCTCAAT
GGAAAACGCACGAGGCGAGGAGCAGCGCAACGACTGGCAGCACAGTGAGTCACAGGTCACTCTGCAGCCG
CATCTGAG
>dme_circ_0008247_junction_seq
CACAGGTCACTCTGCAGCCGCATCTGAGCTTCAAGCTAAGCAAAAACGTAGTAAAT
>dme_circ_0008248
GATAAAGAACTCTTAACAAACACTACATATCAATACGCTATATGTACAAGCTAAAAAGTGAGGGAGCGTT
TAGCTCCTTTGTTCCGATAACGGTGATATCCGACGATATTCGATATAATGATATACATATGATTCATGAC
TAGCACGGCTGCACGTCCTCCGCCGGCGGCAATTGGTTGGGATCATAATAGTAGAGACTGGGCGACCACG
GCCTCTGTGCTCCCGCCTTCAATTCGGGATGAGCAAGTGCCACCGCCTGGCAGCCACATTCCTGGGTCGC
ATCCTTACCCGGACCCTCGCCGGGAAACTCCTGACCCACCGCTACGGCGCGCACCACAACCTGGTGCGGA
TTGGTCACATCCGCATTGGTAATAACTCCGGCCTCGTGACGAAAACTGTAAAATCGGTTCGTCAAATGCC
CATCCAAGTAAATCTGGAAAGTAGAGAAACGTTTGAAAAATGTTTGCCAGTATATGCAGGACGAACTTCT
TCCTCACCTCATATCCTGCTACAGCGCGACAGTCCTCCAATTGCCATTGGACGTAGAGAGCGGTGTTACC
CAATGGCCACACACTCTTAATTTTGACGGGAAAACTATCGCCGGGTCCACCTCTGTTGGTCAAGTCTGTA
GCCTTAAGGTGCTATGGAATTAAAAATAAGGGGTTATTATTAAGGAAATGGATTTTATCAATTGCAGTGG
ATGTCGAACCTTTGGTTTCGCTTTCTTGTTGCCGAGCACACAATCCATGGCATGCATATCAACGATGATG
GAGTCTATAAAGTATTTCTGCAAAAAATAAATAGTAGATATATAAGATAATGAAGGAATCTCGAAGTTTC
TTACCATTTCGCAGAGTGGATCACACATTCCGGAGCGCAGGGTATTGGCAACCGTCTTGCACTCCTCGTC
GCATATGAGAGCGCGACAGTGGCAGCATAGGCATCCTTGTTGTTCTGGGGGACACTCATCATCGCATTCC
TCGCACTTTTCCAACGGGCAATCTTCCGGGCATTCCTCACAATTCAGCGCGCACTCTTCGCATACATCGT
CCTCGCAGGAGTACACATC
>dme_circ_0008248_junction_seq
ATACATCGTCCTCGCAGGAGTACACATCGATAAAGAACTCTTAACAAACACTACAT
>dme_circ_0008249
AAATACTTTATAGACTCCATCATCGTTGATATGCATGCCATGGATTGTGTGCTCGGCAACAAGAAAGCGA
AACCAAAGGTTCGACATCCACTGCAATTGATAAAATCCATTTCCTTAATAATAACCCCTTATTTTTAATT
CCATAGCACCTTAAGGCTACAGACTTGACCAACAGAGGTGGACCCGGCGATAGTTTTCCCGTCAAAATTA
AGAGTGTGTGGCCATTGGGTAACACCGCTCTCTACGTCCAATGGCAATTGGAGGACTGTCGCGCTGTAGC
AGGATATGAGG
>dme_circ_0008249_junction_seq
GACTGTCGCGCTGTAGCAGGATATGAGGAAATACTTTATAGACTCCATCATCGTTG
>dme_circ_0008250
GATCCGGCTCCGTGGTTGCTTTACCATCATCCTTACTTTCGAACTTTTTTACTTCCTCGTCATCCTCACA
ATCACGAAAGAAGTCATCATCCAAGCCATCACCCGAACAGGTTAGTGCATTTCCAATGTCAAAGAATTGT

TCCGGGCACTGACAAGGTTGCATGTTGTCATTTTCCTTGGGAGATTTTGGCTCAG
>dme_circ_0008250_junction_seq
TCATTTTCCTTGGGAGATTTTGGCTCAGGATCCGGCTCCGTGGTTGCTTTACCATC
>dme_circ_0008251
GATATCGGTCTCAGGAATCTGAGGTTCTGCCGGCGGCAGGAAGCACTTGCAGAGGTATTCCTCCAGTTGA
TGAAAGCACTCGACTCGTTGGAGCTCTCTCTTTGACGGGGGTTTTTCGACTGGATCGCCTTCCCCATTGC
CAGCATGTTGGGCCGTACAGCACTCATCGAAGGTTAGGCACGGTGGGCTAGCTTCATCCGCTG
>dme_circ_0008251_junction_seq
AGGCACGGTGGGCTAGCTTCATCCGCTGGATATCGGTCTCAGGAATCTGAGGTTCT
>dme_circ_0008252
ATGGAGAGCACCTGCGAGGGCCACTGCCAGAGTCTGCAGGATCTGCTGGAGCAGTCTGCCCAGCGGCTGG
AGGATCGGGACAAGCGCTGCTTGCAACAATTGCTGCAGTGCGTCCAGCAACTGGATCTGGATACCATGAT
GCCAGGCGATCAGGTG
>dme_circ_0008252_junction_seq
GGATACCATGATGCCAGGCGATCAGGTGATGGAGAGCACCTGCGAGGGCCACTGCC
>dme_circ_0008253
ATTGAGGAGAATCCAACGATGACCAGGTGAAGTATAGCAGGGACTTTCGGCAGGATCGGGGTGCACCGGC
ATATGCAGTACTTGAAAAAGCAGAAACCCaaaaaaaaaCGACGGCAACATTTTTATATACTAAAGTTACA
TAAAAATGGCTGAAAACCAAACCAAAACGACCTCTAGCAAGGGATGTAATACAATGAGCAGGACCGCCCA
TATCCTGGCGCCCCACAATGGGACTGTGGTCCGCCGGACGGATCCTGGCTCCAAACTCAAAGAGAGCTTC
CACACGGAGAAGCACGATATCGAAGACTGGCAAACGATGGCCAAGAACAAGTTGAAGCGCAAGAAGAAAC
TGAGCAGCTCCCTGACTTCCGTTGGGGATACGGTGAATCAGGAGAAGCGCACTCGCATCGAGGGCATCCA
GCTGCATTGTCGCGGCAAATTGCGACCCAAGGAGACCCAATCCTTTGAGGCCGAGAAGGGCCCATCTGCC
GGCTCCCAGAAGCAACTGGGAAAGGCAGAAAAAGATCGAGAACGACCCGTAATAATTGGTGGCGTGACTA
CCACAAATGTGATAGCAGCACCTCCACAAAGGATCAAGAGAAAACTGCAGGAGAACATGGACACCATCCA
GAAGCTAAATGCCCTCACGGAGCAACTGCGTCTGGAGGTAAATGAACTGAAGTCCAGTCTGATCACAGAA
CGCGGTGCAGTGAGAGCCTTAAGGGTAAGTCCATTAATATACATATATACCAATAAGGAACGTGATAAGC
CCATTTCATTTTACCTTAATCGCACCATTTTATATTGCATAATGTTGTGAAACTTTTTAAATTGAGTTAT
CAAATACAAATACATTCGTTTTAAAAGTATTCGTGCACTGAAAACATATTTGACCTTTCATGAAAGTGAC
GATTGGCAATCAGTAAACTCGGAAATAAATATACATGCAATCAGTTTGTTCAAGATTACATTTAAATATT
TCTGGTTTCATCTAGATATAAAATATGATAGAATAGTCACACGTTTGATATTAAAACCCATTTCGATCTG
ATCTTTTGAATTTAAAGAGACATTAAAAAATGCAATCTATCGCTTTTTTATTTCAACCCAGGGGCTCAGA
ATGACGCAGATAGTCGTCGGTGGAAGAGTGAAGTGAAGAAGTTGCAGCATACGCTGGAAATGGCCAAGAA
GAGCAACGGTATAAAGAAGGCCATCGAATCGGGCCCAGAATCGAGTGGTGCACATGTGACCGCCGATGGC
CTCATCAACTATGAAATCCAAAGACTGACTAACGAGATTGCCGTGCTGAAGGAAGCGAATCGAAACAAGG
AGGAGAAGACCCAGGGTAAGTGGACGCCACATCAACACAATCATCCGCCTCACATTGCATGCTGCATGCG
ATAGTTAGTCCCGCCGCTTTGTTACCGCCAAATTGTAGTTGTGGGTCAAGTGCTCCCAGGCCAGCACAAT
GCATGCCATCCCGATCCCCACCAGCCACAGAGCGAATACGGCGTGGAAATGCTGCAGTTTGAGGACCACC
GGTTCATCATTGCCACTCCCGCGTCGACGGTTTACCACCTTTACTCCAGACTTAATAAAGGCGGAGTTCA
TGATGCGATCCCAGTATCGCTCGAATCCAAACTCATGCGAACTCCGGATCAGTGAGTCCAGGAAACCCAA
GTAGGGTGAACCGTAGGGGACGATATAAACGGCGTGAAACGGCACCAGGCAGCTGTTCATCAGGTGGAAA
AGCGGACGACCCAGGTGCATGTGCACACGGCTGTTCACCTGGAAACGGGCGATGTGATACTTCTCCAAAT
AGGCAAATCTGCGAAAGTAGATTTTATGGAATTACAGCAATTAATCCACGTACACGCATACCTTATGTCA
TTGTTCCTCATCATTTCGTACATCTGGGTGTCGGAAACTTCCAGCATTTGTTCCCGAATTCTGGACTCGT

GCTTGTGTCCCAGGGTTAGGAAGTGTTGGATATGTTTTACGTGCCTTGGACGAATGATGATGTGGTAGTG
GGACCTAGCCAACGCACCCAATTGGTTTATATCGGGTAGATAAGGCTGGAACACCATCATGCTGGTCAGA
TTTCCCTTGAATGCCGAGCATATAAGTAGTCCAAAGTAAAGCCAGAAGATCAGAAACAATCGGATCGAGT
GGTTTGGCGGAATCTGGGTCATGGGCATAGTTAGGGCTCCGGCGAAAAGTTGCATGGGAACCCTACCCAC
TTCAGATTTCAGGATGTAGCAGAAGACATTCGCCACCAGAACAGAAACCAGAATTAGGGCCCAAACTAAT
GAGCCAAATGAGCGAAATATGTTCCAGAAAGTGGGAGCAGTCTTGGCTTTCGGCACAATCACGCAAAGAT
CATCACGCGTGTGGGATACCGTGCTTTCCGCCTGTTTCCTGAAGGTATCCGGAACCAAGAAACGAATGTT
CATGGCCACATCCACGTACTCCTTGGCCACCTCAAGGAAACATATGTCCGAGCTGAGATTGTGGTTGTTA
TACGAGTGCGGACGCGTTATCATCATGGTCGCGTTCAGTCGCTCAGCCACATAGGCCGCCAGCAGGCCAT
CGGTCCCCAAGATAGTTCCCTGTCTCCAGAAGATTGCCCTCACATCGTCCTTGTAGATGCACATTCTCAG
CGGCTTCCTCTGCATATTGGGTATGGTCTTTGGGAAAAGCTGCTCTAAAGTTGGAACCTCGTTTGGTTTA
TTGTCCACTGGTATCAAATAGTTGTCCATAAACGGGTTGTACCGATAGGCATTCAGTCGTCCATCTCGCC
AGTAGAGGATCACGATGTTCAGTAGCCATATCTTCCAGAATTGCCGGAAACTGGCCCGCATCCAAGCATC
GGAAACGGTATCCGCATCTCTGACCAAGAGAAAAACATGCGATCGATGCTTGGCAGCCGATTTCTTCCGG
ATAAGTGAAAGCTCCAATGGTTGTGATATGTTTGTGGTGATCATCACCATGTACATTAGTATGCCATCAT
CCTTGACCGCCTCCAGTGGTGTATTTTGATTCCTGAAAGTAAAATATTCGAATATTTGTGGTTATTATAT
GTTATCAGTATTGTTATGGGATTAAATGATCAACTTGCCTGAGAGATATGTACGATATGTCGCACTTGGT
CACCTCTTTGAGTATTTGACCCGTATGCTCGTGGGAGGTTTGATTCTCGAAGTAGACGAACAGGACCTTA
ATCCGAGCCTTGCAGAGGATCTGACTCATCTTGACCATCACGATCGAACTTTTCTTCAAATCTAACGGAA
TGTGATTCTCCCGTGTCCCGGCCAAATTGACCAGGATTGCCAGGAGCAGAATGTGTTTTAGCCACTGGAT
GGACATTGCAATGCCAACTGTTTGAGCAGAGGGATGACACCATTGTTTTTATACCCAGAGCCAATTTCGA
TATAGCAATTTCCCTAATGCTTGCCATATCGGCAAAGGGCGTTAAGCAAATATTATGCTATATCTTCTGC
ACTTTTATACCCTGTAAAAGGAACATTGCGAGTTACTGCCTACAGATTGCATTACCTAATTAACACAGAA
ATATTCTTTTCCATACTGTTTTAATTTCAGGTTTTAGTAACCCTCTATAGTTCCCAAGATTGTGCGATGA
ATTTTAAGTAGGCATATAACTCAATAAAGTGAAAGTCTTTAGGTGTTTGCTATTTGTAAAATTTTCGttt
tttttaattttttttttATGAAGGGTATGCGATTCTTCGACTGTGGCAAGAACTTTCCTTTCCCGTTCTTT
ATGTAGCATTTTTTGTGTGTTTTTGTGTCTCGTTTGGTTAATGGACGTACGCGACCTAATCGCAAAGTGC
AAACTAACAGCATGGATTAACTTGCTTCTGGTATCTCCCGCTGCTTGGAGCCCCGTATCCAAAAAGTTGG
CAACACGCAAACATGTATGATTCGACCATTTCTTGCAGATTATGGGGCAGGCTGATAGACTCAAGGCTGC
CGATATGCGGCAATTGAAGAACGCGTACGAAAACCGATTGACTCACATACAGAAATCAGCCAAAATTGAA
ATAACACGTTTGGTAAGTTGTGCGTAGCGGCTGCTAACGGGTCACCAAAAGTTGGCTCAAAAGAAGTGGT
TGTGAATggggggggggggggggAAGCGTTACGGAACGAAACCCGGCGGTTCAGAAGCGGACGGGCAACT
TTTCATACCACCATTGAAATTCATACATGCATTTTGAATTTGCACAGCAAGAAAACAGGTCATATTCAAC
TAGCTGCTATAAAGTACCCATATTATAAGCCTTATATCATTTTGATTTACTGGTCATATATTCGTATAAA
CATTTTCATTTTTCGTGATGTTATTACTAGAGTTTAGTTTCAATCAGTAAGACCATATACATATAAACAT
TTCTTTTTGCACTTACACCAAACTAATGGGATATAGTTACCTACCATTTGTTCTCATTAAAAACATACAC
AACAAGTTGTGTATTCCCATTAATGGTATAAACATTTAATAACGCTGAATTGAGCCACCAACATGGGCTT
GTATAAATATTAGACCAATTCTGGTTGATTATTGAACAGTTTTACAGGTGGATGAACACGTATTGCTCAT
ATATCTCTAAACTCCATATGTTTAGTATGAATCTAACCAATCTCTGCTACTTATTATTCCAGTTTAGAGG
AAATAAAGACCAAAGAACGTAATATAGGACAACTGAGGAAGGACTTGCTGGCACTGCAGAGTCCCAAGAA
GCTGAAGCAGAATGAAGCCAAAGTGCCAGCGACGACGAAACCCAAAACGAACCCCAACGAAACGAGCCAA
AAGCCAAAGAAACCCACTACGAAGGCGGCGGGAGAAACAAAAGCTAAAACCGAACCAGATACAGATACAG
ATATATCCGAGCAAAATCCCATACGCCAGGCTCAAGTTACACCTGAGG
>dme_circ_0008253_junction_seq

ATACGCCAGGCTCAAGTTACACCTGAGGATTGAGGAGAATCCAACGATGACCAGGT

>dme_circ_0008254

GGACCACAGCACTACTTCAACCAAGTGCTGGGAAATCGCTCCAAGTTCTTCAGTTCCCTGGCCCAAAGCT
GCAACCTAAAGGGAGGAGAGCTCAAGTCCAATCCCACAACTGCCGAAGAAATGGAAATCTTTATGGCGCT
TTCCCTGCTTATGTGTGACCTGAAGCTGGAGCACCTATCGGATTACTGGAGCACTAACATG

>dme_circ_0008254_junction_seq

CCTATCGGATTACTGGAGCACTAACATGGGACCACAGCACTACTTCAACCAAGTGC

>dme_circ_0008255

GTCTGCTAGCGCCGGATAGCCGTCCAAGGGTTCCGACTTGATGTCCACACTGCGTCGCTTCACCGGCGAC
CGGTGATCGTCCTGACGCGGCTTAAGTTGCTGCTTGCGCTTTCTTCCGGAGCTGGACATTTCGTTTGGCT
GCGTTTTCTCTTCCAAATAAT

>dme_circ_0008255_junction_seq

TTTGGCTGCGTTTTCTCTTCCAAATAATGTCTGCTAGCGCCGGATAGCCGTCCAAG

>dme_circ_0008256

TTTAAGCGGAGAACAGCTTAGATTTCATTGAAGCAGGATAGAGATACAGATGCAAAACCTATTTGTAAAA
TTCAAAAGAAGAGAAAACCACTTTACTTAAAACAGACCAAAACAGCCCAGATTCGCCCGCTCTGCCTTGG
ACTGTGGGCACAAACGTTCCGGCCCGCTGACTTTTTCAACACCAATCAGTACAAAATAATTTCCATTTCA
ATGTTGGGAGCCCGAAACAAGATCGCGGCGGAGCTCTCCATGCTGCGAGTCCAAGGCGTTTGTGTCCCCA
ACCAAGGCGACTGCGAATTGAAGGCCTCTGAAATTATAATTCAAATTCAATTTTTTAATTTAAATTTAAA
GCGTAACGACGACGATTCGACCAAGTTCGAATATTATTTTTAACCTCTTTTATTTAAGCCGAAACATGAT
TCGTCGTTCTAAAAACAATGAAAAGAAAGCAACCAAGCACTATGTatttttttaatttctccattgatct
aatttacaatttaatttgcaatttttttgTGATACTTTGCTATATTTTTAAACCTGGTATG

>dme_circ_0008256_junction_seq

ACTTTGCTATATTTTTAAACCTGGTATGTTTAAGCGGAGAACAGCTTAGATTTCAT

>dme_circ_0008257

GTATCAcaaaaaattgcaaattaaattgtaaattagatcaatggagaaattaaaaaaatACATAGTGCTT
GGTTGCTTTCTTTTCATTGTTTTTAGAACGACGAATCATGTTTCGGCTTAAATAAAAGAGGTTAAAAATA
ATATTCGAACTTGGTCGAATCGTCGTCGTTACGCTTTAAATTTAAATTAAAAAATTGAATTTGAATTATA
ATTTCAGAGGCCTTCAATTCGCAGTCGCCTTGGTTGGGGACACAAACGCCTTGGACTCGCAGCATGGAGA
GCTCCGCCGCGATCTTGTTTCGGGCTCCCAACATTGAAATGGAAATTATTTTGTACTGATTGGTGTTGAA
AAAGTCAGCGGGCCGGAACGTTTGTGCCCACAGTCCAAGGCAGAGCGGGCGAATCTGGGCTGTTTTGGTC
TGTTTTAAGTAAAGTGGTTTTCTCTTCTTTTGAATTTTACAAATAGGTTTTGCATCTGTATCTCTATCCT
GCTTCAATGAAATCTAAGCTGTTCTCCGCTTAAACTGACGTCCAGTAAGTAACAAACGTTTTGCTTACAC
TTaaaaaaaaTGCAAAATTCTGCGCGACAA

>dme_circ_0008257_junction_seq

aaaaaaaaTGCAAAATTCTGCGCGACAAGTATCAcaaaaaattgcaaattaaattg

>dme_circ_0008258

gatcaatggagaaattaaaaaaatACATAGTGCTTGGTTGCTTTCTTTTCATTGTTTTTAGAACGACGAA
TCATGTTTCGGCTTAAATAAAAGAGGTTAAAAATAATATTCGAACTTGGTCGAATCGTCGTCGTTACGCT
TTAAATTTAAATTAAAAAATTGAATTTGAATTATAATTTCAGAGGCCTTCAATTCGCAGTCGCCTTGGTT
GGGGACACAAACGCCTTGGACTCGCAGCATGGAGAGCTCCGCCGCGATCTTGTTTCGGGCTCCCAACATT
GAAATGGAAATTATTTTGTACTGATTGGTGTTGAAAAAGTCAGCGGGCCGGAACGTTTGTGCCCACAGTC
CAAGGCAGAGCGGGCGAATCTGGGCTGTTTTGGTCTGTTTTAAGTAAAGTGGTTTTCTCTTCTTTTGAAT
TTTACAAATAGGTTTTGCATCTGTATCTCTATCCTGCTTCAATGAAATCTAAGCTGTTCTCCGCTTAAAC

TGACGTCCAGTAA
>dme_circ_0008258_junction_seq
GTTCTCCGCTTAAACTGACGTCCAGTAAgatcaatggagaaattaaaaaaatACAT
>dme_circ_0008259
GTGCGATCTTATTTCTGGGATTTTCCACTATCATTTACATTTTGGGGTTCCTTTTGTTTTTGGCTTATCA
CTTCGGTGCCAATTCACTTGGCAAAAACACATAACAAAACTCACGCTGCCTCTTTCTACGCAAAATACAA
ATAACTTTGGATTGGCTGAGGATATCTGGAAGTGTGGGAGGCTTGGGATGGCTTAAATAATTTACATACA
TATTAATATTAACAATTAATAAGTAATGCTAATAATGAAAATAAG
>dme_circ_0008259_junction_seq
AATAAGTAATGCTAATAATGAAAATAAGGTGCGATCTTATTTCTGGGATTTTCCAC
>dme_circ_0008260
CTACAAATGCGAACTGCAAACATGTTTAATTATTCTTAATGTGTAATTGCGAAATGGCTTTAAAAAATTC
TAATTTAAACATTCTTGAATTGTACATAGAAGGGAACAACGCTAAAATGTTCAAATAAAATTTTCTAAAA
ACGAGAAATCGATAAACAATAAGAATAAGATCATAAGAAGATCAGCACTTG
>dme_circ_0008260_junction_seq
AATAAGATCATAAGAAGATCAGCACTTGCTACAAATGCGAACTGCAAACATGTTTA
>dme_circ_0008261
GTTCTTCTTCCTCTAATCGGGCTTTTCCTTCGCTTAAGTCCTCTTTCCATTTGGTTTTCCTTGTATTTCA
GCCAGGCATCCATTGAGTGTTGTACTGGTTGCAGTTTGTCGATTGGCTGGCTGTCTCTATAAAGATTGCG
CATTGAAGTTGACAGGGGAGTTTTGGAGTTTGGGGGAGAT
>dme_circ_0008261_junction_seq
CAGGGGAGTTTTGGAGTTTGGGGGAGATGTTCTTCTTCCTCTAATCGGGCTTTTCC
>dme_circ_0008262
ATAAAGATATCGATTAAAATTTCTGCTACAAAAATAAATATATATGTAACGCATATTGTAAATGTTCTAA
GTTAAGTGACATAAATCAAATATTTGTGTAAAGTTTAATATTTTAATACGCGTCGAAGTACAACGAGTGA
GGCTACAGAAGAGCACACACTAAAGTGGAG
>dme_circ_0008262_junction_seq
CTACAGAAGAGCACACACTAAAGTGGAGATAAAGATATCGATTAAAATTTCTGCTA
>dme_circ_0008263
ATCAAGGTGGACGAGAACGGCAAGATAGTGGACGCCAAGTTCAAGACCTTCGGCTGTGGATCGGCCATTG
CCAGCAGCTCCCTGGCCACCGAGTGGGTGAAGGGCAAGTCCATCGACGAGGCCGGAAAGCTGAAGAATAC
GGACATCGCCAAGGAGCTGCGTCTGCCGCCCG
>dme_circ_0008263_junction_seq
ATCGCCAAGGAGCTGCGTCTGCCGCCCGATCAAGGTGGACGAGAACGGCAAGATAG
>dme_circ_0008264
GTATCCATTGCATTCGATTGATTAATTTCTAGCTCGATATAATAGTTCAGAGGCAGAGCCAAACGATCAC
TTAAAGGCACAATTAACAGACAATATCGAATTAAATTTATTATATTATATAAGCAATGCCTATACTACCT
CTTTGCTCAGCTCATTTCGTCTGCCCTGGAATTGCGGCCCTAAGACTACACATATGATACTACGGATCTA
TTTACAATTTCGTATATCGTTCAAGTTGGCTGGTGAATG
>dme_circ_0008264_junction_seq
GTATATCGTTCAAGTTGGCTGGTGAATGGTATCCATTGCATTCGATTGATTAATTT
>dme_circ_0008265
CCAGTAGAATCCCTCTCCGACGATTTCCTATTCCCCATGACCACATCCCGACTCTCGTATGCATCAAGTC
CCGAATCCGATTTGGAACTGAGTCCCGGCCCATCCGCATCATTGTCGCTTGGCAATTTGCCGCAGTTGGT

TAAAGCGAGCTCAGATCCATCGATTGCCACTAATCAGGATAACTTGGATAGGGATAGAGACATAATTGGT
GAAGGACTACCACCTCCATATACGGTAAGAGGTCGaaaaaccaaccaaccaaacaaacaaacaaacatac
aaaacaaaaaaaaactcaaacTAAGAAATGGCATGCGCTTACACTaaaatcaatcaaaagaaaaaacaacc
aaaaacTTGGGAAATTTCTAAGCGTCCATCCAGTTCTCATCAAAATAACAATGGTCAACAAAAATAAAGA
AAATATTACGTGAAATTCAAAAGAGACATTCTAAAATCAATATTTTCCATAATAGCTCTCATGAAAGTTT
ATTTTTGCTGACTAGTGTGCTAATCTAATTCACCACCCACTCACGAACCCCTTGCAATGCACCTGCTGCA
GGGTACCCTACGACCATGCTGTCCCCGCGAATCCCAATCGCCGCCAGACCATGGACTCCAGCAAGTACAG
CATCTACGGCACCAATGTGCCACCACAGCAGCAGCAGCCCGGCGTTGGAGGAGATACGGCTGCAGTGAGA
CCGCAATCTCTGTACGGGATTAATGCTCCCGATCTGCCGCCCCGCATCGATCGTCAGTCCAAGCCGGGCG
ATATACCACTGAACACCTCGGGCTCATCGTCGCGAAATGGCACCCTGGGTCGAAGTGCCCAGGAGCGTCT
GTTTGGCAAGGCTGTGGTGCAGGACGATGTCCAGGCGGAGTACATAACCCGAAACGCTCTGGTCGGGTCT
GGAGCTGCAGAAACCCTGGATCGCcagcagcagcagcagcagcagACTCATGCATCCTTGGAGCGCCAAG
CGCGTTTGAATGCGCAGCTAAAGGCCAACGGAGTAGGCGCCGGAGGAGGTGGTGCATCCACCTACGACAG
TGTGTCCAGCTACGACTCGTACAACAATACACAAATGGCCATGCAAAACCTGGGCCGACTAGGTCCCAAT
GCACCCGACGATCTCAAATCAGTGCCAAATGCAAAGTGGTCGCCCCTTGCCTCCGACGGGTCAGTCGCAC
GAGTACGGTCGCACTCCGCACGATCATCGCAGCTTCGGAGGACCCAACGATCTGAACCGGCAGAGCAGTC
CGGGCAGGCCGCACTACCATGACATGAATGCGTCCCGTAATATCGGGTATGCATGATGAAAATTCATTAC
TTTGGGGGAACGCCCTCGAAAAATGTATAAATTGCGAGGCTCAGTCAGAAATAAAAACATACTTCTTACT
TTCTTTTTCTCCTGCGCCGTCGTTATTATCTGAAATCACTCGCATGCCTCTTTATCGACTGTGTCGTAAA
TATTCCTTTGTATCGTTCTGAATAAATGCAATATGATTATGTGTATATCCACCTGTGTCTCTGGCCCATT
AGACGCTATCATTTACATGCGCCCCATTTGAAGCACTTGCATATGATGCACTCGCTCTCACCTTCGTCCT
CGTTGTCGCTCTCACCTTCGCCCTCGTCCTTGCCCCTTCACATGTCCTGCGGCCTTCAGCTGACACAGCA
GCAGCTGCTACGCCTCTCCACCGGCGGCTTCGATCGCCGACCCATTGCTGCACCACGTCTGCATGCCCTG
ATGGCACCACTGCCACCGCCAGCTCTGATGTCCAGTGCCTCGGCCTACGATACACTCGTCTAAGCAACGA
AATCGAAATCGAAACTATTTTATAGATACACTACGACTTTTTATATGCGATACTGCACTGGAAACATTCC
TTTAGTACATGTTTTTACTTTCATAGTTTTTAGAAAGTCGATCTAATTGAAATTGTTTTTATTTTACATT
TTACCTTCAAGTTAATGATTAAAGCCTTTAGTGTTCTCAATGATCTGTATTGATACCTTCGACCATGTAA
ATAGTTAACTCTTTTGCGAGCTCCAACCCGAAGTGCAATAAACGATACTGATTAAAACCCGATTAACCCA
GATTTTTGTATGCCGTCTAAGCACTATGAATATTTATCATATTTCTACCACACATATCCTTATAATTACA
TCAACTTTTTTCTCTTTTTTGCTTATGTATTGTGCTCGCCATCATCAACTGTTTGTCCCCAAAAACAAAA
CTCAATCTCACACACCCAAAACCACTGCCACCCATAACGCAATTGTATATCACCGAATCCACAATCCCGA
AACCATTTGAATCAACAATCCATCAGTGTCTAAGCAACGAAATCGAAATCGAAACTATTTTATAGATACA
CTACGACTTTTTATATGCGATACTGCACTGGAAACATTCCTTTAGTACATGTTTTTACTTTCATAGTTTT
TAGAAAGTCGATCTAATTGAAATTGTTTTTATTTTACATTTTACCTTCAAGTTAATGATTAAAGCCTTTA
GTGTTCTCAATGATCTGTATTGATACCTTCGACCATGTAAATAGTTAACTCTTTTGCGAGCTCCAACCCG
AAGTGCAATAAACGATACTGATTAAAACCCGATTAACCCAGATTTTTGTATGCCGTCTAAGCACTATGAA
TATTTATCATATTTCTACCACACATATCCTTATAATTACATCAACTTTTTTCTCTTTTTTGCTTATGTAT
TGTGCTCGCCATCATCAACTGTTTGTCCCCAAAAACAAAACTCAATCTCACACACCCAAAACCACTGCCA
CCCATAACGCAATTGTATATCACCGAATCCACAATCCCGAAACCATTTGAATCAACAATCCATCAGTCGG
AATAACTCCGTAACGCAAGCGGATTATACAAAGCTACCGAAGACTGCGCCACATGGAGTAGTTCCTCCGC
CGAATGTGAGCAATGGCCAGGGCCAGATGAATGGCAGCGGAACGCCGAGCAGCAATGGCAGTGGACCCTT
CAAGCCGGTGCCACCACCCAAACCCAAAAACTATAGGCCACCGGTGCAAAGTGGTGGTAGCAGTGGCAGC
GGAGGCACAACTCCTTGGGAGAATGGGGTAAGTTTCATCTTCTCTCTTTCTTAGACCTTCCACTCATCAT
ATACTCCCTTTTCAGGGACTCTGGTTCGCCCCGTTCTCCGAATGGCTTCTATTATCCACCAACGCCTTCG

CATCATCATTACGGCCAGCAGGCGACCCCGGGTTCACCCAGCAATGGCCACATGCAGCCACCACCACCGC
AGCAGCAGCAGCCCACCTACGGCGGAAGTAATGGCAACTATGGCCAGGCACCACCACCACAATCCTATCC
GCAGGCCAATGGCTACAATGGGAATGGGCATCACTACAACGGGGGCAGCGGCACAGGACCCTATATTGCA
CCACATCGCGGCATGCCACCGCCAATAGGGAAACCTGCCGCCTCACACGCCCGAACGTCATGCTCTTGAC
TTGGCCGGAAGTCGGGAGCAGCGCGGTTCGGCCTTCGAACTGTATCGTAAACCGCAAATCGGAGCTACCG
CCGGGCACCATCACAACATGAGGGTGAGTCCATAAGTCCATTAGATACCCGTAGCTCCCTGAATTGCTCT
CCCATTGCTCTCCCAAAAGGATATCCTCCTTTGCCaaaagtaaaaaacaaaaaatgaaatcaaacacacg
aaaaTATACAGAACTCTTATTGATTCCCCACTCCCAATTTTCAACATTTGTGTTAACGAAAAATGTTTTA
TATTCTTGTTAAATGCCATTTTTGTACTTTACAATatcaaacattcagtcagcgaaacaatcagtcaatc
aatcaatcaatcaTTATGCCAATCTCTTCATACTTTCATGCGGTTGATGGTCATTACAAATTATTCGATG
CAAAATTCACGAAAAACGCACAACGAAAACAGATCGCATATATTCCATAAATTTAATAATTATTGTATTT
TGCAATAACCAAAATTATTAACCAAACCATAAATTAAAGCTTAACAAAGTTTGTAAAAACCAAATCTAAG
GTTGTTGAGTTGAGCGCTTCACGCACATATaaaaaatgaaaaaaaaaaaacgaaaataaaaCATTTTTCG
GGTTAACTTCGACTTGCTATTTTAGTTTACAGTACAAAGATTCGTATCCACATTTCTCTACTTTCGAACT
TCAACTTGTATTACTATTTAATATGATCATGCTTTTAGCTGGTTTCTTAAATATAACAACTCTCAATACA
TTTCTCAGGTATTTGAAAGTTAATCTTGAATCTAACTATGGTTTTGGGTAAATTCAACATTTCTAAATAA
AAACCATAACTAACAAAAAACTACTTTAAAATAAAGTTGAAATATTTGGTAAATGGTATGTTTATTCTAC
AGTTTTGGTTAATATGCAAAGTATTTAATATAAATATATGAATACATTTTACATGTGCGCTTCTTGTGTG
TCGTAAGCGAAAGTTTTGCAGGAATAAATTGCAAGTTAGACATGTTTGTTCAATATTTTTACCTCCCTTA
TTTAACTGATGAAATGTACCGCCGCGAATTAAAACATATTCTGGTGGACATTTTTATAAAAACAAATTGA
TTGCTAAAGTTCAGAACCCAAACTTGAATTCACTTCACGAATACATTTaaaaaaaaaaaCTCCTTGCTAA
TTGCACACAAAAATTTCGAATCACATAATAAATCTTTAGCGAAATGGAGCCGTACGATGAGCGCTACGAT
GATTACTATAACATGCCACCCCCTGCCGCCCATCCGTCCCAAGGGCATCATATGCAGCGATCCCGATCAG
CTCCTCGTTATCCACACGAGCGACCTCCTCACGCCCAAGATCCGAACTACTACGGCCACTATGGAACGTC
CAGGGGACATAGTCAGCCGCGCCAGCAATATTCGCAGCATCACCAACAGCAGTATTACGATGATCATGGG
TTGGAGATGGGTCCACCGCCATTGCCGCCACATAAGAAGAAGAAATCGGTGCTAAAGAGCCCACTGGTGG
CATTAAAGAATGCACTGCTTAAGAGTACGAGGCCTCTAAGGAGAATGAACAGCATGGTGGAACCGGAGAG
GAAACCGAAGGGACTTCGACGCCAGCAATCGATGCTGGAGAGGGGTGTGCAAAGACCCTACTACCCGGAT
GAGTATCCAACGTATCCAGCTGGCTTTGAGGAGCGTGCCCATGGTGGACAAGGCATGATGCAACCACATT
CACAAGATGCCTACTATCAAAGGGGTGGTCACTACGCGCCGCAACAGCAGGAGATGATGAACAGCACCTA
TCAGAATCTGGAGGGTGAGGATATCTATGGGAATATCGGCAACACAGTTCCCAGAATGCCGCATCACCAG
GACAATGGTTACGGATATGACCAATATGATCTCTATGCGAATCGAGCGTGCATCGATCTCGAAAGAAGGC
AAGCAGAGGCAGCTGCAGCTTCTGGCGGACGAAATGGAAGGAGAATTGTAAGGCGGCACAGCACCACTAC
TGCAGATCGTGGAGGAAATCCCGGACCGCCAAGGAGGCCTATGATCAGTCCTGGTTATGAACAAGATCCG
CAAGAGATCTACCAGACCCGCAACGGAGCCTACATGCTGCCCGATCAGCGAAGGGCGCCCAGTTCGGAGG
TGATGACCCGTAGACGATTCTATCCGGGGGCTGCCAATGAGCAGACTGAGGAGGAACCGCTCTACCAATC
ACGACGCGAAATGCAGCGCGAAATGCAGAGAAATCATCTGTACCAATCGAAACGAGAAATGCAGGAGAGG
ATTAGCCAGGGCAAGAGGGATATGGAGCGAGAATTTAGCCCCCAAAGCAGCAGCAGCCAGTCGGAAGCCT
CCAATTCGGAGGCAATATACCAGAGTCGGCGGGAGGCCAAGGAAAGTGCTCTGAAAACGAGGTCACAACT
GAGGGATCAGATCTATCAGACTAGACGGGAAGCCCTGGACAGCATGGCTGAACCGATCTATGTATCCAAA
AGAGGTATGGGCAGACCGGCCCCAATCTATGAGACCCGGGAAGAGAGTATCCTCCAGTCCCGAGAAAATG
AAACAGATGAAAAGAAGGAAAAGGAGTGCAAAACCGAGCAGATTCAGGTGGAGATTAATGCTCAACCGGA
AGAACAGATCGAAGATTCGACCTTAAGTCGTTCGGACCTGCAGAAATCTTCGGACACGGTGATAGAAAAT
CCAGCTAGAGCTCCTCTTGTGCAGGACGAAGTAGATGATGACGAAGAGGAGGAGGTTCAGGATCAGCGTG

ATCCCGCTGAGAGCTCTGCGGATCAGACAGAGCAGCCAACAGCTATTGAAAATAATCTGCACAATGAAGG
AATGGTGGAGCAATCACAGAATGATTCGGATGACGTATTCGAAGCAGCAGATAAGGTTTCTCCCCTGGCC
CCTCCACCAGCTCCTGTTCAACCGCCGACCCCGCGATCCGGTAGAGCTCCCTTCCACATTTCGAATATCC
TTAAGCGAACAGCACCTCCGCCGAGTTCCCCAATCGGTGACAGCTGTACCTCCATCGAAACCCAGTACAC
ATCACAAGCCAGCTTGCCAGTGGGTCCACCCAATGCGACCAGTACGCCGTTTAGTAGCAGCATGTCATTG
CCCATCGCCGGACCAGTAAATAATCAACCTTCGGCCAACGGACGCTTTCCCACTCTGCCCCGTGAACCCA
GCACCTCGCGCGGATTTTTTGACTCCAACGGTGGCACTTTGGCGGATAAGCTGTGGCACGTCTCACTGCA
AATCCCGCCGGGAGCCATTCCGGCCGGAGTACGACAAGAGATCTACTTCACCGTGAGCGACCCGCGAATG
GGACAAGCTGTCGGCGGTCCTCCGCTGGACATGGAAAATGG
>dme_circ_0008265_junction_seq
GCGGTCCTCCGCTGGACATGGAAAATGGCCAGTAGAATCCCTCTCCGACGATTTCC
>dme_circ_0008266
GTACCCTACGACCATGCTGTCCCCGCGAATCCCAATCGCCGCCAGACCATGGACTCCAGCAAGTACAGCA
TCTACGGCACCAATGTGCCACCACAGCAGCAGCAGCCCGGCGTTGGAGGAGATACGGCTGCAGTGAGACC
GCAATCTCTGTACGGGATTAATGCTCCCGATCTGCCGCCCCGCATCGATCGTCAGTCCAAGCCGGGCGAT
ATACCACTGAACACCTCGGGCTCATCGTCGCGAAATGGCACCCTGGGTCGAAGTGCCCAGGAGCGTCTGT
TTGGCAAGGCTGTGGTGCAGGACGATGTCCAGGCGGAGTACATAACCCGAAACGCTCTGGTCGGGTCTGG
AGCTGCAGAAACCCTGGATCGCcagcagcagcagcagcagcagACTCATGCATCCTTGGAGCGCCAAGCG
CGTTTGAATGCGCAGCTAAAGGCCAACGGAGTAGGCGCCGGAGGAGGTGGTGCATCCACCTACGACAGTG
TGTCCAGCTACGACTCGTACAACAATACACAAATGGCCATGCAAAACCTGGGCCGACTAGGTCCCAATGC
ACCCGACGATCTCAAATCAGTGCCAAATGCAAAGTGGTCGCCCCTTGCCTCCGACGGGTCAGTCGCACGA
GTACGGTCGCACTCCGCACGATCATCGCAGCTTCGGAGGACCCAACGATCTGAACCGGCAGAGCAGTCCG
GGCAGGCCGCACTACCATGACATGAATGCGTCCCGTAATATCGGGTATGCATGATGAAAATTCATTACTT
TGGGGGAACGCCCTCGAAAAATGTATAAATTGCGAGGCTCAGTCAGAAATAAAAACATACTTCTTACTTT
CTTTTTCTCCTGCGCCGTCGTTATTATCTGAAATCACTCGCATGCCTCTTTATCGACTGTGTCGTAAATA
TTCCTTTGTATCGTTCTGAATAAATGCAATATGATTATGTGTATATCCACCTGTGTCTCTGGCCCATTAG
ACGCTATCATTTACATGCGCCCCATTTGAAGCACTTGCATATGATGCACTCGCTCTCACCTTCGTCCTCG
TTGTCGCTCTCACCTTCGCCCTCGTCCTTGCCCCTTCACATGTCCTGCGGCCTTCAGCTGACACAGCAGC
AGCTGCTACGCCTCTCCACCGGCGGCTTCGATCGCCGACCCATTGCTGCACCACGTCTGCATGCCCTGAT
GGCACCACTGCCACCGCCAGCTCTGATGTCCAGTGCCTCGGCCTACGATACACTCGTCTAAGCAACGAAA
TCGAAATCGAAACTATTTTATAGATACACTACGACTTTTTATATGCGATACTGCACTGGAAACATTCCTT
TAGTACATGTTTTTACTTTCATAGTTTTTAGAAAGTCGATCTAATTGAAATTGTTTTTATTTTACATTTT
ACCTTCAAGTTAATGATTAAAGCCTTTAGTGTTCTCAATGATCTGTATTGATACCTTCGACCATGTAAAT
AGTTAACTCTTTTGCGAGCTCCAACCCGAAGTGCAATAAACGATACTGATTAAAACCCGATTAACCCAGA
TTTTTGTATGCCGTCTAAGCACTATGAATATTTATCATATTTCTACCACACATATCCTTATAATTACATC
AACTTTTTTCTCTTTTTTGCTTATGTATTGTGCTCGCCATCATCAACTGTTTGTCCCCAAAAACAAAACT
CAATCTCACACACCCAAAACCACTGCCACCCATAACGCAATTGTATATCACCGAATCCACAATCCCGAAA
CCATTTGAATCAACAATCCATCAGTGTCTAAGCAACGAAATCGAAATCGAAACTATTTTATAGATACACT
ACGACTTTTTATATGCGATACTGCACTGGAAACATTCCTTTAGTACATGTTTTTACTTTCATAGTTTTTA
GAAAGTCGATCTAATTGAAATTGTTTTTATTTTACATTTTACCTTCAAGTTAATGATTAAAGCCTTTAGT
GTTCTCAATGATCTGTATTGATACCTTCGACCATGTAAATAGTTAACTCTTTTGCGAGCTCCAACCCGAA
GTGCAATAAACGATACTGATTAAAACCCGATTAACCCAGATTTTTGTATGCCGTCTAAGCACTATGAATA
TTTATCATATTTCTACCACACATATCCTTATAATTACATCAACTTTTTTCTCTTTTTTGCTTATGTATTG
TGCTCGCCATCATCAACTGTTTGTCCCCAAAAACAAAACTCAATCTCACACACCCAAAACCACTGCCACC

CATAACGCAATTGTATATCACCGAATCCACAATCCCGAAACCATTTGAATCAACAATCCATCAGTCGGAA
TAACTCCGTAACGCAAGCGGATTATACAAAGCTACCGAAGACTGCGCCACATGGAGTAGTTCCTCCGCCG
AATGTGAGCAATGGCCAGGGCCAGATGAATGGCAGCGGAACGCCGAGCAGCAATGGCAGTGGACCCTTCA
AGCCGGTGCCACCACCCAAACCCAAAAACTATAGGCCACCGGTGCAAAGTGGTGGTAGCAGTGGCAGCGG
AGGCACAACTCCTTGGGAGAATGGGGTAAGTTTCATCTTCTCTCTTTCTTAGACCTTCCACTCATCATAT
ACTCCCTTTTCAGGGACTCTGGTTCGCCCCGTTCTCCGAATGGCTTCTATTATCCACCAACGCCTTCGCA
TCATCATTACGGCCAGCAGGCGACCCCGGGTTCACCCAGCAATGGCCACATGCAGCCACCACCACCGCAG
CAGCAGCAGCCCACCTACGGCGGAAGTAATGGCAACTATGGCCAGGCACCACCACCACAATCCTATCCGC
AGGCCAATGGCTACAATGGGAATGGGCATCACTACAACGGGGGCAGCGGCACAGGACCCTATATTGCACC
ACATCGCGGCATGCCACCGCCAATAGGGAAACCTGCCGCCTCACACGCCCGAACGTCATGCTCTTGACTT
GGCCGGAAGTCGGGAGCAGCGCGGTTCGGCCTTCGAACTGTATCGTAAACCGCAAATCGGAGCTACCGCC
GGGCACCATCACAACATGAGG
>dme_circ_0008266_junction_seq
TACCGCCGGGCACCATCACAACATGAGGGTACCCTACGACCATGCTGTCCCCGCGA
>dme_circ_0008267
CCAGTAGAATCCCTCTCCGACGATTTCCTATTCCCCATGACCACATCCCGACTCTCGTATGCATCAAGTC
CCGAATCCGATTTGGAACTGAGTCCCGGCCCATCCGCATCATTGTCGCTTGGCAATTTGCCGCAGTTGGT
TAAAGCGAGCTCAGATCCATCGATTGCCACTAATCAGGATAACTTGGATAGGGATAGAGACATAATTGGT
GAAGGACTACCACCTCCATATACGGTAAGAGGTCGaaaaaccaaccaaccaaacaaacaaacaaacatac
aaaacaaaaaaaaactcaaacTAAGAAATGGCATGCGCTTACACTaaaatcaatcaaaagaaaaaacaacc
aaaaacTTGGGAAATTTCTAAGCGTCCATCCAGTTCTCATCAAAATAACAATGGTCAACAAAAATAAAGA
AAATATTACGTGAAATTCAAAAGAGACATTCTAAAATCAATATTTTCCATAATAGCTCTCATGAAAGTTT
ATTTTTGCTGACTAGTGTGCTAATCTAATTCACCACCCACTCACGAACCCCTTGCAATGCACCTGCTGCA
GGGTACCCTACGACCATGCTGTCCCCGCGAATCCCAATCGCCGCCAGACCATGGACTCCAGCAAGTACAG
CATCTACGGCACCAATGTGCCACCACAGCAGCAGCAGCCCGGCGTTGGAGGAGATACGGCTGCAGTGAGA
CCGCAATCTCTGTACGGGATTAATGCTCCCGATCTGCCGCCCCGCATCGATCGTCAGTCCAAGCCGGGCG
ATATACCACTGAACACCTCGGGCTCATCGTCGCGAAATGGCACCCTGGGTCGAAGTGCCCAGGAGCGTCT
GTTTGGCAAGGCTGTGGTGCAGGACGATGTCCAGGCGGAGTACATAACCCGAAACGCTCTGGTCGGGTCT
GGAGCTGCAGAAACCCTGGATCGCcagcagcagcagcagcagcagACTCATGCATCCTTGGAGCGCCAAG
CGCGTTTGAATGCGCAGCTAAAGGCCAACGGAGTAGGCGCCGGAGGAGGTGGTGCATCCACCTACGACAG
TGTGTCCAGCTACGACTCGTACAACAATACACAAATGGCCATGCAAAACCTGGGCCGACTAGGTCCCAAT
GCACCCGACGATCTCAAATCAGTGCCAAATGCAAAGTGGTCGCCCCTTGCCTCCGACGGTCAGTCGCAC
GAGTACGGTCGCACTCCGCACGATCATCGCAGCTTCGGAGGACCCAACGATCTGAACCGGCAGAGCAGTC
CGGGCAGGCCGCACTACCATGACATGAATGCGTCCCGTAATATCGGGTATGCATGATGAAAATTCATTAC
TTTGGGGGAACGCCCTCGAAAAATGTATAAATTGCGAGGCTCAGTCAGAAATAAAAACATACTTCTTACT
TTCTTTTTCTCCTGCGCCGTCGTTATTATCTGAAATCACTCGCATGCCTCTTTATCGACTGTGTCGTAAA
TATTCCTTTGTATCGTTCTGAATAAATGCAATATGATTATGTGTATATCCACCTGTGTCTCTGGCCCATT
AGACGCTATCATTTACATGCGCCCCATTTGAAGCACTTGCATATGATGCACTCGCTCTCACCTTCGTCCT
CGTTGTCGCTCTCACCTTCGCCCTCGTCCTTGCCCCTTCACATGTCCTGCGGCCTTCAGCTGACACAGCA
GCAGCTGCTACGCCTCTCCACCGGCGGCTTCGATCGCCGACCCATTGCTGCACCACGTCTGCATGCCCTG
ATGGCACCACTGCCACCGCCAGCTCTGATGTCCAGTGCCTCGGCCTACGATACACTCGTCTAAGCAACGA
AATCGAAATCGAAACTATTTTATAGATACACTACGACTTTTTATATGCGATACTGCACTGGAAACATTCC
TTTAGTACATGTTTTTACTTTCATAGTTTTTAGAAAGTCGATCTAATTGAAATTGTTTTTATTTTACATT
TTACCTTCAAGTTAATGATTAAAGCCTTTAGTGTTCTCAATGATCTGTATTGATACCTTCGACCATGTAA

ATAGTTAACTCTTTTGCGAGCTCCAACCCGAAGTGCAATAAACGATACTGATTAAAACCCGATTAACCCA
GATTTTTGTATGCCGTCTAAGCACTATGAATATTTATCATATTTCTACCACACATATCCTTATAATTACA
TCAACTTTTTTCTCTTTTTTGCTTATGTATTGTGCTCGCCATCATCAACTGTTTGTCCCCAAAAACAAAA
CTCAATCTCACACACCCAAAACCACTGCCACCCATAACGCAATTGTATATCACCGAATCCACAATCCCGA
AACCATTTGAATCAACAATCCATCAGTGTCTAAGCAACGAAATCGAAATCGAAACTATTTTATAGATACA
CTACGACTTTTTATATGCGATACTGCACTGGAAACATTCCTTTAGTACATGTTTTTACTTTCATAGTTTT
TAGAAAGTCGATCTAATTGAAATTGTTTTTATTTTACATTTTACCTTCAAGTTAATGATTAAAGCCTTTA
GTGTTCTCAATGATCTGTATTGATACCTTCGACCATGTAAATAGTTAACTCTTTTGCGAGCTCCAACCCG
AAGTGCAATAAACGATACTGATTAAAACCCGATTAACCCAGATTTTTGTATGCCGTCTAAGCACTATGAA
TATTTATCATATTTCTACCACACATATCCTTATAATTACATCAACTTTTTTCTCTTTTTTGCTTATGTAT
TGTGCTCGCCATCATCAACTGTTTGTCCCCAAAAACAAAACTCAATCTCACACACCCAAAACCACTGCCA
CCCATAACGCAATTGTATATCACCGAATCCACAATCCCGAAACCATTTGAATCAACAATCCATCAGTCGG
AATAACTCCGTAACGCAAGCGGATTATACAAAGCTACCGAAGACTGCGCCACATGGAGTAGTTCCTCCGC
CGAATGTGAGCAATGGCCAGGGCCAGATGAATGGCAGCGGAACGCCGAGCAGCAATGGCAGTGGACCCTT
CAAGCCGGTGCCACCACCCAAACCCAAAAACTATAGGCCACCGGTGCAAAGTGGTGGTAGCAGTGGCAGC
GGAGGCACAACTCCTTGGGAGAATGGGGTAAGTTTCATCTTCTCTCTTTCTTAGACCTTCCACTCATCAT
ATACTCCCTTTTCAGGGACTCTGGTTCGCCCCGTTCTCCGAATGGCTTCTATTATCCACCAACGCCTTCG
CATCATCATTACGGCCAGCAGGCGACCCCGGGTTCACCCAGCAATGGCCACATGCAGCCACCACCACCGC
AGCAGCAGCAGCCCACCTACGGCGGAAGTAATGGCAACTATGGCCAGGCACCACCACCACAATCCTATCC
GCAGGCCAATGGCTACAATGGGAATGGGCATCACTACAACGGGGGCAGCGGCACAGGACCCTATATTGCA
CCACATCGCGGCATGCCACCGCCAATAGGGAAACCTGCCGCCTCACACGCCCGAACGTCATGCTCTTGAC
TTGGCCGGAAGTCGGGAGCAGCGCGGTTCGGCCTTCGAACTGTATCGTAAACCGCAAATCGGAGCTACCG
CCGGGCACCATCACAACATGAGG
>dme_circ_0008267_junction_seq
TACCGCCGGGCACCATCACAACATGAGGCCAGTAGAATCCCTCTCCGACGATTTCC
>dme_circ_0008268
CCAGTAGAATCCCTCTCCGACGATTTCCTATTCCCCATGACCACATCCCGACTCTCGTATGCATCAAGTC
CCGAATCCGATTTGGAACTGAGTCCCGGCCCATCCGCATCATTGTCGCTTGGCAATTTGCCGCAGTTGGT
TAAAGCGAGCTCAGATCCATCGATTGCCACTAATCAGGATAACTTGGATAGGGATAGAGACATAATTGGT
GAAGGACTACCACCTCCATATACGGTAAGAGGTCGaaaaaccaaccaaccaaacaaacaaacaaacatac
aaaacaaaaaaaaactcaaacTAAGAAATGGCATGCGCTTACACTaaaatcaatcaaaagaaaaaacaacc
aaaaacTTGGGAAATTTCTAAGCGTCCATCCAGTTCTCATCAAAATAACAATGGTCAACAAAAATAAAGA
AAATATTACGTGAAATTCAAAAGAGACATTCTAAAATCAATATTTTCCATAATAGCTCTCATGAAAGTTT
ATTTTTGCTGACTAGTGTGCTAATCTAATTCACCACCCACTCACGAACCCCTTGCAATGCACCTGCTGCA
GGGTACCCTACGACCATGCTGTCCCCGCGAATCCCAATCGCCGCCAGACCATGGACTCCAGCAAGTACAG
CATCTACGGCACCAATGTGCCACCACAGCAGCAGCAGCCCGGCGTTGGAGGAGATACGGCTGCAGTGAGA
CCGCAATCTCTGTACGGGATTAATGCTCCCGATCTGCCGCCCCGCATCGATCGTCAGTCCAAGCCGGGCG
ATATACCACTGAACACCTCGGGCTCATCGTCGCGAAATGGCACCCTGGGTCGAAGTGCCCAGGAGCGTCT
GTTTGGCAAGGCTGTGGTGCAGGACGATGTCCAGGCGGAGTACATAACCCGAAACGCTCTGGTCGGGTCT
GGAGCTGCAGAAACCCTGGATCGCcagcagcagcagcagcagcagACTCATGCATCCTTGGAGCGCCAAG
CGCGTTTGAATGCGCAGCTAAAGGCCAACGGAGTAGGCGCCGGAGGAGGTGGTGCATCCACCTACGACAG
TGTGTCCAGCTACGACTCGTACAACAATACACAAATGGCCATGCAAAACCTGGGCCGACTAGGTCCCAAT
GCACCCGACGATCTCAAATCAGTGCCAAATGCAAAGTGGTCGCCCCTTGCCTCCGACGGGTCAGTCGCAC
GAGTACGGTCGCACTCCGCACGATCATCGCAGCTTCGGAGGACCCAACGATCTGAACCGGCAGAGCAGTC

CGGGCAGGCCGCACTACCATGACATGAATGCGTCCCGTAATATCGGTGAGTAGCTCTATATGCCATAGTT
TATTCAAAACGAACTAACATATTTCATATCATTCGACAGATCCACGCAATGGCACACCACAGCGTCCTTC
GAATCTGGGCCTGGAAAGCAGCCCACGCAAGCCTTTGGTGGAGACAAAAACGGATTACGGCAAATACAGG
>dme_circ_0008268_junction_seq
GACAAAAACGGATTACGGCAAATACAGGCCAGTAGAATCCCTCTCCGACGATTTCC
>dme_circ_0008269
GGTGATCGAACCACATGGGAGTACCACACGGTGGCGGTGACTCGGGTGCCGGGCTATGGATTCGGCATCG
CCGTTTCCGGTGGACGTGATAATCCGCACTTCGCCAACGGCGATCCCTCGATAGCAGTTAGCGATGTGCT
GAAAGGTGGCCCCGCCGAGGATCGATTGCAGGTAAGTACCCTCGCGATGATTCAACAGAAGTTACTGACC
CTGTAGGAAGTAAACCTTTCCAATGCATTTTGAATTATTATCTGACAGGAAACTAATTGTTGTTCCCGAA
TAAGTCCGACATGCTGAATAGACCCCCATGCAGGCTGATCAAATAAAGCATATAAGAACTAATCATCCAC
TGCTCCAAACGAAAAACGGGCATTATAAATTCCCGACATGCTGCTGACAATTGACATCGTTAATTTTGCG
CTTAGAACCTATACTTTTATATACCCACTTCTACAAATATAAACCCGAAATGCGCACACTTCAATACTTC
AAACTCACGTAGAGTCGGTCCCCAAAAAGAAATGGTATTTAATTAAACCAACGTCTCGGCAACTCGACAA
CTCGGTCCCTCTGAAGCTGTTTCTCTCTTGGCGATAATAAAAATTCCAAAGCGTTTTTCAATAGACAGCG
CAGAGGCGGCCCTAGTGATTACGAACTTGTCATAATCttttttttCCCAAGAAACTATTTACCCTGGCCC
AGACATGCAGAGAACTATTTTCACGTTGTCCCTTACCTGCGACTGCCGGAGGATTTCAAAAGGAAAATCC
GCTGCCGAGGCTGGAAAATTGTTGGCCAAGTGAGCGGAGACAAGGGGAAATTAGCTGCAGCCCAGCACAT
GTATTTCTGAAAAGTTGGCTAAGAGCATGTGGTCAAAGTTTCGCTGTTACGCAGCTCAGTAACTAGTACA
CACTCAGAAAAAATATTACTTCGCATTGAGACATTCAAAAATAAGttttttttCTATTACCATCCTAACC
AGGATTACAAACAACTTACCTACCTAATTTTTAATGAAATAAATCAAAACTTTTATATTTTGTTGACTTG
CGAAACACATTTTTCCTCAGCACATAACAGGGTTTTTCCAAACCCAACTTCTGGCATTCCAAGGCGCATT
TTCCGCATTTTTGGACGGCTTAATCGTTAAGCCGCTCCGAGCTCCGAGTTCCGACCAGACCAGACCCAAT
GGTTCCATCCATGGCGAACCGAAAGTAAAACGCTGCTCAGGTTGACTTTGTTCGGCTGCCGCTTCTTTCT
GCACTCGTAGACAACTTAATTACGGTTATGTTTCGCCTCAGACATGCAACATAATTTGTATCATTAGGCA
CTTTGTCTGCCGCGACGAGAGCAGAGCAACACACACTTATTGTATCTGTATCTAAAGTATAACATTCTTG
TCATTAATTCGTCATGTGCTATTTACTATAAAATTTTTACTTTTCATTCCTCCACTTCGGTGTTCCGTTC
CAAGAACGGACCGTAATTGTTCAGGCCTGGCCTGTACAAATAAACCAAGTACCGCCAGAGATATTCCTCA
GAAATCCATGGAAGCCGCTTATAGCAGACGCCTCGATTCTCCATTACTTCCAGTCTAGTCATGCCGAAGA
ACTCAGACAATTGTCGGTTTATTAGCGTATGTTTTCAATCCGAGTCTAACCTGATTTCACTTCAAGTTTC
TGAGGGGGATTGAGAGTGAGAAGTGTGATCTTTACCTGTCCGATCGCATTGCACTCAATGATCCGAAACG
AAATATATCTGCAGCTGATGTAGCAGGTGTGCTACTGCTTTCAATACCCTGTACTCAACTGTGACACTGA
GTAAATGCAATCAGGTCCACGATAACCCTATTTATTTCATTAATATATATATATATTTATTTAGGTCAACAT
GATAGTGATTTTAATTATTGAATAGCTGATATGATAACTATGTAAAGTTACCTTCAAACTATTCAAAGCA
TAAGAATATATTTCCTGGGTATTTCCAAGTTGCGACTATCACAAAATGTTTCCTTAATTATGGCTGCAGG
TCTAATGACTGTGTAAAAATGGACAAGGTCGCTTCTAATGACCTGCATATAAACAGAAACTTCTGTTCTC
CTAGTTTGGTCACGATTCGGTCATTAGGGCAGGGTTTACGTTCTACCCGACAATTACTTAGCCAAGCCAA
GTGATCCATATTATTATGTCTGGCACTAAAACCATTTCTATTATTCCTCTTTGCAGAAGTCAACGATCGG
ATAATCTCGGTGAACGGCGTCTCTCTGGAGAACGTGGAATATGCTACCGCGGTGCAGGTGTTACGCGATA
GTGGCAACACGGTGCAATTGGTGGTCAAGCGTCGCGTGCCCCTCAATCCCATTAATGCTGCAGGTGCAGT
GCAGCATCAGCATTCACACAGTCTCAGTTCGGTTGGTCTAATGGCCAATGGAAGTGGAGGAGTGGCGCCA
ACCCCCATTACGAGTCTGAGTCAACCCAACTCGCTGAACTCTTCCCTAGTGCAGAATGCCAGCAGTGGTC
AGCCCATCAAGGTGACCCTCACCAAAGGCGGAAAGAAAGATGACTATGGTGTGGTGCTCGGCTGCCGGCT
GTTTGTTAAGGAGATCTCCTCTAAAGCTCGCGAGCAACTAAATGCCAATGGTTATAGCTTGCAAGAGGGT

GACATCATCACCCGCATCCACAATACTAACTGCGGGGATACGATGAGTCTTAAGGAGGCGAAAAAGATCA
TTGATGGCTGCAAGGAGCGATTGAACCTAGTGGTTCTAAGGGACATTACCAACCAAACAGCTGTTAGCCA
ATTGAATCTGAACAATTCAGCCAGCCACCAGGCGTCGGGAAATATATACGCCACGCATCAGCCTCAGGTA
TCCGGATGTAGCAGCAGTAACAACAATCTTGAAGATCCATATCTGCCGGGCGGAGCCAGTTACTCCTCGC
AGAATCTGTACGTGCAACCGCCAACTCGCACCTCCAATGGTCCCAATATCAATGGCAATGGCCTGAACGA
CGAAAAGAGTAATCTTACGCCGCGAGGTCGCTCCCGGGGCCCCATAATGGATGGGGTATCCCTGCAGCAA
CTGGATAGACCCGTAACACCGACTCGGGGCAGGAGTGCAGCCATCGACGAGCCGCCGCGTCCACCACCAC
CAAGGGGATCGAGCGGAGGAGCAGCTCAAGAGGATTTCTACAGCTCTCGCAGGCAATTGTACGAGGAGCG
TCAGAGTGCCGAACCGAGATTCATTTCCTTCCAGAAGGAAGGAAGCGTAGGCATCCGGCTAACCGGCGGC
AATGAAGCTGGTATTTTTGTGACTGCCGTACAACCAGGATCTCCAGCCTCGCTGCAGGGTCTAATGCCTG
GCGATAAGATCCTCAAGGTCAACGACATGGACATGAACGGAGTGACGCGCGAGGAGGCTGTTCTCTTCCT
GTTGAGTCTCCAGGATCGCATCGATCTGATTGTGCAGTACTGCAAGGAGGAATACGACGAGGTGGTGACC
AACCAACGTGGCGACTCCTTCCACATCAAGACACATTTCCACTGCGATAATCCTTCCAAAGGCGAGATGG
CCTTCAAGGCGGGCGATGTTTTCCGGGTGATCGATACCCTGCACAATGGTGTGGTCGGATCCTGGCAGGT
GCTGAAGATCGGTCGGGGTCACCAGGAGATGCAGCGCGGCGTAATACCGAACAAGTCGCGGGCTGAAGAG
CTGGCCACTGCTCAGTTCAATGCCACCAAGAAGGAGATGAATGCAAATGAATCGAGAGGCAACTTCTTCC
GGCGACGACGGTTCGACGCACCGACGCTCGAAGAGTCTGTCCCGCGAGAACTGGGACGATGTGGTCTTCT
CCGATAGCATTTCCAAATTCCCCGCCTACGAGCGTGTCGTTCTGCGCCATCCAGGCTTTGTCCGACCCGT
AGTGCTCTTCGGACCCGTATCCGATTTGGCCCGCGAGCGATTGGCCAAAGATTTCCCCGATAAGTTTTCG
ACTCCACTGCAGGATGACGACAAGTCAGCCGCAACTAGTGGAAAGTGCCGCATCGTAAGGCTCTCGAATA
TACGCGATGTGATGGATCGTGGAAAGCACGCCCTGCTGGACATTACGCCCAATGCAGTGGATCGTCTCAA
TTACGCGCAGTTCTATCCGGTGGTGATCTTCCTGAAGACAGACAGCAAGCACGTGATCAAGCAGCTGCGT
CACGGACTTCCCAAGGCGGCACACAAGAGCTCCAAGAAGCTGCTGGAGCAGTGCCAGAAACTGGAGCGCG
TCTGGTCACACATCTTTAGCACCCAGATCGCGTTGAGCGACGAGGAGTCCTGGTACCGAAAGTTGCGCGA
TTCGATCGATCTGCAGCAGAGCGGCGCCGTGTGGATGTCCGAGTCTAAGGGTGAGTCTTTTCGTTTTCAC
GTCCGGATTTTAAGTCCATCCTAGTTGTTGTCTGAGTATTGTCCAATGTAGTTAGTCCTTAAGTCTTATT
AAAACCAAAGCATCTATTGGATGTCGTGGCTCCATATGATAACTTCGTAATGTCTAACTTAACGATGTCC
TAACTGTCCAAGTAGGTGATGGATTAATAAATGTAGAGAATTATGTTATAGGAGAGAAATATTTAATTCG
AAAGTTTTAAGTTTCCATTAACCACATAGAAACCTTGTTTTGTTTACAAAATTTATGTAGGCATATTGCG
AGAATGTCTTTTCAAAATAAATGATTCTATTAGATCAATTAACGTGTGAACACTAATGAACATGAAAACT
TTAGAAAACAATATTAAAGAAAGGAATGTTTTTCAGTTTATTTGATATAGTTTTAGTTATACCGATGATA
TCCCAGCCCAATAGGGTTTTCCAATATTAAATAATCTTATTTATCGAATGGACTCCTTAACTAATTTGAA
ACCCCTATAAATCTATAATTTCAACAAATGGTTATGGTCAATATTCTAATTTTTACTTTCGATAATTTTT
CTTATTCGTTTTTTAAAACACACAACCACACACACTTACAAACATGCCCATGATTGTTGTCAACGAACCG
TATaaaaataaaaacaaaaaCATATTAACAGTTCCCCGATTCCCCCACCGAAATGTTATTCCCCCCCTAC
ATCGCCCAGCCATGCAAGTTAGCTTGTTGCCGCCCCATATCATCTACCTCAGTGCCACGCTACTCGCAGG
ATATGAATCTGAATCTAAACCAGAATCCAAGTAAATCCTATTCACTAATCCAGAACCAGGCACATACCCC
TCCGCCGCCACGCCCACCGCCCGTGGGTTATGCCCACAGCCAGAGTTTGGCCGAAAAGCCCCGAGATCGC
AGGTCTTATACGATGGTGCCGATCATAAAGCCCGCTTCTTCGATGCCCAGTGGTCTGGCACCGACCGTTG
AGCCTGCTGCTCCCATGATGCCACTAGACGATTGTGTTCTGGCCATGGATGGGTCCAACTCCCAGTATCA
TAGTTCTCAGCCAAATCTAATGAGTCAGAGTTATTTGAACTCGAATTTAAATGGCGGCAGTACGAGTGCC
GTGGATCAAACGAATCACTCAAACGCCATGAGGGGCTTTCAGCCCGGACCATCGGTAGTCAGCCGCTATG
GATTGCGGTCCAATGCCCAGGTGGAACGTAGGACCACTCCCTACTACTACCACGAGCTGCTGCTGCAGAA
GAATCTCTTCGATTCCAGTCTGAATCTTCTGCCCcagcagcagcagcagcatcagccggatctggccaag

agcaagaacagcaatgtgcagctggagcagcaacaacagcagcagcagcagcatcagaagcatcttgcag
atgcatcgaatgcatcCAAAAGGGCACCGATGGCCAAGCCACAGCAGCGTTTGATTAGCAATGGTCAGGG
CAGGAGCAACTACTCCTCCTCGGGGTCACTGAGCAATCTTCTGGGCAATGGCCATGGGCTGAGCAATGGT
CACGAGAGCAGCGATGGCACTTTGGTGGATGAGGGCAGCTTTACACTGGCCTTCAATAATCTCACCAATC
AAATAGCCAGTCTCAATAATTCAAGTAGTCTAAACAATAATAATTGTAGCACCAGCAGGAGCATCAAcaa
caacagcaacagccataatattaatgccaacaacaacaatagccatgttaataatgcTGATTCCAACAAC
AATGTTGGCCAAAGTAACGAATGCATTGCATGAGAATCGGTGTTAATTTAGTTGAAAAGGTCCAGAAAAC
GAGATCTAATTTATAGATCTGATTTATTCCGATCTTGGGCCACAAGACTTTTCAACTGAATCGTATCTCC
TCTATCCTTCCTATCAGCCCCTTCTTTGTGTAATTATCGCATGTAGTTGTTTGTACTAAATTATTCATAA
GACCCAAATCTTCACATGTAGCATGATCAAGAACAATAATATTGCGTGTAGCCCGCTATTCGTTGAATAT
GTATGTACATATAAATGTGTGTAATCGTGAAGCTAAAAGCATGTAGAGGGATCACAACTACCACATAATC
CTCCAACAGCACGAAAATTGTCATTCCAGGGAATGCTTAAAGTACAATATTTAATCTCAAGAGATCTAAG
ATCTGCATTTATACGAATTTGAGTACTTTTATCTTCTCACATTTCCACTTTGTACTCCCAACGAATGACA
GTTTTCCAACTCCAAACGTACTCATTACCAATTCTATCGAACCAATTTGACATACATATTTAGGCTTAAG
TATTTACGCAAAAATCGCCATAAAAATTTTTGCCAATATTTTTATAAAGTTGCTAACTAAATGTTTTTCG
TTTCATTTGTTTCTTTTATTTACATACCAAAACCAAATATATATCATCATGGTCATTCCCCGCTTAATCA
CCCGTAAAACCCGATGCATATAATcaaacaaaccaaacaccacaaaaataatcaaaatgtccttgtccaa
aactaaaaacaaaaaccaaacaacataatatactcaaaatgccaaataTCTTGTGTTCGTCCACACATTA
CGTATCCAAATGCTTCGCAATCGAACGCCTTTCAATCAACCGAATCGTATCGAATCAATCATATCTCTTT
CAATGTTTCATAAACGTTTCTTGTTGCTGTTCTTGCGTTTAAATTGACAACAATTGCaaaaaaaaaTGT
TGTTCGCTCACAACGCGCTAAATTTTGAACATTCGaaaaccaaacaaaaaaccaaaaaaaaaacTATCGC
TAAAAATCAATTAACTTTTGCCAAACAATTGCGCTCAATCCTGCAAACTACCAAACCCCAGCCAGTAGAA
TCCCTCTCCGACGATTTCCTATTCCCCATGACCACATCCCGACTCTCGTATGCATCAAGTCCCGAATCCG
ATTTGGAACTGAGTCCCGGCCCATCCGCATCATTGTCGCTTGGCAATTTGCCGCAGTTGGTTAAAGCGAG
CTCAGATCCATCGATTGCCACTAATCAGGATAACTTGGATAGGGATAGAGACATAATTGGTGAAGGACTA
CCACCTCCATATACGGTAAGAGGTCGaaaaaccaaccaaccaaacaaacaaacaaacatacaaaacaaaa
aaaactcaaacTAAGAAATGGCATGCGCTTACACTaaaatcaatcaaaagaaaaaacaaccaaaaacTTG
GGAAATTTCTAAGCGTCCATCCAGTTCTCATCAAAATAACAATGGTCAACAAAAATAAAGAAAATATTAC
GTGAAATTCAAAAGAGACATTCTAAAATCAATATTTTCCATAATAGCTCTCATGAAAGTTTATTTTTGCT
GACTAGTGTGCTAATCTAATTCACCACCCACTCACGAACCCCTTGCAATGCACCTGCTGCAGGGTACCCT
ACGACCATGCTGTCCCCGCGAATCCCAATCGCCGCCAGACCATGGACTCCAGCAAGTACAGCATCTACGG
CACCAATGTGCCACCACAGCAGCAGCAGCCCGGCGTTGGAGGAGATACGGCTGCAGTGAGACCGCAATCT
CTGTACGGGATTAATGCTCCCGATCTGCCGCCCCGCATCGATCGTCAGTCCAAGCCGGGCGATATACCAC
TGAACACCTCGGGCTCATCGTCGCGAAATGGCACCCTGGGTCGAAGTGCCCAGGAGCGTCTGTTTGGCAA
GGCTGTGGTGCAGGACGATGTCCAGGCGGAGTACATAACCCGAAACGCTCTGGTCGGGTCTGGAGCTGCA
GAAACCCTGGATCGCcagcagcagcagcagcagcagACTCATGCATCCTTGGAGCGCCAAGCGCGTTTGA
ATGCGCAGCTAAAGGCCAACGGAGTAGGCGCCGGAGGAGGTGGTGCATCCACCTACGACAGTGTGTCCAG
CTACGACTCGTACAACAATACACAAATGGCCATGCAAAACCTGGGCCGACTAGGTCCCAATGCACCCGAC
GATCTCAAATCAGTGCCAAATGCAAAGTGGTCGCCCCTTGCCTCCGACGGGTCAGTCGCACGAGTACGGT
CGCACTCCGCACGATCATCGCAGCTTCGGAGGACCCAACGATCTGAACCGGCAGAGCAGTCCGGGCAGGC
CGCACTACCATGACATGAATGCGTCCCGTAATATCGGTGAGTAGCTCTATATGCCATAGTTTATTCAAAA
CGAACTAACATATTTCATATCATTCGACAGATCCACGCAATGGCACACCACAGCGTCCTTCGAATCTGGG
CCTGGAAAGCAGCCCACGCAAGCCTTTGGTGGAGACAAAAACGGATTACGGCAAATACAGG
>dme_circ_0008269_junction_seq

GACAAAAACGGATTACGGCAAATACAGGGGTGATCGAACCACATGGGAGTACCACA

>dme_circ_0008270

GGTGATCGAACCACATGGGAGTACCACACGGTGGCGGTGACTCGGGTGCCGGGCTATGGATTCGGCATCG
CCGTTTCCGGTGGACGTGATAATCCGCACTTCGCCAACGGCGATCCCTCGATAGCAGTTAGCGATGTGCT
GAAAGGTGGCCCCGCCGAGGATCGATTGCAGGTAAGTACCCTCGCGATGATTCAACAGAAGTTACTGACC
CTGTAGGAAGTAAACCTTTCCAATGCATTTTGAATTATTATCTGACAGGAAACTAATTGTTGTTCCCGAA
TAAGTCCGACATGCTGAATAGACCCCCATGCAGGCTGATCAAATAAAGCATATAAGAACTAATCATCCAC
TGCTCCAAACGAAAAACGGGCATTATAAATTCCCGACATGCTGCTGACAATTGACATCGTTAATTTTGCG
CTTAGAACCTATACTTTTATATACCCACTTCTACAAATATAAACCCGAAATGCGCACACTTCAATACTTC
AAACTCACGTAGAGTCGGTCCCCAAAAAGAAATGGTATTTAATTAAACCAACGTCTCGGCAACTCGACAA
CTCGGTCCCTCTGAAGCTGTTTCTCTCTTGGCGATAATAAAAATTCCAAAGCGTTTTTCAATAGACAGCG
CAGAGGCGGCCCTAGTGATTACGAACTTGTCATAATCttttttttCCCAAGAAACTATTTACCCTGGCCC
AGACATGCAGAGAACTATTTTCACGTTGTCCCTTACCTGCGACTGCCGGAGGATTTCAAAAGGAAAATCC
GCTGCCGAGGCTGGAAAATTGTTGGCCAAGTGAGCGGAGACAAGGGGAAATTAGCTGCAGCCCAGCACAT
GTATTTCTGAAAAGTTGGCTAAGAGCATGTGGTCAAAGTTTCGCTGTTACGCAGCTCAGTAACTAGTACA
CACTCAGAAAAAATATTACTTCGCATTGAGACATTCAAAAATAAGttttttttCTATTACCATCCTAACC
AGGATTACAAACAACTTACCTACCTAATTTTTAATGAAATAAATCAAAACTTTTATATTTTGTTGACTTG
CGAAACACATTTTTCCTCAGCACATAACAGGGTTTTTCCAAACCCAACTTCTGGCATTCCAAGGCGCATT
TTCCGCATTTTTGGACGGCTTAATCGTTAAGCCGCTCCGAGCTCCGAGTTCCGACCAGACCAGACCCAAT
GGTTCCATCCATGGCGAACCGAAAGTAAAACGCTGCTCAGGTTGACTTTGTTCGGCTGCCGCTTCTTTCT
GCACTCGTAGACAACTTAATTACGGTTATGTTTCGCCTCAGACATGCAACATAATTTGTATCATTAGGCA
CTTTGTCTGCCGCGACGAGAGCAGAGCAACACACACTTATTGTATCTGTATCTAAAGTATAACATTCTTG
TCATTAATTCGTCATGTGCTATTTACTATAAAATTTTTACTTTTCATTCCTCCACTTCGGTGTTCCGTTC
CAAGAACGGACCGTAATTGTTCAGGCCTGGCCTGTACAAATAAACCAAGTACCGCCAGAGATATTCCTCA
GAAATCCATGGAAGCCGCTTATAGCAGACGCCTCGATTCTCCATTACTTCCAGTCTAGTCATGCCGAAGA
ACTCAGACAATTGTCGGTTTATTAGCGTATGTTTTCAATCCGAGTCTAACCTGATTTCACTTCAAGTTTC
TGAGGGGGATTGAGAGTGAGAAGTGTGATCTTTACCTGTCCGATCGCATTGCACTCAATGATCCGAAACG
AAATATATCTGCAGCTGATGTAGCAGGTGTGCTACTGCTTTCAATACCCTGTACTCAACTGTGACACTGA
GTAAATGCAATCAGGTCCACGATAACCCTATTTATTTCATTAATATATATATATTTATTTAGGTCAACAT
GATAGTGATTTTAATTATTGAATAGCTGATATGATAACTATGTAAAGTTACCTTCAAACTATTCAAAGCA
TAAGAATATATTTCCTGGGTATTTCCAAGTTGCGACTATCACAAAATGTTTCCTTAATTATGGCTGCAGG
TCTAATGACTGTGTAAAAATGGACAAGGTCGCTTCTAATGACCTGCATATAAACAGAAACTTCTGTTCTC
CTAGTTTGGTCACGATTCGGTCATTAGGGCAGGGTTTACGTTCTACCCGACAATTACTTAGCCAAGCCAA
GTGATCCATATTATTATGTCTGGCACTAAAACCATTTCTATTATTCCTCTTTGCAGAAGTCAACGATCGG
ATAATCTCGGTGAACGGCGTCTCTCTGGAGAACGTGGAATATGCTACCGCGGTGCAGGTGTTACGCGATA
GTGGCAACACGGTGCAATTGGTGGTCAAGCGTCGCGTGCCCCTCAATCCCATTAATGCTGCAGGTGCAGT
GCAGCATCAGCATTCACACAGTCTCAGTTCGGTTGGTCTAATGGCCAATGGAAGTGGAGGAGTGGCGCCA
ACCCCCATTACGAGTCTGAGTCAACCCAACTCGCTGAACTCTTCCCTAGTGCAGAATGCCAGCAGTGGTC
AGCCCATCAAGGTGACCCTCACCAAAGGCGGAAAGAAAGATGACTATGGTGTGGTGCTCGGCTGCCGGCT
GTTTGTTAAGGAGATCTCCTCTAAAGCTCGCGAGCAACTAAATGCCAATGGTTATAGCTTGCAAGAGGGT
GACATCATCACCCGCATCCACAATACTAACTGCGGGGATACGATGAGTCTTAAGGAGGCGAAAAAGATCA
TTGATGGCTGCAAGGAGCGATTGAACCTAGTGGTTCTAAGGGACATTACCAACCAAACAGCTGTTAGCCA
ATTGAATCTGAACAATTCAGCCAGCCACCAGGCGTCGGGAAATATATACGCCACGCATCAGCCTCAGGTA
TCCGGATGTAGCAGCAGTAACAACAATCTTGAAGATCCATATCTGCCGGGCGGAGCCAGTTACTCCTCGC

AGAATCTGTACGTGCAACCGCCAACTCGCACCTCCAATGGTCCCAATATCAATGGCAATGGCCTGAACGA
CGAAAAGAGTAATCTTACGCCGCGAGGTCGCTCCCGGGGCCCCATAATGGATGGGGTATCCCTGCAGCAA
CTGGATAGACCCGTAACACCGACTCGGGGCAGGAGTGCAGCCATCGACGAGCCGCCGCGTCCACCACCAC
CAAGGGGATCGAGCGGAGGAGCAGCTCAAGAGGATTTCTACAGCTCTCGCAGGCAATTGTACGAGGAGCG
TCAGAGTGCCGAACCGAGATTCATTTCCTTCCAGAAGGAAGGAAGCGTAGGCATCCGGCTAACCGGCGGC
AATGAAGCTGGTATTTTTGTGACTGCCGTACAACCAGGATCTCCAGCCTCGCTGCAGGGTCTAATGCCTG
GCGATAAGATCCTCAAGGTCAACGACATGGACATGAACGGAGTGACGCGCGAGGAGGCTGTTCTCTTCCT
GTTGAGTCTCCAGGATCGCATCGATCTGATTGTGCAGTACTGCAAGGAGGAATACGACGAGGTGGTGACC
AACCAACGTGGCGACTCCTTCCACATCAAGACACATTTCCACTGCGATAATCCTTCCAAAGGCGAGATGG
CCTTCAAGGCGGGCGATGTTTTCCGGGTGATCGATACCCTGCACAATGGTGTGGTCGGATCCTGGCAGGT
GCTGAAGATCGGTCGGGGTCACCAGGAGATGCAGCGCGGCGTAATACCGAACAAGTCGCGGGCTGAAGAG
CTGGCCACTGCTCAGTTCAATGCCACCAAGAAGGAGATGAATGCAAATGAATCGAGAGGCAACTTCTTCC
GGCGACGACGGTTCGACGCACCGACGCTCGAAGAGTCTGTCCCGCGAGAACTGGGACGATGTGGTCTTCT
CCGATAGCATTTCCAAATTCCCCGCCTACGAGCGTGTCGTTCTGCGCCATCCAGGCTTTGTCCGACCCGT
AGTGCTCTTCGGACCCGTATCCGATTTGGCCCGCGAGCGATTGGCCAAAGATTTCCCCGATAAGTTTTCG
ACTCCACTGCAGGATGACGACAAGTCAGCCGCAACTAGTGGAAAGTGCCGCATCGTAAGGCTCTCGAATA
TACGCGATGTGATGGATCGTGGAAAGCACGCCCTGCTGGACATTACGCCCAATGCAGTGGATCGTCTCAA
TTACGCGCAGTTCTATCCGGTGGTGATCTTCCTGAAGACAGACAGCAAGCACGTGATCAAGCAGCTGCGT
CACGGACTTCCCAAGGCGGCACACAAGAGCTCCAAGAAGCTGCTGGAGCAGTGCCAGAAACTGGAGCGCG
TCTGGTCACACATCTTTAGCACCCAGATCGCGTTGAGCGACGAGGAGTCCTGGTACCGAAAGTTGCGCGA
TTCGATCGATCTGCAGCAGAGCGGCGCCGTGTGGATGTCCGAGTCTAAGG
>dme_circ_0008270_junction_seq
GGCGCCGTGTGGATGTCCGAGTCTAAGGGGTGATCGAACCACATGGGAGTACCACA
>dme_circ_0008271
GACTCGGACATCCACACGGCGCCGCTCTGCTGCAGATCGATCGAATCGCGCAACTTTCGGTACCAGGACT
CCTCGTCGCTCAACGCGATCTGGGTGCTAAAGATGTGTGACCAGACGCGCTCCAGTTTCTGGCACTGCTC
CAGCAGCTTCTTGGAGCTCTTGT
>dme_circ_0008271_junction_seq
TGCTCCAGCAGCTTCTTGGAGCTCTTGTGACTCGGACATCCACACGGCGCCGCTCT
>dme_circ_0008272
GGAAAAGGAAAACGATCGCAATCGTCGCGCTGTTAGTTATGGCGGTGACGCCACCTCCTCCCCCCATTCG
GCCACCATACCCCGCGCCGCCCCCCGTCGGGTGGCCTTGATGAAGGATCCACCGCCACCGCCGCCACCCA
AGCCGCAATTGGGTTCCAAAGCGGATTCTGTATCGCATTTGGCCAGCAAATATCCCCAATCCGAGTACAA
TCTCATACAGAAAATCGACTCGAACAGCACACTCACTGCTCCGGCGCAATATCAACCGCAGAATTTCTAC
GCTAATACTGGCACCATATCCTCTACGAATGGCTACGGATCGCTGTTATGTCACGCCAGTTCGACGACCA
GTCCGCTGGCGGTCCGGAAGAGGGACAAACTGCTTCATCGCTTCAGCGATGCCGCCACCTTGGGCCGGAA
GCTCAAGAAGAAGAAGAACACGAATCGCACCTGCAGATCCATGACGGAGGCCATTGAGATGCTGGCCGAT
CCCGTCATCGAAGATGAATTCTTTG
>dme_circ_0008272_junction_seq
GATCCCGTCATCGAAGATGAATTCTTTGGGAAAAGGAAAACGATCGCAATCGTCGC
>dme_circ_0008273
TTCAAACCACAATTTTTGGAACCATAAGAATTGTTCGCACTTACGAAATAGTGCTAATGGGTTATCCTTT
AACCAGGCGCTTCAGATTGGTAAAGAGCCGCAACGTGAAGTGGTAGATCATTTCCACAAGACTCAAGATG
GAGAACCCCATGAATAGACCGAAAATGCCACCGCAATTCGCCAGAAACTCGGTCATTCCGTAAAGCTCCG

ACCGCTTTGAAGTGATGAACTGACTCTGCTTGAAGTAAATGGACAAGCGGGACATCTGGGAGCCGGGATA
CTCCTTCAGGAACTCAGTGTCGCCCTCAGCCACTAGCATTTCTTCCAAATCAAAGTTGGCCTGGGAAATC
TCCGTGTTGTAGACGAGGGATGTGCATGCTGGCATGCAGTTACAGGCAGATTCCACGCTCCGCGAATTTT
CTCGGCCAGCGTTCAGCGCTTTTACTCTTTTGAATTCCCTGACTAACAACTCCCTTTCTGCCCGATCGTA
GCAGTGGATCTTGTCCTCTCCGCATACAGGCATGTCCACGTTTCTGGGCATCGAGAACTTAACGCATCCG
CACTTGGTCAGCGTGAAGTTGGCCAAGCACTCCAG
>dme_circ_0008273_junction_seq
TCAGCGTGAAGTTGGCCAAGCACTCCAGTTCAAACCACAATTTTTGGAACCATAAG
>dme_circ_0008274
GCACCAATACTTCCGCCAAAACCTATTATCTTAAGTCCGTGAAAAAGCGGGTGACCACGTGTTTCCAGCA
GTTGCGCGATAAACTCCAGTCATCCGGTTCCTTTCGCAAGAGTTCCTCCAGCTGCCTCAACCAGATCTTC
GTAAG
>dme_circ_0008274_junction_seq
CCAGCTGCCTCAACCAGATCTTCGTAAGGCACCAATACTTCCGCCAAAACCTATTA
>dme_circ_0008275
GTTCCACTGGTTAAAATCGTTGGCGTGGGCGTCATTTCGTGGCGTCTCTTCATTATGTTCATGCCGTTAA
ATGGATGCACAAAGATGCCCAAATGGCGGTTTTCAGTCGGTTCGGTTTTCTGGCTTTGGGTTCTGCAGCT
TGGCTGATGCCCAGGTGGAGCCAAATTGATTGG
>dme_circ_0008275_junction_seq
GATGCCCAGGTGGAGCCAAATTGATTGGGTTCCACTGGTTAAAATCGTTGGCGTGG
>dme_circ_0008276
GATCATCGTCAATATCGGATCGGTGGACATTATGCGCGGCAAGCCTTTGGTTCAGATCGAGCACGATTTT
CGGCTACTGATCAAGGAGATGCACAATATGCGATTGGTGCCGATTCTAACAAATCTTGCACCGCTGGGCA
ACTATTGTCACGATAAGGTATTATGTGACAAAATCTACCGATTCAACAAGTTTATCCGAAGCGAATGCTG
TCACCTAAAG
>dme_circ_0008276_junction_seq
TATCCGAAGCGAATGCTGTCACCTAAAGGATCATCGTCAATATCGGATCGGTGGAC
>dme_circ_0008277
ACAGGAGTGTATGTCGATGACCTTTAGGTGACAGCATTCGCTTCGGATAAACTTGTTGAATCGGTAGATT
TTGTCACATAATACCTTATCGTGACAATAGTTGCCCAGCGGTGCAAGATTTGTTAGAATCGGCACCAATC
GCATATTGTGCATCTCCTTGATCAGTAGCCGAAAATCGTGCTCGATCTGAACCAAAGGCTTGCCGCGCAT
AATG
>dme_circ_0008277_junction_seq
CTGAACCAAAGGCTTGCCGCGCATAATGACAGGAGTGTATGTCGATGACCTTTAGG
>dme_circ_0008278
GATTCTTGGCCTGGCTCCCATCGCCGTCCAGCAGCTGAGGAACCTCAATGTCCAGGAACACGTTTCCTAC
AGTCTGCTCAATGAGGCCAAGATCCCAACACCTCGGTGAGTAAACCCAATCGGAATTATCAAAATCGCGG
GCAAACAACCAACAATTTGCGTCAAAGTCGCAGTCCAAAGTTCAATGAAAGTGCCGCGTTGTCGTTGCGT
CGTCTGTTTCCTTTGGCTTATGTGCGTTTTGGACTTTGCCTGAACTTAATAATATGCATTGAGTATAGCC
GACTACGGGGTAAACAAACCAGAGAAACGCAGCCGCCGATGATAAGGGTGATAACAAGTTTTATACTATA
ACACACTTTGATCGGCGTAAAGTGACAAGGGTCAGTGACGTGGGCTTTGATAAGATGGAAGCGGCATTAG
TTGACGTCTATCATAATTAAACTAACTAAATATACATAAACTTTGGGTAAAGAAAATATATTTTAATGTT
TAAAATACCTGTAATTTAAATTTAAGCAATTGCAAAGCTAGAATATTTTGATTTCCATTTCGTTTGATAA
ATTGAGATTATTAGAATCAAAAGATAGTAGTTGCTTTTGCTATTATTCAGGTAAAATCAGGCGACTGATT

TATTTTCTAAATGAGGGCTTGTCATCTCTCGAATGACCGAATAACCTTGTCTCAACTTTCAACCTTTGTG
GTCTACTGATAACACGATTTACGTGAAATGTTCCTATCAACAATCAATCAGGAACATCGTAAAGAGCGCA
CCCCCAAAATTATTTCGCGTACTGAGTGATTCATTTTTTTGCAGGTTTCGCTGTTGCCAAGAACGGCAAG
GAGGCCAACGATATCGCCACCAAGCTGAAGACCGATAACCTGGTGCTGAAGGCTCAGGTTTTGGCCGGAG
GACGAGGCAAAGGAACCTTTAAGAATGGCCTGAAGGGAGGTGTGCGAGTGGTCTACGAGTCCCCAGACCG
CTGAAGAACTTTCAAGCAAAATGATCGATCAGCTTCTGGTCACCAAGCAAACCGGAGCAGCCGGTCGCAT
CTGCAAAAAGGTGATGGTTGCCGAAAGGAAGTTCCCCCGCCGTGAGTTCTACTTCGCCGTGATGATGGAG
CGTGCCTTTAACGGGTCCCGTGCTGATCGCCTCCAAGGAGGGTGGTGTTGATATTGAGGAGGTGGCTGCC
AGCAGTCCAGATGCCATTTTGTACGAACCCATCGATATAGGCACTGGCTTGACCAGTGAACAGGCCGAAA
AGATCGTCAAGAAGGTTGGTCTGGGCGGCGATGGCGAGGACACACATGTCCAGATGCTGTTGAATCTGTA
TGACCTGTTCGTCAAGAAGGATGCCCTCTTGGTTGAGATCAACCCCTATGCCGAGGATGCCATGTCCGGC
T
>dme_circ_0008278_junction_seq
CCCTATGCCGAGGATGCCATGTCCGGCTGATTCTTGGCCTGGCTCCCATCGCCGTC
>dme_circ_0008279
GCTGAGGAACCTCAATGTCCAGGAACACGTTTCCTACAGTCTGCTCAATGAGGCCAAGATCCCAACACCT
CGGTGAGTAAACCCAATCGGAATTATCAAAATCGCGGGCAAACAACCAACAATTTGCGTCAAAGTCGCAG
TCCAAAGTTCAATGAAAGTGCCGCGTTGTCGTTGCGTCGTCTGTTTCCTTTGGCTTATGTGCGTTTTGGA
CTTTGCCTGAACTTAATAATATGCATTGAGTATAGCCGACTACGGGGTAAACAAACCAGAGAAACGCAGC
CGCCGATGATAAGGGTGATAACAAGTTTTATACTATAACACACTTTGATCGGCGTAAAGTGACAAGGGTC
AGTGACGTGGGCTTTGATAAGATGGAAGCGGCATTAGTTGACGTCTATCATAATTAAACTAACTAAATAT
ACATAAACTTTGGGTAAAGAAAATATATTTTAATGTTTAAAATACCTGTAATTTAAATTTAAGCAATTGC
AAAGCTAGAATATTTTGATTTCCATTTCGTTTGATAAATTGAGATTATTAGAATCAAAAGATAGTAGTTG
CTTTTGCTATTATTCAGGTAAAATCAGGCGACTGATTTATTTTCTAAATGAGGGCTTGTCATCTCTCGAA
TGACCGAATAACCTTGTCTCAACTTTCAACCTTTGTGGTCTACTGATAACACGATTTACGTGAAATGTTC
CTATCAACAATCAATCAGGAACATCGTAAAGAGCGCACCCCCAAAATTATTTCGCGTACTGAGTGATTCA
TTTTTTTGCAGGTTTCGCTGTTGCCAAGAACGGCAAGGAGGCCAACGATATCGCCACCAAGCTGAAGACC
GATAACCTGGTGCTGAAGGCTCAGGTTTTGGCCGGAGGACGAGGCAAAGGAACCTTTAAGAATGGCCTGA
AGGGAGGTGTGCGAGTGGTCTACGAGTCCCCAGACCGCTGAAGAACTTTCAAGCAAAATGATCGATCAGC
TTCTGGTCACCAAGCAAACCGGAGCAGCCGGTCGCATCTGCAAAAAGGTGATGGTTGCCGAAAGGAAGTT
CCCCCGCCGTGAGTTCTACTTCGCCGTGATGATGGAGCGTGCCTTTAACGGGTCCCGTGCTGATCGCCTC
CAAGGAGGGTGGTGTTGATATTGAGGAGGTGGCTGCCAGCAGTCCAGATGCCATTTTGTACGAACCCATC
GATATAGGCACTGGCTTGACCAGTGAACAGGCCGAAAAGATCGTCAAGAAG
>dme_circ_0008279_junction_seq
TGAACAGGCCGAAAAGATCGTCAAGAAGGCTGAGGAACCTCAATGTCCAGGAACAC
>dme_circ_0008280
GTTCCTTTGCCTCGTCCTCCGGCCAAAACCTGAGCCTTCAGCACCAGGTTATCGGTCTTCAGCTTGGTGG
CGATATCGTTGGCCTCCTTGCCGTTCTTGGCAACAGCGAAACTGCAAAAAAATGAATCACTCAGTACGCG
AAATAATTTTGGGGGTGCGCTCTTTACGATGTTCCTGATTGATTGTTGATAGGAACATTTCACGTAAATC
GTGTTATCAGTAGACCACAAAGGTTGAAAGTTGAGACAAGGTTATTCGGTCATTCGAGAGATGACAAGCC
CTCATTTAGAAAATAAATCAGTCGCCTGATTTTACCTGAATAATAGCAAAAGCAACTACTATCTTTTGAT
TCTAATAATCTCAATTTATCAAACGAAATGGAAATCAAAATATTCTAGCTTTGCAATTGCTTAAATTTAA
ATTACAGGTATTTTAAACATTAAAATATATTTTCTTTACCCAAAGTTTATGTATATTTAGTTAGTTTAAT
TATGATAGACGTCAACTAATGCCGCTTCCATCTTATCAAAGCCCACGTCACTGACCCTTGTCACTTTACG

CCGATCAAAGTGTGTTATAGTATAAAACTTGTTATCACCCTTATCATCGGCGGCTGCGTTTCTCTGGTTT
GTTTACCCCGTAGTCGGCTATACTCAATGCATATTATTAAGTTCAGGCAAAGTCCAAAACGCACATAAGC
CAAAGGAAACAGACGACGCAACGACAACGCGGCACTTTCATTGAACTTTGGACTGCGACTTTGACGCAAA
TTGTTGGTTGTTTGCCCGCGATTTTGATAATTCCGATTGGGTTTACTCACCGAGGTGTTGGGATCTTGGC
CTCATTGAGCAGACTGTAGGAAACGTGTTCCTGGACATTGAGG
>dme_circ_0008280_junction_seq
GTAGGAAACGTGTTCCTGGACATTGAGGGTTCCTTTGCCTCGTCCTCCGGCCAAAA
>dme_circ_0008281
CTATCTACTTTTGGGCATCGGGAATCTCGAAGTTCACGTCCATCTTCATTTCGCGGGCCAGTTTGACGAT
TTGCGCCAAGTGCACGGCGAGATCGGCAGCCTTATCTAGATCATCGCGGGCCAGAATCTTCAGTCCAGAT
GTACGAATCAGTTCGCGGGCCTCCTTGACTTTGGTTCCCTGCAATGCAAACCAGTTTTGTTTAGCATATC
TCAGCAAGTCAGATAAATGCATCCATGAAAACTCACCTGAAGACGCACAACCACGGGCATGTTCAGATTC
AGATCCTTGGTGGCGGAGATGATACCCTCGGCAATGACATCACAACGCATAATACCACCGAAGATG
>dme_circ_0008281_junction_seq
ATCACAACGCATAATACCACCGAAGATGCTATCTACTTTTGGGCATCGGGAATCTC
>dme_circ_0008282
GGTCCGAGTACAGGCCGTGCGAGCCGTCGGAGCCTTCATCCTCTACCACGACAAGGAGAACGAGACGGCC
ATCCACAAGCACTTCGCTGATATGCTGCCCCGCATGATCCACATCACTGGCGAGACCATCGAGGCCCAGG
ATGATCAGAGTCTGCTAAAGCTCCTGATTGAGATGACCGAGAATTGCCCGAAGTTCCTGCGTCCCCAGCT
GGAGTTCATTTTTGAG
>dme_circ_0008282_junction_seq
GCGTCCCCAGCTGGAGTTCATTTTTGAGGGTCCGAGTACAGGCCGTGCGAGCCGTC
>dme_circ_0008283
GTTTATTGGTGATGCCGGAGAAGTCATGGATATGCTGCTGGTCAATCACACCGAgggggggggAACTAGCT
GATGATGATCCGCAGACCTCCTATCTCATCACTGCCTGGGCACGTATGTGCAAAATCCTTGGCAAGCAAT
TTGAGCAATATCTGCCCCTCGTTATGGGACCAGTGATGCGAACGGCCACCATGAAGCCTGAGGTGGCGAT
GCTGGACAACGACGAGGTGGAGGACATCGACGGTGATGTGGACTGGTCTTTCATTAATCTTGGCGAGCAG
CAGAACTTCGCCATTCGCACGGCTGGCATGGACGACAAGGCTTCGGCCTGTGAGATGCTGGTTT
>dme_circ_0008283_junction_seq
AAGGCTTCGGCCTGTGAGATGCTGGTTTGTTTATTGGTGATGCCGGAGAAGTCATG
>dme_circ_0008284
GTTCTACTTCCACGACGGTGTCCGCACAGCTGCTGCTGAGTCGCTGCCTTACCTTCTCGACTGCGCAAAA
ATTAAGGGTCCACAGTATCTGGAGGGCATGTGGATGTTCATTTGTCCGGAGCTGCTCAAGGTAATCGTCA
CGGAACCGGAGCCAGACGTGCAGTCGGAGCTGCTGAATTCGCTGGCAAAATGCATTGAGACATTGGGTCC
GAATTGCCTTAACGAGGATGCCATGAAACAAGTTCTCGAGATTATAAACAAGTAT
>dme_circ_0008284_junction_seq
ACAAGTTCTCGAGATTATAAACAAGTATGTTCTACTTCCACGACGGTGTCCGCACA
>dme_circ_0008285
GTATCTGGAGGGCATGTGGATGTTCATTTGTCCGGAGCTGCTCAAGGTAATCGTCACGGAACCGGAGCCA
GACGTGCAGTCGGAGCTGCTGAATTCGCTGGCAAAATGCATTGAGACATTGGGTCCGAATTGCCTTAACG
AGGATGCCATGAAACAAGTTCTCGAGATTATAAACAAGTAT
>dme_circ_0008285_junction_seq
ACAAGTTCTCGAGATTATAAACAAGTATGTATCTGGAGGGCATGTGGATGTTCATT
>dme_circ_0008286

GTTCTTACCCGCCTTTGAACAGGTGGCTCCGCACTTTGTCAAGCTACTGGAGCCCAGCCGACCTGTAGCT
GATCGGCAGTGGGGCTTATGCGTGTTCGATGATCTGATCGGTGAGTTGGTTTTCTTAATTGAGTTTTGCA
GTTCAATAGTACCGCCGATACCTTCAAACATCAAATCATTTGTGCCACCATAAAACTAAAGACTTGTATT
CTCATTTACCTACAGAATTTTGTGGCCCCGCTTGTGCTCCATACCAGCAGATCTTTACGCCAGCCCTCGT
CCAGTACGTGTGCGATAAGGCCCCAGAAGTGCGCCAGGCGGCTGCCTACGGATGCGGGGTGCTCGGCCAA
TTCGCTG
>dme_circ_0008286_junction_seq
GGATGCGGGGTGCTCGGCCAATTCGCTGGTTCTTACCCGCCTTTGAACAGGTGGCT
>dme_circ_0008287
GATCATTCCACTGCTGGTGCAGGTCATCAACGATCCCAAAGCCCGCGAAATCGAGAACATCAGTCCGACA
GAGAATGCGATCTCGGCGTTTGCTAAGATCCTCAAGTACAACAACTCGGCGCTGAGCAACGTGGACGAGC
TGATCGGTGTTTGGTTCTCATGGCTGCCGGTGTCCGAGGATTCGGAAGAAGCCGCCCACATCTATGGCTA
CCTATGCGACTTGATTGAAGGCAACCATCCG
>dme_circ_0008287_junction_seq
ATGCGACTTGATTGAAGGCAACCATCCGGATCATTCCACTGCTGGTGCAGGTCATC
>dme_circ_0008288
ATGTGGGCGGCTTCTTCCGAATCCTCGGACACCGGCAGCCATGAGAACCAAACACCGATCAGCTCGTCCA
CGTTGCTCAGCGCCGAGTTGTTGTACTTGAGGATCTTAGCAAACGCCGAGATCGCATTCTCTGTCGGACT
GATGTTCTCGATTTCGCGGGCTTTGGGATCGTTGATGACCTGCACCAGCAG
>dme_circ_0008288_junction_seq
TGGGATCGTTGATGACCTGCACCAGCAGATGTGGGCGGCTTCTTCCGAATCCTCGG
>dme_circ_0008289
GTCCGACAGAGAATGCGATCTCGGCGTTTGCTAAGATCCTCAAGTACAACAACTCGGCGCTGAGCAACGT
GGACGAGCTGATCGGTGTTTGGTTCTCATGGCTGCCGGTGTCCGAGGATTCGGAAGAAGCCGCCCACATC
TATGGCTACCTATGCGACTTGATTGAAGGCAACCATCCGGTCATTCTTGGGGCAAACAACGGCAATCTGC
CGCGCATTGTCTCCATCATCGCTGAATCCTTCTGCACCAAGGTGGTTGAGGCGCAGTCCGCCACCGGCAC
TCGTATGCTCACTATCGTCAAACAAGTGGAATCGAATCCGGAGGTAATGGCTGCTTGTGCCAGCACCTTG
AGCCCGGAACAGCAGCAGGCGCTGCAGGATGCCTACCGGGAGCTGGCAAACGTGGCTCCCGCCTAAGATA
TCGCAATCCCAATCCTCTTCAGACCAAGTCCATTCAT
>dme_circ_0008289_junction_seq
CAATCCTCTTCAGACCAAGTCCATTCATGTCCGACAGAGAATGCGATCTCGGCGTT
>dme_circ_0008290
GGTATTAAAGGCCGCGATGGCGGAGGAGGAAAGCGAATTCCTGGAGGCCTATGCTGGCATCAGAACAGCC
TACAAAGCGGCCATGACTCAGGTGGACCTGGCCGTAAGTCACGAGGAGCAGGAATCCCCCGGCCAGGCCA
TTGTGGCCTATGAGCTGGCCCTGCGTATGATAGAGGACACCTTCGGCATTCCAGTAGGTCTTCCCAACAA
GATCGACACCGTGCAGGCGGAGTGGAACGACGCCTGTGCGCTGATCCAAAAGCTCAAGAGCGCCGAAACG
GAATTGAGATACCGCCTCAAAGTTCTGCGATCCCAGAAACAATCCATTGATGATTCAGCCGTGGAGGCCA
CGGAGGAAAGTCGAGCAGAGATGGATACAAAAAGGCCGCCGTTGCTGGCGGAAAACCCCTCCACGCAATA
TGGCATAGCCAATGCTAGCGGAGCACCAAAAACCTACAGAGAGCTCGCTGCCGGCTTGCGGGAGCTCTTG
GCTGTTCGGGACGCCAAAGTCTTGCTCGATGAGCTCTTCCGTGCACAGGTCAAAATGTACCGAATCGAAG
CCAGTGGATCAGTAACCACCATAAGCGGCTCGTCAACTATGTCATTGGTAATGTGCACCGTGGGTGGCAA
ATGGAAGTACCTCAGTGGCATCTACTTCATTCAGTGCTCCATGCCAAATGAGGGGACTGCAGGGATTTGG
CTTTACCCCCTGGTTCCCTCGATCACAAACTGCTACCAAACAGAGTACGGAGCCTTTATTTTTCCTGATA
TGGAGTGCCAGCAGCCGGGCAATGCATTTGGTTTGATGCTAACGAAGGAGGGCCAGACATCGCGCACGGA

GGACGAACTGGAGGATCTTCAGCAGTTCTTTCTTGACCTGTTGGAGGCCGTACTCGCTGGCACGGTAGTG
CAGCTGAAATCGCCGACCTCCCAACGTGCTGGCCTAGCTTCAGATACCGTTTCCGGGTCGGAGCAGGTGT
CCAGACACATTGTAAGTGCGGCTGATTTCATTGCTAGTAACCTGGTACGGGGCGCCGAGAAGACAGGAGG
CTTTATGCTTAGGAGTACTCCGTATATCATTTCCAAGATGACGCCAGCCTCGATGGATGCTCAGGTGCCC
AGCTCCGTCCAAACTTCAGGGTGAGTTAGTTCCAAATAGGGAAGAGCTGTATTTAGGGCGGAAGTATGGT
AGCTTCCGAATGAGCATGTAACGATAAAATTAGAAGATAAGATTCGAAGTCGAAGATGTGATCGTCACTA
CACTTCCACCTCGATCGGAGTTTGTGCTTGTGTTTGATAATCATGTTAATCAATGGTTTGGAAATGTGCA
GATTACATATAAGATAGATCTAATGTATCTTATGTTGTAACCTTTTGTTCATTATAGGTTGAGGTGGCCC
AAAAAGTGACGCACGCCGCAGCCGGAATGACAGGATGGATAGCTGGAAAGGTGGGCACTGCTTCAATGGC
AGTCGGCCGCTACCTGGCTCCCCACATTCAGGAGCAAGGCTCTAAACTGCTGCAGAAGGGATTTGGCTAC
GACACCAGCGAGGCTAATAGCACCATGGAGGGCGCCATGACCATCGCGGCAGGAGCCGTGGAAGGAGTGA
GCACCGTATTCGATGGCCTCGAGACTTCCGCCAAGATCCTAGGCTCCAGCCTCAGTGAAAACTCCGTCAA
GATAATCGAACACAA
>dme_circ_0008290_junction_seq
AAAACTCCGTCAAGATAATCGAACACAAGGTATTAAAGGCCGCGATGGCGGAGGAG
>dme_circ_0008291
TAATATCCAGGCGCTCGAACGGGTTCACTTTTAAACAGCCACGGATTATATCGCTGAAGCACTGGTACTG
AGGATCTGGCGGAAGCATGTAATTGGCGTTAATGATCCGCAGCTTTCCGCCATCCTCGTAGGGATGCTTG
CGGTAGCACAGGAAGTAGAGAATGCATCCGAGAGCCCATATGTCCACTTTGGGCCCAATCGGGTTGTTGG
ACCAAGTGTCGAGCATCTCAGGGGATCGG
>dme_circ_0008291_junction_seq
CCAAGTGTCGAGCATCTCAGGGGATCGGTAATATCCAGGCGCTCGAACGGGTTCAC
>dme_circ_0008292
TGATTTGCTTGTTGGCCACCAGTACGTTCTTGGCCAAGCGCGGTCCTATTAGCAGCGTTTTGGGTGCCGT
CGTTATGCCGCTGGTGGAAGTAGTTCCACTATTACTGGTGCTACTGGCTAATCGCAACAG
>dme_circ_0008292_junction_seq
TACTGGTGCTACTGGCTAATCGCAACAGTGATTTGCTTGTTGGCCACCAGTACGTT
>dme_circ_0008293
GGGTTTCGCACATGCCATACAATTGCTCCGCACAGGTGCCGGCCACAACGAAGTCATCGGGTGCGTTGGG
GCAGCCGATCAGGTCCATGTTGCAGATAAAAGGCTCCATGGTCTTGGGATCCAGGCCAGCAACGACCGGC
TCAATGAAGTACGGGCCAAAGCGGTGCTCGTACAGAAAGCTGGACATCATGGCCGAGAAGGGCTTGGGAC
ACATTTCACG
>dme_circ_0008293_junction_seq
CCGAGAAGGGCTTGGGACACATTTCACGGGGTTTCGCACATGCCATACAATTGCTC
>dme_circ_0008294
ATGATGGTATGGCGGCAGAAAGCGGTTCGGTGTAAACCTCAGAGTAAGCAGAACTTTTGAGAGGACTTTC
GGCCGGAAGGGACTCGATGGGCATTATATGAAGGTCTAGAGGTCCTTTGAGCGACCAATTGTGTGTCTCA
GCGAGGGCTGCAAGGCCCTCCAGCACCATAG
>dme_circ_0008294_junction_seq
AGGGCTGCAAGGCCCTCCAGCACCATAGATGATGGTATGGCGGCAGAAAGCGGTTC
>dme_circ_0008295
GGTGACCGGCAAAGTCATCTGTGACTAGATTCGGCTCGCCGCCCGGCAGACCGGCACATACGGGGCAGAC
CACCTCTAGACTGGTCTCCGTGTGCTCGGCGGAGACATGCTCCAGCAGGGTGGCGTCGCTGAAGCCCATC
TTCTTGCAGTAGGGACAGGTGAAGCTCTGCGGCTGGTCGGAGGCTAGCATCTCGCCGCCAAAGTACAGCT

CGATATCGGAGCGGGTGAGGATGCACTGCATGGGATGCTCGACCAAATGGCGCGTGGAG
>dme_circ_0008295_junction_seq
GGGATGCTCGACCAAATGGCGCGTGGAGGGTGACCGGCAAAGTCATCTGTGACTAG
>dme_circ_0008296
CTTGGTGGATTGGACTCCCGTCAAAATCGGAAATAAAAGGCACTACCCACCCACATACACACACAAACAC
ACTTCTGCCAACTCTCCTCCCTCAACCGCACAAGCGCCGCAGTTGCACCTCGACTGAATTCACGATTTTA
CACTGCACAGGTGCAGTGCTGCGCAAAACAAAACGCAAGACTCTCGGATCCGAACTCCGTGGCTTTGGGC
TTTTCCTGCCTTATTTCCCGTGCGCACATATGTACACATTCACATGTATTCATG
>dme_circ_0008296_junction_seq
CATATGTACACATTCACATGTATTCATGCTTGGTGGATTGGACTCCCGTCAAAATC
>dme_circ_0008297
GTGATGAACTACTTCAATCTGAAAGATGTGGTCAATAGCACTTTGAATGCGGACGGTGATAGTGCCCTGC
ATGTGGCGTGCCAGCAAGACCGAGCGCACTACATACGACCTTTGCTGGGTATGGGATGCAATCCAAATCT
GAAGAATAATGCCGGAAATACCCCACTGCATGTGGCCGTCAAGGAGGAGCACTTAAGCTGCGTGGAAAGC
TTCCTTAATGGAGTGCCAACCGTACAATTGGACCTCTCGCTAACGAACGACGATGGTCTGACGCCTTTGC
ACATGGCCATTCGACAGAACAAGTACGATGTGGCCAAGAAACTGATCAGCTATGATCGGACCTCGATTAG
CGTGGCCAACACAATGGATGGTAATAATGCACTCCACATGGCAGTTCTGGAGCAAAGTG
>dme_circ_0008297_junction_seq
CTCCACATGGCAGTTCTGGAGCAAAGTGGTGATGAACTACTTCAATCTGAAAGATG
>dme_circ_0008298
CGTAATTGTGTCGAGGAAACGCCGACGAACCGGGTCCTCTGCCAATAGCAGCAGTTCGGGAACAGAAAGC
TCTAATAACTCGCTGGATCTGCCCAAAACGCTGGGCTTGGCGCAGCCACCAAATGGCTTGCCAAACCTTT
CACAGCATGATCAGACTATAAGTGAGGAGTTCGGACGCGAAAAGCATCTAAATGAATTTATTGCTTCCGA
AGATTTCCGCAAACTTATCGAGCACAACTCTTCTGATCTCGAGAAGATCTGCCAG
>dme_circ_0008298_junction_seq
CTCTTCTGATCTCGAGAAGATCTGCCAGCGTAATTGTGTCGAGGAAACGCCGACGA
>dme_circ_0008299
GTTCTTCGAGGAGGATGAGGACGGGGAAACCGTGTGGGAGGCATACGCAAAGTTCCGCGAATCGGATGTA
CACCACCAATATGCCATTGTGTGCCAGACGCCTCCGTACAAAGATAAGGACGTGGACCGCGAGGTCAACG
TGTACATCGAGCTCATTCGTCCCTCCGACGATGAGCGCTCATTCCCGGCGCTGCCCTTCCGCTACAAGCC
ACGGAGCG
>dme_circ_0008299_junction_seq
CTGCCCTTCCGCTACAAGCCACGGAGCGGTTCTTCGAGGAGGATGAGGACGGGGAA
>dme_circ_0008300
TTCTCACAGGATCAGCACGGCTCGCGGTTTATCCAACAGAAGTTGGAGCGGGCCACCGCCGCCGAGAAGC
AAATGGTGTTCAGCGAGATCCTGGCGGCAGCCTATAGCCTGATGACCGATGTCTTTGGCAACTATGTCAT
CCAGAAGTTCTTTGAGTTCGGCACTCCCGAGCAGAAGAACACGCTGGGCATGCAGGTCAAGGGTCATGTG
CTGCAGCTGGCGCTGCAAATGTATGGCTGCCGAGTGATTCAGAAGGCTCTGGAGAGCATCTCGCCGGAGC
AGCAGCAGGAAATCGTCCACGAACTGGACGGACATGTGCTGAAATGCGTCAAGGATCAGAATGGCAATCA
TGTGGTGCAAAAGTGCATTGAGTGCGTGGACCCCGTGGCGCTGCAG
>dme_circ_0008300_junction_seq
TGAGTGCGTGGACCCCGTGGCGCTGCAGTTCTCACAGGATCAGCACGGCTCGCGGT
>dme_circ_0008301
CTACGCGATCTCGCTAACCACATTGTGGAGTTCTCACAGGATCAGCACGGCTCGCGGTTTATCCAACAGA

AGTTGGAGCGGGCCACCGCCGCCGAGAAGCAAATGGTGTTCAGCGAGATCCTGGCGGCAGCCTATAGCCT
GATGACCGATGTCTTTGGCAACTATGTCATCCAGAAG
>dme_circ_0008301_junction_seq
TGTCTTTGGCAACTATGTCATCCAGAAGCTACGCGATCTCGCTAACCACATTGTGG
>dme_circ_0008302
GTCATCCCGGCATTCCTCGATCACACGGGCTCCTTGCTGATGGGAGGACCTCGCACCGGGACGCCGATGC
GTCTGGTTAGCCCCGCccccgttctggtgcccccgggcgctacccgtgccggcccccccgcccccgcaGGG
GCCACAGCTGTATCAGCCGCAGCCGCAGACGGCCCAACAGAATCTCTACTCGCAGCAGAATGGATCCAGT
GTCGGAGG
>dme_circ_0008302_junction_seq
CGCAGCAGAATGGATCCAGTGTCGGAGGGTCATCCCGGCATTCCTCGATCACACGG
>dme_circ_0008303
CAACTGTTTCGCTCGCAGAATCCGGGCCTagcagcagttgccacaaatgcagcggccgcagcagcagccg
cagcagctgccacatcggcagcgagtgctgcggcagcggtgggcgcaccacccgttcccaacggatcgct
gcagcagtcgcagcagcaacagcagcagcagcaacaacagcagcagcaacaacagaTGCACATGGCGGCC
GCGTCGCAACAATTTTTGGCCGCCCAGCAGCAGGCGCAAAATGCGGCCTATGCCGCCCAACAGGCCACGT
CCTACGTCATCAATCCGGGCCAGGAGGCTGCCCCGTATATGGGCATGATTGCCGCCGCCCAGATGCCGTA
CTATGGCGTAGCACCATGGGGCATGTATCCGGGCAATCTGATTCCGCAACAGGGAACGCAGCCGCGCCGC
CCCCTCACCCCCTCGCAGCAGGGTGCCGAGAATCAGCCGTATCAGGGTGAGATATATAGCCAGGACATTA
TCATCCATCCCGCACACTAGCCGCCGTGTTAATGTCGCACACAATGAAGCATTCATCGTATGTACATACA
TAGCGGCACTGGTGGCACCATATGATGGTGCTGTACATATGACAGGTGTCAATTGACTGTAACAAAGGTT
TCACACTCGCGGTCACGGCTAAAGTTAAACTTAGTTAAAGAATTCATCGTACAGTAGCCATCTCAACGTA
TTAAAATGCTATTTTAATGGTAGGAATCAGAAAAACATGAGCTAATTGCATATTAAACGGTTTATTTAGT
AGGGATCACAACTGGAGATTTGTAAATTCaaaaaaaaaaaaaaaaaCTACTATGATGTAAAGCTCCCCTT
GTAATTCTAACCATAACTATTTAATTTGACCTGAAACGTTGTATATTTATGCTCTGTCTCTCTTCTTTTC
TCtttttttttATACTCACATCATCAACAACACGCTTCTATAACCAACAAACAATGAACAAAACCAAACA
AAAATCTAACCCCCTGTACAAAAACGAAATCCGAAACGTGTAAAATACCGTGACCATCGTGCTACTACTG
CTGCTTCTGCAGGGTCATCCCGGCATTCCTCGATCACACGGGCTCCTTGCTGATGGGAGGACCTCGCACC
GGGACGCCGATGCGTCTGGTTAGCCCCGCccccgttctggtgcccccgggcgctacccgtgccggccccc
cgcccccgcaGGGGCCACAGCTGTATCAGCCGCAGCCGCAGACGGCCCAACAGAATCTCTACTCGCAGCA
GAATGGATCCAGTGTCGGAGG
>dme_circ_0008303_junction_seq
CGCAGCAGAATGGATCCAGTGTCGGAGGCAACTGTTTCGCTCGCAGAATCCGGGCC
>dme_circ_0008304
GATAAAAGTGACGATGGGAAATAATTTAGTCATAAATTATGTATTATTATGTAGGAAGAGGTTTATTTAA
AAAGGTTATATACCTTTTACCCTTAAATTGCTGGCACTGAAATTCAGTTTTCGCTCGCCAGCACCAACGA
TAAGCTAACGCAATTCGCTTAACTATTTACGAGTATATCGCCCAGTCACAAACAACAAAAATGTA
>dme_circ_0008304_junction_seq
TCGCCCAGTCACAAACAACAAAAATGTAGATAAAAGTGACGATGGGAAATAATTTA
>dme_circ_0008305
GTAAGTTCAACTTCCTTTCCTTCAAGGCAACGAATACAAAACGAATGCAATCGAATGGCAGCTAAGTTTC
AAGTAGACTCCTCTATTTTATATATCAGCATACACAAACATCTCTTATATATACATATGTAGCCTTTAGC
AATATCGAAAGCATTCTCGTTTTTAATTATAACAAAACACAAGATTCGTCAACAGACGACTCGAAAATTG
TGTCAGGCCGATGCGAGAGTTTTTTGTTGTTGTTTTGCGGTTTGCCCTGATAAGCGAACAATTTCTATAA

CATTTCCTGTGTTGCCCGATAAAAGCAGTGAACAAAATGTGGCCCTGATAAGGGCAGAACATATTGCAAA
GTTCACTCCCTTCTTCTGAGAGGCATATGAAATTTAGCTTGAGCCTTGCATATTAGATTTTCGAACCCGT
AGTTTCTTCACACACATATAAAATATTCAGGCTGTGGAATGGAATGAATGACGATGAGGAGGAGCAGCAG
CAGCAGTGGGCTGGATTCATATGAACAAGAGCAGCGTATGATGCAAAAGGAGGAAACTTTCCCCAGACTT
TGGTTTGCCGGGGAAAATTGAATTTGCTGCTATTGACTTTGGGGGAAAGAACACCGACAGCGAAACATAG
CAAAACTTTCATGCGAGCATAGGCAACACACCAATGAAACAAAGaaaaaaaattacaaaaaatgtcaaag
cagaaggaaaaagttaaaactgaaaagcaaaaattgcaaaacacaaatCGAGAACTGATGTCTGGTAGAG
AGTATGCAttttttttgtgtgtattttccgttttttgGCTTCCAAAACATTCCCCAttttgttgttttgg
tttgtttttgtttCAGCTAGCTATGCCGCGCCAACATTCAATAAAAATTCAATTTGCAATTGAATTTGTT
TTCACTCGATATCCTTCCATTTCTCTGCTATCCCCTCCGTCTCCCTCTTGCTGACTGTCCCTCTCTTTCT
GACAGCATTGCAACTTGGTAAACATTGCAAGAATCGCCAATAATATTATACATATCCCCAACAACCAATT
CACCATGCATGCATGAGTGCAAAGAGTTTGCTACGATAGTGCCAATCAAGTGATATATTAACCGCAATCC
ATTTAATAAAATTGTTCAAATGAACAAGAGGCTTGTTGTTTATGCTCtgtgtgtgtgtgtgtgtgtgtgt
gtgtgtgGAGAATATTCAAAAATCTACCAAGTTATATGAATAGTTTATATTTTGAACCGCTAACCCAAAT
CGGTTAACAACTTTATATCCGATGCACAAAATATGCTGTCTAGGCCGCAGACCGACCATTCACTTTTCAT
ATCGCTCACTACTCAAATCTAAGCCCACACATCAGCATCATATTTATTTCATCAGTTTGTTTTGGCAATT
TTAATCCCAACAGAACATTGTCAGTTTGCTTAAATTATAAATGCCTTTCGCCATATTTCAGTTTGGTTAA
AAAAGCCACAGAACTCGCATACGGCAGCCAAGTGGGTGGTGGGTGGTGAATGGGCTGAATTCGGAGGATG
AGAAGCTTATCGTTTGCTGCCGGCTTTGCTGCGGTTTTTACCAGTTTCCGTTCTCGCGTGGCTTATCGAT
TAAATTCATATCATTGACACGTCATAAACAAAAAATACAATGGTCGGGGTAGCAGAGataaactaaaaat
aaataaatatatacatatataaaaatGGTAGAAACTGAGCGAGAGCAGGATTGTGTGGCCATGGGTTTCT
GCCTCGATTTGTGTGGTTGGGAAAACCTTTTAGACCAGGTCCAAACGACTAAGTGAATTAGCCCAAAGCG
AAAACGATATAGGTGaaacaaaaaagcggcagcgggaaaaaaaacaaaaaagtggcagcgggaaaaaaac
aaaaaagtggcagcggaaaaaataacaTTAATAAATAGAATTTTTTTGTTATAACTAACGGTCTTGTCCA
GGAATCTGGATCCTGGACTaaaaaaaaaaaaaaCAGGTTCCCTTGCTCGCAACCCTTTTTCTTGTCAATA
TGCAAATGGGTACaaaaagcaaaaaaataacaaaatGTGGGGGACAAGAGGCAAAAGTGAGCAAAAGTCG
CGCAAATTAAAGGCGAAAATGATGATAATGGAAGCAGTCGCCTCGTTGTCACTCCGACCGACTCCCTTCC
TGAAAAGCAGTCACGTTAAACGGAAATGGCAATGTCTAGACTGTGGACTTTTTGGACCCTAGTCCTTCGT
CGTTGCAGTTTGGGTGGG
>dme_circ_0008305_junction_seq
AGTCCTTCGTCGTTGCAGTTTGGGTGGGGTAAGTTCAACTTCCTTTCCTTCAAGGC
>dme_circ_0008306
gtaagttttatcCATCACCGGAGAATCGAGAACGAAGACCACCACCTCCATTCGGGTTTAGCACCCTGTT
CCTCGGATGTTGCGAGCACATGTGTAATGGTTTTTATTCTCGGtttttttttttATTACACTATCAAGTG
CATTTTGGGGAATCTACTTACAAAAATTACATTTCCGTGGCTTTTAAACTGAGTCAAAACTTTAATGCTT
GGGCGAAGCACTTGTACGAAAGTATTTTGTGTACTGAAGATATTTTTATTACTTTTCAAATGGAACATTT
CCTTAATTTAGTTTTTATTTGTTCGCGCTACTTATTGAAAACAATATTTTTTAATTGAAATTTAATGGAT
CTGGAATTTTCGTATCTCATTTTCATAAACATTTTAATATGCCTGCTTGTAAATGTAGTATCATAAAAGT
ACAATTTAATAACCAAAACAATGAACTCAAGACTGCGAAAAACACCTTGAACTATATTTGCCTTACCATA
TTACACCACTAGGCCTGAACTATAAACATTTTCAAAAGTATCATTATTAACATACGTGAAACATTCGCGC
ACATGCCGTAAATTTCAGGCCTTATTTTGTACTCATGCTTTTAGGGTATTTTGTATTAACAGCCAGTTCG
ATATTCGTTTTATATATGTATTCATATACATTCAATCGAAAAGATTTCATTTGGTGTCAAGGTGTTCTGG
GTAATAGGTAATTATTTTCATCATTGTTAAAAAGAATCCCAAAAATATGCAAAAACTCTGGGTCACCTTT
CTAGTTTACTTTTTTTGTGTTCAATATTTAATTTTTGAGTTGATTTCCCCCGAGGTTTTCAGTTTTCAGC

CACATAATTAATGTCTACTTAAGTGAAATGACAGTCGGTCAGTCGCTCTGATTTTCCTCGATCCCTACCG
CCCACCGCGCCCCCTATTTTCCACCCAGCTAGTTTAATTACTCACTAAGCAGACTATATAGCAACAGCGA
AATCGGGCGAGTAGTGCGCAGTCAACAGTGTCTGCTAAAAATATCTCATCACCGCACACACCTGCACGCC
GGACAGCCCGATATATTCGTTCGCTGCGCTGAGG
>dme_circ_0008306_junction_seq
CCCGATATATTCGTTCGCTGCGCTGAGGgtaagttttatcCATCACCGGAGAATCG
>dme_circ_0008307
TCGCACTCCTGGTTCACGTCAACCATCACCTGCAGAGGAGTCCCAGCCACGTCCCCCCAATCTACTCTTT
CCTCCACTGCCCTTCAATCACATGCTCATGGATCATGGCCAAGGCATGGGAGGCGGCTTGGGCGGAGTTG
TTGGATCTGGCAACGGAGTCGGCGGTGGCAGCGGCGGAGGCGGGGCAGGCGGCGCTTATGCGGCCCACCA
GCAGATGGCCGCCCAGATGAGTCAATTGCAACCGCCGATGATGAACGGCGTTGGCGGCGGAATGCCAATG
GCAGCACAGGTAGGATGGTTAAGTTTAAGTGAACAAAAGTTTAATAGATAAGATCAAAATTTTATAAATT
TAATTAGAACTTTAAATAGTTAATTTCAATTAAGAAGTGTAATAACTAATTGTAAATTCGACTTTACAGT
CACCAATGTTGAATCACCAGGCAGCTGGACCCAATCACATGGAATCTCCCGGAAATCTCTTGCAGCAGCA
AAATTTTGATGTTCAGG
>dme_circ_0008307_junction_seq
TTGCAGCAGCAAAATTTTGATGTTCAGGTCGCACTCCTGGTTCACGTCAACCATCA
>dme_circ_0008308
ATTACCACATCCACTGCCAAGAAATTGTGGGAGAAATCCGATGGCAAGGGCGTATCCTCGAGCACTCCCG
GTGGACCGTTGCATCCCCTGCAGATCCCCGGCATCGGGGATCCCTCCTCCGTGTGGAAGGATCACACCTG
GTCCACACAGGGCGAGAATATATTGGTGCCGCCCCCCTCGCGAGCCTACGCCCATGGAGGCGCCTCCGAT
ACTTCAAACAGCGGCAATGCGGGCATACTGAGGGTGAGTAGTAGACAACTTTCAACTCGGTCCAGACTCC
GACTAAAGACCCCAGACTAGACCGCTCTAAACTCTAGATTGGTTGGACAAAACTGGTTTTCTCGCTATGT
TCTTCTCGCCTCTCTTCTCTTGACCGCAGAATTTTTTCAAGTTATGATTACTGAAACTGAAATTTTTCCA
GTTCAAGGTTGGGTTCTTTCATTTTTGCATAGACTTTCACTCGATCCACAGTGTGTAGTGTGTTGTTAGC
CAAGACAAGAAGTGGAGTTTTCCAGTTTTCATCGTGACTGGCAAACGGTTGAGGAAATTATGACTGGTTC
TGTGTATAATAGACATGGCTAGTCATTCttttttttttttCCTTACGAAGGTTATTCAATAGAGGTATTA
ACATTATTCAAAACTATAACCAATTTTTAAGTTGTAAGGCCTACGATTATATGTAATTAGATGAAGAAAT
CCATTTCCTTTCTGTTTTGCATTACATTACTTAATTAAGAGCTCTTAGCAATTACATATTGATGTCAGTT
AAGGAAATAATTGCCTTAGTTTCGTATTATTTAGTAATATATTACTTGCGAGCATATGCTAATAATTGGC
TTGCTTGTTAATTGTTAAGAGTCAAACCTTGAATATTAAATTATCGAAAAGTGTTGACCCACAAAATTCG
TATCAATTACCTGAAATATTTACCGGCCGGGTCTTAACCCACATTTTTTCGTTGTACTTCACCTGCAATT
GCCATATCTTCGTATTCATGTGTGACATAAAGCTGCAAAGTGATTGTTGTGCGTCTGCCCACGGTTTTCG
TATTTCCATTTCCATTTACATTATTTATTATTAAGTTTTAATTGTTATTTATGGCAATGGGGAAAGCTTT
TTTCTCCTCGTGAGGCGGCCCACCTGCGTTTAACGCCTTTTTCAGTTGACGCAACGAAAATACGACGACA
CTTTGAGTGAGTTAAATTGGAATTTGTGCAGTTATGTTGAAAGAGTTATGCTCAAAAGTTGTAGGAGGAG
TGCTGCTCGGTGCCTAAGCTTGAATTCAAAGACAATTTGTATTGGTTAAGCAAGTTAGCAAGTAATTGGA
ATAAAGCTTTATGATGCAACTAGATTTGATCTAGAATTCGACTATTATTGCGTTCATCAAGGTCTAGGTC
TATTCTTAAAGCGTACTTAAATATACAATTCCAATTATTGCAATTTCTGGGCCACTTCCTCTTATCAAGC
TGCAGTACTTAAACAACATTTAGCCGAAAACAAATGCATGAGAGACCAGCAACTAGCTTTTAGGACTAAC
AAAAGAAAAAGAAACCAGTCTGCGGTTTTCTCTCCCTCTCTCGCTCTCGCACTTATATCTATATCTCACT
CTCCGCTGTGGCAGCCGGCTGGTTTCTCAGTTTCTTTAttttttttttGTCCACTGACGCTGTTTTGCAA
TTTCATTGCCGGTTGGCCCAAACATGTTTCAATGCCAAACGTAATAACTGTTATGATGTAAGTGGCAACA
ACAACAGCAGCCAGCCAGCGAGCAGAAGAGCCACAGAAGCAGCGATAAAAATCACCTGCATCAAGATCAG

CTCGAAGAAATCGATACTCATTCCGGCTTCAGTTTGAGTTGTATGAGATTGCAACGCTGATTACAAGGAA
CTTTAGATCATGTCTGAAACAAACAATATAAATGTATAAATAAATTAAGTAAAAGGAAGTAAGCAGTAGT
TAGAAACATAATTCAATAGAATTATTGGGCTGATGAAATTGAATTGTAAAGttttttttttATATATTA
TGTTGTATTTGCTATACACATTTTGATTAGATTTCGTTAGATATTGCATTACAGCGTGGGTTTCAGTTGG
TTTTTTGTATTACCACTAttttttttGTTGCTACTGCGGCTGGAGGTTTCTTTTTTATATGGCTCTGTTT
GTTATTGTACATTTTGTTTGAAATTAAAGTTGCGAAAACATTGAAAGGCGAAAAGCGCTGCTAAAAATAA
TTCGAGGCAGGCAAAAGTGGACAGCCAAGTGACTCACCTGACTGGcacacacacacacacacacacacac
TGATTGCTGCTTCTTCTTTTGCCACCTGCCAGGTGACCCAGACTACTGCTAACCCATTACAACCGACGCC
CCTTCCCCTTTCAGCGCCCTTTCCCGCTTACCACTCTGACCTTTTTGGCTGCCGATGCCTCATTTTTTGT
TGGTTTTCCCGCCTCTTTGCCCTCTCTCGCTCCACTGGTGGCCGCACTTTCTTTTGTCTTGAAAAGTGAA
AATTTTGGGTCACCGAAAGCGGAGCAGCGGAGCTAATGGGATTGTATGAACATACTACATTGTGCTAGGT
CAGACATGACTCTTAGAATTCGGGGGCCAGTGGAAAGTTGCCAATGAATACCTGACCTCGGTCAAACTAG
ACGCTCGCTCTACAAAATGATGAAAAGTGAGAGTGTGAAATCTAAGCTTTGAATTCGTTATAAATTCAGC
TGGACTGTAATTGGAACCCCAAAAAAGTACTAGATTTGTGGCTAGCCAAAATTAAGAGAAGAGTTATATA
AAAATGAAGATAATCCTTTTATGCTTGGCTGGCGTCATGACTGGGTTTGTTTACCAGCCAGATGGTTGGT
AATCACCGACAGAATTCTAAAATATACGTAGCTAAGCTATATGACTAATACAGGTAGTCAAAAAGATCTC
AAAGTGTATGACGTAAGCTGCTTAGAAATTATAGAAATTACAAGCTTTTAGTAGCAATGCACTTTGCATT
ACAGTTGGATTAACAGTAATTAATTCCTTCGCTTTGGATATTTGTTTCCCCAGCTGCTTTGGCTGAGAGC
GCTAAACTTTTGTTCTGCTCCCAAGCTTGTTTCTCTGAAGTCACATGTATTGTTAATAATGCGGTGTGAA
TGACCTGGCAATGAAATTATAGCTGGGAAATTACATACGAGCACACATGCATTTGACGCTAGAAAGAAAA
GAGGCAAAACAATGTGCAGAGTAGAGTGTAGTAAAAATCACTGTGCTTGCAGAGTCATCGACTTGGGTGC
AGTGGGTTAAGAGCGATGAATGAATGGAAGCTGCCGTGCCGAGATAAATGCATTAATTAATTTATCAGCA
TGACTGACTCACCATACTACATACAGTGGCAAACATTTCCAGTTTGATCTAAAGTTTGGCTCGACCATTT
TATTTGCATGTTAAACGATGCTCGTTTCGCTGATTTTATGCAGATGCTGTGTTTAATTTATGAACCGCTG
GTCAGCTGGAGTGCTGACGTCACGAGTCATTTGGAAACAAGTTTGCTGCTTAAGTCTTTTGTTTTACCCG
GTGTTTGGCCAATAGCTGCAAAACTGTGCAGTTGGTAGTAATATTATGACGGCAAATTAGGCTCCACTAT
TCTTAGATTAGTTTTAATTTATAATTTAAAGTTTGAGATTTTTTATTTCAAATAAATTGCTTTATTCCTT
CTGTTCTAAATACTTTTTAGTACACTTAACAGTTTGAGAATCTGTGATAAGAAAAAGGTTTATGCCACGG
CTAAGTGGTGAATACAGTTACTCACAAACTTTCCACAGTTCAAAATTATATTATTACAGTTTGAGACTGC
ACTGTTAAATAACATGCTGTTAAGATCCTTTGTTTCTTTTAAACTGTGAAATCTATAGCTTAAATAATTA
AAAACAAATATTTATTATATTTTTTAACTTTCATTTTATTCTAGTTCGCACTCCTGGTTCACGTCAACCA
TCACCTGCAGAGGAGTCCCAGCCACGTCCCCCCAATCTACTCTTTCCTCCACTGCCCTTCAATCACATGC
TCATGGATCATGGCCAAGGCATGGGAGGCGGCTTGGGCGGAGTTGTTGGATCTGGCAACGGAGTCGGCGG
TGGCAGCGGCGGAGGCGGGGCAGGCGGCGCTTATGCGGCCCACCAGCAGATGGCCGCCCAGATGAGTCAA
TTGCAACCGCCGATGATGAACGGCGTTGGCGGCGGAATGCCAATGGCAGCACAGGTAGGATGGTTAAGTT
TAAGTGAACAAAAGTTTAATAGATAAGATCAAAATTTTATAAATTTAATTAGAACTTTAAATAGTTAATT
TCAATTAAGAAGTGTAATAACTAATTGTAAATTCGACTTTACAGTCACCAATGTTGAATCACCAGGCAGC
TGGACCCAATCACATGGAATCTCCCGGAAATCTCTTGCAGCAGCAAAATTTTGATGTTCAGG
>dme_circ_0008308_junction_seq
TTGCAGCAGCAAAATTTTGATGTTCAGGATTACCACATCCACTGCCAAGAAATTGT
>dme_circ_0008309
ATTACCACATCCACTGCCAAGAAATTGTGGGAGAAATCCGATGGCAAGGGCGTATCCTCGAGCACTCCCG
GTGGACCGTTGCATCCCCTGCAGATCCCCGGCATCGGGGATCCCTCCTCCGTGTGGAAGGATCACACCTG
GTCCACACAGGGCGAGAATATATTGGTGCCGCCCCCCTCGCGAGCCTACGCCCATGGAGGCGCCTCCGAT

ACTTCAAACAGCGGCAATGCGGGCATACTGAGGTCCCCGCGATTCGACTTGCGCCAAAGTGGTTGAATAT
GTTTTCAGTGGCTCGCCCACCAACAAAGATAGCTCGCTTTCCGGATTGGAACCGCATTTGCGGAATCTAA
AGTTTGACGACAACGATAAGGTAAGGTCTTAAAcgatgaaaatagaaatagaatagatagaaaatcaaat
agaatcgataTTAATAATAACAATACCTTGTTTTCAATTACAGTCACGCGACGATAAGGAGAAAGCAAAC
TCTCCGTTTGACACAAACGGTTTGAAGAAAGACGATCAGGTCACAAACTCAAATGGTGTTGTCAACGGCA
TTGACGATGACAAGGGCTTCAAG
>dme_circ_0008309_junction_seq
CGGCATTGACGATGACAAGGGCTTCAAGATTACCACATCCACTGCCAAGAAATTGT
>dme_circ_0008310
TCCTGTAGAGTGCTGAGTAGCAAGTTTCCATTACAATAAACCTCCATATAGATCCTACAGCCGTCGCGTG
CCTTGGTCATTCTGGGCACAGGCTGGCAGCTGAGTGAAACAAGGGTTGTGTTCTTATAGTGCGGCAGCAG
GGGCGTGGGCCGCAGGATGTCACCGAAGTAATACAGG
>dme_circ_0008310_junction_seq
CCGCAGGATGTCACCGAAGTAATACAGGTCCTGTAGAGTGCTGAGTAGCAAGTTTC
>dme_circ_0008311
AACCCGGACAAATGGAAGTACAATCCGCCGATGAATCCGGCCAATGCCGCTCCTGGCGGTCCACCGGGAA
ATGGCAGTAATGGTGGGCCCGGCGCCATTGGAACCATTGGCATGGGCAGCGGATTGGGTGGTGGTGGCGG
CGGCGGAGCTGGCGGCGGAAATAATGGCGGCTCTGGTACGAATGGCGGTCTGCATCATCAATCGATGGCC
GCTGCAGCTGCGAATATGGCAGCCATGCAACAGGCGGCGGCGTTGGCCAAGCACAATCACATGATATCAC
AGgcagcagccgcagttgcagcccagcaacaacatcagcatccacaccagcagcatccccagcagcagca
gcaacagcagcaggcgcagaaccaggggcatccacatcaccttatgggcggtggcaatggactgggcaac
ggcaatggattgggcatacaacatcccggccagcaacagcagcagcagcagcaacaacagcagcagcaac
aTCCCGGCCAGTACAACGCGAATCTGCTTAACCATGCGGCTGCCTTGGGTCACATGTCATCTTATGCCCA
ATCGGGTGGCAGCATGTACGACCATCATGGTGGAGCCATGCACCCGGGAATGAACGGCGGCATGCCCAAG
CAACAGCCATTGGGTCCACCCGGAGCCGGAGGACCCCAGGACTATGTCTACATGGGTGGCCAGACCACTG
TGCCCATGGGAGCCGCAATGATGCCGCCACAGAATCAATATATGAACAGCTCTGCTGTTGCAGCTGCCAA
TCGGAATGCAGCGG
>dme_circ_0008311_junction_seq
GTTGCAGCTGCCAATCGGAATGCAGCGGAACCCGGACAAATGGAAGTACAATCCGC
>dme_circ_0008312
TTTGTGTATATTTTCATCATTAAAACGTCGTGTAAATTAAAATTCATCCTAATTATAATTAATAAACTAC
ATTAATAAACGGAAATTCCACAATTCTCAACAAGAAATCTGTAAAAATTCAAGTTTTAACCAATTCTCAT
ACGTAATTAAATAAATAAAGTTCAGTTTTGTTAAGTCCCAAAAACAACAGCTGTGCAACTGTGTAAAATA
TTATTCATAATTTTGTGTAAATTTATGTaaaaaaaaaaaaaaaaaCAAACCAACCACGACAACCAAACCA
AACGAAGCAAATTCAATTTTTTTGATCAAAAAGTGTGTGAAAAATCTCAGCAAAGCCTGTAAAATAAATA
TTAAATATATTAAGCAAAAAGTGTGGTTTCTTTTACAACAaaataaataaaagcaaaaaacaagtgtaaa
tctgaagcgaaaattaaaaaaaaaTTATATGTAACATTTGTGAATATTTCCGAGCGAATAGTGTTGCAAA
ACGCGCGTGTGGTTCTTTGTGCTGCAAGTTAAAATACAATTCAAGTTGGCAATACGCGCAAAATTGTCAG
CTGCGATAGCTAGGAAAAGCCTCCAAAATTGAGCTCCTAACCGCGCCCACAATTGCCATATCGACGCCCT
Cgccgcagcagcaacaccaacagcagcagcagcagcagcagcagcaactctatcagcaacatcaacagca
gcagcagcaacattacggtccaccaccgccctactttcaacagctacaccagcaacaccaacagcagcag
caacaacagcagcagcagcaacaccagcaacacatGAAGTTTTTGGGTGGTAACGATGATCGCAATGGCC
GCGGAGGCGTCGGCGTTGGCACGGATGCCATTGTAGGATCTCGAGGTGGCGTCTCTCAGGATGCCGCCGA
TGCAGCTGGTGCCGCCGCAGCCGCCGCCGTCGGCTATGTCTTCCAGCAGCGTCCATCGCCTGGTGGGGTT

GGCGTCGGCGTGGGCGGAGTGGGTGGCGGTGTGCCAGGGGTCGGAGCCGTAGGCTCGACCTTGCACGAGG
CCGCCGCCGCCGAGTACGCCGCCCACTTTGCCCAGAAGCAACAGCAGACCCGATGGGCGTGCGGCGACGA
CGGCCATGGGATCGATGAACCCGGACAAATGGAAGTACAATCCGCCGATGAATCCGGCCAATGCCGCTCC
TGGCGGTCCACCGGGAAATGGCAGTAATGGTGGGCCCGGCGCCATTGGAACCATTGGCATGGGCAGCGGA
TTGGGTGGTGGTGGCGGCGGCGGAGCTGGCGGCGGAAATAATGGCGGCTCTGGTACGAATGGCGGTCTGC
ATCATCAATCGATGGCCGCTGCAGCTGCGAATATGGCAGCCATGCAACAGGCGGCGGCGTTGGCCAAGCA
CAATCACATGATATCACAGgcagcagccgcagttgcagcccagcaacaacatcagcatccacaccagcag
catccccagcagcagcagcaacagcagcaggcgcagaaccaggggcatccacatcaccttatgggcggtg
gcaatggactgggcaacggcaatggattgggcatacaacatcccggccagcaacagcagcagcagcagca
acaacagcagcagcaacaTCCCGGCCAGTACAACGCGAATCTGCTTAACCATGCGGCTGCCTTGGGTCAC
ATGTCATCTTATGCCCAATCGGGTGGCAGCATGTACGACCATCATGGTGGAGCCATGCACCCGGGAATGA
ACGGCGGCATGCCCAAGCAACAGCCATTGGGTCCACCCGGAGCCGGAGGACCCCAGGACTATGTCTACAT
GGGTGGCCAGACCACTGTGCCCATGGGAGCCGCAATGATGCCGCCACAGAATCAATATATGAACAGCTCT
GCTGTTGCAGCTGCCAATCGGAATGCAGCGG
>dme_circ_0008312_junction_seq
GTTGCAGCTGCCAATCGGAATGCAGCGGTTTGTGTATATTTTCATCATTAAAACGT
>dme_circ_0008313
GCAAACCAACGCCAAATTGTCATCATGATGTGTGTTGGACTGTTCTAAAAGGAACACTTGTCATTAGCCC
GGCTAATGACAATACAGAAGAAGAGAATATCATTGGGAACATTTGGAGATGTTCTGTTTTCCAATAGATC
TGCAAGTTTGAGTCTCataaacaaaagaccttaaataaaaatttaaataaaataaaagattattaaaaaa
aaaaCTGTCTTGTGGCAATCAAAAAATTAATACAATAACacaagagagaatggtatagtccagtttcccg
actatcagatacccgttactcagctagtgggagtggtggagcgaaatttcaaaattcttttggcatgtcg
atGTAAGTTGGCAGTATAAATACAATAATAAAAACACAAATATCAAAACCttttttttATCAATCTTCTG
ATCACGCCCATTAACGCATAGAATACTGGTTACAGTATAACCTCTAAATCTTTTTCGGCAGAGTTATTAA
TTGGATATCGGCATTATACCTTTGCAATAATGTTTTCTATAATGTACACTTTTCTTTCTAGATACAAACG
AGCAAAACATCGACCAATAAACATCCCACTCTGTAAAGGAATTTCAAAGG
>dme_circ_0008313_junction_seq
ATCCCACTCTGTAAAGGAATTTCAAAGGGCAAACCAACGCCAAATTGTCATCATGA
>dme_circ_0008314
GGTTTATTTAAACTTGTATACGATTGAAATTTCTGCAATCAAATCAAATTTGTTGTTACAATAAAGTTCA
GTTCTACTTCGGAACTTGAAATCTTCCCCAAATATGTTATTTATGTGTTATTTATTTGGTAATTTCAATC
CAAGTTGAAATCGTTTTCCGAGCTATGTGTCGTCATTACAGTGCAGTGCAGTTGTAAACGGTTTGTCCGA
TTTCTCAGCAATAAATCGGAATATATATGTAGACAAAGTGTGTTGTTTTAGGAGGGGAAG
>dme_circ_0008314_junction_seq
ACAAAGTGTGTTGTTTTAGGAGGGGAAGGGTTTATTTAAACTTGTATACGATTGAA
>dme_circ_0008315
CATCGAAGAACGTCGCCCAGTGGGCCGACCGTCGGCTGGATCCGTCAACCTGAACATATCGCGCACGGGA
CGCGGCCTGGGAAGACCCAAGAAGCGGGCTGTCGAGTCCAATGGCGATGGGGAGCCACAAGTGCCCAAGA
AACGCGGTCGTCCACCACAGAACAAGTCGGG
>dme_circ_0008315_junction_seq
GCGGTCGTCCACCACAGAACAAGTCGGGCATCGAAGAACGTCGCCCAGTGGGCCGA
>dme_circ_0008316
GTTCCAAGACGTAATCAAGATGTTCTCCAGCTTGATGTCCCCGTGGCAGATCTTCTGTTTGTGGCACTGA
TTCAGGGCACACAAAATTTGGAACGTTATCCACTTTTTCTCCAATACGGTGAGAAATGGTCGTGTGGATA

CGCGATCATAGAGACTGTGCTTCACATACTCCCGCATTAT
>dme_circ_0008316_junction_seq
GACTGTGCTTCACATACTCCCGCATTATGTTCCAAGACGTAATCAAGATGTTCTCC
>dme_circ_0008317
GCGCTCGGTACTTCTTCGGCTCCCCATCCTTGCCGCCCTTGAACGCGGGTAGAAAGCCCAGTTTCTTCCA
AGTTTCATCCTTCGAAAGGCCCGAGGGTCGCTTGGCCTTTTTTGCTTGCGACATTTTTACATACGGTTTG
TGTTAAATTACGTGACAGT
>dme_circ_0008317_junction_seq
TACGGTTTGTGTTAAATTACGTGACAGTGCGCTCGGTACTTCTTCGGCTCCCCATC
>dme_circ_0008318
GCCGCCAAGCCCACCAAGCACCTGAACCCCTCTCTGCACACCGCTGACAAGCAGTTCCTCCAGGAGGTTG
GATTCATCAACAGTGCCGCCGATCATCTGGCTGAGATGGCCAAGCCCTCGAATGTCCTGATGCTGCGCGT
TTCCGTCGATGGAGTGGCCAAGGCTCATGGCGAGAAGTCCGTGGCCGTGGAGGAGGCCAACAAATTGCTG
TCGGCCGCTATCAGCCGCCTGCTTGCTGCCTCCCAGAAGTCCAGCGACTCGGTTCTGTTTGTCCAGACCA
CGGAGAAGGACGTGGCAGCATCGC
>dme_circ_0008318_junction_seq
ACCACGGAGAAGGACGTGGCAGCATCGCGCCGCCAAGCCCACCAAGCACCTGAACC
>dme_circ_0008319
GATTGGTGGTAGCCGCTGGCTGAGCAACAAAAGTTGGTAAGGAGCCAACGAAAGGTGGAAAGCCTGATGG
CTTCATGCTGGGCACGCCACCTTGGTCCACAGATCCAAATATGTCGAATGGGTCATTGTTTATTTTTGGT
TGCGGCTGCTGCAGTGGCGGTGGTGGCAG
>dme_circ_0008319_junction_seq
GCGGCTGCTGCAGTGGCGGTGGTGGCAGGATTGGTGGTAGCCGCTGGCTGAGCAAC
>dme_circ_0008320
GGAAAGCAAAAGGAAAACTGGCAGCGTTTTCCTCTTGCAGCTGAGAAAAACCGCAGGACGCAGCTCAGCA
GCAGCGTCGCAGTCGTGTGCGTTGTGCATAAGaaaaaagaagaaaaaaaaTCCTGTAATATTGCAGAAAA
AACCGTAATTCGCCACTTGAAACGAAGGAAAAACGAGGAAAAGCAACTGCGTCGTGCACCATTTTGTGAT
ATATTTTTGCCCTCCTCCTTCTTCAACGATCCCTGTGCAATTCTCTGTTAAAGAAAAAATTAAAATAATT
ACTAGCAACCAATTGAATTTCTGCGAGACCAACATAACACATAAAAATTTCCAACTACTGTGATTTGAAC
AAAGCTCTAAGTTTTTGATTCAAGCCTGTGTAATTTCCACCCACAACGAATTGAAAAA
>dme_circ_0008320_junction_seq
TAATTTCCACCCACAACGAATTGAAAAAGGAAAGCAAAAGGAAAACTGGCAGCGTT
>dme_circ_0008321
GAAAAAACCGTAATTCGCCACTTGAAACGAAGGAAAAACGAGGAAAAGCAACTGCGTCGTGCACCATTTT
GTGATATATTTTTGCCCTCCTCCTTCTTCAACGATCCCTGTGCAATTCTCTGTTAAAGAAAAAATTAAAA
TAATTACTAGCAACCAATTGAATTTCTGCGAGACCAACATAACACATAAAAATTTCCAACTACTGTGATT
TGAACAAAGCTCTAAGTTTTTGATTCAAGCCTGTGTAATTTCCACCCACAACGAATTGAAAAAGTAAGTG
AATTCAAATGAGAAAAAGAAAATTATAATTTATTTCATTTTCCTCTTCGCTCGCCTACGTTGCTTACTCA
CCACTAGTCGATTCCCCACGCTCTCTCTCTCTTCACCACAGTCACCAATACACACGGGCTCGCCTACGTC
ACTTTGTCGTGTGTGTGCTTAGACTAGCGTTGCCATGTCGCGCGAATTACGAAGACAGGAACAATAAATA
AATAGCAGTTAAATAAATAGTAGAAAATGCCATACAAAATATATGGGAATTGACTTTAAATTACATTTTT
GAGTTTTCaaaataaaaactaaattttaaatacttaaattataaaaaaattaattaaaaaatgaaaTAGT
TTCCACATTTCTTGAAGAATATACTGTTAACAGCAGTTAACGAAGTGTAAGCAGAAGGAACAAGTACAAT
GTTATGCATTAAGAATATGAATCCCTAATCTCATTTCATTCAGTGCAGCTGTTCAAGTTTTTTCTATAGT

TCTTTGCCAAGAAAATATTACGACTTGCCAAAAACCAAAACCCCAACCACAAAATGTAGACTTTGAAGCG
AAGGACGTCTCTGCTGATAAGTGAAAAATAAATGACTGATAGCTCAAGTACAAACATGCACATAGTGATA
TGCGTATATACATGTGCGTGCATTTGTGTGTATATACCCTTCAGCTATCTCCAAATTTAAATAACATTTT
CCTCTTTATCGCAGTGACCGAAGAAATATCTTTATCTGCACACTCCACAATTTTAATATTATTTTTGCCT
GCAGTGCGTGACTTGAATTTTTTGCTCACTCACTCTTTCGTATGACTCATAGACCCGCATAAAAGCAAGA
CGAGTTCGAATTATCTACGGATTTGGGATTACGGTTCTTATCGGCGAGTGCATAGGGGGTTCTGGCTGCG
ATGGCAACCCTGATTGTTGGACAGTCGCCCAGCTGACtttgtttttccaacttttcttggtgactcactt
gcctttttgctgtttttctttttCGTTAACTCCAGCTCACCTTGTAACAATTATAAAAAGTACATATTTG
CTCGTGttttttttttcggtataagtttttttcctttgcattcgtatttttCTGTGCTGACGCTTTACAT
AAAGAATTATCTCGTTTTTTCGTATCACCTAGAGGCACGTTTTCTGGCATATGCACATTTTGATTATAGT
CGGCAATCAAGCACTTTTAGTGACGGAATTAAACGAAATGACGACTTTCTTGAAATTTCTGCCGTCTATT
TCGCCATTTCCAGTCGAATTTTCGTCGAGATAACAGTGAAAAAAAGATAGGAAACGAATCAAGCGCTAAT
TATTTGTGATTGGTTCGGTAAACAAAAATGTTTTTGAGTGAATCAGAAAGAGCGAGTCAAATAGTCGATG
ATATCGAGTAGGAAACACTTGAACAAATTTAAGCACTTTTCAAGGTGGATTCCTCTAAGTTTGGCAACGT
AAAATTTGGTTTTTCTACGCCGAATTAAAAACTTTAAGTAAAAATCTTGTTAAATGGAATTGTTAATACA
ACAAATTCTAAATTGGAATTGTTCTTAAGCAGCGATTATTTTAATTGATTTGtttttttttttATCAAAG
TTCATAGATTGAAATAGTATTTGCTTATCACGCTAGTGATTCATGGATCGATATTGTTTGTCACGATTGT
TTGAGAACTTTCCCGATCCCGGATGACTAATCCCCTTGTTGGCAAAAATCGCTTATAATTCTTGGCAAGT
TGAGCAACAACAACACGTGTCGTTGTCGCTAATATCTAAAATTATATAAATATGTATGTTCTCGCGAAAA
ACACACAAAAAGCAGCGCGTTCGTTCAAACAAGTTTTGGTCGTTTGAAAAACAAAAACAATTTGTTTTGA
TGACGGCTCGTTTCGAATTGTGTTAATATACATAAAAACAAACAAATTATACCAACACTCTCATGTATGT
ATCGTATATAAAACCAGCCCTGCCTTTCATTTCCCTCGTCGTTTTATTCTACCTTTACTTTTTGTTGTGG
CTAAATGGTTTTATATAAACAAGTAAAAATTGATAAGCGAACGAGACCGTTGGACGCGTGAACGGCCGCG
GCCTCATTTTCAGTTGTTTTCTTTGGTTATAGGTCGCGTGTAAATGGATGCCGCACACCCGTGCACCCGT
ACACCGCCCAAAAAACCCGATGCCGACCATCCACCCACCATATGGCCGGGCATACGATCGAAAACTTCGG
ATCGGCTCGTACATTTCGGGCCTTTCATTATCTTTGCTTGCCGTTTTCGAAATGTTTTGTGCATAGCGCT
ATTTTTAGCACTCTAATCCCCTATACTTGAtcttctttttgtcggctgtcgttgttttctctcttgtctt
tttttctcgttttAAGTTATGCAATTTTGTTTTGTCTGTCGGTCCCTTTAGAATAACAGTTGTGATCGAA
ATACTAATAGAATTTAAAGTTTTTAATATTTTCGTAAAGTTGTTTGTTTTAAAATCATTTATCTTGAATT
ATAGATGAAAGACAAAACATTACATGGTGGtttttttttATCAAGCAAGCGATACTAATTTAATGACCGC
AGCTGTTTCTTTTGGGGACAGACTGTACTGCTTCGATTTCGATCTTGATTAAATCCCATTGAAAATAGGC
ATTTAGAATGCACATATATCGGCTTTTTTTGGGAGCACAATTACCGCGTCCAAATGCGGGCATAGATTTC
AATGTAAATTAATTTGGTATCTGTATAAATTTTCTTTTCTTCATCAATCAATTTCGACACATGTACTTTA
TTCACAGAATTTTCTTTATCTAATGTGAAAGACATCGCTGGTATACATAAATAAATTTCTATATGTTTTG
TTTATTGTCTGGCAGAAAAGCAAAACGGAAATAGCTGAAAAACAAAAATACCGCCAGTATATTTAGGTCA
AATAATAAATTATCTGTATTTTATACATGTGAGTGCGAATCCTTGAACACAAGTCCTTGTCAACGAATTG
AGGCCTTGATCGTTACCAACTGTTCGACTTTAATGCAATTTTAATTTCCTGGCTTATGTAGTACATGTGT
ATGTGTACTTATGAGGAAACCACAAAAACGAATAAAAACAAGAAATAATATAATACGTTGAATCGAACCG
CAAAGAGAAGAGATAAAAGCCACCAACAACAACTGGCTCACCTTGTGGTCAAGAGCTAAAATTTTCTTTC
CATCCAACATCGATTTTCCCATTGCAACGTCGCCCGTATAACCTTTCTTTTGCCTCCCCAATATTGTGTT
ATCTATTTTCTTGCCAGTTTGTTTTTCCTTTGATTTCAATTTTCTCCAATTTGCTGTACAGGTTTTTTGT
GTGTGGGAATCGATTTAGGCGCTCGATTGGCTTTAGTGTTCGCCCCCCTCCCACAAATTAAAGTTGTGTG
CCCGAATAAAAAAGGAATAAGGAGAATAAGTGAAAAGCTCCTCTCGTCGTCAGGAAATTTAAATCCGATG
CGGTGTTGTTGCTGCTGTTGGTTTTTCCATGGTTTTTTCCTTTATTTTGCCAATGCCTGTGGCGCGCGCA

TATTCACCACATCTGGCAACCATGGCGACCGTTAAACTCGCTCCCCCGCCCACACACAGAAACCGTTAGC
CAGACAAAATGAAAAGCGAAACTTTTTGGCCCAGCCTTTTCTTTTGTCCGTTTCCTTGCTCCCgatgatg
atgatgatgatgatggtggtgatgatgCCTCGCCCTGTTCTTTGTCTCCTGTCTTGGTCTCCAGTTCATT
GCCTCTTTCTTCTTGCAACTGAAGAAAAGGAGAAGGAATAAAAGAAGCGAATTTGTAATGCATAAATCAA
TAAAATCAACGATGCACACACGATGCTGTTCTTGCTGTTTCAATATCAACGCCCTCGCCACCGCCCAGCA
TCAAGTCATCAACTAAACTTATCTTGGCTCTGACTCAGCCGCAGTCATAGCCTCCTCATCTCATATCGTC
TTATTACGAATGATAATACCCTTCTCGATTCCACTGACAAAGTTCAAGCGCCAACGGCAAAGTTCAATAG
CACAAAATGGCAGTTGAAAAAGTATATATTTTCCTTATTACAGTCGCACTTCCCTATAGAAAAAGCTAAA
AAACTGGATAATTATTCTATTCCACTTGTTTTGAATACAATCGGTATTTGCAGTCTTCCCATTTTTAATT
GAAATTTCGAGGTTTTTAGGTACCGATCCTGATTTAAATAAAAAGTGTATATATAAATAGTGATTAAACC
AAAAAGGAAGTTCTTTTCGTATTTCCATTGTGTTTAACAGCCGAAAAACCATAATGGCGAATGTCAAGTT
CAAGCTAGCATGGCTTCACTGTATGACTGAAATTCTTCAACTCCATCCCATGACTCAAGGCTTCCGCATC
ACTCATTGTTTTTGTTTTCGCATTTTCCGTCAATCTCTCCATCTGTCAGCCATTCATTCAAATGACCATT
GTCTACTACAGTCGAGAGTTGATTTTCAAAGCTCGGCGAAGAAATAGAGTCTTTGAACGGCCTTCGCTTT
TCTGCACATAAAGCCAAAGCCAGATCTCTTTCGGTTAGAGAGAGATACTTCTTCTTTGTCTCGAAATCTA
TATATGTATGTGTATATAAGTATATCTTTCCTTCTTTCTGTTTGTTTTGCTCCTTTGTGCACTTACGTTT
TTTGTGAATTCTATAAAACTGGTTTCTTTTCGGCGGCGATGTGTGCGGATTGTTAAATCGCGACCATAAC
ACCAGAGCTACGAACTCCATTCCATTCCATTTCCTTTTGACTGGTGCTCACAGGCTGAGGTTCAGATTGA
GACTCAGTCTTACAGTGTTTGTTGTCTGCTTCAGTGATTTGATTAGCCCATAAAGCCGAGACGTTGAGGT
ACAGTGAACATAGCGCTGGGTTTTTTAAACTAATTTACTTATTTGTTTCGTCATCTATTCTCACTGCTTT
GCACAACTTACCCCTGAGTTTCGCCCGCATTTTTCATTTTCCACACTGTATCGTAAATTGAGTTTGACCC
GCTTTTGATGGGAAAGGTTGACACTCATTGCAGTTCGATAAATTTTATACTATAAAATAACTATAAACAG
CCTTACAAAGTTGAACAAAATTAGTTTTACTAAATAGTAAACTCTTATATATCTATGTATATTTTTACCA
TAGCCCACATGATGCACTGTAATTCTTATGCTACTATTGTCGCAATATCAATTTGCCAATTAGTTACGCT
TCAAACGTTGGATAATATCCAGTACTTCGCTGTGATACTTCTCCTCCTCTTTCTCCGCACTCTTCTCGGT
TTCTTCTGGCTCCCCTGCAGCGTTGACGTTGCCCCATATGATGATGATCTTCATCATCGCGGTGGTTGGT
TTCTGTCGGTCTCTTGGGTTTGTCAACTATACTTCTTCATCTCCGTTTCTTTACTTCTCCTCCTTCTCCT
TCAATTTTGGACTCTCCCCTATTCCAGGGAAGGCAATCTCCAGCCAAGAGAGCTGGCTACTCATGTGGAC
GTCGCTCTTCTGAAGCGTTTCATATTTCATTTTCCCATGCGATTTCCATTAATCTTCCATGTGTGTAAAA
AGGCAACCAAAAAACTACTTTATAACCATTTTAGGGATTACTAGGGGGTGCACGGCAACGAATATTATTT
ACCGCCGACTACAAAATGGATAATATAATAAAAAGTACTGTAAATAAAGCTTCATAAATTATCATTAAAT
TTTTTTAAAGCAAAGGTGTCCAAACACAGTTTTAGATTACCAAGGATAAATGGTTTAGCAGGCATTCTTT
TTCAGTGTAGTTGGGCATTGTAACTGCACAACTTGCCGTTCGTTTTCCTCTTTTCCTCTTATTTGTTGCT
TTTGTTGACCTGTAGCTGGCAGTGAAGAAATAGCAGAAACAGCAGAAGGAAAGAGAGCAAAAGTTGTGGT
GACTTTCGCTGCACGAAGAGCGGAGTTTTGCACACTTCTGCGAGAGCCTCCTCCGTTCCCTCATCTTTAG
CGTTCCCCTTCAACACCTTTTATCTGCTCCCCTTTTTAGTCTCCTTCCTTTCCGGCCAACGCCTGCTCCT
TTTATTTTTTACTTAATGGCCTTTGGCTGCTGCTGCACACACACTTGCACATAATTCATAAAAAATGGCT
CAAGTGAATTATTGGCGCTCgtaaattcttataaatgaattgtaaattcttataaatgaattTTATTAAT
ATATGTATGGTCAGTGGTACAAAAACCGGGTTCGTTCTGGTTAAATGCAATTCAGATGTGCATATGTATG
TGTGTAAATATGTGCCTTTATGGCAGAATTGGTTAATTAATTACATGTGCTCGAATGAAATCGCTTCAAT
CAATGTCTGCTTCAGATCAGACGGCAACAAAAGATGAATCGATGTCTGGCGAGGATCAGTTACTTGAGCT
AGCCAGGTGTTAAATTTAACATAAATGTTAAGAATCATATTCAAATATCTTAAATGATTTCGAAGTGTTT
TTATTTATCTTCATTTCAAAAGTTCCATGAAATAAAATACATAAAACAAACAACATTATGCTAATACGAA
GAAAGAATTTTATTCGAATCATATGGATTTCCAAGAATGTTGATATAAACACATGAAATTACGGGGAAAA

TGCATTTGAAATACGTTTATCGATAGATCGATGAGCAATGCGCTGCAACTTTGATTGCCTTTCTCTTCGG
CCAATATACAAGACCCAGAATCCAAATCATTTTAAATACTTGAAGACCACAACTAATGACATTACGGCTG
GTCTGTACTTAAAGTGTGACAATCTGTTTATTGCTACCATTTGCTTTGCTTCTTGTGTATTTTTGTGTGT
TTTCTCCTCCTCATCGTGGTTTAATGGACGCCATTGTTGTTGACTTTTGTGGTTCGTTCTTCGGGTCCTC
GCTTCTCCTCTTCTTTGTCTGGGTCCAAAAGAACCCGGGTTCAAGGTGAAATCAGTTCATGTCCGCAGGG
CAGCCTGTCGGTCGTCCGTTGATTATTTGTATCCTGCCACGCAGGCGATCAGTCTACAGTAAAACCTCCC
TGTAGCGAACCCTTTTCGAAGTTAATAATGGGTAAAAGCGGTATTTCAAATTTAGATGCCTTATTAGATA
GTTCTTGGCTGCAAAACTATCATCATTTTCTAGTAATTCTAATCTTGTTTATTCTATTAATGCTATTCGG
TCTGAACTGTaaaaaaaaaaCGATAAGTACCGTGTCCGTCTGTCCAGTTTTAACCCTCCAGTGCCTCATC
CCTATTCGCTCTGTCTTTTCCCGTAAACATTCAAATCAATAGGTTGTTTGCAACAACCCTCAGTTTTTGA
ACTTGTACTCACTTCCCGGGCATGTGCATGTGGAGTTTTTTGACCTATCGCACAACTTTGGTCGCCGCTT
CAACATTTTGTTGTTTCCAGAGCTGTGTAattttgaacaggtgtttcccagcgtttcatttttgtttttt
ggttgtggtttggtttACCAAACATACTATACACGTTAATCCTAAGCTGCCCTGCCCCTCAATGTCTGGG
ATCAGTCCTTTCATTCATTTTCGAGGGCGTTGTTCAGGGTTTTATTCATTTTCGCAACAGAACCAAAATC
GCCGGAAACATGACCTCTCGAGGCGATCATTTATAATGGCAAAAATAATACTCCTTCAGTCTGAAAGGAG
ATTTCTGTAGTCTTAAATGTAGTCGTCTCTCGGTCGTTCATAGATTGTAATTTATTTTGATTAATGATGG
GCGGCAACGTCTCTCGCTCTCCTCTTGGCCTCCTATTTCTTGGCATCTTCTTTCTTCCCCAGTGTTCCGT
TAGTTTTATATTTTGTTTTCAATATTTATATTTTGTTCATCAACTCGTATTTTAAACCAATTTCATTGGC
ACGCTCTACTGTTGTATTATACGTAGTATCCACTGTGTGTATCTAAAACATTGATTGATTATTTTAACCA
CTTTAAAGTATTTTGTTGGGAAAACATCTTAATTAACCTCTTTAAAACGGGAGGTATAAGATGTGATTTC
CTCAGTTGGAACTATAAGTTTTTGTACCCACACTTGGACTTTACAATAGAATATAGGGTCAATCACACTC
AATATAAACCAGTTATCATATTAGCCGGGCTTTAAAGTATAGCTAATGGGGCCTGGTTTAGTTGGCGAGG
TGAGTTGAGGGTTAAGACTGTGAGCTCAGCTGCCTGACCGGAAGCTCCGCGCATTCTTGCGACTTGCGTT
TTGAGCTTAAGCTTAAGCCCCTGAGCTCAACTCGGTTCTAAATTGAGCCGAGCAGCCATCTTGATTCTCA
CTCTCTTTCTCTCGTTGTCTCTTCTCTTTTGGTTTGTGCCTCCTTTTGCGCACATTTTAATAAAGTTTAA
TAATTCAATGGGTGATGTTGTCTCTTTGTTGCATTTCATCAGCATATGTTTGTTGGTTTTACTGTTGCTG
CTCCTTTATCTTTATCGTTGTTGTCTGGTCCGCTTTACGCGCTCATTAATATGCAAATGTGAGTCAGAGC
GGTTTATGTTTGTTGTGGTTGTTCCTGGAGTTATACATGGATCTTTTTATTTCTTTATATTCTTTTTACC
TTTTATACTGCCCGTCATCATCATCATCTTCGTCGTCATGTCCGTTCCTCGAGCCTTTCCTGTTGTAAAT
TCAGTATTTGAATGCAGGTCAACAGCACATTAACCTGCGTCCTTGTAATTTGTTTGCTATCGTCCGCTTT
CATATCAGTGGATAGCGGAAAGTGAGTGGTAGTCGAATATATGATACCCCACAGCGATACCCGATCCTGA
AAACCCCAATCAATTTGACTCGTTCATGTTTGACTTGCATGATTAATGGACATGAGCCACCACACCTGAA
GAAATAAGCTAAATAAGAGCTGAATTATTGAATAAATGGAATTATTTCATACGCGTATGTACATGTTTGT
AAAATATACACGTTTTGCTATGCATAATGCACATCCAGATAAGCTGTCCCTCAATAAAAATGCATAATAC
CGGGGAATGCGATTTATGAACGGTTTCTTCTCACCTCCCGCGATTTCTATTCTGTTATCGCTTTCGACTT
GCGTAATAATGCCTTTTGCAATGGGGAAACCACGCGCTAATTTTTGCCTCTCTTGCTAATTGTTTTGCAC
TTAGCACTTGCatttattttatttttcttatattatttttttaCTTATGCAATTTACTTGAATGCGCATTT
TTCACTCTGCTGCGTAAGAGAGCAAACAAACAATGACGTCACGCGCTTAAGTGATGTCGTCATAAAGTTT
GCGCGAAAGAATCATATGATTTCCTATTTGCTTTAATGACCAACACTTTAACACACGAAAGCAGAACGAC
ACACGTTCGCTAGATAACAAAACGAATTCGCTTAAAAATAGCAGAGGTCGTTAAGTTGTCGGAGGGGGGT
GAGTGGGATGGGAAGGAGGCATACAAAACAAAATAAATAAACCCAATACATATGCAAAGCTTTTATTCTT
GATATGTATaaaaaaaaaCTTTGGCACAGCAGCCGCGCGTGCTTGTTGACGTATGTACATACTAACGTAC
ATATATTTTTGTATGCTCACGTACAGTGaaaaaaaaCCGACAGTCCAAACGATTCTTTTAGATATTTTT
ATGTAATTATTGACCTAAGAAACATGTTTTTGTATTTGATACACTACCTTTTACTGTTTCATCGTTGGTG

TGATATTCTATGCAAATGCTTGAAGAATAGTTTAATAGATTTTTCTGTGCACATAAAGAGTCAAAGAGAG
GGAGAGAGATATTCGCCAGCCAATTTGTTGTGTGGGCAATTTTTCCTTTTATTGTTGACATTTTGATTAG
CCCACCTCCAATATATAAAATGCATGTGTAACTACGTATGTGTGTGTGGGGGAGCGCGAAAGAGGATGCG
AGACGGCCAGCTGTTTTGACTTTGCAATTAAATGACAACAGCAGGAAATCAAGCCAACATATGTACATAC
AAACACACTTGAACATCGCACATTGTTTGCTCTGCGCTCCACACCGCAACTATAAATCAAAATAAATTTT
CTCATACAAGCAAAAGTTGCGTTGAAATAAAGGCAAAGCTAGGAAAATTATTTATGGCTGCGCCCCACTA
CACAAAAACAACACCAATACACTGAATTCAGTCGATTGTATGTACAAACCTTAGTAAATGCAATGCCAGT
TTAAACATATGTCACTTTTTCTCCCCCCTTTCTCCATCAAGCTCAAACTCACCACAGCAAATGTTGCGCT
CTCaaaaaaaaaTACTCCCCATGCTACGTTGTAAATGTAAAAGTTGCTGAGGCCATATTTTGTTTGCTTG
TAGTTTACATGCGCACATCTCTAAAACCTGTCTATGTATTGAGCAAAAATGTTGGTTGTTTTGGCTACAG
TTGTGCAATGGTTTATTGTGTTGAAGAGGAACCCCCCTCAGCTTAAGCCATTTATTAGTTTGTAATATAC
GACAGCAGAGCTATGTAGCTTTCTCACCACTAAGGTCAAATTTTGTATAACAGAATATCGCTCTCTTTAT
TGCACACAGGCGCACGCACACATGCACACATCCGCTCACGCATTCAGAGCTTCGTGTGCCCATTTTGATT
CTCGCCCCGAGCTCCAAAGTCGCAATGAAAATAACAGTAAATGTTATTGTGCTTGTGCTCAATtctcctc
tctctctctctctcgcccttttaccttttttctctcgttctctctcATTCCTTCTTTGTTGACCATGTAA
CTGATGGTTCAATCTCCCACCTCTTTTTATTGTTGTTGTTGGTGGATGATGGCTTTTACCATTAGGTTTT
GACTTTACTTCTACACTGCGGGAAATGAAACGATTAAACTGTAAGAAATTCTAAAAACTTAATTTTATAT
GTTTTAAGAGATCCATTTTAGTTCTTAACTAAAATCTTTTTTAATTAATTATTTTAGTTACTTTAAATTA
ACTGAATACATTTGTGTATACGTTGGGAAGTGTCAGAAACCAGTTTATATAATTTATTCAGTTTGAATTT
CACAACATATTGCAAAACGATTAAGGTACAAGGGAGTGCGTACTTTGAACTGCGCCATAAAAAGATGTAT
CGTTCAGTGTATCTCTCGGATTTCGCCCATAACTCTCTCTTACTTTGTTTTTGATGGATGTGCAGCTCGG
CACGTCGACTTTTCGACTTTGAGAAAGTAAACTGATCGCGATAAATCGATACTGGCCAGGTATTTTCTGT
TAAATCGATCGACATAAACAATTGGATACTTGGGGAATATATATGGCACTAGTTTGAACCGTTAGGTGTG
ACTGCTTTATATTCATTAAGCATAAAACCAAACTCGTTTAGCTTACTACCTCTTACTACGGCTCGTTCTC
AGCTTGCTGGCGAAACTGGTTTAGGCCCGACTTTGTTCCCTCTCTCGCAGATGAACCTATAGTGTGTATA
TCTGTGTGCGATATGCTTGACAGCCTTGAAGAGAGCGAGCGGATAAATGAAAAACAACATAGTGTTTACT
TGTTATGTGGTTTCTGCTGTTGTTGCTAAATTTTTTATACAGCATACATTTGTATTTTGTGTGTGTGATT
GTGGGTCTTGTTTTTATTGAACGTATTGCAATGCAATTTAGTGCGACATAGTTGCAGATCATCAATATGA
AACAACATAATCCATTTTAATTGTTTCCATATGAGGAAACTCATAAGCAATAATCAATAGATGATACTAA
AATACTATATTTCAAATGATGATATTTCCATGCACCACTCTGAAGCTTTTATTAGCATCGTGCAGATTAT
GGATTTCTGAAAATTGAAAAATTACATTATGCTATGATAAACCTTTTACAAGGAGAATTTACTAATTTTG
CCCTATCTTTATTCGTTTTACAGGCTCGTACTCTGGATTATTTACGGCAGTCACGAAGCAAAAAACATAA
GCGCAGCCTAATAATTGGACGAACTGATTAAACAACAGTTAAAACAAGGCCCGCAAAAACAACGGCGGAT
CAACAGTTgctagtcgcaagttgcaaatcgctagttgcacgttgccaatTTGAAATTGCACGCTTCCACG
ACTGAGAGCAGGAAGGAAAGGAATCGCTGCCGCAGCAGGAAGCAGACGAAACTTCGCTGGGCGAAAGTAA
AAGCAGGGTTGGGAAATAAAGCAAATCAAACAAACAAGAAATCGACCGCACAGACCCACAAAGGCGCTTT
AGCCAGCCATGGAACGTGAATCTAACCCAATGCAACCCATGTGCCGCTCCGGTTGCGGCTTCTATGGTAA
TCCAGCCACAGATGGTCTCTGCTCCGTATGCTATAAGGGACTCGCTTAGAAAAAAGCAACAGCCACCTGT
GAGCAGCACACCTGTCTCCGTTCCAAGCCCACAACCTAGCCCCACATTCAGTCCGGCCATCGCAATCACC
AACACCGCACAGCCCACAGTCACCAGTTTACAACAGCCACACAACGATGTCAAAGAGGGTGAGCAGAGTT
ACGGAGATGTTTGCAGAGGAGTAGTTCCAGTTTCCACTATCAAGATTAGTTGTAATTTCAAATTTAAAAA
CGTCTTTCAGTCTTCTTTAAAAGCTATATTTTGTACTCCTGAAAAGCGGTCCTCTTAGCTTAGTAAGCGT
TTCTTGAAATTAGAAACTGGAACTAGTCTAGTAGAATAGTAACTAAGATCCTTGCATGTTACATACTTAT
GGAACTTTTTCAAATGATGATACTTCCATGCACCACTCTGAAGCTTTTATTAGCATTGTGCAGATTATGG

ATTTCTGAATAATGAAAAATTTCATAATTTAATACATGACTGCTTTGATTTGCACGAAAACCACATAGGC
TCGGCGTTTTGTGCCCTGTTTAGTATATTTTTACATTCAGTCAGTGATAATTACTTTTGACTTTCGGACA
TAAATTCAGTGTAATCTTAAGTGAAATTGTTGAAACAATTTGTTGTTTGTGCAAATGAACATTAGGGGAA
ATCAAGAAGTTGGCTTGCGGTGTCGATTTAGTGATCTCTGTAACTTTTGTGTCCGTGTGGATGAGTTTAC
TATCCTGATGATCCTGCCACCAATTACTCCGCCGCATTTGTAAATAAGGTAAAGTGTCCACGGTCTCTAT
CCGCCAGTGACCACTCCAAGAAGCTGCCGCTTCAGTTATACAATAATCTTAGAACGACTTAGAGCGGGGA
TTCTGAGATTTCGTTTCAGTTTTAAAGTAGAGTACCATTTTCCTTTGATCTTGTTCGATAATTGCGCTAT
TTTCTTCTTAGTTAATGTTTGATCTGTAAGCGATCTCAGAAGCCCTTATTCCTGCAAGAAATCGTTCGGT
TTAATGTCACTTATTTGTCAATTCCATTCGGTTTCAGTTTGGCAGTAGCTTCTGCTCGGAATCAAAATTT
TCCTATATTTCGGCCCTTATTTCAAAATTAAAAGTCTTTCCAAATTGTTAATATGGTAGTTACCATTGTT
GCCTCTCCGCATAAAAAAACTGGTCGTGTGGTACCTCCCAAAAGTACCCCAGTTGCCGACAAATCTCGAT
CCGTTTCCACGCTTCTATCGACTCATAAGCGAAAGGGGCCGATGAGCCTTGGAATCAAGCGTGCCGCAGC
CGGCGCGCTAACGAGTCTTAGCGGACCGATAGCCGCCGGACCTGTGGGCCTTGGCCTGGTCACTGGAACG
GATAGGAATGGATTCCCGCAACACATTGCGGCCTCAAAGGGTACTGAATGTTCTTACTGGGTCTACCGGC
GTCGCCCAGGCGCACTAACCTCGTTTCCCACCTACACATCTCTCCTATACTTCGATGCGATGTTGCAGCA
CAAGCGACAGCAACCCGTCCAGCAGCTCCGATCCCGCCGCCACGCTCATACCTCCGGGCAGGGACATCAG
CAGCACCCGCTTCTGCCTCCGCTCGCCTTGCTGCCAAGGTGCCTGGTGCCCCAGTCGTCGGATTGGTCTC
ACACACCGGAAGCAGCAACATAAGCCAACCTCCGTCAGTCACCAAGAAGAGCTCCCTGTCCAGCCGTAAG
CTTGGAGGTGGCAAAACCACAGATTCCAGCATCAAGCGGCGCGTAAAGTTCTCGGCAAATGTGCACACAT
TCCCTTCCCTTACAAACACCAAGAAGTCCACCCGGATTGGCGAGCTACAGCTGAAGAAGAAGGTAAAGAA
GCTGAAGCGGATCCGATCGCGTCGGGAGGGCGGAAGTGGTGAGACAGCGCGTGATCTCCTTAACTCAAGT
GGTATAGCCGCAAGTAATCTGGACATTTACAAACACGGCGTTATCGTTGATGTGGAACCAGTTAAAACAC
CAGAACCACCACGTGTTCTGCGCCTAAACGTTGTGGGCAAATCATCGCCGGCGACGGGAAAATCTGCATT
AATAACAGCGCTGCGGCGTAAGGCTAAAAGATCAGCAGAACAGGAAGAAAAGTCCAAAGAATCTATAGCT
ACTGCTACAGGTAGTAGGACCGGAAAGCCCAAGACAAAGACGATCAGAAAAAGTGGTGGTAGCAGCGCGA
AGACTCTGCTCGCATCCAGCTCTCATTTGGTGGATAGTAGGGCTGGCTTAGAGGGGAGGAAGAAATCAGT
AGTAACGGCGGCAACTGCCAGTCAGCGGGTTAGTCATAAACAAAAACAAAATGGGCGGCGAGGCCATGGA
AAAATCTGAAGGCGGCGATCAGCCGGAGAGTGCTGGCGAGAAGGAGCAGGTGCATCAAGGAGCAGACCAA
CAGGTTCAACGGGAAAAGCGTCGTCGAAGTGTGTCTCCTGATGGGGGCGATGTAGGCAGTGTAAAGATGG
CTGATGTGGTTTTAGAAGGCGCATCGGATGTTGCCGTAACTAAGAAGCGGGTAAACATTAACCACAACAC
ATCATCATCGCATCGCCACTCTTGTTTTCCTCACAAAATAGATTCTCACTCGGCCGATTCGATACGTATT
CTTGGCAACAGTTGTACAGTTGACGGTGCTCTGCTAAAGCCGAAAAAACGCCACTCAACGCCACACAGCG
AATGCTAATACATTTTGATGAAACAGTCGAAATTGTTTAGTCTAATAAAAGAGGAAATAATATACTAAAG
CAAAGTTCTTCAAGGCTTTTTGAATGCAGATCGAGGCGGCGGACGAGAATGTATAGTTGTGGTTTAAATA
ATAGATGCTGTGTTTCCTGTGTTTCAATCTACAATTAAATTAAGAGATTAATAGAGATTTTCCATTCCCT
TTTTGATTTGTAAAGCTCTCGCCCTTCACTATTATTTTGCGCTCAGCTATGGAAAAGCTTTCTTTCTGTT
GGAAAATGGCTAGCAAATCTGTTGATGCTATCATATATTTTTGTCATGTGATTGAAATTCCGTTTTTGTT
TTATTTCCGTTTCCGCAGGAAAATCACCGAAGAAGCCGCCGCTGCTGCCAAAGTCAACAGCGAAGCCATC
ACATCGGCGACTGGTCCAAACACTTCCACACAAGCAGCCGCCTCCGCCAACGAGGAGGATGACAAGGACA
AGGAAGACGATAAGGATGCCAAAAAGAAGAAGAACCGATGTGGCGAGTGCCGCAAGAAAGTTGGCCTGAC
CGGGTTTCCAGTGTCGCTGCGGCGGTCTGTACTGCGCCGTGCATCGTTATTCTGATAAACATAACTGCAC
ATTCGATTATAGAGAGCATGGCGCACAGGAAATCCGACGCAACAATCCGGTTGTCGTTGGCGAGAAGATT
CAAAAGATTTGAATTTCGATATTGTGCCAGTAACAGTAACAGTAGCCGTAGAATGATAATAACCAACTAC
ACATGATAAGAGTACatatatatatatatGCTGCGCTATAACAAGGAAagcaacaagaaaaacaacacca

gcagaaagcataaacacacaccaCACAAAATCACACAGAGTAATGCTAAATGAATCGATAAAAACTGTAA
TTGTAATTTAATTATAATGTAAACAGCAATTTCAAGCAACAATAGTTCAGAGAAACGATAACCAAATACC
CCGCAAAAATGAAGCAACAACTGGAACAACAATTTCGATGCTCAACAACGTTAAGCTAGCAACGTCCAGC
AGCAAGCTGCGGCAGCAACTTGCCACAATGCACCAGGAAGCAGCGTTCAGCGGCCAACACCTTTGAACCA
GACGCGTGACAGCGTTAGCAAGTGCAACAACAATCACCGCCGCGTTTGTGGAATACGCAGAGCAAACGCA
GCAACATCAACAACAACCAGCACAACCGATTGCCTTGGTCGCCTGCTGAGCTGGGCCGaaaaaaaaaCAG
TCTTGAAGAAAACACCTTTCATCGATCAACGTTGAAATAGTTTGTTATTCCAAATACAATTTGAAAATTG
TTTGTATTTAGCTAAAAATCCAacaaacaacacacggccacatttgcaacgacaacaagcacacaacaaa
caacaaacCTTTGTTTCATGTTATCAAACACCGCAACAATCTTTTAGTAAGACAATGTGTGTCATTTGTT
TTTGTAATATGTTGGAGTGTATTAAATaaaaaaaaaaTTCAAAATGCAAAGTATgaaaagaaattttaag
aaaaatttaacaaacccaaattcacatacaaaacaaaaaaaCCAACCAAGCAACCAACTCTTTCTCAATT
CTTAATTATGAGCTATTAAATGaaaaaagaaaaaaaCTAATATATATTAATTAATTATGTATATGTATTa
aaaaagaaaaagcaagaaaaaaaatatgcaaaattaagcgtaaaaaaacaaaGCCCTCACCACaaaaaaaa
aaaatgtaaaaaaaaCCTGAAGAAAAGAAACAAAGAATAACGCAGTTGTGTAGAGCTCCCTAAAAAGTTT
GCTAAGAATCCACGACCACGCTAGACACGAGCgagagagagtatagctatatagagagagagagagagag
cggagaAGCGCGATTAGTGTAAAGCAACGTGGAGCATAACAACTCCCCTATACATAGAAATCTAAATATA
ATTATAATTAATTAGTGAATCAAAGCAGGCGAATGCAGTGCAAGCGGCGTTTTTTCTTCGTTTTACTTTT
TGCCCTCCTGTCCCTCTAACATTAAATTGAATTGATTTTAATTTTCTGATTTGCGATGAATtatatatat
atatatgcataatattaactatatctacatctatatatGTATGGTAGGATATGTAAATCGTGTGAAGCAT
ACACACATATAGTTTCTTTGGGTATATGCACATTAAGGCGaaaaacaaaagataaaaaccattaaaaaaG
CATATTCTATCtatatatatatataGCGGTTAcaaagaaaattcagcaaaaatggcaaacgaaaaatgca
aaaaatacaataaatATATATGGTTCgtctttagatttttattgctttaagcgttatttagtttttcttt
gtttttattaAAACACACATGCGTGTACACAACAAAACAACCAATCCCTTTTTAATTTAGCTTTCTCCAT
TCTGTAGTACATAATTTACAATTTAATTCAAATCAATTACACCTATGCAAATAAATTTACATTTAGAAAT
AAATTGTGTTGTTAACCCTAcaaacaaaaaaaatacaaaaatgaaaaaaaaaaaaaatcaaaacaaaTGTTG
TAAGTAAAACTAAACTTACCAATAACAAAGAGGAAATAATGCAATAAATTTGATTCTACAATTAAAATTC
CCGCAAGACCAAAACCAATCTAATAAGTACATTTTTGGCACAAAGAACTGAGAAGGAATCGTTTTCGCAC
CACAGCAACAAAACAAAAGCCGACACCAAACTCGCCTTTAACCAATCGCCTTCCCATTGGGAAAACTTTT
CGCGAGATCGAATCTATCTAGAGGTGTTACCAAATCCCAGGCAACACTCCTAGTTATACGTTGATCTGGG
GAATCTCGGGGTCAAAGCGGGTCCATAGGGACCTTCGTTGTGCACAGAAGATTCTGGACAAGAGCATTGA
GCACAGCAATCCAGCAATAGTGAAGTCCAACCACGTTAGGGTGTGTTTAGTCAGAACATTTGAACTGTAC
AAAATCAGAGATGAAAAATGCAACCTAGAAACGAACTTCAATATAATTTTAAATGAAAGACGAGACCTAA
AAATGTACAGAGCCAGAGATAAAATAGATGGAAAAATATTGGGGATGTGAAAAACTGCAATTAATCAACA
AGAACAGCTAAATGTCATTTTAATTTTAATTTGTCCCCCGCAATGATAAAGGCAACACTCTACATATACG
TATATCATAATTTAATTGAAAACTATAAAATTAATTAGAAAAATTGTATACATACACGAACACACAATAT
GGATTTTAAGAATTAACGAACAAGCGTACCACAATTTTAAGCCGTTTTAAAACTGATTTACTAATAAAAT
TTGAAGATGATTATAAACTTCGAATCGATTTAAAACCCATTTCTATAATTTTTGCATAGTTGTAGTTTTC
AGTTACAAAAATTTACAATGCTTTAGTGTGCTTTTAGTTGAAATTGTGTTTATATATTTAACGAAAGAGT
AAAGTGAAAAGGCAAATAATAATTATAACTAAGCGTAATAAATTTTTATTATTATTAACCAGAACGTTTA
TTCTAGTAGTTCTTTATTGTATTACCAGATTAATTATTATTATTTTGTTGTGTGCACCTCTTAGTCACTT
TTGCGCTTATAAGCTAATCAAAACAACTCGCAAGCAATCACTTCCACACCGTGTAcaatcccaatcccaa
tcctgatcccaaatcccCCAGCCTGAGAATCCCAGTTCCGCCAAAATCTACTTGTGTTAAAATCTTTTGC
AATTGCTAGCCGAATTTGATAATTGTTTGTCTCTTACTTTGGATATGTGAAGTGTTTTGTTGACAGAAGG
AGCAAAGATGGAACGAAATTAGTTGATTTAAGCGTATGAATGGGATTTAATGAATATGAATATGAATGCA

TCCAATATCTAAAACTCTATGCGTATATAAATATTGTACTAATGTGTAATTGTAATTAAATGCGTCGGAT
GAGAATATCTGCAGCTGAAGCAGAAGTTGGGTGGAGAAACAGTGAACATATATATTTAACACAGAAACGG
TAAAGCTAGAAAATCTAAATAGAAATAGATATATACTTATACCTAAACATACATGAGCCTCAGATCCGGG
CGTATATAAGAGCAACAACTATTTTAATTGCAATTTCAAGCAACAATAGTTCAGAGAAACGATAACCAAA
TACCCCGCAAAAATGAAGCAACAACTGGAACAACAATTTCGATGCTCAACAACGTTAAGCTAGCAACGTC
CAGCAGCAAGCTGCGGCAGCAACTTGCCACAATGCACCAGGAAGCAGCGTTCAGCGGCCAACACCTTTGA
ACCAGACGCGTGACAGCGTTAGCAAGTGCAACAACAATCACCGCCGCGTTTGTGGAATACGCAGAGCAAA
CGCAGCAACATCAACAACAACCAGCACAACCGATTGCCTTGGTCGCCTGCTGAGCTGGGCCGaaaaaaaa
aCAGTCTTGAAGAAAACACCTTTCATCGATCAACGTTGAAATAGTTTGTTATTCCAAATACAATTTGAAA
ATTGTTTGTATTTAGCTAAAAATCCAacaaacaacacacggccacatttgcaacgacaacaagcacacaa
caaacaacaaacCTTTGTTTCATGTTATCAAACACCGCAACAATCTTTTA
>dme_circ_0008321_junction_seq
TGTTATCAAACACCGCAACAATCTTTTAGAAAAAACCGTAATTCGCCACTTGAAAC
>dme_circ_0008322
GCTCGTACTCTGGATTATTTACGGCAGTCACGAAGCAAAAAACATAAGCGCAGCCTAATAATTGGACGAA
CTGATTAAACAACAGTTAAAACAAGGCCCGCAAAAACAACGGCGGATCAACAGTTgctagtcgcaagttg
caaatcgctagttgcacgttgccaatTTGAAATTGCACGCTTCCACGACTGAGAGCAGGAAGGAAAGGAA
TCGCTGCCGCAGCAGGAAGCAGACGAAACTTCGCTGGGCGAAAGTAAAAGCAGGGTTGGGAAATAAAGCA
AATCAAACAAACAAGAAATCGACCGCACAGACCCACAAAGGCGCTTTAGCCAGCCATGGAACGTGAATCT
AACCCAATGCAACCCATGTGCCGCTCCGGTTGCGGCTTCTATGGTAATCCAGCCACAGATGGTCTCTGCT
CCGTATGCTATAAGGGACTCGCTTAGAAAAAAGCAACAGCCACCTGTGAGCAGCACACCTGTCTCCGTTC
CAAGCCCACAACCTAGCCCCACATTCAGTCCGGCCATCGCAATCACCAACACCGCACAGCCCACAGTCAC
CAGTTTACAACAGCCACACAACGATGTCAAAGAG
>dme_circ_0008322_junction_seq
ACAACAGCCACACAACGATGTCAAAGAGGCTCGTACTCTGGATTATTTACGGCAGT
>dme_circ_0008323
gaaaaacaacaccagcagaaagcataaacacacaccaCACAAAATCACACAGAGTAATGCTAAATGAATC
GATAAAAACTGTAATTGTAATTTAATTATAATGTAAACAGCAATTTCAAGCAACAATAGTTCAGAGAAAC
GATAACCAAATACCCCGCAAAAATGAAGCAACAACTGGAACAACAATTTCGATGCTCAACAAC
>dme_circ_0008323_junction_seq
TGGAACAACAATTTCGATGCTCAACAACgaaaaacaacaccagcagaaagcataaa
>dme_circ_0008324
AATATGCtttttaatggtttttatcttttgtttttCGCCTTAATGTGCATATACCCAAAGAAACTATAT
GTGTGTATGCTTCACACGATTTACATATCCTACCATACatatatagatgtagatatagttaatattatgc
atatatatatatataATTCATCGCAAATCAGAAAATTAAAATCAATTCAATTTAATGTTAGAGGGACAGG
AGGGCAAAAAGTAAAACGAAGAAAAAACGCCGCTTGCACTGCATTCGCCTGCTTTGATTCACTAATTAAT
TATAATTATATTTAGATTTCTATGTATAGGGGAGTTG
>dme_circ_0008324_junction_seq
ATTTAGATTTCTATGTATAGGGGAGTTGAATATGCtttttaatggtttttatctt
>dme_circ_0008325
TGTTGCCTTTATCATTGCGGGGGACAAATTAAAATTAAAATGACATTTAGCTGTTCTTGTTGATTAATTG
CAGTTTTTCACATCCCCAATATTTTTCCATCTATTTTATCTCTGGCTCTGTACATTTTTAGGTCTCGTCT
TTCATTTAAAATTATATTGAAGTTCGTTTCTAGGTTGCATTTTTCATCTCTGATTTTGTACAGTTCAAAT
GTTCTGACTAAACACACCCTAACGTGG

>dme_circ_0008325_junction_seq
TGTTCTGACTAAACACACCCTAACGTGGTGTTGCCTTTATCATTGCGGGGGACAAA
>dme_circ_0008326
GCTGGgggatttgggatcaggattgggattgggattgTACACGGTGTGGAAGTGATTGCTTGCGAGTTGT
TTTGATTAGCTTATAAGCGCAAAAGTGACTAAGAGGTGCACACAACAAAATAATAATAATTAATCTGGTA
ATACAATAAAGAACTACTAGAATAAACGTTCTGG
>dme_circ_0008326_junction_seq
TAAAGAACTACTAGAATAAACGTTCTGGGCTGGgggatttgggatcaggattggga
>dme_circ_0008327
CTCACCGACGAGGTGGTGCGCAAGAAATCTGAATACCAAAAACATTTGGAGGGCTACAAGGCGCTGCGCA
CGCGCTTCGAGGAGAACTACATAAAGGGTGAGTGAGAAATGAAGGGTATATCGGGTTGCCAAGTGGCAAT
CCAACCGAATATGGCACAATGAACTGAGTTTCTGAGTTGGTATGATATGATTGAAATTGAAATTTTAGCA
CCCAGTCGCAGTGGCCGCAAGCTGGACGATGTGCGTGACAAGTATCAGAAGGCCTGCCGCAAATTGCATC
TCACACACAACGAGTACGTGCTGTCCATCACGGAAGCCATCGAAGTGGAGAAGGACTTTCGCAACGTCTT
GCTGCCGGGACTGCTGGAGCATCAGCAGTCCGTTCAGGAGAGCTTCATCCTGCTGTGGCGCAACATCCTG
CAGGAGGCGGCCCAGTATGGCGACCTCACGGCCGACAAGTACAAGGAGATCCAGAAGCGCATTGACACTG
TGATAGGGAGCATCAATCCGACCGAGGAGTACGGCGAGTTCACCGAGAAATACAAGAACCTCTCCCACAA
CACCGCTGCTCTTCCAGTTCGATGAGACGCTCATCCAGGATATTCCCGGCAAATTGCAGTCGAGCACATT
GACAGTGGACAACCTCACGGTGGACTGGCTGCGTAATCGTCTCCAGGAACTGGAGGGAGCCGTCAGGGAT
TGCCAGGAGAAGCAGATGAAGATGATCGAGCATGTGAATGGTGGCTCGCCGGTGGccaatggcagcatta
tatccaacggcagcaacacatccaacggcaTTCAGTCCAACAAGGATAGCCTATGGCCGCCAGTCGAAGG
ACCTTAATGCACTGCGCTGCCAGGAGAAGCAGAAACAGAAGCTGGTGGACATGATCAAGTGCGCCCTGAA
CGAGGTGGGATGCGAGGAGCTGCCCTCCGGTTGCGACGATGACCTCACCCTGGAGCAGAACTTCATCGAG
AATGGCTATAACAACGAACAGCAGG
>dme_circ_0008327_junction_seq
GAGAATGGCTATAACAACGAACAGCAGGCTCACCGACGAGGTGGTGCGCAAGAAAT
>dme_circ_0008328
GCAGCCTGATCTCCAAATCCTGGCGATCCTACATGGATGAACTGGACCACCAGGCCAAACAGTTCAAGTT
CAACGCGGAGCAGCTGGAGGTCGTCTGCGATAAGTTGACCCACCTCTCGCAGGACAAACGGAAGGCCAGA
AAGGCCTACCAGGAGGAGCATGCCAAGATCGCAGCACGCCTGAATCATG
>dme_circ_0008328_junction_seq
GCCAAGATCGCAGCACGCCTGAATCATGGCAGCCTGATCTCCAAATCCTGGCGATC
>dme_circ_0008329
CTGCCTGCGCGAGCGTATCGTGCGCGCCTTCCTCATCGAGGAGCAGAAGATCGTCAAGGCCCTGAAGAGC
CAGCGCGAGGCGCTCGTCAAGCCGGTGAAGGCCCCCAAGGCCAAGCCCGAGCCCAAGAAGAAGCCCGCTG
CTGGAGCcaagggaaccaaggccggtgccggcaaggtcaccaaggg
>dme_circ_0008329_junction_seq
aggccggtgccggcaaggtcaccaagggCTGCCTGCGCGAGCGTATCGTGCGCGCC
>dme_circ_0008330
GAGAAGTTGAAGGGTATCACCCCCTCCCGCCCCAGCGAGCGCCCCCGCATGTCCAAGCGCCTGAAGACCG
TGTCCAGGACCTACGGTGGAGTGCTGTGCCACAGCTGCCTGCGCGAGCGTATCGTGCGCGCCTTCCTCAT
CGAGGAGCAGAAGATCG
>dme_circ_0008330_junction_seq
GCCTTCCTCATCGAGGAGCAGAAGATCGGAGAAGTTGAAGGGTATCACCCCCTCCC

>dme_circ_0008331
GCTAATGAAATTGGACACATCCGAAGACCTCGCCCGATGTGCGTGGGTGCTGGCGCATCGCCTTTGAATC
GAATTCAGAGACATGGTGAGTCACTGACCAGAGGCCAAGAAACCCGCGTAGGAGTTCGGTGTCATGTGAG
CAGAGATCAGTCAACAAACACAACATTCAACGGAAGGCGAAATTACAGCATAGAGCAGACGACACATATC
CACAGACAGGTTGCACCATCGAGGAGCTCCATATGGAGGCCACTGATCAGGAGGTCATGCTGGAGGGCAG
GGAAGACCTCATGTCCACATCGGCGACATCGTCCACAAATGGCGGGGACACTAATGGTTGCGGCAAAAAA
TTGGGTGAGTTTTCCATTGCGCTTAAAGCTGCTCACTATATCGGTAGTTTTGGATCCATCCCTTTTCCCT
TTTAAGTATTTTTACTATCTTATAACTGAATATTCAGACGTACATAATTTGAAAATATTTCAATTTCAAT
ACTTCATGCGTTCGTTCAAGCAGAACTAAATGTATGAAAAAGGGACTCGAGTATTACCCAGTATTACATA
AGCTGCTGAATGCTTTTTGCAACTAAATACTAAAATGCTCAAAATGGTCAAGGACGGCAGCTTGGCAGCT
TAATAAGCAATTCAATGGAATTTAAGATCAGAAAATGCAGCCATATAGGCTACTGCCCACACGTTTATAT
GGAAGCAGTATCAATAACTCCACACCCTGGACATCTGTCCGCTGACATATGCATTTAACTCTCCCTTTTG
ATTTCCCGCCCCACACTACTCCGCTCAATTGAAGCAATTCATGCTAATTAGAAATTTTATTGCATTTACT
GCACTTATTGTTAATTGTAGGTCCGTAACCTCTCCGGATCCCACACCGTACGTGACCAAGCAGCGAGTGA
CGCGTCCAACGCCTCCCGCTCCTTCAAAGAATCCGATGCAATTCGTCCAGATCAAGCCCTGCAACCTCTA
CCAGACCGCGCAGGAGCAGCTGaagaaggccgaggaggtcaagaagctgaaggaggtgaagaaggAGGAG
CCAGAGGAGTGGCAGAAC
>dme_circ_0008331_junction_seq
gaaggAGGAGCCAGAGGAGTGGCAGAACGCTAATGAAATTGGACACATCCGAAGAC
>dme_circ_0008332
GCTAATGAAATTGGACACATCCGAAGACCTCGCCCGATGTGCGTGGGTGCTGGCGCATCGCCTTTGAATC
GAATTCAGAGACATGGTGAGTCACTGACCAGAGGCCAAGAAACCCGCGTAGGAGTTCGGTGTCATGTGAG
CAGAGATCAGTCAACAAACACAACATTCAACGGAAGGCGAAATTACAGCATAGAGCAGACGACACATATC
CACAGACAGGTTGCACCATCGAGGAGCTCCATATGGAGGCCACTGATCAGGAGGTCATGCTGGAGGGCAG
GGAAGACCTCATGTCCACATCGGCGACATCGTCCACAAATGGCGGGGACACTAATGGTTGCGGCAAAAAA
TTGGGTGAGTTTTCCATTGCGCTTAAAGCTGCTCACTATATCGGTAGTTTTGGATCCATCCCTTTTCCCT
TTTAAGTATTTTTACTATCTTATAACTGAATATTCAGACGTACATAATTTGAAAATATTTCAATTTCAAT
ACTTCATGCGTTCGTTCAAGCAGAACTAAATGTATGAAAAAGGGACTCGAGTATTACCCAGTATTACATA
AGCTGCTGAATGCTTTTTGCAACTAAATACTAAAATGCTCAAAATGGTCAAGGACGGCAGCTTGGCAGCT
TAATAAGCAATTCAATGGAATTTAAGATCAGAAAATGCAGCCATATAGGCTACTGCCCACACGTTTATAT
GGAAGCAGTATCAATAACTCCACACCCTGGACATCTGTCCGCTGACATATGCATTTAACTCTCCCTTTTG
ATTTCCCGCCCCACACTACTCCGCTCAATTGAAGCAATTCATGCTAATTAGAAATTTTATTGCATTTACT
GCACTTATTGTTAATTGTAGGTCCGTAACCTCTCCGGATCCCACACCGTACGTGACCAAGCAGCGAGTGA
CGCGTCCAACGCCTCCCGCTCCTTCAAAGAATCCGATGCAATTCGTCCAGATCAAGCCCTGCAACCTCTA
CCAGACCGCGCAGGAGCAGCTGaagaaggccgaggaggtcaagaagctgaaggaggtgaagaaggAGGAG
CCAGAGGAGTGGCAGAACCGTAAGTCCATATCACATATGTATACAAATTAGCGGTATACAGCCGCTCGAT
AAATGGGCTGTGAATCGTAGCACTGATGATTTTTTAGGCCTTTAAAAAGGGCTAGACTAAAGCTAAACTG
TTTAGACCTCTTACAATCATGCGATAGTCTGAATATTGTGAAAACAGTTTATTCATGCGTTAGTGTTTAA
GGAAGTGCCTCATTAAGCTCGTCAGGTCTACTAGAATTATTTATCTAACCTAATCTACAAATTTACCCGG
GCTCTCTTAGTAAAAACGAATTCAAGAATCGGCTTCCGAAACTGGTCTTTCTCTTTTTTTATTGTTGGTT
TTCGTTGGCTAGCCGTTGGAAACCTATCGCCCCGGATAAACTAAAGCGAAATCACTTGGGCATGGTACAT
AGCACATGGCTCGTTATGGTCGCTCCAGTTTCCAATGCACGTCGCCCGTTAGCGGACAAAAAGATATTGC
ATTGCATAATCACTATTACATTTTGTTGATAGTTTTTTCCTCGTCGCCTGCTTTTTGCCCACTTCTATTT
TCCTTGCACAATATTGTGTGTTACATGCAAATTTGCTGCTGCCATAACCCTGGAAAAGAGTTGCCTTTGA

CGTGACTGTACCTAACTAATTACCGAATCAACCATTTTGATGCAGGCGCTGCATTTAAAGTGCCTTTTGG
CGTTTAATCAATGTTCCATGGGCATACGGTTTTTCGAAATTTCAGAGGTTCCCAGACGAAACTTTGAGTT
ACGGATGTGCGCCAAAAGAAAAAGGGAAGAAATCCATTAAAATGCGCATGGCGTTTTGAAACTGGGCAAC
CAACTTTGTTCGGAAAGGAAGATCAAATGCCGCTTAGTCTCATTCGTGTATAAATTGCTCTCTTTTCGCT
TAATCGTCTAGTGCAAAAACCTTTTAAATTTAAATTTAGCAATCCGAGTTGCAGATCTATTGAAACCATC
GCGGTCGACCGAAAGCTTTTTACCTGTAAGTCAAACCAGGAAACAAGAGAGAGATTAAAAACCTTTAATA
TAGTATCAAAAATATTTTTTGCCAGATAAATTAAACAAAAACATCTGGTTTTTTAGACGTAGAGTAAGAT
CTCTCTGTGTAATCTAAACAGTTGCACATCACTCCGATTCATTAAAAAATGGATCTTAAATTCTTTACTG
CGGTAATGACTGCCATGAAACTGAGCGAAGTTTACGCATGGTTCGGAAACATTCCACCTTTGTCACGTAG
TTCCGCAGCCGCGTCAAAACTTTTTAATTGCCCTCAAAAGAAACTTACTCCGGCTCGTCCACTTCCCCAG
TGCGAATAACACAATGACAATTTACTCTTACACATTTCCTCTTTTCGTTTTCTATACAAACTTTCGCTAC
CTTTCTGCCCAAAACAAAACATGGCCAACAAAACAACCCGGTGACAATCAACTAATAACAAATGGAAAAT
ACACAAACAGGAACCTTGACAATTGGAAATCATCGAGACGAAAACGTGTCGAACACATCATCGATCGAGC
GGTGGAGACCAAGAAACTCGAGCTGGAGGAGCATGATCGGTTGCGCCGAAAGTCGAAAACATTCACCGAA
ATGATGGAGGAGAGGGGCTGAAAGGGGCGGACCTCGGGGGCGTGCCAAGCTGGCTTCCCTGGCCGTATAC
AACGAGGATGAGACGAACGACCTGAGTGATTTGGGGATCGGAACCAGCAGTGCCAGCGGAAAGAGCAGCC
TGTCCGGGGACTATGACAATAACAGTGTTATG
>dme_circ_0008332_junction_seq
CGGGGACTATGACAATAACAGTGTTATGGCTAATGAAATTGGACACATCCGAAGAC
>dme_circ_0008333
GCTAATGAAATTGGACACATCCGAAGACCTCGCCCGATGTGCGTGGGTGCTGGCGCATCGCCTTTGAATC
GAATTCAGAGACATGGTGAGTCACTGACCAGAGGCCAAGAAACCCGCGTAGGAGTTCGGTGTCATGTGAG
CAGAGATCAGTCAACAAACACAACATTCAACGGAAGGCGAAATTACAGCATAGAGCAGACGACACATATC
CACAGACAGGTTGCACCATCGAGGAGCTCCATATGGAGGCCACTGATCAGGAGGTCATGCTGGAGGGCAG
GGAAGACCTCATGTCCACATCGGCGACATCGTCCACAAATGGCGGGGACACTAATGGTTGCGGCAAAAAA
TTGGGTGAGTTTTCCATTGCGCTTAAAGCTGCTCACTATATCGGTAGTTTTGGATCCATCCCTTTTCCCT
TTTAAGTATTTTTACTATCTTATAACTGAATATTCAGACGTACATAATTTGAAAATATTTCAATTTCAAT
ACTTCATGCGTTCGTTCAAGCAGAACTAAATGTATGAAAAAGGGACTCGAGTATTACCCAGTATTACATA
AGCTGCTGAATGCTTTTTGCAACTAAATACTAAAATGCTCAAAATGGTCAAGGACGGCAGCTTGGCAGCT
TAATAAGCAATTCAATGGAATTTAAGATCAGAAAATGCAGCCATATAGGCTACTGCCCACACGTTTATAT
GGAAGCAGTATCAATAACTCCACACCCTGGACATCTGTCCGCTGACATATGCATTTAACTCTCCCTTTTG
ATTTCCCGCCCCACACTACTCCGCTCAATTGAAGCAATTCATGCTAATTAGAAATTTTATTGCATTTACT
GCACTTATTGTTAATTGTAGGTCCGTAACCTCTCCGGATCCCACACCGTACGTGACCAAGCAGCGAGTGA
CGCGTCCAACGCCTCCCGCTCCTTCAAAGAATCCGATGCAATTCGTCCAGATCAAGCCCTGCAACCTCTA
CCAGACCGCGCAGGAGCAGCTGaagaaggccgaggaggtcaagaagctgaaggaggtgaagaaggAGGAG
CCAGAGGAGTGGCAGAACCGTAAGTCCATATCACATATGTATACAAATTAGCGGTATACAGCCGCTCGAT
AAATGGGCTGTGAATCGTAGCACTGATGATTTTTTAGGCCTTTAAAAAGGGCTAGACTAAAGCTAAACTG
TTTAGACCTCTTACAATCATGCGATAGTCTGAATATTGTGAAAACAGTTTATTCATGCGTTAGTGTTTAA
GGAAGTGCCTCATTAAGCTCGTCAGGTCTACTAGAATTATTTATCTAACCTAATCTACAAATTTACCCGG
GCTCTCTTAGTAAAAACGAATTCAAGAATCGGCTTCCGAAACTGGTCTTTCTCTTTTTTTATTGTTGGTT
TTCGTTGGCTAGCCGTTGGAAACCTATCGCCCCGGATAAACTAAAGCGAAATCACTTGGGCATGGTACAT
AGCACATGGCTCGTTATGGTCGCTCCAGTTTCCAATGCACGTCGCCCGTTAGCGGACAAAAAGATATTGC
ATTGCATAATCACTATTACATTTTGTTGATAGTTTTTTCCTCGTCGCCTGCTTTTTGCCCACTTCTATTT
TCCTTGCACAATATTGTGTGTTACATGCAAATTTGCTGCTGCCATAACCCTGGAAAAGAGTTGCCTTTGA

CGTGACTGTACCTAACTAATTACCGAATCAACCATTTTGATGCAGGCGCTGCATTTAAAGTGCCTTTTGG
CGTTTAATCAATGTTCCATGGGCATACGGTTTTTCGAAATTTCAGAGGTTCCCAGACGAAACTTTGAGTT
ACGGATGTGCGCCAAAAGAAAAAGGGAAGAAATCCATTAAAATGCGCATGGCGTTTTGAAACTGGGCAAC
CAACTTTGTTCGGAAAGGAAGATCAAATGCCGCTTAGTCTCATTCGTGTATAAATTGCTCTCTTTTCGCT
TAATCGTCTAGTGCAAAAACCTTTTAAATTTAAATTTAGCAATCCGAGTTGCAGATCTATTGAAACCATC
GCGGTCGACCGAAAGCTTTTTACCTGTAAGTCAAACCAGGAAACAAGAGAGAGATTAAAAACCTTTAATA
TAGTATCAAAAATATTTTTTGCCAGATAAATTAAACAAAAACATCTGGTTTTTTAGACGTAGAGTAAGAT
CTCTCTGTGTAATCTAAACAGTTGCACATCACTCCGATTCATTAAAAAATGGATCTTAAATTCTTTACTG
CGGTAATGACTGCCATGAAACTGAGCGAAGTTTACGCATGGTTCGGAAACATTCCACCTTTGTCACGTAG
TTCCGCAGCCGCGTCAAAACTTTTTAATTGCCCTCAAAAGAAACTTACTCCGGCTCGTCCACTTCCCCAG
TGCGAATAACACAATGACAATTTACTCTTACACATTTCCTCTTTTCGTTTTCTATACAAACTTTCGCTAC
CTTTCTGCCCAAAACAAAACATGGCCAACAAAACAACCCGGTGACAATCAACTAATAACAAATGGAAAAT
ACACAAACAGGAACCTTGACAATTGGAAATCATCGAGACGAAAACGTGTCGAACACATCATCGATCGAGC
GGTGGAGACCAAGAAACTCGAGCTGGAGGAGCATGATCGGTTGCGCCGAAAGTCGAAAACATTCACCGAA
ATGATGGAGGAGAGGGGCTGAAAGGGGCGGACCTCGGGGGCGTGCCAAGCTGGCTTCCCTGGCCGTATAC
AACGAGGATGAGACGAACGACCTGAGTGATTTGGGGATCGGAACCAGCAGTGCCAGCGGAAAGAGCAGCC
TGTCCGGGGACTATGACAATAACAGTGTTATGGGTGAGTTCCAACCAACGAAGCACATTGCCATAGTAGC
ATGGACCTATTTAGCACCCATTTCTAGTACCAGTTTCTCCGTCTTAATTTTTTTGTCGTTATTAAGACTC
ATTTATGGTGTCACTGCGCTTATAAGCTATTACAGTAAATTAATAATTAAATCTAATTTGTATTTTGCTG
CCACTCAAGCTCAGACTTATTGGAAAGTGTTGCAAGCAATGCGATGCGACACAAATAGATTATTAATCAA
ATGCCCGCACATGCACCACCTTATTGTGGTTATCAAAATACCAATCATAAGTAATAATCAATGCACCACA
AACAATCGAAAATGTGCAGACGATACAAATATTTCCCTTAAGATTGAAACTGATCGAAAGATGTAGCTTA
GGATTGATAAGGATTAGGTGCGCTGGTCGCCACACCTCAAAATAATCAGTTTTGAACTGaaaaaaaaaaT
GCTTGGAAACGGGGATCCTGTAGATTTAAGTGGAAAATATTGAATAAGAGCATTTTAGCAAACGCTAAGG
AAATACAATTTGAGTTTGATAAATAGAACAGCTGTGAAAGGGAAAGAATTACAATCGATGGAGTGCACAA
AACATTTACAGAACCTACAAATATAGGATAATATTTATTCAATTATTCTTATCAAGTTTTATTAATATAG
GTACAGAAACGCTTTCTTCTATCTCTTTTATATTTATAAGGAATACGCTTTAAAAAGATACACTACTACA
ACTAGTGTGTTAGTGGATCCTCATATTTCCATTACCATTTAAGTATTTATCCAAAATTTTTAATAAAAAT
GACAAATTCTATTACCTCCACAGGAGCGACCATGCGGCGGAGCTGGATAAGGCGATTGGAGCAGCTAGCT
CTGTAGCTGCTGGAGTAACCGCCCAAAACGTGGATGAGCAGCTGAATCACATAAACCGCAACGGTAGTAA
TGGGAACCACGGGAACGGAGCGGCTGTTGGCCAGACAGGACCATCAAATTCCTCCAAGTCAGCAGGGCGG
CAATACATCTCCTCGCCAGGATACGATACGTCCTCCAGTAACGCGCAAGCCAGTTCTCCGGATCTCTGTG
AATACACCTACGAAGGAGCCATCCAGGACTACAAGCAAAGAGTTCAACGGGCCAGCAGCAATGGCAATGG
AAATGCCAACGGCAAGTTGATTGGAGAACATATTGCTTATCCCACGAGGCGAGGCTCTAAAATTGAGGAT
CGATTGAGTGGTTTTGAGGTGACATCGCCAAGCGACACGCAGGAGGGTGTAGAGAAGCAAAAGGTGGACG
TGCCCAAGGTTGACATATCCAAACGCAAGGAGATCTTCGAGCAGGCCAAGGCGGAAGTCTCAAACGGTGG
TGCTCCAGCTGCCCCAAAACTCGTATTTCGTGATGGTCTTACCAATGGAAATTCAGCTACCACTGCTCCC
AAATCGGAGGTGAGGCGTCTTTCTGGTGACATCTCCAGCATTCGAGATCGCATGCAGAGTTTGGAACAGC
AGCGGAAGGCTTTCAGTTCCAGCAAGAGTGTGGATGTGCCGGTGCCGCCCCTTAAACAGCGTCTGAATAG
TCTGCAGCAGTCCGTTGCCAAAGAGGAGCAGAAGAAGCCACCGTTGGTGGCGCTCATCGATGCCAGACAA
CTGGAGATCATGAGGGGAGAAGAGGAACGTATGCGCCAGCAGCAACAAAAAGAGAAGCATTCACCAAGCC
AAACTACATTGTGTGCACCTAAGCCAGCTCCTCCTGCCCTTATTATTGAGGAACCGCCGGTGGCAGACAC
CAACGACGACAGCGGCATTCAGGAGGACACCGCTGATGAACTGCAGCAGCAGCAGCAACTCAACGCAGCC
ATCGCCGCCTTGGCGCTCGAGGAGCGCCAGTTGGAGGAAGCAGCCAATGCGGTCAACCAAATTGAAGCGG

AGTTCGATGAGCTGACTGATCTGCATCCCTCGCCATTGCCACCATCTCCAGCTCTTCCGGCGGTTCAACA
AAGCCTCCAACAACCGACAGTCGCTCAGGTTCCAGCACAAGCGGTCAATCAGGCGGCTGCTCCTCCATTG
CGGGACATGGAATTTAGT
>dme_circ_0008333_junction_seq
TCCTCCATTGCGGGACATGGAATTTAGTGCTAATGAAATTGGACACATCCGAAGAC
>dme_circ_0008334
GAACGGACAAATCCCAAGCTTGTCGCACCAGCGGCGATGGCATGGAGCAACCGGAAAACAGTGGAGCGGC
CAAAACAGAGAGCGAAATCCCAGCGAGTGCTGATGTGGACAACAAGGTAAAGAGGCCACAATTATCGCGA
AAAGACAGCTCCGTTGTCCAGGAGGCCGTCATGGGCAGGAAGACATCAACTAGTAGCGGCCACTCCTCAA
ATTTGCCCCTTCCTGGCTTGCATACCCTG
>dme_circ_0008334_junction_seq
TTTGCCCCTTCCTGGCTTGCATACCCTGGAACGGACAAATCCCAAGCTTGTCGCAC
>dme_circ_0008335
GTAAGCTCGCTCCACACCACCATGGCCGACATGGTGGCGGTCAGAACGCACATGGTCTTGAGGAACGGAG
CCTTTAGCAGGCACTCCCAGTACCACTGCAGCGAGGCGCTGTAGCAGATCCTCTCCAGCTGCGTGCGCTG
GCGCGGAAACTCAGATTTGAAGCGCCGGTCCGAAGAG
>dme_circ_0008335_junction_seq
CTCAGATTTGAAGCGCCGGTCCGAAGAGGTAAGCTCGCTCCACACCACCATGGCCG
>dme_circ_0008336
GTCCGTAACCTCTCCGGATCCCACACCGTACGTGACCAAGCAGCGAGTGACGCGTCCAACGCCTCCCGCT
CCTTCAAAGAATCCGATGCAATTCGTCCAGATCAAGCCCTGCAACCTCTACCAGACCGCGCAGGAGCAGC
TGaagaaggccgaggaggtcaagaagctgaaggaggtgaagaaggAGGAGCCAGAGGAGTGGCAGAACCG
TAAGTCCATATCACATATGTATACAAATTAGCGGTATACAGCCGCTCGATAAATGGGCTGTGAATCGTAG
CACTGATGATTTTTTAGGCCTTTAAAAAGGGCTAGACTAAAGCTAAACTGTTTAGACCTCTTACAATCAT
GCGATAGTCTGAATATTGTGAAAACAGTTTATTCATGCGTTAGTGTTTAAGGAAGTGCCTCATTAAGCTC
GTCAGGTCTACTAGAATTATTTATCTAACCTAATCTACAAATTTACCCGGGCTCTCTTAGTAAAAACGAA
TTCAAGAATCGGCTTCCGAAACTGGTCTTTCTCTTTTTTTATTGTTGGTTTTCGTTGGCTAGCCGTTGGA
AACCTATCGCCCCGGATAAACTAAAGCGAAATCACTTGGGCATGGTACATAGCACATGGCTCGTTATGGT
CGCTCCAGTTTCCAATGCACGTCGCCCGTTAGCGGACAAAAAGATATTGCATTGCATAATCACTATTACA
TTTTGTTGATAGTTTTTTCCTCGTCGCCTGCTTTTTGCCCACTTCTATTTTCCTTGCACAATATTGTGTG
TTACATGCAAATTTGCTGCTGCCATAACCCTGGAAAAGAGTTGCCTTTGACGTGACTGTACCTAACTAAT
TACCGAATCAACCATTTTGATGCAGGCGCTGCATTTAAAGTGCCTTTTGGCGTTTAATCAATGTTCCATG
GGCATACGGTTTTTCGAAATTTCAGAGGTTCCCAGACGAAACTTTGAGTTACGGATGTGCGCCAAAAGAA
AAAGGGAAGAAATCCATTAAAATGCGCATGGCGTTTTGAAACTGGGCAACCAACTTTGTTCGGAAAGGAA
GATCAAATGCCGCTTAGTCTCATTCGTGTATAAATTGCTCTCTTTTCGCTTAATCGTCTAGTGCAAAAAC
CTTTTAAATTTAAATTTAGCAATCCGAGTTGCAGATCTATTGAAACCATCGCGGTCGACCGAAAGCTTTT
TACCTGTAAGTCAAACCAGGAAACAAGAGAGAGATTAAAAACCTTTAATATAGTATCAAAAATATTTTTT
GCCAGATAAATTAAACAAAAACATCTGGTTTTTTAGACGTAGAGTAAGATCTCTCTGTGTAATCTAAACA
GTTGCACATCACTCCGATTCATTAAAAAATGGATCTTAAATTCTTTACTGCGGTAATGACTGCCATGAAA
CTGAGCGAAGTTTACGCATGGTTCGGAAACATTCCACCTTTGTCACGTAGTTCCGCAGCCGCGTCAAAAC
TTTTTAATTGCCCTCAAAAGAAACTTACTCCGGCTCGTCCACTTCCCCAGTGCGAATAACACAATGACAA
TTTACTCTTACACATTTCCTCTTTTCGTTTTCTATACAAACTTTCGCTACCTTTCTGCCCAAAACAAAAC
ATGGCCAACAAAACAACCCGGTGACAATCAACTAATAACAAATGGAAAATACACAAACAGGAACCTTGAC
AATTGGAAATCATCGAGACGAAAACGTGTCGAACACATCATCGATCGAGCGGTGGAGACCAAGAAACTCG

AGCTGGAGGAGCATGATCGGTTGCGCCGAAAGTCGAAAACATTCACCGAAATGATGGAGGAGAGGGCAAG
TACAACTCCCAGTTCGCAACTAGTGTCTAACGTGCTGAAAGTGTTGATTGCACAAAATCGAGTCCTGCCA
CGGACGACACGTTCTTTTGAAATGCTTGATACAAGTGAATCCAAATCCAAGTGCTATTATCCAAGTGAAA
TGAATATTGCGGTGTTTCTTTGCGAAAATCGCTATAAGATATCAGACTACATAGTAATCTTTTACATTGT
TTGTCATCCTAAACCACCCGATTTCATTTTCAGCACCTGTGGTGCACCTCCTGAATGTAACTTTAACTTT
ATTAAAACACATAAATTTCCCCACAGGGGCTGAAAGGGGCGGACCTCGGGGGCGTGCCAAGCTGGCTTCC
CTGGCCGTATACAACGAGGATGAGACGAACGACCTGAGTGATTTGGGGATCGGAACCAGCAGTGCCAGCG
GAAAGAGCAGCCTGTCCGGGGACTATGACAATAACAGTGTTATGGGTGAGTTCCAACCAACGAAGCACAT
TGCCATAGTAGCATGGACCTATTTAGCACCCATTTCTAGTACCAGTTTCTCCGTCTTAATTTTTTTGTCG
TTATTAAGACTCATTTATGGTGTCACTGCGCTTATAAGCTATTACAGTAAATTAATAATTAAATCTAATT
TGTATTTTGCTGCCACTCAAGCTCAGACTTATTGGAAAGTGTTGCAAGCAATGCGATGCGACACAAATAG
ATTATTAATCAAATGCCCGCACATGCACCACCTTATTGTGGTTATCAAAATACCAATCATAAGTAATAAT
CAATGCACCACAAACAATCGAAAATGTGCAGACGATACAAATATTTCCCTTAAGATTGAAACTGATCGAA
AGATGTAGCTTAGGATTGATAAGGATTAGGTGCGCTGGTCGCCACACCTCAAAATAATCAGTTTTGAACT
GaaaaaaaaaaTGCTTGGAAACGGGGATCCTGTAGATTTAAGTGGAAAATATTGAATAAGAGCATTTTAG
CAAACGCTAAGGAAATACAATTTGAGTTTGATAAATAGAACAGCTGTGAAAGGGAAAGAATTACAATCGA
TGGAGTGCACAAAACATTTACAGAACCTACAAATATAGGATAATATTTATTCAATTATTCTTATCAAGTT
TTATTAATATAGGTACAGAAACGCTTTCTTCTATCTCTTTTATATTTATAAGGAATACGCTTTAAAAAGA
TACACTACTACAACTAGTGTGTTAGTGGATCCTCATATTTCCATTACCATTTAAGTATTTATCCAAAATT
TTTAATAAAAATGACAAATTCTATTACCTCCACAGGAGCGACCATGCGGCGGAGCTGGATAAGGCGATTG
GAGCAGCTAGCTCTGTAGCTGCTGGAGTAACCGCCCAAAACGTGGATGAGCAGCTGAATCACATAAACCG
CAACGGTAGTAATGGGAACCACGGGAACGGAGCGGCTGTTGGCCAGACAGGACCATCAAATTCCTCCAAG
TCAGCAGGGCGGCAATACATCTCCTCGCCAGGATACGATACGTCCTCCAGTAACGCGCAAGCCAGTTCTC
CGGATCTCTGTGAATACACCTACGAAGGAGCCATCCAGGACTACAAGCAAAGAGTTCAACGGGCCAGCAG
CAATGGCAATGGAAATGCCAACGGCAAGTTGATTGGAGAACATATTGCTTATCCCACGAGGCGAGGCTCT
AAAATTGAGGATCGATTGAGTGGTTTTGAGGTGACATCGCCAAGCGACACGCAGGAGGGTGTAGAGAAGC
AAAAGGTGGACGTGCCCAAGGTTGACATATCCAAACGCAAGGAGATCTTCGAGCAGGCCAAGGCGGAAGT
CTCAAACGGTGGTGCTCCAGCTGCCCCAAAACTCGTATTTCGTGATGGTCTTACCAATGGAAATTCAGCT
ACCACTGCTCCCAAATCGGAGGTGAGGCGTCTTTCTGGTGACATCTCCAGCATTCGAGATCGCATGCAGA
GTTTGGAACAGCAGCGGAAGGCTTTCAGTTCCAGCAAGAGTGTGGATGTGCCGGTGCCGCCCCTTAAACA
GCGTCTGAATAGTCTGCAGCAGTCCGTTGCCAAAGAGGAGCAGAAGAAGCCACCGTTGGTGGCGCTCATC
GATGCCAGACAACTGGAGATCATGAGGGGAGAAGAGGAACGTATGCGCCAGCAGCAACAAAAAGAGAAGC
ATTCACCAAGCCAAACTACATTGTGTGCACCTAAGCCAGCTCCTCCTGCCCTTATTATTGAGGAACCGCC
GGTGGCAGACACCAACGACGACAGCGGCATTCAGGAGGACACCGCTGATGAACTGCAGCAGCAGCAGCAA
CTCAACGCAGCCATCGCCGCCTTGGCGCTCGAGGAGCGCCAGTTGGAGGAAGCAGCCAATGCGGTCAACC
AAATTGAAGCGGAGTTCGATGAGCTGACTGATCTGCATCCCTCGCCATTGCCACCATCTCCAGCTCTTCC
GGCGGTTCAACAAAGCCTCCAACAACCGACAGTCGCTCAGGTTCCAGCACAAGCGGTCAATCAGGCGGCT
GCTCCTCCATTGCGGGACATGGAATTTAGT
>dme_circ_0008336_junction_seq
TCCTCCATTGCGGGACATGGAATTTAGTGTCCGTAACCTCTCCGGATCCCACACCG
>dme_circ_0008337
GAAAAAAGCACGAGAGGAAAAAATGAAAGAAGCCATGGGGAGAAGCGCAGCATTTTTGGAGTATATGGCC
ATGCCAGTCGCACTTTAGGTGTACAGACGAATGTATATTTTATATGCATTTTCAAAAATATAAAGGTAAA
GAACTAAATAAATTCATATTTTTAGACAGGCGATGGCTGCTGCACTTAATGGTAGCAACGATGGGCCGGG

AACACGCTGCGTATACGCAACGTGGTGGCGACTCCATTATGCAACGCACATAATCATCGCTAATGTGAAA
TTCTAATTTCATTTGCTGTACTCATTAATAACTATAATTACGCTAGCTGGCAGCAGCAGTGGAAACCAAC
AAGCGGCTAAAAAAACCACCAGCTGTGCTTCCTTCAGCTTTAACTCCTTCCTGATTTTCTGTTGACTCGA
CACAAATAGGCGTTCTCGTCTGATATTTATAACAAACACACGCAGAACACATCATATGTAGGCTAAACAA
AAGACAGAACTCTAAGCCCAGTCAACGAAATCTAAAGCCAAAATCCAGAGCAGCTTACTCCACTCGTCTG
AAAGCAGACGAACACTTAAGTGCTCGACAATTTAATACCCTTGAAGTTATACCCATAGTTTTGAGGAAGA
GTCTAGGAAGGTCATACAATTTCTTCAGAACCACATTCCATTTTATACCTTAAAAAAACTGTATTTGCTG
CTGAGTAACTATTATTTTCCTAAAATTGGCATATATTATTTAAGACCACAAATTTAGATTATGattctat
ttgtttttttttctgtgcagcattctattcgtttttttctgtgcagcatGAGTGGCAAGCTCAGTGACATC
CTGAAGCTACCCTTAAAAAATGAGGCTCGTAAAAACCGAGCAAATATGGGTTGCCTTGATGGTTGCTCTG
GGGTATGAGATGTGGGGTTTTGGGGTTGGGGTTGGTTCTGGCTCACAATGAGCATAAATTATGCTCACAA
AGGCAAGATGCTGGAGGAGGAACTGGGGGAACGGGGGCTTTCTGGCGGGAGCTTTTCAGGCGCCAACGAC
ATTCACGGCAAACACTCGTTCCAGTCCTATGGCACTCGTTTACTTTGCCATCTTGCAAACCAACTGACGT
CCTGCTGATCTTTGTCGGCCAACAATGGAGAACCCTTTCTTTTTCCTGGATATGCTGCCACCTCTCATGC
TCCCCCTTCCTTTGGTCCCTTTTTCGATCCGCATCCTGGGGGTCAGTGGGCGGCCTTTTCGTATCGCGTG
CCTCGTTATTGATTTTCGCTTTCATTTTTCATGTGGCATGCGACGGGCAACAGGAAGAACAAGCAGAGCA
ACAACCAATAATTGTACGCCAGCCACGCCGCTTACATATGCGGCAAAAGTTTTCCTCATTTTATATTTGA
CGAGTAGGATGAACGCACCGAAAACTACTGGACAGGAGCAAACATAGGGACGGAAGGTTCATTCGACAGA
AGACGACAGCCAAGTCATCCGAAAACGTGCCGCCAAAGTCAATGAGGTCGCACCTCGCCCACTTCCTCCT
CCCGCTCCTGCGACCTCCTGTCCCTCATCCTCGTTTGTTGTTTCGCTTTCTGGCCCGGAGCATTCAGCTT
TCAGTTCACATCAGCCTCTCATCCGCGGCGTCCCCATTCACATTTGCCAGTTGGTTAAATATGCGTCGTG
TTTACCTTTTTGCCAGCAAATGTCCTTGCCGGCGGCTTTAAAGTCCTGTGCTGTGCTCATAATACAGTCT
GGCTACAAGGAGTTGTCCGCCTTCTGAAACCCCCTCACCACCTCGCAGCACCCCTCTCATTTCCCCTCCT
TTATGGCTTATGACATTTGCGCTTTTGTTGTATTTGCATTCGTGTCGATGTTGCCTTCATTTCGCCTCCT
TTGTCAACTTCCGCTAATGCTAAGCCCCGCCCCATCCGAGTGCCACACCCCCTACTTATCCCTACGGTTG
AGAGCGGGGGCGTAACAAAAATGCCCAGTGACTCCAGTGACTCTGGGTTCCGGATTCCGGAATCCAGACA
GTTATCAACAGCCAGGCTGACCCACACTCGCCAGTTATCCGCACTTAGCCTACTCCATTGTTCATTTGAT
TTTATTTTAGTTGCCCCATTTTTTGGTGCTTTCCGGGTGGATGTTCTCCTGCCCCGATTTCaatatatac
acacacatatatatacaatatataAACTCGTACACCCGTATATACCTTTTTCCTGGGCCACTGTGTGTTT
TTGTCAACCCATTTGGGCTGCCTGCAATTATGAATTTGGGACAATTGTTCGGTGTGCGCTCGGATGGCTC
CATGGTGGCTTTGATTTGAGCAAAGAGTTGCCCGGCATGATTAGGGGATTTATTTCTAAAGGACTTGTCC
ATTTGGTTAGTTCATACCCAACAGATATGCCACATAGTATAACACTGAACAAGTGAACAATCGGACGAAT
TGAGTGACATATCGTTTTATCTGATGACACCATTACTGATTGATGAACACTTATCACTTGGATTGCATCC
CTATGACAAGTGGCATTGGGTACACTCATTACGGCTATATAACCACGCGAAGTTGGCAACTTTGTGCTTA
GCATTTGGAAATCCAGAATTTAGTCACGATTCAAACAGGTTCGGTTTTCGAATGAGTCACCATTTTGGAT
CTCAACCTGTCGTAGGTGATCACACACCTCTCATATTCTCCGCTCTCGAGGCCATTACTCACCCATTTTG
TGGACAAATCCAtttttttttAACATTTTCCTCGGCGTGGAAAAGTGTTTTCCAACCAGTTGCCGTACTT
CAAGACGCGCTGACAAGTCAAACAAATGAAACATTTTCGGATTAGCAATGTCAATGTTGCTTCGGAAATG
TCGGAATAAATTGCCATTAGCCTTTGTCATCAAAAATTGAGGATGCCCGAGTGAGTGGTGGTGGTACGGT
ATCCTTGTCTGCAGGCAGGCGAAGATTTATCGCACATGATATTTGTCAAAACGCAGATAGAGATACACAA
GCACACACTCGCCGATAGATACAAAACTGCTGTCAGCGGATTTGTGTTGAGCCTATGAAAAGACGATAAA
TGAGCCTGAAATGAGCAAACAAAGACGATAGGCCAACAAATGAGCGGGGGATTTTTGGTCCTCAAAAACG
GTTCCTATCGGCATTTGTGGCTCTTTAAAAATGATTCCACACCAGGAATGATGGTATTTCATTATTATAC
TTTGTCTTAAGGTTTGTAATAAACAGTCGTGCTTATGAAAATAGTCGAatctatataaataattaataat

atatatataaaaatatatatatatatatatatatatatatatatatatatatatatatatatatatgttt
ataAACTTCCTCAACATGTAATTCTTGGAATACTTTATCAGTTAGCGGATTCATTTTTTGATAAGTGCAC
TTAGGACCCGAAACTGGGCAAAACTTAAAGTTTTATCAGCGTCGAGGCGTCTGAGACACGGCCGAGTCAA
CAAAAACAAAACAATTAAGCGAATGGCGGTGGGAGTGGGAATTCGTGATagagagagagagagagagGGA
GACGGAGCAAAGTGCAACACATAACGCAAACTATTTACTGATAAGAATATAAATACACACACATGTACGC
GGGAGAGCACTTAAAACGGCTGAGCAAACACTTGCAAACCCAGCGACGAGCACTCGACAGCTTGTAGATA
AAGTATCTGCCAGATACTCTCGctctcccacacactctctcctgctcccgctcccactccttgtattgct
ctcccgctcccgctcTTGCTGGCAAGTGTTTGAATCTCGTAGATACTTTGGCCTGCCTGCCTCGAAACCC
ATTTCGTTTTTAATTGTTTTCAGTTTTTGAATTTAGTTGTATTTGATTTTCACGTACTCGCGCACTGTCG
TCGCAGAAGTGTGCGTTATTTTAGAAGTTTTAATTTGGATTCGTTCAAGTCTCAAGTGAGTGTCGTGTCG
TTAAGTGCTGGGAATCATCGCCACTGACAGTTTGTTATTTGAGAGCCCCACAAATTGGTTCCCCACCCAA
GCCCCAATCTATCTCCTCGTTAAAAAAAGTGTTTCGTGCTTTAATGTTACACATATGTACAATATATGTA
TGTGGAACTGGCGCCCGAGCAGGGACCTTCTATGCAGATATGTGTACAATGTACATTAAAGAGCCGCTCG
AGAACTGCTTATCGGGCAGCAGCACATGCAAACAACCCAGGCCTTTCAATTAGGCAACAAAACATAAATC
TTGTGCTCTGACAATGGGATGCGGGTCGCGTGTCTCCCCTTTAAAAGGCAAAAAAGCAACCCAACTATAG
TTCGAACTTACTTTCCAAACCTCTGATGTCATGCTTATTCCCTTTCATATTTTGTCTACTCACTTCTCAT
TCGCCTGCAAACAAGTAGCTCACGAAAAGTCATAATATCGTCATAACTCCAGGGGCGTTGGGCCTTCGAG
TCACCATCGCCCCCCTTTAATTTATTTAGTTTGTAGTAAACCCCATCGTTTGTGCTTCCCATCTACATAC
ATCTCTGGTCCGTGCTCCTTATCGTCAAACCAAAAAGTAACGAAAATAAACCTTATCGCCGTCGTCTCCG
CTCGAGTGATAACTGTGAGTCCGACAGGGAGATAAGTTGGCCAGGCCCTATGTATATTTGTATGTAGGCA
TACGACTACGACGACTGACTGAGTAAATGGAATATTTTTTCATTTGCCCAGCGTTCTTTTTTCCACTTTT
CACATAGGAACTTTGATGATAAACCGAGAAATCGGTTCTTGCAGGCTTCCACTAGATGTCCACTGAACTC
GATGGATACCGCACATATGCCACATGGCATATATTGAAAGCTGTTCCAATATGTTGTATACCACACACTA
TATGCCACATACATACATGTATTCAACTTGTGCCTAGAAGTTAGATAACACATTTCATGCACACAAGTAT
GTATATGCAACCTGTTCCAAGGCGGACCTCCCCCATTTCGTACGCCCACTTTCGCCATTCGAACGACTTG
AAATGAATATTGTGCCGTCGGCGGAATATCCTTGGGCTGATTGGAGAGTGAATTTCATGCTGCAGCTGTT
TCCAATTCATCACCATTAACACATTTTCGGCTTGAGTGCATCAATGCCGCTTTCTTGTTGTTTGGCGCTC
ACGGCTTTCGTTCTAATCATCGTCACAGCGAAAAGAATGAACGATAGAAAAGTATTTGGCTGCAAGTTTC
CCAAATCCCATTTGGCCCGCTGCATTTTTCAAGGACACACAAAAAGTGAAAAATGTCGAGCACTTTGCGT
GCTTGCCTGCCATGTGGCATGTGGCATGGTGCATCTCTGGGGACTTTCGGTGGCGGCACACTTCTCGGAG
GTTTGGGACTGAGCGACCTGCAGCTGAAATTTGAATTTGCGACTTTTATTCAGAATTCGAGACGCTCGCC
TCGAATGAGAATTCTTCTACGAACGTCAGCGAAACACAATAAATAGCCGATCGAAAAGCATTAGGTGAAT
TAATTGAAAAACTCAAATGCCAAGTCGAGGCGCAAAAGCAACTGGTTCTCTCACAATAAACGAAAACCCG
TTTTATAGCGCAGGCCATCGCATCTCCTGCTAAATCGATCTTAGTTTCTTGATTTTTGTATTTGTAGTCG
CACTAATTTCTTAAGTAAATGCAAATTTGTGCGACCTACCTGTTTTGTTCTCTGTTTTGTACTGCGCTGA
CGACAGGTGCTGTGATTACACTTCGACAAATAGTAGATACTTAAGTGATTATATAATAGATTGTTTGATA
TTGGAAAATTGTTAGCTAGAGAAGTAGTGCGCCTTAATGATGCATAGAAATAAAGTAATACTTTCGCATT
AAATGATATAACTTAATGCTTCTACACTTCAATTTTTGTTCTTGTTCCTGTGCAGCGCATGCCATACAGT
TGCAAACGCGTATCTATCAGTACGCATTCTGCCGTGTATCTATATATCTGGGCGCATGCTTAGCATTGAA
CGACGGTGGCTTTTGCCATCTCTTCGATTTATTGCTCATTTTGTTTTGTGGTCGGTGCTAAGCGGGCACG
AACAACAGCAAAATGAGTaaaaaaaaaCTTTTTCAATGGTCAAGGTCGTTTGCAGTCAAATCGAATAGCC
ACAACGCACACTTGAGCCCCAGCACTACCAACAGAAATAACATATAAAAATGTTTGTATAATTAAACGCC
ATTTTAAACAAATAAACGCGCGTCGTCAGTCGTTGATGCAGATTTTGTTGTTGCTGTTTCTGTTGGTCGC
GCTTCGGTTTTTGTGTCGTCGCATTTTTAATTAGCAATGAAATCATAATAAAATAAAACATCTCATGCAT

AATTGAGTTGCAAGTTGGGGCTTCATTGCCATGCCATTATGaaaaaaaaaaaacaaaaCTGGCTCAGGTA
TTCATAAGTAACTAAAACTGCTTCGTGGGTACGGACTCGTTTAGATAGGTCTTAGGTGGAACAAATTTAA
ATTAACGATTCATAAGCGCAGTAGAGTGAGTTATTATTAGCATCGCGGTACGGCAGGATTTCTCAACATT
TTAAATTGGCCAGCATTATGTCGAACTCAACGGATTAAGTAATCAGTATAATCAGTATTATAGTAACAAA
TAAAATAGGTAGCTGTAAGATCCTAAATATAAGGTGTAGGTACCGAGTTTTCGCAGACCTTCTAACCTCA
TACACACACACGGGATCCTTTCTTATAGGGGGCCTGCTTTCAAAACTATTTCTGCGGACTCCTGTAATTC
ATCCTGTCGTCTGCTTTCCATTATTACACGTTCTCAACGCTCACAAGATTGCTTCAAACCGATCTGTCTC
TATCCCACATCTGCCTGCCCCGGTATCGTTTTGATATGTGAATGCCTAACACGATCCACGCTTTTGAAAG
ATCCGCCCAGTCCAACCCGAATCCCGCCACGCCCCTTTTTTCCACCAATCTCGCAAGATGCCTTGCTCGT
TTTGTAAATAGCTTTTTTGAAGACGAAAGATCAAGACAAATCAAATCAAATCGATCCAATTTAAATGTTT
GTGCCTATCTAAATGTGTGTGTGTTTTACTACTCGAAAAATGCATTGCCACTCTGACAACTGTATCTATA
TCTACAAGTTTAATGCGAAGAACATACCTATATATTGCCTAAATGTTTACTGTATGTATATTTATTATTG
CTGCATAATTCACTCATAAATAtttttttctttttctttttttCATATAAACGTTaaaaaaaaaaaCTCGC
ACATTCCACCAcaaaaccaccaaacaaaaactaaaaataaaaaactaaatgaaattaaaactgaaaCATA
GGAAGGATCGTAAATAGAGTATCAATATATGTGCATATGCATATATAGCGACGTACACGAACTGTCTCTG
TCACTGATAGCGATCGATAGCTGTACCTCCATGTGTGTGCATGAGTGGCAGCATCAGTAGCGTACCCAAG
GACCAAGGACCTCATCCACATAGCCCATCAATCTCACAGATGCCAGTGGCGGATTCCCCCCGATAAAGGC
ATCGAATCGAATTACATCGCATCCATTGGACGAGGACGAGGACTAGGACTAAAGGACAGTGACTGACGCA
TCCTgcgctcgtttgatcgttcgttcgctcgctcgctcgctctatcgctctttcgctcgcacgcACCaaa
aaaaccaaacagaaataccagaaacaataccaagcaagccaaaaaccaaataatacaaaGCTAAACAGAC
GCCTCATTACCAAACATCGAACGTTTCGTTGTCGTTTAACaaaaaaaaaaaaaaaaaaaaCCAGAAGCAC
AGcaaccaaattcaattcaatcaaatcaagtccaagccaatccaatccaatccaagatccaattaaatta
aatcaagccgaatccaCGAGACATAGAAACTGAAAAGTAAACAGTAAGATCAACAGATCAACGAAACCCA
AAGCAAAAAGCTAAAGCAATCATCAGTCAAAGTCGATAACCAAAACCAGCAACATTCAGTTAAAAATGGC
CGAAGACGCTACCATGGAGACATGTCCAAGTCCTGAAACCGGCGACTCACGCAAAAGGCCACTCGATTCC
GATCCGGAAAATGAACAAACTAAACGCTCACATTTCAGTTCCG
>dme_circ_0008337_junction_seq
CAAACTAAACGCTCACATTTCAGTTCCGGAAAAAAGCACGAGAGGAAAAAATGAAA
>dme_circ_0008338
GAACCTTGACAATTGGAAATCATCGAGACGAAAACGTGTCGAACACATCATCGATCGAGCGGTGGAGACC
AAGAAACTCGAGCTGGAGGAGCATGATCGGTTGCGCCGAAAGTCGAAAACATTCACCGAAATGATGGAGG
AGAGGGCAAGTACAACTCCCAGTTCGCAACTAGTGTCTAACGTGCTGAAAGTGTTGATTGCACAAAATCG
AGTCCTGCCACGGACGACACGTTCTTTTGAAATGCTTGATACAAGTGAATCCAAATCCAAGTGCTATTAT
CCAAGTGAAATGAATATTGCGGTGTTTCTTTGCGAAAATCGCTATAAGATATCAGACTACATAGTAATCT
TTTACATTGTTTGTCATCCTAAACCACCCGATTTCATTTTCAGCACCTGTGGTGCACCTCCTGAATGTAA
CTTTAACTTTATTAAAACACATAAATTTCCCCACAGGGGCTGAAAGGGGCGGACCTCGGGGGCGTGCCAA
GCTGGCTTCCCTGGCCGTATACAACGAGGATGAGACGAACGACCTGAGTGATTTGGGGATCGGAACCAGC
AGTGCCAGCGGAAAGAGCAGCCTGTCCGGGGACTATGACAATAACAGTGTTATGGGTGAGTTCCAACCAA
CGAAGCACATTGCCATAGTAGCATGGACCTATTTAGCACCCATTTCTAGTACCAGTTTCTCCGTCTTAAT
TTTTTTGTCGTTATTAAGACTCATTTATGGTGTCACTGCGCTTATAAGCTATTACAGTAAATTAATAATT
AAATCTAATTTGTATTTTGCTGCCACTCAAGCTCAGACTTATTGGAAAGTGTTGCAAGCAATGCGATGCG
ACACAAATAGATTATTAATCAAATGCCCGCACATGCACCACCTTATTGTGGTTATCAAAATACCAATCAT
AAGTAATAATCAATGCACCACAAACAATCGAAAATGTGCAGACGATACAAATATTTCCCTTAAGATTGAA
ACTGATCGAAAGATGTAGCTTAGGATTGATAAGGATTAGGTGCGCTGGTCGCCACACCTCAAAATAATCA

GTTTTGAACTGaaaaaaaaaaTGCTTGGAAACGGGGATCCTGTAGATTTAAGTGGAAAATATTGAATAAG
AGCATTTTAGCAAACGCTAAGGAAATACAATTTGAGTTTGATAAATAGAACAGCTGTGAAAGGGAAAGAA
TTACAATCGATGGAGTGCACAAAACATTTACAGAACCTACAAATATAGGATAATATTTATTCAATTATTC
TTATCAAGTTTTATTAATATAGGTACAGAAACGCTTTCTTCTATCTCTTTTATATTTATAAGGAATACGC
TTTAAAAAGATACACTACTACAACTAGTGTGTTAGTGGATCCTCATATTTCCATTACCATTTAAGTATTT
ATCCAAAATTTTTAATAAAAATGACAAATTCTATTACCTCCACAGGAGCGACCATGCGGCGGAGCTGGAT
AAGGCGATTGGAGCAGCTAGCTCTGTAGCTGCTGGAGTAACCGCCCAAAACGTGGATGAGCAGCTGAATC
ACATAAACCGCAACGGTAGTAATGGGAACCACGGGAACGGAGCGGCTGTTGGCCAGACAGGACCATCAAA
TTCCTCCAAGTCAGCAGGGCGGCAATACATCTCCTCGCCAGGATACGATACGTCCTCCAGTAACGCGCAA
GCCAGTTCTCCGGATCTCTGTGAATACACCTACGAAGGAGCCATCCAGGACTACAAGCAAAGAGTTCAAC
GGGCCAGCAGCAATGGCAATGGAAATGCCAACGGCAAGTTGATTGGAGAACATATTGCTTATCCCACGAG
GCGAGGCTCTAAAATTGAGGATCGATTGAGTGGTTTTGAGGTGACATCGCCAAGCGACACGCAGGAGGGT
GTAGAGAAGCAAAAGGTGGACGTGCCCAAGGTTGACATATCCAAACGCAAGGAGATCTTCGAGCAGGCCA
AGGCGGAAGTCTCAAACGGTGGTGCTCCAGCTGCCCCAAAACTCGTATTTCGTGATGGTCTTACCAATGG
AAATTCAGCTACCACTGCTCCCAAATCGGAGGTGAGGCGTCTTTCTGGTGACATCTCCAGCATTCGAGAT
CGCATGCAGAGTTTGGAACAGCAGCGGAAGGCTTTCAGTTCCAGCAAGAGTGTGGATGTGCCGGTGCCGC
CCCTTAAACAGCGTCTGAATAGTCTGCAGCAGTCCGTTGCCAAAGAGGAGCAGAAGAAGCCACCGTTGGT
GGCGCTCATCGATGCCAGACAACTGGAGATCATGAGGGGAGAAGAGGAACGTATGCGCCAGCAGCAACAA
AAAGAGAAGCATTCACCAAGCCAAACTACATTGTGTGCACCTAAGCCAGCTCCTCCTGCCCTTATTATTG
AGGAACCGCCGGTGGCAGACACCAACGACGACAGCGGCATTCAGGAGGACACCGCTGATGAACTGCAGCA
GCAGCAGCAACTCAACGCAGCCATCGCCGCCTTGGCGCTCGAGGAGCGCCAGTTGGAGGAAGCAGCCAAT
GCGGTCAACCAAATTGAAGCGGAGTTCGATGAGCTGACTGATCTGCATCCCTCGCCATTGCCACCATCTC
CAGCTCTTCCGGCGGTTCAACAAAGCCTCCAACAACCGACAGTCGCTCAGGTTCCAGCACAAGCGGTCAA
TCAGGCGGCTGCTCCTCCATTGCGGGACATGGAATTTAGT
>dme_circ_0008338_junction_seq
TCCTCCATTGCGGGACATGGAATTTAGTGAACCTTGACAATTGGAAATCATCGAGA
>dme_circ_0008339
GAAGGATCGTAAATAGAGTATCAATATATGTGCATATGCATATATAGCGACGTACACGAACTGTCTCTGT
CACTGATAGCGATCGATAGCTGTACCTCCATGTGTGTGCATGAGTGGCAGCATCAGTAGCGTACCCAAGG
ACCAAGGACCTCATCCACATAGCCCATCAATCTCACAGATGCCAGTGGCGGATTCCCCCCGATAAAGGCA
TCGAATCGAATTACATCGCATCCATTGGACGAGGACGAGGACTAGGACTAAAGGACAGTGACTGACGCAT
CCTgcgctcgtttgatcgttcgttcgctcgctcgctcgctctatcgctctttcgctcgcacgcACCaaaa
aaaccaaacagaaataccagaaacaataccaagcaagccaaaaaccaaataatacaaaGCTAAACAGACG
CCTCATTACCAAACATCGAACGTTTCGTTGTCGTTTAACaaaaaaaaaaaaaaaaaaaaCCAGAAGCACA
Gcaaccaaattcaattcaatcaaatcaagtccaagccaatccaatccaatccaagatccaattaaattaa
atcaagccgaatccaCGAGACATAGAAACTGAAAAGTAAACAGTAAGATCAACAGATCAACGAAACCCAA
AGCAAAAAGCTAAAGCAATCATCAGTCAAAGTCGATAACCAAAACCAGCAACATTCAGTTAAAAATGGCC
GAAGACGCTACCATGGAGACATGTCCAAGTCCTGAAACCGGCGACTCACGCAAAAGGCCACTCGATTCCG
ATCCGGAAAATGAACAAACTAAACGCTCACATTTCAGTTCCG
>dme_circ_0008339_junction_seq
CAAACTAAACGCTCACATTTCAGTTCCGGAAGGATCGTAAATAGAGTATCAATATA
>dme_circ_0008340
GAAGGATCGTAAATAGAGTATCAATATATGTGCATATGCATATATAGCGACGTACACGAACTGTCTCTGT
CACTGATAGCGATCGATAGCTGTACCTCCATGTGTGTGCATGAGTGGCAGCATCAGTAGCGTACCCAAGG

ACCAAGGACCTCATCCACATAGCCCATCAATCTCACAGATGCCAGTGGCGGATTCCCCCCGATAAAGGCA
TCGAATCGAATTACATCGCATCCATTGGACGAGGACGAGGACTAGGACTAAAGGACAGTGACTGACGCAT
CCTgcgctcgtttgatcgttcgttcgctcgctcgctcgctctatcgctctttcgctcgcacgcACCaaaa
aaaccaaacagaaataccagaaacaataccaagcaagccaaaaaccaaataatacaaaGCTAAACAGACG
CCTCATTACCAAACATCGAACGTTTCGTTGTCGTTTAACaaaaaaaaaaaaaaaaaaaCCAGAAGCACA
Gcaaccaaattcaattcaatcaaatcaagtccaagccaatccaatccaatccaagatccaattaaattaa
atcaagccgaatccaCGAGACATAGAAACTGAAAAGTAAACAGTAAGATCAACAGATCAACGAAACCCAA
AGCAAAAAGCTAAAGCAATCATCAGTCAAAGTCGATAACCAAAACCAGCAACATTCAGTTAAAAATGGCC
GAAGACGCTACCATGGAGACATGTCCAAGTCCTGAAACCGGCGACTCACGCAAAAGGCCACTCGATTCCG
ATCCGGAAAATGAACAAACTAAACGCTCACATTTCAGTTCCGGGTAAGTTGACCAaaatccaatccaaat
ccaaatcaaaattcaaaatcaaattcaaattcaaatcgaaatcaatatcgaaatcgaaaCTCAATTCATT
TCGCCAGCAGAAGGAAGAAATTGAAATATATATCTCAAATCGCTCTTGCATATCCCCATTATCGTGCTCT
CTCTTTTTCTCTCTGtcaatctcaatctcgatctcaatctcagtttcaatatcaatctTAAGCTCCTCAA
CTCGACGCTCTGTGTATATCTATCTCTCGTTCGTTATCTATTTATCTATCCATTGCTTTTTTATCTCCGA
AGCGTATAAATAACTGATTCAATTTGTTTGCCCAACTTTTCTGCCGATCGTGCCGTTTCCCTTCTTTATT
ATGTGGTCAtgttgttgttgttgttgttgttATCACAATATATCCCCCCGATATGCGTACCTACATATAT
CTATGTATATATATACTTAGTATCTATGATTTTCTTACTTTTTTCTTTTAGTCGTTTGTCGCTCGTGTTA
TTTATTGTTGCTGTTGTCGTTTGTTATTCAGATTCAATTAATCAGCACGAGAAATTCATTTGCAGTTTGC
CCCCAATACGTTCGATGTCAGTTTTGcaaccgaacaaacgaaccaacgaacgaacgaacaGGCAGCAGAC
TTTGATTCGTAATTAAGCCAGATATGGCGTATACGCCCCGAGGGCCCCCTCTACACGTACACAGCTACAG
TGTACATCTTAAAATGACAACTGGCATCTGTTATATGGCATACAGCGGTATCTAGTACCTAGTGTAGTAT
CTGCCTGGCTGGCGGCTGTATCTTTGCGCAGGATTTGGCGCCGAGACATTGTGGGCCCAGTTTGACCCAA
TTTTGGGCATGACCTCCGCCTGCAATCTCCTGCAGCTTCATCAATGGCCTGCAATTAGATACAAGTGCTG
TCCGTGCACGTCCCTGAACGTCCAAACGAATGGCATTTGAAAAGCTCCTCCCTGCTTCACTCATCCGACA
AACTGAACTGCAGTCGGGACAGCCGAGGGGGTCACAAAAATTCATATGGAGATTAGCATTTAAAAGAAGA
AGCTCCCAATCCGTGAGCTCCATTGCCAAACCCATACTGCctgtctgtcagtcagtctgtcggtcagtcg
gtctgACCACAAGCTATTAACTAGTTGCTTTGCATGATTTGTTCGGCTCAAAACCAGCTGGCAAGCTCCA
AGGTGAGCATGCCACTACGCAATCGAAGTACCTGTCGTCATAGCCACGAAAATAGCAAAAGGTGTAAGTG
TTAGAGTCGCACTGTCGCCCCCGCTGGCTTTTGGTATTTAGACGGATAGCAGTGGCTAGAGCCAGAAGCG
TGTAAACGTGTTTTCGAGTTTCCTAATTGTCAATTGGAAATTATGAAGGGTAGATTAGTCCTCGGTTTTC
GTTCATCACAGCAGGCTCTCTCAAATACGTCAAGTGTTAAAATCCCaaaaaaaaaaaTATCTACAGAAAA
TAAAACAAAATTCCAGTGTTGTCTTAATGTTGCATCAAAAATTTTAAGTTTTATCTCGTGAATTGGAATG
CGGAAGCTTTGTTCGAGTAAGTTCAAGTTCGTTAAATAGGCAAATATAACTTATTTCCATATACTCCACT
CCATTCATTAAAGCAGACACTATATTATATGCTCGGGGACATCCATAGTTAATGGGTTAGTTCTGCCTAA
GAATTTGTTTGCTTATGAACAATGCCATTTTATCAGCTGTTTGTTTGGCCAGTTTCCATTCAAACCGATG
GCATTATAGATGAGCCCAGATAAATAGATTCGTTACATGTATGTGTACAGCTGGGCATTTGTAATTAATT
AGCCACCATTGAGGCTGCCTGTTTGCTGGACAGAGAACAGGTCCATGTCATATGGTTTTGGGTATTTACT
CACGTCGTCGTTTTATTAGTTTTCTGGCAATTTATTTGCTTAGCTGCCTTGACAGTCatttgtttttgt
gtgctatctatttaatttttttcagtttttttcttatttATGACTGTCATGGCTGGAATTCTGTGTAGCTGG
TTGGTGAGAGAGCAGCGCAAAGTTTTTGAGATGCACAGAGAAAAATAATCTTCTTAAATTCTAATAAGTA
TTTAAGGTGTAAGGTTTTAAAGTTTAATCCAAAGAAGTTAGGAAATTCAAACAACTTTTTGTCAACAATT
CTTTTAAGCAAGAATTCAACTCTTTACCATATACTTAAtttttttttGACTTATTGTTTTCCGTGTAGCC
TGGACTAGAAATGCGTCAGTTGGCGGGGCTGCTGCAAAGGAAATGCAGTAAAATCGAGGAAAATCGGGGC
GGTGGGTCAGCGTGGAGGTTTTGGACGACTGGTGGCGTGGCTTATGTAATCAGTACTGGCAGGGCAGCTG

GAAAAGCGTGCGAATAGTTGGGGGTGTATACTGTAGCGAACTCTAGTTCGCCGTAAGAGAGAGAAGCGAG
AGCTGCTCTCGGCAAACGTTGACAGCATGGCAAGCGGTTGACCTACTTTGATGACAATGCGGGTGCGTAC
GtgtatctgtatctgtatctgtGTGTGCCTGTGGTCGCTCCGCTGCCATCGCTGCTTGCCTGCCTGCCTT
TGCTCGGTGCTTTTCGTCTAGGAAGGGTCCTGCGCAGTAGGACGACTCGAACTCCTCGAACGCGGCGACC
ATTCAGCTGCACTGCAGCATTTGGCGCCATTgcagctgccacagcagccgatgcagccgcagcagccgca
AACGCAGCAGCAACGTCCTTGAGTGGTGGGTCCTGTAGAACTGGGTTGGTTGCCTCGATGGCAGGCTGAC
TGACTGCCTGGCTGGCTAGATGCAGCAGCTGAGAATGTGgtatggcagaatggcagtatggcagtgtggc
aTGGTTGAATCTCAAGTGCGTTTACTGCCTAGGGGCTGTGTGCTCTCTCGCACTCTCACTGTTTTGGCCA
CAATCTGAACTGATATCTCTGTGTCGTGTTTTTGGGTTACCTTATTCTTAGGTGCATTCCATTGTTGCGG
CTCGAGTGGCCAGGCATTTGGTGTGACTGCACTTGTAGAGCATCTCGAGTGGCCTACTAATAACTCAAGG
GGCCAGGCAGTTTGACATTCTTTTCGGGCCAGTGATTAGGACAAGCCCGGCTTGCCTCAATAATTGGGTC
ATCTGAAGTCCTTATATTACGCATACGCAGCGTGGGTCAGTCCCAGTGGTAGCACAACTTGAACTCACAA
CACTTTTCTCGTATTTCACAAGCTCTGTTAGTTGTTCCTATCTTGAATAATTGTAATTGTGTAATCTTAA
AGATTAAAACTAACAACGGGGCAGACTAACTCGCTTTGATCCCTCTTCTCGCCATTAATATTATATGCCT
CTAACCGAATTTGGCTACTACTTCACCGTTTTAAATCGCATTTTATATAATCTCAAGGGCTTTAAGCATA
ACTTCCCGTTGTCATTTATGGATCGCTTGGCAAATCGTTTGGTTTAGCTTCCACATTGAATTCTCATCGT
TTTGGTGGGTGAAAACGTGACACGTTCACTCTTTTCGCCGCTGGCTTATCACGTTGGCTTATTCCGTATC
ACATGTTGCCACAGCAGTTTCTATTTTCGACACACACGCCGCCCCCTTTTGTGTCTTCGCCCCCTGTTTG
TGTATTGCTCTCTCCCCGCCGCTCTTTGGCGTTGATAATGAACCGAGTGACATGGAGTGAAGTGAGGCAA
TCATCGGCGGGCCAGCATTAGATCATATATACAGACAGAAACGACGACGACatcatcatgatgatcatga
tcatcatCGTACACTCTGACTCATGAACCTCACTCGCCTCACTTGCACTGGACAGACTCTCCGGATATCT
CTCGCTCTCGCTTGCTCGCTCGCTCCCTCGCCCGTCAGTAGATTCTCCAATTCTCCGGCTCTCTGGCAAA
CTCTATGCTTGAGTCGCGGCTCCGTCTTCGCATCGCACGGAATTCTTCAGTGCGGTTTGGTTGCTCGTTT
CGTGCGCTCGCTCGATCGCTCGCTGTTGCTCGTTTTCCACACACGAATTCCCAGCTAACGCGCAGCCATT
TTGACAGTGATTTTATTGGTTTGGCTCTTTTGGCCAGCAGCATGTTGTTCGCCAGAACCACCTCGTCCAC
GTCGATCAGTCCGCCCGCGATGATGATgcagcagcaccagcagcagcagcagcagcaggatcctcagaag
ctgggacagcaattccatcagcagccagcatttcagctacagcaAAGCTTCTGTACGTTTCTCGGTTAAG
TGTAACTCACTGTGTTCACCTGTTGTTGTTTTGTTGACCTTGGTAATTAGCGTGTGCATTAATAATTATA
GGGTTTATCTCATTCGGTATTTGCCAGGAGCTTTGCTAACTGTATATGCCATCACCTGAGCCCTCCTCGG
CGGGCACAAATACAGACAACCACGTACACCGCGGCGTATGCGTAATATTTCGATATTAATGTTAGCCAAC
GTAACGCGGCAATTAACGTCTTGTCATCTTGGTTTATCTAGCGTTTCAAGGTCGCCTCTTGGTTTGTTAT
TCTCTCGACTGAAGTCTTAGGCTCGACACACACACTTTTCCATTGAAATTAGCATTTGAAAATCGCTGAA
AAGCCTAAGTCAAAGCCAAAGTAAAACCAAAGCGAACCCCGAACCAAAACCCACACAGAGCAATGTATTC
AGTTCAATGCCAGTCATAATGATTATTCAAAAAAGCACTAAGCAATCGACAAGTCGAGTTGAAGTTTGAC
CTCAGCTCAAGCTGGGATCGGATTGAATCGGATCGGGCTTTATATTATCAGTTGATTCTCAAAATCAATT
TCGAGTTACACAGTTGTTCGTAATTAACTCAAGAGTCACGCGGCCTCTCTGTCTGTTTTTTATTTTTGAG
CTCTCAAGTACTCGTCGTACTTAGGGCTCTAACAAAAGTATAAAATAAGAAATACAAAATTGCCGTCTTG
AAAATGCGGGAAGCAAAACGGCGGCAAAACGTAACCTTGAAAAGCGATTTGAATGTGGGCAAACATGTCT
CACTCTATCTCTGCGATTGTGAATGCCtgtgtgtatgtgtatgtgcgtgtgtgtTCGTTTGAAAGAGCCA
GCCAAAAATAAGTCAAGACACTCGTGGCCATGCCCAAAAACTGCTCATGTTGCTCGGGCTCTGAACAAAA
CGAATAAAAAGAGACCACAGATAAATAGAAATAAAAACACACATATTATATCCCTCGAAAACAGCTCCAA
AAAGGGGAGACGGATTTTGGGGATTTTACTTGTATTTTTCTTGGCAAGAGACTTCTTACTCTTCCTTTGT
TGCTCAACCGTCGTCGTGATTTTTTTGGCATTTTGGCGAAAATGTCCGAAAAATGTGTTATTTATGCGTG
GCACAAGTTCGCATTTCCACGGCGCATCCAGTTCCAACCAGTAGCTTTTTTTAACTTCGAATCCATTGTG

TAATCTCGGAGTTTGGCTCTGGCCAACCAGTTGTTAATTTGACTGCCATTATTTGGCAAGACTCACGGGT
CTGGGCAGGTGTTTTGGTGGGCATTTCATCTTCTACTGGCAGCCAGACTTCTCAATGGCGAGAGCTATAA
ATCAATTTTAAGAGCCGAAAGGGTTTCGGTTTGTGTGAAAGATGATCTCACAAAGGTATTCAGAAACGCC
CACTTTGCAtgtgtttttttcagatattcgtttgtttccgtttcagtttgttgtcgaagttagttttttt
tCCCTCTCGAGTCAGATGACATATGTATATGTACCTATTCAAACTTGAATTTAGTTATTAATTTGTGCCT
TCAGATGATTAGTGAAAATTACTGTGCATCAATTAAACAAAAATATTGTTGCGCCTCATTTAGTGCTTGT
GTGGTTCAGTTTATTAGAATGGAACCAAGGTCTTTTGGTTCGAATCAAATAGGAGTAGATTTTGGTTATT
GGAGAAGATGGTTATTGTGTGTTCTAGGCAGAGATACCATCCCATCATCATTTTCAGCTTCTGCCCAATC
ATCAGCAGACCTTATCAACTGGCTTCCGCTGTTTCATCAATTAAAATGCTTTCAATTCGCTCAATTTCAT
ATATGACCATGATATTTGTTACAGCTTTTAATACATCCGACCACTGTGTTGCATATTACcgtttttatta
tcgtttttctgtttgtttcttctttttttgttcttttttttAAACCATTATCGTTCATGCATATAAATGT
GCTTGTAATTATCGAAAGCCAACCATAGCGCAAATATAATTATAGATACTACACAAAATGTAAAAATAAA
TTGATGTGCATGCCGCACAGCAGAACGATAAAGTTAAAAACGAAATGAAAGCCAACCCACAAAGTATATA
AAAAAGAACATTTTCAATAATCACGAACAGTTGTTTTTGTTGTTGTCCCCACTTAAAGCGTGCGATTAGC
AATAATAGAAAAAGTCGCGTATAACTTTTAACAATCCAGCAGCAGCAGCAGCATTTCCACAAAACCAATG
AGAAAACTCTTAACCGAACTCAACTCAAATGTGATAATTCCAAGCAAGCAAAGCAGTTAAAATATGTTGT
GATAAAGCTACGCAAGTAATGACTAAAATATAAAACATATAGAAAAGCCGAAAGTTATTAAACAAACCAA
AAACGACTGAGATCAAATCGAGGAAAACAAATGCAACACATTAGCAGAAGATCATCACAAAATATCGCCG
TCCTGTCATCGAGCCAATCTTCAAGTATTATAGCATCTACAGCACCTGCAACATCTCctctgaattctga
actgtgaactgtgaactctgacctctgagctcAACTCTACAGCTGAATGGTAAAGCATTGGCATTTTTTT
CTAAATGTCTTCCATAGCATTTCTGGATGTTGTGTAACAATCGTCATTAACAAACGCCATCGTACATGGA
CATACTCGTACTATCTCTCTATATCGCCATAGCACCAATTGTCATCCAACTACTCATGTCTGTAAGCGAA
TATGTATCATAAAGTATTTTTGTTCCGATGCGAGCGATATAAGTTCCATCCAAGACAGATGCAATCCTGC
AATCGACAACAAATCAATCGATACCTTTGCTGAAGCTTGCTGCTGCCGCTTTCTCTGTCTCTGTCTGTTT
CTCACTCCGCCTTCTACCTATACAAACtatatatatatatatatatatatatatatatatatatatatat
ataGTCGCAGTCGCATCGTAATCGTTGTTGTTGTTATCGTACAAAATATCTAACAAGAAATCATCAAGAA
ACCAAAAAAATGAACAGGCACCAGAACAGAAGAACACTAAAATCGCCATCCAACAACACCAATATCTAAT
CAATATCGAGCTATCGTCAGCTATCGAATCAAATCGAACTAAATCAAGTCATCCGGAAGTCGAGCGACCT
ACTAAACCACTGCGAAGTCACCACTCAATCAGCCCGATCTATATATCTCTATATGTTAACTTACAACCAC
CGCATCATCATGAAGAGCATTATACCAAACAAATCGTAGaaaaaaaaagaaaaGAATAGCAACAAATGCA
CAGTCCAAAGCCTTACAAACTCGATTGCACATTAAACTCGATGTGTATCTATAATGAATATGCAATTCAG
ATATCGATGGTGAGCGAGGGGGAAAGAGACAGAAAGCGAAGGTCCTTGAAGTGCTCTATCTACCTATCTA
TCTCTCTCCCGCTCGCgtgtgtgtgtgtgtgATCAGAGGAGAGCGAAACCTATGAAAAGCCAGCTTTATC
TAAACAAATTTTCCATTTCTTTTTCTTTCGTTTATTGAACAAAGTTTAATAATTGGAAAGCGGCGCGTAG
GTTGAATCAAAACACTGAACAGTTAAgaaaaaaactaaagcccaaattgcaaattacgaaatataataaa
aaattgaaaaCAAATAACGCATAAatcagttgatcagtggatcagttgaATAAGGAACGAAACGAGTACA
ATCGATTCAAGGCAGCATCAATTCCGTCAAACAAACAAAACCGAAGACTGAACGGGAACGACAACAATTC
CAAGGCAATGGAGAGTATTATGAAGGTGGCCATGGACAAGGCGGCCGAGCAGCTGATTCAGCAATTTGGT
TTTGATTACTTGCAGCAGCAGCTGCAGTTGCAACATCAGAACCAACACAacagcagcccacagcagccac
agcaCCAGCAACTAGAACCAGAAAACGAACATCTCACATACCAATATCAGCAATCAAAGCCCACACATAT
GCAGCAATTAGCTTGTAACTATCAGCCACGACATTCCACAACCACGTCGTCGCCCTCATCCACACATTCG
CTGGCcagcggcggcggcagcagcagtaacagcagcaacagcagcagcagcgacagcagcagcatcaaca
taagccacatcagcaacataagcaacatcagcaacatcggcaacatcagcaacagcaatcatagcaacgc
tgcaTACTCATTAGCCGTTCATAGCTATCAAAAGCAAATAGAAAGCCCGGCTAATCCCAGTCATGTTCCG

CATCATCAGATGGATCTATCGCCCCTGAGTGAGAATGGTTCGCCAAATGGGACACCAGGTGCCCAGACGC
CGACTgcaacagcgagtggcaacacggcagcagcactcgcatcagcagcagcagcagcagcagcagcaac
atcaggaggcaacggcagcagcatcaccaactgcaacagcaataacagcagcagcagcagcaacgcgcaa
cagcaactgcagcTGGGCAACTATAAGACTAACTCCTGCTGGTGTTACG
>dme_circ_0008340_junction_seq
TATAAGACTAACTCCTGCTGGTGTTACGGAAGGATCGTAAATAGAGTATCAATATA
>dme_circ_0008341
GGATCGTAAATAGAGTATCAATATATGTGCATATGCATATATAGCGACGTACACGAACTGTCTCTGTCAC
TGATAGCGATCGATAGCTGTACCTCCATGTGTGTGCATGAGTGGCAGCATCAGTAGCGTACCCAAGGACC
AAGGACCTCATCCACATAGCCCATCAATCTCACAGATGCCAGTGGCGGATTCCCCCCGATAAAGGCATCG
AATCGAATTACATCGCATCCATTGGACGAGGACGAGGACTAGGACTAAAGGACAGTGACTGACGCATCCT
gcgctcgtttgatcgttcgttcgctcgctcgctcgctctatcgctctttcgctcgcacgcACCaaaaaaa
ccaaacagaaataccagaaacaataccaagcaagccaaaaaccaaataatacaaaGCTAAACAGACGCCT
CATTACCAAACATCGAACGTTTCGTTGTCGTTTAACaaaaaaaaaaaaaaaaaaaaCCAGAAGCACAGca
accaaattcaattcaatcaaatcaagtccaagccaatccaatccaatccaagatccaattaaattaaatc
aagccgaatccaCGAGACATAGAAACTGAAAAGTAAACAGTAAGATCAACAGATCAACGAAACCCAAAGC
AAAAAGCTAAAGCAATCATCAGTCAAAGTCGATAACCAAAACCAGCAACATTCAGTTAAAAATGGCCGAA
GACGCTACCATGGAGACATGTCCAAGTCCTGAAACCGGCGACTCACGCAAAAGGCCACTCGATTCCGATC
CGGAAAATGAACAAACTAAACGCTCACATTTCAGTTCCG
>dme_circ_0008341_junction_seq
CAAACTAAACGCTCACATTTCAGTTCCGGGATCGTAAATAGAGTATCAATATATGT
>dme_circ_0008342
GGATCGTAAATAGAGTATCAATATATGTGCATATGCATATATAGCGACGTACACGAACTGTCTCTGTCAC
TGATAGCGATCGATAGCTGTACCTCCATGTGTGTGCATGAGTGGCAGCATCAGTAGCGTACCCAAGGACC
AAGGACCTCATCCACATAGCCCATCAATCTCACAGATGCCAGTGGCGGATTCCCCCCGATAAAGGCATCG
AATCGAATTACATCGCATCCATTGGACGAGGACGAGGACTAGGACTAAAGGACAGTGACTGACGCATCCT
gcgctcgtttgatcgttcgttcgctcgctcgctcgctctatcgctctttcgctcgcacgcACCaaaaaaa
ccaaacagaaataccagaaacaataccaagcaagccaaaaaccaaataatacaaaGCTAAACAGACGCCT
CATTACCAAACATCGAACGTTTCGTTGTCGTTTAACaaaaaaaaaaaaaaaaaaaaCCAGAAGCACAGca
accaaattcaattcaatcaaatcaagtccaagccaatccaatccaatccaagatccaattaaattaaatc
aagccgaatccaCGAGACATAGAAACTGAAAAGTAAACAGTAAGATCAACAGATCAACGAAACCCAAAGC
AAAAAGCTAAAGCAATCATCAGTCAAAGTCGATAACCAAAACCAGCAACATTCAGTTAAAAATGGCCGAA
GACGCTACCATGGAGACATGTCCAAGTCCTGAAACCGGCGACTCACGCAAAAGGCCACTCGATTCCGATC
CGGAAAATGAACAAACTAAACGCTCACATTTCAGTTCCGGGTAAGTTGACCAaaatccaatccaaatcca
aatcaaaattcaaaatcaaattcaaattcaaatcgaaatcaatatcgaaatcgaaaCTCAATTCATTTCG
CCAGCAGAAGGAAGAAATTGAAATATATATCTCAAATCGCTCTTGCATATCCCCATTATCGTGCTCTCTC
TTTTTCTCTCTGtcaatctcaatctcgatctcaatctcagtttcaatatcaatctTAAGCTCCTCAACTC
GACGCTCTGTGTATATCTATCTCTCGTTCGTTATCTATTTATCTATCCATTGCTTTTTTATCTCCGAAGC
GTATAAATAACTGATTCAATTTGTTTGCCCAACTTTTCTGCCGATCGTGCCGTTTCCCTTCTTTATTATG
TGGTCAtgttgttgttgttgttgttgttATCACAATATATCCCCCCGATATGCGTACCTACATATATCTA
TGTATATATATACTTAGTATCTATGATTTTCTTACTTTTTTCTTTTAGTCGTTTGTCGCTCGTGTTATTT
ATTGTTGCTGTTGTCGTTTGTTATTCAGATTCAATTAATCAGCACGAGAAATTCATTTGCAGTTTGCCCC
CAATACGTTCGATGTCAGTTTTGcaaccgaacaaacgaaccaacgaacgaacgaacaGGCAGCAGACTTT
GATTCGTAATTAAGCCAGATATGGCGTATACGCCCCGAGGGCCCCCTCTACACGTACACAGCTACAGTGT

ACATCTTAAAATGACAACTGGCATCTGTTATATGGCATACAGCGGTATCTAGTACCTAGTGTAGTATCTG
CCTGGCTGGCGGCTGTATCTTTGCGCAGGATTTGGCGCCGAGACATTGTGGGCCCAGTTTGACCCAATTT
TGGGCATGACCTCCGCCTGCAATCTCCTGCAGCTTCATCAATGGCCTGCAATTAGATACAAGTGCTGTCC
GTGCACGTCCCTGAACGTCCAAACGAATGGCATTTGAAAAGCTCCTCCCTGCTTCACTCATCCGACAAAC
TGAACTGCAGTCGGGACAGCCGAGGGGGTCACAAAAATTCATATGGAGATTAGCATTTAAAAGAAGAAGC
TCCCAATCCGTGAGCTCCATTGCCAAACCCATACTGCctgtctgtcagtcagtctgtcggtcagtcggtc
tgACCACAAGCTATTAACTAGTTGCTTTGCATGATTTGTTCGGCTCAAAACCAGCTGGCAAGCTCCAAGG
TGAGCATGCCACTACGCAATCGAAGTACCTGTCGTCATAGCCACGAAAATAGCAAAAGGTGTAAGTGTTA
GAGTCGCACTGTCGCCCCCGCTGGCTTTTGGTATTTAGACGGATAGCAGTGGCTAGAGCCAGAAGCGTGT
AAACGTGTTTTCGAGTTTCCTAATTGTCAATTGGAAATTATGAAGGGTAGATTAGTCCTCGGTTTTCGTT
CATCACAGCAGGCTCTCTCAAATACGTCAAGTGTTAAAATCCCaaaaaaaaaaaTATCTACAGAAAATAA
AACAAAATTCCAGTGTTGTCTTAATGTTGCATCAAAAATTTTAAGTTTTATCTCGTGAATTGGAATGCGG
AAGCTTTGTTCGAGTAAGTTCAAGTTCGTTAAATAGGCAAATATAACTTATTTCCATATACTCCACTCCA
TTCATTAAAGCAGACACTATATTATATGCTCGGGGACATCCATAGTTAATGGGTTAGTTCTGCCTAAGAA
TTTGTTTGCTTATGAACAATGCCATTTTATCAGCTGTTTGTTTGGCCAGTTTCCATTCAAACCGATGGCA
TTATAGATGAGCCCAGATAAATAGATTCGTTACATGTATGTGTACAGCTGGGCATTTGTAATTAATTAGC
CACCATTGAGGCTGCCTGTTTGCTGGACAGAGAACAGGTCCATGTCATATGGTTTTGGGTATTTACTCAC
GTCGTCGTTTTATTAGTTTTCTGGCAATTTATTTGCTTAGCTGCCTTGACAGTCatttgtttttttgtgtg
ctatctatttaatttttttcagtttttttcttatttATGACTGTCATGGCTGGAATTCTGTGTAGCTGGTTG
GTGAGAGAGCAGCGCAAAGTTTTTGAGATGCACAGAGAAAAATAATCTTCTTAAATTCTAATAAGTATTT
AAGGTGTAAGGTTTTAAAGTTTAATCCAAAGAAGTTAGGAAATTCAAACAACTTTTTGTCAACAATTCTT
TTAAGCAAGAATTCAACTCTTTACCATATACTTAAtttttttttGACTTATTGTTTTCCGTGTAGCCTGG
ACTAGAAATGCGTCAGTTGGCGGGGCTGCTGCAAAGGAAATGCAGTAAAATCGAGGAAAATCGGGGCGGT
GGGTCAGCGTGGAGGTTTTGGACGACTGGTGGCGTGGCTTATGTAATCAGTACTGGCAGGGCAGCTGGAA
AAGCGTGCGAATAGTTGGGGGTGTATACTGTAGCGAACTCTAGTTCGCCGTAAGAGAGAGAAGCGAGAGC
TGCTCTCGGCAAACGTTGACAGCATGGCAAGCGGTTGACCTACTTTGATGACAATGCGGGTGCGTACGtg
tatctgtatctgtatctgtGTGTGCCTGTGGTCGCTCCGCTGCCATCGCTGCTTGCCTGCCTGCCTTTGC
TCGGTGCTTTTCGTCTAGGAAGGGTCCTGCGCAGTAGGACGACTCGAACTCCTCGAACGCGGCGACCATT
CAGCTGCACTGCAGCATTTGGCGCCATTgcagctgccacagcagccgatgcagccgcagcagccgcaAAC
GCAGCAGCAACGTCCTTGAGTGGTGGGTCCTGTAGAACTGGGTTGGTTGCCTCGATGGCAGGCTGACTGA
CTGCCTGGCTGGCTAGATGCAGCAGCTGAGAATGTGgtatggcagaatggcagtatggcagtgtggcaTG
GTTGAATCTCAAGTGCGTTTACTGCCTAGGGGCTGTGTGCTCTCTCGCACTCTCACTGTTTTGGCCACAA
TCTGAACTGATATCTCTGTGTCGTGTTTTTGGGTTACCTTATTCTTAGGTGCATTCCATTGTTGCGGCTC
GAGTGGCCAGGCATTTGGTGTGACTGCACTTGTAGAGCATCTCGAGTGGCCTACTAATAACTCAAGGGGC
CAGGCAGTTTGACATTCTTTTCGGGCCAGTGATTAGGACAAGCCCGGCTTGCCTCAATAATTGGGTCATC
TGAAGTCCTTATATTACGCATACGCAGCGTGGGTCAGTCCCAGTGGTAGCACAACTTGAACTCACAACAC
TTTTCTCGTATTTCACAAGCTCTGTTAGTTGTTCCTATCTTGAATAATTGTAATTGTGTAATCTTAAAGA
TTAAAACTAACAACGGGGCAGACTAACTCGCTTTGATCCCTCTTCTCGCCATTAATATTATATGCCTCTA
ACCGAATTTGGCTACTACTTCACCGTTTTAAATCGCATTTTATATAATCTCAAGGGCTTTAAGCATAACT
TCCCGTTGTCATTTATGGATCGCTTGGCAAATCGTTTGGTTTAGCTTCCACATTGAATTCTCATCGTTTT
GGTGGGTGAAAACGTGACACGTTCACTCTTTTCGCCGCTGGCTTATCACGTTGGCTTATTCCGTATCACA
TGTTGCCACAGCAGTTTCTATTTTCGACACACACGCCGCCCCCTTTTGTGTCTTCGCCCCCTGTTTGTGT
ATTGCTCTCTCCCCGCCGCTCTTTGGCGTTGATAATGAACCGAGTGACATGGAGTGAAGTGAGGCAATCA
TCGGCGGGCCAGCATTAGATCATATATACAGACAGAAACGACGACGACatcatcatgatgatcatgatca

tcatCGTACACTCTGACTCATGAACCTCACTCGCCTCACTTGCACTGGACAGACTCTCCGGATATCTCTC
GCTCTCGCTTGCTCGCTCGCTCCCTCGCCCGTCAGTAGATTCTCCAATTCTCCGGCTCTCTGGCAAACTC
TATGCTTGAGTCGCGGCTCCGTCTTCGCATCGCACGGAATTCTTCAGTGCGGTTTGGTTGCTCGTTTCGT
GCGCTCGCTCGATCGCTCGCTGTTGCTCGTTTTCCACACACGAATTCCCAGCTAACGCGCAGCCATTTTG
ACAGTGATTTTATTGGTTTGGCTCTTTTGGCCAGCAGCATGTTGTTCGCCAGAACCACCTCGTCCACGTC
GATCAGTCCGCCCGCGATGATGATgcagcagcaccagcagcagcagcagcagcaggatcctcagaagctg
ggacagcaattccatcagcagccagcatttcagctacagcaAAGCTTCTGTACGTTTCTCGGTTAAGTGT
AACTCACTGTGTTCACCTGTTGTTGTTTTGTTGACCTTGGTAATTAGCGTGTGCATTAATAATTATAGGG
TTTATCTCATTCGGTATTTGCCAGGAGCTTTGCTAACTGTATATGCCATCACCTGAGCCCTCCTCGGCGG
GCACAAATACAGACAACCACGTACACCGCGGCGTATGCGTAATATTTCGATATTAATGTTAGCCAACGTA
ACGCGGCAATTAACGTCTTGTCATCTTGGTTTATCTAGCGTTTCAAGGTCGCCTCTTGGTTTGTTATTCT
CTCGACTGAAGTCTTAGGCTCGACACACACACTTTTCCATTGAAATTAGCATTTGAAAATCGCTGAAAAG
CCTAAGTCAAAGCCAAAGTAAAACCAAAGCGAACCCCGAACCAAAACCCACACAGAGCAATGTATTCAGT
TCAATGCCAGTCATAATGATTATTCAAAAAAGCACTAAGCAATCGACAAGTCGAGTTGAAGTTTGACCTC
AGCTCAAGCTGGGATCGGATTGAATCGGATCGGGCTTTATATTATCAGTTGATTCTCAAAATCAATTTCG
AGTTACACAGTTGTTCGTAATTAACTCAAGAGTCACGCGGCCTCTCTGTCTGTTTTTATTTTTGAGCTC
TCAAGTACTCGTCGTACTTAGGGCTCTAACAAAAGTATAAAATAAGAAATACAAAATTGCCGTCTTGAAA
ATGCGGGAAGCAAAACGGCGGCAAAACGTAACCTTGAAAAGCGATTTGAATGTGGGCAAACATGTCTCAC
TCTATCTCTGCGATTGTGAATGCCtgtgtgtatgtgtatgtgcgtgtgtgtTCGTTTGAAAGAGCCAGCC
AAAAATAAGTCAAGACACTCGTGGCCATGCCCAAAAACTGCTCATGTTGCTCGGGCTCTGAACAAAACGA
ATAAAAAGAGACCACAGATAAATAGAAATAAAAACACACATATTATATCCCTCGAAAACAGCTCCAAAAA
GGGGAGACGGATTTTGGGGATTTTACTTGTATTTTTCTTGGCAAGAGACTTCTTACTCTTCCTTTGTTGC
TCAACCGTCGTCGTGATTTTTTTGGCATTTTGGCGAAAATGTCCGAAAAATGTGTTATTTATGCGTGGCA
CAAGTTCGCATTTCCACGGCGCATCCAGTTCCAACCAGTAGCTTTTTTTAACTTCGAATCCATTGTGTAA
TCTCGGAGTTTGGCTCTGGCCAACCAGTTGTTAATTTGACTGCCATTATTTGGCAAGACTCACGGGTCTG
GGCAGGTGTTTTGGTGGGCATTTCATCTTCTACTGGCAGCCAGACTTCTCAATGGCGAGAGCTATAAATC
AATTTTAAGAGCCGAAAGGGTTTCGGTTTGTGTGAAAGATGATCTCACAAAGGTATTCAGAAACGCCCAC
TTTGCAtgtgtttttttcagatattcgtttgtttccgtttcagtttgttgtcgaagttagttttttttCC
CTCTCGAGTCAGATGACATATGTATATGTACCTATTCAAACTTGAATTTAGTTATTAATTTGTGCCTTCA
GATGATTAGTGAAAATTACTGTGCATCAATTAAACAAAAATATTGTTGCGCCTCATTTAGTGCTTGTGTG
GTTCAGTTTATTAGAATGGAACCAAGGTCTTTTGGTTCGAATCAAATAGGAGTAGATTTTGGTTATTGGA
GAAGATGGTTATTGTGTGTTCTAGGCAGAGATACCATCCCATCATCATTTTCAGCTTCTGCCCAATCATC
AGCAGACCTTATCAACTGGCTTCCGCTGTTTCATCAATTAAAATGCTTTCAATTCGCTCAATTTCATATA
TGACCATGATATTTGTTACAGCTTTTAATACATCCGACCACTGTGTTGCATATTACcgtttttattatcg
tttttctgtttgtttcttctttttttgttcttttttttAAACCATTATCGTTCATGCATATAAATGTGCT
TGTAATTATCGAAAGCCAACCATAGCGCAAATATAATTATAGATACTACACAAAATGTAAAAATAAATTG
ATGTGCATGCCGCACAGCAGAACGATAAAGTTAAAAACGAAATGAAAGCCAACCCACAAAGTATATAAAA
AAGAACATTTTCAATAATCACGAACAGTTGTTTTTGTTGTTGTCCCCACTTAAAGCGTGCGATTAGCAAT
AATAGAAAAAGTCGCGTATAACTTTTAACAATCCAGCAGCAGCAGCAGCATTTCCACAAAACCAATGAGA
AAACTCTTAACCGAACTCAACTCAAATGTGATAATTCCAAGCAAGCAAAGCAGTTAAAATATGTTGTGAT
AAAGCTACGCAAGTAATGACTAAAATATAAAACATATAGAAAAGCCGAAAGTTATTAAACAAACCAAAAA
CGACTGAGATCAAATCGAGGAAAACAAATGCAACACATTAGCAGAAGATCATCACAAAATATCGCCGTCC
TGTCATCGAGCCAATCTTCAAGTATTATAGCATCTACAGCACCTGCAACATCTCctctgaattctgaact
gtgaactgtgaactctgacctctgagctcAACTCTACAGCTGAATG

>dme_circ_0008342_junction_seq
cctctgagctcAACTCTACAGCTGAATGGGATCGTAAATAGAGTATCAATATATGT
>dme_circ_0008343
GCCAACCATAGCGCAAATATAATTATAGATACTACACAAAATGTAAAAATAAATTGATGTGCATGCCGCA
CAGCAGAACGATAAAGTTAAAAACGAAATGAAAGCCAACCCACAAAGTATATAAAAAAGAACATTTTCAA
TAATCACGAACAGTTGTTTTTGTTGTTGTCCCCACTTAAAGCGTGCGATTAGCAATAATAGAAAAAGTCG
CGTATAACTTTTAACAATCCAGCAGCAGCAGCAGCATTTCCACAAAACCAATGAGAAAACTCTTAACCGA
ACTCAACTCAAATGTGATAATTCCAAGCAAGCAAAGCAGTTAAAATATGTTGTGATAAAGCTACGCAAGT
AATGACTAAAATATAAAACATATAGAAAAGCCGAAAGTTATTAAACAAACCAAAAACGACTGAGATCAAA
TCGAGGAAAACAAATGCAACACATTAGCAGAAGATCATCACAAAATATCGCCGTCCTGTCATCGAGCCAA
TCTTCAAGTATTATAGCATCTACAGCACCTGCAACATCTCctctgaattctgaactgtgaactgtgaact
ctgacctctgagctcAACTCTACAGCTGAATGGTAAAGCATTGGCATTTTTTTCTAAATGTCTTCCATAG
CATTTCTGGATGTTGTGTAACAATCGTCATTAACAAACGCCATCGTACATGGACATACTCGTACTATCTC
TCTATATCGCCATAGCACCAATTGTCATCCAACTACTCATGTCTGTAAGCGAATATGTATCATAAAGTAT
TTTGTTCCGATGCGAGCGATATAAGTTCCATCCAAGACAGATGCAATCCTGCAATCGACAACAAATCAA
TCGATACCTTTGCTGAAGCTTGCTGCTGCCGCTTTCTCTGTCTCTGTCTGTTTCTCACTCCGCCTTCTAC
CTATACAAACtatatatatatatatatatatatatatatatatatatatatatatataGTCGCAGTCGCATC
GTAATCGTTGTTGTTGTTATCGTACAAAATATCTAACAAGAAATCATCAAGAAACCAAAAAAATGAACAG
GCACCAGAACAGAAGAACACTAAAATCGCCATCCAACAACACCAATATCTAATCAATATCGAGCTATCGT
CAGCTATCGAATCAAATCGAACTAAATCAAGTCATCCGGAAGTCGAGCGACCTACTAAACCACTGCGAAG
TCACCACTCAATCAGCCCGATCTATATATCTCTATATGTTAACTTACAACCACCGCATCATCATGAAGAG
CATTATACCAAACAAATCGTAGaaaaaaaaaagaaaaGAATAGCAACAAATGCACAGTCCAAAGCCTTACA
AACTCGATTGCACATTAAACTCGATGTGTATCTATAATGAATATGCAATTCAGATATCGATGGTGAGCGA
GGGGGAAAGAGACAGAAAGCGAAGGTCCTTGAAGTGCTCTATCTACCTATCTATCTCTCTCCCGCTCGCg
tgtgtgtgtgtgtgATCAGAGGAGAGCGAAACCTATGAAAAGCCAGCTTTATCTAAACAAATTTTCCATT
TCTTTTTCTTTCGTTTATTGAACAAAGTTTAATAATTGGAAAGCGGCGCGTAGGTTGAATCAAAACACTG
AACAGTTAAgaaaaaaaactaaagcccaaattgcaaattacgaaatataataaaaaaattgaaaaCAAATAA
CGCATAAatcagttgatcagtggatcagttgaATAAGGAACGAAACGAGTACAATCGATTCAAGGCAGCA
TCAATTCCGTCAAACAAACAAAACCGAAGACTGAACGGGAACGACAACAATTCCAAGGCAATGGAGAGTA
TTATGAAGGTGGCCATGGACAAGGCGGCCGAGCAGCTGATTCAGCAATTTGGTTTTGATTACTTGCAGCA
GCAGCTGCAGTTGCAACATCAGAACCAACACAacagcagcccacagcagcccacagcaCCAGCAACTAGAA
CCAGAAAACGAACATCTCACATACCAATATCAGCAATCAAAGCCCACACATATGCAGCAATTAGCTTGTA
ACTATCAGCCACGACATTCCACAACCACGTCGTCGCCCTCATCCACACATTCGCTGGCcagcggcggcgg
cagcagcagtaacagcagcaacagcagcagcagcgacagcagcagcatcaacataagccacatcagcaac
ataagcaacatcagcaacatcggcaacatcagcaacagcaatcatagcaacgctgcaTACTCATTAGCCG
TTCATAGCTATCAAAAGCAAATAGAAAGCCCGGCTAATCCCAGTCATGTTCCGCATCATCAGATGGATCT
ATCGCCCCTGAGTGAGAATGGTTCGCCAAATGGGACACCAGGTGCCCAGACGCCGACTgcaacagcgagt
ggcaacacggcagcagcactcgcatcagcagcagcagcagcagcagcagcaacatcaggaggcaacggca
gcagcatcaccaactgcaacagcaataacagcagcagcagcagcaacgcgcaacagcaactgcagcTGGG
CAACTATAAGACTAACTCCTGCTGGTGTTACG
>dme_circ_0008343_junction_seq
TATAAGACTAACTCCTGCTGGTGTTACGGCCAACCATAGCGCAAATATAATTATAG
>dme_circ_0008344
GCGCAAATATAATTATAGATACTACACAAAATGTAAAAATAAATTGATGTGCATGCCGCACAGCAGAACG

ATAAAGTTAAAAACGAAATGAAAGCCAACCCACAAAGTATATAAAAAAGAACATTTTCAATAATCACGAA
CAGTTGTTTTTGTTGTTGTCCCCACTTAAAGCGTGCGATTAGCAATAATAGAAAAAGTCGCGTATAACTT
TTAACAATCCAGCAGCAGCAGCAGCATTTCCACAAAACCAATGAGAAAACTCTTAACCGAACTCAACTCA
AATGTGATAATTCCAAGCAAGCAAAGCAGTTAAAATATGTTGTGATAAAGCTACGCAAGTAATGACTAAA
ATATAAAACATATAGAAAAGCCGAAAGTTATTAAACAAACCAAAAACGACTGAGATCAAATCGAGGAAAA
CAAATGCAACACATTAGCAGAAGATCATCACAAAATATCGCCGTCCTGTCATCGAGCCAATCTTCAAGTA
TTATAGCATCTACAGCACCTGCAACATCTCctctgaattctgaactgtgaactgtgaactctgacctctg
agctcAACTCTACAGCTGAATGGTAAAGCATTGGCATTTTTTTCTAAATGTCTTCCATAGCATTTCTGGA
TGTTGTGTAACAATCGTCATTAACAAACGCCATCGTACATGGACATACTCGTACTATCTCTCTATATCGC
CATAGCACCAATTGTCATCCAACTACTCATGTCTGTAAGCGAATATGTATCATAAAGTATTTTTGTTCCG
ATGCGAGCGATATAAGTTCCATCCAAGACAGATGCAATCCTGCAATCGACAACAAATCAATCGATACCTT
TGCTGAAGCTTGCTGCTGCCGCTTTCTCTGTCTCTGTCTGTTTCTCACTCCGCCTTCTACCTATACAAAC
tatatatatatatatatatatatatatatatatatatatatatatataGTCGCAGTCGCATCGTAATCGTTG
TTGTTGTTATCGTACAAAATATCTAACAAGAAATCATCAAGAAACCAAAAAAATGAACAGGCACCAGAAC
AGAAGAACACTAAAATCGCCATCCAACAACACCAATATCTAATCAATATCGAGCTATCGTCAGCTATCGA
ATCAAATCGAACTAAATCAAGTCATCCGGAAGTCGAGCGACCTACTAAACCACTGCGAAGTCACCACTCA
ATCAGCCCGATCTATATATCTCTATATGTTAACTTACAACCACCGCATCATCATGAAGAGCATTATACCA
AACAAATCGTAGaaaaaaaaaagaaaaGAATAGCAACAAATGCACAGTCCAAAGCCTTACAAACTCGATTG
CACATTAAACTCGATGTGTATCTATAATGAATATGCAATTCAGATATCGATGGTGAGCGAGGGGGAAAGA
GACAGAAAGCGAAGGTCCTTGAAGTGCTCTATCTACCTATCTATCTCTCTCCCGCTCGCgtgtgtgtgtg
tgtgATCAGAGGAGAGCGAAACCTATGAAAAGCCAGCTTTATCTAAACAAATTTTCCATTTCTTTTTCTT
TCGTTTATTGAACAAAGTTTAATAATTGGAAAGCGGCGCGTAGGTTGAATCAAAACACTGAACAGTTAAg
aaaaaaactaaagcccaaattgcaaattacgaaatataataaaaaaattgaaaaCAAATAACGCATAAatc
agttgatcagtggatcagttgaATAAGGAACGAAACGAGTACAATCGATTCAAGGCAGCATCAATTCCGT
CAAACAAACAAAACCGAAGACTGAACGGGAACGACAACAATTCCAAGGCAATGGAGAGTATTATGAAGGT
GGCCATGGACAAGGCGGCCGAGCAGCTGATTCAGCAATTTGGTTTTGATTACTTGCAGCAGCAGCTGCAG
TTGCAACATCAGAACCAACACAacagcagcccacagcagccacagcaCCAGCAACTAGAACCAGAAAACG
AACATCTCACATACCAATATCAGCAATCAAAGCCCACACATATGCAGCAATTAGCTTGTAACTATCAGCC
ACGACATTCCACAACCACGTCGTCGCCCTCATCCACACATTCGCTGGCcagcggcggcggcagcagcagt
aacagcagcaacagcagcagcagcgacagcagcagcatcaacataagccacatcagcaacataagcaaca
tcagcaacatcggcaacatcagcaacagcaatcatagcaacgctgcaTACTCATTAGCCGTTCATAGCTA
TCAAAAGCAAATAGAAAGCCCGGCTAATCCCAGTCATGTTCCGCATCATCAGATGGATCTATCGCCCCTG
AGTGAGAATGGTTCGCCAAATGGGACACCAGGTGCCCAGACGCCGACTgcaacagcgagtggcaacacgg
cagcagcactcgcatcagcagcagcagcagcagcagcagcaacatcaggaggcaacggcagcagcatcac
caactgcaacagcaataacagcagcagcagcagcaacgcgcaacagcaactgcagcTGGGCAACTATAAG
ACTAACTCCTGCTGGTGTTACG
>dme_circ_0008344_junction_seq
TATAAGACTAACTCCTGCTGGTGTTACGGCGCAAATATAATTATAGATACTACACA
>dme_circ_0008345
GCGCAAATATAATTATAGATACTACACAAAATGTAAAAATAAATTGATGTGCATGCCGCACAGCAGAACG
ATAAAGTTAAAAACGAAATGAAAGCCAACCCACAAAGTATATAAAAAAGAACATTTTCAATAATCACGAA
CAGTTGTTTTTGTTGTTGTCCCCACTTAAAGCGTGCGATTAGCAATAATAGAAAAAGTCGCGTATAACTT
TTAACAATCCAGCAGCAGCAGCAGCATTTCCACAAAACCAATGAGAAAACTCTTAACCGAACTCAACTCA
AATGTGATAATTCCAAGCAAGCAAAGCAGTTAAAATATGTTGTGATAAAGCTACGCAAGTAATGACTAAA

ATATAAAACATATAGAAAAGCCGAAAGTTATTAAACAAACCAAAAACGACTGAGATCAAATCGAGGAAAA
CAAATGCAACACATTAGCAGAAGATCATCACAAAATATCGCCGTCCTGTCATCGAGCCAATCTTCAAGTA
TTATAGCATCTACAGCACCTGCAACATCTCctctgaattctgaactgtgaactgtgaactctgacctctg
agctcAACTCTACAGCTGAATGGTAAAGCATTGGCATTTTTTTCTAAATGTCTTCCATAGCATTTCTGGA
TGTTGTGTAACAATCGTCATTAACAAACGCCATCGTACATGGACATACTCGTACTATCTCTCTATATCGC
CATAGCACCAATTGTCATCCAACTACTCATGTCTGTAAGCGAATATGTATCATAAAGTATTTTTGTTCCG
ATGCGAGCGATATAAGTTCCATCCAAGACAGATGCAATCCTGCAATCGACAACAAATCAATCGATACCTT
TGCTGAAGCTTGCTGCTGCCGCTTTCTCTGTCTCTGTCTGTTTCTCACTCCGCCTTCTACCTATACAAAC
tatatatatatatatatatatatatatatatatatatatatatataGTCGCAGTCGCATCGTAATCGTTG
TTGTTGTTATCGTACAAAATATCTAACAAGAAATCATCAAGAAACCAAAAAAATGAACAGGCACCAGAAC
AGAAGAACACTAAAATCGCCATCCAACAACACCAATATCTAATCAATATCGAGCTATCGTCAGCTATCGA
ATCAAATCGAACTAAATCAAGTCATCCGGAAGTCGAGCGACCTACTAAACCACTGCGAAGTCACCACTCA
ATCAGCCCGATCTATATATCTCTATATGTTAACTTACAACCACCGCATCATCATGAAGAGCATTATACCA
AACAAATCGTAGaaaaaaaaagaaaaGAATAGCAACAAATGCACAGTCCAAAGCCTTACAAACTCGATTG
CACATTAAACTCGATGTGTATCTATAATGAATATGCAATTCAGATATCGATGGTGAGCGAGGGGGAAAGA
GACAGAAAGCGAAGGTCCTTGAAGTGCTCTATCTACCTATCTATCTCTCTCCCGCTCGCgtgtgtgtgtg
tgtgATCAGAGGAGAGCGAAACCTATGAAAAGCCAGCTTTATCTAAACAAATTTTCCATTTCTTTTTCTT
TCGTTTATTGAACAAAGTTTAATAATTGGAAAGCGGCGCGTAGGTTGAATCAAAACACTGAACAGTTAAg
aaaaaaactaaagcccaaattgcaaattacgaaatataataaaaaattgaaaaCAAATAACGCATAAatc
agttgatcagtggatcagttgaATAAGGAACGAAACGAGTACAATCGATTCAAGGCAGCATCAATTCCGT
CAAACAAACAAAACCGAAGACTGAACGGGAACGACAACAATTCCAAGGCAATGGAGAGTATTATGAAGGT
GGCCATGGACAAGGCGGCCGAGCAGCTGATTCAGCAATTTGGTTTTGATTACTTGCAGCAGCAGCTGCAG
TTGCAACATCAGAACCAACACAacagcagcccacagcagccacagcaCCAGCAACTAGAACCAGAAAACG
AACATCTCACATACCAATATCAGCAATCAAAGCCCACACATATGCAGCAATTAGCTTGTAACTATCAGCC
ACGACATTCCACAACCACGTCGTCGCCCTCATCCACACATTCGCTGGCcagcggcggcggcagcagcagt
aacagcagcaacagcagcagcagcgacagcagcagcatcaacataagccacatcagcaacataagcaaca
tcagcaacatcggcaacatcagcaacagcaatcatagcaacgctgcaTACTCATTAGCCGTTCATAGCTA
TCAAAAGCAAATAGAAAGCCCGGCTAATCCCAGTCATGTTCCGCATCATCAGATGGATCTATCGCCCCTG
AGTGAGAATGGTTCGCCAAATGGGACACCAGGTGCCCAGACGCCGACTgcaacagcgagtggcaacacgg
cagcagcactcgcatcagcagcagcagcagcagcagcagcaacatcaggaggcaacggcagcagcatcac
caactgcaacagcaataacagcagcagcagcagcaacgcgcaacagcaactgcagcTGGGCAACTATAAG
ACTAACTCCTGCTGGTGTTACGGTGAGTCTGATTAAGAACAAACAAACAAACAAACGCAATCAGCATCAG
CATAGAACTATCAAATCAAAAGTTGCGCACAACTTCCTCTACTACACCATACATAAAATGTACACCACTT
CTCTATATAACTATCTATAACTATCGAGCTCGCTACAGCCAACAAATATCAACCAACCAGCCAACAACAG
CATCAATCACCACGTCGCCTCATTCTAAATGCCGCCAGCAACTTAAAAGCTGAACCaaaaaaaaaaaaaa
TGTACAAAGAAAAAAGTATCAAGAACGCCCAAATGTCAAACGAACTGAACTGAACGCTGAACAAGAAGCC
ACAAGCTATAAATGAATTATCCATTAACATTGCGTTGAAAGTTTTGATAGAAAATAATCCAAACATCATC
AGCATCCACCAATCCAGCACACATTTCGATACACAAGAAATAAAACCAAACCAACAAACACTCAtaaaaa
aaaaactaagcagctaaaacagaaacaagcagccagctaaaaaaaaatccaaaaaaaaaagaaatatata
tatatatatatataaaaaaCTCATATGCTAATCGTACGCTTAAATGTGGGCGTCCATTTCAATTTCGTTT
GTGCCTTAAAATATTATGTTTTGTGTATACACGACTTTCCTTCAGCATTCCCTAGTGATTCATGAGTGTT
GCATAAAGATATTCGCTTTGGTTTTTTTAAATAGGATTTCGTTTGCTCTTTTGTGTGTTTCAATTGAACG
AAGAACCAATTGGTTATGTTTTATTGGCAAATCGTATCAAGATTTAATACCTATGCCTATTAAAGTTCAC
TTCGAGTGTCCTTAAAGCTCTTTCCGGTGCAGGAAATTAAACATACTTGTCGCCTGCAGGATCCAATCGT

TTAATCGCGTGCCACATGCCCCCGCATTACGCATACGTCGCGTGGTACGCGACAAGCACTTGCTGCTCGG
GCTTTGCTTTAACAATTTGCGTGTTGCAGATTGCTCCATGGCATTTATTGTGGTTTAGGGttggagtggg
tagagtggagtggagtggagtggagtggaatggaatggaAAGGGTGGTCCTGACTGTGTGCCAGCTTGTC
GTTAGCCTAGTTTCCCGTCCAATCCTGCACACAATGCATACGAAATTAGTCTCCAGTCTGCCTGCTGCCA
TCATCCTGCAAGCTCTGCCACCCCGCCACCCTGCCACGCCCACTTGCAGCTCCATGGGCGGTCTCTGGCC
AGTGTGCTGCAAGTGTTATTTATGACAGTCACATAATTTAAGCTGCTGCACAGTGTCAACAGCAACGCAG
CTTCGCAACAGCGTTCTTGGCCTGTCCTTTGTGGCCCATAAAAACGCTAAGCAAACGACTTTATGTTTTG
TTTTTCTGTCTTCCTGCATATTATTTACCATGGCCATTTTGCAACTCTCGCTTATTTACGAGTATTTTGC
ATTGCAGTAGAATTTCGTAAAAGAATTTCACCAATAGCACTCTATTCATACGGTATAAGTGATAGATAAC
TGACAGATGCGTCCGGCAATTGGTTCAGTGGCGGAAATTGGCCAGCATGGTGCTAAATATGTGGTCGCAA
TTCGCTTTCGTAGCGAATTCTTTGCGAACCGCATTTTCACACATGCATGCAAAGCAGGGCAAATGGTCCA
ATATTCCCCCAGCCCAAGCGAATACCCATCCCCCGATACCTCATACCACCCAATGTCAGTGCATCGTcgt
gtgtgtgtgcgtgtgtgCGCGCTGTATCTAATTAGAGAAATACTTTTGAAGGACACTCTGGCGCTGAGTG
GCAACGGGACCGACCAAGACGCCGACAACGTCAGCGTATGGCACACATTTCACCGATGCCGTAAGGAACT
GGGTGGTGGTGGTGGTGCCTCGGGGGGCGGATGGTGGCTAAATTTAAACGAACCGAGTGCGCCCAAGTGG
CATCAGCAGAGTCTTGGGCTGCCACAAGTCGCCATGCTGCACGATGGCAGGCAGTCAGGTGCAACGCGCT
TAGATCATTCCATGCCATGCCCGTTCCATTCTATTCCATCTCGTTGTTTTGCTAGCCAATGCGCTTGTGT
AATAGAGCGTCTCCGCTTGACACATTTTATTAATTGAAATTTGTTTGTTTACTTACTTACAACTGGCTGC
CGTCGGTTGTCTTATGTCGTTTGTCGGCGACTTGAGGCTGTGTGACGCAGATGATATCACTCATACGCAG
GGAGGTCCCACTGCATTCACATGCATTCGCCACATTCCCATCACTTAGCCGTGTAATGCCGTGCACTGCC
TTCAGATCCCAACTTATAATCATTGTTTCTGTCTTTTTCTTCTTCTTTCCATTTCAGGTGAGTCAGTTTG
TTCTGGAATTGAGGTTGAAATTGAAAATAATAACAATAATCATATTCATCATGGTAAGCTTTGTCACACT
TTCATTAAATACCATTAAATCCATCTTTGTCTAGCCAAACGAACGACTAATTCAAGATTAATACCGACTA
AAGCGTAGCTCTATATCGTTTTCTGTATCCGTAACTAGATCGCTGTCTGTTTTTAATGCCGCTGCCGCTG
ACGAAAGCGCGCTTTTTGAACCTGAATTCGGACGGACTAAAAACGAAAACTGATTATGCGACCCAACTGA
CAACGGAGCACATTAACCTTCGACCCTTTGACCAAACTTATGCTTTGACTTACatttttttcgtatttat
ttcgtttctttttttttcgcttttGTGTGTTGCCCAAGTAATTTGCGGGGCTTTTTCTTTCGGCCAAGCCA
ACGACCTTCGGAGAAGAAGTTAAAACGGGCTTTCATATGTTTCGTGTGTCGCCAGCATTGTCTGTTCCAT
CTCATCTCGAGCGCATATCTTCCTCATGGCTTGCCCATTACAAAAACCACAGAAAACAGACCACAGTCGA
TacaccacgtccacgcccacgcccacgcccacaccaccataaccaatccatgcccacacacaGCAGTGAA
CCATGCTAATCCACTAAACCAAGTCGAAATCGAAAACTATCTATCTGAAAACTATAACCAAACTAACCGA
GTTGAAAACTCAACTTTACGAACGTCCCTCGTTGTGAGCGTCCAGCCGAACGAACAAACGATCGACGCTG
AAATGCTGTTATTATTTTTAATTTGTAACCTGCCGAATGAGCTAGAAACTAGATCTAACCAACAATAATA
ACCAGATAACTAGATTGTATGCAGCAGCCAAGGGCGCACACAGACTAACTAACCCTGTAACCTGTACCAA
AAACCTCCTAATCTATTGACGAAAAAAAGAATAGTAGCCCAATCCAAGAGAATCTCCCGAGACTAATCCA
TTGGTCGCCCTGTGAACTCCTCTCGATTCTTTCATTTCAGGGCGAGACAACGTATCACATGAAAATACTG
GTGCCCGCGGTGGCCTCCGGGGCCATCATTGGCAAAGGTGGCGAGACGATCGCCTCCCTGCAGAAGGACA
CGGGTGCTAGGGTCAAGATGTCCAAGTCGCACGACTTCTACCCAGGGCACCACCGAACGCGTGTGCCTGA
TCACCGGCTCCACGGAGGCTATCATGGTCGTGATGGAGTTCATCATGGACAAGATCCGCGAGAAGCCCGA
CCTGACCAACAAGATCGTCGATACCGACTCAAAACAGACACAGGAGCGCGACAAGCAGGTCAAGATCCTG
GTGCCCAACTCGACCGCCGGCATGATCATTGGCAAGGGCGGTGCCTTCATTAAACAGATCAAGGAGGAGA
GCGGCTCCTATGTCCAGATCTCGCAGAAGCCCACGGATGTGTCGTTGCAGGAGCGCTGCATCACCATCAT
AGGCGACAAGGAGAACAATAAGAACGCCTGCAAGATGATCCTCTCGAAGATCGTCGAAGATCCCCAGTCG
GGCACCTGCCTGAACGTATCCTACGCGGACGTCAGCGGCCCGGTGGCCAACTTTAATCCGACCGGCTCCC

CGTACGCCACCAATCAGAATGCCATCAACTCGAGCACCGCTTCGTTGAACTCCACGTTGGGCACCACGAT
CGGCGGTGCAAACTCGGCGGCCAGCCTGCTAGTCAACGGCACCGGCATCAATTTGTCCATCAATCTGGGC
TCACCCAATCCGGCGCCCAATCTAGCGGTTGCCACACAGCTGCTCGAGCATATTAAGGGTTGCCATGCGC
GGCTCTGGCTATTCGGAGACCGTAACGAACGAAGTTGTCGCCGCTCTGAGCGTGCTGGCCAAGTACGGCG
TCCTTGGAATGGGCGTGGGTGTGTCGCACACCAACGGCGCCCACTCAACGCTGGGCAACTTCCTGGGCGT
AACGACGCTGGACCAGCAGACAGCCGCTGCCGCATCTGCGGCCACCGCCAGCAATGTGTTCGGTGCCGTT
GGCCAGGTAAATCTGGAGCAATATGCCGCAGCGGTGGCCTCTGCAGCTGCCGCCAGCCGGCCGACGCAGT
CGCAACTGGACGCTGCCGCAGTGCAATTCGATCCATTCCGCCATTTGGGCTCGGCAACGGCGCCGGCCGC
CACGCCCGTCTCACTGAACAACAATAGCTTTGGATTGACGGCGACCACGGGAACGGCGACCACGGCGCAG
CTGGGCGGTCTAAGCAAGAGTCCCACTCCCGGTGACCTTAGCTCCAAGGACTCGAAAAACGTCGAGGTGC
CGGAAGTGATTATCGGCGCTATCCTAG
>dme_circ_0008345_junction_seq
CCGGAAGTGATTATCGGCGCTATCCTAGGCGCAAATATAATTATAGATACTACACA
>dme_circ_0008346
GATACTACACAAAATGTAAAAATAAATTGATGTGCATGCCGCACAGCAGAACGATAAAGTTAAAAACGAA
ATGAAAGCCAACCCACAAAGTATATAAAAAAGAACATTTTCAATAATCACGAACAGTTGTTTTTGTTGTT
GTCCCCACTTAAAGCGTGCGATTAGCAATAATAGAAAAAGTCGCGTATAACTTTTAACAATCCAGCAGCA
GCAGCAGCATTTCCACAAAACCAATGAGAAAACTCTTAACCGAACTCAACTCAAATGTGATAATTCCAAG
CAAGCAAAGCAGTTAAAATATGTTGTGATAAAGCTACGCAAGTAATGACTAAAATATAAAACATATAGAA
AAGCCGAAAGTTATTAAACAAACCAAAAACGACTGAGATCAAATCGAGGAAAACAAATGCAACACATTAG
CAGAAGATCATCACAAAATATCGCCGTCCTGTCATCGAGCCAATCTTCAAGTATTATAGCATCTACAGCA
CCTGCAACATCTCctctgaattctgaactgtgaactgtgaactctgacctctgagctcAACTCTACAGCT
GAATGGTAAAGCATTGGCATTTTTTTCTAAATGTCTTCCATAGCATTTCTGGATGTTGTGTAACAATCGT
CATTAACAAACGCCATCGTACATGGACATACTCGTACTATCTCTCTATATCGCCATAGCACCAATTGTCA
TCCAACTACTCATGTCTGTAAGCGAATATGTATCATAAAGTATTTTTGTTCCGATGCGAGCGATATAAGT
TCCATCCAAGACAGATGCAATCCTGCAATCGACAACAAATCAATCGATACCTTTGCTGAAGCTTGCTGCT
GCCGCTTTCTCTGTCTCTGTCTGTTTCTCACTCCGCCTTCTACCTATACAAACtatatatatatatatat
atatatatatatatatatatatatatataGTCGCAGTCGCATCGTAATCGTTGTTGTTGTTATCGTACAA
AATATCTAACAAGAAATCATCAAGAAACCAAAAAAATGAACAGGCACCAGAACAGAAGAACACTAAAATC
GCCATCCAACAACACCAATATCTAATCAATATCGAGCTATCGTCAGCTATCGAATCAAATCGAACTAAAT
CAAGTCATCCGGAAGTCGAGCGACCTACTAAACCACTGCGAAGTCACCACTCAATCAGCCCGATCTATAT
ATCTCTATATGTTAACTTACAACCACCGCATCATCATGAAGAGCATTATACCAAACAAATCGTAGaaaaa
aaaagaaaaGAATAGCAACAAATGCACAGTCCAAAGCCTTACAAACTCGATTGCACATTAAACTCGATGT
GTATCTATAATGAATATGCAATTCAGATATCGATGGTGAGCGAGGGGGAAAGAGACAGAAAGCGAAGGTC
CTTGAAGTGCTCTATCTACCTATCTATCTCTCTCCCGCTCGCgtgtgtgtgtgtgtgATCAGAGGAGAGC
GAAACCTATGAAAAGCCAGCTTTATCTAAACAAATTTTCCATTTCTTTTTCTTTCGTTTATTGAACAAAG
TTTAATAATTGGAAAGCGGCGCGTAGGTTGAATCAAAACACTGAACAGTTAAgaaaaaaaactaaagccca
aattgcaaattacgaaatataataaaaaattgaaaaCAAATAACGCATAAatcagttgatcagtggatca
gttgaATAAGGAACGAAACGAGTACAATCGATTCAAGGCAGCATCAATTCCGTCAAACAAACAAAACCGA
AGACTGAACGGGAACGACAACAATTCCAAGGCAATGGAGAGTATTATGAAGGTGGCCATGGACAAGGCGG
CCGAGCAGCTGATTCAGCAATTTGGTTTTGATTACTTGCAGCAGCAGCTGCAGTTGCAACATCAGAACCA
ACACAacagcagcccacagcagccacagcaCCAGCAACTAGAACCAGAAAACGAACATCTCACATACCAA
TATCAGCAATCAAAGCCCACACATATGCAGCAATTAGCTTGTAACTATCAGCCACGACATTCCACAACCA
CGTCGTCGCCCTCATCCACACATTCGCTGGCcagcggcggcggcagcagcagtaacagcagcaacagcag

cagcagcgacagcagcagcatcaacataagccacatcagcaacataagcaacatcagcaacatcggcaac
atcagcaacagcaatcatagcaacgctgcaTACTCATTAGCCGTTCATAGCTATCAAAAGCAAATAGAAA
GCCCGGCTAATCCCAGTCATGTTCCGCATCATCAGATGGATCTATCGCCCCTGAGTGAGAATGGTTCGCC
AAATGGGACACCAGGTGCCCAGACGCCGACTgcaacagcgagtggcaacacggcagcagcactcgcatca
gcagcagcagcagcagcagcagcaacatcaggaggcaacggcagcagcatcaccaactgcaacagcaata
acagcagcagcagcagcaacgcgcaacagcaactgcagcTGGGCAACTATAAGACTAACTCCTGCTGGTG
TTACGGTGAGTCTGATTAAGAACAAACAAACAAACAAACGCAATCAGCATCAGCATAGAACTATCAAATC
AAAAGTTGCGCACAACTTCCTCTACTACACCATACATAAAATGTACACCACTTCTCTATATAACTATCTA
TAACTATCGAGCTCGCTACAGCCAACAAATATCAACCAACCAGCCAACAACAGCATCAATCACCACGTCG
CCTCATTCTAAATGCCGCCAGCAACTTAAAAGCTGAACCaaaaaaaaaaaaaaTGTACAAAGAAAAAAGT
ATCAAGAACGCCCAAATGTCAAACGAACTGAACTGAACGCTGAACAAGAAGCCACAAGCTATAAATGAAT
TATCCATTAACATTGCGTTGAAAGTTTTGATAGAAAATAATCCAAACATCATCAGCATCCACCAATCCAG
CACACATTTCGATACACAAGAAATAAAACCAAACCAACAAACACTCAtaaaaaaaaaactaagcagctaa
aacagaaacaagcagccagctaaaaaaaaatccaaaaaaaaaagaaatatatatatatatatatataaaa
aaCTCATATGCTAATCGTACGCTTAAATGTGGGCGTCCATTTCAATTTCGTTTGTGCCTTAAAATATTAT
GTTTTGTGTATACACGACTTTCCTTCAGCATTCCCTAGTGATTCATGAGTGTTGCATAAAGATATTCGCT
TTGGTTTTTTTAAATAGGATTTCGTTTGCTCTTTTGTGTGTTTCAATTGAACGAAGAACCAATTGGTTAT
GTTTTATTGGCAAATCGTATCAAGATTTAATACCTATGCCTATTAAAGTTCACTTCGAGTGTCCTTAAAG
CTCTTTCCGGTGCAGGAAATTAAACATACTTGTCGCCTGCAGGATCCAATCGTTTAATCGCGTGCCACAT
GCCCCCGCATTACGCATACGTCGCGTGGTACGCGACAAGCACTTGCTGCTCGGGCTTTGCTTTAACAATT
TGCGTGTTGCAGATTGCTCCATGGCATTTATTGTGGTTTAGGGttggagtgggtagagtggagtggagtg
gagtggagtggaatggaatggaAAGGGTGGTCCTGACTGTGTGCCAGCTTGTCGTTAGCCTAGTTTCCCG
TCCAATCCTGCACACAATGCATACGAAATTAGTCTCCAGTCTGCCTGCTGCCATCATCCTGCAAGCTCTG
CCACCCCGCCACCCTGCCACGCCCACTTGCAGCTCCATGGGCGGTCTCTGGCCAGTGTGCTGCAAGTGTT
ATTTATGACAGTCACATAATTTAAGCTGCTGCACAGTGTCAACAGCAACGCAGCTTCGCAACAGCGTTCT
TGGCCTGTCCTTTGTGGCCCATAAAAACGCTAAGCAAACGACTTTATGTTTTGTTTTTCTGTCTTCCTGC
ATATTATTTACCATGGCCATTTTGCAACTCTCGCTTATTTACGAGTATTTTGCATTGCAGTAGAATTTCG
TAAAAGAATTTCACCAATAGCACTCTATTCATACGGTATAAGTGATAGATAACTGACAGATGCGTCCGGC
AATTGGTTCAGTGGCGGAAATTGGCCAGCATGGTGCTAAATATGTGGTCGCAATTCGCTTTCGTAGCGAA
TTCTTTGCGAACCGCATTTTCACACATGCATGCAAAGCAGGGCAAATGGTCCAATATTCCCCCAGCCCAA
GCGAATACCCATCCCCCGATACCTCATACCACCCAATGTCAGTGCATCGTcgtgtgtgtgtgcgtgtgtg
CGCGCTGTATCTAATTAGAGAAATACTTTTGAAGGACACTCTGGCGCTGAGTGGCAACGGGACCGACCAA
GACGCCGACAACGTCAGCGTATGGCACACATTTCACCGATGCCGTAAGGAACTGGGTGGTGGTGGTGGTG
CCTCGGGGGGCGGATGGTGGCTAAATTTAAACGAACCGAGTGCGCCCAAGTGGCATCAGCAGAGTCTTGG
GCTGCCACAAGTCGCCATGCTGCACGATGGCAGGCAGTCAGGTGCAACGCGCTTAGATCATTCCATGCCA
TGCCCGTTCCATTCTATTCCATCTCGTTGTTTTGCTAGCCAATGCGCTTGTGTAATAGAGCGTCTCCGCT
TGACACATTTTATTAATTGAAATTTGTTTGTTTACTTACTTACAACTGGCTGCCGTCGGTTGTCTTATGT
CGTTTGTCGGCGACTTGAGGCTGTGTGACGCAGATGATATCACTCATACGCAGGGAGGTCCCACTGCATT
CACATGCATTCGCCACATTCCCATCACTTAGCCGTGTAATGCCGTGCACTGCCTTCAGATCCCAACTTAT
AATCATTGTTTCTGTCTTTTTCTTCTTCTTTCCATTTCAGGTGAGTCAGTTTGTTCTGGAATTGAGGTTG
AAATTGAAAATAATAACAATAATCATATTCATCATGGTAAGCTTTGTCACACTTTCATTAAATACCATTA
AATCCATCTTTGTCTAGCCAAACGAACGACTAATTCAAGATTAATACCGACTAAAGCGTAGCTCTATATC
GTTTTCTGTATCCGTAACTAGATCGCTGTCTGTTTTTAATGCCGCTGCCGCTGACGAAAGCGCGCTTTTT
GAACCTGAATTCGGACGGACTAAAAACGAAAACTGATTATGCGACCCAACTGACAACGGAGCACATTAAC

CTTCGACCCTTTGACCAAACTTATGCTTTGACTTACatttttttcgtatttatttcgtttcttttttttc
gcttttGTGTGTTGCCCAAGTAATTTGCGGGGCTTTTTCTTTCGGCCAAGCCAACGACCTTCGGAGAAGA
AGTTAAAACGGGCTTTCATATGTTTCGTGTGTCGCCAGCATTGTCTGTTCCATCTCATCTCGAGCGCATA
TCTTCCTCATGGCTTGCCCATTACAAAAACCACAGAAAACAGACCACAGTCGATacaccacgtccacgcc
cacgcccacgcccacaccaccataaccaatccatgcccacacacaGCAGTGAACCATGCTAATCCACTAA
ACCAAGTCGAAATCGAAAACTATCTATCTGAAAACTATAACCAAACTAACCGAGTTGAAAACTCAACTTT
ACGAACGTCCCTCGTTGTGAGCGTCCAGCCGAACGAACAAACGATCGACGCTGAAATGCTGTTATTATTT
TTAATTTGTAACCTGCCGAATGAGCTAGAAACTAGATCTAACCAACAATAATAACCAGATAACTAGATTG
TATGCAGCAGCCAAGGGCGCACACAGACTAACTAACCCTGTAACCTGTACCAAAAACCTCCTAATCTATT
GACGAAAAAAAGAATAGTAGCCCAATCCAAGAGAATCTCCCGAGACTAATCCATTGGTCGCCCTGTGAAC
TCCTCTCGATTCTTTCATTTCAGGGCGAGACAACGTATCACATGAAAATACTGGTGCCCGCGGTGGCCTC
CGGGGCCATCATTGGCAAAGGTGGCGAGACGATCGCCTCCCTGCAGAAGGACACGGGTGCTAGGGTCAAG
ATGTCCAAGTCGCACGACTTCTACCCAGGGCACCACCGAACGCGTGTGCCTGATCACCGGCTCCACGGAG
GCTATCATGGTCGTGATGGAGTTCATCATGGACAAGATCCGCGAGAAGCCCGACCTGACCAACAAGATCG
TCGATACCGACTCAAAACAGACACAGGAGCGCGACAAGCAGGTCAAGATCCTGGTGCCCAACTCGACCGC
CGGCATGATCATTGGCAAGGGCGGTGCCTTCATTAAACAGATCAAGGAGGAGAGCGGCTCCTATGTCCAG
ATCTCGCAGAAGCCCACGGATGTGTCGTTGCAGGAGCGCTGCATCACCATCATAGGCGACAAGGAGAACA
ATAAGAACGCCTGCAAGATGATCCTCTCGAAGATCGTCGAAGATCCCCAGTCGGGCACCTGCCTGAACGT
ATCCTACGCGGACGTCAGCGGCCCGGTGGCCAACTTTAATCCGACCGGCTCCCCGTACGCCACCAATCAG
AATGCCATCAACTCGAGCACCGCTTCGTTGAACTCCACGTTGGGCACCACGATCGGCGGTGCAAACTCGG
CGGCCAGCCTGCTAGTCAACGGCACCGGCATCAATTTGTCCATCAATCTGGGCTCACCCAATCCGGCGCC
CAATCTAGCGGTTGCCACACAGCTGCTCGAGCATATTAAGGGTTGCCATGCGCGGCTCTGGCTATTCGGA
GACCGTAACGAACGAAGTTGTCGCCGCTCTGAGCGTGCTGGCCAAGTACGGCGTCCTTGGAATGGGCGTG
GGTGTGTCGCACACCAACGGCGCCCACTCAACGCTGGGCAACTTCCTGGGCGTAACGACGCTGGACCAGC
AGACAGCCGCTGCCGCATCTGCGGCCACCGCCAGCAATGTGTTCGGTGCCGTTGGCCAGGTAAATCTGGA
GCAATATGCCGCAGCGGTGGCCTCTGCAGCTGCCGCCAGCCGGCCGACGCAGTCGCAACTGGACGCTGCC
GCAGTGCAATTCGATCCATTCCGCCATTTGGGCTCGGCAACGGCGCCGGCCGCCACGCCCGTCTCACTGA
ACAACAATAGCTTTGGATTGACGGCGACCACGGGAACGGCGACCACGGCGCAGCTGGGCGGTCTAAGCAA
GAGTCCCACTCCCGGTGACCTTAGCTCCAAGGACTCGAAAAACGTCGAGGTGCCGGAAGTGATTATCGGC
GCTATCCTAG
>dme_circ_0008346_junction_seq
CCGGAAGTGATTATCGGCGCTATCCTAGGATACTACACAAAATGTAAAAATAAATT
>dme_circ_0008347
GCAGCAGCAGCAGCATTTCCACAAAACCAATGAGAAAACTCTTAACCGAACTCAACTCAAATGTGATAAT
TCCAAGCAAGCAAAGCAGTTAAAATATGTTGTGATAAAGCTACGCAAGTAATGACTAAAATATAAAACAT
ATAGAAAAGCCGAAAGTTATTAAACAAACCAAAAACGACTGAGATCAAATCGAGGAAAACAAATGCAACA
CATTAGCAGAAGATCATCACAAAATATCGCCGTCCTGTCATCGAGCCAATCTTCAAGTATTATAGCATCT
ACAGCACCTGCAACATCTCctctgaattctgaactgtgaactgtgaactctgacctctgagctcAACTCT
ACAGCTGAATGGTAAAGCATTGGCATTTTTTTCTAAATGTCTTCCATAGCATTTCTGGATGTTGTGTAAC
AATCGTCATTAACAAACGCCATCGTACATGGACATACTCGTACTATCTCTCTATATCGCCATAGCACCAA
TTGTCATCCAACTACTCATGTCTGTAAGCGAATATGTATCATAAAGTATTTTTGTTCCGATGCGAGCGAT
ATAAGTTCCATCCAAGACAGATGCAATCCTGCAATCGACAACAAATCAATCGATACCTTTGCTGAAGCTT
GCTGCTGCCGCTTTCTCTGTCTCTGTCTGTTTCTCACTCCGCCTTCTACCTATACAAACtatatatatat
atatatatatatatatatatatatatatatataGTCGCAGTCGCATCGTAATCGTTGTTGTTGTTATC

GTACAAAATATCTAACAAGAAATCATCAAGAAACCAAAAAAATGAACAGGCACCAGAACAGAAGAACACT
AAAATCGCCATCCAACAACACCAATATCTAATCAATATCGAGCTATCGTCAGCTATCGAATCAAATCGAA
CTAAATCAAGTCATCCGGAAGTCGAGCGACCTACTAAACCACTGCGAAGTCACCACTCAATCAGCCCGAT
CTATATATCTCTATATGTTAACTTACAACCACCGCATCATCATGAAGAGCATTATACCAAACAAATCGTA
GaaaaaaaaagaaaaGAATAGCAACAAATGCACAGTCCAAAGCCTTACAAACTCGATTGCACATTAAACT
CGATGTGTATCTATAATGAATATGCAATTCAGATATCGATGGTGAGCGAGGGGGAAAGAGACAGAAAGCG
AAGGTCCTTGAAGTGCTCTATCTACCTATCTATCTCTCTCCCGCTCGCgtgtgtgtgtgtgtgATCAGAG
GAGAGCGAAACCTATGAAAAGCCAGCTTTATCTAAACAAATTTTCCATTTCTTTTTCTTTCGTTTATTGA
ACAAAGTTTAATAATTGGAAAGCGGCGCGTAGGTTGAATCAAAACACTGAACAGTTAAgaaaaaaactaa
agcccaaattgcaaattacgaaatataataaaaaattgaaaaCAAATAACGCATAAatcagttgatcagt
ggatcagttgaATAAGGAACGAAACGAGTACAATCGATTCAAGGCAGCATCAATTCCGTCAAACAAACAA
AACCGAAGACTGAACGGGAACGACAACAATTCCAAGGCAATGGAGAGTATTATGAAGGTGGCCATGGACA
AGGCGGCCGAGCAGCTGATTCAGCAATTTGGTTTTGATTACTTGCAGCAGCAGCTGCAGTTGCAACATCA
GAACCAACACAacagcagcccacagcagccacagcaCCAGCAACTAGAACCAGAAAACGAACATCTCACA
TACCAATATCAGCAATCAAAGCCCACACATATGCAGCAATTAGCTTGTAACTATCAGCCACGACATTCCA
CAACCACGTCGTCGCCCTCATCCACACATTCGCTGGCcagcggcggcggcagcagcagtaacagcagcaa
cagcagcagcagcgacagcagcagcatcaacataagccacatcagcaacataagcaacatcagcaacatc
ggcaacatcagcaacagcaatcatagcaacgctgcaTACTCATTAGCCGTTCATAGCTATCAAAAGCAAA
TAGAAAGCCCGGCTAATCCCAGTCATGTTCCGCATCATCAGATGGATCTATCGCCCCTGAGTGAGAATGG
TTCGCCAAATGGGACACCAGGTGCCCAGACGCCGACTgcaacagcgagtggcaacacggcagcagcactc
gcatcagcagcagcagcagcagcagcagcaacatcaggaggcaacggcagcagcatcaccaactgcaaca
gcaataacagcagcagcagcagcaacgcgcaacagcaactgcagcTGGGCAACTATAAGACTAACTCCTG
CTGGTGTTACG
>dme_circ_0008347_junction_seq
TATAAGACTAACTCCTGCTGGTGTTACGGCAGCAGCAGCAGCATTTCCACAAAACC
>dme_circ_0008348
GTTTAATAATTGGAAAGCGGCGCGTAGGTTGAATCAAAACACTGAACAGTTAAgaaaaaaactaaagccc
aaattgcaaattacgaaatataataaaaaattgaaaaCAAATAACGCATAAatcagttgatcagtggatc
agttgaATAAGGAACGAAACGAGTACAATCGATTCAAGGCAGCATCAATTCCGTCAAACAAACAAAACCG
AAGACTGAACGGGAACGACAACAATTCCAAGGCAATGGAGAGTATTATGAAGGTGGCCATGGACAAGGCG
GCCGAGCAGCTGATTCAGCAATTTGGTTTTGATTACTTGCAGCAGCAGCTGCAGTTGCAACATCAGAACC
AACACAacagcagcccacagcagccacagcaCCAGCAACTAGAACCAGAAAACGAACATCTCACATACCA
ATATCAGCAATCAAAGCCCACACATATGCAGCAATTAGCTTGTAACTATCAGCCACGACATTCCACAACC
ACGTCGTCGCCCTCATCCACACATTCGCTGGCcagcggcggcggcagcagcagtaacagcagcaacagca
gcagcagcgacagcagcagcatcaacataagccacatcagcaacataagcaacatcagcaacatcggcaa
catcagcaacagcaatcatagcaacgctgcaTACTCATTAGCCGTTCATAGCTATCAAAAGCAAATAGAA
AGCCCGGCTAATCCCAGTCATGTTCCGCATCATCAGATGGATCTATCGCCCCTGAGTGAGAATGGTTCGC
CAAATGGGACACCAGGTGCCCAGACGCCGACTgcaacagcgagtggcaacacggcagcagcactcgcatc
agcagcagcagcagcagcagcagcaacatcaggaggcaacggcagcagcatcaccaactgcaacagcaat
aacagcagcagcagcagcaacgcgcaacagcaactgcagcTGGGCAACTATAAGACTAACTCCTGCTGGT
GTTACG
>dme_circ_0008348_junction_seq
TATAAGACTAACTCCTGCTGGTGTTACGGTTTAATAATTGGAAAGCGGCGCGTAGG
>dme_circ_0008349

GTTTAATAATTGGAAAGCGGCGCGTAGGTTGAATCAAAACACTGAACAGTTAAgaaaaaaactaaagccc
aaattgcaaattacgaaatataataaaaaattgaaaaCAAATAACGCATAAatcagttgatcagtggatc
agttgaATAAGGAACGAAACGAGTACAATCGATTCAAGGCAGCATCAATTCCGTCAAACAAACAAAACCG
AAGACTGAACGGGAACGACAACAATTCCAAGGCAATGGAGAGTATTATGAAGGTGGCCATGGACAAGGCG
GCCGAGCAGCTGATTCAGCAATTTGGTTTTGATTACTTGCAGCAGCAGCTGCAGTTGCAACATCAGAACC
AACACAacagcagcccacagcagccacagcaCCAGCAACTAGAACCAGAAAACGAACATCTCACATACCA
ATATCAGCAATCAAAGCCCACACATATGCAGCAATTAGCTTGTAACTATCAGCCACGACATTCCACAACC
ACGTCGTCGCCCTCATCCACACATTCGCTGGCcagcggcggcggcagcagcagtaacagcagcaacagca
gcagcagcgacagcagcagcatcaacataagccacatcagcaacataagcaacatcagcaacatcggcaa
catcagcaacagcaatcatagcaacgctgcaTACTCATTAGCCGTTCATAGCTATCAAAAGCAAATAGAA
AGCCCGGCTAATCCCAGTCATGTTCCGCATCATCAGATGGATCTATCGCCCCTGAGTGAGAATGGTTCGC
CAAATGGGACACCAGGTGCCCAGACGCCGACTgcaacagcgagtggcaacacggcagcagcactcgcatc
agcagcagcagcagcagcagcagcaacatcaggaggcaacggcagcagcatcaccaactgcaacagcaat
aacagcagcagcagcagcaacgcgcaacagcaactgcagcTGGGCAACTATAAGACTAACTCCTGCTGGT
GTTACGGTGAGTCTGATTAAGAACAAACAAACAAACAAACGCAATCAGCATCAGCATAGAACTATCAAAT
CAAAAGTTGCGCACAACTTCCTCTACTACACCATACATAAAATGTACACCACTTCTCTATATAACTATCT
ATAACTATCGAGCTCGCTACAGCCAACAAATATCAACCAACCAGCCAACAACAGCATCAATCACCACGTC
GCCTCATTCTAAATGCCGCCAGCAACTTAAAAGCTGAACCaaaaaaaaaaaaaaTGTACAAAGAAAAAAG
TATCAAGAACGCCCAAATGTCAAACGAACTGAACTGAACGCTGAACAAGAAGCCACAAGCTATAAATGAA
TTATCCATTAACATTGCGTTGAAAGTTTTGATAGAAAATAATCCAAACATCATCAGCATCCACCAATCCA
GCACACATTTCGATACACAAGAAATAAAACCAAACCAACAAACACTCAtaaaaaaaaaactaagcagcta
aaacagaaacaagcagccagctaaaaaaaaatccaaaaaaaaaagaaatatatatatatatatataaa
aaaCTCATATGCTAATCGTACGCTTAAATGTGGGCGTCCATTTCAATTTCGTTTGTGCCTTAAAATATTA
TGTTTTGTGTATACACGACTTTCCTTCAGCATTCCCTAGTGATTCATGAGTGTTGCATAAAGATATTCGC
TTTGGTTTTTTTAAATAGGATTTCGTTTGCTCTTTTGTGTGTTTCAATTGAACGAAGAACCAATTGGTTA
TGTTTTATTGGCAAATCGTATCAAGATTTAATACCTATGCCTATTAAAGTTCACTTCGAGTGTCCTTAAA
GCTCTTTCCGGTGCAGGAAATTAAACATACTTGTCGCCTGCAGGATCCAATCGTTTAATCGCGTGCCACA
TGCCCCCGCATTACGCATACGTCGCGTGGTACGCGACAAGCACTTGCTGCTCGGGCTTTGCTTTAACAAT
TTGCGTGTTGCAGATTGCTCCATGGCATTTATTGTGGTTTAGGGttggagtgggtagagtggagtggagt
ggagtggagtggaatggaatggaAAGGGTGGTCCTGACTGTGTGCCAGCTTGTCGTTAGCCTAGTTTCCC
GTCCAATCCTGCACACAATGCATACGAAATTAGTCTCCAGTCTGCCTGCTGCCATCATCCTGCAAGCTCT
GCCACCCCGCCACCCTGCCACGCCCACTTGCAGCTCCATGGGCGGTCTCTGGCCAGTGTGCTGCAAGTGT
TATTTATGACAGTCACATAATTTAAGCTGCTGCACAGTGTCAACAGCAACGCAGCTTCGCAACAGCGTTC
TTGGCCTGTCCTTTGTGGCCCATAAAAACGCTAAGCAAACGACTTTATGTTTTGTTTTTCTGTCTTCCTG
CATATTATTTACCATGGCCATTTTGCAACTCTCGCTTATTTACGAGTATTTTGCATTGCAGTAGAATTTC
GTAAAAGAATTTCACCAATAGCACTCTATTCATACGGTATAAGTGATAGATAACTGACAGATGCGTCCGG
CAATTGGTTCAGTGGCGGAAATTGGCCAGCATGGTGCTAAATATGTGGTCGCAATTCGCTTTCGTAGCGA
ATTCTTTGCGAACCGCATTTTCACACATGCATGCAAAGCAGGGCAAATGGTCCAATATTCCCCCAGCCCA
AGCGAATACCCATCCCCCGATACCTCATACCACCCAATGTCAGTGCATCGTcgtgtgtgtgtgcgtgtgt
gCGCGCTGTATCTAATTAGAGAAATACTTTTGAAGGACACTCTGGCGCTGAGTGGCAACGGGACCGACCA
AGACGCCGACAACGTCAGCGTATGGCACACATTTCACCGATGCCGTAAGGAACTGGGTGGTGGTGGTGGT
GCCTCGGGGGGCGGATGGTGGCTAAATTTAAACGAACCGAGTGCGCCCAAGTGGCATCAGCAGAGTCTTG
GGCTGCCACAAGTCGCCATGCTGCACGATGGCAGGCAGTCAGGTGCAACGCGCTTAGATCATTCCATGCC
ATGCCCGTTCCATTCTATTCCATCTCGTTGTTTTGCTAGCCAATGCGCTTGTGTAATAGAGCGTCTCCGC

TTGACACATTTTATTAATTGAAATTTGTTTGTTTACTTACTTACAACTGGCTGCCGTCGGTTGTCTTATG
TCGTTTGTCGGCGACTTGAGGCTGTGTGACGCAGATGATATCACTCATACGCAGGGAGGTCCCACTGCAT
TCACATGCATTCGCCACATTCCCATCACTTAGCCGTGTAATGCCGTGCACTGCCTTCAGATCCCAACTTA
TAATCATTGTTTCTGTCTTTTTCTTCTTCTTTCCATTTCAGGTGAGTCAGTTTGTTCTGGAATTGAGGTT
GAAATTGAAAATAATAACAATAATCATATTCATCATGGTAAGCTTTGTCACACTTTCATTAAATACCATT
AAATCCATCTTTGTCTAGCCAAACGAACGACTAATTCAAGATTAATACCGACTAAAGCGTAGCTCTATAT
CGTTTTCTGTATCCGTAACTAGATCGCTGTCTGTTTTTAATGCCGCTGCCGCTGACGAAAGCGCGCTTTT
TGAACCTGAATTCGGACGGACTAAAAACGAAAACTGATTATGCGACCCAACTGACAACGGAGCACATTAA
CCTTCGACCCTTTGACCAAACTTATGCTTTGACTTACatttttttcgtatttatttcgtttcttttttt
cgcttttGTGTGTTGCCCAAGTAATTTGCGGGGCTTTTTCTTTCGGCCAAGCCAACGACCTTCGGAGAAG
AAGTTAAAACGGGCTTTCATATGTTTCGTGTGTCGCCAGCATTGTCTGTTCCATCTCATCTCGAGCGCAT
ATCTTCCTCATGGCTTGCCCATTACAAAAACCACAGAAAACAGACCACAGTCGATacaccacgtccacgc
ccacgcccacgcccacaccaccataaccaatccatgcccacacacaGCAGTGAACCATGCTAATCCACTA
AACCAAGTCGAAATCGAAAACTATCTATCTGAAAACTATAACCAAACTAACCGAGTTGAAAACTCAACTT
TACGAACGTCCCTCGTTGTGAGCGTCCAGCCGAACGAACAAACGATCGACGCTGAAATGCTGTTATTATT
TTTAATTTGTAACCTGCCGAATGAGCTAGAAACTAGATCTAACCAACAATAATAACCAGATAACTAGATT
GTATGCAGCAGCCAAGGGCGCACACAGACTAACTAACCCTGTAACCTGTACCAAAAACCTCCTAATCTAT
TGACGAAAAAAAGAATAGTAGCCCAATCCAAGAGAATCTCCCGAGACTAATCCATTGGTCGCCCTGTGAA
CTCCTCTCGATTCTTTCATTTCAGGGCGAGACAACGTATCACATGAAAATACTGGTGCCCGCGGTGGCCT
CCGGGGCCATCATTGGCAAAGGTGGCGAGACGATCGCCTCCCTGCAGAAGGACACGGGTGCTAGGGTCAA
GATGTCCAAGTCGCACGACTTCTACCCAGGGCACCACCGAACGCGTGTGCCTGATCACCGGCTCCACGGA
GGCTATCATGGTCGTGATGGAGTTCATCATGGACAAGATCCGCGAGAAGCCCGACCTGACCAACAAGATC
GTCGATACCGACTCAAAACAGACACAGGAGCGCGACAAGCAGGTCAAGATCCTGGTGCCCAACTCGACCG
CCGGCATGATCATTGGCAAGGGCGGTGCCTTCATTAAACAGATCAAGGAGGAGAGCGGCTCCTATGTCCA
GATCTCGCAGAAGCCCACGGATGTGTCGTTGCAGGAGCGCTGCATCACCATCATAGGCGACAAGGAGAAC
AATAAGAACGCCTGCAAGATGATCCTCTCGAAGATCGTCGAAGATCCCCAGTCGGGCACCTGCCTGAACG
TATCCTACGCGGACGTCAGCGGCCCGGTGGCCAACTTTAATCCGACCGGCTCCCCGTACGCCACCAATCA
GAATGCCATCAACTCGAGCACCGCTTCGTTGAACTCCACGTTGGGCACCACGATCGGCGGTGCAAACTCG
GCGGCCAGCCTGCTAGTCAACGGCACCGGCATCAATTTGTCCATCAATCTGGGCTCACCCAATCCGGCGC
CCAATCTAGCGGTTGCCACACAGCTGCTCGAGCATATTAAGGGTTGCCATGCGCGGCTCTGGCTATTCGG
AGACCGTAACGAACGAAGTTGTCGCCGCTCTGAGCGTGCTGGCCAAGTACGGCGTCCTTGGAATGGGCGT
GGGTGTGTCGCACACCAACGGCGCCCACTCAACGCTGGGCAACTTCCTGGGCGTAACGACGCTGGACCAG
CAGACAGCCGCTGCCGCATCTGCGGCCACCGCCAGCAATGTGTTCGGTGCCGTTGGCCAGGTAAATCTGG
AGCAATATGCCGCAGCGGTGGCCTCTGCAGCTGCCGCCAGCCGGCCGACGCAGTCGCAACTGGACGCTGC
CGCAGTGCAATTCGATCCATTCCGCCATTTGGGCTCGGCAACGGCGCCGGCCGCCACGCCCGTCTCACTG
AACAACAATAGCTTTGGATTGACGGCGACCACGGGAACGGCGACCACGGCGCAGCTGGGCGGTCTAAGCA
AGAGTCCCACTCCCGGTGACCTTAGCTCCAAGGACTCGAAAAACGTCGAGGTGCCGGAAGTGATTATCGG
CGCTATCCTAG
>dme_circ_0008349_junction_seq
CCGGAAGTGATTATCGGCGCTATCCTAGGTTTAATAATTGGAAAGCGGCGCGTAGG
>dme_circ_0008350
GATGGATCTATCGCCCCTGAGTGAGAATGGTTCGCCAAATGGGACACCAGGTGCCCAGACGCCGACTgca
acagcgagtggcaacacggcagcagcactcgcatcagcagcagcagcagcagcagcagcaacatcaggag
gcaacggcagcagcatcaccaactgcaacagcaataacagcagcagcagcagcaacgcgcaacagcaact

gcagcTGGGCAACTATAAGACTAACTCCTGCTGGTGTTACG
>dme_circ_0008350_junction_seq
TATAAGACTAACTCCTGCTGGTGTTACGGATGGATCTATCGCCCCTGAGTGAGAAT
>dme_circ_0008351
GAATTTGATGGTCCTGTCTGGCCAACAGCCGCTCCGTTCCCGTGGTTCCCATTACTACCGTTGCGGTTTA
TGTGATTCAGCTGCTCATCCACGTTTTGGGCGGTTACTCCAGCAGCTACAGAGCTAGCTGCTCCAATCGC
CTTATCCAGCTCCGCCGCATGG
>dme_circ_0008351_junction_seq
AATCGCCTTATCCAGCTCCGCCGCATGGGAATTTGATGGTCCTGTCTGGCCAACAG
>dme_circ_0008352
GGTGAGTCAGTTTGTTCTGGAATTGAGGTTGAAATTGAAAATAATAACAATAATCATATTCATCATGGTA
AGCTTTGTCACACTTTCATTAAATACCATTAAATCCATCTTTGTCTAGCCAAACGAACGACTAATTCAAG
ATTAATACCGACTAAAGCGTAGCTCTATATCGTTTTCTGTATCCGTAACTAGATCGCTGTCTGTTTTTAA
TGCCGCTGCCGCTGACGAAAGCGCGCTTTTTGAACCTGAATTCGGACGGACTAAAAACGAAAACTGATTA
TGCGACCCAACTGACAACGGAGCACATTAACCTTCGACCCTTTGACCAAACTTATGCTTTGACTTACatt
tttttcgtatttatttcgtttcttttttttcgcttttGTGTGTTGCCCAAGTAATTTGCGGGGCTTTTTC
TTTCGGCCAAGCCAACGACCTTCGGAGAAGAAGTTAAAACGGGCTTTCATATGTTTCGTGTGTCGCCAGC
ATTGTCTGTTCCATCTCATCTCGAGCGCATATCTTCCTCATGGCTTGCCCATTACAAAAACCACAGAAAA
CAGACCACAGTCGATacaccacgtccacgcccacgcccacgcccacaccaccataaccaatccatgccca
cacacaGCAGTGAACCATGCTAATCCACTAAACCAAGTCGAAATCGAAAACTATCTATCTGAAAACTATA
ACCAAACTAACCGAGTTGAAAACTCAACTTTACGAACGTCCCTCGTTGTGAGCGTCCAGCCGAACGAACA
AACGATCGACGCTGAAATGCTGTTATTATTTTTAATTTGTAACCTGCCGAATGAGCTAGAAACTAGATCT
AACCAACAATAATAACCAGATAACTAGATTGTATGCAGCAGCCAAGGGCGCACACAGACTAACTAACCCT
GTAACCTGTACCAAAAACCTCCTAATCTATTGACGAAAAAAAGAATAGTAGCCCAATCCAAGAGAATCTC
CCGAGACTAATCCATTGGTCGCCCTGTGAACTCCTCTCGATTCTTTCATTTCAGGGCGAGACAACGTATC
ACATGAAAATACTGGTGCCCGCGGTGGCCTCCGGGGCCATCATTGGCAAAGGTGGCGAGACGATCGCCTC
CCTGCAGAAGGACACGGGTGCTAGGGTCAAGATGTCCAAGTCGCACGACTTCTACCCAGGGCACCACCGA
ACGCGTGTGCCTGATCACCGGCTCCACGGAGGCTATCATGGTCGTGATGGAGTTCATCATGGACAAGATC
CGCGAGAAGCCCGACCTGACCAACAAGATCGTCGATACCGACTCAAAACAGACACAGGAGCGCGACAAGC
AGGTCAAGATCCTGGTGCCCAACTCGACCGCCGGCATGATCATTGGCAAGGGCGGTGCCTTCATTAAACA
GATCAAGGAGGAGAGCGGCTCCTATGTCCAGATCTCGCAGAAGCCCACGGATGTGTCGTTGCAGGAGCGC
TGCATCACCATCATAGGCGACAAGGAGAACAATAAGAACGCCTGCAAGATGATCCTCTCGAAGATCGTCG
AAGATCCCCAGTCGGGCACCTGCCTGAACGTATCCTACGCGGACGTCAGCGGCCCGGTGGCCAACTTTAA
TCCGACCGGCTCCCCGTACGCCACCAATCAGAATGCCATCAACTCGAGCACCGCTTCGTTGAACTCCACG
TTGGGCACCACGATCGGCGGTGCAAACTCGGCGGCCAGCCTGCTAGTCAACGGCACCGGCATCAATTTGT
CCATCAATCTGGGCTCACCCAATCCGGCGCCCAATCTAGCGGTTGCCACACAGCTGCTCGAGCATATTAA
GGGTTGCCATGCGCGGCTCTGGCTATTCGGAGACCGTAACGAACGAAGTTGTCGCCGCTCTGAGCGTGCT
GGCCAAGTACGGCGTCCTTGGAATGGGCGTGGGTGTGTCGCACACCAACGGCGCCCACTCAACGCTGGGC
AACTTCCTGGGCGTAACGACGCTGGACCAGCAGACAGCCGCTGCCGCATCTGCGGCCACCGCCAGCAATG
TGTTCGGTGCCGTTGGCCAGGTAAATCTGGAGCAATATGCCGCAGCGGTGGCCTCTGCAGCTGCCGCCAG
CCGGCCGACGCAGTCGCAACTGGACGCTGCCGCAGTGCAATTCGATCCATTCCGCCATTTGGGCTCGGCA
ACGGCGCCGGCCGCCACGCCCGTCTCACTGAACAACAATAGCTTTGGATTGACGGCGACCACGGGAACGG
CGACCACGGCGCAGCTGGGCGGTCTAAGCAAGAGTCCCACTCCCGGTGACCTTAGCTCCAAGGACTCGAA
AAACGTCGAGGTGCCGGAAGTGATTATCGGCGCTATCCTAG

>dme_circ_0008352_junction_seq
CCGGAAGTGATTATCGGCGCTATCCTAGGGTGAGTCAGTTTGTTCTGGAATTGAGG
>dme_circ_0008353
GGCGAGACAACGTATCACATGAAAATACTGGTGCCCGCGGTGGCCTCCGGGGCCATCATTGGCAAAGGTG
GCGAGACGATCGCCTCCCTGCAGAAGGACACGGGTGCTAGGGTCAAGATGTCCAAGTCGCACGACTTCTA
CCCAGGGCACCACCGAACGCGTGTGCCTGATCACCGGCTCCACGGAGGCTATCATGGTCGTGATGGAGTT
CATCATGGACAAGATCCGCGAGAAGCCCGACCTGACCAACAAGATCGTCGATACCGACTCAAAACAGACA
CAGGAGCGCGACAAGCAGGTCAAGATCCTGGTGCCCAACTCGACCGCCGGCATGATCATTGGCAAGGGCG
GTGCCTTCATTAAACAGATCAAGGAGGAGAGCGGCTCCTATGTCCAGATCTCGCAGAAGCCCACGGATGT
GTCGTTGCAGGAGCGCTGCATCACCATCATAGGCGACAAGGAGAACAATAAGAACGCCTGCAAGATGATC
CTCTCGAAGATCGTCGAAGATCCCCAGTCGGGCACCTGCCTGAACGTATCCTACGCGGACGTCAGCGGCC
CGGTGGCCAACTTTAATCCGACCGGCTCCCCGTACGCCACCAATCAGAATGCCATCAACTCGAGCACCGC
TTCGTTGAACTCCACGTTGGGCACCACGATCGGCGGTGCAAACTCGGCGGCCAGCCTGCTAGTCAACGGC
ACCGGCATCAATTTGTCCATCAATCTGGGCTCACCCAATCCGGCGCCCAATCTAGCGGTTGCCACACAGC
TGCTCGAGCATATTAAGGGTTGCCATGCGCGGCTCTGGCTATTCGGAGACCGTAACGAACGAAGTTGTCG
CCGCTCTGAGCGTGCTGGCCAAGTACGGCGTCCTTGGAATGGGCGTGGGTGTGTCGCACACCAACGGCGC
CCACTCAACGCTGGGCAACTTCCTGGGCGTAACGACGCTGGACCAGCAGACAGCCGCTGCCGCATCTGCG
GCCACCGCCAGCAATGTGTTCGGTGCCGTTGGCCAGGTAAATCTGGAGCAATATGCCGCAGCGGTGGCCT
CTGCAGCTGCCGCCAGCCGGCCGACGCAGTCGCAACTGGACGCTGCCGCAGTGCAATTCGATCCATTCCG
CCATTTGGGCTCGGCAACGGCGCCGGCCGCCACGCCCGTCTCACTGAACAACAATAGCTTTGGATTGACG
GCGACCACGGGAACGGCGACCACGGCGCAGCTGGGCGGTCTAAGCAAGAGTCCCACTCCCGGTGACCTTA
GCTCCAAGGACTCGAAAAACGTCGAGGTGCCGGAAGTGATTATCGGCGCTATCCTAG
>dme_circ_0008353_junction_seq
CCGGAAGTGATTATCGGCGCTATCCTAGGGCGAGACAACGTATCACATGAAAATAC
>dme_circ_0008354
GGCGAGACAACGTATCACATGAAAATACTGGTGCCCGCGGTGGCCTCCGGGGCCATCATTGGCAAAGGTG
GCGAGACGATCGCCTCCCTGCAGAAGGACACGGGTGCTAGGGTCAAGATGTCCAAGTCGCACGACTTCTA
CCCAGGGCACCACCGAACGCGTGTGCCTGATCACCGGCTCCACGGAGGCTATCATGGTCGTGATGGAGTT
CATCATGGACAAGATCCGCGAGAAGCCCGACCTGACCAACAAGATCGTCGATACCGACTCAAAACAGACA
CAGGAGCGCGACAAGCAGGTCAAGATCCTGGTGCCCAACTCGACCGCCGGCATGATCATTGGCAAGGGCG
GTGCCTTCATTAAACAGATCAAGGAGGAGAGCGGCTCCTATGTCCAGATCTCGCAGAAGCCCACGGATGT
GTCGTTGCAGGAGCGCTGCATCACCATCATAGGCGACAAGGAGAACAATAAGAACGCCTGCAAGATGATC
CTCTCGAAGATCGTCGAAGATCCCCAGTCGGGCACCTGCCTGAACGTATCCTACGCGGACGTCAGCGGCC
CGGTGGCCAACTTTAATCCGACCGGCTCCCCGTACGCCACCAATCAGAATGCCATCAACTCGAGCACCGC
TTCGTTGAACTCCACGTTGGGCACCACGATCGGCGGTGCAAACTCGGCGGCCAGCCTGCTAGTCAACGGC
ACCGGCATCAATTTGTCCATCAATCTGGGCTCACCCAATCCGGCGCCCAATCTAGCGGTTGCCACACAGC
TGCTCGAGCATATTAAGGGTTGCCATGCGCGGCTCTGGCTATTCGGAGACCGTAACGAACGAAGTTGTCG
CCGCTCTGAGCGTGCTGGCCAAGTACGGCGTCCTTGGAATGGGCGTGGGTGTGTCGCACACCAACGGCGC
CCACTCAACGCTGGGCAACTTCCTGGGCGTAACGACGCTGGACCAGCAGACAGCCGCTGCCGCATCTGCG
GCCACCGCCAGCAATGTGTTCGGTGCCGTTGGCCAGGTAAATCTGGAGCAATATGCCGCAGCGGTGGCCT
CTGCAGCTGCCGCCAGCCGGCCGACGCAGTCGCAACTGGACGCTGCCGCAGTGCAATTCGATCCATTCCG
CCATTTGGGCTCGGCAACGGCGCCGGCCGCCACGCCCGTCTCACTGAACAACAATAGCTTTGGATTGACG
GCGACCACGGGAACGGCGACCACGGCGCAGCTGGGCGGTCTAAGCAAGAGTCCCACTCCCGGTGACCTTA
GCTCCAAGGACTCGAAAAACGTCGAGGTGCCGGAAGTGATTATCGGCGCTATCCTAGGTGCGCGTCCATA

TTATTATCCATACTAAATTGCAAAGAACCAATCAACAGAGAACCAACCACACCGCAATTTTCTTTACCAA
TCCCAATCCCAAATGCCCCACCAAATCCACACGTTTGAACCCACGCACCTGTGAAATTCCCGGGCGGGAT
TATATATTCCACCAACCAGAAACCCACTCCCGTTACCGATCCCAATCCCAAGCCCCTATTTTTTAATTTG
GCCCTAGAGCCCATTAGACATAGAAACAAAAGCCAAGCAGACACAGGCACATCATTGCAGACTCATTCCA
GAGAGAGAAAAGAGGGTACGACTTATTGAGAGAGATATCCCATTCTAAGCATACAACGCATTCCGTGAGA
CATTCCGATAGACATTCGCAAAGGCTTTCCCAATAAATTCATTGACACTTATAGCGAAATTTCTAGTCAA
GTTCTTGCCTCTCACCCATCCGCCAGCATATatgatccatgatccatgatccGTCTTCGATATTGCACCT
ATTGCACCGTGTAAACATCCGAAATAATTTCTCCGAAATCACGAAAAACCCAACCCAGAAAATTCGCACA
GAACAGCAGCATCCGATAGCTTAGGATTATAAGGCAGTAATTAGGTTTAGCAGTTAGGTTGTGCATTGAG
TAGATTGAATAAAACACATTCACTTTTCCATGTTGTTTAGGCCTAACGAATTTGTTCGATATACTTACGT
GTAGCTGGGCGGATCGCCCAGTTGGCTAGTGCAATTCGTGGGTAACCATGCGTAAAATATAATTACATTG
TTTTTTCTATTTAAATACATTCTTTGTTACGCAGTTGATTTTACCTCAGTGTCAGTTGTAAGGTCGTTAG
GTCATTAGGTTAGGCCCACCGTGACCCCTTTTGCAGCCAATTGACCATTGTTCtttggtttgattttgtt
ttatttttattttttGGCGTCCTTAACACATTAGCGCTATACACGCCCAAAACCTAatatgcatatatat
atatatatatatatatatatatatatatatattgatatatgatatatacatatattactattatagccac
atatattacccatactatcatttaatatCTTGAAAATATTTAACATAAATCTGATGCAAATATTCCAGGT
CCGAGCGGTCGTAGTTTAGTTGAAATTCAACATGTTTCTGGCGCAAATGTGCAAATTTCCAAAAAGGGCA
TATTCGCACCTGGCACTAGAAATCGCATTGTAACCATAACTGGTCAGCCGAGTGCCATTGCCAAAGCGCA
ATATCTAATTGAACAGAAAATCAACGAGGAGGAGACAAAGAGGGCGCGACAAATTCCATTAACCACTGTT
GTGAATTaaaataaaaaaaacaaaaaGTACCATAACTAATATCCGAGTAAAgcaacaaaaccagcaatct
taacagaacatcaaacaaacaggcaaaaagcaaagcaacaacaaaCAAACTACCCCATACCCTCGACGGT
TTTCCTCTAAAACTTGTTAATTTCTTTGAGAAACGAGAGgaaaatttaaaaacctaaaactaaaaagaaa
tgaaatgaaatgagaagggattacaaaaaGGAAACACCAATTGTTATGCTTGCAGCTCAGACTTAAGCAG
TTAAAAGCGAAAATAATAATAATAACGTATTGCAAAAATCCATAGATTTTGTTCCAAGGCCTTCACCTCA
AGAGAtttgctactaacgcccctttgcctactaacgcccctttAAGAAAGTGAAAAACGAGATATGCGAAGT
AGTGAAAAATCTGTAAAAATACTATATGCGTACTACCTTCTCAGCTATACATTGTTGCATTTGTTTGTCA
CGATCGTTAAAACGTAAAAACCGAAAACAAGTTATATTGCCAATGGCCAATTGCCAGTTAGACACAAACT
AGTTGCGAAAGAAGAGCGTTTGGGTTTTTGGTTTGTTTGTGATGCGTATGTTTGCGTTGCGTGTTTTGTT
TTTGGTACTCCCAACCAACTTATACGATACGATACGTTACATGGGGCATATATACATATGTATATGTACA
CAGTTGTTGAGTCTTGTTTATATatattatctacacacatatgcccatacatatatatatatacatatGC
CACCACCCACTTACACACTTACACACCGccccccccccccaaaaaaaaaaaaCACACACCTAATTATTAGC
ATTATGTTGAGCTAAACGAAAATCAAATGTATAATGAGCGAGCGTTTTCAGTGAAAACTTCAGGTGATTC
TTACCCTCGAAATAAG
>dme_circ_0008354_junction_seq
TTCAGGTGATTCTTACCCTCGAAATAAGGGCGAGACAACGTATCACATGAAAATAC
>dme_circ_0008355
GCATACAACGCATTCCGTGAGACATTCCGATAGACATTCGCAAAGGCTTTCCCAATAAATTCATTGACAC
TTATAGCGAAATTTCTAGTCAAGTTCTTGCCTCTCACCCATCCGCCAGCATATatgatccatgatccatg
atccGTCTTCGATATTGCACCTATTGCACCGTGTAAACATCCGAAATAATTTCTCCGAAATCACGAAAAA
CCCAACCCAGAAAATTCGCACAGAACAGCAGCATCCGATAGCTTAGGATTATAAGGCAGTAATTAG
>dme_circ_0008355_junction_seq
TAGCTTAGGATTATAAGGCAGTAATTAGGCATACAACGCATTCCGTGAGACATTCC
>dme_circ_0008356
ATATTGCGCTTTGGCAATGGCACTCGGCTGACCAGTTATGGTTACAATGCGATTTCTAGTGCCAGGTGCG

AATATGCCCTTTTTGGAAATTTGCACATTTGCGCCAGAAACATGTTGAATTTCAACTAAACTACGACCGC
TCGGACCTGGAATATTTGCATCAGATTTATGTTAAATATTTTCAAGatattaaatgatag
>dme_circ_0008356_junction_seq
TAAATATTTTCAAGatattaaatgatagATATTGCGCTTTGGCAATGGCACTCGGC
>dme_circ_0008357
GGAAACACCAATTGTTATGCTTGCAGCTCAGACTTAAGCAGTTAAAAGCGAAAATAATAATAATAACGTA
TTGCAAAAATCCATAGATTTTGTTCCAAGGCCTTCACCTCAAGAGAtttgctactaacgccctttgccta
ctaacgccctttAAGAAAGTGAAAAACGAGATATGCGAAGTAGTGAAAAATCTGTAAAAATACTATATGC
GTACTACCTTCTCAGCTATACATTGTTGCATTTGTTTGTCACGATCGTTAAAAC
>dme_circ_0008357_junction_seq
TGCATTTGTTTGTCACGATCGTTAAAACGGAAACACCAATTGTTATGCTTGCAGCT
>dme_circ_0008358
GATTTTGTTCCAAGGCCTTCACCTCAAGAGAtttgctactaacgccctttgcctactaacgccctttAAG
AAAGTGAAAAACGAGATATGCGAAGTAGTGAAAAATCTGTAAAAATACTATATGCGTACTACCTTCTCAG
CTATACATTGTTGCATTTGTTTGTCACGATCGTTAAAACGTAAAAACCGAAAACAAGTTATATTGCCAAT
GGCCAATTGCCAGTTAGACACAAACTAGTTGCGAAAGAAGAGCGTTTGGGTTTTTGGTTTGTTTGTGATG
CGTATGTTTGCGTTGCGT
>dme_circ_0008358_junction_seq
GTTTGTGATGCGTATGTTTGCGTTGCGTGATTTTGTTCCAAGGCCTTCACCTCAAG
>dme_circ_0008359
GCGAGCGTTTTCAGTGAAAACTTCAGGTGATTCTTACCCTCGAAATAAGGTAAATATTTTACTACACCAA
CCCCCTCCTCACAGCCACCACAGACAAAAACACTTGCTATGGCTAATACTGCAACTGCAAACCGGCTAGT
GAAAAATACAACTTCAAAGAGTTAAGCaaacaaaaaaaaataaaaacataaaaaGAGTGACTTTGTTGAC
CTTTTCGTGATTTCGTTAAAGTTTTCTGTTCCATCTGTTCTCTGTTGTTGCCCCACTCCCTCTAATTGCT
TATCGCTGTTAatatatatatatacatatataAACTTTCCACCTACGATTCTACAGTCTGACCTCTGTAT
ATGAACACTTTCGGAACGCTACAGTTGATGTTGATCATGATAAT
>dme_circ_0008359_junction_seq
CGCTACAGTTGATGTTGATCATGATAATGCGAGCGTTTTCAGTGAAAACTTCAGGT
>dme_circ_0008360
AAGTGGTTGACTGATTGACTGGCTGACTGTCTGATTGAGTTGATTTTGATTCGTCATGATAATGTTGTAG
TAGACTGATTTGGTTTGGAAACACGATtgggttagtggtggtgcggcgcttgtgggtgggtggatgggtg
ggtggtaatggtggttgcggtggtcattgggatggtgCGCGGAACGCTCCAAAAATAAACAAGAAAATAA
ACTTTGCAGGTTTTCATTGTTGGTTTTGTAGTCAAATTTTATTTAAATTTCGTTTCATCTTCGTTTTATT
TATCGAAAAGAAGTGTTATTCTAAATTATATGTGTAAGGAATAAAGTAAATAAGAAAAATTTCATTACAA
GTAAGGCATTTCTCTCTCTGTAGGTGAGTGGTGTTCGTATGGGTACTTTTATCCTTGTGTATTTTGTGTG
TATATGAACAGGGAGTTTTTTAAATGTGATGTTAACATCTTTTGTCTTTACACTGCGTTTCTAGATAGAT
ATGTACATATAATCGTTATGAAATGTTCGTTGAAACAATGTTTAATAATTGCACAATACTTAAAATTAAT
AAGGGAAATTGTTTGGTGGATTGTTTGTTTTGACGTATGAAGAAACCCCTAAATGAATGCTTTAATGTGA
ATATTTTACAAAGAAATAATTGTTTTGCTAAGGCTTTTTATGTTGTTTTCGTAAAttttttttttttttt
tttAAGATATTTGTGATGCATTAGGTGAGAAAATGTAAGGCTTAAAATTAGAATGTAAGAAACAAATGTG
CTAAATTTACCAAAAAAATAGTGGTTAACAAACGCAAAATGACAATGAAAAAAATAAGTAAAAAGGTAAG
AAGTGCAGTTCTCAGACTGGCAACTAACAAATCGCAATGTGttttttttttCATAAATAATATGTATACA
TAAAACAAAAGTTATAAGGAGTAACAAATCGGGAGCACAAAATCATAACCAAAAGGAGACTCACGTGCGC
GCGCAACACATTAATACCACTCGATCCGATCCGATCGAATTGAATCGAATCGGATCGGACTGTATTTAAA

ACTAAACAACATTATCATGATCAACATCAACTGTAGCGTTCCGAAAGTGTTCATATACAGAGGTCAGACT
GTAGAATCGTAGGTGGAAAG
>dme_circ_0008360_junction_seq
GTCAGACTGTAGAATCGTAGGTGGAAAGAAGTGGTTGACTGATTGACTGGCTGACT
>dme_circ_0008361
AAAATAAACTTTGCAGGTTTTCATTGTTGGTTTTGTAGTCAAATTTTATTTAAATTTCGTTTCATCTTCG
TTTTATTTATCGAAAAGAAGTGTTATTCTAAATTATATGTGTAAGGAATAAAGTAAATAAGAAAAATTTC
ATTACAAGTAAGGCATTTCTCTCTCTGTAGGTGAGTGGTGTTCGTATGGGTACTTTTATCCTTGTGTATT
TTGTGTG
>dme_circ_0008361_junction_seq
GTACTTTTATCCTTGTGTATTTTGTGTGAAAATAAACTTTGCAGGTTTTCATTGTT
>dme_circ_0008362
GTTCCAATCACTCCGTTTCCCAGAAATGTCCGATTTGCGAGCCAATAGAGTAGATCCCCCCGCGTGGCTG
AGGGGGCGTGACGAAgggaagatgggaaaatgggaaaatggAGCTACTCGGGTGTCGAACCGATCGGATG
AGAGGTCAGATCCGATCCCATCGGTTCAAAGGACCCACCCGCGTCCCGCTTTCGACGT
>dme_circ_0008362_junction_seq
GGACCCACCCGCGTCCCGCTTTCGACGTGTTCCAATCACTCCGTTTCCCAGAAATG
>dme_circ_0008363
GCTACTCGGGTGTCGAACCGATCGGATGAGAGGTCAGATCCGATCCCATCGGTTCAAAGGACCCACCCGC
GTCCCGCTTTCGACGTGTTTATGCTTGTTTGGATTTTATTGTTTAAATGCGTTTGTTATTTGCT
>dme_circ_0008363_junction_seq
TATTGTTTAAATGCGTTTGTTATTTGCTGCTACTCGGGTGTCGAACCGATCGGATG
>dme_circ_0008364
TATTTAATATTTTACTAATGTTGTATGCTTCTTGTTGTGGCTGCTGAATGATCGTCTCCGCAGCCGAATT
GTTTCGGCCGATCGAGTTTTCCGATAGAATTTTATACATACAATGCTATAATGAGGTTATATAATGATTC
AGTTAGTTCTCCGACCTATCAAATACCAGCGGGGTTCGCATCAAATCGCAAACATATTCGCAACATCTTC
CATGTATGTTGGTATACGTACAGGTATACCATATGCATATGGGGTGTATGTG
>dme_circ_0008364_junction_seq
TATACCATATGCATATGGGGTGTATGTGTATTTAATATTTTACTAATGTTGTATGC
>dme_circ_0008365
GCATTTGAAAATCGCTATGGTAAAAGTTCACACATAACCACGGTATAAAATAGGTTTTCTTCATTTCGCT
CAATTTCATTTAAAGAGGTATAGATTTGAACaaaaaaaaaaaaccaaaaaccaaaaCGTACGACAAGTAA
CAGAAAAATAGTCTAGTGAAATCTTTAAACAATTAAATCAAAAGGTATCCCTACAAGAAGATTCTTTAAA
GTAGCTCATTCAA
>dme_circ_0008365_junction_seq
AGAAGATTCTTTAAAGTAGCTCATTCAAGCATTTGAAAATCGCTATGGTAAAAGTT
>dme_circ_0008366
GTTTTAATAAATCGAGAAAAACATAATTTTTGCAATTAAACAAAACGATAAGAAATTCTGTACAATTGCG
AACGAAAAGTATGCAAACAGTTACTCCTCTCTACATACATATATATGCATAGCTATATAACCCCAAGTGA
ATCATACTCGAACaaaaaaaatgaaaacgaaaatgaaaaagaaaattaataagaaaatgaacaagaTTATA
ATGCAGATTGCGACTAAAACTTTACG
>dme_circ_0008366_junction_seq
TAATGCAGATTGCGACTAAAACTTTACGGTTTTAATAAATCGAGAAAAACATAATT
>dme_circ_0008367

TTCCAGATGCCGAAATACAGCAAGGCTTGGGTATGGGAATCTTGGATTTGCGACCTGGGAGGGGTGTCGA
CGGTTTCTCCACCGACTCCGGCAGCGAAGTCTTCATCATCTGACTGCTAGCTTTGCGCTCCTCTGCAATG
ATCTGCTCACAAGTGTCCATCAAAGCCTTCGAGTGAGCTGCTCTGACACTGCCAATGGGTGAGCTTGGCC
TGTTTGTGATAACATAATTTTTATTATGCTTTTCACACTTTTTCACCTCCTCCTCACTGATGTCCTGAAA
TCCAG
>dme_circ_0008367_junction_seq
TCCTCCTCACTGATGTCCTGAAATCCAGTTCCAGATGCCGAAATACAGCAAGGCTT
>dme_circ_0008368
GGCAAATAGTAGAGATCGATGATGTTCTCGCCCTGCTGCTCACTGCTCAATTGCAGTTGCATATTCTCAT
CGGGCAGGACTTCGCGCAAAACCTGCTTTACATCCTCCGCGGAGCCAATGCCGCTG
>dme_circ_0008368_junction_seq
TACATCCTCCGCGGAGCCAATGCCGCTGGGCAAATAGTAGAGATCGATGATGTTCT
>dme_circ_0008369
GGAAAATACAACAGAACAGGCGTTAACCCAGGAAATAAGCGATTTGCTAGACGACGAGATCATGAAACTG
GAGAATTGACATCCCATAATCTACACGATTGGGTGTTTAAAATTCCCCAGATTTCGGAGTGGTATGTGAT
CTCTCCAGCGCTTTTGCGACACTAGAATAGCTCAGAAAATAACCAAGCTGTTCATCGACCTTAAATGGCT
CTCTCAAATTAATGTATATCGAA
>dme_circ_0008369_junction_seq
TGGCTCTCTCAAATTAATGTATATCGAAGGAAAATACAACAGAACAGGCGTTAACC
>dme_circ_0008370
GATCAACGAAGCTCTTGAATTGGCGCTGCAAGCTATTGATCGCGAGGCGACAACCAAGCCAATGGACAAG
GCACAACATCCACAGAAGCGGGAACAGAAGGAGGAGCTGGGAGGTAAGCCGGAGGAGAAGGAGCCAATTT
GCGAGGAGCAGCACGACGAAGAGTCCAACGAGCAGGGGGAAACACACAACGAGGGTGGGGAAATGCCAAA
TAAAACCGATAAGCAAATGCAGGGGGGACGAGAGCCCATCTATGAGAAT
>dme_circ_0008370_junction_seq
GGGGGGACGAGAGCCCATCTATGAGAATGATCAACGAAGCTCTTGAATTGGCGCTG
>dme_circ_0008371
GATCAACGAAGCTCTTGAATTGGCGCTGCAAGCTATTGATCGCGAGGCGACAACCAAGCCAATGGACAAG
GCACAACATCCACAGAAGCGGGAACAGAAGGAGGAGCTGGGAGGTAAGCCGGAGGAGAAGGAGCCAATTT
GCGAGGAGCAGCACGACGAAGAGTCCAACGAGCAGGGGGAAACACACAACGAGGGTGGGGAAATGCCAAA
TAAAACCGATAAGCAAATGCAGGGGGGACGAGAGCCCATCTATGAGAATGTCAGCTCGACTATATCTATG
CATGAAGTAGATAATGAACAGATAGCAACTATGACGATAACCACACAGACCCAAGCGACGCGAATAAGTC
ACAGGAGAAGCATTCCAAGCCAGCAAACCAGCAACAGTAACGAGAATAATCAAAGCACTAATCAAACCAG
CAACAGCAGGAATCAAAAAGAGAGCAGCACAGCAGCAACATCGGCAGCGAACCCCGTGCCACCAACAACA
CCGGGGGAACAAGCAGAGCCCTACTACCAAGTGCCGAAGGCCACGGAGCCCTACTACGATGCCCCCAAGC
ATTTAAGGCCGGTGCCGGTATACGAAAATGTCGAGATCTTTTACTCGGGCCTGGAGATTAGTCAGGGATC
TGTGGGAGCGCCAGTAGGGCTGATGGAGCCACCCAAGGAAAAGCCACCACCACCGCCCACCGAGAGTCCG
ATTCCGCTGGACGAGGGCCATGACGACGAGCTGGATGGACTGGGCAACAGTCTGGGCCACAACACAGACA
CCTGGTCATCGGACAACACCTACGAGACCATATCGAACGGCACTCGCCGGCACCTTCAGGGACAACCGGA
ACCCGCCCAACCCTCGCCGCCCATCAAGAGGATGAACTCGACCAAACGGATCAAGAAGGAGTTGCGCAAC
AAGCGCTCCAGCTTCCTGGGCATCGAAAACGACGGCGATCTTGACGACATGGAGACCTATCTCGAGCTAA
CAGTGGCCCCGCCTCCGGATATGGCGCAGCTCTTGCAGGAGGAGCGGCGACTGGAGAAGCAGCTGTACAT
CAAGGCGGGACTCTGCGACAGTTCCGACACAG
>dme_circ_0008371_junction_seq

GCGGGACTCTGCGACAGTTCCGACACAGGATCAACGAAGCTCTTGAATTGGCGCTG

>dme_circ_0008372

GATCAACGAAGCTCTTGAATTGGCGCTGCAAGCTATTGATCGCGAGGCGACAACCAAGCCAATGGACAAG
GCACAACATCCACAGAAGCGGGAACAGAAGGAGGAGCTGGGAGGTAAGCCGGAGGAGAAGGAGCCAATTT
GCGAGGAGCAGCACGACGAAGAGTCCAACGAGCAGGGGGAAACACACAACGAGGGTGGGGAAATGCCAAA
TAAAACCGATAAGCAAATGCAGGGGGGACGAGAGCCCATCTATGAGAATGTCAGCTCGACTATATCTATG
CATGAAGTAGATAATGAACAGATAGCAACTATGACGATAACCACACAGACCCAAGCGACGCGAATAAGTC
ACAGGAGAAGCATTCCAAGCCAGCAAACCAGCAACAGTAACGAGAATAATCAAAGCACTAATCAAACCAG
CAACAGCAGGAATCAAAAAGAGAGCAGCACAGCAGCAACATCGGCAGCGAACCCCGTGCCACCAACAACA
CCGGGGGAACAAGCAGAGCCCTACTACCAAGTGCCGAAGGCCACGGAGCCCTACTACGATGCCCCCAAGC
ATTTAAGGCCGGTGCCGGTATACGAAAATGTCGAGATCTTTTACTCGGGCCTGGAGATTAGTCAGGGATC
TGTGGGAGCGCCAGTAGGGCTGATGGAGCCACCCAAGGAAAAGCCACCACCACCGCCCACCGAGAGTCCG
ATTCCGCTGGACGAGGGCCATGACGACGAGCTGGATGGACTGGGCAACAGTCTGGGCCACAACACAGACA
CCTGGTCATCGGACAACACCTACGAGACCATATCGAACGGCACTCGCCGGCACCTTCAGGGACAACCGGA
ACCCGCCCAACCCTCGCCGCCCATCAAGAGGATGAACTCGACCAAACGGATCAAGAAGGAGTTGCGCAAC
AAGCGCTCCAGCTTCCTGGGCATCGAAAACGACGGCGATCTTGACGACATGGAGACCTATCTCGAGCTAA
CAGTGGCCCCGCCTCCGGATATGGCGCAGCTCTTGCAGGAGGAGCGGCGACTGGAGAAGCAGCTGTACAT
CAAGGCGGGACTCTGCGACAGTTCCGACACAGGGTTAGTCGATATAAGTTTTTGCATAGCCTGTAAGAGC
AACGATGGGTGTGGAAGAAAACCGATACCAAAAGCCAACACGGCATAAAACCGAAAGACGAGAGTAATGA
GCAAATCACCAGCATGGAAAGAAGGGGGTTAGGGTGTCGACTGGGCCAGAGGGTATAGGGTGGCCCCCAT
CTACCCCGCGAGGTTAAGTATAAATAATCCGTTTAGTGTGCGTGACCCATGGAGCTGTCAAAGCTTTGAC
TGCAATTTTCTCCCAATTTTGTGTTTAAAGAACTAACGCAAACGAATCATACCGATAAAAACGTAACAAA
AGTATTTTATGAGATTATATGGCAAAATGAGTACGTTTTATATACTCATAGATGGAAGTACATAAATAGT
TATTGAGCGAAGTATGAAAAGTTTAGaaaaaaaaaCCTATAATGTCTGGACACAAATGCGGGTACTAAAC
TTTACGTTAAttttttttgttttATGGATTAGCCTTTTCCATTGCTCCTAAATATACATAGTTGACTTTT
GCTGTCAATATTAACCACACAATTTGTCTAATTCTATCGAAGGGTGAAAGCCGCGATTCTGGAGTTTCTG
AAAACCACTCTCGCCAGAGCAGTGAGCACTACACGAACTCCTCTGAGGAGAACGATACACAGTCAGAGGC
CACACCGCCGCCATTGCCTCCACCACCATCGACCGCCCAAGTGGGTGAAGTCATATACCAAAATGAGAGC
CTCCTGGCTGCCCAGACGCCGCTTCTCCAGACGGTAAAAGGAAACTCCGCCGAGTCCTGGACCGAATCAG
CAACGGCCGCAGCGGCGGCCACGCAATCATTGAGCGAAGCCACAGCAACGGCCAATGCCAAGATGCAGTC
CATCGAGGATAAGATCCGGGAGCAGGGAGAGATGTTGCGGGTGGAACGCGAGCTACTGCACTTCTCGGCA
AGAGGAACTGAAACGGCAACGTGAAAATCTTGTGCTTCGAGAAAATATTGCTCGACGAGAGTTGCAACAC
GGAGCCAAGATGCTGATGTCGAACAACCGGCGCTCACTGCAGGATCTGCACCACGGTCTAGGAATCGGAA
ACGGAATGCTCAGTGCCTTCCAGCCACCGCAACACCACCAGGTTCAAATGCCACCACCGCATCCGCAACA
GATTTACGCCAATGTGCCGCAGCAGCAGCAACAGATGGTTCTGCATGCATATCACCAGATGGACACGGAC
TACCGCAAGTCCATGTCGGATCTAAACGAGTTCTCCAACTGCCTGATACTGCCGCCAACGCCGCCCACAA
AGCCATTGAGGGCTATGCAATTGAACGCCAATGGCCATGGCCTGGAGCCAGATTACGCTGTTAGCACGAG
GCAGCGCCAACCACCGGCTCCGTACGGTGGCTCTCTGGTAAAGATTGCTGGAGCACCAATGCCGCCACGG
CTCCCCGGTCAAGGATATCCCATTCAAGCGCCGACAGCGCCAGCATACCATCACCAGTCGGCCCAAAACT
TAAGCAATATGTCCAGAAACACATTGCTCGCTTTGAGTGCCACCCCCAAACCAAAATACGCGGACGGATG
GGTGCAGGTGCAGCAGCGGAAGAGTTATGACAGCCAACAGACCAGCGATGCGGCTTGGCTGGCCGCTCAG
CAGCAAAAGCGCAAATCCATGCCTGACTACGGCGGAGCACTCTACAATAACAACCACTGGCTACTGCAGG
AGGCGGAGCAGCGACGCATCGAGCAACTTAATCGGCGATCTATACCAGCCAGCAAGTCGATGGGCAAACC
GCTGCCCGATTCCATCATACAAACTCTTACGGAGCGGGTCCAGAGCAAAGGAATCGGCGATCGAAAGCGG

CTTCGATAGCAACGGGAACTATAGCCAGGTAAATGGCAACAACATCTACCAGCAGCAACAGCAGCAGAAG
AATGTGACGAATGGCAGCAGCATCAATGGCAACAGCAGCCAGGGGCAGGAGAAGGTGCTCAGCGTTAGTG
GCAAGAAGAAGTGCTCCCATTGCGGCGACGAATTAG
>dme_circ_0008372_junction_seq
AAGTGCTCCCATTGCGGCGACGAATTAGGATCAACGAAGCTCTTGAATTGGCGCTG
>dme_circ_0008373
GTGGCGTCGCTGCTCGCGACCGTAGCCGGGAACGTCGCAATTGCCGGGTCTACATTTCGAACATTCCATA
CGACTACCGTTGGCAGGATCTGAAGGATCTGTTCCGCCGCATCGTCGGCTCCATTGAGTACGTCCAGCTG
TTCTTCGATGAGAGCGGCAAGGCTCGCGGCTGTGGCATCGTAGAGTTCAAGGATCCGGAGAACGTACAGA
AGGCCTTGGAGAAAATGAACCGCTATGAGGTGAATGGCCGCGAACTGGTGGTCAAGGAGGATCACGGCGA
GCAGCGCGATCAATACGGACGCATTGTGCGAGATGGTGGTGG
>dme_circ_0008373_junction_seq
ACGGACGCATTGTGCGAGATGGTGGTGGGTGGCGTCGCTGCTCGCGACCGTAGCCG
>dme_circ_0008374
GCGGGGCCACTGTTAGCTCGAGATAGGTCTCCATGTCGTCAAGATCGCCGTCGTTTTCGATGCCCAGGAA
GCTGGAGCGCTTGTTGCGCAACTCCTTCTTGATCCGTTTGGTCGAGTTCATCCTCTTGATGGGCGGCGAG
GGTTGGGCGGGTTCCGGTTG
>dme_circ_0008374_junction_seq
GCGGCGAGGGTTGGGCGGGTTCCGGTTGGCGGGGCCACTGTTAGCTCGAGATAGGT
>dme_circ_0008375
GGAACACGAGAGATTTGCGACGTGTGTGACACCACATTGTTCAACTACCATTGGACTTGCCGCAAGTGCG
GCTTTGGAGTGTGTCTGGACTGCGTTAAAGATCGCAAGGAGGGATTGCGACTGCGGCGAGCGGAGAACGC
TGCCCAAAAGGGATGCGATGAGTACCACTGGCTGCTCTGCAGCGACCCCAGTGGCCCACAGGAGCACGTC
CTTACCGAGCTGATGCTAACACAGATCATAGCCGGAGATGCCCTCAACGTCTTGGGCAGGCTGCTGCACG
AGGTTCGTACTTTGTGGCAGGTGCCACAGGTCTGCGGCTGCCTTCTGAGCAAGCAAGCGATTGAAGATGC
ACAATCAAAGGAAGTGATCCAGGATATGATTAAGGAGTCTCAGCTGAAGCAGCACACCAGCTACTCGTCG
CTGGCCTCCGAGCAGAAGGTGCACCAGCAACAGCGCCTCGATCAACTGCACGCCACGAAGTTGGAGTTTG
CCAGAGAGCTAGGTGTAGATTATGTTCCAG
>dme_circ_0008375_junction_seq
AGAGAGCTAGGTGTAGATTATGTTCCAGGGAACACGAGAGATTTGCGACGTGTGTG
>dme_circ_0008376
GAACTCAAAGATGATGTCCAAGCTGGCCCACAAGGCTGGTACACTAAATAGCACATTCGCTCCTGTTTGG
ACCATACTGCGAACATCGGCAGAGAAGCTCTCCACGCTCCACCTGCAGATGGTGCAAAAGCTGACTGAGC
TG
>dme_circ_0008376_junction_seq
GCAGATGGTGCAAAAGCTGACTGAGCTGGAACTCAAAGATGATGTCCAAGCTGGCC
>dme_circ_0008377
CTTGCTCTCTAGCTTCTCGGCATCCTTGTGCGAGCCGTTGTCCTTGCGCAGCTTCTCCAGCTCCTGGACC
TTGCTGGCGTACAGATCGCGCAACTTTTGCACTGCCACCGTCGACGTCTGGATGGCTTGGACACACTCAA
GAGTCTGCGATTCCTCCTCCTTGACGGATTTG
>dme_circ_0008377_junction_seq
CTGCGATTCCTCCTCCTTGACGGATTTGCTTGCTCTCTAGCTTCTCGGCATCCTTG
>dme_circ_0008378
GGTGCGCACGAAGCCGGGAGCCAGCTCGTGTCCCTTCCAACTTGTCAGCTACTGGAAGTGTGAGTCCAGC

TACACGGCCCTTAAAGTGGACTACAAGTACAATAACCACGCCATGGCCAGTGCCTCGCCCCTTCTGAATG
TCACGCTCTCGGTGCCCGTCAATGGATCTGTGCGCAATGTCCAGTCAAAGCCGCATTCTGCCTGGGTTGG
GCGAATCGAATCGTTTGGTGTGGAACTTTACGGACATCTCGCAGAATTCACAAGACGGCGGAGTGGGGAC
GCTGCGGGCACGCCTGGAGCTTAACGATGGTCCCTCTACACCAGCACTACTGGCCACTCAGTTCAACTGC
GAGGGCACCACACTTTCGGGCATCGAGTTTGAGCTCCAGGGCAGCGGCTATCGCTTGTCGCTGGTCAAAC
GGCGCTTCGTTTCTG
>dme_circ_0008378_junction_seq
TCGCTGGTCAAACGGCGCTTCGTTTCTGGGTGCGCACGAAGCCGGGAGCCAGCTCG
>dme_circ_0008379
GTTTAATTCCGTTTGTCTGACAAGCGCACCCTATATAAAAACACACAACTCGCCCATATATACACATCCA
TACTGTTTTTGCTAATAAACAGTCAACTTTCGGTGATCAGTTTCAATTCGCCACCCAGCACAGTGGTACG
AGATTCCTTGAAATGCCAGTTGGAATTTCCAAAAATTTTAACACAATCTTCATGGCTTTGTTTACAACTT
GGGCATATAAACAAATATGAATCAGTGGCGTTTCCAATAGTTACCAATCTTATCGAACTCGGCCATCCAC
TTGTCCCACTGTGCATTTAGTCAATTCAAGTTGATAGTACCCAT
>dme_circ_0008379_junction_seq
TTAGTCAATTCAAGTTGATAGTACCCATGTTTAATTCCGTTTGTCTGACAAGCGCA
>dme_circ_0008380
GAACTGGGACGAACTACGGGCAAATTGTACGGAATCGGCGGACACCTCGAGTGGCTGCAGTGCAGGCGGT
ACGCCACGACGTGGCCCTGTTTGGTAGATCGACTCTGGGGGCAACTGGGTCGTCGGCGCCAGAGACGCAG
ATGATGATGATGATGCCCCAAAAGGCGTGCGCGGCGTGACCGGAAAGGCGGGCGTTTCTGGCCTACTGGT
GGGACTAGGCGTCATCTGCTGTTGGCCATTGGTATTTGGGTTGCTCACTCTCTTCGTACTG
>dme_circ_0008380_junction_seq
ATTTGGGTTGCTCACTCTCTTCGTACTGGAACTGGGACGAACTACGGGCAAATTGT
>dme_circ_0008381
GCTGGTACTCCTATTGCCGGCAATGGCGAGGTGGACTTGGATGTCGATGTGGATGCGGAGAGATCGCGAC
GAGTGCGCGTGAGTGTCTCCTCCATGTGGTTCTGGCCATAAACGGCGCCCAGTTGCTCCAACGGCTCCAA
CTGCTGGCGTTTGTGGGTCGTTTCCAAGCT
>dme_circ_0008381_junction_seq
GCTGGCGTTTGTGGGTCGTTTCCAAGCTGCTGGTACTCCTATTGCCGGCAATGGCG
>dme_circ_0008382
GGAGGTTAAGgtgtgtgtgttgtgtgtgtgtttatgtttatgttgtTAATGCTTTAGAGTGTTTAGAGCTAC
AGACGTTACAGTTCAAAAGGCAAAAATACTACGGAAGTGGATGGACTGGTCGGTCGGTGGGCGGCTGAGA
TCACCACCCACGCACGCTGCCCTGGAAAAGTCTGGTTACGATATACGATACTTGTGTTCTCATAGCGATT
GCAATATGTTTAATTACGCACACATCTACTACGAATACATTAATAAGCCCACCAATATACATATGTAT
>dme_circ_0008382_junction_seq
TAATAAGCCCACCAATATACATATGTATGGAGGTTAAGgtgtgtgtgttgtgtgtgtg
>dme_circ_0008383
CTGTGTGATTACAAGAAAAGCGCAAAGAAAGCTCTCGCCCAGTCGAAAGTCGaatagcaatagcagtggc
atccacaacagcagaagcagcagcaacaacaacaggcagtagcaacaaccgcaggcaggagcaacaacaa
cagtgcggcagcagcatctgcagcaacaacagtgcggcagcagcatgcTGAACTCGAATGCCACCGGCGG
CAACAATCATGGGGGCAGCAGCCGTCGGCCCTTTACCCGCAATTCTTCATCGGTCCGCTCGCAACGCGGC
GGTGGTGGCGGGCTGGGCGGTCGCATGGTATACTCGCCACACACACATCCGCACGTCcagcagcagcaac
aacaacaacagcagcaacagcaacaggggcagcagcggtccagcaataacggcaacaacaacctgtggct
gggcaacagtccgtggtgcaacgtcaatttaaacggtggcaacagcggcaacaacaataacaacggtggc

aacaacaacaataacaacggtggcaacatcagcagcaacaacaacagcaacaatgtcaataacaaagATG
CAAACCCAAATTGG
>dme_circ_0008383_junction_seq
caataacaaagATGCAAACCCAAATTGGCTGTGTGATTACAAGAAAAGCGCAAAGA
>dme_circ_0008384
catacatacaatattttattgctggttttggTGGTAAATTTGCATTGTTAACGGCCGCGGCCAGACGTGC
CCCGCTTCTTTAACACACACGTCTCTCAAACGAATACCGATCATCAAGTGTTTCCCCTATTGACTCTCGT
ATATAGCAACACGTAAAAACCAATATATAGCGATACTGATACCGAAAGCAACTAAACCGAAAGTGTATAC
TAAATACATCCCCGTATGTACCACATAAGACTGCCATCTGCCCCTTGCGTTGCCGTGCCGTGCCGTTGAC
ACTTTTGTATCTGGCCATATTCGTATCTGTATCTCAAACTCACTGACCAACTACTGCAAGCGCAAATGCA
AATGTAATCTGCAAAATGCAAACTGCACACCTGAATCTCAGCGCAAGTCTTAAACGTTTAATCCTCGAAC
TGCCTACAATACCAACCAACCTATCAGCCATCCTGAGCCAATCAACCAACCAACCTATCAACGGTCTACC
GCAACTGGCAAACCAAACAGCTTTAGTTTGTAAGCTTTAATTAtttttttttttttttttGTAAACCTGT
TAATTATACAAACAATCAAAGACTGAATAATTATTTGTAATTGTCCTCTGTTCTCCTAATGGTTCCCAAA
TACGGTCGATCTCTAACCCATCAATTTAAGCTATATACTATATACGACAACACGactttgtcacactgtt
tgttttactttgcacatgttcgttttCTATTTTACGCCTAGTATTTACTATATTACCTACCTATAGAGca
cacacacacacacccacaacacaTGGCCCATGTACGTTAAGTGTAACCCTCTTTAACTGTCGCTCTGT
TGATCTGGAATCTTTCGATTGTACTAACTGCCCGCCGGGCGATAAGTGATAAGTTTGTAATCAGTCATCC
GCCCCTCGACCTCGATCCATACATATCCACCAAACCCGAAGCCGAAAGCAGAATAGAAACCGAAACCCAA
AGCGCTACACACaaaaaaaaaaaCAGGGCCACTAATATTGTTTTTGTTATGTTGACACATTCATGTAAAA
CACATAAATATGTATTAGGTCAATTGATAAGTCCTCGTCCTCACACAGAATTTGTTAGCTTTCGGTCCAT
ATCCAAAtcctcaggtctcaaaggaatgctcgctgggaagaaattaacgtcgaatgaagaagtgatcgcg
aaaagtgacgcctatttgaaagcaaaggagaaatcctactacataaatggtattgaaaaattgaaGCGAA
CTTTGTTGAATGAAAAACTTAAATTTGGCAGaaaaaaaaaTGTTTACTGTGGTAGGTCGGGGACTTTTTA
ATGGACCTGTTATGTATATGTGGGAGGGGCGGCTTAACGATGGGTTATTGTGTTTAACAACTTAAAATGG
AAACGCTGAAAGTGTAAAATTTATCGTTACCACATATCATGGCAGACGGAGTCGGCGGAGTTAGTTCACA
TATACACATACATACATATGTACTACCTATGTACCCGTAACTGTTAACTTtatatctatatctatatata
tcgtatctgtatttgtatcggCAGCTTTTCCACATACCCCCTATACATGAATTTATATACATTTATATCG
TACTTATACCTATAGTTGAAAAACTAACCACGCACGTATCTCATCTCAGTTCCATATAATTGAAAACCTA
TTAAAGCTACACTCTTTAAACAAACTGAAAAATTGTTGCTGTTTTATTTATTACGATTCCAATAAATATT
TACGTATATCCACACATAAACACACCAACACGtatatatacatatcaatatatactcacatttatatata
taaATTTTATTATCCTTGCCTAAGTAAGTATTTGGCCTACTAGAGAAACCATAAATGTTTTGTTATTGAG
CATGTTGGCTTGTTGGTTGTTTGCTTTCTGTTCTGGAGCGCGGGTCCGGCTTAGAATACTTCTTCCtaaa
caaataataataaaaaactcaaaaatgtaatatcaaataccaaaaattaaaatcCACAAACATCTAGAGC
GTAAAAAACCGTTTTCCGTTTTCGATTATAATCTGAGTTGGTGGCATCACGAACTTGAACGAACTCAACT
TTTTGATTAAAATACAAAATATCAGCTAATAGTTATTGTAACTAAATATACCTGATATGATTTATCAATT
TCTCAAGAATTCGCGGAAGTAAAGTAAATTTTCTTGAGTGATTCGGGCCTAGaaaaaaaaGCTCTTTTTA
GGACAAAAGATAATGCTATACAATAATGCTTTCATTTCTACCCTTGAGTCGCCTGTATTTGTAAAACAAA
AATGTTCAATTATGATCAGTTTTGGATGGTCCCAATCGTATTCAGATTGCAAACGGAACCGGGATGCATA
GTTAAGTGTTCAAGCGGCCACTTAATAAGCAAATCGTCTCGTTTCTAATTCCGCCCGCAGCCTGTGTGAT
TACAAGAAAAGCGCAAAGAAAGCTCTCGCCCAGTCGAAAGTCGaatagcaatagcagtggcatccacaac
agcagaagcagcagcaacaacaacaggcagtagcaacaaccgcaggcaggagcaacaacaacagtgcggc
agcagcatctgcagcaacaacagtgcggcagcagcatgcTGAACTCGAATGCCACCGGCGGCAACAATCA
TGGGGGCAGCAGCCGTCGGCCCTTTACCCGCAATTCTTCATCGGTCCGCTCGCAACGCGGCGGTGGTGGC

GGGCTGGGCGGTCGCATGGTATACTCGCCACACACACATCCGCACGTCcagcagcagcaacaacaacaac
agcagcaacagcaacaggggcagcagcggtccagcaataacggcaacaacaacctgtggctgggcaacag
tccgtggtgcaacgtcaatttaaacggtggcaacagcggcaacaacaataacaacggtggcaacaacaac
aataacaacggtggcaacatcagcagcaacaacaacagcaacaatgtcaataacaaagATGCAAACCCAA
ATTGG
>dme_circ_0008384_junction_seq
caataacaaagATGCAAACCCAAATTGGcatacatacaatattttattgctggttt
>dme_circ_0008385
ACCTAGCCCACTCATCGCCACCTCGCGACAGTTGCCAACTTCCACGCGCAACGCCTGCCGCGAGCAGTAG
AACCATCGAAGCGACCTCCCACTCGATCCAATTGGGAATTTTACACCTGGGACAAGTGGCAGCTGCTGGG
AACCAAGAGACATGTCTTCGTCGGCCATTTTCGTTCTGGACGTGAAGGGCAAGG
>dme_circ_0008385_junction_seq
ATTTTCGTTCTGGACGTGAAGGGCAAGGACCTAGCCCACTCATCGCCACCTCGCGA
>dme_circ_0008386
GACCCCCCATTGATAAAAAACACAATCAGTTTACAATGCCACCCTTAGTAGATATTACCACTTCAACAAAC
GCCGTCGTTCTAGGCGGTGGGCAGGAAGGTGAGCTTTTCGTTAAGCTGCAGCTGGGTTTTGATGAGCTGG
GAGAGCGTGATGACCAGCAGAAGGTTGCGTACGTTGGCGTTGAACATCTGAGTGAACTGCTC
>dme_circ_0008386_junction_seq
TGGCGTTGAACATCTGAGTGAACTGCTCGACCCCCCATTGATAAAAAACACAATCAG
>dme_circ_0008387
GTTACCAACTGTTTCTGTGTGCCGCACAAGGAGCACGATGACCAGGTGGAGGCAGAGCTGAGCTACGCCC
TGGACATGTACGACCTCAACCGCAAGGTGAACTCCAACGAGAGCGTGGTAGGCTGGTGGGCCACCGGCAA
CGACG
>dme_circ_0008387_junction_seq
GTAGGCTGGTGGGCCACCGGCAACGACGGTTACCAACTGTTTCTGTGTGCCGCACA
>dme_circ_0008388
GCGATTGTGTACGAATAAACGCCAAATTATGTGGTGTTGCACGTGTTTGGTTTTCACTTCACTGCTACTG
CGCGGCTGCTTTCTGATTAAATCGTTAAAAGTTAAATGAGTTCATCGTTAGCCTATCGGCGTGTGGATCG
AAATT
>dme_circ_0008388_junction_seq
TTAGCCTATCGGCGTGTGGATCGAAATTGCGATTGTGTACGAATAAACGCCAAATT
>dme_circ_0008389
CCAATTTCTTCTCGCCGCCGCCGATCATGAGCAGGTCACTCAATGGCAAGGCCATGACGATGGCTACGTG
TACTTAGATGCCCCCGCAAAAAACCAGTGTGGGCGTGATTTGGAGTTGATTCCCAATCGACACGCACGTC
GAATCTGCTTGGCGTGGCAGCTTCTAATCTCTACGGTCGCACCGCTTCCCCTCCTGCACATCAACTTTCA
CTGGCGGCTTTATTTTCAGCTCGCTGTTATGGATTTTGCGTCCGTTTTTGGTAAACTTGCGGCGCCGTCT
GCGATGATTATTCGG
>dme_circ_0008389_junction_seq
TGCGGCGCCGTCTGCGATGATTATTCGGCCAATTTCTTCTCGCCGCCGCCGATCAT
>dme_circ_0008390
GACCTGCTGGGCGTATTGGTGGTTGGAAAACTTCGCCTCCAGCGGGAAAGTGTATGTGTTCAACCAGTCC
AGAAGAGGCATGTCTAGTCCCAGTCCCAGTTGCGCAAACTGCGGGGCGTGTATGTGGCAGTCGACGAAGC
CCGGCATGAGGAACTGGTAGTCCGACAGGTGAACCTCGGTCAGACCCTTGGCGTGGGCCGGATTGCTGCT
TGCCCACGCATGGTAGTCTT

>dme_circ_0008390_junction_seq
TTGCTGCTTGCCCACGCATGGTAGTCTTGACCTGCTGGGCGTATTGGTGGTTGGAA
>dme_circ_0008391
GATCTTCCGTAACATGCTCTTGTCCAACGAATATTGATTGGGTATATGAGCAGCTTAGCTCGAAAATGGC
AGCCAACAACGGTAACAAATTCTCAATGGAGCACGATTCCGAGGGTTGTGATGAGGTGGACTTTATAGTG
GCCACGCACAACAATAACAACGATTACGAGGATTTGGGCAGCGTGAGTCAGGCGGTGATCAACACCAAAG
TAGCTgcagcagcagcaaccgcagcagcagcaACACCAAACAACGAACCAAACAGCAATACATTGAAAAA
AGCCAAGGAGCGTCGCACCCTCTTCCATTTCGGAAGCAATAAGAAGCTGAGTCAGAGCAAGTCACAGGAG
AGCCAGGAGGCGGGCAGCAAGGATGCTACGCCGGCGACAACTGCTGCTCCATTGCCGCCGGTGCCAATCG
GTACGCCACCGCGACAGCACAAGTTCGTGAAGAGCAACAGCTTGGCCAGATTGCTGGGCAACACCTACAA
TGCCAAGAAGTTTGAGAAGCAGGAGCAAAAGCGTCTGGCCTCCGGATCCGAGGGCGGCAAATTCAACACC
TACAGTGGAAGGCGTGGCCGTGCGGGTCCCTATTTGGAGCGATTCAAGCGGGTGTCCAAGGAGGACGGCG
ATGTGGCTGGCGAGGATGACACGGTGAGGGTCACGAACGTCATAACCCTGACGACGGACTCGCGGGACTT
GCTCTACGGCAGCCGGCAGGAGCATGTGGGTCGCACTGGGGGCTATGACCAGAACGATCAGCTCACCTCG
AAGGCGTATCGCACGCTCACCCGCAGCTTGGGCAAACTCTGGAGGCGCACACACAGCGTGGACATTAGCA
CACCCGATCCGGAGTTCAAGGTCTCGTACCTGGGCAACGTCCTGACCGGTTGGGCCAAGGGCAGGTGAGG
GTTGTGTGGAGAAACAGCTGAATACGCTGTGGCGGAACTACACCCAGCACTCCAAGCCGGACGTGATCAT
GCGCCTGAAGGTGTGCGCCTCCGGCTTGAAGGCCACCACCCGGCAGCACGGACTCACGGAGTACTGGGCC
CATAGGATCACCTACTGTTGCGCACCGAAGAACTATCCGCGGGTCTTCTGCTGGATCTACCGCCACGAGG
GCAGGAAGCTGAAGCACGAGCTCCGCTGCCATGCGGTGCTCTGCAGCAAGGAGAAGATTGCCCAGGACAT
TTGCGATACCCTTAGG
>dme_circ_0008391_junction_seq
TGCCCAGGACATTTGCGATACCCTTAGGGATCTTCCGTAACATGCTCTTGTCCAAC
>dme_circ_0008392
GATCGAGGACACCAATGAGCCGGAGATGATGCCGTGCTGCCAGAAGGACTCCCTCTATCCGGCCATGACG
CTGGGCAGGCGTCGCTGTCGTCGCGGCCACTCCATTCGGCGAACGGGCAAGATACAGTCCTTCTCGCCCT
GCTGCAGTTCGCACATGGCAAAGGAGTTGCCGCAGGAAGAGACCAAGACGATGGCGGCGGCGGGCAGTTC
CGCCAATGATGGCTCCGATTCAGACGACTTCGAGAAGCTGCTTAAGTTCGATACGACTTTGAGTAATGAG
TTGTTGCC
>dme_circ_0008392_junction_seq
ATACGACTTTGAGTAATGAGTTGTTGCCGATCGAGGACACCAATGAGCCGGAGATG
>dme_circ_0008393
GTTCCAGGAGAAGGAGCGACCGGGTCAGCAGCCGCTGATCAGCGAGGCAAGCGTTACGCTGGCCAAGATT
GCGACCGTCAAGCCGCAGGTACCACCAAAGCCGGCTCCACGTCGCTCGCTCAGTCTCAACGCGGTGGCCA
CCGGCGCCTCAGCGGGCTCATCCGCTGGTAAGGCTCCGGGCACCGCTGTCTGAGCGAAATGCTTTAATTA
CAACGGAAACCC
>dme_circ_0008393_junction_seq
CGAAATGCTTTAATTACAACGGAAACCCGTTCCAGGAGAAGGAGCGACCGGGTCAG
>dme_circ_0008394
GTCAAGTCCGGCACTATCTTCGACAACGTCCTGATCACGGACGACGTGGAGCTGGCCGCTAAGGCTGCTG
CCGAAGTCAAGAACACCCAGGCGGGCGAGAAGAAAATGAAGGAGGCCCAGGATGAGGTGCAGCGCAAGAA
GGACGAGGAGGAGGCGAAAAAGGCTTCCGACAAGgacgatgaggacgaggatgatgacgatgaggagaag
gatgatgaATCTAAGCAAGACAAAGATCAAAGCGAACACGACGAATTGTAAGGACGTCTAGGGAAAATTA
CG

>dme_circ_0008394_junction_seq
ATTGTAAGGACGTCTAGGGAAAATTACGGTCAAGTCCGGCACTATCTTCGACAACG
>dme_circ_0008395
GATtcatcatccttctcctcatcgtcatcatcctcgtcctcatcgtcCTTGTCGGAAGCCTTTTTCGCCT
CCTCCTCGTCCTTCTTGCGCTGCACCTCATCCTGGGCCTCCTTCATTTTCTTCTCGCCCGCCTGGGTGTT
CTTGACTTCGGCAGCAGCCTTAGCGGCCAGCTCCACGTCGTCCGTGATCAGGACGTTGTCGAAGATAGTG
CCGGACTTGACCTGCCAGAGATCGAAGCCCAGGGTGCAGATCTCCTTGCGCAGATACAGCTTGTCGTCGG
GCACATACTCGGGATTGGCGATCTCGGGATGCTCCCAGGCGCCCTTGTAGTTGGGGTTGTCCAGCTGCTT
TGGCTGCCACTCGCCCTTGAACTCAGGGTTGTCGATCATCGGTGGCTCCCACTCGCCGTCCATCTCATCG
TCCCAATCCTCAGGCTTTGTGGCATCTGGGTCGGGAATGTGCTCTGGTTTGTCCCAGTCCTCGGGCTTCT
TGTCATCGGGATCGGGAATGGTGGCCTACAGAGATTTACGTTGTTGTTTAATAACAATGAGCCAAACCGG
TTTTTCGATGGGCTAAAGTCTGATTTGGGCGGGGATTTACAACTTACCCTATCGTCCCAGTCTTCGGGCT
TGGTGGCGGTGGGATCCTTGATCTTCTTGGGGGCCAGGAAGTCCCAGTCGTCCTCCAGGTTGCCAGACTC
CACCTTCTCGTTGTCGATGAGCACCTCGTACGTGTTGTCGGGTCTGACAATCAGCGTGTAGAAGTGGGTG
TACACGTCATCTTTGCAGCGGATGTCTTTGCTGATTAGGTGGTTCTTGCCCTTGTAGCTGAAGATCACAT
GGACCTTCTTGGTGCCGGGTCCGCAGATGTCCGGGCCGAACATGATCTCGTACGGCGACTCGCCGTGCAT
GTCGGTCTGGTCGAGGGAGCAGTCGAACAGCTTCACATAGCCGCCGCCGCAGTCGATATTCTGCTCGTGC
TTCACGGAGAACTGCACCACGAGGGGCTTGTCCTCGTTGGAGAAGCCATCAAACTTGCGGCTGGCCGCGT
AGAATCGAGCATCCTGAGAGGTCTGAATGCCTATATAATGTGTGTCCACATCAATACGAATGCGCGCCGC
GCGGAGGTGGGGGGCGAGAAAAAATGTGTATACAGTTACTATAGCTACTCGAAAACGTACACGTAGTGGT
TTttcttcttcttcctcttcttcttcGTGCCCATGTTCTCCGCATACACGGGCACATACAAATGCAGATA
ACACGCCGACGAAAAATAAAAATGGCAGAACGAGCGAAGAGAAAGATTTGGAAAAGGGAGTGACGACACA
TCGACACATATACGCTACCTTTGTCGGCCTCAGCATCGTTGTAGAAGGTACCGGGGGTGAGGACGAATTT
TCCGAACTCCTTGCCAGGATGTTTGCTGTAGATCCACGTATCCTCCCAGTTTTCTACGGGAACAAGCGGG
GGAGTGCTTTTATTATTCTTCTATTTTTATTGTCTCTCCTTTCGTTATTCATTGAAGGGGAGCGGAGGAC
CACATGGCGGCCATAGAACGCGATGACTACCACTTACCGTTGTCGAAATTCTCCTTCAGATAAACCTCGG
CACTAATAAAGCCGACTGTCGCCAGCAACACTATCACTGTTTTGCACCACATCAT
>dme_circ_0008395_junction_seq
ACACTATCACTGTTTTGCACCACATCATGATtcatcatccttctcctcatcgtcat
>dme_circ_0008396
GCTGCTTTGGCTGCCACTCGCCCTTGAACTCAGGGTTGTCGATCATCGGTGGCTCCCACTCGCCGTCCAT
CTCATCGTCCCAATCCTCAGGCTTTGTGGCATCTGGGTCGGGAATGTGCTCTGGTTTGTCCCA
>dme_circ_0008396_junction_seq
GGTCGGGAATGTGCTCTGGTTTGTCCCAGCTGCTTTGGCTGCCACTCGCCCTTGAA
>dme_circ_0008397
GTCCATGTGATCTTCAGCTACAAGGGCAAGAACCACCTAATCAGCAAAGACATCCGCTGCAAAGATGACG
TGTACACCCACTTCTACACGCTGATTGTCAGACCCGACAACACGTACGAGGTGCTCATCGACAACGAGAA
GG
>dme_circ_0008397_junction_seq
TACGAGGTGCTCATCGACAACGAGAAGGGTCCATGTGATCTTCAGCTACAAGGGCA
>dme_circ_0008398
GGGGAGCGGAGGACCACATGGCGGCCATAGAACGCGATGACTACCACTTACCGTTGTCGAAATTCTCCTT
CAGATAAACCTCGGCACTAATAAAGCCGACTGTCGCCAGCAACACTATCACTGTTTTGCACCACATCATG
TGAACGATCCCGCACCACTTTTTGGACTGACTCTTCCAACTCCGATGGTTGTCCGAACTCGGTTAAAGTT

AAGTACTAAATGCCGGCAAGAAAGTGCTATTTATTTATTGCATCTAAATCGTGCGTGTCGGAGCGGGACG
GCTTGTTACTGAAATTGGTTTGCTGGAACGTTTTCATTGGCCCAAATCAGGCCAATCAGGGATGTCTACG
TATTCTTTTTCACCGGCCAGGGCTGATTTCGATAAGCAATCGCATGCTGCCCTGTCGCTATCGATTGCTT
GAGCTGGAATTAGAAACACTAAAGTGACCGTTTGCtaattaaattaaattataaaatatataaaattaat
atattCTGGCATACAATTAATCACTTGCAGCAGGCTAAGCTAAGAAAACATAAAATTACTCCAATAACAA
TTCTTCTACTACTTAACAATTGTAGTAGTTACAGGAAGAACAGTTGTGCTTCCAGAACATTCCTAAGCTT
TTTAACGGCTTAAGGGTTTTGAAAATTTTAACTCACACTTACATCACATGTTAACAATTAAGTTTTCACC
CGATAAAGTTCATAGTATTTAAAAAGTTAGAATGTTAATTATACATTATAATACAAATCAAAAACAAATA
AAGGAATTCCAAAACTTTGCACCTGTATTTCGTTAATTTTAGGAACTTTTTTATCTATAAGTGCTAACTT
TTACATTTGACTTTATTACATTAAAAAATTACAATTTTCGCTGGAAATGTattatattaataaaaaagat
aaatagccagaaatagaaaagtgaaattaaattaattaaaaCAAAAAAGCTTTGGTATTTATACCAATAG
ACCTTCTAAGGTATTTTTTAAGACCTAGCTTTGTGGCAGATACGGTAAGTTCGTGTCGAGCCCACGTGTT
GAGCCAATCTTATCAAACGCCGATTCGAATGCGGCATTAAACAAATGGATTCCCTTAACAACGGATGGTT
CAGCGAACTGCAGGCCGATCTCTGGCCCGGTCAGTCATTCTCCCTGAAAGTCAAGGAGGTCATCCACAAG
GAGAAGTCCCGGTTCCAAGACATCCAGATCGTTGAAAC
>dme_circ_0008398_junction_seq
GGTTCCAAGACATCCAGATCGTTGAAACGGGGAGCGGAGGACCACATGGCGGCCAT
>dme_circ_0008399
TTGAATGGCATCAACACCCAGGGGGAGAATATCGCCGACAATGGTGGCGTCAAGGAGTCATACATCGCCT
ACCGCAGATGGGCTGAGAAGCACGGACCGGAAGCAAAGCTTCCGGGATTGGACTACACCCCGGAGCAGAT
GTTCTGGGTGGCAGCCGGACAGACTTGGTGTGCTAAG
>dme_circ_0008399_junction_seq
GGCAGCCGGACAGACTTGGTGTGCTAAGTTGAATGGCATCAACACCCAGGGGGAGA
>dme_circ_0008400
GAGTTACGGAAAAAACCTTCACCTTCAAGGGCGTAAACGTGCTGGATCCGTCGAAGAAGTACGAGGTCAC
ACTGAACTTCCTCCACGAGGTGGATCCCGAGAAGGTGACCAGCAAGAACATTGGCCGCTGCCTGGAATTC
ACAATACCCAAGAAGGCGGCCGGTCCCTACTGGTCCTCGCTGACCACGGACAAGACCAAGTTGCATTTCC
TAAAAGCCAACTTTGCCAAGTGGCGCGATGAGTCCGACGACGAGGAGG
>dme_circ_0008400_junction_seq
TGGCGCGATGAGTCCGACGACGAGGAGGGAGTTACGGAAAAAACCTTCACCTTCAA
>dme_circ_0008401
GGTGACCAGCAAGAACATTGGCCGCTGCCTGGAATTCACAATACCCAAGAAGGCGGCCGGTCCCTACTGG
TCCTCGCTGACCACGGACAAGACCAAGTTGCATTTCCTAAAAGCCAACTTTGCCAAGTGGCGCGATGAGT
CCGACGACGAGGAGG
>dme_circ_0008401_junction_seq
TGGCGCGATGAGTCCGACGACGAGGAGGGGTGACCAGCAAGAACATTGGCCGCTGC
>dme_circ_0008402
GGTGACCAAAAAGACAACAGCATGTTTGGAAATTTCCTTAACAGCCCTGGTGGCGATTGGAACAACAAGT
TCGACGATTTCAACGTCGATGACGAGGAGGAGGACTCGGATGACAACATCCCAAGTCTGTCCCAGAACGA
CGAGGATGACGAGGAGGGCGGCGAGG
>dme_circ_0008402_junction_seq
GACGAGGATGACGAGGAGGGCGGCGAGGGGTGACCAAAAAGACAACAGCATGTTTG
>dme_circ_0008403
GGTGACCAAAAAGACAACAGCATGTTTGGAAATTTCCTTAACAGCCCTGGTGGCGATTGGAACAACAAGT

TCGACGATTTCAACGTCGATGACGAGGAGGAGGACTCGGATGACAACATCCCAAGTCTGTCCCAGAACGA
CGAGGATGACGAGGAGGGCGGCGAGGGTGATAAGGAGAAGAAGCCAGCTGCCTAGACGGTCGCTCAGATG
GTGTCTCGCAATTTGGCGTAGTCGTAGCGTTAGGCGTGGCTCTTATACCATAAGTCAGCACACGATCACA
ACACTCACTCCTCACATACACACCAGAAAAGAAAAAGAAGAAACACTCAATCACTCATAAAACATCCACA
AACGCATGCGTACATGAAACACTATTCCAATCGAAGAAAACCACATTTAATATACCTGAATATTATGCGT
ATTCAGAGGAGAGAT
>dme_circ_0008403_junction_seq
GAATATTATGCGTATTCAGAGGAGAGATGGTGACCAAAAAGACAACAGCATGTTTG
>dme_circ_0008404
CTTTCCTCTGGTTGTTAAATTCTAGCGTAAAACTTATGGTTTCCCTTGTCGGCGCCAAATCATCTTGTTC
CTTGTGTTGCTTTTTGTTAACATCTCTCCTCTGAATACGCATAATATTCAGGTATATTAAATGTGGTTTT
CTTCGATTGGAATAGTG
>dme_circ_0008404_junction_seq
AATGTGGTTTTCTTCGATTGGAATAGTGCTTTCCTCTGGTTGTTAAATTCTAGCGT
>dme_circ_0008405
TGCCCTTGCTGCGCCGCTACTTCAACTGGGAGCTCCAGCCGCTCCATAGTGTGACTGTGGGTGGGATCTT
CTCCGCCGGCGCCTTTTTCTGTGCAGGAGCCTTGCAGGAACGAATAAAGGTAAGCAGGAATAATAAGATA
CCTCACCTGATCGTTTATTAACCCGAAATATTCGAAGAATTCCCCGCCCCAAACAGTGAATATAGCTTGG
CAACTGCCCCAGTTCCTGCTGCTCATGATGGGCGAATTACTGCTTTCCATTCCGGG
>dme_circ_0008405_junction_seq
TGGGCGAATTACTGCTTTCCATTCCGGGTGCCCTTGCTGCGCCGCTACTTCAACTG
>dme_circ_0008406
TTAACTACAAGGTAGAGCTGTATGATCCGCGCTCTAATGGATTCATGCCCTCGTCGCCCGGAATCGGCAT
GCATGTGGAAGTACGCGACAGCGACGACAAGATTGTGCTGTCCCGCGTGTACAGCTCACAGGGCCGCATC
TCGTTCACCTCGCACACTCCTGGCGAGCACGTCATCTGCATGTTCTCGAACAGCACCGCGTGGTTCAGTG
GTGCCCAGCTGCGTGTTCACCTGGACATCCAGGTGGGAGAGCACGCTATCGACTACGCCCATGTGGCGCA
GAAGGAGAAACTGACTGAGCTGCAGCTGCGCATCCGCCAGCTACTTGACCAGGTGGAGCAGATCACCAAG
GAGCAGAACTACCAGCGATACCGCGAGGAGCGGTTCCGTCACACCAGCGAGAGCACCAACTCCCGCGTGC
TCTGGTGGTCGCTGGCCCAGACCGTCGTCTTGGTTTGCATGGGCTTCTGGCATCTATTTAACCTGTTCCA
CACTTGTGTGTACATTAACGAGCAGTGTTTGTGTGTGTGCTGTCTTTATATAAAGAAAATATCTGCCCTT
GGCCTTATCAATGATAAGATTAATGGCTTGCCAGACCCGTGAGTCAGACTACAAACGACTTACGGCCATG
TCGGGGTGTGTAGAAGAGTTGGCCTGGTTGTGACTGTCAATACAAGTGCTTACATAGGCACACAAGTGTG
GGGGAAACAACGCACACACACATACAACGAATCTCGCAACATACGATATACGATAAGTTGTGATGTATAT
ATTAATATATATATCCATTCCGAAACATCCACAAACAGACAAGACCAGACCGATATACCCTAGCACATTG
GGAACGGAGCTTCGAACTGAGCTCGGTAAAACGGGGCAAGGTTGGGGCATTATCGCATCACATGCATATG
ACATCAGCGCAACACTGCGTATACGTAATCTGGCGATAGTACTGATTGAATGAATTTAATGTCAGGCTCT
ACTAACCGTAGTTCTCCCTTCGTTTTCGTCCCCTTTTCCCCGCAAACAGGTAAACAAAAAATCTACTGTG
CGAAATGCACGAAGAAGTGCTCCGGCGAGGTGCTCCGCGTGGCGGACAACCACTTCCACAAGGCCTGCTT
CCAGTGCTGCCAGTGCAAGAAGTCCCTGGCCACCGGCGGATTCTTCACAAAGGACAACGCCTACTATTGC
ATACCGGACTATCAGCGGCTGTACGGCACCAAGTGCGCAAATTGCCAGCAGTATGTGGAGGGCGAGGTGG
TCAGCACCATGGGCAAGACCTATCACCAGAAGTGCTTCACCTGCTCCAAGTGCAAGCAGCCCTTCAAGTC
GGGCAGTAAGGGTGACCAACACCGGAAAGGAGGTGCTTTGCGAGCAATGCGTCACGGGTGCTCCAGTGTC
GCCCAGTCGCCAGGCGACGGGTGGAGGCGTCTCCTCGCCAGCTCCTCCGGCGGAGAGTCCGACGAGAGCC
ACTGCCCACCAGCAGCACGGCAGTGTGATCTCTCATAAGGCGCACCTCAAGGAGGACTACGATCCCAATG

ACTGTGCCGGCTGTGGGGAGCTGCTGAAGGAGGGCCAGGCACTGGTGGCGTTGGACCGGCAGTGGCATGT
CTCTTGTTTCCGGTGCAAGGCCTGCCAGGCGGTGCTCAATGGGGAGTACATGGGCAAAGACGCGGTGCCC
TACTGCGAGAAGTGCTACCAGAAGGGATTCGGGGTGAAGTGTGCCTACTGCAGTCGCTTCATTAGCGGCA
AGGTGCTCCAGGCGGGCGACAACCACCACTTCCATCCGACCTGTGCCCGCTGCACAAAGTGCGGCGATCC
CTTCGGCGACGGCGAGGAGATGTACCTGCAGGGCAGTGCCATCTGGCATCCGCGATGCGGTCCGGGTCCC
TCCGAGTCCGGCATAATTTTGAACGGCGGCGGAGGCACTTCGTCGGTGGTCGGAGGTGCCTCCAATGGCA
ACTTCACAGACACTGAATGCGACCGGATGAGCTCCAGTGCCCTTAGCGAGATGGGTAAGTAGTCGGATCT
ACAGATGATAAACCGCTGGTACTGAGATAATTCTCGAGACTCCAACACAACAATCCCGAGTTTAAAGAGG
CTGCTGAGCTTGCAACTAATTTGAATGCTGCAAAATTCACGAAAGGCACACGCATTTAAAATCAAATTTC
ATACTAGAATGGGTGATATTTTAGAACTATTTTAGAACCTTATATATTATATTTATATAGGCACCCTGAA
AATATCACCCATCTCAATTTACCACCCCCTTTTTGCACCTACACACAGCACTTATGATTTTATAATTTCA
GTTACTTGCATTTTAGTTGTTTTGAGCTTGCGTTTTTATTTTTTCGTTCGTTTTCAGTACATCCGCTCCA
GAACTCCGAGCTTTAATGGTTCACTTTATTCCTCTAGCCGCAAGGTTTGTTTTATAATTTTACTTTATGA
TTTCCCCTCATTTTCTGCCATCGTTTTTGAAAATACTAAAACCGAAAAGAAAAACACACGGCACTGCATG
TATTTAAACTTTGAAATGAGAGCCCAGGACTAATCCCACTTAATTTGCAGCCACTACCGAACGGTGAGTC
CGGGTCTGATACTCCGGGAGTACGGACGACCCAATGCCGAGGACATCTCGCGCATCTACACCTACAGCTA
TCTGACGGATGCGCCGCACTATCTGCGCAAGCCGATCGATCCGTATGACAAGACGCCGCTGTCGCCGCAC
TTCCATCGGCCCTCCTCATACGCGACCACCGCCAGCAATGCGGGATCCGTGGCCGGAAGCCGCCCGCCGT
CGCGTCCCCACTCGCGGACCCGCAGCGCCATGAAGGTGCTGGTGGACGCCATCCGCTCGGAGACGCCGCG
TCCCAAGAGTCCTGGCATGAACAACGAGGAGCCGATTGAACTGTCTCACTATCCGGCCGCCAAGAAGCCG
CCGCCAGGTGAGCAGCCCAAGATCGAGCGGGATGACTTTCCCGCTCCGCCGTATCCCTACACGGATCCGG
AGCGTAGGCGGCGATACAGTGATACCTACAAGGGAGTGCCCGCCTCCGATGATGAGGACGAGAATGTGGA
GAATGGCAAGCCGAATGGCAAGGTCAAGAATGGCGAGGAGCAGCAGACGCCTTCAACGCGAAGCCGAACA
GCTGGAAAAGCTTAACTCGGGCATCGGATCGGCGATTGCCAAGGACCTCAAGGAGCACGCCAAGTACAGG
AAGTGGAAGCAGAACAATTTGGATCCGCGCAACGCATCCAGGACGCCCTCCGCTTCCAAGGAGCCACTCT
ACAAATTGAGGATACGAATCGCCCATTGGAGCATCGCCATCGCGCAATCTGGACCACCAGAAGCCCTTCT
ACGAGGACGAGATGTTCGATCGCTCCACCAGCTACCGTGGCTCGCTTGGCAAATCGCTTGGAAACGCGCC
CAGCTACAATGGGTAAGTGCTGCCTGTTGCtcagatcggcatagatcgcatcagatccgatccgatccga
tcagatcagattatatcCTTGGTGCCGGGCTGTCGGTTATGTAGCTTATAGCTTACCCACTACCACACGA
ACCTATCCGCTTATATGTAATCCCAAAATAGTTACGACGGTATTTGTTTCTTGTTTCTCTCACTTTCCAA
TGCCGCCACTGGTTTGCTCACCTGGATGAACTAACAACTGTTTCGCATATGTCTCAACTGGTTGGTCTGC
TGCTTCATTGCGTGTCTGAAAACCCAATCCCACCCATCCACCCATCCTGCGCCACTCCTAACAACTATGT
GTTTGCTATTGTTGCCCTGACACCTGACAACAAATAACTCAAAAAACTAACACCCATCCACACTTGACCA
ACTAAAACTCGAACATTTGCATCATGAACATATCAACATCACTTGAACACCATCAACATGTACATCTACA
TCCACATCTGCTCAGTGGTGAGTGCGCTGCGCCATGTGCCCAAGCCAGGATACGGGCTAGCACCACGCTC
CCACACATTCAGCTCCACCACATCCGCTGCGGCCACGATGCATGGTGCCACTGTAAGACCGCTTTCGTTG
TTGCTATGCCGTAGAGTCCTTTGAGTCCCCGTCTTGTGTTTGGATGCCCGTTGGTATCACCTGTAACTTG
CACTGGTTTTTCGTTTATTATTGTTGCATGTGTCCCTCAATTTGATCAGAAAACTAATCCCATGCCCTTT
TCGAAAACAGGCTTCTGTGGACTCGATCACCGAGGGCGATAGGCGAGCTCTGATGGGCGGCGACCTGCCC
GCCTCGAGCACTTACTCAGGAGCACTCTCCTACCACTATCCGCAGGCCGGACTCATCCGGCGCAGTCTGC
CCAACATGTCCCATTCGATAATTAGTTGCGCTAACGCAAAATTGTAACTGAGAACAACTATATGAAAGGA
ACATAAAATCCCAATAAAAAACGAAGTGTATACAGACAGATGATATATGAAATTAGTTAAACATTGCATG
TCCCCGGCATGTAGGCTTTTTATACGCAGTGCTATGGCAATTTAAACGCAAACATAAACTGGACTCTTTT
TCCAAATTACACCTTAATTCAACCATGTAACGGGCTACAGAACCGAGAAATGCATCAAAGAATAAGAAAT

TAATGTACCTACAGCAGAATCACACAGCAAAATGCGAAACATTAGATGTGTGGCATCAAATTTACGCTAA
ATCATTACGCAAattttatttattactaaatattttatttatttatttaaATAGAATATGTCAAAGAATA
AACCAGAGATAATAAAACAGGACAAAAGCAACACTCTGACTTTGATCTGGCACTTTTTCTCATGACGTGT
CGGCGAGCTTAACTTTATTAAAGAACTACTCGAAAAAGTCACTGCAAATAGCCGAAAATGCGCGACCAAT
TCATCAGCCTGGCCCTGATTCTGTGCGTCCTGCACAGCGCCTGCGGCTTGTACTTCCACATATCGGAGAC
GGAGCGCAAGTGCTTCATCGAGGAGGTTCCCGACGAGACAACTGTGATTGG
>dme_circ_0008406_junction_seq
AGGTTCCCGACGAGACAACTGTGATTGGTTAACTACAAGGTAGAGCTGTATGATCC
>dme_circ_0008407
GTAAACAAAAAATCTACTGTGCGAAATGCACGAAGAAGTGCTCCGGCGAGGTGCTCCGCGTGGCGGACAA
CCACTTCCACAAGGCCTGCTTCCAGTGCTGCCAGTGCAAGAAGTCCCTGGCCACCGGCGGATTCTTCACA
AAGGACAACGCCTACTATTGCATACCGGACTATCAGCGGCTGTACGGCACCAAGTGCGCAAATTGCCAGC
AGTATGTGGAGGGCGAGGTGGTCAGCACCATGGGCAAGACCTATCACCAGAAGTGCTTCACCTGCTCCAA
GTGCAAGCAGCCCTTCAAGTCGGGCAGTAAGGGTGACCAACACCGGAAAGGAGGTGCTTTGCGAGCAATG
CGTCACGGGTGCTCCAGTGTCGCCCAGTCGCCAGGCGACGGGTGGAGGCGTCTCCTCGCCAGCTCCTCCG
GCGGAGAGTCCGACGAGAGCCACTGCCCACCAGCAGCACGGCAGTGTGATCTCTCATAAGGCGCACCTCA
AGGAGGACTACGATCCCAATGACTGTGCCGGCTGTGGGGAGCTGCTGAAGGAGGGCCAGGCACTGGTGGC
GTTGGACCGGCAGTGGCATGTCTCTTGTTTCCGGTGCAAGGCCTGCCAGGCGGTGCTCAATGGGGAGTAC
ATGGGCAAAGACGCGGTGCCCTACTGCGAGAAGTGCTACCAGAAGGGATTCGGGGTGAAGTGTGCCTACT
GCAGTCGCTTCATTAGCGGCAAGGTGCTCCAGGCGGGCGACAACCACCACTTCCATCCGACCTGTGCCCG
CTGCACAAAGTGCGGCGATCCCTTCGGCGACGGCGAGGAGATGTACCTGCAGGGCAGTGCCATCTGGCAT
CCGCGATGCGGTCCGGGTCCCTCCGAGTCCGGCATAATTTTGAACGGCGGCGGAGGCACTTCGTCGGTGG
TCGGAGGTGCCTCCAATGGCAACTTCACAGACACTGAATGCGACCGGATGAGCTCCAGTGCCCTTAGCGA
GATGGGTAAGTAGTCGGATCTACAGATGATAAACCGCTGGTACTGAGATAATTCTCGAGACTCCAACACA
ACAATCCCGAGTTTAAAGAGGCTGCTGAGCTTGCAACTAATTTGAATGCTGCAAAATTCACGAAAGGCAC
ACGCATTTAAAATCAAATTTCATACTAGAATGGGTGATATTTTAGAACTATTTTAGAACCTTATATATTA
TATTTATATAGGCACCCTGAAAATATCACCCATCTCAATTTACCACCCCCTTTTTGCACCTACACACAGC
ACTTATGATTTTATAATTTCAGTTACTTGCAttttagttgttttgagcttgcgtttttattttttcgttc
gttttcaGTCACTACCGAACGGTGAGTCCGGGTCTGATACTCCGGGAGTACGGACGACCCAATGCCGAGG
ACATCTCGCGCATCTACACCTACAGCTATCTGACGGATGCGCCGCACTATCTGCGCAAGCCGATCGATCC
GTATGACAAGACGCCGCTGTCGCCGCACTTCCATCGGCCCTCCTCATACGCGACCACCGCCAGCAATGCG
GGATCCGTGGCCGGAAGCCGCCCGCCGTCGCGTCCCCACTCGCGGACCCGCAGCGCCATGAAGGTGCTGG
TGGACGCCATCCGCTCGGAGACGCCGCGTCCCAAGAGTCCTGGCATGAACAACGAGGAGCCGATTGAACT
GTCTCACTATCCGGCCGCCAAGAAGCCGCCGCCAGGTGAGCAGCCCAAGATCGAGCGGGATGACTTTCCC
GCTCCGCCGTATCCCTACACGGATCCGGAGCGTAGGCGGCGATACAGTGATACCTACAAGGGAGTGCCCG
CCTCCGATGATGAGGACGAGAATGTGGAGAATGGCAAGCCGAATGGCAAGGTCAAGAATGGCGAGGAGCA
GCAGACGCCTTCAACGCGAAGCCGAACAGCTGGAAAAGCTTAACTCGGGCATCGGATCGGCGATTGCCAA
GGACCTCAAGGAGCACGCCAAGTACAGGAAGTGGAAGCAGAACAATTTGGATCCGCGCAACGCATCCAGG
ACGCCCTCCGCTTCCAAGGAGCCACTCTACAAATTGAGGGTAAGATATTGATCGTTATAAGAAAATTGAA
AAATGATGATAATGAAAATTGATCGTTATAAGAAAATTGAAAAATGTATCTTCTAGTTCGTGCTCGGTGT
ATTTTGCACTTGAAGAAATCTATTATTATTTGTTTTAAAAATAGAACGATAAGATAGCAAAACATATGGT
TATAGTATATAGTATAGTGTAGTAATACTAATCCTATGACATCTTGATTACACAGAATACGAATCGCCCA
TTGGAGCATCGCCATCGCGCAATCTGGACCACCAGAAGCCCTTCTACGAGGACGAGATGTTCGATCGCTC
CACCAGCTACCGTGGCTCGCTTGGCAAATCGCTTGGAAACGCGCCCAGCTACAATGGGTAAGTGCTGCCT

GTTGCtcagatcggcatagatcgcatcagatccgatccgatccgatcagatcagattatatcCTTGGTGC
CGGGCTGTCGGTTATGTAGCTTATAGCTTACCCACTACCACACGAACCTATCCGCTTATATGTAATCCCA
AAATAGTTACGACGGTATTTGTTTCTTGTTTCTCTCACTTTCCAATGCCGCCACTGGTTTGCTCACCTGG
ATGAACTAACAACTGTTTCGCATATGTCTCAACTGGTTGGTCTGCTGCTTCATTGCGTGTCTGAAAACCC
AATCCCACCCATCCACCCATCCTGCGCCACTCCTAACAACTATGTGTTTGCTATTGTTGCCCTGACACCT
GACAACAAATAACTCAAAAAACTAACACCCATCCACACTTGACCAACTAAAACTCGAACATTTGCATCAT
GAACATATCAACATCACTTGAACACCATCAACATGTACATCTACATCCACATCTGCTCAGTGGTGAGTGC
GCTGCGCCATGTGCCCAAGCCAGGATACGGGCTAGCACCACGCTCCCACACATTCAGCTCCACCACATCC
GCTGCGGCCACGATGCATGGTGCCACTGTAAGACCGCTTTCGTTGTTGCTATGCCGTAGAGTCCTTTGAG
TCCCCGTCTTGTGTTTGGATGCCCGTTGGTATCACCTGTAACTTGCACTGGTTTTTCGTTTATTATTGTT
GCATGTGTCCCTCAATTTGATCAGAAAACTAATCCCATGCCCTTTTCGAAAACAGGCTTCTGTGGACTCG
ATCACCGAGGGCGATAGGCGAGCTCTGATGGGCGGCGACCTGCCCGCCTCGAGCACTTACTCAGGAGCAC
TCTCCTACCACTATCCGCAGGCCGGACTCATCCGGCGCAGTCTGCCCAACATGTCCCATTCGATGCTGGT
GCACGAGCCGGCCAAGATCTATCCCTATCACCTACTGCTAATCACAAACTACCGTCTGCCCTCGGACGTG
GATCGCTGTAACCTAGAGGGCTGGTGCACGAGCCGGCCAAGATCTATCCCTATCACCTACTGCTAATCAC
AAACTACCGTCTGCCCTCGGACGTGGATCGCTGTAACCTAGAGGTAAGTTATGAAGCCAACTTCACAGAC
AGAACGCAAATAACGCTCTCTATCACCACAGCGCCATCTGTCGGACATCGAATTCGAACACATCTTGCAG
TGCGCCAGGTCGGAGTTCTATCGGCTACCCCAGTGGCGACGCAACGAGCTGAAGCGGCGTGTGAAGCTCT
TCTAGGAGGCAAGTGGAAGGCGCAGCATGCGAGGATAGCAACCCAAGTGGAGGAGGAGGCTGTTTGCGAC
GAAACCAAAAGAATTCAATTTACCTTAACAGCAGATGGCTATAACTCACGTGGCAGGAGATGAGAATCAA
TATATAAAATGAAAACGAAACGGGAAACTATGCTAATTTCACAGCCAGTTTTGAAACACGCTTTAAATAG
TTGATGCAAGTGAAACCAACTAAACTAAATTTAAATACATAGTTACATTTACAGTGCGGAGCAACTAGCC
GGCAGCTCCTGTTTATTTTTTTAAGACGCCATCTGTCGGACATCGAATTCGAACACATCTTGCAGTGCGC
CAGGTCGGAGTTCTATCGGCTACCCCAGTGGCGACGCAACGAGCTGAAGCGGCGTGTGAAGCTCTTCTAG
GAGGCAAGTGGAAGGCGCAGCATGCGAGGATAGCAACCCAAGTGGAGGAGGAGGCTGTTTGCGACGAAAC
CAAAAGAATTCAATTTACCTTAACAGCAGATGGCTATAACTCACGTGGCAGGAGATGAGAATCAATATAT
AAAATGAAAACGAAACGGGAAACTATGCTAATTTCACAGCCAGTTTTGAAACACGCTTTAAATAGTTGAT
GCAAGTGAAACCAACTAAACTAAATTTAAATACATAGTTACATTTACAGTGCGGAGCAACTAGCCGGCAG
CTCCTGTTTATTTTTTTAAGAATTAGTTGCGCTAACGCAAAATTGTAACTGAGAACAACTATATGAAAGG
AACATAAAATCCCAATAAAAAACGAAGTGTATACAGACAACTCTGACTTTGATCTGGCACTTTTTCTCAT
GACGTGTCGGCGAGCTTAACTTTATTAAAGAACTACTCGAAAAAGTCACTGCAAATAGCCGAAAATGCGC
GACCAATTCATCAGCCTGGCCCTGATTCTGTGCGTCCTGCACAGCGCCTGCGGCTTGTACTTCCACATAT
CGCAGACGGAGCGCAAGTGCTTCATCGAGGAGGTTCCCGACGAGACAACTGTGATTGGTTAACTACAAGG
TAGAGCTGTATGATCCGCGCTCTAATGGATTCATGCCCTCGTCGCCCGGAATCGGCATGCATGTGGAAGT
ACGCGACAGCGACGACAAGATTGTGCTGTCCCGCGTGTACAGCTCACAGGGCCGCATCTCGTTCACCTCG
CACACTCCTGGCGAGCACGTCATCTGCATGTTCTCGAACAGCACCGCGTGGTTCAGTGGTGCCCAGCTGC
GTGTTCACCTGGACATCCAGGTGGGAGAGCACGCTATCGACTACGCCCATGTGGCGCAGAAGGAGAAACT
GACTGAGCTGCAGCTGCGCATCCGCCAGCTACTTGACCAGGTGGAGCAGATCACCAAGGAGCAGAACTAC
CAGCGATACCGCGAGGAGCGGTTCCGTCACACCAGCGAGAGCACCAACTCCCGCGTGCTCTGGTGGTCGC
TGGCCCAGACCGTCGTCTTGGTTTGCATGGGCTTCTGGCATCTATTTAACCTGTTCCACACTTG
>dme_circ_0008407_junction_seq
GGCATCTATTTAACCTGTTCCACACTTGGTAAACAAAAAATCTACTGTGCGAAATG
>dme_circ_0008408
GTTTCAGCTCGCAGTCCATTAGCGGGAGATAGGTTACGTCCTCCACATCGTCACGCCGCCCATCGATGCG

AGTGAATGGCTCCATCGGCACCAGGGCGCTGAAATGGCCACGTGTGTAGCCCAGTGCTATCGGTGACTTG
GTGCAAAAGTTTTGATCCCAGAACAAAGGCAA
>dme_circ_0008408_junction_seq
AAAAGTTTTGATCCCAGAACAAAGGCAAGTTTCAGCTCGCAGTCCATTAGCGGGAG
>dme_circ_0008409
GAGGAGAATCAGCAACAACTGCAGCAGCCAAACGTGGATACCGTCAGCGTCAATAACTCGTTCAATAAGA
AGCACATTTACCAACTGGGTAAGAGTTTTTAATGTGATTTCTCTGGGTATGACTAATTGATATTCACTTT
CCAGGTTCTTCGGAAACCATCAACAACTGTGATACGCTGCAAGAGCGTCAGGAGCGCCGCCAGCGTCAAA
TCCGACGACAGGTGGACTGGCAGTGGCTGAATGCGTGCCTGGGCGTCG
>dme_circ_0008409_junction_seq
CAGTGGCTGAATGCGTGCCTGGGCGTCGGAGGAGAATCAGCAACAACTGCAGCAGC
>dme_circ_0008410
GGCGTTCGTTTCTGCAGCGTTCTCCGAGGATTCGGCAGGTGCACAAAGTTCTCCGCATTCTCTTCGCTTT
CCTGGTCATTGATCGACATCGATTTGCTAATTGTGGGAGGGGGACGGTTCTCCTCATTCGAG
>dme_circ_0008410_junction_seq
GGGAGGGGGACGGTTCTCCTCATTCGAGGGCGTTCGTTTCTGCAGCGTTCTCCGAG
>dme_circ_0008411
GTGGTTCCTGTTTAAAGACAATTGATTCCGACATAAACAACTACTCGGCTTCAAATCTATCGACCGCAGA
ACAAGCCAAATGTGATACACAATATCAAAAGTCAACATCAGACCACCTGTTATTATTTCCTGCTCG
>dme_circ_0008411_junction_seq
CAGACCACCTGTTATTATTTCCTGCTCGGTGGTTCCTGTTTAAAGACAATTGATTC
>dme_circ_0008412
GCCTTCCGAATTAGAGTTCTCCGATGCACTGCCTGCTGGATTCGGTACATCGCCTCTCTCCGCACCGCTC
ATGATCATTGAGCGCACGGCGTTCCAGTCGATCAGTAGTCTCTCCAAAGCCTTTCTGGCGAACTTCGGTG
GCTCCAGATCATGGTCCGGCATATCATTATCAATTTCGGCTGCGGAGGTCTTCCGATTTGATATCACTGA
ATTGTTTCCGGTCGCTCCGGTGGCAGCGGTGCTAACATTGCGAACCCTTACACTAAAGCGGTACTGTCGC
TGCTCTACAATCTGGCGTCCAACCGTTTGAATAAGGAAAAGAAGAATCGATTCACCCTTGGAGTTAAATT
TGGTGACCAAATCTGTGCAGGATACTAGTTTGCACAGCAGTGCGAAGCGCTTTGTGAGATTTCCAGCGAT
CAGAGCCTTACACTTGCATTTCTCCCAACAATCACAGTAAGCAGTCGGCGCCGTACGCTTCAACTTGCAG
TCGTGTCCTTTGTGGCAGACTCTCGCGCATTCCGTGCAGCAGCACAGGGATCCAGTGAGGCCGCATGTCT
TGCACTCGAAAAT
>dme_circ_0008412_junction_seq
TGAGGCCGCATGTCTTGCACTCGAAAATGCCTTCCGAATTAGAGTTCTCCGATGCA
>dme_circ_0008413
AGTTCCATGCTCTCCAACATCAAGTTGGGGTCCCCTACCTACACCTTCGACCTGGCTCAGCGGCGGGAGC
ATGCTTTGACAATCCTTCAGCAGATGTGCGTAAGTCCAGCCCTTCGTCCGTACCTCTGCCATATGCTGAG
CACTAAGGATGCGCAGGGCCAAACGCCGTTTATGCTATCCG
>dme_circ_0008413_junction_seq
CAGGGCCAAACGCCGTTTATGCTATCCGAGTTCCATGCTCTCCAACATCAAGTTGG
>dme_circ_0008414
ATGATATCTCAAAGATAATTCCGTCCTCCTCCCAGAGTTCCATGCTCTCCAACATCAAGTTGGGGTCCCC
TACCTACACCTTCGACCTGGCTCAGCGGCGGGAGCATGCTTTGACAATCCTTCAGCAGATG
>dme_circ_0008414_junction_seq
GCATGCTTTGACAATCCTTCAGCAGATGATGATATCTCAAAGATAATTCCGTCCTC

>dme_circ_0008415
GATATCATCTTCAATGCTGTCTGAGACATTCTGCGAAGAGCTGGCGCTCAGATTACTAGCCGCCGGTGTT
TCAGCTGGCCAGGGGATGTAGGCATGGTCTTCGTTGTTTGTAGCCATTGGCTGGCTGCCAGACTGTTCAG
CCTGATCGCTATTTATTAGACGATTCATCATGTCGCGCAGATTG
>dme_circ_0008415_junction_seq
TAGACGATTCATCATGTCGCGCAGATTGGATATCATCTTCAATGCTGTCTGAGACA
>dme_circ_0008416
GATATCATCTTCAATGCTGTCTGAGACATTCTGCGAAGAGCTGGCGCTCAGATTACTAGCCGCCGGTGTT
TCAGCTGGCCAGGGGATGTAGGCATGGTCTTCGTTGTTTGTAGCCATTGGCTGGCTGCCAGACTGTTCAG
CCTGATCGCTATTTATTAGACGATTCATCATGTCGCGCAGATTGGTACGATTATCCCTGCTCGTCGATGA
GGCGGAGCCGGCAGCGGATGAACTCATAGTTGCAAAGCTGGAGTTTTCATTGCTCGAAGACGCACTTGCT
CCAAATGCGATGCTGCTAACGTAAGCGCTTGTCGAGACAGTTGGAATGGAACGCGCCACGGAGAGTCCCA
CTAGTGATTTCTTCTCGACACCACCGCTGGGAGAGTCTGGTGGGGAATCCTTGTTTGAGGATGGGGCGCA
CATAATTACGCAAGCGTGGAATATGTTTCGGGCTCCATCGCAACGTTCTTCTACGACAAGAGCCGTATCC
GCTTGATCCTCCCTCTCCAGGCGACCGAGGGCGGCAAAGCTATTCTTGACATCGCACCGCAAGATGTGCG
GCATAAGCTTTTGAGTATCAAAGAGCAAAGCAGTCATGCATACCTTTGACTTCAAATTCACGCCGGAGAG
CATGGCCGGCAGGGAGATTGTGGACATAGTGATTGACTTCACCGGAGGAAGATCAAACCATTGCGGATCC
TTGATCGAGCCAAGGCAATCCTTGGCCAAGGGATATAGGGCTCCATTGCCATCACGAAGCACAATG
>dme_circ_0008416_junction_seq
GGCTCCATTGCCATCACGAAGCACAATGGATATCATCTTCAATGCTGTCTGAGACA
>dme_circ_0008417
GGCGGAGGTGGCATGTCGATGCGATCTGTTGACTCCTTGCTGTTTGAGAAGGATTGTCCATTCTTACTAC
TTCCAGTGCTGGGCATTGCCGTGGACTTTGGCAGATCTTTATCCGGAGTAATTCCATGGGCATTAAGATT
ATTGCCGGCTGCTTGGGATTTGTCTACGCCAGAGTTCGTGTTAATATTTATGATTTTCATGCGGCACACA
TCGGTGAAG
>dme_circ_0008417_junction_seq
GATTTTCATGCGGCACACATCGGTGAAGGGCGGAGGTGGCATGTCGATGCGATCTG
>dme_circ_0008418
AAAAAAGAGCCATCCCCCATGTTGTCCCCCATTTGGATATCTGAAGAATTGCAGCCATGGCCAGAGAAAA
GTTCCGTAAGGTTCAAGACCATAGGAGCTTTGTATTCGGAGTTCATTGCCTTGTCAGAGAGCGGAGATTT
GTACCAGTGGCGCTGGTCAGATGCAGAGCCTTATAAGTCGGAGG
>dme_circ_0008418_junction_seq
TCAGATGCAGAGCCTTATAAGTCGGAGGAAAAAAGAGCCATCCCCCATGTTGTCCC
>dme_circ_0008419
TCTCTCAACGTTTTGTGACATGAAAATTCAACGAGGACACTGTGTACATGAATAGTTGAAAAATTAAAAT
GGTTTCCATGCAATTTGTTTTGCAACCGCTACCGGGCTCGGACGATCAGTTTATTGAAAGG
>dme_circ_0008419_junction_seq
GGGCTCGGACGATCAGTTTATTGAAAGGTCTCTCAACGTTTTGTGACATGAAAATT
>dme_circ_0008420
TTTTAAAAACATGCATCGGATACATGACGACGAGTATTCCGAGGATGCCAAGGAATCGGACTTTAAGAGT
TCGATTCAGAAGGAGATATCAGTGAAGTCGAGACACCAAATTTCCATACCAGACATATGCTCAGATGCTG
TTAAGAACAATTGTTTTCCGCCGACTGGCTTTCTCAATAACTCGATCACCTTTGCCTCACATGTAGTTAA
GTGCAGCTCACCGGCCTCGAGCATCGATGAATCCTCAACGGCGGCCCAGCAGCACGAATCCAATCCACAC
ATGCATATACAGGGTCAGCATATGATGGCTCCCGTTCCCGATCTGGGTTTCTACAATGTGCCCGAGTTCA

TATCCGAGCAGGAGAAGCTCATGTTCTCCGATGCAGAGAGATTCTTGCGGTCGAAGGATAATGAGGTGTG
CAACAATGATTTCAGCTATATGCACGATGAGTTCGCCATGCGGAAGTACTATCATCCGTTATTTGCTCAT
GGCATATGCCGTTGGCCTGGTTGCGAAATGGATTTGGAAGACATCACATCGTTTGTCAAGGCATCTGAAT
ACGGAACATGGTTTGGACGATCGATCAACAGCACAGGCACGGGTTCAAATGCAAGTTGTCTCCCAGCTGG
AATCCCACCTGCAAAAAGAACGAGATCGCTTGCAGGCAATGATGCATCACTTGTATTTGTCCAAGCAGCT
TTTGTCTCCCACCAAAATCGACAGGAAGGGACGTGCCCGGAAGAGAGGGAAAGTTTTGCCGGAGTCCGCT
TACCGTAAATAGCATAGGCCGACCCATCCGACAAACAAATTCACCGAGTCCTCTGAATCTTCCAATGGTT
AATTCCACCAACTTGTGCTCGATCAAAAAGAGAAACCACGACAAAAATACATTTTCCATAAATGGGGGTA
AGTTTGGGAAAAATTTACTATTACTGTATAAAAATAATAATGGTTTTTGGATATAGGATTACCCTATATG
CTTGAAAGAGCAGGTCTTGATGTGCAACAGGGTAAGTAAATTATGTAGACTGATCGTCTTGATGACTATT
CGTCTTGTGCTTCTGGAAGACATATAATAAGAATTTAAATTTGCAGAAATCCATCGAAACAGAGAGTTTT
ATAAGAATGCTGATGTACGACCGCCTTTTACTTATGCTTCCCTCATAAGACAGGTAATTTTAAAAACCAA
TTTCCTAATATAACATTAAtttattttttttatatatattttatttataGGCTATAATTGACTCCCCTGA
CAAGCAGTTAACCCTAAACGAAATCTACAACTGGTTCCAAAACACATTTTGCTACTTCCGGCGCAACGCA
GCTACGTGGAAGG
>dme_circ_0008420_junction_seq
TTCCGGCGCAACGCAGCTACGTGGAAGGTTTTAAAAACATGCATCGGATACATGAC
>dme_circ_0008421
ATTAACTGCACCGGATGACGTGGCATTTTATTTGTAACATATTTAAAATTAAATCAAACATTTAAAGAAT
GTTTAATTTAATTTACACTGAATTCATTTAGCACTGGGCCTTTAGTAGGGGTTAAATCTTATATGCGCCA
AGTTGTTAAATATTCATAACTTTTGGGGAAAGTATCATCTCAATGATAGTGCGGGAAAATTAAGCTGAAA
TTAAATAGGGCGTAATAAATAGAAATCCTTAAACTATCACTCTGATTCGGAGTTTTACAGGAATGGCGGC
ATATGAGTAAAGATAGTTTTATTGAAATGACTCCTAATGACAGACGAAAAAGAGATTATTTCTGTGTATT
TGCATATGCCCATCTCTTGGGTAAAGGATCAATGGTAATGATTATAAATTTCAGTTTTAAAAACATGCAT
CGGATACATGACGACGAGTATTCCGAGGATGCCAAGGAATCGGACTTTAAGAGTTCGATTCAGAAGGAGA
TATCAGTGAAGTCGAGACACCAAATTTCCATACCAGACATATGCTCAGATGCTGTTAAGAACAATTGTTT
TCCGCCGACTGGCTTTCTCAATAACTCGATCACCTTTGCCTCACATGTAGTTAAGTGCAGCTCACCGGCC
TCGAGCATCGATGAATCCTCAACGGCGGCCCAGCAGCACGAATCCAATCCACACATGCATATACAGGGTC
AGCATATGATGGCTCCCGTTCCCGATCTGGGTTTCTACAATGTGCCCGAGTTCATATCCGAGCAGGAGAA
GCTCATGTTCTCCGATGCAGAGAGATTCTTGCGGTCGAAGGATAATGAGGTGTGCAACAATGATTTCAGC
TATATGCACGATGAGTTCGCCATGCGGAAGTACTATCATCCGTTATTTGCTCATGGCATATGCCGTTGGC
CTGGTTGCGAAATGGATTTGGAAGACATCACATCGTTTGTCAAGGCATCTGAATACGGAACATGGTTTGG
ACGATCGATCAACAGCACAGGCACGGGTTCAAATGCAAGTTGTCTCCCAGCTGGAATCCCACCTGCAAAA
AGAACGAGATCGCTTGCAGGCAATGATGCATCACTTGTATTTGTCCAAGCAGCTTTTGTCTCCCACCAAA
ATCGACAGGAAGGGACGTGCCCGGAAGAGAGGGAAAGTTTTGCCGGAGTCCGCTTACCGTAAATAGCATA
GGCCGACCCATCCGACAAACAAATTCACCGAGTCCTCTGAATCTTCCAATGGTTAATTCCACCAACTTGT
GCTCGATCAAAAAGAGAAACCACGACAAAAATACATTTTCCATAAATGGGGGTAAGTTTGGGAAAAATTT
ACTATTACTGTATAAAAATAATAATGGTTTTTGGATATAGGATTACCCTATATGCTTGAAAGAGCAGGTC
TTGATGTGCAACAGGGTAAGTAAATTATGTAGACTGATCGTCTTGATGACTATTCGTCTTGTGCTTCTGG
AAGACATATAATAAGAATTTAAATTTGCAGAAATCCATCGAAACAGAGAGTTTTATAAGAATGCTGATGT
ACGACCGCCTTTTACTTATGCTTCCCTCATAAGACAGGTAATTTTAAAAACCAATTTCCTAATATAACAT
TAAtttattttttttatatatattttatttataGGCTATAATTGACTCCCCTGACAAGCAGTTAACCCTA
AACGAAATCTACAACTGGTTCCAAAACACATTTTGCTACTTCCGGCGCAACGCAGCTACGTGGAAGG
>dme_circ_0008421_junction_seq

TTCCGGCGCAACGCAGCTACGTGGAAGGATTAACTGCACCGGATGACGTGGCATTT
>dme_circ_0008422
GCTAAAACATACTCCCACACAACACATACCAATTTACAAACAGCTTGAGAACTTACAATCGCAAGACAGC
CCAGCTTACAATGATCAAAGGAGAGGAAAAGGATTCGAAGATGCTCAATAGACTTATGCACACCGCGAGC
TGCTTCACTACTTAAGTACGAGTATGGCGAGTCCATAAGGCGTAGGACGAGTGGAGGACGGGGAAAGGAT
CAAGATCACAAATACAGAACAGCAATCATTTTATAAATTCAGCTATATTTGATACAGAACGAAATGTTAT
AGT
>dme_circ_0008422_junction_seq
TATTTGATACAGAACGAAATGTTATAGTGCTAAAACATACTCCCACACAACACATA
>dme_circ_0008423
GTGGTCGGTAGTCGGGTGACCATTGCAGACGCCCAGTTTAATGTTCTGCACACCTGCCTAATTTTGGATG
CAGTGGGTCACTGTGCCAGATTTATTGGTGATACCTTCGACAGGCATATATTTTTAAGTCTACACAAAGT
GCTTCTCAAGG
>dme_circ_0008423_junction_seq
TTAAGTCTACACAAAGTGCTTCTCAAGGGTGGTCGGTAGTCGGGTGACCATTGCAG
>dme_circ_0008424
GTTGCACTGCCGGCGGGCGTCTGCATTTTGGCTGCCACAGTGCTGGTGGACTCGTTCTTTTGGCGCCGCC
TGCTTTGGCCCGAGGGTGAGGTGCTATGGTACAACACCGTACTCAATAAGAGCTCCAACTGGGGTACCTC
GCCTTTTCTATG
>dme_circ_0008424_junction_seq
CCAACTGGGGTACCTCGCCTTTTCTATGGTTGCACTGCCGGCGGGCGTCTGCATTT
>dme_circ_0008425
GGCGCTCTCACCGTCGTCCTTGATGTTCACCTTCTTCCACGGCAGCACACAGATGCCACAGCACTTTTCC
ATGCCACTCAAGTTCTTCTCCGCCTCCCTCATGTCCGCGTTGATCCGGTCCATGCCCTCCTCAATGCGGT
CCAACTGCTCGCCCTGGTCATCCAGGGCCACCAGCGTGCGGATTCCAGCCTCCTTGCTTTCGTCCATGAG
GGCGAGCATCCGACGC
>dme_circ_0008425_junction_seq
TTCGTCCATGAGGGCGAGCATCCGACGCGGCGCTCTCACCGTCGTCCTTGATGTTC
>dme_circ_0008426
GCAGCAATAGTGTGCCTATTGACCGCAGTTCGCTGCTGTTGAAGTTTGATCCCCTCCTTGGAGCCCCTGT
TCCCGTTAATCATCCGAATCAGCAGGAGCAGGCGCTGCAGAATATTCTGGGATCGAACCAGCACCAGAAC
CGGCTCCTTAGCCCAACATTGGAGGAGCACGAAACAAGTGACGGGAACCAGTCTTTTGGAGTAATATCGA
GTGCCAAAGATACAGCCAAGAAATGGGATTTTAAGCCACCTGTGGATAGAACAAAAGGTAAGCCCTCCCT
GATTTTAGTTTTTACTACTAGAGTAGTATTTAGTTTCTGTAACCTGGATCAGTACCACTAGTTGATCGCT
GATGTGACCGGTTTTATTAGCAAGGAAACTCTGTATTATAAATCTTTATGGTATAGCTTGTAAGCTATAA
CAAGTAGAAAGAAGGAGAGCtttaatttttacttaatataatttaattttaaacttaagtttattttat
ttgCTAATATAGGAAAGACCTGGTTACTATTTAAACAGGTGTAATAACAACAGATGCTATAATTTGGTGA
TTTTTTAAATGTTAACATAGCAAACGAACATTCCATAGTTAAGGCACACTATACATGGCGGTCCTTCCTT
GTGTAATTATATCTGTATCTGTAATATTACAGATTTTATAGTaaacaagagagaatgctgtagtcgagtt
ccccgactatcagatacccggtattcggctagtataaatgcgaactcgaaatttcataatttttctggga
tatcgatagatattggggaataaaattatagaaaaaagtaaagtttgttcaaaagtgagggcggctttag
ggcgttagaggggacgtggcaaaccatttttttcattgcaaaccgatagaaatttacaagactaataaaa
ttataaaaaatttgaaaaatggttcaaaagcatgtgcgtggcagttttgtgcggtttgtgagcgtaagag
tgtacgtggaaaaatttggatgagtctttgtctctagaatctgtatgatgaatctcaaccttctagcttt

aatagttcctgagatctcgacgttcatacagacagacggacatggctagatcgactcggctattgatcct
gatcaagaatagaaattcattatatagttcttctagtatacccttttactctacgagtaacgggtataaT
AATCCGGCATTTTCGGTGCTGTTAAATATTTTGTTCACTTAAGGAAAGCATTAAGGGCATTTCTTTAAAA
GATTACACGATCAATTACGATAGGTGTAATTTCGCGATGGTGTAATAAACATTTTTGTGTAATTTGATTT
TTTACCGTCAGAGAATTTAATACTGGGTTTTGATGTTAATTTTGTATTTTTTTCTATCTTAATTTTTGCA
TACAAATAACAGGTCTTCAGGAACTTCGCATAATTAGCATCATACAGAGCTTTATTAATATTGTAAAACC
CATTTCTCCTTTCCAGCCGGTGTCATTGAGGCATATGAGAAGGCAATTGCAGAGCTCATTAGTGAGAAGG
AGCAGCAAGCACAGCTTCACGAACGGCAGTTGCAGGAAGTCCAGGCAGACCGGGACGCAAATTACCATCA
CTTAACATCGTTGGAGACGACATTTTCCGATCTGCATGTGGAAATACGAGAAAAGCAAAGAGATGACCTC
GCAGCTTAAAAGTAACGAGGAATCGCTTCTGGCAGAGCGAAAGCAGATGATGGACAATCTGCGGTTGCAG
GAACAGCGCTACGACAAGATGAAGAACCATGCCATGCAGCAGCTGGAAATGTGCCAACAAAAAGCTGGAC
ACCTACTCGAGGGAGCATGCCGACGAGACGAAAAAACTTAAAGCTTTACTTAAGAAGGAGGAGATCTCGC
GGGTCTCGATGACGGAGCAGCTGCAACAGAAGTCCCGCGAAAACGCTGATCTGCTCAAGATCTGCGAAGA
GCTCATCTATGGCAAGGGACAAGG
>dme_circ_0008426_junction_seq
AAGAGCTCATCTATGGCAAGGGACAAGGGCAGCAATAGTGTGCCTATTGACCGCAG
>dme_circ_0008427
GCCAAATAGAGGGGCATGTAATTACATTAATTATGATGTTGCTCATCAAACTGGTAACTTTGGCTGCTGG
TCCGCCGTCCAGCAGTGCCGGACAAATAAAAGTAGAACAGCCGCCAACACAAATCAGATGGGGGGACATT
ACCCACTAGATCTTAACAAAAACAAAATAAATTGTTGAATTCGAGTAGAGAAATTATTAATCAAGATTAT
GATAGCGGACCAATTTGCGTATAAGATTTGTGAAATCAATTTAGATAAATTATCTTGGTTTGCCATAGAC
CTGGACGATTACGACGATATGCCAGCCTTAGTTGAGAATTCTAGTTAGCCTGCCTGTGCTTTGGGTTCTG
CTTGGTGTTTTACTAGGTTTAGGTACGTTTAATTGTGAACAGCAAACTGGGTTTTGACTATTCAGTAT
>dme_circ_0008427_junction_seq
AGCAAACTGGGTTTTGACTATTCAGTATGCCAAATAGAGGGGCATGTAATTACATT
>dme_circ_0008428
ttaaatttttatttAAATTATTTTGCATGTACTTAATCTCATTTGCGCGATAAGTGTTATTAAGATAAAT
CTTCAACAATAAGATCTTTTAAGTAGGCCACGAAACTGTTTATATTTTGCTTGTTATTATTATGATGCCG
GCCCTTCAAAAAATCGGATATAATTTTTATATG
>dme_circ_0008428_junction_seq
TCAAAAAATCGGATATAATTTTTATATGttaaatttttatttAAATTATTTTGCAT
>dme_circ_0008429
TTGTTTCCTGTTGGTATTGCACAGGGTATGCTGTCAGAGCGGCAACGATGAGTCCTGGCTGAGTCCGGAT
CCCGCCTGGTCGGACCTGGTTAACGATATCAATCAGCCCGGTGAATTGTGCCAGTGTCCTACGTGTCCAG
ATGTGTCTCCAGTGGCCATGGAGGATGCCGTGGCACTCACATACTTCAAGAAGTTCGTCAATCTGCTGTT
CCAGCGCAAGCGTTTTCAGG
>dme_circ_0008429_junction_seq
CTGCTGTTCCAGCGCAAGCGTTTTCAGGTTGTTTCCTGTTGGTATTGCACAGGGTA
>dme_circ_0008430
CCGCCTTACCACGAAATTGCGCTGAATTATAATTATTATCGAGTGCTTACTACCTAGGAGTGATTACATT
GAGAAACCAGGACAATGAGAACGGAGGCAGCGGTCAGTTGTTTCCTGTTGGTATTGCACAGGG
>dme_circ_0008430_junction_seq
AGTTGTTTCCTGTTGGTATTGCACAGGGCCGCCTTACCACGAAATTGCGCTGAATT
>dme_circ_0008431

TTCGTCGGCCTTCAGCCCGTCTTTGTCCTTGTGCTGCGAGTTGAGCTGCTTCCAAGCGATCTCCTCCTTA
TCGTGGATCTTGAGTTCCATGTACAGTGACTTGAGCTTGGGCTCTGTTAAGCGCTGTAACCGAGGTTAGT
ACGGTTAAATATGAGATATCTTATGGTGTCCACTTACATTCTGCGCCTTGGCCCACACGAGATTCAACTT
GGCCATGCGAAATGGCCGCTGGATGCTCTTAAAATCCGGATCATACTTCTCCTGCTTTACTTGCTGAAAA
TGCGGG
>dme_circ_0008431_junction_seq
CTCCTGCTTTACTTGCTGAAAATGCGGGTTCGTCGGCCTTCAGCCCGTCTTTGTCC
>dme_circ_0008432
GGGTGGGTAATCGCTCCAAGTATCTACTCCATTATTATTATTCTATCTCAGTACCAGTGCGTCCTATTTT
AGGTAAATTAAAGGCAGGGGTCCCACTTGACGAGCCACTTGTTTAAATTGTTTATTAAGCACACCGGCGG
TCGTTAGTGGGTTGTGTTTAGGCCAATGATATTTAAATGGCGGTCGGG
>dme_circ_0008432_junction_seq
GGCCAATGATATTTAAATGGCGGTCGGGGGGTGGGTAATCGCTCCAAGTATCTACT
>dme_circ_0008433
GAAGGTCTCGAACAGGTGTGGTCCATGGCCAGCTGTCTCCGGATGCTGGACAATGATTCGAACCGCTCCC
GACTTTGTGCCATAGGCAATTTCAATCCAATTGCCACTTATGCTCGAGGTTTTTGGGGTAAGGTAGACCG
AGATGGCCGTGATAGGGTCTAGTGTGACATCTTTGTAGAGCTCGGTAACCA
>dme_circ_0008433_junction_seq
GTGACATCTTTGTAGAGCTCGGTAACCAGAAGGTCTCGAACAGGTGTGGTCCATGG
>dme_circ_0008434
CTGAGTCCGTTGGCGGTGAAGCCCAGTCCCAAGCCAACGAGCATGGATCAACAGAAGGCAATGGCGGCAG
CTCTGGGGCATCAGACGGCGGTACCCGGTGGACTTAAGCCAGAAGACGACCTCGTTCCAAAGTTTGGAGG
TCTTCCCGAGTTCGATGAGACGCAAATACCGGAACTAGCTCCTTTAAGTACCGATATGGATAGCTCAACT
GAAATCCAACCATTCGGATTCGGATGTTTTTGTCGAGTGTTTATCGCTTCATAGCGAGCGCTACACCGGA
GATCGATCGGAGTTAGAGGCATATTCAAGTGCCCTTAATGATGTTTTGGAGCAGCAGCTGTCCATGACCT
CAGTTTCTACACTGGGAGCTGAATTCAACGATCCTTTGGCAGTTAGCCAGCACAACAACGTCAGCTATGA
AGCCATGGATGTGGATGACCTAAACGAAACCATGGAAATGTTAAAGGACGTCTTGAATGCGGAAGACCGT
CATCTTATGCAAACGCATATTCTCAGCGTAACCCAGTCAAAACCAGAGAATAATTTTGTGCACTATCATC
CAAATCAGGAGCAAAATAGGGTTGAACTAGACCAGTTAGCCAAACACTCTAAACCCAATAGTGATGCAAA
GGAATCAGAATCATTGGTAGAATCAGTACTGTCTGAGGAGTCTGGTACACAATCCTTTGAGCCGAAGCTC
ACCTTATCTGCCTTGGACTCTACGAAAGACTTAGCTAAGCATCAAGAGGAAATGCAAACAAATACAAAAG
ATATATTTCAGAAAGAGAAGCACCACCCGGTCAGTAGTGCTCAGGTGAAGGACGCACAAAATCCTAAGGA
GCCTGAAAAGGCCTTAAATCTCAAAGAGGCTTCTTCAGAACTGTCCACAAAATCAGCAAAATCTCTAGAA
TACCCTAATCAAGAGAAGCAATTTTCGGTGGCAGTAGAATCAATGGAGAGACCATGCACTGCCATCAGCC
TTGAGCAAAATGCATCCCCAGCTATGGAGGCAACTGAAGACAGGACTGTGGAGGAGCTTGATCAAATGGA
TGTGGATGAATCCATGAAAGTGGATGATGCCACAGTGCTTAACTCATGTCGGCAGGAGCAAATTAATGCT
CATTCTCCCGTTTCAAGCAAATTTTTCTTAAAACTCTGCGTTCAAAATGATACGGATTGTCAAGATAATA
AAATGGATCAAAAAACTGCAGGAAAAGAAAGTGCATTAAAGATGACTGAAATAGAGCTGCCTGTAGAATC
TGTCCCATCAGCTTCGCTCTCACCGCCGACACCCGCACTTCAAGCAAAAATTATCGAACTTTCCCTTTCC
CCGCCCATACCTACACATCGCCCAAAAACAGCGGAGGAACTAGAGTTCCTGGCTTTGAGGGAGCTGCCTT
TGCCTGATGGTGTCTCGACGGCGGGGAAGGTGGAATGTAAGCAGCTATCACACGGCCTGGTACCCATTGT
GGCAGTCTTACCAACGCCACTTTACTTGGATCTGAAAGGGAATACAACCACTGCAAGCCTTCCAGAACCG
ACTTCGCCCAGTTTCCCCATCAAAGAACCTGCTGAAAAACAGTCCCACTTTCCACGCTCACCGCGTGCTC
AGCTCGAGCAAAAGGACATGCCCTATTTGGAAGAGGCTGTGGTAGAACGTTCATCACATCGCAAGCAAAG

CGATTTCGGAGTCATAGCGAAATCACCAATAGTCTTTGAGTTAACACAACCGAGTCCTGAGAAGCAGCAC
CTCAACGAAACCTTGCCAATGAGCGAGATTTCTCCCACTTCGTCAAATATGACCTTTGACAGCGGAAGCA
TTACGGAGGACAAATCACAGAAGGGTACAGAGAATATTGAGCCTTCTTCCACCCTTGCCGTAAGTCTGGG
ATCGGTTGATGATAAGCAGCGGCGCACTTTCTCGGTGGTTCAGCCCACGAAGGAAGAGGAGGAGGCCAAA
TCGCGTCGGACTTTCTGTATGGAAAATGAGAACCCTCGACGCACCTTCTGCATGCAGCAGAATGCTTCTC
CAGCGGTGGAGGCTACCGAAGACACCGGGGCTGATGAAGTTATGGATGTGGATATCTCCATGAGGGCAGA
TGAAGTAGCCGTGATGAAGTCGCATCAGCAAACTTGGAAGGAACGCCAAGCACCAATTTCAAATTCACCA
CCAATTCCTACTCACCAACGTTTAAATAGACCTCCTGAGGCGATTTCTCCAGTGTCCCCATTGGGAAATG
GCACCGTTGTGTTGGAGGAGCAAACTCTCTCTGCTAAAGAGCAAGCCACGCTAAGTGCCAGCGATGAGAA
GGACGATGTTTTCTTGGAGCACTTTGGTGCAATTTCCCCAGTTTCGGATGACATGTTTAAAACCAGCAGC
GCTTCCAACAATCAAGCAAAGTTTAGGGTCAATGCTGATGAAGAAACCGGAGGCAAAGGCGCTTCTGCGA
TGATCAGGAAGAGAAGTGGTACAGCTGGCGAGCAGGAAGAGATCTTTGATGCCGAATTCCATGATGGCGT
CAACAACCAAAACCG
>dme_circ_0008434_junction_seq
TCCATGATGGCGTCAACAACCAAAACCGCTGAGTCCGTTGGCGGTGAAGCCCAGTC
>dme_circ_0008435
GGTCCGCTCAGCAGCCAGCTCGGCAGCGTTTCTTTTGGTGTATTGCACAAAAGCGCACTGCTGGCGTGGC
ACCAGCGCAATTGATCGAATTTCGCCGAACTGGTAGAACTGATCTCGCAGCTCGGGTTCCGTGATTTCTT
CAGGAAG
>dme_circ_0008435_junction_seq
GCTCGGGTTCCGTGATTTCTTCAGGAAGGGTCCGCTCAGCAGCCAGCTCGGCAGCG
>dme_circ_0008436
GGTAAGCATCCTTTGAACCATGATGTTAAACTTGATGTTTATTTTGGCACTCGTAAAGGTAAAGTCCCCT
CACTCTTTGAACCCCAATTGAGATTGCAGTCTCGGTCAAGAGTTGTTTCGAATGTAAAATAGGAATAAAC
AAAAGAAAAAACACAACAACCAAATAGTAAATAATGCAAACAACACGCGCAGCTTGTTCATATAAAAGTT
GTGCAACAAGCGCCCCGCGCTGTCTGCGCTATTTGCGTTCAACTGTCATGATAACAAAAATAAAACTATC
AACTTTCAACGAGTAAAATTAACAACTATAGCCGGCATTTTCGTTTTCTTTTTTAC
>dme_circ_0008436_junction_seq
TAGCCGGCATTTTCGTTTTCTTTTTTACGGTAAGCATCCTTTGAACCATGATGTTA
>dme_circ_0008437
GGTAAGCCAACATATCCAAAACTGGGATTTGGGTCACATCCGCGGAACTTTGGATCATTGATCGCTTTGT
GGAAACCATATAAGTTAACGAATGCATTTGCATAATGCCGGGCGCACCGAGCAAATCATAATCATAATCA
TTACGCATACGCAATGGTGTCACTTTACTGCTCAACATTTCAATTTCTCACATATTTTACAGCCATAAAT
TTTATGCGATTTTGCTGCACACAATTTGCGTGACTTTTGTCAATAACTTGCTCTCATATCGCACTGTCTC
ATTATGAGCAAgtgtgtgtgtgtgtgcgtatatatatacaaatat
>dme_circ_0008437_junction_seq
gtgtgtgtgcgtatatatatacaaatatGGTAAGCCAACATATCCAAAACTGGGAT
>dme_circ_0008438
GGAACCCATTGAGCTGAAGAAGCGACTGGAGGAAGTGCGTCTGCTGGAATCGCGACGCTCCGCCCCGTCTG
GCAGACCAGGATCGCGACACGGACTTTGCCAGACTCGCCGACATGAGTGGCGAACGCCGCAGAACCACCA
CACACAGTATTGAGGTGCCATCGCAGCTGACGGCGCAGCACAAAGTGAGTACAGACGCTAAGTTTCTCAA
ATATAGTATAGTAAACCAGCATAGGAAACAGAAATCATGCCAGATTAATCACAATTCATGATGGTTGGCC
CCAGGAATAGAGAAACCTAATGGCGAAACCTCTTGGTTTAAGCTTAAGGTAAACACTCAGAATATCTGTT
TACCTGAGTAAGAACGGCCAATCATTTTGCCGGTCCTACCCGGTTTTTAATCGCAGTTTTATTCGTTCGT

TTTGTGTGCTTCGACTTTGGCTTACGGCTTTTGCATAAATATGCAACGATTGGCGTCTGTGGAGACTGGG
CCATATCAATAATTTATAAGTGGCGACAAATTAGCGGAACCGGCCACCAAGTCACGGACATCGACCGATC
CATGTATAATATCTATAATAAAATTGTGTCTCCGTCTTGTCGGCCAGCTGCGCCCTCGACCGGCCACGCC
CAGCTATTTAGCATAGACGCCGCTTTAACTGCACTTACATAAAGAGTTCTCAGCAGCACCCCCGCGTTTC
GCTGACTTTCACAATGCTCCATTCCACTGGGACGACAGCTGATTGCAAAATAATTCAAATTTTCAATTAC
TACCCGCTCAGACGTCGCGACGACTACGTCGAACTTAAAGACGGCGGCGACGACAAAAAGTACAAAATAC
GAAACACTAACATTTGATTACGCAGGCAGGCGGTCGGGGGCCGGGTGTCATAGTCTAAGTGAGCGCTCTA
ATCAAACGGGGAACTTTTATTTCTGACCGGACTGGGTATCCTGAATTCTTTTGTAATTCCAAGCAAATTA
AATGTGACCAAGCAAATCAAATTAGCGTCCCTTTTGTAGTGTTACCATGCAACGGAAAATGAGGTCAGAA
GTAGGTTCATTTTGGGCAAAAACATATCTGTCTATGCATGCATATATCACAGATATCACAGCTAGTCCCT
TGGCCCCATTAATGTGACGACAAGTGTAGAGCATTAAATTTCCGACTTACAATTGGAGCGTTTGTCACTC
GCCACTGATGGCTGCGTATTCCATTTCAATTTCAATTGATAAGCCTCGCGCGCCGCAGTCGAAACTATTA
GTCAAGTGTGCAAAAAGCAGCAACATCAGCGCTGAATGCCGGCCACGTTCTCAATTGataataaaaataa
taacaataataatcaaaataaaGAATGAAAAAGAGCAAACTTGGGAGCTGAAAACAAAAAGCGCAGGCAG
GCGGCAAAATATGGCTCGACATGTCCAGCCAGCCAGGGGTGTGCGGTTCAAAGGAGGTCGCGTTGCGGCG
GAGAGGGGTTTGGTCACTTGAATTGCGTCGCAAATGCTAACCGGCCATCCAAAGTCGAAACAAAAAACAG
CTGGAAAAACTAAAACTACAGTCATGCGGCATTGTTGCTGTTCCGCCTGCAACTGTTGCTGCTGGTGTTG
CAAGTGGCAACTATAGCTTATGTTGCTGTTGCCGAAAAAAATGGAAAACTAGGAAATACAGAGTTGCTTC
TGCTGATTCGGCTTCATTGTGTCGTTCGTTGTAATAAAATTCAAATAAAATTCAATTGAGTTCAGCGCGA
GTCGCTGGGCCAGTTTAATTTAATTAATTACAATGCCGGCGAGCGCGAGATGCGGCATGTGGCATGTGGC
ACATTGTACATACATAGGTGGGTAGGTGGGGCGAAGGGAACGCCACCCTGTAGTTGTGAAACTGAAACCA
AACAAGGAGACCATTTGCCGGTGGTTTTGCCGCAGTTTCAGTTAGTTTAGTTTATCATTGCCCGTTGCCC
CTTCGTTTTTATTTCATTGTGCTGttttttttttttGTTATTGTTTCGAAAGCCGCCTGCGAATGCGGAAG
TATTTTTCCCGCTCCGCACACAGATACGCAAATAAGCTGTAAGCACAAAGCTCTCGCGATTTCGAGTCTG
CGTAtgtgtgtgtgtgcttgtgtgggtgtgtgtgtgcatctgtgtgcgtgtCTATCGCCGCCATATTCGT
TATGTTTTGATATGCCACATCTGGGCAGCGGGAAAGAGGGAGACGGCACTCACACACACGTGTAATGATA
ACAACTCGCTCACCTTGCACGGAACCGCTCCCAAAgcccccgggcacgcccctgcccccccctgcccTACC
ATCGAGTGACACGCGCTTCTCGCGTAATGCGACGTAATATCAAATGCCCACCCCCCTATAAATACCCCCT
CATTCCGCACACCCCCGCACCTACAACTGGTCAGATATGTGATTTTCTCGCACGCGGAATTTGGGTTTTT
AATGCGCTTTTTTTATTATTATTAATTTGCATAGTTTAGCAAGCGAGCTGATAGTTTGAGTAAACAGTTG
TGTAGTTAATTCGCCGGGTAAACAAGTTGTGGTTTGAATAATATTGGCTAATATTCGTTTGATTCATACC
ATTGAATGAATTTGATCTTGGAAAATCCAGTTGCACACAACCGTTCATTAAGCAGCATTTCATTTTATCG
CAGTGCAATTGCACTTGACAGACAAAGAACGCATAATGATACAGCAGAAATATAAACAAAATAATTAAAT
CaaaaaaaaaaagaaaaagaaaaagaaCAAATGCAGGCGAATTAAATGCGGAAAAAAGTGGTAATAAATA
TAATAAGCGCGTAATTATCGCGTAAATGTCAATTTACGCGGCGAATGCGTTGCATCAGTGCTAATTTTTA
ACGCTCTAGTAGACGACAGACCCCATCTATATGGAACATACCTCCCTCCCCCTTCCCACAAAAAAAGCGC
CCCCCCAGAACCCCGATCCTCACGCGTTCGACTAATAAAAGCGTGTAAGCGTCAATTGGCCAGGTTACTC
AGTTAAAGTTAACTatgttgttgttgtcgttgttttgttttcattcttttttttttCTTGTTGTTATTACT
GCTGTTGTAATTGCAATGATTAACAACAGCAACCGCCGCCGGGGGAGCAAAGTAcccccccccccccATTT
GGCACCCCAACCCCCGTCGTTTTCCAGCTGCATTCGCCTGTGTTGCTTATTGCAAAAACAAACAATATGG
GAAAACTGGCAGCGTGCAAagaaaagcaaaagaacagcacaaaaacacacagaccccaacaacaacaaca
acaGCAGGAATAATAATATAAATAATTTTACAAACAAGTTTACCGTTGcaaaacaaaatagcgcaaaaaa
atgaagaacgaaatcaacaaaaaatactcaacaacaaaatcagaacaacaaaaacaaGTTGTAAATTAAT
TATCAACAAGTTCCACTTGGGCAGGAAAAACAGAAAAAAATTGTGTCTTAAAGTCGATTAGCCAAATTGA

CTTGGTCTTTAACTAGTGTTATTAGTCATGGCTAATATCGATTTGATACCGATTTCCGATGCAAATTGGC
TGAGAATCATTCTCGCTACCTTTTGGCAGAAAAGTTCAGTGACGAAGGCTGCATAAACAACGACGTTGGC
GGTGACGTCAAGAAATTAGCAATAGAAAACGGGGAAAGCGCACGACAGAAATGAAAACAAATTCCATATA
AAAAGCGGGTTCGGGTGATGGAAAAACAGAATTCAAATCCAAGTTGGCCGGCAGCGGCAATTAGCCGAGA
CTTGAAAAATATTTACCAGTTGtttttctatttttttttttttttttgttctttttttGTATTCATTTTGT
CTCTGTATTGTCTCGTATTTAATTTAGCTTTGTTTTCTACGActgctgctgctgctgctgctgGCAAGAC
TATGTACAAATATTATTATGAGCGACTGTTTGTCTTGCCCTCAAAAATACGCCGAGAGTCGAGAGCAGAA
CCGCTCGCAAAAAGAGGGGaaaaaaaaTAATACTTAATTTCAGTACAATAAAAAGTCAAGTCGAGCGCAA
AAGTGGGTCAAAATTATTATTGAAGCAGAACAAAAACAAAGCCAGCAGCAAGGCAACGATATTGGCAATG
CAGCAACAAAATAAGTTGTAGGAGTGcaggggggtgtgcggtggcaggggggggagggggATGGTTCAGCA
AGAGGAAAGAGAACAAAATTTATAATAATTTTCTAAGCGACGGCCCCTCGACGGCAAATTATTGAAATGC
ATTCGCATTTCGCATTCACCCGACCAGGAGAGACACTCCAATACCAGGGACCATATAACCTATATGTATA
TACtaccatgctatgccataccataccataccatGTGGACCCCCTTCTTCACGGAATTCGACCCCTCCAT
ACCCTATATACATAAGCAGGGGTATCCATTGCCCATGCACCGTTCCAATGAACGTAAACACAATTTCCCA
ATGTTCTGCACGAATAGTTTGTTTGATCGTAAAATTTTATTTCTGCATTCAAATCGCAGCGCTAAACAAA
ATGCAAAAAGAGCGAAGAACGCGAAAAAAGCCGAAATGCCATTGCAGTTCGTGCACAGCGAGAAAAATTT
GAGATTTCATGTTTAAATTGTACAATACAATATAACAGGGTAATAATTAAATAAATAGCCATTAAATAAT
TTAGCATACTATGCTTGAATAGATTTGACGATTTGACTGCTGCTGCCATTTGAATTCTTCTTGAGATATT
TCTGCATTTTTGCCAGTGTACTTCACATTTTCCGTTTGGGCCTGTCTTTACCCTTTTCATTCTGCTTATG
ATTTGGCCAAAGAATGAGTTTCGTGCCGCGAAGGAATCCGAATCGATGCGGTTTGGCGGAAGGGTCATGA
ATTCCCTCGAAAAGTGGACGTTTTTAGTTGGCCAAGTGTTGTCTTGTCTTGTGGTCAGTGGGTTTTGCAA
AATGGTCAGATCGTAATTTGTAAAGCAAATAATGCGAATTTCATGCAAGACATTTGCTCCCTTTAATCTG
GCATTCAATTTAAATTTGATCTCCATTTGAACTGCATTTCCCAGAGCATTATTTATGCCCAAATGCATTA
ATAGGCTGTGCCAGAAAAGCTCCATTTCCAACGCACACACACACCTTCTGCTGTGTGAAAAATCATATAG
ACGGCGATGTGTTGACGTTCAATGGGCGAAACAATCTGTTGAAAAGCGCATTGAGTGGCGGATGGATGCA
GAAAAGGGGCCAATGGGCGCATATTCGTACCATTTTCGTCAGCTAGTAAATATTTAGTGGCTCCGGCTAC
TTGTGTTGTCCACTTGGAAATGGGcacacacacaccacacactcgcacacacactcgcacacacTTCTAG
CTAACTAGCAAATAAATAAAAAGCGTAGATCGCGAGTGCTGGGGCGCGAAAACAGCGAGAACTAAAACAG
CAACATGATCAACAGTTGCTCATAAATTAAAGTTCGCCATCGTTTGGTGTGCCGATTTTTTGAATCGCAG
GCAATAAAGTTTAACGTGCACACTTATGACCAACCGCAAAAAGGAGGCCGGCGGAAGGGGGCGGGGCCAG
AGATCGGAGTACCAACTATATAGTACAGTAGTATGCAGTATGCAGAAGTGTGCACGCAACATTTGCACTG
CGGCCAAAACGCAAATTTCGAAGAACAAAAATTAGATTGAATTGTTTCTCCTGCTGCTGTCGCTGCAATT
GCTGGCCAATGCCAAGGAAATGGCCAAAATAAGGGAGTGGTggggggggggggggTAGAGGGGTTTTAAA
GGGGCCCATAATGACCGACCTGGTCTAGGCAATTTGATTGAGTTGTTTCCAATTGAAATGGCAAAAACGC
TGCGGGTGTTGGCACTGATGTCTGACAGCAAATGACTGCGAAACCACTTCGGACTCCGACTCAAAAGTCA
TTTGGCCAGGCCGAAAGGCAGTCCAAATTCAACTTCAAGTCCAAACCGCAGTGGTGGAAGAGCTCTGTAA
TTTCCAGTTACAGTTACAGATTTCTAGCCTGCCAGCTAGCCGATTCAGCTGTGATTTGGCTTATTTCGCC
GGGCCTGCGGtggccagaattgcccgaatggccagaatggcaagaatggcAATTGCATTGCAGTTCCTTC
AACTTCAACTTCGAAGTTGGAGCCACATGCAGCGGTTCTTGAGGCCGTGTCAATTAGAGTTTGGGGCTGG
ACTTGGCACTGGGACTGggatctgggctttggatctgggttttgggtctgAGTCCGGTGTCCTGGGCCCC
CAAGGCGCTGCCATTCCACTTCCATTTGCATTCGGTGACCCAGAAGATTCCGTCAAATTATCGTCTCACG
TTGGCTTTGGCTAATTCGAATGGCAATGGGCAATGGGCAACGCCTTCACCTTGACTTGGTCAAAATCAGG
CACTTGGCTAACTAAGGCTAAGCTCGGTTTTCCCTCGCTTTTCCCACAGCGCTTTACTCTGCTTCGGCTA
ATTAAAGTGTCCGCTTGTTTGTCGCGATTTGTTAATTGAATTAAATGTGTTGGTCAAGCGAGCGGGACAT

CAAAATGCAGGCGATGACTCCACTTTGATGGGGGCATTGGGACTGTCTTTCAGAGTGCTGGTATCGTGTA
CGTGTGTGCAACCCATTTAGCGAGAGTGCACTGTGCACTGCTGCAGTGGCCACTTAACTGTAAAGGCAAA
CGCACGTACAACTGCCGCCAGCTTTTGCGGCTTCGACTGGGCCAAAACGTACATACATATGGCGGCAGAT
CGGTTCGCATGGTGGGCCAATGGCGAGGATTTTAATTGAAATGGACATTGTTAATTGATTAGCAATGTTG
AAAGCATTTTAGCAAAGAGACAGCTATTATTTTTAGTGTTCCCTCAGGATTTATTCGCTCACGTTGTCTT
TGTCTCGGAACCTAATGATTAGAATTTGAAAACGTTTGTGATCAAGTGCTAAACTAAAATATAGTGTTCA
GATAACATGACACATACTTTAAAATCATTTCTTAAAGCGTAATTTAGGTAATTCATGGTTATTACAATGG
TGTAAGGTTCCTTCTCTTTAAAGCCAGATTTTGTATATAAATGTTATTGCCAAGTTCCTTTAATGTCAGA
TTTGTAGCTGCCATTTACCCACTGTGATCTCACGAACACAGTGGGCAATTCCCATGGTCTGCTGCCGGCT
GGTCAGTCAAGTACCGCTAAATTGTTGCAGCAGGAAAGCGCAAGAACACCAACGGCAGCGAGTGcaactg
caacaactgcaacaactgcaGCAGCGACGCTTCCTGACGCTTGGCGCTCTGCATGCTAATCAGCTGGAGG
CGACACACGAGCGGCCCACATACTCGCATGTATGGGTTTGAGTGCTTTGCCAGTGGCAGGTGTGTGTCAG
TCCCACTTCAGCTCCATCCCCATCCGCATCCCCTTTCCACATTGACACATGTGAACCCCCTTTCCGTGTC
TAGACCAATGCCCATGGCCCGGTGACAACTGGCAGCTTGTTACGCCCTTTCGACTGTCACACGCTTATTG
CTCTTGTCACTAactgctggttgttggtagttgttggctgttggttgttgcctgttggtgttgTCGCTTT
CGAGTGGGATGATTGGCGGAGAGGAAATCCCCTCATTCTCTCGCTGGCCAACTTTTCAAATTGAATTTCG
CCGATTTATCAATTTCCCGAAAAACACAAACAATAGAAAGTGggaaatggcaatggaaatggaaatggCT
GCCGACAGCACCGGCAGCGACAACATTGGCCATGGCTAATGTCGTTTTGGGCCAGGTACGACGGTACTAC
GGCAACGAGACGAGTACAAGTACAGTAGGCAACCGATGGCAAGGTGCAGTGCCCGGGGGAAAAACCCCAG
GCCACTTTGAAGTTACTTCGCTGCTGCCATGTCGCGCCTGCCGCTTTTCCGCTTTTCTCCTCTGGCAAAA
GAGCCCAAGGAAAAGCCGCTCATGTCCGCGCATTTCTAATAAGCCCACGGGGCAcacctcaacgcaactc
aactcagcgcggttcaattcaattcaattcaactcggctcggttcaattcaGGCGCTCAAGTTCAAGTTG
AGGCCTCCTGTCGCCAACATCGCAAATAGGGAAGGAAAATCGAGGTAAAAACAAACGCCAAATGGCTGAA
ACAGAATCGACATTGGCGCGGAATTAATACGATTTTTATTCATGGCAACTGCATGGGGAAAAAAGATTTC
AAAAGTTGCTTTAGTCCTTTTATGATGAAATGTTGTGTGAGTCCCATTTCAGTAACTGATGACATTTGAC
ACACGAAATGGGATACAATTAAAATCTCTATGACCATACACATTTTTCTTTTCCTATTTTCTCAGTGTAA
AGGACAGCGACCAGAGACATTAAGTGGGAGGGGATTGAAAGGGAGGGGAACTGGGCTCATGGGGCGATGG
GGCGATAGCCAATCTGCTTTGGCCGCCGGCTGGCTTCCGTTGCCGGAATGTGGTGACAATGTCTTCTTGG
GCTTTTCTCCGCCTCTACCTTTTTGGCTTAGACTTGGGCTTTAGTATTGGTATTTGTATTGGCTTTTTCT
ACTGGCTTCTGCTGTTGCCACAGACAATTGCATTGTTGTTGCCTCCTTGGCCGTTGTCGCAATGCTAGTA
ATTTGTGCAAATGAGCCTCTCAAAAGGCGTGGAGCCTTTGGGACACGAATTCAAGGGAGGGGGGGTAGAT
GGAAGAAATCCGAGGGGCTATCAAGAAAAAACAAACCCAAATAGACAAAAAATGTTTAAAGCGCCATTCG
GCTATGTAAACGATGTGGGCCCCGCCGAAAATGAACAACAAGAAAGGAAAGCTGTTAAATGTTTTTCCCC
ACATTTTCCATTTTCCAGTGCTTCGTTTTCAGATTTTTGCCATAGTTCAGATGGCTTCGTTCCTGCTTTT
TTCCTTCTATTGCGGCAATAAAACGAAATGATAGTACCGTTTTGGCTTTAAATGTTAATTGAGTTGAGTT
GGCCAATCCCTAGATTTAAAGCGCCAACAAATCCAGCCATTTAACGAATCGCGCACAGCTCCAGCTAAAC
TAATCAGCGAATAGCACGTGCTCCGCGCGAATGCCCAAAAAACAGATAAATAATGGCCTCGCCATAAAAA
ACTACGCACCGCGCTGagaaaaaaaaaagagaaaaagaaaTAGCAATTGCAGCATAACTATTTACTACGC
CTACTCTGACGTTTTGCTATCGTTATCTGTGAAAAATTAGCAGCAGGTCCCGGTCTGAATCAAAATAAAC
CGAAAGTAAACAACTGGCGAGCGGGAAAGAAGCTCACGCATACGGCCATAAATGGGGATGATAAAGCGGC
CGGCACAAGACGAGTTGGCCAAAAACACGCCTGCGCCATTGAGTTAACAGCCGCCTCCCCAAAACCATAc
cccctcccccccccccACAACACCGACGTTGATAAAGCCCCCGAGAAATACGCACACACATTTTACTGT
TACGCATCGCGTTCGTTTTCGATTTAATGCCGCCGCGTATTTCACCTGCAAAAATTTCGGTATTCCGCTG
TTTGTTTTGTTTACCTCCACAAATTTCCGTCTGGCCTGCTGGCGCTACTCGGTGGCTTTAAGGCCACCAC

CACATCCCCAAAAGTCGGGCTTTTGTTTGCTTAACTCAATTTTATTGTTCGCCATTCGCaaaaaataaaa
aataaaataaaaaaaCACAAAACAGCCGAAACGGGCTGAGGCGAAAAAGAGCAAAAATTATGTGTGTCAG
CGGCGGGCAGGGCAACAAAATACGAAATACACATAAATGAAACCTCCAAATTACCAGGGACAACAGCAGA
AAGAGGTCGCCTTCTTTAAATAAAATAAACAAATAAAAGCTGAAAACGAAATGCCATGGCATATGGGAAA
AACTCCCAGATAGCCAAAAGCGTGGGTCAAATTGAGTCACCTCACGCAATGCAAAAcaacagagaaacaa
ctaaaaaactaacaacaacatcgacaccttgaaacaacaacagctaacaaCAATCCCAGGCACTTGTCTG
ATTTTCGGCCTGTCCaaaaataaaatcaaaataaaaaaCACACACACTCACACGATTCGTCTGGCAACGT
CATGGGCAAAAATATATACTTATAGTACGAGTATAATAATAATAGACGaaaaaacaacaaatcgtaaaaa
aaacgaaacaaaaaatgtaggcacaaaaaTATTGAATAATAAAAATGCGTTGGAGTGTGGCGATTTGTTTT
GATTTGATTTCAATTCGCTTATGGTAAATGGAAGATTTTGAATATATTTTGAATATTGCGCATACGCGCC
GTTGGCCGCTGTACTGCAAACTGGTTAAGTTGTGAAACCAGAGCGCTTCCTGTTTGTATGCCATAAAAAT
ACCGAAGCTGTTAGCATCGATCCACTTTGGGGCCAAAACACTTGTCATTACACGATTTCCTTTTGTCCAA
ATTGTATTTACCTTCAGTGGGTTTCTTGGCTCTTCGCCTTACCTTTCTTTTTCGAACGAACATTTCCGCT
TTTATTGTTACTGTTGACATTGGCTTGAATAAGCGCAGATATGGGCCATAGAATCTTGGCTCTTATCGTG
CAAACTGATAAGCTAAAAGCTCTTTCACAAAGCACACAAAAACCTCAATGAAGTTGCTGGCACAAAAAAA
TACAACAAACAACAATCGCTCTAATCACAGAAATCAGCCAACGACTTTTGGTTCTTTTTGTTCGCTTTTT
GTGTTGTTTTCGGGTTGCGGGGCATTTATTTTTTGCCTGCGTATAAATTACACTTGAAAATTGTTTAGAT
TTTGATTGGTGATATCTGTGGGGCCtttttgttttttttttttttttttttttttttgttttGGTAGATT
GCAGGAGTCCACGGAGTCGCTTTGTCTCCGGCTAGCTCGCACTTGCGCTTTCCCTAGCCAATTTTTTGGT
GAAAGTCATCGTTTCCCCCGAAAACAAAAGCGAATATCGAGCACGTGGCTAATTTCATAAGCTTTAATtg
caaacttcaaactacaaactgaaaactgcaaaccgcaCTGCAAATCAAGTCGAAATGGAGCTGCAGCCAG
CGAAATTCATGCAATTTTCACTAATTTCGCCGGCACATGCAAACAACCGACTGACTCTCTGGGCTGGCAT
ATCATATCGCATCTCTCACTTCCTGACACCGCAATGTAATTTTGCTTTCAACACCTCTTTTTTCTTTCGT
GTTTCGCTCCTCGACTGACTGTTGCCGTGTTGATTGCGGCCAGCAAACGTGGCCAAATAATGGCCAAGAC
AGCAGAAAAAACACAGCATTTCCCAGGCTCAAGAGCCCCGAAATCGGGCGCGAATATATCGCATTTCAAT
CACGCGCTCTGGGGCAATTTACGTGCGGCCACGCAGCAGCGAGTAGGCCGAAAAGGCATTCAAAAGACGC
GGTCAAAGTTGCGGCAGTTGCAGCAgcagcatcaacaatgcaacatcaacattgcaacTGCAACCGCTCG
CTTCCAGTTTATTAAAGTTTTTCCAGCTCAGCGCAATGGGGCGGAAAGCAAACGGCCTAACGGTACGGAT
TGTGCGCATTAAAATGGCCACATCGTGATGGCCAGGCCAAAACCACCTTTCCCTCAACCCTGCCCAAccc
cccccTCCCAGCAACCCACTCCTGACTAACCGACAGGCACGCGCCTTTTGCCGGCCGGCTAGCAGGAAAA
TGAGGGGGGAAAATCGAGCGCAGCGAGCACTGATTCTCCACGGATGGGGGAAATGCGCGATTTATGCGCG
AATTTACTTTTATGCGATTAGCAACGATCTGCGGCAGAAATTTATCGTTGAGTTTAGTAAATTTAATTGG
GACGGAAATCCAGGCAAAAGGTGATGGTGCCAGCGCTAGTGCTGTTGCATTAGACAAAAGGCTAAAACCT
GGTAGCCGAGTAATAAAGTTGTACGGCCCAGCACAGCTCTTTTGGCCAAGGAGGCAGCATTTGGCTAAGT
GCAGACTTGGCCGCGATTGCTCGCGGCTTTTCACATTGCACAATGGTGATAGTGAGTCGGTTGGCAGGCA
CGAGTTGGTTGtgttttctgttttctgttttctgtttCGTATTTTGTGCTTTGGCCCAAAGCAGATGGCC
AAAAGATAAGCACAGTTGAGGAGTTCTGACCGAAATTCTGAGCATCAATAATTGCTTGCTTCACTAGCAC
TCCATCTTCTGAGGAAACCGCCACCTGCTTTTCAACTTTAATCCTGGTACGCAGCAAGTGCTGGAATCCT
TTGAAGAGTCTTAAACCACTTGTCTCAATAGCCGCCATTTGGCTTAAATGATTTGGCTAGAAGGGGGGTA
GCACTCTGCTTGATTATGGCAAATATGCCAAATCAGTTTTTCCGTTTCAAATGCTTTGAATTTATGAGAG
CGTCGCGTCTTTATGGTCATCCCCCGCCATGGTCATATGGTCATTTGGCCAGCGAAGGTTGCGCCAAGGT
TAGCCAACTTAAAGCCACCTTAAAGCCATCTTGAAACTGCATGGAAAGCCATCGGAACTGGGAAATCTAC
GGGCTGATATTCATTTGACGTCTGTTCGAAAAATGTGAGGCCATCGGAAGGAGGCCAATTAATGACGAAA
CAATAACACACACACAATGGGCGGCGGAATTTGGTGGCCACAGAAATTAGTTAAATGGAAAAATAGTTTT

GCGATGTTTTGCATTGAAATGCCGCTCGAAGCGATTATCGCTGAAAAGACCTCGACGGATTGTTGTTGCT
GTTTTGCGAATTACAACAACGATGGGCGGTCGCTTTGATGTGTCTAAGCAAATGCCGCCAATGTCTGTGT
GTGTATATCTTTATCGCCCAGTTTCTAGCTTCTCTATATGTAGATGACTGATAAAATCAAGAATGATAAT
CTGGGTCAATGTTTGAAGCGGGAATTGAGTTGTTTAAAATGTGTTATATATAGAACTAGGCCATGAGTAC
AGCTCTGTTTCAATGCAACGAAGAGGTGCTTTCTTACAAATAATATATATTATTTATTTACTAAATCCTC
TGTTTTTCCCATCTTAttttttttGCAGGTTCCCGGAAACGTCCGCCCAGCGATCACCAGGACTACGGC
AACTATCTGGAAACATGCCGTGCCGCCGAGATTCTGTCATCGATGAAGTTGCAGAGTCCGCATGGCTGTA
GGTCATCCTCCTTTTCCGACCCTGTCACACCCCCTTTTCGATCACGCCTACATATAAGCTAATTAATTCT
ATCCATTTCACCCCAGCAATGGCCGACAAGTGCTCGAGTCCCGGCAGCAGCTCTTCGGCTTCCTGGAGCT
CCGGTTCCCCGTCGCCGCCATTGAGTGACGACGGCCACGCCCACCACAGCCCACACAATATCATGTCGCC
CCACGATGCGGACAACGCACGCACACGCACAGCATCCGTGTCCACGTCGGACGAGGGCATTGTCATCGAC
TACAAGGAGGAGCGCAAGAAAAAG
>dme_circ_0008438_junction_seq
CGACTACAAGGAGGAGCGCAAGAAAAAGGGAACCCATTGAGCTGAAGAAGCGACTG
>dme_circ_0008439
GTTCCCGGAAACGTCCGCCCAGCGATCACCAGGACTACGGCAACTATCTGGAAACATGCCGTGCCGCCGA
GATTCTGTCATCGATGAAGTTGCAGAGTCCGCATGGCTGTAGGTCATCCTCCTTTTCCGACCCTGTCACA
CCCCCTTTTCGATCACGCCTACATATAAGCTAATTAATTCTATCCATTTCACCCCAGCAATGGCCGACAA
GTGCTCGAGTCCCGGCAGCAGCTCTTCGGCTTCCTGGAGCTCCGGTTCCCCGTCGCCGCCATTGAGTGAC
GACGGCCACGCCCACCACAGCCCACACAATATCATGTCGCCCCACGATGCGGACAACGCACGCACACGCA
CAGCATCCGTGTCCACGTCGGACGAGGGCATTGTCATCGACTACAAGGAGGAGCGCAAGAAAAAG
>dme_circ_0008439_junction_seq
CGACTACAAGGAGGAGCGCAAGAAAAAGGTTCCCGGAAACGTCCGCCCAGCGATCA
>dme_circ_0008440
GGTCATCCTCCTTTTCCGACCCTGTCACACCCCCTTTTCGATCACGCCTACATATAAGCTAATTAATTCT
ATCCATTTCACCCCAGCAATGGCCGACAAGTGCTCGAGTCCCGGCAGCAGCTCTTCGGCTTCCTGGAGCT
CCGGTTCCCCGTCGCCGCCATTGAGTGACGACGGCCACGCCCACCACAGCCCACACAATATCATGTCGCC
CCACGATGCGGACAACGCACGCACACGCACAGCATCCGTGTCCACGTCGGACGAGGGCATT
>dme_circ_0008440_junction_seq
ATCCGTGTCCACGTCGGACGAGGGCATTGGTCATCCTCCTTTTCCGACCCTGTCAC
>dme_circ_0008441
GGTGAGTTTCCCCATGACACTTGAAAATCTGGTTAACAGTAAGTCGTAAATTATATGATTGCCACCTCCC
CCATAATTCTGACTTTCTGGGTGTGCCCCTTGTCGTGTGTCTGTTAAtttttttgttttttttttgtttt
tgtGAAAATCACAATTCTAAATGAGACAGGAAACAAGGAGCGTAAAAACTGCTCAACCAG
>dme_circ_0008441_junction_seq
ACAAGGAGCGTAAAAACTGCTCAACCAGGGTGAGTTTCCCCATGACACTTGAAAAT
>dme_circ_0008442
GTTCAAACCGAGTCCGAGAGCACTTCACCCGACGAAGTGGAGCTCAATTCTGCCACTGAGATATCCACCG
ACTCTGAGTTTGACAACGATGAGATTATACGCCAGGCGCCCAAAATCTTCATCGATGACACCCATCTAAG
GAAGCCCACCAAGGTTCAGGATCAAGTCCACCATGATCGGACCCAATGCAGCTTCCGCCGGACTCCATCA
GAAGCAGTTGGCGGCGCGTGAGAAGGGCGGCAGCTACCTCCAGAAGTACCAACCACAACCGCCACTGCCA
CAGTTTAAACCGTTGGTCCAGGTGGATCCCACCCTGCTCATTGGCAGCCAGCGCGCTCCTCTTCAGAATC
CACGGCCAGGAGACTACTTGCTAAACAAGACGGCCAGTACGGAGGGTATCGCCTCAAAAAAGAGCCTGGA
GCTAAAAAAGCGCTATCTGCTGGGTGAGCCGGCCAATGGCAATAAGATCCAGAAGTCCGGATCCACTTCA

GTGCTGGATTCACGCATTCGCAGCTTCCAGTCGAACATATCGGAGTGCCAGAAGCTTTTGAATCCCAGCA
GCGACATAAGTGCCGGCATGCGAACCTTCCTCGATCGCACAAAGTTGGGCGAAGGCAGCCAGACGACACC
CGGACAGACGAACGAACTAATCCGTTCCGCCACCAGCAATGTGATTAACGATCTGCGCGTGGAGCTTCGG
ATACAGAAAACTGGCTCCAGCCACTCCACGGACAACGAGAAGGAAAACGTTTTCGTGAACTGTAAGAACG
AGCTGAACAAGGGGATGGAATACACGGATGCGGTCAATGCCACGCTGCTGGACCAGCTGGCCAGAAAAAG
TTCACCCACCACGCCGACGAATAAGACGGTGGTCGAGGTTATTGACCTGGTTACACCTGAGAAGCCAATT
GACATTATCGATCTAACGGCACTGGAAACGCCGAAAAAGCAGTTGGTCGATGGTAGCGCCATGGATGTAG
ATGAACGCCTCACACCCGATAGCAACAAAATCAGCGAACTGCAGCAGGAAGTGAAGGAGGAACCCAAGCC
GGATGTCTCTAGGGATGTGAAAGAATGCATACCAGATATACTGGGACACATTAAGGAGGGAACGGGATCG
AAGGAGCCAGGTGGAGAGGACCAACAGAGCCTGCTGGAGCAGTCGGACGAAGAGAAGCGCGACTCACCGG
AAAAGGATGTGGCCGAACATGAGCTTTATGAACCGGACAGTGTGCAGATCCAGGTGCCCAATATCCCATG
GGAAAAAAGCAAGCCGGAGGTCATGTCTACCACCGGCAGCAGTGGCTCCATCTGCTCAAGCTCAGACTCT
TCTAGTATTGAAGACATCCAGCACTACATTTTGGAGTCCACAACTAGTCCAGATACTCAGACAGTTGGCG
GAAAGCACAATGTGCCCCGTTTGGAGGTGCACGACACAAGTGGTGCCCTGATGCAGGTGGACAGCCTGAT
GATTGTGAACGGAAAGTATATTGGGGATCCCGAGGATGTCAAGTTCTTGGATATGCCGGCCAATGTTATT
GTTCCGCCAGCACCGGCGCTTAAAACGAATGAGCTGGATATGGAGGATGACCAAGAGGCGGAGGCGGAAC
CAGTAACTGCTACTCCGGAGCCGGTGGAATGTACGGTCATCGAGGCTGAGCGCCGTGTTACTGCTCCCCC
TCCTTTGCCAGAGATGGGTCCACCCAAACTGAAGTTCGATAGCAAAAATGAGAACAAGATCGAGAGCTTG
AAGAATCTTCCGTTGATCGTAGAGAGCAATGTGGAGCACAGTCAGGCAGTGAAACCCATTACTCTTAACT
TAAGCAATCTGGCCAGGACGCCAGATACACCAACCACGCCCACGGCGCACGATAGCGATAAAACACCCAC
TGGGGAAATTCTCTCGCGAGGATCTGACTCAGAAACCGAGCACACTGGCACTGGTCAGGTACTAACGGAG
ACGGAACTCTCCGACTGGACGGCCGACGACTGTATCTCGGAGAACTTTGTTGACTTGGAGTTCGCGCTTA
ACTCTAACAAGGGTACGATAAAACGGCGCAAGGATCGACGACGCAGTGGAGCAAGCAAACTTCCCAGTGG
CAACGAGGTAATCCACGAGCTGGCCAGGCAGGCGCCAGTGGTGCAAATGGATGGAATTCTTAGTGCCATC
GACATTGATGACATAGAGTTCATGGACACGGGTTCGGAGGGTTCTTGTGCTGAAGCTTATTCCGCAACAA
ATACAGCTCTCATTCAGAATAGAGGTTACATGGAGTACATCGAGGCGGAGCCGAAAAAGACGACCCGCAA
GGCAGCTCCACCATCCAGTTACCCAGGAAATTTACCGCCTTTAATGACGAAGCGGGACGAGAAACTGGGC
GTTGATTACATTGAGCAGGGGGCGTACATAATGCACGATGATGCAAAGACGCCTGTGAATGAGGTGGCTC
CTGCCATGACCCAGTCGCTAACTGACTCAATCACGCTCAATGAACTGGATGATGACAGTATGATAATATC
CCAAACCCAGCCAACGACAACGGAGGAAAGTGAGGCACTGACGGTGGTCACCAGTCCACTTGACACGTCC
TCGCCCAGGGTTCTCGATCAATTTGCATCCATGTTGGCGGCGGGAAAAGGTGACTCCACACCCAGTAGCT
CAGAGCAACAACCAAAGACGTCTACGGTGACGAGCAGCAGCACTGGGCCCAACTCCTCGACAACAGGAAA
CGTCTCGAAGGAGCCGCAGGAGGAGGACCTGCAAATCCAGTTTGAGTATGTTCGAGCACTGCAGCAGCGG
ATATCGCAGATCAGCACCCAACGGCGTAAGAGCTCTAAGGGAGAGGCACCTAACCTGCAGCTAAACAGTA
GCGCACCTGTGATAGAATCAGCCGAGGATCCGGCCAAGCCCGCAGAGGAGCCTCTGGTCTCAATGCGACC
GCGGACCACCAGCATTTCCGGAAAGGTACCGGAGATACCCACACTTAGCAGCAAGCTGGAAGAGATAACC
AAAGAACGCACTAAGCAAAAGGATCTGATTCACGACCTAGTCATGGACAAGTTGCAGTCGAAGAAGCAGC
TAAACGCTGAGAAGCGTCTGCACCGGAGTCGACAGCGCAGTTTGCTGACCAGTGGCTATGCCAGTGGCTC
CAGCCTTAGTCCGACGCCCAAGCTGGCTGCTGCTTGCAGTCCGCAGGATTCCAACTGCTCTAGCCAAGCG
CACTACCACGCCTCCACGGCGGAGGAGGCCCCGAAGCCGCCGGCGGAAAGGCCGTTGCAGAAGTCCGCCA
CGTCCACCTATGTGTCGCCTTATCGCACTGTCCAAGCGCCCACACGTAGTGCTGATCTCTATAAGCCGCG
CCCCTTCAGCGAACACATCGATTCGAACGCTCTGGCGGGTTACAAGCTCGGCAAGACGGCCTCGTTTAAT
GGCGGCAAGTTGGGCGACTTTGCGAAACCCATTGCCCCGGCGAGAGTTAACCGAGGAGGAGGTGTCGCGA
CCGCGGATATAGCCAATATTTCCGCGTCGACGGAGAACCTAAGAAGCGAGGCCAGGGCCAGGGCTCGTCT

TAAGTCTAACACAGAGCTGGGCCTTAGTCCCGAGGAAAAGATGCAGCTAATACGTTCAAGATTGCACTAC
GACCAAAACAGATCTCTGAAGCCGAAGCAACTGGAGGAGATGCCATCCGGGGATCTGGCGGCACGTGCCC
GCAAAATGAGTGCCTCGAAGAGCGTCAATGATCTGGCCTACATGGTGGGACAGCAGCAGCAGCAGCAGGT
TGAGAAGGATGCCGTGCTCCAAGCCAAGGCGGCTGACTTTACATCCGATCCCAATTTGGCGTCCGGTGGT
CAGGAGAAGGCAGGCAAAACTAAGTCCGGACGCAGGCCAAAGGATCCGGAGCGGCGTAAGAGTCTCATAC
AGTCGTTGTCCAGCTTCTTCCAAAAGGGATCTGGATCCGCGGCCTCCAGTTCCAAGGAGCAGGGCGGCGC
TGTGGCTGCCGTCCACTCTGAACAGTCAGAGCGACCAGGCACCAGCAGCAGCGGCACGCCCACAATATCG
GATGCGGCGGGTGGAGGCGGAGGAGGAGGTGGCGTCTTCAGCAGATTCCGCATCTCGCCCAAGTCCAAGG
AGAAGTCAAAGGGTCAGTGATTGTACCAAATATATAATTGTAATGTGGTTTTAGTCGGTGTTGGTGTAAC
CAAGTGTTGTCTGCCTCATGGAGATTGGCTAGTCCAAGCTATCGATTCACACGACCTCATCTAGCATATA
AGTTATATTAACTGCCGAGGACTCGATGTCTGGTGTAATAAGTGTGCCATTTATAACATATGAAATTCTT
TTAATCATTTTGCAGTCTTGCTTTGATCTGAGGAATTTCGGTTTTGGTGTAAGGACTTTTAACTATTCTA
AACTCTATTTACCACACACTTTCGAGTTTTCCAAATTTCTTACACTCTCAACAGCTTAGTCTTATTATTT
GGCATCCGTTACATTTGGTTTATTTTTTATCTAATCGCACATTTCTTCATCTGAGTGGGCTTTTTGTCTG
TTTGCTGTCCTATTAACACAATAAAGTTGCCACAatttttcttatgatttcttttcatttactttattttt
tgttttcttttaccctttgcAGCAAAAGTAATCTAAGTCTTAACATTTAAGATTGACTGCTGAATTATTT
CTTTTGCCTCAAAAATAACATATATGTTTTCTTAGTTTAATATCTATTAGGAGCGCATGGGTTTCAGGGA
AACCTTACGGACATAAGGATATTCAGTTGTGTGTTTTAAACTTTAAGTGTTTATGGCTTAAGTACTTGGC
AGCTTTTTGTTTAATGGGAATTAGTTTAAGAAGTGTTATTTATGATTTAAGCATAATGGATATTTTGCAT
GATTGGTTTCCCTGGATTCCCGGTTATGGTAATCAATAAAAGTTATGTGTTAAGTTTTTTGCCTTTGGAC
ATTTTGCACGTTACATGTACTATATTTTAGTAAATAACAAGCTGTTTCTAATAAGATTTTGAATATTGTT
TATTTTTCCATACGTTTTTCTCGACCTCTTTTTTTCCTCCTCTGAAAAACACTCAATCATTTAAACCGAT
ATATATTCTGATACCTCCTAACGCCATGCTATACTAGGGACAAGGATATGCTGGTCTGCAATGCAGCATC
TCCAGCAGGAGCCACATCCGCATCACAGAAAAATCACTCGCAAGAGTATCTGAACACCACGAACAACAGT
CGCTATCGAAAGCAAACGAACACTGCGAAACCGAAACCCGAATCGTTCTCTTCATCCAGTCCGCAGCTCT
ATATACACAAGCCCCACCACCTGGCCGCAGCTCATCCCAGTGCCCTGGACGACCAGACACCACCACCCAT
ACCGCCTCTTCCACTGAATTATCAGAGATCCGATGGATGAGAGCTACGCTAACGAGACACGAGAGCATAA
GAAGCAACGTGCCATATCGAAGGCTTCACGACAAGCTGAGCTCAAGCGATTGCGAATCGCTCAAGAGATT
CAGCGGGAACAGGAGGAGATCGAGGTGCAACTGAAGGATCTGGAGGCACGCGGCGTGCTTATTGAGAAGG
CCTTGCGAGGCGAGGCGCAGAATATTGAAAAGAACTGGACAAAAGCTCTGCCGATGTGGCCGCCGAGGGA
GCAATTCTCAACGAGATGCTGGAAATTGTCGCCAAGCGAGCCGCCCTACGACCCACAGCCTCCCAGCTCG
ACCTCACGGCAGCGGGATCAGCATCCACGTCCGCCGAGGCAACGGGCATTAAGCTGACGGGACAACCGCA
TGACCACGAAGAATCGATCGATTTGAATAGCCAACTTTCCCTGGAGATTGCTGGTCTGCGAAGTGGTGCA
TGGACTGTTGAGCCTAAATACTTAAGATGAAACAAGGGAACAGGGGAGAGCGTACCAAAAAGGCAGCTGA
ATATACAGAATTAACAATACTAAAACCCGTGAGCTGTAACCGAAACGAGCAACTAACTATTCAAAGTGGA
TAAGCTGGCTAAGAACAATTTCAATATTTATATAAGTAGAGAGCCTAACTAATATGGGAGAAGCGCGCAA
GGTTAATGTAAAAACAGGCAGAGATGAAATCAAAACCAAAAAACTAATTTCGCAATTTAAAAG
>dme_circ_0008442_junction_seq
CCAAAAAACTAATTTCGCAATTTAAAAGGTTCAAACCGAGTCCGAGAGCACTTCAC
>dme_circ_0008443
GTTCAAACCGAGTCCGAGAGCACTTCACCCGACGAAGTGGAGCTCAATTCTGCCACTGAGATATCCACCG
ACTCTGAGTTTGACAACGATGAGATTATACGCCAGGCGCCCAAAATCTTCATCGATGACACCCATCTAAG
GAAGCCCACCAAGGTTCAGGATCAAGTCCACCATGATCGGACCCAATGCAGCTTCCGCCGGACTCCATCA
GAAGCAGTTGGCGGCGCGTGAGAAGGGCGGCAGCTACCTCCAGAAGTACCAACCACAACCGCCACTGCCA

CAGTTTAAACCGTTGGTCCAGGTGGATCCCACCCTGCTCATTGGCAGCCAGCGCGCTCCTCTTCAGAATC
CACGGCCAGGAGACTACTTGCTAAACAAGACGGCCAGTACGGAGGGTATCGCCTCAAAAAAGAGCCTGGA
GCTAAAAAAGCGCTATCTGCTGGGTGAGCCGGCCAATGGCAATAAGATCCAGAAGTCCGGATCCACTTCA
GTGCTGGATTCACGCATTCGCAGCTTCCAGTCGAACATATCGGAGTGCCAGAAGCTTTTGAATCCCAGCA
GCGACATAAGTGCCGGCATGCGAACCTTCCTCGATCGCACAAAGTTGGGCGAAGGCAGCCAGACGACACC
CGGACAGACGAACGAACTAATCCGTTCCGCCACCAGCAATGTGATTAACGATCTGCGCGTGGAGCTTCGG
ATACAGAAAACTGGCTCCAGCCACTCCACGGACAACGAGAAGGAAAACGTTTTCGTGAACTGTAAGAACG
AGCTGAACAAGGGGATGGAATACACGGATGCGGTCAATGCCACGCTGCTGGACCAGCTGGCCAGAAAAAG
TTCACCCACCACGCCGACGAATAAGACGGTGGTCGAGGTTATTGACCTGGTTACACCTGAGAAGCCAATT
GACATTATCGATCTAACGGCACTGGAAACGCCGAAAAAGCAGTTGGTCGATGGTAGCGCCATGGATGTAG
ATGAACGCCTCACACCCGATAGCAACAAAATCAGCGAACTGCAGCAGGAAGTGAAGGAGGAACCCAAGCC
GGATGTCTCTAGGGATGTGAAAGAATGCATACCAGATATACTGGGACACATTAAGGAGGGAACGGGATCG
AAGGAGCCAGGTGGAGAGGACCAACAGAGCCTGCTGGAGCAGTCGGACGAAGAGAAGCGCGACTCACCGG
AAAAGGATGTGGCCGAACATGAGCTTTATGAACCGGACAGTGTGCAGATCCAGGTGCCCAATATCCCATG
GGAAAAAAGCAAGCCGGAGGTCATGTCTACCACCGGCAGCAGTGGCTCCATCTGCTCAAGCTCAGACTCT
TCTAGTATTGAAGACATCCAGCACTACATTTTGGAGTCCACAACTAGTCCAGATACTCAGACAGTTGGCG
GAAAGCACAATGTGCCCCGTTTGGAGGTGCACGACACAAGTGGTGCCCTGATGCAGGTGGACAGCCTGAT
GATTGTGAACGGAAAGTATATTGGGGATCCCGAGGATGTCAAGTTCTTGGATATGCCGGCCAATGTTATT
GTTCCGCCAGCACCGGCGCTTAAAACGAATGAGCTGGATATGGAGGATGACCAAGAGGCGGAGGCGGAAC
CAGTAACTGCTACTCCGGAGCCGGTGGAATGTACGGTCATCGAGGCTGAGCGCCGTGTTACTGCTCCCCC
TCCTTTGCCAGAGATGGGTCCACCCAAACTGAAGTTCGATAGCAAAAATGAGAACAAGATCGAGAGCTTG
AAGAATCTTCCGTTGATCGTAGAGAGCAATGTGGAGCACAGTCAGGCAGTGAAACCCATTACTCTTAACT
TAAGCAATCTGGCCAGGACGCCAGATACACCAACCACGCCCACGGCGCACGATAGCGATAAAACACCCAC
TGGGGAAATTCTCTCGCGAGGATCTGACTCAGAAACCGAGCACACTGGCACTGGTCAGGTACTAACGGAG
ACGGAACTCTCCGACTGGACGGCCGACGACTGTATCTCGGAGAACTTTGTTGACTTGGAGTTCGCGCTTA
ACTCTAACAAGGGTACGATAAAACGGCGCAAGGATCGACGACGCAGTGGAGCAAGCAAACTTCCCAGTGG
CAACGAGGTAATCCACGAGCTGGCCAGGCAGGCGCCAGTGGTGCAAATGGATGGAATTCTTAGTGCCATC
GACATTGATGACATAGAGTTCATGGACACGGGTTCGGAGGGTTCTTGTGCTGAAGCTTATTCCGCAACAA
ATACAGCTCTCATTCAGAATAGAGGTTACATGGAGTACATCGAGGCGGAGCCGAAAAAGACGACCCGCAA
GGCAGCTCCACCATCCAGTTACCCAGGAAATTTACCGCCTTTAATGACGAAGCGGGACGAGAAACTGGGC
GTTGATTACATTGAGCAGGGGGCGTACATAATGCACGATGATGCAAAGACGCCTGTGAATGAGGTGGCTC
CTGCCATGACCCAGTCGCTAACTGACTCAATCACGCTCAATGAACTGGATGATGACAGTATGATAATATC
CCAAACCCAGCCAACGACAACGGAGGAAAGTGAGGCACTGACGGTGGTCACCAGTCCACTTGACACGTCC
TCGCCCAGGGTTCTCGATCAATTTGCATCCATGTTGGCGGCGGGAAAAGGTGACTCCACACCCAGTAGCT
CAGAGCAACAACCAAAGACGTCTACGGTGACGAGCAGCAGCACTGGGCCCAACTCCTCGACAACAGGAAA
CGTCTCGAAGGAGCCGCAGGAGGAGGACCTGCAAATCCAGTTTGAGTATGTTCGAGCACTGCAGCAGCGG
ATATCGCAGATCAGCACCCAACGGCGTAAGAGCTCTAAGGGAGAGGCACCTAACCTGCAGCTAAACAGTA
GCGCACCTGTGATAGAATCAGCCGAGGATCCGGCCAAGCCCGCAGAGGAGCCTCTGGTCTCAATGCGACC
GCGGACCACCAGCATTTCCGGAAAGGTACCGGAGATACCCACACTTAGCAGCAAGCTGGAAGAGATAACC
AAAGAACGCACTAAGCAAAAGGATCTGATTCACGACCTAGTCATGGACAAGTTGCAGTCGAAGAAGCAGC
TAAACGCTGAGAAGCGTCTGCACCGGAGTCGACAGCGCAGTTTGCTGACCAGTGGCTATGCCAGTGGCTC
CAGCCTTAGTCCGACGCCCAAGCTGGCTGCTGCTTGCAGTCCGCAGGATTCCAACTGCTCTAGCCAAGCG
CACTACCACGCCTCCACGGCGGAGGAGGCCCCGAAGCCGCCGGCGGAAAGGCCGTTGCAGAAGTCCGCCA
CGTCCACCTATGTGTCGCCTTATCGCACTGTCCAAGCGCCCACACGTAGTGCTGATCTCTATAAGCCGCG

CCCCTTCAGCGAACACATCGATTCGAACGCTCTGGCGGGTTACAAGCTCGGCAAGACGGCCTCGTTTAAT
GGCGGCAAGTTGGGCGACTTTGCGAAACCCATTGCCCCGGCGAGAGTTAACCGAGGAGGAGGTGTCGCGA
CCGCGGATATAGCCAATATTTCCGCGTCGACGGAGAACCTAAGAAGCGAGGCCAGGGCCAGGGCTCGTCT
TAAGTCTAACACAGAGCTGGGCCTTAGTCCCGAGGAAAAGATGCAGCTAATACGTTCAAGATTGCACTAC
GACCAAAACAGATCTCTGAAGCCGAAGCAACTGGAGGAGATGCCATCCGGGGATCTGGCGGCACGTGCCC
GCAAAATGAGTGCCTCGAAGAGCGTCAATGATCTGGCCTACATGGTGGGACAGCAGCAGCAGCAGCAGGT
TGAGAAGGATGCCGTGCTCCAAGCCAAGGCGGCTGACTTTACATCCGATCCCAATTTGGCGTCCGGTGGT
CAGGAGAAGGCAGGCAAAACTAAGTCCGGACGCAGGCCAAAGGATCCGGAGCGGCGTAAGAGTCTCATAC
AGTCGTTGTCCAGCTTCTTCCAAAAGGGATCTGGATCCGCGGCCTCCAGTTCCAAGGAGCAGGGCGGCGC
TGTGGCTGCCGTCCACTCTGAACAGTCAGAGCGACCAGGCACCAGCAGCAGCGGCACGCCCACAATATCG
GATGCGGCGGGTGGAGGCGGAGGAGGAGGTGGCGTCTTCAGCAGATTCCGCATCTCGCCCAAGTCCAAGG
AGAAGTCAAAGG
>dme_circ_0008443_junction_seq
TCGCCCAAGTCCAAGGAGAAGTCAAAGGGTTCAAACCGAGTCCGAGAGCACTTCAC
>dme_circ_0008444
AGTTCCAAGGTGGCGCTGGCCTTTAAAAAGCAGGCTGCCTCCGAAAAGTGCCGCTTCTGTAAGCAAACCG
TCTACCTGATGGAGAAGACCACCGTGGAGGGATTGGTTCTGCATCGCAATTGCCTTAAGTGCCACCACTG
CCACACCAACTTGCGTCTGGGAGGCTACGCCTTTGATCGGGACGATCCGCAGGGCCGATTTTACTGCACC
CAACACTTCCGGTTGCCACCCAAACCGCTGCCGCAGCGCACCAACAAAGCCAGGGAAATCCGCTGCCGCT
CAACCCGCCTCGCCTGCTGTACCACCAACTGCGGGATCCGTACCCACTGCAGCTGCCACATCGGAGCATA
TGGACACCACTCCACCCAGGGACCAGGTGGATCTACTGGAGACCTCGCGAGCAAATGCCTCTGCCGATGC
CATGTCCGATGATGAAGCCAATGTTATCGATGAGCACGAATGGTCTGGTCGCAACTTCTTGCCCGAGTCC
AACAACGATTCCCAATCAGAGCTATCCAGTTCAGATGAATCGGATACGGAATCGGATTCGGAGATGTTTG
AGGAGGCGGATGATTCGCCGTTTGGTGCTCAGACCCTCCAGCTGGCGTCGGATTGGATTGGAAAGCAATA
CTGTGAGGACAGTGATGATTCTGACGATTTCTACGACTCAAGTGAAGGTATTGCGGGGTGGGTCATATTT
AACTATTTTCTCTCTTGGCGATGGGGAGACTAACCTTCTCATTATTCTCATTTATTTTCCTTGACTCTCT
TACCATATTCTTGAAATCTCCTTCTCATGGTGTTGGTTTCATTCTTGTCCTTGCAAATCCATAAATATTT
CAATTATTAGAAACTCAAGTTAAACGTAGACAATAAAGAAAACATGGCGGACAGAAGCTCTCTGAAATCG
GGCAATTCCTTTGAGTCCGCGCGCAGCCAGCCGTCTACGCCCCTGTCCACGCCCACTCGTGTCGAGATGG
AGCAACTGGAGCGAAATGCTCCACGCAAGTTTAGCAGCGAAATCGAGGCAATCAGCGAGAAACTTTACCA
CATGAACAACATGGTAAAGATGAACAAGGACCTCGAGGTGCTGGCCAAGGAAAATCTGGTCAAGAGCGAC
ATCCTGCGCAAGCTCACGCTGAAGGAGAAGTGGCTGGCGGAGAATGCGGCCATAGCAGCAGGCCAAAAAG
TGACTCCGACTCCTAGTGCGACTGCTCCTGGGCTTCAACCCAAGTCGAAGTTCGATGAAAAGTTCGAAAA
GGTAGTGAGTCCACCTCAGCCGGTTGTCGAACCCAAACCCAAGCCCGTAATCGATTTCAATTTGGATGAG
TTAAAACCACGTAAACCGAACTTTGAGGAGCGACCCAAGGAGCAGCTGCCCAGGCCTGAAAGTTTGAAAA
AACCTCCACAACAAAAACCGAAGGGCAGCAGCACAAACGTGAGCCGCTCCAACAGCTTGAAGAGTAATGC
CAGCAACGGGAGTCCAAAGGTGAAAAAAGCTCCTATTTCAAATAACAGCAAAATGCAAATTGAAGGAATC
CTTGGTACCTTGAGAAAGATTCAGAGCCAGAACAGTAGCGATCAGGATGAAGATATGGATGTAGATGAGG
ATGTGGAAAGAAAGCCAAATAAAGAGCTCAACAGCAAGTTGAAGGAAATCCAAGCCAGCAGCTTTGCCGG
CACAATGGACCATATTAAATCCCAGCTGACTATGCCAACGGTTAGTGCGCAGGCACCACCCTCCATGGAT
CTTTCCAAGTACTTTCCCAACCAGAAGCAAGAGAAGAGCTCCACCAGCAGCACAAACAAGAATCAGGTTA
CCCTGAAGGATGTAAATCTGGCCAAGTACTTCCCCAGCAGTCCAGCTCCACAACGGAGAACCGTGGAAAC
GGTGGCCGATCGACTAAAGAAGTCACAGACTGAGGCTGCTCTAGCTAAAACCAAGCTACTGGAGGATCAG
GccaataaccaggccgaaaagaccaagaaagaggtcgaaaagGAAGGAGAGTCTAAAAAAATCACGAAAA

AGGTGGCAGACTCCAAAGCAGTACCTCCAAAACGGCAGGCCTCGTTAGACACCTTTAGCTTAAGGGAGCA
CCAAATGGATGGAGCATTAGACCTGACCAAAAAGAAGGGTCCCACCAAAGCGAGCGCTGGTGTTAAAAAA
CCAGCCAAATCAGGGAGTACCACATCCGTGACAAAAGCCACGGCCACATCCAAAGGAAAGACCATTAAGA
TTGTAAAGAAAATCGTACCCAAAGGAACCAAAGCTAAAAAGGCAGCAGAAGCGGCCCAAGAATCCGCAGT
AGTAGAGGCTCCGCCAGAAAAGAAACCTCCAAAAGATGAAGCAGAGCGGATATTGGACGAAATTCTGGGA
GATGGTGAGTATCGTTCACCCAGCTCCGAGTATCAGCGCTTGTTCCAGGACGAAAAGTCGCCTAGCGATT
TGTCCGATAACATAGATCGGATACTCGAGGAATCCGGGTTGGATGTGGAATTGGGTCTGCCAAAACGCAG
CAGTAAGAAACTGGTAAAAACCAAATCCCTGGGCGAGGGTGACTTTGACATGAAGCCTTCAAAAGAAAGA
CTTACTGGAGTACAGAACATCCTTAAGCGATTTGAGTCAATGAGTTCAGTCACCTCACAGAACAGTGATG
AACAGGCGGCGTTCAAATTACGTCGCATGGAGTCCACCACCAGTAATCTGAGCAGCTTGACCCGTTCCAG
GGAATCACTTGTCTCCGTCAGCGATTCAATGAGTGATTTGGAGAAAACCATGGATTACCTCCGTAATGAA
TGGCGCAATGAGGCCACCAATTTTCTGCAAAAGAAGCGTGATAAATTCTATGCCAAAAAGGAGGAGCAAG
AAAAGGAGTCTAAAATTCTGGCTAAACCTGATCCCTTAGACAATCTTCCTGTTCAATATCGCGACTCCAA
GTTGGCCAAGTTCTTTGGCTTGGCCGCTAGCAAGTCACCAGAAAACCGAAAGTCCCCTATTAAGAAGAAG
AAATCTCCCTCTAAAACGCCAAAGGTGACCAAAGCAAACAATTCACTGGAGGAGCTGGCCAAGATTAGTA
ATGTTCGCCAGACCAAGAAGGCACAGCCGAAGACTCTAAAGCCTGTAGAAGTAAAACCTTTAAAGCCAGC
CAGTCCAGTTCCAGATGATTTTGAGATTCTTGATCTGCTGGAAAAAGCCACGGAGGCCAAAGAACTGGAA
CGCTCGAAGACCAAGAGTCCAGCCGTAGAATCTATCAGTCAGACGCCCAAAGAAGCTATAGTAGAAATTT
CCTTACCAGTGGAAGACATTAAAAATCTACCCAAAACCGGGTGTGATAAGTCCTCAAACAGCTCTCGCCG
TGGTTCCCAATCTAGTTTGATCATGTCGCGACGACACTCGGAGATTTCTCTAAACGAGAAACTCAACCAG
GACGCACTCGCAGCCTTGAACCAAATAGAAAAAGAAAGAGAGGCGGAGCAAGTGGACGAACTTTTCCAGA
GCATGGTGGAGGAAATGGAGCAGGAACCGCAGCCCACAGCTATTGTCGAACCTCCAGAAGAAGACATCGA
TGCAGATTCCTTGTGCACAACAATTAGCAAGAGTCCCAGTGCACAGCCCGTTACGGTGGTCAAACGTGGC
AGTTCCGAGGATCAGAGCATAGAGAAGCTTTTCAGCCACTTTTCCGACGAGATGCTGGTGAATGTGGAGT
TTGACTCTAACGATGAGCTTGTAGGAATTACACCAAGGGCAACACTCGTTTCCCGAAACACAGAAGATCG
GGACTATCTGGATAAACTAGAGTCTTTGGAGCGCGATGAGGAGACTTTCCAACCGGTTGTTGGGGAAAAA
TTCATACAGGAAAATGTCCAGGACGAAGTGGACGGATTGCATTTCCCATCACGCCCACAACGGAGACCCA
AAAGCAGTTCATCTTCCAGTGAACCATCACTTCCTGTGGCTCCTCAAAGGTTGaaaaaaaaGCTATCGAA
ATTAGATCCGGAGGATATGCCTCCTTCTGTACAGGATCTATTACAACAGGTTTACCAGAAGAACATCCAA
CCTGAATTAGTGGAAGTAATTCCAGTTGAAGGCAAACAAACTTTAAGGTTCCCCAGCATGTTGGCGGAAG
AAGATGTAGACGAAGTGGATCACTCTAAAGAAGGAATCAAGAAAATTGAAACGGCTCCAGAAGAGGTTCG
TAAGGTAACCGAGCCGGAGGATGTTGCTCGGGTAATTCCAAGTCCAATCAAACCGTCAATAAGTCAGAGC
AACTCCCTCAAGAGCGAAAATTCCTCTGGCAGCAGTTTAGTGGAAATACCAAAAATAATTACACCACCCA
AGTCGAGTAGCAAGGAAAACTCTTCGGATTGGGACATGGAGAAGCTGCCCGCATCACCCATGCCACGACG
AAGACTTCTGCCAAATCAAACGCCATATAAAGCCCCAAGCGTGGCTAGTAAGGAGAGCTCCTTAGAGTGG
GACATGGAGAAGCTACCCAACAGTCCCATGTTGCCAAGGAGGAACAAGATGCGAGCAATCTCACCTAGTA
CCAACCCTGTGCAACTTCTCAACAATTTACCCTCCGATGTAGATGACGAGGCGGCACAGAGACGCCTTAT
CGAGGATTTCGAGCAGGAGAGACGTCAGGCTCTGATCAAGCGGGATGAGAACTTCGAGGCCATTGCGGCG
GAGCAACGCAGGCGCGACTCCTTACAGAGCAGCAGTAACTCGAGCAGCAAACGCAGTTTACCACCGCCCA
CGCCGCCCATGATGGCATCGCGACGCGGTACAACTCAAGACACAAATCGCACCCAGGATACCGCATCGCG
GCACGAGGGAACGCCGCCCATGTTCAAGAAGCTGGATGTCGATGGCAGCGGTACATCAATGGATTCCACC
TCCTGCTCCACGCGGCGCAGTTCGTTTGCATTTATAGAGTTACAGGATAACAAACCAGTGATTGTGCCCA
TGCCCAAGAAACTGAAGTTGCCAAAGCCGGAGCCACCTAGGTTCGTACCCGAGCCAGTGGCCACTGATGA
GCCTGTGCCCGAGGTTTTTCAGGGTCGCGCTTGGCCCAAGACACAGTTGGAAGGAGAGGTCGATCTAGGC

GATTCGGATAATGAAGATGAAACAGAAAAGCTAAAGAAACAGCTGCCCGAATACGCTCGTTCGGACTCTC
CTCCTTCGGCTGCATTCAAGAATCGCAAGTGGCCGGATGGGAAAACAGTTTTTGATAAACGAGCCGAATC
TCTTGAGGAGGAAGATATCTTCGAAGGATTACTGTCCCCAAGGAAAAGAGGTTCTCAAAGATTCATGGAC
AAGCCGCGCTCTCAATCGCCACAGCCTTTCAAACCGCTGGCCAACAGCTCCAGGAAAAGCTCAAAGTCTT
TTAGCGACCTTAAGAAAGGACCCTCCTTGCAATCTCTGTCGGCGCAATCCAGCCAGGACACGGACACACT
GTCCACCACCACAACAGTAGCCACAGCTCGTCCTGCTAGCTATGCCAACTACGAGGACCCCATGGATGCC
AGTACCCAAGCTTTGCTGGATCGAAGCAAACGGTTACACAACCGCAAAAGAGATTTCGTAAACGAGCGAG
TAGTGGAGCGCAACCCCTATATGAGGGATGTGCTTAGGAGCACGGATCGCCGTGATTACGACGACGTGGA
TGAAGATCTGACTAGCTACAGGCCAAGACATTATGCCAGCTCCACGCTAAATCGTTTCCCCAACACCACA
ATAAGAAAAAGCAACAACTACGATTACCTCAGTCCCAGCAGTGATTATTTGAGCAGGAGAAGCTACATAC
CGAGCGCCAGTGCAACCAGCAGTTACTATCCATCGACCACGCGTAGCTCCCATTTGAGTGACCTGTTCCG
ACGACGCAGCCCCGCCAGCGGAACTGTATCCGCACTTTCCGGCTACGGCAACAAAGAGTCGTGCGTTATC
TCATAGTTGTTAGCCCTAAACCATTGACCAAAGCAGCCTTCTCTTTTCATTTATATTTTACCCCGTTGTG
GCCGCAGACGACGCCTCAACTACTATCTCTACTGAGAGCCAACCCAACGTCAGCGAAACCAACCCCACAA
ACAGACCCCTGTACCCTGACCAAACCGATCCGACCCGACCCGAACCCCCATTCAAGACACTATAGGTCCC
CGATAAGCAGACCAAGAATACccaaccaaccaaccaaccaatcaaccaTGCTAGACTGCCCGCCCGCCTG
TCTGCTCTCGATGTGCCACGCCTTCAAGTTCAAGTTCAAGTCCAGTCCAAGTCCTCCAATTAGCCAATGA
CTAGATCGGGCTGGCCTTAGATCGAGTTGGCCACTTGATTGAAAGTAAATGCACCTGGGTACGATCTACG
AAGGTAAATTCAAGCACAGCCGTCCGCGAAATGTTGCGCTAACCCCGAACTCAAGCAGCCCGAAAAAAGC
CGAAAGTTTGTAGTCTCTAAACCTAGAAAATACTGAAAACTGAATACCGATTGTGTTGTGTCGTTCTGTT
GCAGTCCTTACAATATAAACAGGCCTAACCCCCAGCTAATCCTGGCCAGCGTTTGTTTTGTCCTTCAGCG
TTCCTTTCGTTTCCCTTCTGGATCGTACCTAAAGGCATGACGCCGCTGTTTTTTTGCTGTTTAACTCACT
ACTCCGACTAATTAGCATGTGCGAACCAAAGGTGTTTGCTGTACATAAAACGCCGCCTACGGGTCGCTAC
TGATCCCGACTCCCATTGCTTTTGTTTTACGTTCGTTTAGTTACCTGCTGTCCTGTCCTAACGCACACTA
ATTCGTTTGTGTATTCGTAACTCCACTTGCAGGGTTCAAACCGAGTCCGAGAGCACTTCACCCGACGAAG
TGGAGCTCAATTCTGCCACTGAGATATCCACCGACTCTGAGTTTGACAACGATGAGATTATACGCCAGGC
GCCCAAAATCTTCATCGATGACACCCATCTAAGGAAGCCCACCAAGGTTCAGGATCAAGTCCACCATGAT
CGGACCCAATGCAGCTTCCGCCGGACTCCATCAGAAGCAGTTGGCGGCGCGTGAGAAGGGCGGCAGCTAC
CTCCAGAAGTACCAACCACAACCGCCACTGCCACAGTTTAAACCGTTGGTCCAGGTGGATCCCACCCTGC
TCATTGGCAGCCAGCGCGCTCCTCTTCAGAATCCACGGCCAGGAGACTACTTGCTAAACAAGACGGCCAG
TACGGAGGGTATCGCCTCAAAAAAGAGCCTGGAGCTAAAAAAGCGCTATCTGCTGGGTGAGCCGGCCAAT
GGCAATAAGATCCAGAAGTCCGGATCCACTTCAGTGCTGGATTCACGCATTCGCAGCTTCCAGTCGAACA
TATCGGAGTGCCAGAAGCTTTTGAATCCCAGCAGCGACATAAGTGCCGGCATGCGAACCTTCCTCGATCG
CACAAAGTTGGGCGAAGGCAGCCAGACGACACCCGGACAGACGAACGAACTAATCCGTTCCGCCACCAGC
AATGTGATTAACGATCTGCGCGTGGAGCTTCGGATACAGAAAACTGGCTCCAGCCACTCCACGGACAACG
AGAAGGAAAACGTTTTCGTGAACTGTAAGAACGAGCTGAACAAGGGGATGGAATACACGGATGCGGTCAA
TGCCACGCTGCTGGACCAGCTGGCCAGAAAAAGTTCACCCACCACGCCGACGAATAAGACGGTGGTCGAG
GTTATTGACCTGGTTACACCTGAGAAGCCAATTGACATTATCGATCTAACGGCACTGGAAACGCCGAAAA
AGCAGTTGGTCGATGGTAGCGCCATGGATGTAGATGAACGCCTCACACCCGATAGCAACAAAATCAGCGA
ACTGCAGCAGGAAGTGAAGGAGGAACCCAAGCCGGATGTCTCTAGGGATGTGAAAGAATGCATACCAGAT
ATACTGGGACACATTAAGGAGGGAACGGGATCGAAGGAGCCAGGTGGAGAGGACCAACAGAGCCTGCTGG
AGCAGTCGGACGAAGAGAAGCGCGACTCACCGGAAAAGGATGTGGCCGAACATGAGCTTTATGAACCGGA
CAGTGTGCAGATCCAGGTGCCCAATATCCCATGGGAAAAAAGCAAGCCGGAGGTCATGTCTACCACCGGC
AGCAGTGGCTCCATCTGCTCAAGCTCAGACTCTTCTAGTATTGAAGACATCCAGCACTACATTTTGGAGT

CCACAACTAGTCCAGATACTCAGACAGTTGGCGGAAAGCACAATGTGCCCCGTTTGGAGGTGCACGACAC
AAGTGGTGCCCTGATGCAGGTGGACAGCCTGATGATTGTGAACGGAAAGTATATTGGGGATCCCGAGGAT
GTCAAGTTCTTGGATATGCCGGCCAATGTTATTGTTCCGCCAGCACCGGCGCTTAAAACGAATGAGCTGG
ATATGGAGGATGACCAAGAGGCGGAGGCGGAACCAGTAACTGCTACTCCGGAGCCGGTGGAATGTACGGT
CATCGAGGCTGAGCGCCGTGTTACTGCTCCCCCTCCTTTGCCAGAGATGGGTCCACCCAAACTGAAGTTC
GATAGCAAAAATGAGAACAAGATCGAGAGCTTGAAGAATCTTCCGTTGATCGTAGAGAGCAATGTGGAGC
ACAGTCAGGCAGTGAAACCCATTACTCTTAACTTAAGCAATCTGGCCAGGACGCCAGATACACCAACCAC
GCCCACGGCGCACGATAGCGATAAAACACCCACTGGGGAAATTCTCTCGCGAGGATCTGACTCAGAAACC
GAGCACACTGGCACTGGTCAGGTACTAACGGAGACGGAACTCTCCGACTGGACGGCCGACGACTGTATCT
CGGAGAACTTTGTTGACTTGGAGTTCGCGCTTAACTCTAACAAGGGTACGATAAAACGGCGCAAGGATCG
ACGACGCAGTGGAGCAAGCAAACTTCCCAGTGGCAACGAGGTAATCCACGAGCTGGCCAGGCAGGCGCCA
GTGGTGCAAATGGATGGAATTCTTAGTGCCATCGACATTGATGACATAGAGTTCATGGACACGGGTTCGG
AGGGTTCTTGTGCTGAAGCTTATTCCGCAACAAATACAGCTCTCATTCAGAATAGAGGTTACATGGAGTA
CATCGAGGCGGAGCCGAAAAAGACGACCCGCAAGGCAGCTCCACCATCCAGTTACCCAGGAAATTTACCG
CCTTTAATGACGAAGCGGGACGAGAAACTGGGCGTTGATTACATTGAGCAGGGGGCGTACATAATGCACG
ATGATGCAAAGACGCCTGTGAATGAGGTGGCTCCTGCCATGACCCAGTCGCTAACTGACTCAATCACGCT
CAATGAACTGGATGATGACAGTATGATAATATCCCAAACCCAGCCAACGACAACGGAGGAAAGTGAGGCA
CTGACGGTGGTCACCAGTCCACTTGACACGTCCTCGCCCAGGGTTCTCGATCAATTTGCATCCATGTTGG
CGGCGGGAAAAGGTGACTCCACACCCAGTAGCTCAGAGCAACAACCAAAGACGTCTACGGTGACGAGCAG
CAGCACTGGGCCCAACTCCTCGACAACAGGAAACGTCTCGAAGGAGCCGCAGGAGGAGGACCTGCAAATC
CAGTTTGAGTATGTTCGAGCACTGCAGCAGCGGATATCGCAGATCAGCACCCAACGGCGTAAGAGCTCTA
AGGGAGAGGCACCTAACCTGCAGCTAAACAGTAGCGCACCTGTGATAGAATCAGCCGAGGATCCGGCCAA
GCCCGCAGAGGAGCCTCTGGTCTCAATGCGACCGCGGACCACCAGCATTTCCGGAAAGGTACCGGAGATA
CCCACACTTAGCAGCAAGCTGGAAGAGATAACCAAAGAACGCACTAAGCAAAAGGATCTGATTCACGACC
TAGTCATGGACAAGTTGCAGTCGAAGAAGCAGCTAAACGCTGAGAAGCGTCTGCACCGGAGTCGACAGCG
CAGTTTGCTGACCAGTGGCTATGCCAGTGGCTCCAGCCTTAGTCCGACGCCCAAGCTGGCTGCTGCTTGC
AGTCCGCAGGATTCCAACTGCTCTAGCCAAGCGCACTACCACGCCTCCACGGCGGAGGAGGCCCCGAAGC
CGCCGGCGGAAAGGCCGTTGCAGAAGTCCGCCACGTCCACCTATGTGTCGCCTTATCGCACTGTCCAAGC
GCCCACACGTAGTGCTGATCTCTATAAGCCGCGCCCCTTCAGCGAACACATCGATTCGAACGCTCTGGCG
GGTTACAAGCTCGGCAAGACGGCCTCGTTTAATGGCGGCAAGTTGGGCGACTTTGCGAAACCCATTGCCC
CGGCGAGAGTTAACCGAGGAGGAGGTGTCGCGACCGCGGATATAGCCAATATTTCCGCGTCGACGGAGAA
CCTAAGAAGCGAGGCCAGGGCCAGGGCTCGTCTTAAGTCTAACACAGAGCTGGGCCTTAGTCCCGAGGAA
AAGATGCAGCTAATACGTTCAAGATTGCACTACGACCAAAACAGATCTCTGAAGCCGAAGCAACTGGAGG
AGATGCCATCCGGGGATCTGGCGGCACGTGCCCGCAAAATGAGTGCCTCGAAGAGCGTCAATGATCTGGC
CTACATGGTGGGACAGCAGCAGCAGCAGCAGGTTGAGAAGGATGCCGTGCTCCAAGCCAAGGCGGCTGAC
TTTACATCCGATCCCAATTTGGCGTCCGGTGGTCAGGAGAAGGCAGGCAAAACTAAGTCCGGACGCAGGC
CAAAGGATCCGGAGCGGCGTAAGAGTCTCATACAGTCGTTGTCCAGCTTCTTCCAAAAGGGATCTGGATC
CGCGGCCTCCAGTTCCAAGGAGCAGGGCGGCGCTGTGGCTGCCGTCCACTCTGAACAGTCAGAGCGACCA
GGCACCAGCAGCAGCGGCACGCCCACAATATCGGATGCGGCGGGTGGAGGCGGAGGAGGAGGTGGCGTCT
TCAGCAGATTCCGCATCTCGCCCAAGTCCAAGGAGAAGTCAAAGG
>dme_circ_0008444_junction_seq
TCGCCCAAGTCCAAGGAGAAGTCAAAGGAGTTCCAAGGTGGCGCTGGCCTTTAAAA
>dme_circ_0008445
GATCGCGAACGGGGAGATATTCCGTCGGCATTGCCGCGCACGGCGGACGAGCAGTTCAGCGATCGGATTA

AAAACATGGAGCAGCGGATGACGGGACGTGGTGGCCTTGGTGGTGACAAGAAGCCCAAGGATCTGATGCG
GGCTATCGGCAAGATCGACTCGAACGATTGGAATGTACGCGAGATCGAGAAGAAGATCGAGCTGTCGAAG
AAGACGGAGATCCACGGGCCTAAGGGCCGCGAGAAGGTTCCCAAGTGGAGCAAGGAGCAGTTCCAGGCGC
GACAGCACAAGATGTCCAAGCCGCAACGCCAGGATTCGCGTGAGGCGGAAAAGTTCAAGGACATCGACCA
GACTATCCGCAACTTGGACAAGCAGCTAAAGGAGGGCCACAATCTGGATGTGGGCGAGCGGGGACGCAAC
AAAGTGGCCTCCATTGCCGGTCAGTTTGGCAAAAAGGATGAGGCCAATTCGGATGAGAAGAACGCGGGCA
GCAGCAATGCCACCACCAACACCAACAACACAGTCATACCCAAATCTGTGAGTAATTTTGTGTTCATCCA
CCACCTTCTTGTATCAATTGTTTTGTTTGGTTTCTATTGTTATCGGTTTCCCCTGTGCtattttatattt
gcttttatatttatgtatactatatttaatatttttgcTTACCTTCATGGTTGCTTCCTGTGGTTGCTGT
AGTCGATGATGTTGTTAGCGTGCTTTAAGCAAAAGTCTAGGCAATTTTCCCACAAACACTCATAGTCATC
AAGCTACATATCCACTCATTTGTACATGACACCCATCGAATGCCAAGCAAATGTTTATGTGTTATGTCGT
TAATTGTTAATCATTGTCATCTTCAAATTTCTTCTAATACTTATCCTTCTAATAATACAGAAGTTCCAAG
GTGGCGCTGGCCTTTAAAAAGCAGGCTGCCTCCGAAAAGTGCCGCTTCTGTAAGCAAACCGTCTACCTGA
TGGAGAAGACCACCGTGGAGGGATTGGTTCTGCATCGCAATTGCCTTAAGTGCCACCACTGCCACACCAA
CTTGCGTCTGGGAGGCTACGCCTTTGATCGGGACGATCCGCAGGGCCGATTTTACTGCACCCAACACTTC
CGGTTGCCACCCAAACCGCTGCCGCAGCGCACCAACAAAGCCAGGGAAATCCGCTGCCGCTCAACCCGCC
TCGCCTGCTGTACCACCAACTGCGGGATCCGTACCCACTGCAGCTGCCACATCGGAGCATATGGACACCA
CTCCACCCAGGGACCAGGTGGATCTACTGGAGACCTCGCGAGCAAATGCCTCTGCCGATGCCATGTCCGA
TGATGAAGCCAATGTTATCGATGAGCACGAATGGTCTGGTCGCAACTTCTTGCCCGAGTCCAACAACGAT
TCCCAATCAGAGCTATCCAGTTCAGATGAATCGGATACGGAATCGGATTCGGAGATGTTTGAGGAGGCGG
ATGATTCGCCGTTTGGTGCTCAGACCCTCCAGCTGGCGTCGGATTGGATTGGAAAGCAATACTGTGAGGA
CAGTGATGATTCTGACGATTTCTACGACTCAAGTGAAGGTATTGCGGGGTGGGTCATATTTAACTATTTT
CTCTCTTGGCGATGGGGAGACTAACCTTCTCATTATTCTCATTTATTTTCCTTGACTCTCTTACCATATT
CTTGAAATCTCCTTCTCATGGTGTTGGTTTCATTCTTGTCCTTGCAAATCCATAAATATTTCAATTATTA
GAAACTCAAGTTAAACGTAGACAATAAAGAAAACATGGCGGACAGAAGCTCTCTGAAATCGGGCAATTCC
TTTGAGTCCGCGCGCAGCCAGCCGTCTACGCCCCTGTCCACGCCCACTCGTGTCGAGATGGAGCAACTGG
AGCGAAATGCTCCACGCAAGTTTAGCAGCGAAATCGAGGCAATCAGCGAGAAACTTTACCACATGAACAA
CATGGTAAAGATGAACAAGGACCTCGAGGTGCTGGCCAAGGAAAATCTGGTCAAGAGCGACATCCTGCGC
AAGCTCACGCTGAAGGAGAAGTGGCTGGCGGAGAATGCGGCCATAGCAGCAGGCCAAAAAGTGACTCCGA
CTCCTAGTGCGACTGCTCCTGGGCTTCAACCCAAGTCGAAGTTCGATGAAAAGTTCGAAAAGGTAGTGAG
TCCACCTCAGCCGGTTGTCGAACCCAAACCCAAGCCCGTAATCGATTTCAATTTGGATGAGTTAAAACCA
CGTAAACCGAACTTTGAGGAGCGACCCAAGGAGCAGCTGCCCAGGCCTGAAAGTTTGAAAAAACCTCCAC
AACAAAAACCGAAGGGCAGCAGCACAAACGTGAGCCGCTCCAACAGCTTGAAGAGTAATGCCAGCAACGG
GAGTCCAAAGGTGAAAAAAGCTCCTATTTCAAATAACAGCAAAATGCAAATTGAAGGAATCCTTGGTACC
TTGAGAAAGATTCAGAGCCAGAACAGTAGCGATCAGGATGAAGATATGGATGTAGATGAGGATGTGGAAA
GAAAGCCAAATAAAGAGCTCAACAGCAAGTTGAAGGAAATCCAAGCCAGCAGCTTTGCCGGCACAATGGA
CCATATTAAATCCCAGCTGACTATGCCAACGGTTAGTGCGCAGGCACCACCCTCCATGGATCTTTCCAAG
TACTTTCCCAACCAGAAGCAAGAGAAGAGCTCCACCAGCAGCACAAACAAGAATCAGGTTACCCTGAAGG
ATGTAAATCTGGCCAAGTACTTCCCCAGCAGTCCAGCTCCACAACGGAGAACCGTGGAAACGGTGGCCGA
TCGACTAAAGAAGTCACAGACTGAGGCTGCTCTAGCTAAAACCAAGCTACTGGAGGATCAGGccaataac
caggccgaaaagaccaagaaagaggtcgaaaagGAAGGAGAGTCTAAAAAAATCACGAAAAAGGTGGCAG
ACTCCAAAGCAGTACCTCCAAAACGGCAGGCCTCGTTAGACACCTTTAGCTTAAGGGAGCACCAAATGGA
TGGAGCATTAGACCTGACCAAAAAGAAGGGTCCCACCAAAGCGAGCGCTGGTGTTAAAAAACCAGCCAAA
TCAGGGAGTACCACATCCGTGACAAAAGCCACGGCCACATCCAAAGGAAAGACCATTAAGATTGTAAAGA

AAATCGTACCCAAAGGAACCAAAGCTAAAAAGGCAGCAGAAGCGGCCCAAGAATCCGCAGTAGTAGAGGC
TCCGCCAGAAAAGAAACCTCCAAAAGATGAAGCAGAGCGGATATTGGACGAAATTCTGGGAGATGGTGAG
TATCGTTCACCCAGCTCCGAGTATCAGCGCTTGTTCCAGGACGAAAAGTCGCCTAGCGATTTGTCCGATA
ACATAGATCGGATACTCGAGGAATCCGGGTTGGATGTGGAATTGGGTCTGCCAAAACGCAGCAGTAAGAA
ACTGGTAAAAACCAAATCCCTGGGCGAGGGTGACTTTGACATGAAGCCTTCAAAAGAAAGACTTACTGGA
GTACAGAACATCCTTAAGCGATTTGAGTCAATGAGTTCAGTCACCTCACAGAACAGTGATGAACAGGCGG
CGTTCAAATTACGTCGCATGGAGTCCACCACCAGTAATCTGAGCAGCTTGACCCGTTCCAGGGAATCACT
TGTCTCCGTCAGCGATTCAATGAGTGATTTGGAGAAAACCATGGATTACCTCCGTAATGAATGGCGCAAT
GAGGCCACCAATTTTCTGCAAAAGAAGCGTGATAAATTCTATGCCAAAAAGGAGGAGCAAGAAAAGGAGT
CTAAAATTCTGGCTAAACCTGATCCCTTAGACAATCTTCCTGTTCAATATCGCGACTCCAAGTTGGCCAA
GTTCTTTGGCTTGGCCGCTAGCAAGTCACCAGAAAACCGAAAGTCCCCTATTAAGAAGAAGAAATCTCCC
TCTAAAACGCCAAAGGTGACCAAAGCAAACAATTCACTGGAGGAGCTGGCCAAGATTAGTAATGTTCGCC
AGACCAAGAAGGCACAGCCGAAGACTCTAAAGCCTGTAGAAGTAAAACCTTTAAAGCCAGCCAGTCCAGT
TCCAGATGATTTTGAGATTCTTGATCTGCTGGAAAAAGCCACGGAGGCCAAAGAACTGGAACGCTCGAAG
ACCAAGAGTCCAGCCGTAGAATCTATCAGTCAGACGCCCAAAGAAGCTATAGTAGAAATTTCCTTACCAG
TGGAAGACATTAAAAATCTACCCAAAACCGGGTGTGATAAGTCCTCAAACAGCTCTCGCCGTGGTTCCCA
ATCTAGTTTGATCATGTCGCGACGACACTCGGAGATTTCTCTAAACGAGAAACTCAACCAGGACGCACTC
GCAGCCTTGAACCAAATAGAAAAAGAAAGAGAGGCGGAGCAAGTGGACGAACTTTTCCAGAGCATGGTGG
AGGAAATGGAGCAGGAACCGCAGCCCACAGCTATTGTCGAACCTCCAGAAGAAGACATCGATGCAGATTC
CTTGTGCACAACAATTAGCAAGAGTCCCAGTGCACAGCCCGTTACGGTGGTCAAACGTGGCAGTTCCGAG
GATCAGAGCATAGAGAAGCTTTTCAGCCACTTTTCCGACGAGATGCTGGTGAATGTGGAGTTTGACTCTA
ACGATGAGCTTGTAGGAATTACACCAAGGGCAACACTCGTTTCCCGAAACACAGAAGATCGGGACTATCT
GGATAAACTAGAGTCTTTGGAGCGCGATGAGGAGACTTTCCAACCGGTTGTTGGGGAAAAATTCATACAG
GAAAATGTCCAGGACGAAGTGGACGGATTGCATTTCCCATCACGCCCACAACGGAGACCCAAAAGCAGTT
CATCTTCCAGTGAACCATCACTTCCTGTGGCTCCTCAAAGGTTGaaaaaaaaGCTATCGAAATTAGATCC
GGAGGATATGCCTCCTTCTGTACAGGATCTATTACAACAGGTTTACCAGAAGAACATCCAACCTGAATTA
GTGGAAGTAATTCCAGTTGAAGGCAAACAAACTTTAAGGTTCCCCAGCATGTTGGCGGAAGAAGATGTAG
ACGAAGTGGATCACTCTAAAGAAGGAATCAAGAAAATTGAAACGGCTCCAGAAGAGGTTCGTAAGGTAAC
CGAGCCGGAGGATGTTGCTCGGGTAATTCCAAGTCCAATCAAACCGTCAATAAGTCAGAGCAACTCCCTC
AAGAGCGAAAATTCCTCTGGCAGCAGTTTAGTGGAAATACCAAAAATAATTACACCACCCAAGTCGAGTA
GCAAGGAAAACTCTTCGGATTGGGACATGGAGAAGCTGCCCGCATCACCCATGCCACGACGAAGACTTCT
GCCAAATCAAACGCCATATAAAGCCCCAAGCGTGGCTAGTAAGGAGAGCTCCTTAGAGTGGGACATGGAG
AAGCTACCCAACAGTCCCATGTTGCCAAGGAGGAACAAGATGCGAGCAATCTCACCTAGTACCAACCCTG
TGCAACTTCTCAACAATTTACCCTCCGATGTAGATGACGAGGCGGCACAGAGACGCCTTATCGAGGATTT
CGAGCAGGAGAGACGTCAGGCTCTGATCAAGCGGGATGAGAACTTCGAGGCCATTGCGGCGGAGCAACGC
AGGCGCGACTCCTTACAGAGCAGCAGTAACTCGAGCAGCAAACGCAGTTTACCACCGCCCACGCCGCCCA
TGATGGCATCGCGACGCGGTACAACTCAAGACACAAATCGCACCCAGGATACCGCATCGCGGCACGAGGG
AACGCCGCCCATGTTCAAGAAGCTGGATGTCGATGGCAGCGGTACATCAATGGATTCCACCTCCTGCTCC
ACGCGGCGCAGTTCGTTTGCATTTATAGAGTTACAGGATAACAAACCAGTGATTGTGCCCATGCCCAAGA
AACTGAAGTTGCCAAAGCCGGAGCCACCTAGGTTCGTACCCGAGCCAGTGGCCACTGATGAGCCTGTGCC
CGAGGTTTTTCAGGGTCGCGCTTGGCCCAAGACACAGTTGGAAGGAGAGGTCGATCTAGGCGATTCGGAT
AATGAAGATGAAACAGAAAAGCTAAAGAAACAGCTGCCCGAATACGCTCGTTCGGACTCTCCTCCTTCGG
CTGCATTCAAGAATCGCAAGTGGCCGGATGGGAAAACAGTTTTTGATAAACGAGCCGAATCTCTTGAGGA
GGAAGATATCTTCGAAGGATTACTGTCCCCAAGGAAAAGAGGTTCTCAAAGATTCATGGACAAGCCGCGC

TCTCAATCGCCACAGCCTTTCAAACCGCTGGCCAACAGCTCCAGGAAAAGCTCAAAGTCTTTTAGCGACC
TTAAGAAAGGACCCTCCTTGCAATCTCTGTCGGCGCAATCCAGCCAGGACACGGACACACTGTCCACCAC
CACAACAGTAGCCACAGCTCGTCCTGCTAGCTATGCCAACTACGAGGACCCCATGGATGCCAGTACCCAA
GCTTTGCTGGATCGAAGCAAACGGTTACACAACCGCAAAAGAGATTTCGTAAACGAGCGAGTAGTGGAGC
GCAACCCCTATATGAGGGATGTGCTTAGGAGCACGGATCGCCGTGATTACGACGACGTGGATGAAGATCT
GACTAGCTACAGGCCAAGACATTATGCCAGCTCCACGCTAAATCGTTTCCCCAACACCACAATAAGAAAA
AGCAACAACTACGATTACCTCAGTCCCAGCAGTGATTATTTGAGCAGGAGAAGCTACATACCGAGCGCCA
GTGCAACCAGCAGTTACTATCCATCGACCACGCGTAGCTCCCATTTGAGTGACCTGTTCCGACGACGCAG
CCCCGCCAGCGGAACTGTATCCGCACTTTCCGGCTACGGCAACAAAGAGTCGTGCGTTATCTCATAGTTG
TTAGCCCTAAACCATTGACCAAAGCAGCCTTCTCTTTTCATTTATATTTTACCCCGTTGTGGCCGCAGAC
GACGCCTCAACTACTATCTCTACTGAGAGCCAACCCAACGTCAGCGAAACCAACCCCACAAACAGACCCC
TGTACCCTGACCAAACCGATCCGACCCGACCCGAACCCCCATTCAAGACACTATAGGTCCCCGATAAGCA
GACCAAGAATACccaaccaaccaaccaaccaatcaaccaTGCTAGACTGCCCGCCCGCCTGTCTGCTCTC
GATGTGCCACGCCTTCAAGTTCAAGTTCAAGTCCAGTCCAAGTCCTCCAATTAGCCAATGACTAGATCGG
GCTGGCCTTAGATCGAGTTGGCCACTTGATTGAAAGTAAATGCACCTGGGTACGATCTACGAAGGTAAAT
TCAAGCACAGCCGTCCGCGAAATGTTGCGCTAACCCCGAACTCAAGCAGCCCGAAAAAAGCCGAAAGTTT
GTAGTCTCTAAACCTAGAAAATACTGAAAACTGAATACCGATTGTGTTGTGTCGTTCTGTTGCAGTCCTT
ACAATATAAACAGGCCTAACCCCCAGCTAATCCTGGCCAGCGTTTGTTTTGTCCTTCAGCGTTCCTTTCG
TTTCCCTTCTGGATCGTACCTAAAGGCATGACGCCGCTGTTTTTTTGCTGTTTAACTCACTACTCCGACT
AATTAGCATGTGCGAACCAAAGGTGTTTGCTGTACATAAAACGCCGCCTACGGGTCGCTACTGATCCCGA
CTCCCATTGCTTTTGTTTTACGTTCGTTTAGTTACCTGCTGTCCTGTCCTAACGCACACTAATTCGTTTG
TGTATTCGTAACTCCACTTGCAGGGTTCAAACCGAGTCCGAGAGCACTTCACCCGACGAAGTGGAGCTCA
ATTCTGCCACTGAGATATCCACCGACTCTGAGTTTGACAACGATGAGATTATACGCCAGGCGCCCAAAAT
CTTCATCGATGACACCCATCTAAGGAAGCCCACCAAGGTTCAGGATCAAGTCCACCATGATCGGACCCAA
TGCAGCTTCCGCCGGACTCCATCAGAAGCAGTTGGCGGCGCGTGAGAAGGGCGGCAGCTACCTCCAGAAG
TACCAACCACAACCGCCACTGCCACAGTTTAAACCGTTGGTCCAGGTGGATCCCACCCTGCTCATTGGCA
GCCAGCGCGCTCCTCTTCAGAATCCACGGCCAGGAGACTACTTGCTAAACAAGACGGCCAGTACGGAGGG
TATCGCCTCAAAAAAGAGCCTGGAGCTAAAAAAGCGCTATCTGCTGGGTGAGCCGGCCAATGGCAATAAG
ATCCAGAAGTCCGGATCCACTTCAGTGCTGGATTCACGCATTCGCAGCTTCCAGTCGAACATATCGGAGT
GCCAGAAGCTTTTGAATCCCAGCAGCGACATAAGTGCCGGCATGCGAACCTTCCTCGATCGCACAAAGTT
GGGCGAAGGCAGCCAGACGACACCCGGACAGACGAACGAACTAATCCGTTCCGCCACCAGCAATGTGATT
AACGATCTGCGCGTGGAGCTTCGGATACAGAAAACTGGCTCCAGCCACTCCACGGACAACGAGAAGGAAA
ACGTTTTCGTGAACTGTAAGAACGAGCTGAACAAGGGGATGGAATACACGGATGCGGTCAATGCCACGCT
GCTGGACCAGCTGGCCAGAAAAAGTTCACCCACCACGCCGACGAATAAGACGGTGGTCGAGGTTATTGAC
CTGGTTACACCTGAGAAGCCAATTGACATTATCGATCTAACGGCACTGGAAACGCCGAAAAAGCAGTTGG
TCGATGGTAGCGCCATGGATGTAGATGAACGCCTCACACCCGATAGCAACAAAATCAGCGAACTGCAGCA
GGAAGTGAAGGAGGAACCCAAGCCGGATGTCTCTAGGGATGTGAAAGAATGCATACCAGATATACTGGGA
CACATTAAGGAGGGAACGGGATCGAAGGAGCCAGGTGGAGAGGACCAACAGAGCCTGCTGGAGCAGTCGG
ACGAAGAGAAGCGCGACTCACCGGAAAAGGATGTGGCCGAACATGAGCTTTATGAACCGGACAGTGTGCA
GATCCAGGTGCCCAATATCCCATGGGAAAAAAGCAAGCCGGAGGTCATGTCTACCACCGGCAGCAGTGGC
TCCATCTGCTCAAGCTCAGACTCTTCTAGTATTGAAGACATCCAGCACTACATTTTGGAGTCCACAACTA
GTCCAGATACTCAGACAGTTGGCGGAAAGCACAATGTGCCCCGTTTGGAGGTGCACGACACAAGTGGTGC
CCTGATGCAGGTGGACAGCCTGATGATTGTGAACGGAAAGTATATTGGGGATCCCGAGGATGTCAAGTTC
TTGGATATGCCGGCCAATGTTATTGTTCCGCCAGCACCGGCGCTTAAAACGAATGAGCTGGATATGGAGG

ATGACCAAGAGGCGGAGGCGGAACCAGTAACTGCTACTCCGGAGCCGGTGGAATGTACGGTCATCGAGGC
TGAGCGCCGTGTTACTGCTCCCCCTCCTTTGCCAGAGATGGGTCCACCCAAACTGAAGTTCGATAGCAAA
AATGAGAACAAGATCGAGAGCTTGAAGAATCTTCCGTTGATCGTAGAGAGCAATGTGGAGCACAGTCAGG
CAGTGAAACCCATTACTCTTAACTTAAGCAATCTGGCCAGGACGCCAGATACACCAACCACGCCCACGGC
GCACGATAGCGATAAAACACCCACTGGGGAAATTCTCTCGCGAGGATCTGACTCAGAAACCGAGCACACT
GGCACTGGTCAGGTACTAACGGAGACGGAACTCTCCGACTGGACGGCCGACGACTGTATCTCGGAGAACT
TTGTTGACTTGGAGTTCGCGCTTAACTCTAACAAGGGTACGATAAAACGGCGCAAGGATCGACGACGCAG
TGGAGCAAGCAAACTTCCCAGTGGCAACGAGGTAATCCACGAGCTGGCCAGGCAGGCGCCAGTGGTGCAA
ATGGATGGAATTCTTAGTGCCATCGACATTGATGACATAGAGTTCATGGACACGGGTTCGGAGGGTTCTT
GTGCTGAAGCTTATTCCGCAACAAATACAGCTCTCATTCAGAATAGAGGTTACATGGAGTACATCGAGGC
GGAGCCGAAAAAGACGACCCGCAAGGCAGCTCCACCATCCAGTTACCCAGGAAATTTACCGCCTTTAATG
ACGAAGCGGGACGAGAAACTGGGCGTTGATTACATTGAGCAGGGGGCGTACATAATGCACGATGATGCAA
AGACGCCTGTGAATGAGGTGGCTCCTGCCATGACCCAGTCGCTAACTGACTCAATCACGCTCAATGAACT
GGATGATGACAGTATGATAATATCCCAAACCCAGCCAACGACAACGGAGGAAAGTGAGGCACTGACGGTG
GTCACCAGTCCACTTGACACGTCCTCGCCCAGGGTTCTCGATCAATTTGCATCCATGTTGGCGGCGGGAA
AAGGTGACTCCACACCCAGTAGCTCAGAGCAACAACCAAAGACGTCTACGGTGACGAGCAGCAGCACTGG
GCCCAACTCCTCGACAACAGGAAACGTCTCGAAGGAGCCGCAGGAGGAGGACCTGCAAATCCAGTTTGAG
TATGTTCGAGCACTGCAGCAGCGGATATCGCAGATCAGCACCCAACGGCGTAAGAGCTCTAAGGGAGAGG
CACCTAACCTGCAGCTAAACAGTAGCGCACCTGTGATAGAATCAGCCGAGGATCCGGCCAAGCCCGCAGA
GGAGCCTCTGGTCTCAATGCGACCGCGGACCACCAGCATTTCCGGAAAGGTACCGGAGATACCCACACTT
AGCAGCAAGCTGGAAGAGATAACCAAAGAACGCACTAAGCAAAAGGATCTGATTCACGACCTAGTCATGG
ACAAGTTGCAGTCGAAGAAGCAGCTAAACGCTGAGAAGCGTCTGCACCGGAGTCGACAGCGCAGTTTGCT
GACCAGTGGCTATGCCAGTGGCTCCAGCCTTAGTCCGACGCCCAAGCTGGCTGCTGCTTGCAGTCCGCAG
GATTCCAACTGCTCTAGCCAAGCGCACTACCACGCCTCCACGGCGGAGGAGGCCCCGAAGCCGCCGGCGG
AAAGGCCGTTGCAGAAGTCCGCCACGTCCACCTATGTGTCGCCTTATCGCACTGTCCAAGCGCCCACACG
TAGTGCTGATCTCTATAAGCCGCGCCCCTTCAGCGAACACATCGATTCGAACGCTCTGGCGGGTTACAAG
CTCGGCAAGACGGCCTCGTTTAATGGCGGCAAGTTGGGCGACTTTGCGAAACCCATTGCCCCGGCGAGAG
TTAACCGAGGAGGAGGTGTCGCGACCGCGGATATAGCCAATATTTCCGCGTCGACGGAGAACCTAAGAAG
CGAGGCCAGGGCCAGGGCTCGTCTTAAGTCTAACACAGAGCTGGGCCTTAGTCCCGAGGAAAAGATGCAG
CTAATACGTTCAAGATTGCACTACGACCAAAACAGATCTCTGAAGCCGAAGCAACTGGAGGAGATGCCAT
CCGGGGATCTGGCGGCACGTGCCCGCAAAATGAGTGCCTCGAAGAGCGTCAATGATCTGGCCTACATGGT
GGGACAGCAGCAGCAGCAGCAGGTTGAGAAGGATGCCGTGCTCCAAGCCAAGGCGGCTGACTTTACATCC
GATCCCAATTTGGCGTCCGGTGGTCAGGAGAAGGCAGGCAAAACTAAGTCCGGACGCAGGCCAAAGGATC
CGGAGCGGCGTAAGAGTCTCATACAGTCGTTGTCCAGCTTCTTCCAAAAGGGATCTGGATCCGCGGCCTC
CAGTTCCAAGGAGCAGGGCGGCGCTGTGGCTGCCGTCCACTCTGAACAGTCAGAGCGACCAGGCACCAGC
AGCAGCGGCACGCCCACAATATCGGATGCGGCGGGTGGAGGCGGAGGAGGAGGTGGCGTCTTCAGCAGAT
TCCGCATCTCGCCCAAGTCCAAGGAGAAGTCAAAGG
>dme_circ_0008445_junction_seq
TCGCCCAAGTCCAAGGAGAAGTCAAAGGGATCGCGAACGGGGAGATATTCCGTCGG
>dme_circ_0008446
GCTTGTAACCCGCCAGAGCGTTCGAATCGATGTGTTCGCTGAAGGGGCGCGGCTTATAGAGATCAGCACT
ACGTGTGGGCGCTTGGACAGTGCGATAAGGCGACACATAGGTGGACGTGGCGGACTTCTGCAACGGCCTT
TCCGCCGGCGGCTTCGGGGCCTCCTCCGCCGTGGAGGCGTGGTAGTGCGCTTGGCTAGAGCAGTTGGAAT
CCTGCGGACTGCAAGCAGCAGCCAGCTTGGGCGTCGGACTAAGGCTGGAGCCACTGGCATAGCCACTGGT

CAGCAAACTGCGCTGTCGACTCCGGTGCAGACGCTTCTCAGCGTTTAGCTGCTTCTTCGACTGCAACTTG
TCCATGACTAGGTCGTGAATCAGATCCTTTTGCTTAGTGCGTTCTTTGGTTATCTCTTCCAGCTTGCTGC
TAAGTGTGGGTATCTCCGGTACCTTTCCGGAAATGCTGGTGGTCCGCGGTCGCATTGAGACCAGAGGCTC
CTCTGCGGGCTTGGCCGGATCCTCGGCTGATTCTATCACAGGTGCGCTACTGTTTAGCTGCAGGTTAGGT
GCCTCTCCCTTAGAGCTCTTACGCCGTTGGGTGCTGATCTGCGATATCCGCTGCTGCAGTGCTCGAACAT
ACTCAAACTGGATTTGCAGGTCCTCCTCCTGCGGCTCCTTCGAGACGTTTCCTGTTGTCGAGGAGTTGGG
CCCAGTGCTGCTGCTCGTCACCGTAGACGTCTTTGGTTGTTGCTCTGAGCTACTGGGTGTGGAGTCACCT
TTTCCCGCCGCCAACATGGATGCAAATTGATCGAGAACCCTGGGCGAGGACGTGTCAAGTGGACTGGTGA
CCACCGTCAGTGCCTCACTTTCCTCCGTTGTCGTTGGCTGGGTTTGGGATATTATCATACTGTCATCATC
CAGTTCATTGAGCGTGATTGAGTCAGTTAGCGACTGGGTCATGGCAGGAGCCACCTCATTCACAGGCGTC
TTTGCATCATCGTGCATTATGTACGCCCCCTGCTCAATGTAATCAACGCCCAGTTTCTCGTCCCGCTTCG
TCATTAAAGGCGGTAAATTTCCTGGGTAACTGGATGGTGGAGCTGCCTTGCGGGTCGTCTTTTTCGGCTC
CGCCTCGATGTACTCCATGTAACCTCTATTCTGAATGAGAGCTGTATTTGTTGCGGAATAAGCTTCAGCA
CAAGAACCCTCCGAACCCGTGTCCATGAACTCTATGTCATCAATGTCGATGGCACTAAGAATTCCATCCA
TTTGCACCACTGGCGCCTGCCTGGCCAGCTCGTGGATTACCTCGTTGCCACTGGGAAGTTTGCTTGCTCC
ACTGCGTCGTCGATCCTTGCGCCGTTTTATCGTACCCTTGTTAGAGTTAAGCGCGAACTCCAAGTCAACA
AAGTTCTCCGAGATACAGTCGTCGGCCGTCCAGTCGGAGAGTTCCGTCTCCGTTAGTACCTGACCAGTGC
CAGTGTGCTCGGTTTCTGAGTCAGATCCTCGCGAGAGAATTTCCCCAGTGGGTGTTTTATCGCTATCGTG
CGCCGTGGGCGTGGTTGGTGTATCTGGCGTCCTGGCCAGATTGCTTAAGTTAAGAGTAATGGGTTTCACT
GCCTGACTGTGCTCCACATTGCTCTCTACGATCAACGGAAGATTCTTCAAGCTCTCGATCTTGTTCTCAT
TTTTGCTATCGAACTTCAGTTTGGGTGGACCCATCTCTGGCAAAGGAGGGGGAGCAGTAACACGGCGCTC
AGCCTCGATGACCGTACATTCCACCGGCTCCGGAGTAGCAGTTACTGGTTCCGCCTCCGCCTCTTGGTCA
TCCTCCATATCCAGCTCATTCGTTTTAAGCGCCGGTGCTGGCGGAACAATAACATTGGCCGGCATATCCA
AGAACTTGACATCCTCGGGATCCCCAATATACTTTCCGTTCACAATCATCAGGCTGTCCACCTGCATCAG
GGCACCACTTGTGTCGTGCACCTCCAAACGGGGCACATTGTGCTTTCCGCCAACTGTCTGAGTATCTGGA
CTAGTTGTGGACTCCAAAATGTAGTGCTGGATGTCTTCAATACTAGAAGAGTCTGAGCTTGAGCAGATGG
AGCCACTGCTGCCGGTGGTAGACATGACCTCCGGCTTGCTTTTTTCCCATGGGATATTGGGCACCTGGAT
CTGCACACTGTCCGGTTCATAAAGCTCATGTTCGGCCACATCCTTTTCCGGTGAGTCGCGCTTCTCTTCG
TCCGACTGCTCCAGCAGGCTCTGTTGGTCCTCTCCACCTGGCTCCTTCGATCCCGTTCCCTCCTTAATGT
GTCCCAGTATATCTGGTATGCATTCTTTCACATCCCTAGAGACATCCGGCTTGGGTTCCTCCTTCACTTC
CTGCTGCAGTTCGCTGATTTTGTTGCTATCGGGTGTGAGGCGTTCATCTACATCCATGGCGCTACCATCG
ACCAACTGCTTTTTCGGCGTTTCCAGTGCCGTTAGATCGATAATGTCAATTGGCTTCTCAGGTGTAACCA
GGTCAATAACCTCGACCACCGTCTTATTCGTCGGCGTGGTGGGTGAACTTTTTCTGGCCAGCTGGTCCAG
CAGCGTGGCATTGACCGCATCCGTGTATTCCATCCCCTTGTTCAGCTCGTTCTTACAGTTCACGAAAAC
>dme_circ_0008446_junction_seq
TCAGCTCGTTCTTACAGTTCACGAAAACGCTTGTAACCCGCCAGAGCGTTCGAATC
>dme_circ_0008447
GTTCATTGAGCGTGATTGAGTCAGTTAGCGACTGGGTCATGGCAGGAGCCACCTCATTCACAGGCGTCTT
TGCATCATCGTGCATTATGTACGCCCCCTGCTCAATGTAATCAACGCCCAGTTTCTCGTCCCGCTTCGTC
ATTAAAGGCGGTAAATTTCCTGGGTAACTGGATGGTGGAGCTGCCTTGCGGGTCGTCTTTTTCGGCTCCG
CCTCGATGTACTCCAT
>dme_circ_0008447_junction_seq
TTTTCGGCTCCGCCTCGATGTACTCCATGTTCATTGAGCGTGATTGAGTCAGTTAG
>dme_circ_0008448

CTGAACAAGGGGATGGAATACACGGATGCGGTCAATGCCACGCTGCTGGACCAGCTGGCCAGAAAAAGTT
CACCCACCACGCCGACGAATAAGACGGTGGTCGAGGTTATTGACCTGGTTACACCTGAGAAGCCAATTGA
CATTATCGATCTAACGGCACTGGAAACGCCGAAAAAGCAGTTGGTCGATGGTAGCGCCATGGATGTAGAT
GAACGCCTCACACCCGATAGCAACAAAATCAGCGAACTGCAGCAGGAAGTGAAGGAGGAACCCAAGCCGG
ATGTCTCTAGGGATG
>dme_circ_0008448_junction_seq
GAACCCAAGCCGGATGTCTCTAGGGATGCTGAACAAGGGGATGGAATACACGGATG
>dme_circ_0008449
AGTTCCAAGGTGGCGCTGGCCTTTAAAAAGCAGGCTGCCTCCGAAAAGTGCCGCTTCTGTAAGCAAACCG
TCTACCTGATGGAGAAGACCACCGTGGAGGGATTGGTTCTGCATCGCAATTGCCTTAAGTGCCACCACTG
CCACACCAACTTGCGTCTGGGAGGCTACGCCTTTGATCGGGACGATCCGCAGGGCCGATTTTACTGCACC
CAACACTTCCGGTTGCCACCCAAACCGCTGCCGCAGCGCACCAACAAAGCCAGGGAAATCCGCTGCCGCT
CAACCCGCCTCGCCTGCTGTACCACCAACTGCGGGATCCGTACCCACTGCAGCTGCCACATCGGAGCATA
TGGACACCACTCCACCCAGGGACCAGGTGGATCTACTGGAGACCTCGCGAGCAAATGCCTCTGCCGATGC
CATGTCCGATGATGAAGCCAATGTTATCGATGAGCACGAATGGTCTGGTCGCAACTTCTTGCCCGAGTCC
AACAACGATTCCCAATCAGAGCTATCCAGTTCAGATGAATCGGATACGGAATCGGATTCGGAGATGTTTG
AGGAGGCGGATGATTCGCCGTTTGGTGCTCAGACCCTCCAGCTGGCGTCGGATTGGATTGGAAAGCAATA
CTGTGAGGACAGTGATGATTCTGACGATTTCTACGACTCAAGTGAAGGTATTGCGGGTGAGTGGGCACAA
AAGGGGAAGTTTAAAAATTAACTAATTTATATTAAACTCTTCTTTGGATTTTTAGATGACGGCAAAGATG
ACACCGAGGGTGAGGAATTCAAGAAGGCCCGCGAATTGAGGCGCCAGGAAGTTCGCCTGCAGCCGTTGCC
CGCCAATCTGCCCACAGATACGGAGACCGAGG
>dme_circ_0008449_junction_seq
AATCTGCCCACAGATACGGAGACCGAGGAGTTCCAAGGTGGCGCTGGCCTTTAAAA
>dme_circ_0008450
CTATCCAGTTCAGATGAATCGGATACGGAATCGGATTCGGAGATGTTTGAGGAGGCGGATGATTCGCCGT
TTGGTGCTCAGACCCTCCAGCTGGCGTCGGATTGGATTGGAAAGCAATACTGTGAGGACAGTGATGATTC
TGACGATTTCTACGACTCAAGTGAAGG
>dme_circ_0008450_junction_seq
CTGACGATTTCTACGACTCAAGTGAAGGCTATCCAGTTCAGATGAATCGGATACGG
>dme_circ_0008451
GATGCCTTCCAAGATCCACTGGCCACCGATGGTGAGGGAGGGGATGCACTTGAGGCCATGGTAGAGAACG
TTTTGCAGGACGACACAGCAAGCGAGGGCAGTCATCCCAGCAGTGACATGTCTCTGGAAAGTCCAGGAAG
TGAAG
>dme_circ_0008451_junction_seq
ATGTCTCTGGAAAGTCCAGGAAGTGAAGGATGCCTTCCAAGATCCACTGGCCACCG
>dme_circ_0008452
GATTAAAAAAGATTCCATCCAGAAAAATGCTGCGAATCGTTGTAACTTTGGCTCTAATTCTGGCCACAGT
CAATGCCACCAATGTCCAGCAGTGTAAGTAGCTAAAAACTCTTTTATTTTGTTGGAAAAACATCAAATCA
TTTTAAATCGACCAGTTTGTTAGATTGGATTCATGATTCTAACAGATTTCTATGACGATGATTCACTTCG
CAGACTTTGTCTCGAAAACTATTGCCAAAAATACGTTCTGAGAGAAATTTAAAGTCCTAACGTGTATCTG
TTGGCCAGGTTGATATGGCTTATCAACAAACAGCTAATCTCTTAGaaaaaaaaaCATAGCCTTCTTTTGA
GCTCACCTTTTGATTCTTCGAAAACATATTTCCGCTTTCAGGCAAAAATAAGCCGTTTCCACTGGATGTG
AATATTAAGGATTGCGAGGAACCGCCGTGCGTTGTGTACAAAGGAACCATAGCAGTCATGGAGGTGCACT
TCCTGGGCA

>dme_circ_0008452_junction_seq
GCAGTCATGGAGGTGCACTTCCTGGGCAGATTAAAAAAGATTCCATCCAGAAAAAT
>dme_circ_0008453
GATTCCATCCAGAAAAATGCTGCGAATCGTTGTAACTTTGGCTCTAATTCTGGCCACAGTCAATGCCACC
AATGTCCAGCAGTGTAAGTAGCTAAAAACTCTTTTATTTTGTTGGAAAAACATCAAATCATTTTAAATCG
ACCAGTTTGTTAGATTGGATTCATGATTCTAACAGATTTCTATGACGATGATTCACTTCGCAGACTTTGT
CTCGAAAACTATTGCCAAAAATACGTTCTGAGAGAAATTTAAAGTCCTAACGTGTATCTGTTGGCCAGGT
TGATATGGCTTATCAACAAACAGCTAATCTCTTAGaaaaaaaaaCATAGCCTTCTTTTGAGCTCACCTTT
TGATTCTTCGAAAACATATTTCCGCTTTCAGGCAAAAATAAGCCGTTTCCACTGGATGTGAATATTAAGG
ATTGCGAGGAACCGCCGTGCGTTGTGTACAAAGGAACCATAGCAGTCATGGAG
>dme_circ_0008453_junction_seq
GTACAAAGGAACCATAGCAGTCATGGAGGATTCCATCCAGAAAAATGCTGCGAATC
>dme_circ_0008454
GATTCCATCCAGAAAAATGCTGCGAATCGTTGTAACTTTGGCTCTAATTCTGGCCACAGTCAATGCCACC
AATGTCCAGCAGTGTAAGTAGCTAAAAACTCTTTTATTTTGTTGGAAAAACATCAAATCATTTTAAATCG
ACCAGTTTGTTAGATTGGATTCATGATTCTAACAGATTTCTATGACGATGATTCACTTCGCAGACTTTGT
CTCGAAAACTATTGCCAAAAATACGTTCTGAGAGAAATTTAAAGTCCTAACGTGTATCTGTTGGCCAGGT
TGATATGGCTTATCAACAAACAGCTAATCTCTTAGaaaaaaaaaCATAGCCTTCTTTTGAGCTCACCTTT
TGATTCTTCGAAAACATATTTCCGCTTTCAGGCAAAAATAAGCCGTTTCCACTGGATGTGAATATTAAGG
ATTGCGAGGAACCGCCGTGCGTTGTGTACAAAGGAACCATAGCAGTCATGGAGGTGCACTTCCTGGGCA
>dme_circ_0008454_junction_seq
GCAGTCATGGAGGTGCACTTCCTGGGCAGATTCCATCCAGAAAAATGCTGCGAATC
>dme_circ_0008455
GATAACAACAATATCAAGAGCATTACGGCCACCACAACGGCAAAGGTTTTGGGCATGAATCTGCCATATG
CCCTACCCGACGAAGTGTCCGATGTGTGCCGCAATCTCCTGTACGGTGCCATCTGTCCCATCGACAAGGA
CGAGGATGTCACGTATCAGTTCAACTTCTATGTGGAGCCTTCGTTCCCGGAAATAACGGCGGATGTCACT
GTCACGTTGAACGATGCGCAGAACGAACCGATCACCTGCTTCGTCGTGAGCT
>dme_circ_0008455_junction_seq
GAACCGATCACCTGCTTCGTCGTGAGCTGATAACAACAATATCAAGAGCATTACGG
>dme_circ_0008456
GAGCATTACGGCCACCACAACGGCAAAGGTTTTGGGCATGAATCTGCCATATGCCCTACCCGACGAAGTG
TCCGATGTGTGCCGCAATCTCCTGTACGGTGCCATCTGTCCCATCGACAAGGACGAGGATGTCACGTATC
AGTTCAACTTCTAT
>dme_circ_0008456_junction_seq
GGATGTCACGTATCAGTTCAACTTCTATGAGCATTACGGCCACCACAACGGCAAAG
>dme_circ_0008457
GGAGACATCCTCGGTGGGATACAGCGGGCAATAGGCGCCGTACTGGAGATTCGGGCACACGGCAGCCACA
TCCGCAGGCAGTTCATATGGCACCGTAATGATTCCGAGTGTGGTGGCCTTTACCAAGGTCGTCAGCTGGG
TGATGTACTTGTCCACGGCGAAATCGATCTCGAACTTCTGTGTCGTTCCCTTCACAATCTGACAGGGTGG
A
>dme_circ_0008457_junction_seq
CGTTCCCTTCACAATCTGACAGGGTGGAGGAGACATCCTCGGTGGGATACAGCGGG
>dme_circ_0008458
GGTGAAGTAGCGGGACTGGACGGCGAAGTGAGAAGTGGGAAAACGGTAGGTGGAAGGCGGGTGGCAGTGG

AGGAGGGGCGTGACCTCCACCTGTGCGAGCCCAGCTGCAGCTTCCCCGTTGGGCAAGCTGGCAAAGTTTA
TTCG
>dme_circ_0008458_junction_seq
CGTTGGGCAAGCTGGCAAAGTTTATTCGGGTGAAGTAGCGGGACTGGACGGCGAAG
>dme_circ_0008459
CCACAACAGCAGCAGCCGCCGGCGCCTGGGAATAAGGCGGCACTAAATGCCGGAGATGCCAGCAAACAGA
ACAGCGGCAATGCGAACGCCGCTGGCGGTGCGAGCAAGCCGAAGGATGCCTCACGCAACGGCGACAAGCA
GCAGGCGGgcacacagcagcaacagccatcgcagg
>dme_circ_0008459_junction_seq
GgcacacagcagcaacagccatcgcaggCCACAACAGCAGCAGCCGCCGGCGCCTG
>dme_circ_0008460
GGACAGGGACGGACGGCGAAGAACCACCAGAACCAGAACCAAAACAAAGCGGATAAACCAGGAGGCACTA
CGTCTGGCGATATGGAAGATCTCCTCGTGGAAGTTCGGCTCGACAATGGCGCCTACTACAAGG
>dme_circ_0008460_junction_seq
CGGCTCGACAATGGCGCCTACTACAAGGGGACAGGGACGGACGGCGAAGAACCACC
>dme_circ_0008461
GCAACATTTGAAGCAGCAGCCGCCAAGCAAAAGACTGATTTGAGTACAAGGAACTAGAAGCAGGAACGCG
AGGTTTCTGCCACTGCACACTGAATTGTGAGCATACCCACCCATACCTAGCTATATCTATAGCCCTAATA
TCTCCATTCTTCCCCCTCAGGAGCCCCAGATAACGCGGCAAACTGCAGTCGACGGGGAAAAGTCGGGAGT
TGAAGGCTGGGCAGCATCAGCATCAGCAGCAGGAGGGACAGGGACGGACGGCGAAGAACCACCAGAACCA
GAACCAAAACAAAGCGGATAAACCAGGAGGCACTACGTCTGGCGATATGGAAGATCTCCTCGTGGAAGTT
CGGCTCGACAATGGCGCCTACTACAAGG
>dme_circ_0008461_junction_seq
CGGCTCGACAATGGCGCCTACTACAAGGGCAACATTTGAAGCAGCAGCCGCCAAGC
>dme_circ_0008462
GCTCTTTAAATTGGCAGTTAGTTATCCATTCGTCtttttttttATGGAGGGGCGGAGAGTTAGTTAAAGA
TACAGTATACAAATGCAAATTACTATCGGTTAATTACAGTTGCAGTACTAGGCACGAAATggtttttcat
tctat
>dme_circ_0008462_junction_seq
CTAGGCACGAAATggtttttcattctatGCTCTTTAAATTGGCAGTTAGTTATCCA
>dme_circ_0008463
GATTAAAATAGCGACCGGATAGCCTGAGATTCGTCGTAGTAACGAAGCGAACCCCTGCGAAAACGGAGGC
CAACTGCTCGACTTAATTGTCGACCACTGTCAATATTAGCGCCATCGCAACTGCTGGGTGTGGGCGAATC
TGCGCCCCGCCACCGGGTAAGACTGTTCCAAGATTCGTTCGCGCTTTCAGAACCCACTCATTTCACTTTG
GCTTTTCTATATGGCACCCAAAGATATCACTCGAATCATATGGCAAAACACACTCACATATCTATAGCCT
TGCTACGCTTTCATCAGCAAATTGCttttcagtttttttcagcttcaatcttttttgtttttcCACCAAGC
TGCCCACTTGAACCTTTTCGCACACAAACACGCAAATTTACCCACAGGGGGTCGGTGTGCTGGTGTGTGC
CTGAGACCAACATTATTATTATGTAGGGGCTGAAAAGTTTCAAGTGGCTGTAGGCCTGCGGGCCACGATA
ATAAATACAAAACGCACAGCGCCGTTTTGGCCACAAGTGTTTGACATTGTTGACTTGGTCCAAAAGAGCC
CCCCAAACGCTAAACTTCACCACCGATTGATTTCTAACGTTATGCGCCACACAAAAGATCCTTGGAAATA
TAATGGCTATGCAATTTCAACACACTGGCCATTGTCCTTAAATGTCCTTGGCTTGTGCACCTGTTGCCGT
TAGCTCGACTTTATACTCATGCCCACACGCCGTTCGTTTTAATGTTCTGTTCGTGGTGCTTATTTTAATT
AAAATTAAGTTTGAGACACCTTAATTGCCATTTTAATAGAACTTTAGTTTTCACTTAGACCAACTCCGTC
TCTATCTATCTCTCTCTCTGTCCGCTAAAACAATATGCATATCCTTTTAAACCACACATTCACGACATTT

TCCTAAAGGCAAATGCAACTTTTGTGATTTCATGGCGTACTAAACCCATTATTCCGACAGTAAGTGAAAT
CAAATGAAACCATTGGAGTTTGTTTTATATAAATATACTAATCTTTAAACCAGTTGACATATGAAATTTG
AAATGAGTTTTCCCCTCACTTCACTACATTACATTATGACTATGTAATGTTTTTTACGATTTTTACCATC
TTTCACTGCGAAAATGCACATTCGAAGTGACCACATTACGCATACGCCACATTGGCGTAACTGAATTAGA
GAAAGTTTTCAAAACAATTAGGATCCAAGTGAGAACGTTGGCTTAGCTTGGCCACAAAATCTCATTAACT
TCTTGCAATAAAGTTAAATAAAGGGACAAACATATGAACTAAGTGACTTATTTTATGTTTGTGAGACGCT
TTAAATTACTGTATGCCTTAATGTTCAATGTCTATGCACAATAAATATCAGAAATCATTAAATGAATGCC
CAAATTGAAAGCACTCTAGCTAATTAATTAGCAAATCAACTATACTTTGTATGTGttttttttttATGTA
TTTTCGCGTGCCGCGGATCAAAGGGTAAAGACCTTAAAAAAGGAAGTGCCCCCTTTGGCAGAAGGGGGCC
CAGTTGAACGAAACTAATTTGAAATTTCATATGTTCGTCTATTTCTCTATCTTTTCTTCTCGGGCGGTAT
TTGTCTACCACCCCATTTGATTATCTCATCCTTTTTTCACTCAATTGCGCCGCACCATTTAATCTGTCTA
CTTGTCTGGCGGTTGAAAAACAACCGAATCAATCAAAAACACTCGATTACTCGAACACCACCTACCACAA
CCACAACCCACCCACACGAAAACTACTAACAGGATTCAGCTCGGGAGACTTGTCTTCCGAGGTAGACGAT
GTCGATCCGAATAGCCTGCCGCCGGCGGCGCGGCCCATCCAAGATCAGCCGACGAAGCCGCCGGTTGCCG
GCGGACCGCCGAATATGCCGCCACCGCCGGCTCCCGGCCAACCGGCCGGAGCTGCTCCGGAGCTGTCGTT
GAGCTTTGGCGCCGGCAAAACCCCGGCGACGGCCGCACCTGCTCCACCACGTGGTGTCAGTGCGCCGACC
AGTCCGGCCAAGTCGCGCGAAAGCCTGCTGCAGCGGGTGCAGAGCTTGACCGGCGCTGCCCGGGATCAGG
GAGCCTCCATTTTGGGGTATGCTTTGGCATTGGCAGGCACCTACACTCAGAAAAATATGGCAGTCAATCA
GTCACATAACATGTAGAGACGGCATCATCAAGTCTTGGTATATATAGTAAACAATCCACTTTATAAGCAA
TGCGTCTCTGTCAATCCATACCATTATGTTACTGTAATTGTTGTCCTTCTATTTACCTGTTGATTGCTTT
TAGTTTAAATATAGTAACCGTTATTATCCTTGGTATTTATAGATGCTATGGGTGATCACTTACAACAACT
CCTTTACTGTAGAACAAGATATACAATTTCAATTCCTTTGTAAACTTTTTAAATAACTATTCTATTACTC
TATTAACAATTCATGGGTGACTTCATCTTTAATGTGAACTTATAAAATATTAAATATTTTACGTTCTATT
AATTGTTTAAGTAGTCAATGAGAAACAGACTCAAAATCCCGAAGTAAACCGTCTTAAATAACTTTAATGG
TTGTGTCTTACCAAATGTTGCAATCTATGAGATTTTTCTGAGTGTAGGGACTGTTTATTGGTCTCTGAGC
ATGGAATGACATCTAACCCAAAACAGGGAGCTGCGGTTCAGAGCGCCACACAACGGGCACCGGCATTCAG
CAAGGACAAGTACTTCACGCTGCTGGTTTTGGATGACCAGAACACGGACTGGTCCAAATACTTCCGGGGC
AGGCGCTTGCACGGCGACTTCGACATCCGAGTGGAGCAGGCCGAGTTTAGGGGTGAGTTATCCAAGACAC
TGAAACAAATTACACTGGTATAtatatatatccataccagtgtataccatataccatataccatatatat
gtatatagtgacgtatttgggtggtccaaaccagccacttccattatttcaaagaaatcagtaatgcact
ctagtaattttccataacgtatcccagctgcgcagcattcgtttatctttggcagcgcagccgttcttgt
aaacatcctaaagcctgacctaagcagatttgactgccctctttcaacgctacctaatcttaagaaccca
agagcgaggctctcccgaaatacaaatattgttcaaatactgaggcttctcctcaatccaatttgcattt
gatttttagtcttaagctgagatccaaagaataaagtcgtgaaactatttctcctaaaaactatttttta
tttcttggcgttgtccttagtcaactgacgggacattagttcgactcataaataaaacaacaattttact
ggcgcagtcggtaggatacaaaagtatccgaaaaaaagaaccttcgagtggaaaataagttaaattttat
agtccagtgctcgaaacatctcccaaaataaattcgtgaaaactcttcaacttggattataattccaatt
cggttatccaataataagtggaagtgaaatacgaaacaaaaatattaagtccaaaggcaactaagtttta
aaaccaacatataaaaataaaaaattaaaacaatatagaattttaataatacaacacaaaaatttacaaa
acaaaaaaaacaaacaagtgaaactagaaagcttaaaaataataataacattgaatccgaaacaaaacaa
aaaaataaaacacaaaagttaaaaattttacaataaaaatgtcacaaccaattattgcgctgagcgacat
aaaccttgccgaagcccgtcggcagcttaaagacattatgccattcaaaggtgatccagaaacccttcac
acctttatcagcagagtggattacgtaatttcgctctaccaaacaaatgatgtccgacaacagaggattc
tactgggagccatcgaaaggaacttggacggacaaattacacgatctttgggacttccgaacatcgaaga

ttggcctacccttaaagcaagactcatcgcggaatttaaaattcaaacaccaaactacaaacttctggag
aacttcagggagacaccatacagaggaagcctaagagcattctgcgaagaagcggagagacgacgtcaat
tactaatttcgaaactacacctggaaggtaaccaatcggattttcttatttatattcagggtattaaaga
atctattaagatactgataaggaaactaccaatacaattattcactattttagcccatcacgatattaca
gacttaagatccttaattaccattgcacaaaatgagggaatttatgaagaacacattaattttgaatttt
atgaaaaaccagaatatcgtaataaaaattcaaattctaaccggaattcgaaaacacaaaaattcaatac
aaatgttcaaactcaaaatcgaccaagttactcacaatattcccaacccttccaacctaattttaatcaa
tacattcaaccatttagacctagctatacacagcagataactaacaacccacccatgtggcacgcaccta
attatttcagacccaaccaatacataaacccacaacccattattcaaaaaaatcatttccaacaatatcc
caacaaagcccaatttccccaaacaacgcattttagaggaaatacatacccctcgactacaacaaccctct
acatataaaaatactaacttcccgattactaaacgactaagaccatcggacagtgaacaaactaaaatgt
ctattgacgaaattagattccaagacgcgcatgaattcgaacaagtccaacctaattattacgagcaaca
gtattttaaccaaaatcaatacaatccgtatcaaaatcatagcttcattaatgaagggcaacaacaagtc
caatctgtacaaattaataacaaacaaaaccaaaataattctgaactaaacgaaaattttcggttaacag
ccccggaaaatacgaatacataaaaatagtatacaaagggcgctcatacaaatgccttctagacacagga
tcaacaattaatatgatcaatgaaaatatattttgtcttcccattcaaaatagtagatgtgaagttttaa
catcaaatggccctattaccttgaacgacttgattatgttacccagaaatagtattttcaaaaaaaccga
accattttatgtgcacagattttctaataattacgatatgctaattggcagaaaattgttgaaaaatgct
caatcagttattaattacaaaaatgatacagttacccttttttgatcaaacatacaaattaattacttcag
aatccgaaagaaaccaaaatttgtatatccaaaggacaccagaatcaattgcaagctcagatcaggaatc
aataaaaaaattagatttttcacagtttcgattagatcacctaaatcaggaggaaacttttaagttaaaa
ggcttgttaaataaatttagaaatcttgaatataaggagggagagaaattaacatttacaaatacaatta
aacacgtactaaatacaacacataactccccaatttattcgaaacaatacccacttgcgcaaacacacga
aatcgaagtagaaaaccaagtacaggaaatgctgaatcagggattaattagggaaagtaattctccatac
aatagtcctacttgggtcgtaccaaagaaaccggatgcttctggcgtaaataagtacagagtagtaattg
attatagaaagctaaatgaaataaccatacctgacagatatccaattccaaatatggacgaaattcttgg
caaactgggtaaatgccaatattttacaacgatcgatctggcaaagggatttcatcaaatagaaatggac
gaagaatcaatttctaaaactgcattctccacaaaagcggtcattacgaataccttcgaatgccatttgg
ccttaggaatgcacccgctacttttcaaaggtgcatgaataatatccttcgaccgttgcttaacaaacac
tgtttggtgtatctggatgatattataatttttttcaacatcccttacagaacatttaaattcaatacaat
tagtttttacaaagcttgcagatgcaaatttaaaattgcaactagacaaatgtgagttcttaaaaaagga
agctaactttcttggtcacatagttacccctgatggtattaaaccaaatcctattaaagttaaagccata
gtttcatacccaattccgacaaaagtaaaagagataagagctttccttggattaacaggttattatcgca
aatttattccaaattacgcagacatagcaaaacccatgaccagctgcttaaaaaaaaggagcaaagatag
atacacaaaaacttgagtacatagaggcattcgaaaaacttaaggctttgataattcgtgacccaatttt
acaattacctgattttgaaaagaaatttgttttaaccacagatgcaagtaacttggccctcggggctgtc
ctttctcaaaacggtcatcctatatcttttattagtagaacacttaacgatcacgaattaaattacagtg
ctatcgaaaaagaattacttgccatagtttgggccacaaaaacttttcgacattatttactaggacgaca
atttctcattgccagtgaccatcaacctcttagatggcttcataacttaaaggaaccgaatgctaagtta
gaaagatggagagttagattaagcgaataccaatttaaaatagattatattaaagggaaagaaaattcag
ttgccgatgcattatcaagaattaaaattgaagaaaatcatcatagtgaagctactcaacatagtgcaga
agaggacaatagcaaccttattcatttaacagaaaaaccaataaattatttcaaaaaacaaataatcttt
attaaatccgataaaaataaagtagagcattcaaaaatattcggtaactccattaccacaattcaatatg
atgtaatgacacttgaaaaggccaaacaaattttactcgatcactttatccatagaaacattaccattta

tattgagagcgatgtagattttgaaattattcaaagagcacacatagaaattattaataccacctacaca
aaagtaattcgcagtcttttcctattaaagaacgttggttcatacgccgaattcaaagaaatcatacttc
aatcacatgaaaaacttttacaccctggtatacagaaaatgacaaaattatttaaagaaaatcacttctt
tccaaatagccaactattaattcagaatataataaacgaatgcaacatatgcaatttggccaaaacagaa
catagaaacaccaaaatgcctttaaaaatcacacccaacccggaacattgccgagaaaaatttgtagtag
atatttattcatctgagggaaaacattacatcagttgcattgatatttattctaaattcgctacacttga
gcaaattaaaactaaggattggatagaatgcagaaacgcattaatgcgcatttttaatcaactaggtaaa
cccaaattattaaaggcagacagagacggagctttctccagtttagctttaaagcgatggcttgaagaag
aagaagtcgaattacagctcaatacagcaaaaaacggggtagcagacgtcgaaagattacacaaaacaat
aaatgaaaaaattcatataatcaattcatctgatgatgaagaagtaaaattaagcaagatagaaacaatc
ctctacacatacaaccataaaattaaacatgacactactggacagacacctgctcaaattttcttatacg
ctgggcatcccatattagacactcaaaaaattaaagagaagtaaaaaatgaatgtgtcaccaatattatt
aaacacttaaatccaatttgtaattttaagccagtacacacgaacgaaataataaaatacatagaaccaa
acacaattgtaacctggaacttaacccaaacaattcttaaccaaaattgccaaaattcaattaataaaat
aaaaatagaaggaaacaaaatgataagagtaacgcaatgcaaaatagaaatcaataatataattttaagt
gaaaatctgttagaaccagaaatagatttgacaccactatacacaccacttaatataacaaaaataaaaa
ttgtaaaacacaacgacattgttgagatgatttcagagaacaatattacactttacatacaaatgatcat
tgtaataatcgcactaattttgttgtactcatatttaagatatgtatcatttaaaccatttatgatgctg
tatgcaaaacttaaaataagaaaaaatcaaaatcaaaacacaccacaacaaacagaaatagaagaaactc
catttcccacactatatccatcaatcccagcccaagtataggcttctctttaagggaaggggagtgacgt
atttgggtggtccaaaccagccacttccattatttcaaagaaatcagtaatgcactctagtaattttcca
taacgtatcccagctgcgcagcattcgtttatctttggcagcgcagccgttcttgtaaacatcctaaagc
ctgacctaagcagatttgactgccctctttcaacgctacctaatcttaagaacccaagagcgaggctctc
ccgaaatacaaatattgttcaaatactgaggcttctcctcaatccaatttgcatttgatttttagtctta
agctgagatccaaagaataaagtcgtgaaactatttctcctaaaaactatttttttatttcttggcgttgt
ccttagtcaactgacgggacattagttcgactcataaataaaacaacaattttactATATATACCAATTG
TATATACCAATAGCAAATGGGAGACTATATCTTAGCATTAAAATATATATACACATCATACCAAGATTTT
CTCCCTGTTGTTAACTGACTTTTTTTCCCACCTGGGTAATTTCCGCTTAATTAGTGCAATGACAGTGACC
AGCAGTCGTGGATCCAACTTCTTAGGCGGGGGATTGGATTGTCCGCTGGCGCTCTACAGAGTAATAGGAC
CATTGAAGGACCCAAGGACGTGGGTGGACAGTTGGATAGGTGGATGGGTGGATGGATAGGTGGCAAGTGG
TCGTAATCCAAGAAGTACTAAAGAGACGGGAGCCTTTGTCCATTTTGCCTTATTTACGTAGCACTGAGCT
CGGAGCTCTGGCGCTCTATTTTCCATTTGTTTATTTCAAGTCGGATTGTTGACTTTACGATGCTGGTGCT
GTTTTTGCAATTGCGTATGTCCGTAGCAATAAAAAACACTTGTAGCTGCGTAAAAATACGAATGCGAATG
CCAAGTATGGCATACTTGTTGTGACTGTCGGGAAGTTAGACGCCATCTGAATTACCAGAGAAGAAGAGTT
TTGCAATGCAAATATAGTGATTTTCATTTAATTCATGTAGGATGTGCATTAGTCATGCCCAAGTGCAGTC
ACACTGCGTGCAATAAAGATTTCACTACCGTTGTaaaaaaaaTATTACGTGCAAGTAAAATATATACAAT
TTTCTGAGTAATCAAAACCATTGATATTTTTACCTATTGAAATCTTAGTTGCCAATTTATGTTGATTCCA
AAAGTATGCAACAAATATTTATGTTTGCTTACAGTGCATCCGCCTGACAACAGATGGCGCTTTGTATCAT
CATTGCTATATGTTTTCATATGTTTTGCATGCCAACATATTTAATCGTTTTTACTTCAAAATAATGAAAA
ATACAACTTACATTCTGTTTTTAAATATATTTTTCGTGTTTAAAAATTACAAAAGTGTTGCCAGAGATTG
GCAATCTAACAGCTACGTAAATTTTCTTTTTATCATAAAATTTATTTTCTTTTTGGAATATCACACCAAA
ACAGGGTGGCACGTTCCTTCCGCCCGGACTTTGTGCTCATTCGTCAGCCGCCGCGCGACGGATCCAGTGA
CTATCGTTCCACGATCTTGGGTTTAAAGTACGGCGGAGTGCCCAGCATTAATTCGCTGCACTCTATCTAT
CAGTTCCAGGTGAGTAGCGTTGGACTTCATATCGGGCTTACTTTACAACTGATCTTCTTTGATACTCTTA

AGGACAAGCCCTGGGTATTTTCGCACCTGCTGCAATTGCAGCGACGCCTCGGACGCGACGGCTTTCCACT
GATCGAACAGACCTTCTTCCCCAATCCACGCGATTTG
>dme_circ_0008463_junction_seq
GACCTTCTTCCCCAATCCACGCGATTTGGATTAAAATAGCGACCGGATAGCCTGAG
>dme_circ_0008464
GGTGAGTAGCGTTGGACTTCATATCGGGCTTACTTTACAACTGATCTTCTTTGATACTCTTAAGGACAAG
CCCTGGGTATTTTCGCACCTGCTGCAATTGCAGCGACGCCTCGGACGCGACGGCTTTCCACTGATCGAAC
AGACCTTCTTCCCCAATCCACGCGATTTGGTAA
>dme_circ_0008464_junction_seq
TTCTTCCCCAATCCACGCGATTTGGTAAGGTGAGTAGCGTTGGACTTCATATCGGG
>dme_circ_0008465
GACGATGCACAGCCTAAAATGCGTTTTAGCCTCTCACCACCGCCGCAGAAAGCCATTACCAGTGCCCGCC
TAACCTCGCCCACTAAAAACAGCCTCAGCAGCAACAACACCATCACCACCCCCGGCAGCAATAATAGAAG
CCCCAGCAGCACCAAAACCACCATGCTGACCCGCAATGGCGGAGGCCACGGGACCAGCCCCACTGCCTCC
GGATCCGGACACGCCCCCTCCGCCACCGCCGCTGCCGACGATGAAGCCACCTCGGATTACAACCAGTGGC
TGCATGCCATGAAACTGGTGGCCCGCCTGCCCGGAGGCACTCCACCCGAGTTCCGACGCAAGGTGAGATT
TCATCTAGTTTGTTTGttttttttttttGATTTAGACAGCATAAGGTCACAGGATATGGGGATATTTAT
TATTTATAACTACATTCCTCTTAAAGTATATATCAAAGATGGGCTTACCAAATTGGCATCCTTTAAAATA
TATTTCCACCATGGATTACTAAATATATGTATATATAATTTTAAAGAAAAATCCCTTCACTCGTTGAATT
ATTTCTTTCAGTTGTGGCTCTCGCTGGCGGACAAGTATCTCAAGTCGAAGAACGTGGACTGGGCGCAGCA
GAGGGAGAAGTGCTTTTGCGAGGAGTGGCGCGAGGACGACGAGGAGCTGGGCATACAAATCGTCAAGGTG
AGCTCCAGTCACGGACAGGTGATCCTCCAGGGAATCATAAATGACTCGTCTTCGTTTAGGACCTGCATCG
CACTGGCTCGAATCTGTGCACCGGTCCCGCGGGCTCCATAAACCAGGCCAAGCTGAAGCGCATCCTACTG
GGCTATGCCCGTTACAACCCCGAGGTGGGCTATTGCCAG
>dme_circ_0008465_junction_seq
TTACAACCCCGAGGTGGGCTATTGCCAGGACGATGCACAGCCTAAAATGCGTTTTA
>dme_circ_0008466
GCAGGACAACTCGTGGGCGTGCTCCTCTGCCTTAGCTCTTAGGTGTGCCATGTCGATGGAACCATTCGAC
AAAATTCTAATAGGCTCCACCTTCATACCCGCCATTTGTGCAGAAGCTGGATTTGTGCCGTGGGCAGAAA
TTGGAATCAGACAAATATTTCTGTGTCCTTCATTTCGGTGTTCATGG
>dme_circ_0008466_junction_seq
TCTGTGTCCTTCATTTCGGTGTTCATGGGCAGGACAACTCGTGGGCGTGCTCCTCT
>dme_circ_0008467
GCTGGAGGAGAAGCCGCCTTGATGCCCTCACCCACCAGGACCATAACCCCCGCCCAGAGTCCGGTGCAGA
CGAAGAGGAAACTGCAGCCCACTGTTTCTACGGAGAATGCAGCCGGAACTACAGCCAAAAAGAAGGAGAA
GCCGGCCAATAGCTCGTTTGTGATCAGCAAGGATGGTGCACCACTGACTGAAGCGCCACCGACCATCATC
GAACCGCAAGCTACGCTCATGGAGGCGGAGACGATTGCCAATATACCCGAGGAGGTTCCTGTTCCCAGTT
ACTCCCAGAAAGACATGCAAGCGGTGGGTGATATTTGCGATCTGCTGATGGGCACCGCCACAACTCCAGC
CCCTGCACCACCGGCACCCACACCTGTTGCTCGCCAGCCCACCAGGGGAAAACTGGATGCCGTTGTTGAA
ACTCCTGAGGAAAAGGATGCCACCAAAGTGATCAAGAAGTTTGTGAACAAGCACAAGACCGCGGATCTGG
TAAATGCCATCAATGAAAGTGCCTCCAAGGCCGCAGCGCCGGTGAGCGCAGTGGCCCCTCCACCCTTCGT
CCGCAAGTGCAGCTTGCAGGATGAGTCCACACTGAATAAATTCAATGCGGAGCGTCGGAAATCGCGAATC
CTAGAAACTGCAGAGAAGTTCCAGCCACCGCCACCAGTTGCTGCAGCACCTCCCGAAAAACCCAAGAAGC
TCAGCATACCGGGTGTCAGTGTGGGCAGTTTTAAGAAGGAGTTCGAGAAGAAGGCCACCAATCCACCGGC

TGCAGAGGGACCTACGCCCGGTGAATTAAGGGCTCAGGAGCAGGTTGCAGCAGCTGCAGCGGCCGCCCAG
GAAGCCGAAACCTTAAGCACCCCGCCACCATCGCCAGTGGTGGCCCAATCGCTAGAGGGCAGCGATTCCA
AGAACTCTGTGGCCTCTATTTCGCTGGACGAGGCTCGCCGCTCCATGGAGAACTCCATTGCCTTGCTGCT
CCAGGCACAAAACGAGTCCAGCAAGGAGGTGGACCAGTTGTGTGCCCAAACGGAGACCATTGGCGTCAGT
GAACCGGCTACTCAGCAGGAGCGCGAGCGTAAGTTGAAGAACGCCCGCGCCATAATCGGAAATGCCATAC
AACCAG
>dme_circ_0008467_junction_seq
GCCATAATCGGAAATGCCATACAACCAGGCTGGAGGAGAAGCCGCCTTGATGCCCT
>dme_circ_0008468
TATTAATTGGCATTTTCACTTGATAATTACAGTAGACACTTATGATTAATCGATGTGTATTTCTTGTTTG
GCTATTGTTTTATCTGGATTTGTGGGACAATATAAGGTTTAGACTAATTAGACAAGGTAATTCACTTGAT
CGGTGTGATCTGTGATCTGTGTTCAGTTGATTGTTTGTTTTGTTTAGCAGACATCAAAAACTACTTGAAA
TAAAACAGG
>dme_circ_0008468_junction_seq
CATCAAAAACTACTTGAAATAAAACAGGTATTAATTGGCATTTTCACTTGATAATT
>dme_circ_0008469
ATGATATATACAGTCTGTCGCTGGCACCCTCCTTGCCTACATTACCCTTCGAGGTCTTCCGTCAGCTTCT
TGATAGTGTGCCGTTATCCACCGACGACGGTGTTTTGCTACGACGCGTGGTAATCGAAGTGGGGGCCATG
CACCTAGTGCTTAATTGCCTCGGCATTTTCTCGCATCAGTCGCACAACAACAGGATTGGAGCACCGATTA
GCGAACCATCGCCAAGTGGAACCAGTAGTGGTAGCGGCACCATTGGCAATGGAAGTGGAAACGGAAGTGT
AAGCGGTACCAAAACTAATGCACCAGAAGAGGCCACACCTAGTGCCACGTCGGACGACAAAAGCCACATG
TACTGGGCCAAGGGCACTGGTTTCGGTACCGGTTCTACTACTCAGTCGTGGAATGTGGAGCAGGCGCTTC
TTCGGCAAAAGAGCGAGGAGGAGCATGTCACAGTGTTGCTGCTTGTTCTAGCCAGTTACATAAATCCGGG
TGACTGTATCCCAAGTGAAATGTGCGGAGAGGACATCCTAGCTTATCATGATGTCCGAGATGTTACCGGC
GAGTTGCCGCCCATCTTCCAAACCCTACTGCAGCAGTCTTGCTTAATCCCCGCCCTCAGCTCCTATCTGC
GCAACGATTCAGTGCTTGATATCACGCGCCACATTCCGCTATACCGTGCCATACTCAAACTTCTGCGTGC
CCTATCGCTGTCTAAGATGCTTGTCTCGTTGCTCCAACCATCTGCCAGTGGCAGTGGGGACAGCACGCCA
CCCATAGTGGAGTTGCTCACAAGCATGAAGACCTGTGTGGATACCTACGCCAAACGGTTAAAGAGTTAAC
AAGAAGTCAAACATAAAGGGACAGACACATCAGTTGACCTTTAACATCGATGATGGCGACGATGAGGGAT
TAGCTTTGCTTATTCCGGATATCCATGAAACCAGTGTGCTGGTGCAAAAAACCACAGATGCTGATGCGCT
AATCAACATGTTGCATACCAACGAAGACGGCAGCGCACAGCTAGAGCCCCAGAGCAAATCAATTGAACAG
CGCTACTTAGAGCTAATGAAGAAGCGCCAGTTCGATACGTTTGACATGATTGTGGAGTCGGATAACAACA
GTTTCCGCTTTGTGGTCTCGCATCACTTCGAAAAAATGGTTCGGCTCGCTGGCGATCGTTACCATCCGTC
GCGGGTCAAGCGACTTGCCCAGGAGGCAGTTACACTGTCCACCAGCCTACCCCTCAGCTTTAGTAGTTCT
GTATTTGTACGCTGTGATACTGATAGATTGGACATCATGAAGGGTACTCATAACTGGTCCGGCGGACACG
CCTTACGCCAACGGGTGCTTTGAGTTTGATGTCTTCTTTCCGCCGGACTACCCTAACCAGCCCATGCTCA
TCAATCTGGAGACGACGGGTCGCCATTCGGTGCGCTTTAACCCAAACCTCTATAATGACGGCAAAGTTTG
CCTGTCAGTTCTCAACACCTGGCATGGTCGGCCTGAGGAGAAATGGAATGCACAGACTTCAAGCTTCCTG
CAGGTACTCGTTTCCATCCAGTCCCTGATCCTCGTTCCCGAGCCCTATTTCAATGAACCGGGCTTTGAAA
GAAGTCGTGGCAGTCCAAGTGGTACCAATTCATCGCGAGAATATAATAGCAATATATATCAGGCCTGTGT
GCGGTGGGCTATGTTGGAGCAAATACGGAGTCCAAGTCAG
>dme_circ_0008469_junction_seq
GTTGGAGCAAATACGGAGTCCAAGTCAGATGATATATACAGTCTGTCGCTGGCACC
>dme_circ_0008470

GAACAAGCAGCAACACTGTGACATGCTCCTCCTCGCTCTTTTGCCGAAGAAGCGCCTGCTCCACATTCCA
CGACTGAGTAGTAGAACCGGTACCGAAACCAGTGCCCTTGGCCCAGTACATGTGGCTTTTGTCGTCCGAC
GTGGCACTAGGTGTGGCCTCTTCTGGTGCATTA
>dme_circ_0008470_junction_seq
ACTAGGTGTGGCCTCTTCTGGTGCATTAGAACAAGCAGCAACACTGTGACATGCTC
>dme_circ_0008471
GGTGTGGCCTCTTCTGGTGCATTAGTTTTGGTACCGCTTACACTTCCGTTTCCACTTCCATTGCCAATGG
TGCCGCTACCACTACTGGTTCCACTTGGCGATGGTTCGCTAATCGGTGCTCCAATCCTGTTGTTGTGCGA
CTGATGCGAGAAAATGCCGAGGCAATTAAGCACTAG
>dme_circ_0008471_junction_seq
AGAAAATGCCGAGGCAATTAAGCACTAGGGTGTGGCCTCTTCTGGTGCATTAGTTT
>dme_circ_0008472
TGTGCCGTTATCCACCGACGACGGTGTTTTGCTACGACGCGTGGTAATCGAAGTGGGGGCCATGCACCTA
GTGCTTAATTGCCTCGGCATTTTCTCGCATCAGTCGCACAACAACAGGATTGGAGCACCGATTAGCGAAC
CATCGCCAAGTGGAACCAGTAGTGGTAGCGGCACCATTGGCAATGGAAGTGGAAACGGAAGTGTAAGCGG
>dme_circ_0008472_junction_seq
ATGGAAGTGGAAACGGAAGTGTAAGCGGTGTGCCGTTATCCACCGACGACGGTGTT
>dme_circ_0008473
ATGATATATACAGTCTGTCGCTGGCACCCTCCTTGCCTACATTACCCTTCGAGGTCTTCCGTCAGCTTCT
TGATAGTGTGCCGTTATCCACCGACGACGGTGTTTTGCTACGACGCGTGGTAATCGAAGTGGGGGCCATG
CACCTAGTGCTTAATTGCCTCGGCATTTTCTCGCATCAGTCGCACAACAACAGGATTGGAGCACCGATTA
GCGAACCATCGCCAAGTGGAACCAGTAGTGGTAGCGGCACCATTGGCAATGGAAGTGGAAACGGAAGTG
>dme_circ_0008473_junction_seq
ATTGGCAATGGAAGTGGAAACGGAAGTGATGATATATACAGTCTGTCGCTGGCACC
>dme_circ_0008474
AGCAAAATGGAAAAGAGCAGCTTCCTGAACTTTAACAACGTAGAAGCGGGTAGCATGGAGTTCCTTACCG
TTGGTGCTGCTGTGGCAGCCAAGGACAAGCGCATAAAAGATGCTAAGAACCAAGCAGCCATGAACAAGGA
CAAGGAGACTCAGACCAGCTCAACGAACTTGGCTTCCAGATATATTCAATGTGGATGAGTTCTTGTTGCA
ATCGGCGAATGCCGTTAATGGTAACCAGTTGGTCATTCCAGAGTATTCTGATATTGTTCTAGCTGGAGAT
GCCACCATCTCGCAGGTTTTGGCCTCGCTTCAGAATGCAGGACATTCTCTTTCGACGCCCTGTATATCGT
TTGATCTGGTACTTCAACAGCGCTCTGATGACACAAGCGAGGCAAAGAAGACCAAAGCTGCCTGCACTGA
GCCGTTGCCATCACCACTGCAACGATTTTCCAGCAGTGGTGGTCTATCTTTACTCGCTCACTACCTGCCC
ACCGTATATCCGGAACACTCAGGTCGCAAGACCACCTTGGTGTTTAGCGAGAAGGAGAAAAGTCCCCCGC
TCTCTGACTGGGTGAAATTGGAACCGAACGACGAGATCTATGAGGATTTGGAGGACACTCTCTGTGAGCC
AGCAGCTAAGCTGGCAACGGTCAGTTCGGTGCCGCAGCACTCGCTGGCCGCATTTGGATTATTCCTGCGC
CTGCCCGCCTACTCTGATGTCTTGCTGCGAGACAAGATGCGGGCGCAGTGTCTTCTCCGCTTAGTGCTCG
GAGTCACGGGAGATGGGGATGGCAG
>dme_circ_0008474_junction_seq
TCGGAGTCACGGGAGATGGGGATGGCAGAGCAAAATGGAAAAGAGCAGCTTCCTGA
>dme_circ_0008475
TTCCTTACCGTTGGTGCTGCTGTGGCAGCCAAGGACAAGCGCATAAAAGATGCTAAGAACCAAGCAGCCA
TGAACAAGGACAAGGAGACTCAGACCAGCTCAACGAACTTGGCTTCCAGTGAGTATTCGCTAGATTTGCC
ATAAATGCATTCTAAACAAAATAGTCTCTTACAGATATATTCAATGTGGATGAGTTCTTGTTGCAATCGG
CGAATGCCGTTAATGGTAACCAGTTGGTCATTCCAGAGTATTCTGATATTGTTCTAGCTGGAGATGCCAC

CATCTCGCAGGTTTTGGCCTCGCTTCAGAATGCAGGACATTCTCTTTCGACGCCCTG

>dme_circ_0008475_junction_seq

ATGCAGGACATTCTCTTTCGACGCCCTGTTCCTTACCGTTGGTGCTGCTGTGGCAG

>dme_circ_0008476

GATATAGTTGGTCGAACTCCATGCGAAAATCTTAATGGTTTGCAAATTATGGCTCCAATGAGCAACGATG
TAAGATTTTCCTGGTTTCCGGGATGGCAGGCGCTAACTCGCGTAAAGAAGTTGATTGTGGCCCTCTCCAG
GCGGATAAACGATTGTTCCTTAGGCAACGTCTTGCTGAACTGGGCGGCATGTGTGTTACAGAGCAACTGC
AGCAGAGGCTTCCAAAATACGGAGCCCTGCATA

>dme_circ_0008476_junction_seq

AGGCTTCCAAAATACGGAGCCCTGCATAGATATAGTTGGTCGAACTCCATGCGAAA

>dme_circ_0008477

GTACTAATATCAAATCCAAATAATTCACATCGAAGACCGCGATCGCCAGCCTGGACCTATATGTTCTATC
CAAATGAGACGCATGTGGATCTGACTATAACGTTGCCCACAGCAATTTTGTTGAAAGAGGTGCAGCTGGT
GCCGCACACAACTAGTTTGGCATCCTGTCCATCTGCGGTGGCTCTAGAGCTGTCTAGAGATTACGGCCTT
GGTTCGATCCCAGTGGGTGCTCCCATGGCCACCACTGGTCTGACCTGCATTAGACTAAAGCTGGCCAAGG
CAGAGGTGGCTACCAGCATTGTTCTGAGGCTGTACAAGACCCAAGGACTGTGGAAATGTGGGTCTCGTAC
AAATCGCTGTGCTTGGTCAGACAATCTTCGGAAATCGCATGCATGGCCTTCGCTCACCGAGTCCGTTCAT
TGACTCCCTAGCAACGTGCTCCTCGCCAGTGCAAACGGCAGTTTTAGCCGCAATGGATGCGGATTCCATG
CTTGAAGATGATGCTTTTGCGAAGACTAGCATTGGCTGGGTTCGCATTCTGTCGCGCTGTTTCCATGTGG
CCGCTTTACAACCAGACAGTAAGCTGGCTGATCTTATCGCCGCCTATCCGGCTGCATATCCCGGCTTTTT
AGAGGCCTGCTGCTCATTGCTAAACATAATGCCAATGATTCCTAGTCTTGCACAGCAACACTTGGAGACC
ATTTTGTTAAAATTAAGTGCATACAACCACGAGATCAGTTTGCAACTTCTTAGGACGCTCCTGTGGCACA
CCACTCCGCAAGGTTTTTAAACTCTCCAGCGATTCGGTGTGTGATCTTATTTATGAAGTAGTTACCGGAT
CGACGGAGCACCTACTGGACAAACTACGCGTGCTTATCGACTGGGTAATGCAATTGCACGACAACTACAG
CAAGCAGGCGCGCTGTAATAACCCACAAAGTGGATTTGTTAAGGTGATTGCTTCGATTCTGTGGAAAGTG
CATACCCAGCAACAATTGCCTGAGTTACCGCTTCTCATTTCATCTTCGCTGTTCGAGACCTGTTTTGAAT
GGGTCTCTTCGCTGGAGCACGATGAGCCACTGAAGAGCAGTTTTGACTCCTTGTTGTGTGCATTGTGCTT
CATTAAGCCAGAACTGTTTAACCAGCTGTTGGTCAAGCTGGGAGTTAAGCTGGAACCTCCAGCTCGAGGT
CAAACTGATGACAGCAAAGTGCAAAGTGGTAGTGCTTGGTTCCGCCGTGAGGGTAGCGAAAACCTTAGTG
TTTTGCTACAGCGTCCCACATTCCTAAAGACTCTAGCCTTGGCCTGCCAGTCCCCTGCAGCAGTTTACCA
GATGCTAGACTCCGGACTTCCAAAGCTATTGGCCCACGCCATTTACGAGTATTGCATGCATCTCATGCCG
GGATCGGAGCCAGTAGCTTCTGCATCTACAGTGCCGGAAGACGAACCGTTAGCCAACAATCAGGCAGCCG
CTGCAGCAGATGACGAGGACTCGTCTCTTACGGACTTTGACAAGGCCGAGGCAAATGTGGTTTATAATAC
TCCACTGCTAAGTAGCTCGAATGTACCCAAAGTGCTAGATTTCTTTGCCGAATGTTGTGCCGAAGGTCCA
ATGCGTGATTGGTTAGG

>dme_circ_0008477_junction_seq

CCGAAGGTCCAATGCGTGATTGGTTAGGGTACTAATATCAAATCCAAATAATTCAC

>dme_circ_0008478

GCATCAATTGGACCCTGCTGGTTGCTAATAGAGCCACCAGGCTGCACCAACTGAAGCACAACATGCAACA
AAATGTCCTTAAGTCGACGCTTGCACGGAATGCCACTCGTCTCGCTTTTGCTGTGCAGCCAGAGCAATAA
CTCATGCAAGTGCTTGCAAATAGTAG

>dme_circ_0008478_junction_seq

AACTCATGCAAGTGCTTGCAAATAGTAGGCATCAATTGGACCCTGCTGGTTGCTAA

>dme_circ_0008479

CTTGGCTAGCTCCAAGTTGATGGATCTACTGGCTCCCATGAGAAATAGTGAACTGTCCAATGTGGCTCAT
CTACAGGCCTTTATCAACAAGCAACGTGAAGAGGAGCTAAGTGGGCTGGAAAACAACAATAAGGTGGTTT
CACTGTATGATGAGTGCATGCTTCTGCAATATCAGATCAATGTTATACG
>dme_circ_0008479_junction_seq
TTCTGCAATATCAGATCAATGTTATACGCTTGGCTAGCTCCAAGTTGATGGATCTA
>dme_circ_0008480
GGCCAAGGTCGGTACGCTGCAGCTCAGCAGAAACCAAAGCGTAGAATTCCAATGTAGGTCCCAGACCCGT
TCCGACCTCATTCTCGTACTGAATTTCCAGCAGTGCCTTCGAGTGCCCAAAGTCCTGTAGGATGTGCTCT
GCCTGTTTGA
>dme_circ_0008480_junction_seq
TCCTGTAGGATGTGCTCTGCCTGTTTGAGGCCAAGGTCGGTACGCTGCAGCTCAGC
>dme_circ_0008481
GCTTATTAATCTCGTTGCCCATGCCAATATTGCTGACATTCACCGTATTCGATGATCCCACCATCAAACT
GAGAGGCTCCTCCAGATTGTGATCCAGAATCTGGGACAGTGTGCACAAATCGAAATCATACAGGGCTGAG
GCATCGTCGGTGTTTTGGCTAACTCCATTATTCTT
>dme_circ_0008481_junction_seq
CGGTGTTTTGGCTAACTCCATTATTCTTGCTTATTAATCTCGTTGCCCATGCCAAT
>dme_circ_0008482
GCTTATTAATCTCGTTGCCCATGCCAATATTGCTGACATTCACCGTATTCGATGATCCCACCATCAAACT
GAGAGGCTCCTCCAGATTGTGATCCAGAATCTGGGACAGTGTGCACAAATCGAAATCATACAGGGCTGAG
GCATCGTCGGTGTTTTGGCTAACTCCATTATTCTTGTTGTAGGTCATAAGAGCGGACACAAATCCGTTGC
TATCGAAGCCATCGGCGAACTTCTCGTATGGTGTCATAGCCTT
>dme_circ_0008482_junction_seq
CGAACTTCTCGTATGGTGTCATAGCCTTGCTTATTAATCTCGTTGCCCATGCCAAT
>dme_circ_0008483
GGCGTCTTGTACGGTCACAACATCAGCTGGAAGAGAGCTGCTAAGGAAAGGCTTTAGAGCTGAAATCACT
ACAGGAGCTATTCCTTCGTGCCATAGCTCAGTCTTCTTGCGCATGAACTTGCTGCTAGATTTGTGAGCCT
TCTTTGTTAACGACCCGCCACCGCTTGCCACTCCAGAAGATGAGGAGCACGAAGAGGTAGCGTTTACACA
GCTGGATGCGGAGCTTGAAGAGCTCTGCTGTTTTTGCACGCCACTGCTACACGAACTGCTGCTGGATGCT
GCTCCCGCAGCCGCTCCCGAAGTCACTTCCTCCTCTACAGGACGATAGTGAATAGTATGCTGCTGCACCC
ATATACTAGCGTTTCCTAAAAGAGTCTCCGACTCGTTGTCCGTCTCAGGTTGTTCACTGACCAGTGGCGA
GAACTGCTTGACCGCCTGATAAACCGTCATGTTATAGGGCAGCACGTGATCCCCAATCGTAAACTGCAAC
TTGTGTCTAAAGCTCGCCTGGGACAACACCACTGCCGCCACATTGTCATCCATATCCTCTTCACTGTCGT
CGTCGGAGTCTGCACGAATGCCACCATAGCCGCGCACCACCAGGTAACGCTCGATGGCCTGGACCATGGC
CAAGGG
>dme_circ_0008483_junction_seq
GCTCGATGGCCTGGACCATGGCCAAGGGGGCGTCTTGTACGGTCACAACATCAGCT
>dme_circ_0008484
GGATAATGGAGTTGTTGAGGAGCAAAATAAATCTATCACTGAAGCCCATTCACAGGCACCAGAAACACAC
GAATCAGCCCAACCTGACAAGGATGCTGGCAAACAATCATTCCTGAAGTCATTTACTACACTTTCGAAAA
TTCAAG
>dme_circ_0008484_junction_seq
TCATTTACTACACTTTCGAAAATTCAAGGGATAATGGAGTTGTTGAGGAGCAAAAT
>dme_circ_0008485

GTCCACAAATTCGGAAATGGATTTGTCTTCTTCGTGCTCCTCCTCGTTTTCTTCTTCATTCCCGTGGTCT
TCAACATCTTCCTCAAAGCTTTCTAGACGAGCCAGGATTTTATTCTCAGCCTTTTGCTTTGACATGGGTG
ATTGTTCCACCATCTCATCCTTAGCATTCTTTGATTCCAGCTCATCGAATGGCTTGATAGCATCAATTTG
AGGAGGTGCTGGATGCAATTCCTCCGTGCTGTTGGACAGGCCACGTTCCTGATCTTTTAATATTGGTGTG
GAACTATTACGACCCTTTTGCAGAGCCAGTTTTAAGGGCGTCTCCGGCTTGGGGGTTAGAATGCGACAAA
CTGGCACCTTCTCACGTCCTGGAGTCTTGGGAAACCGTTTCTTTTCTACCTCTAAGTCATCAGTGTCGTT
GTTGAAGTCCACCGAttttttttGCTCCACAGCAGGAGAACGCACAGCACGAGCTCTTATGCTGGGCAGA
TGTATAACCTCGGTAACATCGATTTTGTTTTGCTTGTCATCATCCATTGAATCATCTGGGTGTACTTCCT
TACTGTCTTCGACATATTTTGGAGCTTCCGACGGAAATACCGGCTCATCGTCGTCGAGGTCTACAAACTC
CAGTTCTTTTGGTTTCTCTACTGGCTCATCGGTTTGTGGCTCTAACTGAACAATCGAATCTTCTGCATCC
AAAAACTCTTGG
>dme_circ_0008485_junction_seq
CGAATCTTCTGCATCCAAAAACTCTTGGGTCCACAAATTCGGAAATGGATTTGTCT
>dme_circ_0008486
GCCACGCACGCTCTCGCGCAAATTGTCGCCACAGCCGCCAGTGGTGGACAAGAAATCAGCCAAACAGAAG
AAGGGGAAGAAGAAGCAAAAGCCCCAGGAGGCATCACCGCTAAAGAGTTCCGATGAGGAGAACCACGGCC
ATCGTATTCGAACAAATGCTGGCTACGTGACGGTGGTCGATGAGCCGCCCACTAAAGTTCCCAAAATCGA
GCTGATTAAGACAAGTTCGGGCATGGTTCGAGTTGAGCCGTGCACACCCAAACAGAAGTATTTCCGCGAG
CTGCCGCCCACACCGAAAATGCATGGGTTCCGCGAGGAGCCAGGACCCTCCGGGATGTCAAGGAAGCGGG
CCAAGCACGCAGCACCAAAGGTCGAACACAATTCCGCCAAACAGGCGGCGCTGCGCTTCAAGGAGCAGAT
CTTTGCTCGCAGATCTTAGGATACATTACATTTAGAGAAAAATTAGTGGAAATGTTAAGTGTACACAATG
CATACTTCAGTTTTTGTTTCTTTTCATTAGCATTTCCCCGATTGAATTTGATTCTATTATTTGTTCTATT
GGTTATTTGTTACGCAAGCCCTTGCTCTTTGCCATATATTATTATTTTGTTCAATTGCACATCACTTAAC
CACACTTAACGCTAGCAAGTTGGTTGTTGATCCAATCAAGATATGAAATCAATTAAGAAATTTTCGAACT
TGTCCCCtttttttttGAGCATCTGAGAAACAGAAAATAAAGCCTACCCATACAAATACAAAATGAAGTC
AATGCAAAAATTGGCGATGCAGCCTACATCCAGGATGAACTGTCTTCGTCGCTATCCTCTTCTGTGGCCA
GCAAATTCAAAGAATTTTCGAAGCGCAAACGCATTTCGGACAGTTTCTCGGATCTTTCAGAAGAACTTGA
GAGAATAGGTTTGCGACAGATGAGGAAACGCATTAAGTTGGAGAAATCGTTCGAAAGAACTCCCAAAAAG
AAGGTCCAGACCAAGAAGCATTTACCACCGGTTCGGAAGAAGGATAGCGTGAAACGTCGCCGCATTATTA
TAGCGAGCCCCAGTGATGAAGAACATGATGGAAAAGTTCAGACCAATGACGACTCAAGTGAGAACAAAAC
AGAGAAGCCCAAAAACCAAAGTAACTGCTCATCCAATGCCAGTAAATTGAGCGAAGCGGCCCAACTGTTA
AACGTCAGTGAGGAAAAATCTTCCATCTCGGAAACCGAGCTGGAAAGATCTCGCCAAGTCGCTCTCAACG
AACTGAACAAATCGGAGCGGTTTAACAAGACAGAAACGCAACTCGATATCAGCGTCATGGAGGTAGACTC
CTCGGATCACGACGAGGAGGAGAAGACAGAGAAGGTTGGAGCCAAGAACAACAGAAGCCTGTACGAAATC
GTGGATTCTGATGACGAGGAAGAGCAAGATCAAGACCAAAGTGATGCAGCTAAACCAGCTGAAAGCGAAA
ACCATTCCGAAATTAAAAAATCTACATCTTTCATGGACCAAATGTCTGGAGCCAAAGGCAACAAAAGCGT
ATACGAAATAATGGATTCTTATGAAACTGAAGACCCTAAGGAGGCTGGTAaaaacgaggagtctgataag
gacaaaccggccgaaaacgggaagtctgataaagacaaacaagccgaaaccgagatgtctgatgaagata
aaccatctgaaaTCAAAAGCCCTTCCACAATCAAAAAATCCATAATTTCAACTGCGGATGAAGAAGCTCT
GCTCGCCGAATTGGCCTCTAGTGATCTAAGCCATTTGGAAAAAATGTTTAACCCTCTACAGAAATCCCGC
CGCCAGTCCTTACATGTCCCCAGTCCCGAGCTGGCAGCCAAGAATCCCAAACTGAGACGCCGTAGTGAAC
GGGTTGAAGTTGGCAATGACTTCTGCCCCTCGCAGTCCTTTGTTGACATGGTGGCCGAGAAAAAGAGGCA
GAAAAACAAGCGCAAGCGCCTGTCGAAAAGCCTATCAGGCGCACCAGAGGATCTTGAGGAAATGGAGATT
AAGCACGAGCGCAAGCGCCTTAAGAGCTCACATGGTGCTTCCACGGATTCAATGGAGGAGGACAACGAAA

ACGAAACAATGACTGTCGCCGAGGAACATCACAGCGACGGGGAAGTCTCGAATGGTGAAGTTCCTATTGA
AGAAAAGCCCACTACTTCATCTGAGAAACCATCTGCGTCGGAATTACCTGAAGAAGGTAATTCAGCGCCA
CCGGCGCTGAAGAAGGATGTAAAGCGCCTACAGGCAGCGCGCCAGGCTGTGAGTCATGCTGTTAATCTTC
TGGCGCCACCAAAAGCCACCGAAGCAGAG
>dme_circ_0008486_junction_seq
GGCGCCACCAAAAGCCACCGAAGCAGAGGCCACGCACGCTCTCGCGCAAATTGTCG
>dme_circ_0008487
GTCTGCACGAATGCCACCATAGCCGCGCACCACCAGGTAACGCTCGATGGCCTGGACCATGGCCAAGGGG
TCGATCTTTACAGTTCCGCCCTTCCACTGACGTAGGTTATTGCACTGCGGATGGCGTTGCAG
>dme_circ_0008487_junction_seq
GGTTATTGCACTGCGGATGGCGTTGCAGGTCTGCACGAATGCCACCATAGCCGCGC
>dme_circ_0008488
GAGCACTCTGATTGGACCTTCCACCAGGACCGGCAGGAAAGTCGTGTACCTTAACAGGAAACTGTTCTAG
TTGCGTGACACAACCATTTAGCTTGGCCACAAAGGCTCCGAAGGCTATCGGCTCGATAGTTGGCATCTGG
CCGACGTTTTGCAGCAGAGGCTCTAATGGAAGGCCAGCGAATACGTGCATAAAGCTGCGCAGACGTGCAT
CGCGCTCAACCAGTCCA
>dme_circ_0008488_junction_seq
CAGACGTGCATCGCGCTCAACCAGTCCAGAGCACTCTGATTGGACCTTCCACCAGG
>dme_circ_0008489
GGACGGGGATGATGATGAGCAAATAATCAACGATTTTGATGCGGAAGATGAGCGTCATGCCAACGGAAAC
CAGGCAAACAACCTACCCATCAACCATCCACTACTTGGTCAGAAAAGTAACGACTTCGATATAGAG
>dme_circ_0008489_junction_seq
TCAGAAAAGTAACGACTTCGATATAGAGGGACGGGGATGATGATGAGCAAATAATC
>dme_circ_0008490
CATCTCCACCAGGGGCCACTTAAAGAAGCCGAAACATTGAACCTAAACACCGCCATTGCCTTAGACCTCG
AAGACGAGTGAATAACTAGTACTAGAGGGATCTAGAATAGAATACGAGCTTACCTACTTAGTCAACGAAT
TTAGCAACCCAGGTGCAGCCACTACCATCATCGGAGCAAATAACTTTGTGGAGTAGCGAGCGCAAAACAT
CAAACCCCAATGACCACACGCCTGGCTCCACAGCGCCAGTTCATCCTCTCGACGCGCTCTGCCATCGTGG
ATGCCACCTCGCCACCTGAGGACTCAGCGGAGGAGCAGCAGCAGCTGACTGTCGAGGTGGAGAGCGCCAC
CAACGGAGTCATTACCAATAGCACCACATCGCCACCGCCGCATGCCGAGCAGTTCGAGTTCTGCACGGAG
GATGGAGTCGTTGTGTCCGCCACACTGGAGGATGTCATGACACAGGGTAAAACTTTTCCCCCTCTTGCAG
AACTGCCAGCTCTTGCAGAAGAAACTAGTCGACGAGGACGACGATTCGACGGGAGTAGGAGCAACGGATG
CCGATCAGCTGGAGGTGATTAGCGTTGTACTGCCCATGGATGCCGAGGACTCGACCAGCGATGCCCATCT
ACACGGGCATGTGGTGAACAGCAGCAGCGACACCATGGGTACCATTACCACCACGGAACCGAATGGGACC
ACCGTCACCCACTCGATACCCATTCACAGTATGGCCGATCTGGCCGCGATCAAGGATGGCGTGGATCTGG
CCCAGCAGGTGGCCAATGGCCAGGTGACCGTGGTGCAGACCACCGAGGACGACGATGGAACGCCCTTCAT
TACCGTGACGGG
>dme_circ_0008490_junction_seq
ATGGAACGCCCTTCATTACCGTGACGGGCATCTCCACCAGGGGCCACTTAAAGAAG
>dme_circ_0008491
GAGCAGGACCACCGCCAGCACTCTTTCAGCAGCAGGCACCGCCGCAGCTAACCCGCTACACGCCAGCAAC
AGCTACAACAGCGGCTACGTTTGTGCCTCCGCAGCAGCAACAGGTTGTCTACAACCCACAACAGCAGCAG
CACTCATCACTGCGACGCAGTTCACGTGGCAAGACGGGTTCTTGTGTGAGTTCGgcagcggcagcacagc
agcagcagcaccatcagcagcagcaacatAGCTCCGCTGCTGCTGCTGTACAGCAGCAATTGCTGCCACC

GCCTGGTACCTATCAATACCAACAGGTGGGCGGCAATACCGTTGTAGTTGCCGTGCGTcatcagcaacag
ttgcaacagcagcagcagcaacagcagcaactggctttacatcatcagcatcagcagcagcagcatcagc
aacagcagcagcagcatcagcagcagcagcatcagcaacagcagcgtgccactctcgtgcagccaggatt
cttgttcagctatcgcagcaatcaaccccagcagcaacagcaaccagcgcagcaAATACATCAGGGCCCC
AAAGTGACGCATAGCAGTGGTAAGAAGATGAAGGAAGAGGAAGAGCAAAATAAGGATGAAAGGACAAGAG
ACCGCAAGCAAATCAAAATCCAGGGTCTCCTCAATCATTTTCCATAATAAGGCTAAAATCAGAGGAGAAC
TTGTAGATTTTCGCGAGGTTCTTGTTTAAATAGGAGATAGTTTGGCGCTTCTGGCCTTAAAGGAATTGAC
AGCACACACACGCTCTATAGGAATAGGGTTAATTGTGGTCCGCTTAAGACGTTGGTCTTTTGTGATTGTC
CCCGTCTAGGGCAGGAACAAATTCTAATGTGATTTGCAAAACAGTAACCCGAAATCTTTTTATGTGGAAC
TCAAAACGACCCTGATCGTGAGTTCACTGTACAAATTCTTGGCAATTGGATCCACTCTTTATTATTGGTG
CTGGCCGTAGTCTAGGTGATAATATAACCCATACTGCACTGaaaaaaaaaCCACACTGTCCAAAATACTC
CAACCTCCCTTACATCTTTCTTCGTCCCTCGCTCGCTCATTCCTGCGTTGCTTTGCTCGCGGTGTCTGCC
GAATAATTTCCATAACTGAACCGAACTGAAACAAAATAATAGTAAACTTAACATTGTTTTGCTCTGTTTT
CTCTACCTCCCGATCTCTGCCTTCGTCTTCGTCTCCGTCTCTTTGTCTGATTAGCAGCTTCGGATGCTTT
AACATATAGTTTGATGGCACAACAACCGCCAAGTGGACCGCCGCATGCAGGTGGACAGCAAATAACGCCA
GG
>dme_circ_0008491_junction_seq
ATGCAGGTGGACAGCAAATAACGCCAGGGAGCAGGACCACCGCCAGCACTCTTTCA
>dme_circ_0008492
GAGAACGACAACAACCTCAACGTGCCACCAACAACGCaaaaaaaaCCAAGAACAACACACCGGCGGTAGC
AGCAGCCAGCACTATCAAGGCGACAACAACAGCAACAACGTCGTCCGGTTATCAACAACGTGCCGCCCCC
CATGGACAGGGGGCGGCGCCAGGCGCTGGAGGCGGCGGTCCGCGTGCCACGCGCAGCAGCAACAGCAACT
CCAGCAACAACGTTGTTGACTACAGCATTATGCCGCACCTCACAACGGTCGCAACAACCACACCACATGC
CTTGACACCGCAACAGCACCAACAACAACGACAACCGCCACAACCTCCGTCGCATCAGCAACAACAAGTA
CACCAACAACAGCAGCCACCTCAATTGTTGCCCACACATCAGTTTGCACACTTGTCGTCCTTTGTGACAC
CGAcagcagcggcagcagcagcagcagcggcGGCCCACTATCCCCCAGCTTCTGCTGCTGCCGCTTACTT
TGCacagcagcaacaccatcagcatcaacatcagcatcagcaacaACACGTGCAGCAGGCACAGGCACCG
CAGCCGCACTATTACCATAGTTCAGTGGCTGTGTTGCATCAACCTCCACCGCACCATTTTACCTCCACAG
GAGCAGGACCACCGCCAGCACTCTTTCAGCAGCAGGCACCGCCGCAGCTAACCCGCTACACGCCAGCAAC
AGCTACAACAGCGGCTACGTTTGTGCCTCCGCAGCAGCAACAGGTTGTCTACAACCCACAACAGCAGCAG
CACTCATCACTGCGACGCAGTTCACGTGGCAAGACGGGTTCTTGTGTGAGTTCGgcagcggcagcacagc
agcagcagcaccatcagcagcagcaacatAGCTCCGCTGCTGCTGCTGTACAGCAGCAATTGCTGCCACC
GCCTGGTACCTATCAATACCAACAGGTGGGCGGCAATACCGTTGTAGTTGCCGTGCGTcatcagcaacag
ttgcaacagcagcagcagcaacagcagcaactggctttacatcatcagcatcagcagcagcagcatcagc
aacagcagcagcagcatcagcagcagcagcatcagcaacagcagcgtgccactctcgtgcagccaggatt
cttgttcagctatcgcagcaatcaaccccagcagcaacagcaaccagcgcagcaAATACATCAGGGCCCC
AAAGTGACGCATAGCAGTGGTAAGAAGATGAAGGAAGAGGAAGAGCAAAATAAGGATGAAAGGACAAGAG
ACCGCAAGCAAATCAAAATCCAGGGTCTCCTCAATCATTTTCCATAATAAGGCTAAAATCAGAGGAGAAC
TTGTAGATTTTCGCGAGGTTCTTGTTTAAATAGGAGATAGTTTGGCGCTTCTGGCCTTAAAGGAATTGAC
AGCACACACACGCTCTATAGGAATAGGGTTAATTGTGGTCCGCTTAAGACGTTGGTCTTTTGTGATTGTC
CCCGTCTAGGGCAGGAACAAATTCTAATGTGATTTGCAAAACAGTAACCCGAAATCTTTTTATGTGGAAC
TCAAAACGACCCTGATCGTGAGTTCACTGTACAAATTCTTGGCAATTGGATCCACTCTTTATTATTGGTG
CTGGCCGTAGTCTAGGTGATAATATAACCCATACTGCACTGaaaaaaaaaCCACACTGTCCAAAATACTC
CAACCTCCCTTACATCTTTCTTCGTCCCTCGCTCGCTCATTCCTGCGTTGCTTTGCTCGCGGTGTCTGCC

GAATAATTTCCATAACTGAACCGAACTGAAACAAAATAATAGTAAACTTAACATTGTTTTGCTCTGTTTT
CTCTACCTCCCGATCTCTGCCTTCGTCTTCGTCTCCGTCTCTTTGTCTGATTAGCAGCTTCGGATGCTTT
AACATATAGTTTGATGGCACAACAACCGCCAAGTGGACCGCCGCATGCAGGTGGACAGCAAATAACGCCA
GG
>dme_circ_0008492_junction_seq
ATGCAGGTGGACAGCAAATAACGCCAGGGAGAACGACAACAACCTCAACGTGCCAC
>dme_circ_0008493
GAGAACGACAACAACCTCAACGTGCCACCAACAACGCaaaaaaaaCCAAGAACAACACACCGGCGGTAGC
AGCAGCCAGCACTATCAAGGCGACAACAACAGCAACAACGTCGTCCGGTTATCAACAACGTGCCGCCCCC
CATGGACAGGGGGCGGCGCCAGGCGCTGGAGGCGGCGGTCCGCGTGCCACGCGCAGCAGCAACAGCAACT
CCAGCAACAACGTTGTTGACTACAGCATTATGCCGCACCTCACAACGGTCGCAACAACCACACCACATGC
CTTGACACCGCAACAGCACCAACAACAACGACAACCGCCACAACCTCCGTCGCATCAGCAACAACAAGTA
CACCAACAACAGCAGCCACCTCAATTGTTGCCCACACATCAGTTTGCACACTTGTCGTCCTTTGTGACAC
CGAcagcagcggcagcagcagcagcagcggcGGCCCACTATCCCCCAGCTTCTGCTGCTGCCGCTTACTT
TGCacagcagcaacaccatcagcatcaacatcagcatcagcaacaACACGTGCAGCAGGCACAGGCACCG
CAGCCGCACTATTACCATAGTTCAGTGGCTGTGTTGCATCAACCTCCACCGCACCATTTTACCTCCACAG
GAGCAGGACCACCGCCAGCACTCTTTCAGCAGCAGGCACCGCCGCAGCTAACCCGCTACACGCCAGCAAC
AGCTACAACAGCGGCTACGTTTGTGCCTCCGCAGCAGCAACAGGTTGTCTACAACCCACAACAGCAGCAG
CACTCATCACTGCGACGCAGTTCACGTGGCAAGACGGGTTCTTGTGTGAGTTCGgcagcggcagcacagc
agcagcagcaccatcagcagcagcaacatAGCTCCGCTGCTGCTGCTGTACAGCAGCAATTGCTGCCACC
GCCTGGTACCTATCAATACCAACAGGTGGGCGGCAATACCGTTGTAGTTGCCGTGCGTcatcagcaacag
ttgcaacagcagcagcagcaacagcagcaactggctttacatcatcagcatcagcagcagcagcatcagc
aacagcagcagcagcatcagcagcagcagcatcagcaacagcagcgtgccactctcgtgcagccaggatt
cttgttcagctatcgcagcaatcaaccccagcagcaacagcaaccagcgcagcaAATACATCAGGGCCCC
AAAGTGACGCATAGCAGTGG
>dme_circ_0008493_junction_seq
AGGGCCCCAAAGTGACGCATAGCAGTGGGAGAACGACAACAACCTCAACGTGCCAC
>dme_circ_0008494
GGTCTTGATATAGGGCAGGATGCTCAGACCCTTCTCAACGGCCTTTTTGGCCAGCAGACCGGCACCCAGC
ATCACCGAAGGATTCGATGTGTTCGTGCAGGATGTGATGGCCGCAATGACAACAGATCCATGATGCAGTT
TGTAG
>dme_circ_0008494_junction_seq
GACAACAGATCCATGATGCAGTTTGTAGGGTCTTGATATAGGGCAGGATGCTCAGA
>dme_circ_0008495
TTGGAGGGCAAACTGAGCCCACTGGTCACATCCACCGATCTGGTGCTGACCATTACCAAGCACCTGCGCC
AGTTGGGTGTGGTGGGAAAGTTTGTGGAGTTTTACGGCCCGGGAGTTGCGGAACTCAGCATTGCTGACAG
GGCCACCATCAGCAATATGTGTCCCGAATACGGAGCCACCGTGGGATACTTCCCCATCGATGAGAATACT
CTTGG
>dme_circ_0008495_junction_seq
ACTTCCCCATCGATGAGAATACTCTTGGTTGGAGGGCAAACTGAGCCCACTGGTCA
>dme_circ_0008496
GTACGCCCAATCCATTTATCATCGTGGTATGGGAATCAGTGCCCACCACACTGTCGGGATACAGAATCTT
GGAGCCATCGGTTGCGTCATTCTCGAACACCACGCGGGCCAAGTACTCCAGATTCACCTGGTGGACAATA
CCGGAGCCAGGTGGCACGATCAGCATGTTGTTGAAGGCCTTGGCTCCCCACTAATTAGGAAAAACAATAT

TATTAAAAATACTATCTGGATAATAAGCTAAGGAAAAGTAACCTTCAGGAAGGTGAAGCGCTCCTTGTTG
CGCTCAAATTCCAGTGACTGGTTCTTGGCCAAAGCATCAGGCGCACGGGCGAAGTCCACCTGGACCGAGT
GATCGATGACCAAGTCGGCGGGGCAGATGGGATTGATTTTCTCCGG
>dme_circ_0008496_junction_seq
CGGGGCAGATGGGATTGATTTTCTCCGGGTACGCCCAATCCATTTATCATCGTGGT
>dme_circ_0008497
CGGCGCCCGGGGGAGATCAGCTGGTTTGCGCGCCCCTTAAAAAACGCCGCTTGCAGCAGTCTTTGGGATC
CGGTATCTGCGAGGGAGCGGTAGAAATCAACTTAGGTGCAGCAAGCTTTGACCACACTCCTCGTCAGGCA
TCCGGAGACTGTGTTGCACAGCGCACTCGATCTAAGACCATTTCGCCGGAGGAGTTGCCAAGCACAAGCA
GCGCTGCGGCAGCACGCCATCACCAGATTCGCCCCAGTGCCAGCTCTACCAGCTTCTCGAGCAGCCATCG
AAACAAACGAAAGGCCAGTATTGGAGGATCAATAGCTGCGGTCGAGACCACTCCGCATAGAGGAACAGCA
GCTGCTCGCGCCGGACGCAGCGGTAACCTGCTAAACTATTACAGAAAAACGCGTAAGGTTAGCCACACAC
GCTCCTCCCAAAAGTCGGAAAAGCAGTCTATACCAGCGGAGGACACTACGGCCAACCGAAACGGATCAGC
CGGATCCGGATCCAGTAGCAAGTCTGGTGTGATACCCAAGAACAGGAAGAGCCTGCGTCATTCGCAGCAG
AACCTGCTGGACACAGAGGCGGCAGAAGTAGAGAGTGCTGCTGCTGAAACTGGAGGTGAACAGCAGTCGG
AGCACGGAAATCTGGACGTGATCGACCAACTGCCGACCCCTGAAGCTGGTCCTAATCAATCAGAGGCATC
AGGAATCCACAACCAGAGTCAATTGGAGGCGGATCGCCAGCCCGTGGTGCaggacgaagacgacgaggag
gacgacgaggaagaggaagaagaggaggaagaggaggtGGGCTTCTACGGGATTGTAAACTCTGCTGGCT
CGTCATACGAGGAAGACACACAAATAGTTGCCGAGGAGGACGAGATCACAACGGAGGAGGAAGTTGACGA
CGAGGACGACGACGAAATCGAGGAAGAAGACCTCTCGGAGAGCGAATTCGCGCAGCAACTGATTGGTGAA
CTTGGTGG
>dme_circ_0008497_junction_seq
CGCAGCAACTGATTGGTGAACTTGGTGGCGGCGCCCGGGGGAGATCAGCTGGTTTG
>dme_circ_0008498
CACTACCAATCTGTCCAACGAAGCACGCGAATGGGAAATCAGAACGGAAAACTGTTAAAAATTGAAGTGA
AAGAGTCAAAATCACAGCTTATGAAATAGTGTTGTTAATAAGAAAGCGACAGCAAATTGAATCCACAGCT
GCTTAGAGACTATTACCATCCTTTAGGCAACGGACCGATTACGAGCAAACATTTCGAGATCAATATTCCA
TTATTGGTTGTGTTGGCATGTTAAGTGTGTTCGATGCCGGTGCGGTTAAGTGTCGTCTATGGCCGAATCC
GTTAAAAGTCAATCGCTCTCTGCATTGACGGAGGGGCAGCACGACGACGGTGGTTCAACAGCAACATCAG
CGACATTGGCTAATAAAACCAGTGGGACCAACACGACAAGTGCATCCAATCACTCGTCCCAGAGGCGACG
CAACAACAACACAAACAGCAACAGAAACAAGAGTCACCACAAAAACGAGAAACGCAACGCACCGGCTACA
TCAGGCCCCGCTGTAAGCTCCTCCGTCGACGTTGTACCCGCCACCTCGACTGCATCTGCAACCTCTACCC
GTCGGTCTCGCAGTCAAGGTCGCCACTCCTCCGCCGCAGCCCTCAGCAGCAAGGAGTCACAAATCCCCAA
GCGCTCCGTGGGAGCCATCCGCTCCCCATCTTCACCAGCATTCAACTCCATACCGGTCGTCAGTGATCAA
TCGCTATCGCCCGGCAGTCGTAAGCGCCAGCTTAACCAGCACAACAACAGTAACAACAAAGCCAGCAGTA
GCCACCAATGAAAAATTGAAGTGAAAGAGTCAAAATCACAGCTTATGAAATAGTGTTGTTAATAAGAAAG
CGACAGCAAATTGAATCCACAGCTGCTTAGAGACTATTACCATCCTTTAGGCAACGGACCGATTACGAGC
AAACATTTCGAGATCAATATTCCATTATTGGTTGTGTTGGCATGTTAAGTGTGTTCGATGCCGGTGCGGT
TAAGTGTCGTCTATGGCCGAATCCGTTAAAAGTCAATCGCTCTCTGCATTGACGGAGGGGCAGCACGACG
ACGGTGGTTCAACAGCAACATCAGCGACATTGGCTAATAAAACCAGTGGGACCAACACGACAAGTGCATC
CAATCACTCGTCCCAGAGGCGACGCAACAACAACACAAACAGCAACAGAAACAAGAGTCACCACAAAAAC
GAGAAACGCAACGCACCGGCTACATCAGGCCCCGCTGTAAGCTCCTCCGTCGACGTTGTACCCGCCACCT
CGACTGCATCTGCAACCTCTACCCGTCGGTCTCGCAGTCAAGGTCGCCACTCCTCCGCCGCAGCCCTCAG
CAGCAAGGAGTCACAAATCCCCAAGCGCTCCGTGGGAGCCATCCGCTCCCCATCTTCACCAGCATTCAAC

TCCATACCGGTCGTCAGTGATCAATCGCTATCGCCCGGCAGTCGTAAGCGCCAGCTTAACCAGCACAACA
ACAGTAACAACAAAGCCAGCAGTAGCCACCAATGTGAGTTGTATTATCTATGTTTTCTTATCAGCCAAAA
TCATTAGTTGCAAATCCTTTCTCTTACTTTGTTGTGGAAGTACTATTTTTAAAGTTGGCTTTCACAAAGT
CGTACAAGTTTGACAACATACGGTTTCAGATTAGCAAAGGCACAGCACACATGGTGAGTTGTATTATCTA
TGTTTTCTTATCAGCCAAAATCATTAGTTGCAAATCCTTTCTCTTACTTTGTTGTGGAAGTACTATTTTT
AAAGTTGGCTTTCACAAAGTCGTACAAGTTTGACAACATACGGTTTCAGATTAGCAAAGGCACAGCACAC
ATGTATGTAATAACCGTCTTCCGAATTGTCTGTTATTAAAAATACACAGATTTAGAAATATAGTGTTAAA
AAATCTGTTTGCTACGTGCGCTAGTTTTGTTTCGGTAGCGATGAAATCTGTATTTTGTATAACAAATTTA
AATAATTTATGTTTATACAAATTAATGAATTAGATATCATTTACACAAGATAACAAAGTATTGAAAAACA
CTATTTTTGTATAAACTTTTTAACTTTTCGAAATTAGTCAAAGTGCGTTTGGTTATTTGTCAAAATTTTA
AAACCATTCAATCGTTCAATTGGCTTCAAAACAAAACAAATATTTACAAACCGAATTGAAGAGCTAATCC
GAACTTTATTTCCAATGCCTTTTTCAGCCGGCGCCCGGGGGAGATCAGCTGGTTTGCGCGCCCCTTAAAA
AACGCCGCTTGCAGCAGTCTTTGGGATCCGGTATCTGCGAGGGAGCGGTAGAAATCAACTTAGGTGCAGC
AAGCTTTGACCACACTCCTCGTCAGGCATCCGGAGACTGTGTTGCACAGCGCACTCGATCTAAGACCATT
TCGCCGGAGGAGTTGCCAAGCACAAGCAGCGCTGCGGCAGCACGCCATCACCAGATTCGCCCCAGTGCCA
GCTCTACCAGCTTCTCGAGCAGCCATCGAAACAAACGAAAGGCCAGTATTGGAGGATCAATAGCTGCGGT
CGAGACCACTCCGCATAGAGGAACAGCAGCTGCTCGCGCCGGACGCAGCGGTAACCTGCTAAACTATTAC
AGAAAAACGCGTAAGGTTAGCCACACACGCTCCTCCCAAAAGTCGGAAAAGCAGTCTATACCAGCGGAGG
ACACTACGGCCAACCGAAACGGATCAGCCGGATCCGGATCCAGTAGCAAGTCTGGTGTGATACCCAAGAA
CAGGAAGAGCCTGCGTCATTCGCAGCAGAACCTGCTGGACACAGAGGCGGCAGAAGTAGAGAGTGCTGCT
GCTGAAACTGGAGGTGAACAGCAGTCGGAGCACGGAAATCTGGACGTGATCGACCAACTGCCGACCCCTG
AAGCTGGTCCTAATCAATCAGAGGCATCAGGAATCCACAACCAGAGTCAATTGGAGGCGGATCGCCAGCC
CGTGGTGCaggacgaagacgacgaggaggacgacgaggaagaggaagaagaggaggaagaggaggtGGGC
TTCTACGGGATTGTAAACTCTGCTGGCTCGTCATACGAGGAAGACACACAAATAGTTGCCGAGGAGGACG
AGATCACAACGGAGGAGGAAGTTGACGACGAGGACGACGACGAAATCGAGGAAGAAGACCTCTCGGAGAG
CGAATTCGCGCAGCAACTGATTGGTGAACTTGGTGG
>dme_circ_0008498_junction_seq
CGCAGCAACTGATTGGTGAACTTGGTGGCACTACCAATCTGTCCAACGAAGCACGC
>dme_circ_0008499
GTGAACAGCAGTCGGAGCACGGAAATCTGGACGTGATCGACCAACTGCCGACCCCTGAAGCTGGTCCTAA
TCAATCAGAGGCATCAGGAATCCACAACCAGAGTCAATTGGAGGCGGATCGCCAGCCCGTGGTGCaggac
gaagacgacgaggaggacgacgaggaagaggaagaagaggaggaagaggaggtGGGCTTCTACGGGATTG
TAAACTCTGCTGGCTCGTCATACGAGGAAGACACACAAATAGTTGCCGAGGAGGACGAGATCACAACGGA
GGAGGAAGTTGACGACGAGGACGACGACGAAATCGAGGAAGAAGACCTCTCGGAGAGCGAATTCGCGCAG
CAACTGATTGG
>dme_circ_0008499_junction_seq
AGAGCGAATTCGCGCAGCAACTGATTGGGTGAACAGCAGTCGGAGCACGGAAATCT
>dme_circ_0008500
gaggacgacgaggaagaggaagaagaggaggaagaggaggtGGGCTTCTACGGGATTGTAAACTCTGCTG
GCTCGTCATACGAGGAAGACACACAAATAGTTGCCGAGGAGGACGAGATCACAACGGAGGAGGAAG
>dme_circ_0008500_junction_seq
GAGGACGAGATCACAACGGAGGAGGAAGgaggacgacgaggaagaggaagaagagg
>dme_circ_0008501
TCAATCGCTCTCTGCATTGACGGAGGGGCAGCACGACGACGGTGGTTCAACAGCAACATCAGCGACATTG

GCTAATAAAACCAGTGGGACCAACACGACAAGTGCATCCAATCACTCGTCCCAGAGGCGACGCAACAACA
ACACAAACAGCAACAGAAACAAGAGTCACCACAAAAACGAGAAACGCAACGCACCGGCTACATCAGGCCC
CGCTGTAAGCTCCTCCGTCGACGTTGTACCCGCCACCTCGACTGCATCTGCAACCTCTACCCGTCGGTCT
CGCAGTCAAGG
>dme_circ_0008501_junction_seq
CCTCTACCCGTCGGTCTCGCAGTCAAGGTCAATCGCTCTCTGCATTGACGGAGGGG
>dme_circ_0008502
GGTCGAAGAACCATCCAAATACGGAGTTGTGCTGTACGATGAGAACGGCTGTATAAAAAACTTTATTGAA
AAGCCACAAGAGTTCGTTAGCAATAAGATCAATGCCGGCATTTACATCTTTAATCCCTCCGTGCTTGACC
GGATCGAAGTTAAGCCCACATCAATAGAGAAGGAGGTGTTCCCTGAAATGACGCAGCAACAGGAGTTGTA
TGCAATGGATTTAACTGGATTCTGGATGGACATCGGACAGCCAAAAGACTTCCTAACCGGTACGACAGAC
AGAATCACTCGTTTGCTCGCTCAATCTTGATTGCCTAATTGGTCTTATCGCTTTGAAAGCAAATGTGGAT
GTCGTTGTTAATTGTGCTGTGCCTTTTTACCCACCCGGCTTTCGGCTTTATCTATTATTGACATTCCTGG
AACTATAGCTAATGTTTGATAGAACGATTTCTTTTTAAATGAAGTGAGGCTCATTAGGCCGATAGTGGGC
GATGGGAGAAACCAAGTAGAAGAGTAAATTGTTTGTTGGGTTCATGTGGATGCGATTCACGACTTATGAA
TACATGGTACGACAGACAGAATCACTCGTTTGCTCGCTCAATCTTGATTGCCTAATTGGTCTTATCGCTT
TGAAAGCAAATGTGGATGTCGTTGTTAATTGTGCTGTGCCTTTTTACCCACCCGGCTTTCGGCTTTATCT
ATTATTGACATTCCTGGAACTATAGCTAATGTTTGATAGAACGATTTCTTTTTAAATGAAGTGAGGCTCA
TTAGGCCGATAGTGGGCGATGGGAGAAACCAAGTAGAAGAGTAAATTGTTTGTTGGGTTCATGTGGATGC
GATTCACGACTTATGAATACATGTACTGGGACGATACATTGCTGTcttacttacttacttacttacttaA
CTGCATAGGATCTAAGTAACGCTCATCAAATATGCGAAGTACACTGGCAATAGTCAGTTTCTTAAATGTT
TTTTGGTCACCATTGAACGTTAAAAGTGAAGGCTATTGAATGGGCTTGAACACATCGACTTTATCGCTTT
ACTTATTGCACCATTTTATTCCTTTTCAGGCATGTGTCTGTATCTAAGCTCGCTGCGCCAGAAGCAATCC
CCCAAGCTGTACACAGGCCCTGGAGTGGTGGGCAACGTGTTGGTGGATCCCACGGCCAAAATCGGCGAGG
GTT
>dme_circ_0008502_junction_seq
GATCCCACGGCCAAAATCGGCGAGGGTTGGTCGAAGAACCATCCAAATACGGAGTT
>dme_circ_0008503
TATGACGATGGCCTACACGGCTACGGCATGGACTCCGGAGCCGCAGCAGCGGCCATGTACGATCCACACG
CCGGTCACCGGCCGCCCGGACTGCAGGGCCTCCCCTCGCACCACTCTCCGCACATGACGCACGCAGCGGC
GGCGGCGGCCACAGTGGGCATGCACGGCTACCATTCGGGGGCCGGGGGTCATGGAACACCTAGTCATGTA
TCGCCGGTCGGTAATCACCTAATGGGCGCAATACCCGAAGTACACAAACGTGATAAGGATGCGATTTATG
AGGTGAGTATAAGTATAGTGTGCATAGTGTGTAAACTAATGGAATTCGGTTTGGCTTCGGCAGAGGTGGC
AACTCTGCCCAAGTGACCCGAGATGATCTTGATGCTAGTCGAATTTAGCTTAGATTCAGATTGGGACTAA
AGTAGGGGGAATGAGGAATGTGGGAAATTTCGACCTAGAAAAATAGCTGTCAAGTTGTTATTGACTAAGC
GCAAAATAAGTAGTTGCTGAGCACTCAACACCATTATTTATATATATTCCATAAAAAAGTCTAGAACTGA
ATCTATTTTATTGCGTGTGGGATTTGTTAAAAGCTATAATAAAATGTTAATTCAATTTCCTTAGGCTGAA
ATCCAGCCTACCCATTCATAATCAGTTGCATATCGATCAAAGTATCCCAACCAATCAATATACAAACGCC
TGCTCTTTATCGATTTATAATTTTTGGCATGTTCGCAGTGAGCCAACCCAATTTGCCACCTGTTTTCACC
TCTGCGCACGCGTAACTTAGTCAAAAGAGGAAAAAATAAACACGAAAGAAGGATCCTTTTTTCAACAGAT
AGAAAGAGATAAAGAGATTGAACAAGAGAGAGGGAGAAATGGGCGTGATCCTCTTGCGTTTTGCGCAAAA
TACAAAATTATGGGTTTGCTTTTGCGGCCGAAACTCGAAGAGAGAAATGTCCGTTTTCCAATTCTACGGT
ATCTTGGGGGTGTGTCAGGTGTTTAGTGACGGACACGGAACATGAACGTGCTTAGGCATTAGGGAGTCCG
AGAACTAAAACCCTaccaaccaaccaaccaaccaTCCGTCCATCAAAAATGAGGTGCAGTTTCTCGCTCT

GCGGTTCGGGTGTTAATTTGCCAAAATTGTGGTTTCTTCTTCATTTGGTTTTTGTGTCTCTCCCTGCAGT
TTTTATTGTTTTAAAGGGTTTTTTATTCTAACCCGATCGTACGTTTTTGTGTCATTGCGTGTCCTGGCAA
TTTTCATGTGTTGCAACTGCTTTGCCAGCTGTCTGAGTGCCTGAAATGCTTATTTGCCCAGTTTTTCATT
TCACATATATATATAtttttttttttGTGCAAATATTTGCTTTGAAAGGGAATTTCATGAATGACTTATG
CTTGACGACTGTTTGATGATTGTCCCTGTCTCGAGTGACTGTTTGAACACGATGATGATGATTATGACTT
CATTGAGCACCTGCCATTATGATTATTATTAttttttttGGCACCTTATTCCCGAATTCAAATTGAAATT
GTGGAAAAGGCAAGACAAAGGAAAAGAGCTCTCTGTTTAACACCTTCTTGGCAGCCAAACTTCAGCTGCT
CTTTTTGCCCGGTCATCGTAAATGAGTGTCAGGCGGttttattttagcctttttcgtttcttttcgtttg
ttttttttttGTTCTGCTCCTGATCCCAGACCAAAAACGCACTCAACTGTAAATGGCTGGAAACTCCGGA
CTCGGACTCTTATTAATAGTTTGTTAAGCGCAGGTCGACACCCCCTACTTTTCGGAGCGGTCtttttttcc
ttttttGTGCCATCTAATGGGTTCTTTTCTGGTCAGAGGGGTCCCGCCTAATTTTGTTCAGGTGCAATTT
GTACAGAAATTGAGAAATTGGTCAAGGGAAAACTGCTCGCCGAGTGGAAATGGAACAAAAAATGGTCAAA
TCATGTTGAGTTGTTGAACTAATCCGTCAGCATGAAGAGGGGGATTATTTATGTGTGTATAAAGGCTAAG
AAGCAGATATAGAAACAAATACAGATGCAGATGGGAAAAGCAGCTGCTCATTTGCATTTAGCTGGACAGG
AAATGTCCTGGGTTTTTGAGGCAATCCAGGGAAATACTTTGATGCAGTGGCATTGAAAATAGTTAGTACT
TAGAAGTAAAGACTATGGCGATTTTCCAACAAATAAAACATATTATCCAATTATGTATGCTTCAAGCGAA
AATTCTCTAATCATTTATCGGTCAGGTAATCTTTATTTATTGCCACTTTCAGGATTTGTACTTGCCCAGA
GCAATCGATATAGACATAGATACAGATCGAGAGACAGCTGCTCATTTGCATTTTGTAGGGCAGGAAGTGT
CCTGAAATTTGACCAACTGATGTATTATGTATTTATTGTGCCATTTTCCATTGCAGAACATCCGCTATTC
CCGCTTTTGGCGCTGATCTTTGAGAAGTGCGAATTGGCCACATGTACGCCGAGGGAGCCCGGTGTGCAAG
GTGGCGATGTCTGTTCGTCGGAATCGTTCAACGAGGATATTGCAATGTTCAGTAAACAGG
>dme_circ_0008503_junction_seq
GAGGATATTGCAATGTTCAGTAAACAGGTATGACGATGGCCTACACGGCTACGGCA
>dme_circ_0008504
CAATCACCCGTGTTCATCGACGACACGGAAAGATTCACCTGTCGGACGCGCACGTTTCGCTTGCCCTTGA
CCTGGAATAGTCGTGTTTCTCCTTGGGCGTTTGTCTCTACATGCTTGAAGCCAGTTCCGACGCCGCCCTG
TTCATAGCGAATGCCTACAACAACATTCAGTTTAATGAAACGTCCAAATCGGTTTGACTACTTTTACCGT
TTTTGAAATAGCTCAGAAACAGCTGGGACTCGTGGTCCTGCACCTCGCGATGTTGAACAGGGCCACCATT
CAGCAAGTCATCCAACTGGACGGTGAGAATAGCTGCAGCTCCGGCCTCATCGGTCGAGGTCTCCAATCCC
AGCCAGAAG
>dme_circ_0008504_junction_seq
GGTCGAGGTCTCCAATCCCAGCCAGAAGCAATCACCCGTGTTCATCGACGACACGG
>dme_circ_0008505
TGTCGAGCATAGCCTGAGTGAGTGGTTTTTGTCCAATGATGTCCACCTTCAGCTTGCCGCTGGCGTCGCT
GACCTTGTAGAGAGAAACCGCTGCGGCATCTGTCCTCTCGAAGGCACTATCTTCGTCAGCAGTTGACTCA
TCGGGCACCTGGTCAGCAGAGCCCGATCCCAGCACGTCGAAGAAGTGCTGCTTATCGGCATCAGTGCTGA
AGTCGTCGACGATCTGAACACGGGCTCGTCCG
>dme_circ_0008505_junction_seq
GTCGACGATCTGAACACGGGCTCGTCCGTGTCGAGCATAGCCTGAGTGAGTGGTTT
>dme_circ_0008506
TCCAAGTCCTCTTGCTCGTATTGAGCAACTTCTTCAACCTTCAAAAATCCACCAGAGCTCAGATGACAGT
GGAAAAGGCGCGACTCCAGCAGTGGAGCTCCATCGTCACCCAAGCTGCGGTCGTACTGGCCTTCGCCGTT
TAATTCCTCCCAGAACTCATCTGGCTCGGCACCCTCCTCCACCTGCTCGAGCTCAACGTCGTCCCAGTAG
TCAGAAAAGCGATCAACCGCTGCTTGTTTTTCGAAGGCAGATGCGCCCAAGCCATTCCAAATGTAGATTT

TGTGGGACTTTCCCGAATGAAGGACGAAGGCATCACTTGAGGCCAAAGATGAGCTGTCGGCGGCCACCTC
ACTAGCATGAACATCACTTTCGACGGTGCCGCGAATGCGGAAAAGCTGCGCCGTCACCGGAAGTGCAGTA
AAGGATGTTAATAACTTCCCCTTAAATATCTTGTAGAAGTGGCGCGGCTCGTGGCCCTGATTGGTCTGGA
CAAGAAGACCATCCTTGGAACCTACTAGACCCTCCTCAAAAGCACGTTTCCTAGCGGCAGCAGAGGCCTT
TACTCCATGCCACACATAGGTTAGAGAACCAGTGTCCCCGTTGTTAGCCTCGTAGTTGTAAGTGAGCACA
TAAGAGGCAAATCCCAAAAGAGGAAGTTTCTCATCGAAGGGAACGGTGCTGACCACGATCTCATCGGAAC
CAGGCTTTGCAACGTACTGTGTGATGGTTTCAATAACGTTCTGACCGTGATCCGGCATGAACCCAAAGGC
ACGACCACCGCTCTTCTTTAGCTGAGTCACCACGGAGTCGATTTCGTCATCGTTTAATAATTCATCGGAG
CCGATACCCAGAGCCGAGCGAATAAGGCGACTATGGGACATTCCAGCATCACGCCAGGTGTCAAAGTACT
GCTTAAATGGGGCGGACTCGGAGCCCTCTACGATGCGGTGAATTTGGGTCCAGGCCGGGTACTTCTTGGT
GCGTAGGAACTCCTGCGCCTTGGCCATGGCGTCCGTCTTCTCCTTCTGAGTGGCGCCTTTTCCGACCCAC
ACAAAAATGCCAGATCCAG
>dme_circ_0008506_junction_seq
CCGACCCACACAAAAATGCCAGATCCAGTCCAAGTCCTCTTGCTCGTATTGAGCAA
>dme_circ_0008507
GATTCCCGATACCAGGGTGCCGAGTGGAGCCCTCTACACCTTTGATGGCAAGTCTGTGGTGGACAGTAGC
CTAGTGCTGCCGCCCAAATTGGATGCCCCGGACAATGCCAACGTCCACCAGCGACATGCCCAGTACGGAT
TGACCCCCTTGGAGCAGCTGGTCC
>dme_circ_0008507_junction_seq
GGATTGACCCCCTTGGAGCAGCTGGTCCGATTCCCGATACCAGGGTGCCGAGTGGA
>dme_circ_0008508
GTCCGCCCCATTTAAGCAGTACTTTGACACCTGGCGTGATGCTGGAATGTCCCATAGTCGCCTTATTCGC
TCGGCTCTGGGTATCGGCTCCGATGAATTATTAAACGATGACGAAATCGACTCCGTG
>dme_circ_0008508_junction_seq
ATTAAACGATGACGAAATCGACTCCGTGGTCCGCCCCATTTAAGCAGTACTTTGAC
>dme_circ_0008509
GGAACGGTGCTGACCACGATCTCATCGGAACCAGGCTTTGCAACGTACTGTGTGATGGTTTCAATAACGT
TCTGACCGTGATCCGGCATGAACCCAAAGGCACGACCACCGCTCTTCTTTAGCTGAGTCACCACGGAGTC
GATTTCGTCATCGTTTAATAATTCATCGGAGCCGATACCCAGAGCCGAGCGAATAAGGCGACTATGGGAC
ATTCCAGCATCACGCCAGGTG
>dme_circ_0008509_junction_seq
ATGGGACATTCCAGCATCACGCCAGGTGGGAACGGTGCTGACCACGATCTCATCGG
>dme_circ_0008510
TCCAAGTCCTCTTGCTCGTATTGAGCAACTTCTTCAACCTTCAAAAATCCACCAGAGCTCAGATGACAGT
GGAAAAGGCGCGACTCCAGCAGTGGAGCTCCATCGTCACCCAAGCTGCGGTCGTACTGGCCTTCGCCGTT
TAATTCCTCCCAGAACTCATCTGGCTCGGCACCCTCCTCCACCTGCTCGAGCTCAACGTCGTCCCAGTAG
TCAGAAAAGCGATCAACCGCTGCTTGTTTTTCGAAGGCAGATGCGCCCAAGCCATTCCAAATGTAGATTT
TGTGGGACTTTCCCGAATGAAGGACGAAGGCATCACTTGAGGCCAAAGATGAGCTGTCGGCGGCCACCTC
ACTAGCATGAACATCACTTTCGACGGTGCCGCGAATGCGGAAAAGCTGCGCCGTCACCGGAAGTGCAGTA
AAGGATGTTAATAACTTCCCCTTAAATATCTTGTAGAAGTGGCGCGGCTCGTGGCCCTGATTGGTCTGGA
CAAGAAGACCATCCTTGGAACCTACTAGACCCTCCTCAAAAGCACGTTTCCTAGCGGCAGCAGAGGCCTT
TACTCCATGCCACACATAGGTTAGAGAACCAGTGTCCCCGTTGTTAGCCTCGTAGTTGTAAGTGAGCACA
TAAGAGGCAAATCCCAAAAGAGGAAGTTTCTCATCGAAGGGAACGGTGCTGACCACGATCTCATCGGAAC
CAGGCTTTGCAACGTACTGTGTGATGGTTTCAATAACGTTCTGACCGTGATCCGGCATGAACCCAAAGGC

ACGACCACCGCTCTTCTTTAGCTGAGTCACCACGGAGTCGATTTCGTCATCGTTTAATAATTCATCGGAG
CCGATACCCAGAGCCGAGCGAATAAGGCGACTATGGGACATTCCAGCATCACGCCAGGTG
>dme_circ_0008510_junction_seq
ATGGGACATTCCAGCATCACGCCAGGTGTCCAAGTCCTCTTGCTCGTATTGAGCAA
>dme_circ_0008511
GTACTCCAGCCATTATGCAATAGACGAGCCAGATATACTAGAATCACTGACCAAGAAGAGCTGGAAACAA
CTACATCGCCATGTCATTGTGCCAGACGCCGACGAGGTGGCGAGGAACACACGCAGTCGATCCGCCAAGC
TGAGAGCAGCCGTCAAAACAAACTAAACGCATGTCAATAAATTCCCTCGGTCTA
>dme_circ_0008511_junction_seq
ACGCATGTCAATAAATTCCCTCGGTCTAGTACTCCAGCCATTATGCAATAGACGAG
>dme_circ_0008512
TGGCGCGGCTCGTGGCCCTGATTGGTCTGGACAAGAAGACCATCCTTGGAACCTACTAGACCCTCCTCAA
AAGCACGTTTCCTAGCGGCAGCAGAGGCCTTTACTCCATGCCACACATAGGTTAGAGAACCAGTGTCCCC
GTTGTTAGCCTCGTAGTTGTAAGTGAGCACATAAGAGGCAAATCCCAAAAGAGGAAG
>dme_circ_0008512_junction_seq
CATAAGAGGCAAATCCCAAAAGAGGAAGTGGCGCGGCTCGTGGCCCTGATTGGTCT
>dme_circ_0008513
GGTTCCAAGGATGGTCTTCTTGTCCAGACCAATCAGGGCCACGAGCCGCGCCACTTCTACAAGATATTTA
AGGGGAAGTTATTAACATCCTTTACTGCACTTCCGGTGACGGCGCAGCTTTTCCGCATTCGCGGCACCGT
CGAAAGTGAT
>dme_circ_0008513_junction_seq
CCGCATTCGCGGCACCGTCGAAAGTGATGGTTCCAAGGATGGTCTTCTTGTCCAGA
>dme_circ_0008514
GGTTCCAAGGATGGTCTTCTTGTCCAGACCAATCAGGGCCACGAGCCGCGCCACTTCTACAAGATATTTA
AGGGGAAGTTATTAACATCCTTTACTGCACTTCCGGTGACGGCGCAGCTTTTCCGCATTCGCGGCACCGT
CGAAAGTGATGTTCATGCTAGTGAGGTGGCCGCCGACAGCTCATCTTTGGCCTCAAGTGATGCCTTCGTC
CTTCATTCGGGAAA
>dme_circ_0008514_junction_seq
GTGATGCCTTCGTCCTTCATTCGGGAAAGGTTCCAAGGATGGTCTTCTTGTCCAGA
>dme_circ_0008515
GCTCATCTTTGGCCTCAAGTGATGCCTTCGTCCTTCATTCGGGAAAGTCCCACAAAATCTACATTTGGAA
TGGCTTGGGCGCATCTGCCTTCGAAAAACAAGCAGCGGTTGATCGCTTTTCTGACTACTGGGACGAC
>dme_circ_0008515_junction_seq
TGATCGCTTTTCTGACTACTGGGACGACGCTCATCTTTGGCCTCAAGTGATGCCTT
>dme_circ_0008516
AGCTCAGATGACAGTGGAAAAGGCGCGACTCCAGCAGTGGAGCTCCATCGTCACCCAAGCTGCGGTCGTA
CTGGCCTTCGCCGTTTAATTCCTCCCAGAACTCATCTGGCTCGGCACCCTCCTCCACCTGCTCGAGCTCA
ACGTCGTCCCAGTAGTCAGAAAAGCGATCAACCGCTGCTTGTTTTTCGAAGGCAGATGCGCCCAAGCCAT
TCCAAATG
>dme_circ_0008516_junction_seq
GGCAGATGCGCCCAAGCCATTCCAAATGAGCTCAGATGACAGTGGAAAAGGCGCGA
>dme_circ_0008517
GGATGTGCTCGGTGGGTATTTTCTCCTCGGGATCTAGAGCCAAAACGGCGGGCGTGAGGGAAAGCGAGAC
CTGGGCCTTTTGGCCAGGTGTCTTGTTTATCCGGAGCTTGCCCACGTCCATGTTCGTCGGCGCCTTCTCC

CATTTCTGGGCAATGTACTTGGGCACCTTCACCAGCCATACGCCTCGCCCAGCATTGGACAGGTCCAG
>dme_circ_0008517_junction_seq
CGCCTCGCCCAGCATTGGACAGGTCCAGGGATGTGCTCGGTGGGTATTTTCTCCTC
>dme_circ_0008518
GGCGAATTGTCTCAAATTCAGGAGTCGAACACATACCGCATGCGCTAGGGGCAGCCATAACCAATCCGAT
CTGCCATTTGGGATGGGGCTCAGAGCCCTGCAGCGAGGATACATACAATGACTAGACGTAAGGTTTATTC
CAGTGCGAAATGTGCAACGAATTCGTGTTGCGTCTTCTCCTAAGTTTAAAG
>dme_circ_0008518_junction_seq
CGTGTTGCGTCTTCTCCTAAGTTTAAAGGGCGAATTGTCTCAAATTCAGGAGTCGA
>dme_circ_0008519
TTATAGCTTTCTTGATATCCCGAGAGCCGAAGATGCTGGGCGCCAGTGACTGTGAGAGACGTTCGTAGAT
GTCCCCAGAGGCGGCCATTCGCCGGAAGTGCTCCTCCTCATCGGATGTGATGTTGCTGTATCGGGAGATG
GCACCCGCACCCTCTGAGTCGACGGTGATGCCCACCACCCTCATGTAAGGAGCACGGACTCCAACGACGG
CTTTCTCGCGACCATCCCGACGCGAGGGTTTGCCCACCTTGCGAATGGAGTATATGCCCTGGATAAGCAC
GCGATTGCCGGGCACCACGCGTTCACAGAGCGAGCGATCGCAGAAGAGCTGCAAGTGGCGGGGAATCTCA
CCCTGTGGCACAAAGTCGGGGAGCTCTTGCAACTTAAGGGTCTGGAAATCGACGCACTTGCACTTGTCCG
GCATGATGAAGAAGGGGTCCAGCGGGCACTTGGGTCTGCCCGCCTGCTCCGTGTTGCACTTCCTGGGTAG
GGCGTAGCCCTCCAAACCTGGATTCACTTTGAGATTCGGTATGACCGTGCTGCAGGACAGGCATTGGATA
GACATCCGCGTAGCCTTGGCACTTATTCCTGAGGCTGCGACAATGATTCCGGCGATCTTAACCAGCTTGG
ATACGCAGTCCGACTTCAACTGTCGTATGTTGGTGGGATTCGCATTGGAGCTCAGAAGAATCTGGATGTC
GTGCATATGTTCCTCGTGCTCCGGCCGGGGAGCTGTGATCTCGTCGGCTACCTCCCGGGCAGCCTCCTCG
AAGATTTCCAAATGCTCCGTCGGCTGCTTGTTCAGCTTGTCAGCCAAGGTCTCATCGAAGCCCACCAAAT
CCTCCATCTCAATCTCCAAGAAG
>dme_circ_0008519_junction_seq
CAAATCCTCCATCTCAATCTCCAAGAAGTTATAGCTTTCTTGATATCCCGAGAGCC
>dme_circ_0008520
GGATGCTAAACGCGGCACGGAATTGGATTACGTAATTGCCCGCAAGAATCCGCTGTTCCAGAAACCAGTC
TACCTCGAGTTATGGGTAAGTTGGAAGATGACTATGGGGATGGCGTTGGCCATGGAGACCCCTAACCCAA
CGGAGCTTATGATTTATGGATCGTACTTTTCGCCAACAGGGTTCTCCGCTATTTGCCATAAGACAAAAAA
TCGTATCTTCCGAGACCACAGAAGGCACCGTTTTCCTCCTGGGACCATTAGATTTTGAGAAGCAAGCCAT
GTACCATCTAACGATACTAGCCAATGTGAGTATTTATAAGATTTAAGATTAATCATAGCTATAAATGAAA
ATATCATTCCATTATGCCCGAAGGATGCTTATGCTGAGCCTGGTCAGGATAGTCGCAATATAGCGGGCAT
GGAGATAGTGGTTATCGTGCAGGATGTTCAGGATCAGCCGCCAGTGTTCACATCTGCTCCCCCAGTGACC
AAACTGCCACCGGGAATACTGCCAGGCGATAAGGTATATCTTTTCCATATCTCTTAGGAACATAAATTCC
AATATCTCTGCCCGCTTAGATATTGCAAGTCCACGCCGAGGATGGCGACAAGGGGAATCCCCGGGAGGTT
CGCTACGGCTTGGTATCCGAGAACAATCCGTTCACCTCGTTTTTCGACATCAACGAAACAAGCG
>dme_circ_0008520_junction_seq
TCGTTTTTCGACATCAACGAAACAAGCGGGATGCTAAACGCGGCACGGAATTGGAT
>dme_circ_0008521
ATCATAAGCCCGGGCTGTCAGGATAAATATATCCGTATCCTCATCATCCGATCCCGCCTGCCGACGTCTT
CTCGTCTTGGCATTCTTCTTGGAACGCAGGTGGATCTTCTCCCTAACGGTCAGCTCACCGG
>dme_circ_0008521_junction_seq
ATCTTCTCCCTAACGGTCAGCTCACCGGATCATAAGCCCGGGCTGTCAGGATAAAT
>dme_circ_0008522

TAGACTCCTTCGGGACTATTTGGCGGATTGAAGCGCACGTACTCGTATCCGTCCTCCGACATGGACTCGT
CGTTTTCGCTGCGGGCTCTTCGGCGAGATGGTTTCGCCAGTGAGATTTCGGGATCCAAAACGCCATCGAA
TCCCTCTGTCTGAGTGCCAGCATCGCAATCCGTCTGAGTTCCTATGGCCATCGACTGGCCGGGAAGACTC
TGCG
>dme_circ_0008522_junction_seq
GCCATCGACTGGCCGGGAAGACTCTGCGTAGACTCCTTCGGGACTATTTGGCGGAT
>dme_circ_0008523
GGTACCGAAGGCAAGAAAGGACCTATGCGGAGCCGGAAAACCCAGTAGATCGCAAAAGGCCTGCAAGAAA
GTCGTCACCAACGGATTCCCAGCCAGAAGCCATGCCGCGACTATCAAGGCGCGATAGCTCCAAAAGGGGT
TCCAGGAAGCAGACATCCTCAGAACCGCCACATAACCGAGTGTCCATCTCGAA
>dme_circ_0008523_junction_seq
CGCCACATAACCGAGTGTCCATCTCGAAGGTACCGAAGGCAAGAAAGGACCTATGC
>dme_circ_0008524
GATCCTGGAGTGCGACGTGGAGAGCGTGATAAGCTGCATTCGTGAGTGTGAGCTGCTCGAGGGGAAGACT
GCCACGCTGAGGTTGCCGCAAAAACTGAAAACAGCGGCGCCCGTGGCGGTTGCGCAGGAGGAGGAAAAGA
AGCGCGATGTTGGCAGACCCAAG
>dme_circ_0008524_junction_seq
AAAGAAGCGCGATGTTGGCAGACCCAAGGATCCTGGAGTGCGACGTGGAGAGCGTG
>dme_circ_0008525
GTTGGCCCGACACGGCATAGAGGTCAGCCGCAGGACCTTGCAGCGCAGGATCAGCGAGATTCGCTCCAAA
GGCATACGGCAACCGAGAGGATCGCCACAGCCGAAGGAGCCCAGCACTTTCGGCCTCAGTGAGGGGGCGA
AAAAAAGGAG
>dme_circ_0008525_junction_seq
GCCTCAGTGAGGGGGCGAAAAAAAGGAGGTTGGCCCGACACGGCATAGAGGTCAGC
>dme_circ_0008526
ATTCACGCCAAGACAGCGACAATCCGGACAAAAGGTATTCCGGACCAGCAGCGACTATGGATAACAAGAG
CGAGAAGGTTACGGTGAACAAGGAGGAGCTGCGCAAGCGCCTGACCCCGGTGCAGTATCAGGTTACCCAG
GAGGCGGGCACCGAGCGACCCTTCACAGGGTTGCTACAACAAACACTACGAGAAGGGCGTGTACCAGTGC
ATCGTGTGCCACCAGGATCTGTTCAGCTCGGAGACCAAGTACGACTCGGGATGCGGATGGCCGGCCTTCA
ACGATGTCCTAGACAAGGGCAAGGTCACGCTGCACCGCGATGCCAGCATACCAGG
>dme_circ_0008526_junction_seq
CGCTGCACCGCGATGCCAGCATACCAGGATTCACGCCAAGACAGCGACAATCCGGA
>dme_circ_0008527
ATTTCCACGCGACCCTTCCAGCTGGTCGTGGGTCGCGTGTGGAAGGGTTCCGCCTTCGGAGGCTGGCGCT
CCGTTTCCGATGTTCCCAAATTGGTGGAGGACTACCTGAAGAAGGATCTGCTGGTGGACGAGTTCATCAC
CCACGAGCTGCCGCTGTCGCAGATCAACGAGGCATTCGATCTGATGCACAAGGGCGAGAG
>dme_circ_0008527_junction_seq
CATTCGATCTGATGCACAAGGGCGAGAGATTTCCACGCGACCCTTCCAGCTGGTCG
>dme_circ_0008528
GGACTCGTCCGAGGACTGGCCACCGGTCATGTTCGACGGCTCAGTGCTTATTCTACGCTTACCGTCTCCA
CTTCGATGCAGTGCGTCCAGGGACTGGCGTGCTTCTTCGTGCAGCGGCAACAGATTTAGGCAATCGCTAT
AGGCCTTGACGGCTTCGGCTATGCGATTCTCCTCTTCGTAGCTGCGACCCAGAGCCACCAGCGTTTCGCC
CAT
>dme_circ_0008528_junction_seq

GACCCAGAGCCACCAGCGTTTCGCCCATGGACTCGTCCGAGGACTGGCCACCGGTC
>dme_circ_0008529
GAACGTCCTTGCCCTGCCGTGCTGCTGGCTTACTTTCTTCTGATTTTTTTCGAGCTGTAATAATGGCGAG
CCCGTCTGGACAACATAATCACGGGTTTAATGCATATAATTTTCTGCATGAACTGGCCCGAAACCACCCA
CCTAAATATCCATGACCAAAGCTGTTCATAGTCATTAGTTGCTCGCTCCGCTCCGCTCTCTCTCTCTATG
TTCTGCTGTGCTGTTTGCCATGAATAATGACTTGCCACTTGATATTGTTAGCAAATAGCGAAAATTATTA
ATGTGCTCGATGGGGGTGCAAGTTGCGGTGGGCGATGGGCATGGCATTTAGGCCTTCGCTAAAGGTCAAC
CAATGCTTTGGCTTTATGGTCGAGTTCGGTTGAATCCGTTTGTAGAGTCAAATGCATTCAGACGGTGGAA
ATCCAATACTCCATAGGTGAATGATTGAGCGGGCTTTGTGAAACTTTGTATATTTTCCAGGGAATACGGA
GTAGCAGCATTGTCCTTATCAAACAGAGGTGGCCAGCTCGGCAGGACATAAATCAACTGCAGTGATACGC
GGTGGCCATAAACTTTTTGACTGATTTTTTGTCACTTGCAGGGCGAAGGCAAAAGAGTGTATATTACGAC
TCAATGGGGCCAACTGATTGTTTATAAATAAATACCATAAACAAAAGGCCATAAAAAAGCCAGACGGCCG
AAAATTGAAATGAATTTTAAAAAGCGGTGAAAGAGAAAATCGAAATCACAGTCATCGGCGGGGTATCTAT
ATTAAAAATGCATTATGAAAAACACGTCTGTTTGTTTCTCCGACGGATGGAATAAAATGCCGTAAAAAAT
GGAGAATTATATGGCTCACATGAATGTACCAATGCATACACTGCAAAAAATACATTGTATAAGGTAAGTT
CAATACATTTATGTTTAAACAATCCCTGAGTTCGCTGCTAAATGAAGGACTATATGAATATTTAAGTATA
AATGCAAAAAGTTCTCAATTTCAATTTCTTTTATATCTAGAGTTTTCTTGAGTGTAAAGCACATATGTCG
ATATACATATAAATATATATACGCTCAAATGCAGCATTCGATTGCAGCTGAAAATAAACATGCATCAAGC
CAACTGCACGCACCGCCGTAAACATGTCGCCTGAGCAACGATTACGGGCTCCGAGTTCCGAGTTCCCCGT
CCCGGGATTTGGGCCACTGGGTAGGGGGTAGGGGGCTACGGGTTACGGCACCCGGCATTCTCCGGTAGCC
AGGACACGGCAACCGAAACCAGGACGACATGCCACTCATTTCAAGTGGCCAAAACCCATCGCCAGCCACC
CACAACTCACCCACTCACGCGTCACTGCATTCAACAAGCAATTGAATAACAACATTTCCATTTAACGCGC
TTCTCGTATGTCGATTAAATTAAAGCTACATACTGTGCGGATTTCCATGGTTTGGATAGCTGAATGGAAA
GCGGAAAAGCGAAAGCCCAGCAGGTGTTATCGTCAATTGGTCTAGTTTTGCCTAAATGCATTAATCTACC
AGCAGCTGGCTGTCTTTTGTGTGAGCGGAATACTCCAACCAAAGCTGTGACATTTTACAGGAATGCATTT
TCCACTTTTGGG
>dme_circ_0008529_junction_seq
TTACAGGAATGCATTTTCCACTTTTGGGGAACGTCCTTGCCCTGCCGTGCTGCTGG
>dme_circ_0008530
GTTATCACTCGAAGAGGAAAGGACACGGCGAAGCTCCAGGATGCGAGGCAAAAGGATTTGAAATTACATT
TTTAATGCCGAGCGGGCAAGAAAAAGAGCGCGAAATGAGCAGCACGTGCGCGCTTTATTTTAAATATTTA
TAAACAAACAACACACGCGCACACCGACCAACACGCTTATCcacacacacacacacacacacacaAATTT
AGGTGACCAAAGTTAAGCCTCGTCAAATTCTATTAAAAATTCATCTTAACGGTTATGCGTTTTGTACAAA
CAGTCGAGCGGCTAAGGAGCCGGGCAAAAAATGTTTGTAAATATTAAAATGAATTTATGTGCAGAGCAAA
AGTTTTTAATTAAGTGAAAAGCGAAAAGCCGAGTGGAAACGATAAAAACGACCGGCCAAACAGGAAGGAA
TCATCGAGAGTGTGAATTAGAGCAGCGAGCTTACATATTTGAAGCGATGTTCGAGCAGCCAGAGCCCAAT
TTATTTGCATACCTAATGCTCGTGCAGTGAGCCTCGGCAAATTGCGAAGCCCAGCGCAGAATACTAACCA
AACAGTTAACCAATAATTGGCCAATCGGATCGGGAGCAGCCAGCTATAACCATGTCCTTCACCGGTCCAA
TCCTAATATGCCTGATCATGGGAGCCATTACCCATGCAAATTCGCACGAGCATGCCGATGGCTTTAAGCC
AG
>dme_circ_0008530_junction_seq
CACGAGCATGCCGATGGCTTTAAGCCAGGTTATCACTCGAAGAGGAAAGGACACGG
>dme_circ_0008531
GATGGTGAAATCCTGCGCGTTCTGGTGAACAGCTCGGCACAAATCAAATGCGATGTGGGCTCCAGCCAGG

CCGATGACAAAGTGCTTCTGGTTGTTTGGTACAAGAATAATTTACCCATTTACAGGTGGGTCAATCTGAG
CACTACTATAGCCCGTCATTCCTTATCCTGTATCTACATCGCTTTAACAATCGCAATTGCTTTGACAGCT
ACGACACACGTGGCGCCCACGCTGGCACCCCATCCCATTGGCGTGACGAGGAGGTCCTTGAAGATCGGGC
CGTATTTCGAACGCACAAGGAGCCGGCGGAATTGATTATAAATCCGGTTAAGGTAACCCATTCACTTTCC
TCTCTCCGTTTCCCCCCACAGCACCGCGCATCGCGAGTGTCAACAGCGCAGCGCTCTATAATTGGAATTT
CATTTCAGGAAAAGGATGCGGGCAACTTTCGGTGTCGCGTGGACTTCAAGCTTTCGCAGACCCGCAACAG
CAATGTCAATTTGGAGGTTGTCGGTGCGTATCCCCAGAAGCGAAATATGTCAGGAAACCAACCTATTTCG
CGTGCCTCGAACTCCCACTTATAATTGATAATGGCCAAATACTTTTAATTAATAATAAAGCATTGTAAAG
GCAGCATCAAATTGGAGTAGAACATCAGAAAATTTTATCTATTAACCATTTTAAACAAGAATCTTAGGAA
ATAttttttttGAAATCTACCTATTTGCACAATAAGATTGAACAACTAAATGTTCAATATTTTGTAGGAA
AATAGCTTTTAATTTATACACAAAGACAATGTACCCAAATAAAAATCAGATATTTTTTGGTTTGTCCAAA
ACAAATCTCAATTAAAAATGTTCCACTTCAAAGAGTTTAAATAAAAAGAGCCATTAATAAATATATACAC
TGCATACTGTCTACGTGCATACAAAATCAACATTCACATAAATAAATAAATATGGCCAGCCAGAAACGAT
TATAAAAGCAATTAATATTTGTTTCGTACCGAacacacacacacacacacacacatacacatatacacac
acGGAGCCAAACGAGTTGCACATAAATTAGCTGAAATGATTTTCGGTTAGCCGCAAAACTTACAATTTAT
TATTCGAGCTATATGAGTAAGAGCGTTGTATAAATTTTACTTTGCATTGCTTTTGACAGTGCCAAATTGT
TTCTGATTTGCGGCTGTTTAAAATCATTGAATGCGATCCGGGATTTGGCGGGTTCTTTAAAAAAGTTAAA
CAGATTTACTTAATGCATAATTAAATAACAATAAATATTTTTCAGTGCCGCCAACGCAGCCGATTATCTT
CAATGAACGCCGCCTGCGAATCGATTCGAGAGCAGGTCCTTATGAAGAAGGTGGCAGCCTGGAAGTCACC
TGCGTTGTTTACGGAG
>dme_circ_0008531_junction_seq
CTGGAAGTCACCTGCGTTGTTTACGGAGGATGGTGAAATCCTGCGCGTTCTGGTGA
>dme_circ_0008532
GTGCGCCCTCTGTTGGTGGAAATCAGCTTCAATAATCAGCCGATGTCAGCGGATCGAAAGTATGAGATCG
AGTGCCAGGCGATCGGATCGAGGCCACCGGCCAAAATCACCTGGTGGATGGGCAATCTCGAGCTGCATGG
CCATAGCCAGAAGGGTTTCGGAGGATGGCAATGTCAGCACATCGGTGCTCTCGATCACACCCACAAGAGA
GGACCATGGCAAGGCTTTATCCTGCAGGGCAACCAATGAACTCGTTCGAAATGGCATTCGAGAAACGGCC
ATGAAGTTGAATGTGTTCT
>dme_circ_0008532_junction_seq
GAAACGGCCATGAAGTTGAATGTGTTCTGTGCGCCCTCTGTTGGTGGAAATCAGCT
>dme_circ_0008533
GGTTTCGGAGGATGGCAATGTCAGCACATCGGTGCTCTCGATCACACCCACAAGAGAGGACCATGGCAAG
GCTTTATCCTGCAGGGCAACCAATGAACTCGTTCGAAATGGCATTCGAGAAACGGCCATGAAGTTGAATG
TGTTCT
>dme_circ_0008533_junction_seq
GAAACGGCCATGAAGTTGAATGTGTTCTGGTTTCGGAGGATGGCAATGTCAGCACA
>dme_circ_0008534
GGATTCGACGGCGGAATGCGGCAGTGGTTCCTCATGGAAATCTTCGATCAGCACAGTGGTCAGCTTCAGG
CCAACATTAGTGCCAAGTTTGCGGCCCTGAGTGTCACGGGTCTGGATGCAGGTCGTCTCTTCCGGATCTA
TGTGTATGCCGTCAATGGACGAGGACGCAGCGATGCCATTGCCCTCGATGGATACACTTTGAAGGCGGCC
GAAAAGCAAACCGGGTTAGTAAAAAACCCTTACATTTTAAAGGTTATATTTTTAAGGCATTAACACTGCG
GAAAAAATGTTCTACTATCCTACGCTTAGATTCATGCAAAGGTCCTCTAAATTAAACATCTAAAAATGTT
ATATAATTTAACGCCATTATAAAAAGTTAGTTCTTTTTCTGAGTGACTTTTGTTTTTTATGATATTTTTA
TGCTAATATGGAGAATTACTCTCATTTCTTCGCCAAACGAAGCGAACTGCATAACTTTATGCGGCGAAAA

GCCGCTGTATCAGTTTCGCAGCAAAGATTGTCCTGGGTTTTATGATTATTATGCCACCTCGAAATTTAAT
TACGCTAAACTGCTCAATGAATTGTAAATTCAGCGACACAAAACTTTCGCCTGCAGTTTTCGGCCAGTAA
ACGCAAATTTTTATGCAGCCCTGCCTCCCACAAAGAAGCGCaaaaaaaaaTCTGAAATGATTAGCATTAA
TGAATTGGGGTTGGAAGAATGCGGATGGCGAGTTTATTTTATTGAGGGTAAGGGGTCTAAACGAAAAAGG
AAATTGTAATGCTAATTAGCCAAAGGAAGAGCTGACAAGAATTTTATTTTCATTTTGGTACATTTCCCTT
TTGGGGAAAGCAAAGGAATGGAAGTCGAATAGCAAGCAGGGCATCTAGAAGGGACAGTCACATCTTGTGT
AAACAACTTGAGCCGACATTTCAATTTTTCATAAAGTCAGTCGGAGCAGCGGCACGGTGGGCGGTGGGGA
AAAGTGCAAAGCAAACACAGCaaacggcagacgggaaaaccagaaaaacggcagagatggaaatcagaaa
aGAAAATTATCTTGGCTGGGTCAAAGCGTAGATGCTCTGAGTAAAGGTTAAGCCTGATTATGATCAGCAT
TTATAGAGGATTCATTCAGTCAACGGAATGATTTGAATCTCGTCTAGAAACATGGAATGAAATAAAATAC
TATTGTAGCATTATTAAACTCTTACCAATACTTCAATCGGATTTACCTTTGCAGGTTGCCTTGACCTCCT
ACAAGGGCAGTGCCCAGAGTCCGGACAACTTTGAGCTGACACCGATCCTGTCGATTGGCATCTTCGTTGG
CATCCTGGTGGCCATCGTGTGCATCGGAATCGGAACAATTGCCGCACTGAAGTTGCGCTCGCACAAGCAT
CAGCAGCAACAGAAATTCGTACATCCCAATGCGAAATTCTCACGTCCGGGCAATTTGCAAATTAAGGACA
AAATCTCATTGCCATTGAGTCACTCCGAGGAGATGTACGACGAGAAGAATCCCGATGTTGTGCCCTACAA
TGAGG
>dme_circ_0008534_junction_seq
AATCCCGATGTTGTGCCCTACAATGAGGGGATTCGACGGCGGAATGCGGCAGTGGT
>dme_circ_0008535
TCAGAATGCTCTTGGCCGGAACATCTTTGATGTTCTTGATGGGTTTCTTCAGCTCGAAGGGGGAGATGAT
GGTCACCTGATGTCCCGCAGCGGCTAATCCCTTGGCCAGGGCAGATCCCACATGGTAATGGGATCGTGCG
GCGGTATGGAGCACCACCAGG
>dme_circ_0008535_junction_seq
TCGTGCGGCGGTATGGAGCACCACCAGGTCAGAATGCTCTTGGCCGGAACATCTTT
>dme_circ_0008536
AAAGCGCCCGAAAGGAACTCAAACTCCGTAATGCCGGTGATGAGGGCTACCTTGTGAAAGTCGCCACGTC
GAGCTGTTTTGAAAGGACTTTCGATGAGGTAACGCTCCTGCCCAAAATCCTTCTCCACAACGATTCTCCA
GTTGAGCACGGGATTCCAGCCGAATTCAAACATGCCATTATAAGTGCTCACATAATCCAAGTAGGGCTTC
CGCCTCATACACTCCACCATTTCCCCAATGGATTCCTGTGGACACTTCAATAAACCAGCCTGACGTTTGG
CCAGTTGTAGATCATTGTCTTTGTACTTAACTGGTCCGTATGGAGCAGCACTCATGCAGATGCCTCGATG
GAATAGACCACGTGACATGGGCGATAGCATGTGCAGAGCTACACTTATACTTCCCGCACTGTAACCCAGA
AGGGTTACACTATCGGGATCACCGCCAAAACGTTGGATGTGCTGCTGGATCCAGCGCAAAGCCAAGACTT
GATCCTTGAGACCAGCATTGCCAGGAGCTTCCTTACTTCCGGTGGCCAGGAATCCCAAACTACCCAGCCG
ATAATTTAGCGATACCAACACGCAATCACGATCCATAAAATGCTCCGGTCCTGCTAGATATTTACTCTGT
CCAGAGAAGACATAGAATCCGCCGGGATGCAGGAACACGATCACAGGTCGGCGGCCCTTCAAATCCTTTG
TATACACATTGAGTCTCAGACAGTCCTCAGATACATCGGTCATATTGTCCCACGGTTGCGGGCACATCGG
TCCATCCTCGCTAGCATCGAAGATTTTCGGGGACCAGGGCTTCACTGGCTGCGGATTCACAAATCGCAAA
TCTCCCAGAGGTGGCTCTGCATACCGAATGCCTCGGAAGGCATGAAAGTTGACACCCAATCTGGAGGTCA
GATTGGCTCCTCGCACCCGTCCCAGTTCGGTGTCCACCTCGATGGAGAGGACCGATGAACTGGCCAGTAG
AACCAGCAGTTCCACTACGAATAACATCAGCATCTTGCCGGTCTACCTTAAACTGATTTGTAAAAGTTTC
CGCTGTCGTTTTTATACTAAATCTTGTGGCTGAAATCGCTTTTGGGGCTTTACCCAGATGCGATTGCAAA
CTGATGTGATGTGCGTTCAGATTCATACCTATACGAGACTATGGTGTGCAGCTGAATAGTCGCTTCAGAT
AGAAACAAAGGAATGCGTGAAACAGCTGCATAAAAGTTCAATTGTTTTACATTTTAAGTTAAAGCTTTAA
AGCTCTTAAAAATTAAAATTTAACTTTAATGTAAATTCAGTTTAATGCCGATCAATAAATATTTATGCAA

TTTTAAAGCATAACTCATCAAGTGCTATAAAATAGATAGAAACACACTAACTTCGTTAGCTTCAAAATGA
AATGATTATTTTCTAATGGCTTACAAATGCGTGACCTAAAGTGCATTGAACAAGATTCATTGCCAAGCTC
AGAAAAATTCCCCGATATGTAATAAAACAATATATATCAGTCTACCAGCCTACTTTGCTACATGACAAGC
CAATTCAAGTGCATTCTGAGCGAAATAAATTCTACTATAAATATATACTTTACATGGGGCTCAAAAATGT
TCTTAAGACGCTTTGTGGAATTTATTATCGACCAAAATCTAGCCAGCGAAACAATAGCTCCTTGGATGCA
TTTCTAACTAGGATCGGCATTAAAGCCGCCAACATTTCCGCGGAAAACGGTGATTTTCCAGCAGCATGGC
CATGGCCCAAAACAGTTTCAATAAGTTGGGGCTACACAACTTTTACCACGCCTTTTGCTTGGCAAAAGGA
TGAGAAGAGGAAGATGCACGGCAAAGAAAATAAAAGCATTTAGCCCGTGGGCGTTGTGGGTGGGTGTGGC
AGGGGCGGTCAATGGGAGTGGGCGTGGCACTCAGTTCGCTGCTGTTAAAAATTTGAACGCGCTGATGGGC
ATTTTTACAACTGAAGCGTTAACTATGTGCAACTTGCAGCAGGAGGGGAAAACGAGGGTCCATGAAAATG
GGAACCAACAAAGAAAAGTTTTCACCACTGAACCGAAACTACAAATACCCTCGGTTGAAACAATAATGAG
ATGATCAAGAAAATTTGAGGTCCTCATAATAAATTTCTTATATATATAGAAGTTCCTAAAGACTGCAAAC
ATTTTTAAACATTTGCAAAGTTTGCGAATTGAAATATGCATAAGCTTGCAAGATCTTAAGATACATTTAT
AATTTTATTGTGTTTCCATTCCGTGAGAGTATACAACTATAAATGAAATGTAAGCAGCTGAAAATAAAAT
AGAAGACCGAGAAAAATAGGAAAGCAACGTGGCATTTTTTCGATAGAAGCTCACAATATTTTCCATTTTA
GTTTACAAGCTTCGAGCCGCAGGCTGAAACCAACCACACAACCACGCTGTAAAATCAAATGAAATTTCCA
TGAGCTTTTCCAACAACACTGAATCGCAGCAAATTCAAAGACAGTTTATGGAAAAATCCGTAAATACCAA
ATATATTTTTGTTGATTTTATTTCAAACAAATACATTAAGAAAATTATGAGAAAATTTCACTGTTTTTAC
TTTGGAGTTGAATTGAAAATGGGAAGGAGTTTTCATGAAACTCTATACCTAATTTATTCATTCTTAATAC
TTAATAAGTATTTTTCTATTATTGCCAGTTAAAAGCTAATTTGTTTGTAAGCACCCTAATTTAAACTGGT
CATGGAAACCAAACTTATTTATCACTTGAGCTTAATTCCGCTTGGCTTTCTGCTTTGGCTTGGAAGTGTC
CTTCTTAATAAATAATTCCTGCAGGAGTCGCCACACTAAACGGATCAGCAGGAAAATACAATAGAGCACG
AATATAATGCCGCCATATAGTATCAGCATTGCGTCCAGATTGTGGTATTGAATGAAATTCAGATCCTGGG
AGGCGCTCCTTAGATACTTGGCACCCTTATGCCGGGACACATGCTCCACCCAATAAACGGCCCGCTCCAA
AGGAGTCTGCTGCTGATCGCGATATCTGTCGGACATGTCGCGCACCCTCTGGGTGGCTTCGGGATTGTTG
ATTATTTCTGAATCGCAGCCAAAAGCTTGGCAGAACTCAATTCCTCGTAATGAACAGTCACCCCGTAGC
CATTCTGCTCGGCACGAGCCATGTTCAGGAACTGATCGCCAAAGATTGGAATGCCCACGAAGGGTTTGCG
ATGGTAAATGGACTCCGTGGTACTGAGCAGACCGCCGTGCGTAATAAAGGCCAGCACATTGTCATGGGCC
AGGATGTCGTCCTGAGGGAACCAGTCCGAGATGAACACATTGGCGGGCTTTCCGGGCAGATCCGTGTCCT
CGAACTTCCACAGCACACGCTGCTTCAGCTGGGCAAAGGTGTCGATCAGCGCCTGGCGTTTTTCCAACGG
CAGTGTTTTGCTCTTCAGATTGGAGCCCATCGAGAAGTAGATAACTCCATGCTCGGCACCCTCAATGAAC
TCCAGAATGTCCTTGGGCAGCGGCTGGCGCTTTCGATTGATGTGCATGCCGCCCACCTCGATCATATTGG
GTGAGTAGGGTCGTGGAAAACTGAGCGATACATGTTGATTGAGCAGCACCAGAGCCGTGTTCTTGCGCAT
ATCGTAAAAGTCCTGCTTGTTATTGGGGAAGTACTTGCGGTAGAGGACCTCCTGGCGCGGCAGATAGAAG
TAGTTGAGGAAGATATATTCGTAGGTGAGGAAGGCCTGGTTGCCCACTCGCTCTACGAGGGACATGCGGT
CACTAAACTTCAGCAGTGCACTGGGAACATACGAGGGCGGTGAAGGAGATCCCACCTAAAATAAAAGGTA
AGAATATATAAATTATGGAAATCCTCTTAGTCACTATGGCAAATAATCACCCACCAGATCGGTGTTCCAG
CTAATAGCTCCAAAGGTGCCCAGTCCTATAAGTGGAGCCTTGAAATGCTCGGCGAATCCGAAGTGAGCTT
CGTTCAGGAACACCTCGGATATTACGGCATCGAAGGTCTGGTTGGATTTCATCAGTTCTATCACAGATGG
TTCCTTCAGCAGCAATTCGGTTATTTCAATACCCATTTCGTGAAAGTTAATGATCTGCTTGATTATGGGC
TCCTTTGAGCTTTGCAAAAGG
>dme_circ_0008536_junction_seq
TATGGGCTCCTTTGAGCTTTGCAAAAGGAAAGCGCCCGAAAGGAACTCAAACTCCG
>dme_circ_0008537

ATTGGAATGCCCACGAAGGGTTTGCGATGGTAAATGGACTCCGTGGTACTGAGCAGACCGCCGTGCGTAA
TAAAGGCCAGCACATTGTCATGGGCCAGGATGTCGTCCTGAGGGAACCAGTCCGAGATGAACACATTGGC
GGGCTTTCCGGGCAGATCCGTGTCCTCGAACTTCCACAGCACACGCTGCTTCAGCTGGGCAAAGGTGTCG
ATCAGCGCCTGGCGTTTTTCCAACGGCAGTGTTTGCTCTTCAGATTGGAGCCCATCGAGAAGTAGATAA
CTCCATGCTCGGCACCCTCAATGAACTCCAGAATGTCCTTGGGCAGCGGCTGGCGCTTTCGATTGATGTG
CATGCCGCCCACCTCGATCATATTGGGTGAGTAGGGTCGTGGAAAACTGAGCGATACATGTTGATTGAGC
AGCACCAGAGCCGTGTTCTTGCGCATATCGTAAAAGTCCTGCTTGTTATTGGGGAAGTACTTGCGGTAGA
GGACCTCCTGGCGCGGCAGATAGAAGTAGTTGAGGAAGATATATTCGTAGGTGAGGAAGGCCTGGTTGCC
CACTCGCTCTACGAGGGACATGCGGTCACTAAACTTCAGCAGTGCACTGGGAACATACGAGGGCGGTGAA
GGAGATCCCACCTAAAATAAAAGGTAAGAATATATAAATTATGGAAATCCTCTTAGTCACTATGGCAAAT
AATCACCCACCAGATCGGTGTTCCAGCTAATAGCTCCAAAGGTGCCCAGTCCTATAAGTGGAGCCTTGAA
ATGCTCGGCGAATCCGAAGTGAGCTTCGTTCAGGAACACCTCGGATATTACGGCATCGAAGGTCTGG
>dme_circ_0008537_junction_seq
CTCGGATATTACGGCATCGAAGGTCTGGATTGGAATGCCCACGAAGGGTTTGCGAT
>dme_circ_0008538
ACAGTCCTCAGATACATCGGTCATATTGTCCCACGGTTGCGGGCACATCGGTCCATCCTCGCTAGCATCG
AAGATTTTCGGGGACCAGGGCTTCACTGGCTGCGGATTCACAAATCGCAAATCTCCCAGAGGTGGCTCTG
CATACCGAATGCCTCGGAAGGCATGAAAG
>dme_circ_0008538_junction_seq
ATACCGAATGCCTCGGAAGGCATGAAAGACAGTCCTCAGATACATCGGTCATATTG
>dme_circ_0008539
TCTCAGACAGTCCTCAGATACATCGGTCATATTGTCCCACGGTTGCGGGCACATCGGTCCATCCTCGCTA
GCATCGAAGATTTTCGGGGACCAGGGCTTCACTGGCTGCGGATTCACAAATCGCAAATCTCCCAGAGGTG
GCTCTGCATACCGAATGCCTCGGAAGGCATGAAAG
>dme_circ_0008539_junction_seq
ATACCGAATGCCTCGGAAGGCATGAAAGTCTCAGACAGTCCTCAGATACATCGGTC
>dme_circ_0008540
GTCTTGGCTTTGCGCTGGATCCAGCAGCACATCCAACGTTTTGGCGGTGATCCCGATAGTGTAACCCTTC
TGGGTTACAGTGCGGGAAGTATAAGTGTAGCTCTGCACATGCTATCGCCCATGTCACGTG
>dme_circ_0008540_junction_seq
CTGCACATGCTATCGCCCATGTCACGTGGTCTTGGCTTTGCGCTGGATCCAGCAGC
>dme_circ_0008541
GTAACGCTCCTGCCCAAAATCCTTCTCCACAACGATTCTCCAGTTGAGCACGGGATTCCAGCCGAATTCA
AACATGCCATTATAAGTGCTCACATAATCCAAGTAGGGCTTCCGCCTCATACACTCCACCATTTCCCCAA
TGGATTCCTGTGGACACTTCAATAAACCAGCCTGACGTTTGGCCAGTTG
>dme_circ_0008541_junction_seq
ATAAACCAGCCTGACGTTTGGCCAGTTGGTAACGCTCCTGCCCAAAATCCTTCTCC
>dme_circ_0008542
GAACTCAAACTCCGTAATGCCGGTGATGAGGGCTACCTTGTGAAAGTCGCCACGTCGAGCTGTTTTGAAA
GGACTTTCGATGAGGTAACGCTCCTGCCCAAAATCCTTCTCCACAACGATTCTCCAGTTGAGCACGGGAT
TCCAGCCGAATTCAAACATGCCATTATAAGTGCTCACATAATCCAAGTAGGGCTTCCGCCTCATACACTC
CACCATTTCCCCAATGGATTCCTGTGGACACTTCAATAAACCAGCCTGACGTTTGGCCAGTTG
>dme_circ_0008542_junction_seq
ATAAACCAGCCTGACGTTTGGCCAGTTGGAACTCAAACTCCGTAATGCCGGTGATG

>dme_circ_0008543

AAATCGATGGAAGGACACTCCAATCAGGGCGTCGCTCAAGAGTTCACCCATACCCTTAAGTGATTTCGGG

TACTCCAGCTTGTCCAAACTTTCCGGCATATATTTTTCCCGGAAAACTCGACTGGCAGCTCGCGATTGCG

TTGAGTTTTGTTCCAAGAGCAACGCAATAGAAGCATAGTGTTCCCAATCCCTATTATAGCGG

>dme_circ_0008543_junction_seq

ATAGTGTTCCCAATCCCTATTATAGCGGAAATCGATGGAAGGACACTCCAATCAGG

>dme_circ_0008544

AAATCGATGGAAGGACACTCCAATCAGGGCGTCGCTCAAGAGTTCACCCATACCCTTAAGTGATTTCGGG

TACTCCAGCTTGTCCAAACTTTCCGGCATATATTTTTCCCGGAAAACTCGACTGGCAGCTCGCGATTGCG

TTGAG

>dme_circ_0008544_junction_seq

TCGACTGGCAGCTCGCGATTGCGTTGAGAAATCGATGGAAGGACACTCCAATCAGG

>dme_circ_0008545

GGTCGTTTCGCAGTGTTCCCGGTCCGGTGCAGTTGTAGTTGCATTTTCGCCAGCGATTTAGACGCCATCT

TCCGACTCCCGACAAGCCAATCACCATGAAGATCTACAAGGATATCATCACCGGCGACGAGATGTTTGCC

GACACCTACAAGATGAAGCTG

>dme_circ_0008545_junction_seq

GTTTGCCGACACCTACAAGATGAAGCTGGGTCGTTTCGCAGTGTTCCCGGTCCGGT

>dme_circ_0008546

GGTCGTTTCGCAGTGTTCCCGGTCCGGTGCAGTTGTAGTTGCATTTTCGCCAGCGATTTAGACGCCATCT

TCCGACTCCCGACAAGCCAATCACCATGAAGATCTACAAGGATATCATCACCGGCGACGAGATGTTTGCC

GACACCTACAAGATGAAGCTGGTGGATGATGTGATCTACGAGGT

>dme_circ_0008546_junction_seq

AGCTGGTGGATGATGTGATCTACGAGGTGGTCGTTTCGCAGTGTTCCCGGTCCGGT

>dme_circ_0008547

GTGATATCAGTGCCCTCGTCGGCCTCCTCGGCGGATGCGTTGGCGCCCTCCAGCTTGATATCGTCACCCT

GGCGGGTGATCAGCTTTCCGTACACCTCGTAGATCACATCATCCACCAGCTTCATCTTGTAGG

>dme_circ_0008547_junction_seq

ACATCATCCACCAGCTTCATCTTGTAGGGTGATATCAGTGCCCTCGTCGGCCTCCT

>dme_circ_0008548

TCCTTCAGGTAGAGGGTGTACGACTTCTTGTCGCCGAAGGCGAAGCACTCCGTCAGTCGGTGGTTAAGCA

CAACATCCACGCCAGACTCCGAGGTGATATCAGTGCCCTCGTCGGCCTCCTCGGCGGATGCGTTGGCGCC

CTCCAGCTTGATATCGTCACCCTGGCGGGTGATCAGCTTTCCGTACACCTCGTAGATCACATCATCCACC

AGCTTCATCTTGTAGG

>dme_circ_0008548_junction_seq

ACATCATCCACCAGCTTCATCTTGTAGGTCCTTCAGGTAGAGGGTGTACGACTTCT

>dme_circ_0008549

GGTGTACGGAAAGCTGATCACCCGCCAGGGTGACGATATCAAGCTGGAGGGCGCCAACGCATCCGCCGAG

GAGGCCGACGAGGGCACTGATATCACCTCGGAGTCTGGCGTGGATGTTGTGCTTAACCACCGACTGACGG

AGTGCTTCGCCTTCGGCGACAAGAAGTCGTACACCCTCTACCTGAAGGACTACATGAAGAAGGTGCTGGC

CAAGCTGGAGGAGAAGTCGCCCGACCAGGTCGACATCTTCAAGACCAACATGAACAAGGCCATGAAGGAC

ATCCTTGGCCGCTTCAAGGAGCTGCAGTTCTTCACCGGCGAATCTATGGACTGCGACGGCATGGTGGCCC

TGGTGGAATACCGCGAAATCAACGGCGACAGCGTGCCCGTGCTCATGTTCTTCAAGCACG

>dme_circ_0008549_junction_seq

GTGCCCGTGCTCATGTTCTTCAAGCACGGGTGTACGGAAAGCTGATCACCCGCCAG
>dme_circ_0008550
GGGCGCCAACGCATCCGCCGAGGAGGCCGACGAGGGCACTGATATCACCTCGGAGTCTGGCGTGGATGTT
GTGCTTAACCACCGACTGACGGAGTGCTTCGCCTTCGGCGACAAGAAGTCGTACACCCTCTACCTGAAGG
ACTACATGAAGAAG
>dme_circ_0008550_junction_seq
CCTCTACCTGAAGGACTACATGAAGAAGGGGCGCCAACGCATCCGCCGAGGAGGCC
>dme_circ_0008551
GGGCGCCAACGCATCCGCCGAGGAGGCCGACGAGGGCACTGATATCACCTCGGAGTCTGGCGTGGATGTT
GTGCTTAACCACCGACTGACGGAGTGCTTCGCCTTCGGCGACAAGAAGTCGTACACCCTCTACCTGAAGG
ACTACATGAAGAAGGTGCTGGCCAAGCTGGAGGAGAA
>dme_circ_0008551_junction_seq
AGAAGGTGCTGGCCAAGCTGGAGGAGAAGGGCGCCAACGCATCCGCCGAGGAGGCC
>dme_circ_0008552
GGGCACTGATATCACCTCGGAGTCTGGCGTGGATGTTGTGCTTAACCACCGACTGACGGAGTGCTTCGCC
TTCGGCGACAAGAAGTCGTACACCCTCTACCTGAAGGACTACATGAAGAAGGTGCTGGCCAAGCTGGAGG
AGAA
>dme_circ_0008552_junction_seq
AGAAGGTGCTGGCCAAGCTGGAGGAGAAGGGCACTGATATCACCTCGGAGTCTGGC
>dme_circ_0008553
AACTGCAGCTCCTTGAAGCGGCCAAGGATGTCCTTCATGGCCTTGTTCATGTTGGTCTTGAAGATGTCGA
CCTGGTCGGGCGACTTCTCCTCCAGCTTGGCCAGCACCTTCTTCATGTAGTCCTTCAGGTAGAGGGTGTA
CGACTTCTTGTCGCCGAAGGCGAAGCACTCCGTCAGTCGGTGG
>dme_circ_0008553_junction_seq
GAAGGCGAAGCACTCCGTCAGTCGGTGGAACTGCAGCTCCTTGAAGCGGCCAAGGA
>dme_circ_0008554
GTTACAATTTTGAAAGTCTTAAATTTATGTTTAAAAACGCTGACAAAAATGCCTGTCTTCGATCAGTGCG
ATTTTATATCTAATTTCTTTTACCGGAGCGCTTATAAATTCGACATTGTATGTTTATTGTTTAATCACA
GCGAGCTAAACAATTCAATTGCGCCACCTATT
>dme_circ_0008554_junction_seq
GCTAAACAATTCAATTGCGCCACCTATTGTTACAATTTTGAAAGTCTTAAATTTAT
>dme_circ_0008555
AGCTAAGTAAAAGAAAGTGTTTGATTTTCAGTTGTACTTTCCGAATTTTTGTTAACTTATTAACGTTTGT
ATTTGTTGAAATGCGTTCGGCATTCATTCCAATTCTATACATACTGAAAGCATGGCATAACTTATGCAAT
AGTCCATATCGAGGAGCATTAGTGTAATTGTAAGCTAAGCGAAAGATTTTGTGCAAAGG
>dme_circ_0008555_junction_seq
AAGCTAAGCGAAAGATTTTGTGCAAAGGAGCTAAGTAAAAGAAAGTGTTTGATTTT
>dme_circ_0008556
AAGCTGTACAAGGAAGTGCCCGCCTACAAGCTGATCACCCCATCGGTGGTCTCCGAGCGTCTGAAGATCC
GCGGCTCCCTGGCCAAGCGTGCTCTCATCGAGCTGCGCGAGAAGGGTACGTTTCCTTACGCTTCCTACCT
TTTCGTTAGCATAAATCCTAATGGCACTCTTCTTTGCTTTCAGGTCTGATCAAGCAGGTCGTGCAGCATC
ATTCCCAGG
>dme_circ_0008556_junction_seq
AAGCAGGTCGTGCAGCATCATTCCCAGGAAGCTGTACAAGGAAGTGCCCGCCTACA

>dme_circ_0008557
CCGCAAAAGACACAAAAGAAGAAGGAGGGATCCGGCGGCGGCAAGGCCAAGAAGAAGAAGTGGTCCAAGG
GAAAAGTCAGGGACAAGCTGAACAACCAGGTGCTGTTCGACAAGGCCACCTACGAGAAGCTGTACAAGGA
AG
>dme_circ_0008557_junction_seq
GCCACCTACGAGAAGCTGTACAAGGAAGCCGCAAAAGACACAAAAGAAGAAGGAGG
>dme_circ_0008558
GTCTCATCGTAAGTTCTCGGCACCCCGCCATGGCTCCATGGCCTTCTACCCCAAGAAGCGCTCAGCTCGC
CATCGCGGTAAGGTTAAGGCCTTCCCCAAGGATGACGCCAGCAAGCCAGTCCATCTGACCTGCTTCATCG
GCTACAAGGCCGGCATGACCCACATTGTGCGCGAGGCCGATCGTCCTGGCTCCAAGTAAGTACCAGCATC
CACCTCTATCAACTAACAACTACTTTCCATGATGAGTATACTAGAAGATTTATGGTTAGAACACATATGA
TTAACCGTGATTATCTCAACTAGGGCGGACTGACCAAGCCCACTCTGTAGATGATTTCTCTTAGGGAATA
CCATTACTCGGTATTAATGGCTTTTCTTTCTCTTGCAGGAGATCAACAAGAAGGAGGTGGTCGAGGCCGT
CACCGTTCTGGAGACCCCGCCCATGATTGTGGTCGGTGCTGTCGGCTACATCGAGACTCCCTTCGGTCTG
CGTGCCCTGGTCAACGTTTGGGCCCAGCATCTCTCCGAGGAGTGCCGTCGTCGCTTCTACAAGAACTG
>dme_circ_0008558_junction_seq
AGTGCCGTCGTCGCTTCTACAAGAACTGGTCTCATCGTAAGTTCTCGGCACCCCGC
>dme_circ_0008559
GTTCTCGGCACCCCGCCATGGCTCCATGGCCTTCTACCCCAAGAAGCGCTCAGCTCGCCATCGCGGTAAG
GTTAAGGCCTTCCCCAAGGATGACGCCAGCAAGCCAGTCCATCTGACCTGCTTCATCGGCTACAAGGCCG
GCATGACCCACATTGTGCGCGAGGCCGATC
>dme_circ_0008559_junction_seq
ATGACCCACATTGTGCGCGAGGCCGATCGTTCTCGGCACCCCGCCATGGCTCCATG
>dme_circ_0008560
GGCCAGCAAGAAGTGGACCGATGATCTCGGCAAGAAGAGCATCGAGAATGACTTCCGCAAGATGCTGCGC
TACTGCAAGGTGATCCGTGTGATTGCCCACTCGCAGGTAAATATCAATTTCAATTATGTATTCCATATTT
CCCGACGTCCTTGGCCTTGTTGCGCCACTGGTGCTGGGTCCTCGGGCTCGGTGCCTTTGGTGCGCCTGGC
CTAAAAAGCATTCGCGCTGTGGTTTTCAATGCACGGTTTAGGGATGCAACGTCGCAGAGCAATCTGCCCC
TCGGGTGGCGGGACTTGATATATTCCAGTAACGGTGCGGAGCCGTTCAGGCGATGATAGCGCGGTTAAAC
CATATCACGTACAGTCCCAATTTCCAATACCACAAGGTGGATGAAACTAACCTGTTGTGTACTTTTTCTC
TATTTGCCTGTAGATCCGCTTGATCAAGCAGCGCCAAAAGAAGGCCCATGTCATGGAGATCCAGCTGAAC
GGCGGCTCCATCGAGGACAAGGTCAAGTGGGCTCGCGAGCATTTGGAGAAGCCCATCCAG
>dme_circ_0008560_junction_seq
TCGCGAGCATTTGGAGAAGCCCATCCAGGGCCAGCAAGAAGTGGACCGATGATCTC
>dme_circ_0008561
GGTCAAGTGGGCTCGCGAGCATTTGGAGAAGCCCATCCAGGTCAGCAACGTCTTCGGCCAGGACGAGATG
ATCGACTGCGTTGGTGTGACCAAGGGTAAGGGTTTCAAGGGTGTCACCTCGCGTTGGCACACCAAGAAGC
TGCCCCGCAAGACGCACAAGGGTCTGCGCAAGGTGGCCTGTATTGGTGCCTGGCATCCGTCGCGTGTGTC
CACCACCGTGGCCCGTGCCGGTCAGAAGGGTTACCACCACCGTACCGAGATCAACAAGAAGATCTACCGC
ATCGGCGCTGGCATCCACACCAAGGACGGCAAG
>dme_circ_0008561_junction_seq
CGCTGGCATCCACACCAAGGACGGCAAGGGTCAAGTGGGCTCGCGAGCATTTGGAG
>dme_circ_0008562
GGTGGCCTGTATTGGTGCCTGGCATCCGTCGCGTGTGTCCACCACCGTGGCCCGTGCCGGTCAGAAGGGT

TACCACCACCGTACCGAGATCAACAAGAAGATCTACCGCATCGGCGCTGGCATCCACACCAAGGACGGCA
AG
>dme_circ_0008562_junction_seq
CGCTGGCATCCACACCAAGGACGGCAAGGGTGGCCTGTATTGGTGCCTGGCATCCG
>dme_circ_0008563
GGCAGAGCGCTTGGTGTGCTTCAGCAGGGACTTGCGCAGGGTGATGATGCGCTTCTTGGAGCCGATGCAG
CAGCCCTTGATCATGACGAAGTCGTTGTTGACCTCACCGTAGTGGGGGAAGCCACCCATGGGCGTGATGC
TCTTGTCGG
>dme_circ_0008563_junction_seq
CCACCCATGGGCGTGATGCTCTTGTCGGGGCAGAGCGCTTGGTGTGCTTCAGCAGG
>dme_circ_0008564
GCATCACGCCCATGGGTGGCTTCCCCCACTACGGTGAGGTCAACAACGACTTCGTCATGATCAAGGGCTG
CTGCATCGGCTCCAAGAAGCGCATCATCACCCTGCGCAAGTCCCTGCTGAAGCACACCAAGCGCTCTGCC
CTGGAGCAGATCAAGCTCAA
>dme_circ_0008564_junction_seq
GCTCTGCCCTGGAGCAGATCAAGCTCAAGCATCACGCCCATGGGTGGCTTCCCCCA
>dme_circ_0008565
GAATATCTAAAACTAAAACACTCATATTGCACCGAGTACACTTTGTTATATATGTTTTACAAGCGCGGAG
GACAACAATATGATGATAATCGAACGAAAATTTGGAAACACAAACAAGTTAAAGGAACGTCAGCGTACCA
GCTGTGGAAATGTGAGTAGTACAGCGAGTTACATACAATGGAAAGCGGTTGGAAAAATATATAAATAACA
TACACCTACCACTCTCACTTTCTGCTTACGGCTTAGGAATTCGACTTAAACTTAGCACGAAAATATTGTT
ATGGTTCGCCATGCAGTGTTTCTGTCCACTGCATCGCATACTGAATCATCTCTGCCACCGATCTTTTCCT
TTTTCTTTTAGAGAGCGTGCTCGTTCAGCAAATGAGTGGAGGTGGACGACGTGTCCCGATCTCCTCCAAT
ATACGCGCCTAAGTGCTTCGCGAGTGAAAGGGACGAACTGCGTCTACTCGATCTACTGCTCCAGCCAGGA
TCCAGTGACGGCATTGGGTGACTTAAGCTGCGGGCCACAAGAGGAGAAGTGAAAAAGTGAATGTGCGAAC
GAAGTTTAATGGGAGCACTCTGCTCCCATGTTTCCATGCTCACTCACCTTTATGTTGAACTGCTTCAGAG
TGCTCTCCAGTGCCATGGCATTGCCCGAGAAGTAGGCAGCGATGGCATTGTGCAGCAGAATCAATTGCTT
GTGCATCACTTTAATCTATCGACGGATAAACGGATTGGATCAATGGTTAGTCTCGCTAAATGCATAGGTA
AGATCCATTTGGCACCACTTACGCGATTCTCGTCGAGAAATTGCATTTTGACGGCCACGTCCGAGCGCAG
CTTCTCATATTGCTCCTTGTGCTGCGCATAGCTGCGCTGGGTCTCCTCAAGAGCGACGGCCGAGGGCGTC
AGCTCCGGCTTGGT
>dme_circ_0008565_junction_seq
CGGCCGAGGGCGTCAGCTCCGGCTTGGTGAATATCTAAAACTAAAACACTCATATT
>dme_circ_0008566
GATGGACAGATACTTGCGCTGCGTCTCGCGCAACTGCTCTATCTGGGCCTCCAGTTCCGAGTCCACGGTC
CGCTGCGACTTGCCCAACTTCTCCAGCATTATCTGTCGCGTACACTTGTACGTCGATATACTCCAGTTCT
TGAGACTGTCGATCTTGGAGGCGCTAGTGCGTATAATGCTGCCGCTCCCGCTGTCCGTGGGGCTCAGGAT
GCCGTTACCGGATG
>dme_circ_0008566_junction_seq
GTGGGGCTCAGGATGCCGTTACCGGATGGATGGACAGATACTTGCGCTGCGTCTCG
>dme_circ_0008567
GGTGCACCTTTTGCCAGTATGAACTGAGTGATGACGACCGGCGCCGCATGAGGTGTGGACATGCCATGCA
CAATCTCTGCTACTTGGTGTTTCGCTATTCCCATAGAAAGTGCCTCGAGTGCGATAAGGTGGTTAATGTC
AATATAGCCGGTGACTT

>dme_circ_0008567_junction_seq
TGGTTAATGTCAATATAGCCGGTGACTTGGTGCACCTTTTGCCAGTATGAACTGAG
>dme_circ_0008568
CCTTTATTTTTCGCAGGAACAGGACCGCCTCACGTCCATCAATGATTCGGTGATCGTAGGTCAGGGCGAT
GTACATCATAGGACGTATCTTGACCTGGTTGGACAATGCAATGATTAAAATACAGCAGGTTGTTAAGCGA
ATGTTGAGGAGTCCTCACCTCTCCCTTGACAGCAATTGGCCGCTCAAAGATGCCGTGCATGCCGAGAATG
GCACTCTGTGGCGGATTGATGATGGGCGTGCCCATCAGGGATCCGAACACTCCGCCATTGCTGATGGTGA
AGGTGCCACCGTCCATGTCCTCCACGGTGATGGCATCACGCCTGGCCTTATCGGCCAGACCGGCCAGTGC
GATCTCGATGTCCGCATAGTTCATGCCCTCCACGTTGCGGATGACGGG
>dme_circ_0008568_junction_seq
TCATGCCCTCCACGTTGCGGATGACGGGCCTTTATTTTTCGCAGGAACAGGACCGC
>dme_circ_0008569
GAACAAGAATAAGCCAGGTGGAATCGCCTGCCGAGCCCTTGCGGTGTACTGTCGAAATAAATAAGATGCG
AGTGGCTGCTACCGATGTTGATCTTGGGATGGGATTTGGGTGGGTCTCCTTAGGATGGGGTAGTGGCG
>dme_circ_0008569_junction_seq
TGGGTCTCCTTAGGATGGGGTAGTGGCGGAACAAGAATAAGCCAGGTGGAATCGCC
>dme_circ_0008570
GATTCTTGATGTCACGCTGTGCAGCCTGTCGCCGGAGAAGTCACACATCCTTAGAATCAGCGCAGTGCGC
ACCCTGAATGGATTCCTGCAGGCGAACGAAACAATCGATGGAGAGAAGCGAACTTTGTTGGTCTCAAAGC
TGCCGGGTTTCCTAGACGGAATTATGGCTTTAGTGCCGGGATCCAAGGCAGCA
>dme_circ_0008570_junction_seq
GGCTTTAGTGCCGGGATCCAAGGCAGCAGATTCTTGATGTCACGCTGTGCAGCCTG
>dme_circ_0008571
GTTGTCGGCTATGTCGCCCTGGTAGTTGGTTTCCTTCAGCAGCTCTTGCAACAGTTGTTCGTCCTCGGCG
TTATCCTCGTCAGATTCGAACAGATCGGATATGTAACGCGGCTTGTCCGGATTTCCGGGCGCATCCTCAT
CATCGCCATCCTCTTCGCTGTCCGTAAGAGGTTCGTCGCTCTTGCCCTCCTGGAAGTGCTGGTACTCGGA
GATCAAAACCTTGAAGATCTTGACGAGAGCCGGAATTGTTACCCACTTCTGGGTTGTAGCTGCCACGGAT
CGAGTACGTCTGCGCGTGTCGGTGGGGTCGTCCACCAGTTCTTTGAATGTTATTGTGGTCAAACGG
>dme_circ_0008571_junction_seq
TTCTTTGAATGTTATTGTGGTCAAACGGGTTGTCGGCTATGTCGCCCTGGTAGTTG
>dme_circ_0008572
ATGAATCCGTAACGCGTGCGCTGGCTGTGAACAGCGGTTGGACGTGGTGGAGAGGCAACGTCATCATCGA
CGTACTGTTCATCATCTTCCTCGTCATTCTCGCCATCTTCCCCTTTCCGCTGACTGTTGGGCTCAAGCTG
TTCCCAGCGAAGAAAAACCAAGAGGAAGATGTCCTCCAACAGCGAGCACAAGGCGCCCAGCTGATGCAGC
TGCCTTAACTGCACGGAAAGCTGTACCAACTTGGTCTTCGTCGTTTGTTCGCCTTGGTCGAGGACCACAT
CGTACACATGACAGAGAGCGGTGTAGTAGCTCAGAATCTCCACCTCCTCCTGGTGGTGACACTTTAGTGC
AGGGGTCAGCTGCTGCCATTTGTAAGTATCTTCAATCAAACGGTGTAGCTCTTGGTGCTTGAAATGAATA
CCATTTTCATGGATCAAGGGCAGAACGCCTGGCGCCCGCAGAAGTGCATCAACTCTTAGCGTTTTGCTTA
TCTCCCGCGGACGCAAGAAATTAAGCAAAGTGGACAGGCGTTGAAAGTCAGCATAGAAAAACCCGTGAAC
TTCCTGCAATTTCTCAGGCACAGCGCTGACATCGAATTCCGCTAGCAATTTTTCCAGGCATCCCAGCTCT
TCCAAAGTTTGCGAGCCCAGAGCAAACTCATTGGCGAGTCGAAGGATAAGCAGGTTATTCCTCAACGTGA
CATCCTGAAAGCACTTAAAGTCCTTGAGATGTTGGCACACGCTGGCTACAAAGTGCTCCCTTTCGTTGAG
AGCCAGCCACAGGACCAAAAGTTTAGTGGGCGCCGCCTCACTACACTGCGCCCGCATGGTCAGTTGCTGC
TCATGCAACAGATCATCCAGGAGTTGTCCATCGCTGCGCGCCAACATGGTCAGCATTAAGTGTTCACTGG

TTATGGAATCAACCACTAGACGAGTCATCAATTTCTGACGAAGTGTAGAGGCCTCCAAAACGCTTGCCGG
AAAATTGGCCAGCACACAAAGGGAACGCGGCGATGCGACATCGCCATCTAGCAGCTGTCGAAGCAAGG
>dme_circ_0008572_junction_seq
TCGCCATCTAGCAGCTGTCGAAGCAAGGATGAATCCGTAACGCGTGCGCTGGCTGT
>dme_circ_0008573
GATTCATTCACAAGACTACAAGAGCGCACCGGAGCAGCAAGGTAGATTTCAGCGCCTGGTGGATGCTATT
AGCGAAGCTCTGTGGAAGCTGGGAGTTTTACAAAAGATAGAACAGTCACTGCTCAAATCGGCGCCATCGA
TCAGCTGCCTGCTAGAGCCTGATCAGCTGCTCCAGCTGGTTCAACTTCACAGCACAGCCAAGAAAAAGGC
CTCCAGCGACGACGAAAGCCGGGAACGCAGCAACCATGCCTCCAGTCTCAACCGGCGGAAGGCTCGAAGG
CAGCGACGGGCGGTCAGCTTTAGTGGAGGAGTAGCTGGAAAAGCATCCGCGGATGGAGGTCTCGCAATGG
AGCAGCTGCGGGCACGAGCTCAATTACTTAGTGGCAGTGGCAGCAGGAAAAATTCTTCG
>dme_circ_0008573_junction_seq
TGGCAGTGGCAGCAGGAAAAATTCTTCGGATTCATTCACAAGACTACAAGAGCGCA
>dme_circ_0008574
GAGCAGCAGACCAAGAAAATCGGACACATCACCGCGAACGAGATCTTGCTGACGTGCGACCAGATGCAGA
GCCCTGCCTTCGCGTACCGGGAACACGCAGAACACTCGCCGCGCGCCATGTACATGCGCGACTGGCGATC
GGCCCTGCGGACGCCCACGTACCGGATTGGGAATCGGACGGTGCGCTTTAACTATCACTTCGCCCTTCTG
ATTGTTTGCGCCTTCGTACTCGTCCTACTCTTCTACTTTGCGCGCCAGGGATCGCACTCCTCCTCCGACG
GCTACTGGCTGCGCCGCAGTTACACGGATGCCCGGAGTCTGGACAATGGTGGTCCAGTGGTCCAGGCCTA
CAATGCCACATATCCGCTCACGTCACCCATGGTGCTGCGTGGCGGTGTGATAAACTACCGGATAGCCATG
ATTGCCGATCTGGACACCAGTTCCAAGGTGAGCAAGGGCGATGGGAGCAGCACGTGGCGTAGCTACCTGA
AGAAGGGCTACCTTACCTACACGGTGGCCAGGTCAGAGATCCAGATTAGCTGGGATGACGGGGCGCCGAT
AGTCCTGGAATCAGCATTTGCCCTTAAGGGCCGCGGAATGGAGCTATCTGAGCTAGTCACCTTCAACGGG
CGCCTGCTCACCTTCGACGACCGAACGGGTTTGATATACGAGATTGTGAATGACAAGCCCATTCCCTGGG
TGATCCTGCTCGATGGAGACGGACACAGTGCCAAGGGCTTCAAGGCCGAATGGGCCACAGTGAAAGAGCA
AACGCTGTATGTGGGCTCCATGGGCAAGGAGTGGACGACAAGTGCGGGTGATTTCGAGAACAATAACCCC
ATGTACGTAAAGGCCATCACTCCGTCCGGCGAGGTGAGGTCGCTCAACTGGGTGGACAATTTCAAGCAGT
TGCGCCTGCAGTCGATGCAAATAACCTGGCCGGGCTACATGATCCACGAGAGTGGAACCTGGTCAGAGGA
AAGGAATCGCTGGTTTTTCCTGCCTAGGCGGTGTTCGAAAGAGAA
>dme_circ_0008574_junction_seq
TCCTGCCTAGGCGGTGTTCGAAAGAGAAGAGCAGCAGACCAAGAAAATCGGACACA
>dme_circ_0008575
GGAGAGTCTTTTCCATTTCGGCCTTCTTTGCAGCGGGTATTTTCGCGCCATGATGAAGTAGTGCCTTGCC
AGCGGATTGGACTCGTCTGGAAATAATAAAATGCAATTCAGATTCATTGAGAACTCTATGGAGATTAATT
TTATCATATATCAGGGTATGTTTTGGAAAGTTAGGTGATAAAAAAGAGGCAAAATATGTCTGGTAAGATA
ACTGATAAGGCAATCTACATTCCTCGCAATGTATAATTTACTTTTACTTTTAACTTAGTTCTGAAAATGT
ATGGCATACTTACTTGAGCTGCGGATCATCATGGTACTTGACAGATCCGTCTTCCTGCTTGAAAGGAGCT
TCGCACTGCACGGCCAGTTTGCTGCGAACCCTTAGATCCGAGATGTTGCTTAGCTGGTGGCAGGTTGGGC
ACAGGAGCAGCACATCGTCGGAGGTGTGAGATTTCATGACCAATGGGAAATGCTTTCTGTATTCCCGCGG
TACCACGTTCTTCCGAATGTACGCATCCCGGTCACCACACACCACGCACTGGTTTTTCTTGATTGTTTGA
TAGAAGCGCCCCACATCTCCGACCGCTCGACCCGCTGGCTCGAAGTTAAGGCGGACAGTGAAGGGCTCTT
CGCTTATATGCGTGCCCAGATTTTGATTGAGATACCAGGACGCCTTGCGCCTGTCAATTGTACACAGGAG
TTCGCCATCGGGCGCCTGCAGCAGGCAGTTATCGTAGAAGTCTTTGGTTCGGGTAGCTATTTGCCGGTAC
GGTTGCTTCTTGGGCACCCATTTATTGCTTTTGGATTGGGTGGAGCCCTTTGAACGAGTTACTCCGCTGC

CGGACGGGTTGAGTGTGAAGCCTTTCGTGAAGTCCACGTCGAGAAAGGGTTCGAATTTGTTGTGCATGCT
ATTGTCGTCCA
>dme_circ_0008575_junction_seq
AATTTGTTGTGCATGCTATTGTCGTCCAGGAGAGTCTTTTCCATTTCGGCCTTCTT
>dme_circ_0008576
GTCGCACAATATTATCCAGAAGGAAGGGGGCGTAATCCAGCCTCACTGATAGCCTTGAGCCCGAGGTGCA
GACCCTAGTATGATCATGCAGCTCCTGGGCCAGCCTGGCACGTTTTCCAGACTTGGAATTCTTGCCCAGC
CACCCGGGAAAGTTAATCTGTCCAGTGAAGTGGCCACACATGTGCTCGCCAGGAAGGACCGAGCTGAAAA
AGGCCTGAGTGGGTAGAAGACTCCAAGCAGAATTCGCACGAATACGCTTTTCAACAAGGTCACCCAAGCT
TAGGGCATCTGCCGTAGCTGCCACCTTTGCCAGGACATCCTTTCTGCAAAGAGTGAAGTTGGTGAATGGG
TACGCCCTTGGCTTAGGTAAACTAGACTTACTTGTTTCCCTGTGGTAGGACCTGCAGATAGTTTTGCTGC
ACAAAGAGCGGGGCCAGGCTGTAGTCGTGAAAGAAAAGG
>dme_circ_0008576_junction_seq
GGCCAGGCTGTAGTCGTGAAAGAAAAGGGTCGCACAATATTATCCAGAAGGAAGGG
>dme_circ_0008577
GGGTGTGCCCGCTGCTTTAGACGTTATGAAGGATTACCATCTACTCCGTGAGGATCTGGACTCGCTGGTC
GAGCTTACATCCTGGCCCGGCAAGAAATCCCCACTGGACGCAGTGGATGGGAGAGTAAAAGCCGCTTTAA
CGCGATCCTACAACAAGGAG
>dme_circ_0008577_junction_seq
CGCTTTAACGCGATCCTACAACAAGGAGGGGTGTGCCCGCTGCTTTAGACGTTATG
>dme_circ_0008578
GTGTCCTGGATAAGGAAGCGCGACCTGCACATCCTGACGGTGGGAATACTAACGTACACAAACGACCAGC
GCTTCCAGTCGCTGCACTCCGAGGGATCCGACGAGTGGACGCTGCGCATCTCATCGCCGCAGCCGCGGGA
TTCGGGCACCTACGAGTGCCAGGTGTCCACGGAGCCGAAGATCAGCCAAGGATTTCGCCTCAACGTTGTG
GGTGTCACGGGCCAAGATCCTGGGCAACGCGGAGCTCTTCATCAAGAGCGGCAGCGACATCAATCTCACC
TGCCTGGCGATGCAGTCGCCGGTGCCGCCATCGTTCATCTACTGGTACAAGGGCAAGCGGGTCATGAATT
ACTCGCAGCGCGGTGGCATCAACGTCATCACGGAGCGTTCCACACGGACCAGCAAGCTGCTCATAGCCAA
GGCCACGCCGGCGGATTCGGGCAACTACACGTGCTCCCCGAGCAGCTCAGG
>dme_circ_0008578_junction_seq
ACTACACGTGCTCCCCGAGCAGCTCAGGGTGTCCTGGATAAGGAAGCGCGACCTGC
>dme_circ_0008579
GGAAAATGCACGAGTGGGTTTTTTGGGGTAGAAGGATGAACTAAGTAACTAAGTCGCCAAGCCGAATCGC
CGAGTTACACCCACACCTTGTTGAACAGATCCATGAAGGAGTCGTAGATCATAAAGGTGATGGCCACATC
CAGGCACACACGACCTAATCTTGGCACGGTGCCCTTGTAGAAGGCGGCGGGACCTTCGTTCTTCAAGATC
TCCACCGCACAGTGGGCAGTGTTCTTGTATTTCGATGCTTCCAGACCCTGCATCCTCGTCTTGACCACAT
CCAGCGGCGTATTACCGAACACAGAGGCTGCTCCGGCGATAGCTCCAAACACTCCAACCACCAACTTGGG
CACTGGCTTCGTGTGATCGTCACCCTT
>dme_circ_0008579_junction_seq
GCACTGGCTTCGTGTGATCGTCACCCTTGGAAAATGCACGAGTGGGTTTTTTGGGG
>dme_circ_0008580
GTAACTAAGTCGCCAAGCCGAATCGCCGAGTTACACCCACACCTTGTTGAACAGATCCATGAAGGAGTCG
TAGATCATAAAGGTGATGGCCACATCCAGGCACACACGACCTAATCTTGGCACGGTGCCCTTGTAGAAGG
CGGCGGGACCTTCGTTCTTCAAGATCTCCACCGCACAGTGGGCAGTGTTCTT
>dme_circ_0008580_junction_seq

TCTCCACCGCACAGTGGGCAGTGTTCTTGTAACTAAGTCGCCAAGCCGAATCGCCG
>dme_circ_0008581
CTATCGCCGGAGCAGCCTCTGTGTTCGGTAATACGCCGCTGGATGTGGTCAAGACGAGGATGCAGGGTCT
GGAAGCATCGAAATACAAGAACACTGCCCACTGTGCGGTGGAGATCTTGAAGAACGAAGGTCCCGCCGCC
TTCTACAAGGGCACCGTGCCAAGATTAGGTCGTGTGTGCCTGGATGTGGCCATCACCTTTATGATCTACG
ACTCCTTCATGGATCTGTTCAACAAGGTGTGGGTGTAACTCGGCGATTCGGCTTGGCGACTTAG
>dme_circ_0008581_junction_seq
AACTCGGCGATTCGGCTTGGCGACTTAGCTATCGCCGGAGCAGCCTCTGTGTTCGG
>dme_circ_0008582
GTTACACCCACACCTTGTTGAACAGATCCATGAAGGAGTCGTAGATCATAAAGGTGATGGCCACATCCAG
GCACACACGACCTAATCTTGGCACGGTGCCCTTGTAGAAGGCGGCGGGACCTTCGTTCTTCAAGATCTCC
ACCGCACAGTGGGCAGTGTTCTT
>dme_circ_0008582_junction_seq
TCTCCACCGCACAGTGGGCAGTGTTCTTGTTACACCCACACCTTGTTGAACAGATC
>dme_circ_0008583
CTATCGCCGGAGCAGCCTCTGTGTTCGGTAATACGCCGCTGGATGTGGTCAAGACGAGGATGCAGGGTCT
GGAAGCATCGAAATACAAGAACACTGCCCACTGTGCGGTGGAGATCTTGAAGAACGAAGGTCCCGCCGCC
TTCTACAAGGGCACCGTGCCAAGATTAGGTCGTGTGTGCCTGGATG
>dme_circ_0008583_junction_seq
CCAAGATTAGGTCGTGTGTGCCTGGATGCTATCGCCGGAGCAGCCTCTGTGTTCGG
>dme_circ_0008584
CATGGATCGCTCCGCCTTCGCGTCCCTTGTCAGCCCGTACCGCCGACGTCCGTGGATGACGGAGCACGGA
GCGGCGGCTGCGGACAGCGGCCAGGTGGGCCTTAAGGGCATCGTCGCCGGCGGTATTACGGGCGGCATCG
AGATCTGCATCACCTATCCCACCGAGTATGTGAAGACACAACTGCAGCTGGACGAGAAGGGAGCGGCGAA
GAAG
>dme_circ_0008584_junction_seq
GCTGGACGAGAAGGGAGCGGCGAAGAAGCATGGATCGCTCCGCCTTCGCGTCCCTT
>dme_circ_0008585
GAGCGCGTGTCCCCTTTCGTATCGAAAAGTGCTAATCAACCGCCGTCGTCCAAAGGCAATCCCGGTGACC
GAGCAAGTCCCCTGTACTTGTAATGCTACACTCTATATAGTGCAATTTAGCTCCAATCAGTGCAATCAGC
ATGCAGTCGATGAAAGCCGATGAACTGCCGGAGAATCCACGGGAGCGATCCAGTCCCCTCTCGTCACTTA
TGTTCTGGTGAGCGTAGAAATTATATCTTTCTGGGCATTTCGAGACTAAATTTTTATTTCTTTTTAATTA
AATGGTTTGTCCAACCATCTCATTGCAAAAAGAATAGAATATATATATAT
>dme_circ_0008585_junction_seq
CATTGCAAAAAGAATAGAATATATATATATGAGCGCGTGTCCCCTTTCGTATCGAAAA
>dme_circ_0008586
GTTTTGCCCTACCCGTTCTGTTTAAAGGCCGCAAGAAGACGCTGGAACAAAAGGATCTATATAGAGCCCT
CAAAGAGCACAAATCGGGTGAGTTCTTTGATTAATTTCCATTCGCAGCTATATTAATGGGTATCATCTTT
AATATATAGATTCCCTGGGAGATCGGTTGTGCGCAGCCTGGGATGAGCAGGTGGCGAAAAATGAAACTCC
ACGTTTGGGTCGCGCCCTGACGAAAGTGTTTGGCTTTCATTTGTTCATTACGGGAGTATTCCTGCTTGCC
CAGGAGTTTTTGACCAAGAGTCACCCAGCCCATTTGCCTGATTGGAGTGATGGCATACTTTGCCGGGAAT
GATCCGGATCGCTCCAAGGCCCAGTTATGGGCAGCGGGTCTTATAGCTGGTTCCGTGTTTAGTGTGTGTA
TCGGTCATCCCTATATGCTGGGTCTACTTCATCTGGGCATGAAGATGCGCATTGGCACTTAGCAGTCTAA
TCTATCGCAAGGCCCTGCGGCTGAGTAGAACCGCTTTGGGTGACACCACAGTGGGTCAGGTGGTCAATCT

GCTGTCCAACGATGTGGGTCGATTTGACTTGGTACTGATCAACGTTCACTACCTGTGGATAGCTCCGTTG
GAACTGATTGCAGTTACCTACCTGATGTATCTCGAG
>dme_circ_0008586_junction_seq
TGCAGTTACCTACCTGATGTATCTCGAGGTTTTGCCCTACCCGTTCTGTTTAAAGG
>dme_circ_0008587
CTGGCCGAGATCCTTTGAGAATCGATTGAGGATGCGTCCCGATGGATTCGTGTTGAAGAAATACATGGCA
GCCCTCGTAATGCCCTGGTACATAGCATTGTGCAGCTGCGTGGAACTGCGCATGGCCATTTTGTAGAAGA
GCATGGTCCGTACAATGGTAAACACCACAACAGCTACATTGAGCGCGGCAAAGTAATACATATCCTGCGG
ATCCG
>dme_circ_0008587_junction_seq
GCAAAGTAATACATATCCTGCGGATCCGCTGGCCGAGATCCTTTGAGAATCGATTG
>dme_circ_0008588
GGAACCCGATGCCAAGTCCCGAGGAAATCCAGTGCCACACATGCGTGTTGTAAATTTTAATGCGCCCAGA
AAACTTGAGAAGAACGACCCAGTTCTGCTTACCATAGATAGAATATGCCAGAAGCGTTTTTCATCAGACC
CAGGAACTCAAGAGAAACTATTTGACCCGAGAAATTGAGGCAAATGTGAAACATATGTGCGAGCTGGAGT
GTTGGCGTTACATTTTTGGTTATTGTTCTCTTTTGACCACGAACTGAAATTGAATTAACCACTCGCGGAG
ACAATACGAGGAACAATCCAAACCAGCTGAGCAATACAATTAAGAAGACAAACGAAACATAACGAACCCA
GTACCATTCCAAGTTAAAGCATTTAGCCCCAGCCTCCACAGAGACAAAGGAGCAACTAATCCTGCTCGTT
CTATGCCACATCCTCAGCAGGTCCCACGCCAGCGGTAAGCAAGATTCTCGTCTACAAAATGTCCCTGGCC
GACATGGGCACAGCCTCTCGATCCGCCGGCGGAGAAGGTGGTAGACACTATGATCTAGCCACGGGCGGAG
CTGGAAGTGGGGGTCATCCAGTGGGCGGCGGTCTGCCCGGAGGCCGCATGACGCACTCCCTCAGCACTCC
TTCCGGGGTGGATGGCACGCCCTCCACACCCCGACATCGCGGCGGCAAAAAGCTGACTGTTCGCATCCAG
ATGCTGGACGACTCGATCACCATGTTCCAAGTACAG
>dme_circ_0008588_junction_seq
CGACTCGATCACCATGTTCCAAGTACAGGGAACCCGATGCCAAGTCCCGAGGAAAT
>dme_circ_0008589
CCATTCACTTTTCGGTATAATTAATAATTTTACTAGCAACACAATATTAAGCTACTTCTGAAGCGGACTT
ATTTCTGCAGACGCCTAGTGGCCTTTCGTATCGGACGACTCTCCGGTGTGGGGGCAAACATGTTGCCGGC
ATTCGCCTGCTGGGCCGGACTGACGCCGCCAAAGTTGAAGCCCCCGGTTGGATTGCTTGCTGGCGGTGCA
GGGGCTGGGCTCCCAAAGACATTCGGGGCTGGAGTGCCGCCAGCGCCACCAAAGCTGAATGGCTTTGCGG
>dme_circ_0008589_junction_seq
GCGCCACCAAAGCTGAATGGCTTTGCGGCCATTCACTTTTCGGTATAATTAATAAT
>dme_circ_0008590
TAAGGGATCACTGGTTGATTACCTGCGATCGCGAGGACGACAGCACATAACCAAAAAGGATCAAATCATT
TTTGCCTAGTAAGCAATATTGTTAGCTTTTGTTTTGAATCCTTACTAATTAACGTTACTCAATAGCGACA
CTGCCTCTGGTATGGAGTATCTGGAAGCCAAAAAGGTTGTCCATCGCGATCTGGCAGCTCGCAATGTCCT
TATATCAGAGGATTGCGTGGCTAAAG
>dme_circ_0008590_junction_seq
CTTATATCAGAGGATTGCGTGGCTAAAGTAAGGGATCACTGGTTGATTACCTGCGA
>dme_circ_0008591
TGATAACTCGCAGACGAGCAGTGGCAGCCAGACGGCGTCCAGTGCCAGCAAGCGATCCAATCGATCCGTG
GGCCGGAGTCATCGAGGTTCGATTGCGCAGGACACAAGGCGACGCTACTACTTTGGCAGCAAGGACATCT
ACTGG
>dme_circ_0008591_junction_seq

CTACTTTGGCAGCAAGGACATCTACTGGTGATAACTCGCAGACGAGCAGTGGCAGC
>dme_circ_0008592
GATCGTCCTTAAATTCGAAACGTATATCAATGCCTTCATCAGCCATGGTACCAGCTTGTGGGTATATACT
TGCGATCGTAAAATATACCTAAATGAACACATACATACGTTAGGCAAAGAGCTACAAAAGCCCATTATGC
TTATGCCCGATGCCGAGCTCTCTGGCTTGCATCTGGCCAATCTCACCCATTTCTCGCCCCACTATCTGCT
GGCCACTCACAGTTCAGCAGGATCCACACGCCTGCTACTGTTGTCATATAAGGATAATGACAACAAGCCA
GGCGAGTGGTTCTATCGAGTGCACAGTTCCGTGCGGATCAACGGATTGGTGAATGCTGTTGCCGTCGCAC
CTTATGCCATGAATGAGTTTTACGTGCAAACAGTGAATAATGGTCACACATACGAGGTGTCGCTTAAAGC
GGACAAGACACTTAAGGTGGAGCGATCTTATGTGCAGCTGCATGAGCCGGCTGACCAGATAGATTGGGTT
ATC
>dme_circ_0008592_junction_seq
GCCGGCTGACCAGATAGATTGGGTTATCGATCGTCCTTAAATTCGAAACGTATATC
>dme_circ_0008593
GTTCCAGAAAAACATCTACGTGGCCTACGCTGATCATCTTAGAGCCATTGCCAAACTGGACAACGCCAGT
CTCATGTACGAACGTGGCGGACAGTTGCAGCAGGCGCTACTTAGTGCGAAACACACTCTCGATTGGCAAC
GTGTTTTGGTGCTGGCCAAAAAGCTGAGTGAACCTCTGGATCAGGTGGCCCAGTCGCTGGTGGGGCCGTT
GCAACAGCAGGGTCGCCACATGGAAGC
>dme_circ_0008593_junction_seq
TGCAACAGCAGGGTCGCCACATGGAAGCGTTCCAGAAAAACATCTACGTGGCCTAC
>dme_circ_0008594
GTGGATACCACCACGCAGCTGGACGAGGATGTTGAGGAGCTTCTGCTTCAGATCATCGACGACTTTGTCG
AGGACACCGTCAAGTCGACGAGCGCCTTCGCCAAGCACCGAAAGTCTAACAAGATCGAGGTGCGCGACGT
GCAGCTGCACTTTGAGCGGAAGTACAACATG
>dme_circ_0008594_junction_seq
GCTGCACTTTGAGCGGAAGTACAACATGGTGGATACCACCACGCAGCTGGACGAGG
>dme_circ_0008595
GAAACATGTCAGCGAGCGAAGAAACAATAGCGGACAGTCAGGTCTCCGAGAATGTGGAGGATGTGGAATT
CGTAGACATGGCGTTCACAAAGGAGTTGCGTAAAGCCACAAAAGATGTGCATAATCTTACTGATGTTCTA
GTGAATGCAAAGATCGCACTTGGCCCTTTCTGATGACGAGGTCTGGTATGATGGACTCCTGGCCTTCTAC
GAACTGTACAAATTCTTTGAGACCCATCTGCCGGAGCGCCTGCTGCCCAAAGAATTCCACAGAACAGCGG
CATTTGAAAGGGATTTTGCCTATTTCTACGGTTCCGATTGGAGGAAGGACTACGAAATTCGCCCGGCGGT
CCAGAAATATCTCGAGCATCTGGAAAAGATTGCCGCACAGAACGAACTCTTGCTGTTCGCCTACTCCTAT
CAGAT
>dme_circ_0008595_junction_seq
TCTTGCTGTTCGCCTACTCCTATCAGATGAAACATGTCAGCGAGCGAAGAAACAAT
>dme_circ_0008596
GGCCAACGATGAATGCAGGTGCTGTGTGAAAGTGCCTCTGTTCCTGGAGAGGGACGGATCCAAGTTGAAT
CAAAGCCGGAACGTGACTTTTGCCGAAAGTAAATTGAGCCACCGCAGCGATAAAAGCTGCCATGCCAACC
GGCGAGTGGCGCTAGGACACTCGTAAAGATAAAGGATCCATGGCTCGGCCGAATCCAGGTGCACCCGTGA
TGAGAGGCGATGACATCTATTCGCGGGTGCACTCCGCCACCCCTCTGCCCCTGCCCCTACACCTGGTCCT
CCTGCTCTTCCTGCTCGTGCTGCAGCTCCTGGCACCGGCTGCAGCGACTCCCGTGAATCCCTGTCCCCAC
AAGGGCATGGCCGAACTGATTCGCAACAGTCCGGTAATTGTCAAGGCCCTCGTCGCACGGATCTTCGCCG
ATGAGCCGGAGGGACCGGTGCAGTCCCTCGCACAGGACACGATTTTAATAACATTAACGCCGGAAACGAT
ATACAAGGGTGCCTCGCTGCTGAAAGTGGCCAACGGCAATACCGATGCCGGGAATCCCATGTCGCCGAGG

GTGGGAGGTCTCATGTCCTGGAGCTCGGGCCATCGCAGCAGAGGTGGCAGCGCTGGATCCGCGGGCAGCG
GTGGCACCTGGAGTGGCGCTGAGTCAGCCTTTCAGTACGAGGGGGCAGTTGAATGCGACCCTCCAGCCAC
TGCGTTGTTTCGATAATAACATGCTGAAAATGCTGCCGGCGGAGTTAATTGCCTTTGGCAAACTGCTGCC
CGCTGCGACGGCATCCACCGGAGGCAGTCCGCAGACCGCACGACAGCTTCAACGACCGCCCGTGGGTGGC
GCCACCGATGTGCTGCAAGTCAGCGTCAACGGTCTGCATCGCTGGACGCCGCAGTTCGAGAATCATATCT
GGAAGCATTTGGGGTAAGCATGCACTTCCGCTTTCTTGTGTGGCTGCACTTAAAAACTGATTAGTTTAAA
ATGGACTTTAAATAAATTAGCTTCCGGTTTTCTAGCAATTATATTAAGCTAGGCAATTACTGCTTTACGA
TTTGTATCATCttttttttCAACACCTTTACATACATTTAATATATTTTAGGACTTGTATATACAATATT
TTCCGAGTGCATCCGCCCAGAAGCCACTCGATTGCCTCCTCCGGCCACCCGAAACCAGGACTGTCCTGGC
CGGGCTTCCGTTTTTGCTTCCGTTAGTTAATCGCATTTCGCTTGTTAGTTGTTGCATTGTTGAGGAAATC
GCTCCGGTCGGATGTTTGTCGAGTATTGGATGAACCCGGCGGGATAGAGTTTCCCAAACTAGAGGCCACT
GGTCAGGCCGGGCTCGGATGTGGCAATCAGCGGTAATTGGCACTGGAGGGCCAGAATCTCCCATCGCTCG
AATATCGCAACATAATTGCATGCGACTGTGCTATTCAAAGCCGATTACATGCCCTGCCTTCCGATGATAT
CCTTAAAGTAATTCAACAGTGAAATGCTGCGAACACTTAAAATTCCGATACTCATTCGGCTTATTCGTCA
GTCTATTGTGACTTCATGAGCCCAGCTGGGGGATTAATCTTTGATGAAGATTGATAGCCATCGTTGACGA
TACCTCCGACTGGATGGGGGtaaaatataaaatagagcaaataaaataaaataaaaaGCAGTATACAAGG
CGGCAACATCTCGATGACGCAGCAGATGTAGCAGATGTGGGCCACTGCCTCCTCCAAAGCCGGCAATCCG
CACCAGCGCCACTCGAGGGAAGCAACACTTGACTTTTGTGTCAAGCGGCCAGGCGCCCACCAAGGATCTG
CCTTGAATTGCGGCAACTGAATGAAAGGACTTTTCGTCCCAACAACAAGCGGCACTAAAATTTGGCACGA
TGATGTCTTAATTTTATGGCTCATAGTCAGGAAGAAAAGGCATTACTTATATGCGCATGAAGAAAAGATG
GAGTACAAAGTGTACTAATAAAAAAGGGCATTTATAAAATTCCTATTTTAAATGCAGTAATATTTATGTA
AACAGTTTCAAGCCCTCTAATTTAAAAAGAATTTTTTGTTGCCAAAAGGTAACCTTTGTGATTACACAAG
CCATTTAAAACTGAAAGAGACAAACTTACATTTAATTTGATTTTCACTGCAAACAATTGACAATCAATTG
CAGGGCTGGGACGATTGGAGCGACTTCACCTTGTGCAGTGTCACCTGCGGTCAGGGGGTGCAGCAGCGTT
TCCGGCGCTGTCTACTCGACAATCCGCTGGTGGACATCAACATGAACATCAATCTGGAGGAGgacgacga
cgacgacgacgaGGATGAACAGGCTCAGGGTGCAGACGATGCGGGCGCAGTTGCCCAACTGTCCAAGGAG
ACGGACGTTTTCGGCGATGGCAACAACGATCTGCTGGCAGATGAGGATGAGAACGAGCTGCTCATAATGC
GAGCAAGTGGCGACGAGTCATTGCATAAATTTGACCAGGCAACGACGACGATTACGCCGCAGACCAGTGT
CCGTGCCCATGCCCATCCGAGCGCCAGTCCCCCGGCACCTGACCCCATGGGAGAGACAAGCGGCGGTAAT
GAACCACCAAGTGATGATGAAGGAGCAGGCGTTGGAGGAGCTGGCGAAAGTGTCAGGTTTGTGCCGAGTG
CGCACAATGACCGGTCTGGAAACGAAGTGGGAGCTGGAGCGGCGACTGGCTCCAGTGCTGGGACCAGGAA
GGCACGTGCCAGCAATCAGCACAAGAAACGCAAGCCCACCAAGAGCGTGGCCCTGTCGACGCTCCTCTGC
GAGGGCTACAACATCGAGCAGCGCAACTGCAACAGTTTCGAGTGCAGCGGATGACATAAGCGATCTGCTA
AAGTTCTACAAGAAGCTGCCGATGGGTGAGGATTTGCCAACTCAAGCTTCGGATGCTTCGCTTGCCCCTT
CCTACCCGGATGCATATTCAAATTTGGACCTCACCACGGCGGGTCTGATGGGCACTGCATTCAGTCCAAC
GCCCTCGACGCCCTACAACATGGGCTACATTCGCAGCTGGAGGAACCTCCTTAACTTCACCCTTATGGTC
ACATTAAGGGCTAAGGAACGACACCAAGAGCACGGCGACCATATTCTCCATAAGAAACGCCACTCACAAT
CTTTATCTGGAGGCCTGCAAGGACGGACTGCGTTTGTATTTGGAGCGGGACAATACGACGGAAATGTTGC
CGGTGAAATTCAATTTATACGATTATCGCTGGCATCAAGTGGCCATCAGGGTGAGAGAGTGTACACATAT
GGGATGGGGTATATGGTGCTCGGAAGGATCCAGGCGGTGGATGTGGGTAGTGGGTTCCCACCTCCGTGCG
ATGGTGATGATTGGCAATCACTGCCTCTGTGTGTGCCTCTGCGTGGGCGAATGGCACTCACTTCGATCTG
ATGGCCATCGACGTGGACACTTTCGTTTATTTGCATTTTCAATGTATTAGCATAAATGTTTGTGCCATGA
AACGCACACTGCCAAACACTTGACACACATGCAAATAGTAGGCAGGCACACAGTTGTGGCAAAGAAAAAA
ATTTCAGCATTTTATGTGCAAATCTATAAAACTTGGAAAAATAAAGTTTTATTTTTAATTTGATTATA

AGCCACGTTGTACATATCTTTTATTTTCTTTCTAAGAATCTGTGGACAATATAATTCGATTATAATGGT
GAAATTTAACATTCATTGCTATATTCTTGACCGCAACTGTATACAGACAGCGTGCACATGAATATCGAAG
GCCCttttttggtttttgctttttACTGGCTACAACGACACACAGCCAAATTGACACTTGGTTCAAGTGG
GCACCATTTGTCATCGACCGTCGGCACATTTCTCACTGTCACTGTATGGGTGCCCACCCATACATATGTA
TGTACGCGCGAGTGTACTAACAATGCACCACTATGTATCCATATATATGTACATATATAGTTTAATTATT
TGCGAATAGCTCACTTTACCATTATTGTTTCGATGTTATTTTCATCCACCGTGCAGCATACAGAACGGCG
ATTTCATATCGATTTACGTCGATTGCGCGTGGACAAACAGTTTCGTTGTCTCCAAGCGACTTTTTACGTT
GCCCGTGGACGCGGATGTCGAAATCGGACGCGGCTTCAAT
>dme_circ_0008596_junction_seq
GGATGTCGAAATCGGACGCGGCTTCAATGGCCAACGATGAATGCAGGTGCTGTGTG
>dme_circ_0008597
GTAGCCATCGGCACTGGTGGCCAGCAGAAGCTTGCTTTGCTCGTTAGTCGTCAGCTGGGTGATCTGATCT
TCCAGTTCTTTTAGCTCGAAGATGGCGTTCTTGGTGCGCAAATCCCACAGCTTGACCGTACCGGCATCAT
CGCCCGAGGCAAAGAGATTTTCATTCAGAACG
>dme_circ_0008597_junction_seq
CGAGGCAAAGAGATTTTCATTCAGAACGGTAGCCATCGGCACTGGTGGCCAGCAGA
>dme_circ_0008598
AGCTTCCGTGGAAACCCCCATCATCCCAGTGCATGGAATCCGATAAAAAGTTTTCACTTTTCACTGCGGC
GCTTGTGTTTTCCCCACGTAGCTTTGGAATTTCCTGCTAACGGCGATGAAAAACTTGACGGAAAAGTGGT
GCTGACAAAAGAGTGTGTATTTGACAAGCAAAGAAAAAATTTCCGTCTTCAAACTTTATTTTGAGCTTTA
AATCGTTTTTACTCGCTCGAGAACTGTGCAAATAATAGCCCCGAATTTCCATTTGGAGTACCTAGAGTAC
ATAGCTGGCAATAGCTTTGGAACCCAAATACTTAACCCACTGCTTGTTTTTGTTTCTGTTTCCGGACAAA
AAAAGCAAACTGGTCTCTGTTAGACAGTTGTACCTCCTCATACCCGTAAATTAAACATTTTTTCGTACAT
ATACACAATTTTAGCGTTAGACAGTTGTACCTCCTCATACCCGTAAATTAAACATTTTTTCGTACATATA
CACAATTTTAGCGCATTTTGGCCTGCATGCGCTGCAACGCACCTTTGCCGTTCTGGTAACGTTGCCCACT
AGACGACTGAAATGGATAGCTTGGAGAACGACCAGGAGGAGCTGCACCACCAGCCGAGGGTCACCTTGGC
GTACGGCAGTGTGGCCACCAGTTCGGGTGGACAGGAAAACCGATTCCCAGTATGCGATGTACTATAATCA
GCAGAGGGATTCGGAGGGCTACATGCTAATGACGCCCTTCCTGTCCAGTTCGCAGAGCCACAGCCACACC
CGTTCGCCGCCCACGTACACCGCCATGGTGGAGCCACGTCGCGAAGAGAGTTCTGGATTTGAGGGCTTGG
CCAGGCGGTGTGACAAGTGCAGTTCGCTGGACGGACTCTGCCGCTGCGAATCGATGGTGGCCAGTAGCAG
TCGCTATGCGGACTCCACACAGAACCAGTCGCATTCGGAGGCTCAGTTCTTGGAGCAGCCCTCATCCTCC
ACAGCGGCGATCGCCAATCCGCCCGCTGTTCATTCCATTCCACGACCCAAGCGAAAGAAGACTGAACCCC
TGACAATGGAGCCAGAGAGTGACAATGACGGCGACGCGGTGGTTGCGTCCGTCAGCGGCACATGGCCTGC
CGGTGCTATAGAAGAGCATGCTATGGACTCTACGGCGGTCGTTTACGAGGTCCCCTCTAGTTCGCTGCAG
CTCAGCAACAGCTCCTTCGAAACCGCCCCAAGTGCCGTAAATGCTTACGATGATGTGACATCGAGTGCCT
CGGACACGGAGCACGGCCAGTTTGCTCATACGCGTACCACCAACACCACGACGACTACGTCTGCCGTTCA
GGCTCACAAGAAGAAGGCCAGCCGCTCCAATAATGCCTCCAACACCTCTTCCAGAGCAGTAGATACGGAT
GCCACGCCAAGCACGAGTGCCCAATCGAGAAAGCGACAGATGAGTCAATTTTCTTATCGCAAATCAAGGG
TTTTGGAGCCGGAAAACGAGAGCAGCGATGATGGCTGGGATTTGCGTGTGCATAATCCCGTTCCACCAAT
TGAGCCCACAGTAACGATTATCCG
>dme_circ_0008598_junction_seq
CAATTGAGCCCACAGTAACGATTATCCGAGCTTCCGTGGAAACCCCCATCATCCCA
>dme_circ_0008599
GTTTTTATATGTATAAAACAATATCTACTCTTTCTTTTATTGCTGGCTTCGTATTCTTTTCTCTTGGCTT

TCGTTTCCAATTCTGTTTAGCCATAATTTTAAAACCAATTCGCTTCATACAAATATTCCTTGATTAAATC
GGTCAGCTAATCAATTATCTGCAGGATTTCCCCATATTCTCGTTG
>dme_circ_0008599_junction_seq
TCTGCAGGATTTCCCCATATTCTCGTTGGTTTTTATATGTATAAAACAATATCTAC
>dme_circ_0008600
TATCATTTTCCACAGACCTTATTTGTTTACGTCCCACGAAATCAATGTCAAGTGCATGCTGCATACACAC
CAATTCTTCTATTTTTCCAACCAACAGCAACTTGTGCGaaaaacttccaaaaaaacaaaaacaaaataaac
gaaaaattaaactgaccacaaaaaataaagttaaaTGCAGACATATGAAATCATAAATGATTGTTCATAA
TTTTAGCTCAAAACGCTGCTGCCAAAAAAAGTTGCCAACGGCCTCGTTTTGGGTTTGG
>dme_circ_0008600_junction_seq
GTTGCCAACGGCCTCGTTTTGGGTTTGGTATCATTTTCCACAGACCTTATTTGTTT
>dme_circ_0008601
TTGATTTTACAAAGTTAATTGAATTATCAAAATTTCGAGTCGCTCGCCAATTAGTTGGAATCGAAATTTA
CACTTAAAAAATTAAACGCTAGTAAGTTATTTCAAAGAAATTGACACGTACACAAGATAAACTGCAATAT
AAAGGTAATTTTTTGTTACAGTTTATAGTTTTGTAACGTGTATATGCCAATTGCTTGGAAATAGTTTACA
TGCGTACAGTTAATTATTTCAAAAAATACTGAAACATATATTTAATTAAGCCCCAACACTTCTCTACACT
GACATCCATACACACTAAGGCCAGCTACACTGGGCAAAAATCGAACACACTCTTGACATTGCAAGTTGCT
CAACACGTTTTGATGGGCGTAAATGAAATAATAACACGCAAACCAATTTTGTTAAATACATACACCTATA
ATATTTAAAAAATTG
>dme_circ_0008601_junction_seq
ACATACACCTATAATATTTAAAAAATTGTTGATTTTACAAAGTTAATTGAATTATC
>dme_circ_0008602
GGTGTATGTATTTAACAAAATTGGTTTGCGTGTTATTATTTCATTTACGCCCATCAAAACGTGTTGAGCA
ACTTGCAATGTCAAGAGTGTGTTCGATTTTTGCCCAGTGTAGCTGGCCTTAGTGTGTATGGATGTCAGTG
TAGAGAAGTGTTGGGGCTTAATTAAATATATGTTTCAGTATTTTTTGAAATAATTAACTGTACGCATGTA
AACTATTTCCAAGCAATTGGCATATACACGTTACAAAACTATAAACTGTAACAAAAAATTACCTTTATAT
TGCAGTTTATCTTGTGTACGTGTCAATTTCTTTGAAATAACTTACTAGCGTTTAATTTTTTAAGTGTAAA
TTTCGATTCCAACTAATTGGCGAGCGACTCGAAATTTTGATAATTCAATTAACTTT
>dme_circ_0008602_junction_seq
TCGAAATTTTGATAATTCAATTAACTTTGGTGTATGTATTTAACAAAATTGGTTTG
>dme_circ_0008603
TTAATTGAATTATCAAAATTTCGAGTCGCTCGCCAATTAGTTGGAATCGAAATTTACACTTAAAAAATTA
AACGCTAGTAAGTTATTTCAAAGAAATTGACACGTACACAAGATAAACTGCAATATAAAGGTAATTTTTT
GTTACAGTTTATAGTTTTGTAACGTGTATATGCCAATTGCTTGGAAATAGTTTACATGCGTACAGTTAAT
TATTTCAAAAAATACTGAAACATATATTTAATTAAGCCCCAACACTTCTCTACACTGACATCCATACACA
CTAAGGCCAGCTACACTGGGCAAAAATCGAACACACTCTTGACATTGCAAGTTGCTCAACACGTTTTGAT
GGGCG
>dme_circ_0008603_junction_seq
CAAGTTGCTCAACACGTTTTGATGGGCGTTAATTGAATTATCAAAATTTCGAGTCG
>dme_circ_0008604
TATGTTTTTATTTCTCATGACAAAAATTGTTATAGGAAAGGATAAACGGGCAGCAAGAATAAGTTGATTT
TACAAAGTTAATTGAATTATCAAAATTTCGAGTCGCTCGCCAATTAGTTGGAATCGAAATTTACACTTAA
AAAATTAAACGCTAGTAAGTTATTTCAAAGAAATTGACACGTACACAAGATAAACTGCAATATAAAGG
>dme_circ_0008604_junction_seq

GTACACAAGATAAACTGCAATATAAAGGTATGTTTTTATTTCTCATGACAAAAATT
>dme_circ_0008605
GGAAATTGAGAGTGGCAATAGTCTACGCTGTTTGAAGCAAGCCAAACAGTCGTTCGAGATGaaaacaac
agaaaacaaatacctttttttataaaaaagattcaaaaaattgtaatttatattgcagtataatttttta
ttatgaaaaataaaaaCGAATACGAAAAGTGTTTGGTCAAGAGATGCATAAAACACTTACGACTTTTCTG
TTTTGGTGAAATTGCTTAAATACTAAGTACTCTTAAAAATTTGTTCATGCCTCTTGATTCAAAATGATGA
TAATTTTACAACGCCCGTTCCAAATACCAACATGTACAACATATGAAAATTGTTAATATTTGAATAGTAT
GTTTTTATTTCTCATGACAAAAATTGTTATAGGAAAGGATAAACGGGCAGCAAGAATAAGTTGATTTTAC
AAAGTTAATTGAATTATCAAAATTTCGAGTCGCTCGCCAATTAGTTGGAATCGAAATTTACACTTAAAAA
ATTAAACGCTAG
>dme_circ_0008605_junction_seq
AATTTACACTTAAAAAATTAAACGCTAGGGAAATTGAGAGTGGCAATAGTCTACGC
>dme_circ_0008606
CTTCTCGCACAGCGGCGGTGCCGCTGGTTACAAGATTGCTCCAATGCCGCCACCAATGCAGCCGCCTCCG
CGGCACAACAGCGCCGGAAGCAGTGGCAGTGGTAGCGGCAGCGGCGGATCTCCGCCATCTGACGAGCAGT
CGGCCATACGCAACTGGCGTCAGGATTGTGTTTACGCCCGCAGTTGCGGGATGAACCAGGGACCGGAACA
GCAGCGGCACAACCGTTCGTCGGGTCAGCGTGTCCAGCAGCAGCCGCGGATTGGCAGTGGACGCTTCGCC
CACCTGCAGGCGGCCCAATTGATGGACGAGTTGCCTGATATGAGGATCGGCCTTTCGCCGCCCAGCGAGA
GTGTACTGCTCCAAAGGCGCCTGCGCCAGCAGCAGCGGGCCAACGATCTGTCTGAGTACTGCGAGCCGGC
CACCGCCAGTGGTTAAGCGGGTCCACTGTGGACAGATAACTCTG
>dme_circ_0008606_junction_seq
GCGGGTCCACTGTGGACAGATAACTCTGCTTCTCGCACAGCGGCGGTGCCGCTGGT
>dme_circ_0008607
GAGTTATCTGTCCACAGTGGACCCGCTTAACCACTGGCGGTGGCCGGCTCGCAGTACTCAGACAGATCGT
TGGCCCGCTGCTGCTGGCGCAGGCGCCTTTGGAGCAGTACACTCTCGCTGGGCGGCGAAAGGCCGATCCT
CATATCAGGCAACTCGTCCATCAATTGGGCCGCCTGCAGGTGGGCGAAGCGTCCACTGCCAATCCGCGGC
TGCTGCTGGACACGCTGACCCGACGAACGGTTGTGCCGCTGCTGTTCCG
>dme_circ_0008607_junction_seq
GACGAACGGTTGTGCCGCTGCTGTTCCGGAGTTATCTGTCCACAGTGGACCCGCTT
>dme_circ_0008608
GTTATCTGTCCACAGTGGACCCGCTTAACCACTGGCGGTGGCCGGCTCGCAGTACTCAGACAGATCGTTG
GCCCGCTGCTGCTGGCGCAGGCGCCTTTGGAGCAGTACACTCTCGCTGGGCGGCGAAAGGCCGATCCTCA
TATCAGGCAACTCGTCCATCAATTGGGCCGCCTGCAGGTGGGCGAAGCGTCCACTGCCAATCCGCGGCTG
CTGCTGGACACGCTGACCCGACGAACGGTTGTGCCGCTGCTGTTCCGGTCCCTGGTTCATCCCGCAACTG
CGGGCGTAAACACAATCCTGACGCCAGTTGCGTATGGCCGACTGCTCGTCAGATGGCGGAGATCCGCCGC
TGCCGCTACCACTGCCACTGCTTCCGGCGCTGTTGTGCCGCGGAGGCGGCTGCATTGGTGGCGGCATTGG
AGCAATCTTGTAACCAGCGGCACCGCCGCTGTGCGAGAAGCTCCCCAGGTAATTGCGCTGCTGGTTGCCA
TG
>dme_circ_0008608_junction_seq
CCAGGTAATTGCGCTGCTGGTTGCCATGGTTATCTGTCCACAGTGGACCCGCTTAA
>dme_circ_0008609
GAAGCTCCCCAGGTAATTGCGCTGCTGGTTGCCATGGTGGCCACCGCCGCCTCCGGTGCTAAACGGCGGG
CGATACTTGCGATAGTCCGCTGTCGGCTGCTGTTCCTGCGAGTGATGGGTGCGATAACGCCAGTTTTCGG
T

>dme_circ_0008609_junction_seq
GATGGGTGCGATAACGCCAGTTTTCGGTGAAGCTCCCCAGGTAATTGCGCTGCTGG
>dme_circ_0008610
GAAGCTCCCCAGGTAATTGCGCTGCTGGTTGCCATGGTGGCCACCGCCGCCTCCGGTGCTAAACGGCGGG
CGATACTTGCGATAGTCCGCTGTCGGCTGCTGTTCCTGCGAGTGATGGGTGCGATAACGCCAGTTTTCGG
TGTGCTGCCTCTGAGGAACTTGATCCGCAAGCTG
>dme_circ_0008610_junction_seq
GCCTCTGAGGAACTTGATCCGCAAGCTGGAAGCTCCCCAGGTAATTGCGCTGCTGG
>dme_circ_0008611
GGTAATTGCGCTGCTGGTTGCCATGGTGGCCACCGCCGCCTCCGGTGCTAAACGGCGGGCGATACTTGCG
ATAGTCCGCTGTCGGCTGCTGTTCCTGCGAGTGATGGGTGCGATAACGCCAGTTTTCGGTGTGCTGCCTC
TGAGGAACTTGATCCGCAAGCTGGTAGCGTGGCACAATGTACCGCTGGTGCTGCTGTCGCTGCTTACGAG
TGTAGGCATTGGACGGCAGCTGTTGCTTGCTGTGCTCATGCAGATTCTCCTC
>dme_circ_0008611_junction_seq
GCTTGCTGTGCTCATGCAGATTCTCCTCGGTAATTGCGCTGCTGGTTGCCATGGTG
>dme_circ_0008612
GTTTTCGGTGTGCTGCCTCTGAGGAACTTGATCCGCAAGCTGGTAGCGTGGCACAATGTACCGCTGGTGC
TGCTGTCGCTGCTTACGAGTGTAGGCATTGGACGGCAGCTGTTGCTTGCTGTGCTCATGCAGATTCTCCT
C
>dme_circ_0008612_junction_seq
GCTTGCTGTGCTCATGCAGATTCTCCTCGTTTTCGGTGTGCTGCCTCTGAGGAACT
>dme_circ_0008613
GCTGTTGCTTGCTGTGCTCATGCAGATTCTCCTCGTCATCGTAGTCGTCGTCCTCGTCGATATCGGCATC
CTCGTCGTTCTGATATGCATCGGGGGCAAAGTTAACAGTGCGGCCGGTCTTGCTGTGGTGACCCGCGGTG
TGGGATAGGGCTACAGGCTTCTCCTCTTCCGCTGACATGCCAAAAGTCGTTTGCATTGTTGTCCCCTCAG
CAGTAGTCGTCGGTTTGGTCACGTTCGAATCATCTTCCTCTGGGTTATCGGTTGGTGGGC
>dme_circ_0008613_junction_seq
TCTTCCTCTGGGTTATCGGTTGGTGGGCGCTGTTGCTTGCTGTGCTCATGCAGATT
>dme_circ_0008614
ATGATTCGAACGTGACCAAACCGACGACTACTGCTGAGGGGACAACAATGCAAACGACTTTTGGCATGTC
AGCGGAAGAGGAGAAGCCTGTAGCCCTATCCCACACCGCGGGTCACCACAGCAAGACCGGCCGCACTG
>dme_circ_0008614_junction_seq
GGTCACCACAGCAAGACCGGCCGCACTGATGATTCGAACGTGACCAAACCGACGAC
>dme_circ_0008615
TGCCAGCAGCATCGACACCAGCAGCATCAGCAACACGCCCACCAACCGATAACCCAGAGGAAGATGATTC
GAACGTGACCAAACCGACGACTACTGCTGAGGGGACAACAATGCAAACGACTTTTGGCATG
>dme_circ_0008615_junction_seq
GACAACAATGCAAACGACTTTTGGCATGTGCCAGCAGCATCGACACCAGCAGCATC
>dme_circ_0008616
GTCTCATAAATTTCAGGTAGCTTTTCATTTAAATCACTAATACTATCCAATAGTTTAACATCATCATCTT
CAATTGCATCGCCAGTTTGCCAGTTGTTAATGTGTGTTGTAGTCATAACTGGTGTTTGCAGCTTGTTATT
ATCGGTGATGGTAGTAGCACTAATATCACTTGATGGTCCTTGCTTCTTAGCCCCATCAACATCATCCTGC
T
>dme_circ_0008616_junction_seq

TTCTTAGCCCCATCAACATCATCCTGCTGTCTCATAAATTTCAGGTAGCTTTTCAT
>dme_circ_0008617
ATAATGCAGCAGCAGGATGAGGAGAAGAAAGAGGAAAAACAGCAGGATGATGTTGATGGGGCTAAGAAGC
AAGGACCATCAAGTGATATTAGTGCTACTACCATCACCGATAATAACAAGCTGCAAACACCAGTTATGAC
TACAACACACATTAACAACTGGCAAACTGGCGATGCAATTGAAGATGATGATGTTAAACTATTGGATAGT
ATTAG
>dme_circ_0008617_junction_seq
ATGATGTTAAACTATTGGATAGTATTAGATAATGCAGCAGCAGGATGAGGAGAAGA
>dme_circ_0008618
ATAATGCAGCAGCAGGATGAGGAGAAGAAAGAGGAAAAACAGCAGGATGATGTTGATGGGGCTAAGAAGC
AAGGACCATCAAGTGATATTAGTGCTACTACCATCACCGATAATAACAAGCTGCAAACACCAGTTATGAC
TACAACACACATTAACAACTGGCAAACTGGCGATGCAATTGAAGATGATGATGTTAAACTATTGGATAG
>dme_circ_0008618_junction_seq
AAGATGATGATGTTAAACTATTGGATAGATAATGCAGCAGCAGGATGAGGAGAAGA
>dme_circ_0008619
GTTTAACATCATCATCTTCAATTGCATCGCCAGTTTGCCAGTTGTTAATGTGTGTTGTAGTCATAACTGG
TGTTTGCAGCTTGTTATTATCGGTGATGGTAGTAGCACTAATATCACTTGATGGTCCTTGCTTCTTAGCC
CCATCAACATCATCCTGCTGTTTTTCCTCTTTCTTCTCCTCATCCTGCTGCTGCATTATCTTATTCTCCT
CCGTCTGCGCATCGTTGCGCATTATGGTGAGCTTAGCAAATTCGCTTTGCTGAGTGTTTTTCTTCATATC
CATATTATCCATAATAAGGTCCTCCCTTATATACCCACC
>dme_circ_0008619_junction_seq
TAATAAGGTCCTCCCTTATATACCCACCGTTTAACATCATCATCTTCAATTGCATC
>dme_circ_0008620
ATAATGCAGCAGCAGGATGAGGAGAAGAAAGAGGAAAAACAGCAGGATGATGTTGATGGGGCTAAGAAGC
AAGGACCATCAAGTGATATTAGTGCTACTACCATCACCGATAATAACAAGCTGCAAACACCAGTTATGAC
TACAACACACATTAACAACTGGCAAACTGGCGATGCAATTGAAGATGATGATG
>dme_circ_0008620_junction_seq
ACTGGCGATGCAATTGAAGATGATGATGATAATGCAGCAGCAGGATGAGGAGAAGA
>dme_circ_0008621
AGGGCGACGACTATCTGTATGAGCAGCAGCAGCGCGTCGATGATAAATTCGGGGAAGAGGAGTTCGAGGA
CGAACCAAAGGAGGAAACTCAAGCTGACAATCTAAAGCTATCGAAAGCTTACGTGGAGCAAGTTGGCGAG
ACCAATGTGGAGAAATCGAAGCCACAGGACGATAACGG
>dme_circ_0008621_junction_seq
AGAAATCGAAGCCACAGGACGATAACGGAGGGCGACGACTATCTGTATGAGCAGCA
>dme_circ_0008622
GACCTGCTCCACTCATCATCATCATCGATGTGGCCAAAAGCCTCCAGACCTTCATCGTCGTCATCTTCCT
CGTGGACGTTCAATGTCTTGAAGCCCCCGCTATTCGGATAAATTGCAGATGCATTGCGAGACGCATTTCG
GGAAGACCGACGGCTATTGGCCCGCTGGCCGTGCATCGAGGACATACGCCCTCCGTAGCCGCACAAGGAG
GAAGCGGGATTGCGCTGCGAGCGATTGCGAGT
>dme_circ_0008622_junction_seq
CGGGATTGCGCTGCGAGCGATTGCGAGTGACCTGCTCCACTCATCATCATCATCGA
>dme_circ_0008623
GACCTGCTCCACTCATCATCATCATCGATGTGGCCAAAAGCCTCCAGACCTTCATCGTCGTCATCTTCCT
CGTGGACGTTCAATGTCTTGAAGCCCCCGCTATTCGGATAAATTGCAGATGCATTGCGAGACGCATTTCG

GGAAGACCGACGGCTATTGGCCCGCTGGCCGTGCATCGAGGACATACGCCCTCCGTAGCCGCACAAGGAG
GAAGCGGGATTGCGCTGCGAGCGATTGCGAGTGTAATGACCCGGCGGCTTGGGATTGTAGTTTTCATCGC
TATAGGCATCCATGTAGGCCTGCGATGGCTG
>dme_circ_0008623_junction_seq
AGGCATCCATGTAGGCCTGCGATGGCTGGACCTGCTCCACTCATCATCATCATCGA
>dme_circ_0008624
TCGGCAAGGGAAATCTCGCAGTTTGCCGAATCTGCTATGGCCGTCAAGCGTGTGGTCCTACAGCATTTCT
CGATGCGCTACGATTACATGAAAGAGCGTCCCAACATCTACCAGCCATCGCAGGCCTACATGGATGCCTA
TAGCGATGAAAACTACAATCCCAAGCCGCCGGG
>dme_circ_0008624_junction_seq
ATGAAAACTACAATCCCAAGCCGCCGGGTCGGCAAGGGAAATCTCGCAGTTTGCCG
>dme_circ_0008625
GCAGTTCCTGGCACGTGCGGCAGTTTTCACCGCGATTCCAGCCGCAATCCTCACCACAGTTGCCCGAAAA
TGGCGGATAACCGCAGAGCAGGATGTAAGCAATGACTCCCAGGGACCACAAATCGCACCTCTTATCGTAA
TA
>dme_circ_0008625_junction_seq
ACCACAAATCGCACCTCTTATCGTAATAGCAGTTCCTGGCACGTGCGGCAGTTTTC
>dme_circ_0008626
GGAGTGGCTGCTGGCGACGAGATGTCCGTAGTGAACTTAATCCCCGAACCCAAATCAAAGTCGCAAATCT
TGATTGGGCACAGCGAATCCGTCTTGACACACAGAATATTCTCCGGCTTGAGATCACGATGGGCAATGCC
CTTCTTGTGCAGAAAATCAAGTCCGGAGGCGATCTCCTTGATTATCTGCGAGGCCTCATGCTCGGAGAAG
CAAATGTGCTCCTGTATGCGCGATAGCAATGGACCACCGTTGATCTTCTCGAATACCAG
>dme_circ_0008626_junction_seq
GACCACCGTTGATCTTCTCGAATACCAGGGAGTGGCTGCTGGCGACGAGATGTCCG
>dme_circ_0008627
ATCAACGGTGGTCCATTGCTATCGCGCATACAGGAGCACATTTGCTTCTCCGAGCATGAGGCCTCGCAGA
TAATCAAGGAGATCGCCTCCGGACTTGATTTTCTGCACAAGAAGGGCATTGCCCATCGTGATCTCAAGCC
GGAGAATATTCTGTGTGTCAAGACGGATTCGCTGTGCCCAATCAAGATTTGCGACTTTGATTTGGG
>dme_circ_0008627_junction_seq
CAATCAAGATTTGCGACTTTGATTTGGGATCAACGGTGGTCCATTGCTATCGCGCA
>dme_circ_0008628
GAATATTCTCCGGCTTGAGATCACGATGGGCAATGCCCTTCTTGTGCAGAAAATCAAGTCCGGAGGCGAT
CTCCTTGATTATCTGCGAGGCCTCATGCTCGGAGAAGCAAATGTGCTCCTGTATGCGCGATAGCAATGGA
CCACCGTTGATCTTCTCGAATACCAG
>dme_circ_0008628_junction_seq
GACCACCGTTGATCTTCTCGAATACCAGGAATATTCTCCGGCTTGAGATCACGATG
>dme_circ_0008629
TCCGGGACAGCTGCTTCTGCCGCTGCTGCGAAAGCAAGCAACAACAATAACAACAACCACCCCCGTGGAT
CTGGGGACAGCGGCATCCGGAGTGGCAGCGGCATTTCCTGCAGCAACACGGACAACAGCTGCAGCCAGAG
TCAGTCAGGGTGAGCAATTTGAGTTTCTAGAACTCTGGGAGTTTGACACTAAATTCCCATAGCTGAGAAG
CAAACTGTGATGCATGAGATATATTTTGGAAACCTTCTGAAGAGATTTCGCATATTTGCGCTTCGTGTGT
CACCAGTTTAATTTTAACTAGACAACATGTTAACAGAATGATGAAATAATTGCAAATATTTGCTCTGACT
GTGAATTTGGCAGCTCTTTCGTATAAATTTGAACTTCTACCTATTGGAAATTCACCTGTATCTCTGTCGT
AATATTGTTGTATTGCCTAAATATAAAGTATATTTCGTAGGCAACAGTTATTTGCATGCCAAAAGAAAAC

AAAAGCCTTATTTAAGCATATAATTATTATTTTGAGAATGTGACAAACGTTCTTTGCTTCTTAAAGTTGT
ACCTATAAAATACGATTTGTAGGATATTTCTGCTAAAATGCAATGGTaaaaaaaaGATATACGTTAATGT
AATATTTTGTATCGGAAGTTATACTTCCTCTAAAGTTCCTAACCATATGTATGTATTATTCTGCTTGTCA
TTGGGTGGGGACTGACTACCAATCAGAGAAAAGACCCGACCATTGCTCCTACGCTTTCATTCAGTGGATT
TCTAGTCAGTTATCGGCAAAGCTGGGCCAGGCAGCACTTTCGTTTCGTTTACCCTTGCTAATCAATCGAA
CGAACTAGGGAGCATTGACTGACTGGGCCACGTTGCGCACCGGGTGCCGCAGTGTATTTCCTTGACGTCA
CACGATGTTCGAGAACATGCATGCTGTCTGCCTGCCGGCGCCCAACCCCACCACCCACTCCGCTTCCCTT
CACCCACCCCGCACAGTCTGTGTGGTGTTTACAGTTGTTTTGTTTCGCCAAGAGAACCCCATTGCATCAT
TCGACTGGGGCCATTAAGCTGAATATAAGTGTGTATGTGCCCTAATTTTAGGGGTATTCCAGCATCTGGA
CTTTATAACCATCACTGGGGGTGTTATTATTGTACACAAGTTCTTAATTATATGTGGGATTTGATATATG
CTATAAAATAACAATGACAAAGATCAACTAAATCCACTAATTAGTCATATTCTAACTGGAGGTTAAACTA
GAAGTTTGTTGTTTTCGTTTAGATAGTGATTATAGTCCATACTTTGTGTTTATCAAAGATAATCTTATGA
AACAGGACAGATTGTTGTGTAAATATGCTAGATACGGTTGGGTTGGACCACTGACATCCACTAATTCCAC
TAATATCCTATAAAATCGGGCATAAGTGAGCTGCTTTGAAAAATCATGAAATCCATATGGGTAAACTGCT
GTACAATAGGAATATATTCACACATACATATGTAGATTTCAGCACCAATAGTTACCCTTTGTAATTAGAC
CAGTTGACTGGTATAAATTCGCATAATATGATTATCAATTTAAGCAATACTAGATTCCAAGCACAAAAGG
GTATTCCTTTTCCCTGCCATTCCACCAACACCACCAAAGCTGCCGGCGAAGTGAGAGAGACGGCACTAGT
ATCTGTGCCAGCTAGCCGAGACCGAGAAATTGAAATTGAGCGTGTGCAGGCAGTTATGGAAATGGAAAAT
GACTCATGATGGCTGATCGATTGAGCGTAATGCACTGACACTCGTGTTCAAATGCGTATTCGTGCCCAAA
ATGCAGCGAACGCGGCGAAATGTGAACAAACTACGATTATAACCTTCATGACGTTTATTGCACTTGGTGT
GACGTTCGTTGCTTGGCCTCCCACACATATAAAATGTGTGCGCCCATTAGGAGTTGTGCTGGAGTGGATT
TTGCAGCTGCAATCAACTGAGTGCAAAACGCTGTCAATTGGAGCCCACCAGCTGGAAACTTGTTTTCCCC
CCAACGATCCATAATAATTTTCCATAATTATCGAGAAGTTTTTCATTCTATCGAGGCCTATCGATAGGAG
GACATCTAGTGGTGCCTACGTGCAGCCTCCTCCGATTCATGAAATCAGCCCAATATATCTCTGTCTCCCT
TGCTCGTTTGCGCAAAAGCCCTGAGAGAGAAATTTATTTTTTAGCTGTGGCAATGCCGCAGTGGAAAAAA
CGTCAGCTGAGGCATATGTGTTGTTTACTGTGCAAAACGAAAACAAAGCAAGTTAAATGTAGTTTTTGTT
TTGCTGTTTGCCCTTTCCACTTGTTTGCTTTTAAACAGAACTCGCGCACATCACACCACCTTCATTGGTA
TGCATTGGCATTGGTGTTGGAGTCTGTCTCTCTCCCTCGCACCTCTGCTCCACGTCTTTCACGACATTTT
CTCGGGCATCGCGCTCTCACACTCCAAAACGTACAAAAACAATGAAACAGGGCTCTCTCTGCACTGAAAG
AAAAATAACTTGTTTACTTCAAATTAGCTAAATATGAAAACATTAGGCTGTTCAGTGAAATACGTTTAGT
TTATGTGCCACTGTAAAACTATGCTCTAACCGTTAAAAATCAATCATATGCCACAACCATATTTCTTCCT
GTGTACTCTCTTTTTACAAGAACTTAATTGCATCATGTAACGCTCTCCCGCACCGGTCTCTTCCTGTGCC
GCTCTCTTAAACTTTAGTCTCTCTCTTTCGACCGCTTCTCGgtttgtcgctttgttattgtttttcctgt
tgctgtttttgttttgcttgcGGCGTGTCGGACACAACCACAAAGCGACTTTTTGTCAAAACTGGTTCTG
TGTCCAATATGATGTGGCTTTATCGCCGCTCTCTTTCCGCCGCCGATGCGTTGGTGTGCGTGGGCTTATT
GGCCAGGTTGTGTGATAACACAGGTATCGGTGGAGGTACAGGTATCAGTATCGGTTACCTCCTTAATCTG
GCCTAAACTAGTGTGTCAATATTTACACCCCTCGAAAGACAAGAACTCGATCGGCAAACACATAGCTAAT
GTTTTCTCTTTGCCATCGAGATCTCAGCAAATATGAGTTTGTATCGACCAACTAACTTGACTCGATGAAT
TGACATGGCCGGTAATTGTTGTGCATTCGATTTGAAACCGGATTTTTGCAACCAGAGGCGTTTCTCTTCC
GGGCTGCTTTTTTAATTTGAGTGTAAGCAAAAAGATTGACTTATTGTTTTTGTTGTCCTATGACGAACGA
CACTCGCAATTATTGAGCAGGCTTCTAACTACGTACATATCTATGCTTGTACATATGTATGTACAAAGTA
TGTTTATCGCTACCCTTGCAAACAGCGCATTATAAACATATACATACATATCTATTTGTTACAACTCTCG
CTCTCGTTTCGGCCGCTCTCTTCGCTCGCCGCCCACCGCTCTTTTTTCGCTCTTCGCGCACCGCGGTAGT
TTGGTTTTCAGTTTTTGTCGCTCCCCCGAAAGCGGGCGCGTGTTTGAATTCGATTCTTTCCTCGTTGGCG

CCGCGCGACGTAGGTCCGCAAAGATTTGTCCGTGtttttccatttctttcaaattcatttttttggccaa
ttggtttttgTCGCCGGTACTAATCTCACATAAACGCGAAATAAATCTAATTCGAAATTCCCGGCCATTT
GATCTGAACAATTTTCCCTGTGCTTCCAATAATAACAATCATTCAGCAGTAAATTGTCGTTTTCGTTTTC
GTCGGTCGAAAAGGGAAAAGTGTACTTCAAATCGATGATGATCCAACCGGAATAGGGTTAAAATTAGCCC
AACATCGTAATTAAACAAACAACTAATTCGAAACTAAAGCAATACCATATCGACGGGGAGCAATATATAT
CGAAGGCAGGCGGTTTTATCTACGCATTGCAAAAAACGTGCCGCCCAGAAGAAGAACAAGCAGCAGAACG
GGCGTTGCTTAGACAAATTGCGTTGCGATTAAAAGGCGaacagcagcagcagcaacaacatcagcggcaa
aaggcagcaaacatgttaggtaaatacagcaacaacaacgatagcaacaAAGTCTAGGAACGAGATGAGA
TGAGAGAACTGTCGCACACACTCAGCCCTGCTCACTCACACCCACATAACGTGATGGGCATGTGCATCTG
TATGTGAGGGGAAACGTTTCGAAATGCCGGTTTTTGCATTTTTGTTATTGTTTTAGTTCCAAGTTTCCTC
TACATTTTACAGGCTGCTAAAAGCAATTACATTTTAGGCTTATCTTTAAACAACTGATTTTTAATTTAAA
AATTAAAGTAACTAAAACGACCTTGTATGCATATAATAACTATGGTAATATATTCCCATTTGGTTATTCT
CTAATTTAATTCCTCATCTTATTTTTTATTGTCATACCTTATGTTCTGGTTCTCTCGCCGTTTTCATACA
TTTTTTCGCTTTTACAATTTCATTTGCTGCGCAATAATTTTCTGCTTCTTTACAGCACACAAACACAGGC
GTGCAGATAACGTTCGTGTGTGATGTGTGCAAGAGTGTTAGTGTCTCTGCCTGCCCGTGAATTCATGGTG
TCAGGAGAAGAAGGTGGTTTAAGaaaaaaaaGAGTGAGTTGTTGGGGTGGCATCTCTTCAGCATTCTAGA
CCATTCGCAAGGGAACGTGTAAATAACATGTATATGAAATGTTTAATTAATTTGCGGAGTTACAAAAGAA
TTCTTTTAACTGATTAAAGTGTGTTTTTAGATGTAGGCTGAATAAAATGTTAACGAAATCTAAATTTTTG
AAATTCCAATTAAAATATTTGCATTTTTAATTGAACAATATTAATATACCTACATGTATGTATGTATGTA
TTAAAGCAGTGACTTATTCTTGCTTAGGATTACATTTTCTGCCGTCACAGCTCTTAGATGTTGAGCACCG
CAAATTGGCAACTTATTCGAAGTGCTCTCTGTCGCACTTTCACCCTGCCATGAAGTAACAGAGAGCGAGA
AAGAAGCGGACCCACCTTATCTTCCTCTTCCTCCCTACGAGCCTACTCCCACTCTTTCGGATCCTCTCCA
CCGCTCTCTAAAAGCTCCTTATGTGCGACCGCATGTTGTTTCTCCTTCCCTTATTTTCGTTTCGCTGGCA
TTATTTGAATTTAAATCGGCAAAGTTAAGTGCCAAAAGCAAACCTTTTGCTTTGCTCGCTTTTTGCGTAG
TTCGTCCGTCTTATCAACCTTTTGTTTATTCGTAAATCGAGTGGCTACCGTGTTATTTTGCGTTTCCTAC
CCAGACATTTGATAACTTATTATGGCACTGGCTTCGATGATGTTTGCGCTTAGCCTTAAAATTGAAATTT
CTCAAGGTTTTCTCTAGACGCTTTCGAATCTTGTTTTGTTTTCAGTTTCGTATTATCTGCTAGCTTTTGT
TTTCTTCTTCTGTGTTTTGCACCTGTACGTGAATGTCCTTCATCTCGGTTAAtttttttttAACTTTAAA
GAACCCCAATTCTAATCCCGATTCCAGTTGGAAGTTTTTTAAGCTATTCGTTAAAAAATGTTTTGCATAC
ATATGTATTTATAAATTGCTAAGTACAATGTGCATTGATTCTTTAAAAATACCTTTGGTATTTTTATACA
TATGTACTTCATAGGGAATTTACTTGGTACCGAACTTACATAAAACTTTACATATGCACATGCATAAATA
AGCATCTGAAAAGCCGGCCGGCAAACCATGAAAATATTTATCTAGGTTTTCAAATTCGTATCAGGTTTGT
TTTGACATAAGTTGGTTTGGCTCTGTAACGATTGCCTAGCCGCTGATGAAATGTAGTAAGGGCATTTCAA
TAAATGCCTACTGTCGTCATCACCTTATCGACTTGCACCTGAAGGGATCGGATGATGTGTTATGCCGACG
TCAAGCACGGACTTTTTGTGCCGGAACTGCGGAAATTGTTTATGCAATTTAAACAAAAATACACACGTCT
TCAGAACACTCACACATATTTTGTTCATCAAAACGAAATGATTTGGCGAAAAATAAGATGCATATATACA
TACATTATTAATTAAATGCGCAGAATATTCGGCAGACTTGTGAGAGGGGAGAGTCCATTTCGATAAAAAA
GAGAAATGCAAAAAGTTTGAACGCGTCTGTTGTTTCTGTTGTCTCGGCGTTTATTTACGCAATTGATAAC
AATTAAAGCTGGCCAACAAATAGGCCAAATTTTATATTTAGAACAGAACGGAAAGCGTGCGACGAATCAG
AAAAGCGTGGGTGGGCGAAAGGGGGAGAAGCAAAGTGTTTATATAAATTGGTCATTCATTTAAAAGACGG
ATAGCAAGAAAATGTTTTTTATAAAATGAATTTTACAATGCCATGGGTATAAAAACAAAAACTAAGATTT
CAATTATAGTAATGTAGGAAAAACTGTACCCTAAGAGTACTTGACTCCATAACTCCAATGAAGCAATACT
TATTTACAATGCAACGTGACTTCATTAGTAGTTCCTTGGTCGAGCAGCTATATTTGCCCAAAGATACCGC
CCTGGTGCAGCCGTCTTGGGCTTTATAAATAGTCGCCCGGCTATTGACAACAATTGAAGAACAGGCACAC

ACCCAATCAAGCTGATCGCCGGTCCTCGGCCCCTTGCTCAGTCCCCTGATCTTGGGGTTAGCTCCAGGCC
TAGCTCTGAACGATTCCAACCAGAAGTTTTCTATATAGCAGCGGCCTGCTTGCGGCTGTTTTCTCATTAT
TTAATTTTACATATTTATGCACACTTGCGGCGACTTGTTTACGACACGCGATTTGCTTTCTACGCAGTTA
GTGCAAAAGTACGAGTATTTCTTATTTTATATTCCCTCATTATTATTTTTATACATTGATAAATGCAGAA
AAAATAGGCATGCAAATAAATATGGCGAATGTTTTCGATAAAAAGCTCATACCATACATACATGGTACTT
GTCAAGCCGATCGCACTCCGATATTAAGCTAGCCTATCCGCTTATTACCAGCTAGAATGCAGAAGTTAAA
GGGTTGGAGCGAATTTATTGTAGTTCGAAACTTTTATTTCTGACATTGTCTTATTTGGTTGTTTTTACAG
AACGGACAGAATGAGCTGACGCGCTACAGCAGCGAGGATGTGTCGGGCAACGAGTCCAGCGAGGCGCCCA
ACATGACCGAGGTGGAACGCCAGGCGGAGCTTAACAGGCACAAGGAGGAGATGCAGAAGAAGCGGCGCAA
GAAACGCATCAGCTCCTCCCTTCATTCCTCAACCTTTCAGGGAGCTGTACAAGCTGACTGGCGAGATTCT
TGGCGAGGGAGCCTACGCATCGGTGCAAACTTGCGTTAACATCTACACCGATCTGGAGTATGCCGTGAAG
GTGATCGATAAGATACCGGGGCATGCGCGTGCACGTGTCTTTCGCGAAGTGGAGACATTCCATCACTGCC
AGGGCCATTTGGGCATCTTGCAACTGATTGAATTCTTTGAGGATGACGAGAAGTTCTACCTGGTATTCGA
GAAGATCAACGG
>dme_circ_0008629_junction_seq
TCTACCTGGTATTCGAGAAGATCAACGGTCCGGGACAGCTGCTTCTGCCGCTGCTG
>dme_circ_0008630
GTGATCGATAAGATACCGGGGCATGCGCGTGCACGTGTCTTTCGCGAAGTGGAGACATTCCATCACTGCC
AGGGCCATTTGGGCATCTTGCAACTGATTGAATTCTTTGAGGATGACGAGAAGTTCTACCTGG
>dme_circ_0008630_junction_seq
TTTGAGGATGACGAGAAGTTCTACCTGGGTGATCGATAAGATACCGGGGCATGCGC
>dme_circ_0008631
GCACAAGGAGGAGATGCAGAAGAAGCGGCGCAAGAAACGCATCAGCTCCTCCCTTCATTCCTCAACCTTT
CAGGGTGAGTATGGGGGTTAGAGAATAGCTGACAATGTGCATTTCCTTAATTGGTAATTTCTTCCACCAG
AGCTGTACAAGCTGACTGGCGAGATTCTTGGCGAGGGAGCCTACGCATCGGTGCAAACTTGCGTTAACAT
CTACACCGATCTGGAGTATGCCGTGAAGGTGATCGATAAGATACCGGGGCATGCGCGTGCACGTGTCTTT
CGCGAAGTGGAGACATTCCATCACTGCCAGGGCCATTTGGGCATCTTGCAACTGATTGAATTCTTTGAGG
ATGACGAGAAG
>dme_circ_0008631_junction_seq
GATTGAATTCTTTGAGGATGACGAGAAGGCACAAGGAGGAGATGCAGAAGAAGCGG
>dme_circ_0008632
GCTCCGCCTGGCGTTCCACCTCGGTCATGTTGGGCGCCTCGCTGGACTCGTTGCCCGACACATCCTCGCT
GCTGTAGCGCGTCAGCTCATTCTGTCCGTCTGTAAAAACAACCAAATAAGACAATGTCAGAAATAAAAGT
TTCGAACTACAATAAATTCGCTCCAACCCTTTAACTTCTGCATTCTAGCTGGTAATAAGCGGATAGGCTA
GCTTAATATCGGAGTGCGATCGGCTTGACAAGTACCATGTATGTATGGTATGAGCTTTTTATCGAAAACA
TTCGCCATATTTATTTGCATGCCTATTTTTTCTGCATTTATCAATGTATAAAAATAATAATGAGGGAATA
TAAAATAAGAAATACTCGTACTTTTGCACTAACTGCGTAGAAAGCAAATCGCGTGTCGTAAACAAGTCGC
CGCAAGTGTGCATAAATATGTAAAATTAAATAATGAGAAAACAGCCGCAAGCAGGCCGCTGCTATATAGA
AAACTTCTGGTTGGAATCGTTCAGAGCTAGGCCTGGAGCTAACCCCAAGATCAGGGGACTGAGCAAGGGG
CCGAGGACCGGCGATCAGCTTGATTGGGTGTGTGCCTGTTCTTCAATTGTTGTCAATAGCCGGGCGACTA
TTTATAAAGCCCAAGACGGCTGCACCAGGGCGGTATCTTTGGGCAAATATAGCTGCTCGACCAAGGAACT
ACTAATGAAGTCACGTTGCATTGTAAATAAGTATTGCTTCATTGGAGTTATGGAGTCAAGTACTCTTAGG
GTACAGTTTTTCCTACATTACTATAATTGAAATCTTAGTTTTTGTTTTTATACCCATGGCATTGTAAAAT
TCATTTTATAAAAAACATTTTCTTGCTATCCGTCTTTTAAATGAATGACCAATTTATATAAACACTTTGC

TTCTCCCCCTTTCGCCCACCCACGCTTTTCTGATTCGTCGCACGCTTTCCGTTCTGTTCTAAATATAAAA
TTTGGCCTATTTGTTGGCCAGCTTTAATTGTTATCAATTGCGTAAATAAACGCCGAGACAACAGAAACAA
CAGACGCGTTCAAACTTTTTGCATTTCTCTTTTTTATCGAAATGGACTCTCCCCTCTCACAAGTCTGCCG
AATATTCTGCGCATTTAATTAATAATGTATGTATATATGCATCTTATTTTTCGCCAAATCATTTCGTTTT
GATGAACAAAATATGTGTGAGTGTTCTGAAGACGTGTGTATTTTTGTTTAAATTGCATAAACAATTTCCG
CAGTTCCGGCACAAAAAGTCCGTGCTTGACGTCGGCATAACACATCATCCGATCCCTTCAGGTGCAAGTC
GATAAGGTGATGACGACAGTAGGCATTTATTGAAATGCCCTTACTACATTTCATCAGCGGCTAGGCAATC
GTTACAGAGCCAAACCAACTTATGTCAAAACAAACCTGATACGAATTTGAAAACCTAGATAAATATTTTC
ATGGTTTGCCGGCCGGCTTTTCAGATGCTTATTTATGCATGTGCATATGTAAAGTTTTATGTAAGTTCGG
TACCAAGTAAATTCCCTATGAAGTACATATGTATAAAAATACCAAAGGTATTTTTAAAGAATCAATGCAC
ATTGTACTTAGCAATTTATAAATACATATGTATGCAAAACATTTTTTAACGAATAGCTTAAAAAACTTCC
AACTGGAATCGGGATTAGAATTGGGGTTCTTTAAAGTTaaaaaaaaaTTAACCGAGATGAAGGACATTCA
CGTACAGGTGCAAAACACAGAAGAAGAAAACAAAAGCTAGCAGATAATACGAAACTGAAAACAAAACAAG
ATTCGAAAGCGTCTAGAGAAAACCTTGAGAAATTTCAATTTTAAGGCTAAGCGCAAACATCATCGAAGCC
AGTGCCATAATAAGTTATCAAATGTCTGGGTAGGAAACGCAAAATAACACGGTAGCCACTCGATTTACGA
ATAAACAAAAGGTTGATAAGACGGACGAACTACGCAAAAAGCGAGCAAAGCAAAAGGTTTGCTTTTGGCA
CTTAACTTTGCCGATTTAAATTCAAATAATGCCAGCGAAACGAAAATAAGGGAAGGAGAAACAACATGCG
GTCGCACATAAGGAGCTTTTAGAGAGCGGTGGAGAGGATCCGAAAGAGTGGGAGTAGGCTCGTAGGGAGG
AAGAGGAAGATAAGGTGGGTCCGCTTCTTTCTCGCTCTCTGTTACTTCATGGCAGGGTGAAAGTGCGACA
GAGAGCACTTCGAATAAGTTGCCAATTTGCGGTGCTCAACATCTAAGAGCTGTGACGGCAGAAAATGTAA
TCCTAAGCAAGAATAAGTCACTGCTTTAATACATACATACATACATGTAGGTATATTAATATTGTTCAAT
TAAAAATGCAAATATTTTAATTGGAATTTCAAAAATTTAGATTTCGTTAACATTTTATTCAGCCTACATC
TAAAAACACACTTTAATCAGTTAAAAGAATTCTTTTGTAACTCCGCAAATTAATTAAACATTTCATATAC
ATGTTATTTACACGTTCCCTTGCGAATGGTCTAGAATGCTGAAGAGATGCCACCCCAACAACTCACTCtt
ttttttCTTAAACCACCTTCTTCTCCTGACACCATGAATTCACGGGCAGGCAGAGACACTAACACTCTTG
CACACATCACACACGAACGTTATCTGCACGCCTGTGTTTGTGTGCTGTAAAGAAGCAGAAAATTATTGCG
CAGCAAATGAAATTGTAAAAGCGAAAAAATGTATGAAAACGGCGAGAGAACCAGAACATAAGGTATGACA
ATAAAAAATAAGATGAGGAATTAAATTAGAGAATAACCAAATGGGAATATATTACCATAGTTATTATATG
CATACAAGGTCGTTTTAGTTACTTTAATTTTTAAATTAAAAATCAGTTGTTTAAAGATAAGCCTAAAATG
TAATTGCTTTTAGCAGCCTGTAAAATGTAGAGGAAACTTGGAACTAAAACAATAACAAAAATGCAAAAAC
CGGCATTTCGAAACGTTTCCCCTCACATACAGATGCACATGCCCATCACGTTATGTGGGTGTGAGTGAGC
AGGGCTGAGTGTGTGCGACAGTTCTCTCATCTCATCTCGTTCCTAGACTTtgttgctatcgttgttgttg
ctgtatttacctaacatgtttgctgccttttgccgctgatgttgttgctgctgctgctgttCGCCTTTTA
ATCGCAACGCAATTTGTCTAAGCAACGCCCGTTCTGCTGCTTGTTCTTCTTCTGGGCGGCACGTTTTTTG
CAATGCGTAGATAAAACCGCCTGCCTTCGATATATATTGCTCCCCGTCGATATGGTATTGCTTTAGTTTC
GAATTAGTTGTTTGTTTAATTACGATGTTGGGCTAATTTTAACCCTATTCCGGTTGGATCATCATCGATT
TGAAGTACACTTTTCCCTTTTCGACCGACGAAAACGAAAACGACAATTTACTGCTGAATGATTGTTATTA
TTGGAAGCACAGGGAAAATTGTTCAGATCAAATGGCCGGGAATTTCGAATTAGATTTATTTCGCGTTTAT
GTGAGATTAGTACCGGCGAcaaaaaccaattggccaaaaaaatgaatttgaaagaaatggaaaaaCACGG
ACAAATCTTTGCGGACCTACGTCGCGCGGCGCCAACGAGGAAAGAATCGAATTCAAACACGCGCCCGCTT
TCGGGGGAGCGACAAAAACTGAAAACCAAACTACCGCGGTGCGCGAAGAGCGAAAAAAGAGCGGTGGGCG
GCGAGCGAAGAGAGCGGCCGAAACGAGAGCGAGAGTTGTAACAAATAGATATGTATGTATATGTTTATAA
TGCGCTGTTTGCAAGGGTAGCGATAAACATACTTTGTACATACATATGTACAAGCATAGATATGTACGTA
GTTAGAAGCCTGCTCAATAATTGCGAGTGTCGTTCGTCATAGGACAACAAAAACAATAAGTCAATCTTTT

TGCTTACACTCAAATTAAAAAAGCAGCCCGGAAGAGAAACGCCTCTGGTTGCAAAAATCCGGTTTCAAAT
CGAATGCACAACAATTACCGGCCATGTCAATTCATCGAGTCAAGTTAGTTGGTCGATACAAACTCATATT
TGCTGAGATCTCGATGGCAAAGAGAAAACATTAGCTATGTGTTTGCCGATCGAGTTCTTGTCTTTCGAGG
GGTGTAAATATTGACACACTAGTTTAGGCCAGATTAAGGAGGTAACCGATACTGATACCTGTACCTCCAC
CGATACCTGTGTTATCACACAACCTGGCCAATAAGCCCACGCACACCAACGCATCGGCGGCGGAAAGAGA
GCGGCGATAAAGCCACATCATATTGGACACAGAACCAGTTTTGACAAAAAGTCGCTTTGTGGTTGTGTCC
GACACGCCgcaagcaaaacaaaaacagcaacaggaaaaacaataacaaagcgacaaacCGAGAAGCGGTC
GAAAGAGAGAGACTAAAGTTTAAGAGAGCGGCACAGGAAGAGACCGGTGCGGGAGAGCGTTACATGATGC
AATTAAGTTCTTGTAAAAAGAGAGTACACAGGAAGAAATATGGTTGTGGCATATGATTGATTTTTAACGG
TTAGAGCATAGTTTTACAGTGGCACATAAACTAAACGTATTTCACTGAACAGCCTAATGTTTTCATATTT
AGCTAATTTGAAGTAAACAAGTTATTTTTCTTTCAGTGCAGAGAGAGCCCTGTTTCATTGTTTTTGTACG
TTTTGGAGTGTGAGAGCGCGATGCCCGAGAAAATGTCGTGAAAGACGTGGAGCAGAGGTGCGAGGGAGAG
AGACAGACTCCAACACCAATGCCAATGCATACCAATGAAGGTGGTGTGATGTGCGCGAGTTCTGTTTAAA
AGCAAACAAGTGGAAAGGGCAAACAGCAAAACAAAAACTACATTTAACTTGCTTTGTTTTCGTTTTGCAC
AGTAAACAACACATATGCCTCAGCTGACGTTTTTTCCACTGCGGCATTGCCACAGCTAAAAAATAAATTT
CTCTCTCAGGGCTTTTGCGCAAACGAGCAAGGGAGACAGAGATATATTGGGCTGATTTCATGAATCGGAG
GAGGCTGCACGTAGGCACCACTAGATGTCCTCCTATCGATAGGCCTCGATAGAATGAAAAACTTCTCGAT
AATTATGGAAAATTATTATGGATCGTTGGGGGGAAAACAAGTTTCCAGCTGGTGGGCTCCAATTGACAGC
GTTTTGCACTCAGTTGATTGCAGCTGCAAAATCCACTCCAGCACAACTCCTAATGGGCGCACACATTTTA
TATGTGTGGGAGGCCAAGCAACGAACGTCACACCAAGTGCAATAAACGTCATGAAGGTTATAATCGTAGT
TTGTTCACATTTCGCCGCGTTCGCTGCATTTTGGGCACGAATACGCATTTGAACACGAGTGTCAGTGCAT
TACGCTCAATCGATCAGCCATCATGAGTCATTTTCCATTTCCATAACTGCCTGCACACGCTCAATTTCAA
TTTCTCGGTCTCGGCTAGCTGGCACAGATACTAGTGCCGTCTCTCTCACTTCGCCGGCAGCTTTGGTGGT
GTTGGTGGAATGGCAGGGAAAAGGAATACCCTTTTGTGCTTGGAATCTAGTATTGCTTAAATTGATAATC
ATATTATGCGAATTTATACCAGTCAACTGGTCTAATTACAAAGGGTAACTATTGGTGCTGAAATCTACAT
ATGTATGTGTGAATATATTCCTATTGTACAGCAGTTTACCCATATGGATTTCATGATTTTTCAAAGCAGC
TCACTTATGCCCGATTTTATAGGATATTAGTGGAATTAGTGGATGTCAGTGGTCCAACCCAACCGTATCT
AGCATATTTACACAACAATCTGTCCTGTTTCATAAGATTATCTTTGATAAACACAAAGTATGGACTATAA
TCACTATCTAAACGAAAACAACAAACTTCTAGTTTAACCTCCAGTTAGAATATGACTAATTAGTGGATTT
AGTTGATCTTTGTCATTGTTATTTTATAGCATATATCAAATCCCACATATAATTAAGAACTTGTGTACAA
TAATAACACCCCCAGTGATGGTTATAAAGTCCAGATGCTGGAATACCCCTAAAATTAGGGCACATACACA
CTTATATTCAGCTTAATGGCCCCAGTCGAATGATGCAATGGGGTTCTCTTGGCGAAACAAAACAACTGTA
AACACCACACAGACTGTGCGGGGTGGGTGAAGGGAAGCGGAGTGGGTGGTGGGGTTGGGCGCCGGCAGGC
AGACAGCATGCATGTTCTCGAACATCGTGTGACGTCAAGGAAATACACTGCGGCACCCGGTGCGCAACGT
GGCCCAGTCAGTCAATGCTCCCTAGTTCGTTCGATTGATTAGCAAGGGTAAACGAAACGAAAGTGCTGCC
TGGCCCAGCTTTGCCGATAACTGACTAGAAATCCACTGAATGAAAGCGTAGGAGCAATGGTCGGGTCTTT
TCTCTGATTGGTAGTCAGTCCCCACCCAATGACAAGCAGAATAATACATACATATGGTTAGGAACTTTAG
AGGAAGTATAACTTCCGATACAAAATATTACATTAACGTATATCttttttttACCATTGCATTTTAGCAG
AAATATCCTACAAATCGTATTTTATAGGTACAACTTTAAGAAGCAAAGAACGTTTGTCACATTCTCAAAA
TAATAATTATATGCTTAAATAAGGCTTTTGTTTTCTTTTGGCATGCAAATAACTGTTGCCTACGAAATAT
ACTTTATATTTAGGCAATACAACAATATTACGACAGAGATACAGGTGAATTTCCAATAGGTAGAAGTTCA
AATTTATACGAAAGAGCTGCCAAATTCACAGTCAGAGCAAATATTTGCAATTATTTCATCATTCTGTTAA
CATGTTGTCTAGTTAAAATTAAACTGGTGACACACGAAGCGCAAATATGCGAAATCTCTTCAGAAGGTTT
CCAAAATATATCTCATGCATCACAGTTTGCTTCTCAGCTATGGGAATTTAGTGTCAAACTCCCAGAGTTC

TAGAAACTCAAATTGCTCACCTGACTGACTCTGGCTGCAGCTGTTGTCCGTGTTGCTGCAGGAAATGCCG
CTGCCACTCCGGATGCCGCTGTCCCCAGATCCACGGGGGTGGTTGTTGTTATT
>dme_circ_0008632_junction_seq
CAGATCCACGGGGGTGGTTGTTGTTATTGCTCCGCCTGGCGTTCCACCTCGGTCAT
>dme_circ_0008633
TTAATATGTCTGCAACCGATTTAATTATATATGTGCAATTTATAATCCTTTTGATTTGCCTTTTAGTTGC
CCTCACGGCGCATTTGTTCACTACATTTAATGCTAAATGTGTTTTCCCCGCCGAGACAACGGGGGAAATT
GAATTCTTATTGAATTATGCACACTTTTAAGGAAGTAAGAAGCTCTACAAGCGAAATTTGTACTTCTAGT
CTTG
>dme_circ_0008633_junction_seq
ACAAGCGAAATTTGTACTTCTAGTCTTGTTAATATGTCTGCAACCGATTTAATTAT
>dme_circ_0008634
GTGTCTTCCGAGTGAGAGCTATGGGTGCACTTCTTCTGCTGTAATCCACCCATTCTGACCGTTCCATCAA
GATCCGTACCAAGGGAACTCGTTTCCTCCTCGGAGTGGCTGGAATGACTGCACTTCTTGCAGCTTGCTCC
TCCTCCACCGCTGCTCAAACGAGCTTCGTAGTTTTTCCGCACTGTGG
>dme_circ_0008634_junction_seq
CGAGCTTCGTAGTTTTTCCGCACTGTGGGTGTCTTCCGAGTGAGAGCTATGGGTGC
>dme_circ_0008635
GAAGTGATGGCTTGAGGTGCGCGCCGTTGTGGCCGCGCTGGAAGCGATGCTTGCCGTGTCGCCCTGCGAC
ATATTCACATTCGGCGGTCCAACAGCTCCATCTATGGCCTTGTACTTTGTGCAGTTCTTCATGGAGTGCT
CCGTGCACTTCTCGTGATAGGAGTTTCCGCAGTCCATGCACCGGTAGCCAACCGGTCCCCAGAGAAGCTT
GGTGCAGTGATTACAGTTGGTGGGCGTAGTGTATGGATGCTTCTCGAAACGATGCGGGTGGAAGTACTTG
TCCTGGTAACCTGCCAGACGACCCTTCATGATGATCTCCAGTGTAGAGCGTTT
>dme_circ_0008635_junction_seq
TCATGATGATCTCCAGTGTAGAGCGTTTGAAGTGATGGCTTGAGGTGCGCGCCGTT
>dme_circ_0008636
GGTACAGCACCGATATCTTTTTCTCCTTTAGCAGGGAGGCAATCGGAAAAGTGCGAACAATCACCTGCTC
ACAGAAGAGGGGATCGCAAGGTGAACCCTTAAAAATCACACGGTAGTTGGTGAGGAAAAGAGCTCCCTCG
GCGGGTATCAGGCACTGGGTTTCATCTTCGCGTCCATCCGGCATAAGGAAGCAACGCAGGTGGTCTGTCA
CCAG
>dme_circ_0008636_junction_seq
GGAAGCAACGCAGGTGGTCTGTCACCAGGGTACAGCACCGATATCTTTTTCTCCTT
>dme_circ_0008637
ATGTATGGTCGCTATACCAAAGATCTAGGAGAATTTGCCAAGGATGAAGCCAGAAAGCTCAAAATACTCG
AAAAGCGACGAAAGCAGGAAGATAAGCAAAGGAATAAGGTAAACTTAAAATGAACGCTGAACTAATAACT
TATAATTTACCCGTGAAATACAATGTTATAAATGCTCTTGTGATTTACTAAAATGCCAACTAAATGAGAA
AACGAAATATTCAAGGTATGCGCACAGCTATTTGGCTAAGTAGATTTGAATGAATTGTGTAGAGTCTTCG
AGTAGACCGTAATTTCCTTTGATATCCGACCGAACGTATCAGTTTATAAAGCAGCAGTGCTGGGAAATAT
TTACAGGGAACTGCTGGGCAAACATTCGACCCGGGCGAAGCGGGTATCATCATGGATCTGGAGGCACACG
GTGGCCCGACTGGGCGAGGATTGGGTATTCCTGGCCCTACTGGGCATCATTATGGCCCTGCTCTCGTTCA
TCATGGACAAGGGCATATCCATATGTACAAACGGCGCGAATTTGGCTGTATCGCGATCTGACGTCACAGC
CTTTTGTTCAGTACATCGCATGGGTCTCACTGCCGGTCTGTTTGATATTGTTCTCAGCCGGCTTTGTCCA
TCTCATCGCACCGCAAAGTATAGG
>dme_circ_0008637_junction_seq

TCCATCTCATCGCACCGCAAAGTATAGGATGTATGGTCGCTATACCAAAGATCTAG
>dme_circ_0008638
ATCGCCGTCCCGTTCCGCCTTGGTGACACCCACTTTGATAAGGAGATAATTAAAGAGTAATACATGTTCC
CGATTTGCCTTTCGTCTGGGCCATTGCTTTGCGTTTTGCCGGTGTTAATTAAGCCTTCACACACTTAAGT
GTGCCACTTGAAATGTACGCATGAGCACTGTCTTATCGCCAGTCGGATTCGGCCAACGGAGTAGACAAAA
GTAAAAAGTGATCTCACAGATAACAGAAAATTCCGAATCGGATTCGGATTCAGtatcagtaacggcagca
gcaacaacagcagcagcattttcccatctgaagtcacaatcgcagtgcatcggcagcagcaTATTCATAA
GAAATCTTTGGCCGCAGCCACATCGGCGGATCAACTCCACGCCACTGGCCAGTGGCCATTGAAGGGAAGT
CGGTCTTGTGACCACGACGTGGTGCTGCAGCTAATTGAATTTAATGCACAAATGAGCCTAAGGAATTTGC
TTTCATAAGTCTGACGAATGCAATTCCTTTACTGCGCCACTTGCATTGTCATCTGTCATCGTCGAGATAT
TTCACTTCCGAGTCGCACACCACTGCCGTCCCAAGATGAAAGCGACCAGGAACGGCGGCCAGCTGCTCAT
CGCTCCTTCGCCGCAGACTGCTGCCCGTCTGCTGCCCCTGCGCACCTACTCCAACGCCTCCTCGCTGGGC
CACCACCATGACAGGAGCACCCTGTCCGACGGCGACGAGGAGGAGGGCGGCCTTGGCTACACCCACACGC
TGGGTGAGTAAAGTACCAGGTTCTAATCATATTCGATTAGCCTTAAAACCACTGCATTGTTTTGATGCTC
ATGCAACTATTCAATAATATGAATAAAGTCATATTTTAAGGCTCACCATATAGTTTATTAACAATTTTGT
AATCGAAACCTCATGTACAGCAGAAACCATCGTATAGAGATTCAAATGTCATTACCTGAGATTTTATAAC
TAGCTAATTTTACCTGAGATGGATGTCACATTCACTTAATATCACTACATAGACATTCATCATTTTATAT
AGTATCGCGGTTTTAAATTCACGTCTGTTGCTTGCGGTTAGACACATTAAGTTCTATATATTGTTTTATT
ACAAAGTGATTAAAGGCACTTTCAACGACAAGTCTTAGCACAAAGAAATTTAAAATGTAATAATTTCCCT
TCGACTATTCATTTCTCTAGCTTCGGAAGCCATAAATTGAGTCCATAAAGTTTTATGGGGGATGGCGTGA
AAATTTACCTGTTATCATAAACATTTTATTGATCGAGAATTGATTGATTGTTTTACAATGTCAATCGGAT
TCATGAGCATAAAGTTTATTATATACCAAAAGTAGAGCCAAATGCTAATCTATTAAGTGGGGTAAAAAAG
CCAAACAAATGTTGACCAAAAATTAAACGATTCGATTAATAAGAAAGCCGTAAATACCCGTGCTAATAGG
AAAATCCATTTATCTATTTAAGGCCCACAATTGTGTTTGCATTAGAACGCCTACGACGACCCAGTTTGTT
TGTTATTAATTGttttttttttATCGTAATTGCTTGGCTGGAAATCCCGAGTTGATAATAAATTGCCGT
CTACTGTATTTTTATTTACATAAACACGTCGATATGGCGCTTAGATTTGGCAATTCCGACCTTGTATTAG
ATGGTGTTGTTTATATAAGGGGATTGCTCGTTTTTAATTAAAATAATCTAATTAATGTTTATAAAAAGTA
ATATATTGCTTTAAAGAACGTATTTCTCATACGCCAAGAGATTTTTCGCGAGGGTATAATTAAGTTCGAT
ACGCCTTTACACACATGTTTCTTGTTTTACTTTTTCCGCTCATGTGATTAACATGTGACTATTTGCTTGT
TTTAACTTTATTTGCTTCGCTTTTTTCTTGATTAATTTTTGTCATGTGCGGTGACACATGTTGCTCAACA
TAGCAATGAGATGATAATGATATTAAGCTGTAGGATAATTTGCAAGCATTTTGTTGAGAAGTTCCAAAAC
TCCCACGGGCCAGACAAAAGCTTCACCTTGACTTCTCAAATCTATTCTCAACCAGATTGTGTATTGATTT
GGTTTTTTCTATACTCCAGATATATCCAACGCAAACCAAATCATTAAAAATTTGTTCAGACACGGCAATT
AACACCTTGTGCAAAAATGAATGTAGAGAATGTACTCCATTATATTGTATATTGAAACCGTAGAAGACGC
TTAACTATGGGATCGTTTCTCTTGTCAGCTGTAATTTAGAACTACTGACTCAAACATGCAATTAGTTACT
ACTACTAGTCTAGGTGGTAACTTTTTATTTAATGGCAATTAATGACTGCCTCAAAGAAGGCATTATCCAC
TTGGATTTGACGGGCTCTTATCGGCGCAATATTAAACCATCCATCAAAATCGAATCGTTATCACTAATCA
CTTAATATTATTTGACCGTTTCAGAATTTCCCATAAAAAAATGTCCATTACTTTGGTGCTAGCTCTATAG
ATGATGCTTTAATAGGTCATAGAGATATACTTTCTTGCTATGGTCATAAAGTTACTAAGCCAAATTTATA
TTTCTTTATAGCCAGTTATTTACACTTTGGCCCTAACTCTTTATCTTTTTGCCATGGATATAAGAAATTT
ACGGTCACATGCACCCAGAAGCTAAGCACGATATTTACTTAAAGATTTTGTTCAATTTTTAATTTCGTTC
GGCTGCTTCTGTCCCCCATCCTGAATAATTTCTAACCGCGCCTCAGGTGTTGTTGCTATTTGGACCCTTA
TCGCGGATTTTGCGGCTCGAAAGGTGTTTCTAGCACCATCTGAGATGCTGGCTCTCCAGTCGCCACACGG
TTTTAGCCAAGATCACTTTGTTTTATCGCAAGCTTGCTAATTTCTTTCATATGAGTTCGAAAATGGCGTT

TCACAAAAAAGTACGTAAATTATCATTTCATTCGGACAAATATCTCTGCAACTGTTTTTGCGCCATGAAA
CACAAAAGACAAACTTAGGGAATAATTATTATACTAAAGTTAATTATATGCGTTGTGACAGATGGTTTCC
TTGATTCGGAATGTTTGAATTTATGGTAAATATCTTTCAAGACCCATTATGTCAATTCAAAATGTTCAAC
TAAAACAAATTTGAATCGGACTATGTGATGAGATGAATTATGTAAATATAAACAATAATATAAGATATTA
AGATACAATCATACAAATACAACTATGAGAACTACTTATAAATTAACAGACACAAAATGCCGCACTGCTT
AATGGGTCAAGCAGTGCGCTGATATCTAACAGCTCGCGACAAAAGATAAGCTTTCAGTCTCTttataccc
gttactcgtaatttaaaagggtatactagacttcttaagatgaaaacgtttccgaccatagacctagtat
atatactcttgatcaggatcaatagccgtatcgatctggccctacccgtctgtttttttccgttcgtacga
acgtcgagatctccgaaactataaaagctaaaagtttgagattaagtatacagattctagaaccataaac
atgtaacatgttcccacgtccCCatttattaattttaatattttttttcattttCCACCGATATTACAGAA
AATGTTGAAATCTTTCACTTTTCCAGCTGagtgacagacagtgacagtgacagtgacagtgacagtcagA
TACCCatctgatagtacctcgaccatagcattctctcttgtttTTAATAGGTTTCTCTTAAATTCCTATT
CCTATAGCAATTTCCCGAAAGTCATTAAAGTTCAAGATTATGGTCTGAGTACTCGATTTACGCGCAGACC
AACAATCTATACCTATTTAGGGTTGCCATTATCATTATCGCAAATTAGGGGTAGAAGTTGTAGATTGACT
AAGACAATACGGGCCAGAAGAGCCGGTACACTTTAATACTTAAATGTGATAAATCCAACGGATTCTTGAA
GACATGTGACCCAATGCCTAGCCTACACATTGAGACTATTAAAGCAAGTAATCGCAATTTATGCGGCTTA
AGTGATCCGTCACAGTGGGTCGAGCCATGCCAATATCTTGCAAGAAAGCTGGAGTGACTAATGAGCATCA
GTCATTATTTTCTAGAAAGAAAATAGTGAGGATTATTACTTTTCAGTGCATAATTGGCAATAGCAATGGA
ATATCTACATATTTAAAAACAATTTTCAATGGCAATCCTCGATGGAACCTATGATTGACCTAGACAATAA
GACCATGCCCACTGTGCACCGCACTTCGTGTCATCCAAGCGCTTGGCATTTTCACTTTTTTGCCGTTGGA
CCCGTCCACAGATTATTAAATTTATGAAATTCTATTGATGTTTACACGCAAACTTTGACCGCTCGAGAGT
CAGTGGATGGATATACTACATACAAATGTGGGAACTATATGTACAAGTATCCGCAAACTTGAGACTGCTG
AAAGAGCAGACGAGTGCGTCGGTGTCTGTGTACTAAATTTAATGGAATCTTGAATTTAAGCGGGACTGGA
GCGTTCTATCTTCATGACACTATTCAGTTTGACTTTAGACACTGCTCGACTCACATGCAGTCGGGAATTG
AATTCGCAAACGGATTTCAAATACGGGAAATGAGCAAAAATATCTGTGCCATGGATGTGACGAAACCGTA
TCGATCCCATCTTTGATACGAGTAGTGCGCCATCGATTGCGTTCAACTGCTATAAACCCATTAAAAACCC
ATTTGAACTGACAACAGCACAGTATGGTTTATTTCGGCGATCGGCAACGCGATCGCAATCGCGATCGGAG
TAATCAGAAAGTCGAGCGAATCATTCACGATGAAGAGTTTGGCGAGGAGAACGTCGAGTTGGTGGACTCG
GAGTGGGCGGACTTCGAGAAGTTCATTTGCCAGTTGCGCAAGCGGCGGAACAGTGCTATGTCCATGGAGG
AGGAACTGCGTCACGTGCAGCGGCATCCGAAGATCAAGTCGCACGCCTTCTATCCGTGCCCGCCGCCAGC
TGAAAATGCTCGGGATTCGGACTCCTCGGACGATGATGATCCCATCGGATACATCGATACTCTTG
>dme_circ_0008638_junction_seq
GATCCCATCGGATACATCGATACTCTTGATCGCCGTCCCGTTCCGCCTTGGTGACA
>dme_circ_0008639
ATCGCCGTCCCGTTCCGCCTTGGTGACACCCACTTTGATAAGGAGATAATTAAAGAGTAATACATGTTCC
CGATTTGCCTTTCGTCTGGGCCATTGCTTTGCGTTTTGCCGGTGTTAATTAAGCCTTCACACACTTAAGT
GTGCCACTTGAAATGTACGCATGAGCACTGTCTTATCGCCAGTCGGATTCGGCCAACGGAGTAGACAAAA
GTAAAAAGTGATCTCACAGATAACAGAAAATTCCGAATCGGATTCGGATTCAGtatcagtaacggcagca
gcaacaacagcagcagcattttcccatctgaagtcacaatcgcagtgcatcggcagcagcaTATTCATAA
GAAATCTTTGGCCGCAGCCACATCGGCGGATCAACTCCACGCCACTGGCCAGTGGCCATTGAAGGGAAGT
CGGTCTTGTGACCACGACGTGGTGCTGCAGCTAATTGAATTTAATGCACAAATGAGCCTAAGGAATTTGC
TTTCATAAGTCTGACGAATGCAATTCCTTTACTGCGCCACTTGCATTGTCATCTGTCATCGTCGAGATAT
TTCACTTCCGAGTCGCACACCACTGCCGTCCCAAGATGAAAGCGACCAGGAACGGCGGCCAGCTGCTCAT
CGCTCCTTCGCCGCAGACTGCTGCCCGTCTGCTGCCCCTGCGCACCTACTCCAACGCCTCCTCGCTGGGC

CACCACCATGACAGGAGCACCCTGTCCGACGGCGACGAGGAGGAGGGCGGCCTTGGCTACACCCACACGC
TGG
>dme_circ_0008639_junction_seq
GGCGGCCTTGGCTACACCCACACGCTGGATCGCCGTCCCGTTCCGCCTTGGTGACA
>dme_circ_0008640
GTATATTGAGATATTCCGCAGCTCGATTGCTGAAATGAAGAGGGCCACAGGCGCCGGTGGCGGTGTCGGA
GGACGCCCTGGCCCTTATGACATACGTGATCGTGGTGCCAACCGTGGTGGCAATGATTTCGGCGGAGGGC
GCAATGGATTGGGGCAACAATGGCAACTTTGGCGTTGGTGCCAACAACATGCTGGGCTTCAACAATCTGC
CAAGCCTGATGAACTCGGGCAACTTTGGAAACAACCAAGGTGGAAATAATGGAAATTTTGGAAATAACTC
TGGACCCAGCAATTTCGGCAACTTTGGTGGCGGCAACAACGGTGGAAATTCCGGCAACTTTGGAAACGAC
AGTGGTAACAGCGGAAACTTTGGAAACTTCGGCAATAATGGAGGCGGAAACTTCGGCGGCAACAACAACG
GCGGCGGTGGTTTCAACAGTGGCAACAACTTCAATTCTCCAGGTGGCGTTAATAATTTCGGCAACAATGG
CGGCTCCAATTTTGGCGGCAATGGTGGAGGAGGTTTCAATAACGGCGGAAACTTCGTATCCTCGAGTGGT
GTTGGCAACTTTGGCCCGATCGGCGGCGGTCGCAACAATAACAACGGAAATTTCGGAAACTCTGGATTCG
GTAACTTTGGAGGAAACAACAATGTCGGCTCGAACTTCGGAGGAGGCAACAACGGCGGTGGCGGCGGATT
CAACAATGGCTCGAACTTCGGCGGCGGCAACTCAATGGGCGGTGGTGGCAATTTCGGTCCCATCGGCGGC
GGTCGGGGCAACGACATCGAGTATTATACAATCCACATGCGCGGACTTCCCTACACTTCATTTGAAAACG
ACGTTTTTAAGG
>dme_circ_0008640_junction_seq
ACTTCATTTGAAAACGACGTTTTTAAGGGTATATTGAGATATTCCGCAGCTCGATT
>dme_circ_0008641
GTAGCTAGTGTCGCTGTAACGTGTATATTGTGTTCTGTGTTTTTAAACTAGTGTTCTCCGCTTAGGCATC
GTACACGGACTTCAGGGCCAATTCGGGCTTGTCGTAGCCCAGTTCCTCGATGGTTGGGATGCCCAGCTCC
TGCAGTGTGGGGGTGATCTTCTCCAGCAAGTAGGGATAGAGGG
>dme_circ_0008641_junction_seq
ATCTTCTCCAGCAAGTAGGGATAGAGGGGTAGCTAGTGTCGCTGTAACGTGTATAT
>dme_circ_0008642
GAGTAATGGTGCGATCCCACATGCCCGGCAGCGTGCAGATACGGATGTGCTCGGCTCCGTCGAACACCAA
CCACTCATACACCTCGTCCGAGACGCACAGCACATTCCACTTGCGGCAGAGCTCGGCTATCCTCTCTAGC
TCTTTCCGGTTGAAGACCTTTCCAATAGGGTTGTGGGGCGT
>dme_circ_0008642_junction_seq
AGACCTTTCCAATAGGGTTGTGGGGCGTGAGTAATGGTGCGATCCCACATGCCCGG
>dme_circ_0008643
GCTTCCGAAGAGCCTCCGGAAAAAAGAGCAGCTAAGGAAGAGCCATTGTCCAAAAAGAAAACAACAGAAG
CCTTTAAGCCCACCCCTATTCTCAACGAAATAAACGTGGACGACACGGAGATGGGTCAAAGGGAACAAAT
TGAAGAGGAAGCTACTGATCAAGACAAAACCCAGGAGTCGGCTCCAGCTGAAGAGTCTTAGTTATT
>dme_circ_0008643_junction_seq
CGGCTCCAGCTGAAGAGTCTTAGTTATTGCTTCCGAAGAGCCTCCGGAAAAAAGAG
>dme_circ_0008644
GATCGGGCACCATGGAAAGCACAACGAAAGCGATTATAACTGTACTAAGGGTTAACCGCCATAAGCCGGA
GTTCGTCATACCCGCGCTGTCCAATGCCACCATTGAGATACCTGGTGATATTGTCCAGCCCGATTATCTA
CTGCTAACCG
>dme_circ_0008644_junction_seq
GTCCAGCCCGATTATCTACTGCTAACCGGATCGGGCACCATGGAAAGCACAACGAA

>dme_circ_0008645
ACACCAATCCACGGCTATCCTCCACGGCAACGATTACCGTTAGTGTTTTGGATGCCAACGACAATAAGCC
AGTTTTCGAGCAGGAGAGCTATTCTGCATCTGTTTCGGAGGCGGCGCTTCCGGGTCAGTATATCGCCACC
ATTACGGCTCGGGATGTGGACTCCGGAAGTTATGGTGACTCTGGCATTCGGTACAGCTTATCCGGAACCG
GTGCTGAACTTTTCCATGTCAATGAGCAGACAGG
>dme_circ_0008645_junction_seq
AACTTTTCCATGTCAATGAGCAGACAGGACACCAATCCACGGCTATCCTCCACGGC
>dme_circ_0008646
GATACATATAGTTCACATAAATGACGACACTGGACAATTGTTTACTAGGTTTTCAAGCGGATTCAAAATG
TATGTTTCTTGTGTACGTGTTTGCTGAGCCACGAACTACTACAAATATTAATTTTCGTTTAGGCATTGGT
TGAGTTTAGTTTTACAGGACTGGGTTTGCGCGCATCTAAAATGAAACTATTTGACATTGCAATCGCTTGA
AATG
>dme_circ_0008646_junction_seq
CTATTTGACATTGCAATCGCTTGAAATGGATACATATAGTTCACATAAATGACGAC
>dme_circ_0008647
GTGTGGTTAGTGTTTTGCTTGATACTTGCTTAACATGGTTGACCTCAACATCTGGCGTGGTCATGGAGGA
TTGCTGGCGCTCTAGTTGCCGCTGCTTTTCCCGCTCCATTTTCTCCAGGCCCTCTCTGAAAGATTAAGGA
AATTATTACAAGTATCTATCTTTTGTGCATCTTATTGTGGTTCTTTGCACACTCATACCTTATCCAAGCT
GGCAACATTTTCCGTTTGTTTGCATCGATCTCACTGCTAGCCCCGCTACTTCCGCTTTCCGCATGCGTCT
GCTGTGTGTGCTGTGGCGGCAGGTGTGTATGTGGTGTTAGGTGGTGTGGATGTGCATGCGGCTGATGTGC
TGGTGGATGG
>dme_circ_0008647_junction_seq
TGCATGCGGCTGATGTGCTGGTGGATGGGTGTGGTTAGTGTTTTGCTTGATACTTG
>dme_circ_0008648
GTCTACGGTAGCAGCGCTGACCTCAACGTGGCCGTCGATGACGGAGACGCGTCGTTTAACGGAGACGAGA
ATCATAAGGTGTGGAACGGTTCGCAGGACCAGTTGGACAGCATCGGGCCATTAGAGAGTCCCTATGAGTT
GTTTGCGGGAAACACCTCCACCTTGGGAAGGCCTCTACGGGCGCGTCAAATCAGCACGCCCATCGACGTG
CCACCGCCACCAGTGAAGGATGAGCGAAAGACGAAGCGGGACAAAAAGTTAACCAAGTCTAGTGGCAGCC
AAAGCCTTTCCGGCACCCTTATCCGGCCAAAGCCCGTACACCCTGCGCCGCAGCATCGCTCAGGATTCCA
AGGGCCGTCGGGACCTGGGAGCGTGACCTATGGGCACGTTTCTGGACATGGCCTTCACGCCGCCCGTTAC
CATACAATTAGTCATCGCGGCATTCCCCCGGGTTCACACAGCCACCTCACCCACCACCCACCGCCCCACC
CTTCACAGAATCAGGTCCATATGATGCACCACCCCCACATTGGGGGACTACCGCCCATGCAGATTCCCGT
GATGATGCCGCAGCAGTACGCGACTCTTCAGCCGTCGCGCTCAACTGGCAAGAAGAAGAAAAAGGATAAA
AAGAGTGGCGCGGGCGGAGGCGTTCCCGTGGGCATGCCCATAGTACCTCCCATATACGCCTTTCAGCAAC
AGGTCGTGGGTGTTCCAGCTCCCCAATTAGCACAGTCCCTAATCGGAGAGACGCGACCTCTGGGTCATTC
CAGCCGGAAGCTGGCGGCATCAATGGGGAACGGCCTAGACGACTCCGGTAATTCTGGTGCCGAATCGCCA
TCACCAGGTGGCACTGGCATCTACAAG
>dme_circ_0008648_junction_seq
ATCACCAGGTGGCACTGGCATCTACAAGGTCTACGGTAGCAGCGCTGACCTCAACG
>dme_circ_0008649
GTGAACGGCCCATTGCCCTTGATCGGCAGTGCCAAAATATTGCCGTTGATCTCGTAGAGCCCATCGCCAT
GGAGATGGGGCAGAATCAACTCGAAGTCAAAGCGTCGGTTTTGGGTGGAAGCCCGCAGCTTGGTGATCTC
AAAGTTGGAGGCGCCCAGGATGTTCAGCTTCTTGGCTTTTACCGTGAGCCCGGCGGATCCATCCAGAATG
CTCAGATCTCCAATGTAGAGGGGCTCTAGCGGCGGTACGTTCAGTTCCTTAATGCCCTTGGCCAGATAGG

GTCGCAAGTTGTGAACACTGCTCTTCAGGCACTTCGACAGTTCGGGATCGTTGCGATGGCACACCTGGAT
ATAGTCAGCTGAAGAACAAGCTTGATAAGTATACTGGACTATAGTGAACTGTCTTCGTTACTTACGCATA
TTGCCAGCTGAGATGCATGCTACAAAACAGATCAACAGGGAGGCTACGATGAGTTGGAACTGCATTTTGC
GT
>dme_circ_0008649_junction_seq
TACGATGAGTTGGAACTGCATTTTGCGTGTGAACGGCCCATTGCCCTTGATCGGCA
>dme_circ_0008650
GATTGGGGAAAACTTTAACAACTTCGCTGGCTGGCGCATCGTTCGATTACTTTAGCTTACTATAGAGACT
TCACGATTACATTCATGGACAGCTTTAACAACATTTAAACACGATATACATACACATTCAATATTCAAAG
AGAGTACATTTAAAATGCTTCGG
>dme_circ_0008650_junction_seq
CAAAGAGAGTACATTTAAAATGCTTCGGGATTGGGGAAAACTTTAACAACTTCGCT
>dme_circ_0008651
GTAAACGCGTAGGCCATCGGCCAGAAGCCGGTTGGCGCTGGACGAGTACATCGCAATGTACAGTCCAATG
AAGTTGTACATGGCACATGAGATGGTGAAGGCAATCAGAGTCATAATCAATGAGATACTCCCCTGCAGAG
CCGCAAAGAGATCACCCCAATCGTCGAAAACTGCACGCGAACTACAGCTAAATGGATCAATGCTCGGCAG
GAAGTTCATGCAAATGGCCAGCATCGAGGTGATGCCCAAGCCGAAGATTCCCTGCCAGCCAGCAGCTTGC
AGTGGAGCAACATTCGAGGTTTTTAGCTGTTTCTCCTCGCAAACATATTGCAGTCCGTGCAGAATTTCAG
CAATGATGATGAGTAA
>dme_circ_0008651_junction_seq
GCAGAATTTCAGCAATGATGATGAGTAAGTAAACGCGTAGGCCATCGGCCAGAAGC
>dme_circ_0008652
CGTTTTTACCAAGTCCTACAGTTGGGGCTCCACTTTATCCACGGTTAACATGTTCAAATGAGCGACCACT
TGGACGGGCAATCGAACTTGAGCTCCAAGACCAAGAAATCTTTGGTAATGCAGGGAGAACGGATTTAAAA
TAAACTTTAGATTATAATATTCACTGAAATAAAATATTAAATGAATAAGGATGTCGGGATCACTGCCAGC
TACTTTCGTCAAGGGTTTCCACGATGAGGAGAAGGTGCGTCGTATGGAGTACCGCCAACTCGGATCCACG
GGTCTGCACGTGTCTAAATTAGCTATAGGTGGCTCCCCCCTTTGCAATCTCTTCTTTGATGACTATGATC
GCGAGGAGGGCATCCTTATGGTGCAGGAGGCCATTAGATCTGGCATTAACTATATAGACACAGCTCCATT
CTATCGGAGGTGCTGCTTGGCCAGGCCCTAAAGGATGTACCCCGGGAGGCCTATTATATTGCCACTAAAG
TGGCGCGCTACGGGCTGGATCCGAAGAATATGTTTGACTATTCGGCTGACAAAGCTCGGGAGAGTGTGAA
GCGGAGTCTGGAGCGGCTCCAGTTGGACAGGGTGGACATACTACAGGGTTCATGACGTGGACGCGGCACC
CAATCTAGACATAGTGCTGAATGAGACCATACCCGTCCTCGAGGAGTACGTCCAGGCGGGAAAGGCTCGA
TTCATCGGAGTCACCGCCTACGATGTGGACGTGCTGAAGGAGTGTGCCGAGCGGGGCAAGGGTCGCATTC
AGGTGGTGCTCAACTATGCTCGTTACACCCTTTTAGACAACACCTTGCTGCGCTACATGAAGGACTTCCA
GAAAATGGGAGTGGGCGTTGTCTGTGCGGCCGCTCACTCATTGGGACTCTTGAGAAACGCTGGACCACAT
GCATCGCATCCCGGTAGTCAGGAAATCCTGGCCGTGGCCAAACGGGGGGCCGAAATCTGCCAGCAGAGGA
ACGTGGAGCTGGGAAAGCTGGCCATGTACTATACGATGCAACTGGATGGGGCGGCCACCTTCCTCATCGG
CATCCCCAACCGAAAGCTGCTGCGGATTAACCTGGACGCGATCTTCGACGGTCTCACTTCCCACGAACAG
GAAGTGCTGCAGTATTTGCGCGAAAAG
>dme_circ_0008652_junction_seq
GGAAGTGCTGCAGTATTTGCGCGAAAAGCGTTTTTACCAAGTCCTACAGTTGGGGC
>dme_circ_0008653
GTTATGATTCAGCAGCTCCGGCTGTTCCTTGAGCATGCGAGCCCATTCCGTATTCTGCAGAGCCTTGTCA
GTTGGTGCAAAGATGGTCACGTTGTCCAGATCGTCGAACTGATCATCGTAGCCGGAGGCCTCCAGCAATC

GCTGGAAGATGGTCAGGTTCTTTTGCGACATCAGCGAGGTCATGGGCAGAGCCGATTCGGTGGGCAGGAT
GGT
>dme_circ_0008653_junction_seq
GCAGAGCCGATTCGGTGGGCAGGATGGTGTTATGATTCAGCAGCTCCGGCTGTTCC
>dme_circ_0008654
ATTAAAATTGTGATACCAAACATGCTACGGCTGTGGGCCTGCCTGCTCCTCCTGGGATCAATCCAGATCC
AGGCGGTTCCATTCTACGGCGAGTGAGTATCAATCCGACAAGTTTTTCGTGCTGTGTAGTAGCGTTCGTT
TCATGCACATTTCACGTAGTTTGAGTTGTTTGCGTTGCGGGCTAAATGGTTAACATATGTACATCGACAT
CTTCTGATAAGGCATGAACTAAATGTTTTATGTTATCGGAACACAATTGAAATGCCAGGAATGCCTGGGA
CCCACAAAATTGAAATACTTCTCTAATTAAACATATACAATTTCATAGAATCATTGGAACAAAATTTTAC
CTAACAAGCGCATCCCAAATAACTaaaaacggaaaaaaattaaaaaaaaaaaaaCACTTTGAAAGAAATA
CAAAATTAGATCTAAATTGAAACGTTTGATACCATCCATAACAATGTTTATAACAATATTAATGTAAGCC
ACGAGTTAAAAAACAAGTATTTTGCCATTATTGAAGTTTTACAATAACGCTTTCAAATATATTTTTGAAG
ACCAATTGGTAAAGTTTCCAAATAATCGTTAATATTAAGTGCTTAAGTCCCATAGAACTAACCCTAGTAG
ATTTTTCCCATCCGCCCGACCTTTTAAATCTGTCCCAATAAACGAAATGTTGAATTCCAGTTCAAGTTCT
GAAGAGGTTGCCGAATTCGATGACTCCTCGGATTCCACACACGAGTCCTCAGATAACTCCAGGTGAACTA
TAAGTAAACGTAGTTAGCCCAGCTGTTGAGTCCCCAACATTTCCCAGTAGATTTGCTCATTTACAACTAC
TTAACAAAGCAAACATACCGACGAGGTTTACATGTTAAACAACTTAAAAATGCTTAAACAGTTTCAATAT
TTTTTTCACTCACGGGTTCCGCTGGGCCCACTGACACCACATAAAAGTTGGCTAATACCGGGTTCAGTGT
TTTTTATGACATTTTCAAGCCCAACGAGGCACCGAGCCCTGTTTGTTCTTGGAGAAACTTTTCGCCCCGCT
AAACGAAACTGCAAATAAAGTTGACCGAAATGTTTATGGTAGCGAAGGCGCTGAAAGAGCGAAAAGTTCC
CCCGGAAAGAAAGTCCAAAAGCCCGCCGAGTATTGACCTCAATTGATAAACCTTAAACTGAAATTGTTGA
GTCGCAGAGATTGTCTGCCAATAAATCCGCATCGAACTGGGTTCTTTGCCGCCATTGGAAATGTGCAAAG
GCCACGATCTGAGTCTTTGGCttttattatttttttccccctttttttGCGAAAGATACTGCATCGCAAGA
GAACAATGTGCAACCAAAGTGGGGCAACACCGCGGGACTTGGGCAACCAAGATCCACATCTTCTTCCACC
GATCTTCTCCGTTCGCTCCACACTTGGCTGACTTTGCCACCTTTGGGTTCAAGCCGCCGCAGGGCTCATC
TTTGGCACACAAATCTCGAACAGTTTTGGCCCTGAGAGCAATTGACATTTGACACGGCATTAAGTGGCAT
ATTGTGGGTAATGGGTAATGCGCCGGCAAAAGAATGTCTCCGCAAACTCTATAATCTGCCCTCGAATTGG
GTCGCCCACAATTTGGTTCCCCCTTTAACGCTCACAGAATGtttttttttATTCTGCCTGTCACGGCTGC
GCTTAAATTAAATCAGGCAGCCATTTGGTTGGATCCATCTCTGCCCTCGACGTAAACATTTCCCTGGAAA
TTTCTGTGGCCAATGACTTTCGCTCTGGCTTCAAGCGAACAGAATTTAGTTTGTTTGCAAATTATATTCG
ATTCACAGATTGGATACGCAGTCTACAAATATTCCAAGTAGTCTTGAATACCATGATCACAAAACAAATA
CTTCACACATTCATTAATCTTTCACAAAGTACTTAGTCATTGAATTACTGCTAAATGGATATATTTAATA
TCTTATCAAACAACATACAGTTGATATTATATTTTTGCACAATTTTTAAGATGAAGAGTAATTATTCTAA
GATTTCAAAAGTCATTCGCTCTATTGTAAGATTTTTTATTTCTTTATTCAAATCTGTTCATATGAATGTA
TACATTTTGAAATATTATTTGCATTCAATAATCGACGTACAGTAGTTAAAATAATGAAAAAGAAATAAAA
TTTTTGACTTATTTCCAAGTAATATAAAATATTTATGATAACGTTTTGAGTAAATATTCTTGCGAATAAA
TTGTGAATAAATTAAGTGTTCCTTTTTATCAAATATTTTGATGGTTTTTTGATTGATGGACACACAATTG
ATTTAAACTGAGGTGCATTCACTCGCTCTAGCAATATTATAGAAGTGATTTCCAGAGAATCTGACAAGGT
AGAAAGAGaaaaaaaaaaaTCAAACTGGCCAAGCCTCTATGTATTGTAATGTCGGCTTAAACCGCCTGTA
TCTGTAGCTCAGCCAAAGCCGCTTCGAGCCCTGTCGATCACGTCCCGCCTGGATGAGTCATCTTGGACGG
AGGCAGATCTCCAACCAGAAGTGAATCGGATACACAGAAGTACCTCAAAGTCAGCAACCCAACAAATTGT
TTACGTTTCGCCTTTTCGGGCAGTATTGttttattatttatttccagctagtttcgtttcgtatattttt
ttcatttagcacttattatGAGCTTTTTTTAATTAGCTCCCGCGAAGCGAATTTTAATGAGCCGCAGCGA

AGAAAAAAAGATTTGAAGTTGGCTAACGCGTATGTATGTCTTCATAGAAAGTATCTGAGTCAGTTTACAG
ATTTCGAATGCATTGGGAGACGCAGAAAAAACCAAAATCCGTTGACATAAATCTCAGTTGATGGATGACA
AGTGCAGGAACATTTCTCAAGTTTTGTTCGCCTTTTTCAGCGATTGTTTCGGCACTTAAAAAGTTATTTT
CATTTGATTCTAAAAAGAGTTCCACTGACAGGCGCTTAGTATGCGGCTCAGGTAAATGCTGCGCAAAAAA
CCTAAACAATGAGCACGATCAGCATATATACTACATACAGATAGATACATATATTTAGTTTGGTGAGGAT
AGAGTTGAGCTTCTTTTTATGCGAGGTAACAATGGAGGCATATGAATTATTGGGTCCCGGTTCCGTTTCG
ATTCGTTCCGCTCACCAATGGAATTTGCATGAAGGGAacacacacagacacacacacacacacacaTA
TGGGCACACAGTAGCCAGCTAATACTGTATACACTAACCACATCACTCTACACCACGTCCACTCCTACGC
CCACTCCCACCGTTCTGCAGCTTTATGCCACATTTAACCCACCCATTGCCGGCGCATCGCAATCTGTTCG
CGCCCCTGCCCTTCGCACCACTCGCACCGCTCGAGCCCCAAGATCCACTGCATGCCCAGGCCTCGCAGCG
GATGGCGAGCGTCTGGAACGGACCCGCCCCGTTGGCGCCGCAACTCCACTCATCTCACCTGTTGCCCCTC
TTCAGCAGCCTTTATGCCACATTTAACCCACCCATTGCCGGCGCATCGCAATCTGTTCGCGCCCCTGCCC
TTCGCACCACTCGCACCGCTCGAGCCCCAAGATCCACTGCATGCCCAGGCCTCGCAGCGGATGGCGAGCG
TCTGGAACGGACCCGCCCCGTTGGCGCCGCAACTCCACTCATCTCACCTGTTGCCCCTCTTCAGCAGCGG
ATACGACACCGAGTTCGTGCCACTGGAGCACCAGCAGCAGCCGCAGGGACGCCAGGAGACGGCGGCCACC
GTTCCCGCTCAAACCCCCAGCGGTGTGGAGCAGAAGCCGTTCAGGTAAGTGCTCTATATCTCTATAATAT
ACATATATTTAGATATTCGTTTAAAATTTATTAAAATAGAAATCCCAAAACTCTTTTAATTATTTCTGTT
GCAATTGGTTTCTAGTCCaaatctggaataaaatctgaatttaaatctgaaattgaatctTAGAAACGAC
CAATTTATAAACTACTTTGCAAAACAGCAAGAGGTAGCCATTCTTGATCAACTATGCTGATCGCTTAATT
ATAGTGATTTAACAATGCCATAAAAATTGCACTCGCAAAATTAGTATCTTGAAGAATATTATGTGCAAGC
TTAATCTCAGCGTAATTTGAATTGTTACTAAAGAATATTTTAAGTGTCTTAAGCAATTGTTAAATTTTAT
AGTATGGTTTTAATTGCGTTTCCTTACTTCGCAGAATGTGGACACCATCACCACGGATGTGAATTCCCCA
AATCCGGCCATCTTCTTCCAGCAATCGTTCCCCTTCTTTGGCAATGAGTTCTTCAATTCGTTCGGAGGCT
TTGGCTTTGGAGCTGCCCAGGAGCCCTGGTGGAAGGGGGTAAGTGCCATCCATTAAGGGCATTTGCCCCC
ACTTGAATGCTaaaaaaaaaaaaaaaaGTCCCCCAGTTATGGTCACTAATTTGGCGTCTTTCCTAATCGC
CGCACTCGAGTGATTCTGTGGCCAACTTCGCCGTTCTTGGCATTTGGCAATTAAGAGAGCACAATGTCAA
GTTCATACGACCCATTAACTATATGATTGTATGGTATATATATTCTATAATTGTGTAAAGTTGGATGGGT
TATCGCCATCATAGAGGCAATCGAGCATATCAATTAAATGGTAAATACCTACTTAAATAATTTTCCATCC
AAGATTATTCGCAGCTGAGGCTGCTTAATAATTTTCCAGGGCTCGATTGCTGtttttttttttAATTGCC
CAACCAGATATGGCATTGTATATAGGGAAAAGTGAATGAATATATAGATATATAGTGTCTATATTGGATA
AGTGCACAACTGCACGGTTTCTACAGCCGAGCTTGCACTTTTATAAATTGTGAAATCCACTTGTCGTCTT
TTTTGCACTGTGCGATGAACTCGTTACTGCGCGGGCATAGTAAAATGAAAGTCACAGATGTGGCAAATTG
ATAAATTAGCTGCATTACAAACAtatgtatgtatgtatgtatgCGACTGTGTATCTATGGTTCAGTGAGA
AATTTCTAAAATCTACGCACACAATGAGTTCGCATTCAGTAGACTCCTCTCATCGCTGCCATAATTGAAT
TGATGGACTGGCAAAGCTGTTAAGCCAACACACATACGCATTTATATAAAAGTCAGTTTGTATAATATAA
CTATGCTTTCTATGTGCCGGACTCATTGTGCAAATCCATTGGGCAAACAATCGGTTAGCGAGTGCCGATC
AACCGACTAAGTTGGATTGGATTCGGAGTTGTGGATATATAGGAATTTAGGGTTGAATCAAAATTATATA
CATATATGTATAGTGGTATATAGTGCTATATAGTGCATTGACATTTCGATTTGCATGTCTGTTATATGAC
CAATTCATTAGCAGGTTTGGCTATAATTTGTAGAGAACGTAAAGGGAGAATGATCATCATAGATTAAATA
TAGCAACAAGTTATTAGTAATTGCTCATTGAAGATAACTTATAAAACTAATGATTTGAATATATGCCTGA
AATACACTATTTTTAAAGCTTATGTATTTGAGTCTCGAAAAACGATTGCCTTGAATTATATTTGTTCATT
TCGCCAAACTATTACTACATCACATTGTTAACCAATAACCAATTTTTCGCTTTATTCAGAACCCAATGTG
TGCACCGAAAAGGAGGAAGACGAGACTGTGGCCAGTGAAACGGAGGCAGAAGAAACCGTGGACACCTTTG
GCCAGGAACGTGATGGCGTTACCAACGTGGTGCGATCCCCACTCTTTGGCCAG

>dme_circ_0008654_junction_seq
CGTGGTGCGATCCCCACTCTTTGGCCAGATTAAAATTGTGATACCAAACATGCTAC
>dme_circ_0008655
GAAGAGTCGAGTGTGCTGCAGCAAAAGTACCAAAACATCAAGGAACTTAACTCTGAGCAACGCAAGAAGT
TTAATTCTCTAAAGGACCGCTTCATTGATGTACACCGCAAGTTGAAGAACCTCAAGGAGTGCAAGTGCGT
GCTGCTGGAAACGCAGCACGAGTACGCCGCATCCGTTTCCAAATGGCAGGTGGAGATTATCAAAGCTTCG
CAACTGTTGTGCGCAAAGATGGCATCCCTGCAGGCCGAGAATGAAAAACTGAAGCTGAACAATGGAAA
>dme_circ_0008655_junction_seq
ATGAAAAACTGAAGCTGAACAATGGAAAGAAGAGTCGAGTGTGCTGCAGCAAAAGT
>dme_circ_0008656
GCAGCCTTCGGCGGCTCGACAAGGCCGCCATATGGAGGAGGTTGGCGTCCGCTCGGTCCAATCAGTCCGC
TCAGCCACGACGACACTCGAAACCGAGCTGCTGGCCAACCGgtcccagtcccagtcgcagtcccattcgc
agtcGGAGCCGGAACCGCTGTCGGAACCGGAGTCAAGCAGCCCCGGATGCCGCCAGCCGCGTGAATCCTG
CTGGCCTTATCAGATCAGATAGAAACCCGCCGAGGACACACATCCACGAGAGACACACTCATAATGGGCA
CAAAAACCCAACCACAATTGGCCAAAGGGTGAGTGGAAGAGAGAAAGTTAAGTAGGTCCGAAATGCTATC
AAGTGGTCACTTTTCATGGCGATGAATATCAAGCTTATATTGAAAATAGAAATTGGCGTAAATTAAATTG
TTTAAAATGGCACACATTTAGCTAATAAAGATCTATTCTATAAAAACGTTAATATAAGATATTTCAAAAT
AAATCTGAGCCAACGCCATGCGAATTGTATTTTAAAGGAGAGTTGTGCTGTTATTACATTATTTATTAGA
AATATATCCGATTATTTAACTCTAAAGAAAAATAATAAACATAGGAAATGCAGTATAAGATTTTTGTTAG
GTGAGGTCAGGTACGCCCCTTCTTATCGTTTGCTGGATTTTTTTAATACTTGGTCATCCCCAATTCTATG
ATAAATGCAGACCCATATCTGTATTGGTAGATTTACCTTTGATACTAACAATTAAAACAAAATTAATGCG
TTCAGCGATAGAACAAAACTCGACTTACGAATCAATAACCAGTATAAGCACGATGTCCGCTACAAATTGA
TTTTGCTCAAAGCCTTTGGCATTTCAGATTGGGAAATCTCGCTGCCTCGGGCAGCCAAAAAGTTGAACAG
TTGTTACTGATTTCGGCCAGATAGTGACCCAGTTCTTTGGCGTTACTAAACCAATTGCAGGGAGAGACGA
CGCGGAACTCTGACAAAAAGCAGCCCGTTGCTCGATTTTGGCCCTGACACTATTCCGCAAAGG
>dme_circ_0008656_junction_seq
TTTTGGCCCTGACACTATTCCGCAAAGGGCAGCCTTCGGCGGCTCGACAAGGCCGC
>dme_circ_0008657
GCAGCCTTCGGCGGCTCGACAAGGCCGCCATATGGAGGAGGTTGGCGTCCGCTCGGTCCAATCAGTCCGC
TCAGCCACGACGACACTCGAAACCGAGCTGCTGGCCAACCGgtcccagtcccagtcgcagtcccattcgc
agtcGGAGCCGGAACCGCTGTCGGAACCGGAGTCAAGCAGCCCCGGATGCCGCCAGCCGCGTGAATCCTG
CTGGCCTTATCAGATCAGATAGAAACCCGCCGAGGACACACATCCACGAGAGACACACTCATAATGGGCA
CAAAAACCCAACCACAATTGGCCAAAGG
>dme_circ_0008657_junction_seq
CAAAAACCCAACCACAATTGGCCAAAGGGCAGCCTTCGGCGGCTCGACAAGGCCGC
>dme_circ_0008658
GAAGTAGTCACCTGCAGCAATCCGGAACTAGGAGCAAGTGCTCCAGATCCAGTGGATTTAACATGGGGAG
GGAAAAACACTGAGGGACAGCTTACGACGGCCACAAGATCCACTCTGCCAGATAGTCCAATGACAACACA
AAACGATCTAATTACGACAGCCAGCAACTTAGCGACAGCAAAAAAGCCAGCCACAGCAGCCTCAGAGGCA
GTAGTAGATACGgcagaaacagcagaaacagcaCCACCGACTGAGGCAACCAGCTCATGGAGCTCCACGG
CCGTAGCCCGGGGCATGTCATCAGCAGATGGAGCAGGGGAGGCGATAGCAACCAGCCAGTCAGCACAAAT
TGGGCTAACTACTCAGgccacagtacggcaaacagaagcgcagacagcatcaacagcgacacaagccgca
gcagcaacatcagcagcaacatcagcggcaatagcatcagcagcagcagcaacagagaccgcagcagaga
tgctcatatcaacaatttATCCAGCATCAACGGAAACGGATTTGCACAACTCCATTGAGCGAGTAGAGGG

GCAACGTGGAAAGATGGCGAACGCGTTTCATCCGGGTGTCCCTGATTCATTGACCAAGGGCTTCTCCATT
CCGACCTTCCTGCCTCCATTTCCCGTTTTCGCGGCTGCGGATTTGCCAGCTTACCGCGCCGCTGCTGATG
CAGCCGAGGCGGCCAAGTTGGCGGCAGAGGCGGCGGCCCAGGCAGCGGCCGCCAAAACATCCTCGGAAGC
GGTGACAATGTCGCCGGAGGAGCAGCGCCGTCAAATGTTTAATGAGCAGCACTCCTACCTGGCCGCCCAC
CGCGATGGTGGCGATGGGGCGGGGTCGGCGGTGCGGCGCAACCTGACAATGCCCGTGCTAAACATCACCG
CTCAGATGGGAAACCACGCCTACATGCCGTGCCAG
>dme_circ_0008658_junction_seq
GGGAAACCACGCCTACATGCCGTGCCAGGAAGTAGTCACCTGCAGCAATCCGGAAC
>dme_circ_0008659
GTTAACCAAGTCTAGTGGCAGCCAAAGCCTTTCCGGCACCCTTATCCGGCCAAAGCCCGTACACCCTGCG
CCGCAGCATCGCTCAGGATTCCAAGGGCCGTCGGGACCTGGGAGCGTGACCTATGGGCAC
>dme_circ_0008659_junction_seq
GGGACCTGGGAGCGTGACCTATGGGCACGTTAACCAAGTCTAGTGGCAGCCAAAGC
>dme_circ_0008660
CCGCTTGGAGGAGTGTGCCTCTTCTACGTGTCCACCCAGCATCAACCGAATGGAGAGTAGCGAGCGCCAG
CTGGTTCATCTTTTACCCGAAATCATCGCTGGTTTTAAGCCACAATTTGATGTACCTCAGCTAACACCTG
AGCAGTTGGGTTCTTCACTTCAACAATGGCTAGATAAAATGAACGCCTTGGCAGCGGCTCACTTGCAGCA
AGTCTTTGCACTAGTCACCAACATGCAGACCATCCAGGACATTAAGTCCGCGGCCAGAACTAATGGTCGA
CCGGATTTCG
>dme_circ_0008660_junction_seq
GCCAGAACTAATGGTCGACCGGATTTCGCCGCTTGGAGGAGTGTGCCTCTTCTACG
>dme_circ_0008661
GATAAAGCTTCGGATGACAGCGGTTGCGTGCGATGTTAATCTTCACCAGCAGCGAGTGCATCGATCGCTT
GGCATCTAGCAAATTGCCGGTCAGCGTGTGCGAGCGCTTCAAGAGCTCGAATTGTTTGGTGGCCTGTGGA
TTTGCAGTGTGATACGCGGCCACCGGCAACTTGCCTTCGAATAGCCAGTCCTGGTCGTGCTTAAATCTCA
TAGAATCCCGACCCCTCTGCGGATTGCGACAAATGTAGCACACATACTT
>dme_circ_0008661_junction_seq
GATTGCGACAAATGTAGCACACATACTTGATAAAGCTTCGGATGACAGCGGTTGCG
>dme_circ_0008662
TTAACGAGACGGAGCAGACGCTACTCGTGCGCTTTGAGCGCAATCACAAGCTAAAGGTGTCACCTTCGAC
CAGCGGCAGCTTTCAGGAGTGGATGGCCATCAAGTCGGAGCGGCTGAGGCAGCGCCTCAGCAACCGTGTG
CTGCCCGTCTTTGAGCTGGACGAGAAGTGCATGGCCCGCTGGTCGGGGCCGCGTAAG
>dme_circ_0008662_junction_seq
CATGGCCCGCTGGTCGGGGCCGCGTAAGTTAACGAGACGGAGCAGACGCTACTCGT
>dme_circ_0008663
GGCTATACTGATTTATCTGGCCGAGAAGTACGATAAAGATGGCTCCCTTTATCCCAAGGATCCCCAGCAG
AGAGCCGTGATCAATCAGCGCCTGTTTTTCGATCTGAGTACTCTGTACCAGAGCTACGTGTACTACTACT
ATCCCCAGTTGTTCGAGGATGTGAAGAAGCCAGCTGATCCCGATAACCTCAAGAAGATCGATGATGCTTT
CGCTATGTTCAATACTCTGTTGAAGGGTCAGCAGTACGCCGCCCTCAACAAGTTGACTCTGGCCGATTTT
GCGCTTCTGGCCACCGTTTCCACTTTTGAAATATCGGAATATGATTTTGGTAAATATCCGGAAGTGGTTA
GGTGGTACGACAATGCCAAGAAAGTGATACCCGGCTGGGAGGAGAACTGGGAGGGCTGCGAGTACTACAA
GAAATTGTATCTGGGTGCGATTTTGAACAAACAATGACGGG
>dme_circ_0008663_junction_seq
GGTGCGATTTTGAACAAACAATGACGGGGGCTATACTGATTTATCTGGCCGAGAAG

>dme_circ_0008664
CTTCGCCAACTACTACTACCCACAGGTGTTCGCCAAGGCGCCCGCCGATCCAGAGGCCTTCAAGAAGATC
GAGGCCGCCTTCGAGTTCCTGAACACCTTCCTGGAGGGACAGGACTACGCCGCCGGTGACTCCCTTACCG
TAGCCGACATTGCCCTGGTGGCAACCGTGTCCACATTCGAGGTGGCCAAATTCGAGATCAGCAAG
>dme_circ_0008664_junction_seq
CGAGGTGGCCAAATTCGAGATCAGCAAGCTTCGCCAACTACTACTACCCACAGGTG
>dme_circ_0008665
ATCCTTAAGGTCGAGCTTTTCGATAGGAAAAGCACTCAGTATTTCGTGCGTCTGGTGCTCGATGCTATGA
AATACCGACAGGAGCACAATATTGTACGTCCCGACATGATAAACATGCTGATGGAGGCTCGTGGTATCAT
CCAGACGGAGAAGACCAAGGCATCTGCCGTTCGCGAGTGGAGCGATCGCGACATTGTCGCCCAGTGTTTC
GTGTTCTTCTTTGCTGGCTTCGAGACTTCGGCGGTGCTGATGTGCTTCACCGCTCACGAGCTAATGGAGA
ACCAGGATGTGCAGCAGAGGCTATACGAGGAGGTACAGCAAGTGGACCAAGACCTGGAGGGCAAAGAACT
GACCTATGAGGCAATTATGGGCATGAAGTATCTGGATCAGGTCGTCAACGAGGTTCTGCGAAAGTGGCCG
GCGGCAATTGCCGTCGATCGAGAATGCAACAAGGATATCACCTTCGATGTGGATGGCCAGAAAGTCGAGG
TCAAGAAGGGCGATGTCATCTGGCTGCCTACATGCGGCTTCCATCGTGATCCCAAGTACTTTGAGAACCC
TATGAAGTTCGATCCGGAACGTTTTAGCGATGAGAACAAGGAGTCTATTCAGCCATTCACATACTTCCCC
TTTGGCCTGGGTCAACGCAACTGCATTGGGTTCCCGATTCGCTCTACTTGAAGCAAAGGCTGTTATCTAC
TATCTTTTGAAGGACTACCGCTTTGCCCCTGCCAAGAAATCCTGCATTCCACTAGAGCTCATTACTTCCG
GTTTTCAGTTGTCACCCAAAGGCGGCTTTTGGATTAAACTTGTTCAAAGGAACTAATCTTATAAACGATC
AAAATAATAATATGTTTTGTTGAATGTTATGTTTTAAAGAAGTTGACATTATTAAATTTAAACAATTAAT
GGTAAATATTCCCGATTTATTTACTCGTGAGTCGAAAGTCTGTTTGCATTGTTCTGTGAAATAGACAACC
ACATTTTTAATTTGCAATTAGTGTGCGTATGCTATTGTCCAGGCTAACGTGGCAGGCCAGGATTCTAACT
CGTCTTCTGTGTAATAAGATGGCCAGCAAGGGATTGCACAATGAGGACAACAAGTTGGACACTTTCCGTC
GCATCTGTGATGAAATTGCCGGCGAGTCAAGTTACCTGAAGAAAGCGGAGAGACTGCAGCGATTCTTCGA
AAAGGGGAGCAGTGGCAAGGGCTTCAAGGGCGACACCGTGCTCTGGGTGCAGTTCCTCATTCCGGCGGCC
AATCAGCGCGTCTACAATCTGCAGAACAAGGCGCTGATCAAACTCTTTTCGCGCATTTTTAATGCAGATC
AACAGCAAATGCATCTGGATCTAGAGCAGGGATGGCGACGTTTCGGAAACTCTACGCAAGCACTTTGCTG
CTTCTAAGAAACTCAAGCCGCAAAAGCAGAGCAAACTCTATCTGCAGGAGGTGGAGGAAATGCTCCTGCA
CCTGGTGGAGCGTACCAAAGAGGATGAGCAAACAGATCTGCTACAGAAGTTGTGCTCGAAGGCCACTGAC
CTGGATCTGCGCACCTTTATTCGCTTGGTGAAGCACGACTTAAGAATCAATGCCCGAGCCCGGCACATCC
TGGATGCTTTTGGTCCTGATGCCTACCCCGCATATCAGTCTTCCCGCGATTTGGTGGCAATTGTCGAACA
GTTTGCCACCAAAGAAAATAAAAATTATGCTAGTAGTCCCGTTAAGAAGACTAAGAAAACGAGCTCGAGT
GCAATTCAGGTAATGACACCCATATCGCCCATGTTGGCCAGTCCCTGCAAGTCGGTGGAGGACGCCTTCA
AGAAGAGCCCCACTGGACTATATAGCGAGATCAAATACGATGGAGAGCGAGTTCAAATCCACAAGCAGGG
CAACGACTTCAAGTTCTTTAGTCGCAATCTAAAGCCTGTGGTGGATCACAAGATACGGGCTCTGAAAAAA
TATATTCCGCAAGCCTTTCCTGGCGGTGGCGAAATGATACTGGACTCTGAGATCATTCTGGTCGACACTG
ACACAGGAGCACTGCTGCCCTTTGGTTCGTTGGGTGCCCATAAGAAGCAGACTTTCGCCAATGCTGCTGT
ATGCTTATTCGTCTTCGATTGCATACTGTATGATGGCGAGGATTTGACCCAGCTGGCCATTTCGCAAGCG
TCGCGAGATTCTTGAGCAGAATATTCAACCCATTAAATCCCACGTGCAGCTGTCGGAGTCGGAGTTCCTC
AAGACCACGAGGGAACTGGCCGCGATGACGGCCAGGGTGTTACATGCCAATCTGGAGGGTGTGGTCCTAA
AGAGTCCAACCAGCACATATCAGCCCGGCAAAAGGAACTGGCTTAAGGTCAAGAAGGATTACCTATTCGA
CGGAAAGATGGCTGACACCGCGGACTTGGTTGTTTTGGGTGCTTGGTACGGATCAGGGAAGAAAGGTGGC
ACGCTTAGTATTTTTCTCATGGGCTGCTACGATGCGTTTGGGCGTTTGTGGAAGACAGTGACCAAAGTGC
ACTCTGGCTTGGATGATGCTACCAACGCAGAGGTACATAAATCACTAATCAAACTAACCGAACGTGCTGA

TGCCAACGCAATTCCCAGTTGGCTGTTATGCAGTAAGTCCCTTGTACCAGATGTACTGGCCAAGGATCCG
ATGAAAATGCCAGTATGGGAGATTACTGGCGCAGAGTTCACCAAGTCCGATGCCCATACCGCGTCTGGAA
TCTCAGTACGATTTCCCAGGATTACTCGTCGTCGCAGTGATAAATCAGCCAAAGAGGCCAATGATCTGGC
CCATCTAGAAGATCTTTTCGAGGCCTCAAAATCGAATGTAAATGTTGATTTACTTCTGGCCAATTGTAGT
AAAGATGAGACAGCAATGAATAACAAAACAACTCCTGAAAAGGAGAAAAAACTGAATGGCAATCAATCAA
ATGGTAGTAAGAAATTGAAAAGGTCCAATACTGGTTCTGATGACGAAGACGGAGCTCCTGTGCTCAGCAA
ACGCAAGGAGTCCAAAGCGATTCGAAGCTTGTCCCCTTCGCAAAAGGGATTAAAGGATTTTTTTAAGCCC
AGCAAGGAGTTTAAATCAGTTGTGAAAAAGGAACCAAAGGAGGATGAAGGCAATATTGAATCCACTAGCT
CGAAACCCCAGTTGTTCGAAGGATTGGTGGCACACTTTGCCATGGAGAAGGATTCAAGTAAACTGATGCA
GCAGTTTTCACAGCATGGAGGATGCTGTACAACAGAACCACAAAAAGCTGATCTGGTCTTTTATGCGAAT
ACGAACGAGGCATTGGACCTAGAGAAGCTTAGGAACGCTCTATCGTCGTAGCTGCCGGCACTTGAATGTA
AGCTGGCTCCAAAGCTGTCTCGATACTCAGAGTCGTCAACCTTTTCCCCTACACGCCGTGATGATGAAAT
GATGAGTGGTATTCTAAATCGTTTGCACTCAGAAATTTGTACATGCTTGATTAAACCGAACACACATACA
TATGTGTGTATTATTGACAACAGTTTGTTAGTTATATTTTGGACGCTTGGATCGTCAGACTAGGCGCTCG
TTCAACATGCTGTGGGAGTTCTTGGCCCTATTTGCGATCGCTACCGCCTTGTTCTACCGCTGGGCGTCCG
CCAACAATGATTTCTTCAAGGATAGGGGCATCGCCTATGAAAAGCCAGTGCTCTACTTGGGCAACATGGC
CGGCATGTTCCTACGGAAGAGAGCCATGCTGGACATAGTCTGTGATCTCTACACCAAAGGCGGAAGTGGC
AAGGTTAGTTAAATTTATTGATCCGGATTTAATAAGTTAATTACCTAATAAGCATCCGGGTTACTTGCAC
TTGCACTTGAAACTCGATTGGAAGAAGTGTGTATCTACTGGCCTCCAGCATTGCGTACAAGCCTATATAA
AATCGTATGTCAAGGTCAGGCAATCAGTGATCTATCCATACACACATTTAGTGGGTGCTGCTGCGTTCGA
CTACGTTTTGAATAACAAACAATGAACAATTATAGAAATTCAGAAATACTCTATTATATTTTGTAAAGTC
TTTTATATAAAGCAATGTGTAACTGCCAAGATAATTGGGGAAATGTTTGGTTGGTTCTATTAGTTTAATC
TTATCGATTATACGACAGCTTTAAATGTTGTTTGTCGCGGACGGCACTGTGTCCATATTTAGATAGGAAA
AATAGACGATAAAGCGTAATAACGATAAGGTTATCTAAATTATTAATTAATTTCATATTGGGGCAACCCT
ATTTGTGACTGTTTCCTTTTTGTTCCTTTATAAAGATGCACTCTTTGTGTACTCAGAATTTTCAATTGGA
TTTCGTGTGCATATAATTTATTAATATACCaaaaaaaaaTTTACCCCACTATAAATTTATAGTTTTTCAA
AAAGAAATCGAGATGGGTTAATTGAAAATTCGTCGGATAGGTAATGAACAACGCTAGCCATCCATAAACT
CAAATTTCTGTTATCGTTTTCTAAAATTGTTGAGAGCACGATTAATAATTTCTGATTTTATTTATCAGTG
TGTGACTCACAAAAAGCTTATCGGTCATTAACATTAAttttttttttagaatttttttGGTATCTTCGCGC
AGCGACAACCACTTTTAATGGTCCGCGACCCAGATCTTATCAAGCAGATAACCATAAAGGACTTCGATCA
CTTTATCAATCATCGCAATGAGTTTGACACCAGCAGCGATGATGATCCACACGACATGAGTAATCTTTTC
AGCAGCTCGCTGTTCTCCATGCGAGATGCTCGGTGGAAGGATATGCGAAGCACTCTGAGTCCGGCATTTA
CAGGCAGCAAAATGCGTCAGATGTTCCAGCTGATGAACCAGGTGGCCAAGGAGGCCGTGGACTGTCTTAA
GCAGGATGATTCAAGGGTTCAGGAGAATGAACTGGACATGAAGGACTACTGCACACGATTCACCAACGAT
GTGATCGCCTCGACAGCCTTTGGACTGCAAGTGAACTCGTTCAAGGATCGCGAAAATACCTTCTATCAGA
TGGGAAAAAAAGTTGACCACATTTACATTCCTGCAGAACATGAAGTTCATCTTGTTATTCGCATTGAAGAG
TCTTAATAAGG
>dme_circ_0008665_junction_seq
TTATTCGCATTGAAGAGTCTTAATAAGGATCCTTAAGGTCGAGCTTTTCGATAGGA
>dme_circ_0008666
attttttGGTATCTTTGAGCAGCGACAACCACTTTTAATGGTGCGCGACCCAGATCTTATCAAGCAGATA
ACCATAAAGGACTTCGATCACTTTATCAATCATCGCAATGTGTTTGCCACCAGCAGCGATGATGATCCAC
ATGACATGAGTAACCTTTTCGGCAGCTCGCTGTTCTCCATGCGAGATGCTCGGTGGAAGGATATGCGAAG
CACTCTGAGTCCGGCATTTACAGGCAGCAAAATGCGTCAGATGTTCCAGCTGATGAACCAGGTGGCCAAG

GAGGCCGTGGACTGTCTTAAGCAGGATGATTCAAGGGTTCAGGAGAATGAACTGGACATGAAGGACTACT
GCACACGATTCACCAATGATGTGATCGCCTCGACAGCCTTTGGACTGCAAGTGAACTCGTTCAAGGATCG
CGAAAATACCTTCTATCAGATGGGAAAAAAGCTGACCACATTTACATTCCTGCAGAGCATGAAGTTCATG
TTGTTCTTCGCATTGAAGGGTCTTAATAAGGATCCTTAAGGTCGAGCTTTTCGATAGGAAAAGCACTCAG
TATTTCGTGCGTCTGGTGCTCGATGCTATGAAATACCGACAGGAGCACAATATTGTACGTCCCGACATGA
TAAACATGCTGATGGAGGCTCGTGGTATCATCCAGACGGAGAAGACCAAGGCATCTGCCGTTCGCGAGTG
GAGCGATCGCGACATTGTCGCCCAGTGTTTCGTGTTCTTCTTTGCTGGCTTCGAGACTTCGGCGGTGCTG
ATGTGCTTCACCGCTCACGAGCTAATGGAGAACCAGGATGTGCAGCAGAGGCTATACGAGGAGGTACAGC
AAGTGGACCAAGACCTGGAGGGCAAAGAACTGACCTATGAGGCAATTATGGGCATGAAGTATCTGGATCA
GGTCGTCAACGAGGTTCTGCGAAAGTGGCCGGCGGCAATTGCCGTCGATCGAGAATGCAACAAGGATATC
ACCTTCGATGTGGATGGCCAGAAAGTCGAGGTCAAGAAGGGCGATGTCATCTGGCTGCCTACATGCGGCT
TCCATCGTGATCCCAAGTACTTTGAGAACCCTATGAAGTTCGATCCGGAACGTTTTAGCGATGAGAACAA
GGAGTCTATTCAGCCATTCACATACTTCCCCTTTGGCCTGGGTCAACGCAACTGCATTGGGTTCCCGATT
CGCTCTACTTGAAGCAAAGGCTGTTATCTACTATCTTTTGAAGGACTACCGCTTTGCCCCTGCCAAGAAA
TCCTGCATTCCACTAGAGCTCATTACTTCCGGTTTTCAGTTGTCACCCAAAGGCGGCTTTTGGATTAAAC
TTGTTCAAAGGAACTAATCTTATAAACGATCAAAATAATAATATGTTTTGTTGAATGTTATGTTTTAAAG
AAGTTGACATTATTAAATTTAAACAATTAATGGTAAATATTCCCGATTTATTTACTCGTGAGTCGAAAGT
CTGTTTGCATTGTTCTGTGAAATAGACAACCACATTTTTAATTTGCAATTAGTGTGCGTATGCTATTGTC
CAGGCTAACGTGGCAGGCCAGGATTCTAACTCGTCTTCTGTGTAATAAGATGGCCAGCAAGGGATTGCAC
AATGAGGACAACAAGTTGGACACTTTCCGTCGCATCTGTGATGAAATTGCCGGCGAGTCAAGTTACCTGA
AGAAAGCGGAGAGACTGCAGCGATTCTTCGAAAAGGGGAGCAGTGGCAAGGGCTTCAAGGGCGACACCGT
GCTCTGGGTGCAGTTCCTCATTCCGGCGGCCAATCAGCGCGTCTACAATCTGCAGAACAAGGCGCTGATC
AAACTCTTTTCGCGCATTTTTAATGCAGATCAACAGCAAATGCATCTGGATCTAGAGCAGGGATGGCGAC
GTTTCGGAAACTCTACGCAAGCACTTTGCTGCTTCTAAGAAACTCAAGCCGCAAAAGCAGAGCAAACTCT
ATCTGCAGGAGGTGGAGGAAATGCTCCTGCACCTGGTGGAGCGTACCAAAGAGGATGAGCAAACAGATCT
GCTACAGAAGTTGTGCTCGAAGGCCACTGACCTGGATCTGCGCACCTTTATTCGCTTGGTGAAGCACGAC
TTAAGAATCAATGCCCGAGCCCGGCACATCCTGGATGCTTTTGGTCCTGATGCCTACCCCGCATATCAGT
CTTCCCGCGATTTGGTGGCAATTGTCGAACAGTTTGCCACCAAAGAAAATAAAAATTATGCTAGTAGTCC
CGTTAAGAAGACTAAGAAAACGAGCTCGAGTGCAATTCAGGTAATGACACCCATATCGCCCATGTTGGCC
AGTCCCTGCAAGTCGGTGGAGGACGCCTTCAAGAAGAGCCCCACTGGACTATATAGCGAGATCAAATACG
ATGGAGAGCGAGTTCAAATCCACAAGCAGGGCAACGACTTCAAGTTCTTTAGTCGCAATCTAAAGCCTGT
GGTGGATCACAAGATACGGGCTCTGAAAAAATATATTCCGCAAGCCTTTCCTGGCGGTGGCGAAATGATA
CTGGACTCTGAGATCATTCTGGTCGACACTGACACAGGAGCACTGCTGCCCTTTGGTTCGTTGGGTGCCC
ATAAGAAGCAGACTTTCGCCAATGCTGCTGTATGCTTATTCGTCTTCGATTGCATACTGTATGATGGCGA
GGATTTGACCCAGCTGGCCATTTCGCAAGCGTCGCGAGATTCTTGAGCAGAATATTCAACCCATTAAATC
CCACGTGCAGCTGTCGGAGTCGGAGTTCCTCAAGACCACGAGGGAACTGGCCGCGATGACGGCCAGGGTG
TTACATGCCAATCTGGAGGGTGTGGTCCTAAAGAGTCCAACCAGCACATATCAGCCCGGCAAAAGGAACT
GGCTTAAGGTCAAGAAGGATTACCTATTCGACGGAAAGATGGCTGACACCGCGGACTTGGTTGTTTTGGG
TGCTTGGTACGGATCAGGGAAGAAAGGTGGCACGCTTAGTATTTTTCTCATGGGCTGCTACGATGCGTTT
GGGCGTTTGTGGAAGACAGTGACCAAAGTGCACTCTGGCTTGGATGATGCTACCAACGCAGAGGTACATA
AATCACTAATCAAACTAACCGAACGTGCTGATGCCAACGCAATTCCCAGTTGGCTGTTATGCAGTAAGTC
CCTTGTACCAGATGTACTGGCCAAGGATCCGATGAAAATGCCAGTATGGGAGATTACTGGCGCAGAGTTC
ACCAAGTCCGATGCCCATACCGCGTCTGGAATCTCAGTACGATTTCCCAGGATTACTCGTCGTCGCAGTG
ATAAATCAGCCAAAGAGGCCAATGATCTGGCCCATCTAGAAGATCTTTTCGAGGCCTCAAAATCGAATGT

AAATGTTGATTTACTTCTGGCCAATTGTAGTAAAGATGAGACAGCAATGAATAACAAAACAACTCCTGAA
AAGGAGAAAAAACTGAATGGCAATCAATCAAATGGTAGTAAGAAATTGAAAAGGTCCAATACTGGTTCTG
ATGACGAAGACGGAGCTCCTGTGCTCAGCAAACGCAAGGAGTCCAAAGCGATTCGAAGCTTGTCCCCTTC
GCAAAAGGGATTAAAGGATTTTTTTAAGCCCAGCAAGGAGTTTAAATCAGTTGTGAAAAAGGAACCAAAG
GAGGATGAAGGCAATATTGAATCCACTAGCTCGAAACCCCAGTTGTTCGAAGGATTGGTGGCACACTTTG
CCATGGAGAAGGATTCAAGTAAACTGATGCAGCAGTTTTCACAGCATGGAGGATGCTGTACAACAGAACC
ACAAAAAAGCTGATCTGGTCTTTTATGCGAATACGAACGAGGCATTGGACCTAGAGAAGCTTAGGAACGCT
CTATCGTCGTAGCTGCCGGCACTTGAATGTAAGCTGGCTCCAAAGCTGTCTCGATACTCAGAGTCGTCAA
CCTTTTCCCCTACACGCCGTGATGATGAAATGATGAGTGGTATTCTAAATCGTTTGCACTCAGAAATTTG
TACATGCTTGATTAAACCGAACACACATACATATGTGTGTATTATTGACAACAGTTTGTTAGTTATATTT
TGGACGCTTGGATCGTCAGACTAGGCGCTCGTTCAACATGCTGTGGGAGTTCTTGGCCCTATTTGCGATC
GCTACCGCCTTGTTCTACCGCTGGGCGTCCGCCAACAATGATTTCTTCAAGGATAGGGGCATCGCCTATG
AAAAGCCAGTGCTCTACTTGGGCAACATGGCCGGCATGTTCCTACGGAAGAGAGCCATGCTGGACATAGT
CTGTGATCTCTACACCAAAGGCGGAAGTGGCAAG
>dme_circ_0008666_junction_seq
TCTCTACACCAAAGGCGGAAGTGGCAAGatttttttGGTATCTTTGAGCAGCGACAA
>dme_circ_0008667
CAAGTGGACCAAGACCTGGAGGGCAAAGAACTGACCTATGAGGCAATTATGGGCATGAAGTATCTGGATC
AGGTCGTCAACGAGGTTCTGCGAAAGTGGCCGGCGGCAATTGCCGTCGATCGAGAATGCAACAAGGATAT
CACCTTCGATGTGGATGGCCAGAAAGTCGAGGTCAAGAAGGGCGATG
>dme_circ_0008667_junction_seq
CAGAAAGTCGAGGTCAAGAAGGGCGATGCAAGTGGACCAAGACCTGGAGGGCAAAG
>dme_circ_0008668
GACCCTTCAATGCGAAGAACAACATGAACTTCATGCTCTGCAGGAATGTAAATGTGGTCAGCTTTTTTCC
CATCTGATAGAAGGTATTTTCGCGATCCTTGAACGAGTTCACTTGCAGTCCAAAGGCTGTCGAGGCGATC
ACATCATTGGTGAATCGTGTGCAGTAGTCCTTCAT
>dme_circ_0008668_junction_seq
TGGTGAATCGTGTGCAGTAGTCCTTCATGACCCTTCAATGCGAAGAACAACATGAA
>dme_circ_0008669
GAGCCGGGCTGCTACGGCATCCTAACCATTGATGTGAAAACTGGTGCACAGAAAAGTGTTCTCTTTGTTC
CTCGCTTTCCCGATGAGTACGGCACCTGGATGGGCGAACTGCTTGGGCTGCAGGAGTTCAAGGCCATGTA
CGAGGTGGACGAGG
>dme_circ_0008669_junction_seq
TTCAAGGCCATGTACGAGGTGGACGAGGGAGCCGGGCTGCTACGGCATCCTAACCA
>dme_circ_0008670
TGAAAACAAAACAAGAAACAAAAAATCGAAGAGGGGAAATCGTTGAACCGAGGAGGTGGAGGCGAATGCT
TGCCAAGAAGTATAAAGAGGAATTATAAGCACTAAGTTTCGACCTTGAATCAGttttttttttGTTCGTC
CACAATGCAGTCCGACCGAATGGAccagccagccagccagccagAGAAATATCTGCAAGAAAATCAGCGA
AATATATTAGTTTGTCCCTAAAACTCCTTACCATTCATCAGCTATTCAAACGTCCGCTTCCCGTCATTTT
AAGGATGTG
>dme_circ_0008670_junction_seq
GTCCGCTTCCCGTCATTTTAAGGATGTGTGAAAACAAAACAAGAAACAAAAAATCG
>dme_circ_0008671
GACTGCTCGTGGTGCTGCAGTTGTGTGATATTACTTTTTACGCTTGTCGGCTGGCTTGAGGATTTGGAAC

GAGCACATCAGCGTATCGTCCACGGACATCATGGCGCCGGCATTGTCGAACTCGCCGCAGTAGTTGGGCG
CCGAGAACAGGGTGACCAGCATGCGTTTGGCAAAGAACTCGTACCCATCCTCGACGACTTGATGGGCTCG
GCAGATGAGATCGAACTCGTGCTTCTGCAAAAACTTGGCCACCACCTCGGCACCGAAGGTGAAGCTAACG
CCGCGGTCGTTTTCGCCCCAGCCCATGGTATCCTTATCGGGATCGGACCACAGGAGATCGCACAGCAGTC
CCTGGTCGGGCACATCGGTTGGCCGCATAATGCGACGGATCTGCTCCATGGAGGTCAAATCGGGACTGAG
ACCACCGTGGCAGCAGAAGATCTTCTCGTCGACAATGGCCGCCACTGGCAGGCAGTTGAAGCAGTCCGTG
AATGTCTTCCACAACTTGATGCTGTAGCGACGCTTGCATTCGTCGTAGAATCCGTATATGCGATTAATGC
TGGCGCATTCGTGGTTGCCGCGCAGCAGGAAGAAATTCTCCGAGTATTTGATCTT
>dme_circ_0008671_junction_seq
GGAAGAAATTCTCCGAGTATTTGATCTTGACTGCTCGTGGTGCTGCAGTTGTGTGA
>dme_circ_0008672
GTGTGGATCTGGTGCACTAGTGCTGCTGTTGGGTGTCGAGGTTACCGCGAACGATCGTTGGTAGCGCCGA
AATAGCCTCGAAATCTTTAATTTCCGAAATCTATTTCTGCTAAATACTTTTCACAGCTAACAAGTATGAA
AATTTCTTCGTATGCT
>dme_circ_0008672_junction_seq
AACAAGTATGAAAATTTCTTCGTATGCTGTGTGGATCTGGTGCACTAGTGCTGCTG
>dme_circ_0008673
GGTCGGCCTCCACTTTTTTGGCCAGCGCATCGACGGCCTCCTTGGTGGGCAGGGTTTTGAGGATAACCAC
CACATCGGCTACATTGTTGCCCGTCAGTATGGTGCCCTTCTTGAAGGAGCCAACCTGACGCACTTCCTCC
AGTTGCTGTGGAATAATCAGAACAGTTTAAACATGCAAATCACAAGGAGTTGTGTAATGTACTCACACAA
GTTGTCAGATCCCCGGGTGCAACCACCAGATTGTCGAGAACAGCCTGCACCTTGGTCACTAGATTGCCAA
TAGCCGTCTGCTCGCTGGGAGTGGGACTCAG
>dme_circ_0008673_junction_seq
CCGTCTGCTCGCTGGGAGTGGGACTCAGGGTCGGCCTCCACTTTTTTGGCCAGCGC
>dme_circ_0008674
GTTCCTCGTTGGTCGCGCCATTGACCACCACAACGTCGAACTGACCGAAGAACTCAGAGGTCTTCTCCTT
CAAGGGCTCGCGGTCGGCGGAGATGTCCACCATGGGATTGAGAGCACGTGCCCGTGTCAATGATGCTTCG
GCCCGGTTGGTGTTCAGCGATTCACGGGGGACAAGGAATTGTGAACAAAAGTCCTCCTCGGTTACGTCCT
TGTCATCCAGCAGCTTCACGGAGTTCACTCCGGACAGGATGATGTTCTTGGTTATCTCGGCGCCCAGGCC
GCACAGTCCAGCGATGAGAATCTTGGCCGTGCGAAGGCTTAAATCGAAAATATATTTAGCGCATTATAG
CGCCATTGCGGAATGCCGGTTTTTTTGCACTCACCGTTTCTGAGATTCCAGACCCCAGAGTCGGATTTGT
CTGTCGTACAGCTCGTTCTCCGCCTCAGTGAGCTCCACGGCTGTCTCGCTGGTGTCCATATCCACGACCA
TTATTTATTTATCAAGTTTAAAAT
>dme_circ_0008674_junction_seq
ACCATTATTTATTTATCAAGTTTAAAATGTTCCTCGTTGGTCGCGCCATTGACCAC
>dme_circ_0008675
GCTGCAGGAGGACTCCACCGGAGTGCTCTTCGAGTGCGATGTGGAAACCACCGACGGTGGTCTTGTCAAG
GACATCACCGTGATGAAGAAGGCCGAGAAGCGCCGCCTGAAGCTCGTCTGGCGCAACATCATCGCCTTCG
GTTACCTCCATCTGGCTGCCCTCTACGGAGCCTACCTCATG
>dme_circ_0008675_junction_seq
GGCTGCCCTCTACGGAGCCTACCTCATGGCTGCAGGAGGACTCCACCGGAGTGCTC
>dme_circ_0008676
GATACTACATGATTTTGATGCCGATTGCTTGCTTCATCATCCCGACCGTGGTGCCCATGTACGCCTGGGG
TGAATCCTTCATGAACGCCTGGTTTGTGGCCACCATGTTCCGCTGGTGTTTCATTCTGAACGTAACCTGG

CTGGTGAACAGTGCTGCCCACAAGTTCGGTGGCCGTCCCTACGACAA
>dme_circ_0008676_junction_seq
ACAAGTTCGGTGGCCGTCCCTACGACAAGATACTACATGATTTTGATGCCGATTGC
>dme_circ_0008677
AAATCGATGAAGGCGGTAGTGAAGTTTAGCGAGTATTTGCCAAACTCGGCCGTCTTGTAGTCCCAGGGGA
AGACGTGATGGTAGTTATGCCATCCCTCTCCAAAGGCCAAGATAGCCACGGAGATGTTCTCCGAAGGATT
GATAAATCTAAAAAGAACAAACCTCTGTAAGTACACATCCATAAGTTGTTCAAACTCTAAGCACTTACTT
GTCGTAGGGACGGCCACCGAACTTGTGGGCAGCACTGTTCACCAGCCAGGTTACGTTCAGAATGAAACAC
CAGCGGAACATGGTGGCCACAAACCAGGCGTTCATGAAGGATTCACCCCAGGCGTACATGGGCACCACGG
TCGGGATGATGAAGCAAGCAATCGGCATCAAAATCATGTAG
>dme_circ_0008677_junction_seq
GCAAGCAATCGGCATCAAAATCATGTAGAAATCGATGAAGGCGGTAGTGAAGTTTA
>dme_circ_0008678
TTATCATCAACGCTTTATCTTAGAATTAATTAAATGGGCTTCTGCTCTGTTAGGAAATATGTTCCGCGGA
CATTTGCAAAATGGAAATTTATGTTTCTTTGCTTTACTTGAATTTGATTGATTTTCTGCTAAACCCTCCC
ATGATTGGCCCTACGCTCAACCTGCCTATTCGCTTTTTTTGTGTGTAATGACAGCATCCTCAATCTCCTC
CTTGGGCTGGTCCACATCGCCCCATCCCCACGTGGCGTGAGTGCCGTCACCGGTACGCTTCACACGCTTC
TTTATGATATCCGTGGACACCGTCTTGAGGTCGTAGGCCCAGCCGATCTTGGCGAAGAAATCGATGAAGG
CGGTAGTGAAGTTTAGCGAGTATTTGCCAAACTCGGCCGTCTTGTAGTCCCAGGGGAAGACGTGATGGTA
GTTATGCCATCCCTCTCCAAAGGCCAAGATAGCCACGGAGATGTTCTCCGAAGGATTGATAAATCTAAAA
AGAACAAACCTCTGTAAGTACACATCCATAAGTTGTTCAAACTCTAAGCACTTACTTGTCGTAGGGACGG
CCACCGAACTTGTGGGCAGCACTGTTCACCAGCCAGGTTACGTTCAGAATGAAACACCAGCGGAACATGG
TGGCCACAAACCAGGCGTTCATGAAGGATTCACCCCAGGCGTACATGGGCACCACGGTCGGGATGATGAA
GCAAGCAATCGGCATCAAAATCATGTAG
>dme_circ_0008678_junction_seq
GCAAGCAATCGGCATCAAAATCATGTAGTTATCATCAACGCTTTATCTTAGAATTA
>dme_circ_0008679
GAAATATGTTCCGCGGACATTTGCAAAATGGAAATTTATGTTTCTTTGCTTTACTTGAATTTGATTGATT
TTCTGCTAAACCCTCCCATGATTGGCCCTACGCTCAACCTGCCTATTCGCTTTTTTTGTGTGTAATGACA
GCATCCTCAATCTCCTCCTTGGGCTGGTCCACATCGCCCCATCCCCACGTGGCGTGAGTGCCGTCACCGG
TACGCTTCACACGCTTCTTTATGATATCCGTGGACACCGTCTTGAGGTCGTAGGCCCAGCCGATCTTGGC
GAAGAAATCGATGAAGGCGGTAGTGAAGTTTAGCGAGTATTTGCCAAACTCGGCCGTCTTGTAGTCCCAG
GGGAAGACGTGATGGTAGTTATGCCATCCCTCTCCAAAGGCCAAGATAGCCACGGAGATGTTCTCCGAAG
GATTGATAAATCTAAAAAGAACAAACCTCTGTAAGTACACATCCATAAGTTGTTCAAACTCTAAGCACTT
ACTTGTCGTAGGGACGGCCACCGAACTTGTGGGCAGCACTGTTCACCAGCCAGGTTACGTTCAGAATGAA
ACACCAGCGGAACATGGTGGCCACAAACCAGGCGTTCATGAAGGATTCACCCCAGGCGTACATGGGCACC
ACGGTCGGGATGATGAAGCAAGCAATCGGCATCAAAATCATG
>dme_circ_0008679_junction_seq
GAAGCAAGCAATCGGCATCAAAATCATGGAAATATGTTCCGCGGACATTTGCAAAA
>dme_circ_0008680
TCCCAGGGGAAGACGTGATGGTAGTTATGCCATCCCTCTCCAAAGGCCAAGATAGCCACGGAGATGTTCT
CCGAAGGATTGATAAATCTAAAAAGAACAAACCTCTGTAAGTACACATCCATAAGTTGTTCAAACTCTAA
GCACTTACTTGTCGTAGGGACGGCCACCGAACTTGTGGGCAGCACTGTTCACCAGCCAGGTTACG
>dme_circ_0008680_junction_seq

GGCAGCACTGTTCACCAGCCAGGTTACGTCCCAGGGGAAGACGTGATGGTAGTTAT
>dme_circ_0008681
TTATCATCAACGCTTTATCTTAGAATTAATTAAATGGGCTTCTGCTCTGTTAGGAAATATGTTCCGCGGA
CATTTGCAAAATGGAAATTTATGTTTCTTTGCTTTACTTGAATTTGATTGATTTTCTGCTAAACCCTCCC
ATGATTGGCCCTACGCTCAACCTGCCTATTCGCTTTTTTTGTGTGTAATGACAGCATCCTCAATCTCCTC
CTTGGGCTGGTCCACATCGCCCCATCCCCACGTGGCGTGAGTGCCGTCACCGGTACGCTTCACACGCTTC
TTTATGATATCCGTGGACACCGTCTTGAGGTCGTAGGCCCAGCCGATCTTGGCGAAGAAATCGATGAAGG
CGGTAGTGAAGTTTAGCGAGTATTTGCCAAACTCGGCCGTCTTGTAGTCCCAGGGGAAGACGTGATGGTA
GTTATGCCATCCCTCTCCAAAGGCCAAGATAGCCACGGAGATG
>dme_circ_0008681_junction_seq
TCCAAAGGCCAAGATAGCCACGGAGATGTTATCATCAACGCTTTATCTTAGAATTA
>dme_circ_0008682
TTTATATATATTAGTTCCTTGATAATTCTTGTTGTTTTTGGCTTACGCTAAATACTAAAATCTTTCATTT
GCAGGTATTTCtttttctttgaagcggaaagttttcctccatttgtatcatttttttcttttagatttttta
ACTGAAAATTTCTCTCAAATTACAAAAGTGATTTCGAGATAACAGTGAATGAAAATCTCCAATTGCCATC
TATTGTACATAAATATCCAGGTATTAAGCAATTATATGCGTGTATAAATACAAAGTTAGTTTTGCGTCTC
CCCATAAAAAGAGAGCGAAATAAAAACATTTCCGGAGCTGGTTTAACACAGACGGTATAAAGTTTTTCGA
GATACAACAAACAGTTATCATCAACGCTTTATCTTAGAATTAATTAAATGGGCTTCTGCTCTGTTAGGAA
ATATGTTCCGCGGACATTTGCAAAATGGAAATTTATGTTTCTTTGCTTTACTTGAATTTGATTGATTTTC
TGCTAAACCCTCCCATGATTGGCCCTACGCTCAACCTGCCTATTCGCTTTTTTTGTGTG
>dme_circ_0008682_junction_seq
CAACCTGCCTATTCGCTTTTTTTGTGTGTTTATATATATTAGTTCCTTGATAATTC
>dme_circ_0008683
TTATCATCAACGCTTTATCTTAGAATTAATTAAATGGGCTTCTGCTCTGTTAGGAAATATGTTCCGCGGA
CATTTGCAAAATGGAAATTTATGTTTCTTTGCTTTACTTGAATTTGATTGATTTTCTGCTAAACCCTCCC
ATGATTGGCCCTACGCTCAACCTGCCTATTCGCTTTTTTTG
>dme_circ_0008683_junction_seq
CGCTCAACCTGCCTATTCGCTTTTTTTGTTATCATCAACGCTTTATCTTAGAATTA
>dme_circ_0008684
GGGTTTAGCAGAAAATCAATCAAATTCAAGTAAAGCAAAGAAACATAAATTTCCATTTTGCAAATGTCCG
CGGAACATATTTCCTAACAGAGCAGAAGCCCATTTAATTAATTCTAAGATAAAGCGTTGATGATAACTGT
TTGTTGTATCTCGAAAAACTTTATACCGTCT
>dme_circ_0008684_junction_seq
TTGTATCTCGAAAAACTTTATACCGTCTGGGTTTAGCAGAAAATCAATCAAATTCA
>dme_circ_0008685
GCAAAGAAACATAAATTTCCATTTTGCAAATGTCCGCGGAACATATTTCCTAACAGAGCAGAAGCCCATT
TAATTAATTCTAAGATAAAGCGTTGATGATAACTGTTTGTTGTATCTCGAAAAACTTTATACCGTCTGTG
TTAAACCAGCTCCGGAAATGTTTTTATTTCGCTCTCTTTTTATGGGGAGACGCAAAACTAACTTTGTATT
TATACACGCATATAATTGCTTAATACCTGGATATTTAT
>dme_circ_0008685_junction_seq
TATAATTGCTTAATACCTGGATATTTATGCAAAGAAACATAAATTTCCATTTTGCA
>dme_circ_0008686
TTCCTTGATAATTCTTGTTGTTTTTGGCTTACGCTAAATACTAAAATCTTTCATTTGCAGGTATTTCttt
ttctttgaagcggaaagttttcctccatttgtatcatttttttcttttagatttttttaACTGAAAATTTCTC

TCAAATTACAAAAGTGATTTCGAGATAACAGTGAATGAAAATCTCCAATTGCCATCTATTGTACATAAAT
ATCCAGGTATTAAGCAATTATATGCGTGTATAAATACAAAGTTAGTTTTGCGTCTCCCCATAAAAAGAGA
GCGAAATAAAAACATTTCCGGAGCTGGTTTAACACAGACGGTATAAAGTTTTTCGAGATACAACAAACAG
TTATCATCAACGCTTTATCTTAGAATTAATTAAATGGGCTTCTGCTCTGTTAGGAAATATGTTCCGCGGA
CATTTGCAAAATGGAAATTTATG
>dme_circ_0008686_junction_seq
GCGGACATTTGCAAAATGGAAATTTATGTTCCTTGATAATTCTTGTTGTTTTTGGC
>dme_circ_0008687
CAATTATATGCGTGTATAAATACAAAGTTAGTTTTGCGTCTCCCCATAAAAAGAGAGCGAAATAAAAACA
TTTCCGGAGCTGGTTTAACACAGACGGTATAAAGTTTTTCGAGATACAACAAACAGTTATCATCAACGCT
TTATCTTAGAATTAATTAAATGGGCTTCTGCTCTGTTAGGAAATATG
>dme_circ_0008687_junction_seq
ATGGGCTTCTGCTCTGTTAGGAAATATGCAATTATATGCGTGTATAAATACAAAGT
>dme_circ_0008688
AAATATCATATGTATAGGTGAAAGTGTCTTTTAGTGTTCTAACATCGATCGTTTATTATATAGTTTATAT
ATATTAGTTCCTTGATAATTCTTGTTGTTTTTGGCTTACGCTAAATACTAAAATCTTTCATTTGCAGGTA
TTTCtttttctttgaagcggaaagttttcctccatttg
>dme_circ_0008688_junction_seq
tttgaagcggaaagttttcctccatttgAAATATCATATGTATAGGTGAAAGTGTC
>dme_circ_0008689
GGCATTTATAACTCCCCTTAAATTATTTCTCCGCCACTCACACCTTCAGCCTATTTTATGCATATTTGTG
GAACAATGGAAAACAATTAAATATTGAGAACCGTTCGTAATGGAGTCGTGAAGCCAGAAAAGTTGACCCC
AATAAGGGAATTGG
>dme_circ_0008689_junction_seq
GAAAAGTTGACCCCAATAAGGGAATTGGGGCATTTATAACTCCCCTTAAATTATTT
>dme_circ_0008690
GTATTAGATGCAGGTTCTTGTTAGAACTCTAGGGCTAGGGCCATTTGTCCGTGTTTGTTAAATTCCTCGG
AATGCTTTAAATCCGTGCGACTGCGTCGTCTTCCTAGACAATAACTGATAAGTGTTGTTGCGCGCTATTG
TTGCTAAATATACGTATTTTAATGTTGGCATTCCAAAAAACGTAATTGTAATCTAGTT
>dme_circ_0008690_junction_seq
TTCCAAAAAACGTAATTGTAATCTAGTTGTATTAGATGCAGGTTCTTGTTAGAACT
>dme_circ_0008691
CAAAGCTAAAACCCAATTGTTATTTCAACGACAATCGCATGAATTGAAAATGCTCAAAATACCTAAAGTA
AACCCCTAAATCGTATAATCAACTAAACATAGCGAGAATATATAGTCAAAACCATATATGTAGGACCCTG
GCGATTGGTCAAAACCAAAGTCCGTCTAAATTAGCCAACATATAGTGCATCGATATCAGACTAACCAAAT
TACg
>dme_circ_0008691_junction_seq
GCATCGATATCAGACTAACCAAATTACgCAAAGCTAAAACCCAATTGTTATTTCAA
>dme_circ_0008692
TTGCTCAACTTGGAAAAGCGCCAGGGCGTGGCAGGAAAATCAACAACCCTTCGCGCCATATGGCTTGACT
CACTGCGGCAACTTCAGCGCGTGTGGACACTTTCATTTCCCTAATTAACAGTCCAAACGGGAAATTCGAA
GCCaaaaaccgaaaccgaaaatcaaaaaatccaaaagcaaaacgcaaaaatcAACGCGTAGGAATTGTAC
AATAAATAATACGAAAAATAAAACCCCTACGCGAGTGACCAACGTATAATTTAAAGCTCGTGGCCACCAA
AATGCGAGTGGCCAATTGGTTGTACGTACCATTTTGGCAACAACCACAGCTGCGAGCCAGAACATCAAAC

AGTCGGCCAAGATGCGAGCGTTGAAAACATCATCATCAGgaaataaattcgggaaaaatgaataaaaata
taaatataaagcgatatgaataaataCATGAACTAGCACATAGCCAAAGCGATATTCTAAATGAGTGCTA
GGGGGCAAAAAAGGCATAGGACATAGGATTTTACCATTTTCCCAACACTTAGTATTGACGGGCATTGCGG
GGTACTGAGGGTATGGTATTGGACCGAAGATGCCGACCAGAGCGGAATATACATATCGAGATATAGATTC
TGATATGGCGGCTGTATGCTAAAAGGCATTTAAAGCGAATGACATTCTTTCGCCCAGCTGTTGTCATATA
AATCACAGCTTTTAAACAGGGTGTGTCTTTAGAGTTTGGTTACTTAAATTTCAAATTTAAGATGATAAAG
TTTCATAGAAATTAAAGTTAAAAAAGTTCAATTCTATTTAAAAATCAAGGTCATTGAAATTTTAAAATAT
ATTTTAATTGGTTATAAAAAATTTGCTACTTTGCAATTCTCAATTTCAATTGCCTTAACAGAATGATGAT
ATATTTTGAAGTATATAAAACATTTTTTAATAAACGAATACTAACTTAACATTATTAAATGGCTAATTGT
TGACCACATCATAGCCCAGTCCCTTAAAACATAAACAGTGATTTATAACACGATAAAAAGACACACCCTA
TTCCATTCGCATAAATGTAGGTAAAACACGCACAACATCAACAACTCAACTTCTGCGTCAGTAAATAAAC
AAATATTGACTCAAATCGATACAAATGCCAGCGGCAACAACTAGATAATGCACAAAAGTGATTATAATCG
GGGCAACaaaaaaaaaaaaaGAAGAAGAAAAGCGAGCCCATGTTTTTCCAAGCTTTTAATAATGTGTTCT
TTTCTTTGttttttttttCCTTCTTTCCTTTCTCGATGGAACATTTATGGCACAGTTTGCTCAACTTGGA
AAAGCGCCAGGGCGTGGCAGGAAAATCAACAACCCTTCGCGCCATATGGCTTGACTCACTGCGGCAACTT
CAGCGCGTGTGGACACTTTCATTTCCCTAATTAACAGTCCAAACGGGAAATTCGAAGCCaaaaaccgaaa
ccgaaaatcaaaaaatccaaaagcaaaacgcaaaaatcAACGCGTAGGAATTGTACAATAAATAATACGA
AAAATAAAACCCCTACGCGAGTGACCAACGTATAATTTAAAGCTCGTGGCCACCAAAATGCGAGTGGCCA
ATTGGTTGTACGTACCATTTTGGCAACAACCACAGCTGCGAGCCAGAACATCAAACAGTCGGCCAAGATG
CGAGCGTTGAAAACATCATCATCAGgaaataaattcgggaaaaatgaataaaaatataaatataaagcga
tatgaataaataCATGAACTAGCACATAGCCAAAGCGATATTCTAAATGAGTGCTAGGGGGCAAAAAAGG
CATAGGACATAGGATTTTACCATTTTCCCAACACTTAGTATTGACGGGCATTGCGGGGTACTGAGGGTAT
GGTATTGGACCGAAGATGCCGACCAGAGCGGAATATACATATCGAGATATAGATTCTGATATGGCGGCTG
TATGCTAAAAGGCATTTAAAGCGAATGACATTCTTTCGCCCAGCTGTTGTCATATAAATCACAGCTTTTA
AACAGGGTGTGTCTTTAGAGTTTGGTTACTTAAATTTCAAATTTAAGATGATAAAGTTTCATAGAAATTA
AAGTTAAAAAAGTTCAATTCTATTTAAAAATCAAGGTCATTGAAATTTTAAAATATATTTTAATTGGTTA
TAAAAAATTTGCTACTTTGCAATTCTCAATTTCAATTGCCTTAACAGAATGATGATATATTTTGAAGTAT
ATAAAACATTTTTTAATAAACGAATACTAACTTAACATTATTAAATGGCTAATTGTTGACCACATCATAG
CCCAGTCCCTTAAAACATAAACAGTGATTTATAACACGATAAAAAGACACACCCTATTCCATTCGCATAA
ATGTAGGTAAAACACGCACAACATCAACAACTCAACTTCTGCGTCAGTAAATAAACAAATATTGACTCAA
ATCGATACAAATGCCAGCGGCAACAACTAGATAATGCACAAAAGTGATTATAATCGGGGCAACaaaaaaa
aaaaaaGAAGAAGAAAAGCGAGCCCATGTTTTTCCAAGCTTTTAATAATGTGTTCTTTTCTTTGtttttt
ttttCCTTCTTTCCTTTCTCGATGGAACATTTATGGCACAGTTCCAGGAGGCCATAACATCGAATAATAA
TATCTGAATTAAGAGCAGCAGAACTAAAAAACCAACCACTTGGCCGAAACAAACTAAAAATAATTCGAAG
CCCGAGAAATGTTAAAAATAAACCCAAAAGAAATCAACGAATATTTTTCGGAGGCAAAACAAGTCGGCGG
CAACCGCAGCAGCCGCGGTGGCAAAGAATATGAACAGAATTAAAAGGCAGCTGCTTGGAGGCTAGGCTAC
CaaaaaataaatattaaaaaaaaaaCCCAATCAGCGAGCAGAAGCAGCAAAAGCGAATTAACACaaaaaa
tatcaaaaagtaaaatagaaaataGAAGCCGCACAACAGTAAATAGCCGAAAAAATTGTCGTCCAGCCGA
GGTAGCCTCTCCATCAGCGGCGGAGCAGGCGGAAAAGCTACGGAAAATCAACGGAAAAGCGAGGCCATCA
CAAGAGCTCGCGTGGGCGGCGTTCGCTGGCAAGGACAGCGGTTGGTCGGTTGGTCGTTGACGTTGGCAGC
GTCTGAAACTGAGGCGGATTCTGAAGCGGAGGAGCGGCGACGTTGTCGTGGCCACACGGTTTACGCTCCA
CGGTCCGCGGGCCACTTACGCGAATCAATTTCCGTTGCGGTGGCCGTTTCAGTTTTGGTTTCAACGTCGC
CGGCGCCACGCGTGTCGTGATTTCCCAATGGCGCTTCCTCCAGTGGGTGTTTCAACCTAAAAGTGCGATA
ATAAAATACGGATCTACCACACAGTGTTACTAAAAAGATAGAGAAAGAGATAGAAAGAGAGCGCTACAAG

CCAAAGATAAGTTATAAATTATTGTAAAATTAAAACCAGACAACGAGACTCATGTTGTTGTGCGACGGAC
TTGGCCCGGAGCCACCGCGGCAAAGGCATCGAAATCGAACCAGTGCCGCCAGGATTAGAAAAAGACCGAA
ATGCTGCTGCGGCGATGGCGGCAGCGGCAATCAGGCGGAGCAGCCCGGCGGGATAGTTAGCAACCCAATT
AGTTATGGCCAAAGTCTGACAACATTGGCGCGGGTCACGGCGGCCGCACTGACCACGGCGGCCATGCTGC
ACACAACGAATGCCCTGGCTGCCACCGGCTCATCCAGCGCATCCAACTCTTCCACCGGCGGAATAGCACT
GCCGCTGGGCACTGCCACACCTGCCACACACGAACTGAATGCCACACAGCCGTTTGGCGGCTCGGGTCTG
AACTTCAACGAAAGCGGCGCAGGATTAAGTGACCATCATCATCATCAACAACACAATCCCGACGAGGATT
GGCTGGACAACATCGTTTGGGTGTTCAAGGCCTTTGTCATGCTGCTCATCATCATTGCGGCCATCTGCGG
CAATCTGCTTGTTATTATTTCTGTGATGCGTGTTAGAAAATTAAGG
>dme_circ_0008692_junction_seq
TTCTGTGATGCGTGTTAGAAAATTAAGGTTGCTCAACTTGGAAAAGCGCCAGGGCG
>dme_circ_0008693
TATTGAAGTCCCGTCTTGCAATGTACATAAATTGAATTGCCTAATGATTGTCGTAACTCCCGCCAACACA
CTGTAATTTCACTTGTTAAAGTTTACTATTTTTGTTAGGTTTTAAGTGTGTATGCTCTTTGAATTGTTTA
TATTTTTGTCTAATCATTTTCGCATAAAGAATCGTAGCATTCCAACAAAAATTAACAATTGCCTACCTTG
TGTTACTTTCGTCTGCTCCCCTGGTTTTCTTTCGTTAGGCGTTTCACCAGTTCGCACAAGTATTAAACAA
TCCCCGCCCCCTGTTTTCCG
>dme_circ_0008693_junction_seq
TTAAACAATCCCCGCCCCCTGTTTTCCGTATTGAAGTCCCGTCTTGCAATGTACAT
>dme_circ_0008694
TTAAATATCGTCCAAGCGAGCAAACCTACTCTAAAGTGTGTGTTTTAGTATTGAAGTCCCGTCTTGCAAT
GTACATAAATTGAATTGCCTAATGATTGTCGTAACTCCCGCCAACACACTGTAATTTCACTTGTTAAAGT
TTACTATTTTTGTTAGGTTTTAAGTGTGTATGCTCTTTGAATTG
>dme_circ_0008694_junction_seq
GTTTTAAGTGTGTATGCTCTTTGAATTGTTAAATATCGTCCAAGCGAGCAAACCTA
>dme_circ_0008695
GAGCATACACACTTAAAACCTAACAAAAATAGTAAACTTTAACAAGTGAAATTACAGTGTGTTGGCGGGA
GTTACGACAATCATTAGGCAATTCAATTTATGTACATTGCAAGACGGGACTTCAATACTAAAACACACAC
TTTAGAGTAGGTTTGCTCGCTTGGACGATATTTAACTAGTTATTGTTTTGGCTACGCTGGGTGGCTCCGA
GATACACGCATCCAGGCTCTCAACGTAGATTAGATATTTTGTGGTTTAATTGTGGTTTAT
>dme_circ_0008695_junction_seq
GATATTTTGTGGTTTAATTGTGGTTTATGAGCATACACACTTAAAACCTAACAAAA
>dme_circ_0008696
TTAAATATCGTCCAAGCGAGCAAACCTACTCTAAAGTGTGTGTTTTAGTATTGAAGTCCCGTCTTGCAAT
GTACATAAATTGAATTGCCTAATGATTGTCGTAACTCCCGCCAACACACTGTAATTTCACTTG
>dme_circ_0008696_junction_seq
TCCCGCCAACACACTGTAATTTCACTTGTTAAATATCGTCCAAGCGAGCAAACCTA
>dme_circ_0008697
GTTATTGTTTTGGCTACGCTGGGTGGCTCCGAGATACACGCATCCAGGCTCTCAACGTAGATTAGATATT
TTGTGGTTTAATTGTGGTTTATGTATATATAGACGCATACACAGCGGATATTGCAGGAGGTGGAGACGCG
CCGCACGTTGCGCCGCCGCCAGCTTAAATTATGAATGaaacaaaattttggacaaaaatcaaaaacaaaa
caataaacAGGATCTAATGGCGCGAGTCCCTAGGGTAGAATTCGGCCAGGATGGAGGCCGGGATACGCTT
GAGCATCTCCTTGGGGAAGATACGCAGCAGCTGCCAGCCAATGTCCAGGGATTCGAAGACAGTGCGGTTC
TCATAGTTGCCCTGCAGGTACAGAGGAAAGTTTTGTAACAAACCCACTTAATATCAAGCGCGTTCAACTC

ACCTGCGAGATGAAGTTCTTCTCGAACTTGGTCAAGAACTCCAGATACAGCAGATCGTCGGGCGTCAGGG
CCTCCTCACCCACCACAGCCTTCATGGCCTGCACGTCCTTGCCGATGGCGTAGCAAGCGTACAGCTGGTT
GGACACGTCAGAGTGGTCCTTACGGGTCATGCCCTCGCCGATGGCAGACTTCATCAAACGCGACAGCGAT
GGCAGCACGTTGACTGGTGGGTAGATCTGTCTGTTGTGCAGCTGACGATCGACGTAGATCTGGCCCTCGG
TAATGTAACCGGTCAAATCGGGAATGGGATGG
>dme_circ_0008697_junction_seq
GTAACCGGTCAAATCGGGAATGGGATGGGTTATTGTTTTGGCTACGCTGGGTGGCT
>dme_circ_0008698
GATACAGCAGATCGTCGGGCGTCAGGGCCTCCTCACCCACCACAGCCTTCATGGCCTGCACGTCCTTGCC
GATGGCGTAGCAAGCGTACAGCTGGTTGGACACGTCAGAGTGGTCCTTACGGGTCATGCCCTCGCCGATG
GCAGACTTCATCAAACGCGACAGCGATGGCAGCACGTTGACTGGTGGGTAGATCTGTCTGTT
>dme_circ_0008698_junction_seq
CGTTGACTGGTGGGTAGATCTGTCTGTTGATACAGCAGATCGTCGGGCGTCAGGGC
>dme_circ_0008699
GCAGATCGTCGGGCGTCAGGGCCTCCTCACCCACCACAGCCTTCATGGCCTGCACGTCCTTGCCGATGGC
GTAGCAAGCGTACAGCTGGTTGGACACGTCAGAGTGGTCCTTACGGGTCATGCCCTCGCCGATGGCAGAC
TTCATCAAACGCGACAGCGATGGCAGCACGTTGACTGGTGGGTAGATCTGTCTGTTGTGCAGCTGACGAT
CGACGTAGATCTGGCCCTCGGTAATGTAACCG
>dme_circ_0008699_junction_seq
GTAGATCTGGCCCTCGGTAATGTAACCGGCAGATCGTCGGGCGTCAGGGCCTCCTC
>dme_circ_0008700
GGCATGGTCAGAATGGGGATCTGGGTGATGGATCCGTTGCGTCCCTCCACACGACCGGCACGCTCGTAGA
TGGTGGCCAAATCGGTGTACATGTAACCGGGGAAACCACGACGACCGGGCACCTCCTCACGGGCAGCAGA
CACCTCACGCAGAGCCTCGGCGTACGAGGACATGTCG
>dme_circ_0008700_junction_seq
CAGAGCCTCGGCGTACGAGGACATGTCGGGCATGGTCAGAATGGGGATCTGGGTGA
>dme_circ_0008701
GTGCTCGAGGTGAGCGGCTCCAAGGCCGTGGTCCAGGTGTTCGAGGGCACCTCGGGCATCGATGCCAAGA
ACACGCTTTGCGAGTTCACCGGCGACATTCTGCGCACGCCCGTGTCGGAGGATATGTTGGGCCGTGTGTT
CAACGGTTCCGGCAAGCCCATCGACAAGGGACCCCCAATTCTGGCTGAGGATTTCCTGGACATCCAGGGC
CAGCCCATCAACCCCTGGTCGCG
>dme_circ_0008701_junction_seq
AGGGCCAGCCCATCAACCCCTGGTCGCGGTGCTCGAGGTGAGCGGCTCCAAGGCCG
>dme_circ_0008702
GTGCTCGAGGTGAGCGGCTCCAAGGCCGTGGTCCAGGTGTTCGAGGGCACCTCGGGCATCGATGCCAAGA
ACACGCTTTGCGAGTTCACCGGCGACATTCTGCGCACGCCCGTGTCGGAGGATATGTTGGGCCGTG
>dme_circ_0008702_junction_seq
CCCGTGTCGGAGGATATGTTGGGCCGTGGTGCTCGAGGTGAGCGGCTCCAAGGCCG
>dme_circ_0008703
GGTCGTGGGTGTTCCAGCTCCCCAATTAGCACAGTCCCTAATCGGAGAGACGCGACCTCTGGGTCATTCC
AGCCGGAAGCTGGCGGCATCAATGGGGAACGGCCTAGACGACTCCGGTAATTCTGGTGCCGAATCGCCAT
CACCAGGTGGCACTGGCATCTACAAG
>dme_circ_0008703_junction_seq
ATCACCAGGTGGCACTGGCATCTACAAGGGTCGTGGGTGTTCCAGCTCCCCAATTA

>dme_circ_0008704
GTGTGGACTCCCTGATGCTTGTGGAGGAGGGCTTCGAAGTTGTGTCCGTAGATGCCTCTGATAAGATGTT
GAAGTACGCCCTCAAGGAGCGCTGGGCCCGGCGAAATGAAGCTGCCTTCGATAAGTGGGGTCATTGAGGA
AGCCAACTGGTTGACACTGTACGACGACATTCAGGAGCACATTCAGGATGGTTTCGATGCCGTCATTTGC
TTGGGCAACTCCTTTGCCCACTTGATGGACGGGTTCGGGGATCAGCGGGAGCACAAGCAGGCTATTGGCA
ACTTCGAAAAGTGCCTCAAGCCTGGAGGCGTCCTGCTTATCGATCATCGCAACTACGACAATATCCTGGA
AACGGGAGCTACGCCTGCCAAGAGCATCTACTATAAT
>dme_circ_0008704_junction_seq
TACGCCTGCCAAGAGCATCTACTATAATGTGTGGACTCCCTGATGCTTGTGGAGGA
>dme_circ_0008705
GTGTGGACTCCCTGATGCTTGTGGAGGAGGGCTTCGAAGTTGTGTCCGTAGATGCCTCTGATAAGATGTT
GAAGTACGCCCTCAAGGAGCGCTGGGCCCGGCGAAATGAAGCTGCCTTCGATAAGTGGGGTCATTGAGGA
AGCCAACTGGTTGACACTGTACGACGACATTCAGGAGCACATTCAGGATGGTTTCGATGCCGTCATTTGC
TTGGGCAACTCCTTTGCCCACTTGATGGACGGGTTCGGGGATCAGCGGGAGCACAAGCAGGCTATTGGCA
ACTTCGAAAAGTGCCTCAAGCCTGGAGGCGTCCTGCTTATCGATCATCGCAACTACGACAATATCCTGGA
AACGGGAGCTACGCCTGCCAAGAGCATCTACTATAATGACGAGTCACACGGCGGACATCAAGACATCCGT
GCTCTTCTACTGCGGTAAGCCCGCCCTGGTGTCGATGGACTACCTGATCGCTGGCAACAAGTTGACCAGT
GAGTTCCGTCTGTCCTACTATCCCCACGGGCTGAAACGATTCCAGGAGATTCTAGCCGAGATCTTCTCCG
CCAAAGCCAAGCACCAGTTGTATGGTGACTTCAAGGAGATGAGCCAG
>dme_circ_0008705_junction_seq
GTATGGTGACTTCAAGGAGATGAGCCAGGTGTGGACTCCCTGATGCTTGTGGAGGA
>dme_circ_0008706
GCAGCACTCCGAATCCAATCCTAGCCAGCAGCCATGGCTGAGAATGAACCGCTGAACGACCTGCAGAGCC
AGGTGAATGGTCGTATCCTGCCCGCCGCCAATGGACATACAGCATCCGCGACCAACGGATTGGTGCATGC
GAATGGCAAGGATGCAATGAAGCCGTCAGCTCCCGTTCAGAATGGGAATGGCAACGCGAACGGGACACAC
ATCGAGGCGCCCTGCTGTCGGGACATACACGGCAAGGAGCCACCGGACGGAGGAGCACGCGCCTGGCTGG
TCATGGTGAGCGCCTTCCTTTGCAACGGCATCATCTTTGGGTTCATCAACACCTACGGCGTTATCCATTC
GCTGCTGACGGATAGGCTTACGAAGCTCGGCGATCCGGAGGCGTCCAGCAAAGCGGGGTGAGTGTGTCAC
GGAAATCGGGTATCGAGTATGGGGTATGGAACTTGGGGATGTTTCCCCCATCCATCCGTACACGAAAGCA
ATTTGTCTATAATCCCTTCAGCCCCTGGCGCATCTATTCGGAATTTATGGACGGCTGCTGCCGGCGTTGA
TTGTAATTATGCAGGCAGGGCAAATGAGATTAGATGGGAACTGGGCAGTTGTTTTTTGCGACTACTATCT
CTTTTGGGCACGGACCTGGCCAACCACTCCAGAGAACAAATCATATTTTATGGCCTCTGATTGCTGACCG
GGGGGTCTCAGACTTTGATTTGCAAAAGCCTAGAGGTTCAATCGTCAAAGATTCCCCATTAGTTGATAAT
ATTGTCACATCTGACTTGCATTTAATGGCATCCACAGGCAAAGATTGTACTATCACCACTTGAAGTTGCC
TTGAAATATATAAAGGAGATCTTATATTTACGAATTTAAATTTGATTAGATGATGACGGACTTCATACGC
AATAATACTTTGCCAATTTTTATTTACATTTATTAATATTTATTTCGAATCTAACGGACACTCGGACATG
GCAACGCACATTGGCGTCTCCCATCCGCCAGCTCTTGGGTTCAATAAAACGTTGTAGTACGTAGTACGAA
CGAAGTGACGATATAGGAATCCAACGTAGCGACGTCAAGTGAATTAAATTGCTAAAAGTTGATTGGATCA
CAAAGCATTATTCAACAGACTTCGAGTGCGAAGAGCACGAAGACAGCGACGTCGCATAGAGTTGGAGCGG
AAAATCCGTAGGGTTCTCCGCCAGAAGTGCATCATTGTTTTTTGGCCACCTTAACTGGAGCACCGGGCAG
AGCCACCCTCAACCAGAAACAACTGAAACAAACGAGCAGGGTTCATACCACAGCATTTGTCAAGGTTGTG
CCGCTTGGCCTGCATTACGCATACGCAACGTGGGACGGCTAACGCATTGGGGGCACGGCCGGCGTTTTTA
AGACGAGCTGGTCGAATTGGCCAAGCCGACCGGCAGAAGACGAAAGCCACCAGCCAGTGACCCAGTTACT
TGGTCGACTGACGGCGCTGCATCGGGGAAGCCCCTCAAGTGCTCCGCCTGACAGCGATCCTCCTCCGACC

GAACGGCAGCCAGATGCACTCATCTAGGGCTAAGGCTAGAGCTAAGCCAAGACTGGCTGCCAGTTCAGTT
CCGCAATATGATTAAAACCTGACGTAATCCGAACCCGAACCCCAGCTCTTGTGTGCCATATTTTCCTTTT
CCTCCTCTTCCTCCGCCCACTTTTATCGCGGTGTCGATTCGTCAATTAATTGTCAGCCTTTGTCAATTAC
ACACCTCCGTAGGCACATCAGCGCGACTTGGGGGAGCTTAGTGGACGCTGGTTGGACCATTGGTTCATCC
GCGCGGATCGGAGGTCAAGATCAATCATCCAATCGAGTGTCAATCTAAGTGCTATCCCACGATGCAATCC
GCCTGCAGATTGCGTCAGATATTGCATGAGATCGCATCGGGTTGCCTACCTAACTATGAATATGAGGCGT
CCTATAAATATTCCGATCTATTTTCGCCAGACGCCGGCGGAGCCATGCAACCCGGCAGATTCCCCTTCCG
TACGGATTCGTTTTTAGCACCGATTTTTGGCACCGTCCATTAGGCTATTATCTAATGCCTCTCCTCGCCA
TTGACTGCTGGCTGTGCAGTGAGAGAGTATCGTCGTCGACGTCTCTATCTGTGGCATTGACATTGGACTG
GCCGGCTCTTGCCGAGATTGCAGTGGAATATCGTTCTGCGCATGTGGGCGAGAGTTACGTGCCATCTCAC
GGCCCCATTCCTCAATGGGACGTGTCTCGGCGAACGGTCAATAGGTATCCATGAGGCATCTGGAGTGGGT
CAAGTGTTATTTTGGGACATGTGCGTTGCACTTTCTGTTCTATTGTATTTCTACCATATTATATACTATG
TGGAATTTAAGATAGCTGAGGAGCGTACTTCTTATACACTGAAATGAAAATGAAAATGGAAAGAAATAAA
CAAATCTTTGGATTAAAGTTCAAGGGAGGTTAGCTTATGAATGGTGTGCATATCGCCATTAAAAAAGCCA
TTCCGACGTAGATTGTGACAACAAATTCGGACGGACACGCGAACTGAATCGAGGGCTTATCTCCAGTTCC
GGCGTATAGAAGTATTTGAAATTCTCCGCGATTGGAACTACAAGTCCGCTTAGTTTTGAGGGGTCATAGA
CTTCTAATTGCGTGCTATAAATATAATCTATACGGTTTCAATTAGTCGTAAATAAAGCGATAATTGCTTT
GGAAGCGCTACCTGCTCCGGTCCACTGTCCATGTCCCAGGCAATGGACCATTGGGACGCTGGCGCGGCTG
CAGCTGCACAACTGCGTTATCACGGCATTTGATCATCCTCGCAGGCGGGCAACAAAGAATTAACCCAATT
CTGTCAGTCGGACATCAATTGGAAGTGCCAAAGTGGAACAAGCGAAAGTGTTTACGCCACGAAAACCATT
GGATTTCCCCCACTTCTTCAGCCAGAGTTCAGCGGAAAATTATTGACGACGAAAATCGAATCTTTGAGCG
GCGTGCGACAAGATGCATTTGGTCTTCCAACCCCATCAACCGCCAGCGGATCGGGTTTCCAGCTCGCACA
CTCTGTTCGACGCAGTCGGGCATCCACCTGGAAGTTGCAATGAAACACCTGGGATGGCGGCCCAACTCTA
GACATGGTTTGCATGGCCTCACTTCACAATCGACCGCAAGTGCGTGAGATTCGATTTGTCAATTGAAATT
GGTCCAAGTGAGTCGAGCAGACAGTTGGTCAACAAGATCGGGATTAGGTAGTTGCCTCACGGCAAATAAA
TGCACAGGAATCCATCTAGAAACAATATATATATAAACAATATTAACAATATAAATTTTGATAAGCAAGA
AAACCAAATATTGTGCAAGACGTGAGATTGCAAATCAACATTCCTTCACATCCACATATTGAAATGATCT
TTACTTAAGATGAAAGGTATTTAATATTTCAACATAGCTTTAAAAATGGGTCAAAATGTAGCTGTATTTA
AGGAAGCACACCTTCTGGATTTCACAAAGTTTCTTGTTTTCACACAGATTACTTAATTTGGATATCAAGA
ATACTTAATCATTGTAGAAATTACTTTCAGCCTCTGATTGGCGCCCTGGCCATCGGCACCACATTCCTCT
TCTCCACAATGGCTGGCTGTCTGACGGACAAGATTGGCCTGCGGCTGACCACGTTCGCCGGCGGAGTCCT
CTCCGCGGGAGGACTGCTGCTGTCCTCGTTTTGCACGGAGAACATCGGCGCCCTGTACTTCACCTACGGG
ATCATGTTCGGCCTGGGTGCCGCCCTTGCCTACACGCCCACATTGGCCATCCTGGGCCACTATTTCAAGC
GGTACCTGGGAAAGGTGAGTGGCTTTGTGACCGCCGGCTCCAGCGTTTTCACTGTGATACTGCCGCCCTG
TCTGGACAAGCTGCTCGGTAGCTATGGTCTGGAGGGGACCCTGCGGGATAATGAGCCTCGTTTCCGCCTT
TATTATCCTTTGCTCCTTCGTGTACAAGCCACTGCATCCGCCACCGGAGCCGCCGAAGAAGAAGCCCGGC
AGATCGCGCATCAATCTCTTCCTACGCTCGATCATCAACGTGGAGATCTGGAAGCGTAAACGCTTCGTCA
TCTGGGCACTTTGTGTGCCGCTCGCCTTGTTCGGCTACTTTGTGCCCTATGTGCACATGATGCAGTTCGT
GAAAACCACTTTTCCGGGAGAGGATGTCAACCTGCCGGTCATGTGTATCGGCATCACCTCCGGCATCGGG
CGTCTGATTTTCGGAGTCATTGCCGATATGCCGGGTGTGAACCGTATGTACCTGCAACAATTGTCCTTGG
TGTCCATTGGCCTGGTTACCTTGCTGCTGCCGCTCACCAACTCGTACGTCATCCTCGTCTCGTTCACCCT
GGTCATGGGTCTTTTCGACGGCTGCTTCATTTCGCTGCTGGGCCCAATAGCCTACGAGATCTGTGGCCCC
TCGGGCGCCACACAGGCCATCGGCTTTCTGCTAGGACTCAGTTCTATTCCGCTCACAGTGGGTCCACCAG
TGGCGGGCATGATCTTCGACAGCACGAACTCCTACACATTGCCGCTGATCCTCGCCGGACTCCCCCCACT

ACTTGGCTCCTCGCTCTTGTTCCTCATCAAGTGCGTCAAGGAGGAGGAGAACGGGGTCAGTGCGCCGCGA
GCGGTGGTACTGGCCAATGGAGACATCGAGATTGCCGGCAACGGCCATTATCGGTCGGCTGGTAAGTATT
AGGTTTTCCTACTCCTACCCAATCCCTTTTAAACTAGGTTAATTGATTTGCCACTTTGTAACAGGCACCA
AGTCCAGTGCACCGCTGATGGGTGCCACGAATGGTATCAATGGAAGCGCACCAGCGTCCCCAACTATCCA
GAGCAATGGACACGTGGACAACGGAACTGG
>dme_circ_0008706_junction_seq
GCAATGGACACGTGGACAACGGAACTGGGCAGCACTCCGAATCCAATCCTAGCCAG
>dme_circ_0008707
GTTCCGTTGTCCACGTGTCCATTGCTCTGGATAGTTGGGGACGCTGGTGCGCTTCCATTGATACCATTCG
TGGCACCCATCAGCGGTGCACTGGACTTGGTGCCTGTTACAAAGTGGCAAATCAATTAACCTAGTTTAAA
AGGGATTGGGTAGGAGTAGGAAAACCTAATACTTACCAGCCGACCGATAATGGCCGTTGCCGGCAATCTC
GATGTCTCCATTGGCCAGTACCACCGCTCGCGGCGCACTGACCCCGTTCTCCTCCTCCTTGACGCACTTG
ATGAGGAACAAGAGCGAGGAGCCAAGTAGTGGGGGGAGTCCGGCGAGGATCAGCGGCAATGTGTAGGAGT
TCGTGCTGTCGAAGATCATGCCCGCCACTGGTGGACCCACTGTGAGCGGAATAGAACTGAGTCCTAGCAG
AAAGCCGATGGCCTGTGTGGCGCCCGAGGGGCCACAGATCTCGTAGGCTATTGGGCCCAGCAGCGAAATG
AAGCAGCCGTCGAAAAGACCCATGACCAGG
>dme_circ_0008707_junction_seq
GCAGCCGTCGAAAAGACCCATGACCAGGGTTCCGTTGTCCACGTGTCCATTGCTCT
>dme_circ_0008708
GCCACACAGTGCAGGCGACTGCCCTTTTATATGTACTATACTCGTTGTGAACGCGCCATTAATGCGCTGG
GTATTAAAACACACACCGTATCACCGTATGACCGACCGTATGAGATATCAATCATAAATTCGTAGAATTT
ACACAAGCATATAGCATATTTTTGGATAATCGATTGTAAAGCAGGATGTAGAGTAGCAGCCTAGTGGAAT
AAGACAAGGAGTGTGTGTTAAGCGCCTAATTGTCATTGTTGACTTAAGGAAAGTGAAGTGTGGATAAACA
ATTGTTCAATGCCAATGAAAAAAGCATGAGTTAAACTGTATACAATATCTAG
>dme_circ_0008708_junction_seq
CATGAGTTAAACTGTATACAATATCTAGGCCACACAGTGCAGGCGACTGCCCTTTT
>dme_circ_0008709
TCAACAATGACAATTAGGCGCTTAACACACACTCCTTGTCTTATTCCACTAGGCTGCTACTCTACATCCT
GCTTTACAATCGATTATCCAAAAATATGCTATATGCTTGTGTAAATTCTACGAATTTATGATTGATATCT
CATACGGTCGGTCATACGGTGATACGGTGTGTGTTTTAATACCCAGCGCATTAATGGCGCGTTCACAACG
AGTATAG
>dme_circ_0008709_junction_seq
ATTAATGGCGCGTTCACAACGAGTATAGTCAACAATGACAATTAGGCGCTTAACAC
>dme_circ_0008710
ATATTGTATACAGTTTAACTCATGCTTTTTTCATTGGCATTGAACAATTGTTTATCCACACTTCACTTTC
CTTAAGTCAACAATGACAATTAGGCGCTTAACACACACTCCTTGTCTTATTCCACTAGGCTGCTACTCTA
CATCCTGCTTTACAATCGATTATCCAAAAATATGCTATATGCTTGTGTAAATTCTACGAATTTATGATTG
ATATCTCATACGGTCGGTCATACGGTGATACGGTGTGTGTTTTAATACCCAGCGCATTAATGGCGCGTTC
ACAACGAGTATAG
>dme_circ_0008710_junction_seq
ATTAATGGCGCGTTCACAACGAGTATAGATATTGTATACAGTTTAACTCATGCTTT
>dme_circ_0008711
GTTAAACTGTATACAATATCTAGGTTAATGTCGATGTGCAGCTTGAAGGGCCATTAATTTGCAATCGTAC
CACTTTTGATACACAATAAATGTATTTTTGTTATACTACAAATTTACAATCGACGGAATCGTGGGTTACG

GCAT
>dme_circ_0008711_junction_seq
CAATCGACGGAATCGTGGGTTACGGCATGTTAAACTGTATACAATATCTAGGTTAA
>dme_circ_0008712
GACATGTCCGAGATTTCCATAGAGGAGACCCCATTGGATTCGTTTTTCGATTCCAAATCAATTTCGATTT
CACGTTGCGGGGTGGGGCTGGGACTTTGTATGCGCTGTGAACGCAACTGGTCGGAACGCTTCAGTCTGGA
TGCATGTGGAGTCAGTGCACCGATCACTTCCGGCTCCTTAGGTTGCGAGCTAG
>dme_circ_0008712_junction_seq
ACTTCCGGCTCCTTAGGTTGCGAGCTAGGACATGTCCGAGATTTCCATAGAGGAGA
>dme_circ_0008713
GTATCGATGGGTTCGTGCAGCTGGGCAAAGTGCAGCTTGTGTTCGGTGAGTCCGCCCTTTGGACGATCGC
GGGTGATGACACCACGGCTACATAGAAAGCATCTATAAGTATCAGGATGTATTTTGATTATATATCGACC
TACCTATCCACCATCCAGATGCGGGCCTTGGCCTCCTCCTCGGTGAGACCCTCCACCTTCATGGCCATCA
GGCACAGGTTGGCAATACCAAGAGCCGCTTCTCCGGCGCCCAGGAACAACAGCGTGTTATCCTTCAGCTG
GGTCTTCTTGATCTTTAGCGAGGCCAGCAGACCAGCCACGGCCACCGACGCGGTTCCTTGAATATCGTCG
TTGAAGGTGCAGAAGGAGTCGCGGTATTTGGACAACAGCCTGAAGGCATTGGCGTTGGCAAAGTCCTCGA
ACTGGATTAGGCAGTTTTGACCAAAGCGACGAACGCAGGCATGCATGAACTCATCGATGAACTCATC
>dme_circ_0008713_junction_seq
CATGCATGAACTCATCGATGAACTCATCGTATCGATGGGTTCGTGCAGCTGGGCAA
>dme_circ_0008714
GGCATTGGCGTTGGCAAAGTCCTCGAACTGGATTAGGCAGTTTTGACCAAAGCGACGAACGCAGGCATGC
ATGAACTCATCGATGAACTCATCGTACAGATCTCCAGTGGCCCTGCGTTCGCGCAGACCGATGTACAGGG
GATCCTCCAGGATGGATTCG
>dme_circ_0008714_junction_seq
GTACAGGGGATCCTCCAGGATGGATTCGGGCATTGGCGTTGGCAAAGTCCTCGAAC
>dme_circ_0008715
GTGGCCCTGCGTTCGCGCAGACCGATGTACAGGGGATCCTCCAGGATGGATTCGGTATTGGTGCCCACAT
CCAAGGTGATGGGCAGGCACTGCGATGGCTTAATGCCCGCCAAGGCCGTATACAGGGACAGTTTGCCCAC
GGGTATACCCATTCCGTTGGCGCCCAGATCTCCCA
>dme_circ_0008715_junction_seq
CCCATTCCGTTGGCGCCCAGATCTCCCAGTGGCCCTGCGTTCGCGCAGACCGATGT
>dme_circ_0008716
AATAGCGCCATCCGTGGCATTACCCAGAAATTCGGCCATATTCTGAGGCAAGGGATCCGGCGTGTCCTTT
ACTTGGATGCCTCCGATCTCAATGGCAGCAGGAACATTTGGTCGGATGGGACCCTCACTGGGTGCGTGGG
AGGCGAAGAAAATCAACGACGTG
>dme_circ_0008716_junction_seq
GTGGGAGGCGAAGAAAATCAACGACGTGAATAGCGCCATCCGTGGCATTACCCAGA
>dme_circ_0008717
GTTTGATGAGGATGGCAGTGGATCGATTAATATGACCGAGTTCCTGCTTAAACTAAGGGTAAGATGCACA
ATACATCTTATTTTGTACATACCATTCATAAGCTATAAACTATTCCATTCCATTGAAATATATTATTTTC
CCATACTTAGCCCCCCATGCCGCAGAGTCGTTTGAATATTATTGATCAGGCGTTCAACAAAATGGATCGT
GATGAAGATGGAGTAATTACCATTCAAGATCTTAAGAACGTGTACTCTGTAAAAGAACATCCCAAG
>dme_circ_0008717_junction_seq
CGTGTACTCTGTAAAAGAACATCCCAAGGTTTGATGAGGATGGCAGTGGATCGATT

>dme_circ_0008718
GGCGTTGCAACAGGCAAATATCATGGTGCCATATTGACAAACTCGGAGACATGGGAGGCAAAGTCGTTGC
AGGCTGGACGCAAATCTGGCGATTTATTGGCACCCATAATTTATTGCCCCGAATTGCATTTCTCGGAATT
CGCTTCGGCCATTGCTGATATGAAAAACTCGGTGGCGG
>dme_circ_0008718_junction_seq
ATTGCTGATATGAAAAACTCGGTGGCGGGGCGTTGCAACAGGCAAATATCATGGTG
>dme_circ_0008719
GTTGCAAAAAGTGCTCATGGAGCGGCGGGAGGAGTTGGACAAGCGAGTTTCCTTCATAGAGGAACTTGAT
CGAGAACTGGAGGCCACCAAACTTCACAATTTGGCCATGAAAGATTGGTTTAAACAGCAGAAAATGCTGG
CCAAACAGCGCAAAAACGAAATCATGGAAAG
>dme_circ_0008719_junction_seq
AACAGCGCAAAAACGAAATCATGGAAAGGTTGCAAAAAGTGCTCATGGAGCGGCGG
>dme_circ_0008720
GGTGATGTGCATCCTGGGTGGCAGCTATGTGGAACGACGCGTGGGTCCTGACCAGAATTGTGTGCGAAGA
ATGCTGTACGGGATCTGTAAGGCATACCGATAATCGGAGTGCCACAGACATCTATAAAGAACACCTTCCC
TTAGTGGATTCCTATATAGATTTCCGTAGCATCTCCAGAAACATCACCAATCTCTCTTTTATATCCCATT
TTATAATACCAGGCAGCATCCTGACCACTTCTTTGATTGGGGTCATCATCCTGGCTAATTGTGATGACAA
AATACTAGTACTT
>dme_circ_0008720_junction_seq
TAATTGTGATGACAAAATACTAGTACTTGGTGATGTGCATCCTGGGTGGCAGCTAT
>dme_circ_0008721
gaaacaaaaacacaacttaaactaagaacagacaaataacaaaaTAACAACTATGCCACGTACATTAGAG
TACATTGAAATTAGAGTTAAAACCGAATTGTTTGCCGTGAAATTCGGAGCCCCAGAAATTCCGAACCTAA
AATGTTATCGAGAAACGCAGACGCAGGCGCTTGCTGAAGCTAGTGAAAACTTTGGTATTAAC
>dme_circ_0008721_junction_seq
TGAAGCTAGTGAAAACTTTGGTATTAACgaaacaaaaacacaacttaaactaagaa
>dme_circ_0008722
TTGACTATATAGTGGCTGCGTTCATTGACCTTTTCGCGAAGATGGAATGATTACTGGCCCGGAGCTATTC
GAAATTGCCTCGCGTATAGCAGCTTATTACAGTTGAGAGTTATTTGTGGTTATATGGAATCGTTCGTTAT
AGCTAG
>dme_circ_0008722_junction_seq
GTTATATGGAATCGTTCGTTATAGCTAGTTGACTATATAGTGGCTGCGTTCATTGA
>dme_circ_0008723
AAAATGTCCGCATCGACGGACCAGGATCCCGTGGAACTAATGCTGAAGAAGACCGGCTGCATAGAGCTTC
ACTACAAGGTGCAGGAGTGTATCGCCGAGACGGGCGACTGGAGGGCTTGCCAGGAGAAGG
>dme_circ_0008723_junction_seq
GGCGACTGGAGGGCTTGCCAGGAGAAGGAAAATGTCCGCATCGACGGACCAGGATC
>dme_circ_0008724
GAAACGATGCTGCCTACAATAAGTTTGAGAAACTGCTTGAGTGCAACAAGGAGATCTATTGGATTACGAG
CGGTTACCTCTACGATAAAAATGCCGAATATCTTGATTTTATTGATGATACAGCGATCTCCGACAATATG
ATATTAGTGCTATTCAGCGCCAT
>dme_circ_0008724_junction_seq
ATATGATATTAGTGCTATTCAGCGCCATGAAACGATGCTGCCTACAATAAGTTTGA
>dme_circ_0008725

GAAACGATGCTGCCTACAATAAGTTTGAGAAACTGCTTGAGTGCAACAAGGAGATCTATTGGATTACGAG
CGGTTACCTCTACGATAAAAATGCCGAATATCTTGATTTTATTGATGATACAGCGATCTCCGACAATATG
ATATTAGTGCTATTCAGCGCCATGATTAATGGCTAACAATCCGATGCTGCTGCTACAAATACTCGCCTAT
AATGTTGAATGCATTGTGAAAGCGTTTTCCGAGGGCATGATCGAAATTGCCCAAAATATTCAGGAAAACG
AGAAGATTTCGGCAGAAAGGATGCTGACAT
>dme_circ_0008725_junction_seq
AAGATTTCGGCAGAAAGGATGCTGACATGAAACGATGCTGCCTACAATAAGTTTGA
>dme_circ_0008726
GGATTCTCGATTTCAATATGAAGCGCCAAAAATGATTGCAGCGTTTGCATCGTAGTCTTGAAAAGGTTTT
TTTCATTGATTTAATTAGAGCCACTGAAAGCGGTTGGCCAGCCAGAAAAACGATAAAAGGGATTCCATAC
CAGTCTGCGCCCAGCGCCCCCCGAAACGCGGTAGTACAGAAATATCAAAAAAAGAACCACAGCCGGAAAA
GGGCAAAAGTTTTATTCCGACGACGACGAAGAGCAGAGGACAGCAGCAGACAGATTGAAAAACAGCGAAA
TGGACAAAAATCTGATGATGCCGAAGCGTTCGCGCATCGATGTCAAGGGCAACTTTGCCAACGGACCGCT
GCAAGCACGCCCACTGGTGGCCCTACTCGATGGACGCGACTGCTCCATCGAGATGCCCATCCTAAAGGAT
GTGGCCACGGTGGCCTTTTGCGATGCACAAAGCACCTCCGAAATACACGAGAAGGGTCCTCAACGAGGCA
GTGGGAGCTCTGATGTGGCACACCATCATCTTGACAAAGGAGGATCTTGAGAAGTTCAAAGCTTTACGCA
TTATCGTGCGCATCGGCAGCGGCACAGACAACATTGATGTAAAGGCGGCGGGCGAACTGGGCATCGCCGT
GTGCAACGTTCCCGGCTACGGAGTCGAGGAGGTGGCGGACACGACGATGTGCCTCATCCTCAACCTCTAC
CGGCGGACATACTGGCTAGCGAACATGGTGCGCGAGGGCAAGAAGTTCACCGGACCCGAGCAAGTGCGGG
AGGCTGCGCATTGTAAGTCATTGGCCAAAAACGATATGTAAAATCAGTGAAATTCTACGGCATTGGTATT
GATTGAAAATAGTTATTCTTTTAAGGGCATATAATGCTTAAGCTCCCCGCGAAGGTAGCAGAAAAATTTT
AGAAGCAAAGGAAAAGTCAGTTGATATTATTTATGCTAGTCACGTACATAGATTACAGATAGTATAAAAC
CTACATACAATATATTTACAAACATAAAAGGAAAACGATTTGTCTATTTCCATTTGAGTTGGACGATTGC
GAATTGTATTGATAAAAACAAGTCACCATGACATTTGTATAGCTTGGTTTTTAACGAATGCTTTCATTTG
CCATGGATGTCTGATAAAATAGATTGTTTTCATATTTACCAGATTATTATTTCACCGATTGAATTCAGTG
GCTACAGCTCATAAATATAGCGTACGCTTATTGCCGTATTCAAATTACTTCATCCAATCCAAATGCAAAT
TGCGTGTTTCAAAATGCATGATTTGCATTTAATCAAGACTTTCAGCTAGTTATAAATAATAGAGGAGCTA
GAACGCACATATATGAGCAGCATTAATATTTAATCGATACCACCAAGTATACCTTTGAATGCATCTAACT
GGAACAACACCATCTGCTTGTTTTCTCAACGCAGGGGCTGTGCACGCATCCGCGGCGACACCTTGGGTCT
GGTGGGACTGGGCCGCATTGGTAGCGCCGTGGCCCTGCGGGCTAAGGCGTTCGGCTTCAACGTCATCTTC
TACGATCCCTACCTGCCCGATGGCATCGACAAGTCGCTGGGCCTAACTCGCGTCTACACGCTGCAGGACC
TGCTTTTCCAGTCCGATTGCGTCTCACTGCATTGCACGCTCAACGAGCACAACCATCATTTAATCAATGA
ATTCACAATTAAACAGGATGCGACCTGGTGCATTTCTGGTGAACACCGCACGCGGCGGTCTGGTCGATGA
CGAGACCCTGGCGTTGGCGCTGAAGCAGGGAAGGATAAGAGCTGCCGCCCTGGACGTTCACGAAAACGAA
CCTTACAATGTATTTCAAGGGCGCACTGAAGGATGCCCCAAATCTGATTTGCACACCGCACGCCGCCTTC
TTCAGCGACGCGTCCGCAACGGAGCTGCGCGAAATGGCAGCCACCGAGATCCGGCGGGCGATCGTCGGCA
ATATTCCAGACGTGCTGAGGAATTGCGTCAACAAGGAGTACTTCATGCGCACGCCGCCTGCCGCTGCCGC
CGGGGGCGTGGCGGCGGCTGTTTATCCCGAAG
>dme_circ_0008726_junction_seq
GGCGTGGCGGCGGCTGTTTATCCCGAAGGGATTCTCGATTTCAATATGAAGCGCCA
>dme_circ_0008727
GGATTCTCGATTTCAATATGAAGCGCCAAAAATGATTGCAGCGTTTGCATCGTAGTCTTGAAAAGGTTTT
TTTCATTGATTTAATTAGAGCCACTGAAAGCGGTTGGCCAGCCAGAAAAACGATAAAAGGGATTCCATAC
CAGTCTGCGCCCAGCGCCCCCCGAAACGCGGTAGTACAGAAATATCAAAAAAAGAACCACAGCCGGAAAA

GGGCAAAAGTTTTATTCCGACGACGACGAAGAGCAGAGGACAGCAGCAGACAGATTGAAAAACAGCGAAA
TGGACAAAAATCTGATGATGCCGAAGCGTTCGCGCATCGATGTCAAGGGCAACTTTGCCAACGGACCGCT
GCAAGCACGCCCACTGGTGGCCCTACTCGATGGACGCGACTGCTCCATCGAGATGCCCATCCTAAAGGAT
GTGGCCACGGTGGCCTTTTGCGATGCACAAAGCACCTCCGAAATACACGAGAAGGGTCCTCAACGAGGCA
GTGGGAGCTCTGATGTGGCACACCATCATCTTGACAAAGGAGGATCTTGAGAAGTTCAAAGCTTTACGCA
TTATCGTGCGCATCGGCAGCGGCACAGACAACATTGATGTAAAGGCGGCGGGCGAACTGGGCATCGCCGT
GTGCAACGTTCCCGGCTACGGAGTCGAGGAGGTGGCGGACACGACGATGTGCCTCATCCTCAACCTCTAC
CGGCGGACATACTGGCTAGCGAACATGGTGCGCGAGGGCAAGAAGTTCACCGGACCCGAGCAAGTGCGGG
AGGCTGCGCATTGTAAGTCATTGGCCAAAAACGATATGTAAAATCAGTGAAATTCTACGGCATTGGTATT
GATTGAAAATAGTTATTCTTTTAAGGGCATATAATGCTTAAGCTCCCCGCGAAGGTAGCAGAAAAATTTT
AGAAGCAAAGGAAAAGTCAGTTGATATTATTTATGCTAGTCACGTACATAGATTACAGATAGTATAAAAC
CTACATACAATATATTTACAAACATAAAAGGAAAACGATTTGTCTATTTCCATTTGAGTTGGACGATTGC
GAATTGTATTGATAAAAACAAGTCACCATGACATTTGTATAGCTTGGTTTTTAACGAATGCTTTCATTTG
CCATGGATGTCTGATAAAATAGATTGTTTTCATATTTACCAGATTATTATTTCACCGATTGAATTCAGTG
GCTACAGCTCATAAATATAGCGTACGCTTATTGCCGTATTCAAATTACTTCATCCAATCCAAATGCAAAT
TGCGTGTTTCAAAATGCATGATTTGCATTTAATCAAGACTTTCAGCTAGTTATAAATAATAGAGGAGCTA
GAACGCACATATATGAGCAGCATTAATATTTAATCGATACCACCAAGTATACCTTTGAATGCATCTAACT
GGAACAACACCATCTGCTTGTTTTCTCAACGCAGGGGCTGTGCACGCATCCGCGGCGACACCTTGGGTCT
GGTGGGACTGGGCCGCATTGGTAGCGCCGTGGCCCTGCGGGCTAAGGCGTTCGGCTTCAACGTCATCTTC
TACGATCCCTACCTGCCCGATGGCATCGACAAGTCGCTGGGCCTAACTCGCGTCTACACGCTGCAGGACC
TGCTTTTCCAGTCCGATTGCGTCTCACTGCATTGCACGCTCAACGAGCACAACCATCATTTAATCAATGA
ATTCACAATTAAACAGGATGCGACCTGGTGCATTTCTGGTGAACACCGCACGCGGCGGTCTGGTCGATGA
CGAGACCCTGGCGTTGGCGCTGAAGCAGGGAAGGATAAGAGCTGCCGCCCTGGACGTTCACGAAAACGAA
CCTTACAATGTATTTCAAGGGCGCACTGAAGGATGCCCCAAATCTGATTTGCACACCGCACGCCGCCTTC
TTCAGCGACGCGTCCGCAACGGAGCTGCGCGAAATGGCAGCCACCGAGATCCGGCGGGCGATCGTCGGCA
ATATTCCAGACGTGCTGAGGAATTGCGTCAACAAGGAGTACTTCATGCGCACGCCGCCTGCCGCTGCCGC
CGGGGGCGTGGCGGCGGCTGTTTATCCCGAAGGTAAACTACAAATGATATCAAATCAAGAAAAGTAGAGA
GACAGAGACAGGCGAGCAAGGGAGCGTTATTACAAGTaaaaaaaaaaacaaatcgaaacaaaaGGTAAAC
TACAAATGATATCAAATCAAGAAAAGTAGAGAGACAGAGACAGGCGAGCAAGGGAGCGTTATTACAAGTa
aaaaaaaaaacaaatcgaaacaaaacaaaacaaaaaaTATGAGAAGAACATAAGAAATCAACCAACAAAA
TCCAAGCAAAAATACAATACCAGAAATCGTTAAAAAGAAAAacacacacataccttacacacacacacac
acacaccataccatagcacacccacacaagcacacgcacacgcacacaGATGTAGCAAGCATCCCAAACA
TACTTCTATTCTTTCTCTGACTCATTCGAAAGTATATATATATAGACGACGGGAGTGGAGCGCGCCAGAG
AGAGTATATAACATTTGAAGCACGATTCAAGAACACACGCTATAAGAGTTGATATATAAGTTACACAGTT
TAAGCtatacacatatatagatctatatatatgcatatatatatttatatatgtatatatatataaatatgt
atatatataaatatataGTGGCTAAGCGCTGCACCGAGATCAGAATGTTTGACGTCGATGAGGAGGATTA
GAACGCAGCCGATGTTGGCCAAAAGTTACGTTTATCCAATATGTATCTGTGTACGTATATGCATTGTTTC
GGCCCTTCTAGAACAGAGAACGCATTCAAAAGCATCTCTGAAATATGTTTAGAGCGTTTTCGGCGAGTTT
CCAAACTACGTCGCTATCGAACTTAGTAAGAATTCCTTTAACTGAAGTTCAAGTAAGCTAGATTTCTTCA
GATTAATTCAAAACAGCGTTTACGTGGCCAATTGTCTTCTCCTTAGTCTACAACAAATACATACGTGCAT
ATTTAGTGACATAGTGAATATACAGATAAACGAATAAGTTTCAAGATAATTTACACATGAAAACAAGGTG
AAAGTAAACAATATGATGGATTATTGTTGTTGATGTTTCGCAACTGACGTCCCTTCATCCCTCCCTCCCA
AGAATCCCTCACTAAGTATCCGGCTTTAACTTTAACCGAAACTAGAAACAATCAGCGAAAAAGTATGTAA
AGTAATTAATTATGTTCTGTGTATTTAGCCATGTATTAAATGTATTTACAAATTAATATTATTACCGAAA

AACCAAGCAAACGATTCCAATACGACTTACTCCCCACGTCAACACATTTTGTAAGCTTAAGAATAAAATT
GGTTACCTTATTTTGCTCCATTCATTGCTGACTTTCAATTGTTAAATGGTTCGAATAAGACGAGAGAACA
CATCCCAGAGGACACAGTGTTTAACTGAAACTATTATTCTGTTGCTCAAGCTCTGCTCTAATTTACAATA
TGATCTCATCATTCAAGTTTATTTTCGCCTTACGTACATTCTTGTGTTTTCGATTGGCTACAACAAACAG
GCAAACAGCAAGGAAGTTAATACCTAAAGCatatacatatatatataaatatatatatGAAGTAAAAGTA
AATAAAATAGATGACAACTCGGCTCGTTAAATTCAACAACATCTATAAATAGAAAGGAGAAAACTACAAT
TAAATTAACGCATAGTCTATGTACATTTAAAATATAACAGAAATTATATTAAAGCGAAACAGAAAGAACT
ACaaaaatgctaaaaaagaaagaatacaaaaaaaGATAACACATGTAATATTAATGAACCTAGATTTACC
TTAACGATAAAAGGCAACTGAACCACGATGATAACAAGGATATAATCGTCAGCGAGCGGCACATTAAACA
TTGTTAGCGTGTAAATTGTAATTTTATTTAACGAGAATATATATTTACATGTAAACGAAAGTGCGCAACA
ATTGTAAGACTATCATTTTGTACAATTTTGCAATTTAAGTTTAAAACCGAGAAGCTTCAAGCAGTTTGAA
TGGACTCCTCATCCTTCTCCCTATCCACCTTATTTATCTTCAAGTCTATCCGATAACGCTCAAAAGATAT
ATACATACAAACCCAAATCCGATTCGATCCcttttttactcttttttttctttcttctggttttatgtttct
tttGTATCGGTTTTAAGGACAAACATTATGTAATATTTCAAACATTTACCAGCACAAAGCATACACACGC
ATACACACAAACCATagacacagatgcagatagagagcagatgcagatacagatacagatacagatactc
acacatcaaacacacacccacgcacacacacGCGATCCTATATAGCAAGCTGCAACAACAAATGAGCAAA
GTAAAGcataaataagcataagcgtaaaagtaaaaaattaaacgtaaaagtaatgagacaaaagaataaa
ATACTGTTATTTTGTAATGTATAAAAGGATATTTTTAATAACTGTGAAAGCTAAATAAAATTAAAAGCGT
AACAATTGACGATATGAAGTAACAAAAACAAAAGTTAAGACGGAGAAGTTGATTTGATgaaaaaccaaga
aaacctttccagaaaaaaagaacacgaaaagaaaaaaaaacaaaagataaaacaaataaaacaaaaTCTT
TAATGAACCCATTATTGTTTTAACTTGCCAATTGGAAGCTGTTAAATTCTCGTCCTCGTTTCCTATTTTG
TGTGACAGTGTTTGTTTGCAAGACTGTTTGTTTGAAATGTTATATTGCCCCGGCAATTTGTTTAAAATCC
TGGGATGCTGTGATATGGCATCATTGAGTGTCTGCGAGCAGCAGAGCAGAGAGCACACACATTAAGTTTA
AACTATTAAGGCCTGGCCAAAGTCAGCAATGAATGGAAATGCCGATAGCCGTACTATTTCTCGTAGCCTT
AGCCAATTTGTATCAATAGCCCTAAGGAGAACGCACCGCACCGCCCCCGCCCAAAGGGATTGATTGAGTT
CATCCGTACTATTCGTATACCGTTTACATAAGGGGCAATGCGCCAGTTTCTCTTCTCAATCATAATTCAT
CACTAAAACCAAATCCCCCAGACTTCGCTCATGTTTAGATCCCCTGAATACACAGACTCGGCAGCTCAGA
ACCCGATAAACACATAATaccaaccaaccaaccaaccaaccaaccaaacAGCAACATTTCATAATCTTTA
CCTAACTttcaaccaatatcaaataccaaataccaaataccaataccaaccaaaatCGAAATACAATCCA
GGATTAAATGGCGGCTATTATACAGGGCGCACTGCATCACCGGGCACACAGCACCACACCGCACGATGGC
CCCCACAGTACAACCAACCTGGGCAGCACCGTAGGAGGCGGTCCGACGACAGTCGCTCAagctgccgctg
cagctgtggccgctgcagcagccgccgcCCTCCTGCCGTCGCCGGTTCCGTCGCACTTGTCGCCCCAGGT
CGGCGGATTGCCGCTTGGAATT
>dme_circ_0008727_junction_seq
CCAGGTCGGCGGATTGCCGCTTGGAATTGGATTCTCGATTTCAATATGAAGCGCCA
>dme_circ_0008728
GGATTCTCGATTTCAATATGAAGCGCCAAAAATGATTGCAGCGTTTGCATCGTAGTCTTGAAAAGGTTTT
TTTCATTGATTTAATTAGAGCCACTGAAAGCGGTTGGCCAGCCAGAAAAACGATAAAAGGGATTCCATAC
CAGTCTGCGCCCAGCGCCCCCCGAAACGCGGTAGTACAGAAATATCAAAAAAAGAACCACAGCCGGAAAA
GGGCAAAAGTTTTATTCCGACGACGACGAAGAGCAGAGGACAGCAGCAGACAGATTGAAAAACAGCGAAA
TGGACAAAAATCTGATGATGCCGAAGCGTTCGCGCATCGATGTCAAGGGCAACTTTGCCAACGGACCGCT
GCAAGCACGCCCACTGGTGGCCCTACTCGATGGACGCGACTGCTCCATCGAGATGCCCATCCTAAAGGAT
GTGGCCACGGTGGCCTTTTGCGATGCACAAAGCACCTCCGAAATACACGAGAAGGGTCCTCAACGAGGCA
GTGGGAGCTCTGATGTGGCACACCATCATCTTGACAAAGGAGGATCTTGAGAAGTTCAAAGCTTTACGCA

TTATCGTGCGCATCGGCAGCGGCACAGACAACATTGATGTAAAGGCGGCGGGCGAACTGGGCATCGCCGT
GTGCAACGTTCCCGGCTACGGAGTCGAGGAGGTGGCGGACACGACGATGTGCCTCATCCTCAACCTCTAC
CGGCGGACATACTGGCTAGCGAACATGGTGCGCGAGGGCAAGAAGTTCACCGGACCCGAGCAAGTGCGGG
AGGCTGCGCATTGTAAGTCATTGGCCAAAAACGATATGTAAAATCAGTGAAATTCTACGGCATTGGTATT
GATTGAAAATAGTTATTCTTTTAAGGGCATATAATGCTTAAGCTCCCCGCGAAGGTAGCAGAAAAATTTT
AGAAGCAAAGGAAAAGTCAGTTGATATTATTTATGCTAGTCACGTACATAGATTACAGATAGTATAAAAC
CTACATACAATATATTTACAAACATAAAAGGAAAACGATTTGTCTATTTCCATTTGAGTTGGACGATTGC
GAATTGTATTGATAAAAACAAGTCACCATGACATTTGTATAGCTTGGTTTTTAACGAATGCTTTCATTTG
CCATGGATGTCTGATAAAATAGATTGTTTTCATATTTACCAGATTATTATTTCACCGATTGAATTCAGTG
GCTACAGCTCATAAATATAGCGTACGCTTATTGCCGTATTCAAATTACTTCATCCAATCCAAATGCAAAT
TGCGTGTTTCAAAATGCATGATTTGCATTTAATCAAGACTTTCAGCTAGTTATAAATAATAGAGGAGCTA
GAACGCACATATATGAGCAGCATTAATATTTAATCGATACCACCAAGTATACCTTTGAATGCATCTAACT
GGAACAACACCATCTGCTTGTTTTCTCAACGCAGGGGCTGTGCACGCATCCGCGGCGACACCTTGGGTCT
GGTGGGACTGGGCCGCATTGGTAGCGCCGTGGCCCTGCGGGCTAAGGCGTTCGGCTTCAACGTCATCTTC
TACGATCCCTACCTGCCCGATGGCATCGACAAGTCGCTGGGCCTAACTCGCGTCTACACGCTGCAGGACC
TGCTTTTCCAGTCCGATTGCGTCTCACTGCATTGCACGCTCAACGAGCACAACCATCATTTAATCAATGA
ATTCACAATTAAACAGGATGCGACCTGGTGCATTTCTGGTGAACACCGCACGCGGCGGTCTGGTCGATGA
CGAGACCCTGGCGTTGGCGCTGAAGCAGGGAAGGATAAGAGCTGCCGCCCTGGACGTTCACGAAAACGAA
CCTTACAATGTATTTCAAGGGCGCACTGAAGGATGCCCCAAATCTGATTTGCACACCGCACGCCGCCTTC
TTCAGCGACGCGTCCGCAACGGAGCTGCGCGAAATGGCAGCCACCGAGATCCGGCGGGCGATCGTCGGCA
ATATTCCAGACGTGCTGAGGAATTGCGTCAACAAGGAGTACTTCATGCGCACGCCGCCTGCCGCTGCCGC
CGGGGGCGTGGCGGCGGCTGTTTATCCCGAAGGTAAACTACAAATGATATCAAATCAAGAAAAGTAGAGA
GACAGAGACAGGCGAGCAAGGGAGCGTTATTACAAGTaaaaaaaaaaacaaatcgaaacaaaaGGTAAAC
TACAAATGATATCAAATCAAGAAAAGTAGAGAGACAGAGACAGGCGAGCAAGGGAGCGTTATTACAAGTa
aaaaaaaaaacaaatcgaaacaaaacaaaacaaaaaaTATGAGAAGAACATAAGAAATCAACCAACAAAA
TCCAAGCAAAAATACAATACCAGAAATCGTTAAAAAGAAAAacacacacataccttacacacacacacac
acacaccataccatagcacacccacacaagcacacgcacacgcacacaGATGTAGCAAGCATCCCAAACA
TACTTCTATTCTTTCTCTGACTCATTCGAAAGTATATATATATAGACGACGGGAGTGGAGCGCGCCAGAG
AGAGTATATAACATTTGAAGCACGATTCAAGAACACACGCTATAAGAGTTGATATATAAGTTACACAGTT
TAAGCtatacacatatatagatctatatatatgcatatatatatttatatatgtatatatatataaatatgt
atatatataaatatataGTGGCTAAGCGCTGCACCGAGATCAGAATGTTTGACGTCGATGAGGAGGATTA
GAACGCAGCCGATGTTGGCCAAAAGTTACGTTTATCCAATATGTATCTGTGTACGTATATGCATTGTTTC
GGCCCTTCTAGAACAGAGAACGCATTCAAAAGCATCTCTGAAATATGTTTAGAGCGTTTTCGGCGAGTTT
CCAAACTACGTCGCTATCGAACTTAGTAAGAATTCCTTTAACTGAAGTTCAAGTAAGCTAGATTTCTTCA
GATTAATTCAAAACAGCGTTTACGTGGCCAATTGTCTTCTCCTTAGTCTACAACAAATACATACGTGCAT
ATTTAGTGACATAGTGAATATACAGATAAACGAATAAGTTTCAAGATAATTTACACATGAAAACAAGGTG
AAAGTAAACAATATGATGGATTATTGTTGTTGATGTTTCGCAACTGACGTCCCTTCATCCCTCCCTCCCA
AGAATCCCTCACTAAGTATCCGGCTTTAACTTTAACCGAAACTAGAAACAATCAGCGAAAAAGTATGTAA
AGTAATTAATTATGTTCTGTGTATTTAGCCATGTATTAAATGTATTTACAAATTAATATTATTACCGAAA
AACCAAGCAAACGATTCCAATACGACTTACTCCCCACGTCAACACATTTTGTAAGCTTAAGAATAAAATT
GGTTACCTTATTTTGCTCCATTCATTGCTGACTTTCAATTGTTAAATGGTTCGAATAAGACGAGAGAACA
CATCCCAGAGGACACAGTGTTTAACTGAAACTATTATTCTGTTGCTCAAGCTCTGCTCTAATTTACAATA
TGATCTCATCATTCAAGTTTATTTTCGCCTTACGTACATTCTTGTGTTTTCGATTGGCTACAACAAACAG
GCAAACAGCAAGGAAGTTAATACCTAAAGCatatacatatatatataaatatatatatGAAGTAAAAGTA

AATAAAATAGATGACAACTCGGCTCGTTAAATTCAACAACATCTATAAATAGAAAGGAGAAAACTACAAT
TAAATTAACGCATAGTCTATGTACATTTAAAATATAACAGAAATTATATTAAAGCGAAACAGAAAGAACT
ACaaaaatgctaaaaaagaaagaatacaaaaaaaGATAACACATGTAATATTAATGAACCTAGATTTACC
TTAACGATAAAAGGCAACTGAACCACGATGATAACAAGGATATAATCGTCAGCGAGCGGCACATTAAACA
TTGTTAGCGTGTAAATTGTAATTTTATTTAACGAGAATATATATTTACATGTAAACGAAAGTGCGCAACA
ATTGTAAGACTATCATTTTGTACAATTTTGCAATTTAAGTTTAAAACCGAGAAGCTTCAAGCAGTTTGAA
TGGACTCCTCATCCTTCTCCCTATCCACCTTATTTATCTTCAAGTCTATCCGATAACGCTCAAAAGATAT
ATACATACAAACCCAAATCCGATTCGATCCcttttttactctttttttctttcttctggttttatgtttct
tttGTATCGGTTTTAAGGACAAACATTATGTAATATTTCAAACATTTACCAGCACAAAGCATACACACGC
ATACACACAAACCATagacacagatgcagatagagagcagatgcagatacagatacagatacagatactc
acacatcaaacacacacccacgcacacacacGCGATCCTATATAGCAAGCTGCAACAACAAATGAGCAAA
GTAAAGcataaataagcataagcgtaaaagtaaaaaattaaacgtaaaagtaatgagacaaaagaataaa
ATACTGTTATTTTGTAATGTATAAAAGGATATTTTTAATAACTGTGAAAGCTAAATAAAATTAAAAGCGT
AACAATTGACGATATGAAGTAACAAAAACAAAAGTTAAGACGGAGAAGTTGATTTGATgaaaaaccaaga
aaacctttccagaaaaaaagaacacgaaaagaaaaaaaaacaaaagataaaacaaataaaacaaaaTCTT
TAATGAACCCATTATTGTTTTAACTTGCCAATTGGAAGCTGTTAAATTCTCGTCCTCGTTTCCTATTTTG
TGTGACAGTGTTTGTTTGCAAGACTGTTTGTTTGAAATGTTATATTGCCCCGGCAATTTGTTTAAAATCC
TGGGATGCTGTGATATGGCATCATTGAGTGTCTGCGAGCAGCAGAGCAGAGAGCACACACATTAAGTTTA
AACTATTAAGGCCTGGCCAAAGTCAGCAATGAATGGAAATGCCGATAGCCGTACTATTTCTCGTAGCCTT
AGCCAATTTGTATCAATAGCCCTAAGGAGAACGCACCGCACCGCCCCCGCCCAAAGGGATTGATTGAGTT
CATCCGTACTATTCGTATACCGTTTACATAAGGGGCAATGCGCCAGTTTCTCTTCTCAATCATAATTCAT
CACTAAAACCAAATCCCCCAGACTTCGCTCATGTTTAGATCCCCTGAATACACAGACTCGGCAGCTCAGA
ACCCGATAAACACATAATaccaaccaaccaaccaaccaaccaaccaaacAGCAACATTTCATAATCTTTA
CCTAACTttcaaccaatatcaaataccaaataccaaataccaataccaaccaaaatCGAAATACAATCCA
GGATTAAATGGCGGCTATTATACAGGGCGCACTGCATCACCGGGCACACAGCACCACACCGCACGATGGC
CCCCACAGTACAACCAACCTGGGCAGCACCGTAGGAGGCGGTCCGACGACAGTCGCTCAagctgccgctg
cagctgtggccgctgcagcagccgccgcCCTCCTGCCGTCGCCGGTTCCGTCGCACTTGTCGCCCCAGGT
CGGCGGATTGCCGCTTGGAATTGTGAGTAGCCAATCG
>dme_circ_0008728_junction_seq
GCCGCTTGGAATTGTGAGTAGCCAATCGGGATTCTCGATTTCAATATGAAGCGCCA
>dme_circ_0008729
GTTGTGACTTGGGGTCCACCTCCTCCTGCGAGGTATTAGTGTCCGTGCTCTCCGACTGAATCAGCAGCAT
GCTGGCATTTAGCGCTGGCGTGGCATTGTTGAAAGCTGCCGGGGATCCGAGGGGCTTGAATGCAGCGGAT
TCATTGCCAAATTCCGCACTAGCTGTCTTGTTGGCCGA
>dme_circ_0008729_junction_seq
ATTCCGCACTAGCTGTCTTGTTGGCCGAGTTGTGACTTGGGGTCCACCTCCTCCTG
>dme_circ_0008730
ATTTACGTTAGAGAACACCAACTTGTGGTTCGACGAGTAAATCACCGTGGGACGATCAGAGCAGGCAAAC
ACATTAGTGGTAGATAAGGATCGGAAGGTGCGGAGCGTGGTTGGTTGTGTACCGAGAGTGACTTTCTTCT
TG
>dme_circ_0008730_junction_seq
TTGTGTACCGAGAGTGACTTTCTTCTTGATTTACGTTAGAGAACACCAACTTGTGG
>dme_circ_0008731
CTTGTTCTCGTGATAATGGAAGATGATAATCCTTCCAACCTTCGGCTCCGGTTCTTCTGGAATTACAAGT

GATGTGGCCACCACATAGTAAGTGTTGGGATCATCGCCCAGTTTGGCGGACATTAGAGAGGATATAGTTT
CGGGTGCAACGAATTGGTGGGCGTGCAGAACCTCGAACG
>dme_circ_0008731_junction_seq
AATTGGTGGGCGTGCAGAACCTCGAACGCTTGTTCTCGTGATAATGGAAGATGATA
>dme_circ_0008732
CTCTCAATTAGATCTCCATCGATGAATCCCTCGCTGGGTTCAACTTTGCTGTTGATTTGGAAATTGCGGT
AGTACGTGTGCTCAATCTTGCCCACCGACTTGATTATTTTCTTCAGCCGCTCCTCCAGGCCGTGCAGGAA
GTCGTAGAAGTCCTGCGGTATCTGGGTTACAATGCCAATGGCTCCGTTGCATG
>dme_circ_0008732_junction_seq
GTTACAATGCCAATGGCTCCGTTGCATGCTCTCAATTAGATCTCCATCGATGAATC
>dme_circ_0008733
GGCATTTGCGATTCGCAGCGAAGAGCAGAATCTTCTGATTGAGCGCATCCTGGTCCTGGTGCGGAACGTT
CTGCAAGTGCCAGCGAATCCGGAGGCCGAGTGCCGGGCGGACAATGATGCCAGTCTGCACGACCAGGTCA
TTTGGGCGTTGCATCAAACTGGAATGCTGGACTTGGTGCTGTTTGTCATCTCCTCGCCGGATGAGGAGCA
GTTCCATTTGCACGGACTGGAGATTGTTTGCCTGCTTTTCAGGGAACAGGAGTGCCGAATCCCTGGCGGA
CGCCTCCCTGCAGCGCAGTCTGAGCGAAAAGCAGCGGGACCAGCAGGAGCTCCTGGCCGCACGACGACGG
GAGCGTGCCCGCCGTCAGGCTCGTCCTCCACCAGGGCGGCATTCCCGTTTCGGCGGAACCTATGTCATTC
GGAACATGAAGTCCGTTTCGGATCGGGATGTGATATGCCACCAGGCTCTGGAGCGCGTGTCATCGATCGA
TTTTGACCGCGAGAAGCAGCAGCAGAAGCGCTCCCACCGACACATCCAGGAGGAGGCGCAGGTGACCCGT
CGCAGTGCGTTCACCGTTAGGGTGAGTCCCGGTCTGCTCGTAACCCACAGCCATATCCATTTGTCTGTGG
CCACTTCCTGCACTTTGGCTCCGGGCCTTGTTCGCATTGTGCTGTTGCTGCTGGAGCTGGTGTGGCCATT
AGCATCGGAATTGTTATGGCCACCAACGGAGTGGTGGAGCCACATCCCGGCCATTGATTTTCGATTTTAC
GCTGCCACGAGGACAGCCGTATCCGAGAGATCCAAGCGGAGCGAGCGAGCAGTCGAGCAGAGAGCGTATA
AAATAATATTCAATAATAAATGAAGCCATTTCCTTTTGGCCCTTTCGGTTCGCCCTCCCATCCCGGTCTT
TTTCCACCCAGCAGCCTGTAATTCCTGCTGTTTTATAGAATCACACAAACGATATTGGCTGCTAGGGGCT
TCCAGACTCTCGGGTTCCGCCTGACTTGGCCGGGCTAAACAATATCTTGGCGCCGGGCGTCTCTGTTCCC
GTTTCCCCTAATGGCCACCAGGTGCAGCTATTTTATGCTTCTCCGCTAACCAACAAACTACTTAAAGGAT
CTGCCACCACGCCCGCAGGCCAAGATTCCCGGGCTGCCGCTATCCTGGCCTTCTTCAACAGTTACAGTAA
ATGCATTCGGTTCCGGTCTTCTTTTTGGTCACAAAAATATATACCCTAGTTGGCGGGAGAATGTCCaaaa
aaaaaCTACAACTATATTGGTAAAAAGTAAGACATTTAATGCATAAATGTTGTGTGAGCCACGTTATGCT
CAAGTCTGCACCACTTATAACAATGATTGATAGCCACGAATTGAACTTTGTATCCGACATCTCCTCTTCA
TCTCTTCCCCAAGCCTAGAGCATTTGTAATTCGCCTGGGAAGTTCTGGTTTCTTCCTCAATTTGTGGCAT
GAAGCATAAAAAGCGGAGCCGAGATAAAACTGCAGCAGCAACTTGGCGGCGCCATCGGCATTTTCTGCAT
CGCCATTGCCATCGGCATCGACTTTGGGATCTCTGGCCGACGAGGCATGCAATTTTCAAGTAAAATCCTC
AAAAGGTTTTTATCCGCTGCTGCGATTTGGAACTGGCTGGCAAACATTTTTGCTTGTTAGTCGTCCATTG
CTTGGCCTGGTTTATGGGACAAGATGCTTAGCCACATAGTCGGTTGGTTGGCCAAATCTTTAGGCGCCCA
TAAATTGATGGAGCAGAACAAATGCGACCGGGGCGCCGCCGGTGGCCGTGATTAGACAACTCGGAATGTC
CCATTCCGACCGCAAACGAATGGCCAAAGTCAGAGGAGCAAGGCACGTAGGCGGAATAGGCGAAAGTTCA
TCAATGTCACGTGCTGAAGCTGGAGCACCGTGCTCCCCAACAATTTGTTAGCCAACCACAAGTGCATGTT
GCAATAAGCCGACTAAGGGGCCTGGCCATTTTTTGTTCAGTTTTGCCTCCATTTTGGCATAAATTAATCC
GCGTCGGCATCGATGCGTTGGATTTGTGTTTCGCCTGGTGGCAGAGATTGCTCCACGCTCGACAGGATTG
GGTAtgggtgggtcgcttgctggcctgcctgggtggctggttgcctggttggttggctggttggctggtt
ggctggttgggtgcATCTTTTAGCCAGCTTTGCACAAATAAATTGGCCAGGCGAAATTTATTGCATCAGC
GCTGCCAAATGCCGTTGCAGAGTCCGCATTTGTTATATAAACTGATAGAGGGCGTTCGCTTTCAATCAAT

ATTATTGTGGTATAACCATTTTTCATGTCTTTTGCCCATCCTTTCACCCACCCACCCAATCTGCGCCTTG
AACGTTTTAGCTTTTGGATCCCCCTCTCACAGTTTTTCGTACTTTTCCTGGCTTTTGCAGGATTGTGCCT
GCGCGAATACTGCATCGAGGTACTGCGCTCCGCTTACAACACTCTGGTGAGGCAGGTGCGTCGGGTGCTG
GAACGCAACGCCGGATCGTCGAGTCACGATGACTCCTATCTATTGTGGGCCATACGATTTTTCATGGAGT
TCAATCGTTTGAGTGGTCTACAATTGCAGTTAGTCAGGGTGAGTGGCATTCAAGTGCTGGCACTTGCAAT
CGAACTGGGTACATTTATAAACTATTTAAGTCACACAATTTTGAAATGGCATTTTAATGAACTTCTTTCA
GTATAAAAGATTCCTAAGATTCTAAACTTATATTTTAATATATTATATTAAGTAGACATAAGCATCGTCA
TCCTTGGCATCACTGACATATGATTTCCGTTTGGCAATGTCATTGCGTAAGAGATTTCTGGAAATTGTGC
GACTTGCCCCAGATATTCAACTGGATGCCGCCTAACGTGTTCAAATTAATTGCCTCCAAATTTCAAAGGA
TACACAGACGAGTTTTCACAAGTGATATGATGATGATGCTGATGAATGAGCTCTGCTTTCAGTGGCTTCA
TGAAGCGCTGACCTCGTCCTCCGGCCAAGTCAGCAGATATGCAAATCTCCGGCGCACGAGCTACACTTCC
AAACCGCCTTCATCAAGGCTTTTAATCAAATTACATTGCAACACTCTTCAGATGAAGGAAATTTCCCTAA
ACTTTTATTTCGTTGGCCACTCCCAATTTGAACATTTTTCCTCAAAGAGGACAACACACTGCTGCTGAAA
TTTATAACGCAGCAACTGTGCAGTAAAAACTGGGAAGTTCAACAAGTGCCTTGCCATTTAAATTAATTGT
AAGCTAGTGTAAACTATTCATTCACATGTAAGAAAGTTAAAACTGCATCGAACTGCAATTGGCAGTAGGA
TCGAGTTCGAGTTTAACTAGACTCTACTAGATTCTCTAAAGTAACCACAACTTTAAATTCAATTTTCTTT
TATTTGTTTATAGCTCGCAAGCGCTTGCAATAAACTTAAAATTGATTTCGTTGAAATGTTTTCGACAGCA
AATAAGCTGCGAATACTTGTTTATCTGTCGAAAGGACAATCCATCATCTGAGCTGTTGAAGTGTGAAAAG
TAGAGACCGCTTATAAGGGGTATTTTCAATAGTTTCCATTTGTTGTTATTCTTTCTTTTTTACAAGAAAA
GGGGAAAATGCGCAGCAAATAAACTTTAGTGTGATGACAATTTCCATTTGGCTCTTAGCACTTTGCCCTA
AAATGTTCCAGCAAAGTGTCAATGGGAAATCTAAAGCATTTTCCCAGCATTTCCCGCAAGAATTTTTTCT
GCGGTCATCACACAATTTGTGTGTCATTCCTTGGTGGCCCGACATCAGCGTTTCGTCACTAGAGTTTTAT
TGCATTACTTTTTGGCGGGGGACAACAAGCACATGTACAAATCAAAGTCATGACGCCAATTGTTGACCCA
CCAAACTTGACCACTTTACAACCACTGCAAGTTCCACTCCTCCATAAAGTTCTTAGCCAAAGTTGTCAAA
GGAACTATACTGATATCGTGATTTTTAGACACTAGAAATAAAATAGTTATGAAGTGAAGACCATATATCA
CACATATATAGTTTTATGCCCATAGAAGAAGTCAGAAAACACATAGGGGGAATTTGTAGTAGAACAATTT
AATTACTATAAATTAACATTATTATATGAATTAGATGACAATACTTTTTCAGGAAGCTCTGTTGTTCAGT
GTATATATCCCCCCTTGTCGGTATTTCTGGCGTTGTGTTGTTGCTTTCATTCTTGTCCAGCATGCTGTTA
ATAAAATTTTGCGTTCTTTATGCTGTCATCCGGCTGACGTTGCTCCTCCTCCGCATGCGGATCCGGCTCC
TACTCCAGTTCCAGCTCCTGCTCCGTTTTGGTCTCTTTCGCTGGCTGTGTGGTGGGAAATTTCTTTAAAA
TTGTTTTGGTTACTTTCAAATTAGTTTCTGAATGACAACAATGAAGCTTTGCAAAAATGTTGTCATTCCG
TTGGCGCAAAGTTTTCCCAGATCCTACCCTTTTTTCCCCTTTTCCGCCGCTTGGCGACGACTCCCTCGCA
CTATTCTAAGTTGCTTGGTTCTTCTACTGGAGCTCCCAACTCCTAGAACTGCGGCTCCTGCTCTCTTATT
CATTTTCAGCAATGTCCCTTCGCCCTTTATCAGAGTTTTTCGTCTCATTTTTGCACTTATATATAGTAAG
CATCCTATGTACAATATATACTCGCCGACCATAAAACTTTCTGTTTATTTCCTCAAATGCAATTTGACGC
TGCCTCCGCACCGCCCTGAACTTTCTTTATCGTCTGCCATTTTCTACTGATTTTGCACTATATAAAGCAT
GGCATTGCTGTAATGTATGGGCTTTCCGATGGGCAAACTTTTCCAGCAGCCAGCAACCCGAAAAAAAGTG
AAATTGAGTTTGTCATTTTGTTTATGCGAGTACACGTACACAGCATTGATACAGAAATCAATCGCTGAAG
TCTGCGAATGATCAGGAATTTGCAAAAGTACAAGGATCCCCTATAAGGCACAAATTTGATTGATGAAATC
ATAAAATTAAGTCCTAAGAAACTTATTAAACTTTTGAAAATGGATAACCATATAAAGTATTGATTTCCAA
GAGTGGTCAGGATATGCAATATGATATTACAAATATTAAGTGGTGAAATATTTCGGAGTCCTTTGTTTAG
CTCGCAATATTTTTGCCACTTGAAACATCAATATTTTATCCCATTTTGTATGAGTGCAGCCAGTTGAAGC
CCAAATCTCAATTAGTTTATTTGCTTTGCACAGTTTCTCGTAAAAAGTTTGTGCATCAATCAGTGTTTTT
CCGTTTGTTCACTTTGCCACATTATCGTGCTGCTGTTCATTGCACTTTTATTGATTTTATAATTTATTTA

TTTAGGTTCCTATTGCTGCTGCTGCCGCACATCTGTAATTAATTTGTTTTTAATGCCTCTTCAAATGCTC
TCGACGTTGTCGCAGAACTTTAATGGTGGTTGCCACCGACGTAGTGCTGCTAATTTCGCCCAAGTTGCAG
GCAAATGCTGCAGCCACCGGAAGTGGGCGGCTTAGTGGGGCGTGGTTGGCCGGTGGGTGGCCACTGGGAG
ACGTGCGGCATGTCAACGCCCAGTGGTACCCGCATCCTTGGGGGGAGGAAAGGACAAGCAAAACTTAGGC
TGGCTGGCGCTCAATTCTAGCGCTACTTAAAAATTAATTTTGTTAATGAGTTTTTCGCTCTGGCAGTTGC
AAGTGGCAGACGGCAAGCTGCTTACCTGTTTTGCTGCTCCATGCTGCCAACTTGGGCCAACTTTAATGCA
GAATTCTACTACTTAAATATGCACTCAAACACAACGATTCACACCATACACGATGCCATGTGCACTGAAA
ACAAATAGTGGGGTAGTTACTAGGCCCGAAAGCCTTGCACTCATCATTACTATAATTCtttttttttGTT
ACACTAGTTTATTTAAATAGTTATATTTTATTTAATTGTTAAGTAATTGGGTATCTTCATTGTCTGGCTG
TGTACACGAGTGTTATGTAACTCTGTTGGCTTTTTCCATTTCATTCGCAGGGTAAGTCTCCTCCGTTCCA
GCTCCAGGTCCTGCCCCATCCTTGCATCCTGATCCCGGTGGCCACCTAAGCCGAGCGTACAGTGTGTGTG
CGGGTCTGTTGTTGTTTGCTTTGGCGCTGTAAGTGCACTTTTGGGGGCACTCTTATGCCGCTCGACGATT
GCCTTGGATTAAACTGGGTGCCTGGCTCTTGGCACTGGGCTCCCAGTACTCAAGCCAAGCTCCGTACATC
CTTTAAGCCCGCCGGAATGATGGAGAAACTTCAAATGAACAGCGAGCTTTGCCGGGGATTAGCTTGAAAT
GTCTGGCACAAAAGGTTTCCTTGATCTTTTTTCTGAAGATTGTAGGTAAATGCCACACCAATTAGATAGA
TCAAAAGTGGGAATCAAAGTGGCAAGAAGCTTATTATTCTGTGTTAAAGATAGTTGGAAACTAACAGCTT
CATTACCATTAACCGGAAATAAACACTTATGTCAAAGCCCAACTGATTCCGTTCACACCAAGGATATTGG
GACTGGCCACAAGTAATGAAGGGCGTTTATAATTACACACATGTGTGTTGAGTCTCCTTGTGGCTGTCAG
ATCGTCAGGCTCATTACAATGCGGCACACACCATCTTTCGACCCTGCCTCGCCACTTTACTTGCAATCAA
GAGCTCCAACGTCTGCCAAGTTAATGCTTACCAATTTTTTAATTAAAGGACATTCCGGGAATTGCTCCAA
AGCCTGCTGGCACTGCAGAAGCTAAAGGATGACAACAACGCGCGAGCCCTTTTCGATATGTTATTGAACA
ACGTGTGCTATGTCCTGGAGTATCGGGAGACGGTGCTGCATTTGTTAATGAACTACAATGAGGCGCACAG
CACAAAAGTGAGTACCCGAGCGAATAACCATTCCAACTATGCACACACATTCCGATTGAAAATAACTTGC
CCTTCCGGCCATGGTAAAACCCTCGCACCCTCGTTGCCACTACCAAATACCCACTACCATACTCATCACG
AACTGAATTTGGAAACCATCTAGCGGCCTGGCTGCATTTCTGATAGATAACGGCCACGACCCCGTGGCAT
CTGGAAATAAGCAAGCCAAACACACATCCTCTAATGGGATTACTACGAGCACTCGATGTAGTGGCTCTGC
CCCCTACCGAAATATGCACTGCAGGCTGTCAGGATATCGCCGCTTCTGCCAGGATCTCTCGCCCCGACAA
TCAGGCATTGTTTGTCTTGGCGGCATGCCTTTGTCTTGAGTCCTGCTCTGTTTGCTCGGCTATTTGCACA
GATGTCTGCTGGGTCGCCAAAGCTCACTATTAGTGCTGGCAAACCAGTGGTTTCCGCTTTCAGTGCAGCA
AAATCTCAATGGAAATTTAAATGAAGCTATACAAGTATGATAGCTAAGTTTCTAGACTTATCTAGTGGGT
CTTAAAAAGTCAGCAGTACATTTTAATGGCTATCAATTGTATTTGAATACAGTTTTAATAATGTTTCAAT
ATACTTTATGACCTATATTCATATAGGCCAGCCTATTTATTTATTTGGAAGTAATTATAAAAGTTACAGT
TTTTTGGCCCTTTGTAGCTGTCCTTGAGTGGACAACACTATCCCCGAAGAGAGTTCAGTGAATGTGGTTT
GTTTATAAGCGCGTGTGTTCCCCCAAAAGCCGGCATCTCCAAATGAACTAGTTGCACATTCCTCGCGAGG
CGGCGATGGGAGTTGGCTACTAGCTTAATTGATTTTACAAGCATGTGCTGTATGCGTGCCCTGGCATAAA
AATTATGCCATTTCCCACAAGCCAAACCATGTTTTTCAGTGTTTTTATTTGAGGGCAAATCGTGCACCTT
GGCTGCATCCTAATTGCCGAGTTAACACCGCGTACTCCGCACGATAGCTGAATCGAGATTATTTCAATCG
AAACGTGTGCCACAGTGCGGCTACTTGATAAATCGCTGCAGAACATTGAATATAGTGCTGCTCCAACAGA
TTCAGCACGATTTTCTCGATTTACTCGGAGGTGTAGAGTGCTTTAAACTCATTACCTACATTTGATCGAG
TTCATCTGGCAGATTGGGTTCATTGACACAGAATAAACATGATATACACGTATGTATTTATTTCCAGCAA
TTGGTTAGGGACCAAAGAATATAATGGTACGTTTGCATTTCTCGGTGCATGCTTGCTTTTAAAAACTCTT
TTAAAAAAATGAATGCATATGCATTGGCAAACCCTTTATAAATGAACCCCACTGCGCAATCCATTTATAT
AATTGCCAATTTTTAGTCCCGAACATGAAGAGTTAGACTTGAATATTTCTAAATGGCTGCACAAATTCGC
CTGTCAAACAAACTCGACTCCGAGTGCTCTGTGTCGAGTGATACCTGCAATAAATGCCTGAAAATTTATT

TCTGTGTCACAGACTCCGTGACAGTGGCAAAAAGATACCATGGGTCGGCAAAGTTTTCTAGCCTGTCGGT
CTGCATTTAATTAAATTATCTATCTCTAAGATATAACAACCTTTTCTACTACAGTCCACAAAAGTTCTGA
GTACACATTTCTTTAAGCTCAGAATTCACTTTAATTATGAAGTTTTCGCAGAGAATTTATCATGATCGTT
ATCTTTATGAAAATTTTGAAATTCTTGGAATTTTTGATAGACGTAAGGTGCTTTGCTGGAAAAATAAATC
AAGGATATTCCCTATTTCCATATTGTATGGTAATTTTATTAAATAAATTCTGTTTTGGTCTACAAATCGA
TTTGTGAATAGAAAAGCAAAATAACTAAAAAGCAAATCTTGCCTCCAGAGTTTAAAATTATTGTTTATAT
TTAATTATTATGTGTTATTTTAAATACCTTCGCAACCAGCAGATGTTTTTATATTCATTGGTTTGTTGTT
TTCTTTATTGAAGTGTTAGATATTGGCATTTTAAATTTTGATTAACGTACAAAAATATGGTATGTTACAT
AAACAAACATAATCCCTTAAGCTGTACATATGTTTTCAACCCAATTCAACCAATTACCCGACGCTTCAAA
AGGCAATAAATAAAAGTATAAATATATATATATTTTCATCAGCTAGCATAGTATCATAATCGAATCCTAG
TACGTTCCACTGTATCGAATGACACATAAACAGATAAACAAATTGTTGTGTGCCTGTTGGGTTTTACTGT
CGACTATCAACGCATCACATCAGCCAGTTGGATGGATCCTAGTTAGTCGGTCTGAGCAGAGCAGTCTGTT
GGTTGGGGCAATGGCAATTTCAAAGCTTTGACTAAATTAATTTAGCGCGCAAACATGAAATTGACAGAGA
TTCGCCGGGGAGAGATGGCTTGGTTCAGGAAACTCGTTGCGATGCcagatacaatacgcagatacagata
cagatacagatacaTTTACTCAACTCGCGTGTAGCTGTGGGTTGTTGATGGGCCAGCAACTGTATGGCTT
AATTGGAGCATGATTAATGCGGAGCCAGTTGAAAATAGTTGGTTTATACAATTACATCGATTAAACATTA
AGTTGAACAGCTTTCAATTAGGCAGGCCAATTGGCAATCATGTAAATTTCGCTGCCTTTTGAACTGCAAT
CAGTGTTTTGAGTGTCTGACAGATTAAAGATTTTTCGGTGTCAAGATGAATAATTTATGACCCCGCCAGG
CTGACGTATCCGCTATCTCGCAGATTAATGCCACCCAGTTTACCGCTGATTTGTTGGATATGAGTAGTGC
TGCCTCCACTTAACACAACAAAGACGTGAAAATCTACCAAACTGAAGCAATGAAAATTATTCATTCATGT
AATAGATCCAAAATTTTGCTACATACAATACACATTTTGTTTGAAATGTTAAAAAATTAAGGAATATGGG
GTAATCAAATATTTTTTTGTAAGCAAATCTGGCCGTTGAATGGCATAAAGTATCTACAAAATGACCAGAA
CGGCAACACTAGCGTGGAGCTGCAATTGACGCCCCATCAGTCATTGTTGTTGTGGCTGTCATAATTAATA
ATATTTTCATTATAAACGTCTTATGCCAACAACAAGAGTGGGAACCCAAATAGTCGGTCCGTCTGTGGGT
GTTTTGGGCGCAATTACATTTTATCATTTATTCAACATGCGCTCAGGCTCGGCGCGAAGACAATCTCCAT
CTTCTTGTTCCCCGATCCCCTGTCCCCAACCCCTAACTCTGAGTACTTCTGAGCTCTGACCAGCCCCAAA
AACACCCAAGTGTTCTAATTAATTCTGTATGTTCAACCTGCTGCTGCTTGAACTTCTTCATCCCATTTTC
CCTGGCATTTTGCGGCTGTTGGCGTCCATTTAATCAGCGTAAACGCGAATATCAAATCAGGCGCAACAAC
AACAGCGGCATAGGCGAAAGCAGCCAGAAGTTACAAAACCGCGTGCATACAAATTAATTATAAATAATTT
ATTAACGATTTATCTTTGTCCTGCTGCCAGGCTTTGCTGGTCACATGTGCTTGAGATTGAGTATGTGTGC
AGAGCGAAAAAAGGGGGTGTTGAGCTAGAGCTTATATTCGAAAATCCTTAAAAATCCTTTACAATAAAGA
AGTTTCCTAGTTAAAACTACAAATATCAAGAAAATTAAGTTTTGTCTTTATTAACACTATCATAATAATG
ATAAAAATCGATTTGTTAAATCCAAATTCTTAAGTTTAACCTTTTTCTGTCAGTGTAGAATGTAAATAGG
AGTGGAAATAAATGGGTGGAGTTGACTCGATTTGCACCTGCGCATCATTAGGGCGGTGTAGGTGGGCAAT
CGATTTGGATCCGCTTGTAAAATGGTTTGTGGCATGCAAGATGTTGTGCAACTATCAAACCGCCCACCCG
CCTATTCGCTGCGGTTACTTGGCGTTGTGGTCAGGTGGCACCAGCATCCGCATTCGTATCTGCAGCAGCA
GAACCACCACCACCACCACCGCCGCCATCATCTTCTTCAACCAATGGCAGTGTTGCTCAAATGTCTGATG
AGAAAGTAGCCAAAAGTAGGCTTTATAGTACCCTTGTTTCTAAATCTATGAACATTAAGTTCTTAATAAA
TTTAAAAAAATTTAGTTGGATGTATGTTTCCCTTAATGTGTAGTTTGTATCCTATTGGACCATTCTATAT
TAAACAATTATACTGATCAAAAGGATTCTATGGATCCACTCGATTTGCTGAATTGACATCACAGTTCCCA
GCTACCAGCAGTTTTTGCATCTCGCCTGCTATTAATGCGTCCACGGACCACGGACATCTTTGCGGTAACA
TGGCCGTCATCGTGGCTACGCTTGATTCATTTCACATTTCAGTTATCATCAAATAAGCACCACACACTGC
CCGCAAACAGGACAGAAGCTTCGATCTGGATACGGAATACGGAATAGGGGGACATGGGCGAACACATAGC
ACCACTAAAATTTCAAATCCACATGCACCACACCACACGACACCACActccctgcccaatcccaatccca

atccccatccccatcccattcccaatcccaatccTTGCAGTCAACTGAGCGCCTTTATCATCAATTGCCA
GTGCCCCCTTGGATTCCTCCTGCTGTCAGTTGTGGTCACTGCTGACAGTTCTTGATCAATACAAATGGCC
AAATATCTTGGAATTGGCTGGTGATTGCTAACTGGTTTCTATTCATCGGCTGCTTTATGCGGCTGTTGAA
CCTGGCTTGTTTAGTTCTATTATAGACGTTTAATTTATTGGACCCCTAATTGGGTAGAGCAACTCGGGTT
GCATTGTCAGGGCCAAAGTATAGGATTTGATTGGTGCTTAGTTAGTATATGATATATATTCTACTATCAT
ATTCTACCTATTACAATAAGTGTTTTTTATATATGTATAAAGTTTATATAACAAGGATATGTTGTCAATA
AAAGCCAAGTATCCCTTGCTAAAAATAACATAACAAACATACATACAAACTTATCTAATGACTTCAAATT
GTCAAACGCCTATGAGATTTTTGGCACCTATCACCTAGGAAACCAAATCTTTAACTGTTAGGGAAAGGAA
CCTACGGTTTGGCCCATGAAAATAATATTGAAGTTTTGACTTCGCTCAAAAACATGCTTTTGATTTTTAT
TGAACCTCGCAGAACTACGCGTGCTTTTGAATTATTTGCCTGTTTAATTTCGCTTAATGTCTATCCGTTT
ACGCCGTCAATTCCTGCAGGAATCCGGCGGGTCACTGAATGCCGGATAACCACTTTCATTACAAATTCAT
TTCCCCGGTCGccggctcccgactcccgtctgccgactcccgtctgccgactcccATGATCAGCTCGCCA
GCTGCGTTGTGGCACATTGACCGCATTCGAAAACGTCGATGATGCTGGATGCTCCTGCAGTGAGGCTCCC
GTGGGGCATTAAAATTCGCAATGAATTTGCATAAATTTTTCGGCGGCAACACTGTGACCTTTTTGGAAAC
ACTTCACCATTTTCATCGCCAACGCAATGTTGCCAATTTTATGGACCTGGGATCTGGCAATTAGAGTGAA
CGTTGAAGAACTTTAATATGCATTCATTTCACGCTGGAaaacaagagagaattatatagtccagttcctc
gactatcagCAGATGGAGCTTTATCTCATTGTAAATCTTGAAAATATCTCAAAGTGTATATCCGTTTTTA
TACGATAAAATTCAAAAATATACCTTTCAATACCAATCGACGTGCATTGCTGTGTCATTCTGCCTAGCAA
ACCTGCTCTCTATTAGGGCCCGCCTACTTTGCATACGCTTCCTGTTTGGAATTGTCCTTTTCGGTGACTC
GCGCGATTTTCATTATTCAAACGACAATCAATCATTTTAACAAATTGACGTGCACAATGACGTTGGCTTT
TATTTTTTGGGTACGGGTTGCCTAAAAACAAAGCGGACAGCTGATTTTAGCAGTTTGGCGGCTGTCTTCT
GCCGCGTTTTCCttttttttttCCAATTTTGTTGGCTGCCTTTTCACATTTTGAGGGTAATTTGCTAAT
TGGAGGCGTCATCGGCTTCGACAGAAAAGGCAACGCCACATCATCGCTCAGCCCAAAATCGAGTTGGCTT
AGTTAGTCAGTCATGGTGGGGAACTTTTCGTCCTGCCCGATGAATGGGTCGAATGGCATTGGGCGGGGCA
GCTGTTGGCCCTTTTTAATGTAGTCGATGATATTCAATAAGTATTTTTGGGACGGTCGAAAGGTTATTGA
TGTTGGACTTTGATTTATTCCTACGAACATTCTCGTAAAAGTATAAATTCTACACTGTCAATGAATTTAT
TTCATTTCTAAGGCATACATATCAAAGCAAAATATTTTTGAGCAATGAATGCGTTTATTTGTGTTGGGCA
GAGGATATCTATCAATCAATTTAAATTTACTGGATTATTTATTTATATAAATTTATCAAATTTCTAAAAC
TGTTCAAACTGTGTTGAGAGTCTATTAAAACTAAAATATGAGTGTGCCAAACCATTTAACACCTGGTCTT
CCACCTTATGGTAACCCCCAAAATTTATTAATTACTCCGTGTGTGGCATGAATATAAATATTTATTTATA
AATTGAATTCAACCCCAATGAAATTCAGTTGAAAAAATTAAGTATTTGTCACATTTAATTGAATTGAAGC
AATGTGGCATGCATTTTTCATATAGATACGGGGCCAACATGCAACAAAATTCAATTTGGTTACGTGTGCA
CACATATAATGAAGAATTTAATTACAATATATATTCATTTTGTTAACTAACCTCTGCACTCCAACTCCAC
AGGGGTTTTTCTGCGCGATGTGGTGGAAACGGCCAACGTTTTCATTAAAATGATGGAGCGCTTCTGCCAG
GACTCTGTGGTGGTGCAAGACAAGAAGCGAGGTCGTGGCGCCTCCCGAAAGAAGAAGCAGGCGGCGACCA
AGTCCAAGCCACCAGCTGCACCACAGCCCACTGAGGGTAAGACCCAGATGAAAAATAAAATAAAGTAGTC
AGTTGAAGTTTTTCACGCCCAGGGTTATGTTATGATTTTCGAGTAACTAAGCTTTAATTTAAAAATAATT
ACGAACATTTTCCTGAATTTTCTGCTTGAAGTCAACATCAATCTGTCCTTTATATAGATTCTGTTTAATG
TTTTATAAATAAATGGCCGCATTAAATAACTTTATTTAAGCCTGCTGGTCAGCACCCGCTGTGCAGGCCA
TAAAATACACCCTAAATTGCCAATAAAATTTAAAACGAATTGCAAACAGAAGCAAAGAAAAGGACCATGC
ACAAAAAACAATTGTCGTCTATGCTATGACCGTTGAAGATTTATGTGCTTGATCGTGAAATTTAATTGAC
TGAAAATATGGaaaaaaaaGTACAGAAAATGTATAGAAGCTTGTCCCAGCAACGATTCTATTTCATTCAA
TTTTAACGACTGGGACTTTTATGTATTTTCTACTTCGTGTGTGCGTATTATTTTCATGGTACACAATTGC
ATGCGACCTTAGCTAACGCAGCCGCAAACTACTTCATTCTCTCACTCGCGGAAAATGTGGAAAACTTTCA

CCAGTCGGTCCACCAGGTTTCCGTTGTTTTTCCGCAGCTTTTAACCTTTTCAAAAGGCTCAGTTGTGCGT
TTAGTTCTTGTTATTTTCCCAACATACGATGTGATAAAGGCTGGAAGACTATAATTGCGTTAGTTAACAT
TTATTTGAATTTATGGAGTTTTACTTAACATGGTCCTTAAAACTTAACATTATGGTATTGACAGATTTTA
AGAACTATTAGGTTAGTTACTACACACCAAAGTCACATTTCTCTGAATGGAATTTAAATATTTGCTAAAA
TAAGCCGAGCCAAGCTTGTAAGAGTATCTTAAAGTCGCATATTTTCCTGGGTGTCCAGAGCCCATTGTAT
TCCCAACCATCTTTTGTTTTTGCACACTTTGTCGCGTTGGGAAAACGACGCACCACTGAGCGTGATTTCC
GGGCAGTGGAAACTGGCCTTTGGAGTTTTCCACAACAGTTTGCAACTGCATCATACTGCGTGGACTGGCC
ATTTTTGTGCCCTCCGATTTGGTCCTGTTGGCCAGGAGGTGCACATAGGGCAAGGATATGGGAATATTAA
CACGAAGGTCGTGGCTGACGTGAACAAGATTAATTTTATTTTTTTGCAACAACCTTCAATTGTGTTAATT
TCTGACATATTTATTTGTATAGCTGCGGTCGTGTAGCTGTGCTATTGTTTTTGGGCCACGCACACGCGTT
CAGCGCATCAATTTTTCAACAATTAATTTATAAATATTTGTCCTTGCCGCCGAGCGAAAAACATGACAAT
CGCCACGAGGAACAAATATTTCCGTGTTCCTCCTCTTTTGGCTTGAAAGAGTTAAGCGTGCGATAAGCAA
AAAAGATTTAATTGTGTAACGTGTAAAATAAGCATAAATTAAATGAATAATAAAATTATGATAATAGTGA
AGCAGTTTGATCATTTTGCCTTGCCCAACAATTTAGCAAGAATTTCGATATTCGAAAAATTTCATAAAAG
ATTTAAATGCATATCATTTAATTTTTTATTGTTTAAATTGCTTTTGAAAATAATCTAAAAATTGCGCGtc
ttttatttaacgtataattattttaaaaaatttatttattttttcacttattaGACTGATTTAAAAAATA
AATGAGAAGAAAATACTTTGCAGTAATCATCGTCAGACATCAACCATTGATTTACAATTAATATGCATTG
TTAACAGGTCGGAAATGGGCAAAAAATGAGAGAACAATTGTTTAAATGTGTTTCTTTTTTGCAAGCAGAA
AATTACAATTCATAGCTGTTACCAGTCTTTAAACTTTAAATAATTTAATGAAATCATAGGAACTGTAATG
TAAATTATGTATGAATATAACTATTAAGCTGCTTTTTCATTCTCTAAAATATAAAGTTATGCTTTGTAAG
TGATCAAACGTAATCAATATGCCTGACAGCGATTTTAAAAAATCGAATTCGAGTTATAGAAAATCATTTT
ATAGCCATTTTATTTCCAACACATTTTTAAATTTCATTTAAATGCTCACATTGTATTTGGtttttttttt
tGTCACTAGCAAATGGCTGACAAGGATTATTAGATATTGGGTTATAATTACCACTGCCTGTGGTATAATT
AATTCTATTGCTCCGATATTTACGTTACGACTAATTTTCCTCTTTGATTATCCATTTGCAAAACAATGTC
GAATCATAAATAATTTATTAATGCATAATTGCAGGGAAGAATTGTCGAGCAAATGGGCTGAACTGGCAAC
GGAAGTGTGCAGCCTGCTGTCCACGGAATTGGAAATGCCAGAGGATGAGCAGCCCCTGCCCTTCGATGCC
GCATCCGAGAAATCCATTGATGATCAGCGGTGAGTTAGTTGCAACTATTTTTATTCCTTCCTTACTTTCT
TCCTTCCTTGTACTGCATCTGACCCCCTGAAACGAACCTATTATTACCAGTTCGATTTCCAGTGGCACAA
CGTGTCCTTATTATGTTTCTTGGTCTTTCTCCACTTTCAGCGAGGATTGCATGATTCGCATAAATAAACT
GCTTCGTTCCGAGAAACTGGACCAGGCCATCGCCCTTCTTCGTGCTGCCAG
>dme_circ_0008733_junction_seq
AGGCCATCGCCCTTCTTCGTGCTGCCAGGGCATTTGCGATTCGCAGCGAAGAGCAG
>dme_circ_0008734
GGGTTTTTCTGCGCGATGTGGTGGAAACGGCCAACGTTTTCATTAAAATGATGGAGCGCTTCTGCCAGGA
CTCTGTGGTGGTGCAAGACAAGAAGCGAGGTCGTGGCGCCTCCCGAAAGAAGAAGCAGGCGGCGACCAAG
TCCAAGCCACCAGCTGCACCACAGCCCACTGAGGGTAAGACCCAGATGAAAAATAAAATAAAGTAGTCAG
TTGAAGTTTTTCACGCCCAGGGTTATGTTATGATTTTCGAGTAACTAAGCTTTAATTTAAAAATAATTAC
GAACATTTTCCTGAATTTTCTGCTTGAAGTCAACATCAATCTGTCCTTTATATAGATTCTGTTTAATGTT
TTATAAATAAATGGCCGCATTAAATAACTTTATTTAAGCCTGCTGGTCAGCACCCGCTGTGCAGGCCATA
AAATACACCCTAAATTGCCAATAAAATTTAAAACGAATTGCAAACAGAAGCAAAGAAAAGGACCATGCAC
AAAAAACAATTGTCGTCTATGCTATGACCGTTGAAGATTTATGTGCTTGATCGTGAAATTTAATTGACTG
AAAATATGGaaaaaaaaGTACAGAAAATGTATAGAAGCTTGTCCCAGCAACGATTCTATTTCATTCAATT
TTAACGACTGGGACTTTTATGTATTTTCTACTTCGTGTGTGCGTATTATTTTCATGGTACACAATTGCAT
GCGACCTTAGCTAACGCAGCCGCAAACTACTTCATTCTCTCACTCGCGGAAAATGTGGAAAACTTTCACC

AGTCGGTCCACCAGGTTTCCGTTGTTTTTCCGCAGCTTTTAACCTTTTCAAAAGGCTCAGTTGTGCGTTT
AGTTCTTGTTATTTTCCCAACATACGATGTGATAAAGGCTGGAAGACTATAATTGCGTTAGTTAACATTT
ATTTGAATTTATGGAGTTTTACTTAACATGGTCCTTAAAACTTAACATTATGGTATTGACAGATTTTAAG
AACTATTAGGTTAGTTACTACACACCAAAGTCACATTTCTCTGAATGGAATTTAAATATTTGCTAAAATA
AGCCGAGCCAAGCTTGTAAGAGTATCTTAAAGTCGCATATTTTCCTGGGTGTCCAGAGCCCATTGTATTC
CCAACCATCTTTTGTTTTTGCACACTTTGTCGCGTTGGGAAAACGACGCACCACTGAGCGTGATTTCCGG
GCAGTGGAAACTGGCCTTTGGAGTTTTCCACAACAGTTTGCAACTGCATCATACTGCGTGGACTGGCCAT
TTTTGTGCCCTCCGATTTGGTCCTGTTGGCCAGGAGGTGCACATAGGGCAAGGATATGGGAATATTAACA
CGAAGGTCGTGGCTGACGTGAACAAGATTAATTTTATTTTTTTGCAACAACCTTCAATTGTGTTAATTTC
TGACATATTTATTTGTATAGCTGCGGTCGTGTAGCTGTGCTATTGTTTTTGGGCCACGCACACGCGTTCA
GCGCATCAATTTTTCAACAATTAATTTATAAATATTTGTCCTTGCCGCCGAGCGAAAAACATGACAATCG
CCACGAGGAACAAATATTTCCGTGTTCCTCCTCTTTTGGCTTGAAAGAGTTAAGCGTGCGATAAGCAAAA
AAGATTTAATTGTGTAACGTGTAAAATAAGCATAAATTAAATGAATAATAAAATTATGATAATAGTGAAG
CAGTTTGATCATTTTGCCTTGCCCAACAATTTAGCAAGAATTTCGATATTCGAAAAATTTCATAAAAGAT
TTAAATGCATATCATTTAATTTTTTATTGTTTAAATTGCTTTTGAAAATAATCTAAAAATTGCGCGtctt
ttatttaacgtataattattttaaaaaatttatttattttttcacttattaGACTGATTTAAAAAATAAA
TGAGAAGAAAATACTTTGCAGTAATCATCGTCAGACATCAACCATTGATTTACAATTAATATGCATTGTT
AACAGGTCGGAAATGGGCAAAAAATGAGAGAACAATTGTTTAAATGTGTTTCTTTTTTGCAAGCAGAAAA
TTACAATTCATAGCTGTTACCAGTCTTTAAACTTTAAATAATTTAATGAAATCATAGGAACTGTAATGTA
AATTATGTATGAATATAACTATTAAGCTGCTTTTTCATTCTCTAAAATATAAAGTTATGCTTTGTAAGTG
ATCAAACGTAATCAATATGCCTGACAGCGATTTTAAAAAATCGAATTCGAGTTATAGAAAATCATTTTAT
AGCCATTTTATTTCCAACACATTTTTAAATTTCATTTAAATGCTCACATTGTATTTGGttttttttttG
TCACTAGCAAATGGCTGACAAGGATTATTAGATATTGGGTTATAATTACCACTGCCTGTGGTATAATTAA
TTCTATTGCTCCGATATTTACGTTACGACTAATTTTCCTCTTTGATTATCCATTTGCAAAACAATGTCGA
ATCATAAATAATTTATTAATGCATAATTGCAGGGAAGAATTGTCGAGCAAATGGGCTGAACTGGCAACGG
AAGTGTGCAGCCTGCTGTCCACGGAATTGGAAATGCCAGAGGATGAGCAGCCCCTGCCCTTCGATGCCGC
ATCCGAGAAATCCATTGATGATCAGCGGGTGAGTTAGTTGCAACTATTTTTATTCCTTCCTTACTTTCTT
CCTTCCTTGTACTGCATCTGACCCCCTGAAACGAACCTATTATTACCAGTTCGATTTCCAGTGGCACAAC
GTGTCCTTATTATGTTTCTTGGTCTTTCTCCACTTTCAGGGTGAGTTAAAAATGCCATTCCCTAGTCTCT
GAACCCTGTTGCTAGGGCTCCAGACGTAATGCCTTTGTAAATCCCGCTTTTTAATAACTTGACATTGACC
GGGGCACGGAAAAGGTACGAAAGCAATTCGCATAATAAATTAACTCTAGTCAAAAATGGGCACaaaaaaa
aaaaaaaTGCGCTTGGTCGTCCTGTGTCCTTTAATGCAACTTCACTGGCATTTATCGAATTTTATTTTCA
TCCTGTTTATTTTTTTATATCCTTTCTCTGCGCTTTTGCGCGATGCCTTTCGCTGCCCTGCGTTGCTCTT
AAATTAAATAGTTTTTATTTATTGTTAAGGCAGAGCACGCTGGCACCCCGGTAACTACAGATAATGGCAG
TGCCCGGAATTTCTTGTCCCTGCTCTCCCTTTTTGCCCCCACAGATAATAAGAAGAGTCCTTGCGCTGTC
CTGTGCATTTTGCACAGACAATTTCTCGGTGCGCGGTTTTTTGTCCCAGCCGCCTGCAATTTTTCAGTCA
ACAAACCGCAGAGTTGGCAAGGTGAggttgatggaggttggtggatggaggatggtggatggaACCAGGT
CAGAGAACCTCCTCTGCGCCTTTCAATTTAATTGCCCGCTCAATTATGTAAAGCCCATTGAAGACGCGCT
CAAGAAATGTTTATTGTTTGCTCGGGGAAAATTGCGTGAAAATAATTTATCTTTTTCGCGTCAACATTTA
AATAGGGCCGTGCCGACAGCACCCGCACAGAAACATCAGCAGATGTCGCCGGGTGTCAAAGGCTGGCTGG
TTGTGCTGTGCTATGCCAGAGCAATCGAAAACGTGGCTTAAATATTTCCAAGTCATTTAATTACAACCTC
ATTAAAATTTTCCAcctgctgcctgcctgcctgcttgcctgTTGGTTCTGGTCGGAGTACTCGTGTCTCA
AAGGAATCCAATCGATGGCATCTGCAAATAGGAGGAGACCGAGATGCTGGAGGCTCCAGCTCCTACTCTT
CGCTCTGTGCAAATTGATTGGATATATTCGAGGAAAGTGTTGCCAGTGAGATGGCTCCACAgtgtgtgtg

tgtgtTTGTAGAGGCTTTCTCGAAATTTATGTACATTTTAATTGTTGCCAACGAAAATTGTTGCATTAAA
ATGAATACAATTTTTGCTGTGCGACTCGGCTGCTGCAGTGGCAGCTTAAAAAGGTCTAGACGCTTTTTGT
GCTAATAAATTAAAATGCGATATGCATATAGAGCACTACCGAAGCTTGCATCCACCGCCTACCGCTCACT
GCCCACGCCCCCGACCACGCCCTCCCATGGGAACCCACTCCTTTGTCTGGGCATTGTCTGGAACTTTTTT
GTGCCCCGTTGCCTAGCACTTTTTGCCACTCACTTTATGTCGCATACCCTACAAAAGAGTCCCACCAATT
GGATTCGATCTTGCTTAAAAAAATGATAGACTTAGATAAGTTTTTATTTTACATTTAACAAAGTATATTC
TGTACTAAGAAGAAGGTTTTATTAAAGTTGACATTTATACAATCTTTTCGATTGTTTTAGATTGATTGGT
TTGGTCTAATAAGTAGACGTTGAATCTACAGCTATATTTTTGTTGCTTCCATTAGCCTATTTATGATTTT
CTACAAATTATACAAGTTTGTCATAGCGCCAGTTCATTAATCTTTAAATACAAATACGTGTCGTATCCAT
TGAGAATGCCAAGCATGAGAGTATATTATATATTTTAGATGCCTCTGTTTCTCATTTCACTCTTTTCCAT
GAGGACGCTTTTGGGTAAGTAAAATATTGAAAACAAACTGGAGTTTGTGGGCCCGACATGCAGACATTCG
TAATTTGGGTCTGGTGTGGCATGTGTGTAAGTGCATTGAGGATGATGTGTGCCGGGGCGTGTGTGTCTGT
CCAGAATTTTTAAATACTTGCCAAGTCACACTATTAGGGGTTTTTTCATTTAAGTTGAGCGAGAGGCAAA
AGAGGAAAAATTTGTAGCTTTTCTGGGTTTTCCTGCTCCGGCTGCTTTGGCTTTCGCCGGCGTTGGCTTA
ATGGGTGCCCAGAAATGTGTGCTTGGaaaaaaaaGCAAACTTTTAAGTGAAGTAAATGCGCAAAATGTTT
ATTCAAACTTCACTTTGGCTGCAACTTTTAGAACCCCCCTCCATCCCACATCCTCTCTCACCATCCACGC
TGCCCCGCCCACTTGGGCCGCCTTTTAACTAAGCTTATTTAAGCGTCTGCTGATGCGCTTATTTAGCATA
GCAACGGCAAGCTTCGCTTTCGCCTTCCATTTCGCCTTTGCACGTTTCTTGTTGAtttttttttGACTGG
AAATAAAGAGTATTTTCAGGTTTTAAGTTGGTGTCCAAACTCATAGTGTTTAGTGGTGAAAATAACCCAT
TTAGAGTTTTAAAGATCACACAATAAAATACGGTCTGAAAAATAATATCAAGCTGTAAATAAAATTAAAT
ACAttttttttttAACGTGTTTATTCAAATGCAAATTGTTTTGGTCTAAAAATTTTTGCATTTTAATCTAA
TAGCCACGCTATCTATTTTTAATTGTCCATCAGGGGACATTTCTTCTCTCTGCTAATACTCGAAATTAAA
ATTGTATGCTGCTTTGGAAAATTTCAAACATTTCATTATGGCATATTATAAATTTAATCACAGAGTTTTT
GGTAAAAACACCTGGAATTTGAGCCGACTTAATCCATTGCATTGGCCAGAATATCGATTCATAATTAATC
CAGTTTCATTCAATGTGATTAAAAAATGTTTTGCCTCTTGCATCAGGAACTTTTATCATTGAAATAAATA
GTTTATCTGCTGGCGCGCACAGCTCAACAATATTGAATATAATCGAACTATAAAGCCAAAAGCTCCAAAC
CGCTGGCCCGATGGTTTTCATAGATTTTTCATGGTACATAATTAACCAATATTCGCCCCTGACCAGCATA
GTGGCACACTAAAAACCGCTAATTATCGCCACGCCACATCGCTGTAAAAGTTGCACGCAAAATATTGTCG
CATAACTTTTAAATAAGCGCAGGCAAATGTTGATCCAAAGCCAAAAATTTTTCCCACCCACCCATGGATA
GGTTTCTGTGTGCTCTGTTGCACATTGTCCAATCCCTATGGTGGCAACTAACGAGCTCCGAAAATGAGAC
CCGCAGGATGGCAGGCATGGAACATAGAGCACATAGCGTATACGCCATGGATGACGCAGGACAATATGCA
TGGGGGCGTGGCCATGGGCAAGTCAGTCAATTTATTGGCTGGGCGACAGCGGGAAACATAAGTTGCACTA
TCGCCTACAAGCACGACCACAAATAAGCATAAATATGTCGCTTAGAGGGACTTACTCCACATTCCTTACC
GACTGACTGCCTGCCTGCCACTGATATGGCCAAGAACCTCTCGCTTTCCACTGCCACAAGGTCGTTCGAT
GACACTATTAGAAAAAATGTACAGTTTTGGGTGTCTGTAAAAGAGAACAAGTTGATAAAAATTATTGTAA
AGAAAAGAAAGATTAAAATTGTGTTCACATGTTATTAGGATATTAAATACAAAATTCAACTAATATTGGA
AATTTAAATGGCAAATATTTaaaaaaaaaTCCTGCATTTTTGAGCTTACATTTATTAAACGTTTAAGCTT
TCCTATTATTAATTATATAGTATGTTTTATATTTTAAATGATTTCTAGGCTAATTCGAGTGTCGTAACCA
AGTTTTGCTCTATCGCTTTCTCGTTATGTTTTTGCTGTGCGTTGCATATTAAGCTGCTTTCAAATATTAA
ATTAACAGCGGCAACGTTGCGAAAAGCCTGCAACGTTGCATCATCACAGCAGTGGGAAGGGATTATATTG
TGTGTGACATCGAAAAAAGGGAAAAATAGAAAGAAGGCAGGACGCACACAACATGTGAAATGTTGGGCAA
GTGCAACGGGTGGTGTATCCTGACGCAATTTTAAACGCAACAATTTCAGTTGCCGGGGCTAAAAGCCTAT
TGCCTATTGTTGGAGCTTACAttttttttttaattttttGTGGGAAATTGTTACCTAAAGCGAACTATTTTG
TTGCTACCAATGCGCTAGCTTTTATGTGTTTTGATTATTTTGGAATGGAGAATTAAATGAAAAATGACAT

TTACATGAAGTTCTAAAAGCTTAGTTGCGGATTGGATCAATAAATCAAAAAACTAGAAACCTAGCCGAAA
TAAAAGCAAATGAATGGAAAGACCTATAATAACGAATAATCAACACCAATTGAACATGGTGGCAAATGAT
GAAGCTTTAGAACTATATATTCAAATAAAAAGACGAAATAAATAGATAGATATAGTAAATTGTGCTACGT
AAACTTTTCTTTAAACCATTACATTTATTTAGTCAGGCTTTAGTAAAGGTATCCCGGTTAATACATCAAT
TTCCGCTGCATTCCCAACTTTAATGAAATATATTTTACTGCTTTTGTTCTAACGCATCCCTTGTTGTGTA
TTCCCCAGGGAAGTTTGGCCGGAGAACGAAGTGTTCGGTGCAATTTCCGCTGCACCGGAAGACGAGCTGC
TGCTCCTGCGTGAGATTTTCATGTCCAACATTACAACAG
>dme_circ_0008734_junction_seq
GAGATTTTCATGTCCAACATTACAACAGGGGTTTTTCTGCGCGATGTGGTGGAAAC
>dme_circ_0008735
CGCACAAGCACCTCATTGTGGGGCGAATGGATGGGCGTGCTGCACGGCGATGAGATCGAATACTTCTTTG
GCCAGCCGCTGAACAACTCCCTGCAGTATCGACCTGTGGAGCGTGAGCTGGGCAAGCGTATGCTCAGTGC
GGTCATCGAGTTTGCTAAGACGGGGAAATCCCGCTCAGGATGGCGAGGAGTGGCCCAACTTCTCCAAGGA
GGATCCCGTCTACTATATTTTCAGCACCGACGATAAGATCGAGAAATTGGCCAGGGGTCCTTTGGCGGCT
CGCTGCTCGTTCTGGAATGATTACTTGCCAAAAGTCAGGAGTTGGGCAGG
>dme_circ_0008735_junction_seq
ACTTGCCAAAAGTCAGGAGTTGGGCAGGCGCACAAGCACCTCATTGTGGGGCGAAT
>dme_circ_0008736
GCACTTACTTCTTGCTGTACGATTTCATCGATTACTTCGATAAGGACGATGCCACGGCCCTGCCACGGGA
CAAATACCTGGAAATTATGAACAATATTTTTGGCAAGGCAACGCAAGCGGAACGCGAGGCCATCATTTTC
CAGGGTAATTATTAAATACGCACTGGCATTCTATGATGAATAAGCAACTGCAAAAGACAGATGCACTGAA
GAAAATTTATTTTATTTTTGAAATTATGCATACCAATTCGCATTTCAAGGCCTTAGGaatattatattat
aatataatatataatgtattaaaatatCACATTCCCCGTAGTATCTTATAGAATTTTGTTGATTTATACT
TATGTATATTGCGAATATTTTTTAAAGTACACATCCCCCGAAGTGCATTAGTGCAACGTGCAACTGCTCC
GTGTCACCAGCAGCATCGCTGCATGTGCTAAACATGTTAAATGCGACGTCAACGGAGACGGCTGGCTGAT
TGAATTGCATTTAGTGGCTTTATTCATTTACTCTGCCCTGTCGCCGGTTAATTTCATTTCGCGGCATTCT
GCGTCAGCACTGGATATGCATTTGACAGTTAAGTCCGGACAAAATCCGGAATTAGCTGGGCCAGGATCCT
GTAGAGCTTTTAAAAGATCCAATTTCTCTGAAACAGTCCATTGGTTATTGGTTAATTGGACAGCCAACTT
CATTTTGTAGATGACTACGGACTTCGTTCCCATTGAGTTGGCAAAAATTATGATAGTTGCTTAGAAACAT
TTGCCACACTAATATCTTATATTATATTACAGTAACTGGCGGGTCTTATGATTTATTTTAAAACTTTATG
GACGTAATGTACTGATCATTAATTTAAATTATTCCAAATTAGAGAGGGATATATTGAGGTAAACTTTTTT
CTTCTTTTTCAGTTATACCAGTTGGGAAGGCAATCCTGGCTATCAGAACCAGCAGCAAATCGGACGCGCC
GTGGGCGATCACTTCTTCACCTGCCCCACCAACGAGTATGCCCAGGCTCTGGCGGAGCGAGGCGCTTCCG
TGCACTACTACTACTTTACACACGGTGAGTACCACATCGAGGGTGCCCACTGTATCCTGGCGAAATGGCC
AGAACAGCATGGCACCCATTAAATATTTCTCAGGCTTTGACACGTAAGATGGCGTTGAAGCGCGTGCGTC
CAATCGGAATGCGGTCAAATGGGGCGCAAGTGACGCAACGGCGCAAAAATCCACGCTGAAGTCACTGCAA
CGCAGGCAACTGCAATTTGCGTGTCATTAAAATTGCACACAAGGCTAATGGCAGGCAGCAGTCCGGCTCA
GGCCCAAAGTGGATAACTGTCGCAAGTATTCGACACTATGAAAATGTTTGCTCAAGTCCAGAGAATTTCT
TTTTTACAATCTTAAGCCAttttttttGAAAGTTGAGCCAACTGAAAAGCGTGAATAAACTATGCGCTTG
CAGATACCTTTCGAGTGTCGAGTTCAAGCTGACTCTTTCAAAAGGTATTCAATTAAATTAAAAAAGCTGA
ATTTCTCTCGATGTACTCCACAGTGGCTGCATTTTCGATGGAAGCAGTTACGGCTCCGTGATATGTCAAC
GCTACACTGTGAACGTTAATTATTAATGTCTGAATGATGGCGGGGCGTTTTCAAAAGCCGAAGTATATTA
CTAAGGGTTGAACCTTCTGAAAGCAGAGCTTGCGGCTAATCAGAGACATAGAAAGTGAAAGTACTTTAGG
CAGAATGGGGTCAGGAGCATACCCTAGTCAATTGCATTTGACGGAGAATAACTTGAACATGAATTCAAGT

TAGCGTTATTCAATGGTGTATAATTATTCATGCTTGTAAAGGTTAAAGTTAAAGTTCGTTGATAAACTAA
TTAACGAAAAATACTCAGACTTGCGTAATGTTTTCTTCGAGCTGCATCGAAATTaaaaaaaaaTTGTAAT
TATAGGCAATTTCTAAAGAGCTAGAAGTAAGTTATGTGCTACCGATATTACTGGACAATTAATCCGTCAA
CCAAGAAATTACTGTATTTCTTATGATTGTGATGATTGAAAAATCATTGACACTGAATGAAGATATCATC
CCTGTCCTTTGTCGTGTGCAATTTTGCGTAGCGTGTCCGACCACTTCGTGTGAATTTTTCAATTAATCAA
GCAATTTGTTGAAGACAGCTACAACGCGTGCAATCCGTTGATTGGACTTCATAATGTCACACCTATGGGG
GCAAGGGTCGCTGTCGCAGTTGTTCTTGCTGTTGTTAAGCTAGCAGGCAACCTTGGCCGCCATAATTATG
GTAACCAAAAGTTTCACGCAACTCACGCCCACCCAAGAGTGAAGAACCCTCGCAAAGACACACATACTAA
GAAACGCCCCACGTTGGGCGCCAATTAAACTGTACGCACACGCACAGTTCTCAGGATGTTGGCTGCGGGC
AGACACAACAACTATACAACTACAAAGTAGCTAAGAGGGAACAGGGCAAGAAATCGTTACTGGATCACGA
CCCTACGAAAGGGGCCAGGAGCATGGGAAAGCGGAGTCGACTAATAAAACTTCAATTTACGAGGTCACGG
CCCGCATGCCCTGCACGTGTGGGGCATAATTTTAGTCAGTTATTTGTTTAAACGACTTTAGCTGCGGTGC
CTGCTGCCGTCGATTGATATCGGCTGCTTGCAGACCCAGTTGGATCGGCCTAATGATTAACAAATACGCA
GCTCTTACAATGCGTATACACAGAGTGGTCTGTATTATGTATTGATTTTCGTTCGCCGGCTTCGATTCCT
TGAATATACACAAACTAATGGCTGCGATATCGTCTACCATATTGACCGATAATAAGTGGTAATTTGGCCG
AACGAATCACTGCAGTAGGCAAATCAATATTAAGCATACGCAGCGTAGAAATTAGTGGGCGGTACTCTTA
CGACACATATATCTCTGGTGTTGGGAGGTTTGAGTTTTCCTAAGGACGAAAATGCACGAAAAAACTATAT
ATTTCCATTTTCATTGCAATCAATATGTTTCCACATCACTCGTTAGTTTATCAAATAGCCAATATGCTCT
TTACTTTTGATGTTCGGTTAATATTTACGCCAGTTTACAGTGATAATCAAACTGTTTTACCCGTCGAGTT
GTGGCTCCTTCCGGCTGATTGCACAATATGCAGAATTTAGTGCGTGCGGTGTGCCAGTTTGACTGCATTG
TTTATACCAAATCGCCCGTTAATAATCCAATAGACCAGCAGCGCCAGCAGTTGACAGGCAGAAACTGACC
GGTTGTTGCCACCCCCGAAATGGAAATGAGAGCAAATGAAATTAGTTTTAATTACAGCCGTCAAGTTGCA
GCAGCACAACTCGTAACTCACTTTGCCCATAAACGGGTACGCGATGGGGCGACGAAGCATGGCAAATGGG
GTCGCCAGCTGCTAAAGTGCCTGCCAGATTGTCTATCTGCTTGGCTGGTGACCAGGGGGTCATCCCCATT
CCCCACTGGCCGCATGCTTGACTGACCAGCGTTCCTCCCATTGTCCTCCCATATTTTAGCCCCCCTCCCA
GATCCTGTTTCTGTTTCATTTCGCCATTCGCCGCTGACTACTGCAGCGTGCAACGTGCCTGGCACTTAAA
TAAGTTTTCCTTCTCCACTGTTCCTCCTGCTTTTCCTGCTCCTACTGGTTGTACTCCACTCCACTTTACT
CCACTGTAGTTCGCTACTCCTCTCGACTTTCGACTGTGGCGCTTCTCATTATGACCTACGAGTCCAAGTT
TCACTGCTGTTTGCAATTTTGCGCTTAAATCAAAAAAGTTTAGTTCTCCGCTCCGGCTGCGTACAATTTT
TCATTATTTGCACGAAAAGGCGTAAGGACTGCAGGACTTTTATGTTATGTGTGTACGTACTACTTTATAT
AGGGGACGTGTGTCCTGACAGCCAACGACCAAGTCGCATAAATTTCCAACAACTTGGCAGGAGCAGAAGG
AGCAAAAGCAGCGGCCCGCATTACCAGAATTACCCATGGCATATCCCGGACACTATTCATTATGCGACTT
GGCACTTGGCTGCCAGCCTGTCAAGGTGCAGCTCAAGCAGAGCCCGACTGTGGCCAACTCAAGTTGAATG
GTTAAGTGCTTagccctcagctttcagttctcagttctcagccctcagctctcggctttcagctcttagc
tGGGCGTAATGGTCAGCAGAATCGTATAGGCAGGTTTTCCTTTTTCGCTTTGCATAAGATTGCATTTTAC
AAGAGGATTGCCTAATTAGCATTTAGGCTGGTGTTGCTGGCCAGAGAATGAAAATCAGCACGATGGTAGC
AAAGAGTACGGACGGCTAGTAGCTTTAAACGTGCCTTTGATATTGTTACATTTAAGTATCAGTATAAGCC
GCATACAAAGTGAGTTCTAAAAAAATTTCGAGCTTAGATATCTAAGAACTAAAACTAAAATAATGTATAT
GTGGCTTTCATTATTGTCATAGTGCCAAGCATTTATGTGCCGATGGAAATATTTGGGTGTTAATTTTCTT
AGGCATTATGGGTATATGCCCCTGCAGATGTGGTCCATGGTATAGCATGGCGTATACGCAACGTGGTCGC
CCGTGCATGTACCCAGTGCAACAGCCTGTCCTGCGATTCCGCATCCCCAGATGCAGCTCCAGTGCAAACT
GCTGTGAGATACGTGAAATGGCGCCACACGAGGGCATTTAATGCCGCTGCCTGTCAAGGGCGACAGAGTT
TTGGGACTGAAATGCTGAAACTCAGAGCTGGCAGCCGACCAGGCGTTTACTTATTTATTCATTCGttttt
tttttCGGCGTTCCAGTGCGCTGCATGCTGCGACAATATTTACCATTAACAGCGAACGCCAGCGAGCAGG

CGAGCGAAATCAAATAAATAAATAAAAAGGGTAGATGTTTATCTGCATAATTTCTGGCGGAGTTTACGAC
AGCGTGGCGCTCTGCTCTCATTTATCGACGGTCCCATTTTCACGGCATTTTCTTTGGCTTTTCTAGCCAT
GTTCAATTTCTTATTTGGATTCTTTTGCTTTTTTGCAGCCGCACAAGCACCTCATTGTGGGGCGAATGGA
TGGGCGTGCTGCACGGCGATGAGATCGAATACTTCTTTGGCCAGCCGCTGAACAACTCCCTGCAGTATCG
ACCTGTGGAGCGTGAGCTGGGCAAGCGTATGCTCAGTGCGGTCATCGAGTTTGCTAAGACGGGGAAATCC
CGCTCAGGATGGCGAGGAGTGGCCCAACTTCTCCAAGGAGGATCCCGTCTACTATATTTTCAGCACCGAC
GATAAGATCGAGAAATTGGCCAGGGGTCCTTTGGCGGCTCGCTGCTCGTTCTGGAATGATTACTTGCCAA
AAGTCAGGAGTTGGGCAGG
>dme_circ_0008736_junction_seq
ACTTGCCAAAAGTCAGGAGTTGGGCAGGGCACTTACTTCTTGCTGTACGATTTCAT
>dme_circ_0008737
GTTTCTGCCCCGAATTATTTTATATTTATTTTCGGCGCTTGACAACCGTCGACGCCGCTACCACTTGGTT
TTGGGTTACTCTTTTTATGCCCGGCGCGGACTTCGTTTGATGCGCTTTGCGTCATTTTGGTTCGAGCAGT
TTGGTTACTTTTGTGTAGCAGTACGAGTGGAATTTAAAAGTAAACCACTCTTGCAACTGG
>dme_circ_0008737_junction_seq
TTTAAAAGTAAACCACTCTTGCAACTGGGTTTCTGCCCCGAATTATTTTATATTTA
>dme_circ_0008738
GACATTTATGTAGAGGCAGTCCTCGGACACGTTGGTGTTGGGGTTCCAGATCTCCTCGCCGGAGAAGCCG
GGGAAGTACTCGTAACTAGGTGGCGGAAGGGAGGAAGGGAGGAGGAAAGGAAAAGAGCGAGAGGAGAGTT
CTTGATTAGCTGGTGACCCATATGCAAATCAAAGGCTGTTTGTGCCCCATCTGGAATTCGGTCAAGCCAA
TTACGGCAAACTAACCAGCTGTGTTATTTATTTTTATTCGCTCTCTCTTCCGTTTTGCCCACTTCACTCC
AGCACTGGGATTCATTTCCCCACaaaaaaaaaaaaaCACTGGCACACTCACATACGCCCCGTTGTCCCGG
GCTCAGTCCCCCACAATTTGACATAGTTATTCCGCTTTATAAATGTTGAATCGCTCGAATCGATATCCCT
ACGCATTCGTCGAAAGCTCAGCATTTGTCTGTCCGTCTCCATAGATACCTTTGTATATTCCTGTATGCTG
GTGGATGTGTGTATGTGTGTGCTTAGGTGTGTTTGGCTTCGTGTCCATTAAAATTAACTTTGCCGTTTCG
CATAAAAGTAATTTATCACAGAGGCGGAGAGAAAAGACGATTTCTGTTTCCTGCGCCAGCTGCCCCATTC
GCCTTTATCACTATATTTTTCTAATTTCCTTTTGCCGCTTTCCTTTTGGGGTGCGGAAAAACTTTGCTTC
TCTATTTCAAGAGCGAAAGTATGGCAAACCAAATGAGTTTGATTAAATAAATGGAAGAGGAATGGGTAAA
ATGTTTTTATTTCCTCTGTATCTCTTCGATTTTCTATATACACACTTGCAAAAAGTTTCAAAAAGAGAAA
CATCAAACGGCATATTCTTTTATGCAAATTCCTGCTAACCAACAGTGTTAGCCATTGCAATCTCTACCAT
TAGTAAATTTGCAAAACTCTCTGTTAAGTTCCGAATATAATTATATATTTCAACTACATCCATCGTACGT
ATCTCTCTACAAAAAATATACATTTTCCTTCCTTGAATGGCAAACAATGTGCGATTTCAATTGGCAGGGT
CAACAAGCTGAACAAAGTTTCCAATTGGCTTTTTCCGTTTTGACCGGCTTTTTGCATGCGGCATGACGTC
ACTGGCAACGGTTAGGTCAACAGCGAGCTGCCGCTGCAGCCGGGCTGCGATTCTCCTTTCTAGGTCAGAT
GCAAAAGTGGAACGTTCCAGCCCTCCCCCTCTGAGCCACAAATACCCGCTAAGTAAGTGATGACAAATCT
CTCGTATTTTCGAAAAAATATTACCCCCTTTATTCGCTGTCTTTGTCAAAAACCCCAGATAACGTCATTT
GATAGATCGATTTGCTCTTTAGATCTTATGAAACTATCTTATCTTGAAATATTTGTGTAACTACTTTTTG
AAAACACACTTATAATTCTGACTAATTGTTGGCAAAACCCTTGCACATTTGATTACTTTTGTAATAATAT
GGTTAAAACTTGGATTTCAACTTACCGCTCTTGGACGCAGGTGGCGGATAACCGCGTGGCGTCGAGGACG
CCGTGCCATGGCTCCGCGGGAACCGGCTTTCGGAAGCGCAGGTCCTCGACGGGCGGCTTGGCGTAGGGGA
TGCCCGTGTAGACATGCACCTCCCTGCCCTGCACCGTCACGGAGCGACCGCGTACAGGTCCGGAGGATGT
CTGCACGACCAGGCGATCGATGACGCCGCAGACGCCGGACAGGTGCAGTGATAGCACCAGCACCAGGGGC
AGCGGGATGGTCAGAGGCAGGGGCAAGGACATGGGCAGGACTCTGCTCTGCCGACAGGAGATGGCCATGA
CGCGGATGCGCAGGGCGTCGTTGGCTGGCTAAAGCG

>dme_circ_0008738_junction_seq
CGCAGGGCGTCGTTGGCTGGCTAAAGCGGACATTTATGTAGAGGCAGTCCTCGGAC
>dme_circ_0008739
TTCAAACACATCCGCAGACGGAGAAGAAAGGCCAAGCAGACGGAGTGGGCTCTGCAAAAGTGCAACAGCT
GCTCTGAAAACCGAAACGAATAGAGGACGCCAACTGAAAACCATTTCAAACTCTAAGCCAAGTCCAATAA
AGCCGAGACCCGATTCGAGTGGCGAGTACCGAGCAACCAGTACCCAGTTTCCAGTTCCCAGATTCCGGAT
TCCAAAGCTGCTGCGGCGGGAGAATGTGTAGTGCAACCCACGGGCGGCAGCTTCAAGTGGCAATTGTGGC
AGCAGAGGCGAACTCAACCGGATAATAAGTTGTGGATGCGGCTTGTAGCGCCGGGCAGGCATCCCGAATC
CGCCAGACACACCACTTCCGTACCGAGTTACCCAGCGAGCAGAAATCGCGAATCGCGAACAGCCAGAGAA
CCAGAGACAAGTGGTCAGTGCCAGTGACGTCAGGCCAAAGTAGGCCACCACCACCCATCGCTACCGCTTT
AGCCAGCCAACGACGCCCTGCGCATCCGCGTCATGGCCATCTCCTGTCGGCAGAGCAGAGTCCTGCCCAT
GTCCTTGCCCCTGCCTCTGACCATCCCGCTGCCCCTGGTGCTGGTGCTATCACTGCACCTGTCCGGCGTC
TGCGGCGTCATCGATCGCCTGGTCGTGCAGACATCCTCCGGACCTGTACGCGGTCGCTCCGTGACGGTGC
AGGGCAGGGAGGTGCATGTCTACACGGGCATCCCCTACGCCAAGCCGCCCGTCGAGGACCTGCGCTTCCG
AAAGCCGGTTCCCGCGGAGCCATGGCACGGCGTCCTCGACGCCACGCGGTTATCCGCCACCTGCGTCCAA
GAGCGG
>dme_circ_0008739_junction_seq
GTTATCCGCCACCTGCGTCCAAGAGCGGTTCAAACACATCCGCAGACGGAGAAGAA
>dme_circ_0008740
TTTGCGTGTTTGTGCCCTGGTGCAGTTTCGCTTGCCGCATTTTGAACTGCTTTACCAGCTTTACATGCCG
GGCACTCAACTCATGCCGCGCCTTTGCCGCTTCGTTGTGAAAGCGCACGCGGCAGAATTTGCAAATCTGA
GTCTTTCGGTCCTTGGAG
>dme_circ_0008740_junction_seq
GCAAATCTGAGTCTTTCGGTCCTTGGAGTTTGCGTGTTTGTGCCCTGGTGCAGTTT
>dme_circ_0008741
GTACGACAAGTTCCGGGCAGACTACGTCCGCAATCCGCACCAACTGGCCGTGGACTGCAACGATGATCCG
CTGTCGCAGTCGACGCAAAGCGTGTGGAATCAGTACTTTAGCGACCAGGACCTATTCGCCGTCATCCGGC
AGGATGTGGTGCGCACTTTCCCGGGTGTGGACTTCTTCCGTAAGCCATTGGTGCAGAATGCAATGGTCAA
CATACTG
>dme_circ_0008741_junction_seq
GGTGCAGAATGCAATGGTCAACATACTGGTACGACAAGTTCCGGGCAGACTACGTC
>dme_circ_0008742
TAATGGTGAACTCGGCCTTCAATGCTGGCTCATCAAAGCAGGGAAAAGCCTGGCGAGCGTAGGTGGGTTC
GAACTTGGAGGTGGCGATCCACTTGCGGGTCTCATCCTCCTTCACATACGACGAACTGTACAGTCCCACA
ATCTTGTTGGCCATCGATCCTTCGAATCCAATGTGCAGCCTTACTGTCCTACCTTTTGTCAGTGGTTCGT
TCAGCTGGAATATTAAAAATTCCCTCACAGCATCTACTGTTGTCTCCAGGATCTCCAAAG
>dme_circ_0008742_junction_seq
TCTACTGTTGTCTCCAGGATCTCCAAAGTAATGGTGAACTCGGCCTTCAATGCTGG
>dme_circ_0008743
ACGGCATCTACGCCCAAGTACTCAACGAAACTAGCAAATCCTTCGTTTAGCCACAGATCGTTCCACCAGT
TCATGGTTACTTATGGAAGATAATTGGAGTTACGGTTAAGAATACGAATTCCGATCTATGCAAACCGCCA
TTTAGCTTACCCAAGTTTCCAAACCACATGTGAGCGAATTCGTGGGCAATTACGCTGGCGATGCGCTGCT
TGTTGGTGGCGGAACTCGTGGCTTCGTCGTACAAAAGCGATGTTTCCCTGTAGGTGACCAATCCCCAGTG
CTCCATGGCGCCCGAGACGAAGTCGGGAATAGCAGCCATATCGAGTTTGGGCAGCGGATAAGCGATTTGG

AAGTAGTCAATGTAGTACTCGATGACTCCCTTGCCAATGGTCACAGCCAGATCCACTTTGTCGAGTTGCT
CCGGAGTGGCGTAGACACTCATGGAGAAGGTTTCTCCAATGCCCTTAGTGTCAATGCTCACCTGCTTGTA
GGCGAAGTCGGAGACAATAAAGCAGGCCAAGTAAGTGCTCATGGGAACACTTTTTGCAAACGTGACCTCC
TGGAAGGCTCCCTGGCTGACACTCGAGTCCACGTTCATGTTGGACAGGGCATGGTAATCCTCCCCAGAAG
GATGCACTAGAGTAATGG
>dme_circ_0008743_junction_seq
TCCCCAGAAGGATGCACTAGAGTAATGGACGGCATCTACGCCCAAGTACTCAACGA
>dme_circ_0008744
ATCCACTTTGTCGAGTTGCTCCGGAGTGGCGTAGACACTCATGGAGAAGGTTTCTCCAATGCCCTTAGTG
TCAATGCTCACCTGCTTGTAGGCGAAGTCGGAGACAATAAAGCAGGCCAAGTAAGTGCTCATGGGAACAC
TTTTTGCAAACGTGACCTCCTGGAAGGCTCCCTGGCTGACACTCGAGTCCACGTTCATGTTGGACAGGGC
ATGGTAATCCTCCCCAGAAGGATGCACTAGAG
>dme_circ_0008744_junction_seq
TAATCCTCCCCAGAAGGATGCACTAGAGATCCACTTTGTCGAGTTGCTCCGGAGTG
>dme_circ_0008745
TTTCCAAACCACATGTGAGCGAATTCGTGGGCAATTACGCTGGCGATGCGCTGCTTGTTGGTGGCGGAAC
TCGTGGCTTCGTCGTACAAAAGCGATGTTTCCCTGTAGGTGACCAATCCCCAGTGCTCCATGGCGCCCGA
GACGAAGTCGGGAATAGCAGCCATATCGAGTTTGGGCAGCGGATAAGCGATTTGGAAGTAGTCAATGTAG
>dme_circ_0008745_junction_seq
ATAAGCGATTTGGAAGTAGTCAATGTAGTTTCCAAACCACATGTGAGCGAATTCGT
>dme_circ_0008746
CGAATTCGTGGGCAATTACGCTGGCGATGCGCTGCTTGTTGGTGGCGGAACTCGTGGCTTCGTCGTACAA
AAGCGATGTTTCCCTGTAGGTGACCAATCCCCAGTGCTCCATGGCGCCCGAGACGAAGTCGGGAATAGCA
GCCATATCGAGTTTGGGCAGCGGATAAGCGATTTGGAAG
>dme_circ_0008746_junction_seq
TTTGGGCAGCGGATAAGCGATTTGGAAGCGAATTCGTGGGCAATTACGCTGGCGAT
>dme_circ_0008747
CTCTCACCCGGGCAGCTGTGCCGGCTCCAGCTTCCGGATATTTGGCGAAGAATTGCTCCATCTCCTCCAG
CTTGGTTTGGGTGCTAAAGCGGGCAGTGATCGAGGGAATCAGGTTGCCCAAATAACGCTCGTTGAGTCCG
AATCGGTCCACCAGTCGCTGCCAGTTCTCCCGGACATAGTCCCAAACCAGGGACTCACCAACTGGATTCG
CAGAGATGTAGGTAAGGCAGGTGAAGTAGTCCTGACCCCTCACGTATTCCTCGTTCCACGCCAGGTCAAT
GTAGCGCTGCAAAATCCAGGGAATCTGGATGGCAGACAGGCCATACATCAGTTTGGACTTCTCGCTGGCA
TCGGATTCGTTGACGAACAGCTCCCAGACTGCATCCCAATCCTCCTGGCTTCCAACGGACTGTATTCCGT
AGTAGTAGACAGTTTCACGGACATCGGCCTTTGGACGATCCTCCGGCTTGGCCAGCCATGCGTTGAACTG
CTCTCCGGCTTCAGTGAGACAGGATTCCAGACCCAGAGAACAGGCGGCACTCAAAGCAGTGACACGAAGG
CGGCTAGAGATGCAGGGGAATGATATAAAAAATACACACATCTCTTAAAGAATCTACCCACTTATCCAAA
TGATCCTCGCCGACTGTCCAGGTGAGTGCGGTATAGATGGGCTCAATCAAAGCTGTGGCGTACTTCTTGT
ACTTGGCATAGGTGCTGGTGTAGTAGAGTGTTCTCTTAAGCGAAG
>dme_circ_0008747_junction_seq
GTGTAGTAGAGTGTTCTCTTAAGCGAAGCTCTCACCCGGGCAGCTGTGCCGGCTCC
>dme_circ_0008748
CTCCCAGACTGCATCCCAATCCTCCTGGCTTCCAACGGACTGTATTCCGTAGTAGTAGACAGTTTCACGG
ACATCGGCCTTTGGACGATCCTCCGGCTTGGCCAGCCATGCGTTGAACTGCTCTCCGGCTTCAGTGAGAC
AGGATTCCAGACCCAGAGAACAGGCGGCACTCAAAGCAGTGACACGAAGGCGGCTAGAGATGCAGGGGAA

TGATATAAAAAATACACACATCTCTTAAAGAATCTACCCACTTATCCAAATGATCCTCGCCGACTGTCCA
GGTGAGTGCGGTATAGATGGGCTCAATCAAAGCTGTGGCGTACTTCTTGTACTTGGCATAGG
>dme_circ_0008748_junction_seq
GTGGCGTACTTCTTGTACTTGGCATAGGCTCCCAGACTGCATCCCAATCCTCCTGG
>dme_circ_0008749
GTACAAGAAGTACGCCACAGCTTTGATTGAGCCCATCTATACCGCACTCACCTGGACAGTCGGCGAGGAT
CATTTGGATAAGTGGGTAGATTCTTTAAGAGATGTGTGTATTTTTTATATCATTCCCCTGCATCTCTAGC
CGCCTTCGTGTCACTGCTTTGAGTGCCGCCTGTTCTCTGGGTCTGGAATCCTGTCTCACTGAAGCCGGAG
AGCAGTTCAACGCATGGCTGGCCAAGCCGGAGGATCGTCCAAAGGCCGATGTCCGTGAAACT
>dme_circ_0008749_junction_seq
TCGTCCAAAGGCCGATGTCCGTGAAACTGTACAAGAAGTACGCCACAGCTTTGATT
>dme_circ_0008750
GTGAAGTAGTCCTGACCCCTCACGTATTCCTCGTTCCACGCCAGGTCAATGTAGCGCTGCAAAATCCAGG
GAATCTGGATGGCAGACAGGCCATACATCAGTTTGGACTTCTCGCTGGCATCGGATTCGTTGACGAACAG
CTCCCAGACTGCATCCCAATCCTCCTGGCTTCCAACGGACTGTATTCCGTAGTAG
>dme_circ_0008750_junction_seq
GCTTCCAACGGACTGTATTCCGTAGTAGGTGAAGTAGTCCTGACCCCTCACGTATT
>dme_circ_0008751
GAATTTTGTAGGATAGTTGGTTGGCATCCGCCAGGGCGAAGGCATCGTTCAAAAGATGGCCACGATCGGC
GATTTCGAATCGAGCGGGACTGGTGGTTAGCTGCTGGATGAGCAGGGCCCACAGGCTCTCCTCGTAGTTA
ACCCGATAGTAGCCCGTTTGGTTGACATTCAACTTGATCCACTGCACCTCGCTGGGCACGGCAATACCCA
CAGAGTCCACATCGTAGTCGTATATGAAGCTGTTCTCTGAACCATCATCAGCAAACCAGGTAATCGGAAC
ACTCCATTTGTATCCGTATGCGCTATCTGAAGGGGCTTCCTCGTAACTGGCTGGATTGGAGAGGAATCGC
TGCTGGGTAACCTTAAAACTACCATCTGCAACCTTGGAGACATTCAACACCGGATAACCCATCTGTTCGG
TCCAGGTGAGCATCAGTTTCTTGATATCCAAGTCAGTTACCACAGCTTCGACTTCGGTGAGGAAATCGTC
GGTCACCGTGTTATTGAACTGATGCTTGACCAGGTAATTGGTCACCGCCTCCTCAAACTTTTCGGCGCCC
ACAAGTGTCTCCAGCATGCGGATCACGGATCCTCCCTTTTCGTAACTGATGGTGTCGAATATGGCCGTAA
TTTCATCGGGGCTTTCTACCTTCTGGACAATCGGATGGGATGATAGCTTGGCATCGTATAACAACACGGG
TTGCAGGGCCACGATTTGGAATTGCTCCAGCTGATTGGTAAATATAAGATATTTTCTTTGAGGATTCAAA
TCGGTATTATCCATTTCCTTACCATTCCCCAGTCCGGGTGCACAGCATTGACTCCCTTGTACTGCATGTA
GCGGGCGAATCCCTCGTTCAGCCACAGATCGTTCCACCACTTCATGGTGACCAGGTTTCCGAACCACTGG
TGTGCAATCTCATGGGCCAGTGTTCCGGCTATCGATTGTTTGTTCGCTGTGGAACTGTAACTGGGATCGT
ACAGCAGAGCGGTCTCTCTGTAAGTCACCAATCCCCAGTGCTCCATGGCCCCAGATGCAAAGTCGGGAAT
GGCGGCCATGTCCAGTTTGGTCAGCGGATAGGGCACCTTATAG
>dme_circ_0008751_junction_seq
TTTGGTCAGCGGATAGGGCACCTTATAGGAATTTTGTAGGATAGTTGGTTGGCATC
>dme_circ_0008752
GATTGGCGGCTATGTACTGCACACAGGTGAAATAGTCCTGGGAGCGAACGATGCTCTCATCACTGGAGGC
CTGTGCCAGGAAGTCGAACAGATACTGGCTGTTCCGTACGCCAGACAATCCGTACATCAGCTTCAGTTTC
TCGCTGGCATCGGTTTCGGCCACAAAGAGTTGGAACAACTGATCCCAGCTCGATTGGCTGGTGGACTGCT
GCATGCCGTAGTAG
>dme_circ_0008752_junction_seq
GCTGGTGGACTGCTGCATGCCGTAGTAGGATTGGCGGCTATGTACTGCACACAGGT
>dme_circ_0008753

GTAAGATATCAGATCCAGTGCCACGGAGTAGTTAAGCTGTCCGGCAGCGGCCAGGGTATTGGCATCGTTC
AGCAGATGAGCGCGATCGGCGGTGCTGAAGGTTTCCCGGGAGTTCTTCAGGGCCGATATGAGTTCCGCCC
ACTGTTCGCTGCCGTAGTTCACCCGG
>dme_circ_0008753_junction_seq
CCACTGTTCGCTGCCGTAGTTCACCCGGGTAAGATATCAGATCCAGTGCCACGGAG
>dme_circ_0008754
GCTAAAGATAACTAACACGCAAAATGCCCCACCAAGAGAGAAAATCGATGTGGTTCTTTGGCGGATTGGC
GAGCGTAGGTGCGGCCATGGTGACCCATCCGCTCGACCTCATCAAGGTCACGCTGCAGACGCAACAGGGT
CACCTGTCGGTGGCCCAGTTGATCCCTAAACTCGCCCGCGAACAGGGAGTACTCGTCTTCTACAACGGCC
TCTCCGCCTCAGTGTTGCGCCAATTGACATACTCCACTGCGAGATTCGGGGTGTATGAGGCCGGAAAGAA
GTACGTCAATACGGACAGCTTTGGCGGCAAGGTGGCCCTGGCAGGTGCATCCGGTCTCGTCGGAGGAATC
GTTGGAACTCCGGCTGACATGGTCAATGTGCGCATGCAGAACGACGTAAAACTACCGCCGCAACAACGAC
GCAA
>dme_circ_0008754_junction_seq
TAAAACTACCGCCGCAACAACGACGCAAGCTAAAGATAACTAACACGCAAAATGCC
>dme_circ_0008755
GTATCGATTGGCATCGCGGCTAATTCAAGGTGCCAAACTTGAGACGCAATTGCTCCAGGAACACAAAGGT
GATGATCGTGTGTGGACCCAGACGCACAAATGCCGGCACATAGCCCTTAAAGAAGCCGAGGGGACCCAGT
TTCGCCGTGTGCTTCACGATGTCCCACAGGCCATTGAACTCGCCGGGTTTCGCGTTCATGGACCGCGTCT
TCAGCACATCCAACGGTTGGGTGAGAGTGGTGGCTATCGTTCCGGCCACCAGGGAGGCGGTGAAG
>dme_circ_0008755_junction_seq
CGTTCCGGCCACCAGGGAGGCGGTGAAGGTATCGATTGGCATCGCGGCTAATTCAA
>dme_circ_0008756
TTATGTTCTACACGGTGAACGAATTGCTGCTGGAATGTTGCATTGCTCAAGGGAATCTGCCAGCCAGCCA
AGGAGAATTATTGCCTAGTTAAGGTATCGATTGGCATCGCGGCTAATTCAAGGTGCCAAACTTGAGACGC
AATTGCTCCAGGAACACAAAGGTGATGATCGTGTGTGGACCCAGACGCACAAATGCCGGCACATAGCCCT
TAAAGAAGCCGAGGGGACCCAGTTTCGCCGTGTGCTTCACGATGTCCCACAGGCCATTGAACTCGCCGGG
TTTCGCGTTCATGGACCGCGTCTTCAGCACATCCAACGGTTGGGTGAGAGTGGTGGCTATCGTTCCGGCC
ACCAGGGAGGCGGTGAAG
>dme_circ_0008756_junction_seq
CGTTCCGGCCACCAGGGAGGCGGTGAAGTTATGTTCTACACGGTGAACGAATTGCT
>dme_circ_0008757
GATTCCGGTGACTTCCGCAATCCTATGGACTTCTTTGAGAAGTTCTTTGGCGCTGGATTCGGAGGTAGTG
GCGGTGGACGCAGGCGCGAGAGGCGTGGCAAGGACGTGGTGCACCAGATGTCCGTACAGCTGGAGGAGCT
GTACAATGGAGCGACCCGCAAGCTGCAGCTCCAGAAGAAT
>dme_circ_0008757_junction_seq
GACCCGCAAGCTGCAGCTCCAGAAGAATGATTCCGGTGACTTCCGCAATCCTATGG
>dme_circ_0008758
GGTATTGGAAGTGCACATTGAGAAGGGAATGCGGGATGGCCAGAAGATCGTGTTCACGGGAGAGGGCGAT
CATGAGCCGGAATCTCAGCCGGGCGATATTATTATTCTTTTGGACGAGAAGGAGCACTCCACATTCGCGC
ACGCCGGACAGGATTTAATGATGAAGATGCCGTTGCAACTTGTCGAAGCGCTCTGCGGATTCCAGCGAAT
CGTTAAAACGCTCGATGATCGCGATCTGATTGTGTCCACACAGCCCGGCGAG
>dme_circ_0008758_junction_seq
TCTGATTGTGTCCACACAGCCCGGCGAGGGTATTGGAAGTGCACATTGAGAAGGGA

>dme_circ_0008759
GATATATGCACTATGTCTATAACGCGTGGTTGTTTGTAAACCAACATTTTTCTTTATGTTTATGTTGCGT
TGGATTGTCAAAGAATCGAaaacggaaactgaaactaaactaaactgaaattgaaaTTAGCCGCCACTAG
AACCGTATCTAAAGTCCGCGCCAGCGAACACGTCTAGGAACAAGTTTAA
>dme_circ_0008759_junction_seq
CAGCGAACACGTCTAGGAACAAGTTTAAGATATATGCACTATGTCTATAACGCGTG
>dme_circ_0008760
GTTATTGATGTGGTTCTCCGAGGCGCATCGCTGGCCGGGATAGACGTTGTCCGGGAAATTCTTGCCGGCC
TTCATTAGGCAGTTGATGAAGTCCATCGTGAGCGACTCGCGCGTGTACTCCACCTGATAGGAGTTGGCCT
GAATGTGCTCGATGGCGCAGGCATGCACCTTATTGCCATAGCACTCGTTCGGTCCGTGGTGGCAGGTGAA
CGTCACTTCGGAGCCCTGG
>dme_circ_0008760_junction_seq
GCAGGTGAACGTCACTTCGGAGCCCTGGGTTATTGATGTGGTTCTCCGAGGCGCAT
>dme_circ_0008761
GGAAGAAGCGATGGAACTTCGAGGCTATGGCATAGATTACACCCGGCTCCGATTTTGTGGTGGTGATATC
GGCCACAAACTGCAACTGATCGCGATCGAAGGCCAATACCGATTGCTGGTTTGTCGTTGGATTCCACGAG
GCGATGGCTGTTTCACTCAGGGGACTGAAGATCAGGCTGCTGTCGAACGGGGATACGGCGAGGCCAATGC
CCTGGGAGCTCTTCTTGCCCACCAGCTTCACATCCAGCATTTTGCCATCTGGCAAAGGACCCGCACGCAG
GACATTCTTATGCACGGAGAAAACTCTGTAAAATAAAGAGATACAATTAGATATTACTAATTTTTGCTTT
AAAATCTAAACCTATCATAGCCTATAGCCTATTTACTTTTTTAATTATGATCGTCCATGTATATTTTAAT
GAGAAAGCTAACATAACTCGAAGATATTTGAATTAATTTAATGATCATTATTTCAACAGTTAAGCTAACA
TTGAAACTCAAAAGGCAATGTAAGCCATTACAGATAATCATACCTTCCTAAAGCTACGTGACCAATTACC
TTAAGTCAATAAAGGTATTTCTATGTTTTTTGTGGGTAATTTGCAACTGAATGAACTGGTTCGGTTTTCT
ACTCAAAAATCGAATTACCTATCAAATTTTTTACTGTTTAAAGTTGACTCACCTATCCGTGGCCAATGGC
TGGAAGTAAACCACACCGGTGCGCTCATCGAAAGTCAAACCGACCACTCCATCCATCAGAACAAAGCGGT
GCTCATGAATCTCGGACTGTGCGAAGTCCGGATCAGGATACATGGCTGGATGGGATACCCGCCAGGTGAC
GTCCTTTCCACT
>dme_circ_0008761_junction_seq
ATACCCGCCAGGTGACGTCCTTTCCACTGGAAGAAGCGATGGAACTTCGAGGCTAT
>dme_circ_0008762
CTGAATTGAATGTAATTGTGCGAGTATTTTTGTAAATGTATTGTCCGCCGGGGGTCCCAGAACCGAAAAA
ATGACTTACCACACACCAACGAAATGTTAAGTGCAAGCAATCCACTCAATTATTTTTTGTCTAAGCATAC
AGCAACGATGTTGAAGCATTTAAATG
>dme_circ_0008762_junction_seq
ACAGCAACGATGTTGAAGCATTTAAATGCTGAATTGAATGTAATTGTGCGAGTATT
>dme_circ_0008763
GGTGACTATCAACAAGACCTTCGACATGGACCACAACACGGAGACACTGAAGCGCCTCTCGAACGCCAAC
GAGAAACCCAATAACCAAGTGAAACTGGCCAATGTGAACACCACGGCCCTGCCGCCTGGCAACATCAAGT
GCAATATTTTAAAGGGCTCGCGTTGAGGAGGAGCTGGGTGCCGGAGGCAACGCCAAGCTTACCAATCAGA
TGTTCGTACCGGCCGCCcacagcagcaacatgcacacgagcaacacaaacagcagcaacaACCATGCACA
CAGCGATGGACCGGATTCCCTGAACGCCACCTACATATCAGTGG
>dme_circ_0008763_junction_seq
TCCCTGAACGCCACCTACATATCAGTGGGGTGACTATCAACAAGACCTTCGACATG
>dme_circ_0008764

TTGCCGGTTCGGAGCGGTTAGACAATTTCAAATTACAGGGAGAATCTCAGAAAACCGGAGTGGAGGAGTG
CCAGAGTACCGGACCACCGGAGCAACGGAATTCCGGAATACCGGAGTGCCGGAGAGTTCCGGCCTTCTGT
ATAATCATGTATGTACATATCGCTTAGACCGCTAATTAGGACTACTTAGGTCTAGTTAAATACACG
>dme_circ_0008764_junction_seq
GGACTACTTAGGTCTAGTTAAATACACGTTGCCGGTTCGGAGCGGTTAGACAATTT
>dme_circ_0008765
GGTTGTTTCTGCTTTGTTGTCTTGTCCTGTGTCTTGtcgtatatcgtatatcgaatatcgtatatAGTTT
TTCTCAATCGAACGGGAAGAAGGCAACCCGATGTGCCAGCCAGTCAATGCTTGCTTCTTTTGTTTGTTGG
TTCTATAATTATTATTACATATTACAGTTAATTCATAATTAAAGTTAAACAAATACTTATACTCTATTGT
ATTGTGTAATATATGAAACAttttagctgtatttttttgttttgttttgtaagtatttttgtttgatttct
tctgtttttttttCCTATCTTTCCTATCTCTCCGACATCCTGTGCTCTACTGACGCGTTAAACAATTACC
TCGCATAATGTATGGGACTACATTAGAAAAACAAATTAGTTTCGACACACAGACAACGTGAGCATTGCAC
AGCAGTTAGTTTGGCAAATGTTGTTTGCCACGTTTCCCTGCAATGTTGTCTTGTGGTTTTGCACGGGTTG
AAgggttgtttggtgggcggttggttg
>dme_circ_0008765_junction_seq
GAAgggttgtttggtgggcggttggttgGGTTGTTTCTGCTTTGTTGTCTTGTCCT
>dme_circ_0008766
GAAAAAGGAAGATTTAAGTAAAAAATGAAAAACGTTACAGTTAAGTGAAAAGTTAACACTGTTGGTGCGG
TTTTGCATGGCGGATCGCTCGATTGCTCGATCTCTGGTCGCTTGGTTGCTTGATTGCCTGATTGATTGCT
TTGTTCGGATGTCGGTGGATAGTAGATGGATATAGTGGCTCTACTGGTGTTGTTCTCACCAAGGACCCTC
GGTCACCGGTTGTATCG
>dme_circ_0008766_junction_seq
CAAGGACCCTCGGTCACCGGTTGTATCGGAAAAAGGAAGATTTAAGTAAAAAATGA
>dme_circ_0008767
GTTACGAAAGTAATATTACATAATTAACGGGTACAAATGCGGGGAGTGGGAACCGAAACACCCGATCTGC
GTACTTGGCATTAATTTATTCAACAGATCTAACTCGGTGCTTAAGCTACAGCTCCCCGCACTTTTATTTT
TGTGGTCTGGTTGGTTAGTTTTAGAATTTCAAGTTGTAGTTGTAGAGTAGTAACGGAATAGGAAATTATA
AAGTCTAAGAATATATTGTTATTAGGGTTTTAATATAAAGTGGGGGCACAAAATTATTGCTATAATTGTA
TGGTAAGTATATGTAAAGGTATACAAAAATTTTGTTTATATCAAAAAAATTTGTTTACACATTTAAACAT
CATAATAATCGGTATATTAATTAATAAAAATATAGCATAAAATTTATATAAAAACTTTTCTTTCTGGGAT
AACTTTAATATAAATGTCAATAAatttttttcttatcttgcctagattttttttgtttggttttctttgTA
TACAGACAACTTGCTAAAAACATTGAGAAACATGCAATTTCTTTGTAAAACAATTTACTCAAAGTTTATG
ATTAAAGGT
>dme_circ_0008767_junction_seq
AATTTACTCAAAGTTTATGATTAAAGGTGTTACGAAAGTAATATTACATAATTAAC
>dme_circ_0008768
GTAATATTACATAATTAACGGGTACAAATGCGGGGAGTGGGAACCGAAACACCCGATCTGCGTACTTGGC
ATTAATTTATTCAACAGATCTAACTCGGTGCTTAAGCTACAGCTCCCCGCACTTTTATTTTTGTGGTCTG
GTTGGTTAGTTTTAGAATTTCAAGTTGTAGTTGTAGAGTAGTAACGGAATAGGAAATTATAAAGTCTAAG
AATATATTGTTATTAGGGTTTTAATATAAAGTGGGGGCACAAAATTATTGCTATAATTGTATGGTAAGTA
TATGTAAAGGTATACAAAAATTTTGTTTATATCAAAAAAATTTGTTTACACATTTAAACATCATAATAAT
CGGTATATTAATTAATAAAAATATAGCATAAAATTTATATAAAAACTTTTCTTTCTGGGATAACTTTAAT
ATAAATGTCAATAAatttttttcttatcttgcctagattttttttgtttggttttctttgTATACAGACAA
CTTGCTAAAAACATTGAGAAACATGCAATTTCTTTGTAAAACAATTTACTCAAAGTTTATGATTAAAGGT

GTATGTAAATGTACAAATGTATATATAATTGGTATACAGTCAAATTGTCGACGCACATTGACAACATTTG
ATAGGATAATTATTAGCAAAAAGTTCATTTCAAGGTTCTGCTAAATTTCAAATTCAAAGATATACGAATT
ATAAGAG
>dme_circ_0008768_junction_seq
AAATTCAAAGATATACGAATTATAAGAGGTAATATTACATAATTAACGGGTACAAA
>dme_circ_0008769
ACTTTATAATTTCCTATTCCGTTACTACTCTACAACTACAACTTGAAATTCTAAAACTAACCAACCAGAC
CACAAAAATAAAAGTGCGGGGAGCTGTAGCTTAAGCACCGAGTTAGATCTGTTGAATAAATTAATGCCAA
GTACGCAGATCGGGTGTTTCGGTTCCCACTCCCCGCATTTGTACCCGTTAATTATG
>dme_circ_0008769_junction_seq
CTCCCCGCATTTGTACCCGTTAATTATGACTTTATAATTTCCTATTCCGTTACTAC
>dme_circ_0008770
ataagaaaaaaatTTATTGACATTTATATTAAAGTTATCCCAGAAAGAAAAGTTTTTATATAAATTTTAT
GCTATATTTTTATTAATTAATATACCGATTATTATGATGTTTAAATGTGTAAACAAATTTTTTTGATATA
AACAAAATTTTTGTATACCTTTACATATACTTACCATACAATTATAGCAATAATTTTGTGCCCCCACTTT
ATATTAAAACCCTAATAACAATATATTCTTAGACTTTATAATTTCCTATTCCGTTACTACTCTACAACTA
CAACTTGAAATTCTAAAACTAACCAACCAGACCACAAAAATAAAAGTGCGGGGAGCTGTAGCTTAAGCAC
CGAGTTAGATCTGTTGAATAAATTAATGCCAAGTACGCAGATCGGGTGTTTCGGTTCCCACTCCCCGCAT
TTGTACCCGTTAATTATG
>dme_circ_0008770_junction_seq
CTCCCCGCATTTGTACCCGTTAATTATGataagaaaaaaatTTATTGACATTTATA
>dme_circ_0008771
GTGTGTTCAAACAATAACAGTTTAGTTAACAATTAAAAAAAGGCACAAGTTGCAATTGCGCAAAAGGCCA
AAAACACTGGCGTTTCACACAGTTTCACATAATTGGCACATGCTCCTCTCTtgtgtttcatgtttctttg
tttgttgttatgtctggggtttctttctgttcttctggttgtttGTTTGCTGCCCTTGCGTTTGAGGTAT
TTGTCATCTTTGGAAAGTCTGTTGAAGGCAACCAagttgttgttgatgttgttctggttgtt
>dme_circ_0008771_junction_seq
agttgttgttgatgttgttctggttgttGTGTGTTCAAACAATAACAGTTTAGTTA
>dme_circ_0008772
GCTACAACAACCAGTGGGACGGACAGGGATCATACGGCGGCTATGGCGGCGGCTACGGCGGATATGGTGC
CGGTGGTTATGGCGACTACTATGCCGGCGGCTACTATAATGGATATGACTACGGTTATGG
>dme_circ_0008772_junction_seq
ACTATAATGGATATGACTACGGTTATGGGCTACAACAACCAGTGGGACGGACAGGG
>dme_circ_0008773
GAAACTGTTTGTCGGTGGTCTGAGCTGGGAAACGACTGAGAGTAAGTATAAAACTAAAACGAAGTAAATA
AACAAATGCTAACAAGTTGCTTTTCTATTATTTCAGAGGAACTCCGCGATCACTTCGGCAAATATGGCGA
GATCGAGAGCATCAATGTCAAGACAGATCCCCAGACCGGTCGGTCCCGAGGATTCGCCTTCATCGTGTTT
ACAAACACCGAGGCCATTGACAAAGTCAGCGCCGCGGATGAGCACATAATCAACAGCAAGAAGGTCGATC
CCAAGAAGGCCAAGGCCAGGCACGGCAAGATCTTTGTCGGCGGCCTCACCACAGAGATCAGCGATGAGGA
GATTAAGACCTACTTTGGACAGTTCGGCAATGATCGTCGAGGTGGAGATGCCATTCGACAAGCAAAAGTC
GCAGCGCAAAGGATTCTGCTTCATCACCTTCGATTCGGAGCAGGTGGTGACAGATTTGCTGAAGACGCCC
AAGCAGAAGATCGCCGGCAAGGAGGTCGATGTTAAGCGTGCGACGCCCAAGCCGGAGAACCAGATGATGG
GCGGTATGCGTGGTGGACCACGCGGTGGTATGCGCGGCGGACGCGGTGGCTACGGAGGACGAGGTGGGCT
ACAACAACCAGTGGGACGGACAGGGATCATACGGCGGCTATGGCGGCGGCTACGGCGGATATGGTGCCGG

TGGTTATGGCGACTACTATGCCGGCGGCTACTATAATGGATATGACTACGGTTATGG
>dme_circ_0008773_junction_seq
ACTATAATGGATATGACTACGGTTATGGGAAACTGTTTGTCGGTGGTCTGAGCTGG
>dme_circ_0008774
GTGGAGATGCCATTCGACAAGCAAAAGTCGCAGCGCAAAGGATTCTGCTTCATCACCTTCGATTCGGAGC
AGGTGGTGACAGATTTGCTGAAGACGCCCAAGCAGAAGATCGCCGGCAAGGAGGTCGATGTTAAGCGTGC
GACGCCCAAGCCGGAGAACCAGATGATGGGCGGTATGCGTGGTGGACCACGCGGTGGTATGCGCGGCGGA
CGCGGTGGCTACGGAGGACGAGG
>dme_circ_0008774_junction_seq
GCGGACGCGGTGGCTACGGAGGACGAGGGTGGAGATGCCATTCGACAAGCAAAAGT
>dme_circ_0008775
GTGGAGATGCCATTCGACAAGCAAAAGTCGCAGCGCAAAGGATTCTGCTTCATCACCTTCGATTCGGAGC
AGGTGGTGACAGATTTGCTGAAGACGCCCAAGCAGAAGATCGCCGGCAAGGAGGTCGATGTTAAGCGTGC
GACGCCCAAGCCGGAGAACCAGATGATGGGCGGTATGCGTGG
>dme_circ_0008775_junction_seq
AGAACCAGATGATGGGCGGTATGCGTGGGTGGAGATGCCATTCGACAAGCAAAAGT
>dme_circ_0008776
GAATCAAACGCCAGCTATGGTCATGGATGCGACCCAATCGCAGCAGCCTTCGCCGCAATCTGTGGGACGC
GAGTTTGTCCGCCAGTACTACACGCTGTTGAACAAGGCGCCCAACCACTTGCATCGCTTCTATAACCACA
ACTCGTCGTACATCCATGGAGAGTCCAAGCTGGTGGTCGGTCAGCGGGAGATCCACAACCGAATCCAGCA
GCTGAACTTCAACGATTGCCACGCGAAGATCAGCCAGGTTGATGCCCAGGCCACTTTGGGCAACGGTGTG
GTG
>dme_circ_0008776_junction_seq
CCAGGCCACTTTGGGCAACGGTGTGGTGGAATCAAACGCCAGCTATGGTCATGGAT
>dme_circ_0008777
CTTACATTCTTTTAGTTTTTGGATTCACAACGAAGATTTGCACAGAATTTCGGTATATTTTGGCACGAAC
TGGCTGGAAAAAGCGAGCCTAACGCAATTAAACTAAGAAGGGACTCAGGACCGTTGACGCAACTCTTTAC
ATCCTATCTTTCTTTCCTTCTTTTTTGTTTGTAGTTCGACGCCCCGAGAGTTGCGCTCAGGAACATTTAA
AGGACAGTTGACGTTTTATGTTGCTCCAACTACTTTATCTTAGTGTATGAGTGTGTGATTTGCGTGTGTT
TAATTGCATGAGATGTTCTTGAATGTAATTGATTGATTGCATTGTGGAAgaagtcgatgtttttaacgtt
gggttgttgctgctgttcttgatg
>dme_circ_0008777_junction_seq
cgttgggttgttgctgctgttcttgatgCTTACATTCTTTTAGTTTTTGGATTCAC
>dme_circ_0008778
TTCGATGACTACGATCCCGTTGACAAAATCATCCTTCAGAAGACCCACTCCATCAAGAACAAGACCCTGG
ACGTGAAGAAGGCTATTGCCAAGCAGGATATGGATCGACAGGGCGGAGGTGGCGGACGCGGAGGTCCTCG
AGCTGGCGGTCGCGGTGGTCAGGGTGACCGCGGCCAGGGAGGCGGTGGCTGGGGAGGCCAGAACAGACAG
AACGGTGGGGGCAACTGGGGCGGAGCTGGCGGCGGCGGAGGATTCGGCAACAGCGGCGGTAACTTTGGAG
GCGGTCAGGGCGGCGGCTCTGGCGGTTGGAATCAGCAAGGCGGAAGCGGAGGTGG
>dme_circ_0008778_junction_seq
GGAATCAGCAAGGCGGAAGCGGAGGTGGTTCGATGACTACGATCCCGTTGACAAAA
>dme_circ_0008779
GTCCTCGAGCTGGCGGTCGCGGTGGTCAGGGTGACCGCGGCCAGGGAGGCGGTGGCTGGGGAGGCCAGAA
CAGACAGAACGGTGGGGGCAACTGGGGCGGAGCTGGCGGCGGCGGAGGATTCGGCAACAGCGGCGGTAAC

TTTGGAGGCGGTCAGGGCGGCGGCTCTGGCGGTTGGAATCAGCAAGGCGGAAGCGGAGG
>dme_circ_0008779_junction_seq
GTTGGAATCAGCAAGGCGGAAGCGGAGGGTCCTCGAGCTGGCGGTCGCGGTGGTCA
>dme_circ_0008780
GTGGCGGACGCGGAGGTCCTCGAGCTGGCGGTCGCGGTGGTCAGGGTGACCGCGGCCAGGGAGGCGGTGG
CTGGGGAGGCCAGAACAGACAGAACGGTGGGGGCAACTGGGGCGGAGCTGGCGGCGGCGGAGGATTCGGC
AACAGCGGCGGTAACTTTGGAGGCGGTCAGGGCGGCGGCTCTGGCGGTTGGAATCAGCAAGGCGGAAGCG
GAGG
>dme_circ_0008780_junction_seq
GTTGGAATCAGCAAGGCGGAAGCGGAGGGTGGCGGACGCGGAGGTCCTCGAGCTGG
>dme_circ_0008781
GTTGTATCGGCCGTTGTTGCGAATGAAGGCATCTACCACCGGCGCAAACAGATTGCACTTGACAATGTGT
CTGTAAGTAAGGTAAGTTGGTTatattgtttaaccataagagtttttggggacgcttacctaTTGTAAAA
CTCATCTTTAAGTGCAATTATCTTGCGCAGTAATCGCAGAGCGCCGAGGACAAGGAACGTATGTGTGCTC
TTCATTAGCACCAGTATTCGCTTGAGGAGATCCTTttgcaacaaaaagtttttaatg
>dme_circ_0008781_junction_seq
ATCCTTttgcaacaaaaagtttttaatgGTTGTATCGGCCGTTGTTGCGAATGAAG
>dme_circ_0008782
GCAACAGACACAGCCCGAGATCGCGGAGCTACAACAGCAGCTGAGCAGCGTGGAAGCGCCTCAAAGCCAG
GAGTTGGAGCTGTCACAatcggccgccgccagtgcatcgcccacttcgtcatcctcgtctctggaagcct
cgacgtcctcttcatcggcgtcatcgtcttcatcctc
>dme_circ_0008782_junction_seq
cttcatcggcgtcatcgtcttcatcctcGCAACAGACACAGCCCGAGATCGCGGAG
>dme_circ_0008783
GCTTGCACGGCTGCAGCACTCCCATCTACACACTGCTTAAGCCGAGCCATAAGAAAAGGGCAGCAATTCC
TTGTTAAAAGAATGCTGCGTCGTCGCCCGAGCCTAATGGAATATCCGGCTTCCAACGGATTCCTGCCACT
TGCCAATGCCATTATTCAGGGCGAGATGTGCATCATTGACTTGCTGCTCTCGGCCGGTTGCAGCGTCCAC
ATTGGAGATCCGGGAAGCGGCCGGACCCCGCTGCACGTTGGTGACCGCACTAGTCTAAATTGCCAGTTAT
TAACGAGTTTTAAACCTCACAGCTGGCTTTTTACTACGGTCACCTGCCGTCCGCTCGCATTCTGCTGAAC
AAGAAGGCCCGCCTGGAGGCGACGGACAGTAACGGCATGACTCCCGCCCACTGCGCCGTTGATGCCAATC
AACTGGAAATAGTCAAGTTCGCTCTCGAAGCGGGAGCGAATGCTGAGGCCAGAGACGTCTGCGGCTGGAC
CCTTCTTATGCGCGCAGGTAGGACTAGAAGGACTGAAACTGCTTAAATCGAATTTAAATTCGCACCTTGC
TTGCAGTGGTTATGAACGCTAGCATGGAGCTCATCAAAGTCCTTGTTACGCACGGCGCAGACTTGGCGGC
TTTGGATGGTGTTGGCAAAACTTGCACTGACTTGGCGAAACTTTATCACAGACAGGAGGCAAAGGATTAC
TTTGACGAGGTGCAAAAGTTTAGGCTGGAGAAATCAGTCGCGACCGCAGACGCAGTGACTGCAATAACGA
GG
>dme_circ_0008783_junction_seq
CGCAGACGCAGTGACTGCAATAACGAGGGCTTGCACGGCTGCAGCACTCCCATCTA
>dme_circ_0008784
ATGGCATGTGGATGCGGAGGATGGGCGTGAGGATGCTGACTGGGTCCTGGGCCATGCAATCCGCCATTTG
ACATTGTCATGGCATGCTTCCGCTGTAGTGTGTTCGGACCCATAGGCCGCTCCCCTTCATTGAGGTCAAG
ACCTGCCACCATTTGGGCGATCTCACGCTCCTTCTTTATATCTGG
>dme_circ_0008784_junction_seq
CGATCTCACGCTCCTTCTTTATATCTGGATGGCATGTGGATGCGGAGGATGGGCGT

>dme_circ_0008785

GCTGGGACTGTGGATGATGATAACTTGTATAGTGCAACGGCAGGAAGGTCATCATCGTCAAAAACAACAA
CTAAAGCAACGACATCACAGGCAATAGTGGCAACAACAACAGCAGCGTTAGCAATCACAAATCTTGGTAG
TAGCAAAAATCTAGAGACACCCAACCAAACAGAAACAGAGGCGACACCATCATCGTCATCAACAGCGTCA
TCAACCAAAGCGAAATTCCAACTAAAGCCCATTTTGAAAGTGCACGAGGCCAGCAAAACCCGCATTATTC
AGGTGCAACCCAAACATCTGGAAGAGCGGCCAAAGCTCAGATTGCAATTGCCCAAGGCGATGGGAAATGA
GGAGGAGCAGAAGCAAGAGGATGAGGAGCAGGAGAATGTTGAGGCTGAGCCCGTACCCATTCTGCCAAAG
AAAACGGCGCCCAAGTCGCCTCAGAATGCCAATTATGTGCCTGTGTACAACAATAAGCTGGTTAGCAACT
ACGAGAACATGGAGTTTGGCAAGCCAATCCCCGAAAGTAAAATCCCTCACAGCAATGCAGCCATTACCGA
TCCCGAGGAGACGCCCATGCTAATGCAACTAAAGCTGTTCAAGGAACAGCAGGACCAGCAGAAGCAACAA
CAGGAAAACGAAGAGGAGGACCTGACACCAACAGCGGAAAGTAAACCCATTCCAGGCGAGGAGGATATGG
GGGCTCCAAAGGTTATAAAAGACCAGAAATTAGAATCGAACGAGGAGGAGCAGGAGCTGGAGGATGAGGA
TGACAGCACGGCTTGCGAGGAGTACACAGATGATGATATAGATGAAGCTCTGGCTCAAGATGATGAGGAT
GAAGCTGTCGTCGAGGCAGTAGAATCCCAGGAACTGGCTGAGGAGCGGGAACTGCAGCAGATATATGTTA
ACGAGCCCATCACACCCACGGACCAACAATTCGACGAGAGTCACTATCTGCCCATGACGCCCAAGAAAGT
GGAACTGGGTCAGCCGGGTGTCCTGACCTTAGTTTCCGCGACGTACGCCGAGGAGGAGGAGGAGAATCAC
TATGTAGAGATGACAAAGGGCATACAGGACGAGGATTGTAGAAGCAACTACGAGATAATGTGTTTGGCTA
GCACCAGTTCGAGTGCCTTCAAGGTGACCACCAAAACGGAGCCTGTCTACATGGAACTGGCAGGGGTAAA
GGCATCATCCGCAGTGCCAGCCACCGAAGAGGCCCACTGCTCTTCGGGCCGTTCCACTTTAAAGAAGTGC
AAGAAATCGGGCACTGAAACCCTGAAGAAGAAGACCAAGAAGCGACAGGGCAAGGACATGCCCGATATAC
TTAAGCCGGCCAAAAGTGCCCTGGCCTTGGCCAGCGATAGTTCGGATGCGGATGACGAGAGCAGCCGACA
GCAGTTAGAGGCTAAGAAACTGCGCTCCAGGTCACGTTTCAGTTTATCGGACACATTTAGGCCAGCTTCT
TACTACTTGGGAGCCTCAACGCCTCTCAACAATTATGCAGAGAGCTCGGATAGTGAGATATTGCCACCAC
CACCCATTCCCGCTTCACCACCGCCCATGGAGGAGCTGAAAACGGAGGAGATCTTCTCCTCCGAGCACTA
TGATACTGTGAAAAGAAAGGATAGCAATGGCAAAGTGAATCTATCGTACGAGCAATTGCCCAAGATGCAT
GCCAGCAATACGTCCCTGAATCTGCCGAAAGCTGTGCCCAGTCCTACTCTGAAAAGTAGTCGTCTCTCCT
TGCCTGACCATTTCACCAAGGTGCGGATGACACCGCAGAGATTATTGGTTCCAGCCACACCGCCGCAGCA
GCATCACGCCAGGACATTATCCGACTCCAACTATAGTTGCCAGCTGACCGATACAAGTTCCCGAACATCC
TCGGATTTGGATCTATATCGCAGGAGGGGTCAACAGCAGAGGTGTACCAGCAATAATTCTCTGCCCTTAA
GCAGTGAAAACGAGTCCGTGGAATTCCGCCAGAGATCGGACTCCGAATTGGATCGCCAGCGTTCGAGGAG
ACCACTTTCCCAAGAATCCATCAGTGAGATTGAGTCGTTGAGCGAACAGTTTGAGGAAACCCTAAGTAGT
CATGAATTGGACACGTACCTGAGCCATTTGCATCTGTCCACCGGTCAGGCCACGCCCACCAGTCACCTGC
TGAATGATGCAAATGCGGCAGCCGGAGCCGATATCCTGACCAATCTCATCAAGCCACCGAAAACATTCCG
GAATGCCGAGGACGAGGAGCAGCATTTCTATGGTAACATACACTTTCTCTCCTCCACGGACTCAATTCAG
CAGTTGGGTGAAGCTGGAGCAACGGCATTACGGATTATCCACAGTCGCAATAACAGCAATATATCCACCC
AGTCGGCTCCTTATTACTACTCCGAGCTGCCAGCACCGCCGCCCTTGAACAATCAGCGGGATATCCATGC
ACATGGGCTGAACATTGCCCACATACATAATCCCATCGATCGGCATCAGTTCAATGTGGACGCACTGGTG
GACAAGGATCAGGCCATCGATAGCAAGAACATCTATTGTGGGCAGAAGGACAACAACGAGAAGCTGAGCA
ACAAGAATCTCAAGCTGAACAAATCTGTGGATCTGCCGGCAGAGATGGAGGGCAGTGGTAGCAGTGGTGG
TGCACTAAACCCGGTGGTCCATTCATATCTCACAACTACTAAAAACACAAATCTCGCTCGTAAAACGATT
GGTGGTAGCAATCCGTCGGATGATGGCAATCCTAACGAAGATGGTGCTGGTGAAAACACGGAGGAGGCTG
GCAAGACCCCGTCGAATAATGTCCATACCAACCTTTCAGTTTTAGGTGGCGAGCTGCTCTGGGAGGAGGA
TGCTCTGTGGCGGGAGAGCTTGCGTCGGGTGTCCCAGCGGCATGCCAGATCCTTGGACGATCTGGACAGG
ATTACGGCTGCTCCACCAATTTCTAGTACGCCCAAGGCAAAACTGAGCCGCGAGGTGACCTATGTGAATG

ACAGCTTGAAGCCGCGCCAGCATCCGCACATAACCAACGAGCATGATGTCTATGTGCAACTGATGGACAC
TACGTCCTCCTCGCACGACCACGACCAAACGGATTCCGATGTCTACGAGGTCCTGCGGGAGGAGACCGCC
TCGAATTTGTCACACAAATCAAACGAACTGGATCGCGAGACCATCCGTCAGTGGGATGCCATGTCGAGTG
GCCTAATGAAGAGCAACCACAGCGGAGTTGAGGCTACAGGAGCTAGCTCTGCCCACCTGCCCCTAACCAG
CAGTGTGCGCTCCATAGTTCAGCAGTTGAACAATAGTTCCATGGATGATGCCAATGGCAATCCCATGGCG
ACGGGCAAAAAGTCAAGAGCGAACAACAATAATTAGTGAGGCTAGCACCGCGGAAAATGCAACCTCCTCG
TTTCCGTTCCGAAAAATATATGTGTGCAATCATTTCCGTAGTTTCAATTTTAGCTTTGCATTTTGCAATT
TTGCAACTTTTGATTTTGCACTCTCCAAAATATTTATGTCACTCTGTTTGCCCGTCATAATTTTCGATT
AGGTAGTGCTCTatttgttttgttgactgtcactgttgctgttgttgttgctgttattgtcattattgtt
gtgttATGTTGTTTTGTTTAATTAATCAACTG
>dme_circ_0008785_junction_seq
tATGTTGTTTTGTTTAATTAATCAACTGGCTGGGACTGTGGATGATGATAACTTGT
>dme_circ_0008786
GCCTCGATTTCCGTGCGCAATGTGAACAATCTGCCGGTGGGAAATCTGACGGTGAAGCCGGATCCCTCGG
ATGTCCATCAGGAGTCCGCTTTTGCACCCTACTATGGTGGAGCGGCGGAAACTAAGTACGCCAAGAATAC
GGTGCTCACGGATCGCGGTCTGTATGCCGCAAACGGATCTGGATTGGCCACATCCACGCCCCAAAATCAT
CAGCAGTTCCGGATGCGGCACACCGGAAGTAGGGCGGAAATCGATATGCTGGAACGCGAGACAAGCAGTC
AGATCAGGGGTGAGTTCATGTCGGGTTAAAACTTAAGCGCATTTCAGAATCTTTCCAAAATTTGACATAC
CTCACTCAATTCTGTGAGTAAGCAGTCTTAAACCTATTGTCCGTCCATACAATTAAAATGTTACGAGAGC
ATGAAAAATGAAACTCAATGAAATGCTTCTCACCGAATTTGCTACTTTGGGGGCAGGAACGAACGAAAGC
TTAACATATGTTTACACAACCCACAGAACTTGGATGTCTCGGCCGGGGATTTGCTGAGTCGCACCCACGA
GGAGCTGGTCCTTTTGCTGATTCAGTTGAGGAGACAGAGTAGCCAGACTGCCAGGGCCATCGAGCAGTGC
TGCAGCGATATCCATGATGTGCAGGTGGATAAACAAGTGCATTTTATGTTTATCTTCGGGAGGTTTTGGC
TAACTTCTGGTGCTGGTTCTGTGACTAAATCTATTTTAAGCCCATCTCACTGTTTAAAATATTATTTTAA
GTAGCCCCACTAACAATGCATATATGTAATCCTTCACAGGAATCGCCTGCGAAGTGCCGAGGGTTTGACC
AGAGCCGAGAGTATCCAGCGTTTGGACTACTTGAAGCAGCACCTTTTGGACCTGGAGCGGCACTACGAGA
AGAGCAAGCCGCTGGTCAATTTGGTGGACAACATGGTCAAGTTGGGCTCACTGTATCGCAACGATGCAAA
CGGCAGGGTCCAACCAGCCACACTGGATCGCCTGGAGTTCAATCAGCGGATGCAGGAGCAGCAAATGCTG
CAAGAGGAGCAGCAGCAGTGGGAGCGTCTCAGTCCCAATCAGGCAGAGTTGCAGGGCCAAGGTGCGTGAG
CTCTACCAACTGGATCAGCTGCTGCAGGAGGAGTCGGGCACTTTGCAGAGTTTGCAGCGGGACAAGGAAG
ATCTCGAGCGAGCCTTGGGAGGATTGAGGGCTCGCATCCACGACAGCAATGCCACGCCCATGGCCCTGGA
GGCGGCCAAGAAGCAGCAGCACATCCTGGAGCGTGAGCTGTCGCGGGTTCATCAGCTTCTGGCCGAGAAC
TCTAAG
>dme_circ_0008786_junction_seq
TCATCAGCTTCTGGCCGAGAACTCTAAGGCCTCGATTTCCGTGCGCAATGTGAACA
>dme_circ_0008787
GCCTCGATTTCCGTGCGCAATGTGAACAATCTGCCGGTGGGAAATCTGACGGTGAAGCCGGATCCCTCGG
ATGTCCATCAGGAGTCCGCTTTTGCACCCTACTATGGTGGAGCGGCGGAAACTAAGTACGCCAAGAATAC
GGTGCTCACGGATCGCGGTCTGTATGCCGCAAACGGATCTGGATTGGCCACATCCACGCCCCAAAATCAT
CAGCAGTTCCGGATGCGGCACACCGGAAGTAGGGCGGAAATCGATATGCTGGAACGCGAGACAAGCAGTC
AGATCAGGGGTGAGTTCATGTCGGGTTAAAACTTAAGCGCATTTCAGAATCTTTCCAAAATTTGACATAC
CTCACTCAATTCTGTGAGTAAGCAGTCTTAAACCTATTGTCCGTCCATACAATTAAAATGTTACGAGAGC
ATGAAAAATGAAACTCAATGAAATGCTTCTCACCGAATTTGCTACTTTGGGGGCAGGAACGAACGAAAGC
TTAACATATGTTTACACAACCCACAGAACTTGGATGTCTCGGCCGGGGATTTGCTGAGTCGCACCCACGA

GGAGCTGGTCCTTTTGCTGATTCAGTTGAGGAGACAGAGTAGCCAGACTGCCAGGGCCATCGAGCAGTGC
TGCAGCGATATCCATGATGTGCAGGTGGATAAACAAGTGCATTTTATGTTTATCTTCGGGAGGTTTTGGC
TAACTTCTGGTGCTGGTTCTGTGACTAAATCTATTTTAAGCCCATCTCACTGTTTAAAATATTATTTTAA
GTAGCCCCACTAACAATGCATATATGTAATCCTTCACAGGAATCGCCTGCGAAGTGCCGAGGGTTTGACC
AGAGCCGAGAGTATCCAGCGTTTGGACTACTTGAAGCAGCACCTTTTGGACCTGGAGCGGCACTACGAGA
AGAGCAAGCCGCTGGTCAATTTGGTGGACAACATGGTCAAGTTGGGCTCACTGTATCGCAACGATGCAAA
CGGCAGGGTCCAACCAGCCACACTGGATCGCCTGGAGTTCAATCAGCGGATGCAGGAGCAGCAAATGCTG
CAAGAGGAGCAGCAGCAGTGGGAGCGTCTCAGTCCCAATCAGGCAGAGTTGCAGGGCCAAGGTGCGTGAG
CTCTACCAACTGGATCAGCTGCTGCAGGAGGAGTCGGGCACTTTGCAGAGTTTGCAGCGGGACAAGGAAG
ATCTCGAGCGAGCCTTGGGAGGATTGAGGGCTCGCATCCACGACAGCAATGCCACGCCCATGGCCCTGGA
GGCGGCCAAGAAGCAGCAGCACATCCTGGAGCGTGAGCTGTCGCGGGTTCATCAGCTTCTGGCCGAGAAC
TCTAAGGTATCCAAG
>dme_circ_0008787_junction_seq
TCTGGCCGAGAACTCTAAGGTATCCAAGGCCTCGATTTCCGTGCGCAATGTGAACA
>dme_circ_0008788
GCCTCGATTTCCGTGCGCAATGTGAACAATCTGCCGGTGGGAAATCTGACGGTGAAGCCGGATCCCTCGG
ATGTCCATCAGGAGTCCGCTTTTGCACCCTACTATGGTGGAGCGGCGGAAACTAAGTACGCCAAGAATAC
GGTGCTCACGGATCGCGGTCTGTATGCCGCAAACGGATCTGGATTGGCCACATCCACGCCCCAAAATCAT
CAGCAGTTCCGGATGCGGCACACCGGAAGTAGGGCGGAAATCGATATGCTGGAACGCGAGACAAGCAGTC
AGATCAGGGGTGAGTTCATGTCGGGTTAAAACTTAAGCGCATTTCAGAATCTTTCCAAAATTTGACATAC
CTCACTCAATTCTGTGAGTAAGCAGTCTTAAACCTATTGTCCGTCCATACAATTAAAATGTTACGAGAGC
ATGAAAAATGAAACTCAATGAAATGCTTCTCACCGAATTTGCTACTTTGGGGGCAGGAACGAACGAAAGC
TTAACATATGTTTACACAACCCACAGAACTTGGATGTCTCGGCCGGGGATTTGCTGAGTCGCACCCACGA
GGAGCTGGTCCTTTTGCTGATTCAGTTGAGGAGACAGAGTAGCCAGACTGCCAGGGCCATCGAGCAGTGC
TGCAGCGATATCCATGATGTGCAGGTGGATAAACAAGTGCATTTTATGTTTATCTTCGGGAGGTTTTGGC
TAACTTCTGGTGCTGGTTCTGTGACTAAATCTATTTTAAGCCCATCTCACTGTTTAAAATATTATTTTAA
GTAGCCCCACTAACAATGCATATATGTAATCCTTCACAGGAATCGCCTGCGAAGTGCCGAGGGTTTGACC
AGAGCCGAGAGTATCCAGCGTTTGGACTACTTGAAGCAGCACCTTTTGGACCTGGAGCGGCACTACGAGA
AGAGCAAGCCGCTGGTCAATTTGGTGGACAACATGGTCAAGTTGGGCTCACTGTATCGCAACGATGCAAA
CGGCAGGGTCCAACCAGCCACACTGGATCGCCTGGAGTTCAATCAGCGGATGCAGGAGCAGCAAATGCTG
CAAGAGGAGCAGCAGCAGTGGGAGCGTCTCAGTCCCAATCAGGCAGAGTTGCAGGGCCAAGGTGCGTGAG
CTCTACCAACTGGATCAGCTGCTGCAGGAGGAGTCGGGCACTTTGCAGAGTTTGCAGCGGGACAAGGAAG
ATCTCGAGCGAGCCTTGGGAGGATTGAGGGCTCGCATCCACGACAGCAATGCCACGCCCATGGCCCTGGA
GGCGGCCAAGAAGCAGCAGCACATCCTGGAGCGTGAGCTGTCGCGGGTTCATCAGCTTCTGGCCGAGAAC
TCTAAGGAAACTGGAGCAGACAGTGGCGGGAAATGCTCGCCTGGAGCAGGAGTTACTCCTTCTCCGTCAG
AAGGTACAAGCCGTTCGAGGATCCACCACCAATGGAATGAACGGTGATGGCTCTCACATCAATGGTGACC
AAACTGCCGCCGTTTTGGCCTCGGAATTGGAGCGAGTGCAGTCCCTGGTGGGCGATATGCAAAGACAACG
CCACGAACTCAGCTCCGCAGTTCGCCAGCTGACGGAGAACTCGACCAGGTTGTACCAGGAGATTGGTAAC
AAGGAGATGAACGGCGGTGGCTCCACCAATGGCAGTCTGAAGAAGCGAAGCAACTCAACTAGTTGGACGG
AAACCGATTTGGATGCTAATATGCTGCGATGCGGATCCCGCCAGCAGTTGAATGATTCCACTTTGAACCT
ATCCACTCCCCTCTATGTGGACACCAATAGTTCGACCAAACTGAGTGACTACAATCGATACAATGGCAGT
AGCGATGCCATCGAGATGAGTGGAGTGGACAGTGATGGTTTCCTGGACAGCAATCCATTCGCGCTGGGCT
TGGAGAAGCAGGAGATTAAGACAGTGAGGATTGTCAAGCGAGAGTCTGAACGTCGGAATCGCGATCGCAG
TGAAAGGGGTCTAAGCAACTCCATTCAGAATCTGGATCAGGTCCTGGAGGAGGAGCAATATGCCCAACAG

CAGAGGGAACAGCAGCTGTACGCTCAAAACTTGGAAGAACCGATGACCAACGGGCACCACAGTCGATCCA
AGTCATTGCCCCGAAACTACAGTGAGCCCCCGAAACAACGACACAGTCGTCATCTGAATGGTGGCAAGCA
GAATGGTCACCACTATAATGGTGGCTACGATTACGATCGGAACAGCAACTACGAGCATCAGCCGCCACCA
CCACCGGCACCACAGAGCAATGGTCATCACCACAATCATCAGCAAAGGGAGCAGCGGGAGCACCTTAATC
CACTGGCCAATGCCTATTTCGCCAAGCAGCTCCAGCAGCAGGCGAATCCCTCGAGGGACAGTGCCCGCGT
GGCCTTGCGGACCAAGACCGATTCCCTGCAGAGCCTCAACAAAAGCCTTACGGACATCAGTCCGGAGCCA
GTGTTCCAGAGCGTTGCTGCCCGCCAGATCATCAACGAAATGTCCGCCGGTTCGGCATCGGAGGACACCG
AGAAGGTGGTGGAGAAGGTGCCACCGCACCACAAGCATCGCAGAGCGGTTCCAAGGGAGAAGAGGCGACA
CTACACTGCGCCAAACAATGTCAATCAGAAAGCCATGGAAAAGGTGCAGGCCGAAAACGATATGAATCGA
AATGAACACCAACTGGAGAGCTCGCGATGATTTGGATATGGAGGTGGCCCTGCGACCGCGAATGAATGCT
CCTGATGTCGTTCGTTCTGCTTTGGGACAGGGCGAAAAGATATCCGAGAACACCATAGACAATTTGCTGC
TGGCACCGAACAAAATAGTCATACCCGAGCGTTACATACCTGAAACAGTGAGAAATTAATAATCGttttt
ttttATTCAATTTATTAATCTATTTAAATTTAAATAGACGCCCGAGCTGTCGCCGGAGGAAAAGAAGCGT
CGTCAGGAGAAGGTCGAGTCCATCAAGAAAATGCTGGCTGAGGCGCCCATTAGCAGCAATGTAAGCCTCC
CAGACTCAGTCCATATATCCATATATATTCATTTAAAATAATTCAATTCCTTCAGGAAAACGAAAGCCTG
CCCCCGAGCAAAATCAACGCCGAGAAGAAGCAGCGCGAGCACCTGCTGCAACTCAACCAAATCCTGGCCC
AACAGGTGATGCAAGTCAGCAAGATCGTGGCCG
>dme_circ_0008788_junction_seq
GTGATGCAAGTCAGCAAGATCGTGGCCGGCCTCGATTTCCGTGCGCAATGTGAACA
>dme_circ_0008789
GCCTCGATTTCCGTGCGCAATGTGAACAATCTGCCGGTGGGAAATCTGACGGTGAAGCCGGATCCCTCGG
ATGTCCATCAGGAGTCCGCTTTTGCACCCTACTATGGTGGAGCGGCGGAAACTAAGTACGCCAAGAATAC
GGTGCTCACGGATCGCGGTCTGTATGCCGCAAACGGATCTGGATTGGCCACATCCACGCCCCAAAATCAT
CAGCAGTTCCGGATGCGGCACACCGGAAGTAGGGCGGAAATCGATATGCTGGAACGCGAGACAAGCAGTC
AGATCAGGGGTGAGTTCATGTCGGGTTAAAACTTAAGCGCATTTCAGAATCTTTCCAAAATTTGACATAC
CTCACTCAATTCTGTGAGTAAGCAGTCTTAAACCTATTGTCCGTCCATACAATTAAAATGTTACGAGAGC
ATGAAAAATGAAACTCAATGAAATGCTTCTCACCGAATTTGCTACTTTGGGGGCAGGAACGAACGAAAGC
TTAACATATGTTTACACAACCCACAGAACTTGGATGTCTCGGCCGGGGATTTGCTGAGTCGCACCCACGA
GGAGCTGGTCCTTTTGCTGATTCAGTTGAGGAGACAGAGTAGCCAGACTGCCAGGGCCATCGAGCAGTGC
TGCAGCGATATCCATGATGTGCAGGTGGATAAACAAGTGCATTTTATGTTTATCTTCGGGAGGTTTTGGC
TAACTTCTGGTGCTGGTTCTGTGACTAAATCTATTTTAAGCCCATCTCACTGTTTAAAATATTATTTTAA
GTAGCCCCACTAACAATGCATATATGTAATCCTTCACAGGAATCGCCTGCGAAGTGCCGAGGGTTTGACC
AGAGCCGAGAGTATCCAGCGTTTGGACTACTTGAAGCAGCACCTTTTGGACCTGGAGCGGCACTACGAGA
AGAGCAAGCCGCTGGTCAATTTGGTGGACAACATGGTCAAGTTGGGCTCACTGTATCGCAACGATGCAAA
CGGCAGGGTCCAACCAGCCACACTGGATCGCCTGGAGTTCAATCAGCGGATGCAGGAGCAGCAAATGCTG
CAAGAGGAGCAGCAGCAGTGGGAGCGTCTCAGTCCCAATCAGGCAGAGTTGCAGGGCCAAGGTGCGTGAG
CTCTACCAACTGGATCAGCTGCTGCAGGAGGAGTCGGGCACTTTGCAGAGTTTGCAGCGGGACAAGGAAG
ATCTCGAGCGAGCCTTGGGAGGATTGAGGGCTCGCATCCACGACAGCAATGCCACGCCCATGGCCCTGGA
GGCGGCCAAGAAGCAGCAGCACATCCTGGAGCGTGAGCTGTCGCGGGTTCATCAGCTTCTGGCCGAGAAC
TCTAAGGAAACTGGAGCAGACAGTGGCGGGAAATGCTCGCCTGGAGCAGGAGTTACTCCTTCTCCGTCAG
AAGGTACAAGCCGTTCGAGGATCCACCACCAATGGAATGAACGGTGATGGCTCTCACATCAATGGTGACC
AAACTGCCGCCGTTTTGGCCTCGGAATTGGAGCGAGTGCAGTCCCTGGTGGGCGATATGCAAAGACAACG
CCACGAACTCAGCTCCGCAGTTCGCCAGCTGACGGAGAACTCGACCAGGTTGTACCAGGAGATTGGTAAC
AAGGAGATGAACGGCGGTGGCTCCACCAATGGCAGTCTGAAGAAGCGAAGCAACTCAACTAGTTGGACGG

AAACCGATTTGGATGCTAATATGCTGCGATGCGGATCCCGCCAGCAGTTGAATGATTCCACTTTGAACCT
ATCCACTCCCCTCTATGTGGACACCAATAGTTCGACCAAACTGAGTGACTACAATCGATACAATGGCAGT
AGCGATGCCATCGAGATGAGTGGAGTGGACAGTGATGGTTTCCTGGACAGCAATCCATTCGCGCTGGGCT
TGGAGAAGCAGGAGATTAAGACAGTGAGGATTGTCAAGCGAGAGTCTGAACGTCGGAATCGCGATCGCAG
TGAAAGGGGTCTAAGCAACTCCATTCAGAATCTGGATCAGGTCCTGGAGGAGGAGCAATATGCCCAACAG
CAGAGGGAACAGCAGCTGTACGCTCAAAACTTGGAAGAACCGATGACCAACGGGCACCACAGTCGATCCA
AGTCATTGCCCCGAAACTACAGTGAGCCCCCGAAACAACGACACAGTCGTCATCTGAATGGTGGCAAGCA
GAATGGTCACCACTATAATGGTGGCTACGATTACGATCGGAACAGCAACTACGAGCATCAGCCGCCACCA
CCACCGGCACCACAGAGCAATGGTCATCACCACAATCATCAGCAAAGGGAGCAGCGGGAGCACCTTAATC
CACTGGCCAATGCCTATTTCGCCAAGCAGCTCCAGCAGCAGGCGAATCCCTCGAGGGACAGTGCCCGCGT
GGCCTTGCGGACCAAGACCGATTCCCTGCAGAGCCTCAACAAAAGCCTTACGGACATCAGTCCGGAGCCA
GTGTTCCAGAGCGTTGCTGCCCGCCAGATCATCAACGAAATGTCCGCCGGTTCGGCATCGGAGGACACCG
AGAAGGTGGTGGAGAAGGTGCCACCGCACCACAAGCATCGCAGAGCGGTTCCAAGGGAGAAGAGGCGACA
CTACACTGCGCCAAACAATGTCAATCAGAAAGCCATGGAAAAGGTGCAGGCCGAAAACGATATGAATCGA
AATGAACACCAACTGGAGAGCTCGCGATGATTTGGATATGGAGGTGGCCCTGCGACCGCGAATGAATGCT
CCTGATGTCGTTCGTTCTGCTTTGGGACAGGGCGAAAAGATATCCGAGAACACCATAGACAATTTGCTGC
TGGCACCGAACAAAATAGTCATACCCGAGCGTTACATACCTGAAACAGGTAAGTAGAGTCAACCTGGAAT
ACATTAGGTCTCCTACCAGATGATAATCCTTGTGTTAAGTTCCAATCGATACCCGAAAATAAAGAACCAA
TGCAATGCCGTGCAAGTTGAGTTTATGTTCCATGTTTATGTTCTTACTAATATGCTCTCTCGAAATCGAA
CATATAGACCCTTGTGTTATTTATCTATACGTATATATATACCTAAGCGTTTATTCAGTGTAATCTAACC
ATGCCAGAAAACCCATCCATTTCAAACTGTCAACTCTATGCTCTTCTAATCTCTATATATGTATCTTCTT
TCAACTCTTTCTGATTTCTAGATGATTAATGATAAAACATATCATATGCATACCATCTAATCTAAAGCGT
TAAGCCCCCGTTCGCATAAGAACTCAATGATCATGCCCCCCCACCTCATTGCGTGCGTAATCACCGGCGT
CGTCCAAAGTTCATAACCCCTTCTCACAATGACACACCTCCAAACACACAATCTAGACACTCTCAACAGT
CAATCATACATCTCAAAACCTGTGGCCCACATTGCCCTCTCATGCATTCGCATCTGCACCCGACCAGATT
ATGTATAATTAATTATAAATTAAGTAACCATCGATGAGAGTTCTATGAACTTCCGCAAGATTGCATATTT
CACATATTGCGCATACGCAATGTTAGCAGACCCAGAAAGCATATCAAGCTGAGAATTCTCAGGAGTTTCT
AATTTGTTTTAAATAAATATTTCAATAATCATATTGTGAATAATTCGTATTAGACCATATTTATTTAAGA
GATGATTTTGTATGTTATTGAGGTTGAAAGGCCCTAGGAAGGTATATATTTATTATAAAACTAACACTCA
ATTTACGTTAATCTTATAATTGTATAATTTTTGCATTCGTTTAATTCTACATGTTTATTTGTTTGTTTTG
CATATTTTTCTAGGAAATCCCACTAGTCACAACTAATTTGCAAGTAGCTTGTCGAGTTGTTTCTGTTCAC
CTTTTGTGTTTTACATACCTATGCATTGCATTTAACTTTTGTTCACGTTGTGTGtttttttttGCCAATC
TGAGAGGGTTCGCATAACGATACGCGGTAAATGAGTTTGCTGTTCGAAACATACCAAACATATATATGCA
TAACTAGTACGATACACCTAACTATACAGTAACTACAGCAAACAAGAACACACGCGAAGTTGAGATTTAC
AGCATGCTAAACTCACATAAAAAACTCACCCAAATCAAACGCAACAGCTCACAACAAGAATACTaaaaaa
aaaaacagaaaaaaagagaaaaaaCGACAACAAGAATAAACTGCAACGCAATAGcaacaagaacaagtac
aacaacaacaacaagcacaagaacaacaagaacaacaacaGATATACGATTACGTAAGTCCAG
>dme_circ_0008789_junction_seq
caacaGATATACGATTACGTAAGTCCAGGCCTCGATTTCCGTGCGCAATGTGAACA
>dme_circ_0008790
GAATCGCCTGCGAAGTGCCGAGGGTTTGACCAGAGCCGAGAGTATCCAGCGTTTGGACTACTTGAAGCAG
CACCTTTTGGACCTGGAGCGGCACTACGAGAAGAGCAAGCCGCTGGTCAATTTGGTGGACAACATGGTCA
AGTTGGGCTCACTGTATCGCAACGATGCAAACGGCAGGGTCCAACCAGCCACACTGGATCGCCTGGAGTT
CAATCAGCGGATGCAGGAGCAGCAAATGCTGCAAGAGGAGCAGCAGCAGTGGGAGCGTCTCAGTCCCAAT

CAGGCAGAGTTGCAGGGCCAAGGTGCGTGAGCTCTACCAACTGGATCAGCTGCTGCAGGAGGAGTCGGGC
ACTTTGCAGAGTTTGCAGCGGGACAAGGAAGATCTCGAGCGAGCCTTGGGAGGATTGAGGGCTCGCATCC
ACGACAGCAATGCCACGCCCATGGCCCTGGAGGCGGCCAAGAAGCAGCAGCACATCCTGGAGCGTGAGCT
GTCGCGGGTTCATCAGCTTCTGGCCGAGAACTCTAAG
>dme_circ_0008790_junction_seq
TCATCAGCTTCTGGCCGAGAACTCTAAGGAATCGCCTGCGAAGTGCCGAGGGTTTG
>dme_circ_0008791
GAATCGCCTGCGAAGTGCCGAGGGTTTGACCAGAGCCGAGAGTATCCAGCGTTTGGACTACTTGAAGCAG
CACCTTTTGGACCTGGAGCGGCACTACGAGAAGAGCAAGCCGCTGGTCAATTTGGTGGACAACATGGTCA
AGTTGGGCTCACTGTATCGCAACGATGCAAACGGCAGGGTCCAACCAGCCACACTGGATCGCCTGGAGTT
CAATCAGCGGATGCAGGAGCAGCAAATGCTGCAAGAGGAGCAGCAGCAGTGGGAGCGTCTCAGTCCCAAT
CAGGCAGAGTTGCAGGGCCAAGGTGCGTGAGCTCTACCAACTGGATCAGCTGCTGCAGGAGGAGTCGGGC
ACTTTGCAGAGTTTGCAGCGGGACAAGGAAGATCTCGAGCGAGCCTTGGGAGGATTGAGGGCTCGCATCC
ACGACAGCAATGCCACGCCCATGGCCCTGGAGGCGGCCAAGAAGCAGCAGCACATCCTGGAGCGTGAGCT
GTCGCGGGTTCATCAGCTTCTGGCCGAGAACTCTAAGGTATCCAAG
>dme_circ_0008791_junction_seq
TCTGGCCGAGAACTCTAAGGTATCCAAGGAATCGCCTGCGAAGTGCCGAGGGTTTG
>dme_circ_0008792
GAGGATGAAGACCTCGATGGTGATGGAGTGAAAAATGACGAAGACGAGGATCTGGATGGAGATGGCAAAG
TAAATCACGGTAAGAATAATTATAGCATAAAAAGTCTTGAATTATTATAAAAcatttttttatttgaactt
gtttttttattttCAGAGGACGAAGATCTTGATGGAGATGGCGTGAAGAATGAAAAGGACGACGATTTGGA
TGGCGATGGTACCAACAATTCTC
>dme_circ_0008792_junction_seq
TTGGATGGCGATGGTACCAACAATTCTCGAGGATGAAGACCTCGATGGTGATGGAG
>dme_circ_0008793
GTCCTTCGACAGCAGCATATCCTGCTTTAAGGTGCCCAACGTGGATCAACCTGTGGTCTATGCCCCCGTC
TCCGAGCTCACCGACTACGACGATGTGAGGAGCTACCAGGAGGCGGCCAAGCGGTCCATGGAGAAGGTTC
TCAAG
>dme_circ_0008793_junction_seq
CAAGCGGTCCATGGAGAAGGTTCTCAAGGTCCTTCGACAGCAGCATATCCTGCTTT
>dme_circ_0008794
ATCAACCCAATGAACTCCTATCTTCGCGGCATTCCGAAGCTGCTGGCAGGCCCTAATGGGCCGTAGATGG
CATGTGGATGCGGAGGATGGGCGTGAGGATGCTGACTGGGTCCTGGGCCATGCAATCCGCCATTTGACAT
TGTCATGGCATGCTTCCGCTGTAGTGTG
>dme_circ_0008794_junction_seq
TGTCATGGCATGCTTCCGCTGTAGTGTGATCAACCCAATGAACTCCTATCTTCGCG
>dme_circ_0008795
AATGTGGAGCTAAATTCACTGGGGCAGATCTTTAGCAACCAGGAGCATGAGAAGGATTCAGAGGATTCTG
CCTCGAATTGTCAGCTAGAGGAGAAGGATCAATCAAATTCGGAGGTGGTGAAGGAGCAGG
>dme_circ_0008795_junction_seq
TCAAATTCGGAGGTGGTGAAGGAGCAGGAATGTGGAGCTAAATTCACTGGGGCAGA
>dme_circ_0008796
CTTTCCTATTGGATCATATCATGAAACCGAGGCCAACCTATTTGGTTTACGAAATGGACGAGGGTAGAGT
GTCCATAGCCACCGATCTGAGTCGTATTCCCAGCGAACTACTTGGCCTGCCCAGTCGAGTGTTTGCCGCT

CAGCTGGAGGACTCCGTACCCGAGGAGCTTGAGACTCAAGGTGCAGAACTCACGGTCAAG
>dme_circ_0008796_junction_seq
GACTCAAGGTGCAGAACTCACGGTCAAGCTTTCCTATTGGATCATATCATGAAACC
>dme_circ_0008797
GTAAACAGTATAGATTCAATATGATCGGAATATATTTGCTGATCGCTGCAGTCACGCTGCTATATGTCTA
TCTCAAGTGGACATTTAGCTACTGGGATCGCAAGGGATTCCCATCGACGGGAGTGAGCATACCGTTCGGT
GCCCTGGAATCCGTGACAAAGGGAAAGCGATCCTTCGGTATGGCCATATACGATAT
>dme_circ_0008797_junction_seq
GATCCTTCGGTATGGCCATATACGATATGTAAACAGTATAGATTCAATATGATCGG
>dme_circ_0008798
GATTCAATATGATCGGAATATATTTGCTGATCGCTGCAGTCACGCTGCTATATGTCTATCTCAAGTGGAC
ATTTAGCTACTGGGATCGCAAGGGATTCCCATCGACGGGAGTGAGCATACCGTTCGGTGCCCTGGAATCC
GTGACAAAGGGAAAGCGATCCTTCGGTATGGCCATATACGATAT
>dme_circ_0008798_junction_seq
GATCCTTCGGTATGGCCATATACGATATGATTCAATATGATCGGAATATATTTGCT
>dme_circ_0008799
GATTCAATATGATCGGAATATATTTGCTGATCGCTGCAGTCACGCTGCTATATGTCTATCTCAAGTGGAC
ATTTAGCTACTGGGATCGCAAGGGATTCCCATCGACGGGAGTGAGCATACCGTTCGGTGCCCTGGAATCC
GTGACAAAGGGAAAGCGATCCTTCGGTATGGCCATATACGATATGTACAAGTCGACAAAGGAACCGGTGA
TCGGTTTGTATCTCACATTGAGACCGGCACTTCTCGTCCGAGATGCCCAACTGGCCCACGATGTGTTGGT
CAAGGATTTTGCCAGTTTTCACGATCGTGGCGTGTATGTGGATGAAAAGAACGATCCCATGTCCGCGAGT
TTGTTCCAAATGGAAGGAGCCAGCTGGCGGGCCTTGCGCAACAAGCTGACGCCCTCATTCACATCCGGCA
AACTGAAGGCCATGTTCGAGACCTCAGATTCGGTTGGAGACAAGTTGGTGGACAGCATTAGGAAGCAACT
GCCTGCGAACGGCGCCAAGGAGCTGGAACTCAAGAAGCTAATGGCTACCGTAAGTTGAAGCGATTAGATT
AGCAAATATCAATTGCGTTGCAATAATGTAGCTAATTATTTGAGTTTTACAAATACTAAACTTATTTTTT
GAACAACCAATTCACAGTTTCAGACTGCGATGCGATTATAAATCATGTCATTTCATCCGAACTGAGAACT
TGCAATAGTTTTGCTGTCTTATGAAAAAAATGAACATACTAAACTAATGTAACGTAACGCATTCGAAATT
TATGGCCGTTGTTAATTGTATTTAAACAGATACCCCTTATCTGATAAACAAACTAATGAAGTGTACTTCA
TTTATTTCTTTTGTTAGATACGCCATCGACATTATTGCCACGACTATTTTTGGACTGGATGTTGATAGCT
TTGCGGATCCCAACAACGAATTCCAGATTATTAGCAAAAAAGTGAATC
>dme_circ_0008799_junction_seq
TTCCAGATTATTAGCAAAAAAGTGAATCGATTCAATATGATCGGAATATATTTGCT
>dme_circ_0008800
GGCGTCAGCTTGTTGCGCAAGGCCCGCCAGCTGGCTCCTTCCATTTGGAACAAACTCGCGGACATGGGAT
CGTTCTTTTCATCCACATACACGCCACGATCGTGAAAACTGGCAAAATCCTTGACCAACACATCGTGGGC
CAGTTGGGCATCTCGGACGAGAAGTGCCGGTCTCAATGTGAGATACAAACCGATCACCGGTTCCTTTGTC
GACTTG
>dme_circ_0008800_junction_seq
ACCGATCACCGGTTCCTTTGTCGACTTGGGCGTCAGCTTGTTGCGCAAGGCCCGCC
>dme_circ_0008801
CTCCTTGGCGCCGTTCGCAGGCAGTTGCTTCCTAATGCTGTCCACCAACTTGTCTCCAACCGAATCTGAG
GTCTCGAACATGGCCTTCAGTTTGCCGGATGTGAATGAGGGCGTCAGCTTGTTGCGCAAGGCCCGCCAGC
TGGCTCCTTCCATTTGGAACAAACTCGCGGACATGGGATCGTTCTTTTCATCCACATACACGCCACGATC
GTGAAAACTGGCAAAATCCTTGACCAACACATCGTGGGCCAGTTGGGCATCTCGGACGAGAAGTGCCGGT

CTCAATGTGAGATACAAACCGATCACCGGTTCCTTTGTCGACTTG
>dme_circ_0008801_junction_seq
ACCGATCACCGGTTCCTTTGTCGACTTGCTCCTTGGCGCCGTTCGCAGGCAGTTGC
>dme_circ_0008802
GATTATTAGCAAAAAAGTGAATCGTAACAACATTGAAGATATTATTCGTGGAACATCTAGTTTCCTGTAT
CCCGGGTAAGTTGAAGGTGGACTTTATAGACTTTATAATTAAACATAAAACCTATATCTTTCAACCATTT
GCAGGCTGGAGAAATTCTTTGTGAAAATCGGCTGGAAACAGGAGGCCACCGAAAGAATGCGCGAGTTGTC
CAATCGAACCGTCGATCTCAGGGAACAGAACAATATAGTTCGCAAGGATCTGCTGCAACTTCTGCTGCAG
CTGCGGAATCAGGGAAAGATCAATACCGATGACAATATTTGGTCGGCGGAAAGCACTAAGAATGGTGTTA
AGTCCATGTCCAAGGACTTGATTGCCGGCCAGCTATTCCTCTTCTATGTTGCCG
>dme_circ_0008802_junction_seq
GGCCAGCTATTCCTCTTCTATGTTGCCGGATTATTAGCAAAAAAGTGAATCGTAAC
>dme_circ_0008803
ACTAGGAGCTACTTTTTGAGAGACAGACGCACGGGAACACCTGATTTGGGCATCAGCGGTATTCCATACA
CGCCGAACTCAATCTCACACTTTTCCTTCCGGATCTCCAGATCGAAGTTCGACAAAACCTTGGCAATCGC
TATCTTCACATTCACCTTTCCCATGCGGGCGCCTGGAAAATAGACGGAAATTTCTTAGTTTCCATGGGCA
ATTGGAAAGAGATCGGTGGGATATAGTGGATTTGAGTATTACTTATCTGAAGTATTCAAACTCCCATTTC
ACCCTGTTCATGTGGAACCTTTCTTCTTACCGATACACATCCTGGGTCCCTCGCCGAATGGCAAATAGGC
CGCCTGGGTGTAGTCTTTACCATTCTCCAGATACCGTTCCGGTTTGTAGGCAAGCGGATCGGGGAAGTAC
TCCTCATCGCGGTGCATTCCGATGAGGGAGATTATGATGGGAGTACCCTTTTGGATCACAAGTTTGCTAT
CGGGCACAGGATAGTCCTTGGTGCAGATTCTATTCAGCAGTGGCAGAGCGGGATATTTGCGAGCAGTCTC
TGTTAGAAAGTATACAATATTAAACCATTTATaaaaaaaaTATGGAAAATATATATGAAAGAACTGCCAT
ATATTCCCATCAAAACTTACCGAGTATACACGCCTCCAGATACTTCATGTCCGAGATGGCGTCGTATGTC
AGCTTTCCGCCATGCTTTTCGATGGCACTGCGAACATCCTCCTTAGCCTTTTCCATCACTTCAGGATTTT
GGGTTAGCTCGTATAGGG
>dme_circ_0008803_junction_seq
TCAGGATTTTGGGTTAGCTCGTATAGGGACTAGGAGCTACTTTTTGAGAGACAGAC
>dme_circ_0008804
ATTATGATGGGAGTACCCTTTTGGATCACAAGTTTGCTATCGGGCACAGGATAGTCCTTGGTGCAGATTC
TATTCAGCAGTGGCAGAGCGGGATATTTGCGAGCAGTCTCTGTTAGAAAGTATACAATATTAAACCATTT
ATaaaaaaaaTATGGAAAATATATATGAAAGAACTGCCATATATTCCCATCAAAACTTACCGAGTATACA
CGCCTCCAGATACTTCATGTCCGAGATGGCGTCGTATGTCAGCTTTCCGCCATGCTTTTCGATGGCACTG
CGAACATCCTCCTTAGCCTTTTCCATCACTTCAGGATTTTGGG
>dme_circ_0008804_junction_seq
GCCTTTTCCATCACTTCAGGATTTTGGGATTATGATGGGAGTACCCTTTTGGATCA
>dme_circ_0008805
GCTTAAGCAAAACCGCCGCCCAGCAACAACAAGAAACCCATTTAATGAgtgtgtgtgtgtgtgAGCAGAA
AATTATGCCAACAGAATTCTAAAAAATATATATGTATATATTTAAATAACCACTAAAAATCAAGAGTAAG
CTCCGAAACGTGTTTATATTAAAATTTTTTACCCATTTGAACTTTAAACAACTTTTCGACTGCGCCGCAC
GGTTTTCATTGAATTCTCATACGAATTCCAATTAAGTGTTGTGTGTGTGCGTCCAATTGGCATGAATTGATG
ACGTGACGTGAtcgctctcttcgctctctccctctcttTCTTCGGcacacacacatacacacacTACGCT
CTTTAATCGCGCCGCTCTCTTCGCGCTCTTTTTATTTGTTTATTTTGATTGTTGTTATATATATATTCTA
ATTTACCGTTTTTACAATCGTCTTTAGCTTAAGCAAAACCGCCGCCCAGCAACAACAAGAAACCCATTTA
ATGAgtgtgtgtgtgtgtgAGCAGAAAATTATGCCAACAGAATTCTAAAAAATATATATGTATATATTTA

AATAACCACTAAAAATCAAGAAATCCGCACAAGGCTTAAGCAAAACCGCCGCCCAGCAACAACAAGAAAC
CCATTTAATGAgtgtgtgtgtgtgtgAGCAGAAAATTATGCCAACAGAATTCTAAAAAATATATATGTAT
ATATTTAAATAACCACTAAAAATCAAGAAATCCGCACAAGTTTCGCTCAAAACAACAACACAATCATATA
TACTACCCATaaaaaaaaaacaaacccataaaaaaGTGCAAAGCAACCAGGAAAATAAAGTAAAACAGTG
CCAGTTAACAACAGCAAACGGCGATCAACAAATCCCACCGGCAAAATCAACAATTTTTTAAATCGCAATC
AACAAGGAGCAACAAATTAGCCAGCTATTTCCCATTGCAAACATTTGGCGGGGTAAATATTATTAACAAT
TACGAGTAGGTGTACTTGATTGAATGAAAAATGTCATGTTTTTTAAGTTCCTAAATTCGATTCGAATCTA
ATCGGCAATTGATTGAAATATTCTAAAAGTCAAATTTTGTTTCGTCACGCGATTAAAAAAGTAATTGAAG
CAGTGCTTTCCTACACATCAATTAAAATTTAAATTAAATGGTATTTTATGTTGATTACAACAACAttttt
tttAGTCGCCACAATAAAATATTCATTCTTCCGTGAAATATTTCCAATATAAATTGCTATTGCTATATTT
ATAAAAATAAGCTTTGATAAAAGTTAAGAGGTTTAATAGAAACTAGAGTTAGGTTCTTTTATTTATATTG
GTGTTTAGAATTTTAGATAGGAAAGCTGGACTTTTTGTTGTGTTAAAGTAGTCACATCTATTGAATTTAC
GACCCAAATTTTAATACAACCGATAGGGAATGTGATTTATAACCTTATTCGAGTAATTCCTAGTTTCACT
TTCAAGATCAAAACGACTAGAATCATAAAGTCGGTCACGTTCGGCGCTGCAGTGTCGCTCCAATAGATGA
TCTTTCAGTTCGTGTGGGTTTTGATTAGTCCGTTTTGGTGTGTTACTTGTCATGGGAGTGGGCAGGGGAG
ATCTTAATTAGAGCAGCGCTGATAATGGCTCTAAAAATAGAGCCCCAGCGCCACACTGATAGCAGAGCTT
GAAGCGTGTGTCGTTTGCGTGGAGCTCATTTCTTTCGTTCTGTGTTTGTTTGCTCCCCCGATTTTCGTAT
GCGATCCACTAAGGTCGATTTTGTTGCCATATTTTGCCAGCGATCTCGTATACGCACCGTTTTCAGATGC
TTTTGAATATTCTCTTGACATCAGTATTGACATTGAAATGTGATTAGGTTTAATTTTCATAAATGGGTTG
ATTCGCCACTCGGTTAGCTGTCGAAAGAGGCTTTTTTATTCCCCATTTACAGATAAGGCTTTTATCTATT
CGCAATGTGCCAAGGTACAACAGCAGCTGATGGCCCAAAAAAAGTGTACATATAAGCCTATACACACATC
TGTATAGTCGTCCTATGTGCTTGCGATTGTGACTTTTATCATATCTTGAGTGTTTGCTAGCCACATTGGT
GGAACGTGCCAGTTGATAACAAGAAAGTGTGCGAAAGTTTGTCTAAAAAGCTGTAATTGAAGCAGGGAAA
CCGGGAAGTCAATTAATTTTGAGAGACAACATTAAACGTATTCTTCGTATTCTAGTATTCTTCAAGAATT
AGTAAAGCTCTTACTAATAAGCTCGTTTGAGAATGACGATAAGTTATATCGATAATGTCGATTTTACCGA
TAACCGAAAAACGTTAGTAATGGAAATTTAATGATCATTAAATGAGAGCCACTACATATTGTTTTGTTTA
GAAAGATATAGTCGGAATACCAGTAGTTATTTCTTAGTATTATTTTGTGATTTTTAAATATTCTACTCAG
ATGTGTAGAACTTTTTTTCGAACGCTTCTTTTTGCTTAATTTATTTCGTTTTTCGCGCCGACCCCAAAAA
CGTTAGTAATGCAAATCGATTGCGTTTGCGAAGTCAGATGATCCCATCCATGATTTCCATGCGTCAGTTG
TCCCAGCTGTCAATCGAAGATCGCCGTTTGGGGGCTCTACGTTCCTGGTTGACTTCGCCATCGGTCGAGT
GGAATCGAATCGTTCGTTTGCTCTTTAATTATTGTAATGAATTCAATAAACATTGCAGGGCCTCTGCCGT
TCATTTGCATTTGCGTTCGCACTCGCTGCATTCTGAACTATTGCACATTGCCTGGTCGGGCCTCTCCTTC
TCtatatagcgatatatatgtatatgtatatgtatatataaatatatagaaatctacatatgtattcatt
tatatatatGTAATTCTCTATTTCTCTTAGTTGCTCTAGAAGCGCGTGATTCTCTCGCAGATCCACGTGC
CGCCAGATAACTGCACTTTCAATAAGCTTTTATTTGAATAAGCTTCCGCGATAATTGACTTAATAAATTA
TTAAATATTAACAGCGTAGAAAAAACCAGAATGCAATGACTGACATGGCATCTCCTTGTGGATTTTCTAA
TGCCAAGTTAATAGGAAAATCATGCAAAATAGCTGGAAAGTGCATTAGGGCCCGACTGAGCTCACAAAAA
TGTCTTAATATATCATTTTCAATCACTGCATTACCAGATTCCTAAGTCTTAGTCACTTTCCAATGATTAA
TATATACACATATAAATTATTGTTATTTATTTCCCAAAACTAACTTTAATATCAAGCAAAAACGCAGTGG
TACAGAAAACCAACAATTAAAGCACAGTACAAATAATATGAAAAAGAGCGCAGATTGAAAGTGCCACAAA
AAAGATTTGTTGTCCAAGGTTTATGCTTGGCATTGTAAAGGGGTTATTGTGGCGTTGCTTGGGGTCAGAG
GTTTGGAGGGTGCCCACCGCGATAAGCAGCTGGTCGCCACAGAGGGAGAGGGATACAAACAGATAGAAAC
GGGTGCCGGCGAGTGGAGCTGCTGCTCCACATGGCAACAGTTGCTGGCTGGCGGGGAGTGCATAAATATG
ATGTGCATTGCTTTCGTTTCAGCTCTTTATCGCTTCTTTATCTTTGGGGTTGCGTGATAAGAACGCACTC

GGAAAAAAAAGCGACAGCACAAAGTGTCGACAGGGAATTATTGGGAATTATTACAGGGAGTTGCGAAGTTG
AAAACCTTTAAAGTGAAATTAGAAAAGTGAATCAATTATATTTTTTATTCAACAAAAAAGGAAGCTTAAC
TTAACACAAAAACACTTAAAGCCCGATCAAACTTTTACTGTAAGATTATAATAATAATACCAGACCAATA
TAATTTTAAGTCGAttttttttCAATATTAGGCTTAACTTTCCTTTAACCGGAAGTTCGCCCATGTACCG
TTTTAACCTGAAGTTATAACTAGTCCCTTGGCAAGCCGAACTCGTCGCTCTCGCGTTATATTGCTTTATC
AGCAGGTTAGTCCATGACGAGTTGATATTGCTGGGCATAATTCACTTTCGAAGGCAAAGGTCGTTCGGTT
TTATGGAGGGTGACACAGCACAGCACACACATTTTGAGATCCCCGGCACACCGGAGTGGCCGACAAGTGG
GTTCGATGTGGCCTCGTCATCGTCACCGCGCCATCGTCATTATTATGATCATCATGGGGTCGCGAGGTAC
TTGAGATCATGGGCCTGGAACCGAACCTGGGCTTTGGGATTGATAccgtcgccgttgtcgtcgtcgtcgt
cgtcgccgccgtcCTCTTCATCTCGAAGTCGGCTCATTAGGCAGGGTAGACCGGACCAGATTGGTTCAGA
CCATACCAGTTGCTGTGCTGCTCGAATACCTACCTCCTCATCGACGACCAGCTGTCAGTCttgcttttta
ttttctctgtttttggtttttttcttttcgtttttcgatttttttcttcttttttttttttGGCCGAGCAT
CGGGCTTTTCTCGCATTGACTTACTTTGCTTTATAATCCTGTTTGACGGGTTGTGCTGCTGGCTGCTGGG
GTGTAGATCTCTTTTACTTCCACATTGCTGAACCCGGTCATGTGTGATGAGAGGGGCAGGTACAGCGTTG
AGCAGCAGCATGGGAGGAAGGGTGAGGAAGGGCCATACCTGAGATTCAGGGGCACCACGAGATGGTCCAG
TCTCAAGGCACAAGAAATCACATGGTTCGTTCGCCGAAGTCGAGGGTTCTTGCAATCAAGGAGCATCTGG
CGGACACCTTTCTGGTTGGCCAAGTGATCTTGAGAGCGCAGCTCCCCGCCTGATAAGCACTGAGTTAGCA
GGGACGTGGGCAAAAGTAGGGCTCTCTAGGAGTAGTTAGGAACTATTaaaaaaaaGCAATAGAAGATATT
AAATGTAATGGAATGCTATCATTTCAAATATGTACAAATCTGTTACCAAACTTCTGTCATAAGAACTGTG
AGTTTAAATGACTAATGCATCCCTATGGTTTTACAACTCAAAAATCGATTAAAAGCTATTTTGAATTTAG
TTATTTTTAATTATAATCCATTTAAGTAGACTTTACCTAAACGTCGCCTGTCTGATTCCCCCTGAACTAT
ATAAAGTCGACGCCCCTGAATTGTCGACTATAGCTTGGCTTATCATCTGATTGGCGAGGCCAAGTCAATC
CGGAACAGCTGCTCAAAGGCGGCCTAGCAGCTACGATTTTTAGTAGGGCGCCAACGAGTGGCACTAACTT
AACTGATAGCCGAACTATTTTCTATACGCCCGCTGAAGTGGGAAATAATACTCATACGCCTATATGTACG
TGTAAGAACCATACGGCGGAACTCATGAGAAAAGTCGGAGGAGAGCGCACTATTGAAAGGCGTTACGTAA
GATGTGTGGGTTTATTTTTGTTTTGTTCGCTCTGTTGATCTTTATTTATTTCGTTTTGCTCCTCATTCTT
CCCTTTTCTTACCACTGATTCTGTTTTAATTTTTATAAAATCTTTTTATTGCCATCTTCGCTTAGTTCGC
CCTGCctttgttttgttttattatttttttaagatctgctttttcttcggtttttttgtttttctcacttgc
tttcgttcggcttttGGCTCTATCTTTTAATCTATATGTATAAATATATTTTTGAGGACCCCCACGTTTA
TCGGCGTATATCTAGTTATAGAATTTCTCAACTTGTTGCGCGTCGTCACAAAGCTTGAAAAATTGGACCA
CCTGAAGATTTGTACCGAGCGGAAATTTTGACTTTGCTTGTTCGTGACATTGAAATACATACATACGTAC
GTGGAATGCTGTGCTAATTTCTTTTTGGGGTACCTTCCCCCAAATTTCCTAAAATCATTGTGTAATTTTT
GAAAATACACCGATTATTTTCGTAGCCCATTTCCACATCCCACGCCGCCGTGATCTTTAAAGCTGATTTA
TGCTTTATTTGCCCAGTTTGAGGAAAGAGCCAAAACCAACGCGATTCTGAATCACGCTATAATTTTTGAT
GTGATACTCAGACTTTTGCGTTGTTTGCATTTGCATTAGGAATATTATCTGATATTATCGCTAGTATCCT
ATTAACATAATCTAGCAGGTTTCAGCCGATCCATTGAATTCCCCTCTAATATCGAGCGATCAAAGTCAAA
ACACCTACGTCTTATATGCGTCACTGGATAATCTAATGTAAGAAAAATATGTTTGTCGAGAACCTGTCTC
AAAAAACCTCAACAATCAACCGACTTTCAAAGTAAGCTGTGACGCAGTAGATCAAAAGTATTTCGCATCG
GACTATATATATTGAGTTAATCCCATTCGCAATCCAATACTTTTTCATCTGTCTTGTTATTGtttacatt
tttttctacctatgcttttctattttttttgtatttttgttttGCTTATTGCCCCTGGTTATTTTCAAATA
ATGACGTTCTTAATATTATTAAATGTTATTGCATATTATTTAATGCTTTACTCACGACTTTGCGACGCAA
TTTCTCTTCGTGAAAGTaaaaaaaaaaaTAGATTAAATTTAAGTGCGAAATGCAAATAGCAAGTGCTCAT
TTACTCAAAATAGTTGCCCGTTGGCAATTAGAATTTTACACTATTTTAATGGACATTGCGGTGTAGACTA
TACTGATACTCAGTGTCTAAAAATATTATGATGTTTTCTCTAAAACAATCCTGATTTATTTAATGGTTAA

TCTAATTTAAAGCTTTCTAAAAACGTTGAGTATACTATTTTAAGGCCTGCAGATATAATCATACATTTTA
AAGATACTTTTTCTAAAAATAAATTCAAAAGAATAAATTATGATATGCCAACCACACTTACTATAAACAA
ATTCTTTTGTTCACGGAGCAGAAAACAATATGTATTAAAATCAGTTTAAAATAAAGATGTTGAAACTGAG
AAGAGGAGTTTGGAGCGAAGAGAAAGCGATGATACAATATATTGACCCCAAAAGCAAATCAATGAACTTT
CGTTAAATGAGTGCTCCGAAAATTCCAGATGCAGATACATAGGGAATATCTGAGTCGATCATTGGCGAGC
TTAGAGTTACTGGTAGTTTTGTGCGAGTGtattttttgggtttctttttttgacagccccagtgtatttgc
tttgatttttttttgttgagttttgttgttgttgCTAGTGGCTTAGGTCAGGTTGGAATGTTTCGGTAAG
TTTTTGTTTTGCGTCTGGACTGGGTTTTTCTTCTAttttttttttttCTCCTTTGACGGGGCACTTGAGTG
CGACTCATTGGCATTCGCATTGCGATTATTTGCGGCCGTAAACCTTTCGCCGGGCAGATATTATGCAATG
ACAAGCGACGACGCGCGACCCTATCTAAATTATTTCGTTATTTAAATACACAACACAATAAAAACGCAGC
ACGAAAAATTCCGAGAAGGAAATAATGAGGTGACGTTTGGATTGCTCTCAGGTAGGCAAATGTTTAGGTA
GCCAATAAATGAACTTCCGTGTTCTCAAGGTAATTCTTTGCCGTCGCTGTTGGATTCTCTTCATTTCCCC
TGCTTTTCCGCTCTCTCGCAGTCTATATTTTTTGGCCAAGATAAATTATTATAAAACGCCGAATAAAAGT
GTGCCTCGTACGATCCGCACACAGATACTCGCTGTCGTTTCtgtgtgtgtgtgAGTTTTCTATTTGTT
TACAATTTCAAGTTGAAGAGAAGAAAAGCTTTTTGCGCTCTTAAGTGGACATTATAAGCTGACAAACATT
TCGAtttttttttttttACAAATTTTCTTAAGTATATATAATTTTTTGCATTTTTAATGAGAACTTTTAG
TAATATTTAAGAAAGTAATTTTTTGTAGTTATCGTTTATAAGTGATTAATAGTTTAGTTAGTTTAGTCTA
CTCATTGGGTTTCTTGTTTTATGATTCTTCGTTTTTGATTAATGAATCGTTCATTATAATTAATTTTGAT
TATTTCTTAACAGAAGGTAACCTTTTAAATTTGAGTACCTGAAGTGCATTTCAATATATTAGCTCGATGA
TAACATTTTTAACATTTGCGTACCTTATGAATTCAAATTATAAATTTCATCAGACTTTTGTCATTATCAG
TTGGCAATAATTCTATGAAATGTATTCCTTAAATATAGAGTGACACTTGGCATTCCCAGACCAAATTATA
CTTGATAATACCTCAGATAAATGTCTTCAGTTCCAAGAAGTTCATTGATAATGTTTTTTATACCAAAAAG
AGCCAATGTTTGGGTTAAAATTGTACCAGACAACTGCTATCAAAGTGCGATGCTCGTTGAGCTTCAATGA
CTTATCAGTCCTCTCATTCCAGACAGAGTCAATAGACCTATATATTGGAGTAGATTAAATATATATGTAT
GTAGGAACCTACAGAGAGAGACTTGTCAAACTTTTGATTATCAATACTTGTAGTATCATCAATTATTAGG
AAATATATGTATAACTTCGGTCGCCGCCGTGCTAACTGAAATCATTTCAATTATATACTACAGCTCGTAT
TCCAGCTGTGAGCTAAATTTAGTCATAAAGGCATTTATAGATTTTATTCAAACTAATTGATTTCCATTCC
TAGGCaaaaaaaaaGAAACAATAAAATTGTAGAGTACTGAAAGGCAAAGAAAATGTGCGAGGCATTTAAA
CAAGATCATAACCTACATTATCGTAACGCTATTGCGTTTGCCTTAGTTTCCTCTCTTATTTGTATTGGGT
CTGCTGATAACGCTCTCTTACTTTCATTTAAGAGCGATTTTCTTCAATACGTACTaaaaaaaaGAATTTA
CATTTGTTCGATTTGCATTGAAAAATAATCAAACTACATTTTCTACGATTATTATTTATAGTGCTGTCGC
AAAGTTTTTCACATTAAGTACTCCTAATCAAATGGTATACGGGTACTATAAATCATATACGGGTACAAGA
AATTGTTCACCCTGAGTAAAATATAATTTTTATAAGGAAACGTTATAGTAATGGGTGTGTGCTCTCTCCa
aaaaaaaaGGTAAAGATTAATTTAATCGTCTACTGAAAAATCGATTTGTCAATTTGTTGAACGACCCAAG
ATCATTCGGAAATCGTTTTCCACATTTCGAAGCACGAAAACAATCAAAATATAATTTCGAGCGAGACGAT
TGATAGAGAGACAAGATGACTTGACAAAATTACACAAAAATGCTATAGTAATTTCAATCCAAGGTTAAAA
TTCGACATATATGTATGAGTACTCTGAACAAGATATATAGTCATGTACAACTTTAAATGATCAAAGTAAT
TTTAAATTAATCTTTAGTAGCTTGATTAGATTTGTATTTAAATTATTAATATAATTAAGATATATTTTAA
TTGGCTTTCTTTTAGGATTGATTGCGAAGCTTTCACATTCGCTTCGATATCTTTTGGGCTGGCGAGGATT
GCTTGAAGAGCCGCAAAGCTGCAACTGCACAACGAACTTTTCTACCCCATGATGGACGGCTACGCGCAGG
AATGGCCCAGGCTGACCCACACAGATAACGGCCTGGCCATGGACCAGCTGGGCGGGGATCTGCCCCTGGA
CGTGGGCTTCGAGCCACAGACCCGGGCCAGATCCAACACATGGCCATGTCCGCGTCCCGAAAACTTTGTG
GAGCCCACCGACGAGTTGGACAGTACAAAGGCCAGCAATCAGCAGTTGGCCCCAGGAGGACTCACAGCAG
GCTATACAGAATGCGAATGCAGCCAAGAAGAACTCATCGCGTCGCAATGCATGGGGAAATCTATCCTATG

CGGATCTCATCACGCATGCCATTGGATCGGCCACCGACAAACGATTGACACTGAGTCAGATTTACGAGTG
GATGGTCCAGAATGTGCCATATTTCAAGGACAAGGGCGATTCGAATAGCAGTGCCGGATGGAAG
>dme_circ_0008805_junction_seq
CGATTCGAATAGCAGTGCCGGATGGAAGGCTTAAGCAAAACCGCCGCCCAGCAACA
>dme_circ_0008806
GTCAAAACACCTACGTCTTATATGCGTCACTGGATAATCTAATGTAAGAAAAATATGTTTGTCGAGAACC
TGTCTCAAAAAACCTCAACAATCAACCGACTTTCAAAGTAAGCTGTGACGCAGTAGATCAAAA
>dme_circ_0008806_junction_seq
AAGTAAGCTGTGACGCAGTAGATCAAAAGTCAAAACACCTACGTCTTATATGCGTC
>dme_circ_0008807
GATTGATTGCGAAGCTTTCACATTCGCTTCGATATCTTTTGGGCTGGCGAGGATTGCTTGAAGAGCCGCA
AAGCTGCAACTGCACAACGAACTTTTCTACCCCATGATGGACGGCTACGCGCAGGAATGGCCCAGGCTGA
CCCACACAGATAACGGCCTGGCCATGGACCAGCTGGGCGGGGATCTGCCCCTGGACGTGGGCTTCGAGCC
ACAGACCCGGGCCAGATCCAACACATGGCCATGTCCGCGTCCCGAAAACTTTGTGGAGCCCACCGACGAG
TTGGACAGTACAAAGGCCAGCAATCAGCAGTTGGCCCCAGGAGGACTCACAGCAGGCTATACAGAATGCG
AATGCAGCCAAGAAGAACTCATCGCGTCGCAATGCATGGGGAAATCTATCCTATGCGGATCTCATCACGC
ATGCCATTGGATCGGCCACCGACAAACGATTGACACTGAGTCAGATTTACGAGTGGATGGTCCAGAATGT
GCCATATTTCAAGGACAAGGGCGATTCGAATAGCAGTGCCGGATGGAAG
>dme_circ_0008807_junction_seq
CGATTCGAATAGCAGTGCCGGATGGAAGGATTGATTGCGAAGCTTTCACATTCGCT
>dme_circ_0008808
GAACTCCATACGTCACAATCTGTCGCTGCACAACCGCTTTATGAGGGTCCAAAACGAGGGCACCGGCAAG
TCATCCTGGTGGATGCTCAACCCGGAGGCCAAGCCCGGCAAGTCTGTGCGCCGCCGTGCCGCTTCCATGG
AGACGTCCCGGTACGAGAAGCGGCGCGGCAGGGCCAAGAAGCGGGTGGAGGCACTGCGTCAGGCGGGCGT
GGTGGGCCTCAACGATGCCACGCCCTCGCCCAGCAGCAGCGTCAGCGAGGGGCTGGATCACTTTCCCGAG
AGTCCGCTCCACAGGTGGCGGTGGCTTCCAATTATCGCCCGATTTCCGGCAACGCGCCTCATCCAATGCC
AGTTCCTGCGGACGCCTGAGCCCCATTAGGGCGCAGGATCTTGAGCCCGACTGGGGATTCCCCGTTGACT
ACCAGAACACAACGATGACGCAGGCCCACGCCCAGGCGCTCGAGGAGCTGACGGGCACAATGGCGGATGA
GCTGACGCTGTGCAACCAGCAGCAGCAAGGGGTTCAGTGCCGCCTCGGGACTTCCCTCTCAGCCCCCGCC
CCCGCCCTatcagccgccgcagcatcaacaggcgcagcagcagcaacagcagcagTCGCCCTACGCCCTC
AACGGCCCCGCCTCCGGCTACAACACGCTGCAGCCGCAGTCGCAGTGCCTGCTGCACCGGTCCCTCAATT
GCAGTTGCATGCACAATGCAAGAGATGGGTCTCTCGCCGAACTCAGTAACCACAACAATGTCGCCCGCCT
ATCCAAACAGCGAGCCCTCATCGGACTCCCTGAACACGTACAGCAACGTGGTGCTCGATGGTCCGGCGGA
CACTGCGGCACTGATGGTgcagcagcagcagcagcagcagcaacagcaacagcTGTCCGCCAGCTTGGAA
GGCTGCTGCAGCAGGAGATGAGCTACGGCGGCCTGCTGGACATCAATATACCGCTGGCCACGGTCAACAC
GAACCTGGTCAACAGCAGCAGTGGGCCCCTGAGcatcagcaacattagcaacctcagcaacataagcagc
aattccggcagcagtctcagtctgaatcagttgcaggctcagctgcaacagcagcagcagcagcagcagg
cgcagcaacagcagcaggcgcagcagcaacaacagcagcatcagcagcaccagcaacagttgctgctaaa
taataacaacaacagcagcagcagCCTGGAATTGGCAACACAAACGGCTACCACAAATCTGAATGCTCGG
GTTCAGTACTCACAGCCCAGCGTGGTGACCTCGCCACCATCCTGGGTGCACTAGATCCACTGTGGGTCGA
TCGTAATCCAAATCGCAAGGATGATCGTTATAGCCGGTAGTCGTcgttgttgtgtgtgtgcgtgtgtgtt
gtttgtgtCGCCATTGGAATGCTTTAAACGCTAATTCTAAGAACCGTAGTGTAAAGAATTGTAAGCAGCC
GCAGCTGGTTGGGAAACTGGAAACGAGGATTCGGATGCGGAGTCGATGTCTTTATCGGGTATGCAGCAGC
TGCAGGATCAGTGAGCCGAAAGCGGGAGCCGCAGCTGGACCGTCACTAAAAAGTTTACAAA

>dme_circ_0008808_junction_seq
GCTGGACCGTCACTAAAAAGTTTACAAAGAACTCCATACGTCACAATCTGTCGCTG
>dme_circ_0008809
GCACATCTGTGTTACACTTTGGTATGCATGTGAAACATTTGGTTAACTAAAACATATATCTACGCAATTT
AAAAGGCGATCTATTAAATTTCAAGGGGCAAGCTTTTCGTTTTTAAATTTAATTCCTATATTTAAATGGT
GGGCGTTCACTAGTGATTGATTTATGCTTACCATTATCGATAATATTATTAA
>dme_circ_0008809_junction_seq
TGCTTACCATTATCGATAATATTATTAAGCACATCTGTGTTACACTTTGGTATGCA
>dme_circ_0008810
GAATATCGATGCGAGGCTTAGACACAAGGCTCTACGTAGTTGCCCGGAAACAGACCGGTAACACCATCCA
TGACGCCCTCCCACCAGCCGTCGTCGTTTTTCTTGAGCACGTACAGCACTGAGCTTTCCTGGAAGCTCAG
CTCGTCGTCCTTGTCGGCATAGTAGTCGTAAATGGCTACCACTGGAATGTTTGTAAGAATTAGTAAGGTA
TATTGTTAGTTGCTCCAATCAGCCTACCCTTCTCAATGAAGTTCTTGGGCACCCAGCCGGGTAAGTTCTG
ATCCTCGGGCACTATGGGCGCCAGCTGCGGTCCCGTCGATGTGCGTGGTCGTCCGAAGTCTTGATGCTCA
TCCTCCGGCGGCGGCGGAGGTGGCAAGGGAGGCGACTGTGAGCCGGGACGAGCGAAGTTCAAGCTGAA
>dme_circ_0008810_junction_seq
AGCCGGGACGAGCGAAGTTCAAGCTGAAGAATATCGATGCGAGGCTTAGACACAAG
>dme_circ_0008811
GGTGGAAACCCCGCCCAGATTCTCGCTGAAATTCGAGCAAATCTATCTTTCCAAGCCAACACATTGGGAA
AAGGATGGTTCGCCCAGTCCTATGATGCCGAACGAGGCCCGCCTGCGAAATCTAACTTACTCCGCTCCTC
TCTACGTGGACATTACGAAAACCAAGAATGTCGAGGGCCTGGATCCC
>dme_circ_0008811_junction_seq
AACCAAGAATGTCGAGGGCCTGGATCCCGGTGGAAACCCCGCCCAGATTCTCGCTG
>dme_circ_0008812
GATACCCATTATGATTGTGTTTCGAGCATTGGGCTTCGTGGCCGATCGCGATATTCTGGAGCATATCATC
TATGACTTCGACGATCCCGAGATGATGGAAATGGTGAAACCTTCTCTGGACGAGGCTTTTGTAGTCCAAG
AGCAGAATGTTGCCTTGAATTTCATTGGAGCTAGAGGAGCACGTCCCGGAGTCACAAAAGACAAGCGTAT
AAAGTACGCTAAGGAAATTTTGCAAAAGGAAATGCTGCCCCACGTTGGAGTATCCGATTTCTGCGAGACC
AAGAAGGCATACTTTTTGGGCTACATG
>dme_circ_0008812_junction_seq
CAAGAAGGCATACTTTTTGGGCTACATGGATACCCATTATGATTGTGTTTCGAGCA
>dme_circ_0008813
TTCGTCGTCGTTTTCAGGAAGGGTAATGGTCTTGCCAATAACCACATCGTCGCCAGATACACGAATTCCT
GGGGCAATAATTCCATCATCGTCTAACTTGTCGTAGTGAGCATTTCTCATTCCTTGGCAGGTGCCGCGAT
GAGGCTTCTCGAAGTTCTCTTCCTGATCACCGACACGTTTGTTCTCCGAATCCTTGTATGATCGGTAGAA
CACAGAGCGGAAGAATCCACGCTCCACAGCGGATGCATTCAGAATGACAGAATCCTCCTGGTTGTAGCCA
GTGTAGCAGAGAATAGCCACGATTGAATTGATGCCAGCTGGTAGTTCTCGGAAACGCAGGTACTCCATGG
AACG
>dme_circ_0008813_junction_seq
CTCGGAAACGCAGGTACTCCATGGAACGTTCGTCGTCGTTTTCAGGAAGGGTAATG
>dme_circ_0008814
GACAATAACAACGAGACTCTGGATGCTGTTGTGCCGCCACCTCCACCGCCAGAAAGGAACAGTTCTTATT
TGATCATGTCACAGCAGAAACTGCGAGGCAGCACTGGAAACGCGACCTCGTCCATGGGCCTACTGAAAAC
GGCCACCAGCAATCAGGCCGCAGCTGCCGTAGAGATTAAAAAGGCATCTCTTCTCAACACTCAAACTAAT

AATAACAACAATTTGAGTGGGAGCTTAAACAACAACACCATGCATGGATCACCACTCAGCGCCATGGAGC
TAAATGCCGCCTATGTTTCGGCCACGGGAACAGGAATAATTGGAGGATTGGGCACACCGCCTCCGCCGCC
ACCGTTGATGCAAAGGGACAACAAGCGGGTGAGCTTCCATGATGAAGAGAATAATTTTGTGAGCGGGAAT
AGCCAGCAACAACAGCTGCAGCAGCAATATATTATGGATAATTACGGTATGGAGCATCAGGATTTGGACA
CTATCAGAGAGGATACGAGTGAGATCCTCGTACGCGTCGACTGGCGGAGAAACAGCAGCAGCAACAGCAA
CAACGGGCTGGAGATGCGGTGCCCGAAAAGCTGTCGTTCAAGGAGAAGATGAAGATGTTTGCCCTGGAGT
CGGGGGAAGATAAGACGCCCAAGGACAAACTGAAAATATCGCGAGCCCAACGAGATATAGACGCGGTGCA
CTAAGAAGAAGGGATCGTTGCTTAATGCTATGTACATACAGACCAGAATCCACAAAGCTTACACTTCAAT
ATATTGGGGGCCAAGTTGTTACGTTAGCTCTACATAACAAAACACACACATAGATATATAGGTCATGTTA
TATATGTCGGAACACTTTAGAAACAAATAAACCTAACGAATTTGCTTAGCAAGGCAATAACAATCCATAG
TTATCGCGTTTCAGAGTTAATAACCATACACAAAATGTTAACTGATTTCAATTAGTGAGAATGTCAGTGT
ATATCATTATAG
>dme_circ_0008814_junction_seq
GTGAGAATGTCAGTGTATATCATTATAGGACAATAACAACGAGACTCTGGATGCTG
>dme_circ_0008815
GATATTATGAAACTGGATCGATAAAACCTCGAGCTATAGGTGGTTCAAAGCCACGAGTAGCTACAACCCC
GGTTGTGCAAAAAATTGCAGATTACAAACGGGAATGTCCCAGCATATTTGCGTGGGAAATACGAGATCGA
CTGCTATCGGAACAAGTTTGCAATAGTGATAACATTCCAAGTTGTAAGTTATATTTTGCGCACATAATAA
TATTTCTGCACGTGctaataaaagttctaacataccattttaagcgttttcgatccttaaaaatatatat
gtatattcatgatcaggatctaggaccagtccgtacctctctttctgtcttacgttaaaaattttagaag
tttaaagctgaaagattgtttcaaactaactttctaatagagccttatagctttaagctatttatagttg
ttttcctgttctcttaaaaactctgatctgactaataaaagttctaacataccattttaagcgttttcga
tccttaaaaatatatatgtatattcatgatcaggatggatctaggaccagtctgtacctctctttctgtc
ttaagttaaaaattttagaagtttaaagctggaagattgtttcaaacgaattttctaatagagccttata
gctttaagctatttatagttgttttcctgttctcttaaaaactctgatTAATGATACAgttactcgtaga
gtaaaatggtatactggattcgttgaaaataacattacaggtagaagacagcgttcgcgattttataaag
tatatATTCCTAAACAgtccatacaaacgctgtgatctcgggaagtaaaaaagctgagatattatgcatg
cagattctagTAAAGCAGAAATGTGTCATTACAGAGTCATTACACAGAAGCCAAAACGTCAGGTCCATCC
TTTGAAAATGTTGTTCAATATTTTTATTGTTTTCATTGCAATTTTTATCGGTACACCAAATGAATATT
GAAAATCTGTTAAAAATCATGCAACTGATAGAATGAAACCTACATCGTATATGTACACAAAACTAacccg
ctactcgtagagcaaaagggtaatcttgattcgtcgaaatgacgtgtaactggtagatctgaccatgccc
gtctgtccgtccgtatggccgtatgaaacgctaataaaagttaagataaggcatgcagattccagaggca
aaaacgcagcgtaagtttgtttaacgatgttgccacgcccatagcccacaaaaaactcccacgctcacac
ttgtacaaatttaaaaaattctctTTTGTTTAAattagaatcaccagctgagtcgatctggcatgcccgt
cagtcGCCCGCTTGTAACACTGGATCGATCTAATCATGACTACCTGTCCATTACTCTGTATAAACGTCGG
AATCTCGGAAACCATATTATTTGGGCGGTTACAGTACTATTAATGAGCTAAATTTTGAAAATATCAATTT
TCAATAACGCAAAGGAAGCTAGCTTCGGAAATATGAAGTTTTGAGATATTTTGCGTTTGAGAAAGGGCGT
CACGAGAGAAATCCCAGTTTAAGATGGCTTTAATAGTTTCAAAGGCTATAAGCAAGGGTGTACAAGGATT
AAATAATTTTGGGACAAACCCAATTCGTTCTTTTTAAAATAGATTATGAAAAGGATATTTTCGACAGTCT
CATGGCATTTTCTGGGGTTTATTTGTTTTTCGATAGTATGCTCTAGTTATAATAATCAGTATAAAAACAT
GTCCATTATTCAGGTAAATAAGGGTACATTTTTTCTTTTTAGGTTCTTAGCTAGGTTAGGATTAGGTTTT
GTGGCTTAATGACTTGTATCGTTTTTAAATCCGGATGTATGGAAGCAAGTACACAGTAAAGTAATGATAT
GTATTAATGATATGTGCTCACAATTATCTCCAATCATTGCCCCTACGAGTTTGTATATACATTAATTACA
ATAAAAATTCCCATTTTAGTaaaaaaaaaaCAAGAAAGAacccgatactcagctagtgtgaatgcgaacg

cgaaatttcatcatttttctgggatcagaacagtcacacccacactcttgaacaatttttaactttttttc
tcactttattccccaatatcAATGTTGatttttaatcgttttattgtttgtcttgccaatttctatcggt
ataccaaaaacactttcgccacgccccttcttacaccaacaaaacgctcaaaacggatctatcgatatca
caaaaaaataatgaaatttcgctttcgtatttagtctgatagtcggggaactcgactatagcattctctc
tctgtttTTGGTAAGTTAATGGCCTTACTAACGTTTCAGTAGTTTCTATATGCATAAGATCCGAATGCCT
CCTAGTCTATTTCGTATCTACTAACCAATGATCGTAAGACTGCGTATGATTAAAGAGAACTTGCTTAGCA
TCTGTTTTGCGAGTAGCCAAATTTGTTTGTTGGAAGAACCTCTTTACGGCGTTATTGGTGTCCATATTGC
AAGTGCTGTCCATTGTGCAACACTAACAAATACATTTCTCCCCTGGCTTCTAATTATTACTTATGTTATT
TTGTTTAGCATCTATTCTATATTTAATCCTATATGCCATTCACAAAATATTTATTTAAGTTTCCATTACC
AGAAAAGCACAAAAAGCATATATACACCGGCTGTATTTGAGTAATTTTATGTCTACTTGGTCCAACCTAC
ACATATAGCGTTGGCGCTTTATCAAGTGAAAATGTTGACGGTTGACGCTGAGGtatcgatataccagaaa
aatgatgaaatttcgcgttcgaattcacactagctgagtaactggtatctgatagcagggaaactcgacg
atagcattcgctcgtgtttTTTACACCCTTTACTCttttttttttCTAAAGAAGTCCGTCCCCATATTTT
ATACTTCGATTATACTTCTGGTTCAGTTTATTTGATGTCCTTTGATGTTTTGTTCACAAATGTGGGCGAG
TAATTCATTATGGCTATGTCTCATCCCAACGTCTATGTATGTACTTCTGTTAAATAAAGAACAGCGTTTG
GCTTACGGCCCAAGCACGTGCCAGCACTTTAGTCGACTGTGAAAGTGGCATATTTCATGTCTCCTAATGA
CGTTGTTCACGTATTCACAAATCTGTATAAAGTTAGCTTATAAGAACATGTACAATCCAATAAACAAAAC
TTTTAAATTTAGAATTAAATATATCGCGAGGAAACGTTAAGAATAAGTTTTTTTATGTTTTATTGTTTAT
TACCTGTTAGTCACCTAACAAGAAGGTGACTTTTATTTGTTGGAAAAAACCACAATATATAACCCGTTAT
ATATAAAACTACTACTAGtactagattcgttgaaaaatatgtaacaggtagaaagaagcgtttccgacca
atagtcaagtcgatctagccttgtccgtatgttcgtgtgtccgtctgacagtctgtccgtccgtatgaac
gtcgagtgctcaggaactataaaagctagaaggttgagattcagaatacaaattcattaagacataccaa
accacccgaaactgtcacgcccacacttttgaatttttgtaaatttctgtcggtttgccaaaaaacGAAC
CGCCTAAACCTTATTGAATTTAACTGGCTTACACATATGTTATCAGTTAAAAAGCAATATCATTAACAAA
TAAGAAAGAACTCTTTAGTCGAGTTTTTTAACCGCATACGTTACTAGTTGAAGTGCTCGCAATATTTTTT
AAATTATTGTAGGTATTTCAATTTCAAGTTTGTAGGCGTTAGAGTTACTAGAACTTGCATGTATAATCTC
AAATTATTAGCTGTGCCACACTCTCCAGATTCGTTGATATGTATACCCTTTTACTCTACGATTaacgctt
cctccaacctgttacatatttcagcgaatgtagtataccctattgctttacgaataatgCTATGAATAGA
ATAGGGGTCTAGAATCGACAACCGAGCTATCATAATAATAAGAATAGTATACTAGATTTCTGTGTATATA
TCTTTCTCGCTTTCAAAATCGGATTGGCTAAAATATTTGTTAGCCAATCGAGAAGCAGAAAGGAAGAATG
GGGGTTGGCTCAAAAAGTTACGCACCCATTTCAAGTTTGGTTAGAAAGCATGAAAGAAGTAGGAAATACT
TTTGGCCTTCAGTAATTTAAAACGCTCTAAGTCATCATACTCAGTATATGTGTACTATTTATGTACATAA
TACTGTTCGAAGGCTCGTACGCACATCCTTTTAGGCCTTGCATTCGCAAGCGTCAGTAAATAAGACGTTG
GTGTCTGTTGGCTTGCATGTGTTTCAGTACACATCGTTTTCCCATTTGGAAATGTACATATTGTGTTCAT
GTATTTTATTGTACGCAGTATACTGGGATTATTTGCAGGGTTTCATCTATTAATCGAGTCTTACGTAACC
TGGCCTCACAAAAGGAGCAGCAAGCTCAGCAACAAAACGAATCCGTTTATGAAAAGCTTCGCATGTTTAA
TGGCCAAACGGGCGGATGGGCATGGTATCCAAGCAATACAACGACGGCACATTTGACGCTACCACCAGCA
GCTTCCGTTGTGACATCTCCTGCAAATTTATCAGGACAGGCCGATCGGGATGATGTTCAAAAAAGAGGGC
AAGTAGTAATATTAGATACAAATATATATAATAATGCACAAAATTTGCTCATATGATTAACACAAACAAT
TAATTAAATATATTAGGGATCAACATTTTAGAAATTTCCTTCTGTCCAATTTCTTTGATCAGAATATTGT
ACATTGAAAGAAAGGGCCCTGTGTTAAATAAAATGTTTCCGACTCTAAAGAGGGTTGAAAACAATTGCAA
GTATTTTGTCACAGATGATCAAACATCAGGGCAAACAAATAGTTAATTTGTTGGTCATTTGTTATTTGCC
ATTAAGTTCATTGAATTCTGTTCCAGCTTCTGATACGTTGAATTGGGTTTTCTTCAATCAATAAATTGAG
CTAGCATATGTCTTCATTATCAGTAAGCATTAACCGACCCTTTAATAACATTTAATGTCGTTTCTTTGTG

ACGAATAAAATTAAATCATTTCAGAAAAGGAAATACGTTTGTTAGTATAATTAATGAAAATGCTTAGCCT
ACTACCAACTATTTTAAAATTCAATTCATTTTCTAATCCTACTTCAAACAAAAAAGGCAGCTACCATTAG
CAATAAATATATAGTTTTAACAAGTATATCGAAATAATGATTTATATAATTTTTACTCGTATAGTAAAAG
GTGCGTATACGAAGTATTTTATACCACTGCACAAGCCGATAGTTGCTACGCCCAGCTTTGCGATCACAAA
CATTTTCATACATAATAATATTTTACGAATCGTATGAAATGCAATTGTCGTTGTCAACAGTGGAAAAAGG
CAATGTCTGGCCGTATTAAATGTATTATTGATCAAAGTCGCTAACTTGGTTAATGTAGTCAAATCCGTCT
GTTCGTATAAGCGGTTACGAATGGTTTTGTATGTCAAGACACTGGTGTTTTTCCTTCTTTTTGAAGTACA
TCTTAAAACAAAAATTTATAATATTGTTCTCAATATTTGTTTTAGGAATTACAATTTTCAGTAGAAGTTT
CGCATACAAACTCTCACGATAGTACATCGGATGGAAACTCTGAACATAATTCATCCGGGGACGAAGACTC
TCAAATGCGGTTGCGCCTAAAAAGGAAGTTACAGCGCAATCGGACATCATTTTCTAATGAGCAAATTGAC
AGTCTTGAAAAAG
>dme_circ_0008815_junction_seq
AATGAGCAAATTGACAGTCTTGAAAAAGGATATTATGAAACTGGATCGATAAAACC
>dme_circ_0008816
GATATTATGAAACTGGATCGATAAAACCTCGAGCTATAGGTGGTTCAAAGCCACGAGTAGCTACAACCCC
GGTTGTGCAAAAAAATTGCAGATTACAAACGGGAATGTCCCAGCATATTTGCGTGGGAAATACGAGATCGA
CTGCTATCGGAACAAGTTTGCAATAGTGATAACATTCCAAGTTGTAAGTTATATTTTGCGCACATAATAA
TATTTCTGCACGTGctaataaaagttctaacataccattttaagcgttttcgatccttaaaaatatatat
gtatattcatgatcaggatctaggaccagtccgtacctctctttctgtcttacgttaaaaattttagaag
tttaaagctgaaagattgtttcaaactaactttctaatagagccttatagctttaagctatttatagttg
ttttcctgttctcttaaaaactctgatctgactaataaaagttctaacataccattttaagcgttttcga
tccttaaaaatatatatgtatattcatgatcaggatggatctaggaccagtctgtacctctctttctgtc
ttaagttaaaaattttagaagtttaaagctggaagattgtttcaaacgaattttctaatagagccttata
gctttaagctatttatagttgttttcctgttctcttaaaaactctgatTAATGATACAgttactcgtaga
gtaaaatggtatactggattcgttgaaaataacattacaggtagaagacagcgttcgcgatttttataaag
tatatATTCCTAAACAgtccatacaaacgctgtgatctcgggaagtaaaaaagctgagatattatgcatg
cagattctagTAAAGCAGAAATGTGTCATTACAGAGTCATTACACAGAAGCCAAAACGTCAGGTCCATCC
TTTGAAAATGTTGTTCAATATTTTTTATTGTTTTTCATTGCAATTTTTATCGGTACACCAAATGAATATT
GAAAATCTGTTAAAAATCATGCAACTGATAGAATGAAACCTACATCGTATATGTACACAAAACTAacccg
ctactcgtagagcaaaagggtaatcttgattcgtcgaaatgacgtgtaactggtagatctgaccatgccc
gtctgtccgtccgtatggccgtatgaaacgctaataaaagttaagataaggcatgcagattccagaggca
aaaacgcagcgtaagtttgtttaacgatgttgccacgcccatagcccacaaaaaaactcccacgctcacac
ttgtacaaatttaaaaaattctctTTTGTTTAAattagaatcaccagctgagtcgatctggcatgcccgt
cagtcGCCCCGCTTGTAACACTGGATCGATCTAATCATGACTACCTGTCCATTACTCTGTATAAACGTCGG
AATCTCGGAAACCATATTATTTGGGCGGTTACAGTACTATTAATGAGCTAAATTTTGAAAATATCAATTT
TCAATAACGCAAAGGAAGCTAGCTTCGGAAATATGAAGTTTTGAGATATTTTGCGTTTGAGAAAGGGCGT
CACGAGAGAAATCCCAGTTTAAGATGGCTTTAATAGTTTCAAAGGCTATAAGCAAGGGTGTACAAGGATT
AAATAATTTTGGGACAAACCCAATTCGTTCTTTTTAAAATAGATTATGAAAAGGATATTTTCGACAGTCT
CATGGCATTTTCTGGGGTTTATTTGTTTTTCGATAGTATGCTCTAGTTATAATAATCAGTATAAAAACAT
GTCCATTATTCAGGTAAATAAGGGTACATTTTTTCTTTTTAGGTTCTTAGCTAGGTTAGGATTAGGTTTT
GTGGCTTAATGACTTGTATCGTTTTTAAATCCGGATGTATGGAAGCAAGTACACAGTAAAGTAATGATAT
GTATTAATGATATGTGCTCACAATTATCTCCAATCATTGCCCCTACGAGTTTGTATATACATTAATTACA
ATAAAAAATTCCCATTTTAGTaaaaaaaaaaCAAGAAAGAacccgatactcagctagtgtgaatgcgaacg
cgaaatttcatcatttttctgggatcagaacagtcacacccacactcttgaacaatttttaactttttttc

tcactttattccccaatatcAATGTTGatttttaatcgttttattgtttgtcttgccaatttctatcggt
ataccaaaaacactttcgccacgccccttcttacaccaacaaaacgctcaaaacggatctatcgatatca
caaaaaaataatgaaatttcgctttcgtatttagtctgatagtcggggaactcgactatagcattctctc
tctgtttTTGGTAAGTTAATGGCCTTACTAACGTTTCAGTAGTTTCTATATGCATAAGATCCGAATGCCT
CCTAGTCTATTTCGTATCTACTAACCAATGATCGTAAGACTGCGTATGATTAAAGAGAACTTGCTTAGCA
TCTGTTTTGCGAGTAGCCAAATTTGTTTGTTGGAAGAACCTCTTTACGGCGTTATTGGTGTCCATATTGC
AAGTGCTGTCCATTGTGCAACACTAACAAATACATTTCTCCCCTGGCTTCTAATTATTACTTATGTTATT
TTGTTTAGCATCTATTCTATATTTAATCCTATATGCCATTCACAAAATATTTATTTAAGTTTCCATTACC
AGAAAAGCACAAAAAGCATATATACACCGGCTGTATTTGAGTAATTTTATGTCTACTTGGTCCAACCTAC
ACATATAGCGTTGGCGCTTTATCAAGTGAAAATGTTGACGGTTGACGCTGAGGtatcgatataccagaaa
aatgatgaaatttcgcgttcgaattcacactagctgagtaactggtatctgatagcagggaaactcgacg
atagcattcgctcgtgtttTTTACACCCTTTACTCttttttttttCTAAAGAAGTCCGTCCCCATATTTT
ATACTTCGATTATACTTCTGGTTCAGTTTATTTGATGTCCTTTGATGTTTTGTTCACAAATGTGGGCGAG
TAATTCATTATGGCTATGTCTCATCCCAACGTCTATGTATGTACTTCTGTTAAATAAAGAACAGCGTTTG
GCTTACGGCCCAAGCACGTGCCAGCACTTTAGTCGACTGTGAAAGTGGCATATTTCATGTCTCCTAATGA
CGTTGTTCACGTATTCACAAATCTGTATAAAGTTAGCTTATAAGAACATGTACAATCCAATAAACAAAAC
TTTTAAATTTAGAATTAAATATATCGCGAGGAAACGTTAAGAATAAGTTTTTTTATGTTTTATTGTTTAT
TACCTGTTAGTCACCTAACAAGAAGGTGACTTTTATTTGTTGGAAAAAACCACAATATATAACCCGTTAT
ATATAAAACTACTACTAGtactagattcgttgaaaaatatgtaacaggtagaaagaagcgtttccgacca
atagtcaagtcgatctagccttgtccgtatgttcgtgtgtccgtctgacagtctgtccgtccgtatgaac
gtcgagtgctcaggaactataaaagctagaaggttgagattcagaatacaaattcattaagacataccaa
accacccgaaactgtcacgcccacacttttgaatttttgtaaatttctgtcggtttgccaaaaaacGAAC
CGCCTAAACCTTATTGAATTTAACTGGCTTACACATATGTTATCAGTTAAAAAGCAATATCATTAACAAA
TAAGAAAGAACTCTTTAGTCGAGTTTTTAACCGCATACGTTACTAGTTGAAGTGCTCGCAATATTTTTT
AAATTATTGTAGGTATTTCAATTTCAAGTTTGTAGGCGTTAGAGTTACTAGAACTTGCATGTATAATCTC
AAATTATTAGCTGTGCCACACTCTCCAGATTCGTTGATATGTATACCCTTTTACTCTACGATTaacgctt
cctccaacctgttacatatttcagcgaatgtagtataccctattgctttacgaataatgCTATGAATAGA
ATAGGGGTCTAGAATCGACAACCGAGCTATCATAATAATAAGAATAGTATACTAGATTTCTGTGTATATA
TCTTTCTCGCTTTCAAAATCGGATTGGCTAAAATATTTGTTAGCCAATCGAGAAGCAGAAAGGAAGAATG
GGGGTTGGCTCAAAAAGTTACGCACCCATTTCAAGTTTGGTTAGAAAGCATGAAAGAAGTAGGAAATACT
TTTGGCCTTCAGTAATTTAAAACGCTCTAAGTCATCATACTCAGTATATGTGTACTATTTATGTACATAA
TACTGTTCGAAGGCTCGTACGCACATCCTTTTAGGCCTTGCATTCGCAAGCGTCAGTAAATAAGACGTTG
GTGTCTGTTGGCTTGCATGTGTTTCAGTACACATCGTTTTCCCATTTGGAAATGTACATATTGTGTTCAT
GTATTTTATTGTACGCAGTATACTGGGATTATTTGCAGGGTTTCATCTATTAATCGAGTCTTACGTAACC
TGGCCTCACAAAAGGAGCAGCAAGCTCAGCAACAAAACGAATCCGTTTATGAAAAGCTTCGCATGTTTAA
TGGCCAAACGGGCGGATGGGCATGGTATCCAAGCAATACAACGACGGCACATTTGACGCTACCACCAGCA
GCTTCCGTTGTGACATCTCCTGCAAATTTATCAGGACAGGCCGATCGGGATGATGTTCAAAAAAGAGGGC
AAGTAGTAATATTAGATACAAATATATATAATAATGCACAAAATTTGCTCATATGATTAACACAAACAAT
TAATTAAATATATTAGGGATCAACATTTTAGAAATTTCCTTCTGTCCAATTTCTTTGATCAGAATATTGT
ACATTGAAAGAAAGGGCCCTGTGTTAAATAAAATGTTTCCGACTCTAAAGAGGGTTGAAAACAATTGCAA
GTATTTTGTCACAGATGATCAAACATCAGGGCAAACAAATAGTTAATTTGTTGGTCATTTGTTATTTGCC
ATTAAGTTCATTGAATTCTGTTCCAGCTTCTGATACGTTGAATTGGGTTTTCTTCAATCAATAAATTGAG
CTAGCATATGTCTTCATTATCAGTAAGCATTAACCGACCCTTTAATAACATTTAATGTCGTTTCTTTGTG
ACGAATAAAATTAAATCATTTCAGAAAAGGAAATACGTTTGTTAGTATAATTAATGAAAATGCTTAGCCT

ACTACCAACTATTTTAAAATTCAATTCATTTTCTAATCCTACTTCAAACAAAAAAGGCAGCTACCATTAG
CAATAAATATATAGTTTTAACAAGTATATCGAAATAATGATTTATATAATTTTTACTCGTATAGTAAAAG
GTGCGTATACGAAGTATTTTATACCACTGCACAAGCCGATAGTTGCTACGCCCAGCTTTGCGATCACAAA
CATTTTCATACATAATAATATTTTACGAATCGTATGAAATGCAATTGTCGTTGTCAACAGTGGAAAAAGG
CAATGTCTGGCCGTATTAAATGTATTATTGATCAAAGTCGCTAACTTGGTTAATGTAGTCAAATCCGTCT
GTTCGTATAAGCGGTTACGAATGGTTTTGTATGTCAAGACACTGGTGTTTTTCCTTCTTTTTGAAGTACA
TCTTAAAACAAAAATTTATAATATTGTTCTCAATATTTGTTTTAGGAATTACAATTTTCAGTAGAAGTTT
CGCATACAAACTCTCACGATAGTACATCGGATGGAAACTCTGAACATAATTCATCCGGGGACGAAGACTC
TCAAATGCGGTTGCGCCTAAAAAGGAAGTTACAGCGCAATCGGACATCATTTTCTAATGAGCAAATTGAC
AGTCTTGAAAAAGGGTAACAAAGTGTTGTATTTTAACTAGGTCTTAATAAATTACCTCACCACATAACAC
TTCATTATACTAATATAACTAATATATTTTATAAAAAAATACCATTAGTTTTAGAAATAAAGAGCTAAGG
AAAAAAATACCTGTATTGGTAATAAAAGTATATCGATTTTCAAAAATCGTCAAATTCTGTGAATGAATTT
CGAGCGAATATTTAAAACAACAATTACAACGGATTTTGAGAGGAAGTGTTCTAAAACTAATTTTCGCTTG
ACAAACTCGACACTCGATTTTGCTGATAGACCTTTAAATCTACGGTTTACATTTAGATTAAAAATGTTTC
ATGATGAGTCCACATATATTCTGGTTAACATATATATACATGTATTAAAAGAATGGTTTTATGGTACACA
AATATCGTTTTGTTTAGTGCCGCGACGTAGAGAAACGCCATATAAAAACTCTTTATAACTGTATTTTCAT
ATTCTCGATACTATcgataatcagcgataataataattataataattgataataaatGTACATATGTACA
TATATATACACTAAATATGTAGGGTCAGTCAATTGCGTAACAAACTGAAAAACTTAATTTCATTTTTTAA
TTGCTCGCATTTTTGCTGTTTATAACGTCAAAATATATTCGTTTTACAGTAAAACATACCATAAGGTCTA
TTTTAGCATTAATCACTAATTTATATCTGAACTTTTATCGACGTGCATTACAGAATTTGAAAGAACACAT
TATCCCGATGTTTTTGCGCGAGAAAGGCTTGCTGATAAAATTGGTTTGCCAGAGGCACGTATTCAG
>dme_circ_0008816_junction_seq
AATTGGTTTGCCAGAGGCACGTATTCAGGATATTATGAAACTGGATCGATAAAACC
>dme_circ_0008817
GATATTATGAAACTGGATCGATAAAACCTCGAGCTATAGGTGGTTCAAAGCCACGAGTAGCTACAACCCC
GGTTGTGCAAAAAATTGCAGATTACAAACGGGAATGTCCCAGCATATTTGCGTGGGAAATACGAGATCGA
CTGCTATCGGAACAAGTTTGCAATAGTGATAACATTCCAAGTTGTAAGTTATATTTGCGCACATAATAA
TATTTCTGCACGTGctaataaaagttctaacataccattttaagcgttttcgatccttaaaaatatatat
gtatattcatgatcaggatctaggaccagtccgtacctctctttctgtcttacgttaaaaattttagaag
tttaaagctgaaagattgtttcaaactaactttctaatagagccttatagctttaagctatttatagttg
ttttcctgttctcttaaaaactctgatctgactaataaaagttctaacataccattttaagcgttttcga
tccttaaaaatatatatgtatattcatgatcaggatggatctaggaccagtctgtacctctctttctgtc
ttaagttaaaaattttagaagtttaaagctggaagattgtttcaaacgaattttctaatagagccttata
gctttaagctatttatagttgttttcctgttctcttaaaaactctgatTAATGATACAgttactcgtaga
gtaaaatggtatactggattcgttgaaaataacattacaggtagaagacagcgttcgcgattttataaag
tatatATTCCTAAACAgtccatacaaacgctgtgatctcgggaagtaaaaaagctgagatattatgcatg
cagattctagTAAAGCAGAAATGTGTCATTACAGAGTCATTACACAGAAGCCAAAACGTCAGGTCCATCC
TTTGAAAATGTTGTTCAATATTTTTTATTGTTTTTCATTGCAATTTTTATCGGTACACCAAATGAATATT
GAAAATCTGTTAAAAATCATGCAACTGATAGAATGAAACCTACATCGTATATGTACACAAAACTAacccg
ctactcgtagagcaaaagggtaatcttgattcgtcgaaatgacgtgtaactggtagatctgaccatgccc
gtctgtccgtccgtatggccgtatgaaacgctaataaaagttaagataaggcatgcagattccagaggca
aaaacgcagcgtaagtttgtttaacgatgttgccacgcccatagcccacaaaaaactcccacgctcacac
ttgtacaaatttaaaaaattctctTTTGTTTAAattagaatcaccagctgagtcgatctggcatgcccgt
cagtcGCCCGCTTGTAACACTGGATCGATCTAATCATGACTACCTGTCCATTACTCTGTATAAACGTCGG

AATCTCGGAAACCATATTATTTGGGCGGTTACAGTACTATTAATGAGCTAAATTTTGAAAATATCAATTT
TCAATAACGCAAAGGAAGCTAGCTTCGGAAATATGAAGTTTTGAGATATTTTGCGTTTGAGAAAGGGCGT
CACGAGAGAAATCCCAGTTTAAGATGGCTTTAATAGTTTCAAAGGCTATAAGCAAGGGTGTACAAGGATT
AAATAATTTTGGGACAAACCCAATTCGTTCTTTTTAAAATAGATTATGAAAAGGATATTTTCGACAGTCT
CATGGCATTTTCTGGGGTTTATTTGTTTTTCGATAGTATGCTCTAGTTATAATAATCAGTATAAAAACAT
GTCCATTATTCAGGTAAATAAGGGTACATTTTTTCTTTTTAGGTTCTTAGCTAGGTTAGGATTAGGTTTT
GTGGCTTAATGACTTGTATCGTTTTTAAATCCGGATGTATGGAAGCAAGTACACAGTAAAGTAATGATAT
GTATTAATGATATGTGCTCACAATTATCTCCAATCATTGCCCCTACGAGTTTGTATATACATTAATTACA
ATAAAAATTCCCATTTTAGTaaaaaaaaaaCAAGAAAGAacccgatactcagctagtgtgaatgcgaacg
cgaaatttcatcatttttctgggatcagaacagtcacacccacactcttgaacaatttttaactttttc
tcactttattccccaatatcAATGTTGatttttaatcgttttattgtttgtcttgccaatttctatcggt
ataccaaaaacactttcgccacgccccttcttacaccaacaaaacgctcaaaacggatctatcgatatca
caaaaaaataatgaaatttcgctttcgtatttagtctgatagtcggggaactcgactatagcattctctc
tctgtttTTGGTAAGTTAATGGCCTTACTAACGTTTCAGTAGTTTCTATATGCATAAGATCCGAATGCCT
CCTAGTCTATTTCGTATCTACTAACCAATGATCGTAAGACTGCGTATGATTAAAGAGAACTTGCTTAGCA
TCTGTTTTGCGAGTAGCCAAATTTGTTTGTTGGAAGAACCTCTTTACGGCGTTATTGGTGTCCATATTGC
AAGTGCTGTCCATTGTGCAACACTAACAAATACATTTCTCCCCTGGCTTCTAATTATTACTTATGTTATT
TTGTTTAGCATCTATTCTATATTTAATCCTATATGCCATTCACAAAATATTTATTTAAGTTTCCATTACC
AGAAAAGCACAAAAAGCATATATACACCGGCTGTATTTGAGTAATTTTATGTCTACTTGGTCCAACCTAC
ACATATAGCGTTGGCGCTTTATCAAGTGAAAATGTTGACGGTTGACGCTGAGGtatcgatataccagaaa
aatgatgaaatttcgcgttcgaattcacactagctgagtaactggtatctgatagcagggaaactcgacg
atagcattcgctcgtgtttTTTACACCCTTTACTCttttttttttCTAAAGAAGTCCGTCCCCATATTTT
ATACTTCGATTATACTTCTGGTTCAGTTTATTTGATGTCCTTTGATGTTTTGTTCACAAATGTGGGCGAG
TAATTCATTATGGCTATGTCTCATCCCAACGTCTATGTATGTACTTCTGTTAAATAAAGAACAGCGTTTG
GCTTACGGCCCAAGCACGTGCCAGCACTTTAGTCGACTGTGAAAGTGGCATATTTCATGTCTCCTAATGA
CGTTGTTCACGTATTCACAAATCTGTATAAAGTTAGCTTATAAGAACATGTACAATCCAATAAACAAAAC
TTTTAAATTTAGAATTAAATATATCGCGAGGAAACGTTAAGAATAAGTTTTTTTATGTTTTATTGTTTAT
TACCTGTTAGTCACCTAACAAGAAGGTGACTTTTATTTGTTGGAAAAAACCACAATATATAACCCGTTAT
ATATAAAACTACTACTAGtactagattcgttgaaaaatatgtaacaggtagaaagaagcgtttccgacca
atagtcaagtcgatctagccttgtccgtatgttcgtgtgtccgtctgacagtctgtccgtccgtatgaac
gtcgagtgctcaggaactataaaagctagaaggttgagattcagaatacaaattcattaagacataccaa
accacccgaaactgtcacgcccacacttttgaatttttgtaaatttctgtcggtttgccaaaaaacGAAC
CGCCTAAACCTTATTGAATTTAACTGGCTTACACATATGTTATCAGTTAAAAAGCAATATCATTAACAAA
TAAGAAAGAACTCTTTAGTCGAGTTTTTTAACCGCATACGTTACTAGTTGAAGTGCTCGCAATATTTTTT
AAATTATTGTAGGTATTTCAATTTCAAGTTTGTAGGCGTTAGAGTTACTAGAACTTGCATGTATAATCTC
AAATTATTAGCTGTGCCACACTCTCCAGATTCGTTGATATGTATACCCTTTTACTCTACGATTaacgctt
cctccaacctgttacatatttcagcgaatgtagtataccctattgctttacgaataatgCTATGAATAGA
ATAGGGGTCTAGAATCGACAACCGAGCTATCATAATAATAAGAATAGTATACTAGATTTCTGTGTATATA
TCTTTCTCGCTTTCAAAATCGGATTGGCTAAAATATTTGTTAGCCAATCGAGAAGCAGAAAGGAAGAATG
GGGGTTGGCTCAAAAAGTTACGCACCCATTTCAAGTTTGGTTAGAAAGCATGAAAGAAGTAGGAAATACT
TTTGGCCTTCAGTAATTTAAAACGCTCTAAGTCATCATACTCAGTATATGTGTACTATTTATGTACATAA
TACTGTTCGAAGGCTCGTACGCACATCCTTTTAGGCCTTGCATTCGCAAGCGTCAGTAAATAAGACGTTG
GTGTCTGTTGGCTTGCATGTGTTTCAGTACACATCGTTTTCCCATTTGGAAATGTACATATTGTGTTCAT
GTATTTTATTGTACGCAGTATACTGGGATTATTTGCAGGGTTTCATCTATTAATCGAGTCTTACGTAACC

TGGCCTCACAAAAGGAGCAGCAAGCTCAGCAACAAAACGAATCCGTTTATGAAAAGCTTCGCATGTTTAA
TGGCCAAACGGGCGGATGGGCATGGTATCCAAGCAATACAACGACGGCACATTTGACGCTACCACCAGCA
GCTTCCGTTGTGACATCTCCTGCAAATTTATCAGGACAGGCCGATCGGGATGATGTTCAAAAAAGAGGGC
AAGTAGTAATATTAGATACAAATATATATAATAATGCACAAAATTTGCTCATATGATTAACACAAACAAT
TAATTAAATATATTAGGGATCAACATTTTAGAAATTTCCTTCTGTCCAATTTCTTTGATCAGAATATTGT
ACATTGAAAGAAAGGGCCCTGTGTTAAATAAAATGTTTCCGACTCTAAAGAGGGTTGAAAACAATTGCAA
GTATTTTGTCACAGATGATCAAACATCAGGGCAAACAAATAGTTAATTTGTTGGTCATTTGTTATTTGCC
ATTAAGTTCATTGAATTCTGTTCCAGCTTCTGATACGTTGAATTGGGTTTTCTTCAATCAATAAATTGAG
CTAGCATATGTCTTCATTATCAGTAAGCATTAACCGACCCTTTAATAACATTTAATGTCGTTTCTTTGTG
ACGAATAAAATTAAATCATTTCAGAAAAGGAAATACGTTTGTTAGTATAATTAATGAAAATGCTTAGCCT
ACTACCAACTATTTTAAAATTCAATTCATTTTCTAATCCTACTTCAAACAAAAAAGGCAGCTACCATTAG
CAATAAATATATAGTTTTAACAAGTATATCGAAATAATGATTTATATAATTTTTACTCGTATAGTAAAAG
GTGCGTATACGAAGTATTTTATACCACTGCACAAGCCGATAGTTGCTACGCCCAGCTTTGCGATCACAAA
CATTTTCATACATAATAATATTTTACGAATCGTATGAAATGCAATTGTCGTTGTCAACAGTGGAAAAAGG
CAATGTCTGGCCGTATTAAATGTATTATTGATCAAAGTCGCTAACTTGGTTAATGTAGTCAAATCCGTCT
GTTCGTATAAGCGGTTACGAATGGTTTTGTATGTCAAGACACTGGTGTTTTTCCTTCTTTTTGAAGTACA
TCTTAAAACAAAAATTTATAATATTGTTCTCAATATTTGTTTTAGGAATTACAATTTTCAGTAGAAGTTT
CGCATACAAACTCTCACGATAGTACATCGGATGGAAACTCTGAACATAATTCATCCGGGGACGAAGACTC
TCAAATGCGGTTGCGCCTAAAAAGGAAGTTACAGCGCAATCGGACATCATTTTCTAATGAGCAAATTGAC
AGTCTTGAAAAAGGGTAACAAAGTGTTGTATTTTAACTAGGTCTTAATAAATTACCTCACCACATAACAC
TTCATTATACTAATATAACTAATATATTTTATAAAAAAATACCATTAGTTTTAGAAATAAAGAGCTAAGG
AAAAAAATACCTGTATTGGTAATAAAAGTATATCGATTTTCAAAAATCGTCAAATTCTGTGAATGAATTT
CGAGCGAATATTTAAAACAACAATTACAACGGATTTTGAGAGGAAGTGTTCTAAAACTAATTTTCGCTTG
ACAAACTCGACACTCGATTTTGCTGATAGACCTTTAAATCTACGGTTTACATTTAGATTAAAAATGTTTC
ATGATGAGTCCACATATATTCTGGTTAACATATATATACATGTATTAAAAGAATGGTTTTATGGTACACA
AATATCGTTTTGTTTAGTGCCGCGACGTAGAGAAACGCCATATAAAAACTCTTTATAACTGTATTTTCAT
ATTCTCGATACTATcgataatcagcgataataataattataataattgataataaatGTACATATGTACA
TATATATACACTAAATATGTAGGGTCAGTCAATTGCGTAACAAACTGAAAAACTTAATTTCATTTTTTAA
TTGCTCGCATTTTTGCTGTTTATAACGTCAAAATATATTCGTTTTACAGTAAAACATACCATAAGGTCTA
TTTTAGCATTAATCACTAATTTATATCTGAACTTTTATCGACGTGCATTACAGGGCACGTATTCAGGTTT
GTCTTTCAGTTTTGTTTAATTATATTCAGTTTGTAGACCTTTTTTTGCAGAGCTCAATTTATTTATATTT
TTGTAAATTATGTCAAACTAGCAATTAACTAATTTAAAATATTTTAATCGACCCTTAATGTTTTGGGCGG
CACATACGAAAATGTATTTCATTTATTGTTGTAAACTATACTACTTAAACATACACACTTTCACGTTTTC
TTCGTTTTAATATTTCTGGGTTTCATAACTAGAATTGCGCAAGCAGATTCAAATTGTAATATATGAAAAA
ATTATAATATTGCTACGCCCAGTTTAGCGCACATAAAGATTTTATATATAAATAAATTACCCTTCCCTGC
TAATCCGGAACACATTAATTGTAAGcgttattcgtagagtaatagggtgtactagattagttgaaaataa
tgttacaggcagtagatgtaacgtcgagatctcggaaactataagagctagaaagttgagattcagcata
caaattatagacccatgttgccacgtccactctaacgcccacaaactgcacaaaactgcacaaaactgcc
acgcccaaatttgggaaaattgttggatatttttcaaatttttatgggtcgtgtaaatttctatcgatt
tgccacgcctactctaggtccctaaagccgtcaaacctgtacgcccacacttttgaacaatgtttaaatt
gtttctcattttattccccaatatctatcgatatcccagaaaaattacgaaatttcgcgttcgtatttac
actagctaaggaactcgactatagcattctctctggttatacccgttactcgtagagtaagagggtatac
tagaaagtacgtttccgaccatacaaagtatatataatttatatcaggatcaatgccgagtcggtctagc
catgtccgtctgtccgtatggagagtggacgagtgaccacaacatcattactgttgtggccgaaccaact

accgcgcgcgcagttgagagcatagctcctgtgccgtcctggaacttctccaatgcacggtggcaattgt
tcaaggaggaaatggtgagtagagcagccgagctcccggaaaacttctcagagtcgtcgttggaccagca
agtttcgaccctgcgcagtacagtacataatgtatgtgatattgcgatggaaagaaagttgactcaatcg
cccagcaggagagctcgttggtggtccggagacttcgtcgcctactccaagacggaaggcgtcgtgatga
cgatgccgctgtactgcttgtagctcaggcgtgcctcagccaattggctgattgggagggtgaaaatgga
tgactggaaacgcttcgtgggagatcatgccgacgacccatgggggcgcgtctacaagatttgccgaggc
cgcagaaagtgcacggagattggatgcctccacgtgaatggcgagctgatcactgattggggtgactgtg
cacgagtgctcctccgcaatttcttcccagtcccttcccttcccagtcggagtccgaagcaccgactgcc
atcgcggaggaagtcccaccggccctcgaagtattcgaggttgatttatgtgttgcccggttgaagagca
tgcgctctcccggcttggacggcatcaatggcactatctgcaaggcagtctggcgcgccatacctgagca
cctagcatcgatgttttccctatgcatccgattgggagctgaaacgtggattcccgctggaggagaacga
ctggcttcacggcgaagacattgcgtgtctaagctgggagcagaggaagacacgcctagaggagtgttta
ttccagagttggcagaacagatgggacgacgaaagcgaaccaggacgggtgacgcataggtttatgccat
ccgtcactctcgcctatcgggatccaagttttggattctcgatgaggacgtctttcctgctttcaggcca
cgggtcgttcagtgcagcgataccactgcttgcgcatgtggaggtccatatgaggactggatgcacatat
tgtgcgcttgccccctatatgcagatttgcgggaccttgatggacttggagtgcagcgccttggcgaaaa
cacgccacaagttcggagggatccgaaagtcatacatAtatgaacgttgagatctcagaaactattaaag
cttgaaggttgatattcagcatacagaatacgcagtgcaagtttgttgccacgcgcactctaacgcaaca
aaccgaacaaatctgtcacgcccacacttttaaaaaatgtgttgatattctttgccgcgcacactcgacg
ccctaaaaccggccaaaactgtaacgcccacattttaaaccatgtttcgatgtttgttcataattttatt
agtcttgtaaatttctaccgaattgccaaacaactttttgccgcgcccactttaacgtcctcaagccgcc
caaaactaccacgcccacatttttgaaaaattggtagatatatttttataattttattagtcgtcacaat
ttctgtcgacttgccaaacaactttttgccacgcccacactaacgccctaaagccgccgaaccgatcacg
cccacaatttggaacaacttttaagtttttttctcattttattccccaataaatttcccgttcgcattcac
accatctgagtaacgggcatcgatagtccgggaactcgactatagcattctctcttgtttACCTAATATT
TTCATATCATCCTCGCACTACGTTTTGCTGAGTAACCGCTATTCGGCCGATTCTAAGCTAGCTAGTCTGC
ATAATAATTTCCCAAGTTTCGTTACATTACAGGTAGATTATATGTATTTCGGAACGACCGACCCATTATA
TAACAAAACTGCCTaaaaaactgaaaaattaaaaaaaaaaaCGGTACCCTTGTTTTTTTGTTATACTCG
GTATAATCCAAATGTTATAGAAAAATATAGAAAATATATTCTTCAAGGCTATATAAGCTCAGCTTGCCGA
AGCTTTAGCTTCCTTTCTTAAtataccccttactagtatggtactagattaattgaaatgtatttaacag
gtacaaaaatgcgactccaaccatggatgtatatatatacatttacattAAATTATTTCTTCAGCCTAAT
TTTAACTTCCTAACTCTTAAGGCTCCCGAAATCACTGCATTCTTACAGACGTAAAGATGAAATGGCACAA
ATacagacggacatgaccagatcgactcggctaataatcccgtatatgaatacacatatatgtgtaCTTT
AAAATCAACTTATCAGTATTTTCGCAACACATGCATAAGAGTATAATAAAATAACTTACCTCTGTGCGTT
ATTTTAATAACTGTATCTTGCAAAATAATTGTAAAACATTTTAACTCGAGGCGTCAAGTATAAGCAGCGG
AATGGGCGGTTTAGAAGTCATCCCTCAACTGCTTTTCACCCCTAAAGCTGTGTTGTTTTGTCGTCAAAGT
GAGATGAAATATTTATCGTTCCGCTCTTGATTATTTGTTTAAGTGAATAAAAACAATTTGACTGTAAAAA
TACTTGATGCTTCTTGTGATTTTTCGCATTGAGTGGTAATAACGGGGGCGAAGCTTGGATTTACCAAGCT
TGAAGGGCTTGGTCGGAAGCCGGAGAGGGAGCTGCAACAATAACGCTGGGTAATAAATGTCATGGGGAAA
ATATATTAAAGCAAAAAGTATTGTAATACGTTTTCACCCCTTTGTCATCTTGAATGCTTTGGCTCACTTT
ATTTCGCTTGTGTTATGTTACAAGTAAAACCGCATATAAAGCGTGTATTTCTCCTAACCTAAACTGCGTG
ACAAAAGAAATATGAAAGTTCATTAGACTGCAAGtttttttttTTCaaaaaaaaaaaaCATTGTTATGCTC
TTTCCACTTTAGTCATAATAAAAACCAATTCCAACAGCATAATAATCCCGTTCCTTACCTTAGTCTACGT
ACATGTGGCTGTGGGTGCCACTACTAAAATTTATTTTTTGTAATCAGTAAATGCAACCAAATCGTATTCT

GAAAATATTTAAACACCCATTTTAAAGTTTTTAACATAAAAGTAGCCAACTGCTATTTTCCGCTAAACT
CTTCACAATCTACATAATAAAGAGCCCTAAATCAGAGGTTTCATTGATTCAGCGGAACTGACAAAATGAC
ACACAAGTTACGTGTTTACCTACGAACAACAACAAAATATTTGGTACACGAAATTCATTCATTAAAAACA
AAGaaacaaagagaatgctatagtcgagttccccgactatcaggtactatcaggaagatgggcgaagggc
gaaattctataattttctggcataccgatagatattggcaaataagatgagaataaatttaaaaaaaaat
gttaaaaaaaacataggataaagggcacagtatttttggtataccgataaaaattggcaggacaaacaac
aaaacaaaggaaaatcaaaacatttgagcgtttggcaaatcaaacaaaatttaaaagactaataaaaaag
tgaaaaaaattttaacacatttttaaagtatgggcgtggcagtttttatcgctttgtgggtgttagagtg
ggcgtggcaacataagtaaacaaaattgcgtctttgtctctggaatctgtatgcttaatctcaaccttta
agcttttgcagttcctgagatctagacgctattgatgctgatcaaaaaaatatatatttatatggtcga
aaacgttttcttctgcctgttacatagatttaaacgaatctagtatacgcttttagtctacgaataacTT
AATTTGTTTGCGACTTGTGTTACGTAAGAAGCGGTGTGGTCAAACTTTTGATAACTTTAGAAGACTAATA
TAAAAAATATTAAATAATTTTACAAAGTGCAAAATTtggggaaaaaaacttttgctgcgtctatgtctct
ggaatctgcactcgtttcaacttattatcttttatagtttctaaaatcttgacggacaggcggacgtaca
tggccagattgactcggcttttgatcctgatcaacaaaatagatagatgtatatattgtagggtcggaaa
cgctaccttctacctgtaacatacttcttaacgaacctggtaACGGCTATAAAAAACTTCTTAGTAAATA
CATACTAAATTTGAAGTAATTTTAAGAAGCTTCTGATAAGCTGATTATTATAAGTGCTTaaaaaaaaGTC
GATTTAGCGGATTTCTATGGCAGTCTAGTAACTAAAAATTTGTAGCTACATACATTATTTAGCTTAATTA
ttttcttttttttttagtttaaatagttaattaactatattatattattaaaatatattttttttcagaaat
attaAGTTCGTTTAATATATAAAAATCAATAACCTTCATCGTGAAAAATTTTTAATAGGGTTTGGTTTTC
AAACCGACGAGCTAAATGGCGCCGAGAAGAAAAAATGCGAACTCAGAGACGATCGGCCGATACCGTGGAC
GGCAGTGGTCGAACCAGCACGGCAAATAATCCTTCAGGAACAACTGCATCTTCCTCCGTCGCAACGTCAA
ACAACTCGACTCCAGGGATTGTGAACTCAGCAATCAACGTTGCGGAACGAACATCATCTGCATTAGTTAG
TAATAGCCTTCCCGAGGCTTCAAATGGACCAACTGGTTTTGGGTGGTGAAGCTAATACTACACACACCAG
CTCTGAAAGCCCACCCCTTCAGCCAGCGGCACCGCGGCTACCCTTAAATTCTGGATTCAACACCATGTAC
TCATCTATTCCACAACCGATTGCAACGATGGCTGAAAATTACAA
>dme_circ_0008817_junction_seq
CGATTGCAACGATGGCTGAAAATTACAAGATATTATGAAACTGGATCGATAAAACC
>dme_circ_0008818
GGTTTCATCTATTAATCGAGTCTTACGTAACCTGGCCTCACAAAAGGAGCAGCAAGCTCAGCAACAAAAC
GAATCCGTTTATGAAAAGCTTCGCATGTTTAATGGCCAAACGGGCGGATGGGCATGGTATCCAAGCAATA
CAACGACGGCACATTTGACGCTACCACCAGCAGCTTCCGTTGTGACATCTCCTGCAAATTTATCAGGACA
GGCCGATCGGGATGATGTTCAAAAAAGAGGGCAAGTAGTAATATTAGATACAAATATATATAATAATGCA
CAAAATTTGCTCATATGATTAACACAAACAATTAATTAAATATATTAGGGATCAACATTTTAGAAATTTC
CTTCTGTCCAATTTCTTTGATCAGAATATTGTACATTGAAAGAAAGGGCCCTGTGTTAAATAAAATGTTT
CCGACTCTAAAGAGGGTTGAAAACAATTGCAAGTATTTTGTCACAGATGATCAAACATCAGGGCAAACAA
ATAGTTAATTTGTTGGTCATTTGTTATTTGCCATTAAGTTCATTGAATTCTGTTCCAGCTTCTGATACGT
TGAATTGGGTTTTCTTCAATCAATAAATTGAGCTAGCATATGTCTTCATTATCAGTAAGCATTAACCGAC
CCTTTAATAACATTTAATGTCGTTTCTTTGTGACGAATAAAATTAAATCATTTCAGAAAAGGAAATACGT
TTGTTAGTATAATTAATGAAAATGCTTAGCCTACTACCAACTATTTTAAAATTCAATTCATTTTCTAATC
CTACTTCAAACAAAAAAGGCAGCTACCATTAGCAATAAATATATAGTTTTAACAAGTATATCGAAATAAT
GATTTATATAATTTTTACTCGTATAGTAAAAGGTGCGTATACGAAGTATTTTATACCACTGCACAAGCCG
ATAGTTGCTACGCCCAGCTTTGCGATCACAAACATTTTCATACATAATAATATTTTACGAATCGTATGAA
ATGCAATTGTCGTTGTCAACAGTGGAAAAAGGCAATGTCTGGCCGTATTAAATGTATTATTGATCAAAGT

CGCTAACTTGGTTAATGTAGTCAAATCCGTCTGTTCGTATAAGCGGTTACGAATGGTTTTGTATGTCAAG
ACACTGGTGTTTTTCCTTCTTTTTGAAGTACATCTTAAAACAAAAATTTATAATATTGTTCTCAATATTT
GTTTTAGGAATTACAATTTTCAGTAGAAGTTTCGCATACAAACTCTCACGATAGTACATCGGATGGAAAC
TCTGAACATAATTCATCCGGGGACGAAGACTCTCAAATGCGGTTGCGCCTAAAAAGGAAGTTACAGCGCA
ATCGGACATCATTTTCTAATGAGCAAATTGACAGTCTTGAAAAAG
>dme_circ_0008818_junction_seq
AATGAGCAAATTGACAGTCTTGAAAAAGGGTTTCATCTATTAATCGAGTCTTACGT
>dme_circ_0008819
GGTTTCATCTATTAATCGAGTCTTACGTAACCTGGCCTCACAAAAGGAGCAGCAAGCTCAGCAACAAAAC
GAATCCGTTTATGAAAAGCTTCGCATGTTTAATGGCCAAACGGGCGGATGGGCATGGTATCCAAGCAATA
CAACGACGGCACATTTGACGCTACCACCAGCAGCTTCCGTTGTGACATCTCCTGCAAATTTATCAGGACA
GGCCGATCGGGATGATGTTCAAAAAAGAGGGCAAGTAGTAATATTAGATACAAATATATATAATAATGCA
CAAAATTTGCTCATATGATTAACACAAACAATTAATTAAATATATTAGGGATCAACATTTTAGAAATTTC
CTTCTGTCCAATTTCTTTGATCAGAATATTGTACATTGAAAGAAAGGGCCCTGTGTTAAATAAAATGTTT
CCGACTCTAAAGAGGGTTGAAAACAATTGCAAGTATTTTGTCACAGATGATCAAACATCAGGGCAAACAA
ATAGTTAATTTGTTGGTCATTTGTTATTTGCCATTAAGTTCATTGAATTCTGTTCCAGCTTCTGATACGT
TGAATTGGGTTTTCTTCAATCAATAAATTGAGCTAGCATATGTCTTCATTATCAGTAAGCATTAACCGAC
CCTTTAATAACATTTAATGTCGTTTCTTTGTGACGAATAAAATTAAATCATTTCAGAAAAGGAAATACGT
TTGTTAGTATAATTAATGAAAATGCTTAGCCTACTACCAACTATTTTAAAATTCAATTCATTTTCTAATC
CTACTTCAAACAAAAAAGGCAGCTACCATTAGCAATAAATATATAGTTTTAACAAGTATATCGAAATAAT
GATTTATATAATTTTTACTCGTATAGTAAAAGGTGCGTATACGAAGTATTTTATACCACTGCACAAGCCG
ATAGTTGCTACGCCCAGCTTTGCGATCACAAACATTTTCATACATAATAATATTTTACGAATCGTATGAA
ATGCAATTGTCGTTGTCAACAGTGGAAAAAGGCAATGTCTGGCCGTATTAAATGTATTATTGATCAAAGT
CGCTAACTTGGTTAATGTAGTCAAATCCGTCTGTTCGTATAAGCGGTTACGAATGGTTTTGTATGTCAAG
ACACTGGTGTTTTTCCTTCTTTTTGAAGTACATCTTAAAACAAAAATTTATAATATTGTTCTCAATATTT
GTTTTAGGAATTACAATTTTCAGTAGAAGTTTCGCATACAAACTCTCACGATAGTACATCGGATGGAAAC
TCTGAACATAATTCATCCGGGGACGAAGACTCTCAAATGCGGTTGCGCCTAAAAAGGAAGTTACAGCGCA
ATCGGACATCATTTTCTAATGAGCAAATTGACAGTCTTGAAAAAGGGTAACAAAGTGTTGTATTTTAACT
AGGTCTTAATAAATTACCTCACCACATAACACTTCATTATACTAATATAACTAATATATTTTATAAAAAA
ATACCATTAGTTTAGAAATAAAGAGCTAAGGAAAAAAATACCTGTATTGGTAATAAAAGTATATCGATT
TTCAAAAATCGTCAAATTCTGTGAATGAATTTCGAGCGAATATTTAAAACAACAATTACAACGGATTTTG
AGAGGAAGTGTTCTAAAACTAATTTTCGCTTGACAAACTCGACACTCGATTTTGCTGATAGACCTTTAAA
TCTACGGTTTACATTTAGATTAAAAATGTTTCATGATGAGTCCACATATATTCTGGTTAACATATATATA
CATGTATTAAAAGAATGGTTTTATGGTACACAAATATCGTTTTGTTTAGTGCCGCGACGTAGAGAAACGC
CATATAAAAACTCTTTATAACTGTATTTTCATATTCTCGATACTATcgataatcagcgataataataatt
ataataattgataataaatGTACATATGTACATATATATACACTAAATATGTAGGGTCAGTCAATTGCGT
AACAAACTGAAAAACTTAATTTCATTTTTTAATTGCTCGCATTTTTGCTGTTTATAACGTCAAAATATAT
TCGTTTTACAGTAAAACATACCATAAGGTCTATTTTAGCATTAATCACTAATTTATATCTGAACTTTTAT
CGACGTGCATTACAGAATTTGAAAGAACACATTATCCCGATGTTTTTGCGCGAGAAAGGCTTGCTGATAA
AATTGGTTTGCCAGAGGCACGTATTCAG
>dme_circ_0008819_junction_seq
AATTGGTTTGCCAGAGGCACGTATTCAGGGTTTCATCTATTAATCGAGTCTTACGT
>dme_circ_0008820
GGTTTCATCTATTAATCGAGTCTTACGTAACCTGGCCTCACAAAAGGAGCAGCAAGCTCAGCAACAAAAC

GAATCCGTTTATGAAAAGCTTCGCATGTTTAATGGCCAAACGGGCGGATGGGCATGGTATCCAAGCAATA
CAACGACGGCACATTTGACGCTACCACCAGCAGCTTCCGTTGTGACATCTCCTGCAAATTTATCAGGACA
GGCCGATCGGGATGATGTTCAAAAAAGAGGGCAAGTAGTAATATTAGATACAAATATATATAATAATGCA
CAAAATTTGCTCATATGATTAACACAAACAATTAATTAAATATATTAGGGATCAACATTTTAGAAATTTC
CTTCTGTCCAATTTCTTTGATCAGAATATTGTACATTGAAAGAAAGGGCCCTGTGTTAAATAAAATGTTT
CCGACTCTAAAGAGGGTTGAAAACAATTGCAAGTATTTTGTCACAGATGATCAAACATCAGGGCAAACAA
ATAGTTAATTTGTTGGTCATTTGTTATTTGCCATTAAGTTCATTGAATTCTGTTCCAGCTTCTGATACGT
TGAATTGGGTTTTCTTCAATCAATAAATTGAGCTAGCATATGTCTTCATTATCAGTAAGCATTAACCGAC
CCTTTAATAACATTTAATGTCGTTTCTTTGTGACGAATAAAATTAAATCATTTCAGAAAAGGAAATACGT
TTGTTAGTATAATTAATGAAAATGCTTAGCCTACTACCAACTATTTTAAAATTCAATTCATTTTCTAATC
CTACTTCAAACAAAAAAGGCAGCTACCATTAGCAATAAATATATAGTTTTAACAAGTATATCGAAATAAT
GATTTATATAATTTTTACTCGTATAGTAAAAGGTGCGTATACGAAGTATTTTATACCACTGCACAAGCCG
ATAGTTGCTACGCCCAGCTTTGCGATCACAAACATTTTCATACATAATAATATTTTACGAATCGTATGAA
ATGCAATTGTCGTTGTCAACAGTGGAAAAAGGCAATGTCTGGCCGTATTAAATGTATTATTGATCAAAGT
CGCTAACTTGGTTAATGTAGTCAAATCCGTCTGTTCGTATAAGCGGTTACGAATGGTTTTGTATGTCAAG
ACACTGGTGTTTTTCCTTCTTTTTGAAGTACATCTTAAAACAAAAATTTATAATATTGTTCTCAATATTT
GTTTTAGGAATTACAATTTTCAGTAGAAGTTTCGCATACAAACTCTCACGATAGTACATCGGATGGAAAC
TCTGAACATAATTCATCCGGGGACGAAGACTCTCAAATGCGGTTGCGCCTAAAAAGGAAGTTACAGCGCA
ATCGGACATCATTTTCTAATGAGCAAATTGACAGTCTTGAAAAAGGGCACGTATTCAGGTTTGTCTTTCA
GTTTTGTTTAATTATATTCAGTTTGTAGACCTTTTTTTGCAGAGCTCAATTTATTTATATTTTTGTAAAT
TATGTCAAACTAGCAATTAACTAATTTAAAATATTTTAATCGACCCTTAATGTTTTGGGCGGCACATACG
AAAATGTATTTCATTTATTGTTGTAAACTATACTACTTAAACATACACACTTTCACGTTTTCTTCGTTTT
AATATTTCTGGGTTTCATAACTAGAATTGCGCAAGCAGATTCAAATTGTAATATATGAAAAAATTATAAT
ATTGCTACGCCCAGTTTAGCGCACATAAAGATTTTATATATAAATAAATTACCCTTCCCTGCTAATCCGG
AACACATTAATTGTAAGcgttattcgtagagtaatagggtgtactagattagttgaaaataatgttacag
gcagtagatgtaacgtcgagatctcggaaactataagagctagaaagttgagattcagcatacaaattat
agacccatgttgccacgtccactctaacgcccacaaactgcacaaaactgcacaaaactgccacgcccaa
atttgggaaaattgttggatatttttttcaaatttttatgggtcgtgtaaatttctatcgatttgccacgc
ctactctaggtccctaaagccgtcaaacctgtacgcccacacttttgaacaatgtttaaattgtttctca
ttttattccccaatatctatcgatatcccagaaaaattacgaaatttcgcgttcgtatttacactagcta
aggaactcgactatagcattctctctggttatacccgttactcgtagagtaagagggtatactagaaagt
acgtttccgaccatacaaagtatatataatttatatcaggatcaatgccgagtcggtctagccatgtccg
tctgtccgtatggagagtggacgagtgaccacaacatcattactgttgtggccgaaccaactaccgcgcg
cgcagttgagagcatagctcctgtgccgtcctggaacttctccaatgcacggtggcaattgttcaaggag
gaaatggtgagtagagcagccgagctcccggaaaacttctcagagtcgtcgttggaccagcaagtttcga
ccctgcgcagtacagtacataatgtatgtgatattgcgatggaaagaaagttgactcaatcgcccagcag
gagagctcgttggtggtccggagacttcgtcgcctactccaagacggaaggcgtcgtgatgacgatgccg
ctgtactgcttgtagctcaggcgtgcctcagccaattggctgattgggagggtgaaaatggatgactgga
aacgcttcgtgggagatcatgccgacgacccatgggggcgcgtctacaagatttgccgaggccgcagaaa
gtgcacggagattggatgcctccacgtgaatggcgagctgatcactgattggggtgactgtgcacgagtg
ctcctccgcaatttcttcccagtcccttcccttcccagtcggagtccgaagcaccgactgccatcgcgga
ggaagtcccaccggccctcgaagtattcgaggttgatttatgtgttgcccggttgaagagcatgcgctct
cccggcttggacggcatcaatggcactatctgcaaggcagtctggcgcgccatacctgagcacctagcat
cgatgttttccctatgcatccgattgggagctgaaacgtggattcccgctggaggagaacgactggcttc

acggcgaagacattgcgtgtctaagctgggagcagaggaagacacgcctagaggagtgtttattccagag
ttggcagaacagatgggacgacgaaagcgaaccaggacgggtgacgcataggtttatgccatccgtcact
ctcgcctatcgggatccaagttttggattctcgatgaggacgtctttcctgctttcaggccacgggtcgt
tcagtgcagcgataccactgcttgcgcatgtggaggtccatatgaggactggatgcacatattgtgcgct
tgccccctatatgcagatttgcgggaccttgatggacttggagtgcagcgccttggcgaaaacacgccac
aagttcggagggatccgaaagtcatacatAtatgaacgttgagatctcagaaactattaaagcttgaagg
ttgatattcagcatacagaatacgcagtgcaagtttgttgccacgcgcactctaacgcaacaaaccgaac
aaatctgtcacgcccacacttttaaaaaatgtgttgatattctttgccgcgcacactcgacgccctaaaa
ccggccaaaactgtaacgcccacattttaaaccatgtttcgatgtttgttcataattttattagtcttgt
aaatttctaccgaattgccaaacaactttttgccgcgcccactttaacgtcctcaagccgcccaaaacta
ccacgcccacatttttgaaaaattggtagatatatttttataattttattagtcgtcacaatttctgtcg
acttgccaaacaactttttgccacgcccacactaacgccctaaagccgccgaaccgatcacgcccacaat
ttggaacaacttttaagtttttttctcattttattccccaataaatttcccgttcgcattcacaccatctg
agtaacgggcatcgatagtccgggaactcgactatagcattctctcttgtttACCTAATATTTTCATATC
ATCCTCGCACTACGTTTTGCTGAGTAACCGCTATTCGGCCGATTCTAAGCTAGCTAGTCTGCATAATAAT
TTCCCAAGTTTCGTTACATTACAGGTAGATTATATGTATTTCGGAACGACCGACCCATTATATAACAAAA
CTGCCTaaaaaactgaaaaattaaaaaaaaaaaCGGTACCCTTGTTTTTTTTGTTATACTCGGTATAATC
CAAATGTTATAGAAAAATATAGAAAATATATTCTTCAAGGCTATATAAGCTCAGCTTGCCGAAGCTTTAG
CTTCCTTTCTTAAtataccccttactagtatggtactagattaattgaaatgtatttaacaggtacaaaa
atgcgactccaaccatggatgtatatatatacatttacattAAATTATTTCTTCAGCCTAATTTTAACTT
CCTAACTCTTAAGGCTCCCGAAATCACTGCATTCTTACAGACGTAAAGATGAAATGGCACAAATacagac
ggacatgaccagatcgactcggctaataatcccgtatatgaatacacatatatgtgtaCTTTAAAATCAA
CTTATCAGTATTTTCGCAACACATGCATAAGAGTATAATAAAATAACTTACCTCTGTGCGTTATTTTAAT
AACTGTATCTTGCAAAATAATTGTAAAACATTTTAACTCGAGGCGTCAAGTATAAGCAGCGGAATGGGCG
GTTTAGAAGTCATCCCTCAACTGCTTTTCACCCCTAAAGCTGTGTTGTTTTGTCGTCAAAGTGAGATGAA
ATATTTATCGTTCCGCTCTTGATTATTTGTTTAAGTGAATAAAAACAATTTGACTGTAAAAATACTTGAT
GCTTCTTGTGATTTTTCGCATTGAGTGGTAATAACGGGGGCGAAGCTTGGATTTACCAAGCTTGAAGGGC
TTGGTCGGAAGCCGGAGAGGGAGCTGCAACAATAACGCTGGGTAATAAATGTCATGGGGAAAATATATTA
AAGCAAAAAGTATTGTAATACGTTTTCACCCCTTTGTCATCTTGAATGCTTTGGCTCACTTTATTTCGCT
TGTGTTATGTTACAAGTAAAACCGCATATAAAGCGTGTATTTCTCCTAACCTAAACTGCGTGACAAAAGA
AATATGAAAGTTCATTAGACTGCAAGtttttttttTTCaaaaaaaaaaaaCATTGTTATGCTCTTTCCACT
TTAGTCATAATAAAAACCAATTCCAACAGCATAATAATCCCGTTCCTTACCTTAGTCTACGTACATGTGG
CTGTGGGTGCCACTACTAAAATTTATTTTTTGTAATCAGTAAATGCAACCAAATCGTATTCTGAAAATAT
TTAAACACCCATTTTAAAGTTTTTTAACATAAAAGTAGCCAACTGCTATTTTCCGCTAAACTCTTCACAA
TCTACATAATAAAGAGCCCTAAATCAGAGGTTTCATTGATTCAGCGGAACTGACAAAATGACACACAAGT
TACGTGTTTACCTACGAACAACAACAAAATATTTGGTACACGAAATTCATTCATTAAAAACAAAGaaaca
aagagaatgctatagtcgagttccccgactatcaggtactatcaggaagatgggcgaagggcgaaattct
ataattttctggcataccgatagatattggcaaataagatgagaataaatttaaaaaaaaaatgttaaaaa
aaacataggataaagggcacagtatttttggtataccgataaaaattggcaggacaaacaacaaaacaaa
ggaaaatcaaaacatttgagcgtttggcaaatcaaacaaaatttaaaagactaataaaaaagtgaaaaaa
ttttaacacatttttttaaagtatgggcgtggcagtttttatcgctttgtgggtgttagagtgggcgtggc
aacataagtaaacaaaattgcgtctttgtctctggaatctgtatgcttaatctcaacctttaagcttttg
cagttcctgagatctagacgctattgatgctgatcaaaaaaatatatattttatatggtcgaaaacgttt
tcttctgcctgttacatagatttaaacgaatctagtatacgcttttagtctacgaataacTTAATTTGTT

TGCGACTTGTGTTACGTAAGAAGCGGTGTGGTCAAACTTTTGATAACTTTAGAAGACTAATATAAAAAAT
ATTAAATAATTTTACAAAGTGCAAAATTtggggaaaaaaacttttgctgcgtctatgtctctggaatctg
cactcgtttcaacttattatcttttatagtttctaaaatcttgacggacaggcggacgtacatggccaga
ttgactcggcttttgatcctgatcaacaaaatagatagatgtatatattgtagggtcggaaacgctacct
tctacctgtaacatacttcttaacgaacctggtaACGGCTATAAAAAACTTCTTAGTAAATACATACTAA
ATTTGAAGTAATTTTAAGAAGCTTCTGATAAGCTGATTATTATAAGTGCTTaaaaaaaaGTCGATTTAGC
GGATTTCTATGGCAGTCTAGTAACTAAAAATTTGTAGCTACATACATTATTTAGCTTAATTAttttcttt
ttttttagtttaaatagttaattaactatattatattattaaaatatattttttttcagaaatattaAGTT
CGTTTAATATATAAAAATCAATAACCTTCATCGTGAAAAATTTTTAATAGGGTTTGGTTTTCAAACCGAC
GAGCTAAATGGCGCCGAGAAGAAAAAATGCGAACTCAGAGACGATCGGCCGATACCGTGGACGGCAGTGG
TCGAACCAGCACGGCAAATAATCCTTCAGGAACAACTGCATCTTCCTCCGTCGCAACGTCAAACAACTCG
ACTCCAGGGATTGTGAACTCAGCAATCAACGTTGCGGAACGAACATCATCTGCATTAGTTAGTAATAGCC
TTCCCGAGGCTTCAAATGGACCAACTGGTTTTGGGTGGTGAAGCTAATACTACACACACCAGCTCTGAAA
GCCCACCCCTTCAGCCAGCGGCACCGCGGCTACCCTTAAATTCTGGATTCAACACCATGTACTCATCTAT
TCCACAACCGATTGCAACGATGGCTGAAAATTACAA
>dme_circ_0008820_junction_seq
CGATTGCAACGATGGCTGAAAATTACAAGGTTTCATCTATTAATCGAGTCTTACGT
>dme_circ_0008821
GAATTACAATTTTCAGTAGAAGTTTCGCATACAAACTCTCACGATAGTACATCGGATGGAAACTCTGAAC
ATAATTCATCCGGGGACGAAGACTCTCAAATGCGGTTGCGCCTAAAAAGGAAGTTACAGCGCAATCGGAC
ATCATTTTCTAATGAGCAAATTGACAGTCTTGAAAAAGGGTAACAAAGTGTTGTATTTTAACTAGGTCTT
AATAAATTACCTCACCACATAACACTTCATTATACTAATATAACTAATATATTTTATAAAAAAATACCAT
TAGTTTTAGAAATAAAGAGCTAAGGAAAAAAATACCTGTATTGGTAATAAAAGTATATCGATTTTCAAAA
ATCGTCAAATTCTGTGAATGAATTTCGAGCGAATATTTAAAACAACAATTACAACGGATTTTGAGAGGAA
GTGTTCTAAAACTAATTTTCGCTTGACAAACTCGACACTCGATTTTGCTGATAGACCTTTAAATCTACGG
TTTACATTTAGATTAAAAATGTTTCATGATGAGTCCACATATATTCTGGTTAACATATATATACATGTAT
TAAAAGAATGGTTTTATGGTACACAAATATCGTTTTGTTTAGTGCCGCGACGTAGAGAAACGCCATATAA
AAACTCTTTATAACTGTATTTTCATATTCTCGATACTATcgataatcagcgataataataattataataa
ttgataataaatGTACATATGTACATATATATACACTAAATATGTAGGGTCAGTCAATTGCGTAACAAAC
TGAAAAACTTAATTTCATTTTTTAATTGCTCGCATTTTTGCTGTTTATAACGTCAAAATATATTCGTTTT
ACAGTAAAACATACCATAAGGTCTATTTTAGCATTAATCACTAATTTATATCTGAACTTTTATCGACGTG
CATTACAGAATTTGAAAGAACACATTATCCCGATGTTTTTGCGCGAGAAAGGCTTGCTGATAAAATTGGT
TTGCCAGAGGCACGTATTCAG
>dme_circ_0008821_junction_seq
AATTGGTTTGCCAGAGGCACGTATTCAGGAATTACAATTTTCAGTAGAAGTTTCGC
>dme_circ_0008822
gtttctaaaatcttgacggacaggcggacgtacatggccagattgactcggcttttgatcctgatcaaca
aaatagatagatgtatatattgtagggtcggaaacgctaccttctacctgtaacatacttcttaacgaac
ctggtaACGGCTATAAAAAACTTCTTAGTAAATACATACTAAATTTGAAGTAATTTTAAGAAGCTTCTGA
TAAGCTGATTATTATAAGTGCTTaaaaaaaaGTCGATTTAGCGGATTTCTATGGCAGTCTAGTAACTAAA
AATTTGTAGCTACATACATTATTTAGCTTAATTAttttcttttttttttagtttaaatagttaattaacta
tattatattattaaaatatattttttttcagaaatattaAGTTCGTTTAATATATAAAAATCAATAACCTT
CATCGTGAAAAATTTTTAATAGGTTTGGTTTTCAAACCGACGAGCTAAATGGCGCCGAGAAGAAAAAATG
CGAACTCAGAGACGATCGGCCGATACCGTGGACGGCAGTGGTCGAACCAGCACGGCAAATAATCCTTCAG

GAACAACTGCATCTTCCTCCGTCGCAACGTCAAACAACTCGACTCCAGGGATTGTGAACTCAGCAATCAA
CGTTGCGGAACGAACATCATCTGCATTAGTTAGTAATAGCCTTCCCGAGGCTTCAAATGGACCAACTGGT
TTTGGGTGGTGAAGCTAATACTACACACACCAGCTCTGAAAGCCCACCCCTTCAGCCAGCGGCACCGCGG
CTACCCTTAAATTCTGGATTCAACACCATGTACTCATCTATTCCACAACCGATTGCAACGATGGCTGAAA
ATTACAA
>dme_circ_0008822_junction_seq
CGATTGCAACGATGGCTGAAAATTACAAgtttctaaaatcttgacggacaggcgga
>dme_circ_0008823
GGTTTGGTTTTCAAACCGACGAGCTAAATGGCGCCGAGAAGAAAAAATGCGAACTCAGAGACGATCGGCC
GATACCGTGGACGGCAGTGGTCGAACCAGCACGGCAAATAATCCTTCAGGAACAACTGCATCTTCCTCCG
TCGCAACGTCAAACAACTCGACTCCAGGGATTGTGAACTCAGCAATCAACGTTGCGGAACGAACATCATC
TGCATTAGTTAGTAATAGCCTTCCCGAGGCTTCAAATGGACCAACTGGTTTTGGGTGGTGAAGCTAATAC
TACACACACCAGCTCTGAAAGCCCACCCCTTCAGCCAGCGGCACCGCGGCTACCCTTAAATTCTGGATTC
AACACCATGTACTCATCTATTCCACAACCGATTGCAACGATGGCTGAAAATTACAA
>dme_circ_0008823_junction_seq
CGATTGCAACGATGGCTGAAAATTACAAGGTTTGGTTTTCAAACCGACGAGCTAAA
>dme_circ_0008824
aaaggaagcgttatcgacATTGGGGAGTATGTATATTCTTGGATATTTCTGTATTTTCCTTCGTCTGTCT
TATTCAATTCAAATGACAACCTTTTTACGACTCCCACCTTAAAAAACCAATAAAACAACAAAAACTTTTT
ATATTTTTTAATTGGTTTATTATTTGTCCTGCCATG
>dme_circ_0008824_junction_seq
TAATTGGTTTATTATTTGTCCTGCCATGaaaggaagcgttatcgacATTGGGGAGT
>dme_circ_0008825
GAGCAAAGAATTAATTGTGTTCTATTATCTGTATTATTCTGTATTATTATATCTGTAAAAATATATTTTG
ATATTGATTTCAGGCTATATCTAATAACATGTAATAAAAATGAAATGTATTATACTTTGATGGAATTTTT
CATTGAACCTATAATTGTATCGAAAACTTTCTCACATTATATTCCTTTGGGGGCTCTTTAAAAAAAGTGA
GAAAAATTAATATCGAATCCTGACAATGTGTTATATTGTATATAGGTTCTGCGTAG
>dme_circ_0008825_junction_seq
TGTTATATTGTATATAGGTTCTGCGTAGGAGCAAAGAATTAATTGTGTTCTATTAT
>dme_circ_0008826
GAATCCATAAAAGTTTGAGCTACTACTGACAGGCAGGAATCCACTATGTTCGATTTATGGATGTCAAAGA
CAAAATTTGGATTTTTAATTAAGTTCACCCAAAACCTACGAATACAATAATATTCTTAACAAAAATTTGT
CTAAGGAAATATTTATACTAACCTCAATGGTAAGCTGTTACTTTTCCAAGTATGCACAACTTCTGGATCA
GTAATACCGTGCAATTGAGCTTGATCGTCAAGAAAATCAAACATGTACTTTATAGCTAATGGAAGTGCCG
ATCCACGGTGAGCGGTGCTAAATATCGTTTCAAAGAGATCATCTACAAATTTCTGAAGCGTTCCTTTAGT
AGCCAATAGCCTGGTTAAGTAAATCTCTGACACTAATTTGTTAACCCGTTCCCCTTCTTTTTGCATATCA
CTATCATGATGTTTTACTAGATGCCA
>dme_circ_0008826_junction_seq
CACTATCATGATGTTTTACTAGATGCCAGAATCCATAAAAGTTTGAGCTACTACTG
>dme_circ_0008827
TTCCTGATGCTGTTGTGGCTAATGATGCCAACTCCTCAACCGTAGCATTTATTGCGCCGGGACCTCCACA
ACATCCGGTGACTAATGTGATGTACGTCGGAGTTACATACACAAATAATTCACCGTACCGCAGTGAAATT
CCCGCAGTTGCATCCCGCTCCCTCGAAAAGACTAAAATGTTTCAGATTGCTTCCTCGGCAGTTACAACTG
GAACGAGAACTTTTATAAACTCATACGCACGTGAAACATATTTTGTCAACTATGTTTATGGATTCAGTTC

TGAGCGATTTTCTTACTTTCTAACAACACAGTTAAAGCACAGTCACCATTCTTCTCCTAAAGAGTACATA
ACGAAACTCGTACGAATATGTCAGGAAGACTCGAACTATTATTCTTACACTGAAATTCCAGTGGAATGTA
TAAGTGATGCTCAAGGCGGCACTAAATTCAACTTGGTTCAGGCTGGATTCTTGGGAAAACCTAGCTCAGA
CCTGGCTCAAAGCTTAGGTATATCTATTCAAGATGATGTCCTCTTTGCTGTTTTTTCAAAAGGAGAAGGG
AATACACCAACGAACAATTCTGCACTCTGCATTTACTCATTAAAATCTATTCGTCGAAAGTTTATGCAAA
ACATAAAATCCTGTTTTAACGGAAGTGGCATGAGGGGCCTGGACTTTATATCACCCAGCATGCCATGTGT
TCTAACGGTAAGTATAGTTTTATTGACTACTTTAGCTTCAAAGTAAAATCCTTTTAAAAAGTAATGTCTT
ATTGTAAAGTGCTCAGTGTTTACTTAATTATTTCTAAGCCCTTTAAAGTTAATTCCCACGTTTCGTCAAA
Ttatatatatatatttttatatatatatatatacatgtattatatatatttatatttatatttatattta
tatttatatttatatttatatttatatttatatttatatttatatttatatttatatttatatttatatt
tatatttatatttatatttatatttatatttatatttatatttatatttatatttatatttatatttata
tttatatttatatttatatttatatttatatttatatttatatttatatttatatttatatttatattta
tatttatatttatatttatatttatatttatatttatatttatatttatatttatatttatatttatatt
tatatttatatttatatttatatttatatttatatttatatttatatttatatttatatttatatttata
tttatatttatatttatatttatatttatatttatatttatatttatatttatatttatatttatattta
tatttatatttatatttatatttatatttatatttatatttatatttatatttatatttatatttatatt
tatatttatatttatatttatatttatattttattttattttattttattttattttattttattttta
tttttattttattttattttattttatttatatttatatttatatttatatttatatatatatatttta
tatttatatttatatttatatttatatttatatttatatatatatttatactggaaatattattttatgT
gatacactagattcgttgcaaagtgtgtaacagccagaaagaagcgtttcagaccatgtaaagtatatat
ttattgatcaaattaatagacgagttgaaatggtcatttccatatgaacgcctcgatctcaggaactata
aaagccagagattaagcaggtagaatccaaagaaatagacgcagcccaagttGATTTTTTAAATGTTGCA
AAGCAATATCTTACGGAGTTTTGAGAAATGTCTGAAAAATTccgttactagtagagtaaaagggtatact
aaattcttagaaaatgtgcttccttgtgtgtgacgtgaccatataaattatataatttttgatcaggatc
aacagccgagtcgatctggaccgtgtgtccgtccgtatgaacgtcgtggtcttaaaaactataatcggac
aaaactgacacgttaaaaaattgttttgatgtttgtattagtcttgtgaattttgtatcgaacgccaaaa
aattgtccaaaactgtcacgcccacacttttaaaaaaatggttttcccaatttttacagatatcccagag
aatattgaaatctttcacactttcagctaagtaacgagtatctgatagtcggggaacccgactagcattt
tctcttgtttttttttatgaagagtttttttttagtagagtaaaagggtatactaaattcttagaaaatg
tgcttccttgtgtgtgacgtgaccatataaattatataatttttgatcaggatcaacagccgagtcgatc
tggaccgtgtgtccgtccgtatgaacgtcgtggtcttaaaaactataatcggacaaaactgacacgttaa
aaaattgttttgatgtttgtattagtcttgtgaattttgtatcgaacgccaaaaaattgtccaaaactgt
cacgcccacacttttaaaaaaatggttttcccaatttttacagatatcccagagaatattgaaatctttc
acactttcagctaagtaacgagtatctgatagtcggggaacccgactagcattttctcttgttttttttt
atgaagaCATCCAGTTTTAGAATTAGCATTCGTGGAAAAGGCAATTTAAAATAATTTATTCTCGCATATA
ATGAGCTAAAAATATTATTTAAAGGTACTTCTTTATCTATAGGCGTGGCTATTCGTTATTCGTATGCCAA
TATGACTGTCCTTTTACTTCAGCTATTATGCTCTTCGGAATCTCATATCCTAAGAATTATTTTATCTTAG
CAAGAAAATGAAATATCAGTTGCTTATTGGTAATTTTTTTGGATGCTGACAAATTGATTTCAGAGTAAGT
ATAGTTTTATTGACTACTTTAGCTTCAAAGTAAAATCCTTTTAAAAAGTAATGTCTTATTGTAAAGTGCT
CAGTGTTTACTTAATTATTTCTAAGCCCTTTAAAGTTAATTCCCACGTTTCGTCAAATtatatatata
tttttatatatatatatatacatgtattatatatatttatatttatatttatatttatatttatatttat
atttatatttatatttatatttatatttatatttatatttatatttatatttatatttatatttatattt
atatttatatttatatttatatttatatttatatttatatttatatttatatttatatttatatttatat
ttatatttatatttatatttatatttatatttatatttatatttatatttatatttatatttatatttat

atttatatttatatttatatttatatttatatttatatttatatttatatttatatttatatttatattt
atatttatatttatatttatatttatatttatatttatatttatatttatatttatatttatatttatat
ttatatttatatttatatttatatttatatttatatttatatttatatttatatttatatttatatttat
atttatatttatatttatatttatatttatatttatatttatatttatatttatatttatatttatattt
atatttatatttatattttttattttttattttttattttttattttttattttttattttttattttttat
ttttattttttattttttatttatatttatatttatatttatatttatatatatatttatatttatatttat
atttatatttatatttatatttatatatatatttatactggaaatattattttatgTgatacactagatt
cgttgcaaagtgtgtaacagccagaaagaagcgtttcagaccatgtaaagtatatatttattgatcaaat
taatagacgagttgaaatggtcatttccatatgaacgcctcgatctcaggaactataaaagccagagatt
aagcaggtagaatccaaagaaatagacgcagcccaagttGATTTTTTAAATGTTGCAAAGCAATATCTTA
CGGAGTTTTGAGAAATGTCTGAAAAATTccgttactagtagagtaaaagggtatactaaattcttagaaa
atgtgcttccttgtgtgtgacgtgaccatataaattatataatttttgatcaggatcaacagccgagtcg
atctggaccgtgtgtccgtccgtatgaacgtcgtggtcttaaaaactataatcggacaaaactgacacgt
taaaaaattgttttgatgtttgtattagtcttgtgaattttgtatcgaacgccaaaaaattgtccaaaac
tgtcacgcccacacttttaaaaaaaatggttttcccaatttttacagatatcccagagaatattgaaatct
ttcacactttcagctaagtaacgagtatctgatagtcggggaacccgactagcattttctcttgtttttt
tttatgaagagtttttttttagtagagtaaaagggtatactaaattcttagaaaatgtgcttccttgtgt
gtgacgtgaccatataaattatataatttttgatcaggatcaacagccgagtcgatctggaccgtgtgtc
cgtccgtatgaacgtcgtggtcttaaaaactataatcggacaaaactgacacgttaaaaaattgttttga
tgtttgtattagtcttgtgaattttgtatcgaacgccaaaaaattgtccaaaactgtcacgcccacactt
ttaaaaaaaatggttttcccaatttttacagatatcccagagaatattgaaatctttcacactttcagcta
agtaacgagtatctgatagtcggggaacccgactagcattttctcttgtttttttttatgaagaCATCCA
GTTTTAGAATTAGCATTCGTGGAAAAGGCAATTTAAAATAATTTATTCTCGCATATAATGAGCTAAAAAT
ATTATTTAAAGGTACTTCTTTATCTATAGGCGTGGCTATTCGTTATTCGTATGCCAATATGACTGTCCTT
TTACTTCAGCTATTATGCTCTTCGGAATCTCATATCCTAAGAATTATTTTATCTTAGCAAGAAAATGAAA
TATCAGTTGCTTATTGGTAATTTTTTTGGATGCTGACAAATTGATTTCAGAAACTGCAAACCATTGGAGA
GGACTTCTGTGGATTAGATGTAAACTCACCTTTAGGAGGAGAAACCCCCATTACTTCAGTACCGGTGGCC
ATGTTTAACACAAAGTTAACTTCTGTTGCTGCTACGAGTACAAGCGGGTATACCGTTGTCTTCGTGGGGA
CTAGCGATGGATTTCTTAAGAAAGTTGTAATCGAGTCTTCATCTATTGCCAACGAGTATGCTAGTTTTGC
AGTAGATTTAGGGTCTGAAATTAATCGGGATATGCAGTTTGACAACCAAAACCTTTATATATATGTTATG
TCCAAAACAAAAGTATCCAAGGTTAAAGTATTTGATTGCTCTGATTACAAGACATGTGGAGATTGCTTAG
GTGCTAGAGATCCATATTGTGGCTGGTGCTCATTGGAAAATAAATGTAGCCCACGCTCCAACTGCCAAGA
CGACGCAAACGATCCTCTCTATTGGGTTAGCTATAAAACAGGGAAATGTACAACAATAACAAGTGTTGTG
CCACACCAGCTACAACGTACTACAGCTCGAACCTTAGAGCTAATCATAGATCATTTGCCTCAGCTGAAAG
AGAATTTAATATGTGCTTTTACTACAGAGGATAAAGCCCTTTTCACTAATGCTACAAAAAAACGAAATGG
CGTAAACTGCACGACACCACGAACGGATATGCTACCCCAAATTGAACAAGGGAAACATCACTTTACTGCC
AAACTATCTGTTCGCACCAGAAATGGACCCGACTTAGTGTCCACGGATTTTACTTTCTTTGATTGCAGTA
CGCACTCTTCTTGTACACGTTGTGTGTCCTCGGAGTTTCCATGTGATTGGTGTGTTGAAGCTCACAGGTG
CACACACGACACAGCTGAAAACTGCCGGAACGATATTTTGGTAACAGGTGTTAGCCGGATAGGTCCTAGT
TACAGATCAGGTCCCGGATTTTGTCCAACGATCAACGCTACAGGTGATGGAAGTGAAGTACTTGTCGCTG
GTGGTACAAGCAAATCCATTAAAGTAAAAGTTCATATTATTGGGCAATTTATAGTTCAAACGCGGTTTGT
TTGTCAATTTAATATTGAGGGACGTGTAACTAGTTTAAACGCTCAGTTGTTGGGTGACACCATTTACTGC
GATAGCATGGAATTTCAGTATACTTCTAGATCGCCCAATTTGACAGCAACTTTCGCTGTAATATGGGGCG
GGTCGAAGCCACTTGACAATCCTCATAATATACATGGTGAGTTGTATTTAGAATAAGATTTTTAAAATTT

TACTCTTAAAATTTTATGTTTTGCAGTTGTTATATATCGATGTCGAGAAATGGCCGACAGCTGTGGTATA
TGCTTGGCCCTATCTGAAAAGTACAATTGCGGATGGTGCTCGTCTACTAATACATGCGAAGTTGAAGAAC
AATGTAATAAAAACAAAGAGGGGAAAACTGATTGGTTAAATCGAAGTGAAATTTGCCCAAACCCTGAAAT
TCATACATTCCAACCAAAAACAGGTCCCTGGGAAGGAGGAACAAATATAACAATACGAGGAATTAATTTA
GGCAAAAACTATAACGATATTTATTCAGGAGTTCGAATAGCTGGTATAAATTGTATGCCGTTTCCTCAAT
TTTATATTGACACTAAGCAAATAGTATGTACCGTGGATAGTCCTGGAGAACAGATGTATAGAAATGGCAA
AATAGTGGTACAAATTGGGGACTACCGTGGTGAGTCCAAAGAGGACTACGAGTTCGTTGACCCGAAAATT
TTGGATTTTAATCCAAAGTTTGGTCCAACTTCAGGTGGAACCGAAATACATATAACTGGAAAGCACTTAA
ATGCCGGCTCTCGTATACAGGCATCCATAAACGATCACTTACCGTGTAAAATACTAAGCACAGATTCTTC
TCAAGCAATATGTCGCACTTCTGCTTCCCCTGGTATTATTGAAGGAAGACTAAAAATGTCTTTTGATAAT
GGGCCCCGAGAGTTTAATGACTACAATTTTAAATATGTACTGGATCCTACAGTTGAACACGTTAGTTCTG
GACCAAGCGGGCAAATAAAAGTTCCAAAAGGAATACCAGCAGGCGGTATACGAATTTCTGTAACGGGTAC
CCAATTTACCAGTATACAAAATCCCAATATTTATGTAGTGTATAATGGAGAGATGTATGCAAGCCCATGT
CGGGTGCAATCGGATACGGAAATGGAATGTGCATCTCCAGTTGTTGATGTGGACAGCCATGTTATTGAAG
CGGAAAGGCCAATTCTTCTTGAATATGGGTTCCTAATGGACAATGTTTTGCGTGTTCAAAATTTATCTAA
GGTTCATAACAACCATTTCGAGCTTTACCCAAATCCAGAGTATTTTATATTCGAAGAAAGAGTTAAGTAC
TTTAAGAGTGAATACTTAACCATAAATGGCCGAAATTTGGATCGCGCTTGCAAAGAAAGTGATGTAGAAG
TAAAGATAGGAAATGGATTTTGCAATATTACATCACTTTCAAGACAACAACTAACATGTAGACCTCCTTC
TGAAGCAACAGCAACCAAAAGCATGAATGGACCTGAAGTAATTGTTCGTATTGGAACTTCGCTAGAGTAT
CGTATCGGTATACTTAGCTACGAATCGTCTAACATCATTTTGGATTGGGGAGAAAATGTAATTTTTGCTG
TAATAGCCACAATTGTTATCTTGCTGCTGATTTTTGTTGCTTTGCTTGTGGCTTATAAAAAGAAAAGTTC
GGAATCGAGCCGAGTTCTTCGAAACATGCAGGAGCAGATGGACATTCTGGAGCTACGAGTCGCAGCTGAA
TGTAAAGAGGCCTTTGCCGAACTCCAAACTGAAATGACTGATCTCACAGGCGATCTTACTTCTGGAGGCA
TTCCATTTTTGGATTACCGGTCATATGCAATGAAGATTCTATTTCCAAACCATGAAGACCACATTGTACT
ACAATGGGAACGTCCTGAGCTTCTTCGGAAAGAAAAAGGCTTACGTATTTTTGGCCAACTAATCATGAAT
AAAACTTTCCTCCTGCTTTTTATACGAACCCTGGAGTCCAACCGATACTTCTCAATGCGAGAACGAGTTA
ATGTGGCCTCCTTAATAATGGTCACTCTGCAATCTAAACTAGAATATTGCACAGATATATTAAAAACTTT
ATTGGGAGACTTGATCGAAAAATGTATAGAAGGAAAGAGTCATCCAAAGCTCTTACTACGACGAACTGAA
AGTGTGGCAGAAAAAATGTTAAGTGCTTGGTTTACATTCCTTCTTTACAAGTTTTTGAAGGAATGTGCTG
GCGAGCCTTTATATATGCTTTTCCGAGCTGTGAAAGGACAAGTAGACAAGGGTCCTGTTGACGCTTGTAC
ACATGAGGCAAGATACTCCCTTAGTGAGGAAAAACTTATACGACAGTCTATTGATTTTCGACCAATGAAC
GTTTATGCAAGCATTATACAGCAGCCAATATTTTGCAATAACTTAGATATGTTACCTTCTCACACCGAAA
ATGTATCTGTTAAAGTATTGGATTGCGACACAATTGGACAAGTGAAAGAAAAATGCTTGGAAACCATTTA
TAGAAACATTCCATCAAGCCAAAGACCTCGAAAGGATGACTTAGATTTAGGTAAGTAAAGCTAGACTTAA
TAGTAACCCAAAGTCGTAAAAATATGATTTGTCCACTCAAATGTTTGTCCGGCCAAATGAACGTAAGGAT
TGGATAATAAGCTTGATGATGGTCATACTGATGTATTCTGATGTATTCTTAGGAAGTTTTGTATCATTTT
TTGACCAAGCCAACACAAAACGGTTTTGTAACTAAATTAAAATTAAATTGTATACAAAACAGCCTCGTGA
GCAGTGAATCCGTACATATTGAAAGGCGGCGGTGGAGAATAATTTGCTTTAATAAAAATCATTGTAATTT
TTTGTATGTAGTGTCCGTGATTTGATGAATTTGTGAATGACATTGCAGTAACCACTATTCAATCAAATTG
GACCGCAAACCATTTGAATTTAAATTTTAAATAAAATAGAAAGCGATGATTTTTCAATGTGTTCGATTAA
CTGTATTGATATAACAATGTGTGTTAAACTTTCCAAACTTTCTTAATAAATATATATATGGTTTTTCGAA
GTTAGATACTTATCTTGTGCTTCCACTAAGGTGTCTCAAATTCCATAAAAACTAAAAAGGCTGACAAGCT
GTAAGTAAACTTATTGTTTTATTTTTATAAATAGAAGCCTTTTTTGTTCACACTATATATGTAAAGGTCA
GGTATCTCAACAAGTTTGATTCGTTAATATACAGAAACAGAATATTGCtttttttttATCTATGAAGGGT

TTTCATTTGACTTTTCATAATAATTTCTGTTTTAGAAGTGGAGAACAGGCGCTACTGGGCGAGTAATTCT
GTACGATGAGGACGTAACATCAAAAACGGAGAGCGAATGGAAAAAGCTCAACACTTTGCAGCACTATAAT
GTTCCGGATGGAGCCGGGCTAAGCTTAGTACCAAAGCAAAGTTCCATTTATAACTTCAGTATTTTATCGG
ACAAAAATGAAAAATCGCACAAGGTATGAAACTCTTAATATATCGAAATACACCTCCTCCTCTCCGACAT
TTAGCCGCGCAGGAAGTCCTCTTAACAATGATATGCATGAAAATGGTCTGAGATACTGGCATCTAGTAAA
ACATCATGATAGTGATATGCAAAAAGAAGGGGAACGGGTTAACAAATTAGTGTCAGAGATTTACTTAACC
AGGCTATTGGCTACTAAAGGAACGCTTCAGAAATTTGTAGATGATCTCTTTGAAACGATATTTAGCACCG
CTCACCGTGGATCGGCACTTCCATTAGCTATAAAGTACATG
>dme_circ_0008827_junction_seq
GGCACTTCCATTAGCTATAAAGTACATGTTCCTGATGCTGTTGTGGCTAATGATGC
>dme_circ_0008828
GTTTTATTCATGATTAGTTGGCCAAAAATACGTAAGCCTTTTTCTTTCCGAAGAAGCTCAGGACGTTCCC
ATTGTAGTACAATGTGGTCTTCATGGTTTGGAAATAGAATCTTCATTGCATATGACCGGTAATCCAAAAA
TGGAATGCCTCCAGAAGTAAGATCGCCTGTGAGATCAGTCATTTCAGTTTGGAGTTCGGCAAAGGCCTCT
TTACATTCAGCTGCGACTCGTAGCTCCAGAATGTCCATCTGCTCCTGCATGTTTCGAAGAACTCGGCTCG
ATTCCGAACTTTTCTTTTTATAAGCCACAAGCAAAGCAACAAAAATCAGCAGCAAGATAACAATTGTGGC
TATTACAGCAAAAATTACATTTTCTCCCCAATCCAAAATGATGTTAGACGATTCGTAGCTAAGTATACCG
ATACGATACTCTAGCGAAGTTCCAATACGAACAATTACTTCAGGTCCATTCATGCTTTTGGTTGCTGTTG
CTTCAGAAGGAGGTCTACATGTTAGTTGTTGTCTTGAAAGTGATGTAATATTGCAAAATCCATTTCCTAT
CTTTACTTCTACATCACTTTCTTTGCAAGCGCGATCCAAATTTCGGCCATTTATG
>dme_circ_0008828_junction_seq
AGCGCGATCCAAATTTCGGCCATTTATGGTTTTATTCATGATTAGTTGGCCAAAAA
>dme_circ_0008829
GCAAAAATTACATTTTCTCCCCAATCCAAAATGATGTTAGACGATTCGTAGCTAAGTATACCGATACGAT
ACTCTAGCGAAGTTCCAATACGAACAATTACTTCAGGTCCATTCATGCTTTTGGTTGCTGTTGCTTCAGA
AGGAGGTCTACATGTTAGTTGTTGTCTTGAAAGTGAT
>dme_circ_0008829_junction_seq
ACATGTTAGTTGTTGTCTTGAAAGTGATGCAAAAATTACATTTTCTCCCCAATCCA
>dme_circ_0008830
ACAACAACTAACATGTAGACCTCCTTCTGAAGCAACAGCAACCAAAAGCATGAATGGACCTGAAGTAATT
GTTCGTATTGGAACTTCGCTAGAGTATCGTATCGGTATACTTAGCTACGAATCGTCTAACATCATTTTGG
ATTGGGGAGAAAATG
>dme_circ_0008830_junction_seq
AACATCATTTTGGATTGGGGAGAAAATGACAACAACTAACATGTAGACCTCCTTCT
>dme_circ_0008831
GGATCCAGTACATATTTAAAATTGTAGTCATTAAACTCTCGGGGCCCATTATCAAAAGACATTTTTAGTC
TTCCTTCAATAATACCAGGGGAAGCAGAAGTGCGACATATTGCTTGAGAAGAATCTGTGCTTAGTATTTT
ACACGGTAAGTGATCGTTTATGGATGCCT
>dme_circ_0008831_junction_seq
CACGGTAAGTGATCGTTTATGGATGCCTGGATCCAGTACATATTTAAAATTGTAGT
>dme_circ_0008832
CTATAAAACAGGGAAATGTACAACAATAACAAGTGTTGTGCCACACCAGCTACAACGTACTACAGCTCGA
ACCTTAGAGCTAATCATAGATCATTTGCCTCAGCTGAAAGAGAATTTAATATGTGCTTTTACTACAGAGG
ATAAAGCCCTTTTCACTAATGCTACAAAAAAACGAAATGGCGTAAACTGCACGACACCACGAACGGATAT

GCTACCCCAAATTGAACAAGGGAAACATCACTTTACTGCCAAACTATCTGTTCGCACCAGAAATGGACCC
GACTTAGTGTCCACGGATTTTACTTTCTTTGATTGCAGTACGCACTCTTCTTGTACACGTTGTGTGTCCT
CGGAGTTTCCATGTGATTGGTGTGTTGAAGCTCACAGGTGCACACACGACACAGCTGAAAACTGCCGGAA
CGATATTTTGGTAACAGGTGTTAGCCGGATAGGTCCTAGTTACAGATCAGGTCCCGGATTTTGTCCAACG
ATCAACGCTACAGGTGATGGAAGTGAAGTACTTGTCGCTGGTGGTACAAGCAAATCCATTAAAGTAAAAG
>dme_circ_0008832_junction_seq
GGTACAAGCAAATCCATTAAAGTAAAAGCTATAAAACAGGGAAATGTACAACAATA
>dme_circ_0008833
GTTTACATGATGGTCATACAAGTTATTGCTCCAAGTTAAATTATCTTGAATGGAACGGATAATAAAAATT
GCAAGAATGACACCAACGCAAAGGAAATTTTAATATTTACGTAAAATTTCAAATAAATAAGGGACATACA
AGCGCCTACAAGTAACAATTTCATAAATAAGGTAGCTTTGATAGTCCTGTTTAAGGAGCGATTGTGGAGA
TGAATTTTGTTCTTAGTGTGTTGTTAATAGCAATTCTTAAAGATAAGTATGATTACTGGCTGCCATCGAG
GACAGGTTGTCAAACCCTGGCAGCAAATATATGGTCTGACAAGACGACTGCGCAAAATGAGCCGATAAAT
TTGACTAATGCAAATGCACCGATAAAGAATGCAAAGAATCTGAATTCTACGATTACAAATGTAGCTGCGT
TTGACACCAAATTGAATCACCTATTAGTGGACACCATTACTGGACGAGTCTTCGTTGGTGGTGTTAACAG
GTTGTATCAGTTGTCGCCTGATTTGGAGCTTTCCGAAACTGTGAAAACGGGGCCTCAAAATGATTCCGTC
GAGTGTAGCATTCTTGATTGTCCATTAAATGCAGTTCGCAGTCCGACGGACAATTATAATAAGGTCTTGC
TCATTGATCGTGCAACTTCAAGACTTATTGCGTGTGGATCACTATTTCAAGGTACATGCACAGTTCGTAA
TCTTCAAAATGTCAGCATAATTGAGCATGAAGTTCCTGATGCTGTTGTGGCTAATGATGCCAACTCCTCA
ACCGTAGCATTTATTGCGCCGGGACCTCCACAACATCCGGTGACTAATGTGATGTACGTCGGAGTTACAT
ACACAAATAATTCACCGTACCGCAGTGAAATTCCCGCAGTTGCATCCCGCTCCCTCGAAAAGACTAAAAT
GTTTCAGATTGCTTCCTCGGCAGTTACAACTGGAACGAGAACTTTTATAAACTCATACGCACGTGAAACA
TATTTTGTCAACTATGTTTATGGATTCAGTTCTGAGCGATTTTCTTACTTTCTAACAACACAGTTAAAGC
ACAGTCACCATTCTTCTCCTAAAGAGTACATAACGAAACTCGTACGAATATGTCAGGAAGACTCGAACTA
TTATTCTTACACTGAAATTCCAGTGGAATGTATAAGTGATGCTCAAGGCGGCACTAAATTCAACTTGGTT
CAGGCTGGATTCTTGGGAAAACCTAGCTCAGACCTGGCTCAAAGCTTAGGTATATCTATTCAAGATGATG
TCCTCTTTGCTGTTTTTTCAAAAGGAGAAGGGAATACACCAACGAACAATTCTGCACTCTGCATTTACTC
ATTAAAATCTATTCGTCGAAAGTTTATGCAAAACATAAAATCCTGTTTTAACGGAAGTGGCATGAGGGGC
CTGGACTTTATATCACCCAGCATGCCATGTGTTCTAACGG
>dme_circ_0008833_junction_seq
TCACCCAGCATGCCATGTGTTCTAACGGGTTTACATGATGGTCATACAAGTTATTG
>dme_circ_0008834
GAACACATGGCATGCTGGGTGATATAAAGTCCAGGCCCCTCATGCCACTTCCGTTAAAACAGGATTTTAT
GTTTTGCATAAACTTTCGACGAATAGATTTTAATGAGTAAATGCAGAGTGCAGAATTGTTCGTTGGTGTA
TTCCCTTCTCCTTTTGAAAAAACAGCAAAGAGGACATCATCTTGAATAGATATACCTAAGCTTTGAGCCA
GGTCTGAGCTAGGTTTTCCCAAGAATCCAGCCTGAACCAAGTTGAATTTAGTGCCGCCTTGAGCATCACT
TATACATTCCACTGGAATTTCAGTGTAAGAATAATAGTTCGAGTCTTCCTGACATATTCGTACGAGTTTC
GTTAT
>dme_circ_0008834_junction_seq
CCTGACATATTCGTACGAGTTTCGTTATGAACACATGGCATGCTGGGTGATATAAA
>dme_circ_0008835
GAACACATGGCATGCTGGGTGATATAAAGTCCAGGCCCCTCATGCCACTTCCGTTAAAACAGGATTTTAT
GTTTTGCATAAACTTTCGACGAATAGATTTTAATGAGTAAATGCAGAGTGCAGAATTGTTCGTTGGTGTA
TTCCCTTCTCCTTTTGAAAAAACAGCAAAGAGGACATCATCTTGAATAGATATACCTAAGCTTTGAGCCA

GGTCTGAGCTAGGTTTTCCCAAGAATCCAGCCTGAACCAAGTTGAATTTAGTGCCGCCTTGAGCATCACT
TATACATTCCACTGGAATTTCAGTGTAAGAATAATAGTTCGAGTCTTCCTGACATATTCGTACGAGTTTC
GTTATGTACTCTTTAGGAGAAGAATGGTGACTGTGCTTTAACTGTGTTGTTAGAAAGTAAGAAAATCGCT
CAGAACTGAATCCATAAACATAGTTGACAAAATATGTTTCACGTGCGTATGAGTTTATAAAAGTTCTCGT
TCCAGTTGTAACTGCCGAGGAAGCAATCTGAAACATTTTAGTCTTTTCGAGGGAGCGGGATGCAACTGCG
GGAATTTCACTGCGGTACGGTGAATTATTTGTGTATGTAACTCCGACGTACATCACATTAGTCACCGGAT
GTTGTGGAGGTCCCGGCGCAATAAATGCTACGGTTGAGGAGTTGGCATCATTAGCCACAACAGCATCAGG
AACTTCATGCTCAATTATGCTGACATTTTGAAGATTACGAACTGTGCAT
>dme_circ_0008835_junction_seq
GACATTTTGAAGATTACGAACTGTGCATGAACACATGGCATGCTGGGTGATATAAA
>dme_circ_0008836
GAATAATAGTTCGAGTCTTCCTGACATATTCGTACGAGTTTCGTTATGTACTCTTTAGGAGAAGAATGGT
GACTGTGCTTTAACTGTGTTGTTAGAAAGTAAGAAAATCGCTCAGAACTGAATCCATAAACATAGTTGAC
AAAATATGTTTCACGTGCGTATGA
>dme_circ_0008836_junction_seq
TGACAAAATATGTTTCACGTGCGTATGAGAATAATAGTTCGAGTCTTCCTGACATA
>dme_circ_0008837
GTACATGCACAGTTCGTAATCTTCAAAATGTCAGCATAATTGAGCATGAAGTTCCTGATGCTGTTGTGGC
TAATGATGCCAACTCCTCAACCGTAGCATTTATTGCGCCGGGACCTCCACAACATCCGGTGACTAATGTG
ATGTACGTCGGAGTTACATACACAAATAATTCACCGTACCGCAGTGAAATTCCCGCAGTTGCATCCCGCT
CCCTCGAAAAGACTAAAATGTTTCAGATTGCTTCCTCGGCAGTTACAACTGGAACGAGAACTTTTATAAA
CTCATACGCACGTGAAACATATTTTGTCAACTATGTTTATGGATTCAGTTCTGAGCGATTTTCTTACTTT
CTAACAACACAGTTAAAGCACAGTCACCATTCTTCTCCTAAAGAG
>dme_circ_0008837_junction_seq
GCACAGTCACCATTCTTCTCCTAAAGAGGTACATGCACAGTTCGTAATCTTCAAAA
>dme_circ_0008838
GCGGGATGCAACTGCGGGAATTTCACTGCGGTACGGTGAATTATTTGTGTATGTAACTCCGACGTACATC
ACATTAGTCACCGGATGTTGTGGAGGTCCCGGCGCAATAAATGCTACGGTTGAGGAGTTGGCATCATTAG
CCACAACAGCATCAGGAACTTCATGCTCAATTATGCTGACATTTTGAAGATTACGAACTGTGCAT
>dme_circ_0008838_junction_seq
GACATTTTGAAGATTACGAACTGTGCATGCGGGATGCAACTGCGGGAATTTCACTG
>dme_circ_0008839
TGTGTTGTTAATAGCAATTCTTAAAGATAAGTATGATTACTGGCTGCCATCGAGGACAGGTTGTCAAACC
CTGGCAGCAAATATATGGTCTGACAAGACGACTGCGCAAAATGAGCCGATAAATTTGACTAATGCAAATG
CACCGATAAAGAATGCAAAGAATCTGAATTCTACGATTACAAATGTAGCTGCGTTTGACACCAAATTGAA
TCACCTATTAGTGGACACCATTACTGGACGAGTCTTCGTTGGTGGTGTTAACAGGTTGTATCAGTTGTCG
CCTGATTTGGAGCTTTCCGAAACTGTGAAAACGGGGCCTCAAAATGATTCCGTCGAGTGTAGCATTCTTG
ATTGTCCATTAAATGCAGTTCGCAGTCCGACGGACAATTATAATAAGG
>dme_circ_0008839_junction_seq
CGCAGTCCGACGGACAATTATAATAAGGTGTGTTGTTAATAGCAATTCTTAAAGAT
>dme_circ_0008840
CAATTCTTAAAGATAAGTATGATTACTGGCTGCCATCGAGGACAGGTTGTCAAACCCTGGCAGCAAATAT
ATGGTCTGACAAGACGACTGCGCAAAATGAGCCGATAAATTTGACTAATGCAAATGCACCGATAAAGAAT
GCAAAGAATCTGAATTCTACGATTACAAATGTAGCTGCGTTTGACACCAAATTGAATCACCTATTAGTGG

ACACCATTACTGGACGAGTCTTCGTTGGTGGTGTTAACAGG
>dme_circ_0008840_junction_seq
ACGAGTCTTCGTTGGTGGTGTTAACAGGCAATTCTTAAAGATAAGTATGATTACTG
>dme_circ_0008841
TGTGTTGTTAATAGCAATTCTTAAAGATAAGTATGATTACTGGCTGCCATCGAGGACAGGTTGTCAAACC
CTGGCAGCAAATATATGGTCTGACAAGACGACTGCGCAAAATGAGCCGATAAATTTGACTAATGCAAATG
CACCGATAAAGAATGCAAAGAATCTGAATTCTACGATTACAAATGTAGCTGCGTTTGACACCAAATTGAA
TCACCTATTAGTGGACACCATTACTGGACGAGTCTTCGTTGGTGGTGTTAACAGG
>dme_circ_0008841_junction_seq
ACGAGTCTTCGTTGGTGGTGTTAACAGGTGTGTTGTTAATAGCAATTCTTAAAGAT
>dme_circ_0008842
GTAATCATACTTATCTTTAAGAATTGCTATTAACAACACACTAAGAACAAAATTCATCTCCACAATCGCT
CCTTAAACAGGACTATCAAAGCTACCTTATTTATGAAATTGTTACTTGTAGGCGCTTGTATGTCCCTTAT
TTATTTGAAATTTTACGTAAATATTAAAATTTCCTTTGCGTTGGTGTCATTCTTGCAATTTTTATTATCC
GTTCCATTCAAGATAATTTAACTTGGAGCAATAACTT
>dme_circ_0008842_junction_seq
AAGATAATTTAACTTGGAGCAATAACTTGTAATCATACTTATCTTTAAGAATTGCT
>dme_circ_0008843
GTTTAGCATCTGTGCGGCCGGCCAGAGCATATAGTTGCGGACCAATATAGATAGGTAGCTATCAAGAATG
CGCTGCCGGATTCGGTCGATGGGCTCTCCGTTGGAGAGTGGCACCAAAAAAGACATGGCCATGGTAAAGG
GTGGCGCAAAAAGGGTCTGGTCGACCAGCATTTTG
>dme_circ_0008843_junction_seq
AAAAAGGGTCTGGTCGACCAGCATTTTGGTTTAGCATCTGTGCGGCCGGCCAGAGC
>dme_circ_0008844
GGTGAACCCATTGATGAGCGTGGACCAATTGATACAGACAAGACCGCTGCCATTCATGCTGAGGCTCCTG
AATTCGTTCAGATGTCTGTTGAGCAAGAGATTCTTGTCACTGGAATCAAAGTCGTCGATCTCCTGGCTCC
ATACGCCAAAGGTGGTAAAATTGGGTTGTTTGGAGGTGCCGGTGTGGGCAAAACTGTGTTGATCATGGAG
CTCATTAACAACGTGGCTAAAGCTCATG
>dme_circ_0008844_junction_seq
CTCATTAACAACGTGGCTAAAGCTCATGGGTGAACCCATTGATGAGCGTGGACCAA
>dme_circ_0008845
GGTGAACCCATTGATGAGCGTGGACCAATTGATACAGACAAGACCGCTGCCATTCATGCTGAGGCTCCTG
AATTCGTTCAGATGTCTGTTGAGCAAGAGATTCTTGTCACTGGAATCAAAGTCGTCGATCTCCTGGCTCC
ATACGCCAAAGGTGGTAAAATTGGGTTGTTTGGAGGTGCCGGTGTGGGCAAAACTGTGTTGATCATGGAG
CTCATTAACAACGTGGCTAAAGCTCATGGTGGATACTCG
>dme_circ_0008845_junction_seq
CGTGGCTAAAGCTCATGGTGGATACTCGGGTGAACCCATTGATGAGCGTGGACCAA
>dme_circ_0008846
TATCCACCATGAGCTTTAGCCACGTTGTTAATGAGCTCCATGATCAACACAGTTTTGCCCACACCGGCAC
CTCCAAACAACCCAATTTTACCACCTTTGGCGTATGGAGCCAGGAGATCGACGACTTTGATTCCAGTGAC
AAGAATCTCTTGCTCAACAGACATCTGAACGAATTCAGGAGCCTCAGCATGAATGGCAGCGGTCTTGTCT
GTATCAATTGGTCCACGCTCATCAATGGG
>dme_circ_0008846_junction_seq
TATCAATTGGTCCACGCTCATCAATGGGTATCCACCATGAGCTTTAGCCACGTTGT

>dme_circ_0008847
GTCTTGTCCTTTAGTGAAATAACACCACCCTCGATCATCTCATTGTATAAATCGTTTCCCTCACGAGTGC
GTTCACCAACTCCCGCGAAAACCGAGTATCCACCATGAGCTTTAGCCACGTTGTTAATGAGCTCCATGAT
CAACACAGTTTTGCCCACACCGGCACCTCCAAACAACCCAATTTTACCACCTTTGGCG
>dme_circ_0008847_junction_seq
AAACAACCCAATTTTACCACCTTTGGCGGTCTTGTCCTTTAGTGAAATAACACCAC
>dme_circ_0008848
GGTGGTAAAATTGGGTTGTTTGGAGGTGCCGGTGTGGGCAAAACTGTGTTGATCATGGAGCTCATTAACA
ACGTGGCTAAAGCTCATGGTGGATACTCGGTTTTCGCGGGAGTTGGTGAACGCACTCGTGAGGGAAACGA
TTTATACAATGAGATGATCGAGGGTGGTGTTATTTCACTAAAGGACAAGACCTCAAAG
>dme_circ_0008848_junction_seq
TATTTCACTAAAGGACAAGACCTCAAAGGGTGGTAAAATTGGGTTGTTTGGAGGTG
>dme_circ_0008849
GGTGCCGGTGTGGGCAAAACTGTGTTGATCATGGAGCTCATTAACAACGTGGCTAAAGCTCATGGTGGAT
ACTCGGTTTTCGCGGGAGTTGGTGAACGCACTCGTGAGGGAAACGATTTATACAATGAGATGATCGAGGG
TGGTGTTATTTCACTAAAGGACAAGACCTCAAAG
>dme_circ_0008849_junction_seq
TATTTCACTAAAGGACAAGACCTCAAAGGGTGCCGGTGTGGGCAAAACTGTGTTGA
>dme_circ_0008850
GTCTTGTCCTTTAGTGAAATAACACCACCCTCGATCATCTCATTGTATAAATCGTTTCCCTCACGAGTGC
GTTCACCAACTCCCGCGAAAACCGAGTATCCACCATGAGCTTTAGCCACGTTGTTAATGAGCTCCATGAT
CAACACAG
>dme_circ_0008850_junction_seq
TTGTTAATGAGCTCCATGATCAACACAGGTCTTGTCCTTTAGTGAAATAACACCAC
>dme_circ_0008851
TTAGAGCAACACGGGCACGAGCACCTGGCGGCTCGTTCATTTGCCCATATACGAGAGCCACCTTTGAGGT
CTTGTCCTTTAGTGAAATAACACCACCCTCGATCATCTCATTGTATAAATCGTTTCCCTCACGAGTGCGT
TCACCAACTCCCGCGAAAACCGAG
>dme_circ_0008851_junction_seq
GCGTTCACCAACTCCCGCGAAAACCGAGTTAGAGCAACACGGGCACGAGCACCTGG
>dme_circ_0008852
GATGATCGAGGGTGGTGTTATTTCACTAAAGGACAAGACCTCAAAGGTGGCTCTCGTATATGGGCAAATG
AACGAGCCGCCAGGTGCTCGTGCCCGTGTTGCTCTAACTGGACTGACCGTTGCAGAGTACTTCCGTGATC
AAGAAGGACAGGATGTGTTGCTGTTTATCGACAACATTTTCCGATTTACTCAAGCTGGCTCAGAAGTGTC
CGCTTTGTTGGGTCGTATTCCCTCAGCCGTCGGTTACCAGCCAACCTTGGCTACCGACATGGGTTCTATG
CAGGAGCGTATTACCACCACCAAAAAGGGATCCATTACTTCTGTTCAGGCCATATATGTGCCCGCTGATG
ATTTGACCGATCCTGCTCCTGCTACTACATTCGCTCATTTGGATGCCACCACT
>dme_circ_0008852_junction_seq
TACATTCGCTCATTTGGATGCCACCACTGATGATCGAGGGTGGTGTTATTTCACTA
>dme_circ_0008853
GGAATACGACCCAACAAAGCGGACACTTCTGAGCCAGCTTGAGTAAATCGGAAAATGTTGTCGATAAACA
GCAACACATCCTGTCCTTCTTGATCACGGAAGTACTCTGCAACGGTCAGTCCAGTTAGAGCAACACGGGC
ACGAGCACCTGGCGGCTCGTTCATTTGCCCATATACGAGAGCCACCTTTGAGGTCTTGTCCTTTAG
>dme_circ_0008853_junction_seq

GAGCCACCTTTGAGGTCTTGTCCTTTAGGGAATACGACCCAACAAAGCGGACACTT
>dme_circ_0008854
TAAATCGGAAAATGTTGTCGATAAACAGCAACACATCCTGTCCTTCTTGATCACGGAAGTACTCTGCAAC
GGTCAGTCCAGTTAGAGCAACACGGGCACGAGCACCTGGCGGCTCGTTCATTTGCCCATATACGAGAGCC
ACCTTTGAGG
>dme_circ_0008854_junction_seq
TGCCCATATACGAGAGCCACCTTTGAGGTAAATCGGAAAATGTTGTCGATAAACAG
>dme_circ_0008855
TAATGGATCCCTTTTTGGTGGTGGTAATACGCTCCTGCATAGAACCCATGTCGGTAGCCAAGGTTGGCTG
GTAACCGACGGCTGAGGGAATACGACCCAACAAAGCGGACACTTCTGAGCCAGCTTGAGTAAATCGGAAA
ATGTTGTCGATAAACAGCAACACATCCTGTCCTTCTTGATCACGGAAG
>dme_circ_0008855_junction_seq
CACATCCTGTCCTTCTTGATCACGGAAGTAATGGATCCCTTTTTGGTGGTGGTAAT
>dme_circ_0008856
TTCGGCAATCGCACGCGAGAGTACAGTGGTGGCATCCAAATGAGCGAATGTAGTAGCAGGAGCAGGATCG
GTCAAATCATCAGCGGGCACATATATGGCCTGAACAGAAGTAATGGATCCCTTTTTGGTGGTGGTAATAC
GCTCCTGCATAGAACCCATG
>dme_circ_0008856_junction_seq
GGTAATACGCTCCTGCATAGAACCCATGTTCGGCAATCGCACGCGAGAGTACAGTG
>dme_circ_0008857
GGCCATATATGTGCCCGCTGATGATTTGACCGATCCTGCTCCTGCTACTACATTCGCTCATTTGGATGCC
ACCACTGTACTCTCGCGTGCGATTGCCGAACTGGGTATTTACCCTGCTGTGGATCCACTGGATTCGACTT
CTCGAATCATGGATCCCAACATCATTGGCCAAGAACACTATAACGTTGCCC
>dme_circ_0008857_junction_seq
ATTGGCCAAGAACACTATAACGTTGCCCGGCCATATATGTGCCCGCTGATGATTTG
>dme_circ_0008858
GGCCATATATGTGCCCGCTGATGATTTGACCGATCCTGCTCCTGCTACTACATTCGCTCATTTGGATGCC
ACCACTGTACTCTCGCGTGCGATTGCCGAACTGGGTATTTACCCTGCTGTGGATCCACTGGATTCGACTT
CTCGAATCATGGATCCCAACATCATTGGCCAAGAACACTATAACGTTGCCCGTGGTGTGCAGAAAATCTT
GCAAGACTACAAATCACTTCAGGATATAATAGCCATTCTCGGAATGGATGAGTTGTCTGAGGAAGATAAG
CTCACAGTCGCTCGTGCTCGCAAGATTCAGCGTTTCTTGTCGCAGCCATTCCAA
>dme_circ_0008858_junction_seq
TCAGCGTTTCTTGTCGCAGCCATTCCAAGGCCATATATGTGCCCGCTGATGATTTG
>dme_circ_0008859
GTAGATGGTCGTAGTCCCCAGCTAGAATTGCTGAGAAACCTTTTATAGTTTGCTCCAGGGGCACTAGTTT
ACCAGCATGCCCGGTGAAGACTTCAGCAACTTGGAATGGCTGCGACAAGAAACGCTGAATCTTGCGAGCA
CGAGCGACTGTGAGCTTATCTTCCTCAGACAACTCATCCATTCCGAGAATGGCTATTATATCCTGAAGTG
ATTTG
>dme_circ_0008859_junction_seq
AATGGCTATTATATCCTGAAGTGATTTGGTAGATGGTCGTAGTCCCCAGCTAGAAT
>dme_circ_0008860
GGATATAATAGCCATTCTCGGAATGGATGAGTTGTCTGAGGAAGATAAGCTCACAGTCGCTCGTGCTCGC
AAGATTCAGCGTTTCTTGTCGCAGCCATTCCAAGTTGCTGAAGTCTTCACCGGGCATGCTGGTAAACTAG
TGCCCCTGGAGCAAACTATAAAAG

>dme_circ_0008860_junction_seq
CTAGTGCCCCTGGAGCAAACTATAAAAGGGATATAATAGCCATTCTCGGAATGGAT
>dme_circ_0008861
GTCGCTCGTGCTCGCAAGATTCAGCGTTTCTTGTCGCAGCCATTCCAAGTTGCTGAAGTCTTCACCGGGC
ATGCTGGTAAACTAGTGCCCCTGGAGCAAACTATAAAAGGTTTCTCAGCAATTCTAGCTGGGGACTACGA
CCATCTACCTGAAGTTGCTTTCTACATGGTCGGCCCAATCGAAGAAGTT
>dme_circ_0008861_junction_seq
CTACATGGTCGGCCCAATCGAAGAAGTTGTCGCTCGTGCTCGCAAGATTCAGCGTT
>dme_circ_0008862
ATTTGCTGACTTTGAAGATTTTTCCAGTACGCCTATCTAGGCAGCTTCCTTTGCCAGGCGGTCAGCCTTT
TCTACAACTTCTTCGATTGGGCCGACCATGTAGAAAGCAACTTCAGGTAGATGGTCGTAGTCCCCAGCTA
GAATTGCTGAGAAACCTTTTATAG
>dme_circ_0008862_junction_seq
GCTAGAATTGCTGAGAAACCTTTTATAGATTTGCTGACTTTGAAGATTTTTCCAGT
>dme_circ_0008863
GGTTTCTCAGCAATTCTAGCTGGGGACTACGACCATCTACCTGAAGTTGCTTTCTACATGGTCGGCCCAA
TCGAAGAAGTTGTAGAAAAGGCTGACCGCCTGGCAAAGGAAGCTGCCTAGATAGGCGTACTGGAAAAATC
TTCAAAGTCAGCAAATCTATCATTGAATATTATGCATTGAATTTGTAATAAACTTTGTAAGATCAAATTA
AATTCAATACAAGCGAAGAAAAA
>dme_circ_0008863_junction_seq
AATTAAATTCAATACAAGCGAAGAAAAAGGTTTCTCAGCAATTCTAGCTGGGGACT
>dme_circ_0008864
GGCTTAACTCGACCAGCGCTATACCCATTTGCGGGAGGTCAATATCCTTACCCAATGCTAAGTTCAGATA
TGTCTCAAGTGGCATCATGGTATGTTTTTTAAATCGCTCTTTAAGTTGAAATTAATATAAATTTATTTTT
AGGCACACTCCTTCTGTTTATTCTGCATCTAGTTTTAGAACTCCTTATCCCTCCTCACTACCAATAAATA
CTACACTTGCAAGGTAATATCATGCTTTTGGGAAATTAAAAGTGTATTTAGCCACCCATGCTTCAATAAA
TCTATGATATATGTCTAGGTTTGTGAAAAAGAGCTAATCCATTTTTTGTCTTGATGCAACATACCTACGT
ATTACGCTATGTGTGGCTTGCTTTAAAAAAATTTTTATTCCACCGCTCAACTGTGTGGATTGTTTTTAAT
TTAAAGTACGATTCCGGAACTATGATTACGTGACGAGTTAATCTACAGATTAACACAGAAACCAAATAGT
AATTAAGAAATTatttttaaaatagttttgcttgtaaattttagaaaaatgtaatgataaaaatttagttt
ttttaAGTTTGCTTTCATTTATTGGATCTTGTCGAAAAGGATGTAACAATAAATGTATACCTACGATTTT
TGTAGAGAATTGAGGATCAGTATTAAGGACTCTGCATTCtatatacatacatacatatacatatacatac
atatactcacatactctaactatattcaatattttccacaagtaaatgggtacctatatacatatatatg
tatatatatataggtaggtacccatttgcttgctatatatatatatatatatatatatatatatatatat
atatatatgtgtatgcatatacatacACAAACCACAAAAGAGCATCACACCCACagttttgataaatgtt
ttagtttttatttgtgatttgtattttttttCCCATTGCAAATTATAATGTAACATAATAACGTGCAAAGT
AAAATCAATAAAATTTGTTATAGTTATAAAAACTAGCGAGACACTTGAATATTTGAATTATATTCTATGA
TAACTAAGGGAAAAACTAGTAAGTTTATATTAGTAAACTCGTGTCCTAAGATATTGTAATTTTTAAGTTA
ACACCCCTTTCAACAATAGCTCCCTTAAAATACGTTAACTCGGCAGACCAACTTGCGGAATAACCTCTTA
AATATGTTTGTAACATTGATGTGTGTGGTGGCTAAATTTGCTTTACGTGTCCCAAATTTAATGTTACATT
TCCATATAATAGAATAGAATACCTATTTTTTACACTTTACTTGCAGGTGATTTTCCATTCCGTTTTTCAC
CTAGCCTTTTACCTTCAGTACATGCCACATCGCACCACGTTATAAATGCTCATTCTGCTATTGTGGGGGT
TAGCTCTAAACAAGAATGCGGTGTTCAGGACCCAACAACGAATAATAGATATCCAAG
>dme_circ_0008864_junction_seq

ACCCAACAACGAATAATAGATATCCAAGGGCTTAACTCGACCAGCGCTATACCCAT

>dme_circ_0008865

GCAGAAGTATGATAACTAAAAAAGGTGAAGGATCACAGGTCAAAGAATCAACCGATTCATCTAGCACTAC
TCTTGAAGACGATGACATAAAAGGTAAGTAAATAAAATATGTATTTAACGTTCATTTATAGTAACCAAAT
AAGTGAAGACATAGTAGATCAGGATTCACTATCAACCACTATAGCTTTAGAACAAGACATGGGTTTTTAG
TGTAACGTAGTTACATAGTTTTCATTGGAAGAGGCTTTGTGATCATATTGGAAGTTTAATATTCTGTAAA
GTGGTGTCCCCATTCATTAAGATATAGTTGCTGttatatatttacattatttatttatttattttatggc
aactttaggctgcatgtgatttattattattacaTTTTAACAAATCAACTTAAATTGAAGCATTGTTTAC
AATTTCTTATTAAAGGAATCCATAAATTTCCTCAAGCTTACGAAAACCAGGTAGTTTCCCTAAAACTAGT
GGAAATTACCAGTTTTAACACATTAATTGTAAACCTCATACTTAAATGTATCGTTAATATAATTTTATCT
AATTTTTAAATTGGTGTTAGTATTGTATTTTTTGCACGTCTTCGCTTAGACATACATATACCAAATAATA
AAGTCAATAGAAAGTTTTGCACTTTTATATTGTACAAGAATTTGAAAAATATACGATATACTTCTCTTTA
CTTTCATTCCCATACTTTCATTACTACTTCTCATCGTTATAATTCTTTAATTTAATGCAACAAAATGTAC
CTTCGTTTTGATCTTAAGTGTTATAATTCGGTATACACGAGttttttttCAAAAATCAAAAGTGCTTATA
TATTATAAGATTTCATAAGCTTCGGCTTGAGTAATTGGTTCCATTCCTGTTTTTTATATAACATTTTAtt
atacccgttactcgtagagtaaaaaagtatactacattcgttgacaagtacgtaacacgcagaaggaagc
gtttccgaccatataatgtatattattcttggtcaggatcaatagccgaatcgatttggccatgtctgtc
tgtccgtatggacgtcgagatctcaggaactataaaacccatgttgccacgcccactataacgctcagaa
accacacaaaactgccacgcccacattgaaacattgttggatactttttcataattttattagtcACATT
CCATATGACCGTTTTAAGACATAATTTGTATATTATTTGAAAAGACGAAATTATAGTTAAAAATATATTG
AAAATGGTCTTAGTACTGGTATTTTTAAAACGAAGAAAATATGGAATTAAAGCTAAAATTACGGTTTTAG
ACTTTATAAAAAACTAGTTTTATGCTGAATTGTCGGTATAAAAATATTGTATTACAAATATTTTGAAGTT
GAAGAATGTAGTAAAAAACAGTGTTTCGAGTTTGTTTGAATTGTAAAAAAGTTAATCCGCCATATTTTTG
CCTTTTTTAAAGCCAATAATATGCAAACGGATTCCTGTTTTACATTTATTTCAACCATTAACATTGGGGA
TTTCAAATGCGTATTTTGAATTTATTATAAGTTACAACCAAACTTCATTGCATTAACTTAATAACAGAAA
TTGTATGGTTTAAACTAAATCACAAAAACAAACACATTTCATTATTTTAACGTAGAGGAAATATTGGGAC
AATTGCATTTTCAAATATGACATTTTTGTACATTTTTTTCCTAGAAGATAAAAAAGGAACCGTTGATCGC
AGTACTACAGTCGTATCAAAAGAGCCTGAAGG

>dme_circ_0008865_junction_seq

CTACAGTCGTATCAAAAGAGCCTGAAGGGCAGAAGTATGATAACTAAAAAAGGTGA

>dme_circ_0008866

AAAACAAGAAATAAAACTCCTGAAAAGTGAAATCTGTAGAGCAGTTGAAGATCTTTAGTCGCAATAGATT
TTCTTAAGTGTGCCATCGCGATGGCTGCACCAGCAGCCTGTACGCGTTTTTCGGACAATTACGACATCAA
AGAAGAGTTGGGAAAGGTAAGGCTTTCTGTGTTCTATGTAGGTTATAAATCACATATGTACGCATTTATG
CTATGCACACGCATGTAGGGAAACTAAGTGCTGTTTTTAGGTAATTCGTCGAgtacagtaaaagggtata
ctagatccttgatccttggaaagtatgtatattcttgatcaggatcaatagccgagtccgtctctccgta
tggtcgtcgagatctcagaaactatacaagctagaaggcatacagattttagaaacaaatacgcagcgca
agtttgttgacccatgttgccacatgcgccacgccctcacttttgaaaaatgtgttgatatttttttcaca
tttgtattggtcttgtaaatttctatcgatttgctcaaaaaaaaagtttgccacgcccattctaacgccct
ttaacttctatcgatttgcttaaaagccgccaaccggtcacgtccacactttggaccaatttttaaactt
ttttcttattttattccccaatatctatcgttatcctagaaaaatgatgaaatttcgcgttcccattcac
actagctgagtaactggtatctgatGCCGTTTTTTCAACGGCGATATGGCATATCGTAAGAAATCAGTAG
TAAACATTGACTGTCAAAAAGTTTTCCTGTAAAATGTACTTTCTTTTTGCTAAGTACGCTTTTGGAAGGG
ACCATTCTTTTTGTGTAATATCTTCGTCGTCCCTATCTATTTGCCACACAAGTATCAAATAGTTCTACAG

TAATTAAGTTGTATTTCTTGGTGTTGTGCTATTTAGTAATTAATAAATTTTTACTGCCAAGACGTGGCCA
AAATAATTTTGGAATAGCAGTATTGGCAAAGTGTGTAATTTACAAAAAATATATTAAATAACTTTTAAGA
AGTTTGAGCATGACAGCTGGGACAAATTTTGGGCTTAGAGAGAACCATTTGCTTGCTCGCCAAAAACCTC
TTATTAACATATTTGTACACATGCATGTATGCATATAAGAGATGCTTATAATATATATATATATATAttt
tttttttttGCTTTTAGAGTATGCAACGTGTATATAAATATAATTTACTTACTTATTCTTTAAAGATACG
TTTTTCTGAAGATGTATGATCTACATAAAAGTAGACGAACATGGCCTAATATCACTTTGAACATCTACTA
TACATAATTTTTATATGTACATTCAATCACGGCTTAGACCCCAGTAGTGTTGATGACTTGCAATGTGATC
CTGATAAAGGATGGTTTGACGTCCACGGTAACACAACGAAGAAAAAAATAAAATAGAGTTTAGTAGATAT
ATCAATCCGCATCTGCAAGCAACATTCCATTCCTCGCCCTCCCTACTCTCCAATAACAAATCTTCCAAGT
AATTTATAATTATAATAATTATAAAAACTTGCAGAACAGCAAAAACCGAACTCATGCAATAGACTTTCCT
CTCACTAAAAGCGTAACCTAACAAAACAGTTATTTACACGGACAGTTCAAATCTAGAAACATCACTTCCT
CCAGCGTCACCGACAAAATATATAATATGTTCAAAATGTATTTTATAAACATATATTTTTATAGCCTTTA
AGGCTTATAATCATTGTCATCCGTaaaaaaaaaaCATTAAATACAAATGTATACAACTAATAAATTGTTA
ATATGTACAATTTCAGTGTAAGCATTAACCTGCATAGACTCCTTACCAAATATTTAAATTGCCTATCCCC
AAAAGTGTTTTAAAAATATGAGTTTCTAACAAATGCTTGTAAATGTGGCAATGACTCGACTATTGTTTAT
TGAGATTCATACTTCATCCTTTTCCCTCCGTTCGTTCATACTATAATATGAATATGAATCAAATTTTAAA
GCCGCaatataatatatataatataatatGAATCAAGTTTTAGCCGCAATATATTTTTGTTATATTAGAG
ATGTGTTTTCTGCTGTCGCAACTTAAATATAACCTAAATTTAAATTTAGAGCTTCAATCGCAAAATTTGA
CTATGCCTACATACATTTGCAGTTGTAACCGGTGGTGAACTTTTTGAAGATATCGTTGCACGCGAATTTT
ATTCAGAAGCTGATGCATCACATTGTATTCAACAAATATTGGAATCGGTCAATCACTGCCACCAAAATGG
TGTGGTGCACCGAGATCTGAAACCAGAGAATTTACTATTAGCTAGTAAGGCTAAGGGTGCAGCAGTGAAA
CTCGCTGACTTTGGTCTAGCCATTGAAGTTCAAGGCGATCATCAGGCTTGGTTTGGATTTGCCGGCACAC
CAGGATATCTGTCTCCTGAGGTATTGAAAAAGGAGCCCTATGGGAAATCGGTAGACATATGGGCATGTGG
CAACGCGAACGTGTGGCTTCCGTTGTGCATCGCCAAGAAACCGTAGACTGTCTCAAGAAATTTAATGCGC
GGCGCAAGCTAAAGGGAGCCATACTTACGACAATGTTGGCGACCAGAAATTTTTCGAGGTATGTAACTTA
ACTTAATTAATCTTGACCACATCAATATACTTGGCGTCAACACAAAAATATACAAAAAGAATAATAaaaa
cgctatggccgatttcgttgacaattatatacccgttattcagcttgtaggattgATATAAATATATTTA
ACTTCGTATAAAAAatacccttttactcgtagagtaaaaaggtagactatattgtttgaaaagtatgtaac
aagcagaaagaagcgtttccgagcataaatagaataatattctcgatcaggatcaatagtagagtcgatc
tggaactgtccgtccgtatgaacgttgagatcttaggaactataaaaacaagaaggttgagattacgcaa
cctttattgatccatataaacactctaacgaccacaaaccgtacaaaactgcgacgcccaaacttttgaa
aaatgtgtagatatttttttcacatttgtattagtcttgtaaatttttttatcgatttgcagaaaaaattga
gacctcgttcatacggacatggccagatcgagtcgggtattgatcctgatcgagaatatacagttgcggt
aacaaaatagcaccataagcacattttgtgtttgtcccagcgtttctccattttctgaaactttttcaaa
aaaaataattgttaaaacaaaaaaaaccttcaaatttaggcggtaacaaaaatataactgttcctcgtag
tttgctaaaactaaccaaaaaaaataaaaaaataattcaacaaatggattatatcttaagaactatgttta
aagaatcttaatatttggttgcgggaccgcgcatcgccctggcatagagtccaccaagtcctggcctagc
ttttgaggaataaaaatgaccagaccccaaagatcgtcgttattattgggtttggcttcagaaacctatt
tttgacgtccgcccaaaggttttcgattggattcaagtcgggtgactgagcaggccagttcattccttga
atcgatttctgctttgaccactttcgagctttcttgctcgtgtgttttgagtcgttatcctgttgaaatg
tccaaaccaacggcatttcatcctcggcatatggcagcatcacattttccaggatatctgtgtaaatgtg
ctgatccatgatgccttgaatccaatggatcggacctactacattgtatgaaaagcacgcccataccatg
atgtttgatccccccatgctttaccgccttaaaggtgaagccgaggattatattcagttcgcggtggtagc
cgaacataagacctagagccattcccaccaaacaacacaattttggacattgagggctttcttcgccgtg

tttcgttcttttcgacaaacagcactgctttgcggatcattttgcttgaacaaccgacaatgcgaccaat
ttcagcgtaggttttaccttcagagatcacgtttttaatcaaatttctttttttcgacggtacaatgcttt
ccgtgacccatgactagagaatttttggtcttcgtttggaaaaaattcagttaaaacctttaatacaact
ccttatttcaaaatttttcgaaaaaaacccaaagcaatcactcctattattttaatcagcaaatacgtgg
tcagtgctatttttgttaccgcctcatttcgcgcacttttgcagcaagtgcccaccaaaaaaaagaaccg
ttacattgagagactaaaaatttcttgctcagagagccaacatatggtacttattattcatgcaatctga
ctttaaaaaatataaacatttaataatttttttttaggaaatatacttttcacctgcagtagtgctattat
tttagcggcagctgtatacactttatatggtcggaaacgcttccttttactccacgagtaacgggtataa
TAATTAATTaaaaaaaaaaaacaacaaaaacaaaacATGCACGTACATATTAAGGTTTAATTAAATATTAT
AAGATTTAAACTGATTTTAACTAAAAGTGTATTTAATTTACAGGCAGAAGTATGATAACTAAAAAAGGTG
AAGGATCACAGGTCAAAGAATCAACCGATTCATCTAGCACTACTCTTGAAGACGATGACATAAAAGG
>dme_circ_0008866_junction_seq
CTACTCTTGAAGACGATGACATAAAAGGAAAACAAGAAATAAAACTCCTGAAAAGT
>dme_circ_0008867
GTTTTGATTAGTAGCATCAACTGTAAATAATAGCCCATCTAAGCTTTTTCCGCATACAACACATGTAAAA
CATTGCGGATGATATGGTTTTCCAGTAGCTCTAAGAATTCTCTCTAAAATTGGTTCCATGCAAACAGAGC
ATTTTTCCAGTGTTTGTAAATAATCATATTCGCAATAGG
>dme_circ_0008867_junction_seq
GTTTGTAAATAATCATATTCGCAATAGGGTTTTGATTAGTAGCATCAACTGTAAAT
>dme_circ_0008868
ATAATCCGGCAATTTTGGAACAGCAATTAGAAGCTCTTGCTTACCATAAACTTCAAATGGAAAAAAAGGG
TCTTTTGGGAGTACAAGCTAAGCCAACACAACCTCTCAACAGCTTTACAAAGCCCCTTTCAAAGACTTTA
AGCAAAAGCTTAATATATTCTAATTTAGGTTCTGTTCGCAAAGAAATAGAAACATTAGAACTATTAACAG
ACGAAACTAAGATTTCTGCGAGTACATATTCAAACGTTAATGAAACTGGCAATGGACTCTTCTCATAGCT
CAACGCAAAAGATGCTATCCGTCTGCACTAATTTTATTTCAGACAACGAAAAAGATGAGTTACCGCCACC
ACCTAGTCCAGAGAGCGCTGTGAGTTCTTCATACAGTGAGCTTCGTCACGCCACTTTGGAATTCAACAAA
CCAATTGATTATTTACAAAATAACCAGACAACGAATCCTTTACAAATATATGCAAACCAATATGCGATGC
AGCATGATGCCACAGGCAAGGGTACACAAAATCTGAAGTTCATTACTCTGGAGTATATTTCTGTCTGATA
AACAATCTTATTCATCTAATAGAAGTGCACGACAAAAATTAAGATATGCAATCAGCAAAGCTGAAGAATA
ATTAAATGACTTACGAACGTAGGCAGTTTTTTCCATTGAACTTTTAGTTAACTTGAAATATGTTAATGTA
TATACATTTATTaaaaaaaaaaaaaCAATAAACAATAACTATTTCCACATTTAGAAAGAACTTTAAACGT
TAAATAGATGGATTAACAACATTTTGTGGATTATGATGTACAAATGCATCTATTTCATTCGCTAGTCCGG
CAGGTTTTATTGCCTATATTGTTCCCAAAATCAGAGTgggcatacgtgcatgggcagatagactcggcta
gtgttcctttgcaacaaaaaaatatatacgatcgaaatcgattcttcatatatacatgttacatactttt
caccgaatcttttattcctttttacGATTATAACTTCCAAATGATGTATTAATTGTATATTTGGAACAAA
ATTTTGTTTGCattttataataataatttatataattcataattttttaattttCAGAAGCTCTTCTACA
TATGATTCAATATATGAACCTATAAACCCTCGACCCTGTGTTGCAGATACGCTGCCTCGTGAAAGTTATA
ATCTGCATAATTCATACGTTAATGATAATAATCCAAACATTTCCCATGAATACAATATTTCGAATTCGAT
TGAAGCTAACCAAACACTATACATACATGGTAATGCTAGAACTACATTTTATGACGTGAACAGCATCCAT
AGAAATGATAAGGAAGGTCTAAAAAATTACATTTCAATACCCACCGAACCGGTTCAAGAATTGGAAAACT
ACGGGTAAGTTAATATAATAAATTTTTATCGATTAAAATTAACGTATATTTTATACAGGTAGGTGTGTCA
AATGCAATTCACGTGTACTTGGAGAAAGTAGTGGATGTACAGCAATGGATCAAATATACCACATATTTTG
CTTCACATGTACAGATTGCCAAATTAACTTACAGGGTAGGTGTGTCAAATGCAATTCACGTGTACTTGGA
GAAAGTAGTGGATGTACAGCAATGGATCAAATATACCACATATTTTGCTTCACATGTACAGATTGCCAAA

TTAACTTACAGGGAAAACCTTTCTATGCATTAGACGGCAAACCCTATTGCGAATATGATTATTTACAAAC
ACTGGAAAAATGCTCTGTTTGCATGGAACCAATTTTAGAGAGAATTCTTAGAGCTACTGGAAAACCATAT
CATCCGCAATG
>dme_circ_0008868_junction_seq
CTACTGGAAAACCATATCATCCGCAATGATAATCCGGCAATTTTGGAACAGCAATT
>dme_circ_0008869
GTTTCGTCTGTTAATAGTTCTAATGTTTCTATTTCTTTGCGAACAGAACCTAAATTAGAATATATTAAGC
TTTTGCTTAAAGTCTTTGAAAGGGGCTTTGTAAAGCTGTTGAGAGGTTGTGTTGGCTTAGCTTGTACTCC
CAAAAGACCCTTTTTTTCCATTTGAAGTTTATGGTAAGCAAGAGCTTCTAATTGCTGTTCCAAAATTGCC
GGATTATCTAAAGTTGTTTTTCT
>dme_circ_0008869_junction_seq
TTGCCGGATTATCTAAAGTTGTTTTTCTGTTTCGTCTGTTAATAGTTCTAATGTTT
>dme_circ_0008870
GACAACGTTCATTGTATTCGAATCGGTTTAATTCGCGGATGGTCTGTGTGTTTCTTAATCTAAGAAGTAT
ATACAAAGTGAAATTGGACCAGTGTTGGTAAAGGTAAAAAATACATGTAGTAAAATAAAAATGTGATACA
AATTGTGGCGTTTGCTATTCCCAGGCTATCCCTAAGGGGCCCAGCGTAATGGCCAGGATGAAATACAATA
TCGCTTTGATTGGAATCCTAGCTTCTGTGCTTTTAACAATTGCTGTAAATGCTG
>dme_circ_0008870_junction_seq
GTGCTTTTAACAATTGCTGTAAATGCTGGACAACGTTCATTGTATTCGAATCGGTT
>dme_circ_0008871
TTCCGCGCCTTCGTAATTATATTTGCGTTACCCACTTTCGATGTTGATGTATGTGTAGGGCACATGCCGA
AAACGTCAACCTCATGTTCCGCTTCTATTCCCGATTGAAGCAATGATACAACTGCGCGCTTAATATTCAA
AGAGTAGTCCAAGTCGCTGGAG
>dme_circ_0008871_junction_seq
ATTCAAAGAGTAGTCCAAGTCGCTGGAGTTCCGCGCCTTCGTAATTATATTTGCGT
>dme_circ_0008872
GGCGCGGAACTTAAACAGCTGTTCGCATCGCGAGCAGATAAACAGTGGCTTGGTGTCCGGCAAAGTTAAC
GAAAAAGCTGGCATTACGTCTAGCCTGCTGTTGCAGGCAAACTATATAAAGGAGTCCAGGATTGTAAACC
ACTTAATTGAAAATGTTCAGCTGACAGAAACATATAAGTTTATTGGAAATACTAAAAGAAACTCTGATAT
CAGTGCAAAA
>dme_circ_0008872_junction_seq
TAAAAGAAACTCTGATATCAGTGCAAAAGGCGCGGAACTTAAACAGCTGTTCGCAT
>dme_circ_0008873
GCTGTTCGCATCGCGAGCAGATAAACAGTGGCTTGGTGTCCGGCAAAGTTAACGAAAAAGCTGGCATTAC
GTCTAGCCTGCTGTTGCAGGCAAACTATATAAAGGAGTCCAGGATTGTAAACCACTTAATTGAAAATGTT
CAGCTGACAGAAACATATAAGTTTATTGGAAATACTAAAAGAAACTCTGATATCAGTGCAAAAGTAGTCA
CAATATTAAAACTGAAAAATCCAAGCGGTACCAAGGCAAACTCACCAGGAACTGGTTCTACTGTCAGAAG
CCTAATATTTCAGAGACCAGAAACCTATACCTCCAAAAATATTAACGCCCTCAAAACGATTCTATCTGAT
CTCGTAGATTCAACTGGCGACTATGTGAAAAAAGAAACAGCTAAAAAGTTTGTTGAGTTTATTAG
>dme_circ_0008873_junction_seq
CAGCTAAAAAGTTTGTTGAGTTTATTAGGCTGTTCGCATCGCGAGCAGATAAACAG
>dme_circ_0008874
GGAGTCCAGGATTGTAAACCACTTAATTGAAAATGTTCAGCTGACAGAAACATATAAGTTTATTGGAAAT
ACTAAAAGAAACTCTGATATCAGTGCAAAAGTAGTCACAATATTAAAACTGAAAAATCCAAGCGGTACCA

AGGCAAACTCACCAGGAACTGGTTCTACTGTCAGAAGCCTAATATTTCAGAGACCAGAAACCTATACCTC
CAAAAATATTAACGCCCTCAAAACGATTCTATCTGATCTCGTAGATTCAACTGGCGACTATGTGAAAAAA
GAAACAGCTAAAAAGTTTGTTGAGTTTATTAGGTTGCTACGCCAATCTGATAGTGAGACTTTATTAGAAC
TGGCTGCGTTTCCACATCCAAACAAAGTCTTAGCCCGCAAGGTATATTTGGATGGATTATTTCGGACCAG
TACAGCTGAGTCAGCTAGAGTTATATTAAAACAGCTTTCCAAATTTGATGAGAAGGAGAAATTACTTGCA
ATACTGTCCTTAAACATAGTGAAAAGTGTTGACAAAGAGACCCTCAATCAAGCGGCTTCTCAGCTCTTAC
CTAACGCGCCTAAAGAACTATATATAGCTGTGGGTAACTTAGTTGCTAAATATTGTTTAAAAAATTATTG
TCAAGGACCGGAAATTGATGCCATATCTAAAAAGTTTTCTGATGGCCTTAAGCACTGTAAGCCAAATACT
AAGCGGGAAGAAGAGCGCATTGTGTACATTTTAAAGGGACTAGGAAATGCAAAGAGTTTAAGTGGCAATA
CGGTAGCTGCACTAAGTGAGTGCGCTTCCACAGGACGCTCCAACCGCATACGTGTCGCAGCCTTGCACGC
TTTTTCAAAAGTTAAATGCGAAGAAACCTTGCAATCTAAATCCTTGGAACTTCTTAAGAATCGTAACGAA
GATTCAGAGTTGCGTATTGAAGCTTATTTGTCCGCGATTTCATGCCCTAACGCAGAGGTTGCTAATCAAA
TTTCGGAAATAGTTAATTCCGAAACTGTTAACCAGGTTGGAGGATTTATATCATCTAATTTAAAAGCTAT
CCGAGACTCTACAGACGTCAGCAGAGACCAGCAAAAATACCATTTGGCTAACATTAGGGTTACAAAAACA
TTTCCCGTTGACTACAGACGATACAGTTTTAATAATGAGGTTTCCTATAAGCTTGAATCCCTTGGTGTTG
GCGCCAGCACTGATTACCAAATAATATATTCGCAACATGGATTCTTGCCCCGATCATCTCGTATCAATGT
AACAACAGAATTTTTTGGCACGAACTACAATGTATTTGAGGCAAGCGTCCGACAAGAAAATGTTGAAGAC
GTATTGGAATACTATTTAGGGCCGAAAGGATTAGTAAACAAAGATTTTGACGAAATTGTTAAACTCATTG
AAGTTGGCAATAATGGTGTTGCTGCTGGTGGCCGAGCCCGACGATCAATTGTTGATGACGTTTCTAAAAT
TTCGAAAAAGTATAAAATGTATGGAGTCAAAAAT
>dme_circ_0008874_junction_seq
AAAGTATAAAATGTATGGAGTCAAAAATGGAGTCCAGGATTGTAAACCACTTAATT
>dme_circ_0008875
CTGTTTCTTTTTTCACATAGTCGCCAGTTGAATCTACGAGATCAGATAGAATCGTTTTGAGGGCGTTAAT
ATTTTTGGAGGTATAGGTTTCTGGTCTCTGAAATATTAGGCTTCTGACAGTAGAACCAGTTCCTGGTGAG
TTTGCCTTGGTACCGCTTGGATTTTTCAGTTTTAATATTGTGACTACTTTTGCACTGATATCAGAGTTTC
TTTTAGTATTTCCAATAAACTTATATGTTTCTGTCAGCTGAACATTTTCAATTAAG
>dme_circ_0008875_junction_seq
TTCTGTCAGCTGAACATTTTCAATTAAGCTGTTTCTTTTTTCACATAGTCGCCAGT
>dme_circ_0008876
GCTTCTGACAGTAGAACCAGTTCCTGGTGAGTTTGCCTTGGTACCGCTTGGATTTTTCAGTTTTAATATT
GTGACTACTTTTGCACTGATATCAGAGTTTCTTTTAGTATTTCCAATAAACTTATATG
>dme_circ_0008876_junction_seq
CTTTTAGTATTTCCAATAAACTTATATGGCTTCTGACAGTAGAACCAGTTCCTGGT
>dme_circ_0008877
CAACTAAGTTACCCACAGCTATATATAGTTCTTTAGGCGCGTTAGGTAAGAGCTGAGAAGCCGCTTGATT
GAGGGTCTCTTTGTCAACACTTTTCACTATGTTTAAGGACAGTATTGCAAGTAATTTCTCCTTCTCATCA
AATTTGGAAAGCTGTTTTAATATAACTCTAGCTGACTCAGCTGTACTGGTCCGAAATAATCCATCCAAAT
ATACCTTGCGGGCTAAGACTTTGTTTGGATGTGGAAACGCAGCCAGTTCTAATAAAGTCTCACTATCAGA
TTGGCGTAGCAACCTAATAAACTCAACAAACTTTTTAGCTGTTTCTTTTTTCACATAGTCGCCAGTTGAA
TCTACGAGATCAGATAGAATCGTTTTGAGGGCGTTAATATTTTTGGAGGTATAGGTTTCTGGTCTCTGAA
ATATTAGGCTTCTGACAGTAGAACCAGTTCCTGGTGAGTTTGCCTTGGTACCGCTTGGATTTTTCAGTTT
TAATATTGTGACTACTTTTGCACTGATATCAGAGTTTCTTTTAGTATTTCCAATAAACTTATATG
>dme_circ_0008877_junction_seq

CTTTTAGTATTTCCAATAAACTTATATGCAACTAAGTTACCCACAGCTATATATAG
>dme_circ_0008878
GTTTATTGGAAATACTAAAAGAAACTCTGATATCAGTGCAAAAGTAGTCACAATATTAAAACTGAAAAAT
CCAAGCGGTACCAAGGCAAACTCACCAGGAACTGGTTCTACTGTCAGAAGCCTAATATTTCAGAGACCAG
AAACCTATACCTCCAAAAATATTAACGCCCTCAAAACGATTCTATCTGATCTCGTAGATTCAACTGGCGA
CTATGTGAAAAAAGAAACAGCTAAAAAGTTTGTTGA
>dme_circ_0008878_junction_seq
AAAAAGAAACAGCTAAAAAGTTTGTTGAGTTTATTGGAAATACTAAAAGAAACTCT
>dme_circ_0008879
GTATAGGTTTCTGGTCTCTGAAATATTAGGCTTCTGACAGTAGAACCAGTTCCTGGTGAGTTTGCCTTGG
TACCGCTTGGATTTTTCAGTTTTAATATTGTGACTACTTTTGCACTGATATCAGAGTTTCTTTTAG
>dme_circ_0008879_junction_seq
TTTGCACTGATATCAGAGTTTCTTTTAGGTATAGGTTTCTGGTCTCTGAAATATTA
>dme_circ_0008880
GTAGTCACAATATTAAAACTGAAAAATCCAAGCGGTACCAAGGCAAACTCACCAGGAACTGGTTCTACTG
TCAGAAGCCTAATATTTCAGAGACCAGAAACCTATACCTCCAAAAATATTAACGCCCTCAAAACGATTCT
ATCTGATCTCGTAGATTCAACTGGCGACTATGTGAAAAAAGAAACAGCTAAAAAGTTTGTTGAGTTTATT
AGGTTGCTACGCCAATCTGATAGTGAGACTTTATTAGAACTGGCTGCGTTTCCACATCCAAACAAAGTCT
TAGCCCGCAAGGTATATTTGGATGGATTATTTCGGACCAGTACAGCTGAGTCAGCTAGAGTTATATTAAA
ACAGCTTTCCAAATTTGATGAGAAGGAGAAATTACTTGCAATACTGTCCTTAAACATAGTGAAAAGTGTT
GACAAAGAGACCCTCAATCAAGCGGCTTCTCAGCTCTTACCTAACGCGCCTAAAGAACTATATATAGCTG
TGGGTAACTTAGTTGCTAAATATTGTTTAAAAAATTATTGTCAAGGACCGGAAATTGATGCCATATCTAA
AAAGTTTTCTGATGGCCTTAAGCACTGTAAGCCAAATACTAAGCGGGAAGAAGAGCGCATTGTGTACATT
TTAAAGGGACTAGGAAATGCAAAGA
>dme_circ_0008880_junction_seq
ATTTTAAAGGGACTAGGAAATGCAAAGAGTAGTCACAATATTAAAACTGAAAAATC
>dme_circ_0008881
GCGGTACCAAGGCAAACTCACCAGGAACTGGTTCTACTGTCAGAAGCCTAATATTTCAGAGACCAGAAAC
CTATACCTCCAAAAATATTAACGCCCTCAAAACGATTCTATCTGATCTCGTAGATTCAACTGGCGACTAT
GTGAAAAAAGAAACAGCTAAAAAGTTTGTTGAGTTTATTAGGTTGCTACGCCAATCTGATAGTGAGACTT
TATTAGAACTGGCTGCGTTTCCACATCCAAACAAAGTCTTAGCCCGCAAGGTATATTTGGATGGATTATT
TCGGACCAGTACAGCTGAGTCAGCTAGAGTTATATTAAAACAGCTTTCCAAATTTGATGAGAAGGAGAAA
TTACTTGCAATACTGTCCTTAAACATAGTGAAAAGTGTTGACAAAGAGACCCTCAATCAAGCGGCTTCTC
AGCTCTTACCTAACGCGCCTAAAGAACTATATATAGCTGTGGGTAACTTAGTTGCTAAATATTGTTTAAA
AAATTATTGTCAAGGACCGGAAATTGATGCCATATCTAAAAAGTTTTCTGATGGCCTTAAGCACTGTAAG
CCAAATACTAAGCGGGAAGAAGAGCGCATTGTGTACATTTTAAAGGGACTAGGAAATGCAAAGAGTTTAA
GTGGCAATACGGTAGCTGCACTAAGTGAGTGCGCTTCCACAGGACGCTCCAACCGCATACGTGTCGCAGC
CTTGCACGCTTTTTCAAAA
>dme_circ_0008881_junction_seq
TGTCGCAGCCTTGCACGCTTTTTCAAAAGCGGTACCAAGGCAAACTCACCAGGAAC
>dme_circ_0008882
GTTTGTTGAGTTTATTAGGTTGCTACGCCAATCTGATAGTGAGACTTTATTAGAACTGGCTGCGTTTCCA
CATCCAAACAAAGTCTTAGCCCGCAAGGTATATTTGGATGGATTATTTCGGACCAGTACAGCTGAGTCAG
CTAGAGTTATATTAAAACAGCTTTCCAAATTTGATGAGAAGGAGAAATTACTTGCAATACTGTCCTTAAA

CATAGTGAAAAGTGTTGACAAAGAGACCCTCAATCAAGCGGCTTCTCAGCTCTTACCTAACGCGCCTAAA
GAACTATATATAGCTGTGGGTAACTTAGTTGCTAAATATTGTTTAAAAAATTATTGTCAAGGACCGGAAA
TTGATGCCATATCTAAAAAGTTTTCTGATGGCCTTAAGCACTGTAAGCCAAATACTAAGCGGGAAGAAGA
GCGCATTGTGTACATTTTAAAGGGACTAGGAAATGCAAAGAGTTTAAGTGGCAATACGGTAGCTGCACTA
AGTGAGTGCGCTTCCACAGGACGCTCCAACCGCATACGTGTCGCAGCCTTGCACGCTTTTTCAAAAGTTA
AATGCGAAGAAACCTTGCAATCTAAATCCTTGGAACTTCTTAAGAATCGTAACGAAGATTCAGAGTTGCG
TATTGAAGCTTATTTGTCCGCGATTTCATGCCCTAACGCAGAGGTTGCTAATCAAATTTCGGAAATAGTT
AATTCCGAAACTGTTAACCAGGTTGGAGGATTTATATCATCTAATTTAAAAGCTATCCGAGACTCTACAG
ACGTCAGCAGAGACCAGCAAAAATACCATTTGGCTAACATTAGGGTTACAAAAACATTTCCCGTTGACTA
CAGACGATACAGTTTTAATAATGAGGTTTCCTATAAGCTTGAATCCCTTGGTGTTGGCGCCAGCACTGAT
TACCAAATAATATATTCGCAACATGGATTCTTGCCCCGATCATCTCGTATCAATGTAACAACAGAATTTT
TTGGCACGAACTACAATGTATTTGAGGCAAGCGTCCGACAAGAAAATGTTGAAGACGTATTGGAATACTA
TTTAGGGCCGAAAGGATTAGTAAACAAAGATTTTGACGAAATTGTTAAACTCATTGAAGTTGGCAATAAT
GGTGTTGCTGCTGGTGGCCGAGCCCGACGATCAATTGTTGATGACGTTTCTAAAATTTCGAAAAAGTATA
AAATGTATGGAGTCAAAAATGTGCAAGATCTCAATTTAGATGTGTCATTAAAACTGTTTGGTTCGGAATT
GGCATTTTTGAGCCTAGGCGACAACATACCAAGTTCCCTAGATGACATTATAAACTACTTCTCAACTTCT
TTCGAAAAAGCAAAACAGGAATTATCTTCGTTTGAAAAGCAATTTTCCAGTCACCACTTATTCCTTGATA
CAGACCTCGCTTATCCAACTAGTATTGGAGTACCTTTGGAGCTTGTAGCCCAAGGTTTTGCAGCTACCAA
AGTTGATCTTGCCGTTAGTCTTGATATTAATGCAATTCTAGAACAAAACTGGCAAAAGGCTAAGTATAGA
CTAAAGTTTGTTCCGAGTGTCGATATTAATGCTAACGTTCAGATAGGCTTTAATGCACAAGTACTATCCA
CTGGACTCCGCGTGGTTTCGTCAGCCCATTCTGCCACCGGTAGCGATATTACTGTAGCTGTTATTAGCGA
TGGAGAGGGCTTTAACGTTGACCTCGAGCTACCACGCGAAAAACTTGAGCTTATTAATTTTAATGTTGAC
ACAGAACTATATGTAGCCGAACAAGACAAACAAAAGGCAATTGCCCTAAAGGGTAACaaaaaaaaTAAGA
ATTCTCAACCTAGTGAGATATGCTTTAATCAATTGGAACTTGTTGGATTAAATATTTGCATTAAGAGTTC
AACAAGCTTAAGTGAGGTTCAAGCTGGTAACGGCAATGTTGCAGAGAGAGGACTTTCTGTTTCGGAGAAA
TTTCATTTATCTAGACCATTTAATTTCGCCGTTTACTTAACGACTGAACGCAAATTTACATTCAAAGGAA
TTCACACACAGGAAGCTTTCTCCCAAAAGTGGAAATTAGATTATTCCACTCCTGGATCCAAAGTTTCTCA
CGATACAACTGTTGTATATGAACTCGGAAATAAGCCTAAAACATTTAGTAGATTATCCTTTGATAACTCT
CAATGCCACTTTGCAGTGGAAGGTGGAATAAACAACGACAAGAATGAATTGGTTGTATATGGTCAATACG
AACAAGACAAAGAAATaaaaaaaaagtaaaaTTGGCTTTAGCAAAAACGGAAATGaatacaagccattaat
tgaaatccaagacaataatggaaTCTCAAATAGCATAAATGGTTACCACGCAGACGGAAAAATA
>dme_circ_0008882_junction_seq
AAATGGTTACCACGCAGACGGAAAAATAGTTTGTTGAGTTTATTAGGTTGCTACGC
>dme_circ_0008883
AGCTGAGAAGCCGCTTGATTGAGGGTCTCTTTGTCAACACTTTTCACTATGTTTAAGGACAGTATTGCAA
GTAATTTCTCCTTCTCATCAAATTTGGAAAGCTGTTTTAATATAACTCTAGCTGACTCAGCTGTACTGGT
CCGAAATAATCCATCCAAATATACCTTGCGGGCTAAGACTTTGTTTGGATGTGGAAACGCAGCCAGTTCT
AATAAAG
>dme_circ_0008883_junction_seq
TGTGGAAACGCAGCCAGTTCTAATAAAGAGCTGAGAAGCCGCTTGATTGAGGGTCT
>dme_circ_0008884
CAACTAAGTTACCCACAGCTATATATAGTTCTTTAGGCGCGTTAGGTAAGAGCTGAGAAGCCGCTTGATT
GAGGGTCTCTTTGTCAACACTTTTCACTATGTTTAAGGACAGTATTGCAAGTAATTTCTCCTTCTCATCA
AATTTGGAAAGCTGTTTTAATATAACTCTAGCTGACTCAGCTGTACTGGTCCGAAATAATCCATCCAAAT

ATACCTTGCGGGCTAAGACTTTGTTTGGATGTGGAAACGCAGCCAGTTCTAATAAAG
>dme_circ_0008884_junction_seq
TGTGGAAACGCAGCCAGTTCTAATAAAGCAACTAAGTTACCCACAGCTATATATAG
>dme_circ_0008885
GAAACCTCATTATTAAAACTGTATCGTCTGTAGTCAACGGGAAATGTTTTTGTAACCCTAATGTTAGCCA
AATGGTATTTTTGCTGGTCTCTGCTGACGTCTGTAGAGTCTCGGATAGCTTTTAAATTAGATGATATAAA
TCCTCCAACCTGGTTAACAGTTTCGGAATTAACTATTTCCGAAATTTGATTAGCAACCTCTGCGTTAGGG
CATGAAATCGCGGACAAATAAGCTTCAATACGCAACTCTGAATCTTCGTTACGATTCTTAAGAAGTTCCA
AGGATTTAGATTGCAAGGTTTCTTCGCATTTAACTTTTGAAAAAGCGTGCAAGGCTGCGACACGTATGCG
GTTGGAGCGTCCTGTGGAAGCGCACTCACTTAGTGCAGCTACCGTATTGCCACTTAAACTCTTTGCATTT
CCTAGTCCCTTTAAAATGTACACAATGCGCTCTTCTTCCCGCTTAGTATTTGGCTTACAGTGCTTAAGGC
CATCAGAAAACTTTTTAGATATGGCATCAATTTCCGGTCCTTGACAATAATTTTTTAAACAATATTTAGC
AACTAAGTTACCCACAGCTATATATAGTTCTTTAGGCGCGTTAGGTAAGAGCTGAGAAGCCGCTTGATTG
AGGGTCTCTTTGTCAACACTTTTCACTATGTTTAAGGACAG
>dme_circ_0008885_junction_seq
CAACACTTTTCACTATGTTTAAGGACAGGAAACCTCATTATTAAAACTGTATCGTC
>dme_circ_0008886
TCCCTTTAAAATGTACACAATGCGCTCTTCTTCCCGCTTAGTATTTGGCTTACAGTGCTTAAGGCCATCA
GAAAACTTTTTAGATATGGCATCAATTTCCGGTCCTTGACAATAATTTTTTAAACAATATTTAGCAACTA
AGTTACCCACAGCTATATATAGTTCTTTAGGCGCGTTAGG
>dme_circ_0008886_junction_seq
CTATATATAGTTCTTTAGGCGCGTTAGGTCCCTTTAAAATGTACACAATGCGCTCT
>dme_circ_0008887
AAGTTCCAAGGATTTAGATTGCAAGGTTTCTTCGCATTTAACTTTTGAAAAAGCGTGCAAGGCTGCGACA
CGTATGCGGTTGGAGCGTCCTGTGGAAGCGCACTCACTTAGTGCAGCTACCGTATTGCCACTTAAACTCT
TTGCATTTCCTAGTCCCTTTAAAATGTACACAATGCGCTCTTCTTCCCGCTTAGTATTTGGCTTACAGTG
CTTAAGGCCATCAGAAAACTTTTTAGATATGGCATCAATTTCCGGTCCTTGACAATAATTTTTTAAACAA
TATTTAGCAACTAAGTTACCCACAGCTATATATAG
>dme_circ_0008887_junction_seq
CAACTAAGTTACCCACAGCTATATATAGAAGTTCCAAGGATTTAGATTGCAAGGTT
>dme_circ_0008888
GATTTAGATTGCAAGGTTTCTTCGCATTTAACTTTTGAAAAAGCGTGCAAGGCTGCGACACGTATGCGGT
TGGAGCGTCCTGTGGAAGCGCACTCACTTAGTGCAGCTACCGTATTGCCACTTAAACTCTTTGCATTTCC
TAGTCCCTTTAAAATGTACACAATGCGCTCTTCTTCCCGCTTAG
>dme_circ_0008888_junction_seq
TACACAATGCGCTCTTCTTCCCGCTTAGGATTTAGATTGCAAGGTTTCTTCGCATT
>dme_circ_0008889
GCGGGAAGAAGAGCGCATTGTGTACATTTTAAAGGGACTAGGAAATGCAAAGAGTTTAAGTGGCAATACG
GTAGCTGCACTAAGTGAGTGCGCTTCCACAGGACGCTCCAACCGCATACGTGTCGCAGCCTTGCACGCTT
TTTCAAAAGTTAAATGCGAAGAAACCTTGCAATCTAAATCCTTGGAACTTCTTAAGAATCGTAACGAAGA
TTCAGAGTTGCGTATTGAAGCTTATTTGTCCGCGATTTCATGCCCTAACGCAGAGGTTGCTAATCAAATT
TCGGAAATAGTTAATTCCGAAACTGTTAACCAGGTTGGAGGATTTATATCATCTAATTTAAAAGCTATCC
GAGACTCTACAGACGTCAGCAGAGACCAGCAAAAATACCATTTGGCTAACATTAGGGTTACAAAAACATT
TCCCGTTGACTACAGACGATACAGTTTTAATAATGAG

>dme_circ_0008889_junction_seq
CTACAGACGATACAGTTTTAATAATGAGGCGGGAAGAAGAGCGCATTGTGTACATT
>dme_circ_0008890
AAGTTCCAAGGATTTAGATTGCAAGGTTTCTTCGCATTTAACTTTTGAAAAAGCGTGCAAGGCTGCGACA
CGTATGCGGTTGGAGCGTCCTGTGGAAGCGCACTCACTTAGTGCAGCTACCGTATTGCCACTTAAACTCT
TTGCATTTCCTAGTCCCTTTAAAATG
>dme_circ_0008890_junction_seq
CTTTGCATTTCCTAGTCCCTTTAAAATGAAGTTCCAAGGATTTAGATTGCAAGGTT
>dme_circ_0008891
CAACCTCTGCGTTAGGGCATGAAATCGCGGACAAATAAGCTTCAATACGCAACTCTGAATCTTCGTTACG
ATTCTTAAGAAGTTCCAAGGATTTAGATTGCAAGGTTTCTTCGCATTTAACTTTTGAAAAAGCGTGCAAG
GCTGCGACACGTATGCGGTTGGAGCGTCCTGTGGAAGCGCACTCACTTAGTGCAGCTACCGTATTGCCAC
TTAAACTCTTTGCATTTCCTAGTCCCTTTAAAATG
>dme_circ_0008891_junction_seq
CTTTGCATTTCCTAGTCCCTTTAAAATGCAACCTCTGCGTTAGGGCATGAAATCGC
>dme_circ_0008892
GAGTTTAAGTGGCAATACGGTAGCTGCACTAAGTGAGTGCGCTTCCACAGGACGCTCCAACCGCATACGT
GTCGCAGCCTTGCACGCTTTTTCAAAAGTTAAATGCGAAGAAACCTTGCAATCTAAATCCTTGGAACTTC
TTAAGAATCGTAACGAAGATTCAGAGTTGCGTATTGAAGCTTATTTGTCCGCGATTTCATGCCCTAACGC
AGAGGTTGCTAATCAAATTTCGGAAATAGTTAATTCCGAAACTGTTAACCAGGTTGGAGGATTTATATCA
TCTAATTTAAAAGCTATCCGAGACTCTACAGACGTCAGCAGAGACCAGCAAAAATACCATTTGGCTAACA
TTAGGGTTACAAAAACATTTCCCGTTGACTACAGACGATACA
>dme_circ_0008892_junction_seq
ACATTTCCCGTTGACTACAGACGATACAGAGTTTAAGTGGCAATACGGTAGCTGCA
>dme_circ_0008893
GTTTAAGTGGCAATACGGTAGCTGCACTAAGTGAGTGCGCTTCCACAGGACGCTCCAACCGCATACGTGT
CGCAGCCTTGCACGCTTTTTCAAAAGTTAAATGCGAAGAAACCTTGCAATCTAAATCCTTGGAACTTCTT
AAGAATCGTAACGAAGATTCAGAGTTGCGTATTGAAGCTTATTTGTCCGCGATTTCATGCCCTAACGCAG
AGGTTGCTAATCAAATTTCGGAAATAGTTAATTCCGAAACTGTTAACCAG
>dme_circ_0008893_junction_seq
AATAGTTAATTCCGAAACTGTTAACCAGGTTTAAGTGGCAATACGGTAGCTGCACT
>dme_circ_0008894
GTTTAAGTGGCAATACGGTAGCTGCACTAAGTGAGTGCGCTTCCACAGGACGCTCCAACCGCATACGTGT
CGCAGCCTTGCACGCTTTTTCAAAAGTTAAATGCGAAGAAACCTTGCAATCTAAATCCTTGGAACTTCTT
AAGAATCGTAACGAAGATTCAGAGTTGCGTATTGAAGCTTATTTGTCCGCGATTTCATGCCCTAACGCAG
AGGTTGCTAATCAAATTTCGGAAATAGTTAATTCCGAAACTGTTAACCAGGTTGGAGGATTTATATCATC
TAATTTAAAAGCTATCCGAGACTCTACAGACGTCAGCAGAGACCAGCAAAAATACCATTTGGCTAACATT
AGGGTTACAAAAACATTTCCCGTTGACTACAGACGATACAGTTTTAATAATGAG
>dme_circ_0008894_junction_seq
CTACAGACGATACAGTTTTAATAATGAGGTTTAAGTGGCAATACGGTAGCTGCACT
>dme_circ_0008895
CCAAATGGTATTTTTGCTGGTCTCTGCTGACGTCTGTAGAGTCTCGGATAGCTTTTAAATTAGATGATAT
AAATCCTCCAACCTGGTTAACAGTTTCGGAATTAACTATTTCCGAAATTTGATTAGCAACCTCTGCGTTA
GGGCATGAAATCGCGGACAAATAAGCTTCAATACGCAACTCTGAATCTTCGTTACGATTCTTAAGAAG

>dme_circ_0008895_junction_seq
CTGAATCTTCGTTACGATTCTTAAGAAGCCAAATGGTATTTTTGCTGGTCTCTGCT
>dme_circ_0008896
GAATCGTAACGAAGATTCAGAGTTGCGTATTGAAGCTTATTTGTCCGCGATTTCATGCCCTAACGCAGAG
GTTGCTAATCAAATTTCGGAAATAGTTAATTCCGAAACTGTTAACCAGGTTGGAGGATTTATATCATCTA
ATTTAAAAGCTATCCGAGACTCTACAGACGTCAGCAGAGACCAGCAAAAATACCATTTGGCTAACATTAG
GGTTACAAAAACATTTCCCGTTGACTACAGACGATACAGTTTTAATAATGAG
>dme_circ_0008896_junction_seq
CTACAGACGATACAGTTTTAATAATGAGGAATCGTAACGAAGATTCAGAGTTGCGT
>dme_circ_0008897
GAGACCAGCAAAAATACCATTTGGCTAACATTAGGGTTACAAAAACATTTCCCGTTGACTACAGACGATA
CAGTTTTAATAATGAGGTTTCCTATAAGCTTGAATCCCTTGGTGTTGGCGCCAGCACTGATTACCAAATA
ATATATTCGCAACATGGATTCTTGCCCCGATCATCTC
>dme_circ_0008897_junction_seq
CAACATGGATTCTTGCCCCGATCATCTCGAGACCAGCAAAAATACCATTTGGCTAA
>dme_circ_0008898
AAACGTCATCAACAATTGATCGTCGGGCTCGGCCACCAGCAGCAACACCATTATTGCCAACTTCAATGAG
TTTAACAATTTCGTCAAAATCTTTGTTTACTAATCCTTTCGGCCCTAAATAGTATTCCAATACGTCTTCA
ACATTTTCTTGTCGGACGCTTGCCTCAAATACATTGTAGTTCGTGCCAAAAAATTCTGTTGTTACATTGA
TACGAGATGATCGGGGCAAGAATCCATGTTGCGAATATATTATTTGGTAATCAGTGCTGGCGCCAACACC
AAGGGATTCAAGCTTATAGGAAACCTCATTATTAAAACTGTATCGTCTGTAGTCAACGGGAAATGTTTTT
GTAACCCTAATGTTAGCCAAATGG
>dme_circ_0008898_junction_seq
TTTTGTAACCCTAATGTTAGCCAAATGGAAACGTCATCAACAATTGATCGTCGGGC
>dme_circ_0008899
TATTCCAATACGTCTTCAACATTTTCTTGTCGGACGCTTGCCTCAAATACATTGTAGTTCGTGCCAAAAA
ATTCTGTTGTTACATTGATACGAGATGATCGGGGCAAGAATCCATGTTGCGAATATATTATTTGGTAATC
AGTGCTGGCGCCAACACCAAGGGATTCAAGCTTATAGGAAACCTCATTATTAAAACTGTATCGTCTGTAG
TCAACGGGAAATGTTTTTGTAACCCTAATG
>dme_circ_0008899_junction_seq
AACGGGAAATGTTTTTGTAACCCTAATGTATTCCAATACGTCTTCAACATTTTCTT
>dme_circ_0008900
GTTTTAATAATGAGGTTTCCTATAAGCTTGAATCCCTTGGTGTTGGCGCCAGCACTGATTACCAAATAAT
ATATTCGCAACATGGATTCTTGCCCCGATCATCTCGTATCAATGTAACAACAGAATTTTTTGGCACGAAC
TACAATGTATTTGAGGCAAGCGTCCGACAAGAAAATGTTGAAGACGTATTGGAATACTATTTAGGGCCGA
AAGGATTA
>dme_circ_0008900_junction_seq
GGAATACTATTTAGGGCCGAAAGGATTAGTTTTAATAATGAGGTTTCCTATAAGCT
>dme_circ_0008901
GTTTTAATAATGAGGTTTCCTATAAGCTTGAATCCCTTGGTGTTGGCGCCAGCACTGATTACCAAATAAT
ATATTCGCAACATGGATTCTTGCCCCGATCATCTCGTATCAATGTAACAACAGAATTTTTTGGCACGAAC
TACAATGTATTTGAGGCAAGCGTCCGACAAGAAAATGTTGAAGACGTATTGGAATACTATTTAGGGCCGA
AAGGATTAGTAAACAAAGATTTTGACGAAATTGTTAAACTCATTGAAGTTGGCAATAATGGTGTTGCTGC
TGGTGGCCGAGCCCGACGATCAATTGTTGATGACGTTTCTAAAATTTCGAAAAAGTATAAAATGTATGGA

GTCAAAAATGTGCAAGATCTCAATTTAGATGTGTCATTAAAACTGTTTGGTTCGGAATTGGCATTTTTGA
GCCTAGGCGACAACATACCAAGTTCCCTAGATGACATTATAAACTACTTCTCAACTTCTTTCGAAAAAGC
AAAACAGGAATTATCTTCGTTTGAAAAGCAATTTTCCAGTCACCACTTATTCCTTGATACAGACCTCGCT
TATCCAACTAGTATTGGAGTACCTTTGGAGCTTGTAGCCCAAGGTTTTGCAGCTACCAAAGTTGATCTTG
CCGTTAGTCTTGATATTAATGCAATTCTAGAACAAAACTGGCAAAAGGCTAAGTATAGACTAAAGTTTGT
TCCGAGTGTCGATATTAATGCTAACGTTCAGATAGGCTTTAATGCACAAGTACTATCCACTGGACTCCGC
GTGGTTTCGTCAGCCCATTCTGCCACCGGTAGCGATATTACTGTAGCTGTTATTAGCGATGGAGAGGGCT
TTAACGTTGACCTCGAGCTACCACGCGAAAAACTTGAGCTTATTAATTTTAATGTTGACACAGAACTATA
TGTAGCCGAACAAGACAAACAAAAGGCAATTGCCCTAAAGGGTAACaaaaaaaaTAAGAATTCTCAACCT
AGTGAGATATGCTTTAATCAATTGGAACTTGTTGGATTAAATATTTGCATTAAGAGTTCAACAAGCTTAA
GTGAGGTTCAAGCTGGTAACGGCAATGTTGCAGAGAGAGGACTTTCTGTTTCGGAGAAATTTCATTTATC
TAGACCATTTAATTTCGCCGTTTACTTAACGACTGAACGCAAATTTACATTCAAAGGAATTCACACACAG
GAAGCTTTCTCCCAAAAGTGGAAATTAGATTATTCCACTCCTGGATCCAAAGTTTCTCACGATACAACTG
TTGTATATGAACTCGGAAATAAGCCTAAAACATTTAGTAGATTATCCTTTGATAACTCTCAATGCCACTT
TGCAGTGGAAGGTGGAATAAACAACGACAAGAATGAATTGGTTGTATATGGTCAATACGAACAAGACAAA
GAAATaaaaaaaaagtaaaaTTGGCTTTAGCAAAAACGGAAATGaatacaagccattaattgaaatccaag
acaataatggaaTCTCAAATAGCATAAATG
>dme_circ_0008901_junction_seq
aataatggaaTCTCAAATAGCATAAATGGTTTTAATAATGAGGTTTCCTATAAGCT
>dme_circ_0008902
GGTTTCCTATAAGCTTGAATCCCTTGGTGTTGGCGCCAGCACTGATTACCAAATAATATATTCGCAACAT
GGATTCTTGCCCCGATCATCTCGTATCAATGTAACAACAGAATTTTTTGGCACGAACTACAATGTATTTG
AGGCAAGCGTCCGACAAGAAAATGTTGAAGACGTATTGGAATACTATTTAGGGCCGAAAGGATTAGTAAA
CAAAGATTTTGACGAAATTGTTAAACTCATTGAAGTTGGCAATAATGGT
>dme_circ_0008902_junction_seq
TAAACTCATTGAAGTTGGCAATAATGGTGGTTTCCTATAAGCTTGAATCCCTTGGT
>dme_circ_0008903
GCTTGAATCCCTTGGTGTTGGCGCCAGCACTGATTACCAAATAATATATTCGCAACATGGATTCTTGCCC
CGATCATCTCGTATCAATGTAACAACAGAATTTTTTGGCACGAACTACAATGTATTTGAGGCAAGCGTCC
GACAAGAAAATGTTGAAGACGTATTGGAATACTATTTAGGGCCGAAAGGATTAGTAAACAAAGATTTTGA
CGAAATTGTTAAACTCATTGAAGTTGGCAATAATG
>dme_circ_0008903_junction_seq
GTTAAACTCATTGAAGTTGGCAATAATGGCTTGAATCCCTTGGTGTTGGCGCCAGC
>dme_circ_0008904
TTTAACAATTTCGTCAAAATCTTTGTTTACTAATCCTTTCGGCCCTAAATAGTATTCCAATACGTCTTCA
ACATTTTCTTGTCGGACGCTTGCCTCAAATACATTGTAGTTCGTGCCAAAAAATTCTGTTGTTACATTGA
TACGAGATGATCGGGGCAAGAATCCATGTTGCGAATATATTATTTGG
>dme_circ_0008904_junction_seq
GAATCCATGTTGCGAATATATTATTTGGTTTAACAATTTCGTCAAAATCTTTGTTT
>dme_circ_0008905
GTAAACAAAGATTTTGACGAAATTGTTAAACTCATTGAAGTTGGCAATAATGGTGTTGCTGCTGGTGGCC
GAGCCCGACGATCAATTGTTGATGACGTTTCTAAAATTTCGAAAAAGTATAAAATGTATGGAGTCAAAAA
TGTGCAAGATCTCAATTTAGATGTGTCATTAAAACTGTTTGGTTCGGAATTGGCATTTTTGAGCCTAGGC
GACAACATACCAAGTTCCCTAGATGACATTATAAACTACTTCTCAACTTCTTTCGAAAAAGCAAAACAGG

AATTATCTTCGTTTGAAAAGCAATTTTCCAGTCACCACTTATTCCTTGATACAGACCTCGCTTATCCAAC
TAGTATTGGAGTACCTTTGGAGCTTGTAGCCCAAGGTTTTGCAGCTACCAAAGTTGATCTTGCCGTTAGT
CTTGATATTAATGCAATTCTAGAACAAAACTGGCAAAAGGCTAA
>dme_circ_0008905_junction_seq
TTCTAGAACAAAACTGGCAAAAGGCTAAGTAAACAAAGATTTTGACGAAATTGTTA
>dme_circ_0008906
GTAAACAAAGATTTTGACGAAATTGTTAAACTCATTGAAGTTGGCAATAATGGTGTTGCTGCTGGTGGCC
GAGCCCGACGATCAATTGTTGATGACGTTTCTAAAATTTCGAAAAAGTATAAAATGTATGGAGTCAAAAA
TGTGCAAGATCTCAATTTAGATGTGTCATTAAAACTGTTTGGTTCGGAATTGGCATTTTTGAGCCTAGGC
GACAACATACCAAGTTCCCTAGATGACATTATAAACTACTTCTCAACTTCTTTCGAAAAAGCAAAACAGG
AATTATCTTCGTTTGAAAAGCAATTTTCCAGTCACCACTTATTCCTTGATACAGACCTCGCTTATCCAAC
TAGTATTGGAGTACCTTTGGAGCTTGTAGCCCAAGGTTTTGCAGCTACCAAAGTTGATCTTGCCGTTAGT
CTTGATATTAATGCAATTCTAGAACAAAACTGGCAAAAGGCTAAGTATAGACTAAAGTTT
>dme_circ_0008906_junction_seq
GCAAAAGGCTAAGTATAGACTAAAGTTTGTAAACAAAGATTTTGACGAAATTGTTA
>dme_circ_0008907
CTATTGTTAAATGTATCGAACTTCCATAAATATTTTCGGGAGAATGTTCATCATTTATGAAGAAACCAGC
CTCATACAGACCATTTTCCAATTTTAAATTTTCTTTTATTAAGAAGGTTTGGTGATCTAAAGAAATTCGA
AGCTCAGAGGTTAATGAATTTGTCCCAACATCCGACCATCCGTTTACTGCTACGTGGGCGTTATTAGAAT
TTGACACTTGGAAGTTTTCAAAATTATATCTTTCAATATTATTACTGtttttttttACAACTATTTTTCC
GTCTGCGTGGTAACCATTTATGCTATTTGAGAttccattattgtcttggatttcaattaatggcttgtat
tCATTTCCGTTTTTGCTAAAGCCAAttttacttttttttATTTCTTTGTCTTGTTCGTATTGACCATATA
CAACCAATTCATTCTTGTCGTTGTTTATTCCACCTTCCACTGCAAAGTGGCATTGAGAGTTATCAAAGGA
TAATCTACTAAATGTTTTAGGCTTATTTCCGAGTTCATATACAACAGTTGTATCGTGAGAAACTTTGGAT
CCAGGAGTGGAATAATCTAATTTCCACTTTTGGGAGAAAGCTTCCTGTGTGTGAATTCCTTTGAATGTAA
ATTTGCGTTCAGTCGTTAAGTAAACGGCGAAATTAAATGGTCTAGATAAATGAAATTTCTCCGAAACAGA
AAGTCCTCTCTCTGCAACATTGCCGTTACCAGCTTGAACCTCACTTAAGCTTGTTGAACTCTTAATGCAA
ATATTTAATCCAACAAGTTCCAATTGATTAAAGCATATCTCACTAGGTTGAGAATTCTTAttttttttGT
TACCCTTTAGGGCAATTGCCTTTTGTTTGTCTTGTTCGGCTACATATAGTTCTGTGTCAACATTAAAATT
AATAAGCTCAAGTTTTTCGCGTGGTAGCTCGAGGTCAACGTTAAAGCCCTCTCCATCGCTAATAACAGCT
ACAGTAATATCGCTACCGGTGGCAGAATGGGCTGACGAAACCACGCGGAGTCCAGTGGATAGTACTTGTG
CATTAAAGCCTATCTGAACGTTAGCATTAATATCGACACTCGGAACAAACTTTAGTCTATACTTAGCCTT
TTGCCAGTTTTGTTCTAGAATTGCATTAATATCAAGACTAACGGCAAGATCAACTTTGGTAGCTGCAAAA
CCTTGGGCTACAAGCTCCAAAGGTACTCCAATACTAGTTGGATAAGCGAGGTCTGTATCAAGGAATAAGT
GGTGACTGGAAAATTGCTTTTCAAACGAAGATAATTCCTGTTTTGCTTTTTCGAAAGAAGTTGAGAAGTA
GTTTATAATGTCATCTAGGGAACTTGGTATGTTGTCGCCTAGGCTCAAAAATGCCAATTCCGAACCAAAC
AGTTTTAATGACACATCTAAATTGAGATCTTGCACATTTTTGACTCCATACATTTTATACTTTTTCGAAA
TTTTAGAAACGTCATCAACAATTGATCGTCGGGCTCGGCCACCAGCAGCAACACCATTATTGCCAACTTC
AATGAGTTTAACAATTTCGTCAAAATCTTTG
>dme_circ_0008907_junction_seq
GAGTTTAACAATTTCGTCAAAATCTTTGCTATTGTTAAATGTATCGAACTTCCATA
>dme_circ_0008908
GTCAAAAATGTGCAAGATCTCAATTTAGATGTGTCATTAAAACTGTTTGGTTCGGAATTGGCATTTTTGA
GCCTAGGCGACAACATACCAAGTTCCCTAGATGACATTATAAACTACTTCTCAACTTCTTTCGAAAAAGC

AAAACAGGAATTATCTTCGTTTGAAAAGCAATTTTCCAGTCACCACTTATTCCTTGATACAGACCTCGCT
TATCCAACTAGTATTGGAGTACCTTTGGAGCTTGTAGCCCAAG
>dme_circ_0008908_junction_seq
GGAGTACCTTTGGAGCTTGTAGCCCAAGGTCAAAAATGTGCAAGATCTCAATTTAG
>dme_circ_0008909
GATGACATTATAAACTACTTCTCAACTTCTTTCGAAAAAGCAAAACAGGAATTATCTTCGTTTGAAAAGC
AATTTTCCAGTCACCACTTATTCCTTGATACAGACCTCGCTTATCCAACTAGTATTGGAGTACCTTTGGA
GCTTGTAGCCCAAGGTTTTGCAGCTACCAAAGTTGATCTTGCCGTTAGTCTTGATATTAATGCAATTCTA
GAACAAAACTGGCAAAAGGCTAAGTATAGACTAAAGTTT
>dme_circ_0008909_junction_seq
GCAAAAGGCTAAGTATAGACTAAAGTTTGATGACATTATAAACTACTTCTCAACTT
>dme_circ_0008910
GGAATTATCTTCGTTTGAAAAGCAATTTTCCAGTCACCACTTATTCCTTGATACAGACCTCGCTTATCCA
ACTAGTATTGGAGTACCTTTGGAGCTTGTAGCCCAAGGTTTTGCAGCTACCAAAGTTGATCTTGCCGTTA
GTCTTGATATTAATGCAATTCTAGAACAAAACTGGCAAAAGGCTAAGTATAGACTAAAGTTT
>dme_circ_0008910_junction_seq
GCAAAAGGCTAAGTATAGACTAAAGTTTGGAATTATCTTCGTTTGAAAAGCAATTT
>dme_circ_0008911
GTCACCACTTATTCCTTGATACAGACCTCGCTTATCCAACTAGTATTGGAGTACCTTTGGAGCTTGTAGC
CCAAGGTTTTGCAGCTACCAAAGTTGATCTTGCCGTTAGTCTTGATATTAATGCAATTCTAGAACAAAAC
TGGCAAAAGGCTAAGTATAGACTAAAGTTTGTTCCGA
>dme_circ_0008911_junction_seq
GCTAAGTATAGACTAAAGTTTGTTCCGAGTCACCACTTATTCCTTGATACAGACCT
>dme_circ_0008912
GTTGAGAATTCTTAttttttttGTTACCCTTTAGGGCAATTGCCTTTTGTTTGTCTTGTTCGGCTACATA
TAGTTCTGTGTCAACATTAAAATTAATAAGCTCAAGTTTTTCGCGTGGTAGCTCGAGGTCAACGTTAAAG
CCCTCTCCATCGCTAATAACAGCTACAGTAATATCGCTACCGGTGGCAGAATGGGCTGACGAAACCACGC
GGAGTCCAGTGGATAGTACTTGTGCATTAAAGCCTATCTGAACGTTAGCATTAATATCGACACTCGGAAC
AAACTTTAGTCTATACTTAGCCTTTTGCCAGTTTTGTTCTAGAATTGCATTAATATCAAGACTAACGGCA
AGATCAACTTTGGTAGCTGCAAAACCTTGGGCTACAAGCTCCAAAGGTACTCCAATACTAGTTGGATAAG
CGAGGTCTG
>dme_circ_0008912_junction_seq
CCAATACTAGTTGGATAAGCGAGGTCTGGTTGAGAATTCTTAttttttttGTTACC
>dme_circ_0008913
GACCTCGCTTATCCAACTAGTATTGGAGTACCTTTGGAGCTTGTAGCCCAAGGTTTTGCAGCTACCAAAG
TTGATCTTGCCGTTAGTCTTGATATTAATGCAATTCTAGAACAAAACTGGCAAAAGGCTAAGTATAGACT
AAAGTTTGTTCCGAGTGTCGATATTAATGCTAACGTTCAGATAGGCTTTAATGCACAAGTACTATCCACT
GGACTCCGCGTGGTTTCGTCAGCCCATTCTGCCACCGGTAGCGATATTACTGTAGCTGTTATTAGCGATG
GAGAGGGCTTTAACGTTGACCTCGAGCTACCACGCGAAAAACTTGAGCTTATTAATTTTAAT
>dme_circ_0008913_junction_seq
CGAAAAACTTGAGCTTATTAATTTTAATGACCTCGCTTATCCAACTAGTATTGGAG
>dme_circ_0008914
GCTACCAAAGTTGATCTTGCCGTTAGTCTTGATATTAATGCAATTCTAGAACAAAACTGGCAAAAGGCTA
AGTATAGACTAAAGTTTGTTCCGAGTGTCGATATTAATGCTAACGTTCAGATAGGCTTTAATGCACAAGT

ACTATCCACTGGACTCCGCGTGGTTTCGTCAGCCCATTCTGCCACCGGTAGCGATATTACTGTAGCT
>dme_circ_0008914_junction_seq
TGCCACCGGTAGCGATATTACTGTAGCTGCTACCAAAGTTGATCTTGCCGTTAGTC
>dme_circ_0008915
CTCAAGTTTTTCGCGTGGTAGCTCGAGGTCAACGTTAAAGCCCTCTCCATCGCTAATAACAGCTACAGTA
ATATCGCTACCGGTGGCAGAATGGGCTGACGAAACCACGCGGAGTCCAGTGGATAGTACTTGTGCATTAA
AGCCTATCTGAACG
>dme_circ_0008915_junction_seq
TACTTGTGCATTAAAGCCTATCTGAACGCTCAAGTTTTTCGCGTGGTAGCTCGAGG
>dme_circ_0008916
GCTACCACGCGAAAAACTTGAGCTTATTAATTTTAATGTTGACACAGAACTATATGTAGCCGAACAAGAC
AAACAAAAGGCAATTGCCCTAAAGGGTAACaaaaaaaaTAAGAATTCTCAACCTAGTGAGATATGCTTTA
ATCAATTGGAACTTGTTGGATTAAATATTTGCATTAAGAGTTCAACAAGCTTAAGTGAG
>dme_circ_0008916_junction_seq
CATTAAGAGTTCAACAAGCTTAAGTGAGGCTACCACGCGAAAAACTTGAGCTTATT
>dme_circ_0008917
GCTACCACGCGAAAAACTTGAGCTTATTAATTTTAATGTTGACACAGAACTATATGTAGCCGAACAAGAC
AAACAAAAGGCAATTGCCCTAAAGGGTAACaaaaaaaaTAAGAATTCTCAACCTAGTGAGATATGCTTTA
ATCAATTGGAACTTGTTGGATTAAATATTTGCATTAAGAGTTCAACAAGCTTAAGTGAGGTTCAAGCTG
>dme_circ_0008917_junction_seq
TCAACAAGCTTAAGTGAGGTTCAAGCTGGCTACCACGCGAAAAACTTGAGCTTATT
>dme_circ_0008918
CTTGAACCTCACTTAAGCTTGTTGAACTCTTAATGCAAATATTTAATCCAACAAGTTCCAATTGATTAAA
GCATATCTCACTAGGTTGAGAATTCTTAttttttttGTTACCCTTTAGGGCAATTGCCTTTTGTTTGTCT
TGTTCGGCTACATATAGTTCTGTGTCAACATTAAAATTAATAAGCTCAAGTTTTTCGCG
>dme_circ_0008918_junction_seq
TAAAATTAATAAGCTCAAGTTTTTCGCGCTTGAACCTCACTTAAGCTTGTTGAACT
>dme_circ_0008919
ATAAATGAAATTTCTCCGAAACAGAAAGTCCTCTCTCTGCAACATTGCCGTTACCAGCTTGAACCTCACT
TAAGCTTGTTGAACTCTTAATGCAAATATTTAATCCAACAAGTTCCAATTGATTAAAGCATATCTCACTA
GGTTGAGAATTCTTAttttttttGTTACCCTTTAGGGCAATTGCCTTTTGTTTGTCTTG
>dme_circ_0008919_junction_seq
TTAGGGCAATTGCCTTTTGTTTGTCTTGATAAATGAAATTTCTCCGAAACAGAAAG
>dme_circ_0008920
GAATTCTCAACCTAGTGAGATATGCTTTAATCAATTGGAACTTGTTGGATTAAATATTTGCATTAAGAGT
TCAACAAGCTTAAGTGAGGTTCAAGCTGGTAACGGCAATGTTGCAGAGAGAGGACTTTCTGTTTCGGAGA
AATTTCATTTATCTAGACCATTTAATTTCGCCGTTTACTTAACGACTGAACGCAAATTTACATTCAAAGG
AATTCACACACAGGAAGCTTTCTCCCAAAAGTGGAAATTAGATTATTCCACTCCTGGATCCAAAGTTTCT
CACGATACAACTGTTGTATATGAACTCGGAAATAAGCCTAAAACATTTAGTAGATTATCCTTTGATAACT
CTCAATGCCACTTTGCAGTGGAAGGTGGAATAAACAACGACAAGAATGAATTGGTTGTATATGGTCAATA
CGAACAAGACAAAGAAATaaaaaaaa
>dme_circ_0008920_junction_seq
TACGAACAAGACAAAGAAATaaaaaaaaGAATTCTCAACCTAGTGAGATATGCTTT
>dme_circ_0008921

GTTCAACAAGCTTAAGTGAGGTTCAAGCTGGTAACGGCAATGTTGCAGAGAGAGGACTTTCTGTTTCGGA
GAAATTTCATTTATCTAGACCATTTAATTTCGCCGTTTACTTAACGACTGAACGCAAATTTACATTCAAA
GGAATTCACACACAGGAAGCTTTCTCCCAAAAGTGGAAATTAGATTATTCCACTCCTGGATCCAAAGTTT
CTCACGATACAACTGTTGTATATGAACTCGGAAATAAGCCTAAAACATTTAGTAGATTATCCTTTGATAA
CTCTCAATGCCACTTTGCAGTGGAAGGTGGAATAAACAACGACAAGAATGAATTG
>dme_circ_0008921_junction_seq
TGGAATAAACAACGACAAGAATGAATTGGTTCAACAAGCTTAAGTGAGGTTCAAGC
>dme_circ_0008922
GGTTCAAGCTGGTAACGGCAATGTTGCAGAGAGAGGACTTTCTGTTTCGGAGAAATTTCATTTATCTAGA
CCATTTAATTTCGCCGTTTACTTAACGACTGAACGCAAATTTACATTCAAAGGAATTCACACACAGGAAG
CTTTCTCCCAAAAGTGGAAATTAGATTATTCCACTCCTGGATCCAAAGTTTCTCACGATACAACTGTTGT
ATATGAACTCGGAAATAAGCCTAAAACATTTAGTAGATTATCCTTTGATAACTCTCAATGCCACTTTGCA
GTGGAAGGTGGAATAAACAACGACAAGAATGAATTGGTTGTATATGGTCAATACGAACAAGACAAAGAAA
TaaaaaaaagtaaaaTTGGCTTTAGCAAAAACGGAAATGaatacaagccattaattgaaatccaagacaa
taatggaaTCTCAAATAGCATAAATGGTTACCACGCAGACGGAAAAATAGTTGTaaaaaaaaaCA
>dme_circ_0008922_junction_seq
GACGGAAAAATAGTTGTaaaaaaaaaCAGGTTCAAGCTGGTAACGGCAATGTTGCA
>dme_circ_0008923
GGTTCAAGCTGGTAACGGCAATGTTGCAGAGAGAGGACTTTCTGTTTCGGAGAAATTTCATTTATCTAGA
CCATTTAATTTCGCCGTTTACTTAACGACTGAACGCAAATTTACATTCAAAGGAATTCACACACAGGAAG
CTTTCTCCCAAAAGTGGAAATTAGATTATTCCACTCCTGGATCCAAAGTTTCTCACGATACAACTGTTGT
ATATGAACTCGGAAATAAGCCTAAAACATTTAGTAGATTATCCTTTGATAACTCTCAATGCCACTTTGCA
GTGGAAGGTGGAATAAACAACGACAAGAATGAATTGGTTGTATATGGTCAATACGAACAAGACAAAGAAA
TaaaaaaaagtaaaaTTGGCTTTAGCAAAAACGGAAATGaatacaagccattaattgaaatccaagacaa
taatggaaTCTCAAATAGCATAAATGGTTACCACGCAGACGGAAAAATAGTTGTaaaaaaaaaCAGTAAT
AATATTGAAAGATATAATTTTGAAAACTTCCAAGTGTCAAATTCTAATAACGCCCACGTAGCAGTAAACG
GATGGTCGGATGTTGGGACAAATTCATTAACCTCTGAGCTTCGAATTTCTTTAGATCACCAAACCTTCTT
AATAAAAGAAAATTTAAAATTGGAAAATGGTCTGTATGAGGCTGGTTTCTTCATAAATGATGAACATTCT
CCCGAAAATATTTATGGAAGTTCGATACATTTAACAATAGCTGACCAGTCTTATGCTCTTAAAACTAACG
GGAAAGCAGCTGCTTGGTCCATTGGCAGTGATGGTAGTTTTAATTTCCAAAAACTTGCCGATTCCAACTC
TGCTCGTGCAGGCTCACTTGTGGAAAATGTAGAAATTCA
>dme_circ_0008923_junction_seq
GCTCACTTGTGGAAAATGTAGAAATTCAGGTTCAAGCTGGTAACGGCAATGTTGCA
>dme_circ_0008924
GCTGGTAACGGCAATGTTGCAGAGAGAGGACTTTCTGTTTCGGAGAAATTTCATTTATCTAGACCATTTA
ATTTCGCCGTTTACTTAACGACTGAACGCAAATTTACATTCAAAGGAATTCACACACAGGAAGCTTTCTC
CCAAAAGTGGAAATTAGATTATTCCACTCCTGGATCCAAAGTTTCTCACGATACAACTGTTGTATATGAA
CTCGGAAATAAGCCTAAAACATTTAGTAGATTATCCTTTGATAACTCTCAATGCCACTTTGCAGTGGAAG
GTGGAATAAACAACGACAAGAATGAATTGGTTGTATATGGTCAATACGAACAAGACAAAGAAATaaaaaa
aagtaaaaTTGGCTTTAGCAAAAACGGAAATGaatacaagccattaattgaaatccaagacaataatgga
aTCTCAAATAGCATAAATG
>dme_circ_0008924_junction_seq
aataatggaaTCTCAAATAGCATAAATGGCTGGTAACGGCAATGTTGCAGAGAGAG
>dme_circ_0008925

GCTGGTAACGGCAATGTTGCAGAGAGAGGACTTTCTGTTTCGGAGAAATTTCATTTATCTAGACCATTTA
ATTTCGCCGTTTACTTAACGACTGAACGCAAATTTACATTCAAAGGAATTCACACACAGGAAGCTTTCTC
CCAAAAGTGGAAATTAGATTATTCCACTCCTGGATCCAAAGTTTCTCACGATACAACTGTTGTATATGAA
CTCGGAAATAAGCCTAAAACATTTAGTAGATTATCCTTTGATAACTCTCAATGCCACTTTGCAGTGGAAG
GTGGAATAAACAACGACAAGAATGAATTGGTTGTATATGGTCAATACGAACAAGACAAAGAAATaaaaaa
aagtaaaaTTGGCTTTAGCAAAAACGGAAATGaatacaagccattaattgaaatccaagacaataatgga
aTCTCAAATAGCATAAATGGTTACCACGCAGACGGAAAAATAGTTGTaaaaaaaaaCAGTAATAATATTG
AAAGATATAATTTTGAAAACTTCCAAGTGTCAAATTCTAATAACGCCCACGTAGCAGTAAACGGATGGTC
GGATGTTGGGACAAATTCATTAACCTCTGAGCTTCGAATTTCTTTAGATCACCAAACCTTCTTAATAAAA
GAAAATTTAAAATTGGAAAATGGTCTGTATGAGGCTGGTTTCTTCATAAATGATGAACATTCTCCCGAAA
ATATTTATGGAAGTTCGATACATTTAACAATAGCTGACCAGTCTTATGCTCTTAAAACTAACGGGAAAGC
AGCTGCTTGGTCCATTGGCAGTGATGGTAGTTTTAATTTCCAAAAACTTGCCGATTCCAACTCTGCTCGT
GCAGGCTCACTTGTGGAAAATGTAGAAATTCAGTATAAAAATAAGCAGGTCGGAGGAATAAAGATTATGT
CGAATTTTGATGTTAATAAAATGGATGTCGATGTGGAAATTTCGCGTGAGCAAAAAATTGGGTCCATAAT
CGTTAAATACGAAAGTAATCAGCGACACGCACAGGATTATTCCCTGGAGGCAAGTGCAAAGATTAACAAG
CACTCCATAGATGTTATATCTAAGTGTGATTTTAACGGAAACGTCTATGTTGTCGATAACTCTTTAGTAA
CTAGTTGGGGAACTTTACTATCTGCAAAAGGAGAAATAGGACAACGTTACTCGGCCCAAGACATTAATAT
TAATATTCAAGGCAATGTTCAAATTAGCGGAAAGGACAAAGTTACTCAATGGATACTTAAAGTAATCGGC
ACACCTGACAAAACCAACAGCGACTTTAGGATTAGCCGAGACACCTCAGAGCTTATTAAACTAACTAGCG
AGTCTCAACATCCGCAAGATAAAATATCCTTTGCCAAACTTAACTTAATTGTCAAAAATCAATTAACTGC
CAAAGGCGAGTTTCGTGTAGCCAAAAATGGAAAGGGTGACTTTACAGCCAGTATTGACACTCTGAAGACT
GAACCGAAGCACAAACTAGAAATAGAATCTAAATTTCACATACAGTCTCCAAAGTACGATATTGATGCTT
CCCTAACACTTGATGGAAAAAGGAAAGTGCACTTGAAGTCAGAAAACACTATAGAAAAGTTAAAATTCTC
AACTAAAAACATTGGTGAGGCAAACGACAAAATAATTGCTTTCGAAGCTAATGGAAGTTTAAAAGGCGAA
TTGCGAGGAAATGGGGAAATACAAG
>dme_circ_0008925_junction_seq
GAATTGCGAGGAAATGGGGAAATACAAGGCTGGTAACGGCAATGTTGCAGAGAGAG
>dme_circ_0008926
TTCATATACAACAGTTGTATCGTGAGAAACTTTGGATCCAGGAGTGGAATAATCTAATTTCCACTTTTGG
GAGAAAGCTTCCTGTGTGTGAATTCCTTTGAATGTAAATTTGCGTTCAGTCGTTAAGTAAACGGCGAAAT
TAAATGG
>dme_circ_0008926_junction_seq
TCGTTAAGTAAACGGCGAAATTAAATGGTTCATATACAACAGTTGTATCGTGAGAA
>dme_circ_0008927
TTATCAAAGGATAATCTACTAAATGTTTTAGGCTTATTTCCGAGTTCATATACAACAGTTGTATCGTGAG
AAACTTTGGATCCAGGAGTGGAATAATCTAATTTCCACTTTTGGGAGAAAGCTTCCTGTGTGTGAATTCC
TTTGAATGTAAATTTGCGTTCAGTCGTTAAG
>dme_circ_0008927_junction_seq
GAATGTAAATTTGCGTTCAGTCGTTAAGTTATCAAAGGATAATCTACTAAATGTTT
>dme_circ_0008928
GGAATTCACACACAGGAAGCTTTCTCCCAAAAGTGGAAATTAGATTATTCCACTCCTGGATCCAAAGTTT
CTCACGATACAACTGTTGTATATGAACTCGGAAATAAGCCTAAAACATTTAGTAGATTATCCTTTGATAA
CTCTCAATGCCACTTTGCAGTGGAAGGTGGAATAAACAACGACAAGAATGAATTGGTTGTATATGGTCAA
TACGAACAAGACAAAGAAATaaaaaaaagtaaaaTTGGCTTTAGCAAAAACGGAAATGaatacaagccat

taattgaaatccaagacaataatggaaTCTCAAATAGCATAAATGGTTACCACGCAGACGGAAAAATAGT
TGTaaaaaaaaaCAGTAATAATATTGAAAGATATAATTTTGAAAACTTCCAAGTGTCAAATTCTAATAAC
GCCCACGTAGCAGTAAACGGATGGTCGGATGTTGGGACAAATTCATTAACCTCTGAGCTTCGAATTTCTT
TAGATCACCAAACCTTCTTAATAAAAGAAAATTTAAAATTGGAAAATGGTCTGTATGAGGCTGGTTTCTT
CATAAATGATGAACATTCTCCCGAAAATATTTATGGAAGTTCGATACATTTAACAATAGCTGACCAGTCT
TATGCTCTTAAAACTAACGGGAAAGCAGCTGCTTGGTCCATTGGCAGTGATGGTAGTTTTAATTTCCAAA
AACTTGCCGATTCCAACTCTGCTCGTGCAGGCTCACTTGTGGAAAAT
>dme_circ_0008928_junction_seq
TGCTCGTGCAGGCTCACTTGTGGAAAATGGAATTCACACACAGGAAGCTTTCTCCC
>dme_circ_0008929
GGAATTCACACACAGGAAGCTTTCTCCCAAAAGTGGAAATTAGATTATTCCACTCCTGGATCCAAAGTTT
CTCACGATACAACTGTTGTATATGAACTCGGAAATAAGCCTAAAACATTTAGTAGATTATCCTTTGATAA
CTCTCAATGCCACTTTGCAGTGGAAGGTGGAATAAACAACGACAAGAATGAATTGGTTGTATATGGTCAA
TACGAACAAGACAAAGAAATaaaaaaaaagtaaaaTTGGCTTTAGCAAAAACGGAAATGaatacaagccat
taattgaaatccaagacaataatggaaTCTCAAATAGCATAAATGGTTACCACGCAGACGGAAAAATAGT
TGTaaaaaaaaaCAGTAATAATATTGAAAGATATAATTTTGAAAACTTCCAAGTGTCAAATTCTAATAAC
GCCCACGTAGCAGTAAACGGATGGTCGGATGTTGGGACAAATTCATTAACCTCTGAGCTTCGAATTTCTT
TAGATCACCAAACCTTCTTAATAAAAGAAAATTTAAAATTGGAAAATGGTCTGTATGAGGCTGGTTTCTT
CATAAATGATGAACATTCTCCCGAAAATATTTATGGAAGTTCGATACATTTAACAATAGCTGACCAGTCT
TATGCTCTTAAAACTAACGGGAAAGCAGCTGCTTGGTCCATTGGCAGTGATGGTAGTTTTAATTTCCAAA
AACTTGCCGATTCCAACTCTGCTCGTGCAGGCTCACTTGTGGAAAATGTAGAAATTCAGTATAAAAATAA
GCAGGTCGGAGGAATAAAGATTATGTCGAATTTTGATGTTAATAAAATGGATGTCGATGTGGAAATTTCG
CGTGAGCAAAAAATTGGGTCCATAATCGTTAAATACGAAAGTAATCAGCGACACGCACAGGATTATTCCC
TGGAGGCAAGTGCAAAGATTAACAAGCACTCCATAGATGTTATATCTAAGTGTGATTTTAACGGAAACGT
CTATGTTGTCGATAACTCTTTAGTAACTAGTTGGGGAACTTTACTATCTGCAAAAGGAGAAATAGGACAA
CGTTACTCGGCCCAAGACATTAATATTAATATTCAAGGCAATGTTCAAATTAGCGGAAAGGACAAAGTTA
CTCAATGGATACTTAAAGTAATCGGCACACCTGACAAAACCAACAGCGACTTTAGGATTAGCCGAGACAC
CTCAGAGCTTATTAAACTAACTAGCGAGTCTCAACATCCGCAAGATAAAATATCCTTTGCCAAACTTAAC
TTAATTGTCAAAAATCAATTAACTGCCAAAGGCGAGTTTCGTGTAGCCAAAAATGGAAAGGGTGACTTTA
CAGCCAGTATTGACACTCTGAAGACTGAACCGAAGCACAAACTAGAAATAGAATCTAAATTTCACATACA
GTCTCCAAAGTACGATATTGATGCTTCCCTAACACTTGATGGAAAAAGGAAAGTGCACTTGAAGTCAGAA
AACACTATAGAAAAGTTAAAATTCTCAACTAAAAACATTGGTGAGGCAAACGACAAAATAATTGCTTTCG
AAGCTAATGGAAGTTTAAAAGGCGAATTGCGAGGAAATGGGGAAATACAAGGTACCTTTATATTTAACGC
TCCCGATGGTCGAGTCATCGACGGCAGTATCAATCGTAAGATTTCCACAAATGCAAAAAGTGGCTTATCT
CAAGGCAACATTGACGCGCAGCTTAGTGATACACCTTTTGGCAGTAATAAGAAACGCTCAATTTCACTAA
TCGGAAAGCTTGATCGGCTCAACACAAAAACCAAAGAATTTTCTGCAAACAGTAATTTAGTCTACACGGC
ATTTAATGGAGAAAAGTCGGAGATAAGTTATCAAATTAAGCAGCAACCAAACGGGGATGCTAAAAACATC
GATTTTAGCCTTAAGGCCTATGGAAATCCACTACCCCAACCTTTTGAGATCGCTTTTGCCTTAGGAGATT
ACAGTGCACAGCATGCCGTAGTTAGTATCACAAGTAAGTACGGTGAGATTTTTTCTGTAAGTGCAAATGG
AAACTACAACAATAATCAAGCACTTGAATATGGGCTTCAGGCTAATATTGAAATTCCGAAATCCACCTTG
AAGTCCTTAGAAATAAACAGTCATGGAAAGGTCCTAAAATCTTTAATCGGAAATGAAAACGCTGCATACA
ATGTAGAATTCTTCCTGGATTCTAAAACCAGTCTAGGGCAATATGCTCGTGTCAATACAGTATGGAACGG
CACGGCAAATGATGGCAGCTATGATTTTGAAGCTCAAACTAACAATATGGAGTCTCCATTAAAGTTCAAT
G

>dme_circ_0008929_junction_seq
AATATGGAGTCTCCATTAAAGTTCAATGGGAATTCACACACAGGAAGCTTTCTCCC
>dme_circ_0008930
GCTTTCTCCCAAAAGTGGAAATTAGATTATTCCACTCCTGGATCCAAAGTTTCTCACGATACAACTGTTG
TATATGAACTCGGAAATAAGCCTAAAACATTTAGTAGATTATCCTTTGATAACTCTCAATGCCACTTTGC
AGTGGAAGGTGGAATAAACAACGACAAGAATGAATTGGTT
>dme_circ_0008930_junction_seq
AATAAACAACGACAAGAATGAATTGGTTGCTTTCTCCCAAAAGTGGAAATTAGATT
>dme_circ_0008931
TGTCAATACTGGCTGTAAAGTCACCCTTTCCATTTTTGGCTACACGAAACTCGCCTTTGGCAGTTAATTG
ATTTTTGACAATTAAGTTAAGTTTGGCAAAGGATATTTTATCTTGCGGATGTTGAGACTCGCTAGTTAGT
TTAATAAGCTCTGAGGTGTCTCGGCTAATCCTAAAGTCGCTGTTGGTTTTGTCAGGTGTGCCGATTACTT
TAAGTATCCATTGAGTAACTTTGTCCTTTCCGCTAATTTGAACATTGCCTTGAATATTAATATTAATGTC
TTGGGCCGAGTAACGTTGTCCTATTTCTCCTTTTGCAGATAGTAAAGTTCCCCAACTAGTTACTAAAGAG
TTATCGACAACATAGACGTTTCCGTTAAAATCACACTTAGATATAACATCTATGGAGTGCTTGTTAATCT
TTGCACTTGCCTCCAGGGAATAATCCTGTGCGTGTCGCTGATTACTTTCGTATTTAACGATTATGGACCC
AATTTTTTGCTCACGCGAAATTTCCACATCGACATCCATTTTATTAACATCAAAATTCGACATAATCTTT
ATTCCTCCGACCTGCTTATTTTTATACTGAATTTCTACATTTTCCACAAGTGAGCCTGCACGAGCAGAGT
TGGAATCGGCAAGTTTTTGGAAATTAAAACTACCATCACTGCCAATGGACCAAGCAGCTGCTTTCCCGTT
AGTTTTAAGAGCATAAGACTGGTCAGCTATTGTTAAATGTATCGAACTTCCATAAATATTTTCGGGAGAA
TGTTCATCATTTATGAAGAAACCAGCCTCATACAGACCATTTTCCAATTTTAAATTTTCTTTTATTAAGA
AGGTTTGGTGATCTAAAGAAATTCGAAGCTCAGAGGTTAATGAATTTGTCCCAACATCCGACCATCCGTT
TACTGCTACGTGGGCGTTATTAGAATTTGACACTTGGAAGTTTTCAAAATTATATCTTTCAATATTATTA
CTGtttttttttACAACTATTTTTCCGTCTGCGTGGTAACCATTTATGCTATTTGAGAttccattattgt
cttggatttcaattaatggcttgtattCATTTCCGTTTTTGCTAAAGCCAAttttactttttttttATTTC
TTTGTCTTGTTCGTATTGACCATATACAACCAATTCATTCTTGTCGTTGTTTATTCCACCTTCCACTGCA
AAGTGGCATTGAGAGTTATCAAAGGATAATCTACTAAATGTTTTAGGCTTATTTCCGAGTTCATATACAA
CAGTTG
>dme_circ_0008931_junction_seq
TTATTTCCGAGTTCATATACAACAGTTGTGTCAATACTGGCTGTAAAGTCACCCTT
>dme_circ_0008932
TTTTAAGAGCATAAGACTGGTCAGCTATTGTTAAATGTATCGAACTTCCATAAATATTTTCGGGAGAATG
TTCATCATTTATGAAGAAACCAGCCTCATACAGACCATTTTCCAATTTTAAATTTTCTTTTATTAAGAAG
GTTTGGTGATCTAAAGAAATTCGAAGCTCAGAGGTTAATGAATTTGTCCCAACATCCGACCATCCGTTTA
CTGCTACGTGGGCGTTATTAGAATTTGACACTTGGAAGTTTTCAAAATTATATCTTTCAATATTATTACT
GtttttttttACAACTATTTTTCCGTCTGCGTGGTAACCATTTATGCTATTTGAGAttccattattgtct
tggatttcaattaatggcttgtattCATTTCCGTTTTTGCTAAAGCCAAttttactttttttttATTTCTT
TGTCTTGTTCGTATTGACCATATACAACCAATTCATTCTTGTCGTTG
>dme_circ_0008932_junction_seq
ATATACAACCAATTCATTCTTGTCGTTGTTTTAAGAGCATAAGACTGGTCAGCTAT
>dme_circ_0008933
AAATTCGAAGCTCAGAGGTTAATGAATTTGTCCCAACATCCGACCATCCGTTTACTGCTACGTGGGCGTT
ATTAGAATTTGACACTTGGAAGTTTTCAAAATTATATCTTTCAATATTATTACTGtttttttttACAACT
ATTTTTCCGTCTGCGTGGTAACCATTTATGCTATTTGAGAttccattattgtcttggatttcaattaatg

gcttgtattCATTTCCGTTTTTGCTAAAGCCAAttttactttttttttATTTCTTTGTCTTGTTCG
>dme_circ_0008933_junction_seq
actttttttttATTTCTTTGTCTTGTTCGAAATTCGAAGCTCAGAGGTTAATGAATT
>dme_circ_0008934
AAACCAGCCTCATACAGACCATTTTCCAATTTTAAATTTTCTTTTATTAAGAAGGTTTGGTGATCTAAAG
AAATTCGAAGCTCAGAGGTTAATGAATTTGTCCCAACATCCGACCATCCGTTTACTGCTACGTGGGCGTT
ATTAGAATTTGACACTTGGAAGTTTTCAAAATTATATCTTTCAATATTATTACTGtttttttttACAACT
ATTTTTCCGTCTGCGTGGTAACCATTTATGCTATTTGAGAttccattattgtcttggatttcaattaatg
gcttgtattCATTTCCGTTTTTGCTAAAGCCAAttttactttttttttATTTCTTTGTCTTGTTCG
>dme_circ_0008934_junction_seq
actttttttttATTTCTTTGTCTTGTTCGAAACCAGCCTCATACAGACCATTTTCCA
>dme_circ_0008935
ATATAACATCTATGGAGTGCTTGTTAATCTTTGCACTTGCCTCCAGGGAATAATCCTGTGCGTGTCGCTG
ATTACTTTCGTATTTAACGATTATGGACCCAATTTTTTGCTCACGCGAAATTTCCACATCGACATCCATT
TTATTAACATCAAAATTCGACATAATCTTTATTCCTCCGACCTGCTTATTTTTATACTGAATTTCTACAT
TTTCCACAAGTGAGCCTGCACGAGCAGAGTTGGAATCGGCAAGTTTTTGGAAATTAAAACTACCATCACT
GCCAATGGACCAAGCAGCTGCTTTCCCGTTAGTTTTAAGAGCATAAGACTGGTCAGCTATTGTTAAATGT
ATCGAACTTCCATAAATATTTTCGGGAGAATGTTCATCATTTATGAAGAAACCAGCCTCATACAGACCAT
TTTCCAATTTTAAATTTTCTTTTATTAAGAAGGTTTGGTGATCTAAAGAAATTCGAAGCTCAGAGGTTAA
TGAATTTGTCCCAACATCCGACCATCCGTTTACTGCTACGTGGGCGTTATTAGAATTTGACACTTGGAAG
TTTTCAAAATTATATCTTTCAATATTATTACTGtttttttttACAACTATTTTTCCGTCTGCGTGGTAAC
CATTTATGCTATTTGAGAttccattattgtcttggatttcaattaatggcttgtattCATTTCCGTTTTT
GCTAAAGCCAAttttactttttttttATTTCTTTGTCTTGTTCG
>dme_circ_0008935_junction_seq
actttttttttATTTCTTTGTCTTGTTCGATATAACATCTATGGAGTGCTTGTTAAT
>dme_circ_0008936
TTATCGACAACATAGACGTTTCCGTTAAAATCACACTTAGATATAACATCTATGGAGTGCTTGTTAATCT
TTGCACTTGCCTCCAGGGAATAATCCTGTGCGTGTCGCTGATTACTTTCGTATTTAACGATTATGGACCC
AATTTTTTGCTCACGCGAAATTTCCACATCGACATCCATTTTATTAACATCAAAATTCGACATAATCTTT
ATTCCTCCGACCTGCTTATTTTTATACTGAATTTCTACATTTTCCACAAGTGAGCCTGCACGAGCAGAGT
TGGAATCGGCAAGTTTTTGGAAATTAAAACTACCATCACTGCCAATGGACCAAGCAGCTGCTTTCCCGTT
AGTTTTAAGAGCATAAGACTGGTCAGCTATTGTTAAATGTATCGAACTTCCATAAATATTTTCGGGAGAA
TGTTCATCATTTATGAAGAAACCAGCCTCATACAGACCATTTTCCAATTTTAAATTTTCTTTTATTAAGA
AGGTTTGGTGATCTAAAGAAATTCGAAGCTCAGAGGTTAATGAATTTGTCCCAACATCCGACCATCCGTT
TACTGCTACGTGGGCGTTATTAGAATTTGACACTTGGAAGTTTTCAAAATTATATCTTTCAATATTATTA
CTGtttttttttACAACTATTTTTCCGTCTGCGTGGTAACCATTTATGCTATTTGAGAttccattattgt
cttggatttcaattaatggcttgtattCATTTCCGTTTTTGCTAAAGCCAAttttactttttttttATTTC
TTTGTCTTGTTCG
>dme_circ_0008936_junction_seq
actttttttttATTTCTTTGTCTTGTTCGTTATCGACAACATAGACGTTTCCGTTAA
>dme_circ_0008937
TTACTAAAGAGTTATCGACAACATAGACGTTTCCGTTAAAATCACACTTAGATATAACATCTATGGAGTG
CTTGTTAATCTTTGCACTTGCCTCCAGGGAATAATCCTGTGCGTGTCGCTGATTACTTTCGTATTTAACG
ATTATGGACCCAATTTTTTGCTCACGCGAAATTTCCACATCGACATCCATTTTATTAACATCAAAATTCG

ACATAATCTTTATTCCTCCGACCTGCTTATTTTTATACTGAATTTCTACATTTTCCACAAGTGAGCCTGC
ACGAGCAGAGTTGGAATCGGCAAGTTTTTGGAAATTAAAACTACCATCACTGCCAATGGACCAAGCAGCT
GCTTTCCCGTTAGTTTTAAGAGCATAAGACTGGTCAGCTATTGTTAAATGTATCGAACTTCCATAAATAT
TTTCGGGAGAATGTTCATCATTTATGAAGAAACCAGCCTCATACAGACCATTTTCCAATTTTAAATTTTC
TTTTATTAAGAAGGTTTGGTGATCTAAAGAAATTCGAAGCTCAGAGGTTAATGAATTTGTCCCAACATCC
GACCATCCGTTTACTGCTACGTGGGCGTTATTAGAATTTGACACTTGGAAGTTTTCAAAATTATATCTTT
CAATATTATTACTGttttttttACAACTATTTTTCCGTCTGCGTGGTAACCATTTATGCTATTTGAGAt
tccattattgtcttggatttcaattaatggcttgtattCATTTCCGTTTTTGCTAAAGCCAAttttactt
ttttttATTTCTTTGTCTTGTTCG
>dme_circ_0008937_junction_seq
acttttttttATTTCTTTGTCTTGTTCGTTACTAAAGAGTTATCGACAACATAGAC
>dme_circ_0008938
TAACGTTGTCCTATTTCTCCTTTTGCAGATAGTAAAGTTCCCCAACTAGTTACTAAAGAGTTATCGACAA
CATAGACGTTTCCGTTAAAATCACACTTAGATATAACATCTATGGAGTGCTTGTTAATCTTTGCACTTGC
CTCCAGGGAATAATCCTGTGCGTGTCGCTGATTACTTTCGTATTTAACGATTATGGACCCAATTTTTTGC
TCACGCGAAATTTCCACATCGACATCCATTTTATTAACATCAAAATTCGACATAATCTTTATTCCTCCGA
CCTGCTTATTTTTATACTGAATTTCTACATTTTCCACAAGTGAGCCTGCACGAGCAGAGTTGGAATCGGC
AAGTTTTTGGAAATTAAAACTACCATCACTGCCAATGGACCAAGCAGCTGCTTTCCCGTTAGTTTTAAGA
GCATAAGACTGGTCAGCTATTGTTAAATGTATCGAACTTCCATAAATATTTTCGGGAGAATGTTCATCAT
TTATGAAGAAACCAGCCTCATACAGACCATTTTCCAATTTTAAATTTTCTTTTATTAAGAAGGTTTGGTG
ATCTAAAGAAATTCGAAGCTCAGAGGTTAATGAATTTGTCCCAACATCCGACCATCCGTTTACTGCTACG
TGGGCGTTATTAGAATTTGACACTTGGAAGTTTTCAAAATTATATCTTTCAATATTATTACTGttttttt
ttACAACTATTTTTCCGTCTGCGTGGTAACCATTTATGCTATTTGAGAttccattattgtcttggatttc
aattaatggcttgtattCATTTCCGTTTTTGCTAAAGCCAAttttacttttttttATTTCTTTGTCTTGT
TCG
>dme_circ_0008938_junction_seq
acttttttttATTTCTTTGTCTTGTTCGTAACGTTGTCCTATTTCTCCTTTTGCAG
>dme_circ_0008939
ATACTTGAACGGAATGAAAGTATGCTCGGAAGAACAGCCAATGAGTCAAAAGAATGCGTTAGAGAACCAA
TCCAAGATTGAAAATCAATTACATGATCCGATGAGCCTGGATAAGTGCTTCTAATCGAGACCCAAATGGA
ACGAACAGCCTTTATTAAAAGCTGTCCAAGTTCTTCGATGTATTTTTCATTATCAATATGTTCTTGCTTT
AATTTAGCGATTAAGTAATCGTGGAGTTTTTGTAGAAACTCGGAAGTCTCTGGGGTCTGTGGTAAAATGT
TAATCTGATCAAATAGACTATTTGTTAACTCTGTTGCCTTTTCAAATAGTTTCAGAACTTTAACTTTGTC
ATTAAACTCATTACGAATACTTTCAAATGACGGAAGGCTTGCGACATATTGCTTGAATTCATGGGTTACA
CCTTCAGCGGCATTAACCACAATTTTAATGACTTCCTGAGCAGCGTCGTTTATAGGTTTTACAATTTCTC
CAATAGCTTTTCCATAGTTTTTCAGAGCTGGTCCATATTTCTCAAGCGCAGAAAGGAGATCTTTAAATGA
CTGTGTACAAATCTTGATAAACTCAGTTTTGAGTGTACTTATTAGTTTCTGTACAGTTATAATGAAATCT
TCCCAGGCTTTAAGAAGGGAATCCTTCCAAAGGGCTTGCAATTTATCGTAAATATCAACAATGGACTTCT
GAGCCTTTTCCAATATTTCTGATATTGCCTTGTTTATGTCATCAACAATACCGGCTATTTTTTTAAATAG
GGTGCGTCTAAAAAAGGGAATGCATATATTTTAACGAAAATGTTATACTGAATCATTTACTTACATGCCA
TCAATAATCGGTGCAATTGATTGATCAGCCTCAAGCTCTTGAACAATATCATTTTTATAATCAAGTAATT
TACCCTGCAGTTTGGAAAAATCTGGTCCAGCTTCTTTAGCCAATTTAACAATTTTATCCGTAACTTGACG
AGATTTTTTTAAGCGTTCCTTGATATTTTCAGAAATATATTCACTATCTTTATTTATTTCCGATTCAACA
ATATGCTGTAAAAAATATATATATCATAAATACCAACGAATTTAACTTAATATATCTTACCCAGAAACCA

TTTAGATGATCTTCATTTATAAAGTAGTTTGTTTGTAAAAAGTTTGTTGCATCTAATGCCACTCGGCCAT
TAAATAATTGTTTCCCACTTCCAATGACATCTACTTTAAATTCCTTGCCCAGCTTAACTTCTGCAGTGCC
ATCGATGAAATCTTGACGCTTCAGTATAATCTTTGCAGAATTAGGTTGAACCTCCGATGTAATTTCAATT
GGATTTTTATCAAGAATTTGAACCTTTGTATTAAGCTTTGCATAATGGTTTTGCTTAGAAAAGTCTCCGA
CTATATAAATGATATGTTTATTTGATTCACTTAAAGAGATTTCAAATTTTTCCTTATTTAAAAACGAAAT
CGCCTTAACATCGATTTCACCGTTATTTAATTCCAAGCCAATGTTATACTCATGGGCTTCAATATCCACT
GCAGCGTTACTATTTATTTGGTACTGAAACTGAAGGCCAGCAGACTTAACAGTTGTAATATATGCAATAT
TGAAACCATTTTGAGAACGGGAGTTGCGCAATTCACTGTTTCCAACCACTTTTTGTTCCGGCAGTCGGAA
AATATCAAATATTACTTCGGCGCTTGCGATCTGTTGATTCACGTCTCCATTCAATTGTCCAGAAATACTT
AATGGACGAATAGTTGGATGTTCAAACTTAAGTTTCCCGTTGGCGTTGAATGCAACGCGTTCGGTGCCCG
TTACATCTACAATAGCAGAAAAGCGGGCAACGTCGTCAGGCTTTTGCAACTTGTCAATAAAGAATGAAGC
TGTTTGCTCGTAGCGGCCAAAAAGGCTTCCGTCTGCTGGTATTTTCTGAGTAGTTTCGAACAAAATTTGA
CGTTTCGGGAGTGCTAATTTAATTGTGGACGACGTGGGCTGAACGTTGGCTGTAAATTGGTACTTGAAAT
TATTTCCTGTTATAATAAAGCTATCTAAATGGTACTGATACTTAAGACCTTGACGTGTATTTTGAGACTT
TAATTCAAACTCGTATGGATATCCAAAATCTCTCAGGTCAACAATAATAAGTGTGGTATGTTCCACAGCG
TCCAGTTTTTGTTCATCAATGCTCACAATTGATTGTACTATTAAATTTGTACACTGTCTCTTTTCTTCAC
AAACGGAAAATTTGGTGTTGAATTCCTTGTTTGTGATTTTTAAAGTGCCTAATCCGTTTTTCGATCCTAA
ATTACCCATAAAAGTGTAAGAGAAACCAACTTCCCTATCAGTACCAAAATCAAAGTGTTGCCGGGTAAGC
TTAAAATTGCCACTGAGCGCCTTTCCTTGATACTGCACTTTGCCTTGTCCATCGATAATAGTTTTGTTTA
GTTCTTTTTTAAGAGCATAAGTGGCTGTACCTGAGAAAATTATGCCATTTTCATTCTTAAGTTGAATTTT
AATGTTCTTACTTTGAGCTCGTGCTTCAAGTTCGTAGCCATCGAGCTTTAATTTCTTCGAGCTCCAGTTG
ACAATTATATAAAACTCATCAATGCTGACGTATGAAGCTTCACTATTTATTTTAATGTCCAAATCGGCCA
AGTTTAGAATTTCAAAGAGTACCTTTCCTTTTCGGTCTCCTAAGAGTTCAGCAATGCTCGATATGATAAT
TGGCTTTTCTTTCAACAGATCTAAACGAATATCAACACCTTTTTTGTGGGCCGAATGATAAAATGTTGTG
TCTAATCCGTATGACTTGTCAGTCTGTTTGAATAGTGCAGTGACTACATATGTCGAATCATCCAAAGGTT
TAAAAGTGCGAATATTAACTTTGATATCTTTTACAGAATCAAAGCTGGAATCTATAACATATGCTATATC
TACTTTCTCGGCTCCATAACCTAATGAAGCATCCATTTTTACGTATTGTGCTCCGTACTGTGCGTTAAGT
ACGTACGTTTGTTTACCGGTAAGGTCACCATCTTTTATATTACCTGTTTGTTTCCGATGATATTTACCAT
TGAACTTTAATGGAGACTCCATATTGTTAGTTTGAGCTTCAAAATCATAGCTGCCATCATTTGCCGTGCC
GTTCCATACTGTATTGACACGAGCATATTGCCCTAGACTGGTTTTAGAATCCAGGAAGAATTCTACATTG
TATGCAGCGTTTTCATTTCCGATTAAAGATTTTAGGACCTTTCCATGACTGTTTATTTCTAAGGACTTCA
AGGTGGATTTCGGAATTTCAATATTAGCCTGAAGCCCATATTCAAGTGCTTGATTATTGTTGTAGTTTCC
ATTTGCACTTACAGAAAAAATCTCACCGTACTTACTTGTGATACTAACTACGGCATGCTGTGCACTGTAA
TCTCCTAAGGCAAAAGCGATCTCAAAAGGTTGGGGTAGTGGATTTCCATAGGCCTTAAGGCTAAAATCGA
TGTTTTTAGCATCCCCGTTTGGTTGCTGCTTAATTTGATAACTTATCTCCGACTTTTCTCCATTAAATGC
CGTGTAGACTAAATTACTGTTTGCAGAAAATTCTTTGGTTTTTGTGTTGAGCCGATCAAGCTTTCCGATT
AGTGAAATTGAGCGTTTCTTATTACTGCCAAAAGGTGTATCACTAAGCTGCGCGTCAATGTTGCCTTGAG
ATAAGCCACTTTTTGCATTTGTGGAAATCTTACGATTGATACTGCCGTCGATGACTCGACCATCGGGAGC
GTTAAATATAAAGGTACCTTGTATTTCCCCATTTCCTCGCAATTCGCCTTTTAAACTTCCATTAGCTTCG
AAAGCAATTATTTTGTCGTTTGCCTCACCAATGTTTTTAGTTGAGAATTTTAACTTTTCTATAGTGTTTT
CTGACTTCAAGTGCACTTTCCTTTTTCCATCAAGTGTTAGGGAAGCATCAATATCGTACTTTGGAGACTG
TATGTGAAATTTAGATTCTATTTCTAGTTTGTGCTTCGGTTCAGTCTTCAGAGTGTCAATACTGGCTGTA
AAGTCACCCTTTCCATTTTTGGCTACACGAAACTCGCCTTTGGCAGTTAATTGATTTTTGACAATTAAGT
TAAGTTTGGCAAAGGATATTTTATCTTGCGGATGTTGAGACTCGCTAGTTAGTTTAATAAGCTCTGAGGT

GTCTCGGCTAATCCTAAAGTCGCTGTTGGTTTTGTCAGGTGTGCCGATTACTTTAAGTATCCATTGAGTA
ACTTTGTCCTTTCCGCTAATTTGAACATTGCCTTGAATATTAATATTAATGTCTTGGGCCGAGTAACGTT
GTCCTATTTCTCCTTTTGCAGATAGTAAAGTTCCCCAACTAGTTACTAAAGAGTTATCGACAACATAGAC
GTTTCCGTTAAAATCACACTTAGATATAACATCTATGGAGTGCTTGTTAATCTTTGCACTTGCCTCCAGG
GAATAATCCTGTGCGTGTCGCTGATTACTTTCGTATTTAACGATTATGGACCCAATTTTTTGCTCACGCG
AAATTTCCACATCGACATCCATTTTATTAACATCAAAATTCGACATAATCTTTATTCCTCCGACCTGCTT
ATTTTTATACTGAATTTCTACATTTTCCACAAGTGAGCCTGCACGAGCAGAGTTGGAATCGGCAAGTTTT
TGGAAATTAAAACTACCATCACTGCCAATGGACCAAGCAGCTGCTTTCCCGTTAGTTTTAAGAGCATAAG
ACTGGTCAGCTATTGTTAAATGTATCGAACTTCCATAAATATTTTCGGGAGAATGTTCATCATTTATGAA
GAAACCAGCCTCATACAGACCATTTTCCAATTTTAAATTTCTTTTATTAAGAAGGTTTGGTGATCTAAA
GAAATTCGAAGCTCAGAGGTTAATGAATTTGTCCCAACATCCGACCATCCGTTTACTGCTACGTGGGCGT
TATTAGAATTTGACACTTGGAAGTTTTCAAAATTATATCTTTCAATATTATTACTGtttttttttACAAC
TATTTTTCCGTCTGCGTGGTAACCATTTATGCTATTTGAGAttccattattgtcttggatttcaattaat
ggcttgtattCATTTCCGTTTTTGCTAAAGCCAAttttactttttttttATTTCTTTGTCTTGTTCG
>dme_circ_0008939_junction_seq
acttttttttATTTCTTTGTCTTGTTCGATACTTGAACGGAATGAAAGTATGCTCG
>dme_circ_0008940
GAAATaaaaaaaaagtaaaaTTGGCTTTAGCAAAAACGGAAATGaatacaagccattaattgaaatccaag
acaataatggaaTCTCAAATAGCATAAATGGTTACCACGCAGACGGAAAAATAGTTGTaaaaaaaaaCAG
TAATAATATTGAAAGATATAATTTTGAAAACTTCCAAGTGTCAAATTCTAATAACGCCCACGTAGCAGTA
AACGGATGGTCGGATGTTGGGACAAATTCATTAACCTCTGAGCTTCGAATTTCTTTAGATCACCAAACCT
TCTTAATAAAAGAAAATTTAAAATTGGAAAATGGTCTGTATGAGGCTGGTTTCTTCATAAATGATGAACA
TTCTCCCGAAAATATTTATGGAAGTTCGATACATTTAACAATAGCTGACCAGTCTTATGCTCTTAAAACT
AACGGGAAAGCAGCTGCTTGGTCCATTGGCAGTGATGGTAGTTTTAATTTCCAAAAACTTGCCGATTCCA
ACTCTGCTCGTGCAGGCTCACTTGTGGAAAATGTAGAAATTCA
>dme_circ_0008940_junction_seq
GCTCACTTGTGGAAAATGTAGAAATTCAGAAATaaaaaaaaagtaaaaTTGGCTTTA
>dme_circ_0008941
AAATTCGAAGCTCAGAGGTTAATGAATTTGTCCCAACATCCGACCATCCGTTTACTGCTACGTGGGCGTT
ATTAGAATTTGACACTTGGAAGTTTTCAAAATTATATCTTTCAATATTATTACTGtttttttttACAACT
ATTTTTCCGTCTGCGTGGTAACCATTTATGCTATTTGAGAttccattattgtcttggatttcaattaatg
gcttg
>dme_circ_0008941_junction_seq
attgtcttggatttcaattaatggcttgAAATTCGAAGCTCAGAGGTTAATGAATT
>dme_circ_0008942
gacaataatggaaTCTCAAATAGCATAAATGGTTACCACGCAGACGGAAAAATAGTTGTaaaaaaaaaCA
GTAATAATATTGAAAGATATAATTTTGAAAACTTCCAAGTGTCAAATTCTAATAACGCCCACGTAGCAGT
AAACGGATGGTCGGATGTTGGGACAAATTCATTAACCTCTGAGCTTCGAATTTCTTTAGATCACCAAACC
TTCTTAATAAAAGAAAATTTAAAATTGGAAAATGGTCTGTATGAGGCTGGTTTCTTCATAAATGATGAAC
ATTCTCCCGAAAATATTTATGGAAGTTCGATACATTTAACAATAGCTGACCAGTCTTATGCTCTTAAAAC
TAACGGGAAAGCAGCTGCTTGGTCCATTGGCAGTGATGGTAGTTTTAATTTCCAAAAACTTGCCGATTCC
AACTCTGCTCGTGCAGGCTCACTTGTGGAAAATGTAGAAATTCAGTATAAAAATAAGCAGGTCGGAGGAA
TAAAGATTATGTCGAATTTTGATGTTAATAAAATGGATGTCGATGTGGAAATTTCGCGTGAGCAAAAAAT
TGGGTCCATAATCGTTAAATACGAAA

>dme_circ_0008942_junction_seq
ATTGGGTCCATAATCGTTAAATACGAAAgacaataatggaaTCTCAAATAGCATAA
>dme_circ_0008943
GCATAAATGGTTACCACGCAGACGGAAAAATAGTTGTaaaaaaaaaCAGTAATAATATTGAAAGATATAA
TTTTGAAAACTTCCAAGTGTCAAATTCTAATAACGCCCACGTAGCAGTAAACGGATGGTCGGATGTTGGG
ACAAATTCATTAACCTCTGAGCTTCGAATTTCTTTAGATCACCAAACCTTCTTAATAAAAGAAAATTTAA
AATTGGAAAATGGTCTGTATGAGGCTGGTTTCTTCATAAATGATGAACATTCTCCCGAAAATATTTATGG
AAGTTCGATACATTTAACAATAGCTGACCAGTCTTATGCTCTTAAAACTAACGGGAAAGCAGCTGCTTGG
TCCATTGGCAGTGATGGTAGTTTTAATTTCCAAAAACTTGCCGATTCCAACTCTGCTCGTGCAGGCTCAC
TTGTGGAAAATGTAGAAATTCAGTATAAAAATAAGCAGGTCGGAGGAATAAAGATTATGTCGAATTTTGA
TGTTAATAAAATGGATGTCGATGTGGAAATTTCGCGTGAGCAAAAAATTGGGTCCATAATCGTTAAATAC
GAAAGTAATCAGCGACACGCACAGGATTATTCCCTGGAGGCAAGTGCAAAGATTAACAAGCACTCCATAG
ATGTTATATCTAAGTGTGATTTTAACGGAAACGTCTATGTTGTCGATAACTCTTTAGTAACTAGTTGGGG
AACTTTACTATCTGCAAAAGGAGAAATAGGACAACGTTACTCGGCCCAAGACATTAATATTAATATTCAA
GGCAATGTTCAAATTAGCGGAAAGGACAAAGTTACTCAATGGATACTTAAAGTAATCGGCACACCTGACA
AAACCAACAGCGACTTTAGGATTAGCCGAGACACCTCAGAGCTTATTAAACTAACTAGCGAGTCTCAACA
TCCGCAAGATAAAATATCCTTTGCCAAACTTAACTTAATTGTCAAAAATCAATTAACTGCCAAAGGCGAG
TTTCGTGTAGCCAAAAATGGAAAGGGTGACTTTACAGCCAGTATTGACACTCTGAAGACTGAACCGAAGC
ACAAACTAGAAATAGAATCTAAATTTCACATACAGTCTCCAAAGTACGATATTGATGCTTCCCTAACACT
TGATGGAAAAAGGAAAGTGCACTTGAAGTCAGAAAACACTATAGAAAAGTTAAAATTCTCAACTAAAAAC
ATTGGTGAGGCAAACGACAAAATAATTGCTTTCGAAGCTAATGGAAGTTTAAAAGGCGAATTGCGAGGAA
ATGGGGAAATACAAGGTACCTTTATATTTAACGCTCCCGATGGTCGAGTCATCGACGGCAGTATCAATCG
TAAGATTTCCACAAATGCAAAAAGTGGCTTATCTCAAGGCAACATTGACGCGCAGCTTAGTGATACACCT
TTTGGCAGTAATAAGAAACGCTCAATTTCACTAATCGGAAAGCTTGATCGGCTCAACACAAAAACCAAAG
AATTTTCTGCAAACAGTAATTTAGTCTACACGGCATTTAATGGAGAAAAGTCGGAGATAAGTTATCAAAT
TAAGCAGCAACCAAACGGGGATGCTAAAAACATCGATTTTAGCCTTAAGGCCTATGGAAATCCACTACCC
CAACCTTTTGAGATCGCTTTTGCCTTAGGAGATTACAGTGCACAGCATGCCGTAGTTAGTATCACAAGTA
AGTACGGTGAGATTTTTTCTGTAAGTGCAAATGGAAACTACAACAATAATCAAGCACTTGAATATGGGCT
TCAGGCTAATATTGAAATTCCGAAATCCACCTTGAAGTCCTTAGAAATAAACAGTCATGGAAAGGTCCTA
AAATCTTTAATCGGAAATGAAAACGCTGCATACAATGTAGAATTCTTCCTGGATTCTAAAACCAGTCTAG
GGCAATATGCTCGTGTCAATACAGTATGGAACGGCACGGCAAATGATGGCAGCTATGATTTTGAAGCTCA
AACTAACAATATGGAGTCTCCATTAAAGTTCAATGGTAAATATCATCGGAAACAAACAGGTAATATAAAA
GATGGTGACCTTACCGGTAAACAAACGTACGTACTTAACGCACAGTACGGAGCACAATACGTAAAAATGG
ATGCTTCATTAGGTTATGGAGCCGAGAAAGTAGATATAGCATATGTTATAGATTCCAGCTTTGATTCTGT
AAAAGATATCAAA
>dme_circ_0008943_junction_seq
CAGCTTTGATTCTGTAAAAGATATCAAAGCATAAATGGTTACCACGCAGACGGAAA
>dme_circ_0008944
GACGGAAAAATAGTTGTaaaaaaaaaCAGTAATAATATTGAAAGATATAATTTTGAAAACTTCCAAGTGT
CAAATTCTAATAACGCCCACGTAGCAGTAAACGGATGGTCGGATGTTGGGACAAATTCATTAACCTCTGA
GCTTCGAATTTCTTTAGATCACCAAACCTTCTTAATAAAAGAAAATTTAAAATTGGAAAATGGTCTGTAT
GAGGCTGGTTTCTTCATAAATGATGAACATTCTCCCGAAAATATTTATGGAAGTTCGATACATTTAACAA
TAGCTGACCAGTCTTATGCTCTTAAAACTAACGGGAAAGCAGCTGCTTGGTCCATTGGCAGTGATGGTAG
TTTTAATTTCCAAAAACTTGCCGATTCCAACTCTGCTCGTGCAGGCTCACTT

>dme_circ_0008944_junction_seq
TTCCAACTCTGCTCGTGCAGGCTCACTTGACGGAAAAATAGTTGTaaaaaaaaaCA
>dme_circ_0008945
GACGGAAAAATAGTTGTaaaaaaaaaCAGTAATAATATTGAAAGATATAATTTTGAAAACTTCCAAGTGT
CAAATTCTAATAACGCCCACGTAGCAGTAAACGGATGGTCGGATGTTGGGACAAATTCATTAACCTCTGA
GCTTCGAATTTCTTTAGATCACCAAACCTTCTTAATAAAAGAAAATTTAAAATTGGAAAATGGTCTGTAT
GAGGCTGGTTTCTTCATAAATGATGAACATTCTCCCGAAAATATTTATGGAAGTTCGATACATTTAACAA
TAGCTGACCAGTCTTATGCTCTTAAAACTAACGGGAAAGCAGCTGCTTGGTCCATTGGCAGTGATGGTAG
TTTTAATTTCCAAAAACTTGCCGATTCCAACTCTGCTCGTGCAGGCTCACTTGTGGAAAATGTAGAAATT
CAGTATAAAAATAAGCAGGTCGGAGGAATAAAGATTATGTCGAATTTTGATGTTAATAAAATGGATGTCG
AT
>dme_circ_0008945_junction_seq
TTTTGATGTTAATAAAATGGATGTCGATGACGGAAAAATAGTTGTaaaaaaaaaCA
>dme_circ_0008946
GTAATAATATTGAAAGATATAATTTTGAAAACTTCCAAGTGTCAAATTCTAATAACGCCCACGTAGCAGT
AAACGGATGGTCGGATGTTGGGACAAATTCATTAACCTCTGAGCTTCGAATTTCTTTAGATCACCAAACC
TTCTTAATAAAAGAAAATTTAAAATTGGAAAATGGTCTGTATGAGGCTGGTTTCTTCATAAATGATGAAC
ATTCTCCCGAAAATATTTATGGAAGTTCGATACATTTAACAATAGCTGACCAGTCTTATGCTCTTAAAAC
TAACGGGAAAGCAGCTGCTTGGTCCATTGGCAGTGATGGTAGTTTTAATTTCCAAAAACTTGCCGATTCC
AACTCTGCTCGTGCAGGCTCACTTGTGGAAAATGTAGAAATTCA
>dme_circ_0008946_junction_seq
GCTCACTTGTGGAAAATGTAGAAATTCAGTAATAATATTGAAAGATATAATTTTGA
>dme_circ_0008947
GATATAATTTTGAAAACTTCCAAGTGTCAAATTCTAATAACGCCCACGTAGCAGTAAACGGATGGTCGGA
TGTTGGGACAAATTCATTAACCTCTGAGCTTCGAATTTCTTTAGATCACCAAACCTTCTTAATAAAAGAA
AATTTAAAATTGGAAAATGGTCTGTATGAGGCTGGTTTCTTCATAAATGATGAACATTCTCCCGAAAATA
TTTATGGAAGTTCGATACATTTAACAATAGCTGACCAGTCTTATGCTCTTAAAACTAACGGGAAAGCAGC
TGCTTGGTCCATTGGCAGTGATGGTAGTTTTAATTTCCAAAAACTTGCCGATTCCAACTCTGCTCGTGCA
GGCTCACTTGTGGAAAATGTAGAAATTCAGTATAAAAATAAGCAGGTCGGAGGAATAAAGATTATGTCGA
ATTTTGATGTTAATAAAATGGATGTCGATGTGGAAATTTCGCGTGAGCAAAAAATTGG
>dme_circ_0008947_junction_seq
TGGAAATTTCGCGTGAGCAAAAAATTGGGATATAATTTTGAAAACTTCCAAGTGTC
>dme_circ_0008948
TTTTAAGAGCATAAGACTGGTCAGCTATTGTTAAATGTATCGAACTTCCATAAATATTTTCGGGAGAATG
TTCATCATTTATGAAGAAACCAGCCTCATACAGACCATTTTCCAATTTTAAATTTTCTTTTATTAAGAAG
GTTTGGTGATCTAAAGAAATTCGAAGCTCAGAGGTTAATGAATTTGTCCCAACATCCGACCATCCG
>dme_circ_0008948_junction_seq
TGAATTTGTCCCAACATCCGACCATCCGTTTTAAGAGCATAAGACTGGTCAGCTAT
>dme_circ_0008949
GCTGACCAGTCTTATGCTCTTAAAACTAACGGGAAAGCAGCTGCTTGGTCCATTGGCAGTGATGGTAGTT
TTAATTTCCAAAAACTTGCCGATTCCAACTCTGCTCGTGCAGGCTCACTTGTGGAAAATGTAGAAATTCA
GTATAAAAATAAGCAGGTCGGAGGAATAAAGATTATGTCGAATTTTGAT
>dme_circ_0008949_junction_seq
AGGAATAAAGATTATGTCGAATTTTGATGCTGACCAGTCTTATGCTCTTAAAACTA

>dme_circ_0008950
GTCTTATGCTCTTAAAACTAACGGGAAAGCAGCTGCTTGGTCCATTGGCAGTGATGGTAGTTTTAATTTC
CAAAAACTTGCCGATTCCAACTCTGCTCGTGCAGGCTCACTTGTGGAAAATGTAGAAATTCAGTATAAAA
ATAAGCAG
>dme_circ_0008950_junction_seq
TGTAGAAATTCAGTATAAAAATAAGCAGGTCTTATGCTCTTAAAACTAACGGGAAA
>dme_circ_0008951
GTTTTAATTTCCAAAAACTTGCCGATTCCAACTCTGCTCGTGCAGGCTCACTTGTGGAAAATGTAGAAAT
TCAGTATAAAAATAAGCAGGTCGGAGGAATAAAGATTATGTCGAATTTTGATGTTAATAAAATGGATGTC
GATGTGGAAATTTCGCGTGAGCAAAAAATTGGGTCCATAATCGTTAAATACGAAA
>dme_circ_0008951_junction_seq
ATTGGGTCCATAATCGTTAAATACGAAAGTTTTAATTTCCAAAAACTTGCCGATTC
>dme_circ_0008952
GTTTTAATTTCCAAAAACTTGCCGATTCCAACTCTGCTCGTGCAGGCTCACTTGTGGAAAATGTAGAAAT
TCAGTATAAAAATAAGCAGGTCGGAGGAATAAAGATTATGTCGAATTTTGATGTTAATAAAATGGATGTC
GATGTGGAAATTTCGCGTGAGCAAAAAATTGGGTCCATAATCGTTAAATACGAAAGTAATCAGCGACACG
CACAGGATTATTCCCTGGAGGCAAGTGCAAAGATTAACAAGCACTCCATAGATGTTATATCTAAGTGTGA
TTTTAACGGAAACGTCTATGTTGTCGATAACTCTTTAGTAACTAGTTGGGGAACTTTACTATCTGCAAAA
GGAGAAATAGGACAACGTTACTCGGCCCAAGACATTAATATTAATATTCAAGGCAATGTTCAAATTAGCG
GAAAGGACAAA
>dme_circ_0008952_junction_seq
CAATGTTCAAATTAGCGGAAAGGACAAAGTTTTAATTTCCAAAAACTTGCCGATTC
>dme_circ_0008953
GAAATTCAGTATAAAAATAAGCAGGTCGGAGGAATAAAGATTATGTCGAATTTTGATGTTAATAAAATGG
ATGTCGATGTGGAAATTTCGCGTGAGCAAAAAATTGGGTCCATAATCGTTAAATACGAAAGTAATCAGCG
ACACGCACAGGATTATTCCCTGGAGGCAAGTGCAAAGATTAACAAGCACTCCATAGATGTTATATCTAAG
TGTGATTTTAACGGAAAC
>dme_circ_0008953_junction_seq
TATATCTAAGTGTGATTTTAACGGAAACGAAATTCAGTATAAAAATAAGCAGGTCG
>dme_circ_0008954
GGAATAAAGATTATGTCGAATTTTGATGTTAATAAAATGGATGTCGATGTGGAAATTTCGCGTGAGCAAA
AAATTGGGTCCATAATCGTTAAATACGAAAGTAATCAGCGACACGCACAGGATTATTCCCTGGAGGCAAG
TGCAAAGATTAACAAGCACTCCATAGATGTTATATCTAAGTGTGATTTTAACGGAAACGTCTATGTTGTC
GATAACTCTTTAGTAACTAGTTGGGGAACTTTACTATCTGCAAAAGGAGAAATAGGACAACGTTACTCGG
CCCAAGACATTAATATTAATATTCAAGGCAATGTTCAAATTAGCGGAAAGGACAAA
>dme_circ_0008954_junction_seq
CAATGTTCAAATTAGCGGAAAGGACAAAGGAATAAAGATTATGTCGAATTTTGATG
>dme_circ_0008955
GATTATGTCGAATTTTGATGTTAATAAAATGGATGTCGATGTGGAAATTTCGCGTGAGCAAAAAATTGGG
TCCATAATCGTTAAATACGAAAGTAATCAGCGACACGCACAGGATTATTCCCTGGAGGCAAGTGCAAAGA
TTAACAAGCACTCCATAGAT
>dme_circ_0008955_junction_seq
TGCAAAGATTAACAAGCACTCCATAGATGATTATGTCGAATTTTGATGTTAATAAA
>dme_circ_0008956

GATTATGTCGAATTTTGATGTTAATAAAATGGATGTCGATGTGGAAATTTCGCGTGAGCAAAAAATTGGG
TCCATAATCGTTAAATACGAAAGTAATCAGCGACACGCACAGGATTATTCCCTGGAGGCAAGTGCAAAGA
TTAACAAGCACTCCATAGATGTTATATCTAAGTGTGATTTTAACGGAAACGTCTATGTTGTCGATAACTC
TTTAGTAACTAGTTGGGGAACTTTACTATCTGCAAAAGGAGAAATAGGACAACGTTACTCGGCCCAAGAC
ATTAATATTAATATTCAAGGCAATGTTCAAATTAGCGGAAAGGACAAA
>dme_circ_0008956_junction_seq
CAATGTTCAAATTAGCGGAAAGGACAAAGATTATGTCGAATTTTGATGTTAATAAA
>dme_circ_0008957
GATTATGTCGAATTTTGATGTTAATAAAATGGATGTCGATGTGGAAATTTCGCGTGAGCAAAAAATTGGG
TCCATAATCGTTAAATACGAAAGTAATCAGCGACACGCACAGGATTATTCCCTGGAGGCAAGTGCAAAGA
TTAACAAGCACTCCATAGATGTTATATCTAAGTGTGATTTTAACGGAAACGTCTATGTTGTCGATAACTC
TTTAGTAACTAGTTGGGGAACTTTACTATCTGCAAAAGGAGAAATAGGACAACGTTACTCGGCCCAAGAC
ATTAATATTAATATTCAAGGCAATGTTCAAATTAGCGGAAAGGACAAAGTTACTCAATGGATACTTAAA
>dme_circ_0008957_junction_seq
GGACAAAGTTACTCAATGGATACTTAAAGATTATGTCGAATTTTGATGTTAATAAA
>dme_circ_0008958
GTAATCAGCGACACGCACAGGATTATTCCCTGGAGGCAAGTGCAAAGATTAACAAGCACTCCATAGATGT
TATATCTAAGTGTGATTTTAACGGAAACGTCTATGTTGTCGATAACTCTTTAGTAACTAGTTGGGGAACT
TTACTATCTGCAAAAGGAGAAATAGGACAACGTTACTCGGCCCAAGACATTAATATTAATATTCAAGGCA
ATGTTCAAATTAGCGGAAAGGACAAAGTTACTCAATGGATACTTAAA
>dme_circ_0008958_junction_seq
GGACAAAGTTACTCAATGGATACTTAAAGTAATCAGCGACACGCACAGGATTATTC
>dme_circ_0008959
GGCAAGTGCAAAGATTAACAAGCACTCCATAGATGTTATATCTAAGTGTGATTTTAACGGAAACGTCTAT
GTTGTCGATAACTCTTTAGTAACTAGTTGGGGAACTTTACTATCTGCAAAAGGAGAAATAGGACAAC
>dme_circ_0008959_junction_seq
CTATCTGCAAAAGGAGAAATAGGACAACGGCAAGTGCAAAGATTAACAAGCACTCC
>dme_circ_0008960
GTGCAAAGATTAACAAGCACTCCATAGATGTTATATCTAAGTGTGATTTTAACGGAAACGTCTATGTTGT
CGATAACTCTTTAGTAACTAGTTGGGGAACTTTACTATCTGCAAAAGGAGAAATAGGACAACGTTACTCG
GCCCAAGACATTAATATTAATATTCAAGGCAAT
>dme_circ_0008960_junction_seq
AGACATTAATATTAATATTCAAGGCAATGTGCAAAGATTAACAAGCACTCCATAGA
>dme_circ_0008961
TTTAATAAGCTCTGAGGTGTCTCGGCTAATCCTAAAGTCGCTGTTGGTTTTGTCAGGTGTGCCGATTACT
TTAAGTATCCATTGAGTAACTTTGTCCTTTCCGCTAATTTGAACATTGCCTTGAATATTAATATTAATGT
CTTGGGCCGAGTAACGTTGTCCTATTTCTCCTTTTGCAGATAGTAAAGTTCCCCAACTAGTTACTAAAGA
GTTATCGACAACATAGACGTTTCCG
>dme_circ_0008961_junction_seq
AGAGTTATCGACAACATAGACGTTTCCGTTTAATAAGCTCTGAGGTGTCTCGGCTA
>dme_circ_0008962
TCGCTGTTGGTTTTGTCAGGTGTGCCGATTACTTTAAGTATCCATTGAGTAACTTTGTCCTTTCCGCTAA
TTTGAACATTGCCTTGAATATTAATATTAATGTCTTGGGCCGAGTAACGTTGTCCTATTTCTCCTTTTGC
AGATAGTAAAGTTCCCCAACTAGTTACTAAAGAGTTATCGACAACATAGACG

>dme_circ_0008962_junction_seq

TACTAAAGAGTTATCGACAACATAGACGTCGCTGTTGGTTTTGTCAGGTGTGCCGA

>dme_circ_0008963

TTTAGAATTTCAAAGAGTACCTTTCCTTTTCGGTCTCCTAAGAGTTCAGCAATGCTCGATATGATAATTG
GCTTTTCTTTCAACAGATCTAAACGAATATCAACACCTTTTTTGTGGGCCGAATGATAAAATGTTGTGTC
TAATCCGTATGACTTGTCAGTCTGTTTGAATAGTGCAGTGACTACATATGTCGAATCATCCAAAGGTTTA
AAAGTGCGAATATTAACTTTGATATCTTTTACAGAATCAAAGCTGGAATCTATAACATATGCTATATCTA
CTTTCTCGGCTCCATAACCTAATGAAGCATCCATTTTTACGTATTGTGCTCCGTACTGTGCGTTAAGTAC
GTACGTTTGTTTACCGGTAAGGTCACCATCTTTTATATTACCTGTTTGTTTCCGATGATATTTACCATTG
AACTTTAATGGAGACTCCATATTGTTAGTTTGAGCTTCAAAATCATAGCTGCCATCATTTGCCGTGCCGT
TCCATACTGTATTGACACGAGCATATTGCCCTAGACTGGTTTTAGAATCCAGGAAGAATTCTACATTGTA
TGCAGCGTTTTCATTTCCGATTAAAGATTTTAGGACCTTTCCATGACTGTTTATTTCTAAGGACTTCAAG
GTGGATTTCGGAATTTCAATATTAGCCTGAAGCCCATATTCAAGTGCTTGATTATTGTTGTAGTTTCCAT
TTGCACTTACAGAAAAAATCTCACCGTACTTACTTGTGATACTAACTACGGCATGCTGTGCACTGTAATC
TCCTAAGGCAAAAGCGATCTCAAAAGGTTGGGGTAGTGGATTTCCATAGGCCTTAAGGCTAAAATCGATG
TTTTTAGCATCCCCGTTTGGTTGCTGCTTAATTTGATAACTTATCTCCGACTTTTCTCCATTAAATGCCG
TGTAGACTAAATTACTGTTTGCAGAAAATTCTTTGGTTTTTGTGTTGAGCCGATCAAGCTTTCCGATTAG
TGAAATTGAGCGTTTCTTATTACTGCCAAAAGGTGTATCACTAAGCTGCGCGTCAATGTTGCCTTGAGAT
AAGCCACTTTTTGCATTTGTGGAAATCTTACGATTGATACTGCCGTCGATGACTCGACCATCGGGAGCGT
TAAATATAAAGGTACCTTGTATTTCCCCATTTCCTCGCAATTCGCCTTTTAAACTTCCATTAGCTTCGAA
AGCAATTATTTTGTCGTTTGCCTCACCAATGTTTTTAGTTGAGAATTTTAACTTTTCTATAGTGTTTTCT
GACTTCAAGTGCACTTTCCTTTTTCCATCAAGTGTTAGGGAAGCATCAATATCGTACTTTGGAGACTGTA
TGTGAAATTTAGATTCTATTTCTAGTTTGTGCTTCGGTTCAGTCTTCAGAGTGTCAATACTGGCTGTAAA
GTCACCCTTTCCATTTTTGGCTACACGAAACTCGCCTTTGGCAGTTAATTGATTTTTGACAATTAAGTTA
AGTTTGGCAAAGGATATTTTATCTTGCGGATGTTGAGACTCGCTAGTTAGTTTAATAAGCTCTGAGGTGT
CTCGGCTAATCCTAAAGTCGCTGTTGGTTTTGTCAGGTGTGCCGATTACTTTAAGTATCCATTGAGTAAC
TTTGTCCTTTCCGCTAATTTGAACATTGCCTTGAATATTAATATTAATGTCTTGGGCCGAGTAACGTTGT
CCTATTTCTCCTTTTGCAGATAG

>dme_circ_0008963_junction_seq

GTTGTCCTATTTCTCCTTTTGCAGATAGTTTAGAATTTCAAAGAGTACCTTTCCTT

>dme_circ_0008964

TTTGGCAAAGGATATTTTATCTTGCGGATGTTGAGACTCGCTAGTTAGTTTAATAAGCTCTGAGGTGTCT
CGGCTAATCCTAAAGTCGCTGTTGGTTTTGTCAGGTGTGCCGATTACTTTAAGTATCCATTGAGTAACTT
TGTCCTTTCCGCTAATTTGAACATTGCCTTGAATATTAATATTAATGTCTTGGGCCGAGTAACG

>dme_circ_0008964_junction_seq

TAATATTAATGTCTTGGGCCGAGTAACGTTTGGCAAAGGATATTTTATCTTGCGGA

>dme_circ_0008965

TTAAGTTTGGCAAAGGATATTTTATCTTGCGGATGTTGAGACTCGCTAGTTAGTTTAATAAGCTCTGAGG
TGTCTCGGCTAATCCTAAAGTCGCTGTTGGTTTTGTCAGGTGTGCCGATTACTTTAAGTATCCATTGAGT
AACTTTGTCCTTTCCGCTAATTTGAACATTGCCTTGAATATTAATATTAATGTCTTGGGCCGAG

>dme_circ_0008965_junction_seq

AATATTAATATTAATGTCTTGGGCCGAGTTAAGTTTGGCAAAGGATATTTTATCTT

>dme_circ_0008966

AATTTCAAAGAGTACCTTTCCTTTTCGGTCTCCTAAGAGTTCAGCAATGCTCGATATGATAATTGGCTTT

TCTTTCAACAGATCTAAACGAATATCAACACCTTTTTTGTGGGCCGAATGATAAAATGTTGTGTCTAATC
CGTATGACTTGTCAGTCTGTTTGAATAGTGCAGTGACTACATATGTCGAATCATCCAAAGGTTTAAAAGT
GCGAATATTAACTTTGATATCTTTTACAGAATCAAAGCTGGAATCTATAACATATGCTATATCTACTTTC
TCGGCTCCATAACCTAATGAAGCATCCATTTTTACGTATTGTGCTCCGTACTGTGCGTTAAGTACGTACG
TTTGTTTACCGGTAAGGTCACCATCTTTTATATTACCTGTTTGTTTCCGATGATATTTACCATTGAACTT
TAATGGAGACTCCATATTGTTAGTTTGAGCTTCAAAATCATAGCTGCCATCATTTGCCGTGCCGTTCCAT
ACTGTATTGACACGAGCATATTGCCCTAGACTGGTTTTAGAATCCAGGAAGAATTCTACATTGTATGCAG
CGTTTTCATTTCCGATTAAAGATTTTAGGACCTTTCCATGACTGTTTATTTCTAAGGACTTCAAGGTGGA
TTTCGGAATTTCAATATTAGCCTGAAGCCCATATTCAAGTGCTTGATTATTGTTGTAGTTTCCATTTGCA
CTTACAGAAAAAATCTCACCGTACTTACTTGTGATACTAACTACGGCATGCTGTGCACTGTAATCTCCTA
AGGCAAAAGCGATCTCAAAAGGTTGGGGTAGTGGATTTCCATAGGCCTTAAGGCTAAAATCGATGTTTTT
AGCATCCCCGTTTGGTTGCTGCTTAATTTGATAACTTATCTCCGACTTTTCTCCATTAAATGCCGTGTAG
ACTAAATTACTGTTTGCAGAAAATTCTTTGGTTTTTGTGTTGAGCCGATCAAGCTTTCCGATTAGTGAAA
TTGAGCGTTTCTTATTACTGCCAAAAGGTGTATCACTAAGCTGCGCGTCAATGTTGCCTTGAGATAAGCC
ACTTTTTGCATTTGTGGAAATCTTACGATTGATACTGCCGTCGATGACTCGACCATCGGGAGCGTTAAAT
ATAAAGGTACCTTGTATTTCCCCATTTCCTCGCAATTCGCCTTTTAAACTTCCATTAGCTTCGAAAGCAA
TTATTTTGTCGTTTGCCTCACCAATGTTTTTAGTTGAGAATTTTAACTTTTCTATAGTGTTTTCTGACTT
CAAGTGCACTTTCCTTTTTCCATCAAGTGTTAGGGAAGCATCAATATCGTACTTTGGAGACTGTATGTGA
AATTTAGATTCTATTTCTAGTTTGTGCTTCGGTTCAGTCTTCAGAGTGTCAATACTGGCTGTAAAGTCAC
CCTTTCCATTTTTGGCTACACGAAACTCGCCTTTGGCAGTTAATTGATTTTTGACAATTAAGTTAAGTTT
GGCAAAGGATATTTTATCTTGCGGATGTTGAGACTCGCTAGTTAGTTTAATAAGCTCTGAGGTGTCTCGG
CTAATCCTAAAGTCGCTGTTGGTTTTGTCAGGTGTGCCGATTACTTTAAGTATCCATTGAGTAACTTTGT
CCTTTCCGCTAATTTGAACATTGCCTTGAATATTAATATTAATGTCTTGGGCCGAG
>dme_circ_0008966_junction_seq
AATATTAATATTAATGTCTTGGGCCGAGAATTTCAAAGAGTACCTTTCCTTTTCGG
>dme_circ_0008967
GATATTTTATCTTGCGGATGTTGAGACTCGCTAGTTAGTTTAATAAGCTCTGAGGTGTCTCGGCTAATCC
TAAAGTCGCTGTTGGTTTTGTCAGGTGTGCCGATTACTTTAAGTATCCATTGAGTAACTTTGTCCTTTCC
GCTAATTTGAACATTGCCTTGAATATTAATATTAATG
>dme_circ_0008967_junction_seq
AACATTGCCTTGAATATTAATATTAATGGATATTTTATCTTGCGGATGTTGAGACT
>dme_circ_0008968
TTTGGCAAAGGATATTTTATCTTGCGGATGTTGAGACTCGCTAGTTAGTTTAATAAGCTCTGAGGTGTCT
CGGCTAATCCTAAAGTCGCTGTTGGTTTTGTCAGGTGTGCCGATTACTTTAAGTATCCATTGAGTAACTT
TGTCCTTTCCGCTAATTTGAACATTGCCTTGAATATTAATATTAATG
>dme_circ_0008968_junction_seq
AACATTGCCTTGAATATTAATATTAATGTTTGGCAAAGGATATTTTATCTTGCGGA
>dme_circ_0008969
TTAAGTTTGGCAAAGGATATTTTATCTTGCGGATGTTGAGACTCGCTAGTTAGTTTAATAAGCTCTGAGG
TGTCTCGGCTAATCCTAAAGTCGCTGTTGGTTTTGTCAGGTGTGCCGATTACTTTAAGTATCCATTGAGT
AACTTTGTCCTTTCCGCTAATTTGAACATTGCCTTGAATATTAATATTAATG
>dme_circ_0008969_junction_seq
AACATTGCCTTGAATATTAATATTAATGTTAAGTTTGGCAAAGGATATTTTATCTT
>dme_circ_0008970
[truncated: 444,243 more chars]
